# Extending the Coding Potential of Viral Genomes with Overlapping Antisense ORFs: A Case for the De Novo Creation of the Gene Encoding the Antisense Protein ASP of HIV-1

**DOI:** 10.3390/v14010146

**Published:** 2022-01-14

**Authors:** Angelo Pavesi, Fabio Romerio

**Affiliations:** 1 Department of Chemistry, Life Sciences and Environmental Sustainability, University of Parma, 43124 Parma, Italy; angelo.pavesi@unipr.it; 2 Department of Molecular and Comparative Pathobiology, Johns Hopkins University School of Medicine, Baltimore, MD 21205-2196, USA

**Keywords:** HIV-1, antisense protein, env, amino acid diversity, codon permutation test, nucleotide diversity, primate lentiviruses, symmetric evolution

## Abstract

Gene overprinting occurs when point mutations within a genomic region with an existing coding sequence create a new one in another reading frame. This process is quite frequent in viral genomes either to maximize the amount of information that they encode or in response to strong selective pressure. The most frequent scenario involves two different reading frames in the same DNA strand (sense overlap). Much less frequent are cases of overlapping genes that are encoded on opposite DNA strands (antisense overlap). One such example is the antisense ORF, asp in the minus strand of the HIV-1 genome overlapping the env gene. The asp gene is highly conserved in pandemic HIV-1 strains of group M, and it is absent in non-pandemic HIV-1 groups, HIV-2, and lentiviruses infecting non-human primates, suggesting that the ~190-amino acid protein that is expressed from this gene (ASP) may play a role in virus spread. While the function of ASP in the virus life cycle remains to be elucidated, mounting evidence from several research groups indicates that ASP is expressed in vivo. There are two alternative hypotheses that could be envisioned to explain the origin of the asp ORF. On one hand, asp may have originally been present in the ancestor of contemporary lentiviruses, and subsequently lost in all descendants except for most HIV-1 strains of group M due to selective advantage. Alternatively, the asp ORF may have originated very recently with the emergence of group M HIV-1 strains from SIVcpz. Here, we used a combination of computational and statistical approaches to study the genomic region of env in primate lentiviruses to shed light on the origin, structure, and sequence evolution of the asp ORF. The results emerging from our studies support the hypothesis of a recent de novo addition of the antisense ORF to the HIV-1 genome through a process that entailed progressive removal of existing internal stop codons from SIV strains to HIV-1 strains of group M, and fine tuning of the codon sequence in env that reduced the chances of new stop codons occurring in asp. Altogether, the study supports the notion that the HIV-1 asp gene encodes an accessory protein, providing a selective advantage to the virus.

## 1. Introduction

Most often, new genes are created by the transfer of existing genetic material through various mechanisms, such as exon shuffling, gene duplication, retroposition, lateral gene transfer, and gene fusion or fission [1]. However, in rare cases, new genes are created de novo [2]. This can also occur in genomic regions where a coding sequence is already present via the introduction of point mutations that generate a new start codon and/or that remove existing stop codons in a different reading frame. The resulting genomic region will, therefore, contain two overlapping open reading frames (ORFs): the ancestral or the ‘overprinted’ gene, and the novel or the ‘overprinting’ gene [3]. Typically, the original gene and the new gene can be identified with high accuracy based on their phylogenetic distribution [4]. Indeed, proteins that are the product of genes that are created de novo do not have homologs in other organisms and they are taxonomically restricted [5,6].

Overprinting is quite frequent in viral genomes [7,8]. There are two theories that have been proposed to explain the high abundance of overlapping genes in viral genomes [9]: the gene-compression theory (error-prone viral polymerases and biophysical constraints within the viral capsid drive the creation of overlapping genes that maximize the amount of information encoded in small genomes [10]) and the gene-novelty theory (de novo creation of genes is driven by selective pressure, giving rise to gene products that provide a selective advantage to the virus and become fixed in the population [3]). The high abundance of overlapping genes in viral genomes has allowed the use of statistical and computational methods to investigate the composition bias, structural features, evolution, and potential function of overlapping gene pairs and de novo proteins [3,9,11,12]. These studies showed that composition varies greatly between overlapping and non-overlapping genes, as well as between ancestral and novel genes in overlapping pairs [12]. New genes that emerged more recently evolve rapidly under positive or weakly purifying selection, while older ones evolve more slowly under increasingly stronger negative selection [4]. Interestingly, in some cases, overlapping genes show ‘asymmetric evolution’ whereby the two members of a pair evolve at different rates [13]. Proteins that are encoded by overlapping genes are enriched in high-degeneracy amino acids (arginine, leucine, serine) [12,14], and in amino acids with a high propensity toward structural disorder (arginine, proline, serine) [3,12,15,16]. In the absence of a binding partner, these proteins lack a stable secondary and tertiary structure, rapidly converting among various structural forms. Structural disorder affords proteins that are encoded by overlapping genes greater freedom of sequence evolution without loss of function [3]. The fixation of new genes in the viral population strongly suggests that their products provide a selective advantage [9]. Although most de novo genes encode for accessory proteins, they may promote viral pathogenicity or facilitate virus spread through various mechanisms [3,17,18,19,20,21,22,23].

Most overlapping gene pairs or triads that have been studied to date are encoded in two or three different reading frames of the same DNA strand. This is the case of the env, tat, and rev genes in the human retrovirus HIV-1 [24], and the p13/p30, tax, and rex genes of HTLV-1 [25]. Much less frequent are the cases of overlapping ORFs that are encoded on different DNA strands and in opposite orientations. The most intensely studied is the HTLV-1 basic leucine zipper (bZIP) factor (hbz) gene [26], which is encoded in the pX region of the proviral genome, a so-called ‘gene nursery’ due to the presence of a high number of new genes and overprinting events [11]. The hbz gene generates spliced and unspliced antisense transcripts from the proviral 3′LTR encoding for a 206-aa protein (HBZ) that contributes to HTLV-1 chronic infection [27,28]. The HBZ protein plays a role in the leukemic process following HTLV-1 infection [29,30,31]. Antisense genes are also found in HTLV-2, 3, and 4, which express the antisense proteins, APH-2, 3, and 4, respectively [32], and in the simian T-cell leukemia virus (STLV), which expresses the simian bZIP (SBZ) protein with function that is similar to HBZ [33].

A highly conserved antisense gene (asp) is also present in the minus strand of the HIV-1 genome overlapping the env ORF [34]. This gene was predicted to encode an antisense protein (ASP) of ~190 residues, rich in hydrophobic amino acids, and possibly associated with cellular membranes [34]. Based on its amino acid sequence, ASP presents several conserved features: intracellular N-terminal and C-terminal ends, and two transmembrane (TM) domains at both ends of an extracellular loop [35,36]. For a comprehensive review on the HIV-1 antisense protein ASP see Gholizadeh et al. [37].

The origin and evolution of the ORF-encoding ASP has been the focus of several studies. Cassan et al. reported that an ASP ORF of >150 aa is present almost exclusively in the pandemic HIV-1 strain (group M), and much less frequently in non-pandemic HIV-1 strains (groups O, N, and P) and SIV that is closely related to HIV-1 [38]. This study also found a positive correlation between the presence of the ASP ORF and viral subtype prevalence, suggesting a possible role of ASP in virus spread [38]. Finally, Cassan et al. showed the existence of selective pressure to maintain an intact ASP ORF in the HIV-1 genome by conserving the start codon and by avoiding early stop codons [38].

There are two subsequent reports that analyzed the evolution of the ASP ORF. The first study found a positive correlation between specific amino acid residues in ASP and preferential CCR5 vs. CXCR4 coreceptor usage [39]. The second study described OLGenie, a computer algorithm to study the co-evolution of overlapping genes [40]. When applied to ASP, OLGenie found that mutations that were occurring within this ORF are disproportionately synonymous, indicating an evolutionary effort to maintain the amino acid sequence of ASP [40].

In the present study, we have used a combination of statistical methods to further analyze the nucleotide and codon sequence, the genetic structure, and the evolution of the antisense ORF of HIV-1 strains belonging to group M. Our results support the hypothesis of a recent de novo origin of the HIV-1 antisense gene asp by overprinting of the env gene [38], through the creation of a start codon, the progressive removal of existing internal stop codons, and the evolution of a nucleotide sequence that reduces the likelihood of new stop codons emerging from synonymous mutations in env. These elements, along with the fact that an intact antisense ORF has been conserved in HIV-1 strains of group M for more than 80 years of worldwide spread before the introduction of highly active antiretroviral therapy limiting viral evolution, represent further evidence in support of the notion that this ORF encodes a protein providing a selective advantage to the virus.

## 2. Materials and Methods

### 2.1. Assembly of a Sample Set of 3725 env/asp Overlaps with ‘Intact’ asp and 1735 env/asp Overlaps with ‘Interrupted’ asp

From the Los Alamos HIV Sequence Database (www.hiv.lanl.gov/content/index, accessed on: 12 March 2021), we downloaded a dataset of 7590 aligned sequences of the entire env gene of HIV-1 (7561 sequences from groups M, N, O, and P), SIVcpz, and SIVgor (29 sequences). The mean length of the env sequences is 2574 nt (standard deviation (sd) = 39 nt), while the length of the multiple alignment is 3822 nucleotide positions due to the presence of several insertions and deletions (indels). The aligned region of env that overlaps the antisense ORF encoding ASP (env/asp) is comprised between nucleotide positions 1816 and 2682. The nucleotide positions 2679–2681 (triplet CAT) and 1818–1820 (triplet CTA) correspond, in the reverse frame, to the canonical ATG start codon and the canonical stop codon of the antisense ORF, respectively. In the reference genome sequence of HIV-1 (NCBI accession number K03455), env/asp is comprised between nucleotides 7371–7943. In the reference env sequence, env/asp is comprised between nucleotides 1147–1715. From the dataset, we removed 968 sequences, due to the presence of undetermined nucleotide sites in the env/asp region. Through analysis of the resulting dataset (6622 sequences), we assembled a first sample set of 3725 env/asp overlaps (Supplementary File S1A) fulfilling the following criteria: asp antisense frame was not interrupted by premature stop codons and delimited by an ATG start codon at canonical nucleotide positions 2679–2681 and a stop codon at canonical nucleotide positions 1818–1820. The mean length of the env/asp sequences is 564 nt (sd = 15 nt). All the sequences belong to viral strains from the HIV-1 group M, except one from SIVcpz (isolate MB66; NCBI accession number DQ373063). The complementary dataset with 3725 env sequences outside the asp overlap (as well as outside overlap with other HIV-1-coding sequences) is included in Supplementary File S1B. We also generated a control set of 1753 env/asp overlaps in which the asp ORF is delimited by start and stop codons at canonical positions but that have lost the ability to encode a full-length ASP protein due to the presence of one or more internal stop codons (Supplementary File S2A). The set includes 1231 sequences with asp ORF that is interrupted by 1 internal stop codon, 403 sequences with asp ORF interrupted by 2 internal stop codons, 102 env sequences with asp ORF that is interrupted by 3 internal stop codons, 11 env sequences with asp ORF that is interrupted by 4 internal stop codons, and 6 env sequences with asp ORF that is interrupted by 5 internal stop codons. All the sequences belong to the group M of HIV-1. The complementary dataset with 1735 env sequences outside the overlap with asp and other HIV-1 genes is included in Supplementary File S2B.

### 2.2. Comparative Analysis of the Nucleotide Diversity in the Overlapping and Non-Overlapping Region of env Gene

The sample set of the 3725 aligned env/asp overlaps was used to measure the genetic variability at first, second, and third codon position of the env gene. The measure of genetic variability that we used is the nucleotide diversity (π), which is defined as the number of differences per nucleotide site between two randomly chosen sequences from a population [41]. When more than two sequences are available, the average nucleotide diversity is computed as the arithmetic mean of all the pairwise comparisons. We, thus, carried out a total of (3725 × 3724)/2 pairwise comparisons and calculated the mean nucleotide diversity (π) at the first, second, and third codon positions of the env region that overlapped the antisense ORF-encoding ASP. Being an antisense overlapping coding region, the first, second, and third codon positions of env overlap the second, first, and third codon positions of asp, respectively. By extending the analysis to the non-overlapping coding regions of env, we calculated the mean nucleotide diversity of the first, second, and third codon positions of env non-overlap. We tested the hypothesis that the region of env overlapping asp is subjected to significant evolutionary constraints by using the Student’s *t*-test to compare the mean values of nucleotide diversity at codon positions of env overlap with those at the corresponding codon positions of env non-overlap.

### 2.3. Testing the Hypothesis of Asymmetric/Symmetric Evolution in the Antisense Overlap env/asp

Asymmetric evolution means that—within a given overlapping-gene arrangement—the protein that is encoded in one frame is significantly more variable than that which is encoded in the other frame, because the two proteins evolved under different selection pressures. In the converse scenario, symmetric evolution means that the two encoded proteins are subjected to similar selection pressures. In this case, the number of amino acid substitutions in the protein encoded in one frame is expected to be not significantly different from that in the protein that is encoded in the other frame. We used the sample set of the 3725 aligned env/asp overlapping regions to assess if this sense/antisense overlap evolved in accordance with one or the other model. We first translated each env/asp nucleotide sequence into the corresponding ENV/ASP amino acid sequence. We then carried out a total of (3725 × 3724)/2 pairwise comparisons. For each comparison, we compared the number of amino acid identities and differences that were found in ENV with those that were found in ASP using the contingency-table χ^2^ test [42], with a cut-off value of 3.84 for 1 degree of freedom (*p* < 0.05). A χ^2^ value below the cut-off indicates that the number of amino acid substitutions that are found in ENV is not significantly different from that found in ASP (symmetric evolution).

### 2.4. Assembly of an Enlarged Sample Set of 4362 env/asp Overlaps

We enlarged the sample set of 3725 overlaps (cases with an antisense ORF delimited by canonical ATG and stop codons) to a sample set including 637 additional overlaps, all belonging to the group M of HIV-1. The great majority of them (597 cases) were overlaps in which the antisense ORF starts at the canonical ATG codon, but it is interrupted by an early stop codon that is located upstream of the canonical stop. However, these antisense ORFs are all >450 nt, which is compatible with the expression of an antisense protein of >150 aa with a conserved carboxyl-terminal transmembrane domain [38]. An additional 27 sequences represent cases where the antisense overlap is delimited by a canonical stop codon while lacking a canonical ATG start codon. However, the presence of an alternative downstream ATG allows the expression of an antisense protein of >150 aa and a conserved amino-terminal transmembrane domain. Finally, 12 sequences represent overlaps where the antisense ORF has the canonical ATG start codon but lacks the canonical stop codon, and 1 sequence represents an overlap where the antisense ORF lacks both canonical ATG and stop codons. Nevertheless, these 13 antisense ORFs retain the ability to encode a protein >150 aa due to the conservation of both N- and C-terminal transmembrane domains. The mean length of the antisense ORF nested within the 637 additional overlaps is 524 nt. The accession number and nucleotide sequence of the 637 additional env/asp overlaps are reported in Supplementary File S3. Once we obtained a sample set of 4362 env/asp overlaps (3725 + 637), we assembled a control set including a total of 2260 spurious env/asp overlaps. The great majority of them (2183 cases) belong to the group M of HIV-1, while the remaining ones belong to the groups N, O, and P of HIV-1, SIVcpz, and SIVgor. A spurious overlap consists of an env region overlapping an antisense ORF that is interrupted prematurely by one or more early stop codons and having a length ≤ 450 bp. The lack of a full antisense ORF within env is not rare, as it was found to occur in 23% of the group M sequences that were examined by Cassan et al. [38]. The accession number and nucleotide sequence of the 2260 spurious overlaps are reported in Supplementary File S4.

### 2.5. Detection of Critical Sites in the Antisense Protein by Comparative Analysis of the Amino Acid Composition

From the 4362 env/asp overlaps, we obtained a sample set of 4362 functional antisense proteins via translation of the nucleotide sequence of each antisense ORF. We carried out translation also in the control set of 2260 spurious env/asp overlaps. In this case, translation of an antisense ORF that was interrupted by premature stop codons and thus having lost the ability to encode a full protein (>150 residues) yielded a control set of 2260 ‘non-functional’ antisense proteins. We aligned the functional and non-functional antisense proteins in accordance with the pattern of the multiple alignment of env sequences from Los Alamos HIV Sequence Database (www.hiv.lanl.gov/content/index, accessed on: 5 September 2021) (see paragraph 2.1 above). We then compared the amino acid content of the 4362 functional antisense proteins with that of the 2260 ‘non-functional’ antisense proteins at each position of the alignment. Using the contingency-table χ^2^ test [42], we first compared the amino acid content in accordance with four functional classes: acidic (Asp, Glu), basic (Arg, His, Lys), hydrophobic (Ala, Ile, Leu, Met, Phe, Pro, Trp, Val), and polar (Asn, Cys, Gln, Gly, Ser, Thr, Tyr). In the case of a χ^2^ value that was above the cut-off of significance, we selected only the amino acid positions in which there was a difference in the content of functional amino acids that was higher than 5% or lower than −5%. Following the identification of a significantly different content of a given functional class, we used again the χ^2^ test to identify the specific amino acid(s) within the class that were significantly enriched or depleted in the functional antisense protein.

### 2.6. Assembly of a Dataset of env Sequences Covering a Wide Phylogenetic Range of Primate Lentiviruses

The sample and control datasets we assembled previously contain a very large fraction of env sequences from group M of HIV-1 and a very small fraction from groups N, O, and P of HIV-1 and from SIV infecting African apes (chimpanzee or gorilla). To extend the phylogenetic range, we first extracted from the Los Alamos HIV Sequence Database (www.hiv.lanl.gov/content/index, accessed on: 5 September 2021) a compendium of 95 ENV protein sequences covering a wide spectrum of primate lentiviruses. We then downloaded the corresponding nucleotide sequences from NCBI and selected the coding region of env which is homologous to the env/asp overlap in the reference genome sequence of HIV-1 (accession number K03455, nucleotides 7371–7943). After the exclusion of sequences with undetermined nucleotide sites, we obtained a dataset of 84 env sequences. In addition to 8 sequences from the groups N, O, and P of HIV-1 and 8 sequences from HIV-2, it contains 68 sequences of SIV infecting not only African apes but also several species of the family *Cercopithecidae*. We further enriched the dataset by including 40 env sequences from the various subtypes of group M of HIV-1 (A, B, C, G, H, J, K, F, and CRF01), in accordance with their worldwide prevalence. This dataset (124 sequences) is available as Supplementary File S5, and it was used to assess whether the env/asp overlap had a monophyletic or polyphyletic origin. For this analysis, we utilized the software MEGA (version 11 for macOS, https://www.megasoftware.net/, accessed on: 3 November 2021). First, the set of 124 sequences was uploaded into MEGA and aligned by MUSCLE (Multiple sequence comparison by log-expectation). Next, the phylogeny of the sequences was reconstructed using the maximum likelihood method (bootstrap method with 500 replications, and Kimura 2-parameter model). 

## 3. Results

### 3.1. The Nucleotide Diversity of the env Region Overlapping asp Is Significantly Lower than That in the Non-Overlapping Region

Using the sample set of 3725 aligned env sequences as benchmark (see Materials and Methods), we sought to compare the nucleotide diversity at the first, second, and third codon positions within the portion of the env gene overlapping asp with that, within the portion of the env gene, does not overlap asp. In addition to the asp overlap in the antisense strand, env overlaps two genes in the sense strand: the 3′ end of the vpu gene (87 nt; positions 6225-6311 in reference HIV-1 HXB2 genome, NCBI accession number K03455), and the second exon of the tat/rev genes (279 nt, positions 8376-8654 of HIV-1 HXB2). We excluded these two overlaps in the sense strand from the env non-overlapping region that was in our analyses. The measure of genetic variability we utilized was the nucleotide diversity (π), defined as the number of differences per nucleotide site between two randomly chosen sequences from a population [41]. When more than two sequences were available, the average nucleotide diversity was computed as the arithmetic mean of all pairwise comparisons. The sample set of the 3725 aligned env/asp overlaps was used to measure the genetic variability at the first, second, and third codon positions of the env gene.

**Figure 1:**
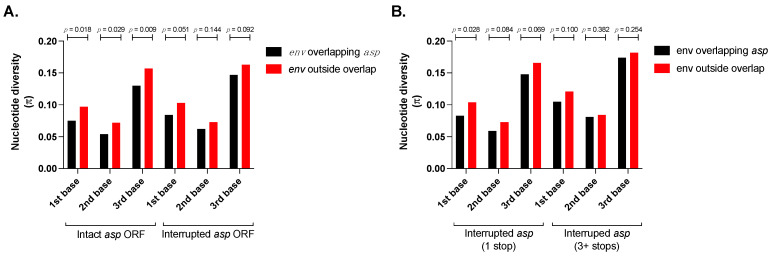
The nucleotide diversity of the env gene in the region of overlap with the asp ORF compared to the region of non-overlap. We assembled a dataset of 3725 env sequences that overlap an ‘intact’ asp ORF, defined as containing start and stop codons at canonical positions and lacking internal stop codons. We also assembled a set of 1735 env sequences that overlap an ‘interrupted’ asp ORF, defined as containing start and stop codons at canonical positions but containing one or more internal stop codons. All the sequences are derived from HIV-1 strains belonging to group M, except for one, which is from the SIVcpz strain MB66 (NCBI acc. number DQ373063). The env sequences from each strain were subdivided into a region of overlap with the asp ORF (env overlapping asp, black bars), and a region of non-overlap with asp (env outside overlap, red bars). The latter does not include the env sequences that overlap with vpu and tat/rev. We found that the nucleotide diversity (π) in the first, second, and third base of each codon in the env region of overlap with asp were significantly lower than the ones in the region of non-overlap in the case of env sequences overlapping ‘intact’ asp (*p* = 0.018, *p* = 0.027, and *p* = 0.009, respectively; Student’s *t*-test), but not in the case of env sequences that were overlapping ‘interrupted’ asp (*p* = 0.051, *p* = 0.144, and *p* = 0.092, respectively; Student’s *t*-test) (panel **A**). From the 1735 env sequences that were overlapping ‘interrupted’ asp ORF, we extracted a subset of 1231 sequences with one internal stop codon in asp, and a subset of 119 sequences with three or more internal stop codons in asp. We found that the nucleotide diversity (π) in in the env region of overlap with asp was significantly different compared to the region outside the overlap only in first base of the 1231 sequences with one stop codon in asp (*p* = 0.028, Student’s *t*-test), while all the other comparisons were not significantly different (panel **B**).

Using the Student’s *t*-test, we calculated the nucleotide diversity (π) in the region of env overlapping asp (positions 1816 to 2682 in the alignment). Within this region, we selected the nucleotide positions without gaps or with a frequency of gaps ≤ 1%. Of the 168 codons that were examined, we found a mean π value of 0.075 at the first base, 0.054 at the second base, and 0.130 at the third base. We then calculated the nucleotide diversity in the portion of env that did not overlap asp, vpu, and tat/rev. It consisted of three subregions that were comprised between positions 151–1815, 2683–3264, and 3634–3822. After the exclusion of nucleotide positions with a frequency of gaps >1%, we found over a total of 509 codons with a mean π value of 0.097 at the first base, 0.072 at the second base, and 0.157 at the third base. The Student’s *t*-test revealed that the mean nucleotide diversity in the region of overlap was significantly lower than that in the region of non-overlap (Figure 1A): 0.075 vs. 0.097 at the first base (*t*-test = 2.08; *p* = 0.018), 0.054 vs. 0.072 at the second base (*t*-test = 1.90; *p* = 0.029), and 0.130 vs. 0.157 at the third base (*t*-test = 2.36; *p* = 0.009).

The region of the HIV-1 genome containing the env/asp overlap coincides, in part, with the highly conserved Rev response elements (RRE; [43]), which could, in part, account for the lower degree of nucleotide diversity of the env sequence overlapping an intact asp ORF compared to env outside the overlap (Figure 1A). To address that possibility, we performed the analysis of nucleotide diversity using a set of 1753 HIV-1 sequences in which the asp ORF contains canonical start and stop codons, but it is interrupted by one or more internal stops and, therefore, lacks protein-coding capacity. We found that the difference of nucleotide diversity in the first, second, and third base of env within the overlap and the env sequence outside the overlap dropped below statistical significance when the asp ORF lacked protein-coding capacity due to internal stop codons (Figure 1A): 0.084 vs. 0.103 at the first base (*t*-test = 1.74; *p* = 0.051), 0.062 vs. 0.073 at the second base (*t*-test = 1.06; *p* = 0.144), and 0.147 vs. 0.163 at the third base (*t*-test = 1.33; *p* = 0.092). These results show that presence of RRE in the env/asp overlap does not entirely account for the lower nucleotide diversity of the env sequence overlapping asp.

Finally, from the set of 1753 sequences with interrupted asp ORF, we extracted two subsets: a subset of 1231 sequences with only one internal stop codon in asp, and another subset of 119 sequences with three or more internal stop codons in asp. We then performed the nucleotide diversity analysis in the two subsets of the sequences with interrupted asp ORF. Our results show that the difference in nucleotide diversity between env within the overlap and env outside the overlap is lower in the subset of sequences with three or more premature stops in the asp ORF (Figure 1B). Indeed, for the first subset of 1231 sequences and the second subset of 119 sequences we found: at the first base 0.083 vs. 0.104 (*t*-test = 1.91; *p* = 0.028) and 0.105 vs. 0.121 (*t*-test = 1.28; *p* = 0.100), respectively; at the second base 0.059 vs. 0.073 (*t*-test = 1.38; *p* = 0.084) and 0.081 vs. 0.084 (*t*-test = 0.30; *p* = 0.382), respectively; and at the third base 0.148 vs. 0.166 (*t*-test = 1.48; *p* = 0.069) and 0.174 vs. 0.182 (*t*-test = 0.66; *p* = 0.254), respectively. It should be noted that, while the *p* values are above the threshold of statistical significance in all the pairwise comparisons (except in the case of sequences with asp ORF with one stop at first base), they are greater in the second subset of 119 sequences with three or more stops than in the first subset of 1231 sequences with only one stop in asp.

### 3.2. Symmetric Evolution of the env/asp Overlap

Given an overlapping-gene arrangement, asymmetric evolution means that the protein that is encoded in one frame is significantly more variable than that which is encoded in the other frame, because the two proteins evolved under different selection pressures. In the converse scenario, symmetric evolution means that the two encoded proteins are subjected to similar selection pressures. In this case, the number of amino acid substitutions in the protein that is encoded in one frame is expected to be not significantly different from that in the protein that is encoded in the other frame.

**Figure 2:**
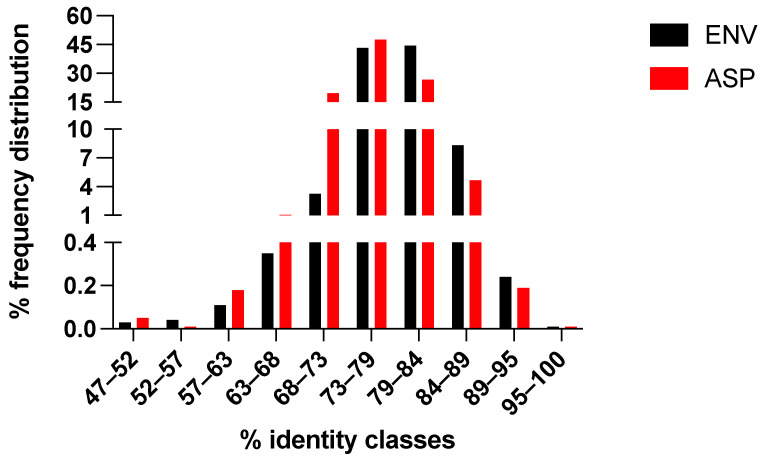
Evolution of the env and asp ORFs in the region of overlap. Using the dataset of 3725 env sequences that overlap an ‘intact’ asp ORF, we first translated each env and asp nucleotide sequence into the corresponding ENV and ASP amino acid sequence. We then carried out a total of (3725 × 3724)/2 pairwise comparisons of ENV and ASP amino acid sequences. Each comparison assessed the number of amino acid identities and differences that were found in ENV (black bars) with those that were found in ASP (red bars) using the contingency-table χ^2^ test [42]. The frequency distribution of the amino acid identity in ENV (black bars) was very similar to that in ASP (red bars), with only 792 cases (0.011%) showing a χ^2^ > 3.84 (1 degree of freedom, *p* < 0.05). The mean amino acid identity of ENV (79.0%; sd = 3.6%) was found to be close to that of ASP (76.7%; sd = 4.3%). These findings indicated the env/asp evolved symmetrically.

The set of 3725 aligned env/asp sequences was also a valuable sample to assess whether the overlap evolved in a symmetric or asymmetric fashion (see the Materials and Methods). After translation of each env/asp nucleotide sequence into the corresponding env/asp amino acid sequence, we carried out a total of (3725 × 3724)/2 pairwise comparisons. For each comparison, the number of amino acid identities and differences in env, as well as those in asp, were included into a 2 × 2 contingency table and analyzed using the χ^2^ test with a cut-off value of 3.84 (1 degree of freedom, *p* < 0.05). A χ^2^ value below the cut-off is a signature of symmetric evolution because the number of amino acid identities in ENV is not significantly different from that in ASP. Over a total of (3725 × 3724)/2 pairwise comparisons, we found only 792 cases (0.011%) with a χ^2^ value that was above the cut-off. This finding indicated that the env/asp evolved symmetrically. Indeed, the mean amino acid identity of ENV (79.0%; sd = 3.6%) was found to be close to that of ASP (76.7%; sd = 4.3%), as well as the frequency distribution of the amino acid identity in ENV was very similar to that in ASP (Figure 2). Symmetric evolution was further confirmed when we carried out a χ^2^ analysis by moving along the overlap with a window scanning of 30 amino acid positions and a step of one position. As found previously, the number of cases with a χ^2^ value above the cut-off was extremely low (data not shown).

### 3.3. Identification of 46 Critical Amino Acid Sites in the Antisense Protein ASP

A previous study by Cassan et al. [38] showed that an ‘intact’ antisense ORF (i.e., with start and stop codons at canonical positions, and without internal stops) is present only in pandemic HIV-1 strains (group M). On the other hand, non-pandemic HIV-1 strains (groups N, O, and P) and closely related SIVcpz and SIVgor strains contain antisense ORFs that are unable to express a protein due to the presence of internal stop codons. This led to the hypothesis that the product of the antisense ORF (ASP) may play a role in pathogenesis or virus spread [38]. On the other hand, the lack in some group M HIV-1 strains of an ‘intact’ asp gene has been explained by the fact that ASP plays an accessory role, which can be lost without fatally compromising HIV-1 replication [38]. However, the expression of ASP in vivo remains a matter of controversy. Thus, we sought evidence that might shed light onto this open question.

**Figure 3:**
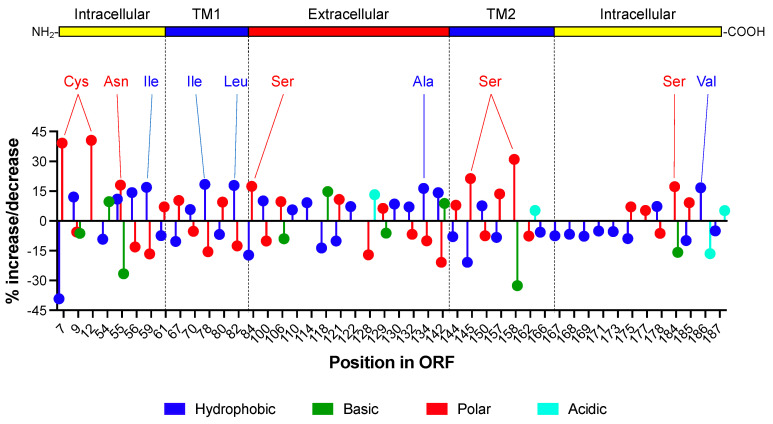
Evolution of the intact and interrupted asp ORFs. We assembled a set of 4362 sequences that contained an intact asp ORF that was able to express a protein >150 aa, and a set of 2260 control sequences where the asp ORF was interrupted by internal stops. The asp ORFs in both datasets were translated into proteins and then we compared the amino acid content in accordance with four functional classes: hydrophobic (Ala, Ile, Leu, Met, Phe, Pro, Trp, Val), polar (Asn, Cys, Gln, Gly, Ser, Thr, Tyr), basic (Arg, His, Lys), and acidic (Asp, Glu) using the contingency-table χ^2^ test [42]. In the case of a χ^2^ value above the cut-off of significance, we selected only the amino acid positions where functional amino acids (internal stop codons in the control set were excluded from the analysis) showed an enrichment that was higher than 5% or a depletion that was lower than –5%. Following the identification of the positions with a significantly higher/lower content of a given class of amino acids, we again used the χ^2^ test to identify the specific amino acid(s) within that class with enrichment that was higher than 5% or depletion that was lower than −5% in ASP that was encoded by intact ORFs. The figure reports the percent enrichment or depletion of hydrophobic (blue bars), polar (red bars), basic (green bars), and acidic (aqua bars) amino acids in ASP that was encoded by intact ORFs compared to interrupted ORFs. Residues that were enriched more than 15% are indicated above the graph. Superimposition of the linear map of ASP allows the localization of each enriched/depleted amino acid with respect to the major functional domains of the protein.

For these studies, we reasoned that if the antisense ORF does not express a protein product regardless of the presence of internal stop codons, then the sequence of ‘intact’ and ‘interrupted’ antisense ORFs should be under the same degree of evolutionary constraint that is imposed by the env ORF on the opposite strand. In other words, if the presence or absence of start and stop codons in the antisense strand does not identify an actual protein-coding ORF, then the ‘intact’ and ‘interrupted’ antisense ORF should both evolve following random genetic drift resulting in no significant difference in codon sequence. To address this hypothesis, we used the sample set of 4362 env sequences with an ‘intact’ antisense ORF (potentially able to express a full-length ASP) as a benchmark and a control set of spurious env/asp overlaps consisting of 2260 env sequences with an antisense ORF interrupted by early stop codons and thus lacking the ability to encode a functional ASP. We performed a comparative analysis, site-by-site, of the ASP amino acid sequence (obtained following translation of the antisense ORFs in the two sets of env/asp overlaps) to identify potential key differences. Using the contingency-table χ^2^ test [42], we detected 46 positions that showed a significant enrichment that was higher than 5% or a depletion that was lower than −5% of amino acid content in the ASP from ‘intact’ antisense ORFs compared to ASP from ‘interrupted’ ORFs. The pattern of enrichment/depletion in the four functional classes of amino acids (hydrophobic, polar, basic, and acidic) along the ASP sequence is shown in Figure 3. Among others, the figure highlights a 40% enrichment of the polar amino acid, Cys in the N-terminal intracellular domain, a 30% enrichment of another polar amino acid (Ser) in the C-terminal transmembrane domain TM2, and an 18% enrichment of the hydrophobic amino acids, Ile and Leu in the N-terminal transmembrane domain TM1. Further details of the comparative analysis are reported in Supplementary Table S1.

These results show that the ‘intact’ and ‘interrupted’ antisense ORFs evolved in different directions and under different selective pressures that resulted in the enrichment/depletion of specific codons in one compared to the other. These differences could include the selective pressure that the sense (env) and antisense (asp) ORFs impose on each other when an ‘intact’ antisense gene leads to the expression of an actual protein, but not in the case of spurious overlaps that do not express an antisense protein. In other words, they could reflect the ability and the inability of ‘intact’ and ‘interrupted’ antisense ORFs, respectively, to express an antisense protein ASP playing a role in the virus life cycle. While formal demonstration that ASP is expressed in vivo will require wet lab studies, the analyses above point in that direction.

### 3.4. Analysis of env Sequences from a Large Dataset of Primate Lentiviruses Suggests a De Novo Origin of the Antisense ORF Encoding ASP

There are two alternative hypotheses that could be envisioned to explain the origin of the antisense ORF. The first one asserts that a functional antisense ORF was originally present in a progenitor of contemporary lentiviruses, and it was subsequently lost in all of its descendants except for most HIV-1 strains of group M due to selective advantage. The alternative hypothesis suggests that a functional antisense ORF originated very recently with the emergence of group M HIV-1 strains from SIVcpz. The study by Cassan et al. [38] favored the latter, but conclusive evidence in support of either hypothesis is still lacking.

**Figure 4:**
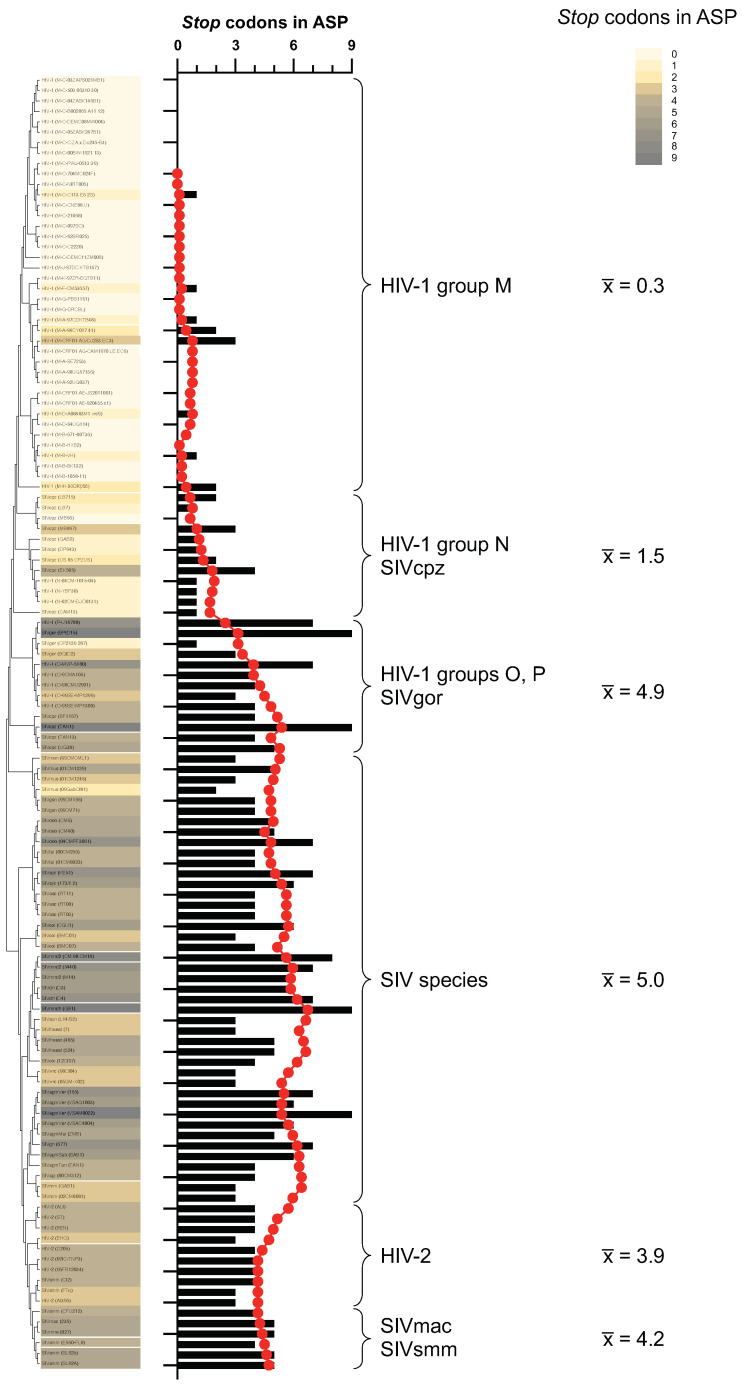
Phylogenetic analysis of internal stops in the asp ORF from primate lentiviruses. We assembled a dataset of 124 sequences spanning the region from the site of the canonical start codon to the site of the canonical stop codon. The dataset includes 40 HIV-1 strains of group M, and 84 HIV-1 strains of groups N, O, and P, HIV-2, and non-human primate lentiviruses (SIV). For group M HIV-1 strains, we included sequences from clades A, B, C, D, F, G, H, J, and K, and two circulating recombinant forms (CRF01_AE and CRF01_AG). The number of sequences representing each clade approximates the worldwide prevalence of the clade itself (except for F, H, J, and K; prevalence < 1%), and it approximates the ratio of intact and interrupted antisense ORF that was reported previously [38]. We counted the number of internal stop codons in each of the 124 antisense ORFs of the dataset. The alignment that was generated with the 124 sequences was utilized to build a maximum likelihood phylogenetic tree (see Methods), which is juxtaposed on to the graph reporting the number of internal stop codons in each antisense ORF (black bars), and the average number of stop codons in the previous 10 sequences moving from the top to the bottom of the cladogram (red dots). The figure also shows the average number of stop codons in six major lentiviral groups in the cladogram.

To address this question, we assembled a dataset of 124 env sequences that were homologous in the region of overlap with asp, which covers a wide range of primate lentiviruses, including the four groups of HIV-1 (M, N, O, and P), HIV-2, and several SIV species infecting African apes and Old-World monkeys (Supplementary File S5). Using the software MEGA [44], we aligned the 124 env sequences and generated a maximum likelihood cladogram (Figure 4). Next, we counted the number of internal stop codons within the antisense ORF of each viral strain and we superimposed these values onto the phylogenetic tree (details of this analysis are reported in Supplementary Table S2). In the case of an old origin of the antisense ORF followed by its progressive disappearance, we should expect to see in the tree a homogeneous distribution of the number of internal stop codons. In contrast, we observed a skewed distribution, with a mean number of internal stop codons ranging around 4.0 to 5.0 in multiple SIV species infecting Old-World monkeys, HIV-2, SIVgor, and HIV-1 groups O and P. The mean number of stop codons decreased to ~1.5 in HIV-1 strain of group N and in SIVcpz. Although in most of these strains the antisense ORF remained unable to express a full-length ASP protein, the SIVcpz strain MB66 contains an ‘intact’ asp gene with no internal stops that is potentially able to express an antisense protein. Of interest, the SIVcpzMB66 genome was previously found to have the highest sequence homology to HIV-1 group M [45]. Finally, the mean number of internal stop codons declined to <1 in HIV-1 group M, thus allowing the expression of a full-length (>150 aa) ASP protein. Of note, the mean number of stop codons in the asp ORF of HIV-1 group M has remained well below 1 (i.e., ~0.3) despite 60–80 years of virus spread among humans throughout the world in the absence of antiretroviral therapy to limit viral evolution, suggesting that the preservation of an intact antisense ORF with protein coding potential might be advantageous to the virus [38].

To further test this hypothesis, we performed a permutation test whereby the order of synonymous codons within the env region overlapping the antisense ORF was randomly rearranged while leaving unchanged both the virus codon bias and the amino acid sequence of the protein. For instance, the TCA codon encoding serine at position 5 in the env reference sequence is swapped with the synonymous AGT codon (Ser) at position 29. We swapped all the synonymous codons of env in the region of overlap with asp. After performing 10,000 permutations, we calculated the percent frequency of outcome sequences in which the rearranged order of the synonymous codons in env yields an antisense ORF that is not interrupted by stops. For this test, we selected a subset of 68 env sequences in which the antisense ORF is delimited by canonical ATG and stop codons, or by alternative ATG and stop codons that are located within 10 codon positions from the canonical ones. The viral strains that were included in this subset of env sequences and the results of the permutation test are reported in Table 1, which is sorted in order of decreasing number of internal stops in the original (not permuted) antisense ORF. We found that 88% of the SIV sequences (29 out of 33) showed an almost null frequency of uninterrupted antisense ORFs (from 0 to 2%). Exceptions were four SIVcpz isolates, yielding a range of frequency of uninterrupted antisense ORF from 10 to 49%. A null frequency was also obtained for HIV-2 (2 sequences) and in HIV-1 group P (1 sequence), but it increased to 8% and 20% in two HIV-1 group N sequences. In the case of eight env sequences from HIV-1 group M strains with an antisense ORF interrupted by stops, we obtained a mean frequency of 13% uninterrupted sequences. In contrast, a remarkably high mean frequency (46.1%; sd = 14.1%) was found in the 22 env sequences from HIV-1 group M strains with an ‘intact’ antisense ORF. Of interest, a similar outcome was obtained with the env sequence from SIVcpzMB66, which yielded 49% of rearranged codon sequences with antisense ORF that were not interrupted by stops.

Altogether, these findings confirm the hypothesis of a recent de novo origin of the asp gene by overprinting of the env gene [38] and suggest the progressive removal of internal stops as a potential path. Further, they support the hypothesis that in the HIV-1 group M the region of env overlapping asp developed and maintained a fine tuning in the order of synonymous codons that favors the appearance and conservation of an antisense ORF without internal stops.

## 4. Discussion

The plus strand of the HIV-1 proviral genome contains nine genes that encode 16 different proteins in three reading frames. This remarkable coding capacity is organized in less than 10 kb, and it involves a high degree of sequence overlap among eight of the nine genes: gag and pol, pol and vif, vif and vpr, vpr and tat, tat and rev, vpu and env, and env and tat/rev. In particular, the HIV-1 genome displays a very high degree of complexity in the region that contains the env gene, which overlaps with vpu in its 5′, and with tat/rev in its 3′. The present study further highlights the remarkable complexity of the HIV-1 genome, as it supports the presence of a tenth gene—asp—which is encoded in the minus (antisense) strand of the genome, and it overlaps env. Our results confirm the prediction that HIV-1 expresses a protein that is translated from an antisense RNA, originally proposed by Miller [34], and, at the same time, it corroborates the conclusions of the study by Cassan et al. [38].

In our study, three lines of evidence support the presence of an antisense ORF in the HIV-1 genome. First, we found that the region of env that overlaps the asp gene shows a lower degree of nucleotide diversity compared to the region of env that does not overlap asp or other HIV-1 genes (Figure 1A). Although the asp ORF was first identified more than 30 years ago and multiple studies have investigated the expression and function of its protein product (for a comprehensive see [37]), part of the HIV-1 scientific community still doubts its protein-coding potential. If asp were a “pseudo-ORF” without the ability to express a protein that affords a selective advantage to HIV-1, then the asp sequence would not be under selective pressure, and it would not affect the nucleotide diversity (i.e., evolution) of env encoded on the opposite strand. However, our results show significantly lower nucleotide diversity in the env region of overlap with asp compared to the region of non-overlap. Our interpretation of this result is that the asp ORF does encode a (non-essential) protein that provides a selective advantage to HIV-1, and, therefore, the nucleotide diversity of the env in the region of overlap is limited by a “tug of war” between the need for env to evolve and escape immune responses and the need for the virus to preserve the asp ORF. At the same time, it is important to acknowledge that the position within the HIV-1 genome of the highly conserved Rev response elements (RRE; [43]) coincides, in part, with the env/asp overlap. This could, in part, account for the lower degree of nucleotide diversity of the env sequence that is overlapping the intact asp compared to env outside the overlap (Figure 1A). However, the difference in nucleotide diversity between the env sequence outside the overlap and the env sequence within the overlap drops below statistical significance when the asp ORF is interrupted due to internal stop codons and lacks protein-coding capacity (Figure 1A). Therefore, the presence of RRE in the env/asp overlap does not entirely account for the lower nucleotide diversity of the env sequence overlapping asp. Moreover, the difference in nucleotide diversity between the env region overlapping asp vs. outside the overlap is smaller (and with higher *p* values) in the case of viral sequences with three or more internal stops compared to the ones with only one internal stop in asp (Figure 1B). A higher number of internal stops suggests that, presumably, the former lost asp-coding capacity and the adaptive conflict between env and asp earlier than the latter, thus allowing more time to normalize the nucleotide diversity of env inside and outside the region of overlap with asp. Altogether, we conclude that the lower nucleotide diversity of env in the region of overlap with intact asp ORF is due to the evolutionary constraints that the two protein-coding ORFs impose on each other.

The ability of the antisense ORF to limit sequence evolution of env in the region of overlap indicates an effort by the virus to conserve the asp. Indeed, the significantly lower nucleotide diversity at each codon position of env in the region of overlap compared to that outside of the overlap reflects the evolutionary constraints that are typical of dual-coding regions [9,12,46]. The extremely low nucleotide diversity at second codon position of env is a consequence of the overlaps with the first codon position of asp, and we know from the genetic code that a substitution in second position causes an amino acid change in 100% of cases and in first position in 95% of cases. In agreement with this, we found that the env/asp overlap evolves according to a symmetric model (Figure 2), because each accepted amino substitution in ENV protein was paired to an accepted amino acid substitution in ASP protein. Symmetric evolution occurs rather frequently in viral overlapping genes, as it was found in 43% (28 out of 65) of sense overlaps [9].

The second line of evidence in our results confirming the existence of a functional antisense ORF within env that is expressing the ASP protein is the divergent codon evolution of asp in viral strains where the ORF is intact (no internal stop codons) compared to viral strains where the ORF is interrupted (one or more internal stop codons) (Figure 3). This indicates that the ‘intact’ and ‘interrupted’ antisense ORF evolved under different selective pressures that resulted in the enrichment/depletion of specific amino acids in one compared to the other. The enrichment of amino acids that were found in the protein that was encoded by intact antisense ORFs raises an interesting question about the evolution of the overlap. That is, was the selection pressure acting on env to determine parallel amino acid changes in asp or vice versa? A further analysis of the datasets we collected could answer the question. Indeed, the wide collection of several thousand env/asp overlaps given in Supplementary Files (4362 overlaps with intact antisense ORF and 2260 overlaps with interrupted antisense ORF) could be used as reference datasets for subsequent evolutionary studies.

Finally, our detailed sequence analyses of a broad range of primate lentiviruses infecting humans, African apes, and Old World monkeys show that their genomes have evolved in such a way as to progressively remove internal stop codons within the asp ORF (Figure 4). Moreover, our permutation tests indicate that the HIV-1 genome has developed a fine-tuning of the codon bias in the region of env overlapping asp that reduces the likelihood of new stop codons emerging in the antisense ORF following synonymous mutations in env (Table 1). The study by Cassan et al. [38] demonstrated that an intact antisense ORF is present only in pandemic HIV-1 strains (group M), and it is absent in all other primate lentiviruses, including non-pandemic HIV-1 groups. Our results point in the same direction and they support the conclusion of a de novo origin of the antisense ORF asp.

In conclusion, the present study shows that the antisense ORF asp of HIV-1 is functional and it encodes a protein that provides a selective advantage to the virus. Indeed, this ORF has been conserved through decades of viral replication and worldwide spread before and after the introduction of antiretroviral therapy in the mid 1990s. Its presence in >80% of high-prevalence HIV-1 strains is not the consequence of happenstance. Rather, this, and previous studies [38], strongly suggest a functional role of the asp product in viral replication. The next challenge will be to provide biological evidence of this and to identify the molecular mechanism(s) that underlie its function. Recent studies may provide indications about future avenues of research aimed at tackling this challenge [35,47]. 

## Figures and Tables

**Table 1:** Test of permutation of synonymous codons of env within the region of overlap with asp using 68 sequences from multiple human and non-human lentiviral genomes. Columns 1–4 report the name of the virus strain, NCBI accession number, the maximum length of the asp ORF within the region of overlap, and the number of internal stop codons in the asp ORF, respectively. For each sequence, we performed 10,000 permutations whereby we randomly swapped the order of all synonymous codons in the env ORF. We then determined the number of stop codons in the asp ORF that resulted from the codon swap in env [column 5; mean (sd)], and the percentage of the 10,000 permutations that yielded an asp ORF without internal stop codons (column 6).

Virus Species (Group-Clade-Isolate)	NCBI acc. #	Codons from ATG to First Stop	Stops between Canonical ATG and Stop	Mean Number (sd) of Internal Stops in the 10,000× Permutated Antisense ORF	Percent of 10,000× Permutated Antisense ORF Not Interrupted by Stops
SIVgor (BPID15)	KP004990	67	9	7.5 (1.6)	0
SIVmnd1 (GB1)	M27470	29	9	7.3 (1.2)	0
SIVagmVer (VSAM0022)	KR862356	19	9	7.0 (1.7)	0
SIVcpz (TAN1)	AF447763	17	9	6.2 (1.5)	0
SIVagmVer (155)	M29975	18	7	7.0 (1.5)	0
SIVgri (677)	M58410	85	7	6.0 (1.3)	0
HIV-1 (P-U14788)	HQ179987	70	7	6.0 (1.1)	0
SIVagmVer (VSAC4004)	KR862336	97	6	6.3 (1.4)	0
SIVagmVer (VSAG1003)	KR862363	18	6	5.7 (1.5)	0
SIVmac (239)	M33262	34	5	5.0 (1.0)	0
SIVagmMal (ZMB)	LC114462	29	5	5.0 (1.3)	0
SIVmne (027)	U79412	34	5	4.9 (1.1)	0
SIVcpz (UG38)	JN91690	54	5	3.9 (1.3)	0
SIVgsn (99CM71)	AF468658	75	4	2.9 (1.3)	2
SIVsmm (CI2)	JX860430	16	4	2.9 (0.8)	0
SIVsmm (CFU212)	JX860407	50	4	4.4 (1.0)	0
SIVagmTan (TAN1)	U58991	90	4	4.4 (1.3)	0
SIVcpz (BF1167)	JQ866001	19	4	3.0 (1.0)	0
SIVcpz (EK505)	DQ373065	117	4	4.0 (1.3)	0
SIVtal (00CM266)	AY655744	116	4	5.0 (1.3)	0
SIVtal (01CM8023)	AM182197	72	4	5.3 (1.2)	0
SIVgsn (99CM166)	AF468659	94	4	3.8 (1.2)	0
SIVsmm (FTq)	JX860414	42	4	4.7 (1.2)	0
HIV-2 (96FR12034)	AY530889	38	4	3.9 (1.0)	0
HIV-1 (M-CRF01-AG-91DJ_263)	JQ715398	67	3	2.1 (1.0)	4
SIVmus (01CM1246)	EF070329	125	3	3.3 (1.5)	2
SIVgor (CP2139.287)	FJ424866	85	3	2.9 (1.2)	1
HIV-2 (EHO)	U27200	42	3	3.5 (0.9)	0
SIVcpz (MB897)	EF535994	127	3	2.9 (1.0)	0
SIVgor (BQID2)	KP004991	176	3	4.1 (1.3)	0
SIVcpz (GAB2)	AF382828	96	2	1.2 (0.9)	24
SIVcpz (US.85.CPZUS)	AF103818	151	2	1.6 (0.9)	10
HIV-1 (M-A-94CY017.41)	AF286237	44	2	1.5 (0.9)	9
SIVmus (09GabOI81)	KF304707	0	2	3.2 (1.2)	0
SIVcpz (LB715)	KP861923	148	2	3.1 (1.0)	0
HIV-1 (M-H-90CR056)	AF005496	108	2	3.1 (0.8)	0
SIVcpz (DP943)	EF535993	145	1	0.7 (0.7)	41
HIV-1 (M-D-A08483M1.vrc9)	HM215358	110	1	0.8 (0.7)	39
HIV-1 (M-F-CM53657)	AF377956	132	1	1.3 (0.9)	21
HIV-1 (N-02CM-DJO0131)	AY532635	136	1	1.2 (0.8)	20
HIV-1 (M-C-C113-E6-2S)	HM639260	136	1	1.5 (1.1)	17
HIV-1 (M-B-VH)	AF146728	113	1	1.4 (0.9)	13
HIV-1 (N-YBF30)	AJ006022	139	1	1.9 (1.1)	8
SIVcpz (LB7)	DQ373064	88	1	1.4 (0.6)	0
HIV-1 (M-A-97CDKTB48)	AF286238	140	1 ^†^	1.6 (0.7)	0
HIV-1 (M-B-1058-11)	AY331295	191	0	0.4 (0.6)	66
HIV-1 (M-C-503-06310-30)	KT183169	171	0	0.4 (0.6)	64
HIV-1 (M-C-03ZAPS021MB1)	DQ369978	182	0	0.4 (0.6)	65
HIV-1 (M-C-04ZASK146B1)	AY772699	187	0	0.4 (0.6)	62
HIV-1 (M-C-92BR025)	U52953	187	0	0.5 (0.6)	60
HIV-1 (M-CRF01-AE-620435_c01)	JX512900	189	0	0.5 (0.7)	55
HIV-1 (M-C-21068)	AF067155	193	0	0.6 (0.7)	53
HIV-1 (M-C-097SO)	MF373199	184	0	0.6 (0.7)	50
HIV-1 (M-G-DRCBL)	AF084936	183	0	0.6 (0.7)	50
SIVcpz (MB66)	DQ373063	182	0	0.6 (0.7)	49
HIV-1 (M-C-C2220)	U46016	183	0	0.7 (0.7)	47
HIV-1 (M-B-HXB2)	K03455	190	0	0.8 (0.8)	41
HIV-1 (M-J-97DC.KTB147)	EF614151	186	0	0.9 (0.8)	38
HIV-1 (M-B-671-00T36)	AY423387	188	0	0.9 (0.8)	36
HIV-1 (M-C-05ZASK247B1)	DQ369994	181	0	0.9 (0.8)	34
HIV-1 (M-G-PBS1191)	MH705134	181	0	1.0 (0.9)	30
HIV-1 (M-D-94UG114)	U88824	188	0	1.0 (0.8)	30
HIV-1 (M-K-97ZR-EQTB11)	AJ249235	180	0	1.2 (0.9)	25
HIV-1 (M-CRF01-AG-CAM1970 LE.EC6)	JQ715394	155	0 ^†,‡^	0.5 (0.7)	59
HIV-1 (M-A-92UG037)	U51190	158	0 ^†^	0.6 (0.7)	53
HIV-1 (M-CRF01-AE-JS2011001)	KM111555	176	0 ^‡^	0.6 (0.7)	49
HIV-1 (M-A-98UG57136)	AF484509	162	0 ^†^	1.1 (0.9)	26
HIV-1 (M-A-SE7253)	AF069670	162	0 ^†^	1.2 (0.9)	22

^†^, viral strains in which the premature stop codon at position 12 from the canonical start codon is followed by a new in-frame start codon after the premature stop. The new start codon re-opens the ASP reading frame, essentially nullifying the premature stop codon. In the case of these sequences, only the premature stop codons downstream of the new start codon are counted. ^‡^, viral strain in which the ASP ORF contains an early stop codon in proximity of the canonical stop. Despite the early stop codon, the ASP ORF still encodes a protein of >150 aa with intact functional domains, and, therefore, this sequence is classified as having zero internal stop codons.

## Data Availability

Data supporting the results reported in this study can be found in the Supplementary Material section and at www.hiv.lanl.gov/content/index (accessed on: 5 September 2021).

## Supplementary Materials

The following information is available at: https://www.mdpi.com/article/10.3390/v14010146/s1. Supplementary File S1A: Sample set of 3725 env sequences overlapping intact asp. Supplementary File S1B: Sample set of 3725 env sequences outside the region of overlap with intact asp. Supplementary File S2A: Sample set of 1735 env sequences overlapping interrupted asp. Supplementary File S2B: Sample set of 1735 env sequences outside the region of overlap with interrupted asp. Supplementary File S3: Sample set of 637 env sequences overlapping asp lacking start and stop codons at canonical positions, but with protein-coding capacity. Supplementary File S4: Sample set of 2260 env sequences overlapping asp lacking protein-coding capacity. Supplementary File S5: Sample set of 124 env/asp overlap sequences from a wide phylogenetic range of primate lentiviruses. Supplementary Table S1: evolution of the intact and interrupted asp ORFs. Supplementary Table S2: list of viral strains used to generate the cladogram in Figure 4.
