# Supplementary material for: Extending the Coding Potential of Viral Genomes with Overlapping Antisense ORFs: A Case for the De Novo Creation of the Gene Encoding the Antisense Protein ASP of HIV-1
Source: Viruses. 2022 Jan 14;14(1):146. doi: 10.3390/v14010146 (PMC8781085; doi:10.3390/v14010146)
Supplement: Supplementary file 1 [file Supplementary File S1B.pdf › Supplementary File S1B.pdf]

Supplementary File S1B.

Supplementary File S1B is a complement of Supplementary File S1A. The latter contains a sample set of 3725 *env/asp* overlaps in which the *asp* ORF, delimited by a start and stop codons at canonical positions, can encode a full-length ASP protein. Supplementary File S1B contains the non-overlapping coding region of the 3725 *env* sequences. It includes the region of *env* outside the antisense overlap with *asp*, as well as the region outside the sense overlap with genes *vpu* and *tat/rev*. The non-overlapping coding region of *env* is reported in the alignment format with gaps and it consists of 2436 nucleotide positions. Considering the 3822 nucleotide positions of the multiple alignment of *env* (see paragraph 2.1 in Materials and Methods), the non-overlapping coding region is comprised between nucleotide positions 151-1815, 2683-3264 and 3634-3822. All sequences belong to the group M of HIV-1, except for that from SIV infecting chimpanzee (isolate cpzMB66; acc. number DQ373063). The non-overlapping coding region of *env* is also reported in the format without gaps.

```
>K03455
GCT---ACA------------------------------------------
GAAAAATTGTGGGTCACAGTCTATTATGGGGTACCTGTGTGG---AAGGAAGCAACC---
ACCACTCTATTTTGTGCATCAGATGCTAAAGCATATGATACAGAG---GTACATAAT----------------
--------------GTTTGGGCCACA---CATGCCTGTGTACCCACAGAC---
CCCAACCCACAAGAAGTAGTATTGGTA---------AATGTGACAGAAAATTTTAAC----------------
--------------------------ATGTGGAAAAATGACATGGTAGAACAGATGCATGAGGATATA---
ATCAGTTTATGGGATCAAAGCCTA---AAGCCATGTGTAAAATTA---
ACCCCACTCTGTGTTAGTTTAAAGTGCACTGATTTGAAGAATGATACTAATACCAATAGT-------------
-------------------------------------------------------------------------
-------------------------AGTAGCGGGAGAATGATAATGGAGAAAGGAGAGATAAAA---
AACTGCTCTTTCAATATCAGCACAAGCATAAGA---------------------------
GGTAAGGTGCAGAAAGAATATGCATTT---TTTTATAAACTTGATATAATACCAATAGATAAT----------
-------------------------------------------------------------------------
----------------------
GATACTACCAGCTATAAGTTGACAAGTTGTAACACCTCAGTCATTACACAG---
GCCTGTCCAAAGGTATCCTTTGAGCCAATTCCCATACATTATTGTGCCCCGGCTGGTTTTGCGATTCTAAAAT
GTAAT---AATAAGACGTTCAAT------GGAACAGGACCATGTACAAAT-----------------------
-GTCAGC---ACAGTACAATGTACACATGGAATTAGGCCAGTAGTATCAACT---
CAACTGCTGTTAAATGGCAGTCTAGCAGAAGAAGAG------------
GTAGTAATTAGATCTGTCAATTTCACGGACAAT---------GCTAAAACCATAATAGTACAGCTGAAC----
-----------ACATCTGTAGAA---------ATTAATTGTACAAGACCCAACAACAATACAAGAAAA-----
-AGAATCCGTATCCAG------AGAGGACCAGGGAGAGCA---TTT------GTTACAATAGGAAAA------
------------ATAGGAAATATG------------AGACAAGCACATTGT---AACATTAGTAGAGCA---
AAATGGAATAACACTTTAAAACAGATAGCTAGCAAATTAAGA---------------GAACAATTTGGA----
--------------------------------AATAATAAAACAATAATCTTTAAG------
CAATCCTCAGGAGGGGACCCAGAAATTGTA---ACGCACAGTTTTAATTGTGGAGGGGAA---
TTTAAGCAGCTCCAGGCAAGAATCCTGGCTGTGGAAAGATACCTAAAGGATCAACAGCTCCTGGGGATTTGGG
GTTGCTCTGGAAAACTCATTTGCACCACTGCTGTGCCTTGGAATGCTAGTTGGAGT-----------------
----------------------------AATAAATCTCTGGAACAGATTTGG------AAT------
CACACGACCTGGATGGAGTGG---GACAGAGAAATTAACAATTACACAAGCTTAATACAC---
TCCTTAATTGAAGAATCGCAAAACCAGCAAGAA----------------------------------------
-----------------------------
AAGAATGAACAAGAATTATTGGAATTAGATAAATGGGCAAGTTTGTGGAATTGGTTTAACATAACAAATTGGC
TGTGGTATATAAAATTATTCATAATGATAGTAGGAGGCTTGGTAGGTTTAAGAATAGTTTTTGCT---
GTACTTTCTATAGTG---------AATAGAGTTAGGCAGGATATTCACCA------
TTATCGTTTGCTGTTAGCTTGCTCAATGCCACAGCCATAGCAGTAGCTGAG---
GGGACAGATAGGGTTATAGAAGTAGTACAAGGAGCTTGTAGAGCT----------------------------
--------ATTCGCCACATA------CCTAGAAGAATAAGACAGGGCTTGGAAAGGATTTTGCTA---TAA
>KF725913
GTT---GCA------------------------------------------
GAAAACTTGTGGGTTACTGTTTACTATGGGGTACCTGTGTGG---AAAGATACAGAG---
```

ACCACCTTGTTTTGTGCATCAGATGCTAAAGCACATGAAGCAGAA---GTACATAAT----------------
--------------GTCTGGGCCACA---CATGCCTGTGTACCCACAGAC---
CCCAACCCACAAGAAATACCTTTGGTA---------AATGTGACAGAAAAGTTCAAC----------------
--------------------------ATATGGAAAAATAACATGGTAGAGCAGATGCAGACAGATATA---
ATCAGTCTATGGGACCAGAGCCTA---AAGCCATGTGTATCATTA---
ACCCCTCTCTGCGTTACTTTAGATTGTAGTCATAACATCACATCAACCAATGCAACC----------------
----
---CTTAAAGAAATAGAAATGGCA---
AACTGCTCTTTCAATACGACCACAGAATTAAGG--------------------------
GATAAGCAACAGAAAGTGTATTCACTT---TTTTATAAACTTGATATAGTAAAACTTAATAAT----------
----

AATAATGGTAGTAAGCAGTTTAGATTAATACATTGCAATACCTCAACCATTACACAG---
GCTTGTCCAAAGGTATCCTTTGAACCAATTCCCATACATTATTGTGCCCCAGCTGGTTTTGCGATTCTAAAGT
GTAAC---GAGGTAGGGTTCAAT------GGATCAGGACTATGCAGGAAT----------------------
-GTCAGC---ACAGTACAATGTACACATGGAATCAGGCCAGTAATATCAACT---
CAATTGCTGTTGAATGGCAGTCTAGCAGAAGGAGAG-----------
GTACAAATTAGATCTGAAAATATCACAAACAAT---------GCCAAAACCATAATAGTGCAATTTAAT----
-----------GAGACTGTAGAA---------ATTAATTGTACCAGGCCTAATAACAATACATTGAGA-----
-AGGGTAAGTATA------------GGACCAGGACGAGCA---TTC------TATGCAAGAGATAGA------
---------ATAATGGGAGATATA-----------AGACAAGCACATTGC---AATGTCAGTAGAGGA---
AAATGGAATCAAACATTACAAAGGGTAGTCTTACAATTAGAA---------------AAACACTTTAAG----
-----------------------------------AACAAAACAATAAAGTTTACT------
GAACCCTCAGGAGGGGACTTAGAAATTATG---ACACACAGTTTTAATTGTGGAGGAGAA---
TTTAAACAGCTCCAGGCACGAGTCCTGGCTGTGGAAAGATACCTAAAGGATCAACAGCTCCTAGGAACATGGG
GTTGCTCTGGAAAACTCATCTGCACCACTAATGTGCCTTGGAACTCTAGTTGGAGT-----------------
----------------------------AATAAATCTATGAATGAGATATGG------GAT------
AACATGACCTGGTTACAATGG---GATAAAGAAATTGATAATTACACACAAATAATATAT---
GGGCTAATTGAAGAATCGCAGATCCAGCAGGAA---------------------------------------
----
AGGAATGAAAAAGATTTGTTGGCATTTGACAAATGGGCAAGTCTGTGGACATGGTTTGACATATCAAACTGGC
TGTGGTATATAAGACTTTTTATAATGATAGTAGGAGGCTTAATAGGATTAAGAATAATTTTTGCT---
GTGCTTTCTATAATA---------AATAGAGTTAGGCAGGGATACTCACCT------
CTGTCGTTTGCTATTAATTGCTTAGATACCCTAGCAATAGCAACAGCTAAC---
TGGACAGATAGGGTTATAGAATTAGTACAAAGCATTTGTAGAGCT----------------------------
--------TTTCTCCACATA------CCTAGAAGAATCAGACAGGGCATCGAAAGGGCTTTGCTG---TAA
>KY968401
GCT---GAA---
GATCAAAAGTGGGTCACAGTCTATTATGGGGTACCTGTGTGG---AAAGAAGCAACC---
ACCACTCTTTTTTGTGCATCAGATGCTAAAGCATATGACACAGAG---GCACATAAT----------------
--------------GTTTGGGCCACA---CATGCCTGTGTACCCACAGAC---
CCTAACCCACAAGAAGTAGTAATGGAA---------AATGTAACAGAGAATTTTAAC----------------
--------------------------ATGTGGAAAAATAACATGGTAGAACAGATGCATGAGGATATA---
ATCAGTTTATGGGATGAAAGCCTA---AAGCCATGTGTAAAATTA---
ACTCCACTCTGTGTTACTTTAAATTGCACTAATTTGAAGAACAATAATACCACCACCACCAATACTGCTGCCT
CTCCTAGTAGT---
---GGGGGAGAAATAAAA---
AACTGCTCTTTCAATATAACCACAGGCATAGGA--------------------------
AAAAAGGTGAGGAGTGAATATGCAATT---TTTAATAAGCTTGATATAGTACCAATAGATAAT----------
----

GATAATACAAGTTATATGTTGATAAGTTGTAACACCTCAGTCATTACACAG---
GCCTGTCCAAAAACTTCCTTTAATCCAATTCCCATACATTATTGTGCCCCGGCTGGTTTTGCGATTCTCAAGT
GTAAT---GATAAGAAATTCAAT------GGAACAGGACCATGTACAAAT----------------------
-GTCAGC---ACAGTACAATGTACACATGGAATTAGGCCAGTAGTATCAACT---
CAAATGCTGTTAAATGGCAGTTTAGCAGAAGGAGAG-----------
GTAATAATTAGATCTGAAAATCTCACGAACAAT---------GCTAAAACCATAATAGTACAGCTGAAT----
-----------GAAACTGTAGAA---------ATTAATTGTACAAGACCCACCAACAATACAAGAAAA-----
-AGTATACATATG-----------GGATGGGGAAGAGCA---TTT------TATGCAACAGGAGAA------
---------ATAATAGGAGATATA-----------AGACAAGCACATTGT---AACCTTAGTGCAGTA---

AAATGGAACAACACTTTAAAACAGATAGTTAGAAAACTAAAA---------------GAACATTTTGAA----
-----------------------------------AATAAAACAATAATTTTTAAA------
CCATCCTCAGGAGGGGACCCAGAAATTACA---ATGTACACCTTTAATTGTGGAGGGGAA---
TTTAAACAGCTCCAGGCAAGAGTCCTGGCTGTGGAAAGATACCTAAGTGATCAACGGTTCCTAGGGATTTGGG
GTTGCTCTGGAAAACTCATCTGCACCACTACTGTACCTTGGAATAATAGTTGGAGT-----------------
----------------------------AATAAATCTCTGGAAACAATTTGG------AAC------
AACATGACCTGGATGCAGTGG---GAGAAAGAAATTGACAATTACACACACTTAATATAC---
ACCTTACTTGAACAATCGCAGAACCAACAAGAA--

AAGAATGAACAAGAATTATTAGAATTGGATAAGTGGGCAAATTTGTGGAATTGGTTTGACATAACAAATTGGC
TGTGGTATATAAAAATATTCATAATGATAGTAGGAGGCTTAGTAGGTTTAAGAATAGTTTTTACT---
GTAATTTCTATAGTG---------AATAGAGTTAGGCAGGATACTCACCA------TTATCATTC-----
TATTTTGCTCAATGCCACAGCTGTAGCAGTAGCGGGG------ACAGAG---
GTAATAGAAGTAGTACAAAGA---TTTGGAGGT------------------------------------
ATCCTCCACATA------CCA---CGAATAAGACAGGGCTTAGAAAGAGCTTTGCAA---TA-
>KY968403
GCT---ACA--
AAAAATTTGTGGGTCACAATCTATTATGGGGTACCTGTGTGG---AAAGAAGCAACC---
ACCACTTTATTCTGTGCATCAGATGCTAAAGCATATGACACAGAG---GTACATAAT----------------
--------------GTTTGGGCCACA---CATGCCTGCGTACCCACAGAC---
CCTAACCCACAAGAAATAAGATTGGAA---------AATGTGACAGAAAATTTTAAC----------------
--------------------------ATGTGGAAAAATAACATGGTAGAGCAAATGCATGAGGATATA---
ATCAGTTTATGGGATGAAAGCCTG---AAGCCATGTGTAAAATTA---
ACCCCACTCTGTGTTACTTTAAATTGCACTAATTTGAACAATGCTACTAATGCCAATATT-------------
----
-------------------------------------AGTCAAAGGATAGAGGGAGAAATGAAA---
AACTGCTCTTTCAATGTCACCTCAAATATAGGA---------------------------
AATAAAATGCAGAAAGAATATGCACTT---TTTTATAAACTTGATGTAGTACCAATAGAAGGT----------
----

GATAACACAAGCTATACTTTGATACATTGTAACACCTCAGTCATTACACAG---
GCCTGTCCAAAGGTAACCTTTGAACCAATTCCTATACATTATTGTACCCCGGCTGGTTTTGCGATTCTAAAGT
GTAAT---GATAATGAGTTCAAT------GGAACAGGACCATGTAAAAAT-----------------------
-GTTAGC---ACAGTACAATGTACACATGGAATTAGGCCAATAGTGTCAACT---
CAACTGCTGTTAAATGGCAGTCTAGCAGAAGAAGGG------------
GTAGTAATTAGATCTGAAAACCTCACGAACAAT---------GCTAAAACCATAATAGTACAGCTGAAT----
-----------GAATCTGTAGTA---------ATTAATTGTACAAGACCCAGCAACAATACAAGAAAA-----
-GGTATACATCTA------------GCATGGGGGAGAACA---ATT------TATGCAACAGGAGAA------
---------ATAATAGGAGACATA------------AGACAAGCACATTGT---AACCTTACCAAAAGA---
CAATGGGAAGACACTTTAAAAAAGGTAGCTATAAAATTACAA---------------GAACAATTTAAA----
-----------------------------------AATAAAACAATAGTCTTTAAC------
CACTCCTCAGGAGGAGACCCAGAAATTGTA---ATGCACACTTTTAATTGTGGAGGGGAA---
TTTAAGCAGCTCCAGGCAAGAGTCCTGGCTGTGGAAAGATACCTAAGGGATCAACAGCTCCTAGGGATTTGGG
GTTGCTCTGGAAAACTCATCTGCACCACTACTGTGCCTTGGAATGCTAGTTGGAGT-----------------
----------------------------AATAAATCTCTGGATGAAATTTGG------AAT------
AACATGACCTGGATACAGTGG---GAGAGAGAAATTGACAATTACACAAACATAATATAC---
ACTTTAATTGAAGAATCGCAGAACCAACAAGAA--

AAAAATGAACAAGACTTATTGGCATTGGATCAATGGGCAAATTTGTGGAATTGGTTCAGTATATCGAATTGGC
TGTGGTATATAAAAATATTCATAATGATAGTAGCAGGCTTAGTAGGTTTAAAAATAGTTTTTTCT---
ATAATTTCTATAGTG---------AATAGAGTTAGGCAGGATACTCACCA------TTGTCATTC-----
TATCTTGCTCGATGCCACAGCTATAGCAGTAGCGGGG------ACAGAG---
ATTATAGAAGTAGCACAAAGGATTGGA---GCT------------------------------------
ATCCTCCATATC------CCA---AGAATAAGACAGGGCTTCGAAAGGGCTTTGCTA---TA-
>AY037269
GCT---GCA---GAA------------------AAT---AAT---AAT---
ACTAATTTGTGGGTCACAGTTTATTATGGGGTACCTGTGTGG---AAAGAAGCAACC---
ACCACTCTATTTTGTGCCTCAGATGCTAAAGCATATGACACAGAG---GTACATAAT----------------
--------------GTTTGGGCCACA---CATGCCTGTGTACCCACAGAC---
CCTGACCCACAAGAAATAAATTTGCCA---------AATGTGACAGAAAATTTTAAC----------------

--------------------------ATGTGGAAAAATAACATGGTAGAACAGATGCATGAGGATATA---
ATCAGTTTATGGGATCAAAGCCTA---AAGCCATGTGTAAAATTA---
ACCCCACTCTGTGTTACTTTAAATTGCACTGATCTGAATGCTACTGAAATCAATATGAATAATGCTAATGCT-
----
-------------ACTGGGACCACTAATACCAATATCACCACCAAAGACATACAAAATATGAAA---
AACTGTTCTTTCAATATCACCGAAGGCATAAAA---------------------------
AATAGAAGGAAGAAACAACATGCAATC---TTTGATAAACTTGATGTAGTACCAATAGAGAAT----------
----

AATAGTACAAGCTATATGTTGGTAAGTTGTAACACCTCAGTCATTACACAA---
GCCTGTCCAAAAGTATCCTTTGAACCAATTCCCATTCATTATTGTGCCCCGGCTGGTTTTGCGATTTTAAAGT
GTAGA---GATAAGAAGTTCAAT------GGAACAGGACCATGTATCAAT-----------------------
-GTCAGC---ACAGTACAATGTACACATGGAATTAGGCCAGTAGTGTCAACT---
CAACTGCTGTTAAATGGCAGTCTGGCAGAAGAAGAG------------
ATAGTAATTAGATCTGAAAATTTCACGGACAAT---------GCTAAAACCATAATAGTACAGTTGAAC----
-----------CAAACTGTAAAA---------ATTAACTGTACAAGACCCAACAACAATACAAGACAA-----
-GGTATACCTATA------------GGACCGTTGGGTTCA---TTT------TATGCAACAACAGAA------
---------ATAATAGGAAATATA------------AGAAAAGCATATTGT---AACGTGAGTAAAAAA---
GATTGGAATGACACTTTAAGCAAGGTAGCTAAAAAATTAGGA---------------GAACAGTGGAAT----
-----------------------------------CAAACAACAATAATCTTTGAT------
CATCCATCAGGAGGGGACCTAGAAATTGTA---ATGCACACTTTTAATTGTGGAGGGGAA---
TTCAAGCAGCTCCAAGCAAGAGTCCTGGCTGTAGAAAGATACCTAAAGGATCAACAGCTCCTAGGGATTTGGG
GTTGCTCTGGAAAGCTCATCTGCACCACTGCTGTGCCTTGGAATGATAGTTGGAGT-----------------
----------------------------AATAAATCTGTGGATTACATCTGG------AGA------
AACATGACCTGGATGCAGTGG---GAGAGAGAAATAGACAATTACACAGACCACATATAC---
CTCTTACTTGAAAATTCGCAGAACCAACAAGAA--

AAGAATGAACAAGAATTATTGGAATTTGATAAGTGGGCAAATTTGTGGAATTGGTTCAACATATCTAACTGGC
TGTGGTATATAAAAATATTCATAATGATAGTAGGAGGCTTGGTAGGCTTAAGAATAGTTTTTACT---
GTACTTTCTATAGTG---------AATAGAGTTAGGAAGGATACTCACCA------
TTATCATTTGCTGTTAGTTTGCTTAATGCTACAGCTATAGCAGTAGCTGAG---
GGGACAGATAGGATAATAGAAGTAGGACAAAGACTTGGTAGAGCT----------------------------
--------ATTCTCCACATA------CCTAGAAGAATAAGACAGGGTTTAGAAAGGCTTTGGTA---TAA
>KY968394
GCT---GCA--
GACCAATTGTGGGTCACAGTCTATTATGGGGTACCTGTGTGG---AAAGAGGCAACC---
ACCACTCTATTTTGTGCATCAGATGCTAAAGCATATGATACAGAG---GCACATAAT----------------
--------------GTTTGGGCTACA---CATGCCTGTGTACCCACAGAC---
CCCCGCCCACAAGAAGTAGTATTGGCA---------AATGTAACAGAAAATTTTAAC----------------
--------------------------ATGTGGAAAAATAACATGGTAGAACAAATGCAYGAAGATATA---
ATCAATTTATGGGATGAAAGCCTA---AAGCCATGTGCAAAATTA---
ACCCCACTCTGTGTTACTTTAAATTGCACTGATGTCACTATTAGTCCTAATACT-------------------
----
----------------------------GCTTGGAGTAATGTGACAATAAGAGAAGAAATGACA---
AACTGCTCTTTCAACATCACCACAAGCATAAGA---------------------------
GACAAGGTGACGAAGGAATTTGCACTT---TTTTATAGAGATGATGTAGTACCAATAGACAAT----------
----

AATTATACTAACTATAGGTTGATAAATTGTAACACCTCAGTCATTACACAG---
GCCTGTCCAAAGGTATCCTTTGAGCCAATTCCCATACATTACTGTGCCCCAGCTGGTTTTGCGATTCTAAAGT
GTAAT---AATAAGACATTTAAT------GGAACAGGACCATGTACAAAT-----------------------
-GTCAGC---ACAGTACAATGTACACATGGAATTAGGCCAGTAGTGTCAACT---
CAACTACTGTTGAATGGCAGTCTAGCAGAAGAAGAG------------
ATAGTAATTAGATCTGAGAATTTCACAGACAAT---------GCTAAAACCATAATAGTACAGTTGAAT----
-----------GAATCTATAGAA---------ATTAATTGTACAAGACCCAACAACAATACAAGAAAA-----
-AGTATACATATA------------GGACCAGGGAGAGCA---TTT------TATGCAACAGGAGAC------
---------ATAATAGGAGATATA------------AGACAAGCACATTGT---AACATTAGTAGAACA---
AAATGGAATAATACTCTACAACTGATAGTTAACAAATTAAGA---------------GAACAATTTAAG----
-----------------------------------AATAAGACAATACTCTTTAAG------
CCATCCTCAGGAGGGGACCCAGAAGTTGTA---ATGCACACTTTTAATTGTGGAGGAGAA---

TTTAAGCAGCTCCAGGCAAGAGTCCTGGCTGTGGAAAGATACCTAAAGGATCAACAGCTCCTGGGGATTTGGG
GTTGCTCTGGAAGACTCATCTGCACCACTGCTGTGCCTTGGAATGCTAGTTGGAGT-----------------
----------------------------AATAAATCTTATGATCAGATATGG------GAT------
AACATGACCTGGATGCAGTGG---GAAAAAGAAATTGACAATTACACAGGTTTAATATAC---
AATTTACTTGAAGAATCGCAAAACCAACAAGAA--

AAGAATGAACAAGACTTATTGGCATTGGATCAGTGGGCAAGTCTGTGGAATTGGTTTGACATAACAAATTGGC
TGTGGTATATAAAAATATTCATAATGATAGTAGGAGGCCTGGTAGGTTTAAGAATAGTGCTTGCT---
GTAATTTCTGTAGTA---------AATAGAGTTAGGCAGGGATACTCACCT------TTATCGTTG-----
TATCTTGCTCAACGCTACAGCCATAGCAGTA---GGA---GGGACAGAG---
CTTATAGAAGTAGTACAAAGAATTTGA---GCT------------------------------------
ATTTGCAATATA------CCA---AGAATAAGACAGGGCTTGGAAAGGGCTTTGCTA---TA-
>KY968407
GCT---ACG--
GAAAATTTGTGGGTCACAGTATATTATGGGGTACCTGTGTGG---AAAGAARCAACC---
ACCACTCTATTTTGTGCATCAGATGCTAAAGCATATGACACAGAG---RCACATAAT----------------
--------------GTTTGGGCCACA---CATACCTGTGTACCCACAGAC---
CCTAATCCACAAGAAGTAAAATTGGAA---------AATGTGACAGAAAATTTTAAC----------------
--------------------------ATGTGGAAAAATGGCATGGTAGAACAGATGYATGAGGATATA---
ATCAGTTTATGGGATGAAAGCCTA---AAGCCATGTGTAAAATTA---
ACCCCACTCTGTGTTACTTTACATTGCACTAATGCTAATGGTACTGTTAACAATAGCAGTAATAGAACTACT-
----
----------------------------AACGCAAGTTTGGAAAAGATGGAGGGAGAAATAAAA---
AACTGCTCTTTCAATATCACCACAGGCAGAGGA---------------------------
AATGAGATGAAGAAAGAATATGCACTT---TTTTATACATTTGATGTAGTACCAATAAATGAT----------
----

GATAATACAAGCTATATGCTGACACATTGTAATACCTCAGTCATTAAACAG---
GCCTGTCCAAAGGTATCTTTTGAACCAATTCCCATACATTATTGTACCCCGGCTGGTTTTGCGCTTCTAAAGT
GTAAT---GATAAGAAGTTCAAT------GGAACAGGACCATGTAAAAAT-----------------------
-GTTAGC---ACAGTACAATGTACACATGGAATTAGGCCAGTAGTGTCAACT---
CAACTACTGTTAAATGGCAGTCTAGCAGAAAAAGAG------------
ATAGTAATTAGATCTGAAAATTTCACAGACAAT---------GCTAAAACCATAATAGTACAGCTGAAT----
-----------AAATCTGTAGTA---------ATTAATTGTACAAGACCCAACAACAATACAAGAAAA-----
-AGTATACCTATA------------GGATTTGGAAGAGCA---TTG------TATGCAACAGGAGAA------
---------ATAATAGGAGATATA-----------AGACAAGCACATTGT---AACCTTAGTACAGAA---
AGCTGGAAAGAAACTTTAAAACAGGTAGCTGAAAAATTAAAA---------------GAACAATTTGGT----
-----------------------------------AATAAAACAATAGTCTTTAAT------
CAATCATCAGGAGGGGACCCAGAAATTACA---ATGCACACTTTTAATTGTGGAGGGGAA---
TTTAAGCAGCTCCAGGCAAGAGTCCTAGCTGTGGAAAGTTACCTAAAGGATCAACAGCTCCTAGGGATTTGGG
GTTGCTCTGGAAAACTCATCTGCACCACTGCTGTACCTTGGAACACTAGTTGGAGT-----------------
----------------------------AATAAATCTTATAATGAGATTTGG------GAT------
AAAATGACCTGGATGGAGTGG---GAAAGAGAAATTGATAATTACACAGGCTTAATATAC---
ACTTTAATTGAGGAATCGCAAAACCAACAAGAA--

AAGAATGAACAAGACTTATTGGCATTAGACAAATGGGCAGATCTGTGGAATTGGTTTAGCATAACTAATTGGC
TGTGGTACATAAGATTATTCATAATGATAGTAGGAGGCTTGATAGGTTTAAGAATAATTTTTACT---
GTACTTTCTATAGTG---------AATAGAGTTAGGCAGGGATACTCACCA------CTATCATTC--
TGCTGTTTTGCTCAATGCCACAGCTATAGCAGTTGCGGGG------ACAGAG---
ATTATAGAAGTAGCACAAAGAATTTGA---GCT------------------------------------
ATTATCCACATA------CCA---AGAATAAGACAGGGCTTAGAAAGGCTTTTGATA---TA-
>DQ383746
GCT---ACA---GAC---------------------------------
ACAAACAAATTGTGGGTCACAGTCTATTATGGGGTACCTGTGTGG---AAAGAAGCAACC---
ACCACTTTATTTTGTGCATCAGATGCTAAAGCACATAATACAGAG---GTGCATAAT----------------
--------------GTTTGGGCCACA---CATGCCTGTGTACCCACAGAC---
CCTAACCCACAAGAAATGGTAATGAAA---------AATGTGACAGAAAACTTTAAC----------------
--------------------------ATGTGGAAAAATAACATGGTAGAACAGATGCATGATGATATA---
ATCAGTTTATGGGATCAAAGCCTA---AAGCCATGTGTAAAATTA---
ACCCCACTCTGTGTTACCCTAAATTGCACTAATTGGAATAGTACTACTAATGCCACTGACATA----------

----
--------------------------ACTACTAATGCCACTGACATGGGAGAAGGAGAAATAAAA---
AAATGCTCTTTC---------CAACCTAGAAGA---------------------------
GAAAAGGGGGTAAAAGAATATGCACTT---TTTCGTAAACTTGATTTAGAACTAATAAATAAG----------
----

ACCAGCAATAATACCAGCTATAGGTTGATAAGTTGTAACACCTCAGTCATAACACAG---
GCATGTCCGAAGGTATCCTGGGAACCAATTCCTATACATTATTGTGCCCCGGCTGGTTTTGCGATTCTAAAGT
GTAAC---AATAAGACGTTCAAT------GGAACAGGACTATGTAGAAAT-----------------------
-GTCAGC---ACAGTACAATGTACACATGGAATTAGGCCAATAGTATCAACT---
CAATTGTTGTTAAATGGCAGCCTAGCAGAAGAAGAG------------
GTAGTAATTAGATCTAAAAATTTCACAAACAAT---------GCTAAAACCATAATAGTACAGCTGAAT----
-----------GCAGCTGTAGAA---------ATTAATTGTACAAGACACGGCAACAATACAAGAAGA-----
-AGCATACATATG------------GGACCAGGGAGAGCA---TTT------TATACAACAGGAGAG------
---------GTAACAGGAGATATA------------AGAAAAGCACATTGT---AAGGTTAGTAAAGCA---
AAATGGGAGAACACTTTAAAACAGATAGGTATAAAATTAAGA---------------GAACAATTTCCA----
-----------------------------------AATAAAACAATAGTCTTTAAT------
CAATCATCAGGGGGGGACCCAGAAATTACA---ATGCACATTTTTAATTGTGCAGGGGAA---
TTTAAGCAGCTTCAGGCAAGAGTCCTGGCTATAGAAAGATACCTAAAGGATCAACAGCTCCTAGGGATTTGGG
GTTGCTCTGGAAAACTCATTTGCACCACTGCTGTGCCTTGGAATGCTAGTTGGAGT-----------------
----------------------------AATAAATCTTTGGATAAAATTTGG------GAT------
AACATGACCTGGATGGAGTGG---GAGAGGGAAATTGATAATTACACAAGCTTAATATAC---
ACTCTACTTGAAGAATCGCAGTACCAGCAGGAA--

AAGAATGAACAAGAATTATTGGAATTGGATAAATGGGCAAGTTTGTGGAATTGGTTTGACATAACAAATTGGC
TGTGGTATATAAAAATATTCATCATGATAGTAGGAGGCTTAGTAGGTTTAAGAATAGTTTTTGCT---
GTACTTTCTATAGTG---------AATAGAGTTAGGCAGGGATACTCACCA------
TTATCATTCGCTGTTAGTTTGATTGATGCCATAGCTATAGCAGTAGCTGAG---
GGGACAGATAGGGTTATAGAAGTAGTACAAAGATTCTGTAGAGCT----------------------------
--------ATCCTCCACATA------CCTACAAGGATAAGACAGGGCTTAGAAAGGGCTTTGCTA---TAA
>KY968396
GCT---GCA--
GATCAATTGTGGGTCACAGTCTATTATGGGGTACCTGTGTGG---AAAGAGGCGACC---
ACCACTCTATTTTGTGCATCAGATGCTAAAGCATATGATACAGAG---GCACATAAT----------------
--------------ATTTGGGCCACA---CATGCTTGTGTACCCACAGAC---
CCCAACCCACAAGAAGTAAACTTGACA---------AATGTGACAGAACGTTTTGAT----------------
--------------------------GCGTGGAAAAATAACATGGTAGAACAAATGCATGAGGATATA---
ATCAGTTTATGGGATCAAAGTCTA---AAACCATGTGTAAAACTA---
ACCCCACTCTGTGTTACTTTAAATTGTACTGATTATAATGGGAATGTGACTGCTAATAATAGTAGCATCAAT-
----
----------------------AATAGTAGTGGGGGAAGAGAAATGGAAAAAGGAGAAATAAAA---
AACTGCTCTTTCAATGTCACCACAAACATAAGA---------------------------
GATAAGGTGCAGAAAGCAAATGCTCTT---TTGTATAAGCTTGATGTAGTACCAATAGATAAT----------
----

GATACTAGCAGCTATAGGTTGATAAGTTGTAACACCTCAGTCATTACACAA---
GCCTGTCCAAAGATAACCTTTGAGCCAATTCCCATACATTATTGTGCCCCAGCTGGTTTTGCAATTCTAAAGT
GTAAA---GATAAGAAGTTCAAT------GGGAAAGGAGACTGTAAAAAT-----------------------
-GTCAGC---ACAGTACAATGTACACATGGAATTAAGCCAGTAGTGTCAACT---
CAATTGCTATTAAATGGCAGTCTAGCAGAAGAAGAG------------
ATAGTAATTAGATCTGACAACTTCTCGGACAAT---------GCTAAAACCATAATAGTACAGCTGAAT----
-----------AAATCTGTAGAA---------ATTAAGTGTACAAGACCCAACAACAATACAAGAAAA-----
-AGTATAAATATA------------GGACCAGGGAGGGCA---TTT------TATGCAACAGGAGAC------
---------ATAATAGGAGATATA------------AGGCAAGCACATTGT---AACATTAGTAAAGCA---
GAATGGAATAACACTTTAAGAAAGGTAGCTATCAAATTAAGA---------------GAACAATTTAAG----
-----------------------------------AATAAAACAATAGCCTTTAAT------
AAATCCTCAGGAGGGGACCCAGAAATTGTA---ATGCACAGTTTTAATTGTGGAGGGGAA---
TTTAAGCAGCTCCAGGCAAGAGTCCTGGCTGTGGAAAGATACCTAAGGGATCAACAGCTCCTAGGGATTTGGG
GTTGCTCTGGAAAACTCATTTGCACCACTACTGTGCCTTGGAATACTAGTTGGAGT-----------------
----------------------------AATAAATCTCAGGATGAGATTTGG------AAT------

AACATGACCTGGATGGAGTGG---GAAAAAGAAATTGACAATTACACAAACTTAATATAC---
ACCTTAATTGAAGAGTCGCAAAACCAGCAAGAA--

AAGAATGAACTAGATTTATTGAAATTAGATCAGTGGGCAAGTTTGTGGAATTGGTTTAGCATATCAAATTGGC
TGTGGTATATAAAAATATTCATAATGATAGTAGGAGGCTTAATAGGTTTAAGAATAGTTTTTACT---
GTACTTTCTATAGTG---------AATAGAGTTAGGCAGGATACTCACCA------TTATCGTTT--
TGCTATCTTGTACAACGCAACAGCAATAGCAGTAGCG------GGAACAGAG---
GTTATAGAAATAGCAAGAAGATTTGTA---GCT------------------------------------
CTTCTCCACATA------CCA---AGAATAAGACAGGGCTTCGAAAGGATTTTGCTA---TA-
>DQ383748
GCT---GTG--
GAACAATTGTGGGTCACTGTCTATTATGGGGTACCTGTATGG---AAAGAAGCTAAC---
ACCACTCTATTTTGTGCATCAGATGCTAAAACATATGAAACAGAG---GCACATAAT----------------
--------------ATTTGGCCACA---CATGCCTGTGTACCCACAGAC---
CCCAACCCACAAGAAGTAGAATTG------------AATGTGACAGAAACTTTTAAC----------------
--------------------------ATGTGGAAAAATAACATGGTAGAACAGATGCATAAGGATATA---
ATCAGTTTATGGGATCAAAGCCTA---AAGCCATGTGTAAAATTA---
ACCCCACTCTGTGTTACTTTAAATTGCAATGATACTGATACCCAGAATATC----------------------
----
----------------------------------ACTAGGGGGATAGAGAAATTAGAAATAAAA---
AACTGCTCTTTCAATATTACCACAAGCATAAGA---------------------------
GATAAAGTGCAGAGAGAATATGCACTT---TTTTATAAACTTGATGTAGTACCAATAGATAAT----------
----

AATAGTACTAGGTATAGGTTGATAAGTTGTAACACCTCAGTCATTACACAA---
GCCTGTCCGAAGGTAACCTTTGAGCCAATTCCTATACATTATTGTGCCCCGGCTGGTTTTGCGCTTCTAAAAT
GTAAG---GATAAGAAGTTTAAT------GGAACAGGACCATGTACAAAT-------------------------
-GTCAGC---ACAGTACAATGTACACATGGAATTAGACCAGTAGTATCAACG---
CAACTGCTGTTAAATGGCAGTCTAGCAGAAGAAGAG------------
ATAGTAATTAGATCTGCCAATTTCTCAGACAAT---------GCTAAAATCATAATAGTACAGCTGAAT----
-----------GAGTCTGTAGTA---------ATTAATTGTACAAGACCCCACAACAACACAAGAAAA-----
-AGTATACATATA-------------GGACCAGGGAGAGCA---TTT------TATACAACAGGAGGA------
---------ATAATAGGAGATATA------------AGACAAGCACATTGT---AACATTAGTAAAGCA---
AAATGGAATGAAACTTTAAAACGGATAACTACAAAATTAGAA---------------GAGCAATTTAAG----
-----------------------------------AATAAAACAGTAGTCTTTAAT------
TCCTCCTCAGGAGGGGACCCAGAAATTGAG---ATGCACAGTTTTAATTGTGGAGGGGAG---
TTTAAGCAGCTCCAGGCAAGAGTCCTAGCTGTGGAAAGATACCTAAAGGATCAACAGCTCCTGGGGATTTGGG
GTTGCTCTGGAAAACTCATTTGCACCACTAATGTACCTTGGAATACTAGTTGGAGT-----------------
----------------------------AATAAAACTTTAAATGATATTTGG------AAT------
AACATGACCTGGATGGAGTGG---GAGAGAGAAATTGACAATTACACAAGCCTGATATAC---
ACCTTAATTGAGGAAGCGCAAAACCAGCAAGAA--

AAGAATGAACAAGAATTATTGGAATTAGATAAATGGGCAAATTTGTGGAATTGGTTTGACATAACACAATGGC
TGTGGTATATAAAAATATTCATAATGATAGTAGGAGGCTTGATAGGTTTAAGAATAGTTTTTTCT---
GTACTTTCCATAGTG---------AATAGAGTTAGGAAGGATACTCACCA------
TTATCGTTTGCTGTTAGCTTGCTCAACGCCGTAGCCATAGCAGTAGCTGGG---
GGGACAGATAGGGTTCTAGAAGTGTTACAAAGAATTGGGAGAGCT----------------------------
--------ATTATCCACATA------CCTACAAGAATCAGACAGGGCTTGGAAAGGGCTTTGTTA---TAA
>DQ383751
ACT---GCA---GAA---------------------GAT------
GCAGGAAGAAAATTGTGGGTCACAGTCTATTATGGGGTACCTGTGTGG---AAAGAAGCAAAC---
ACCACTCTATTTTGTGCATCAGATGCTAAAGCATATGATACAGAG---GTACATAAT----------------
--------------GTTTGGCCACA---CATGCCTGTGTACCCACAGAC---
CCCAGCCCACAAGAAGTATTCTTGAAA---------AATGTGACAGAAGATTTTAAC----------------
--------------------------ATGTGGAAAAATGACATGGTAGAACAGATGCATGAGGATATA---
ATCAGTTTATGGGATCAAAGCCTA---AAGCCATGTGTAAAACTA---
ACCCCACTCTGTGTTACTTTAAATTGCACTGACTACGTAGGGAATACTACTAATGCTGGGAATACTACT----
----
-------------------AATACTACTAGTACTGTAAGCCCTACTGCTAGTACAGGAATGAAA---
AACTGCTCTTTCAATGTCACCACAGACCTAAGA---------------------------

GATAAGGTGAAGAAAAATTATGCACTT---TTTTATAGCCTTGATGTAGTACCAATAGAAAAT----------
----

GAAAATACCAGCTATAGATTGATAAGTTGTAACACCTCAATCATTACACAG---
GCCTGTCCAAAGGTATCCTTTGAGCCAATTCCCATACATTATTGTGCTCCAGCTGGTTTTGCACTTCTAAAGT
GTAAC---AATAAGACATTTAAC------GGAACAGGACCATGTGGAAAT----------------------
-GTCAGC---ACAGTACAATGTACACATGGAATTAGGCCAGTAGTATCAACT---
CAACTGCTGTTAAATGGCAGTCTAGCAGAAGAAGAG-----------
ATAGTAATTAGATCTGCCAATTTCTCAGATAAT---------ACTAAAACCATAATAGTACAGCTGAAC----
-----------AAAGCTGTAAAA---------ATTAATTGTACAAGGCCCAACAACAATACAAGAAGA-----
-AGTATACACATA------------GGACCAGGGAGAGCA---TTT------TATACAACAGGAGAA------
---------ATAATAGGAGATATA------------AGAGATGCACATTGC---AACATTAGTAAAGCA---
GAATGGAATACCACTTTAAGACAGATAGCTATAAAATTAAGA---------------GAACAATTCAAG----
-----------------------------------AATACAACAATAGTCTTTAAT------
AGTTCTTCAGGAGGGGACCCAGAAATTGTA---ATGCACAGTTTTAATTGTGGGGGGGAA---
TTTAAGCAGCTCCAGGCGAGAGTCCTGGCTGTGGAAAGATACCTACAGGATCAACAACTCCTGGGAATTTGGG
GTTGCTCTGGAAAACTCATTTGCACCACTGCTGTGCCTTGGAACAATAGCTGGAGT-----------------
----------------------------AATAGATCTCTGGAGTCTATTTGG------AAT------
AACATGACCTGGATGCAGTGG---GAAAAGGAAATTGACAATTACACAGGCTTAATATAC---
AATTTACTTGAAAAGTCCCAGAACCAACAAGAG--

AAAAATGAACAAGAATTATTGGAGCTCGATAAATGGGCAAACTTGTGGAATTGGTTTAGCATAACAAATTGGC
TGTGGTATATAAGAATATTCATAATGATAGTAGGAGGCTTAGTAGGTTTAAGAATAGTTTTTGCT---
GTAATTTCTATAGTA---------AAGAGAGTTAGGGAGGGATATTCACCT------
TTATCGTTTGCTGTTAGCTTGTTCAACGCTACAGCTATAGCAGTAGCTGAG---
GGGACAGATAGAATTATAGAAGCAGTGCAAGGAGCTTGTAGAGCT----------------------------
--------ATCCTCAACATA------CCTAGAAGAATAAGACAGGGCTTGGAAAGGCTTTTGCTA---TAA
>KY968399
GCT---GTA--
GAACAATTGTGGGTCACAGTCTATTATGGGGTACCTGTGTGG---AGAGAAGCAAAC---
ACCACTTTATTTTGTGCATCAGATGCTAAAGTATATGATACAGAG---GCACATAAT----------------
--------------GTCTGGGCCACA---CATGCCTGTGTACCCACAGAC---
CCCAACCCACAAGAAGTAGAATTAAAA---------AATGTGACAGAAAATTTTAAC---------------
--------------------------ATGTGGAAAAATAACATGGTAGAACAGATGCATAAGGATATA---
ATCAGTTTATGGGATCAAAGCCTA---AAGCCATGTGTAAAATTA---
ACCCCACTCTGTGTTGCTTTACAGTGCACTAATGTTACTAATAAGAATGCTACTAATACCACTAGTGATGCTA
CTAGTACTCCT--
--------------------------------------AGTAGTAGCGAGGAAGTACAAATGAAA---
AACTGCTCTTTCAATGTTACCTCATTCATAACA--------------------------
AATAAGGTGAAGAAAGAATATGCACTT---TTTTATAGTCTTGATGTAGTACCAATAGCTAAT----------
----

GAGAATAACAGCTATAGGTTGATAAGTTGTAATACCTCAATCATTACACAG---
GCCTGTCCAAAGATATCCTTTGAGCCAATTCCTATACATTATTGTGCCCCGGCTGGTTTTGCGATTCTAAAAT
GTAAT---GATAAGAAGTTCAAT------GGAACAGGACCATGTAAAAAT----------------------
-GTCAGC---ACAGTACAATGTACACATGGAATTAGGCCAGTAGTATCAACA---
CAACTGCTGCTAAATGGCAGTCTAGCAGAAGAAGAG-----------
GTGGTAATTAGATCAGCAAATTTCTCAGACAAT---------GCTAAAACCATAATAGTACAGCTGAAA----
-----------GAACCTGTAGAA---------ATTAATTGTACAAGACTCAACAACAATACAAGAAAA-----
-AGGATAACTATG------------GGACCAGGGAGAGTA---TAT------TATACAACAGGAGGA------
---------ATAATAGGAGATATA------------AGAAAAGCACATTGT---AACATTAGTGAAGAA---
AAATGGAATAGCACTTTACAAAAGATAGTTGAAAAATTAGGA---------------GAACAATTTAAA----
-----------------------------------AATAGAACAATAGTTTTTAGT------
AAATCCTCAGGAGGGGACCCAGAAATTGTA---ATGCACAGTTTTAATTGTGGAGGGGAA---
TTTAAGCAGCTCCAGGCAAGAATCCTGGCTGTGGAAAGATACCTAAAGGATCAACAGCTCCTGGGGCTCTGGG
GTTGCTCTGGAAAACTCATCTGCACCACTACTGTGCCTTGGAATGCTAGTTGGAGT-----------------
----------------------------AATAAATCTCTAAGTACAATTTGG------AAT------
AACATGACCTGGATGGAGTGG---GAAAGAGAAATTGACAATTACACAGGCTTAATATAC---
ACCCTAATTGAAGAATCGCAGATCCAGCAAGAA--

AAGAATGAACAAGAATTGTTGGCATTAGATAAATGGGCAAATTTGTGGAATTGGTTTGACATATCAAACTGGC
TATGGTATATAAAAATATTCATAATGATAGTAGGAGGCTTGATAGGTTTAAGAATAGTTTTTTCT---
GTACTTTCTATAGTG---------AATAGAGTTAGGCAGGGGTACTCACCA------TTATCATTTAGT----
-CTTGCTCAACGCCACAGCCATAGCAGTAGCGGGG------ACAGAG---
ATTTTAGAAGTATTACAAAGAATT---ATAGCT------------------------------------
ATTATCCACATA------CCA---AGAATCAGACAGGGCTTGGAAAGGGCATTGCTA---TA-
>KY658686
GCT---ACA--
GAACAATTGTGGGTCACAGTTTATTATGGGGTACCTGTGTGG---AAAGAAGCAACA---
ACCACTTTATTTTGTGCATCAGATGCTAAAGCATATGTTACAGAG---GCACATAAT---------------
--------------ATTTGGGCCACA---CATGCCTGTGTACCCACAGAC---
CCCAACCCACAAGAAGTATTATTGAAA---------AATGTGACAGAAAATTTTAAC---------------
--------------------------ATGTGGGAAAATAACATGGTAGAACAGATGCATGAGGATATA---
ATCAGTTTATGGGATCAAAGCCTA---AAGCCATGTGTAAAATTA---
ACCCCACTCTGTGTCACTTTAAGTTGCACTGATGCCAACAGGACTGAGAATACTAATATCACT---------
----
-------------------------------------AGACAAATGGATAAAGAAGAAATAAAA---
AACTGCTCTTTCAATGTCACCACAAACCTAAGA--------------------------
GATAAGGTGCAGAAAGAATATGCACTC---TTTTATAGACTTGATGTAGTACCACTAGATAAC---------
----

AGTTATAGTAGCTATAGGTTGAAAAGTTGTAACACCTCAGTCATTACACAG---
GCCTGTCCAAAGGTATCCTTTGAGCCAATTCCCATACATTATTGTGCCCCGGCTGGTTTTGCGATTCTAAAGT
GTAAT---GATAAGACATTCAAT------GGAACAGGACCATGTACAAAT----------------------
-GTCAGC---ACAGTACAATGCACACATGGAATTAGGCCAGTAGTGTCAACT---
CATTTGCTGTTAAATGGCAGTCTAGCAGAGGAGGAG-----------
GTAGTAATTAGATCTAAAAATTTCACGGACAAT---------GTTAAAAACATAATAGTACAGCTGAAG----
-----------GAAGCTGTACAA---------ATTAATTGTTCAAGACCCGGCAACAATACAAGAAAA-----
-AGTATACCTATA-----------GGACCAGGGAGAGCA---TTT------TTTAGAACAGGAGAC------
---------ATAATAGGAGATATA-----------AGACAAGCACATTGT---AACCTTAGTAGTGCA---
AAATGGAATGACACCTTAAAACAGGTAGTTACAAAATTAAGA---------------GAACAATATGGG----
--------------------------------AACAATACAATTATTGCCTTTAAT------
CAATCCTCAGGAGGGGACCCAGAAATTATA---ATGCACAGTTTTAATTGTGGAGGAGAA---
TTTAAACAGCTCCAGGCAAGAGTCCTGGCTGTGGAAAGATACCTAAAGGATCAACAGCTCCTGGGGATTTGGG
GTTGCTCTGGAAAACTCATCTGCACCACTGCTGTGCCTTGGAATTCTAGTTGGAGC----------------
----------------------------AATAAATCTTTGAAGACGATTTGG------GAT------
AACATGACTTGGATGCAGTGG---GAAAAAGAAATTAACAATTACACAGACTTAATATAC---
ACCTTAATTGAAGAATCGCAAAACCAACAAGAA---------------------------------------
----
AAGAATGAACAAGAATTATTATCATTAGATAAGTGGGCAAGTTTGTGGACTTGGTTTGATATATCAAATTGGC
TGTGGTATATAAGAATATTTATAATGATAGTGGGAGGCTTGGTAGGTTTAAGAATAGTTTGTGCT---
GTGCTTTCCCTAGTA---------AATAGAGTTAGGCAGGGATACTCACCA------
CTATCATTTGCTGTTAGCTTGTTTAATGCAATAGCTATAACAGTAGCTGAG---
GGAACAGATAGGGTTATAGAAGTAGTACAAAGATTTGTAGAGCT---------------------------
--------ATCCTCAACATA------CCTAGAAGAATAAGACAGGGCTTGGAAAGGGCTTTGCTA---TAA
>KY658683
GCT--
GACCAATTGTGGGTCACAGTCTATTATGGGGTACCTGTATGG---AGAGAAGCAAAC---
ACCACTCTGTTTTGTGCATCAGATGCTAAAGCATATAAGACAGAG---GTACATAAT---------------
--------------GTTTGGGCCACA---CATGCCTGTGTACCTACAGAC---
CCTAGCCCACAAGAAGTAGAATTGAAA---------AATGTGACAGAAAATTTTAAC---------------
--------------------------ATGTGGAAAAATGACATGGTAGAACAAATGCATAAGGATGTA---
ATCAGTTTATGGGATGAAAGCCTA---AAGCCATGTGTACAATTA---
ACCCCACTTTGTGTTACTTTACAATGCRCTAATTGGACAGCTGATAATAATACTAAT---------------
----
----------------------------AGTACTATCAGGAGGGAAATGGAGAGAAACATAAAA---
AACTGCTCTTTCAATATCACCACAGAGGTACAA--------------------------
GATAAGAGGAAAAAAGCCTATGCACTC---TTTGATAGTCTTGATATAGTACCAATAGATAAT---------
----

AATAATACAAACTATATGTTAATAAATTGTAATACCTCAGTCATTACTCAA---
GCCTGTCCAAAGGTAACTTTTGAACCAATTCCAATACATTATTGTGCCCCGGCTGGTTTTGCGATTCTAAAGT
GTAAG---GATAAAAATTTCAAT------GGAACAGGACCCTGTACAAAT-----------------------
-GTCAGT---ACAGTACAATGTACACATGGATTAGGCCAGTAGTGTCAACT---
CACCTGTTGTTAAATGGCAGTCTAGCAGAAGAAGAG------------
ATAGTAATTAGATCTGAAAATTTCACAAACAAT---------GCTAAAACTATAATAGTACAGCTGAAT----
-----------GAAACAGTAGAA---------ATTAATTGTACAAGACCCAGCAACAACACAAGAAAG-----
-AGCATACACATA------------GGGCCAGGGAGAACA---ATG------TATGCAACAGGACAA------
---------ATAATAGGAGATATA------------AGACAAGCACATTGT---AACATTAGTAGCACA---
AAATGGAACAACACTTTAGGAAAGGTAGTTAAAAAATTAAGG---------------GAACAATTTAAT----
-----------------------------------AAAACAAAAATAATATTTCAG------
CCATCCTCAGGAGGGGACCCAGAAATTGTG---ATGCACACTTTTAATTGTGCAGGGGAA---
TTTAAGCAGCTCCAGGCAAGAGTCCTGGCTGTGGAAAGATACCTAAAGGATCAACAGCTCCTGGGATTTGGG
GTTGCTCTGGAAAACTCATTTGCACCACTTCTGTGCCTTGGAATGCTAGTTGGAGT-----------------
----------------------------AATAAATCCCTGAACGCAATTTGG------GAT------
AACATGACCTGGATGGAATGG---GATAGAGAAATTAACAACTACACAAACATAATATAC---
AGCTTGCTTGAAAAATCGCAAAACCAACAAGAA--

AAGAATGAACAGGAATTGTTGGAATTGGATAAGTGGGCAAATTTGTGGAATTGGTTTGACATATCAAACTGGC
TGTGGTATATAAAAATATTCATAATGATAGTAGGAGGCTTAGTAGGTTTAAGAATAGTTTTTGCT---
ATACTTTCTATAGTG---------AATAGAGTTAGGCAGGGATACTCACCA------
TTATCATTCGCTGTTAGTTTGCTTAATGCTATAGCTATAGCAGTAGCTGAG---
GGAACAGATAGGATTATAGAAATAGTACAAAACATTGGTAGGGGT----------------------------
--------ATCCTCCATATA------CCCAGAAGAATACGCCAGGGACTTGAAAGGGCTTTGCTA---TAA
>MH078530
GTT---ACA--
GGACAATTGTGGGTCACAGTCTATTATGGGGTACCTGTGTGG---AAAGAAGCAAAA---
ACCACCCTATTTTGTGCATCAGATGCTAAGGCATATGATACAGAG---GCACATAAT----------------
--------------GTTTGGGCCACA---CATGCCTGTGTACCCACAGAC---
CCCAACCCACAAGAATTAAAATTGGAA---------AATGTGACAGAAAATTTTAAC----------------
--------------------------ATGTGGAAAAATGACATGGTAGAACAAATGCATGAGGATATA---
ATCAGTTTATGGGATCAAAGCCTA---AAGCCATGCGTAAAATTA---
ACCCCACTCTGTGTTATTTTAAGTTGCAGCAATTTAGTTAGTACTAACAATTTAAATAGTACTAATAACAGTA
CCAGTAATAAC--
-------AGTACCAGTAATAACAGTACCAGTGGGGAAGACTTAGACATGAATGAAGAACTAAAA---
AACTGCTCTTTCAATATCACCACAGGCATAGGA---------------------------
AATAAGATGAGGAGAGACTATGCAATT---TTTAATAAACTTGATATAGTACCAATAGATGGTGAT-------
----

GGTAGTAATAATAATACAAGCTATATGTTGATAAATTGTAATACCTCAGTCATTACACAG---
GCTTGCCCAAAGGTAACATTTGAACCAATTCCCATACATTATTGTGCCCCGGCTGGTTTTGCGATTCTACAAT
GTAAG---GACAAGAAGTTCAAT------GGATCAGGATTATGTAAAAAT-----------------------
-GTCAGC---ACAGTACAATGTACACATGGATTAGGCCAGTAGTGTCAACT---
CAACTGCTGTTAAATGGCAGTCTAGCAGAAGAAGAG------------
GTAGTAATTAGATCTGAAAATATTTCAGACAAT---------GTTAAAACCATAATAGTACAACTGAAA----
-----------GAAGCTGTAAAA---------ATTAATTGTACAAGACCCAACAACAATACAAGAAAA-----
-AGCATACATATG------------GGACCAGGAGCAGCG---CTC------TATGCAACAGGAGAA------
---------ATAATAGGGGATATA------------AGACAAGCACATTGT---AACCTTAGTAAAACA---
GAATGGTATAAAACTTTGAGACAGATAGCTGCAAAATTAAAA---------------GAACAATTTCAG----
-----------------------------------AATAAAACAATAGTTTTTAAT------
CACTCCGCAGGAGGGGACCCAGAAATTGTA---ACACACAGTTTTAATTGTGCAGGGGAA---
TTCAAACAGCTCCAGGCAAGAGTCCTGGCTGTGGAAAGATACCTAAGGGATCAACAGCTCCTGGGATATGGG
GTTGCTCTGGAAAACTCATCTGCACCACTGCTGTGCCTTGGAATAGTAGTTGGAGT-----------------
-------------------------AGGAATAAATCTCTAGATTATATTTGG------GGC------
AATATGACCTGGATGGAGTGG---GAAAGAGAGATTGACAACTACACAGATTTGATATAC---
ACCCTAATTGAAGACTCACAGATCCAGCAAGAA--

AAGAATGAAAAAGATTTATTGGAACTCGATAAATGGGCAAGTTTGTGGAATTGGTTTGACATAACAAACTGGC
TGTGGTATATAAGACTATTCATAATGATAGTAGGAGGCTTGATAGGTTTAAGAATAGCTGTTACT---
GTAATTTCTATAGTG---------AATAGAAGTAGGCAGGGATACTCACCA------

TTATCGTTTGCTGTTAGCTTGCTTAATGCCATAGCCATAGCAGTAGCTGAG---
GGAACAGATAGGGTTATAGAGATAGGACAAAGCATAGGTAGAGCT----------------------------
--------ATCCTCAACATA------CCTAGAAGAATAAGACAGGGTGTAGAAAGAGCTTTGATA---TAA
>KY658685
GCT---ACA--
GAAAAATTGTGGGTCACAGTCTATTATGGAGTACCTGTGTGG---AAAGAGGCAAAC---
ACCACTCTATTTTGTGCATCAGATGCTAAATCATATATTACAGAG---GTACATAAT----------------
--------------GTTTGGGCCACA---CATGCCTGTGTACCCACAGAC---
CCTAGCCCACAAGAAGTAAAATTGGAA---------AATGTGACAGAAGACTTTGAC----------------
--------------------------ATGTGGAAAAATAACATGGTAGAACAGATGCATAAGGATATA---
ATCAGTCTATGGGATGAAAGCCTA---AAGCCATGTGTAAAGTTA---
ACCCCACTTTGTGTTACTTTAGATTGCATTGATGTTCCCACGAATTGCACTAATACCGGT-------------
----
----------------------AATTGCACTAACTGGAAAATAAAGGAGGGGGGAGAACTAAAA---
AACTGCTCTTTCAATATCACCACATACAGAACA---------------------------
GATAAAATGCAGAAAGGAAATGCATTG---TTTTATAACCTTGATATAATAAAAATAGATAATGAA-------
----

GGTAATAATAGCTATAGGTTGCTAAGTTGTAACACCTCAGTCATTACACAG---
GCATGCCCAAAGATATCCTGGGAACCAATTCCAATACATTATTGTACCCCGGCTGGTTTTGCGCTTCTAAAAT
GTAAT---GATAAGAACTTCAAT------GGAACAGGACCATGTAAAAT------------------------
-GTCAGC---ACAGTACAATGTACACATGGAATTAGGCCAGTAGTATCAACT---
CAATTGTTGTTAAATGGCAGTCTAGCAGAAGGAGAC------------
ATAATAATTAGATCTGAAAATATCACGGACAAT---------GCTAAAACCATAATAGTACAGCTGAAT----
-----------AAAACTGTAAGC---------ATTACGTGTCAAAGACCCAACAACAATACAAGGAAA-----
-AGTATAAATATA------------GGACCAGGGAGGGCA---ATT------TATGCCACAGGACAA------
---------ATAATAGGAAATATA------------AGACAAGCATATTGT---AACATTAGTAATGCA---
ACGTGGCTAAACACGTTACAACAGGTAGCTAAAAAATTACAA---------------GAACATTTT-------
-----------------------------------AATAAAACAATAGTCTTTAAT------
CAATCCTCAGGAGGGGACCCAGAAATTGTA---ATGCATAGTTTTAATTGTGGAGGGGAA---
TTTAAGCAGCTCCAGGCAAGAGTCCTGGCTGTGGAAAGATACCTACATGATCAACAGCTCCTAGGGCTTTGGG
GTTGCTCTGGAAAACTCATCTGCACCACTGCTGTGCCTTGGAATGATAGCTGGAGT-----------------
----------------------------AATAAATCTCAGGATGACATTTGG------GGA------
AACATGACCTGGATACAGTGG---GAAAGAGAAGTCAATAATTACACAAAATTAATATAC---
AATCTAATTGAAATATCGCAAAACCAGCAGGAA--

CAGAATGAACAAGAATTGTTGGAATTGGATAAATGGGCAAGTTTGTGGAATTGGTTTGATATAACAAACTGGC
TATGGTATATAAAAATATTCATAATGATAGTAGGAGGCTTAATAGGATTAAGAATAGTTTTTACT---
GTACTTTCTATAATA---------AATAGAGTTAGGAAGGGATACTCACCA------
TTATCGTTCGCTATTAGCTTGCTTAACGCCATAGCCATAGCAACAGCTGAG---
GGGACAGATAGGATTATAGAAGGAGTACGAAGGATTCTTAGAGCT----------------------------
--------TTTCTCCATATA------CCTAGAAGAATCAGGCAAGGATTCGAAAGGGCTTTATTA---TAA
>MH078533
GGT---GCA--
GAACAGTTATGGGTCACAGTCTATTATGGGGTGCCTGTGTGG---AAAGAGGCAACC---
ACCACTCTATTTTGTGCATCTGATGCCAAGGCATATAAGGATGAG---GCACATAAT----------------
--------------GTTTGGGCCACA---CATGCCTGTGTACCCACAGAC---
CCCAACCCACAAGAAATGATATTGGAA---------AATGTGACAGAAAATTTTAAC----------------
--------------------------ATATGGAAAAATGATATGGTAGAACAGATGCATCAGGATATA---
ATCAGTTTATGGGATAGCAGCCTA---AAGCCATGTGTACAGTTA---
ACCCCATTGTGTGTCACTTTAAATTGCACTGAGTTGAATAATACTACTAAGGTGAACAGGACTAGTATTAATA
CT---
---GAAATAGACTTGAAA---
AACTGTTCCTTCAATGTCACCTCAAACATAAGA---------------------------
GATAAATATCAGAAAGAATATGCAATG---TTGTATAAGCTTGATTTGGTACCAATAGATGGC----------
----

GAGAATGCCAGCTATAGATTAATAAGTTGTAATACCTCAGTCATAACACAG---
GCCTGTCCAAAGATAACCTTTGAGCCAATTCCCATCCACTATTGTGCCCCGGCTGGTTTTGCGATTCTAAAGT
GCAAC---AATAAAACATTTAAT------GGAAAAGGACCATGTACAAAT-----------------------

-GTCAGT---ACAGTACTATGCACACATGGAATTAGACCAGTAGTATCAACT---
CAACTGCTGTTAAATGGTAGTCTAGCAGAAGAGGAA------------
ATAGTAATTAGATCTGAAAATTTCACAGACAAT---------GCCAAAACCATAATAGTACAGTTAAAT----
-----------GAAACTGTAGAA---------ATTAATTGTACAAGACCCAACAACAATACTAGGAAA-----
-AGTATTCATATA------------GGACCAGGGAGAACA---TTT------TATGCAACAGGAGAC------
---------ATAATAGGAGACATA------------AGACAAGCACATTGT---AACATTAGTGCAGTA---
AAATGGAATAACACCCTAAGACAAGTAGCTAAAAAATTAAGT---------------GAACAATTTAAT----
-----------------------------------AATAGCATAATAATCTTTAAG------
AACTCTTCAGGAGGAGACCCAGAAATTACA---ACACACAGCTTTAATTGTGGAGGGGAA---
TTTAAGCAGCTCCAGGCAAGAGTCCTGGCTGTAGAAAGATACCTACGGGATCAACAGCTCCTGGGAATTTGGG
GTTGCTCTGGAAAACTCATCTGCACCACTACTGTGCCTTGGAATAAAACTTGGAGT-----------------
----------------------------AATAGAACTTTCAAGGAAATTTGG------GAC------
AACATGACCTGGATGCAGTGG---GAAAGAGAAATTGATAACTACACAGGCTTAATATAC---
ACTTTAATTGAAGAATCGCAGAAACAGCAAGAA--

AAGAATGAACATGAATTATTGGCATTAGATAAATGGGCAAGTTTGTGGAGTTGGTTTGACATAACAAACTGGC
TGTGGTATATAAGAATATTCATAATGATAGTAGGAGGCTTGATAGGATTAAGAATAGTTTTTACT---
GTACTTTCTATAGTG---------AATAGAGTTAGGAAGGGATACTCACCA------
TTATCGTTCGCTGTTAGCTTGTGTAATACTATAGCCATTGCAGTAGCTGAG---
GGGACAGATAGGATTATAGAAATATTACAAAGAACTAGTAGAGCT----------------------------
--------ATTATAAACATA------CCTAGAAGAATAAGACAAGGCCTTGAAAGGCTTTTGATA---TAA
>AY037268
GCT---ACA--
GAACAATTGTGGGTCACAGTCTATTATGGGGTACCTGTGTGG---AAAGAAGCAAAC---
ACCACTCTATTTTGTGCATCAGATGCTAAAGCATATGATACAGAG---GCACATAAT----------------
--------------GTTTGGCCACA---CATGCCTGTGTACCCACAGAC---
CCCAACCCACAAGAAGTAGAATTGGTA---------AATGTGACAGAAGAATTTAAC----------------
--------------------------ATGTGGAAAAATAACATGGTAGAACAGATGCATGAGGATATA---
ATCAGTTTATGGGATCAAAGCCTA---AAGCCATGTGTAAAATTA---
ACTCCACTCTGTGTTACTTTAAATTGCACTAATTGGATGAGGACAAATAATACCTTAAATAATACCTCAAATA
ATGCC--
----------ACTAATCAGGATATAACTAGAATCAATAATACGATGGAGAACGGAGAAGTAAAA---
AACTGCTCTTTCAATATCACCACAAACATGAAA---------------------------
GACAAGGTGCAGAAAAAATATGCACTT---TTTTATGACCTTGATGTAGAACCAATAGACAAT----------
----

AGTAGTTATAGGATAAAAAGTTGTAACACCTCAGTCATTACACAG---
GCCTGTCCAAAAACATCCTTTGAGCCAATTCCTATACATTATTGTGCCCCGGCTGGTTATGCGATTCTAAAGT
GTAAG---GATAAGAATTTCAAT------GGAACAGGAAATTGTAAAAAT-----------------------
-GTCACC---ACTGTACACTGTACACATGGAATTAGGCCAGTAGTATCAACT---
CAACTGCTGTTAAATGGCAGTCTAGCAGAAGAAGAC------------
ATAGTAATTAAATCTGCTAACCTCTCAGATAAT---------ACTAAAACCATAATAGTACAGCTGAAT----
-----------GAATCTATAGCA---------ATTAATTGTACAAGACCTGGCAACCGTACAAGAAGA-----
-AGGATAACTCTA------------GGACCAGGGAGAGTA---TAT------TATACAACAGGAGAA------
---------ATAATGGGAGATATA------------AGAAAAGCACATTGT---AACCTTAGTAAAGCA---
GACTGGAATAACACTGTAAGAAAGATAGCTATTAAATTAAGA---------------GAAAATTTC-------
-----------------------------------AATAAAACTATAGTCTTTAAT------
CAATCCTCAGGAGGGGACCTAGAAATTGTA---ATGCACATTTTTAATTGTGGAGGGGAA---
TTCAAACAGCTCCAGGCAAGAGTCCTGGCTGTGGAGAGATACCTAAGGGATCAACAGCTCCTGGGGATTTGGG
GTTGCTCTGGAAAACTCATTTGCACCACTGCTGTGCCTTGGAATACTAGTTGGAGT-----------------
----------------------------AATAAATCTCTAAATGATATTTGG------AAT------
TACACGACCTGGATGCAATGG---GAAAAAGAAATTGACAATTACACAAGCATAATATAC---
AGCTTAATTGAAGAATCGCAAACCCAACAAGAA--

AAGAATGAACAAGAATTATTGGCATTGGATAAATGGGCAAGTTTGTGGAATTGGTTTGAAATATCAAACTGGC
TGTGGTATATAAAAATATTCATAATGATAGTAGGAGGCTTGATAGGTTTAAGAATAGTTTTTACT---
GTACTGACTATAGTG---------AATAGAGTTAGGCAGGGATACTCACCA------
TTATCGTTAGCTGTTAGCTTGTTCAATGCAGTAGCCATAGCAGTAGCTGAG---
GGAACAGATAGGATTATAGAAATAGCACAAAGAATCGGTAGAGCT----------------------------
--------ATCCTCAACATA------CCTACAAGGATAAGACAGGGCTTGGAAAGGGCTTTGCTA---TAA

```
>AY037282
GCT---ACA------------------------------------------
CAACAATTGTGGGTCACAGTCTATTATGGGGTACCTGTATGG---AAAGAAGCAAAC---
ACCACTCTATTTTGTGCATCAAATGCTAAAGCATATGATACAGAG---GTACATAAT----------------
--------------GTTTGGGCCACA---CATGCCTGTGTACCCACAGAC---
CCTAACCCACAAGAATTAGTATTGAAA---------AATGTGACAGAAAATTTTAAC----------------
--------------------------ATGTGGAAAAATAACATGGTAGAACAGATGCATAAGGATATA---
GTTGATTTATGGGATCAAAGCCTA---AAGCCATGTGTAAAATTA---
ACCCCACTCTGTGTCACTATGGATTGCACTGATTATAAAAATGCTACCAATACCACTAGTGGTAGT-------
-------------------------------------------------------------------------
----------------------ACCAATACCACTAGCTATGCTACCGGGGGAGAAGAAATAAAA---
AACTGCTCTTTCAACATCTCCACAAGCATAAGA---------------------------
AATAAGGTACAGAAAGAATATGCACTT---TTTTATAGCCTTGATGTAATACCAATAGATAAT----------
-------------------------------------------------------------------------
----------------------
AGTAATACCTCCTATACTTTGAGAAGTTGTAACACCTCAATCATTACACAG---
GCCTGTCCAAAGGTATCCTTTGAGCCAATTCCTATACATTTTTGTGCCCCGGCTGGTTTTGCGATTCTAAAAT
GTAAT---GATAAAAAATTCAAT------GGAACAGGACCATGTAAAAT------------------------
-GTCAGC---ACAGTACAATGTACACATGGAATTAGGCCAGTAGTATCTACA---
CAATTGCTGTTAAATGGCAGTCTAGCAGAAGAAGAG------------
GTAGTAATTAGATCTGCCAATTTCTCGGACAAT---------ACTAAAACCATAATAGTACAGCTGAAT----
-----------GAATCTGTAGAA---------ATTAACTGTACAAGGCCCAACAACAATACAAGGAAA-----
-AGTATACATATA------------GGACCAGGGAGAGCA---TTT------TATACAACAGGACAA-------
---------GTAATAGGAGATATA-----------AGACAAGCACATTGT---AACATTAGTAGAGAA---
AAATGGAATAACACCTTAAACCTGATAGTTAGCAAACTAAGA---------------GAACAAGTCAAA----
-----------------------------------AACAAAACAATAGCCTTTAAG------
CAAGCCTCAGGAGGGGACCCAGAAATTGTG---ATGCACACTTTTAATTGTGGAGGGGAA---
TTTAAGCAGCTCCAGGCAAGAGTCCTGGCTATAGAAAGATACCTAAAGGATCAACAGCTCCTAGGAATTTGGG
GGTGCTCTGGAAAACTCATTTGCACCACTGCTGTGCCTTGGAATGCTAGTTGGAGT-----------------
----------------------------AATAAATCTTATAATGATATTTGG------GAT------
AACATGACCTGGATGCAGTGG---GAGAGAGAAATTGATAATTACACAGGCTTAATATAC---
ACTTTAATTCAAATATCGCAGATCCAGCAAGAA----------------------------------------
-----------------------------
AAGAATGAACAAGAGTTATTGGAATTGGACAAATGGGCAAGTTTGTGGAATTGGTTTGACATAACACAGTGGC
TGTGGTATATAAGATTATTCATAATGATAGTAGGAGGCTTAGTAGGGTTAAGAATAATTTTTACT---
GTACTTTCTATAGTG---------AATAGAGTTAGGCAGGGATACTCACCA------
TTATCATTCGCTGTTAGTTTGTTTAATACCATAGCTATAGCAGTAGCTGAG---
GGGACAGATAGGGTTATAGAAGGAGTACAAAGACTTTGTAGAGCT----------------------------
--------ATTCTCCACATA------CCTAGAAGAATAAGACAAGGCTTAGAAAGGGCTTTACTA---TAA
>FJ952154
ACT---GCA------------------------------------------
GACAAATTGTGGGTCACAGTCTATTATGGGGTACCTGTGTGG---AAAGAAGCAACT---
ACCACTCTATTTTGTGCATCAGATGCTAAAGCATATGATACAGAG---GTACATAAT----------------
--------------GTTTGGGCCACA---CATGCCTGTGTACCTACAGAC---
CCCAGCCCACAAGAAGTATTATTGGAA---------AATGTGACAGAAGATTTTAAC----------------
--------------------------ATATGGAAAAATAACCTGGTAGAACAGATGCATGAGGATATA---
ATCAGTTTATGGGATCAAAGCCTA---AAGCCATGTGTAAAATTA---
ACCCCACTCTGTGTTAGTTTAAAGTGCACTGATTTGAAGAATGATACTAATACCAATAGT-------------
-------------------------------------------------------------------------
-------------------------AGTAGCGGGAGAATGATAATGGAGAAAGGAGAAATAAAA---
AACTGCTCTTTCAATATCGCCGCAAACATAAGA---------------------------
GATAGGGTGCAGAAAGAACATGCATTT---TTTTATAAACTGGATATAGTACCAATAGATAAT----------
-------------------------------------------------------------------------
----------------------
GATACCAGTAGCTATAGATTGATAAGTTGTAACACCTCAGTCATTAAACAG---
GCCTGTCCAAAGGTATCCTTTGAACCAATTCCCATACATTATTGTGCCCCGGCTGGTTTTGCGATTCTAAAGT
GTAAT---AATAAAACGTTCAAT------GGATCAGGGCCATGTACAAAT-----------------------
-GTCAGC---ACAGTACAATGTACACATGGAATTAGGCCAGTAGTGTCAACT---
CAACTGCTGTTAAATGGCAGTCTAGCAGAAGAAGGG------------
GTAGTGATTAGATCTGAAAATTTCACAGACAAT---------GCTAAAAACATAATAGTACAGCTGAGT----
```

-----------GTATCTATAAAG---------ATTAATTGTACAAGACCCAACAACAATACAAGAARM-----
-AGTATAYCAATA------------GGACCAGGGAGAGCA---TTT------TATGCAACAGGACAA------
---------ATAATAGGAGATATA------------AGACAAGCACATTGT---AACATTAGTAGAACA---
GAGTGGAATAACACTTTACAAGAGGTAGCTAAAAAATTAGAA---------------GAACAATTTAAG----
-----------------------------------AATAAAACAATAGTCTTTAAT------
CAATCCTCAGGAGGGGACCCAGAAATTGTA---ATGCACAGTTTTAATTGTGGAGGGGAA---
TTTAAGCAGCTCCAGGCAAGAGTCCTGGCTGTGGAAAGATACCTAAGGGATCAACAGCTCCTAGGGATTTGGG
GTTGCTCTGGAAAACTCATTTGCACCACTGCTGTGCCTTGGAATATTAGTTGGAGT-----------------
----------------------------AATAAAAGTTTGGATGACATTTGG------GGG------
AACATGACCTGGATGCAGTGG---GAGAGAGAAATTGACAATTACACAGACTTAATATAC---
ACCTTACTTGAAGAATCGCAAAACCAACAAGAA---------------------------------------
----
AAGAATGAACAAGAATTACTGGAATTAGATAAGTGGGAAGTTTGTGGAATTGGTTTAGCATAACAAATTGGC
TGTGGTATATAAAAATATTCATAATGATAGTAGGAGGCTTGGTAGGTTTAAGAATAGTTTTTACT---
GTAATTTCTATAGTG---------AATAGAGTTAGGCAGGGATACTCACCA------
TTATCGTTTGCTGTTAGTTTGCTTAATGCCACAGCTGTAGCAGTAGCTGAG---
GGGACAGATAGGATTATAGAAGTAGTACAAAGAATTGGTAGAGCT---------------------------
--------GTTCTCCACATA------CCTACAAGAATAAGACAGGGCTTGGAAAGGGCTTTGCTA---TAA
>DQ676886
ACC---
GAGCAATTGTGGGTCACAGTCTATTATGGGGTACCTGTGTGG---AAAGAAGCAACC---
ACCACTCTATTTTGTGCATCAGATGCTAAAGCATATGTTACAGAG---AAGCATAAT----------------
--------------GTTTGGGCCACA---CATGCCTGTGTACCCACAGAC---
CCCAATCCACAAGAGGTAGAATTGAAA---------AATGTGACAGAAGATTTTAAC----------------
--------------------------ATGTGGAAAAATAACATGGTAGAACAGATGCATGRAGATATA---
AYCAGTTTATGGGAACAAAGCATG---AAGCCATGTGTCAAATTA---
ACCCCACTCTGTGTTACTTTAAATTGTACTGATGTTAAAGAGAGAAATGCTAATGATACCACTAGTACTACTA
GTCCC---
----------CCTAATACTAGTACCCCTAGTAATAGTAGTCTGGAAAATAATAGTGACATAAAA---
AACTGCTCTTTCAATATCACCACAAGCATAAGA--------------------------
GATAACATAAAGAAAGAACATGCACTT---TTCTATGCACTTGATGTAGTACCAATAGATAAT----------
----

AGTAGTAGCTATAGGTTGGYAAGTTGTAACACCTCAGTCATTACACAG---
GCCTGTCCAAAGATATCCTTTGAGCCAATTCCCATACATTATTGTGCCCCGGCTGGTTTTGCGATTCTAAAGT
GTAAC---CAGAAGGAGTTCAAT------GGAACAGGACCATGTACAAAT-----------------------
-GTCAGT---ACAGTACAATGTACACATGGAATCAGGCCAGTAGTATCAACT---
CAATTGCTGTTAAATGGCAGTCTAGCAGAAGAAGAT------------
GTAGTAATTAGATCTGCCAATTTCACAGACAAT---------ACTAAAACCATAATAGTGCAGCTGAAT----
-----------GAATCTATAGTA---------ATTAATTGTACAAGACCCAACAACAATACAAGAAAA-----
-AGCATACATATG------------GGACCAGGGAAAGCA---TTT------TTTGCAACAGGGGAT------
---------ATAATAGGAGATATA------------AGACAAGCACATTGT---AACCTTAGCAGAACA---
GAATGGAATAAAGCCTTAAACAGGATAGTTACAAAATTAGGA---------------GAACAATTTAAAAAT-
--------------------------------AGTACCGAAACAATAACCTTTACG------
CAATCCTCAGGAGGGGACCCAGAAATTGTA---ATGCACAGTTTTAATTGTGGAGGGGAA---
TTCAAGCAGCTCCAGGCAAGAGTCCTGGCTGTGGAAAGATACCTAAGGGACCAACAGCTCCTGRGGATTTGGG
GTTGCTCTGGAAAACTCATCTGCACCACTACTGTGCCTTGGAACACTAGTTGGAGT-----------------
----------------------------AATAAAACTTATGATAAGATTTGG------AAT------
AACATGACATGGATGCAGTGG---GAAAAAGAAATTGACAATTACACAGACCTAATATAC---
ACCTTAATTGAGGAATCGCAGAACCARCAAGAA---------------------------------------
----
AAGAATGAACAAGACTTATTGGAATTAGATAAGTGGGCAAGTTTGTGGAATTGGTTTGACATATCTCAATGGC
TGTGGTATATAAAAATATTCATAATGATAGTAGGAGGCTTGATAGGTTTAAGAATAGTTTTTGCT---
GTGCTTTCTATAGTG---------AATAGAGTTAGGCAGGGATACTCACCA------
TTGTCATTTGCTGTTAGCTTGCTCAACGCCACAGCTATAGCAGTAGCTGAA---
GGGACAGATAGGATTATAGAAGTAGTGCAAAGAGCTTGTAGAGCT---------------------------
--------ATTCTCCACATA------CCTAGAAGGATAAGACAGGGCTTGGAAAGAGCTTTGCTA---TAA
>AF042100
GCT---GCA---------------------------------------
GAACACTTGTGGGTCACAGTCTATTATGGGGTACCTGTGTGG---AGAGAGGCAACC---

ACCACTCTATTCTGTGCATCAGATGCTAGAGCATATGATACAGAG---GTACATAAT---------------
--------------GTTTGGGCCACG---CATGCCTGTGTACCCACAGAC---
CCCAACCCACAAGCAGTAGCATTAGCA---------AATGTGACAGAAAATTTTAAT----------------
--------------------------ATGTGGAAAAATAACATGGTAGAACAGATGCATGAGGATATA---
ATCAGTTTATGGGATCAAAGCCTA---AAGCCATGTGTAAAATTA---
ACCCCACTCTGTGTTACTTTAAATTGCACTGATGTGAAGAATGCTACTAATACCAATAGTACTAATACC----
----
-------------------AATAATAGTAGCAGGGAAATGATTGAGGAAGAAGGAGAAATAAAA---
AACTGCTCTTTCAATATTACCACAAGAATAAGA--------------------------
AATAAGGTGCAGAAAGAATATGCACTT---TTTTATAAACTTGATGTAGTACCAATAGATAATGAT-------
----

AGAACTAATAATACCAGCTATAGGTTGGTAAGTTGTAATACCTCAGTCATTACACAG---
GCCTGTCCAAAAGTATCCTTTGAGCCAATTCCCATACATTATTGTGCCCCGGCTGGTTTTGCGATTCTAAAGT
GTAGA---GATAAGAAGTTCAAT------GGAACAGGACCATGTAAAGGT----------------------
-GTCAGC---ACAGTACAATGCACACATGGAATTAGGCCAGTAGTATCAACT---
CAACTGCTGTTAAATGGCAGTCTAGCAGAAGAAGAG-----------
GTAGTAATTAGATCTGAAAATTTCACAAACAAT---------GCTAAAACCATAATAGTACAGCTAAAC----
-----------GAAGCTATAGCT---------ATTAATTGTACAAGACCCAGCAACAGTACAGGACAA-----
-AGTATACGTATA------------GGACAAAGGAGAGCA---TTT------TATGCAACAGGGAAA------
---------ATAATAGGTGATATA-----------AGACATGCACATTGT---AACATTAGTGGAGCA---
AAATGGGATAACACCTTACAACAGATAGTTAATTTCTTAAAA---------------GAACAATTTGGG----
---------------------------------AATTATAAAACAATAGTCTTTAAT------
CAATCCTCAGGAGGCGACCCAGAAATTGTA---ACACACAGTTTTAATTGTGGAGGGGAA---
TTTAAGCAGCTCCAGGCAAGAGTCCTGGCTCTGGAAAGATACCTAAGGGATCAACAGCTCCTGGGGATTTGGG
GTTGCTCTGGAAAACTCATTTGCACCACCACTGTGCCTTGGAATGCTAGTTGGAGT-----------------
----------------------------AATAAATCTCTGGATCAGATTTGG------AAT------
AACATGACCTGGATGCAGTGG---GAAAGAGAAATTAACAATTACACAAGCTTAATATAC---
ACCTTGATTGAAGAATCGCAAAACCAACAAGAA---------------------------------------
----
AAGAATGAACAAGAATTATTGGAATTAGATAAATGGGCAAATTTGTGGAATTGGTTTGACATAACAAAATGGC
TGTGGTATATAAAAATATTCATAATGATAGTAGGAGGCTTGGTAGGTTTAAGAATAGTTTTTACT---
GTACTTTCTATAGTG---------AATAGAGTTAGGCAGGGATACTCACCA------
TTATCGTTTGCTGTTAACTTGTTCAATGCTATAGCCATAGCAGTAGCTGAG---
GGGACAGATAGGGTTATAGAAGTATTACAAAGAGCTTATAGAGCT----------------------------
--------ATTCTCCACATA------CCTAGAAGAATAAGACAGGGCTTGGAAAGGGCTTTGCTA---TAA
>AF042101
GCT---GCA---
GACAAATTGTGGGTCACAGTCTATTATGGGGTACCTGTGTGG---AAAGAAGCAACC---
ACCACTCTATTTTGTGCATCAGATGCTAAAGCATATGATACAGAG---GTACATAAT---------------
--------------GTTTGGGCCACA---CATGCCTGTGTACCCACAGAC---
CCCAACCCACAAGAAGTAGTATTGAAA---------AATGTGACAGAAAATTTTAAC----------------
--------------------------ATGTGGAAAAATAACATGGTAGAACAAATGCATGAGGATATA---
ATCAGTTTATGGGATCAAAGCCTA---AAGCCATGTGTGAAATTA---
ACCCCACTCTGTGTTACTTTAAATTGCACTGACTACTCAAAGAATGCTACTAATACCACTAGTAGTAGCGAG-
----
----------------ACAGTGATGGAGAAGGGAGAAAAAGTGATGGAGAAGGGAGAAATAAAA---
AACTGCTCTTTCAATATCACCACAAACATAAGG--------------------------
GGTAAGGTGCAGAAAGAATATGCACTT---TTTTATAAACTTGATGTAGTACCAGTAGATAAG---------
----

GACAATACTAGTTATAAGTTGATAAGTTGTAACACCTCAGTCATTACACAG---
GCCTGTCCAAAGGTATCCTTTGAGCCAATTCCCATTTATTATTGTGCCCCGGCTGGTTTTGCGATTCTAAAGT
GTAAA---GATGAAAAGTTCAAT------GGAAAAGGACTATGTACAAAT----------------------
-GTCAGC---ACAGTACAATGTACACATGGAATTAGGCCAGTAGTATCAACT---
CAACTGCTGTTAAATGGCAGTCTAGCAGAAGGAGAG-----------
GTAATAATTAGATCTGAAAATATCACAAACAAT---------GCTAAAACCATAATAGTACAGCTGAAA----
-----------GATCCTGTAGAA---------ATTAATTGTACAAGACCCAACAACAATACAAGAAAA-----
-AGTATACATATA------------GGACCAGGGAGAGCA---TTT------TATGCAACAGGAGAT------
---------ATAATAGGAAATATA-----------AGGCAAGCATATTGT---ACCCTTAATAGAGCA---

AGGTGGAATGACACTTTAAAACAGATAGCTGAAAAATTAGGA---------------GAACAATTTAAG----
-----------------------------------AATAAAACAATAGTATTTAAT------
CAATCCTCAGGAGGGGACCCAGAAATTGTA---ATGCACAGTTTTAATTGTGGAGGGGAA---
TTCAAGCAGCTCCAGGCAAGACTCCTGGCTGTGGAAAGATACCTAAAGGATCAACAGCTCCTGGGAATTTGGG
GTTGCTCTGGGAAACTCATTTGCACCACTACTGTGCCTTGGAATGCTAGTTGGAGT-----------------
----------------------------AATAAATCTCTGGATAAGATTTGG------AAT------
AACATGACCTGGAGGGAGTGG---GAAAAAGAAATTGACAATTACACAGACTTGATATAC---
ACCTTAATTGAACAATCGCAGAACCAGCAAGAA--

AAGAATGAACAAGAATTATTAGAATTAGATAAATGGGCAAGTTTGTGGAATTGGTTTGACATAACCAATTGGC
TGTGGTATATAAAAATATTCATAATGATAGTAGGAGGCTTGATAGGCTTAAGAATAGTTTTTATT---
GTACTTTCTGTAGTG---------AATAGAGTTAGGCAGGGATACTCACCA------
TTATCGTTTGCTGTTAGCTTGCTCAATGTCACAGCCATAGCAGTAGCTGAG---
GGGACAGATAGGGTTATAGAAGTATTACAAAGAATTTATAGAGCT----------------------------
--------ATTATCCACATA------CCTACAAGAATAAGACAGGGCTTTGAAAGGGCTTTGCTA---TAA
>AF538304
GCT---ACA--
GAAAAGTTATGGGTCACAGTCTATTATGGGGTACCTGTGTGG---AAAGAAGCAACT---
ACCACTCTATTTTGTGCATCAGATGCTAAAGGATATGAGACAGAG---GCACATAAT----------------
--------------GTTTGGGCCACA---CATGCCTGTGTACCCACAGAC---
CCCAACCCACAAGAAATAGTATTGGAA---------AATGTGACAGAAAACTTTAAC----------------
--------------------------ATGTGGAAAAATAACATGGTAGAACAAATGCATGAGGATATA---
ATCAGTTTATGGGATCAAAGCCTA---AAGCCATGTGTAAAATTA---
ACCCCACTCTGTGTTACTTTAAATTGCACTGATGCTAATACCACTAGTAGTACTAATACCACTAATGGT----
----
----------------ACTAATCCCACTATTAGTGGCAGCAGAATGGAAAAAGGAGAAATAAAA---
AACTGCTCTTTCAACGTCACCACAAACATGAGA---------------------------
GATAAGATGCAGAGAGCTTTTGCACTT---TTTTATAAACTTGATGTAGTACCAATAGATAAGAAT-------
----

AATGATAACAATAATACCAGCTATAGGTTGATAAGTTGTAATACCTCAGTCATTACCCAA---
GCCTGTCCAAAAGTATCCTTTGAGCCAATTCCCATACATTATTGTGCCCCGGCTGGTTTTGCGATTCTAAAAT
GTAAT---GATAAGAAGTTCAAT------GGAACAGGACCATGTACAAAT-----------------------
-GTCAGC---ACAGTACAATGTACACATGGAATTAGGCCAGTAATATCAACT---
CAACTACTGTTAAATGGCAGCCTAGCAGAAGAGGTG------------
GTAGTAATTAGATCTAGCAATTTCACAGACAAT---------ATTAAAAACATAATAGTACAGCTGAAC----
-----------GAACCTGTAGAA---------ATTAATTGTACAAGACCCAATAATAATACAAGAAAA-----
-AGTATACATATA------------GCACCAGGGAGAGCA---TTT------TATGCAACAGGAGAA------
---------ATAATAGGAGATATA------------AGACAAGCATATTGC---AACATTAGTAGAGAA---
AAATGGAATAACACTTTAAAACAGATAGTTGAAAAATTAAGA---------------GAACAATATGTG----
-----------------------------------AATAAAACAATAGTCTTTAAT------
CAATCCTCAGGAGGGGACCCAGAAATTGTA---ATGCACAGTTTTAATTGTGGAGGGGAA---
TTTAAGCAGCTCCAGGCAAGAGTCCTGGCTGTGGAGAGATACCTAAAGGATCAACAGCTCCTGGGGATTTGGG
GTTGCTCTGGAAAACTCATCTGCACCACTGCTGTGCCTTGGAATTCTAGTTGGAGT-----------------
----------------------------AATAAATCTCTGGATAAGATTTGG------GAT------
AACATGACCTGGATGGAGTGG---GAAAGAGAAATTGACAATTACACAGGCTTAATATAC---
ACCTTAATTGAACAATCACAAATCCAGCAAGAA--

AAGAATGAACAAGAATTATTGGAATTGGATCAATGGGCAAGTTTGTGGAATTGGTTTGACATAACAAAATGGC
TGTGGTATATAAAAATATTCATAATGATAGTAGGAGGCTTGGTAGGTTTAAAAATAGTTTTTTCT---
GTGCTTTCTATAGTG---------AATAGAGTTAGGCAGGGATACTCACCA------
TTGTCATTTGCTATTAGCTTGCTTAATGCTACAGCCATAGCAGTAGCTGAG---
GGGACAGATAGGGTTATAGAAGTATTGCAAAGAGCTTATAGAGCT----------------------------
--------ATTATCCACATA------CCTAGAACAATAAGACAGGGCTTGGAAAGGGCTTTGCAA---TAA
>AF538306
GCT---GCA--
GAACAGTTATGGGTCACAGTCTATTATGGGGTACCTGTGTGG---AAGGAAGCAACG---
ACCACGCTATTTTGTGCATCAGATGCTAAAGCATATGATCCAGAG---GTACATAAT----------------
--------------GTTTGGGCCACA---CATGCCTGTGTACCCACAGAC---
CCCAACCCACAAGAAGTAGTATTGGTA---------AATGTGACAGAAAACTTTAAC----------------

--------------------------ATGTGGAAAAATGACATGGTAGAACAGATGCAGGAGGATGTA---
ATCAGTTTATGGGATCAAAGCCTA---AAGCCATGTGTAAAATTA---
ACCCCGCTCTGTGTTACCTTAAATTGCAATAATGCTAAGCTCAATAATACTAATACCACT-------------
----
----------------------------AAGGAATGGGAAAAGATGGAGGAAGGAGAAATGAAA---
AACTGCTCTTTCAACACCACCACAAGCATAAGA---------------------------
GATAAGATGCAGAAAGAATATGCGCTT---TTTTATAAACTTGATGTAGTACAAATAGATAATAAC-------
----

AATAACAATACTAATACCAGCTATAGGTTGATAAATTGTAACACCTCAGTCATTACCCAG---
GCCTGTCCAAAGGTATCCTTTGAGCCAATTCCCATCCATTATTGTGCCCCGGCTGGTTTTGCGATTCTAAAAT
GTAAC---AATAAGACGTTCAAT------GGAACAGGACCATGTACAAAT-----------------------
-GTCAGC---ACAGTACAATGTACACATGGAATTAGGCCAGTAGTATCAACT---
CAACTGCTGTTAAATGGCAGTCTAGCAGAAGATGAG------------
GTAGTAATTAGATCCAGCAATTTCACGGACAAT---------GTTAAAACCATAATAGTACAGCTGAAC----
-----------CAATCTGTAGCA---------ATTAATTGTACAAGACCTAACAACAATACAAGAAAA-----
-AGTATACATATA------------GGACCAGGGAGAGCA---TTT------TATGCAACAGGAGAT------
---------ATAATAGGAAATATA------------AGACATGCACATTGT---AACATTAGTAGAAAA---
GAGTGGAATAACACTTTAAAATTGATAGTTGAAAAATTAAGA---------------GAACAATTTGGA----
--------------------------------GAGAACAAGACAATAGTCTTTAAT------
CACTCCTCAGGAGGGGACCCAGAAATTGTA---ACGCACAGTTTTAATTGTGGAGGGGAA---
TTTAAACAGCTCCAGGCAAGAGTCCTGGCTGTGGAAAGATACCTAAAGGACCAACAGCTCCTAGGGATTTGGG
GTTGCTCTGGAAAACTCATCTGCACCACTACTGTACCTTGGAATGCTAGTTGGAGT-----------------
----------------------------AATAAATCTCTGAATAAGATTTGG------GAT------
AACATGACCTGGATGGAGTGG---GACAAAGAAATTAGCAATTACACAAACTTAATATAC---
ACCTTAATTGAAGAATCGCAGAACCAGCAAGAA--

AAGAATGAACAAGAATTATTGGAATTGGATAAATGGGCAAATTTGTGGACTTGGTTTGACATAACAAATTGGC
TGTGGTATATAAAAATATTCATAATGATAGTAGGAGGCTTGGTAGGTTTAAGAATAGTTTTTACT---
GTACTTTCTATAGAG---------AATAGAGTTAGGCAGGGATATTCACCA------
TTATCGTTTGCTGTTAGCTTGCTTAATGCCACAGCCATAGCAGTAGCTGAG---
GGGACAGATAGGATTATAGAAGTATTACAAGCAAGTTATAGAGCT----------------------------
--------ATTATCCACATA------CCTAGAAGAATAAGACAGGGCTTGGAAAGGGCTTTGCTA---TAA
>AF042103
GCG---GCA--
GAACCATTGTGGGTCACAGTCTATTATGGGGTACCTGTGTGG---AGAGAAGCAACC---
ACCACTCTATTTTGTGCATCAGATGCTAAAGCATATGATACAGAG---GTACATAAT----------------
--------------GTGTGGCCACA---CATGCCTGTGTACCCACAGAC---
CCCAGCCCACAAGAAATACCATTGGAA---------AATGTGACAGAAAAGTTTAAC----------------
--------------------------ATGTGGAAAAATGACATGGTAGAACAGATGCATGAGGATATA---
ATTAGTTTATGGGATCAAAGCCTA---AAGCCATGTGTAAAATTA---
ACCCCACTCTGTGTTACTTTAAATTGCACTGATGCTACTAAAACCAATAATAGTAGCGGGGGTACTAAA----
----
-------------------ACCAATAATAGTAGCGGGGGAGCGGTGGGGGAAGGAGAAATAAAA---
AACTGCTCTTTCAATATCACCACAAGCATGAGA---------------------------
GATAAGGTGCAGAAACAATATGCTCTT---TTTTATAAACTTGATGTAGTACCAATAGACAAT----------
----

AATAGTACTAATACCAGCTATAGGTTGATAAGTTGTAACACCTCAGTCATTACACAG---
GCCTGTCCAAAGGTATCTTTTGAGCCAATTCCCATACATTATTGTGCCCCGGCTGGTTTTGCGATTCTAAAGT
GTAAA---GATAAGAAGTTCAAT------GGAAAAGGACTATGTAAAAAT-----------------------
-GTCAGC---ACAGTACAGTGTACACATGGAATTAGGCCAGTAGTATCAACT---
CAACTGCTGTTAAATGGCAGCCTAGCAGAAGAAGAG------------
GTAGTGATTAGATCTGACAATTTCACGAACAAT---------GCTAAAACCATAATAGTACAGCTAAAA----
-----------GAATCTGTAAAA---------ATTAATTGTACAAGACCCAACAACAATACAAGAAAA-----
-AGTATAACTATA------------GGACCAGGGAAAGCA---TTT------TATGCAACAGSAGAA------
---------ATAATAGGAGACATA------------AGACAAGCACATTGT---AACCTTAGTAGAGTA---
GACTGGAATGAGACTTTAAGACAGATAGCTATAAAATTAGGA---------------GAACAATTTAAG----
-----------------------------------AAGAACACAATAGTCTTTAAT------
CCATCCTCAGGAGGGGACCCAGAAATTGTA---ATGCACAGTTTTAATTGTGGAGGGGAA---

TTTAAGCAGCTCCAGGCAAGAGTCCTGGCTGTGGAAAGATACCTAACGGATCAACAGCTCCTGGGGATTTGGG
GTTGCTCTGGAAAACTCATTTGCACCACTGCTGTGCCTTGGAATGCTAGCTGGAGT-----------------
----------------------------AATAAATCTCTAGAGAAGATTTGG------AAT------
AACATGACCTGGAGGGAGTGG---GAAAGAGAAATTCACAATTACACAAAATTAATATAC---
TCCTTAATTGAAGAATCGCAAAACCAACAAGAA--

AAGAATGAACAAGAATTATTGGAATTAGACAATTGGGCAAGTTTGTGGAATTGGTTTGACATAACAAAATGGC
TGTGGTATATAAAAATATTCATAATAGTAGTAGGAGGCTTGGTAGGTTTAAGAATAGTTTTTGCT---
GTAATTTCTATAGTG---------ACTAGAGCTAGGCAGGGATACTCACCA------
TTATCGTTTGCTGTTAGCTTGCTCAATGCCACAGCCATCGCAGTAGCTGAG---
GGGACAGATAGAGTTCTAGAAGTATTACAAAGAGCTTATAGAGCC----------------------------
--------ATTCTCCACATA------CCTAGAAGAATAAGACAGGGCCTCGAAATGGCTTTGCTA---TAA
>AF042104
GCT---GCA--
GACAAATTGTGGGTCACAGTCTATTATGGGGTACCTGTGTGG---AGAGAAGCAGCC---
ACCACTCTATTTTGTGCATCGGATGCTAAAGCATATGATACAGAG---ATGCATAAT----------------
--------------GTTTGGGCCACA---CATGCCTGTGTACCCACAGAC---
CCCAACCCACAAGAAGTAGTATTGGGA---------AATGTGACAGAAAATTTTAAC----------------
--------------------------ATGTGGAAAAATAACATGGTAGAACAGATGCATGAGGATATA---
ATCAGTTTGTGGGATCAAAGCCTA---AAGCCATGTGTAAAATTA---
ACCCCACTCTGTGTCTCTTTAAATTGCACTGATTATGTGGGGAATGCTACTAATACCACT-------------
----
-------------------------AGTAGTAGCGAGGAAGCGATGGAGAGAGGAGAAATAAAA---
AACTGCTCTTGCAATGTCACCACAAGCATGAGA---------------------------
GATAAGGTGCAGAAAGAATATGCATTT---TTTTATAAACTTGTTGTAGTACCAATAGATAAT----------
----

AATAATAATAATACCAGCTATAGGTTGATAAGTTGTAACACCTCAGTCATTACACAG---
GCCTGTCCAAAGATATCCTCTGAACCAATTCCCATACATTATTGTGCCCCGGCTGGTTTTGCGCTTCTAAAGT
GTAAC---AATAAGACGTTCAAT------GGAAAAGGACCGTGTACATAT-----------------------
-GTCAGC---ACAGTACAGTGTACACATGGCGTTAAGCCAGTAGTATCAACT---
CAACTGCTGTTATATGGCAGTCTAGCAGAAGAAGAG------------
GTAGTAATTAGATCTGACAATTTCACAGACAAT---------GCTAAAACCATAATAGTACAGCTGAGA----
-----------GACCCTGTACAA---------ATTAATTGTACAAGACCCGCCAACAATACAAGAGAA-----
-AGTATACACATA------------GGACCAGGGAGAGCA---TTT------TATGCAACC---GAC------
---------ATAATAGGAGATATA------------AGACAAGCACATTGT---AACAGTAGTAGAGCA---
GAATGGATAAAAACTTTACAACAGGTAGTTACAAAATTAAAA---------------AAACAATTTGGG----
--------------------------------AATAATAAAACAATAGTCTTTAAC------
CCATCCTCAGGAGGGGACCCAGAAATTGTA---ATGCACATTTTTAATTGTGGAGGGGAA---
TTTAAGCAGCTCCAGGCAAGAGTCCTGGCTGTGGAAAGATACCTAAGGGATCAACAGCTCCTGGGAATTTGGG
GTTGCTCTGGAAAACTCATTTGCACCACTGCTGTGCCTTGGAATGCTAGCTGGAGT-----------------
----------------------------AATAAATCTCTGGATCAGATTTGG------AAT------
AACATGACCTGGATGCAGTGG---GAAAGAGAGATTGACAATTACACAAGATTAATATAC---
ACCTTAATTGAAGAATCGCAGAACCAACAAGAA--

AAGAATGAACAAGACTTATTGGTATTAGATAAATGGGCAAGTTTGTGGAATTGGTTTGACATAACAAGTTGGC
TGTGGTATATAAAAATATTCATAATGGTAGTAGGAGGCTTAGTAGGTTTAAGAATAATTTTTACT---
GTGCTTTCTATAGTA---------AATAGAGTTAGGCAGGGATACTCACCA------
TTATCGTTTGCTATTAGCTTGTACAATGCCACCGCCACAGCAGTAGCTGAG---
GGGACAGATAGAGTTATAGAAGTATTACAAAGAGCTGGTAGAGCT----------------------------
--------GTCCTCCACGTA------CCTAGAAGAATAAGACAGGGCTTCGAAAGGGCTATGCTA---TAA
>AF042105
GCT---GAA---GAA------------------------------------
GACAAATGGTGGGTCACAGTCTATTATGGGGTACCTGTGTGG---AGGGAAGCAACC---
ACCACCCTATTTTGTGCATCAGATGCTAAAGCATATGATACAGAG---GCACATAAT----------------
--------------GTTTGGGCCACA---CATGCCTGTGTACCCACAGAC---
CCCAGCCCACAAGAAATAGCATTGGAA---------AATGTGACAGAACATTTTAAC----------------
--------------------------ATGTGGAAAAATAACATGGTAGAACAGATGCATGAGGATATA---
ATTAGTTTATGGGATCAAAGTCTA---AAGCCATGTGTAAAATTA---
ACCCCACTCTGTGTTACTTTAAATTGCACTGATTTGGAGACTAAGAATACTACTATTACC-------------

----
-------------------------AATAGTAGCCGGGAAAAGATAGAAAAAGGAGAAATAAAA---
AACTGCTCTTTCAATATCGACACAGACATGGGT---------------------------
GCCAAGGCGCAGAAACACTCTGCAGTT---
TTTAATAAACTTGATGTAGTATCAATAGATAATGATAATAAAACT----------------------------
----
ACTGATAAAAGTAATGCACAAGGTAATAATAGTAAAACTATTACCAACTATAGGTTGATAAGTTGTAACACCT
CAAAAATTACACAG---
GCCTGCCCAAAGATATCCTTTGAGCCAATTCCCATACATTATTGTGCCCCGGCTGGTTTTGCGATTCTAAAGT
GTAGA---GAGGAGGACTTCAAT------GGAACAGGACTATGTAAAAAT-----------------------
-GTCAGC---ACAGTACAGTGTACACATGGAATTAGGCCAGTAGTATCAACT---
CAACTACTGTTAAATGGCAGTCTAGCAGAAAAAGAG------------
GTAGCAATTAGATCTGCCAATTTCATGGACAGT---------AATAAAAACATAATAGTACAGCTGAAC----
-----------GAATCTGTAAAA---------ATTAGTTGTATAAGACCCAACAACAATACAAGAAAA-----
-AGTATGACTCTA------------GGACCAGGAAAGGTA---TTC------TATACAACAGGA---------
---------ATAACAGGAGATATA------------AGAAAAGCACATTGT---AACATTAGTAGAAAA---
GAATGGAATAAAACTTTAGAACGGATAGCTATAAAACTAGGA---------------GAACAATTTAAG----
-----------------------------------AATAAAACAATAGTCTTTAAA------
CCATCCGCAGGAGGGGACCCAGAAATTAAA---ATGCACAGTTTTAATTGTGGAGGGGAA---
TTTAAGCAGCTCCAGGCAAAAGTCCTGGCTGTGGAAAGATACCTAATGGATCAACAGCTCCTGGGAATTTGGG
GTTGCTCTGGAAAACTCATTTGCAACACTGCTGTGCCTTGGAATGCTAGCTGGAGT-----------------
-------------AATAGTAGTAAG---ATACATTCTCTAGAAGAGATTTGG------GGG------
AACATGACCTGGATGCAGTGG---GAAAAAGAAATTCGCAATCACACAAAATACATATAC---
TCCTTACTTGAAGAATCGCAGAACCAACAGGAC--

AAGAATGAACAAGAACTATTGGAATTGGATCACTGGGCTAGTTTGTGGAATTGGTTTGACATAACAAACTGGC
TATGGTATATAAAAATATTCATAATGGTAGTAGGAGGCTTGATAGGTTTAAGAATAGTTTTTGCT---
GTACTTTCTATAGTA---------AATAGAGTTAGGCAGGGTTACTCACCA------
TTGTCGTTTGCTGTTAGCTTGTTCAATGCCACCGCCATAGCAGTAGCTGAG---
GGGACAGATAGAGTTTTAGAAGTATTACAAAGAGTTTATAGAGCT----------------------------
--------ATTCTCCACATA------CCTAGAAGGATAAGACAGGGCCTAGAAATGGCTTTGCTA---TAA
>AF538302
GCT---ACA--
GAAAAATTGTGGGTCACAGTCTATTATGGGGTACCTGTGTGG---AAAGAAGCAACC---
ACCACTCTATTTTGTGCATCAGATGCTAAAGCATATGCTACAGAG---GTACATAAT----------------
--------------GTTTGGCCACA---CATGCCTGTGTACCCACAGAC---
CCCGACCCAAAAGAAGTAGAATTAAAA---------AATGTAACAGAAAATTTTAAC----------------
--------------------------ATGTGGAAAAATAACATGGTAGATCAGATGCATGAGGATATA---
ATCAGTTTATGGGATCAAAGTCTA---AAGCCATGTGTAAAATTA---
ACTCCACTCTGTGTTACTTTACATTGCACCGATAATATCACTAGTAATGCTAATACCACTACTAGT-------
----
-------------------CCTCCTACCACTAGTAGTGTAGAGGAGGAGAAAGGAGAAGTAAAA---
AACTGCTCTTTCAATGTCACCTCAGGTATAAGA---------------------------
GATAAGGTGCAGAAAGAATATGCACTT---TTTTATAAACTTGATGTAGTACCAATAAATAAT----------
----

GATAGTAGAAGCTATAGGATGATAAGTTGTAACACCTCAGTCATTACACAG---
GCCTGTCCAAAGGTATCCTTTGAGCCAATTCCCATACATTATTGTGCCCCGGCTGGTTTTGCGATTCTAAAAT
GTAAT---GATAAGAAGTTTAAT------GGAACAGGACCATGTACAAAT-----------------------
-GTCAGC---ACAGTACAATGTACACATGGAATTAGGCCAGTAGTATCAACT---
CAGCTGCTGTTAAATGGTAGTCTAGCAGAAGAAGAG------------
GTAGTGCTTAGATCTGAAAATTTCACGAACAAT---------GCTAAAACCATAATAGTACAACTGAAA----
-----------AAACCTGTAAAC---------ATTACCTGTATAAGACCCAGCAACAACACAAGAAAA-----
-AGTATAAATATA------------GGACCAGGAAGAGCA---TTT------TATGCAACAGGAGAA------
---------ATAATAGGAGACATA------------AGACAAGCACATTGT---AACATTAGTAGAGCG---
GAATGGAATAACACTTTAAAACAGATAGTTAAAAAATTAAGA---------------GAAAAATTTGAG----
-----------------------------------AACAAAACAATAATCTTTAAT------
CAATCCTCAGGAGGGGACCCGGAAATTGTA---ATGCACAGTTTTAATTGTGGAGGGGAA---
TTCAAGCAGCTCCAAGCAAGAATCCTGGCTGTGGAAAGATACCTAAAGGATCAACAGCTCCTGGGAATTTGGG
GTTGCTCTGGAAAACTCATCTGCACCACTAATGTGCCTTGGAATGCTAGTTGGAGT-----------------

-----------------------------AATAAATCTCTGAGTCAGATTTGG------GAA------
AACATGACCTGGATGGAGTGG---GAAAGAGAAATTAACAATTACACAGAAGAAATATAC---
AACTTAATTGAAAAATCACAGAACCAGCAAGAA--

AAGAATGAACAAGAATTATTGGAATTAGATAAATGGGCAAGTTTGTGGAATTGGTTTGACATATCAAAATGGC
TGTGGTATATAAAAATATTCATAATGATAGTAGGAGGCTTGATAGGTTTAAGAATAGCTTTTACT---
GTACTTTCTATAGTG---------AAGAGAGTTAGGCAGGGATATTCACCA------
TTGTCGTTTGCTGTTAGCTTGCTCAACGCCACAGCCATAGCAGTAGCTGAG---
GGGACAGATAGGATTATAGAAATAATACAAAGAGCTTTTAGGGCT-----------------------------
--------ATTCTCCACATA------CCTACAAGAATAAGACAGGGCTTGGAAAGGGCTTTGCTA---TAA
>AY624305
GCT---ACA---
GAACAATTGTGGGTTACAGTCTATTATGGGGTACCTGTGTGG---AAAGAAGCAACC---
ACCACTCTATTTTGTGCATCAGATGCTAAAGCATATGATACAGAG---GTACATAAT----------------
--------------GTTTGGGCCACA---CATGCCTGTGTACCCACAGAC---
CCCAACCCACAAGAAATAATATTGAAA---------AATGTGTCAGAATATTTTGAT----------------
--------------------------ATGTGGAAAAATAACATGGTAGAACAAATGCATGAGGATATA---
ATCAGTTTATGGGATCAAAGCCTA---AAGCCATGTGTAAAATTA---
ACCCCACTCTGTGTTACTTTAGAGTGCACCAATGCTAAAAGTATCAATGCTACTAGTACCAATAATAATAACA
GT---
-------------ACCAATAATAGTAGTAGTTGGAAACTGCCGGAGGAGCCAGGAGAAATAAAA---
AATTGCTCTTTCAATATCACCACAAACATGAGA---------------------------
GATAAGATGCAGAAAGAATATGCAATT---
TTTTATAACCTTGATGTAGTACCAATAGATGATAATAAGAATAATAATAGTAGTAAT----------------
----
AATAAGAATAATAATAGTAGTAATAACACATATAGGTTATATAGGTTGATAAGTTGTAACACCTCAGTCATTA
CACAG---
GCCTGTCCAAAGGTATCCTTTCAGCCGATTCCCATACATTATTGTGCCCCGGCTGGTTTTGTGATTCTAAAAT
GTAAT---GATAAAAGGTTCAAT------GGAAGTGGACCATGTACAAAT-----------------------
-GTTAGC---ACAGTACAATGTACACATGGAATCAGGCCAGTAGTGTCTACT---
CAACTGCTGTTAAATGGCAGTCTAGCAGAAGAAGAG-----------
GTAATAGTTAGATCTGCCAATTTCACGGACAAT---------GCTAAAACTATAATAGTACATCTGAAT----
-----------GAATCTGTAGAA---------ATTAATTGTACAAGACCCAACAACAATACAAGAAGA-----
-GGTATATATATA------------GGACCAGGGAGAGCA---ATT------TATACAACAGACAAA------
---------ATAGTAGGAGATATA------------AGAAAAGCATATTGT---ACTATTAGTAAAGAA---
AATTGGAATAAAACTTTAGCACAGATAGTTATAAAATTAAGA---------------GAACAATTAAAGAAC-
--------------------------------GATACTAAAACAATAGTCTTCAAG------
CAATCCTCGGGAGGGGACCCAGAAATTGTA---ACACATAGTTTTAATTGTGGAGGGGAG---
TTTAAGCAGCTACAGGCAAGAGTCCTGTCTGTGGAAAGATACCTAAGGGATCAACAGCTCCTGGGGATTTGGG
GTTGCTCTGGAAAACTCATCTGCACCACTACTGTGCCTTGGAATGCTAGTTGGAGT----------------
-----------------------------AATAAATCTCTGGAGAAGATTTGG------GAT------
AATATGACCTGGATGGAGTGG---GAAAAAGAAATTGACAATTACACAGACCTAATACAC---
ACCTTACTTGCAGAATCGCAAAACCAACAAGAA--

AAGAATGAACAAGAATTATTGGCATTAGATAAGTGGGCAAGTTTGTGGAATTGGTTTGACATAACACAATGGC
TGTGGTATATAAAAATATTCATAATGATAGTAGGAGGCTTGATAGGTTTAAGAATAATTTTTGCT---
GTACTTTCTATAGTG---------AATAGAGTTAGGCAGGGATACTCACCA------
TTATCATTGGCTGTTAGCTTGCTCAACGCTACAGCTATAGCAGTAGCTGAG---
GGGACAGATAGGGTTATAGGAGTAGTACAAAGAGCTTGTAGGGGC-----------------------------
--------ATCCTCCACATA------CCTAGAAGAATAAGACAGGGCCTGGAGTGAGCTTTGATA---TAA
>JN002002
GCT---ACA---
GAAAAATTGTGGGTCACAGTCTATTATGGGGTACCTGTGTGG---AAGGAAGCAACC---
ACCACTCTATTTTGTGCATCAGATGCTAAAGCATATGATACAGAG---GTGCACAAT----------------
--------------GTCTGGGCCACA---CATGCCTGTGTACCCACAGAC---
CCTGACCCACAAGAAGTAGTATTGGAA---------AATGTGACAGAAGATTTTAAC----------------
--------------------------ATGTGGAAAAATAACATGGTAGAACAGATGAATGAGGATATA---
ATCAGTCTATGGGATCAAAGCCTA---AAGCCATGTGTAAAATTA---
ACCCCACTCTGTGTTACTTTAAATTGCACTGATGATTTGAGGAATGATACTAATACCAATAATAAT-------
----

----------------------AGTAGTGAGAGAGTGATAGAGATAGAGAAAGGAGAAATAAAA---
AACTGTTCTTTCAATGTCACCACAAGCATAAGA---------------------------
AATAAGATGCAGAAAGAATATGCATTT---TTTAATAGACTTGATGTAGTAAAAATAGATAAT----------
----

GATAATACTAGCTATAGGTTAATAAATTGTAATACCTCAGTCATTACACAG---
GCATGTCCAAAGGTATCCTTTGAGCCAATTCCCATACATTATTGTGCCCCGGCTGGTTTTGCGATTCTAAAGT
GTAAC---AATAAGATGTTCAAT------GGAACAGGAGCATGTACAAAT-----------------------
-GTCAGC---ACAGTACAATGTACACATGGAATTAGGCCAGTAGTGTCAACT---
CAACTGCTGTTAAATGGCAGTCTAGCAGAAAAAGAG------------
GTAGTAATTAGATCTGAAAATTTCGCAAACAAT---------GCTAAAACCATAATAGTACAGCTAAAT----
-----------GAATCTGTAGTA---------ATTAATTGTACAAGACCCAACAACAATACAAGAAAA-----
-AGTATACATATA------------GCACCAGGGAGAGCA---TTT------TATGCAACAGGGGAA------
---------AAAATAGGAGATATA------------AGACAAGCACATTGT---AACCTTAGTGAAGCA---
AAATGGAATAACACGCTAAAACAGATAGTTATAAAATTAAGA---------------GAACAATTTGGG----
-----------------------------------AATAAAACAATAGTCTTTAAT------
CAATCCTCTGGAGGGGACCCAGAAATTGTA---ATGCACAGTTTTAATTGTGGAGGGGAA---
TTTAAGCAGCTCCAGGCAAGAGTCCTGGCTGTGGAAAGATACCTAAGGGATCAACAGCTCCTAGGGATTTGGG
GTTGCTCTGGAAAACTCATTTGCACCACCTCTGTGCCTTGGAATGCTAGTTGGAGT-----------------
----------------------------AATAAAACTCTAGAAAACATTTGG------GAT------
AACATGACCTGGATGCAGTGG---GAAAGAGAAATTAGCAATTACACAGACATAATATAC---
ACCTTACTTGCAGAATCGCAGAACCAACAAGAA--

AAGAATGAACAAGAATTATTAGAATTAGATAAGTGGGCAAGTTTGTGGAATTGGTTTGACATAACAAACTGGC
TGTGGTATATAAAAATATTCATAATGATAGTAGGAGGCTTGGTAGGTTTAAGAATAGTTTTTGCT---
GTAATTTCAATAGTG---------AATAGAGTTAGGCAGGGATACTCACCA------
TTATCGTTGGCTGTTAGCTTGCTCAATGCCACAGCCATAGCAGTAGCTGAG---
GGGACAGATAGGGTTATAGAAGTAGTACAAGGAGCTTGTAGAGCT----------------------------
--------ATTCGCCACATA------CCTAGAAGAATAAGACAGGGCTTGGAAAGGATTTTGCTA---TAA
>JN002009
GCT---ACA--
GAAAAATTGTGGGTCACAGTCTATTATGGGGTACCTGTGTGG---AAAGACGCAAAC---
ACCACTCTATTTTGTGCATCAGATGCTAAAGCATATGATACAGAA---GTACATAAT----------------
--------------GTTTGGGCCACA---CATGCCTGTGTACCCACAGAC---
CCCAACCCACAAGAAGTAGTATTGGGG---------AATGTGACAGAAGAATTTGAC----------------
--------------------------TTGTGGAAAAATTACATGGTAGAACAGATGCATGAGGATGTA---
ATCAACCTATGGGATCAAAGCCTA---AAGCCATGTGTAAAATTA---
ACCCCACTCTGTGTTACTCTAAATTGCACTGATTATGAGAGAAATGCTACTATGAAAAATGATACT-------
----
-------------------ACTAATACTAATACCAAGAATAGTAGTAAGTGGGGGGAAATGAAA---
AACTGCTCTTTCAAAATCTCCACAAACATAAAA---------------------------
GATAAGACACAGAGAGAATATGCACTT---TTTTATAAACTTGATGTAGTACCAATAAATAAT----------
----

GATAGTACTAGCTATAGGTTAATAAATTGTAATACCTCAGTCATTACACAG---
GCCTGTCCAAAGGTATCCTTTGAGCCAATTCCCATACATTATTGTACCCCGGCTGGTTTTGCGATTCTAAAGT
GTAAC---AATAAGACATTCAGT------GGAAAAGGGCCATGTACAAAT-----------------------
-GTCAGC---ACAGTACAATGTACACATGGAATTAGGCCAGTAGTGTCAACT---
CAACTGCTGTTAAATGGCAGTCTAGCAGAAGAAGGG------------
GTAGTAATTAGATCTAGCAATTTCTCGGACAAT---------GCTAAAACCATAATAGTACAGCTGCAG----
-----------AAACCTATAAAA---------ATTAATTGTACAAGACCACAC---AATACAAGAAAA-----
-AGTATACATATA------------GGACCAGGGAGAGCA---TTT------TATGCAACAGGAGAT------
---------ATAGTAGGAGATATA------------AGAGAAGCACATTGT---AACCTTAGTAGAACA---
GATTGGAATAATACTTTAAAACAGGTAGTTGAAAAATTAAGA---------------TTTGGG----------
-----------------------------------AATAAAACAATAGTCTTTAAA------
AAATCCTCTGGAGGGGACCCAGAAATTGTA---ATGCACAGTTTTAATTGTGGAGGGGAA---
TTTAAGCAGCTCCAGGCAAGAGTCCTGGCTGTGGAAAGATACCTAAGGGATCAACAGCTCCTGGGGATTTGGG
GTTGCTCTGGAAAACTCATTTGCACCACTTCTGTGCCTTGGAATGCTAGTTGGAGT-----------------
----------------------------AATAAATCTCTGGAAAAAATTTGG------GAT------
AACATGACCTGGATGGAGTGG---GAAAAAGAAATTAACAATTACACAGGGATAATATAC---

AACTTACTTGAAGAATCGCAAAACCAACAAGAA--

AAGAATGAACAAGAATTATTGGAATTAGATAAATGGGCAAGTTTGTGGAATTGGTTTGACATAACAAAATGGC
TGTGGTATATAAAAATATTCATAATGATAGTAGGAGGCTTAATAGGTTTAAGAATAGTTTTTGCT---
GTATTTTCTATAGTG---------AATAGAGTTAGGCAGGGATATTCACCA------
TTATCGTTTGCTGTTAGCTTGCTCAATGCCACAGCCATAGCAGTAGCTGAG---
GGGACAGATAGGGTTATAGAAGTAGTACAAGGAGCTTGTAGAGCT---------------------------
--------ATTCGCCACATA------CCTAGAAGAATAAGACAGGGCTTGGAAAGGATTTTGCTA---TAA
>GU367406
GCT---GCA--
GGAACGTTGTGGGTCACAGTCTATTACGGAGTACCTGTGTGG---AAAGAAGCAACT---
ACCACTCTATTTTGTGCATCAGATGCTAAAGCATATCATACAGAG---GTACATAAT----------------
--------------GTTTGGGCCACA---CACGCCTGTGTACCCACAGAC---
CCCGACCCACAAGAAATACTATTGGAA---------AATGTGACAGAATATTTTAAT----------------
-------------------------ATGTGGAAAAATAACATGGTAGACCAAATGCAGGAAGATATA---
ATCAGTATATGGGATCAAAGCCTA---AAGCCATGTGTAAAATTA---
ACCCCACTCTGTGTTACTTTAAATTGCACTAATTTGAGGAATATCACTGTG---------------------
----
---------------------------------GGGAATAATACTCAGGGAGAAGAAATGAAA---
AACTGCTCTTTCAATATCACCACAGTAGTAAGA--------------------------
GATAAGGTAAAGAAAGAGTATGCTCTT---TTTTATAAACTTGATATAGTACCAATAGATAATACAATA----
----

GATAATACTACCAATAGTACCACATATAGGTTGATAAATTGTAATACCTCAGTCATTACACAG---
GCCTGTCCAAAGGTATCCTTTGAACCAATTCCCATACATTATTGTGCCCCGGCTGGTTTTGCGATTCTAAAGT
GTAAC---AATAAGACATTCAAT------GGAAAAGGACCATGTACAAAT----------------------
-GTCAGT---ACAGTACAATGTACACATGGAATTAGGCCAGTAGTGTCAACT---
CAACTACTGTTAAATGGCAGTCTAGCAGAAGAAGAG-----------
GTAGTAATTAGATCTGACAATTTCTCGGATAAT---------GCTAAAATCATAATAGTGCAGCTGAAC----
-----------AAATCTGTAGAA---------ATTAATTGTACAAGACCCAACGACAATAGAAGAAAA-----
-AGTATACAAGTA------------GGACCAGGGAGAGCA---CTT------TATGCAACAGGACAA------
---------ATAATAGGAGATATA------------AGACAAGCACATTGT---AACATTAGTAAAGCA---
AATTGGACTAACACTTTACAAAAGATAGTTAAAAAATTAAAA---------------GAACAATTTAAA----
----------------------------------AATAAAACAATAGTCTTTAAT------
CCACCCTCAGGAGGGGACCTAGAAATTGTA---ATGCACAGTTTTAATTGCGGAGGGGAA---
TTTAAGCAGCTCCAGGCAAGAGTCCTGGCTGTGGAAAGATACCTAAAGGATCAACGGCTCCTAGGGATTTGGG
GTTGCTCTGGAAAACTCATCTGTCCCACTGCTGTGCCTTGGAATGCTAGTTGGAGT----------------
----------------------------AATAAATCTCTGGAGGTGATTTGG------AAT------
AACATGACCTGGATGGAGTGG---GAAAGAGAAATTGACAATTACACAAGCTTAATATAC---
ACCTTAATTGAAGAATCGCAAACCCAACAAGAA--

AAGAATGAACTAGAGTTATTGGAATTAGATAAGTGGGCAAGTTTGTGGAATTGGTTTGACATAACAAAATGGC
TGTGGTATATAAAAATATTCATAATGATAGTAGGAGGCTTGATAGGTTTAAGAATAATTTTTGCT---
GTACTTTCTATAGTG---------AATAGAGTTAGGCAGGGATACTCACCA------
TTGTCATTTGCTATTAGCTTGCTCAACGCCACAGCTATAGCAGTAGCTGAG---
GGAACAGATAGGGTTATAGAAGTAGGACAAAGAATTTGGAGAGCT---------------------------
--------TTTCTCCATATA------CCTAGAAGAATAAGACAAGGCTTCGAAAGGGCTTTGCTA---TAA
>GU367395
GCT---GCA--
GAGAAGTTGTGGGTCACAGTCTATTATGGGGTACCTGTGTGG---AAAGAAGCAACC---
ACCACTCTATTTTGTGCATCTGATGCTAAAGCATATGAGACAGAG---GTGCATAAT----------------
--------------GTCTGGGCCACA---CATGCCTGTGTACCCACAGAC---
CCCAACCCACAGGAAGTAGTATTA------------AATGTGACAGAAAATTTTAAC----------------
-------------------------ATGTGGAAAAACAACATGGTAGAACAAATGCATGAGGATATA---
ATCAGTTTATGGGATGAAAGCCTA---AAGCCATGTGTAAAATTA---
ACCCCACTCTGTGTTACTTTAAATTGCACTGATGTGAAGAATAATGTTACTGCTGAT----------------
----
------------------------------AATACCACTACTAGTCCTTTAGGAGAAATAAAA---
AACTGCTCCTTCAAAATCACCACAAGTATAAAA--------------------------
GATAAGAAGCAAGAAGAATATGCATTT---TTTTATAAACTTGATGTAGTGCCAATAGATAAT---------

----

GATAATACTAGCTATAGGTTGATAAATTGTAACACCTCAGTCATTACACAG---
GCCTGTCCAAAGGTATCATTTGAGCCAATTCCCATACATTATTGTGCCCCGGCTGGTTTTGCGATTCTAAAGT
GTAAC---AATAAAACGTTCAAT------GGAAAGGGACCATGTACAAAT-----------------------
-GTCAGC---ACAGTACAATGTACACATGGAATTAGGCCAGTAGTGTCAACT---
CAACTGCTGTTAAATGGCAGTCTAGCAGAGGAAGAG------------
ATAGTGATTAGATCTGCCAATTTCACGGACAAT---------GCTAAAACCATAATAGTACAGCTGAAT----
-----------GAATCTGTAGTA---------ATCAATTGTACAAGACCCAACAACAATACAAGGAAA-----
-AGTATACATATA------------GGTCCAGGGAAAGCA---TTT------TATGCAACAGGAGAA------
---------GTAGTAGGAAATATA------------AGACAAGCATTTTGT---ACCCTTGATAAAACA---
AAATGGGAGAACACTTTAAGACAGATAGCTATAAAGTTAAGA---------------ACACAATTTGAG----
-----------------------------------ACTGAGACAATAGTATTTAAT------
CAATCTACAGGAGGGGACCCAGAAATTGTA---ATGCACAGTTTTAATTGTGGAGGGGAA---
TTTAAGCAGCTCCAGGCAAGAGTCCTGGCTGTGGAAAGATACCTAAGGGATCAACAGCTCCTAGGGATTTGGG
GTTGCTCTGGAAAACTCATTTGCACCACTGCTGTGCCTTGGAATACTAGTTGGAGT-----------------
----------------------------AATAAAAATCTAACTGACATTTGG------AAT------
AACATGACCTGGATGGAGTGG---GAAAAAGAAATTGATAACTACACAAGTGTAATATAC---
AATTTACTTGAAGAATCGCAGAACCAACAAGAA--

AAGAATGAACAAGAATTATTAGCATTGGATAAGTGGGCAAGTTTGTGGAATTGGTTTGACATAACAAATTGGC
TGTGGTATATAAAAATATTCATAATGATAGTAGGAGGCTTGATAGGCTTAAGAATAGTTTTTGCT---
GTACTTTCTATAGTG---------AATAGAGTTAGGCAGGGATACTCACCA------
TTATCATTGGCTGTTAGCTTGCTCAACACCACAGCAATAGCAGTAGCTGAG---
GGGACAGATAGGGTTATAGAAATAGTACAAAGAGCTGGTAGAGCT----------------------------
--------ATCCTCCACATA------CCTAGAAGAATAAGACAGGGCTTAGAAAGGGTTTTGCTA---TAA
>FJ653258
ACT---GCA--
GAACAATTGTGGGTCACAGTCTATTATGGGGTACCTGTGTGG---AGAGATGCAAAC---
ACCACTCTATTTTGTGCATCAGATGCTAAAGCACATGATACAGAG---GTACATAAT----------------
--------------GTTTGGGCGACA---CATGCCTGTGTACCCACAGAC---
CCCAACCCACAAGAAGTAGTATTGGCA---------AATGTGACAGAAAATTTTAAC----------------
--------------------------ATGTGGAAAAATAACATGGTAGACCAAATGCATGAGGATATA---
ATCAATTTATGGGATCAAAGCCTA---AAGCCATGTGTAAAACTA---
ACCCCACTCTGTGTTACTTTAAATTGCACTGATGTCAATAGTACTAGTACCAACACTACTAATACC-------
----
-------------------AATAGTAGTAGTTGGGGGGAAAAAGTGGAAAGAGGAGAAATAAAA---
AACTGCTCTTTCAATATCACCACAAGCATAAAA---------------------------
GATAAGGTACAGAAAGAATATGCACTC---TTTTATAAACTTGATGTAGTATCAATAGATAAT----------
----

AGTAGTACTAGCTATAGGTTGATAAGTTGTAACACTTCAGTCCTTACACAA---
GCCTGTCCAAAGATAACCTTTGAGCCAATTCCCATACATTATTGTGCCCCGGCTGGTTTTGCGATTATAAAGT
GTAAT---GATAAAAGGTTCAAT------GGAACAGGATCATGCAAAAAT-----------------------
-GTCAGC---ACAGTACAATGTACACATGGAATTAAGCCAGTAGTATCAACT---
CAACTACTGTTAAATGGCAGTTTAGCAGAAGAAGAG------------
GTAGTAATAAGATCTGAGAATTTCACAAACAAT---------GCTAAAACCATAATAGTACAGCTGAAT----
-----------GAAACTGTAGAA---------ATTAAGTGTACAAGACCCGGCAACAATACAAGAAAA-----
-AGTATATATATA------------GGACCAGGGAGAGCA---GTT------CATGTAACAGACAGA------
---------ATAATAGGAGATATA------------AGACAAGCACATTGT---AACATTAGTAGGACA---
CAATGGAAAAACACTTTAAAAAGGATAGCAATAAAATTAAGA---------------GAACAATTTAGG----
-----------------------------------AATAAAACAATAGCCTTTAAT------
AGATCCTCAGGAGGGGACCCAGAAATTGTG---ATGCACAGTTTTAATTGTAGAGGGGAA---
TTTAAGCAGCTCCAGGCAAGAGTCCTGGCTGTAGAAAGATACCTAAGGGACCAACAGCTCCTGGGAATTTGGG
GTTGCTCTGGAAAACTCATCTGCACCACTGCTGTGCCTTGGAATAATAGTTGGAGT-----------------
----------------------------AATAAGTCTCATACAGATATTTGG------AAT------
AACATGACCTGGATGGAGTGG---GAGAGAGAAATTGACAATTACACAGGCTTAATATAC---
AACTTACTCGAACAAGCACAAAACCAGCAAGAA--

AAGAATGAACAAGAATTGTTGGAATTGGATCAATGGGCAAATTTGTGGAATTGGTTTGACATAACAAAGTGGC

TGTGGTATATAAAAATATTCATAATGATAGTAGCAGGCTTGGTAGGTTTAAGAATAGTTTTTGCT---
GTACTTTCTATAGTG---------AATAGAGTTAGGCAGGGATACTCACCA------
TTATCGTTTGCTGTTAGCTTGCTTGATACCATAGCCATAGCAGTAGCTGAG---
GGGACAGATAGGGTTATAGAAATAGTACAAAGAGCTTGTAGAGCA----------------------------
--------ATTCTCCACATA------CCTAGAAGAATAAGACAGGGCTTGGAAAGGGCTTTGCTA---TAA
>MN485973
GCT---GCA--
GAHAACTTGTGGGTCACAGTTTATTATGGGGTACCTGTGTGG---AAAGAAGCAACC---
ACCACTCTCTTTTGTGCATCAGATGCTAAAGCATATGACACAGAG---GCACATAAT----------------
--------------GTTTGGGCCACA---CATGCCTGTGTACCCACAGAC---
CCTAGTCCACAAGAAGTAAGATTGGAA---------AATGTGACAGAAAATTTTAAC----------------
--------------------------ATGTGGAAAAATAATATGGTAGAACAGATGCAGGAGGATATA---
ATCAGTTTATGGGATCAAAGCCTA---AAGCCATGTGTCAAATTA---
ACCCCACTCTGTGTTACGTTAAATTGCACTGAGTTGAATGTTAATAGTACAGATAGTAATAGT----------
----
-------------------------AGCAGTATAGGTAATAGTAATATGAAAGGGGAAATAAAA---
AACTGCTCTTTTAATATCACCACAAGAATAGGA---------------------------
AATAGGCTGCAGAAAGATTATGCACTT---TTTAATAAGCTTGATGTAGTACCAATAAATAAT----------
----

GATAATGAAAGCTATATGTTAATAAATTGTAATACCTCAGTCATTACACAA---
GCCTGTCCAAAAGTATCCTTTGAACCAATTCCCATACATTATTGTGCCCCGGCTGGTTTTGCGCTTCTGCAGT
GTAAT---GATAAGAAGTTCAAT------GGAACAGGACCGTGCAAAAAT-----------------------
-GTCAGC---ACAGTACAATGTACACATGGAATTAGGCCAGTAGTGTCAACT---
CAACTGCTGTTAAATGGCAGTCTAGCAGAAGAAGAG------------
ATAGTAATTAGATCTGAAAATTTTACGAACAAT---------GTTAAAACCATAATAGTACAGCTGAAT----
-----------GAAACTGTAGAA---------ATTAATTGTGTAAGACCCAACAACAATACAAGAAAA-----
-AGTATACATATG------------GGACTGGGGAGAGCA---TTT------TATGCAACAGGAGAC------
---------ATTATAGGAAATATA------------AGACAAGCACATTGT---AACCTTAGTAAAGAA---
GACTGGGGGAAGACTCTAGAACGGGTAAGAATAAAATTAGAA---------------GAACAATATAAG----
-----------------------------------AATAAAACAGTAGTCTTTAAT------
CAATCCTCAGGAGGGGACCAAGAAATTGTA---ATGCACACTTTTAATTGTGGAGGGGAA---
TTTAAGCAGCTCCAGGCAAGAGTCCTAGCTGTGGAAAGATACCTAAAGGATCAACAGCTCCTGGGGATTTGGG
GTTGCTCTGGAAAACTCATTTGCACCACTGCTGTGCCTTGGAATGTTAGCTGGAGT-----------------
----------------------------AATAAATCTATGGATAARATTTGG------GAT------
AACATGACCTGGATGGAGTGG---GAGAGGGAAATTRAYAATTACACAGGCTTAATATAT---
ACCTTACTTGAAGAATCACAAAACCAACAAGAA--

AAAAATGAACAAGAATTAYTGGAATTGGATAAGTGGGCAAGTTTGTGGAAYTGGTTTGACATAACAAAATGGC
TGTGGTATATAAAAATATTCATAATRRTAGTAGGAGGTTTAGTAGGTTTAAGAATAGTYTTTACT---
GTACTTTCTATAGTG---------AMGAGAGTTAGGCAGGGATATTCACCA------
TTATCGTTGGCTGTTAGTCTGYTTAATGTTACAGCTATAGCAGTAGCTGAG---
GGGACAGATAGGGTTATAGAAGTAGTACAGAGGATTTTTAGAGCT----------------------------
--------ATTATCCACATA------CCTACAAGAATAAGACAGGGCTTAGAAAGGCTTCTGCTA---TAA
>MN485981
GCT---GCA--
GGAAATCTGTGGGTCACAGTATATTATGGGGTACCTGTGTGG---AAAGAAGCAACC---
ACCACTTTATTCTGTGCATCAGATGCTAAAGCATATGACACAGAG---GCACATAAT----------------
--------------GTTTGGGCCACA---CATGCCTGTGTGCCCACAGAC---
CCTAATCCACAAGAAATAGTAATAGAA---------AATGTGACAGAAAACTTTAAC----------------
--------------------------ATGTGGAAAAATGACATGGTAGACCAGATGCATGAGGATGTA---
ATCAGTTTATGGGATCAAAGCCTA---AAGCCATGTGTGAAATTA---
ACCCCACTCTGTGTTATTTTAACTTGTACTAATGTGCAAAATCGTACTAATGCGAATAATTATACT-------
----
--GTGGAAGAGGGGGTAGGAATAAAG---
AACTGCTCTTTCAATATCACCACAACAACAGGA---------------------------
AATAAGAGRAAGACGGAYTASSCACTT---TTTAATAGYTTTGATGTAGTACCAATARWTAATAYA-------
----

AATAATAAAAGCTATATGTTGACAGCTTGTAACACYTCAGTTGTCACMCAA---

GCCTGTCCAAAGATATCCTTTRAACCAATTCCCWTMCATTATTGTGCCCCGGCTGGTTTTSCAATTYTAAAAT
GTAWT---AWTAAGMCATTTAAT------GGAMCAGGACCATGTAAAAT------------------------
-KTCASC---ACAATMCAATSCACACATGGGATTAGGCCAGTAGTATCAAYT---
CAGTTGYTGTTAAATGSCAGTYTASCAGAAGGAGAG------------
ATAATAATTAGATCTGAAAATCTCACGAGCAAT---------ACTAAAAACATAATAGTACAGTTAAAT----
-----------GAAACTATACAA---------ATTAATTGTACAAGACCCAACAACAATACAAGAAAA-----
-AGTATAAACATA------------GGACCAGGAAGAACA---TTT------TATGCAACAGGAGGA------
---------ATAATAGGAAATATA------------AGACAAGCACATTGT---AACATTAGTGAAGCA---
CAATGGAACAAAACTTTAGGACAGATAGTGAAAAAATTACAA---------------GCACATTTTGAG----
-----------------------------------AATAAAACAATAATCTTT---------
AAATCCTCAGAAGGGGAMCCAGAAGTTGTA---ATGSACACTTTTAATTGTGGAGGGGAA---
TTTAAGCAGCTCCAGGCAAGAGTCCTGGCTGTGGAAAGATACCTAAAGGATCAACAGCTCCTGGGGATTTGGG
GATGCTCTGGAAAACTCATTTGCACCACTACTGTGCCTTGGAATGCTAGCTGGAGT-----------------
----------------------------AATAAATCTTATGAACATATTTGG------CAA------
AACATGACCTGGATGCAGTGG---GAAAAAGAAATTGACAATTACACAGGCTTAATATAC---
ACTTTACTAGAAAAATCACAGATCCAGCAAGAA--

CAGAATGAACAAGAATTATTGGCATTGGATAAGTGGGCAAGTTTGTGGAATTGGTTCGACATAACACAATGGC
TGTGGTATATAAAAATATTCATAATGATAGTAGGGGGCATAGTAGGTTTAAGAATAGTTTGTAGT---
GTACTTTCTATAGTG---------AATAGAGTTAGGCAGGGATACTCACCA------
TTGTCATTCGCTGTTAGCTTGCTTAATACAACAGCTATAGCAGTAGCTGAG---
GGAACAGATAGGATTATAGAAATAGCACAAAGACTTTTTAGAGCT----------------------------
--------ATCATCCACATA------CCTAGAAGAATAAGACAGGGCTTCGAAAGGGCTTTGCTA---TAA
>MN485993
GCT--
AAAGATTTGTGGGTCACAGTTTATTATGGGGTACCTGTGTGG---AAAGAAGCAACC---
ACCACTCTATTTTGTGCATCAGATGCTAAAGCATATGACACAGAG---GTACATAAT----------------
--------------GTTTGGCCACA---CATGCCTGTGTACCCACAGAC---
CCCAAACCACAAGAAATATTATTGGAA---------AATGTAACAGAACCTTTTAAC----------------
--------------------------ATGTGGAAAAATAACATGGTAGAACAAATGCATGAAGACATA---
AAAAGTTTATGGGATCAAAGTCTA---AAGCCATGTGTAAAATTA---
ACCCCACTCTGTGTTACTTTAAAGTGTACTAATGTGACCAATACAAGCAAGGTTGACAATGCTACA-------
----
---AGAGAAGAACTAAGA---
AACTGCTCTTTCAATGTCACCACAGTTATAAGA---------------------------
GATAAGAAAACTAAAGAACATGCATTA---TTTTATAGATCTGATATAGTACCAATAGATGATGGG-------
----

AATAATGCCAGTTATAGGTTGATAAATTGTAATACATCAGTCATTACCCAG---
GCATGTCCAAAGGTGACCTTTGAACCAATTCCCATCCATTATTGTGCCCCAGCTGGTTTTGCGATTTTCAAAT
GTAAT---GACAAGAGGTTTAAT------GGAACAGGACCATGTAAAAT------------------------
-GTAAGC---ACAGTACAGTGTACTCATGGAATTAGGCCAGTGGTATCAACT---
CAACTGCTGTTAAATGGCAGTCTAGCAGAAGAGGAG------------
ATAGTAATTAGATCTGAAAATTTCACAAACAAT---------GCTAAAACCATAATAGTACAGCTGAAC----
-----------GCATCTGTAGAA---------ATTAACTGTACAAGACTCAACAACAACACAAGAAGA-----
-GGCATACATATA------------GGACCAGGACAAGCA---TTT------TATACAACAGGAGAT------
---------ATAGTAGGAGATATA------------AGACAAGCACACTGT---AACATTAGTAGAGGA---
AAATGGAATAACACTTTAAATCAGGTAGCTAAAAAATTAAGA---------------GAAATATTTAGG----
-----------------------------------AATAAAACAATATCCTTTAAG------
CAACCCTCAGGAGGGGACGCAGAAATTGTA---ATGCACAGTTTTAATTGTGGAGGGGAA---
TTTAAGCAGCTCCAGGCGAGAGTCCTGGCTGTGGAAAGATACCTAAAGGATCAACAGATCCTGGGAATTTGGG
GCTGCTCTGGAAAACTCATTTGCACCACTGCTGTGCCTTGGAATAATAGTTGGAGT-----------------
----------------------------AATAAATCTTATGAAGATATTTGG------GAT------
AACATGACCTGGATGCAATGG---GAAAGAGAAATTGACAATTACACAGACTCAATATAC---
AAATTAATTGAAATATCGCAAAACCAACAAGAA--

AAGAATGAACAAGAGTTGCTGGAATTGGATAAATGGGCAAGTTTGTGGAATTGGTTTGACATAACAAACTGGC
TGTGGTATATAAAAATATTCATAATGATAGTAGGAGGCCTGGTAGGTTTAAGAATAGTTTTTACT---
GTACTTTCTATAGTA---------AATAGAGTTAGGCAGGGATATTCACCA------
TTGTCGTTGGCTGTTAGCTTGCTCAACGCCACAGCCATAGCAGTAGCTGAG---

GGGACAGATAGGGTTGTAGAAGCATTACGCAGAGCTTTTAGAGCT----------------------------
--------ATCCTCCACATA------CCTACAAGAATAAGACAGGGCTTGGAAAGGCTGTTGCTA---TAA
>MN485996
GCT---GTA-----------------------------AAC---ACA---
GACAAATTGTGGGTCACAGTCTATTATGGGGTACCTGTGTGG---AAAGAAGCAACC---
ACCACTCTATTTTGTGCATCAGATGCTAGAGCATATGATAAAGAG---GCACATAAT---------------
--------------GTTTGGGCCACA---CATGCCTGTGTACCCACAGAC---
CCTGACCCACAAGAAGTAAAATTGGGA---------AATGTGACAGAAAATTTTAAT---------------
--------------------------GTGTGGAAAAATAACATGGTAGAACAGATGCAGGAGGATATA---
ATTAGTTTATGGGATCAAAGTCTA---AAGCCATGTGTAAAATTA---
ACCCCACTCTGTGTCACTTTAAATTGCACTAATGAGATTGCTGCGAATCATACCAACTATAAT---------
----
---GAGACTATAGGAGAAATGAAA---
AACTGCTCTTTCAATATTACCACACACATAAGG--------------------------
GACAAGATACAGAAAGAGTATGCACTT---TTTTATAAACTTGATATAGTACCAATAGATAGT---------
----

GAAAATGATAGCTATAGGTTGATAAGTTGTAATACCTCAGTCATTACACAG---
GCCTGTCCAAAGATATCCTTTGAGCCAATTCCCATACATTATTGTGCCCCGGCTGGTTTTGCGATTCTAAAAT
GTAAT---AATAAGACATTTAGT------GGAAAAGGATCATGTAACAAC----------------------
-GTCAGT---ACAGTACAATGTACACATGGAATTAGGCCAGTTGTGTCAACT---
CAACTGCTGTTAAATGGCAGTCTAGCAGAAGAAGAG-----------
GTAGTAATTAGGTCTGAGAATTTCACAAACAAT---------GCTAAAACTATAATAGTACAGCTGAAG----
-----------AATTCTATACAA---------ATTAATTGTACAAGACCCAACAACAATACAAGAAAA-----
-AGTATACATATG------------GGACCAGGGGCAGCA---TTT------TATGCAACAGGAGAC------
---------ATAATAGGAAATATA-----------AGACAAGCACATTGT---AACCTTAGTAGAACA---
CAATGGGATGACACTTTARAAAAGATAGTTAAAAAATTAAGA--------------GAACAATTTGGG----
--------------------------------AATAACAAAACAATAGTCTTTAAG------
CAATCTGCAGGAGGGGACCCAGAAATTGTA---ATGCACAGTTTCAATTGTGGAGGGGAA---
TTTAAGCAGCTCCAGGCAAGAGTCCTGGCTGTGGAAAGATACCTAAAGGATCAGCAGCTCCTAGGGATTTGGG
GCTGCTCTGGAAAACTCATTTGCACCACTGCTGTGCCTTGGAATGCTAGTTGGAGT----------------
----------------------------AATAAGTCTCAGGATGATATTTGG------AAC------
AACATGACCTGGATGGAGTGG---GAAAGAGAAATTGAGAATTACACAGACCTAATATAC---
ACCCTAATTGCAGACTCGCAAAACCAACAAGAA---------------------------------------
----
AAGAATGAAAAAGACTTATTAGAACTTGATAAGTGGGCAAGTTTGTGGAATTGGTTTAATATAACAAACTGGC
TGTGGTATATAAAAATATTCATAATGATAGTAGGAGGCTTAGTAGGTTTAAGAATAGTTTTTACT---
GTATTTTCTATAGTG---------AACAGAGTTAGGCAGGGATACTCACCA------
TTGTCATTCGCTGTTAGCTTGCTCAATGTCACAGCCATAGCAGTAGCTGAG---
GGGACAGATAGAATTATAGAAGCAATACAAAGAGCTGGAAGAGCT---------------------------
--------ATCCTCCACATA------CCTAGAAGAATAAGACAGGGCTTAGAAAGAAKTTTGCTA---TAA
>MN485998
GCT---ACA--
GAAAAGTTGTGGGTCACAGTTTATTATGGGGTGCCTGTGTGG---AAAGAAGCAACC---
ACCACTCTATTTTGTGCATCAGATGCTAAAGCATATAAAACAGAG---GCACATAAT---------------
--------------GTCTGGGCTACA---CATGCCTGTGTACCAACAGAC---
CCCAACCCACAAGAAATAAATTTAGAA---------AATGTCACAGAAAACTTCAAC---------------
--------------------------ATGTGGAAAAACAACATGGTAGAACAAATGCATGAGGATATA---
ATCAGTTTATGGGATCAAAGCTTA---AAGCCATGTGTAGAATTA---
ACCCCACTCTGTGTTACTTTAAATTGCATTAATGTGACAAAAAGTTGCCTTAATAATACTTCTGATCCGACTA
ACAATTGCACT--
-------------------------AGTAATAATTACACGGTAGAACTGGATAACGGAATAAAA---
AACTGTTCTTTCAATATCACAACAAAAATGGAA--------------------------
GATAAGAAAAAGAAAGAATATGCATTT---TTTAATGAACTTGATATAGTACCAATAGATAAT---------
----

GACACCACTAGCTATAGGTTAATAAGTTGTAACACCTCAGTCATTACACAG---
GCCTGTCCAAAGATCTCCTTTGAACCGATTCCCATACATTATTGTGCCCCGGCTGGTTTTGCGATTCTACAGT
GTAAA---GATAGGGATTTCAAT------GGGACAGGACTATGTAAGAAT----------------------
-GTCAGC---ACAGTACAATGCACACATGGAATTAAACCAGTGGTATCAACT---

CAACTGCTGTTAAATGGCAGTCTAGCAGAAGAAAAG-----------
GTAGTAATAAGATCTGAAAATTTCACAGACAAT---------GTTAAAATCATAATAGTGCAGCTGAAA----
-----------GACCCTGTAAAC---------ATTACTTGTATAAGACCCAACAACAATACAAGAAGA-----
-AGTATACATATA------------GGACCAGGGAGAGCA---TAT------TATGCACCAGGAGAA------
---------ATAATAGGAGATATA------------AGAAAAGCACATTGT---AACATTAATGGGACA---
AAGTGGAATAATACTTTAAAACAGATAGCTAAACAATTAGGG--------------GAGCAATTCAAG----
-----------------------------------AATAAATCAATACGATTTCAG------
CCACACTCAGGAGGAGACCTAGAAATTGTA---ATGCACAGTTTTAATTGTGGAGGGGAA---
TTTAAGCAGCTCCAGGCAAGAGTCCTGGCTGTGGAAAGATACCTACAGGATCAACAGCTCCTAGGAATTTGGG
GTTGCTCTGGGAAACTCATTTGCACCACTGCTGTGCCTTGGAATAATAGCTGGAGT----------------
----------------------------AATAAAAATATGACACAAATTTGG------GAC------
AGCATGACTTGGATGCAGTGG---GATAAAGAAATTAGTAATTACACACAATTAATATAC---
ACCTTAATTGAAGAATCGCAAAACCAACAAGAA---

AAGAATGAAAAAGACTTATTGGAATTGGGTAAATGGGACAGTTTGTGGAATTGGTTTAGCATAACACATTGGC
TGTGGTATATCAAAATCTTTATAATGATAGTAGGAGGCTTAGTAGGTTTAAGAATAGTTTTTACT---
GTACTTTCCTTAGTG---------AATAGAGTTAGGCAGGGATACTCACCA------
ATCTCGTTGGCTGTTAGCTTGTTTAATGCCATAGCCATAACAGTAGCTGAG---
GGGACAGATAGAATCATAGAAGTAGCACAAAGAGCTTTTAGGGCC---------------------------
--------ATCATCCACATA------CCTAGAAGAATAAGACAGGGCTTGGAAAGGGCTTTGCAA---TAA
>MN486009
GCT---GCA---
GAGCAATTGTGGGTCACAGTCTATTATGGGGTACCTGTGTGG---AAAGAAGCAACC---
ACCACTCTATTTTGTGCATCAGATGCCAAAGCATATGATACAGAA---GTACACAAT----------------
--------------GTTTGGGCCACA---CATGCCTGTGTACCCACAGAC---
CCCAGCCCACAAGAAGTAAGATTGAAA---------AATGTGACAGARGATTTTAAC----------------
-------------------------ATGTGGAAAAATGACATGGTAGAACAAATGCATGAGGATGTA---
ATCAGTTTATGGGATCAAAGCCTA---AAGCCATGTGTAAAACTA---
ACCCCACTCTGTGTTACTTTAAATTGTACTGATTATCAGAAGAATAGTACTAATAGCAGTAATAGTACTAATG
TTACTAATAGC--
-------------------AGTAATAGTGCTCACGCTCTCACTGCTTCTCCTGGAGAAATACAA---
AATTGCTCTTTCAATATCACCACAAACATAGGA--------------------------
GACAAGATGCAGAAAACATATGCACTT---
TTTTATAAGCTTGATGTAGTACCAATAGATGATAATTATAATAATAAT------------------------
----
AGTGCTAATAATAATGAGAGCTATAGGGACTATAGGTTGATAAGTTGTAACACCTCAGTCATTACACAA---
TCCTGTCCAAAAATATCCTTTGAGCCAATTCCCATACATTATTGTGCCCCGGCTGGTTTTGCAATTCTAAAAT
GTAAC---AATGACACATTCACT------GGAACAGGGCCATGTACAAAT----------------------
-GTCAGC---ACAGTACAATGTACTCATGGAATTAGGCCAGTAGTATCAACT---
CAACTGCTGTTAAATGGCAGTCTAGCAGAAAGAGAG-----------
ATAGTAATTAGATCTGAAAATCTCACAGACAAT---------GCTAAAAACATAATAGTACAACTGAAA----
-----------GATGCTGTGCAA---------ATTAATTGCACAAGACCCAACAATAATACAAGAAAA-----
-AGTATACACATA------------GGACCAGGGAGCACA---TTT------TATGCAACAGGGGAA------
---------ATAATAGGAGATATA------------AGACAGGCACACTGT---AACCTTACTGCAGCA---
GATTGGAATAATACTTTAAGAGAGATAGTTAAAAAATTAGGA--------------GAACAATTTGGG----
-----------------------------------AATAAAACAATAAAGTTTGAG------
CAACCCTCAGGAGGGGACCCAGAAATTGTA---CTGCACAGTTTTAACTGTGGAGGAGAA---
TTTAAGCAGCTCCAGGCAAGAGTCCTGGCTGTGGAAAGATACCTACAGGATCAACGGTTCCTGGGGATTTGGG
GTTGCTCTGGAAAACTCATTTGCACCACTGCTGTGCCTTGGAATAGTAGTTGGAGT----------------
----------------------------AATAGAACTTACAGTGACATTTGG------GAT------
AACATGACCTGGATGCAATGG---GAGAAAGAAATTAGCCAATACACAGACACAATATAC---
AACTTAATTTCCGAATCGCAGAACCAACAAGAA---

AAGAATGAACATGACTTATTAGAATTAGATAAATGGGCAAATTTGTGGAATTGGTTTGACATAACAAACTGGC
TGTGGTATATAAAAATATTCATAATAATAGTAGGAGGCTTGGTAGGCTTAAGAATAGTTTTTGCT---
GTATTTTCTATAGTG---------AGCAGAGTTAGGCAGGGATACTCACCA------
TTGTCGTTTGCTGTTAGCTTGCTTAACGCCACAGCAATAGCAGTAGCTGAG---
GGAACAGATAGGATTATAGAAATAGTACAAAGGACTGGTAGAGCT---------------------------
--------ATTATCCACATA------CCAGTAAGGATAAGACAAGGCTTAGAAAGGCTTTTGCTA---TAA
>MN486010

ACT---GCA--
GGACAATTGTGGGTCACAGTCTATTATGGGGTACCTGTGTGG---AAAGAAGCAACC---
ACCACTCTATTTTGTGCATCAGATGCTAAAGCATATAAGACAGAG---GCACATAAT----------------
--------------GTTTGGGCTACA---CATGCCTGTGTACCCACAGAC---
CCCAACCCACAAGAAGTACCATTGAAA---------AATGTGACAGAAAATTTTAAC----------------
--------------------------ATGTGGAAAAACAACATGGTAGAACAAATGCATGAGGATATA---
ATCAGTTTATGGGATCAAAGCTTA---AAGCCATGTGTAGAATTA---
ACCCCACTCTGTGTTAATTTAACCTGCACTGATGATTTAAATTGCACTAGTGCCAATATTACTCAAAATTGCT
CTAGTGCCAATAAAACTGGTAATACT---
-------ACCAGTTCTAATACTACCTCTCCTCCTTGGAGTATACTGGATAAAGGGGAAATAAAA---
AACTGTTCTTTCAATATC---ACAACAATGGGA---------------------------
GATATGAAGAGGAAAGAATATGCATTT---TTTAATAGTCTTGATATAGTACCAATAGATAACAAT-------
----

AATAATAATAGCTATAGGTTAATAAGTTGTAACACCTCAGTCATTACACAG---
GCCTGTCCAAAGATATCCTTTGAGCCAATTCCCATACATTATTGTACCCCGGCTGGTTTTGCGATTCTACAGT
GTAAT---GATGAGGATTTCAAT------GGGACAGGACCATGTAAAAT------------------------
-GTCAGC---TCAGTACAGTGTACACATGGAATTAAACCAGTGGTATCAACT---
CAACTACTGTTAAATGGCAGTCTAGCAGAAAAAAAC------------
GTAGTAATAAGATCTGAAAATTTCACAAACAAT---------GCTAAAATCATAATAGTGCAGCTGAAA----
-----------GACCCTGTAAAC---------ATTACTTGTATAAGACACAACAACAATACAAGAAGA-----
-AGTATACATATA------------GGACCAGGGAGAGCA---TAT------TATACACCAGGACAA------
---------ATAATAGGAGATATA------------AGAAAAGCATATTGT---AACATTAGTGGACA---
AATTGGACTGATGCTTTAAAACAGGTAGTTGAAAAATTAGGG---------------AAACAATTCGAT----
-----------------------------------AATAAAACAATAATATTTAAG------
CCACACGCAGGAGGAGACCCAGAAATTGTA---ATGCACAGCTTTAATTGTGGAGGGGAA---
TTTAAGCAGCTCCAGGCAAGAGTCCTGGCTGTGGAAAGATACCTAAAGGATCAACAGCTCCTAGGAATTTGGG
GTTGCTCTGGAAAACTCATTTGCACCACTGCTGTGCCTTGGAATACTAGCTGGAGT---------AAT-----
----------------------------AATAAAAATCTGACACAAATTTGG------GAC------
AACATGACTTGGATGCAGTGG---GATAAAGAAATTAGTAATTACACACAATTAATATAC---
ACCTTAATTGAAGAATCGCAAAACCAACAAGAA--

AAGAATGAAAAAGACTTATTAGAATTGGATAAATGGGACAGTTTGTGGAGTTGGTTTACCATAACAAATTGGC
TGTGGTATATCAAAATCTTTATAATAATAGTAGGAGGCTTAGTAGGTTTAAGAATAGTTTTTACT---
GTACTTTCTATAGTA---------AATAGAGTTAGGCAGGGATATTCACCA------
ATCTCGTTGGCTGTTAGCTTGTTTAATGCCATAGCCATAGCAGTAGCTGAG---
GGGACAGATAGGATTATAGAAGCAGCACAAAGAGCTTTTAGAGCT----------------------------
--------AYTCTCCACATA------CCTAGAAGAATAAGACAGGGCTTGGAAAGGGCTTTGCAA---TAA
>MN486013
GCA---GAA--GAACGA---
TGGGTCACAGTCTATTATGGGGTACCAGTGTGG---AAAGAAGCAACC---
ACCACTTTATTTTGTGCATCAGATGCTAAAGCATATGATACGGAG---GTACATAAT----------------
--------------GTTTGGGCCACA---CATGCCTGTGTACCCACAGAT---
CCCAACCCACAAGAAGTGGAACTGGTG---------AATGTGACAGAAAATTTTAAC----------------
--------------------------ATGTGGAAAAATAGCATGGTAGATCAGATGCATGAGGATATA---
ATCAGTTTATGGGACCAAAGTCTA---AAGCCATGTGTAAAGTTA---
ACCCCACTCTGTGTTACTTTAAATTGCACTAATATCAATAATACCACCAGTAATAATACC-------------
----
-------------------------ACTAGTGGATTAAGGAGAATGGAGCCAGAAGAGATGAAA---
AACTGCTCTTTCAATATCACCACAACCATAACA---------------------------
GATAAGATAAAGAAATACCGTGCCCTT---TTTTATAAAGATGATGTAGTACAAATAAATAGTAATGAG----
----

AATAATAATACTAGTTATCCTAGCTATAGATTGATCAGTTGTAATACCTCAGTCATTACACAG---
GCCTGTCCAAAGGTAACCTTTGAGCCAATTCCCATACATTATTGTGCCCCGGCTGGTTTTGCGCTTCTAAAGT
GTAAA---GATAAGAATTTCAAT------GGAACAGGACCATGTACAAAT------------------------
-GTCAGC---ACAATACAATGTACACATGGGATTAGGCCAGTAGTATCTACT---
CAACTGCTGTTAAATGGCAGTCTAGCAAAAGACGAG------------
GTAGTACTTAGATCTGAAAATTTCATGAACAAT---------GCTAAAACCATAATAGTACAGCTGGAT----
-----------AAACCTGTAGTA---------ATTAACTGTACAAGACCCAACAACAATACAAGACAA-----

-GGTATACATCTG------------GGACCAGGGAAAGCA---TTT------TATGTAACAGGAGAC------
---------ATAATTGGAGATATA------------AGACAAGCACATTGT---AACATTAATGGTACA---
CAATGGAATAATACTTTACAAAAGATAAAAGAAAAATTAAAA---------------GAACAATTTAAA----
------------------------------------AATAAAACAATAGCCTTTAAT------
CACTCCTCAGGAGGGGACCCAGAAATTGTG---ATGCACACCTTTAATTGTGGAGGGGAA---
TTTAAGCAGCTTCAGGCAAGAGTCCTGGCTGTGGAAAGATACCTAAAGGATCAACAGCTCCTGGGGATCTGGG
GCTGCTCTGGAAAACTCATCTGCACCACTACTGTGCCCTGGAATGATACTTGGAGT-----------------
-----------------------------AACAAAACTCGGGAACAAATTTGG------GAT------
AACATGACTTGGATGCAGTGG---GAGAGAGAAATTGACAATCACACAGGCATAATATAC---
AAATTACTTGAAGAATCGCAAAACCAACAAGAA---

AAGAATGAACAAGAATTATTAGAATTGGATAAATGGGCAAGTTTGTGGAATTGGTTTGATATAACAAACTGGC
TGTGGTATATAAAAATATTCATAATAATAGTAGGAGGCTTGGTAGGTTTAAGAATAGTTTTTATT---
GTACTTTCTCTAGTG---------AATAGAGTTAGGCAGGGATACTCACCA------
TTATCGTTGGCTGTTAGCTTGCTCAACACCACAGCCATAACAGTAGCTGAG---
GGGACAGATTGGGTTATAGAAGTAGTACAAAGAGCTTTTAGAGCT----------------------------
--------ATCCTGCGTATA------CCTGTAAGAATAAGACAGGGCTTGGAAAGGGCTTTGCTA---TAA
>MN486014
GCT---GCA---
GATCAGTTGTGGGTCACAGTCTATTATGGGGTACCTGTGTGG---AAAGAAGCAACC---
ACCACTCTATTTTGTGCATCAGATGCTAAAGCATATGGTACAGAG---GCACATAAT----------------
--------------GTTTGGGCCACA---CATGCCTGTGTGCCCACAGAC---
CCGAGTCCACAAGAACTAGTATTGGGA---------AATGTAACAGAAAATTTTAAC----------------
-------------------------ATGTGGAACAATAGCATGGTAGAACAGATGCATGAAGATATA---
ATCAGCCTATGGGATCAAAGCCTA---AAGCCATGTGTAAAATTA---
ACCCCACTCTGTGTCACTTTAAGCTGCACTAATGCTAACTTTACGAATGCTACTTTTGAGAGT----------
----
----------------------GGCAATACTACTATGGAAACAATGGAGGGAGGAGAAGTAAAA---
AACTGCTCTTTCAATGTCACCACAGGCATAGGA---------------------------
AATAAATTACAGAAAACATATGCACTT---
TTTTATAAACTAGATATAGAACGAGTATCTGATAATGATACTAAAGAG-------------------------
----
TATGATAATTATACCAACTATAGGATGATACATTGCAACACCTCAGTCATTACACAG---
GCCTGTCCAAAGATATCCTTTGAACCAATTCCCATACATTATTGTGCCCCGGCTGGTTTTGCGATTCTAAAGT
GTAATAATGAGACGACATTCAAT------GGAACAGGACCATGCACAAAT----------------------
-GTCAGC---ACAGTACAATGTACACATGGAATTAGGCCAGTGGTATCAACT---
CAACTGCTGTTAAATGGCAGTCTAGCAAAAGAAGAG------------
GTAAAGATTAGATCTCAAAATTTCTCGAACAAT---------GCTAAAATTATAATAGTACAGCTGAAC----
-----------ACATCTGTACCC---------ATTAATTGTACAAGACCAGGTAACAATACAAGAAAA-----
-AGTATACATATG------------GGACCAGGGAAAGCA---TTT------TTTGCAACAGGAGAA------
---------ATAATAGGAGATATA------------AGACAAGCACATTGT---AACGTTAGTAGACAA---
GCCTGGAATAAAACTTTAGAACAGGTAGCAGGAAAATTAAGA---------------GAACAATTTAGG----
------------------------------------AATAAAACAATAGTCTTTAAG------
CAACCTTCAGGAGGGGATCCAGAAATTGTA---ATGCACAGTTTTAATTGTGGAGGGGAA---
TTTAAGCAGCTCCAGGCAAGAGTCCTGGCTGTGGAAAGATACCTAAAGGATCAACAGCTCCTAGGAATTTGGG
GTTGCTCTGGAAAACTCATCTGCACCACTGCTGTGCCTTGGAACGATAGTTGGAGT-----------------
-----------------------------AATAGGTCCTTGGATTATATTTGG------AAT------
AATATGACCTGGATGGCTTGG---GAAAAAGAAATTGACAATTACACAAGCATAATTTAT---
GGGTTAATTGTAGAATCGCAGAACCAGCAAGAA---

AAGAATGAAAAAGACTTATTAGAATTAGATAAATGGGCAAGTTTGTGGACTTGGTTTGACATAACAAAGTGGC
TGTGGTATATTAAAATATTCATAATGATAGTAGGAGGCTTGGTAGGTTTAAGAATAGTTTTTACT---
GTGCTTTCTATAGTT---------AATAGAGTTAGGAAGGGATACTCACCA------
CTATCGTTTGCTGTTAGCTTGCTCAATACTACAGCTATAGTAGTAGCTGAA---
GGGACAGATAGGATTATAGAAGTATTACAAAGAGCTTATAGGGCT----------------------------
--------ATTTGCCACATA------CCTACAAGAATAAGACAGGGCTTAGAAAGGCTTTTGCTA---TAA
>MN486022
GCT---GTA---
GAACAATTTTGGGTCACAGTCTATTATGGGGTGCCTGTGTGG---AGAGACGCAAAC---
ACCACTCTATTCTGTGCGTCAGATGCTAAAGCATATGATACAGAG---GTACATAAT----------------

--------------GTTTGGGCCACA---CATGCCTGTGTACCCACAGAC---
CCCAACCCACAAGAAGTGGTAATGGGC---------AATGTGACAGAAAATTTTAAC----------------
-------------------------ATGTGGAAAAATAACATGGTAGAACAGATGCATGAGGATGTA---
ATCAGTTTATGGGATCAAAGCTTA---AAACCATGTGTAAAATTA---
ACCCCACTCTGTGTTACTCTAAATTGTACTGATTATAAGGGGAATTTGACTGGGAATTCGACC----------
----
----------------------GGCACCAATAATAGTAGCATCATGGAGGGAGGAGAAATAAAA---
AATTGTTCTTTCAATATCACCACAAACATAAGA-------------------------
GATAAGAGACAGAAAGAGTATGCACTT---
TTGTATAAACTTGATTTAGTACAAATAGATAATAGTGGAAATAATAAC-------------------------
----
AGTAGCAATAGCAGTTATGACAGTTATAGGTTAATAAGTTGTAATACCTCAGTCATTACACAG---
GCCTGTCCAAAGGTATCCTTTGAGCCAATTCCCATACATTTTTGTACCCCGGCTGGGTTTGCGATTCTAAAAT
GTAAC---AATAAGAGGTTCAAT------GGAACAGGACCATGTACAAAT----------------------
-GTCAGC---ACAGTACAATGTACACATGGGATTAAGCCAGTAGTATCAACT---
CAACTACTGTTAAATGGCAGCCTAGCAGAAGAAGAT------------
GTAGTAATTAGATCTGAAAATATCTCGGAYAAT---------GCTAAAAACATAATAGTACAACTGAAA----
-----------GAGCCAGTAGAA---------ATTAATTGTACAAGACCCAACAACAATACAAGAAAA-----
-GGTATACATATG------------GGACCAGGGCAAGCA---TTC------TATGCAACAGGAGCA------
---------ATAATAGGAGATATT------------AGACAGGCACATTGC---AACCTGAGTTGGACA---
AAGTGGAATAAAACTTTGGGCAACATAGCTGGAAAATTAAGA---------------GAACACTTTGGG----
------------------------------------AATAAAACAATAGTCTTTCAG------
CGACACGCAGGAGGGGACCCAGAAGTTGAA---ATGCACAGCTTTAATTGTAGAGGGGAA---
TTTAAGCAGCTCCAGGCAAGAGTCCTGGCTCTGGAAAGATACCTAAAGGATCAACAGCTCCTGGGGATTTGGG
GTTGCTCTGGAAAGCTCATCTGCACCACTGCTGTGTCTTGGAATGAAAGTTGGAGT-----------------
-----------------------------AATAAATCTCAGGAGCAAATTTGG------AAT------
AACATGACTTGGATGGAGTGG---GATAGAGAAATTAGAAAATACACAGGAAAAATATAC---
TCCTTAATTGAAGAAGCGCAGACCAAACAAGAA--------------------------------------
----
ATGAATGAAAAAGAGTTATTGGAATTGGATAAATGGGCAAGTTTGTGGACTTGGTTTGACATAACTAAATGGC
TGTGGTATATAAAAATATTCATAATGATAGTAGGAGGCTTAGTAGGTTTAAGAATAGTTTTTACT---
GTACTTTCTATAGTA---------AATAGAGTTAGGAAGGGATACTCACCC------
TTGTCGTTCGCTGTTAGCTTGCTTAATGCCACGGCCATAGCAGTAGCTGAG---
GGAACAGATAGGATTATAGAAGTAGCACARAGGGTTGGGAGAGGC---------------------------
--------ATCCTCCACATA------CCTAGAAGAATAAGACAGGGCCTCGAAAGGGCTTTGCAA---TAA
>DQ313249
GCT---ACA---
GAAAATTTGTGGGTCACCGTCTATTATGGGGTACCTGTGTGG---AAAGAAGCAACC---
ACCACTCTATTTTGTGCATCAGATGCCAAAGCATATGATACAGAG---GTACATAAT----------------
--------------GTCTGGGCCACA---CATGCCTGTGTACCCACAGAC---
CCCAACCCACAAGAAATGGAATTGAAA---------AATGTGACAGAAAATTTTAAC----------------
-------------------------ATGTGGAAAAATAACATGGTAGAACAGATGCATGAGGATATA---
ATTAGTTTATGGGATCAAAGCCTA---AAGCCATGTGTAAAATTA---
ACCCCACTCTGTGTTACTTTAAATTGCACTGATTTGAGAAATGCTACTAATACCACTAGT-------------
----
-----------------------------AGTAGCGGGGAAACGATGGAGGGAGGAGAAATGAAA---
AATTGCTCTTTTAATATCACCACAAGCATAAGA-------------------------
GATAAGCTGCAGAAAGTATATGCACTT---TTTTATAAACTTGATGTAACACCAATAGAAAAT----------
----

GATACTACTAGCTATAGGTTGATAAGTTGTAACACCTCGGTCATTACACAG---
GCCTGTCCAAAGATATCCTTTGAGCCAATTCCCATACACTATTGTGCCCCGGCTGGTTTTGCGATTCTAAAGT
GTAAG---GATACAAAGTTCAAT------GGAACAGGACCATGTACAAAC----------------------
-GTCAGC---ACAGTACAATGTACACATGGAATTAAACCAGTAGTATCAACT---
CAACTGCTGTTAAATGGCAGTCTAGCAGAAGAAGAG------------
GTAGTAATTAGATCCAGCAATTTCACGGACAAT---------ACTAAAGTCATAATAGTGCAGCTGAAT----
-----------AACTCTGTAGAA---------ATCAATTGTACAAGGCCCAACAACAATACAAGAAAA-----
-AGTATACCTATA------------GGACCAGGCAGAGCA---TTT------TATACAACAGGAGAA------
---------ATAATAGGAGATATA------------AGACAAGCACATTGT---AACCTTAGTGGAGCA---
AAATGGAATGACGCTTTAAAACAGATAGTTACAAAATTAAGA---------------GAACAATTTAAG----

------------------------------------AATAAAACAATAATCTTTAAT------
CAATCCTCAGGAGGGGACCCAGAAATTGTA---ACGCACAGTTTTAATTGTGGAGGGGAA---
TTTAAGCAGCTCCAGGCAAGAGTCCTGGCTGTGGAAAGATACCTAAGGGATCAACAGCTCCTGGGGATTTGGG
GTTGCTCTGGAAAACTCATTTGCACCACTACTGTGCCTTGGAATACTAGTTGGAGT-----------------
------------------------------AATAAATCTGTGGATTACATTTGG------AAA------
AACATGACCTGGATGCAGTGG---GAAAAAGAAATTGATAATTACACAAGCTTAATATAC---
ACCTTAATTGAAGAATCGCAATACCAGCAAGAA---

AAGAATGAACAAGAATTATTGGAATTAGATAAATGGGCAAGTTTGTGGAATTGGTTTGACATAACAAACTGGC
TGTGGTACATAAAATTATTCATAATGATAGTAGGAGGCTTGGTAGGTTTAAGAATAGTTTTTGCT---
GTACTTTCTATAGTG---------AATAGAGTTAGGCAGGGATACTCACCA------
TTATCGTTCGCTGTTAGCTTGTTCAATGCCACAGCCATAGCAGTAGCTGAG---
GGGACTGATAGGGTTATAGAAATATTACAAAGAGCTTTTAGAGCT-----------------------------
--------ACTCTCCACATA------CCTACACGAATAAGACAGGGCTTGGAAAGGGCTTTGCTA---TAA
>L07421
GCT---GTA---
GAACAATTGTGGGTTACAGTCTATTATGGGGTACCTGTGTGG---AAAGAAGCAACC---
ACCACCCTATTTTGTGCATCAGATGCTAAAGCATATGACACAGAG---GTTCATAAT----------------
--------------GTTTGGGCCACA---CATGCCTGTGTACCCACAGAC---
CCTAGCCCACAAGAAATAGTATTGGAA---------AATGTGACAGAAAATTTTAAT----------------
--------------------------ATGTGGAAGAATAACATGGTAAAACAGATGCATGAGGATATA---
ATCAGTTTATGGGATCAAAGCCTA---AAGCCATGTGTAAAATTA---
ACCCCACTCTGTGTTACTTTAAATTGCACTAATTGGAAGAGTGCTAATAGTACCAGTAAT-------------
----
------------------------------ACCAATAGTAGTTTGGAGGAGAAAGGAGAAATAAAA---
AACTGCTCTTTCAATGTCACCACAAGTATAAAA---------------------------
AATAAGGTACAGACAGAATATGCACTT---TTTTATAAACTTGATGTAATGCCAATAGAGGAT----------
----

GATAATACAAGCTATACATTGATAAATTGTAACACCTCAGTCATTACACAA---
GCCTGTCCAAAGGTATCCTTTGAACCAATTCCCATACATTATTGTACCCCAGCTGGTTTTGCGATTCTAAAGT
GTAAT---GATAAGAAGTTCAAT------GGAACAGGACCATGTAAAAAT-----------------------
-GTCAGC---ACAGTACAATGCACACATGGAATTAAGCCAGTAGTGTCAACT---
CAACTGCTGTCAAATGGCAGTCTATCAGAAGGAGAG------------
GTAGTAATTAGATCTGAAAATTTCACTGACAAT---------GCTAAAACCATAATAGTACAGCTGAAT----
-----------AAATCTGTAGTA---------ATTAATTGTACAAGACCCAACAACAGTAAAAGAAAA-----
-ACTCTACATATG------------GGACCGAAGAGGGCA---TTT------TATGCAACAGGAGAC------
ATAGGAGGATATACAGGATATATA------------AGACAAGCACATTGT---AACATTAGTAGAGCA---
AAATGGAATGACACTTTAAAACAGATAGTTAAAAAATTAAAA------------GAACAATTTGGGAGG----
------------------------------------AATAAAACAATAGTCTTTAAG------
CAATCCTCAGGAGGGGACCCAGAAATTGTA---ATGCACAGCTTTAATTGTGGAGGGGAA---
TTTAAGCAGCTCCAGGCAAGAGTCCTGGCTGTGGAAAGATACCTAAAGGACCAACAGCTCCTAGGGATTTGGG
GTTGCTCTGGAAAACTCGTTTGCACCACTACTGTGCCTTGGAATGCTAGTTGGAGT----------------
------------------------------AATAAATCCCTGAATCAGATTTGG------GAT------
AACATGACCTGGATGGAGTGG---GAGAGAGAAATTGACAATTACACAGGCTTAATATAC---
AGATTAATTGAAGAATCGCAGAACCAACAGGAA---

CAGAATGAACAAGACTTATTGAAATTGGATACGTGGGCAAGTTTGTGGAATTGGTTTAGCATAACAAAATGGC
TGTGGTATATAAAAATATTCATAATGATAGTAGGAGGTTTAATAGGTTTAAGAATAATTTTTGCT---
GTACTTTCTATAGTG---------AATAGAGTTAGGCAGGGATACTCACCA------
TTATCATTTGCTGTTAGCTTGCTTAATACAACTGCTATAGTAGTAGCTGAG---
GGGACAGATAGGGTTATAGAAGTAGTACAAAGAATTGGTAGAGCT----------------------------
--------GTTCTCCACATA------CCTAGAAGAATAAGACAGGGCTTCGAAAGGGCTTTGCTA---TAA
>DQ313250
GCT---ACA---
GAAAAATTGTGGGTCACAGTCTATTATGGGGTACCGGTATGG---AAAGAAACAGAC---
ACCACTTTATTTTGTGCATCAGATGCTAAAGCATATGACAGAGAG---GTACATAAT----------------
--------------GTTTGGGCCACA---CATGCCTGTGTACCCACAGAC---
CCCAACCCACAAGAAGTAGTATTGGAA---------AATGTGACAGAAAATTTTAAC---------------
--------------------------ATGTGGAAAAATAACATGGTAGAACAGATGCAGGAGGATATA---

ATCAGTTTATGGGATCAAAGCCTA---AAGCCATGTGTAAAATTA---
ACCCCACTCTGTGTTACTTTAAATTGCACTGCTCCGAATGTTACCAATACCAATAATAGTACT----------
----
-------------------------AATACCAATAATAGTAGTTTGGACGAAGGAGAAATGAAA---
AACTGCTCTTTCAACATCACCACAAGCATAAAA---------------------------
GATAAGATACAGAGAGAATATGCACTT---TTTTATAGACTTGATATAGTACCAATAGATGGT----------
----

AGTAATAGCAGCTATAGGTTGACAAAGTGTAACACCTCAGTCATTACACAG---
GCCTGTCCAAAGGTGACCTTTGAGCCAATTCCCATACATTATTGTGCCCCGGCTGGTTTTGCGATTCTAAAGT
GTAAC---GATAAAAAGTTCAAT------GGAACAGGACCATGTAAAAAT-----------------------
-GTCAGC---ACAGTACAATGTACACATGGAATTAGGCCAGTAGTATCAACT---
CAACTGTTGTTAAATGGCAGTCTAGCAGAAGAAGAG------------
GTAATAATTAGATCTGAAAATTTCTCGGACAAT---------GCTAAAAACATAATAGTACATCTAAAT----
-----------GAATCTGTAGAA---------ATTAATTGTACAAGACCCAGCAACAATACAAGAAAA-----
-AGTATACATATG------------GGACCAGGAGGAGCA---ATT------TATGCAACAGGAAAA------
---------ATAATAGGAGATATA------------AGACAAGCACATTGT---AACATTAGTGAAAAA---
AAATGGGGAGAAGCTTTAGAAAGGATAGTTAAAAAATTAAGA---------------AAACAATAT-------
-----------------------------------AACAACACAATAATCTTTACT------
CAACCCTCAGGAGGGGACCCAGAAATTGTA---ATGCACAGTTTTAATTGTGGAGGGGAA---
TTTAAGCAGCTCCAGGCAAGAGTCCTGGCTGTGGAAAGATACCTACAGGATCAGCAGCTCCTGGGGATTTGGG
GTTGCTCTGGAAAACTCATTTGCACCACTACTGTGCCTTGGAATGCTAGTTGGAGT-----------------
----------------------------AATAAATCTCTGGAAAAGATTTGG------AAT------
AACATGACCTGGATGGAGTGG---GAAAAAGAAATTGACAATTACACAAACTTAATATAC---
ACCTTAATTGAAGAATCGCAGAACCAACAAGAA--

AAAAATGAACAAGAATTATTGGAGTTGGGCAAGTGGGACAGTTTGTGGAGTTGGTTCGACATATCACAATGGC
TGTGGTATATAAAAATATTCATAATGATAGTAGGAGGTTTGGTAGGTTTAAGAATAGTTTTTGCT---
GTACTTTCTATAGTA---------AATAGAGTTAGGCAGGGATATTCACCA------
TTATCGTTTGCTGTTAGCTTGCTCAATGCCACAGCTATAGCAGTAGCTGAG---
GGGACAGATAGGGTTATAGAAGTAGTACAAAGAGCTGGGAGAGCT----------------------------
--------ATTCTCCACATA------CCTAGAAGAATAAGACAGGGCGCGGAAAGGGCTTTGATA---TAA
>DQ313253
GCT---GTA--
GAACAATTGTGGGTCACAGTCTATTATGGGGTACCTGTGTGG---AAAGAAGCAACC---
ACCACTCTATTTTGTGCATCAGATGCTAAAGCATATGATACAGAG---GTACATAAT----------------
--------------GTTTGGGCCACA---CATGCCTGTGTACCCACAGAC---
CCCAACCCCCAAGAAATAGTAATGGAA---------AATGTGACGGAAAACTTTAAC----------------
--------------------------ATGTGGAAAAATAACATGGTAGAACAGATGCACGAGGATATA---
ATCAGTTTATGGGATCAAAGCCTA---AAACCATGTGTAAAATTA---
ACTCCACTTTGTGTTACTTTAAATTGCACTGATTTGGGGAATGGCCCTGATTTGGGGAATACT----------
----
-------------------------AATAGTAGCGAGAAACCAATGGAAAAAGGAGAAATAAAA---
AACTGCTCTTTCAAAATCACCACAAGAATAAGA---------------------------
GATAAGATGCAAAAAGAATATGCAGCT---TTTTATAAACTTGACCTGGTACAAATAGATAATGATGATAAT-
----

AATACCAGCTATAGTAATACCAGCTATAGGTTATTAAGTTGTAACACCTCAGTCATTACACAG---
GCCTGTCCAAAGGTATCCTTTGAGCCAATTCCCATACATTATTGTGCCCCGGCTGGTTTTGCGATTCTAAAGT
GTAAT---GATAAAAAGTTCGAT------GGAACAGGACCATGTACAAAT-----------------------
-GTCAGC---ACAGTACAGTGTACACACGGAATTAGGCCAGTAGTATCAACT---
CAACTGCTGCTAAATGGCAGTCTAGCAGAAGAGGAG------------
ATAGTAATTAGATCTGAAAATTTCACGAACAAT---------GCTAAAACCATAATAGTACAGCTGAAA----
-----------GAAGCTGTAGAA---------ATTAATTGTACAAGACCCAACAACAATACAAGAAAA-----
-AGTATACATATA------------GGACCAGGGAAGGCA---TTG------TATACAACAGGAGAA------
---------ATAATAGGAAAGATA------------AGACAAGCATATTGT---AACATTAGTGAAGCA---
AAATGGAACAACACTTTAAGACAGATAGCTATACAATTAGGA---------------AAACAATTTGGG----
-----------------------------------AATAAGACAATAATCTTTAAT------
CAATCCTCAGGAGGGGACCCAGAAATTGTA---ATGCACAGTTTTAATTGTGGAGGGGAA---
TTTAAACAGCTCCAGGCAAGAGTCCTGGCTGTGGAAAGATACCTAAAGGATCAACAGCTCCTGGGAATTTGGG

GTTGCTCTGGAAAACTCATTTGCACCACTGCAGTGCCTTGGAATGCTAGCTGGAGT-----------------
----------------------------AATAAATCTCTGGAAAAGATTTGG------AAT------
AACATGACCTGGATGCAGTGG---GAAAAAGAAATTGACAATTACACGAGCTTAATATAC---
ACCTTAATTGAAGAATCGCAAATCCAACAAGAA--

AAGAATGAACAAGAATTATTGGAATTAGATAAATGGGCAAGTTTGTGGAATTGGTTTGACATAACAAAATGGC
TGTGGTATATAAAAATCTTCATAATGATAGTAGGAGGCTTGGTAGGTTTAAGAATAATTTTTACT---
GTACTTTCTGTAGTG---------AATAGAGTTAGGCAGGATACTCACCA------
TTGTCATTGGCTGTTAGCTTGCTCAACGCCACCGCCATAGCAGTAGCTGAG---
GGGACAGATAGGTCTATAGAGGGATTACAAAGAGCTTTTAGAGCC----------------------------
--------TTTCTCCACATA------CCTACAAGAATAAGACAGGGCGCAGAAAGAGCTTTTCTA---TAA
>DQ313252
GCT---GCA--
GAACAATTGTGGGTCACAGTCTATTATGGGGTACCTGTGTGG---AAAGAAACCACC---
ACCACTCTATTTTGTGCATCAGATGCTAAAGCATATGATACAGAG---GTACATAAT----------------
--------------GTTTGGCCACA---CATGCCTGTGTACCCACAGAT---
CCCAACCCGCAAGAAGTACTATTGGAA---------AATGTGACAGAAAATTTTAAC----------------
--------------------------ATGGGGAAAAATAACATGGTAGAACAGATGCAAGAGGATATA---
ATCAGTTTATGGGATCAAAGCATA---AAGCCATGTGTAAAATTA---
ACCCCACTCTGTGTTACTTTAAATTGCACTGATTGGGGGTATACTACTAATACCACTAGT-------------
----
----------------------------GATAGCAGGACAGGGATGGAGAAAGGAGAAATGAAA---
AACTGCTCTTTCAATATCACCACAAGCATAAGA---------------------------
GATAAGGTGCAGAAAGAATATGCGCTT---TTTTATAAACTTGATGTAGTACCAATAGATGAC----------
----

AATGATAATACCAGCTATAGGTTGATAAGTTGTAACACCTCAGTCATTACACAG---
GCCTGTCCAAAGGTATCCTTTGAGCCAATTCCCATACATTATTGTGCCCCGGCTGGTTTTGCGATTCTAAAGT
GTAAC---GATAAGAAGTTCAAT------GGAACAGGACCATGTACAAAT-----------------------
-GTCAGT---ACAGTACAATGCACACATGGAATTAGGCCAGTAGTATCAACT---
CAACTGCTGTTAAATGGCAGTCTAGCAGAAGAAGAG------------
GTAGTAATCAGATCTGTCAATTTCACAGACAAT---------ACTAAAACCATAATAGTACAACTGAAT----
-----------GAATCTGTAGAA---------ATTAATTGTACAAGACCCGGCAATAATACAAGAAAA-----
-AGTATACATATA------------GGACCAGGGAGAGCA---TTT------TATGCAACAGGAGAA------
---------ATAATAGGAGATATA------------AGACAAGCACATTGT---AACATCAGTAAAACA---
AAATGGAATGACACTTTAAGCCGGATAGTTAAAAAATTAAGA---------------GAACAATTTGAG----
-----------------------------------AATAAAACAATAGCTTTTAAG------
CGACACTCAGGAGGGGACCCAGAAATTGTG---ATGCACAGCTTTAATTGTGGAGGGGAA---
TTTAAGCAGCTCCAGGCAAGAGTCCTAGCTGTGGAAAGATACCTAAAGGATCAACAGCTCCTGGGGATTTGGG
GTTGCTCTGGAAAACTCATCTGCACCACTGCTGTACCTTGGAATGCTAGTTGGAGT-----------------
----------------------------AATAAATCTCTGAATCAGATTTGG------GAT------
AACATGACCTGGATGGAGTGG---GAAAGAGAAATTAACAATTACACAAGCTTAATATAC---
ACCTTAATTGAAGAATCGCAGAACCAACAAGAA--

AAAAATGAACAAGAATTATTGGAATTAGATAAGTGGGCAAGTTTGTGGAATTGGTTTGACATAACAAACTGGC
TGTGGTATATAAGAATATTCATAATGGTAGTAGGAGGCTTGATAGGTTTAAGAATAGTTTTTACT---
GTACTTTCTATAGTG---------AATAGAGTTAGGCAGGATACTCACCA------
TTATCGTTTGCTATTAGCTTGCTCAATGCCACAGCCATAGCAGTAGCTGAG---
GGGACAGATAGGGTTATAGAAGTAGTACAAAGAGCTTATAGAGCT----------------------------
--------ATTATCCACATA------CCTGTACGAATAAGACAGGGCTTGGAAAGGTTTTTGCTT---TAA
>EU191616
GCT---ACA--
GAACAATTGTGGGTCACAGTCTATTATGGGGTACCTGTGTGG---AAAGAAGCAACC---
ACCACTTTATTCTGTGCATCAGATGCTAAAGCATATGATACAGAG---GTACATAAT----------------
--------------GTTTGGCCACA---CATGCCTGTGTACCCACAGAC---
CCCAACCCACAAGAAGTAGGATTGGAA---------AATGTGACAGAAAATTTTAAC----------------
--------------------------ATGTGGAAAAATAACATGGTAGAACAGATGCATGAGGATATA---
ATCAATTTATGGGATGAAAGCCTA---AAGCCATGTGTAAAATTA---
ACCCCACTCTGTGTTACTTTAAATTGCTCTGAGTTGAAGAAGAATGTTAGTTCTGCCAATAATACTGTTGGTT
CTGCC--

----------AATAATACTGATAGTGATGCCAATAGTACTAGTTGGATGAGAGAAGAAATAAGA---
AATTGCTCTTTCAACGTCACCACAAATATAAGA---------------------------
GATAAGATACAGAAAGAATATGCACTT---TTTTCTAAGCTTGATGTAGTACCAATAGGTGAT----------
----

GATAAGAATAGTACCAGCTATAGGTTGATAAATTGTAACACCTCAGTCATTACACAG---
GCCTGTCCAAAGGTATCCTTTGAGCCAATTCCCATACATTATTGTGCCCCAGCTGGTTTTGCGATTCTAAAGT
GTAAA---GATAAGAAGTTCAAT------GGAACAGGACCATGTAAAAAT-----------------------
-GTCAGC---ACAGTACAATGCACACATGGAATTAGGCCAGTGGTATCAACT---
CAACTGCTGTTAAATGGCAGTCTAGCAGAAGAAGAG------------
GTAGTAATTAGATCTAAAAATTTCACGAATAAT---------GCTAAAATCATAATAGTACAGCTGAAC----
-----------GAATCTGTAGAA---------ATTAATTGTACAAGACCCAACAACAATACAAGGAAA-----
-GGTATACATATA------------GGACCAGGAAGAGCA---TTT------TATGCAACAACAGAA------
---------ATAACAGGAGATATA------------AGACAAGCACATTGT---AACATTAGTGGAGAA---
AAATGGAATAGGACTTTAGAAAAGATAGTTACCAAATTAAGA---------------GAACAATTTGAG----
-----------------------------------AATAAAACAATAATCTTTAAT------
CAATCCTCAGGAGGGGACCCAGAAATTGTA---ATGCACAGTTTTAATTGCGGAGGGGAA---
TTTAAGCAGCTCCAGGCAAGAATCCTGGCTGTGGAAAGATACCTAAGGGATCAACAGCTCCTAGGGATTTGGG
GTTGCTCTGGAAAGCTCATCTGCACCACTACTGTGCCTTGGAATGTTAGTTGGAGT-----------------
----------------------------AATAAATCTCTGGATCAGATTTGG------GAA------
AACATGACCTGGATGGAATGG---GAAAGAGAAATTGGCAATTACACAAGCTTAATATAC---
AACTTAATTGAAGAATCGCAGAACCAACAAGAA--

AAGAATGAAAAAGAATTATTGGAATTGGATAAGTGGGCAAGTTTGTGGAATTGGTTTGACATATCAAATTGGC
TGTGGTATATAAGAATATTCATAATGATAGTAGGAGGCTTGGTAGGTTTAAGAATAGTTTTTGCT---
GTATTTTCTATAGTG---------AATAGAGTTAGGCAGGGATACTCACCA------
TTATCATTTGCCATTAGCTTGCTCAACGCTACAGCTGTAGCAGTAGCTGAG---
GGGACAGACAGGGTTATAGAAGTAGCACAAGGAGCTTTTAGAGCT----------------------------
--------ATTCTCCACATA------CCTAGAAGAATAAGACAGGGCTTGGAAAGGTGTTTGATA---TAA
>MH746266
GTT---ACA--
CCAGAATTGTGGGTCACAGTCTATTATGGGGTACCTGTATGG---AAAGAAGCAACT---
ACCACTCTATTTTGTGCATCAGAGGCTAAAGCATATGATACAGAG---GTACATAAT----------------
--------------GTTTGGCCACA---CATGCCTGTGTACCCACAGAC---
CCTAGCCCACAAGAAATAGTATTGGAA---------AATGTGACAGAAAATTTTAAT----------------
--------------------------GTGTGGAAAAATGACATGGTAGATCAGATGCATGAGGATATA---
ATCAGTTTATGGGATCAGAGCCTA---AAACCATGTGTAAAGTTA---
ACTCCACTATGTGTCACTTTAAATTGCACTAATTTGACTATTGGCAATAATACCAAT----------------
----
-------------------------AGTACGGAAGAAATGAAAAACTGGGCAAGAGAAATGAAA---
AACTGCTCTTTCAATGTCACCACAGAATTAAGA---------------------------
GACAAAACAAAGAAAGAATATGCACTG---TTTTATAGACTTGATGTAGTACCAATAAATGAT----------
----

AATGATACAAGCTATACATTGATAAATTGTAACACCTCAACCATTACACAG---
GCCTGTCCAAAGGTATCCTTTGAACCAATCCCCATACATTTTTGTGCCCCAGCTGGTTTTGCGATTCTAAAAT
GTAAT---AATAAGACATTCAGT------GGAAAAGGACCATGTAATAAT-----------------------
-GTCAGC---ACAGTACAATGTACACATGGAATTAGGCCAGTAGTGTCAACT---
CATCTGCTGTTAAATGGTAGTCTAGCAGAAGAAGAG------------
ATAGTAATTAGATCTGAAAATATCACAGACAAT---------ACTAAAACCATAATAGTGCAGTTGAAT----
-----------GAAACTATAGAA---------ATCAATTGTACAAGACCCAACAACAATACAAGAAAG-----
-AGTATAAACATA------------GGGCCAGGGAGAGCA---TTT------TATGCAACAGGAGCA------
---------ATAACAGGAAATATA------------AGACAAGCACATTGT---AACCTTAGTGGATCA---
GCCTGGAATAAAACCTTACAAAAGGTAGTCATAAAATTAAGA---------------GAACAATTTAGG----
-----------------------------------AATAAAACAATAATTTTTAAG------
CCACCCTCAGGAGGGGATCCAGAAATTGTA---ATGCACACCTTTAATTGTGGAGGGGAA---
TTCAAACAGCTCCAGGCAAGAGTCCTGGCTGTGGAAAGATACCTAAAGGATCAACAGCTCCTAGGGATTTGGG
GTTGCTCTGGAAAACTCATTTGCACCACTGCTGTGCCTTGGAATGCTACTTGGAGT-----------------
----------------------------AATAAAAATCTGAGTCAGATTTGG------GAC------
AACATGACCTGGATGCAGTGG---GAGAGAGAAATTGACAATTACACAAGCATAATATAC---

AACTTAATTGAAGAATCGCAGAACCAGCAAGAA--

AAGAATGAACAGGAATTGTTGGAATTGGATAAGTGGTCAAGTCTGTGGAATTGGTTTAACATAACAAATTGGC
TGTGGTATATAAAAATATTCATAATGATAGTAGGAGGCTTGATAGGCTTAAGAATAGTTTTTACC---
ATACTTTCTCTAGTA---------AATAGAGTCAGGCAGGGATACTCACCA------
TTATCATTGGCTATTAGTTTGCTTAATGCCACAGCTATAGCAGTAGCTGAA---
GGGACAGATAGGATCATAGGAATAGTACAAGGAGCTTGCAGAGCT----------------------------
--------ATACGCCACATA------CCTAGAAGAATAAGACAGGGTGCAGAAAGGCTTTTGCTA---TAA
>MH746265
GCT---ACA--
GAAGATTTGTGGGTCACAGTCTATTATGGGGTACCTGTGTGG---AGAGAAGCAAAC---
ACCACTCTATTTTGTGCATCAGAGGCTAGAGCATATAAAACAGAG---GCACATAAT----------------
--------------GTTTGGGCCACA---CATGCCTGTGTACCCACAGAC---
CCCGACCCACAAGAAGTGCGATTGGAA---------AATGTGACAGAAAATTTTAAT----------------
--------------------------ATGTGGAAAAATAACATGGTTGATCAGATGCATGAGGATGTA---
ATCAGTTTATGGGATCAAAGCCTA---AAGCCATGTGTAAAATTA---
ACCCCACTCTGTGTTACTCTAAATTGCACTAATTTGAACAATAGTAATAGTCAGAATAAT-------------
----
----------------------------ACTACTATAGAGGGAGGAGCAAAAGGAGAAATGAAA---
AACTGCTCTTTCAACACTTCCACAAGCATACAG---------------------------
GATAAAAGGCAGAAACAGTATGCACTA---TTTTATAAACTTGATCTAGTACAAATAAATGAAGAT-------
----

GATAATGCCAGTTATACATTGATAAGTTGTAACACCTCAGTCCTTACACAA---
GCCTGTCCAAAGGTATCCTTTGAACCAATTCCTATACATTATTGTACCCCGGCTGGTTTTGCGATTCTAAAGT
GTAAT---GATACAAAGTTCAAT------GGAACAGGGCCATGTAAAAT------------------------
-GTCACC---TCAGTACAATGTACACATGGAATTAGGCCAGTAGTATCAACT---
CAACTGCTGTTAAATGGCAGTCTAGCAGAAGAAGAA------------
ATAGTAATTAGATCTGCCAATTTCTCAGACAAT---------GCTAAAACCATAATAGTACAGCTAAAT----
-----------GAATCTGTAGTA---------ATTAATTGCACAAGACCCAACAACAATACAAGGAAA-----
-AGTATACATATA------------GGACCAGGGAGAGCA---TTT------TATGCAACAGGAGAC------
---------ATAATAGGAGACATA------------AGACAAGCACATTGT---AACATTAGTAGATCA---
CAATGGAATAAAACTTTAGAACAGATAGTTACAAAATTAAGA---------------GAACAATTTGGG----
-----------------------------------AATAAAACAATAGTCTTTAAC------
CAATCCTCAGGAGGGGATCCAGAAATTGTA---ACACACAGTTTTAATTGTGGAGGGGAA---
TTTAAGCAGCTCCAGGCAAGAGTCCTGGCTGTGGAAAGATACCTAAGGGATCAACAGCTCCTGGGGATTTGGG
GTTGCTCTGGAAAACTCATCTGCACCACTGCTGTGCCTTGGAATACTAGTTGGAGT-----------------
----------------------------AATAAAACTCAGACAGAGATTTGG------GAT------
AACATGACCTGGATGCAGTGG---GACAGAGAAATCCACAATTACACACACTTAATATAC---
TCCTTAATTGAAGAATCGCAGAACCAGCAAGAA--

AAGAATGAACAAGAATTATTGGAATTTGATAAATGGGCAAATTTGTGGAATTGGTTTGACATATCAAAGTGGC
TATGGTACATAAAAATATTCATAATGATTGTAGGAGGCTTGATAGGTTTAAGAATAGTTTTTACT---
GTACTTTCTATAGTG---------AATAGAGTTAGGCAGGGATACTCACCA------
TTGTCATTTGCTACCAGCTTGCTCAACGCCACAGCCATAGTAGTAGCTGAG---
GGGACAGATAGGATTATAGAATTAGCACAAAGAATTTGTAGAGCT----------------------------
--------ATTCGCCACATA------CCTAGAAGAATCAGACAGGGCGCGGAAAGAGCTTTAATA---TAA
>MH746264
GCA---GCA--
GAAAAATTGTGGGTCACAGTCTATTATGGGGTACCTGTATGG---AAAGAAGCAACC---
ACCACTCTATTTTGTGCATCAGATGCTAAAGCATATGATACAGAG---GTACATAAT----------------
--------------GTTTGGGCCACA---CATGCCTGTGTACCCACAGAC---
CCCAATCCACAAGAAATAGTAATGGAA---------AATGTGACAGAAGATTTTAAC----------------
--------------------------ATGTGGAAAAATAACATGGTAGAACAGATGCATGAGGATATA---
ATCAGTTTATGGGATCAAAGCCTA---AAACCATGTGTAAAGTTG---
ACCCCACTCTGTGTTACTTTAAATTGCACTGATTGGAGGAATGCTACTAATACT-------------------
----
---ACTTTGGAAGGAGAAGTAAAA---
AACTGCTCTTTCAATATAGCCACAAGCATAAAA---------------------------
GATAAGATGCAGAGAGAATATGCACTT---TTTTATAAATATGATGTAGTACAAATAGATGAGGATAAGAAT-

----

GGTACTAAGGATAATACTAGCTATACTAGCTATAGGTTGATAAATTGTAACACCTCAGTCATTACACAG---
GCTTGTCCAAAGGTATCCTGGGAGCCAATTCCCATACATTATTGTACCCCGGCTGGTTTTGCGATTCTAAAGT
GTAAT---AATGAGACGTTCAGT------GGAAAGGGACCATGTACAAAT----------------------
-GTCAGC---ACAGTACAATGTACACATGGAATTAGGCCAGTGGTGTCAACT---
CAACTGCTGTTAAATGGCAGTCTAGCAAAAACAGAG-----------
GTAATAATTAGATCTGAAAATTTCACAGACAAT---------ACTAAAACCATAATAGTACAGCTGGAG----
-----------GAGGCTATAGTA---------ATTAATTGTATAAGACCTAGCAACAATACAAGAAAA-----
-AGTATACCTATA------------GGACCAGGGAGAGCA---TTC------TATACAACAGGAGAC------
---------ATAATAGGAGACATA------------AGACAAGCACATTGT---AACCTTAGTAGAACA---
GGATGGAAGGACGCCTTACAAAAGATAGCTAAAAAGTTAGGA---------------AACCAATATAAT----
---------------------------------AAAACAAAAATAGTCTTTAAT------
CAATCCTCAGGAGGGGACCCAGAAGTTGTA---ATGCACACTTTTAATTGTGGAGGAGAA---
TTTAAGCAGCTCCAGGCAAGAGTCCTGGCTGTAGAAAGATACCTAAAGGATCAGCAGCTCCTAGGAATTTGGG
GATGCTCTGGAAAACTTATTTGCACCACTGCTGTGCCTTGGAATGCTAGTTGGAGT-----------------
---------------------------AATAAATCTCTGGATACAATTTGG------AAT------
AACATGACCTGGATGCAGTGG---GAGAAAGAAATTGACAATTACACAAGCTTAATATAC---
ACTTTAATTGAAGAATCGCAGAACCAACAAGAA---------------------------------------
----
AAGAATGAGCAAGAATTATTAGAATTAGATAAGTGGGCAAGTCTGTGGAATTGGTTTAGCATAACAAAATGGC
TGTGGTATATAAAAATATTCATAATGATAGTAGGAGGCTTAATAGGTTTAAGAATAGTTTTTGCT---
GTACTCTCGCTAGTG---------AATAGAGTAAGGCAGGGATACTCACCA------
TTGTCATTTGCTGTTAGCTTGCTTAATACAACAGCTATAGCAGTAGCTGAG---
GGGACAGATAGGATTATAGAAGTAGCACAAAGGGTTTTTAGAGCT---------------------------
--------TTTCTCCATATA------CCTAGAAGGCTGAGACAGGGCTTGGAAAGGGCTTTACTA---TAA
>MH746261
GCT---ACA--
GAACAATTGTGGGTCACAGTCTATTATGGGGTACCTGTGTGG---AAAGAAGCAAAC---
ACTACTCTATTCTGTGCATCAGATGCTAAAGCATATAAAACAGAG---GTTCATAAT----------------
--------------GTTTGGGCCACA---CATGCCTGTGTACCCACAGAC---
CCCAGCCCACAAGAAGTACTATTGGCG---------AATGTGACAGAAAATTTTGAC----------------
-------------------------ATGTGGAAAAATAACATGGTAGAACAGATGCATGAGGATATC---
ATCAGTTTATGGGATCAGGGCCTA---AAGCCATGTGTAAGATTA---
ACCCCACTCTGTGTTACTTTAAATTGCACTGATTATGAGGGGAATACTACTAGTGCCAATACTAGTAAGGGGG
GTAATGGTACT---
----------------AGTACCAAGAATACTACTTGGGACACGATGGAGAAAGGAGAAATAAAA---
ATCTGCTCTTTCAATGTCACCACAGACGTGGAA--------------------------
GATAGGAGAAAGAAAGAACATGCACTT---
TTTTATAAACTTGATATAGAACAAATGGAGAATAATAATAATGGTAGCTAT---------------------
----
AATAATAATAATAATGGTAGCTATAAGAACTATAAGTTGATAAGTTGTAAATCCTCAACCCTTACACAG---
GCTTGTCCAAAGATGACTTTTGAGCCAATTCCCATACATTATTGTGCCCCGGCTGGTTATGTGATTCTAAAAT
GTAAC---AATGAGACATTCAAT------GGAACAGGACCATGTACAAAT----------------------
-GTCAGC---ACAGTACAATGTACACATGGAATTAAGCCAGTAGTGTCAACT---
CAGCTACTATTAAATGGCAGTCTAGCAAAAGGAGAT-----------
GTAGTAATTAGATCTCAAAATATTACAAATAAT---------GCTAAAATCATAATAGTACAGCTGAAT----
-----------GAGTCTGTAGAA---------ATTAATTGTACAAGACCCAACAACAATACAAGAAAA-----
-GGTATACGTATA------------GGACCAGGGAGAGCA---TTT------TTAGCAGCAGAAAAA------
---------ATAATAGGAGATATA------------AGGAAAGCACATTGT---AATATTAGTGGAACA---
AAATGGACTGAGACTTTAGGAAAGATAGTTAGAAAATTAAGA------------GAACACCACTTTAAG----
---------------------------------AACAAAACAATAGTCTTTAAT------
CACTCCTCAGGAGGGGACCCAGAAATTGTA---ATGCACAGTTTTAATTGTGGAGGGGAA---
TTTAAACAGCTCCAGGCAAGAGTCCTGGCTGTGGAAAGATACCTACAGGATCAACAGCTCCTAGGGATTTGGG
GTTGCTCAGGAAAACTCATCTGCACCACTGCTGTGCCTTGGAATGTTAGTTGGAGT-----------------
---------------------------AATAAAAGTGAGGACCGGATTTGG------AAT------
AACATGACCTGGATGCAGTGG---GAAAGAGAAATTGACAATTACACAGGCATAATATAC---
AACTTAATTGAAAAATCACAGAAACAACAAGAA---------------------------------------
----
GAGAATGAACAAGAATTATTGGCATTGGGTAAGTGGGACAGTCTGTGGAGTTGGTTTGACATAACAAACTGGC

TGTATTATATAAAAATATTCATAATAATAGTAGGAGGCTTGATAGGTTTAAGAATAGTTTTTACT---
GTACTTTCTATAGTG---------AATAGAGTTAGGCAGGGATACTCACCC------
TTATCATTTGCTGTTAGCTTGCTTAATACCACAGCCATAGTAGTAGCTGAG---
GGGACAGATAGGGTTATAGAAGTAGTAAGAGGAATTTTTAGAGCT----------------------------
--------ATCATCCACATA------CCTACAAGAATAAGACAGGGCTTCGAAAGGGCTTTGCTA---TAA
>MH746254
GCT---GCA---
GAACAATTGTGGGTCACAGTCTATTATGGGGTACCTGTGTGG---AGAGAAGCAAAC---
ACCACTCTATTTTGTGCCTCAGATGCTAAAGCATATGATACAGAG---GTACATAAT----------------
--------------GTCTGGGCCACA---CATGCCTGTGTACCCACTGAC---
CCCAACCCACAAGAAATAGTATTGGAA---------AATGTGACAGAAAATTTTAAC----------------
--------------------------ATGTGGAAAAATAACATGGTGGAGCAGATGCATGAGGATATA---
GTCAGTATATGGGACCAAAGCCTA---ACACCATGTGTAAAATTA---
ACCCCACTCTGTGTTACCTTAAATTGCACTGATGATTTGGAGAACCTTACTAGTACCAAT-------------
----
-------------------------AATACTAGTTTGGGAAACATAGAGAAAGGAGAAATAAAA---
AACTGCTCTTTCAATATCACCACAAGTATAAGA--------------------------
GATAGGAAGCAGAGAGAATATGCACTT---
CTCTATAGACTTGATGTAGTACCAATAGAGAATAATAATAGTACA----------------------------
----
AATAGTAGTTATAGCAGCTATAGGATGATACATTGTAATACCTCAGTCATTACCCAG---
GCCTGTCCAAAAGTATCCTTTGAACCAATTCCCATACATTATTGTGCCCCGGCTGGTTTTGCGATTATAAAGT
GTAAT---GATAAGAAGTTCAAT------GGAACAGGACCATGTAGAAAT-----------------------
-GTCAGC---ACAGTGCAGTGTACACATGGAATTAGGCCAGTAGTGTCAACT---
CAGCTGCTGTTAAATGGCAGTCTAGCAGAAAAAGAG-----------
ATAGTGATTAGATCTGAAAATTTCACAAACAAT---------GCCAAAACTATACTAGTACAGCTAAAT----
-----------GTAGCTGTACCA---------ATTAATTGTACAAGACCCAGCAACAATACAAGTAAA-----
-AGTATACATCTA------------GGATCAGGGAGAGCA---CTT------TATGCAACAGAACGA------
---------ATAATAGGAGATACA------------AGACAAGCACATTGT---AATCTTAGTGAAAAC---
GCATGGCATAACACTTTAGAGCAAGTAGCTAAAAAATTAAGA---------------GAACAATAT-------
-----------------------------------AATAACACAATAGTTTTCACA------
CAATCCTCAGGAGGGGACCTAGAAATCATG---ACGCACATGTTTAATTGTGGAGGAGAA---
TTTAAACAGCTCCAGGCAAGAGTCCTGGCTGTGGAAAGATACCTAAAGGATCAACAGCTCCTAGGGATTTGGG
GTTGCTCTGGAAAACTCATTTGCACCACTACTGTGCCTTGGAACTCTAGTTGGAGT----------------
-----------------------------AATAGAACTCAGGATGATATCTGG------AAT------
CACACGACCTGGATGGAGTGG---GAAAGAGAAATTGATAATTACACAGAATTAATATAC---
TCCTTAATTGAAAAATCGCAGAACCAGCAAGAA---------------------------------------
----
AAGAATGAACTAGAATTATTGGAATTGGATAAGTGGGCAAGTTTATGGAATTGGTTTGATATAACAAATTGGC
TGTGGTATATAAAATTGTTCATAATGATAGTAGGAGGCTTGATAGGTTTAAGAATAGTTTTTGCT---
GTGCTTTCTATAGTG---------AACAGAGTTAGGCAGGGGTACTCACCA------
TTGTCATTTGCTGTCAGCTTGCTTAACGCCACTGCTATAGCAGTAGCCGAG---
GGGACAGATAGGATCATAGAAGTAGTAAGAAGGACTTTTAGAGCT---------------------------
--------GTTCTGCACATA------CCTACAAGAATAAGACAGGGCCTTGAAAGAGCTTTGCTA---TAA
>DQ358809
GCG---CAA---
CAATTGTGGGTCACAGTATATTATGGGGTACCTGTGTGG---AAAGAAGCAACC---
ACCACTCTATTTTGTGCATCAAATGCTAAAGCATATGACACAGAG---GTACATAAT----------------
--------------GTTTGGGCCACA---CATGCCTGTGTACCCACAGAC---
CCTAACCCACAAGAAGTAGTATTGGAA---------AATGTGACAGAAAATTTTAAC----------------
--------------------------ATGTGGAAAAATAACATGGTAGAACAAATGCATGAAGATATA---
ATCAGCTTATGGGATCAAAGCCTA---AAGCCATGTGTAAAATTA---
ACCCCACTCTGTGTTACCTTAAATTGCACTGATGTAAATAAAAAGAATGGTACTAGCAACAAT----------
----
-------------------------ACTAAGGATTGGGAAAAGATGAAGGAGGGAGAAATAAAA---
AACTGTTCTTTCAATGCCACCACAAGCATAAGA--------------------------
GATAAGGTGCGGAAAGAATATGCACTT---TTTTATAGACTTGATGTGGCACCAATGAAAACA----------
----

GATAATACAAGCTATACATTGATAAATTGTAACACCTCAGTCATTACGCAG---

GCCTGTCCAAAGGTATCCTTTGAACCAATTCCCATACATTATTGTGCCCCGGCTGGTTTTGCGATTCTAAAGT
GTAAT---GATAAGAAGTTCAAT------GGAACAGGACCATGTACAAAT----------------------
-GTCAGC---ACAGTACAATGTACACATGGAATTAGGCCAGTAGTGTCAACT---
CAACTACTGTTAAATGGCAGTCTAGCAGAAGAGGAG------------
GTAGTAATTAGATCTGAAAACTTTACAAACAAT---------GCTAAAACCATCATAGTACAGCTGAAT----
-----------GAATCTGTACAA---------ATTAATTGTTCAAGGCCCAACAACAATACAAGAAAA-----
-AGTATACATATA------------GGACCGGGGAGAGCA---TTT------TATGCAACAGGAGAC------
---------ATAATAGGAGATATA------------AGACAAGCACATTGT---AATCTTAGTACAGAA---
CAATGGGAAAAAACTTTAAAGCAGATAGATACAAAATTAAAA---------------GAACAATTC-------
-----------------------------------AATAAAACAATAATCTTTAAT------
CAATCCTCAGGAGGGGATCCAGAAATTGTA---ATGCACAGTTTTACTTGTGGAGGGGAA---
TTTAAGCAGCTTCAGGCAAGAGTCCTGGCTGTGGAAAGATACCTAAAGGATCAACAGCTCCTGGGGCTTTGGG
GTTGCTCTGGAAAACTCATTTGCACCACTGCTGTGCCTTGGAATGCTAGTTGGAGT-----------------
----------------------------AATAAAAGTCTGGAACAAATTTGG------GAT------
AACATGACCTGGATGGAGTGG---GAGAGAGAAATTGACAATTACACAAGCTTAATATAT---
ACCTTAATTGAAGAATCACAAAACCAACAAGAA--

AAGAATGAAAAGGAATTATTGGAATTGGATGAGTGGGCAAGCCTGTGGAATTGGTTCAGCATAACAAAATGGC
TGTGGTATATAAAAATATTCATAATGATAATAGGAGGCTTAATAGGTTTAAGAATAGTTTTTGCT---
GTACTTTCTATAGTA---------AACAGAGTTAGGCAGGGATACTCACCA------
TTATCGTTCGCTGTTAGCTTGCTTAATGCCATAGCTGTAGCAGTAGCTGAG---
GGGACTGATAGGATTATAGAAATAGGACAAAGAATTTGTAGAGGT---------------------------
--------ATCCTCAACATA------CCTAGAAGAATAAGACAGGGCTTAGAAAGGGCTTTGCTA---TAA
>DQ358810
GCT---ACA--
CAATTGTGGGTTACAGTCTATTATGGGGTGCCTGTGTGG---AAAGAAGCAACC---
ACCACTCTATTTTGTGCATCAGATGCTAAAGCATATGACACAGAG---GTACACAAT----------------
--------------GTTTGGGCCACA---CATGCCTGTGTACCCACAGAC---
CCTAACCCACAAGAATTAAAATTGGTA---------AATGTGACAGAATATTTTAAC---------------
-------------------------ATGTGGAACAATAGCATGGTAGAACAGATGCATGAAGATATA---
ATCAGTCTATGGGATGAAAGCCTA---AAGCCATGTGTAGCATTA---
ACCCCACTCTGTGGTGCTGCAAAGTGCACTGATGTGGGAAATGATACTACCATTAATGGTGCTAAT-------
----
------------------AACGCTAATAGAACAATAGAGGATAAGATGGGGGAACTAATAAAA---
AGCTGCTCTTTCAATATCACCACAAGCATAGGA--------------------------
GATAAGAGGCAGAAAGAATATGCACTT---CTTTATAAATATGATATAACGTCAATAGAGAAT---------
----

AATAGTACAAGCTATACATTGATAAATTGTAATACCTCAGTCATTAAACAA---
GCCTGTCCAAAGGTATCCTTTGAACCAATTCCCATACATTATTGTGCCCCGGCTGGTTTTGCGATTCTAAAAT
GTAAG---GACAAGAAATTCAAT------GGAACAGGACCATGTACAAAT----------------------
-GTCAGC---ACAGTACTATGTACACATGGAATTAAGCCAGTAGTGTCAACT---
CAACTGTTGTTAAATGGCAGTCTAGCAGAAGAAGAC------------
ATAGTAATTAGATCTGAAAATTTCACGAACAAT---------GCTAGAACCATAATAGTACACCTGAAT----
-----------GAATCTGTACAA---------ATTAATTGTACAAGACCCAACAACAATACAAGAAGA-----
-AGTATACATATG------------GGACCAGGGAGAGCA---TGG------TATACAACAGGAGAA------
---------ATAATAGGGGATATA------------AGACAAGCATATTGT---AACATTAGCGAAAAA---
ATTTGGGCACAAACTATAAAAAGGGTAGCTATAAAATTAAAA---------------GAACAATTTAAT----
-----------------------------------ACTACAAAAATAGTTTTTAAT------
CAATCCTCAGGAGGGGACCCAGAAATTGTA---ATGCACAGTTTTAATTGTGGAGGGGAA---
TTTAAGCAGCTCCAGGCAAGAGTCCTGGCTGTAGAAAGATACCTAAAGGATCAACAGCTCCTAGGAATTTGGG
GTTGCTCTGGAAAACTCATTTGCGCCACTGCTGTGCCTTGGAATACTAGTTGGAGT----------------
----------------------------AATAAATCTTATGAAGAGATTTGG------GAT------
AACATGACTTGGATGCAGTGG---GAGAAAGAAATTGACCAGTACACAAACACAATATAC---
ACCTTAATTGAAGAATCGCAACGCCAACAAGAA--

AAGAATGAACAAGAATTATTAGCATTGGATTCGTGGGCAAGTTTGTGGAATTGGTTTAGCATATCAAAATGGC
TGTGGTATATAAAAATATTCATAATGATAGTAGGAGGCTTAATAGGTTTAAGAATAGTTTTTACT---
GTACTTTCTATAGTG---------AATAGAGTTAGGCAGGGATACTCACCA------
TTGTCATTCGCTGTCAGCTTGCTTAATGCCACAGCTATAGCAGTAGCTGAG---

GGGACAGATAGGATTATAGAAGTAGTACAAAGAATTGGTAGAGCT----------------------------
--------ATTCTCCACATA------CCTAGAAGAATAAGACAGGGCTTTGAAAGGGCTTTGCTA---TAA
>JN692435
GCA---GAA---
AAGTTGTGGTTCACATTCTATTATGGGGTACCTGGGAGA---AAAGATGCAACC---
ACCATTCTATTCTGTGCATCAGATGCTAAAGCACATCACACAGAG---GCACATAAT----------------
--------------ATTTGGCCACA---CATGCCTGTGTACCCACAGAC---
CCTAACCCACAAGAAGTAAAATTGGAA---------AATGTGACAGAAAATTTTAAC----------------
--------------------------ATGTGGAAAAATAACATGGTAAAACAGATGCATGAGGATATA---
ATCAATTTATGGGATCAAAGCCTA---AAACCATGTGTAAAATTA---
ACCCCACTCTGGGTTACTTTAAATTGCAGTGATTACAAGGGTACTACTACCAATAACAGTACGGAT-------
----
----------------------ACTAACAATAATAGTAGTAAGGTGGAGGGAGGAGAAATGAAA---
AACTGTTCTTTCAATGTAACCACAGGAATAGGA---------------------------
AATAAAATGCAGAAAGAATATGCAATT---TTGTATAAACTTGATGTAACACAAATAGATAAT----------
----

ACAAGCTATACATTGATAAATTGTAACACCTCAGTCATTACACAG---
GCCTGTCCAAAGACATCCTTTGTACCAATTCCCATACATTATTGTACCCCGGCTGGTTTTGCGATTCTACAGT
GTAAT---GATAAAAAGTTTAAT------GGAACAGGAACATGTAAAAAT-----------------------
-GTCAGC---ACAGTACAGTGTACACATGGAATCAAGCCAACAGTGTCAACT---
CAACTACTGTTAAATGGCAGCCTAGCAGAA---GAT------------
GTAATACTTAGATCAGAAAATTTCTCAGACAAT---------GCTAAAACCATAATAGTACAGCTGAAG----
-----------GACCCTGTAGAA---------ATTAATTGTACAAGACCCAACAACAATACAAGAAGA-----
-AGTATAAATATA------------GGACCGGGGAGAGCA---TTT------TATGCAACAGGAGCA------
---------GTAATAGGAAATATA------------AGACAAGCACATTGT---AACATTAGTAGAGAA---
AAATGGAATAACACTTTAACACAGGTAGTTGAAAAATTAAGA---------------GAACAATTTAAG----
-----------------------------------AATAAAACAATATCCTTTCAT------
CAATCCTCAGGAGGGGACCCAGAAATTATA---ATGCACACTTTTAATTGTGGAGGGGAA---
TTCAAGCAGCTCCAGGCTAGAGTCCTGGCTGTGGAAAGATACCTAAAGGATCAACAGCTCCTAGGGATTTGGG
GTTGCTCTGGAAAACTCATTTGCAACACTAATGTGCCTTGGAATACTAGTTGGAGT-----------------
----------------------------AATAAATCTCTGGACGACATTTGG------AAT------
AACATGACCTGGATGCAGTGG---GACAAAGAAATTGACAATTACACAAAATTAATCTAC---
ACCTTAATTGAAGAATCGCAGAACCAGCAGGAA--

AAGAATGAACAAGAATTATTGGAATTGGATAAGTGGGCAAGTTTGTGGAATTGGTTTGATATATCACAATGGC
TGTGGTATATAAGAATATTCATAATAATAGTGGGAGGTTTAGTAGGTTTAAGAATAGTTTTTGCT---
GTGCTTTCCATAGTG---------AATAGAGTTAGGCAGGGATACTCACCA------
TTATCATTTGCTGTTAGTTTGCTTAACGCCACAGCTATAGCAGTAGCAGAG---
GGGACAGATAGGATTATAGAAGTAGTACAAATAACTGGTAGAGCT----------------------------
--------GTTATCCACATA------CCTAGAAGAATAAGACAGGGCTTCGAAAGGGCTTTGCTA---TAA
>JN692440
GCT---ACA--
AAAAAATTGTGGGTTACAGTCTATTATGGGGTACCTGTGTGG---AAAGAAGCAAAC---
ACTACTTTATTTTGTGCATCAGATGCTAAAGCTTATGATACAGAG---GCGCATAAT----------------
--------------GTTTGGCCACA---CATGCCTGTGTACCTACAGAC---
CCCAACCCACAGGAAATAGTATTGGCA---------AATGTGTCAGAATATTTTGAC----------------
--------------------------ATATGGAGAAACAACATGGTAGAACAAATGCATGAGGATATA---
ATCAGCTTATGGGATCAAAGCCTA---AAGCCATGTGTAAAATTA---
ACCCCACTCTGTGTCACTTTAAACTGCACTGATTTGGGGAATGATACTAATACCAACAAT-------------
----
-------------------------AGTAGTGATGTAGGAGACATGGAGAAAGGAGAAATGAAA---
AACTGCTCTTTCAATATCACCACAGAAGTGAAA---------------------------
GATAAAATGAAAAAAGAATATGCAACT---TTCTATAGACTTGATGTAGTACTAATAGATAAGGAAAATAAG-
----

AGTGATAATACTAGTAATACTCGCTATAGGTTGATAAGTTGTAACGCCTCAGTCATTACACAG---
GCCTGTCCAAAGATATCCTTTGAGCCAATTCCCATCCATTATTGTGCCCCGGCTGGTTTTGCGATTCTAAAGT
GTAAT---AATAAGACATTCAAT------GGAAAAGGATCATGTACAAAT-----------------------
-GTTAGC---ACAGTACAATGCACACATGGAATTAGGCCAGTAGTATCAACT---

CAACTGCTATTAAATGGCAGTTTAGCAGAAGAAGAG------------
GTAATAATCAGATCTGACAATTTCTCGAACAAT---------GCTAGAACCATAATAGTACAGCTGAAT----
-----------GAAGCTGTACAA---------ATTAATTGTACAAGACCCAACAACAGTACAAGAAAA-----
-GGTATACATATA------------GGACCAGGGAAAGCA---GTT------TATGCAACAGAAAAA------
---------ATAATAGGAAACATA------------AGAAAGGCACATTGT---AACATTAGTGAATCA---
AGATGGAACAACACTTTAAAACAGATAGTTATAAAATTAAGA---------------GAACAATTTAAG----
-----------------------------------AATAAAACAATAAAATTTAAC------
CACTCCTCAGGAGGGGACCCAGAAATTGCA---ATGCACAGTTTTAATTGTGGAGGGGAA---
TTTAAGCAGCTCCAGGCAAGAGTCCTGGCTCTTGAAAGATACCTAAAGGATCAACAGCTCCTGGGAATGTGGG
GTTGCTCTGGAAAACTCATCTGCCCCACTGCTGTGCCTTGGAATGCTAGTTGGAGT-----------------
----------------------------AATAGATCTCTGAATAGCATTTGG------GAT------
AACATGACCTGGATGGAGTGG---GAAAGAGAAATTGACAATTACACAGACTTGATATAC---
ACTTTAATTGCAGAATCGCAGAACCAACAAGAA--

AAGAATGAACAAGAATTATTGGAATTAGATAAATGGGCAAGTTTGTGGAATTGGTTTAGCATAACACATTGGC
TGTGGTATATAAAAATATTCATAATGATAGTAGGAGGCTTGATAGGTTTAAGAATAGTTTTTGCT---
GTACTATCTATAATA---------AATAGAGTTAGGCAGGGATACTCACCA------
TTATCGTTGGCTGTTAGCTTGCTTGATACCATAGCCATAGTAGTAGCTGAG---
GGGACAGATAGGATTATAGAAGGGTTACAAAGGGTTTTTAGAGCT---------------------------
--------ATTCTCCACATA------CCTACAAGGATAAGACAGGGATTTGAAAGGGCTTTGCTA---TAA
>JN692443
GCT---ACA--
GAACAATTGTGGGTCACAGTCTATTATGGGGTACCTGTGTGG---AAAGAAGCAAAC---
ACCTCTCTATTTTGTGCATCAGATGCTAAAGCATATAGCACAGAG---GTACATAAT----------------
--------------GTTTGGGCCACA---CATGCCTGTGTACCCACAGAC---
CCTAACCCACAAGAAGTAGTATTGAAA---------AATGTGACAGAAAGTTTTAAC----------------
-------------------------ATGTGGAAAAATAACATGGTAGAACAGATGCATGATGATATA---
ATTAGTTTATGGGATGAAAGCCTA---AAGCCATGTGTAAAATTA---
ACCCCACTCTGTGTTACTTTAAAGTGCAGTAATGTGAATTTAAAGTGCAGTAATGAGAATTTAAATTGC----
----
------------------AGTAATGACAATGCTAGTAGGATAGAAATGGAGAGAGAAATAAAA---
AACTGCTCTTTCAACATCACCACAAGCATACAA---------------------------
AATAAGAATAGTAAAGAATATGCACTT---TTTTATAAACTTGATGTAGTACCACTAGATAAGAAT-------
----

GATAATACAAGCTATATGTTAATAAATTGTAACACCTCAGAAATTACACAG---
ACCTGCCCAAAGGTATCCTTTGAACCAATTCCCATACATTATTGTGCCCCAGCTGGTTTTGCGATTCTAAAGT
GTAAT---GATAAGAAATTCAAT------GGAACAGGACCATGTAAAAAT----------------------
-GTCAGC---ACAGTACAGTGTACACATGGAATTAAGCCAGTAGTGTCAACT---
CAACTGCTGTTAAATGGCAGTCTAGCAGAAGAAGAG------------
GTAGTGATTAGAGCTGAAAATTTCACAGACAAT---------ACTAAAACCATAATAGTACAGCTGAAC----
-----------GAAACTGTAGTA---------ATTAATTGTATAAGACCCCACAACAATACAAGAAAA-----
-AGTATACCGGTG------------GTA---TGGAGGACA---TTA------TATGCAACAGGGGCT------
---------ATAACAGGAAATATA------------AGACAAGCACATTGT---AACCTTAACAGAACA---
CAATGGAACAAAACTTTAGGACAGATAGTAACAAAATTAAGG---------------GAACAATTTGGA----
-----------------------------------GATAAAACAATAATCTTTACC------
CAATCCTCAGGAGGGGACCCAGAAATTGTA---ATGCACAGTTTTAATTGTGGAGGGGAA---
TTTAAGCAGCTCCAGGCAAGAGTCCTGGCTGTGGAAAGATACCTACGGGATCAACAGCTCCTAGGGATTTGGG
GTTGCTCTGGAAAACTCATTTGCACCACTGCTGTGCCTTGGAATGCTAGTTGGAGT-----------------
----------------------------AATAAAACTTATAATGACATTTGG------AAT------
AACATGACCTGGATGGAGTGG---GAAAGAGAAATTAACAATTATACAGGCTTAATATAC---
ACTTTAATTGAAGAATCGCAGAACCAACAAGAA--

AAGAATGAAAAAGAATTATTGGAATTAGATAAGTGGGCAAGTTTGTGGAATTGGTTTGACATAACAAAATGGC
TGTGGTATATAAAAATATTCATAATGATAGTAGGAGGCTTAATAGGTTTAAGAATAGTTTCTGCT---
GTATTTTCTATAGTA---------AATAGAGTTAGGCAGGGATACTCACCT------
TTGTCATTCGCTGTTAATTTGCTTAATGCCACAGCTATAGCAGTAGGTGAG---
GGAACAGATAGGATTATAGAAGGATTACAAAGACTGGGTAGAGCT---------------------------
--------ATTCTCCACATA------CCAAGAAGAATAAGACAGGGCGCAGAAAGGGCTTTGCAA---TAA
>JN692444

GCT---ACA--
GAACAATTGTGGGTCACAGTTTATTATGGGGTACCTGTGTGG---AAAGAAGCAACT---
ACCACTCTATTTTGTGCATCTGATGCTAAAGCATATGATACAGAG---GCACATAAT----------------
--------------GTTTGGGCCACA---CATGCCTGTGTACCCACAGAC---
CCTGCCCCACAAGAAATAGAATTAAAA---------AATGTAACAGAAAATTTTAAC----------------
--------------------------ATGTGGAAAAATGACATGGCAGAACAAATGCATGAAGATATA---
ATCAGTTTATGGGATCAAAGCCTA---AAGCCATGTGTAAAATTA---
ACCCCACTCTGTGTTACTTTAAATTGCACTGATATCCCTGCCAATAAGAATAGT-------------------
----
-------------------------------AGTGTGAATACCACTAGTTTAGGAGAGATGAAA---
AACTGCTCTTTCAATATCAGCACAAAAATAAAA---------------------------
GATAAGGTAGAAAAGGTCTGGGCAACT---TTTTATAATCTTGATATAGTACAAATAGAAAAT----------
----

AATAATAGTTATATGCTGACAAGTTGTAATACCTCAGTCCTTACACAG---
GCCTGTCCAAAGATGACCTTTGAACCAATTCCCATACATTATTGTGCCCCGGCTGGTTTTGCGATTCTGAAGT
GTAAC---GATAAGAAGTTCAAT------GGAACAGGACCATGTAAAAAT-----------------------
-GTCAGC---ACAGTACAATGTACACATGGAATTAGGCCAGTAGTGTCAACT---
CAACTACTGCTAAATGGCAGTCTAGCAGAAGAAGAG------------
GTGATAATTAGATCTGAAAATATCATGGACAAT---------ACTAAAACCATAATAGTACAGCTAAAT----
-----------GAAACTGTGCAA---------ATTAATTGTTCAAGACCCAACAACAATACAAGAAAA-----
-AGTATACATATA------------GCACCAGGGAGAGCA---TTT------TATGCAACAGGAGGT------
---------ATAATAGGAGATATA------------AGACAAGCACATTGT---AACATTAGTGAAGCA---
GCTTGGAATAAAACTTTAGAACGAGTAGTTATAAAATTAAGA---------------GAACATTTTAAA----
-----------------------------------AACAAAACAATAAACTTTACA------
CAACCCTCAGGAGGGGATCCAGAAATTGTA---ACGCACAGTTTTAATTGTGGAGGGGAA---
TTTAAACAGCTCCAGGCAAGAGTCCTGGCTGTGAAAAGATACCTAAAGGATCAACAGCTCCTAGGGATTTGGG
GTTGCTCTGGAAAACTCATCTGCACCACTGCTGTGCCTTGGAATGCTAGTTGGAGT-----------------
----------------------------AATAAATCTAGGGATCAAATTTGG------GAG------
AACATGACCTGGATGGAGTGG---GAAAGAGAAATTGACAATTACACAGAATCAATATAC---
AGCTTAATCGAAAAAGCGCAAAACCAGCAAGAA--

AAGAATGAACAAGACTTATTATCATTAGATCAGTGGGCAAGTTTGTGGAATTGGTTTAGTATAACAAAATGGC
TGTGGTATATAAAAATATTCATAATGATAGTAGGAGGCTTGATAGGTTTAAGAATAATTTGTGCT---
GTACTTTCTATAGTA---------AATAGAGTTAGGCAGGGATACTCACCA------
TTATCATTGGCTGTTAGCTTGCTCAACGCCACAGCAATAGCAGTAGCTGAG---
GGGACAGATAGGATTATAGAAGTAGTACAAAGGGCAGGTAGAGCT----------------------------
--------ATTCTTCACATA------CCTAGAAGAATAAGACAGGGCTTGGAAAGGGCTTTGCTA---TAA
>JN692445
GCA---AAA---GAA------------------------------------
GAAAAATTGTGGGTCACAGTCTATTATGGGGTACCTGTGTGG---AAAGAAGCAACT---
ACCACTCTGTTTTGTGCATCAGATGCTAAAGCTTATGTCACAGAG---ATGCATAAT----------------
--------------GTTTGGGCCACA---CATGCCTGTGTACCCACAGAC---
CCCAATCCACAAGAGGTAGTACTGGGA---------AATGTGACAGAAAATTTTAAC----------------
--------------------------ATGTGGAAAAATAACATGGTAGAACAGATGCATGAGGATATA---
ATCAGCTTATGGGATCAAAGCTTA---AAGCCATGTGTAAAATTA---
ACCCCACTCTGTGTTACTTTAGAATGTAATGATGCAAAACTTAACAGTACAAAAACTAACAGTACAACTAAC-
----
----------------AGTACTGATCCTAACAATAGTAACTTGGGGATAGAGGGAGAAATAAAA---
AACTGCACTTTCAATGTCACCAGCATGGGA---------------------------------
AAGAGTAGGAAAGAAAATGCAATT---TTTAATAAACTTGATATAGTACCAATAGAGGATGATAGTAAT----
----
----AATAGTACTAGTAATAGTAGTAAAAGTTATATGTTGACAAGTTGTAACACCTCAGTCATTACACAA---
GCCTGTCCAAAGGTATCCTTTGAACCAATTCCCATACATTATTGTACCCCAGCTGGTTTTGCGATTCTAAAAT
GCAAT---GATAAGAAGTTCAAT------GGATCAGGACCATGTACAAAT-----------------------
-GTCAGC---ACAGTACAATGTACACATGGAATTAGGCCAGTAGTGTCAACT---
CAATTACTATTAAATGGTAGTCTAGCAGAAGAAGAG------------
GTAATGATTAGATCTCAAAATTTTTCAGACAAT---------GCTAAAACCATAATAGTACAGCTGAAT----
-----------GACACTGTACAA---------ATTAATTGTACAAGACCCCACAACAATACAAGAAAA-----
-AGTATACGTATA------------GGACCAGGGGCAGCA---TTT------TATGCAACAGGAGAT------

---------ATAATAGGAGATATA------------AGACAAGCACATTGT---AACATTAGTAGAGCA---
GAATGGAACAAAACTTTAAAATTTATAGTAGCAAAATTACAA--------------GAACAATTTAAC----
-----------------------------------ATAACAAAATTAAATTTTAGG------
AACTCCTCAGGAGGGGATCCAGAAATTGTA---ATGCACAGCTTTAATTGTGCAGGGGAA---
TTTAAGCAGCTCCAGGCAAGAGTCCTGGCTGTGGAAAGATACCTAAGGGATCAACAGCTCCTGGGGATTTGGG
GTTGCTCTGGAAAACTCATCTGCACCACTGCTGTGCCTTGGAATACTAGTTGGAGT----------------
----------------------------AATAGATCTCTGAATATTATTTGG------GGT------
AACATGACATGGATGGAGTGG---GAAAGAGAAATTGACAATTACACAGACTTAATATAC---
AACTTGCTTGAAGAATCGCAGAACCAACAGGAA--

AAGAATGAACAAGAATTATTAGAATTGGATAAGTGGGCAGACTTGTGGAGTTGGTTTGACATAACACACTGGC
TATGGTATATAAGATTATTCATAATGATAGTAGGAGGCTTAATAGGTTTAAGAATAGTTTTTGCT---
GTACTTTCTATAGTA---------AATAGAGTTAGGAAGGGATACTCACCA------
TTGTCATTCGCTGTTAGCTTGCTTAACGCCACAGCTATAGCAGTAGCTGAG---
GGAACAGATAGGGTTATAGAAGTAGTACAAAGAGCTGGTAGAGCT---------------------------
--------ATTCTCAACATA------CCTAGAAGAATAAGACAGGGCTTAGAAAGAGCTTTGCTA---TAA
>JN692447
GCT---GCA---
GACCATTTGTGGGTCACAGTCTATTATGGGGTACCTGTGTGG---AAAGAACCCCCC---
ACCACTCTATTTTGTGCATCAGATGCTAAAGCACATTATACAGAA---ATGCATAAT----------------
--------------GTCTGGGCCACA---CATGCCTGTGTACCCACAGAC---
CCCAACCCACAAGAGGTAGTATTGGAA---------AATGTGACAGAAAATTTTAAC---------------
--------------------------ATGTGGAAAAATGACATGGTAGAACAGATGCATGAGGATATA---
AAAAGTTTATGGGATCAAAGCCTA---AAGCCATGTGTAAAATTA---
ACCCCACTCTGTGTTACTTTAAATTGCACTGATTGGGAGAATAAGAATATTACGAATAATAAT---------
----
---------------------AGTAGCATGAATACAAATATTATAGAGAAAGGAGAAATAAAA---
GATTGCTCTTTCAATATCACCACAAGCATAAAA--------------------------
GATAAGGTGCAGAAAGTATATGCACAA---
TTTTATAAACTTGATGTAACACCAATAGGTGATGATAATAATAGTAAT-------------------------
----
AATAATGGTAGTAGAAACTATACAAACTATAGATTGATAAATTGTAATACCTCAGTCATCACACAG---
GCCTGTCCAAAGGTGTCTTTTGAACCAATTCCCATACATTATTGTGCCCCGGCTGGTTTTGCGATTCTAAAAT
GTAAT---GATAAGAAGTTCAAT------GGAACAGGACCATGTAAAAAT----------------------
-GTCAGC---ACAGTACAATGTACACATGGAATTAGGCCAGTAGTGTCAACT---
CAACTACTGTTAAATGGCAGTCTAGCAGAGGAAGAG-----------
GTAGTAATTAGATCTAAAAACTTCACAAATAAT---------GCTTACACCATAATAGTACAGCTAAAT----
-----------GAGACTGTACAA---------ATTAATTGTTCAAGACCCGGCAATAATACAAGAAAA-----
-AGTATACATCTA------------GGACCAGGGAGCGTA---TTT------TAT---ACAGGAGAA------
---------ATAATAGGAGATATA------------AGACGAGCATATTGT---AACATTAGTGGAAAA---
GCCTGGAATAAAACTTTAAATCAGATAGCCACAAAACTAAGA--------------GAACAATTTGAG----
-----------------------------------AATAAAACAATAATCTTTCAA------
CCATCTTCAGGAGGGGACCCAGAAATTGTA---ATGCATAGTTTTAATTGTGGAGGAGAA---
TTTAAACAGCTCCAGGCAAGACTCCTGGCTGTGGAAAGGTACCTAAGGGATCAACAGCTCCTAGGGCTTTGGG
GTTGCTCTGGAAAACTCATTTGCACCACCGCTGTGCCTTGGAACTCCAGTTGGAGT----------------
----------------------------AATAAATCTCTGGATACAATTTGG------GAT------
AACATGACCTGGATGCAGTGG---GATAAAGAAATTGACAATTACACAAACTTAATATAC---
TCTTTACTTGAAGAATCGCAGTCCCAGCAAGAA--

AACAATGAAAAGAAATTATTAAAATTGGATCAGTGGGCAAGTTTATGGAATTGGTTTGATATAACAAACTGGC
TGTGGTATATAAAAATATTCATAATGATAGTAGGAGGCTTGATAGGTTTAAGAATAGTTTTTACT---
GTACTTTCTATAGTG---------AATAGGGTTAGGCAGGAATACTCACCT------
TTGTCATTTGCTATTAGCTTGCTTGATGCCACAGCCATTGCAGTAGCTGGG---
GGGACAGATATAGTTATAGAAGCAGCAAGAATAATTTTTAGAGCT---------------------------
--------ATTATCCACATA------CCTACAAGAATAAGACAGGGCTTCGAAAGGGCTTTGCTA---TAA
>EF637056
GCA---GAA--
CAATTGTGGGTCACAGTCTATTATGGGGTACCTGTGTGG---AAAGAAGCAACC---
ACCACTTTATTTTGTGCATCAGATGCTAAAGCATATGAAACAGAG---GTACATAAT---------------
--------------GTTTGGGCCACA---CATGCCTGTGTACCCACAGAC---

CCTAACCCACAAGAAGTAGTATTGGGA---------AATGTGACAGAAAATTTTAAT----------------
--------------------------ATGTGGAAAAATAACATGGTAGACCAGATGCATGAGGATGTA---
ATCAGTTTATGGGATGAAAGCTTA---AAGCCATGTGTACAATTA---
ACCCCACTCTGTGTCACTTTAGATTGTACTAACTATAATATTACTAGTAAAACTAATAGAACTAAT-------
----
----------------------GAAAATGATATTAGTTTGGAAATGGGAGAGGGAGAACTAAAA---
AACTGCTCTTTCAATGTCACCACAGGTATAAAA--------------------------
AATAAGGTAAAAAAGGAGTATGCAATT---TTTAATAGACTTGATGTAGTACCAATAGATAGC---------
----

AGTAATAGCAATAGTAGCTATATGATAATAAATTGTAACACCTCAGTCATTACCCAG---
GCCTGTCCAAAGGTATCCTTTGAACCAATTCCTATACATTATTGTACCCCGGCTGGTTATGCGATTCTAAAGT
GTAAT---GATAAAACGTTCAAT------GGAACAGGACCATGTAAAAAT----------------------
-GTCAGT---ACAGTACAATGTACACATGGAATCAGACCAGTAGTGTCAACT---
CAACTGTTGTTAAATGGCAGCTTAGCAGAAGAAGAC-----------
ATAATAATTAGATCTGAAAATCTCACAAATAAT---------GCTAAAAACATAATAATACAGCTGAAT----
-----------GAAACTATAAAA---------ATTAATTGTACAAGACCCAACAACAATACAAGAAAA-----
-AGTATACATTTC------------GCACCTGGGCAGGCA---TTT------TATGCAACAGGAGAT------
---------ATAATAGGAGATATA------------AGACAAGCACACTGT---AACATTAGTGGACAA---
GATTGGAACAGAACCTTAACACGGATAGCTAAAAAATTAAAA---------------GAACAATTT-------
-----------------------------------AATAAATCAATACAATTTACA------
AAACACTCAGGAGGAGACCCAGAAATTGAA---ATGCATAGTTTTAATTGTGGAGGGGAA---
TTTAAGCAGCTCCAGGCAAGAGTCCTGGCTGTGGAAAGATACCTAAGGGATCAACAGCTCCTAGGAATTTGGG
GATGCTCTGGAAAACTCATTTGCACCACTTCCGTGCCTTGGAATACTAGTTGGAGC-----------------
----------------------------AATAAAACTTGGAATGAGATTTGG------AAT------
AACATGACATGGATGCAATGG---GAAAAAGAAATTGACAATTACACAGGCATAATATAC---
ACCTTACTTGAAGAATCGCAAAACCAACAAGAA---------------------------------------
----
AAGAATGAACAAGAATTATTACAATTAGACTCATGGGCAAATTTGTGGAGTTGGTTGGACATATCAAACTGGC
TGAAGTATATAAAAATATTCATAATAATAATAGGAGGCTTAATAGGTTTAAAAATAGTTTTTACT---
GTAATTTCTATAGCA---------AATAGAGTTAGGCAGGGATACTCACCC------
CTATCGTTCGCTGTCAGCTTGCTTAATGCCACAGCAATTGCAGTAGCTGAG---
GGGACAGATAGAATTATAGAAGTAGTACAAATAGCAGGTAGAGCT---------------------------
--------ATTCTCAATATA------CCTAGAAGAATAAGACAGGGCTTAGAAAGGGCTTTACTA---TAA
>EF637054
ACT---GAA---GAA------------------------------------
GACAAATTGTGGGTCACAGTCTATTATGGGGTACCTGTGTGG---AAAGAAGCAACC---
ACCACTTTATTTTGTGCATCAGATGCTAAAGCATATGACACAGAG---GCACATAAT----------------
--------------GTTTGGGCCACA---CATGCCTGTGTACCCACAGAC---
CCTGACCCACAAGAAGTAGTATTGGAG---------AATGTGACAGAAAACTTTAAC---------------
--------------------------ATGTGGAAAAATAGCATGGTAGAACAGATGCAGGAGGATATA---
ATCAGTTTGTGGGATCAAAGCTTA---AAGCCATGTGTAAAATTA---
ACTCCACTCTGTATTACTTTAAATTGCTCTAATACAGAGAGAAATGTTACAGGTATCAAGGCA---------
----
----------------------GAGAAAAATATTACAGATAATAGTATAAAGGAAGAAATAAAA---
AACTGCTCTTTCAATGTC---ACAAGCATAGGG--------------------------
AATAAGGTGCGGAAAGAACATGCAATT---TTTAATAAACTTGATGTAGTACCAATAAATGATAAGACA----
----

AATAATAATACAAACTATACAAACTATATGTTGATAAGTTGTAACACCTCAGTCGTCACACAG---
GCCTGTCCAAAGGTAACCTTTGAACCAATTCCCATACATTATTGTGCCCCGGCTGGTTTTGCGATTCTAAAGT
GTAAT---GATAGGAAATTCAAG------GGATCAGGACCATGTAAAAGT----------------------
-GTCAGT---GTAGTACAATGTACACATGGAATTAAGCCAGTAGTGTCAACT---
CAACTGCTGTTAAATGGCAGTTTAGCAGAAGAAGAG-----------
GTAATAATTAGATCTGAAAATTTCACGAACAAT---------GCTAGAACCATAATAGTACAGCTGAAT----
-----------GGAACTATACAA---------ATTAATTGTACAAGACCCAACAACAATACAAGAAAA-----
-AGTATAAATATG------------GGACCAGGGAGAGCA---TTT------TATGCAACAGGAGAT------
---------ATAATAGGAGATATA------------AGACAAGCACATTGT---AACATTAGTAGAAAA---
GACTGGAACAAAACTTTAGAACAGATAGTTAGAAAATTAAGG---------------GAACAATTTGGT----
-----------------------------------AATAAAACAATAAGCTTTAAG------

CAATCCGCAGGAGGGGACCCAGAAATTGTA---ATGCACAGTTTTAATTGTGGAGGGGAA---
TTTAAGCAGCTCCAGGCAAGAGTCCTGGCTGTAGAAAGATACCTACAGGATCAACAGCTCCTAGGGATTTGGG
GTTGCTCTGGAAAACTCATCTGCACCACTACTGTGCCTTGGAATACTAGTTGGAGT-----------------
----------------------------AACAGGTCTTTGGATAACATTTGG------CAG------
AACATGACTTGGATGGAGTGG---GAGAGAGAAATTGGCAATTACACAAAATTCATATAC---
ACCTTACTTGAAGAATCGCAGAACCAACAGGAA--

AATAATGAAAAAGAATTATTAGAATTGGATAAGTGGGCAAGTTTGTGGAATTGGTTTACCATAACAAACTGGC
TGTGGTATATAAAAATTTTCATAATGATAGTAGGAGGCTTAATAGGTTTAAGAATAGTTTTTGCT---
GTACTCTCTATAGTG---------AATAGAGTTAGGCAGGGATACTCACCC------
TTGTCATTCGCTGTTAGTTTGTTTGATGCAATAGCTATAGCAGTAGCTGAG---
GGGACAGATAGAGTGATAGAAGTGTTACAAAGAATTGGCAGAGCT----------------------------
--------TTTCTTAACATA------CCTACAAGAATAAGACAGGGCTTAGAAAGGGCTTTACTA---TAA
>EF637051
GCT---GCA--
GGGAAGTTGTGGGTCACAGTTTATTATGGGGTACCTGTGTGG---AAAGAAGCAACC---
ACCACTCTATTTTGTGCATCAGATGCTAAAGCATATGAAGAAGAG---GCACATAAT----------------
--------------GTTTGGGCCACA---CATGCCTGTGTACCCACAGAC---
CCTAACCCACAAGAAGTAGTATTGGAA---------AATGTGACAGAAAATTTTAAC----------------
--------------------------ATGTGGAAAAATAATATGGTAGAACAGATGCATGAGGATATA---
ATCAGCTTATGGGATCAAAGCCTA---AAGCCATGTGTAAAATTA---
ACCCCACTCTGTGTTACTTTAAATTGCACTGACTTGACAAATAAAACTACCCCTACCAAT-------------
----
----------------------------AGTAGTCTTGAGAAAGGGATGGGGGGAGAAAGAAAA---
AACTGCTCTTTCAATATCACCACAGGCATAAAA---------------------------
AATAAGGTGCAGAAAGAATATGCTACT---TTTTATAAACTTGATGTAGTACCAATAGATAAT----------
----

GATAATACAAGCTATATGTTGATAAATTGTAATACCTCAGTCATTACACAG---
GCCTGTCCAAAGGTAACCTTTGAACCAATCCCAATACATTATTGTGCCCCGGCTGGTTTTGCGATTCTAAAGT
GTAAT---GATAAGGGGTTCAAT------GGAACAGGGCCATGTAAAAAT------------------------
-GTCAGC---ACAGTACAATGTACACATGGAATTAGGCCAGTAGTATCAACT---
CAATTGCTATTAAATGGCAGCCTATCGGAAGAAGGG------------
GTAGTAATTAGGTCTGAAAATCTCACAAACAAT---------GCTAAAACCATAATAGTACAGCTGAAC----
-----------GAATCTGTAGAA---------ATTCATTGTATAAGACCTGGTAATAATACAAGCAAA-----
-AGTATACACATG------------GGACCGGGGCGAGCA---TTT------TATGCAACAGGAGAA------
---------ATAATAGGAGATATA------------AGACAAGCACATTGT---AATATTAGTAAAACA---
AAATGGACAACACTTTAAACCAGATAGTTACAAAATTAAGA----------------GAACAATTT-------
-----------------------------------AATAAAACAATATCCTTTAAT------
CACTCCTCAGGAGGGGACCCAGAAATTGTA---CTACACAGTTTTAATTGTAGAGGGGAA---
TTTAAGCAGCTCCAGGCAAGAGTCCTGGCTGTGGAAAGATACCTAAGGGATCAACAGCTCCTAGGGATTTGGG
GTTGCTCTGGAAAACTCATTTGCACCACTGCTGTACCTTGGAATACTAGTTGGAGT-----------------
----------------------------AATAAATCTCTGGATACCATTTGG------GGT------
AACATGACTTGGATGCAGTGG---GAGAAAGAAATTGACAATTACACAGGCTTAATATAC---
AACTTACTTGAGGAATCGCAAAACCAACAAGAA--

AAGAATGAACAAGAATTATTGGCATTGGATAACTGGGCAAATTTGTGGAATTGGTTTGACATATCAAATTGGC
TATGGTATATAAAAATATTCATAATGATAGTAGGAGGCTTAGTAGGCTTAAGAATAGTTTTCACT---
GTGCTTTCTATAGTA---------AATAGAGTTAGGCAGGGATATTCACCA------
TTGTCATTCGCTGTTAATTTGCTTAATACTACAGCTATAGCAGTAGCTGAG---
GGGACAGATAGGATTATAGAAGGATTACAAAGAATTTGTAGAGCT----------------------------
--------ATTATCCACATA------CCTAGAAGAATAAGACAGGGCGCAGAAAGGATTTTGCTA---TAA
>EF637050
GCT---CCA--
AATAACTCGTGGGTCACAGTCTATTATGGAGTGCCTGTGTGG---AAAGAAGCAACT---
ACCACCCTATTCTGTGCATCAGATGCTAAAGCATATGACACAGAG---GCACATAAT----------------
--------------GTTTGGGCAACA---CATGCCTGTGTACCCACAGAC---
CCTAACCAGCAGGAAGTAGAATTGGAA---------GATGTAACAGATAATTTTGAC----------------
--------------------------ATGTGGAAAAAGAGCATGGTAGACCTGATGAATGAAGAGATA---
GTCAGTGTATGGGATCAAAGCGTA---AAGCCATGTGTAAAATTA---

GCCCCACTCTGTGTTACTCTAAATTGCACTAATTTGAATAATAGTGTTGATAAGATCAATACT----------
----
-------------------------AGTAGTAGTAGTGAGAACGAGATAAAGGAAGAAATAAAA---
AACTGCTCTTTTAATATCACCACAAGCAGAAGA---------------------------
AATAAATTGCGGAAAGAATATGCACTT---
TTTTATAAGCTTGATATAGTACCAATAGATAATGATAAAATAAGC----------------------------
----
AGTGATACAGGTAATACAAGCTATGCAAGCTATACATTGATAAGTTGTAACACCTCAATCATTACCCAA---
GCCTGTCCAAAGGTATCCTTTGAACCAATTCCCATACATTATTGTACCCCGGCTGGTTTTGCGATTCTAAAAT
GTAAT---GAGAAGAACTTCACT------GGAACAGGACCATGTAAAAAT----------------------
-GTCAGT---ACAGTACAATGTACACATGGAATTAGGCCAGTAGTGTCAACT---
CAATTGCTGTTAAATGGCAGTCTAGCAGAAGGAGAG-----------
ATAGTAATTAGATCTGAAAATTTCACGGACAAT---------GCTAAAACCATAATAGTACAGCTGAAT----
-----------GAATCTGTAGTA---------ATTAATTGTACAAGACCCAACAACAATACAAGAAAA-----
-AGTATACATATG------------GGACCAGGGAGAGCA---TGG------TTCACAACAGGAGAA------
---------ATAATAGGAAATATA------------AGAGAAGCACATTGT---AACCTTAGTAGAACA---
AAATGGAGTAACACTTTAAAACAGATAGCTATAAAATTAGGA---------------AACTACTTTGAC----
-----------------------------------AACAAAACAATAATCTTTACT------
AACTCCTCAGGAGGGGACCCTGAAATTGTA---ACTCACAGTTTTATTTGTGGAGGGGAG---
TTTAAGCAGCTCCAGGCAAGAGTCCTAGCTGTAGAAAGATACCTAAAGGATCAACAGCTCCTAGGGATTTGGG
GTTGCTCTGGAAAACTCATCTGCACCACTACTGTGCCTTGGAATAATAGTTGGAGT----------------
-----------------------------AATAAATCTCTAGATTATATTTGG------AAT------
AACATGACTTGGATGCAGTGG---GAAAAAGAGATTGACAATTACACAGAGGTAATATAC---
AACTTAATTGAAAAATCGCAAAACCAGCAAGAA--

AAGAATGAACAAGACTTATTGGAATTGGATAAGTGGGACAGTTTGTGGAATTGGTTTGACATAACAAATTGGC
TGTGGTATATAAAAATATTCATAATGATAGTAGGAGGCTTAATAGGATTAAGAATAGTTTTTACT---
GTACTTTCTATAGTA---------AATAGAGTTAGGAAGGGATACTCACCA------
TTATCGTTCGCTGTTAGTTTGCTTAATGCCACAGCTATAGCAGTAGCTGAG---
GGGACAGATAGAATTATAGAAGTAGTACAAAGAACTGGTAGAGCA---------------------------
--------ATTCTCCACATA------CCTAGAAGAATAAGACAGGGCTTAGAAAGGGCTTTGCAA---TAA
>EF637048
GCT---AAT---
GATCAATTGTGGGTCACAGTCTATTATGGGGTACCTGTGTGG---AAAGACGCAAAA---
ACCACTCTATTTTGTGCATCAGATGCTAAAGCATATGACACAGAG---GTACATAAT----------------
--------------GTGTGGGCCACA---CATGCCTGTGTACCCACAGAC---
CCTAACCCACAAGAAGTAGTATTGGGA---------AATGTGACAGAAAATTTTAAC---------------
--------------------------ATGTGGAAAAATGACATGGTAGAACAGATGCATGAGGATATA---
ATCAGTTTATGGGATCAAAGCCTA---AAGCCATGTGTAAAATTA---
ACCCCACTCTGTGTCACTTTAGATTGTAGCCATAATGTTGATAGTATCAATGGTAGCCATAGTGTTACTACTA
TC--
----------AATGGGACCAATAATGTTACTACTACCCTTGCGCAGGAGGAGAGGGAAATAAAA---
AACTGCACTTTCAATATCACAACAGGCTTAAAA-------------------------
GATAGGGTGAAACAAGACTATGCACTT---TTTAATAGCCTTGATGTAGTACCAATAGGCAAG---------
----

AACAATACAAGCTATATGTTGGCAAGCTGTAACACCTCAGTCATTACACAG---
GCCTGTCCAAAGGTAACCTTTGAACCAATCCCCATACATTATTGTGCCCCGGCTGGTTTTGCGATTCTAAAGT
GTAAT---AATAAGACATTTAAT------GGAACAGGGCCATGCACAAAT----------------------
-GTCAGC---ACAGTACAATGTACACATGGAATTAAGCCAGTAGTATCAACT---
CAACTGCTGTTAAATGGCAGTCTAGCAGAAGAAGAG-----------
ATAGTAATTAGAACTGAAAATTACACAAACAAT---------GCTAAAATCATAATAGTACAGCTGAAT----
-----------GAATCTGTAATA---------ATTAATTGTACAAGACCCAACAACAATACAAGAAAA-----
-AGTATACCAATG------------GGACCGGGGAGAGCC---ATG------TATGCAACAGGAGGC------
---------ATAGTAGGAAATATA------------AGACTAGCACATTGT---AACATTAGTGGAGAA---
AAGTGGAATGACACTTTAAGACGGATAGCTATAAAATTACAT---------------AAACAATTT-------
-----------------------------------AATAAAACAATAGTCTTTAAT------
CAATCCTCAGGAGGAGACTCAGAAATTATA---ATGCACACTTTTAATTGTGCAGGGGAA---
TTTAAGCAGCTCCAGGCAAGAGTCCTGGCTGTGGAAAGATATCTAAGGGATCAACAGCTCCTAGGAATTTGGG
GTTGCTCTGGAAAACTCATCTGCACCACTAATGTGCCTTGGAACACTAGTTGGAGT----------------

----------------------------AGCAAATCAGAGAAAGACATTTGG------AAT------
AACATGACCTGGATGGAGTGG---GAGAAAGAAATTGACAACTACACAGACTTAATATAC---
AACTTACTTGAAAAATCGCAAATCCAGCAAGAA--

AAGAATGAACAAGACTTATTGTCATTGGATAAGTGGACAAATTTGTGGTCTTGGTTTGACATAACAAACTGGC
TGTGGTATATAAGAATATTCATAATGATAGTAGGGGGTTTAATAGGTTTAAGAATAGTTTTTACT---
GTATTTTCTATAGTG---------AATAGAGTTAGGCAGGGATACTCACCA------
TTATCATTCGCTGTTAGTTTGCTTAATGCAACAGCTGTAGCAGTAGCTGAG---
GGACAGATAGGATTATAGAAGTAGCACAAGGATTTGGTAGAGCT-----------------------------
--------ATCCTTCACATA------CCTAGAAGAATAAGACAGGGATTAGAAAGGGCTTTGCTA---TAA
>EF637047
GCT---GCA--
GAACAATTGTGGGTCACAGTTTATTATGGGGTACCTGTGTGG---AAAGAGGCAACT---
ACCACTCTATTTTGTGCATCAGATGCTAAAGCATATGACACAGAG---GCACATAAT----------------
--------------GTCTGGGCCACA---CATGCCTGTGTACCCACAGAC---
CCTGATCCAAATGAAATAGTATTGGGA---------AATGTGACAGAAAATTTTAAC----------------
--------------------------ATGTGGAAAAACAACATGGTAGAACAGATGCATAAGGATATA---
ATCAGTCTATGGGATGAGAGCCTA---AAGCCATGTGTGAAATTA---
ACCCCCCTCTGTGTCACTTTAAATTGCACTGATGTGTGTGACAATAATACTCAA-------------------
----
-------------------------------ACTAATTGTTCTACTATTAATTCAGAAATGAGA---
AACTGCTCTTTCGATATTGACACAAAAAATAGGA-----------------------------
AATATACGAAAAGAACATGCAATT---TTTAATAAACTTGATATAGTACCAATAGAGCAAGAAGAACCGCAT-
----
-------------------------AGAAGATATATGTTGAGAAATTGTAACACCTCAGTCATTACGCAG---
GCCTGCCCAAAGACATCCTTTGAACCAATTCCTATACATTTCTGTGCCCCGGCTGGTTTTGCGATTCTAAAGT
GTAAT---AATAAAACGTTCAAT------GGAAAAGGACCATGTACAAAT-----------------------
-GTCAGC---ACAGTACAATGTACACATGGAATTAGGCCAGTAGTGTCAACT---
CAACTGCTGTTAAATGGCAGTCTAGCAGAAGAAGAG------------
GTAATAATTAGGTCTGAAAATTTCACGGACAAT---------GTTAAAAACATAATAGTACAGCTAAAT----
-----------GAGTCTGTAGAA---------ATTAATTGTACAAGACCCAACAACAATACAAGAAAG-----
-AGCATACATATG------------GGATGGGGAAGAGCA---TTT------TATGCAACAGGAAAG------
---------ATCATAGGAAATATA------------AGACAAGCATATTGC---AACATTAGTAGAACA---
AAATGGAGTGACACTTTAAAAAGAATAGCTATAAAATTAAGA---------------GAACAATTTAAG----
-----------------------------------AATAAAACAATAGTTTTTAAT------
TCATCCTCGGGAGGGGACCCAGAAATTGTA---ACACATAGTTTTATTTGTGGAGGGGAA---
TTTAAGCAGCTCCAGGCAAGAATCCTGGCTGTGAAAAGATACCTAAAGGATCAACAGCTCCTAGGGATATGGG
GTTGCTCTGGAAAACTCATCTGCACCACTGCTGTGCCTTGGAATACTAGCTGGAGT-----------------
----------------------------AATAAATCTCAGGATAACATTTGG------AAT------
AACATGACCTGGATGGAGTGG---GAGAGAGAAATAGACAATTACACAAATACAATATAC---
AGGTTACTTGAAGAATCGCAGAACCAACAAGAC--

AAGAATGAACAAGAGTTATTAGAATTGGATAAGTGGGCAAGTTTGTGGAGTTGGTTTAGCATAACAAACTGGC
TGTGGTATATAAGAATATTCATAATGATAGTAGGGGGCTTAGTAGGTTTAAGAATAGTTTTTACT---
GTGATTTCAATAGTG---------AATAGAGTTAGGAAGGGATACTCACCA------
TTATCATTCGCTGTTAGTTTGCTTAATGTCACAGCTATAGTAGTAGCTGAG---
GGACAGATAGACTTATAGAAGCAGCACAAGGAGTTGGTAGAGCT-----------------------------
--------ATTATCCACATA------CCTACAAGAATAAGACAGGGCTTAGAAAGGGCTTTGCTA---TAA
>EF637046
GCT---ACA---AAA------------------------------------
GAACAATTGTGGGTCACAGTCTATTATGGGGTACCTGTGTGG---AAAGAAGCAACT---
ACCACTTTATTTTGTGCATCAGATGCGAAAGCATATGAAACAGAG---ATGCATAAT----------------
--------------GTTTGGGCCACA---CATGCCTGTGTACCCACAGAC---
CCTAATCCACAAGAAGTAGTATTGGAA---------AATGTGACAGAAAATTTTAAC----------------
--------------------------ATGTGGAAAAATGACATGGTGGACCAGATGCATGAGGATATA---
ATCAGTTTATGGGATCAAAGCCTA---AAGCCATGTGTGAAATTA---
ACCCCAATCTGTGTTATTTTACATTGCACTAATTATACTAATACTAGTGCTAATACCTTTAGTAGTCCT----
----
-------------------CTTACCAATGATAGCAGGTGGGAAGATATGAAGGAAGAAATAAAA---
AACTGCTCTTTCAATATCACCACAGGCATAAAA---------------------------

GATAAGGTGAAAAAAGAACATGCACTT---TTTAATAGACTTGATGTAGTACCAATAGATAATGCA-------
----

GAGAATGGTAGTAGGACAAGCTATATGTTAAGAAATTGTAACACCTCAATGATTACACAG---
GCCTGTCCAAAGGTATCCTTTGAACCAATTCCCATACATTATTGTGCCCCGGCTGGTTTTGCGATTCTAAAGT
GTAAT---AATAAAACATTCAAT------GGATCAGGGCCATGTAAAAAT----------------------
-GTCAGC---ACAGTACAATGTACACATGGAATTAGGCCAGTAGTGTCCACT---
CAACTGCTGTTAAATGGCAGCCTAGCAGAAGAGGAG-----------
ATAGTAATTAGATCTAAAAATTTCACGGACAAT---------GCTAAAATTATAATAGTACAACTGAAT----
-----------ACATCTGTAGAA---------ATTAAGTGTACAAGACCCAACAACAATACAAGAAAA-----
-AGTATACATATG------------GGATGGGGGAGAGCA---TTT------TATGCAACAGGAGAA------
---------ATAATAGGAAATATA------------AGACAAGCACATTGT---AATATTAGTGGAACA---
AATTGGACTAACACTTTAAGGCAGGTAGCTCAAAAATTAGAA---------------GAATTTAAT-------
-----------------------------------AATAAAACAATAGTCTTTAGT------
CCATCTTCAGGAGGGGACCCAGAAATAGCA---ATGCACAGTTTTAATTGTGGAGGGGAA---
TTTAAGCAGCTCCAGGCAAGAGTCCTGGCTGTGGAAAGATTCCTAAGGGATCAACAGCTCCTAGGGATTTGGG
GTTGCTCTGGAAAACTCATTTGCACCACTGCTGTGCCTTGGAATGCTAGTTGGAGT-----------------
---------------------------AATAAATCTTTGAGTGAGATTTGG------GAT------
AACATGACCTGGATGGAGTGG---GAAAAAGAAATTGACAATTACACAGGCTTAATATAC---
TCCTTACTTGAAGACTCGCAAAACCAACAAGAA---------------------------------------
----
AAGAATGAACAAGACTTATTGGCATTGGATAAGTGGGCAAGTTTGTGGAATTGGTTTAACATATCAAACTGGC
TGTGGTATATAAAAATATTCATAATGATAGTAGGAGGTTTGATAGGTTTAAGAATAGTTTTTACT---
GTGCTTTCTGTAGTA---------AATAGAGTTAGGCAGGGATACTCACCA------
TTATCGTTCGCTATTAGTTTGCTTAATTACACAGCTATAGCAGTAGCTGAG---
GGGACAGATAGGATCATAGAAGTAGTACAAAGAACTGGTAGAGCT----------------------------
--------ATTCTCCACATA------CCTAGAAGAATAAGACAGGGCTTAGAAAGGGCTTTGCAA---TAA
>JN692450
GCT---ACA---
GAACAGTTGTGGGTCACAGTCTATTATGGGGTACCTGTGTGG---AAAGAAGCAACC---
ACCACTCTATTCTGTGCATCAGATGCTAAAGCATATGAAACAGAG---GTACATAAT---------------
--------------GTTTGGGCTACA---CATGCCTGTGTACCCACAGAC---
CCTAACCCACAAGAAGTAGTATTAGCA---------AATGTGACAGAAGATTTTAAC---------------
--------------------------ATGTGGAAAAATAACATGGTGGAACAGATGCATGAGGATGTA---
ATCAGCTTATGGGATCAAAGCCTA---AAGCCATGTGTAAAATTA---
ACCCCACTCTGTGTTACTTTACATTGCACTAATTGGAATGGTACAAATACCACTAGTTCTAAT---------
----
-------------------------ACCACCACTATTGAGGATAAAATGAGGGAAGAAATACAA---
AACTGCTCTTTCAACATAACCACAGGGATAGGA--------------------------
AAAAAGGTGAGAAAAAAACTATGCATTT---TTCAATAAACTTGATATAGTACCAATAGAGGATGAT-------
----

GATAATAATAATACTAGCTATATGTTGATAAATTGTAACACCTCAGTCATTACACAG---
GCCTGTCCAAAGGTATCCTTTGAACCAATTCCTATACATTTTTGTGCCCCGGCTGGTTTTGCGATTCTAAAGT
GTAAT---AATAAAACGTTCAAT------GGAACAGGACCATGTAATAAT----------------------
-GTCAGC---ACAGTACAATGTACACATGGAATTAGGCCAGTAGTGTCAACT---
CAACTGTTGTTAAATGGTAGTTTGGCAGAAGAAGAA-----------
ATAATAATTAGATCTGAAAATATCTCAGACAAT---------GCTAAAAACATAATAGTACAGCTGAAA----
-----------GAAACTGTGCAA---------ATTAATTGTACAAGACCCAACAACAATACAAGGAAA-----
-AGTATACCTATG------------GGATTTGGGAGAGCA---TTT------TATGCAACAGGAGAC------
---------ATAATAGGAGATATA------------AGACAAGCACATTGT---AACATTAGTAGAACA---
AAATGGAATAATACTTTAAAACAAATAAGTAGAAAATTAAGA---------------GAATATTTTAAT----
-----------------------------------CATAATACAACAATCTTTGCT------
AATGCTTCAGGAGGAGACCCAGAAATTATA---ATGCACAGTTTTAATTGTGGAGGAGAA---
TTTAAGCAGCTCCAGGCGAGAGTCCTGGCTGTGGAAAGATACCTAAGGGATCAACAGCTCCTAGGGATTTGGG
GTTGCTCTGGAAAACTCATTTGCACCACTTCTGTGCCTTGGAATGATAGTTGGAGT----------------
---------------------------AAAAGCAATGTAACTACAATCTGG------GAT------
AACCTGACCTGGATGCAGTGG---GAGAGAGAAATTGACAATTACACAGGCTTAATATAC---
ACCTTACTTGAAGAATCGCAGAACCAACAAGAA---------------------------------------
----

AAGAATGAACAAGATTTATTGGCCTTGGATAAGTGGGCAAATTTGTGGAATTGGTTTAACATAACAAATTGGC
TGTGGTATATAAAAATATTCATAATGATAGTAGGAGGCTTAATAGGATTAAGAATAATTTTTACT---
GTACTTTCTATAGTG---------AATAGAGTTAGGCAGGGATACTCACCA------
TTATCATTAGCAGTTAGTTTGCTCAACGCCACAGCTATAGCAGTAGCTGAG---
GGGACAGATAGGGTAATAGAAATAGTACAAAGAATTGGCCGAGCT----------------------------
--------ATTCTCCACATA------CCTAGAAGAATAAGACAGGGCTTAGAAAGGGCTTTGCTA---TAA
>JN692451
GCT---GCA--
GATCAATTGTGGGTCACAGTCTATTATGGGGTACCTGTGTGG---AAAGAAGCAACC---
ACCACTCTATTTTGTGCCTCAGATGCTAAAGCATATGACACGGAG---GCACATAAT----------------
--------------GTCTGGGCCACA---CATGCCTGTGTACCCACAGAC---
CCTAACCCACAAGAAGTAGTATTGACA---------AATGTAACAGAAAAGTTTAAC----------------
--------------------------ATGTGGAAAAATGACATGGTGGAACAGATGCAGGAGGATGTA---
ATCAGTTTATGGGATGAAAGCCTA---AAGCCATGTGTAAAACTA---
ACCCCACTCTGTGTTACTTTAAACTGCACTGATTTTAATACTACTAATACCAATAATAGTACTGCTTCTAATA
CC---
-------------AATAATAGTACTAGTACTAGTTGGAAAGAGATGGAGGCGGGAGAAATAAAA---
AACTGCTCTTTCAATGTCACTGCAAGCATAGAT---------------------------
AAGAAGGTGCGAAAAGACTATGCACTT---TTTAATAAACTTGATGTAGTACCAATAGATAAT----------
----

GATAGTAATAATACAAGCTATATGTTGATAAATTGTAACACCTCAGTCATTACACAG---
GCTTGTCCAAAGATATCCTTTGAACCAATTCCCATACATTATTGCACCCCAGCTGGTTTTGCGATTCTAAAGT
GTAAT---GATAAGAAGTTCAAT------GGAACAGGACCATGTAAAAT------------------------
-GTCAGC---ATAGTACAATGTACACATGGAATTAGGCCAGTAATGTCAACT---
CAGCTGCTGTTAAATGGCACCTTAGCAGAAGAAGAG------------
GTAGTAATTAGATCTGAAAATATTTCAGACAAT---------GTTAAAACCATAATAGTACAGCTAAAT----
-----------GAAACTGTAGAA---------ATTAATTGTACAAGACCCAACAACAATACAAGAAAG-----
-AGTATACAGCTG------------GTAGGGGGGAGAACA---CTT------TATGCAACAGGAAAA------
---------ATAATAGGAGAGATA------------AGACAAGCACATTGT---AACATTAGTGCAACA---
AAGTGGACTAACGCTTTAAACCAGACAGTTACAAAATTAAGA---------------GAACAATTT-------
-----------------------------------AATAAAACAATAATCTTTACT------
CAATCTTCAGGAGGGGACCCAGAGATAACA---ATGCACACTTTTAATTGTGGAGGGGAA---
TTTAAACAGCTCCAGGCAAGAGTCCTGGCTGTGGAAAGATACCTAATGGATCAACAGCTCCTAGGGATTTGGG
GTTGCTCTGGGAAACTCATTTGCCCCACTACTGTGCCTTGGAATACTAGTTGGAGT-----------------
----------------------------AATAAATCTTATCAGGCTATTTGG------AAC------
AACATGACATGGATGCAATGG---GAAAAAGAAATTGACAATTACACAGAATTAATATAC---
AGCTTACTTGAAGAATCGCAAAACCAACAAGAA--

AAGAATGAACAGGAATTATTGGAATTGGATAAGTGGGCAAGTTTGTGGAATTGGTTCTCTATAACAAATTGGC
TGTGGTATATAAAAATATTCATAATGATAGTAGGAGGCTTAATAGGTTTAAGAATAGTTTTTACT---
GTACTTTCTATAGTA---------AATAGAGTTAGGCAGGGATACTCACCC------
TTATCGTTCGCTGTTAGTTTGCTTAATTTCATAGCTATAGCAGTAGCTGAA---
GGGACAGATAGGATTATAGCAGTAGTACAAAGAGCTGGTAGAGCT----------------------------
--------TTTCTCAACATA------CCAAGGAGAATAAGACAGGGACTCGAAAGGGCTTTGCTA---TAA
>JN692452
GCT---GAA--
AAAGATTTGTGGGTCACGGTCTATTATGGGGTGCCTGTGTGG---AAAGAAGCAACC---
ACCACCCTATTTTGTGCATCAGATGCTAAAGGCTATAATACAGAG---GTACATAAT----------------
--------------GTTTGGCCACA---CATGCCTGTGTACCCACAGAC---
CCTAACCCACAGGAACTAGAATTGAAA---------AATGTAACAGAAGACTTTGAC----------------
--------------------------ATGTGGAAAAATAATATGGTAGAACAGATGCATGAGGATATA---
ATTAGTTTATGGGATCAAAGCCTA---AAGCCATGTGTAAAATTA---
ACTCCACTCTGTGTTACTTTAAATTGTATTAATTGGACTAGTACCAATATCAATAGTAGTAATGTG-------
----
----------------------TGGGCCAATAGTAGTTTGAAATTTAAGGAGGGAGAAATAAAA---
AACTGCTCTTTCAATATCTCCACAAGCATAGGA---------------------------
AATAAGATGCAGAAAAACTATGCACTT---TTCAATAAACTTGATGTAGTTCCAATAGATAAT----------
----

ACAAGTTATATGTTGGTAAATTGTAACACCTCAGTCATTACACAG---
TCTTGTCCAAAAGTATCCTTTGAACCAATTCCCATACATTATTGTGCCCCGGCTGGTTTTGCGATTCTAAAGT
GTAAT---GATAAAAAATTCAAT------GGAAAAGGACAATGTAAAAAT-----------------------
-GTTAGC---ACAGTACAATGTACACATGGAATTAAGCCAGTAGTATCAACT---
CAACTACTGTTAAATGGCAGCCTAGCAGAAGAAGAG------------
ATAGTAATTAGATCTGAAAACATCACAAACAAT---------GCTAAAACCATAATAGTACATCTGAAT----
-----------AAATCTGTAGAA---------ATTAATTGTACAAGACCCAACAACAATACAAGAAAA-----
-GGTATACATATA------------GGACCGGGGAGAACA---TTT------TATGCAACAGGACAA------
---------ATAATAGGAGATATA------------AGACAGGCACATTGT---AACATTAGTGGAGGA---
AAATGGAATAACACTTTAAAACAGATAGTTATAAAATTAGGA---------------GAACAATTTAAG----
-----------------------------------AACAAAACAATAGTCTTTAAT------
CAGTCCTCAGGAGGGGACCCAGAAATTGTA---ATGCACAGTTTTAATTGTGGAGGAGAA---
TTCAAGCAGCTCCAGGCAAGAGTCCTGGCTGTGGAGAGATACCTACAGGATCAACAGCTCCTAGGAATTTGGG
GGTGCTCTGGAAGACTCATTTGCACCACTAATGTGCCTTGGAATAGGACTTGGAGT-----------------
----------------------------AATAAATCACGTGAGGACATTTGG------GGT------
AACATGACTTGGATGCAGTGG---GAGAAGGAAATTGACAATTACACAGACATAATATAC---
ACATTACTTGAAACATCGCAACACCAGCAAGAA--

AAGAATGAACAAGAATTATTGGAATTAGATAAATGGGCAAGTTTATGGACTTGGTTTGACATATCAAATTGGC
TGTGGTATATAAGAATATTCATAATGATAGTAGGAGGCTTAGTAGGTTTAAGAATAGTTTTTGCT---
GTACTTTCTATAGTG---------AATAGAGTTAGGCAGGGATACTCACCA------
TTATCGTTCGCTGTTGGTTTGCTCAACGCCACAGCTATAGCAGTAGGTGGG---
GGGACAGATGGGATTATAGAAGTAGTAAGAAGACTTTGTGGAGCT----------------------------
--------GTGCTCAACATA------CCTACAAGAATAAGACAAGGCTTAGAAAGGGCTTTGCTA---TAA
>JN692453
GCA---GAA---
AATTTGTGGGTCACAGTCTATTATGGGGTACCTGTGTGG---AAAGAAGCAACC---
ACCACTCTATTTTGTGCATCAGATGCTAAAGCATATGACACAGAG---GCACATAAT----------------
--------------GTTTGGCCACA---CATGCCTGTGTACCCACAGAT---
CCTAACCCACAGGAAGTGGAATTGGAA---------AATGTGACAGAAAATTTTAAC----------------
--------------------------ATGTGGAAAAATGACATGGTAGAACAGATGCAGGAGGATATA---
ATCAGTTTATGGGATCAAAGCCTA---AAGCCATGTGTAAAATTA---
ACCCCACTCTGTGTTACTTTAAATTGCACTGATGTAAAGAATAATGGTACCAACAATAGTAGTATTGTT----
----
----------------AATATTACCACTAGTAATAATAGGGAAGAGATGAAGGGAGAAATGAAA---
AACTGCTCTTTCAATGTCACCACAAGAATAGGA---------------------------
GGTAAAATGCAGAAAGAATATGCAGTT---TTCTATAAACTTGATGTAGTACCAATAGATAAT----------
----

GATAATACAAGCTATACATTGATAAGTTGTAACACCTCAGCCACTACACAT---
GCCTGTCCAAAGATATCCTTTGAACCAATTCCAATACATTATTGTGCCCCGGCTGGTTTTGCGATTCTAAAGT
GTAAA---GATAAGAAGTTCAAT------GGAACAGGACCATGTAAAAAT-----------------------
-GTCAGC---ACAGTACAATGTACACATGGAATTAGGCCAGTAGTGTCAACT---
CAATTGCTGTTGAATGGTAGTCTAGCAGAAGAAGAG------------
ATAGTAATTAGATCTGAAAATTTCTCGGATAAT---------GCTAAAACCATAATAGTACAGTTGAAC----
-----------AAATCTGTGGAA---------ATTAATTGTACAAGACCCAATAACAATACAAGAAGA-----
-AGTATACCTATA------------GGACCAGGGAGAGCA---TTG------TATACAACAGGAGAA------
---------ATAATAGGAGATATA------------AGACAAGCACATTGT---AACGTTAGTAAACAA---
AATTGGACAGAAACTTTAAGACAGATAGCTAGAAAATTAAAA---------------GAACAATTTAAC----
-----------------------------------AGCACAAACATAATCTTTAAG------
CAATCCTCAGGAGGGGACCCGGAACTAGTA---ATGCACAGTTTTAATTGTGGAGGGGAA---
TTTAAGCAGCTCCAGGCAAGAGTCCTGGCTGTGGAAAGATACCTAAGAGATCAACAGCTCCTGGGGATTTGGG
GTTGCTCTGGAAAACTCATCTGCACCACTGCTGTGCCTTGGAATAATAGTTGGAGT-----------------
-------------------------AAGAATAAATCTCTGGACAACATTTGG------AAT------
AACATGACCTGGATGCAGTGG---GAGAGAGAAATTGACAATTACACAAACTTAATATAC---
ACTTTGCTTGAAGAATCGCAGAACCAACAAGAA--

AAGAATGAACAAGAATTATTGGAATTGGATAAGTGGGCAAGTCTATGGAATTGGTTTGATATAACAAACTGGC
TGTGGTATATAAGGATATTCATAATGATAGTAGGAGGCTTAATAGGTTTAAGAATAGTTTTTACT---
GTACTTTCTATAGTG---------AATAGAGTTAGGAAGGGATACTCACCA------

TTATCATTCGCTGTTAGTTTGCTTAATGCCATAGCTATAGCAGTAGCTGAG---
GGGACAGATAGGGTTATAGAAGTAGTACATAGGGCTTGTAGAGCT-------------------------
--------ATTCTCCACATA------CCTAGAAGAATAAGGCAGGGCTTAGAAAGGGCTTTAATA---TAA
>JN692457
GCT---GAA---AAA------------------------------------
GACAACTGGTGGGTCACAGTCTATTATGGGGTACCTGTGTGG---AAAGAAGCAACC---
GCCACTCTATTTTGTGCATCAGATGCTAAAGCATATGATACAGAG---GCACATAAT----------------
--------------GTTTGGGCCACA---CATGCCTGTGTACCCACAGAC---
CCTAACCCACAAGAATTAAAAATGGAA---------AATGTGACAGAAAATTTTCAC----------------
-------------------------ATGTGGAAAAATAGCATGGTAGAACAGATGCAGGAGGATATA---
ATCAGTTTATGGGATCAAAGCCTA---AAACCATGTGTAAAATTA---
ACCCCACTTTGTGTTACTTTAAATTGCAGTAATAGTGTAAATGCCACTATTAGTGCTAGTGAGAAC-------
----
----------------------AGAACTTTTGATTGGGAACAAGGGATGGAAAAAGAAATACAA---
AACTGCTCTTTCAATGTAACCACAAGCATAGGA---------------------------
CAAAAAGTGCGGAAGGAACATGCACTT---TTTAATAGACTTGATATAGTGCCAATAGATAAT----------
----

AGTACAAACACAAGCTATATATTGATAAGTTGTAACACCTCAGTCATTACACAA---
GCCTGTCCAAAGATATCCTTTGAACCAATTCCCATATATTATTGTGTCCCGGCTGGTTTTGCGATTCTCAAGT
GTAAT---GATAAGAAGTTCAAT------GGAACAGGACCATGTAAGAAT----------------------
-GTCAGC---ACAGTACAATGCACACATGGAATTAGGCCAGTAGTGTCAACT---
CAACTGCTGTTAAATGGCAGTTTAGCAGAAGAAGAG------------
GTAATAATTAGATCTGAAAATATCACAGACAAT---------ACCAAAAACATAATAGTGCAGTTGAAT----
-----------GATACCGTACAA---------ATTAATTGTACAAGACCCGGCAACAATACAAGAAGG-----
-AGTATATCTATG------------GGACCGGGGAGAGCA---TTT------TATACAACAGGAGAC------
---------ATAATAGGAAATATA------------AGACAAGCACATTGC---ACCCTTAGTAGAGCA---
CACTGGAACAACACTTTAAAACAGATAGTTATAGCATTGAAA---------------AAACACTTT-------
-----------------------------------AATAAAACAATAAAATTTAAT------
CAATCCGCAGGAGGGGACCCAGAAATTGTA---ATGCACAGTTTTAATTGTGGAGGGGAA---
TTTAAACAGCTCCAGGCAAGAGTCCTTGCTGTGGAAAGATACCTACGGGATCAACAGCTCCTAGGGATTTGGG
GTTGCTCTGGAAAACTCATTTGCACCACTGCCGTGCCTTGGAATATTAGTTGGAGT-----------------
-------------------------AACAGGACAAATTTGACTGCAATTTGG------AAT------
AACATGACCTGGATGCAGTGG---GAGAGAGAAATTGACAATTACACAAACATAATATAC---
AACCTACTTGAGGAATCGCAAGTCCAACAGGAA---------------------------------------
----
AAGAATGAACAAGAATTATTGGAATTGGACAAATGGGCAAGCTTGTGGACTTGGTTTGACATAACAAATTGGC
TGTGGTATATAAGAATATTCATAATAATAGTAGGAGGCTTAATAGGTTTAAGAATAGTTTTTACT---
GTACTTTCTATAGTG---------AATAGAGTTAGGCAGGGATACTCACCA------
TTGTCATTCGCTGTTAGTTTGTTTAATGCCACAGCTATAGTAGTAGCTGAG---
GGGACAGATAGAGTAATAGAAGTAGTACAAAGAATTGGCTTAGCT-------------------------
--------TTTCTCCACATA------CCTAGACGAATAAGACAGGGCCTCGAAAGGGCTTTGCTA---TAA
>FJ195090
GCT---GCA---GAA------------------------------------
GAAAAATTGTGGGTCACAGTCTATTATGGGGTGCCTGTGTGG---AAAGAAGCAAAC---
ACCACTCTATTTTGTGCATCAGATGCTAAAGCATATAGCACAGAG---GCACATAAT----------------
--------------GTTTGGGCCACA---CATGCCTGTGTACCCACAGAC---
CCCAACCCACAAGAAGTAGTATTGGAA---------AATGTGACTGAAAATTTTAAC----------------
-------------------------ATGTGGAAAAATGACATGGCAGAACAGATGCATCAGGATATA---
ATCAGTTTATGGGATGAAAGCCTA---AAGCCATGTGTAAAATTA---
ACCCCACTCTGTGTTACTTTAAATTGCACTGAATTGAGTATTACTACTTGTACCAATAGTAGTACCAATAGTA
GCAAT---
----------AGTGCTGGTAGTAATAATACTTGTATAGATGAACAGATGAGGGGGGAAATAAAA---
AACTGCTCTTTCAATATCACCACAAGCATAAAG---------------------------
AATAAAATGCAGAAGGAATATGCACTT---TTTTATAAACTTGATATAGCACCAATAGATGAT----------
----

GATAATACAAGCTATACATTGATAAGTTGTAACACCTCAGTCATTACACAG---
GCCTGTCCAAAGACATCCTTTGAACCAATTCCCATACATTATTGTATCCCGGCTGGTTTTGCGATTCTAAAGT
GTAAT---GATAAGAAATACAGT------GGAACAGGACCATGTAAAAAT----------------------

-GTCAGC---ACAGTACAATGTACACATGGAATTAGGCCAGTAGTGTCAACT---
CAACTACTGTTAAATGGCAGTCTAACAGAAGAAGAC------------
ATAGTAATTAGATCTGAAAATTTCACAGACAAT---------GCTAAAATTATAATAGTACACCTGAAC----
-----------GAGTCTGTAGAG---------ATTAATTGTACAAGACCCAACAACAATACAAGAAAA-----
-AGTATACATATG------------GGATGGGGAGAGCA---TTT------TATGCAACAGGAGAA-------
---------ATAATAGGAGATATA------------AGACAAGCACATTGT---AACCTCAGTAAAGCA---
AAGTGGGAAACCACTTTAAAAAGGGTAGCTATAAAATTAAGA---------------GAACAATAT-------
-----------------------------------AACAAAACAATAGCCTTTAAT------
AAATCCTCAGGAGGGGACCCAGAAATTGTA---ATGCACACTTTTAACTGTGGAGGGGAG---
TTTAAGCAGCTCCAGGCAAGAGTCCTGGCTGTGGAAAGATACCTAAGGGATCAACAGCTCCTGGGAATTTGGG
GTTGCTCTGGAAGACTCATTTGCACCACTGCTGTGCCTTGGAATGCTAGTTGGAGT-----------------
----------------------------AATAAATCTCTAAGTAATATTTGG------GAT------
AACATGACCTGGATGGAGTGG---GAAAGAGAAATTGGCAATTACACAGAATTAATATAC---
AGTTTAATTGAAGAATCGCAGAACCAACAGGAA--

AAGAATGAACAAGAATTATTGGCATTGGACAAGTGGGCCGATTTGTGGAATTGGTTTGACATATCAAAATGGC
TGTGGTATATAAGACTATTCATAATGATAGTAGGAGGCTTAATAGGTTTAAGAATAGTTTTTACT---
ATAATTTCTTTAGTG---------AATAGAGTTAGGCAGGATACTCACCA------
TTATCGTTCGCTGTTAATTTGTATAATACTATAGCTATAGCAGTAGCTGAG---
GGACAGATTGGGTTATAGAAGTAGTACAAAGACTTGGCAGAGCT-----------------------------
--------TTTATCCACATC------CCTAGGAGAATAAGACAGGGCTTAGAAAGAATTTTGCTA---TAA
>JN692459
GCT---ACA--
CAACAATTGTGGGTCACAGTCTATTATGGAGTACCTGTGTGG---AAAGAAGCAACC---
ACCACTCTATTTTGTGCATCAGATGCTAAAGCATATGAGACAGAG---GCACATAAT----------------
--------------GTTTGGGCCACA---CATGCCTGTGTACCCACAGAC---
CCTAATCCTCAAGAAATAAACCTGGCA---------AATGTGACAGAAAATTTTAAC----------------
--------------------------ATGTGGAAAAATAACATGGTAGAACAGATGCATGAGGATATA---
ATCAGTTTATGGGACCAAAGCCTA---AAGCCATGTGTAAAATTA---
ACCCCACTCTGTGTTGTTTTAAATTGCACTGATGCGCAAGATGGTATTATTGCAAAAAATGATAATAGT----
----
-------------------ACTACTAATAGGACTTTTAAGGAAGGGATGAATAAAGAAATAAGA---
AACTGTTCTTTCAATATCTCCACAGGCATAGGA---------------------------
AACAAAGTAAGGGAAGATTATGCACTT---TTTAATCTGCTTGATGTAGTACCAGTAGAAAAT----------
----

AATAATAGAAGCTATATATTGAAAAATTGTAACACCACAGTCATTACACAG---
GCATGTCCAAAAGTATCCTTTGAACCAATTCCCATACATTATTGTGCCCCGGCTGGCTTTGCGATTTTAAAAT
GTAAT---AATAATACGTTCAGT------GGAACAGGGCCATGTACAAAT-----------------------
-GTCAGT---GTAGTACAATGTACACATGGAATTAGGCCAGTAATATCAACT---
CAACTGCTGTTAAATGGCAGTCTAGCAAAAGATGAG------------
GTAGTAATTAGATCTGAAAATTTCACCAACAAT---------ATTAAAAACATAATAGTACAGCTGAAC----
-----------AAAACTGTAGTA---------ATTAATTGTACAAGACCCGGCAACAATACAAGAAGA-----
-AGTATACATATA------------GCCCCAGGGAGAGCA---TTT------TATGCAAGAGGAGAT-------
---------ATAATAGGAGATATA------------AGACAAGCACATTGT---AACCTTAGTAAAGCA---
GACTGGGTAGGGCTCTAACAAAGGTAGCTAAAAAAATTAAGA---------------GAAAAATTTAAA----
-----------------------------------AATAAAACAATAATTTTTACA------
AATTCCTCAGGAGGGGACCCAGAAATTGTA---ATGCACAGTTTTAATTGTGGAGGGGAA---
TTTAAGCAGCTCCAGGCAAGAGTCCTAGCTGTGAAAAGATACCTAAGGGATCAACAGCTCCTAGGGATTTGGG
GTTGCTCTAGAAAACTCATTTGCACCACTGCTGTGCCTTGGAATACTAGCTGGAGT-----------------
----------------------------AATAAAAATCTAAGTGCAATTTGG------GAT------
AATATGACCTGGATGCAGTGG---GAGAAAAAAATTGACAATTATACAGAACTAATATAC---
TCCTTACTTGAAAAATCGCAAAACCAACAAGAA--

AAGAATGAGCAAGAATTATTGAAATTGGATAAGTGGGCAAACTTGTGGAACTGGTTCGACATATCAAAATGGC
TGTGGTATATAAAAATATTCATAATGATAGTAGGAGGCTTAGTAGGTTTAAAAATAGTTTTTACT---
GTACTTTCTATAGTA---------AATAAAGTTAGGAAGGATACTCACCA------
TTGTCATTCGCCATTAGCTTGCTTAATGCTACAGCTATAGCAGTAGCTGAG---
GGACAGATAGGATCATAGAAGCAGTACAAAGAATTGGTAGAGCT-----------------------------
--------ATTCTTCACATA------CCCAGAAGAATCAGACAGGGCTTAAAAAGGCTTTTACTA---TAA

>JN692460
GCT---ACA--
AAACCATTGTGGGTCACAGTCTATTATGGGGTACCTGTGTGG---AAAGAAGCAACC---
ACCACTCTATTTTGTGCATCAGACGCTAAAGCATATGACACAGAG---GCACATAAT----------------
--------------GTTTGGGCCACA---CATGCCTGTGTACCCACAGAC---
CCTAACCCACAAGAAGTAGTGTTGGAA---------AATGTGACAGAAAATTTTAAC----------------
--------------------------ATGTGGAAAAATAACATGGTAGAACAGATGCATGAGGATGTA---
ATCAGTTTATGGGATCAAAGCTTA---AAGCCATATGTAAAGTTA---
ACCCCACTTTGTGTTACTCTAAATTGCAGTAATGTAATTTGCACTGGGAATAGCACTAGTAATAGTAATTGTA
GTAGC--
-------GCTGGTATTGATACTGGTATGAAAAATAGTAGCGCTGGTACTGATACTGGTATGAAA---
AACTGCTCTTTCAACATCACCACACCCATAAGA---------------------------
GGTAGGGTGCGAAAGGAATATGCAATT---TTTGACAAACTTGATGTAGTGCCAATAAATGAAAATAAT----
----

AATAATGATAGTAAGGCAGAATATATGTTGGTAAATTGCAACACTTCAGTCATTACACAG---
GCCTGTCCAAAGATATCCTTTGAACCAATCCCCATACATTATTGTGCCCCGGCTGGTTTTGCGATTCTACAAT
GTAAT---GAAAAGACGTTCAAT------GGAACAGGACCATGTAAAAAT-----------------------
-GTCAGC---ACAGTACAATGTACACATGGAATTAGGCCAGTGGTGTCAACT---
CATCTGCTGTTAAATGGCAGTCTGGCAGAAGAAGAG------------
ATAGTAATTAGATCCGAAAATCTCACAAACAAT---------GCTAAAACCATAATAGTACAGCTGAAC----
-----------GAAACAGTAGAC---------ATTAATTGTACAAGACCCAACAACAATACACAAAGA-----
-AGTATAAATATA------------GGACCAGGGAGAGCA---TGG------TATACAACAGGAGCT------
---------ATAATAGGAGATATA------------AGACAAGCATATTGT---ACAGTTAATAAAACA---
AAATGGGAAAACACTCTAAAACAGATAGTTATAAAATTAGCA---------------AAACAATTTCCT----
-----------------------------------AATAAAACAATAGGCTTTAAG------
CAACCCTCAGGAGGGGACCCAGAAGTTACA---ATGCACACTTTTAATTGTGGAGGAAAA---
TTTAAGCAGCTCCAGGCAAGAGTCCTGGCTGTAGAAAGATACCTAAGGGATCAACAGCTCCTTGGGATTTGGG
GTTGCTCTGGAAAACTCATTTGCACCACTTCTGTGCCTTGGAATAATAGTTGGAGT-----------------
-------------------------AATAAATCTCTAGATGACATTTGGAAA------ACA------
AACATGACCTGGATGGAGTGG---GAGAGAGAAATTGACAATTATACAGTCGAAATATAC---
AAATTACTTGAAGAATCGCAGAACCAACAAGAA--

AAGAATGAACAAGACTTATTGGCATTGGATAAGTGGGCAGGTCTGTGGAATTGGTTTAGCATAACAAATTGGC
TGTGGTATATAAAAATATTCATAATGATAGTAGGAGGCTTAATAGGTTTAAGAATAGTTTTTACT---
GTACTG------------------AATAGAGTTAGGCAGGGATACTCACCA------
TTGTCATTCGCTGTTAGCTTGCTTAATACCATAGCTATAGCAGTAGCTGAG---
GGGACAGATAGGATCATAGAAGGAGCACAAACCATTGGTAGAGCC----------------------------
--------ATTCTCCACATA------CCTAGAAGAATAAGACAGGGCGCAGAAAGGGCTTTGCAA---TAA
>JN692463
ACT---GCA---GAA------------------------------------
CAACTATTGTGGGTCACAGTCTACTATGGGGTACCTGTGTGG---AAAGAAGCAACC---
ACCACTCTATTTTGTGCATCAGATGCTAAAGGATATGATACAGAG---GTACATAAT----------------
--------------GTTTGGGCCACA---CATGCCTGCGTACCCACAGAC---
CCCAACCCACAAGAAGTAGTATTGGAA---------AATGTGACAGAAAGGTTTGAC----------------
--------------------------ATGTGGAAAAATAACATGGTAGAGCAGATGCATGAGGATGTA---
ATCAGTTTATGGGATCAAAGCCTA---AAGCCATGTGTAAAATTA---
ACCCCACTCTGTGTTACCTTAAATTGCAGTAATGCTAATTTCACTAATTCTAATAGTACTAAT----------
----
-------------------------AATAATAGTAGTATTAAGATGGGGGAGGGAGAAGTAAAA---
AACTGCTCGTTCAATATCACTGCAGGCATAAGA---------------------------
GATAAGATACAGAAAGAATATGCACTG---TTATATAAACTTGATATAGTACCAATAGATAAT----------
----

AGTAGTACTAGCTATAGGTTAATAAGTTGTAACACCTCAGTCATTACACAG---
GCCTGTCCAAAGGTAACCTTTGAGCCAATTCCTATACATTATTGTGCCCCAGCTGGTTTTGCGATTCTAAAGT
GTAAT---GATAAGCGGTTCAAT------GGAACAGGACAGTGTAAAAAT-----------------------
-GTCAGC---ACAGTACAGTGTACACATGGAATTAAGCCAGTAGTATCAACT---
CAATTCCTGTTAAATGGCAGTCTAGCAGAAGAAGAG------------
GTAGTAATTAGATCTGAAAACATCACAGACAAT---------GGTAAGAACATCATAATACAGCTGAAC----

-----------GAAACTGTAATA---------ATTAACTGTACAAGACCCGGCAACAATACAAGAAAA-----
-AGTATACATATA------------GGACCAGGAAGAGCA---TTT------TATGCAACAGGAGAC------
---------ATAATAGGAGATATA------------AGACAAGCACATTGT---AACCTTAGTAGAGAA---
GCTTGGAATAACACTTTATACAAGGTAGTTAAAAAATTAAGA---------------GAACAATTT-------
-----------------------------------AATAAAACAATAATCTTTAAC------
TCATCCTCAGGAGGGGACCAAGAAGTTGTA---ATGCACAGTTTTAATTGTGCAGGGGAG---
TTTAAGCAGCTCCAGGCAAGAGTCCTGGCTGTGGAAAGATACCTACAGGATCAACAGCTCCTAGGGATTTGGG
GTTGCTCTGGAAGGCTCATCTGCACCACTAATGTGCCTTGGAATTCTAGTTGGAGT-----------------
----------------------------AATAAATCTCTAAATTATATTTGG------GAT------
AACATGACTTGGATGGAATGG---GAAAGAGAAATTGATAATTACACAGGATTCATATAC---
ACTTTACTTGAAGAATCGCAGAACCAACAAGAA---

AAGAATGAACAAGACTTATTGGAATTAGATAAATGGGCAAGTTTGTGGAATTGGTTTAGCATAACAAACTGGC
TGTGGTATGTAAAAATATTCATAATGATAGTAGGAGGCTTGATAGGTTTAAGAATAATTTTTACT---
GTACTTTCTATAGTG---------AATAGAGTTAGGCAGGGATACTCACCA------
TTATCGTTGGCTGTTAGCTTGCTCAACGCCACAGCCATAGCAGTAGCTGAG---
GGGACAGATAGGATTATAGAAGTATTACAAAGACTTTTTAGAGCT----------------------------
--------ATTCTCAACATA------CCTAGAAGGATAAGACAGGGCCTCGAAAGGGCTTTGCTA---TAA
>JN692467
GCT---GCA--
GAAAAGTTGTGGGTCACAGTTTATTATGGAGTGCCTGTGTGG---AAAGATGCAAAC---
GCCACTCTATTTTGTGCATCAGAGGCTAAATCAGTTGACACAGAG---GCACATAAT----------------
--------------GTTTGGGCCACA---CATGCCTGTGTACCCTCAGAC---
CCAAACCCACAGCAGATAGTCTTGGGG---------AATGGGACAGGATCTTTTAACTA--------------
--------------------------TGTGAGAAAAACAACATGCTGGAGCAGATGCAGGTGGTTAGG---
ATCAGTTTATGGGATCAAAGAGAA---AAGCCAAGAGCAAAAGGA---
ACTACGGTATGTGTTACTCTTTTGGCACCAATGGTGGAATACTACTGCGAATACTACTGCCAATAC-------
----
-------------TACAGACAAT--ATTATTCGGGTGGAAAAAATGGAGGAGGGAGAAATAAAA---
AACTGCTCTTTCAATCTCTCCTCAAACATAGGA--------------------------
AATAGGAGGCATAGAGAATATGCACTT---TTTTATAGACTTGATGTAATGTCAATAGCTAAT----------
----

GATAGTACAAGCTATACACTGATAAATTGCAACACCTCAGTCATTACACAG---
GCCTGTCCAAAGATAACTTTTGAACCAATTCCCATACATTATTGTGCCCCAGCTGGTTTTGCGATTCTAAAGT
GTAAT---GATAAGAAATTCAAT------GGATCAGGACCATGTACAAAT-----------------------
-GTCAGC---ACAGTACAATGTACACATGGAATTAGGCCAGTAGTGTCAACT---
CAACTATTGTTAAATGGCAGTCTAGCAGAAGAAGAA------------
GTAGTAATTAGATCTGAAAATTTCACGGACAAT---------GCTAAAAACATAATAGTACAGCTGAAA----
-----------GAAACTGTAGAA---------ATAAATTGCACAAGACCTAATAACAATACAAGAAAA-----
-AGTATACATCTA------------GGGCCAGGTAAAGTA---TTG------TATGCAACAGGGGAA------
---------ATAATAGGAGATATA------------AGAAAAGCACATTGT---AGCCTTAATAGAACA---
AAATGGAATAACACTTTAGAACAGATAATTAAAAAACTAGGA---------------GAACAATTTAAG----
-----------------------------------AATAAAACAATAACCTTTAAG------
CCTTCCTCAGGAGGGGACCCAGAAATTGAA---ACACACAGTTTTAATTGTGCAGGGGAA---
TTTAAGCAGCTCCAGGCGAGAGTCCTGGCTCTGGAAAGATACCTAAAGGATCAACAGCTCCTAGGGATTTGGG
GTTGCTCTGGAAAACTCATCTGCACCACTGCTGTGCCTTGGAATGATAGTTGGAGT-----------------
----------------------------AATAAATCTTTGAATGACATTTGG------GAT------
AAATTGACCTGGATGGAGTGG---GAGAAAGAAATTGATAATTACACAGGCCTAATATAC---
ACCTTACTTGAAGAATCGCAAAACCAGCAAGAA---

AAGAATGAACAAGATTTATTGCAATTGGATAAGTGGGACAGTTTGTGGAATTGGTTTGACATAACAAGTTGGC
TGTGGTACATAAAAATATTCATAATGATAGTAGGAGGCTTAATAGGTTTAAGGATAGTTTTTGCT---
GTATTTTCTATAGTG---------AGTAGAGTTAGGCAGGGATATTCACCA------
TTATCGTTCGCTATTAGTTTGCTTAATGCCACAGCTATAGCAGTAGCTGAA---
GGGACAGATAGGATTATAGAAATAGTGCAAAGATTTTGTAGAGCT----------------------------
--------ATTCTCCACATA------CCTACAAGAATAAGACAGGGCCTCGAAAGGGCTTTGCTA---TAA
>JN692475
GCT---GCA--
GAACAATTATGGGTCACAGTCTATTATGGAGTACCTGTGTGG---AAAGAAACAACC---

ACCACTTTATTTTGTGCCTCAGATGCTAGAGCATATGACACAGAG---GCACATAAT----------------
--------------GTTTGGGCCACA---TCTGCCTGTGTACCCACAGAC---
CCTAGCCCACAAGAAGTAGTAATGGAA---------AATGTGACAGAAAACTTTAAC----------------
--------------------------ATGTGGAAAAATAACATGGTAGAACAGATGCATAAGGATATA---
ATCAGTTTATGGGATCAAAAGCTA---AAGCCATGTGTAACATTA---
ACCCCATTCTGTGTTACTTTAAATTGCACTAATGCTAATAGTACTCATGCTAATAAGACCAATACC-------
----
----------------------ACTGCTCCTACTACTACCACTGCTGTAGGTGAAGAAATAAAA---
CACTGCTCTTTCAATGTCACAGATAACATAGGA------------------------------
AAGGTACGAACAGAACGTGCATAT---TTTAATAAGCTTGATGTAGTACCAATAGATAATGATAAT-------
----
-------------AATAATGGTAGTGGAAGATATATGTTGATAAGTTGTAACACCTCAGTCATTACACAG---
GCCTGTCCAAAGATATCCTTTGACCCAATTCCCATACATTATTGTGCCCCGGCTGGTTTTGCGATTCTAAAAT
GTAAT---GATAAAATGTTCAAT------GGAACAGGACCATGTACAAAT-----------------------
-GTCAGC---ACAGTACACTGTACACATGGAATTAGGCCAGTAGTATCAACC---
CAGTTGTTATTAAATGGCAGTCTAGCAGAAGAAGAG------------
ATAGTAATTAGATCTGAAAATTTCACGAACAAT---------GCTAAAACCATAATAGTACAGTTGAAC----
-----------AAAACTGTAGTA---------ATTAATTGCACAAGACCCAACAACAACACAAGAAAA-----
-AGTATACATATG------------GGACCAGGGAAATCA---TTT------TATGCAACAGGAGAT------
---------ATAATAGGAGATATA------------AGACAAGCACATTGT---AATGTTAGTAGCATG---
AAGTGGACTAACACCTTACAACAGATAGTGATAAAATTAGCA---------------
GAACAATTTAATATAACAGGA------CAA------------------GATAATAAAACAATAAGCTTTAAT-
-----CGATCCGCAGGAGGGGACCCAGAAATTGTA---ATGCACAGTTTTAATTGTGGAGGGGAA---
TTTAAGCAGCTCCAGGCAAGAGTCCTAGCTGTGGAAAGATACCTAAAGGATCAACAGCTCCTAGGGATTTGGG
GTTGCTCTGGAAAACTCATCTGCACCACTAATGTGCCTTGGAATACTACCTGGAGT-----------------
----------------------------AAGAAATCTCTGAATGAAATTTGG------AAT------
AACATGACCTGGATGCAGTGG---GAAAGAGAAATTGATAATTACACAGGCTTAATATAC---
AACTTACTAGAAGAATCGCAAATCCAACAAGAA--

AAGAATGAAAAAGACTTACTGGAATTGGATAAGTGGGCAAATTTGTGGAATTGGTTTGACATATCAAACTGGC
TGTGGTATATAAGAATATTCATAATGATAATAGGAGGCTTAATAGGTTTAAGAATAGTCTTTACT---
GTACTCTCTATAGTA---------AGAAGAGTTAGGCAGGGATACTCACCA------
TTGTCATTCGCTATTAATTTGCTGAATTGCACAGCTATAGCAGTAGCTGAG---
GGGACAGATAGGATTATAGAAGTATTACAAAGAACTGGTAGAGCT----------------------------
--------ATTTGCAACATA------CCTAGAAGAATAAGACAGGGCTTAGAAAGGGCTTTGCAA---TAA
>FJ195091
GCT---GCA--
GACAAATTATGGGTCACAGTTTATTATGGGGTGCCTGTGTGG---AAAGAAGCAACC---
ACCACTCTATTTTGTGCATCAGATGCTAAAGGATATGATACAGAA---GTACATAAT----------------
--------------GTGTGGGCCACA---CATGCTTGTGTACCCACAGAC---
CCTAACCCACAGGAAATGGTATTGGCA---------AATGTGACAGAGAATTTTAAC----------------
--------------------------ATGTGGAAAAATGACATGGTGGACCAGATGCATGAGGATATA---
ATCAGTTTATGGGATCAATTCTTA---AAGCCATGTGTAAAATTA---
ACCCCACTCTGTGTTACTTTAAATTGTTCTACTTTGAGTACAAAGTGCAATAATACT----------------
----
-------------------------------GGTACTAATAATAGCCGTTGCGAAGAAATGAAA---
AACTGCACTTTTAATATCACCACAGGCATAGGA---------------------------
GGTAAGATGAAAAAAGAATATGCACTT---TTTTATACAGGTGATATAGTATCAATAGAGGGA----------
----

GGTAATAAGACTAGCTATATGTTGATAAATTGTAATACCTCAGTCATTAAACAG---
GCCTGTCCAAAGGTAACCTTTGAACCAATTCCTATACATTATTGTGCCCCGGCTGGTTTTGCGATTTTAAAGT
GTAAT---GATAAGAAGTTTAAT------GGATCAGGACCATGTACAAAT-----------------------
-GTCAGC---ACAGTACAATGTACACATGGAATTAGGCCAGTAGTGTCAACT---
CAACTGTTGTTAAATGGCAGTCTAGCAGAAGAAGAT------------
ATAGTAATTAGATCTGAAAATTTCACGGACAAT---------GCTAAAAGCATAATAGTACAGCTGAAT----
-----------AAAACTGTAGTA---------ATTAATTGTACAAGGCCTAACAACAATACAAGAAAA-----
-AGTATACATATG------------GGATGGGGAAGAACA---TTT------TATGCAACAGGAGAC------
---------ATAATAGGAGACATA------------AGACAAGCACACTGC---AATCTCAGTAGAGCA---
GACTGGAACAGCACTTTACAACAAATAACTGCAAAATTAAAA---------------GAACAATTTAAG----

---------------------------------AATCAAACAATAGTTTTTAAT------
CAATCCTCAGGAGGGGACCCAGAAATTGTA---AAGCACAGTTTTAATTGTGGAGGGGAA---
TTCAAGCAGCTCCAGGCAAGAGTCCTGGCTGTGGAAAGATACCTAAAGGATCAACAGCTCCTAGGGATCTGGG
GTTGCTCTGGAAAACTCATTTGCACCACTAATGTGCCTTGGAATAATAGTTGGAGT--------------
---------------------------AATAGAAACTTGAGTAAGATTTGG------GAT------
AACATGACCTGGATGCAATGG---GAAAAAGAAATTGACAATTACACAGACATAATATAC---
CAATTGCTTGAAAAATCGCAAAACCAGCAAGAA-------------------------------------
----
AAGAACGAACAAGAATTATTGGAATTGGATAAATGGGAAGTTTATGGAATTGGTTTGACATAACAAACTGGC
TATGGTATATAAAAATATTCATAATGATAGTAGGAGGCTTAATAGGTTTAAGAATAGTTTTTGCT---
GTATTTTCTATAGTG---------AATAAAGTTAGGCAGGGATACTCACCA------
TTGTCGTTCGCCATTAATTTGCTTAACACAATAGCTATAGCAGTAGCTGAG---
GGGACAGATAGAATTATAGAAGTAGGGCAAAGAATTGGTAGAGCT-------------------------
--------ATCATTCACATA------CCTAGAAGAATAAGACAAGGCGCAGAAAGGGCTTTGCAG---TAA
>FJ195088
GCT---ACA--
GAAAAATTGTGGGTCACAGTCTATTATGGGGTACCTGTGTGG---AAAGAAGCAACC---
ACCACTCTATTTTGTGCATCAAATGCTAAAGCATATGACACAGAG---GTACATAAT-------------
--------------GTTTGGGCCACG---TATGCCTGTGTACCCGCAGAC---
CCTAATCCACAAGAAGTAGTACTGGAA---------AATGTGACAGAATACTTTAAC--------------
-------------------------ATGTGGAAAAATACAATGGTAGAACAAATGCATGAGGATATA---
ATCAGTTTATGGGATCAAAGCCTA---AAGCCATGTGTAAAATTA---
ACTCCGCTCTGTGTTACTTTAAATTGTATTAATGCTAATAAGACTGCTGACAATAGT-------------
----
---------------------------AGTAGTAGTGGGGAAAATCTGAAGGAAGAAATAAAA---
AACTGCTCTTTCAATATCACCACAAACAGAAGA--------------------------
ACTAAGGTACAAAGAGAGTATGCATTT---TTTAATAGACTTGATGTAGTACCAATAGATAAT----------
----

AGTAATACAAGCTATATGTTGATACATTGTAACACCTCAGTCATTACACAG---
GCCTGTCCAAAGATATCCTTTGAACCAATTCCCATACATTATTGTACCCCAGCTGGTTTTGCGATTCTCAAGT
GTAAT---GATAAAAAGTTCAAG------GGAACAGGACCATGTACAAAC---------------------
-GTCAGT---ACAGTACAATGTACACATGGGATTAAGCCAGTAGTGTCAACT---
CAACTGCTGTTGAATGGCAGTCTAGCAGAAGGAGAG-----------
ATAGTAATCAGATCTGAAAATTTCACGAACAAT---------GCTAAAACCATAATAGTACAGCTGAAC----
-----------AAGTCTGTAGAA---------ATTAATTGTACAAGACCCAACAACAATACAAGAAAA-----
-AGTATACATCTA------------GGATGGGGGAGAGCA---TTT------TATGCAACAGGAGAA------
---------ATAATAGGAAATATA------------AGACAAGCATCTTGT---AACCTTAGTGGAACA---
GACTGGGAGAGCACTTTAAAACAGATAGTAGAAAAGTTAAGA--------------GAACAATTTAAA----
---------------------------------AATAAAACAATATCCTTTAAT------
CAATCCTCAGGAGGGGACCCAGAAATAGTA---ATGCACAGCTTTAATTGTGGAGGGGAA---
TTTAAGCAGCTCCAGGCAAGAGTCCTGGCTGTGGAAAGATACCTAAGGGATCAACAGCTCCTAGGGATTTGGG
GTTGCTCTGGAAAGCTCATCTGCACCACTACTGTGCCTTGGAATGCTAGCTGGAGT--------------
---------------------------AATAAATCTCTGAATATGATTTGG------GAT------
AACATGACCTGGATGGAGTGG---GAGAAAGAAATTAACAATTACACAGGCTTAATATAC---
AACTTACTTGAAGAATCGCAAAACCAACAAGAA-------------------------------------
----
AAGAATGAACAAGAATTATTGGAATGGGATAAGTGGGCAAATTTGTGGAATTGGTTTGACATAACAAACTGGC
TGTGGTATATAAAAATATTCATAATGATAGTAGGAGGCTTAGTAGGTTTAAGAATAGTTTTTACT---
GTACTTTCGATAGTG---------AATAGAGTTAGGAAGGGATACTCACCA------
CTGTCATTCGCTGTTAGTTTGCTTAATGCCACAGCTATAGCAGTAGCTGAA---
GGGACAGATAGGATTATAGAAATAGTACAAAGGACTGGTAGAGCT-------------------------
--------ATTCTCCACATA------CCTACAAGAATAAGACAGGGCTTAGAAAGGGCTTTGCTA---TAA
>FJ195089
GCT---AAA---GAA------------------------------
GAAGAAGATTTGTGGGTTACAGTCTATTATGGGGTACCTGTGTGG---AAAGAAGCATCC---
ACCACTCTATTTTGTGCATCAGATGCTAAAGCATATGACACAGAG---GCACATAAT-------------
--------------GTTTGGGCCACA---CGTGCCTGTGTACCCACAGAC---
CCTAAACCACAAGAAATAGTAATGGAA---------AATGTGACAGAAAATTTTAAC--------------
-------------------------ATGTGGAAAAATAACATGGTAGAACAGATGCATGAAGATATA---

ATCAGTTTATGGGATGAAAGCCTA---AAGCCATGTGTAAAATTA---
ACCCCAATTTGTGTAACTTTAAAATGCACTAATGTGACGTTGAATAATAACAGTACAGAG------------
----
----------------------------AATAATAACAGTACTATAGACTTGGAAGAGGTAAGA---
AACTGCTCTTTCAATATAACAAGAGGCATGGGA-------------------------
GGAAAGGTGCGGAAGGACTATGCCATT---TTTAATAAACTTGATATAGTACCAATAGGTGGT----------
----

AGTAATAATACAAGTTATATGTTAATAAATTGTAACACCTCAGTCATTACACAG---
GCCTGTCCAAAGGTATCCTTTGAACCAATTCCCATACATTATTGTGCCCCGGCTGGTTTTGCGATTGTAAAGT
GTAAT---GATAAAAGTTTCAAT------GGAACAGGACCATGTACAAAT----------------------
-GTCAGT---ACAGTACAATGTACACATGGAATTAGGCCAGTAGTATCAACT---
CAACTGCTGTTAAATGGCAGTTTAGCAGAGGGAGAT-----------
GTAATAATTAGATCTGAAAATTTCACTAATAAT---------GCTAAAACCATAATAGTACAGCTGAAT----
-----------GAAACTGTAGAA---------ATTATTTGTATAAGACCCCACAACAATACAAGAAGG-----
-AGTATACATATG------------GGATGGGGGAAAACG---TTC------TATGCAACAGGAGAA------
---------ATAATAGGAGATATA------------AGACAAGCACATTGT---AACATTAGTTTTACA---
AAATGGAATAACACTTTAAAACAGATAGTTAGAAAATTAAGG--------------GAACAATTT-------
-----------------------------------AATAAAACAATAATCTTTAAT------
TCATCCTCAGGAGGAGACCTAGAAATTGTA---ATGCACACTTTTAATTGCGGAGGGGAA---
TTTAAGCAGCTCCAGGCAAGAGTCCTGGCTGTGGAAAGATACCTACAGGATCAACAGCTCCTAGGGATTTGGG
GTTGCTCTGGAAAACTCATCTGCACCACTGCTGTGCCTTGGAATACTAGTTGGAGT-----------------
----------------------------AATAAGTCTTATAATCAGATTTGG------AAT------
AACATGACCTGGATGCAGTGG---GAGAAAGAAATTGACAATTACACACAACAAATATAC---
ACCTTACTTGAAGAATCGCAGTTCCAACAAGAA---------------------------------------
----
CACAATGAACAAGAATTATTGCAATTGGATAAATGGGCAAGTTTGTGGAATTGGTTTAGTATAACACAATGGC
TGTGGTATATAAAAATATTCATAATGATAGTAGGAGGCTTAGTAGGTTTAAGAATAGTTTTTACT---
GTAATTTCTTTAGTG---------AATAGAGTTAGGCAGGGATACTCACCA------
TTGTCATTCGCTGTTAGTTTGCTTAATGCCACAGCTATAGCAGTAGCTGAG---
GGGACAGATAGGGTGATAGAAGTATTACAAAGAATTGGCAGAGCA---------------------------
--------GTACTCCACATA------CCTAGAAGAATAAGACAGGGCTTAGAACTAGCTTTACAA---TAA
>JN692479
GCT---GCA---------------------------------------
GAACAGTTGTGGGTTACAGTCTATTATGGGGTACCCGTGTGG---AAAAATGCAACC---
ACCACTCTATTTTGTGCATCGGATGCTAAGGCATATCATCCAGAG---GCACATAAT----------------
--------------ATTTGGGCCACA---CATGCCTGTGTACCCACAGAC---
CCTAACCCACAAGAAATAAACTTGCAG---------AATGTGACAGAATATTTTAAC----------------
--------------------------ATGTGGAATAATAACATGGTAGAACAGATGCATGAGGATATA---
ATCAGTTTATGGGATCAAAGCCTA---AAGCCATGTGTAAAATTA---
ACCCCGCTCTGTGTCACTTTAAATTGCACTGATGCTAACACTACTACTAGTACCAATAGTAGTAGG-------
----
-------------------ACCAATAGTAGTGAAAAACTAAAAATGGAGAAGGGAGAAATGAAA---
AACTGCTCTTTCAATATCACCGAAAGCATAAAA-------------------------
AATAGGGTGAAGAAAGAATATGTACTT---TTTGATAGACTTGATATAGTCCAACTAGATAGTGAT-------
----

GATAATACAAGCTATGCATTAAGAAGTTGTAACACCTCAGTCATTACACAG---
GCCTGTCCAAAGATATCCTTTGAACCAATTCCCATACATTATTGCACCCCAGCTGGTTTTGCGATTCTAAAGT
GCAAT---GATAAAAACTTCAAG------GGATCAGGACCATGTACAAAT----------------------
-GTCAGC---ACAGTACAATGTACACATGGAATTAAACCAGTAGTGTCAACT---
CAACTACTGTTAAATGGCAGTCTAGCAGAAGGAGGA---------
GAAGTAGTACTTAGGTCAAAAAAATTTCACAGACAAT---------ACTAAAACCATAATAGTACAGCTGCAT-
--------------AACAATGTAACA---------ATTAATTGTCTAAGACCTAACAACAATACAAGAAAA--
----AGTATACACATA------------GCACCAGGGAGAGCA---TTT------TATGCAACAGGAGCT---
------------ATAATAGGAGATATA------------AGACGAGCACACTGT---AACATCAGTAAAAAA-
--GCTTGGAATGACACTTTAAGACTGGTAGCTAACAAACTAAGA---------------GAACAATTTAAT--
-------------------------------------AATAAGATAATAGTCTTTAAC------
CACTCCTCAGGAGGAGACCCAGAAATCGTA---ATGCACACTTTTAATTGTGGAGGGGAA---
TTTAAGCAGCTCCAGGCTAGAGTCCTGGCTATAGAAAGATACCTAAAGGATCAACAGCTCCTAGGAATTTGGG

GTTGCTCTGGAAAACTCATTTGCACCACTAATGTGCCTTGGAATACTAGTTGGAGT-----------------
----------------------------AATAAATCTCTGGACAGCATTTGG------GGT------
AACATGACCTGGATGGATTGG---GATAGAGAAATTAGCAAGTACTCACAATCAATATAC---
ACCTTAATTGAAAAATCGCAAAACCAGCAGGAA--

AAGAATGAACAAGAATTATTAGAATTAGATAAGTGGGCAAGTTTGTGGAATTGGTTTGACATATCAAATTGGC
TGTGGTATATAAAAATCTTCATAATGATAGTAGGAGGTTTAGTAGGCTTAAGAATAGTTTTTGCT---
GTACTTTCTATAGTA---------AATAGAGTTAGGCAGGGATACTCACCA------
TTATCATTTGCTGTTAATTTGCTTAATACCACAGCTATAGCAGTAGCAGAG---
GGGACAGATACGATTATAGAAGTAGCACGGACAATTGGTAGAGGT----------------------------
--------TTTCTCCACGTA------CCTAGAAGAATAAGACAGGGCTTAGAAAGGGCTTTGCTA---TAA
>HQ377443
GCT---GAA---GAA------------------------------------
GACAAATTGTGGGTCACAGTCTATTATGGAGTACCTGTGTGG---AAAGAAGCAACC---
ACCACTCTATTTTGTGCATCAGAGGCTAAAGCATATGATACAGAG---GTACATAAT----------------
--------------GTTTGGGCCACA---CATGCCTGTGTACCCACAGAC---
CCTAGCCCACATGAACAAGTATTGGGA---------AATGTGACAGAAAACTTTAAC----------------
-------------------------ATGTGGAAAAATAACATGGTAGACCAGATGCATGAAGATATA---
AAGAGTTTATGGGATCAAAGCTTA---AAGCCATGTGTAAAATTA---
ACTCCACTCTGTGTTACTTTAAATTGCTCTAATTATTTGAAGAATGTTACAAATACCACCAGTACC-------
----
----------------------AACAATATTACTGGTTTGGGAAATATGGTGGGAGAAATAAAA---
AACTGCTCTTTCAATGTCACTACAGGCATAGGA--------------------------
AACAAGGTAAAGAAAGAACATGCACTT---TTTAATAGACTTGATATAGTAAAATCAGACAAT----------
----

GATAGTACAAGCCATATATTGGTAAATTGTAACACCTCAGTCCTTACACAA---
GCCTGTCCAAAGGTATCCTTTGAACCAATTCCCATACATTATTGTGCCCCGGCTGGTTTTGCGATTCTAAAGT
GCTCT---GATCCAAAGTTTAAT------GGAACAGGACCATGTACAAAT-----------------------
-GTCAGT---ACAGTACAATGTACACATGGAATTAGGCCAGTAGTATCAACT---
CAATTGCTGCTAAATGGCAGTTTAGCAGAAGAAGAG-----------
GTAATAATTAGATCTGAAAATATCTCAAACAAT---------GCCAAAACCATAATAGTACAGCTGAAC----
-----------AGCTCTGTAGAA---------ATTAACTGTACAAGACCCAATAACAATACAAGAAAA-----
-AGTATACATATA------------GGACCAGGGAAAGCA---TTT------TATGCAACAGGAGAT------
---------ATAATAGGAGATATA------------AGACAAGCACATTGT---AACATTAGTAGCACA---
GCTTGGAACAACACTTTAAAAAAGATAGTCGAAAAATTAAGA---------------GAACACTTT-------
-----------------------------------AATAAAACAATAATCTTTAGT---
CCTCCTGTCACAGGAGGGGACCCAGAAATTGTA---ATGCACAGTTTTAATTGTGGAGGGGAG---
TTTAAACAGCTCCAAGCAAGAGTCCTGGCTGTGGAAAGATACCTAAAGGATCAACAGCTCCTAGGGATTTGGG
GTTGCTCTGGAAAACTCATTTGCACCACTGCTGTGCCTTGGAATACTAGTTGGAGT-----------------
----------------------------AGTAAAACTCATGATGAAATTTGG------AGT------
AACATGACCTGGATGCAGTGG---GAGAGAGAAATAGACAATTACACAGACATAATATAT---
GGGTTACTTGAGAAATCGCAAAACCAGCAAGAA--

AAGAATGAACAGGAATTATTGGAATTGGATAAGTGGACAAGTTTGTGGAGTTGGTTTGACATAACAAATTGGC
TGTGGTATATAAAAATATTCATAATGATAGTAGGGGGCTTAGTAGGTTTAAGAATAGTTTTTACT---
GTACTTTCTATAGCG---------AATAGAGTTAGGCAGGGATACTCACCA------
TTGTCGTTCGCTATTAGCTTGCTCAATACCACAGCAATAGTAGTAGGTGAG---
GGGACAGATAGAATTATAGAAGCTTTGCAAAGAGCTGGTAGAGCT----------------------------
--------ATTCTCCATATA------CCTAGAAGAATAAGACAAGGCTTAGAAAGGGCTTTGCTA---TAA
>HQ377487
GCT---ACA---------------------------------------
GAGGAATTGTGGGTCACAGTCTATTATGGGGTACCTGTGTGG---AAAGAAGCAACC---
ACTACTTTATTTTGTGCATCGGATGCTAAAGCATATGGCACGGAG---GTACATAAT----------------
--------------GTTTGGGCCACA---CATGCCTGTGTACCCACAGAC---
CCCAACTCACAAGAAATAGTATTGGAA---------AATGTAACAGAAAATTTTAAC----------------
-------------------------ATGTGGAAAAATAACATGGTAGAACAGATGCATGAAGATGTA---
ATCAGTTTATGGGATCAAAGCCTA---AAGCCATGTGTAAAATTA---
ACCCCACTCTGTGTTACTTTAAATTGCACTGATTGGAAGAAAAATGGTACTGATGTCAATAATGCTAGTGCTA
CAACCCCTCCTACGTCTACGTCTACCACCAGT---

----AGTGCTTTGAGAAATAGGGCGGAAGAAATAGATTTGAAAAATATGACGGGAGAAATAAAA---
AACTGCTCTTTCAATATCTCCACAAGCATAAAG------------------------
AATAAGATACAAAAACAGTATGCACTG---TTTAATATACTTGATGTAGTGCCAATAGAGAAT---------
----

AATACTACAGAATATATGTTGATAAATTGTAACACCTCAGTCATTACACAG---
GCCTGTCCAAAGGTATCCTTTGAACCAATTCCCATACATTATTGTGCCCCGGCTGGTTTTGCGATTCTAAAGT
GTAAT---AATAAGAAGTTCAAT------GGAACAGGACCATGTACAAAT----------------------
-GTCAGT---ACAGTACAATGTACACATGGAATTAAGCCAGTAGTGTCAACC---
CAACTGCTGTTAAATGGCAGTCTAGCAGAAGAAGAT------------
GTAGTAATTAAATCTGAAAACTTCACGAACAAT---------GCTAGAGCCATAATAGTACAGTTGAAT----
-----------GAAACTGTAGGA---------ATTAATTGTACAAGACCCGGCAACAATACAAGAAAA-----
-AGTATACCTATA------------GGACCGGGGAGAGCA---TTT------TATGCAACAGGAGAT------
---------GTAATAGGAGATGTA------------AGAAAAGCACATTGT---AACATTAGTGGAGGA---
AAATGGGAAGAAACTTTAAAAAGGATAGCTACAAAATTCACA---------------GAACAATTTAAT----
----------------------------------AAGAGCACAATAATATTTAAA------
CAATCCTCAGGAGGGGACCCAGAAATTGTA---ATGCACAGTTTTAATTGTGGAGGGGAA---
TTTAAGCAGCTCCAGGCAAGAGTCCTAGCTGTGGAAAGATACCTAAGGGATCAACAGCTCCTAGGGATTTGGG
GTTGCTCTGGAAAACTCATTTGCACCACTAATGTGCCTTGGAATAGTAGTTGGAGT-----------------
---------------------------AATAAATCTCTGGATAAAATTTGG------GAT------
AACATGACCTGGATGGAGTGG---GAGAAAGAAATTGACAATTACACAGGCTTAATATAC---
AACTTACTTGAAGAATCGCAGAACCAGCAAGAA-------------------------------------
----
AAGAATGAACAAGAATTATTAGCATTGGATAAATGGGCAAGTTTGTGGAATTGGTTTGACATAACAAACTGGC
TGTGGTATATCAGAATATTCATAATGATAGTAGGAGGCTTAGTAGGTTTAAGAATAGTTTTTCT---
GTGCTTTCTATAGTA---------AATAGAGTTAGGAAGGGATACTCACCC------
TTATCATTCGCTATTAGTTTACTTAATGCCACAGCTATTGCAGTAGCTGAG---
GGAACAGATAGGGTTATAGAAGTAGTACAAAGAACTGGTAGAGCT---------------------------
--------ATTCTCAACATA------CCTAGAAGAATAAGACAAGGATTTGAAAGAGCTTTGCTA---TAA
>JN687739
GCT---CAC--
GAACAATCGTGGGTCACAGTCTATTATGGGGTACCTGTGTGG---AAAGAAGCAACC---
ACCACTCTATTTTGTGCATCAGATGCTAAAGCATATGACACAGAG---GTACATAAT---------------
--------------GTTTGGGCCACA---CATGCCTGTGTACCCACAGRC---
CCTAATCCACAAGAAGTAGTATTGGGA---------AATGTGACAGAAAATTTTAAC---------------
-------------------------ATGTGGAAAAACAACATGGTAGAACAGATGCATAAGGATATA---
ATCAGTTTATGGGATGAAAGCCTA---AAGCCATGTGTAAAAATA---
ACCCCAATCTGTGTTACTTTAAATTGCATTAATTGGAATAGTACCAATAGTACCAGTAAT------------
----
------------------------GCCACTAGTAGGGTGGGAAACATAGTGGGAGAAATAAAA---
AACTGCTCTTTCAATGTCACCACAGGCATAGGA-------------------------
GATAAGGTGAAAAAAGACTATGCGCCT---TTTAATATAATTGATATAGTACCAATAGATAAT---------
----

GTAAGTTTTATGTTGATAAATTGTAACACCTCAGTCATTACACAA---
GCCTGTCCAAAGGTATCCTTTGAACCAATTCCCATACATTATTGTGCCCCGGCTGGTTTTGCGATTCTAAAGT
GTAAT---AATAAGGAATTCAAT------GGAACAGGACCATGTAAAAAT----------------------
-GTCAGC---ACAGTACAGTGTACACATGGAATTAAGCCAGTAGTGTCAACT---
CAACTGCTGTTAAATGGCAGTCTATCAGAAGGGGAG------------
GTAATAATTAGATCTGAAAATTTCACAAACAAT---------GCTAAAACCATAATAGTACAGCTGAAT----
-----------GAAACGGTAGAC---------ATTAATTGTACAAGACCCAGCGACAATACAAGAAAG-----
-AGTATACATATG------------GGATGGGGGAGAGCA---TTT------TATGCAACAGGAGAA------
---------ATAACAGGAGATATA------------AGACAAGCACATTGT---AACATCAGTAGAGCA---
AAATGGAACAATACCTTAAGACAGATAGCTATAAAATTAAGA---------------GAACAATTTAGG----
----------------------------ACTAATATTACTACAATAATCTTTAAT------
CGATCCTCAGGAGGGGACCCAGAAATTGTA---ACACACAGTTTTAATTGTGCAGGGGAG---
TTTAAGCAGCTCCAGGCAAGAGTCCTGGCTGTGGAAAGATACCTAAGGGAACAACAGCTCCTAGGACTTTGGG
GTTGCTCTGGGAAAATCATTTGCACCACCGCTGTGCCTTGGAATGCTAGTTGGAGT-----------------
---------------------------AATAAATCTCTGAATGAAATTTGG------AAT------
AACATGACCTGGATGGAGTGG---GAGAGAGAGATTGACAATTACACAGGCTTAATATAC---

AGCTTACTTGAAGAATCGCAAAACCAACAAGAC---------------------------------------
----
AAGAATGAAAAAGAATTATTAGAATTGGATAAGTGGGCAAATTTGTGGAATTGGTTTGACATAACAAACTGGC
TGTGGTATATAAAAATATTCATAATGATAGTAGGAGGCTTAATAGGTTTAAGAATAATTTTTACT---
GTACTTTCTATAGTG---------AATAGAGTTAGGCAGGGATATTCACCA------
TTGTCTTTCGCTGTTAGCTTGCTTAATACAACAGCTATAGCAGTAGGTGAA---
GGGACAGATAGGGTTCTAGAAGGGTTACAAAGAATTGGTAGAGCT----------------------------
--------TTTCTCCACATA------CCTAGAAGAATAAGACAGGGCTTAGAAAGGGCTTTGCTA---TAA
>JF680907
GCT---GCA-----------------------CTA---GCA---GCA---
CCAAACTTGTGGGTCACAGTCTATTATGGGGTACCTGTGTGG---AAAGAAGCAACC---
ACCACCCTGTTTTGTGCATCAGATGCTAAAGCATACAAAACAGAG---GTACATAAT----------------
--------------GTCTGGGCCACG---CATGCCTGTGTACCCACAGAC---
CCCAACCCACAGGAAGTATTATTAGAA---------AATGTGACAGAAAATTTTAAC----------------
--------------------------ATGTGGAAAAATAACATGGTAGACCAGATGCATGAGGATATA---
ATCAGTTTATGGGATCAAAGCCTA---AAACCATGTGTAAAATTA---
ACCCCACTTTGTGTTACTTTAAATTGCACTGATGATTTGAAGAATGATACTGGTGCCCAGAATACTAGTAAT-
----
----------------AGTGCCACTAATAGTAGTTGGGGACATATAGAGAAAGGAGAAATAAAA---
AACTGCTCTTTCAATGTCACCACAAGTATAAGA--------------------------
GGTAAGATAAAGAAGGAACATGCTCTC---TTTTATAGACTTGATGTAGTACCAATAAAAGAA----------
----

AATAGTGATAATACTAGTTATAGATTGATAAGTTGTAATACCTCAGTCATTACCCAA---
GCCTGTCCAAAGGTAACCTTTGAGCCAATTCCCATACATTATTGTGCCCCGGCTGGTTTTGCGATTCTAAAGT
GTAAC---AATAAGACGTTCAAT------GGAACAGGACCATGCACAAAT----------------------
-GTCAGC---ACAGTACAATGCACACATGGAATTAAGCCAGTGGTGTCAACT---
CAACTGCTGTTAAATGGCAGTCTAGCAGAAAAGGAG-----------
GTAGTGATTAGATCTGAAAATTTCACGAACAAT---------GCTAAAACCATAATAGTACAGCTGAAT----
-----------GAATCTGTAGCA---------ATTAATTGTACAAGACCCAACAATAATACAAGAAAA-----
-AGTATACATATA------------GCACCAGGGAAAGCA---TTT------TATGTAACAGGAGAG------
---------ATAATAGGAGATATA------------AGACAAGCACATTGT---AACATTAGTAGAAAA---
CAATGGAATAAAACTTTAGAAAAAATAGTTGAAAAATTAAGA---------------GAACAATTTAAT----
-----------------------------------AACACAAAAATAATCTTTGAG------
CCATCTTCAGGAGGGGACCCAGAAATTGTG---ACTCACAGTTTTAATTGTGGAGGAGAA---
TTCAAACAGCTCCAAGCAAGAGTCCTGGCTGTGGAAAGATACCTAAGAGATCAACAGCTCCTAGGGATATGGG
GTTGCTCTGGAAAACTCATTTGCACCACTAACGTGCCTTGGAACACTAGTTGGAGT----------------
-------------------------AAGAATAAATCTCTGGATGACATTTGG------CAA------
AATATGACCTGGATGGAGTGG---GAGAGAGAAATTGACAATTACACAAGCTTAATATAT---
AACTTACTTGAAGAATCGCAGAACCAACAAGAA---------------------------------------
----
AAGAATGAACAAGAATTATTAGAATTGGATAAGTGGGCAAGTTTGTGGAATTGGTTTACCATAACAAATTGGC
TGTGGTATATAAAAATATTCATAATGATAGTAGGAGGCTTGGTAGGTTTAAGAATAGTTTTAGCT---
GTACTTTCTATAGTA---------AATAGGGTTAGGCAGGGATACTCACCA------
GTATCATTTGCTGTTAGCTTGCTTAATGCCACAGCAATAGCAGTAGCTGAA---
GGAACAGATAGGATTGTAGAAATAGCACAAAGAATTTTTAGAGCT---------------------------
--------TTTCTTCACATA------CCTACAAGAATAAGGCAGGGCTTAGAAAGGGCTTTACTA---TAA
>KU749389
GCT---ACA---
GATAACTTGTGGGTCACAGTCTATTATGGGGTACCTGTGTGG---AAAGAAGCAACC---
ACTACTCTATTTTGTGCATCAGATGCTAAATCATATGACAAAGAG---GTACATAAT----------------
--------------GTTTGGGCCACA---CATGCCTGTGTACCCACAGAC---
CCCAACCCACAAGAAGTAGAATTGGAA---------AATGTGACAGAATATTTTAAC----------------
--------------------------ATGTGGAAAAATAACATGGTAGAACAGATGCATGAGGATATA---
ATCAGTTTATGGGATCAAAGCCTA---AAGCCATGTGTAAAATTA---
ACCCCACTCTGTGTTACTTTAAACTGCACTGATTATAATGGTACTAGTACTAATACCACTAGTAATGCCACTA
ATACCACCAAC--
-------------ACTGCCTCCCCCAATGTTGCCACTGATAAGATAGAGAGACAAGAAATGAAA---
AACTGCTCTTTCAATGTCACCGCAGACACAAGA--------------------------
GATAAGATAAAAAAAGAATATGCAGTT---TTCTATAAACTTGATGTAGAGCCAATAAATAAT----------

----

GATAGTACCAGCTTTAGATTGATAAGTTGTAATACCTCAGTCATTACACAG---
GCCTGTCCAAAGGTATCCTTTGAGCCAATTCCCATACATTATTGTGCCCCGGCTGGTTTTGCGATTCTAAAGT
GTAAA---GATAAGATGTTCAAT------GGAAAAGGACTATGTAAAAAT-----------------------
-GTCAGC---ACAGTACAATGTACACATGGAATTAGGCCAGTAGTCTCAACT---
CAACTGCTGTTAAATGGCAGTTTAGCAGAAGAAGAG------------
GTAATAATTAAATCTGAAAATTTCACAAACAAT---------GCCAGAACTATAATAGTACAGCTGAAT----
-----------GAATCTGTAACA---------ATTAATTGTACAAGACCCAACAACAATATAAGAAAA-----
-GGTATACATTTA------------GGACCAGGGAGTGTA---TTT------CATGTACACCAAAAA------
---------GTAATAGGAGATATA------------AGGAAAGCATATTGT---AACATTAGTAGAGCA---
GAATGGAATAAAACTTTAGAACAAATAGTAAAAAAATTAAGA---------------GAACAATTTAAG----
-----------------------------------AATAAAACAATAGTCTTTAAC------
CAATCCTCAGGAGGGGACCCAGAAATTGTA---ATGCACAGTTTTAATTGTGGAGGGGAA---
TTTAAGCAGCTCCAGGCAAGAGTCCTGGCTGTGGAAAGATACCTAAGGGACCAACAGCTCCTAGGAATTTGGG
GTTGCTCTGGAAAACTCATCTGCACCACTACTGTGCCTTGGAATGCTAGTTGGAGT-----------------
----------------------------AATAAATCTCTGGAAGATATTTGG------GAC------
AACATGACCTGGATGGACTGG---GAAAAAGAAATTGATAATTACACAGGCATAATATAC---
TCTTTGCTTGAAAAATCGCAAAACCAACAAGAA--

AAGAATGAACAAGATTTATTGGCACTAGATAAATGGGCAAATTTATGGAATTGGTTTGACATATCAAATTGGC
TGTGGTATATAAAAATATTCATAATGATAGTAGGAGGCTTGATAGGTTTAAGAATAGTTTTTACT---
GTACTTTCTATAGTG---------AATAGAGTTAGGCAGGGATACTCACCA------
TTGTCATTCGCTGTTAGCTTGCTCAATACCATAGCCATAGTAGTAGCTGAG---
GGGACAGATAGGGTCATAGAAATATTACAGAGGGCTTTTAGAGCT----------------------------
--------ATCCTTCACATA------CCTACAAGAATAAGACAGGGCTTGGAAAGGGCTTTGCTA---TAA
>KT427690
GCT---GAT--
GAAAACTTGTGGGTCACAGTCTATTATGGGGTACCTGTGTGG---AAAGAAGCAACC---
ACCACTCTATTTTGTGCATCTGATGCTAAAGCAGTTGATAAGGAG---GTACATAAT----------------
--------------GTTTGGGCCACA---CATGCCTGTGTACCCACAGAC---
CCTAATCCACAAGAAATAGTATTGGGA---------AATGTGACAGAAAATTTTAAC----------------
-------------------------ATGTGGAAAAACAACATGGTAGAACAAATGCATGAGGATGTA---
ATCAGCTTATGGGACCAAAGTCTA---AAGCCATGTGTAAAATTA---
ACCCCACTCTGTGTGACTTTAGAGTGCAGTAATATAACAGGTAATATT-------------------------
----
-------------------------------------AGTGAGGAAATGAGGACAGAAATAAAA---
AACTGCTCTTTCAATATCACCACAAGAATACAG---------------------------
GATAAGGTGCAAAAACAGAATGCAATT---TTCAACACACTTGATCTAGTACAAATAGAGAAT----------
----

AATAGTAGCTATATGTTGATAAATTGTAACACCTCAGTCATTACACAG---
GCATGTCCAAAGGTAACTTTTGAGCCAATTCCCATACATTATTGTGCCCCGGCTGGTTTTGCGATTCTAAAAT
GTAAT---GATAAGAAATTCAAT------GGAACAGGAACATGCACCAAT-----------------------
-GTTAGC---ACAGTACAATGTACACATGGCATTAGGCCAGTAGTGACAACT---
CAACTGCTGTTAAATGGCAGTCTAGCAGAAGAAGAA------------
GTAATAATCAGATCTGAGAACATCACGAACAAT---------GTTAAAACCATAATAGTACAGTTGAAC----
-----------GAATCTGTAGAG---------ATTAATTGTACAAGACCCAGCAACAATACAAGAAAA-----
-GGTATACATATG------------GGATTTGGGAGAGCA---TTT------TATGCAACAGGAGAA------
---------ATAATAGGAGATATA------------AGACAGGCACATTGC---AATCTTAGTGGTGCA---
CAATGGGAAAGAACTTTAAAACAGGTAGCAAAGAAGTTGAGA---------------GACCAATTTAAT----
-----------------------------------AATAAAACAATAATCTTTAAT------
CAATCTGCAGGAGGGGACCTAGAAATTGTA---ACACACACTTTCAATTGTGGAGGGGAA---
TTTAAACAGCTCCAGGCAAGAATCCTGGCTGTAGAAAGATACCTAAAGGATCAACAGCTCCTAGGGCTTTGGG
GCTGCTCTGGAAAACTCATCTGCACCACTACTGTGCCTTGGAACTCTAGTTGGAGT-----------------
----------------------------AACAAATCTTATGATATAATTTGG------GAG------
AACATGACCTGGATGCAGTGG---GAAAAAGAGATTAGCAAGTACTCAGGCATAATATAT---
GGGTTAATCGAAGACTCGCAGATCCAGCAGGAA--

AAGAATGAACAAGAATTATTGGCATTGGACAAATGGGCAAGTCTGTGGAATTGGTTTAACATATCAAACTGGC

TGTGGTATATCAAAATATTCATAATGATAGTAGGAGGCTTGATAGGCTTAAGAATAGTTTTTGCT---
GTGCTTTCTATAGTA---------AATAGAGTTAGGAAGGGATACTCACCT------
TTGTCATTAGCTGTTAGTTTGCTTAATGCCACAGCTATAGCAGTAGCTGAA---
GGGACAGATAGGGTTATAGAACTAGTGCAAAGAATTTTTAGAGCT----------------------------
--------TTCCTCCACATA------CCTAGAAGAATAAGGCAGGGCCTCGAAAGGGCTTTACTA---TAA
>KT427688
ACT---AAG--
GAAGATTCGTGGGTCACAGTCTATTATGGGGTACCTGTGTGG---AAAGAAGCAACC---
ACCACTCTATTTTGTGCATCAGATGCTAAAGTATATGACACAGAG---ATGCATAAT----------------
--------------GTTTGGGCCACA---CATGCCTGTGTACCCACAGAT---
CCTAGCCCACAAGCAATACATTTGGAA---------AATGTAACAGAAAATTTTAAC----------------
--------------------------ATGTGGAAAAACAACATGGTAGAACAGATGCATGAGGATATA---
ATCAGCCTATGGGATCAAAGCCTA---AAGCCATGTGTAAAATTA---
ACCCCACTCTGTGTTGCTTTAAATTGCACTGATGTTTCTAATACCAATGCTACTAGTAAT-------------
----
----------------------------AATAGTATAGAAGGGAAGATAAGAGAAGAAATGAGA---
AACTGCTCTTTCAATGTCACCACAAACATAGGA---------------------------
AATAAGATGCACAAAGAATATGCACTT---TTGTATAAGCTTGATATAGTATCAATAGATAAT----------
----

GATAATACAAGTTATACATTGATAAATTGTAATACCTCAGTCATTACACAG---
GCCTGTCCAAAGGTATCTTTTGAACCAATTCCCATACATTATTGTGCCCCGGCTGGTTTTGCGATTCTAAGGT
GTAAT---AATCAGACATTTAAT------AGAACAGGACCCTGTACAAAT-----------------------
-GTCAGC---ACAGTACAATGTACACATGGAATTAGACCAGTAGTGTCAACT---
CAACTACTGTTAAATGGCAGTCTAGCAGAAGGAGAT------------
GTAGTAATTAGATCTGAAAATTTCACAAACAAT---------GCTAAAACCATAATAGTACAGCTAAAT----
-----------GTATCTGTAGAA---------ATTAATTGTACAAGACCAGGCAACAATACAAGAAGA-----
-AGTATACCTCTA------------GGACCAGGGAGAGCA---TTT------TATGCAACAGGGAAC------
---------ATAATAGGAGATATA------------AGAAAAGCACATTGT---AACATTAGTAAAGAC---
AAATGGGAGAGCACTTTAAAACAGGTAACCATAAAATTAAAA---------------GAACAATTTGGG----
--------------------------------AGGAACAAAACAATAGTCTTTAAG------
CCACACACAGGAGGGGATCCAGAAGTTGTA---ATGCACACTTTTAATTGTGGAGGAGAA---
TTTAAGCAGCTCCAGGCAAGAGTCCTGGCTGTGGAAAGATTCCTAAGGGATCAACAGCTCCTAGGGATTTGGG
GTTGCTCTGGAAAACTCATTTGCACCACTACTGTGCCTTGGAATGCTAGTTGGAGT-----------------
----------------------------AATAGATCTCTAGAAATGATCTGG------GAT------
AATCTGACCTGGATGGATTGG---GAGAGAGAAATTGGCAATTATACAAACACAATATAC---
AACTTACTTGAACAATCGCAGAACCAACAAGAA--

AAGAATGAACAAGACTTATTGGCGTTGGATAAATGGGCAAGTTTGTGGAATTGGTTTGACATATCAAAGTGGC
TGTGGTATATAAAAATATTCATCATGATAGTAGGAGGCTTGATAGGCTTAAGAATAGTTGTTGCT---
ATACTTTCATTAGTA---------AATAGAGTTAGGCAGGGATACTCACCC------
TTATCATTGGCTATTAATTTGCTTAATACCACAGCTATAGCAGTAGCTGAA---
GGGACAGATAGGATTATAGAAATAGTACAAAGAATTTGTAGAACT----------------------------
--------ATTCTCCACATA------CCTAGAAGAATAAGACAAGGCTTAGAAAGACTTTTGCTA---TAA
>KT427686
GCC---ACA--
GAAAAATTATGGGTCACAGTCTATTATGGGGTACCTGTGTGG---AAAGAAGCAAAC---
ACCACTTTATTTTGTGCCTCAGACGCTAAAGCATATTCCACAGAG---GCACATAAT----------------
--------------GTTTGGGCCACA---CATGCTTGTGTACCCACAGAC---
CCTAACCCACAAGAAATGGTATTGAAA---------AATGTGACAGAAAATTTTAAC----------------
--------------------------ATGTGGAAAAATAACATGGTAGAACAGATGCATGAGGATGTA---
ATCAGTTTATGGGATCAAAGCCTA---AAGCCATGTGTAAAATTA---
ACCCCACTCTGTGTTACTTTAAATTGTACTCCTGCACACTGTATAAATGGTACTTTGAACAATAATGAGTCGC
TATGTAATACA--
----------------------------ACCAATAACAGTACTTTTTATAGTAGTGACATGAGG---
AACTGCTCTTTCAATTTCACCACAAGCATA------------------------------
GGTAAGATGCAAAAAGAATATGCAGCT---
TTTCATATATCTGATTTAGTATCAATAGATGATAGTAGTGCAAATAGTACAGATAAA----------------
----
AGTACAAATAGTAGTACAAATAGTACAGATAATACAAAATATTATAAGTTAATAAGTTGTAATGCCTCAGTCG

TTACACAG---
GCCTGTCCAAAGATATCCTTTGAACCAATTCCCATACATTATTGTACCCCGGCTGGTTTTGTGCTTCTAAAGT
GTAAG---GATAAGAATTTCAAT------GGATCAGGACCATGTACAAAT----------------------
-GTCAGC---ACAGTACAATGTACACATGGAATTAGGCCAGTAGTGTCAACT---
CAACTGCTGTTAAATGGCAGTCTAGCAGAAGAAGAC-----------
ATAGTAATTAGATCTGAAAATTTCTCAAACAAT---------GCTAAAACCATAATAGTACAGCTGAAC----
-----------CAATCTGTAATA---------ATTAATTGTACAAGACCCAACAACAATACAAGAAAA-----
-AGTATACATATG-----------GGACCAGGAAAAGCA---TTT------TTTGCAACAGGAGAC------
---------ATAATAGGAGACATA-----------AGACAAGCACATTGT---AACATTAGTGGGACA---
CAATGGAACAATGCTTTACAACAGATAGCTATAAAATTAGGA--------------GAACAATTTAAT----
----------------------------------AAAACAAAAATAGTCTTTAAT------
CAGTCATCAGGAGGGGACCCAGAAGTTGTG---ATGCACACTTTTAATTGTGGAGGGGAG---
TTTAAGCAGCTCCAGGCAAGAGTCCTGGCTGTGGAAAGATACCTAAAGGATCAACAGCTCCTAGGAATTTGGG
GTTGCTCTGGGAGACTCATTTGCACCACTGCTGTGCCTTGGAATACTAGTTGGAGT----------------
---------------------------AATAGGTCTCTGGATGAGATTTGG------AAT------
AACATGACCTGGATGGATTGG---GAGAGAGAAATTGACAATTATACAGGCTTAATATAC---
TCCTTAATTGAAAAATCGCAAAACCAACAGGAA-------------------------------------
----
AAGAATGAACAAGAATTATTAGAATTGGATAAGTGGGACAGTTTGTGGAGTTGGTTCAGCATAACACAATGGC
TGTGGTATATAAAAATATTCATAATGATAGTAGGAGGCTTAATAGGTTTAAGAATAATTTTTGCT---
ATACTTTCTATAGTG---------AATAGAGTTAGGCAGGGATACTCACCA------
TTGTCATTCGCTATTAGTTTGTTTGATACTATAGCTATAGCAGTAGCTGGG---
GGGACAGATAGGGTTATAGAAGTAGCACAAAGAGTCTGTAGGGCT---------------------------
--------ATCCTCCACATC------CCTGTAAGAATAAGACAAGGCTTAGAAAGGCTTTTGGTA---TAA
>KT427685
GCT---AAA--
GAAAAATATTGGGTCACAGTCTATTATGGGGTACCTGTATGG---AAAGAAGCAACT---
ACCACTCTATTTTGTGCATCAGATGCTAAAGCATATGACCCAGAG---GCACATAAT---------------
--------------GTCTGGGCCACA---CATGCCTGTGTACCCACAGAT---
CCTAACCCACAAGAAGTAGTATTGGAA---------AATGTGACAGAAAATTTTAAC---------------
-------------------------ATGTGGAAAAATGACATGGTAGAACAGATGCATGAGGATATA---
ATCAGTTTATGGGATCAAAGCCTA---AAGCCATGTGTAAAATTA---
ACCCCGCTCTGTGTCATCTTAAATTGCACTAATTCTACAAATAGCAATGCTACTGTCACTTCTAAT-------
----
---AATAGGAAGGAAGAAATGAAA---
AACTGCTCTTTCTACATCTCCACAGGCATAGAA-------------------------GAGAAGAAG---
AAAGACTATGCACTT---TTTAATAGACTTGATTTAGTACCAATAAAAAAT---------------------
----
-------------AATGATAGTTATATGTTAGTAAGCTGCAACACCTCAGTCCTAACACAG---
GCATGTCCAAAGACAACCTTTGAACCAATTCCCATACATTATTGTGCCCCGGCTGGTTTTGCGATTTTAAAGT
GTAAT---GATACAAAGTTCAAT------GGAACAGGACTATGTAGAAAT----------------------
-GTCAGC---ACAGTACAATGTACACATGGAATTAGGCCAGTAGTGTCAACT---
CAACTGCTATTAAATGGCAGTTTATCAAAAGGAGAG-----------
GTAATGATTAGATCTGAAAATATCTCAAACAAT---------GCTAAAACCATAATAGTACAGCTAAAT----
-----------GAACCTGTAAAA---------ATTAATTGTACAAGACCCGGCAACAATACAAGAAAA-----
-AGTATACATATG-----------GGATGGGGAAGAGCA---TTT------TATGCAACAGGAGAC------
---------ATTATAGGAGATATA-----------AGACAGGCACATTGT---AACCTCAGTAAAGGA---
GAATGGAACAACACATTAACACAGGTAGTTAAAAAATTAAAA-----------GAACTAGAACTATTTAAG-
----------------------------------AATAAAACAATAATCTTTGAT------
CAACCCTCAGGAGGGGACTCAGAAGTTGTA---CTGTTCAATTTTAATTGTGGAGGGGAA---
TTTAAACAGCTCCAGGCAAGAGTCCTGGCTGTGGAAAGATACCTAATGGATCAACAGCTCCTAGGGATGTGGG
GTTGCTCTGGAAAACTCATTTGCAACACTAATGTGCCTTGGAATAATACTTGGACTAAGGGT---
AATAGAACTTCA-----------------------------AATCTAACAAAAATTTGG------GAT----
--AACATGACCTGGATGGAATGG---GAGAGAGAAATTGACAATTACACAAACTACATATAC---
ACTTTACTTGAAGAAGCGCAGAACCAACAAGAA-------------------------------------
----
AAGAATGAACAAGAGTTATTAGAATTGGATAAGTGGGCAAGTTTGTGGAATTGGTTTAGCATAACAAACTGGC
TGTGGTATATAAAATTATTCATAATGATAGTAGGGGGCTTAATAGGTTTAAGAATAGTTTTTACT---
GTAATTTCTATAGTG---------AATAGAGTTAGGCAGGGATACTCACCA------
TTGTCATTCGCTGTTAGTTTGCTTAATGCCACAGCTATCGCAGTAGCTGAG---

GGGACAGATAGGGTAATAGAAGCAGTACAAGGATTTGGTAGAGCT----------------------------
--------GTCCTCCACATA------CCTAGAAGAATAAGACAAGGATTAGAAAGGGCTTTGCTA---TAA
>KT427683
GCT---ACA---
CAATTGTGGGTCACAGTCTATTATGGGGTACCTGTGTGG---AAAGAAGCAACC---
ACCACTCTATTTTGTGCATCAGATGCTAAAGCAGTTGATAAAGAG---GTACATAAT----------------
--------------GTTTGGGCTACA---CATGCTTGTGTACCTACAGAT---
CCTAGTCCACAAGAAGTATTATTGGAA---------AATGTGACAGAAAATTTTAAT----------------
--------------------------ATGTGGAAAAATGACATGGTAGAACAGATGCATCAGGATGTA---
ATCAGTTTATGGGATGAAAGCCTA---AAGCCATGTGTAAAATTA---
ACCCCACTCTGTGTTACTTTAAACTGCACTGATTATTATGGAAATAATACTAGTGAAAGAAATCATAGT----
----
---GATATGAAGGGAGAAATGAAA---
AATTGCTCTTTCAATATCACTAACAGAATAAGA---------------------------
AATACGATGCACAAAGAATATGCACTT---TTATATAAACTTGATTTAGAACCACTAGGAAGG----------
----

GATAATACAAGCTATACATTGATAAACTGTAACACCTCAGTCATTACACAG---
GCCTGTCCAAAAGTAACCTTTGAACCAATTCCAATACATTATTGTACCCCGGCTGGTTTTGCGATTCTAAAGT
GTAAT---GATAAGAAGTTCAAT------GGAACAGGACCATGTAAAAAT-----------------------
-GTTAGT---ACAGTACAATGCACCCATGGAATTAAGCCAGTGGTGTCAACT---
CAACTGCTGTTAAATGGCAGTCTAGCAGAAGAAGAG------------
ATAGTAATCAGATCTGAAAATATTACAAACAAT---------GCTAAAACTATAATAGTACACCTGAAG----
-----------GAAGCTGTACAA---------ATTAATTGTACAAGACCCAACAACAATACAAGAAAA-----
-AGTATACATATG------------GGATGGGGAAGAGCA---TTT------TATGCAACAGGAGAA------
---------ATAATAGGAGACATA-----------AGACGAGCCTATTGT---AACATTAGTAAGGCA---
CAATGGGAAAACACTCTAAACCAGACAGTTGAAAAACTAAGA---------------GAACAATTTCCT----
-----------------------------------AATAAAACCATAATTTTTAAT------
CAATCCTCAGGAGGGGACCCAGAAATTGTA---ATGCACAGTGTTAATTGTGGAGGGGAA---
TTTAAGCAGCTCCAGGCAAGAGTCCTGGCTGTGGAAAGATACCTACAGGATCAACGGCTCCTAGGGCTTTGGG
GTTGCTCTGGAAAACACATTTGCCCCACTGCTGTGCCTTGGAATGCTAGTTGGAGT-----------------
----------------------------AATAAATCTCTAGACCAGATTTGG------AAC------
AACATGACTTGGATGCAGTGG---GAGAAAGAAATTGACAATTACACACAACTGATATAC---
AACTTAATTGAAGAATCACAGACCCAGCAGGAA--

AAGAATGAAAAAGAATTATTGGAATTGGATAAGTGGGCAGACTTGTGGACCTGGTTTAGCATAACAAAATGGC
TGTGGTACATAAGATTATTCATAATGATAGTAGGAGGCTTAATAGGTTTAAGAATAGTTTTTGCT---
GTAATTTCTATAGTG---------AATAGAGTTAGGCAGGGATACTCACCA------
TTATCGTTCGCTGTTAGCTTGCTTAATACTATAGCTATAGCAGTAGCTGAG---
GGGACAGATAGGGTTATAGAAGTAGTACAAAGAACTGGTAGAGCT----------------------------
--------ATCCTACACATA------CCTAGAAGAATAAGACAAGGCTTAGAAAGGATTTTGCTA---TAA
>KT427682
GTT---ACA--
TCACAATTATGGGTCACAGTCTATTATGGGGTACCTGTGTGG---AGAGAAGCAAAC---
ACCACTCTATTTTGTGCATCAGATGCTAAAGCATATGACACAGAA---AAACATAAC----------------
--------------GTTTGGGCCACA---CATGCCTGTGTACCCACAGAT---
CCTAACCCACAGGAAATAGTTTTAGAA---------AATGTGACAGAAGAATTTGAT----------------
--------------------------GCATGGAAAAATGACATGGTAGAACAAATGCACAAGGATATA---
ATTAGTTTATGGGATGAAAGTCTA---AAGCCATGTGTAAAATTA---
ACCCCGCTCTGTGTTGTTTTAAATTGTACTAACTGGAATGCTAATGCTACCAATGGGAATGCTACTGCTACCA
ATGGGAATGGTACTGCTACCAATGAG---------------------------------------
ACTAATCCTGCTAACAGTACTAATCCTGCTAACAGTAGTAAGGTGGAAAATTTGGGGGAAGACGAGGTAAAA-
--AACTGCTCTTTCCATACCAATAACAGGATGAGA---------------------------
AAGGGAATGCAGACAGAATATGCACTT---TTTTATAAGCTTGATGTAATGTCAATAGATAAT----------
----

AACAGTTACACATTGGTAAATTGCAACACCTCAGTCATTAAACAG---
ACCTGCCCAAAGGTATCCTTTGAACCAATTCCCATACATTATTGTGCCCCGGCTGGTTTTGCGATTCTAAAGT
GTAAT---GATAAGGAGTTCAAT------GGAAAAGGACCATGTAAAAAT-----------------------
-GTCAGC---ACAGTGCAATGTACACATGGAATTAGGCCAGTAGTGTCAACT---

CAACTACTGTTAAATGGCAGTCTAGCAGAAAAAGAG------------
ACGGTAATTAGATCCAAAAATTTCTCAAACAAT---------GCTAACACCATAATAGTTCAGCTGAAT----
-----------GAAACTATAATA---------ATTAATTGTACAAGACCCCACAACAATACAAGAAAA-----
-AGTATACATTTG------------GGACCAGGGGCAGTA---TTA------TATGCAACAGGAAAA------
---------ATAATAGGAGACATA------------AGACAAGCACATTGC---AACCTTAGTAAAGCA---
GGTTGGCAGAAAACTTTAAAACAGGTAGCTATAAAATTAAGA---------------GGAAAATTTGAG----
-----------------------------------AATAAAACAATAGCTTTTAAT------
CACTCCTCAGGAGGGGACCCAGAAATTGTA---ATGCATACTTTTAATTGTGGAGGGGAA---
TTCAAGCAGCTTCAGGCAAGAGTGCTGGCTCTAGAAAGATACCTAAGGGATCAACAGCTCCTAGGAATTTGGG
GCTGCTCTGGAAAACTCATCTGCCCCACTGCTGTGCCTTGGAATGCTAGTTGGAGT-----------------
----------------------------AATAAATCTCTAGAAGCGATTTGG------GAT------
AACATGACCTGGATGGAATGG---GAAAGAGAAATTAGCAATTACACAAACTACATATAC---
ACCTTAATTGAAGAATCGCAGAACCAACAGGAA--

AAGAATGAACAAGAATTATTGGAATTGGATAAATGGGCAAATTTGTGGAATTGGTTTTCCATATCAAACTGGC
TGTGGTATATAAAAATATTCATAATGATAGTAGGAGGCTTAATAGGTCTAAGAATAGTTTTTGCT---
GTACTTTCCATAATA---------AATAGAGTTAGGCAGGGATACTCACCA------
TTGTCGTTCGCTGTTAGTTTGATTAATACTATAGCCATAGCAGTAGCTGAG---
GGAACAGATAGAGTTTTAGAAGTAGCACAAAGACTTGTTAGAGCC----------------------------
--------ATTCGCCATATA------CCTAGAAGAATAAGACAGGGTGCAGAAAGGGCTTTGCAA---TAA
>KT427680
GCC---ACA--
GACAGATTGTGGGTCACAGTCTATTATGGGGTACCTGTGTGG---AAAGAAGCAACC---
ACCACTCTATTTTGTGCATCAGATGCTAAAGCAATTGAAACAGAA---GTACATAAT----------------
--------------GTTTGGGCCACA---CATGCCTGTGTACCCACAGAC---
CCTAAGCCACAAGAAGTAAGATTGGTA---------AATGTAACAGAAGATTTTAAT----------------
--------------------------ATGTGGAAAAATAACATGGTAGAACAGATGCATGAGGACATA---
ATCAGTTTATGGGATGAAAGCCTA---AAACCATGTGTAAAATTA---
ACCCCACTCTGTATTAGTTTAAATTGCACCGATTATAATGGTACTACT-------------------------
----
-------------------------AATAATAGTACATTGGGAGACATGGTGGGAGAAATAAAA---
AACTGCTCTTTCAAGTTAAGTCAAGGGATA------------------------------
GGTAAGATGCAGAAAAACAATGCACTT---TTTTATAAACTTGATTTAATGCAAATAAATGAGGAA-------
----

AATAATACAAGATATACATTGATAAACTGTAATACCTCAATCATTAAACAG---
GCCTGTCCAAAGGTATCCTTTGAACCAATTCCCATACATTATTGCGCCCCGGCTGGTTTTGCGATTCTAAAGT
GTAAT---GATAAGAAGTTCAAT------GGAACAGGACCATGTAAAAAT-----------------------
-GTCAGC---ACAGTACAATGTACACATGGAATTAGACCAGTAGTGTCAACT---
CAATTACTGTTAAATGGCAGCCTAGCAGAAGAAGAG------------
CTAGTAATAAGATCTGAAAATTTCACGGACAAT---------ACTAAAAACATAATAGTACAGCTGAAC----
-----------GAAACTGTAAAA---------ATTAATTGTACAAGACCCAACAACAATACAAGAAAA-----
-AGTATAAGTATA------------AGACCAGGGGCAACA---TTT------TATGCAACAGGAGAC------
---------ATAATAGGAGATATA------------AGACAGGCATATTGT---ACACTTAATGGGACA---
GAATGGGAAAAAACTTTAACACAGATAACTGAAAAATTAGGA---------------GAACAGTTTAAT----
-----------------------------------ACAGCAAATATAGTCTTTAAT------
CAATCTACAGGAGGAGACCCAGAAGTTGTA---ATGCATAATTTTAATTGTGGAGGGGAA---
TTTAAGCAGCTCCAGGCAAGAGTCCTGGCTGTAGAAAGATACCTGCAGGATCAGCAGCTCCTAGGGATTTGGG
GTTGCTCTGGAAAACTCATCTGCACCACCTCTGTGCCTTGGAATGCTAGTTGGAGT-----------------
----------------------------AATAAATCTCTAGATAGCATTTGG------GGT------
AACATGACCTGGAGACAATGG---GAGAAAGAAATAGATAATTACACACAAGAAATCTAC---
AACTTACTTGAACAATCGCAAAACCAACAAGAA--

AAGAATGAACAAGAATTACTAGAATTAGATAAATGGGCAAGTTTGTGGAATTGGTTTGACATATCAAACTGGC
TGTGGTATATAAGAATATTCATCATAGTGGTAGCAGGCTTAGTAGGTTTAAGAATAGCTTTTATT---
GTATTTTCTATAGTC---------AATAGAGTCAGGCAGGGATACTCACCA------
CTATCATTCGCTATTAGTTTACTTAATGCCACAGCTTTTGCAGTAGCTCAG---
GGGACAGATAGGATTATAGAAGTAGCACAAATAATTGGGAGAGGC----------------------------
--------ATCCTCAATATA------CCTAGAAGAATAAGACAGGGCTTAGAAAGGCTTTTGA--------
>KT427675

ACT---GCA--
GGACAATTGTGGGTCACAGTCTACTATGGGGTACCTGTGTGG---AAAGAAGCAACC---
ACCACTCTATTTTGTGCATCAGATGCTAAAGGATATGATACAGAG---GTACATAAT----------------
--------------GTTTGGGCCACG---CATGCCTGTGTACCTACAGAC---
CCCAACCCACAAGAAGTAGTATTGGAA---------AATGTGACAGAAAAGTTTAAC----------------
--------------------------ATGTGGAAAAATAACATGGTAGAGCAGATGCATGAGGATATA---
ATCAGTTTATGGGATCAAAGCCTA---AAGCCATGTGTAAAGTTA---
ACCCCACTCTGTGTTACCTTAAATTGCACTGATGTTACTAATGTTAATGCCACTAGTGGTAATACTAAT----
----
-------------GCCACTAGTAGTAATACTACTATAACAGATAATATGGAGGGAGAAATAAAA---
AACTGCTCGTTTAATATCACCTCAGGCATAAGA---------------------------
GATAAGGTACAGAAAGAATATGCACTG---TTATATAAACTTGATGTAGTACCAATAGATAAT-----------
----

AGTAATACTAGCTATAGGTTAATAAGTTGTAACACCTCAGTCATTACACAG---
GCCTGTCCAAAGGTAACCTTTGAGCCAATTCCCATACATTACTGTGCCCCGGCTGGTTTTGCGATTCTAAAGT
GTAAT---GATAAGAAGTTCAAT------GGAACAGGACAGTGTAAAAAT-----------------------
-GTCAGC---ACAGTACAATGTACACATGGAATTAAGCCAGTAGTATCAACT---
CAACTGCTGTTAAATGGCAGTCTAGCAGAAGAAGAG------------
GTAGTAATTAGATCTGAAAACTTCACGAACAAT---------GCTAAGACCATAATAGTACAGCTGAAC----
-----------AAAACTGTAATA---------ATTAATTGTACAAGACCTAACAACAATACAAGAAAA-----
-AGTATAAATATA------------GGACCAGGGAGAGCA---TTT------TATACAACAGGAGAC------
---------ATAATAGGCGATATA------------AGACAAGCACATTGT---AACCTTAGTAAAGAA---
GATTGGGAAGACACTTTAGATAAGGTAGCTAAAAAATTAAGA---------------GAACAAGTTGGG----
--------------------------------AATAAAACAACAATAATCTTTAAT------
GCATCCTCAGGAGGGGACCCAGAAATTGTA---ATGCACAGTTTTAATTGTGGAGGGGAA---
TTTAAACAGCTCCAGGCAAGAGTCCTGGCTGTGGAAAGATACCTAAAGGATCAACAACTCCTAGGGATCTGGG
GCTGCTCTGGAAAGCTCATCTGCGCCACTAATGTGCCTTGGAATTCTAGTTGGAGT-----------------
----------------------------AATAAATCTCTACAGTATATTTGG------AAT------
AACATGACCTGGATGGAATGG---GAAAAAGAAATTGATAATTACACAGAATTCATATAC---
ACTTTACTTGAAGAATCGCAGAACCAACAAGAA--

AAGAATGAACAAGACTTATTGGAATTAGATAAATGGGCAAATTTGTGGAATTGGTTTAGCATAACAAACTGGC
TGTGGTATATAAAAATATTCATAATGATAGTAGGAGGCTTGATAGGTTTAAGAATAATTTTTACT---
GTACTTTCTATAGTG---------AATAGGGTTAGGAAGGGATATTCACCA------
TTATCGTTGGCTATTAGCTTGCTCAACGCCACAGCAGTAGCAGTAGCTGAA---
GGGACAGATAGGGGTATAGAAATATTACAAAGAATTGTTAGAGCT----------------------------
--------GTTATCCACATA------CCTAGAAGGATAAGACAGGGCCTCGAAAGGGCTTTGCAA---TAA
>KT427671
GCT---AAA--
GAAGATTGGTGGGTCACAGTATACTATGGGGTACCTGTGTGG---AGAGAAGCAACC---
ACCACTTTATTTTGTGCATCAAATGCTAAAGCATATGACCCAGAG---GCGCATAAT----------------
--------------GTTTGGGCCACA---CATGCCTGTGTACCCACAGAC---
CCTGACCCACAAGAAATAGCATTGGAA---------AATGTAACAGAAAATTTTAAC----------------
--------------------------ATGTGGAAAAACAACATGGTAGAACAGATGCATGAGGATATA---
ATTAGTTTATGGGATGAAAGTCTA---AAACCCTGTGTAAAATTA---
ACCCCACTCTGTGTTACTTTACATTGTACTGATTGGAATGGTTGGAATGCTACTTCCAACACAAACACGACCC
AGTCTAGCACT--
----------------ACCCCCTCTAATAGTAGTATCTGGGAAAACATAAGGGGAGAAGTAAAA---
AACTGCTCTTTCAATATCTCTACAAGCACAGAA---------------------------GAGAAAAG---
AAAGAATATGCAACT---TTTTATACACTTGATGTAGTAAAAATAAATAATGAT-------------------
----
----------------AGTAGTTATAGGTTGATAAGTTGTAACACCTCAGTCCTTACACAG---
GCTTGTCCAAAGACAACCTTTGAACCAATTCCCATACATTATTGTGCCCCGGCTGGTTTTGCGATTCTAAAGT
GTAAT---GATAAAAAGTTTAAT------GGAACAGGACCATGTACAAAT-----------------------
-GTCAGC---ACAGTACAATGTACACATGGAATTAAGCCAGTAGTGTCAACT---
CAATTGCTGTTAAATGGCAGTCTAGCAGAAGAAGAG------------
ATAATAATTAGATCTGAAAATATCACGAATAAT---------GCTAAAACAATAATAGTACAGCTGAAC----
-----------GAAACTGTAGTG---------ATAAATTGTACAAGACTAGGCAACAATACAAGAAGA-----
-AGTATACATATG------------GGACCAGGGAGAGCA---TTT------TACACAACAGGAGAG------

---------ATAATAGGAGATATA------------AGAAAAGCATACTGT---AAAATTAATGCAGCA---
GCATGGACAGACACTTTAAGACAGGTAGCTATAAAATTAAGA--------------GAACAATTT-------
----------------------------------AATAATACCATAGTCTTTCAG------
AATTCCTCAGGAGGGGACCCAGAAATTGTA---ACGCACAGTTTTAATTGTGGAGGGGAA---
TTTAAGCAGCTCCAGGCAAGAGTCCTGGCTATAGAAAGATACCTAAAGGATCAACAGCTCCTAGGGATTTGGG
GTTGCTCTGGAAAACTCATCTGCACCACCTCTGTGCCCTGGAACACTAGTTGGAGT----------------
-------------------------AAAAATAAAACTCTAGAGGATATTTGG------AAT------
AACATGACCTGGATGCAGTGG---GAGAGAGAAATTGATAATTACACAGACTTAATATAT---
AACTTAATTGAAAAATCGCAAAATCAGCAGGAA---------------------------------------
----
AAGAATGAACAAGAATTGTTGGAATTGGATAAGTGGGCAAGTTTGTGGAATTGGTTTGATATAACAAACTGGC
TGTGGTACATAAAAATATTCATAATGATAGTAGGAGGCATAGTAGGTTTAAGAATAATTTTTAGT---
GTACTTTCTATAGTG---------AATAGAGTTAGGCAGGGATACTCACCA------
TTATCATTCGCTGTTAGTTTGTTTAATGCCATAGCTATAGCAGTAGCTGAG---
GGGACAGATAGAATTATAGAAGTAGCACAAAGAGTCGGTAGAGCT---------------------------
--------ATTCTCCACATA------CCTACAAGAATAAGGCAGGGTTTAGAAAGGTCTTTGCTG---TAA
>KT427745
GCT---ACA---
AAACCATTGTGGGTCACAGTCTATTATGGGGTACCTGTGTGG---AAAGAAGCAACC---
ACCACTCTATTCTGTGCATCAGATGCTAAAGCATATGACACAGAG---GCACATAAT---------------
--------------GTTTGGGCCACA---CATGCCTGTGTACCCACAGAC---
CCTAACCCACAAGAAGTAAGGTTGGAA---------AATGTGACAGAAAATTTTAAC---------------
--------------------------ATGTGGAAAAATGACATGGTAGAACAGATGCATGAAGATGTA---
ATCAGTTTATGGGATCAAAGCTTA---AAGCCATGTGTAAAGTTA---
ACCCCACTCTGTGTTACTCTAAATTGCAGTGATGTAACTTGCAATAATAGTAGTTGT---------------
----
--GATGGGAAGGAACAAATGACA---
AACTGCTCTTTCAACATCACCACACCCATAAGA--------------------------
GGTAGTAGGAAAAAAGAACATGCAATT---TTTGATAAACTTGATGTAGTGCCAATAGATAATGATAAT---
----

AATAGGAATAATACAGAATATATGTTGGCAAATTGCAACACCTCAGTCATTACACAG---
GCCTGTCCAAAGATATCCTTTGAACCAATCCCCATACACTATTGTGCCCCGGCTGGTTTTGCGATTCTACAGT
GTAAT---GAAAAAAATGTTCAAT------GGAACAGGACCATGTAAAAAT---------------------
-GTCAGC---ACAGTACAATGCACACATGGAATTAGGCCAGTGGTGTCAACC---
CATCTGCTGTTAAATGGCAGTCTGGCAGAAGAAGAG-----------
ATAGTAATTAGATCTGAAAATATCACAAACAAT---------GTTAAAACCATAATAGTACAGCTGAAC---
-----------GAAACAGTAGTA---------ATTAATTGTACAAGACCCAATAACAATACAAGAAAA----
-AGTATAAATATG------------GGACAAGGGAGAGCA---TGG-----TATGCAACAGGAGAT------
---------ATAATAGGAGATATA------------AGACAAGCACATTGT---AATGTTAGTGAAACA---
GCATGGAACAACACTCTAAAACAGATAGTTATAAAATTAGCA--------------AAACTATTTCAT----
----------------------------------AATAAAACAATAGTCTTTAAT------
CAATCCTCAGGAGGGGACCCAGAAGTTACA---ATGCACACTTTTAATTGTGGAGGGGAA---
TTTAAACAGCTCCAGGCAAGAGTCCTGGCTGTAGAAAGATACCTAAGGGATCAACAGCTCCTTGGGATTTGGG
GTTGCTCTGGAAAACTCATTTGCACCACCGCTGTGCCTTGGAATGATAGTTGGAGT----------------
-------------------------AATAAATCTATAGATGACATTTGGAAT------TCA------
AACATGACCTGGATGGAGTGG---GAGAGAGAAATTGACAATTATACAGGCATAATATAC---
AAATTACTTGAAGAATCGCAAACCCAACAAGAA---------------------------------------
----
AAGAATGAACAAGACTTATTGTCATTGGATAAGTGGGCAAGTCTGTGGGATTGGTTTAGCATAACAAACTGGC
TGTGGTATATAAAAATATTCATAATGATAGTAGGAGGCTTAATAGGTTTAAGAATAGTTTTTACT---
GTACTTTCTATAGTG---------AATAGAGTCAGGCAGGGATACTCACCA------
TTGTCATTCGCTGTTAGCTTGTTGAACGCCACCGCTATAGCAGTAGCTGAG---
GGGACAGATAGGATCATAGAATTAGCACGAACCATTGGTAGAGCC---------------------------
--------ATTCTCCACATA------CCTAGAAGAATAAGACAGGGCGCAGAAAGGGCTTTGCAA---TAA
>KJ849788
GCT---GCT---
CAACAATGGTGGGTCACAGTCTATTATAAAGTACCTGTATGG---AGAGAAGCAGAC---
ACCACTTTATTTTGTGCATCAGATGCTAAAGCATATGATAAGGAG---GTACATAAT---------------
--------------GTTTGGGCCACA---CATGCCTGTGTACCCACAGAC---

CCCAGCCCACAAGAAGTGGTATTGGAA---------AATGTGACAGAAGAGTTTAAC----------------
--------------------------ATATGGAAAAATAATATGGTAGAACAAATGCATAAGGATATA---
ATTAGTTTATGGGATGAAAGCCTA---AAACCATGTGTAAAGTTA---
ACCCCACTCTGTGTTACTCTAAATTGCACTGACTTGAAGAATATTAGT------------------------
----
--AGTACAGATAAAGGAGAAATAAAA---
AACTGCTCTTTCAAGGTCACCACAAGCATAAAA--------------------------
GATAAGGAGGTGAAAGAATATGCTCTT---TTCTATAAACTTGATGTAGTACAAATAAATGATGATGATAGT-
----

AATACAAGCTCTAGTAATTATACAAACTATAGGTTGATAAGTTGTAACACCTCAGTCATTACACAG---
GCCTGTCCAAAGGTAACTTTTGAGCCAATTCCCATACATTATTGTGCCCCGGCTAGTTTTGCGATTCTAAAGT
GTAAC---AATAAGACATTCAGT------GGAAAAGGCACATGTAACAAT----------------------
-GTCAGT---ACAGTACAATGCACACATAGAATTAGACCAGTAGTATCAACT---
CAACTGCTGTTAAATGGCAGTCTAGCAGAAGAAGCT-----------
ATAGTGCTTAGATCTGACAATTTCACAAATAAT---------GCTAAAACCATAATAGTACAGTTGAAA----
-----------GAACCTGTAAAA---------ATTAATTGTACAAGACCCTTCAACAATACAAGAAAA-----
-GGTATACATATA------------AGACCAGGGAGAGCA---TTT------TATACAACAGGAGAC------
---------ATAATAGGAGACATA------------AGACAAGCACATTGT---AATATTAGTGGAGCA---
GAATGGACTAAAACTTTAGAACAGGTAGCTACAAAATTAAGA---------------GAACAATTTGAA----
----------------------------------AATAAAACAATAGCCTTTAAT------
CCCTCCTCAGGAGGGGACCCAGAGATTGTA---ACACACAGTTTTAATTGTGGAGAGGAA---
TTTAAGCAGCTCCAGGCAAGAGTCCTGGCTGTGGAAAGATACCTAAAGGATCAGCAGCTCCTGGGGATTTGGG
GTTGCTCTGGAAAATTCATCTGCACCACTTCTGTGCCTTGGAATGCTAGTTGGAGT----------------
-------------------------GGGAATAGATCTCTGCCAGACATTTGG------GAT------
AACATGACCTGGATGCAGTGG---GAAAAAGAAATTGATAATTACACAGACATAATATAC---
AGCTTACTTGAAAAATCGCAGAACCAACAGGAG---------------------------------------
----
AAAAATGAACAAGAATTATTGGAATTGGATAAATGGGCAAGTTTGTGGAATTGGTTTGACATAACAAATTGGC
TGTGGTATATAAAAATATTCATAATGATAGTAGGAGGCTTGGTAGGTTTAAGAATAGTTTTTACT---
GTACTTTCTGTAGTA---------AATAGAGTTAGGCAGGGATATTCACCA------
TTATCGTTCGCTGTCAGCTTATTCAACACCACAGCTGTAGTAGTAGCTGAA---
GGAACAGATAGGGTTATAGAAGTAGCACAAAGAATTTTTAGAGCT---------------------------
--------ATTATCAACATA------CCTAGAAGAATAAGACAGGGCTTGGAACGTGCTTTGCTA---TAA
>KT427740
GCT---ACA--
GAACCACTGTGGGTCACAGTCTATTATGGGGTACCTGTGTGG---AAAGAAGCAACC---
ACCACTTTATTTTGTGCATCAGATGCTAAAGCATATGACACAGAG---GTACATAAT----------------
--------------GTTTGGGCCACA---CATGCCTGTGTACCCACAGAC---
CCTAGCCCACAGGAAGTACTATTGGAA---------AATGTAACAGAAGATTTTAAC---------------
--------------------------ATGTGGAAAAATAGCATGGTAGAACAGATGCAGGAAGATGTA---
ATCAGTTTATGGGATCAAAGCCTA---AAGCCATGTGTAAAATTA---
ACCCCACTCTGTGTTACTTTAAATTGCACCAATAATGTTACTACTACCAATAGTAATAGTACT---------
----
-------------------------------------AGAATGGAAGGAAAAGAAGAGATAAAA---
AACTGCTCTTTCAATATAACCACAAGCATAAAC--------------------------
AATAAGGTAAAACTAGACTATGCACTT---TTTAATACGCTTGATGTAGTGCCAATAGATACT---------
----

GATAATACAAGCTATATGTTAACAAGTTGTAACACCTCAGTCATTACCCAG---
GCCTGTCCAAAGGTATCCTTTGAACCAATTCCCATACATTATTGTACCCCGGCTGGTTTTGCGCTTCTAAAGT
GTAAT---GATAAGAAATTCAAT------GGATCAGGACCCTGTACAAAT----------------------
-GTCAGT---ACAGTACAATGTACACATGGAATTAGGCCAGTAGTGTCAACT---
CAGCTGCTGTTAAATGGCAGTCTAGCAGAAGAAGAG-----------
ATAGTAATTAGATCTGAAAATTTCACAAACAAT---------GCTAAAACCATAATAGTGCATCTCAAC----
-----------ACAACAGTAAAT---------ATTACTTGTGTAAGACCCAACAATAATACAAGAAAA-----
-AGTATACATATA------------GGACCAGGAAGAGCA---TTT------TATGCAACAGGACAA------
---------ATAATAGGAGATATA------------AGAAAAGCACATTGT---AACCTTAGCAAAGCA---
GACTGGGAAGACACTTTAAAAAGGGTAGCTAAAAAATTAAGG---------------GAACACTTT-------
----------------------------------AGTAAAACAATAAGCTTTAAG------

CAGTCCTCAGGAGGGGACCCAGAAGTTGTA---ATGCACAGTGTTAATTGTGGAGGGGAA---
TTTAAACAGCTCCAAGCAAGAGTCCTGGCTGTGGAAAGATACCTAAGAGATCAACAGCTCCTAGGGATTTGGG
GTTGCTCTGGAAAACTCATTTGCACCACTAGTGTACCTTGGAATGCTAGTTGGAGT-----------------
----------------------------AATAAAACTGTAGAAGAGATTTGG------GAT------
AACATGACCTGGATGCAGTGG---GAGAAAGAAATTGACAATTACACAGGTTTAATATAC---
AACTTACTTGAAGAATCGCAAAACCAACAAGAA---

AAAAATGAACAAGAATTATTGGCATTGGATCAGTGGGCAAATTTATGGAATTGGTTTGACATAACAAAATGGC
TGTGGTATATAAAAATATTCATAATGATAGTAGGAGGCCTAGTAGGTTTAAGGATAATTTTTACT---
GTGCTTTCTATAGTA---------AATAGAGTTAGGAAGGGATACTCACCA------
TTATCATTCGCTGTCAGTTTGCTTAATGCTACAGCTATAGCAGTAGCTGAG---
GGGACAGATAGGATTATAGAAGTAGTACAAAGAATTGGTAGAGCT----------------------------
--------ATCCGCAACGTA------CCTAGAAGAATAAGACAGGGCTTTGAAAGAAGTTTACTA---TAA
>KT427739
GCT---ACA---
AAACAATTGTGGGTCACAGTCTATTATGGGGTACCTGTGTGG---AAAGAAGCAACC---
ACCACTCTATTTTGTGCATCAGATGCTAAAGCATATGACACAGAG---GCACATAAT----------------
--------------GTTTGGGCCACA---CATGCCTGTGTACCCACAGAC---
CCTAACCCACAAGAAGTAGTGTTGGAG---------AATGTGACAGAAAATTTTAAC----------------
--------------------------ATGTGGAAAAATAACATGGTAGAACAGATGCATGAGGATGTA---
ATCAGTTTATGGGATCAAAGCTTA---AAGCCATGTGTAAAGTTA---
ACCCCACTCTGTGTTACTCTAAATTGCAGTGATGTAACTTGCACTGGAAATAGCACTAATAGTAGTGCTAGTA
ATAGCACTAAT--
-------------------AGTAGTTGTAATGGACAGGAGGGGACATTGAAGGGAGAAATGAAA---
AACTGCTCTTTCAACATCACCACACCCATAAGA---------------------------
GGTAGGGTGCGAAGCGAATATGCAATT---TTTGATAAACTTGATGTAGTGCCAATAAATGAT----------
----

AATAACAATAATAATAGTGAGTATATGTTGATACATTGCAACACCTCAGTCATTACACAG---
ACCTGTCCAAAGATATCCTTTGATCCAATCCCCATACATTATTGTACCCCGGCTGGTTTTGCGATTCTAAAGT
GTAAT---GAAAAGACGTTCAAT------GGAACAGGACCATGTAAAAAT-----------------------
-GTCAGC---ACAGTACAATGTACACATGGAATTAGGCCAGTGGTGTCAACT---
CATCTGCTGTTAAATGGCAGTCTGGCAGAAGAAGAG-----------
ATAGTAATTAGATCTGAAAATCTCACAAACAAT---------GCTAAAACCATAATAGTACAGCTGAAC----
------------AAAACAGTACAA---------ATTAATTGTACAAGACCCAACAACAATACAAGAAGA-----
-AGTATACATATA------------GGACCAGGGAGAGCA---TGG------TATACAACAGGAGCT------
---------ATAATAGGAAATATA------------AGACAAGCACATTGT---AACGTTAGTAAAACA---
GAATGGGAAGACACTCTAAATAAGATAGCTATAAAATTAGCA---------------GAACAATTTCCT----
-----------------------------------AAGGGAACAATAGCCTTTAAA------
CAATCCTCAGGAGGGGACCCTGAAGTTACA---ATGCACACTTTTAATTGTGGAGGGGAA---
TTTAAGCAGCTCCAGGCAAGAGTCCTGGCTGTGGAAAGATACCTAAGGGATCAACAGCTCCTTGGGATTTGGG
GTTGCTCTGGGAAACTCATTTGCACCACCTCTGTGCCTTGGAATAATAGTTGGAGT-----------------
-------------------------AATAAGTCTCTAGATGACATTTGGAAA------ACA------
AACATGACCTGGATGGAGTGG---GAGAGAGAAATTGACAATTATACAGGCATAATTTAC---
AAATTACTTGAAGAATCGCAGAACCAACAAGAA---

AAGAATGAACAAGACTTATTGGCATTGGATAAGTGGACAAGTCTGTGGAATTGGTTTAGCATAACAAACTGGC
TGTGGTATATAAAAATATTCATAATGATAGTAGGAGGCTTAATAGGTTTAAGAATAGTTTTTTCT---
GTACTTTCTATAGTG---------AATAGAGTTAGGCAGGGATACTCACCA------
CTGTCATTTGCTGTTAGCTTGCTTAATGCCACAGCTATAGCAGTAGCTGGG---
GGGACAGATAGGATCATAGAAATAGCACAAGGCATTGGTAGAGCC----------------------------
--------ATTCTCCACATA------CCTAGAAGAATAAGACAGGGCTTAGAAAGGGCTTTGCAA---TAA
>KT427738
GCT---GTA---
AATAAGACGTGGGTCACAGTCTATTATGGGGTGCCTGTGTGG---AAGGAAGCAACC---
ACCACTTTATTTTGTGCATCAGATGCTAAAGCATATGAAACAGAG---GCTCATAAT----------------
--------------ATTTGGGCCACA---CATGCCTGTGTACCCACAGAC---
CCTAACCCTCAAGAAATAGAATTGAAA---------AATGTGACAGAAGATTTTAAC----------------
--------------------------ATGTGGAAAAATAACATGGTAGACCAAATGCATGAGGATATA---
ATCAGTTTATGGGATCAAAGCCTA---AAGCCATGTGTAAAATTA---

ACCCCACTCTGTGTCACTTTAGATTGCAAAAATATAAGTAGAAATGCCACTGCTGWAAGTAAAAT-------
----
-------------------ACCACTAGTGTAAATAATACTATTAAGGAGGATGGAGACATGAAA---
AACTGCTCTTTCAATGTCACCACAATAGGAAGA---------------------------
AGCAAGGTAAAGACAGAATATGCATTT---TTTAATAAACTTGATATAGTACCAATAGATAAT---------
----

AGTGATACACAATATACATTGATAACCTGTAACACCTCAGTCATTACACAG---
GCCTGTCCAAAGATATCCTTTGAACCAATTCCCATACACTATTGTGCCCCGGCTGGATTTGCGATTCTAAAGT
GTAAT---GATAATGACTTCAAT------GGAACAGGAAAATGTACAAAT----------------------
-GTCAGC---ACAGTACAATGTACACATGGAATTAAGCCAGTAGTGTCTACT---
CAATTACTGTTAAATGGTAGTTTAGCAGAAAAAGSA-----------
GTAGTAATTAAATCTGAAAATTTCACAAACAAT---------GCTAAGAATATAATAGTGCAGCTACCG----
-----------GCCCCTGTAGTA---------ATTAATTGTACAAGACCCAACAACAATACAAGAAAA-----
-GGTATACATATG------------GGACCAGGGCAGACA---TTC------TATGCAACAGGACAA------
---------ATAATAGGAGACATA------------AGAAAAGCACATTGT---ACCATTAATAAGAAC---
AGTTGGAACAACACTTTAGGACAGGTAGCTRAAAAATTAAGA---------------GAACAATTTAAG----
----------------------------------AATAAAACAATAATCTTTAAT------
CAGTCCTCAGGAGGGGACCAAGAAATTATA---ACGCACAGCTTTAATTGTGGAGGGGAA---
TTTAAACAGCTCCAGGCTAGAGTCCTGGCTGTGGAAAGATACCTAAGGGATCAACAGCTCCTAGGAATTTGGG
GTTGCTCTGGAAAACTCATCTGCAACACTAATGTGCCTTGGAATAGTAGTTGGAGT----------------
----------------------------AATAAATCTCTGAGTACAATCTGG------GAT------
AACATGACCTGGATGGAATGG---GACAAAGAAATTAACAATTACACAAGCATAATATAC---
ACCTTAATTGAAAAATCGCAGATCCAGCAGGAA---------------------------------------
----
AAGAATGAACAAGAATTATTGTCATTGGATGAGTGGGCAAGTTTGTGGAATTGGTTTGACATAACTAGATGGC
TGTGGTATATAAAAATATTCATAATGATAGTAGGAGGAATAGTAGGTTTAAGAATAGTTTTTACT---
GTAATTTCTATAGTG---------AATAGAGTTAGGCAGGGATACTCACCA------
TTGTCATTTGCTGTTAGTTTGCTTAATGCTACAGCTATAGCAGTAGCTGAG---
GGGACAGATAGGATTATAGAAGTACTAAGAATAATTGGGAGAGGT---------------------------
--------TTTTTCCACATC------CCTAGAAGAATTAGACAGGGCTTTGAAAGAGCTTTGCTA---TAA
>KT427735
GCT---ACA---
GAAAAATTGTGGGTCACAGTATATTATGGAGTACCTGTGTGG---AAAGAAGCATCC---
ACCACTCTATTTTGTGCATCAGATGCCAAAGCATATGACACAGAG---GTACATAAT---------------
--------------GTTTGGGCCACA---CATGCCTGTGTACCCACAGAC---
CCTAACCCACAAGAAATAGTATTGGAA---------AATGTGACAGAAAATTTTAAC---------------
--------------------------ATGTGGAAAAATGACATGGTAGAACAGATGCATGAGGATATA---
ATCAGTTTATGGGATCAAAGCCTA---AAGCCATGTGTAAAATTA---
ACTCCACTTTGTGTTACTTTAAATTGCACTGGGGTTGAGATAAATAAGATAAGTAATACCAGT---------
----
----------------------AGTAGTAGTAATTTTACTATGGATGGTAAAGATGAGTTAACG---
AACTGCTCTTTCAATGTCGCCTCAATCATAAGA---------------------------
GATAAGGTGAGGAAAGAATATGCACTT---TTTTATAAATCTGATGTAGTACCAATAGAAAAT---------
----

AATAAAACCTATACATTAATACATTGTAACACCTCAGTCATTACACAG---
GCCTGCCCAAAGGTATCCTTTGAACCAATTCCCATACATTATTGTACCCCTGCTGGTTTTGCGATTCTAAAGT
GTAAT---GATAAGAAGTTCAAT------GGAACAGGACCATGTACAAAT----------------------
-GTCAGC---ACAGTACAATGTACACATGGAATTAGGCCAGTAGTGTCAACT---
CAGTTACTGTTAAATGGCAGCCTAGCAGAAGAAGAG-----------
GTAGTAATCAGATCTGAAAATTTCACAAACAAC---------GCTAAAACCATAATAGTACAGCTGAAA----
-----------GAGTCTGTAGTG---------ATTAATTGTACAAGGCCCAACAACAATACAATAAAA-----
-AGGATACATATA------------GGACCAGGGAGAGCA---TTC------TATGCAACAGGACAA------
---------ATAATAGGAGACATA------------AGAAAAGCACATTGC---AACATTAGTGGTACA---
GAATGGGACAAAACTTTAAAACAGGTGGCTAGGAAACTCAGT---------------GCACAGTTTGGA----
----------------------------------AATAAAACAATAGCCTTCAAA------
AACTCCTCAGGAGGGGACCCAGAAATTGTA---ATGCATAGTTTTAATTGTGGAGGGGAA---
TTTAAGCAGCTCCAGGCAAGAGTCCTGGCTGTGGAAAGATACCTACAGGATCAACAGCTCCTAGGAATTTGGG
GTTGCTCTGGAAAACTCATTTGCACCACCGCTGTGCCTTGGAATGCTAGTTGGAGT----------------

----------------------------AATAAAAATCTGAGTCAGATTTGG------GAT------
AACCTGACCTGGATGGAGTGG---GAAAGAGAAATTGACAATTATACAGACTTAATATAT---
AACTTAATTGAAGTATCGCAGAACCAGCAGGAA--

AAGAATGAACAAGAATTATTGGAATTGGATAAGTGGGCAAGTTTGTGGARTTGGTTTGATATAACAMAATGGC
TGTGGTATATAAAAATATTCATAATGATAGTAGGAGGCTTAATAGGTTTAAGAATAGTTTTTGCT---
GTACTRTCCATAGTG---------AATAGAGTTAGGCAGGGATATTCACCA------
TTATCGTTCGCTGTTAGTTTGCTTAATGCCAYAGCTATAGCAGTAGCTGAG---
GGGACAGATAGGATTATAGAAGTAGCACAAAGAATTGGTAGGGCT----------------------------
--------TTTCTCCACATA------CCTAGAAGAATAAGACAAGGCTTAGAAAGGATTTTGCTA---TAA
>KT427734
GCT---GCA------------------------TCA---GAA---TCA---GAT---
TTGTGGGTCACAGTTTATTATGGGGTACCTGTGTGG---AAAGAAGCAACC---
ACCACTCTGTTTTGTGCATCAGATGCTAAAGCATACAAAACAGAA---ATGCATAAT----------------
--------------GTCTGGGCTACA---CATGCCTGTGTACCCACAGAC---
CCCAACCCACAGGAAGTATTCTTGGAA---------AATGTGACAGAAGAGTTTAAC----------------
--------------------------ATGTGGAAAAATAACATGGTAGAACAGATGCATGAAGATATA---
ATCAGTTTATGGGATCAAAGCCTA---AAGCCATGTGTACAATTA---
ACCCCACTCTGTGTTACTCTCAATTGCACTAATCCTCAGCTTAATTGCACTGATTCTAAG-------------
----
-------------------------TGTAAGAACATTACGATTACAGTAGAAGGAGATGTAAAA---
AACTGCTCTTTCAATAGCACCACAGAGATAAGA---------------------------
GACAAGTGGCGGAAAGAATATGCTCTC---TTACATAAACTTGATATAGTACAAATAGAGAAT----------
----

AATCAGACTGGTAACTATAGTAGTTATAGATTGATAAGTTGTAATACCTCAGTCATTACTCAA---
GCCTGTCCAAAGGTAACATTTGAGCCAATTCCCATACATTATTGTGCCCCGGCTGGTTTTGCGATTCTAAAGT
GTAGA---GATAAAAGGTTCAAT------GGAACTGGAATATGTAGAAAT-----------------------
-GTCAGC---ACAGTACAATGTACACATGGAATTAAACCAGTAGTGTCAACT---
CAACTGCTGTTAAATGGCAGTCTAGCAGAAGAAGGG------------
ATAGTACTTAGATCCGAAAACTTCTCGAACAAT---------GCTAAAACCATAATAGTACAGCTGCAC----
-----------ACACCTGTACAA---------ATTAATTGTACAAGACCTGGCAACAATACAAGAAAA-----
-AGTATACATATA------------GGACCAGGGAGAGCA---TTC------TATGCAACAGGAGAC------
---------ATAATAGGAGATATA------------AGAGAAGCACATTGT---AACCTTAGTGAGACA---
CAGTGGAAGAACACTTTACAAAACATATCTGAAGAATTAAGA---------------AAACAATAT-------
-----------------------------------AATAAGACAATAATCTTTAAT------
CAATCCTCAGGAGGGGACCCAGAAATTGTA---ATGCACAGTCTTAATTGTGGAGGGGAA---
TTTAAACAGCTCCGGGCAAGAATCCTGGCTGTGGAAAGATACCTGGCGGATCAACAGCTCCTAGGGATTTGGG
GTTGCTCTGGAAAACTCATTTGCACCACTGCTGTGCCTTGGAACACTAGTTGGAGT-----------------
----------------------------AATAAATCTTTAGACTACATTTGG------GGA------
AACATGACCTGGATGCAGTGG---GAAAGAGAAATTAACAATTACACAGGCTTAATATAC---
AACCTAATTGAAGAATCGCAGAACCAGCAGGAA--

AAGAATGAACAAGAATTGTTAGAATGGGATAAGTGGGCAAACTTGTGGAATTGGTTTGACATAACAAACTGGC
TGTGGTACATAAAAATTTTCATAATGATAGTAGGAGGCTTGGTAGGGTTAAGAATAGTTTTTGCT---
GCACTTTCAATAATA---------AATARAGTTAGGCAGGGATACTCACCA------
ATATCATTTGCTGTTAGCTTGTTTGATGCCACAGCAATAGCAGTAGCTGAA---
GGGACAGATAGAATTATAGAAGCAGCACAAAGAATTTTTAGAGCT----------------------------
--------TTTCTTCACATA------CCAGTAAGAATAAGGCAGGGCTTAGAAAGGGCTTTACTA---TAA
>KT427733
GCT---ACA--
GAACAATTGTGGGTCACAGTCTACTATGGGGTACCTGTGTGG---AGAGATGCAAAC---
ACCACGCTATTTTGTGCATCAGATGCTAAAGCATATGAAAGAGAA---GCACATAAT----------------
--------------GTTTGGGCCACA---CATGCCTGTGTACCCACAGAC---
CCCAACCCTCAAGAAGTAAAATTGGAA---------AATGTGACAGAAAAGTTTAAT----------------
--------------------------ATGTGGAAAAACAATATGGTAGAACAAATGCATGAGGATATA---
ATCAGTTTATGGGATGAAAGTCTA---AAGCCATGTGTAAAATTA---
ACCCCTCTCTGTGTTACTTTAAATTGCAGTGATTTGAATAATACTAATTTGAATAATACTAATAGC-------
----
-------------------------AACAATAGTAGACATGATATGGAACAAGGAGAAATGAAA---

AACTGCTCTTTCAATGTCACCAACCAGATAAAA--------------------------
AATAAGTTACAGAAGGAATATGCACTT---TTGTATAAGTCTGATCTGATGGCAGTAGATAAT----------
----

AAGAGTTATACGTTGATAAATTGTAACACCTCAGTCATTACACAA---
GCCTGTCCAAAGGTATCCTGGGAACCAATTCCAATACATTTTTGTGCCCCGGCTGGTTTTGCGATTCTAAAGT
GTAAT---GATAATAAGTTTAAT------GGAACAGGACCATGTAAAAAT----------------------
-GTTAGC---ACAGTACAATGTACACATGGAATTAAGCCAGTAGTGTCAACT---
CAACTGCTGTTAAATGGTAGTTTAGCAAAGGGAGAA---------
GAGGTAGTAATTAGAGTTGAAAACTTCACAGACAAT---------GCTAAAACCATAATAGTACAGCTGAAT-
--------------CAATCTGTATGG---------ATTAATTGTACAAGACCCAACAACAATACAAGAAAA--
----AGTATACATATA------------GGACCAGGGAAAGCA---TTC------TATGCAACAGGAGAC---
------------ATAATAGGAGACATA------------AGACAAGCACATTGT---AACCTTAGCAGCGAC-
--GATTGGAATAAAACTTTAGATTGGATAGTCAAAGAAGTAAGA--------------
AAACAATTTCAATTA--------------------------------GATAATAAAACAATAATCTTTAAT-
-----GGGTCCTCAGGAGGGGACCCAGAAATTGTA---TTTCACACTTTTAATTGTGGAGGGGAA---
TTTAAACAGCTCCAGGCTAGAGTCCTGGCTGTGGAAAGATACCTAAAGGATCAACAGCTCCTGGGAATTTGGG
GTTGCTCTGGAAAACTCATTTGCACCACTACTGTGCCTTGGAATGCTAGTTGGAGC----------------
----------------------------AATAGATCTCTGAAAACAATTTGG------GAC------
AACATGACCTGGATGGAGTGG---GACAGAGAAGTTAACAATTACACCGGCCTAATATAC---
ACCTTACTTGAAGAATCGCAGAATCAGCAGGAA---------------------------------------
----
AAGAATGAACAAGAATTATTGGAACTGGATAAGTGGGCAAGTTTGTGGACTTGGTTTGACATAACAAATTGGC
TGTGGTATATAAGATTATTCATAATGATAGTAGGAGGTTTAGTAGGTTTAAGAATAGTTTTTACT---
GTACTTTCTATAGTG---------AATAGAGTTAGGCAGGGATACTCACCA------
TTGTCATTTGCTGTTAGCTTGTATAACGCCATAGCTATAGCAGTAGCTGAG---
GGAACAGATAGAATTTTAGAAGTAGTACAAATAATTGGGAGAGCT---------------------------
--------TTCCTGCACATA------CCTAGAAGAATTAGACAGGGCTTAGAAAGGGCTTTGCTA---TAA
>KJ849803
GCT---ACA---
GAACAATTGTGGGTCACAGTCTATTATGGGGTACCGGTGTGG---AGAGAAGCAACC---
ACCACTCTATTTTGTGCATCAGATGCTAAAGGATATGACACAGAG---GCACATAAT----------------
--------------ATTTGGGCCACA---CATGCCTGTGTACCCACAGAC---
CCTAACCCACAGGAAATAAACTTGGAC---------AATGTAACAGAAAATTTTAAT---------------
--------------------------ATGTGGAAAAATAACATGGTAGAACAGATGCATGAGGATATA---
ATTAGTTTATGGGATCAAAGCTTA---AAGCCATGTGTAAAATTA---
ACCCCACTCTGTGTTACTTTAAATTGCACTGATTGGAACGCCAACGACACTGCTGCCAACGCC----------
----
----------------------ACTGTCACAAATAGTAGTTTGAGTATGAAGGGAGAAATGAAA---
AACTGCTCTTTCAATATCACTGAAAGCATAGGA-------------------------
AATAAGAGGCGAAAAGAATATGCACTG---TTTAATAAACTTGATGTAATATCAATAGATAAT----------
----

AATGATAATACAAGCTATATGATAATAAACTGTAACACCTCAGTCATTAGACAG---
GCCTGTCCAAAAGTAACCTTTGAACCAATTCCCATACATTATTGTGCCCCGGCTGGTTTTGCGATTCTAAAGT
GTAAT---GATAAGGATTTCAAT------GGAACAGGACCATGCAAAAAT----------------------
-GTCAGC---ACAGTACAATGTACACATGGAATTAGGCCAGTAGTGTCAACT---
CAACTGCTGCTAAATGGCAGTTTAGCAGAA---GAG------------
GTAGTAATTAGATCTGAAAATTTCACAAACAAT---------GCTAAAACTATAATAGTACAGCTAAAT----
-----------AAAACTGTAGTA---------ATTAATTGTACAAGACCCAACAACAATACAAGAAAA-----
-AGTATAAATATT------------GGACCAGGGAGAGCA---TTC------TATGCAACAGGAGAC------
---------ATAACAGGAGACATA------------AGAAGGGCACATTGT---ACCCTTAGTAGGGAA---
GATTGGAATAACACTCTAAGACAGATAGCTAAAGTATTAAGA---------------GAACAATTTGGG----
---------------------------------GAGAATAAAAAGATAGCCTTTAAT------
CAGTCCTCAGGAGGGGACCCAGAAATTATA---CTGCACACTTTTAATTGTGGAGGGGAA---
TTTAAACAGCTCCAGGCTAGAGTCCTGGCTGTGGAAAGATACCTACAGGATCAACGGCTCCTAGGGATTTGGG
GTTGCTCTGGAAAACTCATTTGCCCCACTGCAGTGCCTTGGAATGCTAGTTGGAGT----------------
----------------------------AATAAATCTATGGATGACATTTGG------AAT------
AACATGACCTGGATGGAGTGG---GACAGGGAAATTAATAATTACACAAGATTAATATAT---
AGCTTACTTGAAGAATCGCAAAACCAACAAGAC---------------------------------------

----
AAGAATGAAAAAGAATTATTAGAATTGGATAAGTGGGCAAGTTTGTGGAATTGGTTTGACATATCTAATTGGT
TGTGGTATATAAAAATATTCATAATGATAGTAGGAGGTTTAGTAGGTTTAAGAATAGTGTTTACC---
ATACTTTCTATAGTG---------AATAGAGTTAGGCAGGGATACTCACCA------
TTGTCATTCGCTGTTAGCTTGCTTAATGCCACAGCTATAGCAGTAGCTGAG---
GGGACTGATAGAATTATAGAAGTAGGACAAGCAATTGGTAGAGCT----------------------------
--------ATTATCCACATA------CCCAGGAGAATCAGACAGGGCTTAGAAAGGACTTTGCTA---TAA
>KT427730
GCT---ACA--
GACAATTTGTGGGTCACAGTCTATTATGGGGTACCTGTGTGG---AAAGAAGCATCC---
ACCACTCTATTTTGTGCATCAGATGCTAAAGCATACGACACAGAG---GCACATAAT----------------
--------------GTTTGGGCCACG---CATGCCTGTGTACCCACAGAC---
CCTAACCCACAAGAAATAAGATTGGAA---------AATGTGACAGAAAATTTTAAT----------------
--------------------------ATGTGGAAAAATAACATGGTAGAACAAATGCAGGAGGATATA---
ATCAGTTTATGGGATCAAAGCCTA---AAGCCATGTGTAAAATTA---
ACCCCACTCTGTGTTACTTTAAATTGCACTGATTATAAAAATAATAGTACCACTAATATT-------------
----
---ACTGAAAATATAGAAATGAAA---
AACTGCTCTTTCAATGTCACCAACAGAATAAGA---------------------------
AACACATTACAGAAAGAATATGCTGTT---
TTTTATAAACTTGATATAGAACAAATAGATAAGGATAATGTAAGCGAT-------------------------
----
GCAGGCAATAAAAGCTATGAAAGCTATGTGTTGACAAGTTGTAACACCTCAGTCATTACACAG---
GCCTGTCCAAAGATATCCTTTGAACCAATTCCAATACATTATTGTGCCCCGGCTGGTTTTGCGATTCTAAAGT
GTAAT---GATAATAAGTTCAAT------GGAACAGGACCATGTACAAAT-----------------------
-GTTAGC---ACCGTACAGTGTACACATGGAATTAGGCCAGTAGTGTCAACT---
CAACTACTGTTAAATGGCAGTTTAGCAGAAGGAGGA---------
GAGGTAGTACTTAGATCTGAAAATTTCACAAACAAT---------GCTAAAACCATTATAGTACAGCTGAAG-
--------------GAACATGTAAGA---------ATTAATTGTACAAGACCTAATAACAATACAAGAAAA--
----AGTATACATTTC------------GGACCAGGACAAGCA---TTA------TATGCAACAGGAGAT---
------------ATAATAGGAGATATA------------AGACAAGCACATTGT---AACCTTAGCAGGGAA-
--GATTGGAATAAAACTTTAAGACAAATAGTTCCAAAATTAAGA---------------GAGCAATTTGGG--
-------------------------------------AATAAAACAATAATCTTTAAT------
CAGGCCTCAGGAGGGGACCCAGAAATTGTA---ATGCACAGTTTTAATTGTGGAGGGGAA---
TTCAAACAACTCCAGGCTAGAGTCCTGGCTGTGGAAAGATACCTAAAGGATCAACAGCTCCTAGGGATATGGG
GTTGCTCTGGAAAACTCATCTGCACCACTACTGTGCCTTGGAATACTAGTTGGAGT-----------------
----------------------------AATAAATCTCTAGAACAGATTTGG------AAT------
AACATGACCTGGATGGAGTGG---GACAGAGAAGTTAGCAATTACACAAAGTTAATATAC---
TCCTTAATTGAAGAATCGCAGAACCAACAGGAA--

AAAAATGAACTAGAATTATTGGAATTGGATAAGTGGGCAAGTTTGTGGAATTGGTTTGAAATATCAAACTGGC
TGTGGTATATAAAAGTATTCATAATGATAGTAGGAGGTTTAGTAGGTTTAAGAATAGTTTTTGCC---
ATATTTTCTATAGTG---------AATAAAGTTAGGCAGGGATACTCACCA------
TTATCATTTGCTGTTAGTTTGTTTAATGCCATAGCTATAGCAGTAGCTGAG---
GGGACAGATAGGATTATAGAAGTAATAAGAGCAATTGGTAGAGGC----------------------------
--------TTTCTCCATATA------CCTAGAAGAATCAGACAGGGCTTAGAAAGGGCTTTAATA---TAA
>KT427728
GCT---ACA--
GAACAGTTGTGGGTCACAGTCTATTATGGGGTACCTGTGTGG---AAGGAAGCAACC---
ACCACTCTATTTTGTGCCTCAGATGCTAAAGCAGTTGACACAGAG---GCACATAAT----------------
--------------GTTTGGGCCACA---CATGCCTGTGTGCCCACAGAT---
CCTGACCCACAAGAAATAAAATTGAAA---------AATGTGACAGAAAAATTTAAC----------------
--------------------------ATGTGGAAAAATAACATGGTAGAACAGATGCATGAGGATATA---
ATCAGCTTATGGGATCAAAGCCTA---AAGCCATGTGTAAAATTA---
ACCCCACTCTGTGTTACTTTAAATTGCACTGATCTGAGGAATGCTACTGGTGCTAATAGCACTGGTATTAATG
GCACTAATGGT--
----------------------ACTAGTAACAGTAGTGTGAAATATGTGGGGGGGACATGAAA---
AACTGCTCTTTCAATATCACCACAAACATAGAA---------------------------
AATAAGATGAAGAAAGAACGTGCACTT---TTTTATACATCTGATGTAGTAAGAATAGATGAC----------
----

AATGACAATGGTACAAGTTATAAATTAACAAGCTGTAACACCTCGGTTGTTACACAA---
GCTTGTCCAAAGATATCCTTTACTCCAATTCCCATACATTATTGTGCCCCGGCTGGATTTGCGATTCTAAAGT
GTAAT---GATAAGAAGTTCAAT------GGATCAGAAATATGTACAGAT-----------------------
-GTCAGC---ACAGTACAATGTACACATGGAATTAAGCCAGTAGTGTCAACT---
CAACTGCTGTTAAATGGCAGTCTAGCAGAAGAAGAG------------
ATAGTAATTAGATCTGAAAATCTCACAAACAAT---------GCTAAAACCATAATAGTACAGCTGAAC----
-----------AGGTCTATAGCA---------ATTAATTGTACAAGACCCAACAACAATACAAGAAAA-----
-GGTATCCATATG------------GGACCGGGAAAAACA---TTT------TATGCAACAGGAGAA------
---------ATAATAGGAGATATA------------AGACAAGCACATTGT---AACCTTAGTAGAACA---
CAATGGAATGAAACTTTAGGACAAATAGTAGACAAATTAAGA---------------GAACACTTTCCT----
-----------------------------------AATAAAACAATAATTTTTAAC------
CAATCCTCAGGAGGGGACCCAGAAATTGTA---ATGCACAGTTTTAATTGTGGAGGGGAA---
TTTAAGCAGCTCCAGGCAAGAGTCCTGGCTGTGGAAAGATACCTAAGGGATCAACAGCTCCTAGGAATTTGGG
GTTGCTCTGGGAAACTCATTTGCACCACTGCTGTGCCTTGGAATAATAGCTGGAGTAGTGAT-----------
-------------------AGAACT---AATGAAAATCTCAGTCAGATCTGG------GAT------
AATATGACCTGGATGCAATGG---GAGAAAGAAATTGACAATTACACAGGCTTAATCTAC---
AATTTACTTGAAAAATCGCAAATCCAACAAGAA--

AAAAATGAACAAGACTTATTGGAATTGGATAAGTGGGCAAGTTTGTGGAATTGGTTTGACATCTCAAATTGGC
TGTGGTATATAAGATTATTCATAATAATAGTAGGAGGCTTAGTAGGTTTAAGGATAGTTTTTAGT---
GTAATTTCCTTAGTG---------AATAGAGTTAGGCAGGGATACTCACCA------
TTATCATTCGCTATCAGCTTGTTTAACGCCATAGCCATAGCAGTAGCTGAG---
GGGACAGATAGGGTTATAGAAGTAGTACTAAGAATTGGAAGAGCT----------------------------
--------ATTCGCCACATA------CCTACAAGAATAAGACAGGGCTTCGAAAGGGCATTGCTA---TAA
>KT427727
GCT---GCA--
GAAAATTTGTGGGTCACAGTCTATTATGGGGTACCTGTGTGG---AAAGAAGCAACC---
ACCACTCTATTTTGTGCATCAGATGCTAAAGCACATAACACAGAA---GCACATAAT----------------
--------------GTTTGGGCCACA---CATGCCTGTGTACCTACAGAC---
CCTAACCCACAAGAAGTAAGATTGAAA---------AATGTGACAGAAAATTTTAAC----------------
--------------------------ATGTGGAAAAATAACATGGTAGAACAAATGCAGGAGGATATA---
ATCAGTTTATGGGATCAAAGCCTA---AAGCCATGTGTAAAGTTA---
ACCCCAATCTGTGTTACTTTAGAGTGCAAAAATGCTACTTTTAATTGCACAAATTTGAGAGACAATACCACTA
GTGAA--
-------------AATTGCACAAATGTGAGAAACGATACTTATATCGCTAGTGGGGAAATGAAA---
AACTGCTCTTTCAATGTTACCACAAACATAAAA---------------------------
AATAAGATGAAAAATGAATATGCACTT---TTTTATAGCCTTGATATAGAAAAAATAGATAAT---------
----

ACAAGCTATACATTGAGAAATTGTAACACCTCAGTCATTACACAG---
GCCTGTCCAAAGGTATCGTTTGAACCAATTCCCATACATTATTGTGCCCCGGCTGGTTTTGCGATTCTAAART
GTAAT---GAMAACAAGTTCART------GGAAMAGGRCCATGTAAMAAT-----------------------
-GTCAGC---ACAGTACAATGTACACATGGAATTAAGCCAGTAGTGTCAACT---
CAACTGCTGTTAAATGGCAGTTTAGCAGAAGAARGA---------
GAGGTAGTAATTAGATCTGAAAATCTCACAAACAAT---------GCTAAAACCATAATAGTACAGCTGAAG-
--------------AACCCTGTAAAA---------ATAAATTGTACAAGACCCAATAACAATACAAGAAGA--
----AGTATACMTMTA------------GGACCAGGGRSAGCA---TTC------TATGCAACAGGAGAC---
------------ATAATAGGAGACATA------------AGGCAAGCACATTGT---AACCTTAGTAGGAAA-
--GATTGGAATAACACTTTAAGAGAGATAGCTATAAAACTAAGA---------------GAACAATTTAAG--
-------------------------------------AATAGAACAATAATCTTTAAT------
CAGTCCTCAGGAGGGGACCCAGAAATTGTA---CTGCACACCTTTAATTGTGGAGGGGAA---
TTTAAACAGCTCCAGGCTAGAGTCCTGGCTGTGGAAAGATACCTAAAGGATCAACAGCTCCTAGGGATTTGGG
GTTGCTCTGGAAAACTCATTTGCACCACTACTGTGCCTTGGAATACTAGTTGGAGT-----------------
----------------------------AATAAATCCTWTGACCARATTTGG------ART------
AACATGACCTGGATGGAGTGG---GACAGAGAAGTTAGCAATTACACAAAATTAATATAC---
AMCTTAATTGAAGAATCGCAGAACCAACAGGAG--

AAGAATGAACAAGAATTGTTGGAATTGGATAAGTGGGCAAGTTTGTGGAATTGGTTTGATATAACAAATTGGC
TGTGGTATATAAGAATATTCATAATARTAGTAGGAGGATTAGTAGGTTTAAAAATAGTTTTTGCW---

ATACTYTCTATAGTR---------AATAGAGTTAGGCAGGGATACTCACCA------
TTGTCATTGGCTGTTAGTTTGCTTAACGCCACAGCTATAGCAGTAGCTGAG---
GGGACAGATAGGATTATAGAAGCATTACAAGCAGTTGGTAGAGCT----------------------------
--------TTTCTCCACATA------CCTAGAAGAATTAGACAGGGCTTCGAAAGGGCTTTGCTA---TAA
>KT427725
GCT---GTA--
GAACAATTCTGGGTCACAGTTTATTATGGAGTACCTGTGTGG---AAAGAAGCAACC---
ACCACTCTATTTTGTGCATCAGATGCTAAAGCATATGATACAGAG---GTACACAAT----------------
--------------GTTTGGGCCACA---CATGCCTGTGTACCCACAGAC---
CCCAACCCACGAGAAATAGTATTRGRA---------AATGTTACAGAAAACTTTAAC----------------
--------------------------ATGTRGAAAAATGACATGGTGGAACAGATGCATCAGGATATA---
ATCAGTTTATGRGATGAAAGCCTA---AAGCCATGTGTAAAATTA---
ACCCCACTCTGTATTACTTTAAATTGCACTAATTATTCAAATAGTACTARTAAT-------------------
----
----------------------------GTCACTATTGATGAGGMGGCGCGGGGAGAAATAAAA---
AACTGTTCTTTCAATATCACCACAAACATAAGA---------------------------
GATAAAGTGAAGAAAGAATATGCACTT---TTTTATACAATGGATGTAGTACAAGTAAAGGATGAGGAT----
----
----------AATAATAATAGAAATAGTACCAGCTAT------------
AGTAACACCTCAGTCATTACACAG---
GCCTGTCCAAAGGTATCCTTTGATCCAATTCCCATACATTATTGTGCCCCGGCTGGTTTTGCGATTCTAAAGT
GTAAC---AATAAGACGTTCAAT------GGATCAGGACCATGTACAAAT-----------------------
-GTTAGC---ACAGTACAATGCACACATGGAATTAAGCCAGTAGTGTCAACT---
CAACTACTGTTAAATGGCAGTCTGGCAGAAGAAGAT------------
GTAGTAATTAGATCTGCCAACTTCTCAAATAAT---------GCTAAAGTCATAATAGTACAGCTGAAT----
-----------GAATCTGTAGCA---------ATAAATTGTACAAGACCTAACAACAATACAAGAAAA-----
-GGTATACATATA------------GGACCAGGGAGAGCA---TTT------TATGCAACAGGAGAG------
---------ATAATAGGAAATATA------------AGACAAGCACATTGT---AACATTAGTGAAGTA---
CAATGGAACAAAACCTTAAAACTGATAGTTGGAAAACTAAAA---------------GAACAATTT-------
-----------------------------------AACAAGACAATAATCTTTAAT------
CACTCCTCAGGGGGGGACCCAGAAATTGTA---ATGCACACTTTTAACTGTAAAGGGGAA---
TTTAAACAGCTCCGGGCAAGAGTCCTGGCTATAGAAAGATACCTACAGGATCAACAGCTCCTAGGGATTTGGG
GTTGCTCTGGAAAACTCATCTGCACCACTGCTGTGCCTTGGAATACTAGTTGGAGT-----------------
----------------------------AATAAAACTCAAAATGACATTTGG------GAG------
AACATGACCTGGATGCAATGG---GAAAGAGAAATTGAGAATCACACAGGCACAATATAC---
ACCTTACTTGAAGAATCGCAGAACCAACAAGAA--

AAGAATGAACAAGAATTATTGGAATTGGATAAGTGGGCAAGTTTGTGGAATTGGTTTGACATAACACAATGGC
TGTGGTATATAAAAATATTCATAATGATAGTAGGGGGCTTGATAGGTTTAAGAATAATTTTTGCT---
GTGCTTTCTATAGTG---------AATAGAGTTAGGCAGGGATACTCACCA------
TTGTCATTTGCTGTTAGCTTGCTYAATGCCACAGCAATTGCAGTAGCTGAA---
GGGACAGATAGGGTCATAGAAGTAGCACAAAGAGCTTGTAGAGCT----------------------------
--------ATCCTCCACATA------CCTAGAAGAATAAGACAAGGCCTTGAAAGAATTTTAGTG---TAA
>KT427724
GCT---AAA---CAA------------------------------------
GAAGGGTCGTGGGTCACAGTCTATTATGGGGTACCTGTGTGG---AGAGAGGCAACC---
ACCACTCTATTTTGTGCATCGGATGCTAAAGCATATAAACCAGAA---GCACATAAT----------------
--------------GTGTGGGCCACA---CATGCCTGTGTACCCACAGAC---
CCTAACCCACAAGAATTACCATTGGAA---------AATGTGACAGAAAATTTTAAC----------------
--------------------------ATGTGGAAAAATAATATGGTAGACCAAATGCATGAGGATATA---
ATTAGTTTATGGGATCAAAGCCTA---AAGCCATGTGTAAAATTA---
ACCCCACTCTGTGTTATTTTAAAGTGCACTAATTTGACAATTAATGCCACTGCTAATGCGAATAATGCC----
----
-------------------------ACTGCTCCAACTATTGGTTCAATAGAGGAAGAAATGAAA---
AACTGCTCTTTCAATGTCTCCGCAGGCATAGGA---------------------------
AAACGGGTGCGAAAAGAATATGCACTT---
TTTAATAAATTTGATGTGATACCAATAGATAATGATAAGAAGAAT----------------------------
----
AATAGTACAACATCTAATAGTACAGACTATATGTTGGTAAATTGTAACACCTCAGTCATTACACAG---
GCTTGTCCAAAGACATCCTTTGAACCAATTCCCATACATTATTGTGCCCCGGCTGGCTTTGCGATTCTAAAGT

GCAAT---GAGAAGAACTTTAAT------GGAACAGGGCCATGTAAAAAT-----------------------
-GTCAGT---ACAGTACAATGTACACATGGAATTAGACCAGTAGTGTCAACC---
CAACTGCTGTTAAATGGTAGTTTAGCAGAAGATGAG------------
GTAATGGTTAGATCTAAAAATTTCTCAGACAAT---------ACTCAAACTATAATAGTACAGCTAAAT----
-----------AATACTGTAAAA---------ATGAATTGTACAAGACCCGGCAACAATACAAGAAAA-----
-AGTGTACATATG------------GGACCTGGGGGCGCA---ATT------TATGCAACAGGACAA------
---------ATAATAGGAGATATA------------AGAAAAGCACACTGT---AACATTAGTAGGGCA---
GATTGGATCGGAGCTTTAAATCAGGTAGCTGTAAAATTAAAA---------------GAACAATTTAAG----
-----------------------------------AATAAAACAATAAAATTTGAC------
AAATCCTCAGGAGGGGACCCAGAAGTTGAA---ATGTTCAATTTCAATTGTGGAGGGGAA---
TTTAAACAGCTCCAGGCTAGAGTCCTGGCTGTGGAAAGATACCTACAGGATCAACAGCTCCTAGGGATTTGGG
GTTGCTCTGGAAAACTCATCTGCACCACTAATGTGCCTTGGAACTCTACTTGGAGT-----------------
----------------------------AATAAATCTATAAATGACATTTGG------GGT------
AACATGACCTGGATGGAGTGG---GACAGGGAAATTCAAAATTACACAGGATTAATATAC---
ACCTTAATTGAAGAATCGCAAATCCAACAGGAA--

AAGAATGAACTAGACTTATTGAAATTGGATGAATGGGCAAGTTTGTGGAATTGGTTTGACATATCAAAATGGC
TGTGGTATATAAAAATATTCATAATGATAGTAGGAGGCATAGTAGGTTTAAGAATAGTTTGTACT---
GTACTTTCTATAGTA---------AATAGAATTAGGCAGGGATACTCACCA------
TTGTCATTCGCTATTAGTTTGCTTAATACCACAGCTGTAATAGTAGCAGAA---
GGAACAGATAGGATTATAGAAGCAGTACAAAGAATTGGCAGAGCT----------------------------
--------ATCCTCCACATA------CCTAGAAGGATCAGACAGGGCTTAGAAAGGGTTTTGCTA---TAA
>KT427723
GCT---ACA--AAG------
TGGGTCACAGTCTATTATGGGGTACCTGTGTAC---AAAGAAGCAACC---
ACCACTCTATTCTGTGCATCAGATGCTAAAGCATATGTCTCAGAG---GCCCATAAT----------------
--------------GTTTGGGCCACA---CATGCCTGTGTACCCACAGAC---
CCTAACCCACAAGAAACAAGGTTGGAG---------AATGTGACAGAAAATTTTAAC----------------
--------------------------ATGTGGAAAAATAACATGGTAGAACAGATGCATGAGGATGTA---
ATCAGTTTATGGGATCAAAGCTTA---AAGCCATGTGTAAAGTTA---
ACCCCACTCTGTGTTACTCTAAATTGCCATAATGTAACTTGCAACAGTACTAGTAACAGCAATAGTACT----
----
----------------------AGTAATAGCAATAATAGTTGTTCTGGGAAGGGAGAAATGACA---
AACTGCTCTTTCAACATCACCACACCCATAAAA---------------------------
GGTAGGGTGCGAAAAGAATATGCAATT---TTTGATAGACTTGATGTAGTGCCAATAGGTAAT----------
----

AATAATAATAATGATAGTGGTGCAGAATATATGTTAGTACATTGCAATACCTCAGTCATTACACAG---
GCCTGTCCAAAGATAACCTTTGAACCAATCCCCATACATTATTGTGCCCCGGCTGGTTTTGCGATTCTAAAGT
GTAAT---GAAAAGAAGTTCAAT------GGAACAGGACCATGTAAAAAT-----------------------
-GTCAGC---ACAGTACAATGCACACATGGAATTAGGCCAGTGGTGTCAACA---
CAGCTGCTGTTAAATGGCAGTCTGGCAGAAGAAGAG------------
ATAGTAATTAGATCTGAAAATCTCACAAACAAT---------GCTAAAATCATAATAGTACAGCTGAAT----
-----------GAAACAGTAGAA---------ATTAATTGTACAAGACCCAACAACAATACAAGAAAA-----
-AGTATACCTATA------------GGACCAGGGAGAGCA---TGG------TATGCAACAGGAGAT------
---------ATAATAGGAGATATA------------AGACAAGCACATTGT---AATGTTAGTGGAACA---
AAATGGGAGGAAACTCTAAAACAGATAGTTATAAAATTAGCA---------------AAACAATTTCAG----
-----------------------------------AATAAAACAATAGCCTTTAGG------
CAACCCTCAGGAGGGGACCCAGAAGTTACA---ATGCACACTGTTAATTGTGGAGGGGAA---
TTTAAGCAGCTCCAGGCAAGAGTCCTGGCTGTAGAAAGATACCTACAGGATCAGCAGCTCCTAGGGATTTGGG
GTTGCTCTGGAAAACTCATTTGCACCACTTCCGTGCCTTGGAATGCTAGTTGGAGT-----------------
----------------------------AATAAAAATCTGACTCAGATTTGG------GAT------
AACATGACCTGGATGGAGTGG---GAAAGAGAAATTGACAATTACACAGGCTTAATATAC---
AACTTACTTGAAGAATCGCAGAACCAACAAGAA--

AAGAATGAACAAGAATTATTAGCATTGGATAAATGGGCAAGTTTGTGGAATTGGTTTGACATATCAAACTGGC
TGTGGTATATAAAAATATTCATAATGATAGTAGGAGGCTTAATAGGTTTAAGAATAGTTTTTACT---
GTGCTTTCTATAGTA---------AATAGAGTTAGGCAGGGATATTCACCA------
TTATCATTTGCTATTAGTTTGCTTAATGCCACAGCTATAGCAGTAGCTGAG---

GGGACAGATAGTATTATCAGAGCAGTACAAATAGTAGGTAGAGCT---------------------------
--------ATTCTCCACATA------CCTAGAAGAATAAGACAGGGCTTAGAAAGAGCTTTGCTA---TAA
>KT427720
GCT---AGA---
GGACAATTGTGGGTCACAGTCTATTATGGGGTGCCTGTGTGG---AAAGAAGCAACC---
ACCACTCTATTCTGTGCATCAGATGCTAAAGCATATAGCACAGAG---GCACATAAT---------------
--------------GTTTGGGCCACA---CATGCCTGTGTACCCACAGAC---
CCTAACCCACAAGAAGTAGTATTGGAA---------AATGTGACAGAAAATTTTAAC---------------
--------------------------ACGTGGAAAAATGACATGGTGGAACAGATGCATGAGGATGTA---
ATCAGTTTATGGGAGCAAAGCCTA---AAGCCATGTGTAAAATTA---
ACCCCACTCTGTGTTACTCTAAACTGCACTGATTATAATAATACCAAT------------------------
----
-------------------------AATAGTACTTTGGATAAGAATATGAAGGGAGAAATAAAA---
GAATGCTCCTTCAATATCACCACAAGCATAAAA--------------------------
GATAGGGTACAAAAACAAACTGCACTT---
TTTAATAGACTTGATATAGTACCAATAGATGATAATAATAGTACA---------------------------
----
AACAATAATAGTGCAAACTATATGTTGATAAGTTGTAATACCTCAGTCATTACACAG---
GCCTGTCCAAAGATAACCTTTGAACCAATTCCTATACATTATTGTGCCCCGGCTGGTTTTGCGATTCTAAAGT
GTAAT---AACAAAACGTTCAAT------GGAAAAGGACCATGTACAAAT----------------------
-GTCAGC---ACAGTACAATGTACACATGGAATTAAGCCAGTAGTGTCAACT---
CAACTGCTGTTAAATGGCAGTTTAGCAGAAGAAGAG-----------
ATAGTAATTAGATCTGAAAATTTCTCGGATAAT---------GCAAAAACCATAATAGTACAGCTGAAC----
-----------CAATCTGTAGTA---------ATTAATTGTACAAGACCCGGCAACAATACAAGCAAA-----
-AGTATAAATATG---------GTAGGG---GGAAAA------TTC------TTTGCAACAGGAAGA------
---------ATAATAGGAAATATA------------AGACAAGCACATTGT---AACCTTAGTGAAACA---
GACTGGAGAAAAACTATAAAACAGGTAAAGGAAAAATTACAC---------------AGACAATAT-------
-----------------------------------AATAAAACAATAATCTTTGAT------
CACTCCTCAGGAGGGGACCCAGAAGTTATA---CTGCACACTTTTAATTGTGGAGGGGAA---
TTTAAGCAGCTCCAGGCAAGAGTCCTAGCTGTGGAAAGATACCTAAAGGATCAACAGCTCCTAGGGATTTGGG
GCTGCACTGGAAAACTCATTTGCACCACTGCTGTGCCTTGGAATAATAGTTGGAGT----------------
----------------------------AATAAATCTCTGGATGAAATTTGG------AAT------
AACATGACCTGGATGGAGTGG---GAGAAGGAAATTGACAATTACACAGGCATAATATAC---
AGATTACTTGAAGAATCGCAGAACCAACAAGAA---------------------------------------
----
AAAAATGAACAAGACTTATTGGCATTGGATAAGTGGGCAAGTTTGTGGAATTGGTTTGACATAACAAAATGGC
TGTGGTATATAAGAATATTCATAATGATAGTAGGAGGCTTAATAGGTTTAAGAATAGTTTTTACT---
GTACTTTCTATAGTG---------AATAGAGTTAGGAAGGGATACTCACCA------
TTGTCGTTCGCAGTTAGCTTGCTTAATGCCACAGCAATAGCAGTAGCTGAG---
GGAACAGATAGGGTTATAGAAGTAATACAAAGAATTGGTAGAGGC--------------------------
--------TTTCTTCATATA------CCTAGAAGAATAAGACAGGGCTTAGAACGAGCTTTGCTA---TAA
>KT427716
GCA---
GAGCAGTTGTGGGTCACAGTCTATTATGAGGTACCTGTGTGG---AAAAAAGCAAAC---
ACCACTCTATTTTGTGCATCAGATGCTAAAGCATATCAAACAGAA---GCACATAAT---------------
--------------GTTTGGGCTACA---CATGCCTGTGTACCTACAGAC---
CCTAACCCTCGAGAGGCAAAATTGGAA---------AATGTGACAGAATATTTTAAC---------------
--------------------------ATGTGGAAAAATAACATAGTAGAACAAATGCATGAAGATATA---
ATCAGTTTATGGGATCAAAGCCTT---AAGCCATGTGTAAAATTA---
ACCCCACTCTGTGTTACTTTAAATTGCATTAATGCAACTATTAAGAATAAGACAAATAAGAATGCAACTATTA
GGAATAAGATA---
-------------------AATAATAATGCGACAGAACAGCTAGAGCAGCCAAGAAACATGCAA---
AACTGCTCTTTCAACATCTCCACAATAAGA--------------------------------
AGGAGCAAGAATAACTATGCAATT---TTTTCTAGTCTTGATATAGTACCAACAGATGAG------------
----
-------------GATAATAGTAGTACAAGCTATATGTTGAGAAGTTGTAACACCTCAGTCATTACTCAG---
GCATGTCCAAAGATATCCTTTGAACCAATTCCCATATATTATTGTGCCCCGGCTGGTTTTGCGATTCTAAAAT
GTAAT---GATAATAATTTCAAT------GGATCAGGACCATGTAAAAAT----------------------
-GTCAGC---ACAGTACAATGTACACATAGGATTAAGCCAGTGGTGTCAACA---
CAACTGCTGTTAAATGGCAGTCTAGCAGAGGGAGGA---------

GATGTAATAATTAGATCTGAAAATTTCACAAACAAT---------GCTAAAACCATAATAGTACAGCTGAAT-
--------------GAATCTGTAACA---------ATTAATTATACCAGACCTAACAACAATACAAGAAAA--
----AGTATACATCTA------------GGAGCAAGGAGAGCA---CTC------TATACAACA---GAC---
------------ATAGTAGGAGACATA------------AGAAAAGCATATTGT---ACCATTAATGAGACA-
--GAGTGGAAAAACACTTTAGAAAAGGTAGCTAAAAAATTAAAA---------------GAACAATTTAAT--
-------------------------------------ACGTCACAAATAGCCTTTAAT------
CAGTCCTCAGGAGGGGACTTAGAAATTACA---ATGCACACTTTTAATTGTAGAGGGGAA---
TTTAAACAGCTCCAGGCTAGAGTCCTAGCTGTAGAAAGATACCTAAGGGATCAACAGCTCCTAGGAATTTGGG
GTTGCTCTGGAAAACTCATTTGCACCACTACTGTGCCTTGGAATACTAGTTGGAGT-----------------
----------------------------AATAAATCTAGGGAAGACATTTGG------AAT------
AACATGACCTGGATGGAATGG---GACAGAGAAATTAACAAATACACAAGAGAAATATAC---
TCATTGCTTGAAAAATCGCAGAACCAACAGGAA--

AAGAATGAACAAGACTTATTGGAATTGGACAAGTGGGCAAATTTGTGGAATTGGTTTGACATATCAAGATGGC
TGTGGTATATAAAAATATTCATAATGATAGTAGCAGGTTTAGTAGGTTTAAGAATAGTTTGTTTC---
ACACTTTCTTTAGTG---------AATAGAGTTAGGCAGGGATACTCACCA------
TTGTCATTTGCTGTTAGTTTGCTTAATGCAACAGCTATAGCAGTAGCTGAG---
GGGACAGATAGGCTTATAGAAGCAGCAAGAAGAATTGGGCTAGCT----------------------------
--------GTTCTCCACATA------CCTAGAAGAATCAGACAGGGCTTAGAAAGGGCTTTGCTA---TAA
>KJ849767
GCT---ACA--
GAAAAATTATGGGTCACAGTCTATTATGGAGTACCTGTGTGG---AAAGAAGCAACC---
ACTACTCTATTTTGTGCATCAGATGCTAAGGCATATCATACAGAA---GCACATAAT----------------
--------------GTTTGGGCCACA---CATGCCTGTGTACCCACAGAC---
CCTAACCCTCAAGAAATAAATTTGGAA---------AATGTGACAGAAAATTTTAAT----------------
--------------------------ATGTGGAAAAATAACATGGTGGAACAAATGCATCAGGATATA---
ATTAGTTTATGGGATGAAAGCCTA---AAGCCATGTGTAAAATTA---
ACCCCACTCTGTGTTACTTTAAAATGCACAGATTTCAATGGTAACGGTACTAACACCAGTAGCACACAGCCTA
CTACC--
-------ACTAGTAGTACCAGTAATAGTAACATTAGTTTGGAAATGGAAGTAGGAGAAATAAAA---
AACTGCTCTTTCAATGTC---ACAAGAGGAGGA---------------------------
GACAAGTATCAGAAAGAATATGCACTT---TTTTACAAACTTGATTTAGTAACAACCAATGAT----------
----

AAGGATAATACAAGCTATACATTGATAAATTGTAACACCTCAGTCATTACACAG---
GCCTGTCCAAAGGTATCCTTTGAACCAATTCCCATACATTATTGTGCCCCGGCTGGTTTTGCGATTCTAAAGT
GTAAT---GAGAAAAACTTCACT------GGAACAGGACCATGTAAAAAT-----------------------
-GTCAGC---ACAGTACAATGTACACATGGAATTAAGCCAGTAGTATCAACT---
CAACTACTGTTAAATGGCAGTTTGGCGGAAGAAGAG------------
GTAGTACTTAGATCTGCAAATTTCTCAGACAAT---------ACTAAAACCATAATAGTACAGCTGACA----
-----------AAACATGTAAAT---------ATTACTTGTATGAGGCCCAACAACAATACAAGAAGA-----
-GGTATACATATA------------GGACCAGGGAGAGCA---TTC------TATGCAGCAGGAGAC------
---------ATAATAGGAAACATA------------AGACAAGCACACTGT---AACCTTAGTAGAGTA---
GAATGGAATAACACTTTAAAACAGATAGCTATAAAATTAAAA---------------GAACAATTT-------
-----------------------------------AATAAAACAATAGTCTTCAAT------
CAGTCCTCAGGAGGGGACCCAGAAATTGTA---ATGCATAGTTTTAATTGTGGAGGGGAA---
TTTAAACAGCTCCAGGCCAGAATCCTGGCTGTGGAAACATACCTAAGGGATCAACAGCTCCTGGGAATTTGGG
GTTGCTCTGGAAAACTCATTTGCACCACTACTGTGCCTTGGAATAGTAGTTGGAGT-----------------
----------------------------AATAAAACTCTGAATACCATTTGG------GAT------
AACATGACCTGGATGCAGTGG---GACAAAGAAATTAGCAATTACACAGGCATAATATAC---
ACTTTACTTGAAGAATCGCAGAACCAACAGGAA--

GACAATGAGAGAAAATTATTGGAATTGGATAAGTGGGACAGTTTGTGGAATTGGTTTAGCATAACAAACTGGC
TGTGGTATATAAAAATATTCATAATGATAGTAGCAGGCTTAGTAGGTTTAAGAATAGTTTGTACC---
ATACTTTCTATAGTG---------AATAGAGTTAGGCAGGGATACTCACCA------
TTGTCGTTTGCTGTTAGTTTGCTTAATGCCACAGCTGTAGCAGTAGCTGAG---
GGGACAGATAGGATTATAGAAGTAGCACAAATACTTGGTAGAGCT----------------------------
--------TTTATCCACATA------CCTAGAAGAATCAGACAGGGCTTCGAAAGGGCTTTGCTA---TAA
>KT427847

GTT---GCA--
GGACAATTGTGGGTCACAGTCTATTATGGGGTACCTGTATGG---AAAGAAGCAACC---
ACCACTCTATTTTGTGCATCAGATGCTAAAGCATATGACACAGAG---GTCCATAAT----------------
--------------GTATGGGCCACA---CATGCCTGTGTACCCACAGAC---
CCTAACCCACAAGAAGTAGTATTGGGA---------AATGTGACAGAAAATTTTAAC---------------
--------------------------ATGTGGAAAAATGACATGGTAGAACAAATGCATGAGGATGTA---
ATCAGTTTATGGGATCAAAGCCTA---ACGCCATGTGTAAAATTA---
ACCCCACTCTGTGTTGCTTTAAATTGCTCTAGACCACTCTTAAATTGTACTAATAATACTACCAAAAGTGAG-
----
-------------------------AATTGCACTAATTGGGAAAAGATGGGGGAAGAAATAAAA---
AACTGCTCTTTCAATATCACCACAAACATGAAA------------------------------
AAGGTGCGAACAGAAGCTGTACTG---TTTAATGCACTTGATATAGTACCAATAGATAAT-------------
----
-------------------------ACAAGCTATATGTTGAGACATTGTAACACCTCAGTCATTAAACAG---
GCCTGTCCAAAAGTATCCTTTGAACCAATTCCCATACATTATTGTGCCCCAGCTGGTTTTGCGATTCTAAAGT
GTAAT---AATAAGGAGTTCAAT------GGAACAGGAGCATGTACAAAT----------------------
-GTCAGT---ACAGTACAATGTACACATGGGATTAGGCCAGTAGTGTCAACT---
CAACTACTGTTGAATGGCAGTCTAGCAGAAGGAGAG-----------
GTGGTAATTAGGTCTGAAAATTTCACAAACAAT---------GCTAAAATCATAATAGTACAGCTGAAC----
-----------GAAACTGTACAA---------ATTAATTGCACAAGACCCAACAACAATACAAGAAAG-----
-AGTATACATCTA------------GGATCAGGGAGAGCA---TTT------TATGCAACAGGAGAC------
---------ATAATAGGAGATATA------------AGACAAGCACATTGT---AACATTAGTAAAACA---
AAATGGGAAAACACTTTAAAAAAGGTAGCTAGAAAATTATAT--------------AAACAATTT-------
------------------------------------AATAAAACAATAGCCTTTACG------
CAATCTTCAGGAGGGGACCCAGAATTGGTA---ATGCACACTTTTAACTGTGGAGGGGAA---
TTTAAGCAGCTCCAGGCAAGAGTCCTAGCTGTGGAAAGATACCTAAGGGATCAACAGCTCCTAGGGATTTGGG
GTTGCTCTGGAAAATTCATTTGCACCACTACTGTGCCTTGGAATAGTAGTTGGAGT----------------
----------------------------AATAGATCTCTGGACACGATTTGG------GAT------
AACATGACCTGGATGGAATGG---GAGAGAGAAATTAGCAATTACACAAATGTAATATAC---
ACTTTACTTGAAGAATCGCAGAACCAACAAGAA---------------------------------------
----
AAGAATGAACGAGAATTATTAGAATTGGACAAGTGGGCAAATTTGTGGAATTGGTTTGACATAACAAACTGGC
TGTGGTATATAAAAATATTCATAATGATAATAGGAGGCTTAATAGGTTTAAGAATAGTTTTTACT---
GTACTGTCTATAGCG---------AATAGAGTTAGGCAGGGATACTCACCA------
TTGTCGTTCGCTATTAGTTTGCTTAATGCCACAGCTATAGCAGTAGCTGAG---
GGGACAGATAGGGTTATAGAAGTAGTACAGAGAGCTGGTAGAGCT---------------------------
--------GTTCTTCACGTA------CCTAGAAGAATAAGACAGGGCCTCGAAAGGCTTCTATTA---TAA
>KT427853
GCT---ACA--
GAACAATTGTGGGTCACAGTCTATTATGGGGTACCAGTGTGG---AAAGAAGCAACC---
ACTACTCTATTTTGTGCATCAGATGCTAAAGCATATCAAGCAGAA---GCACATAAT----------------
--------------ATTTGGGCCACA---CATGCTTGTGTACCCACAGAC---
CCTAGCCCCCAAGAAGTAAAATTGGAA---------AATGTGACAGAAAATTTTAAC---------------
--------------------------ATGTGGGAAAATAACATGGTAGAACAAATGCATGAGGATATA---
ATCAGTTTATGGGATCAAAGCCTA---AGACCATGTGTAAAGTTA---
ACCCCACTCTGTGTTACTTTAAATTGCTCTAATGCTACTTATTCCGATAGTAGTAGTGGTACCAGTACTGATG
CCCCTGCTACC---
-------------------------AGTAGTAGTAGTTGGGGAAAAGAAATAGATAACTGGAAA---
AACTGCTCTTTCAATGTCTCAAGAAGCATAGGA-----------------------
AGAAGATGGGTACAGAAGGAATATGCACTT---TTTAATAAACTTGATGTAGTGTCAATAGATAAT-------
----

AAAACACAATATATGTTGACAAATTGTAACACCTCAGTCATTACACAG---
GCCTGTCCAAAGATATCCTTTGAACCAATTCCCATATATTATTGTGCCCCGGCTGGTTTTGCGCTTCTAAGGT
GTAAT---GATAACAAGTTCAAT------GGAACAGGACCATGTACAGAT----------------------
-GTCAGC---ACAGTACAATGTACACATGGAATCAGGCCAGTAGTATCAACT---
CAACTGCTGTTAAATGGCAGCTTGGCAGAAGGAGAA---------
GGTGTAATACTTAGATCAAAAAATTTCACAAAAAAT---------ACAGACATCATAATAGTACAGCTGAAT-
--------------GAATCTGTAGGA---------ATTAATTGTACAAGACCCAGTAACAATACAAGGAGA--
----AGTATACATCTA------------GGACCAGGGAGGGTA---ATC------TATGCAACAGGACAA---

------------ATAACAGGAGATATA------------AGACAAGCACATTGT---ACACTTAGTGGGGAA-
--AAATGGAATAAAACTTTAAAACAGATAGCTACCAAATTAAGA---------------GAACAATTTGAG--
-------------------------------------AATAAAACAATAGTCTTTAAT------
CAGTCCTCAGGAGGGGACCCAGAAGTTGTA---ATGCACACTTTTAATTGTGGAGGAGAA---
TTTAAACAGCTCCAGGCTAGAGTCCTGGCTGTGGAAAGATACCTAAAGGATCAACAGCTCCTAGGGATTTGGG
GTTGCTCTGGAAAACTCATTTGCACCACTACTGTGCCTTGGAATACTAGTTGGAGT-----------------
----------------------------AATAAATCTTTGGATAAGATTTGG------AAT------
AACATGACCTGGATGGAGTGG---GACAGAGAAATTAACAATTACACAAGCTTAATATAC---
ACCTTAATTGAAGAATCGCAGAACCAGCAGGAA--

AAGAATGAACAAGAATTATTGGAATTGGATAATTGGGCAAGTTTGTGGAATTGGTTTGACATATCAAACTGGC
TGTGGTATATAAAAATATTCATAATGATAGTAGGAGGTTTAATAGGTTTAAGAATAGTTTTTACC---
ATACTTTCTATAGCG---------AGTAGAGTTAGGCAGGGATACTCACCA------
TTATCATTCGCTGTTAGTTTGCTCAATGCCACAGCTATAGCAGTAGCTGAG---
GGGACAGATAGAATTATAAGAGTAGTACAAATAATTGGTGGAGCT----------------------------
--------TTGCTTCACATA------CCTAGAAGAATCAGACAGGGATTAGAAAGGGCTTTGCTA---TAA
>KT427709
GCT---GCA--
GACAAGTTGTGGGTCACAGTCCATTATGGGGTACCTGTGTGG---AAAGAGGCAACC---
ACTACTCTATTCTGTGCATCAGATGCTAAGGCATATGACACAGAA---GTACATAAT----------------
--------------GTCTGGCCACA---CATGCCTGTGTACCCACAGAT---
CCTGACCCACAAGAATTAGAATTAAAA---------AATGTGACAGAAGATTTTAAC----------------
--------------------------ATGTGGAAAAATAACATGGTAGAACAGATGCATGAGGATATA---
ATCAGTTTATGGGATCAAAGCCTA---AAACCATGTGTAAAATTA---
ACCCCACTCTGTGTCACTTTAAGGTGTAATAATACCAAT----------------------------------
----
----------------------------AGTAGTGGGGAGGGAGTATTTAAAGGAGAAATGAAG---
AACTGCTCTTTCAATATCACCACTAGCATGAGA---------------------------
GATAAGGTACAAAAACAATATGCACTT---TTTTATACACTTGATATAGTACCAATAGATAAT----------
----

ATTAGCTATAGGTTGATAAGTTGTAACACCTCAGTCATTACACAG---
GCCTGTCCAAAGGTATCCTTTGAGCCAATTCCCATACATTATTGTGCCCCGGCTGGTTTTGCGCTTCTAAAAT
GTAAT---GATAAAAAGTTCGAT------GGAAAAGGGAAATGTAACAAT-----------------------
-GTTAGC---ACAGTACAATGTACACATGGAATTAAGCCAGTAGTATCAACT---
CAGCTGCTGTTAAATGGCAGTTTAGCAGAAGAAGAG------------
ATAGTAATTAGATCTGAAAATCTCACGAACAAT---------GCTAAAAGCATAATAGTACAGCTGAAT----
-----------AAGACTGTAACA---------ATTAATTGTATAAGGCCCAACAACAATACAAGAAGA-----
-AGTATAACTATA------------GGACCAGGAAGAGCA---TTT------TATGGAACA---GAT------
---------ATAATAGGAGATATA------------AGACAAGCACATTGC---AACCTTAGTAGTAAA---
GACTGGAATAATACTTTAAGACAGATAGCTAACAAATTAAAA---------------GAACAATTTATT----
-----------------------------------AATAAAACAATATATTTTAAT------
CAATCCTCAGGAGGGGACCCAGAAATTGTA---ATGCACAGTTTTAATTGTGGAGGGGAA---
TTTAAGCAGCTCCAGGCAAGAGTCCTGGCTGTGGAAAGATACCTAAGGGATCAACAGCTCCTAGGAATTTGGG
GTTGCTCTGGAAAACTCATCTGCACCACTGCTGTGCCTTGGAATGCTAGTTGGAGT-----------------
----------------------------AATAAATCTTTTGGGCAAATTTGG------AAT------
AACATGACCTGGATGGAGTGG---GAGAAAGAAATTGACAATTACACAGGCATCATATAT---
AACTTACTTGAGGAATCGCAGAACCAGCAGGAA--

AAGAATGAACAAGACTTATTGGAATTGGATAAGTGGGACAGTTTGTGGAATTGGTTTGACATAACAAAATGGC
TGTGGTATATAAAAATATTCATAATGATAGTAGGAGGCTTAATAGGTTTAAGAATAGTTTTTGCT---
GTATTTTCCATAGTA---------GATAGAGTTAGGCAGGGATACTCACCT------
TTGTCATTGGCTGTTAGTTTGCTTAATACCACAGCTATAGCAGTAGCTGAG---
GGGACAGACAGGATTATAGAAGTACTACAAAGAATTTTTAGAGCT----------------------------
--------ATTTGCAACATA------CCTAGAAGAATAAGACAGGGCTTAGAAAGGGCTTTGCAA---TAA
>KT427702
GCA--
GAACAGTTGTGGGTTACAGTCTATTATGGGGTACCTGTGTGG---AAAGAAGCAACC---
ACCACACTATTCTGTGCATCAGATGCTAAAGGTTATAAGACAGAG---GTACATAAT----------------
--------------GTTTGGGCCACA---CATGCATGTGTACCCACAGAT---

CCCAACCCACAGGAAGTAGAATTGACA---------AATGTGACAGAAAATTTTAAC----------------
--------------------------ATGTGGAAGAATAACATGGTAGAGCAGATGCATGAGGATATA---
ATCAGTTTATGGGATCAAAGCTTA---AAGCCATGTGTAAAACTA---
ACCCCACTCTGTGTTACTTTAAATTGTGCTAATATTACT---------------------------------------
----
----------------------------ACGAGTATGAATGTTACTGAAAATGGAGAAATGAAG---
AACTGCTCTTTCAACATCACCACAAGCATAAAA---------------------------
GATAAGATGCAGAAAGCATATGCTCTT---TTTTATAGATATGATATGGTACAAATAGATAATGAT-------
----

AGTACCAATAGTACCAATAGTACCAACTATAGGTTGATAAGTTGTAACACCTCAGTCATTACACAG---
GCATGTCCAAAGGTATCCTTTGAGCCAATTCCAATACATTATTGTGCCCCGGCTGGTTTTGCGATTCTAAAAT
GTAGA---GATAAAAAGTTCAAT------GGAAAAGGAGCATGTAAAAAT----------------------
-GTCAGC---ACAGTACAATGCACACATGGAATTAAGCCAGTAGTATCAACT---
CAACTGCTGTTTAATGGCAGTCTAGCAGAAGAAGAG-----------
GTAGTAATTAGATCTGAAAATATCTCAGACAAT---------GCTAAAACCATAATAGTACAGCTGAAG----
-----------GAACCTGTAGAG---------ATTAATTGTACAAGACCCAACAACAATACAAGAAAA-----
-GGTATACATATA------------GGACCAGGGAGAACA---TTT------TATGCAACAGGAGAA------
---------ATAATAGGAAATATA------------AGACAAGCACATTGT---AACCTTAGCAGCACA---
AAATGGAATAACACTTTATGGGAGATAGTTAAAAAATTAAGA---------------GAACAATTTAGG----
-----------------------------------AATAAAACAATAGTCTTTAAT------
AAATCCTCAGGAGGGGACCCAGAAATTGCA---CATATCACTTTTAATTGTGGAGGGGAA---
TTTAAGCAGCTCCAGGCAAGAGTCCTGGCTGTGGAAAGATACCTACAGGATCAACAGCTCCTGGGAATTTGGG
GTTGCTCTGGAAAACTCATTTGCCCCACTAATGTGCCTTGGAATATTAGTTGGAGT-----------------
----------------------------AACAAATCTCTGGATAACATTTGG------GGT------
AACATGACCTGGATGCAGTGG---GAGAAAGAAATTGACAATTACACAGGCTTAATATAT---
AACTTAATTGAAGAATCGCAGAACCAGCAGGAG---------------------------------------
----
AAAAATGAACAGGAATTGTTGGAATTGGATAAATGGGCAAGTTTGTGGAGTTGGTTTAGCATAACAAAATGGC
TGTGGTATATAAAAATATTCATAATGATAGTAGGAGGCTTGATAGGTTTAAGAATAGTCTTTACT---
GTACTTTCTATAGTA---------AATAGAGTTAGGCAGGGATACTCACCA------
TTATCGTTTGCTGTTAGCTTGCTCAATGCAATAGCCATAGCAGTAGGTGAA---
GTAACAGATAGGGTTCTACAAGGATTACAAAGGGCTTTTAGGGCT---------------------------
--------ATTCTCCACATA------CCTGTCAGAGTAAGACAAGGCTTGGAAAGGGCTTTGCTA---TAA
>KT427700
GCT---ACA---
GAAAAATTGTGGGTCACAGTCTATTATGGGGTACCTGTGTGG---AAAGAAGCAACC---
ACCACTCTATTTTGTGCATCAGATGCTAAGGCATATCAAACAGAA---GCACATAAT----------------
--------------GTTTGGGCCACT---CATGCTTGTGTACCCACAGAC---
CCTAACCCTCAAGAAGTAAAATTGGAG---------AATGTGACAGAAAATTTTAAC----------------
--------------------------ATGTGGAAAAATAACATGGTAGACCAAATGCATGAGGATATA---
ATCAGTTTATGGGATCAAAGCCTA---AAGCCATGTGTAAAATTA---
ACCCCACTCTGTGTTACTTTAACCTGCACTAATTTGAACCATACCAGCAATGGTACTAGAGCT----------
----
-------------------------AATAATGATAGTTTGGGAAATATGGAAGGAGAAATGAAA---
AACTGCTCTTTCAATGTCACCAGAAGCATAGGG---------------------------
AATAAGTTACGGAAAGAATATGCACTT---TTGTATAAACTTGATATAGTACCAATAAATAAC----------
----

AGTAATACTAGTTATATATTGATAAATTGTAACACCTCAGTCATTACACAG---
GCCTGTCCAAAGATATCCTTTGAACCAATTCCCATACATTATTGTACCCCGGCTGGTTTTGCGATTCTAAAGT
GTAAT---GATCATAAGTTCAAT------GGAACAGGACCATGTACAAAT----------------------
-GTCAGC---ACAGTACAATGTACACATGGAATTAGGCCAGTAGTATCAACT---
CAGCTGCTGTTAAATGGCAGTTTAGCAAAAGGAGAG-----------
GTAGTAATTAGATCTGAAAATTTCACGAACAAT---------GCTAAAACCATAATAGTACAATTGAAG----
-----------GACCCTGTGAAA---------ATTAATTGTACAAGACTCAGTAACAATACAAGAAAA-----
-AGTATACCTATA------------GGACCAGGGAGAGCC---TTC------TATGCAACAGGAGAC------
---------ATAATAGGGGACATA------------AGACAAGCACATTGT---AACCTTAGTATGGGA---
GAATGGAATAACACTTTAAAACAGATAGTTGAACAATTAAGA---------------AAACAATTTAAG----
-----------------------------------AATAAAACAATAGTCTTTAAC------

TCGTCTTCAGGAGGGGACCCAGAAATTGTA---ATGCACACGTTTAATTGTGGAGGGGAA---
TTTAAACAGCTCCAGGCTAGAGTCCTGGCTCTGGAAAGATACCTACAGGATCAACAGCTCCTAGGAATTTGGG
GTTGCGCTGGAAAACTCATCTGCACCACTACTGTGCCTTGGAATACTAGTTGGAGT-----------------
----------------------------AATAAATCTCTGGACATGATTTGG------AAA------
AACATGACCTGGATGGAGTGG---GAAAGAGAAATTGATAATTACACAGGCTTAATATAC---
ACCTTACTTGAACAATCGCAGAACCAGCAGGAA--

AAGAATGAACAAGAATTATTGGAATTGGATAAGTGGGCAAGTTTGTGGACTTGGTTTGACATAACACACTGGC
TGTGGTATATAAAAATATTTATAATGATAGTAGGAGGTTTAGTAGGTTTAAGAATAGTTTTTACC---
ATACTTTCTATAGTG---------AATAGAGTTAGGCAGGGATACTCACCA------
TTGTCATTTGCTGTTAGTTTGCTTAATGCCACAGCTATAGCAGTAGCTGAG---
GGGACAGATAGGGTTATAGAATTCCTACAAAGAATTGGTAGAGCT----------------------------
--------TTTCTCCACATA------CCTAGAAGAATCAGACAGGGCTTAGAAAGGGCTTTACTA---TAA
>KT427699
GCT---ACA--
GAAAAGTTGTGGGTCACAGTCTATTATGGGGTACCTGTGTGG---AAAGAAGCAACT---
ACCACTCTATTTTGTGCCTCAGATTCTAAAGCATATGAGACAGAG---GTACATAAT----------------
--------------GTTTGGGCCACA---CATGCCTGTGTACCCACAAAC---
CCCAAACCACAAGAAGTAGTATTGGAA---------AATGTGACAGAAGACTTTAAC----------------
--------------------------ATGTGGAAAAATAATATGGTAGAACAGATGAATGAGGATATA---
ATCAGTTTATGGGATCAAAGCCTA---AAGCCATGTGTAAAATTA---
ACCCCACTCTGTGTCACTTTAAATTGCACGGATGCTACTACTTTTAGTAATGCTACAGGG-------------
----
----------------------AATGTTACTTTGACCAATAGTACTGTTTTAGGAGAAATGAAA---
AACTGCTCTTTCAATATCACCACAGTCATAAGA---------------------------
GATAAGGTTAAGAAAGAATATGCACTG---TTTTACAAGCTTGATGTAATACCAATAGATAAT----------
----

AATACTAGAAACTATAGGTTGATACATTGTAACTCCTCAGCCATTACACAG---
GCCTGTCCAAAGGTATCATTTGATCCAATTCCCATACATTATTGCACCCCAGCTGGTTTTGCGATGCTAAAGT
GTAAT---GACAAGCAATTCAGT------GGAAAGGGACCATGTAAAAAT-----------------------
-GTTAGC---ACAGTACAGTGTACACATGGAATTAGGCCAGTAGTGACAACT---
CAACTGCTGTTAAATGGCAGTCTAGCAGAAGAGGAT------------
GTAGTAATTAGATCTGACAATTTCACAGACAAT---------ACTAAAACCATAATAGTACAGTTAAAT----
-----------GAAAGTGTTGTA---------ATTAATTGTACAAGACCCTCCAACAATACAAGAAAA-----
-AGGATATATGTA------------GGACCAGGGAGAGCA---GTG------TATGCAACAGACGCT------
---------ATAATAGGAGATATA------------AGACAAGCACATTGT---AACCTTAGTAGCCTA---
CAATGGAATAAAACCTTAAACAAGATATTTAAAAAATTAAAA---------------GAAAAATTCGAG----
-----------------------------------AATAAAACAATAGTCTTTAGG------
CCATCTGCAGGAGGGGACCCAGAGATTGTA---ATGCACAGTTTTAATTGTAGAGGGGAA---
TTTAAGCAGCTCCAGGCAAGAGTCCTGGCTGTGGAAAGATACCTAAGGGATCAACAGCTCCTAGGGATTTGGG
GTTGCTCTGGAAAACTCATCTGCACCACTACTGTGCCTTGGAATACTAGTTGGAGT-----------------
----------------------------AATAAATCTCTGGATAAGATTTGG------GAT------
AACATGACCTGGATGCAGTGG---GAAAAAGAAATTGATAATTACACAGGCTTAATATAT---
AACTTACTTGAAGAATCGCAGATCCAACAAGAA--

AAGAATGAACAAGAATTGTTGGAATTGGATAAATGGGCCAGTTTGTGGACTTGGTTTGACATAACACAATGGC
TGTGGTATATAAAGATATTCATAATGATAATAGGAGGCTTGATAGGTTTAAGAATAGTTTTTGCT---
GTACTTTCTATAGTA---------AATAGAGTTAGGCAGGGATATTCACCA------
TTATCGTTGGCTGTTAGCTTGCTCAATGCCACAGCCATAGCAGTAGCTGAG---
GGRACAGATAGGATTATAGAAGTAGYACAAAGAATTGGTAGAGCT----------------------------
--------TTTCTCCACATA------CCTAGAAGAGTGAGACAGGGCTTAGAAAGGGCTTTGCTA---TAA
>KT427692
GCT---CAA---
AAGGTCACAGTCTATTATAAGGTACCTGTGTAC---AAAGAAGCAAAC---
ACCACTCTATTCTGTGCATCAGATGCTAAAGCATATGACACAGAG---GCCCATAAT----------------
--------------GTT---GCCACA---CATGCCTGTGTACCCACAGAC---
CCTAACCCACAAGAAACAAGGTTGGAG---------AATGTGACAGAAAATTTTAAC----------------
--------------------------ATGTGGAAAAATAACATGGTAGAACAGATGCATGAGGATGTA---
ATCAGTTTATGGGATCAAAGCTTA---AAGCCATGTGTAAAGTTA---

ACCCCACTCTGTGTTGCTCTAAATTGCCATAATGTAACTTGCAACAGTACTAGTAACAGCAATAGTACT----
----
----------------------AGTAATAGCAATAATAGTTGTCCTGAGAAAGGAGAAATGACA---
AACTGCTCTTTCAACATCACCACACCCATAAAA--------------------------
GGTAAGGTGCAAAAAGAATATGCAATT---TTTGATAGACTTGATGTAGTGCCAATAAGTAAT----------
----

AATAATAATAATGATAATGGTACAGAATATATGTTGATACATTGCAATACCTCAGTCATTACACAG---
GCCTGTCCAAAGATAACCTTTGAACCAATCCCCATACATTATTGTACCCCGGCTAGTTTTGCGATTCTAAAGT
GTAAT---GAAAAGAAGTTCAAT------AGAACAGGACCATGTAAAAAT----------------------
-GTCAGC---ACAGTACAATGCACACATGGAATTAGGCCAGTAGTGTCAACA---
CATCTGCTGTTAAATGGCAGTCTGGCAGAAGAAAGG-----------
ATAGTAATTAGATCTGAAAATCTCACAAACAAT---------GCTAAGACCATAATAGTACAGCTGAAT----
-----------GAAACAGTAGAA---------ATTAATTGTACAAGACCCAACAACAATACAAGAAAA-----
-AGTATAACTATA------------GGACCAGGGAGAGCA---TGG------TATGCAACAGGAGAT------
---------ATAATAAGAAATATA-----------AGACAAGCACATTGT---AATGTTAGTGAAAAA---
AAATGGGAAGAAACTCTAAAACAGATAGTTATAGAATTAGCA--------------AAACAATTTCAG----
-----------------------------------AATAAAACAATAGCCTTTAAT------
CAACCCTCAGGAGAGGACCCAGAAGTTACA---ATGCACACTGTTAATTGTGGAGGGGAA---
TTTAAGCAGCTCCAGGCAAGAGTCCTGGCTGTAGAAAGATACCTAAGGGATCAACAGCTCCTGGGGATTTGGG
GTTGCTCTGGAAAACTCATTTGCACCACCTCTGTGCCTTGGAATGATAGTTGGAGC----------------
-------------------------AATAAATCTCTAGATGACATTTGGCAC------TCA------
AACATGACTTGGAGGGAGTGG---GAGAAAGAAATTGACAATTATACAGACATAATATAC---
AAATTACTTGAAGAATCGCAGACCCAACAAGAA--

AAGAATGAACAAGACTTATTGGCATTGGATACGTGGGAAAGCCTGTGGAATTGGTTTAGTATAACAAATTGGC
TGTGGTATATAAAAATATTCATAATGATAGTAGGAGGCTTAATAGGTTTAAGAATAGTTTTTACT---
GTACTTTCTATAGTG---------AATAGAGTTAGGCAGGGATACTCACCA------
TTGTCATTCGCTGTTAGCTTGCTTAATGCAACAGCTATAGCAGTAGCTGAG---
GGGACAGATAGGATCATAGAATTAGCACAAGCCATTGGTAGAGCC---------------------------
--------ATTCTCCACATA------CCTAGAAGAATAAGACAGGGCGCAGAAAGGGCTTTGCAA---TAA
>KT427792
GCT---ACA---
GAACAATTGTGGGTCACAATATATTATGGGGTACCTGTGTGG---AAAGACGCAAAC---
ACCTCTCTATTTTGTGCATCAGATGCTAAAGCATATGACACAGAG---GTACATAAT----------------
--------------GTTTGGGCCACA---CATGCCTGTGTACCCACAGAC---
CCTAAACCACAAGAAATAGAATTGGTA---------AATGTGACAGAAAATTTTAAC---------------
--------------------------ATGTGGAAAAATAACATGGTAGAACAGATGCATGAGGATATA---
ATCAGTTTATGGGATCAATTCCTA---AAACCATGTGTAAAACTA---
ACCCCACTCTGTGTCACTTTAAATTGTAATAATTGGAATGGGACCAAGAATGACACCAAT------------
----
--AAGACATGGGAGGGAGAAATAAAA---
AACTGCACTTTCAATGTCACATCAGGAGTGGGA-------------------------
GATAAAAGGAAGAAAGAATATGCACTT---TTTAATGCACTTGATGTAGTACCCATAGATAAA----------
----

AACAATAGTACAGAATATATGTTGATAAGTTGTAACACCTCAGTCATAACACAG---
GCCTGTCCAAAGATATCCTTTGAACCAATTCCTATACATTATTGTGCCCCGGCTGGTTTTGCGATTCTAAAGT
GTAAT---GATATAGAGTTCAAT------GGAACAGGACAATGTAAAAAT----------------------
-GTCAGC---ACAGTACAGTGTACACATGGAATTAAACCAGTAGTATCAACT---
CAACTACTGTTAAATGGCAGCCTATCAAAGGGAGAG-----------
GTAGTAATTAGATCTGAAAATTTCACGAGCAAT---------GACAAAACCATAATAGTACAGCTGAAT----
-----------AAATCTGTAGAA---------ATTAACTGTACAAGGCCCGGCAACAATACAAGAAGA-----
-GGTATACATCTT------------GGACCTGGGCAAGCA---ATA------TATGCAACAGGACAA------
---------ATAATAGGAGATATA-----------AGACAAGCACATTGC---AACCTTAGTAAAACA---
GAATGGGAAACAGCTTTAGAACAGACAGCTATACAATTGAGA--------------AAACAATTTGGC----
-----------------------------------AACAAGACAATAATCTTTAGG------
AATCACTCAGGAGGGGACCCAGAAATTACA---ATGTTCACTTTTAATTGTGGAGGGGAG---
TTTAAGCAGCTCCAGGCAAGAGTCCTGGCTGTGGAAAGATACCTAAAGGATCAACAGCTCCTAGGGATCTGGG
GTTGCTCTGGAAAACTCATTTGCACCACTACTGTACCTTGGAATGCTAGTTGGAGT----------------

```
---------------------------AATAAATCTTATAATCAGATTTGG------GAT------
AACATGACTTGGATGGAGTGG---GAGAAAGAAATTAACAATTATACAGGCTTAATATAT---
AGCTTAATTGAAGAATCACAAAACCAGCAGGAA-------------------------------------
----------------------------
ATAAATGAAAAAGAATTATTGGAATTGGATAACTGGGCAAATCTGTGGAATTGGTTTGACATATCAAATTGGC
TGTGGTATATAAAAATATTCATAATGATAGTAGGAGGCTTAATAGGATTAAGAATAATTTGTGCT---
GTGCTTTCTATAGTG---------AATAGAGTTAGGCAGGGATATTCACCA------
TTGTCATTCGCTGTCAGCTTGCTTAACACCACAGCTATAGTAGTAGCTGAG---
GGGACAGATAGGATTATAGAAATAACACAAAGAATTTGTAGGGGT--------------------------
--------ATTCGCAACATA------CCTAGAAGAATAAGACAGGGCTTCGAAAGGGCTCTGCTA---TAA
>KT427791
GCT---GCA---------------------------------------
GATGACTTGTGGGTCACAGTCTATTATGGGGTACCTGTGTGG---AAAGAAGCAACC---
ACCACTCTATTTTGTGCATCAGAGGCTAAAGCATATAAAACAGAA---GTACATAAT---------------
--------------GTTTGGGCTACA---CATGCCTGTGTGCCCACAGAC---
CCCAAACCACAAAAAGTAGTATTAAAA---------AATGTAACAAAAAAATTTTAAT---------------
-------------------------ATGTGGAAAAATAACATGGTARACCAGATGCATGAGRATATA---
ATCAGTTTATRGRATCAAAGCCTA---AAGCCATGTGTAAAATTA---
ACTCCACTCTGTGTTACTTTAAATTGCACTRACCCAAARATTAATAARACTRATAATRGTACCCCA-------
----------------------------------------------------------------------
-----------------------------------------CTGAATGATACTGGTGTAAAA---
AACTGCTCTTTCTACATCACCTCGGGCATAAGA--------------------------
GATAAGGTGCAGAAAGAACATGCACTT---
TTTTATACAATTGATTTAGTACCAATAGATAAAGATAATGATAAT--------------------------
---------------------------------------------------
ACTAGCGGTAGTGGTAATATTAGTTATAGGTTGATCAGTTGTAACACCTCAGTCATCACACAA---
GCCTGTCCAAAGGTAACCTTTGAGCCAATCCCCATACATTATTGTGCCCCGGCTGGGTTTGCGATTCTAAAGT
GTAAT---AATAAGACATTCAAT------GGAACAGGACCATGTACAAAT----------------------
-GTCAGC---ACAGTCCAATGTACACATGGAATTAAACCAGTGGTATCAACT---
CAACTGCTGTTAAATGGCAGTTTAGCAGAAGAAGAG-----------
GTAGTAATTAGATCTGAAAATATCACGGACAAT---------GCTAAAAACATAATAGTACAGCTGAAT----
-----------GAAACTGTAGAA---------ATTAATTGTACAAGACCCAACAACAATACAAGAAGA-----
-GGTATACATATA-----------GGACCAGGGAGGGTA---TTT------TATACATCAAAC---------
---------ATAGTAGGAGATATA-----------AGAAAAGCATATTGT---AACATTAGTAGAGAA---
AAATGGAATAACACTTTAAATAAGATAGTTAACAAATTAAGA-------------GAGCAATTTGTG----
----------------------------------AATAAAACAATAGTCTTTAAT------
CAATCCTCAGGAGGGGACCCAGAAATTGTA---ATGCACAGTTTTAATTGTGGAGGGGAA---
TTTAAGCAGCTCCAGGCAAGAGTCCTAGCTGTGGAAAGATACCTACAGGATCAACGGCTCCTAGGGCTTTGGG
GGTGCTCTGGAAAAATCATCTGCACCACTGCTGTGCCTTGGAATGCTAGTTGGAGT----------------
---------------------------AATAAATCTCTAAATGATATTTGG------AAT------
AACATGACCTGGATGGAGTGG---GAGAAAGAAATTGACAATTACACAGGGTTAATATAC---
ACTTTAATTGAAGAATCGCAAACCCAACAAGAA-------------------------------------
----------------------------
AAAAATGAAAAGGACTTATTGGAATTGGATAAATGGGCAAGTTTGTGGAATTGGTTTGACATAACAAACTGGC
TGTGGTATATAAAAATATTCATAATGATAGTAGGAGGCTTAGTAGGTTTCAGAATAATTTTTGCA---
GTATTTTCTTTAGTA---------AATAGAGTTAGGCAGGGGTATTCACCA------
CTATCATTTGCTGTCAGTCTGCTTAACGTCACAGCTATAGCAGTAGCCGAA---
GGGACAGATAGGGTTATAGAAATAGTACAAAGAGCTTGGAGAGGT--------------------------
--------ATTCTCCATATA------CCTAGAAGAATAAGACAGGGCTTTGAAAGGGCTTTGCTG---TAA
>KT427787
GCT---AAA---------------------------------------
GACAATTTGTGGGTCACAGTCTATTATGGGGTACCTGTGTGG---AAAGAAGCAAAC---
ACCACTCTATTTTGTGCATCAGATGCTAAAGCATATAAGAAAGAG---GCACATAAT---------------
--------------GTTTGGGCCACA---CATGCCTGTGTACCCACAGAC---
CCCAACCCACAAGAAGTAGCATTGGAA---------AATGTGACAGAAAATTTTAAC---------------
-------------------------ATGTGGAAAAATAACATGGTAGAACAGATGCATGAAGATATA---
ATCAGCTTATGGGATCAAAGCCTA---AAGCCATGTGTAAAATTA---
ACCCCACTCTGTGTTACTTTAAATTGTACTGATTTTGATACTACTACTAATACCAATAATACC----------
----------------------------------------------------------------------
-------------AATAGTAGTAGCGAGGTAAAAATGGATGAAACAATGAAAGGAGAAATAAAA---
```

AATTGTTCTTTCAACATCACCACAAGCATAAAA---------------------------
AATAAGCTGCAGAAAGAATATGCACTC---TTTTATAAACTTGATGTAACACCAATAGAGAAT----------
----

GAGAATACTAGCTATAGGATAATAGGTTGTAACACCTCAGTCATTACACAG---
GCCTGTCCAAAGATATCCTTTGAGCCAATTCCCATACATTATTGTGCCCCGGCTGGTTTTGCGATTCTAAAGT
GCAGA---GATAAGAAGTTCAAT------GGAACAGGACCATGTAGAAAT-----------------------
-ATCAGC---ACTGTACAATGTACACATGGAATTAGGCCAGTAGTATCAACT---
CAACTGCTGTTAAATGGCAGCTTAGCAGAAGAAGAT------------
GTAGTAATTAGATCTGCTAATTTCACAAATAAT---------GCTAAAACCATAATAGTACAGCTGAAC----
-----------GCATCTGTAGAA---------ATTAATTGTACAAGACCAGGCAACAATACAAGAAAA-----
-AGTATACATATA------------GGACCAGGGAAGGCA---TAT------TATGTAACAGGAAGC------
---------ATAACAGGAGATATA------------AGACAAGCACATTGT---AACATTAGTAAAGCA---
AAATGGAATGATACTTTAAGGCAGATAGCTAGAAAATTAAAA---------------GAACAATTTGAG----
-----------------------------------AATAAAACAATAGTCTTTAAT------
CAATCCTCAGGAGGGGACCCAGAAATTGTA---ATGCATACTTTTAATTGTGGAGGGGAA---
TTTAAGCAACTCCAGGCAAGAGTCCTGGCTGTGGAAAGATACCTACAGGATCAACAGCTCCTGGGGATCTGGG
GTTGCTCTGGAAGACTCATCTGCACCACTGCTGTGCCTTGGAATGTTAGTTGGAGT-----------------
----------------------------AATAAAACTCTGGATCAAATTTGG------GGG------
AACATGACCTGGATGGAGTGG---GAAAGAGAAATTGACAATTACACAGGCTTAATATAC---
ACCTTAATTGAAGAATCGCAGAACCAGCAGGAA--

AAGAATGAACAAGACTTATTGGCATTAGATAAATGGGCAAGCTTGTGGAATTGGTTTGACATAACAAATTGGC
TGTGGTATATAAAAATATTCATAATGATAGTAGGAGGCTTGATAGGTTTAAGAATAGTTTTTACT---
GTACTTTCTATAGTG---------AATAGAGTTAGGCAGGGATACTCACCA------
TTATCGTTTGCTGTTAGCTTGCTCAATGCCACAGCCATAGCAGTAGCTGAG---
GGGACAGATAGGGTTATAGAAATAGTACAAAGAATTGGTAGAGCT----------------------------
--------ATACTCCACATA------CCTAGAAGAATAAGACAGGGCTTGGAAAGGGCTTTGCTA---TAA
>KT427786
GCT---GTA--
GAACAATTGTGGGTCACAGTCTATTACGGGGTACCTGTGTGG---AAAGAAGCAACC---
ACCACTTTATTCTGTGCATCAGATGCTAGGGCATATCACACAGAG---GTACATAAT----------------
--------------GTTTGGGCCACA---CATGCCTGTGTGCCCACAGAC---
CCTAGCCCACAAGAAGTAAAATTGGAA---------AATGTGACAGAAAATTTTAAT----------------
--------------------------ATGTGGAAAAATGACATGGTAGAACAAATGCATGAGGATATA---
ATCAGTTTATGGGATCAAAGCCTA---AAACCATGTGTAAAATTA---
ACCCCACTCTGTGTTGTTTTAACTTGCACTGATGCTAATTTCACTACTAATGGTACTAGTAGCAATAATAATA
CTAGTGGCAAT--
----------------------------AGTAGTAGTAGTATTCTGGAAGATATGAAAATGAAA---
AACTGCTCTTTCAATGTCACCACAGGCATAGGA---------------------------
AATAAGATGCAGAAAGAATATGCACTT---TTTCATAAGCTTGATGTAATACCAATAGATGGTAAT-------
----

AATAGCACAAATTATACATTGATAAGTTGTAACACCTCAGTCATTACACAG---
GCCTGTCCAAAGGTATCCTTTGAACCAATTCCCATACATTATTGTGCCCCGGCTGGTTTTGCGATTCTAAAAT
GTAGA---GATACGAAGTTCAAT------GGAACAGGACCATGTTCAAAT-----------------------
-GTCAGC---ACAGTACAATGTACACATGGAATTAAGCCAGTAGTGTCAACT---
CAACTACTGTTAAATGGCAGTCTAGCAGAAGAAGAA------------
GTAGTAATTAGATCTCAAAACTTCTCGGACAAT---------GGGAAAATCATAATAGTACATCTGAAT----
-----------GAATCTGTAGCA---------ATTAATTGTACAAGACCCGGCAACAATACAAGAAGA-----
-AGTATAACCTTC------------GGACCGGGGAAAGCC---TTT------TATGCAACAGGAAAC------
---------ATAATAGGAGATATA------------AGACAAGCACATTGT---AACATTAGTAGGGTC---
CAATGGAATAATACTTTAAAACTGATAGTCACAAAATTAAGA---------------GAACATTGG-------
-----------------------------------AATAACACAATAATATTTAAT------
CACCCCTCAGGAGGGGACCCAGAAATTGTA---ATGCACAGTTTTAATTGTGGAGGGGAA---
TTTAAGCAGCTCCAGGCAAGAGTCCTGGCTGTAGAAAGATACCTAAGGGATCAACAGCTCCTAGGGATTTGGG
GTTGCTCTGGAAAACTCATCTGCACCACTGCTGTGCCTTGGAATACTAGTTGGAGT-----------------
----------------------------AATAAAAATCTGACTACGATTTGG------GAC------
AACATGACCTGGATGGAGTGG---GAGAGAGAAATTGACAATTACACAGGCTTAATTTAC---
ACTTTACTTGAAGAATCGCAAAACCAACAAGAA--

AAGAATGAACAAGAGCTATTGGCATTAGATTCATGGGCAAGTTTGTGGAATTGGTTTGACATAACAAAATGGC
TGTGGTATGTAAAAATATTCATAATGGTAGTAGGAGGTTTAATAGGTATAAAAATAGTTTTTACT---
GTGCTTTCTATAGTG---------AATAGAGTTAGGCAGGGATACTCACCA------
CTGTCATTGGCTGTTAGTTTGCTTAATGCCACAGCTATAGCAGTAGCTGAA---
GGGACAGATAGGGTTATAGAAGTAGTACTCAGAGTTTGTAGAGCT----------------------------
--------ATTTACCACATA------CCTAGAAGAATAAGACAAGGCTTAGAAAGAACTTTACTG---TAA
>KT427784
GCT---ACA--
AACCAATTGTGGGTCACAGTTTATTATGGGGTACCTGTATGG---AAAGAAGCAACC---
ACCACTTTGTTTTGTGCATCAGATGCTAAAGCATATGACACAGAG---GTACATAAC----------------
--------------GTTTGGCCACA---CATGCCTGTGTACCCACAGAC---
CCCAACCCACAAGAAGTAATATTGGAG---------AATGTGACAGAAAATTTTAAC----------------
--------------------------ATGTGGACAAATAACATGGTAGAGCAGATGCATCAGGATATA---
ATCAGTTTATGGGATGAAAGTCTA---AAGCCATGTGTAAAATTA---
ACCCCACTCTGTGTTACTTTAAATTGCAACACTCCAAATTTTAATGCCACTTCAAATTGTAATAGTACC----
----
-------------GGAAGTGCTAGTACCAATTGTAGTTTGGGAGATATGAAAGAAGAAATGAAA---
AACTGCTCATTCAATATCACCACAAGCATAAGA---------------------------
GATAAGAGGCGTGAAGAATATGCACTT---TTTTATAAAAATGATATAGTATCACTAGGTAAT----------
----

AGCAGCTATACATTGATAAACTGTAATACCTCAGTCATTACACAG---
GCTTGTCCAAAGGTATCCTTTGAACCAATTCCCATACATTATTGTACCCCGGCTGGTTTTGCGATTCTAAAGT
GTAAT---GATAAAAAGTTCAAT------GGGACAGGACCATGTAGAAAT-----------------------
-GTCAGC---TCAGTACAATGTACACATGGAATTAGGCCAGTAGTATCAACT---
CAGCTACTGTTAAATGGCAGTCTAGCAGAAGAAGAA------------
GTAGTAATTAGATCCCAAAACATCACGAACAAT---------GCTAAAACCATAATAGTACACCTGAAT----
-----------GAATCTGTACAA---------ATAAATTGTACAAGACCTGGCAACAATACCAGAAAA-----
-AGTATAAACATA------------GGACAAGGAGGAGCA---TGG------TATGCAACGGGAGCC------
---------ATAATAGGAGATATA------------AGACAAGCACATTGC---AACATTAGTGGAGCA---
GCCTGGAACAAAACTTTAAAACAGATAGTTATAAAATTAAGA---------------GAACAATTTGGG----
-----------------------------------AACAAAACAATAATCTTTAGT------
CCATCCTCAGGAGGAGACCCAGAAATTGTA---ATGCACACTTTTAATTGTGGAGGGGAA---
TTCAAGCAGCTCCAGGCAAGAGTCCTGGCTGTAGAAAATTACCTAAAGGATCAACAGCTCCTGGGGATTTGGG
GATGCTCTGGAAAACTCATTTGCACCACTGCTGTGCCTTGGAATGCTAGTTGGAGT-----------------
----------------------------AATAAAACTCTAAAGCAGATTTGG------GAC------
AACATGACCTGGATAGACTGG---GAGAGAGAAATTGACAATTATACAGACATAATATAT---
TCCCTAATTGAAGAATCGCAGAACCAACAGGAA--

AAGAATGAACAAGACTTATTGGCACTTGATAAATGGGCAAGCTTGTGGAGTTGGTTTGACATATCACAATGGC
TGTGGTACATAAGAATATTCATAATGATAGTAGGAGGCTTAATAGGTTTAAGAATAGTTTTTACT---
GTACTTTCTATAGTG---------AATAGAGTTAGGCAGGGATATTCACCA------
TTATCGTTCGCTGTTAGTTTGCTTAATGCCATAGCTATAGCAGTAGGTGAG---
GGGACAGATAGGATTATAGAAGCAACACAAAGATTTGGTAGAGCT----------------------------
--------ATTCTCAACATA------CCTAGAAGAATAAGGCAGGGCTTTGAAAGGGCTTTGTTA---GAA
>KT427781
GCT---GCA--
GAAAATTTGTGGGTCACAGTCTATTATGGGGTACCTGTGTGG---AGAGAAGCAAAC---
ACCACTCTATTTTGTGCATCAGATGCTAAAGCATATGACACAGAG---GCACATAAT----------------
--------------GTTTGGGTCACA---CATGCCTGTGTACCCACAGAT---
CCCAACCCACAAGAAATAAATTTGGAA---------AATGTCACAGAAAATTTTAAT----------------
--------------------------ATGTGGAAAAATAACATGGTAGAACAGATGCATGAGGATATA---
ATCAGTTTATGGGATGAAAGCCTA---AAGCCATGTGTAAAGTTA---
ACCCCACTCTGTGTTACTTTAAATTGCACCAATACTACTAGTACCAACAATAGTAGTTTAAATGCTACTACTA
CC---
----------------------------CCTAGTAGTATGGGAAACATGAAGGAAGACATGAAA---
AACTGCTCTTTCAATACCACCACAAGCATAAAA---------------------------
GATAAACAGAGGAAAGAATATGCACTT---TTTTATAAACTTGATGTAGTATCAATAGAAAAT----------
----

AATAGTACAAGCTATATGTTGATAAATTGTAACACCTCAGTCATTACACAG---
GCCTGTCCAAAGACATCCTTTGAACCAATTCCCATTCATTATTGTGCCCCGGCTGGTTTTGCGATTCTAAAAT
GTAAT---GATAAGAAGTTCAAT------GGAACAGGACCATGCACCAAT-----------------------
-GTCAGC---ACAGTACAATGCACACATGGAATTAGACCAGTAGTGTCAACT---
CAGCTGCTGTTAAATGGCAGTCTAGCAGAAGAAGAG------------
GTAGTAATTAGATCTGAAAATCTCACAAACAAT---------GCTAAAACCATAATAGTACAGTTGAAG----
-----------GAAGCTGTAGTA---------ATTAATTGTACAAGACTCGGCAACAATACAAGAAAA-----
-AGCATACATATG------------GGATTTGGGAGAACA---TTG------TATGCAACAGGAGAA------
---------ATAATAGGAGATATA------------AGAAAAGCACATTGT---AACATTAGTAGAACA---
CAATGGAACAGCACCTTAAAACAGATAGCTAAAAAATTGGAA---------------GAGCAATTTAAA----
-----------------------------------AACAAGACAATAGTCTTTAAT------
CAAACCTCAGGAGGGGACCCAGAAGTTACA---ATGCTCACTTTTAATTGTGGAGGGGAA---
TTTAAGCAGCTCCAGGCAAGAATCCTGGCTGTGGAAAGATACCTAAAGGATCAACAGCTCCTAGGGATTTGGG
GTTGCTCTGGAAAACTCATCTGCACCACTAATGTGCCTTGGAATGTTAGTTGGAGC-----------------
----------------------------AATAAATCTTATAATGACATTTGG------GGT------
AACATGACCTGGATGGAATGG---GAGAGGGAAATTAGCACTTACACAAACACAATATAC---
ACTTTAATTGAACGATCGCAAAATCAGCAAGAA--

AAGAATGAACAAGAATTATTGGAATTGGATAAGTGGGCAAGTTTGTGGAGTTGGTTCGACATAACAAACTGGC
TGTGGTATATAAAAATATTCATAATGATAGTAGGAGGCTTAGTGGGCTTAAGAATAGTTTTTACT---
GTAATTTCTATAGTG---------AATAGAGCTAGGCAGGGATACTCACCA------
GTATCATTAGCTGTTAGTTTGCTTAACGCTATAGCCATTGCAGTAGCTGAA---
GGGACAGATAGGATTATAGAAGTAATACAAAGAACTTGTAGAGCC----------------------------
--------ATTCTCCATATA------CCTAGAAGAATAAGACAGGGTTTAGAAAGGACTTTGCTA---TAA
>KT427656
GCT---GAA---GAA------------------------------------
GAAAAAATGTGGGTCACAGTCTATTATGGGGTACCTGTGTGG---AAAGAGGCAACT---
ACCACTCTATTTTGTGCATCAGATGCTAAAGCATATAGCACAGAG---GCACATAAT-----------------
--------------GTTTGGGCCACA---CATGCCTGTGTACCCACAGAC---
CCTAATCCACATGAAGTAGTATTGAAA---------AATGTGACAGAAAATTTTAAC----------------
--------------------------ATGTGGAAAAATGACATGGTAGAACAAATGCATGAGGATGTA---
ATCAGTCTATGGGATCAAAGCCTA---AAGCCATGTGTAAAACTA---
ACCCCACTCTGTGTCACTTTAAATTGCACTAATATCACTAGGAGCACAACTAATAGTAGCAGGAACGCA----
----
-------------------ACTGATAGTAACAATGCCACTAATATAGATGAAGAAGACATGAAA---
AACTGCTCTTTCAATATC---ACAGACATAGGA---------------------------
AACAAACAACGGAGGGATTATGCAATT---TTTAATAAACTTGATATAGTACCAATAGATAAT----------
----

AATAGCACAAACTATATGTTAATAAGTTGTAACACCTCAGTCGTTACACAG---
GCCTGTCCAAAGATATCCTTTGAACCAATTCCCATACATTATTGTGCCCCGGCTGGTTTTGCGATTCTAAAAT
GTAAT---GATTCACAGTTCAAT------GGAACAGGACCATGTAAAAAT-----------------------
-GTCAGC---ACAGTACAATGTACACATGGAATTAGACCAGTAGTGTCAACC---
CAACTGCTGTTAAATGGCAGTTTATCAGAAGGAGAG------------
GTAATGATTAGATCTGAAAATCTCACTAACAAT---------GCTAAAAACATAATAGTACAGCTGAAT----
-----------AAAACTGTAGTA---------ATTAATTGTATAAGACCCGGCAACAATACAAGAAAA-----
-AGTATAAATATG------------GGTCCGGGAAGAGTA---TTT------TATGCAACAGGACAA------
---------ATAATAGGAGATATA------------AGACAAGCACATTGT---AATATTAGTGGAAAA---
GAATGGCACAACACTCTAGGAAACATAACTGCAAAATTAAGA---------------GAAAAATTT-------
-----------------------------------AATAAAACAATAATCTTTAAT------
CAATCCTCAGGAGGAGACCAAGAAATTGTA---AAATTCACTTTTAATTGTGGAGGGGAA---
TTTAAGCAGCTCCAAGCAAGAGTCCTGGCTGTGGAAAGATACCTAGAGGATCAACAGCTCCTAGGGATTTGGG
GTTGCTCTGGAAAACTCATCTGCACCACTGCTGTGCCTTGGAATAGTAGTTGGAGTAAAAAA---CAT-----
----------------------------AAAACAAATCTGAGCACTATTTGG------GAT------
AACATGACCTGGATGGAGTGG---GAGAGAGAAATTGATAATTATACAGGATTAATATAC---
CAATTACTTGAAGAATCGCAGACCCAACAAGAA--

CAGAATGAACAAGATTTATTGGCATTGGATAAGTGGGCAAGTTTGTGGAATTGGTTCAGCATAACAAAATGGC
TGTGGTATATAAAAATATTCATAATGATAGTAGGAGGCTTAATAGGTTTAAGAATAGTTTTTACT---

GTACTTTCTATAGTA---------AATAGAGTTAGGCAGGGATATTCACCA------
TTGTCATTTGCTACTAGTTTGCTTAATGCTACAGCTGTAACAGTAGCTGAG---
GGAACAGATAGAGTGATAGAAATAGTACAAAGGGCTGGCAGAGCT----------------------------
--------ATTCTCCACATA------CCTAGAAGAATAAGACAGGGCTTAGAAAGGGCTTTGCTA---TAA
>KT427650
GCT---AAA---GAA------------------------------------
GAACAATTGTGGGTCACAGTTTATTATGGGGTACCTGTGTGG---AAAGAAGCAACC---
ACCACTCTATTCTGTGCATCTGATGCTAAAGCATATAAGACAGAG---GCACATAAT----------------
--------------GTTTGGGCCACA---CATGCCTGTGTACCCACAGAC---
CCTAATCCACAAGAAATAGTATTGAAA---------AATGTGACAGAAAATTTCAAC----------------
-------------------------ATGTGGAAAAATAACATGGTAGATCAGATGCATGAGGATATA---
ATCAGTTTATGGGATCAAAGTCTA---AAGCCATGTGTAAAATTA---
ACCCCACTCTGTGTTACTTTAAATTGCACTAATGCAACTGCTACTAGGGTTAATACCACT-------------
----
-------------------GTTGGGTATACCACTGTTGTGCCGAAAACGACGGCAGAAATAAAA---
AATTGCTCTTTCAATGTC---ACAAGCATAGGA--------------------------
AACAAGAACCGGAAAGAATATGCACTT---TTTAATAGCCTTGATGTAGTGCCAATAGATAAT----------
----

AATAATACAGAATATATGTTGGTAAGTTGTAACACCTCAGTCATTACACAA---
GCCTGTCCAAAGGTATCCTTTGAACCAATTCCCATACATTATTGTGCCCCGGCTGGTTTTGCGATTCTAAAGT
GTAAC---GATAAGAAGTTCAAT------GGAAAAGGACCATGTACAAAT----------------------
-GTCAGC---ACAGTACAATGTACACATGGAATTAGGCCTGTAGTGTCAACT---
CAACTACTGTTAAATGGCAGTTTATCAGAAGGAGAC------------
ATAATAATTAGGTCTGAAAATATTACAAACAAT---------GCTAAAACCATAATAGTGCAGCTGAAT----
-----------GAAACTGTACAA---------ATTAATTGCACAAGACCCAACAACAATACAAGGAAA-----
-AGTATACACATG------------GGACCGGGAAAAGCA---TTT------TATGCAACAGGAGAC------
---------ATAATAGGAAATATA------------AGACAAGCATACTGT---ACCCTTAATGGAACA---
AAATGGAATAATACTTTAAAACAAATAGTTACAAAGTTGAGA---------------GAAAACTTT-------
-----------------------------------AATAAAACAATAAATTTTAAT------
CAATCCTCAGGAGGGGACCCAGAAGTTGTA---ATGTTCAATTTTAATTGTGGAGGGGAA---
TTTAAGCAGCTCCAGGCCAGAGTCCTGGCTGTGGAAAGATACCTAAGGGATCAACAGCTCCTAGGGATTTGGG
GTTGCTCTGGAAAACTCATCTGCAACACTGATGTGCCTTGGAATGCTAGTTGGAGT-----------------
-----------------------------AATAGAAATCTGACAAGTATTTGG------AAA------
AACATGACCTGGATGCAGTGG---GAAAGAGAAATTGACAATTACACAGAATTAATATAC---
AGTTTACTTGAAGCATCGCAAAACCAACAAGAA--

CAGAATGAACAAGAATTATTGGCATTGGATAAGTGGGCAAATTTGTGGAATTGGTTTGATATAACAAAATGGC
TGTGGTATATAAAAATATTCATAATGATAGTAGGAGGCTTAATAGGTTTAAGAATAGTTTTTACT---
GTACTTTCTATAGTG---------AATAGAGTTAGGAAGGGATACTCACCA------
TTGTCATTGGCTATTAGTTTGCTTAATGCCACAGCTATAGCAGTAGCTGAG---
GGGACAGATAGAGTGATAGAAGTAGTACAAGCAATTGGCAGAGTT----------------------------
--------ATCCTTCACATA------CCTAGAAGAATAAGACAGGGCTTAGAAAGGGCTTTAATA---TAA
>KJ849811
GCT---GCA---GAA------------------------------------
GAACAATTGTGGGTCACAGTTTATTATGGGGTACCTGTGTGG---AAGGAAGCAAAC---
ACCACTCTATTTTGTGCATCAGATGCTAAAGCATATAACACAGAG---AGCCATAAT----------------
--------------GTTTGGGCCACA---CATGCCTGTGTACCCACAGAC---
CCCAACCCACAAGAACTAGTATTGAAA---------AATGTGACTGAAAATTTTAAC----------------
-------------------------ATGTGGAAAAATAACATGGCAGAACAAATGCATCAGGATATA---
ATCAGTTTATGGGATGAAAGCCTA---AAGCCATGTGTAAAATTA---
ACCCCACTCTGTGTCACTTTAAATTGCACTGATTTGAATTGTAGTAATAGTACTATTAATAAG----------
----
-------------------------ACTTCTTGTATAGATCAACAGATGAAGGGGGAAATAAAA---
AACTGTTCTTTTAATATCACCACAAGCATAAAA--------------------------
AATAGGATGCATAAGGAATATGCACTT---TTTTATAAACTTGATGTAACACAAATAGATAAT----------
----

ACAAGCTATACATTGGTAAGTTGTAATACCTCAGTCATTACACAG---
GCCTGTCCAAAGACATCCTTTGAACCAATTCCTATACATTATTGTACCCCGGCTGGTTTTGCGATTCTAAAAT

GTAAT---GATAAAAAATACAAT------GGAACAGGACCATGTACAAAT----------------------
-GTCAGC---ACAGTACAATGTACACATGGAATTAGGCCAGTGGTGTCAACT---
CAACTACTGTTAAATGGCAGTCTAGCAGAAGAAGAG-----------
ATAGTAATTAGATCTGAAAATCTCACAGACAAT---------GCTAAAATTATAATAGTACACCTGAAC----
-----------GAGTCTGTACAA---------ATTAATTGTACAAGACCCAACAACAATACAAGAAAA-----
-AGTATACATATG------------GGATTTGGGAGAGCA---TTT------TATGCAACAGGAGAA------
---------ATAATAGGAGATATA------------AGACAAGCACATTGT---AACCTCAGTAGAACA---
AAATGGATAACCACTTTAGGAAAGGTAGCCACAAAACTAAGA--------------AAAATATAT-------
---------------------------------AACAGAACAATAGTCTTTAAA------
AATTCCTCAGGAGGGGACCCAGAAATTGTA---ATGCACACTTTTAACTGTGGAGGGGAG---
TTTAAGCAGCTCCAGGCAAGAGTCCTGGCTGTGGAAAGATACCTAAGGGATCAACAGCTCCTGGGAATTTGGG
GTTGCTCTGGAAAACTCATTTGCCCCACTGCTGTGCCTTGGAATGCTAGTTGGAGT----------------
---------------------------AATAAAAACTTAAGTAGCATATGG------GAT------
AACATGACCTGGATGGAGTGG---GAAAAAGAAATTGACAATTACACAACAGAAATATAT---
AATTTACTTGAAGAATCGCAATACCAACAGGAA---------------------------------------
----
AAGAATGAACAAGACTTATTGGCTTTGGATAAGTGGGCAAATTTGTGGAGTTGGTTTGACATATCAAATTGGC
TGTGGTATATAAAAATATTCATAATGATAGTAGGAGGCTTAATAGGTTTAAGAATATTTTTTACT---
ATAATTTCTTTAGTG---------AATAGAGTTAGGCAGGGATACTCACCA------
TTATCGTTCGCTATTAATTTGTTTAATACTGTAGCTATAACAGTAGCTGAG---
GGGACAGATTGGATTATAGAAATAGTACAAAGACTTGGCAGAGCT--------------------------
--------ATTATCCACATA------CCTAGAAGAATAAGACAAGGCTTAGAAAGAATTTTGCTA---TAA
>KJ849825
GGT---TCA--
GACAATTTGTGGGTCACAGTCTATTATGGGGTACCTGTGTGG---AAAGAAGCAACC---
ACCACTCTGTTCTGTGCATCAGATGCTAAAGCAACTGACCCAGAG---GTACATAAT---------------
--------------ATTTGGGCCACA---CATGCCTGTGTACCCACAGAC---
CCTGATCCACAAGAGATAAAATTGAAA---------AATGTGACAGAAAATTTTAAC---------------
-------------------------ATGTGGAAAAATAACATGGTAGAACAGATGCATGAGGATATA---
ATTAGTTTATGGGATCAAAGTCTA---AAGCCATGTGTAAAATTA---
ACCCCACTCTGTGTTACTTTACATTGCTCCAATTACACTGGTAACAGTACTAATACT---------------
----
---------------------------AATAGAGACTTTAAGAGAAATGGAGGGGAAATACAA---
AACTGTACTTTCAATGTCACCACAAGCGCAAGA-------------------------
GATAAGATGAGGGAAGAATATGCACTT---TTTTACAGACTTGATATAGAACCAATAAATACA----------
----

GATAAGACAAGATATACAAAGATAAACTGTAACACCTCAGTCATTACACAG---
GCCTGTACAAACGTAAATTTTGATCCAATTCCCATACATTATTGTGCCCCGGCTGGTTTTGCGATTCTAAAGT
GTACG---GATAAAAATTTCAAT------GGAACAGGACCATGTAAAAAT----------------------
-GTCAGC---ACAGTACAGTGTACACATGGAATTAAGCCAGTAGTGTCAACT---
CAACTGCTGTTAAATGGCAGTCTATCAGAAGGAGAC-----------
ATAGTAATTAGATCTGAAAATATCACAAACAAT---------GCTAAAACCATAATAGTACAGCTAAAT----
-----------ACATCTGTAAAA---------ATTAATTGTACAAGACCCAACAACAATACAAGCAAA-----
-AGTATACATATA------------GGACAGGGAAGAGCA---TGG------TCTGCAACAGGAAGA------
---------ATAATAGGAGATATA------------AGACAGGCACATTGT---AACCTTAGCGCAGAA---
CAATGGAATAAAACTTTAGGACTGGTAGCTAAAAAATTACAA--------------GAACAATTTAAA----
----------------------------AATAAATCAATAGCAATAGCCTTTCAG------
CCGCACTCAGGAGGGGACCCAGAAATTGTA---ATGCACAGTTTTAATTGTAGAGGGGAA---
TTTAAGCAGCTCCAGGCAAGAGTCCTGGCTGTAGAAAGATACCTAAAGGATCAACAGCTCCTGGGAATTTGGG
GTTGCTCTGGAAAACTCATTTGCACCACTTCTGTGCCTTGGAATACTAGTTGGAGT----------------
---------------------------AATAAAAATCTGAGTGCAATTTGG------GAC------
AACATGACTTGGATGCAGTGG---GAGAGAGAAATTGACAATTACACAGACTTAATATAC---
ACATTGCTTGAAGAATCGCAGAACCAACAAGAA---------------------------------------
----
AAAAATGAACAAGAATTATTGGAATTGGATAAGTGGGCAAGTTTGTGGAATTGGTTTAGCATAACAAACTGGC
TGTGGTATATAAAAATATTCATAATAATAGTAGGGGGCTTAATAGGTTTAAGGATAGTTTTTACT---
GTACTTTCTATAGTG---------AATAGAGTTAGGCAGGGATACTCACCA------
TTGTCATTCGCTGTTAGTTTGTACAACGCCATAGCTATAGCAGTAGCTGAG---

GGGACAGATAGGGTTATAGAAGTAGTACAAAGATTTTGTAGAGCT----------------------------
--------ATTCTCCACATA------CCTACAAGACTAAGACAGGGCTTAGAAAGGCTTTTGCTA---TAA
>KT427775
GCT---ACA--
GAAAAATTGTGGGTCACAGTCTATTATGGAGTACCTGTGTGG---AAAGAAGCAACC---
ACCACTCTATTTTGTGCATCAGATGCTAAAGCATATCACCCAGAG---GCACATAAT----------------
--------------ATTTGGGCCACA---CATGCCTGTGTACCCACAGAT---
CCTAACCCACAAGAAGTAGTATTGGAA---------AATGTGACAGAAAATTTTAAC----------------
--------------------------ATGTGGAAAAATTACATGGTAGAACAGATGCAAGAGGATATA---
ATCAGTTTATGGGATCAAAGCCTA---AAGCCATGTGTAAGATTA---
ACCCCACTCTGTGTTAGTTTGAAGTGCACTGATTTGAAGAATGATACTAATACCAATAGT-------------
----
-------------------------AGTAGCGGGAGAATGATAATGGAGAGAGGAGACATGAAA---
AACTGTTCTTTCAATATCACCAATAACATAGAA---------------------------
GACAAGAGGCATAAAGAAAATGCACTG---TTTTATAAACTTGATGTAGTACAAGTAAACAGT----------
----

AGTGATAATACAAGCTATAGATTGATAAGTTGTAACACCTCAGTCGTTACACAG---
GCCTGCCCAAAGATATCCTTTGAACCAATTCCCATACATTTCTGTGCTCCAGCTGGTTTTGCGATTCTAAAGT
GTAAT---AATAATACGTTCAAT------GGATCAGGACCATGTACAAAT-----------------------
-GTCAGC---ACAGTACAATGTACACATGGAATTAGGCCAGTAGTGTCAACT---
CAACTGTTGTTAAATGGCAGTCTAGCAGAAGGAAAC---------
AAGACAGTAATTAGGTCTCAAAATTTCTCAAACAAT---------GCTAAAATCATAATAGTACAGCTGAGG-
--------------AAACCTGTAGAA---------ATTAATTGTACAAGACCCAATAACAATACAAGCAAA--
----GGTATACATATR------------GGACCGRGGARATCA---TTT------TATGCAACAGGAGCA---
------------ATAATAGRGGATATA------------AGGAAAGCTTATTGT---AACATTAGTAAAAAA-
--GAATGGAATGACACTTTAAAAGARATAGTTAAAGAATTAAGG---------------GAACAATTTAAC--
-------------------------------------AATAAAACAATAATCTTTAAT------
CAGTCCTCAGGAGGGGACCCAGAAATTGTA---ATGCACAGTTTTAATTGTGGAGGGGAA---
TTTAAGCAGCTCCAGGCTAGAGTCCTGGCTGTGGAAAGATACCTAAAGGATCAACAGCTCCTAGGAATTTGGG
GTTGCTCTGGAAAACTCATTTGCACCACTAATGTGCCTTGGAATACTAGTTGGAGT-----------------
----------------------------AATAAATCTCTAGACACAATTTGG------GAT------
AACATGACCTGGATGCAGTGG---GACAAAGAAATTAGCAATTACACAAGCTTAATATAC---
TCATTAATTGAAGAATCGCAAATCCAACAGGAA--

AAAAATGAAAAGGAGTTATTGGAATTGGATAAGTGGACAAGTTTGTGGAGTTGGTTTGACATATCAAACTGGT
TATGGTATATAAAAATATTCATAATGATAATAGGAGGTTTAGTAGGGTTAAGAATAGTTTTTACT---
GTACTTTCCTTAGTG---------AATAGAGTTAGGCAGGGATACTCACCA------
TTATCATTTGCTGTCAGCTTGCTTAATGCCATAGCTATAGCAGTAGCAGAG---
GGGACAGATAGGCTTATAGCAGTAATACAAACAGTTGGTAGAGCT----------------------------
--------CTTCTTCACATA------CCTAGAAGAATAAGACAGGGATTAGAAAGGGCTTTGCTA---TAA
>KT427769
GCT---GCA---
AACCTGTGGGTCACAGTATATTATGGGGTGCCTGTATGG---AAAGATGCAACC---
ACCACTCTATTTTGTGCATCAGATGCTAAAGCATATGACACAGAG---GTACATAAT----------------
--------------GTTTGGGCCACA---CATGCCTGTGTACCCACAGAC---
CCTAACCCACAGGAAATAGTATTGGAA---------AATGTGACAGAAAATTTTAAC----------------
--------------------------ATGTGGAAAAATAACATGGCAGAACAGATGCATGAGGATATA---
ATTGAATTATGGGATCAAAGCCTA---AAGCCATGTGTAAAAATA---
ACCCCACTCTGTGTTGTTTTAAACTGCACTAATTTGAATAGTACTAGCACCAATAGTACG-------------
----
----------------------------AATAGTACTAGTCAGAATGATTGGGGAGAAATGAAA---
AACTGCTCTTTCAATATCACCACAGGAATAGGA---------------------------
GATAAGAAGCAGCAAGAATATGCACTT---TTTTATACACTTGATATGGTATCATTAGGTAAT----------
----

GATAATACAAGCTATACGTTGAAAAATTGTAACACCTCAGTCATTACACAG---
GCTTGTCCAAAAGTATCTTGGGAACCAATTCCTATACATTATTGTACCCCGGCTGGTTTTGCGATTCTAAAGT
GTAAT---GATAAGAAGTTCAAT------GGAAAAGGACCATGTACAAAT-----------------------
-GTCAGC---ACAGTACAATGTACACATGGAATTAGGCCAGTAGTGTCAACT---

CAACTACTGTTGAATGGCAGTCTAGCAGAAGGGGAG------------
GTAGTAATTAGATCTGAAAACTTTACAGACAAT---------GCTAAAACCATCATAGTACAACTGAAC----
-----------GAAACTATAAAG---------ATTAATTGTACAAGACCCAACAACAATACAAGAAAA-----
-AGTATACATCTA------------GGAATGGGGAGAGCA---ATT------TATGCAACAGGAGGC------
---------ATAGTAGGAAATATA------------AGACAAGCACATTGT---AACATATCTAGAGTA---
GAATGGAATAACACTTTAAAATATATAGCTAAAAAGTTAGAA---------------GAACAGTTTAAA----
-----------------------------------AATAAAAGTATAAGCTTTAAG------
CCATCCTCAGGAGGAGACCCAGAAATTGTA---ATGCACACCTTTAATTGTGGAGGAGAA---
TTTAAGCAGCTCCAGGCAAGAATCCTGGCTGTGGAAAGGTACCTAAGGGATCAACAGCTCCTAGGGATTTGGG
GATGCTCTGGAAAACTCATTTGCACCACTGCTGTGCCTTGGAATAATAGTTGGAGT-----------------
----------------------------AATAAAACTTGGGATAAGATTTGG------AAT------
AACATGACCTGGATGGAGTGG---GAAAGAGAAATTGACAATTACACAGGCTTAATATAT---
AGCTTACTTGAAGAATCGCAGAACCAACAGGAC--

AAGAATGAACAAGAATTATTGGAATTGGATAAGTGGGCAAGTCTGTGGAATTGGTTTGATATAACAAAATGGC
TGTGGTATATAAAAATATTCATAATGATAGTAGGAGGCTTAATAGGTTTAAGAATAGTTTTTACT---
GTACTTTCTATAGTG---------AATAGAGTTAGGCAGGGATACTCACCA------
TTGTCATTCGCTGTCAGTTTGCTTAATGCCACAGCTATAGCAGTAGCTGAG---
AGGACAGATAGGATTATAGAAGTAGTACAAAGAATTTATAGAGGC----------------------------
--------ATTCTCCACATA------CCTAGACGAATAAGACAGGGCTTAGAAAGGGCTTTGCTA---TAA
>KT427767
GCT---GCA--
GAACAATTGTGGGTCACAGTCTATTATGGGGTACCTGTGTGG---AAAGAAGCAGAC---
ACCACTCTCTTTTGTGCATCAGATGCTAAAGCATATGATACAGAG---GTACATAAT----------------
--------------GTTTGGGCCACA---CATGCCTGTGTACCCACAGAC---
CCCAACCCACAAGAAGTAGAATTGGGA---------AATGTGACAGAAAACTTTAAC----------------
--------------------------ATGTGGAAAAATAACATGGTAGAACAGATGCAAGAGGATATA---
ATCAGTTTATGGGATCAAAGCTTA---AAGCCATGTGTGAAGTTA---
ACCCCACTCTGTGTTACTTTAATGTGGAATTATGTGGGGAATAAGACTAATGCCACTAGTGCTAATACC----
----
-------------------ACTGCCATAGTGCTAATATCATATAGAGACAAAGGAGAAATAAAA---
AACTGCTCTTTCATGTCACCT--
AAGGTGGTGAAAGAATATGCACTT---TTTTATAAACTTGATTTAGTACCATTAGATAAT-------------
----
----AATACTAGTAATAATAATAGTATTAGATATAAG---------------ACCTCAGTCACTACACAG---
GCCTGTCCAAAGGTATCCTTTGAGCCAATTCCCATACATTATTGTGCCCCGGCTGGTTTTGCGATTCTAAAAT
GTAGA---RAGGAGGAKTTTAAT------GGAACAGGACCATGTAATAAT-----------------------
-ATCAGC---ACAGTACAATGTACACATGGAATTAGGCCAGTAGTATCGACT---
CAACTGCTGTTGAATGGCAGTCTAGCAGCAAAAGAG------------
GTAATAATCAGATCTGCCAATCTCACAGACAAT---------ACTAAGACCATAATAGTACAACTGAAC----
-----------AAAGCTGTAAGA---------ATTAATTGTACAAGACCCAACAACAATACAAGAAAA-----
-AGTATACGTATCCAG------ATAGCACCAGGGAGAGCA---TTT------TATGCAACAGGA---------
---------AAAATAGGAGATATA------------AGAAGAGCACATTGT---AACATTAGTAGAGCA---
GATTGGAACGAAACTTTAGGACAGGTAGCTAGCAAATTAAGA---------------GAAAAATTTGGA----
--------------------------------AATAATAGAACAATAGTCTTTAAT------
CACTCCTCAGGGGGGGACCCAGAAATTGTA---ATGCACAGCTTTAATTGTGGAGGGGAA---
TTTAAGCAGCTCCAGGCAAGAGTCCTGGCTGTGGAAAGATACCTACAGGATCAACAGCTCCTGGGGCTTTGGG
GTTGCTCTGGAAAACTCATTTGCACCACTRCTGTACCTTGGAATACTAGTTGGAGT-----------------
----------------------------AATAAATCTCTGGATYWCATTTGG------GRS------
AACATGACCTGGATGCARTGG---GAAAAGGAAATTRACAAYTACACAGGCWTAATATAC---
AACTTAATAGAAAAATCGCAAAAYCAACAAGAA--

AAGAATGAACAAGAATTATTGGAATTAGATAAATGGGCAAGTTTGTGGAATTGGTTTGACATAACAAAATGGC
TGTGGTATATAAAAATATTCATAATGATAGTAGGAGGCATGGTAGGTTTAAGAATAGTTTTTACT---
GTACTTTCTATAGTG---------AATAGAGTTAGGAAGGGATACTCACCA------
CTATCGTTTGCTGTTAGCTTGTTCAACGCCATAGCCATAGCAGTAGCTGAG---
GGGACAGATAGGATTTTAGAGGCATTTCAAACAGCTTTTAGAGCT----------------------------
--------ATTATCCACATA------CCTAGAAGAATAAGACAGGGCTTGGAAAGGCTTTGCTA---TAA
>KT427764

GCG---GCA---
GAAGACTTATGGGTCACAGTATATTATGGGGTACCTGTGTGG---AAAGAAGCAACT---
ACCACACTATTTTGTGCATCAGATGCTAAAGCATATAGCCCAGAA---GCACACAAT---------------
--------------GTTTGGGCCACA---CATGCTTGTGTACCCACAGAT---
CCTAACCCACAAGAAGTAGTATTGACA---------AATGTGACAGAAAATTTTAAC---------------
--------------------------ATGTGGAAAAATAACATGGTAGAACAGATGCATGAGGATATA---
ATCAGTTTATGGGATCAAAGCCTA---AAACCATGTGTAAAATTA---
ACCCCACTCTGTGTTACTCTAAATTGCACTGATTTAAACAATACTACTGCTACCAATAGTACT---------
----
-------------------------AATACCAATAATACTATTAAGGAGAGGGAAGAAATGAAA---
AACTGCTCTTTCAATATCACCACAAGCATAAAA--------------------------
AGTAAAATGCAGAAAGAATTTGCACTT---CTGTATAAGCTTGATATAGTACCAATAGATAAT---------
----

AATAATAATAATACAAACTATATGTTGATAAATTGTAACACCTCAGTCATTACCCAA---
GCCTGTCCAAAGACAAACTTTGAACCAATTCCTATACATTATTGTGCTCCGGCTGGTTTTGCGATTCTAAAT
GTAAT---GATAAGACGTTCAAT------GGAACAGGACCATGTAAAAT-----------------------
-GTCAGC---ACAGTACAATGTACACATGGAATTAGGCCAGCAGTGTCAACC---
CAACTGCTGTTAAATGGCAGTCTATCAGAAGGAGAG-----------
GTAATGATCAGGTCTGAAAATTTCACAAACAAT---------GCTAAAATCATAATAGTACAACTGAAC----
-----------GAAACTATAGAA---------ATCAATTGTACAAGACCCAACAACAATACAAGAAAA-----
-AGTATACATATG------------GGATGGGGGAGAGCA---TTT------TATGCAACAGGAGAA------
---------ATAATAGGAGACATA------------AGAAAATCACATTGC---AACATTAGTGCAGAA---
AAATGGAGAACCACTTTAAAAAAGGTAGAGGAAAAATTAAAA---------------GAACAATTTCAG----
-----------------------------------AACAAAACAATATTATTTAAT------
GGATCCTCAGGAGGGGACCCAGAAATTGTA---ACGCACAGTTTTAATTGTGGAGGGGAG---
TTTAAGCAGCTCCAGGCAAGAGTCCTGGCTGTGGAAAGATATCTAGGGGATCAACAGCTCCTAGGGATTTGGG
GTTGCTCTGGAAAACTCATTTGCACCACTAATGTGCCTTGGAATTCTAGTTGGAGT----------------
----------------------------AATAAAAATCTGACACAGATCTGG------GAT------
AACATGACTTGGATGGAGTGG---GAGAGAGAAATTGACAATTACACACAAGTAATATAC---
AACTTGATTGAAGAATCGCAGAACCAACAGGAA---------------------------------------
----
AAGAATGAACAAGAATTATTGGCATTAGATAAGTGGGCAAGTTTGTGGAATTGGTTTAGCATAACAAACTGGC
TGTGGTATATAAAAATATTCATAATGATAATAGGAGGCTTAGTAGGTTTAAAAATAGTTTTTGCT---
GTATTTTCTATAGCG---------AATAGAGTTAGGCAGGGATACTCACCA------
TTGTCATTCGCTATTAGTTTGTTTAATGCCACAGCTATAGCAGTAGCTGAA---
GGAACAGATAGGGTTATAGAAGCAGCACAAAGAATTGGTATAGCT---------------------------
--------ATCTTCCATATA------CCTAGAAGAATAAGACAGGGGTTAGAAAGGCTTTTGCTA---TAA
>KT427761
GCT---AAA---AAA-----------------------------------
GAACAATTGTGGGTCACAGTATATTATGGGGTACCTGTGTGG---AAAGAGGCAACC---
ACCACTCTATTCTGTGCATCAGATGCTAAAGCATATGTTACAGAG---AAACATAAT---------------
--------------GTTTGGGCCACA---CATGCCTGTGTACCCACAGAC---
CCCAATCCACAAGAAATAGTACTGGGA---------AATGTGACAGAAAATTTTAAC---------------
--------------------------ATATGGAAAAATGACATGGTAGAACAGATGCATGAGGATATA---
ATCAGTTTATGGGATCAAAGCCTA---AAGCCCTGTGTAAAATTA---
ACCCCACTTTGTGTTACTTTAAATTGCACTGATTATAATACCACTAGCAATAATACCGCT------------
----
----------------------------AGTGGGATGGGACAGATGGAGTTAGGAGAAATAAAA---
AACTGCTCTTTCAACATCACCACAAACATAAAA--------------------------
AGGAAGATGCAGAAAGAATATGCACTT---
TTTTATAAGCTTGATGTAGTGCCAATAGATGATGATAAGAATAAT---------------------------
----
ACACATAATAATAGCAGCTATAGCAGCTATAGGTTGATAAGTTGTAACACCTCAGTCATTACACAG---
GCCTGTCCAAAAGTATCTTTTGAGCCAATTCCTATTCACTATTGTGCCCCAGCTGGTTTTGCGATTCTAAAGT
GTAAT---GATAAGAAGTTCAAT------GGAACAGGATCATGTAAAAT-----------------------
-GTCAGT---ACAGTACAATGTACACATGGAATTAAGCCAGTAGTGTCAACT---
CAACTGTTGTTAAATGGCAGTTTAGCAGAAGAAGAG-----------
ATAGTACTTAGATCTGAAAATATCACAAACAAT---------GCTAAAACCATAATAGTGCAGCTGAAC----
-----------AAAACTGTAGCA---------ATTAATTGTACAAGACCCAACAACAACACAAGAAGA-----

-GGTATACATGTA------------GGACCAGGGAGAGCA---CTT------TATACAACA---GAC------
---------ATAATAGGAGATATA------------AGACAAGCACATTGT---AACATTAGTAAAAAA---
CAATGGGAAAACACTTTAATACAGATAGGTGCAAAATTAAAT---------------GAAACATTTAGA----
-----------------------------------AAGGAAACAATAATCTTTAAT------
CAATCCTCAGGAGGAGACCCAGAAATTATA---ATGCACAGTTTTAATTGTGGAGGGGAA---
TTTAAGCAACTCCAGGCAAGAGTCCTGGCTGTGGAAAGATACCTAAAGGATCAACAGCTCCTGGGGATTTGGG
GTTGCTCTGGAAAACTCATTTGCACCACTAATGTGCCTTGGAATGTTAGTTGGAGT-----------------
----------------------------AATAAATCGCTAAATGAGATTTGG------AAT------
AACATGACCTGGATGGAATGG---GAAAAAGAAATTGATAATTACACAAGCTTAATATAT---
AACTTAATTGCAGACTCGCATATCCAACAAGAA--

AAGAATGAACAAGAATTATTAGAATTAGATAAGTGGACAAATTTGTGGAATTGGTTTGACATAACACGGTGGC
TGTGGTATATAAAAATATTCATAATAATAGTAGGAGGCTTGGTAGGTTTAAGAATAGTTTTTGCT---
GTACTTTCCTTAGTG---------AATAGAGTTAGGCAGGGATACTCACCA------
TTATCATTGGCTGTTAGCTTGCTCAACGCCACAGCCATAGCAGTAGCCGAG---
GGGACAGATAGAGTTATAGAAGTAGGACAAAGAATTTTTAGAGCT----------------------------
--------TTCCTGCACATA------CCTGTAAGAATCAGACAGGGCTTGGAAAGGGCTTTGCTA---TAA
>KT427757
GCT---GCA--
GGACAATTGTGGGTCACAGTATATTATGGGGTACCTGTGTGG---AAAGAAGCAAAC---
GCCTCTCTATTTTGTGCATCAGATGCTAAAGCATATGACACAGAA---GTACATAAT----------------
--------------GTTTGGGCCACA---CATGCCTGTGTACCCACAGAC---
CCTGACCCACAAGAAATAAATTTGGTA---------AATGTGACAGAAAATTTTAAC----------------
--------------------------ATGTGGAAAAATAACATGGTAGAACAGATGCATGAGGATATA---
ATCAGTTTATGGGAACAATACCTA---AAACCATGTGTAAAACTA---
ACCCCACTCTGTGTCACTTTAAATTGTATTAATGGGAATGTGACCAATACTAGAACTGAGACCAATAATGCGA
CC---
----------------------------ACTCAGGGCCCACTCTCATTGGAAGGAGAAATAAAA---
AACTGTACTTTCAATGTCACCACAGGAGTGGAA---------------------------
AATAAAAAGAGGAAAGAATATGCACTT---TTTAATAGTCTTGATGTAGTACCCATAGATGGT----------
----

AACAGTAATAGTACAGAATATATGTTGATAAGTTGTAATACCTCAGTCATAACACAG---
GCCTGTCCAAAGATATCCTTTGAACCAATTCCTATACATTATTGTGCCCCGGCTGGTTTTGCGATTCTAAAGT
GTAAT---GATATAGAGTTCAAT------GGAACAGGACTATGTAAAAAT-----------------------
-GTCAGC---ACAGTACAGTGTACACATGGAATTAGACCAGTAGTAACAACT---
CAACTACTGCTAAATGGCAGCCTTTCAAAGGGAGAG------------
ATAGTAGTTAGATCTGAAAATTTCACAAAGAAT---------GATAAAACCATAATAGTACAGTTAAAT----
-----------AAATCTATAGAA---------ATTAACTGTACAAGACCCGGCAACAATACAAGCAAA-----
-GGTATACATTTT------------GGACCTGGGCAAGCA---ATA------TATGCAACAGAACGA------
---------CTAATAGGAGATACA------------AGAAAAGCACATTGC---ATCCTTAATAAAACA---
GAATGGGAAACAGCTCTAGAACAGACAGCTAAAAAATTAAGA---------------GAAAAATTT-------
-----------------------------------AACAAGACAATAGTCTTTAGA------
AATCACTCAGGAGGGGACCCAGAAATTGTA---ATGTTCACTTTTAATTGTGGAGGGGAG---
TTTAAGCAGCTCCAGGCAAGAATCCTGGCTGTCGAAAGATACCTAAAGGATCAACAGATCCTAGGAATTTGGG
GCTGCTCTGGAAAACTCATCTGCACCACTACTGTACCTTGGAATTCTAGTTGGAGC-----------------
----------------------------AATAAATCTTATGATCAGATTTGG------AAC------
AACATGACTTGGATGGAGTGG---GAGAAAGAAATTAGTAATTATACAGGCTTAATATAT---
ACATTAATTGAAAAATCACAAAACCAGCAGGAA--

ATAAATGAAAAAGAATTGTTGGAATTGGATAAATGGGCAAGTCTGTGGAATTGGTTTGACATATCACATTGGC
TGTGGTATATAAGATTATTCATAATGATAGTAGGAGGCATAATAGGCTTAAGAATAATTTGTGCT---
GTGCTTTCTATAGTG---------AATAGAGTTAGGCAGGGATATTCACCA------
TTGTCATTTGCTATCAGCTTGCTTAATACCACAGCTATAGTAGTAGCTGAG---
GGGACAGATAGGATTATAGAAGTAACACAAAGAATTTGTAGAGCT----------------------------
--------ATTCGCAACGTA------CCTAGAAGAATAAGACAGGGCTTAGAAAGGGCTTTGCTA---TAA
>KT427756
GCT---ACA--
GACAAGTTGTGGGTCACAGTCTATTATGGGGTACCTGTATGG---AAAGAAGCAACC---
ACCACTCTATTTTGTGCATCAGATGCTAAAGCATATGATACAGAG---GTGCACAAT----------------

--------------GTTTGGGCCACA---CATGCCTGTGTACCCACAGAC---
CCTAACCCACAAGAAGTAGTATTGCTG---------AATGTGACAGAAAGTTTTAAC----------------
--------------------------ATGTGGAAAAATAACATGGTAGAACAGATGCATGAGGATATA---
ATTAGTCTATGGGATCAAAGCCTA---AAGCCATGTGTAAAATTG---
ACCCCACTTTGTGTTACTTTAAAGTGCAATGAGACTAATTTGAAGAATGAAACTAATGGAAAT----------
----
-------------------GTTACTATTACAAATGTTACACTGAATGGGGAGGGAGGAATAAAA---
AACTGCTCTTTCAATGTCACCACAGGCCTAAAT---------------------------
GATAAAAGGCAGACAGCATATGCAACT---TTTTATAATCTTGATATAGTACAAATAAATAAA----------
----

ACTAAAGATAATAGTACATATAGGTTAATAAGTTGTAATACCTCAGTCATTACACAG---
GCCTGTCCAAAGATATCCTTTGAGCCAATTCCCATACATTATTGTGCCCCAGCTGGTTTTGCGATCCTAAAGT
GTAAT---GATAAGATGTTCAAT------GGAACAGGACCATGTACAAAT-----------------------
-GTCAGC---ACAGTGCAATGCACACATGGAATTAGGCCAGTAGTAACAACT---
CAACTGCTGTTAAATGGCAGTCTAGCAGAAGAAGAG------------
GTAGTAATTAGATCTGCCAATTTCACAGACAAT---------ACTAAAACCATAATAGTACATCTGAAT----
-----------GAATCAGTAGAA---------ATTAATTGTACAAGACCCAGCAACAATACAAGAAAA-----
-AGTATACATTTT------------GGACCAGGGAAAGCA---TTT------TATGCAACAGACGAA------
---------GTAATAGGAGATATA------------AGAAAAGCACATTGT---AACATTAGCAGAACA---
AAATGGAATACCACTTTACAACGAGTACTTGAAAAACTAAGA---------------CAACATTGG-------
-----------------------------------AATAAAACAATATTCTTTGAA---
CAACCTCCTCCAGGAGGGGACCTAGAAATTGTA---ATGCACAGTTTTAATTGTGGAGGGGAA---
TTTAAGCAGCTCCAGGCAAGAGTCCTGGCTGTGGAAAGATACCTAAAGGATCAACAGCTCATGGGGATTTGGG
GTTGCTCTGGAAAACTCATCTGCACCACTGCTGTGCCTTGGAATACTAGTTGGAGT-----------------
----------------------------AACAAAAGTTATAATGCCATTTGG------GAT------
AACATGACCTGGATGCAGTGG---GAAAAGGAAATTGAGAACTACACAGAAACAATATAC---
ACATTACTTGAAAACTCACAAAACCAACAAGAA--

CAGAATGAAAAGGAACTATTGGAATTAGATAAATGGGCAAGTCTGTGGAATTGGTTTAGCATAACACATTGGC
TGTGGTATATAAAAATATTCATAATGATAGTAGGAGGCTTAATAGGGTTAAGAATAATTTTTACT---
GTACTTTCTATAGTG---------AATAGAGTTAGGCAGGGATACTCACCA------
TTATCGTTGGCTATTAGCTTGTTCAATACCACCGCCATAGCAGTAGCTGAG---
GGGACAGATAGGATTTTAGAAGTATTACAAAGAATTTATAGAGCT----------------------------
--------ATTATCCACATA------CCTGTACGAATAAGACAGGGCTTGGAAAGGGCTTTGCTA---TAA
>KT427755
GCT---GCA--
AAAGGCTTGTGGGTCACAGTCTATTATGGGGTACCTGTGTGG---AGAGAAGCAAAC---
ACCACTTTATTTTGTGCATCAGAKGCTAAAGCTTATCATACAGAA---GTACATAAT----------------
--------------GTTTGGGCCACA---CATGCCTGTGTACCCACAGAC---
CCCAACCCACAAGAAGTAGCATTGGAA---------AATGTGACAGAAAATTTTAAC----------------
--------------------------ATGTGGAAAAATGACATGGTAGAACAGATGCATGAAGATATA---
ATCAATTTATGGGATCAAAGCCTA---AAGCCATGTGTAAAATTA---
ACCCCACTATGTGTTACTTTAAATTGCACTGATGTAAAGAATAGTACCACTAATAATGGTACCAATGGGAATA
GTACCACTAGT--
----------------AATGGTACCAATAGTACTAACATCAAAAATATGARAGAACAAATGAAA---
AACTGCTCTTTCAATACCACCACAAACATAAGA---------------------------
GATAAGATGCAAAAAGAATATGCATTG---TTTTATAAAGTTGATATAGTATCAATAGAAAAT----------
----

GATAATACTAGTTATAGGTTGATTCATTGTAATACCTCAGTCATTACACAG---
TCCTGTCCAAAGGTAACCTTTGAGCCAATTCCCATACATTATTGTGCCCCAGCTGGTTTTGCGATTCTAAAGT
GTAAT---AATAAGGAGTTCAAG------GGGACAGGGCCTTGTACAAAT-----------------------
-GTCAGC---ACAGTACAATGTACACATGGAATTAAACCAGTAGTGTCAACT---
CAACTGCTGTTAAATGGAAGTCTAGCAGAAGGAAGA------------
GTAGTAATTAGGTCTAGCAATTTCTCAGACAAT---------ACTAAAAACATAATAGTACAGCTGAAC----
-----------AAAACCATAGAA---------ATTAATTGTACAAGGCCCAACAACAACACCAGGAAA-----
-AGTATACACATA------------GGACCAGGGAGAGCA---TTT------TATGCAACAGGAGAC------
---------ATAATAGGAGATATA------------AGACAAGCACATTGT---AACATTAGTAGAATA---
CAATGGAATGAAGCTTTAAAAGGGGTAGCTGAAGAATTAAGT---------------AAACAATTTAAG----

```
-----------------------------------AATAAAACAATAGCCTTTAAT------
AAATCCTCAGGAGGGGACCCAGAAATTGTA---GCACACAGTTTTAATTGTGGAGGGGAA---
TTTAAACAGCTCCAGGCAAGAGTCCTGGCTGTGGAAAGATACCTAAGGGATCAACAGCTCCTAGGGATTTGGG
GTTGCTCTGGAAAACTCATCTGCACCACTGCTGTGCCTTGGAACACTAGTTGGAGT-----------------
----------------------------AATAGATCTCTAGATGCTATTTGG------AAT------
AACATGACCTGGATGGAATGG---GAAAGAGAAATTGACAATTACACAGACTTAATATAC---
ACCTTACTTGAAGAATCACAAAACCAGCAAGAA----------------------------------------
-----------------------------
AAGAATGAGCAAGACTTATTGGCCTTGGATAAGTGGGCAAGTTTGTGGAATTGGTTTAGTATATCAAACTGGC
TGTGGTATATAAGAATATTCATCATGATAGTAGGAGGCTTGGTAGGTTTAAGAATAGTT-TTATT---
ATACTTTCTGTGGTG---------GATAGAGTTAGGCAGGGATACTCACCA------
TTGTCATTCGCTATAAGCTTGTTTAATGCTACAGCTATAACAGTAGCTGAG---
GGGACAGATAGAATTATAGAAGTAGCACGAGCAATTTTTAGAGGT----------------------------
--------ATTCTCAACATA------CCTAGAAGAATAAGACAGGGCTTAGAAAGGGCTTTGCTA---TAA
>KT427754
GCC---AAG------------------------GAG---CCC---AAA---
GAGCAATTGTGGGTCACAGTATATTATGGGGTACCTGTGTGG---AGAGAAGCAACC---
ACCACTCTATTTTGTGCATCAGATGCTAAAGCATATGAAACAGAG---GTACATAAT----------------
--------------GTCTGGGCCACA---CATGCCTGTGTACCCACAGAC---
CCTAACCCACAAGAAATAGTATTGGAA---------AATGTGACAGAAAATTTTAAC----------------
--------------------------ATGTGGAAAAATAACATGGTAGAACAAATGCATGAGGATGTA---
ATCAGTTTATGGGATGAAAGCCTA---AAGCCATGTGTAAAATTA---
ACCCCGCTCTGTGTTACTTTAATTTGCAAGGATAGTGTTGATAGCACCAATAGTAGCACTAGC----------
-------------------------------------------------------------------------
-------------------ATCAATAATAGTTTGGTGGAAGGAATGACAATGGAAGGAATGACA---
AACTGTTCTTTCAATATCACCTCAAAAATAAGA---------------------------
GATAAGGTACAGAAAGAATATGCACTA---TTTTATAATTCTGATGTAGTACCAATAGATAATGGG-------
-------------------------------------------------------------------------
----------------------------
ACAAGCTATATGTTGAGAAGCTGTAATACCTCAGTCATTACACAG---
GCCTGTCCAAAGGTAACCTTTGAACCAATTCCTATACATTATTGTACCCCGGCTGGTTTTGCGATTCTAAAGT
GTAAT---GATAAGAAGTTCAAT------GGAACAGGTCCATGTAAAAT------------------------
-GTCAGC---ACAGTACAATGTACACATGGAATTAGGCCAGTTGTGTCAACT---
CAACTACTGTTAAATGGCAGTCTAGCAGAAGAAGAG------------
ATAGTAATTAGATCTGAAAATTTTACGAACAAT---------GCTAAAACCATAATAGTACAGTTGAAT----
-----------GAAACTGTGGAA---------ATTAATTGCACAAGGCCCAACAACAATACAAGCAAA-----
-GGTATACATATA------------GGGCCGGGAAGAATA---TTT------TATGCAACAGAAAGA------
---------ATAATAGGAGATATC------------AGACAAGCACATTGT---AACATTAGTAAAACA---
AAATGGGAAAAAACTTTAGAAAAAGTAGCTAGTAAATTAAGA---------------GAACAATATATT----
-----------------------------------AATAAAACAATAATTTTTAAT------
CAATCCTCAGGAGGGGACCCAGAAATTACA---ATGCACACTTTTAATTGTGGAGGGGAA---
TTTAAGCAGCTCCAGGCAAGAGTCCTGGCTGTGGAAAGATACCTAAAGGATCAACAGCTCCTAGGGATTTGGG
GTTGCTCTGGAAAACTCATTTGCACCACTTCTGTGCCTTGGAATGCTAGTTGGAGT-----------------
----------------------------AATAAATCTTACAATGAGATTTGG------AAC------
AACATGACCTGGATACAATGG---GAGAGAGAAATTGACAATTACACAAGCTTAATATAC---
ACTTTAATTGAAGACTCGCAAAACCAACAAGAA----------------------------------------
-----------------------------
AAGAATGAAAAAGAATTATTGGAATTGGATAAGTGGGCAAGTTTGTGGAGTTGGTTTGATATAACACAATGGC
TGTGGTATATAAGACTATTCATAATGATAGTAGGAGGCTTAATAGGTTTAAGAATAGTTTTTACT---
GTACTTTCTATAGTG---------AATAGAGTTAGGCAGGGATACTCACCA------
TTATCATTGGCTATTAGCTTGTTTAATGCAATAGCTATCGCAGTAGCTGAG---
GGGACAGATAGGATTATAGAAGCAGTACAAAGAGCTGGTAGAGCT----------------------------
--------ATCCTTCACATA------CCTAGAAGAATAAGACAGGGCTTTGAAATAGCTTTGCTA---TAA
>KT427750
GCT---AAG------------------------------------------
GAACAATTGTGGGTCACAGTATATTATGGGGTACCTGTGTGG---AAAGAAGCAACC---
ACCACTCTATTCTGTGCATCAGATGCTAAAGCATATGACACAGAG---GTACATAAC----------------
--------------GTTTGGGCCACA---CATGCCTGTGTACCCACAGAT---
CCTAACCCACAAGAAATAGGCTTGGAA---------AATGTGACAGAAAATTTTAAC----------------
--------------------------ATGTGGAAAAATAACATGGTAGAACAGATGCATGAGGATGTA---
```

ATCAGTTTATGGGATCAAAGCCTA---AAGCCATGTGTAAAATTG---
ACCCCACTCTGTGTTACCTTAAATTGCACTAATGTGACAAATGAAACAATAGCCAAACAAAGTAACAAAACTG
CACAAGGA--
-------------CAAGGTAACAAAACTGCATACGTTGATAAAGAGATGCAGGGAGAAATAAAA---
AACTGCTCTTTCAATGTCACCAAAGGCATAGAA-------------------------
AATAGGAGGCAGAAAGAATATGCATTT---TTTTATAAAACTGATGTAGTCAAGATAGATAAT---------
----

GATACTACAAGCTATACGTTGATCAATTGTAACACCTCAGTCATCACACAG---
GCCTGCCCAAAGGTATCTTTTGAACCAATTCCCATACATTTTTGTACCCCTGCTGGTTTTGCGCTTCTAAAAT
GTAGA---GATAAAAAGTTCAAT------GGAACGGGACCATGTACCAAT----------------------
-GTCAGC---ACAGTACAATGTACACATGGAATTAGGCCAGTAGTGTCAACT---
CAACTACTGTTAAATGGCAGTCTAGCAGAAGAAGAG-----------
ATAGTAATTAGATCTGAGAACCTGACGAACAAT---------GCTAAAACTATAATAGTACAGCTGAAA----
-----------GAAGCTGTAGAA---------ATTAATTGTACAAGGCCCAACAACAATACCAGAAAA-----
-AGTATACATTTA------------GGATGGGGGAGAGCA---TTT------TATGCAACAGGAGAA------
---------ATAATAGGGGATATA------------AGACAAGCATATTGT---ATCCTTAATAAAACA---
AAATGGATAAAAACTTTAGAACAGATAGCTACAAAATTAAGG--------------GAAAAATTTAAT----
----------------------------------AATAAAACAATAATTTTTAAT------
AATGCCTCAGGAGGGGATCCAGAAATTGTA---ATGCACACTTTTAATTGTGGAGGGGAA---
TTTAAGCAGCTCCAGGCAAGAGTCCTGGCTGTAGAAAGATACCTAAAGGATCAACAGCTCCTAGGGATTTGGG
GTTGCTCTGGAAAACTCATTTGCACCACTGCTGTACCTTGGAATGCTAGTTGGAGT-----------------
----------------------------AATAAAACTTTGGATAAAATTTGG------AAT------
AACATGACCTGGATGGAGTGG---GAGAAAGAAATTGACAATTACACAAGCTTAATATAT---
ACCTTAATTGAAGAATCGCAGAACCAGCAGGAA---

AAGAATGAACAAGAATTATTAGAATTGGATGCATGGGCAAGTTTGTGGAATTGGTTTGACATAACAAAATGGC
TGTGGTATATAAAAATATTCATAATGATAGTAGGAGGCTTAATAGGTTTAAGAATAGTTTTTACT---
GTACTTTCTATAGTG---------AATAGAGTTAGGCAGGGATACTCACCA------
TTATCATTCGCTGTTAGTTTGCTTAATGCCTTTGCTATAGCAGTAGCTGAA---
GGGACAGATAGAGTTATAGAAGTAGTACAAAGACTTTGTAGAGGT---------------------------
--------ATCCTCCACATA------CCTAGAAGAATAAGACAGGGCTTAGAAAGAGCTTTGCTA---TAA
>KT427830
GCT---GGA---
GAACAATTGTGGGTCACAGTCTATTATGGGGTACCTGTATGG---AAAGAAGCAACC---
ACCACTCTATTTTGTGCGTCAGATGCTAAAGCATATGATACAGAG---GTACACAAT----------------
--------------GTTTGGGCCACA---CATGCCTGTGTACCCACAGAC---
CCCAGTCCACAAGAAGTCATATTGAAA---------AATGTGACAGAAAATTTTAAC----------------
-------------------------ATGTGGAAAAATGACATGGTAGAACAGATGCAGCAGGATATA---
ATCAGTTTATGGGATCAGAGCCTA---AAGCCATGTGTAAAACTA---
ACCCCACTCTGTGTTACTTTAACTTGCACTGATGTTGAAGATAAAACTAATGCCACTAGTAGT---------
----
-------------AATAGTACCACTAGTGGTAATAGTACTACTTGGGAGAAGGGAGAAATGAAA---
AACTGCTCTTTCAATGTCACCACAGGCATAAGA-------------------------
GATAAGACAACGAGAGAACATGCACTT---
TTCTATAAACTTGATATAGTACAAATAGATAACAGTAGTAGCAACTATAGC---------------------
----
AACAGTAGCAACAATAGTAGCAACTATAGTAACTATAGGTTGATAAATTGTAATACCTCAGTCATTACACAG-
--
GCCTGTCCAAAGGTATCCTTTGATCCAATTCCCATACACTATTGTGCCCCGGCTGGTTTTGCGATTTTAAAGT
GCAAA---GATAAGCAGTTCAAT------GGAACAGGACCATGTAAAAAT----------------------
-GTCAGT---ACAGTGCAGTGTACACATGGAATTAAGCCAGTAGTATCAACT---
CAACTGTTATTAAATGGCAGTTTAGCAGAAGAAGAG-----------
ATAATAATTAGATCTAGCAATTTCACAGACAAT---------GCTAAAGTCATAATAGTGCAGCTGAAC----
-----------GAATCTGTACAA---------ATAAATTGTACAAGACCCAACAACAATACAAGAAAA-----
-AGTATAAATATA------------GGGCCAGGGAGAGCA---TTT------TATGCAACAGGAGAT------
---------ATAATAGGAGATATA------------AGACAAGCACATTGT---AACATTAGTGGAACA---
CAATGGAACAGAACTCTACAACAGGTAGCTAAAAAGTTAAGA--------------GAACAATTTAAT----
----------------------------------AATAAGAGCATAACCTTTAAT------
CAATCCTCAGGAGGGGACCCAGAAATTGTA---ATGCACAGTTTTAATTGTGGAGGGGAA---

TTTAAGCAGCTCCAGGCAAGAGTCCTGGCTGTAGAAAGCTACCTAAGGGATCAACAGCTCCTAGGGATTTGGG
GTTGCTCTGGAAAACTCATCTGCACCACTGCTGTGCCTTGGAATGCTAGTTGGAGT-----------------
----------------------------AATAAATCTCTAGATGACATTTGG------AAT------
AACATGACCTGGATGCAATGG---GAAAAAGAAATTGACAATTATACAGGCTTAATATAC---
ACCTTAATTGAACAATCGCAAAACCAACAAGAA--

AAAAATGAACAAGAATTATTAGAATTAGATAAGTGGGCAAATTTGTGGAGTTGGTTTAACATAACAAATTGGC
TGTGGTATATAAGAATATTCATAATGATCATAGGAGGCTTGATAGGTTTAAGAATAGTTTTTGCT---
ATACTCTCTGTAGTA---------AATAGAGTTAGGCAGGGATACTCACCT------
TTGTCATTTGCTGTTAGCTTGCTTAATGCCACAGCTATTGCAGTAGCTGAG---
GGGACAGATAGGATCATAGAAGTAGCACAAAGAATTGGTAGGGCT----------------------------
--------ATCCTCCACATA------CCTGTACGAATAAGACAGGGCTTGGAAAGGGCTTTGCTA---TAA
>KT427829
GCT---GCA--
GGACAATTTTGGGTCACAGTCTACTATGGGGTACCTGTGTGG---AAAGAAGCAACT---
ACCACTCTATTTTGTGCATCAGATGCTAAAGGATATGATACAGAG---GCACATAAT----------------
--------------GTTTGGGCCACA---CATGCCTGTGTACCCACAGAT---
CCCAACCCACAAGAAGTAGTATTGGGA---------AATGTAACAGAAAATTTTAAC----------------
--------------------------ATGTGGAAAAATAACATGGTAGAGCAGATGCATGAGGATATA---
ATCAGCTTATGGGATCAAAGCCTA---AAGCCATGTGTAAAGCTA---
ACCCCACTCTGTGTTACTTTAAATTGCACTGATGTTAAGAATGTTGGTGTTAATGCCACTAGTACTCCTCCTT
CTAGTACTCTT--
---AGTCCTGTGAGGGATATGAAA---
AACTGCTCATTTAATGTCACTTCAGGAATAAGA---------------------------
GATAAGGTACAGAGAGAATATGCACTT---TTTTATAAACTTGATATAGTACCAATAGATAAT----------
----

GATAATAATAGTAATAGCTATAGGTTAATAAGTTGTAACACCTCAGTCATTACACAG---
GCCTGTCCAAAGGTAACCTTTGAGCCAATTCCCATACATTATTGTGCCCCGGCTGGTTTTGCGATTCTAAAGT
GTAAC---AATAAGACGTTCAAT------GGAACAGGACAGTGTACCAAT-----------------------
-GTCAGC---ACAGTACAGTGTACACATGGAATTAGGCCAGTAGTATCAACT---
CAACTGCTGTTAAATGGCAGTCTAGCAGAAGAAGGG------------
GTAGTAATTAGATCTGAAAACTTCACGAACAAT---------GCTAAGACCTTAATAGTACAGCTGGAC----
-----------GAAACTATAAAA---------ATTAATTGTACAAGACCCAACAATAATACAAGAAAA-----
-AGTATACATATA------------GGACCAGGGAGAGCA---TTT------TTTGCAACA---GAC------
---------ATAATAGGAAATATA------------AGACAAGCACATTGT---AACCTCAGTAAAACA---
GACTGGAATAACACTTTAAAAAAGGTAGCTGAAAAATTAGGA---------------AAACAATTTAGG----
-----------------------------------AATAAAACAATAAGCTTTAAG------
CCATCCTCAGGAGGGGACCCAGAAATTGTA---ATGCACAGTTTTAATTGTGCAGGGGAA---
TTTAAGCAGCTCCAGGCAAGAGTCCTGGCTGTGGAAAGATACCTAAGGGATCAACAGCTCCTAGGGATTTGGG
GTTGCTCTGGAAAGCTCATCTGCACCACTAATGTGCCCTGGAATTCTAGTTGGAGT-----------------
-------------------------AATAATAGATCTCTAGAGGATATTTGG------GGT------
AACATGACCTGGATGGAATGG---GAAAGAGAAATTGATAATTACACAGGCTACATATAC---
AGTTTACTTGAAGAATCGCAGAACCAGCAAGAA--

AAGAATGAACAAGACTTATTGGCATTAGATAAATGGGCAAGTCTGTGGAATTGGTTTAGCATAACAAACTGGC
TGTGGTATATAAGAATATTTATAATGATAATAGGAGGCTTGATAGGTTTAAGAATAGTTTTTGCT---
GTACTTTCTATAGTG---------AATAGAGTTAGGCAGGGATACTCACCA------
TTATCGTTGGCTGTTAGCTTGCTCAACGCCACAGCCATAGCAGTAGCTGAG---
GGGACAGATAGGGTTATAGAAGTAATACAAAGAATTGTTAGAGCT----------------------------
--------ATTATCCACATA------CCTACAAGGATAAGACAGGGACTCGAAAGGGCTTTACTA---TAA
>KT427824
GCT---GCA--
GAAAACTTGTGGGTTACAGTCTATTATGGGGTACCTGTGTGG---AAAGAAGCAACC---
ACCACTCTATTTTGTGCATCAGATGCTAAAGCTTATAGTACGGAG---GCACATAAT----------------
--------------GTTTGGGCCACG---CATGCCTGCGTACCCACAGAC---
CCCAACCCACGAGAACAACTATTGGGA---------AATGTGACAGAAGATTTTAAC----------------
--------------------------ATGTGGAAAAACAGCATGGTAGAACAGATGCAGGAGGATATA---
ATCAGTTTATGGGATCAAAGCCTA---AAGCCATGTGTAAAGTTA---
ACCCCACTCTGTGTTACTTTAAACTGCAGTGAACCTAAGTGCAGTAAGAATGGTACTAATAGCACTRGTACTA

GTAATACTAMTGGTRCTAGTAATACTAGTGCTAGT-----------------------------------
----GGTAATRRTACTAGTGCTAATAGTACTAGTTTCACTTGTGAAAGAACAGATGAAATAAAA---
AATTGCTCTTTCAATATCACAACAAGCATAAGA------------------------
AACACGGTGCGGAAAGAATATGCAATA---TTTAATAGCCTTGATGTAGTACAAATAGATGATAAT-------
----

GATAATACTAGCTATAGGTTAATAAGTTGTAACACCTCAGTCATTACACAG---
GCCTGTCCAAARATATCCTTTGAGCCAATTCCCATACATTATTGTACCCCGGCTGGTTTTGCGCTTCTAAAAT
GTAAT---GATAAGGGGTTCAGT------GGAAAAGGAACATGTAAAAAT----------------------
-GTCAGC---ACAGTACAATGCACACATGGAATTAGACCAGTAGTGTCAACT---
CAACTACTGTTAAATGGCAGCCTAGCAGAAGAAGAT-----------
GTAGTAATTAGATCTGCCAATTTCACAGATAAT---------GCTAAAACCATAATAGTACAGCTAAAC----
-----------AAGTCTGTAGAA---------ATAATTTGTGTGAGACCCAACAATAATACAAGAAAA-----
-AGTATACATATA------------GGGCCAGGGAGCACA---TAT------TTTGCAACAGGAGAT------
---------ATAATAGGAGATATA------------AGACAAGCACATTGT---AATATTACTAGATCA---
AAATGGACTGAAACTTTGCGCCAGCTAGCAAGAAAATTAGGT---------------GAACAATTTCAA----
--------------------------------AATATGACAAAAATAAACTTTACT------
GCATCCTCAGGAGGGGACCCAGAAGTTGTG---ATGCACAGTGTTAACTGTGGAGGGGAA---
TTTAAGCAGCTCCAGGCAAGACTCCTGGCTGTGGAAAGATACCTACAGGATCAACAGCTCCTAGGGCTTTGGG
GTTGCTCTGGAAAACTCATTTGCACCACTACTGTGTCTTGGAATACTAGTTGGAGC-----------------
----------------------------AATAAAAATCTGAGCCAAATTTGG------GAG------
AACATGACCTGGATGCAGTGG---GAAAAAGAAATTAGCAATTACACAGAACAAATATAC---
ACCTTACTTGAACAATCACATAACCAGCAAGAA--

AGMAATGAACAAGAATTATTGGCATTGGATAAGTGGGACAGCCTGTGGAACTGGTTTGACATAACACATTGGC
TGTGGTATATAAAAATATTCATAATGATAGTAGGAGGCTTGATAGGTTTAAGAATAATTTTTGCT---
GTACTTTCTATAGTG---------AATAGAGTTAGGCAGGGATACTCACCA------
TTATCGTTKGCTGTTAGCTTGCTTAATATCACAGCTATAGCAGTAGCTGAG---
GGGACAGATAGGGTTATAGAAGTAGGACAAAGAGCTTTTAGAGCT---------------------------
--------GTTCTCCACATA------CCTAGAAGAATAAGACAGGGCTTTGAAAGGATTTTGCTA---TAA
>KJ849790
GCT---GCA---
AGAAATTTGTGGGTTACAGTCTATTATGGGGTGCCTGTGTGG---AAAGAAGCAACC---
ACCACCCTATTTTGTGCATCAGATGCTAACTCCTTGGATACAGAG---GCACATAAT---------------
--------------GTTTGGGCCACA---CATGCCTGTGTACCCACAGAC---
CCCAACCCACAGGAAGTAGACTTGGGA---------AACATGACAGAAGATTTTAAC---------------
-------------------------ATGTGGAAGAATAACATGGTAGAACAGATGCATGAGGATGTA---
ATCAGTTTATGGGATCAAAGCCTA---AAGCCATGTGTGAAATTA---
ACCCCACTCTGTGTTACTTTAAAATGCACTAATGTAACGATTAATAATACCAAGAATGATACTAAT-------
----
---------------------ACCACTCAGGGTCCTCTTGCAAGTATGGAGAGAGAAATGACA---
AACTGCTCTTTCAATATCACCACAAAAGTAGGA------------------------
AATAAGATGCAGAAAGAATATGCACTT---TTTTATAAACTTGATGTAGTACCAATAGATAAT---------
----

GAAAGCTATACCTTGATAAGTTGTAACACCTCAGTCATTACACAG---
GCCTGTCCAAAGGTATCCTTTGAACCAATTCCCATACATTATTGTGCCCCGGCTGGTTTTGCGCTTCTAAAAT
GTAAG---GATAAAAAATTCAAT------GGGACAGGAACATGTAGAAAT----------------------
-GTCAGC---ACAGTACAATGTACTCATGGAATTAAGCCAGTGGTGTCAACT---
CAACTACTGTTAAATGGCAGTCTAGCAGAGGATGAG-----------
GTAATAATTAGATCTGAAAATTTCACAAACAAT---------GCTAAAATCATAATAGTACAACTGAAT----
-----------GAATCTGTATAT---------ATTAATTGTACAAGACCCAACAACAATACAAGAAAA-----
-AGTATACATCTA------------GGATGGGGGAGAGCA---TTT------TACGCAACAGGGGAA------
---------ATAATAGGAGATATA------------AGACAAGCACATTGT---AACATTAGTAGAAGC---
CAATGGAATAAAACTTTAGAACAGGTAGCTAAAAAATTAAGA--------------GAACAATTT-------
----------------------------------AACAAAACAATAATCTTTAAT------
CATTCCTCAGGAGGGGACCTAGAAATTGTG---ACTCATAGTTTTAATTGTGGAGGGGAA---
TTTAAGCAGCTCCAGGCAAGAGTCCTGGCTGTGGAAAGATACCTAAGGGATCAACAGCTCCTAGGGATTTGGG
GTTGCTCTGGAAAACTCATCTGCACCACTGCTGTGCCTTGGAATGATAGTTGGAGT----------------
----------------------------AATAAATCTCTGGGGGATATTTGG------GAT------

AACATGACATGGATGCAGTGG---GAGAAAGAAATTAACAATTACACAAGCGAAATATAC---
CGCTTAATTGAAAAGGCGCAAAACCAACAAGAA---

CAGAATGAACAAGAATTATTGGAATTGGATTCGTGGGCAAGTTTATGGAATTGGTTTAGCATATCAAAATGGC
TGTGGTATATAAAAATATTCATAATGATAGTAGGAGGCTTAGTAGGATTAAGAATAGTTTTTACT---
GTACTTTCTATAGTG---------AATAGAGTCAGGCAGGGATACTCACCC------
TTATCATTCGCTGTTAGTTTGCTTAATGCCACAGCTATAGCAGTAGCTGAG---
GGGACAGATAGAATTTTAGAAGTAGTGCAAAGACTTTGTAGAGCT---------------------------
--------ATTCTCAACATC------CCTAGAAGAATAAGACAGGGTTTGGAAAGGGCTTTGCTA---TAA
>KT427819
GCT---ACA--
GAAAATTTGTGGGTCACAGTCTATTATGGGGTACCTGTGTGG---AAAGAAGCATCC---
ACCACTTTATTTTGTGCATCAGATGCCAAAGCAGTTAAAACAGAG---GTGCATAAT----------------
--------------ATTTGGGCCACA---CATGCCTGTGTACCCACAGAC---
CCTAATCCTCAAGAAATGGAATTGGAA---------AATGTGACAGAAAATTTTAAC---------------
-------------------------ATGTGGAAAAATAACATGGTAGAACAGATGCATGAGGATATA---
AAAAATTTATGGGATCAAAGCCTA---AAGCCATGTGTAAAATTA---
ACCCCATTCTGTGTTACTTTAAATTGCACTAATTTGAATCATACCAATACTGATAATAGTAGTAATAAT----
----
---GAAGGGATACAGGAAATGAGA---
AACTGCTCTTTCAATATCACCAATAGCATAAAA--------------------------
AATAAGATGCAGAAAGAATATGCACTT---TTGTATAGACTTGATATAGTACCAATAAAAGAT----------
----

GATAATACAAGTTATACATTGATAAACTGTAACACCTCAGTTATTACACAG---
GCGTGCCCAAAGGTAACCTTTGAACCAATTCCCATACATTATTGTGCCCCGGCTGGTTTTGCGATTCTAAAGT
GTAAA---GATAGGGAGTTCAAT------GGAACAGGACCATGTACAAAT----------------------
-GTCAGC---ACAGTACAATGTACACATGGAATTAAGCCAGTAGTATCAACG---
CAACTGTTGTTAAATGGCAGTCTATCAGAAGAAAAG-----------
GTAATAATTAGATCTGAAAATTTAACAAACAAT---------GCTAAAAACATAATAGTACAGCTGCCT----
-----------AAACCTGTAACA---------ATTAATTGTACAAGACCCAACAACAATACAAGAAAA-----
-GGTATACATATG------------GGATGGGGGAGAACA---TTT------TATGCAACGGGAGCA------
---------ATAATAGGAGATATA-----------AGACAAGCACATTGT---AACCTTAACAGTACA---
GAATGGAACAACACTTTAAAACAAGTAGCTGACAAATTAAGA---------------GAACACTTTAAT----
-----------------------------------AATAAAACAATAGTCTTTAAC------
CACTCATCAGGAGGGGACCCAGAAATTGAA---ATGCACATGTTTAATTGTGGAGGGGAA---
TTTAAGCAGCTCCAGGCAAGAGTCCTGGCTGTGGAAAGATACCTAAAGGATCAACAGCTCCTAGGGATTTGGG
GTTGCTCTGGAAAACTCATTTGCACCACTGCCGTGCCTTGGAATACTAGTTGGAGT-----------------
-----------------------------AATAAATCTCTAGACAATATTTGG------AAC------
AACATGACCTGGATGCAGTGG---GAGAGAGAAATTGACAATTACACAGGCCTCATATAC---
AACTTACTTGAAGAATCGCAATACCAACAGGAA---

AAGAATGAACAGGACCTATTGGCATTGGATAAGTGGGCAAGTTTGTGGAATTGGTTTGACATAACAAAATGGC
TGTGGTATATAAAAATATTCATAATGATAGTAGGAGGCATAGTAGGTTTAAGAATAGTTTTTACT---
GTACTTTCTATTGTA---------AATAGAGTTAGGCAGGGATACTCACCA------
TTGTCGTTCGCTGTTAGTTTGCTTAATGCCATAGCTATAGCAGTAGCTGGG---
GGGACAGATAGGATTATAGAAATAGTACGAGGAATTTGTAGAGCT---------------------------
--------ATTCTCCACATA------CCTAGAAGAATAAGACAGGGCTTAGAAAGGGCTTTGCTA---TAA
>KJ849796
GCT--
GACCAATGGTGGGTTACAGTCTATTATGGGGTACCTGTGTGG---AAAGAAGCAAAC---
ACCACTCTATTTTGTGCATCAGATGCTAAAGCATATGACACAGAG---GCACATAAT----------------
--------------GTTTGGGCCACA---CATGCCTGTGTACCCACAGAC---
CCTAGCCCACAAGAAATAGTAATGGCA---------AATGTGACAGAAAATTTTAAT---------------
-------------------------GTGTGGAAAAATACCATGGTAGAACAGATGCATGAGGATATA---
ATCAGTATATGGGATCAAAGCCTA---AAGCCATGTGTAAAATTA---
ACCCCACTCTGTGTTATTTTAAATTGTAGTGATGCAGCTAACAAGGTCAATGGCACTAGTACTAATAGTACAG
CCAGTAAT--
----AGCACAACTGGCAACGCCACTGGTACTACTAATAAGTTAGACATGAAAGGAGAAATAAAA---
AACTGCTCTTTCAATATCACCACAGGCATAGGA--------------------------

AACAAGATGAAAAGAGAATATGCACTT---TTTAATAGACTTGATGTAGTACAAATAGATAAA---------
----

AATAATACTATAGAGTATATGTTGGAAAATTGTAACACCTCAGTCATTAGACAG---
GCCTGTCCAAAGACATCCTTTGAACCAATTCCCATACATTTTTGTGCCCCGGCTGGTTTTGCGATTCTAAAGT
GTAAT---GATAAGAAGTTCAAT------GGAACAGGACTATGTACAAAT----------------------
-GTCAGC---ACAGTACAATGTACACATGGAATTAAGCCAGTAGTGTCAACT---
CAACTACTGTTAAATGGCAGCTTAGCAGAGGAAGAG-----------
GTAATAATTAGATCTGAAAATCTCACGAACAAT---------GTTAAAACCATAATAGTACAGCTAAAT----
-----------GAAACTGTAGAA---------ATTACTTGTACAAGACCCAATAACAATACAAGAAAA-----
-AGTATACATATG------------GGATTGGGAAGAGCA---TTT------TATGCAACAGGAGAT------
---------ATAATAGGAGATATA------------AGACAAGCACATTGT---AACATTAGTAGAGCA---
AAATGGAATAACACTTTAACAAACATAGCTATAAAATTAAGA---------------GAAAAATTT-------
----------------------------------AATAGAACAATAATCTTTAAT------
CAATCCTCAGGAGGGGACCCAGAAATTGTA---ATGCACACTTTTAATTGTGGAGGGGAA---
TTTAAGCAGCTCCAGGCAAGAGTCCTGGCTGTGGAAAGATACCTACAGGATCAACAGCTCCTAGGGATTTGGG
GTTGCTCTGGAAAACTCATCTGCACCACTAATGTGCCTTGGAATAATAGTTGGAGTAAGAAT----------
---------------------------AAAACAGATCTAAAAGAAATTTGG------GAT------
AACATGACCTGGATAGAGTGG---GAGAGAGAAATTGACAATTACACAAAAGAAATACAC---
AAATTACTTGAAGAATCGCAGAACCAACAAGAA---

AAGAATGAACAAGAATTATTGGCATTAGATAAATGGGCAAATTTGTGGAATTGGTTTGACATAACAAGATGGC
TGTGGTATATCAAAATATTCATAATGATAGTAGGAGGCTTAATAGGTTTAAGAATAGTTTTTACT---
GTACTTTCTATAGTG---------AATAGAGTTAGGCAGGGATACTCACCA------
TTATCATTCGCTGTTAGTTTGCTTAATACCACAGCTATAGCAGTAGCAGAA---
GGGACAGATAGGGTAATAGAATTAGCACAAAGGCTTGGCAGAGCT---------------------------
--------TTTCTCCACATA------CCTAGAAGAATAAGACAGGGCTTCGAAAGGGCTTTAGTA---TAA
>KT427815
GCT---AAG--
GAGCAATCGTGGGTCACAGTCTATTATGGGGTGCCTGTGTGG---AGAGAAGCAACC---
ACCACTCTGTTCTGTGCATCAGATGCTAAAGCATATGCTAAAGAA---GTACATAAT---------------
--------------GTTTGGGCCACC---CATGCCTGTGTACCCACAGAC---
CCCAACCCACAAGAAGTACGATTGGAA---------AATGTGACAGAAAATTTTAAC---------------
-------------------------ATGTGGAAAAATAACATGGTAGAACAAATGCATGAGGATATA---
ATCAGTCTATGGGATCAAAGCCTG---AAACCATGTGTAAAATTA---
ACCCCACTCTGTGTTACTTTAAACTGCAGAAATTATACGGGGGATACTAATACTACCACTTCCAATACTACCA
CTCCCACTAGT---
--ATGGGAGAAGTGAAA---
AACTGCTCTTTCGATATCACCACAAACATAAGA--------------------------
GATAAGGTGCGGAAAGAACATGCACTT---TTTTATACATATGATTTAGTAAAAATAGTTGATAGTGAT----
----

AGTGATAATAGTAGTGATAATAATAGTTATAGGTTGATAAGTTGTAACACCTCAGTCATTACCCAA---
GCCTGTCCAAAAGTAACCTTTGAACCAATTCCCATACATTACTGTGCCCCGGCTGGTTATGCGATTCTAAAGT
GTAAT---GATACAAAGTTCAAT------GGATCAGGACCATGTCAAAAT----------------------
-GTTAGC---ACAGTACAATGTACACATGGAATTAAGCCAGTAGTGTCAACT---
CAACTGCTGTTAAATGGCAGTCTAGCAGAAGAAGAC-----------
ATAGTAATTAGATCTGCAAATATTTCAGACAAT---------ACTAAAACCATAATAGTACAGCTGAAA----
-----------GAACCTGTACAA---------ATTAATTGTACAAGACCCAACAATAATACAAGGAAA-----
-AGTATAAATATA------------GGACCAGGGGCA------TTC------TATACAACAGGAAGC------
---------ATAATAGGAGATATA------------AGACAGGCATATTGT---AATCTTAGTATGACA---
AAATGGAATAACACTTTAGGACAGATAGTTAACAAATTAAGA--------------
GAACAATTTAAGGAACAATTT------GAG--------------------AATAAGACAATACAATTTAAT-
-----CAGTCCTCGGGAGGAGACCCAGAAATTGTA---ATGCACAGTTTTAATTGTGGAGGGGAA---
TTTAAGCAGCTCCAGGCAAGAGTCCTAGCTGTGGAAAGCTACCTAAAGAACCAACAGCTCCTGGGAATTTGGG
GTTGCTCTGGAAAACTCATCTGCACCACTAATGTGCCTTGGAATACTAGTTGGAGT----------------
---------------------------AATAGAACTAGAAAAAAAAATTTGG------AAT------
AACATGACCTGGATGCAATGG---GAAAAAAAAATTGACAATTACACAGGCGTAATATAC---
AATTTACTTAAAATATCACAGAACCAACAAGAA---

AAGAATGAACAAGAACTACTAAAATTAGATAAATGGGCAAGTTTGTGGACTTGGTTTGACATATCAAACTGGC
TGTGGTATATAAGACTATTCATAATAATAGTAGAAGGCTTAATAGGTTTAAGAATATTTTTACT---
GTACTTTCTATAGTG---------AATAGAGTTAGGCAGGGATACTCACCA------
ATATCGTTTGCTGTTAGTTTGCTTAATACCACAGCTGTAACAGTAGCTGAG---
GGAACAGATAGAGTGATAGAAGCAGTGCAAAGAGTTTTTAGAGCT----------------------------
--------ATCCTTCACATA------CCTACAAGAATAAGACAGGGCTTGGAAAGGGCTTTGCAA---TAA
>KJ849807
GCT---AAA--
GACAATTTGTGGGTCACAGTCTATTATGGGGTACCTGTGTGG---AAAGAAGCCATC---
ACCACTCTATTCTGTGCATCAGATGCTAGAGCATATGATACAGAG---GCGCATAAT----------------
--------------GTTTGGGCCACA---CATGCCTGTGTACCCACAGAC---
CCCAACCCACAAGAAGTAGCATTGGTA---------AATGTGACAGAAAATTTTAAC----------------
--------------------------ATGTGGAAAAATAACATGGTAGATCAAATGCAGGAGGATATA---
ATCAGTTTATGGGATCAAAGTCTA---AAGCCATGTGTAAAACTA---
ACCCCACTCTGTGTTACGTTAAACTGCAGCAATTATAATAATACTAAGACCAATGATACTAAGACCAATAATG
AA---
----------ACAATGGGTAATAATAATAGTGAAGGGAGGTTAATGGAGCCAGAAGAGATAAAA---
AACTGCTCTTTCAATATCTCCTCAAGCATAAGG---------------------------
GATAAGAGGCTGAAAGAATATGCACTT---TTTTATAAGCTTGATGTAACACCAATAGGGAAT----------
----

GATAATGCAACTTATATGTTGACAAATTGTAACACCTCAGTCATTACACAG---
GCCTGTCCAAAGGTATCCTTTGAACCAATTCCCATACATTATTGTGCCCCGGCTGGTTTTGCGATTCTAAAGT
GTAGA---GATAAAAAGTTCAAT------GGATCAGGACTATGTCACAAT-----------------------
-GTCAGC---ACAGTACAATGCACACATGGAATTAGGCCAGTAGTGTCAACT---
CAATTGCTGTTAAATGGCAGTCTAGCAGAAGAAGAG------------
ATAGTAATTAGATCTGAAAATCTCACAAACAAT---------GCTAAAACCATAATAGTACAGCTGAAA----
-----------GAACCTGTAAAC---------ATTACCTGTGCAAGACCCAACAACAATACAAGAAAA-----
-AGCATAAGTATG------------GGAGTAGGGAGAGCA---TGG------TTTATGAAAGGAGAT------
---------GTAATAGGAAATATA------------AGACAAGCACATTGT---AATATTAGTGGAAAA---
CAATGGAATGACACCTTATATCAGGTAGCTAAAAAATTACGT---------------GAACAGTTTAAG----
-----------------------------------AATAAAACAATAGTCTTTAAT------
CAATCTGCAGGAGGGGACCTAGAAATTGTA---ATGCACACGTTTAATTGTGGAGGGGAA---
TTTAAGCAGCTCCAAGCAAGAGTCCTGGCTATAGAAAGATACCTAAAGGATCAACAGCTCATGGGAATTTGGG
GTTGCTCTGGAAAACTCATCTGCACCACTAATGTGCCTTGGAATGCTAGTTGGAGT-----------------
----------------------------AACAAAAATTTGAGTGCTATTTGG------GAT------
AACCTGACCTGGATGCAGTGG---GAAAGAGAAATTGACAATTACACAGAATTAATATAC---
ACCTTACTTGAAGACTCGCAAAACCAGCAAGAA--

AAGAATGAACAGGACTTATTGGCATTAGATAAATGGGCAAGTTTGTGGAATTGGTTCAACATAACTAACTGGC
TGTGGTATATAAAAATATTCATAATGATAGTAGGAGGCTTGGTAGGCTTAAGGATAATTTTTTCT---
GTACTTTCTATAGTA---------AATAGAGTTAGGCAGGGATATTCACCA------
TTGTCATTTGCTGTTAGCTTGCTCAACGCCACAGCCATAGCAGTAGCTGAG---
GGGACAGATAGGATTATAGAAGCAGTACAAGGAGCTGGTAGAGCT----------------------------
--------ATTCTACATATA------CCTGTAAGAATAAGACAAGGCTTGGAAAGGGCTTTACTA---TAA
>KT427805
GCT---GAA---GAA------------------------------------
GACAAATTGTGGGTCACAGTCTATTATGGAGTACCTGTGTGG---AAAGAAGCAACC---
ACCACTCTATTTTGTGCTTCAGATGCTAAAGCATATGACACAGAG---GTACATAAT----------------
--------------GTTTGGGCCACC---CATGCCTGTGTACCTACAGAT---
CCTAACCCACAAGAAAARGAGTTGGAA---------AATGTGACAGAAAATTTTAAC----------------
--------------------------ATGTGGAAAAATAACATGGTAGAGCAAATGCATGAGGATATA---
ATCAGTTTATGGGATCAAAGCCTA---AAGCCATGTGTAAAGTTA---
ACCCCACTATGTGTTACTTTAAACTGCTCTAATTCGATTAGAGCGATAGGCCCTAATGAGACT----------
----
-------------GTGCTTAATGAGACTACGCTTAATGGGACATGGATAAGGGAAGAAATGAAA---
AACTGCTCTTTCAATGTCACCACAGACATAGGA---------------------------
GGAAAGGTGAAGAAAGACTATGCACTT---TTTAATAMACTTGATGTAGTAAAARTAGATRAT----------
----

GATAGCTATACATTGATAAATTGTAACACCTCAGTCATTAGACAG---
GCCTGTCCAAAGGTATCCTTTGAACCAATTCCCATACATTATTGTACCCCAGCTGGTTTTGCGATTCTAAAGT
GTAAT---GATAAGAAGTTCAAT------GGATCAGGACCATGTAGAAAT----------------------
-GTCAGT---ACAATACAATGTACACATGGAATTAAGCCAGTAGTGTCAACT---
CAACTATTGTTAAATGGCAGTTTAGCAGAAGAAGAA-----------
ACAATAATTAGATCTGAAAATTTCACGGACAAT---------ACTAAAAACATAATAGTACAACTGAAT----
-----------CAAMCTGTARAA---------ATTAAGTGTATAAGACCCCACAACAATACAAGRAGA-----
-AGTATACATATG------------GGATGGGGGAGAGCA---TTA------TATGCAACAGGAGAW------
---------ATAAYAGGAGACMYA------------AGACAAGCACATTGT---AACATTAGTGGAAMA---
AWTTGGARCACTACTTTARRGCAGATAGYTRRAAAATTARAA---------------GAACAATAC-------
----------------------------------AATAAAACAATAATCTTTGCT------
CCGTCCTCAGGAGGGGACCCAGAAGTTGTA---ATGCACACTTTTAACTGTGGAGGGGAA---
TTTAAACAGCTCCAGGCAAGAGTCCTGGCTGTGGAAAGATACCTACAGGATCAACAGCTCCTAGGAATTTGGG
GTTGCTCTGGAAAACTCATCTGTACCACTGCTGTGCCTTGGAATACTAGTTGGAGC-----------------
----------------------------AATAGATCTCTGGATAGCATTTGG------GAG------
AATATGACCTGGATGCAGTGG---GAGAAAGAAATTGACAATTACACAGAAYTAATATAC---
ACATTACTTGAAAAATCGCAGAACCAACAAGAA---------------------------------------
----
AAGAATGAACAAGAWTTATTAGGRCTGGATAAGTGGGCAAGTTTGTGGAATTGGTTTRACATAACAAATTGGC
TGTGGTATATAAGACTATTCATAATGATAGTAGGAGGCTTAATAGGTTTAAGAATAGTTTTTACT---
GTACTTTCTATAATA---------AATAGAGTTAGGAAGGGATACTCACCA------
TTATCATTCGCTGTTAGTTTGCTTAATGCTACAGCTATAGCAGTAGGTGAG---
GGAACAGATAGGGTGATAGAAATAGCACAAAGAGCTAGCAGAGCT----------------------------
--------ATTATCCATATA------CCTAGAAAAATAAGACAAGGCTTCGAAAAGGCTTTGCTA---TAA
>KJ849808
GCT---ACA---
GAAAAGTTGTGGGTCACAGTCTATTATGGGGTACCTGTGTGG---AAAGAAGCAAAC---
ACCACTCTATTTTGTGCATCAGATGCTAAAGCAGTTGACACAGAG---GTACATAAT----------------
--------------GTTTGGGCCACA---CATGCCTGTGTACCCACAGAC---
CCTAGCCCACAGGAAGTAGTATTGGAG---------AATGTGACAGAAAATTTTAAC---------------
-------------------------ATGTGGAAAAATAACATGGTAGAACAAATGCATGAGGATATA---
ATCAGTCTGTGGGATCAAAGCCTA---AAGCCATGTGTAAAATTA---
ACCCCACTCTGTGTTACTTTAAATTGCACTAATTGGACGGTAAATAGCACTGGGAAGGTTACAGTC-------
----
-------------------GATGGTAATAATACTAGTTGGGGAAATATGACGGAAGAAGTAAGA---
AACTGCTCTTTCAATGTCACCACAAGCATAGGA--------------------------
AATAAGATGCATAAAGAATATGCACTT---TTTTATAAACTTGATCTAATGCCAATAAATGAT----------
----

GATAATGTTACAAAATATATATTAATAAATTGTAATACCTCAGTCATTACGCAG---
GCCTGCCCAAAGGTATCCTTTAAACCAATTCCCATACATTATTGTACCCCGGCTGGTTTTGCGCTCCTAAAAT
GTAAT---GATAAAAAAATTCAAT------GGAACAGGACCATGTAAAAAT----------------------
-GTTAGC---ACAGTACAATGTACACATGGAATTAAGCCAGTAGTGTCAACT---
CAACTACTGTTAAATGGCAGTCTAGCAGAAGAGGAG-----------
ATAGTGATTAGGTCAGAAAATTTCACGGACAAT---------GCTAAAACCATAATAGTACAACTGAAT----
-----------GAAACTATAGAA---------ATTAATTGTACAAGACCCGGCAACAATACAAGAAGA-----
-AGTATAACTATA------------GGACCGGGGAGAGCA---TTT------TATGCAACAGGAGAC------
---------ATAATAGGAGATATA------------AGAAAAGCATATTGT---ACTCTTAATAGAACA---
AAGTGGGAAGACATTTTAGGAAAGGTAGCTAGAAAATTAGAA---------------GAACAATTTGAG----
--------------------------------AATAAAACAACAATAATCTTTAAT------
AGCTCCTCAGGAGGGGACCTAGAAATTGTA---ATGCACAGTTTCAATTGTGGAGGGGAA---
TTTAAGCAGCTCCAGGCAAGAGTCCTGGCTGTGGAAAGATACCTACAGGATCAACAGCTCCTAGGGATTTGGG
GTTGCTCTGGAAAACTCATCTGCACCACTACTGTGCCTTGGAATACTAGCTGGAGT-----------------
----------------------------AATAAATCTTATGCAGACATTTGG------GAC------
AACATGACCTGGATGCAGTGG---GAGAAAGAAATTGGCAATTACACAGACATAATATAC---
ACCTTACTTGAAGGAGCGCAAAATCAACAAGAA---------------------------------------
----
AAGAATGAACAAGAATTATTGCAATTAGATACGTGGGCAAGTTTGTGGAATTGGTTTAGCATATCAAATTGGC
TGTGGTATATAAGAATATTCATAATGATAGTAGGAGGCTTAATAGGTTTAAGAATAGCTTGTACT---
GTACTTTCTATAGTG---------AATAGAGTTAGGCAGGGATACTCACCA------

TTATCGTTCGCTGTTAGTTTGCTTAATGCCACGGCTATAGCAGTATCTAAA---
GGGACAGATAGGGTTATAGAAGTATTACAAATAATTGGTAGAGGG----------------------------
--------ATTCTCCACATA------CCTAGAAGAATAAGACAGGGCTTAGAAAGAATTGTGCTA------
>KT427799
GCT---ACA--
GAACAATTGTGGGTCACAGTCTATTATGGAGTGCCTGTGTGG---AAAGATGCAAAT---
ACCACCTTATTTTGTGCGTCAGATGCTAAAGCATATGACACGGAG---GTGCATAAT----------------
--------------GTTTGGGCCACA---CATGCCTGTGTACCCACAGAC---
CCTAGTCCACAGGAAATAGTATTGAAA---------AATGTGACAGAAAATTTTAAC----------------
--------------------------ATGTGGAAAAATAACATGGTAGAACAGATGCATAAGGATATA---
ATCAGTTTATGGGATGAAAGCCTA---AAACCATGTGTAAAATTA---
ACCCCACTCTGTGTTACCTTGAATTGCTCTAATTACAATAGTACCAAT-------------------------
----
----------------------------AGTACAATAGATCCCAATATGGAGGGAGCAATAAAA---
AATTGCTCTTTCAATGCTACCACAGGTATACAA---------------------------
AATAAAATGAAGAAAGAATATGCACTG---TTTTATAGCCTTGATATAGTACAAATAGAAAGTGAAAATAAA-
----

AGCAATAAAAGCTATATGTTAAGAAGTTGCAATACCTCAGTCATTACACAG---
GCCTGTCCAAAGGTAACCTTTGAACCAATTCCCATACATTATTGTGCCCCGGCTGGTTTTGCGATTCTAAAGT
GTAAT---GATAAGAAATTCAAT------GGAACAGGACCATGTAAAAAT-----------------------
-GTCAGC---ACAGTACAGTGTACACATGGAATCAGGCCAGTAGTGTCAACC---
CAACTGCTGTTAAATGGCAGTCTAGCAGAAGAAGAG------------
ATAATAATTAGATCTGAAAATATCACAAACAAT---------GCTAAAACCATAATAATACAGCTGAAC----
-----------AGGTCTATAGAA---------ATTAATTGTACAAGACCCAACAACAACACAAGAAAG-----
-AGTATACATATG------------GGATGGGGGAGAGCA---TTT------TATGCAACAGGAGAT------
---------ATAATAGGAGACATA------------AGACAAGCACATTGT---AACCTTAGTGGAACA---
AAATGGAATAACACTTTATACCAGATAGCTAGAAAATTACGA---------------GAACACTTT-------
-----------------------------------AACAACACAATAGTCTTTAAT------
CAATCCTCAGGAGGGGACCCAGAAATTGTA---ATGCATACTTTTAATTGTGGAGGGGAA---
TTCAAGCAGCTCCAGGCAAGAGTCCTAGCTGTGGAAAGATACCTAAAGGATCAACAGCTCCTGGGGATTTGGG
GTTGCTCTGGAAGACTCATTTGCACCACTGCTGTGCCTTGGAATGCTAGTTGGAGT-----------------
----------------------------AATAAATCTCTAAATGAAATTTGG------GAT------
AACATGACCTGGATGCAGTGG---GAGAGAGAAATTGACAATTACACAAACTTAATATAC---
ACCTTAATTGAAGAGTCGCAATACCAGCAAGAA--

AAGAATGAACAAGAATTATTGGAATTGGATAAATGGGCAAGTTTGTGGAGTTGGTTTAACATAACAAACTGGC
TGT--------------------------GTAGGAGGCTTAGTAGGTTTAAGAATAATTTTTGCT---
GTGCTTTCTATAGTG---------AAAAGAGTTGGCAGGGATACTCACCA------
TTATCGTTCGCTGTTGGTTTGCTTGGTGCCACAGCTATCGCAGTAGCTGGG---
GGGACAGATGGGATTATAGAAGCAGTAAGAGGACTTTGTGGAGCT----------------------------
--------ATTCTCAACATA------CCTGGAAGAATAAGACAGGGCTTAGAAAGAGCTTTGCTA---TAA
>KT427667
GCT---GCA--
GAAAATATGTGGGTCACAGTCTATTATGGGGTACCTGTGTGG---AAAGAAGCTGAC---
ACCACTCTATTTTGTGCCTCAGATGCTAAAGCATATGACACAGAG---GCACATAAC----------------
--------------GTCTGGGCCACA---CATGCCTGTGTGCCTACAGAC---
CCTAACCCACAAGAATTAGACTTGATA---------AATGTGACAGAAAATTTTAAC----------------
--------------------------ATGTGGAAAAATAACATGGTAGAACAGATGCATGAGGATATA---
ATCAGTTTATGGGATCAAAGCCTA---AAGCCATGTGTAAAATTA---
ACCCCACTCTGTGTTATTTTAAATTGCACTGATTTGACTAATACTACTTGCATTAATATGACTAATGCTTGCA
CT---
----------------------------AGGAATATTTCGAAAGAAATGGAGGGAAAAATAAAA---
GCCTGCTCATTTAATATCACCACAAGCAACATA------------------------
AGTAATAAAAAGAAAAAAGAATATGCACTT---
TTTAATAGGCTTGATGTGGTACAAATAGATCAAAGTAATAAT-------------------------------
----
AGCAGCAGCTACAATAGCAGCAGCTATATATTGAGACATTGTAACACCTCAGTCATTACACAA---
ACCTGTCCAAAGATATCCTTTGAACCAATTCCCATACATTATTGTACCCCGGCTGGTTTTGCGCTTCTGAAAT
GTAAG---GATAAAAATTTCAAT------GGAACAGGACTGTGTAAAAAT-----------------------

-GTCAGT---ACAGTACAATGTACACATGGAATTAAGCCAGTAGTGTCAACT---
CAACTGTTGTTAAATGGCAGTCTAGCAGAAGAAGAC------------
ATAGTGATTAAATCTGAAAATTTCAGTGAAAAC---------ACTAAAACCATAATAGTACAGCTAAAT----
-----------GAACCTGTAGAA---------ATTAATTGTACAAGACCCAACAACAATACAAGAAAA-----
-GGTATACATACA------------GCATGGGGAAGAATA---CTT------TATGCAACAGGAGAT------
---------ATAATAGGAGATATA------------AGACAAGCATACTGT---ACCCTTAATGGAACA---
AAATGGAATGAAACCTTAGGAAAGATAGCTCAAAAATTATAT---------------AAACAATAT-------
-----------------------------------AATACTACAATAATCTTTAAT------
CCATCATCAGAAGGGGACCCAGAAATTGTA---ATGCATAATTTTAATTGTGGAGGAGAA---
TTTAAGCAGCTCCAGGCAAGAGTCCTGGCTGTAGAAAGATACCTAGGGGATCAACAGCTCCTAGGAATTTGGG
GATGCTCTGGAAAACTCATTTGCACCACCTCTGTGCCTTGGAATACTAGTTGGAGT-----------------
----------------------------AATAAAAGTCAAAGTGAGATTTGG------GGG------
AACATGACCTGGATGCAGTGG---GAAAAAGAAATTGACAATTACACAAGTGTAATATAC---
ACCTTGCTTGAGGAATCACAGAACCAGCAAGAA--

CAAAATGAAAAGGACTTATTGGAATTGGATAAGTGGGAAAGTTTGTGGAATTGGTTCAGCATTACAAACTGGC
TGTGGTATATAAGACTATTCATAATGATAGTAGGAGGCTTGATAGGTTTAAGAATAATTATTACT---
GTATTTTCTATAGTA---------AATAAAGTTAGGAAGGGATACTCACCA------
TTATCGTTCGCTGTTAGTTTGTTTGATGCCATAGCTATAGCAGTAGCTGAG---
GGGACAGATAGAATTATAGAAGCAGTACATAGATTGGGTAGAGCT----------------------------
--------ATTCTCCACATA------CCTAGAAGAATAAGACAGGGCTTCGAAAGGCTTTACTA---TAA
>KT427794
GCT---AAA--
GAAGATTCATRGGTCACAGTCTATTATGGGGTACCTGTGTGG---AAAGAAGCAACC---
ACCACTCTATTTTGTGCATCAGATGCTAAAGCATATGACCCAGAG---GTACATAAT----------------
--------------GTTTGGGCCACA---CATGCTTGTGTACCCACAGAC---
CCTAATCCACAAGAAGTAAATTTGAAA---------AATGTGACAGAAGACTTTAAT----------------
--------------------------ATGTGGAAAAACAACATGGTAGAACAGATGCATGAGGATATA---
ATCAGCTTATGGGATCAAGGCCTA---CAGCCATGTGTAAAATTA---
ACCCCATTCTGTGTCACTCTAGAATGCAGTRATATTACTAATAATATTACTAATAATAAC-------------
----
-------------------------AGCAKAAGTGATGAAATAAATATGAAGGAAGAATTAAAA---
AATTGTTCATTTAATATCACCACACAGRTAACA---------------------------
AAGAAGGTGCAAAAAGAATWTGCAATT---TTTAATAAACTTGATATAGTACCAATAGAAART----------
----

RATAACAAGAGCTATATGTTGATAAATTGTAATACCTCAGTCATTACACAG---
GCTTGTCCAAAGACATCCTTTGAACCAATTCCCATACATTATTGTACCCCGGCTGGTTTTGCGATTCTAAAGT
GTAAY---GATAAAAAGTTCAAT------GGAACAGGACCATGTACAAAC-----------------------
-GTCAGC---ACAGTACAATGTACACATGGAATTAGGCCAGTAGTGTCAACT---
CAATTGCTGTTAAATGGCAGTCTAGCAGAAGAAGAT------------
GTAGTAATCAGGTCTGAAAATTTCTCGAACAAT---------GCTAAAAACATATTAGTACAGCTGAAT----
-----------GCCACTGTAGAA---------ATTAATTGTACAAGACCCAACAACAACACAAGAAAG-----
-AGCATACATGTA------------GCATGGGGAAGAACA---TTA------TATGCAACAGGAGAC------
---------ATAATAGGAGATATA------------AGACAAGCACATTGT---AACCTTAGTAAAACA---
GGCTGGGAAAAAACTTTAAAACAGATAGTTACAAAATTAGGA---------------GAACAATTTAGG----
--------------------------------AAAAATACAACAATAAAATTTAAT------
CAATCCACAGGAGGAGATCCAGAAATTGTA---ACACATACTTTTAACTGTGGAGGGGAA---
TTTAAGCAGCTCCAGGCAAGAGTCCTGGCTGTAGAAAGATACCTAAGGGATCAACAGCTCCTAGGAATTTGGG
GCTGCTCTGGAAAACTCATCTGCACCACTAATGTACCTTGGAATAGTAGTTGGAGT-----------------
----------------------------AATAAAACTCTAAGCCAGATTTGG------GAT------
AACATGACCTGGATACAGTGG---GAGAGAGAAATTGACAATGTCACAGAGATAATATAC---
AACTTACTTGAAGAATCGCAGAACCAGCAAGAA--

AAGAATGAACAAGAATTATTAGAATTGGATAAGTGGGCAAATTTGTGGAATTGGTTTAGCATAACAAAATGGC
TGTGGTATATAAAAATATTCATAATGATAGTAGGAGGCTTAGTAGGTTTAAGAATAGTTTTTGCT---
GTGCTTTCTCTAGTG---------AATAGAGTTAGGCAGGGATACTCACCA------
TTATCGTTGGCTGTTAGTTTGCTTAATGCCATAGCAATAGTAGTAGCTGAG---
GGAACAGATAGGATTATAGAAATAGTACAAAGAGGTGGAAGAGCT----------------------------
--------ATCCTCCACATA------CCTAGAAGAATAAGACAGGGCTTAGAAAGGGCTTTGCTA---TAA

>KU749390
GCT---ACA--
GATAGATTGTGGGTCACAGTCTACTATGGGGTACCTGTGTGG---AAAGAAGCAATC---
ACCACTCTATTTTGTGCATCAGATGCTAAAGCTTATGATACAGAA---GTGCACAAT----------------
--------------GCTTGGGCCACA---CATGCCTGTGTGCCCACAGAC---
CCCAACCCACAAGAAGTAAGATTGGAA---------AATGTGACAGAAAACTTTAAT----------------
--------------------------GTGTGGAAAAATAACATGGTAGACCAGATGCATGAGGATATA---
ATCAGTTTATGGGACCAAAGCCTA---AAGCCATGTGTAAAATTA---
ACTCCACTCTGTGTTACTTTAAATTGCACTGATCTGAATGCTAATAATACCGCTAGTGGCCAGAATGCTACTA
ATGTCACTAGT--
----------------------AATAGTCCTATCTGGGGGGGGATAGTAAGAGGAGAAGTAAAA---
AACTGCTCTTTCAATGTCACCACAGCCATTAAAGATAAGGTG------
AAGAAAGAATATGAAAAGGTGAAGAAAGAATATGCATAT---
TTCTATAATCTTGATGTAGTACAAATAACTGAG--

AATGCTAATAATAAGAGCTATAGGTTGGCAAGCTGTAACACCTCAGTCATTACACAG---
GCCTGTCCAAAGATATCCTTTGATCCAATTCCCATACATTATTGTGCCCCGGCTGGTTTTGCGATTCTAAAGT
GTAAC---AATAAGACATTCGAT------GGAAAAGGACCATGTAACAAT-----------------------
-GTCAGC---ACAGTACAATGTACACATGGAATTAAGCCAGTAGTGTCAACT---
CAACTGCTGTTAAATGGCAGTCTAGCAGAAGAAGGG------------
GTAGTACTTAGATCTGAAAATTTCACGGAAAAT---------ACTAAAACCATAATAGTGCAGCTGGCA----
-----------ACGCCTGTAAAA---------ATTAATTGTACAAGGCCCAACAATAATACAAGAAAA-----
-AGTATACACATA------------GGACCAGGGAGAGCA---TTT------TATGGAACA---GAA------
---------ATAATAGGAGATATA------------AGACGAGCACATTGC---AACATTAGTAGAGCT---
GACTGGAATGATACTTTAAGAAAGGTAGTTGATAAATTAAGA---------------GAAAAATATAAA----
-----------------------------------AATAAAACAATAATCTTTAAG------
AGTTCCTCAGGAGGGGACCCAGAGATTGTA---ATGCACAGCTTTAATTGTGGAGGGGAA---
TTTAAACAGCTCCAGGCAAGACTCCTGGCTGTGGAAAGATACCTAATGGATCAACAGCGCCTAGGGATTTGGG
GTTGCTCTGGAAGACTCATTTGCACCACTAATGTGCCTTGGAATGCTAGTTGGAGT-----------------
----------------------------AATAAAACTTATAATGAAATTTGG------CAG------
AACATGACCTGGAGGGATTGG---GAAAGAGAAATTGACAACTACACAGGATTAATATAT---
GACTTACTTGAAAAATCGCAAAACCAACAAGAA--

AAGAATGAACAAGAATTATTGGAATTGGATAAATGGGCAAGTTTGTGGAATTGGTTTGATATAACACACTGGC
TGTGGTATATAAAAATATTTATAATGGTAGTAGGGGGCTTGATAGGCTTAAGAATAGTTTTTACT---
GTACTTTCTATAGTG---------AATAGAGTTAGGCAGGGATACTCACCT------
TTATCGTTTGCTGTTAGCTTGCTCAATGCCACAGCCATAGCAGTAGCTGAG---
GGAACAGATAGGGTCATAGAAGTAGTACAAAGAATTTTTAGAGCT----------------------------
--------ATCCTCAATATA------CCTACACGAATAAGACAGGGCTTGGAAAGGGCTTTGCTA---TAA
>KX181912
GCT---ACA--
GAACAATTGTGGGTCACAGTATATTATGGAGTACCTGTGTGG---AAAGAAGCTACC---
ACCACTCTATTTTGTGCATCAGATGCTAAAGCATATGACACAGAG---GTACATAAT----------------
--------------GTTTGGCCACA---CATGCCTGTGTACCCACAGAC---
CCTAACCCACAAGAAGTAGAATTGGAC---------AATGTGACAGAAAACTTTAAC----------------
--------------------------ATGTGGAAAAATAACATGGTAGACCAAATGCATGAGGATATA---
ATCAGTTTATGGGATCAAAGCCTA---AAGCCATGTGTAAAATTA---
ACCCCACTTTGTGTTACTTTAAAATGCACTGATGTGAATAATACCAGTAATAGTACCAATAGT----------
----
-------------------------AATAGTAGTAAAACAATAGAGGAGGTGGGAGAAATAAAA---
AACTGCTCTTTCAATGTCACAACAAGCATAAGG---------------------------
GATAGGCAGAAGAAAGAATATGCGCTT---TTTTATAGACTTGATGTAGTACCAATAGATACA----------
----

GATAATACAAGCTATACATTGATAAATTGTAACACCTCAGTCATTACACAG---
GCCTGTCCAAAGGTATCCTTTGAACCAATTCCCATACATTATTGTGCCCCGGCTGGTTTTGCGATTCTAAAGT
GTAAG---GATAAAAAGTTCAAT------GGAACAGGACCATGTAAAAAT-----------------------
-GTCAGC---ACAGTACAATGTACACATGGAATTAGGCCAGTAGTGTCAACT---
CAACTGCTGTTAAATGGCAGTCTAGCGGAAGAAGAT------------
GTAGTAATTAGATCTGAAAATATCACGAACAAT---------GCTAAAACCATAATAGTGCAGCTGAAT----

-----------GAAACTGTAGAA---------ATTAATTGTACAAGGCCCAACAATAATACAAGAAGA-----
-AGTATACATTTT------------GGACCTGGGAGAACA---TTG------TATGCAACAGGAGAA------
---------ATAATAGGAGATATA------------AGACGAGCACATTGT---ACCATTAATGCAACA---
AAATGGAACAACACTTTAGAAAAGATAGTTAGAGAATTAAGA------------AAACTAGAACTATTTAAG-
-----------------------------------AATAAAACAATAGTCTTTAAT------
CAAGTCTCAGGAGGGGACCCAGAGATTGTA---ATGCACAGTTTTAATTGTGGAGGGGAA---
TTTAAGCAGCTCCAGGCGAGAGTCCTGGCTGTGGAAAGATACCTAAAGGATCAACAGCTCCTAGGGATTTGGG
GCTGCTCTGGAAAACTCATTTGCACCACTGCTGTGCCTTGGAATACTAGTTGGAGT-----------------
----------------------------AATAAATCTCTGGATCAAATTTGG------GAG------
AACATGACCTGGATGCAGTGG---GAGAGAGAAATTGACAATTACACAGACTCAATATAC---
AAATTAATTGAACAATCACAGAACCAGCAAGAA--

AAAAATGAACAAGACTTATTGGCATTGGATACATGGGCAAGTTTGTGGAATTGGTTTGACATAACAAGATGGC
TGTGGTATATAAAAATATTCATAATGATAGTAGCAGGCTTAATAGGTTTAAGAATAGTTTTTACT---
GTACTTTCTATAGTG---------AATAGAGTTAGGCAGGGATACTCACCA------
TTGTCATTGGCTGTTAGTTTGCTTAATGCTACAGCTATAGCAGTAGCTGAA---
GGGACAGATAGGGTTATAGAAGTAGCACAAAGAATTGTTACAGCT----------------------------
--------TTTCTCAACGTA------CCTAGAAGAATAAGACAGGGCTTAGAAAGAGCTTTGCTA---TAA
>KX181906
GCT---GAA---GAA------------------------------------
GACCAATGGTGGGTTACAGTCTATTATGGGGTACCTGTGTGG---AAAGAAGCAAAC---
ACCACCCTATTTTGTGCATCAGATGCTAAAGCACATGACACAGAG---GCACATAAT----------------
--------------GTTTGGGCCACA---CATGCCTGTGTACCCACAGAC---
CCTAACCCACAAGAAGTAGTATTA------------AATGTAACAGAAAATTTTAAC----------------
--------------------------ATGTGGAAAAATAACATGGTAGAACAAATGCATGAGGATGTA---
ATCAGTCTATGGGATCAAAGCCTA---AAGCCATGTGTAAAATTA---
ACCCCAATCTGTGTGACTTTAAATTGCACTAATGTGAATACCACAAGTAATAATAGCAGT-------------
----
-------------ACTGGGAACTACAATAATAGTTGGGAAGGGGTGGAGGAGGGAGAATGGAAA---
AACTGCTCTTTCAATATCACTGCAAGCTTAGGG---------------------------
GATAAAGTGCAGAAAGAATATGCAATT---TTTAATAAATATGATGTAGTACCAATAGATAATGAT-------
----

AATAATAGAACCTATATGTTGATAAATTGTAACACCTCAGTCATTACACAG---
GCCTGTCCAAAGACATCCTTTGAACCAATTCCCATACATTATTGTACCCCGCCTGGTTTTGCGCTTCTAAAGT
GTAAT---AATAAGACGTTCAAT------GGAACAGGACCATGTACCAAT-----------------------
-GTCAGC---ACAGTACAATGTACACATGGAATTAAGCCAGTAGTGTCAACT---
CAGCTACTGTTAAATGGCAGTTTATCAGAAGAAGGG------------
GTAATAATTAGATCTGAAAATATCACGGACAAT---------AGTAAAACCATAATAGTACAGCTGCAT----
-----------AAACCTGTAAAA---------ATTAATTGTATAAGGCCCAACAACAATACAAGAAAG-----
-GGTATACATATG------------GGACCAGGGAGAACA---TTT------TATGCAAGAGGAGAA------
---------ATAATAGGAAATATA------------AGATCAGCATATTGT---AACATTAGTAGAGCA---
AAATGGAACGACACCTTAAAAGAGATAGCTGAAAAATTAAAG---------------GACCACTTTAAG----
-----------------------------------AACAAGACACTAATCTTTAAT------
CACTCCTCAGGAGGGGACCCAGAAATTGTA---ATGCACACTTTTAATTGTGGAGGAGAA---
TTTAAGCAGCTCCAGGCGAGAGTCCTGGCTGTGGAAAGATACCTAAGGGATCAACAGCTCCTGGGGATTTGGG
GTTGCTCTGGAAAACTCATCTGCACCACTGCTGTGCCTTGGAATACTAGTTGGAGTACTAGG-----------
-------------------TGGAAG---AATAGGTCTCTAAGTGACATTTGG------GAG------
AACATGACCTGGATGGAGTGG---GAGAGAGAAATTGACAATTACACAGACTTTATATAC---
ACCTTGCTTGAAGAATCACAGAACCAACAAGAA--

AAGAATGAAAAAGACTTATTGGAATTGGATAAGTGGGCAAGTTTGTGGAATTGGTTTGACATAACAAACTGGC
TGTGGTATATAAAATTATTCATAATGATAGTAGGAGGTTTAATAGGTTTAAGAATAGTTTTTACT---
GCACTTTCTATAGTG---------AATAGAGTTAGGCAGGGATACTCACCA------
TTATCATTCGCTGTTAGCTTGTTTAATGCCATAGCTATAGCAGTAGCTGAG---
GGAACAGATAGGATAATAGAAGCAGTACAAAGAATTGGCAGAGCT----------------------------
--------ATTCTCCACATA------CCTAGAAGAATAAGACAAGGCTTAGAAAGGGCTTTGCTA---TAA
>KX181900
GCT---GAA---GAA------------------------------------
GACAAATTGTGGGTCACAGTTTATTATGGGGTACCTGTGTGG---AAAGAAGCAACC---

ACCACTCTATTTTGTGCATCAGATGCTAAAGCATATGACACAGAG---GTACATAAT----------------
--------------GTTTGGGCCACA---CATGCCTGTGTACCCACAGAC---
CCTAACCCACAAGAAGTAGTATTGGAA---------AATGTGACAGAAAATTTTAAC----------------
--------------------------ATGTGGAAAAATAACATGGTAGAACAGATGCATGAAGATGTA---
ATCAGTTTATGGGATCAAAGCCTA---AAGCCATGTGTAAAATTA---
ACCCCACTCTGTGTTACTTTAGAGTGCGAGAATGTGACAAAAAATACT-------------------------
----
-------------------------------TCGGGAGAGATACAGGTGAAGGAGGAACTAAAA---
AACTGCTCTTTCAATGTAACCACAGGCATAGGG---------------------------
AAAAAGGTGCGGAAAGAAAATGCAATT---
TTTGATAGCCTTGATATAGTACCAATAGATGAGGATAATAATAATGAG-------------------------
----
ACTAATAGAAGCTATATGTTGGTAAATTGTAATACCTCAGTCATTACACAG---
GCCTGTCCAAAGGTATCCTTTGAACCAATTCCCATACATTATTGTGCCCCGGCTGGTTTTGCGATTCTAAAGT
GTAAC---AATAAGACATTCAGT------GGGACAGGACCATGTAAAAAT----------------------
-GTCAGC---ACAGTACAGTGTACACATGGAATTAGGCCAGTAGTGTCAACT---
CAACTGTTGTTAAATGGCAGTTTAGCAGAAAAGGAG------------
GTAATAATTAGATCTGAAAATTTCACAAACAAT---------GCTAAAACCATAATAGTACAGCTGAAT----
-----------GAAACTGTACAA---------ATTAACTGTTCAAGACCCAACAACAATACAAGAAAG-----
-AGTATACATATG------------GGATGGGGAAGAGCA---TTT------TATGCAAGAGGAGAC------
---------ATAATAGGAGATATA------------AGGCGAGCACATTGT---AACATTAATGAAGCA---
CAGTGGAACAAAACCTTAGGACAGATAGTAGAAAAATTAAAA---------------GAACAATTT-------
-----------------------------------AATAAAACAATAATCTTTAAT------
CAATCCTCAGGAGGGGACCCAGAAATTGTA---ATGCACACTGTCAATTGTGGAGGGGAA---
TTTAAGCAGCTCCAGGCAAGAGTCCTGGCTGTGGAAAGATACCTAAGGGATCAACAGCTCCTAGGGATTTGGG
GTTGCTCTGGAAAACTCATCTGCACCACCTCTGTGCCTTGGAATTCTAGTTGGAGT-----------------
---------------------------AATAAATCTCTGGATGCTATTTGG------GAT------
AACATGACCTGGATGGAGTGG---GAAAGAGAAATTGACAATTACACAGGCTTAATATAC---
ACATTGCTTGAAGAATCGCAGAACCAACAAGAA---------------------------------------
----
AAGAATGAAAAGGAATTATTGGAATTAGATAAGTGGGCAAGTTTATGGACTTGGTTTAGCATAACAAACTGGC
TGTGGTATATAAAAATATTCATAATGATAGTAGCAGGCTTAGTAGGTTTAAGAATAGTTTTTACT---
GTACTTTCTGTAGTG---------AATAGAGTTAGGCAGGGATACTCACCA------
TTATCGTTCGCTGTTAGTTTGTTTGATGCCATAGCTATAGCAGTAGCTGAG---
GGGACAGATAGGGTGATAGAAGTAGCACAAAGAATTGGCAGAGCT----------------------------
--------ATCCTTCACATA------CCTACAAGAATAAGACAAGGCTTAGAAAGGGCTTTACAA---TAA
>KX181901
GCT---AAA---GAA-------------------------------------
GATGAATTTTGGGTCACAGTCTATTATGGGGTACCTGTGTGG---AAAGAAACAACC---
ACCACTCTATTTTGTGCATCAGATGCTAAAGCATATGACACAGAG---GCACATAAT----------------
--------------ATTTGGGCCACA---CATGCCTGTGTACCCACAGAC---
CCTGACCCACAAGAAGTAGAGTTGGTA---------AATGTGACAGAAAATTTTAAC----------------
--------------------------ATGTGGAAAAATAACATGGTAGAACAGATGCATGAGGATATA---
ATTAGTTTATGGGATCAAAGCCTA---AAGCCATGTGTAAAATTA---
ACCCCACTCTGTGTTATCTTAAATTGTACTAATTACAATGCTACAAAAACAAATACCACTAGT----------
----
----------------------AATAATAGCAGTGAGTGGGAAAATATGAGGGGAGAAATACAA---
AACTGCTCTTTCAATATCACCACAGGCATAGAA---------------------------
AAAAAGATGCGGAGAGAATATGCAATT---TTTAATAAACTTGATATAGTACCAATAAATAAT----------
----

AATAATACAAGCTATATGTTGGTAAATTGTAATACCTCAGTCATTAGACAA---
GCCTGTCCAAAGACAACCTTTGAACCAATTCCCATACATTATTGTACCCCGGCTGGTTTTGCGCTTCTAAAGT
GTAAA---GATAAGGAGTTCAAT------GGATCAGGACCATGTACAAAT----------------------
-GTCAGC---ACAGTACAATGTACACATGGAATTAGGCCGGTAGTATCAACT---
CAACTGCTGTTAAATGGCAGTTTAGCAGAAGGAGAG------------
ATAATAATTAGATCTGAAAATATCACAAACAAT---------GGTAAAACCATAATAATACAGCTGAAC----
-----------GAAGCTGTAGAA---------ATTAATTGTACAAGACCCAACAACAATACAAGAAAA-----
-GGTATACATATG------------GGATGGGGAAAAGCA---TTT------TATGCAACAGGAGAA------
---------ATAATAGGAAATATA------------AGACAAGCACATTGT---AACATTAGTGAAGCA---

AGATGGAACAATACTTTAAAAGGGATAGTTACAAAATTAAGA---------------GAAAAATTTAAT----
-----------------------------------AATAAAACAATAATCTTTAAT------
CAATCCTCAGGAGGGGACCCAGAAGTTGTA---ATGTTCCATTTTAATTGTGGAGGGGAA---
TTTAAGCAGCTCCAGGCAAGAGTCCTGGCTGTGGAAAGATACCTAAGGGATCAACAGCTCCTAGGGATTTGGG
GTTGCTCTGGAAAACTCATCTGCACCACTGCTGTGCCTTGGAATTCTAGTTGGAGT-----------------
----------------------------AATAAAAATCTGAGTACAATTTGG------GAT------
AACATGACCTGGATGGAATGG---GAGAGAGAAATTGACAATTACACAGGCTTAATATAC---
AAGTTACTAGAAGAATCGCAGAACCAGCAAGAA--

AAGAATGAACAAGAATTATTGGCATTGGATAAGTGGGCAAATTTGTGGAATTGGTTTGACATATCAAAATGGC
TGTGGTATATAAGAATATTCATAATGATAGTAGGAGGCTTAATAGGTTTAAGAATAATTTTTGCT---
GTACTTTCTATAGTG---------AATAGAGTTAGGCAGGGATACTCACCA------
TTATCATTCGCTGTCAGTTTGCTTAATGCCACAGCTATAGCAGTAGCTGAG---
GGGACAGATAGAGTGATAGAACTAGCACAGAGGTTGGGCAGGGGT----------------------------
--------ATTCTCCACATA------CCTACAAGAATAAGACAGGGCTTAGAAAGGGCTTTGCTA---TAA
>KX181891
GCT---ACA--
GACAATTTATGGGTCACAGTATATTATGGGGTTCCTGTGTGG---AAAGAAGCAAAT---
ACTACTCTATTTTGTGCATCAGATGCTAAAGCTTATGACACGGAA---GCACATAAT----------------
--------------GTCTGGGCCACA---CATGCCTGTGTACCCACAGAT---
CCTAACCCACAAGAAATAGTATTGAAA---------AATGTGACAGAAAATTTTAAT----------------
--------------------------ATGTGGAAAAATAACATGGTAGAACAGATGCATGAGGATATA---
ATCAATTTATGGGACCAAAGCCTA---AAGCCATGTGTAAAATTA---
ACCCCACTCTGTGTTACTTTAAACTGTACTGAT--

----------------------AATAATAGTAGTAATAATATAGACATGAAGGAAGAAATGAAA---
AACTGCTCTTTCAATATCACCACAGGCATAGGA---------------------------
AATAAGATGCAGAAAGAATATGCACTG---TTTAGAACGCTATATATAATACAAATAAAT-------------
----

ACAATCTATACATTGATAAATTGTAACACCTCAGTCATTACACAG---
GCTTGTCCAAAGATATCCTTTGAACCAATTCCTATACATTATTGTGCCCCGGCTGGTTTTGCGATTCTAAAAT
GTAAT---GACAAAAAGTTCAAT------GGAACAGGACCGTGTAGAAAT-----------------------
-GTCAGC---ACAGTACAATGTACACATGGAATTAGACCAGTGGTGTCAACT---
CAACTACTGTTAAATGGCAGCCTAGCAGAAGAAGAA------------
GTAGTAATTAGATCTAAAAATTTTTCTGACAAT---------ACTAAAACCATAATAGTACATCTGAAC----
-----------CACACTGTAAAA---------ATTAATTGTTCAAGACCCGGCAACAATACAAGGAAA-----
-GGTATACACCTG------------GGATGGGGAAGATCA---TTG------TTTGCAACAGAAATA------
---------ATAGTAGGCGATATA------------AGACAAGCACATTGT---AACCTTAGTGGAGCA---
GAATGGGATAACACTTTAAGGCAGGTAGTTGAAAAATTAGGA---------------GAGCAATTTAAC----
-----------------------------------AAGACAACAATAATCTTTAAC------
CACTCCTCAGGAGGGGACCCAGAAGTTGTA---ATGCACAGTTTTAATTGTGGAGGGGAA---
TTTAAGCAGCTCCAGGCAAGAGTCCTGGCTTTGGAAAGATACCTACGGGATCAACAGCTCCTGGGGCTTTGGG
GTTGCTCTGGAAAACTCATTTGCACCACTACTGTGCCTTGGAATGCTAGTTGGAGT-----------------
----------------------------AATAAAACTCTGAAAGACATTTGG------GAG------
AACATGACCTGGATAGAGTGG---GAAAGAGAAATTGGCAATTATACAGAAGTAATATAT---
AACTTACTTGAAAAATCGCAGAACCAGCAAGAA--

AAGAATGAGCAAGAATTATTGGCATTGGATAAGTGGGCAAGTCTGTGGAATTGGTTTGACATATCACATTGGC
TGTGGTATATAAAAATATTCATAATGATAGTAGGAGGTTTAATAGGTTTAAGAATAGTTTTTACT---
GTACTTTCTATAGTG---------AATAGAGTTAGGCAGGGATACTCACCA------
CTATCATTCGCTATTAGTTTGGTTAATACCATAGCTATAGCAGTAGCAGAG---
GGGACAGATAGGGTGATAGAAGCAGCACAAAGATTTGGTAGGGGC----------------------------
--------ATCCTCCACATA------CCTAGAAGAATAAGACAGGGCTTAGAAAGGGCTTTGCTA---TAA
>KX181907
ACT--
GAACAATTGTGGGTCACAGTCTATTATGGGGTACCTGTGTGG---AGAGAAGCAACC---
ACCACTCTGTTTTGTGCATCAGATGCTAAAGCATACAAAACAGAA---GTACATAAT----------------
--------------GTCTGGGCCACA---CATGCCTGTGTGCCCACAGAC---
CCCAACCCACAAGAAATCCCCTTGGAA---------AATGTGACAGAACCTTTTAAC----------------

--------------------------ATGTGGAAAAATGACATGGTAGAACAGATGCATGAAGATATA---
ATCAGCATATGGGATCAAAGCCTA---AAGCCATGTGTAAAATTA---
ACCCCACTCTGTGTTACTTTACATTGCACTGATGTGACGAATGGGAATGGGACTAATGGC-------------
----
----------------------------AGGAATGTTAAATTGGATACGGAAGGAGAAATAAAA---
AACTGCTCTTTCAATGTTACCACAAGCATAAGA---------------------------
GATAAGATGCAAACACAATATTCTCTC---
TTCTATAAACTTGATGTAGTACCACTAGAGACTAATAGTAGTGTTAAA-------------------------
----
GAGGCTAATAGTAGTCAGAACTATAGTGACTATAGATTGATAAGTTGTAATACCTCAGTCATTACCCAG---
GCCTGTCCAAAGGTAACCTTTGAGCCAATTCCCATACATTATTGTGCCCCGGCTGGTTTTGCGATTCTAAAGT
GTAAT---GATAAGAAGTTCAAT------GGAACAGGATCATGTACAAAT-----------------------
-GTCAGC---ACAGTACAATGTACACATGGAATTAGGCCAGTAGTGTCAACT---
CAACTACTGTTAAATGGCAGTCTAGCAGAAGAAGAG------------
GTAGTAATTAGATCTGAAAATTTCACAAACAAT---------GCTAGAACCATAATAGTACAGCTGAAC----
-----------ACATCTGTAGAA---------ATTAATTGTACAAGGCCCAACAACAATACAAGAAAA-----
-AGTATAACTATA-----------GGACCAGGGGCAGCA---TTT------TTT---ACAGGAGAA-------
---------ATAATAGGGGATATA------------AGAAGTGCATATTGT---ACTATTAATGGAACA---
CAATGGAATAACACTTTAAAACAGGTAGTTACAAAATTAAGG---------------GAACAATTTGGG----
--------------------------------AACACCACAACAATAAGCTTTCAA------
CCACCAAGTGGAGGGGACCCAGAAATTGTA---TTTCACAGTTTTAATTGTGGAGGGGAA---
TTTAAACAGCTCCAGGCAAGAGTCCTGGCTGTGGAAAGATACCTAAGGGACCAACAGCTCCTAGGAATTTGGG
GTTGCTCGGGAAAACTCATTTGCACCACTGCTGTGCCTTGGAACGAGAGTTGGAGT-----------------
----------------------------AATAGATCTCTGAATTACATTTGG------GAT------
AACATGACCTGGATACAGTGG---GAAAGAGAAATTGACAACTACACAGAAACAATATAC---
AGCTTACTTGAGAATGCGCAAAACCAGCAAGAA--

AAGAATGAACAAGACTTATTGCAATTGGATAAATGGGCAAGTTTATGGAATTGGTTTAGCATAACACACTGGC
TGTGGTATATAAAACTATTCATAATGATAGTAGGAGGCTTGATAGGTTTAAGAATAGTTTTTGCT---
GTACTTTCTATAGTG---------AATAGAGTTAGGCAGGGATACTCACCA------
ATATCATTGGCTGTTAGCTTGTTTAATGCCACAGCAATAGCAGTAGCAGAA---
GGAACAGATAGAATCATAGAAATAGCACAAAGAATTTTTAGGGCT----------------------------
--------TTTCTCCACATA------CCTAGAAGAATAAGACAGGGCCTGGAAAGGGCTTTACAA---TAA
>KX181910
GCC---ACG--
GAAAAATTGTGGGTCACAGTGTATTATGGGGTACCTGTGTGG---AGAGAAACAACC---
ACCACTCTATTTTGTGCATCAGATGCTAAAGCATATGAAACAGAG---ATGCATAAT----------------
--------------GTTTGGGCCACA---CATGCCTGTGTACCCACTGAT---
CCTAACCCACAAGAAGTAGTATTGGAA---------AATGTGACAGAAAATTTCAAT----------------
--------------------------ATGTGGAAAAATAACATGGTAGAACAAATGCAGGAGGATATA---
ATCAGTTTATGGGATCAAAGCCTA---AAACCATGTGTAAAATTA---
ACCCCACTCTGTGTTACTTTGAATTGCAGTAATGCTAATTTTACCAATGCTAATGCTACT-------------
----
-------------------AAGAATAATGATAGTACAATAAATGAGATGAACAAAGAAATGAAA---
AACTGCTCTTTCAATATCACCACAGGCATAGGA---------------------------
AATAAGATGCAGAAGGAATATGCACTT---TTTTATGTACATGATGTAGTATTAACAAATGAT----------
----

ACAACAACAAGCTATACATTGATAAATTGTAACACCTCAATAATTACACAG---
GCCTGTCCAAAGGTATCCTTTGAACCAATTCCAATACATTATTGTGCCCCAGCTGGTTTTGCGATTCTAAAGT
GTAAT---GACAAGAAATTCAAT------AGAACAGGACCATGTAAAAAT-----------------------
-GTCAGC---ACAGTACAGTGTACACATGGAATTAAGCCAGTAGTGTCAACT---
CAACTACTGTTAAATGGTAGTCTAGCAGAAGAAGAG------------
GTAATAATTAGATCTGAAAATTTTACAAACAAT---------GCTAAAACCATAATAGCACAGCTAAAC----
-----------CAGTCGGTAGTA---------ATTAATTGTGTAAGGCCCGGTAACAATACAAGAAAA-----
-AGTATACATTTA-----------GGACCAGGAAGAGCA---CTT------TATGCAACAGGAAGT-------
---------ATAATAAGAGATATA------------AGAAAAGCATACTGC---AACCTTAATAAGACA---
CACTGGAACAACACATTAAAACAGATAGTTGTAAAATTAAGT---------------GAACAATTTGGC----
-----------------------------------AATAAAACAATAGTCTTTAAT------
AGATCCTCAGGAGGGGACCCAGAAATTGTA---ATGCACAGTTTTAATTGTGGAGGGGAA---

TTTAAGCAGCTCCAGGCAAGAGTCCTGGCTGTGGAAAGATACCTAAAGGATCAACAGCTCCTAGGGATTTGGG
GTTGCTCTGGAAAACTCATCTGCCCCACTGCTGTGCCTTGGAATGCTAGTTGGAGT-----------------
----------------------------AATAAATCTCTAAATGAGATTTGG------GAG------
AACATGACCTGGATGGAGTGG---GACAGAGAAATTAACAATTACACACAAGTAATATAC---
AACTTACTTGAAAAATCGCAGAACCAACAAGAA--

AAGAATGAGCAAGAATTATTAGCATTGGATACGTGGGCAAGTTTGTGGAATTGGTTTGACATATCAAATTGGC
TGTGGTATATAAAAATATTCTTAATGGTAGTAGGAGGCTTAGTAAGTTTAAGAATAGTTTTTTCT---
GTACTTTCTATAGTA---------AACAGAGTTAGGCAAGGATACTCACCA------
TTATCGTTCGCTGTTAGCTTGCTTAATGCCACAGCAATAGCAGTTGCTGAA---
AGAACAGATAGAATTGTAGAAATAATACAAAAGGTTGGCAGAGGT----------------------------
--------ATTCTCCATATA------CCTAGAAGAATAAGACAAAGCTTAGAGAGGGCTTTGCTA---TAA
>KX181911
GCT---GCA---GAA------------------------------------
GAACAATCGTGGGTCACAGTCTATTATGGGGTACCTGTGTGG---AAAGAAGCAACT---
ACCACTCTATTTTGTGCATCAGATGCTAAAGCATATGAAACAGAG---GCACATAAT----------------
--------------ATTTGGGCTACA---CATGCCTGTGTGCCCACAGAC---
CCTAACCCACAAGAAGTAGCATTGGAA---------AATGTGACAGAAAATTTTAAC----------------
--------------------------ATGTGGAAAAATAACATGGTAGAACAGATGCATGAGGATATA---
ATCAGTTTATGGGATCAAAGCCTA---AAGCCATGTGTAAAATTA---
ACCCCACTCTGTGTTACTTTAAATTGCACAAATGTGAAAAATAATACAAGTACCAATAGTAAC----------
----
-------------------------------------TTTAGTAAGATGGAGGGAGACATAAAA---
AACTGCTCTTTTAATATCACCTCAAGCATAGAA---------------------------
AAAAGGGTGCGGGTAGACTATGCAATT---TTTAATAAACTTGATATAGTACCAATAGATAATAAG-------
----

AATAGTACAGAGTATATGCTGATAAATTGTAACACCTCAGTCATTACACAG---
GCCTGTCCAAAGGTATCCTTTGAACCAATTCCCATACATTATTGTGCCCCAGCTGGTTTTGCGATTCTAAAGT
GTAAA---GATAAGGAGTTCAAT------GGAACAGGACCATGTAAAAAT-----------------------
-GTTAGC---ACAGTACAATGTACACATGGAATTAGGCCGGTAGTGTCAACT---
CAGCTGCTGTTAAATGGCAGTTTAGCAGAAGGAGAT------------
ATAATGATTAGATCTGAAAACATCACAGACAAT---------GCTAAAACCATAATAGTACAGCTGAAT----
-----------GAAACTGTAGAA---------ATTAATTGTACAAGACCCAACAACAATACAAGAAAA-----
-AGTATACATATG------------GGACCGGGGAGAGCA---TTT------TATGCAACAGGAGAC------
---------ATAATAGGAGATATA------------AGGAGAGCACATTGT---AACATTAGTAAAGCA---
AAATGGAACAACACTCTAAGACAGGTAGCTGGAAAATTAAGG---------------GAACACTTC-------
-----------------------------------AATAAAACCATAAACTTTACT------
CAATCCTCAGGGGGAGACCCAGAAATTGTA---ATGTTTAGTCTTAATTGTGGAGGGGAA---
TTTAAGCAGCTCCAGGCAAGAGTCCTGGCTGTGGAAAGATACCTAAGAGATCAACAGCTCCTAGGGATATGGG
GTTGCTCTGGAAAACTTATCTGCACCACCTCTGTGCCTTGGAATACTAGCTGGAGT-----------------
----------------------------AGTAAATCTCTGGATACAATTTGG------AAT------
AACATGACCTGGATGCAGTGG---GAGAGAGAAATTGACAACTACACAGGCTTAATATAC---
AACTTACTTGAAGAATCGCAAAACCAACAAGAA--

AAGAATGAACAAGAATTATTGGAATTGGATAAGTGGGCAAATTTGTGGAATTGGTTTGATATAACACAATGGC
TGTGGTATATAAAAATATTCATAATGATAGTAGGAGGTCTAGTAGGTTTAAGAATAGTTTTTACT---
ATACTTTCTATAATA---------AATAGAGTTAGGCAGGGATACTCACCA------
TTATCATTCGCTATTAGTCTGTTTAATGCTACAGCTGTAACAGTAGCTGAG---
GGGACAGATAGGATAATCGAATTAGCACAAAGAATTGGAATAGGT----------------------------
--------CTCCTCCATATA------CCTAGAAGAATAAGACAGGGTTTAGAAAGGGCTTTGCTA---TAA
>KX181895
GCT---ACA--
GAAAAATCATGGGTCACAGTATATTATGGGGTACCTGTGTGG---AAAGAAGCAACC---
ACCACTCTATTTTGTGCATCAGATGCTAAAGCATATGACACAGAG---GCACACAAT----------------
--------------GTTTGGCCACA---CATGCCTGTGTGCCCACAGAC---
CCTAACCCACAAGAAGGTAGTATTGAA---------AATGTGACAGAAAAATTTAAT----------------
--------------------------ATGTGGAAAAATAATATGGTAGAACAGATGCATGAGGATATA---
ATCAGTTTATGGGATCAAAGCTTA---AAGCCATGTGTAAAATTA---
ACCCCACTCTGTGTTACTCTAAATTGCACTAATGTGACTATGCCTTTAAATTGCACTAATAAGAATAGGACTG

CCAAGAGTAAT---
-------ATGACTTGCAATGGCACCAGACAAAACATTACGGGTACTATAGGGGAAGAAATGAAA---
AACTGCTCTTTCAATGCTCCCACAGGAATAGAA--------------------------
AACAAGGTGAAGAGAGAATATGCAATT---TTTTATGCACTTGATATAGTACCAATAAATGCT---------
----

AGTAGTAACAGCTATATGTTGACACATTGTAACACCTCAGTCATTACACAG---
GCATGTCCAAAGATATCCTTTCAACCAATTCCCATACATTATTGTGCCCCGGCTGGTTTTGCGATTCTACAGT
GTAAC---GATGGGAAATTCAAT------GGAACAGGACCATGTAAAAAT----------------------
-GTCAGC---ACAGTACAATGTACACATGGAATTAGACCAGTAGTGTCAACC---
CAACTGCTGTTAAATGGCAGTCTAGCAAAAGAAGAT-----------
GTAATAATTAGATCTCAAAATATCACAAACAAT---------TCTAAAAATATAATAGTACAGCTGAAC----
-----------ATATCTGTAGGA---------ATTAATTGTACAAGACCCGGCAACAATACAAGAAAA-----
-GGAATACATCTA------------GGATGGGGGAGAGCA---TTT------TATGCAACAGGAGAA------
---------ATAGTAGGAGACATA------------AGAAAGGCACATTGT---AACCTTAATAGCACT---
CAATGGGAAAGCACTTTAAAATATGTAGAGGCAAAATTACAA---------------GAACATTTTCCA----
-----------------------------------AATAGAACAATATACTTCAAT------
CAATCCTCAGGAGGGGACCTAGAAATTGAA---ACACACAGTTTTAATTGTGGAGGGGAA---
TTTAAGCAGCTCCAGGCAAGAGTCCTGGCTGTAGAAAGATTCCTAAAGGATCAACAGCTCCTAGGGATTTGGG
GTTGCTCTGGAAAACTCATTTGCACCACTACTGTGCCTTGGAATACTAGTTGGAGT-----------------
----------------------------AATAAATCTCTAGATTACATTTGG------AAT------
AATATGACCTGGATGGAGTGG---GAAAGAGAAATTGGCAATTACACAGACTTAATATAC---
AACCTAATTGAAGAATCGCAAAACCAACAAGAA---------------------------------------
----
AAGAATGAACTAGACTTATTGGAATTGGATAAATGGGCAAGTTTGTGGAATTGGTTTGACATAACAAAATGGC
TGTGGTATATAAAAATATTCATAATGATAGTAGGAGGCTTAGTTGGCTTAAGAATAGTTTTTGCA---
GTGCTTTCTATAATA---------AATAGAGTTAGGAAGGGATACTCACCA------
TTATCATTCGCTATTAGTTTGCTTAATGCTACAGCTATCGTAGTAGCTGAG---
GGGACAGATAGGATTATAGAAGCAGTACTAAGAATTTGTAGAGCT---------------------------
--------ATTCTCCACGTA------CCCACAAGAATAAGACAGGGTTTAGAAAGAGCTTTGCTA---TAA
>KX181897
GCT---GAA---GAA------------------------------------
GAAAAATTGTGGGTCACAGTCCATTATGGGGTACCTGTGTGG---AAAGAAGCAACC---
ACTACTCTATTTTGTGCATCAGATGCTAAAGCATATAAAACAGAG---GCACATAAT---------------
--------------GTTTGGGCCACA---CATGCCTGTGTACCTACAGAC---
CCTAACCCACGAGAAGTACCATTGGAA---------AATGTGACAGAAAATTTTAAC---------------
-------------------------ATGTGGAAAAATAACATGGTAGAACAAATGCATGAGGATATA---
ATCAGTCTATGGGATCAAAGCCTA---AAGCCATGTGTAAAATTA---
ACCCCACTCTGTGTTATTTTAAACTGCAGTAATTTAAATAATAGTACAAATATCACTAGT------------
----
------------------------AGTGATCAAGGTAATTTGGATATGAGGGAGGAAATAAAA---
AACTGCTCTTTCAATATAACCACAGGCATAGGA--------------------------
AAAAAGGTGCGCAAGGACTATGCAATC---
TTTAATAGAATTGATATAGTACCAATAGATGATAATGATAGTAAA---------------------------
----
AATAATAGTAGCAATAATACAAGCTATATGTTAAGAAGTTGTGATACCTCAGTCATTACACAG---
GCCTGTCCAAAGGTAACCTTTGAACCAATTCCCATACATTATTGTACCCCGGCTGGTTATGCGATTCTGAAGT
GTAAT---GATAAGAAATTCAAT------GGAACAGGACCATGTAAAAAT----------------------
-GTCAGC---ACAGTGCAATGTACACATGGAATTAAGCCAGTAGTGTCAACT---
CAACTACTGTTAAATGGCAGTTTAGCAGAAGAAGAA-----------
GTAATAATTAGATCTGAAAATATCACAGACAAT---------GGTAAAAACATAATAGTACAGCTGAAT----
-----------GAAACTGTAAAA---------ATTAATTGTACAAGACCCAACAACAATACAAGGAAA-----
-AGTATACATATG------------GGATGGGGAAGAGCA---TTT------TATGCAACAGGAGCC------
---------ATAATAGGAGATATA------------AGACAAGCACATTGT---AACCTTAGTAGAGCA---
GAATGGAACAAAACTTTAGAAAAAATAGCTATAAAATTAAAA---------------GAAAGAGTTAAT----
-----------------------------------AAAACAAAAATAATCTTTAAT------
CAATCCTCAGGAGGGGACTCAGAAATTGAA---ATGCACAGTTTTAATTGTGGAGGGGAA---
TTTAAGCAGCTCCAGGCGAGAGTCCTGGCTGTGGAAAGATACCTAAGGGATCAACAGCTCCTGGGAATTTGGG
GCTGCTCTGGAAAACTCATCTGCACCACTGCTGTGCCTTGGAATACTAGTTGGAGT----------------
----------------------------AATAAATATCTGAGTTACATTTGG------AAC------

AACATGACCTGGATGCAGTGG---GAGAGAGAGATTGACAATTACACAAACTTAATATAT---
AACTTACTTGAAGAATCGCAAAACCAACAAGAA---------------------------------------
----
AAGAATGAACAAGAATTATTGGAATTGGATAAGTGGGCAAATTTGTGGAATTGGTTTGACATATCAAAATGGC
TGTGGTATATAAAAATATTCATAATGATAGTAGGGGGCTTAATAGGTTTAAGAATAGTTTTTTCT---
GTACTTTCTCTAGTG---------AATAGAGTTAGGCAGGGATACTCACCA------
TTATCATTCGCTGTTAGTTTGCTTAATGCCACAGCTATAGCAGTAGCTGAA---
GGGACAGATAGGGTTATAGAAGTAGTACAAAGAATTGGTAGAGGT---------------------------
--------ATTCTCCACATA------CCTAGAAGAATAAGACAGGGCTTAGAAAGGGCTTTGCTA---TAA
>KX181915
GCT---ACA---
GAAAAGTTGTGGGTCACAGTCTATTATGGGGTACCTGTGTGG---AAAGAAGCAACC---
ACCACTCTATTTTGTGCATCAGATGCTAAAGCATATGATACAGAA---GGACATAAT----------------
--------------GTTTGGGCCACA---CATGCCTGTGTACCCACAGAC---
CCTAAGCCACAAGAAATAGTATTGGAA---------AATGTGACAGAAGAGTTTAAC---------------
-------------------------ATGTGGAAAAATAACATGGTAGAACAGCTGCATGAGGATATA---
ATTAGTTTATGGGATCAAAGCCTA---AAGCCATGTGTAAAATTA---
ACCCCACTCTGTGTTACTTTAAATTGTACTAATAATATCACCATCACCAAGGATAACAGCACTAGTGCTAATA
GCACTAGTTTG---
-------------------ACTAATAGCACTAGTTTGGATAATAGCACTAGTGGAGAAATAAAA---
AACTGCTCTTTCAATGTTACCACAAGTAGAAGT--------------------------
AATAAGATACGGAAAGAATATGCACTT---TTTTATACACTTGACCTAGTACCAATAGATAATAAAACT----
----
TATATAGATAATACAACTAATATAAATTATACAAGCTATACCTTGATACACTGTGACACCTCAGTCATTACGC
AG---
GCCTGTCCAAAGGTATCCTTTGAACCAATTCCCATACATTATTGTACCCCGGCTGGTTTTGCGATTCTAAAGT
GTAAT---GATAAAAAGTTCAAT------GGAACAGGATCATGTAAAAAT----------------------
-GTCAGC---ACAATACAATGTACGCATGGAATTAAGCCAGTAGTGTCAACT---
CAACTGCTGTTAAATGGTAGTCTAGCAGAAGAAGAC-----------
ATAGTAATTAGAACTGAGAATATTTCAAATAAT---------GCTAAAAATATAATAGTACAGCTGAAC----
-----------GCATCTGTAGCA---------ATTAATTGTACAAGACCCAATAACAACACAAGAAGA-----
-AGTATAAATATA------------GGACCAGGGAGAGCA---TTT------TTTACAACAGGACAG------
---------ATAATAGGAGATATA-----------AGACAAGCATATTGC---ATCCTTAATGGGACA---
ATATGGAACAACACTTTAAGACAGGTAGCTAAAAAATTACAA-----------
GAAATATTAGCACAAGCACAATTA------GTT--------------------
AATAAAACAATAGCCTTTGCA---CAACCACCCCCAGGAGGGGACCCAGAAATTGTC---
ATGCACAATTTTAATTGTGGAGGGGAA---
TTTAAGCAGCTCCAGGCAAGAGTCCTTGCTGTGGAAAGATACCTACAGGATCAACAGCTCCTAGGGATTTGGG
GTTGCTCTGGAAAACTCATTTGCACCACTGCTGTGCCTTGGAATGCTAGTTGGAGT-----------------
----------------------------AATAGATCTCTGGATACAATTTGG------AAC------
AATATGACCTGGATGCAGTGG---GAAAAAGAAATTGACAATTATACAGGCTTAATATAC---
ACTTTAATTGAACAATCGCAGAACCAGCAAGAG---------------------------------------
----
AAAAATGAACAAGAATTATTGGAATTGGATAAATGGGCAAGTTTGTGGAATTGGTTTGACATAACACAGTGGC
TGTGGTATATAAAGTTATTCATAATGATAGTAGGAGGCTTAATAGGTTTAAGAATAGTTTTTACT---
GTGCTTTCTATAGTG---------AATAGAGTTAGGCAGGGCTACTCACCA------
TTATCGTTCGCTGTCAATTTGTTTGGTACCATAGCAATAGCAGTAGCTGAG---
GGGACAGATAGGATTATAGAAGCAGGACAAAGACTTTGTAGAGCA---------------------------
--------ATTCTCCACATA------CCTAGAAGAATAAGACAGGGCTTAGAAAGGCTTTTGCTA---TAA
>KX181916
GCT---ACA---
AACTTGTGGGTCACAGTCTATTATGGGGTACCTGTGTGG---AAAGAAGCAACA---
ACCACTCTATTCTGTGCATCAGATGCTAAAACATATGACCCAGAG---GGACATAAT----------------
--------------GTTTGGGCCACA---CATGCCTGTGTACCCACAGAC---
CCTAACCCACAAGAAATAGTATTAGAA---------AATGTGACAGAAAATTTTAAC---------------
-------------------------ATGTGGAAAAATAACATGGCAGATCAGATGCATGAAGATATA---
ATCAGTTTATGGGATCAAAGTCTA---AAGCCATGTGTAAAATTA---
ACCCCACTCTGTGTTACTTTAGAATGCACTAATTATGAGAAGAATAGTAGTTCAGTGAAT------------
----
----------------------------AGTAGTAGTTGGGAAAATATGAAGGAAGAGATAAAA---

AACTGCTCTTTCAATGTCACTAATAGCATAAGA--------------------------
AATAAAAGGCAGAAAGAATATGCACTT---
TTTAATAGTCTTGATGTAGTACCAATAGATCAGAATAAGACTGTT---------------------------
----
AATCATACAAGCTATATGTTGCTAAATTGTAACACCTCAGTCATTACACAG---
GCATGTCCAAAGATATCCTTTGAACCAATTCCCATACATTATTGTACCCCGGCTGGTTTTGCGATTCTAAAGT
GTAAT---GATAAGAAATTCAAT------GGAACAGGACCATGTACAAAT----------------------
-GTCAGT---ACAGTACAATGTACACATGGAATTAGGCCAGTAGTGTCAACG---
CATCTGCTATTAAATGGCAGTTTAGCAGAAGGAGAG-----------
GTAGTAATTAGATCTGAAAATTTCACAAACAAT--------GCTAAAAACATAATAGTACAGCTGAAT----
-----------GAATCTGTAGAA--------ATTAATTGTATAAGACCCAGCAACAATACAAGAAAA-----
-AGTATACATATA-----------GGACCAGGGAGAGCA---TTT------TATGCAATAGGAGGA------
---------ATCATAGGAGATATA-----------AGACAAGCATATTGC---AACCTTAATAGAGGA---
AACTGGGAAAAAACTTTGAAACAGGTAGTTACAAAATTAGGA-----------
GAACAATTAAAATTAAGAGGACAA------TTTAAT--------------
AATAGCACAACAATAGTCTTTAAT------CAATCCTCAGGAGGGGACCCAGAAATTGTA---
ATGCACACTTTTAATTGTGGAGGGGAA---
TTTAAGCAGCTTCAGGCAAGAGTCCTGGCTGTGGAAAGATACCTAAGGGATCAACAGCTCCTGGGGATCTGGG
GTTGCTCTGGAAAACTCATTTGCACCACTACTGTGCCTTGGAATAATAGCTGGAGT----------------
---------------------------AATAAATCTCTGGAAAAGATTTGG------AAT------
AACATGACCTGGATGGAGTGG---GAAAGAGAAATTGACAATTACACAGGATTAATATAC---
AACTTACTTGAAGAATTGCAGAACCAACAAGAA---------------------------------------
----
AAGAATGAAAAAGACTTATTGGAATTGGATAAGTGGGACAGTTTGTGGAATTGGTTCAGTATAACGCAATGGC
TGTGGTATATAAAATTATTCATAATGATAGTAGGAGGCTTAGTAGGTTTAAGAATAGTTTTTACT---
GTACTTTCTATAGTG--------AATAGAGTTAGGCAGGGATACTCACCA------
TTATCATTCGCTATTAATTTGTTTAATGCAATAGCTATAGCAGTAGCTGAG---
GGAACAGATAGGGTTAAAGAAGTAGTACAAACAATTTGGAGAGCT---------------------------
--------TTCCTCCATATA------CCTAGAAGAATAAGACAGGGCGCAGAAAGGTTTTTGCTA---TAA
>MK041584
GCT---GCA---
AGAAATTTGTGGGTCACAGTCTATTATGGAGTACCTGTGTGG---AAAGAAGCAAAC---
ACCACTTTATTTTGTGCATCAGATGCTAAAGCATATGACACAGAA---GCACATAAT---------------
--------------GTTTGGGCCACA---CATGCCTGTGTCCCCACAGAC---
CCTAACCCACAAGAATTGGTATTGGAT--------AATGTGACAGAAGAATTTAAC----------------
-------------------------ATGTGGAAAAATAACATGGTGGACCAGATGCAGGAGGATATA---
ATTAGTTTATGGGATCAAAGCCTA---AAGCCATGTGTAAAATTA---
ACCCCGCTCTGTGTTGCTTTAAGTTGCACCAATGTGACTGCTAATACTACCAATAATAGTACTTCCACT---
----
------------------------AGGAATAGTAATTTAGAACAGAGGGATGGAGATATAAAA---
AACTGCTCTTTCAATATTACCACAGACATAGGA--------------------------
CATAAGACGCGGAAAGACTATGCAATT---TTTAATGCACTTGATATAGTACCAATAGATAATGGAGATAAT-
----

AAAAGTGACTATAATAAAAGTGACTATATGTTGATAAGTTGTAACACCTCAATAGTTACACAA---
GCCTGTCCAAAGGTAACCTTTGAACCAATTCCCATACATTATTGTGCCCCGGCTGGTTTTGCGATTCTAAAGT
GTAAT---AACAAGACATTCAGT------GGAAAAGGACCATGCAAAAAT----------------------
-GTCAGC---ACAGTACAATGTACACATGGAATTAGGCCAGTAGTGTCAACT---
CAACTGCTGTTAAATGGCAGTCTAGCAGAAGAAGAT-----------
ATAGTAATTAGATCTGAAAATTTCACAAACAAT--------GCTAAAAACATAATAGTACAGCTGAAC----
-----------GAATCTGTAGTA--------ATTAATTGTACAAGACCCAACAACAATACAAGGAAA-----
-AGTATACATATG-----------GGACCGGGGAAAGCA---TTT------TATACAACAGGAAGC------
---------ATAATAGGAGACATA-----------AGACAAGCACATTGT---AACATTAGTAGAGCA---
AAATGGAACAACACTTTAAAACAAGTAGTCAGAAAATTAAGA--------------GAACAATTTAAT----
----------------------------------AATAAAACAATAATCTTTACC------
CACTCCTCAGGAGGAGACATAGAGATTGTA---ATGCATAGTTTTAATTGTAGAGGGGAA---
TTCAAGCAGCTCCAGGCAAGAGTCCTGGCTATTGAAAGATACCTAAAGGATCAACAGCTCCTAGGGATGTGGG
GTTGCTCTGGAAAACTCATTTGCACCACTACTGTGCCTTGGAATACTAGTTGGAGC--------AAT-----
---------------------------AATAGATCCCTGGATCAGATTTGG------GAT------
AACATGACCTGGATGGAGTGG---GAGAGAGAGATTGACAATTACACAAGCTTGATATAC---

ACTTTACTTATAGAATCGCAGAATCAACAAGAA---------------------------------------
----
AAAAATGAACAAGACTTAATGGCATTGGACAAGTGGGACAGTTTGTGGAATTGGTTCAGCATAACAATTGGC
TATGGTATATAAAAATATCCATAATGATAGTAGGAGGCTTAGTAGGTTTAAAAATAGTTTTTACT---
ATACTTTCTATAGTG---------AATAGAGTTAGGAAGGGATACTCACCA------
TTATCATTGGCTATTAGTTTGCTTAACGCCACAGCTATAGCAGTAGCAGAA---
GGGACAGATAGGATTATAGAAGTATTACGCAGAATTTATAGAGCT---------------------------
--------TTTATCCACATA------CCTACAAGAATTAGACAGGGCTTAGAAAGGGCTTTGCTA---TAA
>MK041586
GCT---TTA--------------------------------AAGGATATA---
AACAATGCGTGGGTCACAGTACATTATGGGGTACCTGTGTGG---AAAGAGGCAACC---
ACCACTCTATTTTGTGCATCAGATGCTAAAGCATATGACACAGAG---GCACATAAT----------------
--------------GTATGGCCACA---CATGCCTGTGTACCCACAGAC---
CCTAACCCACAAGAAATAGTCTTGGGA---------AATGTGACAGAAGAATTTAAC---------------
--------------------------ATGTGGAAGAATAACATGGTAGAACAGATGCATGAGGATATA---
ATTAGTTTATGGGATCAAAGCCTA---AAACCATGTGTAAAACTA---
ACCCCACTCTGTGTTACTTTAAATTGCACTGATCTGAAGAATGGGACCGGGACTAACAAT------------
----
-------------------------GCTAGTAAGATTGACGAGAAAATGGAGGGAGAAATAAAA---
AACTGCTCTTTCAATGTCACCACAAGCATAGGA--------------------------
AATAAGTTGCAGAAAGAATATGCTCTT---TTTTATAAACTTGATGTACAACAAATAGGGGGT---------
----

GATAATACAAGCTATACATTGATACAGTGTAATACCTCAGTCATTACACAG---
GCCTGCCCAAAGATATCCTTTACACCAATTCCCATACACTATTGTGCCCCGGCTGGTTTTGCGATTCTAAAAT
GCAAC---GATAAGGAGTTCAAT------GGAACAGGACTTTGTCAAAT-----------------------
-GTCAGC---ACAGTACAATGTACACATGGAATTAAGCCAGTAGTGTCAACT---
CAATTGCTGTTAAATGGCAGTCTATCAGAAGGAGAG-----------
GTAGTAATTAGATCTCAAAATTTCTCAGACAAT---------GTTAAAACCTTACTAGTACAGCTGAAT----
-----------GAAACTATAAAA---------ATTAATTGTACAAGACCTAATAACAATACAAGAAAA-----
-AGTATACATCTA------------GGATGGGGAAGAGCA---TTT------TATGCATCAGGAGAA------
---------ATAATAGGAGACATA------------AGACAAGCACATTGT---AACCTTAGTAGCACA---
CAATGGAACACCACTTTACAACGGATAGTCAGAAAATTACAA--------------AAACAGTATGGG----
-----------------------------------AATAAAACAATAATCTTTAAG------
CACCCCGCAGGAGGGGACCCAGAAATTGAG---ATGCACAGCTTTAATTGTGGAGGGGAA---
TTTAAACAGCTCCAGGCAAGAGTCCTGGCTGTGGAAAGGTACCTAAGGGATCAACAGCTCCTGGGGATTTGGG
GCTGCTCTGGAAAACTCATCTGCACCACTAATGTTCCTTGGAATACTAGCTGGAGT----------------
----------------------------AATAAATCTTTAGAGACAATTTGG------AAT------
AACATGACTTGGAGACAGTGG---GAAAGAGAAATTGACAATTACACTGAGTTGATATAC---
TCCTTACTTGAAGAATCGCAAAACCAACAAGAA---------------------------------------
----
AAGAATGAACAAGAGTTATTAGAATTGGATAAATGGGCAAGCTTGTGGAATTGGTTTGACATAACAAGCTGGC
TGTGGTACATAAAAATATTCATAATGATAGTAGGAGGATTAATAGGTTTAAGAATAGTTTTTACT---
GTGCTTTCTATAGTG---------AATAGAGTTAGGCAGGGATACTCACCA------
TTATCATTCGCTGTTAGCTTGCTTGATACCACAGCTATAGCAGTAGCTGAA---
GGGACTGATAGGGTTATAGAAGTAGTACAAAGAGCTGTTAGAGGT---------------------------
--------GTTATCCACATA------CCTAGAAGAATCAGACAGGGCTTAGAAAGGCTTTTGCTA---TAA
>MK041551
GTT---GCT--------------------------------------
GCAAATCAATTGTGGGTCACAGTATATTATGGAGTACCTGTGTGG---AAGGAAGCAACC---
ACCACTCTATTTTGTGCCTCAGATGCTAAAGCATATGACACAGAG---GTACATAAT----------------
--------------GTTTGGCCACA---CGTGCCTGTGTACCCACAGAC---
CCTAACCCACAAGAAGTACATTTGGGA---------AATGTGACAGAAAATTTTAAC---------------
--------------------------ATGTGGAAAAATGACATGGTAGAACAGATGCATGAGGATATA---
ATCAGTTTATGGGAAGAAAGTCTA---AAGCCATGTGTAAAATTA---
ACCCCACTCTGTGTGACTTTAGACTGCACTGAGGTGATTGTGAACCAGACCACAAATGAAACTAGTACCGAT-
----
-------------------------AATGGTAATTGGAATTATACTATGAAGGATGAAATGAAA---
AACTGCTCTTTCAATGTCACCACAAGCATAGGA--------------------------
AATAAGATGCAGAAAGAATATGCACTT---TTTTATAAACTTGATTTAGTATCAACAGATAAT---------

----

AGTAATAAGACAAACTATACAAGCTATACATTGATAAATTGTAACACCTCAGTCATTACACAG---
GCCTGTCCAAAGGTATCCTTTGAACCAATTCCCATACATTATTGTGCCCCGGCTGGTTTTGCGATTCTAAAGT
GTAAT---AATAAGACATTCAAT------GGAAAAGGACCATGTAACAAT-----------------------
-GTCAGC---ACAGTACAATGTACACATGGAATTAGGCCAGTAGTGTCAACT---
CAATTACTGTTAAATGGCAGTCTAGCAAAAGAAGAC------------
ATAGTAATTAGATCCGAAAACTTTACAAACAAT---------GCTAAAACCATAATAGTGCATCTGAAT----
-----------AAATCTGTAGAA---------ATTAATTGTACAAGGCCCAACAACAATACAAGCAAC-----
-AGTATATCTTA-------------GCAGGGGGAAGAGCA---TTG------TATACAACAGGACGA------
---------CTAATAGGAAATATA------------AGACAAGCACATTGT---AACCTAAGTAGAGAA---
GATTGGACAAAACTTTAAAAGGGGTAGTCATAAAATTAAGG----------------GAACAATTCGGC----
--------------------------------AACAATAAAACAATAAATTTTAAC------
CAATCTGCAGGAGGGGATCTAGAGATTACA---ATGCACACTTTTAATTGTGGAGGGGAA---
TTTAAGCAGCTCCAGGCCAGAGTCCTGGCTGTGGAAAGATACCTAAAGGATCAACAGCTCCTAGGGCTTTGGG
GTTGCTCGGGAAAACTCATTTGCACCACTGCTGTGCCTTGGAACACTAGTTGGAGT-----------------
----------------------------AATAAATCTCTGGATACAATTTGG------GAT------
AACATGACCTGGATGCAGTGG---GAGAAAGAAATTGAAAATTACACAGGCTTAATATAT---
AACTTAATTGAAGAATCGCAGAACCAACAAGAC--

AAAAATGAACAAGAATTATTGGAATTGGATAAATGGGCAAATTTGTGGACTTGGTTTGACATAACAAACTGGC
TGTGGTATATAAGATTATTCATAATGATAGTAGGAGGCTTAATAGGTTTAAGAATAATTTTTGCT---
GTAATTTCTATAATA---------AATAGAGTTAGGGAGGGATACTCACCA------
TTGTCATTCGCTGTTAGTTTGCTGAACGCCTTTGCTATAACAGTAGCTGAA---
GGGACAGATAGAATTATAGAAGGAGTACAAAGACTTGGTAGAGGT----------------------------
--------ATCCTCCACATA------CCTAGAAGAATAAGACAGGGCTTAGAAAGGGCTTTGCTA---TAA
>MK041554
GCT---GTA--
GAAAAATTGTGGGTCACAGTCTATTATGGGGTACCTGTGTGG---AAAGAAGCAAAC---
ACAACTCTATTTTGTGCATCAGATGCTAAAGCATATGACACAGAG---GCACATAAT----------------
--------------GTTTGGCCACG---CATGCCTGTGTACCCACAGAC---
CCCAATCCAAAAGAAATAGTCTTGGAA---------AATGTGACAGAAAATTTTAAC----------------
--------------------------ATGTGGAAAAATAACATGGTAGAACAGATGCATGAGGATATA---
ATCAGTTTATGGGATCAAAGCCTA---AAGCCATGTGTGAAATTA---
ACCCCACTCTGTGTTACTTTGAATTGTAGTAATTCTAACTTCAATAAATCCACTAGT----------------
----
-------------------------AGCAAGAATCATAGTAAGGAGATGGAGAGTGAAATAAAA---
AACTGCTCTTTCAATATCACCACAAGCATAAAA---------------------------
GATAAGGTGAAAAAAGAATATGCATTT---
TTTAATAGACTTGATGTAATACCAATAGATAATGAAAATAATGCA----------------------------
----
AGCTCTAATGCAAGCTCTAATGCAAGCTATATGTTGAGAAGTTGTAACACTTCAGTCCTCACACAG---
GCCTGTCCAAAGATATCCTTTGACCCAATTCCCATACATTATTGTACCCCAGCTGGTTTTGCGATTCTAAAGT
GTAAT---GATAAGAAGTTCAAT------GGAACAGGACCATGTAACAAT-----------------------
-GTCAGC---ACAGTACAATGTACACATGGAATTAGGCCAGTAGTGTCAACT---
CAATTACTGTTAAATGGCAGTCTAGCAGAAGAAGGG------------
ATAGTAATTAGATCTGAAAATTTCACGGACAAT---------ACTAAAAACATAATAGTACAGCTGAAT----
-----------GAGTCCGTAGCA---------ATTAATTGTACAAGACCCAGCAACAATACAAGAAAA-----
-AGTATACATATT------------GGATGGGGGAGAGCA---TTC------TATGCAACAGGAGAA------
---------ATAATAGGAAATATA------------AGACGAGCACATTGT---AACCTTAGTAAAACA---
GACTGGAACAACACTTTAGAAAAGATAAGGAAAAAATTAAAT---------------GAACAATTC-------
-----------------------------------AATAGAACAATATTCTTTGAT------
CAATCCTCAGGAGGGGACTTAGAAATTACA---ATGCACCATTTTAATTGTAGAGGGGAA---
TTTAAGCAGCTCCAGGCAAGAGTCCTGGCTGTGGAAAGATACCTAAGGGACCAACAGCTCCTAGGAATTTGGG
GTTGCTCTGGAAAACTCATTTGCACCACTGCTGTGCCTTGGAATACTAGTTGGAGT-----------------
----------------------------AAGAAAAATCTGAGTCAGATTTGG------GAT------
AACATGACCTGGATGGAGTGG---GAGAGAGAAATTGACAATTACACAGCATTAATATAC---
ACATTGCTTGAAGAATCGCAGAACCAACAGGAA--

AAGAATGAACAAGAATTGTTGGAATTGGATAAATGGGCAAGTTTGTGGAGCTGGTTTGACATAACAAACTGGC

TGTGGTATATAAAAATATTCATAATGATAGTAGGAGGCTTAATAGGTTTAAGAATAGTTTTTACT---
GTGATTTCTATAGTG---------AATAGAGTTAGGAAGGGATACTCACCA------
TTGTCATTCGCTGTTAGTTTGCTTAATGCCACAGCAATAGCAGTAGCTGAG---
GGAACAGATAGGATTATAGAAGGAGTGCGAAGAGCTTGTAGAGCA----------------------------
--------TTTCTCCACATA------CCTAGAAGAATAAGACAGGGCCTCGAAAGGGTTTTAGTA---TAA
>MK041558
GCT---ACA---
AGACAATTGTGGGTCACAGTCTATTATGGGGTACCTGTGTGG---AAAGAAGCAACC---
ACCACTCTCTTTTGTGCATCAGATGCCAAAGCATATGACACAGAG---GCACATAAT----------------
--------------GTTTGGGCCACA---CATGCCTGTGTACCCACAGAC---
CCCAACCCACAAGAAATAAAATTGGAA---------AATGTGACAGAAAATTTTACC----------------
--------------------------ATGTGGAACAATAGCATGGTAGAACAGATGCATGAAGATATA---
ATCAGTTTATGGGATGAAAGCCTA---AAGCCATGTGTAAAATTA---
ACCCCACTCTGTGTTACTTTACATTGCACTGATCAAGGGAATACTACT------------------------
----
-------------------------AATGGGACTATAGAAAAAAACATGGAGGGGGAAATAAAA---
AACTGCTCTTTTAATATCACCACAGAAATAGGA--------------------------
AATAAAAGGCAAAAAGAATATGCACTT---TTTTATAAGCTTGATGTAGTACCAATAGATAAGAATAAT----
----

AATAATACAAACTATACTACAAACTATACATTGATAAATTGTAACACCTCAGTCATTACCCAG---
GCCTGTCCAAAGATATCCTTTGAACCAATTCCCATACATTATTGTGCCCCAGCCGGATTTGCGATTCTAAAGT
GTGGA---GATCCAAAGTTCACT------GGAACAGGGCCATGTTCCAAC-----------------------
-GTCAGC---ACAGTACAATGCACACATGGAATTAAGCCAGTAGTGTCAACT---
CAACTGCTGTTAAATGGCAGTCTAGCAGAGGAGGAG-----------
GTAGTACTTAGGTCTGAAAACTTCACAGACAAT---------GTTAAAACCATAATAGTACAGATGAAC----
-----------CAATCTGTAGCA---------ATTCATTGTACAAGGCCCAACAACAATACAAGAAAA-----
-GGTATACATATA------------GGACCGGGAAGAGCA---CTT------TATACAACAGGAGAA------
---------ATAGTAGGAAATATA------------AGACAAGCACATTGT---AACCTTAGTGCAGCA---
CACTGGAACAATGCTTTAAAACAGATAGCTATAAAATTAATA---------------GAACAATTT-------
-----------------------------------AATAAAACAATAATCTTTAAT------
CAGTCCTCAGGAGGGGATCCAGAAATTGTA---ATGCATACTTTTAATTGTGGAGGGGAA---
TTTAAACAGCTCCAGGCCAGAGTCCTGGCTGTGGAAAGATACCTACGGGATCAACAGCTCCTAGGGATTTGGG
GTTGCTCTGGAAAGCTCATCTGCACCACTGCTGTGCCTTGGAATAATAGTTGGAGT----------------
-----------------------------AATAAAACTCTAGAGGACATTTGG------GAA---
GGGAACATGACCTGGATGCAGTGG---GAGAGAGAAATTAGCAACTACACAAACACAATATAC---
ACCTTACTTGAACAATCGCAGACCCAGCAAGAG--

ATAAATGAAAAAAACTTATTAGCATTAGATGAGTGGGCAAGTCTGTGGAATTGGTTCGATATATCAAAGTGGC
TGTGGTATATAAGATTATTCATAATGATAGTAGGAGGCTTAATAGGTTTAAGAATAGTTTTTGCT---
GTATTTTCTATAGTG---------AATAGAGTTAGGCAGGGGTACTCACCA------
TTGTCATTCGCTGTTAGCTTGTTTAATGCCATAGCTATAGCAGTAGCTGAG---
GGGACAGATAGAATTATAGAAGTAGCACAAAGAACTTGTGGAGCT----------------------------
--------GTTCTTCACATA------CCTAGAAGAATAAGACAGGGCTTAGAAAGACTTTTGCTA---TAA
>MK041556
GCT---GTA---
GAAAAATGGTGGGTCACAGTCTATTATGGGGTACCTGTGTGG---AAAGAAGCAACC---
ACAACTCTGTTTTGTGCATCAGATGCTAAAGCATACGACACAGAG---GCACATAAT----------------
--------------ATTTGGGCCACG---CATGCCTGTGTACCCACAGAC---
CCTAACCCACAAGAAGTATTATTGGGA---------AATGTGACAGAAAATTTTAAC----------------
--------------------------ATGTGGAAAAATAACATGGTAGAACAGATGCAGGAGGATATA---
ATCAGTTTATGGGATCAAAGCCTA---AAGCCATGTGTGAAATTA---
ACCCCACTCTGTGTTACTTTAAATTGTACCAATTCTAATTTCACTAATGCTCAGTCC----------------
----
-------------------------ACAAGTAGTAGTTGGGAGGGGATGGATAGGGAAATAAAA---
AACTGCTCTTTCAATATCACCACAAACATAAAA--------------------------
AATAGGCTGAAAAAAGAATATGCACTT---TTTAATGAACTTGATGTAATACCAATAGATAAT----------
----

AGTACCTATATGTTGAGAAGTTGTAACACTTCAATCCTTACACAG---

GCCTGTCCAAAGATATCCTTTGACCCAATTCCCATACATTATTGTACCCCAGCTGGTTTTGCGATTCTAAAGT
GTAAG---GATAAGAAGTTCAAT------GGAACAGGACCATGTAGAAAT------------------------
-GTCAGC---ACAGTACAATGTACACATGGAATTAAGCCAGTAGTGTCAACT---
CAATTGCTGTTAAATGGCAGTCTAGCAGAAGAAGAG------------
GTAGTAATTAGATCTGAAAATTTCACGGACAAT---------ACTAAAAACATAATAGTACAGCTGAAT----
-----------GAATCTGTAACA---------ATTAATTGTACGAGACCCAACAACAATACAAGAAAA-----
-AGTATACATCTT------------GGATTGGGGAGAGCA---TTC------TATGCAACAGGAGAC------
---------ATAATAGGAGATATA------------AGACAAGCACATTGT---AACCTTAGTCTACAA---
CAATGGAACAGAACTTTAGAAAAGATAAAGGCAAAATTACAG---------------GAACAATTTAAT----
-----------------------------------GGAACAAGAATAACCTTTAAT------
CAATCCTCAGGAGGGGACCTAGAGATTATA---AGTCACCATTTTAATTGTAGAGGGGAA---
TTTAAGCAGCTCCAGGCAAGAGTCCTGGCTGTGGAAAGATACCTAAGGGATCAACAGCTCCTAGGAATTTGGG
GTTGCTCTGGGAAAATCATCTGCACCACTGCTGTGCCTTGGAATACTAGTTGGAGT-----------------
----------------------------AATAAAAATCTGAGTCAGATTTGG------GAT------
AACATGACCTGGATGCAGTGG---GAGAGAGAAGTTGGCAATTACACAGACTTAATATAC---
ACATTGCTTGAAGAATCGCAAAACCAACAAGAA--

AAGAATGAACAAGAATTATTGGAATTGGATAAATGGGCAAGTTTGTGGAATTGGTTTGACATAACAAACTGGC
TGTGGTATATAAAAATATTCATAATGATAGTAGGAGGTTTAATAGGTTTAAGAATAGTTTTTACT---
GTACTTTCTATAGTG---------AATAGAGTTAGGAAGGGATACTCACCA------
TTGTCATTTGCTGTTAGTCTGCTTAATGCCACAGCCATAGCAGTAGCTGAG---
GGGACAGATAGGATTATAGAAGTAGCACAAAGAATTTGKAGAGCA----------------------------
--------TTTCTCCACGTA------CCTAGAAGAATAAGACAGGGCTTCGAAAGGGCTTTGCTA---TAG
>MK041562
GCT---ACA--
GAAAACTTGTGGGTCACAGTCTATTATGGGGTACCTGTGTGG---AAAGAAGCAGAC---
ACCACTCTATTTTGTGCATCAGATGCTAAAACACATGATCCAGAA---GTACACAAT----------------
--------------GTTTGGGCCACA---CATGCCTGTGTACCCACAGAC---
CCCAACCCACAAGAAGTATTATTGGAA---------AATGTGACAGAAAATTTTAAC----------------
--------------------------ATGTGGAAAAATAACATGGCAGAACAGATGCATGAGGATATA---
ATCAGCTTATGGGATCAAAGCCTA---AAGCCATGTGTAAAATTA---
ACCCCACTTTGTGTTACTTTAACTTGCACTAATTTGACGACAGCTAATCATACTGGCACTAATTTGACGACAG
CTAATTATACTGGGAAT--
-------ATTAGTAGTAGTAATGATTGGGGGAATATTAGTAGTAGTAATGATTGGGAAACAAAA---
AACTGCTCTTTCAATATCACCACAAGTATAAGA---------------------------
GATGAGGTGAAAAAAGAATATGCACTC---TTTTACAGAAGTGATATAACACCAATAAAGGAG----------
----

GATAATACCAGCTATAGGTTGATAAATTGTAACACCTCAACCATTACACAG---
GCCTGTCCAAAGGTAACCTTTGAGCCAATTCCCATACATTACTGTGCCCCAGCTGGTTTTGCGATTCTAAAGT
GTAAT---GATAAAAAGTTCAAT------GGAACAGGACCATGTACAAAT------------------------
-GTCAGT---ACAGTGCAATGTACACATGGAATTAAGCCAGTGGTGTCAACC---
CAACTGCTGTTAAATGGCAGCTTAGCAGAAGAAGAG------------
GTAGTAATTAGATCTGAGAATTTCACAAACAAT---------GCTAAAACCATAATAGTACAGCTAAAT----
-----------GTATCTATAAAA---------ATTAATTGTACAAGACCCGGCAACAATACAATAAAA-----
-AGRATATCTATA------------GGACCAGGGAGAGCA---TTT------CGGACAAGAGGACAA------
---------ATAATAGGAGATATA------------AGACAAGCCCATTGC---AACATTAGTAGAACA---
CAGTGGAATAACACTTTAAAACAAGTAGTTATAAAGTTAAGA---------------GAACAATTTCCA----
-----------------------------------AATAAGACAATAGTCTTTAAT------
AGATCCTCAGGAGGGGACCTAGAAATTGAA---AGGCACATGTTTAATTGTGCAGGGGAA---
TTCAAGCAGCTCCAGGCAAGAGTCCTGGCTGTGGAGAGATACCTAAAGGATCAACAGCTCCTAGGGATTTGGG
GTTGCTCTGGAAAACTCATCTGCACCACTGCTGTGCCTTGGAATGCTAGTTGGAGT-----------------
----------------------------AATAAATCTCTAGATGAGATTTGG------AAA------
AATATGACCTGGATGGAGTGG---GAAAGAGAAATTGACAATTACACACACATAATATAC---
AACTTAATTGAAGAATCGCAAAACCAACAAGAA--

AAAAATGAACAAGAATTATTAGAATTAGATAAGTGGGCAAGTCTGTGGAATTGGTTCGACATAACAAATTGGC
TGTGGTACATAAAAATATTCATAATGATAGTAGGAGGCTTGATAGGTTTAAGAATAGTTTTTACT---
GTACTTTCTTTAGTA---------AATAGAGTCAGGCAGGGATATTCACCA------
CTGTCATTTGCTGTTAGCTTGCTCAACACCACAGCCATAGCAGTAGCTGAG---

```
GGGACAGATAGGATTATAGAAATATTACAAAGAACTGGTAGAGCT----------------------------
--------ATCCTTCACATA------CCTAGAAGAATAAGACAGGGCTTGGAAAGGGCTTTGCTA---TAA
>MG571981
GCT---GAA------------------------------------------
GATAATTTGTGGGTCACAGTCTATTATGGAGTACCTGTGTGG---AAAGAAGCAACC---
ACCACTCTATTTTGTGCATCAAATGCTAAAGCCTATGACCCAGAG---GCACATAAT----------------
--------------GTTTGGGCCACA---CATGCCTGTGTACCCACAGAC---
CCCAATCCACAAGAATTAGTATTGGGA---------AATGTGACAGAAAATTTTAAC----------------
--------------------------ATGTGGAAAAATGACATGGTAGAACAGATGCATGAGGATATA---
ATCAGTTTATGGGATCAAAGCCTA---AAGCCATGTGTAAAATTA---
ACCCCACTCTGTGTTACTTTAAATTGTACTGATTTGAAGAATGTGAATAGTACTCACATTAATAATAATGGCA
CT-----------------------------------------------------------------------
-------------------------------------------------------GATATGAAA---
AACTGCTCTTTCAATATCACCACAAGTGTAAAA---------------------------
GATAAAATACAGAGAGAATATGCACTT---TTTTATAAACTTGATATAACACCAATAGATGAT----------
-------------------------------------------------------------------------
----------------------
GATAATACAAGCTATATGTTGATAAATTGTAACACCTCAGTCATTACACAG---
GCCTGTCCAAAGGTAACCTTTGAACCAATTCCCATACATTATTGTACCCCGGCTGGTTATGCGCTTCTAAAGT
GTAAT---GATAAAAAGTTCAAT------GGATCAGGACCATGTACAAAT-----------------------
-GTCAGC---ACAATACAATGTACACATGGAATTAGGCCAGTAGTGTCAACT---
CAATTGCTGTTAAATGGCAGTCTAGCAGGAGAAGAG------------
GTAGTAATTAGATCTGAAAATTTCACAGACAAT---------GCTAAAAACATAATAGTGCAGCTGAAT----
-----------GAAACTATAACA---------ATTAATTGTACAAGACCCAACAACAATACAAGAAAA-----
-AGTATACATATG------------GGATGGGGGAGAGCA---TTT------TATGCAACAGGAGAA------
---------ATAATAGGAGACATA------------AGACGAGCACACTGT---AACATTAGTAAAAGG---
GACTGGGAAAACACTTTAAGACTGATAGCTGAAAAATTATAT---------------AAACAATTTAAT----
-----------------------------------AATAAAACAATAGTCTTCCAA------
AAAAGTGCAGGAGGGGACCCAGAAATTGAA---ACACACAGTTTTAATTGCGGAGGGGAA---
TTCAAGCAGCTCCAGGCAAGAGTCCTGGCTGTGGAAAGATACCTAAAGGATCAACAGCTCCTAGGGATTTGGG
GTTGCTCTGGAAAACTTATCTGCACCACTACTGTGCCTTGGAATGCTAGTTGGAGT-----------------
----------------------------AATAAATCTCTGAATGCAATTTGG------GAT------
AACATGACTTGGATGGAGTGG---GAGAGAGAAATTGACAATTACACAAGCTTAATATAC---
ACCTTAATTGAAGAATCGCAGAACCAACAAGAA----------------------------------------
-----------------------------
AAGAATGAACAAGAATTATTGGAATTGGATAAGTGGGCAAGTCTGTGGAATTGGTTCGACATAACAAAATGGC
TGTGGTATATAAAAATATTCATAATGATAGTAGGAGGCTTAGTAGGTTTAAGAATAGTTTTTGCT---
GTACTTTCTATAGTA---------AATAGAGTTAGGCAGGGGTACTCACCA------
TTATCATTTGCTGTTAGTTTGTTTAATGCTTTAGCTATAGCAGTAGCTGAG---
GGGACAGATAGGATTATAGAAGTAGTACAAAGAATCTGTAGAGCT----------------------------
--------ATTATCCACATA------CCTACACGAATAAGACAGGGCTTAGAAAGAGCTTTGCTA---TAA
>MG571996
GCT---TTA------------------------AAG---GAT---ATA---
AACAATGCGTGGGTCACAGTACATTATGGGGTACCTGTGTGG---AAAGAGGCAACC---
ACCACTCTATTTTGTGCATCAGATGCTAAAGCATATGACACAGAG---GCACATAAT----------------
--------------GTATGGGCCACA---CATGCCTGTGTACCCACAGAC---
CCTAACCCACAAGAAATAGTCTTGGGA---------AATGTGACAGAAGAATTTAAC----------------
--------------------------ATGTGGAAGAATAACATGGTAGAACAGATGCATGAGGATATA---
ATTAGTTTATGGGATCAAAGCCTA---AAACCATGTGTAAAACTA---
ACCCCACTCTGTGTTACTTTAAATTGCACTGATCTGAAGAATGGGACCGGGACTAACAATGCT----------
-------------------------------------------------------------------------
----------------------------AGTAAGATTGACGAGAAAATGGAGGGAGAAATAAAA---
AACTGCTCTTTCAATGTCACCACAAGCATAGGA---------------------------
AATAAGTTGCAGAAAGAATATGCTCTT---TTTTATAAACTTGATGTACAACAAATAGGGGGT----------
-------------------------------------------------------------------------
----------------------
GATAATACAAGCTATACATTGATACAGTGTAATACCTCAGTCATTACACAG---
GCCTGCCCAAAGATATCCTTTACACCAATTCCCATACACTATTGTGCCCCGGCTGGTTTTGCGATTCTAAAAT
GCAAC---GATAAGGAGTTCAAT------GGAACAGGACTTTGTCAAAAT-----------------------
-GTCAGC---ACAGTACAATGTACACATGGAATTAAGCCAGTAGTGTCAACT---
```

CAATTGCTGTTAAATGGCAGTCTATCAGAAGGAGAG-----------
GTAGTAATTAGATCTCAAAATTTCTCAGACAAT---------GTTAAAACCTTACTAGTACAGCTGAAT----
-----------GAAACTATAAAA---------ATTAATTGTACAAGACCTAATAACAATACAAGAAAA-----
-AGTATACATCTA------------GGATGGGGAAGAGCA---TTT------TATGCATCAGGAGAA------
---------ATAATAGGAGACATA------------AGACAAGCACATTGT---AACCTTAGTAGCACA---
CAATGGAACACCACTTTACAACGGATAGTCAGAAAATTACAA---------------AAACAGTATGGG----
-----------------------------------AATAAAACAATAATCTTTAAG------
CACCCCGCAGGAGGGGACCCAGAAATTGAG---ATGCACAGCTTTAATTGTGGAGGGGAA---
TTTAAACAGCTCCAGGCAAGAGTCCTGGCTGTGGAAAGGTACCTAAGGGATCAACAGCTCCTGGGGATTTGGG
GCTGCTCTGGAAAACTCATCTGCACCACTAATGTTCCTTGGAATACTAGCTGGAGT----------------
----------------------------AATAAATCTTTAGAGACAATTTGG------AAT------
AACATGACTTGGAGACAGTGG---GAAAGAGAAATTGACAATTACACTGAGTTGATATAC---
TCCTTACTTGAAGAATCGCAAAACCAACAAGAA--

AAGAATGAACAAGAGTTATTAGAATTGGATAAATGGGCAAGCTTGTGGAATTGGTTTGACATAACAAGCTGGC
TGTGGTACATAAAAATATTCATAATGATAGTAGGAGGATTAATAGGTTTAAGAATAGTTTTTACT---
GTGCTTTCTATAGTG---------AATAGAGTTAGGCAGGGATACTCACCA------
TTATCATTCGCTGTTAGCTTGCTTGATACCACAGCTATAGCAGTAGCTGAA---
GGGACTGATAGGGTTATAGAAGTAGTACAAAGAGCTGTTAGAGGT---------------------------
--------GTTATCCACATA------CCTAGAAGAATCAGACAGGGCTTAGAAAGGCTTTTGCTA---TAA
>MG571999
GCT---ACA---
ACAAACTTGTGGGTCACAGTCTATTATGGGGTACCTGTGTGG---AAAGAAGCAACC---
ACCACTCTATTTTGTGCATCAGATGCTAAAGCATATGCCAAAGAA---GTACATAAT----------------
--------------GTTTGGGCCACA---CATGCCTGTGTACCCACAGAC---
CCTAACCCACAAGAAGTACCATTGGAA---------AATGTGACAGAAAATTTTAAC----------------
--------------------------ATGTGGAAAAATAACATGGTAGAACAGATGCATGAGGATATA---
ATCAGTTTATGGGATCAGAGCCTA---AAGCCATGTGTGAAATTA---
ACCCCACTTTGCGTTACATTGAACTGCACTGATGTTACTATGGTTAATAGTACTAGTAATAGTAATAGTACT-
----
-------------------AATAGCAGTAGTAGTATTAATAATGAAATAAAGGGAGAAATGAAA---
AACTGCTCTTTCAATATAACCACAAGCATAAAA--------------------------
AACAAGATGCAGAGAGAATATGCACTT---TTTTATAAATCAGATGTAGTACCAATAGATAAT----------
----

CAGACTAGAGAATATATGTTGACAAATTGTAACACCTCAATCATTACACAG---
GCCTGTCCAAAGGTATCCTTTGAACCAATTCCCATACATTATTGTACCCCAGCTGGTTTTGCGATTCTAAAGT
GTAAT---GATAAAAGGTTCAAT------GGAACAGGACCATGTAAAAAT----------------------
-GTCAGC---ACAGTACAATGTACACATGGAATTAGGCCAGTAGTGTCAACT---
CAACTGCTGTTAAATGGCAGTCTTGCAGAAGAAGAT-----------
ATAATAATTAGATCTGAAAATATTACAAACAAT---------GCTAAAACCATAATAGTACAGCTGAAC----
-----------CAAACTGTACAA---------ATTAATTGTACAAGACCCAACAACAATACAAGAAAA-----
-AGTATACATATG------------GGAATGGGAAGAGCA---TTT------TATGCAACAGGAGAA------
---------ATAATAGGAGATATA------------AGACAAGCACATTGT---AACCTCAGTAGAACA---
GCATGGGACAGCACTTTAATACAGATAGGTAGAAAATTAAGA---------------GAACTATTTAAC----
-----------------------------------AATAAAACAATAGTCTTTAAC------
CAATCTTCAGGAGGGGACCCAGAAATTGTA---ATGTACAGTTTTAATTGTGGAGGGGAA---
TTTAAGCAGCTCCAGGCAAGAGTCCTGGCTGTGGAAAGATACCTAAAGGATCAACAGCTCCTAGGGATTTGGG
GCTGCTCTGGAAAGCTCATTTGCACCACTGCTGTGCCTTGGAATGCTAGTTGGAGT----------------
----------------------------AACAAATCTCTAGATAMAATCTGG------AAT------
AACATGACCTGGATGGAGTGG---GAAAGGGAAATTGACAATTACACAGACTTCATATAC---
ACTTTACTTGAAGAATCGCAGAACCAACAAGAA--

AAAAATGAACAAGACTTATTGGGATTGGATCAGTGGGCAAGTTTGTGGAATTGGTTTGACATAACAAATTGGC
TGTGGTATATAAGAATATTCATAATGATAGTAGGAGGTTTAGTAGGTTTAAGAATAGTTTTTACT---
GTGCTTTCTGTAGTG---------AATAGAGTTAGGCAGGGATATTCACCA------
TTATCGTTCGCTGTTAGTCTGCTTAATGCTATAGCTATAGCAGTAGCTGAG---
GGAACAGATAGGGTTGTAGAAGTAGTACAAAGAGCTGGAAGAGCT---------------------------
--------ATCCTCCATATA------CCTGTAAGAATAAGACAGGGCTTAGAAAGAGCTTTGCTA---TAA
>MG572006

GCT---GAA--
GAAGATAAGTGGGTCACAGTCTATTATGGGGTACCTGTGTGG---AAAGAAGCAACC---
ACCACTCTATTCTGTGCATCAGATGCTAAAGCATATAAAACAGAG---GTACATAAT----------------
--------------GTTTGGGCCACA---CATGCCTGTGTTCCCACAGAC---
CCTAACCCACAAGAAGTGGTATTGGAA---------AATGTGACAGAAAATTTTAAC----------------
--------------------------ATGTGGAAAAATAACATGGTAGAACAGATGCATGAGGATATA---
ATTAGTTTATGGGATCAAAGCTTA---AAGCCATGTGTGACACTA---
ACCCCACTCTGTGTTACTCTAGAGTGCACCAATGTTACTCTAGAGTGCACCAATGTTAGTTGCTCTAATAACT
CCAATAATGGT--
-------------CCAACTTGCTCTAATGACACCAGTGTTATTGCCGAGAGGGGGGAAATAAGA---
AACTGCTCTTTCAATATCACCACAGGCATAGAA---------------------------
CCAAAGGGACGGAAGGATTATGCACTA---TTTAATAGATTGGATGTAATACCAATAGATAAT----------
----

ACAGCAAATGATACAGCAAATGATACAAGGTATATGTTGATAAATTGTAACACCTCAGTCATTACACAG---
GCTTGCCCAAAGATAACCTTTGAACCAATTCCAATACATTATTGTGCCCCGGCTGGTTTTGCGATTCTAAAGT
GTAAC---AATAAGACGTTCAAT------GGAACAGGACCATGTAAAAAT-----------------------
-GTCAGC---ACAGTACAATGTACACATGGAATTAGGCCAGTAGTGTCAACT---
CAACTGCTGCTAAATGGTAGCCTAGCAGAAGAAGAC------------
ATAATAATTAGATCTGAAAATTTCACAGACAAT---------GCTAAGATCATAATAGTACAGCTAAAT----
-----------GAGACTGTAGAA---------ATTAATTGTACAAGACCCAACAACAATACAAGCAAA-----
-AGTATACATCTG------------GGACCTGGGAGAACA---TTT------TATGCAACAGGAAGC------
---------ATAATAGGAGATATA------------AGAAAAGCATATTGT---AACATTAGTATAACA---
CAATGGAACAACACTTTAAAACGGATAGCTGCAAAATTAAAA---------------GAGCAATTTAAT----
-----------------------------------GCTACAACAATCAGCTTTAAT------
TCATCTTCAGGAGGGGACCCAGAAATTACA---ATGCACAGCTTTAATTGTGCAGGAGAA---
TTTAAGCAGCTCCAGGCAAGAGTCCTGGCTGTGGAAAGTTACCTAAGGGATCAACAGCTCCTAGGGATTTGGG
GTTGCTCTGGAAAACTCATCTGCACCACTAATGTGCCTTGGAATGGTAGTTGGAGT-----------------
----------------------------AATAGATCTCTGAACACAATTTGG------CAG------
AACATGACCTGGATGCAGTGG---GAAAAAGAAATTGAAAATTATACAGACTTAATATAC---
AACTTGCTTGAGACATCGCAAAACCAACAAGAA--

AAGAATGAACAAGATTTATTGGCATTGGATAAGTGGGCAAATTTATGGAATTGGTTTGACATATCAAAATGGC
TGTGGTATATAAAAATATTCATAATGATAGTAGGAGGTTTAGTAGGTTTAAGAATAATTTTTACT---
GTACTTTCTATAGTG---------AATAGAGTTAGGCAGGGATACTCACCA------
TTATCGTTTGCTGTTAGTTTGCTTAATACCACAGCTGTAGCAGTAGCTGAG---
GGGACAGATAGGGTGATAGAAACAGCACAAAGAATTGGCAGAGCT----------------------------
--------ATTTGTAACATA------CCTAGAAGAATAAGACAGGGCTTCGAAAGGGCTTTGGTA---TAA
>AY173956
GCT---GCA--
GAACAATTGTGGGTCACAGTCTATTATGGGGTACCTGTGTGG---AAAGAAGCAACC---
ACCACTCTATTTTGTGCATCAGATGCTAAAACATATGATACAGAG---GTACATAAT----------------
--------------GTTTGGGCCACA---CATGCCTGTGTACCCACAGAC---
CCCAACCCACAAGAAATAGAATTGGTA---------AATGTGACAGAAAATTTTAAC----------------
--------------------------ATGTGGAAAAATAACATGGTAGAACAAATGCAGGAGGATATA---
ATCAGTTTATGGGATCAAAGCCTA---AAGCCATGTGTAAAACTA---
ACCCCACTCTGTGTTACTTTAAACTGCACTGATTATACTAATACCACTAATACCACTAATACCAGT-------
----
----------------------AGTACCGTTAGTGGGGAAAAGATGGACAGGGGGGAAATAAAA---
AACTGCTCTTTCAATATCACCACAAACATAAGA---------------------------
AATAAGATGCAGAGAACATATGCACTT---TTTTATAAACTTGATGTAGAACCAATAGATAAA----------
----

AATAAGAATACTACTAGATATAGGTTAATAAGTTGTAACAACTCAGTCATTACACAG---
GCCTGTCCAAAGGTATCCTTTGAGCCAATTCCCATACATTATTGTGCCCCGGCTGGTTTTGCGATTCTAAAGT
GTAAC---AATAAGACGTTCAAT------GGAACAGGACCATGTAATAAG-----------------------
-GTCAGC---ACAGTACAATGTACACATGGAATTAGGCCAGTAGTATCAACT---
CAACTACTGTTAAATGGCAGTCTAGCAGAAGAAGGG------------
GTAGTAATTAGATCTGAAAATTTCACGGACAAT---------GCTAAAACCATATTAGTACAGCTGAAA----
-----------GAACCAGTAGAA---------ATTAATTGTACAAGACCCAACAACAAGGCAAGAAGA-----

-CGTATACGTATA------------GGACCAGGGAGAACA---TTT------TATACAGGAAAA---------
---------ATAGTAGGAGATATA------------AGACAAGCATATTGT---AACATTAGTAGAGCT---
AAATGGAATAACACTTTAAACCAGATAGTTACAAAATTAAGA--------------
GAAATTGAACAATTTAAG---------------------------------AATAAAACAATAGTCTTTAAT-
-----CAATCCTCAGGAGGGGACCCAGAAATTGTA---ATGCACAGTTTTAATTGTAGAGGGGAA---
TTTAAGCAGCTCCAGGCAAGAGTCCTGGCTGTGGAAAGATACCTAAAGGATCAACAGCTCCTGGGGATTTGGG
GTTGCTCTGGAAAACTCATTTGCACCACTGCTGTGCCTTGGAATACTAGTTGGAGT-----------------
----------------------------AATAGATCTCATGATAGCATTTGG------AAT------
AACATGACCTGGATGCAGTGG---GAAAGAGAAATTGACAATTACACAGGCTTAATATAC---
ACCTTAATTGAAGAATCGCAAAACCAACAAGAA--

AAGAATGAACAAGAATTATTGGAATTGGATAAATGGGCAAGTTTGTGGGATTGGTTTAACATAACACAGTGGC
TGTGGTATATAAAAATATTCATAATGATAGTAGGAGGTTTAATAGGTTTAAGAATAGTTTTTGCT---
GTACTTTCTATAGTG---------AATAGAGTTAGGCAGGGATACTCACCA------
TTGTCGTTTGCTGTTAGCTTGCTCAACGCCACAGCCATAGCAGTAGCTGAG---
GGGACAGATAGGGCTATAGACATAGTACAAAGAGCTTATAGAGCT----------------------------
--------ATTATCCACATA------CCTACAAGAATAAGACAGGGCTTGGAAAGGGCTTTGCTA---TAA
>AY779552
GCT---GCA---
GAACAATTGTGGGTCACAGTCTATTATGGGGTACCTGTGTGG---AAAGAAGCAAAC---
ACCACTCTATTTTGTGCATCAGATGCGAAAGCATATAAATCAGAG---GTACATAAT----------------
--------------GTTTGGGCCACA---CATGCCTGTGTACCCACAGAC---
CCCAACCCACAAGAAGTAGTATTGGGA---------AATGTGACAGAAAATTTTAAC----------------
-------------------------ATGTGGAAAAATAACATGGTAGATCAGATGCATGAGGATATA---
ATCAGTTTGTGGGATCAAAACCTA---AAGCCATGTGTAAAATTA---
ACCCCACTCTGTGTTACTTTACATTGCACTGATTTGAGGAATGCTACTAATACCACTGGGAATGCTACT----
----
----------------AATACCAATAGTAGTAGCGGAATGGAGATGGAGGGAGGAGAAATAAAA---
AACTGCTCTTTCAATATCACCACAAGCATAAGG--------------------------
GATAAAGTTCAGAAAGAACATGCACTC---TTTTATAAATTTGATATAGTGCCAATAGATAAT----------
----

GATAATACTAGCTATAGGTTGATAAGTTGTAACACCTCAGTCATTACACAG---
GCTTGTCCAAAGGTATCCTTTGAGCCAATTCCCATACATTATTGTGCCCCGGCTGGTTTTGCGCTTCTAAAGT
GTAAC---GATAAGAGGTTCAAT------GGAACAGGATCATGTAAAAAT-----------------------
-GTCAGC---ACAGTACAATGTACACATGGAATTAGGCCAGTAGTGTCAACT---
CAACTACTGTTAAATGGCAGTTTAGCAGAAGATGAG------------
GTAGTAATTAGATCTGCCAATTTCACAGACAAT---------GCTAAAATCATAATAGTACAGTTGAAC----
-----------AATTCTGTAAGA---------ATTCATTGTACAAGACCCAACAACAACACAAGAAGG-----
-AGTATAAATATA------------GGACCAGGGAGAGCA---TTT------TATACAACAGGAGAC------
---------ATAATAGGAGATATA------------AGAAAAGCACATTGT---AACCTTAGTAAATCA---
GATTGGAACAACGCTTTAAGACAGGTAGCGAGAAAATTAGGA---------------GAACAATTTAAG----
-----------------------------------AATAAAACAATAAACTTTACT------
CGTTCCTCAGGAGGGGACCCAGAAATTGCA---ATGCACAGTTTTAATTGTGGAGGAGAA---
TTTAAGCAGCTCCAGGCAAGAGTCCTGGCTGTAGAAAGATACCTAGGGGATCAACAGCTCCTAGGGATTTGGG
GTTGCTCTGGAAAACTCATCTGCACCACTGCTGTGCCTTGGAATGCTAGTTGGAGT-----------------
----------------------------AATAAATCTCTAGATGACATTTGG------GAT------
AACATGACCTGGATGCAATGG---GATAGAGAAATTAGCAATTACACAAGTAAAATATAC---
CAGTTAATTGAAAAAATCGCAGAACCAACAAGAA--

AAGAATGAACAAGAATTATTAGAATTAGATAAGTGGACAAATTTGTGGAATTGGTTTAAGATATCAAACTGGC
TGTGGTATATAAGATTATTCATAATGATAGTAGGAGGCTTGGTAGGTTTAAGAATAATTTTTGCT---
GTACTTTCTATAGTG---------AATAGAGTTAGGCAGGGATATCCACCA------
TTATCGTTTGCTGTTAGCTTGCTCAACGCCACAGCCATAGCAGTAGCTGAG---
GGGACAGATAGGATTATAGAAGTAGTACAAAGAGCTTATAGAGCT----------------------------
--------ATTCTCCACATC------CCTAGAAGAATAAGACAGGGCTTGGAAAGGCTTTTGCTA---TAA
>GU562135
GCT---GCA---
CAAGATTTGTGGGTCACAGTCTATTATGGGGTACCTGTGTGG---AAGGCAGCAAAC---
ACCACTCTATTTTGTGCATCAGATGCTAAGGCATATGATACAGAG---GTACATAAT----------------

--------------GTTTGGGCCACA---CATGCCTGTGTACCCACAGAC---
CCCAACCCACAAGAAGTAGTATTGGAA---------AATGTGACAGAAGATTTTAAT---------------
-------------------------ATGTGGAAAAATAACATGGTAGAACAGATGCAGGAGGATATA---
ATCAGTTTATGGGATCAAAGCCTA---AAGCCATGTGTAAAATTA---
ACCCCACTCTGTGTTACTTTACGTTGCACTGATTTGGAAAATGTTACTAATACCAATGTTACTAAT------
----
----------------------ACCAATAGAAGTATTGAGGGAAAAATGAAGGGAGAAATAAAA---
AACTGCTCTTTCAATATCACCACAAGCATAAGA--------------------------
GATGAAGTGACAAAACAATATGCACTT---
TTTTATAGGCTTGATGTAGTACCAATAGATAATGATGAAGGTAAA---------------------------
----
AGTAATGTTACTTCCAACTATACTGACTATAGGTTGATAAATTGTAATACCTCAGTCATTACACAG---
GCCTGTCCAAAGGTATCCTTTGAGCCAATTCCCATACATTATTGTGCCCCAGCTGGTTTTGCGATTCTAAGT
GTAAT---GATAACAAGTTCAAT------GGATCAGGGCCATGTACAAAT----------------------
-GTCAGC---ACAGTACAATGTACACATGGAATCAGGCCAGTAGTGTCAACT---
CAACTGCTGTTAAATGGCAGCCTAGCAGAAAAAGAG------------
GTAGTAATTAGATCTAGCAATTTCTCGGACAAT---------GCTAAAATCATAATAGTACAACTGAAT---
-----------AAATCTGTAGAA---------ATTAATTGTACAAGACCCAACAACAATACAAGAAAA----
-AGTATACCTATG------------GGACCAGGGAGAGCA---TTT------TATGCAACAGAAGAC-----
---------ATAATAGGAAATATA------------AGACAAGCACATTGT---AACCTCAGTGGAGTA---
GACTGGATGAGAACTTTAAACCAGGTAAGCGCAAAATTACAA--------------GAACAATTTGGG----
-----------------------------------AATAAAACAATAGTCTTTAAT------
CAATCCTCAGGAGGGGACTCAGAAATTGTA---ACACACAGTTTTAATTGTGGAGGGGAA---
TTTAAGCAGCTCCAGGCAAGAGTCCTGGCTGTAGAAAGATACCTAAGGGATCAACAGCTCCTAGGGATTTGGG
GTTGCTCTGGAAAACTCATTTGCACCACTTCTGTGCCTTGGAATGCTAGTTGGAGT----------------
----------------------------AATAAAACTCAGTCAGAAATTTGG------GGT------
AACATGACCTGGATGCAGTGG---GAGAGAGAAATCAACAATTACACAGAATTAATATAC---
AACCTACTTGAAGAATCGCAGAACCAACAAGAA---------------------------------------
----
AAGAATGAACAAGAATTATTGGAATTGGATAAGTGGGCAAGTTTGTGGAATTGGTTTGACATAACAAATTGGC
TGTGGTATATAAAAATCTTCATAATGATAGTAGGAGGCTTGGTAGGTTTAAGAATAGTTTTTACT---
GTACTTTCTATAGTA---------AATAGAGTTAGGCAGGGGTACTCACCA------
TTGTCATTTGCTGTTAGCTTGCTCAATACCATAGCTATAGCAGTAGCTGAG---
GGGACAGATAGGATTATAGAAGTAATACAAAGGATTTTTAGAGGT---------------------------
--------ATCCTCCACATA------CCTAGAAGAATAAGACAGGGTTTAGAAAGGAGTTTGCTA---TAA
>GU562272
GCT---AAA---
GAACAATTGTGGGTCACAGTCTATTATGGGGTACCTGTGTGG---AAAGATGCAACC---
ACCACTCTATTTTGTGCATCAGATGCTAAAGCATATGATACAGAG---GTACATAAT---------------
--------------GTTTGGGCCACA---CATGCCTGTGTACCCACAGAC---
CCCAACCCACAAGAAGTAGTGATGCCC---------AATGTGACAGAGAATTTTAAC---------------
-------------------------ATGTGGAAAAATAACATGGTAGAACAGATGCAGGAGGATATA---
ATCAGTTTATGGGATCAAAGCCTA---AAGCCATGTGTAAAATTA---
ACTCCACTCTGTGTTACTTTAAATTGCACTGATTGGAAGAATGCTAAAAATGATACTAACACCACTAGTAGTA
GTACT---
----------AATGCCACTGCTGGGAATGCTACTAATAGGGGAATAATGGAGGGAGAAATAAAA---
AACTGCTCTTTCAATATCACCACAGACATGAGA--------------------------
GATAAGATACAGAAACAATATGCACTT---TTTTATAAACTTGATGTAGTACAAATAGATGATAAG------
----

AGTAATAGTACTAATAGCAACTATAGGTTGATAAGTTGTAACACCTCAGTCATTACCCAG---
GCCTGTCCAAAAATAACATTTGAACCAATTCCCATACATTATTGTACTCCTGCTGGTTTTGCGATTATAAAGT
GTAAT---GATAAAAAGTTCAAT------GGATCAGGGCCATGTACAAAT----------------------
-GTCAGC---ACAGTACAATGTACACATGGAATTAGGCCAGTAGTGTCAACT---
CAGCTGCTGTTAAATGGTAGTCTAGCAGAAGAAGAT------------
ATAATAATTAGATCTAGCAATTTCTCAGACAAT---------GCTAAAAATATAATAGTACAGCTGAAT---
-----------AAATCTGTAGAA---------ATTAATTGTACAAGACCCAGCAACAATACAAGAAAA----
-GGTATACATATA------------GGACCAGGGAGTGCA---TTT------TATACAACAGGAGAA-----
---------ATAATAGGAGATATA------------AGACAAGCACATTGT---AACATTAGTAGACAA---
CAATGGGAGGACACTTTAGAAAAAGTAAGTACAAAATTAAAA--------------GAACAATTT-------

----------------------------------AATAAAACAATAGCCTTTAAG------
ACACCCTCAGGAGGGGACCCAGAAATTGTA---ATGCACAGCTTTAATTGTGGAGGGGAA---
TTTAAGCAGCTCCAGGCAAGAGTCCTGGCTGTGGAAAGATACCTAAGGGATCAACAGCTCCTAGGGATTTGGG
GTTGCTCTGGAAAACTCATTTGCACCACTTCTGTGCCTTGGAATGTTAGCTGGAGT-----------------
----------------------------AATAAAACTCAGGATGAGATTTGG------AAA------
AACATGACCTGGATGCAGTGG---GAAAGAGAAATTGACAATTACACAGACTTAATATAC---
AGCTTACTTGAACAATCGCAGAACCAGCAAGAA--

AAGAATGAACAAGAATTATTGGAATTAGATAAATGGACAAGTTTGTGGAATTGGTTTGACATAACAAACTGGC
TGTGGTATATAAAAATATTCATAATGATAGTAGGAGGCTTGATAGGCTTAAGAATAATTTTTGCT---
GTACTCTCTATAATA---------AATAGAGTTAGGCAGGGATACTCACCA------
TTATCGTTAGCTGTTAGCTTGCTCAATACTACAGCTATAGTAGTAGCTGAA---
GGGACAGATAGGGTTATAGAATTTTTACAAAGAGCTTTTAGAGCT----------------------------
--------ATTATCCACATA------CCTAGAAGAATAAGACAGGGCTGCGAAAGGGCTTTGCAA---TAA
>GU562155
GCT---ACA---
GAACCATTGTGGGTCACAGTATATTATGGGGTACCTGTGTGG---AAAGAAGCAACC---
ACCACTCTATTTTGTGCATCAGATGCTAAAGCATATGATACAGAA---GCACATAAT----------------
--------------GTTTGGGCCACA---CATGCCTGTGTACCCACAGAC---
CCCAACCCACAAGAAATAGCATTGGGA---------AATGTGACAGAAAACTTTAAC----------------
--------------------------ATGTGGAAAAATAACATGGTAGAACAGATGCATGAGGATATA---
ATCAGTTTATGGGATCAAAGCCTA---AAGCCATGTGTAACATTA---
ACCCCACTCTGTGTCACTTTGAATTGCAGTGAGGTAAGGAATAATAACAGTACTAATAATACC----------
----
----------------------AATAGTAATGGATGGGGAATGATGGAGAGAGGAGAAATAAAA---
AACTGCTCTTTCAATACCACTACAGCAATGACA--------------------------
GATAAGAAGCAGAAACAATATTCAATT---TTTAATGCACTTGATGTAGTACAAATAGATAAG----------
----

GATAATACTAGCTATAGGTTGATAAGTTGTAACACCTCAGTCATTACAAAG---
GCCTGTCCAAAAGTAACATTTGAGCCAATTCCCATACATTATTGTGCCCCGGCTGGTTTTGCGATTCTAAAGT
GTAAA---GATAAGAAGTTTAAC------GGAACAGGACCATGTAACAAT----------------------
-GTCAGC---ACAGTACAATGTACACATGGAATTAGGCCAGTAGTATCCACT---
CAACTGCTATTAAATGGCAGTCTAGCAGAAGAAGAG-----------
GTAGTAATTAGATCTGAAAATTTCACAAACAAT---------GTGAAAAACATAATAGTACAGCTGAAT----
-----------GAATCTGTAGAA---------ATTAATTGTACAAGACCCAACAACAATACAAGAAAA-----
-AGTATACATATA------------GGACCAGGGAAAGCA---TTT------TACACAACA---GAC------
---------ATAATAGGAGATATA------------AGACAAGCACATTGC---AATATTAGTAGAGCA---
AAATGGAATAACACTTTAAAACAGATAGTTGAAAAATTAGGA---------------GAACAATATAAC----
----------------------------------ATAAGCACAATAGTCTTTCAG------
CCATCCTCAGGAGGGGACCCAGAAATTGTA---ATGCACAGTTTTAATTGTGGAGGGGAA---
TTTAAGCAGCTCCAGGCAAGAGTCCTGGCTGTGGAGAGATACCTAAAGGATCAACAGCTCCTGGGGATCTGGG
GTTGCTCTGGAAAACTCATTTGCACCACTGCTGTGCCTTGGAATGCTAGTTGGAGT----------------
----------------------------AATAAATCCCTGAATGACATTTGG------GAT------
AATATGACCTGGATGGAGTGG---GAAAGAGAAATTGACAATTACACAAAAGAGATATAC---
GATTTAATTGAAAAATCGCAGAACCAACAAGAA--

AAGAATGAACAAGAATTATTGGAATTAGATAAATGGGCAAGTTTGTGGAATTGGTTTGACATAACAAAATGGC
TGTGGTACATAAAAATATTCATAATGATAGTAGGAGGATTGGTAGGTTTAAGAATAATTTTTACT---
GTACTTTCCATAGTA---------AATAGAGTTAGGAAGGGATACTCACCA------
TTATCGTTTGCTATTAGTTTGTTCAACGCCACAGCCGTAGCAGTAGCTGAG---
GGGACAGATAGGGTTATAGAATTTTTACAAAGAGCTTGTAGAGCT---------------------------
--------ATTATCCACATA------CCTAGAAGAATAAGACAGGGCTTTGAAAGGGCTTTGCAA---TAA
>JF320413
GCT---GCA---
GAACAATTGTGGGTCACAGTCTATTATGGGGTACCTGTATGG---AAAGAAGCAAAC---
ACCACTCTATTTTGTGCATCAGATGCTAAAGCATATGAAACAGAG---GTACATAAT----------------
--------------GTCTGGGCCACA---CATGCCTGTGTGCCCACAGAC---
CCTAACCCACAAGAAGTATTATTAGAA---------AATGTGACAGAAAATTTTAAC---------------
--------------------------ATGTGGAAAAATAACATGGTAGAACAGATGCATGAAGATATA---

ATCAGTTTATGGGATCAAAGCCTA---AAGCCATGTGTAAAATTG---
ACCCCACTTTGTGTTACTTTACAATGCACTGATGTGAATGTTACTAGGACTAATGAGACCAAT----------
----
----------------------GATACAAGTAGCTGGAAAAAGATGGATACAGGAGAAATAAAA---
AACTGCTCTTTCAATGTTACCACAGACAGAAGA---------------------------
GATAAGATGCAAAAAGAATATGCATAT---TTCTATAAACCTGATATAGTACAAATAAATGATAGTGAT----
----

ACTGTTAAGAATGAGAGTGACAGTTATAGGTTGATAAGTTGTAACACTTCAGTCATCACACAG---
GCCTGTCCAAAGGTATCCTTTGAGCCAATTCCCATACATTATTGTGCCCCGGCTGGTTTTGCGATTATAAAGT
GTAGA---GATAATAAGTTCAAT------GGAACAGGACCATGTAAAAAT-----------------------
-GTCAGT---ACAGTACAATGCACACATGGAATTAGGCCAGTAGTAGCAACT---
CAATTGCTGTTAAATGGCAGTCTAGCAGAAGAAGAG------------
GTAATAATTAGATCTGAGAATTTTTCTAATAAT---------GCTAAAACCATAATAGTACAGTTGAAT----
-----------AAATCTGTAGAA---------ATTCGTTGTATGAGACCCAACAATAATACAAGAAAA-----
-AGTATAACTATA------------GGACCAGGGAGAGCA---TTC------TATACAACAGGACAA------
---------ATAATAGGAGATATA------------AGACAAGCACACTGT---AACATTAGTGGAACA---
GCCTGGAAAAAAGCTTTAAAACAGACAGTTATAAAATTAGAA---------------GAACAATTTAGG----
-----------------------------------AATAAAACAATAGTCTTTACA------
AATTCCTCAGGAGGGGATCCAGAAGTTGCA---ATGCACACTTTTAATTGTAGAGGAGAA---
TTTAAGCAGCTCCAGGCAAGAGTCCTGGCTGTGGAAAGATACCTAAAGGATCAACAGCTCCTGGGGATTTGGG
GTTGCTCTGGAAAACTCATCTGCACCACTTCTGTGCCTTGGAATGCTAGTTGGAGT-----------------
----------------------------AATAAATCTCTGGATACGATTTGG------AAT------
AACATGACATGGATGGAGTGG---GAAAGAGAAATTGACAATTACACAAAATTGATATAC---
AACTTAATTGAAGAATCGCAAAACCAACAAGAA--

AAAAATGAACAAGAGTTATTGGAATTAGATAAATGGGCAAGTTTGTGGAATTGGTTCACCATAACACACTGGC
TGTGGTACATAAAAATATTCATAATAATAGTGGGAGGTTTGATAGGTTTAAGAATAGTCTTTGTT---
GTACTTGCTATAGTG---------AATAGAGTTAGGCAGGGATACTCACCA------
TTATCGTTTGCTGTTAGCTTGCTTAATGCCACGGCCATAGCAGTAGCTGAG---
GGGACAGATAGGATTATAGAAGGAGTGCGAAGAGCTTGTAGAGCT----------------------------
--------ATCCTCCACATA------CCAACAAGAATAAGACAGGGCTTAGAAAGGGCTTTGCTT---TAA
>GU562266
GTT---GCA--
GAACAATTGTGGGTCACAGTTTATTATGGGGTACCTGTGTGG---AAAGAAGCAACC---
ACCACTCTATTTTGCGCATCAGATGCTAAGGCATATAAGTCAGAG---ATGCATAAT----------------
--------------GTTTGGGCCACA---CACGCCTGTGTACCCACAGAC---
CCCAACCCACAAGAAGTAGAATTGAAA---------AATGTGACAGAAAACTTTAAC----------------
--------------------------ATGTGGAAAAATAACATGGTAGAACAGATGCATGAGGATATA---
ATCAGTCTATGGGATCAAAGCCTA---AAACCATGTGTAAAATTA---
ACCCCACTCTGTGTTACTTTAAATTGCACTGATTTGAAGAATGATACTACCACTAATAATACCACTAATAGTA
CCACTGGGTG---
-GAAAATCGTACTTCCAATAATAGTACCACTAATAGTAGCAAAGGAATGAGGGAAGAAATGAAA---
AACTGCACTTTCAATATCACCACAAGCTTGAGA---------------------------
GATAAGGAACAGAGAACATATGCACTT---TTCTATAAACTTGATGTAGTACAAATAGATAATAAT-------
----

ACTTATAATGGTACTGCTAACTATAGGTTGATAAGTTGTAACACCTCAGTCATTACACAG---
GCCTGTCCAAAGGTATCCTTTGAGCCAATTCCCATACATTATTGCGCCCCGGCTGGTTTTGCGATTCTAAAGT
GTAAG---GATAAGAATTTTAAT------GGAACAGGATCATGTAAAAAT-----------------------
-GTCAGC---ACAGTACAATGTACACATGGAATTAGGCCAGTAGTGTCAACT---
CAACTGCTGTTAAATGGCAGTCTAGCAGAAGAAGAG------------
ATAGTAATTAGATCAGAGAATCTCACAAACAAT---------GCTAGAATCATAATAGTGCAGCTGAAT----
-----------GAAACTGTAGAA---------ATTAATTGTACAAGACCCAACAACAATACAAGAAGA-----
-AGTATTCCTATA------------GGACCAGGGAGAGCA---TGG------TTTGCAACAGGAGAC------
---------ATAACAGGAGATATA------------AGAAAAGCATATTGT---AACATTAGTAGAGCA---
AAATGGGAAAACACTTTAAGACGGGTAGCTAAAAAATTAGGA---------------GAACAATTTAAG----
-----------------------------------AATAAAACAATAGCCTTTAAT------
AGATCCTCAGGAGGGGACCCAGAAGTTGTA---ATGCACAGTTTTAACTGTGGAGGGGAA---
TTCAAACAGCTCCAGGCAAGAGTCCTGGCTGTGGAAAGATACCTAAAGGATCAACAGCTCCTGGGGATCTGGG

GTTGCTCTGGAAAACTCATCTGCACCACTAATGTACCTTGGAATAGTAGCTGGAGT-----------------
----------------------------AATAAATCTCTGAATGAGATTTGG------GAT------
AACATGACCTGGATGGAGTGG---GAAAGAGAAATTAACAATTACACAGGCATAATATAC---
AACTTAATTGAACAATCGCAAACCCAACAAGAA--

AAGAATGAACTAGACTTATTCAAATTAGATCAGTGGGCAAGTTTGTGGAGTTGGTTTAGCATAACAAATTGGC
TGTGGTATATAAAAATATTCATAATAATAGTAGGAGGTTTGGTAGGTTTAAGAATAGTTTTTGCT---
GTGCTTTCTATAGTG---------AATAGAGTTAGGCAGGGATACTCACCA------
CTGTCGTTTGCTGTTAGCTTGCTCAATACCACTGCCATAGCAGTAGCTGAG---
GGGACAGATAGGATTATTGAAGTAGTACAAAGAGTTTTTAGGGCT----------------------------
--------ATTCTCCACATA------CCTAGAAGGATCAGACAAGGTTTAGAAAGGAGTTTATTA---TAA
>AY314061
GCT---ACA--
GAAAAATTGTGGGTCACAGTCTATTATGGGGTACCTGTGTGG---AAAGAAGCAAAA---
ACCACTCTATTTTGTGCATCAGATGCTAAAGCATATAGTACAGAG---GCACATAAT----------------
--------------GTATGGGCCACA---CATGCCTGTGTACCCACAGAC---
CCCAACCCACAAGAAATATTTTTGGAA---------AATGTGACAGAAAATTTTAAC----------------
--------------------------ATGTGGAAAAATGACATGGTAGACCAGATGCATGAGGATATA---
ATCAGTTTATGGGATCAAAGCCTA---AAGCCATGTGTAAAATTA---
ACCCCACTCTGTGTTACTTTAAATTGCACTGATTTAGGGAATACTACTAACACCACTAAT-------------
----
----------------------------AGTAGCTGGGGAAAGATGGAGGGAGGAGAAATAAAA---
AATTGCTCTTTCAATATCACCACAAACATAAGA---------------------------
GATAAGATAAAGAAAGAATATGCACTT---TTTAATAAACTTGATGTAGTACCAATAGCTGAT----------
----

GATAATGATAGTACTAGCTATAGGTTGATAAGTTGTAACACCTCAGTCATTACACAA---
GCCTGTCCAAAGGTATCCTTTGAACCAATTCCCATACATTATTGTGCCCCAGCTGGTTTTGCGATTCTAAAGT
GTAAC---GATAAAAAGTTCAAT------GGAACAGGACCATGTAAAAAT-----------------------
-GTCAGC---ACAGTACAATGTACACATGGAATTAGGCCAGTAGTATCAACT---
CAACTACTGTTAAATGGCAGTCTAGCAGAAGAAGAA------------
GTAGTAATTAGATCTAGCAATTTCACGGACAAT---------GCTAAAACTATAATAGTACAGCTGAAA----
-----------GAAGCTGTAACA---------ATTAATTGTACAAGACCCAACAACAATACAAGAAAA-----
-AGTATACATATA------------CAACCAGGGAGAGCA---TTT------TATGCAACAGGAGAA------
---------ATAATAGGAGATATA------------AGACAAGCACATTGT---AACATTAGTAAAGGA---
GCTTGGAATAACACTTTATTACAGATAGTTAAAAAATTAAAA---------------GAACAATATGGG----
-----------------------------------AATAAAACAATAGCCTTTAAC------
CAATCCTCAGGAGGGGACCCAGAAATTGTA---ATGCACAGTTTTATGTGTGGAGGGGAA---
TTTAAGCAGCTCCAGGCAAGAGTCCTGGCTATGGAAAGATACCTAAAGGATCAACAGCTCCTGGGGATTTGGG
GTTGCTCTGGAAAACTCATCTGCACCACTACTGTGCCTTGGAATACTAGTTGGAGT-----------------
----------------------------AATAAAACTCTGGAACAAATTTGG------GGT------
AACATGACCTGGCTGCAGTGG---GAAAGAGAAATTGATAATTATACAGAATTAATATAC---
AGCTTAATTGAAGAATCGCAAAACCAACAAGAA--

AAGAATGAACAGGAATTATTGGAATTGGATAAATGGGCAAGTTTGTGGAATTGGTTTAGCATAACAAATTGGC
TGTGGTATATAAAAATATTTATAATGGTAGTAGGAGGCTTGGTAGGTTTAAGAATAGTTTTTTCT---
GTACTTTCTATAGTG---------AATAGAGTTAGGCAGGGATACTCACCA------
TTATCGTTCGCTGTTAGCTTGTTTAATGCCACAGCCATAGCAGTAGCTGAG---
GGGACAGATAGGATTATAGAAGTATTACAAAGAATTTATAGAGCT----------------------------
--------ATCCTTCACATA------CCTGTAAGAATAAGACAAGGCTTGGAAAGAGCTTTGCTA---TAA
>GU562033
GCT---ACA--
GAAAAATTGTGGGTTACAGTCTATTATGGGGTACCTGTGTGG---AAAGAGGCAACC---
ACTACTCTATTTTGTGCATCAGATGCTAAAGCATATGATACAGAG---GTACATAAT----------------
--------------GTTTGGGCCACA---CATGCCTGTGTGCCCACAGAC---
CCCAACCCACAAGAAGTAGTATTGGGA---------AATGTGACAGAAAATTTTAAC----------------
--------------------------ATGTGGAAAAATAACATGGTAGAACAGATGCAGGAGGATATA---
ATCAGTTTATGGGATCAAAGCCTA---AAACCATGTGTAAAATTA---
ACCCCACTCTGTGTTACTTTAAATTGCACTGATTGGGGAAATGCTACTAGTACCAATAATAGTAGT-------
----

----------------------GAGGGAAACATAGAAGGAAAAATAAATCAGGGAGAAATGAAA---
AACTGCTCTTTCAATATCACCACAAGCATAAAA---------------------------
GATAAGATGAAGAAAGAATATGCACTT---TTTTATAGCCTTGATGTAGTACCAATAGATAATGATAATAAT-
----

AATACTACTAAAAACTATACCAATTATAGGTTGATAAATTGTAATACCTCAGTCATTACACAA---
GCCTGTCCAAAGGTATCCTTTGAGCCAATTCCCATACATTATTGTGCCCCAGCTGGTTTTGCGATTCTAAAGT
GTAAC---AATAAGACATTCAAT------GGAAAGGACCATGTACGAAT------------------------
-GTAAGC---ACAGTACAATGTACACATGGAATTAGGCCAGTAGTGTCAACT---
CAACTGCTGTTAAATGGCAGTCTAGCAGAAGAAAAG------------
ATAGTAATTAGATCCAGCAATTTCTCGGACAAT---------GCCAAAACCATAATAGTACAGCTCAAT----
-----------AATTCTGTAGGA---------ATTAATTGTACAAGACCCAACAACAAGACAAGAAAG-----
-AGTATACCTATG------------GGACCAGGGAGAGCA---TTT------TATGCAACAGGAGAT------
---------ATAATAGGAGATATA------------AGACAAGCACATTGT---AACCTTAGTAGAAAA---
GAATGGAATAACACTTTAAGCAAGATAGTTATAAAATTAAGA---------------GAACAGTTTGGG----
-----------------------------------AATAAAACAATAGTCTTTAAG------
CAAGCCTCAGGAGGGGACCCAGAAATTGTA---ATGCACAGTTTTAATTGTGGAGGGGAA---
TTTAAGCAGCTCCAGGCAAGACTCCTGGCTGTGGAAAGATACCTACAGGATCAACAGCTCCTAGGGATTTGGG
GTTGCTCTGGAAAACTCATTTGCACCACTGCTGTGCCTTGGAATGATAGTTGGAGT-----------------
----------------------------AATAAATCTCTGGATAAGATTTGG------AAT------
AACATGACCTGGATGGAGTGG---GATAGAGAAATTAACAATTACACAAGTTTAATATAT---
ACCTTAATTGAAAAATCGCAAAACCAACAAGAA--

AAGAATGAACAAGAATTATTGGAATTAGATAAGTGGGCAAGTTTATGGAATTGGTTTGACATAACAAACTGGT
TGTGGTATATAAAAATATTCATAATAATAGTAGGAGGCTTGGTAGGTTTAAGAATAGTTTTTATT---
GTACTTTCTATAGTG---------AATAGAGTTAGGCAGGATACTCACCA------
TTATCATTGGCTGTTAGCTTGCTTAATACCATAACTATAGTAGTAGCTGAG---
GGAACAGATAGGATTATAGAAGTAATACAAAGAACTTGTAGAGCT----------------------------
--------ATTCTCCACATA------CCTACAAGAATAAGACAGGGCTTGGAAAGGGCTTTGCTA---TAA
>GU562058
GCT---ACA--
AAAGATTTATGGGTCACAGTCTATTATGGGGTACCTGTGTGG---AAAGAAGCAACC---
ACCACTCTATTTTGTGCATCAGATGCTAAAGCATATGACACAGAG---GTACATAAT----------------
--------------GTTTGGGCCACA---CATGCCTGTGTACCCACAGAC---
CCTAACCCACAAGAAGTATTCTTGAAA---------AATGTGACAGAATATTTTAAC----------------
--------------------------ATGTGGAAAAATAACATGGTAGAACAAATGCATGAGGATATA---
ATCAGTTTATGGGATCAAAGCCTA---AAGCCATGTGTGAAATTA---
ACTCCACTCTGTGTTACTTTACACTGCACTAATTTGAGGAATGGTACTAATTTGAGGAATGATAGTGATAACA
GTACT--
-------ACTCCCAGTCCTACAACTACCAGTAATATAACCAATATAGAAGAAGGGGAAATAACA---
AACTGCACTTTCAATGTCACCACAACAATAGGA---------------------------
AATAAGATGCAGAAACAATATGCACTT---TTTTATAGACTTGATGTACAAAAAATAGATGAT----------
----

AATGGTAGCTATGCGTTGATAAATTGTAACAGCTCAGTCATTACACAG---
GCCTGTCCAAAGGTATCCTTTGAACCAATTCCCATACATTATTGTGCCCCGGCTGGTTTTGCGATTCTAAAGT
GTAAG---GATAAAAAGTTCAAT------GGAACAGGACCATGTAAAAAT-----------------------
-GTCAGC---ACAGTGCAATGTACTCATGGAATTAGACCAGTAGTGTCAACT---
CAGCTGCTGTTAAATGGCAGTCTGGCAGAAGAAGAG------------
GTAGTGATTAGATCTGAAAATCTCACGAACAAT---------GCTAAAACCATAATAGTACAGCTGAAC----
-----------GAATCTGTAGCA---------ATTAATTGTACAAGACCCAACAACAATACAAGAAAA-----
-AGTATACACATA------------GCACCAGGGAGCGCA---TTT------TATGCAACAGGAGCT------
---------ATAATAGGAGATATA------------AGACAAGCACATTGT---AACATTAGTAGACAA---
CAATGGAATAACACTTTAGAAAAGATAGTTACAAAATTAAGA---------------GAACAATTTGTG----
-----------------------------------AATAAAACAATAATCTTTAAT------
CAATCCTCAGGAGGGGACCCAGAAATTGTA---ATGCACAATTTTAATTGTGGAGGGGAA---
TTTAAGCAGCTCCAGGCAAGAGTCCTGGCTGTAGAAAGATACCTACGGGATCAACAGCTCCTGGGGATTTGGG
GTTGCTCTGGAAAACTCATTTGCACCACTGCTGTGCCTTGGAATGCTAGTTGGAGT-----------------
----------------------------AATAAGTCTCTAGAACAAATTTGG------GAT------
AACATGACATGGATGGAGTGG---GAGAAGGAAATTGACAATTACACAGGCTTAATATAC---

ACCTTAATTGAAGAATCGCAGAACCAACAAGAA--
AAAAATGAACAAGAATTATTGGCATTGGACAAGTGGGCAAGTTTGTGGAATTGGTTTGACATATCAAACTGGC
TGTGGTATATAAAAATATTCATAATGATAGTAGGAGGTTTAGTAGGTTTAAGAGTAGTTTTTACT---
GTACTTTCTATAGTG---------AATAGAGTTAGGCAGGGATACTCACCA------
TTGTCATTTGCTGTTAGTTTGCTTAATGCCACAGCTATAGCAGTAGCTGAG---
GGGACAGATAGAGTTATAGAAATAGTACAAAACATCTGTAGAGCT----------------------------
--------ATTCTCCACATA------CCTAGAAGAATAAGACAGGGCTTAGAAAGGGCTTTGCTG---TAA
>GU562001
GCT---ACA---
GGAAATTTGTGGGTCACAGTCTATTATGGGGTACCTGTGTGG---AGAGAAGCAACC---
ACCACTCTATTTTGTGCATCAAATGCTAAAGCATATGATACAGAG---GCACATAAT----------------
--------------ATTTGGGCCACA---CATGCCTGTGTACCCACAGAC---
CCCGACCCAAAAGAAATTGAATTGACA---------AATGTGACAGAAGATTTTAAT----------------
-------------------------GCGTGGAAAAATGACATGGTAGAACAAATGCATGAGGATATA---
ATTAGTTTATGGGATCAAAGCCTA---AAGTCATGTGTAGAATTA---
ACCCCACTCTGTGTTACCTTACATTGCACTAATAATGTTACTATCAATGCTACT-------------------
----
-------------------------------ATCTATAATAATAGTAGTGGGGCGGGAATGAAG---
AACTGCTCTTTCAATATCACCACAAACATAGAA---------------------------
GATAAGGTGCAGAAAAAACATGCACTG---TTTTATGAACTTGATATAAAACAGATGGATAAT----------
----

ACTAGTTATAGGTTGATAAGTTGTAACACCTCAAAAATTACACAG---
GCCTGTCCAAAGATATCCTTTGAGCCAATTCCTATACATTATTGCGCCCCGGCTGGTTTTGCGATTCTAAAGT
GTAAT---GATAAAAAGTTCAAT------GGAACAGGACCATGTGGAAAT-----------------------
-GTCAGC---ACAGTACAATGTACACATGGAATTAGGCCAGTAGTATCAACT---
CAACTGCTGTTAAATGGCAGTCTAGCAGAAGAAGAT------------
GTAGTAATTAGATCTGAAAACATCACAAACAAT---------GCTAAAACCATAATAATACAGCTGAAA----
-----------GACCCTGTAGAA---------ATTAATTGTACAAGACCCAACAACAATACAAGACAA-----
-GGTATACATATG------------GGACCAGGGAGATCA---TTT------TATACAGCAGGAGCA------
---------ATAATAGGAGATATA------------AGACAAGCACATTGT---AACATTAGTGGAGAA---
AAATGGAATAACACTTTACAACAGATAGTCAAGAAATTAAGA--------------
GAAATTGAGCAATTTAAA--------------------------------AATAAAACAATAGCCTTTAAG-
-----AATTCCTCAGGAGGAGACATAGAAATTGTA---ACACACAGTTTTAATTGTGGAGGGGAA---
TTTAAGCAGCTCCAGGCAAGAGTCCTGGCTGTGGAAAGATACCTAAAGGATCAACAGCTCCTGGGGATTTGGG
GTTGCTCTGGAAAACTCATCTGCACCACTGCTGTGCCTTGGAATACTAGTTGGAGT----------------
-----------------------------AATAGAACTCTGTCTCAGATTTGG------GAG------
AACATGACCTGGATGCAATGG---GAAAGAGAAATTGACAATTACACAGGCTTAATATAC---
ACTTTAATTGAAGAATCGCAGAACCAGCAAGAA--
AAGAACGAACAAGAATTATTGGCATTGGATCAGTGGGCAAATTTGTGGAGTTGGTTTGACATAACAAAATGGC
TGTGGTATATAAAAATATTCATAATGATAGTAGGAGGCTTGGTAGGTTTAAGGATAATTTTTACT---
GCACTTTCTATAGTA---------AATAGAGTTAGGCAGGGATACTCACCA------
TTATCGTTGGCTGTTAGTTTGCTTAATGCTACAGCTATAGCAGTAGCTGAG---
GGGACAGATAGAATTATAGAAGTAGTACAAAGAATTGGTAGAGCT----------------------------
--------ATTCTCCACATA------CCTAGAAGAATAAGACAGGGCTTAGAAAGGAGCTTGCTA---TAA
>GU562080
ACT---ACA---
GAACAATTGTGGGTCACAGTTTATTATGGGGTACCTGTGTGG---AAAGAAGCAACC---
ACCACTCTATTTTGTGCATCAGATGCTAAAGCATATGATACAGAA---GCACATAAT----------------
--------------GTTTGGGCCACA---CATGCTTGTGTACCCACAGAC---
CCCAACCCACAAGAAATATTATTGGGA---------AATGTGACAGAAAGTTTTAAC----------------
-------------------------ATGTGGAAAAATAACATGGTAGAACAGATGCATGAGGATATA---
ATCAGTTTATGGGATCAAAGCCTA---AAGCCATGTGTAAAATTA---
ACCCCACTCTGTGTCACTTTAAATTGCTCTGATTTAGGAAATATTACTAATACCAATACCACT----------
----
-------------------------AGTAGTAGTGGGATAGAGATGGAGAGAGGAGAAATAAAA---
AACTGCTCTTTCAATATCACCACAAGCATAAGA---------------------------
GATAAGATGCAAAAACAATATGCAACT---TTTTATAAACTTGATTTAGTATCAATAGATAAT----------

----
ACTAGCGAATATAGGTTGATAAGTTGTAACACCTCAGTCATTACACAG---
GCCTGTCCAAAAGTAACATTTGAGCCAATTCCCATACATTATTGTGCCCCGGCTGGTTTTGCGATTCTAAAGT
GTAAA---GATAAGAAGTTCAAT------GGAAAAGGACCATGTAACAAT----------------------
-GTCAGC---ACAGTACAATGTACACATGGAATTAGGCCAGTAGTATCAACT---
CAACTGCTGTTAAATGGCAGTCTAGCAGAAGAAGAG-----------
TTAGTAATTAGATCTGCCAATTTCACAGACAAT---------GCTAAAATCATAATAGTACAGTTGAAT----
-----------GAATCTGTAAAA---------ATTAATTGTATAAGACCCAACAACAATACAAGAAAA-----
-AGTATACATATA------------GGACCAGGGAGAGCA---TTC------TATACAACAGGAGAA------
---------ATAATAGGAGATATA------------AGACAAGCACATTGC---AACATTAGTAGAGCA---
ATATGGAATAACACTTTAAAACTGATAGCTAGAAAATTAGGA---------------GAACAATTTGGG----
--------------------------------AATAATAAAACAATAGTCTTTAAT------
CAATCCTCAGGAGGGGACCCAGAAATTGAA---ACGCACAGTTTTAACTGTGGAGGGGAA---
TTTAAGCAGCTCCAGGCAAGAGTCCTGGCTGTGGAGAGATACCTAAAGGATCAACAGCTCCTGGGAATTTGGG
GCTGCTCTGGAAAACTCATTTGCACCACCGCTGTGCCTTGGAATGCTAGTTGGAGT-----------------
----------------------------AATAAATCTCTGAATGACATTTGG------GAT------
AATATGACCTGGATGCAGTGG---GAAAGAGAAATTGACAATTACACAAGCTTAATATAC---
ACTTTAATTGAAGAATCGCAGAACCAACAAGAA--

AAGAATGAACAAGACTTATTGGCATTAGATAAATGGGCAAGTTTATGGAGTTGGTTTGACATAACGAATTGGC
TGTGGTATATAAAAATATTCATAATGATAATAGGAGGATTAGTAGGTTTAAGAATAATTTTTACT---
GTACTTTCTATAGTG---------AATAGAGTTAGGAAGGGATACTCACCA------
TTATCATTTGCTGTTAGCTTGCTTAATGCCACAGCCATAGCAGTAGCTGAG---
GGAACAGATAGGGTTATAGAATTCTTACAAAGAGCTTTTAGAGCT----------------------------
--------TTTATCCATATA------CCTAGAAGAATAAGACAGGGCTTGGAAAGGGCTTTGCAA---TAA
>DQ322223
GCT---ACA---
GAACAATTGTGGGTCACAGTCTATTATGGGGTACCTGTGTGG---AAAGAAGCAACC---
ACCACTCTATTTTGTGCATCAGATGCTAAAGCTTATGATAGAGAA---GCACATAAT----------------
--------------GTTTGGGCCACA---CATGCCTGTGTACCCACAGAC---
CCCCACCCACAAGAAGTAAGATTGGGA---------AATGTGACAGAAAATTTTAAT----------------
-------------------------ATGTGGAAAAATGACATGGTAGAACAGATGCATGAGGATATA---
ATCAGTTTATGGGATCAAAGCCTA---AAGCCATGTGTAAAATTA---
ACCCCACTCTGTGTCACTTTAAAGTGCAATAATACTGCCAATAATAGTAATAGTAATACTACTGCT-------
----
----------------------GGCAATAGTAGTGTAGAGGGAAAGATAAAAGGGGAAATGAAA---
AACTGCTCTTTCAATATCACCACAAGCATAAAA--------------------------
GATAGGGTACAGAAAGAATATGCACTT---TTCTATAACCTTGATATAGTACCAATAGATAAT----------
----

GATAATGATAATACTAGCTATAGGTTGATAAATTGTAATACCTCAGTCATTACACAG---
GCCTGTCCAAAGGTAACCTTTGAACCAATTCCCATACATTATTGTGCCCCAGCTGGTTTTGCGATTCTAAGGT
GTAAC---AATAAGACATTCAGT------GGAAAAGGACCATGTACAAAT----------------------
-GTTAGC---ACAGTACAATGTACACATGGAATTAGGCCAGTAGTGTCAACT---
CAACTGTTGTTAAATGGCAGCCTAGCAGAAGAAGAG-----------
GTAGTAATTAGATCTGACAATTTCACAGACAAT---------GCTAAAACTATAATAGTACAGCTGAAG----
-----------GAAGCTGTAAAC---------ATTACTTGTACAAGACCTGGCAACAATACGAGGAGA-----
-AGTATACCTATG------------GGACCAGGGAGAGCA---TTT------TATACAACAGGAGAY------
---------ATAATAGGAGATATA------------AGACAAGCACATTGT---AACCTTAGTAGAACA---
AGCTGGAATAACGCCTTAAATCAGACAGCTATCAAATTAAGA---------------GAACAATTTAAT----
-----------------------------------AATAAAACAATAATCTTTAAT------
CAATCCTCAGGAGGGGATCTAGAAATTGTA---ATGCACAGTTTTAATTGTGGAGGAGAA---
TTTAAACAGCTCCAGGCAAGAGTCCTGGCTGTGGAAAGATACCTAAAGGATCAACAGCTCCTAGGGATTTGGG
GTTGCTCTGGAAAACTCATTTGCACCACTGCTGTGCCTTGGAATACTAGTTGGAGT-----------------
----------------------------AATAAATCTATGAGTGAGATTTGG------GAT------
AACATGACCTGGATGGAGTGG---GAAAAAGAAATCAATAATTACACAGAACAAATATAC---
AAATTAATTGAAATATCGCAGAACCAACAAGAA--

AAGAATGAACAAGACTTATTAGAATTAGATAAGTGGGCAAGTTTGTGGAATTGGTTCAGCATAACAAACTGGC

TGTGGTATATAAAGATATTTATAATGATAGTAGGAGGCTTGGTAGGTTTAAGAATAGTTTTTACT---
GTACTTTCTATAGTG---------AATAGAGTTAGGCAGGGATACTCACCA------
CTGTCATYTGCTGTTAGCTTGCTTAATACCACAGCTATAGCAGTAGCTGAA---
GGAACAGATAGGGTTATAGAAGTAGGACAAAGAGCTTTTAGAGCT----------------------------
--------GTTATCCACATA------CCTAGAAGAATAAGACAGGGCTTGGAAAGGGCTTTGCAA---TAA
>DQ322227
GCA---GAC---
CAGTTGTGGGTCACAGTCTATTATGGGGTACCTGTGTGG---AAAGAAGCAAAC---
ACCACTTTATTTTGTGCATCAGATGCCAAAGCATATGATACAGAG---GCACATAAT----------------
--------------GTCTGGGCCACA---CATGCCTGTGTACCCACAGAC---
CCAAACCCACATGAACAAGAATTGACA---------AATGTGACAGAAAATTTTAAC----------------
--------------------------ATGTGGAGCAATAACATGGTAGAACAGATGCATGAGGATATA---
ATCAGTTTATGGGATCAAAGCCTA---AAGCCATGTGTAAAATTA---
ACCCCACTTTGTGTTACTTTAAATTGCAATGATGTAAGGAATGCCACTAATGCCAAT----------------
----
----------------------------AGTAGCGAGGGAATGATGGAGAAAGGGGAAATGAAA---
AACTGCTCTTTTAATATCACCACAGCCATGAAC---------------------------AAGGGGGTG---
CAGGAACGTGCACTT---TTTTATAGCCTTGATGTAGTACCAATAGATAAC----------------------
----
----------------AGGACATATAGGTTGATAAGTTGTAACACCTCAAAAGTTACACAG---
GCCTGTCCAAAGATAACCTTTGAGCCAATTCCCATACATTATTGTGCCCCAGCTGGTTTTGCACTTCTAAAGT
GTAGG---GATAAAAATTTCAAT------GGAACAGGACCATGTACAAAT-----------------------
-GTTAGC---ACAGTACAATGCACACATGGAATTAGGCCAGTAGTATCAACT---
CAACTGCTGTTAAATGGCAGTCTAGCAGAAGGAGAG------------
GTAATGATTAGATCTAAAAATTTCACAGACAAT---------GCTAAAACCATAATAGTGCAGCTGAAT----
-----------GAAACTGTAGAA---------ATTAAGTGTACAAGACCTAACAACAATACAAGAAAA-----
-AGTATAACTATG------------GGACCAGGGAAAGCA---TTT------TTTACAACAGGAGAT------
---------ATAATAGGAGATATA------------AGACAAGCACATTGT---AACATTAGTAGAGAA---
AAATGGAATGCAACTTTAGGACAGATAGCTGAAAAATTAAGA---------------GAACAATTTGGG----
--------------------------------GAAAATAAAACAATAGCTTTTAAT------
CGACCCTCAGGGGGGGACCCAGAAATTGTA---ATGCACAGTTTTAATTGTGCAGGGGAA---
TTTAAGCAGCTCCAGGCAAGAGTCCTGGCTGTGGAAAGATACCTACAAGATCAACAGCTCCTAGGAATTTGGG
GTTGCTCTGGAAAACTCATCTGCACCACTGCTGTGCCTTGGAATACTAGTTGGAGT-----------------
----------------------------AATAGATCTCTAAATACAATTTGG------GAA------
AACATGACCTGGATGCAGTGG---GAAAGAGAAATTGATAATTACACAAATACAATATAC---
CAATTACTTGAAAAATCACAGAATCAACAAGAA--

AAGAATGAACAAGAATTATTGGAATTAGATAACTGGGCAAGTTTGTGGAATTGGTTTACCATAACAAATTGGC
TGTGGTACATAAAAATATTCATAATGATAGTAGGAGGCTTGATAGGTTTAAGAATAGTTTTTGCT---
GTACTTTCTATAGTG---------AATAGAGTTAGGCAGGGATACTCACCA------
TTATCATTTGCCATTAGCTTGCTCAATTTCATAGCCATAGCAGTAGGTGAA---
GGGACAGATAGGATTATAGAAGTAGTGCAGACAATTTATAGAGCT----------------------------
--------ATTATCCATATA------CCTAGAAGAATAAGACAGGGCTTAGAAAGGATTTTGCTA---TAA
>DQ322225
GCT---GCA--
GAAAACTTGTGGGTCACAGTCTATTATGGGGTACCTGTGTGG---AAAGAAGCAACC---
ACCACTCTATTTTGTGCATCAGATGCTAAAGCATATGATACAGAG---GCACATAAT----------------
--------------GTTTGGGCCACA---CATGCCTGTGTACCCACAGAC---
CCCAACCCACAAGAAGTGAAATTGGAA---------AATGTGACGGAAAATTTCAAC----------------
--------------------------ATGTGGAAAAATAACATGGAAGAACAGATGCATGAGGATATA---
ATCAGTTTATGGGATCAAAGCCTA---AAGCCATGTGTAAAATTA---
ACCCCACTCTGTGTTACTTTAACTTGCACTAATTTGAATATCACTAATACCAATATCACTAGT----------
----
-------------------------AGTGCTAATATCACTGATGGTAGCGGGGAAGAAATGAAA---
AACTGCTCTTTCAATGTCACCTCAACAACAAGA---------------------------
GATAAGCTGCGGAAAGAATATGCCCTT---TTATATAAAATGGATTTAGTGCCAATAGATAGT----------
----

GATAATACCAGCTATAGGTTAATAAGTTGTAACACCTCAGTCCTCACACAG---
GCCTGTCCAAAGGTGACCTTTGAGCCAATTCCCATACATTATTGTGCCCCGGCTGGTTTTGCGATTCTAAAGT

GTAAT---GATAAGAATTTCACT------GGGACAGGGCCATGTAGAAAT----------------------
-GTCAGC---ACAGTACAATGTACACATGGAATTAGGCCAGTAGTATCAACT---
CAACTGCTGTTAAATGGAAGTCTAGCAGAAGAAGGG-----------
GTAGTAATTAGATCTGCCAATTTCTCAARCAAT---------GCTAAAACCATAATAGTGCAGCTGAAC----
-----------AAGCCTGTAAAA---------ATTAATTGTACAAGACCCAACAACAATACAAGAAAA-----
-AGTATACCTATA------------GGACCAGGGAGAGCA---TTT------TATGCAACAGGAGAC------
---------ATAATAGGAGATATA------------AGACAAGCACATTGT---AAGTTGAGTAAAACA---
CAATGGGATTACACTTTAGAACAGGTAGCTAAAACATTAGAA--------------AAACAATTT-------
-----------------------------------AATAAAACAATAGCATTTAAT------
CGATCCTCAGGAGGGGACTTAGAAATAGTA---ATGCACAGTTTTAATTGTGGAGGGGAA---
TTTAAGCAGCTCCAGGCAAGAGTCCTAGCTGTGGAAAGATACCTAAAGGATCAACAGCTCCTGGGGATTTGGG
GATGCTCTGGAAAACTCATTTGCACCACTGCTGTACCTTGGAATRCTRGTTGGAGT-----------------
----------------------------AATAAATCTTTGGATCAGATTTGG------AAT------
AACATGACCTGGATGGAGTGG---GAAAGAGAAATTGGCAATTACACAAGCTTAATATAC---
ACTCTAATTGAACAATCGCAAAACCAACAAGAA---------------------------------------
----
AAGAATGAACAAGAGTTATTGGAATTGGATAAATGGGCAAGTTTGTGGAATTGGTTTGACATAACAAACTGGC
TGTGGTATATAAGAATATTCATAATGATAGTAGGAGGCTTGATAGGTTTAAGAATAGTTTTTGCT---
GTACTTTCTATRGTA---------AATAGAGTTAGGCAGGGATACTCACCA------
TTATCGTTTGCTGTTAGTTTGCTTAATGCCACAGCTATAGCAGTAGGTGAA---
GGGACAGATAGGGTTATAGAAGGAATACAAAGAGTTGGTRGAGCT---------------------------
--------ATTCTCCACAKA------CCTAGAAGAATAAGACAGGGCTTGGAAAGGGCTTTGCAG---TAA
>DQ322239
GCT---GCA--
RAAAAATTGTGGGTTACAGTCTATTATGGGGTACCTGTGTGG---AAAGAAGCAAAC---
ACCACTCTATTTTGTGCATCAGATGCCAGAGCATATGATACAGAG---GTACATAAC----------------
--------------GTTTGGGCCACA---CATGCCTGTGTACCCACCGAC---
CCCAACCCACAAGAAGTAGTATTGGAA---------AATGTGACAGAAAATTTTAAC---------------
-------------------------ATGTGGAARAATGACATGGTAGAACAGATGCATGAGGATATA---
ATTGGGTTATGGGATGAAAGCCTA---AAGCCATGTGTAAAATTA---
ACTCCACTCTGTGTTACTCTAAATTGCACTGATGAGTTGAGGAATGACACTTGTAGGAATGACACT-------
----
---------------------TGTACTAATAGTAGTACTGAGGATATTAAAGGAGAAATAAAA---
AACTGCTCTTTCAATATTACCACAAACATAAAC-------------------------
GGTAAGATGCAGAAAGAATATTCACTT---TTCTATAAACTTGATGTAGTACCAATAGATAGTGAG-------
----

AATAATGGTACTAACAGCTCCTATAGATTGATAAGTTGTAATACCTCAGTCATTACACAA---
GCCTGTCCAAAGGTGTCCTTTGAGCCAATTCCCATACATTATTGTACCCCGGCTGGTTTTGCGCTTCTGAAAT
GTAAC---AATAAGACATTCAAT------GGAACAGGACCATGTAAAAAT----------------------
-GTCAGC---ACAGTACAATGTACACATGGAATTAAGCCAGTAGTGTCAACT---
CAACTGCTGTTGAATGGCAGTCTAGCAGAAGAAGGG-----------
ATAGTAATTAGATCTGAAAATTTCAGTGATAAT---------GCTAAAACCATAATAGTACAGCTGGCG----
-----------AAACCTGTAAAA---------ATTAAGTGTACAAGACCCAACAACAATACAAGAAGA-----
-GATATACATATA------------GGACCAGGGAGAGCA---TTT------TATGCA---GGAGAA------
---------ATAATAGGAAATATA------------AGAAAAGCTCATTGT---AACCTTAATAGAACA---
GAATGGAGTAACACTTTACAAGAGATAGTTGACAAATTAAGA--------------GCACAATTTGGG----
--------------------------------AAGAATAAAACAATAGTCTTTAAT------
CAATCCTCAGGAGGGGACATAGAAATTGTA---ACGCACAGTTTTTGTTGTGGAGGGGAA---
TTTAAGCAACTTCAGGCTAGAGTCCTGGCTGTGGAAAGATACCTAAAGGATCAACAGCTCCTAGGGATTTGGG
GTTGCTCTGGAAGACTCATTTGCACCACTACTGTGCCTTGGAATGCTAGTTGGAGT-----------------
----------------------------AATAAATCCCTAGATAATATTTGG------AAT------
AACATGACCTGGATGGAGTGG---GAAAGAGAAATTAGCAATTACACAGGCTTAATATAC---
AACTTAATTTCAGAAGCGCAAAACCAACAAGAA---------------------------------------
----
AAAAATGAACAAGAGTTATTGGAATTAGATAAATGGGCAAGTTTGTGGAATTGGTTTGACATAACAAAATGGC
TGTGGTATATAAAAATATTTATAATAATAGTAGGAGGCTTGGTAGGTTTAAGAATAGTTTTTGCT---
GTACTTTCTATAGTA---------AATAGAGTTAGGCAGGGATATTCACCC------
TTATCATTTGCTGTTAGCTTGCTCAATGCCACAGCTATGGCAGTAGCTGAG---

GGGACAGATAGGGTTATAGAAATAGTACAAAGAGCTTGGAGAGCT----------------------------
--------GTTCTTCACATA------CCTGTAAGAATAAGACAGGGCTTGGAAAGGCTTTGTTA---TAA
>JQ403022
GCT---ACA--
GAACAACTGTGGGTCACAGTCTATTATGGGGTACCTGTGTGG---AAAGAAACAAAT---
GCCACTCTATTTTGTGCATCAGATGCTAAAGCATATGAGACAGAG---AAGCATAAT----------------
--------------GTTTGGGCCACA---CATGCCTGTGTGCCCACAGAC---
CCTAACCCACTAGAATTAAAATTGGAA---------AATGTGACAGAAGATTTTAAC----------------
--------------------------ATGTGGAAAAATAACATGGTAGAACAAATGCAGGAGGATATA---
ATCAGTTTATGGGATCAAAGTCTA---AAGCCATGTGTAAAATTA---
ACCCCGCTCTGTGTTACCTTAAATTGCAGTGATGCGGACGTGAATAGGACTGATAAT----------------
----
-------GTGAATGCTAATAATACCAGTAGTAGTGAGCTACCGATGGAGAAAGGAGAACTAAAA---
AACTGCTCTTTCAATACCACCACAAACATAGGA---------------------------
AATAAGATGGAAAAAAACAATGCACTT---TTTAATAGCCTTGATGTAGTACCAATAGATAAG----------
----

AATAAGACCAGCTATATGTTGATAAATTGTAACACCTCAGTCATTACACAG---
ACCTGTCCAAAGATATCCTTTACACCAATTCCCATACATTATTGTGCCCCGGCTGGCTTTGCAATTCTAAAGT
GTAGA---GATAAGCAATTCAAT------GGAACAGGACCATGTAAAAAT-----------------------
-GTTAGC---ACAGTACAATGTACACATGGAATTAAGCCAGTAGTGTCAACT---
CAACTGCTGTTAAATGGCAGCCTAGCAGAAGAAGAG------------
GTAGTAATTAGATCTGAAAATTTCACCAACAAT---------GCTAAAACCATAATAGTGAGTCTAAAT----
-----------GAATCTGTAGAA---------ATTAATTGTACAAGACCCAACAACAATACAAGGAAA-----
-AGTATACATCTA------------GGACCAGGGAGAGCA---ATT------TATGCAACAGGAGAA------
---------ATAATAGGAAATATA------------AGAAAAGCATATTGT---AACCTCAGTGCTACA---
AATTGGCATAACACTTTACAAAAGATAGTTGTAAAATTAAGA---------------GAGCAATTT-------
-----------------------------------AACAAAACAATAATCTTTCAG------
CCATCCTCAGGAGGGGACCCAGAAATTGAA---ATGCACAGTTTTAACTGTGGAGGGGAG---
TTTAAACAGCTCCAGGCAAGAGTCCTGGCTGTGGAAAGATACCTAGGGGATCAACAGCTCCTAGGGATTTGGG
GTTGCTCTGGAAGACTCATCTGCACCACTGCTGTGCCTTGGAACACCAGTTGGAGT-----------------
----------------------------AATAGATCTCTAGATAATATTTGG---------------
AACATGACCTGGATGCAGTGG---GAAAAAGAAATTGACAATTACACAGGCTTGATATAC---
ACCTTACTTGAACAGTCACAGTTCCAGCAAGAA--

AAGAATGAAAAAGAATTATTAGAATTGGATAAGTGGGCAAGTTTGTGGAGTTGGTTTGACATATCAAATTGGC
TGTGGTATATAAAAATATTCATAATGGTAGTAGGAGGCTTGGTAGGTTTAAGAATAGTTTTTACT---
GTACTTTCTATAATA---------AATAGAGTTAGGCAGGGATACTCACCA------
TTATCATTCGCTGTTAGCTTGCTTAATACTACAGCTATAGTAGTAGCTGAG---
GGGACAGATAGGATTATAGAAGTAGTACAAGGAGTTGGTAGAGCT----------------------------
--------ATCCTCCACATA------CCTAGAAGAATAAGACAGGGATTAGAAAGAGCTTTGCTA---TAA
>JQ403023
GCT---GCA---GAA------------------------------------
GAGAATTTGTGGGTCACAGTCTATTATGGGGTACCTGTGTGG---AGAGAAGCAAAT---
GCCACTCTATTTTGTGCATCTGATGCTAGGGCATATGAAACAGAA---AAACATAAT----------------
--------------GTTTGGGCCACA---CATGCCTGTGTACCCACAGAC---
CCCAGCCCAAAAGAAGTAGTATTGGCA---------AATGTGACAGAAAATTTTAAT----------------
--------------------------GCATGGAAAAATAACATGGTAGAACAAATGCAGGAGGATGTA---
ATCAGTTTATGGGATCAAAGCCTA---AAACCATGTGTAAAATTA---
ACCCCACTCTGTGTTACTCTAAATTGCACTGACTACCGTCCTATTACTGAGAATGCCACT-------------
----
----------------------------------GTGGAGAATAGTACTAAAGGAGAAATAAAA---
AACTGCTCTTTCAATATCACCACAAATTTCAGA---------------------------
GATAAGATGCAGAGACAATATGCACTT---
TTTTATAAACTTGATGTAGCACCAATGGAAGAAAATAATACTACA----------------------------
----
GGTAATACTAACTATACTAACTATAGGTTGATAAGTTGTAACACCTCAGTCATTACACAG---
GCCTGTCCAAAGGTATCCTTTGAGCCAATTCCCATACATTATTGTGCCCCGGCTGGTTTTGCGATTCTAAAAT
GTAAT---GATAAGAAGTTCAAT------GGATCAGGACCATGTACAAAT-----------------------
-GTCAGC---ACAGTACAATGTACACATGGAATTAGGCCAGTAGTGTCAACT---

CAACTGTTATTAAATGGCAGTCTAGCAGAAGAAGAT-----------
GTAGTAATTAGATCTGAAAATTTCACAAAAACT---------ATTAAAACCATAATAGTACAGCTGAAC----
-----------GCATCTGTAATG---------ATTAATTGTACAAGACCCAACAACAATACAAGAAAA-----
-GGTATACATATA------------GGACCAGGGAGAGCA---TTT------TATGCAACAGGAGAC------
---------ATAATAGGAGATATT------------AGACAAGCACATTGT---AACCTTAGTGGAGTA---
CACTGGAATAACACTTTAACAAAGATAGTTGAAAAATTAAGA---------------GAACAATTTGGG----
--------------------------------AATAATAAAACAATAATCTTTAAT------
CAATCCACAGGAGGGGACCCGGAAGTTGTA---ACGCACAGTTTTAATTGTGGAGGGGAA---
TTTAAACAGCTCCAGGCAAGAGTCCTGGCTGTGGAAAGATTCCTAAGGGATCAACAGCTCCTGGGAATTTGGG
GTTGCTCTGGAAAACTCATCTGCACCACTGCTGTGCCTTGGAATACTAGTTGGAGT-----------------
----------------------------AATAAAACTTATCATGAGATTTGG------GAA------
AACATGACCTGGATGGAGTGG---GAAAGAGAAATTGACAATTACACAACCACAATATAC---
AACTTAATTGAAAAATCGCAAAACCAACAAGAA--

AAGAATGAACAAGAATTATTGGAACTAGATAAATGGGCAAGTTTGTGGAATTGGTTTGACATAACAAAATGGC
TGTGGTATATAAAAATATTCATAATGATAGTAGGAGGCTTAGTAGGTTTAAGAATAGTTTTTACT---
GTACTTTCTATAGTG---------AATAGAGTTAGGAAGGGATACTCACCA------
TTATCGTTTGCTGTTAGCTTGCTTAATGCCACGGCCATAGCAGTAGCTGAG---
GGGACAGATAGAATTATAGAATTAGCACAAAGAATTTTTAGGGCT----------------------------
--------ATTATTCACATA------CCTAGAAGAATAAGACAGGGCTTGGAAAGGGCTTTGCTA---TAA
>KC797171
GCT---ACA--
GAACAATTGTGGGTCACAGTTTATTATGGGGTACCTGTGTGG---AAAGAAGCAACA---
ACCACCCTATTTTGTGCATCAGAGGCTAAAGCATATAGTACAGAG---GTACATAAT----------------
--------------GTCTGGGCCACA---CATGCCTGTGTACCCACAGAC---
CCCGACCCACAAGAATTAGAATTGAAA---------AATGTGACAGAAAATTTTGAC----------------
-------------------------GCATGGAAAAATAACATGGTAGAACAGATGCATGAGGATATA---
ATCAGTTTATGGGATCAAAGCCTA---CAGCCATGTGTAAAATTA---
ACCCCCCTCTGTGTCACTCTAAATTGCACTAATGCCACTAGCACTGCTGAAAATGCCACCACCACC-------
----
-------------------ACCACAGCCAATAGCACTGTTGGAAAGCCAGATATGGAAATAAAA---
AACTGCTCTTTCAATATCACTGCAAGTAGAAGA---------------------------
GATAAGATACAGAGAGAATATGCACTT---TTTTATAAACTTGATCTAGTACCAATAGAGGAT----------
----

GGTAATAATAATACTAGTTACTATAGATTGATAAGTTGTAACACCTCAGTCCTTACACAG---
GCTTGTCCAAAAGTAACCTTTGAACCAATTCCCATACATTATTGTGCCCCGGCTGGTTTTGCAATTCTAAAAT
GTAAT---GATAAGAAGTTCAAT------GGAAAGGGACCATGTAAAAAT-----------------------
-GTCAGC---ACAGTACAATGTACACATGGAATTAGGCCAGTAGTGTCAACT---
CAGCTGCTGTTAAATGGCAGTCTAGCAGAAGAAGAA-----------
GTAGTAATTAGATCTGACAATTTCACAAATAAT---------GCTAAAACCATAATAGTACAGCTAAAG----
-----------AAATCTGTAATG---------ATTAATTGTACAAGACCCAACAACAATACACTAAAA-----
-AGTATACACATA------------GGACCAGGGAGAGCA---TTT------TATACAACAGGACAA------
---------ATAATAGGAGACATA------------AGACAAGCACATTGT---AACATTAGTAAAAAG---
GATTGGAACAACACTTTACAACAGGTAGTTACAAAATTAGGA---------------
GGACAATTTAGTAACGCA------------------------------------AAAAAAATAGCCTTTAAT-
-----CAATCCTCAGGAGGGGACCCAGAAATTGTA---ATGCATACTTTTAATTGTGCAGGGGAA---
TTTAAGCAGCTCCAGGCAAGAGTCCTGGCTGTGGAAAGATACCTACAGGATCAACAGCTCCTAGGGATCTGGG
GTTGCTCTGGAAAACGCATCTGCACCACTGCTGTGCCTTGGAATGCTAGTTGGAGT-----------------
----------------------------AATAAATCCTATGATCAGATTTGG------AAA------
AACATGACCTGGATGCAGTGG---GAAAAAGAAATTAACAATTACACAGACTTAATATAC---
ACCTTAATTGAAGACTCGCAGAGCCAGCAAGAC--

GATAATGAACAAAAATTACTAGAATTAAATGAGTGGGCAAGTTTGTGGAATTGGTTTAATATAGCAAAATGGC
TGAGGTATATAAAAATATTCATAATGATAGTAGGAGGCCTGATAGGTTTAAGAATAGTTTTTGCT---
GTACTTTCTATAGTA---------AATAGAGTTAGGCAGGGATATTCACCA------
TTGTCATTTGCTGTTAGCTTGTCCGACGCCACAGCGATAGCAGTAGCTGAG---
GGGACAGATAGGGTTATAGAATTAGTACAAAGAGCTTATAGAGCT----------------------------
--------ATTATCCACATA------CCTGCACGAATAAGACAGGGCGCAGAAAGGGCTTTGCTA---TAA
>JQ403025

```
GCT---GCA-----------------------------------------
GAACAAACGTGGGTCACAGTTTATTATGGGGTACCTGTGTGG---AAAGAAGCTAAC---
ACCACTCTATTTTGTGCATCAGATGCTAAAGCATATGATACAGAG---GCACATAAT---------------
--------------GTTTGGGCCACA---CATGCCTGTGTACCCACAGAC---
CCCAGTCCACAAGAAGTAACATTGGGA---------AATGTGACAGAAAATTATAAC---------------
--------------------------ATGTGGAAAAATAACATGGTAGAACAGATGCAAGAGGATGTA---
ATCAGTTTATGGGATCAAAGCCTA---AAGCCATGTGTAAAATTA---
ACCCCACTCTGTGTTACCTTAAATTGCACTGATGTTAAAAGTGGTAATAATACTACCAAT------------
------------------------------------------------------------------------
----------------------------AGAACCTGGGATAGGATAGAGGAAGGAGAAATAAAA---
AACTGCTCTTTCAATATCACCACAAGCATGAGA---------------------------
AAAAAGATGCAGAAAGAATATGCAACG---TTTTATAATCTTGATATAGTACCAATAGATGAT---------
------------------------------------------------------------------------
------------------
AGTGATAATACTAGCTATAGGTTGATAAGTTGTAATACCTCAGTCATTACACAG---
GCCTGTCCAAAGATATCCTTTGAGCCAATTCCCATACATTATTGTGCCCCGGCTGGTTTTGCGATTCTAAAGT
GTAAT---GATAAGAAGTTCAAT------GGAACAGGACCATGTAACAAT----------------------
-GTCAGC---ACAGTACAATGTACACATGGAATTAGGCCAGTAGTATCAACT---
CAACTGCTGTTAAATGGCAGTCTAGCAGAAGAAGAG------------
GTAGTAATTAGGTCAGAGAATATTACAAATAAT--------GCTAAAACCATAATAGTACAGCTGAAA----
-----------GAAGCTGTAGAA--------ATTAATTGTACAAGACCCAACAACAATACAAGAAAA-----
-AGTATAAATATG-----------GGACCAGGGAGAGCA---TTT------TATGCAACAGGAGAC------
---------ATAATAGGAGACATA------------AGACAAGCACATTGT---AACATTAGTTGGACA---
AAATGGAATAAAACTTTACAACAGGTAGTTGACAAATTAAGA--------------GAACACTTTAAA----
----------------------------------AATAAAACAATAGTCTTTAAT------
CACTCCTCAGGAGGGGACCCAGAAATTGTA---ATGCACAGTTTTAATTGTGGAGGGGAA---
TTTAAGCAGCTCCAGGCAAGAGTCCTGGCTGTGGAAAGATACCTAAAGGATCAACAGCTCCTGGGAATTTGGG
GTTGCTCTGGAAAACTCATTTGCACCACTGCTGTGCCTTGGAATACTAGTTGGAGT----------------
----------------------------AATAAATCTCAGAATGAGATTTGG------AAT------
AACATGACCTGGATGGAGTGG---GAAAGAGAAATTGACAATTACACAGGCTTAATATAC---
ACCTTGCTTGAAGAATCGCAGAACCAACAAGAA---------------------------------------
----------------------------
AAGAATGAACAAGAATTATTGGCATTAGATAAATGGGCAAGTTTGTGGAATTGGTTCAACATAACAAATTGGC
TGTGGTATATAAAAATATTCATAATGATAGTAGGAGGCTTAATAGGTTTAAGAATAGTTTTTACT---
GTACTTTCTATAGTG---------AATAGGGTTAGGAAGGGATACTCACCA------
TTATCGTTTGCTGTTAGCTTGTTTAATACCATAGCCATAGTAGTAGCTGAG---
GGAACAGATAGGGTTATAGAAGTAGTACAAAGAGCTGTTAGAGCT---------------------------
--------CTTCTCCACATA------CCTAGAAGAATAAGACAGGGCGCAGAAAGGGCTTTGCTA---TAA
>JQ403026
GCT---ACA-----------------------------------------
AACAATTTGTGGGTCACAGTCTATTATGGGGTACCTGTGTGG---AAAGAAGCAACC---
ACCACTCTATTTTGTGCATCAGATGCTAAAGGATATGACACAGAG---GTACATAAT---------------
--------------GTTTGGGCCACA---CATGCCTGTGTACCCACAGAC---
CCCAACCCACAAGAAGTAGTATTGGGA---------AATGTGACAGAAAATTTTAAC---------------
--------------------------ATGTGGAAAAATAACATGGTAGAACAGATGCATGAGGATATA---
ATTAGTTTATGGGATCAAAGTCTA---AAGCCATGTGTAAAATTA---
ACCCCACTCTGTGTTACTCTAAGTTGCACTGATTTTAATAATACTACTAACACCCCT---------------
------------------------------------------------------------------------
----------------------------ATTAAAGAGATGAAAATGGAGCCAGGAGAAATAAAA---
AATTGCTCTTTCAATATCACCTCAAATATGAGA---------------------------
GATAAAGTGCAAAAAGAATATGCACTT---
TTTTATAAACTTGATATAACACAAATAGAAGGTAATAGTAATAAA---------------------------
-------------------------------------------------------
GGTAATAGTAATAGTACTAGGTATAGGTTGATAAGTTGTAACACCTCAGTCATTACACAG---
GCCTGTCCAAAGGTAACCTTTGAGCCAATTCCTATACATTATTGTGCCCCGGCTGGTTTTGCGATTCTAAAGT
GTAAT---GATAAAAAGTTCAAT------GGATCAGGACCATGTACAAAT----------------------
-GTTAGC---ACAGTACAATGTACACATGGAATTAGGCCAGTAGTATCAACT---
CAACTGCTGTTAAATGGCAGTTTAGCAGAAGAAGAG------------
ATAATAATTAGATCCAGCAATTTCACGGACAAT--------ACTAAAACCATAATAGTACAGCTGAAT----
-----------GAAACTGTAGAA--------ATTAATTGTACAAGACCCAACAACAATACAAGAAAA-----
```

-AGTATACCTATA------------GGACCAGGCAGAGCA---TTT------TATACAACAGGAGAT------
---------ATAATAGGAGATATA------------AGACAAGCACATTGT---AACCTTAGTAGAACA---
AAATGGGAGAATGCTTTAAAAAAGATAGCTATAAAATTAGGA--------------GAACAATTTAAG----
-----------------------------------AATAAAACAATAGTCTTTAAT------
CAATCTTCAGGAGGGGACCCAGAAATTGTA---ATGCACAGCTTTAATTGTAGAGGGGAA---
TTTAAGCAGCTCCAGGCAAGAGTCCTGGCTGTGGAAAGATACCTAAAGGATCAACAGCTCCTGGGGATTTGGG
GTTGCTCTGGAAAACTCATTTGCACCACTGCTGTGCCTTGGAATGCTAGTTGGAGT----------------
----------------------------AATAAATCTCTGGATCAGATTTGG------CAG------
AACATGACCTGGATGGAGTGG---GAAAGAGAAATTGATAATTACACAGATTTAATATAC---
ACTTTAATTGAAAAATCGCAAAACCAACAAGAA--

AAGAATGAACAAGAATTATTGGAATTGGATAAATGGGCAAGTTTGTGGAATTGGTTTGACATAACACAATGGC
TGTGGTATATAAAAATATTCATAATGATAGTAGGAGGCTTGATAGGGTTAAGAATAGTTTTTACT---
GTATTTTCTATAGTA---------AATAGAGTTAGGCAGGGATATTCACCA------
TTGTCGTTCGCTGTTAGCTTGCTTAACGCCACCGCCATAGCAGTAGCTGAG---
GGAACTGATAGGGTTATAGAAGTATTACAAAGAGCTGGTAGAGCT---------------------------
--------ATCCTTCACATA------CCTACAAGAATAAGACAGGGCTTGGAAAGAGCTTTACTA---TAA
>EU023916
GCT---GCA---
GAACAATTGTGGGTCACAGTCTATTATGGGGTACCTGTGTGG---AAAGAAGCAGCC---
ACCACTCTATTTTGTGCATCAGATGCTAAAGCATATGTCCCAGAG---GTACATAAT---------------
--------------GTTTGGGCCACA---CATGCCTGTGTGCCCACAGAC---
CCTAACCCACAAGAAGTAGTATTGGGA---------AATGTGACAGAAAATTTTAAC---------------
-------------------------ATGTGGAAGAATAACATGGTAGAACAGATGCATGAGGATGTA---
ATCAGTTTATGGGATGAAAGCCTA---AAGCCATGTGTAAAATTA---
ACCCCACTCTGTGTTACTTTAAATTGCACTGATTGGAAGAATAATAATAATACTAATACC------------
----
-------------------------ACCAGTAGTAGCTTGGGAAGTATGGAGGGAGAAATAAAA---
AACTGCTCTTTCAATATCTCCACACGTATAAAA--------------------------
AATAAGATACAAAAAGAATATGCACTG---TTTAATAGACTTGATGTAGTATCAACAGACAAT---------
----

GATAATACAAGCTATATGTTGGTAAATTGTAACACCTCAGTCATTACACAG---
GCCTGTCCAAAGGTATCCTTTGAACCAATTCCCATACATTATTGTGCCCCGGCTGGTTTTGCGATTCTAAAGT
GTAAT---GATAAGAAGTTCAAT------GGAAAAGGACCATGTACAAAT----------------------
-GTTAGT---ACAATACAATGTACACATGGAATTAAGCCAGTAGTGTCAACC---
CAACTGCTGTTAAATGGCAGTCTAGCAGAAGGAGAG-----------
GTAATAATTAGATCTGAAAATTTCACAAACAAT---------GCTAAAACCATAATAGTACAGCTGAAT----
-----------GAAACTGTAGAA---------ATTAATTGTACAAGACCTAGCAACAATACAAGAAAA-----
-AGTATAACTATA------------GGACCGGGGAGAGCA---TTT------TATGCAACAGGAGAA------
---------ATAATAGGAGATATA------------AGAAAAGCACATTGT---AACATTAGTAGAGCA---
AAATGGGAAAACACTTTAAAAAAGATAGCTACAAAATTAACA--------------GAACAATTTAAT----
-----------------------------------AAGAGCACAATAATCTTTAAT------
CAATCCTCAGGAGGGGACCCAGAAATTGTA---ATGCACAGTTTTAATTGTGGAGGGGAA---
TTTAAGCAGCTCCAGGCAAGAGTCCTAGCTGTGGAAAGATACCTAAGGGATCAACAGCTCCTAGGAATTTGGG
GTTGCTCTGGAAAACTCATTTGCACCACTAATGTGCCTTGGAATAGTAGTTGGAGT----------------
----------------------------AATAAATCTCGGAATGAAATTTGG------AAT------
AACATGACCTGGATGGAGTGG---GAGAGAGAAATTGACAATTACACAAGCACTATATAC---
AACTTACTTGAAAAATCGCAGAACCAGCAAGAA--

AAGAATGAACAAGAATTATTGGAATTGGATAAGTGGGCAAGTTTGTGGAGTTGGTTTGACATAACACAATGGC
TGTGGTATATAAAAATATTCATAATGATAGTAGGAGGCTTAATAGGTTTAAGAATAGTTTTTACT---
GTACTTTCTATAGTG---------AATAGAGTTAGGCAGGGATACTCACCC------
TTATCATTCGCTATTAGTTTGCTTAATGCCACAGCTATAGCAGTAGCTGAG---
GGGACAGATAGGGTCATAGAAGTAGTACAAAGAACTGGTAGAGCT---------------------------
--------ATTCTTAACATA------CCTACAAGAATAAGACAGGGCTTTGAAAGAGCTTTGCTA---TAA
>JQ403019
GCT---ACA---
ACAGACTTGTGGGTCACAGTCTATTATGGGGTACCTGTGTGG---AAAGAAGCGACC---
ACCACTCTATTTTGTGCATCAGATGCTAAAGCATACAAAACAGAG---GTGCATAAT---------------

--------------GTCTGGGCCACA---CATGCCTGTGTACCCACAGAC---
CCCAACCCACAAGAAGTAATGTTGAAA---------AATGTGACAGAAAATTTTAAC---------------
-------------------------ATGTGGAAAAATAACATGGTAGAACAGATGCATGAGGATATA---
ATCAGTCTATGGGATCAAAGCCTA---AAGCCATGTGTAAAACTA---
ACCCCACTTTGTGTTACTTTAAATTGCACTGATGTTTACAGGAATGATACTAATAACAAT------------
----
-----------------------AGTACTAACTGGGGGAAGATAGAGAGAGGAGAAGTAAAA---
AACTGCTCTTTCAATGTCACCACAAATATAAAG--------------------------GGTAGGGTG---
CAAGAATATGCTGTC---TTTTATAAACTTGATGTGGTACCAATAAAGGAAAATAATGAT------------
----
----AGTAGTAGCTATAGTAGCTATATACTGACACATTGTAATACCTCAGTCGTTAAACAA---
GCCTGTCCAAAAGTAACCTTTGAGCCAATTCCAATACATTACTGTACCCCGGCTGGTTATGCGCTTCTAAAGT
GTAAT---GATAAGAAGTTCAAT------GGAACAGGACCATGTAAAAAT----------------------
-GTCAGC---ACTGTACAATGTACACATGGAATTAGGCCAGTAGTGTCCACC---
CAAATGCTGTTAAATGGCAGTCTAGCAGAAGAAGAG-----------
GTAGTAATTAGATCTGAAAATTTCACGGACAAT---------GCTAAGACCATAATAGTACAGCTGAAG----
-----------GATCCTGTAAAC---------ATTAGTTGTACAAGACCCAACAACAATACAAGAAAA-----
-AGTGTACGTATA------------GGACCAGGGCAAGCA---CTT------TATACAACA---GAC------
---------ATAATAGGAGATATA------------AGACAAGCACATTGT---AACATCAGTAGAAAA---
GACTGGAATAAGACTTTACAACAGATAGTTCCAAAGTTAAGA---------------AAACAATTT-------
----------------------------------AATAACACAATAGTCTTTCAG------
CCATCCTCAGGAGGAGACATAGAAATTGAA---ACTCACAGCTTTAATTGTGGAGGGGAA---
TTTAAGCAGCTCCAGGCAAGAGTCCTGGCTGTGGAAAGATACCTAAAGGATCAACAGCTCCTAGGGATTTGGG
GTTGCTCTGGAAAACTCATTTGCACCACTGCTGTGCCTTGGAACACTAGTTGGAGT----------------
----------------------------AATAAAAGTCTAAATCAGATTTGG------GAT------
AACATGACCTGGATGGAGTGG---GAAAGAGAGATTGACAACTACACAGGCTTAATATAC---
ACCTTAATTGAAGAGTCGCAGAACCAGCAAGAA---------------------------------------
----
AAGAATGAAAAGGAATTATTGGAATTGGACAAGTGGGCAAGTTTGTGGACTTGGTTTGATATAACAAATTGGC
TGTGGTATATAAAAATATTCATAATGATAGTAGCAGGCTTAGTAGGTTTAAGAATAATTTGTGCT---
GTACTTTCTATAGTG---------AATAGAGTTAGGCAGGGTTACTCACCA------
TTATCATTTGCTGTTAGTTTGTTTAATGCCACAGCAATAGCAGTAGCTGAG---
GGGACAGATAGGATTATAGAAGCAGTACGAAGAACTTTTTGGGCT---------------------------
--------TTTATCCACATA------CCTAGAAGAATAAGACAAGGCTTAGAAAGGGCTTTGCTA---TAA
>JQ403029
ACT---GCA--
GAACAATTGTGGGTCACAGTCTATTATGGGGTGCCTGTGTGG---AAAGATGCAACT---
GCCACTCTATTTTGTGCATCAGATGCTAAAGCATATGATAAAGAG---GTACATAAT---------------
--------------GTTTGGGCCACA---CATGCCTGTGTACCCACAGAC---
CCCAACCCACAAGAAATAGTATTGGAA---------AATGTGACAGAAAATTTTAAC---------------
-------------------------ATGTGGAAAAATAACATGGTAGAACAGATGCATGAAGATATA---
ATTAGTTTATGGGATCAAAGCCTA---AAGCCATGTGTAAAATTA---
ACCCCACTCTGTGTTACTTTAAATTGCACTGATGTGGGGAATGATACTGATACTAGGAATGCTAAT-------
----
-------------GTTACTAATGCCACTAATAGTTGGGGAATAATGGAAAAAGGAGAAATAAAA---
AACTGCTCTTTCAATATCACCACAAGTGTAAGA--------------------------
GATAAAGTGCAGAGAGAATATGCATTG---TTTTATAAACTTGATGTAATACCAATAGATAAT---------
----

GATAATAGGACATATAGGTTGATAAGTTGTAATACCTCAGTCATTACACAG---
GCATGTCCAAAGGTATCCTTTGAGCCAATTCCAATACATTATTGTGCCCCGGCTGGTTTTGGGATTCTAAAAT
GTAAC---GATAAAAGGTATAAT------GGAACAGGACCATGTAAAAAT----------------------
-GTCAGC---ACAGTACAATGTACACATGGAATTAGGCCAGTAGTGTCAACT---
CAGCTGCTGTTAAATGGCAGTCTAGCAGAAGAAGAA-----------
ATAGTAATTAGATCTGACAATATCACAGATAAT---------ACTAAAACCATAATAGTACAGCTGAAT----
-----------GACACTGTAGTA---------ATTAATTGTACAAGACCCAACAACAATACAAGAAAA-----
-AGTATACATATA------------GGACCAGGGAGAGCA---TTT------TATACAACAGGACAA------
---------ATAATAGGAGATATA------------AGACAAGCACATTGT---AACATTAGTGGAACA---
AAATGGAATAACACCTTAAAACAGATAGCTGACAAACTAAGA---------------GAACAATTT-------
----------------------------------AATAAAACAATAGTCTTTAAT------

CAATCATCAGGAGGGGACCCAGAGGTTGTA---ATGCACACTTTTAATTGTGGAGGGGAA---
TTTAAGCAGCTCCAGGCAAGAGTCCTGGCTGTGGAAAGATACCTACAGGATCAACAGCTCCTAGGGATTTGGG
GATGCTCTGGAAAACTCATCTGCACCACTACTGTGCCTTGGAATAATAGTTGGAGT-----------------
----------------------------AATAGAACTCTGAATAAGATTTGG------AAT------
AACATGACCTGGATGGAATGG---GAAAGAGAAATTGACAATTACACAAATGAAATATAT---
AACTTACTTGAAGCATCGCAGAACCAACAAGAA--

AAGAATGAACAAGAATTATTGGCATTAGATAAGTGGGCAAGTTTGTGGAATTGGTTTGACATATCAAATTGGC
TGTGGTATATAAAATTATTCATAATGATAGTAGGAGGCTTGATAGGTTTAAGGATAGTTTTTGCT---
GTACTTGCTATAGTA---------AATAGAGTTAGGCAGGGATATTCACCA------
TTGTCATTCGCTGTTAGCTTGCTCAACGCCACAGCTATCGCAGTAGCTGAG---
GGAACAGATAGGGTTATAGAAGTAATACAAAGAGCTTGTAGAGCT----------------------------
--------ATCCTCCACATA------CCTAGAAGAGTGAGACAGGGCTTGGAAAGGGCTTTGCTA---TAA
>JQ403044
GCT---GCA--
GACCAATGGTGGGTCACAGTATATTATGGGGTACCTGTGTGG---AAAGAAGCAACC---
ACCACTCTATTTTGTGCATCTGATGCTAAAGCATATGATACAGAG---GCACATAAT----------------
--------------GTTTGGCCACA---CATGCCTGTGTACCCACAGAC---
CCCAACCCACAAGAATTAGTATTGGAA---------AATGTAACAGAAGATTTTAAC----------------
--------------------------ATGTGGAAAAATAACATGGTAGAACAGATGCAGGAGGATATA---
ATCAGTTTATGGGATCAAAGCCTA---AAACCATGTGTGAAACTA---
ACCCCACTCTGTGTCACTTTAAATTGCATTAATTTAACTACTACTACCACCAGTACTCCTACTACCACC----
----
----------------AGTACTGGGAGTAGTGGTGCCACCCCTAGTAGTAGTGTGGAAATGAAA---
AACTGCTCTTTCAATGCCACCACAAGCAGAAGA---------------------------
GATAAGGAACGGAAAGAATATGCAATT---TTTGATAGGCTTGACATAGTACTAACAGGTGAA----------
----

ACTAATACTAGCTATATGTTGAGAAGTTGTAACACCTCAGTCATTACACAG---
GCCTGTCCAAAGGTATCCTTTGAACCAATTCCCATACATTATTGTGCCCCAGCTGGTTTTGCGATTCTAAAGT
GTAAT---GATAAGAAGTTCAAT------GGAACAGGATCATGTAAAAAT-----------------------
-GTCAGC---ACAGTACAATGTACACATGGAATTAGGCCAGTAGTGTCAACT---
CAACTACTGTTAAATGGCAGTCTAGCAGAAGAAGAT------------
GTAATAATTAGGTCTGAAAACTTCACAGACAAT---------GCTAAAACCATAATAGTACAGCTAAAT----
-----------GAAACTGTAGAA---------ATTAATTGTACAAGACCCAACAACAATACAAGAAAA-----
-AGCATAACTATA------------GGACCAGGGGCAGCA---TTT------TTTGCAACAGGAGGC------
---------ATAATAGGAGATATA------------AGAAAAGCATATTGT---AACATTAGTAGAGCA---
AAATGGAATAACACCTTAAGACAGATAGTTGGCAAATTAAGA---------------AAACAATTCGGG----
-----------------------------------AACAAAACAATAATCTTTACT------
AAACCCGCAGGAGGGGACCCAGAAGTTGTA---ATGCACACTTTTAATTGTGGAGGGGAA---
TTTAAGCAGCTCCAGGCAAGAGTCCTGGCTCTGGAAAGATACCTACAGGATCAACAGCTCCTAGGGATTTGGG
GTTGCTCTGGAAAACTCATTTGCACCACTGCTGTGCCTTGGAATACTAGTTGGAGT-----------------
-------------------------AGTAATAAATCTTTAAATCAGATTTGG------GAT------
AACATGACCTGGATGCAATGG---GAAAGAGAAATTGACAATTACACAGACTTAATATAC---
ATCTTACTTGAAAAATCGCAAAACCAACAAGAA--

AAGAATGAACAAGAATTATTGGAATTGGATAAATGGGCAAGTTTGTGGGATTGGTTTAGCATAACACACTGGC
TGTGGTATATAAAAATATTCATAATGGTAGTAGGAGGCCTGATAGGTTTAAGATTAGTTTTTACT---
GTATTTTCTATAGTG---------AATAGAGTTAGGCAGGGATATTCACCA------
TTATCGTTGGCTGTTAGCTTGCTTAATACCATAGCCATAGCAGTAGCTGAG---
GGAACAGATAGGATTATAGAAGTATTACAAAGAGCTTGTAGA-------------------------------
-----------------------------CG-CGAATAAAACAGGGCGCAAGAAGGGCTTTGCTA---TAA
>KX792621
GCT---GCA--
GAACAACGGTGGGTTACAGTCTATTATGGGGTACCTGTGTGG---AAAGAAGCAACT---
GCCACTCTATTTTGTGCATCAGATGCTAAGGCATATGATACAGAG---GTACATAAT----------------
--------------GTTTGGGCCACA---CATGCCTGTGTGCCCACCGAC---
CCCAACCCACTAGAGGTAGTATTGGAA---------AATGTGACAGAAAATTTTAAC----------------
--------------------------ATGTGGAAAAATAACATGGTAGACCAGATGCATGAGGATATA---
ATTAGTTTATGGATCAAAGCCTA---AAGCCATGTGTAAAATTA---

ACCCCACTCTGTGTTACTTTAAATTGCACTGATGCAAATGTTACAACTGAGAATGCTACTAATACCAGT----
----
----------------------AATAGTAGTGATGGACTGATGATGGAAAAAGGAGAAATAAAA---
AACTGCTCTTTCAATATCACCACAAACATAAGA---------------------------
GATAAGGTGGAGAAACAAAATGTACTT---TTTTATAAACTTGATGTAATACCAATAGATAATAGTAGAAAT-
----

AATAGTAATAGTAGTTATACCAACTATAGGTTGACAAGTTGTAACACCTCAGTCATTACACAG---
GCCTGTCCAAAGGTATCATTTGAGCCAATTCCCATACATTATTGTGCCCCAGCTGGTTTTGCGATTCTAAAGT
GTAAG---GATAAGGAGTTCAAT------GGAACAGGACCATGTAAAAT------------------------
-GTCAGC---ACAGTGCAATGTACACATGGAATTAGACCAGTAGTGTCAACT---
CAACTGCTGTTAAATGGCAGTCTAGCAGAAGAAGAG------------
GTAGTAATTAGATCTGAAAACTTCTCGAACAAT---------GCTAAAACCATAATAGTACAGCTGAAG----
-----------GTACCTGTAGAA---------ATTAATTGTACAAGACCCAATAACAATACAAGAAAA-----
-GGTATACATATA------------GGACCAGGGAGGGCA---TTT------TATGCAACAGGAAGC------
---------ATAACAGGAGATATA------------AGAAAAGCACATTGT---AATGTCAGCAGACTA---
AAATGGAATGACGCTTTGCGACAGGTAGCTAAAAAACTAAGA---------------AACCAATATAAC----
-----------------------------------AGTACAAAGATAGTCTTTAAT------
CACTCCTCAGGAGGAGACCCAGAAATTGTA---ATGCACAGTTTTAATTGTGGAGGGGAA---
TTTAAGCAGCTCCAGGCGAGAGTCCTGGCTGTGGAAAGATACCTAAAGGATCAACAGATCCTAGGGATTTGGG
GCTGCTCTGGAAAACTCATCTGCACCACTACTGTGCCTTGGAATAATAGTTGGAGT-----------------
----------------------------AATAAAACTCTGAATGAGATTTGG------GAT------
AACATGACCTGGATGCAGTGG---GAAAGAGAAATTAGCAATTACACAGAAACAATATAC---
GACTTAATTGAAAAATCGCAAAACCAACAAGAA--

AAGAATGAACAAGAATTATTGGCATTGGATAAATGGGCAAATTTGTGGAATTGGTTTGACATAACAAAATGGC
TGTGGTACATAAAAATATTCATAATGATAGTAGGAGGCTTGATAGGGTTAAGAATAGTTTTTGCT---
GTGCTTTCTATAGTG---------AAAAGAGTTAGGCAGGGATACTCACCA------
TTATCGTTTGCTGTTAATTTGCTTAATACCATAGCTATAGCAGTAGCTGAG---
GGAACAGATAGGATTATAGAAGTAGTACAGAGAACTTGTAGAGCT----------------------------
--------ATTCTCCACATA------CCTAGAAGAATAAGACAGGGCTTGGAAAGGGCTTTGCTA---TAA
>JQ403043
GCT---ACA--
GAAAAATTGTGGGTCACAGTCTATTATGGGGTACCTGTGTGG---AGAGAAGCAACC---
ACCACTCTATTTTGTGCATCAGATGCTAAAGCTTATGATACAGAG---GTGCATAAT----------------
--------------GTCTGGCCACA---CATGCCTGTGTACCCACAGAC---
CCCAACCCACAAGAAATAAAATTGGAA---------ATGGTGACAGAAAATTTTAAC----------------
--------------------------ATGTGGAAAAATAACATGGTAGAACAGATGCATGAGGATATA---
ATCAGTTTATGGGATCAAAGCCTA---AAGCCATGTGTAAAATTG---
ACCCCACTCTGTGTCACTTTAAATTGCACTAATTATAATGGTACTAATACTACTAGTACTAGT----------
----
-------------------------AATAGTACTGATGGTAGATGGGAAAAAGAAGAAATAAAA---
AACTGCTCTTTCAATATCACCGCAGGCATAAGA---------------------------
GATAAGGTGCAGAAAGAACATGCACTT---TTTTATAGTCTTGATATAGTACCAATAGAGAATAATAAGAAT-
----

AATAACAGCAATAGCAACTATAGGTTGAGAAGTTGTAACACCTCAATCATTACACAA---
GCCTGTCCAAAGGTAACCTTTGAACCAATTCCCATACATTATTGTGCCCCGGCTGGTTTTGCGATTCTTAAGT
GTAAC---AATAAAACGTTCAAT------GGAACAGGAACATGTAACAAT------------------------
-GTTAGC---ACAGTACAATGTACACATGGAATTAAGCCAGTAGTATCAACT---
CAACTGCTGCTAAATGGCAGTCTAGCAGAAGAAGAA------------
ATAGTAATTAGGTCTGAAAATATCACAAACAAT---------GCTAAAACCATAATAGTACATCTAAAT----
-----------GAATCTGTAGAA---------ATTAATTGTACAAGACCCAACAACAATACAAGGAAA-----
-AGTATACACATA------------GGACTAGGAAGAACA---TTT------TATGCAACAGGAGAC------
---------ATAATAGGAGATATA------------AGACAAGCACATTGT---AACCTTAGTTTAGCA---
AAATGGAATACCACCTTGAAAAGGGTAGTTGACAAATTAAGA---------------
GAACAATTTAGAGAACAATTT------GAG---------------------AATAAAACAATAGTCTTTAAT-
-----CAATCCTCAGGAGGGGACCCAGAAATTATA---CTGCACACTTTTAATTGTGGAGGGGAA---
TTTAAGCAGCTCCAGGCAAGAGTCCTGGCTGTGGAAAGATACCTAAGGATCAACAGCTCCTAGGATTTGGG
GATGCTCTGGAAAACTCATTTGCACCACTAATGTGCCTTGGAATGCTAGTTGGAGT-----------------

----------------------------AATAAATCTCAGAAGGAAATTTGG------GAG------
AACATGACCTGGATGCAGTGG---GAAAGAGAAATTGACAATTACACAGGCATAATATAC---
AACTTACTTGAAGCATCGCAAAACCAACAAGAA--

AAAAATGAACAAGAATTATTGGCATTAGATAAATGGGCAAGTTTGTGGTCTTGGTTTGACATAACAAATTGGC
TGTGGTATATAAAAATATTCATAATGATAGTAGGAGGCTTGATAGGTTTAAGACTAATTTTTACT---
GTACTTTCTATAGTG---------AATAGAGTTAGGCAGGGATACTCACCA------
TTATCGTTTGCTGTTAGCTTGCTTAATGTCACAGCCATCGCAGTAGCTGAG---
GGAACAGATAGGATTATAGAATTATTGCAAAGAGCTGGTAGAGCT----------------------------
--------ATCCTTAACATA------CCTAGAAGAATAAGACAGGGCGCGGAAAGGGCTTTGGTA---TAA
>JQ403045
GCT---GAA--
GAACAATTGTGGGTCACAGTCTATTATGGGGTACCTGTGTGG---AAAGAAGCAGAT---
ACCACTCTATTTTGTGCATCAGATGCTAAAGCATATGACACAGAG---AAACATAAT----------------
--------------GTTTGGCCACA---CATGCCTGTGTACCCACAGAC---
CCTAACCCACAAGAAATAAGATTGGAA---------AATGTGACAGAAAATTTTAAT----------------
--------------------------ATGTGGAAAAATAACATGGTAGAACAGATGCAGGAAGATATA---
ATCAGTTTATGGGATCAAAGCCTA---AAGCCATGTGTGGAATTA---
ACCCCACTCTGTGTTACTTTAAACTGCACTGATTTGAATACTACTAGAGCCAACAGTACTAGCTCC-------
----
----------------------AATAGTAGTAGTTTTGGATACATGGAGAAAGGAGAAATAAAA---
AACTGCTCTTTCAATGTCACCACAAACATAAGA---------------------------
GATAAGGTGCAGAAAACCTATGCATTT---TTTTATGACCTTGATGTAATACAAATAGATAAT----------
----

AGTAATACTACTTATAGGTTGATAAGTTGTAACACCTCAGTCATTAAACAG---
GCTTGTCCAAAGGTATCCTTTGAGCCAATTCCCATACATTTTTGTGCCCCGGCTGGTTTTGCGATTCTGAAGT
GTAAG---GATAAAAGGTTCAAT------GGAACAGGACCATGTAAAAAT-----------------------
-GTCAGT---ACAGTACAATGTACACATGGAATTAGGCCAGTAGTATCAACT---
CAACTGCTGTTAAATGGCAGTCTAGCAGAAGAAGAG------------
ATAGTAATTAGATCTGAAAATCTTACGGACAAC---------GCTAAAACCATAATAGTGCAGTTGAAT----
-----------GAATCTGTACAA---------ATTAATTGTGCAAGACCCAACAACAATACAAGAAAA-----
-GGTATCCATATA------------GGACCAGGGAGAGCA---TTT------TATACAACAGGAGAA------
---------ATAATAGGAGATATA------------AGGCAAGCACATTGT---AACATTAGTAGAGCA---
AAATGGAACAACACTTTAAGACAGGTAGCTAGAAAATTAAGA---------------GAACAATTTGGG----
-----------------------------------AATGCAACAATAGTTTTTAAT------
CGCTCCTCAGGAGGGGACCCAGAAATTGTA---ATGCACAGTTTTAATTGTGGAGGGGAG---
TTTAAACAGCTCCAGGCAAGAGTCCTGGCTGTGGAAAGATACCTAAAGGATCAACAGCTCCTGGGAATTTGGG
GTTGCTCTGGAAAACTCATTTGCACCACTACTGTACCTTGGAATATTAGTTGGAGT-----------------
----------------------------AATAAATCTCTAAGTGACATTTGG------GAT------
AACATGACCTGGATGGAGTGG---GAAAAAGAAATTGACAATTACACAGGCTTAATATAC---
TCCCTGATTGAAGAATCGCAGAACCAGCAAGAA--

AAGAATGAACTAGAGTTATTGCAATTGGACAAATGGGCAAGTTTGTGGAATTGGTTTGACATATCAAACTGGC
TGTGGTATATAAAAATATTCATAATGATAGTAGGAGGCTTGGTAGGTTTAAGAATAGTTTTTACT---
GTACTTTCTATAGTG---------AATAGAGTTAGGCAGGGATACTCACCA------
TTATCATTGGCTGTTAGCTTGTTCAACGCCATAGCCATAGCAGTAGCTGAG---
GGAACAGATAGGGTTATAGAAGCATTCCAAAGAATTGGTAGAGCC----------------------------
--------CTTCTCCACATA------CCTAGACGCATAAGACAGGGCCTTGAAAGGGCTTTGCTA---TAA
>JQ403046
GCT---GGA---AAA------------------------------------
GACAAATTGTGGGTCACAGTCTATTATGGGGTACCTGTGTGG---AAAGAAGCAACC---
ACCACTCTATTTTGTGCATCAGATGCTAAAGCATATAGCACAGAG---ATGCATAAT----------------
--------------GTTTGGCCACA---CATGCCTGTGTACCCACAAAC---
CCTAACCCACAAGAAGTAGTATTGGAA---------AATGTGACAGAAAATTTTAAC----------------
--------------------------ATGTGGAAAAATAACATGGTAGACCAGATGCATGAGGATATA---
ATCAGTTTATGGGATGAAAGCCTA---AAGCCATGTGTAAAATTA---
ACCCCACTCTGTGTTACTTTACATTGCACTAATGTTACAACTACTGCTACTAGAACTACTAATAGTACAAATA
CTACTGCTAGT--
----------------------------AGTCCTGTCAATAACGAGATGGTGGGAGAAATGAAG---

AACTGCTCTTTCAATATCACCACAGATGTAGGA--------------------------
AAAAAGGCGCGGACACAATATGCAATT---TTTAATACACTTGATGTAGTACCAATAGATAAAGAT-------
----

AATAGTACAAGCTATATGTTGGTAAGTTGTAACACCTCAGCCATTACACAG---
ACCTGTCCAAAGGTATCCTTTGAACCAATTCCCATACATTATTGTACTCCGGCTGGTTTTGCGCTTCTACAGT
GTAAT---GATAAGGAGTTCAAT------GGAACAGGACCATGTAAAAAT----------------------
-GTCAGC---ACAGTACAGTGTACACATGGAATTAGGCCAGTAGTGTCAACT---
CAACTACTGTTAAATGGCAGTTTAGCAGAAGGAGAG-----------
ATAATAATTAGATCTGAAAATATTTCAAACAAT---------GCTAAAACTATAATAGTACAGCTAAAT----
-----------GAAACTGTAGTA---------ATTAATTGTACAAGACCCAACAACAATACAAGAAGA-----
-AGTATACAGATA------------GGACCAGGGAGATCA---TTT------TTTGCAACAGGAGAC------
---------ATAATAGGAGATATA------------AGAAGAGCACATTGT---ACCATTAGGGGACAA---
GAATGGAACAACACTTTAAAGCAGATAGCTATAAAACTAAGA---------------GAACAATTTGTC----
-----------------------------------AATAAAACAATAATCTTTAAT------
CACTCCTCAGGAGGGGACCCAGAAATTGTA---AGGTTCAGTTTTAATTGTGGAGGGGAA---
TTTAAACAGCTCCAGGCAAGAGTCCTGGCTGTGGAAAGATACCTAAGAGATCAACAGCTCCTAGGAATTTGGG
GTTGCTCTGGAAAACTCATTTGCACCACTGCTGTGCCTTGGAATACTAGTTGGAGT-----------------
----------------------------AATAAATCTTTAGATGACATTTGG------GAG------
AACATGACCTGGATGCAGTGG---GAAAGAGAAATTGACAATTACACAGACTTAATATAC---
AACTTACTTGAAAAATCGCAAAACCAGCAAGAA---------------------------------------
----
AAGAATGAACAAGAATTATTGGAATTGGATAAGTGGGCAAGTTTGTGGAGTTGGTTTGACATAACACAATGGC
TGTGGTACATAAAAATATTCATAATGATAGTAGGAGGCTTAATAGGTTTAAGAATAGTTTTTACT---
GTGCTTTCTATAATA---------AATAGAGTTAGGCAGGGTTACTCACCA------
TTATCATTTGCTGTTAGTTTGTTTAATGCCACAGCTGTAGCAGTAGCTGAG---
GGGACAGATAGGCTAATAGAAGCAGTACAAAGACTTGGCATAGCT---------------------------
--------TTTCTTAACATA------CCTAGAAGAATAAGACAGGGCCTTGAAAGAGCTTTAATA---TAA
>EU023919
GCT---GCA---AAA------------------------------------
GAAAACTTGTGGGTCACAGTCTATTATGGGGTACCTGTGTGG---AAAGAAGCAACC---
ACCACCCTATTTTGTGCATCAGATGCTAAAGCATATGATACAGAG---GTACATAAT----------------
--------------GTTTGGGCAACA---CATGCCTGTGTACCCACAGAC---
CCCAACCCACAAGAAATAGAATTAATA---------AATGTGACAGAAAATTTTAAC---------------
--------------------------ATGTGGAAAAATGACATGGCAGACCAGATGCATGAGGATATA---
ATCAGTTTATGGGATCAAAGCCTA---AAGCCATGTGTAAAATTA---
ACCCCACTCTGTGTTACTTTAGATTGCACTAATATCAGTACGAATGGTACTAAT-------------------
----
----------------------------------AGCACTTTGGAAGCAGTAAAAGAAATGACA---
AACTGCTCTTTCAATCTCACCACAAGCAGAAGA-------------------------
GAGAAGATGCAGAAAGAATATGCGCTT---TTTTATAAACTTGATGTAGTACCAATAGATAAG---------
----

AATAAGACCAGATATAGGTTAATAAGTTGTAACACCTCAGTCATTACACAA---
GCCTGTCCAAAGGTATCCTTTGAGCCAATTCCCATACATTATTGTGCCCCGGCTGGTTTTGTGATTCTAAAGT
GTAAT---AATAAAACATTCAAT------GGACTAGGACCATGTAACAAT----------------------
-GTCAGC---ACAGTACAATGTACACATGGAATTAAGCCAGTAGTGTCAACT---
CAACTCCTGCTAAATGGCAGTCTAGCAGAAAAAGAG-----------
GTAGTAATTAGATCTGCAAATTTCACGGACAAT---------GCTAAAATCATAATAGTACAGCTGAAT----
-----------GTATCTGTAGGC---------ATTAATTGTACAAGACTCAACAACAATACAAGAAGA-----
-AGTATAAATATA------------GGACCAGGGAGAGCA---TGG------TATACAACAGGAGAA------
---------GTAATAGGAGATATA------------AGAAAAGCAAACTGT---ACCCTGAGTAGGAAA---
AGTTGGAATGACACTTTACAAAAGGTAGTTGAAAAATTAAGA---------------GAACAATTTGGA----
--------------------------------AAGAATAAAACAATAATCTTTAAG------
CAATCCTCAGGAGGGGACCTAGAAATTGTA---AGTCACAGTTTTAATTGTGGAGGGGAA---
TTTAAGCAGATCCAGGCAAGAGTCCTGGCTGTGGAAAGATACCTAAGAGATCAACAGCTCCTAGGGCTTTGGG
GCTGCTCTGGAAGACTCATCTGCACCACTACTGTGCCTTGGAATACTAGTTGGAGT-----------------
----------------------------AATAAAAATCTGAGTCAGATTTGG------GAT------
AACCTGACCTGGATGCAGTGG---GAAAAAGAAATTGACAATTACACAGATTTAATATAC---
ACTTTAATTACAAAATCACAAATCCAACAAGAA---------------------------------------

----
CAGAATGAAAAAGAATTATTGGAATTGGATAAGTGGGCAAGTCTGTGGAATTGGTTTGATATAACAAAATGGC
TGTGGTATATAAAAATATTCATAATGATAGTAGGAGGCTTGATAGGCTTAAGAATAGTTTTTACT---
GTACTTTCTATAGTA---------AATAGAGTTAGGCAGGGATATTCACCA------
TTATCATTTGCTGTTAGTCTATTTAATGCCACAGCTATAACAGTAGCTGAG---
GGAACAGATAGGGTTATAGAAGTAGTACAAAGAGCTTATAGAGCT----------------------------
--------TTTCTCCACATC------CCTAGACGAATAAGACAGGGCTTTGAAAGGGCTTTACTA---TAA
>EU023922
GCT---GCA--
GGACAATTGTGGGTCACAGTCTATTATGGGGTACCTGTGTGG---AAAGAAGCAACC---
ACCACTCTATTTTGTGCATCAGATGCTAAAGCATATGATACAGAG---GTACATAAT----------------
--------------GTTTGGGCCACA---CATGCCTGTGTACCCACAGAT---
CCCGACCCACAAGAAGTACCATTGGAA---------AATGTGACAGAAAAGTTTAAC----------------
--------------------------GCGTGGAAAAATAACATGGTAGAACAAATGCATGAGGATATA---
ATCAGTTTATGGGATCAAAGCCTA---AAGCCATGTGTAAAATTA---
ACCCCACTCTGTGTTACTTTAAATTGCACTGATGTGAAAAATAATAGTAGTAGTAATGATAGTAGT-------
----
-------------------AAAAGTAATACTAATAATGGTAACTGGGAACAAACGGAGATGAAA---
AACTGCTCTTTCAATGTCACCACAAGCATAAGA---------------------------
GATAAGATACAGAAAGAATATGCAATT---TTTTATAATCTTGATATAGTACCAATAGATAAG----------
----

GATAATACCAGCTATAGGTTGATGAGTTGTAACACCTCAGTCATTACTCAG---
GCCTGTCCAAAGGTATCCTTTGAGCCAATTCCCATACATTATTGTGCCCCGGCTGGTTTTGCGATTTTAAAGT
GTAAA---GATAACAAGTTCAAT------GGAACAGGACTATGTAAAAAT-----------------------
-GTCAGC---ACAGTGCAATGCACACATGGAATTAGGCCAGTAGTATCAACT---
CAACTGTTGTTAAATGGCAGTCTAGCAAAAGAAGAG------------
GTAGTAATTAGATCTGAAAATTTTACAAACAAT---------GCTAAAACCATAATAGTACAGCTGAAT----
-----------GAAACTGTAGAA---------ATTAACTGTACAAGACCCAACAACAATACAAGAAGA-----
-AGTATACCTATA------------GGACCAGGGAGAGCA---TTT------TATGCAACA---GAC------
---------ATAATAGGAGATATA------------AGACAAGCACATTGT---AACATTAGTAGAGCA---
AAGTGGAATAACACTTTAAAACAGATAGTTGACAAATTAAGA---------------AAACAATTTGGG----
--------------------------------AAGAATAAAACAATAGTCTTTAAG------
AAATCCTCAGGAGGGGACCCAGAAATTGTA---ATGCACAGTTTTAATTGTGGAGGGGAA---
TTTAAGCAGCTCCAGGCAAGAGTCCTGGCTGTGGAAAGATACCTAAAGGATCAACAGCTCCTAGGGATTTGGG
GTTGCTCTGGAAAACTCATTTGCACCACTGCTGTGCCTTGGAATGCTAGTTGGAGT-----------------
----------------------------AATAAATCTCTGAATGAGATTTGG------GAT------
AACATGACCTGGATGCAGTGG---GAAAGAGAAATTAATAATTACACAGGCTTAATATAC---
AACTTACTTGAAGAATCGCAGAACCAGCAAGAA--

AAGAATGAACAAGACTTATTGGAATTAGATAAATGGGCAAATTTGTGGAATTGGTTTAACATAACAAACTGGC
TGTGGTATATAAAAATATTCATAATGATAGTAGGAGGCTTGGTAGGATTAAGAATAGTTTTTACT---
GTACTTTCTATAGTA---------AATAGAGTTAGGCAGGGATACTCACCA------
CTATCGTTTGCTGTTAGCTTGCTCAACGCCACAGCCATAGCAGTAGCTGAG---
GGGACAGATAGGGTCATAGAAGTATTACAAAGAGCTTGTAGGGCT----------------------------
--------ATTCTCCACATA------CCTAGAAGAATAAGACAGGGCGCAGAAAGGGCTTTGCTA---TAA
>KX792557
GCT---GCA--
GAGCAATTGTGGGTCACAGTCTATTATGGGGTACCTGTGTGG---AAAGAAGCAAAC---
ACCACTCTATTTTGTGCCTCAGATGCTAAAGCATATAGCAAGGAG---GCACATAAT----------------
--------------GTATGGGCCACA---CATGCTTGTGTACCCACAGAC---
CCTAGCCCACAAGAAGTAGTGTTGGCA---------AATGTGACAGAATATTTTAAC----------------
--------------------------ATGTGGAAAAATAACATGGTAGAACAAATGCATGAGGATATA---
ATTAGTCTATGGGATCAAAGTTTA---AAGCCATGTGTGAAATTA---
ACCCCGCTCTGCGTTACCTTAAATTGCACTGATGTGGTGACGAATGTTAATGGGACCAATACTACT-------
----
-------------------------------------TTAGATATGCAGAAAGAAGAAATAAAA---
AATTGCTCTTTTAATGTC---ACAAATATAACA---------------------------
AATAAAAGGCAGAAGGAATATGCAATT---TTTTATAAACTTGATGTAATGCCAATAGCTGGT----------
----

GATAATGCAAGTTATACACTGATAAATTGTAACACCTCAGTCATTACACAA---
GCCTGTCCAAAGGTATCCTTTGAACCCATTCCCATACATTATTGTGCCCCGGCTGGTTTTGCGATTCTACAGT
GTAAT---AATAAGAAATTCAAT------GGATCAGGACCATGTACAAAT-----------------------
-GTCAGT---ACAGTACAATGTACACATGGAATTAGGCCAGTAGTATCCACT---
CAACTGCTATTAAATGGCAGTCTAGCAGAAGAGGAG------------
GTAATGATTAGGTCTAAAAATTTCTCAGAAAAT---------GCTAACATCATAATAGTACAGCTGAAT----
-----------AAAACTGTAGAA---------ATTAATTGTACAAGACCCAATAACAATACAAGAAGA-----
-AGTATACATATA------------GCACCAGGGAGAGCA---TTC------TATGCAACAGGTGAG------
---------ATAATAGGAAATATA------------AAAAAAGCACACTGT---AACATTAGTGAAACA---
AAATGGAATAACACTTTAAAAAAGATAGTTGCAAAGTTAAGA---------------GAACAATATGAG----
-----------------------------------AATAAAACAATGGTCTTTAAT------
CAATCCTCAGGAGGGGACCCAGAAATTGTA---ATGCACAGTTTTAGTTGTGGAGGGGAA---
TTTAAGCAGCTCCAGGCAAGAGTCTTGGCTGTGGAAAGATACCTAAGGGATCAACAGCTCCTAGGAATTTGGG
GTTGCTCTGGAAAACTCATCTGCACCACTGCTGTGCCTTGGAATGCTAGTTGGAGT-----------------
----------------------------AACAAATCTCTGGATAATATTTGG------CAG------
AACATGACCTGGATGGAGTGG---GAAAGAGAAATTGAAAATTATACAGACTTAATATAT---
AACTTACTTGAAACATCGCAAAACCAGCAAGAA--

AAGAATGAACAAGACTTATTGGAATTGGATAAATGGGCAAATTTGTGGAATTGGTTTGACATATCAAATTGGC
TGTGGTATATAAAAATATTCATAATGATAGTAGGGGGTTTAATAGGTTTAAGAATAGTTTTTACT---
GTGCTTTCTATAGTG---------AATAGAGTTAGGCAGGGATACTCACCA------
TTGTCATTCGCTGTTAGCCTGTTTAATGCCATAGCTATAGCAGTAGCTGAG---
GGGACAGATAGGATTATAAAATTAGTACAAAGAATCTGTAGAGGT----------------------------
--------ATCCTCCATATC------CCTAGAAGAATAAGACAGGGCTTCGAAAGGCTTTGCTA---TAA
>KX792831
GCT---GCA--
GACCAATTGTGGGTCACAGTCTATTATGGGGTACCTGTGTGG---AAAGAAGCAACC---
ACCACTTTATTTTGTGCATCAGATGCTAAAGCATATGATACAGAA---GCACATAAT----------------
--------------GTTTGGGCCACA---CATGCCTGTGTGCCCACAGAC---
CCCAGCCCACAAGAAGTATTCTTGGTA---------AATGTGACAGAAAATTTTAAT----------------
--------------------------ATGTGGAAAAATGATATGGTAGAACAAATGCATGAGGATGTA---
ATTAGCTTATGGGATCAAAGCCTA---AAGCCATGTGTAAAATTA---
ACCCCTCTTTGTGTTACTCTAAATTGCAATGATACTCTGTGGACTAATGTAACTGCTAAT-------------
----
-------------------------------------CAAAGTCAAAGTACGGATGAAATGAAA---
ACCTGCTCTTTCAATATCTCCACATCTGTAAAT---------------------------
GATAAGTTTCAGAAACAATATGCAACC---TTTTATAAACTTGATGTAGAACCAATAGGTAAG----------
----

GATAATACTAGCTATAGGTTGATAAGTTGTAACACCTCAGTCATTACACAG---
GCCTGTCCAAAGATATCCTTTGAGCCAATTCCCATACATTATTGTGCCCCGGCTGGTTTTGCAATTCTAAAGT
GTAAT---GATAAGAAGTTCAAT------GGAACAGGACCATGTAGAAAT-----------------------
-GTCAGC---ACAGTACAATGTACACATGGAATTAAGCCAGTAGTATCAACT---
CAACTGCTGTTAAATGGCAGTTTAGCAGAAGAAGAC------------
ATAGTACTAAAATCTGAGAATATCACAGACAAT---------GTTAAAAACATAATAGTACAGCTGAAC----
-----------AAATCTGTAGAG---------ATTGTATGTATAAGACCCAATAATAATACAAGAAGA-----
-AGTGTGCCTATA------------GGACCAGGGAGGGCA---ATT------TATGCAACAGGACAA------
---------ATAATAGGAGATATA------------AGACAAGCACATTGT---AACATTAGTAGAACA---
GATTGGAATAAAGCTTTGATAAAGGTAGCTATAAAACTAAGA---------------GAACAACTTGGG----
-----------------------------AATGAAACAATAACAATAGTCTTTCAA------
AATTCCTCAGGAGGGGACCCAGAAATTACA---ATGTACAGTTTTAATTGTGGAGGAGAA---
TTTAAGCAGCTCCAGGCAAGAGTCCTGGCTGTGGAAAGATACCTACAGGATCAACAGCTCCTGGGGATTTGGG
GCTGCTCTGGAAAGCTTATTTGCACCACTGCGGTGCCTTGGAATGCTAGTTGGAGT-----------------
----------------------------AATAGATCTCTGAATGAAATTTGG------GAT------
AACATGACCTGGATGCAGTGG---GAAAAAGAAATTGATAATTACACAGGCTTAATATAC---
AACTTACTTGAAAGTTCGCAAAACCAGCAAGAA--

AAGAATGAACAAGAATTATTGGAATTGGACAAATGGGCAAGTTTGTGGAGTTGGTTTAGCATATCAAACTGGC
TGTGGTATATAAGAATATTCATAATGATAGTAGGAGGCTTGATAGGTTTAAGAATAGTTTTTACT---

GTACTTTCTATAGTG---------AATAGAGTTAGGCAGGGCTACTCACCA------
TTATCGTTTGCTATTAGCTTGTTTAATGCCATAGCCATCGCAGTAGCTGAG---
GGGACAGATAGAATTGTAGAAGCCATACAAGGAGCTTTTAGAGCT----------------------------
--------ATTCTCCACATA------CCTAGAAGAATAAGACAGGGCTTGGAAAGGGCTTTACTA---TAA
>JQ403042
GCT---ACA--
GAAAATTTATGGGTCACAGTCTATTATGGGGTACCTGTGTGG---AAAGAAGCAAAC---
ACCACTTTATTTTGTGCTTCAGATGCCAAAGCATATCAATCAGAG---GCACATAAT----------------
--------------GTTTGGGCCACA---CATGCCTGTGTACCCACAGAC---
CCTAACCCACAAGAAGTAAGATTGGAA---------AATGTGACAGAAGATTTTAAC----------------
--------------------------ATGTGGAAAAATAACATGGTAGAACAAATGCATGAGGATATA---
ATCAGTTTATGGGATCAAAGCCTA---AAGCCATGTGTAAAATTA---
ACCCCACTCTGTGTTACTTTAAATTGTACTGATTTGAAAAATACTACCAATACCAATAGTAGTGCT-------
----
----------------------GCTAATACCAATACTAGTAGTTGGAGGGAGATAGAAATGAAA---
AACTGCTCTTTCAATGTCACCACAAGCATAAGA---------------------------
GACAAATGGCATAAAGAATATGCACTT---TTTTATAAGCTTGATGTAATGCCAATAGATAAT----------
----

GATAATGCAAGCTATACTTTGATAAATTGTAACACCTCAGTCATTACACAG---
GCCTGTCCAAAGGTATCCTTTGAACCAATTCCTATACATTATTGTGCCCCGGCTGGTTTTGCGATTCTAAAGT
GTAAT---GATAATAAGTTCAAT------GGAACAGGACCATGTAAAAAT-----------------------
-GTCAGC---ACAGTACAATGTACACATGGAATTAGGCCAGTAGTGTCCACT---
CAACTGCTGTTAAATGGCAGTGTGACAGAAGGAGAC---------
GAGGTAGTAATCAGATCTGAAAATCTCACAGACAAT---------ACTAAAACCATAATAGTACAGCTGAAG-
--------------GAACCTATAAAA---------ATTAACTGTACAAGACCCAACAACAATACAAGAAAA--
----AGTATACATATA------------GGACCAGGGAGAGCA---TTC------TATGCAACAGGAGAC---
------------ATAATAGGAGACATA------------AGACAAGCACATTGT---AACCTTAGTAGGGAA-
--CAATGGAATAACACTTTAAAACAAATAGTAAACAAATTAAGA---------------GAAAATTTTGGG--
-------------------------------------AATACAACAATAGTCTTTAAT------
CAGTCCTCAGGAGGGGACCCAGAAATTGTA---ATGCACAGTTTTAATTGTGGAGGGGAA---
TTTAAACAGCTCCAGGCTAGAGTCCTGGCTGTGGAAGCATACCTAAAGGATCAACAGCTCCTAGGGATCTGGG
GTTGCTCTGGAAAACTCATTTGCACCACTGCTGTGCCTTGGAATACTAGTTGGAGT-----------------
----------------------------AATAAATCTCTGGACAGAATTTGG------AAT------
AACATGACATGGATGGAGTGG---GACAGAGAGATTAACAATTACACAAGCTTAATATAC---
TCCTTAATTGAAGAATCGCAGAACCAACAGGAA--

AAGAATGAACAAGATTTATTGGAATTGGATAAGTGGGCAAGTTTGTGGAATTGGTTTAGCATAACAAATTGGC
TGTGGTATATAAAAATATTCATAATGATAGTAGGAGGTTTAGTAGGTTTAAGAATAGTTTTTACC---
ATACTTTCTATAGTG---------AATAGAGTTAGGCAGGGATACTCACCA------
TTGTCATTAGCTGTTAGTTTGCTTAATGCCACAGCTATAGCAGTAGCTGAG---
GGGACAGATAGGATTATAGAAACAATACAAAGAATTGGTAGAGCT----------------------------
--------ATTATCCACATA------CCTAGAAGAATCAGACAGGGCTTAGAAAGGGCTTTGCTA---TAA
>KX792664
GCT---ACA--
AAACAATTGTGGGTCACAGTCTATTATGGGGTACCTGTGTGG---AAAGACGCCACC---
ACCACTCTATTCTGTGCATCAGATGCTAAAGCACATGATCCCGAG---GTACATAAT----------------
--------------GTTTGGGCCACA---CATGCCTGTGTACCCACAGAC---
CCCAACCCACAAGAAGTATTAATGGAA---------AATGTGACAGAACAATTTGAC----------------
--------------------------ATGTGGCACAATAATATGGTAGATCAAATGCATGAGGATATA---
ATCAGTTTATGGGACCAAAGCCTA---AAGCCATGTGTAAAACTA---
ACCCCACTCTGTGTTACTTTAAATTGTACTATGGTAAAGGTAAATGCTAATGCTCCTAATACCACTACTAGTA
CTCCTAGTACC--
----------------ACTACTAGTGCTCCTAATACCACTGGTAGTGATGAAGGAGACCTGAAA---
AACTGCTCTTTTTACATCACCACAAGCGTAGGA---------------------------GATAAAGTG---
AAAGGATATGGAATT---TTTTCTAGCCTTGATATATTCTCAACAAATAAT----------------------
----
-------------AGTAGTACATATAGGTTGAGACAGTGTAACACCTCAGTCATTACACAG---
GCCTGTCCAAAGGTATCCTTTGAGCCAATTCCCATACATTATTGTGCCCCGGCTGGTTTTGCAATTCTAAAGT
GTAAT---GAGAAAATGTTCAAT------GGAACAGGACGATGTAAAAAT-----------------------

-GTCAGC---ACAGTACACTGTACACATGGAATTAGACCAGTAGTGTCAACT---
CAACTGCTGTTAAACGGCAGTCTAGCAAAAGAGGAG------------
GTAGTAATTAGATCTGAAAATTTTACAAACAAT---------GTTAAAAACATAATAGTGCAGCTGAAT----
-----------GAATCTGTAGAA---------ATTAATTGTATAAGACCCAACAACAATACAAGAAAA-----
-AGTTATCCTGTA------------GGAACAGGGAGAGTA---TTA------TATACAAGAGGA---------
---------ATAATAGGAGATATA------------AGGCAAGCACATTGC---AACATTAGTACAGCA---
AAATGGAATGAAACTGTAGAAAAGGTAATTAAAAAATTATAC---------------AAACAATAT-------
-----------------------------------AATAAAACAATAGATATTAGG------
CCACCCTCAGGAGGGGACCCAGAAATTATA---ATGTTCACTTTTAATTGTGGAGGGGAA---
TTTAAGCAGCTCCAGGCAAGAGTCCTGGCTGTGGAAAGATACCTAAAGGATCAACAGCTCCTAGGGCTTTGGG
GTTGCTCTGGAAAACTCATTTGCACCACTGCTGTGCCTTGGAATGCTAGTTGGAGT-----------------
----------------------------AATAAAACTCATGATGACATTTGG------GAT------
AACATGACCTGGGTGCAGTGG---GAAAGAGAAATTGAGAATTACACAGATTTAATATAC---
AACTTAATTGAAAAATCGCAGAACCAGCAAGAA--

AAGAATGAGCAAGAATTATTGGAATTAGATAAGTGGGCAAATTTGTGGAATTGGTTTGACATAACAAATTGGC
TGTGGTATATAAGAATATTCATAATAATAGTAGGAGGCTTGATAGGTTTAAGAATAGTTTTTGCT---
GTACTTTCTATAGTG---------AATAGAGTTAGGCAGGATACTCACCA------
TTATCATTTGCTGTTGGCTTGCTCAATTTCACTGCTATAGCAGTAGCTGAG---
GGGATAGATAGGATTATAGAAGTAGTACAAAGAGCTTGGAGAGCT----------------------------
--------TTTCTCCACATA------CCTAGAAGAATAAGACAGGGCTTGGAAAGGCTTTGCAG---TAA
>KX792688
GCA--
GAACAACTGTGGGTCACAATCTATTATGGGGTACCTGTGTGG---AAAGAAGCAAAT---
ACCACTCTATTTTGTGCATCAGATGCTAAAGCATATAAGACAGAG---GTGCATAAT----------------
--------------GTTTGGGCCACA---CATGCCTGTGTACCCACAGAC---
CCCAATCCACAAGAAGTAGTATTAGAA---------AATGTGACAGAGTATTTCAAC----------------
--------------------------ATGTGGAAAAATAACATGGTAGAACAGATGCATGAGGATATA---
ATCAATTTATGGGATCAGAGCCTA---AAGCCATGTGTAAAAATA---
ACCCCACTCTGTGTCACTTTAAATTGCACTAATTTGAGGAATGATACTAATACCACCAGGAATGGTACT----
----
-------------------------AATAGCACCATTAATAGTGAGGTAAGAGGGGAAATGACA---
AACTGCTCTTTCAATGTTACCACAACAGTAAGG---------------------------
GGTAGGGTTCAAAAAGAATATGCACTT---TTCTATAGACTTGATGTAGTACCACTAGAGGAT----------
----

AGTAATAATACTGAATTTAGGTTAATAAATTGTAACTCCTCAACCATCACACAG---
ACCTGTCCAAAGGTGTCATTTGAGCCAATTCCTATACATTATTGTGCCCCGGCTGGGTTTGCGATTCTAAAGT
GTAAT---GATAAAAAGTTCAAT------GGAACGGGACAATGTACAAAT-----------------------
-GTCAGC---ACAATACAATGTACACATGGAATTAGGCCAGTAGTGTCAACT---
CAACTGCTGTTAAATGGCAGTCTAGCAGAAGAAGAG------------
GTAGTAATTAGATCTGAAAATTTCACGGAAAAT---------ACTAAAAACATAATAGTACAACTGAAT----
-----------GAATCGGTACAA---------ATTAATTGTACAAGACCCAACAACAATACAAGAAAA-----
-AGTATACAGCTA------------GGACCAGGGGCAGCA---TTC------TATGCAACAGGAGAT------
---------ATAATAGGAGATATA------------AGACAAGCACATTGT---AACCTTAGTGAAGAA---
CAATGGAATAAAACTTTAAAACAGGTAGTTACAAAATTAAAA---------------GAACAATTT-------
-----------------------------------AATAAAACAATAGTCTTTACT------
CAGCCCTCAGGAGGGGACCCAGAAATTGTC---ATGCACAGCTTTAATTGTGGAGGGGAA---
TTTAAGCAGCTCCAGGCAAGACTCCTGGCTGTGGAAAGATACCTAAAGGATCAACAGCTCCTAGGGATTTGGG
GTTGCTCTGGAAAACTCATTTGCACCACTGCTGTGCCTTGGAATACTACTTGGAGT-----------------
----------------------------AATAAATCCCAGACTGATATTTGG------AAT------
AACATGACCTGGATGCAGTGG---GAAAGAGAAATTGACAATTACACAGGCTTAATATAC---
ACCTTACTTACAGAATCGCAAATCCAACAAGAA--

AAGAATGAACAAGAATTATTAGAATTAGATAAGTGGGCAAGTCTGTGGAGTTGGTTTAGCATATCAAATTGGC
TGTGGTATATAAGACTATTCATAATAATAGTAGGAGGCTTAGTAGGGTTAAGAATAATTTTTGCT---
GTACTTTCTATAGTA---------AATAGAGTTAGGCAGGATACTCACCA------
ATATCATTAGCTGTTAGCTTGCTCAATACAATAGCTATAGCAGTAGCTGAG---
GGGACAGATAGGGTTATAGAAGCAATACAAAGAGCTTTTAGAGCT----------------------------
--------TTTCTCCACATA------CCTAGAAGAATAAGACAGGGCTTCGAAAGGCTTTGCTA---TAA

>KX792771
GCT---GCA--
GACCAATTGTGGGTCACAGTTTATTATGGGGTACCTGTGTGG---AAAGAAGCAGAC---
ACCACGCTATTCTGTGCATCAGATGCTAAAGCATATGATACAGAG---GCACATAAT----------------
--------------GTTTGGGCCACA---CATGCCTGTGTACCCACAGAC---
CCCAGCCCACAAGAAGTAAAATTGGGA---------AATGTAACAGAGAATTTTAAC----------------
--------------------------ATGTGGAAAAATAACATGGTAGAACAAATGCATGAGGATATA---
ATTAGTTTATGGGATCAAAGCCTA---AAGCCATGTGTAAAATTA---
ACCCCACTCTGTGTCACTTTGCATTGCTATAATATCACAAGTGTGGGGAATAACACAAGTAATACTACC----
----
-------------ATAAATGATATGAGTAATACTACCATAAATGATATGAGGAGAGAAATGAAA---
AACTGCTCTTTCAATGTCACCACAGACGTAAGC---------------------------
GACAAGAGGAAGATAGCGTATGCCACT---TTTTATAACCTTGACATAGTACAAATAGATAGTGATAATACT-
----

GGTAATAATACATATAGGTTAATAAGTTGTAACACCTCAGTCCTCACACAG---
GCCTGTCCAAAGACAACCTTTGAGCCAATTCCCATACATTATTGTGCCCCAGCTGGTTTTGCGATTCTAAAGT
GTAAT---GATAAGAAGTTCAAT------GGAACAGGACAGTGTAACAAT-----------------------
-GTCAGT---ACAGTACAGTGTACACATGGAATTAAGCCAGTAGTATCAACC---
CAACTGCTATTAAATGGCAGTCTAGCAGAAGAAGAC------------
ATAATAATTAGATCTGCCAATCTCACAGACAAT---------ACTAAAACCATAATAGTACAGCTAAAT----
-----------GAATCTGTAACA---------ATTAATTGTACAAGACCCAGCAACAATACAAGAAAA-----
-GGTATACATATA------------GGACCAGGGAGAGCA---TTT------TATACAACAGGAAGC------
---------ATAATAGGAGATATA------------AGACAAGCACATTGT---AACATTAGTAGAAAA---
GAATGGAATGACACTTTAAAAAAGGTAGCTGAAAAACTAAGA---------------GAACAATTT-------
-----------------------------------AACACAACAATAATCTTTCAA---
CCTCCCTCCCCAGGAGGGGACCCAGAAATTGTA---ACACACAGTTTTAATTGTGGAGGGGAA---
TTTAAGCAGCTCCAGGCAAGAGTCCTGGCTGTGGAAAGATACCTAAAGGATCAACAGCTCCTGGGAATTTGGG
GTTGCTCTGGAAAACTCATCTGCACCACTACTGTGCCTTGGAATGCTAGTTGGAGT-----------------
----------------------------AACAAATCTCAGGATGATATTTGG------GGT------
AACATGACTTGGATGCAGTGG---GAAAGAGAAATTGAAAATTACACAGGCTTAATATAC---
AACTTAATTGAAAAATCGCAGAACCAACAAGAA--

AAAAATGAACAAGAATTATTGGCATTGGATGAATGGGCAAGTTTGTGGAATTGGTTCAGCATAACAAATTGGC
TGTGGTATATAAGAATATTCATAATGATAGTAGGAGGCCTAATAGGATTAAAAATAGTTTTTGCT---
GTAGTTTCTATAGTA---------AATAGAGTTAGGCAGGATACTCACCC------
GTATCGTTGGCTGTTAGCTTGCTTAATACCACTGCTATAGTAGTAGCTGAG---
GGGACAGATAGGATTATAGAAGTAATACAAATAGTTTATAGAGCT----------------------------
--------TTTATCCACATA------CCTAGAAGAATAAGACAGGGCTTTGAAAGGGCTTTGCTA---TAA
>KX792725
AGT---GCA--
GAAAAATTGTGGGTCACAGTCTATTATGGGGTACCTGTGTGG---AAAGAAGCAACC---
ACTACTCTATTTTGTGCATCAGATGCTAAAGCACAGAATCCAGAG---ATGCATAAT----------------
--------------ATTTGGGCCACA---CATGCCTGTGTACCCACAGAC---
CCCAACCCACAAGAAGTAATATTGAAA---------AATTTGACAGAAGAATTTAAC----------------
--------------------------ATGTGGAAAAATAACATGGTAGAACAAATGCATGAGGATATA---
ATCAGTTTATGGGATCAAAGCCTA---AAACCATGTGTGAAATTA---
ACCCCACTCTGTGTTACTTTAAATTGCACTAATGCAGAGAGTTTAAATTGCACTGCCACTAATGGAACT----
----
----------------------AATAATTGTAGTGCTAGCAAGGAACCGATGGAGGAAATGAAA---
AACTGCTCTTTCAACATTACTACAAGTGTGCAA---------------------------
GATAAGAAGCAACAAGAATATGCACTT---TTTTATAAACTTGATATAATACCAATAGATAATAATGAGAAT-
----

GACTCAAATAATACCAACTATACCAGCTATAGGTTGATAAGTTGTAACACCTCAGTCATTACACAG---
GCCTGTCCAAAGATAACCTTTGAGCCAATTCCCATACATTATTGTGCCCCGGCTGGATTTGCGATTCTAAAAT
GTAAG---GATAAGAGGTTCAAT------GGAACAGGACTATGTAAAAAT-----------------------
-GTTAGC---ACAGTACAATGTACACATGGAATTAGGCCAGTAGTATCAACT---
CAGCTGCTGTTAAATGGCAGTCTAGCAGAAGAAGGG------------
GTAGTACTTAGATCTGAAAATTTCACGGACAAT---------GCTAAAAACATAATAGTACAGCTGAAA----

-----------GACCCTGTAAAC---------ATTACTTGCACAAGACCCAACAACAACACAAGAAAA-----
-AGTATAACTATA------------GGACCAGGGAGAGCA---TTT------TATGCAACAGGACAA------
---------GTAATAGGAGATATA------------AGAAAAGCACATTGT---GACCTTAATGGAACA---
GAATGGATAATGCTTTAAAACAGATAGTTGAAGAATTAAGA---------------AAACAATATGGG----
-----------------------------------AATAACATAACAATCTTTAAC------
AGCTCCTCAGGAGGGGACCCAGAAATTGTA---ATGCATAGTTTTAATTGTGGAGGGGAA---
TTTAAGCAGCTCCAGGCAAGAGTCCTGGCTGTGGAAAGATACCTAAGGGATCAACAGCTCATGGGAATTTGGG
GTTGCTCTGGGAAACTCATTTGCACCACTACTGTGCCTTGGAATGCTAGTTGGAGT-----------------
----------------------------AATAAATCTCTAGATAACATTTGG------GAG------
AACATGACCTGGATGCAGTGG---GAAAAAGAAATTGACAATTACACAGATGTAATATAC---
AAATTACTTGAGGAATCGCAAAACCAACAAGAA--

AAAAATGAACAAGAATTATTGGAATTAGATAAATGGGCAAGTTTGTGGAATTGGTTTGACATAACAAGATGGC
TGTGGTATATAAAAATATTCATAATGATAGTAGGAGGCTTGGTAGGTCTAAGAATAGTTTTTGCT---
GTACTTTCTATAGTG---------AATAGAGTTAGGCAGGATACTCACCA------
TTATCGTTTGCTGTTAGCTTGCTTAACGCCACAGCTATTGCAGTAGCTGAA---
GGAACAGATAGGATTATAGAAATAGTACAAAGAGCTGGTAGAGCT----------------------------
--------ATCATCCACATA------CCTAGAAGAATAAGACAGGGAGCAGAAAGAGCTTTGCTA---TAA
>KX792808
GTT---GCA--
ACAAATTTGTGGGTCACAGTTTATTATGGGGTACCTGTATGG---AAAGATGCAGTC---
ACCACTTTATTTTGTGCATCAGATGCTAAAGCATATGATACAGAG---GTGCATAAT----------------
--------------GTTTGGCCACA---CATGCCTGTGTACCCACAGAC---
CCCAACCCACAAGAAATAAGATTGGAA---------AATGTGACAGAAAACTTTAAC----------------
--------------------------ATGTGGAAAAATCAAATGGTTGAACAGATGCATGAGGATATA---
ATCAGTTTATGGGATCAAAGCCTA---AAGCCATGTGTAGAATTG---
ACCCCACTCTGTGTCACTCTAGATTGCACTAATGTGACATATATTAATACTACTAACACCACT----------
----
-------------------------AGTAGTACAGGGGACCCAATAAAGGGAGAAGGAATGAAA---
AACTGCTCTTTCAACATCATCACAAACATACAA---------------------------
AACAAGGTGCAGAAAGAATATGCACTT---TTTTATAATCTTGATATAGTACCAATAGGTAATAAA-------
----

AATAATACTAGCTATAGGTTGATAAGTTGTAATACCTCAGTCATTACACAA---
GCCTGTCCAAAAGTATCCTTTGAGCCAATTCCCATACATTACTGTACCCCAGCTGGTTATGCGCTTCTAAAGT
GTAAT---GATAATACGTTCAAT------GGAACAGGACCATGTAGAAAT-----------------------
-GTTAGC---ACAGTACAATGTACACATGGAATTAGGCCAGTAATATCAACT---
CAACTGCTGTTAAATGGCAGTCTAGCAAAAGGAGAG------------
GTAATGATTAGATCTGAAAATTTTACAAACAAT---------GCTAAAATCATAATAGTACAGCTGAAT----
-----------GAAACTGTAGAA---------ATTAATTGTACAAGACCCAGTAACAATACAAGAAAA-----
-AGTATAAATGTA------------GGACCAGGGAGAGCA---ATT------TATACAACAGGACAA------
---------ATAATAGGAGATATA------------AGACAAGCACATTGT---AATATTAGCAGAGCA---
CAGTGGAATACCACTCTAAAAAGGATAGTTGAAAAATTAAGA---------------AAACAATTTGAA----
-----------------------------------AATAAAACAATAGTCTTTAAA------
CCATCCTCAGGAGGGGACCCAGAAATTGTA---GCACACAGTTTTAATTGTGGAGGGGAA---
TTCAAGCAGCTCCAGGCAAGAGTCCTGGCTGTGGAAAGATACCTACAGGATCAACAGCTCCTGGGGATTTGGG
GTTGCTCTGGAAAACTCATCTGTACCACTGCGGTGCCTTGGAATGTTAGTTGGAGT-----------------
----------------------------AATAAATCTATGGATGACATTTGG------AAT------
AACATGACTTGGATGGAGTGG---GATAAAGAAATTAATAATTACACAGGCTTAATATAC---
ACCTTACTTGAAGAATCACAAAACCAACAAGAA--

AAGAATGAAAAGGAATTATTAGAATTAGATAAGTGGGCAAGTTTGTGGACTTGGTTTGACATATCAAATTGGC
TGTGGTATATAAGAATATTCATAATGATAGTAGGAGGCTTAGTAGGTTTAAGAATAATTTTTGCT---
GTACTTACTATAGTG---------AATAGAGTTAGGCAGGATACTCACCA------
TTGTCATTTGCTGTTAGTTTACTTAATGCTACAGCCATAGCAGTAGCTGAA---
GGGACAGATAGAATCATAGAAATATTACAAAGAGCTTTTAGAGCT----------------------------
--------TTTCTCCACATA------CCTAGAAGAATAAGGCAGGGCTTTGAAAGGTGTTTAATA---TAA
>EU023929
GCT---AAT---GAT---------------------------------ACAGAAAAG---
TGGGTCACAGTCTATTATGGGGTACCTGTGTGG---AGAGAAGCAACC---

ACCACTCTGTTTTGTGCATCAGATGCTACAGCATATAATACAGAG---GTACATAAT----------------
--------------GTTTGGGCCACA---CATGCCTGTGTACCCACAGAC---
CCCAGCCCACAAGAAGTAGCAATGGAG---------AATGTAACAGAAAATTTTAAC----------------
--------------------------ATGTGGAAAAATAACATGGTAGAACAGATGCATGAGGATATA---
ATAAGTTTATGGGATCAAAGCCTA---AAGCCATGTGTAAAATTA---
ACCCCACTCTGTGTCACTCTAAATTGCATTAATTATAATAATACTAAGAATGGTACTAATATTACT-------
----
-------------------AATAGTGCCCCACTGAAGGAGAAAGAAGACATAGGAGAAATAAAA---
AATTGCTCTTTCAATATCACCACAGACCTAAGA--------------------------
GATAAGGTGCGGAAAGAATATGCACTT---TTTTATAAACTTGATGTAACACCAATAGACCCGGATGAGAAT-
----

AATACCCAGGGTAAGAATTATACTAGATATAGGTTGATAAGTTGTAACACCTCAGTCATTACACAG---
GCCTGTCCAAAGGTATCCTTTGAGCCAATTCCCATACATTATTGTGCCCCGGCTGGTTTTGCGATTCTAAAAT
GTAAC---AATGAGCAGTTCAAT------GGAACAGGACCATGTAAAAAT----------------------
-GTCAGC---ACAGTACAATGTACACATGGAATTAGACCAGTAGTATCAACT---
CAACTGCTATTAAATGGCAGTCTAGCAAAAGAAGAG------------
GTAGTAATTAGGTCTGAGAATTTCACAAACAAT---------GCTAAAACCATAATAGTACAGCTGAAA----
-----------GAACCTGTACAA---------ATGAATTGCACAAGACCCAACAACAATACAAGAAAA-----
-AGTATACGTATA------------GGACCAGGGAGTGCA---TTT------TATACAACAGGGGAA------
---------ATAATAGGAGATATA------------AGAAAAGCACATTGT---ACCCTTAATAAAACA---
GAATGGAATAACACTTTAAGCCAGATAGTTAAAAAATTAAGA---------------GAACAATTTAAG----
-----------------------------------AATAGAACAATAATCTTTAAT------
CAATCCGCAGGAGGGGACCCAGAAATTGTA---ATGCACACTTTTAATTGTGGAGGGGAA---
TTTAAGCAGCTCCAGGCAAGAGTCCTGGCTGTGGAAAGATACCTAAAGGATCAACAGCTCCTAGGGATTTGGG
GATGCTCTGGAAAACTCATCTGCACCACTAATGTGCCTTGGAATAGTAGTTGGAGT-----------------
---------------------------AATAAATCTCTGGAAAATATTTGG------AAT------
AACATGACCTGGATGGAATGG---GACAGAGAAATTAATAATTACACAACATTAATACAC---
ACCTTACTTGAAGAATCGCAGAACCAGCAAGAA--

AAGAATGAACAAGAATTGTTGGAATTGGATAAATGGGCAAGTTTGTGGAATTGGTTTGACATATCAAACTGGC
TGTGGTATATAAAAATATTTATAATGATAATAGGAGGCTTGATAGGTTTAAGAATAGTTTTTACT---
GTACTTTCTATAGTG---------AATAGAGTTAGGCAGGGGTACTCACCT------
TTATCATTTGCTATAAGCTTGCTCAACGCCACAGCTGTAGCAGTAGCTGAG---
GGGACTGATAGGATTATAGAAGTACTACAAAGGGCCGGTAGAGCT----------------------------
--------ATTCTCCACATA------CCTACACGAATAAGACAGGGCTTGGAAAGGGCTTTGCTG---TAA
>EU023933
GCT---AAA--
GACAATTTGTGGGTCACAGTCTATTATGGGGTACCTGTGTGG---AAAGAAGCAACC---
ACCACTCTATTTTGTGCATCAGATGCTAAAGCATATCACACAGAG---GCACATAAT----------------
--------------ATTTGGGCCACA---CATGCCTGTGTACCCACTGAC---
CCTAACCCACAAGAGATAGTATTGGAA---------AATGTGACAGAAAATTTTAAC----------------
--------------------------ATGTGGAAAAATAACATGGTAGAACAGATGCATGAGGATATA---
ATCAGTTTATGGGATCAAAGTCTA---AAGCCATGTGTAAAATTA---
ACCCCACTCTGTGTCACTTTACATTGCACTAATATTACTAGGAATGATAGT----------------------
----
-------------------------------------AGGGTGGAAGACATAGGGGAAATAAAA---
AACTGCTCTTTCAATGTAACCACAAGTATAAGA--------------------------
AATAAGCAGCAAAGAGAATATGCATTG---TTTTATAAAACTGATGTAATGTCAATAGCAAAT----------
----

GATACTACAAGCTATACATTAATAAATTGTAACACCTCAGTCATTACACAG---
ACCTGTCCAAAGACAGCCTTTGAACCAATTCCTATACATTATTGTGCCCCGGCTGGTTTTGCGATTTTAAAGT
GTAAT---GATAAAAAGTTCAAT------GGAACGGGACCATGTAAAAAT----------------------
-GTCAGC---GTAGTACAATGCACACATGGAATTAGGCCAGTAGTGTCAACC---
CAACTGCTATTAAATGGCAGTCTAGCAGAAGAAGAG------------
GTAGTAATAAGATCTGAAAATTTCACAGATAAT---------GCTAAAGTCATAATAGTACAGCTGAAT----
-----------GAAATTGTAGTA---------GTTAATTGTACAAGACCCAACAACAATACAAGGAAA-----
-AGTATACCTATA------------GGACCAGGGAGAGCA---TTT------TATGCAACAGGAGAT------
---------ATCATAGGAGATATA------------AGACAAGCACATTGT---AACATTAGTAGAGAC---

AAATGGAATACCACTTTAGGACATGTAGTTACAAAATTAAAA---------------GAACAATTTGGG----
-----------------------------------AATAAAACAATAGTCTTTAAG------
CATTCCTCAGGAGGGGACCCAGAAATTGTA---ATGCACAGTTTCAATTGTGGAGGGGAA---
TTTAAACAGCTCCAGGCAAGAGTCCTGGCTGTGGAAAGATACCTAAAGGATCAACAGCTCCTAGGGATTTGGG
GTTGCTCTGGAAAACTCATTTGCACCACTGCTGTGCCTTGGAATACTAGTTGGAGT-----------------
----------------------------AACAAAACTCTGAACAAGATTTGG------GAT------
AACATGACCTGGATGCAATGG---GAAAGAGAAATTGAAAATTACACAGATGAAATATAC---
AGATTAATTGAACAATCGCAGAACCAGCAGGAA--

AAGAATGAACAAGAATTATTAGCATTGGATGAATGGGCAAATCTGTGGAATTGGTTTGACATAACAAATTGGC
TGTGGTATATAAGAATATTCATCATGGTAGTAGGAGGTTTAATAGGTTTAAGAATAATTTTTGCT---
GTACTTTCTGTAGTA---------AATAGAGTTAGGCAGGATACTCACCC------
TTATCGTTTGCTATTAGCTTGCTCAACGCCACAGCCATAGCAGTAGCTGGA---
GGGACAGATAGGATTATAGAAGCTTTGCAAGGAATTGGTAGAGCT----------------------------
--------ATCCTCAACATA------CCCAGAAGAATAAGACAGGGCTTAGAAAGGGCATTGCTA---TAA
>EU023927
GCT---GCA--
GAACAATTGTGGGTCACAGTCTATTATGGGGTACCTGTGTGG---AAAGATGCAACC---
ACCACTCTATTTTGTGCCTCAGATGCTAAAGCATATGAGACAGAG---GCACATAAT----------------
--------------GTTTGGGCCACA---CATGCCTGTGTACCCACAGAC---
CCCAACCCACAAGAAGTAGTATTGGGA---------AATGTGACAGAAGAATTTAAC----------------
--------------------------ATGTGGAAAAATGACATGGTAGAACAAATGCATGAGGATATA---
ATTAGCTTGTGGGATCAAAGCTTA---AAGCCATGTGTAAAAATA---
ACCCCACTCTGTGTTACTTTAAATTGCACTGATTATGTGAATGGTACCAAGGTAAATGCTACCAATGAGCATG
CTAACAAT---
-------ACCAATAATGATACCAATAGCGGTGACTGGAAAGAGATGGATAAAGGAGAAATAAAA---
GAATGCTCTTTCAATGTCACCACAGTCATAAGA---------------------------
GATAGGATGAAGAAAGAAACTGCACTT---TTTTATAAACTTGATATAGTACCAATAGATGCT----------
----

AATGATAATAACAGCTATAGGTTGATAAATTGTAACACCTCAGCCATTACACAG---
GCCTGTCCAAAGGTATCCTTTGAGCCAATTCCCATACATTATTGTGCCCCAGCTGGTTTTGCGATTCTAAAGT
GTAAC---AATAAGACATTCAGT------GGAAGAGGACCGTGTACAAAT-----------------------
-GTCAGC---ACAGTACAATGTACACATGGAATTAGGCCAGTAGTGTCAACT---
CAACTGCTGTTAATGGCAGTCTAGCAGAAGGAGAT-------------
GTAGTAATCAGATCTGAAGATTTCTCAGACAAT---------GCTAAAACCATAATAGTACAGCTAAAT----
-----------AAAACTGTAGTG---------ATTAATTGTACAAGGCCCAACAACAATACAAGAAGA-----
-AGTATATCTATA------------GGACCAGGGAGAGCA---TTT------TATGCAACAGGAGAG------
---------GTAATAGGAGATATA------------AGACAAGCACACTGT---AACCTTAGTAGGAAA---
GACTGGAATGACACTTTAAAACAGATAGCTGAAAAATTAAGA---------------GAGCAATTTGGG----
--------------------------------AATAATAAAACAATAAATTTTACT------
CAATCCTCAGGAGGGGACCCAGAAATTGTA---ATGCACAGTTTCAATTGTGGAGGGGAA---
TTTAAGCAGCTCCAGGCAAGAGTCCTGGCTGTAGAAAGATACCTAAAGGATCAACAGCTCCTGGGAATTTGGG
GTTGCTCTGGAAAACTCATCTGCACCACTGCTGTGCCTTGGAATACTAGTTGGAGT-----------------
----------------------------AATAAATCTATGGATTCAATTTGG------GAA------
AACATGACCTGGATGCAGTGG---GAGAAAGAAATTAGTAACTACACAGACTTAATATAC---
AACTTAATTGAAAAATCGCAGAACCAACAGGAG--

AAAAATGAACAAGAATTATTGGAATTAGATAAATGGGCAAGTTTGTGGAATTGGTTTAACATAACAAACTGGC
TGTGGTATATAAAAATATTCATAATGATAGTAGCAGGCTTAGTAGGTTTAAGAATAGTTTTTTCT---
GTACTTTCTATAATA---------AGGAGAGTTAGGCAGGATACTCACCC------
TTGTCGTTTGCTGTCAGCTTGTTCAACGCTATAGCTATAGCAGTAGCTGAG---
GGGACAGATAGGATTATAGAAGTAGTACAAAGATTTGGTAGAGGC----------------------------
--------ATTCTCCACATA------CCTACAAGAATAAGACAGGGCTTAGAAAGGGCATTGCTA---TAA
>MH714351
GCT---GCA--
GAAAAGTTGTGGGTCACAGTCTATTATGGGGTACCTGTGTGG---AAAGAAGCAACC---
ACCACTCTATTTTGTGCATCAGATGCTAAAGCATATGAGACAGAG---GTACATAAT----------------
--------------GTTTGGGCCACA---CATGCCTGTGTACCCACAGAC---
CCCAACCCACAAGAAGTAGTACTGGCA---------AATGTGACAGAAAATTTTAAC----------------

--------------------------ATGTGGAAAAATAACATGGTAGAACAGATGCATGAGGATATA---
ATTAGTTTATGGGATCAAAGCTTA---AAACCATGTGTAAAATTA---
ACCCCACTCTGTGTTACTCTAAATTGCACTGATTTGAAGAAGAATGATACTAGTACCAATAGTGATACCACTG
CTAGTACCAAT--
-------------------------ACTAATAGCTGGGAGAAATGGACAAAGGAGAAATAAAA---
AATTGCTCTTTCAATATCACCACAAACATACTA---------------------------
GATAAGAGGCAGAAACATTATGCACTT---TTTTATAAACTTGATGTAACACCAATAGAAAAT----------
----

GAGACTAATAGCTATAGGTTGACAAGTTGTAATACCTCAGTCATTACACAG---
GCCTGTCCAAAAGTATCCTTTGAGCCAATTCCCATACATTATTGTGCTCCGGCTGGTTTTGCGATTCTAAAGT
GTAAT---AATAAGACGTTCAAT------GGAACAGGACCATGTACAAAT-----------------------
-GTCAGC---ACAGTACAATGTACACATGGAATTAGGCCAGTAGTATCAACT---
CAACTGCTGTTAAATGGCAGTCTAGCAGAAGAAGAG------------
GTAGTAATTAGATCTGAAAATTTCACGGACAAT---------GCTAAAATCATAATAGTACAGCTAAAT----
-----------GAATCTGTAGAA---------ATTAATTGTACAAGACCCAACAACAATACAAGAAAA-----
-AGTATAAATATA------------GGACCAGGCAGAGCA---TTC------TATGCAGCAGGAGAC------
---------ATAATAGGAGATATA------------AGACAAGCACATTGT---AATCTTAGTAGAACA---
GAATGGAATAACACTCTAAAACAAATAGTTGGAAAATTAAGA---------------GAACAATTTGGG----
--------------------------------ATAAATAAAACAATAGTCTTTAAT------
CAATCCTCAGGAGGGGACCCAGAAATTGTA---ATGCACAGTTTTAATTGTGGAGGAGAA---
TTTAAGCAGCTCCAGGCAAGAGTCCTGGCTCTGGAAAGATACCTAAAGGATCAACAGCTCCTGGGAATTTGGG
GTTGCTCTGGAAAACTCATCTGCACCACTGCTGTGCCTTGGAATACTAGTTGGAGT-----------------
----------------------------AATAAAAATCTGAGTCAGATTTGG------GAT------
AACATGACCTGGATGCAGTGG---GAAAGAGAAATTGATAATTACACAAACTTAATATAC---
AACTTACTTGAAGAATCGCAAAACCAACAAGAA--

AAAAATGAACAAGAATTATTGGAATTAGATAAATGGGCAAGTTTGTGGAATTGGTTCAGCATAACAAATTGGC
TGTGGTATATAAGAATATTCATAATGATAGTAGGAGGCTTAATAGGTTTAAGAATAGTTTTTACT---
GTACTGGCTATAATA---------AATAGAGTTAGGAAGGATACTCACCA------
TTATCGTTCGCTGTTAGCTTGTTCAACGCCACAGCCATAGCAGTAGCTGAG---
GGAACTGATAGGATTATAGAATTCTTACAAAGAGTTTATAGAGCT----------------------------
--------ATTCGCCACATA------CCTACACGAATAAGACAGGGCTTGGAAAGGGCTTTGCTA---TAA
>MH714324
GCA---GCA--
GAACAATTGTGGGTTACAGTCTATTATGGGGTACCTGTGTGG---AAAGAAGCAAAT---
ACCACTCTATTCTGTGCATCAGATGCTAAAGCATATGAGACAGAG---GTACATAAT----------------
--------------GTTTGGGCCACA---CATGCCTGTGTACCCACAGAC---
CCCAACCCACAAGAAGTAGTATTGGAA---------AATGTGACAGAAAATTTTAAC----------------
--------------------------ATGTGGAAAAATAACATGGTAGAACAAATGCAGGAGGATATA---
ATTAGTTTATGGGATCAAAGCCTA---AAGCCATGTGTAAAGTTA---
ACCCCACTCTGTGTCACTTTAAACTGCACTATTTTAAATTGCACTACTAATTGCACTACTAATAGTACTACTA
ATAGTACTAAT--
----------------------------AGTACTAATAGTACATGGGAAGACAAAGGACTCAGG---
AATTGCTCTTTTTATGCCCCCACAAGTATAAGA---------------------------
GATAAGGTAAAGAAAGAATATGCAATT---
TTTTATAACCTTGATTTAGTACAAATAGATGATGATTTAGAAAATAAG-------------------------
----
ACTAACAAAAATTTTACTAAATATAGGTTGGTAAGTTGTAATACCTCAGTCATCACACAA---
GCCTGTCCAAAGGTAACCTTTGAGCCAATCCCCATACATTATTGTGCCCCGGCTGGTTTTGCGATTCTAAAGT
GTAAC---AATAAAACATTCAAT------GGAACGGGATCATGTAGGAAT-----------------------
-GTCAGC---ACAGTACAATGTACACATGGAATTAGGCCAGTAGTATCAACT---
CAACTGCTGTTGAATGGCAGTCTAGCAGAAAAAGAG------------
GTAGTAATTAGATCTGAAAATTTCACGGCAAAT---------CATAAAACCATAATAGTACAGCTGAAT----
-----------GAATCTGTAGAA---------ATTAATTGTACAAGACCCAACAACAATACAAGAAAA-----
-AGTATAAATATA------------GGACCAGGGAGAGCA---TTT------TATGCAACAGGAGAC------
---------ATAATAGGAGATATA------------AGACAAGCATATTGT---ACCCTTAATAAGACA---
GATTGGAATAGGACTTTAGAAAAGGTAGCTAAAAAATTAAAA---------------GAACAATTTGGG----
--------------------------------GAAAATAAGACAATAGTCTTTAAT------
CAATCCTCAGGAGGGGACCTAGAAGTTTCA---ATGCACATATTTACTTGTGGAGGAGAA---

TTTAAGCAGCTCCAGGCAAGAGTCCTGGCTGTGGAAAGATACCTAAAGGATCAACAGCTCCTGGGAATTTGGG
GCTGCTCTGGAAAACTCATTTGCCCCACTGCTGTGCCTTGGAATGCTAGTTGGAGT-----------------
----------------------------AATAAAACTCTGGATAAGATTTGG------AAT------
AACATGACCTGGATGGATTGG---GAAAGAGAAATTGGCAATTACACAAGCTTAATATAT---
AGCTTAATTGAAGAATCGCAAAACCAGCAAGAA--

AAGAATGAAAAAGACTTATTGGAATTGGACAAGTGGGACAGCTTGTGGAATTGGTTTAGTATAACACAATGGC
TGTGGTACATAAAAATATTCATAATGATAGTAGGAGGCTTGATAGGCTTAAGAATAATTTTTGCT---
GTACTTTCTATAGTG---------AATAGAGTTAGGCAGGGATACTCACCA------
TTATCGTTCGCTGTTAGCTTGCTCAACGCCACAGCAATAGCAGTAGCTGAA---
GGGACAGATAGGGTTATAGAATTATTACAAAGAATTGGTAGGGGT----------------------------
--------ATCCTCCACGTA------CCTAGAAGAATAAGACAAGGATTTGAAAGGGCTTTGCTA---TAA
>MH714343
GCT---GCA--
GGAAATTTGTGGGTCACAGTCTATTATGGGGTACCTGTGTGG---AAAGAAGCAACC---
ACCACTCTATTTTGTGCATCAGATGCTAAAGCATATGGTACAGAG---GTACATAAT----------------
--------------GTTTGGGCCACA---CATGCCTGTGTACCCACAGAC---
CCCAACCCACAAGAAGTAGTATTGGGA---------AATGTGACAGAAGATTTTAAC----------------
--------------------------ATGTGGAAAAATAACATGGTAGAACAGATGCATAAGGATATA---
ATTAGTTTATGGGATGAAAGCTTA---AAACCATGTGTAAAATTA---
ACCCCACTCTGTGTTACTCTAAATTGCACTGATTTGAAGAAGGATAATCTAACTTGCGCTAATTTGACGAAGG
GGAGTAATTGTACCAAT--
-AATAGTAGCGTGGGGAATGATACTAACACCAATAATAGTAGCTGGATAGGAGAAGACATGAGA---
AATTGCTCTTTCAATGCCACTACAAATATAGTA---------------------------
GATAAGGTGCAGCAACAATATGCACTT---TTTTATAAACTTGATGTAGTACAAATAGAAGAA----------
----

GATACAAATAATACTAACTATAGATTGATAAGTTGTGACACCTCAGTCATTACACAA---
GCCTGTCCAAAGGTATCCTTTGAGCCAATTCCCATACATTATTGTGCCCCGGCTGGTTTTGCGATTCTAAAGT
GTAAT---AATAAGACGTTCGAT------GGAAAAGGACCATGTAGAAAT-----------------------
-GTCAGC---ACAGTACAATGTACACATGGAATTAGGCCAGTAGTATCAACT---
CAACTGCTGTTAAATGGCAGTCTAGCAGAGGAAGAG------------
ATAGTAATTAGATCTGAAGATTTCAAGAACAAT---------GCTAAAAACATAATAGTACAGCTGAAT----
-----------AAATCTATAGAA---------ATTAATTGTACAAGACCCAACAACAATACAAGAAAA-----
-AGTCTAAATATA------------GGACCAGGCAGAGCA---GTC------TATGCAACAGGAGAA------
---------ATAATAGGAGATATA------------AGACAAGCACATTGT---AACCTTAGTAGAGCA---
GAATGGAATGACACTTTAAAACAGATAGTTGGAAAATTAGGAGAA---------ATTGAACAATTTAGG----
-----------------------------------AATAAAACAATAGTCTTTAAC------
CAATCCTCAGGAGGGGACCCAGAAATTGTA---ATGCACAGTTTTAATTGTGGAGGGGAA---
TTTAAGCAGCTCCAGGCAAGAGTCCTGGCTCTGGAAGCGTACCTAAAGGATCAACAGCTCCTGGGGATTTGGG
GTTGCTCTGGAAAACTCATTTGCACCACTGCTGTGCCTTGGAATACTAGTTGGAGT-----------------
----------------------------AATAAATCTCTGAATTATATTTGG------GAG------
AACATGACCTGGATGGAGTGG---GAAAGAGAAATTGAGAATTACACAGGCTTGATATAC---
ACTTTAATTGAAGAATCGCAAAACCAACAAGAA--

AAAAATGAACAAGAGTTATTGGCATTAGATAAATGGGCAAGTTTGTGGAATTGGTTTAGCATAACAAATTGGC
TGTGGTATATAAGACTATTCATAATGATAGTAGGAGGCTTGATAGGTTTAAGAATAGTTTTTACT---
GTACTGTCTATAGTG---------AATAGAGTTAGGAAGGGATACTCACCA------
TTATCGTTCGCTGTTAGCTTGCTCAACGCCACAGCCATAGCAGTAGCTGAG---
GGGACTGATAGGATTATAGAATTTTTACAAAGAGCTTTTAGAGCT----------------------------
--------ATTCTCCACATA------CCTACAAGAATAAGACAGGGCTTGGAAAGGGCTTTGCTA---TAA
>MH714329
GCT---ACA--
GAAAAATTGTGGGTCACAGTTTATTATGGGGTACCTGTGTGG---AGAGAAGCAAAT---
ACCACACTATTTTGTGCATCAGATGCTAAAGCATATGATACAGAG---GTACATAAT----------------
--------------GTTTGGGCCACA---CATGCTTGTGTACCCACAGAC---
CCCGAACCACAAGAAGTAGTAATGGGA---------AATGTGACAGAAAATTTTAAC----------------
--------------------------ATGTGGAGTAATAACATGGTAGAACAAATGCATGAGGATGTA---
ATCAGTTTATGGGATCAAAGCCTA---AAACCATGTGTAAAATTA---
ACCCCACTCTGTGTTACTTTAAATTGCACTGATTTGACCAATAGTGACACCACTGATAACACCACC-------

```
-------------------------------------------------------------------------
----------------------------ATTAATAGTAACTGGGAAGGGAAAGGAGAAGTAAAA---
AACTGCTCTTTCAATATCACCACAAGCATAAGA---------------------------
GATAAGGTACAGAAAGGATATGCAACT---TTTTATAATCTTGATATAGTACCAATAGAAGAT----------
-------------------------------------------------------------------------
----------------
GATGATAGGAATAACAGCTATAGGTTGATAAGTTGTAACACCTCAGTCATTACACAG---
GCCTGTCCAAAGATAACCTTTGAACCAATTCCTATACATTATTGTGCCCCGGCTGGTTTTGCGATTCTAAAGT
GTAAT---GAGAAGGAGTTCAAT------GGAACAGGACCATGCACAAAT-----------------------
-GTCAGC---ACAGTACAATGTACACATGGAATTAAGCCAGTGGTATCAACT---
CAACTGCTGCTAAATGGCAGTCTGGCAGAAGGAGAG------------
GTGGTAATTAGGTCTAAAAACTTCACAGACAAT---------ACCAAAACTATAATAGTACAGCTGAAC----
-----------CAATCTGTAGAA---------ATTACATGTGTAAGACCCAATAACAATACAAGAAGA-----
-GGTATAAATATG------------GGACCAGGGAGAGCA---TTT------TATACAATGGGAGAC------
---------ATAATAGGAGATATA------------AGACAAGCACATTGT---AACCTTAGTAGAGCA---
AATTGGACTAACACCTTAAAACAGATAGCTGACAAACTAAGA---------------AACCAATTT-------
-----------------------------------AATAAAACAATAATCTTTAAT------
AGCTCCTCAGGAGGGGACCCAGAAATTACA---ATGCACAGTTTTAATTGTAAAGGGGAA---
TTTAAACAGCTCCAGGCAAGAGTCCTGGCTGTAGAAAGATACCTAAGGGATCAACAGCTCCTGGGGATCTGGG
GTTGCTCTGGAAAACTCATTTGCACCACTTCTGTGCCTTGGAATGCTAGTTGGAGT-----------------
----------------------------AATAAATCTCTGGATCAGATTTGG------AAT------
AATATGACCTGGATAGAGTGG---GAAAGAGAAATTGACAATTATACAGGCTTAATATAC---
ACCTTACTTGAAGAATCGCAAAACCAACAAGAA----------------------------------------
-----------------------------
AAGAACGAACAAGAATTATTGGAATTGGATAAATGGGCAAACTTGTGGAATTGGTTTGACATAACAAACTGGC
TGTGGTATATAAAAATATTTATAATGATAGTAGGAGGCTTAATAGGTTTAAGAATAGTTTTTACT---
GTACTTTCTATAGCA---------AATAGAGTTAGGCAGGGATACTCACCA------
TTGTCGTTTGCTATTAGCTTGCTCAACGCCACAGCCATAGCAGTAGCTGAA---
GGGACAGATAGGGTTATAGAAGTGCTACGCAGAGCTTTTAGAGCT----------------------------
--------GTTATCCACATA------CCTAGAAGAATAAGACAAGGCGCCGAAAGGGCTTTGCTA---TAA
>KU600814
GCT---GCA------------------------------------------
GGAAATTTGTGGGTCACAGTCTATTATGGGGTGCCTGTGTGG---AGGGATGCAAAC---
ACCACTTTATTTTGTGCATCAGATGCTAAAGCATATGATACAGAG---GTACATAAT----------------
--------------GTTTGGGCCACA---CATGCCTGTGTACCCACAGAC---
CCCAACCCACAAGAATTGGTATTGGAA---------AATGTAACAGAAGATTTTAAC----------------
--------------------------ATGTGGAAAAATAACATGGTAGAACAGATGCATGAAGATATA---
ATCAGTTTATGGGATCAAAGCCTG---AAGCCATGTGTAAAATTA---
ACCCCACTCTGTGTTACTTTAAATTGCACTGATGCGAAGAATGATACC-------------------------
-------------------------------------------------------------------------
-------------------------------------------------GAAAAGGTAATGAGA---
AATTGCTCTTTCAATATCACCACAAGCCTAAGA---------------------------
GATAAGGTGCAGAAAGAATATGCGCTT---
TTTTATAGACCTGATGTAGTACCAATAGATAATAATAATATAGATAATATT----------------------
--------------------------------------------
ACTAATAATAATATTACTAGCAACTATAGCAGCTATAGGTTGATAAGTTGTAACACCTCAGTCATCACGCAG-
--
GCCTGTCCAAAGGTATCCTTTGAGCCAATTCCCATACATTATTGTGCCCCGGCTGGTTTTGCGATTCTAAAGT
GTAAT---GAGCAGAATTTCAGT------GGAAAAGGAGAATGTAAAAAT-----------------------
-GTCAGT---ACAGTACAATGTACACATGGAATTAGACCAGTAGTATCAACT---
CAACTGCTGTTAAATGGCAGTCTAGCAAAAGAGGGG------------
GTAATAATTAGATCTGACAATTTCTCGGACAAT---------GCTAAAACCATAATAGTACAACTAAAA----
-----------GACCCTGTAGAA---------ATTAATTGTATAAGACCCGGCAACAATACAAGAAAA-----
-AGTATAACTATA------------GGACCAGGGAGAGCA---TTT------TATACAACAGGAGAA------
---------GTAATAGGAAATATA------------AGACAAGCACATTGT---AACCTTAGTGGAGCA---
CACTGGAATAGCACTTTAAAACAGATAGCTGACAAATTAAGA---------------GGACAATTTAAG----
-----------------------------------AATAGAACAATAGTCTTTAAC------
CACTCCTCAGGAGGGGACCCAGAAATTGTA---ATGCACAGTTTTAATTGTGGAGGGGAA---
TTTAAGCAGCTCCAGGCCAGAGTCCTGGCTGTGGAAAGATACCTGAAGGATCAACAGCTCCTAGGAATTTGGG
GTTGCTCTGGAAAACTCATCTGCCCCACTGCTGTGCCTTGGAATGCTAGTTGGAGT-----------------
```

-------------------------AATAATAAATCTCTGGATTATATTTGG------GGT------
AACATGACCTGGATGGAGTGG---GAAAGAGAAATAGACAATTACACAAGCTTAATATAC---
ACCTTAATTGAAGAATCGCAAAACCAACAGGAA--

AAGAATGAACAAGACTTATTGGCATTAGACGAATGGGCAAGTTTGTGGAATTGGTTTGACATATCAAAATGGC
TGTGGTATATAAAAATATTCATAATGATAGTAGGAGGCTTGATAGGTTTAAGAATAGTTTTTACT---
GTACTTTCTATAGTG---------AATAGAGTTAGGCAGGGATATTCACCA------
TTATCTTTTGCTGTTAGCTTGCTTAACGCCACAGCCATAGCAGTAGCTGAG---
GGGACAGATAGGATTATAGAAGTGTTACAAAGAATTGGTAGAGCT----------------------------
--------ATTCTCCACATA------CCTACTAGAATAAGACAAGGCTTGGAAAGGGCTTTGCTA---TAA
>KU600818
GCT---GTA--
GACCAATTGTGGGTCACAGTCTATTATGGGGTACCTGTGTGG---AAAGAAGCAACC---
ACCACTCTATTTTGTGCATCAGATGCTAAATCATATGATACAGAG---GTACATAAT----------------
--------------GTTTGGGCCACA---CATGCCTGTGTACCCACAGAC---
CCCAACCCACAAGAAATAGAATTGGAA---------AATGTGACAGAAAATTTTAAC----------------
--------------------------ATGTGGAAAAATAACATGGTAGAACAGATGCATGAGGATATA---
ATCAGTTTATGGGATCAAAGCCTA---AAGCCATGTGTAAAATTA---
ACCCCACTCTGCGTCACTTTAAATTGCACTAATTGGGGAAATAGTACTAAGAGTAATAATAATGCTACC----
----
-------------------------GGGAGTAGTAAAGTGGGAAAAATGAATGAAGAAGTAAAG---
AACTGCTCTTTCAATATCACCTCAAGCATAAGA---------------------------
GATAAGGTGCAGAAAGAATATGCAATG---CTTTATAAGTCTGATATAGTACCAATAGGAAAA----------
----

AGTAAGACAAGCTATAGGTTAACAAGTTGTAACACCTCAGTCATTACACAG---
GCCTGTCCAAAGGTATCCTTTGAGCCAATTCCCATATATTACTGTGCCCCGGCTGGTTTTGCGATTCTAAAGT
GTAAA---GATAAGAAGTTCAAT------GGAACAGGACCATGTACAAAT-----------------------
-GTCAGC---ACAGTACAATGTACACATGGAATTAGGCCAGTAGTATCAACT---
CAACTGCTGTTAAATGGCAGTCTAGCAGAAGAAGGG------------
ATAGTAATTAGATCTGAGAATTTCACGAACAAT---------GTTAAAACCATAATAGTACAGCTGAAA----
-----------AGCGCTGTAAAA---------ATTAATTGTACAAGACCAGGCAACAATACAAGAAGA-----
-AGTATACATATA------------GGACCAGGGAGAGCA---TTT------TAT---ACAGGAGAA------
---------ATAATAGGAGACATA------------AGACAAGCACATTGT---ACCATTAGTGAAGCA---
GATTGGAATAACACTTTAAGACAGGTAGCTATCAAATTAAAA---------------GAACAATTT-------
-----------------------------------AATAGAACAATAATTTTTACT---
CAACCCGCAGCAGGAGGGGACCCAGAAATTGTA---CTGCACAGTTTTAATTGTGGAGGGGAA---
TTTAAGCAGCTCCAGGCAAGAGTCCTGGCTGTGGAAAGATACCTAAAGGATCAACAGCTCCTGGGGATTTGGG
GTTGCTCTGGAAAACTCATTTGCACCACTGCTGTGCCTTGGAATGCTAGTTGGAGT-----------------
----------------------------AATAAATCTCTGGATAATATTTGG------GGG------
AACATGACCTGGATGGAGTGG---GAAAGAGAAATTGACAATTACACAGGCTTAATATAC---
ACCTTACTTGAAGAATCGCAGAACCAGCAAGAC--

AAGAATGAACAAGAATTATTGCAATTGGATAAATGGGCAAGTTTGTGGAATTGGTTTGACATAACACACTGGC
TGTGGTATATAAAAATATTCATAATGATAGTAGGAGGCTTGATAGGTATAAGAATAGTTTTTACT---
GTGCTTTCTATAGTG---------AATAGAGTTAGGAAGGGATACTCACCA------
TTATCGTTTGCTGTTAGCTTGTTCAACGCCACATCTATAGCAGTAGCTGAG---
GGAACAGATAGGGTTATAGAAATATTACAAAGAGCTGTTAGAGCT----------------------------
--------CTTCTCCACATA------CCTAGAAGAATAAGACAGGGCGCGGAAAGGGCTTTGCTA---TAA
>KU600815
GCT---ACA--
GGAAACTTGTGGGTCACAGTTTATTATGGGGTACCTGTGTGG---AAAGAAGCCACC---
ACCACCCTATTTTGTGCATCAGATGCTAAAGCATATGATGCAGAG---GCACATAAT----------------
--------------GTTTGGGCCACA---CATGCCTGTGTACCCACGGAC---
CCCAACCCACAAGAAGTACTATTGGTA---------AATGTGACAGAATATTTTAAC----------------
--------------------------ATGGGGAAAAATAACATGGTAGACCAGATGCATGAGGATATA---
ATCAGTTTATGGGATCAAAGCCTA---AAGCCATGTGTAAAATTA---
ACCCCACTCTGTGTCACTTTAAGTTGCACTAATGTTAATATTACTAACAATGCCACAGAG-------------
----
-------------------AACATGAACATTAGTATTAACAATAGGATTAGTGAGCAAATGATG---

AACTGCTCTTTCAATATCACCACAAGCATAGGA--------------------------
GATAAGTATCAAAAAGAATATGCGCTT---CTTTATAGACTTGATGTAGTACCAATAGATGGT----------
----

AGTAATAATAATACTAGCTATAGGTTAATAAGTTGTAACACCTCAGTCCTTACACAA---
GCCTGTCCAAAGCTAACCTTTGAGCCAATTCCCATACATTATTGTGCCCCGGCTGGTTTTGCAATCTTAAAGT
GTAAC---GATAGGAAGTTCAAT------GGAACAGGACCATGTAGAAAT----------------------
-GTCAGC---ACAGTACAATGCACACATGGAATTAGGCCAGTAGTATCAACT---
CAACTGCTGTTAAATGGCAGTCTAGCAGAAGAAGAG-----------
GTAGTAATTAGATCTGTTAATTTCTCAGACAAT---------GCTAAAACTATAATAGTACAGCTGAAT----
-----------GAAACTGTACAA---------ATTAATTGTACAAGACCTGGCAATAACATAAGGAAA-----
-AGTATACCTATA------------GGACCAGGAAGAGCA---TTT------TTTGCAACAGGAGAC------
---------ATAATAGGAGATATA------------AGACAGGCACATTGT---AACCTTAATAGAAAA---
GATTGGAATAACACTTTAAAAAACATAGTTGTAAAATTAAGA---------------AAACAATATGGG----
---------------------------------GAAAATAAAACAATAGTTTTTAAT------
CAATCCTCAGGAGGAGACCCAGAGATTGTA---ATGCACAGCTTTAATTGTGGAGGGGAA---
TTTAAGCAGCTCCAGGCAAGAGTCCTGGCTGTGGAAAGATACCTAAAGGATCAACAGCTCCTGGGGATTTGGG
GTTGCTCTGGAAAGCTCATTTGCACCACTACTGTGCCTTGGAATGCCAGTTGGAGT----------------
-----------------------------AACAAATCTCTGAGTACAATTTGG------AAT------
AACATGACTTGGATGGAGTGG---GAAAGAGAAATTGACAATTACACAGGCATAATATAC---
ACTTTACTCACAGAATCACAAATCCAACAAGAA---------------------------------------
----
AAGAATGAACAAGAATTATTAAAATTAGATAGATGGGCAAGTTTGTGGAATTGGTTTAGCATAACAAATTGGC
TGTGGTATATAAGAATATTCATAATGATAGTAGGAGGCTTGGTAGGTTTAAGAATAGTTTTTGCT---
GTACTTTCTATAGTG---------AATAGAGTTAGGCAGGGATACTCACCA------
TTATCGTTTGCTGTTAGTTTGTTCAATGCCACAGCTATAACAGTAGCTGAG---
GGGACAGATAGAATTATAGAAATAATACAAAGGGCTTATAGAGCT---------------------------
--------ATTATCCACATA------CCTAGAAGAATAAGACAGGGCTTGGAAAGGGTTTTGCTA---TAA
>AY180905
GCT---GCA---
GAAAAATTGTGGGTCACAGTCTATTATGGGGTACCTGTATGG---AAAGATGCAACT---
ACCATTCTATTTTGTGCATCAGATGCTAAAGCATATAAGTCAGAG---GTACATAAT----------------
--------------ATTTGGGCCACA---CATGCCTGTGTACCCACAGAC---
CCCAACCCACAAGAAGTAGTCTTGGGA---------AATGTGACAGAAAACTTTAAC----------------
--------------------------ATGTGGAAAAATGACATGGTAGAACAGATGCATGAAGATATA---
ATCAGTTTATGGGATCAAAGCCTA---AAGCCATGTGTAAAATTA---
ACCCCACTCTGTGTTACTTTAGATTGCACTAATTTGAATATTACTAATATCAGTAGTAATACCACCAATACTA
CT---
-------------ACTACCAGTAGTAATACCAGTAGTACAACAGGGATAGGGTTAGAAATGAAA---
AACTGCTCTTTCAATATCACCACAAGCATAAAT--------------------------ACTAAGGTA---
AAAGACTATGCACTT---TTTTATAAACCTGATATAGTACCAGTTAATAAT---------------------
----
-------ACTAATAATACCAGATATAGGTTGATAAATTGTAACACCTCAGTCATTACACAA---
GCTTGTCCAAAGGTATCCTTTGAGCCAATTCCCATACATTATTGTGCCCCGGCTGGTTTTGCGATTCTAAAGT
GTAAC---AATAAAACGTTCAAT------GGAACAGGACCATGTACAAAT----------------------
-ATCAGT---ACAGTACAATGTACACATGGAATCAGGCCAGTAGTATCAACT---
CAACTGCTGTTAAATGGCAGTTTAGCAGAAGAAGAG-----------
GTAATGATTAGATCTAGCAATTTCTCGAACAAT---------GCTAAAATCATAATAGTACAGTTGAAT----
-----------GAATCTGTAGAA---------ATTAATTGTACAAGACCCAGCAACAATACAAGAAAA-----
-AGAGTAACTCTA------------GGACCAGGGCGAGTA---TGG------TATACAACAGGACAA------
---------ATAATAGGAGATATA------------AGACGAGCACATTGT---AACCTTAGTAGAACA---
CAATGGGATAAAACTTTACAACAGATAACTAAAAAATTAAGA---------------GAACAATTTGGG----
------------------------------------AACAAAACAATAGTCTTTAAT------
CACTCCTCAGGAGGGGACCCAGAGATTGTA---ATGCACAGTTTTAATTGTGGAGGGGAA---
TTTAAGCAGCTCCAGGCAAGAGTCCTGGCTGTGGAAAGATACCTAAAGGATCAACAGCTCCTAGGGATTTGGG
GTTGCTCTGGAAAACTCATTTGCCCCACTGCTGTGCCTTGGAATGCTAGTTGGAGT----------------
-----------------------------AATAAATCTCTAGAGGTGATTTGG------AAT------
AACATGACTTGGATGGAGTGG---GAAAGAGAAATTGACAATTACACAAAAGAAATATAC---
AACTTAATTGAAGAATCGCAGAACCAACAAGAA---------------------------------------
----

AAGAATGAACAAGAACTATTGGAATTGGATAAGTGGGCAAGTTTGTGGAATTGGTTTGAAATAACAAAATGGC
TGTGGTATATCAAAATATTCATAATGATAGTAGGAGGCTTGGTAGGTTTAAGAATAGTTTGTGCT---
ATACTTTCTATAGTA---------AATAGAGTTAGGCAGGGATACTCACCA------
TTGTCATTGGCTGTTAGCTTGCTTAACACCATAGCCATAGCAGTAGCTGAG---
GGGACAGATAGGGTTATAGAAGTAGTACAAAGAGCTTTTAGGGCT----------------------------
--------ATTCTCCATATA------CCTACAAGAATCAGACAGGGACTCGAAAGGGCTTTGCTA---TA-
>EU363827
GCT---GCA--
GAAAACTTGTGGGTCACAGTCTATTATGGGGTACCTGTATGG---AAAGATGCAAAT---
ACCACTCTATTTTGTGCATCAAATGCTAAAGCATACTCGGCAGAA---GTACATAAT----------------
--------------GTCTGGGCTACA---CATGCCTGTGTACCCACAGAC---
CCCAACCCACAAGAAGTAGTTTTGGGA---------AATGTGACAGAAAACTTTAAC----------------
--------------------------ATGTGGAAAAATGACATGGTAGAACAGATGAATGAAGATATA---
ATCAGTTTATGGGATCAAAGCCTA---AAGCCATGTGTAAAATTA---
ACCCCACTCTGTGTTACTTTAGATTGCATTAATTTGAATATTACTAATACCAGCAGT----------------
----
----------------------------GATACCAATAGTATGATAAAGGGAGGAGAAATGAAA---
AACTGCTCTTTCAATGTCACCACAAGCATAAGA---------------------------ACTAAGGTG---
AAAGACTATGCACTT---TTTTATAAACTTGATATAGTAAAAATAGATAATGATAAT----------------
----
-AATAATGCTACTAATACCAGCTATAGGTTGAGACATTGTAACACCTCAGTCATTACACAA---
GCTTGTCCAAAGATATCCTTTGAGCCAATTCCCATACATTATTGTACCCCGGCTGGTTTTGCGCTACTAAAGT
GTAAC---AATAAAACATTCAGT------GGAACAGGACCATGTACAAAT-----------------------
-GTCAGT---ACAGTACAATGTACACATGGAATTAGGCCAGTAGTATCAACT---
CAACTGCTGTTAAATGGCAGTTTAGCAGAAGGAGAG------------
GTAGTAATTAGATCTAGCAATTTCTCGAACAAT---------GCTAAAGTCATAATAGTACAGCTGAAT----
-----------AAATCTGTAGCA---------ATTAATTGTACAAGACCCAGCAACAATACAAGAAAA-----
-AGTATAAACATA------------GGACCAGGGAGAGCA---TGG------TATGCAACAGGACAA------
---------ATAATAGGGGATATA------------AGACAAGCACATTGT---AACATTAGTAGAATG---
CAATGGAATAACACTTTAAGACAGGTAGTTGAAAAATTAAGA---------------GAACAATTTGAG----
-----------------------------------AACAAAACAATAACCTTTAAT------
CAACCTTCAGGAGGTGACCCAGAGATTGTA---ATGCACAGTTTTAATTGTGGAGGGGAA---
TTTAAACAGCTCCAGGCAAGAGTCTTGGCTGTGGAAAGATACCTAAAGGATCAACAGCTCCTAGGGCTTTGGG
GTTGCTCTGGAAAACTCATCTGCACCACTACTGTGCCTTGGAATGTTAGTTGGAGT-----------------
----------------------------AATAAATCTTACAATGAGATTTGG------GAT------
AACATGACTTGGATGCAGTGG---GAAAGAGAAATTGACAATTACACAAAAGAAATATAC---
ACCTTAATTGAACAATCGCAAAACCAACAAGAA--

AAGAATGAACTAGAACTATTGGAATTGGATAAATGGGCAAGCTTGTGGAGTTGGTTTGACATATCAAACTGGC
TGTGGTATATTAAAATATTCATAATAATAGTAGGAGGCTTAATAGGTTTAAGAATAGTTTTTACT---
GTACTTTCTATAGTA---------AATAGAGTTAGGCAGGGATACTCACCA------
TTGTCATTTGCTATTAGCTTGTTTAACACCACAGCCATAGTAGTAGCTGAA---
GGGACAGATAGGGTTATAGAAGTAGCACGAAGAGCTTTTAGGGCT----------------------------
--------ATTCGCAACATA------CCTAGAAGAATCAGACAGGGCTTCGAAAGGTCTTTGCTA---TAA
>AY905493
GCT---GCA--
GAAAACTTGTGGGTCACAGTCTATTATGGGGTACCTGTATGG---AAAGACGCAATT---
ACCACTCTATTTTGTGCATCAGATGCTAAAGCATATGATACAGAG---GTACATAAT----------------
--------------GTTTGGGCTACA---CATGCCTGTGTACCCACAGAC---
CCCAACCCACAAGAAGTAGTCTTGGGA---------AATGTGACAGAAAACTTTAAC----------------
--------------------------ATGTGGAAAAATAACATGGTAGACCAGATGCATGAAGATATA---
ATCAGTTTATGGGATCAAAGCCTA---AAGCCATGTGCAAAACTA---
ACCCCACTCTGTGTTACTTTAAATTGCACTAATTTAAAGAATACTACTAGTACCATGAGT-------------
----
----------------------------AATACCAATGGTACAACAGAGGGAGGAGAAATGAAA---
AACTGCTCTTTCAATATCACCACAAGCACAAGC---------------------------ACTAAGGTG---
ACAGACTATGCACTT---TTTTATAAACTTGATATAATACCAATAGAGAAT----------------------
----
----------GATAATACTAGCTATAGGTTGATAAATTGTAACACCTCAGTCATTACACAG---
GCTTGTCCAAAGGTATCCTTTGAGCCAATTCCCATACATTATTGCACCCCTGCTGGTTTTGCGATTCTAAAGT

GTAAC---AATAAAAAATTCAAT------GGAACAGGACCATGTACAAAT----------------------
-GTCAGC---ACAGTACAATGTACACATGGAATTAGGCCAGTAGTATCAACT---
CAACTGCTGTTAAATGGCAGTTTAGCAGAAGAAGAG-----------
GTAGTAATTAGATCTGTCAATTTCTCGAACAAC---------GCTAAAGTCATAATAGTGCAGTTGAAA----
-----------GAATCTGTAGAA---------ATTAATTGTATAAGACCCAACAACAATACAAGAAAA-----
-AGTATACATATG------------GGACCAGGGCAAGCA---TGG------TTTGCAACAGGAGAC------
---------ATCATAGGAGATATA------------AGACAAGCACATTGT---AACATTAGTAGAACA---
AAATGGAATGACACTTTAAGACAGATAACTAAAAAATTAAGA---------------GAACAATTTGGG----
-----------------------------------AACAAAACAATAATCTTTAAT------
CAATCTGCAGGAGGGGACCCAGAGATTGTA---ATGCACAGTTTTAATTGTGGAGGGGAA---
TTTAAGCAGCTCCAGGCAAGAGTCTTGGCTGTGGAAAGATACCTAAAGGATCAACAGCTCCTAGGGATTTGGG
GTTGCTCTGGAAAACTCATTTGCACCACTGCTGTGCCTTGGAATGTTAGTTGGAGT-----------------
----------------------------AATAAATCTCTGAATGAGATTTGG------AAT------
AACATGACTTGGATGGAGTGG---GAAAGAGAAATTGACAATTACACAAGGGAAATATAC---
ACCTTAATTGAAGAATCGCAGAACCAGCAAGAA---------------------------------------
----
AAGAATGAACTAGAACTATTGGAATTGGATAAGTGGGCAAGTTTGTGGAGTTGGTTTGACATATCAAATTGGC
TGCGGTATATCAGAATATTCATAATGATAGTAGGAGGCCTAGTAGGTTTAAGAATAGTTTTTGCT---
GTACTTTCTATAGTA---------AATAGAGTTAGGCAGGGATACTCACCA------
TTGTCATTAGCTATTAGCTTGCTTAACGCCACAGCCATAGCAGTAGCTGAG---
GGGACAGATAGGGTTATAGAAGTAGTACAAAGAGCTTATAGGGCT--------------------------
--------ATTCTCAACATA------CCTACAAGAATCAGACAGGGCCTCGAAAGGGCTTTGCTA---TAA
>JF932492
GCT---GCA---
GAAAACTTGTGGGTCACAGTCTATTATGGGGTACCTGTATGG---AAAGAAGCAACT---
ACCACTCTATTTTGTGCATCAGATGCTAAAGCATATGATACAGAG---GTACATAAT----------------
--------------GTTTGGGCCACA---CATGCCTGTGTACCCACAGAC---
CCCAACCCACAAGAAGTAGTCTTGGTA---------AATGTAACAGAAAACTTTAAC---------------
--------------------------ATGTGGAAAAATAACATGGTAGAACAGATGCATGAAGATATA---
ATCAGTTTATGGGATCAAAGCTTA---AAGCCATGTGTAAAATTA---
ACCCCACTCTGTGTTACTTTAAATTGCACTGATTTGAGGAATAATACTACTGGTACCAGTAATAAT-------
----
------------------ACCAGTAGCAATACCAGTAGTATAATAGAGGGAAGAGAAATAAAA---
AACTGCTCTTTCAATATCATCACAAGCATAGGA--------------------------ACTACTGTA---
AAAGACTATGCACTT---TTTAATACACTTGATATAGTATCAATAGATAAT---------------------
----
AGTAATACCAGTTATAATACCAGTTATAGGTTGACACATTGTAACACCTCAGTCATTACACAA---
GCTTGTCCAAAGGTATCCTTTGAGCCAATCCCCATACATTATTGTACCCCAGCTGGTTTTGCGATTATAAAGT
GTAAC---AATAAAACATTCAGT------GGAACAGGACCATGTACAAAT----------------------
-GTCAGT---ACAGTACAATGTACACATGGAATTAGGCCAGTAGTATCAACT---
CAACTGCTGTTAAATGGCAGCTTAGCAGAAGAAGAG-----------
GTAGTAATTAGATCTAGCAATTTCACAAACAAT---------GCTAAAATCATAATAGTACAGCTGAAT----
-----------GAATCTGTAGAA---------ATTAATTGTACAAGACCCAACAACAATACAAGGAAA-----
-GGGGTATCTCTA------------GGACCAGGGCGAGTA---TGG------TATACAACAGGACAA------
---------ATAATAGGAAATATA------------AGACAAGCACATTGT---AACATTAGTAGAGCA---
AAATGGAATGACACTTTAAAACAGGTAGCTGGAAAATTAAAA---------------GAACAATTTGGG----
-----------------------------------AACAAAACAATAATCTTTAAT------
CCATCTTCAGGAGGGGACTCAGAGATTGTA---ATGCACAGTTTTAATTGTGGAGGGGAA---
TTTAAGCAGCTCCAGGCAAGAGTCTTGGCTGTGGAAAGATACCTAAAGGATCAACAGCTCCTAGGGATTTGGG
GTTGCTCTGGAAGACTCATTTGCACCACTGCTGTGCCTTGGAATGTTAGTTGGAGT-----------------
----------------------------AATAAATCTTATGATGAGATTTGG------GGT------
AACATGACTTGGATGGAGTGG---GAAAGAGAAATTGACAATTACACAAGAGAAATATAC---
ACCTTAATTGAAAATTCGCAGAACCAGCAAGAA---------------------------------------
----
AAGAATGAACTAGAACTATTGGAATTGGATAAGTGGGCAAGCTTGTGGAATTGGTTTGACATAACAAAATGGC
TGTGGTATATCAAAATATTCATAATGATAGTAGGAGGCTTGATAGGTTTAAGAATAGTTTTTGCT---
GTACTTTCTATAGTA---------AATAGAGTTAGGCAGGGATACTCACCA------
TTGTCATTTGCTATTAGCTTGCTTAACACAACAGCCATAGTAGTAGCTGAG---
GGGACAGATAGGGTTATAGAAGTAGTACAAAGAGCTTTTAGGGCT--------------------------
--------ATTCGCAACATA------CCTACAAGAATCAGACAGGGCCTCGAAAGGTCTTTGCTA---TAA

>AY905497
GCT---ACA---------------------------------------
GAAAAGTTGTGGGTCACAGTCTATTATGGGGTACCTGTATGG---AAAGAAGCAACT---
ACTACTCTATTTTGTGCATCAGATGCTAAAGCATATGTTGCAGAG---GTACATAAT---------------
--------------GTCTGGGCCACA---CATGCCTGTGTACCCACAGAC---
CCCAACCCACAAGAAGTAGTCTTGGGA---------AATGTGACAGAAAACTTTAAC---------------
-------------------------ATGTGGAAAAATGACATGGTAGATCAGATGCATGAAGATATA---
ATCAGTTTATGGGATCAAAGCCTA---AAGCCATGTGTAAAACTA---
ACCCCACTCTGTGTTACTTTAAATTGCACTGATTTGAGGAATACTAATAATACCAGTAATACTACC-------
----
---------------------AATAGTAATAGCAGTAGCCCGACAGAGGGAGGAGAAATGAAA---
AACTGCTCTTTCAATATCACCACAGACATAAAA--------------------------ACTAACGTG---
AAAGACTATGCACTT---TTTTATAAACTTGATCTAGTACAAGCAGAGAGT---------------------
----
----------GATAATACTAGCTATAGGTTAATAAATTGTAACACCTCAGTCATTACACAA---
GCTTGTCCAAAGGTATCCTTTGAGCCAATTCCTATACATTATTGTACCCCGGCTGGTTTTGCGATTCTAAAGT
GTAAC---AATAAAACATTCAAT------GGAACAGGACCATGTACAAAT----------------------
-GTCAGT---ACAGTACAATGTACACATGGAATTAGGCCAGTAGTATCAACT---
CAACTGCTGCTAAATGGCAGTTTAGCAGAAGAAGAG-----------
GTAGTAATTAGATCTAGCAATTTCTCGGACAAT---------GCTAAAATCATAATAGTACAGCTGAAT----
-----------AGTTCTGTAGAA---------ATTAATTGTACAAGACCCAACAACAATACAAGAAAA-----
-AGTATAAATATA------------GGACCAGGGCAGGCA---TGG------TATGCAACAGGACAA------
---------ATAATAGGAGATATA------------AGACAAGCACATTGT---AACATTAGTGGAGAA---
AAATGGAATAGCACTCTAAAACTGATAACTGAAAAATTAAGG---------------GTACAATTTGGG----
----------------------------------AACAAAACAATAATCTTTAAT------
CAATCTTCAGGAGGGGACCCAGAGATTGTA---ATGCACAGTTTTAATTGTGGAGGGGAA---
TTTAAGCAGCTCCAGGCAAGAGTCCTGGCTGTGGAAAGATACCTAAAGGATCAACAGCTCCTAGGGATTTGGG
GTTGCTCTGGAAAACTCATTTGCACCACTAATGTGCCTTGGAATGTTAGTTGGAGT----------------
----------------------------AATAAATCTCTAAATGAAATTTGG------GAT------
AACCTGACTTGGATGGAATGG---GAAAGAGAAATTGGCAATTACACAACACACATATAC---
ACCTTACTTGAACAATCGCAGAACCAACAAGAA---------------------------------------
----
AAGAATGAACTAGAACTATTGGAATTGGACAAGTGGGCAAGCTTGTGGAGTTGGTTTGACATATCAAATTGGC
TGTGGTATATCAGAATATTCATAATGATAGTAGGAGGATTGGTAGGTTTAAGAATAGCTTTTGCT---
GTACTTTCTATAGTG---------AATAGAGTTAGGCAGGGATACTCACCA------
TTGTCATTAGCTATTAGCTTGCTTAACGCCACAGCCATAGCAGTAGCTGAG---
GGGACAGATAGGGTTATAGAAGTAGTACAAAGAGTTTATAGGGCT--------------------------
--------ATTCTCAACATA------CCTACAAGAATCAGACAGGGCCTAGAAAGGGCTTTGCTA---TAA
>AY905495
GCT---ACA---------------------------------------
GAAAAATTGTGGGTCACAGTCTATTATGGGGTACCTGTATGG---AAAGAAGCAACA---
ACCACTCTATTTTGTGCATCAGATGCTAAAGCATATGAAACAGAG---GTACACAAT---------------
--------------GTTTGGGCCACA---CATGCCTGTGTACCCACAGAC---
CCCAACCCACAAGAAGTAGTTTTGGAA---------AATGTGACAGAAAACTCTAAC---------------
-------------------------ATGTGGAAAAATGACATGGTAGAACAGATGCATGAAGATATA---
ATCAGTTTATGGGATCAAAGCCTA---AAGCCATGTGTAAAATTA---
ACCCCACTCTGTGTTACTTTAAATTGCACTAATCTGAATATTACTAATACCAGTAGTAATACCAGTAGT----
----
-------------------ACTAATACCAGTAGTAGTAGTACGACAGGGGGAGGAGAAATAAAA---
AACTGCTCTTTCGATATCACCACTAGCATAGGA--------------------------ACTAAGGTG---
AAAGACTATGCATTT---TTTTATAAACTTGATATAGCACCAATAGGTAATGAT------------------
----
-AATAACAGCTCTAATACCAGCTATAGGTTGATAAGTTGTAACACCTCAGTCATTACACAA---
GCTTGTCCAAAGGTATCCTTTGAGCCAATTCCTATACATTATTGTGCCCCGGCTGGTTTTGCGATTCTAAAGT
GTAAC---AATAAAACGTTCAGT------GGAACAGGACCATGTACAAAT----------------------
-GTCAGT---ACAGTACAATGTACACATGGAATTAGGCCAGTAGTATCAACT---
CAACTGCTGTTAAATGGCAGTTTAGCAGAAGATGAG-----------
GTAGTAATTAGATCTAGCAATTTCACAAATAAT---------GCTAAAGTCATAATAGTACAGCTGAAT----
-----------GAATCTGTAACA---------ATTAATTGTACAAGACCCAACAACAATACAAGAAAA-----
-AGTATACATCTA------------GGACCAGGGCAAGCA---TGG------TATACAACAGGAGAA------

---------ATAATAGGAGATATA------------AGACAAGCACATTGT---AATCTTAGTAGAACT---
AAATGGAATGACACTTTAAGACACATAACTGAAAAATTAAGA---------------GAACAATTTAGG----
-----------------------------------AACAAAACAATAATCTTTAAT------
CCACCTTCAGGAGGGGACCAAGAGATTGTA---ATGCACACTTTTAATTGTGGAGGGGAA---
TTTAAACAGCTCCAGGCAAGAGTCTTGGCTGTGGAAAGATACCTAAAGGATCAACAGCTCCTAGGGATTTGGG
GTTGCCCTGGAAAACTCATTTGCACCACTGCTGTGCCTTGGAATATTAGTTGGAGT-----------------
----------------------------AATAAAAATCTGAGTCAGATTTGG------GAT------
AACATGACTTGGATGGAGTGG---GAAAAAGAAATCAGCAATTACACAAAAGAAATATAC---
ACCTTAATTGAAGAATCGCAGAACCAACAAGAA--

AAGAATGAACTAGAACTATTGGAATTGGATAAGTGGGCAAGTTTGTGGAATTGGTTTGACATATCAAATTGGC
TGTGGTATATCAAAATATTCATAATGATAGTAGGAGGCTTGGTAGGTTTAAGAATAGTTTTTACT---
GTACTTTCTATAGTA---------AATAGAGTTAGGCAGGGATACTCACCA------
TTGTCATTAGCTATTAGCTTGCTTAACGCCACAGCCATAGCAGTAGCTGGG---
GGGACAGATAGGGTTATAGAAATAGCACAAAGAGCTTTTAGGGCT----------------------------
--------ATACTCAACATA------CCTACAAGAATCAGACAGGGCTTTGAAAGGGCTTTGTTA---TAA
>AY905494
GCT---ACA--
GAAAAATTGTGGGTCACAGTCTATTATGGGGTACCTGTATGG---AAAGAAGCAAAT---
ACCACTCTATTTTGTGCATCAGATGCTAAGGTATATGCTACAGAG---GTACACAAT----------------
--------------ATTTGGCCACA---CATGCCTGTGTACCCACAGAC---
CCCAACCCACAAGAAGTAGTCTTGGGG---------AATGTGACAGAAAACTTTAAC----------------
--------------------------ATGTGGAAAAATAACATGGTAGAACAGATGCATGAAGATATA---
ATCAGTTTATGGGATCAAAGCCTA---AAGCCATGTGTAAAATTA---
ACCCCACTATGTGTTACTTTAAATTGCACTAATTTGAATAATACTACTAATACCAGTAGTAATACCAGT----
----
----------------AGTACTAGTACCAGTAGTAGTAGTATGACAGAGGGAGGAGAAATAAAA---
AACTGCTCTTTCAATGTCACCACAAGCATAAAA---------------------------ACTAAGGTG---
AAAGACTATGCATTT---TTTTATAAACTTGATATAGTACCAATAGCTACT----------------------
----
----------GATAATACCAGCTACAGGTTGATAAATTGTAACACCTCAGTCATTACACAA---
GCTTGTCCAAAGGTATCCTTTGAGCCAATTCCCATACATTATTGTGCCCCGGCTGGTTTTGTGATTATAAAGT
GTAAC---AATAAAACGTTCAGT------GGAACAGGACCATGTACAAAT-----------------------
-GTCAGT---ACAGTACAATGTACACATGGAATTAGGCCAGTAGTATCAACT---
CAACTGCTGCTAAATGGCAGTTTAGCAGAAGAAGAG------------
GTAGTAATTAGATCTAGCAATTTCACGGACAAT---------GCTAAAACCATAATAGTACAGCTGAAT----
-----------GAATCTGTAGTA---------ATTAATTGTACAAGACCCAACAACAACACAAGAAAA-----
-AGTATACATCTA------------GGACCAGGGAGAACA---TGG------TATACAACAGGACAA------
---------ATAATAGGAAATATA------------AGACAAGCACATTGT---AACCTTAGTAGAACA---
GGATGGAAGGATACTTTAAAACAGATAACTGAAAAATTAAGA---------------GAACAATTTGGG----
-----------------------------------AACAAAACAATAATCTTTAAT------
CAATCTTCAGGAGGGGACCCAGAGATTGCA---ATGCACAGTTTTAATTGTGGAGGGGAA---
TTTAAGCAGCTCCAGGCAAGAGTCTTGGCTGTGGAAAGATACCTAAAGGATCAACAGCTCCTAGGGATTTGGG
GTTGCTCTGGAAAACTCATTTGCACCACTGCTGTGCCTTGGAATGTTAGTTGGAGT-----------------
----------------------------AATAAATCTCTGAGTGAGATTTGG------AAT------
AACATGACTTGGATGGAGTGG---GAAAAAGAAATTAACAATTACACAAGAGAGATATAC---
ACCTTAATTGAAGAATCGCAGAACCAACAAGAA--

AAGAATGAATTAGAACTATTGGAATTGGACAAGTGGGCAAGCTTGTGGAATTGGTTTGACATAACAAAATGGC
TGTGGTATATCAAAATATTCATAATGATAGTAGGAGGCTTGGTAGGTTTAAGAATAGTTTTTGCT---
GTACTTTCTATAGTA---------AATAAAGTTAGGCAGGGATACTCACCA------
TTGTCATTAGCTATTAGCTTGCTTAACGCCACAGCCATAGCAGTAGCTGAG---
GGGACAGATAGGGTTATAGAAGTAGCACAAAGAGCTTTTAGGGCT----------------------------
--------ATTCTCAACATA------CCTACAAGAATTAGACAGGGCTTTGAAAGGGCTTTGCTA---TAA
>HM215397
GCT---GCA--
GAAAACTTGTGGGCCACAGTCTATTATGGGGTACCTGTATGG---AAAGAAGCAACT---
ACCACTCTATTTTGTGCATCAGATGCTAGAGCATATGATACAGAG---GTACATAAT----------------
--------------ATTTGGCCACA---CACGCCTGTGTACCCACAGAC---
CCCAACCCACAAGAAGTAGTATTGGGA---------AATGTGACAGAAAACTTTAAC----------------

--------------------------ATGTGGATAAATGACATGGTAGAACAAATGCATGAAGATATA---
ATCAGTTTATGGGATCAAAGCCTA---AAGCCATGTGTAAAATTA---
ACCCCACTCTGTGTTACTTTAAATTGCTCTAATTTGAATACTACTAATACC----------------------
----
----------------------------------AGTAGTACAATAGAGGGAGGAGAAATAAAA---
AACTGCTCTTTCAATATCACCACAAGCATAAGC---------------------------ACTAAGGTA---
AAAGACTATGCAATT---TTTAATAAGCTTGATATAGTACCAATAGGTAAT----------------------
----
----------GATACTACCAGCTATATATTAATAAATTGTAACACCTCAGTCATTACACAA---
GCTTGTCCAAAGGTATCCTTTGAGCCAATTCCCATACATTATTGTACCCCGGCTGGTTTTGCGATTCTAAAGT
GTAAC---AATAAAACGTTCCAG------GGAACAGGACCATGTACAAAT-----------------------
-GTCAGT---ACAGTACAATGTACACATGGGATTAGGCCAGTAGTATCAACT---
CAACTGCTGTTAAATGGCAGTTTAGCAGAGGAAGAG------------
GTAGTAATTAGATCTAGCAATTTCTCGAACAAT---------GCTAAAGTCATAATAGTACAGCTGAAT----
-----------AAATCTGTAGAA---------ATTAATTGTACAAGACCCAACAACAATACAAGAAAA-----
-AGTATATCTCTA------------GGACCAGGGAGAGCA---TGG------TATGCAACAGGGCAA------
---------ATAATAGGAAATATA------------AGACAAGCACATTGT---AACCTTAGTAGAACA---
GAATGGAATAACACTTTAAAACAAATAGTTAACAAATTAAGA--------------GAACAATATGGG----
-----------------------------------AACAAAACAATAATCTTTAAT------
CAATCTTCAGGAGGGGACCTAGAGATTGTA---ATGCACAGTTTTAATTGTGGAGGGGAA---
TTTAAGCAGCTCCAGGCAAGAGTCTTGGCTGTGGAAAGATACCTGAAGGATCAACAGCTCCTAGGGATTTGGG
GTTGCTCTGGAAAACTCATTTGCACCACTACTGTGCCTTGGAATAGTAGTTGGAGT-----------------
----------------------------AATAAATCTCTGAGTGAGATTTGG------GAT------
AACATGACTTGGATGGAATGG---GAAAGAGAAATTGCCAATTACACAAGAGAAATATAC---
ACCTTAATTGAAGAATCGCAAAACCAACAGGAA--

AAGAATGAACTAGAATTATTGGAATTGGATAAGTGGGCAAGCTTGTGGAATTGGTTTGACATAACAAAATGGC
TGTGGTATATCAGAATATTCATAATGATAGTAGGAGGCTTAGTAGGTTTAAGAATAGTTTTTGCT---
GTACTTTCTATAGTA---------AATAGAGTTAGGCAGGGGTACTCACCA------
TTGTCATTAGCAATTAGCTTGCTTAACGCCACAGCCATAGCAGTAGCTGAG---
GGGACAGATAGGGTTATAGAAGTAATACAAAGAGTTTATAGGGCT---------------------------
--------ATTCTTAACATA------CCTACAAGGATAAGACAGGGCCTCGAAAGGGCTTTGCTA---TAA
>HQ699990
GCT---GCA--
GAAAATTTGTGGGTCACAGTCTACTATGGGGTACCTGTATGG---AAAGACGCAACT---
ACCACTCTATTTTGTGCATCAGATGCTAAAGCATATGATACAGAG---GTGCATAAT----------------
--------------GTTTGGGCCACA---CATGCCTGTGTACCCACAGAC---
CCCAACCCACAAGAAGTAGTCTTGGGA---------AATGTGACAGAAAACTTTAAC----------------
--------------------------ATGTGGAAAAATGGCATGGTAGAACAGATGCATGAAGATATA---
ATCAGTTTATGGGATCAAAGCCTG---AAGCCATGTGTAAAGGTA---
ACCCCACTCTGTGTTACTTTAAATTGCAATAATACTGTGGGGAATGCTACTGATAGTAAT-------------
----
-------------------------AGTACTACCAGTAGCACGACAGGGGGAGGAGAAATAAAA---
AATTGCTCTTTCAATGTCACCACAAACATAAGA---------------------------ACTGAGGGA---
AGAAACTATGCTCTT---TTCAATGCACTTGATGTAGTACCAATAGGTGAG----------------------
----
----------GATAATACCAGCTATATATTGAGACATTGTAACACCTCAGTCATTACACAA---
GCTTGTCCAAAGGTATCCTTTGAGCCAATTCCCATACATTATTGTGCTCCGGCTGGTTTTGCGATTATAAAGT
GTAAC---AATAAAACATTTGAT------GGAAAAGGGCCATGTACAAAT-----------------------
-GTCAGT---ACAGTACAGTGTACACATGGAATTAGGCCAGTAGCATCAACT---
CAACTGCTGTTAAATGGCAGTCTAGCAGAAGAAGAG------------
GTAGTAATTAGGTCTAGCAATTTCACGGACAAT---------GTTAAAGTCATAATAGTGCAGCTGAAT----
-----------GAATCTGTACAA---------ATTAATTGTACAAGACCCAACAACAATACAATAAAA-----
-GGCATACATCTA------------GGACAAGGGCGAACA---TGG------TTTACAACAGGACAA------
---------ATAATAGGAGATATA------------AGACGAGCACATTGT---AACATTAGTAGTGCA---
AAATGGACTAACACTTTAAAACAGATAGCAGAAAAATTAAGA--------------AAACAATTTGAG----
-----------------------------------GAAAAAACAATAATCTTTACT------
CAATCTTCAGGAGGGGACCCAGAGATTGTA---ATGCATAGTTTCAATTGTGGAGGGGAA---
TTTAAGCAGCTCCAGGCAAGAGTCTTGGCTGTGGAAAGATACCTACAGGATCAACAGCTCCTAGGGATTTGGG
GTTGCTCTGGAAAACTCATTTGCACCACTGCTGTGCCTTGGAATGTTAGTTGGAGT-----------------

----------------------------AATAAATCTCTGGAGAAAATTTGG------AGT------
AACATGACTTGGATGGAGTGG---GAAAGAGAAATTGCTAATTACACAAGAGAAATATAC---
ACCTTAATTGAACAATCGCAGAACCAGCAAGAA--

AAGAATGAACTAGAACTATTGGAATTGGATAAGTGGGCAAGCTTGTGGAATTGGTTTGACATAACAAACTGGC
TGTGGTATATCAAAATATTCATAATGATAGTAGGAGGCTTGGTAGGTTTAAGAATAGTTTTCGCT---
GTACTTTCTATAGTA---------AATAGAGTTAGGCAGGGATACTCACCA------
TTGTCGTTAGCTGTTAGCTTGCTTAACGCCACAGCCATAGCAGTAGGTGAG---
GGGACAGATAGGGTTATAGAAGTGGCACTAAGAGTTTATAGGGCT-----------------------------
--------ATTCTTAATATA------CCTACAAGAGTCAGACAGGGCCTCGAAAGGGCATTGCTG---TAA
>HQ699996
GCT---GCA---
GAAAACTTGTGGGTCACAGTCTATTATGGGGTACCTGTATGG---AAAGAAGCAACT---
ACCACTCTATTTTGTGCATCAGATGCTAAAGCATATGCTACAGAA---GTACATAAT----------------
--------------GTTTGGGCTACA---CATGCCTGTGTACCCACAGAC---
CCTAACCCACAAGAAGTAGTTTTGGAA---------AATGTGACAGAAAACTTTAAC----------------
-------------------------ATGTGGAAAAATAACATGGTAGAACAGATGCATGAAGATATA---
ATCAGTTTATGGGATCAAAGCCTA---AAGCCATGTGTAAAATTA---
ACCCCACTCTGTGTTACTTTAAATTGCACTAATTTGAGTAATACTACTAATGGGAATACTACTGAG-------
----
------------------GCCAGAAATAATACCAGTAGTATGACAGAGGGAGGAGAAATGAAA---
AACTGCTCTTTCAATATCACCACAAGCATAAGA---------------------------ACTCAGGTG---
AAAGACCATGCACTT---TTTTATGAACTTGATGTAGTACCAATAAAGGAG---------------------
----
----GATAATGATAATACCAGTTACAGGTTGAGACATTGTAACACCTCAGTCATTACACAA---
TCTTGTCCAAAGGTATCCTTTGAGCCAATTCCCATACATTATTGTACACCGGCTGGTTTTGCGATTCTAAAGT
GTAAC---AATAAAAAGTTCAAT------GGAACAGGACCATGTACAAAT----------------------
-GTCAGT---ACAGTACAATGTACACATGGAATTAGGCCAGTAGTATCAACT---
CAACTGCTGTTAAATGGCAGTTTAGCAGAAGAAGAG-----------
GTAGTAATTAGATCTAGCAATTTCACGGACAAT---------GCTAAAGTCATAATAGTACAGCTGAAT----
-----------GAAGCTGTAGAA---------ATTAATTGTACAAGACCCAACAACAATACAAGAAAA-----
-AGTATAAATTTA------------GGACCAGGACAAGCA---TGG------TATACAACAGGACAA------
---------ATAATAGGAGATATA-----------AGACAAGCACATTGT---AACCTTAGTAGTACA---
AAATGGAACAACACTTTAAAACAGATAGTTAGAAAATTAAGA---------------GAACAATTTAAG----
-------------------------------------AACAAAACAATAATCTTTGAT------
CAACAGTCAGGAGGGGACCTAGAGATTCAA---ATGCACATTTTTAATTGTGGAGGGGAA---
TTTAAGCAGCTCCAGGCAAGAGTCTTGGCTGTGGAAAGATACCTAAAGGATCAACAGCTCCTAGGGATTTGGG
GTTGCTCTGGAAAACTCATTTGCACCACTGCTGTGCCTTGGAATGTTAGTTGGAGT-----------------
----------------------------AATAAATCTCTGAGTGAGATTTGG------AAT------
AACATGACTTGGATGGAGTGG---GAAAGAGAAATTGACAATTACACAAGAGAAATATAC---
ACCTTAATTGAAGAATCGCAGAACCAACAAGAA--

AAGAATGAACTAGAACTATTGGAATTGGATAAGTGGGTAAGTTTGTGGAATTGGTTTGACATAACAAAATGGC
TGTGGTATATCAAAATATTCATAATGGTAGTAGGAGGCTTGGTAGGTTTAAGAATAGTTTTTACT---
GTACTTTCTATAGTA---------AATAGAGTTAGGCAGGGATACTCACCA------
TTGTCATTAGCTATTAGCTTGCTTAACGCCACTGCCATAGCAGTAGCTGAG---
GGGACAGATAGGATTATAGAAGTAACACAAAGAGCTTTTAGGGCT-----------------------------
--------ATTCTCAACATA------CCTACAAGAATCAGACAGGGCCTCGAAAGGGCTTTGCTA---TAA
>EU131797
GCT---GCA---
GAAAAATTGTGGGTCACAGTCTATTATGGGGTACCTGTATGG---AAAGAAGCAACT---
ACCACTCTATTTTGTGCATCAGATGCTAAAGCATATGCTACAGAG---GTACATAAT----------------
--------------ATTTGGGCCACA---CATGCCTGTGTACCCACAGAC---
CCTAACCCACAAGAAGTAGTCTTGGGA---------AATGTGACAGAAAATTTTAAC----------------
-------------------------ATGTGGAAAAATAACATGGTAGAACAGATGCATGAAGATATA---
ATCAGTTTATGGGATCAAAGCTTA---AAGCCATGTGTAAAATTA---
ACCCCACTCTGTGTTACTTTAAATTGCACTAATTTGAACAATACTGCTAATATCAGTAGT-------------
----
----------------------------AATACCAGTAGTATAACAGAGGGAGGAGAAATGAAA---
AACTGCTCTTTCAATGTCACCACAAGCATAGGA---------------------------ACTAAGAAG---

AAAGACTATGCACTT---TTTTATAAACTTGATGTAGAACCAATAGATAAT---------------------
----
-------------AGTACCAGCTATAGGTTGACAAAATGTAACACCTCAGTCATTACACAA---
GCTTGTCCAAAGGTATCCATTGAGCCAACTCCCATACATTATTGTACCCCGGCTGGTTTTGCGATTCTAAAGT
GTAAC---AATAAAACGTTCAAT------GGAACAGGACCATGTACAGAT----------------------
-GTCAGT---ACAGTACAATGTACACATGGAATTAGGCCAGTAGTATCAACT---
CGACTGCTGTTAAATGGCAGTTTAGCAGAAGAAAAG-----------
GTAGTAATTAGATCTAGCAATTTCACGAACAAT---------GCTAAAATCATAATAGTACAGCTGAAT----
-----------AAATCTGTAGAA---------ATTAATTGTACAAGGCCTAACAACAATACAAGAAAA-----
-AGTATACATCTG------------GGACAAGGGCGAGCA---TGG------TATACAACAGGACAA------
---------ATAATAGGAGATATA------------AGACAAGCACATTGT---AACATTAGTAGAATA---
GAATGGAATAACACTTTAGAACAGATAACTGAAAAATTAAGA---------------GAACAATTTGGG----
-----------------------------------AACCAAACAATAATCTTTAAT------
CAATCTTCAGGAGGGGACCCAGAGATTGCA---ACGCACAGTTTCAATTGTGGAGGGGAA---
TTTAAGCAGCTCCAGGCAAGAGTCTTGGCTGTGGAAAGATACCTAAAGGATCAACAGCTCCTAGGGATATGGG
GTTGCTCTGGAAAACTCATCTGCACCACTGCTGTGCCTTGGAATGTTAGTTGGAGT-----------------
----------------------------AATAAATCTTATAGTGAGATTTGG------GAT------
AACATGACTTGGATGCAGTGG---GAAAGAGAAATTGACAATTACACAAAAGAAATATAC---
ACCTTAATTGAAGAATCGCAGAACCAACAAGAA--

AAGAATGAACTAGAACTATTGGAATTGGATAAGTGGGCAAGTTTGTGGAATTGGTTTGACATAACAAAATGGC
TGTGGTACATCAAAATATTCATAATGATAGTAGGAGGCTTGGTAGGTTTAAGGATAGTTTTTGCT---
GTCCTTTCTATAGTA---------AATAGAGTTAGGCAGGGATACTCACCC------
TTGTCGTTAGCTATCAGCTTGCTTAACGCCACAGCCATAGCAGTAGCTGAG---
GGGACAGATAGGGTTATAGAAGTAGTACAAAGAGCTTGTAGGGCT---------------------------
--------ATTCGCAACATA------CCTACAAGAATCAGACAGGGCCTTGAAAGAGCTTTGCTA---TAA
>EU131791
GCT---GCA---
GAAAACTTGTGGGTCACAGTCTATTATGGGGTGCCTGTGTGG---AAAGAAGCAACT---
ACCACTCTATTTTGTGCATCAGATGCTAAAGCATATGAGACAGAG---GTACATAAT----------------
--------------ATCTGGGCCACA---CATGCCTGTGTACCCACAGAC---
CCCAACCCACGAGAAGTAGTCTTGGGA---------AATGTGACAGAAAACTTTAAC----------------
--------------------------ATGTGGAAAAATAACATGGTAGACCAGATGCATGAAGATATA---
ATCAATTTATGGGATCAAAGCCTA---AAGCCATGTGTAAAATTA---
ACCCCACTCTGTGTTACTTTAAATTGCACTGATGTGAATACTACTGATACCAGTAGTAATACTACTGAT----
----
-------------------ACCAGTAATATTACCAGTAGTATGACAGAGGAAGGAGAAATAAAA---
AACTGTACTTTCAATATCACCACAAGCATAAGA--------------------------ACTACGGTG---
AAAGACTATGCACTT---TTTTATAAATATGATATAGATGCAATAGATAAT---------------------
----
-------------AAGACCAGCTATAGGCTGACAAAGTGTAACAACTCAGTCATTACACAA---
GCTTGTCCAAAAGTATCCTTTGAGCCAATTCCCATACATTATTGTACCCCGGCTGGTTTTGCGATTCTAAAGT
GTAAC---AATAAAACGTTCAGT------GGAACAGGACCATGTACAAAT----------------------
-GTCAGC---ACAGTACAATGTACACATGGAATTAGGCCAGTAGTATCAACT---
CAACTGCTGTTAAATGGCAGTTTGGCAGAAGAAGAG-----------
GTAATAATTAGATCTAGCAATTTCTCAGACAAT---------GCTAAAGTTATAATAGTACAGCTGAAT----
-----------GAATCTGTAGAA---------ATTAATTGTACAAGACCCAACAACAATACAAGAAAA-----
-AGTATACATCTA------------GGACCAGGGCATGCA---TGG------CATACAACAAGACGA------
---------ATAGTAGGAAATATA------------AGACAAGCACATTGT---AACATTAGTAGAGCA---
AAATGGAATAACACTTTAAAACTGGTAACTGAAAAATTAAGA---------------GTACAATTTGGG----
-----------------------------------AACAAAACAATAATCTTTACT------
CAACCTGCAGGAGGGGACTCAGAAGTTGTA---ATGCACAGTTTTAATTGTGGAGGGGAA---
TTTAAGCAGCTCCAGGCAAGAGTCTTGGCTGTGGAAAGATACCTAAAGGATCAACAGCTCCTAGGGATTTGGG
GTTGCTCTGGAAAACTCATTTGCACCACTACTGTGCCTTGGAATGCTAGTTGGAGT-----------------
----------------------------AATAAATCTCTGAATGAGATTTGG------GAT------
AAAATGACTTGGATGGAGTGG---GAAAGAGAAATTAACAATTACACAAAAGAAATATAC---
TCCTTAATTGAAGAATCGCAAAACCAACAAGAA--

AAGAATGAACAAGAACTATTGGCATTGGATAAGTGGGCAAGTTTGTGGAGTTGGTTTAACATATCAAATTGGC
TGTGGTATATCAAAATATTCATAATGATAGTAGGAGGCTTGATAGGTTTAAGAATAGTTTTTGCT---

GTACTTTCTATAGTA---------AATAGAGTTAGGCAGGGATATTCACCA------
TTGTCATTAGCTATTAGCTTGCTTAACGCCACAGCCATAGCAGTAGCTGAG---
GGGACAGATAGAATCATAGAAGTAGTACAAAGAGCTTATAGGGCT----------------------------
--------ATTCTTAACATA------CCTACAAGAATCAGACAGGGCCTCGAAAGGGCTTTGCTA---TAA
>EU131790
GCT---GCA--
GAAAACTTGTGGGTCACAGTCTATTATGGGGTACCTGTATGG---AAAGAAGCAACT---
ACTACTCTATTTTGTGCATCAGATGCTAAAGCATATGAGACAGAG---GTACATAAT----------------
--------------ATTTGGACCACA---CATGCCTGTGTGCCCACAGAC---
CCCAACCCACAAGAAGTAGTATTGGGA---------AATGTGACAGAAAACTTTAAC----------------
--------------------------ATGTGGAAAAATAACATGGTAGACCAGATGCATGAAGATATA---
ATCAGTTTATGGGATCAAAGTCTA---AAGCCATGTGTAAAATTA---
ACCCCACTCTGTGTTACTTTAAATTGCACTAATTTGAATAATACCAGTAGTAAT-------------------
----
-------------------------------ATCAGTAGTACGACAGAGGGAGGAGAAATAAAA---
AACTGCTCTTTCAATGTCACCTCAAGCATAAGA---------------------------ATT---AAG---
AAAGACTATGCACTT---TTTTATAATCTTGATATAGTACCAATAGATAAT----------------------
----
----GATAATGATAATACTAGCTATATGTTGAGAAGTTGTAACACCTCAATCATTACACAA---
GCTTGTCCAAAGGTATCCTTTGAGCCAATTCCCATACATTATTGTACCCCGGCTGGTTTTGCGATTCTAAAGT
GTAAC---AATAAAACATTCAAT------GGAACAGGACCATGTACAAAT-----------------------
-GTCAGT---ACAGTACAATGTACACATGGAATTAGGCCAGTAGTATCAACT---
CAACTGTTGTTAAATGGCAGTTTAGCAGAAGAAGAG------------
GTAATAATTAGATCTAGCAATTTCACGGACAAT---------ACTAAAGTCATAATAGTACAGCTGAAT----
-----------GAATCTGTAGAA---------ATTAATTGTACAAGACCCAACAACAATACAAGAAAA-----
-AGTATAAATATA------------GGACCAGGGCGATCA---TGG------TATACAACAGGACAA------
---------ATAATAGGAGATATA------------GGACAAGCCCATTGT---AACATTAGTGGAGTA---
AAATGGAATAACACTTTAAAACTGATAACTATAAAATTAAGA---------------GAACAATTTGGG----
-----------------------------------AACAAAACAATAATCTTTAAT------
CAATCTGCAGGAGGGGACCCAGAGATTGAA---ATGCACATTTTTACTTGTGGAGGAGAA---
TTTAAGCAGCTCCAGGCAAGAGTCTTGGCTGTGGAAAGATACCTAAAGGATCAACAGCTCCTAGGGATTTGGG
GTTGCTCTGGAAAACTCATTTGCACCACTGCCGTGCCTTGGAATGTTAGTTGGAGT-----------------
----------------------------AATAAATCTCTGAGTGAGATTTGG------AAT------
AACATGACTTGGATGGAGTGG---GAAAGAGAAATTGACAATTACACAAAAGACATATAC---
ACCTTAATTGAAGAATCGCAAAACCAACAAGAA--

AAGAATGAACTAGAACTATTGGAATTGGATAAATGGGCAAGTTTGTGGAATTGGTTTGACATAACAAAATGGC
TATGGTATATCAAAATATTCATAATGATAGTAGGAGGCTTGGTAGGTTTAAGAATAATTTTTGCT---
GTACTTTCTATAGTA---------AATAGAGTTAGGCAGGGATACTCACCA------
TTGTCATTTGCTATTAGCTTGCTTAACGCCACAGCAGCAGCAGTAGCTGAG---
GGGACAGATAGGGTTATAAAAGTAGTACAGAGAGCTTGTTGGGCT----------------------------
--------ATTCGCAACATA------CCTAGAAGAATCAGGCAGGGTCTCGAAAGATCTTTGCTA---TAA
>EU131788
GCT---GCA--
GAAAACTTGTGGGTCACAGTCTATTATGGGGTACCTGTATGG---AAAGAAGCAACT---
ACCACTCTATTTTGTGCATCAGATGCTAAAGCATATGAGACAGAG---GCACATAAT----------------
--------------ATTTGGGCCACA---CATGCCTGTGTACCCACAGAC---
CTCAACCCACAAGAAGTAGTCTTGGAA---------AATGTGACAGAAAACTTTAAC----------------
--------------------------ATGTGGAAAAATGACATGGTAGACCAGATGCATGAAGATATA---
ATCAATTTATGGGATCAAAGCCTA---AAGCCATGTGTAAAATTA---
ACCCCACTCTGTGTTACTTTACATTGCACTGATTTGAAGACAACTAATACCAGTAGT----------------
----
----------------------------AATATCACTAGTACGACAGAGGGAGGAGAAATGAAA---
AACTGCTCTTTCAATATCACCACAAGCATAAAT---------------------------ACTAAGGTG---
AAAGACTATGCACTT---TTTCATAAACTTGATCTAGAATCAATAGGTAAG----------------------
----
----------AAGACTGCCAGCTATAGGTTGATACATTGTAACACCTCAACCATTACACAG---
GCTTGTCCAAAAATATCCTTTGAGCCAATTCCTATACATTATTGTACCCCGGCTGGTTTCGCGATTCTAAAGT
GTAAC---AATAAAACGTTCAAT------GGAACAGGACCATGTACAAAT-----------------------
-GTCAGT---ACAGTACAATGTACACATGGAATTAGGCCAGTAGTATCAACT---

CAACTGCTTTTAAATGGCAGTTTAGCAGAGGAAGAG-----------
GTAGTAATTAGATCCAGCAATTTCACAGACAAT---------GCTAAAGTCATAATAGTACAGCTGAAT----
-----------AAATCTGTACAA---------GTTAATTGTACAAGACCCAACAACAATACAAGAAAA-----
-AGTATACATCTA------------GGACCAGGGCACGCA---TGG------CATACAACAGAACGA------
---------ATAATAGGAAATATA------------AGACAAGCACATTGT---AACCTTAGTAGAACA---
GATTGGAATAACACTTTAAAACTGATAACTGAAAAATTAAGA---------------GTACAATTTGGG----
-----------------------------------GGCAAAACAATAATCTTTAAT------
CAATCTTCAGGAGGGGACCCAGAGATTGCA---ATGCATAGTTTTAATTGTGGAGGGGAG---
TTTAAGCAGCTCCAGGCAAGAGTCCTGGCTGTGGAAAGATACCTAAAGGATCAACAGCTCCTAGGGATTTGGG
GTTGCTCTGGAAAACTCATTTGCACCACTGCTGTGCCTTGGAATACTAGTTGGAGT-----------------
----------------------------AATAAATCTCTGAGTCAGATTTGG------GAT------
AACATGACTTGGATGGAGTGG---GAAAGAGAAATTAGCAATTACACAAGAGAAATATAC---
ACCTTAATTGAAGAATCGCAAAACCAACAGGAA--

AAGAATGAACTAGAACTATTAGAATTGGATAAGTGGGCAAGCTTGTGGAATTGGTTTGGCATAACAAAATGGC
TGTGGTATATCAAAATATTCATAATGATAGTAGGGGGCTTAGTAGGTTTAAGAATAATTTTTGCT---
GTACTTTCTATAGTA---------AATAGAGTTAGGCAGGGATACTCACCA------
TTGTCATTGGCTATTAGCTTGCTTAACGCCACAGCTATAGCAGTAGCTGAG---
GGGACAGATAGGATTATAGAAGTAGCACAAAGAGCTTTTAGAGCT----------------------------
--------TTTCTCCACATA------CCTACCAGAATCAGACAGGGCCTAGAAAGGGCTTTGCTA---TAA
>EU131808
GCT---GCA--
GGAAACTTGTGGGTCACAGTCTATTATGGGGTACCTGTATGG---AAAGAAGCAACT---
ACTACTCTATTTTGTGCATCAGATGCTAAAGCATATGATACAGAG---GTACATAAT----------------
--------------GTTTGGGCCACA---CATGCCTGTGTACCCACAGAC---
CCCAACCCACAAGAAGTAGTCTTGGAA---------AATGTGACAGAAAACTTTAAC----------------
--------------------------ATGTGGAAAAATGACATGGTAGAACAGATGCAGGAAGATATA---
ATCAGTTTATGGGATCAAAGCCTA---AGGCCATGTGTAAAATTA---
ACCCCACTCTGTGTTACTTTAAATTGCTCTGATTGTGTTACTTCAAATTGCTCTAATTTGAATACTACT----
----
----------------AAGACCAATAATAATACCAGTAGTACAATAGGGGGAGGAGAAATGAAA---
AACTGCTCTTTCAATATCACCACAAGCATAAGA---------------------------CCT---AAG---
AAAAACTATGCACTT---TTTTATACACTTGATATACAACCAATAGAGGGA----------------------
----
----------GATAATTCCAGCTATATATTGACAAGTTGTAACACCTCAATCATTACACAG---
GCTTGTCCAAAGATATCCTTTGAACCAATTCCTATACATTATTGTGCCCCGGCTGGTTTTGCGATTCTAAAGT
GTAAC---AATAAAACGTTCAAT------GGAACAGGACCATGTACAAAT-----------------------
-GTCAGT---ACAGTACAATGTACACATGGAATTAGGCCAGTAGTATCAACT---
CAACTGCTGTTAAATGGCAGTTTAGCAGAAGAAGAG-----------
GTAGTAATTAGATCTAGCAATTTCACGAACAAT---------GCTAAAATCATAATAGTACAGCTGAAT----
-----------GAATCTGTAGAA---------ATTAATTGTACAAGACCCAGCAACAATACAAGAAAA-----
-AGTATACAGCTA------------GGACAAGGGCGAGCA---TGG------TATACAACAGGACAA------
---------ATAATAGGAGATATA------------AGAAGAGCACATTGT---AACATTAGTGGGACA---
AAATGGAATAACACTTTAAATCAGATAGTTAAAAAATTAAGA---------------GAACAATTTGGG----
-----------------------------------AACAAAACAATAATCTTTAAT------
CAATCTTCAGGAGGGGACCCAGAGATTGTA---ATGCACAGTTTTAATTGTGGAGGGGAA---
TTTAAGCAGCTCCAGGCAAGAGTCCTGGCTGTGGAAAGATACCTAAAGGATCAACAGCTCCTAGGGATTTGGG
GTTGCTCTGGAAAACTCATTTGCCCCACTACTGTGCCTTGGAATGTTAGTTGGAGT-----------------
----------------------------AATAAATCTCTGAGTGAGATTTGG------AAT------
AACATGACTTGGATGGAGTGG---GAAAGAGAAATTGGCAATTACACAAGTGTAATATAC---
AGCTTAATTGAAGAATCGCAGAACCAACAGGAA--

AAGAATGAACTAGAACTATTGGAATTGGATAAGTGGGCAAGTTTGTGGAATTGGTTTGACATGACAAAATGGC
TGTGGTATATCAAAATATTCATAATGATAGTAGTAGGCTTGGTAGGTTTAAGAATAGTTTTTTCT---
GTACTTTCTATAGTA---------AATAGAGTTAGGCAGGGTTACTCACCA------
TTGTCATTGGTTATTAGCTTGCTTAACGCCACAGCCGTAGCAGTAGCTGAA---
GGGACAGATAGGATTATAGAAGTAGCACAAAGAGCTGTTAGGGCT----------------------------
--------ATTCTCAACATA------CCTACAAGAATCAGACAGGGCCTAGAAAGAGCTTTGCTA---TAA
>EU131789

GCT---GCA--
GAAAACTTGTGGGTCACAGTCTATTATGGGGTACCTGTATGG---AAAGAAGCAACT---
ACCACTCTATTTTGTGCATCAGATGCTAAAGCATATGAGACAGAA---CCACATAAT----------------
--------------ATTTGGGCCACA---CATGCCTGTGTACCCACAGAC---
CCAAACCCACAAGAAGTAGTCTTGGGA---------AATGTGACAGAAAACTTTAAC----------------
--------------------------ATGTGGAAAAATGACATGGTAGAACAGATGCATGAAGATATA---
ATCAATTTATGGGATCAAAGCCTA---ACGCCATGTGTAAAATTA---
ACCCCACTCTGTGTCACTTTAAATTGCACTAAATTGGATACAAATAGTACCAGTAGTAATAGTACCAGTAGTA
AT---
-------------AGTACCAGTAGTAATGATACCAATAGTATGATGGAGGGAGGAGGAATGAAA---
AACTGCTCTTTCAATATCACCACAAGCACAAAT---------------------------GCTAGGGTG---
AGAGACTATGCACTT---TTTCATAAGCTTGATATAGAACCAATGGATAAT----------------------
----
----GAAACTAATACTAACAGCTATAGGATAATAAATTGTAACACCTCAATCATTACACGA---
GCTTGTCCAAAGGTATCCTTTGAGCCAATTCCTATACATTATTGTACCCCGGCTGGTTTTGCGATTCTAAAGT
GTAAC---AATAAAACTTTCAAT------GGAACAGGACCATGTACAAAT-----------------------
-GTCAGT---ACAGTACAATGTACACATGGAATTAGGCCAGTAGTATCAACT---
CAATTGCTGTTAAATGGCAGTTTAGCAGAAGAAGAG-----------
GTAGTAATTAGATCTAGCAATTTCTCGGACAAT---------GCTAAAGTCATAATAGTACAGCTGAAG----
-----------GAATATGTAGAA---------ATTAATTGTACAAGACCCAACAACAATACAAGAAAA-----
-AGTATACATCTA------------GGACCAGGGCAAGCA---TTG------TATGCAACAGGACAA------
---------ATAATAGGAGATATA------------AGACAAGCACATTGT---AACATTAGTGGAGAA---
GAATGGAATAACACTTTAGAACAGATAGTTAAAAAATTAAGA---------------GTACAATTTGGA----
-----------------------------------AACAGCACAATAATCTTTAAT------
CAATCTGCAGGAGGGGACCCAGAGATTGTA---ATGCATAGCTTCAATTGTGGAGGGGAA---
TTTAAGCAGCTCCAGGCAAGAGTCCTGGCTGTGGAAAGATACCTAAAGGATCAACAGCTCCTAGGGATTTGGG
GTTGCTCTGGAAAACTCATTTGCACCACTGCTGTGCCTTGGAATGTTAGTTGGAGT-----------------
----------------------------AATAAATCTCTGAATGAGATTTGG------GAT------
AACATGACTTGGATGGAGTGG---GAAAGAGAAATTAGCAATTACACAAAAGAAATATAC---
AACTTAATTGAAGAATCGCAGAACCAACAGGAA--

AAGAATGAACTAGAACTATTGGAATTGGATAAGTGGGCAAGTTTGTGGAATTGGTTTGACATAACAAAATGGC
TGTGGTATATCAAAATATTCATAATGGTAGTAGGAGGCTTGGTAGGTTTAAGAATAGTTTTTACT---
GTACTTTCTATAGTA---------AATAGAGTTAGGCTGGGATACTTACCA------
TTGTCATTAGCTATTAGCTTGCTTAACGCCACAGCCATAGTAGTAGCTGAA---
GGGACAGATAGGGTTATAGAAGTAGTACAAAGAGCTTTTAGGGCT----------------------------
--------GTTCTCCACATA------CCTAGAAGAATCAGACAGGGCCTCGAAAGAGCTTTGCTA---TAA
>MK053935
GCT---GCA--
GAAGACTTGTGGGTCACAGTCTATTATGGGGTACCTGTATGG---AAAGAAGCAAAT---
ACCACTCTATTTTGTGCATCAGATGCTAAAGCATATGATACAGAG---GCACATAAT----------------
--------------GTTTGGGCCACA---CATGCCTGTGTACCCACAGAC---
CCCAACCCACAAGAAGTAGTCTTGAAA---------AATGTGACAGAAAACTTTAAC----------------
--------------------------ATGTGGAAAAATAACATGGTAGAACAGATGCATGAAGATATA---
ATCAGCTTATGGGATCAAAGCCTA---AAGCCATGTGTAAAATTA---
ACCCCCCTCTGTGTTACTTTAAATTGCACCAATTATAATAATACCAATAGTAATACCAGTAGTACA-------
----
---ACAGAGGGAGGAGAAATAAAA---
AACTGCTCTTTCAATATCACCACAAGCATAAAC---------------------------ACAAAGGTG---
AAAGACTATGCACTT---TTTTATAACCTTGATATAGTACAAATAAGTAAT----------------------
----
----------GATAGTAACAGCTATAGGTTGATAAATTGTAACACCTCAGTCATAACACAA---
ACTTGTCCAAAAAATATCCTTTGAGCCAATTCCCATACATTATTGTGCCCCGGCTGGTTTTGCGATTATACAGT
GTAAC---AATAAAAAAATTCAAT------GGAACAGGACCATGTACAAAT----------------------
-GTCAGT---ACAGTACAATGTACACATGGAATTAGGCCAGTAGTATCAACT---
CAACTGCTGTTAAATGGCAGTCTAGCAGAAGAAGAG-----------
GTAGTAATTAGGTCTAGCAATTTCACGGAAAAT---------GCTAAAATTATAATAGTACAGCTGAAT----
-----------AAATCTGTAGAA---------ATTAATTGTACAAGACCCAACAACAATACAAGAAAA-----
-AGTATACATCTA------------GGACCAGGGCGAGCA---TGG------TATGCAACAGGAGAA------
---------ATAATAGGAGATATA------------AGACAAGCACATTGT---AACATTAGTAGAACA---

```
AAATGGAATAACACTTTAAGACAGATAGTTGAAAAATTAAGA---------------GAACAATTTGGG----
-----------------------------------GACAAAACAATAATCTTTAAT------
CAATCTTCAGGAGGGGACCCAGAGATTGTA---ATGCACAGTTTTAATTGTGGAGGGGAA---
TTTAAGCAGCTCCAGGCAAGAGTCTTGGCTGTGGAAAGATACCTAAAGGATCAACAGCTCCTAGGGATTTGGG
GTTGCTCTGGAAAACTCATTTGCACCACTACTGTGCCTTGGGATGCTAAGTGGAGT-----------------
----------------------------AATAAATCTCAGAGTGAGATTTGG------AAT------
AACATGACTTGGATGGAGTGG---GAAAGAGAAATTGAAAATTACACAAGCACGATATAC---
GCCTTAATTGAAGAATCGCAGAACCAACAAGAA----------------------------------------
-----------------------------
AAAAATGAATTAGAACTATTGGAATTGGATAAGTGGGCAAGTTTGTGGAATTGGTTTGACATAACAAAATGGC
TGTGGTATATCAAAATATTCATAATGATAGTAGGAGGCTTGGTAGGTTTAAGAATAATTTTTGCT---
GTACTTTCTATAGTA---------AATAGAGTTAGGCAGGGATACTCACCA------
TTGTCATTAGCTATTAGCTTGCTTAACGCTACAGCCATAGCAGTAGCTGAG---
GGGACAGATAGGGTTATAGAAGTAGCACAAAGAGCTTTTAGGGCT----------------------------
--------ATTCTTAACATA------CCTACAAGAATCAGACAGGGCCTCGAAAGGGCTTTGCTA---TAA
>DQ990880
GCT---GCA------------------------------------------
GAAAAATTGTGGGTCACAGTCTATTATGGGGTACCTGTATGG---AAAGAAACAACT---
ACCACTCTATTTTGTGCATCAGAGGCTAAAGCATATGCTACAGAG---GTACATAAT----------------
--------------ATTTGGGCCACA---CATGCCTGTGTACCCACAGAC---
CCCAACCCACAAGAAGTAGTCTTGGAA---------AATGTGACAGAAAATTTTAAC----------------
--------------------------ATGTGGGAAAATAACATGGTAGAACAGATGCATGAAGATATA---
ATCAGTTTATGGGATCAAGGCCTA---AAGCCATGTGTAAGATTA---
ACCCCACTCTGTGTTACTTTAAATTGCACTGATTTCAAGAGTAATAGTACCAGTAGT----------------
-------------------------------------------------------------------------
----------------------------AATAACAGTAGTACAACAGAGAAAGGAGAAATAAAA---
AACTGCTCTTTCAATATCACCACAAGCACAGGA---------------------------ACTAAGGTA---
AGAGACTCTGCACTT---TTTAATAAGCTTGATGTATTACCAATAGATAATAGTAGTACCAGCTAT-------
-----------------------------------------------------------------
AATAGTAGTACCAGCAATAGGTATCACAGCTATAGGTTGATACATTGTAACACCTCAAGAATTACACAA---
GCTTGTCCAAAGGTATCCTTTGAGCCAATTCCCATACATTATTGTACCCCGGCTGGTTTTGCGCTCCTAAAGT
GTAAC---AATAAAACGTTCAAT------GGAATAGGACCATGTACAAAT-----------------------
-GTCAGT---ACAGTACAATGTACACATGGAATTAGACCAGTAGTAACAACT---
CAACTGCTGTTGAATGGTAGTTTAGCAGACGAAGAG------------
GTAGTAATTAGATCTAAAAATTTCACAGACAAT---------GCTAAAATCATAATAGTACAGCTGAAA----
-----------CAATCCGTAGAA---------ATTAATTGTACAAGACCCAACAACAATACAAGAAAA-----
-AGGATAACTATG------------GGACCAGGGAGAGTA---TTG------TATACAACAGGACAA------
---------ATAGTAGGAGATATA------------AGAAAAGCACATTGT---AACCTTAGTAGACCA---
CAATGGATGACACTCTAAGACAGGTAGTTGGAAAATTAAGA----------------GAACAATTTGGG----
-----------------------------------AACAAAACAATAATCTTTAAT------
CAATCTGCAGGAGGGGATCCAGAGGTTGTA---ATGCACATTTCTAATTGTGGAGGGGAA---
TTTAAACAGCTCCAGGCAAGAGTCTTGGCTGTGGAAAGATACCTAAAGGATCAACAGCTCCTAGGGATTTGGG
GTTGCTCTGGAAAACTCATTTGCACCACTGCTGTGCCTTGGAATGCTAGTTGGAGT-----------------
----------------------------AATAAATCTCACGATGAGATTTGG------AAT------
AACATGACTTGGATGGAGTGG---GAAAGACAAATTGACAATTATACAGCAGAAATATAC---
ACCTTAATTGAACAATCGCAGAACCAACAAGAA----------------------------------------
-----------------------------
AAGAATGAGTTAGAACTATTGGAATTGGATAAGTGGGCAAGCTTGTGGAATTGGTTTAACATGACACAATGGC
TGTGGTATATTAAAATATTCATAATGATAGTAGGAGGCTTGATAGGTTTAAGAATAATTTTTGCT---
GTACTTTCTATAGTA---------AATAGAGTTAGGCAGGGATACTCACCA------
TTGTCATTGGCTATTAGCTTGCTTAACGCCACAGCCATAGCAGTAGGTGAA---
GGGACAGATAGGATTATAGAAGTAGCACAAAGAGCTTGGAGAGCT----------------------------
--------ATTCTCAACATA------CCTACAAGAATCAGACAGGGCCTCGAAAGAGCTTTGCTA---TAA
>HM215420
GCT---GCA------------------------------------------
GAAAAATTGTGGGTCACAGTCTATTATGGGGTACCTGTATGG---AAAGAAGCAACT---
ACCACTCTATTTTGTGCATCAGATGCTAAAGCATATGATGCAGAG---GTACATAAT----------------
--------------GTTTGGGCCACA---CATGCCTGTGTACCCACAGAC---
CCCAACCCACAAGAAGTAGTCTTGGGA---------AATGTGACAGAAACCTTTGAC----------------
--------------------------ATGTGGAAAAATAACATGGTAGAACAGATGCATGAAGATATA---
```

ATTAGTTTATGGGACCAAAGCCTA---AAGCCATGTGTAAAATTA---
ACCCCACTCTGTGTTGTTTTAAATTGCACTAATCCTACTAATACCAGTAGT----------------------
----
-------------------------------------AGGACCAATAGTACGACAGACATGAAA---
AACTGCTCTTTCGATGTCACCACAAACATACAA---------------------------ACTAAGGTG---
AAAGAATATGCACTT---TTTTATAAGCTTGATGTAATACCAATAGATGAT----------------------
----
-------AATAATAATATCAGCTATAGGTTGAGACATTGTAACACCTCAGTCATTACACAA---
GCTTGTCCAAAGGTATCCTTTGAGCCAATTCCCATACATTATTGTGCCCCGGCTGGTTTTGCGATTATAAAGT
GTAAC---AATAAAATGTTCAAT------GGAACAGGACCATGTACAAAT-----------------------
-GTCAGT---ACAGTACAATGTACACATGGAATTAGGCCAGTAGTATCAACT---
CAACTGCTGTTAAATGGCAGCTTAGCAGAAGAAGAG------------
GTAGTAATTAGATCTCGCAATTTCACGGACAAT---------GCTAGAGTCATAATAGTACAGCTGAAC----
-----------GACTCTGTAGAA---------ATTAATTGTACAAGACCCAACAACAATACAAGAAAA-----
-AGGGTAACTCTA------------GGACCAGGGCGAGTA---TGG------TATACAACAGGACAA------
---------ATAATAGGAGATATA------------AGAAAAGCACATTGT---AACCTTAGTATAGCA---
AAATGGAATAACACTTTAAAAAAGATAACTGAAAAATTAAAA---------------GAACAATTTGGG----
-----------------------------------AACAAAACAATAATCTTTAAG------
CAACCTTCAGGAGGGGACCTAGAGGTTGTA---ATGCACAGTTTTAATTGTGGAGGGGAA---
TTTAAGCAGCTCCAGGCAAGAGTCCTGGCTGTGGAAAGATACCTAAAGGATCAACAGCTCCTAGGGATTTGGG
GTTGCTCTGGAAAACTCATTTGCACCACTGCTGTGCCTTGGAATACTAGTTGGAGT-----------------
----------------------------AATAAATCTCTAAGTCAGATCTGG------GAT------
AACATGACTTGGATGGAGTGG---GAAAGAGAAATTGACAATTACACAAGAGAAATATAT---
ACCTTAATCGAACAATCGCAGAACCAACAAGAA--

AAGAATGAACTAGAACTATTGGAATTGGATAAGTGGGCAAGTTTGTGGAATTGGTTTGACATAACAAAATGGC
TGTGGTATATCAAAATATTCATAATGATAGTAGGAGGCTTGGTAGGTTTAAGAATAGTTTTTGCT---
GTACTTTCTATAGTG---------AATAGAGTTAGGCAGGGATACTCACCA------
TTGTCGTTAGCTATTAGCTTGCTTAACGCCACAGCCATAGCAGTAGCTGAG---
GGGACAGATAGGGTTATAGAAGTAGCACGAAGAGTTTATAGGGCT----------------------------
--------ATTCTTAATATA------CCTACAAGAATCAGACAGGGACTCGAAAGGGCTTTGCTA---TAA
>HQ699995
GCT---GCA--
GAAAACTTGTGGGTCACAGTCTATTATGGGGTACCTGTATGG---AAAGATGCAACT---
ACCACTCTATTTTGTGCATCAGATGCTAAAGCATATAAGACAGAG---GCACATAAT----------------
--------------ATTTGGGCCACA---CATGCCTGTGTACCCACAGAC---
CCCAACCCACAAGAAGTAGTCTTGGAC---------AATGTGACAGAAAACTTTAAC----------------
--------------------------ATGTGGAAAAATGACATGGTAGAACAGATGCATGAAGATATA---
ATCAGTTTATGGGATCAAAGCCTA---AAGCCATGTGTAAAATTA---
ACCCCACTCTGTGTCACTTTAAATTGCACTAACTGGAGCAATACTACTAGTACCAGTAGTAATAATAGTGTCA
GAAAT--
----------AATATCAGTATCAGTAGTACTATCAGTACTACACTAGAGGGAGGAGAAGTAAAA---
AACTGCTCTTTCAATATCACCACAAGCATAGGA---------------------------ACTAAGGTG---
AAAGACTATGCACTT---TTTTATAAACTTGATGTAGCACAAATAGATAAT----------------------
----
----------AGTAGTACCAGCTATAGGTTGATAAAGTGTAACACCTCAACCATTACACAA---
GCTTGTCCAAAAGTATCCTTTGAGCCAATTCCCATACATTATTGTGCCCCGGCTGGTTTTGCGATTCTAAAGT
GTAAC---AATAAAACATTCAAT------GGAATAGGACCATGTACAAAT-----------------------
-GTCAGT---ACAGTACAGTGTACACATGGAATTAGGCCAGTAGTATCAACT---
CAACTGCTGTTAAATGGCAGTTTAGCAGAAAAAGAG------------
GTAGTAACTAGATCTAGCAATTTCACAGACAAT---------GCTAAAGTCATAATAGTACAGCTGAGT----
-----------AAATCTATAGAA---------ATTAATTGTACAAGACCTAACAACAATACAAGAAAA-----
-CGTGTAACTCTA------------GGACCAGGGCGAGTA---TGG------TATTCAACAGGACAA------
---------ATAATAGGAGATATA------------AGACGAGCATATTGT---AACATTAGTGGAACA---
AAATGGAATGACACTTTAAAACTGATAACTGAAAAATTAAGA---------------GAACAATTTGGG----
-----------------------------------AACAAAACAATAGTCTTTAAG------
AAATCTTCAGGAGGGGACCCAGAGATTGAA---ACGCACAGTTTTATTTGTGGAGGGGAA---
TTTAAGCAGCTCCAGGCAAGAGTCCTGGCTGTGGAAAGATACCTAAAGGATCAACAGCTCCTAGGGATTTGGG
GTTGCTCTGGAAAACTCATCTGCCCCACTGCTGCGCCTTGGAATTCTAGTTGGAGT-----------------
----------------------------AATAAATCTCTGAGTGAGATTTGG------GAT------

AACATGACTTGGATGGAGTGG---GAAAGAGAAATTGGCAATTACACAAAACAAATATAC---
ACCTTAATTGAAGAAACGCAGAACCAGCAAGAA--

AAGAATGAACTAGAACTATTGGAATTGGATAAGTGGGCAAGCTTGTGGAATTGGTTTGACATATCAAATTGGC
TGTGGTATATCAAAATATTCATAATGATAGTAGGAGGCTTGGTAGGTTTAAGAATAGTTTTTGCT---
GTACTTTCTATAGTG---------AATAGAGTTAGGCAGGGGTACTCACCA------
TTGTCATTCGCCATTAGCTTGCTTAACGCCACAGCAATAGCAGTAGCTGAG---
GGGACAGATAGGGTTATAGAAATAGCACAAAGAATTTTTGGGGCT----------------------------
--------ATTCTCCACATA------CCTACAAGAATCAGACAGGGCCTCGAAAGAGCTTTGCTA---TAA
>HM215428
GCT---GCA--
GAAAACTTGTGGGTCACAGTCTATTATGGGGTACCAGTATGG---AGAGATGCAAAT---
ACCACCTTATTTTGTGCATCAGATGCTAAAGCATATGATACAGAG---GTACATAAT----------------
--------------GTTTGGCCACA---CATGCCTGTGTACCCACAGAC---
CCCAACCCACAAGAAGTAGTCTTGGGA---------AATGTGACAGAAAACTTTAAC----------------
--------------------------ATGTGGACAAATAACATGGTAGAACAGATGCATGAAGATATA---
ATCAGTTTATGGGACCAAGGCCTA---AAGCCATGTGTAAAATTA---
ACCCCACTCTGTGTTGCTTTAGATTGCACCAATTTGAATACTACTAATACCACTAATTTGAATACTACT----
----
----------------GAGACCAGTAATAGTACCATTAGTCCAACAGAGGGAGGAGAAATGAAA---
AATTGCTCTTTCAATATCACCACAAGCATAGGA---------------------------ACT---AAG---
AAAGACTATGCATAT---TTGTATAAACTTGATATAGTACAGATAGATAATGGT-------------------
----
----GATAATAATAGTAACAGATATAGGTTGATAAGTTGTAACACCTCAGTCATTACACAA---
GCTTGTCCAAAGGTATCCTTTGAGCCAATTCCCATACATTATTGTACCCCGGCTGGTTTTGCGATTCTAAAGT
GTAAC---AATAAAACGTTCAAT------GGAACAGGACCATGTACAAAT-----------------------
-GTCAGT---ACAGTACAATGTACACATGGAATTAGGCCAGTAGTATCAACT---
CAACTGCTGTTAAATGGCAGTTTAGCAGAAGAAGAG------------
GTAGTAATTAGATCCAGCAACTTCACAAACAAT---------GCTGGAGTCATAATAGTACAACTGAAT----
-----------GAATCTGTAGAA---------ATTAATTGTACAAGACCCAGCAACAATACAAGAAAA-----
-AGTATACATCTA------------GGACAAGGGAGAGCA---TGG------TATACAACAGGAAAG------
---------ATAATAGGAGATATA------------AGACAAGCACATTGT---AACATTAGTGGAATA---
AAATGGAATAACACTCTAAAATATATAACTGAAAAATTAAGA---------------GAACAATATGGG----
-----------------------------------AATAAAACAATAATCTTTAAT------
CAGTCTACAGGGGGGGACCCAGAGATTGTA---ATGCACAATTTTAATTGTGGAGGGGAA---
TTTAAACAGCTCCAGGCAAGAGTCTTGGCTGTGGAAAGATACCTAAAGGATCAACAGCTCCTAGGGCTTTGGG
GTTGCTCTGGAAAACTCATTTGCACCACTGCTGTGCCTTGGAATGCTAGTTGGAGT-----------------
----------------------------AATAAAAATCTGAGTGAGATTTGG------AAT------
AACATGACTTGGATGCAGTGG---GAACGGGAAATTGGAAATTACACAAAAGAAATATAC---
AACTTAATTGAAGAATCGCAGAACCAACAAGAA--

AAGAATGAACTAGAACTATTGGAATTGGATAAGTGGGCAAGTTTGTGGAATTGGTTTAACATAACACAATGGC
TATGGTATATCAAAATATTCATAATGATAGTAGGAGGCTTGATAGGTTTAAGAATAGTCTTTGCT---
GTACTTTCTATAGTA---------AATAGAGTTAGGCAGGGATACTCACCA------
TTGTCATTAGCCATTAGCTTGCTTAACGCCACAGCCATAGCAGTAGCTGAG---
GGGACAGATAGGGTTATAGAAGTAGCACAAAGAGCCTGTAGGGCT----------------------------
--------ATTCTTAACATA------CCCAGAAGAATCAGACAGGGCTTCGAAGGGGCTTTGCTA---TAA
>EU363828
GCT---GCA--
GAAAACTTGTGGGTCACAGTCTATTATGGGGTACCTGTATGG---AAAGAAGCAACT---
ACCACTCTATTTTGTGCATCAGATGCTAAAGCGTATGATACAGAG---GTACATAAT----------------
--------------GTTTGGCCACA---CATGCCTGTGTACCCACAGAC---
CCCAACCCACAAGAAGTAGTCTTGGGA---------AATGTGACAGAAAACTTTAAC----------------
--------------------------ATGTGGAAAAATGACATGGTAGAACAGATGCATGAAGATATA---
ATCAGTTTATGGGATCAAAGCCTA---AAGCCATGTGTCAAATTA---
ACCCCAATCTGTGTTATTCTAAATTGCACTAATGCTAACAATACCAATAATAGT-------------------
----
-------------------------------AACAGTAGCACAGCAGAGGGAGGAGAAATAAAA---
AACTGCTCTTTCAATATCACCACAAGCATAAAA---------------------------ACTGTGGTG---
AAAGACTATGCACTC---TTTAATAAACTTGATGTAATGCCAATAGGTAAT----------------------

----
----------GATACTACCAGCTATAGGTTGACAAGTTGTAACACCTCAGTCATTACACAA---
GCTTGTCCAAAGGTATCCTTTGAGCCAATTCCCATACATTATTGTACCCCGGCTGGTTTTGCGCTTCTAAAGT
GTAAC---AATAAAACGTTCAAT------GGAAAAGGACCATGTACAAAT----------------------
-GTCAGT---ACAGTACAATGTACACATGGAATTAGGCCAGTAGTATCAACT---
CAACTGCTGTTAAATGGCAGTTTAGCAGAAGAAGAG-----------
GTAGTAATTAGATCTAGCAATTTCTCGAACAAT---------GCTAGAGTCATAATAGTACAGCTGAAT----
-----------GAATCTGTAGAA---------ATTAATTGTACAAGACCCAACAACAATACAAGGAAA-----
-AGTATAAATCTA------------GGACAAGGGCGAGCA---TGG------TATACAACAGGACAA------
---------ATAATAGGAGATATA------------AGACAAGCACATTGT---AACCTTAGTAGAACA---
AGATGGAATGACACTTTAAAACAGATAACTAAAAAATTAAGA---------------GAACAATTTGGG----
-----------------------------------AACAAAACAATAATCTTTGAT---
CAACCTCCAGGAGGAGGGGACCCAGAGATTATG---ATGCACAGTTTTAATTGTGGAGGGGAA---
TTTAAGCAGCTCCAGGCAAGAGTCTTGGCTGTGGAAAGATACCTAAAGGATCAACAGCTCCTAGGGATTTGGG
GTTGCTCTGGAAAACTCATCTGCACCACTGCTGTGCCTTGGAATGTTAGCTGGAGT----------------
----------------------------AGCAAAACTCAGGAGGAGATTTGG------AAT------
AACATGACTTGGATGCAGTGG---GAAAGAGAAATTGACAATTACACAAAAGAAATATAC---
ACCTTAATTGAAGAATCGCAGAACCAGCAAGAA--

AAGAATGAACTAGATCTATTAAAATTGGATGAGTGGGCAAGCTTGTGGAATTGGTTTGACATAACAAAATGGC
TGCGGTATATCCAAATATTCATAATGATAGTAGGAGGCTTAGTAGGTTTAAGAATAGTTTTTGCT---
GTACTTTCTATAGTA---------AATAGAGTTAGGCAGGGATACTCACCA------
TTGTCATTAGCTATCAGCTTGCTTAACGCCACAGCCGTAGCAGTAGCTGAG---
GGGACAGATAGGGTTATAGAAGTAGCACAAAGAGCTTATAGGGCT---------------------------
--------CTTATCCACGTA------CCTAGAAGAATCAGACAGGGCCTTGAAAGGGCTTTGCTA---TAA
>MF591612
GCT---GCA---
GAAAACTTGTGGGTCACAGTCTATTATGGGGTACCTGTATGG---AAAGAAGCAACT---
ACCACTCTATTTTGTGCATCAGATGCTAAAGCATATGATACAGAG---GTACATAAT----------------
--------------GTTTGGGCCACA---CATGCCTGTGTACCCACAGAC---
CCCAACCCACAAGAAGTAGTCTTGGGA---------AATGTGACAGAAAACTTTGAC---------------
--------------------------ATGTGGAAAAATAACATGGTAGAACAGATGCAGGAAGATATA---
ATCAGTTTATGGGATCAAAGCCTA---AAGCCATGTGTAAAATTA---
ACCCCACTTTGTGTTACTTTAAATTGCACTGATTGGAGGAATCCTAATAATACCAGTGGTAATAATACTGATA
ATACCAGGGGT--
----AATAATACTAGTAATAGCAGTAGTAATATCAGTAGTCCGACAGAGGTAGGAGAAATAAAA---
AACTGCTCTTTCAATATCACCACAAGCATAGGA--------------------------ACTAAGGTG---
ACAGACCATGCAACT---TTTTATAAACTTGATATAGTACCAATAGATAAT---------------------
----
----------GATACTACCAGCTATAGGTTGATAAATTGTAACACCTCAGTCATTACACAA---
GCTTGTCCAAAGGTATCTTTTGAGCCAATTCCCATACATTATTGTACCCCGGCTGGTTTTGCGATTCTAAAGT
GTAAC---AATAAAACATTCAGT------GGAACAGGACCATGTACAAAT----------------------
-GTCAGT---ACAGTGCAATGTACACATGGAATTAGGCCAGTAGTATCAACT---
CAACTGCTGTTAAATGGCAGTTTAGCAGAAGAAGAG-----------
GTAGTAATTAGATCTAGCAATTTCACAAATAAT---------GCTAAAGTCATAATAGTACACCTGAAT----
-----------GAATCTGTAGAA---------ATTAATTGTACAAGACCCAACAACAATACAAGAAAA-----
-AGTATACATCTA------------GGACCAGGGCGAGCA---TGG------TATGCAACAGGAGAA------
---------ATAATAGGAAATATA------------AGACAAGCACATTGT---AACATTAGTAGAACG---
AAATGGAATAACACTTTAAGATGGACAACTGAAAAATTAAGA---------------GAACAATTTGGG----
-----------------------------------AACAAAACAATAATCTTTAAG------
CAATCTTCAGGAGGGGACCCAGAGATTGTA---ATGCACACTTTTAATTGTGGAGGGGAA---
TTTAAGCAGCTCCAGGCAAGAGTCTTGGCTGTGGAAAGATATCTAAAGGATCAACAGCTCCTAGGGCTTTGGG
GTTGCTCTGGAAAACTCATTTGCACCACTGCTGTGCCTTGGAATGTTAGTTGGAGT----------------
----------------------------AACAAATCTCTGAGTGCGATTTGG------GAT------
AACATGACTTGGATGGAGTGG---GAAAGAGAAATTGACAATTACACAAGAGAAATATAC---
GCCTTGATTGAAGAATCGCAGAACCAACAAGAA--

AAGAATGAACTAGAACTATTGGAATTGGATAAGTGGGCAAGCTTGTGGAATTGGTTTGACATAACAAAATGGC
TGTGGTATATCAAAATATTCATAATGATAGTAGGAGGCTTGGTAGGTTTAAGAATAGTTTTTGCT---
GTACTTTCTATAGTA---------AATAGAGTTAGGCAGGGATACTCACCA------

TTGTCATTAGCTATTAGCTTGCTTAACGCCACAGCCGTAGTAGTAGCTGAG---
GGGACAGATAGGGTTATAGAAGTAGCACAAAGAGCTTTTTGGGCT----------------------------
--------GTTCTCCACATA------CCTAGAAGAATCAGACAGGGCTTCGAAAGGCTTTGCTA---TAA
>JF932482
GCT---GCA--
GAAAACTTGTGGGTCACAGTCTATTATGGGGTACCTGTGTGG---AAAGAAGCAACT---
ACCACTCTATTTTGTGCATCAGATGCTAAAACTTATTCTACAGAG---GTACATAAT----------------
--------------GTCTGGCCACA---CATGCCTGTGTGCCCACAGAC---
CCCAACCCACAAGAAGTAGTCTTGGAA---------AATGTGACAGAGAACTTTAAC----------------
--------------------------ATGTGGAAAAATAACATGGTGGAACAGATGCATGAAGATATA---
ATCAGTTTATGGATCAAAGCTTA---AAGCCATGTGTAAAATTA---
ACCCCACTCTGTGTGACGTTACATTGTTCTAATTTGACAACTAATGCCAGCAATAATAACATTAGT-------
----
-------------------GCCAGCTATAATACCAGTAGTCCGATAGGGGGAGAAGAAATAAAA---
AACTGCTCTTTCAACTATACCACAAGCACAAGA---------------------------ACTAAGGCA---
AGAGACCATGCACTT---TTTTATAGTCTTGATGTAGAACCAATAGATGGC----------------------
----
----------------ACCCTCTATAGGTTGAGACATTGTAACACCTCAGTCATTACACAA---
GCTTGTCCAAAGATATCCTTTGAGCCAATTCCTATACATTATTGTACCCCGGCTGGTTTTGCGCTTCTAAAGT
GTAAC---AATAAAACATTCAAT------GGAACAGGACCATGTACAAAT-----------------------
-GTCAGC---ACAGTACAATGTACACATGGAATTAAGCCAGTAGTATCAACT---
CAACTGCTGTTAAATGGCAGTTTAGCAGAAGAAGAG------------
GTAGTAATTAGATCTAGCAATTTCACAGATAAT---------GCTAAAGTCATAATAGTACAGCTGAAG----
-----------AACCCTGTAAAG---------ATTAATTGTACAAGACCCAACAACAATACAAGAAAA-----
-AGTATACATCTA------------GGACCAGGGCAAGCA---TGG------TATACAACAGGACAA------
---------ATAATAGGAGATATA------------AGACAAGCACATTGT---AACCTTAGTGGAATA---
CAATGGAATAACACTTTAACACAGATAACAGAAAAATTAAGA---------------GAACAATTTGGG----
-----------------------------------AACAAAACAATAATCTTTAAT------
CAATCTTCAGGAGGGGACCCAGAGATTGTA---ATGCACAGTTTTAATTGTGGAGGGGAA---
TTTAAGCAGCTCCAGGCAAGAGTCTTGGCTGTGGAGAGATACCTAAAGGATCAACAGCTCCTAGGGATTTGGG
GTTGCTCTGGAAAACTCATTTGCACCACTGCTGTGCCTTGGAATGTTAGTTGGAGT-----------------
----------------------------AATAAATCTCTAAATGAGATTTGG------AAT------
AACATGACTTGGATGGAGTGG---GAAAGAGAAATTGAAAATTACACAAAAGAAATATAC---
ACCTTAATTGAAGAATCGCAGAACCAGCAAGAA--

AAGAATGAACTAGAACTATTGGAATTGGATAAGTGGGCAAGTTTGTGGAGTTGGTTTGACATATCAAACTGGC
TGTGGTATATCAGAATATTCATAATGATAGTAGGAGGCTTGGTAGGTTTAAGAATAGTTTTTGCT---
GTACTTTCTATAGTA---------AATAGAGTTAGGCAGGGATACTCACCA------
TTGTCATTTGCTGTTAGCTTGCTTAACGCCACAGCCATAGCAGTAGCTGAG---
GGGACAGATAGGGTTATAGAAGTAGCACAAAGAGCTTTTAGGGCT----------------------------
--------ATTCTCAACATA------CCTACGAGAATCAGACAGGGCTTCGAAAGGCTTTGCTA---TAA
>HQ699949
ACT---GCA--
GAAAAATTGTGGGTCACAGTCTATTATGGGGTACCTGTATGG---AAAGAAGCAACC---
ACTACTCTATTTTGTGCATCAGATGCTAAAGCCTATGATACAGAG---GTACATAAT----------------
--------------GTCTGGCCACA---CATGCCTGTGTACCCACAGAC---
CCCAACCCACAAGAAGTAGTCTTGAAA---------AATGTGACAGAAAACTTTAAC----------------
--------------------------ATGTGGAAAAATAACATGGTAGAACAGATGTATGAAGATATA---
GTCAGTTTATGGATCAAAGCTTA---AAGCCATGTGTAAAGTTA---
ACCCCACTCTGTGTTATCTTAAATTGCACTGATTTGAAGAATACTACT-------------------------
----
----------------------------AATACCAAAATTACAATAAAGGAAAGAGAAATGACA---
AACTGCTCTTTCAATATCACCACAAGCATAAAA---------------------------ACTAAGGTG---
AAAGACTATGGACTT---TTTTATAAACTTGATGTAATGCCAATAGATAAT----------------------
----
----------GATAATACTAGCTATAGGTTGATAAATTGTAACACCTCAATCATTACACAG---
GCTTGTCCAAAGGTATCCTTTGAGCCAATTCCCATACATTATTGTACTCCGGCTGGTTTTGCGATCCTAAAGT
GTAAC---AATAAAAAGTTCAGT------GGTACAGGACCATGTAAAAAT-----------------------
-GTCAGT---ACAGTACAATGTACACATGGAATTAGGCCAGTAGTATCAACT---
CAACTGCTGTTAAATGGCAGTTTAGCAGAAGAAGAG------------

GTAGTAATTAGATCTAAAAATTTCATGAACAAT---------GCTGAAGTCATAATAGTACAGCTGAAT----
-----------GAATCTGTAGAA---------ATTAATTGTATAAGACCCAACAACAATACAAGAAAA-----
-AGCATACCTCTA------------GGACCAGGGAAAGCA---TGG------TATACAACAGGAGAG------
---------ATAATAGGAGATATA------------AGGAAAGCACATTGT---AACCTTAATAGTACA---
AAATGGTATAACACTTTAAAACTGCTAACTAAAAAATTAAGA---------------GAACAATTTGGG----
-----------------------------------AACAAAACAATAGTCTTTAAT------
CAATCCTCAGGAGGGGACCCAGAAGTTGCA---ACGCACAGTTTTAATTGTGGAGGGGAA---
TTTAAGCAGCTCCAGGCAAGAGTCCTGGCTATAGAAAGATACCTAAAAAATCAACAGCTCCTAGGGATTTGGG
GTTGCTCTGGAAAACTCATTTGCACCACTGCTGTGCCTTGGAATACCAGTTGGAGT-----------------
----------------------------AATAAATCTATGGATGAGATTTGG------GGT------
AACATGACTTGGATGGAGTGG---GAAAGAGAAATTGACAATTACACAAAAGAAATATAC---
ACCTTAATTGAAGAATCGCAGAACCAACAGGAA--

AAGAATGAACAAGAATTATTGGAATTGGATAAGTGGGCAAGTTTGTGGAATTGGTTTAGCATAACAAACTGGC
TGTGGTATATAAAAATATTCATAATGATAGTAGCAGGCTTGGTAGGTTTAAGAATAGTTTTTGCT---
GTACTTTCTATAGTA---------AATAGAGTTAGGCAGGGCTACTCACCA------
TTATCATTAGCTGTTAGCTTGCTCAATGCTACAGCTATAGCAGTAGCTGAG---
GGGACAGATAGGGTTATAGAAGTAGTACAAAGAGCTTATAGAGCT----------------------------
--------ATTCTCCACATA------CCTACAAGAATAAGACAGGGCCTCGAAAGGGCTTTGCTA---TAA
>HM215423
GCT---GCA--
GAAAATTTGTGGGTCACAGTCTATTATGGGGTACCTGTATGG---AAAGAAGCAACC---
ACCACTCTATTTTGTGCATCAGATGCTAAAGCATATGATACAGAG---GTGCATAAT----------------
--------------GTCTGGGCCACA---CATGCCTGTGTACCCACAGAC---
CCCAACCCACAAGAAGTAGTCTTGGGT---------AATGTGACAGAAGATTTTAAC----------------
--------------------------ATGTGGGAAAATAACATGGTAGACCAGATGCATGAAGATATA---
ATTAGTTTGTGGGATCAAAGCCTA---AAGCCATGTGTAAGATTA---
ACCCCACTCTGTGTTACTTTAAATTGCACTAATTGGAATGCTACTAATTTGGGAAATGAT-------------
----
-------------------------ACTAATACCATTAGTATGATAAAGGAAGGAGAAATAAAA---
AACTGCTCTTTCAATATCACAACAAACATAAGA---------------------------ACTAAGGTG---
AGAGATTATGCACTT---TTTTATGCACTTGATGTAGTACCAATAGATAAT----------------------
----
----------------GCTAGCTGGAGGTTGACAAGTTGTAACACCTCAGTCATTACACAA---
GCTTGTCCAAAGGTATCCTTTGAGCCAATTCCCATACATTATTGTACCCCAGCTGGTTTTGCGATTCTAAAGT
GTAAT---GATAAAAAGTTCAAT------GGAACAGGACCATGTACAAAT-----------------------
-GTTAGT---ACAGTACAATGTACACATGGAATTAGGCCAGTAGTATCAACT---
CAACTGCTGTTAAATGGCAGTTTAGCAGAAGAAGAG------------
ATAGTAATTAGATCAAGCAACCTCTCGATAAAT---------CATAAAAACATAATAGTGCAGCTGAAT----
-----------AAATCTGTAGAA---------ATTAATTGTACAAGACCCAACAACAATACATTAAAA-----
-AGTATACAGCTA------------GGACTAGGGCGAGCA---TGG------CATGCAACAGGACAG------
---------ATAATAGGAGATATA------------AGGCAAGCACATTGT---AACCTTAGTGGCACA---
GATTGGAATAACACTTTAAACCTGATAACTGAAAAATTAAGA---------------GAACAATTTGGG----
-----------------------------------AGCAAAACAATAGTCTTTAAG------
CCACACGTAGGAGGGGACCCAGAGGTTGTG---ATGCACAATTTTAATTGTGGAGGGGAA---
TTTAAGCAGCTCCAGGCAAGAGTCCTGGCTATAGAAAGATACCTAAAGGATCAACAGCTCCTAGGGATTTGGG
GTTGCTCTGGAAAACTCATTTGTACCACTGCTGTGCCTTGGAATACTAGTTGGAGT-----------------
----------------------------GATAAATCTTTGGAGGAGATTTGG------GAT------
AACATGACTTGGATGCAGTGG---GAAAAAGAAATTAATAATTACACAAAAGAAATATAC---
ACCCTAATTGAACAATCGCAGAACCAACAAGAA--

AAGAATGAACTAGAATTATTGGAATTGGATAAGTGGGCAAGCTTGTGGAATTGGTTTGACATAACAAACTGGC
TGTGGTATATAAAAATATTCATAATGATAGTAGGAGGCTTAGTAGGTTTAAGAATAGTTTTTGCT---
ATACTTTCTATAGTA---------AATAGAGTTAGGCAGGGTTACTCACCA------
TTGTCATTGGCTGTTAGCTTGCTCAATGCTACAGCTATAGCAGTAGCTGAG---
GGGACAGATAGGATTATAGAAGTAATACAAAGAGCTTATAGAGCT----------------------------
--------ATTCTCCACATA------CCTAGAAGAATAAGACAGGGCCTCGAAAGGAGTTTGCTA---TAA
>EU131805
GCT---GAA--
GACAAGTTGTGGGTCACAGTCTATTATGGGGTACCTGTATGG---AAAGAAGCAACT---

ACCACTCTATTTTGTGCATCAGATGCTAAAGCATATGATACAGAG---GTGCATAAT----------------
--------------GTTTGGGCCACA---CATGCCTGTGTACCCACAGAC---
CCCAACCCACAAGAAGTAGTCTTGGGA---------AATGTGACAGAAAACTTTAAC----------------
--------------------------ATGTGGAAAAATAACATGGTAGAACAGATGCATGAGGATATA---
ATCAGCTTATGGGATCAAAGTCTA---AAGCCATGCGTAAAATTA---
ACCCCAATCTGTGTTACTTTAAATTGTTCTAATTTGAATATTACTACTAATAAATTGAATACTACTAAT----
----
-------------------ACCAGAAGTAATCCCAGTAATATGACAGGGGAAGGAGAAATAAAA---
AACTGCTCTTTCAATATCGCCACAAGCTCAAGT---------------------------
AATAAGGTAAAAAAAGACTATGCACTT---TTTTATAACCTTGATGTAATAAAAGTAGATAAT----------
----

AGTACTGACAGCTATATGTTGAGAAGTTGTAACACCTCAATCATTACACAA---
ACTTGTCCAAAGGTAACCTTTGAGCCAATTCCCATACATTATTGTACTCCAGCTGGTTTTGCGCTTCTAAAGT
GTAAC---AATAAAACGTTCAGT------GGAAAAGGACCTTGTACAAAT-----------------------
-GTCAGT---ACAGTACAATGTACACATGGAATTAAGCCAGTAGCATCAACC---
CAATTGCTGTTAAATGGCAGCTTAGCAGAAGAAGAG------------
ATAGTAATTAGGTCTAGCAATTTCACGAACAAT---------GCAAAAATCATAATAGTACAGCTGAAT----
-----------GAATCTGTAGCA---------ATTAATTGTACAAGACCCAACAACAATACAAGAAAA-----
-AGTATATCTATA------------GGACCAGGGCAAGCA---ATG------TATGCAACAGGACAA------
---------ATAATAGGAGATATA------------AGACAAGCACATTGT---AACATTAGTAGAGAA---
CAATGGAATAACACTTTAAGACAGGTAGCTGGAAAATTAAGA---------------GAACAATTTAAG----
--------------------------------AACAAACAAATAATAATCTTTCAG------
CCATCTTCAGGAGGGGACTCAGAGATTGAA---ACGCACAGTTTTAATTGTGGAGGGGAA---
TTTAAACAGCTCCAGGCAAGAGTCTTGGCTATCGAAAGATACCTAAAGGATCAACAGCTCCTAGGAATTTGGG
GTTGCTCTGGAAAACTCATTTGCACCACTGCTGTGCCTTGGAATACTAGTTGGAGT-----------------
----------------------------AATAAATCTCTGAGTCAGATTTGG------GAT------
AACATGACCTGGATGGAGTGG---GAAAGAGAAATTGACAATTACACAAAAGAAATATAC---
TCCTTAATTGAACTATCGCAGAACCAACAAGAA--

CAAAATGAACTAGAATTATTGAAATTGGATAAGTGGGCAAGTTTGTGGAGTTGGTTTGACATATCAAACTGGC
TGTGGTATATCAAAATATTCATAATGATAATAGTAGGCTTGGTAGTTTTAAGAATAATTTTTGCT---
GTACTTTCTATAGTA---------AATAGAGTTAGGCAGGGATACTCACCA------
TTGTCATTTGCCACTAGCTTGCTTAACGCTACAGCCATAGTAGTAGCTGAG---
GGAACAGATAGGGTTATAGAAGCAGTACAAAGAATTTATAGGGCT----------------------------
--------ATCCTTCATATA------CCTAGAAGAATCAGACAAGGACTCGAAAGGGCTTTGCTA---TAA
>EU131800
GCT---GCA---
GAAAACTTGTGGGTCACAGTCTATTATGGGGTACCTGTATGG---AAAGAAGCAACT---
ACTACTCTATTTTGTGCATCAGATGCTAAAGCATATGATACAGAG---GTACATAAT----------------
--------------ATTTGGGCCACA---CATGCCTGTGTACCCACAGAC---
CCCAACCCACAAGAAGTAGTCTTGGGA---------AATGTGACAGAAAACTTTAAC----------------
--------------------------ATGTGGAAAAATGATATGGTAGAACAGATGCATGAAGATATA---
ATCAGTCTATGGGATCAAAGCTTA---AAGCCATGTGTAAAATTA---
ACCCCACTCTGTGTTACTTTAAATTGCACTGATTTGAAAAATACTACTAATGCCAATAGC-------------
----
----------------------------AATAGCATTAACATGACAGAGGGAGGAGAAATGAAA---
AACTGCTCTTTCAATATCACCACAAGCATAAGA---------------------------ACTAAAGTG---
AGAGGCTATGCACTT---TTTTATAAACTTGATATAGTACCAATAGATAATGAT-------------------
----
-AGTAGGACCAATAATACCAGCTATAGGTTAATAAGTTGTAACACCTCAGTCATTACACAA---
GCTTGTCCAAAGGTATCCTTTGAGCCAATTCCCATACATTATTGTGCCCCGGCTGGTTTTGCGATTCTAAAGT
GTAAC---AATAAAACGTTCAAT------GGAACAGGACCATGTACAAAT-----------------------
-GTGAGT---ACAGTACAATGTACACATGGAATTAGGCCAGTAGTATCAACT---
CAACTACTGTTAAATGGCAGTTTGGCAGAAGACGAG------------
GTTGTAATTAGATCTAGCAATTTCTCGAACAAT---------GCTAAAGTCATAATAGTACAGCTAAAT----
-----------AAATCTGTAGCA---------ATTGATTGTATAAGACCCAACAACAATACAAGGAAA-----
-AGTATACATCTA------------GGACTAGGGCGAGCA---TGG------TATACAACAGGAGAA------
---------ATAATAGGAAATATA------------AGACAAGCACATTGT---AACCTTAGTAGAGAA---
AAATGGAATGACACTTTAAGACAGATAACTGTAAAATTAAGA---------------GAACAATTT-------

-----------------------------------AACAAAACAATAAGCTTTAAA------
CAATCTTCAGGAGGGGACCCAGAGATTGTA---ATGCATAGTTTTAATTGTGGAGGGGAA---
TTTACGCACCTCCAGGCAAGAGTCTTGGCTGTGGAAAGATACCTAAAGGATCCGCAGCTCCTAGGGATTTGGG
GTTGCTCTGGAAAACTCATTTGCACCACTGCTGTGCCTTGGAATGTTAGTTGGAGT-----------------
----------------------------AATAAATCTCTGAGTGAAATTTGG------GAT------
AACATGACTTGGATGGAGTGG---GAAAGAGAAATTGACAATTACACAAAAGAAATATAC---
ACCCTAATTGAGGAATCGCAGAACCAGCAAGAA--

AAGAATGAACTAGACCTATTGGAGTTGGATAAGTGGGCAAGTTTGTGGAATTGGTTTGACATATCAAGATGGC
TGTGGTATATCAAAATATTCATAATGATAGTAGGAGGCTTGATAGGTTTAAGAATAGTTTTTGCT---
GTACTTTCTATAGTA---------AATAGAGTTAGGCGGGATACTCACCA-------
TTGTCATTAGCTATTAGCTTGCTTAACGCCACAGCCATAGCAGTAGCTGAG---
GGGACAGATAGGATTATAGAAGTAGTACAAAGAGTTTATAGGGCT----------------------------
--------ATTCTCAACATA------CCTACAAGAATCAGACAGGGCCTCGAAAGGGCTTTGCTA---TAA
>EU131794
GCT---GCA--
GAAAACTTGTGGGTCACAGTCTATTATGGGGTACCTGTATGG---AAAGAAGCAACT---
ACCACTCTATTTTGTGCATCAGATGCTAAAGCATATGACACAGAG---GTACATAAT----------------
--------------ATTTGGGCTACA---CATGCCTGTGTACCCACAGAT---
CCCAACCCACAAGAAGTAGTCTTGGGA---------AATGTGACAGAAAACTTTAAC----------------
--------------------------ATGTGGAAAAATAACATGGTAGAACAGATGCATGAAGATATA---
ATCAGTTTATGGGATCAAAGCCTA---AAGCCATGTGTAAAATTA---
ACCCCACTCTGTGTTACTTTAAATTGCACTGATTTGAATACTACTAATACCCATAGTACTAATGCC-------
----
----------------------AGTAGTAATATCAGTAGTACGACAGAGGGAGGAGAAATAAAA---
AACTGCTCTTTCAATATCACCACAAGCATAAAA---------------------------ACTAAGGTG---
AAAGACTATGCATTT---TTTAATGAACTTGATATAGTACCAATAGCTAAT----------------------
----
----------GATAGTACCAGCCATAGGTTGATAAATTGTAACACCTCAGTCATTACACAA---
GCTTGTCCAAAGGTATCCTTTGAGCCAATTCCCATACATTATTGTACCCCGGCTGGTTTCGCGCTTCTAAAGT
GTAAC---AATAAAACGTTCAAT------GGAACAGGACCATGTACAAAT-----------------------
-GTCAGT---ACAGTACAATGTACTCATGGAATTAGGCCAGTAGCATCAACT---
CAACTGCTGTTAAATGGCAGTTTAGCAGAAAAAGAG------------
GTAGTAATTAGATCTAGCAATTTCATGGACAAT---------GCTAGAATCATAATAGTACAGTTGAAT----
-----------GAATCTGTAGAA---------ATTAACTGTACAAGACCCAACAGCAATACAAGAAAA-----
-AGTATACATCTA------------GGATCAGGGCGAGCA---TGG------TATACAACAGGACAA------
---------ATAATAGGAGATATA------------AGACAAGCACATTGT---AACCTCAGTAGAATA---
AAATGGATAACACTTTAAAACAAATAACTAAAAAATTAAGA----------------GAACAATTTGGG----
-----------------------------------AACAAAACAATAATCTTTAAT------
CAATCTTCAGGAGGGGACCCAGAGATTGTA---ATGCACAGTTTTAATTGTGGAGGGGAA---
TTTAAGCAGCTCCAGGCAAGAGTCCTGGCTGTGGAAAGATACCTAAAGGATCAACAGCTCCTAGGGATTTGGG
GTTGCTCTGGAAAACTCGTTTGCACCACTACTGTGCCTTGGAATGCTAGTTGGAGT-----------------
----------------------------AATAAATCTTTGAGTGAGATTTGG------GAT------
AACATGACTTGGATGGAGTGG---GAAAGAGAAATTGACAATTACACAAAAGAAATATAC---
ACCTTAATTGAAATATCGCAGAACCAGCAAGAA--

AAGAATGAACTAGAACTATTGGAATTGGATAAGTGGGCAAGTTTGTGGAATTGGTTTGACATAACAAAATGGC
TGTGGTATATCAAAATATTCATAATGATAGTAGGAGGCNNGGTAGGTTTAAGAATAGTTTTTGCT---
GTACTTTCTATAGTA---------AATAGAGTTAGGCAGGGATACTCACCA-------
TTGTCATTAGCTACTAGCTTGCTTAACGCCACAGCCATAGCAGTAGCTGAG---
GGGACAGATAGGGTTATAGAAGTAGCACAAAGAGCTTGGAGGGCT----------------------------
--------ATTCTCAACGTA------CCTAGAAGAATCAGACAGGGCCTTGAAAGAGCTTTGCTA---TAA
>EU131807
GCT---GAA--
GAAAAGTTGTGGGTCACAGTCTATTATGGGGTACCTGTATGG---AAAGAAGCAGAT---
ACCACTCTATTTTGTGCATCAGATGCTAAAGCATATGATACAGAG---GTACATAAT----------------
--------------GTTTGGGCCACA---CATGCCTGTGTACCCACAGAC---
CCCAACCCACAAGAAGTAGTCTTGGGA---------AATGTGACAGAAAACTTTAAC----------------
--------------------------ATGTGGAAAAATAACATGGTAGAACAGATGCATGAAGATATA---
ATCAGTTTATGGGATCAAAGCCTA---AAGCCATGTGTAAGATTA---

ACCCCACTCTGTGTTATTTTAAATTGCACTGATTTGGGAAATACTACTAATACCAGTAGT-------------
----
----------------------------AATACCAGTAGTATAACAGAGGGGGGAGAACTAAAA---
AACTGCTCTTTCAATATCACCACAAGCATAAGA---------------------------ACTAAGGTG---
AAAGACTATGCAATT---TTTAATAACCTTGATGTAGCACCAATAAATAAT----------------------
----
-------AATGATAATACCAGCTATAGGTTGATAAATTGTAACACCTCAGTCATTACACAA---
GCTTGTCCAAAGGTATCTTTTGGGCCAATTCCCATACATTATTGTACCCCGCCTGGTTTTGCGCTTATGAAGT
GTAAC---AATAAAATGTTCAAT------GGAACAGGACCATGTACAAAT-----------------------
-GTCAGT---ACAGTACAATGTACACATGGAATTAGGCCAGTAGTATCAACT---
CAACTGCTATTAAATGGCAGTTTAGCAGAAGAAGAA------------
GTAGTAATTAGGTCCAGCAATTTAACAGACAAT---------GCTAAAATCATAATAGTACAGCTGAAT----
-----------AAATCTGTAAAA---------ATTGATTGTATAAGACCCAACAACAATACAAGAAAA-----
-AGTATAAATCTA------------GGACAAGGGCGAGCA---TGG------TATGCAACAGGACAA------
---------ATAATAGGAAATATA------------AGACAAGCACATTGT---AACCTTAGTAGAACA---
GAATGGACTGACACTTTAAAACAGATAACTGAAAAATTAAGA---------------GAACAATTTGGG----
-----------------------------------AAGAAAACAATAATCTTTAAT------
CAATCCTCAGGAGGGGATCTAGAGATTGTA---ATGCACAGTGTTAATTGTGAAGGAGAA---
TTTAAGCAGCTCCAGGCAAGAGTCTTGGCTGTGGAAAGATACCTAAAGGATCAACAGCTCCTAGGGATTTGGG
GTTGCTCTGGAAAACTCATTTGCACCACTGCTGTGCCTTGGAATGCTAGTTGGAGT-----------------
----------------------------AATAAATCTCTGAGTGAGATTTGG------GAT------
AACATGACTTGGATGGAGTGG---GAAAGAGAAATTGACAATTACACAAAAGAAATATAC---
ACCTTAATCGAACTATCACAGAACCAGCAAGAA--

AAGAATGAACTAGACCTATTGGAATTGGACAAGTGGGCAAGCTTGTGGAATTGGTTTGACATATCAAATTGGC
TGTGGTATATCAAAATATTCATAATGATAGTAGGAGGCTTGGTAGGTTTAAGAATAGTTTTTGCT---
GTACTTTCTATAGCA---------AATAGAGTTAGGCAGGGATACTCACCA------
TTGTCATTGGCTACTAGCTTGCTTAACTCCACAGCCATAGCAGTAGCTGAG---
GGGACAGATAGGGTTATAGAAATAGCACAAAGAGCTTTTAGGGCT----------------------------
--------ATTCTCAATATA------CCTACAAGAATCAGACAGGGCCTAGAAAGGGCTTTGCTA---TAA
>EU131809
ACT---CAA--
GACAATTTGTGGGTCACAGTCTATTATGGGGTACCTGTGTGG---AAAGAAGCAACC---
ACCACTTTATTTTGTGCATCAGATGCTAAAGCATATGACAAAGAG---GTACATAAT----------------
--------------GTTTGGCCACA---CGTGCCTGTGTACCCACAGAC---
CCTAACCCACAAGAAGTAGTATTGGAA---------AATGTGACAGAAAATTTTAAC----------------
--------------------------ATGTGGAAAAATTACATGGTAGGACAGATGCATGAGGATATA---
ATCAGTTTATGGGATGAAAGCCTA---AAGCCATGTGTAAAATTA---
ACCCCACTCTGTGTTACTTTAAATTGCACTGATTGGAGGCATAATACTACTAATACCAATAGT----------
----
----------------------ACCAGTAATGCCACTTTGGACACGATGGAGGGAGAAATAAAA---
AACTGCTCTTTCAATATCACCACAGCAATAAGA---------------------------
AATGAGGTGCGACAAGAATATGCACTT---TTTAATAAACTTGATGTAGTAAATATAGATAAT----------
----

AACAATAGTAACTATATGTTAATAAATTGTAACACCTCATCCATTACACAG---
GTCTGTCCAAAGGTATCCTTTGAACCGATTCCAATACATTATTGTGCCCCGGCTGGTTTTGCGATTCTAAAGT
GTAAT---GATAAAAAGTTCAAT------GGGACAGGACCATGTACAAAT-----------------------
-GTCAGC---ACAGTGCAATGTACACATGGAATTAGGCCAGTAGTGTCAACT---
CAACTGCTGTTAAATGGCAGTCTAGCAGAAAAAGAG------------
ATAATAATTAGATCTGAAAATTTCACAGACAAT---------ACTAAGACCATAATAGTACAACTGAAT----
-----------AACACTGTAAAA---------ATTAATTGTATAAGACCCAGCAACAATACAAGAAAG-----
-AGTATACCTATG------------GGACCAGGAAGAGCA---ATC------TATGCAACAGGAGAA------
---------ATAATAGGAGATATA------------AGACAAGCACATTGT---AACATTAGTATGACA---
GACTGGAACAACACTTTAAGACAGGTAGCAATGAAATTAAAA---------------GGACAATTCAGA----
-----------------------------------AATAAGACAATAGTCTTTAAC------
CAATCCTCAGGAGGGGACCCAGAAATTGTA---ATGCACACAGTTAATCGTGGAGGGGAA---
TTTAAGCAGCTCCAGGCAAGAGTCCTAGCTGTGGAAAGATACCTAAGAGATCAACAGCTCCTAGGGATTTGGG
GTTGCTCTGGAAAACTCATCTGCACCACTGCTGTGCCTTGGAATGCTAGTTGGAGT-----------------
----------------------------AATAAATCTCTGGAAGAAATTTGG------AGT------

AACATGACCTGGATGCAGTGG---GAGAGAGAAATTGATAATTACACAGGCTTAATATAC---
ACTTTACTTGAACAATCGCAGAACCAGCAAGAA---

AAGAATGAACAAGAATTATTGGAATTGAATAAATGGGCAGATTTGTGGAGTTGGTTTGACATAACGAATTGGC
TGTGGTATATAAAATTATTTATAATGATAGTAGGAGGCTTAATAGGTTTAAAAATAGTTTTTACT---
GTATTTTCTATAGTG---------AGTAGAGTTAGGCAGGGATACTCACCA------
TTATCATTCGCTGTCAGCTTGCTTAATACCACAGCCATAGCAGTAGCTGAG---
GGGACAGATAGAATTATAGAAGTAGCACGTAGATTTGTTAGGGCT---------------------------
--------ATTATCAACATC------CCTACAAGAACAAGACAGGGCTTAGAAAGGGCTTTGCTA---TAA
>MH479968
GCT---GCA---
GAAAAATTGTGGGTCACAGTCTATTATGGGGTACCTGTATGG---AAAGAAGCAACT---
ACCACTCTATTTTGTGCATCAGATGCTAGAGCATATGATACAGAG---GTACATAAT----------------
--------------GTCTGGGCCACA---CATGCCTGTGTACCCACAGAC---
CCCAACCCACAAGAAGTAGTCTTGGGA---------AATGTGACAGAAAACTTTAAC---------------
--------------------------ATGTGGAAAAATAACATGGTAGAACAGATGCATGAAGATATA---
ATCAGTTTATGGGATCAAAGCCTA---AAGCCATGTGTAAAATTA---
ACCCCACTCTGTGTTATTTTAAATTGCACTAATGTGGAGAATGCTACTAATACCAATGCTACTAATGCT----
----
-------------------------ACTAATACCAATACTACAACAGAGGGAGGAGAAATGAAA---
AACTGCTCTTTCAATATCACCACAAGCATAAAT--------------------------ACTAAAGTG---
AAAGACTATGCACTT---TTCTATAAATATGATGTAGTATCAATAAATAAG---------------------
----
----------GCTAATACCAGCTATAGGTTGATAAATTGTAACACCTCAGTCATTACACAA---
GCTTGTCCAAAGGTATCTTTTGAGCCAATTCCCATACATTATTGTGCCCCGGCTGGTTTTGCGATTCTAAAAT
GTAAC---AATAAAACATTCAAG------AGAACAGGACCATGTACAAAT----------------------
-GTCAGT---ACAGTACAATGTACACATGGAATTAGACCAGTAGTATCAACT---
CAACTGCTATTAAATGACAGTTTAGCAGAAAAAGAG-----------
GTAGTAATTAGATCTAGCAATTTCACGGACAAC---------GCTAGAGTCATAATAGTACAGCTGAAT----
-----------GAATCTGTAGAA---------ATTAATTGTACAAGACCCAACAACAATACAAGAAAA-----
-CGTATAACTCTA------------GGACCAGGGCGAGTA---TTG------TATGCAACAGGACAA------
---------ATAATAGGAGATATA------------AGACGAGCACATTGT---AACCTTAGTAGAACA---
GAATGGAATAACACTTTAACACAGGTAGCTAAAAAATTAAGA---------------GAACAATTTGGG----
-----------------------------------AACAAAACAATAGTCTTTAAT------
CACTCCTCAGGAGGGGACCCAGAGATTACA---ATGCACAGCTTTACTTGTGGAGGGGAA---
TTTAAGCAGCTCCAGGCAAGAGTCTTGGCTGTGGAAAGATACCTAAAGGATCAACAGCTCCTAGGGCTTTGGG
GTTGCTCTGGAAAACTCATTTGCACCACTGCTGTGCCTTGGAATACTAGTTGGAGT----------------
-----------------------------AATAAATCTCTGAGTGATATCTGG------GAT------
AACATGACTTGGATGGAGTGG---GAAAGAGAAATTAACAATTACACAAAAGTAATATAC---
ACTTTAATTGAAGAATCGCAGAACCAACAAGAA---

AAGAATGAACTAGAACTATTGGAATTGGATAAGTGGGCAAGTTTGTGGAATTGGTTTGACATAACAAAATGGC
TGTGGTATATCAAAATATTCATAATGATAGTAGGAGGCTTGGTAGGCTTAAGAATAGTTTTTGCT---
GTACTTTCTATAGTA---------AATAGAGTTAGGCAGGGATACTCACCC------
TTGTCATTAGCTATTAGCTTGCTTAACGCCACAGCCATAGCAGTAGCTGAG---
GGAACAGATAGGGTTATAGAAATAGCACAAAGAGCTTATAGGGCT---------------------------
--------ATCCTCAACATA------CCTACAAGAATTAGACAGGGCCTCGAAAGGGCTTTGCTG---TAA
>JF932468
GCT---GCA---
GAAAACTTGTGGGTCACAGTCTATTATGGGGTACCTGTATGG---AAAGAAGCAACT---
ACTACTTTATTTTGTGCATCAGATGCTAAAGCATATGGTACAGAG---GCACATAAT----------------
--------------ATTTGGGCCACA---CATGCCTGTGTACCCACAGAC---
CCCAACCCACAAGAAGTAGTCTTGGCA---------AATGTGACAGAAGACTTTAAC---------------
--------------------------ATGTGGGAAAATAACATGGTAGAACAGATGCATGAAGATATA---
ATCAGTTTATGGGATCAAAGCCTA---AAGCCATGTGTAAAATTA---
ACCCCACTCTGTGTCACTTTAAATTGCACTAATGCTAATAACACCAGTAGTACTGATAATACC----------
----
---------------------AGTAGTAATACCAGTACTACGACAGAGGGAGGGGAAATAAAA---
AACTGCTCTTTCAATATCACCACAAGCATAAGA--------------------------ACTACGGTG---
AAAGACTATGCACTT---TTCAATAGTCTGGATATAGAAAAAATAGATAAT---------------------

----
-------------ACTACCAGCTATAGATTGATAAAGTGTAACACCTCAGTCATTACACAA---
GCCTGTCCAAAGATATCCTTTGAGCCAATTCCTATACATTATTGTGCCCCAGCTGGTTTTGCGATTCTAAAGT
GTAAC---AATAAAACATTCAAT------GGAACAGGACCATGTACAAAT----------------------
-GTCAGT---ACAGTACAATGTACACATGGAATTAGGCCAGTAGTATCAACT---
CAACTGCTGTTAAATGGCAGTTTAGCAGAAGAAGAG-----------
ATAGTAATTAGATCTAAAAATTTCACAAATAAT---------GATAAAATCATAATAGTACAGCTGAAG----
-----------GAATCTGTAGAA---------ATTAATTGTACAAGACCCAACAACAATACAAGAAAA-----
-AGTATATCTCTA------------GGACCAGGGCGAGCA---TGG------TATACAACAGGAGAA------
---------ATAATAGGAGATATA------------AGACAAGCACATTGT---AACATTAGTGGAGAA---
AAATGGAATGACACTTTAACAAAGATAGCTAAAAAATTAAGT---------------GAACAATTTGAA----
---------------------------------AACAAAACAATAATCTTTAAT------
CAACATTCAGGAGGGGACCCAGAGATTGTA---ATGCACAGCTTTAATTGTGGAGGGGAA---
TTTAAGCAGCTCCAGGCAAGAGTCTTGGCTGTAGAAAGATACCTAAAGGATCAACAGCTCCTAGGGCTTTGGG
GTTGCTCTGGAAAACTCATTTGCACCACTACTGTGCCTTGGAATGTTAGTTGGAGT----------------
---------------------------AATAGATCTCTGAGTGAAATTTGG------GAT------
AACATGACTTGGATGGAGTGG---GAAAAAGAAATTGACAATTACACACAAGAGATATAC---
ACCTTAATTGAAAAATCGCAGAACCAACAAGAA-------------------------------------
----
AAGAATGAACTAGAACTATTGGAATTGGATAAGTGGGCAAGTTTGTGGAATTGGTTTGACATAACACAATGGC
TGTGGTATATCAAGATATTCATAATGGTAGTAGGAGGCTTGGTAGGTTTAAGAATAGTTTTTGCT---
GTACTTTCTATAGTA---------AATAGAGTTAGGCAGGGATACTCACCA------
TTGTCATTTGCTATCAGCTTGCTTAACGCCACAGCAGTAGCAGTAGCTGAG---
GGGACAGATAGGGTTATAGAAGTAGCACAAAGAGCTTATAGGGCT---------------------------
--------ATTCTCAACATA------CCTACAAGAATCAGACAGGGCCTCGAAAGGGCTTTGGTA---TAA
>JF932470
GCT---ACA--
AAAAACTTGTGGGTCACAGTCTATTATGGGGTACCTGTATGG---AAAGAAGCAACT---
ACCACTCTATTTTGTGCATCAGATGCTAGAGCATATGAGACAGAG---GCACATAAT----------------
--------------ATTTGGGCCACA---CATGCCTGTGTACCCACAGAC---
CCCGACCCACAAGAAATATTCTTGGTA---------AATGTGACAGAAAACTTTAAC---------------
--------------------------ATGTGGAAAAATAACATGGTAGAACAGATGCATGAAGATATA---
ATCAGTTTATGGGATCAAAGCCTA---AAGCCATGTGTAAAATTA---
ACCCCACTCTGTGTTACGTTAAATTGCACTAATTTGAATACTACTAATACCAGTAATACTACC---------
----
----------------------AGTAGTAACAACAGTAGTCTGACAGGGGGAGAAGAAATGAAA---
AACTGCTCTTTCAATATCACCACAAGCATAAAA--------------------------ACTAAGGTA---
AAAGACTATGCACTT---TTTTATAAATATGATGTAGTAAAAATAGATAGTGAAAAT----------------
----
----------GATAATACCAGCTACAGGTTGATAAATTGTAACACCTCAGTCATTACACAA---
GCTTGTCCAAAGATATCCTTTGAGCCAATTCCCATACATTATTGTACTCCGGCTGGTTTTGCAATTATAAAGT
GTAAC---GATAAAATGTTCAAT------GGAACAGGACCATGTACAAAT----------------------
-GTCAGT---ACAGTACAATGTACACATGGAATTAGGCCAGTAGTATCAACT---
CAACTGCTGTTAAATGGCAGTTTGGCAGAAGAAGAG-----------
GTGGTAATTAGATCCAGCAATTTCTCGGACAAT---------ACTAAAGTCATAATAGTACAGCTGAAT----
-----------CAATCTGTAGTA---------ATTAATTGTACAAGACCCAACAACAATACAAGAAAA-----
-AGTATAAATATA------------GGACCAGGGAGAGCA---TGG------TATACAACAGGACAA------
---------ATAATAGGAGATATA------------AGACAAGCACATTGT---AACCTTAGTAAAACA---
GAATGGGATAACACTTTAAAACAGATAGCTGGAAAGTTAAGA---------------AAACAATTTGGG----
---------------------------------AACAAAACAATAATCTTTAAT------
CAATCTGCAGGAGGGGACCCAGAGATTGAA---AGGCACAGTTTTAATTGTGGAGGGGAA---
TTTAAGCAGCTCCAGGCAAGAGTCTTGGCTGTGGAAAGATACCTAAAGGATCAACAGCTCCTAGGGATCTGGG
GTTGCTCTGGAAAACTCATTTGCCCCACTGCTGTGCCTTGGAATACTAGTTGGAGT----------------
---------------------------AATAGAACTCACGATGAGATTTGG------AAT------
AACTTGACTTGGAGGGAGTGG---GAAAGAGAAATTAGCAATTACACAAATGAAATATAC---
ACCTTAATTGAAAAATCGCAGAACCAACAAGAA-------------------------------------
----
AAGAATGAACTAGAACTATTGGAATTGGATAAATGGGCAAGTTTGTGGAACTGGTTTGACATAACACAATGGC
TGTGGTATATCAAAATATTCATAATGATAGTAGGAGGCTTGATAGGTTTAAGAATAATTTTTGCT---
GTACTTTCTATAGTG---------AATAGAGTTAGGCAGGGGTACTCACCA------

TTGTCATTAGCTATTAGCTTGCTTAATGTCACAGCCATAGCAGTAGCTGAG---
GGGACAGATAGGGTTATAGAAATAGTACAAAGAGCTTATAGGGCT----------------------------
--------ATTCTTAACATA------CCTACAAGAATCAGACAGGGCCTAGAAAGGGCTTTGCTA---TAA
>JF932471
GCG---ACA--
GAAAAATTGTGGGTCACAGTCTATTATGGGGTACCTGTATGG---AAAGAAGCAACT---
ACCACTCTATTTTGTGCATCAGATGCTAAAGCACATGCTACAGAG---GCACATAAT----------------
--------------ATTTGGGCCACA---CATGCCTGTGTACCCACAGAC---
CCCAACCCACAAGAAGTAGTCTTGGAA---------AATGTGACAGAAAACTTTAAC----------------
--------------------------ATGTGGAGAAATGACATGGTAGAACAGATGCATGAAGATATA---
ATCAGTTTATGGGATCAAAGCCTA---AAGCCATGTGTAACATTA---
ACCCCACTCTGTGTTACTTTACATTGCACCAATGTGACTGTTACTAATACCGGTAGTACTACCAGGAGTAGAC
CTACCAGAAGT--
-------------------------AGTACTACCAGCAGTGTGACAGGGGGAGAAGAAATGAAA---
AACTGCTCTTTCAATATCACCACAAGCATAGGA---------------------------ACTAAGGTG---
GAAGACTATGCAGTT---TTTTATAAACTTGATATAGAACCAATGAGTAAT----------------------
----
----GATACTAATACTACCAGTTATAGGTTGATAAGTTGTAATACCTCAGTCATTAAACAA---
GCTTGTCCAAAGATATCCTTTGAGCCAATTCCCATACATTATTGTGCCCCGGCTGGGTTTGCGATTCTAAAGT
GTAAC---AATAAAACGTTCAAT------GGAACAGGACCATGTACAAAT-----------------------
-GTCAGT---ACAGTACAATGTACACATGGAATTAAGCCAGTAGTATCAACT---
CAACTGCTGTTAAATGGCAGTTTAGCAGAAAGAGAG------------
GTAGTAATCAGATCTAGCAATTTCACGGACAAT---------ACTAAAATCATAATAGTACAGCTGAAG----
-----------GAACCTGTGGAA---------ATTACTTGTGAAAGAACCAACAACAATACAAGAAAA-----
-AGTATACCTCTA------------GGACCAGGGCGAGCA---TGG------TATACAACAGGACAA------
---------GTAATAGGAAATATA------------AGACAAGCAAGTTGT---AACCTTAGTATAGCA---
GACTGGAATAACACTCTAGGGCAGGTAGCTAAAAAATTAAGA---------------GAACAATTTAGG----
-----------------------------------AACAAAACAATAGTCTTTAAT------
CAATCTTCAGGAGGGGACCTAGAGATTGTA---ATGCACACTTTTAATTGTGGAGGGGAA---
TTTAAGCAGCTCCAGGCAAGAGTCCTGGCTGTGGAAAGATACCTAAAGGACCAACAGCTCCTAGGGATTTGGG
GTTGCTCTGGAAAACTCATTTGCCCCACTGCTGTGCCTTGGAATGCTAGTTGGAGT-----------------
----------------------------AATAGATCTCAGAGTGAGATTTGG------AAT------
AACATGACTTGGATGCAGTGG---GAAAGAGAAATTGACAATTACACAAAAGAAATATAC---
ACCTTAATTGAAGAATCGCAGAACCAACAAGAA--

AAGAATGAACTAGAACTATTGGAATTGGATAAGTGGGCAAGTTTGTGGAATTGGTTTGACATAACAAAATGGC
TGTGGTATATCAAAATATTCATAATGATAGTAGGAGGCTTAGTAGGTTTAAGAATAGTTTTTGCT---
ATACTTTCTCTAGTA---------AATAGAGTTAGGCAGGGATACTCACCA------
TTGTCATTGGCTATTAGCTTGCTTAACACCACAGCCATAGCAGTAGCTGAG---
GGGACAGATAGGGTTATAGAAGTAGTACAAAGAGCTTGTAGGGCT----------------------------
--------ATTCTCAACATA------CCTACAAGAATCAGACAGGGCCTTGAAAGAGCTTTGCTA---TAA
>JF932473
GCT---GAA---
AACTTGTGGGTCACAGTCTATTATGGGGTACCTGTATGG---AAAGAAGCAACT---
ACCACTCTATTTTGTGCATCAGATGCTAAAGCATATGATACAGAG---GCGCATAAT----------------
--------------ATTTGGGCCACA---CATGCCTGTGTACCCACAGAC---
CCCAACCCACAAGAAGTAGTCTTGGGA---------AATGTGACAGAAAACTTTAAC----------------
--------------------------ATGTGGAGAAATGACATGGTAGAACAGATGCATGAAGATATA---
ATCAGTTTATGGGATCAAAGCCTA---AAGCCATGTGCAAAATTA---
ACCCCACTCTGTGTTACCTTAAATTGTACTAATTCGAATACTACTAAGACCAGTAGT----------------
----
----------------------------AATACTAGTAGTACAACAGAGGGAGGAGAAATGAAA---
AACTGCTCTTTCAATGTCACCACAAGCATAAAA---------------------------ACTAAGGTG---
AGAGACTATGCACTT---TTTTATAACCTTGATCTAGTACTAATAGATAATGATAAGGAA-------------
----
----------AATAGTACCAGCTATAGGTTGATAAATTGTAACACCACAGTCATTACACAA---
GCTTGTCCAAAGATATCCTTTGAGCCAATTCCCATACATTATTGTGCCCCGGCTGGTTTTGCGATTCTAAAGT
GTAAC---AGTAAAACGTTCAAT------GGAACAGGACCATGTACAAAT-----------------------
-GTCAGT---ACAGTACAATGTACACATGGAATTAGGCCAGTGGTATCAACT---
CAACTGCTGTTAAATGGCAGTCTAGCAGAAGAAGGG------------

ATAGTAATTAGATCTAGCAATTTCACAGACAAT---------GCTAGAACCATAATAGTACAGCTGAAT----
-----------GAATCTGTAGAA---------ATTAATTGTATAAGACCCAACAACAATACAAGAAAA-----
-AGTATATCTCTA------------GGACCAGGAAAAGCA---TGG------TATACAACAGGACAA------
---------ATAATAGGAGATATA------------AGACAAGCACATTGT---AACCTTAGTAGTACA---
GAATGGAATAACACTTTACAGCAGATAATTGGAAAATTAAGA---------------GAACAATTTGGG----
-----------------------------------AACAAAACAATAATCTTTAAT------
CAATCTTCAGGAGGGGACCCAGAGATTGTG---ATGCACAGTTTTAATTGTGGAGGGGAA---
TTTAAACAGCTCCAGGCAAGAGTCTTGGCTGTGGAAAGATACCTAAAGGATCAACAGCTCCTAGGGATTTGGG
GTTGCTCTGGAAAACTCATTTGCACCACTGCTGTGCCTTGGAATGTTAGCTGGAGT-----------------
----------------------------AATAAATCTCTGGGAGAGATTTGG------AAT------
AACATGACTTGGATGGAGTGG---GAAAGAGAAATTAGCAATTACACAAAAGAAATATAC---
ACCTTAATTGAAGAATCGCAGAACCAACAAGAA--

AAGAATGAACTAGAACTATTGGAATTGGATAAGTGGGCAAGTTTGTGGAATTGGTTTGACATATCAAACTGGC
TGTGGTATATCAAAATATTCATAATGATAGTAGGAGGTTTGGTAGGTTTAAGAATAATTTTTGCT---
GTACTTTCTATAGTA---------AATAGAGTTAGGCAGGATACTCACCG------
TTGTCATTAGCTATTAGCTTGCTTAACGCCACAGCCATAGCAGTAGCTGAG---
GGAACAGATAGGATTATAGAAGTAGTACAAAGACTTTGTAGGGCT----------------------------
--------TTTCTTAACATA------CCTAGAAGAATCAGACAGGGCCTCGAAAGGGCTTTGCTA---TAA
>KM217584
GCT---CAA---
AATTTATGGGTCACAGTCTATTATGGGGTACCTGTGTGG---AAAGATGCAGAC---
ACCACTTTATTTTGTGCATCAGATGCTAAGGCATATGCCACAGAG---GTACATAAT----------------
--------------GTTTGGGCCACA---CATGCCTGTGTACCCCACAGAC---
CCTAACCCACAAGAAGTAGTATTGGGA---------AATGTGACAGAAAATTTTAAT----------------
--------------------------ATGTGGAAAAATAACATGGTAGAACAGATGCAACAAGATATA---
ATCAGTTTATGGGATGAAAGCCTA---AAGCCATGTGTAAAATTA---
ACACCACTCTGTGTTACTTTAAATTGCATTGATTACAAGTCTAATACTACTAATGGAACTAAAACT-------
----
----------------------GCCAGTAATAGTAGCTGGGAATATATGGAGGGAGAAATAAAA---
AACTGCTCTTTTAATATCACCACAAACATAGGA---------------------------
AACAAAGTAAAGAAAGATTATGCACTT---TTTAATAAACTTGATGTAGTACCAATAGATAAT----------
----

AGCTCTCATATGTTGATAAATTGTAACACCTCAGTCATTACGCAG---
ACCTGTCCAAAGGTATCCTTTGAGCCAATTCCAATACATTATTGCACCCCGGCTGGTTTTGCGATTATAAAGT
GTAAT---GATAAAAAAATTCAAT------GGAACAGGACCATGTACAAAT----------------------
-GTCAGT---ACAGTACAATGTACACATGGAATTAGGCCAGTAGTGTCAACT---
CAACTGCTGTTAAATGGCAGTCTAGCAGAAGGAGAT------------
ATAGTGATTAGATCTAAAAATTTCACAAACAAT---------GCTAAAACCATAATAGTACAGCTGAAT----
-----------GAAACTGTAGAG---------ATTAATTGTACAAGACCCACCAACAATACAAGAAAG-----
-AGTATACATATA------------GGACCAGGGAGTTCA---TTA------TATGCAACAGGAGCA------
---------ATAATAGGAGATATA------------AGACAAGCACATTGT---AATGTTAGTTTTACA---
AAATGGAACAACACTTTAAAAAGGGTAGCTATAAAATTAAGA---------------GAACAAGTTGGT----
--------------------------------AATGATACAACAATAGCCTTTCGG------
CAACCCTCAGGAGGGGACCCAGAAATTGTA---ATGCACACATTTAATTGTGGAGGGGAA---
TTTAAGCAGCTCCAGGCAAGAGTCCTAGCTGTGGAAAGATACCTAAGGGATCAACAGCTCCTAGGGATTTGGG
GTTGCTCTGGAAAACTCATCTGCACCACTGCTGTGCCTTGGAATGCTAGTTGGAGT-----------------
----------------------------AATAAATCTGTGGATGAGATTTGG------GAA------
AACATGACCTGGATGCAGTGG---GAGAGAGAAATTGACAATTACACAGAATTAATATAC---
TCTTTACTTGAAAAATCGCAGAACCAGCAAGAA--

AAGAATGAGCAAGAATTATTGGAATTGGATAAGTGGGCAAATTTGTGGAGTTGGTTTGACATAACAAACTGGC
TGTGGTATATAAGAATATTCATAATGATAGTAGGAGGCTTAATAGGTTTAAGAATAGTTTTTGCT---
GTGCTTTCTATAGTG---------AATAGAGTTAGGCAGGATACTCACCA------
TTGTCATTCGCTGTTAGTTTGCTTAATACCACAGCTGTAGCAGTAGCTGAG---
GGGACAGATAGAGTTATAGAATTAGCACAAAGAGCTGGTAGAGCT----------------------------
--------ATCCTCAACATA------CCTAGAAGAATAAGACAGGGCGCAGAAAGAGCTTTGCTA---TAG
>KM217662

GCT---CAA--
AATTTGTGGGTCACAGTCTATTATGGGGTACCTGTGTGG---AAAGAAGCAAAA---
ACCACTTTATTTTGTGCATCAGATGCTAAAGCATATGAAACAGAG---GCACATAAT------------------
--------------GTTTGGGCCACA---CATGCCTGTGTACCCACAGAT---
CCTAACCCACAAGAAGTAGTATTGGCA----------AATGTGACAGAAAATTTTAAC-----------------
---------------------------ATGTGGAAAAATGACATGGTAGAACAGATGCAGGAGGATATA---
ATCAGTTTATGGGAAGAAAGCCTA---AAGCCATGTGTAAAATTA---
ACCCCACTCTGTGTTACTTTAAATTGCACTGATGGGAAAAATAATAATACTGGTAATAGTACCAAT-------
----
-------------AGTACTAACGCCACTGTAAGTAGTTTGGAAAAAACAGTGGGAGAAATAAAA---
AACTGCACTTTCAATGTCACCTCACAAATAAAA----------------------------
AATGGGGCGAGAACAGAACGGGCAATT---TTTAATAGACTTGATGTAGTACCAATAGACAAT----------
----

GATAACTCTAGCTATATGTTAATAAACTGTAACACCTCAGTCATTACACAG---
GCCTGTCCAAAGATATCCTTTGAACCAATTCCCATACATTATTGTACCCCGGCTGGTTTTGCGATTATAAAGT
GTAAT---AATAAGACATTCAAT------GGAAAAGGACCATGTACAAAT-----------------------
-GTCAGT---ACAGTACAATGTACACATGGAATTAAGCCAGTAGTGTCAACT---
CAACTGCTGTTAAATGGCAGTCTAGCAGAAGAAGAC------------
ATAGTAATCAGGTCTGAAAATCTCACGAACAAT---------GTTAAAAACATAATAGTACAACTAAAG----
-----------GAAGCTGTAAAA---------ATTAATTGTTCAAGACCCAACAACAATACAAGAAAG-----
-AGTATAAACTTT------------GGACCGGGGGGAGCA---ATC------TATACAACAGGAGAC------
---------ATAATAGGAGATATA------------AGACAAGCATATTGT---AATATTAGTTTTGAA---
GAATGGAACAACACCTTAACACAGGTAGCTATAAAATTAAGA---------------GAACAATATGGA----
------------------------------------AATAAGTCAATAGTCTTTAAG---
CAACCCCCCCCAGGAGGGGATCCAGAAATTGTA---ATGCACACATTTAATTGTGGAGGGGAA---
TTTAAGCAGCTCCAGGCAAGAGTCCTAGCTGTGGAAAGATTCCTAAGGGATCAACAGCTCCTAGGGATTTGGG
GTTGCTCTGGAAAACTCATCTGCGCCACTGCTGTGCCTTGGAATGCTAGTTGGAGT-----------------
-----------------------------AATAGATCTATGGATGAGATTTGG------AAC------
AATATGACCTGGATGCAGTGG---GAGAGAGAAATTGACAATTACACAGATCTAATATAC---
AGTCTACTTGAACAATCGCAGAACCAGCAAGAA--

AAGAATGAGCAAGAATTATTGGAATTGGATAAGTGGGCAGGTTTGTGGAGTTGGTTTTCCATAACACAATGGC
TGTGGTATATAAGATTATTCATAATGATAGTAGGAGGCTTAATAGGTTTAAGAATAATTTTTGCT---
ATACTTTCTATAGTA---------AATAGAGTTAGGCAGGGATACTCACCA------
TTGTCGTTAGCTGTTAGCTTGCTTAATGTTACAGCTATAGCAGTAGCTGAG---
GGAACAGATAGAATTATAGAAGCAGCACAAAGAATTGGTAGAGCT-----------------------------
--------ATTCTCCACATA------CCTAGAAGAATAAGACAGGGCGCAGAAAGAGCTTTGCTA---TAA
>KM217802
GCT---GTA---
GAACAATTGTGGGTTACAATCTATTATGGGGTACCTGTGTGG---AAAGAAGCAACT---
ACCACTCTATTTTGTGCATCAGATGCTAAAGCATATGATACAGAG---GCACATAAT------------------
--------------GTTTGGGCCACA---CATGCCTGTGTACCTACAGAC---
CCTAACCCACAAGAAGTAGTATTGGAA----------AATGTGACAGAAAATTTTAAC-----------------
---------------------------ATGGGGAAAAATAACATGGTAGACCAGATGCAGGAAGATATA---
ATCAGTTTATGGGATGAAAGCCTA---AAGCCATGTGTAAAATTA---
ACCCCACTCTGTATTACTTTAAATTGCACTACTGTGAATGCGAATGTTAGTGTGGATGGGAATGATATTACCA
ATAGTACTCGG--
-----------------------------GAAAAGCTAAAGGAAGGAATAAAGGGAGAAATAACA---
AACTGTTCTTTCAATATCAGCACAAGCATAGAA----------------------------
AAAAGGATAAAGAGAGGATATGCAATT---
TTTGATAAGCTTGATGTAATGCCAATAGATAATCCAGATAATAGCACA--------------------------
----
AATAATGCAAACTATACACTCTATACAGTGAGAAATTGTAACACCTCAGTCATTACGCAG---
GCCTGTCCAAAGATATCTTTTGAACCAATTCCCATACATTATTGTGCCCCGGCTGGTTTTGCGATTCTAAAGT
GTAAT---GATAAAAAATTCAAT------GGAACAGGACCATGTACAAAT-----------------------
-GTCAGC---ACAGTACAATGTACACATGGAATTAGGCCAGTGGTGTCAACT---
CAACTGCTGTTAAATGGCAGTTTAGCAGAAGAAGAG------------
GTAGTAATTAGATCTGAAAATTTCTCGAACAAT---------GCTAAAACCATCATAGTACAGCTGAAT----
-----------GAAACTATAGTA---------ATTAATTGTACAAGACCCAACAACAATACAAGAAAA-----

-GGTATACATTTT------------GGACCGGGGGGACCA---CTT------TTTACAACA---GAT------
---------ATAATAGGAGATATA------------AGACGAGCACATTGT---AAACTTAATGGAACA---
CAATGGAAAACCGCTTTAGAAAGGATAGCTATAAAATTAAGA---------------GAACAATGG-------
-----------------------------------AATAAAACAATAGCCTTTAAT------
AAATCCTCAGGAGGGGATCCAGAAATTGTA---ATGCACAGTTTTAATTGTGGAGGGGAA---
TTTAAGCAGCTCCAGGCAAGAGTCCTGGCTGTGGAAAGATACCTAAGGGATCAACAGCTCCTAGGGATTTGGG
GTTGCTCTGGAAAACTCATTTGCACCACTGCTGTGCCTTGGAATGCCAGTTGGAGT-----------------
----------------------------AATAAATCTATGAGTCAGATTTGG------GAG------
AACATGACCTGGATGGAGTGG---GAGAGAGAAATTAGCAATTACACAAGCTTAATATAC---
ACCTTAATTGAAGAATCGCAGAACCAACAAGAA--

AAGAATGAACAAGAATTATTGGAATTGGATAAGTGGGCAAGTTTGTGGAATTGGTTTAGCATAACAGAATGGC
TGTGGTATATAAAAATATTCATAATGATAGTAGGAGGCTTGATAGGTTTAAGAATAGTTTTTACT---
GTACTTTCTATAGTG---------AATAGAGTTAGGCAGGGATACTCACCA------
TTATCATTGGCTACTAGCCTGTTTAATACTACAGCTATTGCAGTAGCTGAG---
GGAACAGATAGGATCATAGAAATAGTACAAAGAATTTATAGAGCA---------------------------
--------ATTCTTCACATA------CCTACAAGAATAAGACAAGGCTTAGAAAGGCTTTTGCTA---TAA
>KM217995
GCT---GAG---
GATTTGTGGGTCACAGTCTATTATGGGGTACCTGTGTGG---AAAGAGGCAAAC---
ACCACTTTATTTTGTGCATCAGATGCTAAAGCATATAACACAGAG---GCACATAAT----------------
--------------GTTTGGGCCACA---CATGCCTGTGTACCCACAGAT---
CCTAACCCACAAGAAGTACTATTGGAA---------AATGTGACAGAAAATTTTAAC----------------
-------------------------ATGTGGAAAAATAACATGGTAGAACAGATGCAAGAAGATGTA---
ATCAGTCTATGGGATGAAAGTCTA---AAGCCATGTGTAAGATTA---
ACACCACTCTGTGTTACTTTAAATTGCACTGATTGGAAGTCTAATACTACTCATACTACT-------------
----
----------------------------AATATCAGTAGAACTGATATGGAGGGAGAAATAAAA---
AACTGCTCTTTCAATATCACCACAAAAATAGGA--------------------------
AATAAGATACGAAAAGAATATGCACTT---TTTAATAAACTTGATGTAGTACCAATAGATAAT----------
----

AAGACATCTATGTTGATAAATTGTAACACCTCAGTCATTACGCAG---
GCCTGTCCAAAGGTATCCTTTGAACCAATTCCCATACATTATTGTACCCCGGCTGGTTTTGCTATTATAAAGT
GTAAT---AATAAGACATTCAAT------GGAACAGGACCATGTACAAAT-----------------------
-GTCAGT---ACAGTACAATGTACACATGGAATTAGGCCAGTAGTGTCAACT---
CAACTGCTGTTAAATGGCAGTCTAGCAGAAGGAGAG------------
ATAGTAATTAGATCTGAAAATTTCACAAACAAT---------GCTAAAACCATAATAGTACAGCTGAAT----
-----------GAAACTGTAGAA---------ATTAATTGTACAAGACCCAGCAACAATACAAGAAAG-----
-AGTATACATATA------------GGATGGGGGAGATCA---TTA------TATGCAACAGGAGAC------
---------ATAACAGGAGATATA------------AGACAAGCACATTGT---AATGTTAGTGTAGCA---
AAATGGAATAACACTTTAAGACAGGTAGCTATAAAATTAAGA---------------GAACAATTTAAG----
-----------------------------------AATAAAACAATAGCCTTTAAG------
CAACCCTCAGGGGGGGACCCAGAGATTGTA---ATGCACACATTTAATTGTGGAGGGGAA---
TTCAAGCAGCTCCAGGCAAGAGTCCTAGCTGTGGAAAGATACCTAAGGGACCAACAGCTCCTAGGGATTTGGG
GTTGCTCTGGAAAACTCATCTGCACCACTGCTGTGCCTTGGAATGCTAGTTGGAGT-----------------
----------------------------AATAAATCTGTGGATGAGATTTGG------AAA------
AACATGACCTGGATGCAGTGG---GAGAGAGAAATTGACAATTACACAGAATTAATATAC---
ACTTTACTTGAAAAAATCGCAGAACCAGCAAGAA--

AAGAATGAACAAGAATTATTGGAATTGGATAAGTGGGCAAATTTGTGGAATTGGTTTAGTATAACAAAATGGC
TGTGGTATATAAAAATATTCATAATGATAGTAGGAGGTTTAATAGGTTTAAGAATAATTTTTACT---
GTGCTTTCTATAGTG---------AATAGAGTTAGGCAGGGATACTCACCA------
TTGTCATTCGCTGTTAGTTTACTTAATGCCACAGCTATAGCAGTAGCTGAG---
GGGACAGATAGAATTATAGAATTAGCACAAAGACTTGGTAGAGCT---------------------------
--------ATTCTTAACATA------CCTAGAAGAATAAGACAGGGCACAGAACTAGCTTTGCTA---TAA
>JF932475
GCT---GCA---
GAAAACTTGTGGGTCACAGTCTATTATGGGGTACCTGTATGG---AAAGAAGCAACT---
ACCACTCTATTTTGTGCATCAGATGCTAAAGCATATGATACAGAG---GTACATAAT----------------

--------------GTTTGGGCCACA---CATGCCTGTGTACCCACAGAC---
CCCAACCCACAAGAAGTAGTCTTGGGA---------AATGTGACAGAAAACTTTAAC----------------
--------------------------ATGTGGAAAAATAACATGGTAGAACAAATGCATGAAGATATA---
ATCAGTTTATGGGATCAAAGCCTA---AAGCCATGTGTAAAATTA---
ACCCCACTCTGTGTTATTTTAAATTGCACTAATTTGAATGCTACTAATAATACTACTGATACC----------
----
----------------------AGTAATAATACCATTAGTATGATAGAGGAAGGAGAAATAAAA---
AACTGCTCTTTCAATATCACCACAAACATAAGA---------------------------ACTACGGTG---
AAAGACTATGCATTT---TTTTATAAACTTGATGTAGTACCAATAGATAGGGATAAGAAT-------------
----
----------GATAGTACCAGCTATAGGTTGAGACATTGTAACACTTCAGTCATTACACAA---
GCTTGTCCAAAGGTATCCTTTGAGCCAATTCCCATACATTACTGTGCTCCGGCTGGTTTTGCGATTCTAAAAT
GTAAC---AATAAAACGTTTAAT------GGAACAGGACCATGTACAAAT-----------------------
-GTCAGT---ACAGTACAATGTACACATGGAATTAGGCCAGTAGTATCAACT---
CAACTGCTGTTAAATGGTAGTTTAGCAGAAGAAGAG------------
GTAGTAATTAGATCTAGCAATTTCTCGAACAAT---------GCTAAAGTCATAATAGTACAGCTAAAT----
-----------GAATCTGTAGAA---------ATTGAGTGTACAAGACCCAACAACAATACAAGAAAA-----
-AGTATACAGCTA------------GGACTAGGGCGAGCA---TGG------TATACAACAGGACAA------
---------ATAATAGGAAATATA------------AGAAAAGCATATTGT---AACATTAATGGAACA---
AAATGGAATAACACCTTAAAACAGATAGCTAAAAAACTAAGA---------------GAACAATTTGGG----
-----------------------------------AACAAAACAATAAGCTTTAAT------
CAATCTTCAGGAGGGGACCCAGAGATTGTA---ATGCACAGTTTTAATTGTGGAGGGGAA---
TTTAAGCAGCTCCAGGCAAGAGTCTTGGCTGTGGAAAGATACCTAAGGGATCAACAGCTCCTAGGGATTTGGG
GTTGCTCTGGAAAACTCATCTGCACCACTGCTGTGCCTTGGAATGTTAGTTGGAGT-----------------
----------------------------AATAAATCTATGGATGAGATTTGG------ACT------
AACATGACTTGGATGGAGTGG---GAAAGAGAAATTGAAAATTACACAAAAGAAATATAC---
ACCTTAATTGAAAAATCGCAGAACCAACAGGAA--

AAGAATGAACAAGAACTATTGGAATTGGATAAGTGGGCAAGCTTGTGGAATTGGTTTGACATAACAAACTGGC
TGTGGTATATCAAAATATTCATAATGATAGTAGGAGGCTTGGTAGGTTTAAGAATAGTCTTTGCT---
GTACTTTCTATAGTA---------AATAGAGTTAGGCAGGGATATTCACCA------
TTGTCGTTGGCTATTAGCTTGCTTAACGCCACAGCCATAGCAGTAGCTGAG---
GGGACAGATAGGGTTATAGTAGTAGTACAAAGAACTTGTAGGGGT----------------------------
--------ATCCGCAACGTA------CCTAGAAGAATCAGACAGGGCTTAGAAAGGGCTTTGCTG---TAA
>JF932476
GCT---GCA--
GAAAAGTTGTGGGTCACAGTCTATTATGGGGTACCTGTATGG---AAAGAAGCAACT---
ACCACTCTATTTTGTGCATCAGATGCTAAAGCATATGAGACAGAG---GTACATAAT----------------
--------------ATTTGGGCCACA---CATGCCTGTGTACCCACAAAC---
CCCAACCCACAAGAAGTAGTCATGGGA---------AATGTGACAGAAAACTTTAAC----------------
--------------------------ATGTGGAAAAATAACATGGTAGAACAGATGCATGAAGATATA---
ATCAGTTTATGGGATCAAAGTCTA---AAGCCATGTGTAAAATTA---
ACCCCACTCTGTGTTACTTTAAATTGCACTGATTTGAAGAATACTACTAATACCAGTAGTAATACCAGT----
----
-------------ATTACCAGTATTAGTAATACCAGTATTATAACAGAGGGAGGAGACATAAAA---
AACTGCTCTTTCAATATCACCACCAGCATAGGA---------------------------AATAAGGTG---
AAAGACTATGCACTT---TTTTATAGACTTGATGTAGCACCAATAGATAAT----------------------
----
----------GGTACTACCAGCTATAGGTTGATAAATTGTAACACCTCAGTCATTACACAA---
GCTTGTCCAAAGGTATCCTTTGAGCCAATTCCCATACATTATTGTGCCCCGGCTGGTTTTGCGATTCTAAAGT
GTAAC---AATAAAACGTTCAAA------GGAACAGGACCATGTACAAAT-----------------------
-GTCAGT---ACAGTGCAATGTACACATGGAATTAGGCCAGTAGTATCAACT---
CAACTGCTGTTAAATGGCAGTTTAGCAGAAGAAGAA------------
GTAATGATTAGATCTAGCAATTTCACGAACAAT---------GCTAGAGTTATAATAGTACAGCTGAAG----
-----------GAATCTATAGTA---------ATTAATTGTACAAGACCCAACAACAATACAAGAAAA-----
-AGGGTATCTCTG------------GGACCAGGGCGAGTA---TGG------TATACAACAGGACAA------
---------ATAGTAGGAGATATA------------AGACAAGCACATTGT---AACCTTAGTGGCACA---
GAATGGAATAACGCTTTAAAACAGATAGCTAAAAAATTAAGA---------------GAACAATTT-------
-----------------------------------AACAAAACAATAATTTTTAAG------
CAATCCTCAGGAGGGGACCCAGAGATTGTA---ATGCACAGTTTTAATTGTGGAGGGGAA---

TTCAAGCAGCTCCAGGCAAGAGTCTTGGCTGTGGAAAGATACCTAAAGGATCAACAGCTCCTAGGGCTTTGGG
GATGCTCTGGAAAACTCATCTGCACCACTGCTGTGCCTTGGAATTCTAGTTGGAGT-----------------
----------------------------AATAGATCTCTGCATGAGATTTGG------GAT------
AACATGACTTGGATGGAGTGG---GAAAGAGAAATTGACAATTACACAAGAGAAATATAC---
ACCTTAATTGAAGAATCGCAGAACCAACAAGAA--

AAGAATGAACTAGAATTATTAGAATTGGATAAATGGGCAAGTTTGTGGAATTGGTTTGACATAACAAAATGGC
TGTGGTATATCAAAATATTCATAATGATAGTAGGAGGCTTGGTAGGTTTAAGAATAGTTTTTGCT---
GTACTTTCTATAGCA---------AATAGGGTTAGGCAGGGATACTCACCA------
TTGTCATTAGCTATTAGCTTGCTTAATGCTACAGCTATAACAGTAGCTGAG---
GGGACAGATAGGGTTATAGAAATAGCACAAAGAGCTTTTAGAGCT----------------------------
--------TTACTCCACATA------CCTAGAAGAATCAGACAGGGCTTCGAAAGGGCTTTGCTA---TAA
>JF932477
GCT---GCA--
GAAAAATTGTGGGTCACAGTCTATTATGGGGTACCTGTATGG---AAAGAAGCAAT---
ACCACTTTATTTTGTGCATCAGATGCTAGGGCATATGCTACAGAG---GCACATAAT----------------
--------------ATTTGGCCACA---CATGCCTGTGTACCCACAGAC---
CCCACCCCACAAGAAGTAGTCTTGGAA---------AATGTGACAGAAAACTTTAAC----------------
--------------------------ATGTGGAAAAATGACATGGTAGAACAGATGCATGAAGATATA---
ATCAGTTTATGGGATCAAAGCCTA---AAGCCATGTGTAAAATTA---
ACCCCACTCTGTGTTACTTTAAATTGCACTGATTATTTGGGGAATACTACCAGTAGTAAT-------------
----
-------------------------------AACAGCAGTACAGCAGAGGGAGGAGAAATAAAA---
AACTGCTCTTTCAATATCACTACAAGTATAAGA---------------------------ACTAAGGTA---
AGAGACTATGCACTT---TTTTATAAATTTGATGTAGTACCAATAGGTAAT----------------------
----
----------AATGGTACCAAATATAGGTTGATAGAGTGTAACACCTCAGTCATTACACAA---
GCTTGTCCAAAGGTATCTTTTGAGCCAATTCCCATACATTATTGTACCCCGGCTGGTTTTGCGCTTCTAAAGT
GTAAC---AATAAAACATTCAGT------GGAACAGGACCATGTACAAAT-----------------------
-GTCAGT---ACAGTGCAATGTACACATGGAATTAGGCCAGTAGTATCAACT---
CAACTGCTGTTAAATGGCAGTTTAGCAGAAGAAGAG------------
GTAGTAATTAGATCTAGCAATTTCTCGAACAAT---------GCTAGAGTCATAATAGTACAGCTGAAT----
-----------GAAACTGTAGAA---------ATTAATTGTACAAGACCCAATAACAATACAAGAAAA-----
-AGTATACACCTA------------GGACAAGGGAGAGCA---TGG------TATACAACAGGAGAA------
---------ATAATAGGAGATATA------------AGACAAGCACATTGT---AACCTTAGTAGTACA---
AAATGGAATAACACTTTAAGACAGATAACTGAAAAATTAAGA---------------GAACAATTTGGA----
--------------------------------AACAACAAAACCATAATCTTTAAG------
GAACCTGCAGGAGGGGACCCAGAGATTGTA---ATGCACAGTTTTAATTGTGGAGGGGAA---
TTTAAGCAGCTCCAGGCAAGAGTCTTGGCTGTGGAAAGATACCTAAAGGATCAACAGCTCCTAGGGATTTGGG
GTTGCTCTGGAAAACTCATTTGCACCACTACTGTGCCTTGGAATGCTAGTTGGAGT-----------------
----------------------------AATAAATCTCTAAGTGAGATTTGG------GAT------
AACATGACTTGGATGGAGTGG---GAAAGGCAAATTGAAAATTACACAAAAGAAATATAC---
ACATTAATTGAAGAATCGCAGAACCAACAAGAA--

AAGAATGAACTAGACCTATTAGAATTGGATAAGTGGGACAGTTTGTGGAGTTGGTTTAGCATAACAAAATGGC
TGTGGTATATCAAAATATTCATAATGATAGTAGGAGGCTTGATAGGTTTAAGAATAGTTTTTGCT---
GTACTTTCTATAGTA---------AATAGAGTTAGGCAGGGATACTCACCA------
TTGTCATTAGCTGTTAGCTTGCTTAACGCCACAGCCATAGCAGTAGCTGAA---
GGGACAGATAGGGTTCTAGAAATAGCACAAAGAGCTTGTAGGGCT----------------------------
--------ATTCTCAACATA------CCTAGAAGAATCAGACAGGGCTTCGAAAGGGCTTTGCTA---TAA
>JF932483
GCT---GCA--
GAAAACTTGTGGGTCACAGTTTATTATGGGGTACCTGTATGG---AAAGAAGCAACT---
ACCACTCTATTTTGTGCATCAGATGCTAAAGCATATTCTACAGAG---GTACATAAT----------------
--------------ATTTGGCTACA---CATGCCTGTGTACCCACAGAC---
CCCAACCCACAAGAAGTAGTCTTGGAA---------AATGTGACAGAAAACTTTAAC----------------
--------------------------ATGTGGAAAAATGACATGGTAGAACAGATGCAGGAAGATATA---
ATCAGTTTATGGGATCAAAGCCTA---AAGCCATGTGTAAAATTA---
ACCCCACTCTGTGTTACTTTAAACTGCACTAATTTGAATATTACTAATACCAGTAATAATACC----------
----

```
----------------------AGTAGTACTACCAGTAGTATAATACGGGAAGGAGAAATAAAA---
AACTGCTCTTTCAATATCACCACAAGCATAAAT--------------------------ACTAAGGTG---
AAAGACTATGCACTT---TTTTATAAACTTGATGTAGAACAAATGGGAGGG---------------------
-----------------------------------------------------------------------
----------GATAATACCAGCTATAGGTTGATAAATTGTAACACCTCAGTCATTAAACAA---
GCTTGTCCAAAGGTATCCTTTGAGCCAATTCCCATACATTATTGTGCCCCGGCTGGTTTTGCAATTCTAAAGT
GTAAC---AATAAAACATTCAAT------GGAACAGGACCATGTACAAAT----------------------
-GTCAGT---ACAGTACAATGTACACATGGAATTAGGCCAGTAGTATCAACT---
CAACTGCTGCTAAATGGCAGTTTAGCAGAAGAAGAG-----------
GTAGTAATTAGATCTAGCAATTTCAGTGACAAT---------AAGAAAAACATAATAGTACAGCTGAAG----
-----------GACTCTGTAGAA---------ATTAATTGTACAAGACCCAACAACAATACAAGAAAA-----
-AGTATACATCTA------------GGACCAGGGCGAGCA---TGG------TATGCAACAGGACAA------
---------ATAATAGGAGACATA------------AGACAAGCACATTGT---AACATTAGTGGCACA---
AAATGGAATAACACTTTAAAACAGGTAATTAAAAAATTAAGA---------------GAACAATTTGGG----
-----------------------------------AACAAAACAATAATCTTTAAT------
CAATCCTCAGGGGGGGACCCAGAGATTACA---ACGCACAGTTTTAATTGTGGAGGGGAA---
TTTAAGCAGCTCCAGGCAAGAGTCTTGGCTGTGGAAAGATTCCTAAGGGATCAACAGCTCCTGGGGATTTGGG
GTTGCTCTGGAAAACTCATTTGCACCACTACTGTGCCTTGGAATACTAGTTGGAGT----------------
----------------------------AATAAATCTCTGAATGAAATTTGG------AAT------
AACATGACTTGGATGGAGTGG---GAAAGAGAAATTAGCAATTACACAAAAGAAATATAC---
ACCTTAATTGAAAATTCGCAGAACCAGCAAGAA--------------------------------------
-----------------------------
AAGAATGAACTAGACCTATTAGAATTGGATAAATGGGCAAGTTTGTGGAATTGGTTTGACATAACAAAGTGGC
TGTGGTATATCAAAATATTCATAATGATAGTAGGAGGCTTGGTAGGTTTAAGAATAGTTTTTGCT---
GTATTTTCTATAGTA---------AATAGAGTTAGGCAGGGATACTCACCA------
TTGTCATTAGCTATTAGCTTGCTTAACGCCACAGCCATAGTAGTAGCTGAG---
GGGACAGATAGGGTTATAGAAGTAGCACAAAGAGCTTATAGGGCT---------------------------
--------ATTCTTAACATA------CCTACAAGAATCAGACAGGGCCTCGAAAGGGCTTTGCTA---TAA
>JF932484
GCT---GCA-----------------------------------------
GAAAATTTGTGGGTCACAGTCTATTATGGGGTACCTGTATGG---AAAGAAGCAACT---
ACCACTCTATTTTGTGCATCAGATGCTAAAGCATATGATACAGAG---GTACATAAT----------------
--------------GTTTGGGCCACA---CATGCCTGTGTACCTACAGAC---
CCCAGCCCACAAGAAGTAGTCTTGGAA---------AATGTGACAGAAAACTTTAAC---------------
--------------------------ATGTGGAAAAATGACATGGTAGAACAGATGCATGAGGATATA---
ATCAGCCTATGGGATCAAAGCCTA---AAGCCATGTGTAAAATTA---
ACCCCACTCTGTGTTACTTTAAATTGTACTAATTTGAATACTACTACTACTAATACCACTAGA----------
-----------------------------------------------------------------------
----------------------------ACTACCAATAGTCCGATAGCGGTAGGAGAAATGAAA---
AACTGCTCTTTCAATGTCACCACAAACATACAG--------------------------ACCAGGGTG---
AAAGACTATGCACTT---TTTTATAATCTTGATATAGTACCAATAAAGGATGATAAT----------------
-----------------------------------------------------------------------
----GATAATAATAGTAACACCTATAGGTTGATGAATTGTAACACCTCAATCATTACACAA---
TCTTGTCCAAAAAATATCCTTTGAGCCAATTCCCATACATTATTGTGCCCCGGCTGGTTTTGCGATTCTAAAGT
GTAAC---AATAAAACATTCAAT------GGAAAAGGACCATGCACAAAT----------------------
-GTCAGT---ACAGTACAATGTACACATGGAATTAGGCCAGTAGTATCAACT---
CAATTGCTGTTAAATGGCAGTTTAGCAGAAAAAGAG-----------
GTAGTAATCAGATCTAGCAATTTCACAGACAAT---------GCTAAAGTCATAATAGTACAGCTGAAT----
-----------GAATCTGTAGAA---------ATTAATTGTACAAGGCCCAACAACAATACAAGAAAA-----
-AGTATAAATATA------------GGACCAGGGAGAGCA---TGG------TATGCAACAGGAGAA------
---------ATAATAGGAAATATA------------AGACAAGCATATTGT---AACATTAGTAGAGCA---
AAATGGAATGACACTTTAAAAAAGATAGATCAAAAATTAAGA---------------GAACAATTTGGG----
-----------------------------------AATAAAACAATAACCTTTACT------
CACTCTTCAGGAGGGGACCCAGAGATTGTA---ATGCACAGTTTTAATTGTGGAGGGGAG---
TTTAAGCAGCTCCAGGCAAGAGTCTTGGCTGTGGAAAGATACCTAAAGGATCAACAGCTCCTGGGGATTTGGG
GTTGCTCTGGAAAACTCATTTGCACCACTGCTGTGCCTTGGAATGTTAGTTGGAGT----------------
----------------------------AATAAATCTTACAATCAGATTTGG------GAT------
AACATGACTTGGATGGAGTGG---GAAAGAGAAATTGGCAATTACACAAGAGAAATATAC---
ACCTTAATTGAAGAATCGCAGAACCAACAAGAA--------------------------------------
-----------------------------
```

AAGAATGAACTAGAACTATTGGAATTGGATAAGTGGGCAAGTTTGTGGAATTGGTTTGACATAACAAAATGGC
TGTGGTATATCAAAATATTCATAATGGTAGTAGTAGGCTTAGTAGGTTTAAGAATAATTTTTGCT---
GTATTTTCTATAGTA---------AATAGAGTTAGGCAGGGATATTCACCA------
TTGTCATTTGCTGTTAGCTTGCTTAACGCCATAGCCATAGCAGTAGCTGAG---
GGGACAGATAGGGTTATAGAAGTAGCACAAAGAACTTATAGGGCT----------------------------
--------ATTCTCCACATA------CCTACAAGAATCAGACAGGGCCTCGAAAGGGCTTTGCTA---TAA
>JF932485
ACT---GCA--
GAAAAATTGTGGGTAACTGTCTATTATGGGGTACCTGTATGG---AAAGAAGCAACT---
ACCACTTTATTTTGTGCATCAGATGCTAAAGCATATGATACAGAG---GTACATAAT----------------
--------------GTTTGGGCCACA---CATGCCTGTGTACCCACAGAC---
CCCAACCCACAAGAAGTAGTCTTGGGA---------AATGTAACAGAAAACTTTAAC----------------
--------------------------GTGTGGAAAAATAACATGGTAGAACAGATGCATGAAGATATA---
ATTAGTTTATGGGACCAAAGCCTA---AAGCCATGTGTAAAATTA---
ACCCCACTCTGTGTTACTTTAAATTGCTCCGATGTGAAAAATACTAATAATACCAGTAGT-------------
----
----------------------------AATACCAGTAGTATAACAGAGGGAGGAGAAATAAAA---
AACTGCTCTTTCAATATCACCACAAGCATAAGT---------------------------ACTAAGGTG---
AAAGACTATGCATTT---TTTTATAAACTTGATGTAGTACCAATAAATAAT----------------------
----
----------GATAATGCCAGCTATAGGTTGATAAATTGTAACACCTCAGTCATTACACAA---
GCTTGTCCAAAGGTATCCTTCGAGCCAATTCCCATACATTATTGTACCCCGGCTGGTTTTGCGATTCTAAAGT
GTAAC---AATAAAACATTCAAT------GGAACAGGACCATGTACAAAT-----------------------
-GTCAGT---ACAGTACAATGTACACATGGAATTAGGCCAGTAGTATCAACT---
CAATTGCTGTTAAATGGCAGTTTAGCAGAAGAAGAG------------
GTAGTAATTAGATCTAGCAATTTCACGGACAAT---------GCTAAAATCATAATAGTACAGCTGAAT----
-----------GAATCTGTAGAA---------ATTAATTGTATAAGACCCAACAACAATACAAGAAAA-----
-AGTATACATCTA------------GGACCAGGGCAAGCA---TGG------TATACAACAGGACAA------
---------ATAATAGGAGATATA------------AGACAAGCACATTGT---AACATTAGTAGAGAA---
AAATGGAATAACACTTTAGAACAGGTAGCAAAAAAATTAGGA---------------GAACAATTTGGG----
-----------------------------------AACAAAACAATAGTCTTTACT------
CAATCTTCAGGAGGAGACCCAGAGATTGTA---ATGCACAGTTTTAATTGTGGGGGAGAA---
TTTAAGCAGCTCCAGGCAAGAGTCTTGGCTGTGGAAAGATACCTAAAGGATCAACAGCTCCTGGGGATTTGGG
GTTGCTCTGGAAAACTCATCTGCACCACTGCTGTGCCTTGGAATGCTAGTTGGAGC-----------------
----------------------------AATAAATCTCTGAATGAGATTTGG------AAT------
AACATGACTTGGATGGAGTGG---GAAAGAGAAATTGACAATTACACAAGAGAAATATAC---
AACTTAATTGAAGAATCGCAGAACCAACAAGAA--

AAGAATGAACTAGAACTATTGGAATTGGATAAGTGGGCAAGCTTGTGGAATTGGTTTGACATAACACAATGGC
TGTGGTATATCAAAATATTCATAATGATAGTAGGAGGCTTGGTAGGTTTAAGAATAGTTTTTGCT---
GTACTTTCTATAGTA---------AATAGAGTTAGGCAGGGATACTCACCA------
TTGTCATTGGCTATTAGCTTGCTTAACGCCACAGCCATAGCAGTAGCTGAG---
GGGACAGATAGGGTTATAGAAGTAGTACAAAGAACTTATAGGGCT----------------------------
--------ATTATCCACATA------CCTACAAGAATCAGACAGGGCCTCGAAAGGGCTTTGCTA---TAA
>JF932486
GCT---ACA--
GAAAAATTATGGGTCACAGTCTATTATGGGGTACCTGTATGG---AAAGAAGCAAAT---
ACCACTTTATTTTGTGCATCAGATGCTAAAGCATATAAGGCAGAG---GCACATAAT----------------
--------------GTTTGGGCCACA---CATGCCTGTGTACCCACAGAC---
CCCAACCCACAAGAAGTAGTCTTGGGA---------AATGTGACAGAAAACTTTGAC----------------
--------------------------ATGTGGAAAAATAACATGGTAGAACAAATGCATGAAGATATA---
ATCAGTTTATGGGAGCAAAGCCTA---AAACCATGTGTAGAATTA---
ACCCCACTCTGTGTTACTTTAAATTGCACTAATTGTACTTCAAATTGCACTAGTTTTAATTGTACTTCAAGTT
GCACT--
-------------------AATTTGGGGAATGCTACTAATAACAGTGGTAATACAGAACTAAAA---
AACTGCTCTTTCGACACCACCACAAGCATAGGA---------------------------ACTAAGAGG---
AGGGACCATGCACTT---TTTAATGAACTTGATATAGTACAAATGAATGATGGT-------------------
----
----------AATACCAGCAGCTATAGGTTGATAAGTTGTAACACCTCAGTCATTACACAG---
GCTTGTCCAAAGGTTTCCTTTGAGCCAATTCCCATACATTATTGTGCCCCGGCTGGTTTTGCGATTCTAAAGT

GTAAC---AATAAGACGTTCAAT------GGAACAGGACCATGTACAAAT----------------------
-GTCAGT---ACAGTACAATGTACACATGGAATTAGGCCAGTAGTATCAACT---
CAACTGCTGTTAAATGGCAGTTTAGCAGAAGGAGAG-----------
ATAGTAATTAGATCTAGCAATTTCTCGGAAAAT---------GCTAAAATCATAATAGTACAGCTGAAT----
-----------GAATCTGTAACA---------ATTAATTGTACAAGACCCAACAACAATACAAGAAAA-----
-AGTATATCTATG-----------GGACCAGGGCGAGCA---GTG------TATGCAACAGGACAA------
---------ATAATAGGAGATATA-----------AGACAAGCACATTGT---AACATTAGTAGAAAA---
CAATGGAATAACACTTTAGAACGGATATATAAAAAATTAAAA--------------GAAATATAC-------
----------------------------------AACAGAACAATAATCTTTAAT------
CACTCTTCAGGAGGGGACCCAGAGATTGTA---ATGCATAGTGTTAATTGTGGAGGGGAA---
TTTAAGCAGCTCCAGGCAAGAGTCTTGGCTGTGGAAAGATACCTAAAGGATCAACAGCTCCTAGGGATTTGGG
GTTGCTCTGGAAAACTCATCTGCCCCACTGCTGTGCCTTGGAATGCTAGTTGGAGT----------------
---------------------------AATAAATCCCTGAATGAGATTTGG------AAT------
AATATGACTTGGATGGAGTGG---GAAAGAGAAATTGACAATTACACAAAAGAAATATAC---
ACCTTAATTGAAGAATCGCAGAACCAACAAGAA--------------------------------------
----
AAGAATGAACTAGATCTATTGGAATTGGATAAGTGGGCAAGTTTGTGGAATTGGTTTGACATATCAAACTGGC
TGTGGTATATCAGGATATTCATAATGATAGTAGGAGGCTTGATAGGTTTAAGAATAGTTTTTGCT---
GTACTTTCTATAGTA---------AATAGAGTTAGGCAGGGATACTCACCA------
TTGTCGTTGGCTATTAGCTTGTTTAACGCCACAGCCATAGCAGTAGCTGAA---
GGGACAGATAGGGTTATAGAAGTAGTGCAAAGAGCTTTTAGGGCT--------------------------
--------ATTCTCAACATA------CCTACAAGAATCAGACAGGGCCTAGAAAGGGCTTTGCTA---TAA
>MH479957
GCT---GCA---
GAAAACTTGTGGGTAACAGTCTATTATGGGGTACCTGTATGG---AAAGAAGCAACT---
ACCACTCTATTTTGTGCATCAGATGCTAAAGCATATGATACAGAG---GTACATAAT---------------
--------------GTTTGGGCCACA---CATGCCTGTGTACCCACAGAC---
CCCGACCCACAAGAAGTAGTCTTGGGA---------AATGTGACAGAAAACTTTAAC---------------
-------------------------ATGTGGAAAAATAACATGGTAGAACAGATGCATGAAGATATA---
ATCAGTTTATGGGATCAAAGCCTA---AAGCCATGTGTAAAATTA---
ACCCCACTCTGTGTTACTTTAAATTGCACTGATTGTGTTACTTTAAATTGCACT------------------
----
------------------------GATTATACCAATAGAACGACAGAGGGAGGAGAAATAAAA---
AACTGCTCTTTCAACATCACCACAAGCCTAAAA--------------------------ACTAAAGTG---
ACAGACTATGCATTT---TTTTATAAACTTGATGTAGTACCAATAAGTAAT--------------------
----
----------AATAGTACCAGCTATAGGTTGATAAATTGTAACACCACAGTCATTACACAA---
GCTTGTCCAAAGATATCCTTTGAGCCAATTCCCATACATTATTGTACCCCGGCTGGTTTTGCGCTTCTAAAGT
GTAAC---AATAAAACGTTCAAT------GGAACAGGACCATGTACAAAT----------------------
-GTCAGT---ACAGTACAATGTACACATGGAATTAGGCCAGTAGTATCAACT---
CAACTGCTGTTAAATGGCAGTTTAGCAGAAGGAGAG-----------
GTAGTAATTAGATCTAGCAATTTTACGGACAAT---------GCTAAAATCATAATAGTACAGCTGAAT----
-----------GAATCTGTAGAA---------ATTAATTGTATAAGACCCAACAACAATACAAGAAAA-----
-AGTATACATCTG-----------GGACCAGGGCGAGCA---TTG------TATACAACAGGA---------
---------ATAATAGGAGATATA-----------AGACAAGCACATTGT---AACCTTAGTAGCACA---
AAGTGGAATAACACTTTAAAACAGATAACTAAAAAATTAAGA--------------GAACAATTTGGG----
----------------------------------AATAAAACAATAATCTTTAAT------
CAATCCTCAGGAGGGGACCCAGAGATTGTA---ATGCACAGTTTTACTTGTGGAGGGGAG---
TTTAAGCAGCTCCAGGCAAGAGTCTTGGCTGTGGAAAGATACCTAAAGGATCAACAGCTCCTAGGGATTTGGG
GTTGCTCTGGAAAGCTCATTTGCACCACTGCTGTGCCTTGGAATACTAGTTGGAGT----------------
---------------------------AATAAATCTCTGAGTGAGATTTGG------GAT------
AACATGACTTGGATGGAGTGG---GAAAGAGAAATTAACAATTACACAAGGGAAATATAC---
ACCTTAATTGAACAATCGCAGAACCAGCAAGAA--------------------------------------
----
AAGAATGAACTAGAACTATTGGAATTGGATAAGTGGGCAAGTTTGTGGAATTGGTTTGACATAACAAAGTGGC
TGTGGTATATCAAAATATTCATAATGATAGTAGGAGGCTTGGTAGGTTTAAGAATAATTTTTGCT---
GTACTTTCCATAGTA---------AATAGAGTTAGGCAGGGATACTCACCA------
TTGTCATTAGCTATTAGCTTGCTTAACGTCACAGCCATAGTAGTAGCTGAA---
GGAACAGATAGGGTTATAGAAGTAGCACAAAGAGCTTATAGAGCT--------------------------
--------ATTCGCCACATA------CCTACAAGAATCAGACAGGGTCTCGAAAGGGCTTTGCTA---TAA

>JF932487
GCT---GCA--
GAAAACTTGTGGGTCACAGTCTATTATGGGGTACCTGTATGG---AAAGAAGCAACT---
ACCACTCTATTTTGTGCATCAGATGCTAAAGCATATGAAACAGAG---GTACATAAT----------------
--------------ATTTGGGCCACA---CATGCCTGTGTACCCACAGAC---
CCCAACCCACAAGAAGTATTATTGGAA---------AATGTGACAGAAAATTTTAAC----------------
--------------------------ATGTGGAAAAATGACATGGTAGAGCAAATGCATGAAGATATA---
ATCAGTTTATGGGATCAAAGCTTA---AAGCCATGTGTAAAATTA---
ATCCCACTCTGTGTTAATTTAACTTGCACTAAGTTGAATATTACTAATACCAGTAGTAATACCAGTGGTACCA
ATAATACTACC--
-AGTAGTACTACCAGTAGTCCCAATAGTACTGCCAGTAGTATAACAGAGGGAGGAGATATAAAA---
AACTGCTCTTTCAATATCACCACAAGCATAAGA---------------------------ACTGAGGTG---
AGAGATTATGCATTT---TTTTCTAACCTTGATATAGTACCAATAAATAATGGTAATACC-------------
----
----AGCAATGATAATACCAGCTATAGGTTGATAAATTGTAACACCTCAGTCATTACACAA---
GCTTGTCCAAAGGTATCCTTTGAGCCAATTCCTATACATTATTGTACCCCGGCTGGTTTTGTGATTCTAAAGT
GTAAC---GATAAAAAGTTCAAT------GGAACAGGACCATGTAAAAAT-----------------------
-GTCAGT---ACAGTACAATGTACACATGGAATTAGGCCAGTAGTATCAACT---
CAACTGCTGTTAAATGGCAGTTTGGCAGAAGAAGAG------------
GTAGTAATTAGATCTAGCAATTTCTCGAACAAT---------GCTAAAGTCATAATAGTACAGCTGAAT----
-----------GAATCTGTAGAA---------ATTAATTGTACAAGACCCAACAACAATACAAGAAAA-----
-AGTATACATCTA------------GGACCAGGGCAAGCA---TGG------TATACAACAGGACAA------
---------ATAATAGGAAATATA------------AGACAAGCACATTGT---AACATTAGTAGTATA---
AAATGGAATAAAACTTTAGGACAGATAACTAAAAAATTAAAA---------------GAACAATTTGGG----
-----------------------------------AACAAAACAATAATCTTTAAT------
GCATCCTCAGGAGGGGACCCAGAGATTGTA---ATGCACAGTTTTAATTGTGGAGGGGAA---
TTTAAGCAGCTCCAGGCAAGAGTCCTGGCTGTGGAAAGATACCTAGGGGATCAACAGCTCCTAGGGATTTGGG
GTTGCTCTGGAAAACTCATCTGCACCACTGCTGTACCTTGGAATACTAGTTGGAGT-----------------
-------------------AATAGG---AATCTGAGTGAGATTTGGGATAAC------AAT------
AACATGACTTGGATGAAGTGG---GAAAGAGAAATTGAGAATTACACAAAAGAAATATAC---
ACCTTAATTGAAGAATCACAGAACCAACAAGAA--

AAGAATGAACTAGAACTATTAGAATTGGATAAGTGGGCAAGCTTGTGGAGTTGGTTTGACATAACAAACTGGC
TGTGGTATATCAGAATATTCATAATGATAGTAGGAGGCTTGGTAGGTTTAAGAATAGTTTTTGCT---
GTACTTTCTATAGTA---------AATAGAGTTAGGCAGGGATACTCACCA------
TTGTCATTTGCTATTAGCTTGCTTAACGCCACAGCTATAGCAGTAGCTGAG---
GGAACAGATAGGATTATAGAAGTAGTACAAAGAGCTTATAGAGCT----------------------------
--------ATTCTCCACATA------CCCACACGAATTAGACAGGGCCTCGAGAGGGCTTTGCTA---TAA
>JF932489
GCT---GCA--
GAAAAATTGTGGGTCACAGTCTATTATGGGGTACCTGTATGG---AAAGAAGCAACT---
ACCACTCTATTTTGTGCATCAGATGCTAAAGCATATGATACAGAG---GTACATAAT----------------
--------------GTTTGGGCCACA---CATGCCTGTGTACCCACAGAC---
CCCAGCCCACAAGAAGTAGAATTGAAA---------AATGTGACAGAAAACTTTAAC----------------
--------------------------ATGTGGAAAAATAACATGGTAGAACAGATGCATGAAGATATA---
ATCAGTTTATGGGATCAAAGCCTA---AAGCCATGTGTAAAATTA---
ACCCCACTCTGTGTTACTTTAAATTGCACTAATGTGACCCCTAATGTGACCAGGACTAGT-------------
----
----------------------------GAGAAGAATATCACAATAGAGGCAGGAGACATGAAA---
AACTGCTCTTTCAATATCACCACAAGCATAGGG---------------------------AATAAGGTG---
AAAGACTATGCACTT---TTTAATACACTTGATATAGGACTAATAGAGAAT----------------------
----
-------------ACTACCAACTATAGGTTGATACAGTGTAACACCTCAGCCATTACACAA---
GCTTGTCCAAAGGTAACCTTTGAGCCAATTCCCATACATTATTGTGCCCCAGCTGGTTTTGTGATTCTAAAGT
GTAAA---GATAGAAATTTCAAT------GGAACAGGACCATGTAAAAAT-----------------------
-GTCAGT---ACAGTACAGTGTACACATGGAATTAAGCCAGTAGTATCAACT---
CAGCTGCTGTTAAATGGCAGTTTAGCAGAAGGAGAG------------
GTAGTAATTAAATCTAGCAATTTCTCGGACAAT---------GCTAAAGTCATAATAGTACAGCTGAAT----
-----------GAACCTGTAGTA---------ATTAATTGTACAAGAACCAACAACAATACAAGAAAA-----
-AGCATACATCTA------------GGACCAGGGAGAGCA---TGG------TTTACAACAGGACAA------

---------ATAACAGGAGATATA------------AGAAAAGCACATTGT---ACACTTAACGGTACA---
CAATGGAATAACACTTTAAGACAGATAGCTGAAAAGTTAAAA---------------GAACAATTTGAG----
-----------------------------------AACAGAACAATAATCTTTAAT------
CAATCTTCAGGAGGGGACCCAGAGATTGTA---ATGCACAGTGTTAATTGTGGAGGGGAA---
TTCAAACAGCTCCAGGCAAGAGTCTTGGCTGTGGAAAGATACCTAAAGGATCAACAGCTCCTAGGGATTTGGG
GTTGCTCTGGAAAACTCATTTGCACCACTGCTGTGCCTTGGAATGCTAGTTGGAGT-----------------
----------------------------AATAAAACTCATGACAGGATTTGG------GGT------
AACATGACTTGGATGCAGTGG---GAGAGAGAAATTGAAAACTACACAAATGAAATATAC---
ACCTTAATTGAAGAATCGCAGACCCAACAAGAA--

CGGAATGAACTAGAGCTATTAAAATTGGATGAGTGGGCAAGTTTGTGGAATTGGTTTGACATATCAAAATGGC
TGTGGTATATCAGAATATTCATAATGATAGTAGGAGGCTTGATAGGTTTAAGAATAGTTTTTGCT---
GTACTTTCTATAGTA---------AATAGAGTTAGGCAGGGATACTCACCA------
TTGTCATTAGCTATTAGCTTGCTTAACGCTACAGCCATAGCAGTAGCTGGA---
GGGACAGATAGGGTTATAGAAGTAGTACAAAGAGCTTATAGGGCT----------------------------
--------ATTCTCAACATA------CCTAGAAGAATCAGACAGGGCCTCGAGAGGGCTTTGCTA---TAA
>JF932490
GCT---GCA--
GAAAAGTTGTGGGTCACAGTCTATTATGGGGTACCTGTATGG---AAAGATGCAACT---
ACCACTCTATTTTGTGCATCAGATGCTAAAGCATATGATACAGAG---GTACATAAT----------------
--------------GTTTGGGCCACA---CATGCCTGTGTACCCACAGAC---
CCCAGCCCACAAGAAGTAGTCTTAGAA---------AATGTGACAGAAAACTTTAAC----------------
--------------------------ATGTGGGAAAATAACATGGTAGAACAGATGCATGAAGATATA---
ATTAGTTTATGGGATCAAAGCCTA---AAGCCATGTGCAAAATTA---
ACCCCAATCTGTGTTACTTTAAATTGCACTAATTTGAAGAATAATACTAATGCCAGTAGTAAT----------
----
----------------------ATCAGTAATATCAATAGTACAATAGAGGGAGGAGAAATGAAA---
AACTGCTCTTTCAACATCACCACAAGCATGAAT---------------------------ACTAAGGTA---
AAAGACTATGCACTC---TTTTATAGACTTGATATAGTACCAATAGATGAT----------------------
----
----GATGGTGATAATACCACCTATAGGTTAATAAATTGTAACACCTCAGTCATTACACAA---
GCTTGTCCAAAGGTATCCTTTGAGCCAATTCCCATACATTATTGTACCCCGGCTGGTTATGCGATTCTAAAGT
GTAAC---AATAAAACATTCAAT------GGAACAGGACCATGTACAAAT-----------------------
-GTCAGT---ACAGTACAATGTACACATGGAATTAGGCCAGTAGTATCAACT---
CAACTGCTGTTAAATGGCAGTTTAGCAGAAGAAGAG------------
GTAGTAATTAGGTCTAGCAATTTCTCGGAAAAT---------CATAGAGTCATAATAGTACAGCTGAAT----
-----------AAATCTGTAGAA---------ATTAATTGTACAAGACCCAACAACAATACAAGAAAA-----
-AGTATAAATATA------------GGACCAGGGCAAGCA---TGG------TATACAACAGGACAA------
---------ATAATAGGAGATATA------------AGACAAGCACATTGT---AACATTAGTAGTACA---
GATTGGAATAACACTTTAAAACAGATAACTGGGAAATTAAGA---------------GAACAATATGGG----
-----------------------------------AACAAAACAATAATCTTTACT------
CACCATTCAGGAGGGGACTCAGAGGTTGTA---ATGCACAATTTTAATTGTGGAGGGGAA---
TTTAAACAGCTCCAGGCAAGAGTCTTGGCTGTGGAAAGATACCTAAAGGATCAACAGCTCCTAGGAATTTGGG
GTTGCTCTGGAAAACTCATCTGCCCCACTGCTGTGCCTTGGAATGTTAGTTGGAGT-----------------
----------------------------AATAAATCTCTAAATGAAATTTGG------AAT------
AACATGACTTGGATGGAGTGG---GAAAGAGAAATTAGCAATTACACAGGCATAATATAC---
AACTTAATTGAAGAATCGCAGAACCAACAGGAA--

AAGAATGAACTAGAACTATTGGAATTGGATAAGTGGGCAAGTTTGTGGAATTGGTTTAACATAACAGAATGGC
TGTGGTATATCAGAATATTCATAATGGTGATAGGAGGCTTAATAGGTTTAAGAATAGTTTTTGCT---
GTGCTTTCGGTAGTA---------AATAGAGTTAGGCAGGGATACTCACCA------
TTGTCATTGGCTATTAGCTTGCTTAACGCGACAGCCATAGTAGTAGCTGAG---
GGGACAGATAGGGTTATAGAAATAGCAAGAAGGGCTTTTAGGGCT----------------------------
--------ATTCGCAACATA------CCTACAAGAATTAGACAGGGCCTCGAGAGGTCTTTGCTA---TAA
>JF932491
GCT---GTA--
GAACAATTGTGGGTCACAGTCTATTATGGGGTACCTGTATGG---AAAGACGCAAAT---
ACCACTCTATTTTGTGCATCAGATGCTAAAGCATATGATACAGAG---GTACATAAT----------------
--------------GTTTGGGCAACA---CATGCCTGTGTACCCACAGAC---
CCCAACCCACAAGAAGTAGTCTTGGAA---------AATGTGACAGAAAACTTTAAC----------------

--------------------------ATGTGGAAAAATAACATGGTAGAACAGATGCATGAAGATATA---
ATCAGTTTATGGGATCAAAGTCTA---AAGCCATGTGTAGAATTA---
ACCCCACTCTGTGTCACTTTAAATTGCACTAATTTGAACCAGACTACCAATACCAATAGT-------------
----
----------------------------ACTACCATTAGTCCGAAAGGGGGAGGAGAAATAAAA---
AACTGCTCTTTCAATATCACCACAAGTATAAAA---------------------------ACTAAGGTA---
AAAGACTATGCACTT---TTTTATAACCTTGATATAATAAAAATGGAAGAT----------------------
----
----GGTAATAATAATAATACCTATAGGTTGATAAATTGTAACACCTCAGTCATTAAACAA---
GCTTGCCCAAAGATAACCTTTGAACCAATTCCCATACATTATTGTGCCCCGGCTGGTTTTGCGATTCTAAAGT
GTAAC---AATAAAACGTTCAAT------GGAACAGGACCATGTAAAAAT-----------------------
-GTCAGT---ACAGTGCAATGTACACATGGAATTAGGCCAGTAGTATCAACT---
CAACTGCTGTTAAATGGCAGTTTAGCAGAAGAAGAA------------
ATAGTAATTAGATCTAGCAATTTCACGGACAAT---------GCTAAAATCATAATAATACAGCTGAAT----
-----------GAATCTGTAGCA---------ATTAATTGTACAAGACCCAACAACAATACAAGAAAA-----
-AGTATAACTCTA------------GGACCAGGGAGAGTA---TGG------TATACAACAGGACAA------
---------ATAATAGGAGATATA------------AGACAAGCACATTGT---AACCTTAGTAGAACA---
CAATGGAATAACACTTTAAAACAGATAACTGAAAAATTAAGA---------------GAACAATTTGGG----
-----------------------------------AATAAAACAATAATCTTTAAT------
CAATCTTCAGGAGGGGACCTAGAGATTGCA---ACGCACAGTTTTAATTGTGGAGGGGAA---
TTTAAGCAGCTCCAGGCAAGAGTCCTGGCTGTGGAAAGATACCTACAGGATCAACAGCTCCTAGGGATTTGGG
GATGCTCTGGGAAACTCATCTGCACCACTGCTGTGCCTTGGAATGCTAGTTGGAGT-----------------
----------------------------AATAAATCCCTGGATAAGATTTGG------AAT------
AACATGACTTGGATGGAGTGG---GAGAGAGAAATAGGCAATTACACAGAAAAAAATATAC---
AACCTAATTGAAAAGGCGCAGAACCAGCAAGAA--

AAGGATGAACAAGAATTATTGGAATTGGATAAGTGGGCAAGCTTGTGGAATTGGTTTGACATAACAAAATGGC
TGTGGTATATCAAAATATTCATAATGATAGTAGGAGGCTTGATAGGCTTAAGAATAGTTTTTGCT---
GTACTTTCTTTAGTA---------AGTAGAGTTAGGCAGGGATACTCACCA------
TTGTCATTAGCTATTAGCTTGCTTAATGCAACAGCCATAGCAGTAGCTGAG---
GGGACAGATAGGATTATAGAAATAGCACAAAGAGCTTATAGGGCT----------------------------
--------TTTCTCAACATA------CCTACAAGAATCAGACAGGGTCTTGAAAGAGCTTTGCTA---TAA
>JF932493
GCT---GCA--
GAAAACTTGTGGGTCACAGTCTATTATGGGGTACCTGTATGG---AGAGACACAAAT---
ACCACTCTATTCTGTGCATCAGATGCTAAAGCATATGCTACAGAG---GTACATAAT----------------
--------------GTTTGGGCCACA---CATGCCTGTGTACCCACAGAC---
CCCAACCCACAAGAAGTAGTCTTAGAA---------AATGTGACAGAAAACTTTAAC----------------
--------------------------ATGTGGAAAAATGGTATGGTAGACCAGATGCAAGAAGATATA---
ATCAGTTTATGGGATCAAAGTCTA---AAGCCATGTGTAAAATTA---
ACCCCACTCTGTGTTACTTTAAATTGCACTGATTTGGAAAATACTACTAATGCCAGTAGT-------------
----
----------------------------AATATCAGCAGTACGACAGGGGGAGGAGAGATGAAA---
AACTGCTCTTTCAATATCACCACAAGCATGCAA---------------------------ACTAAGGTG---
AAAGACTATGCACTT---TTTTATAATCTTGATGTAGTACAAATAGATGATAAG-------------------
----
----------GATAATACCAGCTATAGGTTGATAACTTGTAACACCTCAGTCGTTACACAA---
GCTTGTCCAAAAGTATCCTTTGAGCCAATTCCCATACATTATTGTACCCCGGCTGGTTTTGCGCTTCTAAAGT
GTAAC---AATAAAACATTCAGT------GGAACAGGACCATGTACAAAT-----------------------
-GTCAGT---ACAGTACAATGTACACATGGAATTAGGCCAGTTGTATCAACT---
CAACTGCTGTTAAATGGCAGTTTAGCAGAAGAAGAG------------
GTAGTAATTAGATCTAGCAATTTCTCGAACAAT---------GCTGACAATATAATAGTACAGCTGAAT----
-----------AAATCTGTAGTA---------ATTAATTGTACAAGACCCAACAACAATACAAGAAAG-----
-AGTATAAATCTA------------GGACAAGGGCGAGCA---TGG------TATACAACAGGACAA------
---------ATAATAGGAGATATA------------AGACAAGCACATTGT---AACCTTAGTGGAGCA---
GCATGGACTAACACTTTAAGACAGGTAGCTGAAAAATTAAGA---------------AAACAATTTGAG----
-----------------------------------AATAAAACAATAACCTTTACG------
CAATCTTCAGGAGGGGACCCAGAGATTGTC---ATGCACAGTTTTAATTGTGGAGGGGAA---
TTTAAGCAGCTCCAGGCAAGAGTCTTGGCTGTGGAGAGATACCTGAAGGATCAACAGCTCCTAGGGATTTGGG
GTTGCTCTGGAAAACTCATTTGCACCACTACCGTGCCTTGGAATGTTAGTTGGAGT-----------------

----------------------------AATAGATCTCTGGATGAGATTTGG------AAT------
AACATGACTTGGATGGAGTGG---GAAAGAGAAATTGACAATTACACAAGGGAAATATAC---
ACCTTAATTGAAGAATCGCAGAACCAACAAGAA--

AAGAATGAATTAGAACTATTGGAATTGGATAAGTGGGCAAGCTTGTGGAATTGGTTTAGCATAACAAAATGGC
TGTGGTATATCAAAATATTCATAATGATAGTAGGAGGCTTGGTAGGTTTAAGAATAGTTTTTGCT---
GTACTTTCTATAGTA---------AATAGAGTTAGGCAGGGATACTCACCA------
TTGTCATTAGCTATTAGCTTGATTAACGCCACAGCTGTAGCAGTAGCTGAG---
GGGACAGATAGGGTTATAGAAGTAGTACAAAGGGCTTTTAGGGCT----------------------------
--------TTTGTTCACATA------CCGACAAGAATCAGACAGGGCCTCGAAAGGGCTTTGTTA---TAA
>JF932499
GCT---GCA--
GAAAACTTGTGGGTCACAGTCTATTATGGGGTACCTGTATGG---AAAGAAGCAACT---
ACCACTCTATTTTGTGCATCAGATGCTAAGGCATATGATACAGAG---GCACATAAT----------------
--------------ATTTGGGCCACA---CATGCCTGTGTACCCACAGAC---
CCCAACCCACAAGAAGTAGTCCTGGGA---------AATGTGACAGAATATTTTAAC----------------
--------------------------ATGTGGAAAAATAATATGGTAGAACAAATGCATGAAGATATA---
ATCAGTTTATGGGATGAAAGCCTA---AAGCCATGTGTAAAATTA---
ACCCCACTCTGTGTTGCTTTAACTTGCACTGATTTGAATACTACTAATACCAAT-------------------
----
----------------------------ACTACCAGTAGTACGACAGAGGGAGGAGAAATAAAA---
AACTGCTCTTTCAATATCACCACAAGCATAAGA---------------------------ACTAAGGTG---
AAAGACTATGCACTT---TTTTATAAACTTGATATAATACCAATAGATAAT----------------------
----
----------------ACCAGCTATAGGTTAATAAGTTGTAATACCTCAGTCATTACACAA---
GCTTGTCCAAAGGTATCCTTTGAGCCAATTCCCATACATTATTGTACCCCGGCTGGTTTTGCGCTTCTAAAGT
GTAAC---AATAAAACATTCACT------GGAACAGGACCATGTACAAAT-----------------------
-GTCAGT---ACAGTACAATGTACACATGGAATTAGGCCAGTAGTATCAACT---
CAACTACTGTTAAATGGCAGTTTAGCAGAAGAAGGG------------
GTAGTAATTAAATCTAGCAATTTCATGAACAAT---------GCTAAAGTCATAATAGTACAGCTGAAT----
-----------GAATCTGTAGAA---------ATTAACTGTACAAGACCCAACAACAATACAAGAAAA-----
-AGTATACAGCTA------------GGACTAGGGCGAGCA---TGG------TATACAACAGGACAA------
---------ATAATAGGAGATATA------------AGACAAGCACATTGT---AACATTAGTAGAACA---
AGATGGAATAACACTTTAAAACAGATAGCTGAAAAATTAAGA---------------GAACAATTTGGG----
--------------------------------AAGAACAAAACAATAATCTTTAAT------
CAATCTTCAGGAGGAGACCCAGAGATTGTA---ATGCACAGTTTTAATTGTGGAGGGAAA---
TTTAAGCAGCTCCAGGCAAGAGTCTTGGCTGTGGAGAGATACCTAAAGGATCAACAGCTCCTAGGGATTTGGG
GTTGCTCTGGAAAACTCATCTGCACCACTGCTGTGCCTTGGAATGCTAGTTGGAGT-----------------
----------------------------AATAAATCTCTGAGTGAGATTTGG------GAT------
AACATGACTTGGATGGAGTGG---GAAAGAGAAATTGACAATTACACAAAAGAAATATAC---
ACCTTAATTGAAGAATCGCAGAACCAACAAGAA--

AAGAATGAACTAGAACTATTGGAATTGGATAAGTGGGCAAGCTTGTGGAATTGGTTTGACATAACACAATGGC
TGTGGTATATCAAAATATTCATAATGATAGTAGGAGGCTTGGTAGGTTTAAGAATATTTTTTGCT---
GTACTTTCTATAATA---------AATAGAGTTAGGCAGGGATACTCACCA------
CTGTCATTAGCTATTAGCTTGCTTAACGCCACAGCCATCATAGTAGCTGAG---
GGGACAGATAGGGTTATAGAAGTAGCACAAAGAGCTTGGAGGGCT----------------------------
--------TTTCTTCACATA------CCTACAAGAATCAGACAGGGCCTCGAAAGGGCTTTACTA---TAA
>JF932500
GCT---GCA--
GAAAACTTGTGGGTCACAGTCTATTATGGGGTACCTGTATGG---AAAGAAGCAACT---
ACCACTCTATTTTGTGCATCAGATGCTAGAGCATATGATACAGAG---ATACATAAT----------------
--------------ATTTGGGCCACA---CATGCCTGTGTACCCACAGAC---
CCCAACCCACAAGAAGTAGTCTTGGGA---------AATGTGACAGAAAATTTTAAC----------------
--------------------------ATGTGGAAAAATAACATGGTAGAACAGATGTATGAAGATATA---
ATCAGTTTATGGGATCAAAGCCTA---AAGCCATGTGTAAAATTA---
ACCCCACTCTGTGTTACTTTAAATTGCACTAATTTGAATACTACTAATACCAGTAGT----------------
----
----------------------------ACTACCAGTAGTCCGACAGAGGGAGGAGAAATGAAA---
AACTGCTCTTTCAATATCACCACAAGCATAAGA---------------------------ACTAAGATG---

ACAGACTATGCACTT---TTTTATAAACTTGATGTAGTACCACTAGATAAT----------------------
----
----AATACTAATACTACCAGCTATAGGTTGATACATTGTAACACCTCAGTCATTACACAA---
GCTTGTCCAAAGGTATCCTTTGAGCCAATTCCCATACATTATTGTACCCCGGCTGGTTTTGCGATTATAAAGT
GTAAC---AATAAAACGTTCAGT------GGAACAGGACCATGTACAAAT-----------------------
-GTCAGT---ACAGTACAATGTACACATGGAATTAGGCCAGTAGTATCAACT---
CAACTGCTGTTAAATGGCAGTTTAGCAGAAGAAGAG------------
GTAGTAATTAGATCTAGCAATTTCACGGACAAT---------GCTAAAGTCATAATAGTACAGCTGAAT----
-----------AAATCTGTAGAA---------ATTAATTGTACAAGACCCAACAACAATACAAGAAAA-----
-AGTATACCTCTA------------GGACCAGGGCGAGCA---TGG------TTTGCAACAGGACAA------
---------ATAATAGGAGATATA------------AGACAAGCACATTGT---AACCTGAGTAGAGCA---
AAATGGAATGACACTTTAAAACAGATAGTTGAAAAATTGAGA---------------GAACAATTTGGG----
-----------------------------------AACAAAACAATAATCTTTAAT------
CAATCCTCAGGAGGGGATCCAGAGGTTGTA---ATGCACAGTTTTAATTGTGGAGGGGAA---
TTTAAGCAGCTCCAGGCAAGAGTCTTAGCTGTGGAAAGATACCTAAAGGATCAACAGCTCCTAGGGATTTGGG
GTTGCTCTGGAAAACTCATCTGCCCCACTAATGTGCCTTGGAATGTTAGTTGGAGT-----------------
----------------------------AATAAATCTCTGAGTGAGATTTGG------GAT------
AACATGACTTGGATGGAGTGG---GAAAGAGAAATTGGCAATTACACAAACCAAATATAC---
ACCTTAATTGAACAATCGCAAAACCAACAGGAA--

AAGAATGAACTAGAACTATTGGAATTGGATAAGTGGGCAAGTCTGTGGAATTGGTTTAGCATTTCAAACTGGC
TGTGGTATATCAGAATATTCATAATGATAGTAGGAGGCTTGATAGGTTTAAGAATAGTTTTTGCT---
GTACTTTCTATGGTA---------AATAGAGTTAGGCAGGGATACTCACCA------
TTGTCATTAGCTATTAGCTTGCTTAACGCCACAGCCATAGCAGTAGCTGAG---
GGGACAGATAGGATTATAGAAGTAGCACAAAGGGTTTTTAGGGCT----------------------------
--------ATTCTCCACATA------CCTACAAGAATCAGACAGGGTCTTGAAAGAGCTTTGCTA---TAA
>KM218168
GCT---GCA---
AATTTGTGGGTCACAGTCTATTATGGGGTACCTGTGTGG---AAAGATGCAAGC---
ACCACTTTATTTTGTGCATCAGATGCTAAAGCATATGACACAGAG---GTACATAAT----------------
--------------GTTTGGCCACA---CATGCCTGTGTACCCACAGAC---
CCCAACCCACAAGAAGTAGTATTGGAA---------AATGTGACAGAAAATTTTAAC----------------
--------------------------ATGTGGAAAAATAACATGGTAGAACAGATGCATGAAGATATA---
ATCAGTTTATGGATGAAAGCTTA---AAGCCATGTGTAAAATTA---
GCACCACTCTGTGTTACTTTAAATTGCACTGATTTGAAGTCTAATACTACTACTAACATC-------------
----
-------------------------AATAATAGTACTGGGGAAGAGATGAAGGGAGAAATAAAA---
AACTGCTCTTTCAATGTCACCACACAAATAAGA---------------------------
AATGAGATAAGAAAAGAATATGCACTT---TTTAATAAACTTGATGTAGTACCAATAGATGACAAGAAGAAG-
----

AATGATAGCTCTAATAGCTCCACATATATGTTGATAAATTGTAACACCTCAGTCATTACGCAG---
GCCTGTCCAAAGGTATCCTTTGAACCAATTCCCATACATTATTGTACCCCGGCTGGTTTTGCGATTGTAAAGT
GTAAT---GATAAGATGTTTAAT------GGAACAGGACCATGCACAAAT-----------------------
-GTCAGT---ACAGTACAATGTACACATGGAATTAGGCCAGTAGTGTCAACT---
CAACTTCTGTTAAATGGCAGTCTAGCAGAAGAAGAT------------
ATAGTAATTAGATCTGAAAATTTTACGGACAAT---------GCTAAGACCATAATAGTACAGCTGAAT----
-----------GAAACTGTAGAA---------ATTAATTGTACAAGACCCAACAACAATACAAGAAAG-----
-AGTATACATATA------------GGACCGGGGAGATCA---TTC------TATGCAACAGGAGAC------
---------ATAATAGGAGATATA------------AGACAAGCACATTGT---AATGTTAGTGTAGAA---
AAATGGACCAGCACTTTAGAAAGGGTAGCTACAAAATTAAGA---------------
GAACAATTTGAATCTCAA---------------------------------AATAAAACAATAGTCTTTAAG-
--CCACCACCCTCAGGAGGGGACCCAGAAAATATA---ATGCACACATTTAATTGTGGAGGGGAA---
TTTAAGCAGCTCCAGGCAAGAGTCCTGGCTGTGGAAAGATACCTAAGGGATCAACAGCTCCTGGGGATTTGGG
GTTGCTCTGGAAAACTCATCTGCACCACTGCTGTGCCTTGGAATGCAAGTTGGAGT-----------------
----------------------------AATAAATCTGTGGATCAGATTTGG------GAA------
AACATGACCTGGATGCAGTGG---GAAAGAGAAATTGAAAATTACACAGGCTTAATATAC---
ACTTTACTTGAAAAATCGCAGAACCAGCAAGAA--

AAGAATGAACAAGAATTATTGGAATTGGATGAGTGGGCAAGTTTGTGGAATTGGTTTGACATATCAAAATGGC

TGTGGTATATAAAAATATTCATAATGATAGTAGGAGGCTTACTAGGTTTAAGAATAGTTTTTACT---
GTGCTTTCTGTAGTG---------AATAGAGTTAGGCAGGGATACTCACCA------
TTGTCATTCACTGTTAGTTTGCTTAATGCCATAGCTATAGCAGTAGCTGAG---
GGGACAGATAGAGTTATAGAAGTAGCACAAAGACTTGGTAGAGCT---------------------------
--------ATCCTTAACATA------CCTAGAAGAATAAGACAGGGAGCAGAAAGAGCTTTGCTA---TAA
>KM218228
GCT---GAA--
AAGTTGTGGGTCACAGTCTATTATGGGGTACCTGTGTGG---AAAAACGCAAAC---
ACCACCTTATTTTGTGCATCAGAGGCTAAAGCATATAGCTCAGAG---GTACATAAT---------------
--------------GTTTGGGCCACA---CATGCCTGTGTACCCACAGAC---
CCTAACCCACAAGAAGTAGTATTGGAA---------AATGTGACAGAAAATTTTAAT---------------
-------------------------ATGTGGAAAAATGACATGGTAGAACAGATGCAGAAGGATATA---
ATCAGTTTATGGGATGAAAGCCTA---AAGCCATGTGCAAAATTA---
ACCCCACTCTGTGTTACTTTAAATTGCACTGATTGGAAGTCTAATACTACTAATACCACTAAT----------
----
-------------ATTATTAATGCCACTCAAAGTAGTGGGAAAATATGAACGGAGAAATAAAA---
AACTGCTCTTTCAATGTAACCACACAAATAGAA--------------------------
AATAAGGTGCGAAAAGAACAGGCACTT---TTTAATAGTCTTGATATAGTACCAATAGATAAG----------
----

GGTAACTCTACATATATGTTGGTAAATTGTAATACCTCAGTCATTACACAG---
GCCTGTCCAAAGATATCCTTTGAACCAATTCCCATACATTATTGTACCCCGGCTGGTTTTGCGATTATAAAGT
GTAAT---AATAAGACATTCAAT------GGAACAGGACCATGTACAAAT----------------------
-GTCAGT---ACAGTACAATGTACACATGGAATTAGGCCAGTAGTGTCAACT---
CAACTACTGTTAAATGGCAGTCTAGCAGAAACAGAT-----------
ATAGTAGTTAGATCTGAAAATTTCACAAACAAT---------GCTAAAACCATAATAGTACAGCTGAAT----
-----------AAAACTATACAA---------ATTGAGTGTATAAGACCCAACAACAATACAAGAAAG-----
-AGTATACATATA------------AGTCCAGGGAGAGCA---TTT------TATGCAACAGGAGAT------
---------ATAATAGGAGATATA------------AGACAAGCACATTGT---AATGTTAGT---GCA---
AATTGGAATGAAACTTTAAAACAGGTAGCTATAAAATTAAAA---------------GACCAATTTAAA----
-------------------------------AATAAGACAACAATAGTCTTTAAG------
CCACCCTCAGGAGGGGATCCAGAAATTATA---ATGCTCACATTTAATTGTGGAGGGGAA---
TTTAAGCAGCTCCAGGCAAGAGTCCTCGCTGTGGAAAGATACCTAAGGGATCAACAGCTCCTAGGGATTTGGG
GTTGCTCTGGAAAACTCATCTGCGCCACTGCTGTGCCTTGGAATGCTAGTTGGAGT----------------
-----------------------------AATAAATCCATAGATGAGATTTGG------AAT------
AACATGACCTGGATGCAGTGG---GAGAGAGAAATTGACAATTACACTGAATTAATATAC---
TCTTTACTTGAAAAAATCGCAGAACCAGCAAGAA--------------------------------------
----
AAGAATGAGCAAGAATTATTGGAATTGGATCAATGGGCAAATTTGTGGAATTGGTTTGACATATCAAACTGGC
TGTGGTATATAAGAATATTCATAATGATAGTAGGAGGCTTAATAGGTTTAAAAATAGTCTTTACT---
GTATTTTCTATAGTG---------AATAGAGTTAGGCAGGGATACTCACCA------
TTATCATTCGCTGTTAGTTTGCTTAATGCCACAGCTATAGCAGTAGCTGAG---
GGAACAGATAGAGTTAGAGAACTAGCACAAGCAATTGGTAGAGCT---------------------------
--------ATTCTCCACATA------CCTAGAAGAATAAGACAGGGCGCAGAAAGACCTTTGGTA---TAA
>JF932479
GCT---ATA--
GAAAACTTGTGGGTTACAGTCTATTATGGGGTACCTGTATGG---AAAGAAGCAACT---
ACCACTCTATTTTGTGCATCAGATGCTAAAGCATATGATACAGAG---GTACATAAT---------------
--------------ATTTGGGCCACA---CATGCTTGTGTACCCACAGAC---
CCCAACCCACAAGAAGTAGTTTTGGGA---------AATGTAACAGAAAACTTTAAC---------------
-------------------------ATGTGGAAAAATGACATGGTAGAACAGATGCATGAAGATATA---
ATCAGTTTATGGGATCAAAGCCTA---AAGCCATGTGTAAAATTA---
ACCCCACTCTGTGTTATTTTAAATTGCACTAATTATTCGGGAAATAATACCAGGAATAAT------------
----
------------------------------ACCAGTAATACGCTAGAGGGAGGAGAAATGAAA---
AACTGCTCTTTTAATATCACCACAAGCATAGGA--------------------------ACTAAGGTG---
ACAGACTCTGCAATT---TTTAATAAACTTGATATAATACCAATAGAGGGG---------------------
----
----------AATAGTAGCAACTATAGGTTGATAAATTGTAACACCTCAGTTTTTACGCAA---
GCTTGTCCAAAGGTATCCTTTGAACCAATTCCTATACATTATTGTACCCCGGCTGGTTATGCGATTCTAAAGT

GTAAC---AATAAAACGTTCAAT------GGAAAAGGACCATGTACAAAT----------------------
-GTCAGT---ACAGTACAATGTACACATGGAATTAGGCCAGTAGTATCAACT---
CAACTGCTATTAAATGGCAGTTTAGCAGAAGAAGGG------------
GTAGTAATTAGATCTAGCAATTTCACAAACAAT---------GCTAAAGTCATAATAGTACAGCTGAAT----
-----------GAATCTGTAGCA---------ATTGATTGTACAAGACCCAACAACAATACAAGAAAA-----
-AGTATACATCTG------------GGACCAGGACGAGCA---TTG------TACATGTCAGGAGGA------
---------ATAGTAGGAAACATA------------AGACAAGCCCATTGT---AACATTAGTGGAACA---
AAATGGAATGACACTTTAAGACAGATAACTGGAAAATTAAGA---------------GAACAATTTGGA----
------------------------------------AACAAAACAATCATTTTTAAT------
CAACCTGCAGGAGGGGACCCAGAGATTGTA---ATGCACAGTTTTAATTGTGGAGGGGAA---
TTTAAGCAGCTCCAGGCAAGAGTCTTGGCTGTGGAAAGATACCTAAAGGATCAACAGCTCCTAGGGATTTGGG
GTTGCTCTGGAAAACTCATTTGCACCACTGCTGTGCCTTGGAATGCTAGTTGGAGT-----------------
-----------------------------AATAAAAATCTGAGTCAGATTTGG------GAT------
AACATGACCTGGATGGAGTGG---GAAAAAGAAATTAACAATTACACAAAAGAGATATAC---
ACCTTAATTGAAGAATCGCAGAACCAACAAGAA---------------------------------------
----
AAAAATGAACTAGAACTATTGGAATTGGATAAGTGGGCAAGTTTGTGGAATTGGTTTGACATAACAAAATGGC
TGTGGTATATTAAAATATTCATAATGATAGTAGGAGGCTTGGTAGGTTTAAGAATAGTTTTTGCT---
GTACTTTCTATAATA---------AATAGAGTTAGGCAGGGCTACTCACCA------
TTGTCATTTGCTATTAGCTTGCTTAACACAACAGCCATAGTAGTAGCTGAG---
GGGACAGATAGGGTTATAGAAGCAGTACAAAGAGCTTGTAGGGCT---------------------------
--------GTTCTCCACATA------CCTAGAAGAATCAGACAGGGTCTCGAAAGGGCTTTGCTA---TAA
>JF932480
GCT---GCA---
GAAAACTTGTGGGTCACAGTCTATTATGGGGTACCTGTATGG---AAAGATGCAACT---
ACCACTCTATTTTGTGCATCAGATGCTAAAGCATATGATACAGAG---GCACATAAT----------------
--------------ATTTGGGCCACA---CATGCCTGTGTACCCACAGAC---
CCCAACCCACAAGAAGTAGTCTTGGGA---------AATGTGACAGAACACTTTAAC---------------
--------------------------ATGTGGAAAAATGACATGGTAGAACAGATGCATGAAGATATA---
ATCAGTTTATGGGATCAAAGCCTA---AAGCCATGTGTAAAATTA---
ACCCCACTCTGTGTTACCTTAAATTGCACTGATTTGAAGAATACTACTAATAATACCAGT------------
----
-------------------------AGTAATGCCAGTAGTATGACAGAGGGAAGAGAAATGAAA---
AACTGCTCTTTCAATATCACCACAAGCATAAAA--------------------------ACTAAGGTG---
AATGACTATGCACTT---TTTTATAAATATGATATAGAATCAATAGATAAT---------------------
----
----------AGTACTACCAGCTATAGGTTAACAAAGTGTAACACCTCAGTCATTACACAA---
GCTTGTCCAAAGGTATCCTTTGAGCCAATTCCCATACATTATTGTACCCCGGCTGGTTTTGCGATTCTAAAGT
GTAAC---AATAAAACGTTCAAT------GGAACAGGACCATGCACAAAT----------------------
-GTCAGT---ACAGTACAATGTACACATGGATTAGGCCAGTAGTATCAACT---
CAACTGCTGTTGAATGGCAGTTTAGCAGAAGGAGAG------------
GTAGTAATTAGATCTAGCAATTTCACGAACAAT---------GCTAAAGTCATAATAGTACAGCTGAAT----
-----------GAATCTGTAGAA---------ATTAATTGTACAAGACCCAACAACAATACAAGGAAA-----
-AGTATACCTCTA------------GGACCAGGGAAAGCA---TGG------TATACAACAGGACAA------
---------ATAATAGGGGATATA------------AGACAAGCACATTGT---AACATTAGTGGATCA---
CAATGGAATAAAACGTTAATACAGATAACTGAAAAATTAAAA---------------GAACGATTTGAG----
------------------------------------AGCAAAAAAATAATCTTTAAG------
CAACATTCAGGAGGGGACCCAGAGATTGTA---ATGCACCATTTTAATTGTGGAGGAGAA---
TTTAAGCAGCTCCAGGCAAGAGTCTTGGCTGTGGAAAGATACCTAAAGGATCAACAGCTCCTAGGGATTTGGG
GTTGCTCTGGAAAACTCATTTGCACCACTACTGTGCCTTGGAATGCTAGTTGGAGT-----------------
-----------------------------AATAAATCTCTGAGTCAGATTTGG------GAT------
AACATGACTTGGATGGAGTGG---GAAAGAGAAATTGACAATTACACAAAAGAAATATAC---
ACCTTAATTGAAGAATCGCAGAACCAACAAGAA---------------------------------------
----
AAGAATGAACTAGAACTATTGGCATTGGATAAGTGGGCAAACTTGTGGAGTTGGTTTGACATAACTCAATGGC
TGTGGTATATCAGAATATTCATAATGATAGTAGGAGGCTTGATAGGTTTAAGAATAGTTTTTGCT---
GTACTTTCCATAATA---------AATAGAGTTAGGCAGGGATACTCACCA------
TTGTCATTAGCTATTAGCTTGTTTAACGCCACAGCCGTAGCAGTAGCAGAG---
GGGACAGATAGGGTTATAGAAGTAGTACAAGGGGCTTATAGGGCT---------------------------
--------TTTATTCATATA------CCTACAAGAATCAGACAGGGCCTCGAAAGGGCTTTGCTA---TAA

>JF932481
GCT---GCA--
AAAGACTTGTGGGTCACAGTCTATTATGGGGTACCTGTATGG---AAAGAAGCAACT---
ACCACTCTATTTTGTGCATCAGATGCTAGAGCATATGATACAGAG---GTACATAAT----------------
--------------ATTTGGGCCACA---CATGCCTGTGTACCCACAGAC---
CCCAACCCACAAGAAGTAGTCTTGGGA---------AATGTGACAGAAAATTTTAAC----------------
--------------------------ATGTGGAAAAATGACATGGTAGAACAGATGCATGAAGATATA---
ATCAGTTTATGGGATCAAAGCCTA---AAGCCATGTGTAAGATTA---
ACCCCACTCTGTGTTGCTTTAAATTGCACTAATTTGGGAAATACTACTAATATCAAGAGT-------------
----
----------------------------AATACCAGTAGTACGACAGAGGGAGGAGAAATAAAA---
AACTGCTCTTTCAATATCACCACAAGCATAAAA---------------------------ACTAAGGTA---
AAAGACTATGCACTT---TTTTATACACATGATGTAGTATCAATAGATAAT----------------------
----
----------------ACCAGCTATAGGTTGATACATTGTAACACCTCAGTCATTACACAA---
GCTTGTCCAAAAGTATCCTTTGAGCCAATTCCTATACATTATTGTGCCCCGGCTGGTTTTGCGATTCTAAAGT
GTAAC---AATAAAACGTTCAAT------GGAACAGGACCATGTACAAAT-----------------------
-GTCAGT---ACAGTACAATGTACACATGGAATTAGGCCAGTAGTATCAACT---
CAACTGCTGTTAAATGGCAGTTTAGCAGAAGAAGAG------------
GTAGTAATTAGATCTAGCAATTTCACAGACAAT---------GCTAAAATCATAATAGTACAGCTGAGT----
----
----ATACCTCTA------------GGACAAGGGCGAGCA---TGG------TATACAACAGGACAA------
---------ATAATAGGAGATATA------------AGACAAGCACATTGT---AACATTAGTAGAACA---
AAATGGAATAACACTTTGAAACAGATGACTGAAAACTTAAGA---------------GAACAATTTGAG----
-----------------------------------AACACAACAATAATCTTTAAT------
AAATCTTCAGGAGGGGACCCAGAGATTGAA---AAACACAGTTTTAATTGTGGAGGGGAA---
TTTAAGCAGCTCCAGGCAAGAGTCCTGGCTGTGGAAAGATACCTAAAGGATCAACAGCTCCTAGGGATTTGGG
GTTGCTCTGGAAAACTCATTTGCACCACTAATGTGCCTTGGAATAATAGTTGGAGT-----------------
----------------------------AATAAATCTCTGAGTGACATTTGG------AAT------
AACATGACTTGGATGGAGTGG---GAAAGAGAAATTGGCAATTACACAAGAGAAATATAC---
ACCTTAATTGAAGAATCGCAGAACCAACAGGAA--

AAGAATGAACTAGAACTATTGGAATTGGATAAGTGGGCAAGTTTATGGAATTGGTTTGACATATCAAACTGGC
TGTGGTATATCAAAATATTCATAATGATAGTAGGAGGCTTGGTAGGTTTAAGAATAGTTTTTGCT---
GTACTTTCTATAGTA---------AATAGAGTTAGGCAGGGGTACTCACCA------
TTGTCATTAGCTGTTAGCTTGCTTAACGCCACAGCCATAGCAGTAGCTGAG---
GGGACAGATAGGGTTATAGAAGTAGTACAAAGAACTTTTAGGGCT----------------------------
--------ATTCTCAACATA------CCTACAAGAATCAGACAGGGCTTCGAAAGGGCTTTGCTA---TAA
>JF346919
GCT---ACA--
GAAAAATTGTGGGTCACAGTCTATTATGGGGTACCTGTATGG---AAAGATGCAACT---
ACCACTCTATTTTGTGCATCAGATGCTAAAGCATATGATACAGAG---GTACATAAT----------------
--------------GTTTGGGCCACA---CATGCCTGTGTACCCACAGAC---
CCCAACCCACAAGAAGTAGTATTGGGA---------AATGTGACAGAAAACTTTAAC----------------
--------------------------ATGTGGAAAAATGACATGGTAGAACAGATGCATGAAGATATA---
ATCAGTTTATGGGATCAAAGCCTA---AAGCCATGTGTAAAATTA---
ACCCCACTCTGTGTTACTTTAAATTGCACTAATCCTAAAACCAATAGTAGTGTGAATACTACTCCC-------
----
----------------------AGTACTAGTAACAGTAGTATGATAAAGGGAGGAGAGATGAAA---
AACTGCTCTTTCAATGTCACCGCAGACATAAAA---------------------------------
CCTAAGAAAGACTATGCACTC---TTTTATAAACTTGATTTAGAACAAATAGATGATGAGAAGAATAAC----
----
ACCAACAATACCAGCGATAGTAATACCAGCTATAGGTTGATAAAGTGTAACACCTCAGTCATTACACAA---
GCTTGTCCAAAGGTATCCTTTGAGCCAATTCCCATACATTATTGTGCCCCGGCTGGTTTTGCGATTCTAAAAT
GTAAC---AATAAAACGTTCAGT------GGAACAGGACCATGTACAAAT-----------------------
-GTCAGT---ACAGTACAGTGTACACATGGAATTAAGCCAGTAGTATCAACT---
CAACTGCTGTTAAATGGCAGTTTAGCAGAAGAAGGG------------
ATAGTAATTAGATCTAGCAATTTCACGGACAAT---------ACCAAAACCATAATAGTACAACTGAAG----
-----------GACCCTGTAGAA---------ATTAACTGTACAAGACCCAACAACAATACAAGAAAA-----
-AGTATACCTCTA------------GGACAAGGGCGAGCA---TGG------TATACAACAGAAAAC------

---------ATAATAGGAGATATA------------AGACAAGCACATTGT---AACATTAGTGGAGAA---
CAATGGAATAACACTTTACAACAGATAGTTAAGAAATTAAGA--------------
GAAATATACAACAAAACA--------------------------ATATATAACAAAACAATAAGCTTTAAT-
-----CAATCTTCAGGAGGGGACCCAGAGATTGTA---ATGCACAGTTTTAATTGTAGAGGGGAA---
TTTAAACAGCTCCAGGCAAGAGTCTTGGCTGTGGAAAGATACCTAAAGGATCAACAGCTCCTAGGGATTTGGG
GTTGCTCTGGAAAACTCATTTGCACCACTGCTGTGCCTTGGAATGCTAGCTGGAGT-----------------
-------------------------AATAATAGAACTGTAGAGAGCATTTGG------AAT------
AACATGACTTGGATGCAGTGG---GAAAAAGAAATCGCCAATTACACAAAAGAAATATAC---
ACCTTAATTGAAGAATCGCAGAACCAGCAAGAA-------------------------------------
----
AAGAATGAACTAGAACTATTGGAATTGGATAAGTGGGCAAGTTTGTGGAATTGGTTTGACATATCAAACTGGC
TGTGGTATATCAAAATATTCATAATGATAGTAGGAGGCTTAATAGGTTTAAGAATAGTTTTTACT---
GTACTTTCTATAGTA---------AATAGAGTTAGGCAGGGATATTCACCC------
TTGTCATTAGCTATTAGCTTGCTTAACGCTACAGCCATAGCAGTAGCTGAG---
GGGACAGATAGGGTTATAGAAATAGCACAAAGAGCTTTTAGAGCT---------------------------
--------ATTCTCAACATA------CCTACAAGAATCAGACAGGGCCTCGAAAGGGCTTTGGTA---TAA
>JF932498
GCT---GCA---
GAAAACTTGTGGGTCACAGTCTATTATGGGGTACCTGTATGG---AAAGAAGCAACT---
ACCACTCTATTTTGTGCATCAGATGCTAAAGCATATGATATAGAG---GTACATAAT----------------
--------------GTTTGGGCCACA---CATGCCTGTGTACCCACAGAC---
CCTAACCCACAAGAAGTAGTCTTGGAA---------AATGTAACAGAAAACTTTAAC----------------
--------------------------ATGTGGCAAAATGACATGGTAGAACAGATGCATGAAGATATA---
ATCAGTTTATGGGATCAAAGCCTA---AAGCCATGTGTAAAATTA---
ACCCCACTCTGTGTTACTTTAAATTGCACTGATTTGAAGAATACTACTAATAATAACAGT-------------
----
-------------------------AGTAATACAAGTAGTACGACAGAGGGAGAAGAACTAAAA---
AACTGCTCTTTCAATATCACCACAAGTATAAAA--------------------------ACT---
GTGGTGAAAGACTATGCATTT---TTTTATAAACTTGATATAGTACCAATAATAGATAAGGGCAATATC----
----
----------AGTAATGGTAATAATAGCTATAGGTTGATAAGTTGCAACACCTCAGTCATTACACAG---
GCTTGTCCAAAGGTATCCTTTGAGCCAATTCCCATACATTATTGTGCCCCGGCTGGTTTTGCGATTCTAAAGT
GTAAC---AATAGAACGTTCAGT------GGAACAGGACCATGTACAAAT----------------------
-GTCAGT---ACAGTACAATGTACACATGGAATTAGGCCAGTAGTATCAACT---
CAACTGCTGTTAAATGGCAGTTTAGCAGAAGGAGAG------------
GTAATAATTAGATCTAGCAATTTCACGAACAAT---------GCTAGGGTTATAATAGTACAGCTGAAT----
-----------GAATCTGTAGAA---------ATTAACTGTACAAGGCCCAACAACAATACAAGAAAA-----
-GGTATACATCTA------------GGACTAGGGCGAACA---TGG------TATACAACAGGAGAA------
---------ATAATAGGAGATATA------------AGACAAGCATATTGT---AACATTAGTGGCACA---
CAATGGAATAACACTTTAAAACAGGTAGTTGAAAAATTAAGA---------------GAACAATTTGGG----
----------------------------------AACAACAAAACAATAATCTTTAAT------
CAATCTTCAGGAGGGGACCCAGAGATTGTA---ATGCACACTTTTAATTGTAGAGGGGAA---
TTTAAGCAGCTCCAGGCAAGAGTCTTGGCTGTGGAAAGATACCTAAAGGATCAACAGCTCCTAGGGATTTGGG
GTTGCTCTGGAAAACTCATTTGCACCACTGCTGTGCCTTGGAATGCTAGTTGGAGT-----------------
----------------------------AATAAATCCCTAAGTGAGATTTGG------AAT------
AACATGACCTGGATGGAGTGG---GAAAGAGAAATTGAAAATTACACAAGAGAAATATAC---
ACCTTAATTGAAGAATCGCAGAACCAACAAGAA-------------------------------------
----
AAGAATGAACTAGAATTATTGGAATTGGATAAGTGGGCAAGTTTGTGGAATTGGTTTGACATAACAAAATGGC
TGTGGTATATTAAAATATTCATAATGATAGTAGGAGGCTTGGTAGGTTTAAGAATAATTTTTGCT---
GTACTTTCTATAGTA---------AATAGAGTTAGGCAGGGATACTCACCA------
TTGTCATTTGCTATTAGCTTGCTTAACGTCACAGCTATAGCAGTAGCTGAG---
GGGACAGATAGGGTTATAGAAGTAGCACAAAGAGCTTATAGAGTT---------------------------
--------ATTTTCCACATA------CCTACAAGAATCAGACAGGGCCTCGAAAGGGCTTTGCTA---TAA
>JX960597
GCT---TCA---
GACAAATTGTGGGTCACAGTCTATTATGGAGTACCTGTGTGG---AAAGAAGCAACC---
ACCACTCTATTTTGTGCATCAGATAGTAAAGCATATAGTACAGAG---TCACATAAT----------------
--------------GTTTGGGCCACA---CATGCCTGTGTACCCACAGAC---
CCCAACCCACAAGAAATAGAGTTGGGA---------AATGTGACAGAAAACTTTGAC----------------

--------------------------ATGTGGAAAAATAACATGGTAGAACAGATGCATAAGGATATA---
ATCAGTTTATGGGATCAAAGCCTA---CAGCCATGTGTAAAATTA---
ACTCCACTCTGTGTTACTTTAGAGTGCACTGATTGGGATGCTAATAAGACCAATGCTAATATTAGCACTAAT-
----
----------------------GAGAAAGGGAAAATAAAAAACATAGAGAAAGGAGAAATAAAA---
AACTGTTCTTTCAATGTCACCACAAGTATAAGA---------------------------
GGTAGGATGCAGAAACAATATGCACTT---TTTTATACACTTGATGTAGTACCAATAGATGATAATAAT----
----

AGTCCTAATGATAATAATGCTACCAAATATAGGTTGATAAGTTGTAACACCTCAGTTATTACACAG---
GCCTGTCCAAAGGTATCCTTTGAGCCAATTCCCATACATTATTGTACCCCGGCTGGTTTTGCGATTCTAAAGT
GTAGA---GATAAGCAGTTCAAT------GGAACAGGGCCATGTAAAAT------------------------
-GTCAGC---ACAGTACAATGTACACATGGAATTAGGCCAGTAGTGTCAACT---
CAACTGCTGTTAAATGGCAGCCTAGCAGAAAAAGAG-----------
GTAGTAATTAGATCTAGCAATTTCACGAACAGT---------GCTAAAACCATAATAGTACAGCTGACA----
-----------AAAGCAGTGAGC---------ATTACTTGTATAAGACCCAACAACAATACAAGAAAA-----
-AGTATACCTATA------------GGACCAGGGAGAGCA---TTT------TATGCAACAGGGGAA------
---------ATAACAGGAGATATA------------AGACAAGCACTGT---ACCCTTAATAGAACA---
GACTGGATGAACACTTTACAACAGATAGCTAGAAAATTAAGA---------------GTCCAATTTAAG----
-----------------------------------AGTAAAACAATAATCTTTAAT------
CAATCCTCAGGAGGGGACCCAGAAATTGTA---ATGCACAGTTTTAATTGTGGGGGGGAA---
TTTAAGCAGCTCCAGGCAAGAGTCCTGGCTGTGGAAAGATACCTAAGGGATCAACAGCTCCTAGGAATTTGGG
GTTGCTCTGGAAAGCTCATTTGCACCACTGCTGTGCCTTGGAATAGTAGTTGGAGT-----------------
----------------------------AACAAATCTCTAGATGAGATTTGG------AAT------
AATATGACTTGGATGGAGTGG---GAGAGAGAAATTGACAATTACACAAACTTAATATAC---
AGTTTAATTGAAGAATCGCAGAACCAGCAGGAA--

AGAAATGAACAAGAATTATTGGAATTGGACAAATGGGCAAATTTGTGGAATTGGTTTGACATAACACAATGGC
TGTGGTATATAAAAATATTCATAATGATAGTAGGAGGCTTAATAGGTTTAAGAATAGTTTTTGCT---
GTACTTTCTATAGTG---------AATAGAGTTAGGCAGGGATACTCACCA------
TTATCGTTTGCTGTTAGCTTGCTCAACGCCACAGCCATAGCAGTAGCTGAG---
GGAACAGATAGGATTATAGAAGCATTACAAAGAATTGGTAGAGCT----------------------------
--------ATTCTCCACATA------CCTACAAGAATAAGACAGGGCTTGGAAAGGCTTTGCTA---TAA
>JX960598
GCT---ACA--
GAACAATTGTGGGTCACGGTCTATTATGGGGTACCTGTGTGG---AAAGAAGCAACC---
ACCACTCTATTTTGTGCATCAGATGCTAAAGCATATGATACAGAG---GTACACAAT----------------
--------------GTCTGGGCCACA---CATGCCTGTGTACCCACAGAC---
CCCAACCCACAAGAGGTAGTAATA------------AATGTGACAGAAGATTTTAAC----------------
--------------------------ATGTGGGAAAATAACATGGTAGAACAGATGCATGAGGATATA---
ATTAGTTTATGGGATCAAAGCCTA---AAGCCATGTGTAAAATTA---
ACCCCACTCTGTGTTACTATGAATTGCACCCCACTCAATGTCAGCACTGGTAGCAACAGGAATACCACT----
----
----------------------------GATAATTGTAGAATTAGTAATAGCTGTGAAATAAAA---
AATTGCTCTTTCAATGTTACCACAAGCATAAGA---------------------------
GATAAGGTGAAGAAAGAACATGCCCTT---TTTGATACAATGGATCTAGTACCAATAGAAGATGAT-------
----

GAGAGTACTAATACTAGTTATAGATTGACAAGTTGTAACACCTCACTCATTACACAG---
GCCTGTCCAAAGGTATCCTTTGAGCCAATTCCCATACATTATTGTACCCCAGCTGGTTTTGCGATTCTACAGT
GTAAC---GATAAAACATTCAAT------GGAACAGGACCATGTCAAAAT------------------------
-GTCAGT---ACAGTACAATGTACACATGGAATTAGGCCAGTAGTATCAACT---
CAACTACTGTTAAATGGCAGTCTAGCAKAACAAGAG-----------
GTAATAATTAGGTCTAAAAATATCTCGGACAAT---------ACCAAAAACATAATAGTACAGCTGAGA----
-----------ACAGCTGTAAAC---------ATAACTTGTATAAGACCCAATAATAATACAAGAAAA-----
-AGTATAAATATA------------GGACCAGGCAGAGCA---TTT------TATGCAACAGGAGAA------
---------ATAATAGGAGACATA------------AGAAAAGCAAATTGT---ACCCTTAATGGAACA---
CAGTGGGAAAACACTTTAAAACAGATAGCTAGAAAATTAAGA---------------GAACACTTT-------
-----------------------------------AATAAAACAATAGTCTTTAAT------
CAATCCTTAGGAGGGGACCCAGAAATTGTA---ATGCACAATTTCAATTGTAGAGGAGAA---

TTTAAGCAGCTCCAAGCAAGAGTCCTGGCGCTGGAAAGATACCTAAAGGATCAACAGCTCATGGGGATTTGGG
GTTGCTCTGGAAAACTCATTTGCACCACTACTGTGCCTTGGAATACTAGTTGGAGT-----------------
----------------------------AACAAAAGTCTGAATCAGATTTGG------GAT------
AACATGACCTGGTTACAATGG---GAAAGAGAAATTGATAATTACACAGGCGAAATATAC---
CAATTACTTGAAGAATCGCAAAACCAACAAGAA--

AAGAATGAACAAGAATTACTGGAGTTAGATAAATGAGCAAATTTGTGGAATTGGTTTGACATAACACATTGGC
TGTGGTATATCAAAATATTCATAATGATAGTAGGAGGCTTGATAGGTTTAAGAATAGTTTTTACT---
GTACTTTCTATAGTG---------AATAGAGTCAGGCAGGGATACTCACCA------
TTATCGTTGGCTATTACCTTGCTCAACGCCACAGCCATAGGAGTAGCTGAG---
GGGACTGATAGGATTTTAGAAGTATTACAAAGATTTTATAGAGCT----------------------------
--------ATTATCCACATT------CCTACAAGAATTAGACAGGGCTTAGAAAGGGCTTTGCTA---TAA
>KC899011
GCT---GCA--
GAAAACTTGTGGGTCACAGTCTATTATGGGGTACCTGTATGG---AAAGAAGCAACC---
ACCACTCTATTTTGTGCATCAGATGCTAAAGCATATGATAAAGAG---GTACATAAT----------------
--------------GTTTGGGCCACA---CATGCCTGTGTACCCACAGAC---
CCCAACCCACAAGAAATATTCTTGGAA---------AATGTGACAGAAAACTTTAAC----------------
--------------------------ATGTGGGAAAATAACATGGTAGAACAGATGCATGAAGATATA---
ATCAGTTTATGGGATCAAAGCTTA---AAGCCATGTGTAAAATTA---
ACCCCACTCTGTGTTACGTTAAACTGCACCAATGCCACTATTACCAGTAGTGCTAATACCACTGGT-------
----
-------------------GCCAGTAGTCCTAGTCCTAGTCCTAGTACTAGTAGGGAGATGAGA---
AACTGCTCTTTCAATATCACCACAAGCATAAAA---------------------------ACTAAGGTG---
AAAGACTATGCACTT---TTTTATAACTTTGATATAGAACAAATGAAAGAT----------------------
----
----------GGTACTAACAATTATAGGTTGATAAATTGTAACACCTCAGTCATTACACAA---
GCTTGCCCAAAGGTATCCTTTGAGCCAATTCCCATACATTATTGTGCTCCGGCTGGTTTTGCAATTCTAAAGT
GTAAC---GATAAAGCATTCAAT------GGAACAGGACCATGTATAAAT-----------------------
-GTCAGT---ACAGTACAATGTACACATGGAATTAGGCCAGTAGTATCAACT---
CAACTGCTGTTAAATGGCAGTTTAGCAGAAGAAGGG------------
GTAGTAATTAGATCTCGCAATCTCACGAATAAT---------GCTGAAGTCATAATAGTACAGCTGAAG----
-----------GAATCTGTAGAA---------ATTAATTGTACAAGGCCCAACAACAATACAAGAAAA-----
-AGTATACCTCTA------------GGACCAGGGAAAGCA---TGG------TATACAACAGGACAA------
---------ATAATAGGAGATATA------------AGAAAAGCACATTGT---AACCTTAGTAGCACA---
AAATGGAATACCACTTTAGAACTGATAGCTAAAAAATTAGGA---------------GAACAATTTAAA----
-----------------------------------AACAAAACAATAGTCTTTACT------
CAATCCTCAGGAGGGGACCCAGAGGTTGTA---ATGCACAATTTTAATTGTGGGGGGGAA---
TTTAAGCAGCTCCAGGCAAGAGTCTTGGCTGTGGAAAGATACCTAAAGGACCAACAGCTCCTAGGGCTTTGGG
GTTGCTCTGGGAAACTCATTTGCACCACTGCTGTGCCTTGGAATGCCAGTTGGAGC-----------------
----------------------------AATAAATCTCTAGAGGAGATTTGG------AAT------
AACATGACTTGGATGGAGTGG---GAAAGGGAAATTGACAATTACACAAGAGAAATATAC---
ACCTTAATTGAAAATTCGCAGAACCAGCAAGAA--

AAGAATGAACTAGAATTATTGGAATTGGATAAGTGGGCAAGTTTGTGGAATTGGTTTGATATAACAAAATGGC
TGTGGTATATAAAAATATTCATAATGATAGTAGGAGGCTTGGTAGGTTTAAGAATAGTTTTTGCT---
GCACTTTCTATAGTA---------AATAGAGTTAGGCAGGGATACTCACCA------
TTGTCATTAGCTGTTAGCTTGCTCAATACTACAGCTATAGCAGTAGCCGAG---
GGGACAGATAGGGTTATAGAAATAATACAAAGAGCTTATAGAGCT----------------------------
--------ATTCTCCACATA------CCTACAAGAATAAGACAGGGCCTCGAAAGGGCTTTGCTG---TAA
>HQ215556
GCT---GGA---GAC------------------------------------
AATGAGTTGTGGGTCACAGTCTATTATGGGGTACCTGTGTGG---AAGGAGGCAAAC---
ACCACTTTATTTTGTGCATCAAATGCTAAAGCATACCACCCAGAG---GCACATAAT----------------
--------------GTTTGGGCCACA---CATGCCTGTGTACCCACAGAT---
CCTAACCCACAAGAGATAGTATTGGAA---------AATGTGACAGAAAATTTTAAT----------------
--------------------------ATGTGGAAAAATAACATGGTAGAACAGATGCATGAGGATGTA---
ATCAGTTTATGGGATGAAAGCCTA---AAGCCATGTGTAAAATTA---
ACCCCACTCTGTATTGCTTTAAATTGCACTGATTGGAAGTCTACTGGGACCAATAAT----------------
----

```
-------------------------------AGTAGTATGGAAAATATGGAGGGAGAAATAAAG---
AACTGCACTTTCAATATCACCTCAAAAATAGGA---------------------------
AATAAGGTGCGAAAACAATATGCACTT---TTTACTAATATTGATGTAATACCAATAGATAAGAAT-------
-------------------------------------------------------------------------
----------------------
AATAACAATAGTTATATGTTGAAAAATTGTAACACCTCAACCATTACACAG---
GCCTGTCCAAAGATGTCCTTTGAACCAATTCCCATACATTATTGTACCCCGGCTGGTTTTGCGCTTCTGCAGT
GTAAT---GATAAGAAGTTTAAT------GGAACAGGACCATGTACAAAT-----------------------
-GTTAGC---ACAGTACAATGTACACATGGAATTAAGCCAACAGTGTCAACT---
CAACTGCTGTTAAATGGCAGTCTAGCAGAAGGAGAC------------
ATAGTAATTAGATCTGAAAATTTCACAAACAAT---------GCTAAAATCATAATAGTACAGCTGAAT----
-----------GAAACTGTAGAA---------ATTAATTGTACAAGACCCAACAACAAAACAAGAAAA-----
-GGTATATCTCTA------------GGACCAGGGAGATGG---AGA------TCATTATATACAACA------
---AGAGATATAATAGGAGATAAA------------AGAAAAGCATATTGT---AACATTAGTGCAGAA---
AAATGGCACAACACTTTAAGACAGGTAGCTATAAAATTAAGA------------GAACAACTTGGATCTAAG-
-----------------------------------AATAGAACTATAATATTTAAT------
CAATCTTCAGGAGGAGACCCAGAAATTGTA---ATGCACATATTTAATTGTGGAGGGGAA---
TTTAAACAGCTCCAGGCAAGAGTCCTAGCTGTGGAAAGATACCTAAAGGATCAACAGCTCCTAGGGATTTGGG
GTTGCTCTGGAAAACTCATCTGCACCACTAATGTGCCTTGGAATGCTAGTTGGAGT-----------------
----------------------------AATAAATCTATGGAAGAGATTTGG------AAT------
AACATGACCTGGATGCAGTGG---GAGAGAGAGATTGACAATTACACAGGCACTATATAC---
ACTTTACTTGTAGAATCGCAGAACCAGCAAGAA----------------------------------------
-----------------------------
AAGAATGAAAAAGAGTTATTGGAGTTGAATAAATGGACAGATTTGTGGACTTGGTTTGACATAACAAATTGGC
TATGGTATATAAAAATATTCATAATGATAGTAGGAGGCCTAATAGGTTTAAGAATAATTTTTACT---
GTACTCTCTATAGTG---------AATAGAGTTAGGCAGGGATACTCACCA------
TTGTCGTTCGCTGTTAGTTTATTCAATACCATAGCTATAGCAGTAGCTGAG---
GGGACAGATAGAATTCTAGAAATAGCACAAAGATTTGGTAGAGCT----------------------------
--------ATTATTCACATA------CCTAGAAGAATAAGGCAGGGCGCAGAAAAGGCTTGGCTA---TAA
>JF346916
GCT---GCA------------------------------------------
GAAAAATTGTGGGTCACAGTCTATTATGGGGTACCTGTATGG---AAGGAAGCAAAT---
ACCACTCTATTCTGTGCATCAGATGCTAGAGCATATGAGACAGAG---GCACACAAT----------------
--------------ATTTGGCCACA---TATGCCTGTGTACCCACAGAC---
CCCAACCCACAAGAAGTAGTCTTGGGA---------AATGTGACAGAAAACTTTAAC----------------
--------------------------ATGTGGAAAAATAACATGGTAGAACAGATGCATGAGGATATA---
ATCAGTTTATGGGACCAAAGCCTA---AAGCCATGTGTAAAATTA---
ACCCCACTCTGTGTTACTTTAGAATGCACTAATCCGGGAAATAGTACTAGTAAT-------------------
-------------------------------------------------------------------------
-------------------------------AGCAGTAGTACAATGGAGGGAGGAGAAATAAAA---
AACTGCTCTTTCAATATCACCACAAGCATAGGA---------------------------ACTGAGGTG---
AGGGACTATGCATTT---CTTTATAAATATGATATAGAATCAATAGATAAT----------------------
-------------------------------------------------------------------------
-------------AGTACCAGATATAGGTTGACAAAATGTAACAGCTCAGTCATTAAACAA---
GCTTGTCCAAAGGTATCCTTTGAGCCAATTCCCATACATTATTGTACCCCGGCTGGTTTTGCGATTATAAAGT
GTAAC---AATAAAACGTTCAGT------GGAACAGGACCATGTACAAAT-----------------------
-GTCAGT---ACAGTACAATGTACACATGGAATTAGGCCAGTAGTATCAACT---
CAATTGCTGTTAAATGGCAGTTTAGCAGAAGAAGAG------------
GTAGTAATTAGATCTAGCAATTTCTCGGACAAT---------GCTAAAACCATAATAGTACAGCTGAAT----
-----------GCATCTGTAGAA---------ATTAATTGTACAAGACCCAACAACAATACAAGAAAA-----
-AGTATATATCTA------------GGACCAGGGCGCTCA---TGG------CATGCAACAGGACGA------
---------ATAATAGGAAACATA------------AGACAAGCACATTGT---AACCTTAGTAGAGAA---
CAATGGAATATCACTTTAAAACAGATAGCTGAAAAATTAAGA---------------GAACAATTTGGG----
-----------------------------------AACAAAACAATAATCTTTAAT------
AGATCTTCAGGAGGGGACCAAGAGATTACA---ATGCACAGTTTTAATTGTGGAGGGGAA---
TTTAAGCAGCTCCAGGCAAGAGTCTTGGCTGTGGAAAGATACCTAAAGGATCAACAGCTCCTAGGGATTTGGG
GTTGCTCTGGAAAACTCATTTGCCCCACTGCTGTGCCTTGGAATACTAGTTGGAGT-----------------
----------------------------AATAAATCTATGAATGAGATTTGG------AAT------
AACATGACTTGGATGGAGTGG---GAAAGAGAAATTAGCAATTACACAAATGTAATATAC---
ACCTTAATTGAGGGAGCGCAGAACCAGCAAGAA----------------------------------------
```

AAGAATGAACTAGAACTATTGGAATTGGATAAGTGGGCAAGCTTGTGGAATTGGTTTGACATAACAAAATGGC
TGTGGTATATCAAAATATTCATAATGATAATAGGAGGCTTGGTAGGTTTAAGAATAGTTTTTGCT---
GTACTTTCTATAGTA---------AATAGAGTTAGGCAGGATACTCACCA------
GTGTCATTAGCTATTAGCTTGCTTAACGCCACAGCCATAGCAGTAGCTGAG---
GGGACAGATAGGGTTATAGAAATAGTACAAAGAGCTTATAGGGCT----------------------------
--------ATTCTCAACATA------CCTACAAGAATCAGACAGGGCCTTGAAAGGGCTTTGTTA---TAA
>JF932494
GCT---GCA--GAAAAG---
TGGGTCACAGTCTATTATGGGGTACCTGTATGG---AAAGAAGCAACT---
ACCACTCTATTTTGTGCATCAGATGCTAAAGCATATGATACAGAG---GTACATAAT----------------
--------------GTGTGGGCCACA---CATGCCTGTGTACCCACAGAC---
CCCAACCCACAAGAAGTAGTCTTGGGA---------AATGTGACAGAAAACTTTAAC----------------
--------------------------ATGTGGAAAAATAGCATGGTAGACCAGATGCATGAAGATATA---
ATCAGTTTATGGGATCAAAGCCTA---AAGCCATGTGTAAGATTA---
ACCCCACTTTGTGTTACTTTACATTGCACTAATGTGAATAATACTGTGAACACCAGT----------------
----
---GAGTCAGAAATGAAA---
AACTGCTCTTTCAATGTCACCACAAGCATAAAA---------------------------ACTAAGGTG---
ACAGACTATGCATTA---TTTTATAAACTTGATGTAGTATCAATAAATGAA----------------------
----
----------AGTAGTAATAGATATAGGTTGATAAGTTGTAACACCTCAGTCATTACACAA---
GCTTGTCCAAAGGTATCCTTTGAGCCAATTCCCATACATTATTGTGCCCCGGCCGGTTTTGCGATTCTAAAGT
GTAAT---GATAAAAAGTTCAAT------GGATCAGGACCATGTACAAAT-----------------------
-GTCAGT---ACAGTACAATGTACACATGGAATTAGGCCAGTAATATCAACT---
CAACTGCTGTTAAATGGCAGTTTAGCAGAAGAAGAG------------
GTAGTAATTAGATCTAGCAATTTCACAAACAAT---------GCTAAAATCATAATAGTACAGCTGAAT----
-----------CAATCTGTAGAA---------ATTAATTGTACAAGACCCAACAACAATACAAGAAAA-----
-AGCATACATATA------------GGACCAGGGCGAGCA---TGG------TATGCAACAGGAAAT------
---------ATAATAGGAGATATA------------AGACAAGCACGTTGT---ACCCTTAATGGCACA---
AAATGGAATAACACTTTAAAACAGATAGCTGAAAAATTAAAA---------------GAACAATTTGGG----
--------------------------------GAAAACAAAACAATAGTCTTTAAT------
CAATCCTCAGGAGGGGACCTAGAAATTGTA---ACGCACAGTTTTAATTGTGGAGGGGAA---
TTTAAGCAGCTCCAGGCAAGAGTCTTGGCTGTGGAGAGATACCTAAAGGATCAACAGCTCCTAGGGATTTGGG
GTTGCTCTGGGAAACTCATTTGCACCACTACTGTGCCTTGGAATGATAGTTGGAGT-----------------
----------------------------AATAAAACTCTGAAGGAGATTTGG------AAT------
AACATGACTTGGATGGAATGG---GAAAGAGAAATTAACAATTACACAAAAGAAATATAC---
ACCTTAATTGAAGAATCGCAGAACCAACAAGAA--

AAGAATGAACTAGAATTATTGGAATTGGATAAGTGGGCAAGTTTGTGGAATTGGTTTGACATAACAAATTGGC
TGTGGTATATAAAAATATTCATAATGATAGTAGGAGGCTTGGTAGGCTTAAGAATAGTTTTTGCT---
GTATTTTCTATAGTA---------AATAGAGTTAGGCAGGATACTCACCA------
TTGTCATTAGCTGTCAGCTTGCTCAATGCCACAGCTATAGCAGTAGCTGAG---
GGGACAGATAGGATTATAGAAGCAATACAAAGAGCTTATAGAGCT----------------------------
--------ATTCTCCACATA------CCTACAAGAATAAGACAAGGCTTAGAAAGGGCTTTGCTA---TAA
>JX140658
GCT---AAA--
GAAAAATTGTGGGTCACAGTCTATTATGGAGTGCCTGTGTGG---AAAGAAGCAATC---
ACCCCTCTATTTTGTGCATCAGATGCTAAAGCATATGCCACTGAG---GCACATAAT----------------
--------------GTTTGGGCCACA---CATGCCTGTGTGCCCACAGAT---
CCTAACCCACAAGAGGTAGATTTGGAA---------AATGTAACAGAAGATTTTAAC----------------
--------------------------ATGTGGAAAAATAACATGGTAGAACAGATGCATGAGGATATA---
ATCAGTTTATGGGATGAAAGCCTA---AAGCCATGTGTAAAATTA---
ACACCACTCTGTGTTACTTTAAATTGCACTGATTGGAAGCCTAATAATAGTACTACTAATAATAATAAT----
----
----------------------ACCAATAATAGTGTTGCCAATCTGGAGATAGGAGAGGTAAAA---
AACTGCTCTTTCAATATCTCCAAAACAGGA------------------------------
AATAAGGTAGAGAAAGGACAGGCACTT---TTTTATAAACTTGATGTAGAACAAATAAATAAT----------
----

AATGCTAATACAAGTTATGCAAATTATGTGTTAACACATTGTAACACCTCAGTCATTAAACAA---
GCCTGTCCAAAGGTATCCTTTGAACCAATTCCCATACATTATTGTGCCCCGGCTGGTTTTGCGATTTTAAAAT
GTAAG---GATAAGAAGTTCAAT------GGAACAGGACCATGTAAAAAT-----------------------
-GTCAGT---ACAGTACAATGTACACATGGGATTAAGCCAGTAGTGTCAACT---
CAACTGCTGTTAAATGGAAGCCTAGCAGAAGAAGAG------------
GTAGTAATTAGATCTGAAAATCTCACAGACAAT---------GCTAAAACCATAATAGTACAGCTGAAT----
-----------ACATCTGTACCA---------ATTACTTGTACAAGACCCAGCAACAATACAAGAAAA-----
-GGAATATCTCTA------------GGACAGGGGGAGTA---TTT------TATACAACAGGAGAT------
---------ATAATAGGAAATATA------------AGACAAGCACATTGT---AATGTTAATAGAACA---
AGCTGGCATGACACTTTAAAACAGATAGCTACAAAATTAGG---------------GAAAAATGGCCT----
-----------------------------------AATAAGACAATAATCTTTAAT------
CAATCCTCAGGAGGGGACCCAGAAATTGCA---ACACACAGTTTTAATTGTGGAGGGGAA---
TTCAAGCAGCTCCAGGCAAGAGTCCTGGCTGTGGAAAGATACCTACAGGATCAACGGCTCCTAGGGATTTGGG
GTTGCTCTGGAAAACTCATTTGCACCACTACTGTGCCTTGGAATGCTAGTTGGAGT-----------------
----------------------------AATAAATCTCTGAATGACATTTGG------AAC------
AACATGACCTGGATGCAGTGG---GAGAAAGAAATTGACAATTATACAGGCTTAATATAC---
TCCTTAATTGAAGAATCGCAAATCCAACAAGAA--

AAAAATGAACTAGAATTATTGAAATTGGATCAGTGGGCAAGTCTATGGAATTGGTTTGACATAACACGATGGC
TGTGGTATATAAAAATATTCATAATGATAGTAGGAGGTTTAATAGGCTTAAAAATAGTTTTTGCT---
GTGCTTTCTATAGTA---------AATAGAGTTAGGCAGGATATTCACCA------
TTATCATTTGCTGTTAGTTTGCTTAACGCCACAGCTATAGCAGTAGCTGAA---
GGGACAGATAGGATTATAGAAGCAGTACAAAGAATTGGTAGAGCT----------------------------
--------ATTATCCACATA------CCTAGAAGAATAAGACAGGGCCTAGAAAGAATTTTGGTA---TAA
>KX692916
GCT---GCA--
GAAAAATTGTGGGTCACAGTTTATTATGGAGTACCTGTATGG---AAAGAAGCAACT---
ACCACTCTATTTTGTGCATCAGATGCTAGAGCATATGATACAGAG---GTGCATAAT----------------
--------------ATGTGGCCACA---CATGCCTGTGTACCCACAGAC---
CCCAACCCACAAGAAGTAGTCTTGGAA---------AATGTGACAGAAAACTTTAAC----------------
--------------------------ATGTGGAAAAATGACATGGTAGAACAGATGCATGAAGATATA---
ATCAGTTTATGGGATCAAAGCCTA---AAGCCATGTGTAAGATTA---
ACCCCACTCTGTGTTACTTTAAATTGCACTAATGCGACATGGATTACTAATATTACCAGTAGTCATACTACTA
ATGCTACCAGT--
-------------AGTAATAATAATACCACCAGTAGTAATACCAATAGTACAGGAGAAATAAAA---
AAATGCTCTTTCAACATCACCACAAGCATAGGA---------------------------ACTAAGGTG---
AAAGACTATGCACTT---TTTTATAGACTTGATATAGAACCAATGAATAAT----------------------
----
----GATACTAATACTACCAGCTATAGGTTGATAAATTGTAACACCTCAGTCATTACACAA---
GCTTGTCCAAAGGTATCCTTTGAGCCAATTCCCATACATTATTGTGCCCCGGCTGGTTTTGCGATTCTAAAGT
GTAAC---AATAAAACATTCAGT------GGAACAGGACCATGTACAAAT-----------------------
-GTCAGT---ACAGTGCAATGTACACATGGAATCAGGCCAGTAGTATCAACT---
CAATTGCTGTTAAATGGCAGTTTAGCAGAAAAAGAG------------
GTAGTAATTAGATCTAGCAATTTCACGGACAAT---------GCTAAAATCATAATAGTACAGCTGAAT----
-----------AAATCTATAGAA---------ATTAATTGTACAAGACCCAACAACAATACAAGAAAA-----
-CGTGTAACTCTA------------GGACCAGGGCGAGTA---TGG------TATACAACAGGAGAA------
---------ATAGTAGGAGATATA------------AGACAAGCACATTGT---AACCTTAGTAGAAGA---
GACTGGAATGACACTTTAAGACAGATAACTGAAAAATTAAGA---------------GAACAATTTGGG----
-----------------------------------AACAAAACAATAATCTTTAAT------
CAATCTGCAGGAGGGGACCCAGAGATTGTA---ATGCACAGTTTTAATTGTGGAGGGGAA---
TTTAAGCAGCTCCAGGCAAGAGTCTTGGCTGTGGAAAGATACCTAAAGGATCAACAGCTCCTAGGGATTTGGG
GTTGCTCTGGAAAACTCATTTGCACCACTAATGTGCCTTGGAATGTTAGTTGGAGT-----------------
----------------------------AATAAATCTCTGACTGAGATTTGG------GAT------
AACATGACTTGGATGCAGTGG---GAAAGAGAAATTGACAATTACACAAAAGAAATATAC---
ACCTTAATTGAACAATCGCAGAACCAGCAAGAA--

AAGAATGAACTAGAACTATTGGAATTGGATAAATGGGCAAGTTTGTGGAATTGGTTTGACATATCAAACTGGC
TGTGGTATATCAAAATATTCATAATGATAGTAGGAGGCTTGGTAGGTTTAAGAATAGTTTTTGCT---
GTACTTTCTATAGTG---------AATAGAGTTAGGCAGGATACTCACCA------
GTGTCATTAGCTATTAGCTTGCTTAACGCCACAGCAGTAGCAGTAGCTGAG---

GGGACAGATAGGGTTATAGAAGTAGTACATAGAGCCTATAGGGCT----------------------------
--------ATTCTTAACATA------CCTACAAGAATCAGACAGGGCTTCGAAAGGGCTTTGCTA---TAA
>KX692929
GCT--
GAAAACTTGTGGGTCACAGTCTATTATGGGGTGCCTGTATGG---AAAGATGCAACT---
ACCACTCTATTTTGTGCATCAGATGCTAAAGCATATTCTACAGAA---GTGCATAAT----------------
--------------ATTTGGGCTACA---CATGCCTGTGTACCCACAGAC---
CCCAACCCACAAGAAGTAGTCTTGGGA---------AATGTGACAGAAAACTTTAAC---------------
-------------------------ATGTGGAAGAATGACATGGTAGAACAGATGCATGAAGATGAA---
ATCAGTTTATGGGATCAAAGCCTA---AAGCCATGTGTAAAATTA---
ACCCCACTCTGTGTTACTTTAAATTGCACTGATTATTTGGGGAATACTACTAATTTGGGGAATACTAATAATA
CCAATAATGATAACAGTAGTACTACTACGCCC---------------------------------------
-AGTACGACTACTTGTAATACCAGTGGTAATATCAGTAGGACAACAGAGGAAAGAGAAATAAAA---
AACTGCACTTTCAATATCACCACAAGCATACAA--------------------------ACTAAGGTG---
AAAAACTCTGCACTT---TTTTATAAATATGATGTAGTACAAATAAATAATGAT------------------
----
-ACTTATAATGGTACTGCCAGCTATAGGTTGATACATTGTAACACCTCAGTCATTACACAA---
GCTTGTCCAAAGATATCCTTTGAGCCAATTCCTATACATTATTGTGCCCCGGCTGGTTTTGCGATTGTAAAGT
GTAAC---AATAAAACATTCAAT------GGAACAGGACCATGTACAAAT----------------------
-ATTAGT---ATAGTACAATGTACACATGGAATTAGGCCAGTAGTATCAACT---
CAACTGCTGTTAAATGGCAGTTTAGCAGAAGGAGGG-----------
GTAGCAATTAGATCTAGCAATTTCATGGAAAAT---------ACTAAAAACATAATAGTACATCTGAAT----
-----------GAATCTGTAACA---------ATTAATTGTACAAGACCCAACAACAATACAAGAAAA-----
-AGTATAAATATA------------GGACCAGGGCGGGCA---TGG------TATACAACAGGACAA------
---------ATAATAGGAAGTATA------------AGACAAGCACATTGT---AACCTTACTAGAAAA---
GACTGGAATAACACTTTAGAACGGATAGTTGAAAAATTAAGA---------------GAACAATTTGGG----
-----------------------------------AACAAAACAATAATCTTTAAA---
CAACCTATACCGGGAGGGGACCCAGAGACTACA---ATGCACAGTTTTAATTGTGGAGGGGAA---
TTTAAGCAGCTCCAGGCAAGAGTCTTGGCTGTGGAAAGATACCTAAAGGATCAACAGCTCCTAGGGATTTGGG
GTTGCTCTGGAAAACTCATCTGCCCCACTGCTGTGCCTTGGAATGTTAGTTGGAGT-----------------
----------------------------AATAGATCTCATGATGGGATTTGG------AAG------
AACATGACTTGGATGCAGTGG---GAAAGGGAAATTGACAATTATACAGAAGAAATATAC---
ACCTTAATTGAAAGATCGCAAAACCAACAAGAA--

AAGAATGAACTAGAACTATTGGAATTGGATAAGTGGGCAAGTTTGTGGAATTGGTTTGACATAACACAATGGC
TGTGGTATATCAAAATATTCATAATGATAGTAGGAGGCTTGGTAGGTTTAAGAATAATTTTTGCT---
GTGCTTTCTATAGTA---------AATAGAGTTAGGCAGGGATACTCACCA------
TTGTCATTGGCTATTAGCTTGCTTAACTCAACAGCCATAGTAGTAGCTGAG---
GGGACAGATAGGGTTATAGAAGTAGTACAAAGAGCTTATAGGGCT----------------------------
--------ATTCTGAACATA------CCTAGGAGGATCAGACAGGGCCTCGAAAGGCTTTTGCTA---TAA
>KX692941
GCT---GCA------------------------------------
GAAAAATTGTGGGTCACAGTCTATTATGGGGTACCTGTATGG---AAAGATGCAAAT---
ACCACTCTATTTTGTGCATCAGATGCTAAAGGATATAAGACAGAG---GCACACAAT----------------
--------------GTTTGGGCCACA---CATGCCTGTGTACCCACAGAC---
CCCAACCCACAAGAAGTAGTCTTGGCA---------AATTTGACAGAAAACTTTAAC---------------
-------------------------ATGTGGGAAAGTGACATGGTAGAACAGATGCATGAAGATGTA---
ATCAATTTATGGGATCAAAGCCTA---AAGCCATGTGTAAAATTA---
ACTCCACTCTGTGTTACTTTAAATTGCACTAATTTGAATATCACTAGTAATAATACCAGTAGTAGTAATACCA
GT--
----------------------AGTAGTAATACCAGTAGTATAACAGGGGGAGGAGAAATGAAA---
AACTGCTCTTTCAATATCACCACAAGCATAAAT--------------------------ACTAAGGTA---
AAAGACTATGCATTT---TTTTATAACCTTGATATAGTACAAATAAATAATGATAAGACCAGT---------
----
----------GATAATACCAGCTATAGGTTGATAAATTGTAATACCTCAGTCATTACACAA---
GCTTGTCCAAAGATATCCTTTGAGCCAATTCCCATACATTATTGTACCCCGGCTGGTTTTGCGATTATAAAGT
GTAAC---AATGAAACATTCAAT------GGAACAGGACCATGTACAAAT----------------------
-GTCAGT---ATAGTACAATGCACACATGGAATTAGGCCAGTAGTATCAACT---
CAACTGCTGTTAAATGGCAGTTTAGCAGAAAGAGAT-----------
GTAGCAATTAGATCAAAAAATTTCTCAAACAAT---------GCAGAGAACATAATAGTACAACTGAAT----

-----------GAAGCTGTACAA---------ATTAACTGTACAAGACCCAACAACAATACAAGAAAA-----
-AGTATAAATATA------------GGACCAGGGCGAGCA---TGG------TATACAACAGGAGAA------
---------ATAATAGGAAATATA------------AGACAGGCACATTGC---AACATTAGTGGATCA---
AAATGGAATGACACTTTAAAACAGGTAGTTAAAAAATTAAGA---------------GAACAATTTAAG----
-----------------------------------AACAAAACAATAACCTTTGAG---
CAACCTCCTCCAGGAGGGGACCCAGAGATTGTA---ATGCACAATTTTAATTGTGGAGGGGAA---
TTTAAGCAGCTCCAGGCAAGAGTCCTGGCTGTGGAAAGATACCTAAAGGATCAACAGCTCCTAGGGCTTTGGG
GTTGCTCTGGAAAGCTCATTTGCACCACTAATGTGCCTTGGAGTGGTAATTGGAGT----------------
----------------------------AGTAAATCTTATAATGACATTTGG------TAT------
AACATGACTTGGATGCAGTGG---GAAAAAGAAATTGAAAATTACACAGGAGTAATATAC---
ACATTAATTGAAGAATCACAGAACCAGCAAGAA--

AAGAATGAACAAGAACTATTGGAATTGGATAAGTGGGCAAGCTTGTGGAATTGGTTTGACATATCAAACTGGC
TGTGGTACATCAGAATATTCATAATGATAGTAGGAGGATTGATAGGTTTAAGAATAATTTTTGCT---
GTGCTTTCTATAGTA---------AATAGAGTTAGGCAGGGATACTCACCA------
TTGTCATTAGCTATTAGTTTGCTTAACGCCATAGCCATAGCAGTAGGTGAG---
GGGACAGATAGGGTTATAGAAGTAGCGCAAAGAATTTATAGGGCT---------------------------
--------ATTCTCAATATA------CCTAGAAGAATCCGACAGGGCCTCGAAAGGGCTTTGCTA---TAA
>KX692973
ACT---GCA--
GAAAAATTGTGGGTCACAGTCTATTATGGGGTACCTGTATGG---AAAGAAGCAGCT---
ACCACTCTATTTTGTGCATCAGATGCTAAAGCATATGATACAGAG---GTACATAAT----------------
--------------ATTTGGGCCACA---CATGCCTGTGTACCCACAGAT---
CCCAACCCACAAGAAGTAGTCTTGGGA---------AATGTGACAGAAGACTTTAAC---------------
--------------------------ATGTGGAAAAATAACATGGTAGAACAGATGCATGAAGATATA---
ATCAGTATATGGGATCAAAGCCTA---AAGCCCTGTGTCAAATTA---
ACCCCACTCTGTGTTACTTTAAATTGCACTAATTTGGGAAGTACTACTAATACCAATAGT------------
----
----------------------------AATACCAGTAGTACGACGGAGGGAGGAGAAATAAAA---
AACTGCTCTTTCAATATCACCACAAGCATAAGA---------------------------ACTAAGGTG---
AAAGACTATGCACTT---TTTTCTGACCTTGATGTAGTACCAATAGATAAA---------------------
----
----------AATAAGACCAGCTATAGGTTGATAACTTGTAACACCTCAGTCCTTACACAA---
GCTTGTCCAAAGGTAACCTTTGAGCCAATTCCCATACATTATTGTACCCCGGCTGGTTTTGCGATTCTAAAGT
GTAAC---AGTAAAACGTTCAAT------GGAACAGGACCATGTATAAAT----------------------
-GTAAGT---ACAGTACAATGTACACATGGAATTAGGCCAGTAGTATCAACT---
CAACTGCTGTTAAATGGCAGTTTGGCAGAAGAAGAG-----------
GTCGTAATTAGATCTAGCAATTTCACGGACAAT---------ACTAAAATCATAATAGTACAGCTGAAT----
-----------GAACCTGTAAAA---------ATTAATTGTACAAGACCCAACAACAATACAAGAAAA-----
-AGTATACCTCTA------------GGACTAGGGCGAGCG---TGG------TATGCAACAGGACAA------
---------ATAATAGGAGATATA------------AGACAAGCACATTGT---AACCTTACTAGAACA---
GAATGGAATGACATGTTAAAAAAGATAACTGAAAAATTAAGA---------------GAACAATTTAGG----
-----------------------------------AACAAAACAATAATCTTTAAA------
CAATCTTCAGGAGGGGACCCAGAGATTGAA---ATGCACAATTTTAATTGTGGAGGGGAA---
TTTAAACAGCTCCAGGCAAGAGTCTTGGCTGTGGAAAGATACCTAAAGGATCAACAGCTCCTAGGGATTTGGG
GTTGCTCTGGAAAACTCATTTGCACCACTGCGGTGCCTTGGAATGCTAGTTGGAGT----------------
----------------------------AATAAATCTCTGAGTGAGATTTGG------GAT------
AACATGACTTGGATGGAGTGG---GAAAGAGAAATTGACAATTACACAAAAGAAATATAC---
ACCTTAATCGAAAAATCGCAAAACCAACAGGAA--

AAGAATGAACTAGAACTATTGGAATTGGATAAGTGGGCAAGTTTGTGGAATTGGTTTGACATAACAAAATGGC
TGTGGTATATCAAAATATTTATAATGATAGTAGGAGGCTTGGTAGGTTTAAGAATAGTTTTTGCT---
GTGCTTTCTATAGTA---------AATAGAGTTAGGCAGGGATACTCACCA------
TTGTCATTAGCTATTAGCTTGCTTAACGCTACAGCCATAGCAGTAGCTGAG---
GGGACAGATAGGGTTATAGAAGTAGTACAAAGAGCCTATAGGGCT---------------------------
--------ATTCTTAACATA------CCTAGAAGAATCAGACAGGGCTTCGAAAGGGCCTTGCTA---TAA
>KX692981
GCT---GTA--
GACAACTTGTGGGTCACAGTCTATTATGGGGTACCTGTATGG---AAAGAAGCAACT---
ACCACTCTATTCTGTGCATCAGATGCTAAAGCATATGATACAGAG---GTACATAAT----------------

--------------GTTTGGGCCACA---CATGCCTGTGTACCCACAGAC---
CCCAACCCACAAGAAGTATTCTTGGAA---------AAGGTGACAGAAACCTTTAAC---------------
-------------------------ATGTGGAAAAATAACATGGTAGAACAGATGCATGAAGATATA---
ATCAGTTTATGGGATCAAAGCCTA---AAGCCATGTGTAAAATTA---
ACCCCACTCTGTGTTACTTTAAATTGCACTAATTGGAAGAATCCTAATGATACCAGTAGT------------
----
---------------------------AATACCAGTAGTACGATAAAGGGGGGAGAAGTAAAA---
AATTGCTCTTTCGATATCACCACAAGCACAGGA--------------------------ACTAAGGTG---
AGAGACCATGCACTT---TTTTATGACCTTGATATGTTACCAACAAATCAG---------------------
----
----------ACTAATACCAGCTATATGTTGATACATTGTAACACCTCAGTCATTACACAA---
GCTTGTCCAAAGGTATCCTTTGAGCCAATTCCCATACATTATTGTGCCCCGGCTGGTTTTGCGATTCTAAAGT
GTAAC---AATGAAACGTTCAAG------GGAACAGGACCATGTACAAAT----------------------
-GTCAGT---ACAGTACAATGTACACATGGAATTAGGCCAGTAGTATCAACT---
CAACTGCTGTTAAATGGCAGTTTAGCAGAAGGAGAA-----------
GTAGTAATTAGATCTAGCAATTTCACGGACAAT---------GCTAAAGTCATAATAGTACAGCTGAAT----
-----------AAATCTGTAGAA---------ATTAATTGTACAAGACCCAACAACAATACACAAAAA-----
-AGGATATCTCTA------------GGACCAGGGCGAGCA---TGG------CATACAACACAACAA------
---------ATAATAGGAGATATA------------AGACAAGCACATTGT---AACCTTAGTATAACA---
CAGTGGAATAACACTTTAAGACAGATAGCTGAAAAATTAAGA---------------GAACAATTTGAG----
-----------------------------------AACAAAACAATAATCTTTAAG------
CAATCTTCAGGAGGGGACCCAGAGATTGTA---ATGCACAGTGTTAATTGTGGAGGGGAA---
TTTAAGCAGCTCCAGGCAAGAGTCTTGGCTGTGGAAAGATACCTAAAGGATCAACAGCTCCTAGGGATTTGGG
GTTGCTCTGGAAAACTCATTTGCACCACTGCTGTGCCTTGGAATGTTAGTTGGAGT----------------
-------------------------AATAATAAATCTGTGGATTACATTTGG------GGT------
AACATGACTTGGATGGAGTGG---GAAAGAGAAATTGACAATTACACAAGTGAAATATAC---
ACTTTAATTGAAGAATCGCAGAACCAACAAGAA---------------------------------------
----
AAGAATGAACTAGAACTATTGGAATTGGATAAGTGGGCAAGTTTGTGGAATTGGTTTGACATAACAAAATGGC
TGTGGTATATCAAAATATTCATAATGATAGTAGGAGGCTTGGTAGGTTTAAGAATAGTTTTTGCT---
GTACTTTCTATAGTA---------AATAGAGTTAGGCAGGGATACTCACCA------
TTATCGTTAGCTGTTAGCTTGCTTAACGCCACAGCCATAGCAGTAGCTGAG---
GGGACAGATAGGGTCATAGAAATAGTACAAAGAGCTTATAGAGCT---------------------------
--------ATTCTTAACATA------CCTACAAGAATCAGACAGGGCCTCGAAAGGGCTTTGCTA---TAA
>KX693001
GCT---GCA---
GAAAACTTGTGGGTCACAGTCTATTATGGGGTACCTGTATGG---AAAGAAGCAACT---
ACCACTCTATTTTGTGCATCAGATGCTAAAGCATATGATACAGAG---GCACATAAT----------------
--------------GTTTGGGCCACA---CATGCCTGTGTACCCACAGAC---
CCCAACCCACAAGAAATAGTCTTGGAA---------AATGTGACAGAAAACTTTAAC---------------
-------------------------ATGTGGAAAAATGACATGGTAGAACAGATGCATGAAGATATA---
ATCAGTTTATGGGATCAAAGCCTA---AAGCCATGTGTGAAATTA---
ACCCCACTCTGTGTTACTTTAAATTGCACTAATTTGAGTATTAATAATACCAGTAATAATACCAGTAATCAT-
----
-------------------ACCAGTAGTAATACCAGTAGTACGACAGAGGGAGGAGAAATGAAA---
AACTGCTCTTTCAATGTCACCACAAGCATAAGA--------------------------ACTAAGGTG---
AATGACTATGCACTT---TTTTATAGAATTGATATAGAACAAATAAATGGT---------------------
----
-------AGTAATAATAACAGTTATAGGTTGATAAATTGTAACACCTCAGTCATTACACAA---
GCTTGTCCAAAGATATCCTTTGAGCCAATTCCCATACATTATTGTACCCCGGCTGGTTTTGCGATTCTAAAGT
GTAAC---AATAAAACATTCAAT------GGAACAGGACCATGTACAAAT----------------------
-GTCAGC---ACAGTACAATGTACACATGGAATTAGGCCAGTAGTGTCAACT---
CAACTGCTGTTAAATGGCAGTTTAGCAGAAGAAGAG-----------
GTAGTAATTAGATCTAGCAATTTCACGGACAAT---------GCTAAAGTCATAATAGTACAGCTGAAT----
-----------AAATCTGTAGAA---------ATTAATTGTACAAGACCCAACAACAATACAAGAAAA-----
-AGTATAAGTCTA------------GGACCAGGGCAAGCA---TGG------TATACAACAGGAGAG------
---------ATAATAGGAAATATA------------AGACAAGCACATTGT---AACATTAGTAGAACA---
AAATGGAATAACACTTTAGAACAGATAAGTAAAAAATTAAGA---------------GAGCAATTTGGG----
-----------------------------------AATAAAACAATAACCTTTAAG------
CAATCTGCAGGAGGGGACTCAGAGATTGTA---ATGCACAGTTTTAATTGTGGAGGGGAA---

TTTAAGCAGCTCCAGGCAAGAGTCTTGGCTGTGGAAAGATACCTAAAGGATCAACAGCTCCTAGGGATTTGGG
GTTGCTCTGGAAAACTCATTTGCACCACTGCTGTGCCTTGGAATGTTAGTTGGAGT-----------------
----------------------------AATAGATCTCTGAATGAGATTTGG------AAT------
AACATGACTTGGATGGAGTGG---GAAAGAGAAATTGCCAATTACACAAAAGAAATATAC---
ACCTTAATTGAACAATCGCAGAACCAGCAAGAA--

AAGAATGAACTAGAACTATTGGAATTGGATAAGTGGGCAAGCTTGTGGAATTGGTTTGACATAACAAAATGGC
TGTGGTATATCAAAATATTCATAATGGTAGTAGGAGGCTTGGTAGGTTTAAGAATAATTTTTGCT---
GTACTTTCTGTAGTA---------AATAGAGTTAGGCAGGGATACTCACCA------
TTGTCATTTGCTATTAGCTTGTTTAACGCCATAGCCATAGCAGTAGCTGAG---
GGGACAGATAGGATAATAGAAGTAGCACAAAGAACTTATAGGGCT----------------------------
--------ATTCTCAACATA------CCTACAAGAATCAGACAGGGCCTCGAAAGGGCTTTGGTA---TAA
>KX693029
GCT---GCA--
GAAAAATTGTGGGTCACAGTCTATTATGGGGTACCTGTATGG---AAAGACGCAAAT---
ACTACTCTATTTTGTGCATCAGATGCTAAAGCATATGCTACAGAG---GTACACAAT----------------
--------------GTTTGGGCCACA---CATGCCTGTGTACCCACAGAC---
CCCAACCCACAAGAAGTAGTCTTGGGA---------AATGTAACAGAAAACTTTGAC----------------
--------------------------ATGTGGAAAAATGACATGGTAGAACAGATGCATGAAGATATA---
ATCAGTTTATGGGATCAAAGCCTA---AAGCCATGTGTAAAGTTA---
ACCCCACTCTGTGTTACTTTAAATTGCACTAATGTGAGGCCTTTAAATTGCACTGATGGGAAGCCTCCAAATT
GCACTGCT---
-------GTGGAGCCTAGTACCAGTGGTAATAACAGTAGTGCGACAGGGGGAGGAGAAATGAAA---
AACTGCTCTTTCAATGTCACCACAAGCATAAGA---------------------------------
ACTAAGAAAACCTATGCAACC---TTTTATAACCTTGATATAGTAAAAATAAATGATGATAAT----------
----
----------GATAGTACCAGTACCAGCTATAGGTTGATAAACTGTAACACCTCAGTCATTACACAA---
GCTTGTCCAAAGGTATCCTTTGAGCCAATTCCCATACATTATTGTACCCCGGCTGGTTTTGCGATTCTAAAGT
GTAAC---AATAAAACGTTCAAT------GGAAAAGGACCATGTACAAAT-----------------------
-GTCAGT---ACAGTACAATGTACACATGGAATTAGGCCAGTAGTATCAACT---
CAACTGCTATTAAATGGCAGTTTAGCAGAAGGAGAG------------
GTAGTAATTAGATCTAGCAATTTCACAGACAAT---------GCTAAAATCATAATAGTACAGCTGAAT----
-----------GAACCTGTAGTA---------ATTAATTGTACAAGACCCAACAACAATACAAGAAAA-----
-AGTATACCTCTA------------GGACCAGGGCAAGCA---TGG------TATACAACAGGAGAC------
---------ATAATAGGAAATATA------------AGACAAGCACATTGT---AACCTTAGTAGAACA---
GAATGGAATAATACTTTAAGACAGATAACTAAAAAATTAAGA---------------GAACACATTGGG----
-----------------------------------AACAAAACAATAACCTTTAAT------
CAATCTGCAGGAGGGGACCCAGAGATTGTA---ATGCACAGTTTTAATTGTGGAGGGGAA---
TTTAAGCAGCTCCAGGCAAGAGTTTTGGCTGTGGAAAGATACCTAAAGGATCAACAGCTCCTAGGGATTTGGG
GTTGCTCTGGAAAACTCATTTGCACCACTGCTGTGCCTTGGAATGCTAGTTGGAGT-----------------
----------------------------AATAAATCTCTGAGTCAGATTTGG------GAT------
AACATGACTTGGATGGAGTGG---GAAAGAGAAATTAACAATTACACAAAAGAAATATAC---
ACCTTAATTGAACAATCGCAGAACCAACAAGAA--

AAGAATGAACTAGAACTATTGGAATTGGATAAGTGGGCAAGCTTGTGGAATTGGTTTGACATAACAAACTGGC
TGTGGTATATCAGAATATTCATAATGATAGTAGGAGGCTTGGTAGGTTTAAGAATAGTTTTTGCT---
GTACTTTCTATAGTA---------AATAGAGTTAGGCAGGGATACTCACCA------
TTGTCGTTGGCTGTTAGCTTGTTTAACGCCACAGCCATAGCAGTAGCTGAG---
GGGACAGACAGGGTTATAGAAGTAGTACAAAGAGCTTTTAGAGCT----------------------------
--------GTTCTTAACATA------CCTAGAAGAATTAGACAGGGTCTTGAAAGGTCTTTGCTA---TAA
>KX693036
GCT---GCA--
GAAAACTTGTGGGTCACAGTCTATTATGGGGTACCTGTATGG---AAAGAAGCAAAT---
ACCACTCTATTTTGTGCATCAGATGCTAAAGCATATGAGACAGAG---GTACATAAT----------------
--------------GTTTGGGCCACA---CATGCCTGTGTACCCACAGAC---
CCCAACCCACAAGAAGTAGTCTTGGGA---------AATGTGACAGAAAACTTTAAC----------------
--------------------------ATGTGGAAAAATGACATGGTAGAACAGATGCATGAAGATATA---
ATCAGTTTATGGGATCAAAGCCTA---AAGCCATGTGTAAAATTA---
ACCCCACTCTGTGTTACTTTAAATTGCACTAATGCTAATATTACCAGTAGTAATACCAGT-------------
----

-------------------------------------AGTACGACAGAGGGAGGAGAAATAAAA---
AACTGCTCTTTCAATATCACCACAAGCACAAGT---------------------------GCTAAGGTA---
AGAGACTATGCACTT---TTTAATAGACTTGATGTAGCACCAATAAATAAT----------------------
----
----GAAACTAATACTACCAGCTATAGGTTGATACATTGTAACACCTCAATCATTACACAA---
GCTTGTCCAAAGGTATCCTTTGAGCCAATTCCCATACATTATTGTACCCCGGCTGGTTTTGCGCTTCTAAAGT
GTAAC---AATAAAACGTTCAGT------GGAACAGGACCATGTACAAAT-----------------------
-GTCAGT---ACAGTACAATGTACACATGGAATTAGGCCAGTAGTATCAACT---
CAACTGCTATTAAATGGCAGTTTAGCAGAAGGAGAG------------
GTAGTAATTAGATCTAGCAATTTCTCAAACAAT---------GCTAAAGTCATAATAGTACAGCTGAAT----
-----------GAAGCTGTAGCA---------ATTAATTGTACAAGACCCAGCAACAATACAAGAAAA-----
-AGTATATCTCTA------------GGACCAGGGCGAGCA---TGG------TTTGCAAGAGGACAA------
---------ATAATAGGAGACATA------------AGACAAGCACATTGT---AACCTTAGTTTCACA---
AAATGGAATAACACTTTAAATGGGATAACTAAAAAATTAAGA---------------GAGCAATTTGGG----
-----------------------------------AGCGAAACAATAATCTTTGAT------
CGATCTTCAGGAGGGGACCCAGAGATTGAA---ATGCACAGTTTTAATTGTGGAGGGGAA---
TTTAAGCAGCTCCAGGCAAGAGTCTTGGCTGTGGAAAGATACCTAAAGGATCAACAGCTCCTAGGGATTTGGG
GTTGCTCTGGAAAACTCATTTGCACCACTGCTGTGCCTTGGAATGCTAGTTGGAGT-----------------
----------------------------AATAGATCTCTGGATGTGATTTGG------AAT------
AACATGACTTGGATGGAGTGG---GAAAGAGAAATTGACAATTACACAAGAGAAATATAT---
ACCTTAATTGAAGAATCGCAAAACCAACAAGAA--

AAGAATGAATTAGAACTATTGGAATTGGATAAGTGGGCAAGTTTGTGGAATTGGTTTGACATATCAAACTGGC
TGTGGTATATCAGAATATTCATAATGATAGTAGGAGGCTTGGTAGGTTTAAGAATAGTTTTTGCT---
GTATTTTCTATAGTA---------AATAGAGTTAGGCAGGGATACTCACCA------
TTGTCGTTAGCTATTAGCTTGCTTAACGCCACAGCCATAGCAGTAGCTGAG---
GGGACAGATAGGGTTATAGAAGTAGCACAGAGAACTGGTAGAGCA----------------------------
--------GTTCTCCACATA------CCTAGAAGAATCAGACAGGGCCTCGAAAGGGCTTTGCTA---TAA
>KX693575
GCT---GCA--
GAAAACTTGTGGGTCACAGTCTATTATGGGGTACCTGTATGG---AAAGAAGCAACT---
ACCACTCTATTTTGTGCATCAGATGCTAAAGCATATGATACAGAA---GTACATAAT----------------
--------------GTTTGGCCACA---CATGCCTGTGTACCCACAGAC---
CCCAACCCACAAGAAGTGGTCTTGGAA---------
AATGTGACAGAAAACTTTAACATGTGGAAAAATAACATGGTAGAACAGACAGAAAACTTTAACATGTGGAAAA
ATAACATGGTAGAACAGATGCATGAAGATATA---ATCAGTTTATGGATCAAAGCCTA---
AAACCATGTGTAAAATTA---
ACCCCACTCTGTGTTACTTTAAATTGCACTAATTTGGGGAATACTACTAATACCAGTAGTAATACTACTAATA
CCAGAAGTAAT--
----------AATACCAGTAGTAATACCAGTAGTGCGGTAGAGGGAGGAGAAGGAGAAATGAAA---
AACTGCTCTTTCAATATCACCACAAGCACAAGT---------------------------ACTAAGGTA---
AAAGACTATGCACTT---TTTTATAAACTTGATGTAGTACAAATAAAAGGT----------------------
----
----------GATAATACCAGCTATAGGTTGATAAATTGTAACACCTCAACCATTACACAA---
ACTTGTCCAAAGGTATCCTTTGAGCCAATTCCTATACATTATTGTACCCCGGCTGGTTTTGCGATTCTAAAGT
GTAAC---AATAAAAAGTTCAAT------GGAACAGGACCATGTACAAAT-----------------------
-GTCAGT---ACAGTACAATGTACACATGGAATTAGGCCAGTAGTATCAACT---
CAACTGCTGTTAAATGGCAGTTTAGCAGAAGAAGAG------------
GTAGTGATTAGATCTAGCAATTTCTCGGACAAT---------GCTAAAGTCATAATAGTACAGCTGAAT----
-----------AAATCTGTAGAA---------ATTAATTGTACAAGACCCAACAACAATACAAGAAAA-----
-AGTATATCTCTA------------GGACCAGGGCAAGCA---TGG------TTTGCAACAGGACAA------
---------ATAATAGGAGATATA------------AGACAAGCACATTGT---AACATTAGTAGAGCA---
AAATGGAATGACACTTTAAGACAGATATATATAAAATTAAGA---------------GAACAATTTGGG----
-----------------------------------AACAAAACAATAATCTTTAAT------
CGATCTTCAGGAGGGGACCTAGAGATTGTA---ATGCACAGTTTTAATTGTGGAGGGGAA---
TTTAAGCAGCTCCAGGCAAGAGTCTTGGCTGTGGAAAGATACCTAAAGGATCAACAGCTCCTAGGGATCTGGG
GTTGCTCTGGAAAACTCATTTGCACCACTGCTGTGCCTTGGAATGCTAGTTGGAGT-----------------
----------------------------AATAAATCTCTGAGTCAGATTTGG------GAT------
AACATGACTTGGATGGAGTGG---GAAAGAGAAATTGACAATTACACAAAAGAAATATAC---
TCCCTAATTGAAGAATCGCAGAATCAACAAGAA--

AAGAATGAACTAGAACTATTGGAATTGGATAAGTGGGCAAGTTTGTGGAATTGGTTTGACATAACAAAATGGC
TGTGGTATATCAAAATATTCATAATGGTAGTAGGAGGCTTAATAGTTTTAAAAATAGTTTTTACT---
GTACTTTCTATAGTA---------AATAGAGTTAGGCAGGGATACTCACCA------
TTGTCATTAGCTATTAGCTTGCTTGACGCCACAGCTATAGCAGTAGCTGGG---
GGGACAGATAGGGTTATAGAAGTAGTACAAAGAGCTTTTAGGGCT----------------------------
--------ATTCTCAATATA------CCTAGAAGAATCAGACAGGGCCTCGAAAGAGCTTTGCTA---TAA
>KX693068
GCT---GCA--
GAAGACTTGTGGGTAACAGTCTATTATGGGGTACCTGTATGG---AAAGAAGCAACT---
ACCACTCTATTTTGTGCATCAGATGCTAAAGCATATGATACAGAA---GTACATAAT----------------
--------------GTTTGGGCCACA---CATGCCTGTGTACCCACAGAC---
CCCAACCCACAAGAAGTAGTCTTGGGA---------AATGTGACAGAAAACTTTAAC----------------
--------------------------ATGTGGAAAAATGACATGGTAGAACAGATGCATGAAGATATA---
ATCAGTTTATGGGATCAAAGCCTA---AAGCCATGTGTAAAATTA---
ACCCCACTCTGTGTTACTTTAAATTGCTCTAATTTGAGGAATACTACTAATACCAGTGGT-------------
----
----------------------------AATACCAGTAGTATGACAGAGGGAGGAGAAATAAAA---
AACTGCACTTTCAATATCACCAAAGACATAAAA---------------------------GAG---GTA---
CAAGACTATGCATTT---TTTTATACATCTGATATAGTACCAATAGATGAT----------------------
----
----GATAATACCAATACCAGCTATAGGTTGACAAAGTGTAACACCTCAGTCATTACACAA---
GCTTGTCCAAAGGTATCCTTTGAGCCAATTCCCATACATTATTGTGCCCCGGCTGGTTTTGCGATTCTAAAGT
GTAAC---AATAAAACATTTAAT------GGAACAGGACCATGTACAAAT-----------------------
-GTCAGT---ACAGTACAATGTACACATGGAATTAGACCAGTAGTATCAACT---
CAACTGCTGTTAAATGGCAGTTTAGCAGAAGAAGAG------------
GTAGTAATTAGATCTAGCAATTTCACGAACAAT---------GCTAGAGTCATAATAGTACAGCTGAAT----
-----------GAATCTGTAGTA---------ATTAATTGTACAAGACCCAACAACAATACAAGAAAA-----
-AGTATACAGCTA------------GGACAAGGGCGAGCA---TGG------TATACAACAGGAGAA------
---------ATAATAGGAGATATA------------AGACAAGCACATTGC---AACATTAGTAAAGAA---
AATTGGACTAACACTTTAAAACAGATAACAAAAAAATTAAGA---------------GAACAATTTGGG----
-----------------------------------CACAAAACAATAATCTTTAAT------
CGATCTTCAGGAGGGGACCCAGAGATTGAA---ATGCACAGTTTTAATTGTAGAGGGGAA---
TTTAAGCAGCTCCAGGCAAGAGTCTTGGCTGTGGAAAGATACCTAAAGGATCAACAGCTCCTAGGGCTTTGGG
GTTGCTCTGGAAAACTCATTTGCACCACTACTGTGCCTTGGAATGTTAGTTGGAGT-----------------
----------------------------AATAAATCTCTGGGTGAAATTTGG------AAT------
AACATGACTTGGATGGAGTGG---GAAAGAGAAATTGAAAATTACACAAAAGTAATATAC---
ACCTTAATTGAAGACTCGCAAAACCAACAAGAA--

AAGAATGAACTAGACCTATTGGAATTGGATAAGTGGGCAAGTTTGTGGAATTGGTTTGACATAACAAAATGGC
TGTGGTACATCAAAATATTCATAATGATAGTAGGAGGCTTGGTAGGTTTAAGAATAGTTTTTGCT---
GTACTTTCTATAGTG---------AATAGAGTTAGGCAGGGATACTCACCA------
TTGTCATTCGCTATTAGCTTGCTTAACGCCACAGCCATAGCAGTAGCTGAG---
GGGACAGATAGGGTTATAGAAGTACTACAAAGAGCTTGTAGGGCT----------------------------
--------ATTCTCAACATA------CCTACAAGAATCAGACAGGGCCTCGAAAGAGCTTTGCTA---TAA
>KX693091
GCT---GCA--
GAAGACTTGTGGGTCACAGTCTATTATGGGGTACCTGTATGG---AAAGATGCAACT---
ACCACTCTATTTTGTGCATCAGATGCTAAAGCATATGATACAGAG---GTGCATAAT----------------
--------------ATTTGGGCCACA---CATGCCTGTGTACCCACAGAC---
CCCAACCCACAAGAAGTAGTCTTGGAA---------AATGTGACAGAAAACTTTAAC----------------
--------------------------ATGTGGAAAAATGACATGGTAGAACAGATGCATGAAGATATA---
ATCAGTTTATGGGATCAAAGCCTA---AAGCCATGTGTAAAATTA---
ACCCCACTCTGTGTTACTTTAAATTGCACTAATTTGAATGTTACTAATTTGAATACTAATAATACCAGTAATA
ATACCAGTAGA--
----------------AATACCAGTAGTACTATCAGTAGCCCGACAGAGGGAGGAGAAATAAAA---
AACTGCTCTTTCAATATCACCACGAGCATAAGA---------------------------ACTACGGTG---
AAAGGCTATGCATTC---TTTTATAAACTTGATGTAGTACCAATAAAAAATGAGAAG----------------
----
----------GATAATACCAGCTATAGGTTGATAAATTGTAACACCTCAATCATTACACAA---

GCTTGTCCAAAGGTATCCTTTGAGCCAATTCCCATACATTATTGTGCCCCGGCTGGTTTTGCGATTCTAAAGT
GTAAC---AATAAAACATTCAGT------GGAAAAGGACCATGTACAAAT-----------------------
-GTCAGT---ACAGTACAATGTACACATGGAATTAGGCCAGTAGTGTCAACT---
CAACTGCTGTTGAATGGCAGTTTAGCAGAAAAAGAG------------
GTAGTAATTAGATCTAGCAATTTCTCGGACAAT---------GTTAAAGTCATAATAGTACAGCTGAAT----
-----------GAATCTGTAGAA---------ATTAATTGTACCAGGCCCAACAACAATACAAGAAAA-----
-AGGGTAACTCTA------------GGACCAGGGCGAGTA---TGG------TATACAACAGGACAA------
---------ATAATAGGAGATATA------------AGACGAGCACATTGT---AACCTTAGTAGAAAA---
GCATGGAATAAAACTTTAGAACAGGTAACTAAAAAATTAAGA---------------GAACAATTTGGG----
-----------------------------------AACAGAACAATAATCTTTAAG------
CAACCTCCAGGAGGGGACCCAGAGATTGTA---ATGCACAGTTTTAATTGTGGAGGGGAA---
TTTAAGCAGCTCCAGGCAAGAGTCTTGGCTGTGGAAAGATACCTAAAGGATCAACAGCTCCTAGGGATTTGGG
GTTGCTCTGGAAAACTCATTTGCACCACTGCTGTGCCTTGGAATGTTAGTTGGAGT-----------------
----------------------------AATAAATCTATGGAGGAGATTTGG------GAT------
AAAATGACCTGGATGGAGTGG---GAAAAAGAAATTGACAATTACACAAAAGAAATATAC---
GCCTTAATTGAAGAATCGCAGAACCAACAAGAA--

AAGAATGAACTAGACCTATTGGAATTGGATAAGTGGGCAAGTTTGTGGAACTGGTTTAGCATATCAAATTGGC
TGTGGTATATCAAAATATTCATAATGATAGTAGGAGGCTTGGTAGGTTTAAGAATAGTTTTTGCT---
GTACTTTCTATAGTA---------AATAGAGTTAGGCAGGATACTCACCA------
TTGTCATTAGCTATTAGCTTGCTTAACGCCACAGCCATAGCAGTAGCTGAG---
TGGACAGATAGGATTATAGAAGTAGCACAAAGAGCTTGTAGGGCT----------------------------
--------ATTCTCAACATA------CCTAGAAGAATCAGACAGGGCCTCGAAAGGGCTTTGCTA---TAA
>KX693093
GCT---ACA--
GAAAAATTGTGGGTCACAGTCTATTATGGGGTACCTGTATGG---AAAGAAGCAACT---
ACCACTCTATTTTGTGCATCAGATGCTAAAGCATATAGTACAGAG---GTACATAAT----------------
--------------ATTTGGGCCACA---CATGCCTGTGTACCCACAGAC---
CCCAACCCACAAGAAGTAGTCTTGAAA---------AATGTGACAGAAAACTTTAAC----------------
--------------------------ATGTGGAAAAATAACATGGTAGAACAGATGCATGAGGATATA---
ATCAGTTTATGGGATCAAAGTCTA---AAGCCATGTGTAAAATTA---
ACTCCACTCTGTGTTACTTTAGATTGCAATAATGTTACTAATACCAGTAATAATACCAGAAGTGAGAACAATA
CTACTACTACC--
----------------------AATAGTACTATCAGTAGTACAACAGAGGGAGGAAAAATAAAA---
AACTGCTCTTTCAATATCACCACAAGCATAAGA---------------------------ACTACAGTG---
AAAGATTATGCACTT---TTTTATAAATATGATATAGCATCAATAGATAAT----------------------
----
----AAGACCAATAGTACCAGCTATAGGTTGATAAATTGTAACACCTCAGTCATTACACAA---
GCTTGTCCAAAGGTATCCTTTGAGCCAATTCCCATACATTATTGTGCCCCGGCTGGTTTTGCGATTCTAAAGT
GTAAC---AATAAAACATTCAAT------GGAACAGGACCATGTAATGAT-----------------------
-GTCAGT---ACAGTACAATGTACACATGGAATTAGGCCAGTAGTATCAACT---
CAACTGCTGTTAAATGGCAGTTTAGCAGAAAAAGAG------------
GTAATAATTAGATCTAGCAATTTCACAGACAAT---------ACTAAAATTATAATAGTACAGCTGAAT----
-----------AAATCTGTAGAA---------ATTAATTGTACAAGACCCAACAACAATACAAGAAAA-----
-AGTGTAACTCTA------------GGACCAGGGCGAGTG---TGG------TATACAACAGGACAG------
---------ATAGTAGGAGATATA------------AGACGAGCATATTGT---AACATCAGTACAGCA---
AAATGGAATAACACTTTAGTACAGATAACTGAAAAACTAAAA---------------GAACAATATGGG----
--------------------------------AAAGACAAAAAAATAATCTTTAAG---
CAACCAATTCCAGGAGGGGACTCAGAGATTGTA---ATGCACAGTTTTAATTGTGGAGGGGAG---
TTTAAGCAGCTCCAGGCGAGAGTCTTGGCTGTAAAAAGATACCTAAAGGATCAACAGCTCCTAGGGATTTGGG
GTTGCTCTGGAAAACTCATTTGCACCACTACTGTGCCTTGGAATGCTAGTTGGAGT-----------------
----------------------------AATAAATCTCTGGAAAAAATTTGG------GAT------
AATATGACTTGGATGCAGTGG---GAAAGAGAAATTGAAAATTACACAAAAGAAATATAC---
AACTTAATTGAACAATCGCAGAACCAGCAAGAA--

AAGAATGAACTAGATCTATTGGAATTGGATAAGTGGGCAAGTTTGTGGAATTGGTTTGACATAACAAACTGGC
TGTGGTATATCAGAATATTCATAATGATAGTAGGAGGCTTGGTAGGTTTAAGAATAGTTTTTGCT---
GTACTTTCCATAGTA---------AATAGAGTTAGGCAGGATACTCACCA------
TTGTCATTTGCTATTAGCTTGTTTAACGCCACAGCCATAGCAGTAGCTAAG---

GGGACTGATAGGGTTATAGAAATAGCGCGAACAGCTTTTAGGGCT----------------------------
--------TTTATCCACATA------CCTAGAAGAATCAGACAGGGCTTAGAAAGGTCTTTGCTA---TAA
>KX693114
GCT---GCA--
GAAAAATTGTGGGTCACAGTCTATTATGGGGTACCTGTATGG---AAAGACGCAAAT---
ACCACTCTATTTTGTGCATCAGATGCTAAAGCATATGCTACAGAG---GTACATAAT----------------
--------------GTTTGGGCCACA---CATGCCTGTGTACCCACAGAC---
CCCAACCCACAAGAAGTAGTCTTGGGA---------AATGTGACAGAAAACTTTAAC----------------
--------------------------ATGTGGAAAAATGACATGGTAGACCAGATGCAAGAAGATATA---
ATCAGTTTATGGGATCAAAGCCTA---AAGCCATGTGTAAAATTA---
ACCCCAATCTGTGTTACTTTAAATTGCACTGACTTGAAGAATACTAATACCAGT-------------------
----
-------------------------AGTAATACCAGTAGTACGGTAGTGGGAGGAGAAATGAAA---
AACTGCTCTTTCAATATCACCACACACATGAAA---------------------------ACT---
AATGAGAAAAACCATGCACTT---TTTTATGCACTTGATGTAGTACCAATAGATGAT----------------
----
----------------AGTAATACCAGCTTTAGGTTGACACATTGTAACACCTCAGTCATTACACAA---
GCTTGTCCAAAGATATCCTTTGAGCCAATTCCCATACATTATTGTACCCCGGCTGGTTTTGCGATTATAAAGT
GTAAC---AATAAAACGTTCAAT------GGAACAGGACCATGTACAAAT-----------------------
-GTTAGT---ACAGTACAATGTACACATGGAATTAGGCCAGTAGTATCAACT---
CAACTGCTGTTAAATGGCAGTTTAGCAGAAGAAGAG------------
GTAGTAATTAGATCTAGCAATTTCACAGACAAT---------GCTAAAATCATAATAGTACAGCTGAAC----
-----------GAATCTGTAGCA---------ATTAATTGTACAAGACCCAACAACAATACAAGAAAA-----
-AGTATAAATATA------------GGACCAGGGCGAGCA---TGG------TATACAACAGGAGAA------
---------ATAATAGGAAATATA------------AGACAAGCACATTGT---AACATTAGTTTCTCA---
AAATGGAATAACACTTTAAAACTGATAACTGAAAAATTAAGA---------------AAACAATTTGGG----
-----------------------------------AGCAACACAATAGTCTTTAAT------
CAATCTTCAGGAGGGGACCCAGAGATTACA---ATGCACAATTTTAATTGTGGAGGGGAA---
TTCAAGCAGCTCCAGGCAAGAGTCTTGGCTGTGGAAAGATACCTAAAGGATCAACAGCTCCTAGGGATTTGGG
GTTGCTCTGGAAAACTCATTTGCACCACTGCTGTGCCTTGGAATACTAGTTGGAGT-----------------
----------------------------AATAAAACTCATAGTGAGATTTGG------GAT------
AACATGACTTGGATGGAGTGG---GAAAAAGAAATTGCTAATTACACAAAAGAAATATAC---
ACCTTAATTGAAAAATCGCAAAACCAACAAGAA--

AAGAATGAACTAGAACTATTGGAATTGGATAAGTGGGCAAGTTTGTGGAGCTGGTTTGACATATCAAACTGGC
TGTGGTATATCAGAATATTCATAATGATAGTAGGAGGCTTGGTAGGCTTAAGAATAATTTTTGCT---
GTACTTTCTATAGTA---------AATAGAGTTAGGCAGGATACTCACCA------
TTGTCATTGGCTATTAGCTTGCTTAACGCCACAGCCGTAGCAGTAGCTGAG---
GGGACAGATAGGATTATAGAAGTAGCACAAAGAACTTATAGGGCT----------------------------
--------ATTCGCAACGTA------CCTACAAGAATCAGACAGGGCCTAGAAAGAGCTTTGCTA---TAA
>KX693689
GCG---GCA--
GAACAGTTGTGGGTCACAGTCTATTATGGGGTACCTGTATGG---GAAGATGCAACT---
ACCACTCTATTTTGTGCATCAGATGCTAAAGCATATGCTACAGAG---GTACATAAT----------------
--------------GTGTGGGCCACA---CATGCCTGTGTACCCACAGAC---
CCTAACCCACAAGAAGTAGTCTTGGGA---------AATGTGACAGAAAACTTTAAC----------------
--------------------------ATGTGGAAAAATAACATGGTAGACCAGATGCATGAAGATATA---
ATCAGTTTATGGGATCAAAGTCTA---AAGCCATGTGTAAAATTA---
ACCCCACTCTGTGTTACTTTAAATTGCACTGACTTGAGTAATACTACTACCAAT-------------------
----
-------------------------AGTAATATCAGTAGTACATCAGAGGGAGGAGAAATGAAA---
AACTGCTCTTTCAATGTCACCACGGATATA---------------------------------
AAACCTAAGAAAGACTATGCACTT---TTTTATAAACTTGATGTAGAAGCAATAGATGGG-------------
----
-------------------AGTAATACCAGCTATAGGTTGACACATTGTAACACCTCAATCATTACACAA---
GCTTGTCCAAAGGTATCCTTTGATCCAATTCCCATTCATTATTGTACCCCAGCTGGTTATGCGATTCTAAAGT
GTAAT---AATAAAACGTTCAAT------GGAACAGGACCATGTACAAAT-----------------------
-GTCAGT---ACAGTACAATGTACACATGGAATTAGGCCAGTAGTGTCAACT---
CAACTGCTATTAAATGGCAGTTTAGCAGAAGAAGAA------------
GTAGTAATTAGATCTAGCAATTTCTCAGACAAT---------GCTAAGGTCATAATAGTACAGCTGAAT----

-----------ACATCTGTAGAA---------ATTAATTGTACAAGACCCAACAACAATACAAGAAAA-----
-AGTATACATCTA------------GGACAGGGGCGAGCA---TGG------TATGCAACAGGAGAA------
---------ATAATAGGAGATATA------------AGACAAGCACATTGT---AACCTTAGTAGCACA---
AAATGGAATAACACTTTAAAACAGATAGTTGAAAAATTAAGA---------------GAACAATTTGGA----
--------------------------------AACAACAAAACAATAGTCTTTAAT------
CAATCCTCAGGAGGGGACCCAGAGATTGTA---ATGCACAGTTTAATTGTGGAGGGGAA---
TTTAAACAGCTCCAGGCAAGAGTCCTGGCTGTGGAAAGATACCTAAAGGATCAACAGCTCCTAGGGATTTGGG
GATGCTCTGGAAAACTCATTTGCCCCACTGCTGTGCCTTGGAATGTTAGTTGGAGT-----------------
----------------------------AATAAATCCCTGAATGAGATTTGG------GAT------
AACATGACTTGGATGGAGTGG---GAAAGAGAAATTCACAATTATACAAGAGAAATATAC---
AACTTAATTGAAGAATCGCAGAACCAACAAGAA--

AAGAATGAACTAGAACTATTGGAATTGGATAAGTGGGCAAGCTTGTGGAATTGGTTTGACATAACAAAATGGC
TGTGGTATATCAAAATATTCATAATGGTAGTAGGAGGCTTAATAGGTTTAAGAATAGTTTTTGCT---
GTACTTTCTATAGTA---------AATAGAGTTAGGCAGGGGTACTCACCA------
TTGTCGTTTGCTATTAGCTTGCTTAACGCCACAGCCATAACAGTAGCTGAA---
GGGACAGATAGGGTTATAGAAATAGTAAGAAGAGCTTATAGGGCT----------------------------
--------ATTCTCCACATA------CCTACAAGAATCAGACAGGGCCTCGAAAGGGCTTTGCTT---TAA
>KX693720
GCA---GCA--
GAAAACTTGTGGGTCACAGTCTATTATGGGGTACCTGTGTGG---AAAGAAGCAAAT---
ACTACTCTATTTTGTGCATCAGATGCTAAAGCATATGATACAGAG---GTACATAAT----------------
--------------ATGTGGGCCACA---CATGCCTGTGTACCCACAGAC---
CCCAACCCACAAGAAGTAGTCTTGGGA---------AATGTGACAGAAAACTTTAAC----------------
--------------------------ATGTGGAAAAATAACATGGTAGAACAAATGCATGAAGATATA---
ATCAGTTTATGGGATCAAAGCCTA---AAGCCATGTGTAAGATTA---
ACCCCACTCTGTGTTGCTTTACATTGCACTAATTTGACTATTAATACTACCAGTAGTAATTGCACTACTACTA
ATACTACCAGTACTACCAGTAGTAAT---
-TGCACTACTACTAATACTACCAGTAGTAATACCAGTAGTACAACAGAGGGGGGAGAAATGAAA---
AACTGCTCTTTCAATATCACCACAAGCATACAA---------------------------ACTAAGGTA---
AAAGACTATGCACTT---TTCTATAACCTTGATATAATACCAATAGATAAT----------------------
----
----------------AACAGCTATAGGTTGATAAATTGTAACACCTCAATCATTACACAA---
GCTTGTCCAAAGGTATCCTTTGAGCCAATTCCCATACATTATTGTGCCCCGGCTGGTTTTGCGATTCTAAAGT
GTAAC---AATAAAACGTTCAAT------GGAACAGGACCATGTACAAAT-----------------------
-GTCAGT---ACAGTACAGTGTACACATGGAATTAGGCCAGTAGTATCAACT---
CAACTGCTGTTAAATGGCAGTTTAGCAGAAAAAGAG-----------
GTTGTAATTAGATCTAGCAATTTCACAGACAAT---------GCTAAAATCATAATAGTACAGCTGAAG----
-----------GAATCGGTACGA---------ATTGAGTGTGAAAGACCCAACAACAACACAAGAAAA-----
-AGTATACATCTA------------GGACCAGGGAGAGCA---TGG------TATGCAACAGGACAA------
---------ATAATAGGAAATATA------------AGACAAGCACATTGT---AACATTAGTAGAGAA---
AAATGGAATGACACTTTAAAACAGGTAACTAAAAAATTAATA---------------GAACAATTTGGG----
-----------------------------------AACAAAACAATAATCTTCAAT------
CAATCTTCAGGAGGGGACCCAGAGATTGTA---ATGCACAGTTTAATTGTGGAGGGGAA---
TTTAAGCAGCTCCAGGCAAGAGTCCTGGCTGTGGAAAGATACCTAAAGGATCAACAGCTCCTAGGGCTTTGGG
GTTGCTCTGGAAAACTCATTTGCACCACTGCTGTGCCTTGGAATGGTAGTTGGAGT-----------------
----------------------------AATAAATCTCTGAGTGAGATTTGG------GAT------
AACATGACTTGGATGGAGTGG---GAAAGAGAAATTGGCAATTACACACAACAAATATAT---
ACTTTAATTGAAGAATCGCAGAACCAACAAGAA--

AAGAATGAACTAGAACTATTGGAATTGGATAAGTGGGCAAGCTTGTGGAATTGGTTTGACATAACAAAATGGC
TGTGGTATATCAAAATCTTCATAATGATAGTAGGAGGCTTGGTAGGTTTAAGGATAGTTTTTGCT---
GTACTTTCTATAGTA---------AATAGAGTTAGGCAGGGGTACTCACCA------
TTGTCGTTAGCTATTAGCTTGCTTAACGCTACAGCCATAGCAGTAGCTGAG---
GGGACAGATAGGGTTATAGAAGTAACACAAAGAGCTTTTAGGGCT----------------------------
--------ATTCTCAACATA------CCTACAAGAATCAGACAGGGCTTAGAAAGGGCTTTGCTA---TAA
>KX693770
GCT---GCA--
GAAAACTTGTGGGTCACAGTCTATTATGGGGTACCTGTATGG---AAAGATGCAACT---
ACCACTCTATTTTGTGCATCAGATGCTAAAGCATATGCCACAGAG---GTACATAAT----------------

--------------ATTTGGGCCACA---CATGCCTGTGTACCCACAGAC---
CCCAACCCACAAGAAGTAGTTTTGGGA---------AATGTGACAGAAAACTTTAAC---------------
-------------------------ATGTGGAAAAATGACATGGTAGATCAGATGCATGAAGATATA---
ATCAGTTTATGGGATCAAAGCCTA---AAGCCATGTGTAAAATTA---
ACCCCACTCTGTGTTATTTTAAATTGCACTAACTGTACTATTGGAACTTGCACTAATTTGGGGAATACTACCA
GGAATGATACC---
---------------------AATAGGAATAACAGTAGCACGACAGAGGGAGGAGAAATAAAA---
AACTGCTCTTTCGATATCACCACAAGCATAAAT--------------------------ACCAGGGTG---
CAAGACCATGCACTT---TTTAATAAACTTGATGTAGAGCCAACAGATAGT---------------------
----
----------AGTAATATCAGCTATAGGTTGAGAAGCTGTAACACCTCAGTCATTACACAA---
GCTTGTCCAAAGGTATCCTTTGAGCCAATTCCAATACATTATTGTGCTCCAGCTGGTTTTGCGATTATAAAGT
GTAAC---GATAACAAGTTCAAT------GGATCAGGACCATGTACAAAT----------------------
-GTCAGT---ACAGTACAATGTACACATGGAATTAGGCCAGTAGTATCAACT---
CAACTGCTGTTGAATGGCAGTTTAGCAGAAGAAGGG-----------
ATAGTAATTAGATCTAGCAATTTCACAGACAAT---------GCTAAAGTCATAATAGTACAGCTGAAT----
-----------GAATCTGTAGTA---------ATTAATTGTACAAGACCCAACAACAATACAAGAAAA-----
-AGTATAAATCTA------------GGACCAGGACGAGCA---TGG------TATGCAACAGGACAA------
---------ATAATAGGAGATATA------------AGACAAGCACATTGT---AACCTTAGTAGTACA---
ACATGGAATAAAACTTTAGAACAGATAGCTAAAAAATTAAGA---------------GAACAATTTGGG----
-------------------------------AGGAATAAAACAATAACCTTTAAT------
CAATCTTCAGGAGGGGACCCAGAGATTGTA---ATGCACAGTTTTAATTGTGGAGGGGAA---
TTCAAGCAGCTCCAGGCAAGAGTCTTGGCTGTGGAAAGATACCTAAAGGATCAACAGCTCCTAGGGATCTGGG
GTTGCTCTGGAAAACTCATCTGCCCCACTGCTGTGCCTTGGAATACTAGTTGGAGT----------------
----------------------------AATAAATCTCTGAGTCAGATTTGG------GAT------
AACATGACCTGGATGGAGTGG---GAAAGAGAAATTAACAATTACACAAGAGAAATATAC---
AACTTAATTGAAGAATCGCAGAACCAACAAGAA---------------------------------------
----
AAGAATGAACTAGAACTATTGGAATTGGATAAGTGGGCAAGTTTGTGGAATTGGTTTGACATAACAAAATGGC
TGTGGTATATCAAAATATTCATAATGGTAGTAGGAGGCTTGATAGGTTTAAGAATAGTTTTTACT---
GTACTTTCTGTAGTA---------AATAGAGTTAGGCAGGGATACTCACCA------
TTGTCATTAGCTATTAGCTTGCTTAACGCCACAGCCATAGCAGTAGCTGAA---
GGGACAGATAGGGTTATAGAAGTAGCACAAAGAGCTGTTAGGGCT--------------------------
--------ATTCTCAACATA------CCTAGAAGAATCAGACAGGGTCTCGAAAGGGCTTTGCTA---TAA
>KX693223
GCT---ACA---
GAAAAATTGTGGGTCACAGTCTATTATGGGGTACCTGTATGG---AAAGAAGCAACT---
ACCACCCTATTTTGTGCATCAGATGCTAAAGCATATGGTACAGAG---GTACATAAT---------------
--------------ATTTGGGCCACA---CATGCCTGTGTACCCACAGAC---
CCCAACCCACAAGAAGTAGTCTTGGGA---------AATGTGACAGAAAACTTTAAC---------------
-------------------------ATGTGGAAAAATGACATGGTAGAACAGATGCATGAAGATATA---
ATCAGTTTATGGGATCAAAGCCTA---AAGCCATGTGTAAAATTA---
ACCCCACTCTGTGTTACTTTAAATTGCACTAATTTGGAGAAGACTACTAATAATAGTACTAATACGACAGAGG
GAGGTAGTAGT---
----------------------------AATACCAATAGTACAATAGAGGGAGGAGAACTAAAA---
AACTGCTCTTTCAATATCACCACAAGCATAAAA--------------------------ACTAAGGTG---
AGAGGCTATGCACTT---TTTTATAAACTTGATATAGTACCAATAGGTCATGAT------------------
----
----AATAATACTAATACCAGCTATAGGTTGATAAAGTGTAACACCTCAGTCATTACACAA---
GCTTGTCCAAAGGTATCCTTTGAGCCAATTCCCATACATTATTGTGCCCCGGCTGGTTTTGCGATTCTAAAGT
GTAAC---AATAAAACATTCAAT------GGAACAGGACCATGTACAAAT----------------------
-GTCAGT---ACAGTACAATGTACACATGGAATTAGGCCAGTAGTATCAACT---
CAACTGCTGTTAAATGGCAGTTTAGCAGAAGAAGAG-----------
GTAGTAATTAGATCTAGCAACTTCACGAACAAT---------GCTAAAGTCATAATAGTACAGCTAAAT----
-----------AAATCTGTAGAA---------ATTAATTGTACAAGACCCAACAACAATACAAGAAAA-----
-AGTATACCTATA------------GGACCAGGGCGAGCA---TGG------TATGCAACAGGAGAA------
---------ATAATAGGAGATATA------------AGACAAGCACATTGT---AACCTTAGTAGAGCA---
GGATGGAATAACACTTTAAGACTAATAACTGAAAAATTAAGA---------------GAACAATTTGGG----
----------------------------------AACAAAACAATAATTTTTAAT------
CAATCTTCAGGAGGGGACCCAGAGATTGTA---ACGCACAGTTTTAATTGTGGAGGGGAA---

TTTAAGCAGCTCCAGGCAAGAGTCTTGGCTGTGGAAAGATACCTAAAGGATCAACAGCTCCTAGGGATTTGGG
GTTGCTCTGGAAAACTCATTTGCACCACTGCTGTGCCTTGGAATGTTAGCTGGAGT-----------------
-----------------------------AATAAATCTTACAATGAGATTTGG------AAT------
AACATGACTTGGATGGAGTGG---GAAAGAGAAATTGACAATTACACAAAAGAAATATAC---
ACTTTAATTGAAACCTCGCAGAACCAACAAGAA---

AAGAATGAACTAGACCTATTGGAATTGGATAAATGGGCAAGCTTGTGGAATTGGTTTGACATAACACAATGGC
TGTGGTATATCAAAATATTCATAATGATAGTAGGAGGCTTGATAGGTTTAAGAATAATTTTTGCT---
GTACTTTCCATAGTA---------AATAGAGTTAGGCAGGGATACTCACCA------
TTGTCATTGGCTATTAGCTTGCTTAACGCCATAGCCATAGCAGTAGCTGAA---
GGGACAGATAGGGTTATAGAAATAGCACAAAGAGTTTGTAGGGCT-----------------------------
--------ATTCTCAACATC------CCTACAAGAATTAGACAGGGCCTCGAAAGAGCTTTGCTA---TAA
>KX693238
GCT---GCA---
GAAAACTTGTGGGTCACAGTCTATTATGGGGTACCTGTATGG---AAAGAAGCAACT---
ACCACCCTATTTTGTGCATCAGATGCTAAGGCATATGCTACAGAG---GTACATAAT----------------
--------------ATTTGGGCCACA---CATGCCTGTGTACCCACAGAC---
CCCAGCCCACAAGAAGTAGTCTTGGGA---------AATGTGACAGAAAACTTTAAC----------------
--------------------------ATGTGGAAAAATGACATGGTAGAACAGATGCATGAAGATATA---
ATCAATTTATGGGATCAAAGCCTA---AAGCCATGTGTAAAATTA---
ACCCCACTCTGTGTTACTTTAAATTGCACCAATTATTTGGGGAATACTAATGATACCAGTAGTAATAATGCTA
CTAATACCAAT---
AGTAGTAATACTACTGCCAATAACAGTAGTAATACCAGTAATACAACATGGGGAGGAGAAATAAAA---
CTCTGCTCTTTCAATGTCACCACAAGCATAAAA--------------------------ACTAAGGTA---
AAAGACTATGCACTT---TTTAATACACTTGATATAGAACCAATAGAGAAT---------------------
----
----------GATAATACCAGCTATAGGTTGATAAAGTGTAACACCTCAGTTATTACACAA---
GCTTGTCCAAAGATATCCTTTGAGCCAATTCCCATTCATTATTGTACCCCGGCTGGTTTTGCGATTCTAAAGT
GTAAC---AATAAAACATTCAAT------GGAACAGGACCATGTACAAAT----------------------
-GTCAGT---ACAGTACAATGTACACATGGAATTAAGCCAGTAGTATCAACT---
CAACTGCTGTTAAATGGCAGTTTAGCAGAAGAAGAG-----------
GTTGTAATTAGATCTAGCAATTTCACAGACAAT---------GCTAAAGTCATAATAGTACAGCTGAAG----
-----------GAATCTGTAAAA---------ATTAATTGTACAAGACCCAACAACAATACAAGAAAA-----
-AGTATACCTCTA------------GGACCAGGGCGAGCA---TGG------TATACATCAGGACAA------
---------ATAATAGGAGATATA------------AGAAAAGCACATTGT---AACATTAGTCGAGCA---
GAATGGAATAACACTTTAAAACAGGTAGCCAAAAAATTAAGA---------------GGACAATTTGGA----
-----------------------------------AACAAAACAATAGTCTTTGAG------
CGATCTTCAGGAGGGGACCCAGAGATTGCA---ATGCACACCTTTAATTATGGAGGGGAA---
TTTAAACAGCTCCAGGCAAGAGTCTTGGCTGTGGAAAGATACCTAAAGGATCAACAGCTCCTAGGGATTTGGG
GTTGCTCTGGAAGACTCATTTGCACCACTGCTGTGCCTTGGAATGCTAGTTGGAGT----------------
-----------------------------AATAAATCTCTGAGTGAAATTTGG------GAT------
AACATGACTTGGATGGAGTGG---GAAAGAGAAATTGGCAATTACACAAGAGAAATATAC---
AACTTAATTGAAGAATCGCAGAACCAACAAGAA---

AAGAATGAACTAGAACTATTGGAATTGGATAAGTGGGCAAGTTTGTGGAATTGGTTTGACATAACACAATGGC
TGTGGTATATCAAAATATTCATAATGATAGTAGGAGGCTTGGTAGGTTTAAGAATAGTTTTTGCT---
GTACTTTCTATAGTA---------AATAGAGTTAGGCAGGGATACTCACCA------
TTGTCGTTAGCTATTAGCTTGCTTAACTCCACAGCCATAGCAGTAGCTGAA---
GGGACAGATAGAGTTATAGAAGTAGTACAAAGAGCTTATAGGACT-----------------------------
--------ATTCTCCACATA------CCTAGAAGAATCAGACAGGGCCTCGAAAGGATTTTGCTA---TAA
>KX693247
GCT---GCA---
GAAAACTTGTGGGTCACAGTCTATTATGGGGTGCCTGTATGG---AAAGAAGCAACT---
ACCACTCTATTTTGTGCATCAGATGCTAAAGCATATGAGACAGAG---GTACATAAT----------------
--------------GTTTGGGCCACA---CATGCCTGTGTACCCACAGAC---
CCCAGCCCACAAGAAGTAGTCTTGGGA---------AATGTGACAGAAAACTTTAAC----------------
--------------------------ATGTGGAAAAATGATATGGTAGAACAGATGCATGAAGATATA---
ATCAGTTTATGGGATCAAAGCCTA---AAGCCATGTGTAAAATTA---
ACCCCACTCTGTGTTACTTTAAATTGCACTAACTGGGAGAATCCTACTAATACCAGTAGTAACACCAGTGCCA
GTAATAACACC---

----------------AGTACCAGTAGTAACACCAGTAGGACGCTAGAGGGAGGAGAAGTAAAA---
AACTGCTCTTTCAATATCACCACAAGCATAAGA-------------------------ACTAAGGTG---
ACAGACTATGCACTT---TTTTATAAACTTGATGTAGTACCAATAAATAAT---------------------
----
----AATACTAATAATACTAGCTATAGGTTGATAAATTGTAACACCTCAGTCATTACACAA---
GCTTGTCCAAAGGTATCCTTTGAGCCAATTCCCATACATTATTGTACCCCGGCTGGTTTTGCGATTCTAAAGT
GTAAC---AATAAAAGGTTCAAT------GGAACAGGACCATGTACAAAT----------------------
-GTCAGT---ACAGTACAATGTACACATGGAATTAGGCCAGTAGTATCAACT---
CAACTGCTGTTAAATGGCAGTTTAGCAGAAGAAGAG-----------
GTAGTGATTAGATCTAGCAACTTCTCGAACAAT---------GCTAAAGTCATAATAGTACATCTGAAT----
-----------GAATCTGTAGAA---------ATTAATTGTACAAGACCCAACAACAATACAAGTAAA-----
-AGTATACATCTA------------GGACCAGGGCAAGCA---TGG------TATACAACAGGACGA------
---------ATAATAGGAGATATA------------AGACAAGCACATTGT---AACCTTAGTAGTACA---
AAATGGATTGACACTTTAAAACAGGTAGCTAAAAAATTAAGA---------------GAACAATTTGAG----
-----------------------------------AACAAAACAGTAATCTTTAAG------
CACTCTTCAGGAGGGGACCCAGAGATTGTA---ATGCACAGCTTTAATTGTGGAGGGGAA---
TTTAAGCAGCTCCAGGCAAGAGTCTTGGCTGTAGAAAGATACCTAAAGGATCAACAGCTCCTAGGGATTTGGG
GTTGCTCTGGAAAACTCATCTGCACCACTGCTGTGCCTTGGAATGTTAGTTGGAGT----------------
----------------------------AATAAATCTCTGAGTGAGATTTGG------GAT------
AACATGACCTGGATGGAGTGG---GAAAGAGAAATTGACAAGTACACAGGAGAAATATAC---
ACCTTAATTGAAGAATCGCAGAACCAACAAGAA---------------------------------------
----
AAGAATGAACTAGAACTATTGGAATTGGATAAGTGGGCAAGCTTGTGGAATTGGTTTACCATAACAAACTGGC
TGTGGTATATCAAGATATTCATAATGATAGTAGGAGGCTTGGTAGGTTTAAGAATAGTTTTTGCT---
GTACTTTCTATAGTA---------AATAGAGTTAGGCAGGGATACTCACCA------
TTGTCGTTAGCTATTAGCTTGCTTAACGCCACAGCAATAGCAGTAGCTGAG---
GGGACAGACAGGGTTATAGAAGTAGTACAAAGAGCTTATAGGGCT---------------------------
--------TTTGTCCACATA------CCTAGAAGAATCAGACAGGGCCTCGAAAGGGCTTTGCTA---TAA
>KX693268
GCT---GCA---
GAAAACTTGTGGGTCACAGTCTATTATGGGGTACCTGTATGG---AAAGAAGCAACT---
ACCACTCTATTTTGTGCATCAGATGCTAAAGCATATGATACAGAG---GCACATAAT----------------
--------------ATTTGGGCCACA---CATGCCTGTGTACCCACAGAC---
CCCAGCCCACAAGAAGTAGTCTTGGGA---------AATGTGACAGAAAACTTTAAC---------------
--------------------------ATGTGGAAAAATGACATGGTAGAACAGATGCATGAAGATATA---
ATCAGTTTATGGGATCAAAGCCTA---AAGCCATGTGTAAAATTA---
ACCCCACTCTGTGTTACTTTGAAATGCACTAATGCTACTAGTTTGAGGAATGATACTAACACCAATAGTACTA
CTGACACCAAT---
-------------------AGTAATAACAACAATAGTACTATCATTAGTACGGCAGAAATAAAA---
AACTGCTCTTTCAATATCACCACAAGCATAAGA-------------------------ACTCAGGTG---
AAAAACTATGCCCTT---TTTTATACACTTGATCTAATACCAATAGATAATGATGTAGATAATGGT-------
----
ACTACCAATAATGATAATAGGACCAACTATAGGTTGATAAATTGTAATACCTCAGTCATTACACAA---
GCTTGTCCAAAAGTATCCTTTGAGCCAATTCCCATACATTATTGTGCCCCGGCTGGTTTTGCGATTCTAAAGT
GTAAC---AATAAAACGTTCAAT------GGAACAGGACCATGTACCAAT----------------------
-GTCAGT---ACAGTACAATGTACACATGGAATTAGGCCAGTAGTATCAACT---
CAGCTGTTGTTAAATGGCAGTTTAGCAGAAAAAGAG-----------
GTAGTAATTAGATCTAGCAATTTCACAGACAAT---------GCTAGAGTCATAATAGTACAGCTGAAT----
-----------GAATCTGTAAAA---------ATTAATTGTACAAGGCCCAACAACAATACAAGAAAA-----
-AGTATATCTCTA------------GGACCAGGGCAAGCA---TGG------TATGCAGCAGGAGAA------
---------ATAATAGGAGATATA------------AGACAAGCACATTGT---AACGTTAGTAGAACA---
AAATGGAATAACACTTTAGAAAAGATAGTTAAAAAATTAAGA---------------GAACAATTTGGG----
-----------------------------------AACAAAACAATAATCTTTAAT------
CGATCTTCAGGAGGGGACCCAGAAATTGAA---ATGCATAGTTTTAATTGTGGAGGGGAA---
TTTAAGCAGCTCCAGGCAAGAGTCCTGGCTGTGGAAAGATACCTGAAGGATCAGCAGCTCCTAGGGCTTTGGG
GTTGCTCTGGAAGACTCATCTGTACCACTGCTGTGCCCTGGAATGTTAGTTGGAGT----------------
----------------------------AATAAATCTCTGAGTGAGATTTGG------GAT------
AACATGACTTGGATGGAGTGG---GAAAGAGAAATTGACAATTACACAAGAGAAATATAC---
ACTTTAATTGAAGAATCGCAGAACCAACAAGAA---------------------------------------
----

AAGAATGAACTAGAACTATTGGAATTGGATAAGTGGGCAAGTTTGTGGAATTGGTTTGACATATCAAAATGGC
TGTGGTATATTAAAATATTCATAATGATAGTAGGAGGCTTGATAGGTTTAAGAATAGTTTTTGCT---
GTACTTTCTATAGTA---------AATAGAGTTAGGCAGGGATACTCACCA------
TTATCGTTGGCTATTAGCTTGCTTAACGCCACAGCCATAGCAGTAGCTGAG---
GGGACAGATAGGATTATAGAAGTAGCACAAAGAGCTTATAGGGCT----------------------------
--------ATTCTCAACATA------CCTAGAAGAATCAGACAGGGCCTCGAAAGAGCTTTGCTA---TAA
>KX693886
GCT---GCA---
GAAGAATCGTGGGTCACAGTTTATTATGGAGTACCTGTATGG---AAAGAAGCAATT---
ACCACTCTATTTTGTGCATCAGATGCTAAAGCATATGCTACAGAG---GTACATAAT----------------
--------------ATTTGGGCAACA---CATGCCTGTGTACCCACAGAC---
CCCAACCCACAAGAAGTATACTTGGAA---------AATGTGACAGAAAACTTTAAC----------------
--------------------------ATGTGGAAAAATGACATGGTAGAACAGATGCAGGAAGATATA---
AGCAGTTTATGGGATCAAAGCCTA---AAGCCATGTGTAAAATTA---
ACCCCACTCTGTGTTACTTTAAATTGCACTGATTTGAAGAATAAAAATAGTACCAGTAGTAATACCAGT----
----
----------------------AGTAGTAATACCAGTAGTATAATAGAGGGAGGAGAAATGAAA---
AACTGCTCTTTCAATATCACCACAAGCATAGGA---------------------------ACTAAGGTG---
AAAGACTATGCACTT---TTTTATAAACTTGATATAGTACAAACAGATGGT----------------------
----
----------GATAATACCAGCTATAGGTTGATAAGTTGTAACACCTCAGTCATTACACAA---
GCTTGTCCAAAGGTATCCTTTGAGCCAATTCCCATACATTATTGTGCCCCGGCTGGTTTTGCGATTCTAAAGT
GTAAC---AATAAAACGTTCAAT------GGAACAGGACCATGTACAAAT-----------------------
-GTCAGT---ACAGTACAATGTACACATGGAATTAGGCCAGTAGTATCAACT---
CAACTGCTGTTAAATGGCAGTTTAGCAGAAGAAGAG-----------
GTAGTAATTAGATCTAGCAATCTCTCAGACAAT---------ACTAAAGTCATAATAATACAGCTAAAG----
-----------GAATCTGTAGAA---------ATTAATTGTACAAGACCCAACAACAATACAAGAAAA-----
-AGTATACCTCTA------------GGACAGGGGCGAGCA---TGG------TATACAACAGGAGAC------
---------ATAATAGGAGATATA-----------AGACAAGCACATTGT---AACCTTAGTAGGGCA---
CAATGGAATAACACTTTAAAGCAGATAGCTAAAAAATTAGGA---------------GAACAATTTAAG----
-----------------------------------AACAAATCAATAAGCTTTAAT------
CAATCTGCAGGAGGGGACCCAGAGATTGTA---ATGCTCAGTTTTAATTGTGGAGGGAAA---
TTTAAACAGCTCCAGGCAAGAGTCTTGGCTGTGGAAAGATACCTAAAGGATCAACAGCTCCTAGGGATTTGGG
GTTGCTCTGGAAAACTCATTTGCACCACTGCTGTGCCTTGGAATGCTAGTTGGAGT----------------
-----------------------------AATAAAACTCTAGATGAGATTTGG------GGT------
AACATGACTTGGATGGAGTGG---GAAAGAGAGATTGACAATTACACAAATGTAATATAC---
ACTTTAATTGAAGAATCGCAGAACCAACAAGAA---

AAAAATGAACTAGAACTATTGAAATTGGATCAGTGGGCAAGTTTGTGGAATTGGTTTGACATATCAAATTGGC
TGTGGTATATTAGAATATTCATAATGATAGTAGGAGGCTTAATAGGTTTAAGAATAGTTTTTGCT---
GTGCTTTCTATAGTA---------AATAGAGTTAGGCAGGGATACTCACCC------
TTGTCATTAGCTATTAGCTTACTTAACACCACAGCCATAGCAGTAGCTGAG---
GGGACAGATAGGATTATAGAAGTAGCACAAAGAGCTTTTAGGGCT----------------------------
--------ATACTCAACATA------CCTAGAAGAATTAGACAGGGCTTCGAAAGGGCTTTGCTA---TAA
>KX693314
GCT---GCA---
GAAAAATTGTGGGTCACAGTCTATTATGGGGTACCTGTATGG---AAAGAAGCAACT---
ACCACTCTATTTTGTGCATCAGATGCCAAAGCATATGCTACAGAA---GTACATAAT----------------
--------------ATTTGGGCCACA---CATGCCTGTGTACCCACAGAC---
CCCAACCCACAAGAAGTATTCTTGGAA---------AATGTGACAGAAAACTTTAAC----------------
--------------------------ATGTGGAAAAATGACATGGTAGAACAGATGCATGAAGATATA---
ATCAGTTTATGGGATCAAAGCCTA---AAGCCATGTGTAAAATTA---
ACCCCACTCTGTGTTAATTTAAATTGCACTGATTTGAAGAATACTACCAATACCAGTAGT-------------
----
-----------------------------AACACCAGTAGTACGACAGAGGGAGGAGAAATGAAA---
AACTGCTCTTTCAATATCACCACAAGCATAAAA---------------------------ACTAAGATA---
AAAGACTATGCACTT---TTTTATAAACTTGATATAGTACCAATAAATAGTGATAATACCAGACCAATA----
----
AATAGTGATAATACCAGCTATAGTGCTAATACCAGCTATAGGTTGATACATTGTAATACCTCAGTCATTACAC
AG---

GCTTGTCCAAAGGTATCCTTTGAGCCAATTCCAATACATTATTGTACCCCGGCTGGTTTTGCGATTCTAAAGT
GTAAC---GATAGAAAGTTCAGT------GGAACAGGACCGTGTACAAAT-----------------------
-GTCAGT---ACAGTACAGTGTACACATGGAATTAGGCCAGTAGTATCAACT---
CAACTGCTGTTAAATGGCAGTTTAGCAGAAGAAGAG------------
GTAGTAATCAGATCTAGCAATTTCACGAACAAT---------GCTAAAATCATAATAGTACAGCTGAAT----
-----------GAATCTGTAGAA---------ATAAATTGTACAAGACCCAACAACAATACAAGAAAA-----
-AGTATACAGCTA------------GGACCAGGGAAAGCA---TGG------TATACCACAGGGCAA------
---------ATAATAGGAGAGATA------------AGACAAGCACATTGT---AACCTTAGTAGAACA---
AAATGGAATAACACTTTAGAACGGATAACTAAAAAAATTAAGA--------------GAACAATTTGGG----
-----------------------------------AACAAAACAATAATCTTTAAT------
CAATCTTCAGGAGGGGACCCAGAGATTGTA---ATGCATAGTTTTAATTGTGGAGGGGAA---
TTTAAGCAGCTCCAGGCAAGAGTCTTGGCTGTGGAAAGATACCTAAAGGATCAACAGCTCCTAGGGATTTGGG
GTTGCTCTGGAAGACTCATCTGCACCACTGCTGTGCCTTGGAATGTTAGTTGGAGT-----------------
----------------------------AATAAATCTCTGGATGATATTTGG------AAT------
AACATGACTTGGATGGAGTGG---GAAAGAGAAATTGAAAATTACACAAGAGAAATATAC---
ACCTTAATTGAAGAATCGCAGAACCAACAAGAA--

AAGAATGAACTAGAACTATTGGAATTGGATAAGTGGGCAAGTTTGTGGAATTGGTTTGACATAACAAAATGGC
TGTGGTATATCAAAATATTCATAATGATAGTAGGAGGCTTGGTAGGTTTAAGAATAGTTTTTGCT---
GTACTTTCTATAGTA---------AGTAGAGTTAGGAAGGGATACTCACCA------
TTGTCGTTAGCTATTAGCTTGCTTAACGCCACATCCATAGCAGTAGCTGAG---
GGGACAGATAGGGTCATAGAAGTAGCACAAAGAGCTTTTAGGGCT----------------------------
--------CTTGTCCACATA------CCTACAAGAATCAGACAGGGCCTAGAAAGGGCTTTGCTA---TAA
>KX693326
GCT---ACA--
GAACAATTGTGGGTCACAGTCTATTATGGGGTACCTGTATGG---AAAGAAGCAACT---
ACCACTCTATTTTGTGCATCAGATGCTAAAGCATATGAGACAGAG---GTACATAAT----------------
--------------GTTTGGGCCACA---CATGCCTGTGTACCCACGGAC---
CCCAACCCACAAGAAGTAGTTTTGGGA---------AATGTGACAGAAAACTTTAAC----------------
--------------------------ATGTGGAAAAATAACATGGTAGAACAGATGCATGAAGATATA---
ATCAGTTTATGGGATCAAAGCCTA---AAGCCATGTGTAAAATTA---
ACCCCAATTTGTGTTACTTTAAATTGCACTAATGTGAATATTACTGGCAGTAATACTACTGAT----------
----
-------------------ACCCATAGTAATGCCAGTAGTATGACAGTGGGAGGAGAAATGAAA---
AACTGCTCTTTCAACATCACCACAAGCATAAGG---------------------------CCTAAGGTG---
AAAGACTATGCACTT---TTTTATAAATCTGATATAGTACAAATAGATAGTGAT-------------------
----
----------AATAATGATACCTATAGGTTGATAAATTGTAACACCTCAGTCATTACACAA---
ACTTGTCCAAAGGTATCCTTTGAGCCAATTCCAATACATTATTGTACCCCGGCTGGTTTTGCGATGCTAAAGT
GTAAC---AATAAAACGTTCAAT------GGAACAGGACCATGTACAAAT-----------------------
-GTCAGT---ACAGTACAATGTACACATGGAATTAGGCCAGTAGTATCAACT---
CAACTGCTGTTAAATGGCAGTTTAGCAGAAGAAGAA------------
GTAGTAATTAGATCTAGCAATTTCTCAGACAAT---------GCTAAAGTCATAATAGTACAGCTAAAT----
-----------AAATCTGTAAAA---------ATTAATTGTACAAGACCCAACAACAATACAAGAAAA-----
-AGTATACAGCTA------------GGACAAGGGCGAGCA---TGG------TATGCAACAGGAGAA------
---------GTATTCGGAGATATA------------AGACAAGCACATTGT---AACATTAGTGGAACA---
AACTGGACTAATACTTTAAGACAGATAACTGAAAAATTAAGG---------------GAACAATTTGGG----
-----------------------------------AATAAAACAATAAGCTTTACT------
CAACCTGCAGGAGGGGACCCAGAGATTGTA---ACGCACAGTTTTAATTGTGGAGGGGAA---
TTTAAGCAGCTCCAGGCAAGAGTCTTGGCTGTGGAAAGATACCTAAAGGATCAACAGCTCCTAGGGATTTGGG
GTTGCTCTGGAAAACTCATTTGCACCACTGCTGTGCCTTGGAATGTTAGTTGGAGT-----------------
----------------------------AATAAATCTCTGAATGAGATTTGG------AAT------
AACATGACTTGGATGGAGTGG---GAAAGAGAAATTGACAATTACACAAAAGAAATATAC---
ACCTTAATTGAACAATCGCAGAACCAACAAGAA--

AAGAATGAACTAGAACTATTGGAATTGGATAAGTGGGCAAGTTTGTGGAGTTGGTTTGACATATCAAACTGGC
TGTGGTATATCAGAATATTCATAATGATAGTAGGAGGCTTGATAGGTTTAAGAATAGTTTTTGCT---
GTACTTTCTGTAGTA---------AATAGAGTTAGGCAGGGATACTCACCA------
TTGTCATTAGCTATTAGCTTGCTTAACGCCACAGCCATAGCAGTAGCTGAG---

GGGACAGATAGGGTTATAGAAGTAGCACAAAGAGCTTATAGGGCT----------------------------
--------ATTCTTAACATA------CCTAGAAGAATCAGACAGGGCCTCGAAAGGGCTTTGGTA---TAA
>KX693339
GCT---GCA--
GAAAAATTGTGGGTCACAGTCTATTATGGGGTACCTGTATGG---AAAGAAGCAACT---
ACCACTCTATTTTGTGCATCAGATGCTAAAGCATATGATACAGAG---GTACATAAT----------------
--------------ATTTGGGCCACA---CATGCCTGTGTACCCACAGAC---
CCCAACCCACAAGAAGTAGTATTGGGA---------AATGTGACAGAAAACTTTAAC----------------
--------------------------ATGTGGAAAAATAACATGGTAGAACAGATGCATGAAGATATA---
ATCAGTTTATGGGATCAAAGCCTA---AAGCCATGTGTAAAATTA---
ACTCCACTCTGTGTTACTTTAAATTGCACTAATTTGAATAATACCAAAAGTAATACCAGT-------------
----
-------------------------------------AGTACGACAGAGGGAGGAGAAATAAAA---
AACTGCTCTTTCAATATCACCACAAGCATAAAA---------------------------ACTAAGGTG---
AGAGACTATGCACTT---TTTTATAAAGTAGATATATTACCAATAGATAAT----------------------
----
----GATGATAATAATACCAGCTATAGGTTGAGACATTGTAACACCTCAGTCATTACACAA---
GCTTGTCCAAAAGTATCCTTTGAGCCAATTCCCATACATTATTGTACCCCGGCTGGTTTTGCGATTCTAAAGT
GTAAC---AATAAAACATTCAGT------GGAACAGGACCATGTACAAAT-----------------------
-GTCAGT---ACAGTACAATGTACACATGGAATTAGGCCAGTAGTATCAACT---
CAACTGCTGTTAAATGGCAGTTTAGCAGAAGAAGAA------------
ATAGTAATTAGATCTAGCAATTTCACGGACAAT---------GCTAAAATCATAATAGTACAGCTGAAT----
-----------AAATCTGTAAAA---------ATTAATTGTACAAGACCCAACAACAATACAAGAAAA-----
-GGTATACCTATA------------GGACCAGGGCGAGCA---TGG------TACACAACAAAAGAC------
---------ATAATAGGAGATATA------------AGACAAGCACATTGT---AACCTTAGTAGAACA---
GAATGGAATAACACTTTACAACAGGTAGCTAAAAAATTAGGA---------------GAACAATTTAAG----
-----------------------------------AACAAAACAATAAGCTTTGAT------
CAATCTGCAGGAGGGGACCCAGAGATTGAA---ATGCACAGTTTTAATTGTAGAGGGGAA---
TTTAAGCAGCTCCAGGCAAGAGTCTTGGCTGTGGAAAGATACCTAAAGGATCAACAGCTCCTAGGATGTGGG
GTTGCTCTGGAAAACTCATCTGCACCACTGCTGTGCCTTGGAACACTAGTTGGAGT-----------------
----------------------------AATAAATCTCTGAATGAGATTTGG------AAT------
AACATGACTTGGATGGAGTGG---GAAAGAGAAATTAGCAATTACTCAGAACAAATATAC---
ACCTTAATTGAAAAATCGCAGAACCAACAAGAA--

AAGAATGAACTAGAACTATTGGAATTGGATAAGTGGGCAAGCTTGTGGAATTGGTTTGACATAACAAACTGGC
TGTGGTATATCAAAATATTCATAATGATAGTAGGAGGCTTGGTAGGTTTAAGAATAGTTTTTGCT---
ATACTTTCTATAGTA---------AACAGAGTTAGGCAGGGATACTCACCA------
TTGTCATTAGCTATTAGCTTGCTTAACGCCACTGCCATAGCAGTAGGTGAG---
GGGACAGATAGGGTTATAGAAGTAGTACAAAGAGCTTATAGGGCT----------------------------
--------ATTCTCAATATA------CCTAGAAGAATCAGACAAGGCCTCGAAAGGGCTTTGCTA---TAA
>KX693374
GCT---GCA--
GGAAACTTGTGGGTCACAGTATATTATGGGGTACCTGTATGG---AAAGAAGCAACT---
ACCACTCTATTTTGTGCATCAGATGCTAAAGCATATGCTACAGAG---GTACATAAT----------------
--------------GTTTGGGCCACA---CATGCCTGTGTACCCACAGAC---
CCCAACCCACAAGAAGTAGTCTTGGGA---------AATGTGACAGAACATTTTGAC----------------
--------------------------ATGTGGAAAAATAACATGGTAGAACAGATGCATGAAGATATA---
ATCAGTTTATGGGATCAAAGCCTA---AAGCCATGTGTAAAGTTA---
ACCCCACTCTGTGTCACTTTAAATTGCACTAATTTGAATACTACTAACTTGAATATTACTAAAACCAATAATA
CT---
-------------------ATCAGTAGTACTGCCAGTAGTATGGTAGAGGGAGGAGAAATAAAA---
AACTGCTCTTTCAATATCACCACAAGCATAAAA---------------------------ACTACAGTG---
AGAGATTATGCACTT---TTTTATAAACTTGATATAGTACAAATGGATGAT----------------------
----
----GATAATACCAATACCAGCTATAGGATGATAAATTGTAACACCTCAGTCATTACACAG---
GCTTGTCCAAAGGTATCCTTTGAACCAATTCCCATACATTATTGTGCCCCGGCTGGTTTTGCGATTCTAAAGT
GTAAC---AATAAAACATTCAAT------GGAAAAGGACCATGTACAAAT-----------------------
-GTCAGT---ACAGTGCAATGTACACATGGAATTAGGCCAGTAGTATCAACT---
CAACTGCTGTTAAATGGCAGTTTAGCAGAAGAAGAG------------
ATAGTAATTAGATCTAGCAATTTCACAAACAAT---------GCTAAAGTCATAATAGTACAGCTGAGA----

-----------GACGCTGTAGAA---------ATTAATTGTACAAGACCCAATAACAATACAAGAAAA-----
-AGCATACCTCTA------------GGACCAGGGCGAGCA---TGG------TATGCAACAGGACAA------
---------ATAGTAGGAGATATA------------AGACAAGCACATTGT---AACCTTACTACAGCA---
AAATGGGTAAAACTTTAGAACAGATAGCCAAAAAATTAAGA----------------AAACAATTTGGG----
-----------------------------------AACAAAACAATAAGCTTTAAG------
CAATCTTCAGGAGGGGACCCAGAGATTACA---ACGCACAGCTTTAATTGTGGAGGAGAA---
TTTAAGCAGCTCCAGGCAAGAGTCCTGGCTGTGGAAAGATACCTAAAGGATCAACAGCTCCTAGGGATTTGGG
GTTGCTCTGGAAAACTCATTTGCACCACTGCTGTGCCTTGGAATACTAGTTGGAGT-----------------
----------------------------AATAAATCTCTGAGTGACATTTGG------GAT------
AACATGACTTGGATGGAGTGG---GAGAGAGAAATTGACAATTACACAAAGGAAATATAC---
ACCTTAATTGAAAAATCGCAAAACCAACAAGAA--

AAGAATGAACTAGACTTATTGGAATTGGATAAGTGGGCAAGTTTGTGGAATTGGTTTGACATATCAAATTGGC
TGTGGTATATCAGAATATTCATAATGATAGTAGGAGGCTTGATAGGTTTAAGAATAGTTTTTGCT---
GTACTTTCTATAGTA---------AATAGAGTTAGGCAGGATACTCACCA------
TTGTCATTAGCTATCAGCTTGCTTGACGCCGCAGCCGTAGCAGTAGCTGAG---
GGAACAGATAGAGTTATAGAAGGAGCAAGAAGAGCTTTTTGGGCT----------------------------
--------TTTATCCACATA------CCTACAAGAATCAGACAGGGCCTTGAAAGGGCTTTGCTA---TAA
>KX693390
ACT---GCA--
GAAAAATTGTGGGTCACAGTCTATTATGGGGTACCTGTATGG---AAAGAAGCAACT---
ACCACTCTATTTTGTGCATCAGATGCTAAAGCATATAAGACAGAG---GTACATAAT----------------
--------------GTTTGGCCACA---CATGCCTGTGTACCCACAGAC---
CCCAACCCACAAGAAGTAGTCTTGGCA---------AATGTGACAGAAAACTTTAAC----------------
--------------------------ATGTGGAAAAATGACATGGTAGATCAGATGCATGAAGACATA---
ATCAGTTTATGGGATCAAAGCCTA---AAGCCATGTGTAAAATTA---
ACCCCACTCTGTGTTACTTTAAATTGCTCTAATTTGAATATTACTAAAACCAGCTGTAATAGTACCAGTAATA
ATACTGCCAACTGTAATAATACCAGTAGTCCTACCAGC------------------------------
ACTACTACTACCAGCAATACTACTAACAGTAGTGATACCAGCAGTACTACAGAGAGAGAAGAAATAAAA---
AACTGCACTTTCAATATCACCACAAGTATAGGA---------------------------ACTAAGGTG---
GCAGACTATGCACTT---TTCTATAAACTTGATATAGTACCAATAGAAAATGAGAATAAA-------------
----
----AGCATTAGTAATAACAGCTATAGGTTGATACATTGCAACACCTCAGTCATTACACAA---
GCTTGTCCAAAGGTATCCTTTGAGCCAATTCCCATACATTATTGTGCCCCGGCTGGTTTTGCGATTCTAAAGT
GTAAC---AATAAAACGTTCAAT------GGAACAGGGCCATGTACAAAT-----------------------
-GTCAGT---ACAGTACAATGTACACATGGAATTAGGCCAGTAGTATCAACC---
CAACTGCTGTTAAATGGCAGTTTAGCAGAAGGAGAG------------
GTAGCAATTAGGTCTAGCAATTTCTCGGACAAT---------GCTAAGGTCATAATAGTACAGCTGAAT----
-----------GAATCTGTAGAA---------ATTAATTGTACAAGACCCAACAACAATACAAGAAAA-----
-AGTATACCTTTA------------GGGCCAGGGAGAGCA---TGG------TTTGCAACAGGAGAA------
---------GTAATAGGAGATATA------------AGACGAGCACATTGT---AACCTTAGTAGAACA---
CAATGGAACAACACTTTAAAACAGATAGCTAAAAAATTAAGA---------------GAACAATGG-------
-----------------------------------AACAAAACAATAATTTTTAAT------
CAATCCTCAGGAGGGGACCCAGAGATTGTA---ATGCACAGTTTTAATTGTGGAGGGGAA---
TTTAAGCAGCTCCAGGCAAGAGTCTTGGCTGTGGAAAGATACCTAAAGGATCAACAGCTCCTAGGGATTTGGG
GTTGCTCTGGAAAACTCATTTGCACCACTACTGTGCCTTGGAATGCTAGTTGGAGT-----------------
----------------------------AATAAATCCTATAAGTACATTTGG------AAT------
AACATGACTTGGATGGAATGG---GAAAGAGAAATTGACAATTACACAGGACTAATATAC---
ACCTTAATTGAAGAATCGCAGAACCAACAAGAA--

AAGAATGAACTAGAACTATTGGAATTGGATAAGTGGGCAAGTTTGTGGAATTGGTTTGACATAACAAATTGGC
TGTGGTATATCAGAATATTCATAATGATAGTAGGAGGCTTAGTAGGTTTAAGAATAGTTTTTGCT---
GTACTTTCTATAGTG---------AATAGAGTTAGGCAGGATACTCACCA------
TTGTCGTTGGCTATTAGCTTGCTTAACGCCACAGCCATAGCAGTAGGTGAG---
GGGACAGATAGGGTTATAGAAGTAGCACAAAGAACTTATAGGGCT----------------------------
--------ATTCTCAACATA------CCTACAAGAATCAGACAGGGCCTCGAAAGGGCTTTGCTA---TAA
>KX693437
GCT---GCA--
GAAAATTTGTGGGTCACAGTCTATTATGGGGTACCTGTATGG---AAAGATGCAACT---
ACCACTCTATTTTGTGCATCAGATGCTAAAGCATATGATACAGAG---GTACATAAT----------------

--------------GTTTGGGCCACA---CATGCCTGTGTACCCACAGAC---
CCCAACCCACAAGAAGTAGTCTTGGGA---------AATGTGACAGAAAACTTTAAC----------------
--------------------------ATGTGGAAAAATGACATGGTAGAACAGATGCATGAAGATATA---
ATCAGTCTATGGGATCAAAGCATA---AAGCCATGTGTAAAATTA---
ACCCCACTCTGTGTTAAATTAAATTGCACTAATTTGAAAATTAAATTGAATAGTAATAGTACCAGTAGTGATA
ACAGGACTGGC--
----------ACCAATAATACCAGTAGTAATACCAGTAGTATAACAGAGGGAGGAGAAATAAAA---
AACTGTTCTTTCAATATCACCACAAGCATAAAA--------------------------ACTAAGGTG---
CAAGACTATGCATTT---TTTTATAACCTTGATATAGTACAAATAGAAGAT---------------------
----
----GATAATATTAGTAACAGCTATAGGTTGATAAATTGTAACACCTCAGTCATTACACAA---
GCTTGTCCAAAGGTATCCTTTGAGCCAATTCCCATACATTATTGTGCCCCAGCTGGTTTTGCGATTCTAAAGT
GTAAT---AATAAAATGTTTAAT------GGAACAGGACCATGTACAAAT----------------------
-GTTAGT---ACAGTACAATGTACACATGGAATTAAGCCAGTAGTATCAACT---
CAACTGCTGTTAAATGGCAGTTTAGCAGAAGAAGAG------------
GTAATAATTAGATCTAGCAATTTCACAAACAAT---------GCTAGGGTCATAATAGTACAGCTGAAT----
-----------GAATCTGTAGAA---------ATTAATTGTACAAGGCCCAACAACAATACAAGAAAA-----
-GGTATACATATA------------GGACCAGGACAAGCA---TTG------TATGCAACAGGAGAA------
---------ATAATAGGAGATATA------------AGACAAGCACATTGT---AACCTTAGTATCACA---
GGGTGGAATAACACTTTACATAAGATATTCAAAAAATTAAGA---------------GAACAATTTGGG----
--------------------------------GTTAATAAAACAATAATCTTTAAT------
CAATCATCAGGAGGGGACCCAGAGATTGTA---ATGCATAGTTTTAATTGTGGAGGGGAA---
TTTAAGCAGCTCCAGGCAAGAGTCTTGGCTGTGGAAAGATACCTAAAGGATCAACAGCTCCTAGGGATTTGGG
GATGCTCTGGAAAACTCATCTGCACCACTGCTGTGCCTTGGAATGCTAGCTGGAGT-----------------
----------------------------AATAAATCCCTGGATGAGATTTGG------GAT------
AACATGACTTGGATGGAGTGG---GAAAGAGAAATAGCCAATTACACAAAAGAAATATAC---
ACCTTAATTGAAGAATCGCAGAACCAACAAGAA--

AAGAATGAACTAGACCTATTGGAATTGGATAAGTGGGCAAGTTTGTGGAATTGGTTTGACATAACAAACTGGC
TGTGGTATATCAGAATATTCATAATGATAGTAGGAGGCTTGATAGGTTTAAGAATAGTTTTTGCT---
GTACTTTCTATAATA---------AATAGAGTTAGGCAGGGATACTCACCA------
TTGTCATTAGCTGTTAGCTTGTTTAATGCCACAGCTGTAACAGTAGCTGAG---
GGGACAGATAGGGTTATAGAAGTAGTACAAAGAGCTTTTAGAGCT----------------------------
--------TTTCTCAACGTA------CCAAGAAGAATCAGACAGGGCTTCGAAAGGGCTTTGCTA---TAA
>KX693455
GCT---GCA--
GAAAAGTTGTGGGTCACAGTCTATTATGGGGTACCTGTATGG---AAAGAAGCAACT---
ACCACCCTATTTTGTGCATCAGATGCTAAAGCATATGATACAGAG---GTACATAAT----------------
--------------ATTTGGGCCACA---CATGCCTGTGTACCCACAGAC---
CCCAACCCACAAGAAGTAGTCTTGGGA---------AATGTGACAGAAAACTTTAAC----------------
--------------------------ATGTGGAAAAATGACATGGTAGAACAGATGCAAGAAGACATA---
GTCAGTTTATGGGATCAAAGCCTA---AAGCCATGTGTAAGATTA---
ACCCCACTCTGTGTTACTTTAAATTGTACTAATTTGAATACTACTAATACCAGT-------------------
----
-------------------------AGTAATACCAGTAGTACGACAGAGGGAGGAGAAATAAAA---
AACTGCTCTTTCAATATCACCACAAGCATAAGA--------------------------ACTAAGGTG---
AAAGACTATGCACTT---TTTTATAAACTTGATATAGTACAAATAGATAATGAT-------------------
----
----------------ACCAGCTATAGGTTGATAAGTTGTAACACCTCAGTCATTACACAA---
GCTTGTCCAAAGGTATCCTTTGAGCCAATTCCCATACATTATTGTACCCCGGCTGGTTTTGCGATTCTAAAGT
GTAAC---AATAAAACATTCAAT------GGAACAGGACCATGTACAAAT----------------------
-GTCAGT---ACAGTACAATGTACACATGGAATTAGGCCAGTAGTATCAACT---
CAACTGCTGTTAAATGGCAGTTTAGCAGAAGCAGAG------------
GTAGTAATTAGATCTAGCAATTTCACGGACAAT---------ACTAAAGTCATAATAGTACAACTGAAT----
-----------GAATCTGTAGAA---------ATTAATTGTATAAGACCCAACAACAATACAAGAAAA-----
-AGTATACCTCTA------------GGACAAGGGCGAGCA---TGG------TATACAACAGGACAA------
---------ATAATAGGAGATATA------------AGACAAGCACATTGT---AACATTAGTAGAGCA---
AAATGGAATAACACTTTAAGACGGATAACTGAAAAATTAAAA---------------GAACAATTTGGG----
-----------------------------------AGCAAAACAATAATCTTTAAT------
CAATCTTCAGGGGGGGACCTAGAGATTGTA---ATGCACATGTTTAATTGCGGAGGGGAA---

TTTAAGCAGCTCCAGGCAAGAGTCCTGGCTGTGGAAAGATACCTAAAGGATCAACAGCTCCTAGGGATTTGGG
GTTGCTCTGGAAAACTCATTTGCACCACTGCTGTGCCTTGGAATGCTAGTTGGAGT-----------------
----------------------------AATAGAACTCTGAATGAGATTTGG------GAT------
AACATGACTTGGATGGAGTGG---GAAAGAGAAATTGACAATTACACAAAAGAAATATAC---
ACCTTAATTGAACAATCGCAGAACCAACAAGAA---

AAGAATGAACTAGAACTATTAGAACTGGATAAGTGGGCAAGCTTGTGGAATTGGTTTAATATAACAAACTGGC
TGTGGTATATCAAAATATTCATAATGATAGTAGGAGGCTTAATAGGTTTAAGAATAGTTTTTGCT---
GTACTTTCTATAGTA---------AATAGAGTTAGGCAGGGATACTCACCA------
TTGTCATTGGCTATTAGCTTGCTTAATACCACAGCCATAGCAGTAGCTGAG---
GGGACAGATAGGGTTATAGAAGTAGCACGAAGAGTTTATAGGGCT----------------------------
--------GTGCGCAACATA------CCTACAAGAATCAGGCAGGGCCTTGAAAGGGCATTGCTA---TAA
>KX693469
GCT---ACA---
GAAAAATTGTGGGTCACAGTCTATTATGGGGTACCGGTATGG---AAAGAAGCAACT---
ACCACTCTATTTTGTGCATCAGATGCTAAAACATATGGTACAGAA---GTACATAAT----------------
--------------ATTTGGGCCACA---CATGCCTGTGTACCCACAGAC---
CCCAACCCACAAGAAGTAGTTTTGGGA---------AATGTGACAGAAAACTTTAAC----------------
--------------------------ATGTGGAAAAATGACATGGTAGAACAAATGCATGAAGATGTA---
ATCAGTTTATGGGATCAGAGTCTA---AAGCCATGTGTAAAATTA---
ACCCCACTCTGTGTTACTTTAAAGTGTACTAATTTGGAGACTAATAATACCAATAGTAATACCAGTAGTCCGC
CCCCTACTACTACCAGTAGTAGT---
AATACCAGTAGTACGACCACTAGTACTACCAGTAGTTATACCAGGAGTACGACAGAGGGAGGAGAAATGAAA-
--AACTGCTCTTTCAATGTCACCACAAACATAAGA---------------------------------
ACTAAGAAAGAATATGCACTT---TTTTATAACCTTGATGTAGTACAAATAGAAAAT----------------
----
----------------ACGACTGACAGCTATAGGTTAATGAGTTGTAACACCTCAATCATTACACAA---
GCTTGTCCAAAGGTATCCTTTGAGCCAATTCCCATACACTATTGTGCCCCGGCTGGTTTTGCGATTCTAAAGT
GTAAC---AATAAAACATTCAAT------GGAAAAGGGCCATGCACAAAT----------------------
-GTCAGT---ACAGTACAATGTACACATGGAATTAGGCCAGTAGTATCCACT---
CAACTGCTGTTAAATGGCAGTTTAGCAGAAGAAGAG-----------
GTAGTAATTAGATCTAGCAATTTCACGAACAAT---------GCTGAAACCATAATAGTACAGCTGAAT----
-----------GAAACTGTAAAA---------ATTAATTGCACAAGACCCAACAACAATACAAGAAAA-----
-AGTATAAATATA------------GGACCAGGGCAAGCA---TGG------TATACAACAGGACAA------
---------ATAATAGGAGATATA------------AGACAAGCACATTGT---AACATTAGTAAAGAA---
AAATGGGGTAACACTCTAGAACAGATAACTAGAAAATTAAGA---------------GAACAATTTGGG----
----------------------------------AGTGAAAAAATAATCTTTAAT------
CACTCTTCAGGAGGGGACCCAGAGATTGTA---ATGCACAGTTTTAATTGTGGAGGGGAA---
TTTAAGCAGCTCCAGGCAAGAGTCTTGGCTGTGGAAAGATACCTAAAGGATCAACAGCTCCTAGGAATTTGGG
GTTGCTCTGGAAAGCTCATTTGCACCACTGCTGTGCCTTGGAATGCTAGTTGGAGT-----------------
----------------------------AATAAATCCCTGAATGAGATTTGG------AAT------
AACATGACTTGGATGGAGTGG---GAGAGAGAAATTGACAATTACACAAAAGAAATATAT---
ACCTTAATTGAAGAATCGCAGAACCAACAGGAA---

AAGAATGAACTAGAGTTATTGGAACTGGATAAGTGGGCAAGTTTGTGGAATTGGTTTGACATAACAAAATGGT
TGTGGTATATCAAAATATTCATAATGATAGTAGGAGGCTTGGTAGGTTTAAGAATAGTTTTTGCT---
GTACTTTCTATAGTA---------AATAGAGTTAGGCAGGGATATTCACCA------
TTGTCATTAGCTATTAGCTTGCTTAACGCCATAGCCATAGCAGTAGCTGAG---
GGGACAGATAGGGTTATAGAAATAGCACAAAGAACTTGTAGGGCT----------------------------
--------ATTCTCAACATA------CCTACAAGAATCAGACAGGGCCTCGAAAGGGCTTTGCTT---TAA
>KX693475
GCT---GCA---
GGAAAATTGTGGGTCACAGTTTATTATGGGGTACCTGTATGG---AAAGAAGCAACT---
ACCACTCTATTTTGTGCATCAGATGCTAAAGCATATAAGACAGAG---GCACATAAT----------------
--------------ATTTGGGCCACA---CATGCCTGTGTACCCACAGAC---
CCCAACCCACAAGAAGTGGTCTTGGAA---------AATGTAACAGAAAACTTTAAC----------------
--------------------------ATGTGGAAAAATGACATGGTAGATCAGATGCATGAAGATATA---
ATCAGTTTATGGGATCAAAGTCTA---AAGCCATGTGTAAAATTA---
ACCCCACTCTGTGTTACTTTAAATTGCACTAATTTGAATAATACCAGTGTTAATACCACT-------------
----

-------------------------AATGATATCAGTAGTACGCCAGAGCCAGGAGAAATGAAA---
AACTGCTCTTTCGATATCACCACAAGCATAGGA---------------------------ACTAAGGTG---
AAAGACTATGCACTT---TTTAATGAACCTGATATAGTATCAATAAAAGAG----------------------
----
----AATAATGACAGTACCAGCTATAGGTTAATAAATTGTAACACCTCAGTCATTACACAA---
GCTTGTCCAAAGGTATCCTTTGAGCCAATTCCCATTCATTATTGTGCCCCGGCTGGTTTTGCGATTCTAAAGT
GTAAC---AATAAAACGTTCAAT------GGGAAAGGACCATGCACAAT------------------------
-GTCAGT---ACAGTACAATGTACACATGGAATTAGGCCAGTAGTATCAACT---
CAATTGCTGTTAAATGGCAGTTTAGCAGAAGAAGAG------------
GTAGTAATTAGATCTGACAATTTCACAGACAAT---------GCTAAAGTTATAATAGTACAGCTGAAT----
-----------GAATCTGTAGAA---------ATTAATTGTACAAGACCCAACAACAATACAAGAAAA-----
-AGTGTATCTCTA------------GGACCAGGGCAAGCA---TGG------TTTACAACAGGACAA------
---------ATAATAGGAGATATA------------AGAAAAGCACATTGC---AACATTAGTAGTACA---
AAATGGAATAACACTTTAAATCAGATAGTTAAAAAATTAAGA---------------CAACAATTTGGG----
-----------------------------------AACAAAACAATAATCTTTAAG------
CGATCTTCAGGAGGGGACCCAGAGATTGAA---GAGCACAGTTTTAATTGTGGAGGGGAA---
TTTAAGCAGCTCCAGGCAAGAGTCCTGGCTGTGGAAAGATACCTAAAGGATCAACAGCTCCTAGGGATTTGGG
GCTGCTCTGGAAAACTCATCTGCCCCACTGCTGTGCCTTGGAATAGTAGTTGGAGT-----------------
----------------------------AATAAATCTCTGAGTGAAATTTGG------GAT------
AACATGACTTGGATGGAGTGG---GAAAGAGAAATTGACAATTACACAAGAGAAATATAC---
ACCTTAATTGAAGAATCGCAGAACCAACAAGAA--

AAGAATGAACTAGAACTATTGGAATTGGATAAGTGGGCAAGCTTGTGGAATTGGTTTGACATAACAAAATGGC
TGTGGTATATCAAAATATTCATAATGGTAGTAGGAGGCTTAGTAGGTTTAAGAATAGTTTTTACT---
GTACTTTCTATAGCA---------AATAGAGTTAGGCAGGGATACTCACCC------
TTGTCATTTGCTATTAGCTTGCTTAACGCCACAGCCATAGCAGTAGCTGAG---
GGGACAGATAGGGTTATAGAAGTAGTAAGAAGAACTTTTAGGGCT----------------------------
--------ATTCTCAATATA------CCTACAAGAATCAGACAGGGCCTCGAAAGAGCTTTGCTA---TAA
>KX693489
GCT---GCA--
GAAAACTTGTGGGTCACAGTCTATTATGGGGTACCTGTATGG---AAAGAAGCAACT---
ACCACTCTATTTTGTGCATCAGATGCTAAAGCATATGATACAGAG---GTACATAAT----------------
--------------GTTTGGCCACA---CATGCCTGTGTACCCACAGAC---
CCCAACCCACAAGAAGTAGTCTTGGTA---------AATGTGACAGAAAACTTTAAC----------------
--------------------------ATGTGGAAAAATAACATGGTAGAACAGATGCATGAAGATATA---
ATCAGTTTATGGGATCAAAGCCTA---AAGCCATGTGTAAAATTA---
ACCCCACTCTGTGTTACTTTACATTGCAAGAATGCTACTGAGACCAGTAATAATACCAGTAGTACA-------
----
---ACAGAGGGGGAGAAATAAAA---
AACTGCTCTTTCAATATCACCACAAGCATAAAA---------------------------ACTGAGGTG---
AGGGACTATGCATTT---TTTTATAACCTCGATATAATACAAATAGATAAT----------------------
----
----------AGTACTACCAGCTATAGGTTGATAAATTGTAACACCTCAGTCATTACACAA---
GCTTGTCCAAAGGTATCCTTTGAGCCAATTCCCATACATTATTGTGCCCCGGCTGGTTTTGCGATTCTAAAGT
GTAAC---AATAAAACATTCAAT------GGAACAGGGCCATGTACAAAT------------------------
-GTCAGT---ACAGTACAATGTACACATGGAATTAGGCCAGTAGTATCAACT---
CAACTGCTGTTAAATGGCAGTTTAGCAGAAGAAGAG------------
GTAGTAATTAGATCTAGCAATTTCACGAACAAT---------GCTAAAGTCATAATAGTACAGCTGAAT----
-----------GAATCTGTAGTA---------ATTAATTGTACAAGACCCAACAACAATACAAGAAAA-----
-AGTATACATCTA------------GGACCAGGGCAAGCA---TGG------TATGCAACAGGACAA------
---------ATAATAGGAGATATA------------AGACAAGCCCATTGT---AACCTTAGTAGAGCG---
CAATGGAACAACACCTTAAGACTGATAACTGTAAAATTAAGA---------------GAACAATTTAAA----
-----------------------------------AACAAAACAATACGCTTTACT------
CAACCTTCAGGAGGGGACCTAGAGATTGTA---ATGCACACTTTTACTTGTGGAGGGGAA---
TTCAAGCAGCTCCAGGCAAGAGTCTTGGCTGTGGAAAGATACCTAAAGGATCAACAGCTCCTAGGGATTTGGG
GTTGCTCTGGAAAACTCATTTGCACCACTGCTGTGCCTTGGAATACTAGTTGGAGT-----------------
----------------------------AATAAATCTCTGGATGAGATTTGG------CTT------
AACATGACTTGGATGGAATGG---GAAAGAGAAATTGGCAATTACACAAGAGAAATATAC---
ACCTTAATTGAAGAATCGCAGAACCAACAAGAA--

AAGAATGAGCTAGAACTATTGGAATTGGATAAGTGGGCAAGTTTGTGGAATTGGTTTGACATAACAAAATGGC
TGTGGTATATCAAAATATTCATAATGGTAGTAGGAGGCTTGGTAGGTTTAAGAATAGTTTTTGCT---
GTACTTTCTATAGTA---------AATAGAGTTAGGCAGGGATACTCACCA------
TTGTCATTAGCTATTAGCTTGCTTAACGCCACAGCCATAGCAGTAGCTGAG---
GGGACAGATAGGGTTATAGAAGTAGCACAAAGAGCTTATAGGGCT----------------------------
--------ATT------------------ACAAGACTCATACAGGGCCGCGAAAGGGCTTTGCTA---TAA
>KX693491
GCT---GCA--
GAAAACTTGTGGGTCACAGTCTATTATGGGGTACCTGTATGG---AAAGAAGCAACT---
ACCACACTATTTTGTGCATCAGATGCTAAAGCATATGATACAGAG---GTACATAAT----------------
--------------ATTTGGGCCACA---CATGCCTGTGTACCCACAGAT---
CCAAACCCACAAGAAGTAGTCTTGGAA---------AATGTGACAGAAAACTTTAAC----------------
--------------------------ATGTGGAAAAATGACATGGTAGAACAGATGCATGAGGATGTA---
ATCAATTTATGGGATCAAAGCCTA---AAGCCATGTGTAAAATTA---
ACCCCACTCTGTGTTATTATGAATTGCAAGAATTTGACTTGTAATGCCACTGATGGGAGTAATACTACTGCCA
ATTGTAATACC--
-------------------ACTAGTAGTAATGCCAATAGGACGATAGAGGGAGGAGAAATGAAA---
AACTGCTCTTTCAATATCACCACAAGCATAAGA---------------------------ACTAATGTG---
AATGATTATGCATTT---TTTTATAATCTTGATCTAATACAAGTAGATAGTAAT-------------------
----
----------GATAATGCCAGCTATAGGTTGATAAACTGTAACACCTCAGTCATTACACAA---
GCTTGTCCAAAGGTAACCTTTGAGCCAATTCCCATACATTATTGTACCCCGGCTGGTTTTGCGCTTCTAAAGT
GTAAC---AATAAAACATTCAAT------GGAACAGGACCATGCACAAAT-----------------------
-GTCAGT---ACAGTACAATGTACACATGGGATTAGACCAGTAGTATCGACT---
CAACTGCTGTTAAATGGCAGTTTAGCAGAAGAAGAG------------
ATAGTAATTAGGTCTAGCAATTTCTCAAACAAT---------GCTAGAATCATAATAATACAGCTGAAT----
-----------GAATCTGTAGAA---------ATTAATTGTACAAGACCCAACAACAATACAAGAAAA-----
-AGTATAACTATA------------GGACCAGGGAGAACA---TGG------TATACAACAGGACAA------
---------ATAATAGGAGATATA------------AGACAAGCACATTGC---AACCTTAGTAGCACA---
AAATGGAATAACACTTTAAGACAGATAACTAAAAAATTAAGA---------------GAACAATATGGG----
-----------------------------------AACAAAACAATAAAGTTTACT---
CAACCAAACAAAGGAGGGGACCCAGAGATTGTA---ATGCACAGTTTTAATTGTGGAGGAGAA---
TTTAAGCAGCTCCAGGCAAGAGTCCTGGCTGTGGAAAGATACCTAAAGGATCAACAGCTCCTAGGGATTTGGG
GTTGCTCTGGAAAACTCATCTGCACCACTGCTGTGCCTTGGAATACTAGTTGGAGT-----------------
----------------------------AATAGATCCCTGGATGAGATTTGG------AAT------
AACATGACTTGGATGGAGTGG---GAAAAAGAAATTGGTAATTACACAAAAGTAATATAC---
ACCTTAATTGAAGAATCGCAGAACCAGCAAGAA--

AAGAATGAACTAGAACTATTGGAATTGGATAAGTGGGCAAGTTTGTGGAATTGGTTTGACATAACAAACTGGC
TGTGGTATATCAGAATATTCATAATGATAGTAGGAGGCTTGATAGGTTTAAGGATAGTTTTTGCT---
GTACTATCTATAGTA---------AATAGAGTTAGGCAGGGATACTCACCA------
TTGTCGTTAGCTATTAGCTTGCTTAACGCCACAGCCATAGCAGTAGCTGAG---
GGGACAGATAGGATTATAGAAGTAGCACAAAGAGCTTATAGGGCT----------------------------
--------ATTCTTAACATA------CCTACAAGAATCAGACAGGGCCTTGAAAGGGCTTTGCTA---TAA
>KX693496
GCT---ACA--
AAAAACTGGTGGGTCACAGTCTATTATGGGGTACCTGTATGG---AAAGAAGCAACT---
ACCACTCTATTTTGTGCATCAGATGCTAAAGCATATGATACAGAG---GTACATAAT----------------
--------------ATTTGGGCCACA---CATGCCTGTGTACCCACAGAC---
CCCAACCCACAAGAAGTAGTCTTGGGA---------AATGTGACAGAAAACTTTAAC----------------
--------------------------ATGTGGGAAAATAACATGGTAGAACAGATGCATGAAGATATA---
ATCAGTTTATGGGATCAAAGCCTA---AAGCCATGTGTAAAATTA---
ACCCCACTCTGTGTTACTTTAAATTGCACTAAGTTGGAGAATATTACTAATACCAATAGTAATACCAGT----
----
-------------AGTAATACCAGTAGTACTAACAGTAGCACGCCAGAGGGAGGAGAAATGAAA---
AATTGCTCTTTCAATATCACCACAAGCATAAGA---------------------------ACTAAGGTG---
AAAGACTATGCACTT---TTTTATAACCTTGATATAATACCAATAAATAATAAGGAA----------------
----
----------AATAATACCAGATATAGGTTGATAAATTGTAACACCTCAGTCATTACACAA---
GCTTGTCCAAAGGTATCCTTTGAGCCAATTCCCATTCATTATTGTGCCCCGGCTGGTTTTGCGATTCTAAAGT

GTAAC---AATAAAAAATTCAAT------GGAGCAGGACCATGTGAAAAT-----------------------
-ATCAGT---ACAGTACAATGTACACATGGAATTAGGCCAGTAGTATCAACT---
CAACTGCTGTTAAATGGCAGTTTAGCAGAAGAAGAG------------
GTAGTAATTAGATCGAGCAATTTCACGAACAAT---------GCTAAAGTCATAATAGTACAGCTGAAT----
-----------GAATCTGTAGAA---------ATTAATTGTACAAGACCCAACAACAATACAAGAAAA-----
-AGTATACCTCTA------------GGACCAGGGAAAGCA---TGG------TATACAACAGGACAA------
---------ATAATAGGAGATATA------------AGACAAGCACATTGT---AACCTGAGTACAACA---
AAATGGAACAAAATTTTAGAACAGATAACTGAAAAATTAAGA---------------GAACAATTTGGG----
-----------------------------------AACAAAACAATAATCTTTAAT------
CAATCTTCAGGAGGGGACCCAGAGATTGTA---ATGCACAGTTTTAATTGTGGAGGGGAA---
TTTAAGCAGCTCCAGGCAAGAGTCTTGGCTGTGGAAAGATACCTAAAGGATCAACAGCTCCTAGGGATTTGGG
GTTGCTCTGGAAAACTCATTTGCCCCACTGCTGTGCCTTGGAATAGTAGTTGGAGT-----------------
----------------------------AATAAATCTCTGGATGAGATTTGG------AAT------
AACATGACTTGGATGGAGTGG---GAAAGAGAAATTGGCAATTATACAAGAGAAATATAC---
ACCTTAATTGAAGAATCGCAGAACCAACAAGAA--

AAGAATGAACTAGAACTATTGGAATTGGATAAGTGGGCAAGTTTGTGGAATTGGTTTGACATAACAAACTGGC
TGTGGTATATCAAAATATTCATAATGATAGTAGGAGGCTTGGTAGGTTTAAGAATAGTTTTTACT---
GTACTTTCTATAGTG---------AATAGAGTTAGGCAGGGATACTCACCA------
TTGTCATTAGCTATTAGCTTGCTTAACGCCACAGCCATAGCAGTAGCTGAG---
GGGACAGATAGGATTATAGAAGGAGCACAAAGAGCTTATAGGGCT----------------------------
--------ATTCTCAATATA------CCTACAAGAATCAGACAGGGCCTCGAAAGGGCTCTGCTG---TAA
>KX693539
GCT---GTG--
GAAAACTTGTGGGTCACAGTCTATTATGGGGTACCTGTATGG---AAAGAAGCAACT---
ACCACTCTATTTTGTGCATCAGATGCTAAAGCATATGATACAGAG---GCACATAAT----------------
--------------ATTTGGGCCACA---CATGCCTGTGTACCCACAGAC---
CCCAACCCACAAGAAGTAGTATTGGGA---------AATGTGACAGAGAACTTTAAC----------------
--------------------------ATGTGGAAAAATAACATGGTAGAACAGATGCATGAAGATATA---
ATCAGTTTATGGGATCAAAGCCTA---AAGCCATGTGTAAAATTA---
ACCCCACTCTGTGTTACTTTAAATTGCACTGATTTGAGGAATACTACTGGTATTGATAGT-------------
----
----------------------------AATACCAGTAGTACGACAGAGGGAGGAGAAATAAAA---
AACTGCTCTTTCAATATCACCACAAACATAAGA---------------------------ACTAAGGTG---
AAAGACTATGCATTT---TTTTATAAACTTGATTTAGTACAAATAGATAAT----------------------
----
----------GATAATACCAGCTATAGGTTGATAAGTTGTAACACCTCAGTCATTACACAA---
GCTTGTCCAAAGGTATCCTTTGAGCCAATTCCCATACATTATTGTGCCCCGGCTGGTTTTGCGATTCTAAAGT
GTAAC---AATAAAACGTTCAGT------GGAACAGGACCATGTACAAAT-----------------------
-GTCAGT---ACAGTACAATGTACACATGGAATTAGGCCAGTAGTATCAACT---
CAACTGCTGTTAAATGGCAGTTTAGCAGAAAAAGAG------------
GTAGTAATTAGATCTAGCAATTTCGCGGACAAT---------GCGAGAGTCATAATAGTACAGCTGAAG----
-----------GAATCTGTAGAA---------ATTAATTGTACAAGACCCAACAACAATACAAGAAAA-----
-AGTATACATCTA------------GGGCTAGGGCGAGCA---TGG------TATACAACAGGACAA------
---------ATAATAGGAAATATA------------AGACAAGCACATTGT---AACATTAGTAGAGCA---
AAATGGAATAGCACTTTAAAACAGATAACTGAAAAATTAAGA---------------GAACAATTTGGA----
-----------------------------------AACAAAACAATAATCTTTAAT------
CAATCTTCAGGAGGGGACCCAGAGATTACA---ATGCACAGTGTTAATTGTGGAGGGGAA---
TTTAAGCAGCTCCAGGCAAGAGTCTTGGCTGTGGAAAGATACCTAAAGGATCAACAGCTCCTAGGAATTTGGG
GTTGCTCTGGAAAACTCATTTGCACCACTGCTGTGCCTTGGAATGCTAGTTGGAGT-----------------
----------------------------AATAAATCTCTGAGTGAGATTTGG------GAT------
AACATGACTTGGATGGAGTGG---GAAAAAGAAATTGACAATTACACAAGAGAAATATAC---
ACCTTAATTGAAGAATCGCAGAACCAACAAGAA--

AAAAATGAACTAGAACTATTGGAATTGGATAAGTGGGCAAGTTTGTGGAATTGGTTTGACATAACAAAATGGC
TGTGGTATATCAAAATATTCATAATGATAGTAGGAGGCTTGGTAGGTTTAAGAATAGTTTTTGCT---
GTACTTTCTATAGTA---------AATAGAGTTAGGCAGGGATACTCACCG------
TTGTCATTAGCTATTAGCTTGCTTAACGCCACAGCCATAGCAGTAGCTGAG---
GGGACAGATAGGGTTATAGAAGTAGTACAAAGAGTTTATAGGGCT----------------------------
--------ATTCTCGACATA------CCTACAAGAATCAGACAGGGCCTCGAAAGGGCTTTGCTA---TAA

>KP109512
GCT---ACA---
GAACAATTGTGGGTCACAGTCTATTATGGGGTACCTGTGTGG---AAAGAAGCAACC---
ACCACTCTATTTTGTGCATCAGATAGTAAAGCATATGATACAGAG---GCACATAAT---------------
--------------GTATGGGCCACA---CATGCCTGTGTACCCACAGAC---
CCCAACCCACAAGAAATAGTATTGGGA---------AATGTGACAGAAAACTTTAAC---------------
-------------------------ATGTGGAAAAATGACATGGTAGAACAGATGCATGAAGATATA---
ATTAGCTTATGGGATCAAAGCCTA---AAGCCATGTGTAAAATTA---
ACCCCACTCTGTGTTACTTTAAAGTGTACTGATTATAATGGGAATGATATTACTAATGGGAATAAAACTGAGA
AG--
------------------GCTATTGATGGAAATGCTACTATTAGTGAGAAGGGAGAAATAAAA---
AACTGCTCTTTCAATGTCACCACAAATATAAGA--------------------------
AATAAGGTGCAGGGACAATATGCACTT---TTTTATAAACTTGATATAGTACAAATTGATGAT---------
----

AGTGATAATACTACTTATAGGTTGATAAGTTGTAACACCTCAGTTATTACACAG---
GCCTGTCCAAAGACATCCTTTACGCCAATTCCTATACATTATTGTGCCCCGGCTGGTTTTGCGATTCTAAAGT
GTAAT---GATATTAAGTTCAAT------GGAACAGGGCCATGTAAAAAT----------------------
-GTCAGC---ACAGTGCAATGTACACATGGAATTAGGCCAGTAGTATCAACT---
CAACTGCTGTTAAATGGCAGCCTAGCAGAAAAGGAAGTA---
AATGGCAGCCTAGCAGAAAAGGAAGAAGGGGTAGTAATTAGATCTAGCAATTTCACGAACAATGCTAAACCCA
TAATAGTACAGCTGACAGAAGCTGTAAAT---------
ATTACATGTACAAGACCCAACAATAATACAAGAAGA------GGTATACACATA------------
GGACCAGGGAGAGCA---ATT------TATGCAACAGGAGCA---------------ATAATAGGAAAAATA-
-----------AGACAAGCACATTGT---AACCTTAGTAGAACA---
GCATGGAATAACACTTTAAGAAAGATAGCTAAAAAATTAAGA---------------GAACAATTTAAT----
-----------------------------------AATAAAACAATAATCTTTACT------
CACCCCTCAGGAGGGGACCCAGAAATTGTA---ATGCACAGTTTTAATTGTGCAGGGGAA---
TATAAGCAGCTCCAGGCAAGAGTCCTGGCTGTGGAAAGATACCTAAAGGATCAGCAGCTCCTGGGAATTTGGG
GTTGCTCTGGAAAGCTCATCTGCACCACTGCTGTGCCTTGGAATACTAGTTGGAGT-----------------
----------------------------AATAAATCTTATAATAAGATTTGG------GAT------
AATATGACCTGGATGGAGTGG---GAAAGAGAAATTGACAATTACACAGAGTTAATATAC---
AATTTAATTGAACAATCGCAGAACCAGCAGGAG---------------------------------------
----
AAAAATGAACAAGAATTATTGGAATTGGACAGTTGGGCAAGTTTGTGGAGTTGGTTTAGCATATCCCAATGGC
TGTGGTATATAAGAATATTCATAATGATAGTAGGAGGCTTGGTAGGTTTAAGAATAGTTTTTGCT---
GTACTTTCTATAGTA---------AATAGAGTTAGGCAGGGATACTCACCA------
TTACCGTTTGCTGTTAGCTTGCTCAACGCCATAGCCATAGCAGTAGGTGAG---
GGAACAGATAGGGTTATAGAAGCATTGCAGAGAATTGGTAGAGCT---------------------------
--------ATTCTCCACATA------CCTAGAAGAATAAGACAGGGCTTGGAAAGGGCTTTGCTA---TAA
>JF932495
GCT---GCA---
GAAAACTTGTGGGTCACAGTCTATTATGGGGTACCTGTGTGG---AAAGAAGCAACC---
ACCATTCTATTTTGTGCATCAGATGCTAAAGCATATGATACAGAG---GTACATAAT---------------
--------------GTTTGGGCCACA---CATGCCTGTGTACCCACAGAC---
CCCAACCCACAAGAAATGGTCTTGGGA---------AATGTAACAGAAAACTTTAAC---------------
-------------------------ATGTGGGAAAATGACATGGTAGAACAGATGCATGAAGATATA---
ATTAGTTTATGGGATCAAAGCCTA---AAGCCATGTGTAAAATTA---
ACCCCACTCTGTGTTACTTTAAATTGCACTGATTTGAAGAATAATGCTACTACTAATAACAGT---------
----
------------------------------------AGTATAAAGGGGGGAGGAGAACTGAAA---
AACTGCTCTTTCGATATCACCACAAGCATAAAC--------------------------ACTAAGGTG---
AAAGACTATGCATTT---TTTTATAATCTTGATATAGTACAAATAGATAAT---------------------
----
----------GATAATACCAGCTATAGGTTGATAAATTGTAACACCTCAGTCATTACACAA---
GCTTGTCCAAAGGTATCCTTTGAGCCAATTCCCATACATTATTGTACCCCGGCTGGTTTTGCGATTGTAAAGT
GTAAC---AATAAAAAGTTCAAT------GGAACAGGACCATGTACAAAT----------------------
-GTCAGT---ACAGTACAATGTACACATGGAATTAGGCCAGTAGTATCAACT---
CAACTGCTGTTAAATGGCAGTTTAGCAGAAGAAGAG-----------
GTAGTAATCAGATCTAGCAATTTCTCGAACAAT---------GCTAAAGTCATAATAGTACAGCTGAAT----

-----------GAATCTGTAGTA---------ATTAATTGTACAAGACCCAACAACAATACAAGAAAA-----
-AGTATACCTCTA------------GGACCAGGGAGAGCC---TGG------TATACAACAGGAGAA------
---------ATAATAGGAGATATA------------AGACAAGCACATTGT---AACCTTAGTAGCACA---
AAATGGAATAACACTTTAAACCAGATATATAAAAAATTAAAA---------------GAAATATAT-------
-----------------------------------AACAAAACAATAGTCTTTAAT------
CAATCCTCAGGAGGGGACCCAGAGATTGTA---ATGCACACTTTTAATTGTGGAGGGGAA---
TTTAAGCAGCTCCAGGCAAGAGTCCTGGCTGTGGAAAGATACCTAAAGGATCAACAGCTCCTAGGGATTTGGG
GTTGCTCTGGAAAACTCATTTGCACCACTGCTGTGCCTTGGAATACTAGTTGGAGC-----------------
-------------------AATAGA---TCTCAGGATGAGATTTGGAATAAT------GAG------
AATATGACTTGGATGAAGTGG---GAAAGAGAAATTGCCAATTACACAAAAGAAATATAC---
ACCTTAATTGAACAATCGCAGAACCAACAAGAA--

CAGAATGAACTAGACTTATTGAAATTGGATGAGTGGGCAAGCTTGTGGAATTGGTTTGACATATCAAAATGGC
TGTGGTATATAAAACTATTCATAATGATAGTAGCAGGCCTGGTAGGTTTAAGAATAGTTTTTGCT---
GTACTTTCTATAGTA---------AATAGAGTTAGGCAGGGATACTCACCA------
CTGTCATTTGCTGTTAGCTTGCTCAACGCCACAGCCATAGTAGTAGCTGAG---
GGGACAGATAGGGTTATAGAAGTAGTACAAAGAACTTATAGAGCT----------------------------
--------ATTCTCCACATA------CCTAGAAGAATAAGACAGGGCTTTGAAAGGGCTTTGCTA---TAA
>JF932496
GCT---GCA--
GAACCCTTGTGGGTCACAGTCTATTATGGGGTACCTGTATGG---AAAGAAGCAACC---
ACCACTCTATTTTGTGCATCAGATGCTAAAGCATATGATACAGAG---GTACATAAT----------------
--------------GTTTGGGCCACA---CATGCCTGTGTACCCACAGAC---
CCCAACCCACAAGAAGTAGTCTTGGGA---------AATGTGACAGAAAACTTTAAC----------------
--------------------------ATGTGGAAAAATGACATGGTAGAACAGATGCATGAAGATATA---
ATCAGTTTATGGGATCAAAGCCTA---AAGCCATGTGTAAGATTA---
ACCCCACTCTGTGTTACTTTAAATTGCATTGATTATTTTAGGAATACTACT----------------------
----
----------------------------AGTAACAGTAGTAGGATAGAGGGGGGAGAAATGAAA---
AACTGCTCTTCCAATATTACCACAAGCATAAAA---------------------------ACTAAGGTG---
AGAGACTATGCACTT---TTTTATAAACTTGATGTAGTACCAATAAATAATACT-------------------
----
----------GATAATACCAGCTATAGGTTGATAAGTTGTAACACCTCAACCATTACACAG---
GCTTGTCCAAAGGTATCCTTTGAGCCAATTCCCATACATTATTGTACCCCGGCTGGTTTTGCGATTCTAAAGT
GTAAC---AATAAAAAGTTCAAT------GGAACAGGACCATGTACAAAT-----------------------
-GTCAGT---ACAGTACAATGTACACATGGAATTAGGCCAGTAGTATCAACT---
CAACTGCTGTTAAATGGCAGTTTAGCAGAAGAAGAG------------
GTGGTAATTAGATCTAGCAATTTCTCGAACAAT---------GCTAGAGTCATAATAGTACAGCTGAAT----
-----------GAATCTGTAGAA---------ATTAATTGTACAAGACCCAACAACAATACAAGAAAA-----
-AGTATAAATCTA------------GGACTAGGGCGAGCA---TGG------TTTACAACAGGACAA------
---------ATTATAGGAGATATA------------AGACAAGCACATTGT---AACCTTAGTAGCACA---
AAATGGAATAACACTTTAAGACAGATAGCTAAAAAATTAAGA---------------GAACAATTTGGG----
-----------------------------------AACAAAACAATAGTCTTTAAT------
AAATCCTCAGGAGGGGACTTAGAGATTGAA---ATGCACAGTTTTAATTGTGGAGGGGAA---
TTTAAGCAGCTCCAGGCAAGAGTCCTGGCTGTGGAAAGATACCTAAAGGATCAACAGCTCCTAGGGATTTGGG
GTTGCTCTGGAAAACTCATTTGCACCACTGCTGTGCCTTGGAACACTAGTTGGAGT-----------------
----------------------------AAGAGATCTATGGATGATATTTGG------AAT------
AATATGACTTGGATGCAGTGG---GAAAGAGAAATTGAGAATTACACAAAAGAAATATAC---
ACCTTAATTGAACAATCGCAGAACCAGCAAGAA--

AAGAATGAACTAGACTTATTGGAATTGGATAAGTGGGCAAGTTTGTGGAATTGGTTTGACATATCAAATTGGC
TGTGGTATATAAAAATATTCATAATGATAGTAGGAGGCTTGGTAGGTTTAAGAATAGTTTTTGCT---
GTGCTTTCTATAGTA---------AATAGAGTTAGGCAGGGATACTCACCA------
TTGTCATTAGCTGTTAGCTTGCTCAATGCCACAGCAATAGCAGTAGCTGAG---
GGGACAGATAGGGTTATAGAAGTAGTACAAAGAGCTTATAGAGCT----------------------------
--------ATTCTCCACATA------CCTACAAGAATAAGACAGGGCTTAGAAAGGGCTTTGCTA---TAA
>GU647196
GCT---GCA--
GAAAACTTGTGGGTCACAGTCTATTATGGGGTACCTGTATGG---AAAGAAGCAACT---
ACCACTCTATTTTGTGCATCAGATGCTAAAGCATATGAGACAGAG---GTACATAAT----------------

--------------ATTTGGGCCACA---CATGCCTGTGTACCCACAGAC---
CCCAGCCCACGAGAAGTAGTCTTGGAA---------AATGTGACAGAAAACTTTAAC---------------
-------------------------ATGTGGAAAAATGACATGGTAGAACAGATGCATGAAGATATA---
ATCAGTTTATGGGATGAAAGCCTA---AAGCCATGTGTAAAATTA---
ACCCCACTCTGTGTTACTTTACATTGCACTAATTTGGAGAATACTACTAATACCAGTAGT------------
----
----------------------------AATACCAGTAGTATGACAGAGGGAGGAGAAATGAAA---
AACTGCTCTTTCAATATCACCACAAGCATAAAA--------------------------ACTAAGGTG---
CAAGACTATGCACTT---TTTTATAAACCTGATATAGTACAAATAGATAAT---------------------
----
----------AGTAATACCAGCTATAGGTTGATACATTGTAACATCTCAGTCATTACACAA---
GCTTGTCCAAAGGTATCCTTTGAGCCAATTCCCATACATTATTGTACCCCGGCTGGTTTTACGATTCTAAAGT
GTAAC---AATAAAAAGTTCAAG------GGAGCAGGACCATGTACAAAT----------------------
-GTCAGT---ACAGTACAATGTACACATGGAATTAGACCAGTAGTATCAACT---
CAACTGCTGTTAAATGGCAGTTTAGCAGAAGAAGAG-----------
GTAGTAATTAGATCTAGCAATTTCTCGGACAAT---------GCTAAAGTCATAATAGTACAGCTGAAT----
-----------GAATCTGTAGAA---------ATTAATTGTACAAGACCCAACAACAATACAAGAAAA-----
-AGGGTAAGTATA------------GGACCAGGGAGAGCA---TGG------TATACAACAAAACAA------
---------ATAGTAGGAGATATA------------AGACAAGCACATTGT---AACATTAGTAGAACA---
AAATGGAATGACACTTTAAAACAGATAACTGAAAAATTAAGA---------------GAACAATTTGGG----
-----------------------------------AACAAAACAATAATCTTTAAT------
CAATCTTCAGGAGGGGACCCAGAAATTGTA---AGGCACAGTTTTAATTGTGGAGGGGAA---
TTTAAGCAGCTCCAGGCAAGAGTCTTGGCTGTGGAAAGATACCTAAAGGATCAACAGCTCCTAGGGATTTGGG
GTTGCTCTGGAAAACTCATCTGCACCACTGCTGTGCCTTGGAATGCTAGTTGGAGT----------------
----------------------------AATAAATCCCTGAGTCAGATTTGG------GAT------
AACATGACTTGGATGGAGTGG---GAAAGAGAAATTAACAATTACACAAAAGAAATATAC---
ACCTTAATTGAAGAATCGCAGAACCAGCAAGAA---------------------------------------
----
AAGAATGAACTAGACCTATTGAAATTGGATGAGTGGGCAAGTTTATGGAATTGGTTTGACATAACAAAATGGC
TGTGGTATATCAAAATATTCATAATGGTAGTAGGAGGCTTGGTAGGTTTAAGAATAGTTTTTGCT---
GTACTTTCTATAGTA---------AATAGAGTTAGGCAGGGATACTCACCA------
TTGTCATTGGCTATTAGCTTGCTTAACACAACAGCCATAGCAGTAGCTGAG---
GGGACAGATAGGATTATAGAAGTAGCACAAAGAGCTTATAGGGCT--------------------------
--------ATTATCCACATA------CCTAGAAGAGTCAGACAGGGCCTCGAAAGGGCTTTGCTG---TAA
>EU363830
GCT---GCA---
GAAAACTTGTGGGTCACAGTCTATTATGGGGTACCTGTATGG---AAAGAAGCAACT---
ACCACTCTATTTTGTGCATCAGATGCTAAAGCATATGCTACAGAG---GTACATAAT---------------
--------------ATTTGGGCCACA---CATGCCTGTGTACCCACAGAC---
CCCAACCCACAAGAAGTAGTCTTGGAA---------AATGTGACAGAAAACTTTAAT---------------
-------------------------ATGTGGAAAAATGACATGGTAGAACAGATGCATGAAGATATA---
ATCAGTTTATGGGATCAAAGCCTA---AAACCATGTGTAAGATTA---
ACCCCACTCTGTGTTACTTTAAATTGCACTACTTTGAAGAATATTAATAATACCAGTGGT------------
----
----------------------------AATATCAGTAATGTAAAAGAGGAAGGGGAAATAAAA---
AACTGCTCTTTCAATATCACCACAAGCATAAAA--------------------------ACTAAGGTG---
AAAGACTATGCAATT---TTTAATAGACTTGATGTAATACCAATAAGTAAT---------------------
----
----------GATAGTACCAGCTATAGGTTGATAAATTGTAACACCTCAGTCATTACACAG---
GCCTGTCCAAAGGTATCCTTTGATCCAATTCCCATACATTATTGTGCCCCGGCTGGTTTTGCGATTCTAAAGT
GTAAC---AATAAAACGTTCAAT------GGAACAGGACCATGTACAAAT----------------------
-GTCAGT---ACAGTACAATGTACACATGGAATTAGGCCAGTAGTATCAACT---
CAACTGCTGCTAAATGGCAGTTTAGCAGAAGAAGAG-----------
GTAGTAATTAGATCTAGCAATTTCACGAACAAT---------GCTAGAATCATAATAGTACAGCTGAAT----
-----------GAATCTGTAGAA---------ATTATTTGTACAAGACCCAACAACAATACAAGAAAA-----
-GGTATACATATA------------GGACCAGGGAAAACA---TGG------TATGCAACAGGACAA------
---------ATAATAGGAGACATA------------AGACAAGCATATTGT---AACCTTAGTAGCACA---
AATTGGACTAACACTTTAAAACAGATAACTGAAAAATTAAGA---------------GAACAATTTGGG----
-----------------------------------AACAAAACAATAATCTTTAGT------
CGATCCTCAGGAGGGGACCCAGAGATTGTA---ATGCACAGTTTTAATTGTGGAGGGGAA---

TTTAAGCAGCTCCAGGCAAGAGTCTTGGCTGTGGAAAGATACCTAAAGGATCAACAGCTCCTAGGGATTTGGG
GTTGCTCTGGAAAACTCATCTGCACCACTGCTGTGCCTTGGAATGCTAGTTGGAGT-----------------
----------------------------AATAAATCCCTGGATGAGATTTGG------GAT------
AACATGACTTGGATGGAGTGG---GAAAGAGAAATTGAAAATTACACAAGAGAAATATAC---
ACCTTAATTGAAAAATCGCAGAACCAACAGGAG--

AAAAATGAACTAGAACTATTGGAATTGGATAAGTGGGCAAGTTTGTGGAATTGGTTTGACATAACAAACTGGC
TGTGGTATATCAAAATATTCATAATGATAGTAGGAGGCTTGATAGGTTTAAGAATAGTTTTTGCT---
GTACTTTCTATAGTA---------AATAGAGTTAGGCAGGGATACTCACCA------
TTGTCATTAGCTATTAGCTTGCTTAATACCACAGCTATAACAGTAGCTGAG---
GGGACAGATAGGGTTATAGAAGTAGCACAAAGAGCTTATAGAGCT----------------------------
--------TTTATTCACATA------CCTAGAAGAATCAGACAGGGCTTCGAAAGGGCTTTGCTA---TAA
>AY561236
GCT---ACA--
GAACAATTGTGGGTCACAGTCTATTATGGGGTACCTGTATGG---AAAGAAGCAAAC---
ACTGTTCTATTTTGTGCCTCAGATGCTAAAGCATATGGTACAGAG---GTACATAAT----------------
--------------GCTTGGGCCACA---CGTGCCTGTGTACCCACAGAC---
CCCAACCCACAAGAAGTAGTATTGGAA---------AATGTGACAGAAAATTTTAAC----------------
--------------------------ATGTGGAAAAATGGCATGGTAGACCAGATGCATGAAGATATA---
ATTAGTTTATGGGATGAAAGCCTA---AAGCCATGTGTCAAATTA---
ACCCCACTATGTGTCACTTTAAATTGCACGAATGCTAATACCACTAGTAGTGGGAGA----------------
----
-------------------------------GTGATGCCCACAATGAAGCCAGAAGAAATAAAA---
AACTGCTCTTTCAATGTCACCACAAGCATAGGA---------------------------AGTAAGGTA---
CAAGAAACTGCACTT---TTTTATAGACTTGATGTAGAACCAATAGATAAG----------------------
----
----------AATGATAGCAGATATAGGTTGATACATTGTAACACCTCAGTCATTAAACAG---
GCCTGTCCAAAGGTATCCTTTGAGCCAATTCCCATACATTATTGTACCCCGGCTGGTTTTGCGATTCTACAGT
GTAAT---GATAAAAAGTTCAAT------GGAACAGGACCATGTACAAAT-----------------------
-GTCAGC---ACAGTACAATGTACACATGGAATTAGGCCAGTAGTGTCAACT---
CAACTACTGCTAAATGGCAGTCTAGCAGAAGAAGAG------------
ATAGTAATTAGATCTGAGAATTTCACGGACAAT---------GCTAAAACCATAATAGTACAGCTAAAT----
-----------GTTTCTGTAGAA---------ATTAATTGTACAAGACCCAACAATAATACAAGAAAA-----
-GGTATACATCTA------------GGACCAGGGAAAGCA---TTC------TATGCATCAGGAGAC------
---------ATAGTAGGAAACATA------------AGACAAGCACATTGT---ACACTTAATAGAACA---
AAATGGGAGAAAACTTTAGGACAGGTAGCTAACAAACTAAGA---------------GAACAATATAAA----
-----------------------------------AATAAAACAATAATCTTTAAT------
CAATCCTCAGGAGGGGACCTAGAAACTGTA---ATGCATAGTTTTAATTGTGGAGGGGAA---
TTTAAGCAGCTCCAGGCAAGAGTCCTGGCTGTAGAAAGATACCTAAAGGATCAACAGCTCCTGGGAATTTGGG
GTTGCTCTGGAAAGCTCATTTGCACCACTACTGTGCCTTGGAATGCTAGCTGGAGC-----------------
----------------------------AATAAATCTCTAGATATGATTTGG------AAT------
AACATGACCTGGATGGACTGG---GAAAAAGAAATTGACAATTACACAGGCTTAATATAT---
ACCTTAATTGAAGAATCGCAGAACCAACAGGAA--

AAGAATGAAAAAGAATTATTGGAATTGGACAAGTGGGCAAGTCTGTGGACATGGTTTGACATAACAAACTGGC
TGTGGTATATAAGACTATTCATAATAATAGTAGGAAGCTTAGTAGGTTTAAGAATAATTTTTGCT---
GTACTTTCTATAGTG---------AATAGAGTTAGGCAGGGCTACTCACCA------
TTATCATTTGCTGTTAGCTTGCTTAATGTCACAGCTATAGCAGTAGCTGAG---
GGGACAGATAGGATTATAGAAGGAGTACAAAGAGCTTGTAGAGCT----------------------------
--------ATTCGCAACGTA------CCTAGAAGAATAAGACAGGGCTTAGAAAGGGCTTTACTA---TAA
>AY561239
GCT---ACA--
GACAAATTGTGGGTCACAGTCTATTATGGAGTACCTGTGTGG---AAAGAAGCAACC---
ACCACTCTATTTTGTGCATCAGATGCTAAAGCATATGATGCAGAA---GCACATAAT----------------
--------------GTTTGGGCCACA---CATGCTTGTGTACCCACAGAC---
CCCAATCCACAAGAAGTAGTATTGACA---------AATGTGACAGAAAATTTTAAC----------------
--------------------------ATGTGGAAAAATAACATGGTAGAACAAATGCAGGAAGATATA---
ATCAGTTTATGGGATCAAAGCCTA---AAGCCATGTGTAAAATTA---
ACCCCACTCTGTGTTACTTTAAATTGCAGTGATGTTGTGAATACTAATAATACCAATAAT-------------
----

----------------------------AGTAGCGGGGAAGAAATAGGGAGAGGAGAAATAAAA---
AACTGCTCTTTCAAT------ATAAGAGAT------------------------------AGGGGGCGA---
AAAGAATATGCAGTT---TTTTATAAACTTGATGTAGTACAAATAGATAATAAGAATAAT-------------
----
AGGAGTAGGAATAATGGGAGTGCCAGCTATAAATTGATAAGTTGTAACACCTCAGTCATTACACAA---
GCCTGCCCAAAGATATCCTTTGATCCAATTCCCATACATTATTGTACCCCGCCTGGTTTTGCGCTTCTAAAGT
GTAAC---GATAAGAAATTCAAT------GGAACAGGACAATGTACAAAT-----------------------
-GTCAGC---ACAGTACAATGTACACATGGAATTAGGCCAGTAGTATCAACT---
CAGCTGCTGTTAAATGGCAGTCTAGCAGAAGAAGAT------------
GTAGTAATTAAATCTGAAAATTTCGCGAACAAT---------GCTAAAACCATAATAGTACAATTGAAT----
-----------GAAACTGTAAAA---------ATTACTTGTATAAGACCCAACAACAATACAAGAAGA-----
-AGTGTACATCTA------------GGACCAGGGAGGGGA---GCA------ATTTATACAGGGGAT------
---------ATAATAGGAAAAATA------------AGACAAGCATATTGT---AACCTTAGTGAAGCA---
GAGTGGGATAAGACTTTAAAACGGATAGTTACAAAATTAAGA---------------GAGCAATTTAAG----
-----------------------------------AATAAAACAATAGCCTTTAAT------
CAATCCTCAGGAGGGGACCCAGAAATTGTA---ATGCACAGTTTTAATTGTGGAGGGGAA---
TTTAAGCAGCTCCAGGCGAGAGTCCTGGCTGTGGAAAGATACCTAAAGGATCAACAGCTCCTGGGGATTTGGG
GTTGCTCTGGAAAACTCATCTGCACCACCGCTGTGCCTTGGAATGCTAGCTGGAGT-----------------
----------------------------AATAAATCTCTAGATGACATTTGG------CAT------
AACATGACCTGGATGCAATGG---GAGAAAGAAATTAACAATTACACAGGCTTAATATAT---
ACCTTAATTGAAGAATCGCAAAACCAGCAAGAA--

AAGAATGAACAAGAATTATTAGAATTGGATAAATGGGCAAGTTTGTGGAATTGGTTTGACATATCAAATTGGC
TGTGGTATATAAAAATATTCATAATAATAGTAGGAGGCTTGGTAGGTTTAAGAATAGTTTTTACT---
GTGCTTTCTATAATA---------AATAGGGTTAGGAAGGGATATTCACCA------
TTATCGTTTGCTGTTAGCTTGCTTAATGCCACAGCCATAGCAGTAGCTGAG---
GGAACAGATAGGGTCTTAGAAGTATTACAAAGAGCTTACAGAGCT----------------------------
--------ATTCTCCACATA------CCTAGAAGAATAAGACAGGGCTTGGAAAGGGCTTTGCTA---TAA
>HQ377377
GTT---GTA---GGA------------------------------------
CAACAATTGTGGGTCACAGTCTATTATGGGGTACCTGTGTGG---AAAGAAGCTACC---
ACCACACTATTTTGTGCATCAGATGCTAAAGCATATGATACAGAG---GTACATAAT----------------
--------------GTTTGGCCACA---CATGCCTGTGTACCCACAGAC---
CCCAATCCACAAGAAATACTATTGCCA---------AATGTAACAGAAGATTTTGAC----------------
--------------------------ATGGGAAAAAATAACATGGTAGATCAGATGCATGAGGATATA---
ATCAGTTTATGGGATCAAAGCCTA---AAACCCTGTGTAAAACTG---
ACCCCACTCTGTGTCACTTTAAATTGCACTAATATTACTAAATCAGAAAATATTACTCAGGCCAATATTACTA
CGTCAGGGAATATT---
----ACTCAGGTCACTCAGGACACCAATAGTAGTAGGGAAATAATGGGGGACGGGGACATGAAA---
AACTGCTCTTTCGATGTTACCACAGGCATAAGA---------------------------
GATAAGGTGAAAAAAGAATATGCACTT---TTTTATAACCTTGATATAACACAAATAGATAAT----------
----

GACACCTATAGGTTGACAAGTTGTAACACCTCAGTCATTACACAG---
GCCTGTCCAAAGGTATCCTTTGAGCCAATTCCCATACATTATTGTACCCCAGCTGGTTATGCACTTCTGAAGT
GTAAG---GATAAAAAGTTCAAT------GGAACAGGACCATGTACAAAT-----------------------
-GTCAGC---ACAGTACAATGTACACATGGCATTAGGCCAGTAGTATCAACT---
CAACTACTGTTAAATGGCAGTCTAGCAGAAGAAGGG------------
GTAGTAATTAGATCTCAAAATTTCTCAAACAAT---------GCTAAAACCATAATAGTACAGCTGAAG----
-----------GACGCTGTAAAG---------ATTAATTGTACAAGACCCAACAACAATACAAGAAAA-----
-GGTATACATATA------------GGACCAGGGAGAGCA---TTT------TACACAACAGGTGAC------
---------ATAATAGGAGATATA------------AGACAAGCACATTGT---AACCTTAGTAGAGCA---
GAATGGAATAACACTTTAAGCCAGGTAGCTACCGAATTAAGT---------------AAACAATTTGGC----
--------------------------------AATAACAAAACAGTCGTCTTTAAT------
TCATCCTCAGGAGGGGACCTAGAAATTGTA---ATGCACATTGTTAATTGTGGAGGGGAA---
TTCAAGCAGCTCCAGGCAAGAGTCCTAGCTGTGGAAAGATACCTAAGGGATCAACAGCTCCTAGGGATTTGGG
GTTGCTCTGGAAAACTCATTTGCACCACTGCTGTGCCTTGGAATGCTACTTGGAGT-----------------
----------------------------AATAAATCTCAGGAGGAGATTTGG------AAT------
AACATGACCTGGATGCAGTGG---GAAAAAGAAATTGATAATTACACAGCACAAATATAC---
ACATTACTTGAAAATTCACAAATCCAACAAGAA--

AAGAATGAACAAGAATTATTGGCATTAGATAAGTGGGCAAATCTGTGGAGTTGGTTTAGCATAACAAATTGGC
TGTGGTATATAAAAATATTCATAATGATAGTAGGAGGCTTGATAGGCTTAAGAATAGTTTTTGCT---
GTACTTTCTATAGTA---------AATAGAGTTAGGCAGGGATACTCACCA------
TTATCGTTTGCTGTTAGCTTGCTTGATGCCACAGCTATAGCAGTAGCTGAA---
GGAACAGATAGGATTATAGAAATAGCACAAAGAATTGGTAGAGCT---------------------------
--------ATCATCCACATA------CCTAGAAGAATAAGACAGGGCTTCGAAAGGGCTTTGCTA---TAA
>AY586542
GCT---ACA---
GACCAATTGTGGGTCACAGTCTATTATGGGGTACCTGTGTGG---AAAGAAGCAACC---
ACCACTCTATTTTGTGCATCAGATGCTAAAGCATATGATACAGAG---GTACATAAT----------------
--------------GTTTGGGCCACA---CATGCCTGTGTACCCACAGAC---
CCCAACCCACAAGAAGTAGTATTGGGA---------AATGTGACAGAAAATTTTAAC----------------
--------------------------ATGTGGAAAAATAACATGGTAGAACAGATGCATGAGGATATA---
ATCAGTTTATGGGATCAAAGCCTA---AAGCCATGTGTAAAATTA---
ACCCCACTCTGTGTTACTTTAAATTGCACTGATTTGAACAGTACTGCTAGTATCACTAGTAATAAT-------
----
-------------------ATCAGTAGTGGTAGTTGGGGAAAGATGGAGAAAGGAGAAATAAAA---
AACTGCTCTTTCAATATTACCACAGAGATAAGA--------------------------
GATAGGGTGCAAGAAGAATATGCACTT---TTTTATAAACTTGATGTAGTACCAATAGATAATAAAGATAGT-
----

AGTAGTAATAATATCAGCARGAGCTTTAGGTTGATACATTGTAACACCTCAACCATTACACAG---
ACCTGTCCAAAGATAACCTTTGAGCCAATTCCCATACACTATTGTGCCCCGGCTGGTTTTGCGATTCTAAAGT
GTAAC---AATAAGACGTTCAGT------GGAAAAGGACCATGTACAAAT----------------------
-GTCAGC---ACAGTACAATGTACACATGGAATTAAGCCAATAGTGTCAACT---
CAACTGCTGTTAAATGGCAGCCTAGCAGAACAAGAT-----------
GTGGTAATTAGATCTGATAATTTCACAGACAAT---------GCTAAAACCATAATAGTACAGCTGAAC----
-----------GAGCCTGTAAAA---------ATTAATTGTACAAGACCCACCAACAACACAAGAAAA-----
-GGTATACATATA------------GGACCAGGGAGAGCA---GTT------TATGCAACAGAAGAA------
---------ATAATAGGAGATATA------------AGACAAGCACATTGT---AACATTTTTAGAAGA---
GAATGGAATAACACTTTAGGAAAGATAGTTGCAAAATTAAGA---------------GAAACATTTAGG----
-----------------------------------AATAAAACAATAATTTTTAAG------
CAATCCTCAGGAGGAGACCCAGAAATTGTA---ATGCACAGTTTTAATTGTAGAGGGGAA---
TTTAAGCAGCTCCAGGCAAGAGTCCTGGCTGTGGAAAGATACCTAAAGGATCAACAGCTCCTGGGAATTTGGG
GTTGCTCTGGAAAACTCATTTGCACCACTACTGTGCCTTGGAATGCTAGTTGGAGT-----------------
----------------------------AATAAATCTCTAGATAAAATTTGG------AAT------
AACATGACCTGGATGCAGTGG---GAAAAAGAAATTGACAATTACACAAGCATAATATAT---
AACTTAATTGAAGAATCGCAGAACCAGCAAGAA--

AAGAATGAACAAGAATTATTGGAATTAGATAAGTGGTCAAATTTGTGGAATTGGTTCGACATAACAAAATGGC
TGTGGTATATAAAAATATTCATAATGATAGTAGGAGGCTTGGTAGGTTTAAGAATAGTTTTTTCT---
GTAATTTCTCTAGTA---------AAAAGAGTTAGGCAGGGATATTCACCA------
TTATCATTGGCTGTTAGCTTGTTCAACGCCACAGCTATAGCAGTAGCTGAG---
GGGACAGATAGGGTTATAGAAATAGTACAAAGAGGATTTAGAGCT---------------------------
--------TTTCTGCACATA------CCTAGAAGAATAAGACAGGGCTTGGAAAGGGCTTTACTA---TAA
>EU668963
GCT---GAA---
GATAAATTATGGGTTACAGTCTATTATGGGGTACCTGTGTGG---AAAGAAGCAACC---
ACCACTCTATTTTGTGCATCAGATGCTAAAGGATATAACACAGAG---GCACATAAT----------------
--------------GTTTGGGCCACA---CATGCTTGTGTACCCACAGAC---
CCTAACCCACAAGAAGTGGTATTGGAA---------AATGTGACAGAAAATTTTAAC----------------
--------------------------ATGTGGAAAAATAACATGGTAGAACAGATGCATGAGGATATA---
ATCAGTTTATGGGATCAAAGCCTA---AAGCCATGTGTAAAATTA---
ACCCCTCTCTGTGTTGTTTTAAATTGCACTGATCCTCGATTGAATAGAACAGAAAACAAT-------------
----
----------------------------AATACTAGTATCGAAAAGATGGATAAAGACATGAAA---
AACTGCTCTTTCAATATCAGCACAAGCATAAAG--------------------------
AATAAGATGCATAAAGAATATGCACTT---TTGTATAAACTTGATATAATGCCAATAGATAATGATAAT----
----

AATAACACAAACTATACATTGAATACATTGATAAATTGTAACACCTCAGTCATCTCACAG---
GCTTGTCCAAAGATATCCTTTGAACCAATTCCAATACATTATTGTGCCCCGGCTAGTTTTGCGATTCTAAAGT
GTAAT---GAGAAGAAGTACAAT------GGAACAGGACCATGTAGAAAT-----------------------
-GTCAGC---ACAGTACAATGTACACATGGAATTAGGCCAGTAGTGTCAACT---
CAACTGCTGTTAAATGGCAGTCTAGCAGAAGAAGAT-----------
ATAGTAATTAGATCTGAAAATATCACGGACAAT---------GCTAAAACCATAATAGTGCAGTTAAAT----
-----------AAATCTGTAGAA---------ATTCACTGTGTAAGACCCGGCAACAATACAAGGAAA-----
-AGTATACATATA------------GCACCGGGGAGAGCA---TTT------TATGCAACAGGAAAT------
---------ATAATAGGAGATATA------------AGACAAGCACATTGT---AACCTTAGTATAACA---
AATTGGACCAACACTTTATACCAAATAGCTGAAAAATTAAGA---------------GAACAATTTGGG----
-----------------------------------AATAAAACAATAGTCTTTAAT------
CAATCCTCAGGAGGGGACCCAGAAATTGTA---ATGCACAGCTTTAATTGTGGAGGGGAA---
TTTAAGCAGCTCCAGGCAAGAGTCCTGGCTGTAGAAAGATACCTACAGGATCAACAGCTCCTAGGGATTTGGG
GTTGCTCTGGAAAACTCATTTGCACCACTGCTGTGCCTTGGAATATTAGTTGGAGT-----------------
----------------------------AACAAATCTTATAATGACATTTGG------GAT------
AACATGACCTGGATGGAGTGG---GAGAGAGAAATTGACAATTACACAGACTTAATATAC---
ACCCTACTTGAAGAATCGCAGAACCAACAAGAA--

AAGAATGAACAAGAACTATTGGAATTGGATAAGTGGGCAAGTTTGTGGAATTGGTTTAGCATAACAAAATGGC
TGTGGTATATAAAAATATTCATAATGATAGTAGGAGGTTTAATAGGTCTAAGAATAGTTTTTGCT---
ATACTTTCTGTAGCG---------AATAGAGTTAGGCAGGGATACTCACCA------
TTATCGTTTGCTGTTAGTTTGTTTAATGCAGTAGCTATAGCAGTAGCTGAA---
GGAACAGATAGAGTTATAAAAATAGCACAAAGAATCTGTAGA-------------------------------
--------------------------------AGAATAAGACAAAGCTTAGAAAAGATTTAGTA---TAA
>FJ388890
GCT---ACA---
GAAAAATTGTGGGTCACAGTTTATTATGGGGTACCTGTGTGG---AAAGAAGCAACC---
ACCACTCTATTTTGTGCATCAGATGCTAAAGCATATGACACAGAG---GTACATAAT----------------
--------------GTTTGGGCCACA---CATGCCTGTGTGCCCACGGAC---
CCCAACCCAAAGGAAGTAAAATTGGAA---------AATGTAACAGAAGATTTTAAC----------------
--------------------------ATGTGGAAAAATAACATGGTAGACCAGATGCATGAAGATGTA---
ATTAGTTTATGGGATCAAAGCTTA---AAGCCATGTGTAAAATTA---
ACCCCACTCTGTGTCACCTTAAATTGCACTGATGAAGTGAAGAATCTTAATGACACCAAT-------------
----
----------------------------AATAGTAGTACTAGTTGGGATAAAGGAGAAGTAAAA---
AACTGCTCTTTCAACATCACCACAAACATAAGA---------------------------
GATAAGGTGCAAAAAGAATATGCACTT---
TTTTATAAACTTGATGTAGTACCAATAGATGATATTGATAATACTAATGGTACTAGAAAT-------------
----
GGTTCCACTTATAATACTAATGGTACTAGAAATGGTTCCACTTATAATAGCTATAGGTTGATAAGTTGTAATA
CCTCAGTCATCACACAG---
GCCTGTCCAAAAGTAACCTTTGAGCCAATTCCCATACATTATTGTGCCCCGGCTGGTTTTGCGATTCTAAGGT
GTAAC---AATAAGACATTCAAT------GGAACAGGACCATGTAAAAAT-----------------------
-GTCAGC---ACAGTACAATGTACACATGGAATTAAGCCAGTAGTATCAACT---
CAGCTGCTGTTAAATGGCAGTCTAGCAGAAGGAGAT-----------
ATAGTAATTAGATCTGAAAATTTCTCAGACAAT---------GCAAAAACCATAATAGTACAACTGAAT----
-----------GACTCTGTAGTA---------ATTAATTGTACAAGACCCAACAACAATACAAGAAGG-----
-AGTATACATATA------------GGACCAGGGAGAGCA---TTT------TATGCAACAGGAGAA------
---------ATAACAGGAGATATA------------AGAGCAGCACATTGT---AACCTTAGTAGAGCA---
GATTGGAATAAGACCTTAGAGAGGATAGCTATAAAATTAAGA---------------GAACAATTTGGT----
--------------------------------GAAAATAAAACAATAATCTTTAGG---
CAACCACCATCAGGAGGGGACCCAGAAATTACA---ATGCATACTTTTAATTGTGGAGGGGAA---
TTTAAACAGCTCCAGGCAAGGGTCCTGGCTGTGGAAAGATACCTAAAGGATCAACAGCTCCTGGGGATTTGGG
GTTGCGCTGGAAAGCTCATTTGCCCCACTGCGGTGCCTTGGAATGCTAGTTGGAGT-----------------
----------------------------AATAAATCTTTTGATGACATTTGG------GAT------
AACATGACCTGGATGCAGTGG---GAAAGAGAAATTAGTAATTACACAGACTTAATATAC---
TCCTTAATTGAAGAATCGCAGAACCAGCAAGAA--

AAGAATGAACAGGAATTATTGGCACTAGATAAATGGGACAGTTTGTGGAGTTGGTTTAGCATATCAAACTGGC

TGTGGTATATAAAAATATTCATAATGATAGTAGGAGGCTTGATAGGTTTAAGAATAGTTTTTGCT---
GTGCTTTCTATAGCG---------AATAGAGTTAGGCAGGGATACTCACCA------
TTGTCGTTCGCTGTTAGCTTGCTCAACGCCACAGCAATAGCAGTAGCTGAG---
GGAACAGATAGGGTTATAGAAATATTACAAAGAGCTGGTAGAGCT----------------------------
--------ATTCTCCACATA------CCTAGAAGAATAAGACAGGGCTTGGAAAGGGCTTTGCTA---TAA
>FJ388895
GCT---GCA--
GAAAAATTGTGGGTCACAGTCTATTATGGGGTACCTGTGTGG---AAAGAAACAACC---
ACCACTTTATTTTGTGCATCAGATGCTAGAGCATATGATACAGAG---GTACATAAT----------------
--------------GTCTGGGCCACG---CATGCCTGTGTACCCACAGAC---
CCCAGCCCACAAGAAGTAGTATTGGGA---------AATGTGACAGAAAATTTTAAC----------------
--------------------------ATGTGGAAAAATAACATGGTAGAACAGATGCATGAAGATATA---
ATCAGTTTATGGGATCAAAGCCTG---AAGCCATGTGTAAAACTA---
ACCCCACTCTGTGTTACTTTAAATTGCACTAATTATGAAGGGAATGCTACTAAT-------------------
----
----------------------------------ACCACTAAAGGTGAGAAAGGAGAAATAAAA---
AATTGCTCTTTCCAGATCACCACAAGTGTAAGA---------------------------
GATAAGAGGAAAGAAGAATATGCACTT---
TTTTATAGGCATGATGTAATACCAATAGATGATGATAATGCCAGT----------------------------
----
AGGAATAGAAGTAATAATGGTAGTAGTTATATATTGATACATTGTAATACCTCAACCATTACACAG---
GCCTGTCCAAAAGTATCCTTTGAGCCAATTCCCATACATTATTGTGCCCCGGCTGGTTTTGCGATTCTAAAAT
GTAAT---AATAAGACATTCAAT------GGAACAGGACCATGTACAAAT-----------------------
-GTCAGC---ACAGTACAATGTACACATGGAATTAAACCAGTTGTGTCAACT---
CAACTGCTGTTAAATGGCAGTCTAGCAGAAGAAGAG-----------
GTAGTAATTAGATCTGAAAATTTCACAAACAAT---------GCTAAAACCATAATAGTGCAGCTGAAT----
-----------CAATCTGTAGCA---------ATCAATTGTGTTAGACCCAACAACAATACAAGAAAA-----
-GGTATACATGTA------------GGACCAGGGAGAGCA---ATT------TATACAACAGGAAAA------
---------ATAATAGGAGATATA------------AGGCAAGCACATTGT---AACCTCAGTATAGAA---
AATTGGAATAAAACTTTACAACACATAGTTATAAAATTAAGA---------------GAACAATTTGGG----
-----------------------------------AATAAAACAATAGTCTTTAAG------
CAATCCTCAGGAGGGGACCCAGAAATTGTA---ATGCACAGCTTTAATTGTAAAGGAGAA---
TTTAAGCAGCTCCAGGCAAGAATCCTGGCTGTGGAAAGATACCTAAAGGATCAACAGCTCCTAGGGATTTGGG
GTTGCTCTGGAAAACTCATTTGCACCACTTCTGTGCCTTGGAACGCTAGTTGGAGT-----------------
-----------------------------AATAAATCTCAGGAGGAGATTTGG------GAG------
AACATGACCTGGATGCAGTGG---GAAAGGGAAATTGACAATTACACAGACTCCATATAC---
ACCTTACTTGAAAAATCGCAGAACCAGCAAGAA--

AAGAATGAACAAGACTTATTAGAATTGGATAAATGGGCAAGTTTGTGGAATTGGTTTGACATAACAAGCTGGC
TGTGGTATATAAAAATATTCATAATGATAGTAGGAGGCTTGATAGGTTTAAGAATAGTTTTTGCT---
GTACTTTCAATAGTA---------AATAGAGTTAGGCAGGGATACTCACCC------
TTATCGTTTGCTGTTAGCTTGCTTGACGCCACAGCCATCGCAGTAGCTGGG---
GGGACAGATAGGGTTATAGAAATAGTAAGAAGAGCCTTTAGAGCC----------------------------
--------ATTCTCCACATA------CCTACAAGAATAAGACAAGGCTTAGAAAGGGCTTTGCTA---TAA
>FJ388898
AGT---GCA--
GGAAATTTGTGGGTCACAGTCTATTATGGGGTACCTGTGTGG---AAAGAAGCAACC---
ACCACTCTATTTTGTGCGTCAGATGCTAAAGCATATGACAAAGAG---GTACATAAT----------------
--------------GTTTGGGCCACA---CATGCATGTGTACCCACAGAC---
CCCAACCCACAAGAAGTAACATTGGAA---------AATGTGACAGAGGATTTTAAC----------------
--------------------------ATGTGGAAAAATGACATGGTAGAACAGATGCATGAGGATATA---
ATCAGTTTATGGGATCAAAGCCTA---AAGCCATGTGTAAAATTG---
ACCCCACTCTGTGTCACTTTAAATTGCAGTGATATTCCTAAGACCAATAGTAGTAACAAT-------------
----
----------------------------AATAGTAGTGAGGAATCGATAATTGGAGAAGTAAAA---
AATTGCTCTTTCAATATCACCACAAACTTAAAA---------------------------
GATAAGGTGCAGAAAGAATATGCACTT---TTTCATAGACTTGATGTAACACCAATAGACAAT----------
----

ACCAGCTATATGTTAGTAAATTGTAACACCTCAGTCATTACACAG---

GCCTGTCCAAAGGTATCCTTCGAACCAATTCCCATACATTTTTGTGCCCCAGCTGGTTTTGCGATTCTAAAAT
GTAAT---AATAAGAGTTTCGAG------GGAAAAGGACCATGTAAAAAT----------------------
-GTTAGC---ACAGTACAATGTACACATGGAATTAGGCCAGTAGTGTCAACC---
CAACTGCTGTTAAATGGCAGTCTAGCAGAAGAAGAG------------
GTAGTAATTAGATCTGCCAATTTCTCGGACAAT---------GCTAAAATCATAATAGTACAGCTGAAT----
-----------GAATCTGTAGTA---------ATTAATTGTACAAGACCCAGCAACAATACAAGAAAA-----
-AGTATACATCTT------------GGACCAGGGAGTGCA---TTT------TATACAACAGGACAA------
---------ATAATAGGAGATATA------------AGAAGAGCACATTGT---AACGTTAGTAGGACA---
GAATGGAATAATACTTTAAAACAGATAGCTATAAAATTAAGA---------------GAACAGTTTAAA----
-----------------------------------AATAAAACAATAGTCTTTAAT------
CAATCCTCAGGAGGGGACCCAGAAATTGTA---ATGCACACTTTTAATTGTGGAGGGGAA---
TTTAAGCAGCTCCAGGCAAGAGTCCTGGCTGTAGAAAGATACCTAAAGGATCAACAGCTCCTAGGGATTTGGG
GTTGCTCTGGAAAACTCATTTGCACCACTGCTGTGCCTTGGAACACTAGTTGGAGT-----------------
----------------------------AATAAATCTCTGGAGGAGATTTGG------AAT------
AACATGACCTGGATGCAGTGG---GAAAAAGAAATTGACAATTACACAGGCTTAATATAC---
ACCTTAATTGAGGAATCGCAGAACCAGCAAGAA---------------------------------------
----
AAGAATGAAAAAGAGTTATTAGAATTGGATAAGTGGGCAAGTTTGTGGACTTGGTTCGACATATCAAACTGGC
TGTGGTATATAAGAATATTCATAATGATAGTAGGAGGTTTAATAGGGTTAAGAATAGTTTTTGCT---
GTACTTTCTGTAGTA---------AATAGAGTTAGGAAGGGATACTCACCA------
ATATCATTTGCTGTTAGTTTGCTTGACGCCACAGCTGTAGCAGTAGCTGAA---
GGGACAGATAGAATTATAGTAATATTACAAGGAGTTTGTAGAGCT---------------------------
--------ATTCGCCACATA------CCCAGAAGGATAAGACAGGGCTTAGAAAGGCTTTTGCTA---TAA
>FJ388905
AAT---GCA---GAC--------------------------------
AAAGATAAGTTGTGGGTCACAGTCTATTATGGGGTACCTGTGTGG---AAAGAAGCAACT---
ACCACTCTATTTTGTGCATCAGATGCTAAAGCATATGAGACAGAG---GTTCATAAT----------------
--------------GTTTGGGCCACA---CATGCCTGTGTACCCACAGAC---
CCCAACCCACAAGAAGTACCAGTGGAA---------AATGTGACAGAAAATTTTAAC---------------
--------------------------ATGTGGAAAAATAACATGGTAGAACAAATGCATGAGGATGTA---
ATCAGCTTATGGGATCAAAGCTTA---AAGCCATGTGTCAAATTA---
ACCCCACTCTGTGTTACTTTAAATTGTACTGAGCCTACCAACGGTACTATTGATAATACCAACGGTACTACCA
GTGAT---
-------GCTAGTAATAGTACCAACAGTACTAAGACTAGGGTTGTGGAACAGGGAGTAATGAAA---
AATTGTTCTTTCAATATCACCACAAACGTGAGA-------------------------
GATAAGATAAAGAAAGAATATGCGCTT---TTTTATAGCCTTGATGTAGTACCACTAGAAGAT----------
----

GATAATAGTAGCTATAGGTTGATAAGTTGTGACACCTCAGTTGTTACACAG---
GCCTGTCCAAAGGTAACCTTCGATCCAATTCCCATACATTATTGTGCCCCAGCTGGGTTTGCAATTCTAAAAT
GTAGA---GATAAGAAGTTCAAT------GGAACAGGACCATGTAAAAAT----------------------
-GTCAGC---ACAGTACAATGTACACATGGAATTAAGCCAGTAGTGTCAACC---
CAACTGCTGTTAAATGGTAGTCTAGCAGAAGAAGAG------------
GTAGTAATTAGGTCTGAAAATTTTTCGGAAAGT---------GCTAAAACCATAATAGTACAGCTAAAT----
-----------GAATCTGTAGCA---------ATTAATTGTACTAGACCCAACAACAATACAAGAAAA-----
-AGTATACATATA------------GCACCAGGGAGAGCA---TTT------TATGCAACAGATGAT------
---------GTAATTGGAGATATA------------AGACGAGCACATTGT---AATGTTAGTGAAACA---
CAATGGAATAGAGCTTTACAAAAGGTAGCTGAAAAATTAAGA---------------GAACAATTTGGG----
--------------------------------GAAAATAAAACAATAGCCTTTAAC------
CGATCCTCAGGAGGGGACCCAGAAATTACA---ATGCATAGTTTCAATTGTGGAGGGGAA---
TTTAAGCAGCTCCAGGCAAGAGTCCTGGCTGTGGAAAGATACCTACAGGATCAGCAGCTCCTGGGAATTTGGG
GTTGCTCTGGAAAACTCATTTGCGCCACTGCTGTGCCTTGGAATACTAATTGGAGT----------------
----------------------------AGTAAATCTCTGAATGAGATTTGG------GAT------
AACATGACCTGGATGCAGTGG---GAAAGAGAAATTGAAAATTACACAGGTGAAATATAC---
AAACTACTTGCAGAATCGCAGGACCAACAAGAA---------------------------------------
----
AAGAATGAACAAGAATTATTGGAATTAGATAAGTGGGCAAGTTTGTGGAGTTGGTTTAGCATAACAAACTGGC
TGTGGTATATAAGAATATTCATAATGATAGTAGGAGGCTTGATAGGTTTAAGAATAGTTTTTGCT---
ATACTTTCTGTAGTG---------AATAGAGTTAGGCAGGGATACTCACCC------
TTATCATTTGCTATTAGCTTGCTGAATACAATAGCTATTGCAGTAGCTGAG---

GGGACAGATAGGATTATAGAAGTAGCACAAAGAGCTTTTAGAGCT---------------------------
--------ATCCTCCACATA------CCTAGAAGAATAAGACAGGGCTTAGAAAGGTCTTTGCTA---TAA
>FJ388910
GCT---ACA---
GACCAATTGTGGGTCACAGTCTATTATGGGGTACCTGTGTGG---AGAGATGCAAAC---
GCCACTCTATTTTGTGCATCAGATGCTAAAGCATATGATACAGAA---GCACATAAT---------------
--------------GTTTGGGCCACA---CATGCCTGTGTACCCACAGAC---
CCCAGCCCACAAGAATTACAATTGGAA---------AATGTGACAGAAAATTTTAAC---------------
-------------------------ATGTGGAAAAATAACATGGTAGAACAGATGCATGAGGATATA---
ATCAGTTTATGGGATCAAAGCCTA---AAGCCATGTGTGAAATTA---
ACCCCACTCTGTGTTACTTTAAATTGCACTGATGTTTCTCGTAACAACAGTAGT------------------
----
---------------------------------AGTAGTGATAATAGCTGGAGAGAAATAAAA---
AACTGTTCTTTCAATATCACCACAAACATGAGA--------------------------
GATAAGGTACAGAGAGAGTATTCACTT---TTCTATAAGCTTGATATAGTGCCAATAGCTGGTGAT-------
----

AATACAAGCAGCAATAGTAGCTATAGGTTGATAAGTTGTAACACCTCAGTCCTTACACAG---
GCCTGTCCAAAAGTATCTTTTGAACCAATTCCCATACATTATTGTGCCCCAGCTGGATTTGCGATTCTAAAAT
GTAAT---AATAAGACATTCAAT------GGAGCAGGACCATGTACAAAT----------------------
-GTCAGT---ACAGTACAATGTACACATGGAATTAGGCCAGTAGTATCAACT---
CAACTGCTGTTAAATGGCAGTCTAGCAGAAGAAGAG-----------
GTAGTAATTAGGTCTGAGAATTTCACAAACAAT---------GCCAAAACTATAATAGTACATTTGAAT----
-----------GAATCTGTAGCA---------ATTAATTGTACCAGACCCAACAACAATACAAGAAAA-----
-GGTGTACATATG------------GGACCAGGGAAAGTA---TTT------TATACAACA---GAT------
---------ATAATAGGAGACATA-----------AGACAAGCACACTGT---AACCTTAGTAGAGGA---
GCCTGGAATAAAACATTAGGACAAATAGTTAACAAATTAAGA--------------GAACAATTTAAA----
-----------------------------------GATAAAACAATAGTCTTTAAT------
CAATCCTCAGGAGGGGACCCAGAAATTGTA---ATGCACAGTTTTAATTGTGGAGGGGAA---
TTTAAGCAGCTCCAAGCAAGAGTCCTAGCTGTGGAAAGATACCTAGCGGATCAACAGCTCCTGGGGATTTGGG
GCTGCTCTGGAAAACTCATCTGCACCACTAATGTGCCTTGGAATAGTAGTTGGAGT----------------
-------------------------AACAATACAGATCTGGATATCATTTGG------AAT------
AACATGACCTGGATGGAGTGG---GATAGAAAAATTCACAATTACACAGGCTTAATCTAC---
ACCTTAATTCAAGAATCGCAAACCCAACAAGAA---------------------------------------
----
AAGAATGAACTAGATTTATTGAAACTAGATGAATGGGCAAGTTTGTGGAATTGGTTTGACATATCAAAGTGGC
TGTGGTATATAAAAATATTCATAATGATAGTAGGAGGCCTGATAGGTTTAAGAATAGTTTTTGCT---
GTACTTTCTATAGTA---------AATAGAGTTAGGAAGGGATACTCACCA------
TTATCGTTTGCTGTTAGCTTGCTCAACGCCACAGCTATAGCAGTAGGTGAG---
GGAACAGATAGAATTTTAGAAGTATTACAAAGGGCTTATAGAGCT---------------------------
--------ATCCTCAACATA------CCTAGAAGAATAAGACAGGGCTTGGAAAGGGCTTTGCTA---TAA
>FJ388911
GCT---GAA---GAA--------------------------------
AAAGAAAATTCGTGGGTCACAGTCTATTATGGGGTACCTGTATGG---AAAGAAGCAACC---
ACCACTCTATTCTGTGCATCAGATGCTAAGGCATATGACACAGAG---GCACATAAT---------------
--------------GTTTGGGCCACA---CATGCCTGTGTACCCACAAAC---
CCTAACCCACAAGAAGTAAAATTGGAA---------AATGTGACAGAAAATTTTAAC---------------
-------------------------ATGTGGAAAAATAACATGGTAGAACAGATGCATGAGGATATA---
ATCAGTTTATGGGATCAAAGTCTA---AAGCCATGTGTAAAATTA-----------------
GCTTTAGATTGCAATAATAAGACAAATTGCACTGAGTGGAAGAAAAGTAATACT------------------
----
----ACCAATAGTAGTAATTGGGAAATGGAGGAGAAAGAGGAAGTAAAA---
AACTGCTCTTTCAATGTCACCAACATAAAAAAT--------------------------AAGATACAG---
AAAGACTATGCACTT---TTTTATAAACTTGATTTAGCACCAATAGATGAT---------------------
----
-------AGTGATAATAAAAGTTATACATTGATAACTTGTAACACCTCAGTCATTACACAG---
GCCTGCCCAAAGATATCCTTTGAACCAATTCCCATACTTTATTGTGCCCCAGCTGGTTTTGCGATTCTAAAGT
GTAAT---GAGGAAAAAATTCAAT------GGAACAGGACCATGTAAAAAT---------------------
-GTCAGC---ACAGTACAATGTACACATGGAATTAAGCCAGTAGTGTCAACT---
CAACTACTGCTAAATGGCAGTCTAGCAGAAAAAGAA-----------

GTAGTAATCAGATCTGAAAATTTCTCGGACAAT---------GCTAAAACCATAATAGTACAGCTGAAG----
-----------GACCCTGTGAGA---------ATTGATTGTACAAGAGTCAGCAACAATACAAGAAAA-----
-AGTGTACATATG------------GGACCAGGGAAAGCA---TAT------TTTACAACA---GAT------
---------ATAATAGGAGATATA------------AGAAAAGCTCATTGT---AACCTTACTAGGGGG---
GATTGGCATAAAGCTTTAAACCAGACAGCTACAAAATTAAGA---------------GAACAATTTGGG----
-----------------------------------AATAAAACAATAATATTTAAT------
AGATCCTCAGGAGGGGACTTATAAGTTGAA---ATGCACAGCTTTAATTGTGGAGGGGAA---
TTTAAGCAGCTCCAGGCGAGACTCCTGGCTGTGGAAAGATACCTAAGGGATCAACAGCTCCTAGGGATTTGGG
GTTGCTCTGGAAAACTCATTTGCACCACTGCTGTGCCTTGGAATGCTAGTTGGAGT-----------------
----------------------------AATAGATCTGTGGAGGATATTTGG------AAT------
AACATGACCTGGATGGAGTGG---GAGAGAGAAATTGACAATTACACAGAATTAATATAC---
AACTTAATTGAACAATCGCAAAACCAGCAAGAA--

AAGAATGAACAAGAATTATTGGAATTGGATAAGTGGGCAAGTTTATGGAATTGGTTTAGCATAACAAGCTGGC
TGTGGTATATAAAAATATTCATAATGATAATAGGAGGTTTAGTAGGTTTAAGAATAATTTTTACT---
GTACTTTCTATAGTG---------AATAGAGTTAGGCAGGGATACTCACCA------
TTATCATTTGCTGTTAGTTTGTTTAATGCCATAGCTATAGCAGTAGCTGAG---
GGGACAGATAGGGTTATAGAAATAGCACAAAGAATTGGTAGAGCT----------------------------
--------ATCCTCCACATA------CCTAGAAGAATAAGGCAAGGCGCAGAAAGGGCTTTAGTA---TAA
>FJ388912
GCT---GAA--
GAAAACTTGTGGGTCACAGTCTATTATGGGGTACCTGTCTGG---AAAGAAGCAACC---
ACCCCTCTGTTTTGTGCATCAGATGCTAAAGCATATGATACAGAG---GCACATAAT----------------
--------------GTTTGGGCCACA---CATGCCTGTGTACCCACAGAT---
CCTAACCCACAAGAAATATACTTGGAA---------AATGTGACAGAAAATTTTAAC----------------
--------------------------ATGTGGAAAAATGACATGGTAGAACAAATGCATGAGGATATA---
ATCAGTTTATGGGATCAAAGCCTA---AAGCCATGTGTAAAATTA---
ACCCCACTCTGTATTACTTTAAATTGCATTGATGTTAACACCACTAGTACTAACACCACTAGT----------
----
----------------------ACTAACACCACTAGCAGTGGCGAGATGGCAGGAGAAATAAAA---
AACTGCTCTTTCAGTATCACCTCAAGCATAAAA---------------------------
GATAAGGTGCAAAAAGAATATGCAAAT---TTTTATAAACTTGATTTAGTACAAATAGATAATGAT-------
----

AATACTAATGATACCAGATATAGGTTGATAAGTTGTAACACCTCAGTCATTACACAG---
GCCTGTCCAAAAGTATCCTTTGAGCCAATTCCCATACATTATTGTGCCCCGGCTGGTTTTGCGATTCTAAAGT
GTAAT---GATAAGAAGTTCAAT------GGAACAGGACCATGTAAAAAT-----------------------
-GTCAGC---ACAGTACAATGTACACATGGAATTAGGCCAGTAGTATCAACT---
CAACTGCTGTTAAATGGCAGTCTGGCAGAAAAAGAG------------
ATAGTAATTAGATCTGCCAATTTCACGGACAAT---------ACTAAAACCATACTAGTACAGCTGAAT----
-----------AAATCTGTAGAA---------ATTAATTGTACAAGACCCAACAACAATACAAGAAAA-----
-AGTATACATATA------------GGACCAGGGAGAGCA---TTT------TATGCAACAGGAGAT------
---------ATAATAGGAGATATA------------AGACAAGCACATTGT---AACCTTAGTAGAGCA---
GATTGGAATGAAACTTTAAGACAGATAGTTGAAAAATTGAGA---------------GAACAATTTGGG----
-----------------------------------AATAAAACAATAGTCTTTAAT------
CAATCCTCAGGAGGGGACCCAGAAATTGTA---ATGCACAGTTTTAATTGTGGAGGGGAA---
TTTAAGCAGCTCCAGGCAAGAGTCCTGGCTGTGGAAAGATACCTAAGGGATCAACAGCTCCTAGGGATTTGGG
GTTGCTCTGGAAAACTCATTTGCACCACTACTGTGCCTTGGAATGCTAGTTGGAGT-----------------
----------------------------AATAAATCTCTGGATGAGATTTGG------GAA------
AACATGACCTGGATGGAGTGG---GAAAGAGAAATTGACAAGTACACAAATGAAATATAC---
ACCTTAATTGAAAATTCGCAAAACCAACAAGAA--

AAGAATGAACAAGAATTATTGGAATTAGATAAATGGGCAAGTTTGTGGAGTTGGTTTAGCCTAACAAATTGGC
TGTGGTATATAAAATTATTCATAATGATAGTAGGAGGCTTGGTAGGTTTAAGAATAGTTTTTTCT---
GTACTTTCTATAGTG---------AATAGAGTTAGGCAGGGATACTCACCC------
TTATCGTTCGCTATTAGCTTGCTCAACGCCACAGCCATAGCAGTAGCTGAA---
GGGACAGATAGGATTATAGAAGTAGTACTAAGAGTTTATAGAGCT----------------------------
--------ATTATCCACATA------CCTACAAGAATAAGACAGGGCTTGGAAAGGGCTTTGCTA---TAA
>FJ388960

GCT---ACA--
GAAAAATTGTGGGTCACAGTATATTATGGGGTACCTGTGTGG---AAAGAAGCAACC---
ACCACTCTATTTTGTGCATCAGATGCTAAGGCATATGATACAGAG---GTACATAAT----------------
--------------GTTTGGGCCACA---CATGCCTGTGTACCCACAGAC---
CCCAACCCACAAGAAATAGAATTGGAA---------AATGTGACAGAAGATTTTAAC----------------
--------------------------ATGTGGAAAAATAACATGGAAGAACAGATGCATGAGGATATA---
ATCAGTTTATGGGATCAAAGCCTA---AAGCCATGTGTAAAATTA---
ACCCCACTCTGTGTCACTTTAAATTGCACTGATTGGGGGAATAGTACTAATACC-------------------
----
-------------------------------AATAGTAGTAATGGGAATACAGGAGAAATGAAA---
AACTGCTCTTTCAGGGTCTCCACAAGCGTAAAA---------------------------
GATAAGATGCAGAAAGAATCTGCACTT---TTTTATGAACTTGATGTGGTACCAATAGATAAT----------
----

GATAATACTAGCTATAGGTTGATAAATTGTAATACCTCAGTCATTACACAG---
GCCTGTCCAAAGGTAACCTTTGAGCCAATTCCCATACATTATTGTGCCCCGGCTGGTTTTGCGACTCTAAAGT
GTGAC---AATAAGACGTTCAAT------GGAAAAGGACCATGTAAAAAT-----------------------
-GTCAGC---ACAGTACAATGTACACATGGAATTAGGCCAGTAGTATCAACT---
CAACTGCTGTTAAATGGCAGTCTAGCAGAAGAAGGG------------
ATAGTAATTAGATCTGAAAATTTTACAGACAAT---------GCTAAAACCATAATAGTACAGCTGAAG----
-----------GAAGCTGTAGTA---------ATTAATTGTACAAGACCAAACAACAATACAAGAAAA-----
-AGTATAAATATA------------GGACCAGGGAGAGCA---TTT------TATGCAACAGGAGAA------
---------ATAATAGGAGATATA------------AGACAAGCACATTGT---AACCTTAGTAGAGCA---
GCCTGGAATGACACTTTAGGACAGATAGTTATAAAATTAGAA---------------GAACAATTTGGG----
-----------------------------------AATAAAACAATAATCTTTAAT------
CAATCCTCAGGAGGGGATCCAGAAATTACA---ATGCACAGTTTTAATTGTGGAGGGGAA---
TTTAAGCAGCTCCAGGCAAGAGTCCTGGCTGTGGAAAGATACCTACAGGATCAACAGCTCCTGGGGATTTGGG
GATGCTCTGGAAAACTCATTTGCACCACTGCTGTGCCTTGGAATACTAGTTGGAGT-----------------
----------------------------AATAAATCTCAATCAGAGATTTGG------AAT------
AACATGACCTGGATGGAGTGG---GAAAGAGAAATTCACAATTACACAGGCTTAATATAC---
ACTTTAATTGAAGAATCGCAGTACCAACAAGAA--

AAGAATGAACAAGAATTATTGGCACTAGATAAGTGGGCAAGTTTGTGGAATTGGTTTGATATAACAAACTGGC
TGTGGTATATAAAAATATTCATAATGATAGTAGGAGGCTTGGTAGGTTTAAGAATAGTTTTTGCT---
GTACTTTCTATAGTG---------AATAGAGTTAGGCAGGGATACTCACCA------
TTATCGTTTGCTGTTAGCTTGCTCAATGTCACAGCTATTGCAGTAGCTGAG---
GGAACAGATAGGGTTATAGAAGTATTACAAAGAGCTGGTAGAGCT----------------------------
--------ATTCTCCACATA------CCTAGAAGGATAAGACAGGGCTTGGAAAGGGCTTTGCTA---TAA
>FJ388914
GCT---ACA--
GAGAAATTGTGGGTCACAGTCTATTATGGGGTACCTGTGTGG---AAAGAGGCAACC---
ACCACTCTATTTTGTGCATCGGATGCTAAAGCATATGATACAGAG---GTACATAAT----------------
--------------GTTTGGGCCACA---CATGCCTGTGTACCCACAGAC---
CCCAACCCACAAGAAGTAATATTGGAA---------AATGTGACAGAAAATTTTAAC----------------
--------------------------ATGTGGGAAAATGACATGGTAGACCAGATGCATGAGGATGTA---
ATCAGTTTATGGGATCAAAGTCTA---AAGCCATGTGTAAAATTA---
ACCCCACTCTGTGTTACTTTAAACTGCACTGATTTGAAGAATAGTAGTTGGACC-------------------
----
----------------------------------AATAGTAGTTTGAAACTGGAAGGAATGAAA---
AACTGCTCTTTCAATGTCACCCCAAGCATAAGA---------------------------
GATAAGTGGCAAAAAGAATATGCACTT---
TTTTATAAATATGATGTAGTACAAATAGATGATACAAATAATAGT----------------------------
----
ACTAATGGAACTGGTAACTTTACTAGCTATAGATTGATAAATTGTAACACTTCAGTCATTACACAG---
GCCTGTCCAAAGGTAACCTTTGAGCCAATTCCCATACATTATTGTACCCCGGCTGGTTATGCGCTTCTAAAGT
GTAAC---AATAAGACATTCAAT------GGAAGTGGACCATGTACAAAT-----------------------
-GTCAGC---ACAGTACAACGTACACATGGAATTAGACCAGTGGTATCAACT---
CAACTGCTGTTAAATGGCAGTCTAGCAGAAGAAGAC------------
ATAGTAATTAGATCTAAAAATTTCTCGGACAAT---------GCTAAAAACATAATAGTACAGCTGAAT----
-----------GAATCTGTAGTA---------ATTAATTGTACAAGACCCCACAACAATACAAGAAGA-----

-AGTATAAATATA------------GGACCAGGGAGAGCA---TTT------TATGCAACAGGACAA------
---------ATAGTAGGAGATATA------------AGACAAGCACATTGT---AACCTTAGTCAAAAA---
GCATGGAATGACACTTTAAGAATGATAGCTATAAAATTAAAG---------------AAACAATTTGGG----
--------------------------------GAGAATAAAACAATAATCTTTAAT------
CAATCCTCAGGAGGGGACCCAGAAATTGTA---ATGCACAGTTTTATGTGTGGTGGGGAA---
TTTAAGCAGCTCCAGGCAAGAATCCTGGCTGTGGAAAGATACCTACAGGATCAACAGCTCCTGGGAATTTGGG
GCTGCTCTGGAAAACTCATTTGCACCACTAATGTGCCTTGGAATGCTAGTTGGAGT-----------------
----------------------------AATAAATCTTATAATGACATTTGG------AAT------
AACATGACCTGGATGCAGTGG---GAAAAAGAAGTTGATAATTACACAAGCTTAATATAC---
ACTTTACTTGAAGAATCGCAGACGCAACAAGAA--

AAGAATGAACAAGATCTATTGGCACTAGATAAGTGGGACAGTTTGTGGAATTGGTTTAGTATAACAAAATGGC
TGTGGTATATAAAGATATTCATAATGATAGTAGGAGGCTTGGTAGGATTAAGAATAGTTTTTGCT---
GTGCTTTCTATAGTG---------AATAGAGTTAGGCAGGGATACTCACCA------
TTATCGTTTGCTGTTAGCTTGCTCAACGCCACAGCTATTGCAGTAGCTGAG---
GGAACAGATAGGGTTATAGAAGTATTACAAAGAGCTGGTAGAGCT----------------------------
--------ATTCTCCACATA------CCTAGAAGAATAAGACAGGGCTTGGAAAGGGTTTTGCTA---TAA
>FJ388915
GCT---GCA--
GACCAATTGTGGGTTACAGTCTATTATGGGGTACCTGTGTGG---AAAGAAGCAACC---
ACCACTCTATTCTGTGCATCAGATGCTAAAGCATATGATACAGAG---GTACATAAT----------------
--------------GTTTGGGCCACA---CATGCCTGTGTACCCACAGAC---
CCCAACCCACAAGAAATAGTAATGGAG---------AATGTGACAGAAAATTTTAAC----------------
--------------------------ATGTGGAAAAATAATATGGTAGAACAGATGCATGAGGATATA---
ATCAGTTTATGGGATCAAAGCCTA---AAGCCATGCGTAAAATTA---
ACCCCACTCTGTGTTACTTTAAATTGCAGTGATTGGAAAGGGAATGCTACTGCAGGGAATACTACT-------
----
-------------------AATAATAGTAGTAGCTTGGGAAGGTTGGATACAGGAGAAATAAAA---
AACTGCTCTTTCAATATCACAACAAGCATAAGA---------------------------
GATAAGATGCAGAAAGAATATGCGCTT---TTTTATAGACTTGATGTAGTACCAATAGAGGGA----------
----

GATAATACTAGCTATAGGTTGATAAGTTGTAACACCTCAGTCATTACACAG---
GCCTGTCCAAAGGTAACCTTTGAGCCAATTCCCATACATTATTGTGCCCCGGCTGGTTTTGCGATTCTAAAAT
GTAAT---GATAAGAAGTTCAAT------GGAACAGGACCATGTAAAAAT-----------------------
-GTCAGT---ACAGTACAATGTACACATGGAATCAGGCCAGTAGTGTCAACT---
CAACTGCTGTTAAATGGCAGTCTAGCAGAAGAAGAG------------
GTAATAATTAGATCTGAAAATTTCACAGACAAT---------GCTAAAACCATAATAGTACAGCTGAAA----
-----------AACCCTGTAAAC---------ATTACTTGTATGAGACCCAACAACAATACAAGAAAA-----
-AGTATACCTATA------------GGACCAGGGAGAGCA---TTT------TATGCAACAGGAGAC------
---------ATAATAGGAGATATA------------AGACAAGCACATTGT---AACCTTAGTAGAATA---
GAATGGAATAATGCTTTAGGACAGATAGCTGGAAAATTAGGC---------------AAACAATTTGGG----
-----------------------------------ATTAATAACACAATATTTAAG------
CCATCCTCAGGAGGGGACCCAGAAATTGTA---ATGCACAGTTTTAATTGTAAAGGGGAA---
TTTAAGCAGCTCCAGGCAAGAGTCCTGGCTGTGGAAAGATACCTAAGGGATCAACAGCGCCTGGGAATTTGGG
GTTGCTCTGGAAAACTCATTTGCACCACTGCTGTACCTTGGAATACTAGTTGGAGT-----------------
----------------------------AACAAATCTCTAGATTACATTTGG------GGT------
AACATGACCTGGATAGAGTGG---GAAAGAGAAATTGACAATTACACAGAATTAATATAC---
AGATTAATTGAACAATCGCAAAACCAGCAAGAA--

AAGAATGAACAAGAATTGTTGGAATTGGATAACTGGGCAAGTCTATGGAATTGGTTTAGCATAACAAATTGGC
TGTGGTATATAAAAATATTCATAATGATAGTAGGAGGGTTAGTAGGTTTAAGAATAGTTTTTACT---
GTACTTTCTATAGTA---------AGAAGAGTTAGGCAGGGATACTCACCA------
ATATCATTTGCTGTTAGCTTGCTCAACGCCACAGCTGTAGCAGTAGCTGAG---
GGGACAGATAGAATAATAGAAGTAGTACAAAGAATTGGTAGAGCT----------------------------
--------ATTCTCCACATA------CCTACTAGAATAAGACAGGGCTTGGAAAGGCTTTTGCTA---TAA
>FJ388918
GCT---GCA--
GACCAATTGTGGGTTACAGTTTATTATGGGGTACCTGTGTGG---AAAGAAGCAACC---
ACCACTCTATTCTGTGCATCAGATGCCAAAGCATATGATACAGAG---GTACATAAT----------------

--------------GTATGGGCCACA---CATGCCTGTGTACCCACAGAC---
CCCAACCCACAAGAAGTAGTATTGGGG---------AATGTAACAGAAAATTTTAAC---------------
---------------------------ATGTGGAAAAATAACATGGTAGAACAGATGCACGAGGATATA---
ATTAATTTAGGGGATCAAAGCCTA---AAGCCATGTGTAAAATTA---
ACCCCACTCTGTGTTACTTTAAATTGCAGTAATTGGGTAAATACTACTGCAAAGAATACTACTACTGCAGGGA
GT--
-------------ACTACTAATAACAGTAGTAGCTTGGGAAGATTGGATAACGGAGAAGTAAAA---
AACTGCTCTTTCAATATCACAACAAGCATAAGA-------------------------
GATAAGATGCAGAAAGAAAATGCGCTT---TTCTATAAATCTGATGTAGTACCAATAGGGGGAAATAAT----
----

ACTAATACTACTAATACTAGCTATAGGTTAATAAGTTGTAACACCTCAGTCATTACACAG---
GCCTGTCCAAAGGTAACATTTGAGCCAATTCCCATACATTATTGTGCCCCGGCTGGTTTTGCGATTCTAAAAT
GTAAT---GATAAAAAGTTCAAT------GGAAAAGGACCATGTAAAAAC----------------------
-GTCAGT---ACAGTACAATGTACACATGGAATTAGGCCAGTAGTGTCAACT---
CAACTGCTGTTAAATGGCAGTCTAGCAGAAGAAGAA-----------
GTAGTAATTAGATCTGAAAATTTCACAGACAAT---------GCTAAGACCATAATAGTACAGCTGAAT----
-----------GACACTGTAATC---------ATTAATTGTACAAGACCCAGCAATAATACGAGAAGG-----
-AGTATACATATA------------GGACCAGGGAGAGCA---TTT------TATGCAACAGACCAA------
---------ATAATAGGAGATATA-----------AGACAAGCACATTGT---AACCTTAGTAGAACA---
CAGTGGAATAACGCTTTACAAAAGATAGTAGAAAAATTAGGC--------------AAACAATTCACG----
-----------------------------------AATAAGACAATAAGTTTTAAG------
CAATCCTCAGGAGGGGACCCAGAAATTGTA---ATGCACAGTTTTAATTGTGGAGGGGAA---
TTTAAGCAGCTCCAGGCAAGAGTCCTGGCTGTGGAACGATACCTAAAGGATCAACAGCTCCTGGGGATTTGGG
GTTGCTCTGGAAAACTCATTTGCACCACTGCTGTACCTTGGAATACTAGTTGGAGT-----------------
----------------------------AATAAATCTCTAGCACAGATTTGG------GAT------
AACATGACCTGGATAGAGTGG---GAAAGAGAAATTAACAATTACACAGACTTAATATAC---
AACTTACTTGAACAATCGCAAAACCAGCAAGAA---------------------------------------
----
AAGAATGAACAAGAATTGTTGGAATTGGATAAATGGGCAAGTCTGTGGAATTGGTTTGACATAACAAATTGGC
TGTGGTATATAAAAATATTCATAATGATAGTAGGAGGGTTAATAGGTTTAAGAATAGTTTTTACT---
GTACTGTCTATAATA---------AGAAGAGTTAGGCAGGGATATTCACCA------
ATATCATTTGCTGTTAGCTTGCTCAACGCCACAGCTGTAGCAGTAGCTGAG---
GGGACAGATAGAATTATAGAAATATTGCAAAGAGCTGGTAGAGCT---------------------------
--------GTTCTCCACATA------CCTACTAGAATAAGACAGGGCTTGGAAAGGAGCTTGCTA---TAA
>FJ388920
GCT---ACA---
GAAAAATTGTGGGTCACAGTCTATTATGGGGTACCTGTGTGG---AAAGACGCAACT---
GCCACTCTATTCTGTGCATCAGATGCTAAAGCATATGATACAGAA---GTACATAAT----------------
--------------GTTTGGGCCACA---CACGCCTGTGTACCCACAGAC---
CCCAACCCACAAGAACTACACTTGAAA---------AATGTGACAGAAAATTTTAAC---------------
---------------------------ATGTGGAAAAATAACATGGTAGAACAGATGCAGGAGGATATA---
ATCAGTTTATGGGATCAAAGCCTA---AAGCCATGTGTAAAATTA---
ACCCCACTCTGCGTCACTTTAAATTGCACTGACCCTTTAAGGAATAATACTTCAAGGACTAAT---------
----
-------------------------AGTACTACTATCACTAATAGAACTGATGTTGAGATGAAA---
AACTGCTCTTTCAATGTCACCCCAATCATAACA-------------------------
GATAAGAGGCAGAAAGAATATGCACTT---TTTTATAAAGATGATATAGTACCAATAGATGATGAA-------
----

AATAGTACTAATTATGCTACCTATAGGTTAATAAGTTGTAACACCTCAGTCATTACACAG---
GCCTGTCCAAAGGTAACCTTTGAGCCAATTCCCATACATTATTGTGCCCCGGCTGGTTTTGCGATTCTAAAAT
GTAAT---GATAAGACATTCAAT------GGAAGTGGACCATGTACAAAT----------------------
-GTTAGC---ACAGTACAATGTACACATGGAATTAAACCAGTGGTATCAACT---
CAGCTGCTGTTAAATGGCAGTCTAGCAGAAAAAGAG-----------
GTAGTAATTAGATCTGAAAATTTCACGAACAAT---------GCTAAAACCATAATAGTGCAGCTGAAT----
-----------CAATCTGTAGTA---------ATTAATTGTACAAGACCCAACAACAATACAAGAAGG-----
-AGTATACATATA------------GCACCAGGGAGAGCA---TTT------TATGGAACA---GAC------
---------ATAATAGGAGATATA-----------AGACAAGCACATTGT---ACCCTTAGTGGGCAA---
AAATGGAATAACACTCTAAGACTGATAGCTATAAAATTAAAA--------------GAACAATTTGGG----

--------------------------------GAGAATAAAACAATAAAATTCAAT------
CATTCCTCAGGAGGGGACCCAGAAATTGTA---ATGCACAGTTTTAATTGTAGAGGGGAA---
TTTAAGCAGCTCCAGGCAAGAGTCCTGGCTATAGAAAGATACCTAAAGGATCAACAGCTCCTGGGGATTTGGG
GCTGCTCTGGAAAACTCATTTGCACCACTGCTGTGCCTTGGAATAATAGTTGGAGT-----------------
----------------------------AAGAGAAATTATAGTGATATTTGG------GAT------
AACATGACCTGGATGCAGTGG---GAAAAAGAAATTGATAATTACACAGGTTTAATATAC---
ACCTTACTTGAGGAATCGCAAATCCAACAAGAA--

AAGAATGAACAAGAATTATTGGAACTAGATAAATGGGCAAGTTTGTGGAATTGGTTCTCTATAACAAATTGGC
TGTGGTATATAAAAATATTCATAATGATAGTAGGAGGCTTGATAGGTTTAAGAATAGTTTTTGCT---
GTGCTTTCTATAGCG---------AATAGAGTTAGGCAGGGATACTCACCA------
TTGTCGTTCGCTGTTAGCTTGCTCAACGCCACAGCAATAGCAGTAGCTGAG---
GGAACAGATAGGGTTATAGAAGTAGTACAAAGAACTGGTAGAGCT----------------------------
--------ATTCTCCACATA------CCTAGAAGGATAAGACAGGGCTTGGAAAGATCTTTGCTA---TAA
>FJ388923
GTT---ACA--
GGAAGATTGTGGGTCACAGTCTATTATGGGGTACCTGTATGGA-
AAGAAGCAACCACCAGAGTACTCTATTTTGTGCATCAGATGCTAAAGCATATGATGCAGAG---
GTACATAAT------------------------------GTTTGGGCCACA---
CATGCCTGTGTACCCACAGAC---CCCAACCCACAAGAAGTACCATTACAA---------
AATGTGACAGAAAACTTTAAC--
ATGTGGAAAAATAACATGGTAGAACAGATGCATGAGGACATA---ATCAGTTTATGGGATCAAAGCCTA---
AAGCCATGTGTAAAATTA---
ACCCCACTCTGTGTTACTCTAAATTGCACAAATATTACTATTAGTACTAATACCACT----------------
----
-------------------------------AAGAATAGTAGTGGGGAAGAAATGACAATGACA---
AACTGCTCTTTTAATGTCACTCCAATCATACAA---------------------------
GATAAGGTGCAGAAAGAATATGCACTT---TTTTATAAACTTGATGTAGTACCAATAGATAAT----------
----

AGTAATAATAATACTAGCTATAGGTTGATAAATTGTAACACCTCAGTCATTACCCAG---
GCCTGTCCAAAGGTAACCTTTGAGCCAATTCCCATACATTATTGTGCCCCGGCTGGTTTTGCGATTCTAAAGT
GTAAT---GATAAGAAATTCAAT------GGAAGCGGACCATGTACAAAT-----------------------
-GTCAGC---ACAGTACAGTGTACACATGGAATTAGGCCAGTGGTATCAACT---
CAACTGCTGTTAAATGGCAGTCTAGCAGAAGAAGAG------------
GTAGTAATTAGATCTGAAAATTTCACAAACAAT---------GCTAAAACCATAATAGTACAGCTGAAG----
-----------GAGCCTGTAGTA---------ATTAATTGTACAAGACCCAACAACAATACAAGAAAA-----
-AGTATAAGTATA------------GGACCAGGGAGAGCA---TTT------TATGCAACAGGAGAA------
---------ATAATAGGAAATATA------------AGACAAGCACATTGT---AACCTTAGTAGGAAA---
GAATGGAATAACACTTTAGGACAGATAGTTATAAAATTAAAA---------------GAACAATTTGGG----
--------------------------------AAGAATAAAACAATAAGATTTAAT------
CAATCCTCAGGAGGGGACCCAGAAATTATA---ATGCACAGTTTTAATTGTGGAGGGGAA---
TTTAAGCAGCTCCAGGCAAGAGTCCTGGCTGTGGAAAGATACCTAAAGGATCAACAGCTCCTGGGAATTTGGG
GCTGCTCTGGAAAACTCATTTGCACCACTGCTGTGCCTTGGAATGCTAGTTGGAGT-----------------
----------------------------AATAGAACTCAGGAGGAGATTTGG------AAC------
AACATGACCTGGATGCAGTGG---GAAAAAGAAATTGATAATTACACAGGCTTAATATAC---
ACCTTAATTGAAGATTCGCAAATCCAACAAGAA--

AAGAATGAACAAGAATTATTGGCACTAGATAAGTGGGCAAGTTTGTGGAATTGGTTTGATATAACAAAATGGC
TGTGGTATATAAAAATATTCATAATGATAGTAGGAGGCTTGGTAGGTTTAAGAATAGTTTTTGCT---
GTGCTTTCTATAGTG---------AATAGAGTTAGGCAGGGATACTCACCA------
TTATCGTTTGCTGTTAGCTTGCTCAATGCCACAGCTATAGCAGTAGCTGAG---
GGAACAGATAGGGTTATAGAAGTATTACAAAGAGCTGGTAGAGCT----------------------------
--------ATTCTCCACATA------CCTAGAAGGATAAGACAGGGCTTGGAAAGGGCTTTGCTA---TAA
>FJ388963
GCT---ACA--
GAAAAATTGTAGGTCACAGTCTATTATGAGGTACCTGTGTGG---AAAGAAGCAACT---
ACCACTCTGTTTTGTGCATCAGATGCTAAAGCACATAATACAGAG---GCACATAAT----------------
--------------GTTTAGGCCACA---CATGCCTGTGTACCCACAGAC---
CCCAGTCCACAAGGAGTAAAATTG------------AATGTGACAGAAAATTTTAAC----------------

---------------------------ATGTGGAAAATAATATGGTAGACCAGATGCATGAGGATATA---
ATCAGTTTATAGGATCAAAGCCTA---AAGCCATGTGTAAAGTTA---
ACCCCACTCTGTGTCACTTTAAATTGCACTGATGTAAACAAAAATACCACTGACAATGGT---------------
----
--------------------------GCCACAACCAATAGTAGTAGTTTAGGAGGAGACATGAAA---
AACTGCTCTTTCAATGTCACCACAGACAGAATA----------------------------
GATAGGATGAGAAAAGAATATGCACTT---TTTTATAAGCTTGATATAGAACAAATAAATGAAGGT-------
----

AATAAGAGTGATAATAATAGCTATAAGTTGATAAGTTGTAATACCTCAGTAGTCACACAA---
GCCTGTCCAAAGATATCCTTTGAGCCAATTCCCATACATTACTGCGCTCCAGCTAGTTTTGCGATTCTAAAGT
GTAAT---GATAAGAAATTCAAT------GGAACAGGAGCATGTAAAGAT-----------------------
-GTCAGC---ACAGTACAGTGCACACATGGAATTAGGCCAGTAGTATCAACT---
CAACTGCTGTTAAATGGCAGTCTAGCAGAAGAAGAG------------
GTAGTAATTAGATCTGAAAATTTCACCGACAAT---------GTTAAAACCATAATAGTACAGCTGAAT----
-----------GAGTCTGTAGAA---------ATTAATTGTATAAGGCCCAACAACAATACAAGAAAA-----
-AGTATACAGATA------------GGACCAGGGAGAGCA---TTG------TATACAACAGGAGAA------
---------ATAATAGGAGACATA------------AGACAAGCACATTGT---AACATCAGTGGAGAA---
AAATGGAATAAAACCTTATACCAGATAGTTATGAAATTAAGA---------------GAACAATTTGAG----
------------------------------------AACAAAACAATATACTTTAAG------
CCACACTCAGGAGGGGACCCAGAAATTGAA---ACACACAGTTTTAATTGTGGAGGGGAA---
TTTAAGCAGCTCCAGGCAAGAGTCCTGGCTGTGGAAAGATACCTAAAAGATCAACAGCTCCTAAGGATTTAAG
GTTGCTCTAGAAAGCTCATCTGCACCACTGCTGTGCCTTAGAATGCTAGTTGGAGT-----------------
----------------------------AATAAATCTTATGATGACACTTAG------AAG------
AACATGACCTAGATGCAGTAG---GAAAAAGAAATTGAAAATTACACAAACTTAATATAT---
AACTTACTTGAAACATCGCAGAACCAGCAAGAA--

AAGAATGAACAAGACTTATTGGCATTAGATAAGTAGGACAGTTTGTGAAGTTAGTTTAATATAACAAATTGGC
TGTGGTATATAAAAATATTCATAATGATAGTAAGAGGCTTAATAAGTTTAAGAATAGTTTTTACT---
GTACTTTCTATAGTG---------AATAGAGTTAGGCAGGGATATTCACCA------
CTATCATTTGCTGTCAGCTTGCTCAACGCCACAGCTATAGCAGTAGCTGAG---
GGGACAGATAAGATTATAGAAGGAATACAAAGAGTTCTTAGAGCT----------------------------
--------ATCCTCCACATA------CCTAGAAGAATAAGACAGGGCTTAGAGAAGTTTTTGCTA---TAA
>FJ388924
GCT---ACA---
GAGAAATTGTGGGTCACAGTCTATTATGGGGTACCTGTGTGG---AGAGAAGCAACT---
ACCACTCTATTTTGTGCATCAGATGCTAGAGCATATGATACAGAG---GTACATAAT----------------
--------------GTTTGGGCCACA---CATGCCTGTGTACCCACAGAC---
CCCAACCCACAAGAACTACACTTGAAA---------AATGTGACAGAAAATTTTAAC----------------
---------------------------ATGTGGAAAAATAACATGGTAGAACAGATGCAGGAGGATGTA---
ATCAGTTTATGGGATCAAAGCCTA---AAGCCATGTGTGAAATTA---
ACCCCACTCTGTGTCACTTTAAATTGCACTGACTTTTCAAGGAATAAT---------------------------
----
--------------------------------------ACTAATACCAATAGTATTGAGATGAAA---
AACTGCTCTTTCAATGTCACCCCAATCATAAAA----------------------------
GATAAGATGCAGAAAGAATATGCACTT---TTTTATAAATATGATGTAGTACAAATAGATAATAAAAAT----
----

ACTAGTAATACTAACTATACTAGCTATAGGCTGATAAATTGTAACACCTCAGTCATTACACAG---
GCCTGTCCAAAGGTAACCTTTGAGCCAATTCCCATACATTATTGTGCCCCGGCTGGTTTTGCGATTCTAAAGT
GTAAC---AATAAGACATTCAAT------GGAAGTGGACCATGTACAAAT-----------------------
-GTCAGC---ACAGTACAATGTACACATGGAATTAGACCAGTGGTATCAACT---
CAACTGCTGTTAAATGGCAGTCTAGCAGAAGAAGAG------------
GTAGTAATTAGATCTGAAAATTTCACAAACAAT---------GCTAAAACCATAATAGTGCAGCTGAAT----
-----------GAATCTGTAGCA---------ATTAATTGTACAAGACCCAACAACAATACAAGAAGG-----
-AGTATAAATATA------------GGACCAGGGAGAGCA---TTT------TATGCAACAGGAGAA------
---------ATAATAGGAGATATA------------AGACAAGCACATTGT---AACCTTAGTAGTAAA---
GCATGGAATAACACTTTAAGACAGATAGTTATAAAATTAAAA---------------GAACAATTTGGG----
---------------------------------AAGAATAAAACAATAAGCTTTAAT------
CAATCCTCAGGAGGGGACCCAGAAATTGTA---ATGCACAGTTTTAATTGTGGAGGGGAA---

TTTAAGCAGCTCCAGGCAAGAGTCCTGGCTGTGGAAAGATACCTAAAGGATCAACAGCTCCTGGGGATTTGGG
GCTGCTCTGGAAAACTCATTTGCACCACTGCTGTGCCTTGGAATACTAGTTGGAGT-----------------
-----------------------------AATAAATCTTATAATGAGATTTGG------GAA------
AACATGACCTGGATGCAGTGG---GAAAAAGAAATTGATAATTACACAGGCTTAATATAC---
ACCTTAATTGAAGAATCGCAAATCCAACAAGAA---

AAGAATGAACAAGAATTATTGGAACTAGATAAATGGGCAAGTTTGTGGAATTGGTTCTCTATAACAAAGTGGC
TGTGGTATATAAAAATATTCATAATGATAGTAGGAGGCTTGGTAGGATTAAGAATAGTTTTTGCT---
GTACTTTCTATAGTG---------AATAGAGTTAGGCAGGGATACTCACCA------
TTATCGTTCGCTGTTAGCTTGCTCAACGCCACAGCAATAGCAGTAGCTGAG---
GGAACAGATAGGGTTATAGAAGTAGTACAAAGAGCTGGTAGAGCT----------------------------
--------ATTCTCCACATA------CCTAGAAGGATAAGACAGGGCTTGGAAAGGGCTTTGCTA---TAA
>FJ388964
GCT---ACA---
GAACCATTGTGGGTCACAGTCTATTATGGGGTGCCTGTGTGG---AAAGAAGCAACC---
ACCACTTTATTTTGTGCGTCAGATGCTAGAGCATATGACAGAGAA---GTACATAAT----------------
--------------GTTTGGGCCACA---CATGCCTGTGTACCCACAGAC---
CCCAACCCACAAGAAGTAGCATTGGAA---------AATGTGACAGAAGAATTTAAC----------------
--------------------------ATGTGGAAAAATAACATGGTAGAACAAATGCATGAGGATATA---
ATCAGTTTATGGGATCAAAGCCTA---AAGCCATGTGTAAAATTA---
ACTCCACTCTGTGTTACTTTAAATTGTACTGCCAATGTTACTACTACA------------------------
----
--------------------------------------CTAGGAAATATAACTGAAGAAATGAGA---
AACTGTTCTTTTAATATTACCACAAGCATAAGA--------------------------
GATAAGAAGCAGAATGAATATGCACTG---TTTTATAGAGATGATGTAATACCAATAGATAGT----------
----

AATAATACTTCCAATTATAGGTTGATAAATTGCAATACCTCAACCATTACACAA---
GCCTGTCCAAAGGTATCCTTTGAGCCAATTCCCATACATTATTGTGCCCCGGCTGGTTTTGCGATTCTAAAGT
GTAAT---GATAAGAAATTTAAT------GGAAAAGGAGAATGTAAAAAT-----------------------
-GTCAGC---ACAGTACAATGTACACATGGAATTAGGCCAGTAGTGTCAACT---
CAACTGCTGCTAAATGGCAGTCTAGCAGAAGAAGAG-----------
ATAGTAATTAGATCTGATAATTTCACGGACAAT---------ACTAAAACCATAATAGTACATTTGAAT----
-----------GAATCTGTAGAA---------ATTAATTGTACAAGGCCCAACAACAATACAAGAAAA-----
-GGTATACATATG------------GGGCCAGGA---GCA---TTT------TATGCAACAGGAAAG------
---------ATAATAGGAGATATA------------AGAAAAGCACATTGT---AACATTAGTAAAGCA---
AAATGGAATAAAACTTTAGAACAAATAGTTATAAAATTAAGA---------------AAACAATTTGAG----
-----------------------------------AATAAAACAATAGTCTTTAAT------
CAATCCTCAGGAGGGGACCTAGAAATTGTA---ATGCACAGTTTTAATTGTGGAGGGGAA---
TTTAAGCAGCTCCAGGCAAGAGTCCTGGCTGTGGAAAGATACCTACAGGATCAACGGCTCTTGGGGATTTGGG
GTTGCTCTGGAAGGCTCATCTGCACCACTAACGTGCCTTGGAATGTTAGTTGGAGT-----------------
-----------------------------AATAAATCTCTGAATCAGATTTGG------AAT------
AACATGACCTGGATGCAGTGG---GAAAAAGAGATTGACAATTACACAAGCTTAATATAT---
AACTTAATTGAAGAATCGCAGAACCAACAAGAA---

AAGAATGAACAAGAATTATTGGAATTGGATAAGTGGGCAAGTTTGTGGAATTGGTTTAGCATAACAAACTGGC
TGTGGTATATAAGAATATTCATAATAATAGTAGGAGGTTTGATAGGGTTAAGAATAGTTTTTACT---
GTGATTTCTATAGTA---------AGTAGAGTTAGGCAGGGATACTCACCA------
TTGTCGTTTGCTATTAGCTTGTTCAATGCCACGGCTATCGCAGTAGCTGAA---
GGGACAGATAGAGTTATAGAAGTAGTACAAAGAGCTTGCAGAGCT----------------------------
--------ATTCTCCACATA------CCTAGAAGAATAAGACAGGGCTTGGAAAGGTCTTTGCTA---TAA
>FJ388927
GCT---ACT---
GAGAAATTGTGGGTCACAGTCTATTATGGGGTACCTGTATGG---AAAGAAGCAAAC---
ACCATTCTATTTTGTGCATCAGATGCTAAAGCATTTGATACAGAG---GTACATAAT----------------
--------------GTTTGGGCCACA---CATGCCTGTGTACCCACAGAC---
CCCAACCCACAAGAAATGAAATTGGAA---------AATGTGACAGAAAATTTCAAC----------------
--------------------------ATGTGGAAAAATAACATGGTAGAACAGATGCATGAGGATATC---
ATCAGTTTATGGGATCAGAGCCTA---AAGCCATGTGTAAAATTA---
ACCCCACTCTGTGTTACTTTAAATTGCACTAATTTGACCACCAATTTGACTACTGATAATACT----------

```
-------------------------------------------------------------------------
----------------------ACTACTGGTAGGGGTAATATTAGTGGTAGTATGGAAATGAAA---
AACTGCTCTTTCAATATCACCACAGACCTAAGA---------------------------
GATAAGGTGAGGGAAGAATATGCAATT---TTTTATACACTTGATACAGTACCAATAGATAAT----------
-------------------------------------------------------------------------
----------------------------
ACCAGCTATAGGTTGATAAGTTGTAATACCTCAGTCATTACCCAA---
GCCTGTTCAAAGATATCCTTTGAGCCAATTCCCATACATTATTGTGCCCCGGCTGGTTTTGCGATTCTAAAGT
GTAAT---GATAAAAAGTTCAAT------GGAACAGGACCATGTAAAAAT-----------------------
-GTCAGT---ACAGTACAATGTACACATGGAATTAGGCCAGTAGTATCAACT---
CAACTGCTATTAAATGGCAGTCTAGCAGAAGAAGAT------------
GTAGTAATTAGATCTGAAAATCTCACGAACAAT---------GCTAAAACCATAATAGTACAGCTGAAT----
-----------GAAACTGTAGAA---------ATCAATTGTACAAGACCCAACAACAATACAAGAAAA-----
-AGTATACATATA------------GGACCAGGGAGAGCA---TTT------TATGCAACAGGACAA------
---------ATAATAGGGGATATA------------AGACAAGCACATTGC---AACATTAGTAGAACA---
AAATGGAATAATACTTTAGAAAGGATAGTTAAAAAATTAGGA---------------GAACAATTTAAA----
-----------------------------------AATAAGACAATAGTCTTTAAT------
CAATCCTCAGGAGGGGACCCAGAAATTGTA---ATGCACAGTTTTAATTGTGGAGGAGAA---
TTTAAACAGCTCCAGGCGAGAGTCCTGGCTGTGGAAAGATTCCTAAAGGATCAACAGCTCCTAGGGATTTGGG
GTTGCTCTGGAAAACTCATTTGCACCACTGCTGTGCCTTGGAATGCAAGTTGGAGT-----------------
----------------------------AATAAATCTTATAATCAAATTTGG------GAT------
AACATGACCTGGATGGAATGG---GAGAGAGAGGTTAGCAACTACACAAATTTAATATAT---
TCCTTAATTGAAGAATCGCAGAATCAACAAGAA----------------------------------------
-----------------------------
AAGAATGAACAAGAATTATTGGAATTGGATAAATGGGCAAATTTATGGAATTGGTTTAACATATCACATTGGC
TGTGGTATATAAAAATATTCATAATGATAGTAGGAGGCTTGGTAGGTTTAAGAATAATTTTTACT---
GTACTTTCTATAGTG---------AACAGAGTTAGGCAGGGATACTCACCA------
TTGTCGTTGGCTATTAGCTTGCTCAACGCCACGGCCATAGCAGTAGCTGAG---
GGGACAGATAGGGGTATAGAAATATTACAAAGAATTCTTAGGGCT----------------------------
--------ATTCTCCGCATA------CCTACAAGAATAAGACAGGGCTTGGAAAGGGCTGTAGTA---TAA
>FJ388933
GCT---ACA------------------------------------------
GAAAAATTGTGGGTCACAGTATATTATGGAGTACCTGTGTGG---AAAGAAGCAACT---
ACCACCCTATTTTGTGCATCAGATAGTAAAGCATATAGTACAGAA---AAACATAAT----------------
--------------GTTTGGGCCACA---CATGCCTGTGTACCCACAGAC---
CCCAACCCACAAGAAATAAACTTGGAA---------AATGTGACAGAAAATTTTAAC----------------
--------------------------ATGTGGAAAAATAACATGGTAGAACAGATGCATGAGGATATA---
GTCAGTTTATGGGAGCAAAGTCTA---AAGCCATGTGTAAAATTA---
ACCCCACTCTGTGTTACTTTAAATTGCACTGAGGCTACCAGTATCAATGCTACCAGTATCAATAATAATAGC-
-------------------------------------------------------------------------
----------------AGTACCAATGATAGTAGAGGGGAAATGAGAGAGGGAGGAGGCTGGTAT---
AACTGCACTTTCACTATCACCACAGAAACAAAA---------------------------AAAGAATAT---
GCATATTATGCTTCT---TTTGATAATCTAGAAATAGAAAATATAAATACT----------------------
-------------------------------------------------------------------------
-------------------ACCTATAGGTTGATAAGTTGTAACACCTCAAAAGTTACACAG---
GCCTGTCCAAAGGTATCCTTTGAGCCTATTCCCATACATTATTGTGCCCCGGCTGGTTTTGCGATTCTAAAGT
GTAAT---GATAAGAATTTCAAT------GGAACAGGACCATGTACAAAT-----------------------
-GTCACC---GCAGTACGATGAACACATGGAATTAAGCCAGTAGTATCAACT---
CAACTACTGTTAAATGGCAGTCTAGCAGAAGGAGAC------------
GTAGTAATTAGATCTGAAAATTTCACAAACAAT---------GCTAAAACCATAATAGTACAGCTGAAA----
-----------GAATCTGTAGAA---------ATTAAGTGTACAAGACCAAACAACAATACAATAAAA-----
-AGTATACATATG------------GGACCAGGGAAAGCC---TTT------TATACAACAGGACAA------
---------ATAATAGGAAATATA------------AGACAAGCACATTGT---AACGTTAGTTTAGCA---
AAATGGAAAAACACCTTGCAACAGGTAGCTGGAAAATTAAGA---------------GAACAATTTGAG----
-----------------------------------AATAAAACAATAGTCTTTAGG------
CCATCCTCAGGAGGGGACCCAGAAATTGTA---TTTCACAGTTTTAATTGTGGAGGGGAA---
TTTAAGCAGCTCCAGGCAAGAGTCCTAGCTGTGGAAAGATACCTAAAGGATCAACAGCTCATGGGAATTTGGG
GTTGCTCTGGAAAACTCATTTGCACCACTGCAGTACCTTGGAATGCTAGTTGGAGT-----------------
----------------------------AATAAATCTCTAAGTGAAATATGG------GAT------
AACATGACCTGGATGCAGTGG---GACAGAGAAATTAACAATTACACAGGATTAATATAC---
```

TCCTTAATTGAAGAATCGCAGAACCAACAAGAA--

AAGAATGAAAAAGAATTATTAGAATTAGATAAATGGGCAAGTTTGTGGAATTGGTTTGACATAACAAACTGGC
TGTGGTATATATAAAATTATTCATAATGATAGTAGGAGGCTTGATAGGTTTAAGAATAGTTTTTACT---
GTACTTTCTATAGTG---------AATAGAGTTAGGCAGGGATATTCACCA------
TTATCGTTTGCTGTTAGCTTGCTCAACGCCACAGCCATAGCAGTAGCTGAG---
GGAACAGATAGGGTTCTAGAAGTGTTGCAAAGAATTTGTAGAGCT----------------------------
--------ATTTTCAACGTA------CCTACAAGAATAAGACAGGGCTTTGAAAGGGCTTTGCAA---TAA
>FJ388935
GCT---GCA--
GATCAATTGTGGGTCACAGTCTATTATGGGGTACCTGTATGG---AAAGAAGCAAAT---
GCCACACTATTTTGTGCATCAGATGCTAAAGCATATAAGACAGAG---GTACATAAT----------------
--------------GTTTGGGCCACA---CATGCCTGTGTACCCACAGAC---
CCCAACCCACAAGAAGTAAAATTGGAA---------AATGTGACAGAAAATTTTAAT----------------
--------------------------ATGTGGAAAAATAACATGGTAGAACAAATGCATGAGGATATA---
ATCAGTTTATGGGATCAAAGCCTA---AAGCCATGTGTAAAATTA---
ACCCCACTCTGTGTTACTTTAAATTGCTCTAATAAGTTAAATAAGTTAAATGTGAATGAGACT----------
----
----------------------CATACCAATGAGACTTGGAAAGAAGTAGATAACGAAATAAAA---
AACTGCTCTTTCAATATCACCACAAGCATGAGA---------------------------
GATAGGATGCAAAAAGAATATGCACTT---TTTTATAAACTTGATATAGTACAAATAGATGATAATAAT----
----

AGTAATAGTGCCAATTATACCAATTATAGATTAATAAATTGTAACACCTCAGTCATTAAACAA---
GCCTGTCCAAAGGTGTCCTTTGAGCCAATTCCCATACATTATTGTGCCCCGGCTGGTTTTGCGATTCTCAAGT
GTAAT---GATAAGAAGTTCAAT------GGATCAGGACCGTGTACAAAT-------------------------
-GTCAGC---ACAGTACAATGTACACATGGAATTAGACCAGTAATATCAACT---
CAACTGCTGTTAAATGGCAGTCTAGCAGAAGACGAG------------
GTAGTAATTAGATCTAAAAATTTCTCGGAAAAT---------GATGACATCATAATAGTACAGTTGAAG----
-----------GAAGCTGTAAAC---------ATTACTTGCATAAGACCCAACAACAATACAAGAAAA------
-AGTATACGTATA------------GGGCCAGGGGCAGCA---TTT------TATGCAACA---GAC------
---------ATAATAGGAGACATA------------AGACAAGCACATTGC---AAACTTTCTGGAATA---
GCATGGAACAAGACTTTATGGCAGATAGCTAAAAAATTAAGA---------------GAACAATTTAAG----
-----------------------------------AATAAGACAATAAAATTTACT------
CAACACTCAGGAGGGGACCCAGAAATTGTA---ATGCACAGTGTAAATTGTGGAGGGGAA---
TTTAAACAGCTCCAGGCAAGAGTCCTGGCTGTGGAAAGATACCTAAGGGATCAACAGCTCCTAGGGATTTGGG
GTTGCTCTGGAAAACTCATTTGCACCACTGCTGTGCCTTGGAATGCTAGTTGGAGT-----------------
----------------------------AATAAATCTGAGGACCAGATTTGG------GGA------
AACATGACCTGGATGCAGTGG---GAAAGAGAAATTGACAATTACACAGGATTAATATAC---
AATCTACTTGAAAAATCGCAAAACCAACAAGAA--

AAAAATGAACAAGAATTATTAGAATTGGATAAGTGGGCAAGTTTGTGGAATTGGTTTGACATAACAAAATGGC
TATGGTATATAAAAATATTCATAATGATAGTAGGAGGCTTGATAGGTTTAAGAATAGTTTTTGCT---
GTACTTTCTGTAGTG---------AATAGAGTTAGGCAGGGATACTCACCA------
TTGTCATTTGCTGTTAGCTTGCTTAACGCCACAGCCATAGCAGTAGCTGAG---
GGAACAGACAGGGTGATAGAACTAGCACAAAGAATTACTAGAGCT----------------------------
--------ATCCTCAACGTA------CCTAGAAGAATAAGGCAGGGGTTGGAAAGGGCTTTGCTT---TAA
>FJ388936
GTT---ACA--
AAAGGATTGTGGGTCACAGTCTATTATGGGGTACCTGTGTGG---AAAGACGCAACC---
ACCACTCTATTTTGTGCATCAGATGCTAAAGCATATGATACAGAG---GTACATAAT----------------
--------------GTTTGGGCCACA---CATGCCTGTGTACCCACAGAC---
CCCAACCCACAAGAAGTAAAATTGGAA---------AATGTGACAGAAGATTTTAAC----------------
--------------------------ATGTGGAAAAATAACATGGTAGACCAGATGCATGAGGATGTA---
ATCAGTTTATGGGATCAAAGCCTA---AAGCCATGTGTAAAATTA---
ACCCCACTCTGTGTCACTTTAAGTTGCACTGATGATTTGAAGAATAGTACTAAC-------------------
----
-------------------------------ATCACTATTAAGTTGGAGAGAGGAGAAATAAAA---
AACTGCTCTTTCAACATCACCACCAACATAAGA---------------------------
GATAAGGTACAGAAAGAACATGCACTT---TTTTATAAACTTGATGTAGTACCAATAGAAGATGGT-------

----

AATAATAGTAATACTAGCTATAGGTTGATAAGTTGTAACACCTCAGTCGTTACACAG---
GCCTGTCCAAAGATAACCTTTGAGCCAATTCCCATACATTATTGTGCCCCAGCTGGTTTTGCGATTCTAAAGT
GTAAC---AATAAGACATTCAAT------GGAAAAGGACCATGTAAAAAT-----------------------
-GTCAGT---ACAGTCCAATGTACACATGGAATTAGGCCAGTAGTATCAACT---
CAGCTGCTGTTAAATGGCAGTCTAGCAGAAGAAGAG------------
GTAGTAATTAGATCTGAAAATTTCACAAACAAT---------GCTAAAACCATAATAGTACAACTGAAT----
-----------GAATCTGTAGTA---------ATTAATTGTACAAGGCCCAACAACAATACAAGAAGA-----
-AGTATACATATG------------GGACCAGGGAAAGCA---TAT------TATGCAACAGGAGAA------
---------ATAATAGGAGATATA------------AGAGAAGCACATTGT---AACCTTAGTGAAGCA---
GCATGGAATAATACTTTAAGAAAGGTACTTATAAAATTAAAA---------------GGACAATTTGGG----
-----------------------------------AATAAAACAATAACCTTTAAT------
CAATCCTCAGGAGGGGACCCAGAAATTACT---ATGCACAGTTTTAATTGTGGAGGGGAG---
TTTAAGCAGCTCCAGGCAAGAGTCCTGGCTGTGGAAAGATACCTAAAGGATCAACAGCTCCTGGGGATTTGGG
GCTGCTCTGGAAAACTCATCTGCCCCATTGCTGTGCCTTGGAATGCTAGTTGGAGT-----------------
----------------------------AATAGATCTATGGATGACATTTGG------CAG------
AACATGACCTGGATGGAGTGG---GAAAGAGAAATTAGTAATTACACAGACTTAATATAC---
AACTTAATTGAAGAATCGCAAAACCAACAAGAA--

AAGAATGAACAAGAATTATTGGCACTAGATCAGTGGGCAAGTTTGTGGAGTTGGTTTGATATATCAAAATGGC
TGTGGTATATAAGAATATTCATAATGATAGTAGGAGGCTTGATAGGTTTAAGAATAGTTTTTGCT---
GTGCTTTCTATAGCG---------AATAGAGTTAGGCAGGGATACTCACCA------
TTATCGTTCGCTGTTAGCTTGCTTAATGCCACAGCTATAGCAGTAGCTGAG---
GGAACAGATAGGATTATAGAAGTATTACAAAGAGCTGGTAGAGCT----------------------------
--------ATTCTCCACATA------CCTAGAAGAATAAGACAGGGCTTGGAAAGGGCTTTGCTA---TAA
>FJ388937
GGT---GCA--
GAAAACTTGTGGGTTACAGTCTATTATGGGGTACCTGTGTGG---AAAGAAGCAACC---
ACCACTCTATTTTGTGCATCAAATGCTAAAGCATATGAAACAGAG---GTATCTAAT----------------
--------------GTTTGGCCACA---CATGCCTGTGTACCCACAGAC---
CCCAGCCCACAAGAAGTACCATTGGGA---------AATGTGACAGAAAATTTTAAC----------------
--------------------------ATGTGGAAAAATAACATGGTAGAACAGATGCATGAGGACATA---
ATCAGTTTATGGGATCAAAGCCTA---AAGCCATGTGTAAAATTA---
ACCCCACTCTGTGTTACTTTAAATTGCACTGATGTAAATATTACTAGGACCACTAATAGTAGT----------
----
-------------------------ACTGCGAATATTAGTAGGGAGGTAAAAGAAGAAATGAAA---
AACTGCTCTTTCAATATTACCACAAGCTTACAA---------------------------
GATAGAAGGCAGAGAGAATATGCACTT---TTTTATAAACTTGATATAGTACCAATAGATAATAATAATAAA-
----

AGTAATGTTAGTAATGTTAGTACCCACTATAGGTTGATAAGTTGTAACACCTCAATCATTACACAA---
GCCTGTCCAAAGGTATCCTTTGAGCCAATTCCTATACATTATTGTGCCCCGGCTGGTTTTGCGATTCTAAAAT
GTAAG---GATAAAAGGTTCAAT------GGAACAGGACCATGTAAAAAT-----------------------
-GTCAGT---ACGGTACAATGTACACATGGAATTAGGCCAGTAGTATCTACA---
CAACTGCTGTTAAATGGCAGTCTAGCAGAAGAAGAG------------
GTAGTAATTAGATCTGCCAATCTCTCGGACAAT---------GCTAAAGTCATAATAGTACAGCTGAAT----
-----------GAATCTGTAGAA---------ATTAACTGTACAAGACCCAACAACAATACAAGAAAA-----
-AGTATACATATA------------GCACCAGGGAGGGCA---TTT------TATGCAACAGGAGGA------
---------ATAATAGGAGATATA------------AGACAAGCATATTGT---ACCATTAGTAGAGCA---
AAATGGAATAAAACTTTAAGCCAGGTAGTTGGAAAATTAAGA---------------GAGCTATTTAAC----
-----------------------------------AATAAAACAATAGTCTTTAAT------
CACTCCTCAGGAGGGGACCCAGAAATTGTA---ACGCACAGTTTTAATTGTGGAGGAGAA---
TTTAAGCAGCTCCAAGCAAGAGTCCTGGCTGTAGAAAGATACCTAAGGGACCAACAGCTCCTAGGAATTTGGG
GTTGCTCTGGAAAACTCATTTGTACCACTACTGTGCCTTGGAATACTACTTGGAGTAAGAAT---TGG-----
-------------------------ACTACGAAAAATCTAACAGATATTTGG------GAT------
AACCTAACCTGGATGGAGTGG---GAAAGAGAAATTGACAATTACACAGGCTATATATAC---
ACCTTGCTTGAAGACTCGCAAAACCAGCAAGAA--

AAGAATGAACAAGAGTTATTAGAATTAGATAAATGGGCAAATTTGTGGAATTGGTTTGACATAACAAACTGGC

TGTGGTATATAAAAATATTCATAATGATAGTAGGAGGCTTGATAGGTTTAAGAATAGTTTTTACT---
GTACTTTCCATAGTG---------AATAGAGTTAGGCAGGGATACTCACCA------
TTATCGTTTGCTGTTAGCTTGTACAACGCCATAGCCATAGCAGTAGCTGAG---
GGGACAGATAGGATTATAGAAATAGTACAAAGAAGTTTTAGAGCT----------------------------
--------ATACTCCATATA------CCTACAAGAATCAGACAAGGCTTGGAAAGGGCTTTGCTA---TAA
>FJ388939
GCT---ACA--
CAAAAGTTATGGGTCACAGTCTATTATGGGGTACCTGTGTGG---AAAGAGGCAGAC---
ACCACTCTATTTTGTGCATCAGATGCTAAAGCATATGATACAGAG---AAGCATAAT----------------
--------------GTCTGGGCCACA---CATGCCTGTGTACCCACAGAC---
CCCAACCCACAAGAATTACATTTGGAA---------AATGTAACAGAAGATTTTAAT----------------
--------------------------ATGTGGAAAAATAACATGGTAGAACAGATGCATGAGGATATA---
ATCAGTCTATGGGATCAAAGCCTA---AAGCCATGTGTAAAATTA---
ACCCCACTCTGTGTTACTCTAACTTGCACTGATTTGAATAACAGCACTGGTGGGAACCATACT----------
----
-------------------------ACTAATGCCCCAGAGATAGATGGAAGAAAAGAAATGACA---
AACTGCTCTTTCAATGTTACCACAGAAAGGACA---------------------------
GATAAGATGCAGAAAGTATATGCACTT---
TTTATAAACTTGATGTAGTACCAGTAGATAATGAAGATAAGGATAATACTAGCTAT-----------------
----
AGTAATACTAGCTATAGGTTGATAAGTTGTAACACCTCAGTCATTACACAG---
GCCTGTCCAAAGGTATCCTTTGAACCAATTCCAATACATTATTGTGCCCCGGCTGGTTTTGCGATTTTAAAGT
GTAAC---AATAAGACATTCAGT------GGAAAAGGACCCTGCAACAAT-----------------------
-GTCAGC---ACAGTACAGTGTACACATGGAATTAAGCCAGTAGTATCAACT---
CAACTACTGTTAAATGGCAGTCTAGCAGAAGAAGAG-----------
GTAATAATTAGATCTGAAAATTTTACAGACAAT---------ACTAAAACCATAATAGTACAGTTGAAT----
-----------GAAACTATAAAA---------ATTAATTGTACAAGACCCAACAACAATACAAGGAAA-----
-AGTATATCCATA------------GGACCAGGGAGAGCG---TTT------TATGCACATGGAGAA------
---------ATAATAGGAGATATA------------AGAAAAGCATCTTGT---ACCCTTAATGGAACA---
CAATGGAATAACACTTTAAGACTGATAGCTGACAAATTACTA---------------GCACAATATAAT----
-----------------------------------GCATCAAAAATAGCCTTTAAT------
CAATCCTCAGGAGGGGACCCAGAAATTGTA---TTACACAGTTTTAATTGTGGAGGGGAA---
TTTAAACAGCTCCAGGCAAGAGTCCTGGCTGTGGAAAGATACCTAAGAGATCAGCAGCTCCTGGGAATTTGGG
GTTGCTCTGGAAAACTCATCTGCACCACTTCTGTGCCTTGGAATGCTAGTTGGAGT-----------------
-----------------------------AATAAATCTCTGGAGGACATTTGG------GAT------
AACATGACCTGGATGCAGTGG---GAAAAAGAAATTGACAATTACACAAGTGTAATATAT---
GAGTTAATTGAAAAATCGCAGAACCAACAGGAA--

CAGAATGAAAAAGACTTATTGGAATTGGATAAATGGGCAAGTTTGTGGAATTGGTTTGACATAACAAACTGGC
TGTGGTATATAAAAATATTCATAATGATAGTAGGAGGATTGATAGGTTTAAGAATAGTTTTTACT---
GTACTTTCTTTAGTG---------AATAGAGTTAGGCAGGGATATTCACCA------
TTATCGTTGGCTGTTAGCTTGCTCAACGTCACAGCCATAGCAGTAGGTGAA---
GGGACAGATAGGGTTATAGAAGCATTGCAAAGCATTGGCAGAGCT----------------------------
--------ATTCTTAACATA------CCTAGAAGAATAAGACAGGGCTTGGAAAGAGCTTTGCTA---TAA
>FJ388955
GCT---ACA--
AACAACTTGTGGGTTACAGTCTATTATGGGGTGCCTGTGTGG---AAAGAAGCAACC---
ACTACTCTATTTTGTGCATCAGATGCTAGAGCATATAAGACAGAG---GTACATAAT----------------
--------------GTTTGGGCCACA---CATGCCTGTGTACCCACAGAC---
CCCAGCCCACAAGAAATAAAATTGGAA---------AATGTGACAGAAAAGTTTAAC----------------
--------------------------ATGTGGAAAAATAACATGGTAGAACAGATGCAAGAAGATATA---
ATCAGTTTATGGGATCAAAGCCTA---AAGCCATGCGTAAAATTA---
ACTCCACTCTGTGTTACTTTAAACTGCATTAATTTAAACTGCACTAATTGCACTAATTTGAAA----------
----
-------------------------AATAGTAGTGAAGGTATAAAAGATGAAGTAGGAATGAAA---
AACTGCTCTTTCAATATCACCACAAGTAGAAGA---------------------------
GATAAAGTGAAGAAAGAATATGCACTT---TTCTACAACCTTGATATAGTACAAATAAATGATGATGAT----
----

AATGGTAATACTAGTAATACTAGTTATAGGTTGGTAAGTTGCAACACCTCAGTCATTACACAG---

GCCTGTCCAAAGGTAACCTTTGAACCAATTCCCATACATTATTGTGCCCCGGCTGGTTTTGCGATTCTAAAGT
GTAAT---GAGATGGGATTCAAT------GGAACAGGACAATGTAGAAAT----------------------
-GTCAGC---ACAGTACAATGTACACATGGAATTAGGCCAGTAGTATCAACT---
CAATTGCTGCTAAATGGCAGTCTAGCAGAAGGAGAT------------
GTAGTACTTAAATCTGCCAATTTCTCGGACAAT---------GCTAAAACCATAATAGTACAGCTAAAT----
-----------GAAACTGTGAAA---------ATTAATTGTACAAGACCCAGCAACAATACTAGAAAA-----
-AGTATAAGTATA------------GGACCAGGGAGAGCA---TTT------TATACAACAGGGGAA------
---------ATAATAGGAGATATA------------AGACAAGCACATTGT---AACATTAGTGGAGAA---
GAATGGAAGAGAACTTTACAACAGACAGTTAAAAAATTAAAA---------------GAACAATTTAAC----
----------------------------------AATAAGACAATAACCTTTAAG------
CAATCCTCAGGAGGGGACCCAGAAATTGTA---ATGCACAGTTTTAATTGTGGAGGAGAA---
TTTAAACAGCTCCAGGCAAGAGTCCTGGCTATAGAAAGATACCTGAAGGATCAACAGCTCCTAGGGATTTGGG
GTTGCTCTGGAAAACTCATTTGCACCACCGCTGTGCCTTGGAATGCTAGTTGGAGT-----------------
----------------------------AATAAATCTTTGGATCAGATTTGG------GAG------
AACATGACCTGGATGCAATGG---GAGAGAGAAATAGACAATTACACAAGTAAAATATAC---
TCTTTGCTTGAAGAATCGCAAAACCAACAAGAA--

AAGAATGAACAAGAATTATTGGAATTAGATAATTGGGCAAGTTTGTGGAATTGGTTTGATATAACAAACTGGC
TGTGGTATATAAAAATATTCATAATGATAGTAGGAGGCTTGATAGGGTTAAGAATAATTTTTACT---
GTACTTTCTATAGTA---------AATAGAGTTAGGCAGGGATACTCACCA------
TTGTCGTTTGCTGTTAACTTGCTCAACGCCATAGCCATAGCAGTAGCTGAG---
GGAACAGATAGGATTATAGAAGTAGCACAAAGAGCTGGGAGAGCT---------------------------
--------ATTCGCCACATA------CCTGTAAGAATAAGACAAGGCTTGGAAAGGCTTTTGCTA---TAA
>JF683736
GTT---GCA---
GGACAAATGTGGGTCACAGTTTATTATGGGGTACCTGTGTGG---AAAGAAGCAACT---
ACCACTCTATTTTGTGCATCAGATGCTAAAGCATATGATACAGAG---GTACATAAT----------------
--------------GTTTGGGCCACA---CATGCCTGTGTACCCACAGAC---
CCCAACCCACAAGAAATAGTATTGGAA---------AATGTGACAGAAAATTTTAAC---------------
--------------------------ATGTGGAAAAATAACATGGTAGAACAAATGCATGAGGATATA---
ATCAGTTTATGGGATCAAAGCCTA---AAGCCATGTGTAAAATTA---
ACCCCACTCTGTGTTACTCTAAATTGTGCTAATCTGACAACTGCTAGTGGTAATAACACTAGT----------
----
-------------------------AATGATAATGAGGCTATGTTGGCGAAAGAACAAATGAAA---
AACTGCTCTTTCAATACCACCACAAACATAAGA--------------------------
GATAAGATGCAAAAAGCATATGCAATT---TTTGATAAGCTTGATATAGTACCAATAGATAATGAT-------
----

AGAAATGATAATACCAGCTATAGGTTGATAAGTTGTAATACCTCAGCCATTACACAA---
GCCTGTCCAAAGGTATCCTTTGAGCCAATTCCCATACATTATTGTACCCCGGCTGGGTTTGCAATTATAAAGT
GCAAT---GATAAGAAGTTCAAT------GGAACAGGACCATGTACAAAT----------------------
-GTCAGC---TCAGTACAATGTACACATGGAATTAGGCCAGTAGTATCAACT---
CAACTACTGTTAAATGGCAGTCTAGCAGAAGATGAG------------
ATAGTAATTAGGTCTGAAAATTTCTCGGACAAT---------GCTAAAACCATAATAGTACAGCTGAAG----
-----------GAACCTGTAGAA---------ATTAATTGTACAAGACCCAACAACAATACAAGAAAA-----
-AGTATAACTATA------------GGACCAGGGAGAGCA---TTT------TATGCAACAGGAGAC------
---------ATAATAGGAGATATA------------AGACGAGCACATTGT---AACATTAGTGGAGCA---
AAATGGAATGAAACATTAAAAAAGGTAGTTGTCAAATTAAGA---------------GAACAATTCGGG----
----------------------------------AATAAAACAATAATCTTTAAT------
CAATCCTCAGGAGGGGACCCAGAAATTGTA---ATGCACATTTTTAATTGTGGAGGGGAA---
TTTAAGCAGCTCCAGGCAAGAGTCCTGGCTGTGGAAAGATACCTAAAGGATCAACAGCTCCTGGGGATTTGGG
GTTGCTCTGGAAAACTCATTTGCACCACTGCTGTGCCTTGGAATGCTAGTTGGAGT----------------
----------------------------AATAGATCTCAATCAGAAATATGG------AAC------
AACATGACCTGGATGCAGTGG---GAAAGAGAAATTGACAATTATACAGGCATAATATAT---
GACTTAATTGAAAAATCACAGACCCAGCAAGAA--

CAGAATGAACAAGAATTATTGGAGTTGGATAAATGGGACAGTTTGTGGAATTGGTTTAGCATAACAAAATGGC
TGTGGTATATAAAAATATTCATAATGATAGTAGGAGGCTTGATAGGTTTAAGAATAGTTTTTACT---
TTATTTTCTATAGTG---------AATAGAGTTAGGCAGGGATACTCACCA------
TTGTCATTTGCTGTTAGCTTGTTCAACGCCACAGCAGTAGCAGTAGCTGAA---

GGGACAGATAGAGTTCTAGAAGGATTAAGAACAGCTGTTAGAGCT----------------------------
--------GTTCGCCACATA------CCTAGAAGAATAAGACAGGGAGCAGAAATAGCTTTGCTA---TAA
>JF683741
GCT---ACA--
GAAAATTTGTGGGTCACAGTCTATTATGGAGTACCTGTGTGG---AAAGATGCAAAC---
ACCACTCTATTTTGTGCATCAGATGCTAAAGCATATGATACAGAG---GTACATAAT----------------
--------------GTTTGGGCTACG---CATGCGTGTGTACCCACAGAC---
CCCAACCCACAAGAAATGGTATTGGGA---------AATGTGACAGAAAATTTTAAT----------------
--------------------------ATGTGGAAAAATAACATGGTAGAACAGATGCATGAGGATATA---
ATCAGTTTATGGGATCAAAGCCTA---AAACCATGTGTAGAGCTA---
ACCCCACTCTGTATTACTTTAAATTGCACTGATTGGAACGCGACTAACACGAATGTAACTGGA----------
----
-------------------------GTTAATAACACAATGAATATAGATAAGGGAGAAATAAAA---
AACTGCTCTTTCAATATCACCACAAACATAAGA---------------------------
GATAGGGTGGAAAAGAACTATGCACTT---TTTTATACACTTGATATAGTACCAATAGATAAT----------
----

AGTGATAATAGTAGCTATAGATTGATAAGTTGTAACACCTCAATCATTAAACAG---
GCTTGTCCAAAGGTAAGCTTTGAGCCAATTCCCATACATTTTTGTACCCCGGCTGGTTTTGCGATTCTAAAGT
GTAAT---GATAAGAAGTTCAAT------GGAACAGGACAATGTAAAAAT-----------------------
-GTCAGC---ACAGTACAATGCACACATGGCATTAGGCCAGTAGTATCAACT---
CAACTGCTGTTAAATGGCAGTCTAGCAGAAAAAGAG------------
GTAGTAATTAGATCTCAAAATATCTCAAACAAT---------GCTAAAACCATAATAGTACAGCTGAAT----
-----------GAATCTGTAGTA---------ATTAATTGTACAAGACCTGGAAACAATACAAGGAAA-----
-AGTATATCGATA------------GGACCAGGGAGAGCA---TTT------TATGCAACAGGAGAT------
---------ATAATAGGAGATATA------------AGACAAGCACATTGT---ACCATTAATATCACA---
AATTGGAATAACACTTTAAGACAGATAGTTGATAAATTAAGA---------------GAAATTGAACAATTT-
--------------------------------AAGAACAAAACAATAGTCTTTAAT------
CACTCCTCAGGAGGGGACCCAGAAATTGAA---ATGCAAAGCTTTAATTGTAGAGGGGAA---
TTTAAGCAGCTCCAAGCAAGAGTCCTGGCTGTGGAAAGATACCTAAGGGATCAACAGCTCCTGGGGCTTTGGG
GTTGCTCTGGAAAACTCATTTGCACCACCACTGTGCCTTGGAATAATAGTTGGAGT-----------------
-------------------------AGGAATAAAAATCTGAGTCAGATTTGG------GAT------
AACATGACCTGGATACAGTGG---GAAAGAGAAATTGACAATTACACAGACATAATATAT---
AGCTTAATTGCAGAGTCGCAACACCAGCAGGAA--

AAGAATGAAAAAGACTTATTGGAATTGGATAAATGGGCAAGTTTGTGGAATTGGTTTGACATAACAAATTGGC
TGTGGTATATAAAAATATTCATAATGATAGTAGGAGGCTTGGTAGGTTTAAGGATAGTTTTTTCT---
GTACTTTCTATAGTA---------AATAGAGTTAGGAAGGGATACTCACCA------
TTATCGTTCGCTGTTAGCTTGTTCAACGCCACAGCCGTAGCAGTAGCTGAG---
GGAACAGATAGGATTATAGAAGTAGTACAAAGAATTTATAGGGCT----------------------------
--------ATTCTCCACATC------CCTACAAGAATAAGACAGGGCTTAGAAAGAGCTTTGCTA---TAA
>JF683743
GCT---GGA--
GAACAATTGTGGGTCACAGTGTATTATGGGGTACCTGTGTGG---AAAGAAGCAACC---
ACCACTCTATTTTGTGCATCAGATAGTAAAGCATATAGTGCAGAA---GCACATAAT----------------
--------------GTGTGGGCCACA---CATGCCTGTGTACCCACAGAC---
CCCAACCCACGAGAAGTACTATTGGAA---------AATGTGACAGAAAAGTTTAAT----------------
--------------------------ATGTGGAAAAATAACATGGTAGAACAGATGCATGAGGATATA---
ATTGGTTTATGGGAACAGAGTCTA---AAACCATGTGTAAAATTA---
ACCCCACTCTGTGTCACGTTAAATTGCACTGATTATTGTGGGAAGAATACTACCACCAATAGTACTAAT----
----
-------------------ACCTGTAATAGTAGCATGGAAGAAACGGGGGAAGGCGGAATGAAA---
AACTGCTCTTTCAATATCACCACAGAAATAAGA---------------------------
GATAGGGTGAGGAAAGAATATGCACTT---TTTTATAATCTTGATGTAGTACAAATAGATCAT----------
----

AGTGATAATAATAGCTATAGGTTGATAAGTTGTAACACCTCAGTCATGACACAG---
GCCTGTCCAAAGATATCCTTTGAACCAATTCCCATACATTATTGTGCCCCGGCTGGTTTTGCGATTCTAAAAT
GTAAT---GATAAGAAGTTCAAT------GGAACAGGACCATGTAAAAAT-----------------------
-GTCAGC---ACAGTACAATGTACACATGGAATTAGGCCAGTAATATCCACC---

CAACTGCTGTTAAATGGCAGTCTAGCAGAAGAAGAG------------
GTAGTAGTTAGATCTGAAAATTTCACAAACAAT---------GCTAAAACTATAATAGTACAGCTGAAT----
-----------GAATCTGTAGTA---------ATAAATTGTACAAGACCCAACAACAATACAAGAAAA-----
-AGTATACCTATG------------GGACCAGGGAAA------TTT------TATACAACAGGAGAC------
---------ATAATAGGAGATATA------------AGAAAAGCATATTGT---ATCCTTAATAGAACA---
CAATGGAATAACACTTTAAAACAGATAGCTCAAAAATTAGGA---------------AAACAATACAAC----
-----------------------------------AAATCAATAATAGTCTTTAAC------
TCCTCCTCAGGAGGGGACCCAGAAGTTGTA---ACTCACAGTTTTAATTGTGGAGGGGAG---
TTTAAGCAGCTCCAGGCAAGGATCCTGGCTGTAGAAAGATACCTAATGGATCAACGGCTCCTGGGAATTTGGG
GTTGCTCTGGGAGACTCATTTGCACCACTGCAGTGCCTTGGAATACTAGTTGGAGT-----------------
----------------------------AATAAATCTTATGATACAATTTGG------GGT------
AACATGACCTGGATGGAGTGG---GAAAGAGAAATTGACAATTATACAAGAGTAATATAT---
AACTTAATTGAAGACTCGCAGAACCAACAAGAC---

AAAAATGAACAAGAATTATTGGAATTGGATAAATGGGCAAGTTTGTGGAATTGGTTTGACATAACAAAATGGC
TGTGGTATATAAAAATATTCATAATGATAGTAGGAGGCTTAATAGGTTTAAGAATAATTTTTACT---
GTATTTTCTATAGTG---------AGGAGAGTTAGGCAGGGATATTCACCA------
CTATCGTTTGCTGTTAGCTTGTTTAATGCCACGGCCATAACAGTAGCTGGG---
GGGACAGATAGGGTTCTAGAAGTGTTACAAAGAATTTGTAGAGCT----------------------------
--------ATTTGCAACATA------CCTAGAAGAATAAGACAAGGCTTGGAAAGACTTTTGCAA---TAA
>JF683747
ACA---GCA---
GGAAAGTTATGGGTCACAGTCTATTATGGGGTACCTGTGTGG---AAAGAAGCAACT---
ACCACTTTATTTTGTGCATCAGATGCTAAAGGATATGAGACAGAG---GTACATAAT----------------
--------------GTTTGGGCCACA---CATGCCTGTGTACCCACAGAC---
CCCCACCCACAAGAGGTAGTATTGGCA---------AATGTGACAGAAAATTATAAC----------------
-------------------------ATGTGGAAAAATAACATGGTAGAACAGATGCATGAGGATATA---
ATCAGTTTATGGGATCAAAGTCTA---AAGCCATGTGTAAAATTA---
ACCCCACTCTGTGTTACTTTAAATTGCACTGAGTTGAAAAATGGTACTATCAACAAT----------------
----
---AGGACAGAAGGAGAAATGAAA---
AATTGCTCTTTCAATATCACCACAAGCATACGA---------------------------
GATAAGATGCAGAAAGAATATGCACTT---TTTTATAAACTTGATATCATACCAATAGATGGG----------
----

AGTAAGAATGATACTAAGTATAGGTTAATAAGTTGTAATACCTCAGTCCTTACACAG---
GCCTGTCCAAAGATATCCTTTGAGCCAATTCCCATACATTATTGTGCCCCGCCTGGTTTTGCGATTCTAAAGT
GTAAC---AATAAGACATTCGAT------GGAAAGGGACCATGTACAAAT----------------------
-GTCAGC---ACAGTACAATGTACACATGGAATCAGGCCAGTAGTGTCAACT---
CAATTACTGTTAAATGGCAGTCTAGCAGAAGAAGAG------------
GTAGTAATTAGATCTGACAATTTCTCAAACAAT---------GCTAAGGTCATAATAGTACAGCTGAAT----
-----------GAAACTGTACAA---------ATTAATTGTACTAGGCCTAACAACAATACAAGAAAA-----
-GGTATAACTATA------------GGACCAGGGAGAGCA---TTC------TACACAACAGGAGAA------
---------ATAATAGGAAATATA------------AGACAAGCATATTGT---AACCTTAGTAGAAAG---
GCATGGAATAACGCACTAAAACAGATAGTTACAAAATTAAGA---------------ACACCATTTGGA----
-----------------------------------AATAAAACTATAGTCTTTAAA------
CCACCCTCTGGAGGGGACCCAGAAATTGTA---ACACACAGTTTTAATTGTGGAGGGGAA---
TTCAAGCAGCTCCAGGCAAGAGTCCTGGCTGTAGAAAGATACCTAAAGGATCAACAGCTCCTAGGGATTTGGG
GTTGCTCTGGAAAACTCATTTGCACCACTACTGTGCCTTGGAACTCTAGTTGGAGT-----------------
----------------------------AATAAATCTTATGATGATATTTGG------AAT------
AACATGACCTGGATGGAGTGG---GAGAGAGAAATTGACAATTACACAAGCACAATATAT---
ACCTTGCTTGAAGAATCGCAGAACCAGCAGGAA---

AAGAATGAACAAGAATTATTGGCATTAGATAAGTGGGCAAGTTTGTGGAATTGGTTTGACATAACAAATTGGC
TGTGGTATATAAAAATATTCATAATGATAGTAGGAGGCTTGGTAGGCTTAAGAATAGTTTTTGCT---
GTACTTGCTTTAGTA---------AATAGAGTTAGGCAGGGATACTCACCA------
TTATCATTTGCTGTCAGCTTGCTTAACGCTACAGCTATAGCAGTAGCTGAG---
GGGACAGATAGGGTTATAGAAGTAGTACAAAGAATTGGTAGAGCT----------------------------
--------ATTCACAACATA------CCTAGGAGAATAAGACAGGGCTTGGAAAGGGCTTTGCAG---TAA
>JF683751

GCT---ACA---
GACAAATTGTGGGTCACAGTCTATTATGGGGTACCTGTGTGG---AAAGAAGCAACC---
ACCACTCTATTTTGTGCATCAGATGCTAAAGCATATGATACAGAG---GCACATAAT----------------
--------------GTTTGGGCCACA---CATGCCTGTGTACCCACAGAC---
CCCAACCCACAAGAAGTAGTATTGGAA---------AATGTGACAGAACCTTTTGAC----------------
-------------------------ATGTGGAAAAACAACATGGTAGAACAAATGCATGAGGATATA---
ATCAGTTTATGGGATCAAAGTCTA---AAACCATGTGTAAAATTA---
ACCCCACTATGTGTTACTTTAAATTGCACTGATTGGAGTGGTAATAATACAAATAATAGTAGTAATAAT----
----
----------------ACAAATAATAATAGCACAGGATTAGAGATGGAGAAAGAAGAAATAAAA---
AACTGCTCTTTTAATATCACCACAAGCATAAGA---------------------------
GATAAGATACAAAAAGAATATGCACTT---TTTTATAAAGTTGATGTAGTACAAATAGATAAT----------
----

AGTAGTAACAAGTATATGTTAATAAATTGTAACACCTCAGTCATTACACAA---
GCCTGTCCAAAGGTATCCTTTGAGCCAATTCCCATACATTTTTGTGCCCCAGCTGGTTTTGCGATTCTAAAAT
GTAGG---GACAAGAAGTTCAAT------GGAACAGGACCATGTACAAAT------------------------
-GTCAGC---ACAGTGCAATGTACACATGGAATTAGACCAGTAGTGTCAACT---
CAACTGCTGTTAAATGGCAGTCTAGCAGAAGAAGAG-----------
ATAGTAATTAGATCTAAAAATCTTACGGACAAT---------ACTAAAACCATAATAGTACAGCTGAAA----
-----------GAACCTGTAAAC---------ATTACTTGTGAAAGGCCCAACAACAATACAAGGAAA-----
-AGTGTACATATA------------GGACCAGGGAGAGCA---TTT------TATGCAACAGGAGAT------
---------ATAATAGGAAATATA------------AGACAGGCACATTGT---AACATTAGTAAAGCA---
GAATGGAATAACACTCTAGCACAGGTAGTCAAAAAATTAAAA---------------GAACAATTTAAG----
-----------------------------------AATAAAACAATAATCTTTGAG------
CCCCCCTCAGGAGGGGACCTAGAAATTGTA---ATGCACAGTTTTAATTGTGGAGGGGAA---
TTTAAGCAGCTCCAGGCACGAGTCCTGGCTGTGGAAAGATACCTAAAGGATCAACAGCTCCTGGGAATTTGGG
GTTGCTCTGGAAAACTCATTTGCCCCACTACTGTGCCTTGGAATACTAGTTGGAGT-----------------
----------------------------AATAAAAATCTAAGTCAGATTTGG------GAT------
AACATGACCTGGATGCAGTGG---GAAAAAGAAATTGACAATTACACAAACTTAATATAC---
AATTTAATTGAAGAATCGCAGAACCAGCAAGAA---

AAGAATGAACAAGAATTATTGGAATTAGATAAATGGGCAAGTTTGTGGAATTGGTTTAATATAACACAATGGC
TGTGGTATATAAAAATATTCATAATGATAGTAGGAGGCTTAATAGGTTTAAGAATAGTTTTTGCT---
GTACTTTCTATAGTG---------AATAGAGTTAGGCAGGGATATTCACCA------
TTATCGTTGGCTGTTGACTTGCTCAACGCCACAGCAATAGCAGTAGCTGAA---
GGGACAGATAGGGTTATAGAATTATTACAAAGAATTGGTAGAGCT----------------------------
--------ATCCTCCACATA------CCTGTAAGAATAAGACAAGGCTTAGAAAGGCTTTTGCTA---TAA
>JF683753
GCT---TCA---
GACAAATTGTGGGTCACAGTCTATTATGGGGTACCTGTATGG---AGAGAAGCAAAC---
ACCATTTTATTTTGTGGATCAGATGCTAAAGCATATGATACAGAG---GTACATAAT----------------
--------------GTTTGGGCCACT---CACGCCTGTGTACCCACAGAC---
CCCAACCCACAAGAAATAGTACTGAAA---------AATGTGACAGAAAATTTTAAC----------------
-------------------------ATGTGGAAAAATCCCATGGTAGAACAGATGCATGAAGATGTA---
GTCAGTTTATGGGATCAAAGCCTC---AAACCATGTGTAAAATTA---
ACCCCACTCTGTGTCACTTTAAATTGCACTGAAGTAAAGAATAGTACTAATAACAACACCACG----------
----
---------------------TATAACACTGATAACAACACCAGTATTCTGGAAGAAATGAGA---
AACTGCTCTTTCAATGTCACCGCAGGAATAAGG---------------------------
GATAAGATGCAGAGAGAATATGCACTT---TTTTATAAACTTGATGTAGTACCAATAAATAAT----------
----

GATAATAGCAGCTATAGGTTGATAAGTTGTAACACCTCATTCATTACACAG---
GCCTGTCCAAAGGTAACCTTTGAGCCAATTCCCATACATTATTGTGCCCCAGCTGGGTTTGCGATTCTAAAGT
GTAAA---GATAAGAAATTCAAT------GGAACAGGAACATGTACAAAC------------------------
-GTCAGC---ACAGTACAATGCACACATGGAATTAGGCCAGTAGTGTCAACT---
CAATTGCTGTTAAATGGCAGTCTAGCAGAAGAAGAT-----------
GTAGTAATTAGATCTCAAAATATCAGTGACAAT---------GCTAAAACCATAATAGTGCAGCTGAAA----
-----------GACCCTGTACAA---------ATTAATTGTACAAGACCCGGCAACAATACAAGAAGA-----

-AGCATACCTATA------------GGACCAGGGAAAGCA---TTT------TATGCAACAGGAGAC------
---------ATAATAGGAAATATA------------AGACAAGCACATTGT---AACATTAGTAGAACA---
CACTGGGAAAAAACTTTAGAACAGGTAGCTAGAAAATTAGGA---------------GAACATTTTAGG----
-----------------------------------AACAAAACAATAGTCTTTAAT------
CACTCCTCAGGAGGGGACCCAGAAATTGTA---ATGCACAGTTTTAATTGTAGAGGGGAA---
TTTAAGCAGCTCCAGGCAAGAGTCCTGGCTGTGGAAAGATACCTACAGGATCAACAGCTCCTAGGAATTTGGG
GTTGCTCTGGAAAACACATCTGCACCACTAATGTGCCTTGGAATACTAGTTGGAGT-----------------
----------------------------AATAAATCTCTAGATATGATTTGG------GAA------
AACATGACCTGGATGGAGTGG---GAAAGAGAGATAAGCAATTACACAGGCTTAATATAC---
TCCTTAATTGAAGAATCGCAAACCCAACAAGAA--

AAGAATGAACAAGACTTATTGGCATTGGATAAGTGGAACAGTTTGTGGAATTGGTTTAGTATATCAAAATGCC
TGTGGTATATAAAAATATTTATAATGGTAGTAGCAGGCTTGGTAGGTTTAAAAATAGTTTTTGCT---
GTATTTTCTATAGTG---------AATAGATGTAGGAAGGGATACTCACCA------
CTATCGTTTGCTGTTAGTTTGCTCAACCCCATAGCTATAGCAGTAACTGAA---
GGAACAGATAGGGTTATAGAAACAATACAAAGAGGTGGTAGAGCT----------------------------
--------ATTTTCCATATA------CCTGCAAGAATAAGACAGGGCTTAGAAAGGGCTTTGCTA---TAA
>JF683754
GTT---ACA--
GGAAACTTGTGGGTCACAGTCTATTATGGGGTACCTGTGTGG---AAAGAAGCAACC---
ACCACTCTATTTTGTGCATCAGATGCTAAAGCACATGATACAGAG---GCACATAAT----------------
--------------ATTTGGGCCACA---CATGCCTGTGTACCCACCGAC---
CCCAACCCACAAGAAGTACTAATGGAC---------AATGTGACAGAATATTTTAAC----------------
--------------------------ATGTGGAAAAATAACATGGTAGACCAGATGCATGAGGATGTA---
ATCCGTTTATGGGATCAAAGCCTA---AAACCATGTGTGAAATTA---
ACCCCACTCTGTGTCACTTTAAATTGCTCTGATGTTTCGGGGAATGTTACTGATATCAAT-------------
----
-------------------------GGTATTAAAACCAATAGTAATGATAATGATGAAATGAGC---
ACTTGCTCTGTTGCTGCTGCCCCAACCAATGAA---------------------------
AATGAAAAAAAAAATAGAACTTGTTTTT---TTTTATGATCTTGTTGTAATACCAAAAAAGGATAAA-------
----

AATAATACTAATACTACTTATGTGTTGACAAGTTGTAACACCTCATCAATTACTAAG---
GCCTGCCCCACGATAACCTTTGTTCCACTTCTTCTACTTTATTGTGCCGCGGCTGCTTTTGCAATTCTAATAT
GTAAC---AATAAAACTTTCAAT------GGAAAAGGACCATGTACAACT-----------------------
-GTCAGT---ACAGCACTACGTACTACTGGAATTAGGCCAGTAGTGTCAACT---
CAATTGCTGTTAAATGGCAGTCTAGCAGAAGAAGAG------------
GTAATAATTAGATCTGAAAATTTCACAAACAAT---------GCTAAAACCATAATAGTACAGCTGAAT----
-----------GAATCTGTAATA---------ATTGATTGTACAAGACCCAATAACAATACAAGAAAA-----
-GGTGTACATATA------------GGACCAGGGAGTGCA---ATT------TATGCAACAGGAAAC------
---------ATAATAGGAGACATA------------AGAAGTGCACATTGT---AACCTTAGTAGAAAA---
GCATGGAATAACACTCTAAGACTGGTAGCTATAAAATTAAGA---------------GAACAATTTGGG----
--------------------------------AAAAATAAAACAATAAAATTTAAT------
CAATCCTCAGGAGGGGACCCAGAAATTGTA---ATGCACAGTTTTAATTGTGGAGGGGAA---
TTTAAGCAGCTCCAGGCAAGAGTCCTGGCTGTGGAAAGATACCTAAGGGATCAACAGCTCCTGGGGATTTGGG
GCTGCTCTGGAAAACTCATTTGCACCACTGCTGTGCCTTGGAATACTAGTTGGAGT-----------------
----------------------------AATAAATCTTATAATGACATTTGG------AAT------
AACATGACCTGGATGCAGTGG---GAAAAAGAAATTGAGAATTACACAGGCTTAATATAC---
AATTTACTTGAAAAATCGCAGACCCAACAAGAA--

AAGAATGAACAAGAATTATTGGAACTAGATAAGTGGGCAAATTTGTGGAATTGGTTTGATATATCAAATGGCC
TGTGGTATATAAAAATATTCATAATGATAGTAGGAGGCTTAATAGGTTTAAGAATAGTTTTTGCA---
GTGCTTTCTTTAGTG---------AATAGAGTTAGGCAGGGATACTCACCA------
TTATCGTTTGCTGTTAGCTTGCTTAATGCCACAGCAATAGCAGTAGCTGAG---
GGAACAGATAGGATTATAGAAGTATTACAAAGGGCTGGTAGAGCT----------------------------
--------ATTCTCCACATA------CCTAGAAGGATAAGACAGGGCTTGGAAAGGGCTTTGCAA---TAA
>JF683769
GCT---GCA--
GAAGATTTGTGGGTCACAGTATATTATGGGGTACCTGTGTGG---AAAGATGCAACC---
ACCACTCTATTTTGTGCATCAGATGCTAAAGCATATGATGCAGAG---CAACATAAT----------------

--------------GTTTGGGCCACA---CATGCCTGTGTACCCACAGAC---
CCCACCCCACAAGAAGTAGTAATGGGA---------AATGTGACAGAAAATTTTGAT---------------
--------------------------ATGTGGAAAAATAACATGGTGGACCAAATGCATGAAGATATA---
ATTAGTTTATGGGATCAAAGCCTA---AAGCCATGTGTAAAACTA---
ACCCCACTCTGTGTTACTTTAAATTGCACTGAAGTGAACAGGAATAATACTAGGAATAATAGTAGTGCT----
----
-------------------ATAGAGACTAGGAATAATAGTAGTGCTATAGAGATAGAGATGAAA---
AACTGTTCTTTCAATGTCACCACAAACATAAGA--------------------------
GATAGGTTTCAGAAAGAATATGCACTT---TTATATAAGCTTGATATAGTACCAATAGATGAT---------
----

GATAATACCAGCTATAGGTTAATAAGTTGTAATACCTCAGTCATTACACAG---
GCCTGTCCAAAGGTAAACTTTGAGCCAATTCCTATACATTATTGTGCCCCGGCTGGTTTTGCGATTATAAAAT
GTAAT---GATAAAAAGTTCAAT------GGAAAAGGACCATGTGAAAAT----------------------
-GTCAGC---ACAGTACAATGTACACATGGAATTAGACCAGTAGTATCAACT---
CAACTGCTGTTAAATGGCAGTCTAGCAGAAGAAGAG------------
GTGATAATTAGATCTGCCAATTTCTCAGACAAT---------GCTAAAAACATAATAGTACAGCTGAAC----
-----------AAAACTGTACAA---------ATTCATTGTATAAGACCCAACAACAATACAAGAAAA-----
-AGTATACATATA------------GGACCAGGAAGAGCA---TTC------TATGCAACAGGAGAT------
---------ATAATAGGAGATATA-----------AGACAAGCACATTGT---AATATTAGTAAAGCA---
GGATGGAATACCGCTTTAAAACAGATAGCTACAAAATTATAC---------------AACCAATTT-------
----------------------------------AATAAAACAATAATTTTTAAT------
CACTCCTCAGGAGGAGACCCAGAAATTAAA---ATGCTCACTTTTAATTGTGGAGGGGAA---
TTTAAACAGCTCCAGGCAAGAGTCCTAGCTGTGGAAAGATACCTAAAGGATCAACAGCTCCTAGGACTTTGGG
GTTGCTCTGGAAAAATCATCTGCACCACTGCTGTGCCTTGGAATGCTAGTTGGAGT-----------------
----------------------------AATAAATCTCTGGATCAGATTTGG------GAT------
AACATGACCTGGATAAAGTGG---GAAAGAGAAATCGATAATTACACAGAATTAATATAT---
AATTTAATTCAAGAATCGCAGAACCAGCAAGAA---------------------------------------
----
AAGAATGAACAAGACTTATTGGAATTGGATAAATGGGCAAATTTGTGGAATTGGTTTGACATATCAAATTGGC
TGTGGTATATAAAAATATTCATAATGATAGTAGGAGGCTTGATAGGTTTAAGAATAATTTTTACT---
GCACTTTCTATAGTA---------AATAGAGTTAGGAAGGGATACTCACCA------
TTGTCGTTTGCTGTTAGCTTGTTCAACGCCACAGCCATAGCAGTAGCTGAA---
GGGACAGATAGGATTATAGAAGCATTACAAAGATTTGCTAGAGCT---------------------------
--------ATCCTCCACATA------CCTAGAAGAATAAGACAGGGCTTAGAAAGGGCTTTGCTA---TAA
>JF683775
GCT---GCA---
GAGCAATTGTGGGTCATAGTCTATTATGGGGTACCTGTGTGG---AAAGAAGCAACC---
ACCACTCTATTTTGTGCATCAGATGCTAAAGCATATGCTACAGAG---GTACATAAT----------------
--------------GTTTGGGCCACA---CATGCCTGTGTACCCACAGAC---
CCCAACCCACAAGAAGTGAGATTGGAA---------AATGTAACAGAAGAATTTAAC---------------
--------------------------ATGTGGAAAAATAACATGGTAGAACAGATGCATGAGGACGTA---
ATCAGTTTATGGGATCAAAGCCTA---AAGCCATGTGTGAAATTA---
ACCCCACTCTGTGTTACTTTAAATTGCATTAACTTCAATTATACTAAGAATTATACTAAGACC---------
----
----------------------CATCAGAATGATAGTCTAAAGATGGAGATAGGAGAAATAAAA---
AACTGTTCTTTCAATGTCACCACAAACATAAAA--------------------------
GATAAGTTTCAGAAGACATATGCACTT---TTTTATAAATCTGATATAGTACCAATAGATACT---------
----

AATAGTACTAATAGTACCAGCTATACATTGATAAGTTGTAACACCTCAGTCCTTACACAG---
GCCTGCCCAAAAGTATCCTTTGAGCCAATTCCAATACATTATTGTGCCCCGGCTGGTTTTGCGATTCTAAAAT
GTAAT---GATAAGAATTTCACT------GGAATGGGAGAGTGTAGAAAT----------------------
-GTTAGC---ACAGTACAATGTACACATGGAATTAAGCCAGTAATATCAACT---
CAACTGCTGTTAAATGGCAGTCTAGCAGAACAAGAA------------
GTGGTAATTAGATCTGAAAACATCACAGACCAT---------TTGAAAAACATAATAGTACAACTGAAG----
-----------GACCCTGTAAAA---------ATTAATTGTACAAGACCTGGCAACAATACAAGAAGA-----
-AGTATACGTATA------------GGGCCAGGGAGTGCA---TTT------TTTGAAACAGGAGAC------
---------ATAATAGGAGACATA-----------AGACAAGCACATTGT---AACATTAGTGCAGTA---
GAATGGAATGACACCTTAACACAGATAGTGAGAAAATTAGGA---------------GAACAATTTGGG----

------------------------------AAAAATAAGACAATAGCCTTTAGA------
CAATCTTCAGGGGGGGATCCAGAAATCGCA---ATGCACAGTTTTAATTGTGGAGGGGAA---
TTTAAACAGCTCCAGGCAAGAGTCCTGGCTGTGGAAAGATACCTACAGGATCAACAGCTCCTGGGGCTTTGGG
GTTGCTCTGGAAAACTCATTTGCACCACTGCTGTGCCTTGGAATACTAGTTGGAGC-----------------
---------------------------AATAAAAGTCTGGAAAAGATTTGG------AAT------
AACATGACCTGGATGCAGTGG---GAAAAGGAAATTGATAACTACACACAAGAAATATAC---
TATTTAATTGAAAAATCGCAGAACCAGCAGGAA--

AAGAATGAACAAGAATTGTTGGAATTGGATAAATGGGCAAATTTGTGGAATTGGTTTGACATAACAAACTGGC
TGTGGTATATAAAGATATTCATAATGATAGTAGGAGGCTTGGTAGGTTTAAGAATAGTTTTTGCT---
ATACTTTCTATAGTA---------CGTAGAGTTAGGCAGGGATATTCACCA------
TTATCGTTTGCTGTTAGCTTATACAATACCATAGCCATAGCAGTAGCTGAA---
GGGACAGATAGGATTATAGAATTAGCACAAAGAGTTTTTAGAGCT----------------------------
--------TTCTGCAATATA------CCTAGAAGAATAAGACAGGGCTTAGAAAGGGCTTTGCAA---TAA
>JF683778
GTG---GCA---
GGAAATCTGTGGGTCACAGTCTATTATGGGGTACCTGTGTGG---AAAGATGCAACT---
ACCACTCTATTTTGTGCATCAGATGCTAAAGCATATTCTACAGAA---GCACATAAT----------------
--------------GTTTGGGCCACA---CACGCCTGTGTACCCACAGAT---
CCCAGTCCACAAGAATTAGTATTGGAA---------AATGTGACAGAGGAATTTAAC----------------
-------------------------ATGGGAAAAAATAACATGGTAGAACAGATGCATGAGGATATA---
ATTAGTTTATGGGATCAAAGCCTA---AAACCATGTGTAGAATTA---
ACCCCACTCTGTGTTACTTTAAATTGCACTGATGTGAATGCTACAACAACTGCTAGTGATGTGAAT-------
----
----------------------GCTACAACAACTGCTAGTGAAATGGAAAAAGGAGAAATGAAA---
AACTGCTCTTTCAATATCACCACAAATATGAGA--------------------------
GATAGGACACAGAAAGAATATGCAACT---TTTTATAGACTTGATATAGAACCACTAGATAAT----------
----

AGTACTAATGCCACATATAGGTTGATAAGTTGTAACACCTCAGTCATTACACAG---
GCCTGTCCAAAGATATCCTTTGAGCCAATTCCTATACATTATTGTGCCCCGGCTGGCTTTGCGATTCTAAAGT
GTAAT---AATAAGACATTCAAT------GGACTGGGACCATGTAACAAT----------------------
-GTCAGC---ACAGTACAGTGTACACATGGAATTAAGCCAGTAGTATCAACT---
CAACTACTGTTAAATGGCAGTCTAGCAGAAGAAAAG-----------
GTAGTAATTAGATCTGTAAATTTCACTGACAAT---------GCTAAAACCATAATAGTACAGCTGAAA----
-----------GACCCTGTACAA---------ATTAATTGTACAAGGCCCAACAATAATACAAGGAAA-----
-AGTATACATATA------------GCACCAGGGAGAGCA---TTT------TATGCAACAGGGGAA------
---------ATAATAGGAGATATA------------AGAAAAGCACATTGT---AACATTAACAGAACA---
CAATGGAATAACACTCTACAA--

---GGAGAA---
TTTAAGCAGCTCCAAGCAAGAGTCCTGGCTGTAGAAAGATACCTAAGGGATCAACAGCTCCTAGGGATTTGGG
GCTGCTCTGGAAAGCTCATTTGCACCACTGCTGTGCCTTGGAACACTAGTTGGAGC----------------
---------------------------AATAAATCTTATGAGGCTATTTGG------AAT------
AACATGACCTGGATGCAGTGG---GAAAAAGAAATTGATAATTACACAAACATAATTTAC---
TCCTATATTGAAGAGGCACAAAACCAACAAGAA--

AAGAATGAACAAGAATTATTGGAATTGGATAAATGGGCAAGTTTGTGGAATTGGTTTAGCATAACAAACTGGC
TGTGGTATATAAAAATATTCATAATGATAATAGGAGGCTTAATAGGTTTAAGAATAGTCTTTACT---
GTACTTTCTGTAGTG---------AATAGAGTTAGGCAGGGATACTCACCA------
TTATCGTTAGCTGTTAGCTTGTATAATTCCATAGCCATAGCAGTAGCTGAG---
GGGACAGATAGAATTATAGAAGCATTCCAAAGA--
-------------------------------------AGACAGGGCTTAGAAAGGATTTTGCTA---TAA
>JF683785
GCT---ACA---
GATAAAATGTGGGTCACAGTCTATTATGGGGTACCTGTGTGG---AAAGATGCAGAC---
ACCACTTTATTTTGTGCATCAGATGCTAAAGCATATGATAAAGAG---GCACATAAT----------------
--------------GTTTGGGCCACA---CATGCCTGTGTACCCACAGAC---
CCCAACCCACAAGAAGTAGACATA------------AATGTGACAGAAGAATTTAAC---------------
-------------------------ATGTGGAAAAATAATATGGTAGAACAAATGCATGAGGATATA---

ATTAGTTTATGGGATCAAAGCCTA---AAACCTTGTGTAAAATTA---
ACCCCACTCTGTGTCACTTTAAATTGCACTGACTGGAAAAAACCTACTAATGCCACTGGTAATGATACTACCA
CGGCC---
-------TCTCCTACCCCTTCTAATATCACTGATCCTTTGAAGCTAGACAAAGGAGAAATACAA---
AACTGCTCTTTCAAAGTCACCACAAGCATAAGA---------------------------
GATAGGGAAACGAAAGAATATGCACTG---
TTTTATAGACTTGATGTAGCACCAATAAATGAGAGTGCTAGCTCTGCTAATGATAGTACTTGCCCTTCC----
----
AATAGTACTAAGGCTAATAATACTAGCGCTTGTAAGGATAGCTATAGAGAATATAGGTTGATAAATTGTAATA
CCACTACCATTACACAG---
GCCTGTGCAAAGATATCCTTTGAGCCAATTCCCATACATTATTGTGCCCCGGCTGGTGCTGCGCTTCTAAAGT
GTATA---GATAAGAAGTTCAAT------GGACCAGGAACATGTGAGAAT------------------------
-GTCAGC---ACAGTACATTGTACACATGGAATTCGGCCAGTAGTGTCAACT---
CAACTGCTGTTAAATGGCAGTCTAGCAGAAGAAGAC------------
ATAGTAATTAGATCTGAAAATTTCACGAACAAT---------GCTAAAACCATAATAGTACAGCTAAAT----
-----------GAAATTATAAAA---------ATTAATTGTCTAAGACCCAACAATAATACAAGAAAA-----
-AGTGTACCTATA------------GTGCCAGGGGGTGCA---TTT------TATGCAACAGGAGAT------
---------ATAACAGGAGATATA------------AGAAAAGCACATTGT---AACATTAGTGGAGCT---
GACTGGAATAACACTTTAAGTCGAATAGTTAAAAAATTAAAA---------------GAACAATTTAAG----
--------------------------------AATGCAACAACAATAAATTTTAAT------
CACTCCTCAGGAGGGGACCCAGAAATTGTA---ACGCACAGTTTTAATTGTGGAGGGGAA---
TTTAAGCAGCTCCAGGCAAGAGTCCTAGCTGTGGAAAGATACCTAAGGGATCAACAGCTCCTAGGGATTTGGG
GTTGCTCTGGAAAACTCATTTGCACCACTGCTGTGCCTTGGAATACTAGTTGGAGT-----------------
----------------------------AATAAAACTCATGATCAGATTTGG---------------
AACATGACCTGGATGCAGTGG---GAAAAAGAAATTGACAATTACACAGATATAATATAC---
AACTTACTTGAAGGAGCACAAATCCAACAAGAA---

AAGAATGAACAAGACTTATTGGAATTTGATAAGTGGGCAAATTTGTGGAATTGGTTTGACATATCAAAATGGC
TGTGGTATATAAAAATATTCATAATGATAGTAGGAGGCTTAGTAGGTTTCAGAATAATTGTTGCT---
GTGCTTTCTATAGTA---------AATAGAATTAGGCAGGGATACTCACCA------
TTGTCATTGGTTATCAGCTTGTTTAATGCCACAGCTATAGCAGTAGCTGAG---
GGAACAGATAGGGTTATAGAACTAGCACAAAGAGTTTTTAGAGCT-----------------------------
--------TTTCTCCACATA------CCTAGAAGAATAAGACAGGGCTTGGAAAGGCTTTATTA---TAA
>JF683787
GCT---GAA--
AACAAATTGTGGGTCACAGTCTATTATGGGGTACCTGTGTGG---AAAGAAGCAACT---
ACCACTTTATTTTGTGCATCAGATGCTAAATCATATAATACAGAG---GTACATAAT-----------------
--------------GTTTGGGCCACA---CATGCCTGTGTGCCCACAGAC---
CCCAACCCACAAGAAGTATTATTGAAA---------AATGTGACAGAATATTTTAAT-----------------
--------------------------GCATGGAAAAATGACATGGTAGAACAAATGCAGGAGGATATA---
ATCAGTTTATGGGATCAAAGCCTA---AAGCCATGTGTAAAATTA---
ACCCCACTCTGTGTTACTTTAAATTGTACTGATTACAATACCACTAATACCACTAGTAGTAAC-----------
----
-------------------------ACCAATAGTGGTGGAGAGATGAATAAAGGAGAAATAAAA---
AACTGCTCTTTTGATATCCCCTCCAGAATAAAA---------------------------
AATAAAGTGAAGAAAGAATATGCCTTG---GTTTATAAACTTGAAGTAGTGGAAATACATAAT-----------
----

GAAAATAAAAATACTCCATATAGGTTGATAAGTTGGAATACCCCAATCATTACACAG---
GCCTGGCCAAAAATATCCTTTGAACCAATTTCCATTAACTATTGTGCCCCAGCTGGTTTTGCCATTCTAAAGT
GTAAC---CATAAGAAATTCCAT------GGAAAAGGAGAATGTAAAAT-------------------------
-GTCCGC---CCAGTACCATGGACACCTGGAATTAGACCAGTAGTATCAACT---
CAACTGCTGTTAAATGGCAGTCTAGCAGAGGAAGAG------------
GTAGTAATTAGATCTGACAATTTCACAAACAAT---------GCTAAAACCATAATAGTACAGCTGAAG----
-----------GAACCTGTAGTA---------ATTAATTGTACAAGACCCAACAACAATACAAGAAAA-----
-GGTATACATATA------------GGACCAGGGAGAGCA---TTT------TATGCAACAGGACAA------
---------GTAATAGGAGATATA------------AGACAAGCACATTGT---AACCTTAGTAGAGCA---
GCATGGAATAGCACTTTACAGGCAATAGCTAGCAAATTAAGC---------------AAAAAATATAAT----
-----------------------------------AAAACAAAAATAGCCTTTAAT------
CAGTCCTCAGGAGGGGACCTAGAAATTATA---ATGCACACTTTTAATTGTGGAGGGGAG---

TTCAAGCAGCTCCAGGCGAGAGTCCTGGCTGTGGAAAGATACCTAAGGGATCAACAGCTCCTGGGGATTTGGG
GTTGCTCTGGAAAACTCATCTGCACCACTAATGTGCCTTGGAATACTAGTTGGAGT-----------------
----------------------------AATAAATCTCTAGATCAGATTTGG------AAT------
AACATGACCTGGATGCAGTGG---GAGAAAGAAGTTGGCAATTACACAGAACTCATATAC---
ACCTTAATTGAAGAATCACAAAACCAGCAAGAA--

AAGAATGAACAAGAGTTACTGGGATTGGATAAATGGGCAAGTTTGTGGAATTGGTTTGACATAACACATTGGC
TGTGGTATATAAAAATATTCATAATGATAGTAGGAGGCTTGATAGGTTTAAGAATAGTTTTTGCT---
GTACTTTCTATAGTA---------AATAGAGTTAGGCAGGGATACTCACCA------
TTGTCGTTGGCTATTAACTTGCTAGACGTCACAGCCATAGCAGTAGCTGAG---
GGGACAGATAGGATTATAGAGGGATTAAGAAGATTTGGTAGAGGA----------------------------
--------ATCCTTCACATA------CCTAGAAGAATAAGACAGGGCTTGGAAGGGGCTTTGCTT---TAA
>JF683788
GCT---ACA--
GAAAATTTGTGGGTCACAGTCCATTATGGAGTACCCGTGTGG---AAAGAAGCAACC---
ACCACCCTATTTTGTGCCTCAGATGCTAAAGCATATAAAAAGGAG---GCACATAAT----------------
--------------GTTTGGCCACA---CATGCCTGTGTACCCACAGAC---
CCTGACCCACAAGAAGTACTCTTGGAA---------AATGTAACAGAAAATTTTGAC----------------
--------------------------ATGTGGAGAAATGGCATGGTAGATCAGATGCATGAGGATATA---
ATCAGTCTATGGGATCAAAGCCTA---AAGCCATGTGTAAAATTA---
ACCCCACTCTGTGTTACTTTAAATTGCACTAATTTGACCAGCACTAATTTGACCAACAGTAATAAT-------
----
-------------------ACTAGTAATTCAACTAGTGGAGAAGAAATGAGGGCAGAAATGAAA---
AATTGCTCTTTCTATGTCACCACAAACATAAAA---------------------------
AAGAGAGAGAAAAAAGAATATGCACTT---TTTTATAGCCTTGATATAGTACCAATAGATAAT----------
----

GATAATACAAGCTATATGCTGATAAATTGTAACACCTCAGTCATTACACAG---
GCCTGTCCAAAGGTATCCTTTGAACCAATTCCCATACATTTTTGTGCCCCGGCTGGTTTTGCGATTCTCAAGT
GTAAT---GATAAAAATTACAAT------GGAACAGGACCATGTAAGAAT-----------------------
-GTTAGC---ACAGGACAATGTACACATGGAATTAGACCAGTAGTATCAACC---
CACCTGCTGCTAAATGGCAGTCTGGCAGAAGAAGAG------------
GTAGCAATAAGATCTCAAAATTTCTCGAACAAT---------GCTAAAACCATATTAGTACAGCTGAAC----
-----------CAATCTGTAGAA---------ATTAATTGTACAAGACCCAACAATAATACAAGAAAA-----
-AGTATAACAATA------------GGACCGGGGAGAGCA---TTT------TATGCAACAGGAGAC------
---------ATAATAGGAGATATA------------AGAAAAGCACATTGT---AACCTTAATAGAGCA---
GCATGGAATAACACTTTAAGACAGGTAGTTAAGAAATTAAAA---------------GAACAATTTGGA----
-----------------------------------AATAAAACAATAAATTTTACA------
CAACCCTCAGGAGGGGACCCAGAAATTATA---ATGCACACTTTTAATTGTGGAGGGGAA---
TTTAAGCAGCTCCAGGCAAGAGTCCTGGCTGTGGAAAGATACCTAAGAGATCAACAGCTCCTGGGGATTTGGG
GTTGCTCTGGAAAACTCATCTGCACCACTAATGTGCCTTGGAATGCTAGTTGGAGT-----------------
----------------------------AATAGATCTCTAGATCATATTTGG------GGT------
AACATGACCTGGATGGAGTGG---GAGAGAGAAATTGACAATTATACAGACTTAATATAC---
ACCTTACTTGAGGAATCGCAGAACCAGCAAGAA--

AAGAATGAGCAAGAATTGTTGAAATTGGACACATGGGCAAGTTTATGGAATTGGTTTGACATAACAAATTGGC
TGTGGTACATAAAAATATTCATAATGATAGTAGGAGGTTTAGTAGGTTTAAGAATAGTTTTTACT---
GTACTTTCTATAGTG---------AATAGAGTTAGGCAGGGATACTCACCA------
TTGTCATTTGCTATTAGTTTGCTTAATGCCACAGCTATAGCAGTAGCTGAG---
GGGACAGATAGGGTTATAGAAGTAGTACAGAGAATTGGTAGAGCT----------------------------
--------TTTCTCCACATA------CCTAGAAGAATAAGACAAGGCTTCGAAAGGGCTTTGCTA---TAA
>JF683790
GCT---ACA---AAC---------------------------------
AAAGACAACTTGTGGGTCACAGTCTATTATGGGGTACCTGTGTGG---AAAGAAGCAACC---
ACCACTCTATTTTGTGCATCAGATGCTAAGGCTTATGATACAGAA---GTACATAAT----------------
--------------GTTTGGCCACG---CATGCCTGTGTACCCACAGAC---
CCCAACCCACAAGAAATGGAATTAAAA---------AATGTGACAGAAAATTATAAC----------------
--------------------------ATGTGGAAAAATAACATGGTAGAACAGATGCATGAGGATATA---
ATTAGTTTATGGGATCAAAGCCTA---AAGCCATGTGTGAAACTA---
ACCCCACTCTGTGTCACTTTAAATTGCACTGATGTGAAAACTAATAGTACCAACAGTAGT-------------

```
-------------------------------------------------------------------------
----------------------------ACTAAACCCACTGATAGTAGCGGGATAGAAATGAAA---
AACTGCTCTTTTAATATCACCACAAGCATAAGA---------------------------
GATAAGATACAGAAAGAATATGCACTT---TTTTATAAACTTGATGTAGTACAAATAGATGAG----------
-------------------------------------------------------------------------
----------------
GATAAGGATAATACTAGCTATAGGTTGATAAGTTGTAACACCTCAGTCATTACACAA---
GCCTGTCCAAAGGTATCCTTTCAACCAATTCCCATACATTATTGTGCCCCGGCTGGTTTTGCGATTCTAAAGT
GTAAT---GATAAGAGTTTCAAT------GGAACAGGAATATGTACAAAT-----------------------
-GTCAGC---ACAGTACAATGTACACATGGGATTAAGCCAGTAGTATCAACT---
CAATTGCTGTTAAATGGCAGTCTAGCAGAAGAAGAG------------
GTGGTACTTAGATCTGAAAATTTCACAAACAAT---------GCTAAAACCATAATAGTACAGCTGAAA----
-----------GAAGCTGTAGAA---------ATTAATTGTACAAGACCCAGCAACAATACAAGAAGA-----
-AGTATACATATA------------GGACCAGGGAGAGCA---TTT------TATGCAACAGGACAA------
---------ATAACAGGAGATATA------------AGGAGTGCATATTGT---ACCCTTAATGGCACA---
AAATGGAATAACACTTTAAAACTGGTAGCTGAAAAATTAAGT---------------CAACAATTTAGA----
-----------------------------------AATAAAACAATACAATTTAAG------
CACTCCTCAGGAGGGGACCCAGAAATTGTA---ATGCACAGTTTTAATTGTGGAGGGGAA---
TTTAAGCAGCTCCAGGCAAGAGTCCTGGCTGTGGAAAGATACCTAAAGGATCAACAGCTCCTGGGGATTTGGG
GTTGCTCTGGAAAACTCATCTGCACCACCTCTGTGCCTTGGAATACTAGTTGGAGT-----------------
----------------------------AATAGATCTCTGAATGATATTTGG------GAT------
AACCTAACCTGGATGCAGTGG---GAAAAAGAAATTGACAATTATACAGGCTTAATATAT---
GACTTAATTGAGGAATCGCAAAAACAACAAGAA----------------------------------------
-----------------------------
AAGAATGAACAAGAATTATTGGCATTAGACAAATGGGCAAGTTTGTGGAATTGGTTTGACATATCAAACTGGC
TGTGGTATATAAAAATATTCATAATGATAGTAGGAGGCTTGATAGGGTTAAGAATAGTTTTTACT---
GTACTTTCTATAGCA---------AATAGAGTTAGGCAGGATATTCACCA------
TTGTCATTTGCTATTAGCTTGCTCAACGCCACAGCCATAGCAGTAGCTGAG---
GGGACAGATAGGGTTCTAGAAACATTACAAAGAATTGGTAGAGCT----------------------------
--------TTCCTGCACATA------CCTAGAAGAATAAGACAGGGCCTTGAAAGGATTTTACTA---TAA
>JF683794
GCT---GCA------------------------------------------
GATAAATTATGGGTCACAGTCTATTATGGGGTACCTGTATGG---AAAGACGCAACC---
ACCACTTTATTCTGTGCATCAGACTCTAAAGCATATGTAACAGAG---GCACATAAT----------------
--------------GTTTGGCCACA---CATGCCTGTGTGCCCACAGAC---
CCCAACCCACAAGAAGTAGTAATGGA----------AATGTGACGGAAGATTTTAAC----------------
--------------------------ATGTGGAGAAATAACATGGTAGAACAGATGCATGAGGATATA---
ATCAGTTTATGGGATGAAAGCCTA---AAGCCATGTGTAAAATTA---
ACCCCACTCTGTGTCACTTTAAATTGTGTGAATTATAACAATAGTGAGAATGCTAATAGTACGAATACTACT-
-------------------------------------------------------------------------
----------------ACCCCTACCCCTACTAGTAGTGGGAAGATGGAAGAAGGGGAAATAAAA---
AACTGCTCTTTTAATGTTACCACAGTTGTAAGA---------------------------
GATAAAGTGCACAAAGAATATGCACTC---TTTTATAGACTTGATGTAGTAAAAATAGAGAAT----------
-------------------------------------------------------------------------
----------------------
AACACTGATAGATATAGGTTGATAAATTGTAATACCTCAGTCATTACACAG---
GCCTGTCCGAAGGTATCCTTTGAGCCAATTCCCATACATTATTGTGCCCCGGCTGGTTTTGCGATTCTCAAGT
GTAAT---AATGAGACATTCAAG------GGAAAAGGAAAATGTGAAAAT-----------------------
-GTTAGC---ACAGTACAATGTACACATGGAATTAGGCCAGTAGTGTCAACT---
CAACTGCTGCTAAATGGCAGTCTAGCAGAAAAGGAG------------
GTAGTAATTAGATCTGACAATTTCTCGGATAAT---------GCTAAAATCATAATAGTACACCTGAAT----
-----------GAGTCTGTAGAA---------ATTAATTGTATAAGGCCCAACAACAATACAAGAAAA-----
-AGTATACATATA------------GCACCGGGGAGAGCA---TTT------TATACAACAGGACAA------
---------ATAATAGGAGACATA------------AGACAAGCACATTGT---AACATCAGTAGAGCA---
AAATGGAATATCACTTTAAGACAGGTAGTTGCAAAATTAGAA---------------GAAGAATTTCCT----
-----------------------------------AACAAAACAATAGTCTTTAAA------
AACTCCTCAGGAGGGGACCCAGAAATTGTA---ATGCACAGTTTTAATTGTGGAGGGGAA---
TTTAAGCAGCTCCAGGCAAGAGTCCTGGCTGTGGAAAGATACCTACGGGATCAACAGCTCCTAGGGATTTGGG
GTTGCTCTGGAAAACTCATTTGCAACACTGCTGTGCCTTGGAATGCTAGTTGGAGTAGTAGA---
TGGAGTAATAATGGT---------------------AATAGATCATTGGATGACATTTGG------AGT----
```

--AACTTGACCTGGATGCAGTGG---GAAAAAGAAATTGACAATTATACACAAACAATATAC---
TCCTTACTTGAAGAATCGCAGATCCAGCAAGAA---

AAAAATGAACAAGAACTGTTAGAATTGGATAAGTGGGACAGTTTGTGGAATTGGTTTGACATATCAAAATGGC
TGTGGTATATAAAAATATTCATAATAATAGTAGGAGGCTTGATAGGTTTAAGATTAACTTTTGCT---
GTGCTTTCTATAGTG---------AGTAGAGTTAGGCAGGGATACTCACCA------
TTGTCATTTGCTATTAGTTTGTACAACGCTACAGCTATAACAGTAGCTGAG---
GGGACAGATAGGATTATAGAAGTAGCACAAAGAATTTGTAGAGCT---------------------------
--------GTCCTCCACATA------CCTAGAAGAATAAGACAGGGCTTAGAAAGGGCTTTGCTA---TAA
>JF683801
GCT---GCA---
GACAAGTTGTGGGTCACAGTCTATTATGGGGTACCTGTGTGG---AAAGAAGCAACC---
ACCACTCTATTTTGTGCATCAGATGCTAAAGCAGTTGACACAGAG---GTGCATAAT----------------
--------------GTTTGGGCCACG---CATGCCTGTGTACCCACAGAC---
CCTAATCCACAAGAAGTAGTATTGGAA---------AATGTGACAGAAAATTTTAAC----------------
--------------------------ATGTGGAAAAATAACATGGTAGAACAGATGCATGAGGATATA---
ATCAGTTTATGGGATCAAAGCCTA---AAGCCATGTGTAAAATTA---
ACCCCACTCTGTGTTACTTTAAATTGCACTGATGTAAAGAATGGTACTAATGGTAGCATT------------
----
-------------------------AATAGGACAATTGAGGAAGAAATGAAGGGAGAAATAAAA---
AACTGCTCTTTCAATGTCACCAAAAGCATAGGA-------------------------
GATAAGATGCAGAAAGAATATGCACTG---TTTTATAAACTTGATGTAATGCAAATAGATAAT----------
----

GATAATGCAAGCTACACATTGATAAACTGTAACACCTCAGTCATTAGACAG---
GCCTGTCCAAAGGTAACCTTTGAACCAATTCCCATACATTATTGTGCCCCGGCTGGTTTTGCGATTCTAAAGT
GTAAT---GATAAAAAGTTTAAT------GGGACAGGACCATGTAAAAAT----------------------
-GTCAGC---ACAGTACAATGTACACATGGAATTAAGCCAGTAGTGTCAACT---
CAACTGCTATTAAATGGCAGTCTAGCAGAAGAAGAG-----------
ATAGTAATTAGATCTGAAAATTTAACAAACAAT---------GCTAAAACCATAATAGTACATCTGAAC----
-----------GAATCTATAGTA---------ATTAATTGTACAAGACCCAACAACAATACAAGAAAA-----
-GGTATACATATG------------GGATGGGGGAGAGCA---TTT------TATGCAACAGGAGAA------
---------ATAGTAGGGGACATA-----------AGACAAGCACATTGT---AACCTTAGCAGTACA---
CAGTGGAACAACACTTTAAGACAGATAGCTATAAAATTAAGA---------------GAAACATTTAAC----
-------------------------------------AACAAAACAATAGTCTTTAAT------
CACTCCTCGGGAGGGGACCCAGAAATTGTA---ACGCACACTTTTAATTGTGGAGGGGAA---
TTTAAGCAGCTCCAGGCAAGAGTCCTGGCTGTGGAGAGATACCTAAAGGATCAGCAGCTCCTAGGGATTTGGG
GTTGCTCTGGAAAAATCATTTGCACCACTGCTGTGCCTTGGAATGTTAGTTGGAGT-----------------
----------------------------AATAAAACTCTGGAACAGATTTGG------AGC------
AACATGACCTGGATGGAGTGG---GAAAGAGAAATTGACAATTACACAGACATAATATAC---
AGCTTAATTCAAGAATCGCAGAACCAGCAGGAT---

AAAAATGAACAAGAATTATTGGAATTGGATAAGTGGGCAAATTTGTGGAATTGGTTTAACATATCAAACTGGC
TGTGGTATATAAAAATATTCATAATGATAGTAGGAGGCTTAGTAGGTTTAAGAATAGTTTTTGCT---
ATACTTTCTATAATG---------AATAGAGTTAGGCAGGGATACTCACCA------
TTGTCATTCGCTGTTAGCTTGCTTAATGTCACAGCAATAGCAGTAGCTGAG---
GGGACAGATAGGATTATAGAATTAGCACAGAGAATAGGTAGGGCT---------------------------
--------ATTCTCCACATA------CCTAGAAGAATAAGACAGGGCTTAGAAAGGGCTTTGCTA---TAA
>JF683804
GCT---ACA---
GAAGATTTGTGGGTCACAGTCTATTATGGGGTACCTGTGTGG---AAAGAAGCAAAC---
ACCACTCTATTTTGTGCATCAGATGCTAAAGCATATGATACAGAG---GCACATAAT----------------
--------------GTTTGGGCCACA---CATGCCTGTGTACCCACAGAC---
CCCAGCCCACAAGAAGTAGTAATGGCA---------AATGTGACAGAAAATTTTAAT----------------
--------------------------ATGTGGAAAAACAACATGGTAGAACAGATGCATGAGGATATA---
ATCAGTTTATGGGATCAAAGCCTA---AAGCCATGCGTAAAATTA---
ACCCCACTCTGTGTTACTTTAAATTGCACTGATGTGCAGAAGAATAATACTAATACCACT------------
----
-------------------------GTGGGTGGCGGGGGACCAATGGAGTCAGGAGAAATAAAA---
AACTGCTCTTTCAATAGCACCACAAACATCAGA-------------------------

GATAGGTTTCAGAAAGAATATGCACTT---TTTTATAAACATGATGTAGTACCAATAAATGAT----------
----

GATAAGAATAGTACTAGCTATATGTTGATAAGTTGTAACACCTCAGTCCTTACACAG---
GCCTGTCCAAAGATATCATTTGAGCCAATTCCTATACATTATTGTGCACCGGCTGGTTTTGCGATTCTAAAGT
GTAAT---GATAAGAAGTTCAAT------GGAAGCGGACCATGTACAAAT-----------------------
-GTCAGC---ACAGTACAATGTACACATGGTATTAGGCCAGTAGTATCAACT---
CAACTGCTATTAAATGGCAGTCTAGCAGAAGAAGAG------------
GTAGTAATTAGATCTGCCAACTTCTCGGACAAT---------GCTAAAACCATAATAGTACAGCTGAAC----
-----------GAAACTGTAGAA---------ATTAATTGTACAAGACCCAACAGAAATATAAGAAAA-----
-AGGATAACCATG------------GGACCAGGGAGAGTA---TAT------TATACAACAGGAGGA------
---------ATAGTAGGAGATATA------------AGAAAAGCATATTGT---AACATTAGTGTGACA---
AAATGGAATAACACCTTAAAAAAGATAGTTAAAAAATTAAGA---------------GAAAAATTTGGG----
-----------------------------------AATAAAACAATAGTCTTTAAT------
CAATCCTCAGGAGGGGACCCAGAACTGGTA---ATGCACAATTTTAATTGTGGAGGGGAA---
TTTAAGCAGCTCCAGGCAAGAGTCCTGGCTGTGGAAAGATACCTAAGGGATCAACAACTCCTAGGAATTTGGG
GTTGCTCTGGAAAACTCATTTGCACCACTGCTGTGCCTTGGAATGCTAGTTGGAGT-----------------
----------------------------AATAAATCCCTGGGTGAGATTTGG------GAA------
AACATGACCTGGATGGAGTGG---GAAAGAAAAATTGACAATTACACAACATTAATATAC---
ACCTTACTTGAAAAATCGCAGAACCAGCAAGAA--

AAGAATGAGCATGAATTATTGGAATTGGATAAATGGGCAAACCTGTGGAATTGGTTTGATATAACAAACTGGC
TGTGGTATATAAAAATATTCATAATGATAGTAGGAGGCTTGATAGGTTTAAGAATAGTTTTTGCT---
ATACTTTCTATAGTG---------AACAAAGTTAGGCAGGGATACTCACCA------
TTATCATTTGCTGTTAGCTTGTTTAATGCCATAGCCATAGCAGTAGCTGAG---
GGAACAGATAGGATTTTAGAAGCTTTACAGAGAATTGGGAGAGCT----------------------------
--------ATTATCCACATA------CCTAGAAGAATAAGACAGGGCTTGAAAAGGGCTTTGCTA---TAA
>JQ403048
GCT---GCA--
GAACAAATGTGGGTCACAGTCTATTATGGGGTACCTGTGTGG---AAAGAAGCAACC---
ACCACTTTATTTTGTGCATCAGATGCTAAAGCATATGATACAGAG---GTACATAAT----------------
--------------GTTTGGGCCACA---CATGCCTGTGTACCCACAGAC---
CCCAACCCACAAGAAGTAGTATTGCCA---------AATGTGACAGAAGAGTTTAAC----------------
--------------------------ATATGGAAAAATGACATGGTAGAACAGATGCATGAAGATGTA---
ATTAGTTTGTGGGATCAAAGCCTA---AAACCATGTGTAAAACTA---
ACCCCACTCTGTGTCACTCTAAATTGTACTAATTTGAGGAATAATACTACTACCAATAGTACTAATACCAATA
GT---
----------------------AGTAATACCAATAGTAATATAGAGGATAAAGAAGACATAAAA---
AACTGCTCTTTCAATGTAACCACAATCAGGAGA---------------------------
GATAAGGTGCGGAAAGAATATGCAATT---TTTCATGAACTTGATGTAGTACCAATAGATGAT----------
----

AGTAATAGTAGTTATATGTTGAGAAGTTGTAATACCTCAGTCATTACGCAG---
GCCTGTCCAAAAGTAACCTTTGAGCCAATTCCCATACATTACTGTGCCCCGGCTGGTTTTGCGATTCTAAAGT
GTAAC---AATAAAACATTTAGT------GGAAGCGGGCTATGTAGAAAT-----------------------
-GTCAGC---ACAGTACAATGCACACATGGAATTAAGCCAGTAGTATCAACT---
CAACTGCTATTAAATGGCAGTTTAGCAGAAAAGGAG------------
ATAGTACTTAGATCTGCCAATTTCTCGGACAAT---------GCTAAAACCATAATAGTACAATTGAAG----
-----------GAACCTATAAAA---------ATTAATTGCACAAGGCCCAATAACAATACAAGAAAA-----
-AGTATACATATA------------GCACCAGGGAGAGCA---TTC------TATGCAACAGGAGAA------
---------ATAATAGGAAATATA------------AGACAAGCACATTGC---AATATTACTAGGAAA---
GATTGGAATAAAACTCTAGGACAGATAGTTGCGAAATTAAGA---------------GAACAATTTGGG----
--------------------------------AATAATAAAACAATAATCTTTAAT------
TCATCCTCAGGAGGGGACCCAGAAATTGTA---ATGCACAGTTTTAATTGTGGAGGGGAA---
TTCAAGCAGCTCCAGGCAAGAGTCCTGGCTGTGGAGAGATACCTAAAGGATCAACAGCTCCTGGGGATTTGGG
GTTGTTCTGGAAAACTCATTTGCACCACTGCTGTGCCTTGGAATACTAGCTGGAAT-----------------
----------------------------AATAAATCTTATGAGGACATTTGG------AAT------
AACATGACCTGGATGCAATGG---GAGAAAGAAATTGACAATTACACACAAGAAATATAC---
AATTTAATTGAAATATCGCAGAACCAACAAGAA--

AAGAATGAACAAGAACTATTGGAATTAGATAAATGGGCAAGTTTGTGGAATTGGTTTGACATAACAAAATGGC
TGTGGTATATAAAAATATTCATAATGATAATAGGAGGCTTGGTAGGTTTAAGAATAGTTTTTGCT---
GTAATTTCTATAGTA---------AACAGAGTTAGGCAGGGATACTCACCA------
CTGTCATTTGCTGTTAACTTGCTCAACACCACCGCCATAGTAGTAGCTGAG---
GGGACAGATAGAATTATAGAAGTAGTACAAATAATTTTTAGAGCT----------------------------
--------GTTCGCCACATA------CCTAGAAGAATAAGACAGGGCTTGGAAAGAAT-TTGCTG---TAA
>KT124812
GCT---ACA--
AAAAATTTGTGGGTCACAGTCTATTATGGGGTACCTGTGTGG---AAAGAAGCAACC---
ACCACTCTATTCTGTGCATCAGATGCTAAAGCATATGAGACAGAG---GTGCATAAT----------------
--------------GTTTGGGCCACA---CATGCCTGTGTACCCACAGAC---
CCCAACCCACAAGAAGTAGTATTGGGA---------AATGTGACAGAAAATTTTAAC----------------
--------------------------ATGTGGAAAAATGACATGGTAGAACAAATGCATGATGATATA---
ATCAGTTTATGGGATCAAAGCCTA---AAGCCATGTGTAAAATTA---
ACCCCACTCTGTGTTACTTTAAATTGCACTAATGTTAAGACTACTAACACCACTGGTAATGGT----------
----
-------------------CTCACTAATAGTAGCTGGGGAAGGATGGAAGAAGGGGAAATAAAG---
AACTGCTCTTTTAATACCACCACAGATGTAAAA---------------------------
GACAGGATGCAAAAACAATATGCACTA---TTTTATAAACTTGATGTAGTACAGATAGATTCC----------
----

AGCACAAATAACAGCGCTGAATATAGGTTGACAAGTTGTAACACCTCAGTCATTACACAG---
GCCTGTCCAAAGATATCCTTTGAACCAATTCCCATACATTATTGTACCCCGGCTGGTTTTGCGATTATAAAAT
GTAAT---GATAAGAAATTCAAT------GGAACAGGACCATGTACTAAT-----------------------
-GTCAGC---ACAGTACAATGTACACATGGAATTAGGCCGGTAGTATCAACT---
CAATTGCTGTTAAATGGCAGTTTAGCAGAAGAAGAG------------
ATAGTAATTAGATCTGAAAATATAACGAACAAT---------GCTAAAACCATAATAGTACATCTGAAT----
-----------GAATCTGTAGAA---------ATTAACTGTACAAGACCCAACAACAATACAAGCAAA-----
-AGTATACCTATA------------GGACCAGGGGGAGCA---TTT------TACACAACAGGAAGT------
---------ATAATAGGAGATATA------------AGACAAGCACATTGT---AACCTTAGTAGAGCA---
AAGTGGGAGAAAACTTTAGAAAAGATAAGTAGTAAATTAAGA---------------GAACAATTTAAT----
-----------------------------------AATAAAACAATAGTCTTTAAT------
CAATCCACAGGAGGGGACCTCGAAATTGTA---ATGCACAGTTTTAATTGTGGAGGGGAA---
TTCAAGCAGCTCCAGGCAAGAGTCCTGGCTGTGGAAAGATACCTAAAGGATCAACAGCTCCTGGGGATTTGGG
GTTGCTCTGGGAAGCTCATTTGCACCACTGCTGTGCCTTGGAATGCTAGTTGGAGT-----------------
----------------------------AATAAATCTATGGAAGACATTTGG------AAT------
AACATGACCTGGATGGAGTGG---GAAAGAGAAATTGACAATTACACAGGGGTAATATAC---
AGATTACTTGAGAAATCGCAAAACCAGCAAGAA--

AAGAATGAACAAGAATTATTGGAATTGGACAAATGGGACAGTTTGTGGAGTTGGTTTAGCATAACAAACTGGC
TATGGTATATAAGAATATTCATAATGATAGTAGGAGGCTTGGTAGGTTTAAGAATAGTTTTAGT---
GTACTTTCTATAATA---------AATAGAGTTAGGCAGGGATATTCACCA------
ATATCGTTGGCTATTAGCTTGCTCAACGCCACAGCCATAGCAGTAGCTGAA---
GGGACAGATAGGTTTATAGAAATATTACAAAGAATTTGTAGAGGT----------------------------
--------ATCCTTCACATA------CCTACAAGAATAAGACAGGGCTTAGAAAGGCTTTTGCTA---TAA
>KT124752
GCT---ACA--
GAAAAATTGTGGGTCACAGTCTATTATGGGGTACCTGTGTGG---AAAGAAGCAACC---
ACCACTTTATTTTGTGCATCAGATGCTAAAGCATATAGTACAGAG---GTACATAAT----------------
--------------GTTTGGGCCACA---CACGCCTGTGTACCCACAGAC---
CCCAACCCACAAGAAATAGTATTGGGG---------AATGTGACAGAAAATTTTAAC----------------
--------------------------ATGTGGAAAAATAACATGGTAGAACAAATGCATGAGGATATA---
ATCAGTTTATGGGATCAAAGCCTA---AAGCCATGCGTAAAATTA---
ACCCCACTCTGTGTTACTCTAAATTGCACTGATTATTGGAAGAATGAAACTAACACCAATGAGACTAGCATCA
ATGGTACT---
-------------AACACCAATAATAGTAGTAACTGGGAAAAAATGGAGAAAGGAGAAATAAAA---
AACTGCTCTTTCAATATCTCCACAAGCGTAAAA---------------------------
GATAAGATGCAGGAAGTATATGCACTT---TTTTATAAGCTTGATGTAGTACCAATAGATAATGAGAATAGG-
----

AATAATACTAATAGCAACATATATAGGTTAATAAGTTGTAACACCTCAGTCACTACACAA---
GCCTGTCCAAAGGTATCCTTTGAGCCAATTCCCATACATTATTGTGCCCCGGCTGGTTTTGCGATTCTAAAGT
GTAAC---AATGAGACGTTCAAT------GGAACAGGACCATGTACAAAT----------------------
-GTCAGC---ACAGTACAATGTACACATGGAATTAGGCCAGTAGTATCAACT---
CAACTGCTGCTAAATGGCAGTCTAGCAGAAAAAGAG-----------
GTAGTAATTAGGTCTGAAAATTTCACAGACAAT---------GCTAAAACCATAATAGTACAGCTGAAA----
-----------GATCCTGTAGTA---------ATTAATTGTACAAGACCCAACAACAATACAAGAAAA-----
-GGTATACATATA------------GCACCAGGGAGAGCA---TTT------TATGCAACAGGAGAC------
---------ATAATAGGAGATATA------------AGAAAAGCACATTGT---AACCTTAGTAGAGAA---
GATTGGAATAAGACTTTACAACAGGTAGTTGCAAAATTAGGA---------------GAGCAATTTAAG----
-----------------------------------AATAAATCAATAGCCTTTAAT------
CAATCCTCAGGAGGAGACCCAGAAATTGTA---ATGCACAGTTTTAATTGTGGAGGGGAA---
TTCAAGCAGCTCCAGGCAAGAGTCCTGGCTGTGGAAAGATACCTAAAGGATCAACAGCTCCTGGGGATTTGGG
GTTGCTCTGGAAAACTCATTTGCACCACTGCTGTGCCTTGGAATGCTAGTTGGAGC-----------------
----------------------------AATAAATCTCTGGATAGTATTTGG------GAT------
AACATGACCTGGATGGAGTGG---GACAGAGAAATTGACAACTACACAAACATAATATAC---
AATTTACTTGGGGAATCGCAAATCCAACAAGAA--

AAGAATGAACTAGAATTATTGGAATTGGATAAATGGGCAAGTTTGTGGAATTGGTTCAACATAACAGAATGGC
TGTGGTATATAAAAATCTTCATAATGATAATAGGAGGCTTGGTAGGTTTAAGAATAGTTTTTACT---
ATACTTTCTGTAGTG---------AATAGAGTTAGGCAGGGATACTCACCA------
CTATCGTTAGCTGTTAGCTTGTTCAACGCTACAGCCATAACAGTAGCTGAG---
GGAACAGATAGGGTTATAGAAGTAGTACAAAGAACTTTTAGAGCT----------------------------
--------ATTATCCACATA------CCTAGAAGAATAAGACAGGGCTTCGAAAGGGCTTTGCTA---TAA
>KT124755
GCT---GCA---
GACAAGTTGTGGGTCACAGTTTATTATGGGGTACCTGTGTGG---AAAGAAGCAACC---
ACTACTCTATTTTGTGCATCAGATGCTAAAGGATATAATACAGAG---GTACATAAT----------------
--------------GTGTGGGCCACA---CATGCCTGCGTACCCACAGAC---
CCCAACCCACAAGAAATAGAATTGGAA---------AATGTGACAGAAGACTTTAAC---------------
--------------------------ATGTGGAAAAATGGCATGGTAGAACAAATGCATGAGGATGTA---
ATCAGTTTATGGGATCACAGCCTA---AAGCCATGTGTAAAATTA---
ACCCCTCTCTGTGTTACTCTAAATTGCACTGATGTGAAGACAAATGCCACTATTAATGCT------------
----
--AACATGACAGAGGAAATAAAA---
AACTGCTCTTTCAATGTCACCACAAGCATAAGA--------------------------
GATAGGGTACAAAGAGAATATGCATTG---TTTTATAAACTTGATATAGTACCAATAAATGATGATAAG----
----

AACAATAGCAGCAATAATACCAGATATAGGTTGATAAGTTGTAACACCTCAGTCATTACACAG---
GCCTGTCCAAAGGTAACCTTTGAGCCAATTCCCATACATTATTGTGCCCCGGCTGGTTTTGCGATTCTAAAAT
GTAAT---GATAAGGAGTTTGAT------GGAAAAGGATCATGTAACAAT----------------------
-GTCAGC---ACAGTACAATGTACACATGGAATTAGACCAGTAGTATCAACT---
CAACTGTTGTTAAATGGCAGTCTAGCAGAAAAAGGG-----------
GTAATGATTAGATCTGACAATTTAACAGATAAT---------ACTAAAACCATAATAGTACAGCTGAAT----
-----------GAATCTGTAGAA---------ATTAATTGTATAAGACCCAACAACAATACAAGAAAA-----
-AGTATAAATATA------------GGACCAGGGAGAGCA---TTT------TATGCAACAGGAGAA------
---------ATAATAGGAGATATA------------AGACAAGCACATTGT---AACATTAGTGCAAAA---
GCATGGAATGACACTTTAAGACAGGTAGCTGAAAAACTAAGG---------------GAAAAATTC-------
-----------------------------------AATAAAACAATATCCTTTAAT------
CGCTCCTCAGGAGGGGACCCAGAAATTGTA---ATGCACAACTTTAATTGTGCAGGGGAA---
TTTAAGCAGCTCCAGGCAAGAATCCTGGCTGTGGAAAGATACCTAAAGGATCAACAGCTCCTGGGAATTTGGG
GTTGCTCTGGAAAACTCATTTGCACCACTGCTGTGCCTTGGAATGCTAGTTGGAGT----------------
----------------------------AATAAATCTATGGAGCAGATTTGG------GAT------
AACATGACCTGGATGCAGTGG---GAAAAGGAAATTGACAATTACACAAGCTTAATATAC---
TCCTTAATTGAAGAATCGCAGAATCAACAGGAA--

AAGAATGAACTAGAATTATTGGAATTGGACAAATGGGCAAGCTTGTGGAATTGGTTTGACATAACAAAGTGGC
TGTGGTATATAAAAATATTCATAATGATAGTAGGAGGCTTGGTAGGTTTAAGGATAGTTTTTACT---
GTACTTTCTATAGTG---------AATAGAGTTAGGAAGGGATACTCACCA------

TTATCGTTTGCTATTAGCTTGCTCAATGCCACAGCTGTAAGAATAGCCGAA---
GGGACAGATAGAATTATAGAAATAGTCCAAGCAGTTTTTAGAGCT----------------------------
--------ATTCTCCACATA------CCTAGAAGAATAAGACAGGGCTTAGAAAGGCTTTTGCTA---TAA
>KT124757
GCT---GCA--
GATAACCTGTGGGTCACAGTCTATTATGGGGTACCTGTGTGG---AAAGAAGCAACC---
ACCACTCTATTTTGTGCATCAGATGCTAAAGCATATGAGACAGAA---GTACATAAT----------------
--------------GTCTGGGCTACA---CATGCCTGTGTACCCACAGAC---
CCCAACCCACAGGAAGTAGTATTGGGA---------AATGTGACAGAAGATTTTAAC----------------
--------------------------ATGTGGAAAAATAACATGGTAGAACAGATGCATGAGGATATA---
ATCAGTTTATGGGAGCAAAGCCTA---ACGCCATGTGTAAAATTA---
ACCCCACTCTGTGTCACTTTAGAGTGTCACAATGTTACTAGGGATGCTGGGAATGCTACTGCAATTCGTACCA
CGACACCTCCT--
----------------------------TCTATAAATAGTACCGTGGAGGACTCCGACATGAAA---
AACTGCTCTTTCAATATCACCACAAATATGAAA--------------------------
AATAAGATATTTAAGGAATATGCACTT---TTTTATAAACTTGATATAGTACAAGATAATAAT----------
----

ACAGGAAATGATAATACATATACATTGGTACAGTGTGATACCTCAGTCATTACACAT---
GCCTGTCCAAAGGTATCCTTTGAGCCAATTCCCATACATTATTGTGCCCCGGCTGGTTTTGCAATTCTAAAGT
GTAAT---AATAATAGTTTCAAT------GGAACGGGATTGTGTACAAAT----------------------
-GTCAGC---ACAGTACAATGCACACATGGAATTAGGCCAGTAGTGTCAACT---
CAACTGCTGTTAAATGGCAGTCTAGCAGAAAAAGAG------------
GTAAAAATTAGGTCTGAAAATCTCACAAACAAT---------GCTAAAACCATAATAGTGCAGCTGAAT----
-----------AAGACTGTAGTA---------ATTAATTGTACAAGACCCAACAACAATACAAAAAAA-----
-AGTGTTAATATA------------GGACCAGGGAGAGCA---TTT------GATGCAACAGGAAAA------
---------ATAATAGGAGATATA-----------AGACAAGCACATTGT---AACCTTAGTAGAGAA---
GAGTGGAATAACACTCTCGAGTGGGTAGTTGAAAAACTAAAA---------------AGAAAATTTCCA----
------------------------------------AATAAGACAATAGACTTTAAA------
CCATCCTCAGGAGGGGACCCAGAAATTACA---AATCACATCTTTAATTGTGGAGGGGAA---
TTTAAACAGCTCCAGGCAAGAGTCCTGGCTATAGAAAGATACCTAAAGGATCAACAGCTCCTAGGGATTTGGG
GTTGCTCTGGAAAACTCATCTGCACCACTACTGTGCCTTGGAACACTAGTTGGAGT-----------------
----------------------------AATAAATCTCTGAGTTACATTTGG------GAT------
AACATGACCTGGATGGAATGG---GAAAGGGAGATTGACAATTACACAGGCTTGATATAC---
ACGTTACTTGAAGAATCGCAGAACCAGCAAGAA--

AAGAATGAACAAGAATTATTGGAATTGGATAAGTGGGCAAGTTTGTGGAATTGGTTTAGTATAACAAAATGGC
TGTGGTATATAAAAATATTCATAATGATAGTAGGAGGCTTGATAGGTTTAAGAATAGTTTTTGCT---
GTACTTGCTATAGTA---------AATAGAGTTAGGCAGGGATACTCACCC------
TTATCATTTGCTGTTAGCTTGCTTAATACCACAGCAATAACAGTAGCTGAA---
GGGACAGATAGGATTATAGAAGTAGTACAAAGAGCTTTTAGAGCT----------------------------
------------------------------------ATAAGACAGGGCTTGGAAAGGCTTTTGGTA---TAA
>KT124794
GCA---GGA---GAA-----------------------------------
CAAGAACAATTATGGGTCACAGTCTATTATGGGGTACCTGTGTGG---AAAGAAGCAAAT---
ACCACTTTATTTTGTGCATCAGATGCTAAAGCACACCATACAGAG---GCACATAAT----------------
--------------ATTTGGGCCACA---CATGCCTGTGTGCCCACAGAC---
CCCAACCCACAAGAAGTAGTATTGGGA---------AATGTGACAGAAAATTTTAAC----------------
--------------------------ATGTGGAAAAATAACATGGTAGAGCAAATGAATGAGGATATA---
ATCAGTTTATGGGATCAAAGCCTA---AAACCATGTGTAAAATTA---
ACCCCACTCTGTGTTACTCTAAATTGCTCTGATGCTAATACCACTAGTAGTAGTACTACTAGTAGTAAT----
----
-------------------ACCCCTAGTTATGGTATGGGACAGATGGATAAAGGAGAAATAAAA---
AACTGCTCTTTCAACATTACCACAGAGATGCAA--------------------------
GATAAAAGGAAAAAAGACTATGCATTA---TTTTATGCACTTGATATAGTACCAATAGATAATGAT-------
----

AATAATACTAGCTATAGGTTGATAAGTTGTAACACCTCAGTCATTACACAG---
GCATGTCCAAAAGTAACCTTTGAGCCAATTCCCATACATTATTGTGCCCCGGCTGGTTTTGCGATTATACAAT
GTAAT---AATAAAACGTTCAAT------GGAACAGGACCATGTACAAAT----------------------

-GTTAGC---ACAGTACAATGTACACATGGAATTAAGCCAGTAGTATCAACT---
CAACTACTGTTAAATGGAAGTCTAGCAGAAAAGGAG------------
GTAGTAATTAGATCTGAAAATTTCACGGATAAT---------ACTAAAATCATAATAGTGCAGCTGAAT----
-----------GAAACTGTAGAA---------ATTAATTGTACAAGACCCAACAACAATACAAGAAAA-----
-AGTATAAATATA------------GGACCAGGGAGAGCA---TTT------TATGCAACA---GAT------
---------ATAATAGGAGATATA------------AGACAAGCATATTGT---AACATTAGTAGAGCA---
AAATGGAATAACACTTTAAAACAAATAGCTGCTAAATTAAAA---------------ACACAATTCCCA----
-----------------------------------AATAAAACGATAATCTTTAGT------
CCATCCTCAGGAGGGGACCCAGAAATTGTA---ATGCACAGTTTTAATTGCGGAGGGGAA---
TTCAAACAGCTCCAGGCAAGAGTCCTGGCTGTGGAAAGATACCTAAAGGATCAACAGCTCTTGGGGATTTGGG
GATGCTCTGGAAAACTCATTTGCACCACTAATGTGCCTTGGAATACTAGTTGGAGT-----------------
----------------------------AATAAATCTGAAAGTCAGATTTGG------GAT------
AACATGACCTGGATGCAGTGG---GAAAGAGAAATTGACAATTACACAAACTTAATATAC---
AACTTACTTGAAGAATCGCAAACCCAACAAGAA--

ATAAATGAGCAAGAATTATTGTCATTGGATAAATGGGCAAGTTTGTGGAATTGGTTTAACATAACAAATTGGC
TGTGGTATATAAGAATATTCATAATGATAGTAGGAGGCTTGATAGGTTTAAGAATAATCTTTTTT---
GTACTTTCTATAGTG---------AATAGTGTTAGGCAGGGATACTCACCA------
TTGTCGTTGGCTGTTAGCTTGCTCAATGCCACAGCCATAGCAGTAGCTGAA---
GGGACAGATAGGGTTTTAGAAGCAGTACAACGAATTGGGAGAGCT----------------------------
--------TTCCTCCACATA------CCTAGAAGAATAAGACAGGGCTTTGAAAGGGCTTTGCTA---TAA
>KT124800
GCT---ACA--
GAAAAACTGTGGGTCACAGTTTATTATGGGGTACCTGTGTGG---AAAGAAGCAAAC---
ACCACTCTATTTTGTGCATCAGATGCTAAAGCATATAGTACAGAG---GCACATAAT----------------
--------------GTTTGGCCACA---CATGCCTGTGTACCCACAGAC---
CCCAACCCACAAGAAATAGTATTGGGA---------AATGTGACAGAAACTTTTAAC----------------
--------------------------ATGTGGAAAAATCACATGGTAGAACAGATGCATGAGGATATA---
ATTAGTTTATGGGATCAAAGCCTA---AAGCCATGTGTAAAACTA---
ACCCCACTCTGTGTTACTTTAAATTGTACTGATAAATTCAATGCTTCTAATACCTTAAAT-------------
----
-------------------------GGTACTAATAAGAATGTAAGTGAGGAAATAGATATGAAA---
AACTGCTCTTTTAATGCTACTGCGGGAATACAG---------------------------
GGTAAGGTAAAGAAAGAATATGCACTT---TTTTATAACCTTGATATAGTACCCTTAGGGGAT----------
----

AATAATGACACCTATAGGTTGATAAGTTGTAATACCTCAGTCATTACACAG---
GCCTGTCCAAAGGTAACTTTTGAGCCAATTCCCATACATTATTGTACCCCAGCTGGATATGCACTTCTAAAGT
GTAAT---GATAAAAAGTTCAAT------GGAACAGGACCATGTACAAAT-----------------------
-GTCAGC---ACAGTACAATGTACACATGGAATTAAGCCAGTAGTGTCAACT---
CAACTGCTGTTAAATGGTAGTCTAGCAGAAGAAGAA------------
ATAGTAATTAGATCTGAAAATTTCACAAATAAT---------GCTAAAACCATAATAGTGCAGCTGAAT----
-----------GCAACTATAAAA---------ATTAATTGTACAAGGCCTAATAACAACACAAGAAAA-----
-AGTATACATATA------------GCACCAGGAAGAGCA---TTT------TATGCAACAGGACAA------
---------ATAATAGGAGATATA------------AGGAAAGCACATTGT---AACATTAGTGGAGGA---
ACATGGAATAACACTTTAAGAAGGATAGCTGCAAAATTAAGG---------------GAGCAATTTGGG----
--------------------------------CAGAATAAAACAATAGCCTTTAAA------
CAATCCTCAGGAGGGGACCCAGAAATTATA---ATGCACACTTTTAATTGTGGAGGGGAA---
TTTAAGCAGCTCCAGGCGAGAGTTCTGGCTGTGGAAAGATACTTAAGGGATCAACAGCTCCTGGGAATTTGGG
GATGCTCTGGAAAGCTCATCTGCACCACTGCTGTGCCTTGGAATACTAGTTGGAGT-----------------
----------------------------AATAAATCTCTGAATGACATATGG------GAT------
AACATGACCTGGATGGAGTGG---GAAAGAGAGATTGACAATTACACAGGCATAATATAT---
AATCTGCTTGAAAAATCACAAATCCAACAAGAA--

CAGAATGAACAAGAATTATTGGAATTAGATAAATGGTCAAATTTGTGGAATTGGTTTAATATAACACAGTGGC
TGTGGTATATAAAAATATTTATAATGATAGTAGGAGGTTTGATAGGTTTAAGAATAGTCTTTGCT---
GTGCTTGCTATAGTG---------AATAGAGTTAGGCAGGGGTACTCACCA------
TTGTCATTCGCTGTTAGTTTGTTTAATGCCACAGCTGTCACAGTAGCTGAG---
GGGACAGATAGGATTATAGAATTAGTACAAAGACTCTGTAGGGCT----------------------------
--------ATTATTCATATA------CCTGTAAGAGTAAGACAGGGCTTAGAAAGGGCTTTGCTA---TAA

>KT124810
GCT---GCA--
GAACAAACGTGGGTCACAGTCTATTATGGGGTACCTGTGTGG---AAAGAAGCAACT---
ACCACTCTATTTTGTGCATCAGATGCTAAAGCATATGATACAGAG---GCACATAAT----------------
--------------GTTTGGGCCACA---CATGCCTGTGTACCCACAGAC---
CCCAACCCACAAGAAGTAGTATTAGAA---------AATGTGACAGAAAATTTTAAC----------------
--------------------------ATGTGGAAAAATAGCATGGTGGAACAGATGAATGAAGATATA---
ATCAGTTTATGGGATCAAAGCCTA---AAGCCATGTGTAAAGTTA---
ACCCCACTCTGTGTTACTTTGAATTGCTCTAATGTGACG----------------------------------
----
---AATGGAGCTGGGGAAACAAAA---
AACTGCTCTTTTCAGGTCAGCACAGGAGTAAAA---------------------------
GATAAGACGCAAAAGGCATCTGCATTG---TTTTATACAGTTGATTTAGTACCAATAAGTAAT----------
----

ACAAATGGTAGCTATAGGATGATAAAGTGTAATACTTCAGTCATTACACAG---
GCCTGTCCAAAAGTATCCTTTGAGCCAATTCCCATACATTATTGTGCCCCGGCTGGTTTTGCGATTCTAAAGT
GTAAT---GATAAGAAATTCAAT------GGAACAGGACCATGTACAAAT-----------------------
-GTCAGC---ACAGTACAATGCACACATGGAATTAGGCCAGTAGTGTCAACT---
CAACTGCTGTTAAATGGCAGTCTAGCAGAAGAAGAG------------
GTAGTAATTAGATCTGCCAATTTCTCAAATAAT---------GCTAAAACTATAATAGTACAGCTGAAT----
-----------GAATCTGTAATA---------ATTAATTGTACAGGGCCCAACAACAATACAAGAAAA-----
-AGTATACATATA------------GGACCAGGGAAAACA---TTT------TATGCAACAGGAGAA------
---------ATAATAGGAGATATA------------AGGCAAGCACATTGT---AACATTAGTGAAGCA---
GCATGGAGCAAAACTTTAAGCCTGATAACTGAAAAATTAAGA---------------GAACAATTTGGG----
--------------------------------AATAATAAAACAATAGTCTATAAG------
CAAGCCTCAGGAGGGGACCCAGAAATTGTA---ATGCATAGCTTTAATTGTGGAGGGGAA---
TTTAAGCAGCTCCAGGCCAGAGTCCTGGCTGTGGAAAGATACCTACAGGATCAACAGCTCCTAGGGATTTGGG
GTTGCTCTGGAAAACTCATTTGCACCACTGCTGTGCCTTGGAATATTAGTTGGAGT-----------------
----------------------------AATAAAACTCAGAGTGAGATCTGG------GAT------
AACATGACCTGGATGGAGTGG---GAAAGAGAAATTAGCAATTACACAAATTTAATATAC---
TCTTTAATTGAAGAATCGCAGAACCAGCAAGAA--

AAGAATGAACAAGAATTATTGGAATTAGATAAGTGGGCAAGCTTGTGGAATTGGTTTGACATAACAAACTGGC
TGTGGTATATAAAAATATTCATAATGATAATAGGAGGCTTGGTAGGTTTAAGAATAGTTTTTGCT---
GTACTTTCTATAGTG---------AACAGAGTTAGGCAGGGATACTCACCA------
TTATCGTTCGCTGTCAGCTTGCTCAATGCCACAGCTATAGCAGTAGGTGAG---
GGAACAGATAGGGTTATAGAAGTTGTACAAAGAGTTTTTAGAGCT----------------------------
--------TTTCTCCATATA------CCTAGAAGATTGAGACAGGGCTTGGAAAGGGCTTTGCTA---TAA
>KT124749
GCT---ACA--
GAAAACCTGTGGGTCACAGTTTATTATGGGGTACCTGTATGG---AAGGAAGCAACA---
ACCACTCTATTTTGTGCATCAGATGCTAAAGCATATGATAAGGAG---GTACATAAT----------------
--------------GTTTGGGCCACG---CATGCCTGTGTACCCACAGAC---
CCCGACCCACAGGAAGTATCCTTGGGA---------AATGTGACAGAAAATTTTAAC----------------
--------------------------ATGTGGAAAAATAACATGGTAGAACAATTGCATGAGGATGTA---
ATCAGTTTATGGGACCAAAGCTTA---AAACCATGTGTAAAATTA---
ACCCCACTCTGTGTTACTTTAAATTGCACTGATTACCAAGGAAATGGTACTAATACAAAT-------------
----
-------------------AGGACTAATACAAATAGTAGTATAGAAGGAGATGGAGAAATAAAA---
AACTGCTCTTTCAATTACACCACAAACATAAGA---------------------------
GATAAGATACAGAGAACATATGCACTT---TTTTATAAACTTGATGTAGTACCAATAGAAAAA----------
----

AATAGTTCCAGCTATAGGTTGATAAGTTGTAATACCTCAGTCATTACACAG---
GCCTGTCCCAAAGTATCCTTTGAGCCAATTCCCATACATTATTGTGCCCCGGCTGGTTTTGCGATTCTAAAGT
GTAAT---GATAAAAAGTTCAAT------GGAAAAGGGGAATGTAGCAAT-----------------------
-GTCAGT---ACAGTACAATGTACACATGGAATTAGGCCAGTAGTATCAACT---
CAACTACTGTTAAATGGCAGTTTAGCAGAAGAAGAG------------
GTAGTAATTAGATCTGACAATTTCACAAATAAT---------GCTAAAACCATAATAGTACAGCTAAAC----

-----------CAATCTGTAACA---------ATTAATTGTACAAGACCCAACAACAATACAAGAAAA-----
-AGTATACATATA------------GGACCAGGGAGAGCA---TTC------TATGCAACAGGAGAC------
---------ATAATAGGAGATATA------------AGACAAGCACATTGT---AACATTAGTAGAATA---
GAATGGAATAACACTTTAGGACAGATAGTTGGAAAATTAAGA---------------GAACAATTTGAG----
------------------------------------AATAAAACAATAGTCTTTAAC------
CAATCCTCAGGAGGAGACCCAGAAATTACA---ATGCACAGTTTTATTTGTGGAGGGGAA---
TTTAAGCAGCTCCAGGCAAGAGTCCTGGCTGTGGAAAGATACCTACAGGATCAACAGCGCCTGGGGATTTGGG
GTTGCTCTGGAAAACTCATTTGCACCACTGCTGTGCCTTGGAATATTAGTTGGAGT----------------
-----------------------------AATAAATCTCTAGGGGATATTTGG------GAT------
AATATGACCTGGATGCAGTGG---GAAAAGGAAATTGACAATTACACAACCACAATATAC---
GATTTACTTGAGAAATCGCAGAACCAGCAAGAA---

AAGAATGAACAAGAATTATTGGAATTAGACAAATGGGCAAGTTTGTGGAATTGGTTTGACATAACAAATTGGC
TGTGGTATATAAAAATATTTATAATGATAGTAGGAGGCATAGTAGGGTTAAGAATAGTTTTAGCT---
GTACTTTCTATAGTG---------AATAGAGTTAGGAAGGGATACTCACCA------
TTATCGTTTGCTGTTAGCTTGTGTGACGCCATAGCCATAGCAGTAGCTGGG---
GGGACAGATAGGATTATAGAAGGGTTACAAAGATTTTGTAGGGCT----------------------------
--------ATCCTACACATA------CCTGTAAGAATAAGACAGGGCTTGGAAAGAGCTTTGCTA---TAA
>KT124753
GCT---ACA---
GACAAATTGTGGGTCACAATTTATTATGGGGTACCTGTGTGG---AAAGAAGCAAAC---
ACCACTCTATTTTGTGCATCAGATGCTAAAACATATGATACAGAG---GTACATAAT----------------
--------------GTTTGGGCCACA---CATGCCTGTGTACCCACAGAC---
CCCAACCCACAAGAAATAGAAGTGAAA---------AATGTGACAGAAAATTTTAAC---------------
---------------------------ATGTGGAAAAATAACATGGTAGAACAGATGCATGAGGATATA---
ATCAGTTTATGGGATCAAAGCCTA---AAGCCATGTGTAAAATTA---
ACCCCACTCTGTGTTACTTTAACTTGCACTAACGTGAGTGCTACTACTACCAATACTACCACTAGTAACACTA
GTACCACTCCT--
-----------------------------ACCACTAATAGTCGCGAGGGAATGAGGGAAATGAAA---
AACTGCTCTTTCAATATCACCACAAACATAAGA--------------------------
GATAAGATAAAGAAAGAATATGCACTT---TTTTATGAACTTGATATAGTATCAATAGATAAC----------
----

AGTAATACCAGCTATAGGTTGATAAGTTGTAATACCTCAGTCATTACACAG---
GCCTGTCCAAAGATAACCTTTGAGCCAATTCCCATACATTATTGTGCCCCGGCTGGTTTTGCGCTTCTAAAGT
GTAAT---GATAAGAAGTTCAAT------GGAACAGGACCATGTAAAAAT-----------------------
-GTCAGC---ACGGTACAATGTACACATGGAATTAGGCCAGTAGTATCAACT---
CAACTGCTGCTAAATGGCAGTCTAGCAGAAGAAGAG------------
GTAGTAATCAGATCTCAAAACTTCTCAAACAAT---------GCTAAAGTCATAATAGTGCAGCTGAAT----
-----------GAAACTGTAGAA---------ATTAATTGTACAAGACCCAACAACAATACAAGAAAA-----
-AGTATACCTATA------------GGACCAGGAAGGGCA---CTT------TATACAACA---GAA------
---------ATAATAGGAGATATA------------AGAAAAGCATATTGT---AACGTTAGTAGAGCA---
AAATGGTATACCACTCTAAAACAGGTAGCTGAAAAATTAAGA---------------GCACAATTTGAG----
------------------------------------AATAAAACAATAGTCTTTAAT------
CACTCCTCAGGAGGGGACCCAGAAATTGTA---ATGCACAGTGTTAATTGTGGAGGAGAA---
TTTAAACAGCTCCAGGCAAGAGTCCTGGCTGTGGAAAGATACCTAAGGGATCAACAGCTCCTGGGGATTTGGG
GTTGCTCTGGAAAACTCATCTGCACCACTACTGTACCTTGGAATACTAGTTGGAGT----------------
-----------------------------AATAAATCTTATAATCAGATTTGG------GAC------
AACATGACCTGGATGCAGTGG---GAAAGAGAAATTGACAACTACACAAACACAATATAC---
AGGTTAATTGAAGAGTCACAGAACCAACAAGAA---

AAGAATGAACAAGACTTATTGGCATTAGATAAATGGGCAAGTTTGTGGAATTGGTTTGACATAACAAACTGGC
TGTGGTATATAAAAATATTCATAATGATAGTAGGAGGCTTAATAGGTTTAAGAATAGTTTTTACT---
ATACTTTCTATAGTG---------AATAGAGTTAGGCAGGGATACTCACCA------
TTGTCGTTTGCTATTAGCTTGTACAACGCCACAGCCATAGCAGTAGCTGAG---
GGGACAGATAGGATTTTAGAAGTATTACAAAGAATTTATAGAGCT----------------------------
--------TTTCTCCACATA------CCTAGAAGAATAAGGCAGGGCGCGGAAAGGGTTTTGGTA---TAA
>KT124754
GCT---ACA---
GAAAAATTGTGGGTCACAGTCTATTATGGGGTACCTGTGTGG---AAAGAGGCAAAC---

ACTACTCTATTTTGTGCATCAGATGCTAAAGCATATGATACAGAG---GTACATAAT----------------
--------------GTTTGGGCCACA---CATGCCTGTGTACCCACAGAC---
CCCAACCCACAAGAAATAGAATTGATA---------AATGTGACAGAAAATTTTAAT----------------
--------------------------ATGTGGAAAAATAACATGGTAGAACAGATGCATGAGGATATA---
ATCAGTTTATGGGATCAAAGCCTA---AAGCCATGTGTAAAATTA---
ACCCCACTCTGTGTTACTTTAAATTGCACTAACTATAATACTACTAATAATGTTACTCATACCAAT-------
----
----------------------AGTAGTGATAGCGAAATACCGATGGATGAGGGAGAAATAAAA---
AACTGCTCTTTCAATATCACCACAAGCAGAAGA---------------------------
AGTAAGGTGCAGGAAGAATATGCAGTG---TTTTATAAACTTGATGTAGTACCAATAAATGAT----------
----

AATAATAATAATAATAGCTATAGGTTGATAAGTTGTAACACCTCAGTCATTACACAG---
GCCTGTCCAAAGATATCCTTTGAGCCAATTCCCATACATTTGTGTGCCCCGGCTGGTTATGCGATTCTAAAGT
GTAAA---GATAAAAAGTTCAAT------GGAACAGGACCATGTGAAAAT-----------------------
-GTCAGC---ACAATACAATGTACACATGGAATTAAGCCAGTAGTATCAACT---
CAACTACTGTTAAATGGCAGCCTAGCAGAAGAAGAG------------
ATAGTAATTAGATCTGAAAATTTCTCGGACAAT---------GCTAAAACCATAATAGTACAGCTGAAC----
-----------CAATCTGTAGAA---------ATTAATTGTACAAGACCCTACAACAATACAAGAAAA-----
-GGTATACATATG------------GGACCAGGAAGAGCA---TTA------TATATAACAGGAGAA------
---------GTAACAGGGAACATA------------AGACAAGCACATTGT---ACCCTCAATAGAACA---
AAATGGATGAACACTTTAAGTCAGGTAGCTGAAAAATTAAAA---------------GAACAATTT-------
-----------------------------------AATAAAACAATAATATTTAAT------
CAATCCTCAGGAGGGGACCCAGAAATTGTC---ATGCACCATTTTAATTGTAGAGGGGAA---
TTTAAGCAGCTCCAGGCAAGAGTCCTGGCTCTGGAAAGATACCTAAAGGATCAACAGCTCCTGGGAATTTGGG
GATGCTCTGGGAAACTCATTTGCACCACTGCAGTGCCTTGGAATGCTAGTTGGAGT-----------------
----------------------------AATAGAACTCTGGATGACATTTGG------GAT------
AACCTGACCTGGATGCAGTGG---GAAAGGGAAATTAGCAATTACACACAAGTAATATAT---
ACCTTAATTGAAGAATCGCAGAGCCAGCAAGAA--

AAGAATGAAAAAGAATTATTGGAATTGGATAAATGGGCAAGTTTGTGGAATTGGTTTGACATAACAAACTGGC
TGTGGTATATAAAAATATTCATAATGATAGTAGGAGGCCTGATAGGTTTAAAAATAGTTTTTACT---
GTACTCTCTATAGTA---------AATAGAATTAGGCAGGGATACTCACCA------
TTGTCGTTTGCTGTTAGCTTGTTCAATGTCACAGCCATAGCAGTAGCTGAG---
GGGACAGATAGGGTTATAGAAGTAGTACAAGGAGCTTATAGAGCT----------------------------
--------ATTATCCATATA------CCTAGAAGAATAAGACAGGGTGCGGAAAGGATTTTCCTA---TAA
>KC247955
GCT---ACA---
GATCTGTGGGTCACAGTCTATTATGGAGTACCAGTATGG---AAAAACGCAACT---
ACCACTCTATTTTGTGCATCAGATGCCAAAGCATATGAGACAGAG---GCACATAAT----------------
--------------GTGTGGGCCACG---CATGCTTGTGTACCCACAGAC---
CCCAACCCACAAGAAGTAGTAATAGCA---------AATGTGACAGAATATTTTAAC----------------
--------------------------ATGTGGGAAAATGACATGGTAGAACAGATGCATGAAGACATA---
ATCAGTTTATGGGATCAAAGCCTA---AAACCATGTGTAAAATTA---
ACCCCACTCTGTGTCACTTTAAATTGCACTAACATCACTAAGAACAGCACTGATAGTAAC-------------
----
-------------------------AACAATAGTAGTGGGTTGGATATGACAGGGGAAATGAAA---
AACTGCTCTTTCAAGGTCACCACCAACAGAAGA---------------------------
GATAAGATGAAAAAAGAATATGCACTT---TTTTATACATCTGATGTAGTACCCATAGATAAC----------
----

AATAGCTACAGGTTGATAAGTTGTAACACCTCAGTCATTACACAA---
GCCTGTCCAAAGGTATCCTTTGAACCAATTCCCATACATTATTGTACCCCGGCTGGTTTTGCGATTCTAAAAT
GTAAT---GATAAAAAGTTCAGT------GGAAAAGGACCATGTACAAAT-----------------------
-GTCAGT---ACGATACAATGTACACATGGAATTAGGCCAGTAGTGTCAACT---
CAACTGCTGCTAAATGGCAGTCTAGCAGAAGAAGAG------------
ATAATAATTAGATCTAAAAATCTCTCGGACAAC---------ACTAAAACCATAATAGTACATTTAAAT----
-----------GAATCTGTAGAA---------ATTAATTGTATAAGGCCCAACAACAATACAAGAAAA-----
-AGTATACCGATA------------GGACCAGGGAAAGTA---TTT------TATGCAACAGGAGAA------
---------ATAATAGGAGACATA------------AGACAAGCACATTGT---ARCATTAGTGAAGAC---

```
AAATGGAACAAAACTTTAACACAGATAATTGAAAAATTAAAA---------------GAACAATTTAAC----
-----------------------------------AACAAAACAATAATCTTTAAG------
CAACCCTCAGGAGGGGATCCAGAAATTATA---TTGCATACTTTTAATTGTGGAGGGGAA---
TTTAAGCAGCTCCAGGCAAGAGTCCTAGCTGTGGAAAGATACCTAAGGGATCAACAGCTCCTAGGGATTTGGG
GTTGCTCTGGAAAACTCATCTGCACCACTTTTGTGCCTTGGAATACTAGTTGGAGT-----------------
----------------------------AATAAAACTCTGAATCAGATTTGG------GAT------
AACATGACTTGGATGCAGTGG---GAAACAGAAATTGGCAATTACACAAACTTAATATAC---
ACCTTACTTGAAGAATCGCAGAACCAGCAAGAA----------------------------------------
-----------------------------
AAGAATGAACAGGAATTGTTGGAATTGGATAAGTGGAACAGTCTGTGGAATTGGTTTAGCATAACAAATTGGC
TGTGGTATATAAAAATATTCATAATGATAGTAGGAGGCTTGATAGGTTTAAGAATAGTTTTTGCT---
GTACTTTCTATAGTG---------AATAGAGTTAGGCAGGGATACTCACCA------
TTATCATTGGCTATTAGCTTGCTCAACGCCACAGCTATCGCAGTAGGTGAA---
GGACAGACAGGATTATAGAAGTAGCACAAAGAATTGGTAGAGCT----------------------------
--------ATCCTCCATATA------CCTGTAAGAATAAGACAGGGCTTAGAAAGGGCTTTRCTA---TAA
>KT124772
ACT---GCA------------------------------------------
GATCAATTGTGGGTCACAGTCTATTATGGGGTACCTGTGTGG---AAAGAAGCGACC---
ACCACTTTATTTTGTGCATCAGATGCTAAAGCATATGATCCAGAA---GTACATAAT----------------
--------------GTTTGGGCCACA---CATGCCTGTGTACCCACAGAC---
CCCAACCCACAAGAAGTAGTATTGGAA---------AATGTGACAGAAAACTTTAAC----------------
--------------------------ATGTGGAAAAATAACATGGTAGAACAGATGCAGGAGGATATA---
ATCAGTTTATGGGATCAAAGCCTA---AAGCCATGTGTAAAATTA---
ACCCCACTCTGCGTTACTTTACATTGCATTGATATGAATACTACTACCAATAATAAGCCTACCACTACCCCTA
AG-----------------------------------------------------------------------
-------------------CCCACTCAGAATGGCGAAGAACTGATGGAAAAAGGAGAACTAAAA---
AAGTGCTTTTTCAATGTTACTTCAAGCGTAAGG---------------------------
GATAAGGTGCAGCAACAATATGCACAT---TTTTATAAACTTGATTTAGTACGAATAGATGATAGTAATAAT-
-------------------------------------------------------------------------
----------
AGTAGCAGTAATAATAGTAGCAGCTATAGGTTGACAAGTTGTAATACCTCAGTCATTACACAG---
GCCTGTCCAAAGGTATCCTTTGAGCCAATTCCCATACATTATTGTGCCCCTGCTGGTTTTGCGATTCTAAAGT
GTAAC---AATAAGACATTCGAT------GGAAAAGGACCATGTACAAAT--------------------
-GTCAGC---ACAGTACAATGTACACATGGAATTAGGCCAGTAGTATCAACT---
CAACTATTGTTAAATGGCAGTCTAGCAGAGGAAGAG------------
GTAGTAATTAGATCTGACAATTTCTCGAACAAT---------GCTAAAACCATAATAGTACAGCTGAAT----
-----------GAATCTGTAGTA---------ATTAATTGTACAAGACCCAACAATAATACAAGAAAA-----
-AGTATACCTATA------------GGACCAGGGAGAGCA---ATG------TTTACAGCAGGAGAA------
---------ATAATAGGAGATATA------------AGAAAAGCATATTGT---ACCATTAATAGAACA---
GACTGGAATAACACTTTAGAAAAGATAGTTAAAAAATTAAGA---------------GAACAATATGGG----
--------------------------------GTTAATAAAACAATAAGCTTCAGG------
CCCTCCTCAGGAGGGGACCCAGAAATTACA---ATGCACAGTTTTAATTGTAGAGGAGAA---
TTCAAGCAGCTCCAGGCAAGAGTCCTGGCTGTGGAAAGATACCTAAGGGATCAGCAGCTCCTAGGAATTTGGG
GTTGCTCTGGGAAACTCATTTGCACCACTGCTGTGCCTTGGAATGCTAGTTGGAGT-----------------
----------------------------AATAAATCTCTAAATGAAATCTGG------AAT------
AACATGACTTGGATGCAGTGG---GAGAGAGAAATTGACAATTACACAAGCTTAATATAC---
TCCCTAATTGAGGAATCGCAAAACCAACAGGAA----------------------------------------
-----------------------------
AAGAATGAACAAGACTTATTGGAATTAGATAAGTGGGCAAGTTTGTGGAATTGGTTTAACATAACAAATTGGC
TGTGGTATATAAAAATATTCATAATGATAGTAGGAGGCTTGATAGGTTTAAGAATAGTTTTTGTT---
GTACTTTCTATAGTA---------AATAGAGTTAGGAAGGGATACTCACCA------
TTATCGTTTGCAATTAGCTTGTTCAACACCACAGCCATAGCAGTAGCTGAG---
GGACAGATAGGCTTATAGAATTGTTTCAAAGAGTGCTTAGAGCT----------------------------
--------ATCCTGAACATA------CCTAGAAGAATCAGACAGGGCTTAGAAAGGGCTTTGCAA---TAA
>KT124797
GCT---ACA------------------------------------------
GAAAAATTGTGGGTCACAGTCTATTATGGGGTACCTGTGTGG---AGAGAAGCAACC---
ACCACTCTATTTTGTGCATCAGATGCTAAAGCATATAAGCCAGAG---ATACATAAT----------------
--------------GTTTGGGCCACA---CATGCCTGTGTACCCACAGAC---
CCCAGCCCACAAGAAGTACATTTAGAA---------AATGTGACAGAGAACTTTAAC----------------
```

--------------------------ATGTGGGACAATAACATGGTAGAACAGATGCATGAGGACATA---
ATCAGTTTATGGGATCAAAGCCTA---AAGCCATGTGTAAAATTA---
ACCCCACTCTGTGTTACTTTAAATTGTACTGATTATCCAACGAATGATACTAATAGTACT-------------
----
-------------------------AGTAATAGTAGTAACATACTGGAGAAAGAAGAAATAAAG---
AACTGCTCTTTCAATATCACATCAGGAGGCATA------------------------
AGAGATAAGGTGAAAACAGAATATGCAGCT---TTTTATAAACTTGATCTAGTACCAATAGAGGAT-------
----

AGTAATAGTAGTAGTAGTTATAGGATAATAAGTTGCAATACCTCAGTCATTACCCAG---
GCCTGTCCAAAGGTATCCTTTGAGCCAATTCCCATACATTATTGTGCCCCAGCTGGTTTTGCGATTCTAAAGT
GTAAT---GACAAGGAGTTCAAT------GGAACAGGACCATGTAAAAAT-----------------------
-GTCAGT---ACAGTACAATGTACACATGGAATTAAGCCAGTAGTATCAACT---
CAACTACTATTAAATGGCAGTCTAGCAGAAAAAGAG------------
GTAATAATTAGATCAAAAAATTTCTCGGATAAT---------AGTAAAATCATAATAGTGCATCTGAAT----
-----------GAATCAATAACA---------ATTAATTGTACAAGGCCTGGCAACAATACAAGAAAA-----
-AGTATACATATA------------GGACCAGGGAGAGCG---TTT------TATGCAACA---GAT------
---------ATAATAGGAGATATA------------AGACAAGCACATTGT---AACATTAGTAGAACA---
GAATGGAATAAGACTTTACAAAAAATAGTTGATAAACTAGGT---------------AAGAAATTAATA----
--------------------------------AATAAAACAAAAATAATCTTTAAT------
CAATCCTCAGGAGGGGACCCAGAGATTGTA---AAACATAGTTTTAATTGTGAAGGAGAA---
TTTAAGCAGCTCCAGGCAAGAGTCCTGGCTGTGGAAAGATACCTAAAGGATCAACAGCTCCTGGGAATTTGGG
GTTGCTCTGGAAAACTCATCTGCACCACTGCTGTGCCTTGGAATACTAGTTGGAGC-----------------
----------------------------AATAGATCTTATAATGACATTTGG------GAT------
AACATGACCTGGATGCAGTGG---GAAAGAGAAATTGATAATTACACAAGCTTAATATAC---
ACTTTAATTGAAGAATCCCAGAACCAGCAAGAA--

AAGAATGAAAAAGAATTATTAGAATTGGATAAATGGGCAAGTTTGTGGAATTGGTTTGACATAACAAACTGGC
TGTGGTATATAAAAATATTCATAATGATAGTAGGAGGCTTAATAGGTTTAAGAATAGTTTTTACT---
GCACTTTCTATAATG---------AATAGAGTTAGGCAGGGGTACTCACCA------
TTGTCGTTGGCTGTTAGCTTGCTCAACACCACAGCCATAGTAGTTGCTGAG---
GGGACAGATAGGATTATAGAAGTAGCACAAAGAGCTTATAGAGCT----------------------------
--------GTTATCAACATA------CCTGTAAGAATAAGACAAGGCTTGGAAAGGCTTTTGCTA---TAA
>KC248026
GCT---GCA--
GATCAATTGTGGGTCACAGTCTATTATGGGGTACCTGTGTGG---AAAGAAGCAACC---
ACCACTCTATTTTGTGCATCAGATGCCAAAGCATATGATACAGAA---GTACACAAT----------------
--------------GTTTGGGCCACG---CATGCCTGTGTACCCACAGAC---
CCCAGCCCACAAGAAGTAGTATTGGAA---------AATGTAACAGAAAATTTTAAC----------------
--------------------------ATGTGGAAAAATGACATGGTAGAACAAATGCATGAGGATATA---
ATCAGTTTATGGGATCAAAGCCTA---AAACCATGTGTAAAATTA---
ACCCCACTCTGTGTTACCTTAAATTGTACTGCTAATATTAGTAGCAGTACTAATGCTTCCACAACATCTCCT-
----
--GGAGAAATGAGA---
AATTGCTCTTTCAATACCACCACAAACATAAGA---------------------------
GATAAGATACAGAAAGAATATGCACTT---TTTTATAATCTTGATGTAGTACAAATAGGTAAT----------
----

GATAATACTAGCTATAGGTTGGTAAGTTGTAACACCTCAGTCATTACACAA---
GYCTGTCCAAAGATATCCTTTGAGCCAATTCCCATACATTATTGTGCCCCGGCTGGTTTTGCAATTCTAAAGT
GTAAC---AATAAAACGTTCAAT------GGAACAGGGCCATGTACAAAT-----------------------
-GTCAGC---ACAGTACAATGTACACATGGAATTAAGCCAGTAGTATCAACT---
CAACTGCTATTGAATGGCAGTCTAGCAGAAGAAGAT------------
ATAGTAATTAGGTCTGAAAATTTCACAGACAAT---------GCTAAAACCATAATAATACAGCTGAAA----
-----------ACTGCTGTGCAA---------ATTAATTGTACAAGACCCAACAACAATACAAGAAAA-----
-GGTATACACATA------------GGACCAGGGAGAACA---TTT------TATGCAACAGGAGCA------
---------ATAATAGGAGATATA------------AGACAGGCACACTGC---AATCTTAGTGCAACA---
GATTGGAATACTACTTTAGCAAACATAAGTAAAAAATTAGGA---------------GAACAATTTGGG----
-----------------------------------AATAAACCAATAGTGTTTAAT------
CAATCCTCAGGAGGGGACCCAGAAATTGTA---CTGCACAGTTTTAATTGTGGAGGGGAA---

TTTAAGCAGCTCCAGGCAAGAGTCCTGGCTGTGGAAAGATACCTAAAGGATCAACAGCTCCTGGGAATTTGGG
GTTGCTCTGGAAAACTCATTTGCACCACTGCTGTGCCTTGGAATACTAGTTGGAGT-----------------
----------------------------AATAAAACTCAAAGTGAGATTTGG------GAC------
AACAYGACCTGGATGGAATGG---GAGAGAGAAATTGGCAATTACACAAAAATAATATAT---
AACTTAATTGAAGAATCGCAGAACCAACAAGAA--

AAGAATGAACAGGAATTATTAGAACTAGATAAATGGGCAAATTTGTGGAATTGGTTTAACATAACACAATGGC
TGTGGTATATAAAAATATTCATAATTATAGTAGGAGGCTTGGTAGGCTTAAGAATAGTTTTTGCT---
GTATTTTCTATAGTG---------AACAGAGTTAGGCAGGATACTCACCA------
TTGTCGTTTGCTGTTAGCTTGCTTAACGCCACAGCAATAGCAGTAGCTGAG---
GGAACAGATAGGATTATAGAAGTAGTACAAAGGATTGGTAGAGCT----------------------------
--------ATTATCCACATA------CCTGTACGGATAAGACAAGGCTTAGAAAGGTTTTTRCTA---TAA
>KC248015
GCT---ACA--
GAAGAAAGGTGGGTCACAGTCTATTATGGAGTACCTGTGTGG---AAAGAAGCAACC---
ACTACTCTATTTTGTGCATCAGATGCTAAAGCATATGATACAGAG---GTGCATAAT----------------
--------------GTTTGGGCCACA---CATGCCTGTGTACCCACAGAC---
CCCAGCCCACAAGAAATAGTATTGGAA---------AATGTAACAGAAGATTTTAAC----------------
--------------------------GTGTGGAAAAATAGCATGATAGACCAGATGCATGAGGATATA---
ATCAGTTTATGGGATCAAAGCTTA---AAGCCATGTGTAAGATTA---
ACTCCACTCTGTGTTGCTTTAAATTGTACTAATGTTACTTGCACTAATAATAAT-------------------
----
---GMGACAGTAGAAGAAATGAAA---
AACTGTTCTTTCAAAACCCTCACAAGGATAAGA---------------------------
GATAAGACACAGGGATATGAATCTGCTCTTTTTATAAAGATGATATAGTACCAATAGATAAT-----------
----

AGCAATAACTATAGGTTGGTAAGTGGTAACACCTCAGTCATTACACAG---
GCCTGTCCAAAGGTATCCTTTGAGCCAATTCCCATACATTATTGTGCCCCAGCTGGTTTTGCGATTCTAAAAT
GTAAA---GATAAGAAGTTCGAG------GGAAAAGGATCATGTGAAAAT-----------------------
-GTTAGC---ACAGTACAATGTACACATGGAATTAGGCCAGTAGTATCAACC---
CAACTGTTGTTAAATGGCAGCCTAGCAGAAGAGGAG------------
GTAGTAATTAGATCTGAAAATTTCACAAACAAT---------GCTAAAATCATAATAGTGCAGCTAAAA----
-----------GAAGCTGTAAAA---------ATTAATTGTACAAGACCCAATAACAATACAAGAAAA-----
-AGTATACCTATG------------GGACCAGGGAAAGCA---TTT------TATACAACAGGAGAT------
---------ATAATAGGAGATATA------------AGACAAGCACATTRT---AACATTAGTGGAGTA---
GCATGGAATAACACTTTAGGAAAGATAGTGGAAAAACTAAGG---------------AAAAAATTTAAC----
-----------------------------------AATAAAACAATAGTCTTTGAG------
CAATCCTCAGGAGGAGACCCAGAAATTGTA---ATGCACAGTTTTAATTGTGGAGGGGAA---
TTTAAGCAGCTCCAGGCAAGGCTCCTGGCTGTAGAAAGATATATAAAGGATCAACAGCTCCTGGGGATTTGGG
GTTGCTCTGGAAAATTCATTTGCACCACTGCTGTGCCTTGGAATGATAGTTGGAGT-----------------
----------------------------AATAAATCTCTGAGCCAGATTTGG------GAT------
AACATGACTTGGATGCAGTGG---GAGAAAGAAATCAACAATTACACAAATTTAATATAT---
GATTTAATTGAAGAATCGCAAAACCAACAAGAA--

AAGAATGAACAAGAATTATTGTCATTAGATAAATGGGCAAGTTTGTGGAATTGGTTTTCCATATCAAACTGGC
TGTGGTATATAAAACTATTCATAATGATAGTAGGAGGCTTAATAGGCTTAAGAATAGTTTTTGCT---
GTACTTTCTATAGTG---------AACAGAGTTAGGCAGGATACTCACCC------
TTGTCGTTTGCTGTTAACTTACTTGACGCCATAACTATAGCAGTAGCTGAG---
GGAACAGATAGGATTATAGAAGGCTTACAAGGAACTGGTAGAGCT----------------------------
--------TTTCTCCACATA------CCTAGAAGAATAAGACAGGGCCTCGAAAGAGCTTTRCTA---TAA
>KT124802
GCT---GTA--
GAAAATTTGTGGGTCACAGTCTACTATGGGGTACCGGTGTGG---AAAGAAGCAAAT---
ACCACTCTATTTTGTGCATCAGATGCTAAAGCATATGATACAGAA---GTACATAAT----------------
--------------GTTTGGGCCACA---CATGCCTGTGTACCCACAGAC---
CCCAACCCGCATGAATTATTCTTGAAA---------GATGTGAAAGAAGAATTTAAC----------------
--------------------------ATGTGGAAAAATAACATGGTAGAACAGATGCATGAGGATATA---
ATCAGTTTATGGGATGAAAGTCTA---AAGCCATGTGTAAAATTA---
ACCCCACTCTGTGTTACTTTAAATTGCACTGATTATAAACAAACCACCAATAGTACCAATAGTACT-------

----
----------------------------ACTACTAGGGCTACCAATAGTAGTGGAGAAATGAAA---
CAATGCTCTTTCAATAGTACCACAAGCATAAGA--------------------------
GATGTTACAACGAAAGAATCTGCACTT---TTTTATAGACTTGATCTAGTACAAATAGGTAAT----------
----

AATAGTGAGAATAATACTAGCTATAGGTTGATAAATTGTAACACCTCAGTCATTACACAA---
GCTTGTCCAAAGGTAACCTTTGAGCCAATTCCCATACATTATTGTGCCCCGGCTGGTTTTGCGCTTCTGAAGT
GTAAA---GATAAGGAATTCAAT------GGAACAGGACGATGTCACAAT----------------------
-GTCAGT---ACAGTACAATGTACACATGGAATTAGGCCAGTAGTATCAACT---
CAACTACTGTTGAATGGCAGTTTGGCAGAAGGAGAG-----------
GTAGTAATTAGATCTGAAAATCTCACAGACAAT---------GCTAAAACCATAATAGTACAACTGAAT----
-----------GACACTGTAGAA---------ATTAATTGTACAAGACCCAACAACAATACAAGAAAA-----
-AGTATACATATA------------GGACCAGGGAGAGCA---TTT------TATGCAACAGGAGAC------
---------ATAATAGGAGATATA------------AGACAAGCACATTGT---AACATTAGTAGAGCA---
AAATGGAATAACACTTTAAGGCTAGTAGCTGAAAAATTAAGA---------------GGACTCTTTAAC----
-----------------------------------AATAAAACAATAGTCTTTAAT------
CAGTCCTCGGGGGGAGACCCAGAAATTGTA---ATGCACAGTTTTAACTGTGGAGGGGAA---
TTTAAGCAGCTCCGGGCAAGAGTCCTGGCTGTGGAAAGATACCTACAGGATCAACAGCTCCTGGGGATCTGGG
GCTGCTCTGGAAAGTTCATCTGCACCACTGCTGTGCCTTGGAATACTAGTTGGAGT-----------------
----------------------------AATAAAAATATGACGCAGATTTGG------AAT------
GAAACCACCTGGATGCAGTGG---GAAAAAGAAATTGATAAATACACAGGCATAATATAC---
ACCTTACTTGAAGAAGCGCAGATCCAACAAGAA--

AACAATGAACTAGACTTATTGAAATTAGATAACTGGGCAAGTTTGTGGAATTGGTTTGACATAACAAATTGGC
TGTGGTACATAAAAATATTCATAATGATAGTAGGAGGCTTAGTAGGTTTAAGAATAGTTTTTACT---
GTGCTTTCTATAGTA---------AATAGAGTTAGGAAGGGATACTCACCC------
TTATCGTTTGCTGTTAGCTTGCTCAATGCCACAGCCATCGCAGTAGCTGAG---
GGAACAGATAGGATAATAGAAGGGTTACAAAGAATTGGTAGAGCT---------------------------
--------ATTTGCAACATA------CCTAGAAGAATAAGACAGGGCTTAGAAAGAGCTTTGCTA---TAA
>KT124765
GCT---GCA---
GAAAAATTGTGGGTCACAGTCTATTATGGGGTACCTGTGTGG---AGAGAAGCAAAC---
ACCACACTATTTTGTGCCTCAGATGCTAAAGCATATGACACAGAG---GTACATAAT----------------
--------------GTTTGGGCCACA---CATGCCTGTGTACCCACAGAC---
CCCAGCCCACAAGAAATAGTACTGGGA---------AATGTGACAGAAAATTTTAAC---------------
-------------------------ATGTGGAAAAATAACATGGTAGAACAGCTGCATGAGGATATA---
ATCAGTATATGGGATCAAAGTCTA---AAGCCATGTGTGAAACTG---
ACCCCACTCTGTGTCACCTTAAATTGCACTGAAACTAACACCACTAGGTCCAATAATACCGCCACTAGTGCC-
----
----------------------------TCCAATATGACCACTAATGACACTAGTGATATGACA---
AACTGCTCTTTCTATGCCCCCACAGGAATACAA--------------------------
GGTAGATTTCAAAAGGAATATGCTCTT---
TTTTATAAACTTGATGTAGTACAAATAGATAAGGATAGTAATAGTACAACT---------------------
----
AGTAATAGTACAGATAGTAATATTAAAAGCTATATGTTGATAAATTGTAACACCTCAGTCATTACACAG---
GCCTGTCCGAAGGTAACCTTTGAGCCAATTCCCATACATTATTGTGCCCCGGCTGGTTTTGCAATTATAAAAT
GTAAT---GATAATAAGTTCAAT------GGAACAGGACCATGTACAAAT----------------------
-GTCAGC---ACAGTGCAATGTACACATGGAATTAGGCCAGTAGTGTCAACT---
CAACTGCTGTTAAATGGCAGTTTAGCAGAAGGAGAG-----------
GTAATAATTAGATCTGAAAATCTCACAAACAAT---------GCTAAAACCATAATAGTACAGCTGAGC----
-----------GAACCTGTACAA---------ATTAATTGTACAAGACCCAACAACAATACAAGGAGA-----
-AGTATAACTATG------------GGACCAGGGAAAGCA---TTT------TTTGGAACA---GAA------
---------GTAATAGGAGATATA------------AGGCAAGCCCATTGT---AACCTTAGTAGAGCA---
AGCTGGAATAAGACTTTAAAAGAAGTAGCTAAGAAATTATAT---------------ACAGAATTT-------
-----------------------------------AATAGAACAATAATCTTTAAT------
CAATCCTCAGGAGGGGACCTAGAAGTGGTA---ATGCACAGTTTTAATTGTGGAGGGGAA---
TTCAAGCAGCTCCAGGCAAGACTCCTGGCTGTGGAAAGATACCTAAAGGATCAACAAATCCTAGGAATTTGGG
GTTGCTCTGGAAAACTCATCTGCACCACTACTGTGCCTTGGAATGCTTCATGGAGT----------------
----------------------------AATAAATCACAGGAAGAGATATGG------CAG------

AACATGACCTGGATGGAGTGG---GAAAGAGAAATTGATAATTACACAGGCACCATATAT---
AACTTACTTGAAATAGCGCAAAACCAGCAAGAA--

AGGAATGAACAAGAATTATTGGCATTAGATAAGTGGGCAAGTTTGTGGAATTGGTTTGACATAACAAACTGGC
TGTGGTATATAAAAATATTCATAATGATAGTAGGAGGGTTAATAGGATTAAGAATAATTTTTGCA---
GTGCTTTCTATAGTA---------AATAGAGTTAGGCAGGGATACTCACCA------
TTGTCATTGGCTATTAGCTTACTTAATGCCACAGCAATAGCAGTAGCTGAG---
GGGACAGATAGGATTATAGAAATAGCACAAAGAATTGGGAGAGCT----------------------------
--------ATTCTCAACATA------CCTAGAAGAATAAGACAGGGTTTAGAAAGGCTTTGCTA---TAA
>KT124811
GCT---ACA--
AATAACTTGTGGGTCACAGTCTATTATGGGGTACCTGTGTGG---AAAGAAGCAACC---
ACCACTCTGTTTTGTGCATCAGATGCTAGAGCATATGACACAGAG---GTGCATAAT----------------
--------------GTTTGGGCCACA---CATGCCTGTGTACCCACAGAC---
CCTAACCCACAAGAAGTGGTATTGGGA---------AATGTGACAGAAAATTTTGAC----------------
--------------------------ATGTGGAAAAATAAAATGGCAGAGCAAATGCAGGAGGATGTA---
ATCAGTCTATGGGATGAAAGTTTA---AAGCCATGTGTAAAATTA---
ACCCCACTCTGTGTTACTTTAAATTGCACTGATGCCAATCCTAAGCAGAATAGTACTGTT-------------
----
-------------------------AATAATGACACTATTACTTTCTTCAAGGAAGAAATAACA---
AACTGCTCGTTCAATGTCACCACAAGCATAGGA---------------------------
GATAAGAGACATAGAGAATATGCACTT---
TTTCATAAAATTGATCTAATGCCAATAGAGAATATAGCAACAAATAGAACA----------------------
----
AATGGAACAAACACAGCAAACAATACAACAAACTATGCATTGATAAACTGTAACACTTCAGTCATTACACAG-
--
GCTTGTCCAAAGGTATCTTTTGAACCAATTCCTATACATTATTGTACCCCGGCTGGTTTTGCGATTCTAAAAT
GTAAT---GATAAAATGTTCAAT------GGAACAGGACCATGTAAAAAT-----------------------
-GTCAGC---ACAGTACAATGTACACATGGAATTAGGCCAGTAGTATCAACC---
CAGCTGCTGTTAAATGGCAGTCTAGCAGAAGAGGAA------------
ATAGTAATTAGATCTCAAAATCTCACAGACAAT---------GCTAGAACCATAATAGTACACCTAAAC----
-----------CAGTCTGTACAA---------ATTAATTGTACAAGACCTGGCAACAATACCAGAAAA-----
-GGTATACATATG------------GGACCGGGAAGAGTC---TTT------TATGCAACAGGAGAA------
---------ATAACAGGAGATATA------------AGAAAAGCACATTGC---AACCTTAGTGAAACA---
GCATGGAACAATACTTTAACACAGGTAGCTAAAAAATTAAGT---------------AAACAATTTAAC----
-----------------------------------AATAAAACAATAAAATTTAAT------
TCATCCTCAGGAGGAGACCCAGAAATAGTA---ATGCTCAATTTTAATTGTGGAGGGGAG---
TTTAAGCAGCTCCAGGCAAGAGTCCTGGCTATGGAAACATACCTAAAGGATCAACAGCTCCTGGGGATTTGGG
GTTGCTCTGGAAAACTCATTTGCACCACTGCTGTGCCTTGGAATACTAGTTGGAGT-----------------
----------------------------AATAGATCTCTAGAATACATTTGG------GGT------
AACATGACATGGATGGAATGG---GAAAAAGAAATTGACAATTACACAGACACAATCTAT---
GCCTTAATTGAAAAATCGCAGAACCAACAGGAA--

AAGAATGAACAAGAATTATTGGCATTGGATTCATGGGCAAGCTTGTGGAATTGGTTCGATATATCAAATTGGC
TGTGGTATATAAAAATATTCATAATGATAGTAGGAGGCTTAATAGGTTTAAGAATAATCTTTACT---
GTACTCTCTATAGTG---------AATAGAGTTAGGAAGGGATACTCACCA------
TTGTCATTCGCTGTTAATTTGCTTAATACCATAGCTATAGCAGTAGGTGAG---
GGGACAGATAGGGTTATAGAAGCAGCACAAAGACTTGGTAGAGCT----------------------------
--------TTTCTCAACACA------CCTAGAAGAATAAGGCAGGGCGCCGAAATAGCTTTACTA---TAA
>KT124767
GCT---GCA--
GGAAATTTGTGGGTCACGGTATATTATGGGGTACCTGTGTGG---AAAGAAGCAAGC---
ACCACTCTATTTTGTGCATCAGATGCTAAAGCATATGATACAGAG---GTACATAAT----------------
--------------GTTTGGGCCACA---CATGCCTGTGTACCCACAGAC---
CCTGACCCACAAGAAGTAATAGTGGAA---------AATGTGACAGAAAATTTTGAC----------------
--------------------------ATGTGGAAAAATGATATGGTAGAACAGATGCATAAGGATATA---
ATCAGTTTATGGGATGAAAGCCTA---AAGCCATGTGTAAAATTA---
ACCCCACTCTGTGTTACTTTAAATTGCAGTGATGGGAATTAT-------------------------------
----
--ACAGAAACAAAA---

AACTGCTCTTTTAAGACCAACCCAAACCTAAAG---------------------------
GATAAGTCACAGAGAGCATATGCATCT---TTCTATAAACTTGACATAGTACCATTAGAAGAG----------
----

AATAGTACAACCTATAGGTTGATAAGTTGTAACACCTCAAGCATTACACAG---
GCTTGTCCAAAGGTATCCTTTGAGCCAATTCCCATACATCTTTGTGCCCCGGCTGGTTTTGCGATTTTAAAAT
GTAGA---AATGATACGTTCAAT------GGAACAGGACCATGTGACAAT-----------------------
-GTCAGT---ACAGTACAATGTACACATGGAATTAGGCCAGTAGTATCAACT---
CAATTGCTGTTAAATGGCAGTCTAGCAAAAAAAGAG------------
ATAGTAATCAGATCTGAAAACTTCACAGACAAT---------GCTAAAACCATAATAGTACAGCTGAAC----
-----------AAATCTGTAAAC---------ATTACTTGTGTAAGACCCGGTAACAATACAAGGAAA-----
-AGTATACATATA------------GGACCAGGGAGAGCA---TTT------TATGCAACAGGAGAA------
---------ATAATAGGAAATATA------------AGACAAGCACACTGT---AATATTAGTAGACAA---
GCTTGGGAACACACATTAAACGAGACAGCTAAAAAATTAGGA---------------GAACAATTAAAG----
-----------------------------------ATAACAACAATAATCTTTCAA------
CCATCCTCAGGAGGGGACCTAGAAATTGAA---ACACACAGCTTTAATTGTGGAGGGGAA---
TTTAAACAGCTCCAGGCAAGAGTCCTGGCTCTAGAAAGATACCTAAGAGATCAACAGCTCCTGGGAATTTGGG
GTTGCTCTGGAAAACTCATCTGCACCACTGCTGTGCCTTGGAACACTAGTTGGAGT-----------------
----------------------------AATAGATCTCTGGAGTACATTTGG------AAT------
AACATGACCTGGTTACAGTGG---GAAAGAGAAATTGACAATTACACAGACTTAATATAC---
ACCTTAATTGAAGAATCGCAAAACCAACAAGAA--

AAGAATGAAAAAGATTTATTGGAATTGGATAAATGGGCAAGTTTGTGGAATTGGTTTGACATAACAAATTGGC
TGTGGTATATAAAAATATTCATAATGATAGTAGGAGGCTTGATAGGTTTAAGAATAATTTTTGTA---
ATACTTTCTGTAGTG---------AATAGAGTTAGGCAGGGATACTCACCC------
TTATCGTTTGCTGTTAGCTTGCTCAACGCCACAGCAATAGCAGTAGCTGAG---
GGGACAGATAGGGTTATAGAAGTAGGACAAAGAATTCTTAGAGGT----------------------------
--------ATCCTTCACATA------CCTAGAAGAATAAGACAGGGCTTGGAAAGGGCTTTGCAA---TAA
>KY324301
GCT---TCA--
GAGAAGTTGTGGGTCACAGTCTATTATGGGGTGCCTGTGTGG---AAAGAAGCAACC---
ACCACTCTATTCTGTGCATCAGATGCTAAAGCACAGAAGGCAGAG---GTACATAAT----------------
--------------ATTTGGCCACA---CATGCCTGTGTACCCACAGAC---
CCCAACCCACAAGAAATAGTATTGGAA---------AATGTGACAGAAAATTTTAAC----------------
--------------------------ATGTGGAAAAATGACATGGTAGAACAAATGCATGATGATGTA---
ATCAGTTTATGGGATCAGAGCTTA---AAGCCATGTGTAAAATTA---
ACCCCGCTCTGTGTTACCTTAAATTGTATTAAATTCAAAAGTACTAATCTCACTGTTACTAATTCCAGTGAAT
TCAAAAGTACTAATCTCACTGTTACTAATTCCACTGACACTAGT-----------------------------
-------------------------GCTAATGAAACGACAAAGGGAACGGTGGAAGGAATGAAA---
AATTGCTCTTTCAATATCACCACAAGTATAAAA---------------------------
GATAAGGTGCAAAAAGAATATGCGCTT---TTTTATGACCTTGATATAATACCAATAGATAGT----------
----

GGTACGGGCTATAGGTTGATAAGGTGTAACACCTCAATCATTACACAA---
GCCTGTCCAAAGGTATCCTTTGAGCCAATTCCCATACACTATTGTGCCCCAGCTGGTTTTGCGATTCTAAAGT
GTAAC---GATAAGAAGTTCAAT------GGAACAGGACCATGTAAAAAT-----------------------
-GTCAGC---ACAGTACAATGTACACATGGAATTAGGCCAGTAGTGTCAACT---
CAACTGCTATTAAATGGCAGTCTAGCAGAAGAAGAG------------
GTAATAATTAGATCTGCCAATTTCACAAACAAT---------GCTAGAACCATAATAGTACAACTGAAT----
-----------GAATCTGTAGAG---------ATTAACTGTACAAGACCCAACAACAATACGAGAAAA-----
-AGTATACATATA------------GGACCAGGGAGAGCA---TTT------TATGCAACAGGAGAA------
---------GTAATAGGAGACATA------------AGACAAGCACATTGT---AACATTAATAGACAG---
AAATGGAATACTACTATACAACGGATAGCTGAAAAATTAAGA---------------GAACAATTTAGG----
-----------------------------------AATAAAACAATAATCTTTCAA------
AATTCCTCAGGAGGGGATCCAGAAATTGTA---ATGCACACTTTTAATTGTGGAGGGGAG---
TTTAAGCAGCTCCAGGCAAGAGTCCTGGCTGTGGAAAGATACCTACAGGATCAACAGCTCCTAGGGATTTGGG
GTTGCTCTGGAAAACTCATTTGCACCACTGCTGTACCTTGGAATAATAGTTGGAGT-----------------
----------------------------AATAAATCTCAGGAAATGATTTGG------GAG------
AACATGACCTGGATGGAATGG---GAGAAGGAAATTAGCAATTACACACACAAAATATAC---
ACTTTACTTGAAGAATCGCAAAATCAACAAGAA--

AAGAATGAGCAGGACTTATTGTCATTAAATAGCTGGGACAGTTTATGGACTTGGTTCAACATATCAAACTGGC
TGTGGTACATAAAAATATTCATAATGATAGTAGGAGGCTTGATAGGATTAAGAATAGTTTTTACT---
ATACTTTCTATAGTA---------AGGAGAGTTAGGCAGGGATACTCACCA------
TTATCATTCGCTGTTAGCTTGCTCAATACCACAGCCATAGCAGTAGCTGAG---
GGGACAGATAGGGTTATAGAAGTAGTACAAAGAATTGGTAGAGCT----------------------------
--------ATCCTCCATATA------CCTAGAAGAATAAGACAGGGCTTCGAAAGAGCTTTGCAA---TAA
>KY324379
GCT---GCA--
GATAAATTGTGGGTCACAATATATTATGGGGTACCTGTGTGG---AAAGAAGCAACC---
ACCACTTTGTTTTGTGCATCAGATGCTAGAGCATATGATGAAGAG---GCACATAAT----------------
--------------GTTTGGGCCACA---CATGCCTGTGTACCCACAGAC---
CCCAACCCACAAGAAGTAAGATTGGAA---------AATGTAACAGAAAATTTTGAC----------------
--------------------------ATGTGGAAAAATAACATGGTAGAACAAATGCATGAGGATGTA---
ATCAATTTATGGGATCAAAGCTTA---AAGCCATGTGTAAAGTTA---
ACCCCACTCTGTGTTACTTTAAATTGCACTGATGTAGATGGTTATAATGGTAATAAGACCCAAAGTAATAAT-
----
-------------------ACCAATGGTAGTGATGGGAAATTGATAGATCCAGGAGAAGTAAAA---
AACTGCTCTTTCAATATCGCCACAGAGATAAGA---------------------------
GATAGGCAACAGAAGCAATATGCACTC---
TTGTATAAACTTGATGTAGTACCAATAGATGATGATAATAATAAT----------------------------
----
AGCAGTAATAGTAATCATAGTACCTATAGGTTAATAAGTTGTAACATCTCAGTCATTACACAA---
GCCTGTCCAAAGATATCGTTTGAGCCAATTCCCATACATTATTGTGCCCCGGCTGGTTTTGCGATTCTAAGGT
GTAAC---AATGACACGTTCAAT------GGAACAGGGCCATGTACAAAC-----------------------
-GTCAGC---ACCGTGCAATGTACACATGGAATTAAGCCAGTAGTATCAACT---
CAATTGCTGTTAAATGGTAGTCTAGCAAAAGAAGGG------------
GTAATAATTAGATCAAAAAATCTCACAGATAAT---------GCTAATATCATAATAGTACAGCTATCG----
-----------ACAGCTGTAGAG---------ATTACCTGTATAAAAAACAACAATAATACAAGAAAA-----
-AGTATAAATATA------------GGACCAGGGAGAGCA---TGG------TATACAACAGGACAA------
---------ATAATAGGAGAAATA------------AGACAAGCATATTGT---AACATTAGTAGAGGA---
AACTGGAGTAACACTTTAGGACAGATAGTTAAAGAATTACAA---------------AAAAATTTTATC----
-----------------------------------AACAAAACAATAGTCTTTAAA------
CCTTCCTCAGGAGGGGACCCAGAAATTGTA---ATGCATAGTTTTAACTGTGGAGGGGAA---
TTTAAACAGCTCCAGGCGAGAGTCCTGGCTCTGGAAAGATACCTAAAGGATCAACAGCTCCTGGGGATCTGGG
GTTGCTCTGGGAAGCTCATCTGCACCACTAATGTGCCTTGGAATGCTAGTTGGAGT-----------------
----------------------------AATAAATCTTTTAATGATATTTGG------AAT------
AATATGACCTGGATGCAGTGG---GAAAAAGAAATAGATAATTACACAGGCTTAATATAC---
AGTTTAATTGAAGAATCGCAGAACCAACAAGAA--

AAGAATGAACAAGACTTATTGGCATTGGATAAATGGGCAAATTTGTGGAATTGGTTTAGCATAACAAAATGGC
TGTGGTACATAAAAATTTTCATAATGATAGTAGGAGGCTTAGTAGGTTTAAGAATAGTGTGTGCT---
ATACTTTCTATAGTA---------AATAGAGTTAGGAAGGGATACTCACCA------
ATATCGTTAGCTGTTAGCCTGTACAATACTATAGCTATAGCAGTAGCTGAG---
GGGACAGATAGAATTATAGAACTAGGACAAAGAGCTTGGAGAGCT----------------------------
--------TTTCTCCACATC------CCTGTGCGAATAAGACAAGGCTTAGAAAGGGCTTTGCTA---TAA
>KY324630
GCA--
GACAATCTATGGGTAACAGTTTATTATGGGGTACCTGTGTGG---AAGGAAGCGAAT---
ACCACTCTATTTTGTGCATCAGATGCTAAGGCATATGAAACAGAA---ATACATAAT----------------
--------------GTTTGGGCTACA---CATGCCTGTGTACCCACAGAC---
CCCAATCCACAAGAAATAGTATTGGAA---------AATGTGACAGAAAATTTTGAT----------------
--------------------------GTGTGGAGAAATAACATGGTAGAACAAATGCATGAAGATATA---
ATCAGTTTATGGGATCAAAGTCTA---AAGCCATGTGTAAAATTA---
ACCCCACTCTGTGTTACCTTAAACTGCACTGATGTGAATGCCACTAATACTAATAACAGCAGTGAAACTGATG
CCAAAAGTAAT--
-------------------------ATTACTGGGGTAAAGTTGATAGAGAAAGGAGAAATAAAA---
AATTGCTCGTTCAATATCACCTCAGGAATAAAA---------------------------
GACAAGGTGCAGAAAACATATGCTTAT---TTTTATAACCTTGATATAGTACAAATGAAGAAGAT--------
----

GCTAATGATAATACTAGCTATAGGTTGATAAGTTGTAATACCTCAGTCATTACACAG---
GCTTGTCCAAAGACGTCCTTTGAGCCAATTCCAATACATTATTGTGCCCCAGCTGGTTTTGCGATTCTAAAGT
GTAAT---GATAAAAAGTTCATT------GGAAAAGGAGCATGTAAAAT------------------------
-GTCAGC---ACAGTACAATGTACACATGGGATTAGGCCAGTAGTGTCAACT---
CAACTGCTGTTAAATGGCAGTCTAGCTGAAGAAGAC------------
ATAGTAATCAGATCTGACAATTTCACGAATAAT---------GCTAATACCATAATAGTACAACTGAAG----
-----------GAGGCTGTAGAA---------ATTAATTGTACAAGACCCAACAATAATACAAGAAAA-----
-AGTATAAATATA------------GGACCAGGGAGAGCA---TTT------TATGCAACAGGAGAT------
---------ATAATAGGAGATATA------------AGACAAGCACATTGT---AACATTGGTAAGGCA---
AGCTGGATAAAAATGTTAAAACAGGTAGCTAAAAAATTAACA---------------GACCAATTCAAC----
-----------------------------------AATAAAACAATAGCCTTTGAG------
CGATCCTCAGGAGGGGATCCCGAGGTTGTA---TTGCACAGTTTTAATTGTGGAGGAGAA---
TTTAAACAGCTCCGGGCAAGAGTCCTGGCTGTGGAAAGATACCTAAAGGATCAACAGCTCCTAGGGATCTGGG
GTTGCTCTGGAAAGCTCATTTGCACCACTGCAGTGCCTTGGAATACTAGTTGGAGT-----------------
----------------------------AATAAGACTCTGGATCAGATTTGG------CAG------
AATATGACATGGATGCAGTGG---GACAAAGAAATTGATAATTACACAAGCTTAATATAC---
ACCTTAATTGAAGAATCGCAAAACCAACAAGAA--

AAAAATGAACTAGAATTATTGGAATTAGATAAGTGGGCAAATTTGTGGAATTGGTTTGATATATCAAACTGGC
TGTGGTATATAAAAATATTTATAATAATAGTAGGAGGCTTGGTAGGTTTAAGAATAATTTTTGCT---
GTGCTTTCTATAGTA---------AATAGAGTTAGGAAGGGATACTCACCA------
TTATCGTTTGCTATTAGCTTGTTCAATGCCACGGCCATAGCAGTAGCTGAG---
GGAACAGATAGAGTTATAGAAGTAGTACAAAGAGCCTGGAGAGCC----------------------------
--------CTTCTCCACATA------CCTAGAAGAATAAGACAGGGCCTGGAAAGAGCCTTGTTA---TAA
>KY324641
ACT---GCA--
GAAAAATTGTGGGTCACAGTCTATTATGGGGTACCTGTGTGG---AAAGATGCAACC---
ACCACTCTGTTCTGCGCGTCAGATGCTAAAGCATACTCGACAGAG---GCACATAAT----------------
--------------GTTTGGCCACA---CATGCCTGTGTACCCACAGAC---
CCCAATCCACAAGAAGTAGAATTGGAA---------AACATGACAGAAAGTTTTAAC----------------
--------------------------ATGTGGAAAAATAACATGGTAGAACAGATGCAGGAGGATATA---
ATCAGTTTATGGGATCAAAGCCTA---AAGCCATGTGTAAAATTA---
ACCCCACTCTGTGTTACTCTAAACTGTACTGATTATAATACTACTACAACCAATGAT----------------
----
----------------------------ACTAGTAAACTGTTAATGGAGAAAGGAGAAATGAAA---
AATTGCTCTTTCAATATCACCACAAGCATAAGA---------------------------
GATAAGATACAGAATCAATATGCACTT---TTCTATAACTTGATGTAGTACAAATGGATGAT-----------
----

AGCAATAACACATATAGGTTGATAAATTGCAACACCTCAGTCATCACACAG---
GCCTGTCCAAAGGTATCTTTTCAGCCAATTCCCATACATTATTGTACCCCAGCTGGTTTTGCACTTCTAAAGT
GTAAG---GATAAGCAGTTCAAT------GGAACAGGGCCATGTAAGAAT-----------------------
-GTCAGC---ACAGTACAGTGTACACATGGAATTAAGCCAGTAGTATCAACT---
CAACTGCTGCTAAATGGCAGCCTAGCAGAAGAAGAG------------
GTAGTAATTAGATCTTCCAACTTCACGGACAAT---------GCTAAAATCATAATAGTACAGCTGAAT----
-----------GAGTCTGTACAA---------ATTAATTGTACAAGGCCTAGCAACAATACAAGAAAA-----
-AGTATAAACATA------------GGACCAGGGAGAGCA---TTT------TATGCAACAGGAGAT------
---------ATAATAGGAGATATA------------AGACAAGCACATTGT---AATATTTCTAGGGAA---
AAATGGAATAGCACCTTAGAACAAATAGCTACAAAATTAAGA---------------GAACAATTTAAG----
-----------------------------------AATAAAACAATAGAATTTAAA------
CCTTCATCAGGAGGGGACCCAGAAATTGAA---ATGCACAGTTTCAATTGTGGGGGGGAG---
TTTAAGCAGCTCCAGGCAAGAGTCCTGGCTGTGGAAAGATACCTAAAGGATCAACAGCTCCTAGGGATTTGGG
GTTGCTCTGGAAAACTCATTTGCACCACTAATGTGCCTTGGAATAATAGTTGGAGC-----------------
----------------------------AATAAATCTCTAGCTAATATTTGG------AAC------
AACATGACCTGGATGGAGTGG---GAAAGAGAAATTGACAACTACTCAGGCTTAATATAC---
AACTTACTTGAAGAGTCACAGAACCAGCAAGAA--

AAGAATGAGCAAGAGTTATTGGAATTAGATAAGTGGGACAGTTTGTGGAGTTGGTTTAGCATAACAAATTGGC
TGTGGTACATAAAAATATTCATAATGATAATAGGAGGCCTAATAGGTTTAAGAATAGTTTTTATT---

ATACTGTCTATAGCG---------AATAGAGTTAGGCAGGGATATTCACCA------
TTGTCATTTGCTGTTAGCTTGTTTAACGCCATCGCTATCGCAGTAGCTGAG---
GGGACAGATAGGATTATAGAAATAGTACAAAGAGCTGGTAGAGCT----------------------------
--------ATTCTTCACATA------CCTAGAAGAATAAGACAGGGCGCGGAAAGGGCTTTGCAA---TAA
>KY324761
GCT---GCA--
GAACAATTGTGGGTCACAGTATATTATGGGGTACCTGTGTGG---AAAGAAGCAAAC---
ACCACCCTATTTTGTGCATCAGATGCTAAGGCATATGAAACAGAG---GTACATAAT----------------
--------------GTTTGGGCCACA---CATGCCTGTGTACCCACAGAC---
CCCAACCCACAAGAAGTCGTATTGGAA---------AATGTGACAGAAAATTTTAAT----------------
-------------------------GCGTGGAAAAATAACATGGTAGAACAAATGCAGGAGGATATA---
ATCAATTTATGGGATCAAAGCCTA---AAGCCATGTGTACAATTA---
ACCCCACTCTGTGTCACTTTAAATTGCACAGATTTGAAGAATAGTACTGATACTGATACTAAT----------
----
---------------------------ACTACTGTCCCTATTAGCGAGAAAGAAGAACTAAAA---
AACTGCTCTTTCAATATCACCTCAGGCGTAGGA-----------------------
GGTGATAAGATTCACACAGAATATGCATTC---TTTAATAAACTTGATATAGTATCAATAGGTAAT-------
----

AGTAACTCATCTTTTAGGTTGATGCACTGTAACACCTCAGTCATTACACAG---
GCCTGTCCTAAGGTATCCTTTGAGCCAATTCCAATACATTATTGTGCCCCGGCTGGTTTTGCGATTCTAAAGT
GTAAT---GATAAGAAGTTCAAT------GGAAGCGGACCATGTACAAAT----------------------
-GTCAGC---ACAGTACAATGTACACATGGAATTAGGCCAGTAGTGTCAACT---
CAACTGCTGTTAAATGGCAGTCTAGCAGAAGAAGAG-----------
ATAGTAATTAGATCTGAAAATATCTCGGACAAT---------GTCAAAACCATAATAGTACAGCTGAAT----
-----------GAATCTATCCCG---------ATTAAGTGTATAAGACCCAACAACAATACAAGAAAA-----
-AGTATAAATATA------------GGACCAGGGAGAGCA---TTC------TATGCAACAGGAGCA------
---------ATAATAGGAAATATA------------AGACAAGCACACTGT---AACATTAGTGAAAGA---
AATTGGACACGCATCTTAGAACAGGTAGCTATAAAATTACAA---------------GAACACTTTCCG----
--------------------------------AATAAGACAACAATAGCCTTTAAC------
CAATCCTCAGGAGGAGACCCAGAAATTATA---CTGCATAGTTTTAATTGCGGAGGGGAA---
TTTAAGCAGCTCCAGGCAAGAGTCCTGGCTGTGGAAAGATACCTAAAAGATCAGCAGCTCCTAGGGATTTGGG
GGTGCTCTGGAAAACTCATTTGCACCACTGCTGTGCCTTGGAATGCTAGTTGGAGT----------------
---------------------------AATAAATCTCTGGACAAGATATGG------AAT------
AACATGACCTGGATGGAGTGG---GAAAGAGAAATTGAAAATTACACAGGGTTAATATAT---
AACTTAATTGAAAAAATCGCAAAACCAACAAGAA--

AAGAACGAACAAGAATTATTGGAATTGGATAAGTGGGCAGGTCTGTGGAGTTGGTTTGATATATCAAACTGGC
TGTGGTATATAAAAATATTCATAATGATAGTAGGAGGCTTAATAGGTTTAAGAATAGTTTTTGCT---
ATACTTTCTATAGTG---------AATAGAGTTAGGCAGGGATACTCACCA------
TTATCATTGGCTATTAGTTTGCTTAACGCCACAGCAATAGCAGTAGCTGAG---
GGGACAGATAGGATCATAGAATTAGTACAAAGATTTGGCAGAGGT----------------------------
--------ATTCTCAACATA------CCTAGAAGAATAAGACAGGGTTTAGAAAGGATTTTGATA---TAA
>KY324808
GCT---ACA--
GAAAATTTATGGGTCACAGTCTATTATGGGGTACCTGTGTGG---AAAGAAGCAACT---
ACCACTTTATTTTGTGCATCAGATGCTAAAGCATATGATACAGAG---GTACATAAT----------------
--------------GTTTGGGCCACA---CATGCCTGTGTACCCACAGAC---
CCCAACCCACAAGAAGTAAAATTGGAA---------AATGTGACAGAAAATTTTAAC----------------
-------------------------ATGTGGAAAAATAACATGGTAGACCAGATGCATGAGGATATA---
ATCAGTTTATGGGATCAAAGCCTA---AAGCCATGTGTAAAATTA---
ACCCCACTCTGTGTTACTTTAAATTGCAGTGATGCTAACATCACTCGCAGTAATACTACT-------------
----
----------------------ATCAATAATGGTACCTGGAATATGGAAAAAGGAGAAGTAAAA---
AACTGCTCCTTCAATATCACCACAAGCATAAGA--------------------------
GATAAAGTTACAAAAGAATATGCACTC---TTTTATAGACTTGATGTAGTACAAATAGGAGAG----------
----

AATAAAAGTAATACCAGTTATAGATTGATAAATTGTAACACCTCAGAAATTACACAA---
GCCTGCCCAAAGGTATCCTTTGATCCAATTCCCATACATTATTGTGCCCCGGCTGGTTTTGCGATTATAAGGT

GTAAT---AATAAGAAGTTCAAT------GGAACAGGACCATGTACAAAT-----------------------
-GTCAGC---ACAGTACAATGTACACATGGAATTAGGCCAGTAGTATCAACT---
CAACTGCTGTTAAATGGCAGTCTAGCAGAAGAAAAG------------
GTAGTAATTAGATCTGAAAATTTCTCAAACAAT---------GCTAAAACCATAATAGTACATCTAAAA----
-----------GACCCTGTAAAC---------ATTACTTGTACAAGGCCCGGCAACAATACAAGGAAA-----
-AGTATACACATA------------GCACCAGGGAGAGCA---TTT------TATGCAACAGGAAAC------
---------ATAATAGGAGAGATA------------AGACAAGCACATTGT---AATATTAGTGGGGCA---
GAATGGAACAACACTTTAAGTCAGATAGTTCCAATTTTAAGA---------------GAACAATTTGGA----
-----------------------------------AATAAAACAATAGCCTTTCAG------
CCATCCTCAGGAGGGGACCCAGAAATTGTA---ATGCACAGTTTTAACTGTAGAGGGGAA---
TTTAAGCAGCTCCAGGCAAGAGTCCTGGCTATAGAAAGATACCTAAAGGATCAACAGCTCCTAGGAATTTGGG
GTTGCTCTGGAAAACTCATTTGCACCACTGCTGTGCCTTGGAATAATAGTTGGAGT-----------------
----------------------------AATAAATCTGTAGATTACATTTGG------AAC------
AACATGACTTGGATGCAGTGG---GAGAGAGAAATTGACAATTACACAGACCTTATATAC---
AATTTACTTGAAAGATCGCAGAACCAGCAAGAA--

AAAAATGAACAAGAATTATTGGAATTGGACAAGTGGGCAAATTTGTGGACTTGGTTTAACATATCAAACTGGC
TGTGGTATATAAAAATATTCATCATGATAGTAGGAGGTTTAGTAGGTTTAAGAATAGTTTTTGCT---
ATACTCTCTGTAGTG---------AATAGAGTTAGGCAGGGATACTCACCA------
CTGTCGTTGGCTATTAGCTTGCTCAACTCCACAGCTATAGCCGTAGCTGAA---
TGGACAGATAGGCTTATAGAAATTGTACAAAGAGCCTGGAGAGCT----------------------------
--------ATTCTCCACATA------CCTAGAAGAATAAGACAAGGCTTAGAAAGAATTCTGCTA---TAG
>MK115683
GCT---ACA--
GAAAAATTGTGGGTTACAGTCTATTATGGGGTACCTGTGTGG---AGAGAAGCAAAC---
ACCACTCTATTCTGTGCATCAGATGCTAAAGCATATGATACAGAG---GCACACAAT----------------
--------------GTTTGGCCACA---CATGCCTGTGTACCCACAGAC---
CCCAACCCACAAGAAATAGGATTGGAA---------AATGTGACAGAAAATTTTAAC----------------
--------------------------ATGTGGAAAAATAACATGGTAGAACAGATGCATGAGGATATA---
ATCAGTTTATGGGATCAAAGCCTA---AAGCCATGTGTAAAATTA---
ACCCCACTCTGTGTTACTTTGAATTGCACTGATTTGGAGACTAAGAAGACTAATAGCACTAATAATGGTGCTA
ACATCACTGCC--
--GCTGATATGAAA---
AACTGCTCTTTCAATATCACTGCAAGCATAAGA---------------------------
GATAAGGTGCAGAAGGAATATGCGCTT---
TTTTATAAACTAGATATAGTACAAATAGATAATGACAATAATACCAGAGCCAAG-------------------
----
GATAATAATACCAGTGCCAATGCCGGTGCCAGCTATAGGTTGATAAGTTGTAACACCTCAGTCCTCACACAG-
--
GCCTGTCCAAAGGTATCCTTTGAACCAATTCCCATACATTATTGTGCCCCGGCTGGGTTCGCACTTCTAAAAT
GTAGA---GATAAGAAGTTTAAT------GGAACAGGGCCATGTACAAAT-----------------------
-GTCAGC---ACAGTACAATGTACACATGGAATTAGGCCAGTAGTATCAACT---
CAACTGCTGTTAAATGGTAGTCTAGCAGAAGAAGAG------------
ATAGTAATTAGATCTGAAAATATCTCAAATAAT---------GCTAAAACCATAATAGTACAGCTAAAC----
-----------GAAACTGTAGAA---------ATTAACTGTACTAGACCCAACAACAATACAAGAAAA-----
-AGTATACATATA------------GGACCAGGGAGAGCA---TGG------TATGCAACAGGAGAT------
---------ATAATAGGAGATATA------------AGAAAAGCATATTGC---AACATTAGCAGAGAA---
CGATGGAATAAAACACTAGGACAGGTAGCTATAAAATTAAGA---------------GAACAATTTAAG----
-----------------------------------AATAAGACAATAGTCTTTAAT------
GGATCCTCAGGAGGGGACCCAGAAATTACA---ATGATTAGTTTTAATTGTGGAGGGGAA---
TTTAAACAGCTCCAGGCAAGAGTCCTGGCTGTGGAAAGATACCTAAAGGATCAACAGCTCCTGGGGCTTTGGG
GTTGCTCTGGAAAACTCATCTGCAACACTGCTGTGCCTTGGAATACTAGTTGGAGT-----------------
----------------------------AACAAATCACTGAATGCTATTTGG------CAT------
AACATGACCTGGATGGAGTGG---GAAAGAGAAATTGACAATTACACAGACATAATATAC---
AGCTTAATTGAGGCATCACAAACCCAGCAAGAA--

CAGAATGAACAAGAATTACTAGCACTAGATAAATGGGCAAGTTTGTGGAATTGGTTTGACATATCAAACTGGC
TGTGGTACATAAAAATATTCATAATGATAGTAGGAGGCTTGGTAGGTTTAAGAATAGTTTTTACT---
GTACTTTCTATAGTG---------AATAGAGTTAGGAAGGATACTCACCA------
TTATCGTTTGCTGTTAGCTTGCTTAATGCCACAGCCATAGCAGTAGCTGAG---

GGGACAGATAGGATAATAGAAGGATTGCACAGAGCTTGTAGAGCT----------------------------
--------ATTCTCCACATA------CCTAGAAGAATAAGACAAGGCGTGGAAAGGGCTTTGCAA---TAA
>MK115918
GTT---GCA--
GCAAACTTGTGGGTCACAGTCTATTATGGGGTACCTGTATGG---AAGGAAGCAACC---
ACCACTCTATTTTGTGCATCAGATGCTAAAGCATATGATACAGAG---GTACATAAT---------------
--------------GTTTGGGCCACA---CATGCCTGTGTACCCACGGAC---
CCCAACCCACAAGAAATAGTATTGGGA---------AATGTGACAGAAAATTTTGAT---------------
-------------------------GTGTGGAAAAATAACATGGTAGAGCAGATGCATGAGGACATA---
ATCAGTTTATGGGATCAAAGCTTA---AAGCCATGTGTAAAATTA---
ACCCCACTTTGTGTTATTTTAAATTGCACTGATGAAGTCAATGTAGTCAACAGA-------------------
----
------------------------ACTAGCAATGATAGCAATGGAATAACAAGAGAACTAAAA---
AACTGCTCTTTCAATATCACCACAAACATAAAA--------------------------
AATAAGGTACAGAAAGAATATGCACTC---
TTTTATAAACTTGATGTAGTACCAATAGATAATGAAGATAATGATACTAGCCTT-------------------
----
AGTAATAATAGCTATAGGTTGATAAGTTGTAACACCTCAGTCATTACACAG---
GCTTGTCCAAAGGTATCCTTTGAGCCAATTCCCATACATTACTGTACCCCGGCTGGTTATGCGCTTCTAAAGT
GTAAC---AATGAGACATTCAGT------GGAAAAGGGCTATGTACAAAT----------------------
-GTCAGC---ACAGTACAATGTACACATGGAATTAGGCCAGTAGTGTCAACT---
CAACTGCTGTTAAATGGCAGTCTAGCAAAACAGGGG-----------
GTAGTAATTAGATCTCAAAATTTCTCAGACAAT---------GCTAAAACCATAATAGTACAGCTGAAG----
-----------GAACCTGTAGAA---------ATTAACTGTACAAGGCCCAGTAACAATACAAGAAAA-----
-GGTATACATACA------------GGACCAGGGAGCGTA---ATT------TATGCAACAGGAGAA------
---------ATAATAGGAGATATA-----------AGACAAGCACATTGT---AACATTAGTGCAGAA---
AAGTGGAATCAGACTTTAATACAGATAGTTGCAAAATTACAA---------------GAACAATTTGAG----
-----------------------------------AATAAAACAATAGTCTTCAAT------
CAATCATCAGGAGGGGACCCAGAAATTGTA---ACACACAGTTTTAATTGTGGAGGGGAA---
TTTAAACAGCTCCAGGCAAGAGTCCTGGCTGTGGAAAGATACCTACAGGATCAACAGCTCCTGGGGATTTGGG
GTTGCTCTGGAAAACTCATTTGCACCACTGCTGTGCCTTGGAATACTAGTTGGAGC----------------
----------------------------AATAAATCTCTGGAAAAAATTTGG------GAT------
AACATGACCTGGATGGAGTGG---GAAAAAGAAATTGGTAATTACACACAATTAATATAC---
ACTTTAATTGAAGAATCGCAGAACCAGCAAGAA---------------------------------------
----
AAGAATGAACAAGACTTATTGGCACTGGATAAATGGGACAGCTTGTGGAATTGGTTTAGTATAACAAAATGGC
TGTGGTATATAAAAATATTCATAATGATAGTAGGAGGCTTAATAGGGTTAAGAATAGTTTTTACT---
GTACTTTCTATAGTA---------AATAGAGTTAGGCAGGGATATTCACCA------
TTGTCGTTGGCTGTTAGCTTGCTCAACGCCACAGCCATAGCAGTAGCTGAG---
GGGACAGATAGAGTTATAGAAATAGTACAAAGAGCTTGTAGAGCT---------------------------
--------ATCCTTCACATA------CCTGTAAGAATAAGACAAGGCTTAGAAAGGGCTTTGCTA---TAA
>MK116058
ACT---GCA--
GACAATTTGTGGGTCACAGTCTATTATGGGGTGCCTGTGTGG---AAAGATGCAAAC---
ACCACTCTATTTTGTGCATCAGATGCTAAAGCATATGATACAGAG---GTACATAAT---------------
--------------GTTTGGGCCACA---CATGCCTGTGTACCCACAGAC---
CCCAGTCCACAAGAAGTACCATTGCCT---------AATGTGACAGAGAATTTTAAC---------------
-------------------------ATGTGGAAAAATGACATGGTAGAACAGATGCAGGAGGATATA---
ATCAGTTTATGGGATCAAAGTCTA---AAGCCATGTGTAAAATTA---
ACCCCACTCTGTGTTACTTTGAATTGCACTGATGTGGAGATTGATAAAACCAGTGCTAATAAGACCATTAATG
TGAATACTAAT---
---------------------ACCACTAATAGTTGGGAAAGGATGGATCCAGGAGAAATAAAA---
AACTGCTCTTTCAATATCACCACTAACATAAGA--------------------------
GATAGGGTGAAGAATGAATATGCGCTT---TTTTATAAACTTGATGTAGTACAAATAAAAGATAAGAAGGCC-
----

AGCAATACCACCTATAGGCTGATAAGTTGTAATACCTCAGTCATTACACAA---
GCCTGTCCAAAGGTATCCTTTGAGCCAATTCCCATACATTTTTGTGCCCCGGCTGGTTTTGCGATTCTAAGGT
GTAAC---AATAAAACATTCAAT------GGATCAGGACCATGTACAAAT---------------------
-GTCAGC---ACAGTACAATGTACACATGGAATTAAGCCAGTGGTATCAACT---

CAACTACTGTTAAATGGCAGCCTAGCAGAAGAAGAG------------
GTAGTAATTAGATCTGAAAGTATCACGAACAAT---------GCTAAAACCATAATAGTACAATTGAAA----
-----------GAAGCTGTAAAA---------ATTAATTGTACAAGACCCAACAATAATACAAGAAGA-----
-GGTATACATATA------------GGACCAGGGAGAGCA---TTT------TATGCAGCATCAGAT------
---------ATAATAGGAAATATA------------AGACAAGCACATTGT---AACATTAGTAAAGCA---
GAATGGAAGAATGCTTTAAACCAGACAGTTATAAAATTAAGA---------------GAATACTTC-------
-----------------------------------AACAAAACAATAGACTTTAAA------
CCATCCTCAGGAGGGGACCCAGAAATTGTA---ATGCACAGTTTTAATTGTGGAGGAGAG---
TTTAAGCAGCTCCAGGCAAGAGTCCTGGCTGTGGAAAGATACCTAAAGGATCAACAGCTCCTGGGAATTTGGG
GTTGCTCTGGAAAACTCATTTGCACCACTGCTGTGCCTTGGAATAATAGTTGGAGT-----------------
----------------------------AACAGATCTCTGGAGACCATTTGG------GAT------
AACATGACCTGGATGGAGTGG---GAAAGAGAAATTAGCAATGTCACGGACATAATATAC---
AGATTAATTGAAGACTCGCAAAACCAACAAGAA--

AAGAATGAAAAAGACTTATTGGAATTAGATAAATGGGCAAGTCTATGGAATTGGTTTAATATAACAAACTGGC
TGTGGTATATAAAAATATTCATAATGATAGTAGGAGGCTTGGTAGGTTTAAGAATAGTCTTTTCT---
GTAATTTCTATAGTG---------AATAGAGTTAGGCAGGGATATTCACCA------
TTGTCATTTGCTATCAGCTTGCTCAACACCACAGCTATAGCAGTAGCTGAG---
GGGACAGATAGGATTATAGAAGTAGCACAAAGAATTTTTAGAGCT----------------------------
--------TTCCTCCACATA------CCTAGAAGAATAAGACAGGGCTTCGAAAGGGCTTTGCTA---TAA
>MK116212
GCT---GCA--
ACAAAGTTGTGGGTCACAGTCTATTATGGGGTACCTGTATGG---AAAGAAGCAACC---
ACCACTCTATTTTGTGCATCAGATGCCAAAGCATATGATACAGAG---GTACATAAT----------------
--------------GTTTGGCCACG---CATGCCTGTGTACCCACGGAC---
CCCAACCCACAAGAAATATTATTGGAA---------AATGTGACAGAAGAATTTAAT----------------
--------------------------GCATGGAAAAATAACATGGTAGAGCAGATGCATGAGGATATA---
ATCAGTTTATGGGATCAAAGCTTA---AAGCCATGTGTAAAATTA---
ACCCCACTTTGTGTTATTTTGCATTGCACTGATGTAACTAGCAAT----------------------------
----
---GAGACGACAGGAGAACTAAAA---
AACTGCTCTTTCAATATCACCACAAAAATAACA---------------------------
AATAAGGTACAGAAAGAATATGCACTC---TTTTATAAACTTGATGTAGTACCAATAAATAATAAGGATAAT-
----

GATACTAACTTTAATAATAATAGCTATAGGTTGATAAGTTGTAACACCTCAGTTATTACACAG---
GCTTGTCCAAAGGTATCCTTTGAGCCAATTCCCATACATTACTGTACCCCGGCTGGTTATGCGATTCTAAGGT
GTAAC---AATGAGACATTCAGT------GGAAAAGGGCCATGTACAAAT-------------------------
-GTCAGC---TCAATACAATGTACACATGGCATTAGGCCAGTAGTGTCAACT---
CAACTGCTGTTAAATGGCAGTCTAGCAAAACAGGAG------------
GTAGTAATTAGATCTCAAAATTTCTCGGACAAT---------GCTAAAACCGTAATAGTACAGCTGAAG----
-----------GACCCTGTAAAA---------ATTAACTGTATAAGGCCCAATAACAATACAAGAAAA-----
-AGTATACCTGCA------------GGACCAGGGAAAGTA---ATT------TATGCAACAGGAGAA------
---------ATAATAGGAGATATA------------AGACAAGCACATTGC---AACATTAGTGCAACA---
GATTGGAATAATACTTTAGGACAGATAGTTAAAAAATTACAA---------------GAACAATTTGGG----
-----------------------------------AATAAAACAATAGTCTTCAAT------
CAATCGTCAGGAGGGGACCCAGAAATTGTA---ACACACAGTTTTAATTGTGGAGGGGAA---
TTTAAACAGCTCCAGGCAAGAGTCCTGGCTGTGGAAAGATACCTACAGGATCAACAGCTCCTGGGGATTTGGG
GTTGCTCTGGAAAACTCATTTGCACCACTGCTGTGCCTTGGAATGCTAGTTGGAGC-----------------
----------------------------AATAAATCTCTGAATGAAATTTGG------GAT------
ACCATGACCTGGATGGAGTGG---GAAAAAGAAATTAGTAATTACACACAATTAATATAC---
ACTTTAATTGAAGAATCGCAGAGCCAGCAGGAA--

AAGAATGAACAAGAATTATTGGCACTAGATAAGTGGGACAGCTTGTGGAGTTGGTTTAGTATAACAAAATGGC
TGTGGTATATAAAAATATTCATAATGATAGTAGGAGGCTTAATAGGGTTAAGAATAGTTTTTACT---
GTACTTTCTATAGTA---------AATAGAGTTAGGCAGGGATATTCACCA------
TTGTCATTTGCTGTTAGCTTGCTCAATGCCACAGCTATAGCAGTAGCGGAG---
GGGACAGATAGGATTATAGAAGTAGTACAAAGAGCTTGTAGAGCT----------------------------
--------ATTCGCCACATA------CCTGTAAGAATAAGACAAGGCTTAGAAAGGGCTCTTCTA---TAA
>MK086129

GCT---GCA--
AATAACTTGTGGGTCACAGTCTATTATGGGATACCCGTGTGG---AAAGAAGCAACC---
ACCACTCTATTCTGTGCATCAGATGCTAAGGCATATGATGCAGAG---CAACATAAT----------------
--------------GTTTGGGCCACA---CACGCCTGTGTACCCACAGAC---
CCCAACCCACAAGAAGTAGTATTGGAA---------AATGTGACAGAAGATTTTAAC----------------
--------------------------ATGTGGAAAAATAACATGGTAGAACAGATGCATGAGGATATA---
ATCAGTCTATGGGATCAGAGCCTA---AAACCATGTGTAAAATTA---
ACCCCACTCTGTGTTACTTTAGACTGCACTAATATTAACAGTAGTACTAGTACTAATAAT-------------
----
-------------------------AGTAGCCATATTAATATTACTATCATGGAAGAAATGAAA---
AACTGCTCTTTCAATGTTACCACAAACATAAGA---------------------------
GATAAGATAAAAAGAGAATATGCACTT---TTATATAAGCTTGATTTAGTGCCAATAGATAAA----------
----

AATACTAGTAGCTTTAGGTTGATAAGTTGTAACACCTCAACCATTACACAG---
GCCTGTCCAAAAGTATCCTTTGAGCCAATTCCTATACATTATTGTACCCCGGCTGGTTTTGCGATTCTAAAGT
GTAAT---GATAAGCAGTTCAAT------GGAACAGGAAAATGTAGGAAT-----------------------
-GTCAGC---ACAGTGCAATGTACACATGGCATTAGGCCAGTAGTATCAACT---
CAACTGCTGTTAAATGGCAGCCTAGCAGAAGAAGAT------------
ATAGTAATTAGATCTGAGAATTTCAGTAACAAT---------GCTAAAAACATAATAGTACAGCTGAAT----
-----------GAAACTGTAGAA---------ATTAATTGTATAAGACCCAACAACTATACAAGAAAA-----
-CGTATATATATA------------GGACCAGGGAGAGCA---TTT------CATACAACAGGACAG------
---------ATAATAGGAGATATA------------AGACAAGCACATTGT---AATATTAGTGAAGTA---
AATTGGAATAACACTCTAGGAAAGATAGCTAATAAATTAAGA---------------GAGACATTTAAA----
-----------------------------------AACAAAACAATAATCTTTAAG------
CAATCCTCAGGAGGGGACCCAGAGATTGTA---ATGCACAGTTTTAATTGTGGAGGGGAA---
TTTAAGCAGCTCCAGGCAAGAGTCCTAGCTGTGGAAAGTTACCTAAAGGATCAACAGCTCCTGGGAATTTGGG
GTTGCTCTGGAAAACTCATCTGCACCACTGCTGTGCCTTGGAATGCTAGTTGGAGT-----------------
----------------------------AATAAATCTCTAGATCAGATCTGG------GAT------
AACATGACTTGGATGCAGTGG---GATAAAGAAATTAGCAATTACACACAATTAATATAC---
ACCTTAATTGAAGAATCGCAGAACCAACAAGAA--

AAGAATGAACAAGATTTATTGGCATTGGATAAATGGGACGGTTTGTGGAGTTGGTTTTCTATAACAAAATGGC
TGTGGTATATAAAAATATTCATAATAATAGTAGGAGGCTTGATAGGTTTAAGAATAGTTTTCTCT---
GTACTTTCTATAGTG---------AATAGAGTTAGGAAGGATACTCACCA------
TTATCGTTTGCTGTTAGCTTGCTTGATGCCACAGCCATAGCAATAGCTGAG---
GGGACAGATAGGGTTATAGAAGGATACGAAGAGCTTTTAGAGCT-----------------------------
--------ATTATCAACATA------CCTAGAAGAATAAGACAGGGCTTAGAAAGGGCTTTGCTA---TAA
>MN090868
GCT---GCA--
ACAAATTTGTGGGTCACAGTCTATTATGGGGTACCTGTATGG---AAGGAAGCAACC---
ACCACTCTATTTTGTGCATCAGATGCTAAAGCATATGATACAGAG---GTACATAAT----------------
--------------GTTTGGGCCACG---CATGCCTGTGTACCCACGGAC---
CCCAACCCACAAGAAGTATTATTGGGA---------AATGTGACAGAAGATTTTAAT----------------
--------------------------GCATGGAAAAATAACATGGTAGAGCAGATGCATGAGGATATA---
ATCAGTTTATGGGATCAAAGCTTA---AAGCCATGTGTAAAATTA---
ACCCCACTTTGTGTTATTTTGCATTGCACTGATGTCAACAATACTAGAAAT----------------------
----
---GGGATGACAGGAGAACTAAAA---
AACTGCTCTTTCAATATCACCACAAAAATAACA---------------------------
AATAAGGTACAGAAAGAATATGCACTC---
TTTTATAAACTTGATGTAGTACCAATAAATAATAAGGATAATGATACTAGCTTT-------------------
----
AATAATAATAGCTATAGGTTGATAAGTTGTAACACCTCAGTTATTACACAG---
GCTTGTCCAAAGGTATCCTTTGAGCCAATTCCCATACATTACTGTACCCCGGCTGGTTATGCAATTCTAAGGT
GTAAC---AATGAGACATTCAGT------GGAAAAGGGCCATGTACAAAT-----------------------
-GTCAGC---TCAATACAATGTACACATGGAATTAGGCCAGTAGTGTCAACT---
CAACTGCTGTTAAATGGCAGTCTAGCAAAACAGGAG------------
GTAGTAATTAGATCTCAAAATTTCTCGGACAAT---------GTTAAAACCATAATAGTACAGCTGAAG----
-----------ACCCCTGTAAAA---------ATTAACTGTACAAGGCCCAATAACAATACAAGAAAA-----

-AGTATACATGCA------------GGACCAGGGAAAGTA---ATT------TATGCAACAGGAGAA------
---------ATAATAGGAGATATA------------AGACAAGCACATTGC---AACATTAGTGCAGCA---
GAGTGGAATGATACTTTAGGACAGATAGTTACAAAATTACAA---------------GAACAATTTGGG----
-----------------------------------AATAAAACAATAGTCTTCAAT------
CAATCGTCAGGAGGGGACCCAGAAATTGTA---ACACACAGTTTTAATTGTGGAGGGGAA---
TTTAAACAGCTCCAGGCAAGAGTCCTGGCTGTGGAAAGATACCTACAGGATCAACAGCTCCTGGGGATTTGGG
GTTGCTCTGGAAAACTCATTTGCACCACTGCTGTGCCTTGGAATGCTAGTTGGAGC-----------------
----------------------------AATAAATCTCTGAATGAAATTTGG------GAT------
AACATGACCTGGATGGAGTGG---GAAAAAGAAATTAGTAATTACACACAATTAATATAC---
ACTTTAATTGAAGAATCGCAGAGCCAGCAAGAA--

AAGAATGAACAAGAATTATTGGCACTAGATAAGTGGGACAGCTTGTGGAGTTGGTTTAGTATAACAAAATGGC
TGTGGTATATAAAAATATTCATAATGATAGTAGGAGGCTTAATAGGGTTAAGAATAGTTTTTACT---
GTACTTTCTATAGTA---------AATAGAGTTAGGCAGGGATATTCACCA------
TTGTCATTTGCTGTTAGCTTGCTCAATGCCACAGCTATAGCAGTAGCTGAG---
GGGACAGATAGGATTATAGAAGTAGTACAAAGAGCTTGTAGAGCT----------------------------
--------ATTCTCCACATA------CCTGTAAGACTAAGACAAGGCTTAGAAAGAGCTTTGCTA---TAA
>AF490512
GCT---GCA--
GAAAAGTTGTGGGTCACAGTCTATTATGGGGTACCTGTGTGG---AAAGAAGCAACC---
ACCACTCTATTTTGTGCATCAGATGCTAAAGCATATGATACAGAG---GTACATAAT----------------
-------------GTTTGGCCACA---CATGCCTGTGTACCCACAGAC---
CCCAACCCACAAGAAGTACCATTGATA---------AATGTGACAGAAGATTTTAAC----------------
--------------------------ATGTGGAAAAATAACATGGTAGAACAGATGCATGAGGATATA---
ATCAGTTTATGGGATCAAAGCCTA---AAGCCATGTGTAAAATTA---
ACCCCACTCTGTGTTACTTTAAATTGCACTGATTTGAAGAATAATGCTACTAATACCACT-------------
----
----------------------------AGTAGTAACGGGGAAACGATGAAAGGAGAAATGAAA---
AACTGCTCTTTCAATATCACCACAAGAATAAGA---------------------------
GATAAGGTGCAGAAAAAATATGCATAT---TTTTATAACCTTGATATAGTACCAATAAATGAT----------
----

GATAAGAATACTACTAGCTATAGGTTGATAAGTTGTAACACCTCAGTCATTACACAG---
GCCTGTCCAAAGATATCCTTTGAGCCAATTCCCATACATTATTGTGCCCCAGCTGGTTTTGCGATTCTAAAGT
GTAAA---GATAAGAAGTTCAAT------GGAACAGGACCATGTAAAAAT-----------------------
-GTCAGC---ACAGTACAATGTACACATGGAATTAGGCCAGTAGTATCAACT---
CAACTACTGTTAAATGGCAGTCTAGCAGAAGAAGGG------------
GTAGTAATTAGATCTGAAAATTTCACAGACAAT---------GCTAAAACTATAATAGTACAGCTAAAC----
-----------GAAACTGTAGAA---------ATTAATTGTACAAGACCCAACAATAATACAAGAAGA-----
-AGTATACATATA------------GGACCAGGGAGAGCA---ATC------TATGCAACAGGAGAC------
---------ATAATAGGAGATATA------------AGACAAGCACATTGT---AACATTAGTAGAGCA---
ACATGGAATAACACTTTAAAAAAGATAGTTACAAAATTAGGA---------------GAACAATTTGGG----
-----------------------------------AATACAAAAATAGTCTTTAAT------
CACTCCTCAGGAGGGGACCCAGAGATTGTA---ATGCACAGTTTTAATTGTGGAGGGGAA---
TTTAAGCAGCTCCAGGCAAGAGTCCTGGCTGTGGAAAGATACCTAAGGGATCAACAGCTCCTGGGGATTTGGG
GTTGCTCTGGAAAACTCATTTGCACCACTGCTGTGCCTTGGAATACTAGTTGGAGT-----------------
----------------------------AATAAATCTCTGGATGAGATTTGG------AAT------
AAAATGACCTGGATGCAGTGG---GAAAGAGAAATTGACAATTACACAGGCTTAATATAC---
ACCTTAATTGAAGAATCGCAAAACCAACAAGAA--

AAGAATGAACAAGAATTATTGGAGTTAGATAAATGGGCAAGTTTGTGGAATTGGTTTGACATAACAAACTGGC
TGTGGTACATAAGAATATTCATAATGATAGTAGGAGGCTTGGTAGGTTTAAGAATAGTTTTTGCT---
GTATTTTCTATAGTG---------AATAGAGTTAGGCAGGGATACTCACCA------
TTATCGTTGGCTGTTAGCTTGTTTAATGCCATAGCCATAGCAGTAGCTGAG---
GGGACAGATAGGGTTATAGAAGTATTACAAAGAGCTGGTAGAGCT----------------------------
--------ATTCTCCACATA------CCTACAAGAATAAGACAGGGCTTGGAAAGGCTTTTGCTA---TAA
>U43096
GCT---ACA--
GAAAAATTGTGGGTCACAGTCTATTATGGGGTACCTGTATGG---AAAGAAGCAACC---
ACCACTTTATTTTGTGCATCAGACGCTAAAGCATATGACAAAGAG---GTACATAAT----------------

--------------GTTTGGGCCACA---CATGCCTGTGTACCCACAGAC---
CCCGACCCACAAGAAGTAGTATTGGAA---------AATGTGACAGAAGATTTTAAC----------------
--------------------------ATGTGGAAAAATAACATGGTAGAACAGATGCATGAGGATATA---
ATCAGTTTATGGGATCAAAGCCTA---AAGCCATGTGTAAAATTA---
ACCCCACTCTGTGTTACTTTAAATTGCACTGATCTGAAGAATGCTACTAATACCAATAATAGT----------
----
-------------------------AGTTGGACGATGACAGGAGAAATGAAAGGAGAAATAAAA---
AACTGCTCTTTCAATATCACCACAAGCATAAGA---------------------------
GATAAGGTGCAGAAAGAATATGCACAC---TTTTATAAACTTGATGTAGTACCAATAGATAAT----------
----

GACAATACTAGCTATAGGTTGATAAGTTGTAACACCTCAGTCATTACACAG---
GCCTGTCCAAAGGTATCCTTTGAGCCAATTCCCATACATTATTGTACCCCGGCTGGTTTTGCGATGCTAAAGT
GTAAA---GATAAGAGGTTCAAT------GGAAAAGGACAATGTAAAAAC-----------------------
-GTCAGC---ACAGTACAATGTACACATGGAATTAGGCCAGTAGTGTCAACT---
CAACTGCTGCTAAATGGCAGTCTAGCAGAAGAAGAG------------
GTAGTAATTAGATCTGACAATTTCACGGACAAT---------GCTAAAACCATAATAGTACAGCTGAAG----
-----------GAATCTGTAGAA---------ATTAATTGTACAAGACCCAACAATTATACAAGCAAA-----
-AGGATACGTATA------------GGAGCAAGGAGAGCA---TTT------TATACAAAAGGAAAA------
---------ATAATAGGAGATATA------------AGACAAGCACATTGT---AACATTAGTGGAGCA---
AAATGGATAGCACTTTAAGACAGATAGTTAAAAAAATTAAGA---------------GAACGATTTGGG----
-----------------------------------AACAAAACAATAGTCTTTAAT------
CAATCCTCAGGAGGGGACCCAGAAATTGTA---ACGCACAGTTTTAATTGTGGAGGGGAA---
TTTAAGCAGCTCCAGGCAAGAGTCCTGGCTGTGGAAAGATACCTAAGGGATCAACAGCTCCTAGGGATTTGGG
GTTGCTCTGGAAAACTTATTTGCACCACTGCTGTGCCTTGGAATGCTAGTTGGAGT-----------------
----------------------------AATAAATCTATGGATATGATTTGG------AAT------
AACATGACCTGGATGGAGTGG---GAAAGAGAAATTGACAATTACACAAGCTTAATATAC---
ACCTTAATTGAAGAATCGCAGAACCAGCAAGAA--

AAGAATGAACAAGAATTATTGGAATTGAATAAGTGGGAAAATTTGTGGAGTTGGTTTGACATATCAAACTGGC
TGTGGTATATAAAAATATTCATAATGATAGTAGGAGGCTTGGTAGGTTTAAGAATAGTTTTTGCT---
GTACTTTCTATAGTG---------AATAGTGTTAGGCAGGGATACTCACCA------
TTATCATTTGCTGTTAGCTTGCTCAATGCCACAGCTATAGCAGTAGCCGAG---
GGGACAGATAGGGTTATAGAAGTAGTACAAAGAGCTTGGAGAGCT----------------------------
--------ATTCTCCACATA------CCTGTAAGAATAAGACAGGGCTTAGAAAGGGCTTTGCTA---TAA
>U43141
GTT---GCA--
GGAAACTTGTGGGTCACAGTCTATTATGGGGTACCTGTGTGG---AAAGAAGCAACT---
ACCACTCTATTTTGTGCATCAGATGCTAAAGCTTATGATACAGAG---GTACATAAT----------------
--------------GTTTGGGCCACA---CATGCCTGTGTACCCACAGAC---
CCCAACCCACAAGAAGTAGTAATGGGA---------AATGTGACAGAAAATTTTAAC----------------
--------------------------ATGTGGAAAAATGACATGGTAGAACAGATGCAGGAAGATATA---
ATCAGTTTATGGGATCAAAGCCTA---AAGCCATGTGTAAAATTA---
ACCCCACTCTGTGTTACTTTAAAATGCACTGATTATAATAATGCCACTAACAGT-------------------
----
-------------------------------AGTTGGGGAAGGATGGAGAAAGGAGAAATACAA---
AACTGCTCTTTCAAGGTCACCACAAACATAAGA---------------------------
GATAAGGTACAGAAAGAATCTGCACTT---TTTTATAAAACTGATGTAGTACCAATAGATAATAATAAGACT-
----

AGTAATAGGGATAATACTACCAGCTATATGTTGATACATTGTAATAGATCTGTCATTACACAA---
GCCTGTCCAAAGGTATCCTTTGAGCCAATTCCCATACATTATTGTGCCCCAGCTGGTTTTGCGATTTTAAAGT
GTAAT---GATAAAAAGTTCAAT------GGAAAAGGACCATGTAAAAAT-----------------------
-GTCAGC---ACAGTACAGTGTACACATGGAATTAGGCCAGTAGTGTCAACT---
CAACTGCTGTTAAATGGTAGTCTAGCAGAAAAAGAA------------
GTAGTAATTAGATCTGACAATTTCACAGATAAT---------ACTAAAACCATAATAGTACATCTGAAC----
-----------GAATCTGTAGAA---------ATTAATTGTACAAGACCCAACAACAATACAAGAAAA-----
-GGTATACATATA------------GGACCAGGGAGAGCA---GTT------TATACGACAGGAAGA------
---------ATAGTAGGAGATATA------------AGACTAGCACATTGT---AACATTAGTAGAGCA---
AGATGGAATAAAACTCTAAACCAAATATTTAGAAAATTAAGA---------------

GAAATTAGACAATTTGAA---------------------------------AATAAAACAATAGTCTTTAAT-
-----CGATCCTCAGGAGGAGACCCAGAAATTGTA---ATGCACAGCTTTAATTGTGGAGGGGAA---
TTTAAACAGCTCCAGGCAAGAGTCCTGGCTGTGGAAAGATACCTAAGGGATCAACAGCTCCTAGGGATTTGGG
GTTGCTCTGGAAAACTCATCTGCACCACTACTGTGCCTTGGAATGCTAGTTGGAGT-----------------
----------------------------AATAAAACCCTGGATCAGATTTGG------AAT------
AACATGACCTGGATGGAGTGG---GAAAGAGAAATTGACAATTACACAAGCTTAATATAT---
ACCTTAATTGAACAATCGCAAAACCAACAAGAA--

AAGAATGAACAAGAATTATTGGAATTGGATAAGTGGGCAAGTTTGTGGAGTTGGTATGACATATCAAATTGGC
TGTGGTATATAAAAATATTCATAATGATAGTAGGAGGCTTGATAGGTTTAAGAATAGTTTTTGCT---
GTGCTTTCTATAGTG---------AATAGAGTTAGGCAGGGATACTCACCA------
TTGTCGTTTGCTGTCAGCTTGTTCAATACTATAGCTATAGCAGTAGCTGAG---
GGAACAGATAGGGTTATAGAAGTAGTACAAAGAGCTTGTAGAGCA----------------------------
--------ATTCTCCACATA------CCTAGAAGAGTGAGACAGGGCTTGGAAAGGGCCTTGCTA---TAA
>EF514704
GCT---GCA--
GACAAATTGTGGGTCACAGTCTATTATGGGGTACCGGTGTGG---AAAGACGCAAAC---
ACCACTCTATTTTGTGCATCAGATGCTAGAGCATACAAAACAGAG---GCACATAAT----------------
--------------GTTTGGGCCACA---CATGCCTGTGTACCCACAGAC---
CCCAACCCACACGAACTGGCATTGGAA---------AATGTGACAGAAGAATTTAAC----------------
--------------------------ATGTGGAAAAATAACATGGTAGAACAGATGCATGAGGATATA---
ATCAGTTTGTGGGATCAAAGCCTA---AAGCCATGTGTAAAATTA---
ACCCCACTCTGTGTTACTTTAATTTGCTCTAATGCCAATATCACATATACTAATGAGACCATT----------
----
----------------------TCTAGGGATAACATCACTATTATGAAAGAGGGAGAAATAAAA---
AACTGCTCTTTCAATGTCACCTCAGGCATAAGA---------------------------
GATAAGGTACAGAAAGAATATGCACTT---TTTTATAAAGATGATGTAGTACCAATAGATATT----------
----

AATAGGACTAGCTATAGATTGATAAGTTGTAACACCTCAGTCATTACACAG---
GCCTGTCCAAAGATATCCTTTGAGCCAATTCCTATACATTTTTGTACCCCGGCTGGTTTTGCGCTTCTAAAGT
GTAGA---AATAAGACATTCAAT------GGAAAAGGGAGCTGTAACAAT-----------------------
-GTCAGC---ACAGTACAATGTACACATGGAATTAAGCCAGTAGTGTCAACT---
CAACTGCTGCTAAATGGCAGTCTAGCAGAAGAAGAG------------
GTAGTAATTAGATCTGAAAATATCACAGACAAT---------GCTAAAACCATAATAGTACAGCTGAAG----
-----------GAACCTGTGCAA---------ATTAATTGCACAAGACCCGGCAATAATACAAGGAAA-----
-AGTATACATATA------------GGACCAGGAAGGGCA---TTT------TATACAACAGGAGAC------
---------ATAATAGGAGATATA------------AGAAAAGCATATTGT---AACATCAGTAGCGTA---
GACTGGAATAATGCTTTAAATCAGACAGTCAAAAAATTAAGA---------------GAACAATTTGAACAA-
-----------------------------TTTGCAAATAAAACAATAGCTTTTAAT------
CAATCCTCAGGAGGGGACCCAGAAATTGTA---ATGCACAGTTTTAATTGTGGAGGAGAA---
TTTAAGCAGCTCCAGGCAAGAGTCCTGGCTGTAGAAAGATACCTAAAGGATCAACAGCTCCTGGGGATTTGGG
GTTGCTCTGGAAAACTCATTTGCACCACTGCTGTGCCTTGGAATACTAGTTGGAGT-----------------
----------------------------AATAAATCTCAAGATGACATTTGG------AAT------
AACATGACCTGGAATCAGTGG---GAAAAAGAAATTGACAATTACACAAGCGTAATATAC---
ACCTTAATTGAGAAATCGCAAAACCAACAAGAA--

AAAAATGAGCAGGAATTGTTGGAATTGGGCCAATGGACAAATCTATGGAATTGGTTTGATATAACAAACTGGC
TGTGGTATATAAAAATATTCATAATGATAGTAGGAGGCTTGGTAGGCTTAAGAATAGTTTTTACT---
GTACTTTCTATAGTG---------AATAGAGTTAGGCAGGGATACTCACCA------
TTGTCGTTTGCTGTTAGCTTGTTCAACGCCATAGCCATAGCAGTAGCTGAG---
GGGACAGATAGGATTTTAGAAGTATTACAAAGAATTTTTAGAGCT----------------------------
--------ATCCTTCACATA------CCTACAAGAATAAGACAAGGCTTGGAAAGGAGTTTGCTA---TAA
>EF514705
GTT---ACA--
GGAAATTTGTGGGTCACAGTCTATTATGGGGTACCTGTGTGG---AAAGAAGCCACC---
ACCACTCTATTTTGTGCATCAAATGCTAAAGCATATGATACAGAG---GTGCATAAT----------------
--------------GTTTGGGCCACA---CATGCCTGTGTACCCACAGAC---
CCCAATCCACAGGAAGTACCATTGATA---------AATGTGACAGAAAATTTTAAC----------------
--------------------------ATGGGGAAAAATAACATGGTAGAACAGATGCAAGAGGATATA---

ATCAGTCTATGGGATCAAAGTCTA---AAACCATGTGTAAAGTTA---
ACCCCACTATGTGTTACTTTAAAATGCATTGATTATGTTAAGAATATTACTAATGGGGGAATACTACTAATA
ATGGTACTGAAGTG--
AGGGGGAATACTACTAGTAATGATACTACTAATACCACTAGTAGTAGCATGGAAACAGAAATGAAA---
AACTGCTCTTTCAATATTACCACAAGCATGAGA--------------------------
GATAAGATGCAGAAAGAATACGCGCTT---TTTTATAAACTTGATGTAGTACAAATAGGTGGG---------
----

GATAATACTAGTTATAGGTTGATAAGTTGTAACACCTCAGTCATTACACAG---
GCCTGTCCAAAGGTATCTTTTGAGCCAATTCCCATACATTATTGTACCCCGGCTGGTTTTGCGCTTCTAAAAT
GTAAT---GATAAGAAGTTCAAG------GGAACAGGACCATGTAAAAT-----------------------
-GTTAGC---ACAGTACAATGTACACATGGAATTAGGCCAGTAGTATCAACT---
CAATTACTGTTAAATGGAAGTCTAGCAGAAGAAGAG------------
GTAGTAATTAGATCTGTCAATTTCTCGGACAAT---------GCTAAAACCATAATAGTACAACTGAAT---
-----------AAATCTGTAGAA---------ATTAATTGTACAAGACCCAACAACAATACAAGAAAA----
-GGTATACATATA------------GGACCAGGGAGAGCA---TTT------TATGCAACAGGAGAG-----
---------ATAATAGGAGATATA------------AGACAAGCACATTGT---AACATTAGTAAAACA---
AAATGGAATGAAACCTTAAGTCAGATAGTTGGGAAATTGCAA---------------GAACAATTTGAG---
-----------------------------------AATAAAACAATAAATTTTAAC------
CAACCCTCAGGAGGGGACCCAGAAATTGTA---ATGCACAGCTTTAATTGTAGAGGGGAA---
TTTAAGCAGCTCCAAGCAAGAGTCCTGGCTGTGGAAAGATACCTAAAGGATCAACAGCTCCTGGGGATTTGGG
GTTGCTCTGGAAAGCTCATCTGCACCACTGCTGTGCCTTGGAATGCTAGTTGGAGT----------------
----------------------------AATAAATCTCTGACTCAGATCTGG------GAT------
AACATGACCTGGATGGAATGG---GAAAGAGAAATTGAAAATTACACAAGCTTAATATAT---
ACCTTAATTGAAGAATCGCAGAACCAGCAAGAA---------------------------------------
----
AAGAATGAACAAGAATTATTAGAATTAGATAAATGGGCAAGTTTGTGGAATTGGTTTAGCATAACAAACTGGC
TGTGGTATATAAGACTATTCATAATGATAGTAGGAGGCTTGATAGGTTTAAGAATAGTTTTTACT---
GTACTTTCTATAGTA---------AATAGAGTTAGGCAGGGATATTCACCA------
TTGTCGTTTGCTGTTAGCTTGTTCAACGCCACAGCAGTAGCAGTAGCTGAG---
GGGACAGATAGGATTATAGAAAGCTTACAAAGAGTTCTTAGAGCT---------------------------
--------ATCCTCCACATA------CCTAGAAGAATAAGACAGGGCCTTGAAAGGGCTTTGCTA---TAA
>EF514706
GCT---AAA---
GATTTGTGGGTCACGGTATATTATGGGGTACCTGTGTGG---AAAGAAGCAACC---
ACCACTCTATTTTGTGCATCAGATGCTAAAGCACATGCTACAGAG---GTACATAAT---------------
--------------GTTTGGCCACA---CATGCCTGTGTACCCACAGAC---
CCCAACCCACAAGAAGTAGAATTGGAA---------AATGTGACAGAAAATTTTAAC---------------
--------------------------ATATGGAAAAATGACATGGTAGAACAGATGCATGAGGATGTA---
ATCAGTTTATGGGATCAAAGTCTA---AAGCCATGTGTAAAATTA---
ACCCCACTCTGTGTTACTTTACATTGCAATGATAATGTTACTGTAAATGCTACTGGTGGG------------
----
----------------------------GGGAATCGTACTGAAATGGAGAAAGGAGAAATAAAA---
AACTGCTCTTTTAATATCACCACAGGAATAAGA--------------------------
GATAAGGTGCAGAAAGAATATGCATTG---TTCTATAAACTTGATATAATACCAATAGATGATGAGAAT---
----

GATGTTACTAAGAATACTAGTTATAGGTTGAGAAGTTGTAACACCTCAGTCATTACACAG---
GCCTGTCCAAAGGTATCCTTTGAGCCAATTCCCATACATTATTGTACCCCGGCTGGTTTTGCGATTCTGAAGT
GTAAA---GACAGAAATTTCAAT------GGAAGTGGACCATGTAAAAT-----------------------
-GTCAGC---ACAGTACAATGTACACATGGAATTAGGCCAGTAGTATCAACC---
CAACTGCTGTTAAATGGCAGTCTAGCAGAAGGAGGG------------
GTAGCAATTAGATCTGCCAATTTCACAGACAAT---------GCTAAAACCATAATAGTACAACTGGAA---
-----------AAAGCTGTACAA---------ATTAATTGTACAAGACCCAGCAACAATACAAGAAAA----
-AGTATACATATG------------GGACCCGGGGGAGCA---TTT------TATGCAACAGGAGCC-----
---------ATCATAGGAGATATA------------AGACAAGCACATTGT---AACATCAGTAGAGCA---
GAATGGAACAACACTTTGAGTCAGATAGTTGGGAAATTACAA---------------GAACAGTTTAAT---
-----------------------------------AAAACAACAATAGTCTTTCAG------
CCATCCTCAGGAGGGGACCCAGAAGTTGTA---ATGCACAGTTTTAATTGTGGAGGGGAG---
TTTAAGCAGCTCCAGGCAAGAGTCCTGGCTGTGGAAAGATACCTACAGGACCAACAGCTCCTGGGGATTTGGG

GCTGCTCTGGAAAACTCATTTGCACCACTGCTGTGCCTTGGAATAATAGCTGGAGT-----------------
----------------------------AATAAATCTCTGGATCACATTTGG------AAT------
AACATGACCTGGAATCAGTGG---GAAAAAGAGATTGACAATTACACAGGCTTAATATTC---
AACTTACTTGAAGAATCGCAAAACCAACAAGAA--

AAGAATGAACAAGAATTATTGGAATTAGATAAATGGGCAAGTTTGTGGAATTGGTTTAACATAACAAGGTGGC
TGTGGTATATAAAAATATTCATAATGATAGTAGGAGGCTTGGTAGGTTTAAGGATAGTTTTTGCT---
GTGCTTTCTATAGTG---------AATAGAGTTAGGCAGGGATATTCACCA------
TTATCATTTGCTGTTAGCTTGCTCAACGCCACAGCCATAGCAGTAGCTGAG---
GGGACAGATAGGGTTATAGAAGTAGTACAAAGAATTTGGAGGGCT----------------------------
--------ATTCTCCACATA------CCCACAAGAATAAGACAAGGCTTCGAAAGGCTTTTACTA---TAA
>EF514707
ACT---ACT--AAG---
TGGGTCACAGTCTATTATGGGGTACCTGTGTGG---AAAGAAGCAACC---
ACCACTTTATTTTGTGCATCAGATGCTAAATCCTATGATACAGAG---GTGCATAAT----------------
--------------GTTTGGGCCACA---CATGCATGTGTACCCACAGAC---
CCCAGCCCACAAGAAGTAGTATTGGAA---------AATGTGACAGAAAATTTTAAC----------------
-------------------------ATGTGGAAAAATAACATGGTAGAACAGATGCATGAGGATATA---
ATCAGTTTATGGGATCAGAGTTTA---AAGCCATGTGTAAAATTA---
ACCCCACTCTGCGTTACTTTAAATTGCACTGATTATGATAATTCTACTGAGACTGCTCCCGCTAAT-------
----
-------------------AGTAGCTGGACTTACAATAGTAGTAGCTTAAGGGAAGAAGTAAAA---
AACTGCTCTTTCAATGTCACCACAAACATAAGA--------------------------
GATAAGATAAAGACAGATTATGCACTT---TTGTATAAACTTGATATAGTACCAATAGATAATAGT-------
----

AATACTAGTGATAATACCAGTTATAGGCTGATAAGTTGTAACACCTCAGTCATTACACAA---
GCCTGTCCAAAGGTATCCTTTGAACCAATTCCCATACATTATTGTGCCCCGGCTGGTTTTGCGATTCTAAAAT
GTAAT---AATAAGACATTCAAT------GGAACAGGACCATGTACAAAT----------------------
-GTCAGC---ACAGTACAATGTACACATGGAATTAGGCCAGTAGTATCAACT---
CAACTGCTGTTAAATGGCAGTCTAGCAGAGAACGAG-----------
ACAGTAATTAGATCTGAGAATTTCACGAATAAT---------GCTAAAGTCATAATAGTACAGCTAAAT----
-----------GAAACTATAAAA---------ATTAATTGTACAAGACCCAGCAATAATACAAGAAGG-----
-AGTATACATATA------------GCACCAGGGAGAGCA---TTT------TATACAACAGGAGCC------
---------ATAATAGGAGACATA------------AGACAAGCACATTGT---AACATTAGTAGAACA---
GACTGGAGTAACACTTTAAGAAAGGTAGTTGAGAAACTCAGA---------------GCACAATTTAAC----
-----------------------------------AATAAAACAATAATCTTTAAT------
CAATCCTCAGGAGGGGACCCAGAAATTGTA---ATGCACAGTTTTAATTGTGGAGGGGAA---
TTTAAGCAGCTCCAGGCAAGAGTCCTGGCTGTGGAAAGATACCTAAGGGATCAACAGCTCCTGGGGATTTGGG
GCTGCTCTGGAAAAATCATCTGCACCACTGCTGTGCCTTGGAATGATAGTTGGAGT----------------
-----------------------------AATAAATCTTTAGATGTTATTTGG------AAA------
AACATGACCTGGATGGAGTGG---GAAAGAGAAATTGACAATTACACAGACTTAATATAC---
TCCTTACTTGAAAAATCGCAAAACCAACAAGAA--

CAAAATGAACAAGAATTATTGGAATTAGATAAATGGGCAAATTTGTGGAATTGGTTTGATATAACAAAATGGC
TGTGGTATATAAAGATATTCATAATGATAATAGGAGGCTTGATAGGTTTAAGAATAGTTTTTGCT---
GTGCTTTCTATAGTA---------AATAGAGTTAGGCAGGGATACTCACCA------
ATATCGTTGGCTGTTAGCTTACTCAACGCCACATCCATAGCAGTAGCTGAG---
GGAACAGATAGGATTATAGAAGCATTACAGAGAATTGGCAGAGCT----------------------------
--------ATCATCCACATA------CCTAGAAGAATAAGACAGGGCTTAGAAAGGGCTTTGCTA---TAA
>EF514709
GCT---GCA---
GAAAAATTGTGGGTCACAGTCTATTATGGGGTACCTGTGTGG---AAAGAAGCAACC---
ACCACTCTATTTTGTGCATCAGATGCTAAAGCATATGATACAGAG---GCACATAAT----------------
--------------GTTTGGGCCACA---CATGCCTGTGTACCCACAGAC---
CCCAACCCACAAGAAGTAGTATTGAAA---------AATGCGACAGAACCTTTTGAT----------------
-------------------------ATGTGGAAAAATAACATGGTAGAACAAATGCATGAGGATATA---
ATTAGTTTATGGGATCAAAGCCTA---AAGCCATGTGTAAAATTA---
ACCCCACTATGTGTTACTTTAAATTGCASTGATGTTACTTTAAATTSTCATGAATTGAGTAAGAATAGTAMTA
ATAGTAAG---

----AATAGTACTAATACCAATAGTAGTAGCACGAGATTAACGACGGAGGCAGAAGAAATGAAA---
AACTGCTCTTTYAATATYACCACAAGCATAAGA--------------------------
GATAAGATACAAAAAGAATATGCTCTT---TTTTATACAGTTGATRTAGTACCAATAGATAAW---------
----

AATAGTACCAAMTATACATTGATAAATTGTAACACCTCAGTCATTACACAR---
GCCTGTCCAAAGATATCCTTTGAGCCAATTCCCATACATTATTGTGCCCCGGCTGGTTTTGCGATTCTAAAGT
GTAAC---GATAAGAAGTTCAAT------GGAACAGGACCATGTACAAAT----------------------
-GTCAGC---ACAGTGCAATGTACACATGGAATTAGGCCAGTAGTGTCAACT---
CAACTGCTGTTAAATGGCAGTCTAGCAGAAGAAGAG-----------
GTARTACTTAGATCTAAAAATTTTACGGACAAT---------ACTAAAACCATAATAGTACAGCTGAAA----
-----------GAAGCTGTAAAA---------ATTAATTGTACAAGACCCAACAACAATACAAGGAAA-----
-AGTGTACATATA------------GGACCAGGGAGAGCA---TTT------TATGCAACAGGAGAT------
---------ATAATAGGAAATATA------------AGACAAGCACATTGT---AACATTAGTAGAACA---
AAATGGAATAACACTCTAGAACAGATAGCCAAAAAATTAAAA---------------GAACAATTTAAG----
-----------------------------------AATAAAACAATAGTCTTTAAT------
CACTCCTCAGGAGGGGACCTAGAAATTGTA---ATGCACAGTTTTAATTGTGGAGGGGAA---
TTTAAACAGCTCCAGGCAAGAGTCCTGGCTGTGGAAAGATACCTAAAGGATCAACAGCTCCTGGGAATTTGGG
GTTGCTCTGGAAAACTCATTTGCACCACTGCTGTGCCTTGGAATACTAGTTGGAGT-----------------
----------------------------AATAAAAGTCTAAGTCAGATTTGG------GAT------
AACATGACCTGGATGCAGTGG---GAAAAAGAAATTGACAATTACACAAGCWTAATATAC---
ACCTTAATTGAAGAATCGCAGAACCAGCAAGAA---------------------------------------
----
AAGAATGAACAAGAATTATTGGAATTAGATAAATGGGCAAGTTTGTGGAATTGGTTTAATATAACACAATGGC
TGTGGTATATAAAAATATTCATAATGATAGTAGGAGGCTTGATAGGTTTAAGAATAGTTTTTACT---
GTACTTTCTATAGTG---------AATAGAGTTAGGCAGGGATACTCACCA------
TTATCGTTGGCTGTTAGCTTGTTCAACGCCACAGCAATAGCAGTAGCTGAG---
GGGACAGATAGGGTTATAGAATTATTACAAAGAATTGGTAGAGCT---------------------------
--------ATTCTCCACATT------CCTACAAGAATAAGACAGGGCTTGGAAAGGCTTTTGCTA---TAA
>EF514710
GCT---GCA---
GGTCAATTGTGGGTCACAGTCTATTATGGGGTACCTGTGTGG---AAAGATGCAACC---
GCCACTTTATTTTGTGCATCAGATGCTAAAGGATACCATACAGAG---GTACATAAT----------------
--------------GTTTGGGCCACA---CATGCCTGTGTACCCACAGAC---
CCCAACCCACAAGAAATAGTATTGGAA---------AATGTGACAGAAGAATTTAAC----------------
--------------------------ATGTGGAAAAATAACATGGTAGAACAGATGCATGAGGATATA---
ATCAGTTTGTGGGATCAAAGCCTA---AAGCCATGTGTAAAATTA---
ACCCCACTCTGTGTCACTTTAAAATGCACTAATGTCACTAAAAATAATAGTAGTAGTACTACCACTTCT----
----
----------------GCCCCTAACACCACTGCTCAAGGGGAACTGATGGGAGATGGAATAAAA---
AACTGCTCTTTCAATGTTACCGCAGGCATAAGA--------------------------
GATAAGATGCAGAAAGAATATGCACTT---TTTTATAAAGTTGATATAGCACCAATAGATGAT---------
----

AATGGTAGCTATACATTGATAAGTTGTAACACCTCAGTCATTACACAG---
GCCTGTCCAAAAATATCCTTTGAGCCAATTCCCATACATTTTTGTGCCCCGGCTGGTTTTGCGATTCTAAAAT
GTAAC---AATAAGACGTTCAAT------GGAAAAGGGCCATGTAAAAAT----------------------
-GTCAGC---ACAGTACAATGTACACATGGAATTAGGCCAGTAGTGTCAACT---
CAACTGCTGTTAAATGGCAGTCTAGCAGAAGAAGAG-----------
GTAGTAATCAGATCTGACAATTTCACAGACAAT---------GGTAAAGCCATAATAGTACAGCTGAAA----
-----------GAACCTGTGAAA---------ATTGATTGCATAAGACCCAACAACAATACAAGAAAA-----
-AGTATACCCATA------------GGACCAGGAAGGGCA---TTT------TATACAACAGGAGAC------
---------ATAATAGGAGATATA------------AGACATGCCTATTGT---AACCTCAGTAGAGAA---
GAATGGAATAAGGCTTTAAATCAGACAGCCAAAAAATTAGAA---------------GAACAATTTGCA----
-----------------------------------AGTAAAACGATAGTTTTTAAC------
CAATCCTCAGGAGGGGACCCAGAAATTGTA---ATGCACACTTTTAATTGTAGAGGGGAA---
TTTAAGCAGCTCCAGGCAAGAGTCCTGGCTGTGGAAAGATACCTAAAGGATCAACAGCTCCTGGGGATTTGGG
GTTGCTCTGGAAAACTCATTTGCACCACTGCTGTGCCTTGGAATAATAGTTGGAGT----------------
-------------------------AAAAATAAATCTCTGGAGGCCATTTGG------GGA------
AACATGACCTGGAATCAGTGG---GAAAGAGAAATTGACAATTACACAGACTTAATATAC---

TCCTTAATTGAGGAATCGCAAAACCAACAAGAA--

AAGAATGAAAAGGACTTATTGGAATTGGATCAATGGGCAAGTTTGTGGAATTGGTTTAGTATAACAAAATGGC
TGTGGTATATAAAAATATTCATAATGATAATAGGAGGCTTGATAGGTTTAAGAATAGTTTTTACT---
GTACTTTCTATAGTG---------AATAGAGTTAGGCAGGGATACTCACCA------
TTATCGTTCGCTGTTAGCTTGTTCAACGCCATAGCCATAGCAGTAGCTGAG---
GGGACAGATAGGGTTATAGAAGTATCACAAAGAGCTTGTAGAGCT----------------------------
--------ATCCTTAACATA------CCTACAAGAATAAGACAGGGCTTGGAAAGGCTTTTGCTA---TAA
>EF514711
GCT---GCA--
GAAAATTTGTGGGTCACAGTCTATTATGGGGTACCTGTGTGG---AAAGAAGCAACC---
ACCACTCTATTTTGTGCATCAGATGCCAAAGCATATGATACAGAA---GTACACAAT----------------
--------------GTCTGGGCCACA---CATGCCTGTGTACCCACAGAC---
CCCAACCCACAAGAAGTAGAATTGAAA---------AATGTGACAGAAAATTTTAAC----------------
--------------------------ATGTGGAAAAATAACATGGTAGAACAGATGCATGAGGATATA---
ATCAGTTTATGGGATCAAAGCCTA---AAGCCATGTGTAAAATTA---
ACCCCACTCTGTGTTTCTTTACATTGCGCTGATTTGAATAATACTAATAATTTGAATGCTACTAAT-------
----
-------------------ACCACTAGTAGTAGCGGGGGGATGATGGAAAAAGGAGAAATAAAA---
AATTGCTCTTTCAATATCACCACAAGCATAAGA---------------------------
GATAGGGTGCAGAAGGAATATGCATTT---TTTTATAAACTTGATGTAATACCAATAGATAAT----------
----

ACTAGTTATAGGTTGATAAGTTGTAACACCTCAACCATTACACAG---
GCTTGTCCAAAGGTATCCTTTGAGCCAATTCCCATACATTACTGTGCCCCGGCTGGTTTTGCGATTCTAAAGT
GTAAA---GATAAGAAGTTCAAT------GGAACAGGACCATGTAAAAAT-----------------------
-GTCAGC---ACAGTACAATGCACACATGGAATTAAGCCAGTAGTATCAACT---
CAACTACTGTTAAATGGCAGTCTAGCAGAAGAAGAG------------
GTAGTAATTAGATCTGCCAATTTCACAGATAAT---------GCTAAAGTCATAATAGTACAGCTGAAT----
-----------AAATCTGTAGTA---------ATTAATTGTACAAGACCTAACAACAATACAAGAAAA-----
-AGTATACCTATG------------GGACCAGGCAAAGCA---TTT------TACACAACAGGACAA------
---------ATAATAGGGGATATA------------AGACAAGCACATTGT---AACATTAGTAGAGAA---
AAATGGAATGACACTTTAAGACAGATAGTTAAAAAATTAAAA---------------GAACAATTTGGG----
--------------------------------GAAAATAGAACAGTAGTCTTTAAT------
CAATCCTCAGGAGGGGACCCAGAAATTGTA---ATGCACAGTTTTAATTGTGGAGGGGAA---
TTCAAGCAGCTCCAGGCAAGAGTCCTGGCCGTGGAAAGGTACCTAAGGGATCAACAGCTCCTGGGGATTTGGG
GTTGCTCTGGAAAACTCATTTGCACCACTGCTGTGCCTTGGAATACTAGTTGGAGT-----------------
----------------------------AATAAATCTCTGGATTATATTTGG------GAC------
AACATGACCTGGATGCAGTGG---GAAAAAGAGATTGATAATTACACAAGCTTAATATAC---
AACTTACTTGAAGACTCGCAAAACCAGCAAGAA--

AAGAATGAACAAGAATTATTGGAACTAGATAAATGGGCAAGTTTGTGGAATTGGTTCAACATAACAAATTGGC
TGTGGTATATAAGAATATTTATAATGATAGTAGGAGGCTTGGTAGGTTTAAGAATAGTTTTTACT---
ATATTTTCTGTAGTG---------AATAGAGTTAGGCAGGGATACTCACCA------
TTATCGTTCGCTGTTAGCTTGTTCAACGCCACAGCCATAGCAGTAGCTGAG---
GGAACTGATAGGGTTTTAGAACTATTACAAAGAACCTTTAGAGCT----------------------------
--------ATTATCCACATA------CCTACACGAATCAGACAGGGCTTGGAAAGGGCTTTGCTA---TAA
>EF514712
GCT---ACA--
GAAAAACTGTGGGTCACAGTCTATTATGGGGTACCTGTGTGG---AAAGAAGCAGAC---
ACCACTCTCTTTTGTGCATCAGATGCTAAAGCATATGATACAGAG---GCACATAAT----------------
--------------GTTTGGGCCACA---CATGCCTGTGTACCCACAGAC---
CCCAACCCACAGGAATTACTATTGGGA---------AATGTGACAGAAAATTTTACC----------------
--------------------------ATGTGGAAAAATAACATGGTAGAACAAATGCATGAGGATATA---
ATCAGTTTATGGGATCAAAGCCTA---AAACCATGTGTAAAAATA---
ACCCCACTCTGTGTTACTTTAGATTGCATTGATGTAAAGAATGATACTAATACCACTAGTAGTGGCGGG----
----
-------------------AAAGAATCTAATGCCACTAGTAGTAGCGAGGGAGGAGACATGAAA---
AACTGCTCTTTCAATATCACCACAACCATG------------------------------
GATAAGGTGCAGGAAGAACGTGCAATT---

TTTTATAACCTTGATATAGTACCAATGGATGATAATAATAAGACT----------------------------
----
AGAACTGTTAGTAATGAGACTAGTAGGAATAGTATCAGATATAGGCTGATAAGTTGTAACACCTCAACCATTA
CACAA---
GCCTGTCCAAAGATATCCTTTGAGCCAATTCCCATACATTATTGTGCCCCGGCTGGTTTTGCGATTCTAAAGT
GTAAT---GATAAGAAGTTCAAT------GGAACAGGACCATGTACAAAT----------------------
-GTCAGC---ATAGTACAATGTACACATGGAATTAGGCCAGTAGTATCAACC---
CAACTGCTGTTAAATGGCAGTCTAGCAGAAGAAGAG-----------
GTAGTAGTTAGATCTGCCAATTTCACAGACAAT---------ACTAAAACCATAATAGTACAGCTGAAA----
-----------GATCCTGTAGGA---------ATTAAGTGTATAAGACCCTACAACAAGAGAAGAGAA-----
-AGGATACGTATA------------GGAATAGGGAGAACA---TTT------CAGACAACAAGAACA------
---------GTAGATGGAGATATA------------AGAAAAGCATATTGT---ATTCTTAATGGAACA---
AAATGGAACGACACTTTAGCACAGATAGCTAAAAAATTAAAC---------------GAACAATATAAT----
-----------------------------------ACTAGTAAAATAATCTTTAGT------
AGCTCCTCAGGAGGGGACCCAGAAATTGTA---ACGCACAGTTTTAACTGTGGAGGGGAA---
TTTAAGCAGCTCCAGGCAAGAATCCTGGCTGTGGAAAGATACCTAAAGGATCAACAGCTCCTGGGGCTTTGGG
GTTGCTCTGGAAAAATCATTTGCACCACTACTGTGCCTTGGGATGCTGCCTGGAGT-----------------
----------------------------AATAGATCTCTAGATGAAATCTGG------GAT------
AACATGACCTGGATGCAGTGG---GAAAGAGAAATTGAAAATTACACAAGCAAAATATAC---
ACCTTAATTGAAGAATCGCAGATCCAACAAGAA--

AAGAATGAAAAAGAATTATTGGAATTAGATAAATGGGCAAGTCTGTGGAATTGGTTTGAAATATCAAATTGGC
TGTGGTATATAAAAATATTCATAATGATAGTAGGAGGCTTGGTAGGTTTAAGAATAGTTTTTACT---
GTACTTTCTATAGTG---------AATAGAGTTAGGCAGGGATACTCACCA------
TTATCGTTTGCTGTTAGCTTGTTCAATACCATAGCCATAGCAGTAGCTGAG---
GGGACAGATAGGGTTATAGAAGCAGTACAAAGAACTTGCAGAGCT----------------------------
--------ATTCGCCACATA------CCTACAAGAATAAGACAGGGCTTGGAAAGGGCTTTACTG---TAA
>EF514697
GCT---ACA--
GACTTGTGGGTCACAGTCTATTATGGGGTACCTGTGTGG---AAAGAAGCAAAC---
ACCACTCTATTTTGTGCATCAGATGCTAAAGCATATGATACAGAG---GTACATAAT----------------
--------------GTTTGGGCCACA---CATGCCTGTGTACCCACAGAC---
CCCAGCCCACAAGAAGTAGAATTGAAA---------AATGTGACAGAAGATTTTAAC---------------
--------------------------ATGTGGAAAAATAACATGGTAGAACAAATGCATGAGGATATA---
ATCAGTTTATGGGATCAAAGCCTA---AAACCATGTGTAAAATTA---
ACCCCACTATGTGTTACTTTAAATTGCACTGATTGGAATGGTACTGCCACTAGTCCTAATGATAATAGCACTA
ATAG---
-------------------------TAGTATAGAGGAAACAAGATGGAGAAAGGAGAAATGAAA---
AACTGCTCTTTCAACATCACCACAGGCATAAGA--------------------------
GATAAGATACAGAAAGAATATGCACTT---
TTTTATAAACTTGATATGGTACCAATAGATGATGATAAGACTAATAGCAGCCCTACTACAACTACTAGCTCTA
CTCTGACTTCTAGCAGCGTTACTAATAGCAACTCTAACAGCAGTAATACTACTAGCAACTCTAGCCAATATAG
GAACTATAGGATGATAAGTTGTAACACCTCAGTCATTACACAG---
GCCTGTCCAAAGGTATCCTTTGAGCCAATTCCCATACATTATTGTGCCCCGGCTGGTTTTGCGATTCTAAAAT
GTAAT---GATAAGAACTTCACT------GGAAAAGGACCATGTAGCAAT----------------------
-GTTAGC---ACAGTACAATGTACACATGGAATTAGGCCAGTAGTGTCAACT---
CAACTACTGTTAAATGGCAGTCTAGCAGAAGAAGGG-----------
ATAGTAATTAGATCTGACAATTTCTCAAACAAT---------GCTAAAAATATACTAGTACAGCTGAAA----
-----------GAAGCTGTAGTA---------ATCAATTGTACAAGACCCAACAACAATACAAGAAGA-----
-GGTATACATATA------------GGACCAGGGAGGGCA---TTT------TATACAACAGGAGAT------
---------GTAATAGGAGATATA------------AGACAAGCACATTGT---AACATTAGCGGAACA---
AAATGGAATAACACTTTATACCAAATAGCTAAAAAATTAAAA---------------GAACAATATAAT----
-------------------------------GAAACAAACAAAATAATCTTTAAT------
CACTCCTCAGGAGGGGACCCAGAAATTGTA---ATGCACAGCTTTAACTGTGGAGGGGAA---
TTTAAGCAGCTCCAGGCAAGAGTCCTGGCGATAGAAAGATACCTACAGGATCAACAGCTCCTAGGAATTTGGG
GTTGCTCTGGAAGACTCATTTGCACCACTGCTGTGCCTTGGAATGCTAGTTGGAGT-----------------
----------------------------AATAGATCCCTGGATCAGATTTGG------AAT------
AACATGACCTGGATGGAGTGG---GAAAGAGAAATTGACAATTACACAAACATAATATAC---
TCCTTAATTCAAGAATCGCAAAACCAGCAAGAC--

AAGAATGAACAAGAATTATTGGAATTAGATAAATGGGCAAGTTTATGGAATTGGTTTAGCATAACACAATGGC
TGTGGTATATAAAAATATTCATAATGATAGTAGGAGGCTTGATAGGCTTAAGAATAGTTTTTGCT---
GTACTTTCTATAGTG---------AATAGAGTTAGGCAGGGATACTCACCA------
TTATCGTTGGCTGTTAGCTTGCTCAACGCCACAGCCATAGCAGTAGCTGAG---
GGGACAGATAGGGTGCTAGAATTATTACAAAGAATTGGTAGGGGT----------------------------
--------ATCCTCCACATA------CCTAGAAGAATAAGACAAGGCTTAGAAAGGAGTTTGCTA---TAA
>EF514701
GCT---GCA---
GACCAATTGTGGGTCACAGTCTATTATGGGGTACCTGTGTGG---AAAGAAGCAAAC---
ACCACTCTATTTTGTGCATCAGATGCTAAATCATATGATACAGAG---GCTCATAAT----------------
--------------GTTTGGCCACA---CATGCCTGTGTACCCACAGAC---
CCCAGCCCACAAGAAGTAGTACTGAAA---------AATGTAACAGAAAATTTTAAC----------------
--------------------------ATGTGGAAAAATAACATGGTAGACCAAATGCATGAAGATATA---
ATCAGTTTATGGATCAAAGCCTA---AAGCCATGTGTAAAATTA---
ACCCCACTCTGTGTTACGTTAAATTGCACTGATGCGCCAGATGCTAATAATACTACTAACACCACCATTGCT-
----
----------------GGTACTTCCACCACTAATACCCCCATTGTTAGTTCAGGAGAGATAAAA---
AACTGCTCTTTTAATACCACCACAAGCATAAGA--------------------------
GATAAGATAAAGAAAGAATATGCACTC---TTTTATAAACTAGATGTAGTACAAATAGATGAT----------
----

ACTAGCAATAATAGCTATAGATTGATAAGTTGTGATACCTCAGTCATTACACAA---
GCTTGTCCAAAGATATCCTTTGAGCCAATTCCCATACATTATTGTGCCCCGGCTGGTTTTGCGATTTTAAAAT
GTAAT---GATACAAAGTTCAAT------GGAACAGGACCATGTAAAAAT-----------------------
-GTCAGT---ACAGTACAATGTACACATGGAATTAGGCCAGTAGTGTCAACT---
CAATTGCTATTAAATGGCAGTCTAGCAGAAGAAGGA------------
ATAGTAATTAGATCTGAAAATTTCACGGACAAT---------GCGAAAAATATAATAGTACAGCTGAAT----
-----------AAATCTGTGAAC---------ATTACCTGCACAAGACCCAGCAATAATACAAGAAGA-----
-AGTATATCGATG------------GGACCAGGGAGAGCA---ATG------TATGCAACAGGACAA------
---------ATAATAGGAGATATA------------AGAAAAGCACATTGT---AACCTTAGTAAAACA---
GAATGGAATAACACTTTAAAACAGATAGTTGAAAAATTAAGG---------------GAACAATTTGGA----
-----------------------------------AATAAAACAATAGTCTTTAAT------
CATTCTGCAGGAGGGGACCCAGAAATTGTA---ACGCATAGCTTTAATTGTGGAGGGGAA---
TTTAAACAGCTCCAGGCAAGAGTCCTGGCTGTGGAAAGATACCTAAAGGATCAACAGCTCCTGGGGATTTGGG
GTTGCTCTGGAAAACTCATCTGCACCACTGCTGTGCCTTGGAATGCTAGTTGGAGT-----------------
----------------------------AATAAATCTATGGATAAGATTTGG------AAC------
AACATGACATGGATGCAGTGG---GAAAAAGAAATTGACAATTACACAGACTTAATATAC---
AACTTACTTGAGCAATCGCAAAACCAACAAGAA--

ATAAATGAACAAGAACTATTGGAATTAGATAAGTGGGCAAGTTTGTGGAATTGGTTTAATATAACAAACTGGC
TGTGGTATATAAAATTATTCATAATGATAGTAGGAGGATTGATAGGTCTAAGAATAGTTTTTACT---
ATACTTTCTTTAGTG---------AATAGAGTTAGGCAGGGATACTCACCC------
TTATCGTTTGCTGTTAGCTTGCTCAATGCCACAGCTATCGCAGTAGCTGAG---
GGGACAGATAGGATTCTAGAAGCAGTACAAAGAGCTTGTAGAGCT----------------------------
--------TTCCTGCATATA------CCTAGAAGAATAAGACAGGGCTTAGAAAGGCTTTTGCTA---TAA
>FJ694790
GCA---ACA---
GAAAAATTGTGGGTCACAGTCTATTATGGGGTACCTGTATGG---AAAGAAGCAAAC---
ACCACTTTATTTTGTGCATCAGATGCTAAAGCATATGATACAGAG---GTACATAAT----------------
--------------GTTTGGCCACA---CATGCCTGTGTACCCACAGAC---
CCCAATCCACAAGAAGTAGTATTGAAA---------AATGTGACAGAAAATTTTAAT----------------
--------------------------GTGTGGAAAAATAACATGGTAGACCAAATGCAGGAGGATATA---
ATCAGTTTATGGATCAAAGCCTA---AAGCCATGTGTAAAATTA---
ACCCCACTCTGTGTTACTTTAGATTGCATTGATTGCACTAATACCTCAAAGTGCAATACCACTACTAATGCT-
----
----------------ACCAACACCAATAATCCTGCTCCCACAATGGAGACAGGAGAATTAAAA---
AACTGCTCTTTCAATATCACCACAGAAATAAGA--------------------------
GATAAGGTACAGAAGGAATCTGCACTT---
TTTTATAAATCTGATGTAGTACCAATAAAAGATGATAATAATGAGAATAATAGT-------------------
----

ACCAATGGGGATGATAATAGTAGCAGCAAGAAAACTAATACCAGATATAGGTTGATAAGTTGTAACACCTCAG
TCATTACACAG---
GCCTGTCCAAAGATATCCTTTGATCCAATTCCCATACATTATTGTGCCCCGGCTGGTTTTGCGATTCTAAAAT
GTAAC---AATAAGACGTTCAGT------GGAAAAGGACCATGCACAAAT-----------------------
-GTCAGC---ACAGTACAATGCACACATGGAATTAGGCCAGTAGTATCAACT---
CAACTGCTGTTGAATGGCAGTCTAGCAGAAGAAGGT------------
GTAGTGATCAGATCTGAAAATTTCACAGACAAT---------GCTAAAAACATAATAGTACAGCTGAAA----
-----------GAGTCTGTACCA---------ATTAATTGTACAAGACCCAACAACAACACAAGAAAA-----
-GGTTTTCGTATA------------GGACCAGGGAGAGCA---GCC------TTTGCACACGGAAAG------
---------ATAATAGGAGATATA------------AGAAAAGCATATTGT---ACCCTTGATGGAACA---
AAATGGAATAACGCTTTAATTCAGATAGCTAAAAAATTAAGA---------------GGACAATTTGGG----
--------------------------------GAGAATAAAACAATAATCTTTAAT------
CAATCCTCAGGAGGGGACCCAGAAATTGTA---ATGCACAGTTTTAATTGTAGAGGGGAA---
TTTAAGCAGCTCCAGGCAAGACTCCTGGCTGTGGAAAGATACCTAAGGGATCAACAGCTCCTGGGAATTTGGG
GTTGCTCTGGAAAACTCATTTGCACCACTGCTGTGCCTTGGAATGCTAGCTGGAGT-----------------
----------------------------AATAAAAATCTAACTCAGATTTGG------GAT------
AACATGACCTGGATGCAGTGG---GAAAAAGAAATTGACAATTACACATCATTAATATAC---
TCCTTGCTTGAAGAATCGCAAAACCAACAAGAA--

CAGAATGAACAAGAATTATTGGAGTTAGACAAATGGGATAGTCTGTGGAATTGGTTTAGCATAACAAATTGGC
TGTGGTATATAAGACTATTCATAATGATAGTAGGAGGCTTGATAGGCTTAAGAATAGTCTTTACT---
GTGCTTTCTATAGTG---------AATAGAGTTAGGCAGGGATACTCACCA------
TTATCGTTGGCTGTTAGCTTGCTTAATGTCACAGCCATAGCAGTAGCTGAG---
GGGACAGATAGAATTATAGAAGCATTACTAAGAACTTTCAGAGCT----------------------------
--------ATTCTCCACATA------CCTAGAAGAATAAGACAGGGCTTAGAAAGAGCTTTGCTA---TAA
>EU839597
GCA---GAA---
CAGTTGTGGGTCACAGTCTATTATGGAGTACCAGTGTGG---AAAGAAGCAACC---
ACTACTCTATTTTGTGCATCAGATGCTAAAGCATATGACACAGAG---AAGCATAAT----------------
--------------GTCTGGGCCACA---CATGCCTGTGTACCCACAGAC---
CCCAACCCACAAGAAATAATATTGAAA---------AATGTGACAGAAAATTTTAAC----------------
--------------------------ATGTGGAAAAATAACATGGTAGAACAGATGCATGAAGATATA---
ATCAGTTTATGGATCAAAGCCTA---AAGCCATGTGTAAAATTA---
ACCCCACTCTGCGTTACTTTAAATTGCACTAATGCCACTAATGGTACT-------------------------
----
-------------------------------------GTGGAATTGGGGGGAGGAGAAATAAAA---
AACTGTTCTTTCAATATCACCACAGATGGAGGC---------------------------
AGTAGGAGACAGAAAGAATATGCACTG---TTTTATAACCTTGATGTAATACCAATGAATGATGCT-------
----

GATAATAATGATAATACTAGCTATAGATTGATACATTGTAATACCTCAGTCATTACACAA---
GCCTGTCCAAAGGTGTCCTTTGAGCCAATTCCTATACATTATTGTGCCCCGGCTGGTTATGCAATTCTAAAGT
GTAAT---GATAAGGAGTTCAAT------GGAACAGGACCATGTAAAAAT-----------------------
-GTCAGC---ACAGTACAATGTACACATGGAATTAGACCAGTGGTGTCAACA---
CAACTGCTGTTAAATGGTAGCCTAGCAGAAGGAGAG------------
GTAGTAATTAGATCTGAAAATTTCACGAACAAT---------GCTAAAACCATAATAGTACAGTTGAAC----
-----------AGCTCTGTAGAA---------ATTAATTGTACAAGACCCGGCAACAATACAAGAAAA-----
-GGTATACATATA------------GGACCAGGGAGCACA---TGG------TATACAACAGGAGAA------
---------ATAGTAGGAGATATA------------AGACAGGCACATTGT---AACATTAGTAGAGAA---
AAATGGAATAATACTTTAAAACAGGTAGTTGAAAAATTAAGG---------------GAGCAATTT-------
-----------------------------------AATAAAACAATAGTCTTCAAA------
CCATCCTCAGGAGGGGACCCAGAAATTGTA---CTGCACAGTTTTAACTGTGGAGGGGAA---
TTCAAACAGCTCCAGGCAAGAGTCCTGGCTGTAGAGAGATACCTAAAGGATCAACAGCTCCTAGGGATTTGGG
GTTGCTCTGGAAAACTCATCTGCACCACTGCTGTGCCTTGGAACACTAGTTGGAGT-----------------
----------------------------AATAGATCTAGGGATTACATTTGG------GAA------
AACATGACCTGGATGGAATGG---GAAAAAGAAATTGATAATTACACAGAAATAATATAC---
CAATTAATTGAAATATCGCAGAACCAGCAAGAA--

AAGAATGAACAAGAATTGTTGGAATTGGATCAATGGGCAAGTTTATGGAATTGGTTTAGTATAACACAGTGGC
TGTGGTATATAAAAATATTCATAATGATAGTAGGAGGCTTGATAGGTTTAAGAATAGTTTTTGCT---

GTGCTTTCTATAGTG---------AATAGGGTTAGGCAGGGATACTCACCA------
ATATCATTGGCTGTTAGCTTGCTTAATACCACAGCAATAGCAGTAGCTGAG---
GGGACAGATAGGATTATAGAAGGGGTAAGAGTAATTGTTAGAGCT----------------------------
--------TTTCTCCACATA------CCTAGAAGAATAAGACAAGGCTTTGAAAGGGCTTTGCTA---TAA
>EU839596
GCA---GCA--
GAAAAATCGTGGGTCACAGTCTATTATGGGGTACCTGTGTGG---AAAGAAGCAACC---
ACCACTCTATTTTGTGCATCAGATGCTAAAGCATATGAGACAGAG---GTACATAAT----------------
--------------GTCTGGGCCACA---CATGCCTGTGTACCTACAGAC---
CCCAACCCACAAGAAATAGTATTGGAA---------AATGTGACAGAATATTTTAAC----------------
--------------------------ATGTGGAAAAATAACATGGTAGAACAGATGCATGAGGATATA---
ATTAGTTTATGGGATCAAAGCCTA---AAGCCATGTGTAAAATTA---
ACCCCACTCTGTGTTACTTTAAATTGCATTAATGCAAATAATAGTGTCAGTAATTTGACA-------------
----
----------------------------AAATTAGATAAAGAAGAAATAAAAAAAGAAATAAAA---
AACTGTTCTTTC------ACGACACAATTAGGA---------------------------
GATCAGATGCAGAAAGAAAGTGCATTG---TTTTATACATTTGATATAACACCACTAAAAGTTTTTAATGAT-
----

ACTAGTGGTAATGATAATGGTACTTATAGATTGATAAGTTGTAATACCTCAGTCATTACACAG---
GCCTGCCCAAAGGTAAGCTTTGAGCCAATTCCCATACATTATTGTGCCCCGGCTGGTTTTGCGATTCTAAAGT
GCAGA---GAGGAAGGGTACAAG------GGAACAGGACCATGCACAAAT-----------------------
-GTCAGC---ACAGTACAATGCACACATGGAATTAGGCCAGTAGTGTCAACT---
CAACTGCTGCTAAATGGCAGTCTAGCAGAAAAAGAG------------
GTAGTAATTAGATCTGAAAATTTCACGGACAGT---------TGGAAAACCATAATAGTGCAGCTGAAG----
-----------GACCCTGTAGAG---------ATTAATTGTACAAGACCCAACAACAATACAAGAAAA-----
-AGTATACATATA------------GCACCAGGGAGAACA---TTT------TATGCAACAGGAAAA------
---------ATAATAGGAAATATA------------AGGCAAGCACATTGT---AATATTAGTAGAGCA---
AGATGGAATGACACTTTAAGACAGGTAGTTGAAAAATTAAGA---------------AAACAATTTGAG----
-----------------------------------AATAAGACAATAATTTTTAAT------
CGCTCCTCAGGAGGAGACCCAGAAGTTGTA---ATGCACAGTTTTAATTGTGGAGGGGAA---
TTTAAACAGCTCCAGGCAAGAGTCCTGGCTGTGGAAAGATACCTAAAGGATCAACAGCTCCTAGGAATCTGGG
GTTGCTCTGGAAAACTCATTTGCACCACTTCTGTGCCTTGGAACGATAGTTGGAGT-----------------
----------------------------AATAAATCTCTGGATGATATTTGG------AAT------
AACATGACCTGGATGCAGTGG---GAAAAAGAAATTGACAATTACACAGATGAAATATAC---
AGATTAATTGAACAAGCACAAAACCAGCAAGAA--

AAGAATGAACAAGAGTTATTAGAATTGGATAAGTGGGCAAATTTGTGGAATTGGTTTAGTATAACAAATTGGC
TGTGGTATATAAAATTATTCATAATGATAGTAGGGGGCTTAATAGGTTTAAGAATAGTTTTTGCT---
ATACTTTCTATAGTG---------AATAGAGTTAGGCAGGGATACTCACCG------
TTATCATTGGCTGTCAGCTTGCTTGATACTACAGCTATAGCAGTAGCTGAG---
GGGACAGATAGGATTATAGAAATAGTGCAAAGAATTTGTAGAGCT----------------------------
--------ATTCGCCACGTA------CCTACAAGAATAAGACAAGGCCTCGAAAGGGCTTTGCTA---TAA
>FJ817365
GCT---GCT---GAA------------------CAA---GCT---GCA---
GAACCATTGTGGGTCACAGTTTATTATGGGGTACCTGTGTGG---AAAGAAGCAACC---
ACCACTTTATTTTGTGCATCAGATGCTAAAGCACAGAGTACAGAG---GCACATAAT----------------
--------------ATTTGGGCCACA---CATGCCTGTGTACCCACAGAC---
CCCAACCCACAAGAAGTAAAATTGGAA---------AATGTGACAGAAAATTTTAAC----------------
--------------------------ATGTGGAAAAATAACATGGTAGAACAAATGCATGAGGATACA---
ATCAGTTTATGGGATCAAAGCTTA---AAGCCATGTGTAAAATTA---
ACCCCACTCTGTGTTACTTTAGACTGCACTGATGACTTGAGGAATGATACTAATAAT----------------
----
----------------------------AGTACTGCTAACATAGTGGAGAAAGGAGAAATAAAA---
AATTGCTCTTTCAATATCACCACAGGCATGAGT------------------------------
AAGGTGCAGAAGGAACACGCTCTT---TTCTATAGCCTTGATGTAGTACCAATGAATGCA-------------
----
-------------------AGTAATACCCAATATAGGTTGATAAGTTGTAATACCTCAGTCATTACACGA---
GCTTGTCCAAAGATATCTTTTGAGCCAATTCCCATACATTATTGTGCCCCGGCTGGTTTTGCGATTCTAAAGT
GTAAT---GATAAAAATTTTGAA------GGAAATGGAACGTGTAAAAAT-----------------------

-GTCAGC---ACTGTACAATGTACACATGGAATTAAGCCAGTAGTATCAACC---
CAACTGTTGTTAAATGGTAGTCTAGCAGAAGAAGAG------------
GTAGTAATTAGAGCTGACAATATCTCAAGCAAT---------ACTAAAACCATAATAGTGCAGCTGAAT----
-----------GAAACTATAGAA---------ATTAATTGTACAAGACCCAACAACAATACAAGAAAA-----
-GGTATACATATG------------GGATCAGGGAGAGCA---TTT------TATACAACAGGGGAC------
---------ATAATAGGAGACATA------------AGAAAAGCACATTGT---AACATTAGTCGAGTA---
AAATGGGAGAACACTTTAGAACAGATAGTTAGGAAATTAAGA---------------GAAAAAGTGGGG----
--------------------------------AATAATAAAACAATAGTCTTTAAT------
CAATCCTCAGGAGGGGACCCAGAAATTGTA---ATGCACAGTTTTAATTGTGGAGGGGAA---
TTTAAGCAGCTCCAGGCAAGAATCCTGGCTGTGGAAAGATACCTACAGGATCAACAGCTCCTGGGGATTTGGG
GCTGCTCTGGAAAACTCATCTGCACCACTTCTGTGCCTTGGAATAGTAGCTGGAGT-----------------
----------------------------AATAAAAGTGTAGAGGAGATCTGG------GGT------
AACATGACCTGGATACAGTGG---GAAAAAGAAATTGACAATTACACAAAGGAAATATAC---
AACTTAATTGAAATATCGCAGAACCAGCAGGAA--

AAGAATGAACTAGACTTATTGAAATTGGATACATGGGCAAGCTTGTGGAATTGGTTTGACATAACAAATTGGC
TATGGTATATAAAAATATTCATAATGATAGTAGGAGGCTTGGTAGGTTTAAGAATAGTTTTTGCT---
GTACTTTCTATAGTG---------AACAGAGTTAGGCAGGGATACTCACCA------
TTATCGTTTGCTGTTAGCTTGCTCAACGCCACAGCCATAGCAGTAGCTGAG---
GGGACAGATAGGATTATAGAATTTTTACAAAGAGCTGGTAGAGCT----------------------------
--------ATACTTCATATA------CCCGTAAGAATAAGACAGGGTTTGGAAAGGGCTTTGCTT---TAA
>KY658702
ACT---GCA--
GAAGATTTGTGGGTCACAGTCTATTATGGGGTACCTGTGTGG---AAGGAAGCAAAA---
ACCACTCTATTTTGTGCATCAGATGCTAAAGCATATGATACAGAG---GTACATAAT----------------
--------------GTCTGGCCACA---CATGCCTGTGTACCCACAGAC---
CCCAGCCCTCAAGAGTTAGAATTAGTA---------AATGTGACAGAAGATTTTAAC----------------
--------------------------ATGTGGAAAAATAACATGGTAGATCAGATGCATGAGGATATA---
ATCAGTTTATGGGATCAAAGCTTA---AAGCCATGTGTAAAATTA---
ACCCCACTCTGTGTTACTTTAAATTGCACTAATTGGAATGCCACTGCTGCTAAGAATATTACCTCT-------
----
----------------------GATAGAAATAGTAGCTTAACAATAGAAGATGGAGAAGTAAAA---
AACTGCTCTTTCAATATTACCACAAGCATAAGA---------------------------
GATAAGGTGCAGAAAGACTATGCACTT---TTTTATAGACTTGATGTAGTACCAATAGATGAG----------
----

ACCAATGATAAGAATAATAGTAATAGCTATAGATTGATAAATTGTAATACCTCAGTCATTACCCAG---
GCCTGTCCAAAAGTATCCTTTGAGCCAATTCCCATACATTATTGTGCCCCGGCTGGTTTTGCGATTCTAAAGT
GTAAC---AATAAGACATTCAAT------GGAACAGGGCCATGTAAAAAT-----------------------
-GTCAGC---ACAGTACAATGTACACATGGAATTAGGCCAGTAGTGTCAACT---
CAGTTGTTGTTAAATGGCAGTCTAGCAGAA---GAG------------
GTAATAATTAGATCTGAGAATCTCACAGACAAT---------GCTAAAACCATAATAGTACAGCTGAAG----
-----------GAACCTGTAAAT---------ATTACTTGTACAAGACCCGGCAACAATACAAGAAAG-----
-AGTGTACATATA------------GGACCAGGAAGAGCA---TTT------TATGCAACAGGAGAA------
---------ATAATAGGAGATATA------------AGACAAGCACACTGC---AATCTGAGTAGAATA---
AAATGGGAGAACACTTTAAAACAGATAGCTAGAAAATTAGGA---------------GAAAAATTT-------
-----------------------------------AATAAAATAATAGTCTTTAAT------
CAATCCTCAGGAGGAGACCCAGAAATTGTA---TCTCACACGTTTAATTGTGGAGGGGAA---
TTTAAACAGCTCCAGGCGAGAGTCCTAGCTGTAGAAAGATACCTAGGCGATCAGCAGCTCCTAGGGATCTGGG
GTTGCTCTGGAAAACGCATCTGCACCACTAATGTGCCTTGGAATACTAGTTGGAGT-----------------
----------------------------AATAAAACTCATAGTCAGATTTGG------GAC------
AACCTGACCTGGATGCAGTGG---GAAAGAGAAATTCAAAATTACACAGGCACAATATAC---
ATCTTACTTGAAAAGGCACAGAACCAGCAAGAA--

ACAAATGAAAAAGAATTATTAGAATTGGATGAGTGGGCAAATTTGTGGAATTGGTTTGATATATCAAAATGGC
TGTGGTATATAAAAATATTCATAATGATAGTAGGAGGCTTGGTAGGTTTAAGAATAGTTTTAGCT---
GTAATTTCTATAGTG---------AACAGAGTTAGGCAGGGATATTCACCA------
TTATCATTTGCTGTTAGCTTGCTTGATACCATAGCTATAGCAGTAGCTGAG---
GGGACAGATAGGATTATAGAAGCAGTAAGAAGACTTTGTAGGGCT----------------------------
--------TTTCTCAACATA------CCTAGAAGAATAAGACAGGGCGCAGAAAGGGCGTTGGTA---TAA

>AY173960
ATT---AGT--
GGACAATTGTGGGTCACAGTCTATTATGGAGTACCTGTGTGG---AAAGAGGCAACC---
ACCACTCTATTTTGTGCATCAGATGCTAAGGCATATGACACAGAG---GTGCATAAT---------------
--------------GTTTGGGCCACA---CATGCCTGTGTACCCACAGAC---
CCTAACCCACAAGAAGTAGTATTAGAA---------AATGTGACAGAAAATTTTAAC---------------
-------------------------ATGTGGAAAAATAACATGGTAGAACAGATGCATGAGGATATA---
ATCAATTTATGGGATCAAAGCCTA---AAACCATGTGTAAAATTA---
ACCCCAGTCTGTGTTACCTTAAACTGCAATAATTCTAAGTTTATAAATAATCCTAGTGTTAATACTACT----
----
-------------------GGACAAAATAATGCTAGTTTGAGACAGATGGAGGAAGAAATGAAA---
AACTGCTCTTTCAATGTCACCGCAGGCCTAAAA--------------------------
GATAAGGTGCGAACAGAACGTGCATTC---TTTAATAGACTTGATGTAGTACCAATTGAAAAT---------
----

GGTAATACTACAAAATTTATGTTGAGAAGTTGTAACACCTCAGCCATCACACAG---
GCATGTCCAAAAATATCCTTTGAACCAATTCCCATACATTATTGTACCCCAGCTGGTTTTGCGCTTCTAAAGT
GTAAT---GATAAGAAGTTCAAT------GGAACAGGACCATGTAAAAAT----------------------
-GTTAGC---ACAGTACAATGTACACATGGAATTAGGCCAGTAGTGACAACT---
CAACTGCTATTAAATGGCAGTCTAGCAGAAGAAGAA-----------
GTAGTAATTAGATCTGAAAATTTCACAAACAAT---------GCTAAAACCATAATAGTACAGCTGAAT----
-----------GAATCTGTAATA---------ATTAATTGTACAAGACCCAGCAACAATACAAGCAAA-----
-AGTATACAGTTG------------GTAGGGGGGAGTGCA---TTG------TATGCCACAAACAGA------
---------ATAATAGGAGATATA------------AGACGAGCATATTGT---GTCCTTGATAAAGCA---
GCATGGAACAAAACTTTAAAACAGGTAGCAATAAAACTAAGA---------------GAACAATTT-------
-----------------------------------AATAAAACAATAATCTTTAAT------
CAATCCTCAGGAGGGGACCCAGAAATTGTA---ATGCATAGTTTTAACTGTGGAGGGGAA---
TTTAAACAGCTCCAGGCAAGAGTCCTGGCTGTGGAAAGATACCTAAAGGATCAACAGCTCCTAGGGATTTGGG
GTTGCTCTGGAAAACTCATTTGCACCACTGCTGTGCCTTGGAATGCTAGTTGGAGT----------------
----------------------------AATAGATCTCTGGAAGACATTTGG------CAG------
AACATGACCTGGATGCAGTGG---GAGAGAGAAATTGACAATTACACAGAATTAATATAC---
AACTTACTTGAAACATCGCAAAACCAACAAGAC---------------------------------------
----
AAGAATGAACAAGAATTATTGGAATTGGATAAGTGGGCAAATTTATGGAGTTGGTTTGACATAACAAATTGGC
TGTGGTATATAAAAATATTCATAATGATAGTAGGAGGTTTAATAGGCTTAAGAATAGTTTTTACT---
GTATTTTCTATAGTG---------AATAGAGTTAGGCAGGGATACTCACCA------
TTATCATTCGCTGTTAGTTTGTTTAATGCCATAGCTATAGCAGTAGCTGAG---
GGGACAGATAGTATTATAGAAGCAGTACAAAGAATTGGTAGAGCT---------------------------
--------ATTCTCCACATA------CCTAGAAGAATAAGACAGGGCTTAGAAAGGGCTTTGCTA---TAA
>KC595150
GTT---GCA--
GGACAATTGTGGGTCACAGTCTATTATGGGGTACCTGTGTGG---AAAGAAGCAACC---
ACCACTCTATTTTGTGCTTCAGATGCTAAAGCATATGATACAGAG---GTACATAAT---------------
--------------GTTTGGGCCACA---CATGCCTGTGTACCCACAGAC---
CCCAACCCACAAGAAGTAGTATTGGGA---------AATGTGACAGAAAATTTTAAC---------------
-------------------------ATGTGGAAAAATAACATGGTAGAACAGATGCATGAGGATATA---
ATCAGTTTATGGGATCAAAGCCTA---AAGCCATGTGTAAAATTA---
ACCCCACTCTGTGTTACCTTAACTTGCACTGATTTGAGAAATGATACTGATAGTGGTGATAATACC-------
----
-------------------ACCAATAGTAGTAGCTGGGGAAAGATGGAGAGAGGAGAAATAAAA---
AACTGCTCTTTCAATACCACCACAAGCATAGAA--------------------------
GATAAGAGGCAGAAAGAATATGCACTT---TTTTATAAACTTGATGTAGTACCAATAGACAAT---------
----

GATAATAGTAGCTATAGGTTGATAAGTTGTAACACCTCAGTCATTACACAG---
GCCTGTCCAAAAGTATCCTTTGATCCAATTCCCATACATTATTGTACCCCGGCTGGTTTTGCGATTCTAAAGT
GTAAC---GATAAGAAGTTCAAT------GGAACAGGACCATGTACAAAT----------------------
-GTCAGC---ACAGTACAATGTACACATGGAATTAGGCCAGTAGTATCAACT---
CAACTGCTGTTAAATGGCAGTCTAGCAGAAGAAGAG-----------
GTAGTAATTAGGTCTAAAAATTTCACGGACAAT---------ACTAAAACCATAATAGTACAGCTGAAA----

```
-----------AAACCTGTAGAA---------ATTAATTGTACAAGACCCAACAACAATACAAGAAAA-----
-AGTATAAATATA------------GGACCAGGGAGAGCA---TTT------TATACAACAGGAGAC------
---------ATAATAGGAGATATA------------AGACAAGCACATTGT---AACCTTAGTAGAGCA---
AAATGGAATGACACCTTAAAACAGATAGTTGACAAATTAAAA---------------GAGCAATTCAGG----
-----------------------------------AATAAAACAATAGTCTTTAAG------
CAATCCTCAGGAGGGGACCCAGAAATTGTA---ATGCATAGTTTTAATTGTGGAGGGGAA---
TTTAAGCAGCTCCAGGCAAGAGTCCTAGCTGTGGAAAGATACATAAAGGATCAACAGCTCCTGGGGATTTGGG
GTTGCTCTGGAAAACTCATTTGCACCACTGCTGTGCCTTGGAATGCTAGTTGGAGT-----------------
----------------------------AATAAATCTCTGGAAAAGATTTGG------GAT------
AACATGACCTGGATGGAGTGG---GAAAGAGAAATTAACAATTACACAGGTTTAATATAC---
AACTTACTTGAAGAATCGCAGAACCAACAAGAA----------------------------------------
-----------------------------
AAGAATGAACAAGAATTATTGGAATTGGATAAATGGGCAAGTTTGTGGAATTGGTTTGACATAACAAAATGGC
TGTGGTATATAAAAATATTCATAATGATAGTAGGAAGCTTGATAGGTTTAAGAATAGTTTTTACT---
GTACTTTCTATAGTA---------AATAGAATTAGGCAGGATACTCACCA------
TTATCGTTTGCTGTTAGCTTGCTCAATGCCACAGCCATAGCAGTAGCTGAG---
GGAACAGATAGGGTAATAGAAGTATTACAAAGAGCTTATAGAGCT----------------------------
--------ATTCTCCACATA------CCTACAAGAATAAGACAGGGCTTGGAAAGGGCTTTGCTA---TAA
>KC595152
GCT---GCA------------------------------------------
GAAAAATTGTGGGTCACAGTCTATTATGGGGTACCTGTGTGG---AAAGAAGCAACC---
ACCACTCTCTTTTGTGCATCAGATGCTAAAGCATATGATACAGAG---GTACATAAT----------------
--------------GTTTGGCCACA---CATGCCTGTGTACCCACAGAC---
CCCAACCCACAAGAAGTAGTATTGGAA---------AATGTGACAGAAAATTTTAAC----------------
--------------------------ATGTGGAAAAATGACATGGTTGAACAGATGCATCAGGATATA---
ATCAGTTTATGGGATCAAAGTCTA---AAGCCATGTGTAAAATTA---
ACCCCACTCTGTGTTACTTTAAATTGCACTGATGATTGGAAGCATGATACTAATACCACTAGAAATGCT----
-------------------------------------------------------------------------
----------------------ACTAATACCACTAGTAGTAGCGGGAACACGATGGAGATGAAA---
AACTGCTCTTTCAATATCACCACAAGCATAAGA---------------------------
GATAAGGTGCAGAAAGAATATGCACTT---TTTTATAAGCTTGATGTAGTACCAATAGACAATGAT-------
-------------------------------------------------------------------------
----------------
AATAGTAGCAATAGTAGCTATAGGTTGATAAATTGTAACACCTCAGTCATTACACAG---
GCCTGTCCAAAAGTAACCTTCGATCCAATTCCCATACATTATTGTGCCCCGGCTGGTTTTGCGATTCTAAAGT
GCAAT---GATAAGAAGTTCAAT------GGAACAGGACCATGTACAAAT-----------------------
-GTCAGC---ACAATACAATGTACACATGGAATTAGGCCAGTAGTGTCAACT---
CAACTGCTGTTAAATGGCAGTCTAGCAGAAGAAGAG------------
GTAGTAATTAGATCTGAAAATTTCACGGACAAT---------GCTAAAACCATAATAGTACAGCTGAAA----
-----------GAAGCTATAGAA---------ATTAATTGTACAAGACCCAACAACAATACAAGAAGA-----
-AGTATAAATATA------------GGACCAGGGAGAGCA---TTT------TATGCAACAGGAAAA------
---------ATAATAGGAGATATA------------AGACAAGCACATTGT---AACCTTAGTAAAGCA---
AAATGGAGGACAATTTAAAACAGATAGTTAAAAAATTAAGA----------------GAACAATTAGGG----
--------------------------------AATAATAAAACAATAGTCTTTAAC------
CACTCCTCAGGAGGGGACCCAGAAATTGTA---ACGCACAGTTTTAATTGTGGAGGGGAA---
TTTAAGCAGCTCCAGGCAAGAGTCCTGGCTGTGGAAAGATACCTAAAGGATCAACAGCTCCTGGGGATTTGGG
GTTGCTCTGGAAAACTCATTTGCACCACTACTGTGCCTTGGAATGCTAGTTGGAGT-----------------
----------------------------AATAAATCTCTGGATAAAATTTGG------GAT------
AACATGACCTGGATGGAGTGG---GAAAGAGAAATTAGCAATTACACAGGCTTAATATAC---
AACTTAATTGAAGAATCGCAGAACCAACAGGAA----------------------------------------
-----------------------------
AAGAATGAACAAGAATTGTTGGAATTGGATAAATGGGCAAGTTTGTGGAATTGGTTTGACATAACAAAATGGC
TGTGGTATATAAAAATATTCATAATGATAGTAGGAGGCTTGGTAGGTTTAAGAATAGTTTTTACT---
GTACTTTCTATAGTG---------AATAGAGTTAGGCAGGATACTCACCA------
TTATCGTTTGCTGTTAGCTTGCTCAATGCCACAGCCATAGCAGTAGCTGAG---
GGGACAGATAGGGTTATAGAAGTATTACAAAGAGCTTATAGAGCT----------------------------
--------ATTCTCCACATA------CCTACAAGAATAAGACAGGGCTTGGAAAGGGCTTTGCTA---TAA
>KC595154
GCT---GCA------------------------------------------
GACAATTTGTGGGTCACAGTCTATTATGGGGTCCCTGTGTGG---AAAGAAGCAGCC---
```

ACCACTCTATTTTGTGCATCAGATGCTAAAGCATATGATACACAG---GTACATAAT----------------
--------------GTCTCGGCCACA---CATGCCTGTGTACCCTCAGAC---
CCCAACCCACAAGAAGTAGAATTGGAA---------AATGTGACAGAAAATTTTAAC----------------
--------------------------ATGTGGAAAAATAATATGGTAGAACAGATGCATGAAGATATA---
ATCAGTTTATGGGATCAAAGCCTA---AAGCCATGTGTAAAATTA---
ACCCCACTCTGTGTTACTTTAAATTGCACTAGTGTTACTTTAAATAGCACTAATGTTAATGGTACTAATTGTA
AGAATGGTACT---
------------------------GATTGTAAGAATGGTACTGGAATGGAGGGAGTAATGAAA---
AACTGCTCTTTCAATATCACCACAAGCATAAGA---------------------------
GATAAAGTAAAGAAAGAATATGCACTT---
TTTTATGCAGTTGATGTAGTACCAATAGACAATGATAATACAAAT---------------------------
----
AATAGTACTAATAGTAGCTATAGGTTGATAAGTTGTAATACCTCAGTCATTACACAG---
GCCTGTCCAAAAGTATCCTTTGAGCCAATTCCAATACATTACTGTGCCCCGGCTGGTTTTGCGATTCTAAAGT
GTAAA---GATAAGAGTTTCAAT------GGAACAGGACCATGTAAAAAT----------------------
-GTCAGC---ACAGTACAATGTACACATGGAATTAGACCAGTAATATCAACT---
CAACTGCTGTTAAATGGCAGTCTAGCAGAAGGAGAG-----------
GTAATAATTAGATCTGCCAATTTCTCGGACAAT---------GCTAGAACCATAATAGTACAGCTGAAC----
-----------AAATCTATAGCA---------ATTAATTGTACAAGACCCAGCAACAATACAAGAAAA-----
-AGTATACATATA------------GGACCAGGGAGAGCA---TTC------TATGCAACAGGAGAA------
---------ATAACAGGAGATATA------------AGACGAGCATATTGT---AMCATTAGTAGAAAA---
GGATGGAATAACACTTTAAAACAGATAGCTGGAAAATTAAAA---------------GAACAATTTGGG----
------------------------------------AATAAAACAATAATCTTTAAT------
CAATCCTCAGGGGGGGATCCAGAAATTGTA---ATGCACAGTTTTAACTGTGGAGGGGAA---
TTTAAACAGCTCCAGGCAAGAGTCCTAGCTGTGGAAAGATACCTAAAGGATCAACAGCTCCTGGGGATTTGGG
GTTGCTCTGGAAAACTCATCTGCACCACTGCTGTGCCTTGGAATGCTAGTTGGAGT-----------------
----------------------------AATAAAACTCTGAGTGAGATTTGG------AAT------
AACATGACCTGGATGGAGTGG---GAAAGAGAAATTGACAATTACACAGGCTTAATATAC---
ACTTTAATTGAAAAAATCGCAGAACCAGCAGGAA---------------------------------------
----
AAGAATGAACAAGAATTATTGGAATTGGATAAATGGGCAAGTTTGTGGAATTGGTTTGACATAACAAGCTGGC
TATGGTATATAAAAATATTCATAATGATAGTAGGAGGCTTAGTAGGTTTAAGAATAGTTTGTGCT---
GTACTTTCCATAGTA---------AAAAGAGTTAGGCAGGGATACTCACCA------
TTGTCGTTTGCTGTTAGCTTGCTCAATACCACAGCCATAGCAGTAGCTGAG---
GGGACAGATAGGGTTATAGAAGTATTACAAAGAGCTTATAGAGCT---------------------------
--------ATTCTCCACATA------CCTACACGAATAAGACAGGGCTTGGAAAGGCTTTTGCTA---TAA
>KC595157
GCT---GCA---
GACAAATTGTGGGTCACAGTCTATTATGGGGTACCTGTGTGG---AAAGATGCAACC---
ACCACTCTATTTTGTGCATCAGATGCTAAAGCATATGATGCAGAG---GTACATAAT----------------
--------------GTTTGGGCCACA---CATGCCTGTGTACCCACAGAC---
CCCAACCCACAAGAAATAGTATTGGAA---------AATGTGACAGAAGAGTTTAAC----------------
--------------------------GCGTGGAAAAATAACATGGTAGAACAGATGCATGAGGATATA---
ATCAGTTTGTGGGATCAAAGCCTA---AAGCCATGTGTAAAATTA---
ACCCCACTCTGTGTCACTTTAAATTGCACTGATGCTCCTTTAAATGGCAATGATACTTCCCGC---------
----
----------------------------ACTGATAATCTTAAGATGGATGAAGGAGAAATAAAA---
AACTGCACTTTCAATGTCACCACAAACATGAGA---------------------------
GATAAGATGCAGAAAGAATATGCACTT---TTTTATAAACTTGATGTAGTACCAATAGACAATGATAAT----
----

AGTAGCCAGAGTAACTATAGTAGCTATAGGTTGATAAGTTGTAACACCTCAGTCATTAGACAG---
GCCTGTCCAAAGGTATCCTTCGAGCCAATTCCAATACATTATTGTGCCCCAGCTGGTTTTGCGATTCTGAAGT
GTAAC---GATAAGAAGTTCAAT------GGAACAGGAAAATGTACAAAT----------------------
-GTCAGC---ACAGTACAATGTACACATGGAATTAGGCCAGTAGTTTCAACT---
CAACTGCTGTTAAATGGCAGTCTAGCAGAAGAAGAG-----------
ATAGTAATTAGATCTGAAAATTTCACAGACACT---------GCTAAAACCATAATAGTGCAGCTGAAT----
-----------GAATCTATAGTA---------ATTAATTGTACAAGACCCAACAACAATACAAGAAAA-----
-GGTATACATATA------------GGACCAGGGAGCGCA---TTC------TATGCAACAGGAGCA------
---------ATAATAGGAGATATA------------AGACAAGCACATTGT---AACATTAGTGGAGGA---

AAATGGCATAATGCTTTAAAACAGATAGTTAAAAAATTAAAA---------------GAAAAATTTGGG----
--------------------------------AGTAATAAAACAATAGTTTTTAAT------
CAATCCTCAGGAGGGGACCCAGAAATTGTA---ATGCACAGTTTTAATTGTGGAGGGGAA---
TTCAAGCAGCTCCAGGCAAGAGTCCTGGCTGTGGAAAGATACCTAAGGGATCAACAGCTCCTGGGGATTTGGG
GTTGCTCTGGAAAACTCATCTGCACCACTTCTGTGCCTTGGAATACTAGTTGGAGT-----------------
----------------------------AATAAATCACTGGATAAAATTTGG------GAT------
AACATGACCTGGATGCAGTGG---GAAAAAGAAATTGACAATTACACAGGCATAATATAC---
TCCTTACTTGAAGAGTCGCAGAACCAACAAGAA--

AAGAATGAACTAGAGTTATTGGAATTGGATAAATGGGCAAGTCTATGGAATTGGTTTGACATAACAAAATGGC
TGTGGTATATAAGAATATTCATAATGGTAGTAGGAGGCTTAGTAGGTTTAAGAATAGTTTTTGCT---
GTATTTTCTGTAGTG---------AAAAGAGTTAGGCAGGGATACTCACCA------
TTGTCGTTTGCTGTTAACTTGCTCAACGCCGCGGCCATAGCAGTAGCTGAG---
GGAACAGATAGAATTGTAGAAGTAGTACAAAGACTTGGCAGAGCT----------------------------
--------ATTCTCCACATA------CCTACAAGAATAAGACAGGGCTTTGAAAGGGCTTTGCAA---TAG
>KC595160
GCT---ACA--
AAAGAATTGTGGGTCACAGTCTATTATGGGGTACCTGTGTGG---AAAGAAGCAACC---
ACCACTCTATTTTGTGCATCAGATGCTAAAGCATATGATACAGAG---GTACATAAT----------------
--------------GTTTGGGCCACA---CATGCCTGTGTACCCACAGAC---
CCCAACCCACAAGAAATAGAATTGAAA---------AATGTGACAGAAAATTTTAAC----------------
--------------------------ATGTGGAAAAATAACATGGTAGAACAGATGCATGAGGATATA---
ATCAGTTTATGGGATCAAAGCCTA---AAGCCATGTGTAAAATTA---
ACCCCACTCTGTGTTACTTTAAATTGCACTGATTTTAATACCACTAGTTGGAGGAATGCTACTAAT-------
----
-------------------ACCACTAGTAGTAGCGGAGAAAAGATGGAGAGAGGAGAAATAAAA---
AACTGCTCTTTCAATATCACCACAAACATAAGA---------------------------
GATAAGGTACAGAAAGAATATGCACTC---TTTTATAAACTTGATGTAGTACCAATAGGCAAT----------
----

GAGACTGGTAGCTATAGGTTGATAAGTTGTAACACCTCAGTCATTACACAA---
GCCTGTCCAAAAGTATCCTTTGATCCAATTCCCATACATTATTGTGCCCCGGCTGGTTTTGCGATTCTAAAGT
GTAAA---GATAAGAAGTTTAAT------GGAACAGGACCATGTACAATT-----------------------
-GTCAGC---ACAGTACAATGTACACATGGAATTAGGCCAGTAGTATCAACT---
CAACTGCTGTTAAATGGCAGTCTAGCAGAAGAAGAG------------
GTAGTAATTAGATCTGAAAATTTCACGGACAAT---------ACTAAAACCATAATAGTACAGCTGAAT----
-----------GAATCTGTAGTA---------ATTAATTGTACAAGACCCAACAACAATACAAGAAAA-----
-AGTATACATATA------------GGACCAGGAAGAGCA---TTT------TATACAACAGGAGAA------
---------ATAATAGGAGATATA------------AGACAAGCACATTGT---AACCTTAGTAGAGCA---
GAATGGAATAACACTTTACAACAGATAGTTATAAAATTAAGA---------------GAACAATTAGGG----
-----------------------------------AATAAAACAATAATCTTTAAT------
CACTCCTCAGGAGGGGACCCAGAAATTGTG---ATGCACAGTTTTAATTGTGGAGGGGAA---
TTCAAGCAGCTCCAGGCAAGAGTCCTGGCTGTGGAAAGATACCTAAAGGATCAACAGCTCCTGGGGATTTGGG
GTTGCTCTGGAAAACTCATTTGCACCACTACTGTGCCTTGGAATGATAGTTGGAGT-----------------
-------------------------AATAATAAATCTATGGATCAGATTTGG------AAT------
AACATGACCTGGATGGAGTGG---GAAAGAGAAATTGACAATTACACAAGCTTAATATAC---
AGCTTAATTGAAGAATCGCAGAACCAACAAGAA--

AAGAATGAACAAGAATTGTTGGAATTGGATAAATGGGCAAGTTTGTGGAATTGGTTTAGCATAACAAAATGGC
TGTGGTATATAAAAATATTCATAATGATAGTAGGAGGCTTGGTAGGTTTAAGAATAGTTTTTACT---
GTACTTTCTATAGTG---------AATAGAGTTAGGCAGGGATACTCACCA------
TTATCGTTTGCTGTTAGCTTGCTCAATGCCACAGCCATAGCAGTAGCTGAG---
GGGACAGATAGAGTTATAGAAGTATTACAAAGAGCTTGTAGAGCT----------------------------
--------ATTCTCCACATA------CCTACAAGAATAAGACAGGGCTTCGAAAGGGCTTTGGTA---TAA
>KC595161
GGT---GCA--
GAACAATTGTGGGTCACAGTCTATTATGGGGTACCTGTGTGG---AAAGAAGCAACC---
ACCACTCTATTTTGTGCGTCAGATGCTAAAGCATATGATACAGAG---GTACATAAT----------------
--------------GTTTGGGCCACA---CATGCCTGTGTACCCACAGAC---
CCCAACCCACAAGAAGTAGTATTGAAC---------AAGGTGACAGAAAATTTTAAC----------------

--------------------------ATGTGGAAAAATAACATGGTGGAACAGATGCATGAGGATATA---
ATCAGTTTATGGGATCAAAGCCTA---AAGCCATGTGTAAAATTA---
ACTCCACTCTGTGTTACTTTGAATTGCACTGACTTGAAAAATGATACTAACAGTGGTAATGCCACTAGTAATA
AT---
----------------------GCCACTGGTAGTAGCTGGATGATGGAGAAAGGAGAAATAAAA---
AACTGCTCTTTCAATATCACCACAAGCATAAGA--------------------------
GATAAGGTGCAGAAGGAATATGCACTT---TTTTATAAAGTTGATGTAGTACCAATAAAAGGTGAT-------
----

AATAATAGCCATGATAGTAGTAGCTATAGGTTGATAAGTTGTAACACCTCAGTCATTACACAG---
GCCTGTCCAAAAGTAACCTTTGATCCCATTCCCATACATTATTGTGCCCCGGCTGGCTTTGCGATTCTAAAGT
GTAAT---AATAAGACGTTCAAT------GGAACAGGACCATGTACAAAT----------------------
-GTCAGC---ACAGTACAATGTACACATGGAATTAGGCCAGTAGTATCAACT---
CAACTGCTGTTAAATGGCAGTCTAGCAGAAAAAGAG-----------
GTAGTAATTAGATCTGAAAATTTCACAGACAAT---------GCTAAAACCATAATAGTACAGCTGAAC----
-----------ACATCTGTAAAA---------ATTAATTGTACAAGACCCAACAACAATACAAGAAGA-----
-AGTATAAATATA------------GGACCAGGGAGAGCA---TTT------TATGCAACAGGAGCC------
---------ATAATAGGAAATATA------------AGACAAGCACATTGT---AACCTTAGTAGATCA---
GAATGGAATGAAACACTAAGACAAATAGCGGTCAAATTAAGA---------------GAACAATTCGGG----
-----------------------------------AATAAAACAATAGTCTTTAAT------
CAACCTGCAGGGGGGGACCCAGAAATAGTA---ATGCACATTTTTAATTGTAGAGGGGAA---
TTTAAGCAGCTCCAGGCAAGAGTCCTGGCTGTGGAAAGATACCTACAGGATCAACAGCTCCTGGGGATTTGGG
GTTGCTCTGGAAAAATCATTTGCACCACCGCTGTGCCTTGGAATGCTAGTTGGAGT-----------------
----------------------------AATAAATCTCTGAATAAGATTTGG------GAT------
AACATGACCTGGATGGAGTGG---GAAAGAGAAATTGACAATTACACAGGCTTAATATAC---
ACCTTACTTGAACAATCGCAGAACCAACAAGAA--

AAAAATGAACAAGAATTATTGGAGTTGGATAAATGGGCAAGTTTGTGGAATTGGTTTGACATAACAAGCTGGC
TGTGGTATATAAGAATATTCATAATGATAATAGGAGGCTTGGTAGGTTTAAGAATAGTTTTTACT---
GTACTTTCTATAGTG---------AATAGAGTTAGGCAGGGATACTCACCA------
TTATCGTTTGCTGTTAGCTTGCTCAACGCCACAGCCATAGCAGTAGCTGAG---
GGGACAGATAGGGTTATAGAAGTGTTACAAAGAGCTTTTAGAGCT---------------------------
--------ATTCTCAACATA------CCTACAAGAATTAGACAGGGAGCGGAAAGGGCTTTGCTA---TAA
>KC595162
GCT---GCA---
GAAAAGTTGTGGGTCACAGTCTATTATGGGGTACCTGTGTGG---AAAGAAGCAACC---
ACCACTCTATTTTGTGCATCAGATGCTAAAGCATATGGTACAGAG---GTACATAAT----------------
--------------GTTTGGGCCACA---CATGCCTGTGTACCCACAGAC---
CCCAACCCACAAGAAGTAGTATTGAAA---------AATGTGACAGAAAATTTTAAC----------------
--------------------------ATGTGGAAAAATAACATGGTAGAACAGATGCATGAGGATATA---
ATCAGTTTATGGGATCAAAGCCTA---AAGCCATGTGTAAAATTA---
ACCCCACTCTGTGTTACTTTAAATTGCACTGATTTGAAGAATGGTACTTATACCACTAGTACTAAT-------
----
-------------------ACCACTAGTAGTAGCGGGAGACCGATGGAGGAAGGAGAAATAAAA---
AATTGCTCTTTCAATATCACCACAAACATAAGA--------------------------
AATAAGGTGCAGAAAGAACATGCACTT---TTTTATAAACTTGATGTAGTACCAATAGACAAT---------
----

GATAATAGTAATAGTAGCTATAGGTTGCTAAGTTGTAACACCTCAGTCATTACACAG---
GCCTGTCCAAAAGTAACCTTTGAACCAATTCCCATACATTATTGTACCCCGGCTGGTTTTGCGATTCTAAAGT
GTAAA---GATAAGAAGTTCAAT------GGAACAGGACCATGTACAAAT----------------------
-GTCAGC---ACAGTACAATGTACCCATGGCATTAGGCCAGTAGTATCCACT---
CAACTGCTGTTAAATGGCAGTCTAGCAGAAGAAGAG-----------
GTAGTAATTAGATCTGAAAATTTCACGGACAAC---------GCTAAAACCATAATAGTACAGCTGAAC----
-----------GAAACTATAGAA---------ATTAATTGTACAAGACCTAACAACAATACAAGAAAA-----
-AGTATAAATATA------------GGACCAGGGAGAGCA---TTT------TATGCAACAGGAGAA------
---------ATAATAGGAAATATA------------AGACAAGCACATTGT---AACATTAGTAGAGCA---
AAATGGAATAACACTTTAAAACAGATAGTTACAAAATTAAGA---------------GAACAATTAGGG----
-----------------------------------AATAAAACAATAGTCTTTAAG------
CAATCCTCAGGAGGGGACCCAGAAATTGTA---ATGCACAGTTTTAATTGTGGAGGGGAA---

TTTAAGCAGCTCCAGGCAAGAGTCCTGGCTGTGGAAAGATACCTAAAGGATCAACAGCTCCTGGGGATTTGGG
GTTGCTCTGGAAAACTCATTTGCACCACTACTGTGCCTTGGAATGCTAGTTGGAGT-----------------
----------------------------AATAAATCTCTGGATACGATTTGG------GAT------
AACATGACCTGGATGGAGTGG---GAAAGAGAAATTAACAATTACACAAGCTTAATATAC---
ACCTTACTTGAAGAATCGCAGAACCAACAAGAA--

AAGAATGAACTAGAATTATTGGAATTGGATAAATGGGCAGGTTTGTGGAATTGGTTTGACATAACAAAATGGC
TGTGGTATATAAAAATATTCATAATGATAGTAGGAGGCTTGGTAGGTTTAAGAATAGTCTTTACT---
GTACTTTCTATAGTG---------AATAGAGTTAGGCAGGGATACTCACCA------
TTATCGTTTGCTGTTAGCTTGCTCAATGCCACAGCCATAGCAGTAGCTGAG---
GGGACAGATAGGGTTATAGAAGGATTACAAAGAGCTTTTAGAGCT----------------------------
--------ATTCTCCATATA------CCTGTAAGAATAAGACAGGGCTTGGAAAGGGCTTTGCTA---TAA
>KC595163
GTT---GCA--
GAACAATTGTGGGTCACAGTTTATTATGGGGTACCTGTGTGG---AAAGACGCAACC---
ACCACTCTATTCTGTGCGTCAAATGCTAAAGCATATGATACAGAG---GCACATAAT----------------
--------------GTTTGGGCCACA---CATGCCTGTGTACCCACAGAC---
CCCACTCCACAAGAAATAGTGTTGGAA---------AATGTGACAGAAAATTTTAAC----------------
--------------------------ATGTGGAAAAATAATATGGTAGAACAGATGCATGAGGATATA---
ATTAGTTTATGGGATCAAAGCCTA---AAGCCATGTGTAAAATTA---
ACCCCACTCTGTGTTACTTTAATTTGCACTGATTGGGATGGAACTTGTAAAAATGGTACTAATAGTAATAATG
GAACTTGTGAAAATGCTACTAATAGTAATAGTACTACT---------------
AACCATACTAACCATACTACTACTAACCATACTACGGGTAACCATACTGCGGGTGTGGGAAAGATAGAGAAAG
GAGAAATGAAA---AACTGCTCTTTCAATATCACCACAAACATGAGA--------------------------
-GATAAGATGCAGAAAGAATATGCAATG---TTTTATAAGCTTGATTTAGAACTAATAGATAAT---------
----

GATACTGCTAGCTATAGGTTGATAAGTTGCAACACCTCAGTCCTTACACAA---
GCCTGTCCAAAGGTATCCTTTGAACCAATTCCCATACATTTTTGTGCCCCGGCTGGTTTTGCGATTCTAAAGT
GTAAA---GATAAGAAGTTCAAT------GGAACAGGACCATGTACAAAT-----------------------
-GTCAGC---ACAGTACAATGTACACATGGAATTAGGCCAGTAGTATCAACT---
CAACTGCTGTTAAATGGCAGTCTAGCAGAAGAAGAC------------
GTAGTAATTAGGTCTGAAAATTTCACGGACAAT---------GCTAAAACCATAATAGTACAGCTGAAA----
-----------GAAGCTGTAGAA---------ATTAATTGTACAAGACCCAGCAACAATACAAGAAGA-----
-AGTATACATATG------------GGACCAGGAAGAGCA---TTT------TATACAACAGGAGAC------
---------ATAATAGGAGATATA------------AGACAAGCACATTGT---AACCTCAGTAGAGCA---
AAATGGGATAAAACTTTAAGACAGGTAGCTCACAAATTAACA---------------GAACAATTC-------
-----------------------------------AATAAAACAATAGTCTTTAAC------
CACTCCTCAGGAGGGGACCCAGAAATTGTA---AAGCACATGTTTAATTGCGGAGGGGAA---
TTTAAGCAGCTCCAGGCAAGAGTCCTGGCTGTGGAAAGATACCTAAGAGATCAACAGCTCCTGGGGATCTGGG
GTTGCTCTGGAAAACTCATTTGCACCACTGCTGTGCCTTGGAATGCTAGTTGGAGT-----------------
----------------------------AATAAATCTCTGGATAAAATTTGG------GAC------
AACATGACCTGGCAGGAGTGG---GAAAAAGAGATTGACAATTACACAGATTTAATATAC---
ACCTTACTTGAAGAATCGCAGAACCAACAAGAA--

AAGAATGAAAAAGATTTATTGGAATTCGATAAATGGGCAAGTTTGTGGAATTGGTTTGACATATCAAAATGGC
TGTGGTATATAAAAATATTCATAATGATAGTAGGAGGCTTGATAGGTTTAAGAATAGTTTTTACT---
GTACTTTCTATAGTA---------AATAGAATTAGGCAGGGATACTCACCA------
TTATCGTTGGCTGTTAGCTTGCTTAATGCTACAGCCATAGCAGTAGCTGAG---
GGAACAGACAGGATTATAGAAATATTGCAAAGAGCTTATAGAGCT----------------------------
--------ATTCTCCACATA------CCTACAAGAATAAGACAGGGAGCGGAAAGGGCTTTGCTG---TAA
>KC595165
GCT---GCA--
AAAAATTTGTGGGTCACAGTCTATTATGGGGTACCTGTGTGG---AAAGAAGCAAAC---
ACCACTCTATTTTGTGCATCAGATGCTAAAGCATATGATACAGAG---GTACATAAT----------------
--------------GTTTGGGCTACA---CATGCCTGTGTACCCACAGAC---
CCCAACCCACAAGAAATAGCATTGGAA---------AATGTGACAGAAAATTTTAAC----------------
--------------------------ATGTGGAAAAATGACATGGTAGAACAAATGCATGAGGATATA---
ATTAATTTATGGGATCAAAGCCTA---AAGCCATGTGTAAAATTA---
ACCCCACTCTGTGTTACTCTAAATTGCACTGATTGGAATAATACTAATAGTGGTAACACTACTACT-------

----
----------------------AATAGTAGTAACTGGGGAAAGATAGAGACAGGAGAAATAAAA---
AATTGCTCTTTCAATATCACCACAAGCCTGAGA---------------------------
GATAAGATGCAGAAAGAATATGCACTT---TTTTATAAACTTGATGTAACACCAATAGAAGCG----------
----

AATAGTACTAGCTATAGGTTGATAAGTTGTAACACCTCAGTCATTACACAG---
GCCTGTCCAAAGGTATCCTTTGAGCCAATTCCCATACATTATTGTGCCCCGGCTGGTTTTGCGATTCTAAAGT
GTAAA---GATAAGAAGTTTAAT------GGAACAGGACCATGTACAAAT-----------------------
-GTCAGC---ACAGTACAATGTACACATGGAATTAAGCCAGTAGTATCAACT---
CAACTGCTGTTAAATGGTAGTCTAGCAGAAGAAGAG------------
GTAGTAATTAGGTCTGAAAATTTCACAAACAAT---------GCTAAAACCATAATAGTACAGCTAAAA----
-----------GACCCTGTACAA---------ATTAATTGTACAAGACCCGGCAACAATACAAGGAAA-----
-AGTATACATATG------------GGACCAGGGAGAGCA---TGG------TATGCAACAGGAGAC------
---------ATAATAGGAGATATA------------AGACAAGCACATTGT---AACCTCAGTAGAGCA---
AAATGGAATGACACTCTAACACAGGTAGTTGAAAAATTAAGA---------------AAACAATTTGGG----
--------------------------------GAGAATAAAACAATAGCCTTTAAT------
CAATCCTCAGGAGGAGACCCAGAAATTGTA---CTGCACAGTTTTATTTGTGGAGGGGAA---
TTTAAGCAGCTCCAGGCAAGAGTCCTGGCTGTGGAAAGATACCTAAGGGATCAACAGCTCCTGGGGATTTGGG
GTTGCTCTGGAAAACTCATTTGCACCACTGCTGTGCCTTGGAATACTAGTTGGAGT-----------------
----------------------------AATAAATCTCTGGATGAGATTTGG------GAT------
AACATGACCTGGATGGAGTGG---GAAAGAGAAATTGACAATTACACAGGCTTAATATAT---
AACTTACTTGAAAAATCGCAAAACCAACAAGAA--

AAGAATGAACAAGAATTATTAGAATTAGATAAGTGGGCAAGTTTGTGGAATTGGTTTGACATAACAAAATGGC
TGTGGTATATAAAAATATTCATAATGATAGTAGGAGGCTTGGTAGGGTTAAGAATAGTTTTTGCT---
GTACTTTCTATAGTG---------AATAGAGTTAGGCAGGGATACTCACCA------
TTATCGTTTGCTGTCAGCTTGCTTAATGCCACAGCTATAGCAGTAGCTGAG---
GGAACTGATAGGGTTATAGAAGTATTACAAAGAGCTTGTAGAGCT----------------------------
--------ATTATCCATATA------CCTAGAAGAATAAGACAGGGAGCGGAAAGGGCTTTGGTA---TAA
>KC595197
GCT---ACA--
GAAAAACTGTGGGTCACAGTCTATTATGGGGTACCTGTGTGG---AAAGAAGCAACC---
ACCACTCTATTTTGTGCATCAGATGCTAAAGCATATGATAAGGAG---GTACATAAT----------------
--------------GTTTGGGCCACA---CATGCCTGTGTACCCACAGAC---
CCCAACCCACAAGAAGTAGTCTTGGAA---------AATGTGACAGAAGACTTTAAC----------------
--------------------------GTGTGGAAAAATGACATGGTAGAACAAATGCATGAGGATATA---
ATCAGTTTATGGGATCAAAGCTTA---AAACCATGTGTAAAATTA---
ACCCCACTCTGTGTTACTTTAAATTGCACTGATTACTATAACAATGCTACTAATGCAAATAGTAGTAGT----
----
---GTAGAGAAAGGAGAAATACAA---
AACTGCTCTTTCAATATCACCACAAACATAAAA---------------------------
GATAAAGTACAGAAAACTTATGCACTT---TTTTATAAACTTGATGTAGTACCAATAGATAATGCC-------
----

AGCAGCTATAGGTTGATAAGTTGTAATACCTCAGTCCTTACACAG---
GCCTGTCCCAAAGTATCCTTTGAGCCAATTCCCATACATTTTTGTGCCCCGGCTGGTTTTGCGATTCTAAAGT
GTAGA---GATACGAAGTTCAAT------GGAAAAGGAACATGTAAAAAT-----------------------
-GTCAGC---ACAGTACAATGTACACATGGAATTAGGCCAGTAGTATCAACT---
CAACTACTGTTAAATGGCAGTTTGGCAGAAGAAGAG------------
GTAGTAATTAGATCTGACAATTTCACAAATAAT---------GCTAGGACCATAATAGTACAGCTGGAA----
-----------CAATCTGTAAAA---------ATTAATTGTACAAGACCCAACAACAATACAAGAAAA-----
-AGCATACATATA------------GGACCAGGGAAAGCA---TTC------TATGCAGCAAGAGAA------
---------ATAATAGGAGATATA------------AGGCAAGCATTTTGT---ACCATTAATAGAACA---
GAATGGAATAACACTTTAGGACAGATAGTTAAAAAATTAAGA---------------GAACAATTTGGG----
-----------------------------------AATAAAACAATAATCTTTAAC------
TCATCCTCAGGAGGGGACCCAGAAATTGTA---ATGCACAGTTTTAATTGTGGAGGGGAA---
TTTAAGCAGCTCCAGGCAAGAGTCCTGGCTGTGGAAAGATACCTACAGGATCAACAGCGCCTGGGAATTTGGG
GTTGCTCTGGAAAACTCATTTGCACCACTGCTGTGCCTTGGAATACTAGTTGGAGT-----------------
----------------------------AATAAATCTCTGAAGGCTATTTGG------GAC------

AATATGACCTGGATGCAGTGG---GAAAAGGAAATTAGCAATTACACAGGCTTAATATAC---
ACCTTAATTGAAGAATCGCAGAACCAGCAAGAA--

AAGAATGAACAGGAATTATTGGAATTGGACAAATGGGAAAGTTTGTGGAATTGGTTTAACATAACAAATTGGC
TGTGGTATATAAAAATATTTATAATGATAGTAGGAGGCTTAGTAGGTTTAAGAATAATTTTGGCT---
GTACTTTCTATAGTG---------AATAGAGTTAGGAAGGGATACTCACCA------
TTATCGTTTGCTGTTAGCTCGTTTGACGCTATAGCCATAGCAGTAGCTGGG---
GGGACAGATAGGATTATAGAAGGGTTACAAAGAATTTGTAGAGCT----------------------------
--------CTTCTCCACATA------CCTGTAAGAATAAGACAGGGCTTGGAAAGAGCTTTGCTA---TAA
>KC595202
GCT---ACA--
GAAAAATTGTGGGTCACAGTCTATTATGGGGTACCTGTGTGG---AAAGAAGCAACC---
ACCACTCTATTTTGTGCATCAGATGCTAAAGCATATGATACAGAG---GTACATAAT----------------
--------------GTTTGGGCCACA---CATGCCTGTGTACCCACAGAC---
CCCAGCCCACAAGAAGTAGCATTGGAA---------AATGTGACAGAAAATTTTAAC----------------
--------------------------ATGTGGAAAAATAACATGGTAGATCAAATGCAGGAGGATATA---
ATCAGTTTATGGGATCAAAGCCTA---AAGCCATGTGCAAAATTA---
ACCCCACTCTGTGTTACTTTAACTTGCACTGATTTGAATTCTACTAATACCAATAGT----------------
----
----------------------------AGTAATGGGAAAACGATGGACAGAGGAGAAATGAAA---
AACTGCTCTTTCAATATCACCACAAGTATAAGA---------------------------
AATAAGGTGACGAAAGAACATGCACTT---TTTTATAAACTTGATGTAGTACCAATAAAGGAT----------
----

GGTACTAGTAGCTATAGGTTGATAAACTGTAACACCTCAGTCATTACACAG---
GCCTGTCCAAAAGTAGCCTTTGAACCAATTCCCATACATTACTGTGCCCCGGCTGGTTTTGCGATTCTAAAGT
GTAGA---GATAAAAAGTTCAAT------GGAACAGGACCATGTACAAAT-----------------------
-GTCAGC---ACAGTACAATGTACACATGGAATTAGGCCAGTAGTATCAACC---
CAACTGCTGTTAAATGGCAGTCTAGCAGAGGAAGAG------------
GTAGTAATTAGATCTGTCAATTTCACGGACAAT---------GCTAAAACTATAATAGTACAGTTGAAT----
-----------GAATCTGTAGCA---------ATTAATTGTACAAGACCCAACAACAATACAAGAAAA-----
-AGTATACCTATA------------GGACCAGGAAGAACA---TTT------TATGCAACAGGAGAC------
---------ATAATAGGAGATATA------------AGACAAGCACATTGT---AATCTTAGTGGAGAA---
CAATGGAGTAACACATTAAGACAGATAGTTATAAAATTAAGA---------------GAACAATTTGGG----
--------------------------------AATAATAAAACAATAGTCTTTAAT------
CAATCCTCAGGAGGGGACCCAGAAATTGTA---ACACACAGTTTTAATTGTGGAGGGGAA---
TTTAAGCAGCTCCAGGCAAGAGTCCTGGCTGTGGAAAGATACCTAAAGGATCAACAGCTCCTGGGAATTTGGG
GTTGCTCTGGAAAACTCATTTGCACCACTGCTGTGCCTTGGAATTCTACTTGGAGT-----------------
----------------------------AATAAATCTCTGGATAGAATTTGG------GGT------
AATATGACCTGGATGGAGTGG---GAAAGAGAAATTGACAATTACACAGGCTTAATATAC---
ACCTTAATTGAAGAATCGCAGAACCAACAAGAA--

AAGAATGAACAAGAATTATTGGCATTGGATGAATGGGCAAATTTGTGGAATTGGTTTGACATAACAAAATGGC
TGTGGTATATAAAAATATTCATAATGGTAGTAGGAGGCTTGGTAGGTTTAAAAATAGTTTTTGCC---
GTATTTTCTATAGTG---------AATAGAGTTAGGCAGGGATACTCACCA------
TTATCGTTTGCTGTTAGCTTGTTCAATGCCATAGCCATAGCAGTAGCTGAG---
GGGACAGGTAGGGTTATAGAAGTATTACAAAGAGCTTATAGAGCT----------------------------
--------ATTCTCCACATA------CCTAGAAGAATAAGACAGGGCTTGGAAAGGGCTTTGCTA---TAA
>KC595203
GCT---ACA--
GAAAAATTGTGGGTCACAGTCTATTATGGGGTACCTGTGTGG---AAGGAAGCAACC---
ACCACTCTATTTTGTGCATCAGATGCCAAAGCATATGATACAGAG---GTACATAAT----------------
--------------GTTTGGGCCACA---CATGCCTGTGTACCCACAGAC---
CCCAACCCACAAGAAATATCGTTGGAA---------AATGTGACAGAAAATTTTAAC----------------
--------------------------ATGTGGAAGAATAACATGGTAGAACAGATGCATGAGGATATA---
ATCAGTTTATGGGATCAAAGCCTA---AAGCCATGTGTAAAATTA---
ACCCCACTCTGTGTTACTTTAAATTGCACTGATGTGTGGAATAATACTACCAATGGGACTAATATTGCCGAA-
----
-------GGGAATAATGCTATCAAGAATAGTAGCGAGGAAATGATGGAAAAAGGAGAAATAAAA---
AACTGCTCTTTCAATATCACCACAAGCATAAGA---------------------------

GATAAGGTGCAGAGAGAATATGCAACT---TTTTATAAACTTGATGTAGTACCAATAAAAAATGAT-------
----

AATAGTGCTGATAGCAGCTATAGGTTAATAAGTTGTAACACCTCAGTCATTACACAA---
GCCTGTCCAAAAGTAACCTTTGAGCCAATTCCCATACATTACTGTGCCCCGGCTGGTTTTGCGATTCTAAAGT
GTAAT---GAGAAGAAGTTTAAT------GGATCAGGACCATGTACAAAT----------------------
-GTCAGC---ACAGTACAATGTACACATGGAATTAAGCCAGTAGTATCAACT---
CAACTGCTGTTAAATGGCAGTCTAGCAGAAGAAGAG-----------
GTAGTAATTAGATCTCAGAATTTCACGGACAAT---------GCTAAAATCATAATAGTACAGCTGAAG----
-----------GAATCCATAAAA---------ATTAACTGTACAAGACCCAACAACAATACAAGAAAA-----
-GGTATACATATG------------GGACCAGGGAAAGTA---TTC------TATACAACAGGGGAA------
---------ATAATAGGAGATATA------------AGACAAGCACATTGT---AACGTTAGTAAAGCA---
AAATGGAATAACGCTTTAAAACAGATAGCTATAAAATTAAGA------------GAAATTGAACAATTTGAG-
-----------------------------------AATAAAACAATAGTCTTTAAT------
CAATCCTCAGGAGGAGACCCAGAAATTGTA---ACGCATAGCTTTAATTGTAGAGGGGAA---
TTTAAGCAGCTCCAGGCAAGAGTCCTGGCTCTAGAAAGATACCTAAAGGATCAACAACTCCTGGGGATTTGGG
GCTGCTCTGGAAAACTCATTTGCACCACTAATGTGCCTTGGAATACTAGTTGGAGT-----------------
----------------------------AATAAATCTCAAAGTGAGATTTGG------GAC------
AACATGACCTGGATGCAGTGG---GATAAAGAAATTGGCAATTACACACACTTAATATAT---
ACCTTAATTGAAGCCTCACAGAGACAACAAGAG---

AAGAATGAGTTGGACTTACTAAAATTGGATACATGGGACAGTTTGTGGAATTGGTTTGACATATCAAAGTGGC
TGTGGTATATAAAAATATTCATAATGATAGTAGGAGGCTTAATAGGTTTAAGAATAGTTTTTGCT---
GTACTTTCTATAGTG---------AATAGAGTTAGGCAGGGATACTCACCA------
TTATCGTTTGCTGTTAGCTTGCTCAACACTACAGCCATAGTAGTAGCTGAA---
GGGACAGATAGGGTTATAGAAATATTACAAAGAGCTTATAGGGCT----------------------------
--------ATCCTTCACATA------CCTGTAAGAATAAGACAGGGCTTTGAAAGGGCTTTGCTA---TAA
>KC595158
GCT---GCA--
GAAAAATTGTGGGTCACAGTCTATTATGGGGTACCTGTGTGG---AAAGAAGCAACC---
ACCACTCTATTTTGTGCATCAGATGCCAAAGCATATGATACAGAG---GTACATAAT----------------
--------------GTCTGGGCCACA---CATGCCTGTGTACCCACAGAC---
CCCAACCCACAAGAAGTAGTATTGGGA---------AATGTGACAGAATATTTTAAC---------------
--------------------------ATGTGGAAAAATAACATGGTAGATCAGATGCATGAGGATATA---
ATCAGTTTATGGGATCAAAGCCTA---AAGCCATGTGTAAAATTA---
ACCCCACTCTGTGTTACTTTAAATTGCACTGATTTGGAGAATAATAATACTACCACTAAT------------
----
----------------------------AGTAGCTGGGAGAAGATGGAGAAAGGAGAAATGAAA---
AACTGCTCTTTTAATATCACCACAAATATGAGA--------------------------
GATAAGATGCAGAAAGAATATGCACTT---
TTTTATAAGCTTGATGTAGTACAAATAGACACTGAGAATCGTAGC---------------------------
----
TCTAATGGGAACAGTAGCTATAGGTTGATAAGTTGTAACACCTCAGTCATTACACAG---
GCCTGCCCAAAAGTATCCTTTGATCCAATTCCCATACATTATTGTGCCCCGGCTGGTTTTGCGATTCTAAAGT
GTAAT---GATAAGGAGTTCAAT------GGAAGTGAATCATGTACAAAT----------------------
-GTCAGT---ACAGTACAATGTACACATGGAATTAGGCCAGTAGTATCAACT---
CAACTGCTGCTGAATGGCAGTCTAGCAGAAAAGGGG-----------
GTAGTAATTAGATCTGAAAATTTCACGAACAAT---------GCTAAAACCCTAATAGTACAGCTGAAC----
-----------AAATCTATAGCA---------ATCAATTGTACAAGACCCAACAACAATACAAGAAGA-----
-GGTATACATATA------------GGACCAGGGAGAGCA---TTT------TATGCAACA---GAT------
---------ATAATAGGAGATATA------------AGACAAGCACATTGT---AACCTTAGTAGAACA---
GACTGGAATGACACTTTAAAACAGATAGTGGAAAAATTAAGA---------------GAACACTTCAAG----
-----------------------------------AATAAAACAATAGTCTTTAAT------
CAATCCTCAGGAGGGGACCCAGAAATTGTA---ATGCACAGTTTTACTTGTGGAGGGGAA---
TTTAAGCAGCTCCAGGCAAGAGTCCTGGCTGTGGAAAGATACCTAAGGGATCAACAGCTCATGGGAATTTGGG
GTTGCTCTGGAAAACTCATTTGCACCACTACTGTGCCTTGGAATACTAGTTGGAGT-----------------
----------------------------AATAAATCTCAGGATGAAATTTGG------GGT------
AACATGACCTGGATGCAGTGG---GAAAAAGAAATTGACAATTACACAGGCTTAATATAC---
ACCTTACTTGAAGAATCGCAGAACCAGCAAGAA---

AAGAATGAACAAGAATTATTAGAATTGGATAAATGGGCAAGCTTGTGGAATTGGTTTACCATAACAAATTGGC
TGTGGTATATAAAAATATTCATAATGATAGTAGGAGGCTTAGTAGGGTTAAGAATAGTTTTTGCT---
GTACTTTCTATAGTG---------AATAGAGTTAGGCAGGGATACTCACCA------
TTATCGTTGGCTGTTAGCTTGCTCAACGCCACAGCCATAGCAGTAGCTGAA---
GGGACAGATAGGGTTTTAGAAGCATTACAAAGGGCTGTTAGAGCT----------------------------
--------ATTCTCCACATA------CCTAGAAGAATAAGACAGGGCGCGGAAAGAGCTTTAGTA---TAA
>KC595168
GCT---GCA--
GAAAAATTGTGGGTCACAGTCTATTATGGGGTACCTGTGTGG---AAAGAAGCAACC---
ACCACTCTATTTTGTGCATCAGATGCTAAAGCATATGATACAGAG---GTACATAAT----------------
--------------GTTTGGGCCACA---CATGCCTGTGTACCCACAGAC---
CCCAACCCACAAGAAGTGGTATTGGGA---------AATGTGACAGAAGACTTCAAC----------------
--------------------------ATTTGGAAAAATAACATGGTAGAACAAATGCATGAGGATATA---
ATCAGTTTATGGGATCAAAGCCTA---AAGCCATGTGTAAAATTA---
ACCCCACTCTGTGTTACCTTAAATTGCACTAATGCTCGGAGGAATGCTACTAATACCACTGATGCTCGGAGC-
----
-------AATGCTACTAATGCCAATAATAGTAGCAGGGGAATGATGGAGGAAGGAGAAATAAAA---
AACTGCTCTTTCAATATCACCACAAGCATAAGA---------------------------
GATAGGATGCAGAAAGAATATGCACTT---TTTTATAAGCTTGATGTAGTACCAATAGAGAATGAG-------
----

AACAGTAGCAGCTATAGGTTGATAAGTTGTAATACCTCAGTCATTACACAG---
GCCTGTCCAAAAGTAACCTTTGAGCCAATTCCCATACATTATTGTGCCCCAGCTGGTTTTGCGATTCTAAAGT
GTAAC---GATAAGAAGTTTGAG------GGAAAAGGACCATGTACAAAT-----------------------
-GTCAGC---ACAGTACAATGTACACATGGAATTAGGCCAGTAGTATCAACT---
CAACTACTGTTAAATGGCAGTCTAGCAGAGGAAGAG------------
GTAGTAATTAGATCTGACAATATCACGGCCAAT---------ACTAAGACCATAATAGTACAGCTGAAT----
-----------GAATCTATAGAA---------ATTAATTGTACAAGACCCAACAACAATACAAGAAAA-----
-AGTATACCTATA------------GGACCAGGGAGAGCA---TTT------TATGCAACAGGAGAC------
---------ATAATAGGAGATATA------------AGACAAGCACATTGT---AACCTTAGCAGAGCA---
CAATGGAATAACACTTTAAGACAGATAGTCCTAAAATTAAGA---------------GAACACTTTGAG----
-----------------------------------AATAAAACAATAGTCTTTAAT------
TCATCCTCAGGAGGGGACCCAGAAATTGTA---ATGCACAGTTTTAATTGTGGAGGGGAA---
TTTAAGCAGCTCCAGGCAAGAGTCCTGGCTGTGGAAAGATACCTAAAGGATCAACAGCTCCTGGGGATTTGGG
GTTGCTCTGGAAAACTTATTTGCACCACTGCTGTGCCTTGGAATGCTAGTTGGAGT-----------------
----------------------------AATAAATCTCTGAATCAGATTTGG------GAT------
AACATGACCTGGATGCAGTGG---GAAACAGAAATTGACAATTACACAAGCATAATATAC---
ACCTTAATTGAAGAATCGCAGAACCAACAAGAA--

AAGAATGAACAAGATTTATTGGAATTGGATAAATGGGCAAGTTTGTGGAATTGGTTTACCATAACAAACTGGC
TGTGGTATATAAAAATATTCATAATGATAGTAGGAGGCTTGATAGGTTTAAGAATAGTTTTTGCT---
GTACTTTCGATAGTG---------AATAGAGTTAGGCAGGGATACTCACCA------
TTGTCGTTTGCTGTTAGCTTGATCAATGCCATAGCCATAGCAGTAGCTGAG---
GGGACAGATAGGATTATAGAAATATTACAAAGAGCTTGGAGAGCT----------------------------
--------GTTCTCCACATA------CCTAGAAGAATAAGACAGGGCTTAGAAAGGGCTTTGCTA---TAA
>KC595190
GCT---GTA--
GAACAATCGTGGGTCACAGTCTATTATGGGGTACCTGTGTGG---AAAGAAGCAACC---
ACCACTCTATTTTGTGCATCAGATGCTAAAGCATATGATACAGAG---GTACATAAT----------------
--------------GTTTGGGCCACA---CATGCCTGTGTACCCACAGAC---
CCCAACCCACAAGAAATAGTATTGGAA---------AATGTGACAGAAAAATTTAAC----------------
--------------------------ATGTGGAAAAATAACATGGTAGAACAGATGCATGAGGATATA---
ATCAGTTTATGGGAGCAAAGCCTA---AAGCCATGTGTAAAACTA---
ACCCCACTCTGTGTCACTTTAAATTGCACTGATGAGTTGAGGAATAGGAATACTACTAACACTAAT-------
----
-------------------------------------AGTAGTTGGGAGAGAGGAGAAATAAAA---
AACTGCTCTTTCAATATCACCACAAGCATAAGA---------------------------GGTAGGGTG---
CAAGGATCTGCACTC---TTTTATAAACTTGATGTAGTACCAATAGATAAT----------------------
----
----ACTAATAATAATACTAGATATAGGTTGATAAATTGTAACACCTCAGTCATTACACAG---

GCCTGTCCAAAAGTATCCTTTGAGCCAATTCCCATACATTATTGTGCCCCGGCTGGTTTTGCGATTCTAAAGT
GTAAA---GATAAGAAGTTCAAT------GGAACAGGACCATGTAACAAT-----------------------
-GTCAGC---ACAGTACAATGTACACATGGAATTAGGCCAGTAGTGTCAACT---
CAACTGCTGTTAAATGGCAGTCTAGCAGAAGAAGAG------------
GTAGTAATTAGATCTGAGAATTTCACGAACAAT---------GCTAAAATCATAATAGTACAGCTAAAT----
-----------GAATCTGTAGAA---------ATTAATTGTACAAGACCCAACAACAATACAAGAAAA-----
-AGTATACATATA------------GGACCAGGGAAAGCA---TTT------TATGCAACAGGAGAA------
---------ATAATAGGAGATATA------------AGACAAGCATATTGT---AACCTTAGTAGAGTA---
AAATGGAATAACACTTTAAGACAGATAGTTAAAAAATTAAGA---------------GAACAATTTGAG----
-----------------------------------AACAAAACAATAGTCTTTAAT------
CAATCTTCAGGAGGGGATCCAGAAATTGTA---ATGCACAATTTTAATTGTGGAGGGGAA---
TTTAAGCAGCTCCAGGCAAGAGTCCTGGCTGTGGAAAGATACCTAAAGGATCAACAGCTCCTGGGGATTTGGG
GTTGCTCTGGAAAACTCATTTGCACCACTGCTGTGCCTTGGAATACTAGTTGGAGT-----------------
----------------------------AATAAATCTCTGAAGTATATTTGG------GAA------
AATATGACCTGGATGGAGTGG---GAAAGAGAAATTGATAATTACACAAACATAATATAC---
ACCTTAATTGAAGAATCGCAGACCCAACAAGAA--

AAGAATGAAAAAGAATTGTTGGAATTAGATAAGTGGGCAAGTTTGTGGAATTGGTTTACTATATCAAACTGGC
TGTGGTATATAAAAATATTCATAATGATAGTAGGGGGCTTGGTAGGTTTAAGAATAGTTTTTACT---
GTACTTTCTATAGTG---------AATAGAGTTAGGCCGGGATACTCACCA------
TTGTCATTTGCTGTTAGCTTGCTCAATTCCACAGCCATAGCAGTAGCTGAG---
GGGACAGATAGGGTTATAGAAGTATTACAAAGAGCTGGTAGAGCT----------------------------
--------ATTCTCCACATA------CCTAGAAGAATAAGACAGGGCTTGGAAAGGGCTTTGCAA---TAA
>KT200351
GCT---GAA--
GACAAATTGTGGGTCACAGTCTATTATGGAGTACCTGTGTGG---AGAGAAGCAAAC---
ACCACTCTATTTTGTGCATCAGATGCTAAAGCTTATGATACAGAG---GTACATAAT----------------
--------------GTTTGGCCACA---CATGCCTGTGTACCCACAGAT---
CCCAACCCACAAGAAGTAGTATTGGCA---------AATGTGACAGAGAACTTTAAC----------------
--------------------------ATGTGGAAAAATAACATGGTAGAACAGATGCAGGAGGATATA---
ATCAGTCTATGGGATCAAAGCTTA---AAGCCATGTGTAAAATTA---
ACCCCACTCTGTGTTACTTTAAATTGCACTGATAAATGGGGAAATGAAACTAAATGGGTAAATGAAACTATAT
AAAGAAATAGA--
----------------------------ACTATTAACAATAGTAGTATGAAAGGAGAAATAAAA---
AACTGTTCTTTCAATATCACCACAAATTTAAGG---------------------------
GACAAGATGCAGAAAGAATATGCACTT---TTTTATAAACTTGATGTAGTACCAATAGATAATAAT-------
----

AGTAATACTAGCTATAGGTTGATAAGTTGTAACACCTCAGTCATTACACAG---
GCCTGTCCAAAGGTAACCTTTGAGCCAATTCCCATACATTATTGTGCCCCGGCTGGTTTTGCGATTATAAAGT
GTAAT---AATAAGACATTCAAT------GGAACAGGACCATGTAAAAAT-----------------------
-GTCAGC---ACAGTACAATGTACACATGGAATTAGACCAGTAGTGTCAACT---
CAACTGCTACTAAATGGCAGTCTAGCAGAAGAAGAG------------
GTAGTAATTAGGTCTGAAAATTTCACGAACAAT---------GCAAAAACCATAATAGTGCAACTAAAG----
-----------GAACCTGTAGAA---------ATTAATTGTACAAGACCTAACAACAATACAAGAAAA-----
-GGTATACATATA------------GGACCAGGGAAAACA---TGG------TATGCAACAGGAGAA------
---------ATAATAGGAGATATA------------AGACAAGCACATTGT---AACCTTACTATAGAA---
AAGTGGAATAACACTTTACATCAGATAGTTAGAAAATTAAGA---------------GAACAATTTGGG----
-----------------------------------AATAAAACAATAATCTTTCAG---
AATTCACCCTCAGGAGGAGACCCAGAAGTTGTA---ATGCATAGTTTTAATTGTGCAGGGGAA---
TTTAAGCAGCTCCAGGCAAGAGTCCTGGCTGTAGAAAGATACCTAAGGGATCAACAGCTCCTGGGGATTTGGG
GTTGCTCTGGAAAACTCATTTGCACCACTACTGTGCCTTGGAATACTAGTTGGAGT-----------------
----------------------------AATAAAAGTCTGAGTGAGATTTGG------GAT------
AACATGACCTGGATGGAATGG---GAGAGAGAAATTGACAACTACACAGACATAATATAC---
ACCCTACTTGAACAATCGCAAAACCAACAAGAA--

AAGAATGAACAAGAATTATTGGCATTAGATAAGTGGGCAAGTTTGTGGAATTGGTTTGACATATCAAATTGGC
TGTGGTATATAAAAATATTCATAATGATAGTAGGAGGCTTGATAGGTTTAAGAATAGTTTTTACT---
GTACTTTCTTTAGTG---------AATAGAGTTAGGCAGGGATACTCACCA------
TTGTCATTTGCTGTTAACTTGCTTAATACAACTGCTATAGCAGTAGCTGAA---

GGGACAGATAGAATTTTAGAATTATTACAAAGAGCTTTTAGAGCT----------------------------
--------GTTCTTCACATA------CCCAGAAGAATAAGACAGGGCTTTGAAAGGAGCTTGCTA---TAA
>EU786676
GCT---GCA--
GAAGACTTGTGGGTCACAGTCTACTATGGGGTACCTGTGTGG---AAAGAAGCAACC---
ACCACTCTATTTTGTGCATCAGATGCTAAATCATATGATACAGAG---GTACATAAT----------------
--------------GTTTGGGCCACA---CATGCCTGTGTACCCACAGAC---
CCCAACCCACAAGAAGTAGTAATGGAA---------AATGTGACAGAAGAATTTAAC----------------
-------------------------ATGTGGAAAAATAACATGGTAGAACAGATGCATGAGGATATA---
ATCAGTTTATGGGATCAAAGCCTG---AAGCCATGTGTAAAATTA---
ACCCCACTCTGTGTTACTTTAAATTGCACTGATCAGTTGAATGCTAATTATACT-------------------
----
-------------------------------AATAATAATAGCTGGAAAATGGGAGAAATAAAA---
AATTGCTCTTTCAATATCACCACAAGCATAAGT---------------------------
GATAAAGTGCAAAAAAACTATGCATTG---TTTTATAAACTTGATTTAGTACAAATAGACAAT----------
----

GATACTAGTGGGAACTATAGGGTGATAAGTTGCAACACCTCAGTCATTACACAA---
GCCTGTCCAAAAATATCCTTTAAGCCAATTCCCATACATTATTGTGCCCCGGCTGGTTTTGCGATTCTAAAGT
GTAAC---AATAAGACGTTCAAT------GGAACAGGAAAATGTAATAAT-----------------------
-GTCAGC---ACAGTACAATGTACACATGGAATTAGGCCAGTAGTATCAACT---
CAACTGCTGTTAAATGGCAGTCTAGCAGAAGAAGAG------------
GTAGTGATTAGATCTGAAAATATCACAAACAAT---------GCTAAAACAATAATAGTACATCTGAAT----
-----------GAATCTGTAGAA---------ATTAATTGTACAAGACCCAACAATAATACAAGAAAA-----
-AGTATAACTATA------------GGACCAGGGAGGGCA---TTT------TATGCAACAGGAGAT------
---------GTAATAGGAGATATA------------AGACAAGCACATTGT---AACATTAGTAAAGTA---
AAATGGAATAACACTTTAAAAAGGATAGCTGCAAAATTAAGA---------------GAACAATTTATG----
-----------------------------------AATAAAACAATAGTCTTTAAC------
AGCTCCTCAGGAGGGGACCCAGAAATTGTA---ATGCACAGTGTTAATTGTGGAGGAGAA---
TTCAAGCAGCTCCAGGCAAGAGTCCTGGCTGTGGAAAGATACCTAAAGGATCAACAGCTCCTGGGGATTTGGG
GTTGCTCTGGAAAACTCATTTGCACCACCGCTGTGCCTTGGAATACTAGTTGGAGT-----------------
----------------------------AATAAATCTCTGAATATGATTTGG------GAT------
AACATGACCTGGATGGAGTGG---GAAAAAGAAATTGATAATTACACAAACGTAATATAC---
AGCTTAATTGAAGAATCGCAAAACCAACAAGAA--

AAGAATGAACAAGAATTATTGGGATTAGATAAATGGGCAAGTTTGTGGAATTGGTTTGACATATCAAACTGGC
TATGGTATATAAAAATATTCATAATGATAATAGGAGGCTTGGTAGGGTTAAGAATAGTTTTTACT---
GTACTTTCTATAATA---------AATAGAGTTAGGCAGGGATACTCACCA------
TTGTCGTTCGCTGTTAGCTTGTTCAACGCCACAGCCATAGTAGTAGCTGAG---
GGAACAGATAGGGTTCTAGAACTATTACAAAGAATTTATAGAGCT----------------------------
--------ATTATTCACATA------CCTACAAGAATAAGACAGGGCTTGGAAAGGGCTTTGCTA---TAA
>KC595196
ACT---GCA--
GAAAAATTGTGGGTCACAGTCTATTATGGGGTACCTGTGTGG---AAAGAAGCAACT---
ACCACTCTATTTTGTGCATCAGATGCTAGAGCATATGATACAGAG---GTGCATAAT----------------
--------------GTTTGGGCCACA---CATGCCTGTGTACCCACAGAC---
CCCAACCCACAAGAAATAGCATTGAGA---------AATGTGACAGAAGATTTTAAC----------------
-------------------------ATGTGGAAAAATAACATGGTAGAACAGATGCATAAAGATATA---
ATCAGTTTATGGGATCAAAGCCTA---AAGCCATGTGTAAAATTA---
ACCCCACTCTGTGTCACTTTGAATTGCACGGATTTGGGGAATGCTACTAATACCAATAGT-------------
----
----------------------------AGTAACGAGGGAATGATGGAGAAAGGAGAAATAAAA---
AACTGCTCTTTCAATATCACCACAAACATAAGA---------------------------
GATAGGGTGCAGAAAGAATATGCACTT---TTTTATAAACTTGATGTAGTACCAATAGACAAT----------
----

AATAATAATAGCTATAGGTTGATAAGTTGTAACACCTCAGTCATTACACAG---
GCCTGTCCAAAAGTAACCTTTGATCCAATTCCCATACATTATTGTGCCCCGGCTGGCTTTGCGATTCTAAAAT
GTAAA---GATAAGAAGTTCACT------GGAAAAGGGCCATGTACAAAT-----------------------
-GTCAGC---TCAGTACAATGTACACATGGAATTATGCCAGTAGTATCAACT---

CAACTGCTGTTAAATGGCAGTCTAGCAGAAAAAGAG-----------
GTAGTAATTAGATCTGACAATTTCTCGGACAAT---------GCTAAAACCATAATAGTACAACTGAAC---
-----------GAATCTGTAGTA---------ATTAATTGTACAAGACCCAACAACAATACAAGGAAA----
-AGTATCCCTATA------------GGACCAGGGAGAGCA---TTT------TATACAACAGGAGCC-----
---------ATAATAGGAGATATA-----------AGACAAGCACACTGT---AACCTTAGTAGAAAA---
GCATGGGATAACACTTTAAAACAGATAGTAGAAAAATTAAAA--------------GAACAATTCCCG----
-----------------------------------AATAAAACAATAGCCTTTAAT-----
CAATCTTCAGGGGGGGACCCAGAAATTGTA---ATGCACAGTTTTAATTGTGGAGGGGAA---
TTTAAGCAGCTCCAGGCAAGAGTCCTGGCTGTGGAAAGATACCCAAAGGATCAACAGCTCCTGGGATTTGGG
GTTGCTCTGGAAAACACATCTGCACCACTACTGTACCTTGGAATACTAGTTGGAGT----------------
----------------------------AATAAATCTCTGGATAAGATTTGG------GAT------
AACATGACCTGGATGGAGTGG---GAAAAAGAAATTAACAATTACACAAGCTTAATATAC---
ACCTTAATTGAAGAATCGCAGATTCAGCAAGAA---------------------------------------
----
AAGAATGAACAAGAATTATTGGAATTGGATAAATGGGCAAGTTTGTGGAATTGGTTTGACATAACAAAATGGC
TATGGTATATAAAAATATTCATAATGATAGTAGGAGGCTTGATAGGTTTAAGAATAGTTTTTGTA---
GTGCTTTCTATAGTA---------AACAGAGTTAGGCAGGGATATTCACCA------
TTATCGTTTGCTGTTAGCTTGCTCAATGCCACAGCCATAGCAGTAGCTGAG---
GGGACAGATAGGGTTATAGAAGTGTTACAAAGAGCTTATAGAGCT---------------------------
--------ATTCTCCACATA------CCTACAAGAATAAGACAGGGCTTGGAAAGGGCTTTGCTA---TAA
>EU786678
GCT---GCA---
GAAAATTTGTGGGTCACAGTTTACTATGGGGTACCTGTGTGG---AAAGAAGCGACC---
ACCACTCTATTTTGTGCATCAGATGCTAAAGGATATGACACAGAG---GTACATAAT---------------
--------------GTTTGGGCCACA---CATGCCTGTGTACCCACAGAC---
CCCAACCCACAAGAAATAGTATTGGGA---------AATGTGACAGAAAATTTTAAC---------------
--------------------------ATGTGGAAAAATAACATGGTAGAACAGATGCATGAGGATGTA---
ATCAGCTTATGGGATCAAAGCCTA---AAACCATGTGTAAAATTA---
ACCCCACTCTGTGTTACTTTAAATTGCACTAATTGGAAGAATAATACCAATACCAATAGCACTACC-------
----
---------------------ACTAGTAGTCCCGAGAGTCCACTGGAGAAAGGAGAAATAAAA---
AACTGCTCTTTCAATGTCACCTCAGGCATAAGA--------------------------
GATAAGGTGCAGAAAGAATATGCACTT---TTTTATAGACTTGATATAGTACCAATAGATAAT---------
----

GAAAATGATAGTTTTAGGTTGATAAGTTGTAACACCTCAGTCATTACACAA---
GCCTGTCCAAAAATAACCTTTGAACCAATTCCCATACATTATTGKGCCCCAGCTGGTTTTGCGATTCTAAAGK
GTAAT---AATAAGACATTCAAT------GGAACAGGACCATGTACAAAT----------------------
-GTCAGC---ACAGTACAATGTACGCATGGAATTAGGCCAGTAGTATCAACT---
CAATTGCTGTTAAATGGCAGTCTAGCAGAAGAAGAT-----------
GTAGTAATTAGATCTAGCAATTTCTCGGACAAT---------GCTAAAATCATAATAGTACATCTGAAT---
-----------GAAACTGTAAAC---------ATTACTTGTATAAGACCCAACAACAATACAAGAAAA----
-AGCATAAATATA------------GGACCAGGGAGAGCA---TTT------TATACAACAGGAGCT-----
---------ATAACAGGAGATATA-----------AGACAAGCACATTGT---AACCTTAGTACATCA---
CAATGGAATAACACTTTAAAACAGATAGTTGCAAAATTAAGA--------------GGACAATTTGGG----
-----------------------------------AATAGAACAATAGTATTTAAT-----
CACTCCTCAGGAGGGGATCCAGAAATTGTA---ATGCACAGTTTTAATTGTGGAGGAGAA---
TTTAAGCAGCTCCAGGCAAGAGTCCTGGCTGTGGAAAGATACCTAAAGGATCAACAGCTCCTGGGAATTTGGG
GTTGCTCTGGAAAACTCATCTGCACCACTAATGTGCCTTGGAATGTTAGTTGGAGT----------------
----------------------------AATAAAACTTATGACCAAATTTGG------GAT------
AACATGACCTGGATGCAGTGG---GAAAAGGAAATAGACAATTACACAGGCATAATATAC---
AACCTAATTGAAATATCGCAAAACCAACAAGAA---------------------------------------
----
AAGAATGAACAAGACTTATTGGAATTGGATAAATGGGACAGTTTGTGGAGTTGGTTTGACATAACAAACTGGC
TGTGGTATATAAAAATATTTATAATGATAGTAGGAGGCTTGATAGGTTTAAGAATAATTTTTACT---
GTGCTTTCTATAGTG---------AATAGAGTTAGGCAGGGATACTCACCA------
TTATCGTTTGCTATTAGCTTGCTCAACGCCACAGCCATAGTAGTAGGAGAG---
GGAACAGATAGAATTATAGAAGTAGTACAAAGAACTGCTCGAGCT---------------------------
--------ATTCGCAACATA------CCTACAAGAATAAGACAGGGCTTTGAAAGGGCTTTGCTA---TAA
>EU786680

GCT---GCA--
GGAAATTTGTGGGTCACAGTCTATTATGGAGTACCAGTGTGG---AAAGAAGCAACC---
ACCACGCTATTTTGTGCATCAGATGCTAAAGCATATGACACAGAG---GCACATAAT----------------
--------------GTTTGGGCCACA---CATGCTTGTGTACCCACAGAC---
CCCAGCCCACAAGAAATAGTATTGGAA---------AATGTGACAGAAAATTTTAAC----------------
--------------------------ATGTGGAAAAATGACATGGTAGAGCAGATGCATGAGGATATA---
ATCGGTTTATGGGATCAAAGCCTA---AAACCATGTGTAAAATTA---
ACCCCACTCTGTGTTACCTTAAATTGCACTAATACTACTAATATTAATGTC----------------------
----
----------------------------------ACTGATAGTGGTATGATAGGAGAAATAAAA---
AACTGCTCTTTCAATATCACCACAGCAGTAAGA---------------------------
GATAGGGTGCAGAAACAATATGCACTT---TTTTATACACTTGATATAGTACCAATAAGCACTGAG-------
----

AATGAGAATTATACAAGCTATAGGTTGGTAAGTTGTAATACCTCAGTCATTACACAG---
GCCTGTCCAAAAATATCCTTTGAGCCAATTCCCATACATTATTGCGCCCCGGCTGGTTTTGCGATTCTAAAGT
GTAAT---AATCAGACATTCAAT------GGAAAAGGACCATGTAAAAAT-----------------------
-GTCAGC---ACAGTACAATGTACACATGGAATTAGGCCAGTAGTATCAACT---
CAACTACTGTTAAATGGCAGTTTAGCAGAA---GAG------------
GTAGTAGTTAGATCTGAAAATTTCACAAATAAT---------GCCAAAACCATAATAGTGCAGCTAAAT----
-----------GAATCTATAGAA---------ATCAATTGTACAAGACCCAACAACAATACAAGAAAA-----
-AGTATACATATA------------GGACCAGGGAGAGCA---TTA------TATGCACAA---GAC------
---------ATAATAGGAGATATA------------AGACAAGCACATTGT---AACATTAGTAGCACA---
AAATGGAATAACACTCTAGGACAGGTAGTTAAAAAATTAAGA---------------GAACAATTCGGG----
-----------------------------------AACAAAACAATAATTTTTAAT------
CAGACCACAGGAGGGGACCCAGAAATTGTA---ATGCACAGTTTTAATTGTGGAGGGGAA---
TTTAAGCAGCTCCAGGCAAGACTCCTGGCTGTGGAAAGATACCTAAAGGATCAACAGCTCCTGGGGATTTGGG
GTTGCTCTGGAAAACTCATTTGCACCACTGCTGTGCCTTGGAATGCTAGCTGGAGT-----------------
----------------------------AATAAATCTTATGAGCAGATATGG------AAT------
AACATGACATGGATGGAGTGG---GAAAGGGAAATTGACAATTACACAGGCTTAATATAC---
AACCTAATTGAAAGTTCGCAAAACCAGCAAGAA--

AAGAATGAACAAGAGTTGTTGGAATTGGATAAATGGGCAAGTTTGTGGAATTGGTTTGACATCACAAAGTGGC
TGTGGTATATAAAAATATTCATAATGATAGTAGGAGGATTGATAGGTTTAAGAATAGTTTTTACT---
GTACTTTCTATAGTA---------AATAGAGTTAGGCAGGGATATTCACCA------
TTATCGTTTGCTGTTAGCTTGCTCAACACTGTAGCCATAGCAGTAGCTGAG---
GGGACAGATAGGGTTATAGAGGCATTGCAAAGAATTTTTAGAGCT----------------------------
--------ATTACCCACATA------CCTACAAGAATAAGACAGGGATTGGAAAGGGCTTTACTA---TAA
>HQ236609
GCT---GAA--
GAAAAATTATGGGTCACAGTCTATTATGGGGTACCAGTGTGG---AAAGAAGCAACC---
ACCACTCTATTTTGTGCATCAGATGCTAAAGGATATGAAACAGAG---GCACATAAT----------------
--------------GTCTGGGCTACA---CATGCCTGTGTACCCACAGAC---
CCCAGCCCACAAGAAGTAAATTTGATA---------AATGTGACAGAAAATTTTAAC----------------
--------------------------ATGTGGAAAAATGAAATGGTAGAACAGATGCATGAGGACATA---
ATCAGTTTATGGGATCAAAGCCTA---AAGCCATGTGTAGAATTA---
ACCCCACTCTGTGTTACCTTAGATTGCACTGATTTTGGAAATACTACTAATACTACT----------------
----
----------------------------------AGTACAACAATGGAAAAAGGAGAAATGAAA---
AACTGCTCTTTCAATATCACCACAGATATAAGA---------------------------
GATAAAATGAAAAAAGAATATGCACTT---TTTTATAATCTTGATATAGTATCAATAGATAAT----------
----

AATAATAATAATAGTAGTAACTATAGGTTGGTGAGTTGTAATACCTCAGTCATTAAACAG---
GCTTGTCCAAAGGTATCCTTTGAACCAATTCCCATACATTATTGTGCCCCGGCTGGCTTTGCGATTCTAAAGT
GTAAT---GATAATAAGTTTAAT------GGAACAGGACCATGTACAAAC-----------------------
-ATCAGC---ACAGTACAATGTACACATGGAATTAGGCCAGTTGTATCAACT---
CAACTGCTGTTAAATGGCAGTCTAGCAAAAGAAGAC------------
ATAGTAATTAGATCTGAGAATCTCACGGACAAT---------ACTAAAACCATCATAGTACATCTGAAT----
-----------AAATCTATAGAA---------ATCAATTGTACAAGACCCAACAACAATACAAGAAAA-----

-AGTATACATATG------------GGACTAGAGAGAGCA---TTT------TATACAACAGGAGGA------
---------ATAATAGGAAATATA------------AGACAAGCACATTGT---AACATTAGTAGAGAA---
CAATGGAATAACACATTAAGTCAAATAGTTAAAAAATTAGGA---------------GAAAAATTCAAA----
-----------------------------------AATAAAACAATGGTCTTTAAT------
CAATCCTCAGGAGGGGACTTAGAAATTACA---ACACATAGTTTTAATTGTGGAGGGGAA---
TTTAAACAGCTCCAGGCAAGAGTCCTGGCTGTGGAAAGATACCTAAAGGATCAACAACTCCTGGGGATTTGGG
GTTGCTCTGGAAAACTCATTTGCACCACTAATGTGCCCTGGAATGCTAGTTGGAGT-----------------
----------------------------AATAAATCTTATAAAGAAATATGG------GAT------
AACCTGACCTGGATGCAGTGG---GAAAGAGAAATTGACAATTACACACAATTAATATAC---
AACCTGATTGAAGAATCCCAGATCCAACAAGAA--

AAGAATGAAAAAGATTTATTAGAATTTGATAAATGGGCAAGTTTGTGGAGTTGGTTTGACATCTCAAAATGGC
TGTGGTATATAAGAATATTCATAATGATAGTAGGAGGCTTGATAGGTTTAAGAATAATTTTTACT---
GTGCTTTCCATAGTG---------AATAGAGTTAGGAAGGATACTCACCA------
TTATCGTTTGCAATTAGCTTGTTTAATGCCACAGCAGTAGCAGTAGCTGAG---
AGAACAGATAGGGTTATAGAAGTAGTACAAAGAGCTTTTAGAGCT----------------------------
--------ATTCTCCACATA------CCTAGAAGAATAAGACAGGGCTTGGAAAGAGCTTTGCTA---TAA
>FJ817366
GCT---ACA--
AACAATTTGTGGGTCACCGTCTATTATGGGGTACCTGTGTGG---AAAGAAGCAACC---
ACCACTCTATTTTGTGCATCAGAAGCTAAAGCATATGATACAGAG---GTACATAAT----------------
--------------ATTTGGCCACA---CATGCTTGTGTACCCACAGAC---
CCCAACCCACAAGAAATGGAATTGAAA---------AATGTAACAGAAAATTTTAAC----------------
--------------------------ATGTGGAAAAATAACATGGTAGAACAAATGCATCAGGATATA---
ATCAGTTTATGGGATCAAAGCCTA---AAACCATGTGTAAAATTA---
ACCCCACTCTGTGTTACTTTAGATTGCACAAATATCAATAGTACTAATAGTACCAATAATACT----------
----
----------------------AGTAGTAATAGTAAAATGGAAGAGACAATAGGAGTAATAAAA---
AACTGCTCTTTCAATGTCACCACAAACATCAGA---------------------------
GATAAGGTGAAAAAAGAAAATGCACTT---TTTTATAGCCTTGATCTAGTATCAATAGGAAAT----------
----

AGTAATACTAGCTATAGGTTGATAAGTTGTAACACCTCAATCATTACACAG---
GCCTGCCCAAAAGTATCTTTTGATCCAATTCCCATACATTATTGTGCTCCTGCTGGCTTTGCGATTCTAAAGT
GCAGG---GATAAAAAGTTTAAT------GGAACAGGACCATGTAGAAAT-----------------------
-GTCAGT---TCAGTACAATGTACACATGGAATTAGGCCAGTAGTATCAACT---
CAACTGTTGTTAAATGGCAGTCTAGCAGAGGAAGAA------------
ATAGTAATTAGATCTGCCAATCTCACAGACAAT---------GCTAAAACCATAATAATACAGTTAAAT----
-----------GAAACTATACAA---------ATTAATTGTACAAGGCCCAACAACAATACAAGAAGA-----
-AGTATACCTATA------------GGACCAGGGAGAACA---TTT------TATGCAACAGGAGAC------
---------ATAATAGGGGATATA------------AGAAAAGCATATTGT---AACATTAGTGCAACA---
AAATGGAATAACACTTTAAGGCAGATAGCTGAAAAGTTAAGA---------------GAGAAATTT-------
-----------------------------------AATAAAACAATAATCTTTAAT------
CAATCCTCAGGAGGGGATCCAGAAGTTGTA---AGGCACACTTTCAATTGTGGAGGGGAA---
TTTAAGCAGCTCCAGGCAAGAGTCCTGGCTCTGGAAAGATACCTAAAGGATCAACAGCTCCTGGGGATTTGGG
GTTGCTCTGGAAAACTCATTTGTACCACTACTGTGCCTTGGAATGCTAGCTGGAGT-----------------
----------------------------AATAAATCTTATAATCAGATTTGG------AAT------
AACATGACCTGGATGGAGTGG---GAAAGGGAAATTGACAATTACACAAACTTAATATAC---
AACTTAATTGAAGAATCGCAGAGCCAACAGGAA--

AAGAATGAATTAGAGTTGTTACAATTGGATAAATGGGCAAGTCTGTGGAATTGGTTTAGCATAACAAACTGGC
TATGGTATATAAAAATATTCATAATGATAGTAGGAGGCTTGGTAGGGTTAAGAATAGTTTTTACT---
GTACTTTCCATAGTG---------AATAGAGTTAGGAAGGATACTCACCA------
CTATCGTTCGCTGTTAGCTTGCTTAATACCACAGCCATAGTAGTAGCTGAG---
GGGACAGATAGGATTATAGAAGTAATACAAAGAGCTGGTAGAGCT----------------------------
--------ATTCTCCACATA------CCTAGAAGAATAAGACAGGGCTTAGAAAGGCTTTTGCTA---TAA
>GQ372988
GCT---GAA---GTA---------------------------------
CAAGAAAAATTGTGGGTCACAGTCTATTATGGGGTACCTGTGTGG---AAAGAAGCAACC---
ACCACTCTATTTTGTGCATCAAATGCTAAAGCATATGATACAGAG---GCACATAAT----------------

--------------GTTTGGGCCACA---CATGCCTGTGTGCCCACAGAC---
CCTAATCCACAAGAAGTAGAATTGGTA---------AATGTGACAGAAAATTTTAAC----------------
-------------------------ATGTGGAAAAATGATATGGTAGAACAGATGCATGAGGATATA---
ATTAGTTTATGGGATGAAAGCCTA---AAGCCATGTGTAAAAATA---
ACCCCACTCTGTGTTACTTTAAATTGCAGTCCGTATTATACCAATGTTACCAATAATACCAATAATAATACCA
CT---
----------GTTGCCAATACCAATAATAGTAGTGGGTTAGAAGGGATAACGGGAGAACTAAGA---
AACTGCTCTTTCAATGTCACAGAGGAAATAAAC--------------------------
AAGAAGATGCGAAAAGAATATGCAATT---TTTAATGAAATTGATATAGTAAAAATAAGTAAT----------
----

GAAAGCTATATGTTGAAAAATTGTAACACCTCAGTCATTAAACAA---
GCCTGTCCAAAGGTATCCTTTGAACCAATTCCCATACATTATTGTACCCCGGCTGGTTTTGCGATTCTAAAGT
GTAAG---GATAAGGAGTTCAAT------GGAACAGGACCATGTGGAAGT----------------------
-GTCAGC---ACAGTACAATGTACACATGGAATTAGGCCAGTAGTGTCAACT---
CAACTGCTGTTAAATGGCAGCCTATCAGAAGGAGAT------------
ATAATGATCAGGTCTCAAAATTTCACAAACAAT---------GCTAAAACTATAATAGTACAGCTGAAT----
-----------CAATCTATAGAA---------ATTAATTGTACAAGACCCAACAACAATACAAGAAAG-----
-AGTATACATGTA------------GGATGGGGGAGAGCA---TTA------TATACAACAGGACAA------
---------ATAATAGGAGATATA------------AGAAAGGCACATTGT---AACATTAGTCTAACA---
AAGTGGAACAACGCTTTATACAAGATAGCTCTAAAATTAAGA---------------GAACAATTTAAG----
-----------------------------------AATAAAACAATAAAATTTATG------
AACTCCTCAGGAGGGGACCCAGAAATTGTA---ACACACAGTTTTAATTGTGCAGGGGAA---
TTCAAGCAGCTCCAGGCAAGAGTCCTGGCTGTGGAAAGATACCTAAGGGATCAACAGCTCCTAGGGATTTGGG
GTTGCTCTGGAAAACTCATCTGCACCACTGCTGTACCTTGGAATGCTAGTTGGAGT-----------------
----------------------------AATAAATCTCTAAATACAATTTGG------AAT------
AACATGACATGGCAGGAGTGG---GAGAGAGAAATTGACAATTACACAGGCTTAATATAC---
ACCTTGCTTGAAGAATCGCAAAACCAACAAGAA--

AAGAATGAACAAGAATTACTAGAATTGGATAAGTGGGCAAATTTATGGAATTGGTTTGATATATCAAACTGGC
TGTGGTATATAAAAATATTCATAATGATAGTAGGAGGCTTAATAGGTTTAAGAATAGTTTGTGCT---
GTACTTGCTTTAGTG---------AATAGAGTTAGGCAGGGATACTCACCC------
TTATCATTCGCTGTTAGCTTGCTTAATACTACAGCTATAGCAGTAGCTGAG---
GGAACAGATAGGATTATAGAAGTAACACAAAGAATTGGTAGAGGT---------------------------
--------TTTCTCCACATA------CCTAGAAGAATAAGACAGGGTTTAGAAAGAGCTTTGCTA---TAA
>FJ670531
GCT---GCA--
GAACAATTGTGGGTCACAGTCTATTATGGGGTACCTGTGTGG---AAAGATGCAGAT---
GCCATTCTATTTTGTGCATCAGATGCTAGAGGATATGGTACAGAG---GTACATAAT----------------
--------------GTTTGGGCCACA---CATGCCTGTGTACCCACAGAC---
CCCAACCCACAAGAAGTACTATTGGAA---------AATGTGACAGAAGCTTTTAAC----------------
-------------------------ATGTGGAAAAATAACATGGTAGAACAGATGCATGAGGATATA---
ATCAGCTTATGGGATCAAAGCCTA---AAGCCATGTGTAAAATTA---
ACCCCGCTCTGTGTTACTTTAAATTGCACTGATGTGGGGAATGTTACTAATATCAATAATGTTACTAAT----
----
-------------------ATCAAGAATAGTAGCGAGGAACTCATGGAGAAAGGAGAAATGAAA---
AACTGCTCTTTCAATATCACCACAAGCATAAGA--------------------------
GATAAGATGCAGAAGGAATATGCACTT---TTTTATAAACTTGATGTAGTACCAATAGGCAATGAT-------
----

AATAATACTAATAGTAGCTATAGGTTGATAAGTTGTAACACCTCAGTCATTACACAG---
GCCTGTCCAAAAGTATCTTTTGAGCCAATTCCCATACATTATTGTGCCCCGGCTGGGTTTGCGATTCTAAAGT
GTAAT---AATAAAACGTTCAAT------GGAAAAGGACCATGTAGAAAT----------------------
-GTCAGC---ACAGTACAATGTACACATGGAATTAGACCAGTAGTATCAACT---
CAACTGTTGTTGAATGGCAGTCTAGCAGAAAAAGAG------------
GTAGTAATTAGATCTGACAATTTCTCAGACAAT---------GCTAAAACCATAATAGTACAGCTGAAC----
-----------GAATCTATAGCA---------ATTAATTGTATAAGACCCAACAACAATACAAGAAAA-----
-AGTATACATATG------------GGACCAGGAAGAGCG---TTT------TATGCAACAGGAGAT------
---------GTAATAGGAGATATA------------AGAAAAGCATATTGT---ACTGTTAATAGAACC---
AACTGGAACAGAACTTTACAACTGGTAGCTGAAAAATTAGGA---------------GAACAATATAAA----

-----------------------------------AATAAGTCAATAATCTTTAAT------
CACTCCTCAGGAGGGGACCCAGAAATTGTA---ATGCACACTTTTAATTGTAGAGGGGAA---
TTTAAGCAGCTCCAGGCGAGAGTCCTGGCTGTGGAAAGATACCTAAAGGATCAACAGCTCCTGGGGATTTGGG
GTTGCTCTGGAAAACTCATCTGCACCACTACTGTGCCTTGGAATGCTAGTTGGAATAAG--------------
-------------------------AATAATAGATCTCTGGAGGATATTTGG------AAT------
AACATGACCTGGATGGAGTGG---GAAAGAGAAATTGATAATTACACAGGCATAATATAC---
AGCTTAATTGAAGAATCGCAGAACCAGCAAGAA--

AAGAATGAGCAAGAATTGTTGGCATTGGATAAATGGGCAAGTTTGTGGAGTTGGTTTGACATATCACAATGGC
TGTGGTACATAAAAATATTTATAATGATAGTAGGAGGCTTGGTAGGTTTAAGAATAGTTTTTACT---
GTACTTTCTATAGTA---------AATAGAGTTAGGAAGGGATACTCACCA------
TTATCGTTTGCTGTTAGCTTGCTCAATACCATAGCCATAGCAGTAGCTGAA---
GGGACAGATAGGGTTATAGAAATATTACAAAGGGTTGGTAGAGCT----------------------------
--------ATCCTTCACATA------CCTACAAGAATAAGACAAGGCTTGGAAAGGGCTTTGCTA---TAA
>FJ853620
GCT---ACA--
GACAAACTGTGGGTCACAGTCTATTATGGGGTACCTGTGTGG---AAAGAAGCAACC---
ACCACTCTATTCTGTGCATCAGATGCTAAAGCATATGATAAGGAG---ATACATAAT----------------
--------------GTTTGGGCCACG---CATGCCTGTGTACCCACAGAC---
CCCAACCCACAAGAAGTAGCCTTGGAA---------AATGTGACAGAAAATTTTAAC----------------
--------------------------ATGTGGAAAAATAACATGGTAGAACAAATGCATGAGGATATA---
ATCAGTTTATGGACCAAAGCTTA---CAACCATGTGTAAAATTA---
ACCCCACTCTGTGTTACTTTAAATTGCACTGATTACAAGGGAAATGCTACTAATCTA----------------
----
-------------------------------AATAATAGTAGTTTAGAGGAAGGAGAAATAAAA---
AACTGCTCTTTCAAGGTCACCACAAACATAAGA---------------------------
GATAAGTTACAGAAAACATATGCACTT---TTTTATAGACTTGATGTAGTACCAATAAATAAT----------
----

GATAATGATAATAACAGCTATAGGTTGATAAGTTGTAATACCTCAGTCATTACACAG---
GCCTGTCCCAAGGTATCCTTTGAGCCAATTCCCATACATTTTTGTGCCCCAGCTGGTTTTGCGATTCTAAAGT
GTAGA---GATAAGAAGTTCAAT------GGAAAAGGAGAATGTAAAAAT-----------------------
-GTCAGC---ACAGTACAATGCACACATGGAATTAGGCCAGTAGTATCAACT---
CAACTGCTGTTAAATGGCAGTTTAGCAGAGGAAGAG------------
GTAGTAATTAGATCTGACAATATCACAGATAAT---------GCTAAAACCATAATAGTACAGCTGAAA----
-----------CAATCTGTAAAA---------ATTAATTGTATAAGACCCAACAACAATACAAGAAAA-----
-AGTATAAATATA------------GGACCAGGGAGAGCA---TTC------TATGCAGCAGGAGAA------
---------ATAATAGGAGATATA------------AGACAAGCACATTGT---AACATTAGTAGAACA---
GAATGGAATAACACTTTAGGACAGATAGTTGAAAAATTAAGG---------------GAAAAATTTAGC----
-----------------------------------AATAAAACAATAATCTTTAAC------
CAATCTTCAGGAGGGGACCCAGAAATTGTA---ATGCACAGTTTTAATTGTCAAGGGGAA---
TTTAAGCAGCTCCAGGCAAGAGTCCTGGCTGTGGAAAGATACCTACAGGATCAACAGCGCCTGGGGATTTGGG
GTTGCTCTGGAAAACTCATTTGCACCACTGCTGTGCCTTGGAATACTAGTTGGAGT-----------------
----------------------------AATAAATCTCTGGGGGCTATTTGG------GAT------
AATATGACCTGGATGCAGTGG---GAAAAGGAAATTAGCAATTACACAAACTTAATATAC---
AACCTAATTGAAGAATCGCAGAACCAGCAAGAA--

AAGAATGAACAAGAATTGTTGGAATTGGACAAATGGGCAAGTTTGTGGAATTGGTTTAGTATAACAAACTGGC
TGTGGTATATAAGAATATTTATAATGATAGTAGGAGGCTTAGTAGGTTTAAGAATAGTTTTTACT---
GTACTTTCTATAGTG---------AATAGAGTTAGGAAGGGATACTCACCA------
TTATCGTTGGCTGTTAGCTTGTTTGATGCTTTAGCCATAGCAGTAGCTGGG---
GGGACAGATAGGATTATAGAAGGGTTACAAAGAGTTGGTAGAGCT----------------------------
--------ATTCTCCACATA------CCTACAAGAATAAGACAGGGCTTGGAAAGAGCTTTGCTA---TAA
>KT200349
GCT---GCA--
GAAAACTTGTGGGTCACAGTCTATTATGGGGTACCTGTGTGG---AAAGAGGCAACT---
ACCACTCTATTTTGTGCATCAGATGCTAAAGCACATAATCCTGAA---GTACATAAT----------------
--------------GTCTGGGCCACA---CATGCCTGTGTACCCACAGAC---
CCCAACCCACAAGAAGTAGCATTG------------AATGTGACAGAAAATTTTAAC----------------
--------------------------ATGTGGAAAAATAACATGGTAGAGCAAATGCATGAGGATATA---

ATCAGTTTATGGGATCAAAGCTTA---AAACCATGTGTAAAATTA---
ACCCCACTCTGTGTTACTCTAAATTGCACTGATGTTAATACCACTAGT-------------------------
----
----------------------------AGTGGCGAGGAAATGGTGGAAAGAGGAGAAATAAAG---
AACTGCTCTTTCAACATCACCTCAGACATA---------------------------------
AAAATGAAGAAAGAATATGCACTT---TTTTATGACCTTGATGTAATATCAATAGAGAAT-------------
----
-------------------AGTAGTACTAGCTATAGAATAAGAAGTTGTAATACCTCAGTAATTACACAG---
GCCTGTCCAAAGGTAACCTTTGAGCCAATTCCCATACATTATTGTGCCCCGGCTGGTTTTGCGATTCTAAAGT
GTAAA---GATAAGCAGTTCAAT------GGAACAGGAGCATGTAAAAAT-----------------------
-GTCAGC---ACAGTACAATGTACACATGGAATTAGGCCAGTAGTGTCAACT---
CAACTGTTATTAAATGGCAGTCTAGCAGAAGAAGAG------------
GTAGTAATTAGGTCTGAAAATTTCACGAACAAT---------GTTAAAACCATAATAGTGCAACTGAAG----
-----------GAACCTGTAGTA---------ATTAATTGTACAAGACCCAACAACAATACAAGAAGA-----
-AGTATACATATA------------GGACCAGGGAGTGCA---TTT------TATACATCAAAT---------
---------ATAATAGGAGATATA------------AGACAAGCACATTGT---AATGTTAGTAGTACA---
AAATGGAATGACACTTTACGTGAGATAGCTAAAAAATTAAGG---------------AAACAATTTGGG----
-----------------------------------AACAAAACAGTAATCTTTAAT---
CAAACCACAAAAGGGGGGGACCCAGAAATTGTG---ATGTTCACTTTTAATTGTGCAGGGGAA---
TTTAAACAGCTCCAAGCAAGAGTCCTGGCTGTGGAAAGATACCTAAAGGATCAACAGCTCCTAGGGATTTGGG
GTTGCTCTGGAAAACTCATCTGCACCACTGCTGTGCCTTGGAATACTAGCTGGAGT-----------------
----------------------------AATAAATCTTATGATTATATTTGG------GGT------
AACATGACCTGGATGGCGTGG---GAAAGAGAAATTGACAATTACACAAGCTTGATATAC---
ACTTTAATTGAAGAATCGCAGAACCAACAAGAA--

AAGAATGAACAAGAATTATTGGAATTGGATAAGTGGGCAAGTTTGTGGGATTGGTTTAGCATATCAAATTGGC
TGTGGTATATAAGACTATTCATAATGATAGTAGGAGGCTTGGTAGGTTTAAGAATAGTTTTTGCT---
GTACTTTCTATAGTA---------AATAGAGTTAGGCAGGATATTCACCA------
TTGTCATTTGCTGTTAGCTTGCTCAACGCCACAGCCATAGCAGTAGCTGAG---
GGAACAGATAGGATTATAGAAGTAGCTCAAAGAGCTTTTAGAGCT----------------------------
--------ATCCTCCACATA------CCTAGAAGAATAAGACAGGGTGCAGAAAGGGCTTTGATA---TAA
>KT200354
GCT---GCA--
GAACAATTGTGGGTCACAATATATTATGGGGTACCTGTGTGG---AAAGAAGCAACC---
ACTACTCTATTTTGTGCATCAGATGCTAAAGCATATGATACAGAG---GTACATAAT----------------
--------------GTTTGGCCACA---CATGCTTGTGTCCCCACAGAC---
CCCAACCCACAAGAAGTAAAATTGGAA---------AATGTGACAGAAAATTTTAAC----------------
--------------------------ATGTGGAAAAACAACATGGTAGAACAGATGCATGAAGATGTA---
ATTAGTTTATGGGATCAAAGCCTA---AAACCATGTGTAAAATTA---
ACCCCACTTTGTGTTACTTTAGATTGCTTTGATGTGGATACCAATAAAACTAAGAATCAGACTGAT-------
----
-------GGGGGTGGAATAATACTAGTATTGAGGGGGGAAAGATGGAGAGAGGAGAACTGAAA---
AACTGCTCTTTCAACATCTCCACAAGCGTAAAA---------------------------
GATAAGTATAAAAAAGTATATGCACTT---TTTTATAAGGCTGATGTAGTACCAATAGATGGA----------
----

AATAATAATAATAATAATACTAGCTATAGGTTGATAAGTTGTAATACCTCAGTCATTACACAA---
GCATGTCCAAAGATATCCTTTGAGCCAATTCCCATACATTATTGTGCCCCGGCTGGGTTTGCGATTCTAAAGT
GTAAC---AATAACACATTCAAT------GGAACAGGACCATGTAACAAT-----------------------
-GTCAGC---ATAGTGCAATGTACACATGGAATTAAACCAGTAGTATCAACT---
CAACTGCTGTTAAATGGCAGTCTAGCAAAAGAAGAG------------
GTAGTAATTAGATCTGCCAATTTCACAAACAAT---------GCTAAAGTCATAATAGTACAGCTGAAC----
-----------AAGTCAGTAGAA---------ATTAATTGTACAAGACCCAACAACAATACAAGAAAA-----
-AGTATACCTATA------------GGACCAGGGAGAGCA---TTT------TATGCAACAGACAAA------
---------ATAATAGGAGATATA------------AGAAAAGCATATTGT---AAGATTAATGGAACA---
GAATGGAATAAAACTTTAGAACAGGTAGTTGATAAATTAAGA---------------AAACAATTTGGA----
--------------------------------AATAATAAAACAATAAGCTTTAAG---
CCACCACCCTCAGGAGGAGACCCAGAAATTGTA---ATGCACAATTTTAATTGTCAAGGGGAA---
TTTAAACAGCTCCAGGCAAGAGTCCTGGCTCTGGAAGGATACCTAAGGGATCAACAGCTCCTAGGAATTTGGG
GTTGCTCTGGAAAAATCATTTGCACCACTACTGTGCCTTGGAATACTAGTTGGGGT-----------------

-------------------------CGTAATAGATCTTACCAGGAAATTTGG------GAT------
AACATGACCTGGATGCAATGG---GAAAAAGAAATTGGCAATTACACAGACTTAATATAC---
TCCTTGCTTGAAGAATCGCAGACCCAGCAAGAA--

AAGAATGAAAAAGAATTATTGGAATTGGATAAATGGGCAAATTTGTGGAATTGGTTTAACATATCAAATTGGC
TGTGGTATATAAAAATATTCATAATGATAGTAGGAGGCTTAGTAGGTTTAAGAATAGTTTTTGCT---
GTACTTTCTATAATG---------AATAGAGTTAGGCAGGGATACTCACCC------
TTATCGTTTGCTGTCAGCTTGCTCAACGCCACAGCAATAGCAGTAGCTGAG---
GGGACAGATAGGATTATAGAAATATTACAAAGAATTTTTAGAGCT---------------------------
--------GTTCTTCACATA------CCTACAAGAATAAGACAGGGCTTAGAAAGGGCTTTGCTA---TAA
>KT200358
GCT---AAA---
GAAAAATTGTGGGTCACAGTGTATTATGGGGTACCTGTGTGG---AAAGAAGCAACC---
ACCACTCTATTTTGTGCATCAGATGCTAAAGCATATGATACAGAG---GCACATAAT---------------
--------------GTTTGGGCCACA---CATGCCTGTGTACCCACAGAC---
CCCAACCCACAAGAAATAGTAATGGAA---------AATGTAACAGAAGATTTTAAC---------------
--------------------------ATATGGAAAAATAACATGGTAGAACAGATGCAGGAGGATATA---
ATTAGTCTGTGGGATCAAAGCCTA---AAGCCATGTGTAAAACTA---
ACTCCACTCTGTGTTACTTTAAATTGTACTAATACTAACAAGAATGCTACTAACGTCACTAATAGT-------
----
-----------------------------------GATTGGGAAGGAAAGGAAGAAGTAAAA---
AACTGCTCTTTCAATGTCACTGCAGGAATAAAG--------------------------
GATAAGGTACAGAAAGAATATGCACTG---
TTTTATAAACCTGATGTAGTACCCATAAAAGATGATGATACTAAT---------------------------
----
ACCACTAATACCACCTATAACACCTACAGGTTGATAAGTTGTAATACCTCAGTCATTACACAG---
GCCTGTCCAAAGATATCCTTTGAGCCAATTCCCATACATTATTGTGCCCCAGCTGGTTATGCAATTTTAAAGT
GTAAT---AGTAAGACGTTCAAT------GGAACAGGACCATGCAAAAAT----------------------
-GTCAGC---ACAGTACAATGTACACATGGAATTAAGCCAGTAGTATCAACT---
CAACTACTGTTAAATGGCAGTCTAGCAGAAGAAGAC-----------
ATAGTAATTAGATCTGAAAACATCACGAACAAT---------GCTAAAACCATAATAGTACATCTGAAT----
-----------GAATCTGTAATG---------ATTAATTGTACAAGACCCAGTAACAATACAAGAAAA-----
-GGTATACATATA------------GGACCAGGGAGAGCA---TTT------TATGCAACA---GAC------
---------ATAATAGGAGACATA------------AGGCAAGCACATTGT---AACATTAGTAGACCA---
CAGTGGGATATGACCTTAAAACGTATAACCACAAAATTAAGA--------------GGAAAATTTGGG----
--------------------------------GAAAATAAAACAATAATCTTTAAC------
CAATCCTCAGGAGGAGACCCAGAAATTGTA---ATGCACAGTTTTAATTGTGGAGGGGAA---
TTTAAGCAGCTCCAGGCAAGAGTCCTGGCTGTAGAAAGATACCTACAAGATCAACAGCTCCTAAAGATTTAAG
GTTGCTCTGGAAAACTCATTTGCACCACTACCGTGCCTTGGAATGCTAGTTGGAGT-----------------
----------------------------AATAAATCTTTAAATGCGATTTGG------GAT------
AACATGACCTGGAACCAGTGG---GAGAAAGAAATTAGCAAGTACACAGGAGTAATATAC---
ACATTAATTGAAGAATCGCAAAACCAACAAGAA--

AAGAATGAACAAGAATTATTAGAATTAGATAAGTGGGCAAGTTTGTGGAATTAGTTTGATATAACAAGCTAGC
TGTAGTATATAAAAATATTCATAATGATAGTAAGAAGCTTAGTAAGTTTAAAAATAGTTTTTGCT---
GTATTTTCTATAGTG---------AATAGAGTTAGGCAGGGATATTCACCA------
TTATCATTCGCTGTTAACTTGCTCAATGCCACAGCTGTAACAGTAGCTGAG---
GGGACAGATAGGATTATAGAAGCAGTACAAAGAACTTGTAGAGCT---------------------------
--------ATTCTCCACATA------CCTAGAAGAATAAGACAAGGCTTGGAAAGGATTTTACTA---TAA
>FJ853622
GCT---ACA---
GATAAATTGTGGGTCACAGTGTATTATGGGGTACCTATATGG---AAAGAAGCAACT---
ACTACTCTATTTTGTGCATCAGATGCTAAAGCATATGATACAGAG---GTACATAAT---------------
--------------GTTTGGGCCACA---CATGCCTGTGTACCCACAGAC---
CCCAACCCACAAGAAGTACTATTGGAA---------AATGTGACAGAACATTTTAAC---------------
--------------------------ATGTGGAAAAATAATATGGTAGAACAGATGCATGAAGATATA---
ATCAGTTTATGGGATCAAAGCCTA---AAGCCATGTGTAAAATTA---
ACCCCACTCTGTGTTGTTTTAAATTGCACTAGTGTGAATAACACTGCTTATGTG------------------
----
---------------------------------AATAAGACTGAAGAAGAGAAAGAAATGAAA---

AACTGCTCTTTCAATATCACCTCAAGAGTAGGA--------------------------
AAGGGATTGCAGAAAGAGTATGCACTC---TTTTATAAAGTGGATATAGTACCATTAGATATT----------
----

AATAATATTAGTAATACTTATAGGTTGATAAAATGTAATACCTCAGTCATTACACAG---
GCCTGTCCAAAGGTGTCCTTTGAGCCAATTCCCATACATTATTGTGCCCCGGCTGGTTTTGCGCTTCTAAAGT
GTAAC---GATAAGAAGTTCAAT------GGATCAGGACCATGTACAAAT----------------------
-GTCAGC---ACAGTACAATGTACACATGGAATTAGGCCAGTAGTATCAACT---
CAACTACTGTTAAATGGCAGTCTAGCAGAAGAAGAA------------
GTAGTAATTAGATCTGAAAATTTCACGAATAAT---------GCTAAAACTATAATAGTACAGCTGAAG----
-----------GAACCTGTAAAA---------ATTAACTGTACAAGACCCAACAACAATACAAGAAAA-----
-AGTATACACCTA------------GGAGCAGGGAAAGCA---ATT------TATACAACAGGAGCC------
---------ATAATAGGAAATATA------------AGACAAGCACATTGT---AACATTAGTTGGGAA---
GACTGGAATAAAGCTTTAAACCAGACAGCTATAAAATTAAGA---------------GAACAATTTGAG----
-----------------------------------AATAAAACAATAGTCTATCAG------
AATTCCTCAGGAGGGGACTCGGAAATTGTA---ATGCACAGTTTTAATTGCGGAGGAGAA---
TTTAAGCAGCTCCAGGCAAGAGTCCTGGCTATAGAAAGATACCTAAAGGATCAACAGCTCCTAGGGATTTGGG
GTTGCTCTGGAAAACTCATCTGCACCACTGCTGTGCCTTGGAATGCTAGTTGGAGT-----------------
----------------------------AATAAATCTCTAAATGATATTTGG------GAT------
AACATGACCTGGATGGAGTGG---GAAAARGAAATTAGCAATTACACAAGCTTAATATAC---
ACCTTACTTGAAAAATCGCAGAACCAACAAGAA---------------------------------------
----
AAGAATGAACAAGAATTACTGGAATTGGATAAGTGGGCAAATTTGTGGAATTGGTTTGACATAACAAACTGGC
TGTGGTATATAAAAATATTCATAATGATAGTAGGAGGCTTGGTAGGTTTAAAAATAGTTTTTGCT---
GTGCTTTCTATAGTG---------AAAAGAGTTAGGCAGGGATATTCACCA------
TTGTCATTTGCTGTTAGCTTATACAACGCCACAGCTATAGCAGTAGCTGAG---
GGAACAGATAGGGTTATAGAAGTAGTACAAAGCATTGTTAGAGCT---------------------------
--------TTTCTCCACATA------CCTAGAAGAATAAGACAGGGCTTAGAAAGGGCTTTGTTA---TAA
>GU362883
GCT---GCA---
GACAAATTATGGGTCACAGTCTATTATGGGGTACCTGTGTGG---AAAGAAGCAACC---
ACCACTCTATTTTGTGCATCAGATGCTAAAGCATATGATACAGAG---GTACATAAT----------------
--------------GTTTGGGCCACA---CATGCCTGCGTACCCACAGAC---
CCCAAACCACAAGAATTGGTATTGGCA---------AATGTGACAGAAAATTTTAAC---------------
--------------------------ATGTGGAAAAATGACATGGTAGAACAGATGCAAGAGGATATA---
ATCAGTTTATGGGATCAAAGCCTA---AAGCCATGTGTAAAATTA---
ACCCCACTATGTGTTACTCTAAATTGTATAGATTATCAGGGGACAAATACAACTAATGTC------------
----
----------------------------TCTACCCCTACAACAACTGGTGCCCAGGAAGTGAAA---
AACTGTTCCTTCAATATCACCACAAACATAAGA--------------------------
GATAAGATGCAGAAAGAATATGCACTT---TTGTATAAACTTGATCTAGTACAGTTAGATAAT----------
----

AGTAATAACAGTTATAGGTTAATAAGTTGTAACACCTCAATCATTACACAG---
GCCTGTCCAAAAATATCCTTTGAGCCTCTTCCAATACATTATTGCGCCCCGGCTGGTTTTGCTATTCTAAGGT
GTAAC---AATAARATATTCGAT------GGAAGAGGACCATGTAAAAAT----------------------
-GTTACC---ACAGTACGATGTACACATGGAATTAAGCCAGTATTATCAACT---
CAGCTGCTGTTAAATGGCAGTCTCGCAGAAGAAGGG------------
ATAGTAATTAGATCTGAAAATTTCACAAATAAT---------GCTAAAACCATAATAGTACAACTGAAA----
-----------GAGCCAGTAGAA---------ATTAATTGTACAAGGCCCAACAACAACACAAGAAGA-----
-AGTATACATATA------------GCACCAGGGAGAGCA---TTT------TACGCAACAGGAGAA------
---------ATAATAGGAGACATA------------AGGCAGGCACATTGC---AACGTTAGTAGAACA---
GCTTGGAAAACAACTTTGAACCAGACAGCTACAAAATTAAGA---------------GAACACTTTGGG----
-----------------------------------AATAAAACAATAGTCTTTAAT------
CAATCCTCAGGAGGGGACCCAGAAATTACA---ATGCACAGTTTTAATTGCAGAGGGGAA---
TTTAAACAGCTCCAGGCAAGAGTCCTGGCTGTGGAAAGATACCTAAAGGATCAACAGCTCCTGGGGATCTGGG
GTTGCTCTGGAAAGCTCATTTGCACCACTGCTGTGCCTTGGAATGCTAGTTGGAGT-----------------
----------------------------AATAAATCCCAGGATCAGATTTGG------GGT------
AACATGACTTGGATGGAGTGG---GATAGAGAAATTAATAATTACACACAATTAATATAC---
TCCTTAATTGAACAATCGCAGAACCAACAAGAA---------------------------------------

----
ATAAATGAGAAAGACTTATTGGAATTGGATACATGGGCAAATTTGTGGAATTGGTTCAACATATCTAACTGGC
TGTGGTATATAAAAATATTCATAATGATAGTAGGAGGCTTAGTAGGTTTAAGAATAGTTTTTACT---
GTACTTTCTATAATA---------AATAGAGTTAGGCAGGGATACTCACCA------
TTGTCGTTCGCTGTTAGCTTACTCAACGCCGCGGCCATAGCAGTAGCTGAG---
GGGACAGATAGGGTTATAGAAGTAGCACAAGGAGTTGGGAGAGGT----------------------------
--------ATTCTCCACATA------CCTAGAAGAATAAGACAGGGCTTAGAAAGGGCTTTGCTA---TAA
>GU362886
GCT---GAT---------------------------------------
GACAACTTATGGGTCACAGTATATTATGGGGTACCTGTGTGG---AAAGAAGCAACC---
ACCACATTATTTTGTGCATCAGATGCTAAATCATATAGTACAGAG---GCACATAAT----------------
--------------GTTTGGGCCACA---CATGCCTGTGTACCCACAGAC---
CCCAGCCCACAAGAAGTAAAATTAGAA---------AATGTGACAGAGAGATTTAAC----------------
--------------------------ATGTGGAAAAATAAAATGGTAGATCAGATGCACGAAGATATA---
ATCAGTCTATGGGATCAAAGCCTA---AAGCCATGTGTGAAATTA---
ACCCCACTCTGTGTTACTTTAAATTGCACTGAATTGAATAGTACTAAAACC----------------------
----
-----------------------------------AATGTTACACTAGATAGAGAGGAAATGAAA---
AACTGCTCTTTCAATATCACCACAAGCATAAGA--------------------------
GATAAGATACAAAAGGAATATGCACTT---TTTTATAAACTAGATATAGTATCAATAGATGAG----------
----

GATAAGAATAATACATATAGGTTGATAAGTTGTAATACCTCAGTCCTTACGCAG---
GCCTGTCCAAAGGTAACCTTTGAGCCAATTCCAATACATTATTGTGCCCCGGCTGGTTTTGCAATTTTTAAGT
GTAAA---GATAAGAAGTTCAAT------GGAACAGGAGACTGCAAAAAT-----------------------
-GTCAGC---ACAGTACAGTGTACACATGGAATTAGACCAGTAGTGTCAACT---
CAACTGCTGTTAAATGGCAGTTTAGCAGAAGAAGAG-----------
ATAGTAATTAGATCTGAAAATTTCACGGACAAT---------GCTAGAACCATAATAGTACATCTGAAT----
-----------GAATCAGTGCAA---------ATTAATTGTACAAGGCCCAACAACAATACAAGAAAA-----
-GGTATACATATA------------GGACCAGGGAAAACA---TTC------TTTGCAACA---GAC------
---------ATAATAGGAGATATA------------AGACAAGCACATTGT---AACATAAGTAAAACA---
CAATGGGATGACACTTTAGGAAAGATAGTTAAAAAATTAAGA---------------GAACAATATAGG----
-----------------------------------AACAAAACAATAGTCTTTAAT------
CAATCCTCAGGAGGGGACCCAGAAATTATA---ATGCACAGTTTTAATTGTGGAGGGGAA---
TTTAAACAGCTCCAGGCAAGAGTCCTGGCTCTGGAAAGATACCTACAGGATCAACAGCTCCTAGGGATTTGGG
GTTGCTCTGGAAAACTCATCTGCACCACTAATGTGCCTTGGAATACTAGTTGGAGC-----------------
----------------------------AATAAATCTATAGGAGCAATTTGG------GAT------
AACATGACCTGGCAGGAGTGG---GAAAAAGAGATTGACAATTATACAGACATAATATAC---
AGCTTACTTGAAACTTCGCAGAACCAGCAAGAA--

CTAAATGAACAAGAATTATTGAAATTGGACACGTGGGCAAGTTTGTGGAATTGGTTTAGTATATCAAAGTGGC
TGTGGTATATAAAAATATTCATAATGATAGTAGGAGGCTTGATAGGTTTAAGGATAATTTTTACT---
GTGCTTTCTATAGCA---------AATAGAGTTAGGCAGGGATATTCACCA------
TTATCATTTGCTATCAGCTTGTTCAACGCCACAGCTGTAACAGTAGCTGAA---
GGGACAGATAGGATTCTAGAAATAATACAAGTAGTTTTTAGAGCT----------------------------
--------ATCCTCCACATA------CCTAGAAGAATAAGACAGGGCTTGGAAAGGCTTTTGCTA---TAA
>JX140656
ACT---GTA---------------------------------------
CAAAGTTTGTGGGTCACAGTCTATTATGGGGTACCTGTGTGG---AAAGAAGCAACC---
ACCACTCTATTCTGTGCATCAGATGCTAAAGCATATGATACAGAG---GCACATAAT----------------
--------------GTTTGGGCTACA---CATGCCTGTGTACCCACAGAC---
CCCAACCCACAAGAAGTATTATTGGAT---------AATGTGACAGAAAATTTTAAT----------------
--------------------------ATGTGGAAAAATAACATGGTAGAACAGATGCAAGAGGATATA---
ATCAGCTTATGGGATCAAAGCTTA---AAGCCATGTGTAAAATTA---
ACCCCACTCTGTGTCACTTTAAATTGCACTGATGTGACTGAGAATACTGTTGAAAACAATACT----------
----
---ATTAATGGAGAAATAAAA---
AACTGCTCTTTTAATATCTCCACAGAAATAAAA--------------------------
GGTAAGACACAGAGAGAACGTGCATAT---TTTTCTAAACTTGATGTAGTACCAATAGATAATAATGGTAAT-
----

AATGCTAACAATACAAGCTATAGGCTAATACATTGTAACACTTCAGTCATTACACAG---
GCCTGTCCAAAGATATCCTTTGAGCCAATTCCCATACATTATTGTACCCCGGCTGGTTTTGCGCTTCTAAAGT
GTAAT---GACAGGAAGTTCAAT------GGAACAGGACCATGTAGAAAT----------------------
-GTCAGC---ACAGTACAATGTACACATGGAATTAGGCCAGTAGTGTCAACC---
CAACTGCTGTTAAATGGCAGTCTAGCAGAAGAAGAG-----------
GTAGTAATTAGATCTGCCAATTTCACAGACAAT---------GCTAAAACCATAATAGTACAGCTGAAG----
-----------GAACCTGTAAAA---------ATTAACTGTACAAGACCCAACAACAATACAAGAAAA-----
-GGTATACAAATG------------GGACCAGGGAGGGCA---TTT------TATGCAACAGGAGAC------
---------ATAATAGGAGATATA------------AGGCAAGCACATTGT---AACCTTAGTAAAATA---
GAATGGGAGAAAGCTTTAAACCAGACAGTTAGAAAATTAAGG---------------GAACATTTTAAA----
------------------------------------AACAAAACAATAAGCTTTAAT------
TCATCTTCAGGAGGGGACCCAGAAATTGTA---ATGTACACTTTTAATTGTGGAGGGGAA---
TTTAAGCAGCTCCAGGCAAGAGTCCTGGCTGTGGAAAGATACCTAAAGGATCAACAGCTCCTGGGGATTTGGG
GTTGCTCTGGAAAACTCATTTGCACCACTACTGTGCCTTGGAATGATAGTTGGAGT-----------------
-------------------------AATAATAAATCTTTAGATTATATTTGG------AAT------
AATATGACCTGGATGGAATGG---GAAAGAGAAATTGACAATCACACAAACTACATATAC---
ACCTTAATTGAAAAAGCGCAAAACCAACAAGAA---

AAGAATGAACAAGAATTATTAGAATTGGATCAGTGGGCAAGTTTGTGGAATTGGTTTAGTATATCCAATTGGC
TGTGGTATATAAGAATATTCATAATAATAGTAGGAGGCTTAGTAGGCTTAAGAATAGTTATGGCT---
GTACTTTCTGTAGTA---------AACAGAGTTAGGCAGGGATACTCACCA------
TTGTCGTTGGCTGTTAGCTTGCTTAACGCCACAGCCATAGCAGTAGCTGAG---
GGGACAGATAGGGTTATAGAAATAATACAAAGAGCTGGTAGAGCT---------------------------
--------ATTCGCCACATA------CCTACAAGAATAAGACAGGGTTTAGAAAGGGCTTTGCTA---TAA
>GU362881
GGT---GAA---
GACTTGTGGGTCACAGTATATTATGGGGTACCTGTGTGG---AAAGAAGCAAAG---
ACCACTCTCTTTTGTGCATCAGATGCTAAAGCATATGATACAGAA---GCTCATAAT----------------
--------------GTTTGGGCTACA---CATGCCTGTGTACCCACAGAC---
CCCAACCCACAAGAAATACCATTGGAA---------AATGTGACAGAAGATTTTAAC---------------
--------------------------ATGTATAAAAATGACATGGTAAGACAAATGCAGGAGGATGTA---
ATCAGTTTATGGGATCAAAGCTTC---AAGCCATGTGTAAAATTA---
ACCCCACTTTGTGTTACTTTAAATTGTGCTAATGTTACTGATACCAATAGT---------------------
----
--------------------------------------ATCAATGAAATGCGAGAAGAAATAAAA---
AATTGCTCTTTCAATATCACCACAAGTGGAGGA--------------------------
AGTAAGAGGCAGAAAGAATATGCACTT---TTTAATGATCTTGATTTAGTAGGGAGTAGCGAT----------
----

ACTGGTTATAGGTTGATACATTGTAATACCACAGTCATTACACAA---
GCCTGTCCAAAGATAACCTTTGAACCAATTCCAATACATTATTGTGCCCCGGCTGGTTATGCGATTCTAAAGT
GTAAT---GATAAGGAGTTCAAT------GGAACAGGACTATGTAGGAAT----------------------
-GTCAGC---ACAGTACAATGTACCCATGGAATTAAACCAGTAGTGTCAACT---
CAACTACTGCTAAATGGCAGTCTAGCAGAAGAAGAT-----------
ATAGTAATTAGGTCTGAAAATTTCACAGACAAT---------GCTAAAACCATAATAGTGCAGCTAAAA----
-----------GAACAAGTAGAA---------ATTAATTGTACAAGACCTAACAACAATACAAGAAAA-----
-GGTATACATATA------------GGACCAGGGAGAACA---TTA------TATGCAACAGGGGAA------
---------ATAATAGGAGATATA------------AGAAAAGCACATTGT---AACATTAGTAGAGCA---
AAATGGAATAACACTTTACGGAACATAGTTGCAAAATTAAGA---------------AAACAATTTAAA----
------------------------------------AATAAAACAATAGTCTTTAAT------
CAATCCTCAGGAGGGGACCCAGAAATTGTA---ATGCACAGTTTTAATTGTCAAGGGGAA---
TTTAAGCAGCTCCAGGCAAGAATCCTGGCTGTAGAAAGATACCTACAGGATCAACAGCTCCTGGGGATTTGGG
GTTGCTCTGGAAAACTCATTTGCACCACTGCTGTGCCTTGGAATACTAGTTGGAGT-----------------
----------------------------AATAGATCTCAAAGTGACATTTGG------AAT------
AACATGACCTGGATGGAGTGG---GAAAGAGAAATTGACAATTACACAAGCTTAATATAC---
ACTTTAATTGAAAAATCGCAGATCCAGCAAGAG---

AAAAATGAACAAGAATTATTGCAATTAGATAACTGGGCAAGTTTGTGGAATTGGTTTAGCATAACAAACTGGC
TGTGGTATATAAGGATATTCATAATGATAGTAGGAGGCTTGATAGGTTTAAGAATAATTTTTGCT---

GTGCTTTCTATAGTG---------AATAGAGTTAGGCAGGGATACTCACCA------
TTATCGTTTGCTGTAAGCTTGCTTAATGCCACAGCTATAGCAGTAGCTGAG---
GGAACAGATAGGGTTATAGAAGCAGTACAGAGACTTTGTAGAGCT------------------------------
--------ATTCTCCACATA------CCAAGAAGAATAAGACAGGGCTTTGAAAGAGCTTTACTA---TAA
>GU362885
GCT---GTA---
GATCAATTGTGGGTCACAGTCTATTATGGGGTACCTGTGTGG---AAAGAAGCAACC---
ACCACTCTATTCTGTGCATCAGATGCTAAAGCATATGATACAGAG---GTACACAAT------------------
--------------GTTTGGCCACA---CATGCCTGTGTACCCACAGAC---
CCCAGCCCACAAGAAATAATATTGGAA---------AATGTGACAGAAAATTTTAAC------------------
--------------------------ATGTGGAAAAATAACATGGTAGAACAGATGCATGAAGATGTA---
ATCAGTTTATGGATCAAAGCCTA---AAGCCATGTGTAAAATTA---
ACCCCACTCTGTGTTACTTTAGAGTGCACTGACGTGGGGAATACTAGTAATGCCAATAATATTACC-------
----
-------------------AATAGTAGTAGCTGGGGAGAGCCAATGGAGAAAGGAGAAATGAAA---
AACTGCTCTTTCAATGTCACTTCAGGCATAAGA---------------------------
GATAAGATGCAGAGGGAATATGCACTT---TTGTATAAACTTGATGTAGTACCAATAGATAAT----------
----

AGCAYAAATAGTACAAACTATAGATTGATAAATTGTGACACCTCAATCATTACACAG---
ACCTGTCCAAAGGTAAGCTTTGAACCAATTCCCATACATTATTGTACCCCAGCTGGTTTTGCAATGCTAAAAT
GTAAT---GATAAAGAGTTCAAT------GGAACAGGACCATGTAAGAAT----------------------
-GTCAGC---ACAGTACAATGTACACATGGAATTAGGCCAGTAGTGTCAACT---
CAACTACTGTTAAATGGCAGTCTAGCAGAAGGAGAC------------
ATAGTAATTAGATCTGAAAATTTCACTGATAAT---------GCTAAGACCATAATAGTACAGCTGAAG----
-----------GAACCCATAGAA---------ATTAATTGTACAAGACCCTATAGCTATACAACAATA---
AGAAGGATACGTATA------------GGACCAGGGAGAGCA---TTT------ACA---ACAGGA-------
-----------AGCGTAGGAGATATA------------AGACAAGCACATTGT---AACCTTAGTCAGGCA--
-ATATGGAATAAAACTTTAAAAGGATAGTTGAAAAATTAAGA---------------AACCAAGTTGGG---
------------------------------------AATAGAACAATAGCCTTTAAC------
AAATCCTCAGGAGGGGACCCAGAAATTGTA---ATGCACAGTTTTAATTGTGGAGGGGAA---
TTTAAGCAGCTCCAAGCAAGAGTCCTGGCTGTAGAAAGCTACCTAAAGGATCAACAGCTCCTAGGGATTTGGG
GTTGCTCTGGAAAACTCATTTGCACCACTGCTGTGCCTTGGAATGCTAGTTGGAGT-----------------
----------------------------AATAAATCTCTGAATCAGATTTGG------GAT------
AACATGACCTGGATGGAATGG---GAAAGAGAAATTAGCAGTTATACAAATGTAATATAC---
AACTTAATTCAAGAATCGCAGAACCAGCAAGAA--

AAGAATGAACAAGAATTATTGGAATTAGATAAGTGGGCAAGTTTGTGGAATTGGTTTAGCATAACAAAATGGC
TGTGGTATATAAAAATATTCATAATGATAGTAGGAGGGTTAATAGGTTTAAGAATAGTTTTTGCC---
ATACTTTCTATAGTG---------AATAGAGTCAGGAAGGGATACTCACCA------
TTATCATTCGCTGTTAGCTTGCTTAACGTCACAGCTGTAGCAGTAGCTGAG---
GGACAGATAGAATTATAGAAATATTACAAAGAACTGGTAGAGCT-------------------------------
--------ATTCTCAACATA------CCTACAAGAATAAGACAGGGCTTAGAAAGGGCTTTGCTA---TAA
>KC473842
ACT---GCA---
GAGAACTTGTGGGTCACAGTTCATTATGGGGTACCTGTGTGG---AAAGAAGCGAGC---
ACCACTTTATTTTGTGCATCAGATGCTAAAGCATATGACACAGAG---GTACATAAT------------------
--------------GTTTGGCCACA---CATGCCTGTGTACCCACAGAC---
CCTAGCCCACAAGAAGTACTATTGGAA---------AATGTGACAGAAAATTTTAAC------------------
--------------------------ATGTGGAAGAATAACATGGTACAACAAATGCATGATGATATA---
ATCAGTTTATGGATGAAAGCCTA---AAGCCATGTGTAAAATTG---
ACCCCACTCTGTGTTACTTTAAATTGCACTAAAGTAAGCTGCACTAATAGTACTTCTTCTAATAATACTGCTT
CTAATAATACTTTA---
-------------ACGTGCAATGATACTATAGGGGGGCAGGGCTGGGCGAAAGAGGAAATAAAA---
AACTGCTCTTTCAATGCCACCAAAGGAATAGGA---------------------------
GATAAGGTGCAGAAAACATATGCTCTT---TTTAATACGCTTGATGTAATGCAAATAGATGGT----------
----

GATAATACAAGCTATACATTAATAAATTGTAACACCTCAGTCATTAGGCAG---
ACCTGTCCAAAGGTATCCTTTGAACCAATTCCCATACATTATTGTGCCCCGGCTGGTTTTGCGATTCTAAAAT

GTAAT---GATAAGAAGTTCAAT------GGAACAGGACCATGTACAAAT-----------------------
-GTCAGC---ACAGTACAATGTACACATGGAATTAGGCCAGTAGTGTCAACT---
CAATTGCTGTTAAATGGCAGTCTATCTGAGGAAGAG------------
GTAATAATTAGATCTAAAAATCTCTCTGACAAT---------GCTAACACCATAATTATACAGCTGAAT----
-----------AAGACAGTAGTA---------ATTAATTGTACAAGACCCGGTAACAATACAAGAAAA-----
-AGTATATCACTA------------GGACCAGGGAGAGTA---TTT------TATGCAACAGGAGAC------
---------ATAATAGGAGATATA------------AGACAAGCATACTGT---AACATTAGTCAAGCA---
GATTGGAATGAAACTTTAACATGGATAGCCATAAAATTAAGA---------------GAAAAATTTAAC----
-----------------------------------AATAAATCAATAACCTTTAGG------
CCATCCTCAGGAGGGGACCCAGAAATTGTA---ATGCACACGTTTAATTGTGGAGGGGAA---
TTTAAGCAGCTCCAGGCAAGAGTCCTGGCTGTGGAAAGATACCTAAGAGATCAACAGCTCCTAGGAATTTGGG
GCTGCTCTGGAAAGCTCATTTGCACCACTGCTGTGCCATGGAATGCTAGTTGGAGT-----------------
----------------------------AATAAAACATACAATCAGATTTGG------GAT------
AACATGACCTGGATGCAGTGG---GAGAGAGAAATTGACAATTACACATCAATAATATAC---
GACTTGATTGAAAAATCGCAAAACCAACAAGAA--

AAAAATGAACAAGACTTATTGGAATTGGATAAGTGGGCAAGTTTGTGGAATTGGTTTGAGATAACAAGTTGGC
TGTGGTATATAAAAATATTCATAATGATAGTAGGAGGTATAGTAGGTTTAAGAATAATTTTTACT---
GTACTTTCTATAGTG---------AATAGAGTTAGGCAGGGATACTCACCA------
TTGTCATTTGCTGTTAATTTGCTTAATACAACAGCTATAGTAGTAGCTGAG---
GGGACAGATAGGATTATAGAAGTAGCACAAAGAGCTTGGAGAGCT----------------------------
--------TTTCTCCACATA------CCTACTAGAATAAGACAGGGCGCAGAGAGGGTTTTGCTA---TAA
>KT200353
GCT---ACA--
GAAAAGTTATGGGTCACAGTCTATTATGGGGTACCTGTGTGG---AAAGAAGCAAAT---
GCCACTTTATTTTGTGCATCAGATGCTAAAGCATATGATACAGAG---GCTCATAAT----------------
--------------GTTTGGGCCACA---CATGCCTGTGTACCCACAGAC---
CCCGACCCACAAGAAATACCATTGAAA---------AATGTGACAGAAAATTTTAAC----------------
--------------------------ATGTGGAAAAATAATATGGTAGACCAAATGCAGGAAGATATA---
ATCAGCTTATGGGATCAAAGCTTA---AAGCCATGTGTACAATTA---
ACCCCACTCTGTGTTACTTTAAACTGTACTGATGTCACAACTAACAAGACTAGTAATGCTACTCAA-------
----
-------------AATAGCAACAATAGTGAGACAGGGAGAATAATGGAAGAAGGAGAAATAAAA---
AACTGTTCTTTTAATATTTCCACAAACATAGCA---------------------------
GACAAGAGGCAGAAAGAATATGCACTT---TTTTATAAACTTGATGTAGTACCAATAGATGATGGT-------
----

AATAATACTAGTTATAGGATAATAAATTGTAACACCTCAGTCATTACACAG---
GCATGTCCAAAGACATCCTTTGAACCAATTCCCATACATTATTGTGCCCCGGCTGGTTTTGCGATTATCAAGT
GTAGA---GATGAAAAGTTCAAT------GGAACAGGACCATGTAACAAT-----------------------
-GTCAGC---ACAGTACAATGTACACATGGAATAAAACCAGTAGTATCAACT---
CAATTGCTGTTAAATGGCAGTCTAGCAAAAGAAGAA------------
ATAGTAATTAGATCTGAAAATTTCTCAAACAAT---------GCTAAAATTATAATAGTGCAGCTGAAG----
-----------GAACCTGTAGAA---------ATTAATTGTACAAGACCCAATAACAATACAAGAAAA-----
-AGTATACATATA------------GGACCAGGGAGGGCA---TTT------TATGCAACAGGAGAC------
---------ATAATAGGAGATATA------------AGACAAGCACATTGC---AACATTAGTAGAACA---
AAATGGAATAACACTTTACTACAAATAGCTAACAAATTAAGA---------------GAACAATTTAAT----
-----------------------------------AATAAAACAATAGTCTTTAAC------
CAATCCTCAGGAGGGGACCCAGAAATTGTA---ATGCACAGTTTTAATTGTGCAGGGGAA---
TTTAAACAGCTCCAGGCAAGAGTCCTGGCTATAGAAAGATACCTAAAGGATCAACAGCTCCTGGGGATTTGGG
GTTGCTCTGGAAAACTCATTTGCACCACTGCTGTGCCTTGGAATACTAGTTGGAGT-----------------
----------------------------AATAAAAGTCTAGACCATATTTGG------AAT------
AACATGACTTGGATGCAGTGG---GAAAGAGAAATTGACAATTACACAGGCCTCATATAC---
ACCCTACTTGAAGATTCGCAAAACCAACAAGAA--

AAGAATGAACAAGAATTATTGGCATTAGATAAGTGGGCAAATTTGTGGAATTGGTTTGACATATCAAACTGGC
TGTGGTATATAAGAATATTCATAATGATAGTAGGAGGCATGATAGGTTTAAGAATAGTTTTTACT---
GTACTTTCCATAGTA---------AATAGAGTTAGGCAGGGATATTCACCA------
TTATCGTTTGCTATCAGCTTGCTGAATGCCACAGCTATAATAGTAGGTGAG---

GGGACAGATAGGATTCTAGAAGCAATACAAAGAGCTTATAGAGCT----------------------------
--------TTTATCCACATA------CCTAGAAGAATAAGACAGGGCTTAGAAAGGTTTTTGCTA---TAA
>KT200355
GCT---GCA---
GAACAATTGTGGGTCACAGTCCATTATGGGGTACCTGTGTGG---AAAGAAGCAACC---
ACTACTCTATTTTGTGCATCAGATGCTAAAGCATATGATACAGAA---GTACACAAT----------------
--------------GTTTGGGCTACG---CATGCCTGTGTACCCACAGAC---
CCCAACCCACAAGAAATAGAAATGAAA---------AATGTGACAGAAGAGTTTAAC---------------
--------------------------ATGTGGAAAAATAATATGGTAGAACAGATGCAGGAGGATATA---
ATCAGTTTATGGGATCAAAGCCTA---AAGCCATGTGTAAAACTA---
ACCCCACTCTGTGTCACTCTAAATTGCACGGATGTAAAGAGGAATGCTAATATGACTTGTTGGGAA-------
----
----------------------AATGGAACAAATCTGCTGAATAATATTACCAGGGACATGAAA---
AACTGCTCTTTCAACATCACCGCAGGCATAAGA--------------------------
GATAAGGTGCAAAGAGAATATTCACTT---TTTTATAAAGGTGATATAATACCAATAGATAAT----------
----

AGTAATAGCTCCTATAGGGTGATAAGTTGTAACACCTCAGTCATTACACAA---
GCCTGTCCAAAGGTAACCTTTGAACCAATTCCTATACATTATTGTGCCCCGGCTGGTTTTGCGATTCTGAAGT
GTAAT---GAGAAAAAATTTCACG------GGAAAAGGACCATGCAAAAAT----------------------
-GTCAGC---ACAGTACAATGTACACATGGAATTAAGCCAGTAGTATCAACT---
CAACTGCTGTTAAATGGCAGTCTAGCAGAAGAAGAG------------
GTAGTAATTAGATCTGACAATTTCTCAAACAAT---------GTTAAAACCATAATAGTACAGCTGACA----
-----------GAACCTGTAAAT---------ATTACTTGTGTAAGACCTGGCAACAATACCAGAAGA-----
-AGTATACATATA------------GGACCAGGGAAAGCA---TTT------TATACAACAGGAGAA------
---------ATAATAGGAGATATA-----------AGACAAGCACATTGT---AATATTAGTGCACAA---
AAATGGAATAGCACTTTACAAAAGATAGTTGAAAAATTAAGA---------------GCACAATTTAAG----
-----------------------------------AATAAAACAATAATCTTCCAG------
AAACCCTCAGGAGGGGATCCAGAGATTGAA---ACGCACAGTTTTAATTGTGGAGGGGAA---
TTTAAGCAGCTCCAGGCAAGAGTCCTGGCTGTGGAGAGATACCTACAGGATCAACAGCGCCTGGGGCTTTGGG
GCTGCACTGGAAAACTCATCTGCACCACTGCTGTGCCTTGGAATGTTAGTTGGAGT----------------
----------------------------AATAAATCTCTGGATCAGATTTGG------AAT------
AACATGACCTGGATGGAGTGG---GAAAGAGAAATTAATAATTACACAGACTTAATATAC---
ACTTTAATTGAAGACTCGCAAAACCAACAAGAA--

AAGAATGAACAAGACTTATTGGAATTCGATAAATGGGCAAATTTGTGGAATTGGTTTAACATATCAAATTGGC
TGTGGTATATAAAAATATTCATAATGATAGTAGGAGGCTTGATAGGTTTAAGAATAGTTTTTGCT---
GTGCTTTCTATAGTG---------AATAGAGTTAGGCAGGGATACTCACCA------
CTATCGTTTGCTGTTAGCTTGTTTGATGCTATAGCTGTAGCAGTAGCTGAA---
GGGACAGATAGGGTTATAGAAGTAGTACAAAGAGCTTTTAGAGCT--------------------------
--------ATCTACAACATA------CCTAGAAGAATAAGACAGGGTTTGGAAAGGGCTTTACTA---TAA
>KT200356
GCA---GAA--
CAATGGTGGGTCACAATCTATTATGGGGTACCTGTGTGG---AAAGAAGCAACC---
ACCACTCTATTTTGTGCATCAGATGCTAAAGCATACAAGAAAGAG---GTACATAAT----------------
--------------GTTTGGGCCACA---CATGCCTGTGTACCCACAGAC---
CCCAATCCACAAGAAATAAGCTTGGAA---------AATGTGACAGAAAAATTTAAC---------------
--------------------------ATATGGAAAAATGACATGGTAGAACAGATGCATGAAGATATA---
ATTAGTTTATGGGATCAAAGCCTA---AAGCCATGTGTAAAATTG---
ACCCCACTCTGTGTTACTTTAAATTGCACGGAGGTTAATTCAAATAGTACGGGGGGGGCT------------
----
----------------------------AATTTAACTAGTAGCAGAGAAAGTTCAGAAATAAAA---
AACTGCTCTTTCAATATCACCTCAAGCATAAGA--------------------------
GATAAAGTGCAGAAAGAATATGCACTG---ATGTATAAAATTGATGTAGTACCAATAGATAATAATAAT----
----

AATAATACTACTAATAATACTAGATATATGTTGAAAAGTTGTAACACCTCAGTCATTACACAG---
GCCTGTCCAAAGGTATCCTTTGAGCCAATTCCTATACATTATTGTGCCCCGGCTGGTTTTGCAATTTTAAAAT
GTAGA---GATAAGAACTTCAAT------GGAAAAGGAGAATGTAAAAAT---------------------
-GTCAGC---ACAGTACAATGTACACATGGAATTAAACCAGTAGTGTCAACT---

CAACTGCTGCTAAATGGCAGTCTAGCAGAAGGAGAG------------
GTAGTAATTAGATCTGAAAATATCACAAACAAT---------GGTAAAACCATAATAGTACAGCTGGAT----
-----------ACACCTGTGCAA---------ATTAACTGCACAAGACCCAACAACAATACAAGAAAA-----
-AGTATAAATATA------------GGACCAGGGAGAGCA---TTT------TATGCAACAGGAGCA------
---------ATAATAGGCGATATA------------AGACAAGCATATTGT---AATGTTAGTAAAGCA---
GAATGGAATAACACCTTAAAAAAGATAAGTGAAAAATTAAAG---------------AAAGAATTTCAA----
-----------------------------------AATAAAACAATAGTCTTTAAT------
CAATCCTCAGGAGGGGACCCAGAAATTGTA---ATGCACAGTTTTAATTGTGGAGGGGAA---
TTTAAGCAGCTCCAGGCAAGAGTCCTGGCTCTGGAAAGATACCTACGGATCAACAGCTCCTGGGGATTTGGG
GTTGCTCTGGAAAACTCATTTGCACCACTACTGTGCCTTGGAATAATAGTTGGAGTAAT-------------
-------------------------AATAAAACAGAGTTAAGCCAGATTTGG------GAT------
AACATGACCTGGAAGGACTGG---GAAAGAGAAATTGGCAATTATACAAATATAATATAC---
AAATTAATTGAGGAATCGCAAAACCAGCAAGAA---

AAGAATGAACAAGAATTATTGGAATTGGATAAATGGGCAAATTTGTGGAATTGGTTTGATATATCAAACTGGC
TGTGGTATATAAAAATATTCATAATGATAGTAGGAGGCTTGGTAGGTTTAAGAATAGTTTTTACT---
GTACTTTCTGTAGTG---------AATAGAGTTAGGCAGGGATACTCACCA------
TTATCGTTTGCCATTAGCTTGCTCAACACCACAGCCATAGTAGTAGCTGAG---
GGGACAGATAGAGTTATAGAAGTAGTACAAAGAGCTTGTAGAGCT----------------------------
--------ATTCTCAACATA------CCTAGAAGAATAAGACAGGGCTTTGAAAGGGCTTTGCTA---TAA
>KY465967
GCT---GCA---
GAACAATTGTGGGTCACAGTCTATTATGGGGTACCTGTGTGG---AAGGAAGCAACC---
ACCACTTTATTTTGTGCCTCAGATGCTAAAGCATATAAAACAGAG---GCACATAAT----------------
--------------GTTTGGGCCACA---CATGCCTGTGTACCCACAGAC---
CCCAACCCACAAGAAGTAGATTTGGTA---------AATGTGACAGAAAATTTTAAT----------------
--------------------------ATGTGGAAAAATGACATGGTAGAACAGATGCATGAGGATATA---
ATCAGTTTATGGGATGAAAGCCTA---AAGCCATGTGTAAAATTA---
ACCCCACTTTGTGTCACTTTAAAATGCATGAACATCAATTGCACTATGAAGAATAATACTGCTACTGCTACCC
AGTGTAGTAGT--
-------GTTAATACCAATATGAGTTATAGCTATAATAGGAGTGGTCTGGAGACAGATATGAGA---
AACTGCTCTTTCAAAGTCACCACAGCAGTAAAA---------------------------
GATAGGGTAAAGCAAGAATATGCATTG---TTTTATAGGCTTGATATAGAACCAATAGAGGGTGAGCAG----
----

GATAATACCAGCTATAGGCTGATAAATTGTAACACCTCAGCCCAAACACAG---
GCCTGTCCAAAGGTATCCTTTGAGCCAATTCCCATTCATTACTGTGCCCCGGCTGGTTTTGCGATTCTAAAAT
GTAAG---GATAAAAACTTTAAT------GGAACAGGACCATGTAAAAAT-----------------------
-GTCAGC---ACAGTACAATGTACACATGGAATTAGGCCAGTAGTGTCAACT---
CAACTCCTGTTGAATGGCAGCCTAGCAGAAGAAGAT------------
ATAGTACTTAGATCTGAGAATATCTCAGACAAT---------AGTAAAAACATTCTAGTACTGCTGAAC----
-----------AGCTCTGTAGAC---------ATTACTTGTATAAGACCCTCCAACAATACAAGAAAA-----
-AGTATAAGTATA------------GGACCAGGGAGGGCA---TTT------TATGCAACAGGTGAC------
---------ATAATAGGAGATATA------------AGACAAGCACATTGT---AACRTCAGTAAAGCA---
AATTGGACAAACATTTTAAAACAGATAGCTATAAAATTAAAA---------------GAACACTTT-------
-----------------------------------AACAAAACAATAGCCTTCAAA------
AACTCCTCAGGAGGAGACCCAGAAATTGTA---ATGCACACTTTTAATTGTGGAGGGGAA---
TTCAAGCAGCTCCAAGCAAGAGTCCTGGCTGTGGAAAGATACCTAAAGGATCAACAGCTCCTGGGAATTTGGG
GTTGCTCTGGAAAAAATCATTTGCACCACTAGTGTGCCTTGGAATGCTAGTTGGAGT----------------
----------------------------AATAAATCTTATTCAGAGATTTGG------GAT------
AACATGACCTGGAGGGAGTGG---GAAAATGAAATTGACAATTACACAAGCTTAATATAT---
ACCCTAATTGAAGAATCCCATAATCAACAGGAA---

AAGAATGAACAAGAATTATTGGCATTGGATAAGTGGGCAAACCTGTGGAGTTGGTTTAGCATAACAAATTGGC
TGTGGTATATAAAAATATTCATAATGATAGTAGGAGGCTTAATAGGTTTAAGAATAGTTTTTGCT---
GTACTTTCTATAGTG---------AATAGAGTTAGGCAGGGATACTCACCA------
TTATCATTTGCTGTTAGCTTGCTCAACGCCATAGCCATAGCAGTAGCTGAG---
GGAACAGATAGGATTATAGAAATAGGACAAAGAGCTTGTAGAGCT----------------------------
--------ATTCTCAACATA------CCTAGAAGAATACGACAGGGCTTTGAAAGGGCTTTGCTG---TAA
>KT276263

ATT---GCA---GAA------------------------CAA---CCA---
AAGCCATTGTGGGTCACAGTTTATTATGGGGTACCTGTGTGG---AAAGAAGCAGTC---
ACCACTCTGTTCTGTGCATCAGATGCTAAAGCATATGACACAGAG---GTACATAAT----------------
--------------GTTTGGGCCACA---CATGCCTGTGTACCCACAGAC---
CCCAACCCACAAGAAGTAAAATTGGAC---------AATGTAACAGAAAATTTTAAT----------------
--------------------------ATGTGGAAAAATAGCATGGTAGAACAAATGCATGAGGATATA---
ATCAGTTTATGGGATCAAAGTCTA---AAGCCATGTGTAAAATTA---
ACCCCACTCTGTGTTACTTTACATTGCACTAATATAACTAACAGTACC-------------------------
----
--ATAGAGGGAAATGATATGAAA---
AACTGCTCTTTCAATGCCACCACAGCCATAAAA--------------------------
GATAAGAAACAGAAAAATTATGCACTT---
TTTAATACACTTGATATAGTACCAATAAATACAATAGATAGTAAA----------------------------
----
ACAACAAATGATACAGAATATGTATTGATAGGTTGTAACACCTCAGTCATTACACAG---
GCCTGCCCAAAGGTAAACTTTGAACCAATTCCTATACATTATTGTACCCCGGCTGGTTTTGCGCTTCTAAAGT
GTAAT---GAGAAAAAACTTCACT------GGAACAGGACCATGTAGAAAT----------------------
-GTCAGT---ACAGTACAATGTACACATGGAATTAGGCCAGTAGTGTCAACT---
CAACTGCTGTTAAATGGCAGTCTAGCAGAGGAAGGG-----------
ATAGTAATTAGATCTGAAAATTTCACGAACAAT---------GCTAAAACCATAATAGTACAACTGTCT----
-----------GACCCTGTAACA---------ATTAATTGTACAAGACCCAACAACAATACAAGAAAA-----
-AGTGTACCTATA------------GGACCGGGGGCAGCA---ATT------TATGCAACAGGAGCC------
---------ATAATAGGAGATATA------------AGACAAGCACATTGT---AACATTAGTAGAACA---
AAATGGAATGACACTTTAAGAAAAATAGTTGAACAATTAAAG---------------GCAAAATATAAT----
-----------------------------------AAGACCACAATAATCTTTAAG------
CCACCCTCAGGAGGGGACCCAGAAATTACA---ATGCACACTTTCAATTGTGGAGGGGAA---
TTTAAGCAGCTCCAGGCTAGAGTCCTGGCCATCGAAAGATACCTAAAGGATCAACGGCTCCTGGGGATTTGGG
GTTGCTCTGGAAAACTCATCTGCACCACTACTGTGCCCTGGAATACCAGTTGGAGT-----------------
----------------------------AATAAATCACTAACCCAGATTTGG------GAT------
AACATGACCTGGATGGAGTGG---GACAGAGAAATTAACAATTACACAAACCTAATATAC---
AGTTTAATTGAAGATGCGCAGAACCAGCAGGAA--

AAGAATGAAAAAGAATTACTGGAATTGGATAAGTGGGACAGCTTGTGGAATTGGTTTGAAATAACAAAGTGGC
TATGGTATATAAAAATATTTATAATGATAGTAGCAGGTTTAGTAGGTTTAAGAATAATTTTTGCT---
GTGCTTTCTATAGTA---------AATAGAGTTAGGCAGGGATACTCACCA------
TTGTCATTTGCTGTGAGTTTGTTTAATGCTGTAGCCATAGTAGTAGCTGAG---
GGGACAGATAGAATTATAGAAGCAGCACAAATACTTGGTAGGGCC----------------------------
--------ATTCTCCACATA------CCTAGAAGAATAAGACAGGGTTTTGAAAGGGCTTTGCTA---TAA
>KT276266
ACT---GAA---
GACAACTTGTGGGTCACAATCTATTATGGGGTACCTGTGTGG---AAAGAAGCAACC---
ACCACTCTATTTTGTGCATCAGATGCTAAAGCATATGTTACAGAG---GTACATAAT----------------
--------------GTTTGGGCCACA---CATGCCTGTGTACCCACAGAC---
CCTAACCCACGAGAAGTAAACTTAGAA---------AATGTGACAGAAAATTTTAAC----------------
--------------------------ATGTGGAAAAATAACATGGTGGATCAGATGCATGAGGATATA---
ATCAGTTTATGGGATGAAAGCCTA---AAACCATGTGTAAAATTA---
ACCCCACTATGTGTTAGTCTAAATTGCACTGATTGGAAGGCAAATACTAATATCACCATT-------------
----
---ACGGAAGTGAAA---
AACTGCTCTTTCGATATCATCAATAGAATAGGA-----------------------------
AAACAGAAAAGAGACCATGCACTG---TTTTCTGTACTTGATATAGCATTAATAAAGAAT-------------
----
-------------------GATAACACAAGCTACATGATAAGACATTGTAATACCTCAGTCATTAAACAG---
GCCTGTCCAAAGGTATCCTTTGAACCAATTCCCATACATTATTGTGCCCCAGCTGGTTTTGCGATTCTAAAGT
GTAAT---GATAAAAAGTTCAAT------GGATCAGGACAATGTGCAAAT----------------------
-GTCAGC---ACAGTACAATGCACACATGGAATTAAGCCAGTAGTGTCAACA---
CAATTATTGTTAAATGGCAGTCTAGCAGAGGGAGAT-----------
GTAATAATTAGATCTAAGAACTTTTCGAATAAT---------GCTGACATCATAATAGTACAACTAAAT----
-----------GAGTCTGTACCA---------ATTAAATGTATGAGACCCAACAACAATACAAGAAAA-----
-AGTGTATCCTTG------------GGACCAGGGAGAGCA---ATT------TATGCAACAGGA---------

---------GAAGTAGGAGATATA------------AGACAAGCACATTGT---AACATTACTAGAAGC---
ATATGGAAGAATATCTTAGCTCAGGTAAAGGAAAATTAAAA---------------GAACAATTTAAT----
----------------------------------AATACTAACATAACCTTTAAG------
CAACCTTCAGGAGGGGACCCAGAAATTGTA---TACCACAGTTTTAATTGTGGAGGGGAA---
TTTAAGCAGCTCCAGGCAAGACTCCTGGCTGTGGAAAGATACCTAAAGGATCAACAGCTCCTGGGAATTTGGG
GTTGCTCTGGAAAACTCATCTGCACCACTGCTGTGCCTTGGAATAGTAGTTGGAGT----------------
----------------------------AAGAGAAATTTCAGTTATATATGG------AAT------
AACATGACCTGGAATGATTGG---GAAAAAGAAATTGATAATTACACAGACTACATATAC---
ACTTTACTTGAAGAGTCGCAGAACCAACAAGAA---------------------------------------
----
AAGAATGAAAAAGAATTATTGGAATTAGATAGTTGGGCAAGTTTGTGGAATTGGTTTAGCATATCAAAATGGC
TGTGGTATATAAAAATATTCATAATGATAGTAGGGGGCTTAATAGGTTTTAAAATAGTTTTTACT---
ATACTCTCTATAGTG---------AATAGAGTTAGGCAGGGATACTCACCA------
TTGTCATTCGCTGTTAGCCTGTACAATACTATAGCTATAGCAGTAGCTGAG---
GGGACAGATAGGGTTATAGAAATATTACAAAGAATTACTAGGGCT---------------------------
--------ATTCTCCACATA------CCTACACGAATAAGACAGGGTTTGGAAAGGGCTTTGCTA---TAA
>KT276267
GCT---GCA---
GACAACTTGTGGGTCACAGTCTATTATGGGGTACCTGTGTGG---AAAGAGGCAACT---
ACCACTCTATTTTGTGCTTCAGATGCTAAAGCATATGATACAGAA---AAACATAAT----------------
--------------GTCTGGGCCACA---CATGCCTGTGTACCCACAGAC---
CCCAACCCACAAGAAATAGACTTGGAA---------AATGTGACAGAAAATTTTAAC---------------
--------------------------ATGTGGGAAAATGACATGGTAGAACAAATGCATGAGGATATA---
ATCAGCTTATGGGATGAAAGCTTA---AAGCCATGTGCAAAATTA---
ACCCCATTCTGTGTTACTTTAAATTGCACTGATAATTTGAGAAATGAGACTTATAACAAC------------
----
---------------------------------GAAACAATAAGGGAGAAAGGAGAAATGAAA---
AACTGCTCTTTCAATATCACCACAAACATAGGA--------------------------
GACAAGAGGAAGAAGAATGAGTATGCACTTTTTTATAAACTTGATGTAATGCCAATTGATGTAATGCCAATAG
GTGGGGAGGAT---

AATACTAACAATACTACTAACTATACTAACTATAGGTTGATAAATTGTAACACCTCAGTCATTACACAG---
GCTTGTCCAAAGGTAACCTTTGAGCCAATTCCCATACATTATTGTGCCCCGGCTGGTTTTGCGATTCTAAAGT
GTAAA---GATAAAAGTTTCAAT------GGAACAGGACCGTGTAAAAAT----------------------
-GTTAGC---ACAGTACAATGTACACATGGAATTAGGCCAGTAGTGTCAACG---
CAACTGTTACTAAATGGCAGTCTAGCAGAAGAAGGG-----------
GTAGTAATTAGATCTGAAAATTTCACAGACAAT---------GCTAAAAACATAATAGTGCAGCTGACG----
-----------AAACCTGTAGAA---------ATTAATTGTACAAGGCCCAACAACAATACAAGGAAA-----
-AGCATCCATCTA------------GGACCAGGGAAAACA---TTT------TATGCCACAGGAGCC------
---------ATAATAGGAGATATA------------AGACAAGCACATTGT---AACATTAGTAGAGCA---
AAATGGAATGAAACTTTACAAAAGATAGTTATCAAATTACAA--------------GAACAATTTGGG----
-------------------------------AATGATACAACAATAGTCTTTAAT------
CAATCCTCAGGAGGGGACCCAGAAATTGTA---ATGCACAATTTTAATTGTTGGGGGGAA---
TTTAAGCAGCTCCAGGCAAGAATCCTGGCTGTGGAAAGATACCTAAAGGATCAACAGCTCCTGGGAATTTGGG
GTTGCTCTGGAAAACTCATTTGCACCACTGCTGTGCCTTGGAATACTAGTTGGAGT----------------
----------------------------AATAAATCTCTGGAACAGATTTGG------AAT------
AACATGACCTGGATGGAGTGG---GAAAGAGAAATTGACAATTATACAAACTTAATATAC---
ACTTTAATTGAAGAATCGCAGAACCAACAGGAA---------------------------------------
----
AAGAATGAACAAGAATTATTGGAATTGGATAAGTGGGCAAGTTTGTGGAGTTGGTTTGACATAACAAACTGGC
TGTGGTATATAAGAATATTCATAATGATAGTAGGAGGCTTGATAGGTTTAAGAATAGTTTTTGCT---
GTGCTTTCTATAGTG---------AATAGAGTTAGGCAGGGATACTCACCC------
CTCTCGTTTGCTGTTAGCTTACTCAACGCCACAGCAATAGCAGTAGCTGAG---
GGGACAGATAGAATTATAGAAACATCACAAAGAGCTTTTAGAGGT---------------------------
--------TTTCTCCACATA------CCTAGAAGAATAAGACAGGGCTTAGAAAGGATTTTGCAA---TAA
>KU685585
GCT---ACA---
GAAGACTTGTGGGTCACAGTCTATTATGGGGTACCTGTGTGG---AAAGAGGCAAAT---
ACCGCTCTATTTTGTGCATCAGATGCTAAAGCATATAATACAGAA---AAACATAAT----------------
--------------GTCTGGGCCACA---CATGCATGTGTACCCACAGAC---

CCCAACCCACAAGAAATGGACTTGGGA---------AATGTGACAGAAAATTTTAAC----------------
--------------------------ATGTGGAAAAATAACATGGTAGAACAAATGCATGAGGATATA---
ATCAGTTTATGGGATCAAAGCTTA---AAGCCATGTGTAAAATTA---
ACTCCATTCTGTGTTATTTTAAATTGTACTGACCTGAAAAATAATAGTGCCCACAATATT-------------
----
-------------------------------------AGCCTGGAAATGAAAGAAGAAATAAAA---
AACTGCTCTTTCAATATCACCAAAAACATGGAA--------------------------GACAAGAAG---
GAGGAATATGCAATT---TTTTATAAATCTGATATAGTATCAATAGGTGGTAAG------------------
----
----------GGTAATAATAGCTATAGGTTGATAAATTGTAACACCTCAGTCATTAAACAG---
GCTTGTCCAAAGGTATCCTTTGAGCCAATTCCCATACATTATTGTGCCCCGGCTGGTTTTGCGATTTTAAAGT
GTAAA---GAGAAAGGGTTCAAT------GGAAAAGGACAGTGTAAAAAT-----------------------
-GTCAGC---ACAGTACAGTGTACACATGGAATTAGGCCAGTAGTGTCAACT---
CAACTGTTATTAAATGGCAGTCTAGCAGAAGGAGAG-----------
ATAGAAATTAGATCTGAAAATTTCACAAACAAT---------GCTAGATCCATAATAGTGCAGCTGAAT----
-----------AAATCTGTAGAA---------ATTAATTGTACAAGACCCGGCAACAATACAAGAAAA-----
-GGCTTTCATATA------------GGACCAGGGCAAACA---CTT------TATGCAACAGGACAA------
---------ATAATAGGAGATATA------------AGACAGGCACATTGT---AACATTAGTGGAGCA---
GAATGGAAGAAGACTTTACAACAGATAGTTGAAAAATTACAA---------------GAACAATTCAAA----
-----------------------------------AATAAAACAATAGTCTTTAAA------
CAATCCTCAGGAGGGGACCCAGAAGTTGTA---ATGCATACTTTTAATTGTGGGGGGGAA---
TTTAAGCAGCTCCAGGCAAGAATCCTGGCTGTAGAAAGATACCTAAAGGATCAACAGCTCCTGGGAATTTGGG
GTTGCTCTGGAAAACTCATTTGCACCACTGCTGTGCCTTGGAATACTAGTTGGAGT-----------------
----------------------------AATAAATCTCAGGATGAGATTTGG------GGT------
AACATGACCTGGATGCAGTGG---GAAAGAGAAATTGACAATTACACAAGCTTCATATAC---
ACTTTAATTGAAGAATCGCAGAGACAACAAGAA--

AAGAATGAACAAGAATTATTGGAATTGGATAAGTGGGCAAGTTTGTGGACTTGGTTTGACATAACAAAATGGC
TGTGGTATATAAAAATATTCATAATGATAGTAGGAGGCTTGATAGCTTTAAGAATAGTTTTTGCT---
GTACTTTCTATAGTA---------AATAGAGTTAGGCAGGGATACTCACCC------
CTATCGTTGGCTGTTAGCTTATTCAACGCCATAGCAATAGCAGTAGCTGAG------------------
ATAGAACTACCACAAAGAATTTTTAGAGGC-------------------------------------
TTTCTCCACATA------CCTAGAAGAATAAGACAGGGCTTAGAAAGACTTTTGCTA---TAA
>KU685589
GGT---GCT---GTA-----------------------------------
GAGCAGTCGTGGGTCACAGTCTATTATGGGGTACCTGTGTGG---AAAGAGGCAACC---
ACAACTCTATTTTGTGCATCAGATGCTAAAGCATATGATACAGAG---GCACATAAT----------------
--------------GTTTGGGCCACA---CATGCCTGTGTACCCACAGAC---
CCCAACCCACAAGAAATAGACTTGGAA---------AATGTGACAGAAACTTTTAAT----------------
--------------------------GCATGGAATAATGACATGGTAGAACAGATGCATGAAGATATT---
ATCAGCTTGTGGGATCAAAGCCTA---AAACCATGTGTAAAATTA---
ACCCCACTCTGTGTTACTTTAAATTGCATTGATTTTACAGGGAATAGTACTAATGTTAATAGTACTAATGCTA
CT---
-------------------------------------GTTGAAGCCCTTACAGGAGAAATAAAA---
AACTGCTCTTTCAATATCACCACAGACTTAATA--------------------------
GATAAGAARAAGAAAGAACATGCACTT---
TTTTATAAACTTGATATAGAACCAATAGATGGGGCTGGGAACAACTCT-------------------------
----
AGCTACTCTAGCTATAAGTTGATAAATTGTAATACCTCAACCATTGCACAG---
GCCTGTCCAAAGATAACCTTTGAGCCAATTCCCATACATTATTGTGCCCCGGCTGGTTTTGCAATTCTAAAGT
GTAAA---GACAAGAAGTTCCAG------GGAAAAGGAGAATGTAAAAAG-----------------------
-GTCAGT---ACAGTACAATGCACACATGGAATCAGGCCAGTAGTGTCAACT---
CAACTGCTGTTAAATGGCAGTCTAGCAGAAGAAGAC-----------
ATAGTAATTAGATCTCAAAATTTCTCGGACAAT---------AAGAAAATCATAACAGTACAACTAAAG----
-----------GAAGCTGTAAAA---------ATTAATTGTACAAGACCCGGCAACAATACAAGAAAA-----
-AGCATACATATA------------GCACCAGGGAGAGCA---TTG------TATGCAACAGGAGAC------
---------ATAATAGGAGATATA------------AGACAAGCACATTGT---AACCTCAGCAGAACA---
GCTTGGAATACCACGTTAGGTGAGATAGCTAAAAAATTACAA---------------GCACAATTTAAT----
--------------------------------GGAACAACAACAATAAAATTTGAG---
CCACCCAACACAGGAGGGGACCCAGAAATTGTA---ATGCACACTTTTAATTGTGGAGGGGAA---

TTTAAACAGCTCCAGGCAAGAGTCCTGGCTGTGGAAAGATACCTACAGGATCAACGGCTCCTAGGACTTTGGG
GTTGCTCGGGAAAACTCATCTGCACCACTACTGTGCCTTGGAATGCTAGTTGGAGT-----------------
----------------------------AATAAATCTCTGGATGAAATTTGG------AAC------
AACATGACCTGGCAACAGTGG---GAGAATGAAATTGACAATTACACAGGCATAATATAC---
AACTTAATTGAAATATCGCAAAACCAGCAGGAA--

AAGAATGAACAAGAATTGTTGGAATTGGATAAATGGGCGAGTCTGTGGAATTGGTTTGACATAACACAATGGC
TGTGGTATATAAAAATATTCATAATGATAGTAGGAGGTTTGATAGGTTTAAGAATAGTTTTTGCT---
GTACTTTCTATAGTG---------AATAGAGTTAGGCAGGGTTATTCACCA------
CTGTCATTTGCTGTAAGCTTGCTTAACGCCACAGCTATAGCAGTAGCAGAG---
GGGACAGATAGGGCTATAGAAATATTACAAAGAATTTTTAGAGCT----------------------------
--------ATTATCCACATA------CCTACAAGGATAAGACAGGGGTTGGAAAGGGCTTTGCTA---TAA
>KY989949
ACT---GCA--
GCCCAATTGTGGGTCACAGTCTATTATGGGGTACCAGTATGG---AAAGAAGCAACC---
GCTACTCTATTTTGTGCATCAGATGCTAAGGCATTTAAGACAGAG---GTACACAAT----------------
--------------GTTTGGGCCACA---CATGCCTGTGTACCTACAGAT---
CCCAACCCACAAGAAATAATAATGGAA---------AATGTGACAGAAAATTTTAAC----------------
-------------------------ATGTGGAAAAATGACATGGTAGAACAGATGCACGAGGATATA---
ATCAGTTTATGGGATGAAAGCCTA---AAGCCATGTGTAAAATTA---
ACTCCACTCTGTGTTACTTTAAATTGCACTGATTATCAGAATAATACA------------------------
----
---ATAGAGAAGGGAGAAATAAAA---
AACTGCTCTTTCTATACCACCCCAGACATGAGA-------------------------
GATAAGAAGCAGACAGCATATGCACTT---TTTTATAAACTAGATGTAGTACCAATAGATGAT----------
----

AATAGTAATGATACTAATAGTAATAATAGCTATAGGTTGATAAGTTGTAATACCTCAGTCATTACACAG---
GCCTGTCCAAAGGTATCTTTTGAACCAATTCCTATACATTATTGTACCCCGGCTGGTTTTGCGCTCCTAAAAT
GTAAC---AATAAGACATTCAAT------GGAACAGGACCATGTACAAAT-----------------------
-GTCAGT---ACAGTACAATGTACACATGGAATTAGACCAGTAGTGTCAACT---
CAACTGCTGTTAAATGGCAGTCTAGCAGAAGAAGAG-----------
ATAGTAATTAGATCTGAAAACTTTTCGAACAAT---------GCTAAAACCATAATAGTACAGCTGACA----
-----------AAACCTGTAGTA---------ATTAATTGTACAAGACCCAACAACAATACAAGAAAA-----
-AGTATACATATA------------GGACCAGGAAGAGCA---TTT------TATGCAACAGGAGAC------
---------ATAATAGGAGATATA-----------AGACAAGCACATTGT---AACATTAGTGAAAGA---
GAATGGAATGAAACTTTAAGGCAGATAGCTGGAAAATTAAGA---------------GAACAATTTAGG----
-----------------------------------AATAAAACCATAGCCTTTAAC------
AAGTCCTCAGGAGGGGACCCAGAAATTGTA---ATGCACAGTTTCAACTGTGGAGGGGAA---
TTCAAGCAGCTCCAGGCAAGAGTCCTGGCTGTGGAAAGATACCTAAAGGATCAACAGCTCCTGGGAATTTGGG
GCTGCTCTGGAAAACTCATCTGCACCACTAATGTGCCTTGGAATAGTAGCTGGAGT-----------------
----------------------------AATAGATCTCAAGAGGAGATTTGG------AAT------
AACATGACCTGGATGCAGTGG---GAAAGAGAAATTGACAATTACACAGGCTTAATATAC---
AACCTAATTGAAAAATCGCARACCCAGCAAGAA--

ATGAATGAATTAGAATTAATGAAATTGGATAAATGGGCAAATTTGTGGAATTGGTTTGACATAACARACTGGC
TGTGGTATATAAGAATCTTCATAATGATAGTAGGGGGCTTAATAGGTTTAAGAATAGTTTTTACT---
GTACTTTCTATAATA---------AATAGAGTTAGGCAGGGATACTCACCA------
ATATCGTTGGCTGTTAACTTGCTTAATACCACAGCTATAGCAGTAGCTGAG---
GGGACAGATAGAGTTATAGAGTTCTTTCAAAGAGGATTTAGAGCT----------------------------
--------ATTATCCACATC------CCCAGAAGAATAAGACAGGGCTTARAAAGGGCTTTGCTA---TAA
>KY989954
GCT--
GAAGATTCGTGGGTCACAGTCTACTATGGGGTACCTGTGTGG---AAAGAAGCAACC---
ACCACTCTATTTTGTGCATCAGATGCTAAAGGATATAAGACAGAG---GTACATAAT----------------
--------------GTTTGGGCCACA---CATGCCTGTGTACCCACAGAC---
CCCAACCCACGAGAAGTAGCATTGGAA---------AATGTGACAGAAGATTTTAAC----------------
-------------------------ATGTGGAAAAATAACATGGTAGAACAGATGCATGAGGATATA---
ATCAGTTTATGGGATCAAAGCCTA---AAGCCATGTGTAAAATTA---
ACCCCACTCTGTGTCACTTTAAACTGCACTGATGTGGATAATACTACTGAAACTAATGTGGGTAATACTACT-

----
----------------------GGAGCTAATGTCAGTAGCAGCAATGGGGGAGGAGAAATGAAA---
AACTGCTCCTTCAATATGACCACAAACATAAGA---------------------------
GACAAGGTGCAGAAACAATATGCACTT---TTTTATACACTTGATATAGTACCAATAGACAAC----------
----

AGTAGTAACTATAGGTTGATAAGTTGTAATACCTCAATCATCACACAG---
GCTTGTCCAAAAACATCCTTTGAGCCAATTCCAATACATTATTGTACCCCGGCTGGTTTTGCGCTTCTAAAGT
GTAAC---AATAAGACATTCAGT------GGGATGGGACCATGTAAAAT------------------------
-GTCAGC---ACAGTACAATGTACACATGGAATCAGGCCAGTAGTATCCACT---
CAATTACTGTTAAATGGCAGTCTAGCAGAAGAAGAG------------
GTAATACTCAGATCTGCCAATTTCACAGACAAT---------ACTAAAGTCATAATAGTACATTTGAAT----
-----------GAATCTGTAGAA---------ATTAACTGTACAAGACCCAATAACAATACAAGTAAA-----
-AGTATACCTATA------------GGACCAGGGAGAGCA---TTT------TATGCAACAGGTAGG------
---------GTAATAGGAGATATA------------AGACAAGCACATTGT---AACATTAGTGCAGCA---
AAATGGAATACTGCTATAACCAGGATAGTTACAAAATTAAGA---------------GAACAATTC-------
-----------------------------------AATAAAACAATAGTCTTTAAC------
AGCTCCTCAGGAGGGGATCCAGAAATTGTA---ATGCATAGTTTTAATTGTGGAGGAGAA---
TTTAAGCAGCTCCAGGCAAGAGTCCTGGCTGTGGAAAGATACCTACAAGATCAACGGCTCCTAGGGCTCTGGG
GTTGCTCTGGAAAACTCATCTGCACCACTGCAGTGCCTTGGAATTCTAGTTGGAGT-----------------
----------------------------AATAAATCTCTAGATAACATTTGG------CAC------
AACATGACCTGGATGCAGTGG---GAAAAGGAAATTGGGAATTACACAGACTTAATATAC---
ACTTTAATTGAAGCCTCGCAAAACCAACAAGAA--

AAGAATGAACAAGAATTGTTGGAATTGGATAAATGGGCAAGTTTGTGGAGTTGGTTTGACATAACACAATGGC
TGTGGTACATAAAAATATTCATAATGATAGTAGGAGGCTTGATAGGTTTTAGAATAGTTTTTACT---
GTACTTTCTATAGTG---------AATAGAGTTAGGCAGGGATACTCACCA------
TTGTCGTTTGCTGTTAGTCTGCTTAATGCCACAGCTATAGCAGTAGGTGAG---
GGGACAGATAGGGTTATAGAAGCATTACAAAGAGTTTGTTTAGCT----------------------------
--------ATTCTCCATATA------CCTAGAAGAATCAGACAGGGCTTAGAAAGGGCTTTGCTA---TAA
>KY989956
GCT---AAA--
GAACAATTGTGGGCCACAATCTATTATGGGGTACCTGTGTGG---AAGGAAGCAACC---
ACCACTCTATTTTGTGCCTCAGATGCTAAAGCATATGATCCAGAG---GTACATAAT----------------
--------------ATCTGGCCACA---CATGCCTGTGTGCCCACAGAC---
CCCAGCCCACAAGAAATAGAATTGGAA---------AATGTGACAGAAAACTTTAAC----------------
--------------------------ATGTGGAAAAATGATATGGTAGAGCAAATGCAGGAGGATATA---
ATCAATTTGTGGGATCAAAGCCTA---AAGCCATGTGTAAAATTG---
ACTCCACTCTGTGTTACTTTAAATTGCACTGATGTGGGAAATAGTACTAATGACAGTCATAATAGC-------
----
-------------ACTAATGGCAATTATAGTAATTGGGAAAGGATGGAGCCAGGAGAAATAAAA---
AAATGCTCTTTCAATATTACCACAAACATGCAA---------------------------
GATAAGAGGCAGAAAGAATATGCACTT---TTTTATAAACTTGATGTAGTACCAATAGATAATAAT-------
----

GATAATAGTAGCTATAGGTTGATAAGTTGTAATACCTCAGTCATTACACAG---
GCCTGTCCAAAGGTAACTTTTGAGCCAATTCCTATACATTATTGTGCCCCGGCTGGTTTTGCGATTCTAAAGT
GTAAC---ACAAAAGGGTTCAAT------GGAACAGGGCCATGTAGCAAT-----------------------
-GTCAGC---ACAGTACAATGTACACATGGAATTAAGCCAATAGTGTCAACT---
CAACTACTGTTAAATGGCAGTCTAGCAGAAGAAGAG------------
GTAGTCATTAGATCTGAAAATATCACAGACCAT---------CTTAAAAACATCATAGTACAGCTGAAT----
-----------AAGTCTGTAAGC---------ATTACTTGTGTAAGACCCAACAACAATACAAGAAAA-----
-AGTGTACACATG------------GGACCAGGGAGTGTA---TGG------TATGCAACAGGAGAC------
---------ATAATAGGAGATATA------------AGACAAGCCCATTGT---AACATTAGTGAAAAT---
GATTGGCAAAACGCCTTAAAGCAGGTAGCCACAAAATTAAGA---------------GAACAGTTT-------
-----------------------------------AATAAAACAATAAACTTTGCT------
AAACATGCAGGAGGGGACCCAGAAGTTGAA---CTGCACAGTTTTAATTGTGGGGGAGAA---
TTTAAACAGCTCCAGGCAAGAGTCCTAGCTGTGGAGAGATACCTAAAGGATCAACAGCTCCTAGGGATTTGGG
GTTGCTCTGGGAAACTCATCTGCCCCACTGCTGTGCCTTGGAATGCTAGTTGGAGC-----------------
----------------------------AATAAATCTCTGGATGATATTTGG------AAT------

AACATGACCTGGATGGAGTGG---GACAGAGAAGTTAGCAATTACACAAACATAATATAC---
AGTTTAATTGAGCAATCGCAAATCCAGCAAGAA--

AAGAATGAACAAGAATTATTAGAATTGGATAAATGGGCAAGTTTATGGAATTGGTTTGATATATCAAACTGGC
TGTGGTATATAAAAATGTTCATAATGATAGTAGGAGGCTTGATAGGTTTAAGAATAGTTTTTGCT---
ATACTTTCTATAGTA---------AATAGAGTTAGGCAGGGATACTCACCA------
TTATCATTTGCTGTAAGCTTGCTTGATGCTACAGCTGTAGCAGTAGCTGAA---
GGGACAGATAGGATAATAGAAGCAGTACAAACAGTTCTTAGAGCA----------------------------
--------GTTATCCGCATA------CCTACGAGAATAAGACAGGGCCTAGAAAGAGCTTTGCTA---TAA
>KC595171
GCT---GCA--
GGAAATTTGTGGGTCACAGTCTATTATGGGGTACCTGTGTGG---AAAGAAGCAACC---
ACCACTCTATTTTGTGCATCAGATGCTAGAGCATATGATACAGAG---GTACATAAT----------------
--------------GTTTGGCCACA---CATGCCTGTGTACCCACAGAC---
CCCAACCCACAAGAAGTAGTATTGAAA---------AATGTGACAGAAAACTTTAAC----------------
--------------------------ATGTGGAAAAATAACATGGTAGAACAGATGCATGAGGATATA---
ATCAGTTTATGGGACCAAAGCCTA---AAGCCATGTGTAAAATTA---
ACCCCACTCTGTATTACTTTAAATTGCACTGATTATAATGGCACTAGTAATAATGCCACTAGTAATAATGCCA
CT---
----------------------GGGGATAGTTGGGGAATGAAGATGGAAGAAGGAGAAATAAAA---
AACTGCTCTTTCAATATCACCACAAGCATAAGA---------------------------
GATAAGGTGCAGAAAGAATATGCACTT---TTTTATAAACTTGATGTAGTACCAATAGACAAT----------
----

GATAATAGTAGCTATAGGTTGATAAGTTGTAACACCTCAGTCATTACACAG---
GCCTGCCCAAAAGTATCCTTTGAGCCAATTCCCATACATTATTGTGCCCCAGCTGGTTTTGCGATTCTAAAGT
GTAAA---GATAAGAAGTTCAAT------GGAACAGGACCATGTACAAAT-----------------------
-GTCAGC---ACAGTACAATGTACACATGGAATTAAGCCAGTAGTATCAACT---
CAACTGCTGTTAAATGGCAGTCTAGCAGAGGAAGAG------------
GTAGTAATTAGATCTGAGAATTTCACGAACAAT---------GCAAAAATCATAATAGTACAGCTGAAT----
-----------GAATCTGTAGAA---------ATTAATTGTATAAGACCCAACAACAATACAAGAAAA-----
-AGTATACATATA------------GGACCAGGCAGAGCA---TTG------TATACAACA---GAA------
---------GTAGTAGGAAATATA------------AGACAAGCATATTGT---AACCTTAGTAGAGCA---
AAATGGAATGACACTTTAAAACAGATAGCTATAAAATTAAGA---------------GAACAATTTAAG----
-----------------------------------AATAAAACAATAGTCTTTAAT------
CAATCCTCAGGAGGGGACCCAGAAATTGTA---ACGCACAGTTTTAATTGTGGAGGGGAA---
TTTAAGCAGCTCCAGGCAAGAGTCCTGGCTGTGGAAAGGTACCTAAAGGATCAACAGCTCCTGGGGATTTGGG
GTTGCTCTGGAAAACTCATTTGCACCACTGCTGTGCCTTGGAATACTAGTTGGAGT-----------------
----------------------------AATAGATCTCTGGAGCAGATTTGG------AAT------
ACAACGACCTGGATGGAGTGG---GAAAGAGAAATTGATAATTACACAAGCTTAATATAC---
ACCTTAATTGAAGAATCGCAAAACCAACAAGAA--

AAGAATGAACAAGAATTATTGGAATTGGATAAATGGGCAAGTTTGTGGAATTGGTTTGACATAACAAAATGGC
TGTGGTATATAAAAATTTTCATAATGATAGTAGGTGGCTTGGTAGGTTTAAGAATAGTTTTTACT---
GTACTTTCTATAGTG---------AATAGAGTTAGGCAGGGATACTCACCA------
TTATCGTTTGCTGTTAGCTTGCTCAACGCCACAGCCATAGCAGTAGCTGAG---
GGGACTGATAGGGTTATAGAAGTATTACAAAGAGCTTATAGAGCT----------------------------
--------ATTCTCCACATA------CCTACACGCATAAGACAGGGCTTGGAAAGGGCTTTGCTA---TAA
>KC595172
GTG---GCA--
GAACAATTGTGGGTCACAGTCTATTATGGGGTACCTGTGTGG---AAAGAAGCAACC---
ACCACTCTATTTTGTGCATCAGATGCTAAAGCATATGATACAGAG---GTACATAAT----------------
--------------GTTTGGCCACA---CATGCCTGTGTACCCACAGAC---
CCCAACCCACGAGAAATAAGATTGGAA---------AATGTGACAGAAAATTTTAAC----------------
--------------------------ATGTGGAAAAATAACATGGTAGAACAGATGCATGAGGATATA---
ATCAGTTTGTGGGACCAAAGCCTA---AAGCCATGTGTACAATTA---
ACCCCACTCTGTGTTACTTTAAATTGCACTGATGTGGGAAATGGTACTAATAGTGGTAATAATACTAGCACT-
----
----------------------AATAGTAGTAACTGGGAAAAGATGGAAAAAGGAGAAATGAAA---
AACTGCTCTTTCAATGTCACCGCAGGCATACAA---------------------------

GATAGGAGGCAGAAAGAATATGCAATT---TTTTATAAGCTTGATATAGTACCAATAGATAATGATAATACC-
----

AACAATACCAACTTTAGGTTGATAAGTTGTAACACCTCAGTCATTACACAA---
GCCTGTCCAAAGGTATCCTTTGAGCCAATTCCCATACATTATTGTACCCCGGCTGGTTTTGCGATTCTACAGT
GTAAC---GATAAAAAGTTCAAT------GGAACAGGACCATGTAAAAAT-----------------------
-GTCAGC---ACAGTACAATGTACACATGGAATTAGGCCAGTAGTATCAACT---
CAACTGCTGTTAAATGGCAGTCTAGCAGAAGAAGAG-----------
GTAGTAATTAGGTCTGAAAATTTCACGGACAAT---------ACTAAAACCATAATAGTACAGCTGAAC----
-----------AGATCTGTAGAA---------ATTAATTGTACAAGACCCAACAACAATACAAGAAAA-----
-GGTATATATATA------------GGACCAGGGAGAGCA---GTT------TATACAACAGACAAA------
---------ATAATAGGAGATATA------------AGAAAAGCACATTGT---AACATTAGTAGAGTA---
GAATGGAATAACACCTTAAAACAGATAGTTGGCAAATTAAGA---------------GAACAATTTGTG----
-----------------------------------AATAAAACAATAGTCTTTAAT------
AAATCCTCAGGAGGGGACCCAGAAATTGTA---ATGCACAGTTTTAATTGTGGAGGGGAA---
TTTAAGCAGCTCCAGGCAAGAGTCCTGGCTGTGGAAAGATACCTACAGGATCAGCAGCGCCTGGGGATTTGGG
GTTGCTCTGGAAAACTCATTTGCACCACTGCTGTGCCTTGGAATGCTAGTTGGAGT-----------------
----------------------------AATAAATCTCTAAATGAAATTTGG------GAT------
AACATGACCTGGATGGAGTGG---GAAAGAGAAATTGACAATTACACAGGCTTAATATAC---
AACTTACTTGAAGAATCGCAAAATCAACAGGAA---

AAGAATGAACAAGATTTATTGGCATTAGATAAATGGGACAGTTTGTGGAGTTGGTTTAACATAACAAACTGGC
TGTGGTATATAAAAATATTCATAATGATAGTAGGAGGCTTGATAGGTTTAAGAATAGTTTTTACT---
GTACTTTCTATAGTG---------AATAGAGTTAGGCAGGGATACTCACCA------
TTATCGTTTGCTGTTAGCTTGCTCAATGCCACAGCTATAGCAGTAGCTGAG---
GGGACAGATAGGGTTATAGAAGTATTACAAAGAGCTTTTAGAGCT----------------------------
--------ATTCTTCACATA------CCTAGAAGAATAAGACAGGGAGCGGAAAGGGCTTTGCTA---TAA
>KC595174
GCT---GCA---
GAAGACACGTGGGTCACAGTCTATTATGGGGTACCTGTGTGG---AAAGAAGCAACC---
ACCACTCTATTTTGTGCATCAGATGCTAAAGCACATGATACAGAG---GTACATAAT----------------
--------------ATTTGGGCCACA---CATGCCTGTGTACCCACAGAC---
CCCCACCCACAAGAAGTAGAATTGGAA---------AATGTGACAGAAAATTTTAAC---------------
--------------------------ATGTGGAAAAATAACATGGTAGAACAGATGCATGAGGATATA---
ATCAGTTTATGGGATCAAAGCCTA---AAGCCATGTGTAAAATTA---
ACCCCACTCTGTGTTACTTTAAATTGCACTGATGGGAGGAATGATACTAATACCACTAGTGCTAAT-------
----
-------------------ACCAATGATAGTAGCGGGGGACCGATGGAGAAAGGAGAAATAAAA---
AATTGTACTTTCAATATCACCACAAACATAGGA---------------------------GATAAGGTG---
GAAGGATCTGCAATT---TTTAATAAACTTGATGTAGTACCAATAGACAATAAT------------------
----
----------AATAACAGTAGATATAGATTAATAAATTGTAATACCTCAGTCATTACCCAG---
GCTTGTCCAAAAGTATCCTTTGATCCAATTCCCATACATTATTGCACCCCGGCTGGTTTTGCGATTCTAAAGT
GTAAT---GATAAGAAGTTCAAT------GGAACAGGACCATGTAAAAAT-----------------------
-GTCAGC---ACAGTACAATGTACACATGGAATTAGGCCAGTAGTATCAACT---
CAACTGCTGTTAAATGGCAGTCTAGCAGAAGAAGAG-----------
GTAGTAATTAGATCTGAAAATTTCACGAACAAT---------GCTAAAATCATAATAGTACAGCTGAAA----
-----------GAACCTATAAAA---------ATTAATTGTACAAGACCCAACAACAATACAAGAACA-----
-AGTATACCTCTA------------GGACCAGGGAGAGCA---TTT------TATACAACAGGAGAC------
---------ATAATAGGAGATATA------------AGAAAAGCCTATTGT---ACCATTAATAGAGCA---
AAATGGAATAACACTTTAAAACAAATAGTTAGCAAATTAAAA---------------GAACAATTCGGG----
-----------------------------------AATAAAACATTCATCTTTAAT------
CAATCCTCAGGAGGGGACCCAGAAATTGTA---ACGCACAGTTTTAATTGTGGAGGGGAA---
TTTAAGCAGCTCCAGGCAAGAGTCCTGGCTGTGGAAAGATACCTACAGGATCAACAGCTCCTGGGGATTTGGG
GTTGCTCTGGAAAACTCATCTGCACCACTACTGTGCCTTGGAATGCTAGTTGGAGT-----------------
----------------------------AATAAATCTCTGAATGAAATTTGG------AAT------
AACATGACCTGGATGGAGTGG---GAAAGAGAAATTGACAATTATACAAGCTTAATATAC---
ACCTTAATTGAAAAATCGCAGAACCAACAAGAA---

AAGAATGAACAAGAATTATTGGAATTGGATAAATGGGCAAGTTTGTGGAGTTGGTTTGACATAACAAATTGGC

TATGGTACATAAGAATATTCATAATGATAGTAGGAGGCTTGGTAGGTTTAAGAATAGTTTTTGCT---
GTACTTTCTATAGCG---------AATAGAGTTAGGCAGGGATACTCACCA------
TTATCATTTGCTGTTAGCTTGCTCAACGCCACAGCCATAGCAGTAGCTGAG---
GGGACAGATAGGGTTATAGAAGCATTACAAAGAGCTTTTAGAGCT----------------------------
--------ATTATCCACATA------CCTAGAAGAATAAGACAGGGCTTCGAAAGGGCTTTGCTA---TAA
>KC595177
GCT---ACA--
GAAAAATTGTGGGTCACAGTCTATTATGGGGTACCTGTGTGG---AAAGAAGCAACC---
ACCACTCTATTTTGTGCATCAGATGCTAAAGCATATGATACAGAG---GTACATAAT----------------
--------------GTTTGGGCCACA---CATGCCTGTGTACCCACAGAC---
CCCAACCCACAAGAAGTAGAATTGAAA---------AATGTGACAGAAGAATTTAAC----------------
--------------------------ATGTGGAAAAATAACATGGTAGAACAGATGCATGAAGATATA---
ATCAGTTTATGGGATCAAAGCCTA---AAGCCATGTGTAAAATTA---
ACCCCACTCTGTGTTACTTTAAATTGCACTGATGTGGGGAATACTACTAATACCAATAGTAGT----------
----
----------------------------AGCGGGGGAATGATGGAGGAGAAAGGAGAAATAAAA---
AACTGCTCTTTCAATATCACCACAAACATGAGA---------------------------
GATAAGATGCAGAGACAATATGCACTT---TTTTATAAACTTGATGTAGTACCAATAGACAAT----------
----

AATACTAATAGCTATAGGTTGCTAAGTTGTAACACCTCAGTCATTACACAG---
GCTTGTCCAAAAGTAACCTTTGAGCCAATTCCCATACATTACTGTGCCCCGGCTGGTTTTGCGATTCTAAAGT
GTAAA---GATAAGAAGTTCAGT------GGAAAAGGAGAATGTAAAAAT-----------------------
-GTCAGC---ACAGTACAATGTACACATGGAATTAGGCCAGTAGTATCAACT---
CAACTGCTGTTAAATGGCAGTCTAGCAGAAGAAGAG------------
GTAGTAATTAGATCTGACAATTTCTCGAACAAT---------GCTAAAACCATAATAGTACAGCTGAAA----
-----------GAATCTATAGAA---------ATTAATTGTACAAGACCCAACAACAATACAAGAAAA-----
-AGTATACATATA------------GGACCAGGGAGTACA---TTG------TATGCAACA---GAA------
---------ATAATAGGAGATATA------------AGACAAGCACATTGT---AACCTTAGTAGAACA---
AAATGGCATAACACTTTAGGACAGATAGTTAAAAAATTAAAA---------------
GAACAAGAAGAATTCAAG---------------------------------AATAAAACAATAGTCTTCAAT-
-----CAATCCTCAGGGGGGGACCCAGAAATTGTA---ATGCACAGTTTTAATTGTGGAGGGGAA---
TTTAAGCAGCTCCAGGCAAGAGTCCTGGCTGTGGAAAGATACCTAAAGGATCAACAGCTCCTGGGGATTTGGG
GTTGCTCTGGAAAACTCATTTGCACCACTACTGTGCCTTGGAATACTAGTTGGAGT-----------------
----------------------------AATAAATCTCTAGAGCAGATTTGG------GAT------
AACATGACCTGGAGGCAGTGG---GAAAGAGAAATTGACAATTATACAGACTTCATATAC---
AACTTAATTGAAGAATCGCAGAACCAACAAGAA--

AAGAATGAACAAGAATTATTGGAATTGGATAAATGGGCAAGTTTGTGGAATTGGTTTGACATAACAAATTGGC
TGTGGTATATAAAAATATTCATAATGATAGTAGGAGGCTTGGTAGGTTTAAGAATAGTTTTTACT---
GTACTTTCTATAGTG---------AATAGAGTTAGGAAGGGATACTCACCA------
TTATCGTTTGCTGTTAACCTGCTCAATGCCATAGCCATAGCAGTAGCTGAG---
GGGACAGATAGGATCATAGAAGTAATACAAAGAGCTTGTAGAGCT----------------------------
--------ATTCTCCACATA------CCTAGAAGAATAAGACAGGGATTTGAAAGGGCTTTGCTA---TAA
>KC595178
GCT---GCA--
GAAGAGTTGTGGGTCACAGTCTATTATGGGGTACCTGTGTGG---AAAGATGCAAAC---
ACCACTCTATTTTGTGCATCAGATGCTAAAGCATATGATACAGAG---GTACATAAT----------------
--------------GTTTGGGCCACA---CATGCCTGTGTACCCACAGAC---
CCCAACCCACAAGAAGTAGTATTGGAA---------AATGTGACAGAAAATTTTAAC----------------
--------------------------ATGTGGAAAAATAACATGGTAGAACAGATGCAGGAGGATATA---
ATTAGTTTATGGGATCAAAGCCTA---AAGCCATGTGTAAAATTA---
ACCCCACTCTGTATTACTCTAAATTGCACTGATTATTTGAGGAATGATACTAACACCACTAGT----------
----
----------------------------GATAGCGGGGGAAGGATGGAGGGAGGAGAGATAAAA---
AATTGCTCTTTTAATATCACCACAAACATAAGA---------------------------
GATAAGGTGCAGAAAGAATATGCACTT---TTTTATAAACTTGATGTAATACCAATAGAAAAT----------
----

GAGAATACTAGCTATAGGTTGATAAGTTGTAACACCTCAGTCATTACACAG---

GCCTGTCCAAAGGTATCCTTTGAACCAATTCCCATACATTATTGTGCCCCGGCTGGTTTTGCGATTCTAAAGT
GTAAC---GATAAGAAGTTCAAT------GGAACAGGACCATGTACAAAT-----------------------
-GTCAGC---ACAGTACAATGTACACATGGAATTAGGCCAGTAGTATCAACT---
CAACTGCTGTTAAATGGCAGTCTAGCAGAAGAAGAG------------
GTAGTAATTAGATCCAGTAATTTCACGGACAAT---------GCTAAGGTCATAATAGTACAGCTGAAT----
-----------GAATCTGTAGTA---------ATTAATTGTACAAGACCCAACAACAATACAAGAAAA-----
-AGTATACATATA------------GGACCAGGCAGAGCA---TTT------TATACAACAGGAGAA------
---------ATAATAGGAGATATA------------AGACAAGCACACTGT---AATCTTAGTAGAGCA---
AAATGGAATGACACTTTAAAACAGATAGTTATAAAGTTAAAA---------------GAACAATTTGGG----
-----------------------------------AATAAAACAATAGTCTTTAAT------
CAATCCTCAGGAGGGGACCCAGAAATTGTA---ACACACAGTTTTAATTGTGGAGGGGAA---
TTTAAGCAGCTCCAGGCAAGAGTCCTGGCTGTGGAAAGATACCTAAAGGATCAACAGTTCCTGGGGATTTGGG
GTTGCTCTGGAAAACTCATTTGCACCACTACTGTGCCTTGGAATACTAGTTGGAGT-----------------
----------------------------AATAAAACTCTGAATCAGATTTGG------GAT------
AACATGACCTGGATGGAGTGG---GACAGAGAAATTAATAATTACACAAGCTTAATATAC---
ACCTTACTTGAAGAATCGCAAAACCAACAAGAA--

AAAAATGAACAAGAATTATTGGAATTAGATAAATGGGCAAGTTTGTGGAATTGGTTTGACATAACAAATTGGC
TGTGGTATATAAAAATATTCATAATGATAGTAGGAGGCTTGGAAGGTTTAAGAATAGTTTTTGCT---
GTACTTTCTATAGTG---------AATAGAATTAGGCAGGGATACTCACCA------
TTATCGTTCGCTGTTAGCTTGCTCAATGCCACAGCCATAGCAGTAGCTGAG---
GGGACTGATAGGGTTATAGAAGTATTACGAAGAGCTTATAGAGCT----------------------------
--------ATCCTTCACATA------CCTACACGAATAAGACAGGGCTTGGAAAGGGCTTTGCTA---TAA
>KC595195
GCT---ACA--
GACAAATTGTGGGTTACAGTCTATTATGGGGTACCTGTGTGG---AAAGAAGCAAAC---
ACCACTCTATTTTGTGCATCAGATGCTAAAGCATATGATACAGGG---GTACATAAT----------------
--------------GTCTGGCCACA---CATGCCTGTGTACCCACAGAC---
CCCCACCCACAAGAAATAGTATTAACA---------AATGTGACAGAAAATTTTAAC----------------
--------------------------ATGTGGAAAAATAACATGGTAGAACAGATGCATGAGGATATA---
ATCAGTTTATGGGATCAAAGCCTA---AAGCCATGTGTAAAATTA---
ACCCCACTCTGTGTTACTTTAAATTGTACTGATTGGGGAAATACTACTAATAGCACCAGT-------------
----
----------------------------AATAGTGGGGAAATGATAGAGAAAGGAGAAATAAAA---
AACTGCTCTTTCAATGTCACCACAAGCATAAGA---------------------------
GATAAGGTGCGAAAAGAATATGCACTT---TTTTATAAACTAGATGTAGTACCAATAAAAGATGATAAT----
----

AGCAGCCATAATGAAAGCTATAGATATAGGTTGATAAGTTGTAATACCTCAGTCATTACACAG---
GCCTGTCCAAAAGTAACCTTTGATCCAATTCCCATACATTATTGTGCCCCGGCTGGATTTGCGCTTCTAAAAT
GTAAC---AATAAAAAGTTTAGT------GGGAAAGGACCATGTACAAAT-----------------------
-GTCAGC---ACAGTACAATGTACACATGGGATTAGGCCAGTAGTATCAACT---
CAACTATTGTTAAATGGCAGTCTAGCAGAAAAAGAG------------
ATAGTAATTAGATCTGACAACTTCACGGACAAT---------ACTAAAACCATAATAGTACAGCTGAAC----
-----------GAATCTGTAGAA---------ATTAATTGTACAAGACCCAACAACAATACAAGAAGA-----
-AGTATACATTTA------------GGACCAGGGGGAGCC---TTT------TATGCAGCAGGAGAG------
---------ATAATAGGGGATATA------------AGACAAGCACATTGT---AACATTAGTAGAGCA---
AAGTGGAATACCACTTTACAACAGATAGCTGGAAAATTAGGA---------------GAACAATTCGGG----
-----------------------------------AATAAAACAATAGTCTTTAAT------
TACTCCGCAGGAGGGGACCTAGAAATTGTA---ATGCACAGTTTTAATTGTGCAGGGGAA---
TTTAAACAGCTCCAGGCAAGAGTCCTGGCTGTGGAGAGATACCTAAAGGATCAACAGCTCCTGGGGATTTGGG
GTTGCGCTGGAAGACACATTTGCACCACTACTGTGCCTTGGAATGCTAGTTGGAGT-----------------
----------------------------AATAAATCTTATACTGATATTTGG------GAT------
AACATGACCTGGATGCAGTGG---GAAAAAGAAATTGACAATTACACAAACTTAATATAC---
TCCTTAATTGAAGAATCGCAAGTCCAGCAAGAA--

AAGAATGAAAAAGAATTGTTGGAATTGGATAAATGGGCAAGTCTGTGGAATTGGTTTAGCATAACACAATGGC
TGTGGTATATAAAAATATTCATAATGATAGTAGGAGGCTTAATAGGTTTAAGAATAGTTTTTACT---
GTACTTTCTATAGTG---------AATAGAGTTAGGCAGGGATACTCACCA------
TTATCGTTTGCTATTAGCTTGTTTAATGCCATAGCCATAGCAGTAGCTGAG---

GGGACAGATAGGGTTATAGAAGTAGTACAAAGAGTTGTTAGGGCT----------------------------
--------ATTCTCCACATA------CCTAGAAGAATAAGACAGGGCTTGGAGAGGCTTTTGCTG---TAA
>DQ854716
GCT---GCA--
GAAAATTTGTGGGTCACAGTTTATTATGGGGTACCTGTGTGG---AAAGAAGCAACC---
ACCACTCTATTTTGTGCCTCAGATGCTAAAGCGTATGATACAGAG---GTACATAAT----------------
--------------GTTTGGGCCACA---CATGCCTGTGTACCCACAGCC---
CCCAACCCACAAGAAGTAGAACTGAAG---------AATGTGACAGAAAATTTTAAC---------------
--------------------------ATGTGGAAAAATAACATGGTAGACCAAATGCATGAGGATATA---
ATTAGTTTATGGGATCAAAGCCTA---AAGCCATGTGTAAAATTA---
ACCCCACTCTGTGTTACTTTAAATTGCACTGATTATGGGAATGATACTAACACCAATAATAGTAATGCTACT-
----
----------------AACCCCACTAGTAGCAGCGGGGGAATGGAGGGGAGAGGAGAAATAAAA---
AATTGCTCTTTCAATATCACCACAAGCATAAGA--------------------------
GATAAGGTGCAGAAAGAATATGCACTT---TTTTATAGTCTTGATGTAATACCAATAAAAGAT----------
----

GATAATACTAGCTATATGTTGAGAAGTTGTAACACCTCAGTCATTACACAG---
GCCTGTCCAAAGGTATCCTTTGAGCCAATTCCCATACATTATTGTGCCCCGGGTGGTTTTGCGATTCTAAAGT
GTAAT---GATAAAAAGTTCAAT------GGAAAAGGACCATGTACAAAT----------------------
-GTCAGC---ACAGTACAATGTACACATGGAATTAGGCCAGTAGTATTAACT---
CAACTGGTGTTAAATGGCAGTCTAGCAGAAGAAGAG------------
GTAGTAATTAGATCAGACAATTTCTCGGACAAT---------GCTAAAAACATAATAGTACATCTGAAT----
-----------GAATCTGTAGAA---------ATTAATTGTACAAGACTCAACAACAATACAAGGAGA---
AGTATACATGTAGGACAT------GTAGGACCAGGCAGAGCA---ATT------TATACAACAGGA-------
-----------ATAATAGGAAAGATA------------AGACAAGCACATTGT---AACATTAGTAGAGCA--
-AAATGGAATAACACTTTAAAACAGATAGTTACAAAATTAAGA------------GAACAATTTAAG------
------------------------------------AATAAAACAATAGTCTTTAAT------
CAATCCTCAGGAGGGGACCCAGAAATTGTA---ATGCACAGTTTTAATTGTGGAGGGGAA---
TTTAAGCAGCTCCAAGCAAGAGTCCTGGCTGTGGAAAGATACCTAAGGGATCAACAGCTCCTGGGGATTTGGG
GTTGCTCTGGAAAACTCATTTGCACCACTGCTGTGCCTTGGAATACTAGTTGGAGT-----------------
----------------------------AATAAATCTCTGAATCAGATTTGG------GAT------
AACATGACCTGGATGCAGTGG---GAAAGGGAAATTGATAATTACACAAGCTTAATATAC---
ACCTTAATTGAAGAATCGCAAAACCAACAAGAA--

AAGAATGAACAAGAATTATTGGAATTAGATAATTGGGCAAGTTTGTGGAATTGGTTTAGCATAACAAATTGGC
TGTGGTATATAAAAATATTCATAATGATAGTAGGAGGCTTGGTAGGTTTAAGAATAGTTTTTGTT---
GTACTTTCTATAGTG---------AATAGAGTTAGGCAGGGATACTCACCA------
TTGTCGTTTGCTGTTAGCTTGCTCAACGCCACAGCCATAGCAGTAGCTGAG---
GGGACTGATAGGGTTATAGAAGTATTACAAAGAGCTTTTAGAGCT---------------------------
--------ATTCTCCACATA------CCTAGAAGAATAAGACAGGGCTTGGAAAGGGCTTTGCAA---TAA
>KC595182
GCT---GCA--
GAGAAATTGTGGGTCACAGTCTATTATGGGGTACCTGTATGG---AAAGAAGCAAAC---
ACCACTCTATTTTGTGCATCAGATGCTAAAGCATATGATACAGAG---GTACATAAT----------------
--------------GTTTGGGCCACA---CATGCCTGTGTACCCACAGAC---
CCCAACCCACAAGAAATAGAATTGAAA---------AATGTGACAGAAGACTTTAAC---------------
--------------------------ATGTGGAAAAATAACATGGTAGAACAGATGCATGAGGATATA---
ATCAGTTTATGGGATCAAAGCCTA---AAGCCATGTGTAAAGTTA---
ACCCCACTCTGTGTTACTTTAAATTGCACTGATTGGAAGAATGAGAATAACGGCACTGGGAAT----------
----
-------------GCTACTAACGCCCATAATGATAGCTTGACGATGGAGAAGGGAGAAATAAAA---
AACTGCTCTTTTAATGTAACCACAAACATAAGA--------------------------
GATAAGATGCAAAAAGAATATGCACTT---TTTTATAAACTTGATGTAGTACCAATAGATAAT----------
----

GATAATAGTAGCTATAGGTTGATAAGTTGTAACACCTCAGTCATCACACAG---
GCCTGTCCAAAAGTATCCTTTGAACCAATTCCCATACATTATTGTACCCCGGCTGGTTTTGCGCTTCTAAAGT
GTAAT---GATAAAAAGTTCAAT------GGAACAGGAACATGTAGAAAT----------------------
-GTCAGC---ACAGTACAGTGTACACATGGAATTAGACCAGTAGTGTCAACT---

CAACTGCTGTTAAATGGCAGTCTAGCAGAAGAAGAC------------
GTAGTAATTAGATCTGCCAATTTAACGGACAAT---------ACTAAAATCATAATAGTACAGCTGAAT----
-----------GAATCTGTAGTA---------ATTAATTGTACAAGACCCAACAACAACACAAGAAAA-----
-AGTATAACTATA------------GGACCAGGAAGAGCA---TTT------TATGCAACAGGAGAA------
---------ATAATAGGGGATATA------------AGACAAGCACATTGT---AACATTAGTCGAGCA---
CAATGGAATAACACCTTAAAACAGATAGTTAGAAAATTAAGA------------
GAACAATTAGGAGAACAATTCAAG------------------------------
AATAGAACAATAGCCTTTAAT------CAGTCCTCAGGAGGGGACCCAGAAATTACA---
ATGCACAGTTTTAATTGTGGAGGGGAA---
TTTAAGCAGCTCCAGGCAAGAGTCCTGGCTGTGGAAAGATACCTACAGGATCAACAGCTCCTGGGGATTTGGG
GCTGCTCTGGAAGACTCATTTGCACCACTGCTGTGCCTTGGAATGATAGTTGGAGT-----------------
----------------------------AATAAATCTCTGAAGGACATTTGG------GAT------
AACATGACCTGGATGGAGTGG---GATAAAGAAATTCACAATTATACAAGCTTAATATAC---
ACCTTAATTGAAAAATCGCAGAACCAGCAGGAA--

AAGAATGAACAAGAATTATTAGAATTGGATAAATGGGCAAGTTTGTGGAATTGGTTTGACATAACAAAGTGGC
TGTGGTATATAAAAATATTCATAATGATAGTAGGAGGCTTAGTAGGTTTAAGAATAGTTTTTACT---
GTACTTTCTATAGTG---------AATAGAGTTAGGCAGGGGTACTCACCA------
TTGTCGTTTGCTGTTAGCTTGCTTAATGCCATAGCCATAGCAGTAGCTGAG---
GGAACAGATAGGATTATAGAAGTATTACAAAGAGCTTGTAGAGCT----------------------------
--------ATTCTCCACATA------CCTACAAGAATAAGACAGGGCTTGGAAAGGGCTTTGCTG---TAA
>KC595159
GCT---GCA--
GAAAAATTGTGGGTCACAGTCTATTATGGGGTACCTGTGTGG---AAAGAAGCAATC---
ACCACTCTATTTTGTGCATCAGATGCTAAAGCATATGATACAGAG---GTACATAAT----------------
--------------GTTTGGGCCACA---CACGCCTGTGTACCCACAGAC---
CCCAACCCACAAGAAGTAGTATTGGAA---------AATGTGACAGAAAATTTTAAC----------------
--------------------------ATGTGGAAAAATGACATGGTAAAACAGATGCATGAAGATATA---
ATCAGTTTATGGGATCAAAGCCTA---AAGCCATGTGTAAAATTA---
ACCCCACTCTGTGTTACTCTAAATTGCACGGATTTGAGGAATGCTACTAATAATAGTAGC-------------
----
--AGGATGGAGGGAGGAGAAATAAAA---
AACTGCTCTTTCAATATCACCACAAGCATAAGA---------------------------
GATAAGGTGCAGAAAGAATATGCATTT---TTTTATAAACTTGATGTAGTACCAATAGATAAT----------
----

GATAATACTAGCTATAGGTTGATAAGTTGTAACACCTCAGTCATTACACAG---
GCCTGTCCAAAGGTATCCTTTGAGCCAATTCCCATACATTATTGTACCCCTGCTGGTTTTGCGATTCTAAAGT
GTAAA---GATAAGAAGTTCAAT------GGAACAGGACCATGTACAAAT-------------------------
-GTTAGC---ACAGTACAATGTACACATGGAATTAGGCCAGTAGTATCAACT---
CAACTGCTGTTAAATGGCAGTCTAGCAGAAGAAGAG------------
GTAGTAATTAGATCTGAAAATTTCACGGACAAT---------ACTAAAACCATAATAGTACAGCTGAAA----
-----------GAATCTGTAGAA---------ATTAATTGTACAAGACCCAACAACAATACAAGAAAA-----
-AGTATACATATA------------GGACCAGGGAGAGCA---TTT------TATACAACAGGAGAA------
---------ATAATAGGAGATATA------------AGACAAGCACATTGT---AACCTTAGTAGAGCA---
AAATGGGAGAGCACTTTAAAACAGATAGTTGGAAAATTAAGA---------------GAACACTTTGGG----
--------------------------------AATAATAAATCAATAGTCTTTAAT------
CAATCCTCAGGAGGGGACCCAGAAATTGTA---GCGCACAGTTTTAATTGTGGAGGGGAA---
TTTAAGCAGCTCCAGGCAAGAGTCCTGACTGTGGAAAGATACCTAAAGGATCAACAGCTCCTGGGGATTTGGG
GCTGCTCTGGAAAACTCATTTGCACCACTACTGTGCCTTGGAATGCTAGTTGGAGT-----------------
----------------------------AATAAATCTCTGAATGAGATTTGG------GAT------
AACATGACCTGGATGGAGTGG---GAGAGAGAAATTAACAATTACACAGGCTTAATATAC---
ACCCTACTTGAGGAATCGCAGAACCAGCAGGAA--

AAGAATGAACAAGAATTATTGGGATTAGATAAATGGGCAAGTTTGTGGAATTGGTTTGACATAACAAAATGGC
TGTGGTATATAAAAATATTCATAATGATAGTAGGAGGCTTGGTAGGTTTAAGAATAGTTTTTGCT---
GTACTTTTTATAGTG---------AATAGAGTTAGGCAGGGATACTCACCA------
TTATCGTCGGCTGTTAGCTTGCTAAATGCCATAGCCATAGCAGTAGCTGAG---
GGGACAGATAGGATTATAGAAATAGTACAGAGAACTTGTAGAGCT----------------------------
--------ATTCTCCACATA------CCTACAAGAATAAGACAGGGCTTGGAAAGGGCTTTGCTA---TAA

>KC595225
GGT---GCA--
GGAGATTTGTGGGTCACAGTCTATTATGGGGTACCTGTGTGG---AAAGAAGCAACA---
ACCACCCTATTTTGTGCATCAGATGCTAAATCATATTCTACAGAG---GCACATAAT----------------
--------------GTTTGGGCCACA---CATGCCTGTGTACCCACAGAC---
CCCGACCCACAAGAAATGGTATTGAAA---------AATGTGACAGAAAATTTTAAT----------------
--------------------------ATGTGGAAAAATAACATGGTAGAACAGATGCATGAGGATATA---
ATTAGTTTATGGGATCAAAGCCTG---AAGCCATGTGTAAAATTA---
ACCCCACTCTGTGTCTCTTTAAATTGCACTGATTTGAGAAATGATACAAGTAACGCTACTGCTTCC-------
----
----------------------AATCTCAGTAGCGAGGACATAAAGGCAAGAAGAGAAATAAGA---
AATTGCTCTTTCAAAATCTCCACAAATATGAAA---------------------------
GATAAGGCGCAGAGGACATATGCACTT---TTTTATAGTACTGATATAGTACAAACAGAAGGTGATAAC----
----

AATAGCAATTCTAGCTATACTATCTTTAGGTTAATAAGTTGTAACACCTCAAGCCTTACACAG---
GCCTGTCCAAAAATGTCCTTTGAACCAATTCCTATACATTATTGTGCCCCGGCTGGATTTGCGATTCTGAAAT
GTAAC---AATAAAACTTTTAAT------GGAACAGGACCATGTACAAAT-----------------------
-GTCAGC---ACAGTGCAATGTACACATGGAATTAAGCCAGTAGTATCAACT---
CAACTGCTGTTAAATGGCAGTCTAGCAGAAGGAGAG------------
GTAATAATTAGGTCAGCCAATCTCACGGACAAT---------ACTAAAAACATAATAGTACAGCTGGAT----
-----------CAAGCTGTACAA---------ATTAATTGTACAAGACCCAACAATAATACAAGAAAA-----
-AGTATAAATATA------------GGACCAGGCAGAGCA---TTT------TATGCACACGGAGAA------
---------ATAATAGGGGATATA------------AGACAAGCACATTGT---AACCTTAGTAGAACA---
GACTGGAACAGAACTTTAGACAAGATAGTTAGAAAATTAAGA------------GAAAAGAACAATTTAGG-
-----------------------------------AATAAAACAATAATTTTTAAT------
CAATCCTCAGGAGGGGACCCAGAAATTGTA---ATGCACAGTTTTAATTGTGGGGGGGAA---
TTTAAGCAGCTCCAAGCAAGAGTCCTGGCTGTGGAGAGATACCTAGGGGATCAACGGCTCCTGGCAGTTTGGG
GCTGCTCTGGAAAGCTCATTTGCACCACTACTGTGCCTTGGAATACTAGTTGGAGC-----------------
----------------------------AATAGATCTAAAGATTACATTTGG------AAT------
AACATGACCCGGATGGAGTGG---GAAAGAGAAATTGATAATTACACAGGTGTAATATAT---
AGGTTAATTGAGGAATCGCAACTCCAGCAAGAA--

CAAAATGAACAAGACTTATTGAAATTGGATTCATGGGCAAGTTTGTGGAATTGGTTTACCTTATCAAATTGGC
TGTGGTATATAAAAATATTCATAATGATAGTAGGAGGCTTGATAGGCTTAAGAATAATTTTTGTT---
GTACTTTCTATAGTG---------AATAGAGTTAGGCAGGGATACTCACCA------
TTATCGTTCGCTGTTAGCTTGCTTAACGCCACAGCCATAGTAGTAGGTGAA---
GGAACTGATAGAATTCTAGAATTTTTACAAAGAGTTTATAGAGCT----------------------------
--------ATTCGCAACATA------CCTACAAGAATAAGACAGGGCTTAGAAAGGGCTTTGCTA---TAA
>KC595222
GCT---ACA--
GGAAATTTGTGGGTCACAGTCCATTATGGGGTACCTGTGTGG---AAAGAAGCAACC---
ACCACTCTATTTTGTGCATCAGATGCTAAAGCATATGCTACAGAG---GTACATAAT----------------
--------------GTTTGGGCCACA---CATGCCTGTGTACCCACAGAC---
CCCAACCCACGAGAATTAAGATTGGAA---------AATGTGACCGAAAATTTTAAT----------------
--------------------------ATGTGGAAAAATAACATGGTAGAGCAGATGCATGAGGACATA---
ATCAGTTTATGGGATCAAAGTCTA---AAGCCATGCGTGAAATTA---
ACCCCACTTTGTGTTACTTTAAATTGCACAAATGCCAATACCACAAATGCCAATAATACC-------------
----
-------------ACAAGTGCCAATACCACTAATATTAGTAGGGAAATGGAAGGAGAAATAAAA---
AACTGCTCTTTCAACGTAACCACAAACTTAAGT---------------------------
GATAAGTTTCAGAGACAATATGCACTT---TTTTATAAACTTGATGTAGTACCAATAGACAAT----------
----

GGGACTAAGAGCTATAGGTTGATAAATTGTAACACCTCAGTCATTACACAG---
GCCTGTCCAAAAGTATCCTTTGAGCCAATTCCCATACATTATTGTGCCCCGGCGGGTTTTGCGATTCTAAAAT
GTAAC---AATGAGACGTTCAAT------GGAACAGGACCATGTACAAAT-----------------------
-GTCAGC---ACAGTACAATGCACACATGGAATTAGGCCAGTAGTATCAACT---
CAACTGCTGTTAAATGGCAGTCTAGCAGAAAAAGAG------------
ATAGTAGTTAGATCTGAAAATTTCACAAACAAT---------GTTAAAACCATAATAGTACAGCTGAAT----

-----------GAACCTATAGAA---------ATTAATTGTACAAGACCCAACAACAATACAAGAAAA-----
-GGTATATTTATA------------GGTCCAGGGAGAGCA---TTT------TATGCAACAAGA---------
---------ATAGTAGGAGATATA------------AGACAAGCACATTGT---AACCTTAGTAGAGAA---
AATTGGGTAACCACTTTAGACCGGATAGCTAAAAAGTTAAGA---------------GAACAATTAGGG----
--------------------------------AAGAATAAAACCATAGTCTTTACG------
CGGTCCTCAGGAGGGGACCCAGAAATTGTA---ACGCACAGTTTTAATTGTGGAGGGGAA---
TTTAAGCAGCTCCAGGCAAGAGTCCTGGCTGTGGAAAGATACCTAAAGGATCAACAGCTCCTGGGGATCTGGG
GTTGCTCTGGAAAACTCATTTGCACCACTACTGTGCCTTGGAATGCTAGTCGGAGT-----------------
-------------------------AGTAATAGATCTCTAACTGGGATCTGG------GAT------
AACATGACCTGGATGGAGTGG---GAAAGAGAAATTGTCAATTACACAGGCTTAATTTAC---
ACCTTACTTGAAGAATCGCAGATCCAACAAGAA--

AAGAATGAACAAGAATTATTGGAATTGGATAAATGGGCAAGTTTGTGGAATTGGTTTGACATAACAAAATGGC
TGTGGTATATAAGAATATTCATAATAATAGTAGGAGGCTTGATAGGTTTAAGACTAGTTTTTGCT---
ATATTTTCTATAGTG---------AATAGAGTTAGGCAGGGATACTCACCA------
TTATCGTTTGCTGTTAGCTTGTTCAACGCCGCCGCCATAGCAGTAGCTGAG---
GGGACAGATAGGGTTATAGAAGTATTACAAAGAGCTTGGAGAGCT----------------------------
--------ATTCTCCATATA------CCTACAAGAATAAGACAGGGCTTAGAAAGGGCTTTGCTA---TAA
>KC595206
GCT---GCA--
GACAACTTGTGGGTCACGGTCTATTATGGGGTACCTGTGTGG---AAAGAAGCAACT---
ACCACTCTATTTTGTGCATCAGATGCTAAGGCATATGATACAGAG---GTACATAAT----------------
--------------GTTTGGGCCACG---CATGCCTGTGTACCCACAGAC---
CCCAACCCACAAGAAATGAAATTGGGA---------AATGTGACAGAAGATTTTAAC----------------
--------------------------ATGTGGAAGAATAACATGGTAGAACAGATGCATGAGGATATA---
ATCAGTTTATGGGATCAAAGCCTA---AAGCCATGTGTAAAATTA---
ACCCCCCTCTGTGTTACTTTAAATTGCACTGATTGGCAGAATAATACT-------------------------
----
-------------------CAGACCACTAGCAGTGGCAAGACAACGATAGTAGGAGAAATAAAA---
AACTGCTCTTTCAATAGCCCCACAAGCATAAGA---------------------------
GATAAGATGCAAAAAGAAGAGGCACTT---TTCTATAGCCTTGATGTAGTGCCAATAGACAAT----------
----

GATAATAGTAGCTATAGGTTGCGAAGTTGTAACACCTCAGTCATTACACAG---
GCCTGTCCAAAAGTATCCTTTGATCCAATTCCCATACATTATTGTGCCCCAGCTGGTTTTGCGATTCTAAAGT
GTAAA---GATAAGAAGTTCAAT------GGAACAGGACCATGTACAAAT-----------------------
-GTCAGT---ACAGTACAATGTACACATGGAATTAGGCCAGTAGTATCAACT---
CAACTGCTGTTAAATGGCAGTTTAGCAGAGGAAGAA------------
GTAGTAATTAGATCTGCCAATATCTCGGACAAT---------GCTAAGACCATAATAGTACAGCTGAAC----
-----------GAAACGGTAAAA---------ATTAACTGTACAAGACCCAGCAACAATACAAGAAAA-----
-GGTATACATATA------------GGACCAGGGAGAGCA---TTT------TATGCAACAGGAGAA------
---------ATAATAGGAGATATA------------AGACAAGCACATTGT---AACATTAGTGGCACA---
GACTGGGAGAAAACTTTAAAACAGATAGCTGGAAAGTTAACA---------------GAACAATTTAGG----
-----------------------------------AATAAAAAAATAGTCTTTAAT------
CAATCCTCAGGAGGGGACCCAGAAATTGTA---AAACACAGTTTTAATTGTGGAGGGGAA---
TTTAAGCAGCTCCAGGCAAGAGTCTTGGCTGTGGAAAGATACCTAAAAGATCAACAGCTCCTGGGGATTTGGG
GTTGCTCTGGAAAACTCATCTGCACCACTACTGTGCCTTGGAATACTAGTTGGAGT-----------------
----------------------------AATAAATCTCTGGATCAGATTTGG------GAT------
AACATGACCTGGATGGAGTGG---GAAAGAGAAATTGACAATTACACAGAATTAATATAC---
ACCTTAATTGAAAAATCGCAAAACCAACAAGAA--

AAGAATGAACAAGAATTGTTAGAATTGGATAAATGGGCAGATTTGTGGACATGGTTTAGCATAACAAACTGGC
TGTGGTATATAAGAATATTCATAATGATAGTAGGAGGCTTGATAGGTTTAAGAATATTTTTTGCT---
GTACTTTCTATAGTG---------AATAGAGTTAGGCAGGGATACTCACCA------
TTATCGTTTGCTATTAGCTTGCTCAACGCCACAGCCATAACAGTAGCTGAG---
GGGACAGATAGGGTTATAGCAGTATTGCAAAGAGCTTTTAGAGCT----------------------------
--------ATTCTCCATATA------CCTACAAGAATAAGACAGGGCTTGGAAAGGGCTTTGCTA---TAA
>DQ141341
GCT---ACA--
GAACAATTGTGGGTCACAGTCTATTATGGGGTACCTGTGTGG---AAAGAAGCAACC---

ACTACTCTATTTTGTGCATCAGATGCTAAAGCACATGACACAGAG---GTGCATAAT----------------
--------------GTTTGGGCCACA---CATGCCTGTGTACCCACAGAC---
CCCAACCCACAAGAAGTAGTATTGGAA---------AATGTGACAGAAAATTTTAAC----------------
--------------------------ATGTGGAAAAATAACATGGTAGAACAGATGCATGAGGATATA---
ATCAGTTTATGGGATCAAAGCCTA---AAGCCATGTGTAAAATTA---
ACCCCACTTTGTGTTACTTTAAATTGCACTGATTGGAAGAATGCTACTAATACCAATAGTACTAAT-------
----
-------------------ACCAATAGTAGTAGCGAGGGAACGATGGAGGGAGGAGAAGTAAAA---
AACTGCTCTTTCAATATCACCACAAACATAGGG---------------------------
GATAAGGTGCAGAAAGAATATGCACTT---TTTTATAAACTTGATGTAGCACCAATAGAAGATGAT-------
----

AATAGTGATAATAGTAGCTATAGGTTGATAAGTTGTAACACCTCAGTCATTACACAG---
GCCTGTCCAAAAATATCCTTTGAACCAATTCCCATACATTATTGTGCCCCGGCTGGTTTTGCGATTCTAAAGT
GTAAC---AATAAGACGTTCAAT------GGAAAAGGACCATGTACAAAT-----------------------
-GTCAGC---ACAGTACAATGTACACATGGAATTAGGCCAGTAGTATCGACT---
CAACTACTGTTAAATGGCAGTCTAGCAGAAGGAGAA------------
GTAGTAATTAGATCTGAAAATTTCACGAACAAT---------GCTAAAACCATAATAGTACATCTGAAT----
-----------GCATCTGTAGAA---------ATTAATTGTACAAGACCCGGCAACAATACAAGAAGA-----
-AGTATACATATA------------GGACCAGGGAGAGCA---TTT------TATACAACAGGACAA------
---------ATAATAGGAGATATA------------AGACAAGCACATTGT---AACCTTAGTAGAGCA---
CAATGGAATGACACTTTAAAACAGATAGTTGAAAAATTACAA---------------GGACAATTTAGG----
-----------------------------------AATAAAACAATAGTCTTTAAT------
CAACCCTCAGGAGGGGACCCAGAAATTGTA---ATGCACAGTTTTAATTGTGGAGGGGGAA---
TTTAAGCAGCTCCAGGCAAGAGTCCTGGCTGTGGAAAGATACCTAAAGGATCAACAGCTCCTGGGGATTTGGG
GTTGCTCTGGAAAACTCACCTGCACCACTGCTGTGCCTTGGAATGCTAGTTGGAGT-----------------
----------------------------AATAAATCTCTAAATGAGATTTGG------GAT------
AACATGACCTGGATGGAGTGG---GAAAGAGAAATTGACAATTACACAAACTTAATATAC---
AACTTACTTGAAGAATCGCAGAACCAACAAGAA--

AAGAATGAAAAAGAATTATTGGAATTGGATAAATGGGCAAGTTTGTGGAATTGGTTTAGCATAACGAATTGGC
TGTGGTATATAAGAATATTCATAATGATAGTAGGAGGCTTGATAGGTTTAAGAATAATTTTTACT---
GTACTTTCTATAGTG---------AATAGAGTTAGGCAGGGATACTCACCA------
TTATCGTTTGCTGTTAGCTTGTTCAATGCCACAGCCATAGCAGTAGCTGAG---
GGGACAGATAGGATTATAGAAGCAGTACAAAGAGCTTTTAGAGCT----------------------------
--------ATTATCCACATA------CCTACAAGAATAAGACAGGGCTTGGAAAGGGCTTTGCTA---TAA
>DQ141345
GCT---GCA---GAA-------------------------------------
GACAAATTGTGGGTCACAGTCTATTATGGGGTACCTGTGTGG---AAAGAAGCAACC---
ACCACTCTATTTTGTGCATCAGATGCTAAAGCATATGATACAGAG---GTACATAAT----------------
--------------GTTTGGGCCACA---CATGCCTGTGTACCCACAGAC---
CCCAACCCACAAGAAATAGAATTGGAA---------AATGTGACAGAATATTTTAAC----------------
--------------------------ATGGGGAAAAATAACATGGTAGATCAGATGCACGAGGATATA---
ATCAGTTTATGGGACCAAAGTCTA---AAGCCATGTGCAAAATTA---
ACCCCACTCTGTGTTACTTTAAATTGCACTGATTATGGGAGGGAAAATTGCACTAGTAATAATACCAATTGCA
CTAGTAAT---
-------AATACCAATCGCACTAGTAATAATATACAGGAAGGGATAGAGAGGGGAGAAATAAAA---
AACTGCTCTTTCAATGTCAGCACAAGCGGGAGA---------------------------
GATAAGGTACATAAAGAATATGCACTT---TTTTATAACCTTGATGTAGTAAGAATAGAAGAA----------
----

GATAATGATACTACCAGCTATAGGTTGACAAGTTGTAACACCTCAGTCATTACACAG---
GCCTGTCCAAAAGTAACCTTTGAACCAATTCCCATACATTATTGTGCCCCGGCTGGTTTTGCGATTCTAAAAT
GTAAA---GATAAGAAGTTCAAT------GGAACAGGATTATGTCAAAAT-----------------------
-GTCAGC---ACAGTACAATGTACACGTGGAATTAGGCCAGTAGTATCAACT---
CAACTACTGTTAAATGGCAGTCTAGCAGAAGAAGAG------------
GTAGTAATTAGATCTGTCAATTTCTCGGACAAT---------GCTAAAACCATAATAGTACAGTTGAAC----
-----------GAATCTGTAGTA---------ATTAATTGCACAAGACCCAACAACAATACAAGGAAA-----
-AGTATACATATA------------GGACTAGGGAGAGCA---TTT------TATGCAACAGGACAA------
---------ATAATAGGAGATATA------------AGACAAGCACACTGT---AACATTAGTGCAGGA---

AAATGGAATAACACTTTACAACAGATAGTTAAAAAATTAAGA---------------GAACAATTTGGG----
-----------------------------------AATAAGACAATAGCCTTTAAT------
CGATCTTCAGGAGGGGATCCAGAAATTGTA---ATGCACAGTGTTAATTGTGGAGGGGAA---
TTTAAGCAGCTCCAGGCAAGAGTCCTTGCTGTGGAAAGATACCTAAGGGATCAACAGCTCCTGGGGATTTGGG
GTTGCTCTGGAAAGCTCATTTGCACCACTGCTGTGCCTTGGAATGCTAGTTGGAGT-----------------
----------------------------AATAAATCTCTGGATGAGATTTGG------AAT------
AACATGACCTGGATAGAGTGG---GAAAGAGAAATTGACAATTACACAGATCTAATCTAC---
AGCTTAATTGAAAATTCGCAGAACCAGCAAGAA--

AAGAATGAACAAGAATTATTGGAATTGGATAAATGGGCAAGTTTGTGGAATTGGTTTGACATAACAAACTGGC
TGTGGTATATAAAAATATTCATAATGATAGTAGGAGGATTGATAGGTCTAAGAATAGTTTTTACT---
GTACTTTCTATAGTG---------AATAGAGTTAGGCAGGGATACTCACCA------
TTATCATTTGCTGTTAGCTTGCTCAATTTCACAGCCATAGCAGTAGCTGAG---
GGGACAGATAGGGTTATAGAAGCAATACAAGGAGCTTTTAGAGCT----------------------------
--------ATCCTCCACATA------CCCACAAGAATAAGACAGGGCTTGGAAAGGGCTTTGCTA---TAA
>EF531330
ACT---GCA---
AATTTGTGGGTCACCGTCTATTATGGGGTACCTGTGTGG---AAAGAAGCAACC---
ACCACTCTATTTTGTGCATCAGATGCTAAAGCATATGACACAGAG---GTACATAAT----------------
--------------GTTTGGGCCACA---CATGCCTGTGTACCCACAGAC---
CCCAACCCACAAGAAATAGAATTGAAA---------AATGTGACAGAAGAGTTTAAC----------------
--------------------------ATGTGGAAAGATAACATGGTAGAACAGATGCATGAGGATATA---
ATCAGTTTATGGGATCAAAGCCTA---AAACCATGTGTAAAATTA---
ACCCCACTCTGTGTTACTTTAAATTGCACTGATGCCAACAATACAAATACTAATAAT----------------
----
-------------------------------GCAATTGTTAATGAGACCAAAGGAGAGATAAAA---
AACTGCTCTTTCAATATCACCACAAGCGTAAAA---------------------------
GATAAGGTGAAAAAGGAATATTCACTT---
TTTTATAAACTTGATGTAGTACCAATAGAAGATGATAATGATACT----------------------------
----
AAGAATGATAATAGAAACTATAGTAACTATAGGTTGATAAGTTGTAACACCTCAGTCATTACACAG---
GCCTGTCCAAAAATATCCTTTGAACCAATTCCCATACATTACTGCACCCCGGCTGGTTTTGCGCTTCTAAAGT
GTAAC---GATAAGAAATTCAAT------GGAACAGGAAAATGTAACAAT-----------------------
-GTCAGC---ACAGTACAATGTACACATGGAATTAAGCCAGTAGTATCAACT---
CAACTGCTGTTAAATGGCAGTCTAGCAGAAGGAAAA---------
GATGTAATGATTAGATCTAGCAATTTCACAGACAAT---------GCTAAAACCATAATAGTACAACTAAAT-
--------------GAGTCTGTAGTA---------ATTAATTGTACAAGACCCAACAACAATACAAGAAGA--
----GGTATACATATA------------GGGCCAGGGAGAGCA---TTT------TATGCAACAGGA------
------------ATAATAGGAGATATA------------AGACAAGCACATTGT---AACCTTAGTAGGGCA-
--GATTGGAATAGAACCTTAGAACTAATAGTTATCAAATTAAGA---------------
GAAATTGAACAATTCAGG---------------------------------AATAAAACAATAGTCTTTAAG-
-----CAATCCTCAGGAGGGGACCCAGAAATTGTA---ATGCACAGTTTTAATTGTGGAGGGGAA---
TTTAAGCAGCTCCAGGCAAGAGTCCTAGCTGTGGAAAGATACCTAAAGGATCAACAGCTCCTGGGGATCTGGG
GATGCTCTGGAAAACTCATCTGCACCACTGCTGTGCCTTGGAATGCTAGTTGGAGT-----------------
----------------------------AACAAATCTCTGGATGATATTTGG------AAT------
AACATGACCTGGAAGGATTGG---GAAAGAGAAATTGACAATTACACAGGCAAAATATAC---
ACCTTACTTGAAGAAGCGCAAAACCAACAAGAA--

AAGAATGAACAAGACTTATTGGAATGGGATAAATGGGCAAGTTTGTGGAATTGGTTTGACATAACAAAATGGC
TGTGGTATATAAAAATATTCATAATGATAGTAGGAGGCTTGATAGGCTTAAGAATAGTTTTTACT---
GTACTTTCTATAGTA---------AATAGAGTTAGGCAGGGATATTCACCA------
TTATCGTTTGCTGTTAGCTTGTTCAATGCCATAGCCATAGCAGTAGCTGAA---
GGGACAGATAGAGTTATAGGAGTATTACAAAGAGCTTATAGAGCC----------------------------
--------ATTCTCCACATA------CCTAGAAGAATAAGACAGGGCTTGGAAAGGATTTTGCTA---TAA
>EF531333
GCT---ACA--
GAACAATTGTGGGTCACCGTCTATTATGGGGTACCTGTGTGG---AAAGAGGCAACC---
ACCACTCTATTTTGTGCATCTGATGCTAGAGCATATGATACAGAG---GTACATAAT----------------
--------------GTTTGGGCCACA---CATGCCTGTGTACCCACAGAC---
CCCAACCCACAAGAAGTAGTATTGGGA------GCAAATGTGACAGAAAATTTTAAC----------------

--------------------------ATGTGGAAAAATAACATGGTAGAGCAGATGCAGGAGGATATA---
ATCAGTTTATGGGATCAGAGCCTA---AAGCCATGTGTAAAATTA---
ACCCCACTCTGTGTTACTTTACATTGCTCTGATGTGAATACTACTGATGTGAATACTACTGATGTGAATACTA
CTAGGACCCATACC---
-AATGCTAGTCATACCAATACCACTAATAATAGCGGGGAAAGGATGGAGATGGGAGAAATAAAA---
AACTGCTCTTTCAACATCACCACAAATATAAGA---------------------------
GATAAGGTGCAGAAGGAATATGCCCTT---TTTTATAAACTTGATGTAGTACCAATAGACAATGATAAT----
----

AATAGTAGTAATAGTAATAACTATGGGTTGATAAGTTGTAATACCTCAGTCATTACACAG---
GCCTGTCCAAAGGTAACCTTTGACCCACTTCCAATACATTATTGTGCCCCGGCTGGTTTTGCGATTCTAAAGT
GTAAT---GAGAAGACGTTCAAT------GGAACGGGACCATGTAAAAAT-----------------------
-GTCAGC---ACAGTACAATGTACACATGGAATTAGGCCAGTAGTATCAACT---
CAGCTGCTGTTAAATGGCAGTCTAGCAGAAAAAGAG------------
GTAGTAATTAGATCTGAAAATATCACGAACAAT---------GCTAAAACCATAATAGTACAGCTAAAT----
-----------GAATCCATACAA---------ATTGAATGTACAAGACCCAATAACAATACAAGGAAA-----
-GGTATACATATG------------GGACCAGGGAGAGCA---TTT------TATGCAACAGGAGAC------
---------ATAATAGGAAATATA------------AGACAAGCACATTGT---AACATTAATAAAGCA---
CAATGGCATGCCGCTTTAAAACGAGTAGCTGCAAAATTGAGA---------------GAAAAATTCGAG----
-----------------------------------AATAAAACAATAATCTTTAAT------
CAATCCTCAGGAGGGGACCCAGAAATTGTA---ATGCACAGTTTTAATTGTAGAGGGGAA---
TTTAAACAGCTCCAGGTAAGAATCCTGGCTGTGGAATCATACCTAAGGGATCAACAGCTCCTGGGGATTTGGG
GTTGCTCTGGAAAACTCATTTGCACCACTGCTGTGCCTTGGAATACTAGTTGGAAT-----------------
----------------------------AATAGATCTCTGAAATACATTTGG------GAT------
AATATGACCTGGATGGAGTGG---GAAAGAGAAATTGGCAATTACACAGGCCTAATATAC---
AACTTAATTGAAGAATCGCAGAACCAACAAGAA--

AAGAATGAACTAGAATTATTGGAATTGGATAAATGGGCAAGTTTATGGAATTGGTTTGACATAACAAAGTGGC
TGTGGTATATAAAAATATTCATAATGATAGTAGGAGGCTTGGTAGGTTTAAGAATAGTTTTTACT---
GTACTTTCTATAGTG---------AATAGAGTTAGGCAGGGATACTCACCA------
TTGTCGTTTGCTGTTAGCTTGCTCAACGCCATAGCCATAGCAGTAGCTGAG---
GGGACAGATAGGGTTATAGAAATAGTACAAAGAGCCTGTAGAGCT----------------------------
--------ATTCTCAACATA------CCTAGAAGAATAAGACAGGGCTTGGAAAGGATTTTGCTA---TAA
>JX422195
GTT---GCA--
GGAAATTTGTGGGTCACAGTCTATTATGGGGTACCTGTGTGG---AAAGAAGCAACC---
ACCACCCTATTTTGTGCGTCAGATGCTAAAGCATATGACACAGAG---GTACATAAT----------------
--------------GTTTGGGCCACA---CATGCCTGTGTACCCACAGAC---
CCCAGCCCACAAGAAATTTTAATGGAA---------AATGTGACAGAAAATTTCAAC----------------
--------------------------ATGTGGAAAAATAACATGGTAGAACAAATGCATGAGGACGTA---
ATCGGTTTATGGGATCAAAGCCTA---AAGCCATGTGTAAAATTA---
ACCCCACTCTGTGTTACTTTAAATTGCACAGATGTGAAAAGAAATTGCACAGATGTGACAAATAGTACAGATG
TGACAGGAAAT--
-------------------------TGTACTGTGAATAATAATACTGAGGAAGTAGAGATAAAA---
AACTGCACTTTCAATATAACCACAGGAGGAATA------------------------
AGGGATAAGATACAGAAAGAATACGCAATG---TTTTATAAGTATGATATAGTACCAATAAATAATGATAAC-
----

AGTAGCTATAACAATAGCTACAATAGCTATAGGTTGATAAGTTGTAACACCTCAGTCATTACACAA---
GCCTGTCCAAAGATATCCTTTGAGCCAATTCCCATACATTATTGTGCCCCGGCTGGTTTTGCGATTCTAAAGT
GTAAT---AATAAAAAGTTCAAT------GGAACAGGACCATGTAAAAAT-----------------------
-GTCAGC---ACAGTACAATGTACACATGGAATTAGGCCAGTAGTATCAACT---
CAGCTGCTGTTAAATGGCAGTCTAGCAGAAGAAGAG------------
GTAGTAATTAGATCTGCCAATTTCACAGACAAT---------ACTAAAATCATAATAGTTCAACTGAAT----
-----------GAATCTGTAGTA---------ATTAATTGTACAAGGCCCAACAACAATACAAGAAGG-----
-AGTATACATATA------------GGACCAGGGAGAGCA---TTT------TATACAACAGGAAGC------
---------ATAATAGGAGACATA------------AGACAAGCATATTGT---AATCTTAGTAGACCA---
CAATGGAATAAGACCTTAGAGAAGATAAAGGCCAAATTAGAA---------------GAACAATTC-------
-----------------------------------AATAAAACAATAACCTTTAAT------
CACTCCTCAGGAGGGGACCCAGAAATTGTA---ATGCACAGTTTTAATTGTGGAGGGGAA---

TTTAAGCAGCTCCAGGCAAGACTCCTGGCTGTGGAAAGATACCTAGGGGATCAACAGCTCCTGGGGATTTGGG
GTTGCTCTGGAAAGCTCATTTGCACCACTAATGTGCCTTGGAATGCTAGTTGGAGT-----------------
----------------------------AATAAATCTGAGGATGATATTTGG------AAA------
AACATGACCTGGATGGAGTGG---GAGAAGGAAATTGACAATCACACACAATCAATATAC---
AGATTACTTGAAGAATCGCAGCTCCAACAAGAA--

CGGAATGAACAAGATTTATTGCAATTAGATCAATGGGCAAGTTTGTGGAATTGGTTCAGCATAACAAAATGGC
TGTGGTATATAAAAATATTCATAATGATAGTAGGAGGCTTAATAGGTTTAAGAATAGTTTTTACT---
GTATTTACCATAGTG---------AACAGAGTTAGGCAGGGATACTCACCA------
CTGTCGTTTGCTGTTAGCTTGCTCAATTTCATAGCCATAGCAGTAGCTGAG---
GGGACAGATAGGGTTATAGAAGTATTACAAAGAATTTGTAGAGCT----------------------------
--------ATCCTCCATATA------CCGAGAAGGATAAGGCAAGGATTGGAAAGGGCTTTGCTA---TAA
>AY535455
GCT---ACA--
GAAAAGTTGTGGGTCACAGTCTATTATGGGGTACCTGTGTGG---AAAGAAGCAGAC---
ACCACTCTATTTTGTGCATCAGATGCTAAAGCATATGATACAGAG---GTACATAAT----------------
--------------GTTTGGCCACA---CATGCCTGTGTACCCACAGAC---
CCCAACCCACGAGAAATAGAATTGAAA---------AATGTAACAGAAGAGTTTAAC----------------
--------------------------ATGTGGAAAAATAACATGGTAGAACAGATGCATGAGGATATA---
ATCAGTTTATGGGATCAAAGCCTA---AAGCCATGTGTAAAGTTA---
ACCCCACTCTGTGTTACTTTAAATTGCACTGATGTAACGGCTAATAGTACTAATACCACT-------------
----
-------------------------AATATTGATGAGGGAAGGATGGAGCAAGGAGAAATAAAA---
AACTGCTCTTTCAATATTACCACAAACATAAGA---------------------------
GATAGGATACAGGAAGTATATGCACTT---TTTTATAAACTTGATGTAGTACCAATAGGGGATGGTAAGAAT-
----

AATACCAGTAAGAATAATACCAGCTATAGGTTGATAAGTTGTAACACCTCAGTCATCACACAA---
GCCTGTCCAAAGGTAACCTTTGAGCCAATTCCCATACATTATTGTGCCCCGGCTGGTTTTGCGATTCTAAAGT
GTAGA---GATAACAAGTTCAAC------GGGAAAGGGCAATGTAACAAT-----------------------
-GTCAGC---ACAGTACAATGTACACATGGAATTAGGCCAGTAATATCAACT---
CAACTGCTATTAAATGGCAGTCTAGCAGAGGAAGAG------------
ATAGTAATTAGATCTGACAATTTCACAGACAAT---------GCTAAAACCATAATAGTACAGCTGAAC----
-----------AAATCTGTAGAA---------ATTAATTGTACAAGGCCCAACAACAATACAAGAAGA-----
-GGTATTCACCTA------------GGACCAGGGAGAGCA---TTT------TATGCAACAGGAGAC------
---------ATAATAGGAGATATA------------AGACAAGCACATTGT---AATGTTAGTAGAGCA---
ACATGGGAGGAGACTTTAGGACAGATAGCTAGCAAATTAAGA---------------GAACAATTCAAA----
-----------------------------------AATAAAACAATAGTCTTTAAT------
CAATCCTCAGGAGGGGACCCAGAAGTTGTA---ATGCACAGTGTTAATTGTGGAGGAGAA---
TTTAAGCAGCTCCAGGCAAGAATCCTGGCTGTAGAAAGATACCTAAAGGATCAACAGCTCCTGGGGATTTGGG
GATGCTCTGGAAAACTCATTTGCACCACTGCTGTGCCTTGGAATGCTAGTTGGAGT-----------------
----------------------------AATAAATCTCTGGATCAGATCTGG------CAT------
AACATGACCTGGATACAGTGG---GAAAGAGAAATTGATAATTACACAAGCATAATATAC---
AGCTTAATTGAAGAATCGCAAAGCCAACAAGAA--

AAGAATGAACAAGAATTATTGGAATTAGATAAATGGGCAAGTTTGTGGAATTGGTTTGACATAACAAACTGGC
TGTGGTATATAAAAATATTCATAATGATAGTAGGAGGCTTGGTAGGTTTAAGAATAGTTTTTACT---
GTACTTTCTGTAGTG---------AATAGAGTTAGGCAGGGATACTCACCA------
TTATCGTTTGCTGTTAGCTTGCTCAACGCCACAGCCATAGCAGTAGCTGAA---
GGGACAGATAGGGTTATAGAAATATTACAAATAGTTTGTAGAGCT----------------------------
--------ATTTGCCATATA------CCTAGAAGAATAAGACAAGGCTTGGAAAGGGCTTTGCTA---TAA
>KC699018
GCT---GCA--
GAAGACAAGTGGGTCACAGTATATTATGGGGTACCGGTGTGG---AAAGAAGCAACC---
ACCACTCTATTTTGTGCATCAGATGCTAAATCATATGATACAGAA---GTACATAAT----------------
--------------GTTTGGCCACA---CATGCCTGTGTACCCACAGAC---
CCCAACCCACAAGAAATAGTAATGGAA---------AATGTGACAGAAAATTTTAAC----------------
--------------------------ATGTGGAAAAATAACATGGTAGATCAGATGCAGGAAGATATA---
ATCAGTTTATGGGATCAAAGCCTA---AAGCCATGTGTAAAATTA---
ACACCACTCTGTGTTACCTTAAATTGCACTGATGTTAATATCACTAATGCTAATATCAAT-------------

----
----------------------ATGAGTTATGCTAATAAGACCATGGAGCAAGGAGAAATGAAA---
AACTGCTCTTTCAACGTCACCACAGAAATAAGA---------------------------
GATAAGGTGCAGAGACAATATGCGCTT---TTTTATAGACTTGATTTAGTATCAATAGATAAT----------
----

GATAATACTAGCTATAGGTTGATAAATTGTAACACTTCAGTCATTACACAA---
GCCTGTCCAAAGGTATCCTTTGAGCCAATTCCTATACATTATTGTGCCCCGGCTGGTTTTGCGATTCTAAAAT
GTAAC---GATAAGAAGTTCAAT------GGAACAGGGCCATGTAAAAT------------------------
-GTCAGC---ACAGTACAATGCACACATGGAATTAGGCCAGTAGTATCAACT---
CAACTGCTGTTAAATGGCAGTCTAGCAGAAGAAGAG------------
GTAGTAATTAGATCTGAAAATTTCACGAACAAT---------GCTAAAAACATAATAGTACAGCTGAAA----
-----------ACCCCTGTACAA---------ATTACTTGTACAAGACCCAGCAACAATACAAGAAAA-----
-AGTATATCTATA------------GGGCCAGGGAGAGCA---TTT------TATGCAACAGGAGAC------
---------ATAATAGGAGATATA------------AGACAGGCACATTGT---AACCTTAGTAGAGAA---
CTATGGAATAACACTTTAAATCAGACAGCTGCAAAATTAAGA---------------GAAGAATTTGGG----
-----------------------------------AATAAAACAATAATCTTTAAT------
CAATCCTCAGGAGGGGATCCAGAAATTGTA---ATGCACAGTTTTATTTGTGGAGGGGAA---
TTTAAGCAGCTCCAGGCAAGAGTCCTGGCTGTGGAAAGATACCTAAAGGATCAACAGCTCCTAGGGATTTGGG
GTTGCTCTGGAAAACTCATTTGCACCACTACAGTGCCTTGGAATACTAGTTGGAGT-----------------
----------------------------AATAAATCTCTAGATTATATTTGG------AAT------
AACATGACCTGGATGCAGTGG---GATAGAGAAATTAGCAATTACACAAGCTTAATATAC---
ACCCTAATTGAAGAATCGCAGAAACAACAAGAA--

AAGAATGAACAAGAATTATTGGCATTAGATAAATGGGCAAGCTTGTGGAATTGGTTTGACATATCAAACTGGC
TGTGGTATATAAAAATATTCATAATGATAGTAGGAGGCTTGGTAGGTTTAAGAATAGTTTTTGCT---
GTGCTTTCTATAGTG---------AATAGAGTTAGGCAGGGATACTCACCA------
TTGTCATTTGCTGTTAGCTTGCTCAATGTCACCGCTATCGCAGTAGCTGAG---
GGAACAGATAGGGTTATAGAAGTAGTAAGAAGAATTTATAGATCT----------------------------
--------ATTATCCACATA------CCTAGAAGAATAAGACAGGGCTTGGAAAGGGCTTTGCTA---TAA
>KU168261
GCT---GCA--
AAACAAAAGTGGGTCACAGTCTATTATGGGGTACCTGTGTGG---AAAGAAGCAACC---
ACCACTCTATTTTGTGCATCAGATGCTAAAGCGTATGATGCAGAG---GCACATAAT----------------
--------------GTTTGGCCACA---CATGCTTGTGTACCCACAGAC---
CCCAGCCCACAAGAATTAAATTTGGAA---------AATGTGACAGAAAACTTTAAC----------------
--------------------------ATGTGGAAAAATAACATGGTAGAACAGATGCATGAAGATATA---
ATCAGTTTATGGGATCAAAGCATA---AAGCCATGTGTAAAAATG---
ACCCCACTCTGTGTTTCTCTAAATTGCTCTGATAAGATCAATATTAATATTAACGGGACC-------------
----
----------------------AATAGTACCAACCCTAATGTGACGGAGGAAGGAGACATGAAA---
AACTGCTCTTTCAATGCCACCACAGAAATAAGA---------------------------
GATAAGATGAAGAAAGAATATGCACTT---TTTTATAGACTTGATATAGTATCAATAAATAGT----------
----

AGTGATAATACTAGCTATAGGCTGATAAATTGTAATACCTCAACCATTACACAA---
GCCTGTCCAAAGATATCCTTTGAGCCAATTCCCATACATTATTGTGCCCCAGCTGGTTTTGCAATTCTAAAGT
GTAAT---CAGAAGAATTTCAAT------GGAACAGGGCAATGTAGAAAT------------------------
-GTCAGC---ACAGTACAATGTACACATGGAATTAAGCCAGTAGTATCAACT---
CAACTGCTGTTAAATGGCAGTCTAGCAGAA---GAG------------
ATAGTAATTAGATCTGAGAATTTCTCAAACAAT---------GCTAAAAACATAATAGTACAGCTGAAA----
-----------GATCCTGTACAA---------ATTAATTGTACAAGACCCAACAACAATACAAGAAGA-----
-AGTATACGTATA------------GGACCAGGGAGCACA---CTT------TATGCAGAAGGAGAC------
---------ATAATAGGAGATATA------------AGACAAGCACATTGT---AACATTAGTGAAGAA---
AAATGGAATCAAACTTTACAAGAGGTAGCTAAAAAATTATTA---------------GAACAATTTGGG----
-----------------AAGAATAAAACA------GAAACAAAAGTAGCTTTTAAT------
CGATCCTCAGGAGGGGACCAAGAAATTGTA---ATGCATACCTTTAATTGTGGAGGGGAA---
TTTAAGCAGCTCCAGGCAAGAGTCCTAGCTGTGGAAAGATACCTAAAGGATCAACAGCTCCTAGGGATCTGGG
GTTGCTCTGGAAAACTCATTTGCACCACTGCTGTGCCTTGGAATAATAGTTGGAGT-----------------
----------------------------AATAAATCTCTAGAAAAAATTTGG------GAT------

AACATGACCTGGATGGAGTGG---GAAAAAGAAATTGACAATTACACAGAATTAATATAC---
ACTTTACTTTCAGAATCGCAAAACCAACAAGAT---------------------------------------
----
AAGAATGAAAAAGACTTATTAGAATTAGACAAATGGGCAAGCTTGTGGAATTGGTTTAGCATAACAAACTGGC
TGTGGTATATAAAAATATTCATAATGATAGTAGGAGGCTTGATAGGGTTAAGAATAGTTTTTACT---
GTACTTTCTATAGTG---------AATAGAGTTAGGCAGGGATACTCACCA------
TTATCGTTTGCTGTTAGCTTGCTTAATGCTACAGCTATTGCAGTAGCTGAA---
GGGACAGATAGGATTATAGAAGCAGTACAAAGAGCTTTTAGAGCT---------------------------
--------ATCCTCCACATA------CCTAGAAGAATAAGACAGGGCTTGGAAAGGGCTTTGCTA---TAA
>JX140652
GCT---GTA---GAC-----------------------------------
AACAATTTGTGGGTCACAGTCTATTATGGGGTACCTGTGTGG---AAAGAAGCAAAC---
CCCACTCTATTTTGTGCATCAGATGCTAAAGCATATGATACAGAG---GCACATAAT---------------
--------------GTCTGGGCCACT---CATGCCTGTGTACCCACAGAT---
CCCAATCCACAAGAAATAATGTTGAAA---------AATGTGTCAGAAAATTTTAAC---------------
--------------------------ATGTGGAAAAATAACATGGTAGAACAGATGCATGAGGATATA---
ATCAGTTTATGGGATCAAAGTTTA---AAGCCATGTGTAAAATTA---
ACCCCACTCTGTGTCACTCTAAATTGCACTGTTGCCACTAATAGTAGTGGTACTAATACCACC---------
----
-------------------------------------GCTGGTAGTAAGATAGAAGGAGTAAAA---
AACTGCTCTTTCAATGTCACCACAAACATAAGA-------------------------
GATAAGATGCAGAAAGCATATGCACTT---TTTTATGACCTTGATATAGTACCAATAGATAAT---------
----

GATAACTCTAGCTATAGATTGATACATTGTAATACCTCAGTCATTACACAA---
GCCTGTCCAAAGATAACCTTTGAGCCAATTCCCATACATTATTGTGCCCCGGCTGGTTTTGCGATTCTAAAGT
GCAAT---GATAAGAAGTTCAAT------GGAACAGGACGATGTGAAAAT----------------------
-GTCAGC---ACAGTACAATGTACACATGGAATTAGGCCAGTAGTGTCAACT---
CAACTGTTGTTAAATGGCACTCTAGCAGAAGAAGAC-----------
ATAGTAATTAGATCTGAGAATATCACAGACAAT---------ACTAAAACCATAATAGTACAGCTGAAG----
-----------GAACCTGTAGAA---------ATTAAGTGTACAAGACCCAGCAACAATACAAGAAAA-----
-AGTATACATATA------------GGACCAGGAAGAGCA---TTT------TATGCAACAGGAGAA------
---------ATAATAGGAAATATA-----------AGGCAAGCACATTGT---AACATTAGTAAAGGA---
AAATGGGAGAAAACTTTAGAAAAGATAGCTAACAAATTAAGA--------------GAACAATTTAAG----
---------------------------------AACAACACAACAATAGTCTTTAAT------
CAATCTGCAGGAGGGGACCCAGAAATTGTA---ATGCACAGTTTTAATTGTGGAGGGGAA---
TTTAAGCAGCTCCAGGCAAGAGTCCTGGCTGTGGAAAGATACCTAAAGGACCAACAGCTCCTGGGGATTTGGG
GTTGCTCTGGAAAACTCATTTGCACCACTACTGTGCCTTGGAATAGTAGTTGGAGT----------------
----------------------------AATAAATCTCTGAATGACATTTGG------GAT------
AACATGACATGGATGGAGTGG---GAAAGAGAAATTAATAATTACACAGACTTAATATAC---
AGCTTAATTGAAAAATCGCAGAACCAGCAAGAA---------------------------------------
----
CAGAATGAACAAGAATTATTGGCATTAGATAAGTGGGCAAGTTTGTGGAGTTGGTTTGATATAACAAACTGGC
TGTGGTATATAAAAATATTCATAATAATAGTAGGAGGCTTGATAGGTTTAAGAATAGTTTGTACT---
GTACTTTCTGTAGTG---------AATAGAGTTAGGCAGGGATACTCACCT------
TTATCATTTGCTGTTAGCTTGCTCAACGCCACAGCCATAGTAGTAGCTGAG---
GGGACAGATAGGATTATAGAAGCATTACAAAGAGCTGGTAGAGCT---------------------------
--------ATTCTCCACATA------CCTAGAAGAATAAGACAGGGCTTAGAAAGGGCTTTGCTA---TAA
>KC699029
GCT---ACA---
AATAATTTGTGGGTCACAGTCTATTATGGGGTACCTGTGTGG---AAGGAAGCAACC---
ACCCCTCTATTTTGTGCTTCAGATGCTAAAGCATTTGATACAGAG---GTACATAAT---------------
--------------GTTTGGGCCACA---CATGCCTGTGTGCCCACAGAC---
CCCAACCCACAAGAAATAGAATTGAAA---------AATGTGACAGAAAATTTTAAT---------------
--------------------------ATGTGGAAAAATGACATGGTAGAACAAATGCAGGAGGATATA---
ATCAGTTTATGGGACCAAAGCCTA---AAGCCATGTGTAAAATTA---
ACCCCACTCTGTGTTACTTTAAATTGCACTGATCTGATGAATAATACCACTACCAAG---------------
----
--------------------------------AACTTTAGCGATGAAATGAGAGGAGAGATAAAA---
AACTGCTCTTTCAATGTC---ACAAGAATAGGA-------------------------

GATAAGAAACATACAGAATATGCAACT---TTTTATAAACTTGATGTAGTACCAATAAGTGAT----------
----

AGTAATACCAGCTATAGATTGATAAGTTGTAACACCTCAGTCATTACCCAG---
GCCTGCCCAAAGATATCGTTTGAGCCAATTCCCATACATTATTGTGCCCCGGCTGGTTTTGCGATTCTAAAGT
GTAAT---GATAAGAAATTCAAT------GGAACAGGACAATGTACAAAT----------------------
-GTCAGC---ACAGTACAATGTACACATGGAATTAGGCCAGTAGTAACAACT---
CAACTGCTGATAAATGGCAGTCTAGCAGAAGAAGAG-----------
GTAGTAATTAGATCTGCCAATTTCACAGACAAT---------GCTAAAACCATAATAGTACAGCTAAAC----
-----------GAAACTGTACAA---------ATTAATTGTACAAGACCCGGCAACAATACAAGAAGA-----
-AGTATACACTTC------------GGACCAGGGAAAGCT---TGG------TAT---ACAGGAGAA------
---------ATAATAGGAGATATA------------AGACAAGCCCATTGT---AACATTAGTGGAACA---
AAATGGAATGACACTTTAAAACAGATAGTTAGCAAATTAAGA---------------GAACAATTTGGG----
-----------------------------------AATAAAATAATAGTCTTTAAT------
CAATCCTCAGGAGGGGACCCAGAAATTGTA---CTGCACAGTTTTAACTGTGGAGGGGAA---
TTTAAGCAGCTCCAGGCAAGAGTCCTAGCTGTGGAAAGATACCTAAAGGATCAACAGCTCCTGGGGATTTGGG
GCTGCTCTGGAAAGCTCATTTGCACCACTGCTGTGCCTTGGAATGCTAGTTGGAGT-----------------
---------------------------AATAAATCTGAGAATGAGATTTGG------AAC------
AACATGACCTGGATGCAGTGG---GACAAGGAAGTTAGCAATTACACAAGCATAATATAC---
AACTTACTTGAACAATCGCAGAACCAGCAGGAA--

AAGAATGAACAAGAATTATTGGAATTAGATAAATGGGCAAGTTTGTGGAATTGGTTTGACATAACACAGTGGC
TATGGTATATAAAAATATTCATAATGATAGTAGGAGGCTTGATAGGTTTAAGAATAGTTTTTGCT---
GTACTTTCTATAATA---------AATAGAGTTAGGCAGGGATATTCACCA------
TTATCGTTGGCTATTAGCTTGTTCAACGCCACAGCCATAGCAGTAGCTGAG---
GGGACAGATAGGGTTATAGAAGTAGCACAAAGAGCTTTTAGGGCT----------------------------
--------ATTATCCACATA------CCTAGAAGAATAAGACAGGGCGCGGAAAGGGCTTTGATA---TAA
>KC699030
GCT---GTA---
GACCAGTTGTGGGTCACAGTCTATTATGGGGTACCTGTGTGG---AAAGAAGCAACC---
ACCACTTTATTTTGTGCATCAGATGCTAAAGCATATGATACAGAG---ATGCATAAT----------------
--------------GTTTGGGCCACA---CATGCCTGTGTACCCACAGAC---
CCCAACCCACAAGAAGTGTTAATGGAA---------AATGTGACAGAAGAGTTTGAT---------------
--------------------------ATGTGGAAAAATAACATGGTAGAGCAAATGCATGAGGATATA---
ATCAGTTTATGGGATCAAAGCCTT---AAGCCATGTGTAAAATTA---
ACCCCACTATGTGTAACTTTAAATTGCACTGATGACCTGACTAACAGTGCTGCTCAGGGGAACAGTACTAACA
GTACTGCTCAAGGGAGTGGTACTACTAATAGTACTGCT-----------------------------------
-------------------------CCGGTGAATAGAACTATTGAGGAACCGGGAGAAATAAAA---
AACTGCTCTTTCAATGTCACCACAGATAGGAAT--------------------------
GATAAGTACCAAAAAGAATATGCACTT---TTCTATAAACTTGATGTAGTACCAACAGATAAT----------
----

AATAATAGTAGCTATAGATTGATAAGTTGTAATACCTCAGTCATTACACAG---
GCCTGTCCAAAGGTATCCTTTGACCCAATTCCCATACATTATTGTGCCCCGGCTGGTTTTGCGATTCTAAAGT
GTAAT---AATAAGACATTCAGT------GGAAAAGGACCATGTAGAAAT----------------------
-GTCAGC---ACAGTACAATGTACACATGGAATTAAGCCAGTAGTGTCAACT---
CACCTGTTGTTAAATGGCAGCCTAGCAGAAGAAAGG-----------
GTAGTAATTAGGTCGGCAAATTTTTCGAAAAAT---------ACTAAAATCATAATAGTACAGCTGAAC----
-----------GACTCTGTAACA---------ATTAATTGTATAAGACCCAATAACAATACAAGAAAA-----
-GGTATAAATATA------------GGACCAGGGAGAGCA---TGG------TATGCAACAACAGAC------
---------ATAATAGGAGATATA------------AGAAAAGCACATTGT---AACATTAGTGAAACT---
GCTTGGAATAAAACTTTAAAAAAGGTGGTTACAGAATTAGGA---------------AAACAATATAAG----
--------------------------------AATGCAACAGCAATAATCTTTAAG------
CCACCCGTAGGAGGGGACCCAGAAATTGTA---TTTCACAGTTTTAATTGTAGAGGGGAA---
TTTAAACAGCTTCAGGCAAGAGTCCTGGCTGTGGAAAGATACCTAAAGGATCAACAGCTCCTAGGGATTTGGG
GCTGCTCTGGAAAACTCATTTGCAACACTGCTGTGCCTTGGAACACTAGTTGGAGT-----------------
---------------------------AATAGATCTCTGGATTATATTTGG------GGA------
AACATGACCTGGATGGAATGG---GAAAGAGAAATTGACAATTACACAGACTTAATATAC---
AACTTAATTGAAGAATCGCAGAACCAGCAAGAA--

AAGAATGAACAAGAACTATTGGAATTAGATAAGTGGGCAAGTTTGTGGAATTGGTTTAGCATAACACAATGGC
TGTGGTATATAAAAATATTCATAATGATAGTGGGAGGCTTGATAGGTTTAAGAATAGTTTTTGCT---
GTACTTTCTTTAGTG---------AATAGAGTTAGGCAGGGATATTCACCA------
TTGTCATTGGCAATTAGCTTGCTTGATGCCACAGCAATAACAGTAGCTGCA---
GGGACAGATAGGGTTATAGAAGCAGTACAAATACTTTTTAGAGCT----------------------------
--------TTTCTTCATATA------CCTAGAAGAATAAGACAGGGCTTAGAAAGAGCTTTGCTA---TAA
>KC699028
TGT---AGT---GCTGCAGAA------------------------------
GAAGGTACATGGGTCACAGTCTATTATGGGGTACCTGTGTGG---AGAGAAACAACC---
ACTACTCTATTTTGTGCATCAGATGCTAAAGCATATGACACAGAG---AAACATAAT----------------
--------------GTTTGGCCACA---CATGCCTGTGTACCCACAGAC---
CCTAACCCACAAGAAGTAATATTAGTA---------AATGTGACAGAAGATTTTAAT----------------
--------------------------ATGTGGAAAAATAACATGGTAGAACAAATGCATGAGGACATA---
ATCAGTTTATGGATCAAAGCCTA---AAGCCATGTGTGAAATTA---
ACCCCACTCTGTGTTACTTTAAATTGCACTAATTGGAATGGTACTAAGACCAAAGATATTAGTTCC-------
----
----------------------AATGTTAGTAGTTGGGAAAAAATGGAGGAAGGAGAAATAAAA---
AACTGCTCTTTCAATGTCATCACAGGCATAGGA---------------------------
GATAAGATGAAGAAAGAATATGCACTT---TTTTACAAACTTGATGTGATACCAATAGATAAT----------
----

GATAATACAAGCTATACATTGATAAATTGTAACACCTCAGTCATTAAACAA---
GCCTGTCCAAAGGTATCCTTTGAACCAATTCCCATACATTATTGTGCCCCGGCTGGTTTTGCGATTCTAAAGT
GTAAT---GATAAGAAGTTTAAT------GGAACAGGACCATGTACAAAT-----------------------
-GTCAGC---ACAGTACAATGTACACATGGAATTAAGCCAGTGGTGTCAACC---
CAGCTGCTGTTGAATGGCAGTCTAGCAGAAGAAGAG------------
GTAGTAATCAGATCTGAAAATTTCACAAACAAT---------GCTAAAACCATAATAGTACAACTGAAG----
-----------AAAGCTGTAAT---------ATTACTTGTATGAGACCCAACAATAACACAAGAAAA-----
-GGTATACATATA------------GGACCAGGAGGAGCA---ATT------TATGCAACAGGAGCT------
---------ATAATAGGAAATATA------------AGACAAGCACATTGT---AACCTTAGTAGAACA---
GATTGGAATAACACTTTAAAACAGATAGCTATGAAATTACAA---------------GAACAAGTTGGG----
-----------------------------------AATAAAACAATAATTTTTAAT------
CACTCCGCAGGAGGGGACCTAGAAATTGTA---ATGCACAGTTTTAATTGTGGAGGGGAA---
TTTAAGCAGCTCCAGGCAAGAGTCCTGGCTGTGGAAAGATACCTACAGGATCGACAGCTCCTAGGGATGTGGG
GTTGCTCTGGAAAACTCATCTGCACCACTACTGTGCCTTGGAATGTTAGTTGGAGT-----------------
----------------------------AATAAATCTCTGGATAAGATTTGG------AAT------
AACATGACCTGGATGCAGTGG---GAGAGAGAAATTGACAATTACACAAGTGTAATATAC---
AACTTACTTGAGGAATCGCAGAACCAGCAAGAA--

AAGAATGAACAAGAATTATTGGAATTGGATAAGTGGGCAAGTTTATGGAGTTGGTTTAGCATAACACAATGGC
TGTGGTATATAAAAATATTCATAATGATAGTAGGAGGTTTAATAGGTTTAAGAATAGTTTTTACT---
GTACTTTCTATAGTG---------AATAGAGTTAGGCAGGGATACTCACCA------
TTATCATTCGCTATTAGTTTGTTTAATGCTTTAGCTATAGCAGTAGCTGAG---
AGGACAGATAGGGTTATAGAAGTAGTACAAAGAATTGGTAGAGGT----------------------------
--------ATCCTCCACATA------CCTAGAAGAATAAGACAGGGCTTCGAAAGGGCTTTGCAA---TAA
>KC699039
GCT---ACA--
GAACAGTTGTGGGTCACAGTCTATTATGGGGTACCTGTGTGG---AGAGAAGCAAAC---
ACTACTCTATTTTGTGCATCAGATGCCAAAGCACATGACACAGAG---GCACATAAT----------------
--------------GTTTGGCCACA---CATGCTTGTGTACCCACAGAT---
CCCAATCCACAAGAAGTGGACGTGACA---------AATATGACAGAAGATTTTAAC----------------
--------------------------ATGTGGAAAAATAGCATGGTAGAACAGATGCATGAGGATATA---
ATCAGTTTATGGATCAAAGCCTA---AAGCCATGTGTAAAATTA---
ACCCCACTCTGTGTCACTTTAAATTGCACTGATTTTTTGGGGAATACTACTGATGTTTTT-------------
----
-------------------GGGAATAGTACTAACACCACTAGTAGTAGTGGAGGAGAAATAAAA---
AAATGCTCTTTCAATGTCACCACAAGTATAGGA---------------------------
AATAAAATACAGAAAGAATATGCACTT---TTTAATACGCTTGATGTAGTACCAATAATAGAT----------
----

GGTAATGATAATAATAGCTATAGGATGATACATTGTAACACCTCAGTCATTACACAA---
GCCTGTCCAAAGATATCCTTTAAGCCAATTCCCATACATTATTGTGCCCCAGCTGGTTTTGCGATTCTAAAGT
GTAAA---GATAAGAAATTCAAT------GGATCAGGACGATGTGAAAAT-----------------------
-GTCAGC---ACAGTACAATGCACACATGGAATTAGGCCAGTAGTGTCAACC---
CAACTGCTGTTGAATGGCAGTCTAGCAGAAGAAGAG------------
ATAGTAATTAGATCTGAAAATTTCTCAAACAAT---------GCTAAAGTCATAATAGTACAGCTGGAT----
-----------AAATCTGTAGAA---------ATTACTTGTGTAAGGCCCAATAACAATACAAGAAAA-----
-AGTATAAATATG------------GGACCGGGGAGAGCC---TTT------TATACAATGGAGAC-------
---------ATAATAGGAAATATA------------AGGCAAGCACATTGT---AACATTAGTAGAACA---
AATTGGGATATCACTGTAACAAAGATAGCGAAAATATTAAGA---------------GAGAAATTT-------
-----------------------------------AACAAAACAATAAACTTTACT------
AGATCTTCAGGAGGGGATCCAGAAATTGTA---ATGCACAGTTTTAATTGTGGAGGAGAA---
TTTAAGCAGCTCCAGGCAAGAGTCCTGGCTGTGGAAAGATACCTAAAGGACCAACAGCTCCTGGGGATTTGGG
GTTGCTCTGGAAAACTCATTTGCACCACTGCTGTGCCTTGGAATGATAGTTGGAGT-----------------
-------------------------AATAATAAAACTCTGAATGAGATTTGG------GAT------
AACATGACCTGGATGCAGTGG---GAAAGAGAAATTGATAAGTACACAAACTCAATATAC---
AATTTAATTGAAGAATCGCAAATCCAACAAGAA--

AAGAATGAACACGAATTATTGAAATTAGATGAATGGGCAAGTTTGTGGAATTGGTTTACTATATCAAAATGGC
TGTGGTATATAAAAATATTCATAATGATAGTAGGAGGCTTGATAGGTTTAAGAATAGTTTTTGCT---
GTACTTTCTATAGTA---------AATAGAGTTAGGCAGGGATACTCACCA------
TTGTCATTGGCTGTTAGCTTATTCGACGCCGTAGCCATAGCAGCAGCTGAG---
GGGACAGATAGGGTTATAGAAGTATTACAAAGAATTGGTAGAGCT----------------------------
--------ATTCTCCACATA------CCTACAAGAATAAGACAGGGCTTAGAAAGGGCTTTGCTA---TAA
>JX140654
GCT---GCA--
GACCAATGGTGGGTCACAGTCTATTATGGGGTACCTGTGTGG---AAAGAAGCAAAC---
CCCACTTTATTCTGTGCATCAGATGCTAAAACATATAAAACAGAG---GCACATAAT----------------
--------------GTTTGGGCTACA---CATGCCTGTGTACCCACAGAC---
CCCAACCCACAAGAAAAGGTATTGGAA---------AATGTGACAGAAGAATTTAAC----------------
--------------------------ATGTGGAAAAATAACATGGTAGAACAGATGCATGAGGATATA---
ATCAGTTTATGGGATGAAAGCCTA---AAGCCATGTGTAAAATTA---
ACCCCACTCTGTGTTACTTTAAATTGCACTGATGTGTCGATGAATGAAACTAATTCTAAGACCACTCCTAGTA
CCTCTACTCCT--
----------ACTAGTGCCTCTACTACCTATAGTGGGAAAAGATGGAGGATGGAGAAATGAAA---
AATTGCTCTTTCAATGTCACCACAATCCTAAGA---------------------------
GACAAGATGCAGACAGAATTTGCACTT---TTTTATAGACTTGATGTAGTACCATTAGATAAT----------
----

AGTAGTAGTACTAGTTATAGGTTGATAAATTGTAACTCCTCAACCATTACACAG---
GCTTGTCCAAAGATATCCTTTGAGCCAATTCCCATACATTATTGTGCCCCGGCTGGTTTTGCGATTCTAAAGT
GTAAT---GATAAGGGATTCAAT------GGAACAGGAACATGTAGGAAT-----------------------
-GTCAGC---ACAGTACAGTGCACACATGGAATTAAGCCAGTAGTGTCAACT---
CAACTGCTGTTAAATGGTAGTCTAGCAGAAGAAGAG------------
GTAGTAATTAGATCTTCCAATTTCTCAGACAAT---------GCTAAAACCATAATAGTACAACTAAAT----
-----------GAAACTGTAGCA---------ATAAATTGTACAAGACCCGGCAATAATACAAGAAGA-----
-AGTATAACTATG------------GGACCAGGGAAAGTA---TTT------TATACAATGAA----------
---------ATAATAGGAAATATA------------AGACGAGCATATTGT---ACCATTAACGAAACA---
AAATGGAATAGCACTTTACAAAAAATAGCTGCAAAATTAGGA---------------GAACAGTTTAAT----
-----------------------------------AAAACAGAAATAGTCTTTACA------
CACTCCTCAGGAGGGGACCCAGAAGTTGTG---ATGCACAGCTTTAATTGTGGAGGAGAA---
TTTAAACAGCTCCAGGCAAGAGTCCTGGCTGTGGAAAGATACCTAAGGGATCAACAGCTCCTAGGGATTTGGG
GTTGCTCTGGAAGACTCATTTGCACCACTGCTGTGCCTTGGAATAATAGTTGGAGT-----------------
-------------------------AATAATAGATCTGTAGACCAAATTTGG------GAT------
AACATGACTTGGATGCAATGG---GAAAGAGAAATTGACAATTATACAGGAGTAATATAC---
AGATTACTTGAAGAATCGCAGAACCAGCAAGAA--

AAGAATGAACAAGAGTTATTAGAATTGGATAAGTGGGCAAGTCTGTGGAATTGGTTTGACATAACAAGATGGC
TGTGGTATATAAAAATATTCATAATGATAGTAGGAGGCTTGATAGGTTTAAGAATAGTTTTTGCT---
GTAATTTCTGTAGTG---------AATAGAGTTAGGCAGGGATACTCACCA------

TTATCATTTGCAGTCAGCTTGCTCAACGCCACAGCTATAGCAGTAGGTGAG---
GGAACAGATAGGGTGATAGAAGTAGTGCAAGGAGCAGGTAGAGCT----------------------------
--------ATTCTCCATATA------CCTAGAAGAATAAGACAGGGCTTGGAAAGGCTTTTGCTA---TAA
>KC699035
GCT---AAG--
GAACAATTGTGGGTCACAGTCTATTATGGGGTACCTGTGTGG---AAAGAAGCAACC---
ACCACTCTATTCTGTGCTTCAGATGCTAAAGCATATGATACAGAG---GCACATAAT----------------
--------------GTTTGGGCTACA---CATGCCTGTGTACCCACAGAC---
CCCAGCCCACAAGAAATAAGATTGGAA---------AATGTGACAGAAAATTTTAAT----------------
--------------------------ATGTGGAAAAATAACATGGTAGAACAGATGCATGAGGATATA---
ATCAGTTTATGGGATCAGAGCCTA---AAGCCATGTGTAAAATTA---
ACCCCACTCTGTGTTATTTTAAATTGCACTGATCCTACTAATAACACCACTAATACCAACAATTCT-------
----
-------------------------------------TCAAGGTTGGAGGAGGGGGAAATGAAA---
AACTGCTCTTTTCATATCAGCACACGAATAACA---------------------------
AATAGGATGCAGAGAGAATATGCACTT---CTTTATAAACTTGATTTAGTACAGATAAATAAT----------
----

AATAATTATAGATTGGTAAGTTGTAACACCTCAGTCCTTACACAG---
GCCTGTCCAAAAACATCCTTTGAGCCAATTCCCATACATTATTGTGCCCCGGCTGGTTTTGCGATTCTAAAGT
GTAAT---GACAAAAAGTTCAAT------GGAACAGGACCATGTAAAAAT-----------------------
-GTCAGC---ACAGTACAATGTACACATGGAATTAGGCCAGTAGTGTCAACT---
CAACTACTGTTAAATGGCAGTCTAGCAGAAGAAGAT------------
GTAGTAATTAGATCTGAAAACTTCACAAACAAT---------GCTAAAACCATAATAGTACAGCTAAAG----
-----------GAAGCTGTACAA---------ATTAATTGTACAAGACCCAACAACAATACAAGAAAA-----
-AGTATACATCTA------------GGACTAGGAAAAGCA---TTT------TATGCAACA---GAC------
---------ATAATAGGAGATATA------------AGACAAGCACATTGT---AACATTAGTAAAGAA---
AGATGGAATAATACCTTAAAACAGGTAGTTGAAAAATTAAGA---------------GGGCAATTTAAT----
-----------------------------------AATAAGACAATAGTCTTTAAT------
CAATCCTCAGGAGGGGACCCAGAAATTGTA---ATGCACAGTTTTAATTGTGGAGGGGAA---
TTTAAGCAGCTCCAGGCAAGAGTCCTGGCTATAGAAAGATACCTAAAGGATCAACAGCTCCTAGGGATTTGGG
GTTGCTCTGGAAAACGCATTTGCACCACTGCTGTGCCTTGGAATGCGAGTTGGAGT-----------------
----------------------------AATAAATCTATGAATGAGATTTGG------AAT------
AACATGACCTGGATGGAGTGG---GAAAAGGAAATTAGTAATTACACACAAAAAATATAC---
ACCTTAATTGAAAAATCGCAAAACCTGCAAGAA--

AAGAATGAACAGGAACTATTGGAATTAGATAAGTGGGCAAATTTGTGGAACTGGTTTGACATAACAAATTGGC
TGTGGTATATAAGGTTATTCATAATGATAGTAGGAGGCTTAATAGGTTTAAGAATAGTTTTTGCT---
GTACTTTCTATAGTA---------AATAGAGTTAGGCAGGGATACTCACCT------
TTATCGTTGGCTGTTAGTTTGCTTAATACCACAGCTATAGCAGTAGCTGAA---
GGGACAGATAGAATTATAGAAGTAGTAAATAGGATTTTTAGAGCT----------------------------
--------GTTCTCCACATC------CCGAGAAGAATAAGACAGGGCTTTGAAAGGCTTTTGCTA---TAA
>KC699036
GCT---ACA--
GAAAAATTGTGGGTCACAGTCTATTATGGGGTACCTGTGTGG---AGGGAAGCAAAC---
ACCACTCTATTTTGTGCATCAGATGCTAAAGCATATGATCCAGAG---GTACATAAT----------------
--------------GTTTGGGCTACA---CATGCCTGTGTACCCACAGAC---
CCCAACCCACAAGAAATAGACTTAGAA---------AATGTGACAGAAGATTTTAAC----------------
--------------------------ATGTGGAAAAATCCCATGGTAGAACAGATGCATGAGGATATA---
ATCAGTTTATGGGATCAAAGCCTA---ACGCCATGTGTGAAATTG---
ACCCCACTCTGTGTTACTTTAAATTGCATTAATTATATGAAGAATGATACTAATGAGACGACCACT-------
----
----------------------------ATTGGTGAGTTGAAGATGAAGGAAGGAGAAATGCAA---
AACTGCTCTTTCAATATCACCACAAGCATAAAA---------------------------
AATAAGAGGCAAAAAGAATATGCACTT---TTTTATAAACAGGATCTGATGTCAATAGATGAA----------
----

AGTGATAATACAAGCTATATATTGATAAAGTGTAACACCTCAGTCATTACACAG---
GCCTGTCCAAAGGTAACCTTTGAACCAATTCCCATACATTATTGTGCCCCAGCTGGTTTTGCGATTCTAAAAT
GTAAA---GATAATAAGTTTAAT------GGAACAGGACCATGTAAAAAT-----------------------

-GTCAGT---ACAGTACAATGTACACATGGAATTAGGCCAGTAGTGTCAACC---
CACCTGCTGTTAAATGGCAGTCTAGCAAAAGAAGAG------------
GTAGTAATTAGATCTAAAAATCTCACAGACAAT---------ACTAAAATCATAATAGTACAGTTGAAG----
-----------GACCCTGTAGTA---------ATTAACTGTACAAGACCCAACAACAATACAAGAAGA-----
-AGTATACATATA------------GGACCAGGGAGAGCA---TTT------TATGCAACACAAGAC------
---------ATAATAGGAAATATA------------AGACAAGCATATTGT---ATCCTTAATGGAACA---
AAATGGAATGAAACCTTAAACCAGATAGTTAAAAAATTAAAA---------------GAACAATTCAAT----
-----------------------------------AATAAAACTATAGTCTTTAAG------
CCCCCAGCAGGAGGGGACCCAGAAATTATA---AAGCATACTTTTAATTGTGGAGGGGAA---
TTTAAACAGCTCCAGGCAAGAGTCCTGGCTGTGGAAAGATACCTAAAGGATCAACAGCTCCTGGGGCTTTGGG
GATGCTCTGGAAAGCTCATTTGCACCACTGCTGTGCCTTGGAATACTAGTTGGAGT-----------------
----------------------------AATAAATCTCAAACGGAAATTTGG------AAT------
AATATGACCTGGATGGAGTGG---GAAAAAGAAATTGATAATTACACAGGCTTAATATAC---
ACCTTAATTGAAGAATCGCAGAACCAGCAGGAA--

AAGAATGAACAAGACTTATTGGAATTGGATAAGTGGTCAAGTTTGTGGAATTGGTTTGATATATCAAATTGGC
TTTGGTATATAAAAATATTCATAATGATAGTAGGAGGCTTGATAGGTTTAAGAATAGTTTTTGCT---
GTACTTTCTATAGTA---------AAAAGAGTTAGGCAGGGGTACTCACCA------
TTATCGTTTGCTATTAGCTTGCTTAACGCCACAGCCATAGCAGTAGCTGAG---
GGAACAGATAGGATTATAGAAGCAGCACAAAGAGTTTATAGAGCT----------------------------
--------ATCCTCCACATA------CCTAGAAGAATAAGACAGGGCTTAGAAAGAGCTTTGCTA---TAA
>KF716494
GCT---GCA--
CCAGACTTGTGGGTCACTGTCCATTATGGGGTACCTGTGTGG---AAAGAAGCAACC---
ACCACTCTATTTTGTGCATCAGATGCTAAAGCATATGATACAGAG---GTACATAAT----------------
--------------GTTTGGCCACA---CATGCTTGTGTACCCACAGAC---
CCCAACCCACAAGAAGTAGTAATA------------AATGTGACAGAAGATTTTAAT----------------
--------------------------GCATGGAAAAATGACATGGTAGAACAGATGCATGAGGATATA---
ATCAGTTTATGGGATCAAAGCCTA---AAGCCATGTGTAAAATTA---
ACTCCACTCTGTGTTACTTTAAATTGTAGTCATGCTTTAAATATTAATAATAGTCATGCTAATAATAGTAGA-
----
----------------------AGTGGGACCAATAGTACTTGGGAACTACAAAATGAAGTAGCA---
AACTGCTCTTTCAATGCTACCACTATCATAAGA---------------------------
GATAAGTTTAAAAAAGAATATGCACTT---TTTTATAGACTTGATATAGTACCAATAGATGATAAGGAT----
----

AATAGCAACAATACTACCTATAGGTTGATAAATTGTAATACCTCAGTCATTACACAG---
GCTTGTCCAAAGATAACATTTGATCCAATTCCTATACATTATTGTGCCCCGGCTGGTTTTGCGATTCTAAAGT
GTAAT---GATAAGAAGTTCAGT------GGAAAAGGACCATGTAAAAAT-----------------------
-GTCAGC---ACAGTACAATGTACACATGGAATTAGGCCAGTAGTGTCAACT---
CAACTATTGTTAAATGGCAGTCTAGCAGAA---GAG------------
GTAGTAATTAGATCTGACAATTTCACGGACAAT---------GCTAAAAACATAATAGTACAGCTGAAC----
-----------GAGACTGTAGAA---------ATTACATGTATAAGACCCAACAACAATACAAGAAAA-----
-GGTATACATATA------------GGACCAGGAAGAGTG---TTT------TATGCAACAGGAGAC------
---------ATAATAGGAGATATA------------AGACAAGCACATTGT---AACATTAGTAGAGAA---
AAATGGAAGAAAACTTTACAAAAGGTAGTTGAAAAATTAGGA---------------GAACAATTTAAA----
--------------------------------AATAAAACAAACATAGCCTTTAAG------
CCCTCCTCAGGAGGGGACCCAGAAATTGTA---ATGCACAGTTTTAATTGTAGAGGGGAA---
TTTAAGCAGCTCCAGGCAAGAGTCCTGGCTGTGGAAAGATACCTAAGGGATCAACAGCTCCTAGGAATTTGGG
GTTGCTCTGGGAAACTCATTTGCACCACTGCTGTGCCTTGGAATACTAGTTGGAGT-----------------
----------------------------AATAAAAATCTGAGTCAAATTTGG------GAT------
AACATGACCTGGATGCAATGG---GAGAGAGAAATTGACAATTACACAGAAATAATATAT---
AACTTACTTGAAGAATCGCAGAACCAACAAGAA--

AAGAATGAACAAGAATTGTTGGAATTGGATAAGTGGGCAAATTTGTGGACTTGGTTTAGCATAACAAATTGGC
TGTGGTATATAAAAATATTCATAATGATAGTAGGAGGCTTGATAGGTTTAAGAATAGTTTTTGCT---
GTACTTTCTATAGTG---------AATAGAGTTAGGAAGGGATACTCACCA------
TTATCGTTCGCTATCAGTTTGTTTGATGCCATAGCTGTAGCGGTAGCTGAG---
GGAACAGATAGGGTTATAGAAGTAGTACAAAGAATTTGTAGGGCT----------------------------
--------ATCCTCCACATA------CCTGTACGAATAAGACAGGGCTTGGAAAGGGCTTTGATA---TAA

>KF716495
GCT---ACA---------------------------------------
GAACAATTGTGGGTCACAGTCTATTATGGGGTACCCGTGTGG---AAGGATGCAATC---
ACCACTCTATTTTGTGCATCAGATGCTAAAGCATATGGTACAGAG---GCACATAAT---------------
--------------ATTTGGGCAACA---CATGCCTGTGTACCTACAGAC---
CCCGACCCACAAGAAGTAGTATTGGGA---------AATGTGACAGAAGATTTTAAC---------------
-------------------------ATGTGGAAAAATAACATGGTAGAACAGATGCATGAGGATATA---
ATCAGTTTATGGGATCAAAGCTTA---AAGCCATGTGTGAAACTA---
ACGCCACTCTGTGTTACTCTAAATTGCACTGATTGTAATGCCCATAATAGTTCTCTAAGTTGCACT-------
----
-------------GATTATAAGGCCAATGATAGTGTGGAGGCTAGTGATGGCGTGAAAATGAAA---
AACTGCTCTTTCAATATCACCACAAGCATAAAA--------------------------
GATAAGATAAAAAAAGAACATGCACTT---TTTTATAGCCTTGATGTAGTACCAATAGAGGGT---------
----

GATAACTCCAGCTATAGGTTAATAAGTTGTAATACCTCAGTTATTACCCAG---
GCCTGTCCAAAGATATCCTTTGAACCAATTCCCATACATTATTGTGCCCCGGCTGGTTTTGCGATTCTAAAAT
GTAAC---AGTAAGACATTCAAT------GGAACAGGGCCCTGTACAAAT----------------------
-GTCAGC---ACAGTACAATGTACTCATGGAATTAGGCCAGTAGTATCAACT---
CAACTGTTGTTAAATGGCAGCCTAGCAGAAGAAGAG-----------
ATAGTAATTAGATCTGAAAATTTCACGGACAAT---------GCTAAAAATATAATAGTGCAGCTGAAT----
-----------AAATCTGTAGAA---------ATTAAGTGTACAAGACCCAGCAACAATACAATGAAA-----
-AGTATACATATA------------GGACCAGGGAGAGCA---TTT------TATACAACAGAACAG------
---------GTAATAGGAGATATA------------AGAAAGGCACATTGT---AATATTAATGGAACA---
GTATGGAAGGACACTTTAGAACAGCTAGTTACAAAATTAAAA---------------GAACAATTTGGA----
-----------------------------------AATAAAACAATAGTCTTTCAG------
CCCTCCTCAGGAGGGGACCTGGAAATTGTA---ACACATAGTTTTAATTGTGGAGGGGAA---
TTTAAGCAGCTCCAGGCAAGAGTCCTGGCTGTGGAAAGATACCTAAGGGATCAACAGCTCCTGGGGATCTGGG
GTTGCTCTGGAAAACTCATTTGCACCACTACAGTGCCTTGGAATACTAGTTGGAGT----------------
----------------------------AATAAATCTCTGGAGGACATTTGG------GAA------
AACATGACCTGGATGCAGTGG---GAAAAACAAATTGACAATTACACAGATATAATATAC---
GGCTTACTTGAGAAATCGCAGAACCAACAAGAA---------------------------------------
----
AAGAATGAACAAGAATTGTTGGAATTAGACAAATGGGCAAGTTTGTGGAATTGGTTCGACATATCAAACTGGC
TGTGGTATATAAAAATATTCATAATGATAGTAGGAGGCTTAATAGGTTTAAGAATAATCTTTACT---
GTGCTTTCTATAGTG---------AATAGAGTTAGGCAGGGATACTCACCA------
ATATCGTTTGCTGTTAACTTGCTTAATGCCACAGCCATAGCAGTAGCTGAG---
GGGACAGATAGGATTATAGAAGCAATACAAAGAATTGGTACAGCT---------------------------
--------ATTCTACACATA------CCTAGAAGAATAAGACAAGGTGCAGAAAGGGCTCTAATA---TAA
>KC699031
GCT---ACA---------------------------------------
AACAACTTGTGGGTCACAGTCTATTATGGGGTACCTGTATGG---AAGGAAGCAGAC---
ACCACCCTATTTTGTGCATCAGATGCTAAAGCATATGACACAGAG---GCACACAAT---------------
--------------GTTTGGGCCACA---CATGCCTGTGTACCCACAGAT---
CCTAACCCACAAGAAGTAAACTTACCA---------AATGTAACCGAAAATTTTAAC---------------
-------------------------ATGTGGAAAAATAGCATGGTAGAGCAAATGCATGAGGATATA---
ATCAGTTTATGGGATCAAAGCCTA---AAGCCATGTGTAAAATTA---
ACCCCACTCTGTGTTACTTTAAATTGCAGTAATGTTAATGTAACCAAAACCAATAGTACTATAAATATAAAA-
----
----------------------AATACTAAATTGACTGGGGACATAGAGCACGGAGAAGTAAAA---
AACTGTTCTTTCAATGTTACCACAAGCATAAAAGATAGG------------
GTAAGGAAAAATAAGGTAAAGGAAGAATATGCATTA---TTTTATAGACCTGATATCATGCCAATAGATAAT-
----

ATAACTAATACAAGCAGCTACACGTTGATAAATTGTAACACCTCAGTCATTTCACAA---
GCCTGTCCAAAGATATCCTTTGAACCAATTCCCATACATTATTGTGCCCCGGCTGGTTTTGCGATTCTGAAGT
GTAAT---GAGAAGCAGTTCAAT------GGAACAGGACCATGTAAAAAT----------------------
-GTTAGC---ACAGTACAATGTACACATGGAATTAAGCCAGTGGTGTCAACT---
CAACTGCTGTTAAATGGCAGTCTATCAGAAGGAGAG-----------
GTAGTACTTAGATCTAAGAATTTCGCAGACAAT---------GCTAAAATTATAATAGTACAGCTGAAT----

-----------AAATCTGTAGAA---------ATTAATTGTATAAGACCTGGCAATAATACAAGAAAA-----
-AGTATACATATA------------GGACCAGGAAGAGCA---TTC------TATGCAACAGGAAGT------
---------ATAATAGGAGATATA------------AGACAAGCACATTGT---AACATTAGTACAGCA---
AAATGGAATAGCACCCTAAAACAGATAGCTATAAAATTACAG---------------GAGCAATTTGGG----
--------------------------------AGGAATAAAACAATAATATTTAAT------
CCATCCTCAGGAGGGGATCCAGAAATTGTA---ATGCACAGTTTTAATTGTGGAGGGGAA---
TTTAAGCAGCTCCAGGCGAGAGTCCTGGCTGTGGAAAGATACCTAAGGGATCAACAGCTCCTAGGGATTTGGG
GCTGCTCTGGAAAACTCATTTGCACCACTGCTGTGCCTTGGAATGATAGTTGGAGTAGTAGG-----------
----------------------------ACTAAAAATCTAACAGGCATTTGG------GAT------
AACATGACCTGGATGCAATGG---GAGAAAGAAATTGAAAATTACACAGACTTAATATAC---
ACTTTACTTGAAGACTCGCAGAACCAGCAAGAA--

AAGAATGAACAAGACTTATTGGAGTTGGATAAGTGGGCAAATTTGTGGAATTGGTTTGACATATCAAATTGGC
TATGGTATATAAAAATATTCATAATGATAGTAGGAGGCTTAATAGGTTTAAGAATAGTTTTTACT---
GTGCTTTCTATAGTA---------AATAGATGTAGGCAGGGATATTCACCA------
TTATCGTTAGCTGTTAGCTTGTTTAATGCCACAGCAGTAGCAGTAGCTGGG---
GGAACAGATAGGGTTATAGAAGTAGTACAAAGAATTTGTAGAGCT----------------------------
--------ATTCTCAACATA------CCTACAAGAATAAGACAGGGCTTAGAAAGGGCTTTGCTA---TAA
>KC699034
GCT---GCA---
GATCAATTGTGGGTAACAGTCTATTATGGGGTACCTGTGTGG---AAAGATGCAACC---
ACCACTCTATTTTGTGCCTCAGATGCTAAAGCATATGAGACAGAG---GCACATAAT----------------
--------------GTTTGGGCCACA---CATGCCTGTGTACCCACAGAC---
CCCAGCCCACAAGAAATAGTATTGGGA---------AATGTGACAGAAGAATTTAAC----------------
--------------------------ATGTGGAAAAATGACATGGTAGAACAAATGCATGAGGATATA---
ATTAGTTTGTGGGATCAAAGCTTA---AAGCCATGTGTAAAAATG---
ACCCCACTCTGTGTTACTTTAAATTGCACTGATTATAATGGTACCCATGTGAATATTACCGGGGTGGGGACTA
ACAAT--
-------ACCAATAATGATACCAATAGCGGTGACTGGAAGTTGATGGATCCAGGAGAGATAAAA---
AACTGCTCTTTCAATGTCACCACAGTCATAAGA--------------------------
GATAAGATAACGAAAGAAACTGCCCTT---
TTTTATAAACTTGATATAGTACCAATAGGTGATAATGAACCAACAAGT-------------------------
----
AGTAATGAGACTAAAAGCTATAACAGCTATAGGTTGATAAATTGTAACACCTCAGCCATTACACAA---
GCCTGTCCAAAGGTATCCTTTGAACCAATTCCCATACATTATTGTGCCCCGGCTGGTTTTGCGATTCTAAAGT
GTAAC---AATAAGACATTCAGT------GGAAGAGGACCGTGTACAAAT----------------------
-GTCAGC---ACAGTACAATGTACACATGGAATTAGGCCAGTAGTGTCAACT---
CAACTGCTGTTAAATGGCAGTCTGGCAGAAGATGAT-----------
ATAGTAATCAGATCTGAGAATTTCTCGGACAAT---------GCTAAAACTATAATAGTACAGCTGAAG----
-----------AAAGCTGTAGTG---------ATTAATTGTACAAGGCCCAACAATAATACAAGAAGA-----
-AGTATATCTATA------------GGACCAGGGAGAGCA---TTT------TATACAACAGGACAG------
---------GTAATAGGAGATATA------------AGACAAGCACACTGT---AACCTTAGTAGGAAA---
GACTGGAATGACACTTTAAAACAAGTAGCTGAAAAATTAAGA--------------
GAACAATTGAGAGTACAATTT------GGGGAC------------------AATAAAACAATAAAGTTTAAT-
-----CAATCCTCAGGAGGAGACCCAGAAATTGTA---ATGCACAGTTTTAATTGTGGAGGGGAG---
TTTAAGCAGCTCCAGGCAAGAGTCCTGGCTGTAGAAAGATACCTAAGGGATCAACAGCTCCTGGGGATTTGGG
GCTGCTCTGGAAAACTCATCTGCACCACTGCTGTGCCTTGGAATACTAGTTGGAGT-----------------
----------------------------AATAAATCTCTGAATGAAATTTGG------GAT------
AACATGACCTGGATGGAGTGG---GAGAAAGAAATTGATAACTATACAGACTTAATATAC---
AAATTAATTGAAAAATCGCAGAACCAGCAGGAG--

AAAAATGAACAAGAATTATTGGAATTAGATAAATGGGCAGGTTTGTGGAATTGGTTTAACATAACAAACTGGC
TGTGGTATATAAAAATATTCATAATGATAGTAGCAGGCTTAATAGGTTTAAGAATAGTTTTTTCT---
GTACTTTCTATAATA---------AGGAGAGTTAGGCAGGGATACTCACCC------
TTGTCGTTTGCTGTCAGCTTGTTCAACGCCATAGCTATAGCAGTAGCTGAG---
GGGACAGATAGGATTATAGAAGCAATACAAAGATTTGGTAGAGGC----------------------------
--------ATTCTCAACATA------CCTACAAGAATAAGACAGGGCTTAGAAAGGGCATTGCTA---TAA
>KF716496
GCT---GCA---
GAACAATTGTGGGTCACAGTCTATTATGGAGTACCTGTGTGG---AAAGAAGCAAAC---

ACCACTCTATTTTGTGCATCAGATGCTAAAGCATATGATACAGAG---GTACATAAT----------------
--------------GTTTGGCCACA---CATGCCTGTGTACCCACAGAC---
CCCAACCCACAGGAAGTAGTATTGGGA---------AATGTGACAGAAGATTTTAAC----------------
--------------------------ATGTGGAAAAATAACATGGTAGAACAAATGCATGAGGATATA---
ATCAGTTTATGGACCAAAGCCTA---AAGCTATGTGTAAAACTA---
ACCCCACTCTGTGTCACCTTAAATTGCACTGGGTTGAACAATGCTACCAATATCACTAGTAGT----------
----
--AATGGGATGACC---
AACTGCTCTTTCAATATCACCACAAGCTTAAGA---------------------------
GACAAGTGGCAGAAAGTATATGCACTT---TTTTATAAACTAGATCTAGTAAAAATAGATGATGAT-------
----

AATAATACTAGCTATAGATTGATAAGTTGTAATACCTCAGTCATTACACAG---
GCCTGTCCAAAGGTATCCTTTGAGCCAATTCCTATACACTATTGTGCTCCGGCTGGTTTTGCGATTCTAAAAT
GCAAT---GATAAGAAGTTCAAT------GGAACAGGACCGTGTAAAAAT-----------------------
-GTCAGC---ACAGTACAATGTACACATGGAATTAAACCAGTAGTATCAACT---
CAACTATTGTTAAATGGTAGTCTAGCAGAAGAAGAG------------
GTAGTAATTAGATCTGCCAATTTCACAGACAAT---------GCTAAAATCATAATAGTACATCTGAAT----
-----------AAATCTGTGCAA---------ATTAATTGTACAAGACCCAGCAACAATACAAGAAAA-----
-AGTATAAATATA------------GGACCAGGGAGAGCA---TTT------TATACAACAGGAGAA------
---------ATAATAGGAGATATA------------AGACAAGCACATTGT---AACCTTAGTAGAACA---
GATTGGAATAACACATTAAGAAGTATAGCTATAAAATTAAAA---------------GAACAATTT-------
-----------------------------------AATAGAACAATAATCTTTAAA------
AATTCTTCAGGAGGGGACCCAGAAATTACA---ATGCACATGTTTAATGGTGGAGGGGAA---
TTTAAGCAGCTCCAGGCAAGAGTCCTGGCTGTGGAAAGATACCTAAAGGATCAACAGCTCCTGGGAATTTGGG
GCTGCTCTGGAAAACTCATCTGCACCACTGCTGTGCCTTGGAATACTAGTTGGAGT-----------------
----------------------------AATAAAACTCTGAATGAGATTTGG------GAG------
AACATGACCTGGATGCAGTGG---GACAGAGAAATTAATAATTACACAAACTTAATATAC---
ACCCTAATTGAAAAATCGCAGAACCAACAAGAG--

AGGAATGAACAAGAATTATTAGAATTAGATAAGTGGTCAAGTTTATGGAATTGGTTTGACATAACAAGCTGGC
TGTGGTATATAAAAATATTCATAATGATAGTAGGAGGCTTGGTAGGTTTAAGGATAGTTTGTACT---
GTACTTTCTATAGTG---------AATAGAGTTAGGCAGGGATACTCACCA------
TTATCGTTTGCTGTTAGCTTGCTCAACGCCACAGCCATAGCAGTAGCTGAG---
GGAACAGATAGGGTTATAGAAATAGTACAAAGAATTTGTAGAGCT----------------------------
--------ATTATCCACATA------CCTGTAAGAATCAGACAGGGCTTGGAAAGGGCTTTGCTA---TAA
>MH000302
GCT---ACA---
GACTTGTGGGTCACAGTCTATTATGGGGTGCCCGTGTGG---AAAGAAGCAAAC---
ACCACGCTATTTTGTGCCTCAGATTCTAAGGCATATAAGACAGAG---GTACATAAT----------------
--------------GTTTGGCCACA---CATGCCTGTGTACCCACCGAC---
CCCAACCCACAAGAAGTAGACTTAATA---------AATGTAACAGAAAATTTTAAT----------------
--------------------------GCATGGAAAAATAACATGGTAGAACAAATGCATGAGGATATA---
ATCAGTCTATGGGATCAAAGCTTA---AAGCCATGTGTAAAATTA---
ACTCCACTCTGTGTTACTTTAAATTGCACTGATGTT-------------------------------------
----
---GAAATGAAA---
AATTGCTCTTTCAATATTACCACAGGCATAAGA---------------------------
GATAAGATGAAAAAAGAAGAGGCTCTT---TTTTATACACTTGATATAGTACCAATAACTAGC----------
----

TATAGGTTGATAAGTTGTAATACCTCAGTCCTTACTCAA---
GCCTGTCCAAAGGTATCCTTTGAGCCAATTCCCATACATTATTGTGCCCCGGCTGGTTTTGCGATTCTAAAGT
GTAAA---GATAAAAAGTTCAAT------GGAACAGGACCATGTAAAAAT-----------------------
-GTCAGC---ACAGTACAATGTACACATGGAATTAGGCCAGTAGTATCAACT---
CAACTGCTGCTAAATGGCAGTCTA---GAAGAAGAG------------
GTAGTAGTTAGATCTGTCAATTTCTCAGACAAT---------ACTAAAACCATAATAGTACAGCTGAAA----
-----------AAAGCGGTAAAC---------ATTACTTGTGTAAGACCCAATAACAATACAAGAAAA-----
-GATATACATATA------------GGACCAGGGAGAGCA---TTT------TATGCAACAGGAGAA------
---------ATAATAGGAAATATA------------AGACAAGCACATTGT---AACATTAGTGAGACA---

AGCTGGAATAACACTTTAAGAGAGATAGTTGAAAAATTAAAA---------------GAACAGTATCCA----
-----------------------------------AATAAAACAATAGCCTTTAAT------
CAGTCTTCAGGCGGGGACCTAGAAGTTGTA---ATGCACACCTTTAATTGCGGAGGGGAA---
TTTAAGCAGCTCCAGGCAAGAGTCCTGGCTTTGGAAAGATACTTGGAGTATCAACAGCTCCTGGGGATTTGGG
GTTGCTCTGGAAAACTCATCTGCACCACTGCTGTACCTTGGAATGCTAGTTGGAGT-----------------
----------------------------AATAAATCTCAGAGTGATATTTGG------AAT------
AACATGACCTGGATGGAGTGG---GAAAGAGAAATTGACAATTACACAGGTATAATCTAC---
ACCTTGCTTGAAAAATCGCAAAACCAACAAGAA--

AAGAATGAACAGGAATTATTAGAATTGGATAAATGGGCAAGTTTGTGGAATTGGTTTAATATAACAAACTGGC
TGTGGTATATAAAAATATTCATAATGATAGTAGGAGGCTTGGTAGGATTAAGAATAGTTTTTACT---
GTACTTTCTATAGCG---------AATAGAGTTAGGCAGGGATACTCACCA------
TTGTCATTTGCTATTAGCTTGCTCAACGCCACAGCCATAGCAGTAGCTGAG---
GGGACAGATAGGGTTATAGAAGTAATACAAAGAACTGGTAGGGCT----------------------------
--------ATTCTCCATATA------CCTGTAAGAATAAGACAGGGCTTAGAAAGAGCTTTGCTA---TAA
>MH000296
GCT--
GAAAAGTTGTGGGTCACAGTCTATTATGGGGTACCTGTATGG---AAAGAAGCAACC---
ACCACTCTATTCTGTGCATCAGATGCTAAAGGATATGATACAGAG---GTACATAAT----------------
--------------GTTTGGGCCACA---CATGCCTGTGTACCCACAGAC---
CCCAACCCACAAGAAATGCCATTAGAA---------AATGTGACAGAAGAATTTAAC----------------
--------------------------ATGTGGAAAAATAACATGGTAGAACAAATGCATGAGGATATA---
ATCAGTCTATGGGATCAAAGCCTA---AAGCCATGTGTAAAATTA---
ACTCCACTCTGTGTTACTTTAAATTGCACTGATTAT-------------------------------------
----
---GAAATGAAA---
AACTGTTCTTTCAATATCACCACAAGCGGAATA---------------------------
AATAAGAAGCAGAAACAACAGGCAATT---TTTAATAGAATTGACATAGTACCAATAGATAGT----------
----

TATAGATTAATAAGTTGTAATACCTCAGTCATTACACAG---
GCTTGTCCAAAGGTAACCTTTGAGCCAATTCCCATACATTATTGTGCCCCGGCTGGTTTTGCGATTCTAAAGT
GTAAT---AATAAGACATTCAGT------GGGAAAGGACCATGTAAAAAT-----------------------
-GTCAGC---ACAGTACAATGTACACATGGAATTAGACCAGTGGTATCAACT---
CAACTACTGTTAAATGGCAGTTTAGCAGAAGAAGAG------------
GTAGTAATTAGATCTGCCAATTTCTCTGACAAT---------AGTAAAACCATAATAGTGCATCTAAAT----
-----------GAATCTGTAGAA---------ATTAATTGTACAAGACCCAACAACAATACAAGGAAA-----
-AGCATACATTTA------------GGACCAGGGAAAGCA---TTT------TATACAACAGGAGAA------
---------GTGATAGGAGACATA------------AGACAAGCACATTGC---AACATTAGTGAAGCA---
AAATGGAGAAAACTTTATCACAGGTAGTTAAAAAATTAAAA---------------GAACAATTTAAA----
-----------------------------------AATAAAACAATAATCTTTAAT------
CAATCCTCAGGAGGGGACCTAGAAATTGTA---ATGCACAGTTTTAATTGTGGAGGGGAA---
TTTAAGCAGCTCCAGGCAAGAATCCTGGCTGTGGAAAGTTACCTAAAGGATCAACAGCTCCTGGGGATTTGGG
GTTGCTCTGGAAAACTCATCTGCACCACTGCTGTGCCTTGGAATAGCAGCTGGAGT-----------------
----------------------------AATAAAACTCTAAGTAAGATTTGG------GAT------
AACATGACCTGGATGGAGTGG---GAAAGAGAGATTAGCAACTACACAAATGAAATATAC---
AACTTAATTGAAGAATCGCAAAACCAACAAGAA--

AAGAATGAACAAGAATTATTATCATTAGATAAATGGGCAAGTCTGTGGAATTGGTTTGACATAACAAAGTGGC
TGTGGTATATAAAAATATTCATAATGATAGTAGGAGGCTTGATAGGTTTAAGAATAGTTTTTACT---
GTACTTTCTATAGCA---------AATAGAGTTAGGCAGGGATATTCACCA------
TTATCGTTAGCTGTTAGCTTGTTCAACGCCATAGCCATAGCAGTAGCTGAG---
GGGACAGATAGGGTTATAGAAATAATACAAAGAGCTTGTAGAGGG----------------------------
--------ATCCTCCACATA------CCTAGAAGAATCAGACAGGGCTTGGAAAGGGCTTTGCTA---TAA
>MH000299
GCA---GAA--
GATAAACTGTGGGTCACAGTCTATTATGGAGTACCTGTGTGG---AAAGAAGCAACC---
ACTACTCTATTTTGTGCATCAGATGCTAAAGGATATGATACAGAG---GTACATAAT----------------
--------------GTTTGGGCCACA---CATGCCTGTGTACCCACAGAC---
CCCAATCCACAAGAGGTAGTATTGGCA---------AATGTAACAGAAGATTTTAAC----------------

--------------------------ATGTGGAAAAATGACATGGTAGAACAGATGCATGAGGATGTA---
ATCGGTTTATGGGATCAAAGCCTA---AAACCATGTGTAAAATTA---
ACTCCACTCTGTGTTACTTTAAACTGCACTGATTAT------------------------------------
----
---GATATGAAA---
GACTGCTCTTTCAATATCACCACAAACATAAGA--------------------------
GATAAGGTGGAGAGACAAAGAGCAGTT---TTTTATAACCTTGATATAGTACCAATAACTAGC----------
----

TATATAATAAGAAGTTGTAATACCTCAGTCATTACACAG---
GCCTGTCCAAAAGTGTCCTTTGAGCCAATTCCCATACATTATTGTGCCCCGGCTGGTTTTGCGATTCTAAAAT
GTAAC---AATAAGACATTTGAT------GGAAAAGGGCCGTGTAAAAAT----------------------
-GTCAGC---ACAGTACAATGCACACATGGAATTAGACCAACAGTATCAACT---
CAACTGTTGCTAAATGGCAGTCTAGCAGAAGAGAAG-----------
GTAGTAATTAGATCTGAAAATTTCACAAACAAT---------GCAAAAACCATAATAGTACAGCTAAAA----
-----------GAAGCTGTAACT---------ATTACTTGTGTGAGATACAACAACAATACAAGAAAA-----
-AGTATCCACATA------------GGACCAGGGAGAGCA---CTT------TATGCAACAGGAGAG------
---------ATAGTAGGAGATATA------------AGGCAAGCACATTGT---AACATTAGTAAAAAA---
CTATGGAATAAAGCCTTAAGGGAGACAGTTGAAAAATTAAGA---------------GAACGATTTAAT----
-----------------------------------AATAGAACAATAACCTTTAAC------
CATTCTTCAGGAGGAGATCCAGAAATTGTA---CTGCACAGTTTTAATTGTGGAGGGGAA---
TTTAAGCAGCTCCAGGCAAGAGTCCTGGCTGTGGAAAGATACCTACAGGATCAACGGCTCCTAGGGATTTGGG
GTTGCTCTGGAAGACTCATCTGCACCACTGCTGTGCCTTGGAATACTAGTTGGAGT-----------------
---------------------------AATAAATCTCTACATGATATTTGG------GAT------
AACATGACCTGGATGCAGTGG---GAAAAAGAGATTGACAATTACACAGGATTAATATAC---
ACCCTAATTGAAGAATCGCAGAATCAACAAGAG--

AAAAATGAAGAAGACTTATTGGCATTAGATAAATGGGCAAGTTTGTGGAATTGGTTCAACATAACACAATGGC
TGTGGTATATAAAAATATTCATAATGATAGTAGGAGGCTTGATAGGTTTAAGAATAGTTTTTACT---
GTGCTTTCTGTAGTG---------AATAGAGTTAGGCAGGGATACTCACCA------
CTGTCGTTTGCTGTTAGTTTGCTTAATACCACCGCTATAGCAGTAGCTGAG---
GGGACAGATAGAATTATAGAAGTAGTACAAAGAGGTTGTAGAGCT----------------------------
--------ATTCGCAACATA------CCTAGAAGAATAAGACAGGGTCTGGAAAGAGCTTTGCAA---TAA
>KC699002
GCT---GCA---
GAACAATTGTGGGTCACAGTCTATTATGGGGTACCTGCGTGG---AAAGAAGCAACC---
ACCACTCTATTTTGTGCATCAGATGCTAAAGCATATGATACAGAG---GTACATAAT----------------
--------------GTTTGGGCCACA---CATGCCTGTGTACCCACAGAC---
CCCAACCCACAAGAAGTAGTATTGGAA---------AATGTGACAGAAAATTTTAAC----------------
--------------------------ATGTGGGAAAATAACATGGTAGAACAGATGCATAAAGATATA---
ATCAGTTTATGGGATCAAAGCCTA---AAGCCATGTGTAAAATTA---
ACCCCACTCTGTGTTACTTTAAATTGCACTGATGATTTGAGGAATGCTACTAATACCACTAGTAGTAAT----
----
-------------------ACCAATAATAGTAGTTGGGGAACGATAGAGAAAGGAGAAATAAAA---
AACTGCTCTTTCAATATCACCACAGGAATAAGA--------------------------
GATAAGGTACAGAAAGAATATGCACTT---TTTTATAAACTTGATGTAGTACCAATAGATAAT----------
----

AGTACCAAATATAGGTTGATAAATTGTAACACCTCAGTCATTACACAG---
GCCTGTCCAAAAATATCCTTTGAGCCAATTCCCATACACTATTGTGCCCCGGCTGGTTTTGCGATTATAAAGT
GTAAC---GATAAGAAGTTCAGT------GGAAAAGGACAATGTACAAAT----------------------
-GTCAGC---ACAGTACAATGTACACATGGAATTAGGCCAGTAGTGTCAACT---
CAATTGCTGTTAAATGGCAGTCTAGCAGAACAAGAT-----------
GTAGTAATTAGATCTGACAATTTCACAGACAAT---------GCCAAAACCATAATAGTACAGCTGAAC----
-----------AAATCTGTAGCA---------ATTAATTGTATAAGACCCAACAACAACACAAGAAAA-----
-GGTATACATATA------------GGACCAGGGAGAGCA---TTT------TATACAACAGGAGAC------
---------ATAATAGGAGATATA------------AGACAAGCACACTGT---AACCTTAGTAGAACA---
GAATGGAATAACACTTTAAAAAAGATAGCTACAAAATTAAGA---------------GAAACATTT-------
-----------------------------------AATAAAACAATAGTCTTTAAA------
CAATCCTCAGGAGGGGACCCAGAAATTGTG---ATGCACAGTTTTAATTGTGGAGGGGAA---

TTTAAGCAGCTCCAGGCAAGAGTCCTGGCTGTGGAAAGATACCTAAAGGATCAACAGCTCCTAGGGATTTGGG
GTTGCTCTGGAAAACTCATTTGCACCACTGCTGTGCCTTGGAATGCTAGTTGGAGT-----------------
----------------------------AATAAAACTCTACATGAGATTTGG------GAT------
AACATGACCTGGATGCAGTGG---GAAAGAGAAATTGACAATTACACAAACATAATATAC---
AACTTAATTGAAGAATCGCAGAACCAACAAGAA--

AAGAATGAACAAGAATTATTGGAATTAGATAAGTGGGCAAGTTTGTGGAATTGGTTTAGTATAACAAATTGGC
TGTGGTATATAAGAATATTCATAATGATAGTAGGAGGCTTGGTAGGTTTAAGAATAGTTTTTGCT---
GTACTTTCTCTAGTG---------AATAGAGTTAGGCAGGGATACTCACCA------
TTATCATTTGCTGTTAGCTTGCTTAATACCATAGCTATCGCAGTAGCTGAG---
GGGACAGATAGGGTTATAGAAGTAGTACAAAGAGCTTGTAGAGCT----------------------------
--------GTTCTACACATA------CCTGTAAGAATAAGACAGGGCTTGGAAAGGGCTTTGCTA---TAA
>KC699011
GCT---GCA--
GGCAACTTGTGGGTCACAGTCTATTATGGGGTACCTGTGTGG---AAAGAAGCATAC---
ACCACTCTATTTTGTGCATCAGATGCTAAAGCCTATGATACAGAA---GTACATAAT----------------
--------------GTTTGGGCCACA---CATGCCTGTGTACCCACAGAC---
CCCAACCCACAAGAAATAGGATTGGAA---------AATGTGACAGAAAAGTTTAAC----------------
--------------------------ATGTGGAAAAATAACATGGTAGAACAGATGCATGATGATATA---
ATTAGTTTATGGGATCAGAGCCTA---AAGCCATGTGTAAAATTA---
ACCCCACTCTGTGTTACTTTAAATTGCACTGACTACTTTGGGAATACTACTAATACCAATAGTACTCCTAATA
CCAATAGTACA--
--GCAGAAATAAAA---
AACTGCACTTTCAATATCACCACAAACATAAGA---------------------------
GATAAGGTGCAGAAAGAATATGCACTT---TTGTATAAACTTGATATAGTACCAATAGATAGT----------
----

AGTAATACCAGCTATAGGTTGAGAAGTTGTAATACCTCAGTCATTACACAA---
GCCTGTCCAAAGGTAACCTTTGAACCAATTCCCATACATTATTGTGCCCCGGCTGGTTTTGCGATTCTAAAGT
GTAGA---GATAAGAAATTTAAT------GGAAAAGGAGACTGTAAAAAT-----------------------
-GTCAGC---ACAGTACAATGTACACATGGAATTAGGCCAGTAGTATCAACT---
CAACTGCTGTTAAATGGCAGTCTAGCAGAAGAAGGG------------
GTAGTAATTAGATCTGACAATTTCTCGAACAAT---------GCTAAAACCATAATAGTACAGCTGAAG----
-----------GACCCTGTAGAA---------ATTAATTGTATAAGACCCAACAACAATACAAGAAAA-----
-AGTATACATATA------------GGACCAGGAAGAGCA---TTT------TATACAACAGGACAA------
---------ATAATAGGAGATATA------------AGACAAGCACATTGT---AACATTAGTAGAGCA---
AAATGGAATAACACTTTAAGACAGATAGTTGAAAAATTAGGA---------------GAACAATTTGGG----
-----------------------------------AATAGAACAATAATCTTTAAT------
CAATCCTCAGGAGGGGACCCAGAAATTGTA---ATGCACAGTTTTAATTGTGGAGGGGAA---
TTTAAGCAGCTCCAGGCAAGAGTCCTGGCTGTGGAAAAATTCCTAAGGGATCAACAGCTCCTGGGAATTTGGG
GTTGCTCTGGAAGACTCATCTGCACCACTGCTGTGCCTTGGAATGCTAGTTGGAGT-----------------
----------------------------AATAAATCTCTGGAGGAAATTTGG------GAT------
AACATGACCTGGAGGGAGTGG---GAAAAAGAAATTAGCAATTACACAGAATTAATATAC---
TCCTTAATTGAAAAATCGCAAAACCAACAAGAA--

AAGAATGAACAAGACTTATTGGAATTGGATAAATGGGCAGATTTGTGGACTTGGTTTGACATAACACATTGGC
TGTGGTATATAAAAATATTCATAATGGTAGTAGGAGGCTTGATAGGTTTAAGAATAGTTTTTTCT---
GTACTTTCTATAGTG---------AATAGAGTTAGGCAGGGATACTCACCA------
CTATCGTTTGCTGTTAGCTTGCTCAATGCCATAGCCATAGCAGTAGCTGAG---
GGGACAGATAGGGTTATAGAAGTATTGCAAAGAGCTTATAGAGCT----------------------------
--------ATTCTCCACATA------CCTACAAGAATAAGACAGGGCTTGGAAAGGGCTTTGCTA---TAA
>KC699010
GCT---GTA--
GAACAATTGTGGGTCACAGTCTATTATGGGGTACCTGTATGG---AAAGAAGCAGAC---
ACCACTCTATTTTGTGCATCAGATGCTAAAGCATATGATACAGAG---GTACATAAT----------------
--------------GTTTGGGCCACA---CATGCCTGTGTACCCACAGAC---
CCCAACCCACAAGAAGTAGTATTGGAA---------AATGTGACAGAAAAATTTAAC----------------
--------------------------ATGTGGAAAAATAACATGGTAGAACAAATGCAGGAGGATATA---
ATCAATTTATGGGATCAAAGCCTA---AAGCCATGTGTAAAATTA---
ACCCCACTCTGTGTTACTTTAAATTGCACTGATTTGGAAAATGCTACTAATACCAATAGT-------------

----
-----------------------------AGTAGCGGGACAGGGATGGAGAGAGGAGAAATAAAA---
AACTGCTCTTTCAATATCTCCACAATGATAAGA----------------------------
GATAAGGTACAGAGAGAATATGCACTT---TTTTATAACCTTGATGTAGTACAAATAGATAAT----------
----

GATACTAATAGTACCAACTATAGGTTGATAAGCTGTAACACCTCAGTCATTACACAG---
GCCTGTCCAAAAGTAACCTTTGAGCCAATTCCCATTCATTATTGTACCCCGGCTGGTTTTGCGATTATAAAGT
GTAAC---GATAAGAAATTCAAT------GGAACAGGACCATGTACAAAT-----------------------
-GTCAGC---ACAATACAATGTACACATGGAATTAGGCCAGTAGTATCAACT---
CAACTGCTATTAAATGGCAGTCTAGCAGAAGAAGAG------------
GTAGTAATAAGATCTGAGAATTTCACGAACAAT---------GCTAAAATCATAATAGTACAGCTGAAC----
-----------GAATCTGTAGAA---------ATTAATTGTACAAGACCCAACAACAATACAAGAAAA-----
-AGTATACACATA------------GGACCAGGGAGAGCA---TTT------TATGCAACAGGAAAA------
---------ATAATAGGAGATATA------------AGACAAGCACATTGT---AACCTTAGTAGTACA---
AAATGGAATAGAACTTTAAAAAAGATAGCTGTAAAATTAAGA---------------GAACAATTTGAG----
------------------------------------AATAAAACAATAGTCTTTAAT------
AAATCCTCAGGAGGAGACCCAGAAATTGTA---ATGCACAGTTTTAATTGTGGAGGGGAA---
TTTAAGCAGCTCCAGGCAAGAGTCCTGGCTATGGAAAGATACCTAAAGGATCAACAGCTCCTGGGGATTTGGG
GTTGCTCTGGAAAACTCATTTGCACCACTAATGTGCCTTGGAATACTAGTTGGAGT-----------------
-----------------------------AATAAATCTCTGGAAAAGATTTGG------AAT------
AATATGACCTGGATGGAGTGG---GATAGAGAAATTAATAATTACACAAATTTAATATAC---
ACCTTAATTGAAGAATCGCAGAACCAGCAAGAA---

AAGAATGAACAAGAATTATTAGCATTGGATCAATGGGCAAGTTTGTGGAATTGGTTTGACATATCGAACTGGC
TGTGGTATATAAAAATATTCATAATGATAGTAGGAGGCTTGGTAGGTTTAAGAATAGTTTTTGCT---
GTACTTTCTATAGTG---------AATAGAGTTAGGCAGGGATACTCACCA------
TTATCGTTGGCTGTTAGCTTGTTTAATGCCATAGCCATAGCAGTAGCTGAG---
GGGACAGATAGGGTTATAGAAGTATTACAAAGAGCTTATAGAGCT----------------------------
--------ATTCTCCACATA------CCTAGAAGAATAAGACAGGGCTTGGAAAGGGCTTTGCTA---TAA
>KC699009
GCT---GCA---
GAAAAATTGTGGGTCACAGTCTATTATGGGGTACCTGTGTGG---AAAGAAGCAACC---
ACCACTCTATTTTGTGCATCAGATGCTAAAGCATATGATACAGAG---GTACATAAT----------------
--------------ATTTGGGCCACA---CATGCATGTGCACCCACAGAC---
CCCAACCCACAAGAAGTAGTATTGGGA---------AATGTGACAGAAAATTTTAAC---------------
---------------------------ATGTGGAAAAATAACATGGTAGAACAGATGCATGAGGATATA---
ATCAGTTTATGGGATCAAAGCCTA---AAGCCATGTGTAAAATTA---
ACCCCACTCTGTGTTACTTTAAATTGCAGTGATTTTGGGAATAATACTAATACCAATAGT------------
----
-----------------------------AGTAGAGGGGGAATAATGGAGAAAGGAGAAATAAAA---
AACTGCTCTTTCAATGTCACCACAAGCATAAGA----------------------------
GATAAGGTGGAGAAAGAAAATGCTTTA---TTTTATAAACTTGATGTAGTACCAATAGATAAT----------
----

GATAATACTAGTACCAGCTATAGGTTAATAAGTTGTAACACCTCAGTCATTACACAA---
GCCTGTCCAAAGGTATCCTTTGAGCCAATTCCCATACATTATTGTGCCCCGGCTGGTTTTGCGCTTCTAAAGT
GTAAC---AATAAGAAGTTCAAT------GGAACAGGACCATGTACAAAT-----------------------
-GTTAGC---ACAGTACAATGTACACATGGAATTAGGCCAGTAGTATCAACT---
CAACTGCTGCTAAATGGCAGTCTAGCAGAAGAAGAG------------
GTAGTGATTAGATCTGAAAATTTCACAAACAAT---------GCTAAAATCATAATAGTACAGCTGAAC----
-----------GAATCTGTAGAA---------ATTAATTGTACAAGACCCAACAACAATACAAGAAAA-----
-AGTATACATATA------------GGACCAGGGAGAGCA---TTT------TATACAACAGGAAAT------
---------ATAATAGGAGATATA------------AGACAAGCACATTGT---AACATTAGTAGAGCA---
AAATGGAATAACACTTTAAATCAGATAGTTAGAAAATTAAGA---------------GAACAATTTGGA----
------------------------------------AATAAAACAATAGTCTTTAAT------
CAATCCTCAGGAGGGGACCCAGAAATTGTA---ACGCACAGTTTTAATTGTGGAGGAGAA---
TTTAAACAGCTCCAGGCAAGAGTCCTGGCTGTGGAAAGATACCTAAAGGATCAACAGCTCCTGGGAATTTGGG
GTTGCTCTGGAAAACTCATTTGCACCACTACTGTGCCTTGGAATGCTAGTTGGAGT-----------------
-----------------------------AATAAATCTCTAGATCAGATTTGG------GAT------

AACATGACCTGGATGGAGTGG---GAAAGAGAAATTGACAACCACACAGGCACAATATAC---
ACCTTACTTGAAAAAGCGCAAAACCAGCAAGAA--

AAGAATGAACAAGAATTATTGGCATTAGATAAATGGGCAAGTTTGTGGAATTGGTTTGACATAACAAAATGGC
TGTGGTATATAAAAATATTCATAATGATAGTAGGAGGCTTGGTAGGTTTAAGAATAGTTTTTACT---
GTACTTTCTATAGTG---------AATAGAGTTAGGCAGGGATACTCACCA------
CTGTCGTTTGCTGTTAGCTTGCTCAATGCCACAGCCATAGCAGTAGCTGAG---
GGGACAGATAGGGTTATAGGAATATTTCAAAGAGCTTGTAGAGCT----------------------------
--------CTTCTGCACATA------CCTAGAAGAATAAGACAGGGCGCGGAAAGGCTTTTCTA---TAA
>KC699008
GCT---GCA--
GAACAATTGTGGGTCACAGTCTATTATGGGGTACCTGTGTGG---AAAGAAGCAACT---
ACCACTCTATTTTGTGCATCAGATGCTAAAGCATATGATACAGAG---GTACATAAT----------------
--------------GTTTGGGCCACA---CATGCCTGTGTACCCACAGAC---
CCCAACCCACAAGAAGTAGTATTGGGA---------AATGTGACAGAAAATTTTAAC----------------
--------------------------ATGTGGAAAAATAACATGGTAGAACAGATGCATGAAGATATA---
ATCAGTTTATGGGATCAAAGCCTA---AAGCCATGTGTAAAATTA---
ACCCCACTCTGTGTTACTTTAAATTGCACTGATGTTGGGAATGCTACTAATGTCAATGATAGTAAT-------
----
-------------------------------------GGGGGAATAGAGAAAGGAGAAATAAAA---
AACTGCTCTTTCAATATCACCTCAAGTGTAAGA---------------------------
GATAAGGTGCAGAAAGAATATGCACTT---TTTTATAGACTTGATGTAATACCAATAGATAAT----------
----

AAAATTAATAATACTAGATATAGGATGATAAATTGTAATACCTCAGTCATTACACAG---
GCCTGTCCAAAGGTATCCTTTGAGCCAATTCCCATACATTATTGTGCCCCGGCTGGTTTTGCGATTCTAAAGT
GTAAT---GATAAAAAGTTCAAG------GGAAAAGGACAATGTACAAAT-----------------------
-GTCAGC---ACAGTACAATGTACACATGGAATTAGGCCAGTAGTGTCAACT---
CAACTGCTGTTAAATGGCAGTCTAGCAGAAGAAGAG------------
GTAGTAATTAGATCTGACAATTTCACAGATAAT---------GCTAAAACTATAATAGTACATCTGAAT----
-----------GAATCTGTAGAA---------ATTAATTGTACAAGACCCAACAACAATACAAGAAAA-----
-AGTATACATATA------------GGACCAGGGAGAGCA---TTT------TATGCAACAGGAGAA------
---------ATAATAGGAGATATA------------AGACAAGCACATTGT---AACATTAGTGGAGCA---
GAATGGAATAAAACTTTAAAACGGATAGCTAGAAAATTAAGA---------------GAACAATTTGAA----
-----------------------------------AATAAAACAATAGTCTTTAGT------
CCATCCTCAGGAGGGGACCCAGAAATTGTA---ATGCACAGTTTTAATTGTGGAGGGGAA---
TTTAAACAGCTCCAGGCAAGAGTCCTGGCTGTGGAGAGATACCTACAGGATCAACGGCTCCTAGGGATTTGGG
GTTGCTCTGGAAAACTCATCTGCACCACTGCTGTACCTTGGAATACTAGTTGGAGT-----------------
----------------------------AATAAATCTCTGGATAATATTTGG------AAT------
AACATGACCTGGATGCAGTGG---GAAAAAGAAATTAACAATTACACAAATGTAATATAC---
ACCTTAATTGAAGAATCGCAGAACCAACAAGAA--

AAGAATGAACAAGAATTATTGGAATTAGATAAGTGGGCAAATTTGTGGAATTGGTTTACCATAACAAACTGGC
TGTGGTATATAAAAATATTCATAATGATAGTAGGAGGCTTGATAGGTTTAAGAATAGTTTTTGCT---
GTACTTTCTATAGTG---------AATAGAGTTAGGCAGGGATACTCACCA------
TTGTCATTTGCTATTAGCTTGCTCAATGCCACAGCTATCACAGTAGCTGAG---
GGAACAGATAGGGTTATAGAAGTAGTACAAAGAGCTTATAGAGCT----------------------------
--------GTTCGCCACATA------CCTACAAGAGTGAGACAGGGCTTGGAAAGGGCTTTGCAA---TAA
>KC699003
GCT---ACA--
GAAAAATTGTGGGTCACAGTCTATTATGGAGTACCTGTGTGG---AAAGAAGCAACC---
ACCACTCTATTTTGTGCATCAGATGCTAAAGCATATGATACAGAG---GTACATAAT----------------
--------------GTTTGGGCCACA---CATGCCTGTGTACCCACAGAC---
CCCAACCCGCAAGAAGTAGTATTGGGA---------AATGTGACAGAAAATTTTAAC----------------
--------------------------ATGTGGAAAAATAACATGGTAGAACAGATGCATGAGGATATA---
ATCAGTTTATGGGATCAAAGCCTA---AAGCCATGTGTAAAATTA---
ACCCCACTCTGTGTTACTTTAAATTGCACTGATTTGAAGAATGCTACTAATACCACTGATGTTAAT-------
----
-------------------ACCACTAATAGTAGCGTGGAACTGATGGAGAAAGGAGAAATAAAA---
AACTGCTCTTTCAATATCACCACACACATGAGA---------------------------

GATAAGATAGAGAAACAATATGCACTT---TTTTATAAACTTGATGTAGTACCAATAAATAAT----------
----

ACTAGCTATAGGTTGATAAGTTGTAACACCTCAGTCATTACACAG---
GCCTGTCCAAAGATATCCTTTGAGCCAATTCCCATACATTATTGTACACCAGCTGGTTTTGCGATTCTAAAGT
GTAAT---AATAAGAAGTTCAAT------GGAACAGGACCATGTACAAAT-----------------------
-GTCAGC---ACAGTACAATGTACACATGGAATTAGGCCAGTAGTATCAACT---
CAATTGCTGTTAAATGGCAGTCTAGCAGAAGAAGAG------------
GTAGTAATTAGATCTGAAAATTTCACGGACAAT---------GCTAAAACCATAATAGTACAGCTGAAC----
-----------AAATCTGTAGAA---------ATTAATTGTACAAGACCCAACAACAATACAAGAAAA-----
-AGTATAAGCATA------------GGACCAGGAAGAGCA---TTT------TATACAACAGGAGAA------
---------ATAATAGGAAATATA------------AGACAAGCACATTGT---AACCTTAATGGAACA---
GAATGGCATAACACTTTAAAACAGATAGTTGAAAAATTAAGA---------------GAACAATTTGGG----
-----------------------------------AATAAAACAATAATCTTTAAT------
CACTCCTCAGGAGGGGACCCAGAAATTGTA---ATGCACAGTTTTAATTGTGGAGGGGAA---
TTTAAGCAGCTCCAGGCAAGAGTCCTGGCTGTGGAAAGATACCTACAGGATCAACAGCTCCTGGGGATTTGGG
GTTGCTCTGGAAAACTCATTTGCACCACTGCTGTGCCTTGGAATGCTAGTTGGAGT-----------------
----------------------------AATAAATCTCTGAGTCAGATTTGG------GAT------
AACATGACCTGGATGGAGTGG---GATAGAGAAATTAATAATTACACAGGCTTGATATAC---
AACTTACTTGAAAAATCGCAGAACCAGCAAGAA--

AAGAATGAGCAAGAATTATTGGAATTGGACAAATGGGCAAGTTTGTGGAATTGGTTTGACATAACACATTGGC
TGTGGTATATAAAAATATTCATAATGATAGTAGGAGGCTTGGTAGGTTTAAGAATAGTTTTTACT---
GTACTTTCTATAGTG---------AATAGAGTTAGGCAGGGATACTCACCA------
TTATCGTTTGCTGTTAGCTTGTTCAATGCCATAGCCGTAGCAGTAGCTGAG---
GGAACAGATTGGGTTATAGAGATAGTACAAAGAGCTTTTAGAGCT----------------------------
--------ATTCTTCACATA------CCTGTACGAATAAGACAGGGCTTGGAAAGGTGTTTGCTA---TAA
>KC699004
GCT---GCA--
GAAAAATTGTGGGTAACAGTCTATTATGGAGTACCTGTGTGG---AAAGAAGCAACC---
ACCACTCTATTTTGTGCATCAGATGCTAAAGCATATGATACAGAG---GTACATAAT----------------
--------------GTTTGGGCCACA---CATGCCTGTGTACCCACAGAC---
CCCAACCCACAAGAAGTATTATTGGAA---------AATGTGACAGAAACTTTTAAC----------------
--------------------------ATGTGGAAAAATGATATGGTAGAACAGATGCATGAGGATATA---
ATCAGTTTATGGGATCAAAGCCTA---AAGCCATGTGTAAAATTA---
ACCCCACTCTGTGTTACTCTACATTGCACTGATTATAGGAATGCTACTAATAAAACTAAT-------------
----
----------------------------AGTGGCGGGGGAATGAAGGAGGGAGGGGAAATAAAA---
AATTGCTCTTTCAATATCACCACACGCATAAGA---------------------------
GATAAGGTGCAGAAAGAATATGCACTT---
TTTTATAAACTTGATGTAGTACCAATAGATAATGATAATAACAAT----------------------------
----
AATACTAATAATACTAGATATAGGTTAATAAGTTGTAACACCTCAGTCATTACACAG---
GCCTGTCCAAAGGTATCCTTTGAGCCGATTCCCATACATTATTGTACCCCGGCTGGTTTTGCGATTCTACAGT
GTAAA---GATAAGAAGTTCAAT------GGAACAGGACCATGTACAAAT-----------------------
-GTCAGC---ACAGTACAATGTACACATGGAATTAGGCCAGTAGTATCAACT---
CAACTACTGTTAAATGGCAGTCTAGCAGAAGAAGAG------------
GTAGTAATTAGATCTAGCAATTTCTCGGACAAT---------GCTAAAACCATAATAGTACAGCTGAAA----
-----------GAGCCTGTAGTA---------ATTAATTGTACAAGACCCAACAACAATACAAGAAAA-----
-AGTATACATATA------------GGACCAGGGAAAGCA---TTT------TATGCAACAGGAGAT------
---------ATAATAGGAGATATA------------AAGCAAGCACATTGT---AACCTTAGTAGAGCA---
AAATGGAATGAGACTTTAAAACAGATAGCTACCAAATTAAGA---------------GAACAATTTAAG----
-----------------------------------AATAAAACAATAGTCTTTAAT------
CAATCCTCAGGAGGGGACCCAGAAATTGTA---ATGCACAGTTTTAATTGTGGAGGGGAA---
TTTAAGCAGCTCCAGGCAAGAATCCTGGCTGTGGAAAGATACCTAAAGGATCAACAGCTCCTGGGGATTTGGG
GTTGCTCTGGAAAACTCATTTGCACCACTGCTGTGCCTTGGAATGCTAGTTGGAGT-----------------
----------------------------AATAAATCTCTGAATAATATTTGG------AAT------
AACATGACCTGGATGGAGTGG---GACAGAGAAATTAACAATTACACAAGCTTAATATAC---
ACTTTAATTGAAGAATCGCAAAACCAACAAGAA--

AAGAATGAACAGGAATTATTGGAATTGGATAAATGGGCAAGTTTGTGGAATTGGTTTGACATAACAAAATGGC
TGTGGTATATAAAAATATTCATAATGATAGTAGGAGGCTTGGTAGGTTTAAGAATAGTTTTTGCT---
GTACTTTCTATAGTG---------AATAGAGTTAGGCAGGGATACTCACCA------
TTATCGTTTGCTGTTAGCTTGCTCAATGCCACAGCCATAGCAGTAGCTGAG---
GGGACAGATAGGGTTATAGAAATATTACAAAGAGCTGGTAGAGCT---------------------------
--------ATTCGCCACATA------CCTACAAGAATAAGACAGGGCCTCGAAAGGGCTTTGCTA---TAA
>KC699005
GCT---GCA---GAA------------------------------------
CAACAATTGTGGGTCACAGTCTATTATGGGGTACCTGTGTGG---AAAGAAGCAACC---
ACCACTCTATTTTGTGCATCAGATGCTAAAGCATATGATACAGAG---GTACATAAT----------------
--------------GTTTGGGCCACA---CATGCCTGTGTACCCACAGAC---
CCCAACCCACAAGAAGTAGAATTGGAA---------AATGTGACAGAAAATTTTAAT----------------
-------------------------ATGTGGAAAAATAACATGGTAGAGCAGATGCATGAGGATATA---
ATCAGTTTATGGGATCAAAGCCTA---AAGCCATGTGTAAAACTC---
ACCCCACTCTGTGTTACTCTAAATTGTAATAATACTCTAAATTGTACTGATGATTCAAAGAATGCTACTAAA-
----
-------------------ACCAATAGTAGTATTGTGGAAAAGATGAAGTGTGGAGAAATAAAA---
AACTGCTCTTTCAATATCACCACAAGCATAAGA--------------------------
GATAAGATGCAGAAAGAATATGCACTT---TTTTATAGACTTGATATAGTACCAATAGATAATGATAAT----
----

AATAATACTAGCACCAGTTATAGGTTGATAAATTGTAACACCTCAGTCATTACACAG---
GCCTGTCCAAAGGTATCCTTTGAGCCAATTCCCATACATTATTGTGCCCCGGCTGGTTTTGCACTTCTAAAGT
GTAAA---GATAAGACGTTCAAT------GGAACAGGACCATGTACAAAT-----------------------
-GTCAGC---ACAGTACAATGTACACATGGAATTAGGCCAGTAGTGTCAACT---
CAACTGCTGTTAAATGGCAGTCTAGCAGAAGAAGAG------------
GTAGTAATTAGATCTGCCAATTTCACAGACAAT---------GCTAAAACCATAATAGTACAGCTGAAG----
-----------GAACCTGTACAA---------ATTAATTGTACAAGACCCAACAACAATACAAGAAAA-----
-AGTATATCTATA------------GGACCAGGGAGAGCA---TTT------TATACAACAGGAGAT------
---------ATAATAGGAGACATA------------AGGCAAGCATATTGT---AACCTTAGTAGAGCA---
GAATGGAATAACACTTTAAGCCAGATATTTACAAAATTAAGA---------------GAACAATTTAAG----
------------------------------------AATAAAACAATAATCTTTAAT------
CAATCCTCAGGAGGGGACCCAGAAATTATA---TTGCACAGTTTTAATTGTGGAGGGGAA---
TTTAAGCAGCTCCAGGCAAGAGTCCTGGCTGTGGAAAGATACCTAAGGGATCAACAGCTCCTAGGGATTTGGG
GTTGCTCTGGAAAACTCATTTGCACCACTACTGTGCCTTGGAATGCTAGCTGGAGT-----------------
----------------------------AATAAATCTCTAGATGAGATCTGG------GAT------
AACATGACATGGATGGAGTGG---GAAAGAGAAATTGGCAATTACACAAGCATAATATAC---
AACTTAATTGAGAAATCGCAAAACCAACAAGAA---

AAGAATGAACAGGAATTATTGGCATTAGATAAGTGGGCAAGTTTGTGGAATTGGTTTGATATATCAAAATGGC
TGTGGTATATAAAAATATTCATAATGATAGTAGGAGGCTTAATAGGTTTAAGAATAGTTTTTGCT---
GTACTTTCTATAGTG---------AATAGAGTTAGGCAGGGATACTCACCA------
TTATCATTTGCTGTTAGCTTGCTAAATGCCACAGCTATAGCAGTAGGTGAG---
GGAACAGATAGGGTTATAGTAGTAGTACAAAGAATTTATAGAGCT---------------------------
--------TTTCTACACATA------CCTAGAAGAATAAGACAGGGCTTAGAAAGAGCTTTGCTA---TAA
>KC699001
GCT---GCA--
GAACAGCTGTGGGTCACAGTCTATTATGGGGTACCTGTGTGG---AAAGAAGCAACC---
ACCACTCTATTTTGTGCATCAGATGCTAAAGCATATGACACAGAG---GTACATAAT----------------
--------------GTTTGGGCCACA---CATGCCTGTGTACCCACAGAC---
CCCAACCCACAAGAAATAGTATTGGAA---------AATGTGACAGAAAATTTTAAC----------------
-------------------------ATGTGGAAAAATGACATGGTAGAACAGATGCATGAGGATATA---
ATCAGTTTATGGGATCAAAGCCTA---AAGCCATGTGTGAAATTA---
ACCCCACTCTGTGTCACTTTAAATTGCACTGATTTTAGGAATACTACAAATACCACT----------------
----
-------------------------------------GAGGTTACCAATAGAGAAGAAATAAAA---
AATTGCTCTTTCAAGAGCAGCACAAACATAAAA--------------------------
GATAGGACACAGATAGAATATGCATTG---TTTTATAACCTTGATTTAGTACAAATAGATAAAGAGAAT----
----

AATAATACTAATAGCAGCTATAGGTTGATAAGTTGTAACACCTCAGTCATTACACAG---
GCCTGTCCAAAGGTATCCTTTGAACCAATTCCCATACATTATTGTGCCCCGGCTGGTTTTGCGATTCTAAAGT
GTAAA---GATAAGAAGTTCAAT------GGAACAGGACCATGTACAAAT----------------------
-GTCAGC---ACAGTACAATGTACACATGGAATTAGGCCAGTAGTATCAACT---
CAACTTCTGTTAAATGGCAGTTTAGCAGAAGAAGAG-----------
GTAGTAATTAGATCTGAAAATTTCACGAACAAT---------GCTAAAACCATAATAGTACAGCTGAAA----
-----------GAAGCTGTAGAA---------ATTAATTGCACAAGACCTTACAACAATACAAGAAAA-----
-AGTATACATATA------------GGACCAGGGAAAGCA---TTT------TATGCAACAGGAGAT------
---------ATAATAGGAGATCCA------------AGACAAGCATATTGT---ATCCTTAATGGAACA---
AAATGGAATGACGCTTTAAAACAGATAGCTAAAAAATTAGGA---------------GAACAATTTAAG----
----------------------------------AATAAAACAATAATCTTTAAT------
CAATCCTCAGGAGGGGACCCAGAAATTGTA---AAGCACACTTTTAATTGTGGAGGGGAA---
TTTAAGCAGCTCCAGGCAAGAGTCCTGGCTATAGAAAGATACCTAAAGGATCAACAGCTCCTAGGGATTTGGG
GTTGCTCTGGAAAACTCATTTGCACCACTGCTGTGCCTTGGAATACTAGTTGGAGC-----------------
----------------------------AATAAAGCTCTGGATGATATTTGG------AAT------
AACATGACCTGGATGCAGTGG---GAAAGAGAAATTGAAAATTACACAGGCTTAATATAC---
ACCTTAATTGAAGAATCGCAGATCCAACAAGAA---------------------------------------
----
AAAAATGAACAAGAATTATTGAAATTGGATAAATGGGCAAGTTTGTGGAATTGGTTTGACATATCAAATTGGC
TGTGGTATATAAAAATATTCATAATGATAGTAGGAGGCTTGGTAGGTTTAAGAATAGTTTTTGCT---
GTACTTTCTATAGTG---------AATAGAGTTAGGCAGGGATACTCACCA------
TTATCGTTGGCTGTTAGCTTGCTTGATGCCACAGCCATAGCAGTAGCTGAG---
GGGACAGATAGGATTATAGAAGCAGTACAAAGAGCTTGTAGAGCT----------------------------
--------ATTCTCCACATA------CCTACAAGAATAAGACAGGGCTTCGAAAGGGCTTTGCTA---TAA
>AY535425
GCA---GAA---
GAACAATTGTGGGTCACAGTCTATTATGGGGTACCTGTGTGG---AAAGAAGCAACT---
ACCACTCTATTTTGTGCATCAGATGCTAAAGCATATGATACAGAG---GTACATAAT----------------
--------------GTCTGGGCCACA---CATGCCTGTGTACCCACAGAC---
CCCAACCCACGAGAAGTAGTAATGGGA---------AATGTGACAGAAGAATTTAAC---------------
-------------------------ATATGGAACAATAGCATGGTAGAACAGATGCATGAAGATATA---
ATTAATTTATGGGACCAAAGTCTA---AAGCCATGTGTAAAATTA---
ACCCCACTCTGTGTTACTTTAAATTGCACTAACTACAATAGTACTAGTAAT---------------------
----
-------------------------------------GATACTACCAAGGAAACAGGAATAAAA---
AAATGCTCTTTCAATATCACCACCTCAGGCGTA-----------------------
AAAGATAGGTTTACGAAGGAAAATGCACTT---TTGTATACAGCTGATGTAGTACCAATAGATAAT-------
----

AGTAGTATCAACTATAGGTTGATAGGTTGTAACACCTCAGTCATTACCCAG---
GCCTGTCCAAAGGTATCCTTTGAGCCAATTCCCATACATTATTGTGCCCCGGCTGGTTTTGCGATTCTAAAGT
GTAAT---AATGAAACATTCGAT------GGAAAAGGACCATGTACAAAT----------------------
-GTCAGC---ACAGTACAATGTACACATGGAATTAGACCAGTAGTATCAACT---
CAACTGCTGTTAAATGGCAGTCTAGCAGAAACAGAG-----------
GTAGTAATTAGATCTGACAATTTCTCGAACAAT---------GCTAAAACCATAATAGTACAACTGACA----
-----------GAACCTGTAGTA---------ATCAATTGTATAAGACCCAACAACAACACAAGAAGA-----
-AGTATACATATA------------GGACCAGGGAGCGCA---TTT------TATACAACAGGACAA------
---------ATAATAGGAGATATA------------AGACAAGCACATTGT---AACATTAATGGAACA---
CAATGGAATAAAACTTTAAGTCTAATAGTTGATAAATTAAAG---------------AAACAATTTAAT----
----------------------------------AATAAAACAATAATCTTTAGG------
AACTCCTCAGGAGGGGACCCAGAAATTGTA---ATGCACAGTTTTAACTGTGGAGGGGAA---
TTTAAGCAGCTCCAGGCAAGAGTCCTGGCTGTGGAAAGACACCTAGGGGATCAACAGCTCCTGGGAATTTGGG
GTTGCTCTGGAAAACTCATCTGCACCACTGCTGTGCCTTGGAATGATAGTTGGAGT----------------
----------------------------AATAAATCTCTGAAATATATTTGG------GAT------
AACATGACCTGGATGCAGTGG---GAAAAAGAAATTGACACCCACATAGGCACAATATAC---
CAATTAATTGAAGAATCGCAGTACCAACAAGAA---------------------------------------
----
AAGAATGAAAAAGAATTATTGGAACTAGATAAATGGGCAAGTTTGTGGAATTGGTTTGACATAACAAACTGGC
TGTGGTATATAAAAATATTCATAATGATAGTAGGAGGCTTGATAGGTTTAAGAATAGTTTTTACT---
GTACTTTCTATAGTG---------AATAGAGTTGGGCAGGGATACTCACCA------

TTATCGTTGGCTGTTAGCTTGTTCAATGCCATAGCCATAGCAGTAGCTGAG---
GGGACAGATAGGATAATAGAAGTAATACAAAGAGCTTATAGGGCT----------------------------
--------ATTCTCCACATA------CCTAG--
>AY713411
GCT---GCA--
GACCAATTGTGGGTCACAGTCTATTATGGGGTACCTGTGTGG---AAAGATGCAACT---
ACCACTCTGTTCTGTGCATCAGATGCTAAAGCATATGATAAAGAA---GTACATAAT----------------
--------------GTTTGGGCCACA---CATGCCTGTGTACCCACAGAC---
CCCAACCCACAAGAAGTAGTATTGGGA---------AATGTGACAGAAAATTTTAAC----------------
--------------------------ATGGGGAAAAATAACATGGTAGAACAGATGCATGAAGATATA---
ATCAGTTTATGGGATCAAAGCCTA---AAGCCATGTGTAAAATTA---
ACCCCACTCTGTGTTACTTTAAATTGCACTGAGTTGAAGAATAGTACTGAT----------------------
----
-------------------------------------ACCAATTTGGGAACACAAGAAATGAAA---
AACTGCTCTTTCAACATCACCACAAGTGTAAGA---------------------------
AATAAGATGAAGAGAGAATATGCACTT---TTTTATAGTCTTGATATAGTACCAATAGATAAT----------
----

GATAATACTAGATATAGGTTAAGAAGTTGTAATACCTCAATCATTACACAG---
GCCTGTCCAAAGGTATCCTTTGAGCCAATTCCCATACATTTTTGTGCCCCGGCTGGTTTTGCGATTCTAAAGT
GTAAC---AATAAAACGTTCAAT------GGAACAGGACCATGTACAAAT-----------------------
-GTCAGC---ACAGTACAATGTACACATGGAATTAGGCCAGTAGTATCAACT---
CAACTGCTGTTAAATGGCAGCCTAGCAGAAGAAGAG------------
GTAGTAATTAGATCTGAAAATTTCACAAACAAT---------GCTAAAACCATAATAGTACAGCTAAAT----
-----------GAATCTGTAGAA---------ATTAATTGTACAAGACCCAACAACAATACAAGAAAA-----
-AGTATACATATA------------GGACCAGGGAGAGCA---TTT------TATACAACAGGAGAT------
---------ATAATAGGAGATATA------------AGACAAGCACATTGT---AACATTAGTAGAACA---
AACTGGACTAACACTTTAAAAAGGGTAGCTGAAAAATTAAGA---------------GAAAAATTTAAT----
-----------------------------------AATACAACAATAGTCTTTAAT------
CAATCCTCAGGAGGGGACCCAGAAATTGTA---ATGCACAGTTTTAATTGTGGAGGGGAA---
TTTAAGCAGCTCCAGGCAAGAGTCCTGGCTCTGGAAAGATACCTACAGGATCAACGGTTCCTAGGGATGTGGG
GTTGCTCTGGAAAACTCATCTGCACCACTGCTGTGCCTTGGAATGCTAGTTGGAGT-----------------
----------------------------AATAAAAATCTGAGTCAGATTTGG------GAT------
AACATGACCTGGATGGAGTGG---GAGAGAGAAATAAGCAATTACACAGAGATAATATAC---
AGCTTAATTGAAGAATCGCAGAACCAACAAGAA--

AAGAATGAACTAGACTTATTACAATTGGATAAGTGGGCAAGTTTGTGGAATTGGTTCAACATAACAAATTGGC
TGTGGTATATAAAAATATTCATAATGATAGTAGGAGGCTTGATAGGTTTAAGAATAGTTTTTACT---
GTACTTTCTATAGTG---------AATAGAGTTAGGCAGGGATACTCACCA------
TTATCATTCGCTGTCAGTTTGCTTAACGCTACAGCTATAGCAGTAGCTGAG---
GGGACAGATAGGGTTATAGAAGTAGTACAAAGAATTTGGAGAGGT----------------------------
--------ATTCTCCACATA------CCTACAAGAATTAGACAGGGCTTCGAAAGGGCTTTGCTA---TAA
>AF041125
GCT---GCA--
GAAAATTTGTGGGTCACAGTCTATTATGGGGTACCTGTGTGG---AAAGAAGCAACC---
ACCACTCTATTTTGTGCATCAGATGCTAAAGCATATGATACAGAA---GTACATAAT----------------
--------------GTTTGGGCCACA---CATGCCTGTGTACCCACAGAC---
CCCAACCCACAAGAAATAGTATTGGAA---------AATGTGACAGAAAATTTTAAC----------------
--------------------------ATGTGGGAAAATGACATGGTAGAACAGATGCATGAGGATATA---
ATCAGTTTGTGGGATCAAAGCCTA---AAGCCATGTGTAAAGTTA---
ACCCCACTCTGTGTTACTCTAAATTGCACTGATTTGAATGTTACTGATACCACTAATAGTAGC----------
----
----------------------AGGGGAGTGGTGGTGGAAGAAGAAATGAAAGGAGAAATAAAA---
AACTGCTCTTTCAATACCACCACAAGAATAAAA---------------------------
GATAAGGGGCAAAAAGAATATGCATTG---TTTTATAAACTTGATGTAGTACCAATAGGTAAC----------
----

AATAATACTAATACCAGCTATAGGTTGATAAGTTGTAACACCTCAGTCATTACACAG---
GCCTGTCCAAAGGTATCCTTTGAGCCAATTCCCATACATTATTGCGCCCCGGCTGGTTATGCGATTCTAAAGT
GTAAA---GATAAGAAGTTCAAT------GGAACAGGACCATGTACAAAT-----------------------

-GTCAGC---ACAGTACAATGTACACATGGAATTAGGCCGGTAGTATCAACT---
CAACTGCTGTTAAATGGCAGTCTAGCAGAAGAAGAG-----------
GTAGTAATTAGATCTACCAATTTCACGGACAAT---------ACTAAAAACATAATAGTACAGCTAAAT----
-----------GAATCTATAGAA---------ATCAATTGTACAAGACCTAACAACAATACAAGAAAA-----
-AGTATACATATG------------GGACCAGGGAAAGCA---TTT------TATACAACAGGAGAA------
---------ATAATAGGGGATATA------------AGACAAGCACATTGT---AATCTTAGTGAAGCA---
AAATGGAATAACACTTTAGGACAGATAGTTGCCAAATTAAAA---------------GAACAATATAAG----
-----------------------------------AATAAAACAATAGCCTTTAGA------
CCATCCTCAGGAGGAGACCCAGAAGTTGTG---ACGCACAGCTTTAATTGTGGAGGGGAA---
TTTAAGCAGCTCCAGGCGAGAGTCCTGGCTGTGGAAAGATACCTAAAGGATCAACAGCTCCTGGGGATTTGGG
GCTGCTCTGGAAAACTCATTTGCACCACTGCTGTGCCTTGGAATGCTAGTTGGAGT-----------------
----------------------------AATAAATCTCTGGAGGAGATTTGG------AAT------
AACATGACCTGGATGGAGTGG---GAAAGAGAAATTGACAATTACACAAGCTTAATATAC---
ACCTTAATTGAAGAATCGCAGACCCAACAAGAA---

AAGAATGAACTAGAATTATTAAAATTAGATGAATGGGCAAGTTTGTGGAATTGGTTTGACATAACAAAATGGC
TGTGGTATATAAAAATATTCATAATGATAGTAGGAGGCTTGGTAGGTTTAAGAATAGTTTTTGCT---
GTACTTTCTATAATG---------AATAGAGTTAGGCAGGGATACTCACCA------
TTATCGTTTGCTGTTAGCTTGCTCAACGCCACAGCAATAGCAGTAGCTGAG---
GGGACAGATAGGGTTATAGAAGTATTACAAAGAATTTGTAGAGCT----------------------------
--------TTTCTCCACGTA------CCTAGAAGAATAAGACAGGGCTTGGAAAGACTTTTGCTA---TAA
>AY535471
GCT---ACA---
GAAAAATTGTGGGTCACAGTCTATTATGGGGTACCTGTGTGG---AAAGAAGCAACC---
ACCACTCTATTTTGTGCATCAGATGCTAAAGCATATAATACAGAG---GCACATAAT----------------
--------------GTTTGGGCCACT---CATGCCTGTGTACCCACAGAC---
CCCAACCCACAAGAAGTAGTATTGGAA---------AATGTGACAGAAAATTTTAAC---------------
--------------------------ATGTGGAAAAATAACATGGCAGAACAGATGCATGAGGATATA---
ATCAGTTTATGGGATCAAAGCCTA---AAGCCATGTGTAAAATTA---
ACCCCACTATGTGTTACTTTACATTGCACTGATTTGGGGAATGCTACT-------------------------
----
-------------------------------------AATACCATGGAGAAAGGAGAAATAAAA---
AACTGCTCTTTCAATTATACCACAACCATAAGA--------------------------
GATAAGGTGCAGAAAGCATATGCACTC---TTTTATAAACTTGATGTAGTACAAATGGATGAG---------
----

GATAATACCAGCTATAGGTTGATAAGTTGTAACACATCAGTCATTACACAG---
GCCTGTCCAAAGATATCCTTTGAGCCAATTCCCATACATTATTGTGCACCGGCTGGGTTTGCGATTCTAAAAT
GTAAT---AATAAAACATTCAAT------GGAACAGGACCATGTACAAAT-----------------------
-GTCAGC---ACAGTGCAATGTACACATGGAATTAGGCCAGTAGTATCAACT---
CAACTGCTGCTAAATGGCAGCCTAGCAGAAGAAGAG-----------
GAAGTAATTAGATCTGAAAATTTCACGGACAAT---------ACTAAAACTATAGTAGTACAGCTGAAA----
-----------GAAGCTGTAGAA---------ATTAATTGTACAAGACCCAACAACAATACAAGAAAA-----
-GGTATACACATA------------GGACCAGGGAGTGCA---TTT------TATACAACAGGAGAA------
---------ATAATAGGAGATATA------------AGACAAGCACATTGT---AACCTTAGTAGAGCA---
AAATGGAATGACACCTTAAACCAGATAGTTATAAAATTAAGA---------------GAACAATTTAGG----
-----------------------------------AATAAAACAATAGTCTTTAAT------
CACTCCTCAGGAGGGGACCCAGAAATAGTA---ATGCACAGTTTTAATTGTGGAGGGGAA---
TTTAAACAGCTCCAGGCAAGAATCCTGGCTATAGAAAGATACCTAAAGGATCAGCAGCTCCTGGGGATTTGGG
GTTGCTCTGGAAAACTCATTTGCACCACTACTGTGCCTTGGAATGCTAGTTGGAGT-----------------
----------------------------AATAAATCTCAGGATGAGATTTGG------AAA------
AACATGACCTGGATGGAATGG---GATAGAGAAATTAACAATTACACAAACTTAATATAC---
AACTTAATTGAACAATCGCAGACCCAACAAGAA---

AAGAATGAGCAAGAATTATTGGAATTAGATAAATGGGCAAGTTTGTGGAATTGGTTTGACATAACAAACTGGT
TGTGGTATATAAAAATATTCATAATGATAGTAGGAGGCTTGGTAGGTTTAAGAATAGTTTTTACT---
GTGCTTTCTATAGTG---------AATAGAGTTAGGAAGGGATACTCACCA------
CTATCGTTTGCTGTTAGCTTGCTCAACGCCACAGCCATAGCAGTTGCTGAG---
GGGACAGATTGGGTTATAGAAATATCACAAAGAGCTTTTAGAGCT--------------------------
--------GTCCTCAACATA------CCTGTAAGAATAAGACAGGGCTTGGAAAGGGCTTTGCTA---TAA

>AF041130
GCT---ACA--
GAAAAATTGTGGGTCACAGTCTATTATGGGGTACCTGTGTGG---AAAGAAGCAACC---
ACCACTTTATTTTGTGCATCAGATGCTAAAGCATATGACACAGAG---GTGCATAAT----------------
--------------GTTTGGCCACA---CATGCCTGTGTACCCACAGAC---
CCTAACTCACAAGAAGTAAGATTGGAA---------AATGTGACAGAAGATTTTAAC----------------
--------------------------ATGTGGAAAAATAACATGGTTGAACAGATGCATGAGGATATA---
ATCAGTTTATGGGATGAAAGCCTA---AAGCCATGTGTAAAATTA---
ACCCCACTCTGTGTTACTTTAAATTGCACTAATTTGGAGAATGCTACCAATATCAATAGT-------------
----
-------------------------AGTAGTAGTGGGGAACAGCTAAGGGAGGGAGAAATGAAA---
AACTGCTCTTTCAATGTCACCACAAGCATAAAA---------------------------
AATAAGCAGCAGAAAGAATATGCACTC---TTTTATAAACTTGATGTAACACCAATAGATAAT----------
----

GATAATACAAGCTATATGTTGATAAATTGTAACACCTCAGCCATTACACAG---
GCCTGTCCAAAGGTATCCTTTGAACCAATTCCCATACATTATTGTGCCCCGGCTGGTTTTGCGATTCTAAAGT
GTAAG---GATAAGAAGTTCAAT------GGAACAGGACCATGTAAAAAT-----------------------
-GTCAGC---ACAGTACAATGTACACATGGAATTAGGCCAGTAGTGTCAACT---
CAACTGCTGTTAAATGGCAGTCTGGCAGAAGAAGAG------------
GTAGTAATTAGATCTGAGAATCTCACGGACAAT---------GCTAAAACCATAATAGTACAGCTGAAT----
-----------GAAACTGTAGAA---------ATTAATTGTACAAGACCCAACAATAATACAAGAAAA-----
-AGTATACATATA------------GGACCAGGGCAAGCA---TTT------TATGCAACAGGAGAA------
---------ATCATAGGAGATATA------------AGACAAGCACATTGT---AACGTTAGTAGAGCA---
AAATGGACTAACACTTTAAAACAGATAGTTACAAAATTAAGA---------------GAACAATTTGGG----
--------------------------------ACTAATACAAAAATAGTCTTTAAT------
CAATCCACAGGAGGGGACCCAGAAATTGTT---ATGCACAGTTTTAATTGTGGAGGGGAA---
TTTAAGCAGCTTCAGGCGAGAATCCTGGCTGTGGAAAGATACCTACAGGATCAACAGCTCCTGGGAATTTGGG
GTTGCTCTGGAAAACGCATTTGCACCACTGCTGTGCCTTGGAATGCTAGTTGGAGT-----------------
----------------------------AATAAATCTCTGAGTCAGATTTGG------GAT------
AACATGACCTGGATGGAGTGG---GAGAAAGAAATTGACAATTACACAGGTTTAATATAC---
ACCCTAATTGAAGAATCGCAGATCCAACAAGAA--

AAGAATGAGCAAGACTTATTGGAATTAGATAGATGGGCAAGTTTGTGGAATTGGTTTAGCATAACAAACTGGC
TGTGGTATATAAAAATATTCATAATGATAGTAGGAGGCTTGGTAGGTTTAAGAATAATTTTTACT---
GTACTTTCTATAGTG---------AATAGAGTTAGGCAGGATACTCACCA------
TTATCGTTCGCTGTTAGCTTGCTCAATGCTACAGCTATAGCAGTAGCTGAG---
GGGACAGATAGGGTTATAGAAGTAGCACAAAGGGTTTTTAGGACT----------------------------
--------ATACTCCACATA------CCAGTAAGAATAAGACAGGGCTTGGAAAGGGCTTTGCTA---TAA
>AF041131
GCT---GTA--
GAACAATTGTGGGTCACAGTCTATTATGGGGTACCTGTGTGG---AAAGAAGCAACT---
ACCACTCTATTTTGTGCATCAAATGCTAAAGCATATGATACAGAG---GTACATAAT----------------
--------------GTTTGGCCACA---CATGCCTGTGTACCCACAGAC---
CCCAACCCACAAGAAGTAGCATTGGAA---------AATGTGACAGAAGAATTTAAT----------------
--------------------------GTGTGGAAAAATAACATGGTAGAACAGATGCATGAAGATATA---
ATCAGTTTGTGGGATCAAAGTCTA---AAGCCATGTGTAAAATTA---
ACCCCACTCTGTGTTACTTTAAATTGCACTGATTATGTGGGGAATACGAATACTACTAATGCCACTGTAACAG
CAGAA--
----------ATAAAAGAGGGGAGTATGAATACTACTAATGCCACTGAAAGAGGGGAAATAAAA---
AAATGCTCTTTCAATACCACCACAAGTGTAAGA---------------------------
GATAAGGTGCAGAAAGAAGAAGCATTG---TTTTATAAACTTGATGTAATACAAATAGGAAAT----------
----

AGTACTAAATATAGAATGATAAATTGTAATACCTCAGTCATTACACAG---
GCCTGTCCAAAGGTATCCTTTGAGCCAATTCCCATACATTTTTGTGCCCCGGCTGGTTTTGCAATTCTAAAGT
GTAAC---AATAAAACATTCAAT------GGAAAAGGACCGTGTACAAAT-----------------------
-GTCAGC---ACAGTACAATGTACACATGGAATTAGGCCAGTAGTGTCAACT---
CAACTGTTGTTAAATGGCAGTTTAGCAGAAGAAGAG------------
ATAGTAATTAGATCTGACAATATCACAGATAAT---------ACTAAAACCATAATAGTACAGCTGAAT----

-----------AAATCTGTAGAA---------ATTAATTGTACAAGACCCAACAACAATACAAGAAAA-----
-AGTATATCTATA------------GGACCGGGGAGAGCA---TTT------TATGCAACAGGAGAT------
---------ATAATAGGAAATATA------------AGACAAGCATATTGT---AACATTAGTGGAGCA---
ACATGGAATAACACCTTAAGACTGATAGCTAAAAAATTAAGA---------------GAACAATTTGTG----
-----------------------------------AATAAAACAATAGTCTTTAAT------
CAATCCTCAGGAGGGGACCCAGAAATTGTA---ATGCTCACTTTTAATTGTGGAGGGGAA---
TTTAAACAGCTCCAGGCAAGAGTCCTGGCTGTGGAAAGATACCTACAGGATCAACGGCTCCTGGGGATTTGGG
GTTGCTCTGGAAAACTCATCTGCACCACTACTGTGCCTTGGAATACTAGTTGGAGT-----------------
----------------------------AATAAATCTCTGAATCAGATTTGG------AAT------
AACATGACCTGGATGCAGTGG---GAGAGAGAAATTGACAATTACACAAGTGAAATATAC---
TACTTACTTGAAAAATCACAAAACCAACAAGAA--

GAGAATGAAAAAGAATTATTGGAATTAGATAAGTGGGCAAGTTTGTGGAATTGGTTTGACATAACAAATTGGC
TGTGGTATATAAGATTATTCATAATGATAGTAGGAGGTTTGGTAGGTTTAAGAATAGTTTTTGCT---
GTACTTTCTATAGTG---------AATAGAGTTAGGCAGGGATACTCACCA------
TTGTCATTTGCTGTTAGCTTGCTTAATGCCACAGCTATCGCAGTAGCTGAG---
GGAACAGATAGGGTTATAGAAGTAGTACAAAGAGCTGGTAGAGCT----------------------------
--------ACTCTCCACATA------CCTACAAGAGTAAGACAGGGCTTAGAAAGGGCTTTGCTA---TAA
>AF041134
GCT---GCA--
GAAAAATTGTGGGTCACAGTCTATTATGGGGTACCTGTGTGG---AAAGAAGCAGAC---
ACCACTCTATTTTGTGCATCAGATGCTAAAGCATATGATACAGAG---GTACATAAT----------------
--------------GTTTGGGCCACA---CATGCCTGTGTACCCACAGAC---
CCCAACCCACAAGAAGTAGCATTGGGA---------AATGTGACAGAAAATTTTAAT----------------
--------------------------ATGTGGAAAAATAACATGGTAGAACAGATGCAAGAGGATATA---
ATTAGTTTATGGGATCAAAGCCTA---AAACCATGTGTAAAATTA---
ACCCCACTCTGTGTTACTCTAAATTGCACTGATTATGTTACTAACACCACTAGTGGTAATTCTGCTAAC----
----
-------------------ACCACTAGTGGTAGCGGGGGAATGATGGAGAGAGGAGAAATAAAA---
AATTGCTCTTTCAAGATCACCACAAGCATAAGA---------------------------
GATAAGGTGCAGAAAGAATATGCATAT---TTTTATAGCCTTGATGTAATAGCAATAGATAAT----------
----

ACTAGCTATAGGTTAAGAAGTTGTAACACCTCAGTCATTACACAG---
GCCTGTCCAAAGATATCCTTTGAGCCAATTCCCATACATTATTGTGCCCCGGCTGGTTTTGCGATTCTAAAGT
GTAGA---GATAAGAAATTCAAT------GGAACAGGTCCATGTACAAAT-----------------------
-GTCAGC---ACAGTACAATGTACACATGGAATTAAGCCAGTAGTATCAACT---
CAACTGCTGTTAAATGGAAGTCTAGCAGAAGAAGAG------------
GTAGTAATTAGATCCAAAAATTTCTCGGACAAT---------ACTAAAATCATAATAGTACAGCTGAAT----
-----------GAAACTGTAGAA---------ATAAATTGTACAAGACCCAATAACAATACAAGAAAA-----
-GGTATACATATA------------GGACCAGGCAGAGCA---TTT------TATGCAACAGGAGAA------
---------ATAATAGGAGATATA------------AGACAAGCACACTGT---AACATTAGTCAAGCA---
AAATGGAATAACACTTTAAAACAGATAGCTATAAAATTAAAA---------------
GAACAAAAACAATTTAGG---------------------------------AATAAAACAATAGTCTTTAAT-
-----CGATCCTCAGGAGGGGACCCAGAAATTGTA---ATGCACAGTTTTAATTGTGGAGGGGAA---
TTTAAGCAGCTCCAGGCAAGAGTCCTGGCTGTGGAAAGATACCTAAAGGATCAACAGCTCCTGGGGATTTGGG
GTTGCTCTGGAAAACTCATTTGCACCACTAATGTGCCTTGGAATACTAGTTGGAGT-----------------
----------------------------AATAAATCTCTGGATCAGATTTGG------GAT------
AACATGACCTGGATGGAGTGG---GAAAGAGAAATTGATAATTACACAGGCTTAATATAC---
AACTTAATTGAAAAATCGCAAAACCAACAGGAA--

AAGAATGAACAAGAATTATTGGAATTAGATAAATGGACAAGTTTGTGGAGTTGGTTTGACATAACAAACTGGC
TGTGGTATATAAGAATATTCATAATGATAGTAGGAGGCTTGGTAGGTTTAAGAATAATTTTTATG---
GTCTTTGCTGTAGTA---------AAAAGAGTTAGGCAGGGGTATTCACCA------
TTATCGTTCGCTGTTAGCTTGTTTAATGCCATAGCCATAGCAGTAGCTGAG---
GGAACTGATAGGGTTATAGAAGTAATACAAAGAACTTTTAGAGCT----------------------------
--------ATTCTCCACATA------CCTACGCGAATAAGACAGGGCTTTGAAAGGGCTTTGCTA---TAA
>AF041133
GCT---AAA--
GAACAGTTGTGGGTCACAGTCTATTATGGGGTACCTGTGTGG---AAAGAAGCAACC---

ACCACTCTATTTTGTGCATCAGATGCTAAAGCATATGATACAGAG---GTACATAAT----------------
--------------GTTTGGGCCACA---CATGCCTGTGTACCCACAGAC---
CCCAACCCACAAGAAGTAGCATTGGAA---------AATGTGACAGAAAATTTTAAC----------------
--------------------------ATGTGGAAAAATAACATGGTAGAACAGATGCATGAGGATATA---
ATCAGTTTGTGGGATCAAAGCCTA---AAGCCATGTGTAAAATTA---
ACCCCACTCTGTGTTACTTTAAATTGCACTGATTTGAGGAATGCTTCTATTACTACCAATATC----------
----
-------------------------AATGCTACTAATATTACTAGTACCAATAGTGAAATGAAA---
AACTGCTCTTTCAATATCACCACAAACATAAGA---------------------------
GATAAAATGCAGAAAGAATATGCACTT---TTTTATAAACTTGATATAGTACAAATAGGTAAT----------
----

AAAAGTGATAGTTATAGGTTGATAAGTTGTAACACCTCAGTCATTACACAG---
GCCTGTCCAAAGGTAACCTTTGAGCCAATTCCTATACATTATTGTGCCCCGGCTGGTTTTGCGATTCTAAAGT
GTAAC---GATAAGATGTTCAAT------GGAACAGGACCATGTAAAAAT-----------------------
-GTCAGC---ACAGTACAATGTACACATGGAATTAGGCCAGTAGTATCAACT---
CAACTGCTGTTAAATGGCAGTTTAGCAGAAAAAGAG------------
GTAGTAATTAGATCTGCCAATTTCTCGGACAAT---------ACTAAAACCATAATAGTACAGTTAAAC----
-----------GAAACTGTACAA---------ATTCATTGTACAAGACCCAACAACAATACAAGAAAA-----
-AGTATACACATA------------GGACCGGGGAGAGCA---TTT------TATGCAACAGGACAA------
---------ATAATAGGAGATATC------------AGACAAGCACATTGT---AACCTTACTAGAGCA---
AACTGGACTAACACTTTAAAACAGATAGTTGAAAAATTAAGA---------------GAACAATATGAG----
-----------------------------------AATAAAACAATAGTCTTTAAC------
CATTCTTCAGGGGGGGACCCAGAAATTGTA---ATGCACAGTTTTAATTGTGGAGGGGAA---
TTTAAGCAGCTCCAGGCAAGAGTCCTGGCTGTGGAAAGATACCTAAAGGATCAACAGCTCCTGGGAATTTGGG
GTTGCTCAGGAAAACTCATCTGCACCACTGCTGTGCCTTGGAATGCTAGTTGGAGT-----------------
----------------------------AATAAATCTCGGGATGAGATTTGG------GAC------
AACATGACCTGGATGCAGTGG---GAAAGAGAAATTAGCAATTATACAGAGTTAATATAC---
AGCTTAATTGAAGAATCGCAAAACCAACAAGAA--

AAGAATGAACAAGAACTATTGGAATTGGATAAATGGGCAAATTTGTGGACTTGGTTTGACATAACACAATGGC
TGTGGTATATAAAAATATTCATAATGATAGTAGGAGGCTTGATAGGTTTAAGAATAGTTTTTATT---
GTACTTTCTGTAGTG---------AATAGAGTTAGGCAGGGATATTCACCA------
TTATCGTTGGCTGTTAGCTTGCTCAATGCCACAGCCATAGCAGTAGCTGAA---
GGGACAGATAGGGTTATAGAAGTAATACAAAGAACTTTTAGAGCT----------------------------
--------ATTATCCACATA------CCTACAAGAATAAGACAGGGCTTGGAAAGGGCTTTGCTA---TAA
>KC699014
GCT---AAA--
GAACAATTGTGGGTCACAGTCTATTATGGGGTACCTGTGTGG---AAAGAAGCAACC---
ACCACTCTATTTTGTGCATCAGATGCTAAAGCATATGATACAGAG---GTACATAAT----------------
--------------GTTTGGGCCACA---CATGCCTGTGTACCCACAGAC---
CCCAACCCACAAGAAGTAGTATTGGAA---------AATGTGACAGAAAATTTTAAC----------------
--------------------------ATGTGGAAAAATGACATGGTAGAACAAATGCATGAGGATATA---
ATCAGTTTATGGGATCAAAGCCTA---AAGCCATGTGTAAAATTA---
ACCCCTCTCTGTGTTACTTTACATTGCACTGATTATAATGGTACTAAGGCAGGGAATGCTACTAATAAT----
----
-------------GGTACTACTACCCCGAGTAGTATCGAGAAAATGGATAAAGGAGAAATACAA---
AACTGCTCTTTCAATGTCACCACAGGAATAAGG---------------------------
GATAAGAAGAAGCAAGAATATGCACTT---TTTTATAGAATTGATGTAATACCAATAGATGAAGAAAAGAAT-
----

AATACTAGTGATAATACTACTAGCTATAGGTTGATAAATTGCAACACCTCAGTCATTACACAG---
GCCTGTCCAAAGATATCCTTTGAACCAATTCCCATACATTATTGTGCCCCAGCTGGTTTTGCGATTCTAAAGT
GTAAT---GATAAGAAGTTCAAT------GGAACAGGACCATGTAAAAAT-----------------------
-GTCAGC---ACAGTACAATGTACACATGGAATTAGACCAGTAGTATCAACT---
CAATTGCTGTTAAATGGCAGTCTAGCAGAAGAAGAG------------
GTAGTAATTAGATCTGAGAATTTTACGGACAAT---------GCTAAAACCATAATAGTACACCTGAAG----
-----------GAATCTGTAGAA---------ATTAATTGTATAAGACCCAACAACAATACAAGAAGA-----
-GGTATACATATA------------GGACCAGGGCAAACA---TTG------TATGCAACAGGGGAA------
---------ATAATAGGAGATATA------------AGAAAAGCACATTGT---ACCATTAATAGGACA---

CAATGGAATAACACTTTAAAACAGATAGTTACAAAATTAAGA---------------GAACAATTTGGG----
-----------------------------------AATAAAACAATAGTCTTTAAT------
CAATCCTCAGGAGGGGACCCAGAAATTGTA---ACGCACAGTTTTAATTGTGGAGGGGAA---
TTTAAGCAGCTCCAGGCAAGAATCCTGGCTGTGGAAAGATACCTAAAGGATCAACAGATCCTAGGGATTTGGG
GCTGCTCTGGAAAACTCATTTGCACCACTACTGTGCCTTGGAATACTAGTTGGAGT-----------------
----------------------------AATAAATCTCTGAATGACATTTGG------GAG------
AACATGACCTGGATGGAGTGG---GAAAGAGAAATTGATAATTACACAAGCTTAATATAC---
ACCTTACTTGAAGAATCGCAGAACCAACAAGAA---

AAGAATGAACAAGAATTATTGGAATTGGATAAATGGGCAAGTTTGTGGAATTGGTTTGATATAACAAATTGGC
TGTGGTATATAAAAATATTCATAATGATAGTAGGAGGCTTGATAGGTTTAAGAATAGTTTTTACT---
GTACTTTCTATAGTG---------AATAGAGTTAGGCAGGGTTACTCACCA------
TTATCGTTTGCTGTTAGCTTGCTCAACGCCACAGCAATAGCAGTAGCTGAG---
GGGACAGATAGGGTTATAGAAATATTACAAAGAGCTGGTAGAGCT---------------------------
--------CTTCTCCACATA------CCTAGAAGAATAAGACAAGGCTTAGAAAGGGCATTGCTA---TAA
>KC699016
GCT---ACA---
GAACAATTGTGGGTCACAGTCTATTATGGGGTACCTGTGTGG---AAAGAAGCAACC---
ACCACTCTATTTTGTGCATCAGATGCTAAAGCATATGATACAGAG---GTACATAAT----------------
--------------GTTTGGGCCACA---CATGCCTGTGTACCCACAGAC---
CCCAACCCACAAGAAACAGAATTGGAA---------AATGTGACAGAAAATTTTAAC---------------
--------------------------ATGTGGAAAAATAACATGGTAGAACAGATGCATGAAGATATA---
ATCAGTTTATGGGAACAAAGCCTA---AAGCCATGTGTAGAATTA---
ACCCCACTCTGTGTTACTTTAAATTGCACTGATGTTAAGAATAATAATACCATTAATAATGCTACTGCTAAGG
CTAATACTACT--
-AGTACTACTAATAATACTACTAGCAGTAATAGTAGCTTGGGAGAGCAGGAGGGAGAAATAAAA---
AACTGCACTTTCAATGTCACCACAAGCATAAGA--------------------------
GATAAGATGCGGGAAGAATATGCACTT---TTTTATAAACTTGATGTAGTACCAATAAAAAAT----------
----

GATAGTGTCAGCTATATGTTGATAAGTTGTAACACCTCAGTCGTTAAACAG---
GCCTGTCCAAAGGTATCCTTTGAACCAATTCCCATACATTATTGTGCCCCGGCTGGTTTTGCGATTCTAAAGT
GTAAT---GATAAGGAATTCAAT------GGAAAAGGAACATGTAGAAAT----------------------
-GTCAGC---ACAGTACACTGTACACATGGAATTAAGCCAGTAGTATCAACT---
CAACTGCTGTTAAATGGCAGTCTAGCAAAAGAAGAG------------
GTAGTAATAAGGTCTGAGAATTTCACGAACAAT---------GCTAAAACCATAATAGTACAGCTGAAA----
-----------AACCCTGTAGGA---------ATTAATTGTACAAGACCCAACAACAATACAAGAAAA-----
-AGTATACCTATA------------GGACCAGGGAGAGCA---TTT------TATGCAACA---GAT------
---------ATAATAGGAGATATA------------AGACAAGCACATTGT---AACCTTAGTAAGACA---
GAATGGAATAACACTTTACAACAGATAGCTGAAAAATTAGGA---------------GAACAATTTAAT----
-----------------------------------AACGCTACAAAAATAGCCTTTAAT------
AAATCCTCAGGAGGGGACCCAGAAATTGTA---ATGCACAGTTTTAATTGTGGAGGGGAG---
TTTAAGCAGCTCCAAGCAAGAGTCCTGGCTGTGGAAAGATACCTAAAGGATCAACAGCTCCTGGGAATTTGGG
GTTGCTCTGGAAAACTCATCTGCACCACTGCTGTGCCTTGGAATACTAGTTGGAGT-----------------
----------------------------AATAAATCTCTGGACGATATTTGG------GAG------
AACATGACCTGGATGCAGTGG---GATGAAGAAATTAGTAATTACACAAAAGACATATAT---
AAATTAATTGAAAAATCGCAGAACCAACAAGAA---

AAGAATGAACAAGATTTATTGGCATTAGATGAATGGGCAAGTTTGTGGAATTGGTTTAGCATAACAAAATGGC
TGTGGTATATAAAAATATTCATAATGATAGTAGGAGGCTTGATAGGTTTAAGAATAGTTTTTACT---
GTACTTTCTATAGTG---------AATAGAGTTAGGCAGGGATACTCACCA------
ATATCGTTGGCTGTTAGCTTGTTTGATACCATAGCCATAGCAGTAGCTGAA---
GGGACAGATAGGATTATAGAAATATTTAGAAGAGCTTTTAGGGCT---------------------------
--------ATTCTCCACATA------CCTAGAAGAATAAGACAGGGCTTAGAAAGGCTTTTGCTA---TAA
>KC699017
ACT---ATA---
GAAGGCTCGTGGGTCACAGTCTATTATGGAGTACCTGTGTGG---AAAGAAGCAACC---
ACCACTCTATTTTGTGCATCAGATGCTAAAGCATATGACACAGAG---GTACATAAT----------------
--------------GTTTGGGCCACA---CATGCCTGTGTACCCACAGAC---
CCTAACCCACAAGAAATAGTATTGGAA---------AATGTGACTGAAAATTTTAAT---------------

--------------------------ATGGGAAAAAATAACATGGTAGAACAGATGCATGAGGATATA---
ATCAGTTTATGGGATCAAAGCCTA---AAGCCATGTGTGAAATTA---
ACCCCAATCTGTGTTACTTTAAATTGCACTAATCTTGCTAATAATACCAATAGTACTAGTTCC----------
----
-------------------------AATAGTAGTTTGGGAAAGATGGAGGCAGGAGAAATGAAA---
AACTGCTCTTTCAATGTCACCAGTAACATAGGA---------------------------
AATAAGAGGTTTAAAGAATATGCACTT---TTTTATAAACTTGATATAGTACCAATAGATAAT----------
----

ACTAGTACAAGATATACATTGATAAATTGTAACACCTCAATCATTACACAG---
GCCTGTCCAAAGGTATCCTTTGAACCAATTCCCATCCATTATTGTGCCCCGGCTGGTTTTGCGATTCTAAAAT
GTAAA---GATACGAAATTTAAT------GGAACAGGACCATGTAAAAAT-----------------------
-GTCAGC---ACAGTACAATGTACACATGGAATTAAGCCAGTAGTGTCAACC---
CAATTGCTGTTAAATGGCAGTCTAGCAGAAGATGAG------------
GTAGTAATTAGATCTGTAAATTTCTCGGACAAT---------GCTAAAACCATAATAGTACAGCTGAAG----
-----------ACGGCTGTAGAA---------ATTAATTGTACAAGACCCAACAATAATACAAGAAAA-----
-AGTATACCTATA------------GGACCAGGGAGAGCA---TTT------TATGCAACAGGAGAG------
---------ATAATAGGAGATATA------------AGAAAAGCATATTGT---AACATTAGTAGAGCA---
ACATGGAATAACACTTTATCACAGATAGTTACAAAATTACAA---------------GGACAATTTGGG----
-----------------------------------AATAAAACAATAATCTTTAAT------
CAATCCTCAGGAGGGGACCCAGAAATTGTA---ATGCACAGTTTTAATTGTGGAGGGGAA---
TTTAAGCAGCTCCAGGCAAGAGTCCTGGCTGTGGAAAGATACCTACAGGATCGACAGCTCCTAGGAATTTGGG
GTTGCTCTGGAAAACTCATCTGTACCACTTCTGTGCCTTGGAATAATAGTTGGAGT-----------------
----------------------------AATAAATCTATAGATTATATTTGG------AAT------
AACATGACCTGGATGCAGTGG---GAGAAAGAAATTGACAATTACACAGGTTTGATATAC---
ACTTTACTTGAAGAATCGCAGAACCAACAGGAA--

AAGAATGAACAAGAATTATTAGAATTGGATAAGTGGGCAAGCCTGTGGAATTGGTTTAGCATAACAAAGTGGT
TGTGGTATATAAAAATATTCATAATGATAGTAGCAGGTTTAGTAGGTTTAAGAATAGTTTTTACT---
GTACTTTCTATAGTA---------AATAGAGTTAGGCAGGGATATTCACCA------
TTGTCATTTGCTGTTAGTTTGTTTAATGCAATAGCTATAGCAATAGCTGAG---
GGAACAGATAGGGTTATAGAAGTAGCACAAAGAATTTGGAGAGGC----------------------------
--------ATCCTCCACATA------CCTAGAAGAATAAGACAGGGCTTAGAAAGACTTTTAATA---TAA
>KC699012
GCT---GCA--
GAACAATTGTGGGTCACAGTCTATTATGGGGTACCTGTGTGG---AAAGAAGCAACC---
ACCACTCTATTTTGTGCATCAGATGCTAGAGCATATGATACAGAG---GTACATAAT----------------
--------------GTTTGGGCCACA---CATGCCTGTGTACCCACAGAC---
CCCAACCCACAAGAAGTAGTATTGGGA---------AATGTGACAGAAAATTTTAAC----------------
--------------------------ACGTGGAAAAATAACATGGTAGAACAGATGCATGAGGATATA---
ATCAGTTTATGGGATCAAAGCTTA---AAGCCATGTGTAAAATTA---
ACCCCACTCTGTGTCACTTTACATTGCACTGATTTGAGAAATGATACT-------------------------
----
---CAAAGTAAA---
AACTGCTCCTTCAATATCACCACAAGTATAAGA---------------------------
GGTAAGATACAGAAAGAATATGCACTT---TTTTATAAGCTTGATATAGTACCAATAGATAAT----------
----

GATAGTAATAGCTATAGGTTGATAAGTTGTAACACCTCAGTCATTACACAA---
GCCTGTCCGAAGGTATCATTTGAGCCAATTCCCATACATTATTGTGCCCCGGCTGGTTTTGCGATTCTAAAGT
GTAAC---GATAAGAAGTTCAAT------GGAACAGGACTATGTACAAAT-----------------------
-GTCAGC---ACAGTACAATGTACACATGGAATTAGGCCAGTGGTGTCAACT---
CAACTACTGTTAAATGGCAGTCTAGCAGAAGAAGAG------------
GTAGTAATTAGGTCTGAAAATTTCACGAACAAT---------GCTAAAACCATACTAGTACAGTTGAAA----
-----------GATCCTGTAGAA---------ATTAATTGTACAAGACCCTATAACAATACAAGAAAA-----
-AGTATACATATA------------GGACCAGGGAGAGCA---TTT------TATGCAACAGGAGAC------
---------ATAATAGGAGATATA------------AGACAAGCATCTTGT---ACCATTAATAGAACA---
AAATGGAATGACACCTTAAAACAGGTAGTTGACAGATTAAGA---------------GAACAATTTAGG----
-----------------------------------AATAAAACAATAGTCTTTCAA------
CCACCCTCAGGAGGGGACCCAGAAATTGTA---TTGCATAGTTTTAATTGTGGAGGGGAA---

TTTAAGCAGCTCCAGGCAAGAGTCCTGGCTGTGGAAAGATACCTAGAGGATCAACAGCTCCTGGGAATTTGGG
GTTGCTCTGGAAAACTCATTTGCACCACTGCTGTGCCTTGGAATGCTAGTTGGAGT-----------------
----------------------------AATAAATCTCTGAGTAACATTTGG------GAT------
AACATGACCTGGATGGAGTGG---GAAAAAGAAATTGGCAATTACACAAGCTTAATATAC---
ACCCTACTTACAGAATCGCAAAACCAACAAGAA--

AAGAATGAACAAGAATTATTGGCATTAGATAAATGGGCAAATTTGTGGAATTGGTTTAGCATAACAAATTGGC
TGTGGTATATAAAAATATTCATAATAATAGTAGGAGGCTTAATAGGTTTAAGAATAGTTTTTACT---
GTACTTTCTGTAGTA---------AATAGAGTTAGGCAGGGATATTCACCA------
TTATCGTTTGCTGTTAGCTTGCTCAACGCCATAGCCATAGCAGTAGCTGAG---
GGAACAGATAGGGTTTTAGAAGTATTACAAAGAGCTTATAGAGCT----------------------------
--------ATTCTCCACATA------CCTACAAGAATAAGACAAGGCGCGGAAAGGGCTTTACTA---TAA
>KC699020
GCT---ACA---AAA------------------------------------
GAAAACTTGTGGGTCACAGTCTATTATGGGGTACCTGTGTGG---AAAGAAGCAACT---
ACCACTCTCTTTTGTGCATCAGATGCTAAAGCATATAGTAGAGAG---GTACATAAT----------------
--------------GTTTGGGCCACA---CATGCCTGTGTACCCACAGAC---
CCCAACCCACAAGAAGTAATGTTGAAA---------AATGTGATAGAAAATTTTAAC----------------
--------------------------ATGTGGAAAAATAACATGGTAGAACAGATGCATGAGGATATA---
ATCAGTTTATGGATCAAAGCCTA---AAGCCATGTGTAAAATTA---
ACCCCACTCTGTGTCACTTTAAATTGCACTGATTTGAATATTACTAGCACCAATAATAGTAGTAGT-------
----
----------------------TTGAATAAAGAAGAAATAAAAAACTATAAAGGAGAAATAAAA---
AACTGCTCTTTCAAAGTCACCACAGACAGAAAA---------------------------
GATAGGCCGAAGACAGAATATGCACTT---TTTTATAAACTTGATATAGTAAAAATAGATAATGAT-------
----

AGTAGGAATAATATTAGCTATAGGTTGATACATTGTAACACCTCAGTCATTACACAG---
GCCTGTCCAAAGGTAACCTTTGAGCCAATTCCCATCCATTATTGTGCCCCGGCTGGTTTTGCGATTCTAAAGT
GTAAC---AATGAGACGTTCAAT------GGAACAGGACCATGTACAAAT-----------------------
-GTCAGC---ACAGTACAATGTACACATGGAATTAGGCCAGTAGTGTCAACT---
CAACTACTGCTAAATGGCAGTCTAGCAGAAAAAGAT------------
GTAATAATTAGATCTGAAAATTTCACGAACAAT---------GTTAAAACCATAATAGTACAACTGAAG----
-----------AAACCTGTAAAA---------ATTAATTGTACAAGACCCAACAACAATACAAGAAAA-----
-AGTATACCTATA------------GGACCAGGGAAAGCA---TTT-------TATGCAACAGGAGAA------
---------ATAATAGGAGATATA------------AGACAAGCACAATGT---AACCTTAATGGAACA---
GAATGGAATAACACTTTAGAACAGATAGTTAAAAAATTAAAA---------------GAACAGTTTAAG----
-----------------------------------AATAAAACAATAATCTTTAAT------
CAATCCTCAGGAGGAGACCCAGAAATTACA---ATGCACAGTTTTAATTGTGGAGGGGAA---
TTTAAGCAGCTCCAGGCAAGAGTCCTGGCTGTGGAAAGATACCTAAAGGATCAACAGCTCCTGGGGATTTGGG
GCTGCTCTGGAAAACTCATTTGCACCACTACTGTGCCTTGGAATGATAGTTGGAGT-----------------
----------------------------AATAAATCTTATGATAAGATTTGG------AAG------
AACATGACCTGGATGCAGTGG---GAAAGAGAAATCAGTAATTACACAGAACAAATATAC---
ACCTTAATTGAAGAATCGCAGAACCAACAAGAA--

AAGAATGAACAAGAATTATTGGAATTGGATAAGTGGGCAAGTTTGTGGAATTGGTTTGATATATCAAACTGGC
TGTGGTATATAAGGATATTCATAATGATAGTAGGAGGCTTGATAGGTTTAAGAATAGTTTTTGCT---
GTACTTTCTATAGTG---------AATAGAGTTAGGAAGGGATACTCACCA------
TTATCGTTTGCTGTTAGCTTGCTCAACGCCACAGCCATAGCAGTAGCTGAG---
GGGACAGATAGGATTATAGAAGTATTACAAAGAGCTGGTAGAGCT----------------------------
--------ATTCTCCATATA------CCTAGAAGAATAAGACAGGGCTTTGAAAGGGCTTTACTA---TAA
>KC699026
ACT---GCA--
GGAAAGTTGTGGGTCACAGTCTATTATGGGGTACCTGTGTGG---AAAGAAGCAACC---
ACTACTCTATTTTGTGCATCAGATGCTAAAGTTTATGACACAGAG---GTACATAAT----------------
--------------GTTTGGGCCACA---CATGCCTGTGTACCCACAGAT---
CCCAATCCACAAGAAGTAGTATTGGAA---------AATGTAACAGAAAATTTTAAC----------------
--------------------------ATGTGGAAAAATAACATGGTAGATCAGATGCAGGAGGATATC---
ATCAGTTTATGGATCAAAGCCTA---AAGCCATGTGTAAAATTA---
ACCCCCCTCTGTGTCACTTTAAATTGCATTAATGTGAATACTACTAATACCACCAGTAGTACTAAT-------

----
-------------------ACCACTGTTAATAGTTGGGAAAAGATGGATAAAGGAGAAATAAAA---
AACTGCTCTTTCAACATCACCACAAGCGTAAGA---------------------------
GATAAGGTAAAGAAAGAATATGCGCTT---TTTTATAGCCTTGATATAGTACAAATAGATAAT----------
----

GATAATACTAGCTATAGGTTGATAAGTTGTAATACCTCAACCCTTACACAG---
GCCTGTCCAAAGATAACCTTTGATCCAATTCCAATACATTATTGTGCCCCGGCTGGTTTTGCGATTCTAAGGT
GTAAT---AATAAGACATTTGAG------GGAAAAGGACCATGTACAAAC-----------------------
-GTCAGT---ACAGTACAATGTACCCATGGAATTAAGCCTGTAGTGTCAACT---
CAACTGCTGTTAAATGGCAGTCTAGCAGAAGAAGAT------------
ATAGTAGTTAGATCTGACAATTTCACAGATAAT---------ACTAAAACCATAATAGTACAGCTGAAC----
-----------AAATCTATAGAA---------ATTAATTGTACAAGACCCAACAACAATACAAGAAAA-----
-AGTTTAGCTATA------------GGACCAGGGAGAGCA---TTT------TATGCAACAGGAGAT------
---------ATAATAGGAGATATA------------AGAAAAGCACATTGT---AACATTAGTAAAGCA---
GAATGGAATAACACATTAAGACAGATAGCTATAAAATTAAGA---------------GAAAAATATGAG----
-----------------------------------AACAAAACAATAGTTTTTAAT------
CAATCCTCAGGAGGGGACCCAGAAATTATA---ATGCACAGTTTTAATTGTCAAGGGGAA---
TTTAAGCAGCTCCAGGCAAGAGTCCTGGCTATAGAAAGATACCTAAAGGATCAACAGCTCCTGGGAATTTGGG
GTTGCTCTGGAAAACTCATCTGCACCACTGCTGTGCCTTGGAATGATAGTTGGAGT-----------------
----------------------------AATAGATCTCTGAGCGAGATTTGG------AAT------
AACATGACCTGGATGCAGTGG---GAAAAGGAAATTGACAATTACACAGAATTGATATAC---
AGCCTAATTGAAATATCGCAAAACCAACAAGAA--

AAGAATGAACAGGACTTATTGGAATTGGGCAAATGGGACAGTTTGTGGAATTGGTTGGACATAACAAATTGGC
TGTGGTATATAAGAATATTCATAATGATAGTAGGAGGCTTGATAGGTTTAAGAATAGTTTTTACT---
GTACTCTCTATAGTG---------AATAGAGTTAGGCAGGGATACTCACCA------
TTGTCATTTGCTATCAGCTTGCTCAACGCCACAGCTATAGCAGTAGGTGAG---
GGGACAGATAGGGTTATAGAAGTAGCACAAAGAATTGTTAGAGCT----------------------------
--------ATTCTCCACATA------CCTAGAAGAATAAGACAGGGCTTGGAAAGGCTTTTGCTA---TAA
>KC699015
GCT---ACA--
GAACAATTGTGGGTAACAGTCTATTATGGGGTACCTGTGTGG---AAAGAAGCAAAC---
ACCACTCTATTTTGTGCATCAGATGCTAAAGCATATGATACAGAG---GTACATAAT----------------
--------------GTTTGGGCCACA---CATGCCTGTGTACCCACAGAC---
CCCAACCCACAAGAAGTAGTATTGGAA---------AATGTGACAGAAACTTTTAAC----------------
--------------------------ATGTGGAAAAATAATATGGTAGAACAGATGCATGAGGATATA---
ATCAGCTTGTGGGATCAAAGCCTA---AAGCCATGTGTAAAATTA---
ACCCCACTCTGTGTTACTCTAAATTGCAATGATTATGTGAAGAATGATACCAATACCACT-------------
----
-------ATGAGTACCAATAGGAGTGATGACAATATCACTGTGGAGGAGAGAGGAGAAATAAAA---
AACTGCTCTTTCAATATCACCACAGACATGAAT---------------------------
GATAAATGGCAGAAAGAATATGCGCTT---TTTTATAAGCTTGATGTAGTACCAATAGAGAAT----------
----

AATAACATTAGCTATAGGTTAATAAGTTGTAACACCTCAGTCATTACACAG---
GCCTGTCCAAAGATAACCTTTGAGCCACTTCCCATACATTATTGTGCCCCGGCTGGTTTTGCGATTCTAAAGT
GTAGA---GATAAAAACTTCAAT------GGAACAGGACCATGTGCAAAT-----------------------
-GTCAGC---ACAGTACAATGTACACATGGAATTAGGCCAGTAGTATCAACT---
CAACTGCTGTTAAATGGCAGTCTAGCAGAAGACAAG------------
GTAGTAATTAAATCTAGCAATTTCTCGGACAAT---------GCTAAAACCATAATAGTACAGCTGAAA----
-----------GAGTCTGTAAAA---------ATTAATTGTACAAGACCCAACAACAATACATTGAAA-----
-AGTATACGATTT------------GGACCAGGGAGCGCA---TTT------TACACAACAGGAGCC------
---------ATAATTGGAGATATA------------AGACAAGCACATTGT---AACATTAGTAAAACA---
GATTGGAATAACACTTTAAAACAGGTAGTTACAGCATTAAGA---------------
GAACAATTTGGGAATAAC---------------------------------ACAAACACAATAGTCTTTAAT-
-----CGATCCTCAGGAGGGGACCCAGAAATTGTA---ATGCACAGTTTTAATTGTGGAGGGGAA---
TTTAAACAGCTCCAGGCAAGAGTCCTGGCTGTGGAAAGATACCTAAGGGATCAACAGCTCCTAGGGATTTGGG
GTTGCTCTGGAAAACTCATTTGCACCACTGCTGTGCCTTGGAATGCTAGTTGGAGT-----------------
----------------------------AATAAATCTCTGGATATGATTTGG------AAT------

AACATGACCTGGATGCAGTGG---GAAAAAGAAATTGACAATTACACAAGCTTAATATAC---
ACCTTAATTGAAGAATCGCAAAACCAGCAAGAA-------------------------------------
----
AAGAATGAACAAGAATTATTAGAACTAGATAAATGGGCAAATTTGTGGAGTTGGTTTGAGATAACAAACTGGC
TGTGGTATATAAAAATATTCATAATGATAGTAGGAGGCTTGATAGGTTTAAGAATAGTTTTTACT---
GTACTTTCTATAGTG---------AATAGAGTTAGGCAGGGATACTCACCC------
TTATCGTTTGCTATTAGCTTGTTCAACGCCACAGCCATAGCAGTAGCTGAG---
GGGACAGATAGGGTTATAGAAGCAGTACAAAGAACTTATAGAGCT---------------------------
--------ATTATCCACATA------CCCAGAAGAATAAGACAGGGCGCGGAAAGGGCTTTACAA---TAA
>KC699013
GCA---GTA---
GGCCAATTGTGGGTCACAGTCTATTATGGGGTACCTGTGTGG---AAAGAAGCAAAC---
ACTACTCTATTTTGTGCATCAGATGCGAAAGCATATGATACAGAG---GTACATAAT----------------
--------------GTTTGGGCCACA---CATGCCTGTGTACCCACAGAC---
CCCAACCCACAAGAAATAGAATTGGAA---------AATGTGACAGAAAATTTTAAC---------------
-------------------------ATGTGGAAAAATAACATGGTAGAACAGATGCATGAGGATATA---
ATCAGTTTATGGGATCAAAGCTTA---AAGCCATGTGTAAAATTA---
ACCCCACTCTGTGTTACTTTAAATTGCACTAACTTGAATGTTACTAACTTGAATGTTACT-------------
----
---------------------CACGGGAATGATACTTCTGGCAATATTACTAGTGAAATGAAA---
AACTGCTCTTTCAATATCACCACAAGCATAAGA--------------------------
GATAAGATGCAGAAAGAATATGCCCTT---TTGTATAAAGTTGATCTAGTACCAATAGATAAT----------
----

AATGGTAGTAGCAGATATAGCAGCTCTAGGTTGATAAGTTGTAACACCTCAGTCATTACTCAA---
GCCTGTCCAAAGGTATCCTTTGAGCCAATTCCCATACATTTTTGTGCCCCGGCTGGTTTTGCGATTCTAAAGT
GCAAG---GATAAGAAGTTCAAT------GGAACAGGACCATGTACAAAT----------------------
-GTCAGC---ACAGTACAATGTACACATGGAATTAGGCCAGTAGTATCAACT---
CAGCTGCTGTTAAATGGCAGTCTAGCAGAAGAAGAG-----------
GTGGTAATAAGATCTGAGAATTTCACGGACAAT---------GCTAAAACCATAATAGTACAGCTGAAT----
-----------GAATCTATAGTA---------ATTAATTGTACAAGACCCAACAACAATACAAGAAGA-----
-AGGATAACTTTA------------GGACCAGGGAGAGTA---TTT------TATACAACAGGAGAC------
---------ATAATAGGAGATATA-----------AGACGAGCACATTGT---AACCTTAGTGAGACA---
AAATGGAATAACACTTTAAGACAGATAGTTAAAAAATTAAGA--------------GAACAATTTGGG----
------------------------------------AATAAAACAATAGCCTTTAAT------
AAATCCTCAGGAGGGGACCCAGAAATTGTA---ATGCACAGTTTTAATTGTGGAGGGGAA---
TTTAAGCAGCTCCAGGCAAGAGTCCTGGCTGTGGAAAGATACCTAAAGGATCAACAGCTCCTGGGGATTTGGG
GTTGCTCTGGAAAACTCATTTGCACCACGGCTGTGCCTTGGAATACTAGTTGGAGT-----------------
----------------------------AATAAATCTTATGATAACATTTGG------GGT------
AACATGACCTGGATGCAGTGG---GATAAAGAAATTAACAATTACACACAATTAATATAC---
ACCTTAATTGAAGAATCGCAGAACCAACAAGAA-------------------------------------
----
AAGAATGAACAAGACTTGTTGGAATTAGACCAATGGGCAAGTTTGTGGAATTGGTTTGACATATCGCACTGGC
TGTGGTATATAAAAATATTCATAATGATAGTAGGAGGCTTGATAGGTTTAAGAATAGTTTTTGCT---
GTACTTTCTATAGTG---------AACAGAGTTAGGCAGGGATACTCACCA------
ATATCGTTGGCTGTTAGCTTGCTTAATACCCTAGCCATAGCAGTAGCTGAG---
GGAACAGATAGAGTTATAGAAGTATTACAAAGAATTTTTAGGGCT---------------------------
--------ATTATCCACATA------CCTAGAAGAATAAGACAGGGCTTGGAAAGGGCTTTGCTG---TAA
>KC699021
GCT---ACA---
GAACAATTGTGGGTCACAGTCTATTATGGGGTACCTGTGTGG---AAAGAAGCAAAC---
ACCACTCTATTTTGTGCATCAGATGCTAAAGCATATGATACAGAG---GCACATAAT----------------
--------------GTGTGGGCCACA---CACGCCTGTGTACCCACAGAC---
CCCAACCCACAAGAAGTAGAATTGGTA---------AATGTGACAGAAAATTTTAAC---------------
-------------------------ATGTGGAAAAATAGCATGGTGGAACAAATGCATGAGGATATA---
ATCAGTTTATGGGATGAAAGCCTA---AAGCCATGTGTAAAATTA---
ACCCCACTCTGTGTTACTTTAAATTGCACTGATTATTCGAATGCTACTAACACCAACAGTAGTAAT-------
----
-------------ACTACAACCACTAATGGTAATTGGGGACAAATGGAGCCAGGAGAAATAAAA---
AATTGCTCTTTCAATATCACCACAAGCATGATT--------------------------

GATAAGAAGAAAACAGAATATGCATTT---TTTTATAAACTTGATGTAGTACAAATAGATAAT----------
----

ACTAGTTATAGATTGATAAGTTGTAATACCTCAGTCATTACACAG---
GCCTGTCCAAAGACATCCTTTGAGCCAATTCCCATACATTATTGTGCCCCGGCTGGGTTTGCGATTCTAAAGT
GTAGC---GATAAGAAGTTCAAT------GGAACGGACCATGCACAAAT------------------------
-GTCAGC---ACAGTACAATGTACACATGGAATTAGGCCAGTAGTATCAACT---
CAACTGCTGTTAAATGGCAGTCTAGCAGAAGAAGAG------------
GTAGTAATTAGATCTGCCAATTTCTCGGACAAT---------GCTAAAAACATAATAGTACAGCTGAAG----
-----------GAACCTGTAATA---------ATGAATTGTACAAGACCCGGCAACAATACAAGAAAA-----
-GGTATACAGATG------------GGACCGGGGAAAGTA---TTT------TTTGCAACAGGAGAA------
---------ATAATAGGAGATATA------------AGGCAAGCACATTGT---AACATTAGTAGAGAA---
AAATGGAATAAAACGTTAGGACAGATAGTTATAAAATTAAGA---------------GAAGAATTTGGG----
-----------------------------------AACAAGACAATAATCTTTAAT------
CAATCCTCAGGAGGGGACCCAGAAATTGTA---ATGTCTAGCTTTAATTGTGGAGGGGAG---
TTTAAGCAGCTCCAGGCAAGAGTCCTGGCTGTGGAAAGATACCTAAGGGATCAACGGCTCCTAGGGATTTGGG
GTTGCTCTGGAAAACATATTTGCACCACTGCTGTGCCCTGGAATGAGAGTTGGAGT-----------------
----------------------------AATAAATCTTACAATGACATTTGG------GAT------
AACATGACCTGGATGCAGTGG---GAAAGAGAAATTGACAATTACACAAGCTTAATATAT---
ACCCTGATTGAAGAATCGCAGGTCCAGCAGGAA--

AAGAATGAACTAGAGTTAATGGAATTGGATAAATGGGCAAGTCTGTGGAATTGGTTTACCATAACAAATTGGC
TGTGGTATATAAAATTATTCATAATGATAGTAGGAGGATTGATAGGTTTAAGAATAGTTTTTGCT---
GTGATTTCTATAATG---------AATAGAGTTAGGCAGGGATACTCACCA------
TTATCGTTTGCTGTCAGCTTGCTCAACGCTATAGCTATAGTAGTAGCTGAG---
GGGACAGATAGGGTTATAGAAATAATACAAAGACTTTGTAGAGCT----------------------------
--------ATTCTCCACATA------CCTAGAAGAATAAGACAGGGCTTGGAAAGGCTTTGGTA---TAA
>KF695111
GCT---ACA--
GAAAAATTGTGGGTCACAGTCTATTATGGGGTACCTGTGTGG---AGAGAAGCAACC---
ACCACTCTATTTTGTGCATCAGATGCTAAAGCATATGATACAGAG---GCACATAAT----------------
--------------GTTTGGGCCACA---CATGCCTGTGTACCCACAGAC---
CCCAGCCCACAAGAAATAGCATTGGAA---------AATGTGACAGAAGATTTTAAC----------------
--------------------------ATGTGGAAAAATAACATGGTAGAACAGATGCATGAGGATATA---
ATTAGTTTATGGGATCAAAGTCTA---AAACCATGTGTAAAATTA---
ACCCCACTCTGTGTTACTTTAAATTGCACTGATTTGGAGACTAAGAATATTACTGATAAA-------------
----
-------------------------AATAGTAGCTGGGCAAGGATAGAAAAAGGAGAAATAAAA---
AACTGCTCTTTCAATGTCGACACAGACATGGGT---------------------------
GCCAAGGTGCAGATACACTCTGCAGTT---
TTTAATAAACTTGATGTAGTACTAATAGGTAATGCTACAATAAATAATGCTACAATAAATAATGAGACAAGA-
----
AATAATGATAATAATGCAACAGGTAAGAATAATGAGACTATTGCCTACTATAGGTTGATAAGTTGTAACACCT
CAAAAATTACACAG---
GCCTGCCCAAAGATATCCTTTGAGCCAATTCCCATACATTATTGTGCCCCGGCTGGTTTTGCGATTCTAAAGT
GTAGA---GAGGAGGACTTCAAT------GGAACAGGACGATGTAAAAAT------------------------
-GTCAGC---ACAGTACAGTGTACACATGGAATTAGGCCAGTAGTATCAACT---
CAACTGCTATTAAATGGCAGTCTAGCAGAAAAAGAG------------
GTAGTAATTAAATCTGAAAATTTCGCAACCAGT---------GTTAAGAACATAATAGTACAGCTAAAT----
-----------GGGTCTGTAAAA---------ATTAGTTGTATAAGACCCAATAACAATACAAGAAAA-----
-AGTATGACTCTA------------GGACCAGGAAAGGTA---TTC------TATACAACAGGA---------
---------GTAACAGGAGATATA------------AGAAAAGCACATTGT---AACATTAGTAGAAAA---
GATTGGAATAAAACTTTAGAAAGGATAGCTATAAAACTAGGA---------------AAACAATTTGAG----
-----------------------------------AATACAACAATAATCTTTCAA------
CCATCCGCAGGAGGGGACCCAGAAATTGAA---ATGCACAGTTTTAATTGTGGAGGGGAA---
TTTAAGCAGCTCCAGGCAAGAGTCCTGGCTGTGGAAAGATTCCTAATGGATCAACAGCTCCTAGGAATTTGGG
GTTGCTCTGGAAAACTCATCTGCACCACTGCTGTGCCTTGGAATATTAGCTGGAGT-----------------
----------------AATAGTACT---AATATACCTCTAGAAGATATTTGG------GGG------
AACATGACCTGGATGCAGTGG---GAAAAAGGAATTCGCAATCACACAAAATACATATAC---
TCCTTACTTGAAGAATCGCAGAACCAACAGGAC--

AAGAATGAACAAGAATTGTTGGAATTGGATCGCTGGGCTAATTTGTGGAATTGGTTTAACATAACAAACTGGC
TGTGGTATATAAAAATATTCATAATGGTAGTAGGAGGCTTGATAGGTTTAAGAATAGTTTTTGCT---
GTGCTTTCTATAGTA---------AATAGAGTTAGGCAGGGTTACTCACCG------
TTGTCGTTTGCTGTTAGCTTGCTCAATGCCACAGCCATAGCAGTAGCTGAG---
GGGACAGATAGGGTTATAGAAGTAGTACAAGGAGCTTGTAGAGCT----------------------------
--------ATTCGCCACATA------CCTAGAAGAATAAGACAGGGCTTGGAAAGGATTTTGCTA---TAA
>EF033658
GCT---GCA--
AATCAATTATGGGTCACAGTCTATTATGGGGTACCTGTGTGG---AAAGAAGCAACC---
ACCACTCTATTTTGTGCATCAGATGCTAAAGCACATAAGGCAGAG---GTACATAAT----------------
--------------ATTTGGGCCACA---CATGCCTGTGTACCCACAGAC---
CCCAACCCACAAGAATTAGAATTAAAA---------AATGTGACAGAAAATTTTAAC----------------
--------------------------ATGTGGAAAAATAACATGGTAGAACAGATGCATGAGGATATA---
ATCAGTTTATGGGATCAAAGCATA---AAGCCATGTGTAAAATTA---
ACACCACTCTGTGTTACCTTAAATTGCACTGATTGGGAGAATGATACTAATAGTAGTAATGGT----------
----
----------------------ACTAACAATAGATGGGAAAAGATGCAGAAAGGAGAATTAAAA---
AACTGCTCTTTCAATATCTCCACAAACCTAAGA---------------------------
GATAGGGTACAGAAAGAATATGCAAAT---TTTTATAGACTTGATCTAGTACAAATAGATAAT----------
----

AATAATAGTAATAGCTATAGGTTGACAAGTTGTAACGCCTCAGTCATTACACAG---
GCCTGTCCAAAGGTATCCTTTGATCCAATTCCTATACATTATTGTGCCCCGGCTGGTTTTGCGATTCTAAAGT
GTAAC---AATAAGAAGTTCAAT------GGAACAGGACCATGTACAAAT-----------------------
-GTCAGT---ACAGTACAATGTACACATGGAATTAGGCCAGTGGTATCAACT---
CAATTGCTGTTAAATGGCAGTCTAGCAGAAGAAGAG------------
GTAGTAATTAGGTCTGAAAATTTCACGAACAAT---------GTTAAAACCATAATAGTACAGCTGAAA----
-----------GAACCTGTAGTA---------ATTAATTGTACAAGACCCCACATGAGTAAAAGCAGA-----
-AGTATACGTTTA------------GGACCAGGGAGACCA---TTT------TATGCAACAGGAAAC------
---------ATAATAGGAGCTTTA------------AAACAAGCACATTGT---AACCTTAGTAGGGCA---
GCATGGAATAACACCTTAAAACAGATAGTTAAAAAATTAAGA---------------GAGAAATTTGAT----
-----------------------------------AATAAAACAATAGCCTTTAAT------
TCATCCTCAGGAGGGGACCCAGAAATTGTA---TTGCACAGTTTTAATTGTGGAGGGGAA---
TTTAAGCAGCTCCAGGCAAGAGTCCTGGCTGTGGAAAGATACCTAAAGGATCAACAGCTCCTGGGGATTTGGG
GTTGCTCTGGAAAACTCATCTGCACCACTGCTGTGCCTTGGAATACTAGTTGGAGT-----------------
----------------------------AATAAATCTATGAGTGACATTTGG------GAT------
AACATGACCTGGATGGAGTGG---GAAAGAGAAATTGACAACTACACGGACATAATATAC---
ACCTTACTTGAAGAATCGCAATACCAACAAGAA--

AAGAATGAACAAGAATTATTAAAATTAGATAAATGGGCAAGTTTGTGGAGTTGGTTTAGCATAACAAACTGGC
TGCGGTATATAAAAATATTCATAATGATAGTAGGAGGCTTAATAGGTTTAAGAATAGTTTTTGCT---
GTACTTTCTATAGTA---------AATAGAGTTAGGCAGGGACATTCACCA------
TTATCGTTGGCTATTAGCTTGCTCAATGCAACAGCCATAGCAGTAGCTGAG---
GGAACAGATAGGGTTATAGAAATATTACAAAGAGCTTATAGAGCT----------------------------
--------ATTCTCCACATA------CCTACAAGAATAAGACAGGGCGCAGAAAGGGCTTTGCTA---TAA
>AF321080
GCT---ACA--
AAAAAATTGTGGGTCACAGTCTATTATGGGGTACCTGTGTGG---AAAGAAGCAACC---
ACCACTCTATTTTGTGCATCAGATGCTAAAGCATATGTCCAAGAG---GCACATAAT----------------
--------------GTTTGGGCCACA---CATGCCTGCGTACCCACAGAC---
CCCAACCCACAAGAAATAGTATTGGAA---------AATGTGACAGAAAATTTTAAT----------------
--------------------------ATGTGGAAAAATAACATGGTAGAACAGATGCATCAGGATATA---
ATCAGTTTATGGGATGAAAGCTTA---AAGCCATGTGTAAAATTA---
ACCCCACTCTGTGTTACCTTAAAATGCACTAACGTGAATACTACTAATACCAATAATACTAGTTCC-------
----
----------------------AATAGTAGTAGTTGGGGAAGTATGGAGGCAGGAGAAATAAAA---
AACTGCTCTTTCAATGTCACCACAAACATAAGA---------------------------
GATAAGAGGAAGAAAGAATATGCACTC---TTTTATAGACTTAATGTAGTACCAATAGATAAT----------
----

GATAATACAAGTTATACTTTGGTAAATTGTAATACCTCAGAGATTACACAG---
GCCTGTCCAAAGGTATCCTTTGAACCAATTCCTATACATTATTGTGCCCCGGCTGGTTTTGCGATTCTAAAAT
GTAAA---GATAAGGAGTTTAAT------GGAACAGGACCATGTAAAAT-----------------------
-GTTAGC---ACAGTACAATGCACACATGGAATTAGGCCAGTAGTGTCAACC---
CAATTGCTGTTAAATGGCAGTCTAGCAGAAGGAGAG-----------
GTAGTAATTAGATCTGAGAATTTCACGAACAAT---------GCTAAAACCATAATAGTACAGCTGAAT----
-----------GAAACTGTAAAA---------ATTCATTGTATAAGACCCAACAATAATACAAGAAAG-----
-AGTATACATATA------------GCACCAGGGAGAGCA---TTT------TATGCAACAGGAAAT------
---------ATAATAGGAGATATA------------AGACAAGCACATTGT---AACCTTAGTCTAGCA---
GAATGGAATAACACTTTATATCAGATAGTTACAAAATTAAGA--------------GATCAATTTGGG----
----------------------------------AATAAAACAATAAACTTTAAT------
CAATCCTCAGGAGGGGACCCAGAAATTGTA---ATGCACAGTTTTAATTGTGGAGGGGAA---
TTTAAGCAACTCCAGGCAAGAGTCCTGGCTGTGGAAAGATACCTACAGGATCGACAGCTCCTGGGGATTTGGG
GTTGCTCTGGAAAACTCATCTGCACCACTGCTGTGCCTTGGAATAATAGTTGGAGT----------------
---------------------------AATAAACCTCTAGATCAGATTTGG------AAT------
AACATGACCTGGATAGAGTGG---GAGAGAGAAATTGACGATTACACAGGTTTAATATAC---
ACTTTAATTGAAGAATCGCAGAACCAACAAGAA---------------------------------------
----
AAGAATGAACAAGAATTATTGGAATTGGATACATGGGCAAGTTTGTGGGATTGGTTTAGCATAACAAAATGGC
TGTGGTATATAAAAATATTCACAATGATAGTAGGAGGTTTAGTAGGTTTAAGAATAGTTTTTACT---
GTACTTTCTATAGTG---------AATAGAGTTAGGAAGGGATACTCACCA------
TTATCGTTCGCTGTTAGTTTGCTTAATGCCATTGCTGTAGCAGTAGCTGAG---
GGGACAGATAGAATTATAGAAGCAGCCCAAAGAATTTGTAGAGCT---------------------------
--------ATTCTCCACATA------CCCAGAAGAATAAGACAGGGCTTGGAAAGGGCTTTGCTA---TAA
>M26727
GCT---GCA---
GAAAATTTGTGGGTCACAGTCTATTATGGGGTACCTGTGTGG---AAAGAAGCAACC---
ACCACTCTATTCTGTGCATCAGATGCTAGAGCATATGCTACAGAG---GTACATAAT---------------
--------------GTTTGGCCACA---CATGCCTGTGTACCCACAGAC---
CCCAACCCACAAGAAGTAGTATTGGGA---------AATGTGACAGAAAATTTTGAC---------------
--------------------------ATGTGGAAAAATAATATGGTAGAACAAATGCAAGAAGATATA---
ATCAGTTTATGGGATCAAAGCCTA---AAGCCATGTGTAAAATTA---
ACCCCACTCTGTGTTACTTTAGATTGCACTGATGTTAATACCACTAGTAGTAGTTTGAGGAATGCTACT----
----
----------------AATACCACAAGTAGTAGTTGGGAAACGATGGAGAAAGGAGAATTAAAA---
AACTGCTCTTTCAATACCACCACAAGCATAAGA--------------------------
GATAAGATGCAGGAACAATATGCACTT---TTTTATAAACTTGATGTATTACCAATAGATAAG---------
----

AATGATACTAAATTTAGGTTAATACATTGTAACACCTCAACCATTACACAG---
GCCTGTCCAAAGATATCCTTTGAGCCAATTCCCATGCATTATTGTACTCCGGCTGGTTTTGCGATTCTAAAGT
GTAAT---GATAAGAAGTTCAAT------GGAACAGGACCATGTACAAAT----------------------
-GTCAGC---ACAGTACAATGTACACATGGAATTAAGCCAGTAGTGTCAACT---
CAACTGCTGTTAAATGGCAGCCTAGCAGAAGAAGAG-----------
GTAATAATTAGATCTAGCAATTTCACAAACAAT---------GCTAAAATCATAATAGTACAGCTGAAT----
-----------AAATCTGTAGAA---------ATTAATTGTACAAGACCCAACAACAATACAAGAAAC-----
-AGGATATCAATA------------GGACCAGGGAGAGCA---TTT------CATACAACAAAACAA------
---------ATAATAGGAGATATA------------AGACAAGCACATTGT---AACCTTAGTAGAGCA---
ACATGGGAGAAAACTTTAGAACAGATAGCTACAAAATTAAGA--------------AAACAATTTAGG----
----------------------------------AATAAAACAATAGCCTTTGAT------
CGATCCTCAGGAGGGGATCCAGAAATTGTA---ATGCACAGTTTTAATTGTGGAGGGGAA---
TTTAAGCAGCTCCAGGCAAGAGTCCTGGCTGTGGAAAGATACCTAAAGGATCAACAGCTCCTAGGGATTTGGG
GTTGCTCTGGAAAGCTCATTTGCACCACTACTGTGCCTTGGAATGCTAGTTGGAGT----------------
---------------------------AATAAATCTCTGAATGAGATTTGG------GAT------
AACATGACCTGGATGCAGTGG---GAAAGAGAAATTGACAATTACACACACTTAATATAC---
ACCTTAATTGAAGAATCGCAGAACCAACAGGAA---------------------------------------
----
AAGAATGAACAAGAATTATTGGAATTGGATAAGTGGGCAGGTTTGTGGAGTTGGTTTAGCATAACAAACTGGC
TGTGGTATATAAGAATATTCATAATAATAGTAGGAGGCTTGGTAGGTTTAAGAATAGTTTTTGCT---

GTACTTTCTATAGTG---------AATAGAGTTAGGCAGGATACTCACCA------
TTATCGTTTGTCATTAGCTTGCTCAACGCCACAGCTATAGCAGTAGCTGAG---
GGGACAGATAGGGTTATAGAAATAGTACAAAGAGCTTATAGAGCT----------------------------
--------TTTCTCAATATA------CCTAGAAGAATAAGACAGGGCTTGGAAAGGGCTTTGCTA---TAA
>JN034158
GCT---GCA--
GATCAATTGTGGGTCACAGTCTATTATGGGGTACCTGTGTGG---AAGGAAGCAACC---
ACCACTCTATTTTGTGCATCAGATGCTAAAGCATATGATACAGAG---GTACATAAT----------------
--------------GTTTGGCCACA---CATGCCTGTGTACCCACAGAC---
CCCAACCCACAAGAAGTAGTATTGGAA---------AATGTAACAGAAAATTTTAAC----------------
--------------------------ATGTGGAAAAATAACATGGTAGAACAGATGCATGAAGATGTA---
ATCAGTTTATGGGATCAAAGCCTA---AAGCCATGTGTAAAATTA---
ACCCCACTCTGTGTTACTTTACATTGCACTAATAATGTGACAATTATTAATGGTACTAATGCCAATAATAATA
CTACTAATGGTACCAAT--
GCTATTGATGCCAATAATAGTAGTTGGGAAGGAATAGGAAGAATAGATAGAGGAGGAGAAATAAAA---
AACTGCTCTTTCAATATCACCACAAGCCTGAGT---------------------------
GATAAGAGGCAGAAAGAATATGCACTT---TTTTATAAACTTGATGTAGAACCAATAGATAAT----------
----

GATAATACTAGCTATAGGTTGATAAGTTGTAACACCTCAGTCATTACACAG---
GCCTGTCCAAAGGTATCCTTTCAGCCAATTCCCATACATTATTGTGCCCCGGCTGGTTTTGCGATTCTAAAGT
GTAAT---GATAAGAAGTTCAAT------GGAACAGGACCATGTAAAAAT-----------------------
-GTCAGT---ACAGTACAATGTACACATGGAATTAGGCCAGTAGTATCAACT---
CAACTGCTGTTAAATGGCAGTCTTGCAGAAGAAGAC------------
ATAGTAATTAGATCTGCCAATCTCACGGACAAT---------GCTAAAATCATAATAGTGCAGCTGAAT----
-----------GAATCTGTAACA---------ATTAATTGTACAAGACCCAATAACAATACAAGAAGA-----
-GGTATACATCTA------------GGACCAGGAGGAGCA---TTT------TATACAACAGGAGAC------
---------ATAATAGGAGATATA------------AGAAAAGCACATTGT---AACATTAGTAGAGCA---
GAATGGAATAAAACTTTAGACAGGATAGCCAGAAAATTAAGC---------------GAACAATTTAAT----
-----------------------------------AATAAAACAATAGTCTTTAAG------
AACTCCTCAGGAGGGGACCCAGAAATTGTA---ATGCACAATTTTAATTGTAGAGGGGAA---
TTCAAGCAGCTCCAAGCAAGAGTCCTGGCTGTGGAAAGATACCTAAAGGATCAACAGCTCCTAGGGATTTGGG
GTTGCTCTGGAAAACTCATCTGCACCACTGCTGTGCCTTGGAATGATAGTTGGAGT-----------------
----------------------------AATAAATCTCTGAACACGATTTGG------AAT------
AACATGACCTGGATGGAGTGG---GAAAGAGAAATTGACAATTACACACACTTAATATAC---
AACTTACTTGAAGAATCGCAGAACCAACAAGAG--

CAGAATGAACACGACTTATTAAAATTAGATCACTGGGCAAGTTTGTGGAATTGGTTTGACATAACAAATTGGC
TGTGGTATATAAAAATATTCATAATGATAGTAGGAGGCTTGGTAGGTTTAAGAATAGTTTTTGCT---
GTACTTTCTATAGCG---------AATAGAGTTAGGCAGGATACTCACCA------
TTGTCATTTGCTGTTAGTTTGCTTAACGCCACAGCGATAGCAGTAGCTGAG---
GGGACAGATAGGGTTATAGAAGTAGTCCGAAGAGCTTGTAGAGCT----------------------------
--------GTTCTCAACATA------CCTACAAGAATAAGACAGGGCTTGGAAAGAGCTTTGCTA---TAA
>JN034139
GCT---GCA--
GAAAACTTGTGGGTCACAGTTTACTATGGGGTACCTGTGTGG---AAAGAAGCAACC---
ACCACTCTATTTTGTGCATCAGATGCTAAAGCATATGACACAGAG---GCACATAAT----------------
--------------GTTTGGCCACA---CATGCCTGTGTACCCACAGAC---
CCTAACCCACAAGAAGTAGTATTGGAA---------AATGTGACAGAGAATTTTAAT----------------
--------------------------ATGTGGAAAAATAACATGGTAGAACAGATGCATGAGGATATA---
ATCAGTTTATGGGATCAAAGCCTA---AAACCATGTGTGAAATTA---
ACCCCACTCTGTGTTACCTTAAATTGCACTAATGTGAAGAATGTTACTAATGCCAATAGTACTAGT-------
----
-------------------TCCAATATTAGTAGTTGGGAAAGGATGGAGGAAGGAGAAATAAAA---
AACTGCTCTTTCAATGTC---ACAAGCATAGGA---------------------------
AATAAGTATCATAAGGAATATGCACTT---CTTTATAAGCTTGATCTAGTACCAATAGATAAAAAT-------
----

GATACTACAAGTTATACATTGATAAATTGTAACACCTCAGTCATTACACAG---
GCCTGTCCAAAGGTATCCTTTGAACCAATTCCCATACATTATTGTGCCCCAGCTGGTTTTGCGATTCTACAAT

GTAAT---GATAAGAACTTTACA------GGAAAAGGACCATGTAAAAAT----------------------
-GTCAGC---ACAGTACAATGTACACATGGAATTAAGCCAGTGGTGTCAACC---
CAATTACTGTTAAATGGCAGTCTAGCAGAAGAAGGG------------
GTAGTAATTAGATCTGAAAATATCACGGACAAT---------GCTAAAACCATAATAGTACAGCTGAAG----
-----------GACTCTGTAAAA---------ATTAATTGTACAAGACCCAACAATAATACAAGAAGA-----
-AGTATACATCTA------------GGACCAGGGAAAACA---TTG------TATGCAACA---GAC------
---------ATAATAGGAGATATA------------AGACGAGCACATTGT---AACATTAGTGTGGCA---
GACTGGAATAACACTTTACAACAAATAGTTATAAAATTAAAG--------------GAACAATTTCAG----
-----------------------------------AATAAAACAATAATCTTTAAT------
CAATCCTCAGGAGGGGACCCAGAAATTGTA---ATGCACACTTTTAATTGTGGAGGGGAA---
TTTAAGCAGCTCCAGGCAAGAGTCCTGGCTGTGGAGAGATACCTAAGGGATCAACAGCTCCTAGGGATTTGGG
GTTGCTCTGGGAAACTCATTTGCACCACTACTGTGCCTTGGAATGCTAGTTGGAGT-----------------
----------------------------AATAAAACTCTGGATCAGATTTGG------AAT------
AACATGACCTGGATGCAATGG---GAGAGAGAAATTGACAATTACACAGGGTTAATATAT---
ACCTTAATTGAAGAATCGCAGAACCAACAGGAA---------------------------------------
----
AAGAATGAACAAGAATTGTTGGAATTGGATAAGTGGGCAAGTTTGTGGAATTGGTTTGACATAACAAATTGGC
TGTGGTATATAAGATTATTCATAATGATAGTAGGAGGTTTAGTAGGTTTAAGAATAATTTTTACT---
GTACTTTCTATAGTG---------AATAGAGTTAGGCAGGGATACTCACCA------
TTATCATTCGCTGTTAGTTTGCTTGATACCATAGCTATAGCAGTAGCTGAG---
GGGACAGATAGGGTTATAGAAGCAATACAAAGAATTGGTAGAGCC---------------------------
--------ATTCTCCACATA------CCTAGAAGAATAAGACAGGGCTTAGAAAGGATTTTACTA---TAA
>MG902199
GCT---GCA---
GAACAATTGTGGGTCACAGTCTATTATGGGGTACCTGTGTGG---AAAGAAGCAAAC---
ACCACTCTATTTTGTGCATCAGATGCTAAAGCATATGAGACAGAG---AAACATAAT----------------
--------------GTTTGGGCCACA---CATGCCTGTGTACCCACAGAC---
CCCAACCCACAAGAAGTAGCATTAGGA---------AATGTGACAGAAAATTTTAAC---------------
--------------------------ATGTGGAAAAATGACATGGTAGAACAAATGCATAAGGATATA---
ATCAGTCTATGGGATCAAAGCCTA---AAGCCATGTGTAAAATTA---
ACCCCACTCTGTGTTACTTTGAATTGCACTGATTTGGGGAATGCCACAGCTAATGCCACAACTAAT-------
----
---ATAGAGAAAGGAGAACTAAAA---
AACTGTTCTTTCAATGTCACCACAAACATAAGA--------------------------
GATAAGGTACAGAAAGGATATGCAGTT---TTTAATAAACTTGATGTAGTACCAATAGACAAT----------
----

GAAAATAGTAGCTATAGGATAATAAGTTGTAACACCTCAGTCATTACACAG---
GCCTGTCCAAAAGTATCCTTTGAACCAATTCCCATACATTTTTGTGCCCCGGCTGGTTTTGCGATTCTAAAGT
GTAAT---GCTACGAATTTCAAT------GGAACAGGATTATGTAAAAAT----------------------
-GTCAGT---ACAGTACAATGTACACATGGAATTAGGCCAGTAGTATCAACT---
CAACTGCTGTTAAATGGCAGTCTAGCAGAAGGAGAG------------
GTAGTAATTAGATCTGAAAATCTCACTGACAAT---------GCTAAAAATATAATAGTACAGCTGAAA----
-----------GACCCTGTAGAA---------ATTAATTGTACAAGACCCAACAACAATACAATAAAA-----
-AGTATACATATA------------GGGCCAGGGAGAGCA---TTT------TATGCAACA---GAT------
---------ATAATAGGAGATATA------------AGACAAGCACATTGT---AACATTAGTATGAAA---
AAATGGAATAACACTTTAAAACAAATAGTTGAAAAATTAAGA--------------AAACACTGG-------
-----------------------------------AATGATACAATAGTCTTTAAT------
CCATCCTCAGGAGGGGATCTAGAAGTTGTA---ATGCACACCTTTAATTGTGGAGGAGAA---
TTTAAGCAGCTCCAGGCAAGAGTCCTGGCTGTGGAAAGATACCTACAGGATCAACAGCTCCTGGGGATTTGGG
GTTGCTCTGGAAAACTCATTTGCACCACTGCTGTGCCTTGGAATGCTAGTTGGAGT-----------------
----------------------------AATAAATCTCTGAATGAGATTTGG------GAT------
AACATGACCTGGATGCAGTGG---GAAAAAGAAATTGATAATCACACAAGAGAAATATAC---
AAATTAATTGAAAAAATCGCAAAACCAACAAGAA--------------------------------------
----
AAGAATGAACAAGAATTATTAGAATTGGATAAATGGGCAAATTTGTGGAATTGGTTTGACATATCAAATTGGC
TGTGGTATATAAGAATATTCATAATGATAGTAGGAGGCTTGATAGGATTAAGAATAGTTTTTACT---
GTACTTTCTATAGTA---------AATAGAGTTAGGCAGGGATACTCACCT------
TTATCGTTCGCTGTTAGCTTGCTTGACACCATAGCCATAGCAGTAGCTGAG---

GGGACAGATAGGGTTATAGAAATATTACAAAGAATTGGTAGAGCC----------------------------
--------ATTCTCAACATA------CCTACAAGAATAAGACAGGGCTTCGAAAGGGCTTTGCTA---TAA
>MG902157
GCT---CAA---
CAACAATTGTGGGTCACAGTCTATTATGGAGTACCTGTATGG---AAAGAAGCAACC---
ACCACTCTGTTTTGTGCATCAGATGCTAAAGCATATGATAAAGAG---GCACATAAT---------------
--------------GTTTGGGCCACA---CATGCCTGTGTACCCACGGAC---
CCCAACCCACAAGAAGTAATATTGGGA---------AATGTGACAGAAAATTTTAAT---------------
-------------------------ATGTGGAAAAATGACATGGTAGAACAGATGCAGGAGGATATA---
ATCAGTTTATGGGATCAAAGCTTA---AAGCCATGTGTAAAATTA---
ACCCCACTCTGTGTTACTTTAAATTGCACTGATTATAATAAAAAGAATAGCACGAATAGCACGGCAACAGATA
AC---
-------------------ACGAATAGCACGGCAACACCGCCGCCGGCGGGAGGAGAGATAAAA---
AACTGCTCTTTCAATATCACCACAAATATAAGA--------------------------
GATAAGATAAAGAAAGAATATGCACTT---TTTTATACACTTGATATAGTACCAATAGGTGAGGAT-------
----

GATAATACTAGCTATAGGTTAACAAGCTGTAATACCTCAGTCATTACACAA---
GCCTGTCCAAAGATAACCTTTGAGCCAATTCCCATACATTATTGTGTCCCGGCTGGTTTCGCGATTATAAAGT
GCAAC---AATAAAACATTCGAT------GGAAAAGGACCATGTAAAAAT----------------------
-GTCAGC---ACAGTACAATGTACACATGGAATCAGGCCAGTAGTGTCAACT---
CAACTGCTGTTAAATGGCAGTCTAGCAGAAGAGGAG-----------
GTAGTAATTAGATCTGACAATTTCACAGACAAT---------GCTAAAAACATAATAGTACAGCTAAAT----
-----------AGTTCTATAGTA---------ATTAATTGTACAAGACCCAACAACAATACAAGAAAA-----
-GGTATACCTATA------------GGACCAGGGAGAGCA---TTT------TATGCAACAGGAGAC------
---------ATAATAGGAAATATA-----------AGACAAGCACATTGT---AACCTTAATAAAACA---
GATTGGAATAAAGCGCTACAACAAATAGCTGGAAAATTAGGA--------------GCACAGTTTGGG----
--------------------------------AAAGATAAAACAATAATCTTTACA------
CAACCCTCTGGAGGGGACCCAGAGATTGTA---TCACACAGTTTTAATTGTGGAGGGGAA---
TTTAAGCAGCTCCAGGCAAGAGTCCTGGCTGTGGAAAGATACCTAACGGATCAACGGTTCCTAGGGCTTTGGG
GATGCTCTGGAAAACTCATTTGCACCACTTCTGTGCCTTGGAATACTAGTTGGAGT----------------
----------------------------AATAGATCTCTGGATGACATTTGG------AGT------
AACATGACCTGGATGGAGTGG---GAAAAAGAAATTGATAATTACACAGGAATAATATAC---
ACCTTACTTGAACAATCGCAGAACCAACAAGAA--

AAGAATGAACAAGAATTATTGGAATTGGATAAATGGGCAAGCTTGTGGAATTGGTTTGACATAACAAAATGGC
TGTGGTATATAAAAATATTCATAATGGTAGTAGGAGGCTTGGTAGGTCTAAGAATAGTTTTTGCT---
GTGTTTTCTTTAGTA---------AATAGAGTTAGGCAGGGATACTCACCA------
TTGTCATTTGCTATTAGCTTGCTCAATGCCACAGCTATTGCAGTAGCTGAG---
GGGACAGATAGGATTATAGAAGTAGTACAAAGATTTTGTAGAGCT--------------------------
--------ATCCTCCACATA------CCCACAAGAATAAGACAAGGCTTGGAAAGGGCTCTGCAA---TAA
>HM586193
ACT---GCA---
GAACAATTGTGGGTCACAGTCTATTATGGGGTACCTGTGTGG---AAAGAAGCAACC---
ACCACTCTATTTTGTGCATCAGATGCTAAGGCATATGATACAGAG---GTACATAAT---------------
--------------GTTTGGGCCACA---CATGCCTGTGTACCCACAGAC---
CCCAGCCCACAAGAAGTAAACTTGGAA---------AATGTGACAGAAAATTTTAAC---------------
-------------------------ATGTGGAAAAATAACATGGTAGAACAGATGCATGAGGATATA---
ATCAGTTTATGGGATCAAAGCCTA---AAGCCATGTGTAAAATTA---
ACCCCACTCTGTGTTACTTTAAATTGCACTGATTTGGGAAATAGTACTGGGGGAAATAGTACTGGG-------
----
-------------------GGTACAAATACTACCTGGGGAAGGATGGAGAAAGGAGAATTAAAA---
AATTGCTCTTTCAATGTCACCACAAACATAAGA--------------------------
GATAAAGTGAAGAGAGAATATGCAGCT---TTTTATAAGCTTGATCTAATGAAAATAGATAAT---------
----

GATAATGATAGCTATACGTTAATAAGTTGCAATACCTCAGTCATTACACAG---
GCCTGTCCAAAGATATCCTTTGAGCCAATTCCCATACATTATTGTGCCCCGGCTGGTTTTGCSATTCTAAAGT
GTAAT---AATAAACAGTTCAAT------GGAACAGGACCATGTACAAAT----------------------
-GTCAGC---ACAGTACAATGTACACATGGAATTAGGCCAGTAGTATCAACT---

CAACTACTGTTAAATGGCAGTCTAGCAGAAGAAAAG-----------
ATAGTAATTAGGTCTGAAAATTTCATGGACAAT---------GCTAAAACTATAATAGTACAGCTGAAC----
-----------GAATCTATAGAC---------ATTAAGTGTATAAGACCCAACAACAATACAAGAAAA-----
-AGTATATCTATG------------GGACCAGGGAGAGCA---TTT------TTTAATACAGGACAA------
---------ATAATAGGAGATATA------------AGACAAGCACATTGT---AACATTAGTAGAGAA---
AAATGGAATAACACTTTAAAACAGATAGTTGACAAATTAAGA---------------GAAAAATTTGGG----
--------------------------------AAAAATAAAACAATAGTCTTTAAT------
CAATCCTCAGGAGGAGACCCAGAAATTGTA---ATGCACAGTTTTAATTGTGGAGGGGAA---
TTTAAACAGCTCCAGGCAAGAGTCCTGGCTGTGGAAAGATACCTAAGGGATCAACAGCTCCTGGGGATTTGGG
GTTGCTCTGGAAAACTCATTTGCACCACTGCTGTGCCTTGGAATGCTAGTTGGAGT----------------
----------------------------AATAAATCTCTAAATACGATTTGG------GAT------
AACATGACCTGGATGGAGTGG---GAAAGAGAAATTGACAATTACMCAAACTTAATATAT---
AACTTACTTGAAGAATCGCARAACCAACAAGAA--

AAAAATGAACAAGAATTATTGGAATTGGATAAATGGGCAAGTTTGTGGAGTTGKTTTAGCATAACAAACTGGC
TGTGGTATATAAGATTATTYATAATGATAGTAGGAGGCTTGGTAGGTTTAAGAATAGTTTTTGCT---
GTGCTTTCCATAGTA---------AATAGGGTTAGGCAGGGATATTCACCA------
TTATCGTTTGCTGTTAGCTTGTTCAACACCTTAGCCATAACAGTAGCTGGG---
TGGACAGATAGGGTTATAGAAGTAATACAAAGAACTTGTAGAGCT---------------------------
--------ATTCTCCACATA------CCTAGAAGAATAAGACAGGGTGCAGAAAGGCTTTTGCTA---TAA
>HM586198
GCT---GCA--
GAAAAATTGTGGGTCACAGTTTATTATGGGGTACCTGTGTGG---AAAGAAGCAAGC---
GCCACTCTATTTTGTGCATCAGATGCTAAAGCATATTATACAGAA---GTACATAAT----------------
--------------GTTTGGGCCACA---CATGCCTGTGTACCCACAGAC---
CCCGACCCACAAGAAATAAAATTGGAA---------AATGTGACAGAAAATTTTAAC----------------
-------------------------ATGTGGAAAAATAACATGGTAGAACAGATGCAAGAGGATATA---
ATCAGTTTATGGGATCAAAGCCTA---AAGCCATGTGTAAAATTA---
ACCCCACTCTGCGTTACTTTAAATTGCACTGATGTGAATTCTACTAGGACCAATAGTAGT------------
----
-------------------------AGTGGTGATGGGGAACTGATGGAGAAAGGAGAAATGAAA---
AACTGCTCTTTCAATGTCACCTCAAATATAAGA--------------------------
GATAAGATGCAGAAACAATATGCACTT---TTCTATAACCTTGATGTAGTACAAATAGATAAT----------
----

GATACTAACTATAGGTTGACAAGTTGTAACACCTCAGTCATTACACAA---
GCCTGTCCAAAGGTATCCTTTGAGCCAATTCCTATACATTACTGTGCCCCGGCTGGTTTTGCGATCCTAAAGT
GTAAT---AATAGGACGTTTAAT------GGAAAAGGACCATGTAAAAAT----------------------
-GTCAGC---ACAGTACAATGTACACATGGAATAAAACCAGTAGTATCAACT---
CAATTACTGTTAAATGGCAGCCTAGCAGAAGAAGAG-----------
GTAGTAATTAGATCTGACAATTTCTCAAACAAT---------GCTAAAATCATAATAGTACAGCTGAAT----
-----------GAATCTGTAACA---------ATTAATTGTACAAGACCCAACAACAATACAAGAAAA-----
-AGTATACATATA------------GCACCAGGGAGAACA---TTT------TATGCAACAGGAGAT------
---------ATAATAGGAGATATA------------AGGAAAGCATATTGT---AATATAAATGGAACA---
AAATGGCATGACACCTTAATACAGGTAAGCGAAAAATTAAAA---------------GAACAATTT-------
-----------------------------------AATAAAACAATAGTTTTTAAT------
CAATCCTCAGGAGGAGACCCAGAAGTTGTA---ATGCACACTTTTAATTGTGGAGGGGAA---
TTTAAACAGCTCCAGGCAAGAGTCCTGGCTGTGGAAAAATACCTAAAGGATCAACAGCTCCTAGGGATTTGGG
GTTGCTCTGGAAAACTCATTTGCACCACTGCTGTGCCTTGGAATACTAGTTGGAGT----------------
----------------------------AATAAATCTCTAAATGCTATTTGG------GAT------
AACATGACCTGGATGGAATGG---GAAAGAGAAATTGACAATTACACAAACGAAATATAC---
AGATTAATTGAAGGAGCGCAAAACCAACAAGAA--

AAGAATGAACAAGAATTATTGGAATTGGATAAATGGGCAAGTTTGTGGAATTGGTTTAGCATAACCAACTGGC
TGTGGTATATAAGACTATTCATAATGATAGTAGGAGGCTTGATAGGTTTAAGAATAGTTTTTACT---
GTACTTTCTATAGTG---------AATAGAGTTAGGCAGGGATACTCACCA------
TTGTCGTTGGCTGTTAGCTTGCTTAACGCTATAGCCATAGCAGTAGCTGAG---
GGGACAGATAGGGTTATAGAAGTGTTACAAAGAGGTTTTAGGGCT---------------------------
--------ATTCTCCACATA------CCTAGAAGAATCAGACAGGGCGCGGAAAGGGCTTTGAAT---TAA
>HM586210

AAT---GCA---
GAGAACTTGTGGGTCACAGTCTATTATGGGGTACCTGTGTGG---AAAGAAGCAACC---
ACCACTCTATTTTGTGCATCAGATGCTAAGGCATATGATACAGAG---GCACATAAT----------------
--------------GTTTGGGCCACA---CACGCTTGTGTACCCACAGAC---
CCCAACCCACAAGAAATGGTATTGGAA---------AATGTGACAGAAGAATTTGAC----------------
--------------------------ATGTGGAAAAATAACATGGTGGAACAGATGCAGGAGGATATA---
ACAAGTTTATGGGATCAAAGCCTA---AAGCCATGTGTAAAATTA---
ACCCCACTCTGTGTTACTTTAAATTGCACTGATGCAAATGTTACTACTACCAATAGTACTATC----------
----
----------------------AATAGTAGTAGCGGGGAACTGATGGAGGTAGGAGAAATAAAA---
AACTGCTCTTTCAACATCAAAGGAGTAACAAGA---------------------------
GATAAGGTGCAGAAAGAAAATGCATAT---TTCTATAAGCTTGATGTGGTAGAAATAGATGATAGT-------
----

AACAGCAGCTATAGGAGCTATAGGTTGATAAGTTGTAACACCTCAGTCATGACACAC---
GCCTGTCCAAAGGTATCCTTTGAACCAATTCCAATACATTATTGTACCCCGGCTGGTTTTGCGCTTCTAAAGT
GTAAT---GATAAGAAGTTCAAT------GGAACAGGACTATGCAAAAAT----------------------
-GTCAGC---ACAGTACAATGCACACATGGAATTAGGCCAGTAGTATCAACG---
CAACTGCTGTTGAATGGCAGCTTAGCAGAAGAAGAG-----------
GTAGTAATTAGATCTGAAAATTTCACAAATAAT---------GTTAAAAACATAATAGTACAGCTGAAT----
-----------GAATCTGTAGTA---------ATTAATTGTACAAGACCCAACAATAATACAAGAAAA-----
-AGTATACATATG------------GGTCCAGGACAAGCA---TTG------TATACAACAAAC---------
---------ATAATAGGAGATACA------------AGACAAGCACATTGT---AACATCAGTAGGAAG---
GAATGGAATCGCACTTTAAAACTGATAGTTAAAAAATTAAGA---------------GCACAATATGGG----
--------------------------------AGTAATAAAACAATAGTCTTTACC------
CCACCCTCAGGAGGGGACACAGAAATTGTA---ACGCACAGTTTTAATTGTGGAGGGGAA---
TTTAAACAGCTCCAGGCAAGAGTCCTGGCTATAGAAAGCTACCTAAAGGATCAACAGCTCCTGGGAATCTGGG
GTTGCTCTGGGAAACTCATCTGCACCACTACTGTGCCTTGGAATACTAGTTGGAGT-----------------
-----------------------------AATAAATCTCTGAATGAGATTTGG------GAT------
AACATGACCTGGATGCAGTGG---GATAAAGAAATTAACAATTACACAAGTACAATATAC---
ACCTTAATTGAAAAAGCGCAGAACCAACAAGAG--

AAGAATGAACAAGAATTATTGGAATTGGATAAATGGGCAAGTTTGTGGAATTGGTTTGACATAACAAACTGGC
TGTGGTATATAAGGTTATTCATAATGATAGTAGGAGGCTTGGTAGGTTTAAGAATAGTTTTTGCT---
GTACTTTCTTTAGTG---------AATAGAGTCAGGCAGGGATACTCACCA------
TTATCATTGGCCATTAGCTTGCTCAACGCCACCGCCATAGCAGTAGCTGAG---
GGGACAGACAGGGCTATAGACATAATACAAAGAGCTTTTAGAGCT---------------------------
--------ATTCTCCACATA------CCTAGAAGAATAAGACAGGGCTTGGAAAGGAGTTTAGTA---TAA
>FJ653437
ACT---GGA--
CAATTGTGGGTCACAGTCTATTATGGGGTACCTGTGTGG---AAAGAAGCAAAC---
ACCACTCTATTTTGTGCATCAGATGCTAAAGCATATGACACAGAG---ATACATAAT----------------
--------------GTTTGGGCCACA---CATGCCTGTGTACCCACAGAC---
CCCAACCCACAAGAACTACCATTGGTA---------AATGTGACAGAACATTTTAAC----------------
--------------------------ATGTGGAAAAATAACATGGTAGAACAGATGCATGAGGATATA---
ATCAGTCTATGGGATCAAAGCTTA---AAGCCATGTGTAAAAATA---
ACCCCACTCTGTGTCACTTTAAATTGTACTGAGTTAAGGAATGCTACTAATACCACTGAT-------------
----
-----------------------------AGTAGTAAAACCACTAGTGATAGTGGGGAAATGAAA---
AACTGCTCTTTTAACTACACCACAAGCCTAAAA---------------------------GGAAGGATG---
AAGGACTATGCACTT---TTTTATAACCTTGATATAGTACCACTAGATAAT----------------------
----
----------AGTAGTAACACATATAGGTTGATAAGTTGTAACAACTCAGTCATTACACAG---
GCCTGTCCAAAGGTAACCTTTGAACCAATTCCCATACATTATTGTGCCCCGGCTGGTTATGCACTTCTAAAGT
GTAGA---GATAAGAAGTTCAAT------GGAACAGGACCATGTACAAAT----------------------
-GTCAGC---ACAGTACAATGTACACATGGAATTAGGCCAGTAGTGTCAACT---
CAACTACTGTTAAATGGCAGTCTAGCAGAAGAAGAG-----------
GTAGTAATTAGATCTGAAAATTTCACAAACAAT---------GCTAAAACCATAATAGTGCAGCTGAAC----
-----------AAACCTGTAGTA---------ATAAATTGTACAAGACCCAACAACAATACAAGAAGA-----
-GGTATACATATA------------GGACCAGGGAGAGCA---TTA------TATACAACA---GAT------

---------ATAATAGGAGATATA------------AGGCAAGCACATTGT---AACCTGAGTGAAGCA---
CAATGGAATGACACTTTAAAACAGATAGTTAAAAAATTAAGA---------------GAACAGTTTGGG----
-----------------------------------AATAAAACAATAAAATTTAAT------
CAATCCTCAGGAGGGGACCCAGAAATTGTA---ATGCACAGTTTTAATTGTGGAGGGGAA---
TTTAAGCAGCTCCAGGCAAGAGTCCTGGCTGTGGAAAGATACCTAAAGGATCAACAGCTCCTAGGGATTTGGG
GTTGCTCTGGAAAACTCATTTGCACCACTGCTGTGCCTTGGAATACTAGTTGGAGT-----------------
----------------------------AATAAATCATATGAGCAGATTTGG------AAT------
AACATGACCTGGATGGAGTGG---GATAAAGAAATTGAAAAATACACAGAATCCATATAC---
ACCTTAATTGAAATATCGCAAAACCAGCAAGAA--

AAGAATGAACAAGAATTATTGGAATTAGATAAGTGGGTAAGTTTGTGGAATTGGTTTGACATAACAAACTGGC
TGTGGTATATAAAAATATTCATAATGATAGTAGGAGGCTTAATAGGTTTAAGAATAGTTTTTGCT---
GTACTTTCTATAGTA---------AATAGAGTTAGGCAGGGATATTCACCA------
TTATCGTTAGCTATCAGCTTGCTGAATGCCACAGCTATAGCAGTAGCTGAG---
GAGACAGATAGGGTTATAGAAGTATTACAAAGAATTGGTAGAGCT----------------------------
--------TTTCTCCACATA------CCTAGAAGGATAAGACAGGGCTTCGAAAGGGCTTTGCTA---TAA
>HQ595768
ACT---ACA---
GAAAAATTGTGGGTCACAGTCTATTATGGGGTACCTGTGTGG---AAGGAAGCCACC---
ACCACTCTATTCTGTGCATCAAATGCTAAAGCATATGATACAGAG---GCACATAAT----------------
--------------GTTTGGGCCACA---CATGCCTGTGTACCCACAGAC---
CCCAACCCACAAGAATTAATATTAGAA---------AATGTGACAGAATATTTTGAC----------------
--------------------------ATGGGAAAAAAATAACATGGTAGAACAGATGCAAGAGGATATA---
ATTAGTTTATGGGATCAGAGCCTA---AAGCCATGTGTAAAATTA---
ACCCCACTCTGTGTTACTTTAAGTTGCACTGATGTAAATGGTACCAATTCTACTAATATT-------------
----
----------------------------AAAAAAGAGATGGTAATGAATAGAGGAGATATGAAA---
AACTGCTCTTTCAATTTCACCACAAACATAAGA--------------------------
GATAAGGTACAGAAAGGACGTGCGATT---TTTGATAATTATGATATAGTACCAATAGATAAT----------
----

ACTAATAATTCTAGCTTTAGGTTGAGAAGTTGTAATACCTCGGTCATTACACAG---
ACCTGTCCAAAGGTGACCTTTGAACCAATTCCCATACATTATTGTGCCCCGGCTGGTTTTGCGATTCTAAAGT
GTAAT---AATAATACATTCAAT------GGAACAGGACCATGTACAAAT----------------------
-GTCAGC---TCAGTACAATGTACACATGGAATTAGGCCAGTAATATCAACT---
CAACTGCTGTTAAATGGCAGTCTAGCAGAAGGAGAG------------
ATAGTAATTAGATCTGTCAATTTCACAGACAAT---------GCTAAAACCATAATAGTACAACTGAAT----
-----------AAATCTGTAGTA---------ATTAATTGTACAAGACCCAACAACAATACAAGACAA-----
-GGTATACATATA------------GGACCAGGGGGAGCA---TTT------TTTGCAAGAGGAGAC------
---------ATAATAGGAGATCCA------------AGACAAGCACATTGT---AACATTAGTAGAACA---
GGATGGAATAATACTTTAGCACAGGTAGTTAAGAAATTAAGA---------------GAACATTGG-------
-----------------------------------AATAAAACAATAATTTTTAAT------
CAACACTCAGGAGGGGACCCAGAAATTATA---ATGCACAGCTTTAATTGTGGAGGGGAA---
TTTAAGCAGCTCCAGGCAAGAGTCCTGGCTGTAGAAAGATACCTGCATGATCAACAGCTCCTAGGGATGTGGG
GTTGCTCTGGAAAACTCATCTGCACCACTGCTGTGCCTTGGAATACTAGTTGGAGT-----------------
----------------------------AATAGATCTCTGAACTACATTTGG------GGC------
AACATGACATGGATACAGTGG---GAAAAGGAAATTGACAATTACACAACTGAGATATAC---
GGCCTAATTGCAGAATCGCAAAACCAACAAGAA--

AAAAATGAGAAGGAATTATTAGAATTGGATAAATGGGCAAGTTTGTGGAATTGGTTTGACATATCAAATTGGC
TGTGGTATATAAGAATATTCATAATGATAATAGGAGGCTTGGTAGGTTTAAGAATAGTTTTTGCT---
GTAATTTCTATAGTG---------AATAGAGTTAGGCAGGGATACTCACCA------
TTGTCGTTTGCTATTAGCTTGTTTGATGCTACAGCCATAGCAGTCGCTGAG---
GGGACAGATAGGGTTATAGCAATTTTACAAAGGGCATTTAGAGCT----------------------------
--------TTCCTTCATATA------CCTAGAAGAATAAGACAGGGCTTCGAAAGGGCTTTGCTA---TAA
>HQ595771
GTT---TCA---
GAACAGGCATGGGTCACAGTCTATTATGGGGTACCTGTGTGG---AAAGAAGCAGAC---
ACCCCTCTATTTTGTGCATCAGATGCTAAAGCATATGAGACAGAG---GCACATAAT----------------
--------------GTTTGGGCCACA---CATGCCTGTGTACCCACAGAC---

CCCAACCCACAAGAAGTAAAATTGGAA---------AATGTGACAGAAAACTTTAAC---------------
--------------------------ATGTGGAAAAATAATATGGTAGAACAGATGCATGAGGATGTA---
ATCAGTTTATGGGATGAAAGCCTA---AAGCCATGTGTAAAATTA---
ACCCCACTCTGTGTGACTTTAAATTGTACTGATTTGAAGAATGATACTGTCAATAATAAT------------
----
-------------------------ACTAGAGATATTCAAGATGAAATGAAAGAAGAAATAAAA---
AACTGCTCTTTCGATATCGAAACAAGCATAAGA--------------------------
GATAAGAAGCGAAAGGAACATGCACTT---TTTTATACACTTGATATAGTACCAATAGATAAT---------
----

AATAGTACTAATACTACTTATAGGTTGACAAGTTGTAACACCTCAGTCATTAAACAG---
GCATGTCCAAAGGTATCCTTTGAGCCAATTCCCATACATTATTGTGCCCCAGCTGGTTTTGCGATTCTAAAAT
GTAAT---GATACTAAGTTCAAT------GGATCAGGACCATGTAAGAAT----------------------
-GTCAGC---ACAGTACAATGTACACATGGAATTAGGCCAGTAGTATCAACT---
CAGCTGCTGTTAAATGGCAGTTTAGCAGAAGAAGAG-----------
ATAATGATTAGGTCTGAAAACTTCTCGAACAAT---------GCTAAAGTAATAATAGTGCAGCTGAAG----
-----------GAATCAGTACCA---------ATTAATTGTACAAGACTCAACAACAATACAAGAAAA-----
-AGTGTAAGGATA------------GGTCCAGGGGCAACA---TTT------TTTGCAACAGGAGAC------
---------ATAATAGGAGATATA------------AGACAAGCACATTGT---AACATTAGTAGAACA---
GCATGGAGTAAAACTTTAGAACAGGTAGCTAAAAAATTAAAA---------------GAACAATAT-------
---------------------------------AATAAAACAATAATTTTTAAT------
CAATCCTCAGGAGGGGACCCAGAATTAGTA---ACACATAGTTTTAATTGTGGAGGGGAA---
TTCAAGCAGCTCCAGGCAAGAGTCCTGGCTCTGGAAACATACCTAAAGGATCAACAGCTCCTGGGAATTTGGG
GTTGCTCTGGAAAACTCATTTGTACCACTGCTGTGCCTTGGAATGCTAGTTGGAGT----------------
----------------------------AATAGAAATCTGACGAGCATTTGG------GAT------
AACATGACCTGGATGGAATGG---GAAAAAGAAATTGATAATTATACAAGCACAATATAC---
ACCTTACTTGAAGAATCGCAGAACCAGCAAGAA---------------------------------------
----
AAGAATGAACAAGAATTATTGGAATTAGACAAATGGGCAAGTTTGTGGAATTGGTTTAGCATAACAAATTGGC
TGTGGTATATAAGAATATTCATAATGATAGTAGGAGGCTTGATAGGTTTAAGAATAGTCTTTACT---
GTACTTTCTATAGTG---------AATAGAGTTAGGCAGGGATACTCACCA------
TTATCATTTGCTGTTAGCTTGTTCAACGCCATAGCAATAGCAGTAGCTGAG---
GGGACAGATAGGATTATAGAAGTAATACAAGAAATTTTTAGAGCT--------------------------
--------TTTATCCACATA------CCTAGAAGAATCAGACAGGGCTTAGAAAGGGCTTTGCTA---TAA
>HQ595772
GCT---ACA--
GAACAATTATGGGTCACAGTCTATTATGGGGTACCTGTGTGG---AAAGAAGCAACT---
ACCACTCTATTTTGTGCATCAGATGCTAAAGCATATGATACAGAG---GTACATAAT---------------
--------------GTTTGGGCCACA---CATGCCTGTGTACCCACAGAC---
CCCAACCCACAAGAATTAGTATTGGAA---------AATGTGACAGAAAATTTTAAC---------------
--------------------------ATGTGGAAAAATAACATGGTAGACCAGATGCATGAGGATATA---
ATCAGTTTATGGGATCAAAGCCTA---AAGCCATGTGTAAAGTTA---
ACCCCACTCTGTGTCACTTTAAATTGCAGTGATGTAAGGAATGCCAATAATGCCAATAAT------------
----
----------------------------AGTAGCGGGGGAAACATAGAGGTAGGAGAAATAAAA---
AACTGCTCTTTCAATATCACCACAAGCATAGGG--------------------------
AATAAGCTGCAAAACCAATATGCAACT---TTTTCTAAACTTGATGTAGTGCCATTAGATAAT---------
----

ACTAGTAATAGCTATAGGTTGATAAATTGTAACACTTCGGTCATTACACAG---
GCCTGTCCAAAGGTAACCTTTGAGCCAATTCCCATACATTATTGTGCCCCAGCTGGTTTTGTGATTCTAAAGT
GTAAT---GATAAGAAGTTCAAT------GGAACAGGACCATGTAAAAAT----------------------
-GTCAGC---ACAGTACAATGTACACATGGAATTAGGCCAGTAGTGTCAACT---
CAACTGCTGTTAAATGGCAGTCTAGCAGAAGAAGAG-----------
ATAGTAATTAAATCTGAGAATCTCACGGACAAT---------ACTAAAACCATAATAGTACAGCTAAAT----
-----------AAATCTATAGCA---------ATTGATTGCATAAGACCCAACAACAATACAAGGAGA-----
-AGTATACATATG------------GGACCAGGGAAAACA---TTT------TTTGCAGCAGAT---------
---------ATAATAGGAGATATA------------AGACAAGCACATTGT---AACCTTAGTAGAACA---
GACTGGAATAAAACCTTAAGAGGGATAGTTATAAAATTAAAA---------------GAATCATTTGTG----
---------------------------------AATAAAACAATAATCTTTAAT------

CAATCATCAGGAGGGGACCCAGAAATTGTA---CTGCACAGCTTTAATTGTGGAGGGGAA---
TTTAAGCAGCTCCAGGCAAGAGTCCTGGCTGTGGAAAGATACCTACAGGATCAACAGCTCCTAGGGATTTGGG
GGTGCTCTGGAAAACTCATCTGCCCCACTGCTGTGCCTTGGAATACTAGTTGGAGT-----------------
----------------------------AATAAATCTCTGAGTCAGATTTGG------GAC------
AACATGACATGGATGCAGTGG---GAAAAAGAAATTGACAATTACACAAGATTAATATAC---
ACATTAATTGAAGAATCACAATACCAGCAAGAA---

AAGAATGAACAAGAATTATTGGAATTGGATAAATGGGCAAGTTTGTGGAATTGGTTTAGCATATCAAAATGGC
TGTGGTATATAAAAATATTCATAATGATAGTAGGAGGCTTGATAGGTTTAAGAATAATCTTTTTT---
GTACTTGCTATAGTG---------AATAGAGTTAGGCAGGGATACTCACCA------
TTATCGTTTGCTGTTAGCTTGTTCAACGCCATAGCCATAGCAGTAGCTGAG---
GGGACAGATAGAGCTATAGAAATATTACAAAGAGCTTGTAGAGCC----------------------------
--------ATTCGCAACATC------CCTAGAAGAATAAGACAGGGTTTGGAAAGGTCTTTGCTA---TAA
>HQ595774
GCT---ACA---
GAAAAATTGTGGGTCACAGTCTATTATGGGGTACCTGTATGG---AGGGAAGCAAAT---
GCCACCTTATTTTGTGCATCAGATGCTAAAGCATATGATACAGAG---AAACATAAT----------------
--------------GTTTGGGCCACA---CATGCCTGTGTACCCACAGAC---
CCCAACCCACAAGAAATAGAATTAAAA---------AATGTGACAGAAAATTTTAAC----------------
-------------------------ATGTGGAAAAATAACATGGTAGAACAGATGCATGAGGATATA---
ATTAGTTTATGGGAACAAAGCCTA---AAGCCATGTGTGAAATTA---
ACCCCACTCTGTGTTACTTTAGATTGCACTAATATTACTAGCAATAATATTGCTAGCAATAATACTGGCAGTA
ATGTTACTGGCAGTAGTAGTACTAGCAATAATAGT--------------------------------------
----------ATTAGTAGTACCACAAATAGCAGTATTTTAGGACCAATGAAAGATGAAATAAGA---
AACTGTTCTTTCAATATCACCACAAGTATAAGA---------------------------
GATAAGGTACAGAGAGAATATGCATTT---TTATATAAATATGATATAGTACCAATAGAAAAT----------
----

AAAAATAATAATACCAAATATAGGTTGATAAGTTGTAACACCTCAGTCATTACACAA---
GCCTGTCCAAAGGTAACCTTTGAACCAATTCCCATACATTATTGTGCCCCTGCTGGTTTTGCGATTATAAAGT
GTAGA---GATAAGAAATTCAAT------GGAACAGGACCATGTAAAAAT-----------------------
-GTCAGC---ACAGTACAATGCACACATGGAATTAGACCAGTAGTATCAACT---
CAACTGCTGTTAAATGGCAGTCTAGCAGAAGAAGAT-----------
ATAATAATTAGATCTGAGAATATCACAGACAAT---------GCTAAAACCATAATAGTACAGCTGAAC----
-----------GAAACTGTGCAA---------ATTAATTGTACAAGACCAGGCAATAATACAAGAAGA-----
-AGTATAAACATA------------GCACCAGGGAGAGCA---TGG------TATACAACAGGGGAA------
---------GTAATAGGAGATATA------------AGACAAGCACATTGT---AACATTAGTCAAAAA---
GAATGGACTAAAACTTTAAAACAGGTAGCTAGAAAACTAAGA---------------GAACAATTT-------
-----------------------------------AATAGGACAATAGCCTTTAAT------
AAATCCTCAGGAGGGGACCCAGAAGTTGTA---ATGCACACTTTTAATTGTGGAGGGGAA---
TTTAAGCAGCTCCAGGCAAGAGTCCTGGCTGTGGAAAGATACCTAAAGGATCAACAGCTCCTAGGGATTTGGG
GTTGCTCTGGAAAACTCATCTGCACCACTGCTGTGCCTTGGAATACTAGTTGGAGT-----------------
----------------------------AATAAATCTCATGATCAGATTTGG------GAA------
AACATGACCTGGATGCAGTGG---GAAAGAGAAATTGAAAATTACACAAACACAATATAT---
GAATTAATTGAGCTATCGCAGAACCAGCAGGAA---

AAGAATGAACAAGACTTATTGGCATTGGATAAATGGGACAGCTTGTGGAGTTGGTTTGACATAACAAATTGGC
TGTGGTATATAAAAATATTCATAATGATAGTAGGAGGCTTGATAGGTTTAAGAATAGTTTTTTCT---
GTAATTTCTGTAGTG---------AATAGAGTTAGGCAGGGATACTCACCA------
TTATCGTTTGCTGTTAGCTTGCTTAACGCCACAGCCATAGCAGTAGCTGAG---
GGGACAGATAGGATTATAGAAGGAGTACAAAGAGCTTTTAGGGCT----------------------------
--------ATCCTCCACATA------CCTAGAAGAATAAGACAGGGCTTGGAAAGGGCTTTGGTA---TAA
>HM204626
ACT---GCA---
GAAAATTTGTGGGTCACAGTCTATTATGGGGTACCTGTGTGG---AAAGAAGCAACC---
ACCACTCTATTTTGTGCATCAGATGCTAAGGCACATGATACAGAA---GTGCATAAT----------------
--------------GTTTGGGCTACA---CATGCCTGTGTACCCACAGAC---
CCCAACCCACAAGAAATAGGATTGGAA---------AATGTGACAGAAAATTTTAAT----------------
-------------------------GCATGGAAAAATAACATGGTAGAGCAGATGCATGAGGATATA---
ATCAGTTTATGGGATGAGAGCTTG---AAGCCATGTGTAAAACTA---

ACTCCACTCTGTGTTACTTTAAATTGTACAGATTGTAAGGGAAATGGTACGGATACTAACAATTGT-------
----
----------------------ACTAGCAGCAGCAATGTGACAGTGGAGGACGGAGAAATGAAA---
AAGTGCTATTTCAATATCACCACAAGCATAAAA---------------------------
GATAAAAAGCAAAAGGAATATGCACTC---TTTTATAAACATGATGTAGTATCAATAGATAAT----------
----

ACTAGCTATAGGTTGATAAGTTGCAACACCTCAGTCATTACACAA---
GCCTGTCCAAAGGTATCCTTTGAACCAATTCCCATACATTATTA-
GCCCCGGCTGGTTTTGCAATTCTAAAGTGTAGA---GATAAGAAATTCAAT------
GGAACAGGACTATGTAGAAAT------------------------GTCAGT---
ACAGTACAATGTACACATGGAATTAGGCCAGTAGTGTCAACT---
CAACTACTGTTAAATGGTAGTCTAGCAGAAGAAGAG------------
GTAGTAATTAGATCTCAAAATTTCACGGACAAT---------GCTAAAACCATAATAGTACAACTGAAT----
-----------GAATCTGTACAA---------ATTAATTGTACAAGACCCAACAACAATACAAGAAAA-----
-AGTATAAATATA------------GGACCAGGGAGGGCA---TTT------TATGCAACAGGAGAG------
---------GTAATAGGAGATATA------------AGAAAGGCACATTGT---AACATTAGTGGAAAA---
GATTGGAATAACACCTTAGAAAGGGTAGCTGAAAAATTGAGA---------------AAACAATTTGGA----
--------------------------------AACAATAAAACAATAGTGTTTAAC------
TCATCCTCAGGAGGGGACCTAGAAATTGTA---ATGCATAGTTTTAATTGTGGAGGGGAA---
TTTAAGCAGCTCCAGGCAAGAGTCCTGGCTATAGAAAGATACCTAAAGGATCAACAGCTCCTGGGGATTTGGG
GTTGCTCTGGAAAACGCATTTGCACCACTGCTGTGCCTTGGAATACGAGTTGGAGT-----------------
----------------------------AATAAATCTATGAATGAGATTTGG------GAT------
AACATGACCTGGATGGAGTGG---GAAAAGGAAATTAGTAAGTACACACAAACAATATAT---
AGCTTAATTGAACAATCGCAAAGCCAGCAAGAA--

AAGAATGAACAGGAACTATTAGAATTAGATAAGTGGGCAAATTTGTGGAATTGGTTTGACATAACAAATTGGC
TGTGGTATATAAAGTTATTCATAATGATAGTAGGAGGCTTAATAGGTTTAAGAATAGTTTTTGCT---
GTACTTTCTGTAGTG---------AAAAGAGTTAGGCAGGGATACTCACCT------
ATATCGTTGGCTGTTAATTTGCTTAATACCACAGCTATAGCAGTAGCTGAA---
GGGACAGATAGGATTATAGAAGTAGCAAATAGGATTTTTAGAGCT----------------------------
--------TTTCTCCACATC------CCGAGAAGAATAAGACAGGGCTTAGAAAGGCTTTTGCTA---TAA
>HM204627
GTT---GCA--
GGCAACTTGTGGGTAACAGTCTATTATGGGGTACCTGTGTGG---AGAGACGCAAAC---
ACCACTCTATTTTGTGCATCAGATGCTAAAGCATATGATACAGAG---GTACATAAT----------------
--------------GTTTGGGCCACA---CATGCCTGTGTACCCACAGAC---
CCCAACCCACAAGAACTAGTATTGGAA---------AATGTAACAGAAGATTTTAAT----------------
-------------------------ATGTGGAAAAATAACATGGTAGAACAAATGCATGAGGATGTA---
ATCAGTTTATGGGATCAAAGCTTA---AAGCCATGTGTAAAATTG---
ACCCCACTCTGTGTTACGTTAAATTGCACTAAGACCAATAATAGTACGAATGTT-------------------
----
----------------------------------AATAATACCATGGGGGGAGGAGAAATGAAA---
AACTGCTCTTTCAATATCACCTCAGAAATAAGA---------------------------
GATAAGACAAAGAAAGAAAATGCACTT---TTTTATACAATAGATCTAGTACCAATAAATAAT----------
----

GATAGCTATAGGTTGATAAGTTGTAACACCTCAGTCATTACACAG---
GCCTGTCCAAAGATAACCTTTGAGCCAATTCCCATACATTATTGTGCCCCGGCTGGTTTTGCGATTCTAAAGT
GTAAG---GATAAGGATTACAAG------GGAACAGGACCATGTAAAAAT-----------------------
-GTCAGC---ACAGTACAATGTACACATGGAATTAAGCCAGTAGTATCAACT---
CAACTGCTACTAAATGGCAGTCTAGCAGAAGAAGAG------------
GTAGTAATTAGATCTGAAAATATCACAGACAAT---------GCTAAAACCATAATAGTACAGCTGAAG----
-----------GAACCTGTAGTA---------ATTAATTGTACAAGACCCAACAACAATACAAGAAAA-----
-AGTATACCTATA------------GGACCAGGGAGAGCA---TTT------TATGCAACAGGAGAC------
---------ATAATAGGAGATATA------------AGGCAAGCACATTGT---AACCTCAGTAGAGTA---
CAATGGATTAATGCTTTAAACCAGACAGTTAGAAAATTAAGA---------------GAAAAATTT-------
-----------------------------------AATAAAACAATAGTCTTTAAC------
CAATCTTCAGGAGGGGACCCAGAAATTGTA---ATGCACAGTTTTAATTGTGGAGGGGAA---
TTTAAGCAGCTCCAGGCAAGAATCCTGGCTGTGGAAAGATACCTGAAGGATCAACAGCTCCTGGGGATTTGGG

GTTGCTCTGGAAAGCTCATTTGCACCACTACTGTGCCTTGGAATGCTAGTTGGAGT-----------------
----------------------------AATAAAACTCTGAGTCAGATTTGG------GAT------
AACATGACTTGGATGCAGTGG---GAAAAAGAAGTTAGTAATTACACAGGCCTAATATAC---
GCCTTAATTGAAGAATCGCAAACCCAACAAGAA--

AAGAATGAACAAGACTTATTGGCATTAGATAAATGGGCAAGTTTGTGGAATTGGTTCGACATATCAAACTGGC
TGTGGTATATAAAGATATTCATAATGATAGTAGGAGGCTTGATAGGGTTAAGAATAGTTTTTACT---
GTACTTTCTATAGTG---------AATAGAGTTAGGCAGGGATACTCACCA------
TTGTCGTTTGCTGTTACCTTGTTCAATGCAATAGCCATAGCAGTAGCTGAA---
GGGACAGATAGGGTTATAGAAGCAGTCCAAAGAGCTTTTAGAGCT----------------------------
--------TTTCTTCACATA------CCTAGAAGAATAAGACAGGGCGCAGAAAGGGCTTTAATA---TAA
>HM204628
GCT---GAC---
CAGTTGTGGGTCACAGTCTATTATGGGGTACCTGTGTGG---AAAGAAGCAAAG---
ACCACTCTATTTTGTGCATCAGATGCTAAAGCAGTTGAAACAGAA---AAACATAAT----------------
--------------GTTTGGGCCACA---CATGCCTGTGTACCCACAGAC---
CCCAGCCCACAAGAAGTACTTTTAAAA---------AATATGACAGAAAGCTTTAAC----------------
--------------------------ATGTGGAAAAATGACATGGTAGAACAGATGCATGAGGATATA---
ATCAGTTTATGGGATGAAAGCCTA---AAACCATGTGTGACATTA---
ACCCCACTCTGTGTTACTTTAAATTGCAAGAACACTACTAATAACACCAGTAATAGT----------------
----
-------------------------------AGTGGGGGAACGATGGAGGGAGGAGAAATGAAA---
AACTGCTCTTTCAATATCACCACAGACATAAGA---------------------------
GATAAGGTGAAAAAAGACTATGCCCTT---TTCTATAACCTTGATATAGTACAAATAGATAGCAGT-------
----

AGAAGTAATAATAGTACTGAATATAGGTTGATAAGTTGCAATACCTCAGTCATTACACAA---
GCCTGTCCAAAGGTATCCTTTACGCCAATTCCCATACATTATTGTGCCCCGGCTGGTTTTGCGATTGTAAAGT
GTAAC---GACAAAAGATTCAAT------GGAACAGGACCATGTGTAAAT-----------------------
-GTCAGC---ACAGTACAATGTACACATGGAATTAAGCCAGTAGTATCAACT---
CAACTGCTATTAAATGGCAGTCTAGCAGAAGAAGAG------------
GTGGTAATAAGATCTGAAAATTTCACGAACAAT---------GCTAAAATCATAATAGTACAGCTGAAT----
-----------GAATCCATAGAG---------ATTAATTGTACCAGGCCCAACAACAATACAAGAAAA-----
-GGTATACATATG------------GGACCAGGGCAAGCA---ATT------TATGGTTATAAAACA------
GGAGAAATA---ATAGGAAATATA------------AGACAAGCACATTGT---AACATTAGTGAAAAG---
AAATGGAACAACACTTTAAAACTGGTAGTTAAAAAATTAAGA---------------GAGCAATTTGGG----
-----------------------------------AATAAAACAATAGCCTTTAAG------
CAATCCTCAGGAGGGGACCTAGAAATTGTA---ACGCACAGTTTTAATTGTGGAGGGGAA---
TTTAAGCAGCTCCAGGCAAGAATCCTGGCTGTGGAAAGATACCTAAAGGATCAACAGCTCCTGGGGATTTGGG
GTTGCTCTGGAAAACTCATTTGCACCACTACTGTGCCTTGGAATACTAGTTGGAGC-----------------
----------------------------AATAAATCTCTGGATGATATTTGG------GAG------
AATATGACCTGGATGCAGTGG---GATAAAGAAATTAGCAATTACTCACAAATAATATAC---
ACCTTAATTGAAGAATCGCAGATCCAGCAGGAG--

AAGAATGAACAAGAATTATTGGCATTAGATAAATGGGCAAGTTTGTGGAATTGGTTTGACATATCAAAATGGC
TGTGGTATATAAAAATATTCATAATGATAGTAGGAGGCTTAATAGGTTTAAGGATAGTTTTTACT---
GTACTTTCCATAGTG---------AATAGAGTTAGGCAGGGATACTCACCA------
TTATCGTTTGCCATTAGCTTGCTCAACGCCACAGCCATAGCAGTAGCTGAG---
GGGACAGATAGGGTTATAGAAGTGTTACAAAGAGTTTTTAGAGCT----------------------------
--------ATCCTCCACGTA------CCTAGAAGACTAAGACAGGGCTTAGAAAGGATTTTGCTA---TAA
>HM204629
GCA---TCA--
GAAAAGTTGTGGGTCACAGTCTATTATGGGGTACCTGTGTGG---AAAGAAGCAACT---
ACCACTCTCTTTTGTGCATCAGATGCTAAAGCATATGATACAGAG---GTACATAAT----------------
--------------GTTTGGGCCACA---CATGCCTGCGTACCCACAGAC---
CCCAACCCACAAGAAGTAGTATTAAAA---------AATGTGACAGAAGATTTTAAC----------------
--------------------------ATGTGGCAAAATAACATGGTAGAACAGATGCATGAAGATATA---
ATTAGTTTATGGGATCAAAGCTTA---AAGCCATGTGTAAAGTTA---
ACCCCACTTTGTGTTACTTTAAATTGTTCCAATGTTACAAATACCAATGCTACA-------------------
----

-------------------------------AATGCCACTGATAGTAGTTGGAATGACATAAAA---
AACTGCTCTTTCAATGTCACCACAAGCATAAGA---------------------------
GATAGGGTTCAGAAAGAATATGCACTT---TTTAATAGACTTGATGTAGTACCAATAGGTGAT----------
----

AATAATACCACAAAATATAGGTTGATAAGTTGTAACACTTCAGTCATTACACAG---
GCCTGTCCAAAGATATCATTTGAGCCAATTCCCATATATTATTGTGCCCCGGCTGGTTTTGCGATTATAAAGT
GTAAT---AATAAGACATTCAGT------GGAAAAGGAAAATGTAACAAT-----------------------
-GTTAGC---ACAGTACAATGCACACATGGAATTAGGCCGGTAGTGTCAACT---
CAACTACTGTTAAATGGCAGTCTAGCAGAGGAAGAG------------
ATAGTAATTAGATCTGATAATTTCACAGACAAT---------GCTAAAACAATAATAGTACAGCTGAAC----
-----------ACATCTGTACAA---------ATTAATTGTACAAGACCCAACAACAATACAAGAAAA-----
-AGTATAACTATA------------GGACCAGGGAGAGCA---TTT------TATGCAACAGGAGAC------
---------ATCATAGGAGATATA------------AGACAAGCCTATTGT---AACATTAGTAAAACA---
CAATGGAATAACACTTTAAGACAGATAGCTATAAAATTAAGG---------------GAACAATTT-------
-----------------------------------AATAAAACAATAGTCTTTAAT------
CACTCCTCAGGAGGAGACCCAGAAATTGTA---ATGCATAGTTTTAATTGTGGAGGAGAG---
TTTAAGCAGCTCCAGGCAAGAGTCCTGGCTCTGGAAAGATACCTAAGGGATCAACAGCTCCTAGGGATTTGGG
GTTGCTCTGGAAAACTCATCTGCACCACTGCTGTGCCTTGGAATACTAGTTGGAGT-----------------
----------------------------AATAAAAATCTGAGTCAAATTTGG------GAT------
AACATGACTTGGATGCAGTGG---GAAAAAGAAATTGAAAATTACACAGGCTTAATATAT---
GACTTACTTGAAAAATCGCAAAACCAGCAAGAA--

CAGAATGAACAAGAATTATTAGCATTAGATAAGTGGGCAAGTTTGTGGAGTTGGTTTGACTTGACAAACTGGC
TGTGGTATATAAGAATATTTATAATGATAGTAGGAGGCTTGATAGGCTTAAGGATAATTTTTGCT---
GTGCTTTCTGTAGTG---------AATAGAGTTAGGCAGGGATACTCACCA------
TTGTCATTGGCTGTCAGCTTGTTCAACGCCACAGCTATAGTAGTAGCTGAG---
GGGACAGATAGAATTTTAGAAGTATTGCAAAGAATTTATAGAGCA----------------------------
--------TTTATCCACATA------CCTAGAAGAATAAGACAGGGCTTCGAAATTGCTTTGCTA---TAA
>HM204632
GGT---GAA---GAA---------------------------------
CAAGAAAAATTGTGGGTCACAGTCTATTATGGGGTACCTGTGTGG---AAAGAAGCAACC---
GCCACTTTATTTTGTGCATCAGATGCTAAGGCATATGATACAGAG---GTACATAAT----------------
--------------GTTTGGCCACA---CATGCATGTGTACCCACAGAC---
CCCAGCCCACAAGAAGTAGTATTGGGA---------AATGTGACAGAAAACTTTAAC----------------
--------------------------ATTTGGAAAAATGACATGGTAGAGCAAATGCATGAGGATATA---
ATCAGTTTATGGGATCAAAGCCTA---AAACCATGTGTAAAATTA---
ACCCCACTCTGTGTTACTTTAAATTGCACTGATTTCAATAGTACAGCCACTAATAGTAGTGGGAAACAG----
----
-------------------ACGACCACTAATAGTAGTAGTGGGATGGAACAAGGAGAAATGAAA---
AACTGCTCTTTCAATGTCACCACAGACTTAAGA---------------------------
GATAAGATAAAGAAAGAATATGCACTT---TTGTATAAAACTGATATAGTACAAATAGATAACTCA-------
----

GGGAATACTAGCTATAGCTATAGATTGATAAGTTGCAACACATCAACCCTTACACAG---
GCCTGTCCAAAGGTATCCTTTCAACCAATTCCCATACATTATTGTGCCCCAGCTGGTTTTGCGATTCTAAAGT
GTAAT---AATAAGACGTTCAAT------GGAAAAGGACCATGTACAAAT-----------------------
-GTCAGC---ACAGTACAATGTACACATGGAATTAGACCAGTAGTATCAACT---
CAACTACTGTTAAATGGCAGTCTAGCAGAGGAAGAG------------
ATAGTAATCAGATCTGACAACTTCTCAGATAAT---------GCCAAAACTATAATGGTACATCTGAAT----
-----------GAATCTATAAAA---------ATTAATTGTACAAGACCTAGCAACAATACAAGAAAA-----
-AGTATACATATA------------GGACCAGGGAGAGCA---TTT------TATGCAACAGGAGCT------
---------ATAATAGGAGATATA------------AGACAAGCACATTGT---AACATTAGTAAAAAA---
CATTGGTTTGAAACTTTAGAAAAAATAGTTATAAAATTAAGA---------------AAACAATATGGG----
--------------------------------GAAAATAAAACAATAGTCTTTAAA------
CAATCCTCAGGAGGGGACCCAGAAATTGTA---CAACACAGTTTTAATTGTGGAGGGGAG---
TTTAAGCAGCTCCAGGCAAGAGTCCTGGCTGTGGAAAGATACCTACAGGATCAACGGCTCCTAGGGATTTGGG
GTTGCTCTGGAAAACTCATCTGCACCACTGCTGTGCCTTGGAATACTAGCTGGAGT-----------------
----------------------------AACAGATCTCTGGATATGATTTGG------AAT------
AATATGACCTGGATGCAGTGG---GAAAAAGAAATTGACAACTACACACAAATAATATAT---

ACCTTAATTGAACAATCGCAAAACCAACAAGAA--

AAGAATGAGCAAGAATTATTGGAATTAGATAAATGGGCAAGTTTGTGGAATTGGTTTAGCATAACAAACTGGC
TATGGTATATAAGACTATTCATAATGATAGTAGGAGGCTTGATAGGTTTAAGAATAGTTTTTGCA---
GTGCTTGCTATAGTA---------AAAAGAGTTAGGCAGGGATATTCACCA------
TTGTCGTTGGCTGTTAGTTTGCTCAACGCTACAGCTATAGCAGTAGGTGAG---
GGGACAGATAGGGTTATAGAAATAGTACAAAGATTTTGGAGAGCT----------------------------
--------TTTCTCCATATA------CCTAGAAGAGTAAGACAGGGCTTGGAAAGGAGTTTGGTA---TAA
>HM204633
GCT---GCA--
GAGGACTTGTGGGTCACAGTCTATTATGGGGTACCTGTGTGG---AAAGAAGCAACT---
ACCACTCTATTTTGTGCATCAGATGCTAAAGCATATACTACAGAG---GTACATAAT----------------
--------------GTATGGGCCACA---CATGCCTGTGTACCCACAGAC---
CCCAACCCACAAGAAGTAGATTTGGAA---------AATGTAACAGAAAATTTTAAC----------------
--------------------------ATGTGGAAAAATAACATGGTAGAACAGATGAATGAGGATATA---
ATCAGTTTATGGGATCAAAGCCTA---AAGCCATGTGTAAAATTA---
ACTCCACTCTGTGTTACTTTAAATTGCACTGATGCCAATACTACCAAAAGTAGCAATAATACTAATCAT----
----
-------------------ACTAGTGCCCCTAAAGTACTGATGGAGGAACAAGGAGAAATAAAA---
AACTGCTCTTTCAATGTCACTGCAGGCATAAGA---------------------------
GATAAGGTAAAAAAGGAATTTGCTCTC---TTTTATAGTCTTGATATAGTACCAATAGATAGTGATAATACA-
----

AATAAAAATACCATTAATAATACCAGTTATAGGTTGATAAGTTGTAACACCTCAGTCACTACACAG---
GCCTGTCCAAAAGTATCCTTTGAGCCAATTCCCATCCATTATTGTGCCCCGGCTGGTTTTGCGATTCTAAAAT
GTAAA---GATGAGACGTTCAGT------GGAAAAGGACCGTGTAACAAT-----------------------
-GTCAGC---ACAGTGCAATGCACTCATGGAATTAGGCCAGTAGTATCAACT---
CAACTATTGTTAAATGGCAGTCTATCAAAAAGAGAG-----------
GTAGTAATTAGATCTGAGAATTTCACAGACAAT---------GCTAAAAACATAATAGTGCAGCTAAAC----
-----------AAATCTGTAGAA---------ATTAATTGTACAAGACCCAACAACAATACAAGAAAA-----
-AGTATAGGTATA------------GGACCAGGAAGTGCA---ATT------TTTGCAACAGGAGAC------
---------ATAATAGGAGATATA------------AGAAAAGCATATTGT---ACCATTGATAGAAAA---
GATTGGAATAACACTTTAGAACAGATAGTTAAAAAATTAGGA---------------GCACAATTTAAG----
--------------------------------AATATAACAAAAATATCCTTTAAT------
AAATCCTCAGGAGGGGACCCAGAAATTGAA---ATGCACAGTTTTAATTGTGGAGGGGAA---
TTTAAACAGCTCCAGGCAAGAGTCCTGGCTGTAGAAAGATACCTAAAGGATCAACAGCTCCTGGGAATTTGGG
GTTGCTCTGGAAAGCTCATCTGCACCACTGCTGTGCCTTGGAATACTAGTTGGAGT-----------------
----------------------------AATAAATCTTATGATAAGATTTGG------AAT------
AACATGACCTGGATGGAGTGG---GAGAGAGAAATTGACAATTACACAGACATAATATAC---
AACTTAATTGAAATATCGCAGAACCAGCAGGAA--

AAGAATGAACAAGAATTATTGGAATTGGATAAATGGGCAAATTTGTGGAATTGGTTCGACATTTCAAACTGGC
TGTGGTATATAAAAATATTCATAATGATAGTAGGAGGCTTGATAGGTTTAAGAATAATTTTTACT---
GTACTTTCTTTAGTG---------AATAGAGTTAGGCAGGGATACTCACCA------
TTGTCGTTTGCTGTTAGCTTGCTTAATGTCACAGCCATAGCAGTAGCTGAG---
TGGACAGATAGGGTTATAGAAGTAGCACAAAGATTTGGTAGAGCT----------------------------
--------ATTCTCAATATA------CCTAGAAGAATAAGACAGGGCTTGGAAAGGGCTTTGCTA---TAA
>HM204638
GGT---GCA--
GAACATTTGTGGGTCACAGTCTATTATGGGGTACCTGTGTGG---AAAGAAGCAACC---
ACCACTCTATTTTGTGCATCAGATGCTAAGGCATATGACACAGAG---GTACATAAT----------------
--------------GTTTGGGCCACA---CATGCCTGTGTACCCACAGAT---
CCTAATCCACAAGAAATGGTAATGGAA---------AATGTGACAGAAAATTTTAAC----------------
--------------------------ATGTGGAAAAATAACATGGTAGAACAAATGCATGAGGATGTA---
ATCAGTTTATGGGATCAAAGCCTA---AAGCCATGTGTAAAATTA---
ACACCACTCTGTGTTACTTTAAATTGTACTAATTATACTGGTACCAGTAATAGTAGTAGTAATGGTACCAGTA
AT---
----------AGTAGTAGTAATAGTACCAGTAATAGTAGTATAAATATGGAGGGAGAAATAAAA---
AACTGCTCTTTCAATGTCACCTCAAGCATAAAT---------------------------
AAGGGGCTGAAAAAAGAACATGCAATT---TTTAATAGACTTGATGTAGTACCAATAGGTAAT----------

----

GATACTACAAGCTATATGATGATAAACTGTAACACCTCAGTCATTACACAG---
GCCTGTCCAAAGGTATCCTTTGAACCAATTCCCATACATTTTTGTGCCCCGGCTGGATTTGCAATTATAAAGT
GTAAT---GATAAGAAGTTCAAT------GGAACAGGACCATGTAAAAAT-----------------------
-GTCAGT---ACAGTACAATGCACACATGGAATTAGGCCTGTAGTGTCAACT---
CAACTGCTGTTAAATGGCAGCCTAGCAGAAGAAGAT------------
ATAGTAATTAGATCTGAAAATTTCTCAAACAAT---------GCTAAAACCATAATAGTACAGCTGAAC----
-----------GAGACTGTTGTA---------ATTAATTGTACAAGACCCAACAATAATACAAGAAAA-----
-AGTATAAATCTA------------GGACCAGGGAGAGCA---TTT------TTTGCAACAGGAGAA------
---------ATAATAGGAGACATA------------AGACAAGCACATTGT---AACATTAGTAGAGTA---
GCTTGGAACAACACTTTAAGACAGATAGCTAAAAAATTAAGT---------------GAACAGTTTAAT----
--------------------------------AATACTGAAACAATATTCTTTAAG------
GAACACCCAGGAGGGGACCCAGAAATACAA---ATGCACAGTTTTAATTGTGGAGGGGAA---
TTTAAGCAGCTCCAGGCAAGAGTCCTGGCTGTGGAAAGATACCTAGAGGATCAACAGCTCCTAGGGATTTGGG
GTTGCTCTGGGAAACTCATTTGCTCCACTTCTGTGCCTTGGAATGCTAGCTGGAGT-----------------
----------------------------AATAAATCTATAGATACAATTTGG------AAA------
AACATGACCTGGATGGAGTGG---GAAAGAGAAATTGACAATTACACAAAGGAAATATAC---
ACCTTACTTACAGACTCGCAGATCCAACAAGAA--

AAGAATGAAAAGGAATTATTGGAATTGGATAAGTGGGCAGACTTGTGGAGTTGGCTTGACATATCACACTGGC
TATGGTATATAAGAATATTCATAATGGTAGTAGGAGGCTTAATAGGTTTAAGAATAGTTTTTACT---
GTACTCTCTATAGTG---------AATAGAGTTAGGAAGGGATACTCACCA------
CTATCATTCGCTATTAGTTTGCTTAATGCCACAGCTATAGCAGTAGCTGAG---
GGGACAGATAGGATTATAGAAGGATTACAGAGAATTGGTAGAGCT----------------------------
--------ATTCTCAACATA------CCCAGAAGAATAAGACAGGGCGCAGAAAGGATTTGTTA---TAA
>HM204640
GCT---ACA--
GAAAAATTGTGGGTCACAGTCTATTATGGGGTACCTGTGTGG---AAAGAAGCAACC---
ACCACTCTATTTTGTGCATCAGATGCTAAAGCATATAACCCAGAG---GTACATAAT----------------
--------------GTTTGGGCCACA---CATGCCTGTGTACCCACAGAC---
CCTAGCCCACAAGAAGTAGTATTGGGA---------AATGTGACAGAAAAGTTTAAC----------------
--------------------------ATGTGGAAAAATAACATGGTAGAGCAGATGCATGAGGATATA---
ATCAGTTTATGGGATCAAAGCCTA---AAGCCATGTGTAAAATTA---
ACCCCACTTTGTGTTACTTTAAATTGCACTAATTGGAATACTACTGATTTGAATACTACTAACATT-------
----
-------------------AATAACAATAATGGTCGGGAGTACATACAGGAAGGAGAAATGAAA---
AACTGCTCTTTCAATATCACCACAAGCATAAGA---------------------------
GATAAGATGAAGAAAGAATATGCACTT---TTTTATAAACTTGATGTAGTACCAATAGATAAT----------
----

GATAAGACTAGATATAGGTTGATAAATTGTAACACCTCAGTCATTACACAG---
GCCTGTCCAAAGGTAACCTTTGAACCAATTCCCATACATTATTGTGCCCCGGCTGGTTTTGTGATTCTAAGGT
GTAAT---AATAAGACATTCAAT------GGAACAGGACCATGTACAAAT-----------------------
-GTCAGC---ATAGTACAATGTACACATGGAATTAGGCCAGTAGTGTCAACT---
CAACTGCTGTTAAATGGCAGTCTAGCAGAAGAAGAG------------
GTAGTAATTAGATCTGAAAATTTCACGGACAAT---------GCTAAAACCATAATAGTACATCTAAAT----
-----------GAATCTGTAGAA---------ATTAATTGTACAAGACCCAACAACAATACAATAAAA-----
-GGTATACATATA------------GGACCAGGGAAAGCA---TTT------TATACAACAGGAGCC------
---------ATAATAGGAAATATA------------AGACAAGCACATTGT---AACATTAGTAGAGAA---
AAATGGAATATCACTTTAGAACGGATAAAGATAAAATTAAAA---------------GAACAATTTGGG----
--------------------------------AGTAATAAAACAATAGTCTTTAAG------
CAATCCTCAGGCGGGGACCCAGAAATTGTA---ATGCACAGTTTTAATTGTGGAGGGGAA---
TTTAAGCAGCTCCAGGCAAGAGTCCTGGCTGTGGAAAGATACCTAAAGGATCAACAGCTCCTAGGGATTTGGG
GTTGCTCTGGAAAGCTCATTTGCACCACTACTGTGCCTTGGAATGCTAGTTGGAGT-----------------
----------------------------AATAAATCTCTGCATGAAATTTGG------GAT------
AACATGACCTGGATGGAGTGG---GAGAAAGAAATTAGCAATTACACAAGCTTAATATAC---
ACCTTAATTGAAGAATCGCAAAACCAACAAGAA--

AAGAATGAACAAGAACTATTGGCATTGGATAAGTGGGCAAGTTTGTGGAATTGGTTTGACATAACAAACTGGC

TGTGGTATATAAAAATATTCATAATGATAATAGGGGGTTTAGTAGGGTTAAGAATAGTTTTTACT---
GTACTTTCTATAGTG---------AATAGAGTTAGGCAGGGATATTCACCA------
ATATCGTTGGCTGTTAGTTTGCTTAATGCCACAGCTATAGCAGTAGCTGAG---
GGGACAGATAGAGTTATAGAAGCAGTACAAAGAGCTTGTGGAGCT----------------------------
--------ATTCTCCACATA------CCTAGAAGAATAAGACAGGGCTTGGAAAGGGCTTTGCAA---TAA
>HM204641
GCT---GAA---
AATTTGTGGGTCACAGTCTATTATGGGGTACCTGTGTGG---AAAGAAGCAACT---
ACCACTCTATTTTGTGCATCAGATGCTAAAGCTTATGAAACAGAG---GTACATAAT----------------
--------------GTTTGGGCCACG---CATGCCTGTGTACCCACAGAT---
CCTGACCCACAAGAATTAAAATTGGTA---------AATGTGACAGAAAATTTTAAC----------------
--------------------------ATGTGGAAAAATAACATGGTAGAACAGATGCATCAGGATATA---
ATCAGTTTATGGATGAAAGCCTA---AAGCCATGTGTAAAATTA---
ACCCCACTCTGTGTTACTTTAAATTGTACTACTTGGAACTCCACTAGTAATGTTACTAATACT----------
----
----------------------TTTATTAATAGTACTATAGGAAATATACAGGGAGAAATAAAA---
AACTGCTCTTTCAATGTCACCTCAAGCATACAA---------------------------
AATAAGATGCAGAAAGAGTATGCACTT---TTTCATAAAACTGATCTAATGTCAACAGAGGGC----------
----

AATACAAGCTATACACTGACAAGTTGTAATACCTCAGTCATTACACAG---
GCCTGTCCAAAGATATCCTTTGAACCAATCCCCATACATTATTGTGCCCCGGCTGGTTTTGCGATTCTAAAAT
GTAAT---GACAAGGAGTTCAAT------GGAACAGGACCATGTAGTGAT-----------------------
-GTCAGC---ACAGTACAATGTACTCATGGAATTAGGCCAGTAGTGTCAACA---
CAACTGCTGTTAAATGGCAGTCTAGCAGAAGGAGGG------------
ATAGTGATTAGATCTGAAAATCTCACTGATAAT---------GCTAAGACCATAATAGTGCAACTAAAT----
-----------AAAAGTATAAAA---------ATTAATTGTACAAGACCCAACAATAATACAAGAAAA-----
-AGTATACATATA------------GCACCAGGGAGAGCA---TTT------TATGCAACAGGAGAA------
---------ATAATAGGAGACATA------------AGAAACGCACATTGT---AATCTTAGTAGAACA---
GCATGGGGAGAAGCTTTACAACAGATAGTTACAGAATTAAGA---------------AAAAAGTTTGGG----
-----------------------------------AATAAAACAATAAGCTTTAAC------
CAATCCTCAGGAGGGGATCCAGAAGTTATA---ATGCACACTTTTAATTGTGGAGGGGAA---
TTTAAGCAGCTCCGGGCAAGAGTCCTGGCTGTGGAAAGGTACCTACAGGATCAACAGCTCCTAGGGCTTTGGG
GTTGCTCTGGGAAACTTATCTGCACCACTGCTGTGCCTTGGAATACTAGTTGGAGT-----------------
----------------------------AATAAAACTCATGAGGAGATTTGG------GGA------
AACATGACCTGGATGCAGTGG---GAGAGAGAAATTAACAATTACACAGGCTTAATATAC---
ACCTTAATTGAAGAATCAAATATCCAACAAGAA--

AAGAATGAACAGGACTTATTGGCATTAGATAAGTGGGAAAGTTTGTGGAGTTGGTTTAGCATATCAAACTGGC
TGTGGTATATAAGAATATTCATAATGATAGTAGGAGGCTTAATAGGTTTAAGAATAGTTTTTACT---
GTGCTTTCTATAGTG---------AATAGAGTTAGGCAGGGATACTCACCA------
TTGTCATTCGCTATTAGTTTGTTTAATGCCACAGCTATAGCAGTAGCTGAA---
GGAACAGATAGGATCATAGAAGCAGCACAAAGGCTTGGAAGAGCT----------------------------
--------ATTATCAACATA------CCTAGAAGAATAAGACAAGGCTTAGAAAGGGCTTTGCAA---TAA
>HM204642
GCT---AAA--
GAACAATTGTGGGTCACGGTCTATTATGGGGTACCTGTGTGG---AAAGAAGCAACA---
ACCACCCTATTTTGTGCATCAGATGCTAAGGCATATGATACAGAG---GTACATAAT----------------
--------------GTCTGGGCCACA---CATGCCTGTGTACCCACAGAC---
CCCAACCCACAAGAAGTAGTAATGGAA---------AATGTAACAGAAGATTTTAAT----------------
--------------------------GTGTGGAAAAATAATATGGTAGAACAGATGCATGAGGATATA---
ATCAGTTTATGGACCAGAGCCTA---AAGCCATGTGTAAAATTA---
ACCCCACTCTGTGTTACTTTAAATTGCAATAATACTATCTCTGGTAGTAGTAATATCTCTAGTAATACT----
----
-------------------AATAATGGAAGTGTGGAACTAACGATGGAGCCAGGAGAAATAAAA---
AACTGCTCTTTCAATACCACCACAAATGAAAGA---------------------------
AATAAGATGCAGAAAGAATATGCACTT---TTTTATAAACTTGATGTAGTATCAATAGATAAT----------
----

AGTAATACTAGCTATAGGTTGATAAGTTGTAACACCTCAATCATTACACAG---

GCCTGTCCAAAGGTATCCTTTGAGCCAATTCCCATACACTTTTGTGCCCCGGCTGGTTATGCGATTCTAAAGT
GTAAC---AATAAAACATTCAGT------GGAAAAGGACCATGTACAAAT-----------------------
-GTCAGC---ACAGTGCAATGTACACATGGAATTAAGCCAGTGGTGTCAACT---
CAGCTACTGTTAAATGGCAGTTTAGCAGAAGAAGGT------------
ATAGTAATTAGATCTGAAAATATCACAAACCCG---------ACTAAGACCATAATAGTACAGCTGAAC----
-----------GAGTCTGTAGAA---------ATTAACTGTACAAGGCCCAACAACAACACAAGAAGA-----
-GGTATACATGTA------------GGACCAGGGAGAGCA---ATT------TATACAACAGGA---------
---------ATAATAGGAGATATA------------AGGCAAGCACATTGT---AACATTAGTAGAGAA---
AAATGGAACCAAACTTTAGGAAGGATAGCTACAAAATTAGGA---------------GGCATATTT-------
-----------------------------------AATAAAACAATAGTCTTTAAG------
CAACACTCAGGAGGGGACCCAGAAGTTGTA---ATGCACAGTTTTAATTGTAGAGGGGAA---
TTTAAACAGCTCCAGGCAAGACTCCTGGCTGTGGAAAGATACCTAGCGGATCAACAGCTCCTAGGGATTTGGG
GTTGCTCTGGAAAACTCATTTGCACCACTGCTGTGCCTTGGAATGCTAGTTGGAGT-----------------
----------------------------AATAAATCCCTAAATGAGATTTGG------GAC------
AACATGACCTGGATGCAGTGG---GAAAAGGAAATTGACAATTACACAAACACAATATAC---
AGTTTGATTCTAGACTCGCATAACCAACAAGAA--

AAGAATGAACAAGAATTATTGGAATTAGATAAGTGGGCAAGTCTGTGGAATTGGTTTGACATAACAAACTGGC
TGTGGTATATAAAAATATTCATAATGATAGTAGGAGGCTTGGTAGGTTTAAGAATAGTTTGTGCT---
GTATTTTCTATCATG---------AATAGAGTTAGGCAGGATACTCACCA------
TTGTCGTTCGCTGTTAGCTTGCTCAATGCCATAACTATAGCAGTAGCTGAG---
GGGACAGATAGGATTATAGAAGTAGCACAAAGAGCTTATAGAGCT----------------------------
--------TTTCTCCACATA------CCTGTAAGAATAAGACAAGGCTTGGAAAGGGCTTTGCAA---TAA
>HM204643
GCT---GCA--
GAAGATTTGTGGGTCACAGTTTATTATGGGGTACCTGTATGG---AAAGATGCAATC---
ACCACTTTATTTTGTGCCTCAGACGCTAAAGCGTATGATGCAGAG---GTGCATAAT----------------
--------------GTTTGGGCCACA---CATGCCTGTGTACCCACAGAC---
CCCAACCCACAAGAAATAAGATTGGAA---------AATGTGACAGAAAATTTTAAC----------------
--------------------------ATGTGGAAAAATAACATGGTTGAACAGATGCATGAAGATATA---
ATCAGTTTATGGGATCAAAGCCTA---AAGCCATGTGTAAAATTG---
ACCCCACTCTGTGTCACTCTAAATTGCACTGATTTGAACAATACTACTAAAACCAGTAAT-------------
----
----------------------------AATACAGGAGAAGCAGGAGAGGGAGTAGAAATGAAA---
AACTGCTCTTTCAACATCACCACAAGCATGAAA---------------------------
GATAAGATAAAGAAAGAGCATGCACTT---TTTTATACACTTGATATAGTACCAATAGATAAT----------
----

AATGCTAATACTAGCTATAGGTTGGTAAGTTGTAATACCTCAGTCATTACACAG---
GCCTGTCCAAAAGTATCCTTTGAGCCAATTCCCATACATTATTGTGCCCCAGCTGGTTTTGCGATTCTAAAGT
GTAAT---AATAACACGTTCAAT------GGAACAGGACCATGTACAAAT-----------------------
-GTTAGC---ACAGTACAATGTACACATGGAATTAAACCAGTAGTATCAACT---
CAACTGCTGCTAAATGGCAGTTTAGCAGAAGGAGAG------------
GTAATGATTAGATCTGAAAATTTCACAAACAAT---------GCTAGAACCATAATAGTACAGCTGAAC----
-----------AAAACTGTAGAA---------ATTAATTGTACAAGACCCAACAACAATACAAGAAGA-----
-AGTATACCTATA------------GGGCCAGGAAGAGCA---TTT------TATGCAACAGGACAA------
---------ATAATAGGAGATATA------------AGAAAAGCATATTGT---AATATTAGCAGAGCA---
CAGTGGAATAACACTTTAAAACAGATAGTTGGAAAATTAAGA---------------GAACAATTTGGA----
--------------------------------AATAATAAAACAATAATCTTTAAG------
CCACACTCAGGAGGGGACCCAGAAATTGTA---ACACACAGTTTTAATTGTGGGGGGAA---
TTTAAGCAGCTCCAGGCAAGAATCCTGGCTGTGGAAAGATACCTAAAGGATCAACAGCTCCTGGGGATTTGGG
GTTGCTCTGGAAAACTCATCTGTACCACTACTGTGCCTTGGAATACTAGTTGGAGT-----------------
----------------------------AATAAGTCTTTGGATGCAATTTGG------CAA------
AACATGACTTGGATGGAGTGG---GATAGAGAGATTAATAATTACACCAAATTAATATAC---
ACCTTAATTGAAGAATCGCAAAACCAACAAGAA--

AAGAATGAAAAAGAATTATTAGAATTAGGTAAGTGGGACAGTCTGTGGAGTTGGTTTGACATAACAAAATGGC
TGTGGTATATAAAAATATTCATAATAATAGTAGGGGGTTTGGTAGGTTTAAGAATAGTTTTTGCT---
GTACTTTCTATAGTG---------AATAGAGTTAGGCAGGATACTCACCA------
CTGTCATTGGCTGTTAGTTTGCTTAATGCTACAGCCATAGCAGTAGCTGAA---

AGGACAGATAGGATCATAGAAGTATTACAAAGAATTTTTAGGGGT----------------------------
--------ATTCTCCACATA------CCTAGAAGAATAAGGCAGGGCTTGGAAAGAATTTTGCTA---TAA
>HM204646
GCT---ACA--
GAACCATTGTGGGTCACAGTCTATTATGGAGTACCTGTGTGG---AAAGAAGCAACC---
ACCACTCTATTTTGTGCATCAGATGCTAAAGCATATGATACAGAG---GCACATAAT----------------
--------------GTTTGGGCCACT---CATGCCTGTGTACCCACAGAC---
CCCAGCCCACAAGAAGTATTCTTGGAA---------AATGTGACAGAAAATTATAAC----------------
--------------------------ATGTGGAAAAATAACATGGTAGAACAAATGCATGAGGATATA---
ATCAGTTTATGGGATCAAAGCCTA---AAGCCATGTGTAAAATTA---
ACCCCACTCTGTGTTACTTTAAATTGCACTGATGAATTGAGGAATAAAACTGATTGGAGGAATCCA-------
----
----------------------AATCAGACGAATGATACAAAGATAGAGAAAGGGGAAATAAAA---
AACTGCTCTTTTGATACCTCCCCAAGCACAACA---------------------------
GACAAAAAACAGAAAGCATATGCACTT---
TTTTATAAGCTTGATGTAGTACAAATAGATGATGTTAATAATAGT----------------------------
----
ACTAATGCTACTGCTAACTATACTAGTTATAGGTTGATAACTTGTAACACCTCAGTCATTACACAG---
GCCTGTCCAAAGATATCCTTTGAGCCAATTCCCATTCATTATTGTGCCCCAGCTGGTTTTGCGATTCTAAAGT
GCAAT---GATAAGAAGTTCAAT------GGAACAGGGCTATGTAGAAAT-----------------------
-GTCAGC---ACAGTACAATGTACACATGGAATTAGGCCAGTAGTGTCAACT---
CATTTACTGTTAAATGGCAGTCTAGCAGAAGAAGAG------------
GTAGTAATTAGATCTCAAAATTTCACGAACAAT---------GCTAAAACCATAATAGTACAGCTGAAG----
-----------ACACCTGTAAAC---------ATTACTTGTACAAGACCCAGCAACAACACAAGAAAA-----
-GGTATACATATG------------GGACCAGGGAGAGCA---TTT------TATGCAACAGGAGAC------
---------ATAATAGGAGATATA------------AGACAAGCACATTGT---AACATTAGTGAAGGA---
GATTGGGTGAGTGCGTTAAACCAAACAGTTGAAAAATTAAAA---------------GAACAATTTAAG----
-----------------------------------AATAAGACAATACAGTTTCAG------
CCACCCTCAGGAGGGGACCCAGAAGTTGAG---ATGCACACTTTTAATTGTGGAGGGGAA---
TTTAAACAGCTCCAGGCAAGAGTCCTGGCTGTGGAAAGATACCTAAAGGATCAACAGCTCCTAGGGCTTTGGG
GTTGCTCTGGAAAACTCATCTGCACCACTGCTGTGCCTTGGAATAATAGTTGGAGT-----------------
----------------------------AATAAATCTCTGGATAAGATTTGG------AAT------
AACATGACCTGGATGGATTGG---GAAAAAGAAATTGACAATTACACAAACTTAATATAT---
GACTTACTTCAAGAATCGCAGAACCAACAAGAA--

AAGAATGAACAAGAATTATTGGAATTGGATAAATGGGCAAGTCTGTGGAATTGGTTTAGCATAACTAACTGGC
TGTGGTATATAAAAATATTCATAATGATAGTAGCAGGCTTGATAGGTTTAAGGATAGTTTTTGCT---
GTGCTTTCTATAGTG---------AATAGAGTTAGGCAGGGATACTCACCA------
TTGTCATTTGCTATTAACTTGCTCAATACTACAGCTATAGCAGTAGCTGAA---
GGGACAGATAGGGTTATAGAAATAATACAAAGAACTTGTAGGGGC----------------------------
--------ATCCTCCATATC------CCTAGAAGAATAAGACAGGGCTTCGAAAGGGCTTTACTA---TAA
>KY658697
GCT---ACA--
GGACAATTGTGGGTCACAGTTTATTATGGGGTACCTGTGTGG---AAAGAAGCAGAC---
ACCACTTTATTTTGTGCATCAGATGCTAAAGCATATGAACGCGAG---GCACATAAT----------------
--------------GTTTGGGCCACA---CATGCCTGTGTACCCACAGAC---
CCCAACCCACAAGAACTAGTATTGGAA---------AATGTGACAGAAAATTTTAAC----------------
--------------------------ATGTGGAAAAATAACATGGTAGAACAGATGCATGAGGATATA---
ATAAGTCTATGGGATCAAAGCCTA---AAGCCATGCGTAAAATTA---
ACCCCACTCTGTGTTACCTTAACTTGCATTGATTTGAAGAATGGTACTAATACCACT----------------
----
----------------------------AGTGGTATCAAAATAGAGGGAAGAGAAGAAATGGCA---
AACTGCTCTTTCAATGTCACCACAAACATAAGA---------------------------
GATAAGGTGCAGAAAGAACATGCAATT---TTTTATAAGCTTGATGTAGAACCAATGACTAAGAAT-------
----

GATAATACCAGCTTTAGGTTAATAAGTTGTAACACCTCAGAGATTAGACAG---
GCCTGTCCAAAGGTATCCTTTGAGCCAATTCCCATACATTATTGTGCCCCGGCTGGTTTTGCGATTCTAAAGT
GTAAG---AATAAGAATTTCACT------GGAACGGGACCATGTACAAAT-----------------------
-GTCAGC---ACAGTACAATGTACACATGGTATTAGGCCAGTAGTATCAACT---

CAACTGCTATTAAATGGCAGTCTAGCAGAAGGAGAG------------
GTAGCAATTAGATCTAGCAATTTCACAAACAAT---------GCTAAAATCATAATAGTACAGTTGAAT----
-----------GAATCTGTAGCA---------ATTAATTGTACAAGAGTCAACAACAATACAAGAAAA-----
-AGTATACATATG------------GGACTAGGGGGAGCA---TGG------CATACAACAGGGAAT------
---------ATAATAGGAGATATA------------AGAAAAGCACATTGT---AACCTTAGTAGAGAA---
GCATGGAAGAACACTTTAAAACAAATAGTTACAAAATTAGGA---------------GAACAATTTGGG----
--------------------------------AATACTAGCAAAATAATCTTCAAC------
CAATCCTCAGGAGGGGACATCGAAATTGTA---ACGCACAGTTTTAATTGTGGAGGGGAA---
TTTAAGCAGCTCCAGGCAAGAGTCCTGGCTGTAGAAAGATACCTAAGGGATCAACAGCTCCTGGGGATTTGGG
GCTGCTCTGGAAAACTCATTTGCACCACTACTGTGCCTTGGAATGTTAGCTGGAGT-----------------
----------------------------AATAAATCTCTGGATGGGATTTGG---------------
AACATGACCTGGATGGAGTGG---GAAAAAGAAATTGATAATTACACAGGCTTGATATAC---
AAATTAATTGAAGAATCGCAAAACCAGCAGGAA--

AAGAATGAACAAGAATTGTTGGAATTGGATAAATGGGCAAGTTTGTGGAATTGGTTTGACATAACAAGCTGGC
TGTGGTATATAAAAATATTCATAATGATAGTAGGAGGCTTGATAGGTTTAAGAATAGTTTTTACT---
GTGCTTTCTATAGTG---------AGAAGAGTTAGGCAGGGGTACTCACCA------
ATATCATTTGCTGTAAGCTTGCTCAATACCATAGCCATAGCAGTAGCTGAG---
GGGACAGATAGGGTTATAGAAGTAGTACAAAGAGCTTGTGGAGCT----------------------------
--------ATTCTCCACATA------CCTAGAAGAATAAGACAGGGCTTGGAAAGGCTTTGCAA---TAA
>HQ595776
GCT---ACA--
GAGCAATTGTGGGTCACAGTCTATTATGGGGTACCTGTGTGG---AAAGAAGCAACT---
ACTACTCTATTTTGTGCATCAGATGCTAAGGCATATAAAACAGAA---AAACATAAT----------------
--------------GTTTGGGCCACA---CATGCCTGTGTACCCACAGAC---
CCCAACCCACAGGAAATATTATTGAGA---------AATGTAACAGAAAATTTTAAT----------------
--------------------------GTGTGGAAAAATGATATGGTAGAACAGATGCATGAGGACATA---
ATCAGTTTATGGATCAAAGCCTA---AAGCCATGTGTAAAATTA---
ACCCCACTCTGTGTTACTCTAAGTTGCACTGATTTGAATACTACTAGTGCCAATAATGCTACTAGTGCCAATG
GTACT--
-------ATTAGTGCCAATACTAGTGCTAGTGCCAATATTAGTAGTGAGATGGAAATGATGAAA---
AACTGCTCTTTCAATATCACCGCAAGCATACAA---------------------------
GATAAGAAACAGAGAGAATATGCACTT---TTTTATAAACTTGATATAGTACCAATAGATGAT----------
----

AGTAATAATAGTACCAACTATAGATTGATAAGTTGTAACACCTCAGTCATTACACAG---
GCCTGTCCAAAAGTATCCTTTGAGCCAATTCCCATACATTTTTGTGCCCCGGCTGGTTTTGCGATCCTACAAT
GTAAT---AATAAGAATTTCAGT------GGAAAAGGAGACTGTAACAAT-----------------------
-GTCAGT---ACAGTACAATGTACTCATGGAATTAAGCCAGTAGTATCAACC---
CAACTGCTGTTAAATGGCAGCCTAGCAGAAGAAGGG------------
GTAGTAATTAGATCTGAAAATATCTCAAATAAT---------GCTAAGACCATAATAGTACAACTAAAA----
-----------GAGCCTGTACAC---------ATTAATTGTACAAGACCCAGCAACAATACAAGAAAA-----
-GGTATACATATA------------GGACCAGGGAGGGCA---TTT------TATGCAACAGGACAA------
---------ATAACAGGAGATATA------------AGACAAGCACATTGT---AACATCAGTGTAGGA---
AGATGGAATGCCACTTTAGAACAGGTAGCTAACAAATTAAGA---------------GAACATTTTGTT----
-----------------------------------AATAAAACAATAGTCTTTAAT------
CAATCCTCAGGAGGAGACCCAGAAGTTGTA---ATGCATACTTTTAATTGTGGAGGGGAA---
TTTAAGCAGCTCCAGGCAAGAGTCCTGGCTGTGGAAAGATACCTAAGGGATCAACAGCTCATGGGAATTTGGG
GTTGCTCTGGAAAACTCATTTGCACCACTACTGTGCCTTGGAATACTAGTTGGAGT-----------------
----------------------------AATAAATCTCTGAATGACATTTGG------GAG------
AACATGACATGGATGCAGTGG---GAAAGAGAAATTGACAATTACACAGGATTAATATAT---
AACTTAATTGAAGAATCGCAAAACCAACAAGAA--

AAGAATGAACAAGATTTATTGGCATTGGACAAATGGGCAGATCTGTGGAATTGGTTTAGCATAACACATTGGC
TGTGGTATATAAGAATATTCATAATGATAGTAGGAGGTTTAATAGGTATAAGAATAGTTTTTACCTGGTGCCT
TTCTATAGTG---------AATAGAGTTAGGAAGGATACTCACCA------
TTATCGTTCGCCATTAGCTTGCTTAATACTACAGCCATAGCAGTAGCTGAG---
GGGACAGATAGGGTTATAGAAATATTCCAAAGAGCTGGTAGAGGT----------------------------
--------ATCCTCCATATA------CCTAGAAGAATAAGACAGGGCCTGGAAAGAATTTGCTA---TAA
>HQ595778

GCT---ACA---------------------------------------
GAACATTTGTGGGTCACAGTCTATTATGGGGTACCTGTGTGG---AAGGAAGCAAAC---
ACCACTCTATTTTGTGCATCAGATGCTAAAGCATATGATACAGAG---GTGCATAAT---------------
--------------GTTTGGGCCACA---CATGCCTGTGTACCCACAGAC---
CCCGACCCACAAGAAATAGTATTGGAA---------AATGTGACAGAAAATTTTAAC---------------
-------------------------ATGTGGAAAAATAACATGGTAGAACAGATGCATGAGGATATA---
ATCAGTTTATGGGATCAAAGCCTA---AAGCCATGTGTAAAATTA---
ACCCCACTTTGTGTTACTTTAAATTGCACTGACTTGAGTAATACCACTAATAGTAATGGAACGATT-------
----
----------------------ACTAATAGTGGTAATAGAACGATATGGGATAAAGAAATGAAA---
AACTGCTCTTTCAATATCACCACAAAAATAACA--------------------------
GAAAAGGTAAAGAAAGAATATGCACTT---TTTTATGAACTTGATATAGTACCAATAGAAGATGAT-------
----

AACGAGAGCAATAATAGCTATAGGTTGAGAAGTTGTAACACCTCAGTCATTACACAG---
GCCTGCCCAAAGGTATCCTTTGATCCAATTCCCATACATTATTGTGCCCCGGCTGGCTTTGCGATTCTAAAGT
GTAAT---GATAAGAAGTTCAAT------GGAACAGGACCATGTACAAAT----------------------
-GTCAGT---ACAGTACAATGTACACATGGGATTAGGCCAGTAGTATCAACT---
CAACTGCTGTTAAATGGCAGTCTAGCAGAAGAAGAG-----------
GTAATAATTAGATATGAAAATGTCGAGGACGGT---------GCTAAAGCCATAATAGTACAACTAAAT----
-----------GAAACTGTAAAA---------ATTAATTGTACAAGACCCAACAACAATACAAGAAAA-----
-AGTATACATCTA------------GGACCAGGGAGAGCA---TTT------TATGCAACAGGAGAC------
---------ATAATAGGGGATATA------------AGACAAGCACATTGT---AACCTTAATAGAACA---
AAATGGAATGACACTTTAAGACAGATCGGTATGAAATTAGGA---------------GAACAATTTAAG----
------------------------------------AATAAAACAATAGTCTTTAAG------
CCATCCTCAGGAGGAGACCCAGAGATTGTA---ATGCACACTTTCAATTGTGGAGGGGAA---
TTTAAGCAGCTCCAGGCAAGACTCCTGGCTGTGGAAAGATACCTACAGGATCAGCAGCTCCTGGGGATTTGGG
GTTGCTCTGGAAAACTCATTTGCACCACTGCTGTGCCTTGGAATGCTAGTTGGAGT----------------
----------------------------AACAAATCTCTGAATGCTATTTGG------AAT------
AACATGACCTGGATGGAGTGG---GAAAGAGAAATTGACAATTACACAAGCTTAATATAC---
ACCTTAATTGAAACATCGCAAAACCAACAAGAA---------------------------------------
----
AGAAATGAACAAGAATTATTGGAATTGGATAAGTGGGCAAGTTTGTGGAATTGGTTTGACATAACAAACTGGC
TGTGGTATATAAAAATATTCATAATGATAGTAGGAGGCTTGATAGGTTTAAGAATAGTCTTTACT---
GTACTTTCTATAGTA---------AATAGAGTTAGGCAGGGATACTCACCA------
TTATCGTTTGCTGTTAGCTTGCTTAATGCCACAGCCATAGCAGTAGCTGAG---
GGAACAGATAGGGTTATAGAAATATTACAAAGAGTTGTTAGAGCT---------------------------
--------ATCCTCAACATA------CCTACAAGAATAAGACAAGGCTTGGAAAGGCTTTTACTA---TAA
>HQ595779
GCT---ACA---------------------------------------
GAACAATTGTGGGTCACAGTCTATTATGGGGTACCTGTGTGG---AAAGAAGCAACC---
ACCACTCTATTTTGTGCATCAGATGCTAAAGCATATGACACAGAG---GTACATAAT---------------
--------------GTTTGGGCCACG---CATGCCTGTGTACCCACAGAC---
CCTAACCCACAAGAAGTAGAATTGAAA---------AATGTGACAGAAAATTTTAAT---------------
-------------------------ATGTGGAAAAATGACATGGTAGAACAGATGCATGAGGATATA---
ATCAGTTTATGGGATCAAAGCCTA---AAGCCATGTGTAAAATTA---
ACCCCACTCTGTGTTACTTTAGAATGCAGTAATTTGAATACTACCATTATTACCAATACTAGTACC-------
----
----------------------AATAATAGTACTTGGGAAGGGTTGGGGAAAGGAGAAATAAAA---
AACTGCACGTTCAACGTCACCACAGGCAGAAGA--------------------------
AAAGGGATGCAGAAGGAATATGCGTTT---TTTTCTGAACTTGATATAGTACCAATAGAGAAC---------
----

AATAATACAAGCTATACATTGACAAGTTGTAACACCTCAGTCATTACACAA---
GCCTGTCCAAAAGTATCCTTTGAGCCAATTCCCATACATTATTGTACCCCGGCTGGTTATGCGATTCTAAAAT
GTAAG---GATAAGAAGTTCAAT------GGGACAGGACCATGTAAAAAT----------------------
-GTCAGC---ACAGTACAATGCACACATGGAATTAGGCCAGTGGTGTCAACT---
CAATTACTGTTAAATGGCAGTCTAGCAGAAGAAGAG-----------
GTAGTAATTAGATCTGAAAATATCACAAACAAT---------GCTAAAACCATAATAGTACAGCTGAAG----
-----------GAACCTGTAGAA---------ATTAATTGTACAAGACCCCACAACAATACAAGAAAA-----

-AGTATATCTACA------------GGACCAGGGAGAGCA---ATT------TATGCAACAGGACAT------
---------ATAATAGGAGATATA------------AGACAAGCACATTGT---AACATTAGTTCACAA---
AAATGGAATAATACTTTGCGACAGATAGTTGAAAAACTAAGA---------------GTACAATTTGGG----
-----------------------------------AATAAAACAATAGTCTTTAAT------
AAATCCTCAGGAGGGGACCCAGAAATTGTA---ATGCACAGTTTTAATTGTGGAGGGGAA---
TTTAAGCAGCTCCAGGCAAGAGTCCTGGCTGTGGAAAGATACCTGCAGGATCAACAGCTCCTGGGGCTTTGGG
GTTGCTCTGGAAAACTCATTTGCACCACTGCTGTGCCTTGGAATACTAGTTGGAGT-----------------
----------------------------AATAAAACTCTGGATAGGATTTGG------GAT------
AACATGACCTGGATGGAGTGG---GAGAAAGAGATTAACAATTACACAAGTTTAATATAC---
ACCTTAATTGAAGAATCGCAGCACCAACAAGAG--

ATAAATGAACAAGAGTTATTCAGATTGGATACGTGGGCAAGTTTGTGGAATTGGTTTGACATATCAAACTGGC
TGTGGTATATAAAAATATTCATAATGATAGTAGGAGGCTTAGTAGGTTTAAGAATAGTTTTTGCT---
GTACTTTCTATAGTA---------AGAAGAGTTAGGCAGGGATACTCACCA------
GTATCATTGGCTGTTAGTTTGCTTAATGCCACAGCTATAGCAGTAGCTGAG---
GGGACAGATAGGATTATAGAAGTAGTACAAAGAGCCTGTAGAGCT----------------------------
--------ATTGTCCACATA------CCTACAAGAATAAGACAAGGCTTTGAAAGGGCTTTACTA---TAA
>HQ595788
ACT---ACA--
GAACAATTGTGGGTTACAGTCTATTATGGGGTACCAGTATGG---AAAGAAGCAGTC---
ACTACTCTATTTTGTGCCTCAGATGCTAAAGCATATGATACAGAG---GTACATAAT----------------
--------------GTTTGGCCACA---CATGCCTGTGTACCTACAGAC---
CCCAGCCCACAAGAAATAGTATTGGGA---------AATGTGACAGAATATTTTAAC----------------
--------------------------ATATGGGAAAATAACATGGTGGAACAGATGCATGAGGATATA---
ATCAGTTTATGGGATCAAAGCCTA---AAGCCATGTGTAGAATTA---
ACCCCACTCTGTGTTACTCTAAATTGCACTGATGCTGTAAAAAATAGTACCAGT-------------------
----
----------------------------------ACCAGAAATGAAACGAATGGAGAACTAAAA---
AACTGCTCTTTCGATATCAACTCAGGCATAAGA---------------------------
GATAAGATACAGAGAGAATATGCTCTT---TTTTATACACTTGATGTAGTGTCAATAGAGAAT----------
----

GATAATAAAAGCTATAGGATGATAAATTGTAACACCTCAGTGATTAAACAA---
GCCTGTCCAAAGGTATCCTTTGAACCAATTCCTATACATCTTTGTACCCCGGCTGGTTTTGCGCTCCTAAAGT
GTAAC---AATAAGACATTCAAT------GGAAAAGGACCATGTACAAAT-------------------------
-GTCAGC---ACAGTACAGTGTACTCATGGAATTAGACCAGTAGTATCAACT---
CAACTGTTGTTAAATGGCAGCCTAGCAGAAGAACAA------------
ATAGTAATTAGATCAAAAAATTTCTCAAATAAT---------GCTTATACCATAATAGTGCAGCTGAAT----
-----------GAAACGATAAAA---------ATTAATTGTACAAGACCCAACAATAATACAAGAAGA-----
-GGTATACATATA------------GGACCAGGGCAAGCA---TTT------TATACTACA---GAC------
---------ATAATAGGAGATATA------------AGACAAGCACATTGT---AACATCAGTGAAGTA---
AAGTGGAATAAGACTTTACAACAGGTAGCTAGCAAACTAAGA---------------GAGCAATTTAGG----
-----------------------------------AACAAGACAATAATATTTAAT------
CGTACCTCAGGAGGAGACCCAGAAATTGTA---ATGCATAGTTTTAATTGTAGAGGGGAA---
TTTAAGCAGCTCCAGGCAAGAGTCCTGGCTATGGAAAGATACCTAGGGGATCAACAGCTCTTAGGGATTTGGG
GTTGCTCTGGAAAACTCATTTGCACCACTGCTGTGCCTTGGAATGCTAGTTGGAGT-----------------
----------------------------AATAAATCCCTAGAGCAGATTTGG------AAT------
AACATGACCTGGATGCAGTGG---GAAAGAGAAATTGACAATTACACAGGCATAATATAT---
AACTTACTTCAAGAATCGCAAAACCAGCAGGAA--

AAGAATGAACAAGAATTGTTGGAATTGGATAAGTGGGCAAGTTTGTGGAGTTGGTTTGACATAACAAATTGGC
TGTGGTATATAAGAATATTCATAATGATAGTAGGAGGCTTGGTAGGTTTAAGAATAATTTTTGCT---
GTACTTTCTATAGTG---------AATAAGGTTAGGCAGGGATATTCACCA------
ATATCATTGGCTGTCAGCTTGCTCAACGTCACAGCAATAGCAGTAGCAGAG---
GGGACAGATAGGGTTATAGAATTAATACAAAGAGCTGGTAGAGCT----------------------------
--------GTCCTCCACATA------CCTAGAAGAATAAGACAGGGCTTCGAAAGGTGTTTACTA---TAA
>HQ595790
GCT---ACA--
GAAAAATTGTGGGTCACAGTCTATTATGGAGTACCTGTGTGG---AAAGAAGCAACC---
ACCACTCTATTTTGTGCATCAGATGCTAAAGCATATGATACAGAG---GTGCATAAT----------------

--------------GTTTGGGCCACA---CATGCCTGTGTACCCACAGAC---
CCAAACCCACAAGAGATAAAATTGGAA---------AATGTGACAGAAGAGTTTAAC----------------
--------------------------ATGTGGAAAAATAACATGGTAGAACAAATGCATGAGGACATA---
ATCAGTTTATGGGATCAAAGCCTA---AAGCCATGTGTAGAATTA---
ACCCCACTCTGTGTTACCTTAAATTGCACTGATAAAATTGGCAATAATACCAGTAGTGCCAATAATAGT----
----
----------------AGTTGTAATACCAATAGTAGTTGTGTTAAGACGGGGGGAGAAATGAAA---
AACTGCTCTTTCAATATCACCACAGACCTGAGA---------------------------
GACAAGGTACGGAAGGAGCATGCACTC---TTTTATACAATTGATATAGTACCACTAGATGAT----------
----

AATAGTACCAGCTATAGGTTGACAAGTTGTAACACCTCAACCATTAAACAG---
GCCTGTCCAAAGGTATCCTTTGAGCCAATTCCCATACATTATTGTGCCCCGGCTGGTTTTGCGATTCTAAAAT
GTAAT---GATACGGACTTTGAT------GGAAAAGGAAATTGTACAAAT-----------------------
-GTCACT---ACAGTACAATGTACACATGGAATTAGGCCAGTAGTATCAACT---
CAACTGCTGCTAAATGGCAGTTTAGCAGAAAAGGAG------------
GTAGTAATTAGATCTGACAACATCTCGGATAAT---------ACTAAGAACATAATAGTACAACTAAAA----
-----------GAAGCTGTAGAA---------ATTAATTGTACAAGACCCAACAATAATACAAGAAAA-----
-AGGATAACTATG------------GGACCAGGGAGAGTA---TTG------TATGCAACAGGAGAG------
---------ATAATAGGAGACATA------------AGACGAGCACATTGT---AACATTAGTGAAAAA---
GAATGGAATAACGTCTTAAAGCAGATAGTTAAAAAATTAGGA---------------GAAAAGTTTAAA----
-----------------------------------AATAATACAATAGCCTTTAAT------
AAATCCACAGGAGGGGACCTTGAAGTTGTA---ATGCACACTTTTAATTGTGGAGGGGAA---
TTTAAACAGCTCCAAGCAAGACTCCTGGCTGTGGAGAGATACCTAAGGGATCAACAGCTCCTGGGGCTTTGGG
GATGCTCTGGAAAACTCATTTGCACCACTACTGTGCCTTGGAATACTAGTTGGAGT-----------------
----------------------------AATAGATCTCTGGATATGATTTGG------GGG------
AACATGACCTGGATGCAGTGG---GAAAAAGAAATTGACAATTACACAGGAATAATATAC---
CAATTAATTGAAGAATCGCAGAACCAACAAGAA--

AAGAATGAGCAAGACTTGTTGGAATTGGATCATTGGGCAAGTCTGTGGAATTGGTTTGACATAACAAACTGGC
TTTGGTATATAAAAATATTCATAATGATAGTAGGAGGCTTAATAGGTTTAAGAATAGTTTTTACT---
GTACTTTCTATAGTG---------AATAGAGTTAGGGAGGGATACTCACCA------
CTATCGTTTGCTGTTAGCTTGCTTAATGCCACAGCCATAGCAGTAGCTGAG---
GGGACAGATAGGGTTATAGAAGCAATACAAAGAGCTTATAGAGCT----------------------------
--------ATTCTCCACATA------CCTAGGAGGATAAGACAGGGGCTTGAAATAGCTTTGCTA---TAA
>HQ595792
ACA---ACA--
GGAATGTTATGGGTCACAGTCTATTATGGAGTACCTGTGTGG---AAAGAAGCAACT---
ACCACTTTATTTTGTGCATCAGATGCTAAAGGATATAAGACAGAG---GTACATAAT----------------
--------------GTTTGGGCCACA---CATGCCTGTGTACCCACAGAC---
CCCCACCCACAAGAGGTAGTATTGGCA---------AATGTGACAGAAAATTATAAC----------------
--------------------------ATGTGGAAAAATAACATGGTAGAACAGATGCATGAGGATATA---
ATCAGTTTATGGGATCAAAGTCTA---AAGCCATGTGTAAAATTA---
ACCCCACTCTGTGTTACTTTAAATTGCACTGAGTTGAGCAATGTTAATGGTACTATCAACAATAGTACAGGG-
----
----------------GGAGAAATGAAAAATACTATCAACAATAGTACAGGGGGAGAAATGAAA---
AACTGTTCTTTCAATATCACCACAAGCATACGA---------------------------
GATAAGATGCAGAAAGAATATGCACTT---TTTTATAAACTTGATATCACACCAATAGATGGC----------
----

AGTGAGAATGATACTAAGTATAGGTTAATAAGTTGTAATACCTCAGTCCTTACACAG---
GCCTGTCCAAAGATATCCTTTGAGCCAATTCCCATACATTATTGTGCCCCGCCTGGTTTTGCGATTCTAAAGT
GTAAC---AATAAGACATTCGAT------GGAAAAGGACCATGTACAAAT-----------------------
-GTCAGC---ACAGTACAATGTACACATGGAATCAGACCAGTAGTGTCAACT---
CAATTACTGTTAAATGGCAGTCTAGCAGAAGAAGAG------------
GTAGTAATTAGATCTGACAATTTCTCAAACAAT---------GCTAAGGTCATAATAGTACAGCTGAAT----
-----------GAAACTATACAA---------ATTAATTGTACTAGGCCTAACAACAATACAAGAAAA-----
-GGTATAACTATA------------GGACCAGGGAGAGCA---TTC------TACACAACAGGAGGA------
---------ATAATAGGAAATATA------------AGACAAGCACATTGT---AACCTTAGTAGAAAG---
GCATGGAATAACGCAATAAAACAGATAGTTACAAAATTAAGA---------------ACACCATTTGGA----

-----------------------------------AATAAAACTATAGTCTTTGAA------
CCACCCTCTGGAGGAGACCCAGAAATAGTA---ACACACAGTTTTAATTGTGGAGGGGAA---
TTCAAGCAGCTCCAGGCAAGAGTCCTGGCTGTAGAAAGATACCTAAAGGATCAACAGCTCCTAGGGATTTGGG
GTTGCTCTGGAAAACTCATTTGCACCACTACTGTGCCTTGGAATTCTAGTTGGAGC----------------
----------------------------AATAAATCTTATAATGATATTTGG------AAT------
AACATGACCTGGATGGAGTGG---GAAAGAGAAATTGACAATTACACAAGCACAATATAC---
ACCTTGCTTGAAGAATCGCAAAACCAGCAGGAA---------------------------------------
----
AAGAATGAACAAGAATTATTGGCATTAGATAAGTGGGCAAGTTTGTGGAGTTGGTTTGACATAACAAATTGGC
TGTGGTATATAAAAATATTCATAATGATAGTAGGAGGCTTGGTAGGCTTAAGAATAGTTTTTGCT---
GTACTTGCTTTAGTA---------AATAGAGTTAGGCAGGGATACTCACCA------
TTATCATTTGCTGTCAGCTTGCTTAACGCTACAGCTATAGCAGTAGCTGAG---
GGGACAGATAGGGTTATAGAAGTAGTACAAAGAATTGGTAGAGCT---------------------------
--------ATTCTTAACATA------CCTAGGAGAATAAGACAGGGCTTTGAAAGGGCTTTGCAA---TAA
>HQ595794
GCT---ACA---
GAAAAATTATGGGTCACAGTCTATTATGGGGTACCTGTGTGG---AAGGAAGCAACC---
ACCACTTTATTCTGTGCATCAGATGCTAAAGCATATGAGACAGAG---GTACATAAT---------------
--------------GTTTGGGCCACA---CATGCCTGTGTACCCACAGAC---
CCCGACCCACGAGAAGTATCATTGGGA---------AATGTGACAGAACCTTTTAAC---------------
-------------------------ATGTGGAAAAATGACATGGTAGAACAAATGCAGGAGGATATA---
ATCAGTTTATGGGATCAAAGCCTA---AAGCCATGTGTAAAATTA---
ACTCCACTCTGTGTTACTTTAAATTGCACTGATGTGAATGTTAATAGAACCACTGTTAATGGAACCACT----
----
-------------------GATTCGAATACTAATAGCCCTATAGAACTGAAAGGAGAAATAAAG---
AACTGCTCTTTCAATATCACCACAAGCATGAGA--------------------------
GATAAGATGAAGAAAGACTATGCACTT---TTTAATAGACTTGATGTAGTACCAATAGAAAAT---------
----

GATAATACCAGTTATAGGTTGATAAGTTGTAACACCTCAGTCATTACACAG---
GCCTGTCCGAAGGTAACCTTTGATCCAATTCCCATACATTATTGTACCCCAGCTGGTTTTGCGATTCTAAAAT
GTAAC---GATAAGAAGTTCAAT------GGAACAGGAGAATGTAGAAAT----------------------
-GTCAGC---ACAGTACAATGTACACATGGAATTAGGCCAGTAGTATCAACT---
CAACTGCTGTTGAATGGTAGTCTAGCAGAAGAAGAG-----------
GTAGTAATTAGATCTGCCAATTTCTCAGACAAT---------GCTAAAGTCATAATAGTACAGCTAGCA----
-----------AAAGCTGTACAG---------ATTAATTGTACAAGACCCAACAACAATACAAGAAAA-----
-AGTATACCGCTA------------GGACCAGGGAGAGCA---TTT------TATACAACAGGAGCC------
---------ATAATAGGAGATATA------------AGACAAGCATATTGT---ACTATTGGTAATTCA---
ACATGGAAGAAAACTCTAAAAAAGATAGTTGAAAAATTAAGA--------------GAAAAATTTTCT----
-----------------------------------AATAACACAATAATCTTTAAT------
CACTCCGCAGGAGGGGACCCAGAAATTGTA---ATGCACAGTTTTAATTGCAGAGGGGAA---
TTTAAGCAGCTCCAGGCGAGAGTCCTGGCTGTGGAAAGATACCTAAAGGATCAACAGCTCCTGGGGATCTGGG
GTTGCTCTGGAAAACTCATCTGCACCACTGCTGTGCCTTGGAATACTAGTTGGAGT----------------
----------------------------AATAAATCTCTGGATAGCATTTGG------GAT------
AACATGACCTGGATGCAGTGG---GAGAGAGAAATTGACAATTACACAGGCTTAATATAC---
TCCTTGATTGAAAAATCGCAAAACCAACAAGAA---------------------------------------
----
AAGAATGAACAAGAACTATTGGAATTGGATAAATGGGCAAGTTTGTGGACTTGGTTTGACATAACAAATTGGC
TGTGGTATATAAAAATATTCATAATGATAGTAGGAGGCTTAATAGGTTTAAGAATAGTTTTTACT---
GTACTTTCTATAGTG---------AATAGAGTTAGGCAGGGATACTCACCA------
TTATCGTTTGCTGTTAGCTTGCTCAATGCCACAGCTGTAGCAGTAGCAGAG---
GGAACAGATAGGATTATAGAAGGATTTCAAAGATTCTTTAGAGCT---------------------------
--------GTTCTCCACATC------CCTAGAAGAATAAGACAGGGCTTAGAAAGGGCTTTGCTA---TAA
>HQ595803
GCT---ACA---
GACCAATTGTGGGTCACAGTCTATTATGGGGTACCTGTGTGG---AAAGAAGCAACC---
ACCACTCTATTTTGTGCATCAGATGCTAAAACCTATGACCCAGAG---GTACATAAT---------------
--------------GTTTGGGCCACA---CATGCCTGTGTGCCCACCGAC---
CCTGACCCACAAGAAGTATTATTGGAA---------AACGTGACAGAAAATTTTAAC---------------
-------------------------ATGTGGAAGAATAACATGGTAGAACAGATGCATGAGGACATA---

ATCAGCTTATGGGATGAAAGCTTA---AAGCCATGTGTAAAATTA---
ACCCCACTATGTGTTCCTTTAAATTGCACTAAGTGGAAGATTAACAGTACCAATAGTACCAGT----------
----
----------------------AATAGTAATAGTAGTATGAGGACGGAGGAACAAGAGATAAGA---
AACTGCTCTTTCAATATCAGCACAAGAGGATAC------------------------
AATAAGGGACAAAAAGAAGAATATGCAGCT---TTTTATGAATTTGATATAGTACCAATAGATAAT-------
----

GGGACAAGCTATAGGTTGATAAATTGTAACACCTCAGTCATTATACAG---
GCCTGTCCAAAGGTATCCTTTGAACCAATTCCCATACATTATTGTGCCCCAGCTGGTTTTGCGATTCTACGGT
GTAAT---GATCAGAAGTTCAAG------GGAACAGGATCATGTACAAAT-----------------------
-GTCAGT---ACAGTCCAATGTACACATGGAATTAGGCCAGTAGTGTCAACT---
CAACTGCTGCTAAATGGCAGTCTAGCAAAAGAAGAG------------
GTAGTAATTAGATCTGAAAATTTCACAAACAAT---------GCTAAAACCATAATAGTACAGCTGAAG----
-----------GAGGCTGTAAGA---------ATTAATTGTACAAGACCCAATAACAATACAAGAAAA-----
-AGTATACATATG------------GGACCAGGGAAAGCA---TTT------TATGCAACA---GAC------
---------ATAATAGGAGATATA------------AGACAAGCACATTGT---AACCTTAGTGGAGTA---
GAGTGGAATAGCACTTTACAACAGGTAGTTAAAAAATTAAGA---------------GAACAATTTGGA----
-----------------------------------AATAAAACAATAGTCTTTAAC------
CAATCTTCAGGGGGGGACCCAGAAATTGTA---ATGCACAGTTTTAATTGTGGTGGGGAA---
TTTAAGCAGCTCCAGGCAAGAGTCCTGGCTGTGGAAAGATACCTAAAGGATCAACAGCTCCTAGGGATTTGGG
GTTGCTCTGGAAAACTCATCTGCACCACCGCTGTGCCTTGAAATGCTAGTTGGAGT-----------------
----------------------------AATAAAACTCTGGATAAGATTTGG------AAT------
AACATGACTTGGATGAAGTGG---GAGAAAGAAATTGACAATTACACAAGCTTAATATAC---
ACCTTAATTGAAGAATCGCAGAACCAACAAGAA--

AAGAATGAATTAGAATTATTGGAATTGGATAAGTGGGCAAGTTTGTGGAATTGGTTTGACATATCACAATGGC
TGTGGTATATAAAGATATTCATAATGATAGTAGGAGGTTTAATAGGCTTAAGAATAGTCTTTGCT---
GTGCTTTCTATAGTG---------AATAGAGTTAGGAAGGGATACTCACCG------
TTATCGTTTGCTATTAGTTTGTTTAATGCCACAGCTATAACAGTAGCAGAA---
GGGACAGATAGGGTTATAGAAATAATACAAACAGCTTGTAGAGCT----------------------------
--------ATTTGCCACATA------CCTAGAAGAATAAGACAGGGCTTAGAAAGGATTTTGCTG---TAA
>JF680906
GCT---GCA--
CCACAATTGTGGGTCACAGTCTATTATGGGGTACCTGTGTGG---AAAGAAGCAACT---
ACCACTCTATTTTGTGCATCAGATGCTAAAGCATATGAGACAGAG---GTACATAAT----------------
--------------GTTTGGGCCACA---CATGCCTGTGTACCCACAGAT---
CCTAATCCACAAGAAATAACATTGGTA---------AATGTGACAGAAAATTTTAAC----------------
--------------------------ATGTGGAAAAATGACATGGTAGAACAGATGCATGAGGATATA---
ATCAGTTTATGGGATGAAAGCCTA---AAGCCATGTGTAAAACTA---
ACCCCACTCTGTGTTACCTTAAATTGCACTGATAATTTGAACAGTACCTATAATAATACTTGG----------
----
-------------------------GTCAATGGTACTTTAAAAATGGAGAAAGGAGAAATGAAA---
AATTGCTCTTTCAATGTCACCACATTCATAGGG---------------------------AAAAAGGTG---
CAAGAACGGGCATTC---TTTTATAAAGTGATATAGTATCAATAGCTAAT-----------------------
----
----------GATACTACAAGCTATACATTGATAAATTGTAACACCTCAGTCGTTACACAG---
GCCTGTCCAAAGGTATCCTTTGAGCCAATTCCCATACATTACTGTGCCCCGGCTGGTTTTGCGATTCTAAAGT
GTAGA---GATAAAAAGTTCAAT------GGAACAGGACCATGTAAAAAT-----------------------
-GTCAGC---ACAGTGCAATGTACACATGGAATTAGACCAGTAGTATCAACT---
CAACTACTGTTAAATGGTAGTCTAGCAGAAGAAGAG------------
GTAGTAATTAGATCTGAAAATTTCACAAACAAT---------GCTAAAACCATAATAGTACAGCTGAAC----
-----------GTATCTGTAGAA---------ATTAATTGTTCAAGACCCAACAACAATACAAGAAAA-----
-AGTATAAATATA------------GCACAAGGGAGAGCA---ATT------TATGCAACAGGAGAA------
---------ATAATAGGAGATATA------------AGACAAGCACATTGT---AACATTAGTAGAGCA---
AAATGGAATCACACGTTACAACTGATAGTTGAAAAACTAAAA---------------GAACAATTTGGG----
--------------------------------GAGAATAAAACAATAGTCTTTAAT------
CAATCTTCAGGAGGGGACCCAGAAATTGTA---AAGCATAGTTTTAACTGTGGAGGGGAA---
TTTAAGCAGCTCCGAGCAAGAGTCCTGGCTGTGGAAAGATACCTAAAGGATCAACAGCTCCTGGGAATTTGGG
GTTGCTCTGGAAAACTCATTTGCACCACTGCTGTGCCTTGGAATGTTAGTTGGAGT-----------------

----------------------------AATAAGTCACTGGAGGCAATTTGG------CAG------
AACATGACATGGATGGAGTGG---GATAGAGAAATTGACAATTACACAAGCTTAATATAT---
ACCTTAATTGAAGAATCACAAAACCAACAAGAA--

AAGAATGAACAAGAATTATTAGAACTAGATAAGTGGGCAAGTTTGTGGAATTGGTTTGACATAACACAATGGC
TGTGGTATATAAAAATATTCATAATAATAGTAGGAGGCTTGGTAGGCTTAAGAATAGTTTTTGCT---
GTACTTTCTATAGTA---------AATAGAGTTAGGAAGGGATACTCACCA------
TTATCGTTCGCTGTTAGCTTGCTTAATGCCACAGCTATAGCAGTAGCTGAG---
GGGACAGATAGGGTGATAGCAGTATTTCAAAGGGCTTTTAGAGCT----------------------------
--------GTCCTCCGCATA------CCTACAAGAATAAGACAAGGCGCGGAAAGGGCTTTAATA---TAA
>HQ595769
GTT---GCA--
GAACAATTGTGGGTCACAGTATATTATGGGGTACCTGTGTGG---AAAGATGCAACT---
ACCACTCTATTTTGTGCATCAGATGCTAAAGCATATGATACAGAG---GCACATAAT----------------
--------------GTTTGGGCAACA---CATGCCTGTGTACCCACAGAC---
CCCAACCCACAAGAAGTAGTATTGAGA---------AATTTGACAGAAAAGTTTAAC----------------
--------------------------ATGTGGAAAAATAACATGGTTGAACAGATGCATGAGGATATA---
ATTAGTTTATGGGAGCAGAGCCTA---AAGCCATGTGTAAAATTA---
ACCCCACTTTGTGTTACTTTAAATTGCACTGATTATGTAGGGACTAATACCACTAATAGTACTATTACT----
----
----------------AAGACCAATAGTAGTGAATGGAATTGGATGAAGACAGAAGAAATGAAA---
AACTGCTCTTTCAATATCACCACCAACATAAGA---------------------------
GACAAAATGCAGAAAGAATATGCAATT---TTCTATAAACCTGATATAGTATCAATAGATAAT----------
----

AATAGTACTAGCTATAGGATAATAAGTTGTAATACCTCAGTCATTACACAG---
GCCTGTCCAAAAGTATCCTTTGAACCAATTCCCATACATTATTGTGCCCCGGCTGGTTTTGCGATTCTAAAGT
GTAAT---GATAAAAAGTTCAAT------GGAACAGGACCATGTAAAAAT-----------------------
-GTCAGC---ACAGTACAATGCACACATGGAATTAGGCCAGTAGTGTCAACT---
CAACTGCTGTTAAATGGCAGTCTAGCAGAAGAAGAG------------
GTAGTAGTCAGATCTGAAAACTTTACAGATAAT---------GCTAAAACCATAATAGTGCAGCTGAAT----
-----------GAATCTGTAGTA---------ATCAATTGTACAAGACCCAACAACAATACAAGAAAA-----
-AGTATACATATA------------GGACCAGGGAAGGCA---TTT------TATACAACAGGAGAA------
---------GTAATAGGAAATATA------------AGACAAGCACATTGT---AACCTTAGTAGTACA---
AGCTGGAATAACACTTTAAAAAGGGTAGTTCAAAAATTAAGA---------------GAACAATTTAAA----
-----------------------------------AATAAAACAATAGTCTTTAAG------
CAATCCTCAGGAGGGGACCCAGAAATTGTA---ATGCACAGTTTTAATTGTGGAGGGGAA---
TTTAAGCAGCTCCAGGCAAGAGTCCTGGCTGTGGAAAGATACCTAGAGGACCAACAGCTCCTAGGAATTTGGG
GTTGCTCTGGAAAACTCATTTGCACCACTTCTGTGCCATGGAATGTTAGTTGGAGT-----------------
----------------------------AATAAATCTTTTGATCGGATTTGG------AAC------
AACATGACTTGGATGCAGTGG---GAAAGAGAAATTGACAATTACACAAATGAAATATAC---
AGTTTACTTGAGAAATCGCAAAACCAACAAGAA--

AAGAATGAACAAGAATTATTGGAATTAGATAAATGGGCAAGTTTGTGGAATTGGTTTGACATAACAAACTGGC
TGTGGTATATAAAAATATTCATAATGATAGTAGGAGGCTTGATAGGCTTAAGGATAGTTTTTGCT---
GGAATTGCTATAGTG---------AATAGAGTTAGGCAGGGATACTCACCA------
TTGTCGTTGGCTATTAGCTTGTTCAATGCCACAGCTATAGCAGTAGCTGAG---
GGGACAGATAGGATCATAGAAGTAGTACAAAGAGCTTGTAGAGCT----------------------------
--------ATTCTCCACATA------CCTAGAAGAATAAGACAGGGCTTGGAAAGGGCTTTGCTA---TAA
>HQ595770
GCT---GCA--
GATCAATTGTGGGTCACAGTCTATTATGGGGTACCTGTGTGG---AAAGAAACAACC---
ACCACTTTATTTTGTGCATCAGATGCTAAGGCATATGATACAGAG---GTACATAAT----------------
--------------GTTTGGGCCACA---CATGCCTGTGTACCCACAGAC---
CCCAACCCACAAGAAGTAGCATTGGGA---------AATGTAACAGAAGATTTTAAC----------------
--------------------------ATGTGGAAAAATAACATGGTAGAACAAATGCATGAGGATATA---
ATCAGTTTATGGGATCAAAGCCTA---AAGCCATGTGTAAAATTA---
ACCCCACTCTGTGTTACTTTAAACTGCTCTAATGTGACAGCCCAGACTAATGAGACAAAT-------------
----
-------------------------GTGAATAATTCGATGAATACTGCTGCAGCAGACATGAGA---

AACTGCTCTTTCAATATCACCACAAGCATAAGA--------------------------
GATAAGATAAAGAAAGAATATGCACTC---TTTTATAAACTTGATGTAGTACCAATAGATAAT---------
----

GATAATACTAGCTATAGGTTGATAAGTTGTAACACCTCAGTCATTACACAG---
GCCTGTCCAAAGGTATCCTTTGAGCCAATTCCCATACATTATTGTGCCCCGGCTGGTTTTGCGATTTTAAAGT
GTAAT---GATAAAAAATTCAAT------GGAACAGGACCATGTAAAAAT----------------------
-GTCAGC---ACAGTACAATGTACACATGGAATTAGGCCAGTAGTATCAACT---
CAACTGCTGTTAAATGGCAGTCTAGCAGAAGATGAA-----------
GTAGTACTCAGGTCTGCCAATTTCACAGACAAT---------GTCAAAACCATAATAGTACAGCTAAAT----
-----------GAATCTGTAGAA---------ATTAATTGTACAAGACCCAACAACCATACAAGGAAA-----
-AGTATACATATG------------GGATCATGGAGAGCA---GTT------TATACAACAGGAGAA------
---------ATAATAGGAAATCTA-----------AGACAGGCACATTGT---AACCTTAGTAGAGCA---
AAATGGAATGACACTTTAGGACAGATAGTTATAAAATTAAGA--------------GAACAATTTAAG----
----------------------------------AATAAAACAATAAACTTTACT------
CAATCCTCAGGAGGGGACCCAGAAATTGTA---ATGCACAGTTTTAATTGTGGAGGGGAA---
TTTAAGCAGCTTCAGGCGAGAATCCTGGCTGTGGAAAGATACCTAAGGGATCAACAGCTCCTGGGAATTTGGG
GTTGCTCTGGAAAACTCATTTGCACCACTGCTGTGCCTTGGAATACTAGCTGGAGT----------------
---------------------------AATAAAAATTTGAGTGAGATCTGG------GAT------
AACATGACCTGGATGCAATGG---GAAAGAGAAATTGACAATTACACAGAGTTAATATAC---
ACCTTAATTGAAAAATCGCAGAACCAGCAGGAA---------------------------------------
----
AAGAATGAACAAGAATTGTTGGAATTGGATAAATGGGCAAGTTTGTGGAATTGGTTTGATATAACAAAATGGC
TGTGGTATATAAAAATATTCATAATGATAGTAGGAGGCTTGGTAGGTTTAAGAATAGTTTTTACT---
GTACTTTCTATAGTA---------AATAGAGTTAGGAAGGGATACTCACCA------
TTATCGTTTGCTGTTAGCTTGTTTGATACTATAGCTATAGCAGTAGCTGAG---
GGGACAGATAGGATAATAGAAGTAATACAAAGAGCTGGTAGAGCT---------------------------
--------GTCCTCCACGTA------CCTAGAAGAATAAGACAGGGCTTGGAAAGGGCTTTGCTA---TAA
>JF680910
GCT---GAA---ACA---
TGGGTCACAGTCTATTATGGGGTACCTGTGTGG---AAAGAGGCAACC---
ACCACTCTGTTTTGCGCATCAGATGCTAAAGCATATGATACAGAG---GTACATAAT---------------
--------------GTTTGGGCCACA---CATGCCTGTGTACCCACAGAC---
CCCAATCCACAAGAAATAGAATTGAAA---------AACGTGACAGAAGAATTTAAC---------------
--------------------------ATGTGGAAAAACAACATGGTAGACCAAATGCATGAGGATATA---
ATTAGTTTATGGGATCAAAGCCTG---AAGCCATGTGTAAAATTA---
ACCCCACTCTGTGTCACTTTAAATTGCACTGATAAGGTGAATAATACTCGGGCCAGTAATAGTAGTCTT----
----
----------------GTAAATAGTACTCATACTATTAATGAGAGTTTGATAATGGACATGAAA---
AACTGCTCTTTCAATGTCACCACAAGTACAAAA-------------------------
GATAAGATACAGAAAGAATATGCACTC---TTTTATAAACTTGATGTAGTACCAGTAGATAAT---------
----

GATAATACTAGCTATAGGTTGATAAGTTGTAATACCTCAGTCATTACACAA---
GCCTGTCCAAAGGTATCCTTTGAGCCAATTCCCATACATTATTGTGCCCCAGCTGGTTTTGCGATTCTAAAAT
GTAAT---GATAAGAAGTTTGAG------GGAAAAGGAACATGTAGAAAC----------------------
-GTCAGC---ACAGTACAATGTACACATGGAATTAGACCAGTAGTGTCAACT---
CAACTACTGTTAAATGGCAGTCTAGCAAAAGAAGAA-----------
ATAGTAATTAGATCTGACAACATCACAGACAAT---------GCTAAAACCATAATAGTACAGCTAAAG----
-----------GAAGCTGTAATA---------ATTAATTGTACAAGACCCAACAACAATACAAGAAAA-----
-AGTATACATATA------------GCACCAGGAAGAACA---TTT------TATGCAACAGGAGAC------
---------ATAATAGGAGACATA-----------AGACAAGCACATTGT---ACCATTAATGGAACA---
CAGTGGAATAACACCTTAAAAGAAGTAGCTAAAAAATTAAGT--------------GAAAAACTTAAT----
----------------------------------ATCAACCAAATAGTCTTTAAT------
AAATCCTCAGGAGGGGACCCAGAGATTGTA---ACGCACAGTTTTAATTGTGGAGGGGAA---
TTCAAACAGCTCCAGGCAAGAATCCTGGCTGTAGAGAGATATCTGCAGGATCAACAGCTCCTAGGGATTTGGG
GTTGCTCTGGAAAACTCATCTGTACCACTACTGTGCCTTGGAATGCTACTTGGAGT----------------
---------------------------AATAAAACTCTGAATGATATTTGG------GAA------
AACATGACCTGGATGCAGTGG---GAGAAAGAAATTAACAACTACACAGAAATAATATAC---
ACCTTACTTACAGATTCGCAGATCCAACAAGAA---------------------------------------

----
AAGAATGAACAAGAATTATTAGAATTAGATAAATGGGCAAGTTTGTGGAATTGGTTTAATATAACAAACTGGC
TGTGGTATATAAAAATATTCATAATGATAGTAGGAGGTTTGGTAGGCTTAAGAATAGTTTTTGCT---
ATACTTTCTGCAGTA---------AATAGAGTTAGGCAGGATATTCACCA------
TTATCATTTGCTGTTAGCTTGCTCAATGTTATAGCTATAGCAGTAGCTGAG---
GGGACAGATAGGATTATAGAAGTAGCACAAAGAGCTTGGGGAGCT----------------------------
--------TTCATACACATA------CCTAGAAGAATAAGACAAGGATTTGAAAGGGCTCTACTA---TAA
>JF680911
ACT---GCA--
GGAAATTTGTGGGTCACAGTCTATTATGGGGTACCTGTGTGG---AAAGAAGCAACT---
ACCACTCTGTTTTGTGCATCAGATGCCAAAGCATATCATCCAGAG---GCGCATAAT----------------
--------------ATTTGGGCCACA---CATGCCTGTGTACCCACAGAC---
CCTAACCCACAAGAAATAGTACTGGAA---------AATGTGACAGAAAATTTTAAC----------------
--------------------------ATGTGGAAAAACAACATGGTAGAACAGATGCATGAGGATATA---
ATTAGTTTATGGGATCAAAGTCTA---AAGCCATGTGTAAAATTA---
ACCCCAGTTTGTGTTACTTTAGATTGCACTAATGTGAATGTGACAAGCTATTGCACTAATTCGACTAAT----
----
----------------ACTACTGGTTGTAATAGTAGTTTAAAAATGAAAACGGGAGAAATGAAA---
AACTGCTCTTTCAATGTCACCACAGTCATAAAA---------------------------
AATAAGTTACAGAAAGAATATGCACTT---TTTTATAAACTTGATGTAGTATCAATAGATGATGATAATAAA-
----
-
AACTCAACAGATGATGTTAAAAACAGCTCAATCTATACATTGATAAATTGTAACACCTCAACCATTACACAG-
--
GCCTGTCCAAAGACATCCTTTGAACCAATTCCCATACATTATTGTGCCCCGGCTGGTTTTGTGATTCTAAAGT
GTAAT---AATAAGACATTTAGT------GGGACAGGACCATGTAAAAAT-----------------------
-GTCAGC---ACAGTACAGTGTACACATGGAATTAGGCCAGTGGTGTCAACC---
CAACTACTGCTAAATGGTAGTCTAGCAGAAGGAGAA------------
GTAATAATTAGATCTGAGAATTTCGCAGACAAT---------GCTAGAACCATAATAGTACAATTAAAT----
-----------GAAACTGTAAAA---------ATTAATTGTACAAGACCCGGCAACAATACAAGGAAA-----
-AGTATACCCATA------------GGGCCAGGGAGAGCA---TTT------TTCACAACAGGAGCC------
---------ATAATAGGAGATATA------------AGACAAGCACATTGT---AACCTTAGTAGGGAA---
GATTGGAATAAAACTTTAAAAAGGGTAGCTACAAAATTAAGG--------------GAACAATTT-------
-----------------------------------AATAAAACAATAAAATTTGAT------
CAATCCTCAGGAGGGGACCAGGAAATTGTA---ATGCACAGTTTTAATTGTGGAGGGGAA---
TTTAAGCAGCTCCAGGCAAGAGTCCTGGCTGTAGAAAGATACCTAAAGGATCAACAGCTCCTGGGGATTTGGG
GTTGCTCTGGAAAACTCATTTGCACCACTACTGTGCCTTGGAATGCTAGTTGGAGT-----------------
----------------------------AATAAATCTATGGATGACATTTGG------AAT------
AACATGACCTGGATGGAGTGG---GAGAAAGAAATTGACAATTACACAGACTTAATATAT---
AACCTAATTGAAAAATCACAGAACCAGCAAGAA--

AAGAACGAACTAGAATTATTGGAATTAGATAAGTGGGCAAGCTTGTGGAATTGGTTTGACATAACACAATGGC
TGTGGTATATAAAAATATTCATAATGATAGTAGGAGGCTTGGTAGGTTTAAGAATAATTTTTGCT---
GTACTTTCTATAGTA---------AATAGAGTTAGGCAGGATATTCACCA------
CTGTCATTTGCTGTTAGTTTGCTTAATGCCACAGCTATAGCAGTAGCTGAG---
GGGACAGATAGGATTATAGAAGTACTACAAAGAATTTGTAGAGCT----------------------------
--------ATCTACAATATA------CCTAGAAGAATAAGACAGGGCTTAGAAAGGGCTTTGCTA---TAA
>JF680913
GCA---CGA---
CAATTGTGGGTCACAGTCTATTATGGGGTACCTGTGTGG---AAAGAAGCAACC---
ACTACTCTATTTTGTGCATCAGATGCTAAAGCATATGATACAGAG---GTACACAAT----------------
--------------GTTTGGGCCACA---CATGCCTGTGTACCCACAGAC---
CCTAACCCACAAGAAGTAAAATTGGTA---------AATGTGACAGAGGATTTTAAC----------------
--------------------------ATGTGGAAAAATGACATGGTGGAACAGATGCATGAGGATATA---
ATCAGTTTATGGGATGAAAGCCTA---AAGCCATGTGTAAAATTA---
ACCCCACTCTGTGTTACCTTAAATTGCACTGATTGCACTAGTACCTGTAATGGCACTAATTGCACTAATACCT
GTAATAATACT--
-TTGACCAACACCAGTGCTTTGACCAATAATAGTAGTTTAAAAATGGAGAGAGGAGAAATAAAA---
AATTGCTCTTTCAATATCACCACAGGAGGAGGA------------------------
GGAAAGGCGCAGAAAGAAGAATATGCACTT---TTTTATAAACTTGATGTAGTACCAATAAAAGGG-------

----

GAAGGGGATAATACAAGTTATACATTGATAAATTGTAACACCTCAGTCATTAAACAG---
GCCTGTCCAAAGGTATCTTTTGAGCCAATTCCCATACATTACTGTGCCCCGGCTGGTTTTGCGCTTCTAAAGT
GTAGA---GATAAAAAGTTTAAT------GGAACAGGAACCTGTAAAAAT----------------------
-GTCAGC---ACAGTACAATGTACACATGGAATTAGACCAGTAGTATCAACT---
CAACTGCTGTTAAATGGTAGTCTAGCAGAAGAAGAG-----------
GTAATAATTAGATCTGAAAATTTCACAAACAAT---------GCTAAAACCATAATAGTACAGCTGAAT----
-----------GCATCTGTAGAA---------ATTAATTGTACAAGACCCAGCAACAATACAAGAAGA-----
-AGTATACATCTA------------GGACCTGGGGGAACA---TTT------TATGCAACAGGTGAC------
---------ATAATAGGAGATATA------------AGACAAGCACATTGT---AACGTTAGTAGCATA---
AAATGGAACAACACGTTGCAACAGTTAGTTAAAAAACTAAGA---------------GGACAATTTGGG----
-----------------------------------AATAAAACAATAGTCTTTAAT------
CAATCCTCAGGAGGGGACCCAGAAATTGTA---ATGCACACTTTTAATTGTGGAGGGGAG---
TTTAAGCAGCTCCGAGCAAGAGTCCTGGCTGTGGAAAGATACCTACATGATCAACAGCTCCTGGGGATTTGGG
GTTGCTCTGGAAAACTCATTTGCACCACTGCTGTGCCTTGGAATGATAGTTGGAGTAATAAA-----------
-----------------------------ACTTATGAGGAGATTTGGGAAAAC------AAA------
AACATGACCTGGATGAAGTGG---GATAGAGAAATTAGCAATTACACAAAACAAATATAC---
ACTTTAATTGAAGAATCGCAAAACCAACAAGAA--

AAAAATGAACAAGAATTATTGGAATTGGATAAGTGGGCAAGTTTGTGGAATTGGTTCGACATAACAAAATGGC
TGTGGTATATAAGAATATTCATAATAATAGTAGGAGGCTTGATAGGCTTAAGAATAGTCTTTGCT---
GTACTTTCTATAGTG---------AATAGAGTTAGGCAGGGATACTCACCA------
TTATCGTTTGCTGTTAGTTTGCTTAATGCCACAGCTATAGCAGTAGCTGAG---
GGGACAGATAGGGTAATAGAAGTATTACAAAGAGCTTTTAGAGCT---------------------------
--------ATCCTCAATATA------CCTGTAAGAATAAGACAGGGCTTGGAAAGGGCTTTATTA---TAA
>HQ595783
GCT---GCA---
GAACAATTGTGGGTCACAGTCTATTATGGGGTACCTGTGTGG---AAAGAAGCAACT---
ACCACTCTATTTTGTGCATCAGATGCTAAAGCACATGACGTAGAG---GTACACAAT----------------
--------------GTTTGGGCCACA---CATGCCTGTGTACCCACAGAC---
CCCAGCCCACAAGAAGTAGTATTGGAA---------AATGTGACAGAAAATTTTAAC----------------
--------------------------ATGTGGAAAAATGACATGGTAGATCAAATGCATGAAGATATA---
ATAAGTTTATGGGATCAAAGTCTA---AAGCCATGTGTAAAATTA---
ACCCCACTCTGTGTCACCTTAAACTGCACTATGTTCAATACTACTATTCATGACCCTAATAGC----------
----
-------------------------ACTAACGGGAGGAATTTTGAGGGAGTAGAGGGGATAAGA---
AACTGCTCTTTCAATATCACTGCAGGGGTAAAG--------------------------
GATAAGGTGCAGAAAGAATATGCAATT---TTTTATACAAGTGATATAGTACCAATAGATAAT----------
----

AGTAATAATGCCAGCTATAGGTTGATAAATTGTAATACCTCAGTCATTACACAG---
ACCTGTCCAAAGGTATCCTT-
GAGCCAATTCCCATACATTATTGTGCCCCGGCTGGTTTTGCGATTCTAAAGTGTAAT---
GATAAGAAATTCAAT------GGAACAGGACCATGTAAAAAT-----------------------GTCAGC-
--ACAGTACAATGTACACATGGAATTAGGCCAGTAGTGTCAACT---
CAACTGCTATTAAATGGCAGTCTAGCAGAAGAAGAG-----------
GTAGTAATTAGATCTGCCAATTTCTCAAATAAT---------GCTAAAACCATAATAGTACAACTGAAT----
-----------GAATCTGTAGCA---------ATTAATTGTACAAGACCTAACAATAATACAAGAAAG-----
-AGTATACCGATA------------GGACCAGGGAGAGCA---TTT------TACGCAACAGGAGAC------
---------ATAATAGGAGATATA------------AGACAAGCACATTGT---AACATTAGTAAAGCA---
GAATGGAACAAAACTTTAGAACGAATAGCTGTAAAATTAAAA---------------GAACAATTTAGA----
-----------------------------------AATAAAACAATAGTCTATAAT------
CACTCCTCAGGAGGGGACCCAGAAATTGTA---ATGCACAGCTTTAATTGTGGAGGGGAA---
TTTAAGCAGCTCCAGGCCAGAGTCCTGGCTGTGGAAAAATACCTAAAGGATCAACAGCTCCTAGGGATTTGGG
GTTGCTCTGGGAAACTCATTTGCACCACTGCTGTGCCTTGGAATAATAGTTGGAGT----------------
-----------------------------AATAAAACTCAGAGTGAGATCTGG------GAT------
AACATGACCTGGATGGAGTGG---GAAAGAGAAATTAACAATTACACAAGTTTAATATAC---
ACCTTAATTGAAGAATCGCAGAACCAACAAGAA--

AAGAATGAACAAGAATTATTGGAATTAGATAAGTGGGCAAATCTGTGGAATTGGTTTGACATAACAAACTGGC
TGTGGTATATAAAAATATTCATAATGATAGTAGGAGGCTTGGTAGGTTTAAGAATAGTTTTTGCT---
GTACTTTCTCTAGTG---------AATAGAGTTAGGCAGGGATACTCACCA------
TTATCATTTGCTGTTAGCTTGTTCAATACCACAGCTATAGCAGTAGCTGAG---
GGAACAGATAGGGTTATAGAAGTAGTACAAGGAATTTGTAGAGCT---------------------------
--------CTGCTCCACATA------CCTAGAAGAGTCAGACAGGGCTTAGAAAGGGCTTTGCTA---TAA
>JF680919
ACA---CAA--
AATTTATGGGTCACAGTCTATTATGGGGTACCTGTGTGG---AAAGAAGCAACC---
ACCACTTTATTCTGTGCATCAGATGCTAAAGCATATGACAAAGAG---GCACATAAT----------------
--------------GTTTGGGCCACA---CATGCCTGTGTACCCACAGAC---
CCTAACCCACAAGAAGTACTATTGGAA---------AATGTGACAGAAAATTTTAAC----------------
--------------------------ATGTGGAAAAATAACATGGTAGAACAGATGCATGAGGATGTA---
ATCAGTTTATGGGATCAAAGCCTA---AAGCCCTGTGTAAAATTA---
ACCCCACTCTGTGTTACTTTAAATTGCACTAATCCAAATGTTACTGCCAGTAATCATACT------------
----
-------------------------AATGTCAATAATAGTAAGTGGGTGGAAGAAGAAATGAAA---
AACTGCTCTTTCAATATCACCACAAGAATGAGA--------------------------
AATAAGATACAGAGAGAATATGCACTT---TTTTATAAACTTGATATAGCACCAATAGGTAAT----------
----

GATAGTACAAGCCATATATTGATAACTTGTAACACCTCAGTCGTTACACAA---
GCTTGTCCAAAGGTGTCCTTTGAACCAATTCCTATACATTATTGTACCCCGGCTGGTTTTGCGATTCTAAAGT
GCAAT---GACAAGAAGTTCAAT------GGAACAGGACCATGTACAAAT-----------------------
-GTCAGT---ACAGTACAATGTACACATGGAATTAGGCCAGTAGTGTCAACT---
CAACTGCTGTTAAATGGCAGTCTAGCAGAAGAAGAG-----------
GTAGCAATAAGATCTGAAAATTTCACAAACAAT---------GCTAAAATTATAATAGTACAGCTGCAA----
-----------AAATCTGTAAAA---------ATTAATTGTACAAGACACAACAACAGTACAAGAAAA-----
-AGTATACCTCTA------------GGACCAGGGAGAGCA---ATT------TTTGCAACAGGAGAC------
---------ATAATAGGAGATATA------------AGACAAGCACATTGT---AACATTAGTAGAGAA---
GAATGGAATAATACTTTAAAACAGGTAGCTGCAAAGTTAGGT---------------AAACAATTTAAT----
------------------------------------ACTACATCAATAGCTTTTAAT------
CAATCCTCAGGAGGGGACCCAGAAATTATA---ATGCACAGTTTTAATTGTGGAGGGGAA---
TTTAAGCAGCTTCAGGCAAGAGTCCTGGCTGTGGAAAGATACCTAAAGGATCAACAGCTCCTAGGAATTTGGG
GTTGCTCTGGAAAACTCATTTGCACCACTACCGTGCCTTGGAATACCAGTTGGAGT-----------------
---------------------------AATAAAACTATGGATCAGATTTGG------GGT------
AACATGACCTGGATGCAGTGG---GAGAAAGAAATTGACAATTATACACACATAATATAT---
GACTTAATTGAAGAATCGCAGAACCAACAAGAA---------------------------------------
----
CAGAATGAACTAGACTTATTAAAATTGGATAAATGGGCAAGTTTGTGGAATTGGTTTGACATATCAAGTTGGC
TATGGTATATAAAAATATTCATAATGATAGTAGGAGGCTTGGTAGGTTTAAGAATAATTTTTGCT---
ATACTTTCTATAGTG---------AATAGAGTTAGGCAGGGATACTCACCA------
TTGTCGTTTGCTGTTAGCTTGCTTGATACCTTCGCCATAGCAGTAGCTGAG---
TGGACAGACAGGGTTATAGAAGTAGTACAAAGAATTGGTAGAGCT---------------------------
--------GTTCTCCACGTA------CCTAGAAGAATAAGACAGGGTTTTGAAAGGGCTTTGCTA---TAA
>JF680920
GCT---ACA--
AAAGATTTGTGGGTCACAGTTTATTATGGGGTACCTGTGTGG---AAAGAAGCAACT---
ACCACTTTATTTTGTGCATCAGATGCTAAAGCATATGACTCAGAA---GTACATAAT----------------
--------------GTTTGGGCCACA---CATGCCTGTGTACCCACAGAC---
CCCAGCCCACAAGAAATAGACTTGATA---------AATGTGACAGAAAATTTTAAC----------------
--------------------------ATGTGGAAAAATAACATGGTAGATCAAATGCATGAGGATATA---
ATCAGTTTATGGGATCAAAGCCTA---AAGCCATGTGTAAAATTA---
ACCCCACTCTGTGTTGCCTTAAATTGCACAAGTGTGACCAATAGTACCAATAAT-------------------
----
-----------------------------GGTACGGTGGAACAGGAAATGGAGGGAGAACTAAAG---
AACTGCTCTTTTAACATCACCACAGATATGAAA--------------------------
GATAATACACAGAGGATGTCTGCAATA---TTTAATAAGCTTGATGTAGTACCAATAGATGGGAAT-------
----

AATACTACTAGCTATAGGTTGATAAGTTGTAACACCTCACGCATTACACAA---
GCCTGTCCCAAAATAAACTTTGAGCCAATTCCCATACATTATTGTGCCCCAGCTGGTTTTGCGATTATGAAGT
GTAAA---GATAATAAGTTCAAT------GGAACAGGACCATGTAGAAAT-----------------------
-GTCAGC---ACAGTACAATGTACACATGGAATTAGGCCAGTAGTATCAACT---
CAACTGCTGTTAAATGGCAGTCTAGCAGAAGAAGAG------------
GTAGTAATTAGATCTGAAAATTTTACAGACAAT---------GCTAAGACCATAATAGTACAGCTGAAT----
-----------GAAACTGTAGAA---------ATTAATTGTACAAGACCCAACAACAATACAAGAACA-----
-AGTATAACTTTG------------GGACCAGGGAGAGCA---TAT------TTTGCAACAGGAGAC------
---------ATAATAGGAAATATA------------AGACAAGCACATTGT---AACATTAGCAGGGAA---
AAATGGAATAACACTTTAGAAAAGGTAGTTAACAAATTAAAA---------------GAACAATTTGGGCAG-
-----------------------------------AATAAAACAATAGCCTTTCGG------
CCATCCTCAGGAGGGGACCCAGAAATTGAA---ACACACAGTTTTAATTGTGCAGGGGAA---
TTCAAGCAGCTCCAGGCAAGAGTCCTGGCTGTGGAAACGTACCTAAAGGATCAACAGCTCCTAGGGATTTGGG
GTTGCTCTGGAAAACTCATCTGCACCACTGCTGTGCCTTGGAATGCTAGTTGGAGT-----------------
----------------------------AATAAGTCTCTGGAGACGATTTGG------GAT------
AATATGACCTGGATGGAGTGG---GAAAGAGAAATTGACAATTACACAAGCACAATATAT---
AATTTAATTGAAGAGTCGCAAAGCCAACAAGAA--

AAGAATGAACAAGAATTATTGGAATTAGATAAGTGGGCAAATTTGTGGAATTGGTTTAACATAACACAATGGC
TGTGGTATATAAAAATATTCATAATGATAGTAGGAGGCTTGATAGGTTTAAGAATAGTTTTTACT---
GTACTTTCTGTAGTG---------AATAGAGTTAGGCAGGGATACTCACCA------
TTGTCGTTTGCAGTTAGCTTGCTTAATGCCACAGCTATCGCAGTAGCTGAG---
GGGACAGATAGGGTTATAGAAATATTCCAAAGAGCTTTTAGAGGT----------------------------
--------TTCCTCCATATA------CCTAGAAGAATAAGACAGGGCGCGGAAAGGCTTTTACTA---TAA
>HQ595786
GCA---AAT---
AACTTGTGGGTCACAGTCTATTATGGGGTACCTGTGTGG---AAAGAAGCAACT---
ACCACTCTATTTTGTGCATCAGATGCTAAAGCATATGATAAAGAA---GCACATAAT----------------
--------------GTTTGGGCTACA---CATGCCTGTGTACCCACAGAC---
CCCAACCCACAAGAGGTATTCTTGGAA---------AATGTGACAGAAGAGTATAAC----------------
--------------------------ATGTGGAAAAATAACATGGTAGAACAAATGCATGAGGATATA---
ATCAGTTTATGGGATCAAAGCCTA---AAGCCATGTGTAAAATTA---
ACCCCACTCTGTGTTACTTTAACCTGCAATAATACTAATAGTAATAGTACCTTCAAT----------------
----
-------------------------------GCTACTGAGAACAATTGGAAAGAAGAAATAAAA---
AACTGCTCTTTCAATATCACCACAGGCATAAAA---------------------------
GATAAGATTCAGAAACAATATGCACTT---TTTTCTACACTTGATCTAGTACCAATAGAGGCT----------
----

AGTAATACCAGCTATAGGATAGTAAGTTGTAACACCTCAGTCATTACACAG---
GCCTGTCCAAAGATATCATTTGAGCCAATTCCCATACATTATTGTACCCCGGCGGGTTTTGCGATTCTAAAGT
GTAAT---GATAAGCAGTTTAAT------GGGAAAGAAAAATGTAAAAAT-----------------------
-GTCAGC---ACAGTACAATGTACACATGGAATTAGGCCAGTAGTGTCAACT---
CAACTGCTGTTAAATGGCAGTCTAGCAGAAGAAGAG------------
GTAGTAATTAGATCTGAAAATTTCACGAACAAT---------GCTAAAACCATAATAGTGCAGTTAAAG----
-----------GACCTTGTAAAA---------ATTAATTGTACAAGACCCAACAACAATACAAGAAAA-----
-AGTATACATATA------------GGACCAGGGAGAGCA---TTT------TTTGCAACAGGAGAA------
---------ATAATAGGAGAGATA------------AGACAAGCACATTGT---AACATTAGTAAGACA---
CAATGGGATAACACTTTAAAAAAGGTAGCTAAAAAATTAAGT---------------CAACAATTTGGG----
-----------------------------------AATAAATCAATAGCCTTTAAT------
CAATCCTCAGGAGGGGACCTAGAAGTTGTA---ATGCACACCTTTAATTGTGGAGGGGAA---
TTTAAGCAGCTCCAGGCAAGAGTCCTGGCTGTGGAAAGATACCTAAAGGATCAACGGCTCCTAGGGATTTGGG
GTTGCTCTGGAAAACTCATTTGCACCACTACTGTGCCTTGGAATGCTAGTTGGAGT-----------------
----------------------------AATAAATCTTACGATTACATTTGG------GAT------
AACATGACCTGGATGCAGTGG---GAAAAAGAAATTGAAAATTACACAGGCTTAATATAC---
ACCTTAATTGAAGAATCGCAAACCCAGCAAGAA--

AAGAATGAACTAGAATTATTGGAATTAGATAAGTGGGCAAGTTTGTGGAATTGGTTTGACATAACAAATTGGC
TGTGGTATATAAAAATATTCATAATGATAGTAGGAGGCTTAATAGGTTTAAGAATAATTTTTGCT---
GTATTCTCTATAGTG---------AATAAAGTTAGGCAGGGGTACTCACCA------

TTGTCATTGGCTGTTAACTTGCTTAATACCATAGCTGTAGCAGTAGCTGAG---
GGGACAGATAGGATTATAGAAATAGCACAAAGATTGGGTAGAGGT---------------------------
--------ATCCTCCATATA------CCTAGAAGAATAAGACAGGGCTTAGAAAGGAGTTTGATA---TAA
>JF680928
ACT---ACA---
GAACAATTGTGGGTTACAGTATATTATGGGGTACCTGTGTGG---AAAGAAGCAACC---
ACCACTTTATTCTGTGCATCAGATGCTAAAGCACATGATCCAGAA---GAGCATAAT---------------
--------------GTCTGGGCCACA---CATGCCTGTGTACCCACAGAC---
CCCAACCCACAAGAAATAAAATTGACA---------AATGTAACAGAAAATTATAAC---------------
-------------------------ATGTGGAAAAATGACATGGTAGACCAGATGCATGAGGATGTA---
ATTAGTTTATGGGATCAAAGCCTG---AAGCCATGTGTAAAATTA---
ACCCCACTCTGTGTTACTTTAAATTGCTCTGATTATAAGAGAAATATTACTAACACCAATGCTACT-------
----
---------------------CAGACCAATAATAGTAGCGAGGAAACAATAGGGGAAATAAAA---
AACTGTTCTTTTAACGTCCCCACAAACCTAGGA--------------------------
GATAAGATGCAGAAAGAATATGCATTA---
TTTTATAAACTTGATGTAGTACTAATAGATAATGATAATGTTAGTAAT------------------------
----
AATAATGGTAGCCAGAATTATAGAAGCTATAGGTTGATAAGTTGTAATACCTCAATCATTAAACAA---
GCCTGTCCAAAGGTATCCTTTGAACCAATTCCCATACATTATTGTGCCCCGGCTGGTTATGCGATTTTTAAGT
GTGCT---GATACAAAGTTTAGT------GGAAAAGGAACTTGTAACAAT----------------------
-GTCACC---TCAGTACAATGCACACATGGAATTAGGCCAGTAGTATCAACT---
CAATTACTGCTAAATGGCAGTCTAGCAGAAGAAGAG-----------
GTAGTAATTAGATCTGACAATTTCTCAGACAAT---------GCCAAAATCATAATAGTACAGCTGAAT----
-----------GAAACTGTAGTA---------ATCAATTGTACGAGACCCAACAACAATACAAGAAAG-----
-AGTATAACTATA------------GGACCAGGGAGGGCA---TTT------TATGCAACAGGAGAC------
---------ATAATAGGAGATATA-----------AGACAAGCACATTGT---AATGTTAGTGGAGAA---
GATTGGAAAAATGCCTTAACAAGGGTAACTGAAAAATTAAAA---------------GAACAATTTCAG----
-----------------------------------AATAAGACAATAATCTTTAAT------
CAATCTTCAGGAGGGGACCCAGAAATTGTA---ATGCACACTTTTAATTGTGGAGGGGAA---
TTTAAGCAGCTCCAGGCAAGAGTCCTGGCTGTAGAAAGATACCTAAGAGATCAACAGCTCCTAGGAATTTGGG
GTTGCTCTGGAAAACTCATTTGCACCACTACTGTGCCTTGGAATACTAGTTGGAGT-----------------
---------------------------AATAAAACTCAGAAAGACATTTGG------GAT------
AACATGACCTGGAGACAGTGG---GAAACAGAAATTGAAAATTACACAGAAACAATATAT---
AGCTTACTTGAGAAATCGCAGAACCAACAAGAA---------------------------------------
----
AAGAATGAACAAGAATTATTAGAATTGGATAAATGGGCAAGTTTGTGGAATTGGTTTGACATAACAAACTGGC
TGTGGTATATAAAGATATTCATAATGATAGTAGGAGGCTTGGTAGGTTTAAGAATAATTTTTGCT---
GTACTTTCTGTAGTG---------AATAGAGTTAGGCAGGGATACTCACCA------
TTATCGTTTGCTGTTAGCTTGCTCAACGTCACAGCCATAGCAGTAGCTGAG---
GGGACAGATAGGATTATAGAAATATCACAAAGAATTTTTAGAGGT---------------------------
--------GTTCTCCACATA------CCTACAAGAATAAGACAGGGCTTGGAAAGGGCTTTGCTA---TAA
>JF680929
GCT---GCA---GAA------------------------CAA---AAA---
GAGCAAAAGTGGGTCACGGTCTATTATGGGGTACCTGTGTGG---AGAGAAGCAAAA---
ACCACTCTATTCTGTGCATCAGATGCTAGAGCATATGAAAAAGAG---GCACATAAT---------------
--------------GTTTGGGCCACA---CATGCCTGTGTTCCCACAGAC---
CCCGACCCACAAGAAGTACCATTGGAA---------AATGTGACAGAAAATTTTAAT---------------
-------------------------ATGTGGAAAAATAACATGGTAGACCAGATGCAGGAGGATATA---
ATCAGTTTATGGGATCAAAGTCTA---AAACCTTGTGTGAAATTA---
ACTCCACTCTGTGTTACTTTAAATTGCACTAATTTAAATGTTACCAATAGCAATAGTACTAGT---------
----
------------------------TCCAATAGTACTGTGGGAAAGAGGGATGAAGAAATGAAA---
AACTGCTCTTTCAATGTCACCACAAACATAAAC--------------------------
AATAAGATACATAAAGAATATGCTCTT---TTCTATAAACTTGATATAATGCCAATAGATAAT---------
----

GATAATACAAGCTATACATTAATAAGTTGTAACACCTCAGTCATTACACAG---
GCCTGTCCAAAGGTATCCTTTGAACCAATTCCTATACATTATTGTGCCCCGGCTGGTTTTGCGATTCTAAAAT
GTAGG---GATAAAAAGTATAAT------GGAACAGGACCATGTAAAAAT----------------------

-GTCAGC---ACAGTACAGTGTACACATGGAATTAAGCCTGTAGTGTCAACC---
CAACTGCTGTTAAATGGCAGTCTGGCAGAAGAAGAT------------
GTAGTAATTAAATCTCAAAAGTTCTCAGACAAT---------GCTAAAAACATAATAGTCCAGCTGAAT----
-----------GAAACTGTAGAA---------ATTAATTGTACAAGACCCGGTAATAATACAAGAAAA-----
-AGTATTCATATG------------GGACCAGGGAAAGCA---TTT------TATGCAACAGGAGAC------
---------ATAATAGGAGATATA------------AGACAAGCACATTGT---AACCTTAGTAGTACA---
AAATGGAATAACACTCTAAAACAGATAGTTAATAAATTAAGA---------------GAACAAGACATAATT-
--------------------------------GGTAATAGTACAATAAACTTCAAG------
CCACCCTCAGGAGGAGACCCAGAAGTTGTA---ATGCACATTTTTAATTGTGGAGGGGAA---
TTTAAGCAGCTCCAGGCAAGAGTCCTGGCTGTGGAGAGATACCTACAGGGTCAACAGCTCCTAGGGATTTGGG
GCTGCTCTGGAAAAATCATTTGCACCACTAATGTGCCTTGGAATAATAGTTGGAGT-----------------
----------------------------AATAAATCTCTGAATGAAATTTGG------GAT------
AATATGACATGGATGGATTGG---GAGAAAGAAATTAACAATTACACAGAGTTAATATAC---
AGCTTACTTGAAGAATCGCAGAACCAACAAGAT--

AAAAATGAACAAGACTTATTGGAATTGGATAAGTGGGACAGTTTATGGAATTGGTTCAGCATAACAAAATGGC
TGTGGTATATAAAAATATTCATAATAATAGTAGGGGGTTTAATAGGTTTAAGAATAATTTTTACT---
GTACTTTCTATAGTG---------AATAGAGTTAGGCAGGGATACTCACCA------
TTATCATTCGCTGTTAGTTTGTTTAATGCAATAGCTATAGCAGTAGCTGAG---
GGGACAGATAGGATTATAGAAGGAACACAAAGAATTTTTAGGGCT----------------------------
--------ATTCTACACATA------CCTAGAAGAATCAGACAAGGCTTTGAAAGGGCTTTGCAA---TAA
>JF680934
GCG---GCA---
GACATGTGGGTCACAGTCTATTATGGGGTACCTGTGTGG---AAAGAAGCAAAC---
GCCACTCTATTTTGTGCATCGGATGCTAAAGCATATGAACAAGAG---GCACACAAT----------------
--------------GTGTGGGCCACC---CATGCCTGTGTACCCACAGAC---
CCTAACCCACAAGAAGTAAAATTGGAA---------AATGTGACAGAAAATTTTAAC----------------
--------------------------ATGTGGAAAAATAACATGGTAGAACAGATGCAAGAGGACATA---
ATCAGTTTATGGGAGCAAAGCCTA---ACGCCATGTGTAAAATTA---
ACTCCACTCTGTGTTACTTTAAATTGTACTAATGCAACGCTTAATAGTACCAATAGTAGTAAT----------
----
-------------------------AGTACTGGTTGGGGACTGATGAAGGAGGGAGAAATAAAA---
AACTGCTCTTTCAATGTCACCACAGCAATAAGA---------------------------
CAGCAGATAAAGGAGGAATATGCACTC---TTTTATGAATCTGATGTAATACCAATAGGCAAGAAG-------
----

AATGAGACAATCTATATGTTGAGGAATTGTAACACTTCAGTCATTACACAG---
ACCTGTGAAAAGGTATCCTTTGAACCAATTCCCATACATTATTGTGCCCCGGCTGGTTTTGCGATTCTACAAT
GTAAT---GATAAAAAGTTCAAT------GGGACAGGACAGTGTAAAAAT-----------------------
-GTCAGC---ACAGTACAATGTACACATGGAATTAGACCAGTAGTGTCAACT---
CAACTGCTGTTAAATGGCAGTCTAGCAAAAGAAGGG------------
GTAGTAATTAGATCTGAAAATCTCACAAACAAT---------GCTAAAACCATAATAGTACAGCTAAAC----
-----------GAATCTGTACAA---------ATACTTTGTGTAAGACCCCACAACAACACAAGAAAA-----
-AGCATACATATA------------GGACCGGGGAGCGCA---TTC------TATGCAACAGGAGCA------
---------ATAATAGGAGATATA------------AGACAAGCACATTGT---AACCTTAGTTACACA---
AACTGGACTAACACCTTAAAGCAGATAGTTGAAAAGCTAGGA---------------GAACAATTTAAG----
--------------------------------AATAAAACAACAATAGTCTTTAAT------
CAATCCGCAGGAGGGGACCCAGAAATTGTA---ATGCACAGTTTTAATTGTGGAGGGGAG---
TTTAAGCAGCTCCAGGCAAGAGTCCTGGCTATAGAAAGATACCTAAAGGATCAACAGCTCCTAGGGATTTGGG
GTTGCTCTGGAAAACTCATCTGCACCACTAATGTACCTTGGAATAATAGTTGGAGT-----------------
----------------------------AATAAAACTCTAGATCAGATATGG------GAT------
AACATGACCTGGATGGAGTGG---GAGAGAGAAATTAACAATTACACAAGCTTAATATAC---
AACTTAATTGAAGAATCGCAAATCCAACAAGAC--

AAGAATGAACAAGATTTATTGGCATTGGATTCGTGGGCAAGTTTGTGGAGTTGGTTTAGCATATCAAATTGGC
TGTGGTATATAAGAATATTCATAATGATAGTAGGAGGTTTAGTAGGCTTAAGAATAGTTTTTACT---
GTACTGTCTATAGTG---------AATAGAGCTAGGAAGGGATACTCACCA------
TTATCGTTCGCTACTAATTTGCTTGATACCATAGCTATAGCAGCAGCTGAG---
GGGACAGATAGGATTATAGAAGGATTACAGAGACTTGGTAGAGCT----------------------------
--------CTTCTCCACATA------CCTAGAAGAATAAGACAGGGCTTAGAAAGGGCTTTGCAA---TAA

>HQ595801
GCA---GCA--
GAACAATTGTGGGTCACAGTCTATTATGGGGTACCTGTGTGG---AAAGAAGCAACC---
ACCACACTGTTTTGTGCATCAGATGCTAAAGCATATGATACAGAA---GCACATAAT----------------
--------------GTTTGGGCCACA---CATGCCTGCGTGCCCACAGAC---
CCCAACCCACAAGAGGTAAAATTGGAA---------AATGTGACAGAAGATTTTGAC----------------
--------------------------ATGTGGAAAAATAACATGGTAGAACAGATGCATGAGGATATA---
ATCAGTTTATGGGATCAAAGTCTA---AAGCCATGTGTAAAATTA---
ACCCCACTCTGTGTTACTTTAAATTGCACTGATTATAATGGTACTTCTTCCAATAGTACTCATACC-------
----
----------------------AATAATAATTATATTAATGAGACAGAGAAAGGAGAAATAAAA---
AACTGCACTTTCAATGTCACCTCTAGCATAAAA---------------------------
GATAAGGTACAGAAAGAAGCTGCACTT---TTTTATAGTATTGATTTAATATCAATTGAGAAT----------
----

AGGAATGATAGCTATATGTTGAGGAGTTGTAACACCTCAGTCATTACACAA---
GCCTGTCCAAAGATATCCTTTGAGCCAATTCCCATACATTATTGTGCCCCGGCTGGTTTTGCGATTCTAAAGT
GTAAT---AATAAGACGTTCAAT------GGATCAGGACCATGCACAAAT-----------------------
-GTCAGC---ACAGTACAATGTACACATGGAATTAGACCAGTAGTATCAACT---
CAACTGCTGTTAAATGGCAGTCTAGCAGAAGAAAAA------------
GTAATGCTTAGATCAGCAAATTTCAGTGACAAT---------ACTAAAACCATAATAGTACAGCTGAAC----
-----------TACTCTGTAGAA---------ATTAATTGCACAAGACCCAACAACAATACAAGAAAA-----
-AGTATACATATA-----------GGACCAGGGAGAGCA---TTT------TATGCAGCAGAAAAA-------
---------ATAATAGGAAACATA------------AGGCAAGCACATTGT---AACATTAGTAGAACA---
AAATGGAATAACACTTTAGGGAGAATAGTTAAAGAATTAAGA---------------GAACAATATGGG----
--------------------------------AAGAATAAAACAATAGTCTTTAAA------
CACTCCTCAGGAGGAGATCCAGAGATTGTA---ATGCATAGTTTTAATTGTGGGGGGGAA---
TTTAAGCAGCTCCAGGCAAGAATCCTGGCTGTGGAAAGATACCTAGGGGATCAACAGCTCCTAGGAATTTGGG
GTTGCTCTGGAAAACTCATCTGTACCACTGCTGTGCCTTGGAATAGTAGTTGGAGT-----------------
----------------------------AATAAATCTCTGGACAGAATTTGG------TAT------
AACATGACCTGGATGCAGTGG---GAAAGAGAAATTGAAAATTACACAGAGGAAATATAC---
AAACTAATTGAAATATCGCAAAACCAACAAGAA--

AAGAATGAACAAGAATTATTGGAATTGGATAAATGGGCGAATTTATGGAATTGGTTTAACATAACAAATTGGC
TGTGGTATATAAAAATATTTATAATGATAGTAGGAGGCTTGATAGGCTTAAGAATAATTTTTATT---
GTACTTTCTATAGTG---------AATAAAGTTAGGCAGGGATACTCACCA------
TTATCATTTGCTGTTAGCTTGTTCAACGCCACAGCAATAACAGTAGCAGAG---
GGGACAGATAGGGTTATAGAACTAGCACAAAGAACTTTTAGAGCT----------------------------
--------ATTCTCCACATA------CCTACAAGAATAAGACAGGGCTTAGAAAGGCTGTTGCTA---TAA
>HQ595802
GCT---GAG---
AAATTGTGGGTCACAGTCTATTATGGAGTACCTGTATGG---AAAGAAGCAACC---
ACCACTCTGTTTTGTGCATCAGATGCTAGAGCATATGATAGAGAG---GCACATAAT----------------
--------------GTTTGGGCCACA---CATGCCTGTGTACCCACGGAC---
CCCAACCCACAAGAAGTAATATTGGGA---------AATGTGACAGAAAATTTTAAT----------------
--------------------------ATGTGGAAAAATGACATGGTAGAACAGATGCAGGAGGATATA---
ATCAGTTTATGGGATCAAAGTTTA---AAGCCATGTGTAGAATTA---
ACCCCACTCTGTGTTACTTTAAATTGCACTGACTATGTGAGGAATGATACTAAGACTAATATC----------
----
----------------------ACTAGTACCACGGTACCACCGATGGAGAAAGGAGAAATAAAA---
AACTGCTCTTTTAGTATCACCACAAACATAAGA---------------------------
GATAAGATACAGAAAGAATATGCACTT---TTTTATAAACTTGATATAGTGTCAATAGATAAT----------
----

AATAGTACTAGCTATAGGTTAATAAACTGTAATACCTCAGTCATTAAACAA---
GCCTGTCCAAAAGTAACCTTTGAGCCAATTCCCATACATTATTGTGCCCCGGCTGGTTTTGCGATTCTAAAGT
GCAAC---AATAAAACATTCGAT------GGAAAAGGACCATGTAAAAAT-----------------------
-GTCAGC---ACAGTACAATGTACACATGGAATCAGGCCAGTAGTGTCAACT---
CAACTGCTGTTAAATGGCAGTCTAGCAGAAGATGAG------------
GTAGTGATTAGATCTGAAAATTTCACGGAAAAT---------ACTAAAAACATAATAGTACAGCTAAAT----

-----------AGTTCTGTAGTA---------ATTAATTGTACAAGACCCAACAACAATACAAGAAAA-----
-GGTATACATATA------------GGACCAGGGAGAGCA---TTT------TATGCAACAGGAGAT------
---------ATAATAGGAGATATA------------AGACAAGCACATTGT---AACCTTAGTAGAGCA---
AAGTGGATGAAGCACTAAAACAAGTAGTTGAAAAATTAGGA---------------GCACAGTTTAGA----
-------------------------------AACAAGACAACAATAATCTTTAAT------
CAATCCTCGGGAGGGGACCCAGAGATTATA---ACACACAGTTTTAATTGTGGAGGGGAA---
TTTAAGCAGCTCCAGGCAAGAGTCCTGGCTGTGGAAAGATACCTAGCGGATCAGCGGTTCCTAGGCTTTGGG
GCTGCTCTGGAAAACTCATTTGCACCACCTCTGTGCCTTGGAATACTAGTTGGAGT----------------
---------------------------AAGAGATCTCTGGATGACATTTGG------AAT------
AACATGACCTGGATGGAGTGG---GAAAAAGAAATTGACAATTACACAGAAATAATATAC---
TCCTTAATTGAGCAATCGCAGACCCAACAAGAA---------------------------------------
----
AAGAATGAAAAAGATTTATTGGAATTGGACAAATGGGCAAGCTTGTGGAATTGGTTTGACATAACAAATTGGC
TGTGGTATATCAAAATATTCATAATGATAGTAGGAGGCTTGGTAGGTCTAAGAATAGTTTGTGCT---
GTGTTTTCTTTAGTA---------AATAGAGTTAGGCAGGATATTCACCA------
TTGTCATTTGCTATTAGCTTGCTCAATGCCACAGCTATTGCAGTAGCTGAA---
GGGACAGATAGGATTATAGAAGTAGTACAAAGATTTTTAGAGCT----------------------------
--------ATCCTCCACATA------CCCGTACGAATAAGACAGGGCTTGGAAAGGGCTTTGCTA---TAA
>HQ595804
GCT---AAA---
GAAAATTTATGGGTCACAGTCTATTATGGGGTACCTGTGTGG---AAAGAAGCAACT---
ACCACTCTATTTTGTGCATCAGATGCTAAAGCATATAGTACAGAG---AAACATAAT----------------
--------------GTTTGGCCACA---CATGCCTGTGTACCCACAGAC---
CCCAACCCACAAGAATTAAAATTGGTA---------AATGTGACAGAGAATTTTAAC----------------
-------------------------ATGTGGAAAAATAGCATGGTAGAACAGATGCATGAGGATATA---
ATCAGTTTATGGGATCAAAGCCTA---AAGCCATGTGTAGAGTTA---
ACACCACTCTGTGTTTCTTTAAATTGCTCTGAGGTGAAGAATGATAATAGGACCAGTAAT------------
----
---------------------------AATAACACAAAAACAATGGAGGGAGGGGAACTAAAA---
AACTGCTCTTTCAATGTCACCACATACGGGGGA--------------------------
GAAAAAAGAAAGAAAGAACATGCACTT---TTTTATAGTCTTGATGTAATAAAAGAAGGTAAC---------
----

AATGACAGCAGCTATAGGTTGGTAAGTTGTAATACCTCAGTCATAACACAG---
GCCTGTCCAAAGATATCCTTTGAACCAATTCCCATACATTATTGTGCCCCGGCTGGTTTTGCGATTCTAAAAT
GTAAT---GATAAAAAGTTTAAT------GGATCAGGGCCATGTACAAAT----------------------
-GTCAGC---ACAGTACAGTGTACACATGGAATTAAGCCAGTAGTATCAACT---
CAACTGTTGTTAAATGGTAGCCTAGCAGAAGAAGAG-----------
GTAGTAATTAGATCTGCCAATTTTACAGACAAT---------GCTAAAACCATAATAGTACAGCTGAAA----
-----------GAACCTGTACAA---------ATTAATTGTACAAGACCCAACAACAATACAAGAAGA-----
-GGTATTCATATT------------GCACCAGGGAGAGCA---TTT------TATGCAACAGGAGAA------
---------ATAATAGGAGATATA------------AGACAAGCACATTGT---AATATTAGCAAAGGA---
AATTGGAGTAACACTTTAAGACAGGTAGCTGAAAAATTAAGA---------------GAACAATATAAG----
----------------------------------AATAAAACAATAAATTTTACT---
CAACCATCCCCAGGAGGGGACCCAGAAATTGTA---ATGCACAGTTTTAATTGTGGAGGAGAA---
TTTAAGCAGCTCCAGGCAAGAGTCCTGTCTATGGAAAGATACCTAAAGGATCAACAGCTCCTGGGGATTTGGG
GTTGCTCTGGAAAACTCATTTGCACCACCGCTGTGCCTTGGAATACTAGTTGGAGT----------------
---------------------------AATAAATCTTATGATAACATTTGG------GGT------
AACATGACCTGGATGGAGTGG---GAAAGAGAAATTGATAACTACACAAGATTAATATAC---
ACCTTAATTGAAGACTCGCAGATCCAGCAAGAA---------------------------------------
----
AAGAATGAACAAGAATTATTGTCATTAGATGAATGGGCAAGTTTGTGGAATTGGTTTGACATAACAAAATGGC
TGTGGTATATAAAAATATTCATAATGGTAGTAGGAGGCTTGATAGGTTTAAAAATAATTTTTGCT---
GTACTTTCTATAGTG---------AATAAAGTTAGGCAGGATACTCACCA------
TTGTCATTGGCTATTAGCTTATTCAATACAACAGCTGTAGTAGTAGCTGAA---
GGGACAGATAGAATTATAGAAGCAGCACAAAGAGTTTTTAGAGCT---------------------------
--------TTCCTTCACATA------CCTAGAAGAATAAGACAGGGCTTCGAAAGGGCTTTGCTA---TAA
>HQ595806
GCT---GAA---
GACAAATTGTGGGTCACAGTCTATTATGGGGTACCTGTGTGG---AAAGAAGCAACT---

ACCACTTTATTTTGTGCATCAGATGCTAAGTCATATAATACAGAG---GTACATAAT---------------
--------------GTTTGGGCCACA---CATGCCTGTGTGCCCACAGAC---
CCCAACCCACAAGAAGTATTATTGGAA---------AATGTAACAGAATATTTTAAT---------------
-------------------------GCATGGAAAAATGACATGGTAGAACAAATGCAGGAGGATATA---
ATCAGTTTATGGGATCAAAGCCTA---AAGCCATGTGTAAAATTA---
ACCCCACTCTGTGTTACTTTAACTTGCACTGATTACAATACTACTAATACCACTAGTAGTAACACC-------
----
----------------------ACTAGTGGTGGAGTAAAACTGATGGATGAAGGAGAAATAAAA---
AACTGCTCTTTTAATATCACCACAGAAATAAGA--------------------------
GATAAGGTGAAGAGAGAATATGCATTG---TTTTATAAACTTGATATAGTGCAAATAGATAAT----------
----

GAAAATAATAATACTCAATATAGGTTGATAAGTTGTAATACCTCAGTCATTACACAG---
GCCTGTCCAAAAGTATCCTTTGAGCCAATTCCCATACACTATTGTGCCCCAGCTGGTTTTGCGATTCTAAAGT
GTAAC---GATAAGGAATTCAAT------GGAAAAGGAGAATGTAAAAT----------------------
-GTCAGC---ACAGTACAATGCACACATGGGATTAGACCAGTAGTATCAACT---
CAACTGCTGTTAAATGGCAGTCTAGCAGAGGAAGAG-----------
GAAGTAATTAGATCTGACAATTTCACAAACAAT---------GCTAAAACCATACTAGTACAGCTGAAG----
-----------AAACCTGTAGTA---------ATTAATTGTACAAGACCCAGCAACAATACAAGAAAA-----
-GGTATACATATA------------GGACCAGGGAGAGCA---TTT------TATGCAACAGGACAA------
---------GTAATAGGAGATATA------------AGACAAGCACATTGT---AACCTTAGTAGAGCA---
GAATGGAATAGCACTTTACAGGCAATAGCTAGCAAATTAAGC---------------AAAAAATATAAT----
-----------------------------------ACAACAAAAATAGCCTTTAAT------
CAGTCCTCAGGAGGGGACCCAGAAATTGTG---ATGTACAGTTTTAATTGTGGAGGGGAG---
TTCAAGCAGCTCCAGGCGAGAGTCCTGGCTGTGGAAAGATACCTAAGGGATCAACAGCTCCTGGGGATTTGGG
GTTGCTCTGGAAAACTCATCTGCACCACTAATGTGCCTTGGAATACTAGTTGGAGT-----------------
----------------------------AATAAATCTCTAAATCAGATTTGG------AAT------
AACATGACCTGGATGCAGTGG---GAGAAAGAAGTTGGCAATTACACAGAACTCATATAC---
ACCTTAATTGAAGAATCACAAAACCAGCAAGAA--

AAGAATGAACAAGAGTTACTGGGATTGGATAAATGGGCAAGTTTGTGGAATTGGTTTGACATAACACATTGGC
TGTGGTATATAAAAATATTCATAATGATAGTAGGAGGCTTGATAGGTTTAAGAATAGTTTTTGCT---
GTACTTTCTACAGTA---------AATAGAGTTAGGCAGGGATACTCACCA------
TTGTCGTTGGCTATTAACTTGCTAGACGTCACAGCCATAGCAGTAGCTGAG---
GGGACAGATAGGATTATAGAGGGATTAAGAAGATTTGGTAGAGGT----------------------------
--------ATCCTTCACATA------CCTAGAAGAATAAGACAGGGCTTTGAAGGGGCTTTGCTT---TAA
>JF680937
GCT---GAA--
AATTTGTGGGTTACAGTCTATTATGGGGTACCTGTGTGG---AAAGAAGCAACC---
ACCACTCTATTTTGTGCATCAGATGCTAAAGCATATCACACAGAG---GTACATAAT---------------
--------------GTTTGGGCCACA---CATGCCTGTGTACCCACAGAC---
CCCAACCCACAAGAAATTATCTTGACA---------AATGTGACAGAAAATTTTAAC---------------
-------------------------ATGTGGAAAAATAACATGGTAGAACAGATGCATGAAGATGTA---
ATCAGCTTATGGGATCAAAGCCTA---AAGCCATGTGTAAAATTA---
ACCCCACTCTGTGTTACCTTAAATTGCATTAACAATAATGGTACCAATGATATTGGTGCCAATGGTACCAGTG
GTGGTACCGGTGGT---
ACCATTAATAGTACCATTAGTACCACCAATAGTAGTATAGGGAGAAGGGAGGAAGGAGAACTAAAA---
AACTGCACTTTTAACATCACCACAAATATAAGA--------------------------
GATAAGATACAGAAAGAATATGCATTG---TTTTATAGCCTTGATCTAGTACCAATAAAGAATGAT-------
----

AGTACCTATAGGTTGATAAGTTGTAACACCTCAGTCATTACACAA---
GCATGTCCCAAAGTAACCTTTGAGCCAATTCCTATACATTATTGTGCCCCGGCTGGTTTTGCGATTCTAAAGT
GTAAC---GATAAGAAGTTCAAT------GGAACAGGGCCATGTAAAAT----------------------
-GTCAGC---ACAGTACAATGCACACATGGAATTAAGCCAGTAGTATCAACT---
CAGCTGTTGTTAAATGGCAGTCTAGCAGAAGAAGAG-----------
GTAGTAATTAGATCTGAAAATTTTACAAACAAT---------GTTAAAACCATAATAGTACAGCTGAAA----
-----------GAAGCTGTAGAA---------ATTAATTGTACAAGACCCGGCAACAATACAAGAAGA-----
-AGTATACATATA------------GGACCAGGGAGAGCA---TTT------TATGCAACAGGAGCC------
---------ATAATAGGAGATATA------------AGACAAGCACATTGT---AACATTAGTGGAGCA---

AAATGGAATAACACTTTAAAAAAGATAGTTGACAAATTAAGA--------------GAACAATTTGAA----
-------------------------------AATAAAACAATAATAGCCTTTCGG------
CCATCCTCAGGAGGGGACCCAGAAATTGTA---ATGCACAGTTTTAATTGTGCAGGGGAA---
TTTAAACAGCTCCAGGCAAGAGTCCTGGCTGTGGAAAAATACCTACAGGATCAACAGCTCCTGGGAATTTGGG
GTTGCTCTGGAAGACTCATCTGCACCACTGCTGTGCCTTGGAATACTAGTTGGAGT----------------
----------------------------AATAAATCTTTCGATATGATCTGG------AAA------
AACATGACCTGGATGGAGTGG---GAAAAAGAAATTGGCAATTACACAAACTTAATATAC---
TCATTAATTCAAGAATCGCAAATCCAACAAGAA---------------------------------------
----
AAAAATGAACAAGAATTATTAGGACTAGATAAGTGGGCAAGTTTGTGGAATTGGTTTGACATAACAAAATGGC
TGTGGTATATAAAAATATTCATAATGATAGTAGGAGGCTTGGTAGGATTAAGAATAGTTTTTACT---
GTACTTTCTATAGTG---------AATAGAGTTAGGAAGGGATACTCACCA------
TTGTCGTTTGCTGTTAGCTTGTTTAATGCCACCGCTATCGCAGTAGCTGAG---
GGGACAGATAGGGTTATAGAAACATTACAAAGAGCTGGTAGAGCT---------------------------
--------ATCCTCCACATA------CCTAGAAGAATAAGACAGGGCTTCGAAAGAATTTTGCTA---TAA
>D10112
GCT---GCA---
GACAAATTGTGGGTCACAGTCTATTATGGGGTACCTGTATGG---AAAGAAGCAACC---
ACCACTCTATTTTGTGCATCAGATGCTAAAGCATATGACACAGAG---GTACATAAT---------------
--------------GTTTGGGCCACA---CATGCCTGTGTACCCACAGAC---
CCTAACCCACAAGAAGTAGTATTGGAA---------AATGTGACAGAAAATTTTAAC---------------
-------------------------ATGTGGAAAAATAACATGGTAGAACAGATGCATGAGGATATA---
ATCAGTTTATGGGATCAAAGCCTA---AAGCCATGTGTAAAATTA---
ACCCCACTCTGTGTTACTTTAATTTGCACTAATGTAAATAATACTAGGACCAATAGTAGTGAT---------
----
----------------------TGGGACAGGAGGGAAGGAGAAAAGATGAAAGGAGAAATAAAA---
AACTGCTCTTTCAATGTCACCACAAGCATAAGA--------------------------
AATAAGGTGCGGAAAGAATATGCACTT---TTTTATAAACTTGATGTAGTACCAATAGATAAG---------
----

GCTAATACAAGTTATACATTGATACATTGTAACACCTCAGTCATTACACAG---
GCCTGTCCAAAGGTATCCTTTGAACCAATTCCCATACATTATTGTACCCCGGCTGGTTTTGCGATTCTAAAGT
GTAAT---GATAAGAAGTTCAAT------GGAAAAGGACCATGTACAAAT----------------------
-GTCAGC---ACAGTACAATGTACACATGGAATTAGGCCAGTGGTGTCCACT---
CAACTATTGTTAAATGGCAGTCTAGCAGAAAAAGAG-----------
GTAGTAATTAGATCTGAAAATTTCACAAACAAT---------GCTAAAACCATAATAGTACAGCTAAAG----
-----------GAACCTGTAGAA---------ATTAATTGTACAAGACTCAACAACAATACAAGAAAA-----
-AGTATAGCTATA------------GGACCGGGGAGAACA---GTT------TATGCAACAGACAGA------
---------ATAATAGGAGATATA------------AGACAAGCACATTGT---AACCTTAGTAGTACA---
AAATGGAACAACACTTTAAAACAGATAGTTACAAAATTAAAA--------------GAACAATTTGGG----
----------------------------------AATAAGACAATAATCTTTAAT------
CAATCCTCAGGAGGGGACCCAGAAATTGTG---ATGCACAGTTTTAATTGTGGAGGGGAA---
TTTAAGCAGCTCCAGGCAAGAGTCCTGGCTGTGGAAAGATACCTAAAGGATCAACAGCTCCTAGGGATTTGGG
GTTGCTCTGGAAAACTCATCTGCACCACTGCTGTGCCTTGGAATGCTAGTTGGAGT----------------
----------------------------AATAAATCTCTGGATAAGATTTGG------AAT------
AATATGACCTGGATGGAGTGG---GAGAGAGAAATTGACAATTACACAAACTTAATATAC---
ACCTTAATTGAAGAATCGCAGAACCAACAAGAA---------------------------------------
----
AAGAATGAAAAAGACTTATTGGAATTGGATACGTGGGCAAGTTTGTGGAATTGGTTTGACATAACAAATTGGC
TGTGGTATATAAAAATATTCATAATGATAATAGGAGGTTTAATAGGTTTAAGAATAGTTTTTACT---
ATACTTTCTTTAGTG---------AATAGAGTTAGGCAGGGATACTCACCA------
TTGTCATTTGCTGTTAGTTTGTTTGATACCATAGCTATAGCAGTAGCTGAG---
GGAACAGATAGGGTTATAGAAGTAGTGCAAAGAGCTTGTAGAGCT---------------------------
--------ATTCTCCACATA------CCTAGAAGAATAAGACAAGGCTTAGAAAGGCTTTTGCTA---TAA
>Y13716
GCT---ACA---
GAAAAATTGTGGGTCACAGTCTATTATGGGGTACCTGTGTGG---AAAGAAGCAACC---
ACCACTCTATTTTGTGCATCAGATGCTAAAGCATATGACACAGAA---AAACATAAT---------------
--------------GTTTGGGCCACA---CATGCCTGTGTACCCACAGAC---
CCCAACCCACAAGAGGTAGTATTGGGA---------AATGTGACAGAAAATTTTAAC---------------

--------------------------ATGTGGAAAAATAACATGGTAGAACAGATGCATGAGGATATA---
ATCAGTTTATGGGATCAAAGCCTA---AAGCCATGTGTAAAATTA---
ACCCCACTCTGTGTTACTTTAAATTGCACTGATTTGAGGAATGATACTAATACC-------------------
----
-------------------------------AATAATAGTATAATGGAGGGAGGAGAAATGAAA---
AACTGCTCTTTCAATATCACCACAAGCATAAGG--------------------------
GATAAGATGCAGAAAGAATATGCACTT---TTGTATAAACTTGATATAGTATCGATAGGTAGT----------
----

GATAATACTAGCTATATATTGACAGAGTGTAATGCCTCAGTCATTACACAG---
GCCTGTCCAAAGATATCCTTTGAGCCAATTCCCATACATTTTTGTGCCCCGGCTGGTTTTGCGATTCTAAAGT
GTAAC---AATAAGACATTCGAT------GGAAAAGGACCATGCACAAAT-----------------------
-GTCAGC---ACAGTACAATGTACACATGGAATTAGGCCAGTAGTGTCAACT---
CAACTGCTGTTAAATGGCAGTCTAGCAGAAGAAAAG-----------
GTAGTAATTAGATCTGACAATTTCACGGACAAT---------GTTAAAACCATAATAGTACAGCTGAAA----
-----------GAAGCTGTAGAA---------ATTAATTGTACAAGACCCAACAACAATACAAGAAAA-----
-GGTATATACATG------------GGACCAGGGAGAAGA---TTT------TATACAACAGGAAGA------
---------ATAATAGGAGATATA------------AGACAAGCACATTGT---AACATTAGTAAAGAA---
AAATGGAATAACACTTTACACCAGATAGTTATAGAATTAAGG---------------AAACAATTTAGG----
-----------------------------------AATAAAACAATAGTCTTTAAT------
CAATCCTCAGGAGGGGACCCAGAAATTGTA---ATGCACAGTTTTAATTGTGGAGGGGAA---
TTTAAGCAGCTCCAGGCAAGAGTCCTGGCTGTGGAAAGATACCTAAAGGATCAACAGCTCCTAGGGATTTGGG
GTTGCTCTGGAAAACTCATTTGCACCACTACTGTGCCTTGGAATGCTAGTTGGAGT-----------------
----------------------------AGTAAATCTCTGGATAAGATTTGG------AAT------
AACACGACCTGGATGGAGTGG---GAAAGAGAAATTGACAATTACACAAACACAACATAC---
ACCTTAATTGAAGAATCGCAGAACCAACAAGAA--

AAGAATGAACAAGAATTATTGGAATTGGATAAGTGGGCAAATTTGTGGAATTGGTTTGACATAACAAACTGGC
TGTGGTATATAAAAATATTCATAATGATAATAGGAGGCTTAATAGGTTTAAGAATAATTTTTGCT---
GTAATTTCTACAGTG---------AATAGAGTTAGGCAGGGATACTCACCA------
TTATCATTGGCTATTAGCTTGCTTAATACCACAGCTATAGCAGTAGCTGAG---
GGGACAGATAGGGTTATAGAAGTAGTACAAAGAGCTTATAGAGCT----------------------------
--------ATTCTCCACATA------CCTACAAGAATAAGACAGGGCTTGGAAAGGGCTTTGCAA---TAA
>JF706393
GCT---ACA---
GACAAATTGTGGGTCACAGTCTATTATGGGGTACCTGTGTGG---AAAGAAACAACC---
ACTACTCTATTTTGTGCATCAGATGCTAAAGCATATGATACAGAG---GTACATAAT----------------
--------------GTTTGGGCCACA---CATGCCTGTGTACCCACAGAC---
CCCAACCCACAAGAAGTAAGATTGGAA---------AATGTGACAGAAAATTTTAAC----------------
--------------------------ATGTGGAAAAATGACATGGTAGAACAGATGCAGGAGGATATA---
ATCAGTTTATGGGATCAAAGCCTA---AAGCCATGTGTAAAATTA---
ACCCCACTCTGTGTTACTTTAAATTGCACTAAGGCGAATATTACTGGTGAGGTGAAT----------------
----
-------------------------------AATACTATAGGATTGGAAAAGATAGAAATGAAA---
AACTGCTCTTTCAATATCACCACAAGCATAAGA--------------------------
GGTAAGATGCAGAAAGAATATGCACTT---
TTTTATAAACCTGATGTAGTACCAATAGATGATGATAATACCAGC----------------------------
----
CATAGTAATACCAACTATTCCAGCTATAGGTTGATAAGTTGTAACACCTCAGTCATTAGACAG---
GCCTGTCCAAAGGTATCCTTTGAGCCAATTCCCATACATTATTGTGCCCCGGCTGGTTTTGCGATTCTAAAAT
GTAAA---GATAAGAAGTTCAAT------GGAACAGGACCATGTAAAAAT-----------------------
-GTCAGC---ACAGTACAATGTACACATGGAATTAGGCCAGTAGTGTCAACT---
CAACTGCTGTTAAATGGCAGTCTAGCAGAAGAAGAG-----------
GTAGTAATTAGATCTGAGAATCTCACGAACAAT---------GCTAAAATCATAATAGTACAGCTGAAT----
-----------GAATCTGTAGAA---------ATTAATTGTACAAGACCCGGCAACAATACAAGGAAA-----
-TTTATACGTATA------------GGACCAGGGGGACCA---GGG------AGCGCATTTTATACA------
ACAAAAGCAGTAATAGGAGATATA------------AGACAAGCACATTGT---AACCTTAGTGAAGCA---
AAATGGAATAACACTTTAAAACAGGTAGTTAAAAAATTAAGA---------------GAACAATTTGTG----
-----------------------------------GATAAAACAATAGTCTTTAAT------
CACTCCTCAGGAGGGGACCCAGAGATTGTA---ATGCATAGTTTTAATTGTGGAGGGGAA---

TTTAAGCAGCTCCAGGCAAGAGTCCTGGCTGTGGAGAGATACCTAAAGGATCAACAGCTCCTGGGGATTTGGG
GTTGCTCTGGAAAACTCATCTGCACCACTGCTGTGCCTTGGAATGCTAGTTGGAGT-----------------
-------------------------AAAAATAAATCTCTGGATGATATTTGG------AAT------
AACATGACCTGGATGGAGTGG---GAAAGGGAAATTGACAATTACACACAATTAATATAC---
AACTTAATTGAAGAATCGCAGAACCAACAAGAA---

AAGAATGAACAAGAATTATTAGAATTGGATAAATGGGCAAGTTTGTGGAATTGGTTTAGCATATCAAATTGGC
TGTGGTATATAAAAATATTCATAATGATAGTAGGAGGCTTGATAGGTTTAAGAATAGTTTTTACT---
GTGCTTTCTCTAGTG---------AATAGAGTTAGGAAGGGATACTCACCA------
TTGTCGTTTGCTGTTAGCTTGCTCAACGCCACAGCCATAGCAGTAGCTGAG---
GGGACAGATAGGGTTATAGAAGTAGTACAAAGAGCTTGTAGGGCT---------------------------
--------ATTCTCAACATA------CCTAGAAGAATAAGACAGGGCTTGGAAAGGGCTTTGCTA---TAA
>JN786861
GTT---GCA-------------------------------------
GGAAATTTGTGGGTCACAGTCTATTATGGGGTACCTGTATGG---AAAGAAGCAACC---
ACCACTCTATTTTGTGCATCAGATGCTAAAGCATATGATACAGAG---GTACATAAT----------------
--------------GTTTGGGCCACA---CACGCCTGTGTACCCACAGAC---
CCCAACCCACAAGAAGTAGTATTGGTG---------AATGTGACAGAAGATTTTAAC---------------
-------------------------ATGTGGAAAAATAACATGGTAGAACAGATGCATGAGGATATA---
ATTAGTTTATGGGATCAAAGCCTA---AAACCATGTGTAAAATTA---
ACCCTACTCTGTGTTACTTTAAAATGCACTGATTGGAAGAGGAATGCCACTGCTAATGCCACTGTTACCACC-
----
-------------ACTGCTACCCCTAGTAGTAGCGGGAGAACGCTGGAGGGAGGAGAAGTGAAA---
AATCGCTCTTTCAATATCACCACAAACATAAGA--------------------------
GATAAAGTGAAGAAAGAATATGCACTT---TTTTATAGAATTGATGTAGTACCAATAGAAAAT----------
----

GATAAAACTAGATATAGATTGATAAATTGTAATACCTCAGTCATTACACAG---
GCCTGTCCAAAGGTATCCTTTGAGCCAATTCCCATACATTATTGTGCTCCGGCTGGTTTTGCGATTCTAAAGT
GTAAT---GATAAGAAGTTCAGT------GGAAAAGGATCATGTAAAAAT----------------------
-GTCAGT---ACAGTACAATGTACACATGGAATTAGGCCAGTAGTAACAGCT---
CAATTGCTGTTAAATGGCAGTCTAGCAGAAGAAGAG-----------
GTAGTAATTAAATCTGACAATTTCACGGACAAT---------GCTAAAAGCATAATAGTACAGCTGAAT----
-----------GAATCTATAGAA---------ATTAATTGTACAAGACCCAACAACAATACAAGAAAA-----
-AGTATACATATA------------GGACCAGGCAGAGCA---TTT------TATGCAACAGGAGAA------
---------ATAACAGGAGATATA------------AGACAAGCACATTGT---AACATTAGTAGAGCA---
AAATGGAATAAAACTTTAGGACAGATAGTTATAAAGTTAAGA------------
GAACAATTTAGAGAACAATTTAAG------AATAAG---------------
ACAATAGAAACAATAGTTTTTAAT------CAATCCTCAGGAGGGGACCCAGAAATTGTA---
ATGCACAGTTTTAATTGTGGAGGGGAA---
TTTAAGCAGCTCCAGGCAAGAGTCCTGGCTGTAGAAAGATACCTACAGGATCAACAGCTCCTGGGGATTTGGG
GTTGCTCTGGAAAACTCATCTGCACCACCACTGTGCCTTGGAATACTAGTTGGAGT-----------------
----------------------------AATAGATCTCAGGAGGAGATTTGG------GGT------
AATTTGACCTGGATGCAGTGG---GAAAAAGAAATTGATAATTACACAAGCTTAATATAC---
ATCTTAATTGAACAATCGCAAAACCAACAAGAA---

AAGAATGAACAAGAATTATTAGAATTAGATAAATGGGCAAATTTGTGGAATTGGTTTAGTATAACAAACTGGC
TGTGGTATATAAAAATATTCATAATGATAGTAGGAGGTTTGATAGGTTTAAGAATAGTTTTTGCT---
GTACTTTCTATAGTG---------AATGGAGTTAGGCAGGGATACTCACCA------
TTGTCGTTCGCTATTAGCTTGCTTAATGCCATAGCCATAACAGTAGCTGAG---
GGGACTGATAGGGTTATAGAAGTAGTACAAAGAGCTTATAGGGCT---------------------------
--------ATTCTCCATATA------CCTACACGAATAAGACAGGGCTTGGAAAGGGCTTTGGTA---TAA
>AJ535594
GCT---ACA---
GACTTGTGGGTCACAGTCTATTATGGGGTACCTGTGTGG---AAAGAGGCAACT---
ACCACTCTATTTTGTGCATCAGATGCTAAAGCATATGATACAGAG---GCACATAAT----------------
--------------GTTTGGGCCACA---CATGCCTGTGTACCCACGGAC---
CCCAACCCACAAGAAGTAAAATTGGAA---------AATGTGACAGAAAATTTTAAC---------------
-------------------------ATGTGGAAAAATAACATGGTAGAACAGACGCATGAGGATATA---
ATCAGTTTATGGGATCAAAGCCTA---AAGCCATGTGTAAAATTA---

ACCCCACTCTGTGTTACTTTACATTGCGTCAATGTTCCTTATAATGTTTCTAAAACCAATAATAGT-------
----
-------------------AGTGTTTCTAATACCAATAATAGTAGTGGGGAAGGGGAAATGAAA---
AGCTGCTCTTTCAACATCACCACAAGCTTAAGA---------------------------
GATAGGGTTAAGAAAGAATATGCACTT---TTTTATACACTTGATCTAGTACCAATAGATGATGATACT----
----

AATAGCACCAATAATGGCACCAATTATAGGTTGATAAGTTGTAACACCTCAGTCATTACACAG---
GCCTGTCCAAAGGTATCATTTGAACCAATTCCCATACATTATTGTGCCCCGGCGGGTTTTGCGATTCTAAAGT
GTAAG---GAGAAGGATTTCACT------GGAAAAGGACCATGTAAAAAT-----------------------
-GTCAGC---ACAGTACAATGTACACATGGAATTAAGCCAGTAGTGTCAACT---
CAACTACTGTTAAATGGCAGTCTAGCAGAAAAAAAT------------
GTAACAATTAGATCTGAAAATTTCACGGACAAT---------GGTAAAAACATAATAGTACAACTGAAG----
-----------GATGCTGTAAGA---------ATTAATTGTACAAGACCCAATAACAATACAAGAAAA-----
-GGCATACATATA------------GGACCAGGGAGAGCA---TTT------TATGCAACAGGAGGA------
---------ATAATAGGAGATATA------------AGACAAGCACATTGT---AACCTTAGTAGAACA---
AAATGGAATGAAACTTTAGAAAAGATAAAGGAAAAATTAGAA---------------GAACAATTTGAA----
-----------------------------------AATAAAACAATAGTCTTTAAT------
CACTCCTCAGGAGGGGACCCAGAAGTTGTA---AGGCACATTTTTAATTGTGGAGGGGAA---
TTTAAGCAGCTCCAGGCAAGAGTCCTGGCTGTAGAAAGATACCTAAGGGATCAACAGCTCCTAGGGATTTGGG
GTTGCTCTGGAAAACTCATTTGCACCACTACTGTGCCTTGGAATACTAGTTGGAGT-----------------
----------------------------AATAAATCTCTGGATAAGATTTGG------GAT------
AACATGACCTGGATGGAATGG---GAAAGAGAAATTGACAATTACACAGACCTAATATAC---
TCCTTACTTGAAAAATCGCAGAACCAGCAAGAA--

AAGAATGAACAAGAATTATTGGAATTAGATAAATGGGCAAGTTTGTGGAATTGGTTTACTATAACAAACTGGC
TGTGGTATATAAAAATATTCATAATGATAGTAGGAGGCTTAATAGGTTTAAGAATAGTTTTTACT---
GTACTTTCTATAGTG---------AATAGAGTTAGGCAGGGATATTCACCA------
TTATCATTTGCTGTCAACTTGCTTAATACCACAGCTATAGTAGTAGCTGAG---
GGAACAGGTAGGGTTATAGAAGTAGTACAAAGAGCTTTTAGAGCT----------------------------
--------ATCCCCCACGTA------CCTAGAAGAATAAGACAGGGCTTTGAAAAGTGTTTGCTA---TAA
>AJ535607
GCT---ACA---GGA------------------------CAA---TTA---
GAACAATTGTGGGTCACAGTATATTATGGGGTACCTGTGTGG---AAAGAAGCAACT---
ACCACCCTATTTTGTGCATCAGATGCTAAAGCACGTGCTACAGAG---GCACATAAT----------------
--------------GTTTGGGCCACA---CATGCCTGTGTACCCACAGAC---
CCCAACCCACAAGAAGTAGTAATGGGA---------AATGTGACAGAAAATTTTAAC----------------
--------------------------ATGTGGAAAAATAACATGGTAGAACAGATGCATGAGGATATA---
AAAAGTTTATGGGAACAAAGCCTA---AAGCCATGTGTAAAATTA---
ACCCCACTCTGTGTTACTTTAAATTGCACTGATAAGTTGAATGTTACTAAAAATAGTAATGGTACTAAT----
----
-------------------AACAGTAGTAGCAGTGGGAAACCGATGGAAGAAGGAGAAATGAAA---
AACTGCTCTTTTAATATCACCACAGAATTAAGA---------------------------
GATAAGGTGAAGAAAGAATATGCGCTT---
TTTTATAGACTTGATGTAGTACAAATAAATAATGATGATAATCCTACTAAGCCTACTAGCAGT----------
----
GCCAACTCTACTAAAGATAGCTATACCAGCTATAGATTGATAAATTGTAACTCCTCAAGCATTACACAG---
GCCTGTCCAAAAGTATCCTTTGAGCCAATTCCCATACATTATTGTGCCCCGGCTGGTTTTGCGATTCTAAAGT
GTAAC---AATAAGACGTTCAGT------GGAACAGGACCATGTACAAAT-----------------------
-GTCAGT---ACAGTACAATGCACACATGGAATTAGGCCAGTAGTATCAACT---
CAGCTGCTGTTAAATGGTAGTCTAGCAGAAGAAGAG------------
ATAGTAATTAGATCTGAAAATTTCTCGAACAAT---------GCTGAAACCATAATAGTACATCTGAAC----
-----------AAATCTGTAGTA---------ATTAATTGTACAAGACCCAACAACAATACAAGAAAA-----
-AGTATACATATG------------GGACCAGGGAGAGCA---CTT------TATGCAACAGGAGAC------
---------ATAATAGGAGATATA------------AGACAAGCACATTGT---AACATTAGTAAACAA---
GACTGGAATGACACTTTACAGCGGATAGTTGTAAAACTAAGA---------------GAACAATTTGGG----
-----------------------------------AATAATAAAACAGAATTTAAG------
CAATCCTCAGGAGGGGACCCAGAAGTTGTA---ATGCACAGTTTTAATTGTGGAGGGGAA---
TTTAAGCAGCTCCAGGCAAGACTCCTGGCTGTGGAAAGATACCTAGGGGATCAACAGCTCCTAGGGCTTTGGG
GTTGCTCTGGAAAACTCATTTGCACCACTGCTGTGCCTTGGAATACTAGTTGGAGT-----------------

---------------------------AATAAATCCCTGACAACAATTTGG------GAA------
AACATGACCTGGATGGAGTGG---GAAAAAGAAATTGACAATTACACAAATTTAATATAC---
AACTTACTTGAACAATCGCAGAACCAGCAAGAA---------------------------------------
----
AAGAATGAACAAGAATTATTGGAATTGGATAAATGGACAGGTTTGTGGAGTTGGTTTAGCATAACAAATTGGC
TGTGGTATATAAGATTATTCATAATGATAGTAGGAGGCTTGATAGGTTTAAGAATAGTTTTTACT---
GTACTTTCTATAGTG---------AATAGAGTTAGGCAGGGCTACTCACCA------
CTATCATTTGCTGTTAGCTTGCTCAACGCCACAGCTGTTGCAGTAGCTGAG---
GGGACGGATAGGGTTATAGAAGTAGTACAAAGAGCTTGTAGAGCT---------------------------
--------GTTCTTCACATA------CCTAGAAGAATAAGACAGGGCTTAGAAAGGGCTTTGCAA---TAA
>AJ535612
GCT---GTA---
GAACAGTTGTGGGTCACAGTCTATTATGGGGTACCTGTGTGG---AAAGAAGCAAAC---
ACCACTCTATTTTGTGCATCAGATGCTAAAGCATATGATACAGAA---GTACATAAT---------------
--------------GTTTGGGCCACA---CATGTCTGTGTACCCACAGAC---
CCCAACCCACAAGAAGTAGTATTGGCA---------AATGTGACAGAAAATTTTAAC---------------
-------------------------ATGTGGAAAAATAACATGGTAGAACAGATGCATGAAGATATA---
ATCAGTTTATGGGATCAAAGCTTA---AAGCCATGTGTAAAATTA---
ACTCCACTCTGTGTTACTCTAAATTGCACTGATGTTAATGTTACTAAAACCAATAGT---------------
----
---------------------------ACAAGCTGGGGAATGATGGAGAAAGGAGAAATAAAA---
AACTGCTCTTTCAACATCACCTCAAACAGGAAA--------------------------
AACAGGATGCAGAAAGAATATGCACTT---TTTTATAAACTTGATGTAATATCAATAGAAAAG---------
----

AATAGTACTAGCTATAGGTTGATAAGTTGTAATACCTCAGTCATTACACAG---
GCCTGTCCAAAGATAACCTTTGAGCCAATTCCCATACATTATTGTGCCCCGGCTGGTTTTGCGATTCTAAAGT
GTAAT---GATAAGACATTCAAT------GGAACAGGACCATGTAAAAAT----------------------
-GTCAGC---ACAGTACAATGTACACATGGAATTAGGCCAGTAGTATCAACT---
CAACTGCTGTTAAATGGCAGTCTAGCAGAAGGAGAG-----------
GTAGTAATTAGATCTGAAAATTTCTCGGACAAT--------GCTAGAACCATAATAGTACAGCTGAAC----
-----------AAAACTGTACAA--------ATTAATTGTACAAGACCCAACAATAACACAAGGAAA-----
-AGTCTAAATGTA-----------GGACCAGGAAGAGCA---GTG------TATACAACAGGAGAT------
---------ATAATAGGAGATATA------------AGACAAGCACATTGT---AACCTTAGTAGAACA---
GACTGGAATAACACTTTAAAACAGATAGTTATAAAATTAAGA--------------GAACAATTTAGG----
----------------------------------AATAAAACAATAGTCTTTAAT------
CACTCCTCAGGAGGGGACCCAGAAATTATA---ATGCACAGTTTTAATTGTGGAGGGGAA---
TTTAAGCAGCTCCAGGCAAGAGTCCTGGCTGTGGAAAGATACCTAAAGGATCAACAGCTCCTAGGGATTTGGG
GTTGCTCTGGAAAACTCATTTGCACCACTGATGTGCCTTGGAATACTAGTTGGAGT----------------
---------------------------AATAAATCTCTGAGTGATATTTGG------GAT------
AACATGACCTGGATGGAGTGG---GAAAAAGAAATTAGCAACTACACAAGCAAAATATAC---
ACCTTACTTGTAGAATCGCAGAACCAACAAGAA---------------------------------------
----
AAGCATGAACAAGATTTATTGGCATTAGATAAATGGGACAGTTTGTGGAATTGGTTTAACATAACAAACTGGC
TGTGGTACATAAAAATATTCATAATGATAGTAGGAGGCTTGATAGGTTTAAGAATAGTTTTTTCT---
GTACTTTCTATAGTA---------AATAGAGTTAGGCAGGGATATTCACCA------
TTATCGTTCGCCATTAGCTTGCTCAATGCCACAGCTATAGCAGTAGCTGAA---
GGGACAGATAGGATTATAGAAGCATTACAAAGAGCTTATAGAGCT---------------------------
--------GTTCTCCACATA------CCTAGAAGAATAAGACAGGGCTTAGAAAGGGCTTTGATA---TAA
>AJ418531
GCT---ACA---
GAAAACTTGTGGGTCACAGTCTATTATGGGGTACCTGTGTGG---AAAGAAGCAACC---
ACCACTCTATTTTGTGCATCAGATGCTAAAGCGTATGAAACAGAG---GTACATAAT---------------
--------------GTATGGGCCACA---CATGCCTGTGTACCCACAGAC---
CCTAACCCACAAGAAGTAGTATTGGGA---------AATGTGACAGAAAATTTTAAC---------------
-------------------------ATGTGGAAAAATAACATGGTAGAACAGATGCATGAGGATATA---
ATCAGTTTATGGGATGAAAGCCTA---AAACCATGTGTAAAACTA---
ACCCCACTCTGTGTTCCTTTAAATTGCACTAATTGGAAGAATAATACTAACACTAATAACAAT---------
----
---------------------AATAGTACTAAGAACAATACCATAGAGATAGAGGAAATAAAA---

AACTGCTCTTTCATC------ACAAACATAGGG---------------------------
AATAAGATAAAGGAAGAATATGCACTT---TTTCATGAACTTGATGTAGTACAAATAGACAGT----------
----

ACAAGCTATACGTTGAAAAATTGTAACACCTCAGTCATTACACAG---
GCTTGTCCAAAGGTATCCTTTGAACCAATTCCCATACATTATTGTACCCCGGCTGGTTTTGCGATTCTAAAGT
GTAAT---GATAAGAAGTTCAAT------GGAACAGGACCATGTACAAAT-----------------------
-GTCAGC---ACAGTACAATGTACACATGGAATTAAGCCAGTAGTGTCAACT---
CAACTGCTGTTAAATGGCAGTCTAGCAGAAGAAGAG------------
GTAGTAATTAGATCTGAAAATTTCACAAACAAT---------GCTAAAACCATAATAGTACAGCTGAAG----
-----------AACCCTGTAAAA---------ATTAATTGTACAAGACCCCACAACAATACAAGAAAA-----
-AGTATACATATA------------GGACCAGGAAGAGCA---TTT------TATGCAACAGGAGAA------
---------ATAATAGGAGATATA------------AGACAAGCACATTGT---AACCTTAGTAGAGAA---
GATTGGAATAACACTTTAAAACAGATAGTTGAAAAGTTAAAA---------------GAACAATTTGGG----
-----------------------------------AATAAAACAATAGTTTTTAAT------
CGATCCTCAGGAGGGGACGCAGAAATTGTA---ATGCACAGTTTTAATTGTGGAGGGGAA---
TTTAAGCAGCTCCAGGCAAGAGTCCTGGCTGTGGAAAGATACCTAAAGGATCAACAGCTCCTAGGGATTTGGG
GTTGCTCTGGAAAATTCATTTGCACCACTGCCGTGCCTTGGAATGCTAGTTGGAGT-----------------
-------------------------AAGAATAAATCTCTGGATGAGATTTGG------AAT------
AACATGACCTGGATGGAGTGG---GAGAGACAAATTGACAATTACACAAACGAAATATAC---
ACCCTAATTGAACAATCGCAGAACCAACAGGAA--

AAAAATGAACTAGAATTATTGGAATTGGATAAGTGGGCAAGTTTGTGGAGTTGGTTTGACATAACACAATGGC
TGTGGTATATAAAAACATTCATAATGATAGTAGTAGGTATAGTAGGTTTAAGAATAGTTTTTGCT---
GTACTCTCTATAGTG---------AATAGAGTTAGGCAGGATACTCACCA------
TTATCATTGACTGTTAGTCTGCTTAATGCCACAGCTATAGCAGTAGCTGAG---
GGGACAGATAGGGTTATAGAAGTAATACAAAGAGCTTGTAGAGCT----------------------------
--------ATTCTCCACATA------CCTAGAAGAATAAGACAGGGCTTAGAAAGGGCTTTGCTA---TAA
>AJ418494
GCT---ACA--
GAAAACTTGTGGGTCACAGTCTATTATGGGGTACCTGTGTGG---AAAGAAGCAACC---
ACCACTCTATTTTGTGCATCAGATGCTAAAGGATATGACAAAGAG---GCACATAAT----------------
--------------GTTTGGGCCACA---CACGCCTGTGTACCCACAGAC---
CCCAACCCACAAGAACAATTCTTG------------AATGTGACAGAAAATTTTAAC----------------
--------------------------ATGTGGAAAAATGACATGGTAGAACAGATGCATGAGGATATA---
ATTAGTTTATGGGATCAAAGCCTA---AAGCCATGTGTAAAATTA---
ACCCCACTCTGTGTTACTTTAAATTGCACTAATGTCAATACCACTGACATCAGGAATGGTACTAATACC----
----
-------------------ACTAGTAGTAGCAAGGAACTAAAAATGGAGGAAGGAGAAATAAAA---
AACTGCTCTTTCAAGACCACCACAAGTATGAAA---------------------------
GATAAGATGCAGACAGAATATGCATTT---TTTTATAAACTTGATGTAGTACCAATAGATGATAATGAT----
----

AATACCAGCTATGGTAATAACAGCTATAGGTTGATAAGTTGTAATACCTCAGTCATTACACAG---
GCCTGTCCAAAGATTTCCTTTGATCCAATTCCCATACATTATTGTACCCCGGCTGGTTTTGCGCTTCTAAAAT
GTAAT---GAAAAGAAGTTCAAT------GGAACAGGACCGTGCAAAAAT-----------------------
-GTCAGC---ACAGTACAATGTACACATGGAATTAGGCCAGTAGTATCAACT---
CAACTACTGTTAAATGGCAGTCTAGCAGAAGAAGAG------------
GTAGTACTTAGATCTGCCAATTTCTCAAACAAT---------GCTAAAACCATAATAGTGCAATTGAAT----
-----------GAATCTGTAGTA---------ATTAATTGTACAAGGCCCAACAACAATACAAGAAAA-----
-AGTATATCATTT------------GGACCAGGGAGCGCA---ATG------TATGCAACAGGAGCA------
---------ATAATAGGAGATATA------------AGACAAGCACATTGT---AACCTTAGTATAACA---
GAATGGAATAACACTTTAACAAAGATAGTTAAAAAATTAAGA---------------GACCAATTTGGG----
--------------------------------GGGAATAAAACAATAGTCTTTAAT------
CAATCCTCAGGAGGGGACCCAGAAATTGTA---ATGCACAGTTTTAATTGTGGAGGGGAA---
TTTAAGCAGCTCCAGGCTAGAGTCCTGGCTGTGGAAAGATACCTAGGGGATCAACAGCTCCTGGGTATTTGGG
GTTGCTCTGGAAAACTCATCTGCACCACTGCTGTGCCTTGGAATGCTAGTTGGAGT-----------------
-------------------------AATAATAGAAATCTGAGTCAGATTTGG------GAT------
ACCATGACCTGGATGGAGTGG---GAAAGAGAAATTGACAATTACACAGATGAAATATAC---
AGATTAATTGAACAATCACAAAACCAACAAGAA--

AAGAATGAACAAGAATTATTGGAATTGGATAAATGGGCAAATTTGTGGAATTGGTTTAGCATATCAAACTGGC
TGTGGTATATAAAAATATTCATAATGATAGTAGGAGGCTTGATAGGTTTAAGAATAGTTTTTACT---
GTGCTTTCTATAATA---------AATAGAGTTAGGCAGGGATATTCACCA------
CTCTCGTTTGCTGTTAGCTTGCTCAACGCCACCGCTATAGCAGTAGCTGAG---
GGGACAGATAGGATTATAGAAATAGTGCAAAGAATTTTCAGAGCT----------------------------
--------GTTCTCCACATA------CCTGTAAGAATAAGACAGGGCTTAGAAAGGGCTTTGCTA---TAA
>AJ418495
GCT---ACA--
GAAAAATTGTGGGTCACAGTCTATTATGGGGTACCTGTGTGG---AAAGAAGCAACC---
ACCACTTTATTTTGTGCATCAGATGCCAAAGCATATGATAAAGAA---GTACACAAT----------------
--------------GTTTGGGCCACA---CATGCCTGTGTACCCACAGAC---
CCCAACCCACAAGAAGTAGAATTGAAA---------AATGTGACAGAAAATTTTAAT----------------
--------------------------ATGTGGAAAAATAACATGGTAAACCAGATGCATGAGGATATA---
ATCAGTTTATGGGATCAAAGCCTA---AAGCCATGTGTAAAATTA---
ACCCCACTCTGTGTCACTTTAAATTGCACTGATGAGTTGAAGAATGCTACTTTTAGGAGTAATACA-------
----
-------------------ACCACTAATAGTAGCTGGGAAAAGATGGAAAAAGGAGAAATAAAA---
AACTGCTCTTTCAATATCACCACAAGCATGAAA---------------------------
GATAAGGTACAAAAAACTTATGCACTT---TTTTATAAACTTGATGTAGTACCAATAAATGGT----------
----

GATAATACTACTAGTTATAGGTTGATAAGTTGTAACACCTCAGCCGTTACACAG---
GCCTGTCCAAAGGTATCCTTTGAGCCAATTCCCATACATTATTGTGCCCCAGCTGGTTTTGCGATTCTAAAGT
GTAAC---AATAAGACGTTCAAT------AGAACAGGACCATGTACAAAC-----------------------
-GTCAGC---ACAGTACAATGTACACATGGAATTAGGCCAGTAGTGTCAACT---
CAACTGCTGTTAAATGGCAGTCTAGCAGAAGAAGAT------------
GTAGTAATTAGATCTGAGAATATCTCAGACAAT---------GCTAAAACCATAATAGTACAACTAAAG----
-----------GAAGCTGTGGAA---------ATTAATTGTACAAGACTCAACAATAATACAAGAAAA-----
-AGTATACATATG------------GGACCAGGGAGAGCA---TTT------TTTGCAACAGGAGAA------
---------ATAATAGGAGACATA------------AGACAAGCACATTGT---AACATTAGTACAACA---
AAATGGAACAAAACTTTAGGACAAGTAGTTAAAAAATTAAGA---------------GAACACTTT-------
-----------------------------------AACAGAACAATAGCCTTTAAT------
CGATCCTCAGGAGGGGACCCAGAAATTGTA---ATGCACAGTTTTAATTGTGGAGGGGAA---
TTTAAGCAGCTCCAGACAAGAGTCCTGGCTGTGGAAAGATACCTAAAGGATCAACAGCTCCTGGGGATTTGGG
GTTGCTCTGGAAAACTCATCTGCACCACTGCTGTGCCTTGGAATACTAGTTGGAGT-----------------
----------------------------AATAAATCTTATAATCAGATTTGG------GAT------
AATATGACCTGGATGGAGTGG---GAGAGAGAAATTGACAATTACACAGGTCTAATATAC---
AACTTAATTGAAAAATCGCAGAACCAACAAGAA--

AAGAATGAACAAGAACTATTGGAATTAGACAAGTGGGCAAATTTGTGGAGTTGGTTTGATATAACAAACTGGC
TGTGGTATATAAAAATATTCATAATGATAATAGGAGGTTTAGTAGGTTTAAGAATAGTTTTTACT---
GTAATTTCTATAGTA---------ACTAGAGTTAGGCAGGGATACTCACCA------
TTGTCATTTGCTGTTAGCTTGTTTAATGCCACAGCTATAGCAGTAGCTGAG---
GGGACAGATTGGGTTATAGAAGTAATACAAAGAGCTTTTAGAGCT----------------------------
--------TTTATCCACATA------CCTACAAGAGTAAGACAGGGCTTGGAAAGGGCTTTGCAA---TAA
>AJ418519
GCA---GCA--
GAACAATTGTGGGTCACAGTCTATTATGGGGTACCTGTGTGG---AAAGAAGCAACC---
ACCACTCTATTTTGTGCATCAGATGCTAAAGCATATGATACAGAG---GTACATAAT----------------
--------------GTTTGGGCCACC---CATGCCTGTGTCCCCACAGAC---
CCCGACCCACAGGAAATAATATTGGAG---------AATGTGACAGAAGATTTTAAC----------------
--------------------------ATGTGGAAAAATGACATGGTAGAACAGATGCATGAGGACATA---
ATAAGTTTATGGGATCAAAGTCTA---AAGCCATGTGTAAAATTA---
ACCCCACTCTGTGTTACTTTAGATTGCACTGATTTGAGTAATGATACTAATTCAAATTGCACTAGAGCGGGGA
AT---
----------TGTACTCATAACAATAGTAGTAGCGATGAAAAGAGGGCGAGAGAAGAAATAAAG---
AACTGCTCTTTCAACATAACCACAAACATAAGA---------------------------
GATAAGATACAGAGGGAATATGCACTT---TTTTATAAACTTGATCTAGTACCAATAGATAAT----------
----

GATACTATCAGACATAGGTTGATAAGTTGTAACACCTCAGTCATTACACAG---
GCCTGTCCAAAAGTATCCTTTGAGCCAATTCCCATACACTATTGTGCCCCGGCTGGTTTTGCGATTCTAAAAT
GTAAC---GATAAGAAGTTCAGT------GGAAAAGGAACATGTAACAAT----------------------
-GTCAGC---GCAGTACAATGTACACATGGAATTAGGCCAGTAGTGTCAACT---
CAACTGCTGTTAAATGGCAGTCTAGCAGAAGAAGAG-----------
GTAGTAATTAGATCTGACAATATCACAAACAAT---------GCTAAAACCATAATAGTACATCTAAAT----
-----------GAATCTGTAGAA---------ATTAATTGTACAAGACCCAACAACAATACAAGAAAA-----
-AGTATACATATA------------GGACCAGGGAGAGCA---TTT------TATACAACAGGGGGA------
---------ATAATAGGAAATATA------------AGACAAGCACATTGT---AACGTTAGTGAAACA---
AAATGGAAAAAAACTTTAAAACAGGTAGGTGAAAAATTAAGA---------------GAACATTTT-------
----------------------------------AATAAAACAATAGTCTTTAAC------
CAATCCTCAGGAGGGGACCCAGAAATTGTA---ATGCACAGTTTTAATTGTGGAGGGGAA---
TTTAAGCAGCTCCAGGCAAGAGTCCTGGCTGTGGAAAGATACCTAAGGGATCAGCAGCTCCTGGGGATTTGGG
GTTGCTCGGGAAAACTCATCTGCACCACTGCTGTGCCTTGGAATACTAGTTGGGGT----------------
---------------------------AATAAATCTCTGACTGAGATTTGG------GAT------
AACATGACCTGGATGCAGTGG---GAAAAAGAAATTGACAATTACACAAGCTTAATATAC---
ACCTTAATTGAAGAATCGCAGAACCAACAGGAA---------------------------------------
----
AAAAATGAACAAGAATTATTGGGATTGGATAAATGGGCAAGTCTGTGGAATTGGTTTGACACAACACATTGGC
TGTGGTATATAAAAATATTTATAATGATAGTAGGAGGCTTGATAGGTTTAAGAATAGTTTTTACT---
GTACCTTCTTTAGTA---------TATAGAGTTAGGCAGGGATATTCACCA------
TTATCGTTAGCTGTTAGCTTGCTCAATACCATAGCCATAGCCATAGCTGAG---
GGGGCAGATAGGGTTAGAGAAGTGTTACAAAGAGTTTTTAGAGCT---------------------------
--------ATTCTCAACATA------CCTAGACGAATAAGACAGGGCTTGGAAAGGGCTTTGCTA---TAA
>AJ418521
GCT---GAA---
GAACAATTGTGGGTCACAGTCTATTATGGGGTACCTGTGTGG---AAAGAAGCAACC---
ACCACTCTATTCTGTGCATCAGATGCTAAAGCATATGATACAGAG---GTACATAAT----------------
--------------GTTTGGGCCACA---CATGCCTGTGTACCCACAGAC---
CCCAACCCACAAGAAGTAGCATTGGTA---------AATGTGACAGAAAATTTTAAC---------------
--------------------------ATGTGGAAAAATAACATGGTAGAACAGATGCATGAGGATATA---
ATCAGTTTGTGGGATCAAAGCCTA---AAGCCATGTGTAAAATTA---
ACCCCACTCTGTGTTACTTTAAATTGCACTGATGTGAATGCTACTAATGCTGAGAATGCTACTACT-------
----
-------------------CCCACTAGTAGTAGCGGGGGACTGATGGAGAGAGGAGAAATAAAA---
AACTGCTCTTTCAATATCACCGCTAGCATAAGA--------------------------
GATAAGATGCAAAGAGAATATGCATTG---TTTTATAAACTTGATGTAGTACCAATAGATAGG---------
----

GATAATACTAGCTATAGGTTGATAAGTTGTAACACCTCAGTCATTACACAA---
GCCTGTCCAAAGGTATCCTTTGAGCCAATTCCCATACATTATTGTGCCCCGGCTGGTTTTGCGATTTTAAAGT
GTAGA---GATAAGAAGTTCAAT------GGAACAGGACCATGTAAAAAT----------------------
-GTCAGC---ACAGTACAATGTACACATGGAATTAGGCCAGTAGTATCAACT---
CAACCGCTGTTAAATGGCAGCCTAGCAGAAGAAGAG-----------
GTAGTAATTAGATCTGTCAATTTCTCGGACAAT---------GCTAAAACCATAATAGTACAGTTGAAA----
-----------GAAGCTGTAGAA---------ATTAAGTGTACAAGACCCAGCAACAATACAAGAAAA-----
-AGCATACCCATA------------GGACCAGGGAGAGCA---TTT------TATACAACAGGCGAA------
---------ATAATAGGAGATATA------------AGACAAGCACATTGT---AACATTAGTGGAGAA---
AAATGGAATAACACTTTAGGACAGATAGTTAAAAAATTAAAA---------------GAACAATTTGAG----
----------------------------------AATAGCACAATAGTCTTTAAT------
CAATCTTCAGGGGGGGACCCAGAAATTGTA---ATGCACAGTTTTAATTGTGGAGGGGAA---
TTTAAGCAGCTCCAGGCAAGAGTCCTGGCTGTGGAAAGATACCTACAGGATCAACAGCTCCTGGGGATTTGGG
GCTGCTCTGGAAAACTCATCTGCACCACTGCTGTGCCTTGGAATACTAGTTGGAGT----------------
---------------------------AATAAATCTATAGATGCGATTTGG------CAT------
AACATGACCTGGATGGAGTGG---GAAAGAGAAATTGACAATTACACAAACTCAATATAC---
TCCTTACTCGAAAAATCGCAAAACCAACAAGAA---------------------------------------
----
AAAAATGAACAAGAATTATTGGAATTAGATGAATGGGCAAGTTTGTGGACTTGGTTTGACATAACAAACTGGC
TGTGGTATATAAAAATATTCATAATAATAGTGGGAGGCTTGGTAGGTTTAAGAATAGTTTTTGTT---

GTACTTTCTCTAGTA---------AATAGAGTTAGGCAGGGATATTCACCA------
TCATCGTTGGCTGTTAGCTTGCTTAATACCACAGCAATAGCAGTAGCTGAA---
GGGACAGATAGGGTTATAGAAGTATTACAAAGAATTTTTAGAGCT----------------------------
--------TTTATCCACATA------CCTAGGAGAATAAGACAGGGCTTTGAAAGGGCTTTGCTA---TAA
>U36869
GCT---GCA--
GAACAATTGTGGGTCACAGTCTATTATGGGGTACCTGTGTGG---AAAGAGGCAACC---
ACCACTCTATTTTGTGCATCAGATGCTAAAGTATATGATACAGAA---GTACATAAT----------------
--------------GTTTGGGCCACA---CATGCCTGTGTACCCACAGAC---
CCCAACCCACAAGAATTAGTATTGGAA---------AATGTGACAGAAAATTTTAAC----------------
--------------------------GCATGGAAAAATAACATGGTAGAACAAATGCATGAGGATATA---
ATCAGTTTATGGGATCAAAGCCTA---AAGCCATGTGTAAGATTA---
ACCCCACTCTGTGTTTCTTTAAATTGCATTGATGTGGAGAGAAATAATACTAATACCACTAGTACT-------
----
-------------------AACTCTAGTAGTAGCTGGGGAACAATAGAGAAAGGAGAAATAAAA---
AACTGCTCTTTCAATATCACCACAAGCATAAGA---------------------------
GATGAGGTGAAAAAAGAATATGCAACT---TTTTATAAACTTGATGTAGTACCAATAGATAAT----------
----

AGTAATAATAGTTATAGGTTGTTAAGTTGTAATACCTCAGTCATTACACAG---
GCCTGTCCACAGATATCCTTTGTGCCAATTCCCATACATTATTGTGCCCCAGCTGGTTTTGCGATTCTAAAGT
GTAAA---GATAAAAAGTTCCAG------GGAAAAGGACCATGTAACAAT-----------------------
-GTCAGC---ACAGTACTATGTACACATGGAATTAGGCCAGTAGTGTCAACT---
CAACTGCTGTTAAATGGCAGTCTAGCAGAAGAAGAG------------
GTAGTAATTAGATCTGACAATTTCTCGGACAAT---------GCTAATACCATAATAGTACAGCTGAAC----
-----------AAATCTGTAGAA---------ATTAATTGTATAAGACCCAACAACAATACAAGAAAA-----
-GGTATACCTATG------------GGACCAGGGAGAGCA---TTT------TATACAACAGGACAA------
---------ATAATAGGAGATATA------------AGACAAGCACACTGT---AACATTAGTAGAGCA---
CAATGGAACAACACTTTAAAACAGATAGTTATAAAATTAAGA---------------GAACAATTTAAC----
-----------------------------------AATAGCACAATAGTCTTTAAT------
CAATCCTCAGGAGGGGACCCAGAAATTGTA---ATGCACAGCTTTAATTGTGGAGGGGAA---
TTTAAGCAGCTCCAAGCAAGAGTCCTGGCTGTAGAAAGATACCTAAAGGATCAACAGCTCCTGGGGATTTGGG
GTTGCTCTGGAAAACTCATCTGCACCACTGCTGTGCCTTGGAATACTAGTTGGAGT-----------------
----------------------------AACAAATCTCTAAAGGCAATTTGG------GAT------
AACATGACCTGGATGCAGTGG---AAAAGGGAAATCGACAATTACACAGGCTTAATATAC---
ACCTTAATTGAAGAGTCGCAGTACCAACAAGAA--

AGGAATGAAAAAGAATTATTAGAATTAGATAATTGGGCAAGTTTGTGGAATTGGTTTGACATATCAAATTGGC
TGTGGTATATAAAAATATTTATAATGATAGTAGGAGGCTTGGTAGGTTTAAGAATAGTTTTTACT---
GTACTTTCTATAGTG---------AATAGAGTTAGGCAGGGATACTCACCA------
TTGTCATTTGCTGTTAGCTTGCTCAACGCCACAGCCATAGCAGTAGCTGAG---
GGGACAGATAGGATTTTAGAAATATTTCAAAGAGCTTATAGAGCA----------------------------
--------TTTATTCACATA------CCTGTAAGAATAAGACAGGGCTTAGAAAGGGCTTTGCTA---TAA
>U36872
GCT---AGA--
GAAAATTTGTGGGTCACAGTCTATTATGGGGTACCTGTGTGG---AATGAAGCAACC---
ACCACTCTATTTTGTGCATCAGATGCTAAAGCATATGATACAGAG---GTACATAAT----------------
--------------GTTTGGGCCACG---CATGCCTGTGTACCCACAGAC---
CCCAACCCACAGGAAATAAAATTGGAA---------AATGTGACAGAAAATTTTAAC----------------
--------------------------ATGTGGAAAAATAACATGGTAGAACAGATGCATGAGGATATA---
ATCAGTTTATGGGATCAAAGCCTA---AAGCCATGTGTAAAATTA---
ACCCCACTCTGTGTTACTTTACATTGCACTGATGCTAATTGCACTAAGAATTGTACTAATTCGGAAAATGCT-
----
----------------GCCATTAGTAATAGCACAGGAATGATAATGGAAAAAGGAGAAATAAAA---
AACTGCTCTTTCAATATCACCTCAAATATAAGA---------------------------
GATAAGTGGCAGAAAGAATATGCACTT---TTTTATAAACTTGATGTAGTACCAATAGATAAG----------
----

AATAATAATGCCAGCTATAGGTTGATAAGTTGTAACACCTCAGTCATTACACAG---
GCCTGTCCAAAGGTATCCTTTGAGCCAATTCCCATACATTATTGTACCCCGGCTGGTTTTGCGCTGCTAAAGT

GTAAT---GATAAGAAGTTCAAT------GGAACAGGACCATGTACAAAT-----------------------
-GTCAGC---ACAGTACAATGTACACATGGAATTAGGCCAGTAGTATCAACT---
CAATTGCTGCTAAATGGCAGTCTAGCAGAAGAAGAG------------
ATAGTAATTAGATCTGAAAATTTTTCGAACAAT---------GCTAAAACTATAATAGTACAGCTGACA----
-----------AAACCTGTAGTA---------ATTAATTGTACAAGACCCAACAACAATACAAGAAAA-----
-AGTATACCTATA------------GGACCAGGAAGAGCA---TTT------TATGCAACAGGAGAC------
---------AATATAGGAGACATA------------AGACAGGCACATTGT---AACCTTAGTAAAACA---
GACTGGAATAACACTTTAAAACAGATAGTTGAAAAATTAAGA---------------GAAGAATTTGGG----
-----------------------------------AATAAAACAATAGCCTTTAAT------
CAATCCTCAGGAGGGGACCCAGAAATTGAA---ATGCACAGTTTTAATTGTGGAGGGGAA---
TTTAAGCAGCTCCAGGCAAGAGTCCTGGCTGTGGAAAGATACCTAAAGGATCAACAGCTCCTGGGGATTTGGG
GTTGCTCTGGAAAACTCATTTGCACCACTGCTGTGCCTTGGAATACTAGTTGGAGT-----------------
----------------------------AACAAAACTATGCAGGAGATTTGG------GAC------
AACATGACCTGGATGCAGTGG---GAAAAAGAAATTGAAAATTACACAGGCTTAATATAC---
ACATTAATTGAAGAATCGCAGAACCAACAAGAA--

AAGAATGAACAAGACTTATTGGCACTAGATAAATGGGACAGTTTGTGGAATTGGTTTAGCATAACAAAATGGC
TGTGGTACATAAAAATATTCATAATGATAGTAGGAGGCTTAATAGGTTTAAGAATAGTTTTTACT---
GTATTTTCTATAGTG---------AATAGTGTTAGGAAGGGATACTCACCA------
TTATCGTTTGCTATTAGCTTGCTCAACGCTACAGCCATAGCAGTAGGTGAG---
GGGACAGATAGGATTATAGAAGTAGTACAAAGGGCTTGTAGAAGA----------------------------
---------------------------------GAATAGAACAGGGTTTGGAAAGGGCTTTGCTA---TAA
>AJ286342
GCT---GCA--
GAAAATTTGTGGGTCACAGTCTATTATGGGGTACCTGTGTGG---AAAGAAGCAACC---
ACCACTCTATTTTGTGCATCAGATGCTAAAGCATATGACACAGGG---GTACATAAT----------------
--------------GTTTGGGCCACA---CATGCCTGTGTACCCACAGAC---
CCTAACCCACAAGAAGTAGTATTGGAA---------AATGTGACAGAAAATTTTAAT----------------
--------------------------ATGTGGAAAAATAACATGGTAGAACAGATGCAGGAGGATATA---
ATCAGTTTATGGGATGAAAGCCTA---AAGCCCTGTGTGAAATTA---
ACCCCACTCTGTGTTACTTTAAATTGCACTAATTGGAAGAATGTTAATAATACCAATAGTACTAGTCCC----
----
-------------------AATAATAGTACTAGTTGGGGAAAGATGGAGGAAGGAGAAATAAAA---
AACTGCTCTTTCAAGGTCACCACATCCATAAAA---------------------------
AATAAGATGCAGAAAGAATATGCACTT---TTTTATAAACTTGATGTAGTACCAATAGATAAG----------
----

AAAAATACAAGATTTATGTTGATACATTGTAACACCTCAGTCATTACACAA---
GCCTGTCCTAAGGTAACCTTTGAACCAATTCCCATACATTATTGTGCCCCGGCTGGTTTTGCGATTCTAAAGT
GTAAT---GATAAGAAGTTTAAT------GGAAAAGGAACATGTAACAAT-----------------------
-GTCAGC---ACAGTACAATGTACACATGGAATTAGGCCAGTGGTGTCAACC---
CAACTGCTGTTAAATGGTAGTCTAGCAGAAGAAGAG------------
GTAGTACTTAGATCTGAAAATTTCACGGACAAT---------GTTAAAACCATAATAGTACAGCTGAAT----
-----------GAAACTGTAGAA---------ATTAATTGTACAAGACCCGGCAATAATACAAGAAAA-----
-AGGATAGCTATA------------GGACCAGGGAGAGCA---GTT------TATGCAACAAGACAA------
---------ATAATAGGAGATATA------------AGACAAGCACATTGT---AACATTAGTAGACAA---
AAATGGAATAAGACTTTAAAACACATAGTTGCAAAATTAAAA---------------GAACAATTTGGT----
--------------------------------AATAATAAAACAATAGTCTTTAAG------
CAATCCTCAGGAGGGGACCCAGAAATTATA---ATGCACACCTTTAATTGTGCAGGGGAA---
TTTAAGCAGCTCCAGGCAAGAGTCCTGGCTGTGGAAAGATACCTAAGGGATCAACAGCTCCTAGGGATTTGGG
GTTGCTCTGGGAAACTCATTTGCACCACTGCTGTGCCTTGGAATGCTAGTTGGAGT-----------------
----------------------------AATAAATCTCAGGATGAGATTTGG------AAT------
AACATGACCTGGATGCAGTGG---GAGAGAGAAATTGACAATTACACAGATTTAATATAC---
ACCTTAATTGAAAATTCGCAGAACCAGCAAGAA--

AAGAATGAACAAGAATTATTGGAATTGGATAAGTGGGCAAGTTTGTGGAATTGGTTTGACATAACAAAATGGC
TGTGGTATATAAAAATATTCATAATGATAGTAGGAGGTTTAGTAGGTTTAAGAATAGTTTTTACT---
GTACTTTCTATAGTG---------AATAGAGTTAGGCAGGGGTACTCACCA------
TTATCGTTCGCTGTTAGTTTGTTTAATGCAATAGCTATAGCAGTAGCTGAG---

GGGACAGATAGGATTATAGAAATAGTACAAAGAATTGGTAGAGGT----------------------------
--------ATCCTCCACATA------CCAGTAAGAATAAGACAAGGCTTAGAAGGGGCTTTGTTA---TAA
>U36875
GCT---GGA--
GAACAATTGTGGGTCACAGTCTATTATGGGGTACCTGTGTGG---AAAGAGGCAACC---
ACCACTCTATTTTGTGCATCAGATGCTAAATCATATGATACAGAG---GTACATAAT----------------
--------------ATGTGGGCCACA---CATGCCTGTGTACCCACAGAC---
CCCAACCCACAAGAAGTAGTATTGGGA---------AATGTGACAGAAAATTTTAAC---------------
-------------------------ATGTGGAAAAATAACATGGTAGAACAGATGCATGAGGATATA---
ATCAGTTTATGGGATCAAAGCCTA---AAGCCATGTGTAAAATTA---
ACCCCACTCTGTGTTACTTTAAATTGCACTGATAATTTAGATAATGGTACCATTAGTAATGCTACTCATACCA
CTAATAGTAGC---
-AGTGTTAATAATACCACTGGTAATAGCACGAGAAAGCTGGAGATAGACAGAGGAGAAATAAAA---
AACTGTTCTTTCAATATTACCACAAACATAAAA--------------------------
GATAAGATGCAGAAAGCGCATGCACTT---TTTTACAAACTTGATGTAGTACCAATAGATAAT----------
----

AGTACTACCAGCTATAGGTTGATAAGTTGTAACACCTCAGTCATTACACAG---
GCCTGTCCAAAGGTATCCTTTGAACCAATTCCTATACATTTTTGTGCCCCGGCTGGTTTTGCGATTCTAAAGT
GTAAT---AATAAGACGTTCAAT------GGAAAAGGACCATGTAAAAAT----------------------
-GTCAGC---ACAGTACAATGTACACATGGAATTAGGCCAGTAGTATCAACG---
CAACTGCTGTTAAATGGCAGTCTAGCAGAAGATGAG------------
GTAGTAATTAGATCTGAAAACTTCACAGACAAT---------GCTAAAACCATAATAGTACACCTGAAT----
-----------GAATCTGTAGAA---------ATTAATTGTACAAGACCCAACAACAAGACAAAGAGA-----
-AGTTTTCATATA------------GGACCAGGGAGAGCA---TTT------TTAACAGAG------------
---------ATAGTAGGAGATATA------------AGACAAGCATATTGT---AACATTAGTAGAGCA---
AAATGGAATAACACTTTACACCAGGTAGTTAACAAATTAAGA--------------GAACAATTTGGA----
-----------------------------------AATAGAACAATAGTCTTTAAA------
AATTCCTCAGGAGGGGACCCAGAAATTGTA---ACACACATGTTTAATTGTGCAGGGGAA---
TTTAAGCAGCTCCAGGCAAGAATCCTGGCTGTGGAAAGATACCTAAGGGATCAACAGCTCCTGGGAATTTGGG
GTTGCTCTGGAAAACTCATTTGCCCCACTACTGTGCCTTGGAATGCTAGTTGGAGT----------------
----------------------------AATAAAAATCTGAGTCAGATTTGG------GAT------
AACATGACCTGGATGGAGTGG---GAAAGAGAAATTGACAATTACACAAGCTTAATATAC---
ACCTTAATTGAAGAATCGCAGAACCAGCAAGAA---------------------------------------
----
AAGAATGAACAAGACTTATTGGAATTAGATAAATGGGCAAGTTTATGGAATTGGTTTAGCATAACAAATTGGC
TGTGGTATATAAAAATATTCATAATAATAGTAGGAGGCTTGATAGGTTTAAGAATGGTTTTTACT---
GTGCTTTCTATAGTG---------AATAGAGTTAGGCAGGGATACTCACCA------
TTATCGTTTGCTATTAGCTTGTTCAACGCCACAGCCATAGCAGTAGCTGAG---
GGGACAGATAGGGTTATAGAAGTAATACAAAGAGCTTGTAGAGCT---------------------------
--------ATTCTCCACATA------CCTAGAAGAATTAGACAGGGCCTGGAAAGGGCTTTGCAA---TAA
>U36879
GCT---GCA--
GAACAATTGTGGGTCACAGTATATTATGGGGTACCAGTGTGG---AAAGAAGCAACT---
ACCACTCTATTTTGTGCATCAGATGCTAAAGCATATGATACAGAG---GTACATAAT----------------
--------------GTTTGGGCCACA---CATGCCTGTGTACCCACAGAC---
CCCAACCCACAAGAAGTAGTATTGGGA---------AATGTGACAGAAAATTTTAAC---------------
-------------------------ATGTGGAACAATAGCATGGTAGAACAGATGCATGAGGATATA---
ATTAGTTTATGGGATCAAAGCCTA---AAGCCATGTGTAAAATTA---
ACCCCACTCTGTGTTACTTTAAATTGTAGTAATGTAGATAATACTAATAATGATACT----------------
----
----------------------------AATCCCACTAGTAGTAGTGGGGAAGGAGAACTAAAA---
AACTGCTCTTTCAATATCACCACAAGAATAGGA--------------------------
GAAAAGGTTCAGAGAAATTATGCACTT---TTTTATACCCTTGATATAGTACCAATAGATAAG----------
----

GATGAAAATATTACTAAATATAGGTTGATAAATTGTAACACCTCAATCATTACACAG---
GCCTGTCCAAAGGTATCATTTGAACCAATTCCCATACATTATTGTCCCCCGGCTGGTTTTGCGATTCTCAAGT
GTAAT---AATAAGACATTCGAT------GGAAAGGGACGATGTCCAAAT---------------------
-GTCAGC---ACAGTACTATGTACACATGGAATTAGGCCAGTAGTGTCAACT---

CAACTGCTGTTAAATGGCAGTCTAGCAGAAGAAGAG-----------
GTAGTAATTAGATCTGACAATTTCTCGGACAAT---------GCTAAAACCATAATAGTACAGCTGAAG----
-----------GAATCTGTACAA---------ATTAATTGTACAAGACCCAACAACAATACAAGAAAA-----
-AGTATACATATA------------GGACCAGGGGAAGCA---CTT------TTTACAACA---GAT------
---------ATAATAGGAAATATA------------AGACAAGCACATTGT---AACCTTAGTAGAACA---
CAATGGAATAACACTTTAAAACAGATAGCTATAAAATTAAGC--------------GAACAATTTAGG----
----------------------------------AATAAAACAATAGTCTTTAAT------
CAATCCTCAGGAGGGGACCTAGAACTTGTA---ATGCACACCTTTAATTGTGGAGGGGAA---
TTTAAGCAACTCCAAGCAAGAGTCCTGGCTGTGGAAAGATACCTGAGAGATCAACAGCTCCTAGGGATTTGGG
GTTGCTCTGGAAAACTCATTTGCACCACTGCTGTGCCTTGGAATGCTAGTTGGAGT-----------------
---------------------------AATAAATCCCTGGATAACATTTGG------AAT------
AACATGACCTGGATGGAGTGG---GAAAGAGAAATTGACAATTACACAAGCTTAATATAC---
ACCTTACTTGAAAAATCGCGGAACCAACAAGAA---

AAGAATGAACAAGAATTATTGGAATTAGATAAGTGGGCAAGTTTGTGGAATTGGTTTGACATAACAAACTGGC
TGTGGTATATAAAAATATTCATAATGATAGTAGGAGGCTTGGTAGGTTTAAGAATAGTTTTTGCT---
GTACTTTCTATAGTG---------AATAGAGTTAGGCAGGGATACTCACCA------
TTATCATTGGCTGTTAGCTTGTTCAATACAATCGCTATAGCAGTAGCTGAG---
GGGACAGATAGGGTTATAGAAGTAGTACAAAGAGCTTTTAGAGCT----------------------------
--------GTTCTCCACATA------CCTGTAAGAATAAGACAGGGCTTGGAAAGGGCTTTGCTA---TAA
>U36880
GCT---GCA---
GAACAATTGTGGGTCACAGTCTATTATGGGGTACCTGTGTGG---AAAGAAGCAACC---
ACCACTCTATTCTGTGCATCAGATGCTAAAGCATATGCCACAGAG---GTACATAAT----------------
--------------GTCTGGGCCACA---CATGCCTGTGTACCCACAGAC---
CCCGACCCACAAGAAGTAGAATTGGGA---------AATGTGACAGAAAATTTTAAC----------------
-------------------------ATGTGGAAAAACAACATGGTAGAACAGATGCATGAGGATATA---
ATCAGTTTATGGGATCAAAGCCTA---AAGCCATGTGTAAAATTA---
ACCCCACTTTGTGTTACTTTAGATTGCTTTGATGTTAATTTTAATGCTACTAATTCCACTAGCAGTAAGGAT-
----
-------------ACTAATACCAATAATAGTAGAGTGGAAATGATAGAGAAAGGAGAAATAAAA---
AACTGCTCTTTCAATATCTCCACAGACATAAGA--------------------------
GATAAGTTGCAGAAAGAATATGCGCTT---TTTTATAAACTTGATGTAATACCAATAGATAAT----------
----

AGTAATACAAGCTATAGGTTGATAAGTTGTAATACCTCAGTCCTTACACAG---
GCCTGTCCAAAAGTATCCTTTGAGCCAATTCCCATACATTATTGTGCCCCGGCTGGTTTTGCGATTCTAAAGT
GTAAT---AATAAGACGTTCAAT------GGAACAGGACCATGCACAAAT----------------------
-GTCAGC---ACAGTACAATGTACACATGGAATTAGACCAGTAGTATCAACT---
CAACTGCTGTTGAATGGCAGTCTAGCAGAAGAAGAG-----------
GTAGTACTTAGATCTGAAAATTTCACAAACAAT---------GCTAAAACCATAATAGTACAGCTAAAA----
-----------GAAGCTGTAGAA---------ATTAATTGTACAAGACCCAGCAATAATACAAGAAAA-----
-AGTATACCTATA------------GGACCAGGGAGAGCA---TGG------TATGCAACAGGAGAA------
---------ATAATAGGAGATATA------------AGAAAAGCACATTGT---ACCATTAATAGAGCA---
AAATGGAATAACACTTTAAAACAGATAGTCGGAAAATTAGGA--------------GAACAATTTGTG----
----------------------------------AATAAGACAATAGTCTTCAAT------
CACTCCTCAGGAGGGGACCCAGAAATTGTA---ATGCACAGTTTTAATTGTGGAGGGGAA---
TTTAAGCAGCTCCAGGCAAGAGTCCTGGCTGTGGAAAGATACCTAAGGGATCAACAGCTCCTGGGGATCTGGG
GTTGCCCTGGAAAACTCATTTGCACCACTGCTGTGCCTTGGAATGCTAGTTGGAGT-----------------
-------------------------AATAATAGAAATCTGACTGAGATTTGG------GAT------
AACATGACATGGATGGAGTGG---GAAAGAGAGATTGATAATTACACAAGCTTAATATAC---
AACTTACTTGAAGAATCGCAGAACCAGCAAGAA---

AAGAATGAACAAGAATTATTGGAATTAGATAAATGGGCAAGTTTGTGGAGTTGGTTTAGCATAACAAATTGGC
TGTGGTATATAAGAATATTCATAATGATAGTAGGAGGCTTGATAGGTTTAAGAATAGTTTTTACT---
GTACTTTCTATAGTG---------AATAGAGTTAGGCAGGGATATTCACCA------
TTGTCGTTTGCTGTTAACTTGTTTAATGCCACAGCAATAGCAGTAGCTGAG---
GGAACAGATAGGGTTATAGAAGTAATACAAAGAGCTTTTAGAGCT----------------------------
--------ATTCTCCACATA------CCTAGAAGAATAAGACAGGGCTTGGGACTAGCTTTACTA---TAA
>U23487

GCT---GAA--
GAAAAATTGTGGGTCACAGTCTATTATGGGGTACCTGTGTGG---AAAGAAGCAACT---
ACCACTCTATTTTGTGCATCAGATGCCAAAGCACATGGGACAGAG---GTACATAAT---------------
--------------GTTTGGGCCACA---CACGCTTGTGTACCCACAGAC---
CCCAACCCACAAGAAATAGTATTGGAA---------AATGTGACAGAATATTTTGAC---------------
--------------------------ATGTGGAAAAATAACATGGTAGAACAGATGCAGGAGGATGTA---
ATCAGTTTATGGGATCAAAGCCTA---AAACCATGTGTAAAATTA---
ACCCCACTCTGTGTTACTTTAGATTGCACTGATTATGTAGGGAATGCTACTAATACCACTAGCACTAAT----
----
-------------------AATACCGCTAGTGGAAGTTGGGGAGCGATGAGAGGGGAAATAAAA---
AACTGCTCTTTCAATATCACTACAAACATAAGA---------------------------
GATAAGGTGCATAAAGAATATGCACTT---TTTTATAAACTTGATGTAGTACCAATAGAAAAG----------
----

AAGAATACTAGCTTTAGATTGATAAGTTGTAACACCTCAACCATTACACAG---
GCCTGTCCAAAGGTATCCTTTGAGCCAATTCCCATACATTATTGTACCCCGGCTGGTTTTGCGATTCTAAAGT
GTAAC---AATAAGAAGTTTGAT------GGAAAAGGACAATGTACAAAT----------------------
-GTCAGC---ACAATACAATGTACACATGGAATTAGGCCAGTAGTGTCAACT---
CAACTGCTGCTAAATGGCAGTCTAGCAGAAGAAGAG-----------
GTGGTACTTAGATCTGACAATTTCACAGACAAT---------GCTAAAACCATAATAGTACATCTGAAT----
-----------GAATCTGTAGAA---------ATTAATTGTACAAGGCCCAGCAACAATTCAAGAAAA-----
-AGTATATACATA------------GGACCAGGGAGAAGA---TTT------CATGTAACAAGAGCC------
---------GTAACAGGAGATATA------------AGACAAGCACATTGT---AACATTAGTAAAGCA---
AAATGGGAGAAAACTCTAAAACAGATAGTTGAAAAATTAAGA---------------GAAAAATTTGGA----
-----------------------------------AACAAAACAATAATCTTTAAT------
CAATCCTCAGGAGGGGACCCAGAAATTGTA---ACTCATAGTTTTAATTGTGGAGGGGAA---
TTTAAGCAGCTCCAGGCAAGAGTCCTAGCTGTGGAAAGATACCTACAGGATCAACAGCTCCTAGGGATTTGGG
GTTGCTCTGGAAAACTCATCTGCACCACTGCTGTGCCTTGGAATTCTAGTTGGAGT----------------
----------------------------AATAAATCTCTGGAACAGATTTGG------GGT------
AACATGACCTGGATGCAGTGG---GAAAAAGAAATTAACAATTACACAGGCTTAATATAC---
ACCTTAATTCAAGAATCACAGAATCAACAAGAA--

AAGAATGAACAAGAATTATTGGAATTGGATAAGTGGGGAAGTTTGTGGAGTTGGTTTAGCATAACAAACTGGC
TGTGGTATATTAAAATATTCATAATGATAGTAGGAGGCTTAGTAGGTTTAAGAATAGTTTTTGCT---
GTACTTTCTTTAGTG---------AATAGAGTTAGGCAGGGATATCCACCA------
TTATCATTTGCTGTTAGCTTGCTCAATGTTACAGCTATCGCAGTAGCTGAG---
GGGACAGATAGGATTATAGAAGTAGTGCAAAGAGTTGGTAGAGCT---------------------------
--------ATTCTCCACATA------CCTGTTAGAATAAGACAGGGCTTAGAAAGAGCTTTGCTA---TAA
>Y13719
GCT---GCA--
GGAAATTTGTGGGTCACAGTCTATTATGGGGTACCTGTGTGG---AAAGAAGCAACC---
ACCACTCTATTTTGTGCATCAGATGCTAAAGCATATGATACAGAG---GTACATAAT---------------
--------------GTTTGGGCCACA---CATGCCTGTGTACCCACAGAC---
CCCAACCCACAAGAAATAGTAATGCAG---------AATGTGACAGAAAATTTTAAC---------------
--------------------------ATGTGGAAAAATAAAATGGTAGAACAGATGCATGAAGATATA---
ATTAGTTTATGGGATCAAAGCCTA---AAGCCATGTGTAAAATTA---
ACCCCACTTTGTGTTACTTTAAATTGTCAAAGCCTAAAGAATAATACCAATAATACCACT------------
----
-------------------------AATAATAACGGGGTAATACAGGGGGAAGGAGAAATGAAA---
AATTGCTCTTTCAATATCACCACAAGCATAAGA---------------------------
GATAAGGTGCAAAAAGAATATGCACTT---TTTTATAGAATGGATGTAATACCAATAGATAAT----------
----

GATAATACTAGTTATAGGTTGATAAATTGTAATACCTCAGTCATTACACAG---
GCCTGTCCAAAGGTAACCTTTGAGCCAATTCCCATACATTATTGTGCCCCGGCTGGTTTTGCGATTCTAAAGT
GTAAT---AATAAGACGTTCAAT------GGAAAAGGTTCATGTAACAAT----------------------
-GTTAGC---ACAGTACAATGTACACATGGAATTAGGCCAGTAGTATCAACT---
CAACTGCTGTTAAATGGCAGTCTAGCAGAAGAAGAG-----------
ATAGTAATTAGATCAGAAAATTTCATGGACAAT---------GCTAAAATCATAATAGTACAACTGAAT----
-----------GAAACTGTAGAA---------ATTAATTGCACAAGACCCAACAACAATACAAGAAAA-----

-AGTATAAATATA------------GGACCAGGCAGAGCA---TTT------TATGCAACAGGAGAC------
---------ATAATAGGAGATATA------------AGGCAAGCACATTGT---AACATTAGTAGAGCA---
AAATGGAATAACACTTTAGGAAAGATAGTTATAAAATTAAGA---------------GAACAATTTGGG----
------------------------------------AATAAAACAATAGTCTTTACT------
CAATCTGCAGGAGGGGACCCAGAAATTGTA---ATGCACAGTTTTAATTGTGGAGGGGAA---
TTTAAGCAACTCCAGGCAAGAGTCCTGGCTGTGGAAAGATACCTAAAGGATCAACAGCTCCTGGGGATTTGGG
GTTGCTCTGGAAAACTCATCTGCACCACTACTGTGCCTTGGAATACTAGTTGGAGT-----------------
-----------------------------AATAAAAATCTGAGTCAGATTTGG------GAC------
AACATGACCTGGATGCAGTGG---GAAAAAGAAATTGAGAATTACACAGACGTAATATAC---
ACCTTAATTGAACAATCGCAAAACCAGCAAGAA---

AAAAATGAACAAGAATTATTGGAGTTAGATAAATGGGCAAGTTTGTGGAATTGGTTTAGCATAACAAGTTGGC
TGTGGTATATAAAAATATTCATAATGATAGTAGGAGGCTTGATAGGTTTAAGAATAGTTTTTACT---
GTACTTTCTATAGTG---------AATAGAGTTAGGCAGGGATACTCACCA------
TTATCGTTCGCTGTTAGCTTGATCAACGCCATAGCCATAGCAGTAGCTGAG---
GGGACTGATAGGGCTATAGAAATATTACAAAGAATTTGTAGAGCT----------------------------
--------ATTCTCAACATA------CCTACGCGAATAAGACAGGGCTTAGAAAGGGCTTTGCTA---TAA
>AJ286330
GCT---ACA---
GAAGAATTGTGGGTCACAGTCTATTATGGGGTACCTGTGTGG---AAAGAAGCAAAC---
ACCACTTTATTTTGTGCATCAGATGCTAAAGCATATGATACAGAG---GCACATAAT----------------
--------------GTTTGGGCCACA---CATGCCTGTGTACCCACAGAC---
CCCAACCCACAAGAAGTAGTATTAGCA---------AACGTGACAGAATATTTTAAC----------------
--------------------------ATGTGGAAAAGTAACATGGTAGAACAGATGCATGAGGATATA---
ATCAGTTTATGGGATCAAAGCCTA---AAGCCATGTGTAAAATTA---
ACCCCATTCTGTGTTACTTTAAATTGCAGTAATGCTACTTATACCAATAGTAGTGAGAATGCTAAG-------
----
----------------------GCTACTCCTACCCCTAAGACGATAGTGGACCCAGAAATGAAA---
AACTGCTCTTTCAATATCACCACAAGCATGAGA--------------------------
GATAAGATGACGAAAGAACAGGCACTT---TTTTATGCACTTGATGTAGTACCAATGGATAATGATAATGAT-
----

ACTATTAGGGACAATGGTACTGCTAGCTATAGGTTGAGAAGTTGTAACACCTCAGTCGTTACACAG---
GCCTGTCCAAAGATAACCTTTGAGCCAATTCCCATACATTATTGTGCCCCGGCTGGTTTTGCGCTTCTAAAGT
GTAAC---AACAAGACATTCAGT------GGAAAAGGACCATGTACAAAT----------------------
-GTCAGC---ACAGTACAATGTACACATGGAATTAAGCCAGTAGTATCAACT---
CAACTGCTGCTAAGTGGCAGTCTAGCCGAAGGAGAG------------
GTAATACTTAGATCTGACAATTTCACGAACAAT---------GCTAAAACCATAATAGTACAGCTGAGT----
-----------ACATCTGTAATG---------ATTAAGTGTTCAAGACCCAGCAACAATACAAGTAAA-----
-AGTATACATATG------------GGACCCGGGAGAGCA---TTT------TATGCAACAGAAAGA------
---------GTAATAGGAAATATA------------AGACAAGCACACTGT---AACCTTACTAGAGCA---
GATTGGAATAGAACTTTAGAAAAGGTAGTTAAAAAATTAGGA---------------GAACAATTTGAG----
-------------------------------AATAAAACAAGAATAAGCTTTAAT------
CAACCCGCAGGAGGGGACCCAGAAATTGTA---ATGCACAGTTTTAATTGTGAAGGGGAA---
TTTAAACAGCTCCAGGCAAGAATCCTGGCTGTGGAAAGATACCTAAGGGATCAACAGCTCCTGGGGATTTGGG
GCTGCTCTGGAAAACTCATTTGCACCACTGCTGTGCCTTGGAATGCTAGTTGGAGT-----------------
-----------------------------AATAGATCTGTGGATTACATTTGG------GAG------
AACATGACCTGGATGGAGTGG---GAAAAAGAAATTGACAATTACACAAACTTAATATAC---
ACCTTACTTGACCACGCGCAGAACCAGCAAGAA---

AAGAATGAACAAGAGTTATTGGAATTGGATAAATGGGCAAGTCTGTGGAATTGGTTTGACATAACAAAATGGC
TGTGGTATATAAAAATATTCATAATGATAGTAGGAGGCTTGGTAGGTTTAAGAATAGTTTTTGCT---
GTGCTTTCTATAGTG---------AATAGAGTTAGGCAGGGATACTCACCA------
TTATCGTTTGCTATTAGCTTGTTCAACGCCACAGCCATAGCAGTAGCTGAG---
GGGACAGATAGGGTTATAGAAGTAGTACAAAGAGCTTTTAGAGCT----------------------------
--------TTTCTCCACATA------CCTAGAAGAATAAGACAAGGCTTAGAAAGAATTTTGCTA---TAA
>AJ286332
GCT---GGA---
GAACAATTGTGGGTCACAGTATATTATGGGGTACCTGTGTGG---AAAGAGGCAACC---
ACCACTCTATTTTGTGCATCAGATGCTAAAGCATATAGTACAGAG---GTACATAAT----------------

--------------GTTTGGGCCACA---CATGCCTGTGTACCCACAGAC---
CCCAACCCACAAGAAGTAGAATTGGAA---------AATGTGACAGAAAATTTTAAC----------------
-------------------------ATGTGGAAAAATGACATGGTAGAACAGATGCATGATGATATA---
ATCAGTTTATGGGATCAAAGCCTA---AAGCCATGTGTGAAATTA---
ACGCCACTCTGTGTTACTTTACATTGCACTGATCTTAAGGCCAATGGTACTAATGCCACTGATACTAGT----
----
----------------GGCACTGGTACTAATACCACTAGTACTAGCGAGAGAGGAGAACTAAAA---
AACTGCTCTTTCAACATCACCACAAGCATAAGA--------------------------
GATAAGGTGCAGACAGAATATGCAATT---TTTTATAAGCTTGATCTAATACCAATGACTAGTAAT-------
----

CCTACCAGTAATAATACCAGCTTTAGGTTGATAAGGTGTAACACCTCAGTCATTACACAG---
GCCTGTCCAAAGGTATCCTTTGAGCCAATTCCCATACATTATTGTGCCCCGGCTGGTTTTGCGATTCTAAAGT
GTAAA---GATAAGAAGTTCAAT------GGAAAAGGAGAATGTACAAAT----------------------
-GTTAGC---ACAGTACAATGTACACATGGAATTAGACCAGTAGTATCAACT---
CAACTGCTGTTAAATGGCAGTCTAGCAGAAGAAGAG------------
GTAGTAATTAGATCTGAAAATTTCACGAACAAT---------GCTAAAACCATCATAGTACAGCTGAAC----
-----------CAATCTCTAGAA---------ATTAATTGTACAAGACCCAACAACAATACAAGAAAA-----
-CGTATATCTATA------------GGACCAGGGAGATCT---TTT------GTTACAACAGGACAA------
---------ATAATAGGAGATATA------------AGACAAGCACATTGT---AACCTTAGCGGGTCA---
CAATGGAATAACACTTTAAAAAGGATAGTTGAAAAATTAGGA---------------GAACAATTTAAG----
---------------------------------AATGTAACAACAATAGTCTTTAAG------
TCATCCTCAGGAGGGGACCCAGAAATTACA---ACGCACAGTTTTAATTGTGGAGGGGAA---
TTTAAGCAGCTCCAGGCAAGAGTCCTGGCTGTGGAAAGATACCTAGGGGATCAACAGCTACTGGGAATTTGGG
GTTGCTCTGGAAAACTCATCTGCACCACTGCTGTACCTTGGAATGCTAGTTGGAGT-----------------
-----------------------------AATAAATCTCTGAACCAGATTTGG------AAT------
AACATGACCTGGATGCAGTGG---GAAAGAGAAATTGACAATCACACAAGCTATATATAC---
GCCTTACTTGAAAAATCGCAAAACCAACAAGAA---

AAGAATGAACAAGAATTATTGGAATTAGATAAATGGGCAAGTTTGTGGAATTGGTTTGACATAACAAAATGGC
TGTGGTATATAAAAATATTCATAATGATAGTAGGAGGCTTGATAGGTTTAAGAATAGTTTTTGCT---
GTACTTTCTGTAGTG---------AATAGAGTTAGGCAGGGATACTCACCA------
TTATCGTTTGCTGTTAGCTTGCTCAATGCCACAGCCATAGCAGTAGCTGAG---
GGGACAGACAGGGTTATAGAAGTATTACAAAGAATTTTTAGAGCT----------------------------
--------TTTATCCACATA------CCTAGAAGAATAAGACAGGGCTTAGAGAGGGCTTTGCTA---TAA
>AJ286334
GCA---GAA---
CAATTGTGGGTCACAGTCTATTATGGGGTACCTGTGTGG---AAAGAAGCAAAC---
ACCGTTCTATTCTGTGCATCAGATGCTAAAGCATATGTTAAAGAG---GCACATAAT----------------
--------------ATTTGGGCCACA---CATGCCTGTGTACCCACAGAC---
CCCAACCCACAGGAAATAATATTGGCA---------AATGTGACAGAAAATTTTAAC----------------
-------------------------ATGTGGAAAAATGACATGGTGGAACAAATGCATGAGGACATA---
ATCAGTTTATGGGATCAAAGCCTA---AAGCCATGTGTAAAATTA---
ACCCCACTCTGTGTTACTTTAAATTGCACTGATTATGTGAAAAATAAGACCACCCCTAGTACTAAT-------
----
-------------------ATCACTAGTGGTGGTGTGGGAGGGATGGAGAAAGGAGAAATAAAA---
AACTGCTCTTTCAAGACTAGCACAGGCATAAAA--------------------------
GATAGGGCACAGAAAGACTATGCACTT---TTTTATAAGGTTGATATAGTACCAATAGATAATAATAAT----
----

AATACTAATACTAATTATACTAATTATATGTTGATAGGTTGTAACACCTCAGTCATTACACAG---
GCCTGTCCAAAAGTATCCTTTGAGCCAATTCCCATACATTATTGTACCCCGGCTGGTTTTGCGATTCTAAAAT
GTAAT---GATAAGAAGTTCAAT------GGAACAGGACCTTGTAGAAAT----------------------
-GTCAGC---ACAGTACAATGTACACATGGAATTAGGCCAGTAGTATCAACT---
CAACTGCTGTTAAATGGCAGTCTAGCAGAAGAAGAG------------
GTAGTAATTAGATCTAACAATTTCTCGGACAAT---------GCTAAAACCATAATAGTACAGCTAAAT----
-----------GAATCTGTAGTA---------ATTAATTGTACAAGACCCAACAACCATACAAGAAAA-----
-AGTATACACCTA------------GGACCAGGGAGGTGG---TCA------GTTTTGGCAACAAAA------
------GACATAATAGGAGATATA------------AGAAAAGCACATTGT---AACGTTAGTGAAGGA---
CAATGGAATAAAACTTTAAGACAGATAGTTGAAAAATTAAAA---------------GAACAATTTAAG----

-----------------------------------AATAAAACAATAGCCTTTAAT------
CAACCCTCAGGAGGGGACCCAGAAATTGTA---ATGCACAGTTTTAATTGTGGAGGAGAA---
TTTAAGCAGCTCCAGGCAAGAGTCCTGGCTGTAGAAAGGTACCTAAAGGATCAACAGCTCCTGGGAATTTGGG
GTTGCTCTGGAAAACTCATCTGCACCACTGCTGTGCCTTGGAATACTAGTTGGAGT-----------------
----------------------------AATAAATCTGAGAGTGACATTTGG------AAT------
AACATGACCTGGATGGAGTGG---GAAAGAGAAATTGACAATTACACAAATTTAATATAC---
ACCTTAATTGAAGAATCGCAGAACCAACAGGAA--

AAGAATGAAAAAGAATTATTGGAATTGGATAAATGGGCAAATTTGTGGTCTTGGTTTAGCATATCAAACTGGC
TGTGGTATATAAAAATATTCATAATGATAGTAGCAGGTTTAGTAGGTTTAAGAATAGTTTTTGCT---
GTGCTTTCTATAGTA---------AATAGAGTTAGGCAGGGATACTCACCA------
TTGTCATTTGCTGTTAGCTTGTTCGATACCACTGCCATAGCAGTAGCTGAA---
GGGACAGATAGGGTCATAGAAGTATTACAAAGAATTTTTAGAGCT----------------------------
--------ATCCTCCACATA------CCTAGAAGAATAAGACAGGGCTTAGAAAGGCTTTGCAA---TAA
>AJ286336
GCT---TTA--
GGACAATTGTGGGTCACAGTCTATTATGGAGTACCTGTGTGG---AAAGAAGCAACC---
ACCACATTATTTTGTGCCTCAGATGCTAAAGCATATGACACAGAG---GTACATAAT----------------
--------------GTTTGGGCCACA---CATGCCTGTGTACCCACAGAC---
CCCAACCCACAAGAAGTAGTATTGGAA---------AATGTGACAGAAAATTTTAAC----------------
--------------------------ATGTGGAAAAATAACATGGTAGAACAGATGCATGAGGATATA---
ATCAGTTTGTGGGATCAAAGCCTA---AAGCCATGTGTAAAAATA---
ACCCCACTCTGTGTTGCTTTAAATTGTAGTAATTTGAATGATACCAATACTAATACCAACTATACTAAT----
----
-------------------ACCAACAATAGTAGTTGGGGAAAGATGGAAAAAGGAGAGATGAAA---
AACTGCTCTTTCAATGTCACCACAGGCATAAGA---------------------------
AATAAGCTGCAGAAAGAATATGCACTT---TTTTATAAACTTGATGTAGTACCAATAGAGGGT----------
----

GATAATACAAGGTATACATTGATAAATTGTAACACCTCAGTCATTACACAG---
GCCTGTCCAAAGGTATCCTTTGAACCAATTCCCATACATTTTTGTGCCCCAGCTGGTTTTGCGATTCTAAAGT
GTAAT---AATAAGACGTTCAAT------GGAACAGGACCATGTACAAAT-----------------------
-GTCAGC---ACAGTACAGTGTACACATGGATTAGGCCAGTAGTGTCAACA---
CAACTACTGTTAAATGGCAGTCTAGCAGAAGAAGAG------------
GTAGTAATTAGATCTGAAAATTTCACAAACAAT---------GCTAAAACCATAATAGTACAGCTGAAG----
-----------ACAGCTGTAGTA---------ATTAATTGTACAAGACCCAACAACAATACAAGAAAA-----
-GGTATACGTATA------------GGACCGGGGAGAGCA---TTT------TTGGTAACAGACCGA------
---------ATAATAGGAGATATA------------AGACAAGCACATTGT---AACATTAGTGGAAAA---
GATTGGAATGAAGCTTTAACACAGACAGTTGCAAAATTAAGA---------------GAACAATTTGGG----
-----------------------------------AATAAAACAATATTCTTTAAT------
CAACCCTCAGGAGGGGACCCGGAAATTGTA---AGGCACACTTTTAATTGTGCAGGGGAA---
TTTAAGCAGCTCCAGGCAAGAGTCCTGGCTGTGGAAAGATACCTAAAGGATCAACAGCTCCTAGGGATTTGGG
GTTGCTCTGGAAAACTCATTTGCACCACTGCTGTGCCTTGGAATACTAGTTGGAGT-----------------
----------------------------AATAAATCTCTGGATCAGATTTGG------AAT------
AACATGACCTGGATGGAGTGG---GAGAGAGAAATTGACAATTACACAAACTTAATATAC---
ACCTTAATTGAAGAATCGCAGAACCAACAAGAA--

AAGAATGAACAAGATTTACTGGCATTGGATAAGTGGGCAAGTTTGTGGAATTGGTTTGACATATCAAATTGGC
TGTGGTATATAAAAATATTCATAATGATAATAGGAGGTTTAGTAGGTTTAAGAATAGTTTTTGCT---
GTGCTTTCTATAGTG---------AATAGAGTTAGGCAGGGATACTCACCA------
TTGTCATTTGCTATTGGTTTGCTTGATGCCACAGCTATAGCAGTAGCTGAG---
GGGACAGATAGGATTATAGAAGTAGTACAAAGAATTTGTAGAGCT----------------------------
--------ATTATCCACATA------CCTAGAAGAATAAGACAAGGCTTAGAAAGGGCTTTACAA---TAA
>AJ286337
GCT---ACA--
GAAGAGAAGTGGGTCACAGTCTATTATGGGGTACCTGTGTGG---AAAGAAGCAACC---
ACCACTCTATTTTGTGCATCAGATGCTAAAGCATATGATACAGAG---GTACATAAT----------------
--------------GTCTGGGCCACA---CACGCCTGTGTACCCACAGAC---
CCCAACCCACAAGAAGTAGAATTGGAA---------AATGTGACAGAAAATTTTAAC----------------
--------------------------ATGTGGAAAAATAACATGGTAGAGCAGATGCATGAGGATATA---

ATTAGTTTATGGGATCAAAGCCTA---AAGCCATGTGTAAAATTA---
ACCCCACTCTGTGTTACTCTAAATTGCAGTGATGATATTAAGAATAATACTAATACTAATACAACT-------
----
----------------------AGTAGTGGTGGGGAAATGATAGAGGAAAAAGGAGAAATAAAA---
AACTGCTCTTTCAATATCACCTCAGACTTAAAA---------------------------
GATAAGGTGCAGAAAGAATATGCATTG---
TTTTATAAACTTGATGTAGAACCAATAGATGATGATAGTAGGAAT----------------------------
-----------------------------AATAAT---
AATAGTACTAGCAATAGGAGTAATAATATTAATACTAGCTATAGGTTAATAAGTTGTAACACCTCAGTCATTA
AACAG---
GCATGTCCAAAGATATCCTTTGAGCCAATTCCCATACATTATTGTACCCCGGCTGGTTTTGCGATTCTACAAT
GTAAG---GATAAGGGGTTCAAT------GGAACAGGTACATGTACCAAT-----------------------
-GTCAGC---ACAGTACAATGTACACATGGAATTAGGCCAGTAGTATCAACT---
CAACTGCTGTTAAATGGCAGTCTATCAGAAGGAGAG------------
GTAGTACTTAGATCTGCCAATTTCAGTGACAAT---------GCTAAAACCATAATAGTACAGCTGAAG----
-----------GAACCTGTACAA---------ATCAATTGTACAAGACCCAGCAACAACACAAGAAAA-----
-AGTATACGTATA------------GGACCAGGGAGTGCA---TTT------TATGCAACAGGAGAA------
---------GTAATAGGAGATATA------------AGGCAAGCACATTGT---AACCTCAGTAAAGTA---
CAATGGAATGACACTTTAGAACAGATAGTTAAAAAATTAGGA---------------GAACAATTTGAG----
-----------------------------------AATAAAACAATAGTCTTTAAT------
CAGTCAGCAGGAGGGGACCCAGAAATTGTA---ATGCACATTTTTAATTGTGGAGGAGAA---
TTTAAACAGCTCCAGGCAAGAGTCCTGGCTGTGGAAAGGTACCTAAAGGATCAACAGCTCCTGGGAATTTGGG
GTTGCTCTGGAAAACTCATCTGCACCACTACTGTGCCTTGGAATGCTAGTTGGAGT-----------------
----------------------------AATAAATCTCTGGATGAGATTTGG------GAT------
AACATGACCTGGATGCAGTGG---GACAGAGAAATTAGCAATTACACAGAATTAATATAC---
TCCTTGATTGAAAAATCGCAGAACCAGCAAGAA--

AAGAATGAACAAGAATTATTGGCATTGGATAAATGGGCAAGTTTGTGGAGTTGGTTTGACATATCAAATTGGC
TGTGGTATATAAGAATATTCATAATGATAGTAGGAGGCTTGATAGGTTTAAGAATAGTTTTTGCT---
GTGCTTTCTATAGTG---------AATAGAGTTAGGCAGGGATACTCACCA------
TTATCGTTTGCTGTTAGCTTGCTCAATGCCACAGCAATAGCAGTAGCTGAA---
GGAACAGATAGGGTTATAGAAATAGTACAAAGAGCTTTTAGAGCT----------------------------
--------ATTCTCCACATA------CCCACAAGAATAAGACAGGGCTTGGAAAGGGCTTTGCTA---TAA
>AF491740
GCT---AGA--
GAAAATTTGTGGGTCACAGTCTATTATGGGGTACCTGTGTGG---AAAGAAGCAACC---
ACCACTTTATTTTGTGCATCAGATGCTAAAGCATATGATACAGAG---GTACACAAT----------------
--------------GTTTGGGCCACA---CATGCCTGTGTACCCACAGAC---
CCCAACCCACAAGAAGTAGTATTGGAA---------AATGTGACAGAAAATTTTAAC----------------
--------------------------ATGTGGAAAAATAACATGGTAGAACAGATGCATGAGGATATA---
ATTAATTTATGGGATCAAAGCCTA---AAGCCATGTGTAAAATTA---
ACCCCACTCTGTGTTACTTTAAATTGCACTGATGCTAATTTCAATAGCACTGATGTGAAGAATGCTACTAATA
CCACT--
----------GCTTCTAATACCACTAGTAGTAGCGGGGGAATGATGGAGGCAGGAGAAATAAAA---
AATTGCTCTTTCAATATTACCACACGCCTAAGA---------------------------
GATAAGGTGCAGAAAGAATATGCACTT---TTTTATAAACTTGATGTAGTACCAATAGGAAAG----------
----

GATAATACTAGCTATAGGTTGATAAGTTGTGACACCTCAACCGTTACACAG---
GCCTGTCCAAAGGTATCCTTTGAGCCAATTCCCATACATTATTGTACCCCGGCTGGTTTTGCGATTATAAAGT
GTAAT---GATAAGAAGTTCAAT------GGAACAGGAACATGTACAAAT-----------------------
-GTCAGC---ACAGTACAATGTACACATGGAATTAGGCCAGTAGTATCAACT---
CAGTTACTGTTAAATGGCAGTCTAGCAGAGGAAGAG------------
GTAGTAATTAGATCCGAAAATTTCACGAACAAT---------GCTAAAAACATAATAGTACAGCTGAAT----
-----------GAAACTGTAGAA---------ATTAATTGTACAAGGCCCAACAACAATACAAGAAAA-----
-AGTATACCTATG------------GGACCAGGCAAAGCA---TTT------TATGCAACAGGAGAC------
---------ATAATAGGAGATATA------------AGACAAGCACATTGT---AACATTAGTAGAACA---
AAATGGAATAAAACTTTAAAACAGATAGTTATAAAGTTAAGA---------------GAACAATTTGGA----
--------------------------------TATAATAAAACAATAATCTTTAAT------
CAATCCTCAGGAGGGGACCCAGAAATTGTA---ACGCACAGTTTTAATTGTGGAGGGGAA---

TTTAAGCAGCTCCAGGCAAGAATCCTGGCTGTGGAAAGATACCTAAAGGATCAACAGCTCCTGGGAATTTGGG
GCTGCTCTGGAAAACTCATTTGCACCACTGCTGTGCCTTGGAATACTAGTTGGAGT-----------------
-----------------------------AATAAATCTATGGAACAGATTTGG------GAC------
AACATGACCTGGATGGAGTGG---GAAAGAGAAATCAGTAATTACACAGACTTAATATAC---
ACCTTAATTGAAAAATCGCAAAACCAGCAAGAA---

AAGAATGAACAAGAATTATTGGAATTAGATAAATGGGCAAGTTTGTGGAATTGGTTTGACATAACAAATTGGC
TGTGGTATATAAAAATATTCATAATGATAGTAGGAGGCTTGATAGGTTTAAGAATAGTTTTTACT---
GTACTTTCTATAGTG---------AATAGAGTTAGGCAGGGATACTCACCA------
TTATCGTTCGCTGTTAGCTTGCTCAACGCCACGACCATAGCAGTAGCTGAG---
GGAACTGATAGGGTTATAGAAATAGTACAAAGAGCTTATAGAGCT----------------------------
--------ATTATCCACATA------CCTACGCGCATAAGACAGGGCTTGGAAAGGGCTTTGCTG---TAA
>HQ122397
GCT---GCA---
GAAAATTCGTGGGTCACAGTCTATTATGGGGTACCTGTGTGG---AAAGAAGCAACC---
ACCATTCTATTCTGTGCATCAGATGCTAAAGCATATGACACAGAG---GTTCATAAT----------------
--------------GTTTGGGCCACA---CATGCCTGTGTACCCACAGAC---
CCCGACCCACAAGAAATATTATTGCAA---------AATGTGACAGAAGACTTTAAC----------------
--------------------------ATGTGGAAAAATGACATGGTAGAACAGATGCATGAGGATATA---
ATTAGTTTATGGGATCAAAGCCTA---AAGCCATGTGTAAAATTA---
ACCCCACTCTGTGTTACTTTAAATTGCACTGATTTGAAAAATGCTACTAGTACCACTAGT-------------
----
-----------------------------AGTAGCGGGGAAACAATGGGGGGAGGAGAAATGAAA---
AATTGCTCTTTCAATATTACCACAAACATAAAA--------------------------
AATAAAGTGAAGAAAGAATATGCACTT---TTTTATAGCCTTGATGTAATAAAAATAGATAAT----------
----

AGTACTGACTATAGGTTGATAAGTTGTAATACCTCAGTCCTTACACAG---
GCCTGTCCAAAAGTATCCTTTGAACCAATTCCAATACATTATTGTGCCCCGGCTGGTTTTGCGATTCTAAAGT
GTAAC---AATAGGACATTCAAT------GGAACAGGACCATGTAGAAAT-----------------------
-GTCAGC---ACAGTACAATGTACACATGGAATTAGGCCAGTAGTATCAACT---
CAACTGCTGCTAAATGGCAGTCTAGCAGAAGAAGAG------------
GTAATAATTAGATCCGAAAATTTCACGAACAAT---------GCTAAAACCATAATAGTACAACTGAAG----
-----------GAATCTGTAGTA---------ATTAATTGTACAAGACCCAACAACAATACAAGAAGA-----
-AGTATAAGTATA------------GGACCAGGCAGAGCA---TTT------TATGCAACAGGAGAT------
---------ATAATAGGGGATATA------------AGACAAGCACATTGT---AACATTAGTGGAGCA---
AAATGGAATGACACTTTAAAACAGATAGTGATAAAGTTAAGA---------------GAACAATTTAAG----
-------------------------------------AATAAAACAATAGTTTTTAAG------
CAATCCTCAGGAGGGGACCCAGAAATTGTA---CTGCACAGTTTTAATTGTGGAGGAGAA---
TTTAAGCAGCTCCAGGCAAGAGTCCTGGCTGTGGAAAGATACCTAAAGGATCAACAGCTCCTGGGAATTTGGG
GTTGCTCTGGAAAACTCATCTGCACCACTGCTGTGCCTTGGAATACTAGTTGGAGT-----------------
-----------------------------AGTAGATCTGTGGATGATATTTGG------GAG------
AACATGACCTGGATGCAGTGG---GAAAGAGAAATTGATAATTACACAGACATAATATAC---
ACTTTACTTGAAAAATCGCAAAACCAACAAGAA---

AAGAATGAACAGGAATTATTGGAATTAGATAAATGGGCAAGTTTGTGGAATTGGTTTGACATATCAAATTGGC
TATGGTATATAAAAATATTCATAATGATAGTAGGAGGCTTGATAGGTTTAAGAATAGTTTTTTCT---
GTACTTTCTATAGTA---------AATAGAGTTAGGCAGGGATATTCACCA------
TTATCGTTCGCTGTTAGCTTGCTCAATGCCACAGCAATAGCAGTAGCTGAG---
GGAACTGATAGGATTATAGAAGTATTACAAAGAGCTTATAGAGCT----------------------------
--------ATTCTCCACATA------CCTACACGCATAAGACAGGGCTTGGAAAGGCTTTTGCTA---TAA
>JN002029
GCT---ACA---
GAAAAATTGTGGGTCACAGTCTATTATGGGGTACCTGTGTGG---AAGGAAGCAACC---
ACCACTCTATTTTGTGCATCAGATGCTAAAGCATATGATACAGAG---GTACATAAT----------------
--------------GTTTGGGCCACA---CATGCCTGTGTACCCACAGAC---
CCCAACCCACAAGAAGTATTATTGGAG---------AATGTGACAGAAAACTTTAAC----------------
--------------------------ATGTGGAAAAATAACATGGTAGAACAAATGCATGAGGATATA---
ATTAGTTTATGGGATCAAAGCCTA---AAGCCATGCGTAAAATTA---
ACCCCACTCTGTGTTACTTTAAATTGCACTGATTTGAGGAATGCTACTAATACCACTAGT-------------

```
------------------------------------------------------------------------
-------------------------AGTAGCGGGAGAATGACAGTGGAGGGAGGAGAAATGAAG---
AATTGCTCTTTCAATATCACCACAAGTATAAGA--------------------------
GATAAAATGCACAAAGAATATGCATTT---TTTTATAAACTTGATATAGTACCAATAGAAAAG---------
------------------------------------------------------------------------
---------------------
GATAATACTAGCTATAGGTTGATAAATTGTAACACCTCAGTCATTACACAG---
GCCTGTCCAAAGGTATCCTTTAGGCCAATTCCCATACATTATTGTGCCCCGGCTGGTTTTGCGATTCTAAAGT
GTAAT---AATAAGAGGTTCAAT------GGAACAGGACCATGTACAAAT----------------------
-GTCAGC---ACAGTACAATGTACACATGGAATTAGGCCAGTAGTATCAACT---
CAACTACTGTTAAATGGCAGTCTAGCAGAGGAAGAG-----------
GTAGTAATTAGATCCGCCAATTTCTCGGACAAT---------GCTAAAAACATAATAGTACAGCTGAAT----
-----------GAAACTGTAGAA---------ATTAATTGTACAAGACCCAATAACAATACCAGAAAA-----
-AGCATACCTATA------------GGACCAGGCAGAGCA---ATT------TATACAACAGGAGGA------
---------ATAATAGGAGATATA------------AGACAAGCACATTGT---AATCTTAGTAGAGCA---
AAATGGAACGACACTTTAAAACAGATAGCTACAAAGTTAAGA---------------GAACAATTTGGG----
-----------------------------------AATAAAACAATAGTCTTTAAT------
CAATCCTCAGGAGGGGACCCAGAAATTGTA---ACGCACAGTTTTAATTGTGGAGGGGAA---
TTTAAGCAGCTCCAGGCAAGAGTCCTGGCTGTGGAAAGATACCTAAAGGATCAACAGCTCCTGGGGATTTGGG
GTTGCTCTGGAAAACTCATTTGCACCACTGCTGTGCCTTGGAATACTAGTTGGAGT-----------------
----------------------------AATAAATCTCTGGATTATATTTGG------AAT------
AACATGACCTGGATGGAGTGG---GAAAGAGAAATTGATAACTACACAGGCTTAATATAC---
ACCTTAATTGAAGAATCGCAAAACCAACAAGAA---------------------------------------
----------------------------
AAGAATGAACAAGAATTATTGGCATTAGATAAATGGGCAAGTTTGTGGAATTGGTTTGACATATCAAACTGGC
TGTGGTATATAAAAATATTCATAATGATAGTAGGAGGCTTGGTAGGTTTAAGAATAGTTTTTTCT---
GTACTTTCTATAGTG---------AATAGAGTTAGGCAGGGATACTCACCA------
TTATCGTTCGCTGTTAGCTTGCTCAATGCCACAGCCATAGCAGTAGCTGAG---
GGGACAGATAGGGTTATAGAAGTAGTACAAGGAGCTTGTAGAGCT---------------------------
--------ATTCGCCACATA------CCTAGAAGAATAAGACAGGGCTTGGAAAGGATTTTGCTA---TAA
>U36882
GCT---GCA-----------------------------------------
GAACAATTGTGGGTAACAGTCTATTATGGGGTACCTGTGTGG---AAAGAAGCAACC---
ACCACTCTATTTTGTGCCTCAGATGCTAAAGCATATGACACGGAG---GTACATAAT---------------
--------------GTTTGGGCCACA---CATGCCTGTGTACCCACAGAC---
CCTGACCCACAAGAAGTAGCATTGGAA---------AATGTAACAGAAAATTTTAAC---------------
--------------------------ATGTGGAAAAATAACATGGTAGAACAGATGCATGAAGATATA---
ATCAGTTTATGGGATCAAAGCCTA---AAGCCATGTGTAAAAATA---
ACCCCACTCTGTGTTACTTTAAAGTGCACTGATGTCACATTTAATTGCACTGGTACCTCAAATTGCACT----
------------------------------------------------------------------------
----------------GATACCAATAGTAGTAGTCCACTAAAGATGGAAAAAGGAGAGATGAAA---
AATTGCTCTTTCAATGTCACCACAAACATAAGA--------------------------
AATCAGGTGAGGAAAGAATATGCACTT---TTTTATAAACTTGATGTAGTACCAATAAATAAA---------
------------------------------------------------------------------------
---------------------
AATGATACAAATTTTATATTGATACACTGTAATACCTCAGTCATTACACAA---
GCCTGTCCAAAGGTGTCCTTTGAACCAATTCTCATACATTATTGTGCCCCGGCTGGTTTTGCGATTCTAAAGT
GTAAA---GATAAAAAGTTCAAT------GGAACAGGACCATGTAAAAAT----------------------
-GTCAGT---ATAGTACAATGCACACATGGAATTAGGCCAGTAGTGTCAACT---
CAACTGCTGTTAAATGGCAGTCTAGCAGAAGAAGAG-----------
GTGGTAATTAGATCTGAAAATTTCACGAATAAT---------ATTAAAAACATAATAGTACAGCTAAAT----
-----------GAGTCTGTAGAA---------ATTAATTGTACAAGACCCGGCAACAATACAAGAAGA-----
-AGTATAACTATA------------GGACCGGGGAGAGCA---TTC------CATGCAACAGGAAGA------
---------ATAATAGGAGATATA------------AGAAGAGCACATTGT---AACCTTAGTAGAGCA---
CAATGGAATAACACTTTAAAACAGATAGCTACAAAATTAAGA---------------GAACAATTTGGG----
--------------------------------GAAAATAAAACAATAGTCTTTAAT------
CAATCCTCAGGAGGGGACCCAGAAGTTGTA---CTGCACATTTTTAATTGTGGGGGTGAA---
TTTAAGCAGCTCCAGGCAAGAGTCCTGGCTGTGGAAAGATACCTACAGGATCAACGGCTCCTAGGAATTTGGG
GTTGCTCTGGAAGACTCATTTGCACCACTGCTGTGCCTTGGAATGCTAGTTGGAGT----------------
----------------------------AATAAATCTCTGAATGAAATTTGG------GAT------
```

AACATGACCTGGATGGAGTGG---GAGAGAGAAATTGAAAATTATACAGGCTTAATATAC---
AACTTACTTGAAACATCGCAGAACCAGCAGGAA--

AAGAATGAACAAGAATTATTGGAATTGGATAAGTGGCCAAATTTGTGGAATTGGTTTAACATAACAAACTGGC
TGTGGTATATAAAATTATTCATAATGATAGTAGGAGGTTTAATAGGATTAAGAATAGTTTTTACT---
ATACTTTCTATAGTG---------AATAGAGTTAGGCAGGATATTCACCA------
CTATCATTCGCTGTTAGTTTGTTTAATACCACAGCTGTAGCAGTAGCTGAG---
GGGACAGATAGGGTTATAGAAGTAGTACACAGAATTTGGAGAGGT----------------------------
--------ATCCTCCATATA------CCTAGAAGAATAAGACAGGGCTTAGAAAGGGCTTTGCTA---TAA
>DQ207940
GCT---CAA--
CTGTGGGTCACAGTCTATTATGGGGTACCTGTGTGG---AAAGAAGCAACT---
ACCACTCTATTTTGTGCATCAGATGCTAAAGCACATCACACAGAA---GTACATAAT----------------
--------------GTTTGGCCACA---CATGCCTGTGTACCCACAGAC---
CCCAGGCCACAAGAAGTAGTAATGGA----------AATGTGACAGAATATTTTAAT----------------
--------------------------ATGTGGAAAAATAATATGGTAGAACAAATGCATGAGGATATA---
ATCAGTTTATGGGATCAAAGCCTA---AAGCCATGTGTAAAATTA---
ACCCCACTCTGTGTTACTTTAGAATGCACTAATGTTACCAAGAACAGTACTGGCAATAGTAGTGATTTT----
----
-------------------GCCAATAACAGTGCTGTCAATAGTAAGATGGATACAGAAGTAAAA---
AACTGCTCTTTCAATGTTACCACAAGTATAAGG---------------------------
GATAAGATGCAGAAAGAATATGCACTC---TTTTATACAGTTGATATAGTACCAATAGAAAAT----------
----

GATAATAGTAGCTATAGATTGATAAATTGTAATACCTCAGTCATTACACAG---
GCCTGTCCAAAGATATCCTTTGAGCCAATTCCCATACATTATTGTGCCCCGGCTGGTTTTGCGATTCTAAAGT
GTAAT---AATAAGACATTTGAT------GGAAAAGGACAATGTAAAAAT-----------------------
-GTCAGC---ACAGTACAATGTACACATGGAATTAGGCCAGTAGTGTCAACT---
CAACTGCTGTTAAATGGCAGTCTAGCAGAAGAAGAG------------
ATTGTAATTAGATCTGCCAATTTCTCTGACAAT---------GCTAAAGTCATAATAGTACATCTGAAT----
-----------GAAACTGTAAAC---------ATTACTTGTGTACGACCCAACAATAATACGAGAAAG-----
-AGTATATCTATA------------GGACCAGGGAGAGCA---TTT------TATGCAACAGGAAGC------
---------ATAATAGGAGATATA------------AGAAAAGCACATTGT---AACATTAGTAGAGCC---
AAATGGAATGACACTTTACGAAGGGTAGCTATAAAATTAGGA---------------GCACAATTTAAA----
-----------------------------------AATAAAACAATAATCTTTAAT------
CAATCTTCAGGAGGGGACCCAGAAATTGTA---ACGCACAGTTTTAATTGTGGAGGGGAA---
TTCAAGCAGCTCCAGGCAAGAGTCCTGGCTGTGGAAAGATACCTAAGAGATCAACAGCTCCTAGGGATTTGGG
GTTGCTCTGGAAAACTCATTTGCACCACTTCTGTACCTTGGAATTCTAGTTGGAGT-----------------
----------------------------AATAAATCTCTGGATATGATTTGG------GGT------
AACATGACCTGGATGCAGTGG---GAAAAAGAAATTGACAATTATACAGACTTAATATAC---
TCCTTACTTGAAGACTCGCAGATCCAACAAGAC--

AAAAATGAAAAAGAGTTATTGGAATTAGATAAGTGGGCAAATTTGTGGAATTGGTTTGATATATCAAACTGGC
TGTGGTATATAAAAATATTCATAATGATAATAGGAGGCTTGGTAGGTTTAAGAATAATCTTTGCT---
GTATTTTCTATAGTG---------AATAGAGTTAGGCAGGATACTCACCA------
CTATCGTTGGCTGTCAGCTTGCTTAATGCTACAGCTATAATAGTAGCTGAG---
GGAACAGATAGAATTATAGAAGTAGCACAAGGGATTTTTAGAGCC----------------------------
--------ATTATCCACATA------CCTAGAAGAATAAGACAAGGCTTGGAAAGGGCTTTGCTA---TAA
>HQ385456
GCT---ACA--
GACAAATTGTGGGTCACAGTATACTATGGGGTACCTGTATGG---AAAGAAGCAACC---
ACCACACTATTTTGTGCATCAGATGCTAAGGCATATAGTACAGAA---AAGCATAAT----------------
--------------GTGTGGGCCACA---CATGCCTGTGTACCCACAGAC---
CCCAACCCACAAGAAGTAGTATTGCAC---------AATGTGACAGAAAATTTTAAC----------------
--------------------------ATGTGGAAAAATAACATGGTTGAACAGATGCATGAGGACATA---
ATCAGTCTATGGGATCAAAGCCTA---AAGCCATGTGTAAAATTA---
ACTCCACTCTGTGTTACCTTAAATTGCACTAATATAAATAAGAATTTTACTAAAACTAAT-------------
----
----------------------------AATAGTAATATGAGGATGGATCAAGGAGAAATGAAA---
AACTGCTCTTTCAATATTACAACAAACATAGGA---------------------------

GATAAGATAAGGAAAGAATATGCACTT---TTTTATAAACTTGATGTAGTATCAATAGATGATAGTAGTAGT-
----

AATACTAGTAATAAGAATTATAGTAACTATAGGTTAATAAGTTGCAACGCCTCAGCCCTCACACAG---
GCTTGTCCAAAGGTAACTTTTGAACCAATTCCCATACATTATTGTGCCCCAGCTGGTTTTGCGATTCTAAAGT
GTAAT---GATGAGAAGTTCAAT------GGATCAGGACCATGTACAAAT----------------------
-GTCAGC---ACAGTACAATGTACACATGGAATTAAGCCAGTAGTATCAACT---
CAACTGCTGTTAAATGGCAGTCTAGCAGAAAAAGAC-----------
ATAGTAATTAGGTCTGAAAATTTCTCAGACAAT---------GCTAAAACCATAATAGTACAGCTAAAC----
-----------ACATCAGTAGTA---------ATTAATTGTACAAGACCCAATAACAATACAAGGAAA-----
-GGTATACCTATA------------GGACCAGGGAGCGCA---TTT------TATGCAACAGGAGAT------
---------ATAATAGGAGATATA------------AGACAAGCACATTGT---AACATTAGTAGAAAA---
GATTGGAATAATACTTTAAAAGGGATAGTTGAAAAATTGAGA---------------GAACAATTTGGA----
-------------------------------GAAAATAAAACAATAAACTTTACT------
CAATCCTCAGGAGGGGATCCAGAGATTGTA---ACACACACTTTTAATTGTGGAGGGGAA---
TTTAAACAGCTCCAGGCAAGAGTCCTAGCTGTGGAAAGATACCTAAAGGATCAACAGCTCCTGGGAATTTGGG
GTTGCTCTGGAAAACTCATCTGCCCCACTGCTGTGCCTTGGAATGCTAGTTGGAGC-----------------
---------------------------AATAAAAATCTGAGTGATATTTGG------AAT------
AACATGACATGGATGCAGTGG---GAAAGGGAGATTGACAATTACACAGGTGTAATATAC---
AGGTTAATTGAAGAATCGCAGAACCAGCAAGAA---------------------------------------
----
CAGAATCAACTAGAATTATTGGAATTAGATAAATGGGCAAGTTGGTGGAGTTGGTTAGACATATCAAACTGGC
TGGGGTACATAAAAAATATTCATGATGATAGTAGGAGGCTTAATAGGTTTAAAAATAGTTTTTGCT---
GTACTTTCTATAGTG---------AATAGAGTTAGGCAGGGATACTCACCA------
TTGTCGTTTGCTGTTAGCTTGCTTAATACCACAGCAATAGTAGTAGATGAG---
GGGACAGATAGGGTTATAGAAGCAGCACAAAGAGCTTTTAGAGCA----------------------------
--------GTTCTCCACATA------CCTAGAAGAATAAGACAAGGCTTAGAAAGGGCTTTGCTA---TAA
>EU839603
GCT---GCA--
GAACAATTGTGGGTTACAGTCTATTATGGGGTACCTGTATGG---AAAGAAGCAAAC---
ACCACTCTATTTTGTGCATCAGATGCTAAAGCATATGAGACAGAG---GCACATAAT---------------
--------------GTCTGGGCCACA---CATGCCTGTGTACCCACAGAC---
CCCAGCCCACAAGAAATAGTATTGGAA---------AATGTGACAGAAAATTTTAAC---------------
--------------------------ATGTGGAAAAATAACATGGTAGAACAAATGCATGAGGATATA---
ATCAGTTTATGGGATGAAAGCCTA---AAGCCATGTGCAAAATTA---
ACCCCACTCTGTGTTACTTTAAATTGCACTGAGTTGAACAGCACGAATAGTAATAGCACGAGGAATAATAGT-
----
----------------AGTACAGTAATAGTAAAGGGACTAACAGAAGACTGGGGAAAGGTAAAA---
AACTGCTCTTTCAATATCACCACAAGCATAAGA--------------------------
GATAAGGTGACTAAAGAATATGCACTG---TTTTATAGAGTTGATGTAGTACCAATAGCTGAT---------
----

AGTGGTAATAATAATAGCTATAGATTGATAAATTGTAATACTTCAGTCATTACACAG---
GCTTGTCCAAAAGTATCCTTTGAGCCAATTCCCATACATTATTGTGCCCCAGCTGGTTTTGCGATTCTAAAAT
GTAAC---AATAAGGCATTCAAT------GGAACAGGACCGTGTAGAAAT----------------------
-GTCAGC---ACAGTACAATGTACACATGGCATTAGGCCAGTAATATCAACT---
CAACTGCTGTTAAATGGCAGTCTAGCAGAAGAA--------------
GTAATAATTAGGTCTGAAAATTTCACAGACAAT---------GCTAAAACCATAATAGTACAGCTGAAG----
-----------AGCTCTGTAAAG---------ATTAATTGCACAAGATTCAGCAATAATACAAGAAGA-----
-AGTATACATATA------------GGGCCAGGGAGAGCA---TTT------TTTGCAACAACAAAC------
---------ATAATAGGAGATATA------------AGACAAGCACATTGT---AACATTAGTAAAGAA---
GAATGGAATAACACTCTGAGTCAGGTATTTCAAAAATTAAGA---------------GAACAATTGGGG----
-------------------------------AATAATAAAACAATAGTCTTTAAT------
CAATCTTCAGGAGGGGACCCAGAAGTTGTA---ATGCACACTTTTAATTGTGGAGGGGAA---
TTTAAACAGCTCCAGGCAAGAGTCCTGGCTGTGGAAAGATACCTAGTGGATCAACAGCTCCTAGGGATTTGGG
GTTGCTCTGGGAAACTCATCTGCCCCACTGCTGTGCCTTGGAATGCTAGTTGGRGT----------------
---------------------------AATAAATCTCTGGGTGATATTTGG------GAT------
AACATGACATGGATGCAGTGG---GAAAAAGAGATTGACAATTACACAGCAGAGATATAC---
AAATTAATTGAAGAATCACAGAACCAGCAAGAA---------------------------------------
----

AAGAATGAACAAGAATTACTAGAATTGGATAAGTGGGCAAGTTTGTGGAATTGGTTTGACATAACAAAATGGC
TGTGGTATATAAAAATATTCATAATGATAGTAGGAGGTTTAATAGGTTTAAGAATAGTTTTTGCT---
ATACTTTCTATAGTG---------AATAGAGTTAGCAGGGATACTCACCA------
TTATCTTTCGCTGTTAGCTTGCTTAATGCCACAGCTATAGCAGTAGCTGAG---
GGGACAGACAGGATAATAAAAGTAGTACAAAGAATTGGTTGGGCT----------------------------
--------ATCCTCCACATA------CCTAGAAGAATAAGACAGGGCTTGGAAAGGGCTTTGCTA---TAA
>EU839601
GCT---GCA--
GATCAATTGTGGGTCACAGTCTATTATGGGGTACCTGTGTGG---AAAGAAGCAACC---
ACCTCTCTATTTTGTGCATCAGATGCCAGAGCATATGACACAGAG---ATGCATAAT----------------
--------------GTTTGGCCACA---CATGCCTGTGTACCCACAGAC---
CCCAGCCCACAAGAAGTAGTAATGGGA---------AATGTGACAGAAAATTTTAAC----------------
--------------------------ATGTGGAAAAATGACATGGTAGACCAAATGCATGAGGATATA---
ATCAGTTTATGGGATCAAAGTCTA---AAGCCATGTGTAAGATTA---
ACCCCACTCTGTGTCACTTTAAATTGCACTGATGTTAAGGGTAAGACATTT----------------------
----
----------------------------------GATGATAAAAATGAGATAAAAGAAATAAAA---
AATTGCACATTCAATGTCACAGGAAATAGAGGA---------------------------
GATAAGGTACGGAGAGAAAATGCAAAT---TTTTATAACCTTGATATAGTATCAATGGATGAGACT-------
----

AATATTAATAATACTAAATATAGATTGTTACATTGTAATACCTCAGTCATTACACAA---
GCCTGTCCAAAGGTATCCTTTGAGCCAATTCCCATACATTATTGTGCCCCGGCTGGTTTTGCGATTCTAAAGT
GTAAT---AATAAAACATTCAGT------GGTTCAGGACCATGTACAAAT-----------------------
-GTTAGC---ACAGTACAATGTACGCATGGAATTAGGCCAGTAGTGTCAACT---CAA---
CTGTTGAATGGCAGCCTAGCAGAAGAAGAG------------
GTAGCAATTAGATCTGAAAATTTCACGGACAAT---------GATAAAAATATAATAGTACAACTGAAT----
-----------GTATCTGTAACA---------ATTAATTGTACAAGACCCAACAACAATACAAGAACA-----
-AGTATACATCTA------------GGACCAGGGAAAACA---TTT------TTTGCAGGAGGA---------
---------GTAATAGGAGATATA------------AGACAGGCACATTGT---AACCTCAGTGRAATA---
GAATGGAATAACACTTTAAGACAGGTAGCTATAAAATTAAAA---------------GAACAATTT-------
-----------------------------------AATAAAACAATAGTCTTTAAT------
CAAACCACAGGAGGGGACCCAGAAGTTGAA---ATGCACCACTTTGTTTGTGGAGGAGAA---
TTTAAACAGCTCCAGGCAAGAATCCTGGCTGTGGAAAGATACCTAAAGGATCAACAGCTCCTAGGGATTTGGG
GTTGCTCTGGAAAACTCATTTGCACCACTAATGTGCCTTGGAACACTAGTTGGAGT-----------------
----------------------------AATAAATCTCTGAATGATATTTGG------AAC------
AACATGACATGGATGGAGTGG---GACAGAGAAGTTAGCAATTACACACAATTAATATAC---
ACCTTAATTGAAGAGTCACAGAACCAGCAAGAA--

AAGAATGAACAAGAATTATTAGAATTGGATAAATGGGCAAGTTTGTGGAATTGGTTTGACATAACAAACTGGC
TGTGGTATATAAAACTATTCATAATGATAGTAGGAGGCTTGATAGGTTTAAGAATAGTTTGTGCT---
ATACTTTCTATAGTA---------AATAGAGTTAGCAGGGATATTCACCA------
TTGTCATTTGCTGTTAGCTTGCTTAATGTCACAGCTATAGCAGTAGCTGAG---
GGAACAGATAGGATTATAGAAATAATACAAAATACTGGGAGAGCT----------------------------
--------ATTCTCCACATA------CCTGTACGAATAAGACAGGGCTTGGAAAGGGCTTTGCTA---TAA
>MH078551
ACT---GCA--
GACCAATTGTGGGTCACAGTCTATTATGGGGTACCTGTGTGG---AAAGAAGCAACC---
ACCACTCTATTTTGTGCATCGGACGCTAAAGCATATAAGACAGAG---ATGCATAAT----------------
--------------GTCTGGGCAACA---CATGCCTGTGTTCCCACGGAC---
CCCGACCCACAAGAAGTAGTATTGAAA---------AATGTGACAGAAAATTTTAAC----------------
--------------------------ATGTGGAAAAATACCATGGTAGAACAGATGCATGAGGATATA---
ATCAGTCTATGGGATGAAAGTCTA---AAGCCATGTGTAAAATTA---
ACCCCACTCTGTGTTACTTTAGAATGCAATGATACGAGTACTAATGAT-------------------------
----
----------------------------ACCATTGACATGGAGAGGAGGAAAGGAGAACTAAAA---
AACTGCACTTTCAACATCACCACACCAATGCAA---------------------------
GATAGGAGGGAAAAAGAATGGGCATTT---TTTAATGCACTAGATGTAGTAAAACTAAATGAG----------
----

AGTAATAATAGCTATAGATTGATAAGCTGTAATACCTCAGTCCTTAAGCAG---
GCCTGTCCAAAAGTATCCTTTGAGCCAATTCCCATACATTATTGTACCCCGGCTGGTTTTACGATTCTGAAGT
GTAAT---GATGAAGGGTTCAAT------GGAACAGGAACATGTAACAAT----------------------
-GTCAGC---ACAGTACAATGTACACATGGCATTAAGCCAGTAGTGTCAACT---
CAATTGCTGTTAAATGGCAGTCTAGCAGAAAAAGGG-----------
GTGGTAATTAGATCTGAAAATTTCAGTGACAAT---------GCTAAAACCATAATAGTACAGCTCAAG----
-----------GACCCTGTAGAA---------ATTAATTGTACAAGACCCAATAACAATACAAGAAGA-----
-GGTATACATATA------------GGACCAGGGAGAGTA---TTC------TTTGCAACA---GAA------
---------GTAATAGGAGATATA------------AGACAAGCATATTGT---AACATTAATGAAACA---
AAGTGGAATAACACTTTACATCAGATAGCTGAAAAATTAAGA---------------AAGCAATTTGAG----
----------------------------------AATAAAACAATAGTTTTTAAT------
CAATCCTCAGGAGGGGATCCAGAAATTGTA---TTTCACAGTTTTAATTGCGGAGGGGAA---
TTTAAACAGCTCCAAGCAAGAGTCCTGGCTGTGGAAAGATACCTAAAGGATCAGCAGCTCCTAGGGATTTGGG
GATGCTCGGGAAAACTCATCTGCACCACTACTGTGCCTTGGAACACTAGTTGGAGT-----------------
---------------------------AATAAATCATATAATGAGATTTGG------AAT------
AACATGACCTGGATGCAATGG---GAAAGAGAAATTGACAATTATACAAAGGTAATATAT---
ACCTTAATTGAAGAATCGCAGAACCAACAGGAA---------------------------------------
----
AAGAATGAACAAGAATTATTGGCATTGGATAAGTGGGCAAGTTTGTGGAGTTGGTTTAGTATAACAAATTGGC
TGTGGTATATAAAAATATTCATAATGATAGTAGGAGGCTTAATAGGTTTAAAAATAGTTTTTGCT---
GTACTTTCTATAGTA---------AATAGAGTTAGGCAGGGATATTCACCA------
TTATCGTTTGCTGTTAGCTTGCTTAATGCCACAGCAATAGCAGTAGCTGAA---
GGGACAGATAGGATTATAGAGGTAGCACAAAGAATCTTTAGAGCT----------------------------
--------TTCCTCCACATA------CCTAGACGAGTAAGACAGGGCTTGGAAAGGGCTTTGCTA---TAA
>MK303352
GCT---GCA---
GACAACTTGTGGGTCACAGTCTATTATGGGGTACCTGTGTGG---AAAGACACCATC---
ACCACTCTATTTTGTGCATCAGATGCTAAAGCATATGAAACAGAG---GCGCATAAT----------------
--------------GTTTGGGCCACA---CATGCCTGTGTACCCACAGAC---
CCCAACCCACAGGAAATAGTATTGGAA---------AATGTGACAGAAAATTTTAAC----------------
-------------------------ATGTGGGAAAATGGCATGGTAGAACAGATGCATGAGGATATA---
ATTAGTTTATGGGATCAAAGCCTA---AGGCCATGTGTAAAATTA---
ACCCCACTTTGTGTCATCTTAAATTGCACTAATGCTAATACTACTTGTGGTAATAGTAATAGTAGTAATGCTA
ATAATAGTATT--
---AGTGACTGTGAAATGATGAAA---
AACTGCTCTTTTAATATCACCACAAGCACAAAA--------------------------GATAAGGTA---
CAAGATTATGCACTT---TTTTATAACTTAGATGTAATATCAGTAGATAAT---------------------
----
----------AACAGTAGTAGATACAGATTGAGAAGTTGTAACACCTCAGTCATTACACAG---
GCTTGTCCAAAAGTATCCTTTGAGCCAATTCCCATACATTATTGTACCCCGGCTGGTTTTGCGCTTCTAAAAT
GTAAT---GATAAGATGTTCAGC------GGGACAGGACCATGTAAAAAT----------------------
-GTCAGC---ACAGTACAATGTACACATGGAATTAGGCCAGCAGTATCAACT---
CAGTTGCTGTTAAATGGCAGTCTAGCAGAAGAAGAA-----------
ATAGTAATTAGATCAAAAAATTTCACAAATAAT---------GCTAACACCATAATAGTACAGCTAAAT----
-----------GAATCTGTAAAA---------ATTGAGTGTGTAAGACCCAACAACAATACAAGAAAA-----
-AGTATACATATA------------GGACCAGGGAAAGCA---TTT------TATGCAACAGGAGCT------
---------ATAATAGGAAATATA------------AGACAGGCATATTGT---AATCTTAGCAAGACA---
GACTGGACTAACACTTTGAATCAGATAAAAATAAAATTAGGA---------------CAACAATTTAGA----
----------------------------------AATAAAGCAATAGTCTTTAAT------
CACTCCTCAGGAGGGGACCCAGAAATTGTA---ATGCACAGTTTTAATTGTGGAGGGGAA---
TTTAAGCAGCTCCAGGCAAGAATCCTGGCTGTGGAAAGATACCTAAAGGATCAACAGCTCCTGGGGATTTGGG
GTTGCTCTGGAAAACTCATTTGCACCACTACTGTGCCTTGGAATGCTAGTTGGAGC----------------
---------------------------AATAAATCTATGAGTGAGATTTGG------GAT------
AACATGACCTGGATGGAATGG---GAAAAAGAAATTGACAATTACACAGGCTTAATATAC---
ACCTTAATTGAGCAAGCGCAAAACCAACAGGAA---------------------------------------
----
AAGAATGAACAAGAATTATTAGAATTAGATAAATGGGCAAGTTTGTGGAGTTGGTTTTCAATATCAAATTGGC
TGTGGTATATAAGATTATTCATAATAATAGTAGGAGGCTTAGTAGGTTTAAGAATAGTCTTTACT---
GTACTTTCTATAATA---------AATAGAGCTAGGGAAGGATACTCACCA------
CTGTCATTTGCTATTAGTTTGCTAAATGCCACAGCTATAGCAGTAGGGGAG---

GGAACAGATAGGGTTCTAGAAATAGTACAAAGAGGTTTTAGGGCT----------------------------
--------TTTCTCCACATA------CCTAGAAGAATAAGGCAGGGCTTAGAAAGGGCTTTAATA---TAA
>MK303348
GCT---AAA--
GAACAATCGTGGGTCACAGTCTATTATGGGGTGCCTGTGTGG---AAAGAAGCAACC---
ACCACTCTATTTTGTGCATCAGATGCTAAAGCACATAATACAGAG---GTGCATAAT----------------
--------------GTCTGGGCCACA---CACGCCTGTGTACCCACAGAC---
CCCAACCCAAACGAAGTAGAATTAGAA---------AATGTGACAGAAAATTTTAAC---------------
--------------------------ATGTGGAAAAATGACATGGTAGAACAGATGCATGAGGATGTA---
ATCAGTTTATGGGATCAAAGCCTA---AAGCCATGTGTAAAATTA---
ACCCCACTCTGTGTTACTTTAGAGTGCACTAATGCTAATAATACTCAAAATAATAGTAGTACATTTCAAAATA
AT--
-------------------------AGTACTACATGGGAAAGGGTGGAACCAGGAGAAATGAAA---
AACTGCTCTTTTAATATCACCACAGAAACCAAA---------------------------GAGAAGGTG---
AAAGACTATGCACTT---TTTTCTAGCTTAGATGTAGAACAAATAAATAAT---------------------
----
AGAACTAATAGTGATAATAGTACTAACTTTAGATTGATAAGTTGTAACACCTCAGTCATTACACAA---
GCTTGTCCAAAGATATCCTTTGAGCCAATTCCTATACATTATTGTGCCCCGGCTGGGTTTGCGCTCCTAAAAT
GTAAA---GATAAAAGGTTCAAT------GGAACAGGACCATGTAAAAAT----------------------
-GTCAGC---ACAGTACAATGTACACATGGAATTAGACCAGTAGTGTCAACT---
CATTTGCTGTTAAATGGCAGTCTAGCAGAAGAAGAG-----------
ATAGTAATTAGATCTAAAAATTTCTCGAACAAT---------GCTGACATCATAATAATACAGCTGAAT----
-----------GAATCTGTGCAA---------ATTAATTGTACAAGACCTAACAACAACACAAGAAAA-----
-GGTGTACCTATG------------GGACCAGGGAAAACA---TTT------TTCACAACA---GAT------
---------GTAATAGGAAATATA------------AGACAAGCACATTGT---AACATTAGCGGGGCA---
AAATGGAATAACACTTTAAGACAGATAGTTACAAAATTAAAA---------------GAACAATATGGA----
--------------------------------AAAAACAAAACAATAGTCTTTAAT------
CAATCCTCAGGAGGGGACCCAGAGATTACA---CTGCACAGTTTTAATTGTGGAGGGGAA---
TTTAAGCAGCTCCAGGCAAGAGTCCTGGCTGTAGAAAGATACCTAAAGGATCAACAGCTCCTGGGGATTTGGG
GTTGCTCTGGAAAACTCATCTGCACCACTACTGTGCCTTGGAATACTAGTTGGAGC-----------------
----------------------------AATAAATCTATAGATGAGATTTGG------AAT------
AACATGACCTGGATGGAATGG---GAGAAAGAAATTGATAATTACACAAGCTTAATATAC---
ACCTTAATTGAGGATGCGCAAAACCAGCAAGAA--

AAGAATGAACAAGAATTATTAGAATTAGATAAATGGGCAAGTTTGTGGAATTGGTTTTCAATATCAAAGTGGC
TGTGGTATATAAAGATATTCATAATAATAGTAGGAGGCTTAGTAGGCTTAAGAATAGTCTTTACT---
GTACTTTCATTAGTG---------AATAGAGTTAGGAAGGGATACTCACCA------
TTGTCATTTGCTATTAGTTTGCTTAATGCCACAGCTATAACAGTAGGGGAA---
GGGACAGATAGGGTTATAGAAATAGTACAAAGAGGTTTTAGGGCT----------------------------
--------ATCCTCCACATA------CCTAGAAGAATAAGACAGGGCTTGGAAAGGGCTTTATTA---TAA
>MK303350
GCT---GCA--
GACAAAATGTGGGTCACAGTCTATTATGGGGTACCTGTGTGG---AAAGAAGCATCC---
ACCACTCTATTTTGTGCATCAGATGCTAAAGCATATAGTACAGAG---GCGCATAAT----------------
--------------GTTTGGGCCACA---CACGCCTGTGTACCCACAGAC---
CCCAACCCACATGAAGTAGAATTGGAA---------AATGTGACAGAAAATTTTAAC---------------
--------------------------ATGTGGACAAATGACATGGTAGAACAAATGCAGGAGGATGTA---
ATCAGTTTATGGGATCAAAGTCTA---AAGCCATGTGTAAAATTA---
ACCCCACTCTGTGTCACTCTAACTTGTACTGATATGAATACTACTAGTAGCAATAATACTACTAACAAT----
----
-------------------------AGTAGTAGCTGGGGAAGAATGGAACAAGGAGAAATAAAA---
AACTGCTCTTTTAATATCACCACAAGCACAAAT---------------------------GATAGGGTG---
AAAGACTATGCACTT---TTTTATAAACTAGATATAGAACAAATGGAAAAG---------------------
----
----------AATAGTACCAGTTATAAATTAATAAGTTGTAACACCTCAGTCATCACACAG---
GCTTGTCCAAAGATATCCTTTGAGCCAATTCCCATACATTATTGTGCCCCAGCTGGTTTTGCGATTCTTAAAT
GTAAT---GATAAAAAGTTCAAT------GGAACAGGAACATGTCAAAAT----------------------
-GTCAGC---ATAGTACAATGCACACATGGAATTAGGCCAGTAGTGTCAACT---
CAGCTGCTATTAAATGGCAGCCTAGCAGAAGAAGAG-----------
GTAGTAATTAGGTCTGAAAATTTCACAAATAAT---------GCTAAAACCATAATAGTACATCTGAAT----

-----------GAATCTATGGAA---------ATTAATTGTACAAGACCCAACAACAACACAAGGAAA-----
-AATATACATATG------------GGACCAGGGAGAGCA---TGG------TATGCAACAGGAGAG------
---------ATAATAGGAGATATA------------AGACGAGCATATTGT---AACCTTAGCAGTACA---
AAGTGGAATAACACTCTAAAAAAGATAGTTATAAAATTAAGA---------------GAACAATTTGGA----
--------------------------------GAGAACAAAACAATAGTCTTTAAT------
AAATCCTCAGGAGGGGACACAGAAATTGTA---ATGCACAGTTTTAATTGTGGAGGGGAA---
TTTAAGCAGCTCCAGGCAAGAGTCCTGGCTCTGGAAAGATACCTAAGGGATCAACAGCTCCTGGGACTTTGGG
GTTGCTCTGGAAAACTCATCTGCACCACTGCTGTGCCTTGGAATGCTAGTTGGAGC-----------------
----------------------------AATAAATCTATGAATGAAATTTGG------GAG------
AACATGACATGGATGCAGTGG---GAAAGAGAAATTGACAATTACACAGGCTTAATATAC---
ACCTTACTTGAGAAATCGCAAAACCAACAAGAA--

AAGAATGAACAAGAATTATTGGAATTAGATAAATGGGACAGTCTGTGGAATTGGTTTTCAATAACAAACTGGC
TGTGGTATATAAAATTATTTATAATGATAGTAGGAGGCTTAATAGGTTTAAGAATAGTCTTTACT---
GTACTTTCTATAGTA---------AATAGAGTTAGGAAGGATACTCACCA------
TTGTCATTTGCTATTAGTTTGCTTAATGCCACAGCTATAGCAGTAGGGGAA---
GGGACAGATAGGATTATAGAAATAGTGCAAAGAGGTTTTAGGGCT----------------------------
--------ATTCTCCACATA------CCTAGAAGAGTAAGACAGGGCTTAGAAAGGGCTTTGGTA---TAA
>MK303351
GCT---GCA--
GAAAATTTGTGGGTCACAGTCTATTATGGGGTACCTGTGTGG---AAAGAAGCCGCC---
ACCACTCTATTTTGTGCATCAGACGCTAAAGCATATGATACAGAG---GTGCATAAT----------------
--------------GTTTGGGCTACA---CATGCCTGTGTACCCACAGAC---
CCCAACCCACATGAAGTAGTATTGAAA---------AATGTGACAGAAAATTTTAAC----------------
--------------------------ATGTGGACAAATAACATGGTAGAACAGATGCATGAGGATATA---
ATTAGTTTATGGGATCAAAGCTTA---AAGCCATGTGTAAAATTA---
ACCCCACTTTGTGTTATTTTAGATTGCACCACTTTGAATAATAATAATAGTAGTAGCAATAATAGTGTTAGC-
----
-------------------------AATAATAGTACTAGGGTGAAGGACTCAGGAGAAATGAAA---
AACTGCTCTTTTAATATCACCACAAGCACAAAA---------------------------GATAAGGTG---
AAAGACTATGCATTG---TTTTATAACCTAGATATAATATCAATAGATGGTAAT-------------------
----
----------AATAATAATAGTTATAGATTAAAAAGTTGTAATACCTCAGTCATTAAACAG---
GCTTGTCCAAAAGTATCCTTTGAGCCAATTCCCATACATTATTGTGCCCCGGCTGGTTTTGCGCTTCTAAAAT
GTAAT---AATAAGACATTCAAC------GGGACAGGACCATGTAACAAT-----------------------
-GTCAGC---ACAGTACAATGTACACATGGAATTAGGCCAACAGTGTCAACT---
CAGTTACTGTTAAATGGCAGTCTAGCAGAAGAAGAG------------
ACAGTAATTAGATCAAAAAATTTCACGAACAAT---------GCTGAAACCATAATAGTACAGCTAAAT----
-----------GAATCTGTAAAA---------ATTGATTGTGTAAGACCCAACAACAATACAAGAAAA-----
-AGTGTTCACATA------------GGACCAGGGCAAGCA---TTT------TTTGCAACAGGAGAT------
---------ATAATAGGGGATATA------------AGACAAGCACATTGT---AATCTTAGCAGGACA---
AGCTGGGATAATACTTTGAAAAGGATAGCTATAAAATTACAA---------------GAACAATTTGGA----
-----------------------------------AATAAAACAATAGTCTTTAAT------
CACTCCTCAGGAGGGGACCTAGAAATTGCA---ACGCTCAGTTTTAATTGTGGAGGGGAA---
TTTAAGCAGCTCCAGGCAAGAATTCTGGCTGTGGAAAGATACCTAAAGGATCAACAGCTCATGGGGATTTGGG
GTTGCTCTGGAAAACTCATTTGCACCACTACTGTACCTTGGAATGCTAGTTGGAGC-----------------
----------------------------AATAGATCTCTGAGTGAGATTTGG------GAT------
AACATGACCTGGATGCAATGG---GAAAAAGAAATTGACAATTACACAGGCTTAATATAC---
AACTTAATTGAGGAAGCGCAAAACCAGCAAGAA--

AAGAATGAACAAGAATTATTAGAATTAGATAAATGGGCAAGTTTGTGGAATTGGTTTTCAATATCAAATTGGC
TGTGGTATATAAGATTATTCATAATGATAGTAGGAGGCTTAGTAGGTTTAAGAATAGTCTTTACT---
GTACTTTCTATAGTA---------AATAGAGCTAGGAAGGATACTCACCA------
TTGTCATTTGCTATTAGTTTGCTAAATGCCACAGCTATAGCAGTAGGGGAG---
GGGACAGATAGGATTATAGAAATAGTGCAAAGAGGTTTTAGGGCT----------------------------
--------ATTCTCCACATA------CCTAGAAGAATAAGACAGGGCTTAGAAAGGGCTTTAATA---TAA
>MK303353
GCT---ACA--
GAAAAGTTGTGGGTCACAGTCTATTATGGGGTACCTGTATGG---AAAGAAGCAACA---
ACCACACTCTTTTGTGCGTCAGATGCTAAAGCATATAACACAGAG---GTGCACAAT----------------

--------------ATTTGGGCCACA---CATGCCTGTGTACCCACAGAC---
CCCAATCCACAAGAAATAGTATTGGAA---------AATGTGACAGAAAATTTTAAC----------------
--------------------------ATGTGGAAAAATAACATGGTAGAACAGATGCAGGAGGATATA---
ATCAGTTTATGGGACCAAAGCCTA---AAGCCATGTGTAAAATTA---
ACCCCACTTTGTGTTACTTTACATTGTGCTAATGTTACGTGCACTAATGTTAGTAGCTCTGATAATCTTAATA
GCACTGCTACT--
-------------AATAGTAGTAGACCTTGCAATGTTAGTGGAGAAATGATAGAAGAAGTAAAA---
AATTGCTCTTTCAATGTTACAGAAGGAATAAGA---------------------------
AATAAGATACAAAAAGTATATGCAACT---TTTAATACACTTGATATAGTACCAATAGATGGGGGA-------
----
AGAATAATAACTGTGACTAAGACAGATGGTAATAAGACTACAAACTATACGTATGGAGACTATATGTTGGTAA
ATTGTAACACCTCAGTCATTACACAG---
GCCTGTCCAAAGGTAACCTTTGAACCAATTCCTATACATTATTGTGCCCCAGCTGGTTTTGCGATTCTAAAGT
GTAAC---AATAAGACGTTCAGT------GGAAAAGGACCATGTACAAAT-----------------------
-GTCAGT---ACAGTACAGTGCACTCATGGGATTAAGCCAGTAGTATCAACT---
CAACTGCTGTTAAATGGCAGCCTAGCAGAA---GAG------------
GTAGTACTTAGATCTGACAATTTCTCTGAGAAT---------ACTAAAACCATAATAGTACAACTGAAG----
-----------GAATCTGTAAAC---------ATTACTTGTATAAGACCTAACAATAATACAAGGAGA-----
-AGTATACATATG------------GGACCAGGGGCAGCA---ATG------TTTGCAACAGGAGAA------
---------ATAATAGGAGACATA------------AGAAGAGCATATTGT---ACCCTTAATGCAACA---
GTTTGGAATAAAACTTTACAACAGGTAGCCACAAAATTACAA---------------AAACATTTTGAG----
-----------------------------------GAAAAAGGAATAGCCTTCGAT------
CAACCCTCAGGAGGGGACCCAGAAATTACA---ATGCACAGCTTTAATTGTGGAGGGGAA---
TTTAAGCAGCTCCAGGCAAGAGTCCTGGCTGTAGAAAGGTACCTAAGGGATCAACAGCTCCTAGGGATATGGG
GTTGCTCTGGAAAACTCATTTGTACCACTAATGTGCCTTGGAATGCTAGTTGGAGT-----------------
----------------------------AATAAATCTCAGAAGGAGATTTGG------GAT------
AACATGACCTGGATGGAGTGG---GAAAGAGAGATTGACAATTACACAGGCTACATATAC---
AGTTTACTTGAGACATCGCAAAACCAGCAAGAA--

AAGAATGAGCAAGAATTATTGGAATTGGATAAGTGGGCAAATTTGTGGAATTGGTTTGACATATCAAATTGGC
TGTGGTATATAAAAATATTCATAATGGTAGTAGGGGGTTTAATAGGTTTAAGAATAGTTTTTGTT---
GTACTTTCTATAGTG---------AATAGAGTTAGGCAGGGATACTCACCA------
TTGTCATTCGCTGTTAATTTGCTTGATACCATAGCTGTAGCTGTAGCTGGG---
GGGACAGATAGGGTTATAGAAGCAGCACAAAGAATTGGTAGAGCT----------------------------
--------ATTCTCCACATA------CCTAGAAGAATAAGACAAGGCTTAGAAAGAGCTTTACAG---TAA
>MK303354
GCT---GCA---
GGGTTGTGGGTCACAGTCTATTATGGGGTACCTGTATGG---AAAGAAGCAACC---
ACCACTTTATTTTGTGCTTCAGATGCTAAAGCATATGATACAGAG---GCACACAAT----------------
--------------GTGTGGCCACA---CATGCCTGTGTACCCACAGAC---
CCCAACCCACAAGAAGTGGTATTGGGA---------AATGTGACAGAAGATTTTAAC----------------
--------------------------ATGTGGGAAAATAACATGGTAGAACAGATGCAGGAGGATGTA---
ATCAGTTTATGGGATCAAAGCCTA---AAGCCATGTGTGAAATTA---
ACCCCACTCTGTGTCATTTTAAATTGCACTGATTTAAATAATAGCACTGGTTTAAATAATACTCAT-------
----
---ACGGGAGAAATGAAA---
AACTGCTCTTTCAATATCACCTCACCCCTAAGA---------------------------
GATAAGATGCAGAAAGAATATGCACTC---TTCTATAAACTTGATATAGTACCAATAGATGATGATAGAGCT-
----

AATAATACAAGTAATAATACTAGTTATAGATTGATAAGTTGTAACACCTCAGTCATTACACAA---
GCATGTCCAAAGGTATCTTTTACGCCAATTCCCATACACTATTGTGCCCCGGCTGGTTTTGCGCTTATAAAGT
GTAAC---AATAAGACATTCGAT------GGAAGAGGACCATGTACAAAT-----------------------
-GTCAGC---ACTGTACAATGTACTCATGGGATTAGGCCAGTAGTATCAACT---
CAACTGTTGTTAAATGGTAGTCTAGCAGAAGAAGAG------------
GTAGTAATTAGATCTAAAAATTTCACTGATAAT---------GCTAAAAATATAATAGTACAACTGAAT----
-----------AAACCTGTACAA---------ATTAATTGTACAAGACCCAACAATAATACAAGAAAA-----
-AGCATACACATA------------GGACCAGGGAGAGCA---TTC------TATGCAACAGGAGAA------
---------ATAATAGGAAATATA------------AGACAAGCACATTGT---AACATTAGTAGAAGA---
AACTGGACCAACACTTTACAACAGATAGTTGGAAAATTAAGA---------------GAAAAATTTGAG----

----------------------------------AACAAAACAATAGTATTTAAT------
CAATCCTCAGGAGGGGACCCAGAAATTGTA---ATGCATAGTTTTAATTGTGGAGGGGAA---
TTTAAGCAGCTCCAGGCAAGAGTCCTGGCTGTAGAAAGATACCTGAGGGATCAACAGCTCCTGGGGATTTGGG
GTTGCTCTGGAAAACTCATTTGCACCACTGCTGTGCCTTGGAATAATAGTTGGAGT----------------
---------------------------AATAAATCTCTGAAAACGATTTGG------GAT------
AACATGACCTGGATGGAGTGG---GAAAAAGAGATTGACAATTACACAGACTTAATATAC---
AATTTACTTGAAGAAGCGCAAAACCAGCAAGAA---------------------------------------
----
AAGAATGAGCAAGAATTATTGGAATTAGATAAATGGGCAAGTTTATGGAATTGGTTTGACATAACAAAATGGC
TGTGGTATATAAAAATATTCATAATGATAGTAGGAGGCTTGATAGGTTTAAGAATAGTTTTTGCT---
GTACTTTCTATAGTG---------AATAGAGTTAGGCAGGGATACTCACCC------
TTGCCGTTCGCTGTTAACTTGCTTAATACCATAGCTATAGCTGTAGCTGAG---
GGAACAGATAGGGTTATAGAAGCAGTACAAAGAATTGGTAGAGCT---------------------------
--------ATTCTCCACATA------CCTAGAAGAATCAGACAGGGCTTAGAAAGGGCTTTGCTG---TAA
>MK303355
GTT---GCA---
GAAAAATTGTGGGTCACAGTCTATTATGGAGTACCTGTGTGG---AAAAATGCAACC---
ACCACTCTATTTTGTGCATCAGATGCTAAAGCATATGACAAAGAG---ATGCATAAT---------------
--------------GTTTGGGCCACA---CATGCCTGTGTACCCACAGAC---
CCCAACCCACATGAAGAATTCTTGGGA------AGCAATGTGACAGAAAATTTTAAC---------------
-------------------------ATGTGGAAAAATGACATGGTAGAGCAGATGCAGGAAGATATA---
ATTAGTTTATGGGATCAAAGCCTA---AAACCATGTGTAAAATTA---
ACCCCACTTTGTGTTACTTTAAATTGCACTAATGTAAATGTGAATGATACTAATGTAAATTGTACTACTAATA
GTACTACTAATGGAAAT---
-------TGTACTACTAAGGGAAATATTAGTGAATGGGAAAGGGTGGAACAAGGAGACCTAAAA---
AACTGCTCTTTTAATATTACCACAAGCACAGGT--------------------------GATAAGGTA---
AAAAACTATTCAATT---TTTGATAGCCAAGATGTAGTACCAATAAATAAG---------------------
----
----------AATAGTGATAAATATAGATTGATAAGTTGTAACACCTCAGTCATTACACAG---
GCTTGTCCAAAAATATCCTTTGAGCCAATTCCCATACATTATTGTACTCCGGCTGGTTTTGCGCTGCTAAAAT
GTAAC---GATAAGAAGTTCAGT------GGGACAGGGCCATGTAAAAAT----------------------
-GTCAGC---ACAGTACAATGTACACATGGAATTAAGCCAGTAGTATCAACT---
CAGTTGCTGTTAAATGGCAGTCTAGCAGAAGAAGAG-----------
ATAGTAATTAGATCTGAAAATTTCTCGGACAAT--------ACTAAAACCATAATAGTACAGCTGAAT----
-----------GAATCTGTAACA--------ATTAATTGTACAAGACCCAACAACAATACAAGAAAA-----
-AGTATACATATG-----------GGACCAGGGAGAGCA---TTC------TTTGCAGCAGGAGAT------
---------ATAATAGGAAATATA-----------AGAAAAGCACATTGT---AATGTTAGCAAGGAA---
AGCTGGAATAATACTTTGGAACGGATAAAGGAAAAATTAAAA--------------GAACAATTTGGA----
-------------------------------GAAAACAAAACAATAATCTTTCAG------
GAGCACTCAGGGGGGGACCCAGAAGTTGTA---GTGCACACTTTTAATTGTGGAGGGGAA---
TTCAAGCAGCTCCAGGCAAGAGTCCTGGCTGTGGAAAGATACCTAAAGGATCAACAGCTCCTGGGGATTTGGG
GTTGCTCTGGAAAACTCATCTGCACCACTACTGTGCCTTGGAATGCTAGTTGGAGC----------------
---------------------------AATAAATCTTTGGATCAGATTTGG------AAT------
AACATGACCTGGATGCAATGG---GAAAAAGAAATTGACAATTACACAGACTTAATATAC---
ACCTTAATTGAGAAAGCGCAAAACCAACAAGAA---------------------------------------
----
AAGAATGAACAAGATTTATTAGAATTAGATAAATGGGACAGTTTGTGGAATTGGTTTTCAATAACAAATTGGC
TGTGGTATATAAAATTATTCATAATGATAGTAGGAGGCTTAATAGGTTTAAGAATAGTCTTTACT---
GTACTTTCTATAGTA---------AATAGAGTTAGGAAGGGATACTCACCA------
TTGTCGTTTGCTATTAGTTTGCTTAATGCCACAGCTATAGCAGTAGGGGAA---
GGGACAGATAGGATTATAGAAATAATACAAAGAGGCCTTAGGGCT---------------------------
--------ATTCTCCACATA------CCTAGAAGAGTAAGACAGGGCTTAGAAAGGGCTTTGATA---TAA
>GU191372
GCT---GCT---
GATCAACTCTGGGTCACAGTTTATTATGGGGTACCTGTGTGG---AGAGAAGCTACC---
ACCACTCTATTTTGTGCATCAGATGCTAAAGCATATGATACAGAG---AAGCACAAT---------------
--------------GTTTGGGCCACA---CATGCCTGTGTACCCACAGAC---
CCCAACCCACAAGAAATAGTATTGGAA--------AATGTTACAGAAAATTTTAAC----------------
-------------------------ATGTGGAAAAATGACATGGTAGAACAGATGCATGAAGATATA---
ATCAGTTTATGGGATGAAAGCCTA---AAGCCATGTGTAAAATTA---

ACCCCACTCTGTATTACGTTACATTGCACTAATTTGAGTCAAGTTGATAGTTGTAATAATACCAATGGTAATG
GTACAGAT---
-------ATCGTTAGTTGTAATAATAGTATCACTATTGATAGGGGGGAAATAAAAGTAATAAAA---
AACTGCTCTTTCAATATCACCACAAACATAAGA---------------------------
GATAGAGTGAAAAAAGAATATGCAATT---TTTAATAAAATGGATATAGTACCAATAGATGAGGAT-------
----

AATAATACAAGTAATACCAACTATAGGTTGATAAGTTGTAACACCTCAGTCATTACACAG---
GCCTGTCCAAAGATATCCTTTGAGCCAATTCCCATACATTATTGTGCCCCGGCTGGTTTTGCGATTCTAAAGT
GTAAT---AATAAGACATTCAAT------GGATTAGGACCATGTACAAAT-----------------------
-GTCAGT---ACAGTACAATGTACACATGGAATTAGGCCAGTAGTATCAACT---
CAACTATTGTTAAATGGCAGTCTAGCAGAAAAAGAG------------
GTAGTAATTAGATCTGCAAACTTCTCGAACAAT---------GCTAAAGTCATAATAGTACGGCTGAAT----
-----------GAATCTGTAACA---------ATAAATTGTACAAGACCTAACAACAATACAAGAAGA-----
-AGTATAAATATA------------CAACCAGGGAGAGCA---TTT------TTTGCAACAGGAGAC------
---------ATAATAGGAAATATA------------AGACAAGCATATTGT---AATATTAGTGGAGCA---
CAATGGAACAGAACTTTAAATTTGATAGCTAAAAAATTAAAA---------------GAACAATTTAAC----
-----------------------------------AACAAGTCAATAATCTTTAGG------
CAATCCTCAGGAGGGGACCCAGAAATTACA---ATGCACAGTTTTAATTGTAGAGGGGAG---
TTTAAACAGCTCCGGGCAAGAGTCCTGGCTGTGGAAAGATACCTACAGGACCAACAGCTCCTAGGGATTTGGG
GTTGCTCTGGAAAACTCATTTGCACCACTGCTGTGCCTTGGAATGCTAGTTGGAGT-----------------
----------------------------AACAAAACTCAAAATGAGATTTGG------GAA------
AACATGACCTGGATGCAATGG---GAAAGAGAAATTGAAAATTACACAGGCACAATATAC---
AACTTACTTGAAGAATCGCAAAACCAACAAGAA--

AAGAATGAACAAGAATTATTGGAATTAAATAAGTGGGCAAGTTTGTGGAATTGGTTTAGCATATCACAATGGC
TGTGGTATATAAAAATATTCATAATGATAGTAGGAGGCTTGATAGGTTTAAGAATAATTTTTGCT---
GTACTCTCTGTAGTG---------AATAGAGTTAGGCAGGGATACTCACCA------
TTGTCATTTGCTGTTAGCTTGTTTAATGCCATAGCAATTGCAGTAGCTGAG---
GGGACAGATAGGGTTATAGAAGCAGTACAGAGGGCTTGTAGGGCT----------------------------
--------ATCCTCCACATA------CCTAGACGAATAAGACAAGGCTTAGAAAGGGCTTTGCTA---TAA
>JF680936
GCT---GCA---GAA------------------------------------
CAATCATGGTGGGTCACAGTCTATTATGGGGTACCTGTGTGG---AAAGAAGCAAAC---
ACCACTCTATTTTGTGCATCAGATGCTAAAGCTCATATACCAGAG---ATGCATAAT----------------
--------------GTTTGGGCCACA---CATGCTTGTGTGCCCACAGAC---
CCCAGCCCACAAGAATTAGAATTGAAA---------AATGTGACAGAAAATTTTAAT----------------
--------------------------ATGTGGAAAAATAACATGGTAGAACAAATGCATGAGGATATA---
ATCAGTTTATGGGATCAAAGCCTA---AAGCCATGTGTAAAATTA---
ACCCCACTCTGTGTTACTTTAGACTGCCAGGAGGCCAAGGGTATCAATGCTACTAATATCACTAGCAAT----
----
----------------ACTAATAGTAGTAGCATGGACCTGAGGGCGGAACAGGGAGAAATACAA---
AATTGCTCTTTCAATATCACTACAAGCATAAGA---------------------------
GACAAAGTAAAGAAAGAATATGCATTA---CTTTATAAAGTTGATATAGTACCAATAGATAATGAGAATAAC-
----

AATAAAAATAAGACTAATAGTACTAACTATAGGTTGATAAGTTGTAACACCTCAGTCATTACACAG---
GCCTGTCCAAAAATATCCTTTGAACCAATTCCCATACATTTTTGTGCCCCGGCTGGTTTTGCGATTCTAAAGT
GTAAT---GATAAGCGGTTCAAT------GGAACAGGACCATGCACAAAT-----------------------
-GTCAGC---ACAGTACAATGTACTCATGGAATCAAGCCAGTAGTTTCAACT---
CAATTGCTGTTAAATGGTAGTCTAGCAGAAAAAGAG------------
GTAGTGATTAGATCTGAGAATTTCACAAACAAT---------GCTAAAACCATAATAGTACAGCTAAAT----
-----------GAAACTGTAGAA---------ATTAATTGTACAAGGCCCAACAACAATACAAGGAAA-----
-AGTATACCTATA------------GGACCAGGTAGGGCA---TTC------TATGCAACAGGAGAA------
---------ATAATAGGAGATATA------------AGACGAGCACATTGT---AACATTAGTGGAGAA---
AAATGGAATAGAGCTTTAAAACAGGTAGCTGAAAAATTAAGA---------------GAACAATAT-------
-----------------------------------AATAAAACAATAGTCTTTAAT------
CACTCCTCAGGAGGGGACCCAGAAATTGTA---ATGCACACTTTTAATTGTGCAGGGGAA---
TTTAAGCAGCTCCAGGCAAGAGTCCTGGCTGTAGAAAGATACCTACAGGATCAACAGCTCCTGGGGATGTGGG
GTTGCTCTGGAAAACTCATCTGCACCACTAATGTGCCTTGGAATACTAGTTGGAGT-----------------

----------------------------AGTAGATCTCTGGATAATATTTGG------AGT------
AATATGACCTGGATGCAGTGG---GAAAAAGAAATTGACAATTATACAGGCTTAATATAC---
AATCTAATTGAAGAATCGCAGAACCAGCAAGAA--

AAGAATGAACTAGACTTATTGGCACTAGATAAATGGGAAAGTTTGTGGAATTGGTTTAGCATAACAAAATGGC
TGTGGTATATAAAAATATTCATAATGATAGTAGGAGGTTTGATAGGGTTAAGAATAGTTTTTACT---
GTACTTTCTATAGTA---------AACAAAGTTAGGCAGGGATACTCACCA------
TTGTCGTTTGCTGTTAGCTTGTTTAATGCTATAGCCATAGCAGTTGCTGAG---
GGGACAGATAGGCTCATAGAAATAGTGCAAGGAGCTGGTAGAGCT----------------------------
--------ATCCTCCACATA------CCAGTAAGAATAAGACAGGGCTTGGAAAGGGCTTTACTA---TAA
>KF061032
GCA---GCA--
GAACAATTGTGGGTCACAGTCTATTATGGGGTACCTGTGTGG---AAAGAAGCAACC---
ACCACTCTATTTTGTGCATCAGATGCTAAAGCATATGATACAGAG---GCACATAAT----------------
--------------GTTTGGGCCACA---CACGCCTGTGTACCCACAGAC---
CCCAACCCACAAGAAATAGTATTGGAA---------AATGTGACAGAAAATTTTAAC----------------
--------------------------ATGTGGAAAAATAACATGGTAGAACAGATGCATGAGGATATA---
ATTAGTTTATGGGATCAAAGCCTA---AAGCCATGTGTAAAATTA---
ACCCCACTCTGTGTTACTTTAAATTGCACTGATGATTTGAGGAATGGTACTAATAGT----------------
----
----------------------------AGTAGCTGGGGAATGATGGAGAGAGGAGAAATACAA---
AATTGCTCTTTCAATGTCACCACAAGCATAAGT---------------------------
GATAAGATGCAGGAAAACTATGCACTT---TTTTATAAACTTGATATAATAAAAATAGACAAT----------
----

AGTACTAACTATAGGTTGATAAGTTGTAACACCTCAGTCATTACACAG---
GCCTGTCCAAAGGTATCCTTTGAGCCAATTCCCATACATTATTGTGCCCCGGCTGGTTTTGCGATTCTAAAGT
GTAAC---AATAAGAAGTTCAGT------GGAACAGGACCATGTACAAAT-----------------------
-GTCAGC---ACAGTACAATGTACACATGGAATTAGGCCGGTAGTATCTACT---
CAACTGCTGTTAAATGGCAGTCTAGCAGAAGAAGAG------------
GTAGTAATTAGGTCTGAAAATTTCACGAACAAT---------GCTAAAAGCCTAATAGTACAGCTGAAT----
-----------CAATCGGTACAA---------ATTAATTGTACAAGACCCAATAACAATACAAGAAGA-----
-AGTATACATATA------------GGACCAGGGAAAGCA---TTT------TAT---ACAGGAGAA------
---------ATAATAGGAAATATA------------AGACAAGCACATTGT---AACCTTAGCAGAGCA---
AAGTGGATGACACTTTAAAAAGGATAGCTGACAAATTAAGA---------------GAACAATATGAG----
-----------------------------------AATAAAACAATAGTCTTTAGT------
CAATCCTCAGGAGGGGACCCAGAAATTGTA---ATGCACAGTTTCAATTGTGGAGGGGAA---
TTTAAGCAGCTCCAGGCAAGAGTCCTAGCTGTGGAAAGATACCTAAGGGATCAACAGCTCCTGGGGATTTGGG
GTTGCTCTGGAAAAGTCATTTGCACCACTGCTGTGCCTTGGAATGCTAGTTGGAGT-----------------
----------------------------AATAAATCTCTGGATAAGATTTGG------GAT------
AACATGACCTGGATGGAATGG---GAAAGAGAAATTGACAATTACACAGGCATAATATAC---
ACCTTACTTGAAGAATCGCAAATCCAACAAGAA--

AAGAATGAACAAGAATTATTGGAATTAGATAAATGGGCAAGTTTGTGGAATTGGTTTGACATAACAAACTGGT
TGTGGTATATAAAAATATTCATAATGATAGTAGGAGGCTTGGTAGGTTTAAGAATAGTTTTTACT---
GTACTTTCTATAGTG---------AATAGAGTTGGGAAGGGATACTCACCA------
TTATCGTTCGCTGTTAATTTGCTCAATGCCACAGCTATAGCAGTAGCTGAA---
GGGACAGATAGGATTATAGAAGCATTACAAAGAGCTTATAGAGCT----------------------------
--------ATTCTCCACATA------CCTACACGAATAAGGCAGGGCTTGGAAAGGGCTTTACTA---TAA
>KF061031
GCT---GAG--
GAACAAAAGTGGGTCACAGTTTATTATGGGGTACCTGTGTGG---AGAGAAGCAAAC---
ACCACTCTATTTTGTGCATCAGATGCTAAGGCATATGCTACAGAG---GTACATAAT----------------
--------------GTTTGGGCCACA---CATGCCTGTGTACCCACAGAC---
CCCAACCCACAAGAAATAGTATTGAGA---------AATGTGACAGAAAATTTTAAT----------------
--------------------------ATGTGGAAAAATAACATGGTAGAACAGATGCATGAGGATATA---
ATTAGTTTATGGGATCAAAGCCTA---AAGCCATGTGTAAAATTA---
ACCCCACTCTGTGTTACTCTAAAATGCAATAACTATACGGGGGATGTTACTAATAACACGGGGAGTAAT----
----
-------------------ACCACTGGTAGTAGCGGGGGAGTGATGGAGGGAGGAGAAATGAAA---

AATTGCTCTTTCAATATCACCACAAACATAAGA--------------------------
GATAAGGTGCGAAAAGATTATGCACTT---TTTTATAATCTTGATGTAATACAAATAAAAGATAAAAAT----
----

AATAATGAAAGTAATAGTTATAGGTTGAGAAGTTGTAACACCTCAATCATTACACAA---
GCCTGTCCAAAGGTATCCTTTGAGCCAATTCCCATACATTATTGTGCCCCGGCTGGGTTTGCGATTCTAAAGT
GTAAC---GATAAGAAATTCAAT------GGAACAGGACCATGTAAGAAT----------------------
-GTCAGC---ACAGTACAATGTACACATGGAATTAAGCCAGTAGTATCAACT---
CAACTGCTGCTAAATGGCAGCCTAGCAGAAGAAGAG-----------
GTAGTAATTAGGTCTGAAAATTTCACGAACAAT---------GCTAAAATCATAATAGTACAGCTGAAT----
-----------AACACTGTAAAT---------ATTAATTGTATAAGACCCAACAACAATACAAGGAAA-----
-AGTATACCCATG------------GGACCAGGGAAAGCA---TTT------TATGCAACAGGAGAC------
---------ATAATAGGAAATATA------------AGACAAGCACATTGT---AACATTAGTAGGGTA---
CAATGGAATAACACTTTAAAACAGATAGCTAACAAATTAAGA---------------GAACAATTTGGG----
--------------------------------AAGAATAAAACAATAGTCTTTAGT------
CCATCCTCAGGAGGGGACCCAGAAATTGAA---ATGCACAGTTTTAATTGTGGAGGAGAA---
TTTAAGCAGCTCCAGGCAAGAATCCTGGCTGTGGAAAGATACCTGAAGGATCAACAGCTCCTAGGGATTTGGG
GTTGCTCTGGAAAACACATTTGCACCACTACTGTGCCTTGGAATAATAGTTGGAGT-----------------
----------------------------AATAGATCTCTGGATCAGATTTGG------GAT------
AACATGACCTGGATGGAGTGG---GAAAGAGAAATTGACAATTACACAGGATTAATACAC---
ACCTTACTTGAAGAATCGCAGACCCAACAAGAA--

AAGAATGAACAAGAATTATTGGCCTTAGATAAATGGGCAAGTTTGTGGAATTGGTTTGACATATCAAACTGGC
TGTGGTATATAAAAATATTCATAATGATAGTAGGAGGCTTAATAGGTTTAAGAATAGTCTTTACT---
GTACTTTCTATAGTG---------AATAGAGTTAGGCAGGGATATTCACCA------
TTGTCGTTCGCTGTTAGCTTGCTCAACGCCACAGCCATAGCAGTAGCTGAG---
GGGACTGATAGGGTTATAGAAGTATTACGCAGATTTTATAGAGGT----------------------------
--------ATTCTCCACATA------CCTACAAGAATAAGACAGGGCTTGGAAAGGGCTTTGCTA---TAA
>AY835445
GCT---GCA---GAA---------------------------------
CAAGGACAATTGTGGGTCACAGTCTATTATGGGGTACCTGTGTGG---AAAGACGCAAGC---
ACCACTCTATTTTGTGCATCAGATGCTAAAGCATATGATACAGAG---GTACATAAT----------------
--------------GTTTGGGCCACA---CATGCCTGTGTACCCACAGAC---
CCCAACCCACAAGAAGTAGTATTGGGA---------AATGTGACAGAAAATTTTAAC----------------
--------------------------ATGTGGAAAAATAACATGGTAGATCAGATGCATGAGGATATA---
ATCAGTTTATGGGATCAAAGCCTA---AAGCCATGTGTGAAACTA---
ACCCCACTCTGTGTTACTTTAAATTGTACTGATAATATTACTAATACTAATACCAATAGTTCAAAGAAT----
----
-------------------AGTTCTACTCATTCTTATAATAATAGTTTGGAAGGAGAAATGAAA---
AACTGCTCTTTCAATATCACCGCAGGAATAAGG--------------------------
GATAAGGTAAAGAAAGAATATGCACTT---TTTTATAAACTTGATGTAGTACCCATAGAGGAGGAT-------
----

AAGGATACTAATAAAACCACCTATAGGTTGAGAAGTTGTAATACCTCAGTCATTACACAG---
GCCTGTCCAAAGGTAACCTTTGAGCCAATTCCCATACATTATTGTGCCCCGGCTGGTTTCGCAATTCTAAAGT
GTAAT---GATAAGAAATTCAAT------GGAACAGGGCCATGTACAAAT----------------------
-GTCAGC---ACAGTACAATGTACACATGGAATTAGGCCAGTAGTGTCAACT---
CAACTGCTGTTAAATGGCAGTCTAGCAGAAGAAGAG-----------
GTAGTAATTAGATCTGAAAATTTCACGAACAAT---------GCTAAAACCATAATAGTACAGCTTAAT----
-----------GAATCTATAGCA---------ATTAATTGTACAAGACCCAATAACAATACAAGAAGA-----
-AGTATACATATA------------GGACCAGGGAGAGCA---TTT------TATGCAACAGGAGAC------
---------ATAATAGGAGATATA------------AGGCAAGCACATTGT---AACATTAGTAGAACA---
GAATGGAATAGCACTTTAAGACAGATAGTTACAAAATTAAGA---------------GAACAATTGGGGGAT-
--------------------------------CCTAATAAAACAATAATCTTTAAT------
CAATCCTCAGGAGGGGACACAGAAATTACA---ATGCACAGTTTTAATTGTGGAGGGGAA---
TTTAAGCAACTCCAGGCAAGAGTCCTGGCTGTGGAAAGATACCTAAGGGATCAACAGCTCCTAGGGATCTGGG
GTTGCTCTGGAAAACTCATTTGCACCACTAATGTGCCTTGGAATGCTAGTTGGAGT-----------------
----------------------------AATAAATCTCTTAATAACATTTGG------GAA------
AACATGACATGGATGGAGTGG---GAAAGAGAAATTGACAATTACACAGACTTAATATAC---
ATCTTACTTGAAAAATCACAGATACAACAAGAA--

AAGAATGAACAAGAATTATTGGAATTAGATAGTTGGGCAAGTTTGTGGAATTGGTTTGACATATCAAAGTGGC
TGTGGTATATAAAAATATTCATAATGATAGTAGGAGGCCTGGTAGGTTTAAGAATAGTTTTTGCT---
GTGCTTTCTATAGCA---------AATAGAGTTAGGCAGGGATACTCACCA------
TTGTCATTTGCTGTTAGTTTACTCAATACCACAGCTATAGCAGTAGCTGAG---
GGGACAGATAGGGTTATAGAAGTAGTACAAAGAGCTTTTAGAGCT---------------------------
--------ATTCTCCACATA------CCTGCAAGAATAAGACAGGGCTTAGAAAGGGCTTTGCTG---TAA
>AY835444
GCT---GAA---
GAAAAGTTGTGGGTCACAGTCTATTATGGGGTACCTGTGTGG---AAAGAAGCAACC---
ACCACTCTATTTTGTGCATCAGATGCTAAAGCATATAATACAGAG---GTACATAAT---------------
--------------GTTTGGGCCACA---CATGCCTGTGTACCCACAGAC---
CCCAACCCACAAGAAGTAGGATTGGAG---------AATGTGACAGAAAATTTTAAC---------------
--------------------------ATGTGGAAAAACAACATGGTAGAACAGATGCATGAGGATATA---
ATTAGTTTATGGGATCAAAGCCTC---AAGCCATGTGTAAAATTA---
ACCCCACTCTGTGTTACTTTAAATTGCAGTGATTTGAGGAATGCTACTAATACCACTAATCCCACT-------
----
-------------------GTTAGTAGCAGGGTAATAAAGAAGGAAATGATGGGAGAAGTAAAA---
AATTGCTCTTTCAATGTCACCACAGACATAAGA--------------------------
GATAGAATGCAGAAAGTATATGCGCTT---
TTTTATAGACCTGATGTAGTACCAATACAAGACCATACTATAGAA---------------------------
----
AACAATAATACAATAGAAAACAATACTACTTATAGGTTGATAAGTTGTAACACCTCAGTCATTACACAA---
GCCTGTCCAAAGATATCCTTTGAGCCAATTCCCATACATTATTGTACCCCGGCTGGTTTTGCGATTCTAAAGT
GTAAT---GATAAGAAGTTCAAT------GGATCAGGACCATGTACAAAT----------------------
-GTCAGC---ACGGTACAATGTACACATGGAATTAGGCCAGTAGTATCAACT---
CAATTGCTGTTAAATGGCAGTCGAGCAGAAGAAGAA-----------
GTAATAATTAGATCTGAAAATTTTACAAACAAT---------GCTAAAACCATAATAGTGCAGCTGAAT----
-----------AAAACTGTAGAA---------ATTAATTGTACAAGACCCAACAACAATACAAGAAAA-----
-AGTATATCTATA------------GGACCAGGGAGAGCA---TTT------TATGCAACAGGGGAC------
---------ATAATAGGAGATATA------------AGACAAGCACATTGT---AACCTTAGTAGAGCA---
GAATGGAATAAAACTTTAAAATATATAAGTACAAAATTAAGA---------------GAACAATTTGGG----
-----------------------------------AACAAAACAATCATCTTTAAT------
GGATCCTCAGGAGGGGACCCAGAAATTGTA---ACACACAGTTTTAATTGTGGAGGGGAA---
TTTAAACAGCTCCAGGCAAGAGTCCTGGCTATAGAAAGATACCTAAAGGATCAACAGCTCCTAGGGATTTGGG
GTTGCTCTGGAAAACTCATCTGCACCACTGCTGTGCCTTGGAACACTAGTTGGAGT-----------------
----------------------------AATAAATCTTTTAATAAAATTTGG------GAC------
AACATGACCTGGATGGAGTGG---GAAAGAGAAATTGACAATTACACAGGCTTAATATAC---
AACTTACTTGAAGAATCGCAAAACCAACAAGAA---------------------------------------
----
AAGAATGAACAAGATTTATTGGCATTAGATAAATGGGAAAGTTTGTGGAATTGGTTTAGCATAACAAAATGGC
TGTGGTACATAAAAATATTCATAATGATAGTAGGAGGCTTGATAGGTTTAAGAATAGTTTTTGCT---
GTACTTTCTATAGTA---------AATAGAGTTAGGCAGGGATACTCACCT------
TTATCATTTGCTGTTAGCTTGCTTAATGCCACAGCAATAGCAGTAGCTGAA---
GGGACAGATAGGATTATAGAAGTAGTACAGAGAACTTTTAGAGCT---------------------------
--------ATTATTCACATA------CCTAGAAGAATAAGACAGGGCTTGGAAAGGCTTTTGCTA---TAA
>EU839605
GCT---ACA---
GACAACTTGTGGGTCACAGTTTATTATGGGGTACCTGTGTGG---AAAGAAGCAACC---
ACCACTCTATTTTGTGCATCAGATGCTAAGGCATATAAAACAGAG---GTACATAAT---------------
--------------GTCTGGGCCACA---CATGCCTGTGTACCCACAGAC---
CCCAACCCAAATGAAGTAGAATTGGAA---------AATGTGACAGAAAATTTTAAC---------------
--------------------------ATGTGGAAAAATGACATGGTAGAACAAATGCATGAGGATATA---
ATTAGTTTATGGGATCAAAGCCTA---AAGCCATGTGTAAAATTA---
ACCCCACTCTGTGTGACTTTAAATTGCACTAATGTTAATACCACTGATGTTAGGCGGAATGATACCAATAGC-
----
---------------ACTGTTGGCACTAATGATGGTTTAAGAATAAAACAGGGAGAAATAAAG---
AACTGCACTTTCAACATCACCACAAGCACAAAT--------------------------GAGAAGGTG---
AAAGGAAATGCAATT---TTTGATACACTTGATGTAGTACAAGCAGGTGAG---------------------
----

----------AATAATAATAGCTATAGATTGATAAGTTGTAATACCTCAGTCATTACCCAG---
GCCTGTCCAAAGGTCTCCTTTGAGCCAATTCCCATACATTATTGTGCCCCGGCTGGTTTTGCGATTTTAAAGT
GTAGA---GACAAGAAGTTCAAT------GGAACAGGACCATGTAAAAT------------------------
-GTTAGC---ACAGTACAATGTACACATGGAATCAGGCCAGTAGTGTCAACT---
CAATTGTTGTTAAATGGCAGTCTAGCAGAAGAAGAG------------
GTAATAATTAGATCTGAAAATATCACGGACAAT---------GCTAAAACCATAATAGTACAGCTGAAG----
-----------GAAGCTGTGGAA---------ATTGAGTGTGTGAGACCCAACAACAATACGAGAACA---
ACAAGTGTACATATA------------GGACCAGGGCGAGCA---ATT------TATGCAACAGGAGAC----
-----------ATAATAGGTGATATA------------AGACGAGCATATTGT---AACCTTAGTAGAGCA--
-AACTGGACTAACGCTTTAAACCAGACAGCTAGGAAATTAAGA---------------GAACAATTT------
------------------------------------AATAAAACAATAATCTTTAAT------
TCATCCTCAGGAGGGGACCCAGAAATTGTG---ATGTTTAGCTTTAATTGTGGAGGGGAA---
TTTAAACAGCTCCAGGCAAGAGTCCTGGCTGTAGAAAGATACCTAAGGGATCAACAGCTCCTGGGGATCTGGG
GTTGCTCTGGAAAACTCATTTGCACCACCTCCGTGCCTTGGAATGCTAGTTGGAGT-----------------
----------------------------AATAAATCTGTGGATAGAATTTGG------GGG------
AACATGACCTGGATGGAGTGG---GAAAGAGAAATTGACAATTACACAGACCTAATATAT---
AACTTACTTATGGAATCGCAGAACCAGCAAGAA--

AAGAATGAAAAAGAATTATTAGAATTGGATAAGTGGGCAAGTCTGTGGAATTGGTTTGACATAACAAACTGGC
TGTGGTATATAAAAATTTTCATAATGATAGTAGGAGGCTTGGTAGGTTTAAGAATAGTTTTTACT---
GTACTATCTATAGTG---------AATAGAGTTAGGCAGGGATACTCACCA------
CTATCATTTGCTGTTAGCTTGTTTAACGCCACAGCAATAGCAGTAGCTGAG---
GGAACAGATAGAGCTATAGAAATAGTGCTAAGAATTTTTAGGGCT----------------------------
--------ATCCTCCACATA------CCTACAAGAATAAGACAGGGCTTGGAAAGGGCTTTGCTA---TAA
>JF680925
GCT---AAA---GAA---------AAAGAA---GAA---GAA---CAA---
GAAAAGTTGTGGGTCACAGTCTATTATGGGGTACCTGTGTGG---AAAGAAGCAGCC---
ACCACTTTATTCTGTGCATCAGATGCTAAAGCATATGATACAGAG---GTACATAAT----------------
--------------GTTTGGGCCACA---CATGCTTGTGTACCTACAGAC---
CCCAGCCCACAAGAAGTAACATTGAGA---------AATGTGACAGAAAATTTTAAC----------------
--------------------------ATATGGAAAAATAACATGGTAGAACAGATGCATGAGGATATA---
ATCAGTTTATGGGATCAAAGCCTA---AAGCCATGTGTAAAATTA---
ACCCCACTTTGTGTCATTCTACATTGCACTGATAACATTACTCTTATCAATGATAATACTTCC----------
----
----------------------ACGAATGCTACTATGAGGGACGGGATGCAAGGGGAAATAAAA---
AACTGCTCTTTCAATGTGACCACAGGTATAAGA---------------------------
GATAAGGTGCGGAATGTATCTGCAATT---TTTAATACAATTGATATAGTACCAATAGAAGAAAAT-------
----

GGTAATACTAGCTATAGGTTGGTAAATTGTAACACCTCAGTCATTAGACAA---
GCCTGTCCAAAGGTATCCTTTGAGCCAATTCCCATACATTATTGTGCCCCGGCTGGTTTTGCGATTCTAAAGT
GTAAT---GATAAAACGTTCAAT------GGATCAGGACCATGTGAAAAT------------------------
-GTCAGC---ACAGTACAATGTACACATGGAATTAAGCCAGTGGTATCAACT---
CAACTGCTGTTAAATGGCAGTCTAGCAGAAGAAAAG------------
ATAGTACTTAGATCTGAAAATTTCACAAACAAT---------GCTAAAACTATAATAGTACAGCTGAAT----
-----------GAAACAGTAGAA---------ATTAATTGTACAAGACCCAACAACAATACAAGAAAA-----
-GGTATACATATG------------GGACCAGGGGGAGCA---TTC------TATGCAACAGACCAA------
---------ATAATAGGAGATATA------------AGAAGAGCATATTGT---GAAATTAATATAACA---
AAATGGAATGACACTTTAAGAAAAATAGTTGGAGAGCTAAGA---------------GAACAATTTGGG----
--------------------------------AAGAATATAACAGTAACCTTTAAT------
CACTCCTCAGGAGGGGATCCAGAAATTGTA---ATGCACAGTTTTAATTGTAGGGGGGAA---
TTTAAGCAGCTCCAGGCAAGAGTCCTGGCTCTGGAAAGATATCTAAGGGATCAACAGCTCCTGGGAATTTGGG
GTTGCTCTGGAAAACTCATCTGCACCACTGCTGTGCCTTGGAATGCTAGTTGGAGT-----------------
----------------------------AATAAATCTTGGGGTGACATTTGG------GAG------
AACATGACCTGGATGCAGTGG---GAAAAAGAAATTGAAAATTACACAGACTTAATATAC---
AACTTAATTGAGGAATCGCAAAACCAACAAGAA--

AAGAATGAACAAGACTTATTGGCATTAGATAAATGGGACAGTTTGTGGAATTGGTTTAACATAACAAAATGGC
TGTGGTATATAAAAATATTCATAATGATAGTAGGAGGCTTGATAGGTTTAAGAATAGTTTTTACT---
GTACTTTCTATAGTA---------AATAGAGTTAGGCAGGGATATTCACCA------

TTGTCGTTGGCTGTTAGCTTGTTTAATGCCATAGCCATAGCAGTAGCTGAG---
GGGACAGATAGGATTATAGAAGCATTGCAAAGAGCTGGAAGAGCT---------------------------
--------ATCCTCCACATA------CCTAGAAGAATAAGACAGGGCTTAGAAAGGGCTTTGCTA---TAA
>HM030564
GCT---ACA---
GAACAATCGTGGGTCACAGTCTATTATGGGGTACCTGTGTGG---AAAGAAGCAACC---
ACCACTCTATTTTGTGCATCAGATGCTAAAGGATATGATACAGAG---GTACATAAT---------------
--------------GTCTGGGCCACA---CATGCCTGTGTACCCACAGAC---
CCCAGCCCACAAGAAGTAGAATTAAAA---------AATGTAACAGAAAATTTTAAC---------------
--------------------------ATGTGGAAAAACAACATGGTAGAACAGATGCAGGAGGATATA---
ATCAGTTTATGGGATCAAAGTCTA---AAGCCATGTGTAAAATTA---
ACCCCACTCTGTGTTACTTTAAATTGTACTGATTGGGAGAGAAATAATAACAATAATACCAAT----------
----
---------------------AATACCACAGAGAATCTGAAAATGGAGACAGGAGAGATAAAA---
AACTGCTCTTTTAACATCACCACAAGCATCAAA-------------------------
GATAAGAGGCTGAGGGAATATGCACTT---TTTTATAAATCTGATATAGTACCAATAAAAGATAGT-------
----

GAGAACAATATTACTAGGTATAGATTGATAAGTTGTAATACCTCAGTTATTACACAG---
GCCTGCCCAAAAGTAACCTTTGAGCCAATTCCCATACATTATTGTGCCCCGGCTGGTTTTGCGATTCTAAAAT
GTAAT---GATAAAAAGTTCAAT------GGAACAGGACCTTGTACCAAT----------------------
-GTCAGC---ACAGTACAATGTACACATGGAATTAGGCCAGTAGTATCAACT---
CAATTGCTGTTAAATGGCAGTCTAGCAGAAGAAGAG-----------
GTAATAATTAGATCTAAAAATATTACAGACAAT---------ACTAAAATAATAATGGTACATCTAAAG----
-----------GAATCTGTAAAA---------ATTACTTGTACAAGACCCAACAACAATACAAGAAAA-----
-AGTATATCTCTA------------GGACAAGGGAGAGCA---ATC------TATGCAACAGGAGAC------
---------ATAATAGGGGATATA-----------AGGAAAGCACATTGT---AACCTTACTAAAGCA---
CAATGGAATAGCACTTTAAAACAAATAGTTAAGAAATTAAGA--------------GAATACTTTCCG----
-----------------------------------AATAAAACAATAGTATTTAAT------
CAATCCTCAGGAGGGGACCCAGAAATTATG---ATGCACACTTTTAATTGTGGAGGGGAA---
TTTAAACAGCTCCAGGCAAGAGTCCTGGCTTTGGAAAGATACCTAAAGGATCAGCAAATCCTAGGAATTTGGG
GCTGCTCTGGAAAACTCATTTGCACCACCAATGTGCCTTGGAACACTAGTTGGAGT-----------------
---------------------------AATAAATCTCTAAATTATATTTGG------GAG------
AACATGACCTGGATGCAATGG---GAAAGGGAGATTGACAATTACACAGATGTAATATAC---
AAATTAATTGAAGAATCGCAGAACCAGCAAGAA---------------------------------------
----
AAGAATGAATTAGACTTGTTAAAATTGGATGAATGGGCAAGTTTGTGGAATTGGTTTGATATAACAAAATGGC
TGTGGTATATAAAAATATTCATAATGATAGTAGGAGGCTTGATAGGCTTAAGAATAGTTTTTACT---
GTACTTTCTATAGCG---------AATAGAGTTAGGCAGGGATACTCACCA------
TTATCATTCGCTGTTAGCTTGCTTAATGCCACAGCTGTAGCAGTAGCTGAG---
GGGACAGATAGGGTTATAGAAGTATTACAAAGAATTTTTAGAGCT---------------------------
--------ATTCTTCATATA------CCTACAAGAATAAGACAGGGCTTGGAAAGGGCTTTACTA---TAA
>HM030559
GTT---GCA---
GAAGATTTGTGGGTCACAGTTTATTATGGGGTACCTGTATGG---AAAGAAGCAGAC---
ACCACTCTATTTTGTGCATCAGATGCTAAAGGATATGACACAGAG---GTACATAAT---------------
--------------GTCTGGGCCACG---CATGCCTGTGTACCCACAGAT---
CCCAGTCCAAATGAAGTAGTAATGGAA---------AATGTGACAGAATACTTTAAC---------------
--------------------------ATGTGGAAAAATAACATGGTAGAACAGATGCATGAGGATATA---
ATTAGTTTATGGGATCAAAGCCTA---AAGCCATGTGTAAAATTA---
ACCCCACTCTGTGTTACTTTAAACTGCACTGATACTAGGAACAATACCAATAACAGGAACGATACCAATAGCA
CA--
-------------GACAGTACCCCTATCCCTGGTGCTGGAAGAATTGATGATGGAGAAATAAAG---
AACTGCTCTTTCAACATCACCACAAGCACAAGC----------------------------GACAGAGTG---
AAGGGACATGCACTT---TTTTATGGAATGGATGTAGTACCAATAAGTAAT---------------------
----
----------GATAATAGTAGTTATAGATTAATAAGTTGTAATACCTCAGTCATTACCCAG---
GCCTGTCCAAAGGTAACCTTTGAGCCAATTCCCATACATTATTGTGCCCCGGCTGGCTTTGCGATTCTAAAAT
GTAAC---GAGAAGAATTTCAAT------GGAACAGGACAATGTACAAAT----------------------
-GTCAGT---ACAGTACAATGTACACATGGAATTAGGCCAGTAGTGTCAACT---

CAGCTGCTGTTAAATGGTAGTCTAGCAGAAGATGAG------------
ATAGTAATTAGATCTGCAAATTTCTCAGACAAT---------GCAAAAACCATAATAGTACAGTTCAAT----
-----------AAATCTATAGTT---------ATTGATTGTACAAGACCCAATAACAATACAAGAAAA-----
-AGTGTACCTATA------------GGACCAGGGAGAGCA---TTT------TATGCAACAGGAGAC------
---------ATAATAGGAAATATA------------AGAAAAGCATATTGT---AACCTTAGTAAAACA---
GAATGGAATAACACTTTAAAACAGGTAGCTATAAAATTACAA---------------GAACAATTT-------
-----------------------------------AATAAAACAATAGTCTTTAAT------
CAATCCACAGGAGGGGACCCAGAAATTGTA---ATGCACAGTTTTAATTGTGGAGGGGAA---
TTCAAACAGCTCCAGGCAAGAGTCCTGGCTGTGGAGAGATACCTAAGGGATCAACAGCTCCTGGGAATTTGGG
GTTGCTCTGGAAAACTCATCTGCACCACTGCTGTGCCTTGGAATGCTAGTTGGAGT-----------------
----------------------------AATAAATCTATAGACACTATTTGG------GAC------
AATATGACCTGGATGGAGTGG---GAAAAAGAAATTGGCAATTACACAAACTTAATATAC---
AACTTACTTGAAGAATCGCAAAACCAACAAGAG--

AAAAATGAACAAGAATTATTAGAATTGGATAAGTGGGCAAGTTTGTGGAATTGGTTTGATATATCAAACTGGC
TGTGGTATATAAAAATATTCATAATGATAGTAGCAGGCTTGGTAGGTTTAAGAATAGTTTTTACT---
ATACTTTCTATAGTG---------AATAGAGTTAGGCAGGGATACTCACCA------
TTGTCATTTGCTGTTAGCTTATTTAATGCCATAGCTATAGCAGTAGCTGAG---
GGGACAGATAGGATTATAGAAGCAGTACAAAGGGGTGTTAGAGCT----------------------------
--------TTTCTCCACATA------CCCAGAAGAATAAGACAGGGCCTAGAAAGAGCTTTGCTA---TAA
>HM030562
GCT---GAA--
GAGGATTTGTGGGTCACAGTCCATTATGGGGTACCTGTGTGG---AGAGAAGCAACC---
ACCACYCTATTTTGTGCATCAGATGCTAAAGCACATAATACAGAG---GCACATAAT----------------
--------------GTTTGGGCCACA---CATGCCTGTGTACCCACAGAC---
CCCAGCCCACAAGAAGTAAAATTGGAA---------AATGTGACAGAAAATTTTAAC----------------
--------------------------ATGTGGAAAAATAACATGGTAGACCAGATGCATGAGGATATA---
ATCAGTTTATGGGACCAAAGCTTA---AAGCCATGTGTAAAATTA---
ACCCCACTCTGTGTTACTCTAAATTGCACTGATCTAGGGAATAATACTTGTACCAATAGTACCAACAATAGT-
----
-------------GCAGAAAATGGTACTTGTGACAGGACACTGATGGAGAAGGGAGAAATAAAA---
AACTGTTCTTTCAATATCAACACAGTCATACAA---------------------------
GATAAGGTACAAAAACAATATGCACTG---TTTTATAAACTTGATGTAGTATCAATAGATAAT----------
----

GATAATACTAACCATACATATAGATTGATAAGTTGTAACACCTCAGTCATTACACAA---
GCCTGTCCAAAGGTATCTTTTGAGCCAATTCCCATACATTATTGTGCCCCGGCTGGTTTTGCGATTCTAAAGT
GTAAA---GATAAGAAATTCAAT------GGAACAGGACCATGTAGAAAT------------------------
-GTCAGC---ACAGTACAATGCACACATGGAATTAGGCCAGTAGTTTCAACT---
CAACTATTGTTAAATGGCAGTCTAGCAGAAGAAGAG------------
GTAGTAATTAGATCTGAAAATTTCAGTAACAAT---------GCTAAAACCATAATAGTACAGCTGAAC----
-----------AAAGAAGTAAAA---------ATCTATTGTGAAAGACCCAACAACAATACAAGAAAA-----
-GGCATACATATT------------GGACCAGGGAGAGCA---TTT------TATGCTACAGGAGAT------
---------ATAATAGGAGATATA------------AGGCAAGCACATTGT---AACATTAGTAGAGAA---
AATTGGACTGACACTTTGAACCAGACAGTTGCAAAATTAGGA---------------GAACAATTCAAG----
--------------------------------AATATAACAACAATAATCTTTACT------
AACTCCTCAGGAGGGGACCCAGAAATTGTA---AGGCACAGTTTTAATTGTGGAGGGGAA---
TTTAAGCAGCTCCAGGCAAGAGTCCTGGCTGTGGAAAGGTACCTAAGGGATCAACAGCTCCTGGGGATTTGGG
GTTGCTCTGGAAAACTCATTTGCACCACTTCTGTGCCTTGGAATACTAGTTGGAGT-----------------
----------------------------AATAAATCCCTGGATTATATTTGG------GGT------
AACATGACTTGGATGGAGTGG---GAGAGAGAAATTGACAATTACACAGAAGTAATATAT---
GATTTACTTGAAGAATCGCAAAACCAGCAAGAA--

AAGAATGAAAAAGAATTGTTGGAATTGGATAAATGGGCAAATTTGTGGAATTGGTTCGACATAACAAATTGGC
TGTGGTATATAAAAATATTCATAATGATAGTAGGTGGCTTAATAGGTTTAAGAATAGTTTTTTCA---
GTACTTTCTATAGTG---------AATAGAGTTAGGCAGGGATACTCACCA------
TTATCGTTTGCTGTTAGCTTGTTTAATGCCATAGCCATAGCAGTAGCTGAG---
GGGACAGATAGGATTATAGAAATATTGCAAAGAATTGGTAGAGCA----------------------------
--------TTTCTCAACATA------CCTAGAAGAATAAGACAGGGCTTGGAAAGGGCTTTGCAA---TAA
>HM030565

GCT---ACA---GAA---------------------------------
AAAGAAAAATTGTGGGTCACAGTCTATTATGGGGTACCTGTGTGG---AAAGAAGCAAAC---
ACCATTTTATTTTGTGCAACAGATGCTAAAGCATATGTTACAGAG---AAACATAAT----------------
--------------GTTTGGGCCACA---CATGCTTGTGTACCCACAGAC---
CCCAACCCACAAGAAATACAATTGAAG---------AATGTGACAGAAAATTTTAAC---------------
--------------------------ATGTGGAAAAATCACATGGTAGAACAGATGCATGAGGATATA---
ATCAGTTTATGGGATGAAAGCCTA---AAGCCATGTGTAAAATTA---
ACCCCACTCTGTGTCACCTTAAATTGTACTGATTGGAAAAATAATAGTACCAAAAACAATGAC---------
----
----------------------ACTGCAACAGTGGTGCCACTGGTGCCACCAGGAGAAATAAAA---
AACTGCTCTTTTAATGTCACCTCAGACAGGAGA--------------------------
GATAAAATGAAGAAAGAATATGCAACT---TTTTATAATCTGGATATAATAAAAATAGAGGAT---------
----

AATGATAATACTAGCTATAGGCTAATAAGTTGTAACACCTCAGCCATTACACAA---
GCCTGTCCAAAGATATCCTTTGAGCCAATTCCCATACATTATTGTGCCCCGGCTGGTTATGCGATTCTAAAGT
GTAAT---GATAACAATTTTAAT------GGAACAGGACCATGTAAAAAT----------------------
-GTCAGC---ACAGTACAATGTACACATGGAATTAGACCAGTGGTGTCAACT---
CAACTGCTGTTAAATGGCAGTCTAGCAGAAAGCGAG-----------
GTAGTAATTAGATCTGAAAATATCACAGACAAT---------GCTAAAACTATAATAGTACAGTTGACA----
-----------GAACCGGTAGAA---------ATTAATTGTACAAGACCTGGCAACAATACAAGAAAA-----
-AGTATAGGTATA------------GGACCAGGGAGAGCA---TTC------TATACAACAGGAGCT------
---------ATAATAGGAAATATT------------AGACAAGCACATTGT---AACATTAGCAATTCA---
AAATGGAATAACACTTTAAGAAAAATAAGTATAGAACTAAGA---------------AAACAGTTCAAG----
-----------------------------------AATAAAACAATAATCTTTACT------
AGTTCTTCAGGAGGGGACCCAGAAATAGTA---ATGCACAGTTTTAATTGTGGAGGGGAA---
TTTAAGCAGCTCCAGGCAAGAGTCCTGGCTGTGGAAAGATACCTAAAGGATCAACAGCTCCTGGGGATTTGGG
GTTGCTCTGGGAAGCTCATCTGCACCACTAATGTGCCTTGGAATAGCAGTTGGAGT----------------
-----------------------------AATAAAAATTACACTCAGATTTGG------AGT------
AACACGACCTGGTTAGAGTGG---GAAAGAGAAATCGACAATTACACAGGCTTAATATAC---
AACTTACTTGAAGAATCGCAAAACCAACAAGAA--

ATAAATGAAAAAGAATTATTGGCATTAGATAAATGGGCAAGTTTGTGGAATTGGTTTGATATATCAAACTGGC
TGTGGTATATAAAAATATTCATAATGATAGTAGGAGGCTTGGTAGGTTTAAGAATAATTTTTACT---
GTACTTTCTATAATA---------AATAGAGTTAGGCAGGGATACTCACCA------
TTATCGTTTGCAGCTAGCTTGTTTAATGCCATAGCCATAGCAGTAGCTGAA---
GGGACAGATAGGATTATAGAAGCATTGCAAAGAGCTGGTAGAGCT--------------------------
--------ATCCTCCACGTA------CCTAGAAGAATAAGACAGGGCTTCGAAAGGGCTTTGCTA---TAA
>AB428551
GCT---GCA---------------------------------------
GACAAATTGTGGGTCACAGTCTATTATGGGGTACCTGTGTGG---AAAGAAGCACCT---
ACCACTCTATTTTGTGCATCAGATGCTAAAGCATATAAAACAGAG---ATGCATAAT----------------
--------------GTTTGGGCCACA---CATGCCTGTGTACCCACAGAC---
CCCAACCCACAAGAAGTATTATTGGGA---------AATATGACAGAAAATTTTAAC---------------
--------------------------ATGTGGGAAAATAACATGGTAGAACAGATGCAGGAGGATGTA---
ATCAGTYTGTGGGATCAAAGCCTA---AAGCCATGTGTAGAACTA---
ACCCCACTCTGTGTTACTTTAAATTGCACTGATTATAATGGTAATAATACAAATAGTACTCATACCAAT----
----
-------------------AGTAGTAGTAGTGAAGGGGAAAAGCTGGAGAAAGGAGAAATAAAA---
AACTGCACTTTTAATATCACCACCAACATAGGA---------------------------GATAAGCAG---
AGAGACTATGCATTC---TTTTATAAACTTGATCTAGAACCAATAAATAAG---------------------
----
----------GATAATGCTAGCTATAGGTTGATAAGTTGTAACACCTCAGTCATTACACAA---
GCCTGTCCAAAGGTATCTTTTGAGCCAATTCCCATACATTATTGTACCCCGGCTGGTTTTGCGCTTCTAAAGT
GTAAT---GATAAGAAATTCAAT------GGAACAGGGCCATGTACAAAT----------------------
-GTCAGC---ACAGTACAATGTACACATGGAATCAGGCCAGTAGTGTCAACT---
CAACTGCTGTTAAATGGCAGTCTAGCAGAAGAAGAG-----------
GTAGTAATTAGGTCTGCCAATTTCTCGAACAAT---------GGTAAAATCATAATAGTACAGCTAAAT----
-----------GAAACTGTAGAA---------ATTAATTGTACAAGACCCAACAACAATACAAGAAAA-----
-GGTATACAGTTA------------GGACAAGGGAGAGCA---CTT------TATGCAACAGGAGAC------

---------ATAATAGGAGATATA------------AGGCAAGCATATTGT---ACCATTAATGGAACA---
AAATGGAATAAAACTTTAAACCAAATAGCTGAAAAATTAAGA---------------GAACAATTTGGG----
--------------------------------GAGAATAAAACCATAAAATTTCAG------
CCATCCTCAGGAGGGGACCCAGAAGTTGTA---ATGCATAGTTTTAATTGTGGAGGGGAA---
TTTAAGCAGCTCCAGGCAAGAGTCCTGGCTGTGGAAAGATACCTACAGGATCAACAGCTCCTAGGGATTTGGG
GTTGCTCTGGAAAACTCATTTGCACCACTAATGTGCCTTGGAATAGTAGTTGGAGT-----------------
----------------------------AATAAAACTCTGAATCAGATTTGG------GAG------
AACATGACCTGGATGCAGTGG---GAGAGAGAAATTGACAATTACACAAGAGAAATATAC---
AGCTTAATTGAAGAAGCGCAGAACCAGCAAGAA--

AAGAATGAACAAGACTTATTGGAATTGGATAAGTGGGATAATTTGTGGAGTTGGTTTAGCATAACAAAATGGC
TGTGGTACATAAAAATATTCATAATGATAGTAGGAGGCTTGATAGGTTTAAGAATAGTTTTTGCT---
GTACTTTCTATAGTA---------AATAGAGTTAGGCAGGGATACTCACCA------
TTGTCATTGGCTGTTAGCTTGCTCAACGCTACAGCTATAATAGTAGCTGAG---
GGGACTGATAGGATTATAGAAATAGCACAAAGAACTTATAGAGCT----------------------------
--------ATTCTCCACATA------CCTACAAGAATAAGACAGGGCTTGGAAAGGGCTTTGCTA---TAA
>AB289588
GCT---AAA--
GAACAAACGTGGGTCACAATTTATTATGGGGTACCTGTGTGG---AAAGAAGCAACC---
ACCACTCTATTCTGTGCATCAGATGCTAAGGCATATGAACAAGAG---GCACATAAT----------------
--------------GTCTGGCCACA---CATGCCTGTGTACCCACAGAC---
CCCAACCCACAAGAAGTAATATTGGAA---------AATGTGACAGAAAATTTTAAC----------------
--------------------------ATGTGGCAAAATAACATGGTAGAACAGATGCATGAAGATATA---
ATCAGTTTATGGGATCAAAGCCTA---AAGCCATGTGTAAAATTG---
ACCCCACTCTGTGTTACTTTACATTGCACTGATGTCAATAGTAGCGCCACC----------------------
----
----------------------------------AGTTTGGCGATGGAGAAAGGAGAAATGAAA---
AACTGCTCTTTTAATGTTACCACAGAGATAAGA---------------------------
GAGAAGGTGAAGAACGAATATGCAATT---TTTTATAAACTTGATGTAATACCAATAAAGAAT----------
----

AGTGATATTAGTTATAGATTGATACATTGTAATACCTCAACCCTTACACAG---
GCCTGTCCAAAGACATCCTTTGAGCCAATTCCCATACATTTTTGTACCCCGGCTGGTTTTGCGATTCTAAAAT
GTAAT---GATAAGAGGTTCAAT------GGAACAGGACCATGTAACAAT-----------------------
-GTCAGC---ACAGTACAGTGTACACATGGAATTAGGCCAGTAGTGTCAACT---
CAACTGTTGTTAAATGGCAGTCTAGCAGAAGAAGAG------------
GTAGTAATCAGATCTGAAAATTTCACAAACAAT---------GCAAAAACTATAATAGTACAACTGCAT----
-----------GAATCTGTACAA---------ATTAATTGTACAAGACCCAACAACAATACAAGAAAA-----
-AGTATACGTATACAT------AGAGGACCAGGGAGAGCA---TTT------GTTACATCAGGC---------
---------ATACCAGGGGATATA------------AGACAAGCACATTGT---AACCTTAGTAGAGCA---
GAATGGAATAAAATGTTAAAACATGTAGTTACAAAATTAGGA---------------ATGCAATTTAAC----
-----------------------------------AATAGAACAATAAAATTTCAA------
CCATCCTCAGGAGGGGACCCAGAGATTGTA---ACTCACAGTTTTAATTGTGGCGGGGAA---
TTTAAACAGCTCCAGGCAAGAGTCCTGGCTATGGAAAGATACCTACAGGATCAACAGCTCCTAGGGATTTGGG
GTTGCTCTGGAAAACTCATCTGCACCACTAATGTGCCTTGGAACTCTAGTTGGAGT-----------------
----------------------------AATAAATCTATAAATGAAATTTGG------GAT------
AACATGACCTGGATACAGTGG---GACAGAGAGATTCATAATTACACAAACTTAATATAC---
ACTTTACTTGAAGAATCGCAGAACCAGCAAGAA--

AAGAATGAACAGGAACTATTGGAATTGGATAAGTGGTCAAATTTGTGGAATTGGTTTAGCATAACACACTGGC
TGTGGTATATAAAAATATTCATAATGATAGTAGGAGGCTTGATAGGTTTAAGAATAGTTTTTGCT---
GTAATTTCTATAGTG---------AATAGAGTTAGGCAGGGATACTCACCA------
TTATCATTGGCTGTTAGCTTGCTTAACACCACAGCAATAGCAGTAGCTGAA---
GGGACAGATAGGATTATAGAAATAGTGCATAGGGCTTTTAGAGCT----------------------------
--------TTGCTCCACATA------CCTAGAAGAATAAGACAGGGCTTTGAAAGGGCTTTACTA---TAA
>AB289590
GCT---GCA---AAA------------------------CAA---TCA---
GAACAATTGTGGGTCACAGTCTATTATGGGGTACCTGTGTGG---AAAGAAGCAACC---
ACCACTTTATTTTGTGCATCAGATGCTAAGGCATATGATACAGAG---GTACATAAT----------------
--------------GTTTGGGCCACA---CATGCCTGTGTACCCACAGAC---

CCCAACCCACAAGAAGTAGTATTGGAA---------AATGTGACAGAAAATTTTAAT----------------
---------------------------ATGTGGAAAAATAACATGGTAGAACAAATGCATGAGGATATA---
ATCAGTTTATGGGACCAAAGCCTA---AAGCCATGTGTAAAATTA---
ACCCCACTCTGTGTTACTTTAAATTGCACTGATCAACTAACTAATAATACTAATACCACT------------
----
-------------------------AGTAGTCCTAGTAGTTCAATAGAGAAAGGGGAAATAAAA---
AACTGCTCTTTCAATATTACCACAAACATGAGA--------------------------
GATAAGATGCAGAAAGAATATGCACTT---TTTTATAAACTTGATGTAGTTCCAATAAATGATAATGCC----
----

ACTAATACCAACTATACCAACTATAGGTTGATAAGTTGTAACACCTCAGTTATTACACAG---
GCCTGTCCAAAGATATCCTTTGAACCAATTCCAATACATTATTGTACCCCGGCTGGTTTTGCGATTTTTAAGT
GTAAT---GATAAGAAGTTTAAT------GGAACAGGACCATGTAAAAAT----------------------
-GTCAGC---ACAGTACAATGTACACATGGAATTAGGCCAGTAGTATCAACT---
CAATTGTTGTTGAATGGCAGTCTATCAGAAGAAGAG-----------
GTAATAATTAGGTCTGAAAATTTCACGAACAAT---------GCTAAAACTATAATAGTACAGCTGAAT----
-----------GAATCTGTAATA---------ATTAATTGTACAAGACCCAATAACAATACAAGAAAA-----
-GGTATACATATA------------GGACCAGGGAGAGCA---TTT------TATACAACAGGAGAA------
---------ATAGTAGGAAACATA------------AGACAAGCACATTGT---AACATTAGCAAAACA---
CAATGGGAGAGTACTTTAAGAAGGATAGCTATAAAATTAAAA---------------GAACAATATAAG----
-----------------------------------AATAAAACAATAGTATTTCAA------
CCATCCTCAGGAGGGGACCTAGAAATTGTA---ATGCACAGTTTTAATTGTGGAGGGGAA---
TTTAAGCAGCTCCAGGCAAGAGTCCTGGCTGTGGAAAGATACCTAAAGGATCAACAGCTCATGGGGATTTGGG
GTTGCTCTGGAAAACTCATCTGCACCACTGCTGTGCCTTGGAATACCAGTTGGAGT-----------------
----------------------------AATAAATCGCTGAATGAGATTTGG------GAT------
AACATGACCTGGATGGAGTGG---GAAAAAGAAGTTGAAAATTACACAAGCTTAATATAC---
ACCTTAATTGAAAAATCGCAAACCCAACAGGAA---------------------------------------
----
ATAAATGAAAAGGAATTATTGGAATTGGATAAATGGGCAAGTTTGTGGAGTTGGTTTGACATAACGAATTGGC
TATGGTATATAAAAATATTCATAATGATAGTAGGTGGCTTGATAGGTTTAAGAATAGTTTTTACT---
GTACTTTCTATAGTG---------AATAGAGTTAGGCAGGGATACTCACCA------
CTATCGTTTGCTGTTAGCTTGCTTGACACCACAGCCATAATAGTAGCTGAG---
GGGACAGATAGGATTATAGAACTCATACAAAGAGCTTTTAGGGCT---------------------------
--------ATTCGCAATATA------CCTGCAAGAATAAGACAGGGCTTGGAAAGGGCTTTGCTA---TAA
>AB428558
GCT---GCA---
GAACAATTGTGGGTTACAGTCTATTATGGGGTACCTGTGTGG---AAAGAAGCAACC---
ACCACTTTATTTTGTGCATCAGATGCTAAAGCATATGACACAGAG---GCACATAAT----------------
--------------GTATGGGCCACA---CATGCCTGTGTACCCACAGAC---
CCCAACCCACAAGAAGTAAAGTTGGAA---------AATGTGACAGAAGATTTTAAC---------------
---------------------------ATATGGGAAAATAACATGGTAGAACAGATGCATGAAGATGTA---
ATCAGTTTATGGGATGAAAGCTTA---AAGCCATGTGTAAAATTA---
ACCCCACTCTGTGTTACTTTAGACTGCACTGATGTTGGGAATACTACTAATACCAATGGTAGTGAT-------
----
-------------------ACCAATAATATTGATAAGACAAGAATGGAAAAAGGAGAAATAAAA---
AACTGCTCTTTCAATATCACCACAACAATAAGA--------------------------
GATAAGGCGCGGAGAGAAAGCGCACTT---TTTTATAGACTTGATGTAGTACCAATAGATGGTAATGATAAT-
----

ACTAGTAGTAATGCTAGTGCCAGTTATAGGTTAATAAATTGTAACACCACAGTCATTACACAG---
GCCTGTCCAAAGATATCCTTTGAGCCAATTCCCATACACTATTGTACCCCGGCTGGTTTTGCGCTTCTAAAGT
GTAAT---GATAAAGGGTTCAGT------GGAAAAGGAAAATGTACTAAT----------------------
-GTCAGC---ACAGTACAATGTACACATGGAATTAGGCCAGTAGTATCAACT---
CACCTGCTGTTAAATGGCAGTCTAGCAGAAAAAGAG-----------
GTAGTAATTATATCTAAAAATTTCTCAGCTAAT---------ACTGAAACCATAATAGTACAGCTGAAC----
-----------GAATCTATAGTA---------ATTAATTGTACAAGACCCAACAACAACACAAGAAAA-----
-GGTGTACATTTA------------GGACCAGGGCAAGCA---ATA------TATACAACAGGAGCC------
---------ATAATAGGAGATATA------------AGACAAGCATATTGT---AACATTAGTGAAGAA---
AAATGGAATAACACTTTAAAACAGGTAGTTACAAAATTAAGA---------------GAAATATTTAAC----
--------------------------------AATGCAACAACAATATTCTTTAAT------

CAATCCTCAGGAGGAGATCCAGAAATTGTA---ATGCACAGCTTTAATTGTGGAGGGGAA---
TTTAAGCAGCTCCAGGCAAGAGTCCTGGCTGTGGAAAGATACCTAAGGGATCAACAGCTCCTAGGGATTTGGG
GCTGCTCTGGAAAACTCATTTGCACCACTGCTGTGCCTTGGAATACTAGTTGGAGT-----------------
----------------------------AATAAATCTCTAGATGAAATTTGG------GAT------
AACATGACCTGGATAGAGTGG---GAAAGAGAAATAGGCAATTACACAGACTTAATATAC---
AACTTACTTGAAAAATCTCAGAACCAACAAGAA--

AAAAATGAACAAGAATTATTGGAATTGGACAGTTGGGCAAGCTTGTGGAATTGGTTTGACATATCAAAATGGC
TGTGGTATATAAAAWTATTCATAATGATAGTAGGAGGCTTAGTAGGTTTAAGAATAGTTTTTACT---
GTACTTTCTATAGTA---------AATAGAGTTAGGCAGGGATATTCACCA------
CTATCATTTGCTATTAGCTTGCTAAATGCCACAGCTATAGCAGTAGCTGAG---
GGGACAGATAGGATTATAGAAGTAGTACAAAGAGCTTATAGAGCT----------------------------
--------ATTATTCACATA------CCTAGAAGAATAAGACAGGGCTTAGAAAGGGCTTTGCTA---TAA
>AB221126
GCT---TTA--
GAACAATTGTGGGTCACAGTTTATTATGGGGTACCTGTGTGG---AAAGATGCAACC---
ACCACTCTATTTTGTGCATCAGATGCTAAAGCATATGACACAGAG---ATGCATAAT----------------
--------------GTTTGGGCCACA---CATGCCTGTGTACCCACAGAC---
CCTAACCCACAAGAGGTAGTATTAGTG---------AATGTGACAGAAGAATTTAAC----------------
--------------------------ATGTGGAAAAATAACATGGTAGAACAAATGCATGAGGATATA---
ATCAATTTATGGGACCAAAGCCTA---AAGCCATGTGTAAAATTA---
ACCCCACTCTGTGTTACTTTACATTGCACGGATTTGAAGAATGTTACTGCTAACAGTACTGCTAGT-------
----
----------------------AATAGCAGTACTAATTGGAAAAACATAAATGAAGTAATGACA---
AACTGCTCTTTCAATGTCACCACAGGTATAGGA---------------------------
AATAGGATGCAGAGAGAGTATGCACTT---TTTTATAAACTTGATGTAGCACCAACAGGTGCG----------
----

GATAATACAAGCTATACATTGATAAATTGTAACACCTCAGTCATTACACAA---
GCCTGTCCAAAAGTATCCTTTGAACCAATTCCCATACATTATTGTACCCCAGCTGGTTTTGCGCTTCTAAAAT
GTAAT---GATAAAAAGTTCAAT------GGAAAAGGACCATGTAAAAAT-----------------------
-GTCAGC---ACAGTACAATGTACACATGGAATTAGGCCAGTGGTGTCAACT---
CAACTATTGTTAAATGGCAGTCTAGCAGAAGAAGAG------------
GTAGTACTTAGATCTGAAAATTTCACAAACAAT---------GCTAAAACCATAATAGTACAGCTGAAT----
-----------AAAACTGTAGTA---------ATTAATTGTACAAGACCCAACAACAATACAAGAAAA-----
-GGTATACATATA------------GGAGCAGGGAGAGCA---ATT------TATGCAACAGGAGCT------
---------ATAATAGGAGATATA------------AGACAAGCACATTGT---AACCTTAGTGAAACA---
GACTGGAAAAACACTTTAAACAAGACAGTTAGAAAGTTAAAA---------------GAACAATTTAAT----
-----------------------------------CTAACAACAATTGTCTTTACA------
ACACCCTCAGGAGGGGACCCAGAAATTACA---ATGCACAGTTTTACTTGTGGAGGAGAA---
TTTAAACAGCTCCAGGCCAGAGTCCTGGCTGTGGAAAGATACCTAAGGGATCAACAGCTCCTGGGGATTTGGG
GCTGCTCTGGAAAACTCATTTGCACCACTGCTGTGCCGTGGAATACTAGTTGGAGT-----------------
----------------------------AATAAAACTTTCAACCAGATTTGG------GAT------
AACATGACCTGGATGCAGTGG---GAGAGAGAAATTGATAATTACACAGGTTTAATATAC---
AGCTTAATTGAAGAATCGCAAAACCAACAAGAA--

AAGAATGAACAAGAGTTATTGGAATTGGATAAGTGGGCAAGTTTGTGGAATTGGTTTGACATATCAAATTGGC
TGTGGTATATAAGAATTTTCATAATGATAGTAGGAGGCTTAATAGGTTTAAGAATAGTTTTTGCT---
ATACTTTCTATAGTA---------AAAAGAGTTAGGCAGGGATACTCACCA------
ATATTGTTGGCTGTTAGTCTGGTTAATGCCACAGCTGTAGCAGTAGCTGAG---
GGGACAGATAGGATTATAGAAGTAGCACAAAGCATTGGGAGAGGC----------------------------
--------ATTCTCCACATA------CCTACAAGGATAAGACAGGGCCTCGAAAGAGCTTTGCTA---TAA
>AB480697
GCT---ACA--
GAACACTTGTGGGTCACAGTCTATTATGGGGTACCTGTGTGG---AAAGAAGCAAAT---
ACCACTCTATTTTGTGCATCAGATGCTAAATCATATGAGACAGAG---ATGCATAAT----------------
--------------GTTTGGGCCACA---CATGCCTGTGTACCCACAGAC---
CCCGACCCACAAGAAGTAAGCTTGGGA---------AATGTGACAGAAAATTTTAAC----------------
--------------------------ATGTGGAAAAATAATATGGTAGAACAGATGCATGAGGATGTC---
ATTAGTTTATGGGATCAAAGCCTA---AAGCCATGTGTAAAACTA---

ACCCCAATCTGTGTTACTTTAAATTGCACTAATAATACTTGGAGTGGGAATGCTACTAATGTCCCT-------
----
----------------------ACCAATGGGAGTAACTTGGGAGAGATGAAAGAAGAAATGAAA---
AACTGCTCTTTCAATATTACCACACCCATAAAA--------------------------
AAGGAGTTGAAGAAAGAATATGCACTC---TTTTATAACCTTGATATAGTACCAATAGATAAT----------
----

ACCAGTTATGGGTTAAGAAGTTGTAACACCTCAGTCATTACACAG---
TCCTGTCCAAAGGTATCCTTTGAGCCAATTCCCATACATTATTGTACCCCGGCTGGTTTTGCGCTTTTAAAGT
GTAGA---GATAAGAGGTTCAAT------GGAACAGGACCATGCGGAGAT-----------------------
-GTCAGT---ACAGTACTATGTACACATGGAATTAGGCCAGTAGTATCAACT---
CAACTGCTGTTAAATGGCAGTCTAGCAGAAGAAGAA-----------
ATAGTAATTAGATCAGAAAATATCACGGACAAT---------ACTAAAACCATAATAGTACAGCTGACC----
-----------AAATTTGTAGAA---------ATTAAGTGTATAAGGCCCAACAACAATACAATAAAA-----
-GGTATACATATA------------GGACCAGGGAGAGCA---TTT------TATGCAACAGGAAAA------
---------ATAACAGGAGATATA------------AGACAAGCACATTGT---AATGTTAGTAGTAAA---
AATTGGACTAGCACTTTAAAACAGGTAGCTAGAAAATTAGGA---------------GAACACTTTCCA----
-----------------------------------AATAAAAACATAACCTTCAAT------
CACTCCTCAGGCGGGGACCCAGAAATAGTA---ATGCACAGTTTTAATTGTGGAGGGGAA---
TTTAAGCAGCTCCAGGCAAGAGTCCTGGCTGTGGAAAGATACCTAAAGGATCAACAGCTCCTGGGAATTTGGG
GTTGCTCTGGAAAACTCATTTGCACCACTACTGTGCCTTGGAATGCTAGTTGGAGT-----------------
---------------------------AATAAATCTCATGATTTCATTTGG------AAT------
AACATGACCTGGATGCAGTGG---GATAAAGAAATTGACAATTACACAAACTTAATATAC---
AACTTACTTGAAAAAGCGCAAAACCAGCAAGAA--

AAGAATGAACAAGAATTATTGGAATTAGATAAATGGGCAAACTTGTGGAATTGGTTTAACATAACAGAATGGC
TGTGGTATATAAAAATATTCATAATGATAGTAGGAGGCTTGATAGGTTTAAGAATAGTTTTTGCT---
GTACTTTCTGTAGTG---------AATAGAGTTAGGCAGGGATACTCACCC------
TTGCCGTTTGCTGTTAGCTTGCTCAATGCCACGGCCATAGCAGTAGCTGAG---
GGAACAGATAGGATTATAGAATTATTACAAAGAATTGGTAGAGCT----------------------------
--------ATTCTCAACATA------CCTAGAAGAATAAGACAGGGCTTGGAAAGGGCTTTGCTA---TAA
>AB480695
GCT---GCA---GAA-----------------------------------
AAGAAAATGTGGGTCACAGTTTATTATGGGGTACCTGTGTGG---AAAGAAGCCACT---
ACCACTCTATTTTGTGCATCAGATGCTAGAGCATATGATACAGAG---GCCCATAAT----------------
--------------GTTTGGGCCACA---CATGCTTGTGTACCCACAGAC---
CCCAACCCACAAGAAGTAGAATTGACA---------AATGTGACAGAAGATTTTAAC----------------
--------------------------ATGTGGAAAAATAACATGGTAGACCAGATGCATGAGGATATA---
ATCAGTTTATGGGATCAAAGCCTA---AAGCCATGTGTAAAGTTA---
ACCCCACTCTGTGTCTCTTTAAATTGCACAAATATTACCAAAAGTAGTAGCAATACTAATAGCAGCCAGACTA
CTACC--
----------ACCACTAGTAGTACCACTAATGGTAGTAGTACCATAGAGGATGGAGACATGAGA---
AACTGCTCTTTCAATATTTCCACAGATGTGGTA--------------------------
GATAAGGTGAAGAAAGAATATGCATCT---TTTTATAAACTTGATATAGTACAAATAGATAAG----------
----

AAAGATAGTGATAGCTATAGGTTAATAAGTTGTAACGCCTCAGTCGTTACACAA---
GCCTGTCCAAAGATATCCTTTGATCCAATTCCCATACATTATTGTGCCCCGGCTGGTTTTGCGATTCTAAAGT
GTAAC---AATAAGACATTCAAT------GGATCAGGACCATGTAACAAT-----------------------
-GTCAGC---ACAGTACAATGTACACATGGGATTAGGCCAGTAGTATCTACT---
CACCTGTTGTTAAATGGCAGCCTAGCAGAGGAAGAG-----------
GTAGTAATTAGATCTGAAAATTTCACAAACAAT---------GCTAAAACCATAATAGTCCAGCTGAAT----
-----------GAATCTGTAACA---------ATTAATTGTACAAGACCCAGCAACAATACAAGAAAA-----
-AGGATACATATA------------GGGCCAGGGAGAGCA---GTG------TATACAACAGATCAA------
---------ATAACAGGAGATATA------------AGAAAAGCATCTTGT---AATGTTAGTAGAGCA---
GAATGGAATAATGCTTTAAAAAAGGTAGTGGAAAAACTAAGA---------------GAACAATTTGGG----
--------------------------------AATAATAAAACAATAGCCTTTAAT------
CAATCCTCAGGAGGGGACCCAGAAATAGCA---ATGCACATGTTTAATTGTAGAGGGGAA---
TTTAAGCAGCTCCAGGCAAGAGTCCTGGCTGTGGAAAGATACCTAAAGGATCAACAACTCATGGGAATCTGGG
GTTGCTCTGGAAAACTCATCTGCACCACTAATGTGCCTTGGAATAGTAGTTGGAGT-----------------

----------------------------AATAAATCTCAAGATGATATTTGG------AAT------
AACATGACCTGGATGCAGTGG---GAAAAAGAAATTGACAATTACACAGGCATAATATTC---
TCCTTACTTGAAAAATCACAAAACCAGCAAGAA--

CAGAATGAAAAGGACTTGTTAGAATTAGATAAATGGGACAGTTTGTGGAATTGGTTTGACATAACACACTGGC
TGTGGTATATAAAAATATTCATTATGATAGTAGGAGGCTTGATAGGTTTAAGAATAATTTTTACT---
GTACTTTCTATAGTA---------AATAGAGTTAGGAAGGGATACTCACCA------
TTATCGTTTGCTGTTAGCTTGTTCAATACTACAGCAATAGTAGTAGCTGAG---
GGGACAGATAGGATTATAGAATTAGCACAAAGGATTTTTAGAGCT----------------------------
--------TTCATACACATA------CCTAGAAGAATAAGACAGGGCTTTGAAAGGGCTTTGCTT---TAA
>AB287363
GCT---GCA--
GAGCAGTTGTGGGTTACAGTCTATTATGGGGTACCTGTGTGG---AAAGAAGCAGAA---
ACCACTCTATTTTGTGCCTCAGACGCTAAAACCTATGATACAGAG---GCACATAGT----------------
--------------GTTTGGGCCACA---CATGCCTGTGTACCCACAGAA---
CCCAACCCACAAGAAGTAGTATTGGAA---------AATGTGACAGAAAATTTTAAC----------------
--------------------------ATGGGGAAAAATAAAATGGTAGAACAAATGCATGAGGATATA---
ATTAGCATATGGGATCAAAGCCTA---AAGCCATGTGTAAAATTA---
ACCCCACTCTGTGTTACTTTAAATTGCACTGATGAAATTCTCAATAGCACTGAGACTAATAATAGA-------
----
-------------------ACTAATACCAGTACCACTAGTGTACCAGATAGAGAACCAATGAAA---
AACTGCTCTTTCGATGTCACCACAAGCCTAAGA---------------------------
GATAAGATTACAAAAGAATATGCACTT---TTTTATAGACCTGATGTAGTACCAATAGATCAGACTAATAAT-
----

ACATATAATAATACATATAGTACATATAGGTTAATACATTGTAACACCTCAGTCATTACCCAA---
GCCTGTCCAAAGATGTCATTTGAGCCAATTCCCATACATTATTGTGCCCCGGCTGGTTTTGCGATTCTAAAGT
GTAAT---AATAATAGCTTCAAT------GGAACAGGACCATGTACAAAT-----------------------
-GTCAGC---ACAGTACAATGTACACATGGAATTAAGCCAGTAGTGTCAACT---
CAACTGCTGTTAAATGGCAGTCTAGCAGAAAAAGAG------------
GTAGTAATTAGATCTGAAAATTTCACGAACAAT---------GCTAAAACCATAATAGTTCAGCTGAAG----
-----------GACCCAGTAGAA---------ATTAATTGTACAAGACCCGGCTACAACACAATAAAA-----
-AGGGTATCGATA------------GGACCAGGAAGAGGA---AGA------GCAATTGTTGCAACA------
---AAAAAGATAGAAGGAGATACA------------AAAAAAGCATATTGT---ACACTTAATAAAACA---
CAATGGAATGACACTTTAAGACAGGTAGCTGAAAAATTAAGA---------------GAACAATTTGGG----
-----------------------------------AATAAAACAATAGTCTTTAAT------
CAATCCTCAGGAGGGGACCCAGAAATTGTA---ATGCATAGTTTTACTTGTGGAGGGGAA---
TTTAAGCAGCTCCAGGCAAGAGTCCTGGCTATGGAGAGATACCTAAGGGATCAACAGCTCCTAGGGATTTGGG
GTTGCTCTGGAAAACTCATTTGCACCACTGCTGTGCCTTGGAATACTAGTTGGAGT-----------------
----------------------------AATAAATCTCTGGGAGACATCTGG------GAT------
AACATGACATGGATGCAGTGG---GAAAGAGAAATTGACAATTACACAAACTTAATATAC---
ACCTTACTTGAAGAATCGCAAAACCAACAAGAA--

AAGAATGAACAAGAATTACTAGAATTAGATAAGTGGGCAAGTTTGTGGAATTGGTTTGACATAACACGATGGC
TGTGGTATATAAAAATATTTATAATGATAGTAGGAGGCTTGATAGGTTTAAGGATAGTTTTTGTG---
ATACTTTCTATAGTG---------AATAGAGTTAGGCAGGGATACTCACCA------
TTATCATTGGCAATTAACTTGCTTAATACCACAGCTATAGCAGTAGCAGAG---
GGGACAGATAGGGTTATAGAAGTAGTGCAAAGGGCTGGTAGAGCT----------------------------
--------ATTCTCCACATA------CCTAGAAGAATAAGACAGGGCTTAGAAAGGATTTTGCTA---TAA
>AB287364
GCT---AAA--
GAACAATTGTGGGTCACAGTTTATTATGGGGTACCTGTATGG---AAAGAAGCAACC---
ACCACTTTATTTTGTGCATCAGATGCTAAATCATATGATACAGAG---GCACATAAT----------------
--------------GTTTGGGCCACA---CATGCCTGTGTGCCCACAGAC---
CCCAACCCACAAGAAATGTTATTGGTA---------AATGTAACAGAAAATTTTAAC----------------
--------------------------ATGTGGAAAAATAACATGGTAGACCAAATGCATGAGGATGTA---
ATCAGTTTATGGGATCAAAGCCTA---AAGCCATGTGTAAAATTA---
ACCCCTCTCTGTGTTACTCTAAATTGCACTGATCCACAGACTAATACCACTGGTGCTAATACCACT-------
----
-------------------GGTACCACTGGTAATAATACCACTATTAGCAACGGAGAAATGAAA---

AATTGCTCTTTCAATATCACCACAAACATGAGC---------------------------
AATAAGGTAAAGAAAGAATATGCACTT---TTTTATACATTAGATATAGTACCAATAGATGATGAT-------
----

AATAATATTACCAGTACCAGCTATAGGTTGATAAGTTGCAACACCTCAGTTGTTACACAG---
GCCTGTCCAAAAATATCCTTTGAGCCAATTCCCATACATTATTGTGCCCCGGCTGGTTTTGCGATTCTAAAGT
GTAAT---GATAAGAAGTTCAAT------GGAACAGGACCATGTAGGAAT-----------------------
-GTCAGC---ACAGTACAATGTACACATGGAATTAAGCCAGTAGTATCAACT---
CAATTACTGTTAAATGGCAGTCTAGCAGAAGAAGAG------------
GTAATAATTAGATCTGAGAATTTCACAGACAAT---------GCTAAAAACATAATAGTACAGCTGAAT----
-----------AAAACTGTAGTA---------ATTAATTGTACAAGACCCAACATCAATAGAAGAAAA-----
-GGTTTACGTATA------------GGACCGGGGAGAGCA---TTT------TTTACGACAGGAAAC------
---------ATAAAAGGAGATATA------------AAACAAGCATATTGT---AACATTAGTAAAACA---
GACTGGAACAACACTTTAATACTGATAGTTAAAAAATTAAGA---------------GAACAATATGAG----
-----------------------------------AATAAAACAATAGTCTTTAAG------
CAATCCTCAGGAGGAGATCTAGAAATCACA---ATGCACAGTTTTAATTGTGGAGGGGAA---
TTTAAGCAGCTTCAGGCAAGAATCCTGGCTGTGGAAAGATACCTAAAGGATCAACAGCTCCTGGGAATTTGGG
GGTGCTCTGGAAAACTCATTTGCACCACTGCTGTGCCTTGGAATACTAGTTGGAGT-----------------
----------------------------AATAAAACTCAAGAGGAAATTTGG------GGA------
AACATGACTTGGATGCAGTGG---GAAAAAGAAATAGACAATTACACAGGCATAATATAC---
AGACTAATTGAAAAATCGCAAAACCAGCAAGAA--

AAGAATGAACAAGATTTATTAGAATTGGATAAATGGGCAAGTTTGTGGAGTTGGTTTGACATAACAAATTGGC
TGTGGTATATAAAAATATTCATAATGATAGTAGGTGGATTAATAGGTTTAAGAATAATTTTTACT---
GTACTTTCTATAGTG---------AATAGAGTTAGGCAGGGATACTCACCA------
TTGTCATTCGCTGTTAGCTTGTTCAACGCCACAGCCATAGCAGTAGCTGGG---
GGGACAGATAGGATTTTAGAAGGATTGCAAAGAGCTTTTAGGGCT----------------------------
--------ATTCTCCACATA------CCTAGAAGAATAAGACAGGGCTTTGAAAGGGCTTTGATA---TAA
>AB588283
GCT---TTA--
GGACAATTGTGGGTTACAGTCTATTATGGGGTACCTGTGTGG---AAAGATGCAACC---
ACCACTCTATTTTGTGCATCAGATGCTAAAGCATATGACACAGAG---GTACATAAT----------------
--------------GTTTGGCCACA---CATGCGTGTGTACCCACAGAC---
CCTAACCCACAAGAAGTAGTATTGGGA---------AATGTGACAGAAAATTTTAAC----------------
--------------------------ATGTGGAAAAATAACATGGTAGAACAAATGCATGAGGATATA---
ATCAGTTTATGGGATCAAAGCCTA---AAGCCATGTGTAAAATTA---
ACCCCACTCTGTGTTACTTTAAATTGCACTAACTTGAATGATACTGCTAAGAACAGCAGTGTTCAT-------
----
----------------------TACAATAGTAGTCATTGGAAAGAAATAAATGAAGTAATGACA---
AACTGCTCTTTCAATGTCACTGCAAGTATAGGA---------------------------
AATAAGAGGCGGAAAGAGTATGCACTC---TTTTATAAACTTGATGTAGTACCAATAGGTAAT----------
----

GATAATACAAGCTATGCATTGATAAGTTGTAACACCTCAGTCATTACACAG---
GCCTGTCCAAAAATATCCTTTGAACCAATTCCCATACATTATTGTACCCCAGCTGGTTATGCACTCCTAAAAT
GTAAT---GATAAAAAGTTCAAT------GGAACAGGACCATGTAAAAAT-----------------------
-GTCAGC---ACAGTACAATGTACACATGGAATTAGGCCAGTAGTGTCAACT---
CAACTACTGTTAAATGGCAGTCTAGCAGAAGAAGAG------------
GTAGTAATTAGATCTGAAAATTTCACAAACAAT---------GCTAAAACCATAATAGTACAGCTGAAT----
-----------GAAACTGTAATA---------ATTAATTGTACAAGACCCAACAACAATACAAGAAAA-----
-AGTATACAGATG------------GGACCAGGGAAATCA---ATT------TATGCAACAGGAAAT------
---------ATAATAGGAGATATA------------AGGCAAGCACATTGC---AGCCTTAATAGAACA---
GAATGGGAAAACACTTTAACAAAGATAGTTATAAAATTAGCA---------------GAACAATTTAAT----
-----------------------------------ACAACAGAAATTATCTTTAAT------
CAATCCTCAGGAGGGGACCCAGAAATTACA---ATGCACAGTTTTAATTGTGGAGGGGAG---
TTTAAACAGCTCCAGGCCAGAGTCCTGGCTGTAGAAAGATACCTAAAGGATCAACAGCTCCTGGGAATTTGGG
GTTGCTCTGGAAAACTCATTTGCACCACTGCTGTGCCTTGGAATACTAGTTGGAGT-----------------
----------------------------AATAAAACTCTAGATGAGATTTGG------AAT------
AACATGACCTGGACACAGTGG---GAAAGAGAAATTGACAATTACACAGGCGAAATATAC---
AGACTAATTGAAAAATCACAGAACCAACAAGAA--

AAAAATGAACAAGAGTTATTGGAATTGGATAAGTGGGCAAATTTATGGAATTGGTTTGACATAACACATTGGC
TGTGGTACATAAAAATTTTCATAATGATAGTAGGAGGCTTAATAGGTTTAAGAATAGTTTTTACT---
GTACTTTCTATAGTG---------AATAGAGTTAGGCAGGGGTACTCACCA------
TTATCATTCGCTATTAGTCTGCTTAATGCCACAGCTGTAGCAGTAGCTGAG---
GGGACAGATAGGATTTTAGAAGTAGTACAAAGAACTTGGAGGGGT----------------------------
--------ATTCTCCACATA------CCTACAAGGATAAGACAGGGCTTAGAAAGGGCTTTACTA---TAA
>AB588296
GCT---AAA--
GAAAATTTATGGGTCACAGTTTATTATGGGGTACCTGTGTGG---AAAGAAGCAACC---
ACCACTCTATTTTGTGCATCAGATGCTAAAGCATATAGTACAGAG---GCACATAAT----------------
--------------GTTTGGGCCACA---CATGCCTGTGTACCCACGGAC---
CCCAACCCACAAGAAGTAAAATTGGAA---------AATGTGACAGAAAATTTTAAC----------------
-------------------------ATGTGGAAAAATAACATGGTAGACCAGATGCATGAGGATGTT---
ATCAGTTTATGGGATCAAAGTCTA---AAGCCATGTGTAAAATTA---
ACCCCACTCTGTGTTACTTTAAATTGCACTGATTACACTGGCAATGCTACCCTTAAAAATACTAATAATACT-
----
-------------ACCAGTGCCAATAATACTAGCTGGGGAGCGATGGGAACAGGAGAAATAAAA---
GAGTGCCAGTTCAATATCACCTCAAACATAGTAAGG------------------
CTACCACAAAAGGTAACGGGACAAAAAGCTCTT---TTTTATAGCCTTGATATAACACCAATAGAGGGTAAG-
----

GCCAATACCAGTTATATGTTGAGAAGTTGTAACGCTTCAGCCATTACACAA---
GCCTGTCCAAAGATGTCCTTTGAGCCAATTCCCATACACTATTGTGCCCCAGCTGGTTTTGCGATTCTAAAGT
GTAAT---GATACAAAGTTCAAT------GGATCAGGACCATGTAGGAAT-----------------------
-GTCAGC---TCAGTACAATGTACACATGGAATTAGGCCAGTAGTATCAACT---
CAACTGCTGTTAAATGGCAGTCTAGCAAGAGAAGAG-----------
GTAGCAATTAGATCCGAAAATTTCACAAACAAT---------GCTAAAGTCATAATAGTACAGCTGAAA----
-----------GAAGCTGTACAA---------ATTAATTGTACAAGACCCAATAACAATACAAGAAAG-----
-AGTATACACTTA------------GGATTCGGCAGGGCA---ATG------TATGCAACAGGGCAA------
---------ATAATAGGAGATATA------------AGACAAGCACATTGT---AACATTAGTAGAGCA---
AGATGGAATGACACTCTATATCAGATAGTTAAAAAATTAAGA---------------
AAACAATATAATGAAACATTA--------------------------GGTAATAAAACAATAGCCTTTAAT-
-----CATTCCTCAGGAGGGGACCCAGAAATTGTA---ATGCACAGTTTTAATTGTGGAGGGGAA---
TTTAAGCAGCTCCAGGCAAGAGTCCTGGCTGTGGAAAGATACCTAAAGGATCGACAGCTCCTAGGGATTTGGG
GTTGCTCTGGAAAACTCATCTGCACCACTGCTGTGCCTTGGAATACTAGTTGGAGT-----------------
----------------------------AATAAATCTTATGATGACATTTGG------AAT------
AACATGACCTGGATGCAGTGG---GAAAAAGAAATTGATAATTACACAAACTTAATATAT---
ACCTTAATTGAGGAATCGCAAAACCAACAAGAA--

AAGAATGAACAAGAATTATTGGCATTAGATCAATGGGCAAGTTTATGGACTTGGTTTGACATATCAAAATGGC
TGTGGTATATAAAAATATTCATAATGATAGTAGGAGGCTTGATAGGTTTAAGAATAGTTTTTACT---
GTAATTTCTATAGTG---------AATAGAGTTAGGCAGGGATACTCACCA------
CTGTCGTTTGCTGTTAGCTTGCTCAACGCTACAGCCATAGCAGTAGCTGAG---
GGGACAGATAGAATTATAGAAATAGCACAGAGAATTTTTAGAGCT----------------------------
--------GTTCTTCACATA------CCTAGAAGAATAAGACAGGGCTTCGAAAGGGCTTTGCTA---TAA
>AB287367
GCT---GCA--
GAACAATTGTGGGTCACAGTCTATTATGGGGTACCTGTGTGG---AAAGAAGCAACT---
ACCACTCTATTTTGTGCATCAGATGCTAAAGCATATGACACAGAG---GTGCATAAT----------------
--------------GTTTGGGCCACA---CATGCCTGTGTACCCACAGAC---
CCCAACCCACAAGAAGTATTATTGGAA---------AATGTGACAGAAAATTTTAAC----------------
-------------------------ATGTGGAAAAATAACATGGTAGAACAAATGCATGAGGATGTA---
ATAAGCTTATGGGATCAAAGCCTA---AAGCCATGTGTAAAACTA---
ACCCCACTCTGTGTTACTTTAAACTGCACTGATTATAATGTTACTAATACTAATAGTACTACT----------
----
-------------------------ACTAATAGTAGTAACTTGAAAGAGAAGGGAGAAATAAAA---
AACTGCACTTTCAATGTCACCACAATGATAGAA--------------------------
AATAAGCAAAAGAAAGAATATGCACTT---TTTTATAAACTTGATATAGCACCAGTAGGGGGT----------
----

GATAATACAAGCTATGCGTTGATAAATTGTAACACCTCAACCATTACACAG---
GCCTGCCCAAAGGTGTCCTTTGAACCAATTCCCATACATTATTGTACACCGGCTGGTTTTGCGCTTCTAAAGT
GTAAA---GATAAGAATTTCAAT------GGATCAGGACCATGTACAAAT-----------------------
-GTCAGC---ACAGTACAGTGTACACATGGAATTAGGCCAGTAGTGTCAACT---
CAACTACTGTTAAATGGCAGTATAGCAGAAGATGAG------------
ATAGTAGTTCGATCTGAAAATTTCTCAGACAAT---------GCTAAAACCATAATAGTACAGCTGAAC----
-----------AGGACTATACAC---------ATTAATTGTACAAGACCCAACAATAATACAAGAAAA-----
-AGTATACATATA------------GGACCAGGGAGAGCA---TTT------TATGCAACAGGAGAT------
---------ATAATAGGAAATATA------------AGACAAGCACATTGT---AACCTTAGTAGAACA---
GACTGGAATAACACTTTAAAACAAATAGCTGAAAAATTAAAA---------------GAACAATTTGGG----
--------------------------------GAGAATAAAACAATAAAATTTGAG------
CCATCTTCGGGAGGGGACCCAGAAATTACA---ATGCACAGTTTTAATTGTGGAGGGGAA---
TTTAAGCAGCTCCAGGCAAGAGTCCTGGCTATAGAAAGATACCTAAAGGATCAACAGCTCCTAGGGATTTGGG
GTTGCTCTGGAAAACTCATTTGCACCACTACTGTGCCTTGGAATACTTCTTGGAGT-----------------
-----------------------------AATAAAACTTATGAGAAGATTTGG------AAC------
AACATGACCTGGATGCAATGG---GAGAGAGAAATTGACAATTACACAGGCTTAATATAC---
ACTTTAATTGAAGAAGCGCAAAACCAACAAGAA---

AAGAATGAACAAGAATTATTGGAATTAGATAAGTGGGACAATTTGTGGAGTTGGTTTAGCATAACACAATGGC
TGTGGTACATAAAAATATTCATAATGATAGTAGGAGGCTTGATAGGTTTAAGAATAGTTTTTGCT---
GTACTTTCTATAGTA---------AATAGAGTTAGGCAGGGATATTCACCA------
TTGTCATTCGCTGTTAGTCTGTATAATGCAATAGCTATAGCAGTAGCTGAA---
GGGACAGATAGGATTATAGAAGTAGGACAAAGAATTTGGAGAGCT----------------------------
--------TTTCTTCACATA------CCTAGAAGAATAAGACAGGGCTTCGAAAGGGCTTTGCTA---TAA
>AB287368
GCT---GAA--
GAAAAAACGTGGGTCACAGTCTATTATGGGGTACCTGTGTGG---AAAGAAGCAACC---
ACCACTCTATTTTGTGCATCAGATGCTAAAGCATATGTACAAGAA---GTGCATAAC----------------
--------------ATCTGGGCTACA---CATGCCTGTGTACCCACAGAC---
CCCGACCCACAAGAAGTAAAATTGGAA---------AATGTGACAGAATATTTTAAC---------------
--------------------------ATGTGGAAAAATAACATGGTAGAACAGATGCATGAGGATATA---
ATCAGTTTATGGGATCAAAGCCTA---AAGCCATGTGTAAAATTA---
ACCCCACTCTGTGTCACTTTAGAGTGCACTGATTATAATGGGAATCAAACTCAAAATGGGACT----------
----
----------------------AAACATAATAATACCAGAGGGATGAAAAAAGGGGAAATGAAA---
AGCTGCTCTTTCAATGTCACCACAAATATAGGA---------------------------
GGTAAAGTAAAGAAGGAAAATGCACTT---
TTTTATAATCTTGATGTAGTAAAATTACGGGATGGTAGTACACAA----------------------------
----
AGTAATAGTAGTAAAAATGATAGTGGGATATATACATTGACAAATTGTAATACCTCAGTCATTACACAG---
GCCTGTCCAAAGGTATCCTTTGAGCCAATTCCAATACATTATTGTACCCCAGCTGGTTTTGCGCTTCTAAAAT
GTAAA---GATGAGAAGTTCAAT------GGATCAGGACCATGTAAAAAT-----------------------
-GTCAGC---ACAGTACAATGTACACATGGAATTAGGCCAGTAGTATCAACT---
CAGCTGCTGTTAAATGGCAGTCTAGCAGGAAAAGAA---------
GGGATAGTAATTAGGTCTGAGAATTTCACAAACAAT---------GCTAAAACCATAATAGTTCAGCTGAAT-
--------------GAGACAGTAGAA---------ATTAATTGTACAAGACCCAACAACAATACAAGAAAA--
----AGTATACCTATG------------GGACCAGGGAGTGCA---ATT------TATGCAACAGGAGCA---
------------ATAATAGGAGATATA------------AGACAAGCACATTGT---AACATAAGTAGGGAA-
--AAATGGAATAACACTCTAAAGAGGGTAGTTGAAGAATTACGA---------------AAACAATTTCCT--
-------------------------------------AATAAGACAATAGTCTTTAAG---
CAGCCACCCTCAGGAGGGGATCCAGAAATTACA---ACTCACAGCTTTAATTGTGGAGGGGAA---
TTCAAACAGCTCCAGGCAAGAGTCCTGGCTGTAGAAAGATACCTAAGGGATCAACAGCTCCTAGGGATTTGGG
GTTGCTCTGGAAAACTCATCTGCACCACTAATGTGCCTTGGAATACTAGTTGGAGT-----------------
-----------------------------AATAAATCTCTGGATTTCATTTGG------AAT------
AACATGACCTGGATGCAGTGG---GACAGGGAAATTGATAATTACACAAGCTTAATATAC---
ACCTTAATTGAAAATGCGCAGAACCAGCAAGAA---

AAGAATGAACAAGAATTATTGGAATTGGATAAATGGGCAAGTTTGTGGAGTTGGTTTGATATATCAAATTGGC
TGTGGTATATAAGAATATTCATAATAATAGTAGGAGGCTTGGTAGGTTTAAAAATAGTTTTTGCT---

GTACTTTCTATAGTA---------AATAGAGTTAGGCAGGGATACTCACCT------
TTATCATTTGCTATAAGCTTGCTTAACGCCAAAGCAATAGCAGTAGCTGAA---
GGAACAGATAGAATTATAGAAATAGTAAGAAGAATTTTTACAGCT----------------------------
--------ATTCTCAACATA------CCTAGAAGGATAAGACAGGGCTTGGAAAGGCTTTTGCTA---TAA
>AB588311
GCT---ACA--
GAAGATTTGTGGGTCACAGTCTATTATGGGGTACCAGTGTGG---AAAGAAGCAAAC---
ACCACTCTATTTTGTGCATCAGATGCTAAGGCTTATGATACAGAG---GCACATAAT----------------
--------------GTTTGGGCCACA---CATGCCTGTGTACCCACAGAC---
CCCAATCCACAAGAGGTGAAATTGGAA---------AATGTGACAGAAAATTTTAAC----------------
--------------------------ATGTGGAAAAATAACATGGTAGAACAGATGCATGAGGATGTA---
ATCAGCTTATGGGATCAAAGCCTA---AAGCCATGTGTAAAATTA---
ACCCCACTCTGTGTTATTTTAAATTGCACTGAGATCAATGGTACCAATGCTGCCAAT----------------
----
-------------------------------------CTTACCAAAGAAGATACAGATATGAAA---
AATTGCTCTTTCAATGCCACTACAAGTGTGACA---------------------------
GATAAAAAACAGAAAGAATATGCACTA---TTCTATAAACTTGATGTAGTACAAATAGATAAG----------
----

AATAGTAATAGTACCAACTATAGGTTGATAAGTTGTAATACCTCAGTCATTACACAG---
GCCTGTCCAAAAATATCCTTTGAGCCAATTCCCATACATTATTGTGCTCCGGCTGGTTTTGCGCTTCTAATGT
GTAAA---GATAAGAAGTTCAAT------GGAACAGGACAATGTGCAAAT--------------------------
-GTCAGC---ACAGTACAATGTACACATGGAATTAGGCCAGTAGTATCAACT---
CAACTGCTGTTAAATGGCAGTCTAGCAGAGGAAGAA------------
GTAGTAATTAGATCTGAAAATTTCACAAATAAT---------GCTAAAGTCATAATAGTACAGCTAAAT----
-----------GAATCTGTAGCA---------ATTAATTGTACAAGACCCAATAACAACACAAGAAAA-----
-GGTATACATATG------------GGACCAGGGAGGGCA---TAT------TACACAACAGGACAA------
---------ATAATAGGAGATATA------------AGACAAGCACATTGT---AACATTAGTAGAGCA---
AAATGGAATAACACTTTAAAACTGATAGCTGACAAATTAAGA---------------GAACAATTTAGG----
-----------------------------------AATACAACAATAACCTTTAAT------
CAGTCCTCAGGAGGGGATCCAGAAATAATA---ATGCATACTTTTAATTGTGGAGGAGAG---
TTCAAGCAGCTCCAGGCAAGAGTCCTGGCTGTAGAAAGATACCTAAGGGATCAACAGCTCCTAGGAATTTGGG
GTTGCTCTGGAAAACTCATTTGCACCACTGCTGTGCCTTGGAATACTAGTTGGAGT-----------------
----------------------------AATAGATCTCTGGATGACATTTGG------GGG------
AACATGACCTGGATGGAATGG---GAAAGAGAAATTGGCAATTACACAGACCTAATATAC---
AACTTACTTGAAAAAGCGCAGAACCAGCAGGAA--

AAGAATGAGCAAGACTTATTGGAATTGGATAAATGGGCAAGTTTGTGGAATTGGTTTGACATATCAAATTGGC
TGTGGTATATAAAAATATTCATAATGATAGTAGGAGGCTTGATAGGTTTAAGAATAGTTTTTGTT---
ATACTTTCTCTAGTG---------AATAGAGTCAGGCAGGGATACTCACCA------
TTGTCGTTTGCTGTTAGTTTATACAATACTATAGCTGTAACAGTAGCTGAG---
GGGACAGATAGGGTTATAGAACTAGGGTATAGAATTGTTAGAGCT----------------------------
--------ATCCTCCACATA------CCTACAAGAATAAGACAGGGCTTGGAAAGGGCTTTGCTA---TAA
>AB731663
GCT---ACA--
GGACAGTTGTGGGTCACAGTCTATTATGGGGTACCTGTGTGG---AGAGAAGCAAAC---
ACCACTCTATTTTGTGCATCAGATGCTAAAGCATATGAAACAGAG---GCACATAAT----------------
--------------GTTTGGGCCACA---CATGCCTGTGTACCCACAGAC---
CCCAACCCACAAGAAGTAGAATTAAAA---------AATGTAACAGAAGATTTCAAC----------------
--------------------------ATGTGGAAAAATAACATGGTAGATCAGATGCATGAGGATGTA---
ATCAGTTTATGGGATGAAAGCCTA---AAGCCATGTGCAAAGCTA---
ACCCCACTCTGTGTTACTTTAAATTGCACTGAT--

----------------------------AGTGGTGGGGCAAAAATGGAGACAGGAGAAATGAAA---
AACTGCTCTTTCAATATCACCACAGACCTAAGA---------------------------
GATAAGAGGAAGGAAGTATATGCAACT---TTTTATAAACTGGATGTAGTACCAATAGAAAGT----------
----

AATAGTAATAGTAGTAGTAATTATAGGTTGATAAGTTGTAATACCTCAGTCCTTACACAG---
GCCTGTCCAAAGATATCCTTTGAGCCAATTCCCATACATTATTGTGCCCCGGCTGGTTTTGCGATTTTCCAAT

GTAAA---GATAAGAAATTCAAT------GGAACAGGACCATGTACAAAT-----------------------
-GTCAGC---TCAGTACAATGTACACATGGAATTAGGCCAGTAGTGTCAACT---
CAACTGTTGTTAAATGGCAGCCTGGCAGAAGAAGAG------------
ATAGTAATTAGATCTGCCAATTTCTCGGACAAT---------GCTAAAAATATAATAGTACAGCTGAAA----
-----------GAAGCTGTACAA---------ATTAATTGTACAAGACCTAACAACAATACAAGAAAA-----
-GGTATTCATATA------------GGACCAGGGAGAGCA---TTT------TATACAACAGGACAA------
---------ATAGTAGGAGATATA------------AGACAAGCACATTGT---AACCTTAGTAGAACA---
CAATGGAATAACACTTTAAAACAAATAGTTGGAAATTAAGA----------------GAACAATTT-------
-----------------------------------AATAAAACAATAGTCTTTAAT------
CACTCTTCAGGAGGGGACCCAGAAATTGTA---ATGCACAGTTTTACATGTGGAGGGGAG---
TTTAAACAGCTCCAGGCAAGAGTCCTGGCTGTGGAAAGATACCTAAAGGATCAACAGCTCCTGGGGATTTGGG
GTTGCTCTGGAAAACTCATTTGCACCACTGCTGTACCTTGGAATGCTAGTTGGAGT-----------------
----------------------------AATAAATCTCACGATGAGATTTGG------AAT------
AACATGACCTGGATGCAGTGG---GAAAGAGAAATTGATAATTACACAAGCACAATATAC---
ACATTAATTGAAGAATCGCAAAACCAGCAAGAA--

AAGAATGAACAGGATTTATTGGCACTAGATAAATGGGCAAGTTTGTGGAGTTGGTTTGACATAACAAATTGGC
TGTGGTATATAAGAATATTTATAATGATAGTAGGAGGCTTAATAGGTATAAGAATAGTTTTTACT---
GTACTTTCTATAGTG---------AATAGGGTTAGGAAGGGATATTCACCA------
CTATCGTTTGCTGTTAGCTTGCTCGACGCTACAGCAATAGCAGTAGCTGAG---
GGGACAGATAGGATTATAGAAGTATTACAAAGAGCTTATAGAGCT----------------------------
--------ATTCTCCACATA------CCTACAAGAATAAGACAGGGCTTGGAAAGGCTTTGCTA---TAA
>AB731667
GCT---GCA------------------------AAA---CAA---TCA---
CAACAATTGTGGGTCACAGTATATTATGGGGTACCTGTGTGG---AAAGAAGCAACC---
ACCACTTTATTTTGTGCTTCAGATGCTAAGGCATATGATACAGAG---GTACATAAT----------------
--------------GTTTGGGCTACA---CATGCCTGTGTACCCACAGAC---
CCCAACCCACAGGAAGTAGTATTGGAA---------AATGTGACAGAAAATTTTAAT----------------
--------------------------ATGTGGAAAAATAGCATGGTGGAACAAATGCATGAGGATATA---
ATCAGTTTATGGGATCAAAGCCTA---AAACCATGTGTAAAATTA---
ACCCCACTCTGTGTTACTCTAAATTGCACTGATCAACCAACTAATACTACCAATACCACTAGT----------
----
----------------------------GGTCATGGTAGTTTAATGGAGAGAGGGGAAATAAAA---
AACTGCTCTTTCAATGTTACCACAGGCATAAGA---------------------------
GATAAGATGCAAAGAGAATATGCACTT---TTTTATAAACTTGATGTAGTTCCAATAAATGATAAT-------
----

GCTAATAATGCCAGCTATACCAACTATAGGTTAATGAGCTGTAACACCTCAGTTATTACACAG---
GCCTGTCCAAAGGTATCCTTTGAACCAATTCCAATACATTATTGTGCCCCGGCTGGTTTTGCGATTTTAAAGT
GTAAT---GATAAGAAGTTTAAT------GGAACAGGACCATGTAAAAAT-----------------------
-GTCAGC---ACAGTACACTGTACACATGGAATTAGGCCAGTAGTATCAACT---
CAGTTGTTGTTAAATGGCAGTCTATCAGAAGGAGAG------------
GTAATAATTAGGTCTGAAAATTTCACGGACAAT---------GCTAAAACTATAATAGTACAGCTGAAT----
-----------GAATCTGTAACA---------ATTAATTGTACAAGACCCAGTAACAATACAAGAAAA-----
-GGTATACAGATA------------GGACCAGGGAGGGCA---TTT------TATACAACAGGAGAA------
---------ATAATAGGAGACATA------------AGACAAGCACATTGT---AACATTAGCAAAACA---
GAATGGAAAAGTACTTTAACAAAGGTAGCTATAAAATTAAGA---------------GAACAATATAAA----
-----------------------------------AATAAAACAATAGAATTTAAA------
CAATCCTCAGGAGGGGACCTAGAAGTTGTA---ATGCACAGTGTTAATTGCGGAGGAGAA---
TTTAAGCAGCTCCAGGCAAGAGTCCTGGCTCTGGAAAGATACCTAAAGGATCAACAGCTCATGGGAATTTGGG
GTTGCTCTGGAAAACTCATCTGCACCACTGCTGTGCCTTGGAATACCAGTTGGAGT-----------------
----------------------------AATAAATCACTGGGTGAGATTTGG------GAT------
AACATGACCTGGATGGAGTGG---GAAAAAGAAATTGAAAATCATACCAGCCTAATATAC---
ACCTTACTTGAAAAATCGCAGACCCAACAGGAA--

ATAAATGAAAAGGAATTATTGGAATTGGATCAATGGGCAAGTCTGTGGAGTTGGTTTGACATATCAAATTGGC
TGTGGTATATAAAAATATTCATAATGATAGTAGGTGGCTTAATAGGTTTAAGAATAATTTTTACT---
GTACTTTCTATAGTG---------AATAGAGTTAGGCAGGGATACTCACCA------
TTATCGTTGGCTGTTAGCTTGTTTGACTCCACAGCCATAGTAGTAGCTGAA---

GGGACAGATAGGATTGTAGAATTCATACAAAGAGCTTTTAGGGCT---------------------------
--------ATTCGCAATATA------CCTGTAAGAATAAGACAGGGCTTGGAAAGGGCTTTGGTA---TAA
>KF716497
GCT---GCA--
GAACAGTTGTGGGTTACAGTCTATTATGGGGTACCTGTGTGG---AAAGATGCAACC---
ACCACTTTATTTTGTGCATCAGATGCTAAAGCATATGACACAGAA---GTACATAAT---------------
--------------GTTTGGGCCACA---CATGCCTGTGTACCCACAGAC---
CCCAACCCACAAGAAGTATGGTTAGAA---------AATGTGACAGAGAACTTTAAC---------------
--------------------------ATATGGAAAAATAACATGGTAGAACAGATGCATGAAGATATA---
ATCAGTTTATGGGATGAAAGCTTA---AAGCCATGTGTAAAATTA---
ACCCCACTCTGTGTCAACTTAACCTGCACTGATGCTAATACCACTAGTAGTGGTATCAAT------------
----
-------------------------------AGTAGTCATGTAATAGAAGAGGGAGAAATAAAA---
AACTGCTCTTTCAATATTACCACAACATTAAGA--------------------------GAGAAGGTG---
AAGACATACGCACTT---TTTGATAGTCTTGATGTAAGATCAATAGGTAAT---------------------
----
----------AGTACTACTAGTTATAGGATAATAAATTGTAATACTTCAGTCATTACACAG---
GCCTGTCCAAAGATATCCTTTGAGCCAATTCCCATACACTATTGTACCCCGGCTGGTTTTGCGCTTCTAAAGT
GTAAC---GATAAACAGTTCAGT------GGAAACGGAACATGTAACAAT----------------------
-GTCAGC---TCAGTACAATGTACACATGGAATTAGACCAGTAGTGTCAACT---
CAACTGCTGCTAAATGGCAGTCTAGCAGAAGAAGAG-----------
GTAGTAATTAGATCAAAAAATTTCACAGATAAT---------GCTAAAATCATAATAGTACAGCTGAAC----
-----------CAATCTGTAGTA---------ATTAATTGTACAAGACCCAACAACAACACAAGGAAC-----
-AGTGTACATATA------------GGACCAGGGAGAGCA---TTA------TATACAACAAAC---------
---------ATAATAGGAGATATA------------AGACGAGCACATTGT---GATATAAGTAGAGCA---
GAATGGAATAACACTTTAAGACATGTGGTTACAAAATTAAAA---------------GAAAAATTTAAT----
---------------------------------GCAACAACAATAATCTTTAAC------
CAATCCTCAGGAGGAGACCCAGAAACTGAA---AGGCACAGCTTTAATTGTGGAGGGGAA---
TTTAAGCAGCTCCAGGCACGAGTCCTGGCTGTGGAAAGATACCTAAAGGATCAACAGCTCCTAGGGATTTGGG
GCTGCTCTGGGAAACTCATTTGCACCACTGCTGTGCCTTGGAATACTAGTTGGAGT----------------
---------------------------AATAAATCTCTAGATGAGATTTGG------AAA------
AACATGACCTGGATGCAGTGG---GAAAAAGACATTGACAATTACACAGAAATAATATAC---
AGCTTACTTGAGGAATCGCAAAACCAACAAGAA---------------------------------------
----
AAGAATGAACAAGAATTATTGGAATTGGATAGTTGGGCAAGTTTGTGGAATTGGTTTGACATATCAAAATGGC
TGTGGTATATAAGGATATTCATAATGATAGTAGGAGGCTTAGTAGGTCTAAGAATAGTTTTTGCT---
GTGCTGTCTATAGTA---------AATAGAGTTAGGCAGGGATACTCACCA------
GTATCATTTGCTATTAGCTTGCTAAATGTCACAGCTATAGCAGTAGCTGAG---
GGAACAGACAGGATTATAGAAATAGCACATAGAGCTTTTAGAGCT---------------------------
--------GTTATCCACATA------CCTGTAAGAATAAGACAAGGCTTAGAAAGGGCTTTGCTA---TAA
>KF716498
GCT---GTA---GAA---------------------------------
AAAGACAATTTGTGGGTTACAGTCTATTATGGGGTACCTGTGTGG---AAAGAAGCAACC---
ACCACTTTATTTTGTGCATCAGATGCTAAAGCATATGACACAGAG---GCACATAAT---------------
--------------GTATGGGCCACA---CATGCCTGTGTACCCACAGAC---
CCCAACCCACAAGAAGTACGGTTAGAA---------AATGTGACAGAAAACTTTAAT---------------
--------------------------ATATGGGAAAATAACATGGTAGAACAGATGCATGAAGATGTA---
ATCAGTTTATGGGAACAAAGCTTA---AAACCATGTGTGAAATTA---
ACCCCACTCTGTGTTACTTTAAACTGCACTGATGCTGGAAATACTACTGAAGCTGGGAATACTACT-------
----
----------------------AGTACCAACAGTAGTAAAATAGAAGAAAAAGGAGAAATAAAA---
AACTGCTCTTTCAATGTCACCACAACGATAGAC--------------------------
AATAAGGCGCGGAAGGAAAGCGCACTT---TTTTATAGACTTGATGTAGTACCAATAGATGATGATAATAAT-
----

AGTGATAGTAGTACTAGTGCTAGTTATAGGTTAATAAATTGTAACACCTCAGTCATTACACAG---
ACCTGTCCAAAGGTATCCTTTGAGCCAATTCCTATACACTATTGTGCCCCGGCTGGTTTTGCGCTTCTAAAGT
GTAAC---AATAAGACGTTCAGT------GGAAAAGGACCATGCACTAAT----------------------
-GTCAGC---ACAGTGCAATGTACACATGGAATTAGGCCAGTAGTGTCAACT---
CACCTGCTGTTAAATGGCAGTCTAGCAGAAAAGGAG-----------

GTAGTACTTAGATCAAAAAATTTCACAAATAAT---------GCTGAAACCATAATAGTGCAGCTGAAC----
-----------CAATCTGTAGTA---------ATTAATTGTACAAGACCCAACAACAACACAAGAAGA-----
-AGTATACATTTA------------GGACCAGGGCAAACA---TTA------TATGCAACAGGAGAC------
---------ATAATAGGGGACATA------------AGACAAGCACATTGT---AACATTAGTCAAGCA---
GCATGGAATAACACTTTAAGACAGGTGGTTGCAAAATTAAAA---------------GAACAATTTAAT----
-----------------------------------GCAACAACAATAGTCTTTAAT------
CGATCCTCAGGAGGAGACCCAGAAATTGTA---ATGCACAGTTTTATTTGCGGAGGGGAA---
TTTAAGCAGCTCCAGGCGAGAGTCCTGGCTGTGGAAAGATATCTAAGGGATCAACAGCTCCTAGGGATTTGGG
GCTGCTCTGGAAAACTCATTTGCACCACTACTGTGCCCTGGAATACTAGTTGGAGT-----------------
-------------------------AAGAATAGAAATCTAACTGATATTTGG------GAT------
AACATGACCTGGGTACAGTGG---GAGAGAGAAATAGACAATTACACAAAAGAAATATAC---
ACTTTACTTGAAGAAGCCCAGAACCAACAGGAA--

AAGAATGAACAAGAATTATTGGAATTGGATAAGTGGGCAAGTCTGTGGAATTGGTTTGACATAACAAGCTGGC
TGTGGTATATAAAGATATTCATAATGATAATAGGAGGCTTAGTAGGTTTAAGAATAGTTTTTGCT---
GTACTTTCTATAGTG---------AATAGAGTTAGGCAGGGATACTCACCA------
CTGTCGTTTGCCATTAGCTTGCTAAACGCCACAGCTATAGCAGTAGCTGAG---
GGGACAGACAGAATTATAGAAGTAATACAAAGACTTTGTAGAGCT----------------------------
--------ATTCTTCACATA------CCTGTAAGAATAAGACAGGGCTTAGAAAGGGCTTTGCTA---TAA
>AB588201
GCT---ACA--
GAACAATTGTGGGTCACAGTTTATTATGGGGTGCCTGTGTGG---AAAGAAGCAACC---
ACCACTCTATTTTGTGCATCAGATGCTAAAGCATATGATACAGAG---GTACATAAT----------------
--------------GTCTGGGCCACA---CATGCCTGTGTACCCACAGAC---
CCCAACCCACAAGAAGTAGAATTGGTA---------AATGTGACAGAAAATTTTAAC----------------
--------------------------ATGTGGAGAAATGACATGGTGGAACAGATGCATGAGGATGTA---
ATCAGTTTATGGGATCAAAGCCTA---AAGCCATGTGTAAAATTA---
ACCCCACTCTGTGTTACTTTAAATTGCACTGATGATTTGAGTAATGCTACTTTGAGGAATACTATTAATACCA
CTAGTGAGTTGAAGAATGCTATTACTAAT--
----TCCACTAGTAGTAGCGAGAATACTACTAATACCAATAGTAGTATTACAGGAGAAATGAAA---
TACTGTTCTTTCAATATCACCACAGTCATAAGA---------------------------
GATAAGGTGCAAACAGAATATGCACTT---TTTTATAAACTTGATGTAGTGCCAATAGATGAT----------
----

AGTAATACCAGCTATAGGTTAATAAGTTGTAACACCTCAGTCATTACACAA---
GCCTGTCCAAAGGTATCCTTTGAGCCAATTCCCATACATTATTGTGCCCCGGCTGGTTTTGCGATTCTAAAGT
GTAAC---GATAAGAAGTTCAAT------GGAACAGGACCATGTACAAAT-----------------------
-GTCAGC---ACAGTACAATGTACACATGGAATTAGGCCAGTAGTATCAACT---
CAACTGCTGTTAAATGGCAGTCTAGCAGAAGAAGAG------------
GTAGTAATTAGATCTGAAAATTTCACGAACAAT---------GCTAAAACCATTATAGTGCAGCTGAAA----
-----------GAATCTGTACAA---------ATTAATTGTACAAGACCCAGCAACAATACAAGAAGA-----
-AGTATACATATT------------GCACCAGGGAGAGCA---TTT------TATACAACAGGAAAA------
---------ATAATAGGAGATATA------------AGACAAGCACATTGT---AACATTAGTAGTACA---
AAATGGAATAGCACTTTAAGACAGATAGTTAAAAAATTAAGA---------------GAACAATTTGGG----
-----------------------------------AATAAAACAATAAATTTTACG------
CGATCCTCAGGAGGGGATCCAGAAATTGTA---ATGCACAGTTTTAATTGTGGAGGGGAA---
TTTAAGCAGCTCCAGGCAAGAGTCCTGGCTGTGGAAAGATACCTAAAGGATCAACAGCTCATGGGGATTTGGG
GTTGCTCTGGAAAACTCATCTGCACCACTACTGTGCCTTGGAATGCTAGTTGGAGT-----------------
----------------------------AATAAATCTCTGCATGACATTTGG------GAT------
AACATGACCTGGATGCAGTGG---GATAGAGAAATTAACAATTACACAGCTTTAATATAC---
AACTTACTTGAAGAATCGCAGAACCAACAAGAA--

AAGAATGAACAAGAATTATTGGAATTAGATAAATGGGCAAGTTTGTGGAATTGGTTTAACATAACAAACTGGC
TGTGGTATATAAAATTATTCATAATGATAGTAGGAGGCTTGGTAGGTTTAAGAATAGTTTTTGCT---
GTACTTTCTATAGTG---------AATAGAGTTAGGCAGGGATACTCACCA------
TTATCGTTAGCTGTTAGCTTGTTCAACGCCACAGCTATAGCAGTAGCTGAG---
GGGACAGATAGGGTTATAGAAGTATTACAAAGGGTTTATAGAGCA----------------------------
--------ATTCTCCACATA------CCTACAAGAATAAGACAGGGCTTGGAAAGGGCTTTGCTA---TAA
>AB588207

GCT---GCA---
GAACAATTGTGGGTCACAGTCTATTATGGGGTACCTGTGTGG---AAAGAAGCAACC---
ACCACTCTATTTTGTGCATCAGATGCTAAAGCATATGATACAGAG---GTACATAAT----------------
--------------GTTTGGGCCACA---CATGCCTGTGTACCCACAGAC---
CCCAACCCACAAGAAGTAGTATTGGCA---------AATGTGACAGAATATTTTAAC----------------
--------------------------ATGTGGAAAAATGACATGGTAGAGCAGATGCATGAGGATATA---
ATCAGTTTATGGGATCAAAGTCTA---AAGCCATGTGTAAAATTA---
ACCCCACTCTGTGTTACCTTAAACTGCTCTGATAATGTGGAGAATGTTACTATTACCAATGGTAGTCTA----
----
-------------------ATAAATACCAATGGTAGTCTAAAGATAGAGAAAGGAGAAATAAAA---
AACTGCTCTTTCAATATCACCACAAGCATAAAA---------------------------
GATAAGGTGCAGAAAGGATATGCACTT---
TTTTATAGACTTGATGTAGTACCAATAGATGATGATAATAATGAT----------------------------
-----------------------------AATAAT---
AATAATAGTAGGACTAATAATAATAAGACTAATACTAGCTATAGGTTGATAAATTGTAACACATCAGCCATTA
CACAG---
GCCTGTCCAAAGGTATCCTTTGAGCCAATTCCCATACATTATTGTGCCCCGGCTGGTTTTGCGATTATAAAGT
GTAAC---AATAAGACGTTCAAT------GGAACAGGACCATGTAAAAAT----------------------
-GTCAGC---ACAGTACAATGTACACATGGAATTAGGCCAGTAGTATCAACT---
CAACTGCTGTTAAATGGCAGCCTAGCAGAAGAAGAG-----------
ATAGTAATTAGATCTGCCAATTTCTCGGACAAT---------GCTAAAATCATAATAGTACATCTGAAC----
-----------GAATCTGTACAA---------ATTAATTGTACAAGACCCAACAACAATACAAGAAAA-----
-GGTATACATATG------------GGACCAGGGGGAGCA---TTT------TATACAACAGGAGAG------
---------ATAACAGGAGATATA------------AGACAAGCACATTGT---AACCTTAGTATAACA---
CAATGGAATAACACTTTAGAACAGGTAGCTGAAAAATTAAGA---------------GAACAATTT-------
------------------------------------AATAAAACAATAGTCTTTAAT------
CACTCCTCAGGAGGGGACCCAGAAATTGTA---ATGCACAGTTTTAATTGTGGAGGGGAA---
TTTAAGCAGCTCCAGGCAAGAGTCCTGGCTGTGGAAAGATACTTAAAGGATCAACAGCTCCTGGGGATCTGGG
GTTGCTCTGGAAAACTCATCTGCACCACTACTGTGCCTTGGAATGATAGTTGGAGT-----------------
----------------------------AATAAAAATCTAAGTGAGATTTGG------GAT------
AAAATGACCTGGATGCAGTGG---GAAAGAGAAATTGACAATTACACAAGCTTAATATAC---
ACCTTACTTGAAGAATCGCAAAACCAACAAGAC---------------------------------------
----
AAGAATGAACAAGAACTATTGGAATTGGATAAATGGGCAAGTTTGTGGAATTGGTTCAGCATAACAAACTGGC
TGTGGTATATAAAAATATTCATAATAATAGTAGGAAGCTTGGTAGGTTTAAGAATAGTTTTTACT---
GTACTTTCTATAGTG---------AATAGAGTTAGGCAGGGATACTCACCA------
TTATCGTTTGCTGTTAGCTTGCTCAACGCCATAGCCATAGCAGTAGCTGAA---
GGGACAGATAGGATTATAGAAGCATTACAAAGAACTTTTAGAGCT----------------------------
--------ATTCTCCACATA------CCTACACGAATAAGACAGGGCTTGGAAAGGGCTTTGCTA---TAA
>AB588209
GCT---GCA---
GAACAATTGTGGGTCACAGTCTATTATGGGGTACCTGTGTGG---AAAGAAGCAACC---
ACCACTCTATTTTGTGCATCAGATGCTAAAGCATATGACACAGAG---GTACATAAT----------------
--------------GTTTGGGCCACA---CATGCCTGTGTACCCACAGAC---
CCTAACCCACAAGAAGTAGTATTGGGA---------AATGTGACAGAAAATTTTAAC----------------
--------------------------ATGTGGAAAAATAACATGGTAGAACAAATGCATGAGGATATA---
ATCAGTTTATGGGATCAAAGCCTA---AAGCCATGTGTAAAATTA---
ACCCCACTCTGTGTTACTTTAAATTGCACTAATACTACTGATAACAACAGCACTAATACTACT----------
----
-------------------------GATAACAACAGTACTAAGTACAATGATAGTGATATGAAA---
AATTGCTCTTTCAATGTCACCACAAGTATAGGA---------------------------
AATAAGATGCAGAAAGAATATGCACTT---TTTTATAAACTTGATGTAGTACCAATAAGTAAT----------
----

GATAGTACAAGCTTTACGTTGATAAATTGTAACACCTCAGTCATTACACAG---
GCTTGTCCAAAGATATCCTTTGAACCAATTCCAATACATTATTGTACCCCGGCTGGTTTTGCGCTTCTAAAGT
GTAAT---GATAAGAAGTTCAAT------GGAACAGGACCATGCACAAAT----------------------
-GTCAGC---ACAGTACAATGTACACATGGAATTAGGCCAGTAGTGTCAACT---
CAACTACTGTTAAATGGCAGTCTAGCAGAAGAAGAG-----------
ATAGTAATTAGATCTGAAAATTTCACAAACAAT---------GCTAAAACCATAATAGTACAGCTGAAC----

-----------GAAACTGTACAA---------ATTAATTGTACAAGACCCAACAACAATACAAGAAAA-----
-GGTATACATATG------------GGATGGGGGAGAGCA---TTT------TATGCAACAGGAGCT------
---------ATAATAGGAAATATA------------AGACAAGCACATTGT---AACCTTAGTAGTACA---
AAATGGAATAACACTTTAAAACAGATAGCTATAAAATTAAGA---------------GAACAATTT-------
-----------------------------------AATAAAACAATTATCTTTAAT------
CGATCCTCAGGAGGGGACCCAGAAATTGTA---ATGCACAGTTTTAATTGTGGAGGGGAA---
TTTAAGCAGCTCCAGGCAAGAGTCCTGGCTGTGGAAAGATACCTAAAGGATCAACAGCTCCTAGGGATTTGGG
GTTGCTCTGGAAAACTCATTTGCACCACTGCTGTGCCTTGGAATACTAGTTGGAGT----------------
----------------------------AATAAAACTATGGATGAGATTTGG------AAG------
AACATGACCTGGATGGAGTGG---GAGAGAGAAATTGACAATTACACAGGCTTAATATAC---
CACTTAATTGAAGAATCGCAAAGCCAACAAGAA---

AAGAATGAACAAGAATTATTGGAATTGGATAAGTGGGCAAGTTTGTGGAATTGGTTTGACATAACAAAATGGC
TATGGTATATAAAAATATTCATAATGATAGTAGGAGGCTTAATAGGTTTAAGAATAGTTTTTGCT---
GTACTTTCTATAGTG---------AATAGAGTTAGGCAGGGATACTCACCA------
CTATCATTCGCTGTTAGTCTGTTTGATGCCACAGCTGTAGCAGTAGCTGAG---
GGGACAGATAGGGTTCTAGAAGTATTACAAAGGATTTGGAGAGCT----------------------------
--------GTTCTCCACATA------CCTACAAGAATAAGACAGGGCTTAGAAAGGGCTTTGCTA---TAA
>AB588211
GCT---GCA---
GAACAATTGTGGGTCACAGTCTATTATGGGGTACCTGTGTGG---AAAGAAGCAACC---
ACCACTCTATTTTGTGCATCAGATGCTAAAGCATATGACACAGAG---GTACATAAT----------------
--------------GTTTGGGCCACA---CATGCCTGTGTACCCACAGAC---
CCTAACCCACAAGAAGTAGTATTGGGA---------AATGTAACAGAAAATTTTAAC----------------
--------------------------ATGTGGAAAAATGACATGGTAGAACAAATGCATGAGGATATA---
ATCAGTTTATGGGATCAAAGCCTA---AAGCCATGTGTAAAATTA---
ACCCCACTCTGTGTTACTTTAAATTGCACTAATTTACAGAATACTACTAGTAACAAT----------------
----
----------------------------GGTAGTGTTGGGGAAGAGATGAAGGGAGAAATGAAA---
AACTGCTCTTTCAATGTCACCACAAATATAGGA--------------------------
AATAAGATGCAGAAAGAATATGCACTT---TTTTATAAACTTGATGTAGTACCAATAGATAAT----------
----

GATAATACAAGCTATACGTTGATAAATTGTAACAACTCAGTCATTACACAG---
GCCTGTCCAAAGGTATCCTTTGAACCAATTCCCATACATTATTGTACCCCGGCTGGTTTTGCGCTTCTAAAAT
GTAAT---GATAAGAAGTTCAAT------GGAACAGGACCATGTACAAAT-----------------------
-GTCAGC---ACAGTACAATGTACACATGGAATTAGGCCAGTAGTGTCAACT---
CAACTACTGTTAAATGGCAGTCTAGCAGAAGAAGAG------------
GTAGTAATTAGATCTGAAAATTTCACAAACAAT---------GCTAAAACCATAATAGTACAACTGAAC----
-----------GAAACTGTACAG---------ATTAATTGTACAAGACCCAACAACAATACAAGAAAA-----
-AGTATACCTATG------------GGACCGGGAAGAGCA---TTT------TATGCAACAGGAGAT------
---------ATAATAGGAGATATA------------AGACAAGCACATTGT---AACCTTAGTGAAGTA---
AAATGGGTAAACACTTTAGGACAGATAAAGGAAAAATTAAAA---------------GAACAATTT-------
-----------------------------------AATAAAACAATTGTCTTTAAT------
CAATCCTCAGGAGGGGACCTAGAAATTGTA---ATGCACAGTTTTAATTGTGGAGGGGAA---
TTTAAGCAGCTCCAGGCAAGAGTCCTGGCTGTGGAAAGATACTTAAGGGATCAACAGCTCCTAGGGATTTGGG
GTTGCTCTGGAAAACTCATTTGCACCACTGCTGTGCCTTGGAATACTAGTTGGAGT----------------
----------------------------AATAAAACTCTGAGTGAGATTTGG------GAC------
AACATGACCTGGATGGAGTGG---GAGAGAGAAATTAACAATTACACAAATTTAATATAC---
AACTTAATTGAAGAAGCGCAAAACCAACAAGAA---

AAGAATGAACAAGAATTATTGGAATTGGATAAGTGGGCAAGTTTGTGGAATTGGTTTGACATATCAAACTGGC
TGTGGTATATAAGAATATTCATAATAATAGTAGGAGGCTTAATAGGCTTAAGAATAGTTTTTACT---
GTACTTTCTATAGTG---------AATAGAGTTAGGAAGGGATACTCACCA------
TTATCATTCGCTGTTAGTCTGCTTAATGCTACAGCTATAGCAGTAGCTGAG---
GGGACAGATAGGGTTATAGAAGTAGGACAAAGAATTTGGAGAGCT----------------------------
--------GTTCTCCACATA------CCAACAAGAATAAGACAGGGTTTAGAAAGGGCTTTGCTA---TAA
>AB588233
GCT---GCA---
GAAGAAACGTGGGTCACAGTCTATTATGGGGTACCTGTGTGG---AAAGAAGCAACC---

ACCACTCTATTCTGTGCATCAGATGCTAAAGCATATGTACAAGAG---GCACATAAT----------------
--------------GTCTGGGCCACA---CATGCCTGTGTACCCACAGAC---
CCCAACCCACAAGAAGTAAGATTGGTA---------AATGTGACAGAAAATTTTAAC----------------
--------------------------ATGTGGAAAAATAACATGGTAGAACAGATGCATGAAGATATA---
ATCAGTTTATGGGATCAAAGCCTA---AAGCCATGTGTAAAATTA---
ACCCCACTCTGTGTTACTTTAAATTGCACTGATGATTTGAGTAATGCCAGTAATAGCGCCACT----------
----
------------------------AAGAATAACAGTTGGGCGATGGAGACAGGAGAAATGAAA---
AACTGCTCTTTTAATGTTACCACAGAGATAAGA--------------------------
GATAAGGTGAAGAAAGAATATGCACTT---TTTTATAAACTTGATGTAATACCAATAAAGGGTGAT-------
----

AATACTAGTAATACTAGCTACAGATTGATAAATTGTAATACCTCAACCCTTACACAG---
GCCTGTCCAAAGATATCCTTTGAGCCAATTCCTATACATTTTTGTACCCCGGCTGGTTTCGCGATTATAAAGT
GTAAT---GATAAGGATTTCAAA------GGAACAGGACCATGTAAAAAT----------------------
-GTCAGT---ACAGTACAATGTACACATGGAATTAGGCCAGTAGTGTCTACT---
CAACTGCTGTTAAATGGCAGTCTAGCAGAAAAGGAG------------
GTAGTAATCAGATCTGAAAATTTCACAGACAAT---------GCTAAAACCATAATAGTACAGCTGAAT----
-----------AAAACTGTACAA---------ATTCATTGTACAAGACCCAACAACAATACAAGAAAA-----
-GGTATACATATA------------GGACCAGGGAGAGCA---TTA------TTTTATGCAACAGAC------
---------ATAATAGGAGATATA------------AGACAAGCACATTGT---AACCTTAGTAGAGCA---
GACTGGAATGAAACTTTAAGACAGGTAGTTACAAAATTAGGA---------------GAACAATTTGGG----
---------------------------------AATAATAAGACAATAAAATTTCAA------
CCATCCTCAGGAGGGGACCCAGAGATTGTA---ACTCATAGTTTTAATTGTGGAGGGGAA---
TTTAAACAGCTCCAGGCAAGAGTCCTGGCTGTGGAAAGATACCTAGGGGATCAACAGCTCCTAGGGATTTGGG
GTTGCTCTGGAAAACTCATCTGCACCACTAATGTGCCTTGGAACTCTAGTTGGAGT-----------------
---------------------------AATAAATCTCTGGATAAGATTTGG------CAT------
AACATGACCTGGATGCAGTGG---GACAGAGAAATTAATAATTACACAAGCTTAATATAC---
AACTTACTTGAAGAATCGCAGAACCAGCAAGAA---------------------------------------
----
AAGAATGAACAGGAATTATTGGAATTGGATAAGTGGTCAAATTTGTGGAATTGGTTTAACATAACAAAATGGC
TGTGGTATATAAAAATATTCATAATGATAGTAGGAGGCTTAATAGGTTTAAGAATAGTTTTTGCT---
GTACTTTCTATAGTG---------AATAGAGTTAGGCAGGGATACTCACCA------
TTATCATTTGCTGTTAGCTTGCTTAACGCCACAGCAATAGCAGTAGCTGAA---
GGGACAGATAGGATTATAGAAGTAGTACAAAGAGCTTTTAGAGCT----------------------------
--------ATCCTCCACATA------CCTAGAAGAATAAGACAGGGCTTGGAAAGGGCTTTACTA---TAA
>AB480698
ACT---ACA---
GAAAAATTGTGGGTCACAGTCTATTATGGGGTACCTGTGTGG---AAAGAAGCAACT---
ACCACTTTATTTTGTGCATCAGATGCTAAAGCATATGACACAGAG---GCACATAAT----------------
--------------GTTTGGGCCACA---CATGCCTGTGTACCCACAGAC---
CCTAACCCACAAGAAGTAGAATTGGTA---------AATGTGACAGAAAATTTTAAT----------------
--------------------------ATGTGGAAAAATGACATGGTAGAACAGATGCATGAGGATATA---
ATCAGTTTATGGGATCAAAGTCTA---AAGCCATGTGTAAAATTA---
ACCCCACTCTGTGTTACTTTAAATTGCATTGATTGGAAGTCAAATTGCACTAATAATAATGATACCCAGAATA
ATAGTAATAATAGTACCCAGAGTGATAATTGTAGTGGGAATACT-----------------------------
----------AATAATACCCATAGTAATAGTAGTTTGGGAAGAATAGATGCAGGAGAAGTAAAA---
AACTGTTCTTTCAATGTCACCACAGGCATAAGA--------------------------
GATAAGATAAAGAAAGAATATGCACTT---TTCTATACACTTGATATAATGCGAGTAAATAAT----------
----

ACAAGCTATACATTGATAAATTGTAACACCTCAGTCATTAAACAA---
GCCTGTCCAAAGACATCATTTGAACCAATTCCCATACATTACTGTACCCCGGCTGGTTTTGCGATTCTAAAGT
GTAAT---GATAAGAAATTCAAT------GGAACAGGACTATGTAAAAAT----------------------
-GTTAGC---ACAGTACAATGTACACATGGAATTAAGCCAGTAGTGTCAACT---
CAACTGCTGTTAAATGGCAGTCTAGCAGAAGAAGAG------------
ATAATAATTAGATCTGCAAATTTCTCAAACAAT---------GCTAAAAACATAATAGTACAGCTGAAT----
-----------GAATCTATACCA---------ATTAATTGTACAAGACCCCACAACAATACAAGAAGA-----
-AGTATATCTTTT------------ACACCAGGGAGTGCA---TTT------GTTGCAACAGAAAAC------
---------ATAATAGGAGATATA------------AGACAAGCACATTGT---AACATTAGTAGAGAA---

AGATGGAATAACACCTTAGGAAAGATAGTTACAAAATTAAGA---------------GAACAATTTAGG----
-----------------------------------AATAAAACAATAGCCTTTAGG------
CCACCCATAGGAGGGGACCCAGAAATTGTA---ATGCACAGCTTTAATTGTGGAGGGGAA---
TTCAAGCAGCTCCAGGCAAGAGTCCTGGCTGTGGAAAGATACCTAAGGGATCAACAGCTCCTAGGGATTTGGG
GTTGCTCTGGAAAACTCATCTGCACCACTGCTGTGCCTTGGAATACTAGTTGGAGT-----------------
----------------------------AATAAATCTCTAAATACAATATGG------GAC------
AACATGACCTGGATGCAGTGG---GAAAGAGAAATTGACAACTACACAGGCATAATATAC---
AACTTACTTGAAGAATCGCAAAACCAACAAGAA--

AAGAATGAAAAAGACTTATTGGAATTGGATAAGTGGGACAGTTTGTGGAGTTGGTTTGATATATCACAATGGC
TGTGGTATATAAAAATATTCATAATGATAGTAGGAGGTTTAATAGGTTTAAGAATAATTTTTACT---
GTACTTTCTATAGTG---------AATAGAGTTAGGCAGGGATACTCACCA------
TTATCGTTCGCTGTTAGTTTGCTTAATACCATAGCTATAGCAGTAGCTGAG---
GGAACAGATAGGATCATAGAAGCAGTACAAAGAACTTGTAGAGCT----------------------------
--------ATTCTCAACATA------CCTAGAAGAATAAGACAGGGCTTAGAAAGGGCTTTGCTA---TAA
>AB564745
GTT---AAT---GCT---------------------------------
ACAGAACAATTGTGGGTCACAGTTTATTATGGGGTACCTGTGTGG---AAAGAAGCAAAC---
ACCACTCTACTTTGTGCATCAGATGCTAAAGCATATGATACAGAG---GCACATAAT----------------
--------------GTTTGGCCACA---CATGCCTGTGTACCCACAGAC---
CCCAACCCACAAGAAGTAAAAATGGAA---------AATGTAACAGAAAATTTTAAC----------------
--------------------------ATGTGGAAAAATAACATGGTAGAACAGATGCATGAGGATATA---
ATCAGTTTATGGATCAAAGTCTA---AAACCATGTGTAAAATTA---
ACCCCACTCTGCATTACTATGGATTGCACTGATGTCAAGAGGAATGCTAATAGCACCAATACCACCACT----
----
-------------------AGTGTTAATACCACTAGCGGGGGAATGGAGGGAGGAGAAATAAAA---
AACTGCTCTTTCAATATCACCACAAACATAAGA---------------------------
GATAAGGTGCAGCAACAATATGCACTT---TTTGACAAACTTGATATGGTACCAATAAATGATAAT-------
----

AATAATACAAGGACTAATAGCTATAGGTTGATAAGATGTAACACCTCAATCATTACACAG---
GCCTGTCCAAAGATAACCTTTGAGCCAATTCCCATACATTATTGTGCCCCGGCTGGTTTTGCGATTTTAAAGT
GTAAT---GATAAGAAATTCAAT------GGAACAGGACTATGTACAAAC-----------------------
-GTCAGC---ATAGTACAATGTACACATGGAATTAGGCCAGTAGTATCAACT---
CAACTGCTGTTAAATGGCAGTCTAGCAGAAGAAGAG------------
GTAGTAATTAGATCTGAAAATTTCACGGACAAT---------GCTAAAACCATAATAGTTCATCTGAAT----
-----------GAATCTGTAAAA---------ATTAATTGTACAAGACCTCACAAAAATAAAATAAGA-----
-ACGATACATATA------------GGACCAGGGAGAGCA---TTT------ATTGGAACA---GAC------
---------ATAGCAGGAAATATA------------AAACAAGCATATTGT---AACATTAGTGGAGAA---
GAATGGAGAGCACTTTAAAAAAGATAGTTAAAAAATTAGAA---------------GAACAATTTAAT----
-----------------------------------ACTACAAGAATAGCCTTTAAC------
CACTCCTCAGGAGGGGACCCAGAAATAGTA---ATGCACAGTGTTAATTGTGGAGGGGAA---
TTTAAGCAGCTCCAGGCAAGAATCCTGGCTGTGGAAAGATACCTAAAGGATCAACAGCTCCTGGGGATTTGGG
GTTGCTCTGGAAAACTCATTTGCACCACTACTGTGCCTTGGAATGCTAGTTGGAGT-----------------
----------------------------AATAAATCTCTGAATACAATTTGG------AAT------
AACATGACATGGATGGAGTGG---GAAAGAGAAATTGACAATTATACAGGCTTAATATAC---
AACTTAATTGAAAAATCGCAGATCCAGCAAGAA--

AAGAATGAACAAGAATTATTGGAATTGGATAAATGGGCAAGTTTGTGGAATTGGTTTGACATAACAAATTGGC
TGTGGTATATAAAAATATTCATAATGATAGTAGGAGGCTTGATAGGTTTAAGAATAGTTTTTACT---
GTACTTTCTATAGTG---------AATAGAGTTAGGCAGGGATACTCACCA------
TTATCGTTGGCTGTTAGCTTGCTCAACGCCATAGCCATAGCAGTAGCTGAG---
GGGACAGATAGGATTATAGAAGCATCACAAAGAGCTGGTAGAGCT----------------------------
--------ATTCTCAACATA------CCTAGAAGAATAAGACAGGGCTTAGAAAGGGCTTTGCTA---TAA
>AB604946
GCT---GCA--
GAACAATTGTGGGTCACAGTCTATTATGGAGTACCTGTGTGG---AAAGACGCATCC---
ACCACTTTGTTTTGTGCATCAGACGCTAAAGCATATGATACAGAA---GTACATAAT----------------
--------------GTTTGGCCACA---CATGCCTGTGTGCCCACAGAC---
CCCAACCCACAAGAAGTAGCATTGGAA---------AATGTAACAGAAAATTTTAAT----------------

-------------------------ATGTGGAAAAATAACATGGTAGATCAGATGCATGAGGATATA---
ATCAGTTTATGGATCAAAGTCTA---AAGCCATGTGTAAAATTA---
ACCCCACTCTGTGTTACTTTAAATTGCACTGATGATGTGAAGAATCTTACTAAAGTACTAAT----------
----
------------------------GGTAGTACCTGGGAAAAGATGGATAAAGGAGAAATAAAA---
AATTGCTCTTTCGATGTAACCACAAACATAAGA--------------------------
GATAAGGTGCAGAAAGAATATGCACTT---TTTTATAAACTTGATGTAGTACCAATAGAAAAT----------
----

GATAATACTAGCTATAGGATGATAAGTTGTAATACCTCAGTCATTACACAG---
GCCTGTCCAAAAATATCCTTTGAGCCAATTCCCATACATTATTGTACCCCGGCTGGTTATGCGATTCTCATGT
GTAAA---GATAAGAAATTCAAT------GGAACAGGACCATGTACAAAT----------------------
-GTCAGC---ACAGTACAATGTACACATGGAATTAGGCCAGTAGTATCAACC---
CAACTGCTGTTAAATGGCAGCCTAGCAGAAGAAGAG-----------
GTAGTAATTAGATCTGCTAATTTCTCAGACAAT--------GCTAAAACTATAATAGTACAGCTAAAT----
-----------GAATCTGTAAAA--------ATTGATTGTACAAGACCCAACAACAATACAAGAAAA-----
-AGTATACATATA-----------GCACCAGGGAGAGCA---TGG------TATGCAACAGGAGAT------
---------ATAATAGGAGACATA-----------AGACAAGCACATTGT---AACATTAGTAGAACA---
AACTGGACTAATACTTTAACACAGATAGTTGGAAAATTAACA--------------GAACAATTTAAG----
----------------------------------AATAAAACAATAATCTTTAAC------
CACTCCTCAGGAGGGGACCCAGAAATTGTA---ATGCACAGTTTTAATTGTGGAGGGGAA---
TTTAAGCAGCTCCAGGCCAGAGTCCTGGCTGTGGAAAGATACCTACAGGATCAACAGCTCCTGGGGATTTGGG
GTTGCTCTGGAAAACTCATCTGCGCCACTGCTGTGCCTTGGAATGCTAGTTGGAGT-----------------
---------------------------AATAAATCTCAGAGTGAGATTTGG------GAT------
AACATGACATGGATGCAGTGG---GAAAGAGAAATTGACAATTACACAGACTTAATATAC---
CTCTTACTTGAGAAATCACAAAACCAGCAAGAA---------------------------------------
----
AAGAATGAACAAGACTTATTGGAATTGGATAATTGGGCAAATCTGTGGAGTTGGTTTGACATATCAAATTGGC
TGTGGTATATAAGAATATTCATAATGATAGTAGGAGGCTTAATAGGTTTAAGAATAGTTTTTGCT---
GTGCTTTCTATAGTG---------AATAGAGTTAGGCAGGATACTCACCA------
TTATCGTTTGCTGTTAGCTTGTACAACGCCACAGCAATAGCAGTAGCTGAG---
TGGACAGATAGGGTTATAGAAGTAGCACAAAGAGCTTTTAGAGCT----------------------------
--------ATTCTCCACATA------CCTACAAGAATAAGACAGGGCTTGGAAAGGCTTTGCTA---TAA
>D12582
GCT---GCA---
GAACAATTGTGGGTCACAGTCTATTATGGGGTACCTGTGTGG---AAAGAAGCAACC---
ACCACTCTATTTTGTGCATCAGATGCTAAAGCATATGATACAGAG---GCACATAAT----------------
-------------GTTTGGGCCACA---CATGCTTGTGTACCCACAGAC---
CCCAACCCACAAGAAGTAGTATTGGTA--------AATGTGACAGAAAATTTTAAC----------------
-------------------------ATGTGGAAAAATAACATGGTAGAACAAATGCATGAGAATATA---
ATCAGTTTATGGATCAAAGCCTA---AAGCCATGTGTAAAATTA---
ACCCCACTCTGTGTTACTTTACATTGCACTGATTTGAGGAATACTACTAATAACAAT---------------
----
---------------------------AGTAGTATCGAGGAAAAGATGAAGGGAGAAATAAAA---
AATTGCTCTTTCAATGTCACCACAAACATAAGA--------------------------
GATAAGGTGCAGAAAGAATATGCACTT---TTTTATAAACTTGATGTAGTACCAATAGATAATGAT-------
----

AATAATAGTACTAATACATGCTATAGGTTGATAAGTTGTGACACCTCAGTCATTACCCAG---
GCCTGTCCAAAGGTATCCTTTGAGCCAATTCCCATACATTATTGTACCCCGGCTGGGTTTGCGCTTCTAAAGT
GTAAT---AATAAGACATTCAAT------GGAACAGGACCATGTAAAAAT----------------------
-GTCAGC---ACAGTACAATGTACACATGGAATTAGGCCAGTAGTATCAACT---
CAACTGCTGTTAAATGGCAGTCTAGCAGAAGAAGGG-----------
GTAGTAATTAGATCTGAAAATTTCACAGACAAT--------GTTAAAACCATAATAGTACAGCTGAAT----
-----------GAAACTGTAAAA--------ATTAATTGTATAAGACCCAACAACAAGACAAGAAAA-----
-AGGGTAACTATG-----------GGACCAGGGAGAGTA---TAT------TACACAACAGGAGAA------
---------ATAATAGGAGATATA-----------AGACAAGCACATTGT---AACATTAGTAGAGCA---
GAATGGAATAAAACTTTAGAACAGATAGCTAACAAATTAAGA--------------AAACAATTTGAG----
----------------------------------AATAAAACAATAGTCTTTAAT------
CAATCCTCAGGAGGGGACCCAGAAATTGTA---ATGCACAATTTTAATTGTGGAGGGGAA---

TTTAAACAGCTCCAGGCAAGAGTCCTGGCTGTGGAAAGATACCTAAAGGATCAACAGCTCATGGGGATTTGGG
GTTGCTCTGGAAAGTTCATCTGCACCACTGCTGTGCCTTGGAATACTAGTTGGAGT-----------------
----------------------------AATAAATCTTTTAATGAAATTTGG------GAT------
AACATGACCTGGATGGAGTGG---GAAAGAGAAATTAACAATTACACAAACTTAATATAC---
AACTTAATTGAAGAATCGCAGAATCAACAGGAA--

AAGAATGAACAAGACTTATTGGCATTAGATAAATGGGACAGTTTGTGGAATTGGTTTAGCATAACAAAATGGC
TGTGGTATATAAAAATATTCATAATGATAGTAGGAGGCTTGGTAGGTTTAAGAATAGTTTTTACT---
GTACTTTCTATAGTG---------AATAGAGTTAGGCAGGGATACTCACCA------
TTATCGTTTGCTGTTAGCTTGCTCAACGCCACAGCAATAGCAGTAGCTGAG---
GGGACAGATAGGGTTATAGAAATATTACAAAGAACTTATAGGGCT----------------------------
--------ATCCTCCACATA------CCTGTAAAAATAAGACAGGGCTTGGAAAGGGCTTTGCTA---TAA
>AB565497
GCT---ACA--
GAACAATTGTGGGTCACAGTCTATTATGGGGTACCTGTGTGG---AAAGAAGCAAAT---
ACCACTCTATTTTGTGCCTCAGATGCTAAAGCATATGATACAGAG---GTACATAAT----------------
--------------GTTTGGGCCACA---CATGCCTGTGTACCCACAGAC---
CCCAACCCACAAGAGGTAGTATTGGAA---------AATGTGACAGAAAATTATAAC----------------
--------------------------ATGTGGAAGAATAATATGGTAGAACAGATGCATGAGGATATA---
ATCAGTTTATGGGATCAAAGCCTA---AAGCCATGTGTAAAATTA---
ACTCCACTCTGTGTTACTTTAAATTGTACTGCATATAATAATAATACTAATACTACTGCTAAAACC-------
----
----------------------TCTGATAGTGGCTGGGGAACGGTAGTAAATGAAGAAGTAAAA---
AACTGCTCTTTCAATATTACCACAAGCATAAAA---------------------------
GATAAGGTGCAGACAGAATATGCACTT---
TTTTATAAAGCTGATATAGTACAAATAGATAATAGTGATAATATA----------------------------
----
GATAAGGATGATAATAATACTAGATATAATAATACATATAGGTTGATGAATTGTAACACCACAGTCATTACAC
AG---
GCCTGTCCAAAGGTATCCTTTGAGCCAATTCCCATACATTATTGTGCCCCGGCTGGTTTTGCGATTCTAAAAT
GTAAA---GATAAGAAGTTCAAT------GGAACAGGACCATGTAAAAAT--------------------------
-GTCAGT---ACAGTACAATGTACACATGGAATTAGGCCAGTAGTATCAACT---
CAATTACTGTTAAATGGCAGTTTGGCAGAAGAAGAG------------
GTAGTAGTTAGATCCGAAAATTTTACAGACAAT---------GCTAAAAACATAATAGTACAGCTGAAC----
-----------AAATCTGTAGTA---------ATTAATTGTACAAGACCCAACAACAATACAAGAAAA-----
-GGCATACATATG------------GGACCAGGAGGAGCA---CTG------TTTACAACA---GAA------
---------ATAACAGGAAATATA------------AGACAGGCACATTGC---AACATTAGTAGAGCA---
GAATGGAATGATACTTTACAAAAGATAGTTGAAAAATTAGGG---------------GAACAATTTAGC----
--------------------------------AAAAATAACACAATAGCCTTTAAT------
CACTCCTCAGGAGGGGACCCAGAAATTGTA---ATGCACACTTTTAATTGTGGAGGGGAA---
TTTAAGCAGCTCCAGGCAAGAGTCCTGGCTGTAGAAAGATACCTAAGGGATCAACAGCTCCTGGGGATTTGGG
GCTGCTCTGGAAAACTCATCTGCACCACTGCTGTACCTTGGAATGCTAGTTGGAGC-----------------
----------------------------AATAAATCTCAGAGCGAGATATGG------GAT------
AACATGACCTGGATGCAGTGG---GAAAAAGAAATTGACAATTATACAGAATTAATATAC---
ACCTTACTTGAAAAATCGCAGAACCAGCAGGAA--

AAGAATGAACAAGAACTATTGGAATTGGATAAATGGGCAAGTTTATGGAATTGGTTTGACATAACAAACTGGC
TGTGGTATATAAAAATATTCATAATGATAGTAGGAGGCTTGATAGGATTAAGAATAGTTTTTACT---
GTACTTTCTATAGTA---------AATAGAGTTAGGAAGGGATACTCACCC------
TTATCGTTCGCTGTTAGCTTGCTCAACGCCACAGCCATAGCAGTAGCTGGG---
GGGACAGATAGGGTTATAGAAATAGTACAAATAGTTGTTAGAGCT----------------------------
--------ATTCGCCACATA------CCTAGAAGAATAAGACAGGGATTGGAAAGGGCTTTGCAA---TAA
>AB565502
GCT---GCA---
AAATTGTGGGTCACAGTTTATTATGGGGTACCTGTGTGG---AAAGACGCAACC---
ACCACTCTATTTTGTGCATCAGATGCTAAAGCCTATGATACAGAG---GTACATAAT----------------
--------------GTTTGGGCCACA---CATGCCTGTGTACCCACAGAC---
CCCAACCCACAAGAAATGATATTGGAA---------AATGTGACAGAAAACTTTAAC----------------
--------------------------ATGTGGAAAAATAACATGGTAGATCAGATGCATGAAGATATA---
ATCAGTTTATGGGATGAAAGCCTA---AAGCCATGTGTAAAATTA---

ACCCCACTCTGTGTTACTTTAAATTGCACTAATTTGAATGCTACTAATACTAATAGTACGGCT----------
----
------------------------AATATTACCAGTAGTCCAATGGGGGTAGGAGAAATGAAA---
AACTGCTCTTTCAATGTTACCACAAGCATACAA---------------------------ACTAAGGTG---
AAAGACTATGCACTT---TTTTATAAAGTTGATGTAATACCAATAGATAAT---------------------
----
-------------TCTACCAATTATAGGTTAAGAGAGTGTAACACTTCAGTCATTACACAA---
GCTTGTCCAAAGGTATCCTTTGAGCCAATTCCTATACATTATTGCACCCCGGCTGGTTTTGCGATTATAAAGT
GTAAC---AATAAACAGTTCAAT------GGATCAGGGGAATGTAAAAAT----------------------
-GTCAGT---ACAGTACAATGTACACATGGAATTAAACCAGTAGTATCAACT---
CAACTGCTGTTAAATGGCAGTTTAGCAGAAGAAGAT-----------
ATAGTACTTAGATCTAGCAATTTTACAGACAAT---------GCTAAAGTTATAATAGTACAGCTGAAT----
-----------AAAGCTATAGAA---------ATTAATTGTACAAGACCCAACAACAATACAAGAAAA-----
-AGTATACCTATG------------GGACCAGGGCAAGCA---TGG------TTTGCAACAGGAGAT------
---------ATAATAGGAGATATA------------AGACAAGCACATTGT---AATCTTAGTAGCACA---
ATATGGAATGACACTTTAGGACAGATAGTTGAAAAATTAAGA---------------GAACAATTTGGG----
-----------------------------------AACAAAACAATAAGCTTTCAG------
CGATCCTCAGGAGGGGACCCAGAGGTTGTA---ATGCACACTTTTAATTGTAGAGGGGAA---
TTTAAGCAGCTCCAGGCAAGAGTCCTGGCTGTGGAAAGATACCTAAAGGATCAACAGCTCCTAGGGATTTGGG
GTTGCTCTGGAAAACTCATCTGCACCACTTCTGTGCCTTGGAATGCTAGTTGGAGT-----------------
----------------------------AATAAAACTTATGAGCAGATTTGG------GAA------
AACATGACTTGGATGCAGTGG---GAAAAAGAAATTGACAATTACACAGGAGAAATATAC---
AACTTAATTGAAGTAGCGCAAAACCAGCAAGAA--

AAAAATGAACTAGAATTATTGGAATTGGATAACTGGGCAAGCTTGTGGAATTGGTTTGACATAACAAAGTGGC
TGTGGTATATAAAAATATTCATGATGATAGTAGGAGGCTTAATAGGTTTAAGAATAGTTTTTGCT---
GTACTTTCTATAGTG---------AATAGAGTTAGGCAGGGATACTCACCA------
TTGTCATTAGCTGTTAGCTTGCTCAATACCACAGCTGTAGCAGTAGCGGAG---
GGGACAGATAGGGTTATAGAAGTAGTACAAAGGGCTTATAGAGCT---------------------------
--------ATTCTCAACATA------CCTACAAGAATAAGACAGGGCTTAGAAAGGGCTTTGCTA---TAA
>KT961002
GCT---GCA--
GAACAATTGTGGGTCACAGTCTATTATGGGGTACCTGTGTGG---AAAGAAGCAACC---
ACCACTCTATTTTGTGCATCAGATGCTAAAGCATATGATACAGAG---GTACATAAT---------------
--------------GTTTGGGCCACA---CATGCCTGTGTACCCACAGAC---
CCCAACCCACAAGAAATAGGATTGGAA---------AATGTGACAGAAAATTTTAAT---------------
-------------------------ATGTGGAAAAATAACATGGTAGAACAGATGCAGGAGGATATA---
ATCAGTTTATGGGATCAAAGCCTA---AAACCATGTGTAAAATTA---
ACCCCACTCTGTGTTACTTTAGATTGCACTGATGCTGTTGGTACCAATAGTAGTAGCAAG-------------
----
------------------------GATACTAATATCAATAATAGTAATGGGGGAGAAATAAAA---
AACTGCTCATTCAACATCACCACAAACATGAGA--------------------------
GATAAGGTGCAGAAAGAATATACAACT---TTTTATAAACTTGATGTAGTACCAATAGACAATAAT-------
----

AATAATACTAGATATAGGTTGATAAGTTGTAACACCTCAGTCATTACACAG---
GCCTGTCCAAAGGTAACCTTTGAGCCAATTCCCATACATTATTGTACCCCGGCTGGTTTTGCGATTCTAAAGT
GTAGA---GATAAGAAGTTCAAT------GGAAAAGGACCATGTAAAAAT----------------------
-ATCAGC---ACAGTACAATGTACACATGGAATTAGGCCAGTAGTATCAACT---
CAACTGTTGTTAAATGGCAGTCTAGCAGAAGGAGAG-----------
GTAGTAATTAGATCTGAGAATTTCACGGACAAT---------GCCAAAACCATAATAGTACAGCTGAAT----
-----------GAATCTGTACAA---------ATTAATTGTACAAGACCCAACAACAATACAAGAAAA-----
-AGTATACATATA------------GGACCCGGGAGTGCA---TTT------TATACAACAGGACAA------
---------ATAATAGGAAATATA------------AGACAAGCATATTGT---ACCATTAGTGAAGCA---
AAATGGAATAACACTTTAAAAAAGATAGCTATAAAATTAAGA---------------GAACAATTTAAC----
-----------------------------------AATAATACAATAATCTTTAAT------
CACTCCTCAGGAGGGGACCCAGAAATTGTA---ATGCACAGTTTTAATTGTGGAGGAGAA---
TTTAAGCAGCTCCAGGCAAGAGTCCTGGCTGTGGAAAGATACCTAAGGGATCAACAGATCCTGGGGATTTGGG
GCTGCTCTGGAAAACTCATTTGCACCACTGCTGTGCCTTGGAATGCTAGTTGGAGT----------------
----------------------------AATAAATCTCTGAATGAGATTTGG------GAT------

AACATGACATGGATGCAGTGG---GAAAGAGAAATTGACAATTACACAGGCTTAATATAC---
AACTTACTTGAAGAATCGCAGAATCAGCAGGAA--

AAGAATGAACAGGAATTATTGGAATTGGATAAATGGGCAGGTTTGTGGAGTTGGTTTAACATAACAAACTGGC
TGTGGTATATAAGATTATTCATAATGATAGTAGGAGGCTTGATAGGTTTAAGAATAGTTTTTGCT---
GTGCTTTCTATAGTA---------AATAGAGTTAGGCAGGGATATTCACCA------
TTATCGTTTGCTGTTAGCTTGCTCAACGCCATAGCCATAGCAGTAGCTGAG---
GGGACAGATAGGGTTATAGAAGGATTGCAAAGAGCTTTTAGAGCT----------------------------
--------ATTCTCCACATA------CCTAGAAGAATAAGACAGGGCTTGGAAAGGCTTTGCTA---TAA
>AB742153
ACT---GCA--
GAAAAATTGTGGGTCACAATCTATTATGGGGTACCTGTGTGG---AAAGACGCAGTC---
ACCACTCTATTTTGTGCATCAGATGCTAAGGCCTATGATACAGAG---GTGCATAAT----------------
--------------GTTTGGCCACA---CATGCCTGTGTACCCACAGAC---
CCCAGCCCACAAGAAGTAGTATTGGGA---------AATGTGACAGAAAATTTTAAC----------------
--------------------------ATGTGGAAAAATAACATGGTAGATCAAATGCATGAAGATATA---
ATCAGTTTATGGGATCAAAGCTTA---AAACCATGTGTAAAATTA---
ACCCCACTCTGTGTTACTTTAGAGTGCTTTGATTATTGTGGGAATAGTACTGCTAAGAATGATACTAACTGT-
----
----------------AGCAATAATACTGATGACTGGCGACAAGTGGAGACAGGAGAAATAAAA---
AATTGCTCTTTCAATATCACCACAAACATAAGA---------------------------
GATAAGATACAGAAACAATATGCAATT---
TTTAATAGACTTGATATAGTAAAAATAGATGATGGTGAGAAAAACAAAAAG----------------------
----
GATGAGAAAAATAGGACTATTGAGAGCTATAGGATAACAAGTTGTAACACCTCAGTCATTACACAG---
GCCTGTCCAAAGGTATCCTTTGAACCAATTCCCATACATTTTTGTGCCCCGGCTGGGTATGCGATTCTGAAAT
GTAAT---AATAATACATTCAAT------GGATCAGGACCATGTACAAAT-----------------------
-GTTAGC---ACAGTACAATGTACACATGGAATTAGGCCAGTAGTGTCAACT---
CAACTACTGTTAAATGGCAGTCTAGCAGAAGATGAG------------
GCAAAAATTAGATCTGAAAATTTCACAAACAAT---------GCTAAAACCATAATAGTACAGCTACAT----
-----------AAGCCTGTGTAC---------ATTAATTGTACAAGACCCAACAACAATACAAGAAAA-----
-GGTATACATATG------------GGACCAGGGAGAGCA---TTT------TATGGAACA---GAT------
---------ATAATAGGAGATATA------------AGACAAGCACATTGT---AACATTAGTAAAACA---
GCATGGAATAACACTTTAAAACAGATAGTTGCCAAATTAAGA---------------GAACAATTTGGG----
--------------------------------AAAAATAAAACAATAGCGTTTAAT------
CACTCCTCAGGAGGGGACCCAGAAATTGTA---ATGCATACTTTTAATTGTAGAGGGGAA---
TTTAAACAGCTCCAGGCAAGAGTCCTGGCTCTGGAAAGATACCTAGTGGACCAACAGCGCCTGGGGATTTGGG
GCTGCTCTGGAAGACTCATTTGCACCACTGCTGTGCCTTGGAATACTAGTTGGAGT-----------------
----------------------------AATAAATCTTATAATGCAATTTGG------GAA------
AACATGACCTGGATGGAGTGG---GAAAGAGAAATTAACAATTACACAGAGGAGATATAC---
AGATTACTTGAAGAATCGCAAAACCAGCAGGAC--

AAGAATGAACAAGAATTATTGGCATTAGATAAATGGTCAAATTTGTGGAATTGGTTTAACATAACAAAATGGC
TGTGGTATATAAGAATATTCATAATGATAGTAGGAGGCTTGATAGGTTTAAGAATAGTTTTTACT---
GTACTTTCTATAGTG---------AATAGAGTTAGGCAGGGATACTCACCA------
TTATCGTTGGCTGTTAGCTTGCTCAACGCCACCGCCATAGCAGTAGCTGAG---
GGAACAGATAGGATTATAGAAGTCTTACAAAGAGTTGGTAGAGCT----------------------------
--------ATCCTCCACATA------CCTAGAAGAATAAGGCAGGGCGCGGAAAGAGCTTTGGTA---TAA
>AB641836
GCT---GAT--
GAAAAGTTGTGGGTCACAGTCTATTATGGGGTACCTGTGTGG---AAAGAAGCAACC---
ACCACTTTATTTTGTGCATCAGATGCTAAAGCACATAATACAGAG---GCACATAAT----------------
--------------GTTTGGCCACA---CATGCCTGTGTACCCACAGAC---
CCCAACCCACAAGAAATGGTATTGGGA---------AATGTAACAGAAAATTTTAAC----------------
--------------------------ATGTGGAAAAATAACATGGTAGAACAGATGCATGAGGATATA---
ATCAGCTTATGGGATCAAAGCTTA---AAGCCATGTGTAAAATTA---
ACTCCACTCTGTGTTACTTTAAATTGCACTGATGCTAATACCAGAAATAATACCAATAGTAAC----------
----
-------------------------AGCAGTAGTACAGAAATACAACAACAGGGAGAAATAAAA---
AACTGCTCTTTCAATGTCACCACAAACATAAGA---------------------------

GATAAGATAAGTAAGGAATATGCACTG---TTTTATAGGCTGGATGTAGTACAAATAGGGAATGAGAAT----
----

AATACCAATTATACCAACTATAGGTTGATAAATTGTAACACCTCAGAAATTACACAG---
ACTTGTCCAAAGGTATCCTTTGAGCCAATTCCCATACATTATTGTACCCCGGCTGGTTTTGCGCTTCTAAAGT
GTAAT---GATAAGAAGTTCAAT------GGAACAGGAACATGTAAAAAT----------------------
-GTCAGC---ACAGTACAATGTACACATGGAATTAAGCCAGTGGTATCAACT---
CAACTGCTATTGAATGGCAGCTTAGCAGAGGAAGAG-----------
GTAATAATTAGATCTGAAAATTTCACGAACAAT---------GCTAAAACCATAATAATACAGCTGAAT----
-----------GAAGCTGTAGAA---------ATTAACTGTACAAGACCCAACAATAATACAAGAAGA-----
-AGTATACATATA------------GGACCAGGGAGAACA---ATT------TTTGCAACA---GAC------
---------ATAATAGGAGATATA------------AGACAAGCACATTGT---AACATTAGTGGTACA---
AAGTGGAATAAAACTTTAAAACAGATAGCCAAAAAGTTAGGA---------------GATCAATTTGGA----
--------------------------------GAAAATAAAACAATAAGCTTTAAC------
AAATCCTCAGGAGGGGACCCAGAAATTACA---ATGCACAGTTTTATTTGTGGAGGAGAA---
TTTAAACAGCTCCAGGCAAGAGTCCTGGCTGTAGAAAGATACCTACGGGATCAGCAGCTCCTAGGGATTTGGG
GTTGCTCTGGAAAACTCATCTGCACCACTGCTGTGCCTTGGAATGCTAGTTGGAGT-----------------
---------------------------AATAAAAGTCTAAAGGGGATTTGG------GAT------
AACATGACCTGGATGGAGTGG---GAAAAAGAAATTGACAATTACACAGAATTAATATAC---
AGTTTGCTTGAAGAATCGCAAAACCAGCAGGAA---------------------------------------
----
AAGAATGAACAAGAATTATTGGAATTGGACAAGTGGGCAAGTTTGTGGAGTTGGTTTGACATATCAAACTGGC
TGTGGTATATTAGAATATTCATAATAATAGTAGGAGGCTTGATAGGTTTAAGAATAGTCTTTGCT---
GTGCTTTCTATAATC---------AATAGAGTTAGGCAGGGATACTCACCA------
TTGTCATTGGCTATTAGCTTGTACAATACTATAGCTATTGCAGTAGCTGAA---
GGGACAGATAGGGTTATAGAAGTAGCACACAGAATTGGTAGAGCT----------------------------
--------ATTTGCAACATA------CCTAGAAGAATAAGGCAGGGAGCAGAAAGAGCTTTGCAA---TAA
>KU869602
GTT---GTA---
GGAGAATTGTGGGTCACAGTCTATTATGGGGTACCTGTGTGG---AAAGAAGCAACC---
ACCACCCTATTTTGTGCATCAGATGCTAAAGCATATGACACAGAA---GTACATAAT----------------
--------------GTTTGGGCCACA---CATGCCTGTGTACCCATAGAC---
CCCAACCCACAAGAAGTAGTATTAGAA---------AATGTAACAGAAAATTTTAAT---------------
--------------------------GTGTGGAAAAATGACATGGTAGAACAGATGCATGAGGATATA---
ATCAATTTATGGGATCAAAGCCTA---AAGCCATGTGTAAAATTA---
ACTCCACTCTGTGTTACTTTAAATTGTAGTAATTTGAAGAATGATACTAAGGCTAATAATGGTAGTAAT----
----
------------------ACCAATAATAGTAGTGGGAGAGTGGTGGAAAAAGGAGTAATGAAA---
AACTGCTCTTTCAATGTTACCACAAATAGAAGA--------------------------
AATAAGCCTCGGAAAGAATATGCTCTT---TTTTATGAACTTGATGTAACACAAATAGATGAT---------
----

ACAAGCTATATATTGACACATTGTAACACCTCAGTCATTACACAG---
GCCTGTCCAAAGGTATCCTTTGAACCAATTCCCATACATTATTGTACCCCGGCTGGTTTTGCGATTCTGCAAT
GTAAT---GATAAGGAGTTCAAT------GGAAAAGGACCATGTTCAAAT----------------------
-GTCAGC---ACAGTACAATGTACACATGGAATTAGGCCAGTAGTATCAACT---
CAACTGTTGTTAAATGGCAGTTTAGCAGAAGGAGAG-----------
ATAGTAATCAGATCTGAAAATTTCACGGACAAT---------GCTAAAACCATAATAGTACAGCTGAAC----
-----------GCATCTGTAGAG---------ATTAATTGTACAAGACCTAACAACAATACAAGAAAA-----
-GGTATACATCTA------------GGACCAGGGAAAACA---TTT------TTTGCAACAGGAGAA------
---------GTAATAGGAGATATA------------AGACAAGCACATTGT---AACATTAGTAAAGGA---
AAATGGGAAAACACTTTAAGACAACTAGCAAAAAAATTAAGA---------------AAACAATTTGAG----
-----------------------------------AATAGAACAATAGTCTTTAAT------
CAACCCTCAGGAGGGGACCCAGAGATTGTA---ATGCACAGTTTTAATTGCGGAGGGGAA---
TTCAAGCAGCTCCAGGCAAGAGTCCTGGCTGTGGAAAGATACCTAAAGGATCAGCAGCTCCTGGGGATTTGGG
GATGCTCTGGAAAGCTCATCTGCACCACTAATGTGCCTTGGAATGCTAGTTGGAGT-----------------
---------------------------AATAAATCTCTGAATAAGATTTGG------GAC------
AACATGACCTGGATGGAGTGG---GACAGAGAAATTAACAATTACACAAGCTTAATATAC---
ACCTTACTTGAAGAATCGCAGAACCAACAAGAA---------------------------------------
----

AAGAATGAACAGGAATTATTGGAATTGGATAAATGGGCAAGTTTGTGGAATTGGTTTGACATATCAAACTGGC
TGTGGTATATAAAAATATTCATAATGATAGTAGGAGGCTTGGTAGGTTTAAGAATAATTTTTTCT---
GTACTTTCTATAGTA---------AATAGAGTTAGGCAGGGATATTCACCA------
ATATCGTTTGCTGTTAGTTTGCTTAATGCCACAGCTAGAGCAGTAGGTGAG---
GGGACAGATAGGATTATAGAAATAATACAAAGAACTTATAGAGCT---------------------------
--------ATTCTCCACATA------CCTAGAAGAATAAGACAGGGCTTGGAAAGGGCTTTGCTA---TAA
>MN043590
GTT---GTA---------------------------------------
GGAGAATTGTGGGTCACAGTCTATTATGGGGTACCTGTGTGG---AAAGAAGCAACC---
ACCACCCTATTTTGTGCATCAGATGCTAAAGCATATGACACAGAA---GTACATAAT---------------
--------------GTTTGGGCCACA---CATGCCTGTGTACCCATAGAC---
CCCAACCCACAAGAAGTAGTATTAGAA---------AATGTAACAGAAAATTTTAAT---------------
--------------------------GTGTGGAAAAATGACATGGTAGAACAGATGCATGAGGATATA---
ATCAATTTATGGGATCAAAGCCTA---AAGCCATGTGTAAAATTA---
ACTCCACTCTGTGTTACTTTAAATTGTAGTAATTTGAAGAATGATACTAAGGCTAATAATGGTAGTAATACCA
AT---
-------------------------AATAGTAGTGGGAGAGTGGTGGAAAAAGGAGTAATGAAA---
AACTGCTCTTTCAATGTTACCACAAATAGAAGA--------------------------
AATAAGCCTCGGAAAGAATATGCTCTT---TTTTATGAACTTGATGTAACACAAATAGATGAT---------
----

ACAAGCTATATATTGACACATTGTAACACCTCAGTCATTACACAG---
GCCTGTCCAAAGGTATCCTTTGAACCAATTCCCATACATTATTGTACCCCGGCTGGTTTTGCGATTCTGCAAT
GTAAT---GATAAGGAGTTCAAT------GGAAAAGGACCATGTTCAAAT----------------------
-GTCAGC---ACAGTACAATGTACACATGGAATTAGGCCAGTAGTATCAACT---
CAACTGTTGTTAAATGGCAGTTTAGCAGAAGGAGAG-----------
ATAGTAATCAGATCTGAAAATTTCACGGACAAT---------GCTAAAACCATAATAGTACAGCTGAAC----
-----------GCATCTGTAGAG---------ATTAATTGTACAAGACCTAACAACAATACAAGAAAA-----
-GGTATACATCTA------------GGACCAGGGAAAACA---TTT------TTTGCAACAGGAGAA------
---------GTAATAGGAGATATA------------AGACAAGCACATTGT---AACATTAGTAAAGGA---
AAATGGGAAAACACTTTAAGACAACTAGCAAAAAAATTAAGA---------------AAACAATTTGAG----
-----------------------------------AATAGAACAATAGTCTTTAAT------
CAACCCTCAGGAGGGGACCCAGAGATTGTA---ATGCACAGTTTTAATTGCGGAGGGGAA---
TTCAAGCAGCTCCAGGCAAGAGTCCTGGCTGTGGAAAGATACCTAAAGGATCAGCAGCTCCTGGGGATTTGGG
GATGCTCTGGAAAGCTCATCTGCACCACTAATGTGCCTTGGAATGCTAGTTGGAGT-----------------
----------------------------AATAAATCTCTGAATAAGATTTGG------GAC------
AACATGACCTGGATGGAGTGG---GACAGAGAAATTAACAATTACACAAGCTTAATATAC---
ACCTTACTTGAAGAATCGCAGAACCAACAAGAA---------------------------------------
----
AAGAATGAACAGGAATTATTGGAATTGGATAAATGGGCAAGTTTGTGGAATTGGTTTGACATATCAAACTGGC
TGTGGTATATAAAAATATTCATAATGATAGTAGGAGGCTTGGTAGGTTTAAGAATAATTTTTTCT---
GTACTTTCTATAGTA---------AATAGAGTTAGGCAGGGATATTCACCA------
ATATCGTTTGCTGTTAGTTTGCTTAATGCCACAGCTAGAGCAGTAGGTGAG---
GGGACAGATAGGATTATAGAAATAATACAAAGAACTTATAGAGCT---------------------------
--------ATTCTCCACATA------CCTAGAAGAATAAGACAGGGCTTGGAAAGGGCTTTGCTA---TAA
>MN043603
GCT---ACA---------------------------------------
GGAGGATTGTGGGTCACAGTCTATTATGGGGTACCTGTGTGG---AAAGAAGCAACC---
ACCACCCTATTTTGTGCATCAGATGCTAAAGCACATAACACAGAG---GTACATAAT---------------
--------------GTTTGGGCCACA---CATGCCTGTGTACCCACAGAC---
CCCAACCCACAAGAAGTAGTATTGGAA---------AATGTGACAGAAAATTTTAAC---------------
--------------------------ATGTGGAAAAATAACATGGTAGAACAGATGCATGAGGATATA---
ATCAGTTTATGGGATCAAAGCCTA---AAGCCATGTGTAAAATTA---
ACTCCACTCTGTGTTACTTTGAATTGTAGTAATTTGAATAATACTAGTAATTTGAAGAATACTACTGATGCCA
ATACTACTATT--
----------------------ACCAATAACAGTAGTAGAGTAGAGGGGGAAGAAGAAATGACA---
AACTGCTCTTTCAATGTTACCACAAACATAAGA--------------------------
AATAAGGTGCAAAAAGAATATGCTCTT---TTTTATAAACTTGATGTAGTACCAATAGATAAT---------
----

ACAAGCTATACGTTGATACATTGTAACACCTCAGTCATTACACAG---
GCCTGTCCAAAGGTATCCTTTGAACCAATTCCCATACATTATTGTACCCCGGCTGGTTTTGCGATTCTACAAT
GTAAT---GATAAGAAGTTCAAT------GGAACAGGACCATGTACAAAT----------------------
-GTCAGC---ACAGTACAATGTACACATGGAATTAGGCCAGTAGTATCAACC---
CAACTACTGTTAAATGGCAGTCTAGCAGAAGAAGAG-----------
ATAGTAATTAGATCTGAAAATTTCACAGACAAT---------GCTAAAACCATAATAGTACAGCTGAAC----
-----------GCATCTGTAGAA---------ATTAATTGTACAAGACCCAACAATAATTCAAGAAAA-----
-GGTATAGGTATA------------GGACCAGGGAGCACA---TTT------TATGCAACAGGAGAT------
---------ATAATAGGAAATATA------------AGACAAGCACATTGT---AACATTAGTGAAGCA---
AAATGGAACAACACTTTAAAACAGCTAGCTATAAAATTAGGA---------------GAACAATTTGGG----
-----------------------------------AATAAAACAATAGCCTTTAAG------
CAATCCTCAGGAGGGGACCCAGAGATTGTA---ATGCACAGTTTTAATTGTGGAGGGGAA---
TTTAAGCAGCTCCAGGCAAGAGTCCTGGCTGTGGAAAGATACCTAAAGGATCAACAGCTCCTGGGGATTTGGG
GATGCTCTGGAAAGCTCATCTGCACCACTAATGTGCCTTGGAATACTAGTTGGAGT-----------------
----------------------------AATAAATCTCTAAATAGGATCTGG------GAT------
AACATGACCTGGATGGAGTGG---GACAGAGAAATTAACAATTACACAAACTTAATATAC---
TCCTTACTTGAAGAATCGCAGAACCAACAAGAA--

AAAAATGAACAGGACTTATTGAAATTGGATACATGGGCAAGTTTGTGGAATTGGTTTGACATATCACGATGGC
TGTGGTATATAAGAATATTCATAATGATAGTAGGAGGCTTGATAGGTTTAAGGATAATTTTGTCT---
GTACTTTCTATAGTG---------AATAGAGTTAGGCAGGGATACTCACCA------
TTATCGTTTGCTGTTAGTTTGCTTAATGCCACAGCTATAGCAGTAGCTGAG---
GGGACAGATAGGGTTATAGAAATAATACAAAGAGCTTGTAGAGCT----------------------------
--------ATTCTCCACATA------CCTACACGCATAAGACAGGGCTTGGAAAGGGCTTTGCTA---TAA
>JQ316128
GCT---ACA---
GGAGGATTGTGGGTCACAGTCTATTATGGGGTACCTGTGTGG---AAAGATGCAAAA---
ACCACCCTATTTTGTGCATCAGATGCTAAAGCATATAGCACAGAG---GCACATAAT----------------
--------------GTTTGGGCCACA---CATGCCTGTGTACCCGTAGAC---
CCCAATCCACAAGAGGTAGACTTGAAA---------AATGTGACAGAAAATTTTAAC---------------
-------------------------ATGTGGAAAAATAACATGGTAGACCAGATGCATGAGGATATA---
ATCAGTTTATGGGATCAAAGCCTA---AAGCCATGTGTAAAATTA---
ACTCCACTCTGTGTTACTTTGAATTGTACCCATAATGTGACTGGTACTAATGCCACTAACAGTGGTGCTACTA
CCAATAGT---
-------------------ACTACTACTACTACCACTACTAATATTGATGACGGAAACATGAAA---
AACTGCTCTTTCAATGTTACCACAAACATAGGA---------------------------
AATAAGTTGCAGAAAGAATATGCTCTT---TTTTATAAACTTGATGTAGTACCAATAGATAAGGATAATACA-
----

AGCAATAATGATAATACAAGCTATACGTTGATACATTGTAACTCCTCAGTCGTTACACAG---
GCCTGTCCAAAGGTATCCTTTGAACCAATTCCCATACATTATTGTGCCCCGGCTGGTTTTGCGATTCTAAAGT
GTAAA---GATAAGAAGTTCAAT------GGAACAGGACCATGTAAAAAT----------------------
-GTCAGC---ACAGTACAATGTACACATGGAATTAGGCCAGTAGTATCAACT---
CAACTGCTGTTAAATGGCAGTCTAGCAGAAGAAGAC-----------
ATAGTAATTAGATCTGAAAATTTCACGGACAAT---------GCTAAAACCATAATAGTACAGCTGAAC----
-----------GAATCTGTAGAA---------ATTAATTGTACAAGACCCAACAACAATACAAGAAAA-----
-AGTATACATATG------------GGGCCAGGGAGAACA---TAT------TATGCAACAGGGGCC------
---------ATAATAGGAAATATA------------AGACAAGCACATTGC---AACATTAGTGAAGAA---
AAATGGAATAAAACTTTAAACCAGATAGCTACAAAATTAAGA---------------GAACAATATAAT----
-----------------------------------AAAACAAAAATATTCTTTGCT------
CCATCCTCAGGAGGGGACCCAGAAATTACA---ATGCACCGTTTTATTTGTGGAGGGGAA---
TTTAAGCAGCTCCAGGCAAGAATCCTGGCTGTGGAGAGATACCTAGGGGATCAACAGCTCCTGGGGATTTGGG
GATGCTCTGGAAAGCTCATCTGCACCACTAATGTGCCTTGGAATACTAGTTGGAGT-----------------
----------------------------AATAAATCTCTGAATAAGATCTGG------GAT------
AACATGACCTGGATGGAGTGG---GACAGAGAAATTAGCAATTACACAGGCTTAATATAC---
AACTTACTTGAAGAATCGCAGAACCAGCAAGAA--

AAGAATGAACAGGAATTATTGGCACTGGATAAGTGGGCAAGTTTGTGGAGTTGGTTGGACATAACAAAATGGC
TGTGGTATATAAAAATATTCATAATGATAGTAGGAGGCTTGGTAGGTTTAAGAATAATTTTTTCT---
GTACTTTCTATAGTG---------AATAGAGTTAGGCAGGGATACTCACCA------

TTATCGTTTGCTGTTAGTTTGCTTAATGCCATAGCTATAACAGTAGCTGAG---
GGGACAGATAGGGTTATAGAAATAGTACAAAGAGCTTACAGAGCT----------------------------
--------ATTCTCCACATA------CCTACACGAATAAGACAGGGCTTGGAAAGGGCTTTGCTA---TAA
>JQ316132
GCT---GCA---ACA------------------------------------
GGAGGAATGTGGGTCACAGTCTATTATGGGGTACCTGTGTGG---AAAGAAGCAACC---
ACCACACTATTCTGTGCATCAGATGCTAAAGCATATGACACAGAG---GTACATAAT----------------
--------------GTTTGGGCCACA---CATGCCTGTGTACCCATAGAC---
CCCAACCCACAAGAAGTAAGATTGGAA---------AATGTGACAGAAAATTTTAAC----------------
--------------------------ATGTGGAAAAATAACATGGTAGACCAGATGCATGAAGATATA---
ATCAGTTTATGGATCAAAGCTTA---AATCCATGTGTAAAATCT---ACTACATTC-----------------
--AGTATTACTAATAGTAGTAGTAATCATAATACTACTAGCACCAATAGTACTAATACC--------------
----
ACTAGTAGTATTGGGGGAGGGATGGAGGGAGGAGAAATGAAA---
AACTGCTCTTTCAATGTTACCACAAGCATAAGA---------------------------
GATAAGGTGAAGAAAGAATTTGCTCTT---TTTTATAAACTTGATGTAAAACAAATAGATAATACA-------
----

GATAATACAAGCTATACATTGATAAATTGTAACTCCTCAGTCATTACACAG---
GCCTGTCCAAAGGTATCCTTTGAACCAATTCCAATACATTATTGTACCCCAGCTGGTTTTGCGCTTCTACAAT
GTAAT---GATAAAAAGTTCAAT------GGAACAGGACCATGTTCAAAT-----------------------
-GTCAGC---ACAGTACAATGTACACATGGAATTAAGCCAGTAGTATCAACT---
CAACTGCTGTTAAATGGCAGTCTGGCAGAAGGAGAG------------
ATGATAATTAGATCTGAAAATTTCACAAACAAT---------GCTAAAACCATAATAGTACAGCTGAAT----
-----------GAATCTGTAGAA---------ATTAAGTGTATAAGGCCCAACAACAATACAAGAAAA-----
-GGTATACATATA------------GGACCAGGGAGTACA---CTT------TATGCAACAGGAGAA------
---------ATAATAGGAGATATA------------AGACAAGCACATTGT---AACATTAGTAGAAAA---
CAATGGAACAACACATTAACACAGCTAGTTAAAAAATTAAAA---------------GAACAATATGGT----
--------------------------------AATAATAAAACAATAGTCTTTGCT---
CAACCCCCCCCAGGAGGGGACCCAGAGATTGTA---ATGCACAGTTTTAATTGTGGAGGGGAA---
TTTAAGCAGCTCCAGGCAAGAGTCCTGGCTGTAGAAAGATACCTAAGGGATCAACAGCTCCTGGGGATTTGGG
GATGCTCTGGAAAGCTCATCTGCACCACTAATGTGCCTTGGAATACTAGCTGGAGT-----------------
----------------------------AATAAATCTTATCATGAGATCTGG------GAT------
AATATGACCTGGATGCAGTGG---GACAGAGAAATTAACAATTACACAAGCTTAATATAC---
ACCTTACTTGAAGAATCGCAGAACCAGCAAGAA--

AAGAATGAACAGGACCTATTGGCATTCGACAAATGGGCAAGTTTGTGGAATTGGTTTGACATATCAAACTGGC
TGTGGTATATAAGAATATTCATAATGATAGTAGGAGGCTTAATAGGTTTAAGAATAATTTGTTTT---
GTACTTACTATAGTG---------AAGAGAGTTAGGCAGGATATTCACCA------
TTGTCATTTGCTGTTAGTTTGCTTAATGCCACAGCTGTAGCAGTAGCTGAG---
GGGACAGATAGGATTATAGAAGTAGTACAAAGATTTTTTAGAGCT----------------------------
--------ATCCTTAACATA------CCTACAAGAATAAGACAGGGTTTTGAAAGGGCTTTGCTA---TAA
>JQ316127
GCT---ACA--
GAAAAATCGTGGGTCACAGTCTATTCTGGGGTACCTGTGTGG---AAAGAAGCAACC---
ACCACCCTATTTTGTGCCTCAGATGCTGAAGCATATGATACAGAG---GTACATAAT----------------
--------------GTTTGGGCCACA---CATGCCTGTGTACCCATAGAC---
CCCAACCCACACGAAGTAGTATTAGAA---------AATGTGACAGAAAATTTTAAC----------------
--------------------------ATGTGGAAAAATAACATGGTAGAACACATGCATGAGGATATA---
ATCAGTTTATGGATCAAAGCCTA---AACCCATGTGTAAAACTA---
ACTCCACTCTGTGTTACTTTAAAATGTAGTGATGAGAATATTAATAGTAATAGTACCAATAATACT-------
----
-------------AGTACCGATGATAGTAGTGGGGGAGGAGTGATGGAGAAAGGAGAAATGAAA---
AACTGCTCTTTCAATGTTACCACACACCTATAT---------------------------
GATAAGGTGAAAAAAGAATATGCTCTT---TTTTATAGACTTGATGTAGTACCAATAGATAATACA-------
----

GATAATACCAGCTATACGTTGATAAATTGTAACACCTCAACCATTACACAG---
GCCTGTCCAAAGGTATCCTTTGAACCAATTCCCATACATTATTGTACCCCGGCTGGTTTTGCGATTCTACAGT
GTAAT---GATAAGAAGTTCAAT------GGAACAGGACCATGTAAAAAT-----------------------

-GTCAGC---ACAGTACAGTGTACACATGGAATTAGGCCAGTAGTATCAACT---
CAACTGCTGTTGAATGGCAGTCTAGCAGAAGGAGAG-----------
GTAATAATTAGATCTGAAAATTTCACAGACAAT---------GCTAAAACCATAATAGTACAGCTGAAC----
-----------GCATCTGTAGAA---------ATTAATTGTACAAGACCCGGCAACAATACAAGGAGA-----
-AGTATACGTATA------------GGACCAGGGAGAGCA---TTT------TATACAACAGGAGAT------
---------ATAACAGGAGATATA------------AGACGAGCATATTGT---AACATTAGTAGAAAA---
GATTGGGAAAACACTTTAAAACAGCTAGTTATAAAACTAAGA---------------GAACAATTTGGG----
-----------------------------------AATAAAACAATAGCCTTTAAT------
CGATCCTCAGGAGGGGACCCAGAGATTGTA---ATGCACAGTTTTAATTGTGGAGGGGAA---
TTTAAGCAGCTCCAGGCAAGAGTCCTGGCTGTGGAGAGATACCTAAAGGATCAACAGCTCCTGGGGATTTGGG
GATGCTCTGGAAAGCTCATCTGCACCACTAATGTGCCTTGGAATACTAGTTGGAGT-----------------
----------------------------AATAAATCCCTGGATAAGATTTGG------AAT------
AACATGACCTGGATGGAGTGG---GACAGAGAAATTAACAATTACACAAACTACATATAC---
ACCTTACTTGAAGAATCGCAGAACCAACAGGAA--

AAGAATGAACAGGACTTATTGGCATTTGATAAATGGGCAAATTTGTGGAGTTGGTTTGACATATCAAGATGGC
TGTGGTATATAAAAATATTCATAATGATAGTAGGAGGCTTGGTAGGTTTAAGAATAATTTGTTCT---
GTACTTTCTATAGTG---------AATAGAGTTAGGCAGGATACTCACCA------
TTATCGTTTGCTGTTAGTTTGCTTAATGCCACAGCTATAGCAGTAGCTGAG---
GGGACAGATAGGATTATAGAAATATTACAAAGAGTTTATAGAGCT----------------------------
--------ATTCTCCACATA------CCTACAAGAATCAGACAGGGCTTAGAAAGGCTTTGCTA---TAA
>JQ316135
GCT---GCA---ACA------------------------------------
GAAGACTTGTGGGTCACAGTCTATTATGGGGTACCGGTGTGG---AAAGAAGCAACC---
ACCACCCTATTTTGTGCATCAGATGCTAAAGCATATGACACAGAG---GTACATAAT----------------
--------------GTTTGGCCACA---CATGCCTGTGTACCCATAGAC---
CCCAACCCACAAGAAGTAGTATTGGAA---------AATGTGACAGAGAATTTTAAC----------------
--------------------------ATGTGGAAAAATAATATGGTAGAACAAATGCATGAGGATATA---
ATCAGTTTATGGGATCAAAGTCTA---AAGCCATGTGTAAAATTA---
ACTCCACTCTGTGTTACTCTGCATTGTAGTAATTTGAATTGTAGTAATTTGAGTAATACTAATACCAGTACTT
TGAATTGTAGTAATTTGAAT---
----------AATACTAATACCAATAATAGTAGTGGGGGAGTGATGGAGGAAGGAGAAATGAAA---
AACTGCTCTTTCAATGTTTCCACAAACATAAAA---------------------------
AATAAGATGCAAAAAGAATATGCTCTT---TTTTATAAACCTGATGTAATGCCAATAGATAAT----------
----

GATGATACAAGCTATACGTTGATAAATTGTAACACCTCAGTCATTACACAG---
GCCTGTCCAAAGGTATCCTTTGAACCAATTCCCATACATTATTGTGCCCCGGCTGGTTTTGCGATTCTACAGT
GTAAT---GATAAGAAGTTCAAT------GGAACAGGACCATGTTCAAAT------------------------
-GTCAGC---ACAGTACAATGTACACATGGAATTAGGCCAGTAGTATCAACT---
CAACTACTGTTAAATGGTAGTCTAGCAGAAGAAGAG-----------
ATAGTAATTAGATCTAAAAATCTCACAGACAAT---------GCTAAAACCATAATAGTACAGCTGAAC----
-----------AAATCTATAGAG---------ATTAATTGTACAAGACCCGGCAACAATACAAGAAAA-----
-AGTATAAGGATA------------GGACCAGGGAGTACA---TTT------TATGCAACAGGAGAC------
---------ATAATAGGAGATATA------------AGACAAACACATTGT---AACATTAGTGAAGCA---
AACTGGAACAACGCTTTAAAACAGCTAGCTATAAAATTAAGA---------------GAACAATTGGAA----
-----------------------------------AGTCAAACAATAGTCTTTAAT------
CAATCTTCAGGAGGGAACCCAGAGATTGTA---ATGCACAGTTTTAATTGTGGAGGGGAA---
TTTAAGCAGCTCCAAGCAAGAGTCCTGGCTGTGGAAAGATACCTAAGGGATCAACAGCTCCTGGGGATTTGGG
GATGCTCTGGAAAGCTCATCTGCACCACTAATGTGCCTTGGAATACTAGTTGGAGT-----------------
----------------------------AATAAATCTCGGAAGGATATCTGG------GAT------
AACATGACCTGGATGGAGTGG---GACAGAGAAATTAGCAATTACACAACCTTAATATAC---
ACCTTGCTTGAAGAATCGCAGAACCAGCAAGAA--

AAGAATGAACAGGAATTATTGGGATTGGATAAATGGGCAAGTTTGTGGAATTGGTTTGACATAACATACTGGC
TGTGGTATATAAGAATATTCATAATGATAGTAGGAGGCTTGGTAGGGTTAAGAATAATTTGTTTT---
GTGCTTTCTATAGTA---------AATAGAGTTAGGCAGGATACTCACCA------
TTATCGTTTGCTGTTAGTTTGCTTAATGCCACAGCTAGAGCAGTAGCTGAG---
GGGACAGATAGGGTTATAGAAATATTACGAAGAGCTTATAGAGCT----------------------------
--------CTTCTCCACATA------CCTAGAAGAATAAGACAGGGCTTCGAAAGGCTTTGCTA---TAA

>JQ316130
GTT---GCA--
GGAGGATTGTGGGTCACAGTCTATTATGGGGTACCTGTGTGG---AAAGAAACAACC---
ACCACCCTATTTTGTGCCTCAGATGCTAAAGCATATGACACAGAG---GCACATAAT----------------
--------------GTTTGGGCCACA---CATGCATGTGTACCCATAGAC---
CCCAACCCACAAGAAGTAGTATTGGAA---------AATGTGACAGAAAATTTTAAC----------------
--------------------------ATGTGGAAAAATAACATGGTAGAACAGATGCATCAGGATATA---
ATCAGTTTATGGGATCAAAGCCTA---AAGCCATGTGTAAAATTA---
ACTCCACTCTGTGTTACTTTAAATTGTAGTAATTACAATGGGACTTACAATGCGACTAGTACCAATGGG----
----
-------------ACTACTACTAGTAGTCCTACTACTAGTAATGTAGAGGAAGGAGAAATGAAA---
AACTGCTCTTTCAATGTTACCACCACCATAAGA---------------------------
GATAAGATGAAGAAGGAACATGCTCTT---TTTTATAGACTTGATGTAGCACCACTAGAAAAT----------
----

AATAATACAACCTATACGTTGATGAATTGTAACTCCTCAACCCTTACACAG---
GCCTGTCCAAAGGTATCCTTTGAACCACTTCCCATACATTATTGTACCCCGGCTGGTTTTGCGCTTCTACAAT
GTAAT---GATAAAAAGTTCAAT------GGAACAGGACCATGTTCAAAT-----------------------
-GTCAGC---ACAGTACAATGTACACATGGAATTAGGCCAGTAGTATCAACT---
CAACTGCTGTTAAATGGCAGCCTAGCAGAAGAAGAA------------
GTAGTAATTAGATCTGAAAATTTCACAAACAAT---------GCTAAGACCATAATAGTACAGCTGAAC----
-----------GCATCTGTAGAA---------ATTAATTGTACAAGACCCAACAACAATACAAGAAGA-----
-AGTATAAGAATA-----------GGACCAGGGAGCGCA---TTT------TATACAACAGGAGAA-------
---------GTAATAGGAAATATA------------AGACTAGCATATTGT---AACATTAGTAGAGAA---
AAATGGAACAAAACCTTAGAACAGCTAGTTATAAAATTAGGA---------------GAACAATTTAAT----
-----------------------------------AAAACAATAATAACCTTTGAT------
AACTCCTCAGGAGGGGACCCACAGATTGTA---ATGCACAGTTTTAATTGTGGAGGGGAA---
TTTAAGCAGCTCCAAGCAAGAGTCCTGGCTGTGGAAAGATACCTAAAGGACCAACAGCTCCTGGGGATTTGGG
GATGCTCTGGAAAGCTCATCTGCACCACTAATGTGCCTTGGAATACTAGTTGGAGT-----------------
----------------------------AATAAATCTCTAAAGGCTATCTGG------GAT------
AACATGACCTGGATGGAGTGG---GACAGAGAAATTAACAATTACACAAACTTGATACAC---
AACTTACTTGAAGAATCGCAAATCCAACAAGAA--

AAGAATGAACAGGAATTATTGGAATTGGATAAATGGGCAAGTTTGTGGAACTGGTTTAGCATAACACAATGGC
TGTGGTATATAAAAATATTCATAATAGTAGTAGGAGGCTTGGTAGGTTTAAGAATAATCTTTTCT---
GTACTTTCTATAGTA---------AATAGAGTTAGGCAGGGATACTCACCC------
TTATCGTTTGCTGTTAGTTTGCTTAATGCCACAGCTATAGTAGTAGCTGAG---
GGGACAGATAGAGTTATAGAAATATTACAAAGAGCTTATAGAGCT----------------------------
--------GTCCTCCACATA------CCTAGAAGAATAAGACAGGGCTTGGAAAGGGCTTTGCTA---TAA
>DQ295195
GCT---GCA---ACA------------------------------------
GGAGGATTGTGGGTCACAGTCTATTATGGGGTACCTGTGTGG---AAAGAAGCAACC---
ACCACCCTATTTTGTGCATCAGATGCTAAAGCATATGACACAGAG---GTACATAAT----------------
--------------GTTTGGGCCACA---CATGCCTGTGTACCCATAGAC---
CCCAACCCACAAGAAGTAGTATTGCCA---------AATGTGACAGAGAATTTTAAC----------------
--------------------------ATGTGGAAAAATAACATGGTAGAACAGATGCATGAGGATATA---
ATCAGTTTATGGGATCAAAGCCTA---AAGCCATGTGTAAAATTA---
ACTCCACTCTGTGTCACTTTAAATTGTAATATTAATAGTAGTAGTGGTGGTAGTAATACTACTAGTACTAAT-
----
-------------ACTACTACTACTAGTAGTAGTAAAGGAGTGATGGTGGAAGAAGGAATAAAG---
AACTGCTGTTTCATCATTGCCACCACCAGCAGA------------------------
AGTGATAAGGTGAAAAAAGAATATGCTCTT---TTTTATAAACTTGATGTAGTGAAAACAGATAAT-------
----

ACTAGCAATTCAAGCTATACGCTGATGAGTTGTAACTCCTCAGTCGTTACCCAG---
GCTTGTCCAAAGGTATCCTTTGAACCAATTCCCATACATTATTGTACCCCGGCTGGTTTTGCGATTCTACAGT
GTAAT---GATAAGAAGTTCAAT------GGATCAGGACCATGTTCAAAT-----------------------
-GTCAGC---ACAGTACAATGCACACATGGAATTAGGCCAGTAGTATCAACT---
CAACTGCTGTTAAATGGCAGTCTAGCAGAAGAAGAT------------
GTAGTAGTTAGATCTGAAAATTTTACAAACAAT---------GCTAAAATCATAATAGTACAGCTGATT----

-----------ATACTTGTAATA---------ATTAATTGTACAAGACCCAACAACAATACAAGAAAA-----
-AGTATAAGTTTT------------GCACCAGGGAGCACT---ATT------TATGCAACAGGAGGT------
---------ATAATAGGAGATATA------------AGACAAGCACATTGT---AACGTTAGTAAAAT---
GAATGGAATGACACTTTAAAGGAAATAGTTATAAAATTAAGA---------------GAACAATTTGGA----
-----------------------------------AATGAAACAATAAAATTTAAT------
CGATCCTCAGGAGGGGACCCAGAGATTGAA---ATGCACAGTTTTAATTGTGGAGGGGAA---
TTTAAGCAGCTCCCAGCAAGAGTCCTGGCTGTGGAAAGATACCTAAGGGATCAACAGCTCCTGGGGATTTGGG
GATGCTCTGGAAAGCTCATCTGCACCACTAATGTACCTTGGAATACTAGTTGGAGT-----------------
----------------------------AATAAATCTCAGGATGATATCTGG------CAT------
AACATGACCTGGATGGAGTGG---GACAGAGAAATTAACAATTACACAAGCTTAATATAC---
ACCTTACTTGAGGAATCGCAGAACCAGCAAGAG---

AAGAATGAACAGGAATTATTGGAATTGGATAAATGGGCAAGTTTGTGGAGTTGGTTTGACATAACAAACTGGC
TGTGGTATATAAAAACATTCATAATGATAGTAGGAGGCTTGGTAGGATTAAGAATAATTTTTTCT---
GTGCTTTCTATAGTA---------AATAGAGTTAGGCAGGGGTACTCACCG------
TTATCGTTTGCTGTTAGTTTGTTTAATGCCACAGCTATAGCAGTAGCTGAG---
GGGACAGATAGGGTTATAGAGATATTACAAAGAGCTTATAGGGCT----------------------------
--------ATTCTCCACATA------CCTACAAGAATAAGACAGGGCTTGGAAAGGGCTTTGCTA---TAA
>DQ295193
GTT---GCA---
GGAGGATTGTGGGTCACAGTCTATTATGGGGTACCTGTGTGG---AAAGAAGCAACT---
ACCACTCTATTTTGTGCATCAGATGCTAAGGCATATGACACAGAG---GTACATAAT----------------
--------------GTTTGGGCCACA---CATGCATGTGTACCCATAGAC---
CCCAACCCACAAGAAGTAGTATTGGCA---------AATGTGACAGAAAATTTTAAC----------------
--------------------------ATGTGGAAAAATAACCTGGTAGAACAAATGCATGAGGATATA---
ATCAGTTTATGGGATGAAAGCCTA---AAGCCATGTGTAAAATTA---
ACTCCACTCTGTGTTACTTTAAATTGTAGTAATTACAATGGGACTAGTACCAATGGGACTAAT----------
----
-------------------------ATCAATAATAGTAGTAGGATGGAGGAAGGAGAAATGAAA---
AACTGCTCTTTCAATGTTACCACAACCATAAGA--------------------------
GATAGGGTGCAGAAGGAATATGCTCTT---TTTTATAAACTTGATGTAGTACCAATAGATAAT----------
----

GATAATACAAGCTATACATTGATAAATTGTAACTCCTCAACCCTTACACAG---
GCCTGTCCAAAAGTATCCTTTGAACCAATTCCCATACATTATTGTACCCCGGCTGGTTTTGCGATTCTAAAGT
GTAAT---AATAAGACGTTCAAT------GGAACAGGACCATGTTCAAAT-----------------------
-GTCAGC---ACAGTACAATGTACACATGGGATTAGGCCAGTAGTATCAACT---
CAACTGCTGTTAAATGGCAGTCTAGCAGAAGAAGAG------------
ATAGTAATTAGATCTGAAAATATCACAGACAAT---------GCTAAAACCATAATAGTACAGCTGAAC----
-----------GCATCTGTACAA---------ATTAATTGTACAAGACCCAACAACAATACAAGAAAA-----
-AGTATAAGGATA------------GGACCAGGGAGCACA---TTC------TATGCAACAGGGGAC------
---------ATAATAGGAGATATA------------AGACAAGCACATTGT---AACATTAGTGAAAAA---
GAATGGAACAACACTTTACAACAGCTAGYTATAAAATTAAGA---------------GAACACTTTGGG----
-----------------------------------AATAAAACAATAATCTTTAAT------
GAATCCTCAGGAGGGGACCCAGAGATTGTA---ATGCACAGTTTTAATTGTGGAGGGGAA---
TTTAAGCAGCTCCAAGCAAGAGTCCTGGCTGTGGAAAGATACCTAAAGGATCAACAGCTCCTGGGGATTTGGG
GATGCTCTGGAAAGCTCATCTGCACCACTAATGTGCCTTGGAATACTAGTTGGAGT-----------------
----------------------------AATAAAACTCTAGAATATATCTGG------GGT------
AACATGACCTGGATGGAGTGG---GACAGAGAAATTAACAATTACACAGACTTAATATAC---
AACTTACTTGAAAAAATCGCAGAACCAACAAGAA--

AAGAATGAACAGGAATTATTGGAATTGGATAAATGGTCAAGCTTGTGGAATTGGTTTGACATAACACAATGGC
TGTGGTATATAAGAATATTCATAATGATAGTAGGAGGCTTGGTAGGTTTAAGAATAATTTTTTCT---
GTACTTTCTATAGTG---------AATAGAGTTAGGCAGGGATACTCACCA------
TTATCGTTTGCTGTTAGTTTGCTTAATGCCACAGCTAGAGTAGTAGCAGAG---
GGGACAGATAGGGTTATAGAAATATTACAAAGAGCTTATAGAGCT----------------------------
--------ATTATCCACATA------CCTAGAAGAATAAGACAGGGCTTGGAAAGGGCTTTGCTA---TAA
>JQ316126
GCT---GCA---
GGAGGATTGTGGGTCACTGTCTATTATGGTGTACCTGCGGGG---AGAGATGCTGAC---

ACCACCCTATTTTGTGCATCATATGCTGCTGCATCTGACGCCGAG---GCACATAAT----------------
--------------GTTTGGGCCACA---CCTGCCTGTGTACCCATAGAT---
CCCAACCCACAAGAGGTAGTATTGGAA---------AATGTGACAGAAAATTTTAAC----------------
--------------------------ATGTGGAAAAATAACATGGTAGAACAGATGCATGAGGATATA---
ATCAGTTTATGGGATCAAAGCCTA---AAGCCATGTGTACAACTA---
ACTCCACTCTGTATTACTTTGAATTGTAGAAATTGGAATAGAAATAATACTAATGCGAATACTATTACT----
----
----------------------AATAGCAGTAGTGTGAAAGCGATGGAGGCAGACGAAGTGAAA---
AACTGTTCTTTCAATGTTACAAATACAAACATA------------------------
AAAGATAAAAGGCAGAGGGAATATGCCCTT---TTTTATAAATATGATCTAATGCCAATAGAGAAT-------
----

AAGACTAATATAAGCCTCTATACGTTGATACATTGTAACACCTCAGTCATTACACAG---
GCCTGTCCAAAGGTATCCTTTGAACCAATTCCCATACATTATTGTACCCCGGCTGGTTTTGCGATTCTACAGT
GTAAT---GATAAGAAGTTCAAT------GGAGCAGGACCATGTAGAAAT-----------------------
-GTCAGC---ACAGTACAACGTACACATGGAATTAGGCCAGTAGTATCAACT---
CAACTGCTGTTAAATGGCAGTCTAGCAGAAGAAGAG------------
ATAATAATTAGATCTGAAAACTTCTCGGACAAT---------GCTAAAACCATAATAGTACAGCTGAAC----
-----------GTATCTGTAGAA---------ATTAATTGTACAAGGCCCAACAATAATATAGAAAGA-----
-AGGATAAGTATA------------GGACCAGGGAGAGCA---TTT------CGTGCAATAAAAGCT------
---------ATAACAGGAGATATA------------AGAAAAGCACATTGT---AACATTAGTGAAGAA---
AATTGGAATGACACTTTAAAACAGCTAGCTATAAAATTAAGT---------------GAACAATTTGGG----
-----------------------------------AATAAATCAATAGTCTTTACG------
CAACCCTCAGGAGGGGACCCAGAGATTGTA---ATGCACAGTTTTAATTGTGGAGGAGAA---
TTTAAGCAGCTCCAGGCAAGAATCCTGGCTGTGGAAAGATATCTACAGGATCAACAGCTCCTGGGGATTTGGG
GATGCTCTGGAAGGCTCATCTGCACCACTAATGTGCCTTGGAATACTAGTTGGAGT-----------------
----------------------------AATAAATCTCTGAAATATATCTGG------GAT------
AACATGACCTGGATGGAGTGG---GACAGAAAAATTAACAATTACACAAGCTTAATATAC---
ACCTTACTTGAAGAATCGCAAAACCAACAAGAA--

AAGAATGAACAGGAATTATTGGAATTGGATAAATGGGCAAGTTTGTGGAATTGGTTTGACATAACCAACTGGC
TGTGGTATATAAGACTATTCATAATGATAGTAGGAGGCTTGGTAGGTTTAAGAATAATTTTCTTT---
GTACTTTCTTTAGTG---------AATAGAGTTAGGCAGGGATACTCACCA------
TTATCGTTTGCTGTTAGTTTGCTTAATGCCACAGCTATAGCAGTAGCTGAG---
GGGACAGATAGAGTTATAGAAATAGTACAAAGAGCTTTTAGAGCT----------------------------
--------ATTCTCCACATA------CCTACAAGAATAAGACAGGGCTTGGAAAGGGCTTTGCTA---TAA
>DQ295196
GCT---GTA--
GAACCATTGTGGGTCACAGTCTATTATGGGGTACCTGTGTGG---AAAGAAGCAACC---
ACCACCCTATTTTGTGCATCAGATGCTAAAGCATATGACACAGAG---GTACATAAT----------------
--------------GTTTGGGCCACA---CATGCCTGTGTACCCATAGAC---
CCCAACCCACAAGAAGTAGTATTGGAA---------AATGTGACAGAAAATTTTAAC----------------
--------------------------ATGTGGGAAAATAACATGGTAGAACAGATGCATGAGGATATA---
ATCAGCTTATGGGATCAAAGCCTA---AAGCCATGTGTAAAATTA---
ACTCCACTCTGTGTTACCTTAAATTGTAGTAATTATGTGAAGAATGATACTAATACCACTAATAGTAAT----
----
-------------------AATAATGATACCACTAAGGGAGTGAGGGAGGAAGGAGAACTGAAA---
AACTGCTCTTTCAATGTTACCACAAGCATAAGA---------------------------
AATAAGATGAAGCAAGAATATGCTCTT---TTTTATAAACTTGATGTAGAACAAATAGATAAT----------
----

ACAAGCTATACGTTGATAAATTGTAACTCCTCAGTCATTACACAG---
GCCTGTCCAAAGGTATCCTTTGAACCAATTCCCATACATTATTGTACCCCGGCTGGTTTTGCGATTCTACAGT
GTAAT---GATAAGAAGTTCAAT------GGAACGGGACCATGTTCAAAT-----------------------
-GTCAGC---ACAGTACAATGCACACATGGAATTAGGCCAGTAGTATCAACT---
CAACTGCTGTTAAATGGCAGTCTAGCAGAAGAAGAG------------
ATAGTAATTAGATCTGAAAATTTCACGAACAAT---------GCTAAAACCATAATAGTACAGCTGAAC----
-----------GCATCTGTAGAA---------ATTAATTGTACAAGACCCAACAACAATACAAGAAGA-----
-GGTATACATATA------------GGACTAGGGAGAACG---TTT------TATGCAACAGGAGAA------
---------ATAGTAGGAAATATA------------AGACAAGCACATTGT---AACATTAATAAAACA---

AAATGGGACAACACTTTAAGACAGCTAGTTATAAAATTAAAA---------------GAACAAGTTGGG----
-----------------------------------AATAAAACAATAGCCTTTGAT------
CAACCCTCAGGAGGGGACCCAGAGATCGTA---ATGCACAGTTTTAATTGTGGAGGGGAA---
TTCAAGCAGCTCCAGGCAAGACTCCTGGCTGTGGAAAGATACCTAAAGGATCAACAGCTCCTGGGGATTTGGG
GATGCTCTGGAAAGCTCATCTGCACCACTGCTGTGCCCTGGAATACTAGTTGGAGT-----------------
----------------------------AATAAATCTCAGGATGAAATCTGG------CAT------
AACATGACCTGGATGCAGTGG---GACAGAGAAATTAACAATTACACAAGCTTAATATAC---
ACCTTGCTTGAAGAATCGCAGAACCAACAAGAA--

AAGAATGAACAGGAATTATTGGAATTGGATAAATGGGCAAGTTTGTGGAATTGGTTTGACATAACACAATGGC
TGTGGTATATAAAAATATTCATAATGATAGTAGGAGGCTTGATAGGTTTAAGAATAATTTGTTCT---
GTACTTTCTATAGTA---------AATAGAGTTAGGAAGGGATACTCACCA------
CTATCGTTTGCTGTTAGTTTGCTTAATGCCACAGCTATAGCAGTAGCTGAG---
GGGACAGATAGGGTTATAGAAATATTACAAAGGGCTTATAGAGCT----------------------------
--------ATCCTCCACATA------CCTAGAAGAGTAAGACAGGGCTTGGAAAGGGCTTTGCTA---TAA
>AY839827
GCT---GCA---GCA------------------------------------
GGAGGCTTGTGGGTTACAGTCTATTATGGGGTACCTGTGTGG---AAAGAAGCAACC---
ACCACACTATTTTGTGCATCAGATGCTAAAGCATATGACACAGAG---GTACATAAT----------------
--------------GTTTGGGCCACA---CATGCCTGTGTACCCATAGAC---
CCCAACCCACAAGAAGTAAGATTGGAA---------AATGTGACAGAAAATTTTAAC----------------
--------------------------ATGTGGAAAAATAACATGGTAGATCAGATGCATGAGGATATA---
ATCAGTTTATGGGATCAAAGCCTA---AAGCCATGTGTAAAATTA---
ACTCCACTCTGTGTTACTTTACATTGTAGTAATGTGAATGTTACTAATAGTACTACTGCCAGTAAA-------
----
-------------------AATAGTAATAGTAGTGTACCATTAATGGAGGTAGGAGAAGTGAAA---
AACTGTTCTTTCAATGTTACCACAAGTATAAGA---------------------------
GATAAGGTGAAGAAAGAATATGCTCTT---TTTTATAAACTTGATGTAGAAAAAATAGATAAT----------
----

ACAAGTACATTGATAGATTGTAACTCCTCAGCCATTAACACACAG---
GCCTGTCCAAAGGTGTCCTTTGAACCAATTCCCATACATTATTGTACCCCGGCTGGTTTTGCGATTCTACAGT
GTAAT---GATAAGAAGTTCAAT------GGATCAGGACCATGTTCAAAT-----------------------
-GTCAGC---ACAGTACAATGTACACATGGAATTAAGCCAGTAGTAACAACT---
CAACTGCTGTTAATGGCAGTCTAGCAGAAGAAGAA------------
ATAGTAATTAGATCTGAAAATTTCTCGAACAAT---------GCTAAAACCATAATAGTACAGCTGAAT----
-----------GCATCTGTAGAA---------ATTAATTGTACAAGACCCAACAACAATACAAGAAAA-----
-AGTATACCTATG------------GGACCAGGGGGTATG---ATT------TATGCAACAGGAGCC------
---------ATAATAGGAGATATA------------AGACAAGCACATTGT---AACATTAGTAGAGCA---
AAATGGAACAATACTTTAGAACAGCTAGCTAAAAAATTTAGA---------------GAACGATATGGG----
--------------------------------AATAATACAACAATAATCTTTAAT---
CCACAACCCCCAGGAGGGGATCCAGAGGTTGAA---ATGCACAGTTTCAATTGTGGAGGGGAA---
TTTAAGCAGCTCCAAGCAAGAGTCCTGGCTGTGGAAAGATACCTAAAGGATCAACAGCTCCTAGGGATTTGGG
GATGCTCTGGAAAGCTCATCTGCACCACTACTGTGCCTTGGAATACTAGTTGGAGT-----------------
----------------------------AATAAATCTCAGGATGCTATCTGG------AAG------
AACATGACCTGGATGCAGTGG---GACAGAGAAATTAGCAATTACACAGGTTTGATATAC---
TCCTTACTTGAAGAATCGCAGACCCAGCAAGAA--

AAGAATGAACAGGAATTATTGAAATTGGATAATTGGGCAGATTTGTGGAGTTGGTTTGACATAACACGATGGA
TGAAGTATGTAAAAATATTCATGATGGTAATAGGAGGCTTGATAGGTTTAAGAATAATTTTTTAT---
GTGCTTTCTATAGTG---------AATAGAGTTAGGCAGGGATACTCACCA------
TTATCGTTTGCTGTTAGTTTGCTTAATGCTACAGCTAGAGCAGTAGCTGAG---
GGGACAGATAGGCTTATAGAAATATTACAAAGAACTTATAGAGCT----------------------------
--------ATCCTCCACATA------CCTACAAGAATCAGACAGGGCTTGGAAAGGGCTTTGCTA---TAA
>DQ837381
GCT---AAA--
GAGCAGTTGTGGGTCACAGTCTATTATGGGGTACCTGTGTGG---AAAGAAGCCAAC---
ACCACTTTATTTTGTGCATCAGATGCTAAAGCATACAGTACAGAG---GTACATAAT----------------
--------------GTTTGGGCCACA---CATGCCTGTGTACCCACAGAT---
CCCAACCCACAAGAAATAGTATTGGAA---------AATGTAACAGAAAATTTTAAT----------------

--------------------------ATGTGGAAAAATAACATGGTAGAACAGATGCATGAAGATATA---
ATCAGTTTATGGGATCAAAGCCTA---AAGCCATGTGTAAAATTA---
ACTCCACTCTGTGTTACTTTAAAGTGCACTAATAATGTGAATGATACTACCACTAATGGCACT----------
----
----------------------TCTAAGTGGAATGGTGATAATAGCACTATAGGAGAAATAAAA---
AAATGCTATTTCAATACTACCACAGGCATGATA---------------------------
GATAAGATGAAGGAAGAATATGCATTA---
TTTTATAACCTTGATATAGTACCACTAGATAATAGTCTTGCTAAGAAT-------------------------
----
AGTAGTCATAATAAGACCAGCTATGCCAATTATAGGTTGATAAGTTGTAACACCTCAGTCATTAAACAG---
GCCTGTCCAAAGATATCCTTTGAGCCAATTCCAATACATTATTGTACCCCGGCTGGTTTTGCGATTCTAAAGT
GTAAC---GATACAAAGTTCAAT------GGAACAGGACCATGTAAAAAT-----------------------
-GTCAGC---ACAGTACAATGCACACATGGAATTAGGCCAGTAGTGTCAACT---
CAATTACTGTTAAATGGCAGTTTAGCAGAAGAAGAC------------
ATAGTAATTAGATCTGAAAATATCACGAACAAT---------GCTAAAAACATAATAGTACAGCTGAAT----
-----------GAACTTGTAAAA---------ATTAATTGTACAAGACCCAACAACAATACAAGAAAA-----
-AGTATACATATA------------GGACCAGGGAAAGCA---TTT------TATGCAGCGGGAGAC------
---------ATAATAGGAAACATA------------AGACAAGCACACTGT---AACATCAGTAGGAAA---
AGCTGGACTGACACTTTAAAAAAGATAGCTGACAAATTAAGT---------------GAACAATTTGGG----
-----------------------------------AATAAAACAATAGTCTTTAAA------
CCCTCCTCAGGAGGGGACCCAGAAATAGTA---ATGCACAGTTTTACTTGTGGAGGGGAA---
TTCAAGCAGCTCCAGGCAAGAGTCCTAGCTGTGGAGAGATACCTAAGGGATCAACAGCTCCTAGGGATTTGGG
GTTGCTCTGGAAAACTCATCTGCACCACTACTGTACCTTGGAATTCGAGTTGGAAT-----------------
----------------------------AACAGAAACCTGAGTCAGATTTGG------GAT------
AACATGACCTGGATGCAGTGG---GAAAAGGAAATTGACAATGTCACAAGTGTAATATAC---
AGGTTAATTGAAAAATCGCAGAACCAGCAAGAA--

AAGAATGAACAAGAATTATTGGAATTGGATAAATGGGCAAATCTGTGGAATTGGTTTGACATAACAAATTGGC
TGTGGTATATAAAAATATTCATAATGATAGTAGGAGGCTTGATAGGTTTAAGAATAGTCTTTACT---
GTACTTTCTATAGTG---------AATAGAGTTAGGCAGGGATACTCACCT------
TTATCGTTTGCTGTTAGCTTGTTCGACGCCACAGCAATAGCAGTAGCTGAG---
GGGACAGATAGGGTTATAGAAGCAGTACAAAGAGCTGGTAGAGCT----------------------------
--------CTCCTTCACATA------CCTAGAAGAATAAGACAGGGCTTAGAAAGGGCTTTGCTA---TAA
>JQ316134
GCT---ACA--
GAAACATTGTGGGTCACAGTCTATTATGGGGTACCTGTGTGG---AAAGAAGCGACC---
ACCACCCTATTTTGTGCATCAGATGCTAAAGCATATGACACAGAG---GTACATAAT----------------
--------------GTTTGGCCACA---CATGCCTGTGTACCCATAGAC---
CCCAACCCACAAGAAGTAGTATTGGCA---------AATGTGACAGAATATTTTAAT----------------
--------------------------ATGTGGAAGAATAACATGGTAGAACAGATGCATGAGGATATA---
ATCAGTTTATGGGATCAAAGCCTA---AAGCCATGTGTAAAATTA---
ACTCCACTCTGTGTTACTTTGCATTGTAGTAATTGGACGGTTAATTTCACCAAGAATAGTAATACTACT----
----
----------------------TTTAACAGTAGTAGTAGTGATGAAATGATAAAGGAAGTAAAA---
AACTGCTCTTTCAATGTTACCACAACCATAAGA---------------------------
GATAAGGTGCAAAAAGAATATGCTCTT---TTTTATAAACTTGATGTAGTGAAAACAGATAATACT-------
----

AGCAATTCAAGCTATACGCTGATGAGTTGTAACTCCTCAGTCGTTACCCAG---
GCTTGTCCAAAGGTATCCTTTGAACCAATTCCCATACATTATTGTACCCCGGCTGGTTTTGCGATTCTACAGT
GTAAT---GATAAGAAGTTCAAT------GGATCAGGACCATGTTCAAAT-----------------------
-GTCAGC---ACAGTACAATGCACACATGGAATTAGGCCAGTAGTATCAACT---
CAACTGCTGTTAAATGGCAGTCTAGCAGAAGAAGAT------------
GTAGTAGTTAGATCTGAAAATTTTACAAACAAT---------GCTAAAACCATAATAGTACAGCTGAAT----
-----------GCATCTGTAGAA---------ATTAATTGTACAAGACCCCACAACAATACAAGAAAA-----
-AGTATACATCTA------------GGACCAGGGAGCACA---TTT------TTTGCAACAGGAGCC------
---------ATAATAGGAAATATA------------AGACAAGCACATTGT---AACATTAGTGGGACA---
AATTGGAAAAACACGTTAAGACAGCTAGTTATAAAATTAAGA---------------GAACAATTTGGG----
--------------------------------AATAATAAAACAATAGCCTTTAAT------
CAATCCTCAGGAGGGGACCCAGAGATTGTA---ATGCACAGTTTTATTTGTGGAGGGGAA---

TTTAAGCAGCTCCAAGCAAGAGTCCTGGCTGTGGAAAGATACCTAAAGGATCAACAGCTCCTGGGGATTTGGG
GATGCTCTGGAAAGCTCATCTGCACCACTAATGTGCCTTGGAATACTAGTTGGAGT-----------------
----------------------------AATAAATCTCTGAATGATATCTGG------GAT------
AACATGACCTGGATGCAGTGG---GACAGAGAAATTAACAATTACACAAACTTAATATAC---
ACCTTGCTTGAAAAATCGCAGAACCAACAAGAA--

AAGAATGAGCAGGAATTATTGGAGTTGGATAAATGGGCAAGCTTGTGGAATTGGTTTGACATAACACAATGGC
TATGGTATATAAAAATATTCATAATGATAGTAGGAGGCTTGGTAGGTTTAAGAATAATTTTTTCT---
GTAATTTCTATAGTA---------AATAGAGTTAGGCAGGGATATTCACCA------
ATATCGTTTGCTGTTAGTTTGCTTAATGCCACAGCTATAGCAGTAGCTGAG---
GGGACAGATAGGATTATAGAAATATTACAAAGAGCTTATAGAGCT----------------------------
--------ATTCTCCACATA------CCTAGAAGAATAAGACAGGGCTTGGAAAGGGCTTTGCTA---TAA
>MN043605
GCT---GCA---
GAATCGTGGGTCACAGTCTATTATGGGGTACCTGTGTGG---AGAGACGCAAAC---
ACCACCCTATTTTGTGCTTCCGATGCTAAGGCATATGACACAGAA---GCACATAAT----------------
--------------GTTTGGGCCACA---CATGCCTGTGTACCCATAGAC---
CCCAATCCACAAGAGGTAGTATTGGAA---------AATGTGACAGAAAATTTTAAC----------------
--------------------------ATGTGGAAAAATAACATGGTAGAACAGATGCATGAGGACATA---
ATCAATTTATGGGATCAAAGCCTA---AAACCATGTGTAAAATTA---
ACTCCACTCTGCGTTACTTTGAGTTGTAGAGAGTTGAATAAGACTAAAACCAACTGCAGTGGTAATTCAAATA
GTACTCCTACTGCAACCCCTCCTCCA---
-------------------GCCAGTAACTGTAGTGGGAAAGTAATGGAGAAAGTAGAAATGAAA---
AACTGCTCTTTCAATGTTACCACAAACCTAAGA---------------------------
GATAAGATGAAGAAAGAATATGCTCTT---TTCTATAGTAGTGATATAACGCAAATAGATAAT----------
----

ACAAGCTATATGTTGACACATTGCAACACCTCAGTCATTACACAA---
GCCTGTCCAAAGACATCCTTTGAACCAATTCCCATACATTATTGTACCCCAGCTGGTTTTGCGCTTCTACAAT
GTAAT---GATAAGAAGTTTAAT------GGATCAGGACCATGTACAAAT-----------------------
-GTCAGC---ACAGTACAATGTACACATGGAATTAGGCCAGTAGTATCAACT---
CAACTGCTGTTAAATGGCAGTCTAGCAGAAAAAGAG------------
ATAATAATTAGATCTGAAAATATCTCAAACAAT---------GTTAAAACCATAATAGTACAACTGAAC----
-----------GTATCTGTAGAA---------ATTAATTGTATAAGACCCAGCAACAATACAAGGAGA-----
-AGTATAAGTTTT------------GCACCAGGAAGTACA---TTT------TATGCAACAGGAGAT------
---------ATAATAGGAGATATA------------AGACAAGCACATTGT---AACATTAGTGAAGCA---
AAATGGAACAACACTTTAAAAAAGCTAGCTATAAAATTAAGA---------------GAGCAATTTGTG----
-----------------------------------AATAAGACAATAGTCTTTAAT------
CAATCCTCAGGAGGGGACCCAGAAATTGTA---ATGCACAGTTTTAATTGTGGAGGGGAA---
TTTAAGCAGCTCCAAGCAAGAGTCCTGGCTGTAGAAAGATACCTAAAGGATCAACAGCTCCTGGGGATTTGGG
GATGCTCTGGAAAACTCATCTGCACCACTAATGTGCCTTGGAATACTAGTTGGAGT-----------------
----------------------------AATAAATCTCTAGATTATATCTGG------GAT------
AACATGACCTGGATGGAGTGG---GACAGAGAAATTAACAATTACACAAACTTAATATAC---
TCCTTACTTGAGAAATCGCAAAACCAGCAAGAA--

AAGAATGAACAGGAATTATTGGAATTGGATAAATGGGGAAGCTTGTGGAGTTGGTTTGATATATCAAATTGGC
TGTGGTATATAAGAATATTCATAATGATAGTAGGAGGCTTGGTAGGTTTAAGAATAATCTTTTTT---
GTACTTTCTAGAGTA---------AATAGAGTTAGGCAGGGATACTCACCA------
TTATCGTTTGCTGTTAGTTTGCTTAATGCCACAGCTATAGCAGTAGCTGAA---
GGGACAGATAGGGCTATAGAAATAGTACAAAGAGCTTTTAGAGCT----------------------------
--------ATTCTCAACATA------CCTACAAGAATAAGACAGGGCTTGGAAAGGGCTTTGCTA---TAA
>KJ140263
GCT---GAT---
GAATTGTGGGTCACAGTCTATTATGGGGTACCTGTGTGG---AAAGAAGCAACC---
ACCACCCTATTTTGTGCATCAGATGCTAAAGCATATGACACAGAG---GTACATAAT----------------
--------------GTTTGGGCCACA---CATGCCTGTGTACCCATAGAC---
CCCAACCCACAAGAAGTGGAATTGGTA---------AATGTGACAGAAAATTTTAAC----------------
--------------------------ATGTGGAAAAATAACATGGTAGAACAGATGCATGAGGATATA---
ATCAGTTTATGGGATCAAAGCCTA---AAGCCATGTGTAAAATTA---
ACTCCACTCTGTGTTACTTTGAATTGTAGTAATTTGAAGAATGAGACTAATACCAATAGGACTA---------

----
-----------------GTACCAATAGTAGTAGTGGGGAGTGATGGAGGAAGGAGAAATGAAA---
AACTGCTCTTTCAATGTTACCACAAGCATAAAA---------------------------
AATAAGGTGCAAAAAGAATATGCTCTT---TTTTATAAACTTGATGTAGTACAAATAGATAAT----------
----

ACAAGCTATACCTTGATAAATTGTAACTCCTCAATCATTACACAG---
GCCTGTCCAAAGATATCCTTTGAACCGATTCCCATACATTATTGTACCCCGGCTGGTTTTGCGATTCTACAGT
GTAAT---GATAAGAAGTTCAAT------GGATCAGGACCATGTTCAAAT-----------------------
-GTCAGC---ACAGTACAATGTACACATGGAATTAGGCAAGTAGTATCAACT---
CAACTACTGTTAAATGGCAGTCTAGCAGAAGAAGAG------------
GTAGTAATTAGATCTGAAAATTTCACAAACAAT---------GCTAAAACCATAATAGTACAGCTGAAT----
-----------ACATCTGTAGAA---------ATTAATTGTATAAGACCCAACAACAATACAAGAAAA-----
-AGTATACATCTA------------GGACCAGGGAGTACA---ATT------TATGCAACAGGAGAC------
---------ATAATAGGAGATATA------------AGACAAGCACATTGT---AACATTAGTAGAGAA---
AAATGGAACAACACTTTAAGACAGATAGTTATAAAATTAAGA---------------GAACAATTTGGG----
--------------------------------AATAAAACAACAATAGTCTTTAAT------
CAATCCTCAGGAGGGGACCCAGAGATTGTA---ATGCACAGTTTTAATTGTGGAGGGGAA---
TTTAAGCAGCTCCAGGCAAGAGTCCTGGCTGTGGAAAGATACCTAAAGGATCAACAGCTCCTGGGGATTTGGG
GATGCTCTGGAAAGCTCATCTGCACCACTAATGTGCCTTGGAATACTAGTTGGAGT-----------------
----------------------------AATAAATCTCAGAAGGAAATCTGG------GAT------
AACATGACCTGGATGCAGTGG---GACAGAGAAATTAACAATTACACAAGCTTTATATAC---
ACCTTACTTGAAGAATCGCAGAACCAGCAAGAA--

AAGAATGAACAGGAATTATTGGAATTGGATAAATGGTCAAGTTTGTGGAATTGGTTTAACATATCTCAATGGC
TGTGGTATATAAGAATATTCATAATGATAGTAGGAGGCTTGATAGGTTTAAGAATAATTTTTTCG---
GTACTTTCTATAGTA---------AATAGAGTTAGGCAGGGATACTCACCA------
TTATCGTTTGCTGTTAGTTTGCTTCATGCCACAGCTAGAGCAATAGCTGAG---
GGGACAGATAGGGCTATAGAAATATTACAAAGAGCTTATAGAGCT----------------------------
--------ATTCTCCACATA------CCTAGAAGAATAAGACAGGGTTTTGAAAGGGCTTTGCTA---TAA
>KT878030
GCT---TTA--
GAACAATCATGGGTCACAGTCTATTATGGAGTACCTGTGTGG---AAAGAAGCAACC---
ACCATTCTATTCTGTGCATCAGATGCTAAAGCACATGACCCAGAA---GTACATAAT----------------
--------------GTCTGGGCCACA---CATGCCTGTGTACCTATAGAC---
CCCGACCCACAAGAAGTAAGATTA------------AATGTGACAGAAAATTTTAAC----------------
--------------------------ATGTGGAAAAATAACATGGTAGAACAGATGCATGAGGATATA---
ATTAGTTTATGGGATCAAAGCCTA---AAGCCATGTGTAAAATTA---
ACTCCACTCTGTGTTACCTTAAATTGTAGTAATGTGACTCCAAATAATGGGACT-------------------
----
-------------------------AGCAATAATATTACTCTGGAAGGAAGGGAGGAAATAAGA---
AATTGCTCTTTCAATGTTACCACAAACATAAGA---------------------------
GATAAGGTAAAGCAAGAATATGCTCTT---TTTTATAGACTTGATGTAGAACAAATAGATAAT----------
----

ACAACCTATACGTTAGTAAATTGTAACTCCTCAACCGTTACACAG---
GCCTGTCCAAAAGTAACCTTTGAACCAATTCCCATACATTATTGCACCCCGGCTGGTTTTGCGCTTCTACAAT
GTAAT---GATAAGAAGTTTAAT------GGATCAGGACCATGTTCAAAT-----------------------
-GTCAGC---ACAGTACAATGTACACATGGAATTAGACCAGTAGTATCAACT---
CAACTGCTGTTAAATGGCAGTCTAGCAGAAGAAGAG------------
ATAATAATTAGATCTGAAAATTTCTCGAACAAT---------GCTAAAAACATAATAGTACAGCTGAAT----
-----------GCATCTGTAGAA---------ATTAATTGTACAAGACCCAACAACAATACAAGGAAA-----
-AGTATAACGCTA------------GGCCCAGGGAGAGCA---TTT------TATGCAACAGGAGAC------
---------ATAATAGGAGATATA------------AGACAAGCACATTGT---AACATTAGTGAACAA---
AATTGGAATGTCACTTTAAAACAGCTAGCTATAAAATTAAGA---------------GAACAATTTAAG----
-----------------------------------AATAAAACAATATCCTTTCAA------
CCACCCACAGGAGGGGACCCAGAGATTATA---ATGCACAGTTTTAATTGTGGAGGGGAA---
TTTAAACAGCTCCAGGCAAGAGTCCTGGCTATAGAAAGATACCTAAAGGATCAACAGCTCCTGGGGATTTGGG
GATGCTCTGGAAAGCTCATCTGCACCACTACTGTGCCTTGGAATACTAGTTGGAAT-----------------
----------------------------AATAGATCTGTGGACGATATCTGG------CAT------

AACATGACCTGGATGCAGTGG---GACAGAGAAATTAACAATTACACAAACGAAATATAC---
AGATTACTTGAAGAGTCGCAAAACCAACAAGAG---------------------------------------
----
AGGAATGAACAAGACTTATTGGAATTGGATAATTGGGCAGGTTTGTGGAGTTGGCTTAGTCTATCAAAATGGA
TGTGGTATATAAAAATAGTTATACTAATAGTAGCAGGCTTGATAGGTTTAAGAATAATTTTCTTT---
GTACTTTCTATAGTA---------AATAGTGTTAGGAAGGGATACTCACCA------
TTATCGTTTGCTGTTAGTTTGCTTAATACCACTGCTGTAGTAGTAGCTGAG---
GGGACAAATAGAATTATAGAAGTATTACAAAGAGCTTATAGAGCT---------------------------
--------GTCCTCAACGTA------CCTAGAAGAATAAGACAGGGCTTTGAAAGGGCTTTGCTA---TAA
>KT878031
GCT---ACA---
GAGGAAAGGTGGGTTACAGTCTATTATGGGATACCTGTGTGG---AAAGAAGCAACC---
ACCACCCTATTTTGTGCATCAGATGCTAAAGCATATGTCAAAGAG---GCACATAAT----------------
--------------GTTTGGGCCACA---CATGCCTGTGTACCCACAGAC---
CCCAACCCACAAGAAGTAGTATTGGTA---------AATGTGACAGAAAATTTTAAC---------------
---------------------------ATGTGGAAAAATAACATGGTAGAACAGATGCAAGAGGATATA---
ATCAGTTTATGGGACCAAAGTTTA---AAGCCATGTGTAAAATTA---
ACTCCACTCTGTGTTACCATGAATTGTAGTGATTTGAATAAGACTAGTACCAATAATAGTAGT----------
----
-------------------GGGAATAGTACTAATGGGGATGGAATGGAGGAAGGAGGAATGAAA---
AACTGCTCTTTCAATGTTACTACAATTATAAGA--------------------------
GATAAGGTGAAGAACGAATATGCTCTT---TTTTATAAGCTTGATATAGCACAAATGGAGGAG----------
----

AATAGTACAAGCTACACATTGATAAATTGTAATTCCTCAGTCATTACACAG---
GCCTGTCCAAAGGTATCCTTTGAACCAATTCCCATACATTATTGTACCCCGGCTGGTTTTGCGCTTCTAAAAT
GTAAT---GATAAGACGTTCAAT------GGATCAGGACTATGTGAAAAT----------------------
-GTCAGC---ACAGTGCAATGTACACATGGAATAAGGCCAGTAGTATCAACT---
CAACTGCTGTTGAATGGCAGTCTAGCAGAAGAAGAG-----------
ATAGTAATTAGATCTGAGAATTTCACGAACAAT---------GCTAAAACCATAATAGTACAGCTGAAC----
-----------ACAGCTGTAGAA---------ATTAATTGTACAAGACCCAACAACAATACAAGAAAA-----
-AGTATACATATA------------GCACCAGGGAGAGCA---TTT------TATGCAACAGGAGGC------
---------ATAATAGGAGATATA-----------AGACAAGCACATTGT---AACATTAGTAAAGAA---
AAATGGAACGAAACTTTAAAACAGGTAGCTATAAAATTAAGA---------------AAACAATTC-------
------------------------------------AATAAAACAATAGTCTTTAAT------
CAATCCTCAGGAGGGGACCCAGAGATTACA---ATGCATAGCTTTAATTGTGGAGGGGAA---
TTTAAGCAGCTCCAGGCAAGAGTCCTGGCTGTGGAAAGATACCTAAAGGATCAACGGCTCCTGGGGATTTGGG
GATGCTCTGGAAAGCTCATCTGCACCACTAATGTGCCTTGGAATACTAGTTGGAGTAATGGA---ACT-----
----------------------------AATGGATCTCTGGACAATATCTGG------GAT------
AGCATGACTTGGATGGAGTGG---GAGAGAGAAATTGACAATTACACAGGCTTAATATAC---
AACTTACTTGAAGAATCGCAGAACCAGCAAGAA---------------------------------------
----
AAGAATGAACAGGAATTATTGGAATTAGATAAATGGGCAAATTTGTGGAACTGGTTTAGCATAACACAATGGC
TGTGGTATATAAAAATATTCATAATAATAGTAGGAGGCTTGGTAGGATTAAGAATAATTTTTTCT---
GTACTTGCTATAGTG---------AATAGAGTTAGGAAGGGATACTCACCA------
TTATCGTTTGCTGTTAGTTTGCTTAATGCCACAGCTATAGCAGTAGCTGAG---
GGGACAGATAGGGTTATAGAAATATTGCAAAGAGCTTTTAGAGCT---------------------------
--------ATTCTCCACATA------CCTACACGAATAAGACAGGGCTTTGAAAGGGCTTTGCTA---TAA
>KT878032
GCT---AAG---CAA-----------------------------------
GAAAAGTTGTGGGTCACAGTCTATTATGGGGTACCTGTGTGG---AGAGAAGCAAAC---
ACCACCCTATTTTGTGCATCAGATGCTAAAGCATATAAAACAGAG---GTGCATAAT---------------
--------------GTTTGGGCCACA---CATGCCTGTGTACCCATAGAC---
CCCAACCCACACGAAATAAGATTGGAA---------AATGTGACAGAAAACTTTAAC----------------
---------------------------ATGTGGAAAAATAACATGGTAGAACAGATGCATACAGATATA---
ATCAGCTTATGGGATCAAAGCCTA---AAGCCATGTGTAAAATTA---
ACTCCACTCTGTGTTACTTTAAATTGTAGTGAGTTAGACAATACTACTATCAGTACCAATAATACA-------
----
-------------------GAGGACATAAGAAAGGAAATAAAAGAGGAAATGGGAAACTTGAGA---
AACTGCTCTTTCAATGTTACCACATTAGTAAGA--------------------------

GATAGGAAGAAACAAGAATATGCTCTC---TTTTATAAACTGGATATAGCAAAATTAGATAAT----------
----

GATAATAATAGTACAAGTTATACATTGATAAATTGTAACTCCACAACCATCACACAG---
GCCTGTCCAAAGGTATCCTTTGAACCAATTCCCATACATTATTGTACCCCGGCTGGTTTTGCGATTCTACAAT
GTAAT---GATGAAAAGTTCAAT------GGATCGGGACCATGTTCAAAT-----------------------
-GTCAGC---ACAGTACAATGTACACATGGATTAAGCCAGTAGTATCAACT---
CAACTGCTGTTAAATGGCAGTCTAGCAAAAGAAGAG------------
ATAGTAATTAGATCTGAAAATTTCACAGACAAT---------GTTAAAACCATAATAGTACAGCTGAAT----
-----------GAGTCTATAGAA---------ATTAATTGTACAAGACCCAACAACAATACAAGAAAA-----
-AGTATAGGTTTT------------GTACCAGGGGCATCA---TTT------ATTGCAACAGGAGCC------
---------ATAATAGGAGATATA------------AGACAAGCACATTGT---CACATTAATAAAACA---
AAATGGAATAACACTTTAGGACAGATAGCTAAAAAATTAGGA---------------GAACAATTTGGG----
-----------------------------------GGTAAGGACATAGTCTTTGAT------
AACAGCTCAGGAGGGGACCTAGAGATTACA---ATGCACAGTTTTAATTGTGGAGGGGAA---
TTTAAACAGCTCCAGGCAAGAGTCCTGGCTGTGGAAAGATACCTAAAGGATCAACAGCTCCTAGGAATTTGGG
GATGCTCTGGAAAACTCATCTGCACCACTAGTGTGCCTTGGAATACTAGTTGGAGT-----------------
----------------------------AATAAAACTAAGAAGGAAATCTGG------GAT------
AACATGACCTGGATGGAGTGG---GACAGGGAAATTAGCAATTACACAACCTTAATATAC---
TCCTTGCTTGAAGAATCGCAGAACCAACAAGAA--

AAGAATGAACAGGAACTATTGAAATTGGATGACTGGGCAGGTTTGTGGAGTTGGCTTGACATCTCACACTGGC
TGTGGTATATAAAAATATTCATAATGATAGTAGGAGGCTTGATAGCTTTAAGAATAATTTTTGCT---
GTATTTTCTATAATA---------AATAGAGTTAGGCAGGGATACTCATCA------
TTGTCGTTCGCTGTTAGTTTGCTTAATGCTACAGCTGTAACAGTAGCTGAG---
GGGACAGATAGAATTATAGAAATATTACAAAGAGTTTTTAGGGCT----------------------------
--------ATTCTCAATATA------CCTAGAAGAATAAGACAGGGCTTGGAAAGGGCTTTGCTA---TAA
>KT878033
GCT---GCA---ACA------------------------------------
GGAGGAATGTGGGTCACAGTATACTATGGGGTACCTGTGTGG---AAAGAAGCAACC---
ACCACCTTATTCTGTGCATCAAATGCTAAAGCATATATCTCAGAA---GCACATAAT----------------
--------------ATTTGGCCACA---CATGCCTGCGTACCCATAGAC---
CCAGACCCACAAGAAGTAACATTGGAA---------AATGTGACAGAAAATTTTAAC----------------
--------------------------ATGTGGAAAAATAACATGGTAGAACAGATGCATGAGGATATA---
ATCAGTTTATGGGATGAAAGCCTA---AAACCATGTGTAAAATTA---
ACTCCACTTTGTGTTACTTTGAATTGTAGTAATGTAAATACCACTGGTACTAATCTCACT-------------
----
----------------------------AGTAGTAATGAGAGGATGGAGGAAGGAGAAATGAAA---
AACTGCTCTTTCAATGTTACCACAGTCCAAAGA---------------------------
GATAAGGTGAAACAAGAATATGCTCTT---TTTTATAGACTTGATGTACAACAAGTAGATAAT----------
----

ACAAGCTATACATTGATAAATTGTAACTCCTCAACCGTTACACAG---
GCTTGTCCAAAGATATCCTTTGAACCAATTCCTATACATTATTGTACCCCGGCTGGTTTTGCGCTCCTAAAAT
GTAAT---GATAACAAGTTCAAT------GGATCAGGACCATGTTCAAAT-----------------------
-GTCAGC---ACAGTACAATGTACACATGGAATTAAGCCAGTAGTATCAACT---
CACCTGTTGTTAAATGGCAGTCTAGCAGAAGAAGAG------------
ATGGTAATTAGATCTCAAAATTTCTCAAACAAT---------GCTAAAACCATAATAGTACAGCTGAAT----
-----------GCATCTGTAGAA---------ATTAATTGCACAAGACCCAACAACAATACAAGAAGG-----
-AGTGTACATATT------------GCACCAGGGAGTGCA---ATT------TTTGCAACAGGAGAC------
---------ATAATAGGAGATATA------------AGACAAGCATATTGT---AACGTTAGTGCAAAA---
AGCTGGAATAACACTTTAAAACAGCTAGTTGAAAAATTAAGA---------------AAACAATATGGG----
--------------------------------GAAAATAGAACAATAACCTTTAAA------
GAACCATCAGGAGGGGACCCAGAGATTGTA---ATGCACATTGTTAATTGTGGAGGGGAA---
TTTAAGCAGCTCCAGGCAAGAGTCCTGGCTGTGGAAAGATACCTAAAGGATCAACAGCTCCTGGGGATTTGGG
GATGCTCTGGAAAGCTCATCTGCACCACTAATGTGCCTTGGAATACTAGTTGGAGT-----------------
----------------------------AATAAATCTTATCATCAAATCTGG------GAT------
AACATGACCTGGATGGAGTGG---GACAGAGAAATTAACAATTACACAGGCTTAATATAC---
AACTTGCTTGAAGAATCACAGATGCAACAAGAA--

AAGAATGAACAGGACCTATTGAAATTGGATGAATGGGCAAGCCTGTGGAACTGGTTTAGCATATCCCAATGGC
TGTGGTATATAAAAATATTCATAATGATAATAGGAGGCTTGGTAGGTTTAAGAATAATTTTTTCT---
GTACTTTCTATAGTG---------AATAGAGTTAGGCAGGGATACTCACCA------
TTATCGTTTGCTGTTAGTTTGCTTAATACCACAGCTATAGCAGTAGCTGAG---
GGAACAGATAGAATTATAGAAATATTACAAAGAGGTTGTAGAGCT----------------------------
--------ATTATCAACGTA------CCTAGAAGAATAAGACAGGGCTTGGAAAGGCTTTGCTA---TAA
>KT878036
GCT---TTA---GAA---------------------------------
CAAGAAAAGTCATGGGTCACAGTTTATTATGGAGTACCTGTGTGG---AGAGAAGCAACC---
ACCCCCTTATTTTGTGCATCAGAGGCTAAAGCATATGAGACAGAA---GCACATAAT----------------
--------------ATTTGGCCACA---CATGCCTGTGTACCCACAGAT---
CCCAAACCACAAGAAGTAGTATTGGAA---------AATGTGACTGAAAATTTTAAC----------------
--------------------------ATGTGGAAAAATGACATGGTAGAACAGATGCATGAGGATATA---
ATCAGTTTATGGATCAAAGCCTA---AAGCCATGTGTAAAGTTA---
ACTCCACTCTGTGTTTCTTTAAATTGTAGCAATGTGAAAAATGTAACTAATGGCACTCAG-------------
----
----------------------------ACCAGTGGTAGTGTGATGAGGGAAGGAGAAATGAAA---
AACTGTTCTTTCAATGTTACCACAAGCATAAGA---------------------------
GATAAGAAGAAGAAAGAATATGCTCTT---TTTTATAGGCTTGATATAGAACAAATAGACAAT----------
----

ACAAGCAACAGCTATACGCTGATAAATTGTAATTCCTCTACCATTACACAG---
GCCTGTCCAAAGATAAGCTTTGAACCAATTCCCATACATTATTGTGCCCCGGCTGGTTTTGCGATTCTAAAGT
GTAAT---GATAAGAAATTCAAT------GGATCAGGACCATGTTCAAAT-----------------------
-GTCAGC---ACAGTACAATGTACACATGGAATTAAACCAGTAGTATCAACT---
CAACTGCTGTTAAATGGCAGTCTAGCAGAAGATGAC------------
ATAGTAATTAGATCTGAGAATTTCACAAACAAT---------GCTAAAATCATAATAGTACAACTGAAC----
-----------ACATCTGTAGCA---------ATTACTTGTGTAAGGCCCAACAACAATACTAGAAAA-----
-AGCATAACGCTC------------GGACCAGGGAGTGCA---TTT------TATGCAACAGGAGAC------
---------ATAATAGGAGATATA------------AGACAAGCACATTGT---AACATTAGTAGACAA---
AATTGGACTAACACTGTAAGACAGCTAGCTGGAAAATTAAGA---------------GAACAATTTGGG----
-----------------------------------AATAAAACAATATACTTTAAC------
CAATCCTCAGGAGGGGACCCAGAGATTGAA---ATGCTCAGTGTTAATTGTGGAGGGGAA---
TTCAAGCAGCTCCAGGCAAGAGTCCTGGCTGTAGAAAGATACCTAAAGGATCAACAGCTCCTGGGGATTTGGG
GATGCTCGGGAAAGCTCATCTGCACCACTAATGTGCCTTGGAATACTAGTTGGAGC-----------------
----------------------------AATAAATCTCTAAGTGATATCTGG------AAT------
AACATGACCTGGATGGAGTGG---GACAGAGAAATTAACAATTACACAAACTTAATATAC---
AACTTACTTGAAGAATCGCAGAACCAACAAGAA--

AAGAATGAACTAGACTTATTGAAATTGGATGAATGGGCAAGTTTGTGGAATTGGTTTGACATATCAAAATGGC
TGTGGTGTATAAAGATATTCATAATGATAGTAGGAGGCTTGATAGGTTTAAGAATAATTTTTTCT---
GTACTTTCTATAGTA---------AATAGAGTTAGGCAGGGCTACTCACCA------
TTATCATTTGCTGTTAGTTTGCTTAATGCCACAGCTATAGCAGTAGCTGAG---
GGGACAGATAGGATTATAGAACTAGTACAAAGAATTTGTAGAGCT----------------------------
--------TTTCTGCACATA------CCTAGAAGAATAAGACAGGGCTTGGAAAGGCTTTGGTA---TAA
>KT878037
GCT---GCA--
GAACAATTGTGGGTTACAGTCTATTATGGGGTGCCTGTGTGG---AGAGACGCAAAC---
ACCACTTTATTTTGTGCATCAGATGCTAAAGCATATGACACAGAG---GCACATAAT----------------
--------------GTTTGGGCCACA---CATGCCTGTGTACCCACAGAC---
CCCAACCCACAAGAAGTACGGTTGGAA---------AATGTGACAGAAAATTTTAAC----------------
--------------------------ATATGGGAAAATGATATGGTAGAACAGATGCATGAAGATATA---
ATCAGTTTATGGATCAAAGCCTA---AAACCATGTGTGAAATTA---
ACCCCACTCTGTGTTACTTTAAACTGCACTGATGAGGTTGGTAATTCTACTCAAAATAGTAATTTTACC----
----
----------------------------CTTAATAGTAATGAAACAGACAGGAGAGATATGAAA---
AACTGCTCTTTCAATATCACCACATCAGTAAAA---------------------------
GATAAGGTGCGGAAAGAACACGCAATT---
TTTAATAGCCTTGATATAGTACAAATAGATGATGAGAATAAGAAT----------------------------
----

AGTAGTGATAATACTAGTGCCAGATATATGTTAAGACATTGTAATACCACAGTCATTTCACAG---
GCCTGTCCAAAGATATCCTTTGAGCCAATTCCCATACACTTTTGTGCCCCGGCTGGTTTTGCGCTTCTAAAAT
GTAAC---AATAAGACGTTCAGT------GGAAAAGGACCATGCAACAAT----------------------
-GTCAGC---ACAGTACAGTGTACACATGGAATTAGGCCAGTAGTGTCAACT---
CAACTGCTGCTAAATGGCAGTCTAGCAGAAGAAGAG-----------
GTAGTAATTAGATCTAAAAATTTCACAGATAAT---------GCTCAAACCATAATAGTACAGTTGAAC----
-----------AAATCTGTAGTA---------ATTAATTGTACAAGACCTGGCAACAACACAATAAGA-----
-AGTATACATGTA------------GGACCAGGACGAGCA---TTC------TACACACCAGGAACC------
---------ATAATAGGAGATATA------------AGAAAAGCATATTGT---AACATTAGTAAAAAA---
GAATGGAATAACACTTTAAGACAGGTAGTTGAAAAATTAAAA---------------GAAAAATTCAAT----
----------------------------------ATAACAACAATAGTCTTTCAG------
CAATCCTCAGGAGGAGACCCAGAAATTACA---ATGCATAACTTTAATTGTAAAGGGGAA---
TTTAAGCAGCTCCAGGCAAGGGTCCTAGCTGTGGAAAGATACCTAAGGGATCAACAGCTCCTAGGGATTTGGG
GCTGCTCTGGAAAGCTCATCTGCACCACTAATGTACCTTGGAATACTAGTTGGAGT-----------------
----------------------------AATAAAACCCACGATACGATTTGG---------------
AACATGACCTGGGTACAGTGG---GAAAGAGAAATTGACAATTACACAGGCCTAATATAC---
ACCTTACTTGAGGAATCGCAGAACCAACAAGAA---------------------------------------
----
AAGAATGAACAAGAATTATTGGAATTGGATAGTTGGGCAAGTTTGTGGAATTGGTTTACAATATCAAACTGGC
TGTGGTATATAAAGATATTCATAATGATAGTAGGAGGCTTAATAGGTTTAAGAATAGTTTTTGCT---
GTACTTTCCATAGTA---------AATAGAGTTAGGCAGGGATATTCACCC------
CTATCATTGGCTATTAGCTTGCTAAACGTCACAGCTATTGCAGTAGCTGAG---
GGGACAGACAGGATCATAGAAGTAGTACAAAGAGCTTGGAGAGGG----------------------------
--------TTTCTACACATA------CCTGCAAGAATAAGACAGGGCTTAGAAAGGGCTTTGCTT---TAA
>KT878038
GCT---TCA---
GAACAAACGTGGGTCACAGTCTATTATGGGGTACCTGTGTGG---AAAGAAGCAACC---
ACCACCCTATTTTGTGCATCCGATGCTAAAGCATATGTCACAGAG---GCACACAAT----------------
--------------GTTTGGGCCACA---CATGCCTGTGTACCCATAGAC---
CCCAACCCACAAGAAGTAAAATTGGAA---------AATGTGACCGAAAAATTTAAC----------------
--------------------------ATGTGGAAAAATAACATGGTGGATCAGATGCATGAGGATATA---
ATTAGTTTATGGGATGAAAGCCTA---AAGCCATGTGTAAAATTA---
ACTCCAATCTGTGTTACTTTAAATTGTAGTGATTTAAAGAAAACCAATAGTAGTAATGATAATAGTACTGAAA
CCAATAGTAGT---
-------------------------AATGATAATAGTACAGTAGTGGAGGAAGGAGATATGAAA---
AACTGTTCTTTCAATGTTACCACAGCCATACAA--------------------------
AATAAGGTGAAGCAAGAGTATGCTCTT---TTTTATAAACTTGATGTAATGCCAATAGCTGAT----------
----

GATAATTCGAGCTATACATTGATAAACTGTAACTCCTCAAGCATTACACAG---
GCCTGTCCAAAGGTGTCCTTTGAGCCAATTCCCATACATTATTGTACCCCGGCCGGGTATGCGATTCTAAAGT
GTAAT---GATAGGAATTTCAAT------GGAACAGGACCATGCAAGAAT----------------------
-GTCAGC---ACAGTGCAATGTACACATGGAATTAGGCCAGTAGTATCAACT---
CAACTACTGTTAAATGGCAGTCTAGCAGAAGGAGAG-----------
ATAGTAATTAAATCTGAAAATATCACAAACAAT---------GCAAAAACCATAATAGTACAGCTGAAC----
-----------GCATCTGTAGAA---------ATTAATTGTACAAGACCAAACAACAATACAAGAAAA-----
-GGTATACATTTT------------GGACCAGGGAGAACA---ATA------TATACAACAGGAGAG------
---------ATAGTAGGAGATATA------------AGACAAGCACATTGT---AACATTAGTGGAGTA---
CAGTGGAAAAACACTTTGACAAAGCTAGCCAAAAAATTAAGA---------------GAACAATTT-------
----------------------------------AACAAAACAATAAGCTTTAAC------
AAATCCTCAGGAGGGGACCCTGAGATTGTA---ATGCACAGTTTTAATTGTGGAGGTGAA---
TTTAAGCAGCTCCAGGCCAGAGTCCTGGCTGTGGAAAGATACCTAAGGGATCAACAGCTCCTGGGGATTTGGG
GATGCTCTGGAAAGCTCATCTGCACCACTACTGTGCCTTGGAATACTAGTTGGAGT-----------------
----------------------------AATAAATCTCAGAATGAAATATGG------AAC------
AACATGACCTGGATGGAGTGG---GACAGAGAAATTGACAATTACACACAACTAATATAT---
TCCCTACTTGAAAAATCACAGATCCAACAAGAA---------------------------------------
----
AACAATGAAAAGGAGTTATTGGAATTGGATAGTTGGCAAAGTCTGTGGAAATGGTTTGACATATCAAATTGGC
TGGGGTATATAAAAATATTTATAATGATAGTAGGAGGCTTGATAGGTTTAAGAATAATTTGTTCT---
GTACTGTCTATAGTG---------AATAGAGTTAGGAAGGGGTACTCACCA------

TTATCGTTTGCTGTTAGTTTGTTTAATGCTGTAGCTATAGCAGTAGCTGAG---
GGGACAGATAGGATTATAGAAGCATTACAAAGAGCTTTTAGAGCT----------------------------
--------ATCCTCCATATA------CCTAGAAGAATAAGACAGGGCTTGGAAAGGGCTTTGCTA---TAA
>KT878022
GCT---GTA--
GAACAAATGTGGGTCACAGTCTATTATGGGGTACCTGTGTGG---AAAGAAGCAACT---
ACCACCCTATTTTGTGCATCAGATGCTAAAGCATATGTCACAGAG---GCACATAAT----------------
--------------GTTTGGGCCACA---CATGCCTGTGTACCCATAGAC---
CCCAACCCACAAGAAGTAAAATTGGAA---------AATGTGACTGAAAAATTTAAC----------------
--------------------------ATGTGGAAAAATAGCATGGTAGAACAGATGCATGAGGATATA---
ATTAGTTTATGGGATGAAAGCCTA---AAGCCATGTGTAAAATTA---
ACTCCAATCTGTGTTACTTTAAATTGTAGTGATTTGAACATTACTAATACTAATACCAGTAGT----------
----
----------------------AGTAGTAATAATACAATAGGAGATATGAGAGAACAGATGAGA---
AACTGCTCTTTCAATGTTACCACAGCCATACAA---------------------------
AATAAGGTGAAGAAAGAATATGCTCTT---TTTTATAAACTTGATGTAATGTCAATAAATAAT----------
----

GATAGTACAAGCTATACATTGATAAACTGTAACTCCTCAAGCATTACACAG---
GCCTGTCCAAAGGTATCCTTTGAACCAATTCCCATACATTATTGTGCCCCGGCTGGTTATGCTATTCTAAAGT
GTAAT---GATAGAAATTTCAAT------GGAACAGGACCATGTAAAAAT-----------------------
-GTCAGC---ACAGTACAATGTACACATGGAATTAGGCCAGTAGTATCAACT---
CAACTACTGTTAAATGGCAGTCTATCAGAAGGAGAG------------
ATAGTAATTAAATCTGAAAATATCACAGACAAT---------GCAAAAACCATAATAGTACAGCTGAAT----
-----------GAATCTGTAGTA---------ATTAATTGTACAAGACCAAACAACAATACAAGAAGA-----
-AGTATACATGTA------------GGACCAGGGAGCATA---ATG------TATGCAACAGGAGCG------
---------ATAATAGGAGATATA------------AGACAAGCACATTGT---AACATTAGTAAAGAA---
AGGTGGGACAATACTTTGAAACAGATAGCTGAAAAATTAAAA---------------GAAAAATTTAAC----
-----------------------------------AACAGAACAATAAGCTTTAAC------
CAATCCTCAGGAGGGGACCCAGAAATAGAA---ATGCACAGTTTTAATTGTGGAGGGGAG---
TTTAAGCAGCTCCAGGCCAGAGTCCTGGCTGTAGAAAGATACCTAAGGGATCAACAGCTCCTGGGGATTTGGG
GATGCTCTGGAAAGCTCATCTGCACCACTAATGTGCCTTGGAATACTAGTTGGAGT-----------------
----------------------------AATAAATCTCACAATGATATATGG------AAC------
AACATGACCTGGATGGAGTGG---GACAGAGAAATTGACAATTACACACGATTAATATAT---
TCCTTACTTGAAACATCGCAGATCCAACAAGAA--

AAGAATGAAAAGGAGTTATTGGAATTGAATAATTGGGAAAATTTGTGGAGTTGGCTTAACATATCAAGTTGGC
TGGGGTATATAAGAATATTTATAATAATAGTAGGAGGCTTGGTAGGTTTAAAAATAATTTTTTCT---
GTACTCTCTATAGTG---------AATAGAGTTAGGAAGGGATACTCACCA------
TTATCGTTTGCTGTTAGTTTGCTTAATGCCATAGCTATAGCAGTAGCTGAG---
GGGACAGATAGGATTATAGAACTATTACAAAGAGCTTATAGAGCT----------------------------
--------ATCCTCCATATA------CCTACCAGAATAAGACAGGGCTTGGAAAGGGCTTTGCTA---TAA
>KT878023
GCT---GTA------------------------AAC---CAA---TCA---
GAACCATTGTGGGTCACAGTCTATTATGGGGTACCTGTGTGG---AAAGAAGCAAAC---
ACCACTTTATTTTGTGCATCAGATGCTAAGGCATATAGTACAGAG---GTACATAAT----------------
--------------GTTTGGGCCACA---CATGCCTGTGTACCCACAGAC---
CCCAATCCACAAGAAATCCTATTGGAA---------AATGTGACAGAAAATTTTAAC----------------
--------------------------ATGTGGAAAAATAACATGGTAGATCAAATGCATGAGGATATA---
ATCAGTTTATGGGACCAAAGCCTA---AAGCCATGTGTAAAATTA---
ACCCCACTCTGTGTTACTTTAAATTGCACTGATAAACTAAATACCACTAATAGTACTAGT-------------
----
----------------------------GCTACTATTAGTCCAATAGAGAAAGGGGAAATAAAA---
AACTGCTCTTTCAATGTTACCACAAACTTAAGA---------------------------
GATAAGATGAAAAAAGAGTATGCACTT---TTGTATAAACTTGATGTGGTTCCAATAAATGAT----------
----

AGTACTAATAATGACAACTATACCAACTACTATAGGTTGATAAGCTGTGACACCTCAATTATTACACAG---
GCCTGTCCAAAGGTATCCTTTGAACCAATTCCAATACATTATTGTGCCCCGGCTGGGTTTGCGATTTTAAAGT
GTAAT---GATAAAAAGTTTAAT------GGAACAGGACCATGTAAAAAT-----------------------

-GTCAGC---ACAGTACAATGTACACATGGAATTAGACCAGTAGTATCAACT---
CAATTGTTGTTAAATGGCAGTCTATCAGAAAAAGAG------------
GTAATAATTAGGTCTCAAAATTTCTCGGACAAT---------GCTAAAATATAATAGTACAGCTGAAT----
-----------GAATCTGTAATA---------ATTAATTGTACAAGACCCAATAACAATACAAGAAAA-----
-AGTATATCTATA------------GGACCAGGAAGAGCA---GTT------TATGCAACAGGACAA------
---------ATAATAGGAAATATA------------AGACAAGCACATTGT---AACATTAGCAAAACA---
CAATGGGAGAAGACTTTAAAAGGATAGCTATAAAATTACAA---------------GAACAATATAAC----
-----------------------------------AATAAAACAATAGTATTTAAC------
CATTCCTCAGGAGGGGACCCAGAACTTGTA---ATGCACAGTTTTAATTGCGGAGGGGAA---
TTTAAGCAGCTCCAGGCAAGAGTCCTGGCTGTGGAGAGATACCTAAAGGATCAACAGCTCATGGGATTTGGG
GTTGCTCTGGAAGACTCATCTGCACCACTGCTGTGCCTTGGAATACCAGTTGGAGT-----------------
----------------------------AATAAATCACTAGATGACATTTGG------AAT------
AACATGACTTGGATGGAGTGG---GAAAAAGAAATTGAAAATTACACAGGCTTAATATAC---
ACCTTAATTGAGAAATCGCAAAACCAACAGGAA--

ATAAATGAAAAGGAATTATTGGAATTGGATAAATGGACAAATTTGTGGAGTTGGTTTGACATATCAAATTGGC
TGTGGTACATAAAAATATTCATAATAATAGTAGGTGGCTTGATAGGCTTAAGAATAGTTTTTACT---
GTACTTTCTTTAGTG---------AATAGAGTTAGGAAGGGATACTCACCA------
TTATCGTTTGCTGTTAGCTTGCTTGACACCACAGCCATAGTAGTAGCTGAG---
GGGACAGATAGGATTATAGAATTCATACAAAGAGCTTTTAGGGCT----------------------------
--------ATTCGCAATATA------CCTGTAAGAATAAGACAGGGCTTGGAAAGGCTTTGGTA---TAA
>KT878024
GCT---GAA--
GAACAATCATGGGTCACAGTCTATTATGGGGTACCTGTGTGG---AAAGAAGCAACT---
ACCACCCTATTTTGTGCATCAGATGCTAAAGCATATGTTGCAGAG---GCACATAAT----------------
--------------ATTTGGCCACA---CATGCCTGTGTACCCATAAAC---
CCCAACCCACAAGAAATGGTAATGGAA---------AATGTGACAGAAAAATTTAAC----------------
--------------------------ATGTGGAAAAATAACATGGTAGATCAGATGCATGAGGATATA---
ATCAGTTTATGGGATCAAAGCCTA---AAGCCATGTGTAAAATTA---
ACTCCACTCTGTGTTACCTTAAATTGTAGTAATTTGAAAAATAGTACTAATAATAATAAT-------------
----
----------------------------ATTACTATTGAAAAAGGAATGGAGGAGGAAATAAAA---
AACTGCTCTTTCAATGTTACCACAAGCATAAGA---------------------------
AATAAGGTGAAGCAAGAATATGCTCTT---TTTTATAGACTTGATATAGAACAAATAGATAAA----------
----

GATAATTCAAGCTATACGTTGATAAATTGTAACTCCTCAGTCATTACACAA---
GCCTGTCCAAAGATATCCTTTGAACCAATTCCCATACATTATTGTACCCCGGCTGGTTTTGCGATTCTACAAT
GTAAT---GATAAGAAGTTCAAT------GGAACAGGTCCCTGTAAAAT-----------------------
-GTTAGC---ACAGTACAATGTACACATGGAATTAGGCCAGTAGTATCAACT---
CAACTGCTGTTAAATGGCAGTCTAGCAGAAGAAGAG------------
ATAATAATGAGATCTGAAAATTTCTCGGACAAT---------ACTAAAACCATAATAGTACAGCTGAAC----
-----------ACATCTGTAGAA---------ATTACTTGTATAAGACCTAACAATAACACAAGAAAA-----
-GGTATACCTATA------------GGACCAGGGAGAGCA---ATT------TATGCATCAGGAGAC------
---------ATAATAGGAGATATA------------AGACAAGCACATTGT---AACATTACTGAAGCA---
AAATGGAACAACACTTTAAGACAGCTTGTTAAAAAATTAAGA---------------GAACAGTTTGGC----
-----------------------------------AATACCACAATAATCTTTAGC------
AACTCCTCAGGAGGGGACCCAGAGATTGTA---AAGTTCAGTTTTAATTGTGGAGGAGAA---
TTTAAACAGCTCCAGGCAAGAGTCCTGGCTGTAGAAAGATACCTAAAGGATCAACAGCTCCTGGGGATTTGGG
GATGCTCTGGAAAGCTCATCTGCACCACTAATGTGCCTTGGAATACTAGTTGGAGT-----------------
----------------------------AATAAATCTCTGAATTATATCTGG------GAT------
AATATGACCTGGATGCAGTGG---GACAGAGAAATTAACAATTACACAGAACAAATATAC---
ACCTTACTTGAAGAATCGCAAATCCAACAGAA--

AAGAATGAACAGGAGTTATTGGAATTGGATAAATGGTCAAATTTGTGGAGTTGGTTTACCATATCAAACTGGC
TGTGGTATATAAGAATATTCATAATGATAGTAGGAGGCTTGATAGGTTTAAGAATAGTTTTTTCT---
GTACTTTCTATAGTA---------AATAGAGTCAGGCAGGGATACTCACCC------
TTGTCGTTCGCTGTTAGCTTGTTTAATACTATAGCTATAGTAGTAGCTGAG---
GGGACAGATAGGGTTATAGGAATATTACAAAGAACTTATAGAGCT----------------------------
--------GTCCTCCATATA------CCTACAAGAATAAGACAGGGCTTAGAAAGGCTTTGCTA---TAA

>KT878025
GCT---ACA---
GATTTGTGGGTCACAGTCTATTATGGGGTACCTGTGTGG---AAAGATGCAACT---
ACCACCCTATTTTGTGCATCAGATGCTAAAGCATATGTCCCAGAG---GCACATAAT----------------
--------------ATTTGGGCCACA---CATGCCTGTGTACCCATAGAC---
CCCAACCCACAAGAAGTAGTATTGGAA---------AATGTGACAGAAAATTTTAAC----------------
--------------------------ATGTGGAGAAATGACATGGTAGAACAAATGCATGAGGATATA---
ATCAGTTTATGGGATCAAAGCCTA---AAGCCATGTGTAAAATTA---
ACTCCACTCTGTGTTACTTTGAATTGTAGTGATTTAAAAAATAATACTAATATCAATATTACT----------
----
-------------------------------------AGTGAGGAAGCAGGGAAGGAAATGACA---
AACTGCTCTTTCAATGTTACCACAGCTATAAGA---------------------------
GGCAAGGTGCGGAATGAATATGCTCTT---TTTTATAGACTTGATGTAGTATCAATAGATAAT----------
----

ACAAGCTATACATTGATAAATTGTAACTCCTCAGTCATTACACAA---
GCCTGTCCAAAGATATCCTTTGAACCAATTCCCATATATTATTGTGCCCCGGCTGGGTTTGCGATTATACAGT
GTAAT---GATAAGACATTCAAT------GGAACAGGACCATGTACAAAT-----------------------
-GTCAGC---ACAGTACAATGTACACATGGAATTAAGCCAGTAGTATCAACT---
CAACTGCTGTTAAATGGCAGTCTAGCAGAAGAAGAG------------
ATAATAATTAGATCTGAAAATTTCACAGACAAT---------GCTAAAACCATAATAGTACAGCTGAAC----
-----------GAATCTGTAACA---------ATTGATTGTATAAGACCCAACAACAATACAAGAAGA-----
-AGTATACATATA------------GGACCAGGGAGAGCA---TTT------TATGCAACA---GAC------
---------ATAATAGGAGATATA------------AGACAAGCATATTGT---AACATTAGTGGAGAA---
AAATGGAACAGCACTTTACACCAGCTAGCTATAAAATTAAGA---------------AAACAATTTAAT----
-----------------------------------AGTGAAACAATAGTCTTTGAG------
CAATCCTCAGGAGGGGACCCAGAGATTGTA---ATGCACAGCTTTAATTGTGGAGGGGAA---
TTTAAGCAGCTCCAGGCGAGAGTCCTGGCTGTGGAAAGATACCTAAAGGATCAGCAGCTCCTGGGGATTTGGG
GATGCTCTGGAAAGCTCATCTGCACCACTAATGTGCCTTGGAATACTAGCTGGAGT-----------------
----------------------------AATAAATCTCTGGATAATATCTGG------AAT------
AACATGACCTGGATGGAGTGG---GACAGAGAAATTACCAATTACACAAACTTAATATAC---
TCCTTGCTTGAAGAATCGCAGAACCAGCAGGAA--

AAGAATGAACAGGAATTATTGGAATTGGATAAATGGGACAGTTTGTGGAGTTGGTTTAGTATAACAAACTGGC
TGTGGTATATAAAAATATTCATAATGATAGTAGGAGGCTTGATAGGTTTAAGAATAATTTTTTCT---
GTGCTTGCTATAGTA---------AATAGAGTTAGGAAGGGATACTCACCA------
TTATCATTTGCTGTTAGTTTGCTTAACGCCACAGCCATAACAGTAGCTGAG---
GGGACAGATAGGATTATAGAAATAATACAAAGAGCTTATAGAGCT----------------------------
--------ATTCTCCACATA------CCTACAAGAATAAGACAGGGCTTGGAAAGGGCTTTGCTA---TAA
>KT878027
GAT---GCA---GCA------------------------------------
GAAAAAACATGGGTCACAGTTTACTATGGGGTACCTGTGTGG---AAAGACGCAAGC---
ACCACCTTATTTTGTGCATCAGATGCTAAAGCACAAAACAGAGAG---GTGCATAAC----------------
--------------GTCTGGGCCACA---CATGCCTGTGTACCCATAGAC---
CCCAATCCACAAGAAACAGTATTGGAA---------AATGTGACAGAAGATTTTAAT----------------
--------------------------ATGTGGAAAAATGGCATGGTAGAACAGATGCATGAGGATATA---
ATCAGTTTATGGGATCAAAGCCTA---AAGCCATGTGTAAAATTA---
ACTCCACTCTGTGTTACTTTAAATTGTAGGGAGGTGAATACAACTACTAATAGTTCTATTAGTATTAAGAATA
CAACTAGTGCT--
----------GATAATAATTCTACTAATAGTAATAAGACAGTGGAGGAGGATGGAGAAATGAAA---
AACTGCTCTTTCAATGTTACCACAAACATAGGA---------------------------
AATAAAAGGAAAGAAGAATATGCTTTG---TTTTATAAACTTGATGTAGTACAAATAGATAAT----------
----

TCAAGCTATACATTGATAAGTTGTAACTCCTCAGTCGTTACACAG---
GCCTGTCCAAAAGTGTCCTTTGAGCCAATTCCTATACATTATTGTACCCCGGCTGGTTTTGCGATTCTACAAT
GTAAT---GATAAGCAGTTCAAT------GGGTCAGGAAAATGTAATAAT-----------------------
-GTCAGC---ACAGTACAATGTACACATGGAATTAGGCCAGTAGTATCAACT---
CAATTGCTATTAAATGGCAGTCTAGCAGAAGAAGAT------------
ATAGTACTTAAATCTGAAAATTTCACAAACAAT---------GCAAAAACCATAATAGTACAGTTGAAC----

-----------TCATCTGTAGAA---------ATTAATTGTACAAGACCCAGTAACAATACAAGAAAA-----
-AGTATAAGGTTC------------GGACCAGGGAGTGCA---TTT------TTTGCACATGGAGAT------
---------GTAACAGGAGATATA------------AGAAAAGCATATTGT---AACATTAGTAGAGAA---
AGCTGGGAAAATACTTTAAGACAGATAGCTATAAAATTAAGA---------------GAAAAATTTGAG----
-----------------------------------AATAAAACAATAGCCTTTAAT------
AAATCCTCAGGAGGGGACCCAGAGATTGTA---ATGCACAGTTTTAATTGTGGAGGGGAA---
TTTAAGCAGCTCCAGGCAAGAGTCCTGGCTGTGGAAAGATACCTACAGGATCAACAGCTCCTGGGAATTTGGG
GATGCTCTGGAAAGCTCATCTGCACCACTAATGTGCCTTGGAATACTAGTTGGAGT----------------
----------------------------AACAAATCTCAGTATGAAATCTGG------AAT------
AACATGACCTGGAATGATTGG---GACAGAGAAATTGGTAATTACACAAACTTAATATAC---
TCCTTACTTGAAGTATCGCAAATCCAGCAAGAA--

AAGAATGAACAGGAACTATTAGAATTGGATAAATGGGCAAATTTATGGAGTTGGTTTGACATAACACAATGGC
TGTGGTATATAAAAATATTCATAATGATAATAGGAGGCTTGGTAGGTTTAAGAATAATTTTTGCT---
GTACTTTCTATAGTG---------AATAGAGTTAGGCAGGGATACTCACCC------
TTATCATTAGCTATTAGTTTGCTTAACGCCACAGCTAGAGCAGTAGGTGAG---
GGGACAGATAGGGTTATAGAATTAGTGCAAAGAGCTTTTAGAGCT---------------------------
--------ATTCTCCACATA------CCTAGAAGAATAAGACAGGGCTTGGAAAGGGCTTTGCTA---TAA
>MK871374
GCT---GCA--
ACAGGAGGATTGTGGGTCACAGTCTATTATGGGGTACCTGTGTGG---AAAGAAGCAATC---
ACCACCCTATTTTGTGCATCAGATGCTAAAGCATATGAAACAGAG---ATGCATAAT----------------
--------------GTTTGGGCCACA---CATGCCTGTGTACCCATAGAC---
CCCAACCCACTAGAAGTAGAATTGCCA---------AATGTGACAGAAAACTTTAAC---------------
--------------------------ATGTGGAAAAATAACATGGTAGAACAGATGCATGAGGATATA---
ATCAGTTTATGGGATCAAAGCCTA---AAGCCATGTGTAAAATTA---
ACTCCACTCTGTGTTATTTTGAATTGTAGTGATGCAAAGAATACTAGTACCAAT------------------
----
-------------------------AGTACTACGGAGAAAGTAATGGAGGAAGGAGAAATGAAA---
AACTGCTCTTTCAATGTTACCACGAAGATAGCA--------------------------
AATATGGTGAATAATGAACGTGATGTA---TTTGATGTAGTTGATATAGTTCATATAGGTAATAAT-------
----

GATACTACAAGCTATATATTGAGACATTGTAATACCACAGTCATAACACAG---
GCCTGTCCAAAGGTGTCCTTTGAACCAATTCCCATACATTATTGTGCCCCGGCTGGTTTTGCGATTCTAAAGT
GTAGA---GACAAGGGGTTCAAT------GGAACAGGACCATGTAAAAAT----------------------
-GTCAGT---ACAGTACAGTGTACACATGGAATTATGCCAGTAGTATCAACT---
CAGCTGCTGTTAAATGGCAGTCTAGCAGAAGGAGAA------------
ATGGTAATTAGATCTGAAAATTTCACGGACAAT---------GCTAAAAACATAATAGTACAGCTGAAC----
-----------ACATCTGTAGAA---------ATTAATTGTACAAGACCCAACAACAATACAAGAACA-----
-AGTATAACTTTA------------GGACCAGGGAGAGCA---TTT------TTTGCAACAGGAGAT------
---------ATAATAGGAAATATA------------AGATATGCATATTGT---AACATTAGTGAAGAA---
AAATGGAACGACACCTTAAGACAGCTAGTTATAAAATTAAGA---------------
GATCAATTTAAAGATCAATTT------CCA------------------AATCTCACAACAATATACTTTAAT-
-----AGTTCCTCAGGAGGGGACCCAGAGATTACA---ACGCACAGCTTTAACTGTGGAGGGGAA---
TTTAAACAGCTCCAGGCAAGAGTCCTGGCTGTAGAAAGATACCTACAGGATCAACGGCTCCTGGGGATTTGGG
GATGCTCTGGGAAGCTCATCTGCACCACTAGTGTGCCTTGGAATACTAGTTGGAGT----------------
----------------------------AATAAATCTCATGATGAAATCTGG------AAT------
AACATGACCTGGATGGCGTGG---GATAGAGAAATTAACAATTACACACAGGTGATATAC---
GACCTGCTTGAGGAATCACAGATCCAGCAAGAA--

AAGAATGAGCAGGAATTATTGAAATTGGATTCATGGGCAGGTCTGTGGAAGTGGTTGGACATATCAAACTGGC
TGTGGTATATACAAATATTCATGATGATGGTAGGAGGCTTGGTAGGTTTAAGAATAATTCTTTGT---
GTACTTTCTGTAGTG---------AATAGAGTTAGGCAGGGATACTCACCA------
TTACCGTTTGCTGTTAGTTTGCTTAATGCCACAGCTATAGCAGTAGCTGAG---
GGGACAGATAGAGTTATAGAAATATTACAAAGAATTTATAGAGCT---------------------------
--------ATTATCCACATA------CCTACAAGAATAAGACAGGGCTTAGAAAGGGCTTTGCTA---TAA
>MN043576
GCT---GCT--
GAATTGTGGGTCACAGTCTATTATGGGGTACCTGTGTGG---AAAGAAGCAACC---

ACCACCCTATTTTGTGCATCAGATGCTAAAGCATATGACACAGAG---GTACATAAT----------------
--------------GTTTGGGCCACA---CATGCCTGTGTACCCATAGAC---
CCCAACCCACAAGAAGTAGTATTGGAA---------AATGTGACAGAGAATTTTAAC----------------
--------------------------ATGTGGAAAAATAACATGGTAGAACAGATGCATGAGGATATA---
ATCAGTTTATGGGATCAAAGCCTA---AAGCCATGTGTAAAATTA---
ACTCCACTCTGTGTTACTTTGAATTGTAGTAATTTGAAGAATGAAACTACTAAGAATGAAACTAATACCAAT-
----
-------------------------AATAGTAGTGGGAATATGAGGGAGGAAGGAGAAATGAAA---
AACTGCTCTTTCAATGTTACCACAAGCATAAGA---------------------------
GATAAGGTGCAGAAAGAATATGCTCTT---TTTTATAAACTTGATGTAGAACAAATAGATAAT----------
----

ACAAGCTATACATTGATAAATTGTAACTCCTCAGTCATTACACAG---
GCCTGTCCAAAGGTATCCTTTGAACCAATTCCCATACATTATTGTACCCCGGCTGGTTTTGCGATTCTACAGT
GTAAT---GATAAAAAGTTCAAT------GGAACAGGACCATGTTCAAAT-----------------------
-GTCAGC---ACAGTACAATGTACACATGGAATTAGGCCAGTAGTGTCAACT---
CAACTGCTGTTAAATGGCAGTCTAGCAGAAGAAGAG------------
ATAGTAATTAGATCTGAAAATTTCACAGACAAT---------GCTAAAACCATAATAGTACAGCTGAAC----
-----------GCATCTGTAGAA---------ATTAATTGTACAAGACCCAACAACAATACAAGAAAA-----
-AGTATAAGAATA------------GGACCAGGGAGTACA---TTT------TATGCAACAGGAGGC------
---------ATAATAGGAGATATA------------AGACAAGCACATTGT---AACATTAGTGGAGCA---
AAATGGAACAACACTTTAAGACAGCTAGTTATAAAATTAAGA---------------GAACAATTTAGG----
---CTTTAAT------
CAATCCTCAGGAGGGGACCCAGAGATTGTA---ATGCACAGTTTTAATTGTGGAGGGGAA---
TTTAAGCAGCTCCAGGCAAGAGTCCTGGCTGTGGAAAGATACCTAAAGGATCAACAGCTCCTGGGGATTTGGG
GATGCTCTGGAAAGCTCATCTGCACCACTAATGTGCCTTGGAATACTAGTTGGAGT-----------------
----------------------------AATAAATCTCAGAAGGAGATCTGG------GAT------
AACATGACCTGGATGGAGTGG---GACAGAGAAATTAACAATTACACAAACTTAATATAC---
ACCTTACTTGAAGAATCGCAGAACCAACAAGAA--

AAGAATGAACAGGAACTATTGGAATTGGATAAATGGGCAAGTTTGTGGAATTGGTTTGACATATCAAAATGGC
TGTGGTATATAAGAATATTCATAATGATAGTAGGAGGCTTAGTAGGTTTAAGAATAATTTTTCT---
GTACTTTCTATAGTG---------AATAGAGTTAGGCAGGGATACTCACCA------
TTATCGTTTGCTGTTAGTTTGCTTAATACCACAGCTGTAGCAGTAGCTGAG---
GGGACAGATAGGGTTATAGAAATAGTACAAAGAGCTTATAGAGCT----------------------------
--------ATTCTCCACATA------CCTACAAGGATAAGACAGGGCTTTGAAAGGGCTTTGCTA---TAA
>KU896118
GCT---GCA---GCA-------------------------------------
GAAGACTTGTGGGTTACAGTCTATTATGGGGTACCTGTGTGG---AAAGAAGCAACC---
ACCACCCTATTTTGTGCATCAGATGCTAAAGCATATGACACAGAG---GTACATAAT----------------
--------------GTTTGGGCCACA---CATGCCTGTGTACCCATAGAC---
CCCAACCCACAAGAAGTAAGATTGCCA---------AATGTGACAGAAAATTTTAAC----------------
--------------------------ATGTGGAAAAATAACATGGTAGAACAGATGCATGAGGATATA---
ATCAGTTTATGGGATCAAAGCCTA---AAGCCATGTGTAAAATTA---
ACTCCACTCTGTGTTACTTTGAATTGTAGTAATTTGAATACCAATAGTAGTAATGTGAATAGTAGTACT----
----
----------------------AATACCACTAGTAGTAGTGGGGGAGTGATGGAGGAAATGAAA---
AACTGCTCTTTCAATGTTACCACAACCATAAGA---------------------------
AATAAGGTGCAGAAAGAATATGCTCTT---TTTTATAAACTTGATGTAGCACCAATAGATAAT----------
----

GATAATACAAGCTATACATTGATAAATTGTAACTCCTCAACCATTACACAG---
GCCTGTCCAAAGGTATCCTTTGAACCAATTCCCATACATTATTGTACCCCGGCTGGTTTTGCGATTCTACAAT
GTAAT---GATAAGAAGTTCAAT------GGAACAGGACCATGTTCAGAT-----------------------
-GTCAGC---ACAGTACAATGTACACATGGAATTAGGCCAGTAGTATCAACT---
CAACTGCTGTTAAATGGCAGTCTAGCAGAAGAAGAG------------
ATAGTAATTAGATCTGAAAATTTCACAGACAAT---------GCTAAAATCATAATAGTACAGCTGAAC----
-----------GCATCTGTAGAA---------ATTAATTGTACAAGACCCAACAACAATACAAGAAAA-----
-GGTATAGGTTTA------------GGACCAGGGAGCACA---TTT------TATGCAACAGAGGCA------
---------ATAATAGGAGATATA------------AGACAAGCACATTGT---AACATTAGTAGAGCA---

AAATGGAACAACACTTTAAGACAGCTAGCTATAAAATTAAGA--------------GAACAATTTGAG----
----------------------------------AATAAAACAATAGCCTTTAAT------
AAATCCTCAGGAGGGGACCCAGAGATTGTA---ATGCACAGTTTTAATTGTGGAGGGGAA---
TTTAAGCAGCTCCAGGCAAGAGTCCTGGCTGTGGAAAGATACCTAAAGGATCAACAGCTCCTGGGGATTTGGG
GATGCTCTGGAAAGCTCATCTGCACCACTAATGTGCCTTGGAATACTAGTTGGAGT----------------
---------------------------AATAAATCTCTGAATGATATTTGG------GAT------
AACATGACCTGGATGGAGTGG---GACAGAGAAATTAACAATTACACAAACTTAATATAC---
ACCTTACTTGAAGAATCGCAGAACCAACAAGAA---------------------------------------
----
AAGAATGAACAGGAATTATTGGAATTGGATAAATGGGCAAGTTTGTGGAGTTGGTTTGACATATCAAATTGGC
TATGGTATATAAGAATATTCATAATGATAGTAGGAGGCTTGGTAGGTTTAAGAATAATTTTTTCT---
GTACTTTCTATAGTA---------AATAGAGTTAGGCAGGGATACTCACCA------
TTATCGTTTGCTGTTAGTTTGCTTAATGCCACAGCTAGAGCAGTAGCTGAG---
GGGACAGATAGGGTTATAGAAATATTACAAAGAGCTTTTAGAGCT---------------------------
--------ATTCTCCACATA------CCTAGAAGAATAAGACAGGGCTTGGAAAGGGCTTTGCTA---TAA
>MN043598
GCT---GCA--------------------------------------
GCAGAAGACTTGTGGGTTACAGTCTATTATGGGGTACCTGTGTGG---AAAGAAGCAACC---
ACCACCCTATTTTGTGCATCAGATGCTAAAGCATATGACACAGAG---GTACATAAT---------------
--------------GTTTGGGCCACA---CATGCCTGTGTACCCATAGAC---
CCCAACCCACAAGAAGTAAGATTGCCA---------AATGTGACAGAAAATTTTAAC---------------
-------------------------ATGTGGAAAAATAACATGGTAGAACAGATGCATGAGGATATA---
ATCAGTTTATGGGATCAAAGCCTA---AAGCCATGTGTAAAATTA---
ACTCCACTCTGTGTTACTTTGAATTGTAGTAATTTGAATACCAATAGTAGTAATGTGAATAGTAGTACTAATA
CCACTAGTAGT---
------------------------------------AGTGGGGGAGTGATGGAGGAAATGAAA---
AACTGCTCTTTCAATGTTACCACAACCATAAGA--------------------------
AATAAGGTGCAGAAAGAATATGCTCTT---TTTTATAAACTTGATGTAGCACCAATAGATAAT---------
----

GATAATACAAGCTATACATTGATAAATTGTAACTCCTCAACCATTACACAG---
GCCTGTCCAAAGGTATCCTTTGAACCAATTCCCATACATTATTGTACCCCGGCTGGTTTTGCGATTCTACAAT
GTAAT---GATAAGAAGTTCAAT------GGAACAGGACCATGTTCAGAT----------------------
-GTCAGC---ACAGTACAATGTACACATGGAATTAGGCCAGTAGTATCAACT---
CAACTGCTGTTAAATGGCAGTCTAGCAGAAGAAGAG-----------
ATAGTAATTAGATCTGAAAATTTCACAGACAAT---------GCTAAAATCATAATAGTACAGCTGAAC----
-----------GCATCTGTAGAA---------ATTAATTGTACAAGACCCAACAACAATACAAGAAAA-----
-GGTATAGGTTTA------------GGACCAGGGAGCACA---TTT------TATGCAACAGAGGCA------
---------ATAATAGGAGATATA------------AGACAAGCACATTGT---AACATTAGTAGAGCA---
AAATGGAACAACACTTTAAGACAGCTAGCTATAAAATTAAGA--------------GAACAATTTGAG----
----------------------------------AATAAAACAATAGCCTTTAAT------
AAATCCTCAGGAGGGGACCCAGAGATTGTA---ATGCACAGTTTTAATTGTGGAGGGGAA---
TTTAAGCAGCTCCAGGCAAGAGTCCTGGCTGTGGAAAGATACCTAAAGGATCAACAGCTCCTGGGGATTTGGG
GATGCTCTGGAAAGCTCATCTGCACCACTAATGTGCCTTGGAATACTAGTTGGAGT----------------
---------------------------AATAAATCTCTGAATGATATTTGG------GAT------
AACATGACCTGGATGGAGTGG---GACAGAGAAATTAACAATTACACAAACTTAATATAC---
ACCTTACTTGAAGAATCGCAGAACCAACAAGAA---------------------------------------
----
AAGAATGAACAGGAATTATTGGAATTGGATAAATGGGCAAGTTTGTGGAGTTGGTTTGACATATCAAATTGGC
TATGGTATATAAGAATATTCATAATGATAGTAGGAGGCTTGGTAGGTTTAAGAATAATTTTTTCT---
GTACTTTCTATAGTA---------AATAGAGTTAGGCAGGGATACTCACCA------
TTATCGTTTGCTGTTAGTTTGCTTAATGCCACAGCTAGAGCAGTAGCTGAG---
GGGACAGATAGGGTTATAGAAATATTACAAAGAGCTTTTAGAGCT---------------------------
--------ATTCTCCACATA------CCTAGAAGAATAAGACAGGGCTTGGAAAGGGCTTTGCTA---TAA
>MN043588
GCT---GCA---
GGAGGATTGTGGGTCACAGTCTATTATGGGGTACCTGTGTGG---AAAGAAGCAACC---
ACCACCCTATTTTGTGCATCAGATGCTAAAGCATATGACACAGAG---GTACATAAT---------------
--------------GTTTGGGCCACA---CATGCCTGTGTACCCATAGAC---
CCCAACCCACAAGAAGTAGTATTGGAA---------AATGTGACAGAAAATTTTAAC---------------

--------------------------ATGTGGAAAAATAACATGGTAGAACAGATGCATGAGGATATA---
ATCAGTTTATGGGATCAAAGCCTA---AAGCCATGTGTA-AATTA---
ACTCCACTCTGTGTTACTTTGAATTGTAGTAATGTGAATGTGAATGTTACTAAGACCAATAGCAGTAGTACC-
----
-------------------------AGTAGTAATGGGGGACTGATGGAGGAAGGAGAAATGAAA---
AACTGCTCTTTCAATGTTACCACAAACATAAGA---------------------------
AATAAGGTGCAGAAAGAATATGCTCTT---TTCTATAAACTTGATGTAATACCAATAGGTAAT----------
----

ACAAGCTATACATTGATAAATTGTAACACCTCAGTCATTACACAG---
GCCTGTCCAAAGGTATCCTTTGAACCAATTCCCATACATTATTGTACCCCGGCTGGTTTTGCGATTCTACAGT
GTAAT---GATAAGAAGTTCAAT------GGAACAGGACCATGTACAAAT-----------------------
-GTCAGC---ACAGTACAATGTACACATGGAATTAGGCCAGTAGTATCAACT---
CAACTGCTGTTAAATGGCAGTCTAGCAGAAGAAGAG------------
ATAGTAATTAGATCTGAAAATTTCACGGACAAT---------GCTAAAACCATAATAGTACAGCTGAAC----
-----------GCATCTGTAGAA---------ATTAATTGTACAAGACCCAACAACAATACGAGAAAA-----
-AGTACAAGTATA------------GGACCAGGGAGAACA---TTT------TATGCAACAGGAGAC------
---------ATAATAGGAAATATA------------AGACAAGCACATTGT---AACATTAGTGAAGCA---
AAATGGAACAACATTTTAAGACAGCTAGCTATAAAATTAAGA---------------GAACAATTTGGG----
-----------------------------------AATAAAACAATAGTCTTCAAT------
CAATCCTCAGGAGGGGACCCAGAGATTGTA---ATGCACAGTTTTAATTGTGGAGGGGAA---
TTTAAGCAGCTCCAGGCAAGAGTCCTGGCTGTGGAAAGATACCTAAAGGATCAACAGCTCCTGGGAATTTGGG
GATGCTCTGGAAAGCTCATCTGCACCACTAATGTGCCTTGGAATACTAGTTGGAGT-----------------
----------------------------AACAAATCTCAGGATGATATCTGG------CAT------
AACATGACCTGGATGGAGTGG---GACAGAGAAATTGACAATTACACAAACTTAATATAC---
ACCTTACTTGAAGAATCGCAGATCCAACAAGAA--

AAGAATGAACAGGAACTATTGGAATTGGATAAATGGGCAAGTTTGTGGAATTGGTTTGACATATCAAATTGGC
TGTGGTATATAAGAATATTCATAATGATAGTAGGAGGCTTGGTAGGTTTAAGAATAATTTTTTCT---
GTACTTTCTATAGTG---------AATAGAGTTAGGCAGGGATACTCACCA------
TTATCGTTTGCTGTTAGTTTGCTTAATGCCACAGCTAGAGCAGTAGCTGAG---
GGGACAGATAGGGTTATAGAACTATTACAAAGAGCTTATAGAGCT----------------------------
--------ATTCTCCACATA------CCTAGAAGAATAAGACAGGGCTTGGAAAGGGCTTTGCTA---TAA
>KU869580
GCT---ACA--
GAAAAATTGTGGGTCACAGTCTATTATGGGGTACCTGTGTGG---AAAGAAGCAACC---
ACCACCCTATTTTGCGCATCAGAGGCTAAAGCATATGACACAGAG---GTACATAAT----------------
--------------GTTTGGCCACA---CATGCCTGTGTACCCATAGAC---
CCCGACCCACAAGAAGTATTATTGGAA---------AATGTGACAGAAAATTTTGAC----------------
--------------------------ATGTGGAGAAATAACATGGTAGAACAGATGCATGAGGATATA---
ATCAGTTTATGGGATCAAAGCCTA---AAGCCATGTGTAAAATTA---
ACTCCACTCTGTGTTACTTTGAATTGTAGTAATTTGAAGAATGAGACTAATACCAATAATGGGACTAAT----
----
-------------------ACCACTAGTAGTATTGGGGGGGTGATGGAGGAAGGAGAAATAAAA---
AACTGCTCTTTCAATGTTACCACAAGCATAAGA---------------------------
AATAAGATGAAGAAAGAATATGCTCTT---TTTTATAAACTTGATGTAGTATCAATAGATAAT----------
----

GATAATACAAGCTATACATTGATAAATTGTAACACCTCAGTCCTTACACAG---
GCCTGTCCAAAGGTATCCTTTGAACCAACTCCCATACATTATTGTACCCCAGCTGGTTTTGCGATTCTACAAT
GTAAT---GATAAGAAGTTCAAT------GGATCAGGACCATGTACAAAT-----------------------
-GTCAGC---ACAGTACAATGTACACATGGAATTAGGCCAGTAGTATCAACT---
CAACTGTTATTAAATGGCAGTCTAGCAGAAGAAGAG------------
ATAGTAATTAGATCTGAAAATTTCACGGACAAT---------GCTAAAACCATAATAGTACAGCTGAAC----
-----------GCATCTGTAGTA---------ATTAATTGTACAAGACCCAACAACAATACAAGAAAA-----
-AGTATACATTTA------------GGACCAGGGAGCACA---TTT------TATGCAACAGGAGAC------
---------ATAATAGGAGATATA------------AGACAAGCACATTGT---AACATTAGTGGAGGA---
GAATGGAATAACACTTTAAGACAGCTAGTTATAAAATTAAGA---------------GAACAATTTGGG----
--------------------------------AGTAGTAAAACAATAAACTTTACT------
CGATCCTCAGGAGGGGATCCAGAGATTGTA---ATGCACAGTTTTAATTGTGGAGGGGAA---

TTTAAGCAGCTCAAGGCAAGAGTCCTGGCTGTAGAAAGATACCTAAGGGATCAACAGCTCCTGGGGATTTGGG
GATGCTCTGGAAAGCTCATCTGCACCACTAATGTGCCTTGGAATACTAGTTGGAGT-----------------
----------------------------AATAAATCTCTGGATGATATCTGG------GAT------
AACATGACCTGGATGCAGTGG---GACAGAGAAATTAACAATTACACAACTTTAATATAC---
TCTTTACTTGAAGAATCGCAGAACCAACAAGAA---

AAGAATGAACAGGGATTATTGGAATTGGATAAATGGGCAAGTTTGTGGAATTGGTTTGACATAACACGATGGC
TGTGGTATATAAAAATATTCATAATGATAGTAGGAGGCTTGGTAGGTTTAAGAATAATTTTTTCT---
GTACTTTCTATAGTA---------AATAGAGTTAGGCAGGGATACTCACCA------
TTATCGTTTGCTGTTAGCTTGCTTAATGCCACAGCTATAGCAGTAGCTGAG---
GGGACAGATAGGGTTATAGAAATATTACAAAGAGCTTATAGAGCT----------------------------
--------ATTCTCCACATA------CCTACAAGAATAAGACAGGGCTTGGAAAGGGCTTTGCTA---TAA
>MN043589
GCT---GCA--------------------------------------
GCAGGAGGATTGTGGGTCACAGTCTATTATGGGGTACCTGTGTGG---AAAGAAGCAACC---
ACCACCCTATTTTGTGCATCAGATGCTAAAGCATATGACACAGAG---GTACATAAT----------------
--------------GTTTGGGCCACA---CATGCCTGTGTACCCATAGAC---
CCCAACCCACAAGAAGTAGTATTGGAA---------AATGTGACAGAAAATTTTAAC----------------
--------------------------ATGTGGAAAAATAACATGGTAGAACAGATGCATGAGGATATA---
ATCAGTTTATGGGATCAAAGCCTA---AAGCCATGTGTAAAATTA---
ACTCCACTCTGTGTTACTTTAAATTGTAGTGATTTGAAGAATGATACTAGTACCAATAAT-------------
----
----------------------------AGTAGTGGGGGAGTGACGGAAGGAGGAGAAATGAAA---
AACTGCTCTTTCAATGTTACCACAAACTTAAGA--------------------------
GATAAGGTGAAGCAAGAATATGCTCTT---TTTTATAGACTTGATGTAGTACCAATAGATAAT----------
----

ACAAGCTATACATTGATAAATTGTAACTCCTCAGTCCTTACACAG---
GCCTGTCCAAAGGTATCCTTTGAACCAATTCCCATACATTATTGTACCCCGGCTGGTTTTGCGATTCTACAGT
GTAAT---GATAAGAAGTTCAAT------GGAACAGGACCATGTACAAAT-----------------------
-GTCAGC---ACAGTACAATGTACACATGGGATTAGGCCAGTAGTGTCAACT---
CAACTGCTGTTAAATGGCAGTCTAGCAGAAGAAGAG-----------
ATAGTAATTAGATCTGAAAATTTCACGGACAAT---------GCTAAAACCATAATAGTACAGCTGAAC----
-----------GCATCTGTAGAA---------ATTAATTGTACAAGACCCAACAACAATACAAGAACA-----
-AGTATAAGTATA------------GGACCAGGGAGAACA---TTT------TATGCAACAGGAGAC------
---------ATAATAGGAGATATA-----------AGACAAGCACATTGT---AACATTAGTGGAGAA---
AAATGGAACAACACTTTAAGACAGCTAGTTATAAAATTAAGA---------------GAACAATTTGGG----
-----------------------------------AATAAAACAATAGTCTTTAAT------
CAATCCTCAGGAGGGGACCCAGAGATTGTA---ATGCACAGTTTTAATTGTGGAGGGGAA---
TTTAAGCAGCTCCAGGCAAGAGTCCTGGCTGTGGAAAGATACCTAAAGGATCAACAGCTCCTGGGGATTTGGG
GATGCTCTGGAAAGCTCATCTGCACCACTAATGTGCCTTGGAATACTAGTTGGAGT-----------------
----------------------------AATAAATCTCTGAAGTATATCTGG------GAT------
AACATGACCTGGATGGAGTGG---GACAGAGAAATTAACAATTACACAAACTTAATATAC---
ACCTTACTTGAAGAATCGCAGAACCAACAAGAA---

AAGAATGAACAGGAATTATTGGAATTGGATAAATGGGCAAGTTTGTGGAATTGGTTTGACATATCACAATGGC
TGTGGTATATAAGAATATTCATAATGATAGTAGGAGGCTTGATAGGTTTAAGAATAATTTTTTCT---
GTACTTTCTATAGTA---------AATAGAGTTAGGCAGGGATACTCACCA------
TTATCATTGGCTGTTAGTTTGCTTAATGCCACAGCTAGAGCAGTAGCTGAG---
GGGACAGATAGGGTTATAGAACTATTACAAAGAGCTTGTAGAGCT----------------------------
--------ATTTTCAACATA------CCTAGAAGAATAAGACAGGGCTTGGAAAGGGCTTTGCTA---TAA
>KU869596
GCT---GCA---AGA-----------------------------------
GAAGAATCGTGGGTCACAGTCTATTATGGGGTACCTGTATGG---AAAGAAGCAACC---
ACCACCCTATTTTGTGCATCAGATGCTAAAGCATATGACACAGAG---GTACATAAT----------------
--------------GTTTGGGCCACA---CATGCCTGTGTACCCATAGAC---
CCCAACCCACAAGAAGTAGTATTGGAA---------AATGTGACAGAAAATTTTAAC----------------
--------------------------ATGTGGAAAAATAACATGGTAGAACAGATGCATGAGGATATA---
ATCAGTTTATGGGATCAAAGCTTA---AAGCCATGTGTAAAGTTA---
ACTCCACTCTGTGTTACTTTAAATTGTAGAAATGAGACTTTGGAGAATGATACTAATACCACTCATAGTGATT

GGAATGATACT--
----------------GTTACCAATAAGAGTAGTGGGGGAGTGATAAAGGAAAGAGAAATGAAA---
AACTGCTCTTTCAATGTTACCACAAGCATAAGA---------------------------
AATAAGGTGCAGAAAGAATATGCTCTT---TTTTATAAACTTGATGTAGTACCAATAGATGAC----------
----

GATAATACAAGCTATACGTTGATAAATTGTAACACCTCAGTCATTAAACAG---
GCCTGTCCAAAGGTATCCTTTGAACCAATTCCCCTACATTATTGTACCCCGGCTGGTTTTGCGAGTCTACAGT
GTAAT---GAGAAGAATTTCAAT------GGAACAGGACCATGTAAAAAT-----------------------
-GTCAGC---ACAGTACAATGTACACATGGAATTAGGCCAGTAGTATCAACT---
CAACTGCTGTTAAATGGCAGTCTAGCAGAAAAAGAG------------
ATAGTAATTAGATCTGAAAATTTCACGGACAAT---------GCTAAAACCATAATAGTACAGCTGAAC----
-----------GCATCTGTAGAA---------ATTAATTGTACAAGACCCGGCAACAATACAAGTAAA-----
-AGTATAAGGATA------------GGACCAGGGAGCACA---TTT------TATGCAACAGAAAGA------
---------ATAATAGGAGATATA------------AGACAAGCACATTGT---AACATTAGTGGAGTA---
AAATGGAAAAACACTTTAAAACAGCTAGTTCTAAAATTAAGA---------------AAACAATTTGGG----
-----------------------------------AATAAAACAATAGCCTTTAAG------
CAATCCTCAGGAGGGGACCCAGAGATTGTA---ATGCACAGTTTTAATTGTGGAGGGGAA---
TTTAAGCAGCTCCAGGCAAGAGTCCTGGCTGTGGAAAGATACCTAAAGGATCAACAGCTCCTGGGGATTTGGG
GATGCTCTGGAAAGCTCATCTGCACCACTAATGTGCCTTGGAATACTAGTTGGAGT-----------------
----------------------------AATAAATCTCAGGAGGAGATCTGG------AAT------
AACATGACCTGGATGGAGTGG---GACAGAGAAATTAACAATTACACAAACTTAATATAC---
AACTTACTTGAAGAATCGCAGAACCAACAAGAA--

AAGAATGAACAGGAATTATTGGCATTGGATAAATGGGCAAGTTTGTGGAATTGGTTTGAAATATCACACTGGC
TGTGGTATATAAAAATATTCATAATGATAGTAGGAGGCTTGGTAGGTTTAAGAATAATTTTTTCT---
GTACTTTCTATAGTA---------AATAGAGTTAGGCAGGGATACTCACCA------
TTATCGTTTGCTGTTAGTTTGCTTAATGCCACAGCTATAGCAGTAGCTGAG---
GGGACAGATAGGGTTATAGAAATATTACAAAGAGCTTATAGGGCT----------------------------
--------ATCCTTCACATA------CCTACAAGAATAAGACAGGGCTTGGAAAGGGCTTTGCTA---TAA
>KX960971
GCT---ACA--
GGAGGATTGTGGGTCACAGTCTATTATGGGGTACCTGTGTGG---AAAGAAGCAACC---
ACCACCCTATTTTGTGCATCAGATGCTAAAGCATATAAAACAGAG---GCACATAAT----------------
--------------GTTTGGGCCACA---CATGCCTGTGTACCCATAGAC---
CCCAACCCACAAGAAGTAGTATTGCAA---------AATGTGACAGAAAATTTTAAC----------------
--------------------------ATGTGGAAAAATGACATGGTAGAACAGATGCATGCGGATATA---
ATCAGTTTATGGGATCAAAGCCTA---AAGCCATGTGTAAAATTA---
ACTCCACTCTGTGTTACTTTGAATTGTAGTAATTTGAATACTACTAATACCAATAATAGT-------------
----
-------------ACTAGTACCAATAATACTAGTGGGAGAGTGATGGAGGAAGGAGAAATGAAA---
AACTGCTCTTTCAATGTTACCACAACCATAAGA---------------------------
GATAAGGTGCAGAAAGAATATGCTCTT---TTTTATAAGCTTGATGTAGTATCAATAGATAAGAAT-------
----

GATAATACAAGCTATACATTGATAAATTGTAACTCCTCAGTCATTACACAG---
GCCTGTCCAAAGGTATCCTTTGAACCAATTCCCATACATTATTGTACACCGGCTGGTTTTGCGATTCTACAGT
GTAAT---GATAAGCAGTTCAAT------GGATCAGGACCATGTAGAAAT-----------------------
-GTCAGC---ACAGTACAATGTACACATGGAATTAGGCCAGTAGTATCAACT---
CAATTGCTGTTAAATGGCAGTCTAGCAGAAGAAGAG------------
ATAGTAATTAGATCTGAAAATTTCACAAACAAT---------GCTAAAACCATAATAGTACAGCTGAAC----
-----------GCATCTGTAGAA---------ATGAATTGTATAAGACCCAACAACAATACAAGAAAA-----
-AGTATAAGTTTC------------GCACCAGGGAGTACA---TTT------TATGCAACAGGAGAC------
---------ATAATAGGAGATATA------------AGACAAGCACATTGT---AACATTAGTGAAGAA---
AAATGGAATAACACTTTAAAACGGCTAGTTATAAAATTAAGA---------------GAACAATTTGGG----
-----------------------------------AATAAAACAATAATCTTTAAT------
CAATCAGCAGGAGGGGACCCAGAGATTATA---ATGCACAGTTTTAATTGTGGAGGGGAA---
TTTAAGCAGCTCCAGGCAAGAGTCCTGGCTGTGGAAAGATACCTAAAGGATCAACAGCTCCTGGGGATTTGGG
GATGCTCTGGAAAGCTCATCTGCACCACTAATGTGCCTTGGAATACTAGTTGGAGT-----------------
----------------------------AATAAATCTCACGATGTTATCTGG------AAT------

AACATGACCTGGATGCAGTGG---GACAGAGAAATTAACAATTACACAAACTTAATATAC---
ACCTTACTTGAAGAATCGCAGAACCAGCAAGAA--

AAGAATGAACAGGAATTATTGGAATTGGATAAATGGTCAAGTTTGTGGAGTTGGTTTGACATATCAAACTGGC
TGTGGTATATAAAAATATTCATAATGATAGTAGGAGGCTTGGTAGGTTTAAGAATAATTTTTTCT---
GTACTTTCTATAGTG---------AATAGAGTTAGGCAGGATACTCACCA------
TTATCGTTTGCTGTTAGTTTGCTTAATGCTACAGCTATAGCAGTAGCTGAG---
GGGACAGATAGGGTTATAGAAATATTACAAAGAGCTTATAGAGCT----------------------------
--------ATTCTCCACATA------CCTACAAGAATAAGACAGGGCTTGGAAAGGCTTTGCTA---TAA
>KJ140267
GCT---ACA--
GAAAGATTGTGGGTCACAGTCTATTATGGGGTACCTGTGTGG---AAAGAAGCAACC---
ACCACCCTATTTTGTGCATCAGATGCTAAAGCATATGACAGAGAG---GTACATAAT----------------
--------------GTTTGGGCCACA---CATGCTTGTGTACCCATAGAC---
CCCAACCCACAAGAAGTAGTATTGGAA---------AATGTGACAGAAAATTTTAAC----------------
--------------------------ATGTGGAAAAATAACATGGTAGAACAGATGCATGCGGATATA---
ATCAGTTTATGGGATCAAAGCCTA---AAGCCATGTGTAAAATTA---
ACTCCACTCTGTGTTACTTTGAATTGTAGTAATTTGAGGAATGATACTAATACCACTAGT-------------
----
-------------------------AGTAGTCAAGAGGAAGTGATGGAGAGAGAAGGAATGAGA---
AACTGCTCTTTCAATGTTACCACAAACATAAGA---------------------------
AATAAGGTGAAGGAGGAATATGCTCTT---TTTTATAAACTTGATATAGTACAAATAGATAAT----------
----

GATAATACAAGCTATACATTGATAAACTGTAACTCCTCAGTCATTACACAG---
GCCTGTCCAAAGGTATCTTTTGAACCAATTCCCATACATTATTGTACCCCGGCTGGTTTTGCGATTCTACAGT
GTAAT---GATAAGAAGTTCAAT------GGAACAGGACCATGTAAAAAT-----------------------
-GTCAGC---ACAGTACAATGTACACATGGAATTAAGCCAGTAGTATCAACT---
CAACTGCTGTTAAATGGCAGTCTAGCAGAAGAAGAG------------
GTAATAATTAGATCTGAAAATTTCACGAACAAT---------GCTAAAACCATAATAGTACAGCTGAAC----
-----------GCATCTGTAGAA---------ATTAATTGCACAAGACCCAACAACAACACAAGAAAA-----
-AGTATACATCTA------------GGACCAGGGAGTACA---TTT------TTTGCAACAGGAGAA------
---------ATAATAGGAGATATA------------AGACAAGCACATTGT---AACATTAGTGAAGAA---
AAATGGAACAACACTTTAAAACAGCTAGTTATAAAATTAAGA---------------AAACAATTTGGG----
-----------------------------------AATAAAACAATAGTCTTTAAC------
CAATCCTCAGGAGGGGACCCAGAGATTGTA---ATGCACAGTTTTAATTGTGGAGGGGAA---
TTTAAGCAGCTCCAGGCAAGAGTCCTGGCTGTGGAAAGATACCTAAAGGATCAACAGCTCCTGGGGATTTGGG
GATGCTCTGGAAAGCTCATCTGCACCACTAATGTGCCTTGGAATACTAGTTGGAGT-----------------
----------------------------AATAAATCTCTGAATCATATCTGG------GAT------
AACATGACCTGGATGGAGTGG---GACAGAGAAATTAACAATTACACAACCTTAATATAC---
ACCTTACTTGAGGAATCGCAGAACCAACAAGAA--

AAGAATGAACAGGAATTATTGGAATTGGATAAATGGGCAAGTTTGTGGAATTGGTTTGACATATCAAATTGGC
TGTGGTATATAAGAATATTCATAATGATAATAGGAGGCTTGGTAGGTTTAAGAATAATTTTTTCT---
GTACTTTCTATAGTG---------AATAGAGTTAGGCAGGATACTCACCA------
TTATCGTTGGCTGTTAGTTTGTTCAATGCCACAGCTATAGCAGTAGCTGAG---
GGGACAGATAGGGTTATAGATATATTACAAAGAGCTTATAGGGCT----------------------------
--------ATTCTCCACATA------CCTACAAGAATAAGACAGGGCTTAGAAAGGCTTTGCTA---TAA
>KJ140248
GCT---GAT---
GAATTGTGGGTCACAGTCTATTATGGGGTACCTGTGTGG---AAAGAAGCAACC---
ACCACCCTATTTTGTGCATCAGATGCTAAAGCATATGACACAGAG---GTACATAAT----------------
--------------GTTTGGGCCACA---CATGCCTGTGTACCCATAGAC---
CCCAACCCACAAGAAGTAGTATTGAAA---------AATGTGACAGAAAATTTTAAC----------------
--------------------------ATGTGGAAAAATAACATGGTAGAACAGATGCATGAGGATATA---
ATCAGTTTATGGGATCAAAGCCTA---AAGCCATGTGTAAAATTA---
ACTCCACTCTGTGTTACTTTGAATTGTAGTAATTTGAAGAATGAGACTAATACCAATAAT-------------
----
----------------------------AGTAGTGGGAGAGTGATGGAGAAAGTAGAAATGAAA---
AACTGCTCTTTCAATGTTACCACAAACATAAGA---------------------------

GATAAGGTGAAGAAAGAATATGCTCTT---TTTTATAAACTTGATGTAACACAAATAGATAAT----------
----

ACAAGCTATACATTGATAAATTGTAACTCCTCAGTCATTACACAG---
GCCTGTCCAAAGGTATCCTTTGAACCAATTCCCATACATTATTGTACCCCGGCTGGTTTTGCGATTCTACAGT
GTAAT---GATAAGAAGTTCAAT------GGAACAGGACCATGTTCAAAT-----------------------
-GTCAGC---ACAGTACAATCTACACATGGAATTAGGCCAGTAGTATCAAGT---
CAACTACTGTTAAATGGCAGTCTAGCAGAAGAAGAG------------
GTAGTAATGAGATCTGAAAATTTCACGAACAAT---------GCTAAAACCATAATAGTACAGCTGAAC----
-----------GCATCTGTAGAA---------ATTAATTGTACAAGACCCAACAATAATACAAGAAAA-----
-AGTATAGATATA------------GGACCAGGGAGAGCA---ATT------TATGCAACAGGAGAC------
---------ATAATAGGAGATATA------------AGACAAGCACATTGT---AACATTAGTGAAGAA---
AAATGGAATAACACTTTAAGACAGATAGTTATAAAATTAAGA---------------GAACAATTTGGG----
-----------------------------------AATAAAACAATAGTCTTTAAT------
CATTCCTCAGGAGGGGACCCAGAGATTGTA---ATGCACAGTTTTAATTGTGGAGGGGAA---
TTTAAGCAGCTCCAGGCAAGAGTCCTGGCTGTGGAAAGATACCTAAGGGATCAACAGCTCCTGGGGATTTGGG
GATGCTCTGGAAAGCTCATCTGCACCACTAATGTGCCTTGGAATACTAGTTGGAGT-----------------
----------------------------AATAAATCTCAGAAATATATCTGG------GAT------
AACATGACCTGGATGGAGTGG---GACAGAGAAATTAACAATTACACAGGCTTAATATAC---
ACTTTACTTGAAGAATCGCAGAACCAGCAAGAA--

AAGAATGAACAGGAATTATTGGAATTGGATAAATGGGCAAGTTTGTGGAGTTGGTTTGACATATCTCAATGGC
TGTGGTATATAAGAATATTCATAATGATAGTAGGAGGCTTGATAGGTTTAAAAATAATTTTTTAT---
GTACTTTCTATAGTA---------AATAGAGTTAGGCAGGGATACTCACCA------
TTATCGTTTGCTGTTAGTTTGCTTCATGCCACAGCTAGAGCAGTAGCTGAG---
GGGACAGATAGGGTTATAGAAATATTACAAAGAGCTTATAGAGCT----------------------------
--------GTTCTCCACATA------CCTAGAAGAATAAGACAGGGTTTTGAAAGGGCTTTGCTA---TAA
>KJ140252
GCT---GCA--
GGGGGATTGTGGGTCACAGTCTATTATGGGGTACCTGTGTGG---AAAGAAGCAGAC---
ACCACCCTATTTTGTGCATCAGATGCTAAAGCATATGACACAGAG---GTACATAAT----------------
--------------GTTTGGGCCACA---CATGCCTGTGTACCCATAGAC---
CCCAACCCACAAGAGGTAGTATTGGAA---------AATGTGACAGAAAATTTTAAC----------------
--------------------------ATGTGGAAAAATAACATGGTAGAACAGATGCATGAGGATATA---
ATCAGTTTATGGGATCAAAGCCTA---AAGCCATGTGTAAAATTA---
ACTCCACTCTGTGTTACCTTGAATTGTAGTAATTTGAATAATAATGCTACTACCAATAGCAGTAGTGAGAA--
----
---------------------------------------AGTGATGGAGGAAGAAGAAATGACA---
AACTGCTCTTTCAATGTTACCACAAACATAAGA---------------------------
AATAAGGTGCAGAAAGAATATGCTCTT---TTTTATAAACTTGATGTAGTACCAATAGATAA-----------
----

TACAAGCTATACGTTGATACATTGTAACTCCTCAGTCATTACACAG---
GCCTGTCCAAAGGTATCCTTTGAACCAATTCCCATACATTATTGTACCCCGGCTGGTTTTGCGATTCTACAGT
GTAAT---GATAAGAAGTTCAAT------GGAACAGGACCATGTACAAAT-----------------------
-GTCAGC---ACAGTACAATGTACACATGGAATTAGGCCAGTAGTATCAACT---
CAACTGCTGTTAAATGGCAGTCTAGCAGAAGAAGAG------------
GTAATAATTAGATCTGAAAATTTCACGAACAAT---------GCTAAAACCATAATAGTACAGCTGAAC----
-----------GCATCTGTAGAA---------ATTAATTGTACAAGACCCAACAACAATACAAGAAAA-----
-AGTATAAGTATA------------GGACCAGGGAGAACA---TTT------TATGCAACAGGAGAA------
---------ATAATAGGAGATATA------------AGACAAGCACATTGT---AACATTAGTGAAGCA---
AAATGGAATAACACTTTAAGACAGCTAGCTATAAAATTAAGA---------------GAACAATTTGGG----
-----------------------------------AATAAAACAATAGTCTTTAAT------
CAATCCTCAGGAGGGGACCCAGAGATTGTA---ATGCACAGTTTTAATTGTGGAGGGGAA---
TTTAAGCAGCTCCAGGCAAGAGTCCTGGCTGTGGAAAGATACCTAAGGGATCAACAGCTCCTGGGGATTTGGG
GATGCTCTGGAAAGCTCATCTGCACCACTAATGTGCCTTGGAATACTAGTTGGAGT-----------------
----------------------------AATAAATCTCTGAATAATATCTGG------GAT------
AATATGACCTGGATGGAGTGG---GACAGAGAAATTAACAATTACACAAGCTTAATATAC---
ACCTTACTTGAAGAATCGCAGAACCAACAAGAA--

AAGAATGAACAGGAATTATTGGAATTGGATAAATGGGCAAGTTTGTGGAATTGGTTTGACATATCACATTGGC
TGTGGTATATAAGAATATTCATAATGATAGTAGGAGGCTTGGTAGGTTTAAGAATAATTTTTTCT---
GTACTTTCTATAGTG---------AATAGAGTTAGGCAGGGATACTCACCA------
TTATCGTTTGCTGTTAGTTTGCTTAATGCCACAGCTATAGCAGTAGCTGAG---
GGGACAGATAGGGCTATAGAAATAGTACAAAGAGCTTATAGAGCT---------------------------
--------ATTCTCCACATA------CCTACACGAATAAGACAGGGCTTGGAAAGGGCTTTGCTA---TAA
>AJ417425
GCT---GTA---
GAAAAATTGTGGGTCACAGTCTATTATGGGGTACCTGTGTGG---AAAGACGCAACC---
ACCACTCTATTTTGTGCATCAGATGCTAAAGCATATGATACAGAG---GTACATAAT---------------
--------------GTTTGGGCCACA---CATGCCTGTGTACCCACAGAC---
CCCAACCCACAAGAAGTGGAATTGAAA---------AATGTGACAGAAGAGTTTAAC---------------
-------------------------ATGTGGAAAAATAACATGGTAGAACAGATGCATGAGGATATA---
ATCAGTTTATGGGATCAAAGCCTA---AAGCCATGTGTAAAATTA---
ACCCCACTCTGTGTTACTTTAAAGTGCACGAATGTTACTAAGGTCAATGTTACTACAGTCAAT---------
----
-------------------------TATACTAGAGAAATGGAGGAAGTGGAGAACGAAATAAAA---
AACTGCTCTTTTAATATCACCACAAACATAAGA--------------------------
GATAAGGTGCAGAGAGAATATGCACTT---TTTTATAGATTAGATGTAGTACCATTAGATAAT---------
----

GATAATACTAGCTATAGGTTGATAAGTTGTAATACCTCAGTCATTACACAG---
GCCTGTCCAAAGATATCCTTTGAGCCAATTCCCATACATTATTGTGCCCCAGCTGGTTTTGCGATTATAAAAT
GTAAT---GATAAGACATTCAAT------GGAAAGGGACAATGTAGAAAT----------------------
-GTCAGC---ACAGTACAATGTACACATGGAATTAGACCAGTAGTATCAACT---
CAACTGCTGTTAAATGGCAGTCTGGCAGAAGAAGAG-----------
ATAGTAATTAGATCGGAAAATCTCACAGATAAT---------GCTAAAACTATAATAGTACAGCTGAAA----
-----------GAACCTGTAAGC---------ATTAATTGTACAAGGCCCAACAACAATACAAGAAAA-----
-AGTATTAATATA------------GGACCAGGGAGAGCA---TTT------TATGCAACAGGAGAC------
---------ATAATAGGAGATATA------------AGACAAGCACATTGC---AACCTTAATAAAACA---
GATTGGAATAACACTTTAGGACAGGTAGTTAAAAAATTAAGA---------------GAACTATTTAAG----
-----------------------------------AATAAAACAATAGTCTTTAAT------
CGATCCTCAGGAGGGGACCCAGAAATTGTA---ATGCACAGTTTTAATTGCGGAGGGGAA---
TTTAAGCAGCTCCAGGCAAGAGTCCTGGCTGTGGAAAGATACCTAAAGGATCAACAGCTCCTGGGAATTTGGG
GTTGCTCTGGAAAACTCATTTGCACCACTACCGTGCCTTGGAATACTAGTTGGAGT----------------
----------------------------AATAAATCTATGACTGACATTTGG------GAT------
AACATGACCTGGATGCAGTGG---GAAAGAGAAATTGAAAATTACACAGGCTTAATATAC---
ACATTAATTGAAGAATCGCAGAACCAGCAAGAA---------------------------------------
----
AAGAATGAACAAGAATTATTGGCATTAGATAAATGGGCAAGTTTGTGGAATTGGTTTAACATAACTAACTGGC
TGTGGTATATAAAAATACTCATGATGATAGTAGGAGGCTTGATAGGTTTAAGAATAGTTTTTACT---
GTACTTTCTATAGTG---------AATAGAGTTAGGCAGGGATACTCACCA------
ATATCGTTTGCTGTTAGCTTGCTCAACGCCACAGCCATAGCAGTAGCTGAG---
GGGACAGATAGGATTATAGAAATAGTACAAAGAACTGGTAGAGCT---------------------------
--------ATTCTCCACATA------CCTAGAAGAATAAGACAGGGCGCAGAACGGGCTTTGGTT---TAA
>KY820529
GCT---GCA---GCA-----------------------------------
GAAGGATTGTGGGTCACAGTCTATTATGGGGTACCTGTGTGG---AAAGAAGCAACC---
ACCACCCTATTTTGTGCATCAGATGCTAAAGCATATGTCACAGAG---AAACATAAT---------------
--------------GTTTGGGCCACA---CATGCCTGTGTACCCATAGAC---
CCCAACCCACAAGAAGTAGTATTGGAA---------AATGTGACAGAAAATTTTAAC---------------
-------------------------ATGTGGAAAAATAACATGGTAGAACAGATGCATGAGGATATA---
ATCAGTTTATGGGATCAAAGCCTA---AAGCCATGTGTAAAAATA---
ACTCCACTCTGTGTTACTTTAAATTGTAGTGATTATTTGAAGAATGAGACTAATACCAAGAAT---------
----
----------GAGACTAATACCAATAATAGTAGTGGGGGAGTGATGGAGAAAGGAGAAATAAAA---
AACTGCTCTTTCAATGTTACCACAAACATAGGA--------------------------
AATAAGGTGCAGAAAGAATATGCTTTT---TTTTATAAACTTGATGTAGTACCAATAGATAAT---------
----

ACAAGCTATACATTGATAAATTGTAACTCCTCAGTCATTACACAG---
GCCTGTCCAAAGGTATCCTTTGAACCAATTCCCATACATTATTGTACCCCGGCTGGTTTTGCGATTCTACAGT
GTAAT---GATAAGAAGTTCAAT------GGAACAGGACCATGTTCAAAT----------------------
-GTCAGC---ACAGTACAATGTACACATGGAATTAGGCCAGTAGTATCAACT---
CAACTGCTGTTAAATGGCAGTCTAGCAGAAGAAGAG-----------
ATAGTAATTAGATCTGAAAATTTCAAGGATAAT---------GCTAAAACCATAATAGTACAGCTGAAC----
-----------GCATCTGTAGAA---------ATTAATTGTATAAGACCCAACAACAATACAAGAAAA-----
-AGTATAAGGATA------------GGACCAGGGAGTACA---TTT------TATGCAACAGGAGGC------
---------ATAATAGGAAATATA------------AGACAAGCACATTGT---AACATTAGTGAAACA---
AAATGGGAAAACACTTTAAGACAGCTAGTTATAAAATTAGGA---------------GAACAATTTAGG----
--------------------------------AATAAAACAACAATAGTCTTTAAT------
CAATCCTCAGGAGGGGACCCAGAGATTGTA---ATGCACAGTTTTAATTGTGGAGGGGAA---
TTTAAGCAGCTCCAGGCAAGAGTCCTGGCTGTGGAAAGATACCTAAGGGATCAACAGCTCCTGGGGATTTGGG
GATGCTCTGGAAAGCTCATCTGCACCACTAATGTGCCTTGGAATACTAGTTGGAGT-----------------
----------------------------AATAAATCTCATAATGATATCTGG------GAT------
AACATGACCTGGATGGAGTGG---GACAGAGAAATTAACAATTACACAAGCTTAATATAC---
ACCTTACTTGAAGAATCGCAGAACCAGCAAGAA--

AAGAATGAACAGGAATTATTGGAATTGGATAAATGGGCAAGTTTGTGGAATTGGTTTGACATATCAAACTGGC
TGTGGTATATAAAAATATTCATAATAATAGTAGGAGGCTTGGTAGGTTTAAGAATAATTTTTTCT---
GTACTTTCTATAGTA---------AATAGAGTTAGGCAGGGATACTCACCA------
TTATCGTTTGCTGTTAGTTTGCTTAATGCCACAGCTAGAGCAGTAGCTGAG---
GGGACAGATAGGGTTATAGAAATATTACAAAGAGCTTATAGAGCT----------------------------
--------ATTCTCCACATA------CCTAGAAGAATAAGACAGGGCTTGGAAAGGGCTTTGCAA---TAT
>MK577479
GCT---ACA---
GAAAAATTGTGGGTCACAGTCTATTATGGGGTACCTGTGTGG---AAAGAAGCAACC---
ACCACCCTATTTTGCGCATCAGAGGCTAAAGCATATGACACAGAG---GTACATAAT----------------
--------------GTTTGGGCCACA---CATGCCTGTGTACCCATAGAC---
CCCGACCCACAAGAAGTATTATTGGAA---------AATGTGACAGAAAATTTTGAC----------------
-------------------------ATGTGGAGAAATAACATGGTAGAACAGATGCATGAGGATATA---
ATCAGTTTATGGGATCAAAGCCTA---AAGCCATGTGTAAAATTA---
ACTCCACTCTGTGTTACTTTGAATTGTAGTAATTTGAAGAATGAGACTAATACCAATAATGGGACTAATACCA
CT---
-------------------------AGTAGTATTGGGGGGGTGATGGAGGAAGGAGAAATAAAA---
AACTGCTCTTTCAATGTTACCACAAGCATAAGA--------------------------
AATAAGATGAAGAAAGAATATGCTCTT---TTTTATAAACTTGATGTAGTATCAATAGATAAT----------
----

GATAATACAAGCTATACATTGATAAATTGTAACACCTCAGTCCTTACACAG---
GCCTGTCCAAAGGTATCCTTTGAACCAACTCCCATACATTATTGTACCCCAGCTGGTTTTGCGATTCTACAAT
GTAAT---GATAAGAAGTTCAAT------GGATCAGGACCATGTACAAAT----------------------
-GTCAGC---ACAGTACAATGTACACATGGAATTAGGCCAGTAGTATCAACT---
CAACTGTTATTAAATGGCAGTCTAGCAGAAGAAGAG-----------
ATAGTAATTAGATCTGAAAATTTCACGGACAAT---------GCTAAAACCATAATAGTACAGCTGAAC----
-----------GCATCTGTAGTA---------ATTAATTGTACAAGACCCAACAACAATACAAGAAAA-----
-AGTATACATTTA------------GGACCAGGGAGCACA---TTT------TATGCAACAGGAGAC------
---------ATAATAGGAGATATA------------AGACAAGCACATTGT---AACATTAGTGGAGGA---
GAATGGAATAACACTTTAAGACAGCTAGTTATAAAATTAAGA---------------GAACAATTTGGG----
--------------------------------AGTAGTAAAACAATAAACTTTACT------
CGATCCTCAGGAGGGGATCCAGAGATTGTA---ATGCACAGTTTTAATTGTGGAGGGGAA---
TTTAAGCAGCTCCAGGCAAGAGTCCTGGCTGTGGAAAGATACCTAAAGGATCAACAGCTCCTGGGGATTTGGG
GATGCTCTGGAAAGCTCATCTGCACCACTAATGTGCCTTGGAATACTAGTTGGAGT-----------------
----------------------------AATAAATCTCAGAATTATATCTGG------GAT------
AACATGACCTGGATGGAGTGG---GACAGAGAAATTAACAATTACACAAGCTTAATATAC---
ACCTTACTTGAAGAATCGCAGAACCAACAAGAA--

AAGAATGAACAGGAATTATTGGAATTGGATAAATGGGCAAGTTTGTGGAATTGGTTTGACATATCACACTGGC
TGTGGTATATAAAAATATTCATAATGATAGTAGGAGGCTTGGTAGGATTAAGAATAATTTTTTCT---
GTACTTTCTATAGTA---------AATAGAGTTAGGCAGGGATACTCACCA------

TTATCGTTTGCTGTTAGTTTGCTCAATGCCACAGCTATAGCAGTAGCTGAG---
GGGACAGATAGGGTTATAGAACTAATACAAAGAGCTTATAGAGCT----------------------------
--------ATTCTCCACATA------CCTACAAGAATAAGACAGGGCTTAGAAAGGGCTTTGCTA---TAA
>KU869586
GCT---GCA---GCA------------------------------------
GGAGGATTGTGGGTCACAGTCTATTATGGGGTACCTGTGTGG---AAAGAAGCAACC---
ACCACCCTATTTTGTGCATCAGATGCTAAAGCATATGACACAGAG---GTACATAAT----------------
--------------GTTTGGGCCACA---CATGCCTGTGTACCCATAGAC---
CCCAACCCACAAGAAGTAGTATTGGAA---------AATGTGACAGAAAATTTTAAC----------------
--------------------------ATGTGGAAAAATAACATGGTAGAACAGATGCATGAGGATATA---
ATCAGTTTATGGGATCAAAGCCTA---AAGCCATGTGTAAAATTA---
ACTCCACTCTGTGTTACTTTAAATTGTAGTGATTTGAAGAATGATACTAGTACCAATAAT-------------
----
----------------------------AGTAGTGGGGGAGTGACGGAAGGAGGAGAAATGAAA---
AACTGCTCTTTCAATGTTACCACAAACTTAAGA---------------------------
GATAAGGTGAAGCAAGAATATGCTCTT---TTTTATAGACTTGATGTAGTACCAATAGATAAT----------
----

ACAAGCTATACATTGATAAATTGTAACTCCTCAGTCCTTACACAG---
GCCTGTCCAAAGGTATCCTTTGAACCAATTCCCATACATTATTGTACCCCGGCTGGTTTTGCGATTCTACAGT
GTAAT---GATAAGAAGTTCAAT------GGAACAGGACCATGTACAAAT-----------------------
-GTCAGC---ACAGTACAATGTACACATGGGATTAGGCCAGTAGTGTCAACT---
CAACTGCTGTTAAATGGCAGTCTAGCAGAAGAAGAG------------
ATAGTAATTAGATCTGAAAATTTCACGGACAAT---------GCTAAAACCATAATAGTACAGCTGAAC----
-----------GCATCTGTAGAA---------ATTAATTGTACAAGACCCAACAACAATACAAGAACA-----
-AGTATAAGTATA------------GGACCAGGGAGAACA---TTT------TATGCAACAGGAGAC------
---------ATAATAGGAGATATA------------AGACAAGCACATTGT---AACATTAGTGGAGAA---
AAATGGAACAACACTTTAAGACAGCTAGTTATAAAATTAAGA---------------GAACAATTTGGG----
-----------------------------------AATAAAACAATAGTCTTTAAT------
CAATCCTCAGGAGGGGACCCAGAGATTGTA---ATGCACAGTTTTAATTGTGGAGGGGAA---
TTTAAGCAGCTCCAGGCAAGAGTCCTGGCTGTGGAAAGATACCTAAAGGATCAACAGCTCCTGGGGATTTGGG
GATGCTCTGGAAAGCTCATCTGCACCACTAATGTGCCTTGGAATACTAGTTGGAGT-----------------
----------------------------AATAAATCTCTGAAGTATATCTGG------GAT------
AACATGACCTGGATGGAGTGG---GACAGAGAAATTAACAATTACACAAACTTAATATAC---
ACCTTACTTGAAGAATCGCAGAACCAACAAGAA--

AAGAATGAACAGGAATTATTGGAATTGGATAAATGGGCAAGTTTGTGGAATTGGTTTGACATATCACAATGGC
TGTGGTATATAAGAATATTCATAATGATAGTAGGAGGCTTGATAGGTTTAAGAATAATTTTTCT---
GTACTTTCTATAGTA---------AATAGAGTTAGGCAGGGATACTCACCA------
TTATCATTGGCTGTTAGTTTGCTTAATGCCACAGCTAGAGCAGTAGCTGAG---
GGGACAGATAGGGTTATAGAACTATTACAAAGAGCTTGTAGAGCT----------------------------
--------ATTTTCAACATA------CCTAGAAGAATAAGACAGGGCTTGGAAAGGGCTTTGCTA---TAA
>MN043595
GCT---GCA---AGA------------------------------------
GAAGAATCGTGGGTCACAGTCTATTATGGGGTACCTGTATGG---AAAGAAGCAACC---
ACCACCCTATTTTGTGCATCAGATGCTAAAGCATATGACACAGAG---GTACATAAT----------------
--------------GTTTGGGCCACA---CATGCCTGTGTACCCATAGAC---
CCCAACCCACAAGAAGTAGTATTGGAA---------AATGTGACAGAAAATTTTAAC----------------
--------------------------ATGTGGAAAAATAACATGGTAGAACAGATGCATGAGGATATA---
ATCAGTTTATGGGATCAAAGCTTA---AAGCCATGTGTAAAGTTA---
ACTCCACTCTGTGTTACTTTAAATTGTAGAAATGAGACTTTGGAGAATGATACTAATACCACTCATAGTGATT
GGAATGATACT--
----------------GTTACCAATAAGAGTAGTGGGGGAGTGATAAAGGAAAGAGAAATGAAA---
AACTGCTCTTTCAATGTTACCACAAGCATAAGA---------------------------
AATAAGGTGCAGAAAGAATATGCTCTT---TTTTATAAACTTGATGTAGTACCAATAGATGAC----------
----

GATAATACAAGCTATACGTTGATAAATTGTAACACCTCAGTCATTAAACAG---
GCCTGTCCAAAGGTATCCTTTGAACCAATTCCCCTACATTATTGTACCCCGGCTGGTTTTGCGAGTCTACAGT
GTAAT---GAGAAGAATTTCAAT------GGAACAGGACCATGTAAAAAT-----------------------

-GTCAGC---ACAGTACAATGTACACATGGAATTAGGCCAGTAGTATCAACT---
CAACTGCTGTTAAATGGCAGTCTAGCAGAAAAAGAG------------
ATAGTAATTAGATCTGAAAATTTCACGGACAAT---------GCTAAAACCATAATAGTACAGCTGAAC----
-----------GCATCTGTAGAA---------ATTAATTGTACAAGACCCGGCAACAATACAAGTAAA-----
-AGTATAAGGATA------------GGACCAGGGAGCACA---TTT------TATGCAACAGAAAGA------
---------ATAATAGGAGATATA------------AGACAAGCACATTGT---AACATTAGTGGAGTA---
AAATGGAAAAACACTTTAAAACAGCTAGTTCTAAAATTAAGA---------------AAACAATTTGGG----
-----------------------------------AATAAAACAATAGCCTTTAAG------
CAATCCTCAGGAGGGGACCCAGAGATTGTA---ATGCACAGTTTTAATTGTGGAGGGGAA---
TTTAAGCAGCTCCAGGCAAGAGTCCTGGCTGTGGAAAGATACCTAAAGGATCAACAGCTCCTGGGGATTTGGG
GATGCTCTGGAAAGCTCATCTGCACCACTAATGTGCCTTGGAATACTAGTTGGAGT-----------------
----------------------------AATAAATCTCAGGAGGAGATCTGG------AAT------
AACATGACCTGGATGGAGTGG---GACAGAGAAATTAACAATTACACAAACTTAATATAC---
AACTTACTTGAAGAATCGCAGAACCAACAAGAA--

AAGAATGAACAGGAATTATTGGCATTGGATAAATGGGCAAGTTTGTGGAATTGGTTTGAAATATCACACTGGC
TGTGGTATATAAAAATATTCATAATGATAGTAGGAGGCTTGGTAGGTTTAAGAATAATTTTTTCT---
GTACTTTCTATAGTA---------AATAGAGTTAGGCAGGGATACTCACCA------
TTATCGTTTGCTGTTAGTTTGCTTAATGCCACAGCTATAGCAGTAGCTGAG---
GGGACAGATAGGGTTATAGAAATATTACAAAGAGCTTATAGGGCT----------------------------
--------ATCCTTCACATA------CCTACAAGAATAAGACAGGGCTTGGAAAGGGCTTTGCTA---TAA
>KX960978
GCT---GCA---GCA------------------------------------
GGAGGATTGTGGGTCACAGTCTATTATGGGGTACCTGTGTGG---AAAGAAGCAAGC---
ACCACCCTATTTTGTGCATCAGATGCTAAAGCATATGACACAGAG---GTACATAAT----------------
--------------GTTTGGGCCACA---CATGCCTGTGTACCCATAGAC---
CCCAACCCACAAGAAGTAATATTGGAA---------AATGTGACAGAAAACTTTAAC----------------
--------------------------ATGTGGAAAAATAACATGGTAGAACAGATGCATGAGGATATA---
ATCAGTTTATGGGATCAAAGCCTA---AAGCCATGTGTAAAATTA---
ACTCCACTCTGTGTTACTTTGAATTGTAGTAATGTGAAGAATGGTACTAATACCAATGGT-------------
----
-------------ACTAGTACCAATAGTAGTAGTGGGACAGGGATAGAGGAAGGAGACATGAAA---
AACTGCTCTTTCAATGTTACCACAACCATAAGA---------------------------
AATAAGGTGCAGAAAGAATATGCTCTT---TTTTATAAACTTGATGTAGAACAAATAGATAAT----------
----

ACAAGCTATACATTGATAAATTGTAACTCCTCAGCCACAACACAG---GCCTGTCCAAAGGTA----------
----TCCCATACATTATTGTACCCCGGCTGGTTTTGCGATTCTACAATGTAAT---GATAAGAAGTTCAAT--
----GGAACGGGACCATGTTCAAT-------------------------GTCAGC---
ACAGTACAATGTACACATGGAATTAGGCCAGTAGTATCAACT---
CAACTGCTGTTAAATGGCAGTCTAGCAAAAGAAGAA------------
GTAGTAATTAGATCTGAAAATTTCACGAACAAT---------GCTAAAATCATAATAGTACAGCTGAAC----
-----------GCATCTGTAGAA---------ATTAATTGTACAAGACCCAACAACAATACAAGAAAA-----
-AGTATACATTTA------------GGACCAGGGAGAACA---TTT------TATGCAACAGGAGAC------
---------ATAATAGGAGATATA------------AGACAAGCACATTGT---AACATTAGTGAAGCA---
AAATGGGAAAACACTTTAAGACAGCTAGTTATAAAATTAAGA---------------GAACAATTTGGG----
--------------------------------AATAACAAAACAATAGTCTTTAAT------
CAATCCGCAGGAGGGGACCCAGAGATTGTA---ATGCACAGCTTTAATTGTGGAGGGGAA---
TTTAAGCAGCTCCAGGCAAGAGTCCTGGCTGTGGAAAGATACCTAAAGGATCAACAGCTCCTGGGGATTTGGG
GATGCTCTGGAAAGCTCATCTGCACCACTAATGTGCCTTGGAATACTAGTTGGAGT-----------------
----------------------------AATAAATCTCAGGAGGATATCTGG------GAT------
AACATGACCTGGATGGAGTGG---GACAGAGAAATTAACAATTACACAAGTTTAATATAC---
ACCTTACTTGAAGAATCGCAGAACCAGCAAGAA--

AAGAATGAACAGGAATTATTGAAATTGGATGAATGGGCAAGTCTGTGGAATTGGTTTGACATATCACGATGGC
TGTGGTATATAAAAATATTCATAATGATAGTAGGAGGCTTGGTAGGTTTAAGAATAATTTTTTGT---
GTGCTTTCTATAGTG---------AATAGAGTTAGGCAGGGATACTCACCA------
TTGTCGTTTGCTGTTAGTTTGCTTAATGCCACAGCTAGAGCAGTAGCTGAG---
GGGACAGATAGGGTTATAGAAATATTACAAAGAGCTTATAGAGCT----------------------------
--------ATTCTCCACATA------CCTACAAGAATAAGACAGGGCTTGGAAAGGGCTTTGCTA---TAA

>AJ417415
GCT---GCA--
GAAGAATTGTGGGTCACAGTCTATTATGGGGTACCTGTGTGG---AAAGAAGCAACC---
ACCACCCTATTTTGTGCATCAGATGCTAAAGCATATGACACCGAG---GCACATAAT----------------
--------------GTTTGGGCCACA---CATGCCTGTGTACCCATAGAC---
CCCAACCCACAAGAAGTAGTATTGGAA---------AATGTGACAGAAAATTTTAAC----------------
--------------------------ATGTGGAAAAATAACATGGTAGAACAGATGCATGAGGATATA---
ATCAGTTTATGGGATCAAAGCCTA---AAGCCATGTGTAAAATTA---
ACTCCACTCTGTGTTACTTTAAATTGTAGTAATTTGAGGAATAATACTAATACCAACAGTAGTACTATT----
----
-------------------ACCAATAGTAGTAGTGAGGGAATGATGGAGAAAGGAGACATGAGA---
AACTGCTCTTTCAATGTTACCACAACCATAAAA---------------------------
AATAAGGTGAAGAAAGAACATGCTCTG---TTTTATAAACTTGATATAACACCAATAGATAAT----------
----

GATACTACAAGCTATACGTTGATAAATTGTAACTCCTCAGTCGTTACACAG---
GCCTGTCCAAAGATATCCTTCGAACCAATTCCCATACATTATTGTACCCCGGCTGGTTTTGCGATTCTACAGT
GTAAT---GATAAGAAGTTCAAT------GGAACAGGACCATGTAAAAAT-----------------------
-GTCAGC---ACAGTACAATGTACACATGGAATTAAGCCAGTAGTATCAACT---
CAACTACTGTTAAATGGCAGTCTAGCAGAAGAAGAG------------
ATAGTAATTAGATCTGAAAATTTCACGAACAAT---------GCTAAAACCATAATAGTACAGCTGAAC----
-----------ACATCTGTAGAA---------ATTAATTGTACAAGACCCAACAACAATACAAGAAAA-----
-GGTATACATATG------------GGACCAGGGAGGACA---TTT------TATGCAACAGGAGAC------
---------ATAATAGGAGACATA------------AGACAAGCACATTGT---AACATTAGTAAAGCA---
AAATGGGAAAATACTTTAAGACAGCTAGCTATAAAATTAAGA---------------GAACAATTTGGG----
-----------------------------------AATAAAACAATAGCCTTTAAG------
CAAGCCTCAGGAGGGGACCCAGAAATTGTA---ATGCACAGTTTTACTTGTGGAGGGGAA---
TTTAAGCAGCTCCAGGCCAGAGTCCTGGCTGTGGAAAGATACCTAAGGGATCAACAGCTCATGGGGATTTGGG
GATGCTCTGGAAAGCTCATCTGCACCACTAATGTGCCTTGGAATACTAGTTGGAGT-----------------
----------------------------AATAAATCTCTGAATGATATCTGG------AAT------
AACATGACCTGGATGGAGTGG---GACAGAGAAGTTAACAATTACACAAACTTAATATAC---
ACCTTACTTGAAGAATCGCAGAACCAACAAGAA--

AAGAATGAACAGGAATTATTGGAATTGGATAAATGGGCAAGTCTGTGGAATTGGTTTGACATAACAAAATGGC
TGTGGTATATAAAAATATTCATAATGATAGTAGGAGGCTTGGTAGGTTTAAGAATAATTTTTTCT---
GTACTTTCTATAGTG---------AATAGAGTTAGGCAGGGATACTCACCA------
TTATCGTTTGCTGTTAGTTTGCTTAATGCCACAGCTATAACAGTAGCTGAG---
GGGACAGATAGGGTTATAGAAATATTACAAAGAGCTTATAGAGCT----------------------------
--------ATCCTCCACATA------CCTGTAAGAATAAGACAGGGCTTGGAAAGGGCTTTGCTA---TAA
>AJ417431
GCT---ACA--
GAAAACTTGTGGGTCACAGTCTATTATGGAGTACCTGTGTGG---AAAGAAGCAACC---
ACCACTCTATTTTGTGCATCAGATGCTAAAGCATATGATAAAGAA---GTACATAAT----------------
--------------GTTTGGGCCACA---CATGCCAGTGTACCCACAGAC---
CCCAATCCACAAGAAGTAGTATTGGCA---------AATGTGACAGAAAATTTTAAC----------------
--------------------------ATGTGGAAAAACAATATGGTAGATCAGATGCATGAGGATATA---
ATTAGTCTATGGGATGAAAGCTTA---AAGCCATGTGTAAAATTA---
ACCCCACTCTGTGTTACTTTAATGTGTGCCAACGTGAATGTCACTAATAAAGCCAACATGACTGGC-------
----
----------------------CCTAATAATACTAGTTGGGAAAAGATGGAAGGAGAGATAAAA---
AACTGTTCTTTCAATATCACCACAAATATAAAA---------------------------
GATAAGAGAGAGAAAAAAATATGCACTT---TTTTATGCACTTGATCTAGTATCAATGAAGGATAATGCA----
----

GATATTAGTAAAACTAATAATAGCTATAGATTGATACATTGTAATACCTCAACCATTACACAG---
GCCTGTCCAAAGGTATCCTTTGAGCCAATTCCCATACATTATTGTGCCCCGGCTGGTTTTGCGATTCTAAAGT
GTAAC---AATAAGAAGTTCAAT------GGAACAGGACCATGTACAAAT-----------------------
-GTCAGC---ACAGTACAATGTACACATGGAATTAAGCCAGTAGTGTCAACT---
CAACTACTGTTAAATGGCAGTCTAGCAGAAGAAGAG------------
ATAGTAATTAGATCTGAAAATCTCACGGACAAT---------GCTAAAAACATAATAGTACAGCTGAAT----

-----------AAATCTATAGAA---------ATTAATTGTACAAGACCCAACAATAATACAAGACAA-----
-AGTATATCTATA------------GGACCAGGGAGAGCA---CTT------TATACCACAGGACAA------
---------ATAATAGGAGATATA------------AGACAAGCATATTGC---AACCTTAGTAAAGTA---
AGCTGGAATAACACTTTAAAACAGATAGCTGCCAAATTAAGA---------------GAACACTTT-------
-----------------------------------AATAAAACAATAATCTTTAAG------
TCCTCCTCAGGAGGGGACCCAGAAATTGTA---ACTCACAGTTTTAATTGTGGGAGGGAA---
TTTAAACAGCTCCAGGCGAGAATCCTGGCTGTAGAAAGATACCTACAGGATCAACAGCTCCTAGGGATTTGGG
GTTGCTCTGGAAAACTCATCTGCACCACTGCTGTGCCTTGGAACGTTAGTTGGAGT-----------------
----------------------------AATAAATCTCATACAGAAATTTGG------GAT------
AACATGACCTGGATGGAGTGG---GAAAAAGAAATTGACAATTACACAAGCATAATATAC---
ACCTTACTTGAGACATCGCAGAACCAGCAAGAA--

AAGAATGAACAAGAATTATTGGAATTGGATAAGTGGGCAAGTTTGTGGAATTGGTTTAGTATATCAAACTGGC
TGTGGTATATAAAAATATTCATAATGATAGCAGGAGGCTTAATAGGGTTAAGAATAGTTTTTGCT---
GTGCTTTCTATAGTA---------AATAGAGTTAGGCAGGGATACTCACCA------
TTATCGTTTGCTGTTAGCTTGCTTAATACCACAGCAATAGTAGTAGGTGAA---
GGGACAGATAGGATTATAGAAGTAGTACAAAGATTTCTTAGAGCT----------------------------
--------GTTCTTCACATA------CCTAGAAGAATAAGACAGGGCTTGGAAAGGGCTTTGCTA---TAA
>AJ417410
GTT---GTA--
GGAGAAGGATGGGTCACAGTCTATTATGGGGTACCTGTGTGG---AAAGAAGCAAAC---
ACCACCCTATTTTGTGCATCAGATGCTAAAGCATATGACACAGAG---GCACATAAT----------------
--------------GTTTGGGCCACA---CATGCCTGTGTACCCATAGAC---
CCCAACCCACAAGAAGTAGTATTGGAA---------AATGTGACAGAAAACTTTAAC----------------
--------------------------ATGTGGAAAAATAACATGGTAGAACAGATGCATGAGGATATA---
ATCAGTCTATGGGATCAAAGCCTA---AAGCCATGTGTGAAAATA---
ACTCCACTCTGTGTTACTATGAATTGTAGTAATTTGGATGATACTAAAACCAATAATACTAATACCACCAATA
GTACTAAA---
-------GCCAATAATACTAGTGGGGAAGGGATGGAGGAAGGAAAAATGAAAGACTTAATGAAA---
AACTGCTCTTTCAATGTTACCACAAGCATAAGA---------------------------
GATAAGATGCAGAGAGAATATGCTCTT---TTTTATAAACTTGATATAGTACCAATAGATAAG----------
----

GATAATACAAGCTATACGTTGATACATTGTAACTCCTCAGTCATTACACAG---
GCCTGTCCAAAGGCATCCTTTGAACCAATTCCCATACATTATTGTACCCCGGCTGGTTTTGCGATTCTGCAGT
GTAAT---GATAAGAAGTTCAAT------GGGACAGGACCATGTACAAAT-----------------------
-GTCAGC---ACAGTACAATGTACACATGGAATTAGGCCAGTAGTATCAACT---
CAACTGCTGTTAAATGGCAGTCTAGCAGAAAAAGAG------------
ATAGTAATTAGATCTGAAAATTTCACAGACAAT---------GCTAAAACCATAATAGTACAGCTGAAC----
-----------GCATCTGTAGAA---------ATTAATTGTACAAGACCCGGCAACAATACAAGAAGA-----
-AGTATAAGTATA------------GGACCAGGGAGAGCA---TTT------TATGCAACAGGAAGC------
---------ATAATAGGAAATATA------------AGACAAGCACATTGT---AACATTAGTAAGGAA---
AAATGGGAAAACACTTTAAGACAGATAGCTATAAAATTAGGA---------------GAACAATTTAGG----
-----------------------------------GATAAAGCAATAGCCTTTAAT------
CAATCCTCAGGAGGGGACCCAGAGATTGTA---ATGCACAGTTTTATTTGTGGAGGGGAA---
TTTAAGCAGCTCCAGGCAAGAGTCCTGGCTGTAGAAAGATACCTAAAGGATCAACAGCTCCTGGGGATTTGGG
GATGCTCTGGAAAGCTCATCTGCACCACTAATGTGCCTTGGAATACTAGTTGGAGT-----------------
----------------------------AATAAATCTCTGGATACTATCTGG------GAG------
AACATGACCTGGATGCAGTGG---GACAGAGAAATTAACAATTACACAAACTTAATATAC---
ACCTTACTTGAAGAATCGCAGAACCAACAAGAA--

AAGAATGAACAAGAATTATTGGAATTGGACAAATGGACAAGTTTGTGGAGTTGGTTTGACATATCAAACTGGC
TGTGGTATATAAGAATATTCATAATGATAGTAGGAGGCTTGGTAGGTTTAAGAATAATTTTTTCT---
GTACTTTCTATAGTG---------AATAGAGTTAGGCAGGGATACTCACCA------
CTATCGTTTGCTGTTAGCTTGCTTAATGCCACAGCTATAGCAGCAGCTGAG---
GGGACAGATAGGGTTATAGAAATATTACAAAGAGCTTATAGAGCT----------------------------
--------ATTCTCCACATA------CCTACACGAATAAGACAGGGCTTGGAAAGGGCTTTGCTA---TAA
>AJ417413
GCT---GCA---ACA------------------------------------
GGAGGATTGTGGGTCACAGTCTATTATGGGGTACCTGTGTGG---AAAGAAGCAGTC---

ACCACCCTATTCTGTGCATCAGATGCTAAAGCATATGACACAGAG---GTACATAAT----------------
--------------GTTTGGGCCACA---CATGCCTGTGTACCCATAGAC---
CCCAACCCACAGGAAGTAAGATTGGTA---------AACGTGACAGAAGATTTTAAC----------------
--------------------------ATGTGGAAAAATAGCATGGTAGAACAGATGCATGCAGATATA---
ATCAGTTTATGGGATCAAAGCCTA---AAGCCATGTGTAAAATTA---
ACTCCACTCTGTGTTACTTTGAATTGTAGTAATGTGAAGAAGAATGAGACTAGTGCCAATAATAGTAGT----
----
----------------AATACCAATAATAGTAGTGGGAAATGATGGACAAAGGAGGAATGAAA---
AACTGCTCTTTCAATGTTACCACAAACATAGGA---------------------------
AATAAGATGAAGAAAGAATATGCTCTT---TTTTATGAACTTGATGTAGTACAAGTAGATAAT----------
----

ACAAGCTATACATTGAAAAATTGTAACTCCTCAATCATTACACAG---
GCCTGTCCAAAGGTATCCTTTGAACCAATTCCCATACATTATTGTACCCCGGCTGGTTTTGCGATTCTACAGT
GTAAT---GATGGAAAGTTCAAT------GGAACAGGACCATGTTCAAAT-----------------------
-GTCAGC---ACAGTACAATGTACACATGGAATTAGGCCAGTAGTATCAACT---
CAACTGCTGTTAAATGGCAGTCTAGCAGAAGGAGAG------------
GTAGTAATTAGATCTGAAAATTTCACGAATAAT---------GCTAAAACCATAATAGTACAACTGAAC----
-----------GCATCTGTAGAA---------ATTAATTGTACAAGACCCAACAACAATACAAGAAAA-----
-AGTATACATATA------------GGACCAGGGAGTACA---ATT------TATGCAACAGGAGAC------
---------ATAATAGGAGATATA------------AGACAAGCACATTGT---AACATTAGTGGAGCA---
AAATGGGAAAATACTTTAAGACAGCTAGCTATAAAATTAAGA---------------GAAAAATTTGAG----
-----------------------------------AATAAAACAATAGCCTTTAAG------
CAATCCTCAGGAGGGGACCCAGAGATTGAA---ATGCACAGTTTTAATTGTGGAGGGGAA---
TTTAAGCAGCTCCGGGCAAGAGTCCTGGCTGTGGAAAGATACCTAAAGGATCAACAGCTCCTGGGGATTTGGG
GATGCTCTGGAAAACTCATCTGCACCACTAATGTGCCTTGGAATACTAGTTGGAGT-----------------
----------------------------AATAAATCTCAGGAGGAGATCTGG------GAT------
AACATGACATGGATGCAGTGG---GACAGAGAAATTAACAATTACACAAGCTTAATATAC---
ACCTTACTTGAAGAATCGCAGAACCAGCAAGAA--

AAGAATGAACAGGAATTATTGGAATTGGATAAATGGGCAAGTTTGTGGAATTGGTTTGACATAACAAACTGGC
TGTGGTATATAAAAATATTCATAATGATAGTAGGAGGCTTGGTAGGTTTAAGAATAATTTTTTCT---
GTACTTTCTATAGTG---------AATAGAGTTAGGCAGGGATACTCACCA------
TTATCGTTTGCTGTTAGTTTGCTTAATGCCACAGCTAGAGCAGTAGCTGAG---
GGGACAGATAAGGTTATAGAAATATTACAAAGAGCTTGTAGAGCT----------------------------
--------ATCCTCCACATA------CCTAGAAGAATAAGACAGGGCTTCGAAAGGGCTTTAGTA---TAA
>AJ417419
GCT---GCA---GCA------------------------------------
GAAAGAATGTGGGTCACAGTCTATTATGGGGTACCTGTGTGG---AAAGACGCAACC---
ACCACCCTGTTTTGTGCATCAGATGCTAAAGCACAAAACAGAGAG---GTACATAAT----------------
--------------ATCTGGGCCACA---CATGCCTGTGTACCCATAGAC---
CCCAACCCACAAGAAGTAGTATTGGAA---------AATGTGACAGAAAATTTTAAC----------------
--------------------------ATGTGGAAAAATAACATGGTAGAACAGATGCATGAGGATATA---
ATCAGTTTATGGGATCAAAGCCTA---AAGCCATGTGTAAAATTA---
ACTCCACTCTGTGTTACTTTGAATTGTAGTGATGTGAAGAATGGTACTAGTACCAATAGTAGTAGTATT----
----
----------------AGTACCAATAGTAGTAGTGAGACAGTGATGGGGGAAGGAGAAATGAAA---
AACTGCTCTTTCAATGTTACCACAAACATAGGA---------------------------
AATAAGGTGAAGAAAGAATATGCTCTT---TTTTATAAACTTGATGTAGTAAAAATAGAAAAT----------
----

TCAAGCTATACATTGATAAGTTGTAACTCCTCAGTCATTACACAG---
GCCTGTCCAAAGGTATCCTTTGAACCAATTCCCATACATTATTGTACCCCGGCTGGTTTTGCGATTCTACAAT
GTAAT---GATAAGAAGTTCAAT------GGAACAGGACCATGTTCAAAT-----------------------
-GTCAGC---ACAGTACAATGTACACATGGAATTAGGCCAGTAGTATCAACT---
CAACTGCTGTTAAATGGCAGTCTAGCAGAAGAAGAT------------
ATAGTAATTAGACCTGAAAATTTCACAAACAAT---------GCTAAAACCATAATAGTACAGCTGAAC----
-----------GCATCTGTAGAA---------ATTAATTGTACAAGACCCAACAACAATACAAGAAAA-----
-AGTATAAGGATA------------GGACCAGGGAGCGCA---TTT------TATGCAAATGGAGAC------
---------ATAATAGGAGATATA------------AGACAAGCACATTGT---AACATTAGTAAAGCA---

AAATGGGAAAACACTTTAAGACAGCTAGCTATAAAATTAAGA--------------GAACAATTTGTG----
-----------------------------------AATAAAACAATAGCCTTTAAT------
CAATCCTCAGGAGGGGACCCAGAGATTGTA---ATGCACAGTTTTAATTGTGGAGGGGAA---
TTTAAGCAGCTCCAGGCAAGAGTCCTGGCTGTGGAAAGATACCTAAAGGATCAACAGCTCCTGGGAATTTGGG
GATGCTCTGGAAAGCTCATCTGCACCACTAATGTACCTTAGAATACTAGTTGGAGT----------------
----------------------------AACAAATCTCAGGATGATATCTGG------AAT------
AACATGACCTGGATGCAGTGG---GACAGAGAAATTGACAATTACACAAACTTAATATAC---
ACCTTACTTGAAGAATCGCAAATCCAACAAGAA---------------------------------------
----
AAGAATGAACAGGAACTATTGGAATTGGATAAGTGGGCAAGTTTGTGGAATTGGTTTGACATATCAAATTGGC
TGTGGTATATAAGAATATTCATAATAATAGTAGGAGGCTTGGTAGGTTTAAGAATAATTTTTGCT---
GTACTTTCTATAGTG---------AATAGAGTTAGGCAGGGATACTCACCC------
TTATCGTTTGCTGTTAGCTTGCTTAACGCCACAGCTATAGCAGTAGGTGAG---
GGGACAGATAGGGTTATAGAACTATTACAAAGAGCTTATAGAGCT---------------------------
--------ATTATCCACATA------CCTAGAAGAATAAGACAGGGCTTGGAAAGGGCTTTGCTA---TAA
>AF224507
GCT---GCA--
GAAAAATTGTGGGTCACAGTCTATTATGGGGTACCTGTGTGG---AAAGAAGCAACC---
ACCACCCTATTCTGTGCATCAGACGCTAAAGCATATGACACAGAG---GTACATAAT---------------
--------------GTTTGGGCCACA---CATGCCTGTGTACCCATAGAC---
CCCAACCCACAAGAAGTATTTTTGGAA---------AATGTGACAGAAAATTTTAAC---------------
--------------------------ATGTGGAAAAATAACATGGTAGAACAGATGCATGAGGATATA---
ATCAGTTTATGGGATCAAAGCCTA---AAGCCATGTGTAAAACTA---
ACTCCACTCTGTGTTACTTTAAAGTGCACTGATTTGAATGATACTAATACCAATAATAGTAGTACTTCGGAAA
AT--
----------AATACTAATCCCACTATTAGTGGTGGGGAAGGGATGGGGGAAGGAGAGATGAAA---
AACTGCTCTTTCAATGTTACCACAAACATAAGA--------------------------
GATAAGGTGCAGAAAGAATATGCTCTT---TTCTATAAACTTGATATAATACCAATAGATAAT---------
----

ACAAGCTATGCATTGAGACATTGTAACACCTCAGTCATTACACAG---
GCCTGTCCAAAGGTATCCTTTGAACCAATTCCCATACATTATTGTGCCCCGGCTGGTTTTGCGATTCTACAAT
GTAAT---GATAAGAAGTTCAAT------GGAACAGGACCATGTTCAAAT----------------------
-GTCAGC---ACAGTACAATGTACACATGGAATTAGGCCAGTAGTATCAACT---
CAACTGCTGTTAAATGGCAGTCTAGCAGAAGAAGAG-----------
ATAGTACTTAGATCTGAAAATTTCACAAACAAT---------GCTAAAACCATAATAGTACAGCTGAAT----
-----------GCATCTGTAGAA---------ATTAATTGTACAAGACTCAACAACAATACAAGGAAA-----
-AGTATAAGGATA------------GGACCAGGAAGCACA---TTT------TATGCAACAGGAGCC------
---------ATAATAGGAGATATA-----------AGACAAGCACATTGT---AACATTAGTAGAGAA---
AAATGGAATGACACTTTAAAACAGCTAGTTATAAAATTAGGA--------------GAGCAATTTGGG----
---------------------------------AATAGTAACATAATAGTCTTTAAA------
CAATCCTCAGGAGGGGACCCAGAGATTGTA---ATGCACAGTTTTATTTGTGGAGGGGAG---
TTTAAGCAGCTACAGGCAAGAGTCCTGGCTGTGGAAAGGTACCTAAGAGATCAACAGCTCCTGGGGATTTGGG
GATGCTCTGGAAAGCTCATCTGCACCACTAATGTGCCTTGGAATACTAGTTGGAGT----------------
----------------------------AATAAATCTGAGAATGAGATCTGG------GAT------
AACATGACCTGGATGGAGTGG---GACAGAGAAATTAACAATTACACAAACTTAATATAC---
GACCTACTTGAAAAATCGCAGAACCAACAAGAA---------------------------------------
----
AAAAATGAACAGGAACTATTGGAATTGGATAAATGGGCAAGTTTGTGAAATTGGTTTAACATAACAAAATGGC
TGTGGTATATAAAAATATTCATAATGATAGTAGGAGGCTTAGTAGGTTTAAGAATAATTTTCTTT---
GTACTTTCTATAGTG---------AATAGAGTTAGGCAGGGATACTCACCA------
TTATCGTTTGCTGTTAGTTTGCTTAATGCCACAGCTGTAGCAGTAGCTGAG---
GGGACAGATAGGATTATAGAAATATTACAAAGAGCTTATAGAGCT---------------------------
--------ATTCTCAACATA------CCTAGAAGAATAAGACAGGGCTTGGAAAGGGCTTTGCTA---TAA
>AB097870
GCT---ACA---ACT--------------------------------
AAAGAAAAAATCGTGGGTCACAGTCTATTATGGGGTACCTGTATGG---AAAGAAGCAATC---
ACCACTCTATTTTGTGCATCAGATGCTAAAGCATATGATACAGAG---ATACATAAT---------------
--------------GTTTGGGCCACA---CATGCCTGTGTACCCACAGAC---
CCCAACCCACAGGAAGTAGTCTTGGGA---------AATGTGACAGAAAACTTTAAC---------------

--------------------------ATGTGGAAAAATAATATGGTAGATCAGATGCATGAAGATATA---
ATCAGTTTATGGGATCAAAGTCTA---AAACCATGTGTAAAACTA---
ACCCCACTCTGTGTCACTTTAAATTGCATTGATTATGTGAAAAATGCTACTAATACTAATAGTACTGCTACT-
----
----------------AATACCACTAGTACAACCAATAGTACAATAGAGGGAGGAGAAATGAAA---
AACTGCTCTTTCAATATCACCACAAGCATAAAA------------------------------AAGGTG---
AAAGACCATGCACTT---TTTTATAAACTTGATATAGTAAAAATGAAAGATGAT-------------------
----
----AGTGCCAATAATAACAGCTATAGGTTGATAAATTGTAACTCCTCAGTCATTACACAA---
GCTTGTCCAAAGGTATCCTTTGAGCCAATTCCCATACATTATTGTGCCCCGGCTGGTTTTGCGATTCTAAAGT
GTAAC---AATAAAACATTCAAT------GGAACAGGACCATGTACAAAT-----------------------
-GTCAGT---ACAGTACAATGTACACATGGAATTAGGCCAGTAGTATCAACT---
CAACTGCTGTTAAATGGCAGTCTAACAGAAGAAGGG------------
ATAGTAATTAGATCTAGCAATTTCACAGACAAT---------GCTAAAGTCATTATAGTACAGCTGAAT----
-----------GAATCTGTACGA---------ATTGATTGTATAAGACCTAACAACAATACAAGAAAA-----
-AGTATAAATCTA------------GGACCAGGGCAAGCA---TGG------TATACAACAGGAGAC------
---------ATAATAGGAGATATA------------AGACAAGCACATTGT---AACCTTACTAGCAAA---
AATTGGAATAACACTTTAATACAGGTAACTGAAAAATTAAGA---------------GAACAATTTGGG----
-----------------------------------AACAAAACAATAGACTTTAAA------
CAATCCTCAGGAGGGGACCCAGAGATTGTA---ATGCACAGTTTTAATTGTGGAGGGGAA---
TTCAAACAGCTCCAGGCAAGAGTCCTGGCTGTGGAAAGATACCTGAAGGATCAACAGCTCATGGGGATTTGGG
GTTGCTCTGGAAAACTCATTTGCACCACTGCTGTGCCTTGGAATACCAGTTGGAGT-----------------
----------------------------AATAAATCTCTGGATGACATTTGG------AAT------
AACATGACTTGGATGGAGTGG---GAAAGAGAAATTCACAATTACACAGCAGAAATATAC---
ACCTTAATTGAACAATCGCAGAACCAGCAAGAA--

AAGAATGAACTAGAATTATTAAAATTGGATACGTGGGCAAGTTTGTGGAGTTGGTTTGACATATCAAATTGGC
TGTGGTATATAAGAATATTCATAATGATAGTAGGAGGCTTAATAGGTTTAAGAATAGTTTTTGCT---
GTACTTTCTATAGTA---------AATAGAGTTAGCCAGGGGTACTCACCA------
TTGTCGGTTGCTGTTAGCTTGCTCAATACCATAGCTATAGCAGTAGCTGAG---
GGGACAGATAGGATTATAGAAATAGTACAAAGAGCTTATAGAGCC----------------------------
--------ATTCTCCACATA------CCTACAAGAATAAGACAGGGCTTAGAAAGGGCTTTGCTA---TAA
>AY423386
GCT---ACA--
GAAAAATTGTGGGTCACAGTCTATTATGGGGTACCTGTGTGG---AAAGAAGCAACC---
ACCACTCTCTTTTGTGCATCAGATGCTAAAGCATATGAAACAGAG---GTACATAAT----------------
--------------GTTTGGGCCACA---CATGCCTGTGTACCCACAGAC---
CCTAACCCACAAGAACTAGTATTAGAA---------AATGTGACAGAAAATTTTAAT----------------
--------------------------ATGTGGAAAAATAACATGGTAGAACAAATGCATGAGGACATA---
ATCAGTTTATGGGATGAAAGCCTA---AAACCATGTGTGAAATTA---
ACCCCACTCTGTGTTACTTTAAATTGCACTAATATTACTAGTTCCAATAATATTACTGGTTCC----------
----
----------------------AATAATAATAGTAATTTGGAACAGATGACAAGAGAAATAAGT---
AACTGCTCTTTCAATATCACCACAACCATAAAA---------------------------
AATAAGAGACAGAGAGAATTTGCACTG---TTGAGTAAACTTGATATAGTACCAATAGATAAT----------
----

GATAGTTATAGTTATATGTTGATAAATTGTAATACCTCAGTCATTACACAG---
GCCTGTCCAAAGGTATCCTTTCAACCAATTCCCATACATTATTGTACCCCGGCTGGTTTTGCGATTCTAAAAT
GTAAT---GATAAGAAGTTTAAT------GGAACGGGACCATGTAAAAAT-----------------------
-GTCAGC---ACAGTACAATGTACACATGGAATTAGACCAGTGGTGTCAACC---
CAATTACTGTTAAATGGCAGTCTAGCAGAAGAAGAG------------
GTAGTAATTAGGTCTAAAAATTTCACAGACAAT---------ACTAAAACCATAATAGTACAGCTGAAG----
-----------GAATCTGTAGAA---------ATTAATTGTACAAGACCCAACAATAATACAAGAAAA-----
-AGTATACATATA------------GGACCAGGGAGAGCA---TTT------TATGCAACAGGAGAA------
---------ATAATAGGAGATATA------------AGACAAGCACATTGT---AACCTTAGTAGAGCA---
AAATGGAATGACACCTTAAACCAGATAGTTGGAAAATTAAGA---------------GAATTATATAAG----
-----------------------------------AATAAAACAATAGTCTTTAAT------
TCATCCTCAGGAGGGGATCCAGAAATTGTA---ATGCACAGTTTTAATTGTAGAGGGGAA---
TTTAAGCAGCTCCAGGCAAGAGTCCTGGCTATAGAAAGATACCTACAGGATCAACAGCTCCTAGGAATTTGGG

GTTGCTCTGGAAAACTCATTTGCACCACTACTGTGCCTTGGAATGCTAGTTGGAGT-----------------
----------------------------AATAAATCTCTGGATCAGATTTGG------AAT------
AACATGACCTGGATGCAGTGG---GAGAGAGAAATTGACAATTACACAAATTTAATATAC---
ACTCTAATTGAAGACTCGCAGAAACAACAAGAA--

AAGAATGAACAAGAATTATTGGCATTGGATACATGGGCAAGTTTGTGGAATTGGTTTAGCATAACAAATTGGC
TGTGGTATATAAAAATATTCATAATGATAGTAGGAGGTTTAGTAGGGTTAAGAATAGTTTTTATT---
GTACTTTCTATAGTG---------AATAGAGTTAGGAAGGGATACTCACCA------
TTGTCATTCGCTGTTAGTTTGCTTAATGCCATAGCTATAGCAGTAGCCGAG---
GGGACAGATAGGGTTATAGAAACAATACAAAGAGCTTTTAGAGCC----------------------------
--------ATTCTCCACATA------CCTAGAAGAATAAGACAGGGCTTAGAAAGGCTTTTACTA---TAA
>HQ386146
GCT---GAA--
GATCAATTGTGGGTCACAGTATATTATGGGGTACCTGTGTGG---AAAGAAGCAAAT---
ACCCCTCTATTTTGTGCATCAGATGCTAAGGCATATAATACAGAA---GCACATAAT----------------
--------------GTTTGGGCCACA---CATGCCTGTGTACCCACAGAC---
CCCGACCCACAAGAAGTATGGATA------------AATGTGACAGAAAATTTTAAC----------------
--------------------------ATGTGGAAAAATAACATGGTAGAACAAATGCATGAGGATGTG---
ATTAGTTTATGGGATCAAAGCCTA---AAGCCATGTGTAAGATTA---
ACCCCACTCTGTGTTGCTTTAAATTGTACTGATACTAACATCACTAGTACTACTCCCACTAGTACTACTGGTA
CTACCAAT---
-------ACTACTGGTACTACCAGTAGTACTACCGTGAGACCAACAGAGGGAGGAGACATGAAA---
AACTGCTCTTTCAATATTAGCACAAGCATGAAA---------------------------
AGTAGGGCGCAGAAAGAATATGCACTT---TTTTATAAGCTTGATTTAGTACCAATAAATGAT----------
----

ACCACTTATAGGTTGATAAGTTGTAATACCTCAAAAATTACACAG---
GCCCGTCCAAAGATATCCTTTGAGCCAATTCCTATACATTATTGTGCCCCGGCTGGTTTTGCGATTCTAAAAT
GTAGA---GATAAGGAGTTTAAT------GGAACAGGACCATGTAATAAT-----------------------
-GTCAGC---TCAGTACAATGTACACATGGAATTAGGCCAGTAGTATCAACT---
CAATTGCTGTTAAATGGCAGTCTAGCAGAAAAAGAC-----------
ATAGTAATCAGATCTGAAAATTTCACAGACAAT---------ATTAAAAACATAATAGTACAGCTGAAA----
-----------GAACCTGTAATA---------ATTAATTGTACAAGACCCAGCAACAATACAAGCAAA-----
-GGTATACACATG------------GGACCAGGGAGAGCA---TTT------TATGCAACAGGACAA------
---------ATAACAGGAGATATA------------AGAAAAGCATATTGT---AACATTAGTGAAGGA---
AAATGGAATAACACTTTAAAAGATATAGCTAGAAAATTAAGA---------------GAACAATATGGG----
--------------------------------AATGACACAAAAATAAATTTTAAA------
CCATCCTCCGGAGGGGACCCAGAAATTGTA---ATGCACAGCTTTAATTGTGGAGGGGAA---
TTTAAGCAGCTCCAGGCAAGAGTCCTGGCTGTAGAAAGATACCTAAAGGATCAGCAGCTCCTGGGGATTTGGG
GTTGCTCTGGAAAACACATTTGCACCACCGCTGTGCCTTGGAATACTAGTTGGAGT-----------------
----------------------------AATAGATCTCTGCAATATATTTGG------CAA------
AACATGACCTGGATGCAGTGG---GAAAAAGAAATTGACAATTACACAAGCTTAATATAC---
ACTTTAATTGAGGAATCGCAAATCCAACAAGAA--

AAGAATGAAAAAGAATTATCGGAATTAGATAAATGGGCAAGTTTGTGGAATTGGTTCAGCATAACAAATTGGC
TGTGGTATATAAAAATATTCATAATGACAGTAGCAGGCCTGGTAGGTTTAAAAATAGTTTTTTCT---
GTATTTTCTATAGTG---------AATAGAGTTAGGCAGGGATACTCACCA------
TTGTCGTTTGCTATTAGCTTATTCAACGCCATAGCCATAGCAGTAGCTGAG---
GGGACAGATAGGGTTATAGAGATATTACAAAGAACTTTTAGAGCT----------------------------
--------ATCCTCCACATA------CCTAGAAGAATAAGACAGGGCTTGGAAAGGGCTTTGCAA---TAA
>HQ386218
GCT---ACA--
GAAGACACGTGGGTCACAGTCTATTATGGAGTACCTGTGTGG---AAAGAAGCAACC---
ACCACTCTATTTTGTGCATCAGATGCTAAAGCATATAGTACAGAG---GCACATAAT----------------
--------------GTTTGGGCCACA---CATGCCTGTGTACCCACAGAT---
CCCAACCCACAAGAAATAGCATTG------------AATGTGACAGAAAATTTTAAC----------------
--------------------------ATGTGGAAAAACAACATGGTGGAACAGATGCATGAGGATATA---
ATCAGTTTGTGGGATCAAAGCTTA---AAGCCATGTGTAAAATTA---
ACCCCACTCTGTGTTACCTTAAATTGCACTGATCTGAAGAATAATACTAAGAAGAATAATACTACTAAGAATG
AT---

```
-------------ACTAATACCATTGCTAATAGCTGGGAAAAAATGGAAAGGGGAGAAATAAAA---
AATTGCTCTTTCAATATCACCACAAGCATAAAA---------------------------
GATAAAATGAAAAGGGAATATGCACTT---TTGTATAAACTTGATTTAGTACCAATAAAAGATAATAAT----
-------------------------------------------------------------------------
-------------
AATAGCCTTGATAATACTAGCTATAGGTTGATAAATTGTAATACCTCAGTCCTTACACAG---
GCCTGTCCAAAGGTAACCTTTGAGCCAATTCCCATACATTATTGTGCCCCGGCTGGTTTTGCGATTCTAAAGT
GTAGA---GATAAAAAGTTTAAT------GGGACAGGACCATGTAAAGAT-----------------------
-GTCAGC---ACAGTGCAATGTACACATGGAATTAGGCCAGCAGTATCAACT---
CAACTACTGCTAAATGGCAGTCTAGCAGAAGAAGAG------------
GTAGTAATTAGATCTGCCAATTTCACAGACAAT---------CTTAAAAACATAATAGTACAGCTGAAC----
-----------ATATCGGTGGCA---------ATTAATTGTACAAGACCCAACAACAATACAAGAAAA-----
-AGGGTAACTCTA------------GGACCAGGGAGAGTA---TGG------TATACAACAGGAGAA------
---------ATAATAGGAGATATA------------AGAAAAGCACATTGT---AACCTTAGTAGAGCA---
GCATGGAATAACACTCTAAAACAGATAGTTATAAAATTAAGA---------------GAACAATTTGGG----
--------------------------------AATAATAAAACAATAAAATTTCAG------
CCATCCTCAGGAGGGGACCTAGAAATTGTA---ACACATAGTTTTAATTGTGGAGGGGAA---
TTTAAGCAGCTCCAGGCAAGAGTCCTGGCTGTGGAAAGATACCTAAAGGACCAACAGCTCCTGGGGATTTGGG
GATGCTCTGGAAAACTCATTTGCACCACTACTGTGCCTTGGAATGCGAGTTGGAGT-----------------
----------------------------AATAAATCTCTAAATGAAATTTGG------GAA------
AATATGACCTGGATGCAGTGG---GATAAAGAGATTAACAATTACACAGACATAATATAC---
AACTTAATTGAAAAGGCGCAAAACCAACAAGAA----------------------------------------
-----------------------------
AAGAATGAACAAGAATTATTAGAATTAGATAAATGGGCAAATTTGTGGAATTGGTTTGACATATCAAACTGGC
TGTGGTATATAAAAATATTCATAATGATAGTAGGAGGATTAATAGGTTTAAGAATAGTTTTTAGT---
GTACTTTCTATAGTA---------AATAGAGTTAGGCAGGATACTCACCA------
TTATCGTTTGCTATTAGCTTGCTCAATACCATAGCCATAGCAGTAGGTGAG---
GGACAGATAGAGTTATAGAAATAATACAAAGAGCTTGTAGAGCT-----------------------------
--------ATTCTCAACATA------CCTAGAAGAATAAGACAGGGCTTTGAAAGGGCTTTGCTA---TAA
>U34603
GCT---GCA------------------------------------------
GAACAATTGTGGGTCACAGTCTATTATGGGGTACCTGTGTGG---AAAGAAGCAACT---
ACCACTCTATTCTGTGCATCAGATGCTAAAGCATATGATACAGAG---GTACATAAT----------------
--------------GTTTGGCCACA---CATGCCTGTGTACCCACAGAC---
CCCAACCCACAAGAAGTAGTATTGGGA---------AATGTGACAGAATATTTTAAC----------------
--------------------------ATGTGGAAAAATAACATGGTAGACCAGATGCATGAGGATATA---
ATCAGTTTATGGGATCAAAGCTTG---AAGCCATGTGTAAAATTA---
ACCCCACTCTGTGTTACTTTAGATTGCGATGATGTGAATACCACTAATAGTACTACTACCACT----------
-------------------------------------------------------------------------
----------------------AGTAATGGTTGGACAGGAGAAATAAGGAAAGGAGAAATAAAA---
AACTGCTCTTTTAATATCACCACAAGCATAAGA---------------------------
GATAAGGTTCAAAAAGAATATGCACTT---TTTTATAACCTTGATGTAGTACCAATAGATGATGGTAATGCT-
-------------------------------------------------------------------------
-------
ACTACCAAAAATAAAACTACTAGAAACTTTAGGTTGATACATTGTAACTCCTCAGTCATTACACAG---
GCCTGTCCAAAGGTATCATTTGAACCAATTCCCATACATTATTGTGCCCCGGCTGGTTTTACGATTCTGAAGT
GTAAC---AATAAGACGTTTGAT------GGAAAAGGACTATGTACAAAT-----------------------
-GTCAGC---ACAGTACAATGTACACATGGAATTAGGCCAGTAGTGTCAACT---
CAACTGCTGTTAAATGGCAGTCTAGCAGAAGAAGAG------------
GTAGTAATTAGATCTGACAATTTCACGGACAAT---------ACTAAAACCATAATAGTACAGCTGAAT----
-----------GAATCTGTAGCA---------ATTAATTGTACAAGACCCAACAACAATACAAGAAAA-----
-GGTATACATATA------------GGACCAGGGAGAGCC---TTT------TATGCAGCAAGAAAA------
---------ATAATAGGAGATATA------------AGACAAGCACATTGT---AACCTTAGTAGAGCA---
CAATGGAATAACACTTTAAAACAGATAGTTATAAAATTAAGA---------------GAACACTTTGGG----
-----------------------------------AATAAAACAATAAAATTTAAT------
CAATCCTCAGGAGGGGACCCAGAAATTGTA---AGGCATAGTTTTAATTGTGGAGGGGAA---
TTTAAGCAGCTCCAGGCAAGAGTCCTGGCTATGGAAAGATACCTAAAGGATCAACAGCTCCTGGGGATTTGGG
GTTGCTCTGGAAAACTCATTTGCACCACTGCTGTGCCTTGGAATACTAGTTGGAGT-----------------
----------------------------AATAAATCTCTGGATGAGATTTGG------GAT------
AACATGACCTGGATGCAGTGG---GAAAGAGAAATTGACAGTTACACAAACACAATATAC---
```

GACTTAATTGCAGAATCACAGAACCAGCAGGAA---------------------------------------
----
AAGAATGAACAAGAATTGTTGGAATTGGATAAATGGGCAAGTTTGTGGAATTGGTTTAGCATAACAAACTGGC
TGTGGTATATAAGAATATTCATAATAATAGTAGGAGGCTTGGTAGGTTTAAGAATAGTTTTTGCT---
GTACTTTCTATAGTA---------AATAGAGTTAGGCAGGGATACTCACCA------
TTATCGTTTGCTGTTAGCTTGCTCAACGCCACAGCCATAGCAGTAGCTGAG---
GGGACAGATAGGGTTATAGAAGTAGTACAAAGAATTTGTAGAGCT---------------------------
--------ATCCTCCACATA------CCTACACGAATAAGACAGGGCTTGGAAAGGGCTTTGCTA---TAA
>AY970946
GCT---ACA--
GAGAAATTGTGGGTCACAGTTTATTATGGGGTACCTGTGTGG---AAAGAAGCAACC---
ACCACTCTATTTTGTGCATCAGATGCTAAAGCATATGATACAGAG---GTACATAAT---------------
--------------GTTTGGGCCACA---CATGCCTGTGTACCCACAGAC---
CCCAGCCCACAAGAAGTAGTATTGGCA---------AATGTGACAGAAAATTTTAAC---------------
--------------------------ATGTGGAAAAATAACATGGTAGAACAAATGCATGAGGACATG---
ATCAGTTTATGGGATCAAAGCCTA---AAGCCATGTGTAAAATTA---
ACCCCACTCTGTGTTACTTTAGATTGCACTAATTTAAGGAATGCTACTAATACCACTGCT------------
----
-------------------------ACTAATACCACTAGTAGTGGATGGGGAGAAGAAATGACA---
AACTGCTCTTTCAATATCACCACAAGCATAAGA--------------------------
AATAAGGTTCAGCAAGAATATGCACTT---TTTTATAAACTTGATGTAGTACCAATAGATAAG---------
----

AATACTGCTAAATATAGGTTGATAAATTGTAACACCTCAGTCATTACACAG---
GCCTGTCCAAAGGTATCATTTGAGCCAATTCCCATACATTATTGTACCCCGGCTGGTTTTGCGATTCTAAAGT
GTAAT---GATAAGAAGTTCAAT------GGAACAGGACCATGTACAAAT----------------------
-GTCAGC---ACAGTACAATGTACACATGGAATTAGGCCAGTAGTGTCAACT---
CAACTGCTGTTAAATGGCAGTCTGGCAGAAGGAGGG-----------
GTAGTAATTAGATCTGAAAATTTCACGAACAAT---------GCTAAAACCATAATAGTACAGCTGAAT----
-----------GAATCTATAGAA---------ATTAATTGTACAAGACCCAACAACAATACCAGAAAA-----
-AGTATACATATA------------GGACCGGGGAGAGCA---TTC------TATGCAACAGGAGAA------
---------ATAATAGGAGATATA------------AGACAAGCACATTGT---AACATTAGTAGAGGA---
AAATGGAATAACACTTTAAAACAGATAGTTACAAAATTAAAA---------------GAACAATATGGG----
-----------------------------------AATAAAACAATAGTATTTAAG------
TCATCCTCAGGAGGGGACCCAGAAATTGTA---ATGCACAGTTTTAATTGTGGAGGGGAA---
TTTAAGCAGCTCCAGGCAAGAGTCCTGGCTGTGGAAAGATACCTAAAGGATCAACAGCTCCTAGGGATTTGGG
GTTGCTCTGGAAAACTCATCTGCACCACTGCTGTGCCTTGGAATGCTAGTTGGAGT----------------
----------------------------AATAAATCCCTGAATAAGATTTGG------GAT------
AACATGACCTGGATGGAGTGG---GAAAGAGAAATTGAAAATTACCCAAGCTTAATATAC---
ACCTTAATTGAAGAATCGCAGAACCAGCAAGAA---------------------------------------
----
AAGAATGAACAAGAATTATTGGAATTAGATAAGTGGGCAAGTTTGTGGAATTGGTTTGACATAACAAACTGGC
TGTGGTATATAAAAATATTCATAATGATAGTAGGAGGCTTGGTAGGTTTAAGAATAGTTTTTGCT---
GTACTTTCTATAGTG---------AATAGAGTTAGGCAGGGATACTCACCA------
TTATCATTCGCTGTTAGTTTGCTTAATGCCACAGCTATAGCAGTAGCTGAG---
GGGACAGATAGGATTATAGAAGTAGTACAAAGAATTTGTAGAGCT---------------------------
--------ATTCGCCACATA------CCTAGAAGAATAAGACAGGGCTTTGAAAGGGCTTTGCTA---TAA
>GU455480
GCT---GCA--
GAACAATTGTGGGTCACAGTCTATTATGGGGTACCTGTGTGG---AAAGAAACAAAA---
ACCACTCTATTTTGTGCATCAGATGCTAAAGCATATGATACAGAG---GTACATAAT---------------
--------------GTTTGGGCCACA---CATGCCTGTGTACCCACAGAC---
CCCAACCCACAAGAAGTAGTATTGGGG---------AATGTGACAGAAAATTTTAAC---------------
--------------------------ATGTGGAACAATGACATGGTAGATCAGATGCATGAGGATATA---
ATCAGTTTATGGGACCAAAGCCTA---AAGCCATGTGTAAAATTA---
ACCCCACTCTGTGTTACTTTAAATTACACTGATTATTGGAAGAATAATGATACTAATACCACTAGTAGTAATG
CT--
-------------ACTAGTACCACTAGTAGTAACGGAAACACAGAGGAGAAAGGAGAAATAAAA---
AACTGCTCTTTCAATATCACCACAACCATAAGA--------------------------
GATAAGGTGCAGAAAGAATATGCACTT---TTTTATAAACTTGATGTTGTACCAATAGATAATGAT-------

----

AATACCAGCTATAATACCAGCTATAGGTTAATAAGTTGTAACACCTCAGTCATTACACAG---
GCCTGTCCAAAAGTATCCTTTGAGCCAATTCCCATACATTATTGTGCCCCGGCTGGTTTTGCGATTCTAAAAT
GTAAT---GATAAGAAGTTCAAT------GGAACAGGATCATGTAAAAAT----------------------
-GTCAGC---ACAGTACAATGTACACATGGAATTAGGCCAGTAGTATCCACT---
CAACTGCTGTTAAATGGCAGTCTAGCAGAAGAAGGG-----------
GTAATAATTAGATCTGAAAATTTCTCGGACAAT---------GCTAAAACCATAATAGTACAGCTGAAT----
-----------GAAACTATAGAA---------ATTAATTGTACAAGACCCAACAACAATACAAGAAAA-----
-GGTATACATATA------------GGACCAGGGAGAGCA---TTT------TATGCAACAGGAGAA------
---------ATAATAGGAGATATA------------AGACAAGCACATTGT---AACCTTAGTATAACA---
AAATGGAATAACACTTTACAACAGATAGTTGAAAAATTAAGA---------------GAACAATTTAAG----
-----------------------------------AACAAAACAATAGAATTTAAG------
CACTCCTCAGGAGGGGACCCAGAAATTGTA---ATGCACAGTTTTAATTGTGGAGGGGAA---
TTTAAACAGCTCCAGGCAAGAGTCCTGGCTCTGGAAAGATACCTAAGGGATCAACAGCTCATGGGAATTTGGG
GTTGCTCTGGAAAACTCATCTGCACCACTGCTGTGCCTTGGAATGCTAGTTGGAGT----------------
----------------------------AATAAATCTCTGAATGAGATTTGG------GAT------
AACATGACCTGGATGGAGTGG---GAAAGAGAAATTGAAAATTACACAGGCAAAATATAC---
AACTTACTTGAAAAATCGCAGAACCAGCAGGAA---------------------------------------
----
AAGAATGAACAAGAATTATTGGAATTGGATAAATGGGAAAGTTTGTGGAGTTGGTTTAGCATAGTAAATTGGC
TGTGGTACATAAAAATATTCATAATGATAGTAGGGGGCTTGATAGGTTTAAGAATAGTTTTTACT---
GTACTTTCTATAGTG---------AATAGAGTTAGGCAGGGATACTCACCC------
TTATCGTTTGCTGTTAGCTTGCTCAATTCCATAGCCATAGCAGCAGCTGAG---
GGGACAGATAGGATTATAGAAGTATTACAAAGAATTTATAGAGCT---------------------------
--------TTTATCCACATA------CCTACAAGAATAAGACAGGGCTTGGAACTAGCTTTGCTG---TAA
>GU455465
GCT---GAA---GAA------------------------------------
GAAGGAAAGTGGGTCACAGTCTATTATGGGGTACCTGTGTGG---AAAGAAGCAACC---
ACTACTCTATTTTGTGCCTCAGATGCTAAAGCATCTGACACAGAG---GTACATAAT----------------
--------------GTTTGGGCCACA---CATGCCTGTGTACCCACAGAC---
CCTAACCCACAAGAAGTAGTATTGGTA---------AATGTGACAGAAAATTTTAAC----------------
-------------------------ATGTGGAAAAATAACATGGTAGAACAGATGCATGAGGATATA---
ATCAGCTTATGGGATGAAAGCCTA---AAGCCATGTGTAAAATTA---
ACCCCACTCTGTGTTACTCTAAATTGCACTAATTTGAAGATGAATGCTAATAATACCAATAATTCTAGTATGA
AGGCTACTGAT---
----ACCAATAATACTAATGCCAATATTACAGAGTGGAAAAAGATGGAGGAAGGAGAAATAAAA---
AACTGCTCTTTTAATGTCACCACAAGCATAGGA--------------------------
AATAAGATGCAGAAAAACTATGCACTT---TTTTATAGACTTGATGTAGTACCAATGGAGGAG----------
----

GATAATACAAGTTATACATTGATAAATTGTAACACCTCAGTCATTACACAA---
GCCTGTCCAAAGGTATCCTTTGAACCAATTCCCATACATTATTGTACCCCGGCTGGTTTTGCGATTCTAAAGT
GTAAT---GATAAGGATTTCAAT------GGAACAGGACCATGTACAAAT----------------------
-GTTAGT---ACAGTACAATGTACACATGGAATTAGGCCAGTAGTATCAACT---
CAATTGCTGTTAAATGGCAGTCTAGCAGAAGGAGAT-----------
GTAGTAATTAGATCTGAAAATTTCACGGACAAT---------GCTAAAACCATAATAGTACAGCTGAAT----
-----------GAATCTGTAGTA---------ATTAATTGCACAAAACCCAACAACAATCAAAGAAGA-----
-AGGATACGCTTA------------GGACCGGGACCGGGG---AGT------GTATTTTATGCAACA------
---GGAGAAATAATAGGACATATA------------AGACAAGCACAATGT---ACCCTTAGTGGAGTA---
AAATGGAATAACACTTTAAGACAGGTAGTTGAAAAATTAAGG---------------GAACAATTTGGG----
-----------------------------------AATAAAACAATAGCCTTCAAT------
CAATCCTCAGGAGGGGACCCAGAAATTGTA---CTGCACAGTTTTAATTGTGGAGGGGAA---
TTTAAGCAGCTCCAGGCAAGAGTCCTGGCTGTAGAAAGATACCTAAGGGATCAACAGCTCCTAGGGATTTGGG
GTTGCTCTGGAAAAATCATTTGCACCACTTCTGTGCCTTGGAATACTAGTTGGAGT----------------
----------------------------AATAAATCTCTAAAGTACATTTGG------GAA------
AACATGACCTGGATAGAGTGG---GAAAGAGAAATTGACAATTACACAAGCCTAATATAC---
ACCTTAATTGAAGAATCGCAGATCCAACAGGAA---------------------------------------
----
AAAAATGAACAAGAGTTATTGGAATTGGATAAGTGGGCAAGTTTGTGGAATTGGTTTACCATAACAAATTGGC

TGTGGTATATAAAAATATTCATAATGATAGTAGGAGGTTTAGTAGGCTTCAGAATAGTTTTTGCT---
GTGCTTTCTATAGCG---------AATAGAGTTAGGCAGGGATACTCACCA------
ATATCATTTGCCATTAGTTTGCTTAATGCCACAGCTATAGCAGTAGCTGAG---
GGGACAGATAGGATTATAGAAGTAGTACAAAGAGCTTGTAGAGCT----------------------------
--------ATTCTCCACATA------CCTAGAAGAATAAGACAAGGCTTAGAAAGGGCTTTGCTA------
>EU744159
GCT---ACA--
GAAAAATTGTGGGTCACAGTCTATTATGGGGTACCTGTGTGG---AAAGAAGCAACC---
ACCACTCTATTTTGTGCATCAGATGCTAAAGCATATGATACAGAG---GTTCATAAT----------------
--------------GTTTGGCCACA---CATGCCTGTGTACCCACAGAC---
CCCAACCCACAAGAAGTAGTATTGGAA---------AATGTGACAGAAAATTTTAAC----------------
--------------------------ATGTGGAAAAATGGCATGGTAGACCAGATGCATGAGGATATA---
ATCAGTTTATGGGATCAAAGCCTA---AAGCCATGTGTAAAATTA---
ACCCCACTCTGTGTTACTTTAAATTGCACTGATTATTTAGGAAATAATACTAATACCACTAATACT-------
----
----------------------GCTAATAGTAGCGGGAAAATAACGGAAGAAGGAGAGATAAAA---
AACTGCTCTTTCAATATCACCACACCAATAAGA---------------------------
GATAGGTTGAAGAAAGAACATGCACTT---TTTTATAAACTTGATGTAGTGCCAATAGATAAG----------
----

GATAATACTAGCTATAGGTTGATAAGTTGTAACACCTCAGTCATTACACAG---
GCCTGTCCAAAGGTATCCTTTGAGCCAATTCCCATACATTATTGTGCCCCGGCTGGTTTTGCGATTCTAAAGT
GTAAT---GATAAGAAGTTCGAT------GGAAAAGGACCATGTACAAAT-----------------------
-GTCAGC---ACAGTACAATGTACACATGGAATTAGGCCAGTAGTATCAACT---
CAACTGCTGTTAAATGGCAGTCTAGCAGAAGAGGAG------------
GTAGTAATTAGATCTGAAAATTTCACGAACAAT---------GCAAAAACCATAATAGTACAGCTGAAC----
-----------GAATCTGTAGAA---------ATTAACTGTACAAGACCCAACAACAATACAAGAAGA-----
-AGTATACATATA------------GGACCAGGGAGAGCA---TTT------TATGCAACAGGAGAC------
---------ATAATAGGAGATATA------------AGACAAGCACATTGT---AACATTAGTAAAGAA---
AAATGGAGAACACTTTAGAACAGATAGTTAGAAAATTAAGA---------------GAACAATTTAAA----
-----------------------------------AATAAAACAATAGTCTTTGAT------
CAATCCTCAGGAGGGGACCCAGAAATTGTA---ACACACAGTTTTAACTGTGGAGGGGAA---
TTTAAGCAGCTCCAGGCAAGAGTCCTGGCTGTAGAAAGATACCTAAGGGATCAACAGCTCCTGGGGATTTGGG
GTTGCTCTGGAAAACTCATTTGCACCACTGCTGTGCCTTGGAATGCTAGTTGGAGT-----------------
-----------------------------AATAAATCTCTGGATGAAATTTGG------AAT------
AACATGACCTGGATGCAGTGG---GAGAAAGAAATAGACAATTACACAAGCTTAATATAC---
ACCTTAATTGAAGAATCGCAGAACCAACAAGAA--

AAGAATGAACAAGAATTATTAGAATTGGATAAGTGGGCAAGTTTGTGGAATTGGTTTGACATAACAAGCTGGC
TGTGGTATATAAAAATATTTATAATGATAGTAGGGGGCTTGGTAGGTTTAAGAATAGTTTTTGCT---
GTATTTACTATAGTG---------AATAGAGTTAGGCAGGGATACTCACCA------
TTATCGTTGGCTGTTAGCTTGCTCAACGCCACAGCCATAGCAGTAGCTGAG---
GGGACAGATAGGGTTATAGAAATATTACAAAGAGCTTTTAGAGCT----------------------------
--------ATTATCCACATA------CCTACAAGAATAAGACAGGGCCTCGAAAGGGCTTTACAA---TAA
>GU455505
GCT---ACC--
GAGGATTTGTGGGTCACAGTCTATTATGGGGTACCTGTGTGG---AAAGAATCAACC---
ACCACTCTATTTTGTGCATCAGATGCTAAAGCATATGATACAGAG---GTACATAAT----------------
--------------GTTTGGCCACA---CATGCCTGTGTACCCACAGAC---
CCCAGCCCACAAGAAATAGTATTGGAA---------AATGTGACAGAAGAGTTTAAC----------------
--------------------------ATGTGGAAAAATAACATGGTAGAACAGATGCATGAGGACATA---
ATCAGTTTATGGGATCAAAGCTTA---AAGCCATGTGTAAAATTA---
ACCCCACTCTGTGTTACTTTAAATTGCACTGATTGGCCTGTAAATTGCACTGAGGAGAATTGCACTGATTGGA
AGAATACG---
----AATAGCCCTGGTGGGGGGAATGTGAGTAGAGAGGAATTGATGGAGAAAGGAGAAATAAAA---
AACTGTTCTTTCAATATCACCACAAACATAAGA---------------------------
GATAAGATGCAGACAGCATATGCAGCT---TTTTATAAACTTGATATAGTACCAATAGAGGATAAT-------
----

AAGGATAATAATGATAGTACCTATAGGTTGATAAATTGTAACACCTCAGTCATTACACAG---

GCCTGTCCAAAGGTATCCTTTGAACCAATTCCCATACATTATTGTGCCCCGGCTGGTTTTGCGATTCTAAAGT
GTAAT---GATAAGCAGTTCAGT------GGGAAAGACATATGTAAAAAT----------------------
-GTCAGC---ACAGTACAATGTACACATGGAATTAAGCCAGTAGTATCAACC---
CAACTGCTGTTAAATGGCAGTCTAGCAGAAGAAGAG------------
GTAGTAATTAGATCTGACAATTTCACGGACAAT---------GCTAAAACCATAATAGTACAGCTGAAC----
-----------GAATCTGTAGAA---------ATTACTTGTATAAGACCCAACAACAATACAAGAAAA-----
-AGTATACATATA------------GGACCAGGGAGAGCA---CTT------TAT---ACAACAGAA------
---------ATAATAGGAGATATA------------AGAAAAGCATATTGT---ACCATTAATGGAACA---
AAATGGAATAACACTTTACAAAAGATAGTTGAAAAATTAGAA------------
AAGAAATTAGTTGAAGAATTAGGA------AAAAATTTTAAGAAT------
AGAACAAATAACACAATAGCCTTT---AGGCCATCCTCAGGAGGGGACCCAGAAATTGTA---
ATGCACAGTTTTAATTGTGGAGGGGAA---
TTTAAGCAGCTCCAGGCAAGAGTCCTGGCTGTGGAAAGATACCTAAAGGATCAACAGCTCATGGGGATTTGGG
GTTGCTCTGGAAAACTCATTTGCACCACTACTGTGCCTTGGAATGCTAGTTGGAGT-----------------
----------------------------AATAAATCTCTAGATCAGATTTGG------GAT------
AACATGACCTGGATGCAGTGG---GAGAAAGAAATTGAAAATTACACAGGCTTAATATAC---
AACTTAATTGAAGAATCGCAGAACCAGCAGGAA---------------------------------------
----
AAGAATGAACAAGAATTATTGGAATTAGATAAATGGGCAAGTTTGTGGAATTGGTTTAGCATAACAAATTGGC
TGTGGTATATAAAAATATTCATAATAATAGTAGGAGGCTTGATAGGTTTAAGAATAGTCTTTACT---
GTACTTTCTATAGTG---------AATAGAGTTAGGCAGGGATACTCACCA------
TTATCGTTTGCTGTTAGCTTGCTCAATACCATAGCCATAGCAGTAGCTGAG---
GGGACAGATAGGATCATAGAAGTATTACAAAGAGCTGGTAGAGCT----------------------------
--------ATCCTCCACATA------CCTGTAAGAATAAGACAGGGCTTAGAAAGGGCTTTGCTA---TAA
>GU455448
GCA---GCA---
GAAAAATTGTGGGTCACAGTCTATTATGGGGTACCTGTGTGG---AAAGAAGCAAAC---
ACCACTCTATTTTGTGCGTCAGATGCTAAAGCATATAGCACAGAG---GTACATAAT----------------
--------------GTTTGGGCCACA---CATGCCTGTGTGCCCACAGAC---
CCCAACCCACAAGAAATAGAATTGAAA---------AATGTAACAGAAAATTTTAAC----------------
--------------------------ATGTGGAAAAATAGCATGGTAGAACAGATGCATGAGGATATA---
ATCAGTTTATGGGATCAAAGCCTA---AAGCCATGTGTAAAATTA---
ACCCCACTCTGTGTTACTTTAAACTGCACTGATTATTGGGGGAATACTACTAATTTGGGGAATACTACTAATG
GG--
----------AAGAATAATACTGATATTAATATCACGAGTGCTACTAATACCATAGAAATAAAA---
AAATGCTCTTTCAATATCACCACAAGCCTAAGA--------------------------
GATAAGGTGCGGAAAGACTATGCACTT---TTCTATAAACTTGATCTAGTACAACTAAGGGAG----------
----

GGTGATAATACCAGCTATAGGTTGATAAGTTGTAACACCTCAGTCATTACACAG---
GCCTGTCCAAAGGTAACCTTTGAGCCAATTCCCATACATTATTGTGCCCCGGCTGGGTTTGCAATTCTAAAGT
GTAAA---GATAAGAAGTTCAAT------GGGACAGGACCATGTAAAAAT----------------------
-GTCAGC---ACAGTACAATGCACACATGGAATTAGGCCAGTAGTATCAACT---
CAACTGCTGTTAAATGGCAGTCTATCAGAAGAAGGG-----------
GTAATAATTAGATCTGCCAATTTCTCGGACAAT---------GCTAAAACCATAATAGTACAGCTAAAT----
-----------GAATCTGTAATA---------ATTAATTGTACAAGACCCAACAACAATACAAGAAAA-----
-AGTATACCTATA------------GGACCAGGGAGAGCA---TTT------TATGCAACAGGAGAC------
---------ATAATAGGAGATATA------------AGACAAGCACACTGT---AACATTAGTGGAATA---
CAATGGAATAATACTTTAAAACAGATAGCTATAAAATTAAGA---------------GAACAATGGGGG----
--------------------------------AATAATAAAACAATAGTCTTTAAT------
CAATCCTCAGGAGGGGACCCAGAAATTGTA---ATGCACACTTTTAATTGTAGAGGGGAA---
TTTAAGCAGCTCCAGGCAAGAGTCCTGGCTGTGGAAAGATACCTAAAGGATCAACAGCTCCTGGGGATGTGGG
GTTGCTCTGGAAAGCTCATTTGCACCACTGCTGTGCCTTGGAATACTAGTTGGAGT----------------
----------------------------AATAAATCTATGAATGAGATTTGG------GAT------
AAAATGACCTGGATGCAGTGG---GAAAAAGAAATTGACAATTACACAAGCTTAATATAC---
TCTTTAATTGAAGATTCACAGATCCAACAAGAA---------------------------------------
----
AAGAATGAGCAAGAATTATTGGCATTAGATAAATGGGAAAGTTTGTGGACTTGGTTTAACATAACAAACTGGC
TGTGGTATATAAGAATATTCATAATGATAGTAGGAGGCTTGATAGGTTTAAGAATAGTTTTTGCT---
GTACTTTCTATAGTA---------AATAGAGTTAGGCAGGGATACTCACCA------

TTATCGTTTGCTGTTAGCTTGTTCAATGCCATAGCCATAGCAGTAGCTGAG---
GGGACAGATAGGATTCTAGAAGTATTACAAAGAATTTTTAGAGCT----------------------------
--------ATACTCCACATA------CCTACAAGAATAAGACAGGGCTTGGAAAGGCTTTGCTA---TAA
>DQ178989
ACT---GCA--
GAACAATTGTGGGTCACAGTCTATTATGGGGTACCTGTGTGG---AAAGAAGCAACT---
ACTACTCTATTCTGTGCATCAGATGCTAAAGCATATGACACAGAG---GCACATAAT----------------
--------------GTTTGGGCCACA---CATGCCTGTGTACCCACAGAC---
CCTAACCCACAAGAAGTAGTATTGGGA---------AATGTGACAGAAAATTTTAAC----------------
--------------------------ATGTGGAAAAATAACATGGTAGAACAGATGCATGAGGATATA---
ATTAGTTTATGGGATCAAAGCCTA---AAGCCATGTGTAAAATTA---
ACTCCATTATGTGTTACTTTAAATTGCATTAATTTGAATACTACTAATACCAATGCTACTACCAATGCTACT-
----
-------------AATACCCATAGTAATAGTAGTTGGGACAGGATGGAGGTAGGAAAAATAAAA---
AACTGCTCTTTCAATGTCACCACAAGCATAGGA---------------------------
AACAAGATGCAGAAAGAATATGCCCTT---TTTTATAAACTTGATGTAATGCCAATATCTGGT----------
----

GATAATACAAGCTATACATTGATAAATTGTAAAACCTCAACCATTACACAG---
GCCTGTCCAAAGGTATCCTTTGAACCAATTCCCATACATTATTGTGCCCCAGCTGGTTTTGCGATTCTAAAGT
GTAAT---GATAAGAAATTCAAT------GGAACAGGACCATGTACAAAT-----------------------
-GTCAGC---ACAGTACAATGTACACATGGAATTAGGCCAGTAGTGTCAACT---
CAACTGCTGTTAAATGGCAGCCTATCAGAAGGGGAG------------
GTAATAATTAGATCTGAAAATTTCACAGACAAT---------GCTAAAACCATAATAGTACAGCTGAAT----
-----------CAATCTATAACA---------ATTAATTGTACAAGACCCAACAACAATACAAGAAGA-----
-AGTATACCTATA------------GGACCAGGGAGAGCC---TTT------TATGCAACAGGAGAC------
---------ATAATAGGAGATATA------------AGAAAGGCACATTGT---AACATTAGCAGAACA---
GATTGGAATAACACTTTAAAACAGGTAGTTACAAAATTAAGA---------------GAACAATTTAAG----
-----------------------------------AATAAAACAATAATCTTTAAG------
CAATCCTCAGGAGGGGACCCAGAAATTGTA---ATGCACAATTTTATTTGTGGAGGGGAA---
TTTAAACAGCTCCAGGCAAGAGTCCTGGCTGTGGAAAGATACCTAAGGGATCAACAGCTCCTAGGGATTTGGG
GTTGCTCTGGGAAACTCATTTGCACCACTGCTGTGCCTTGGAATACTAGTTGGAGT-----------------
----------------------------AATAAATCTCTGAATGCAATTTGG------GAT------
AACATGACCTGGATGGAGTGG---AAGAGAGAAATTGACAATTACACAAACTTAATATAC---
ACCTTACTTAAAGAATCGCAGAACCAACAAGAA--

AAGAATGAACAAGAATTATTAGAATTGGATAAGTGGACAAATTTGTGGAGTTGGTTTAGCATAACAAATTGGC
TGTGGTATATAAGAATATTCATAATGATAGTAGCAGGCATGGTAGGTTTAAGAATAGTTTTTGCT---
GTAATTTCTATAGTG---------AATAGAGTTAGGCAGGGATATTCACCA------
TTATCGTTGGCTATTAGTTTGCTTAATGCCACAGCTGTAGCAGTAGCTGAG---
GGAACAGATAGAGTTATAGAAGTAGTACAAAGAACTTGTGAAGCT----------------------------
--------ATTCTCCACATA------CCTAGAAGAATAAGACAAGGCTTAGAAAGGCTTTGCTA---TAA
>EU744007
GCT---GCA--
GATAATTTATGGGTCACAATCTATTATGGGGTACCTGTGTGG---AAAGATGCAAAC---
GCCTCTCTATTTTGTGCATCAGATGCTAAAGCATATGATACAGAG---GCACATAAT----------------
--------------GTTTGGGCCACA---CATGCCTGTGTACCCACAGAC---
CCCAACCCACAAGAAATAAAAATGGAA---------AATGTGACAGAAAATTTTAAC----------------
--------------------------ATGTGGAAAAATAACATGGTAGAACAGATGCATGAGGATATA---
ATTAGTTTATGGGATCAAAGCCTA---AAGCCATGTGTAAAATTA---
ACCCCACTCTGTGTTACTTTAAATTGCACTGATCCGAGGAATGATACTAGTAATTTGGGGAATGCTACTAATA
GTAATACT---
----ACTAATGATAATGGTACTGCTAGTAATAGTAGCATAGGAAGAATGAGGGAAGAAATGAAA---
AACTGCTCTTTCAATATCACCACTAGCATAAGA---------------------------
GATAAGGTGCAAAAAGAATATGCACTT---TTGTATAAACTTGATATAGTACCAATAGATACTGATAATCAT-
----

AATAGTACTAAGGATACTACTAGCTATAGGTTGATAAGTTGTAACACCTCAGTCATTACACAG---
GCCTGTCCAAAGATATCCTTTGAGCCAATTCCCATACATTATTGTGCCCCGGCTGGTTTTGCAATTCTAAAGT
GTAAT---AATAGGACATTCAAT------GGAAAAGGACCATGTACAAAT-----------------------

-GTCAGC---ACAGTACAATGCACACATGGAATTAGGCCAGTAGTATCAACT---
CAACTGCTGCTAAATGGCAGTCTAGCAGAAGGAGAG------------
ATAGTAATTAGATCTGACAATTTCACAGACAAT---------GCTAAAACCATAATAGTACAGCTAAAC----
-----------GAAACTGTAAAA---------ATTATTTGTACAAGACCCAACAACAATACAAGAAAA-----
-AGTATATCTATA------------GGACCAGGAAGAGCA---TTT------TATGCAACAGGAGAC------
---------ATAGTAGGAGATATA------------AGAAAAGCACATTGT---AACATTAGTAAAGCA---
AACTGGGGGATCACTTTACGACGGGTAGCTGAAAAATTGAGA---------------GAACACTTTAAA----
-----------------------------------AATAAAGCAATAGCCTTTAAT------
CAATCCTCAGGAGGAGACCCAGAAATTACA---ATGCACAGTTTTAATTGTGGAGGGGAA---
TTTAAGCAGCTCCAGGCAAGAGTCCTGGCTGTGGAAAGATACCTAAAGGATCAACAGCTCCTGGGAATTTGGG
GTTGCTCTGGAAAACTCATCTGCACCACTGCTGTGCCTTGGAATACTAGTTGGAGT-----------------
----------------------------AATAAATCTATGGAGGATATTTGG------GAG------
AACATGACCTGGATGCAGTGG---GAAAAGGAAATTGACAATTATACAAAAGACATATAC---
ACCTTAATTGAAAAAGCGCAAACCCAGCAAGAA---

ATGAATGAACAAGAATTATTGGAATTGGATAAATGGGCAAGTTTGTGGAATTGGTTTGACATATCAAAATGGC
TGTGGTATATAAGGCTATTCATAATGATAGTAGGAGGCTTGGTAGGTTTAAGAATAGTTTTTGCT---
GTACTTAATATAGTG---------AATAGAGTTAGGCAGGGATATTCACCA------
TTATCGTTTGCTGTTAGCTTGTTCGACGCCACAGCAATAGCAGTAGCTGAG---
GGGACAGATAGGATTATAGAAGTAGCACAAAGAGCTTTTAGAGCT----------------------------
--------ATTCTCAACATA------CCTACAAGAATAAGACAGGGCTTGGAAAGGGCTTTGCTA---TAA
>GU455525
GCT---ACA---
GAGCAATTGTGGGTCACAGTCTATTATGGGGTACCTGTGTGG---AAAGAAGCAACC---
ACCACTCTATTTTGTGCCTCAGATGCTAAAGCATATGAGACAGAG---GTACATAAT----------------
--------------GTTTGGGCTACA---CATGCCTGTGTACCCACAGAC---
CCCAACCCACAAGAAATAGTATTA------------AATGTGACAGAAAATTTTAAC---------------
--------------------------ATGTGGAAAAATAACATGGTAGAACAGATGCATGAGGATATA---
ATCAGTTTATGGGATCAAAGCCTA---AAGCCATGTGTAAAATTA---
ACTCCACTCTGTGTTACTTTAAATTGCACTGATGAGTTGGAGAATGTGACTGATACTAATAACAAT-------
----
----------------------AGTAGTAGCGAGGGAATGAGGGAGGAGAAAGGAGAAATAAAA---
AACTGTTCTTTCAATATCACCACAGACCTAAGA--------------------------
GATAAGGTGCAGAAAACATATGCAACG---
TTTTATAAGCTTGATATAGTACCAATAGATAATGGTAATGAGACC----------------------------
----
AAGAATGGTAATGAGACCAAGAATACCAGCTATAGGTTGATAAGTTGTAATACCTCAGTCCTTACACAG---
GCCTGTCCAAAGATATCCTTTGAGCCAATTCCCATACATTATTGTGCCCCGGCTGGTTTTGCGATTCTAAAGT
GTAAC---AATAAGACGTTCAAT------GGAACAGGACCATGTACAAAT-----------------------
-GTCAGC---ACAGTACAATGTACACATGGAATTAGGCCAGTAGTATCAACT---
CAACTACTGTTAAATGGCAGTCTAGCAGAAGAAGAG------------
GTAGTAATTAGATCTGAAAATCTCACGAACAAT---------GCTAAAACCATAATAGTACAGCTAAAG----
-----------GAACCTGTAAAA---------ATTGAGTGTATAAGACCCAACAACAATACAAGAAAA-----
-GGTATACATATA------------GGACCAGGGAGAGCA---TTT------TACACAACAGGACAA------
---------ATAATAGGAGATATA------------AGACAAGCACATTGT---AACATTAGTGGAGCA---
GCATGGAATAATACTTTACACAAAATAGCTAAAAAATTAAAA---------------GAAAAATTTGAA----
-----------------------------------AATAGAGCAATAGTCTTTGAG------
CAATCCTCAGGAGGGGACCCAGAAATTGTA---ACACACAGTTTTAATTGTGGAGGGGAA---
TTTAAGCAGCTCCGGGCAAGGATCCTGGCTGTGGAAAGATACCTAAAGGATCAACAGCTCCTGGGAATTTGGG
GTTGCTCTGGAAAACTCATTTGCACCACCGCTGTGCCTTGGAATACTAGTTGGAGC-----------------
----------------------------AATAAATCTTTGAATGAGATTTGG------GAT------
AACATGACCTGGATGGATTGG---GAAAGGGAAATTGACAATTACACAAGCTTAATATAC---
ACCTTAATTGAAGAATCACAGATCCAACAAGAA---

AAAAATGAACAAGAATTATTGGAATTAGATAAATGGGCAAGTTTGTGGAATTGGTTTACCATAACAAACTGGC
TGTGGTATATAAAAATGTTCATAATGATAGTAGGAGGCTTGGTAGGTTTAAGAATAGTTTTTGTT---
GTGCTTTCTATAGTG---------AATAGAGTTAGGCAGGGATACTCACCA------
TTATCGTTTGCTGTTAGCTTGCTCAATGCCACAGCCATAGCAGTAGCTGAG---
GGGACAGATAGGGTTATAGAAATATTACAAAGAGCTGGTAGAGCT--------------------------
--------ATTCTCCACATA------CCTAGAAGAATAAGACAGGGCTTCGAAAGGGCTTTGCAA---TAA

>EU744052
GCT---GCA---
GAACAATTGTGGGTCACAGTCTATTATGGAGTACCTGTGTGG---AAGGAAGCAACT---
ACCACCCTATTTTGTGCATCAGATGCTAAAGCATATGATACAGAG---GCACATAAT----------------
--------------GTTTGGGCCACA---CATGCCTGTGTACCCACAGAC---
CCCAGCCCACGAGAAATACTATTGGAA---------AATGTGACAGAAAATTTTAAC----------------
--------------------------ATGTGGAAAAATAACATGGTAGAACAAATGCATGAGGATATA---
ATCAGTTTATGGGATCAGAGTCTA---AAGCCATGTGTAAAATTA---
ACCCCACTCTGTGTCACTTTAAATTGCACTGATGATGTGAGGAATGTTAATACTACTACT-------------
----
-------------------------AATAGTAGCGGGAAATTGATGGAGAGAGGAGAAATAAAA---
AACTGCTCTTTCAATATCACCACAAGCATAAGA---------------------------
GATAAGATGCAGAAAGAATATGCACTT---
TTTTATAAACTTGATGTAGTACCAATAGATAATGATCCAATAGAT----------------------------
-----------------------------AATGAG---
AATAATAATGGAAGTAGTACTAATAACACTTATACCAACTATAGGTTGATAAGTTGTAACACCTCAGTCATTA
CACAG---
GCCTGTCCAAAGATATCCTTTCAGCCAATTCCCATACATTATTGTGCCCCGGCTGGTTTTGCGATTCTAAAAT
GTAAT---GATAAGAAGTTCAAT------GGAACAGGACCATGTACAAAT----------------------
-GTTAGC---ACAGTACAATGTACACATGGAATTAAGCCAGTAGTATCAACT---
CAACTACTGTTAAATGGCAGTCTAGCAGAAGAAGAG-----------
GTAATAATTAGATCTGAAAATTTCACGAACAAT---------GCTAAAACCATAATAGTACAGCTGAAT----
-----------GAAACTGTAGAA---------ATTAATTGTACAAGACCCAGCAACAATACAAGAAGG-----
-GGTATACATATA------------GGACCAGGGAGAGCA---TTT------TATACAACAGGAGAA------
---------ATAATAGGAGATATT------------AGACAAGCACACTGT---AACATTAGTGGAACA---
AAATGGAATAACACTTTAAGACAAATAGTTATAAAATTAAGA---------------GAACAATTTGTG----
-----------------------------------AATAAAACAATAGTCTTTAAT------
CGATCCTCAGGAGGGGACCCAGAAATTGTA---ATGCACACTTTTAATTGTGGAGGGGAA---
TTTAAGCAGCTCCAGGCAAGAGTCCTGGCTGTGGAAAGATACCTAAGGGATCAACAGCTCCTGGGAATTTGGG
GTTGCTCTGGAAGACTCATCTGCACCACTGCTGTGCCTTGGAATACTAGTTGGAGC-----------------
----------------------------AATAAATCTCTAGATCAGATTTGG------GAA------
AACATGACCTGGATGGAGTGG---GAAAGAGAAATTGACAATTATACAAACATAATTTAC---
AACTTAATTGAAGAATCGCAAAACCAACAAGAA---------------------------------------
----
AAGAATGAACAAGAATTATTGGAATTAGATAAATGGGCAAGTTTGTGGAATTGGTTTAGCATATCAAGCTGGC
TGTGGTACATAAGAATATTCATAATGATAGTAGGAGGCTTGGTAGGTTTAAGAATAGTTTTTGCT---
GTAATTTCTGTAGTA---------AAAAGAGTTAGGCAGGGATATTCACCA------
TTATCGTTTGCTGTTAGCTTGCTCAACGCCATAGCCATAGCAGTAGCTGAG---
GGGACAGATAGGGTTATAGAAGTATTACAAAGAATTTTTAGAGCT----------------------------
--------ATCCTCCATATA------CCTACCAGAGTAAGACAGGGCTTAGAAAGAGCTTTGCTA---TAA
>U15030
GCT---ACA---
GAAAAATTGTGGGTCACAGTCTATTATGGGGTACCTGTGTGG---AAAGAGGCAACC---
ACCACTTTATTTTGTGCATCAGATGCTAAAGCATATGATACAGAG---GTACATAAT----------------
--------------GTTTGGGCCACA---CATGCCTGTGTACCCACAGAC---
CCCAACCCACAAGAAGTAGTATTGGGA---------AATGTGACAGAAAATTTTAAC----------------
--------------------------ATGTGGAAAAATAACATGGTAGAACAGATGCATGAGGATATA---
ATCAGTTTATGGGATCAAAGCCTA---AAGCCATGTGTAAAATTA---
ACCCCACTCTGTGTTACTTTAGATTGCACTGATGTGAATGTTACTGATACCAATAGTACTACTAAT-------
----
-------------------GCCACTATTGGTAGCTGGGAAAAGATGGAAAAAGGAGAAATAAAA---
AACTGCTCTTTCAATATCACCACAAGCATAAGA---------------------------
GATAAGGGGCAGAAAGAATATGCACTT---TTTTATAGACATGATGTAGTACCCATAAATACG----------
----

ACTAAATATAGATTGATAAGTTGTAACACCTCAGTCATTACACAG---
GCCTGTCCAAAGGTATCCTTTGAACCAATTCCCATACATTATTGTGCCCCGGCTGGTTTTGCGATTCTAAAGT
GTAAC---AATAAGACGTTCAAT------GGAAAAGGACCATGTACAAAT----------------------
-GTCAGC---ACAGTACAATGTACACATGGAATTAGGCCAGTCGTATCAACT---
CAACTGCTGTTAAATGGCAGTCTAGCAGAAGAAGAG-----------

ATAGTAATTAGATCTGAAAATTTCACGAACAAT---------GCTAAAATCATAATAGTACAGCTGAAC----
-----------GTATCTGTAGAC---------ATTAATTGTACAAGACCCAACAACAATATAAGAAAA-----
-AGGATACATATA------------GGACCAGGGAGAGCA---TTT------TATACAACAGGACAA------
---------ATAATAGGAAATATA------------AGACAAGCACATTGT---AACATTAGTAAAACA---
AGATGGAATAATACTTTAAACCAGATAGCTAAAAAATTGAGA---------------GAAAAATTTGGG----
-----------------------------------AATAAAACAATAGTCTTTAAT------
CAATCCTCAGGAGGGGACCCAGAAATTGTA---ATGCACAGTTTTAACTGTGGAGGGGAA---
TTTAAGCAGCTCCAGGCAAGAATCCTGGCTGTGGAAAGATACCTAAAGGATCAACAGCTCCTGGGAATTTGGG
GTTGCTCTGGAAAACTCATCTGCACCACTACTGTGCCTTGGAATGCTAGTTGGAGT----------------
----------------------------AATAAATCTCTGGATATGATTTGG------AAT------
AACATGACCTGGATGGAGTGG---GAGAGAGAAATTGACAATTACACAGGCTTAATATAC---
AACTTAATTGAACAATCGCAAAACCAACAAGAA---

AAGAATGAACAAGAATTATTGGAATTGGACAAATGGGCAAATTTGTGGAGTTGGTTTAGCATAACAAACTGGC
TGTGGTATATAAGATTATTCATAATGATAGTAGGAGGCTTAATAGGTTTAAGAATAGTTTTTGCT---
GTACTTGCGATAGTG---------AAGAGAGTTAGGCAGGGATACTCACCA------
TTGTCGTTTGCTGTTAGCTTGCTCAACGCCACAGCCGTAGCAGTAGCTGAG---
GGGACAGATAGGGTTGTAGAAGTCTTACAAAGAGCTGGTAGAGCT---------------------------
--------GTTCTCCACATA------CCTAGAAGAATAAGACAGGGCTTAGAAAGGGCTTTGCTA---TAA
>L08655
GCT---GCA---
GAACAATTGTGGGTCACAGTCTATTATGGGGTACCTGTGTGG---AAAGAAACAACT---
ACCACTCTATTTTGTGCATCAGATGCTAAAGCATATGATACAGAG---GTACATAAT----------------
--------------GTTTGGGCCACA---CATGCCTGTGTACCCACAGAC---
CCCAACCCACAAGAAGTAGTATTGGAA---------AATGTGACAGAAAATTTTAAC----------------
-------------------------ATGTGGAAAAATAACATGGTAGAACAGATGCATGAGGATATA---
ATCAGTTTATGGGATCAAAGCCTA---AAGCCATGTGTAAAATTA---
ACCCCACTCTGTGTTACTTTACATTGCACTGATTTGAAGAATGCTACTAATACCACTAGTAGTAAT-------
----
------------------ACCAATAGTAGTAGTTGGGGAATGATGGAGAAAGGAGAAATAAAA---
AACTGCTCTTTCAATATCACCACAGCCATAAGA--------------------------
GATAAGGTGCGGAGAGAACATGCACTT---TTTTATAAACTTGATGTAGTACCAATAGATAATGAT-------
----

AGTAAAAATATTACCAGCTTTAGGTTGATACATTGTAACACCTCAGTCATTACACAG---
GCCTGTCCAAAGATATCCTTTGAGCCAATTCCCATACACTATTGTGCCCCGGCTGGTTTTGCAATGCTAAAGT
GTAAC---AATAAGACGTTCAGT------GGAAAAGGACCATGTACAAAT----------------------
-GTCAGC---ACAGTACAATGTACACATGGAATTAGGCCAGTAGTGTCAACT---
CAACTGCTGTTAAATGGCAGTCTAGCAGAAGAAGAT-----------
GTAGTAATTAGATCTGACAATTTCACAGACAAT---------GCTAAAACCATAATAGTACAACTGAAC----
-----------GAATCTGTGGTA---------ATTAATTGTACAAGACCTAACAACAACACAAGAAAA-----
-GGTATACATATA------------GGACCAGGGAGAGCA---GTT------TATACAACAGGAAGA------
---------ATAATAGGAGATATA------------AGACAAGCACATTGT---AACCTTAGTAGAGCA---
AAATGGGAAAACACTTTAAAACAGATAGTTACAAAATTAAGA---------------GAACAATTTAGG----
-----------------------------------AATAAAACAATAGTCTTTAAT------
CAATCCTCAGGAGGGGACCCAGAAATTGTA---ATGCACAGTTTTAACTGTGGAGGGGAA---
TTTAAACAGCTCCAGGCAAGAGTCCTGGCTGTGGAAAGATACCTAAAGGATCAACAGCTCCTAGGGATTTGGG
GTTGCTCTGGAAAACTCATTTGCACCACTGCTGTGCCTTGGAATGCTAGTTGGAGT----------------
----------------------------AATAAATCTCTGGATACGATTTGG------AAT------
AACATGACCTGGATGCAGTGG---GAAAAAGAAATAGACAATTACACAGGCTTAATATAC---
ACCTTAATTGAAGAATCGCAGAACCAACAAGAA---

AAGAATGAACAAGAATTATTGGAATTAGATAAGTGGGCAAGTTTGTGGGATTGGTTTGACATAACAAAATGGC
TGTGGTATATAAAAATATTCATAATGATAGTAGGAGGCTTGGTAGGTTTAAGGATAGTTTTTGTT---
GTACTTTCTATAGTG---------AATAGAGTTAGGCAGGGATACTCACCA------
TTATCATTGGCTGTTAGCTTGCTCAATGCCACAGCTATAGCAGTAGCTGAG---
GGGACTGATAGGGTAATAGAAGTAGTACAAAGAATTTGTAGAGCT---------------------------
--------ATTCGCCACATA------CCTAGAAGAGTAAGACAGGGCTTAGAAAGGGCTTTGCTA---TAA
>JF320008

GCT---ACA--
AAAAACTTGTGGGTCACAGTCTATTATGGGGTACCTGTGTGG---AAAGAAGCAAAT---
ACCACTCTATTTTGTGCATCAGATGCTAAAGCATATGATACAGAG---GTACATAAT----------------
--------------GTGTGGCCACA---CATGCCTGTGTGCCCACAGAT---
CCCGACCCACAAGAAATATTATTGGAA---------AATGTGACAGAAAACTTTAAC----------------
--------------------------ATGTGGAAAAATAACATGGTAGAACAGATGCATGAGGATATA---
ATCAGTTTATGGGATCAAAGCCTA---AAGCCATGTGTAAAATTA---
ACCCCACTCTGTGTTACTCTAAATTGCACTAATGCTAATGTTACTAGTGTTAAT-------------------
----
-------------------------------GGTACGAGCGCTACTAGTAGCGAGGAATTGAGG---
AATTGCTCTTTCAATGTTACCACAGACAGAAGA---------------------------
GATAAGATGAAGACAGAATATGCACTT---CTCTATAGGCTTGATATAGTACAAATAGATAAT----------
----

AGTAGTAGTAGGTTGATAAGTTGTAACACCTCAGTCATTACACAA---
GCCTGTCCAAAGATATCCTTTGAGCCAATCCCCATACATTACTGTACCCCGGCTGGCTTTGCGCTTCTAAAGT
GTAAT---GATAAGAAATTTGAG------GGAAAGGGACAATGTAGAAAT-----------------------
-GTCAGC---ACAGTACAATGTACACATGGAATTAGGCCAGTAGTATCAACT---
CAACTGCTGCTAAATGGCAGTCTAGCAGAAGAAGAG------------
GTAGTAATTAGATCCAGCAATTTCTCGGACAAT---------GCTCAAACCATAATAGTACATCTGAAT----
-----------GAATCTGTACAA---------ATTAATTGTACAAGACCCAACAATAATACAAGAAAA-----
-AGTATAAATATA------------GGACCAGGGAGAGCA---TTC------TATGCAACAGAAGCC------
---------ATAGTAGGAAATATA------------AGACAAGCACATTGT---AACCTCAGTAGAGAA---
TCATGGAAAGACACTTTAGAAAGGGTAGCTAAAAAATTAAGA---------------GAAAAATTTAAG----
-----------------------------------AATAAAACAATAGCCTTCAAT------
CACTCCTCGGGAGGAGACCTAGAAATTGTA---ACGCATAGTTTTAATTGTGGAGGAGAA---
TTTAAGCAGCTCCAGGCAAGAGTCCTGGCTGTGGAGAGATACCTAAGGGATCAACAGCTCCTAGGGATTTGGG
GTTGCTCTGGAAAACTCATCTGCACCACTGCTGTGCCTTGGAATAATAGTTGGAGT-----------------
----------------------------AACAAAACTATGGAGCAAATTTGG------GAT------
AACATGACCTGGATGGAATGG---GATAAAGAGATTAACAAATACACAGGAGTAATATAC---
ACCCTAATTGAAAACTCGCAAACCCAACAAGAA--

AAGAATGAACAAGAATTATTAGCATTGGATAAGTGGGCAAGTTTATGGAATTGGTTTGACATAACAAACTGGC
TGTGGTATATAAAAACATTCATAATGATAGTAGGAGGCTTGATAGGTTTAAGAATAGTTTTTGCT---
GTACTTTCTGTAGTG---------AATAGAGTTAGGCAGGGATATTCACCA------
TTATCATTTGCTGTCAGCTTGCTCAATGTCACAGCTATAGTAGTAGCTGAG---
GGGACAGATAGGATTATAGAAGTAGCACAAAGAGCTGGTAGAGCT----------------------------
--------ATTCTCCACATA------CCTAGAAGAATTAGACAGGGCTTGGAAAGGTTTTTGCAA---TAA
>JF320215
GCT---ACA--
GAGCAATCGTGGGTCACAGTATATTATGGGGTACCTGTATGG---AAAGAAGCAACT---
ACCACTTTATTTTGTGCATCAGATGCTAAAGCATATAAGACAGAA---ATGCATAAT----------------
--------------GTTTGGGCCACA---CATGCCTGTGTACCCACAGAC---
CCCAACCCACAAGAAATAAGATTGGAG---------AATGTAACAGAAAATTTTAAC----------------
--------------------------ATGTGGAAAAATAACATGGTAGAACAGATGCATGAGGATATA---
ATCAGTTTATGGGATCAAAGCTTA---AAGCCATGTGTAAAATTA---
ACCCCACTCTGTGTCACTTTAAGTTGCAAGGATCTCAATGGTACTAAGACCAATAATAGTAGTGTT-------
----
----------------------AGTAACAGTAGCATAGGGGAAGAAATGAAAACAGAGATAAAA---
AACTGCACTTTCAATGTCACTGGAGGCATAAGA---------------------------
GATAAGGTACAGAAAGCCTCTGCATTG---TTTAATTTACTTGATATAGTACCAATAGATGGA----------
----

AGTAATGATAGCTATAGGATAACAAGTTGTAACACCTCCACCATTACACAG---
GCCTGTCCAAAAGTAACCTTTGAGCCAATTCCCATACATTATTGTGCCCCGGCTGGTTTTGCGATTCTACAAT
GTAAT---GATAAAAAGTTCAAT------GGAACAGGACCATGTAAAAAT-----------------------
-GTCAGC---ACAGTACAATGTACACATGGAATTAGGCCAGTAGTGTCAACT---
CAATTACTGTTAAATGGCAGTTTAGCAGAAGAAGAG------------
GTGATAATTAGATCTCAGAATATCTCAGACAAT---------ACTAAAACCATAATAGTACAGCTGAAT----
-----------GAATCTATAGTA---------ATTAATTGTACAAGACCCAACAATAATACAAGAAAA-----

```
-AGTATAAATATA------------GGACCAGGGAGAGCA---TTT------TATGCAACAGGAGAT------
---------ATAATAGGAGATATA------------CGCCAAGCACATTGT---AACATTAGTAGAACA---
AAATGGAATAATACTTTAGAACAGATAGTTAAGAAATTAAGG---------------GAAAATTTTGGG----
-----------------------------------AATAGAACAATAGTCTTTAAT------
ACTTCCTCAGGAGGAGATCCAGAAATTGTA---ATGCACAGCTTTAATTGTGCAGGAGAA---
TTTAAGCAGCTCCAAGCAAGAGTCCTGGCTGTGGAAAGCTACCTAAAGGACCAACAGCTCCTAGGGATTTGGG
GTTGCTCTGGAAAACTCATCTGCACCACTACTGTGCCTTGGAATAATAGCTGGAGT-----------------
----------------------------AATAAACCTCTGAAAGAGATTTGG------GAT------
AACATGACCTGGATGGAGTGG---GAAAAAGAAATTAGCAATTACACAAACACAATATAC---
ACCTTAATTGGAGAAGCGCAAAACCAACAAGAA----------------------------------------
-----------------------------
AAGAATGAACAAGAATTATTGGCATTGGATAAATGGGCAAGTTTGTGGAATTGGTTTGACATAACAAGGTGGC
TGTGGTATATAAAAATATTTATAATAATAGTAGGAGGCTTGATGGGGTTAAGAATAGTTTTTACT---
GTACTTTCTATAGTG---------AATAGAGTTAGGCAGGATACTCACCA------
TTGTCGTTTGCTATTAGCTTGCTCAGCGCCACAGCCATAACAGTAGCTGAG---
GGAACAGATAGGATTATAGAAGCACTTCAAAGAACTGGTAGAGCT----------------------------
--------ATTATCAACATA------CCTAGAAGAATAAGACAGGGCTTGGAAAGGTCTTTGCTA---TAA
>JF320208
GCT---CAG------------------------------------------
GAGCAACTGTGGGTCACAGTGTATTATGGGGTACCTGTATGG---AAAGAAGCAACC---
ACCACTCTATTTTGTGCATCAGATGCTAAAGCATATGATACAGAG---GTACATAAT----------------
--------------GTTTGGGCCACA---CATGCCTGTGTACCCACAGAC---
CCCAACCCACAAGAAATAACATTACCA---------AATGTAACAGAAAATTTTAAC----------------
--------------------------ATGTGGAAAAATAACATGGTAGAACAGATGCATGAGGATATA---
ATCAGTTTATGGGATCAAAGCCTC---AAGCCATGTGTAAAATTA---
ACCCCACTCTGTGTTACTTTAAATTGCACTAAACTTAATGGTACTAATAACCTGACTAAT-------------
-------------------------------------------------------------------------
-------------------------ACTAATAACACTAGTAATATCGGGGGGGAAGAGATGAAA---
AACTGCTCTTTCAATATCACCACAAACGTGAGA---------------------------
GATAAAATGAAAAAAGAATATGCAACA---TTTTATAGCCTTGATATAGTACGAATAGAGGGGAAT-------
-------------------------------------------------------------------------
----------------------
GATAATACTAGTTATAGGTTAGTAAGTTGTAACACCTCAGTCCTCACACAG---
GCCTGTCCAAAAATAACCTTTGAGCCAATTCCCATACATTACTGTGCCCCGGCTGGTTTTGCGATTCTAAAAT
GTAAG---GACAAGAAGTTCAAT------GGAACAGGAGCATGTAAGAAT-----------------------
-GTCAGC---ACAGTACAGTGTACACATGGAATTAGACCAGTAGTGTCAACT---
CAACTGCTGTTAAATGGCAGTCTAGCAGAAGAAGAG------------
GTAGTAATCAGATCTGAAAATATCACTGACAAT---------ACTAAAACCATAATAGTACAGCTGAAA----
-----------GAAGCTGTAAAG---------ATTAATTGTACAAGACCCAACAACAATACAAGGAAA-----
-AGTATACATATA------------GCACCAGGGGGAGCA---TTT------TATGCAACTGGGGAT------
---------ATAATAGGAGATATA------------AGACAAGCACATTGC---AACATTAGTAGAGCA---
GACTGGAATAACACTTTAAAACAGATAGTTAAAAAATTAAGG---------------GAAAATTTC-------
-----------------------------------AATAAAACAATAGTCTTTAAT------
ACCTCCTCAGGAGGAGACCCAGAACTTGTA---ATGCACAGCTTTAATTGTGCAGGGGAA---
TTTAAGCAGCTCCAGGCGAGAGTCCTGGCTGTGGAAAGATACCTAAAGGATCAACAGCTCCTGGGGATTTGGG
GTTGCTCTGGAAAACTCATTTGCACCACTACTGTGCCTTGGAATAATAGTTGGAGT-----------------
----------------------------AATAAATCTTTTAAACACATTTGG------AAT------
AACATGACCTGGATGCAGTGG---GAAAAAGAAATTGATAATTACACAGGCTTAATATAC---
TCCTTAATTGAACAATCGCAAACCCAGCAAGAA----------------------------------------
-----------------------------
ATGAATGAACAAGAATTATTGGCATTGGATAAATGGGCAAATTTGTGGAATTGGTTTGACATAACAAACTGGC
TGTGGTATATAAAAATATTTATAATGATAGTAGGAGGCTTGGTAGGTTTAAGAATAGTTTTTACT---
GTATTTTCTATAGTG---------AATAGAGTTAGGAAGGATACTCACCA------
TTATCGTTTGCTATTAGCTTGCTCAACGCCACAGCCATAGCAGTATCTGAG---
GGGACAGATAGGGTTATAGAAATATCGCAAAGAATTGTTAGAGCT----------------------------
--------ATTATCCATATA------CCTAGAAGAGTAAGACAGGGCTTGGAAAGGCTTTTGATA---TAA
>JF320230
GCT---GCA------------------------------------------
GAAAACTTGTGGGTCACAGTATACTATGGGGTACCTGTGTGG---AAAGAAGCAACC---
ACCACTCTCTTTTGTGCATCAAATGCTAAAGCATATGATACAGAG---AAACATAAT----------------
```

--------------GTTTGGCCACA---CATGCCTGTGTACCCACAGAC---
CCCAACCCACAAGAAATAGTATTGGAA---------AATGTGACAGAAGAATTTAAC----------------
--------------------------ATGTGGAAAAATAACATGGTAGAACAGATGCATGAGGATGTA---
ATCAGTTTATGGGATCAAAGCCTT---AAACCATGTGTAAAGTTA---
ACCCCACTCTGTGTTACTTTAAACTGCACTAATGTTGTTACTGCGAAT-------------------------
----
----------------------------------ATGACTGATGAGACTAGGGGAGAAATAAAA---
AATTGCTCTTTCAATGTCACCTCGGTCCCAGGA---------------------------
GACAAGCCACAGAAAGAATATGCACTG---
TTTTATAGTCTTGATGTAGTACAAATAGGTGATAGTGATAGTAAT----------------------------
----
ACTAGCTATAAGAATAACTATACTAGCTATAGGTTAATAAGTTGTAACACCTCAGTCATTACACAG---
GCCTGTCCAAAGGTATCCTTTGAGCCAATTCCAATACATTATTGTACCCCGGCTGGTTTTGCGATTCTAAAGT
GTAAT---GATAAGGGGTTCAAT------GGAACAGGAAAATGTAACAAC-----------------------
-ATCAGC---ACAGTACAATGCACACATGGAATTAAGCCAGTAGTGTCAACT---
CAACTGCTGTTAAATGGCAGTCTGGCAGAAGGAGAA------------
GTGGTAATTAGATCTGCCAACTTCACAGACAAT---------AGTAAAACCATAATAGTACAGCTAAAT----
-----------GAGTCTGTAGAA---------ATAAATTGTATAAGACCCAACAATAATACAAGAAAA-----
-AGTATCCCTATA------------GGGCCAGGGAGAGCA---TTC------TATACAACAGGAGAG------
---------ATAATAGGAAACATA------------AGACAAGCACATTGT---AACATTAGTAGAGCA---
AAATGGAATAAAACTTTAGAACAGCTAGTTACAAAATTAGGA---------------CACCAATTTGAG----
--------------------------------AATAAAACAATAATAAAATTTAAT------
CAATCCTCAGGAGGGGACCCAGAAATTGGG---ATGCACAGTTTTACTTGTGGAGGAGAA---
TTTAAGCAGCTCCAGGCAAGAGTCCTTGCTATAGAAAGATACCTAAGGGATCAACAGCTCCTGGGGATTTGGG
GTTGCTCTGGAAAACTCATCTGCACCACTGCTGTGCCTTGGAATGCTAGCTGGAGT-----------------
----------------------------AATAAATCTGTAGACCACATTTGG------CAA------
AACATGACCTGGATGCAGTGG---GAAAAAGAAATTGACAATTACACACAAACAATTTAC---
AGTTTACTTGAAGAATCGCAGACCCAGCAAGAA--

AGGAATGAAAAAGAATTATTGGAATTGGATAAGTGGGCAAGTCTGTGGAATTGGTTTGACATAACACAATGGC
TGTGGTATATAAAAATATTCATAATGATAGTAGGAGGCTTGATAGGTTTAAGAATAGTTTTTACT---
GTACTTTCTATAGTA---------AATAGAGTTAGGCAGGGATACTCACCT------
TTATCATTTGCTGTCAGCTTATACAACGCAACAGCTATAGCAGTAGCTGAG---
GGGACAGATAGGATTATAGAAGTAGTACTAAGAGTTGGTAGAGCT----------------------------
--------ATTATCCATATA------CCTACAAGAATAAGACAGGGCTTGGAAAGGCTTTTGCTA---TAA
>JF320013
GCT---ACA--
GAAAAGTTGTGGGTCACAGTCTATTATGGGGTACCTGTGTGG---AAAGAAACAACT---
ACCACTCTATTTTGTGCATCAGATGCTAAAGCATACAAAACAGAG---GTACATAAT----------------
--------------GTTTGGCCACA---CATGCCTGTGTACCCACAGAC---
CCCAACCCACAAGAAATAGTATTGGAA---------AATGTGACAGAATATTTCAAC----------------
--------------------------ATGTGGAAAAATAACATGGTAGAACAAATGCATGAGGATGTA---
ATCAGTTTATGGGATCAAAGTCTA---AAGCCATGTGTAAAGTTA---
ACTCCACTCTGTGTTACCTTAAGTTGCACTGAGTTGAGAAATAATACTAATAATAATAATGCTACA-------
----
-------------------GATAGCACCAATAGCAATACTTCTTTCATCAATAATGAAATGAGA---
AACTGTTCTTTCAATGTCACAGCACCCTTAAGA---------------------------
GATAAGGTAAAGACAGAATATGCACTT---CTTTATAAACTTGATATAACACAAATAAAGGAG----------
----

AATAGTGATAATACCAGCTATAGGTTGATAAATTGTAATACCTCAGTCATTACTCAG---
GCCTGTCCAAAGATATCCTTTGAACCAATTCCCATACATTATTGTACCCCGGCTGGTTTTGCGATTATAAAGT
GTAAT---GATAAGAAGTTCAAT------GGAACAGGACCATGTAAAAAT-----------------------
-GTCAGC---ACAGTACAATGTACACATGGAATTAGGCCAACAGTGTCAACC---
CAGTTACTGTTAAATGGCAGTCTAGCAGAAGAAGAG------------
GTAGTAATTAGATCTGAAAATTTCACAAACAAT---------GCTAAAACTATAATAGTACAGTTGAAC----
-----------AAAAATGTAGTA---------ATTAATTGTACAAGACCCAACAACAATACAAGGAAA-----
-AGTATAAATATA------------GGACCAGGGAGAGCA---TGG------TATGCAACAGGAGAC------
---------ATAATAGGAGATATA------------AGACAAGCACATTGT---ACCATTAGTCAAAAA---
AGCTGGAATAACACTTTACACCTGGTAGTAGAAAAATTACGA---------------GTACAATTAGGG----

------------------------------------AATAAAACAATAGTCTTTAAT------
CAATCCTCAGGAGGGGACCCAGAAATTGTA---ATGCACACCTTTAATTGTGGAGGGGAA---
TTTAAGCAGCTCCAGGCAAGAGTCATGGCTGTGGAAAGATACCTACGGGATCAACAGCTCCTGGGGATTTGGG
GTTGCTCTGGAAAACTCATTTACACCACTGCTGTGCCTTGGAATGCTAGTTGGAGT-----------------
-----------------------------AATAAATCTATAGATGAGATTTGG------AAT------
AACATGACCTGGATGCAGTGG---GAAAAAGAAATTGACAATTACACAAGCTTAATATAC---
ACCTTAATTGAAAAATCGCAAAACCAGCAAGAA---

AAGAATGAGCAAGAATTATTAGAATTGGATAAATGGGCAAGTTTGTGGAATTGGTTTGACATAACAAGCTGGC
TGTGGTATATAAAAATATTCATAATGATAGTAGGAGGCTTAATAGGTTTAAGAATAGTTTTTACC---
ATATTTTCTATAGTA---------AATAGAGTTAGGCAGGGATATTCACCA------
TTATCGTTAGCTGTTAGCTTAGTCAATGCCATAGCCATAGCAGTAGCTGAG---
GGGACAGATAGAGCTATAGAAACATTACAAAGAATTTGTAGGGGC----------------------------
--------ATCCTTAACATA------CCTAGAAGAATAAGACAGGGCTTGGAAAGGGCTTTGCTA---TAA
>JF320018
GCT---GTA---GGACATAAT------------------GCT---TCA---
GAACAATTGTGGGTCACAGTCTATTATGGGGTACCTGTGTGG---AAAGAAGCAACC---
ACCACTCTATTTTGTGCATCAGATGCTAAAGGATATGATCAAGAG---GTACATAAT----------------
--------------GTTTGGGCCACA---CATGCCTGTGTACCCACAGAC---
CCCAACCCACAAGAAATAGTATTGGAA---------AATGTAACAGAGAATTTTAAC----------------
--------------------------ATGTGGAAAAATGACATGGTAGACCAGATGCATGAAGATATA---
ATCAGTTTATGGGATCAAAGCCTA---AAGCCATGTGTAAAATTA---
ACCCCACTCTGTGTTACTTTAAATTGCTCTGATGTTAAGAATAGCACTAATAATAAT----------------
----
--------------------------------GCCACTGGGGTGATGGACAACGGGGAAATGAAG---
AACTGCTCTTTCAATATTACCACAAGTATAAAT---------------------------
GATAAGAGGCAAAAAGAATATGCACTG---TTTTATAAACTTGATATAACACAAATGGATAAT----------
----

GATAATACTAGCTATAGGTTAATAAGTTGTAACACCTCAGTCATTACACAG---
GCTTGTCCAAAGATATCCTTTGAGCCAATTCCCATACATTATTGTGCCCCGGCTGGTTTTGCGATTCTAAAAT
GTAAT---GATAAGAAGTTCAAT------GGATCAGGAACATGTAAAAAT----------------------
-GTCAGC---ACAGTACAATGTACACATGGAATCAGGCCAGTAGTATCAACT---
CAATTGCTGTTAAATGGCAGTCGAGCAGAAGAAGAG-----------
GTAATAATTAAATCTGAAAATCTCACGGACAAT---------ATTAAAACCATAATAGTACAGCTGAAT----
-----------GACTCTGTAAAA---------ATTGATTGTATAAGACCCAATAACAATACAAGAAAA-----
-AGTATACATCTG------------GGACAAGGGAGAACA---TGG------TATGCAACAGGAGAA------
---------ATAATAGGAGATATA------------AGACAAGCACATTGT---AATGTTAGTAATAGC---
GATTGGAATAGAACTTTAATATGGATAGTTAACAAATTAAGA---------------GAACACTTTATA----
------------------------------------AACAAAACAATAGTCTTTAAT------
CAATCCTCAGGAGGAGATCCAGAAATAGTA---CTGCACACTTTTAATTGTGGAGGGGAA---
TTTAAACAGCTCCAGGCAAGAGTCCTGGCTGTGGAAAGATACCTAAAGGACCAACAGCTCCTGGGAATTTGGG
GATGCTCTGGAAAGCTCATTTGCACCACTGCTGTGCCTTGGAATACTAGTTGGAGT----------------
-----------------------------AATAGATCTCTAAATGACATTTGG------GAT------
AACATGACCTGGATGCAGTGG---GAAAAAGAAATTGACAATTACACAAACTTAATATAC---
TCCTTAATTGAAGAATCGCAAAACCAACAAGAA---

AAAAATGAACAAGAATTATTAGCATTGGATAAATGGGCAAGTTTGTGGAGTTGGTTTGACATAACAAACTGGC
TGTGGTATATAAAAATATTCATAATGATAGTAGGAGGCTTGATAGGTTTAAGAATAGTTTTTACT---
GTACTTTCTATAGTA---------AATAGAGTTAGGCAGGGATACTCACCA------
TTGTCATTCGCTATTAGCTTGTTCAACGCCATAGCCATAGCAGTAGCTGAA---
GGGACAGATAGGATTATAGAAATATCACTAGCAGCTTTTTGGGCT--------------------------
--------ATTGTCCACATA------CCTAGAAGAATAAGACAGGGCTTGGAAAGAATTTTGTTA---TAA
>JF320028
GCT---ACA---
TCATTGTGGGTCACAGTATATTATGGGGTACCTGTGTGG---AGAGAAGCAGAT---
ACCACTCTATTTTGTGCATCAGATGCTAAGGCATATGATACAGAG---GTACATAAT----------------
--------------GTTTGGGCCACA---CATGCCTGTGTACCCACAGAC---
CCCAACCCACAAGAAGTAGTATTGGAA---------AATGTGACAGAAAATTTTGAT---------------
--------------------------ATGTGGAAAAATAACATGGTAGAACAGATGCATAAGGACATA---

ATCAGTTTATGGGATCAAAGCCTA---AAGCCATGTGTAAAATTA---
ACTCCCCTCTGTGTTAAGTTAAATTGCACTAATGTTAATTTGAATAGCACTGTGCAG---------------
----
---------------------------GACAATAGTACCAGAATAGAGGAAGGAGAAATAAAA---
AATTGCTCTTTCAATATCACATTAAGAGAGGAG-------------------------
GAGAAGGTGCAGACAGACTATGCACTT---TTTTCTAAACTTGATATAGTAAAATATAACAAT---------
----

GATAGCTATAGGTTAATAGGTTGTAACATCTCAGTCACTAAACAG---
GCCTGTCCAAAGGTATCCTTTGAGCCAATTCCCATACATTATTGTGCCCCGGCTGGTTTTGCGATTCTAAAGT
GTAAT---GATAAGAAGTTCAAT------GGAACAGGACCATGTAAAAAT----------------------
-GTCAGC---ACAGTACAATGTACACATGGAATTAGACCAGTAGTATCAACT---
CAACTGCTGTTAAATGGCAGTCTAGCAGAAGAAGAG-----------
GTAGTAATTAGATCTGCCAATCTCTCGGAAAAT---------GCCAAAATCATAATAGTACACCTGAAT----
-----------AAATCTGTAGTA---------ATTAATTGTACAAGACCCAACAACAATACAAGAAAA-----
-AGTGTACACATA------------GGACCAGGGCAGGTA---CTT------TAT---ACAGGAGAC------
---------ATAATAGGAAATATA------------AGACAAGCACATTGC---AACATTAGTAAAAGA---
GCATGGAATAACACTTTACAACAAATAGTTAGAAAATTACAA---------------GAACAATTTAAG----
-----------------------------------AATAAAACAATAGTCTTTAAT------
CAATCCTCAGGAGGGGACCCAGAAATTGTA---ATGCACAGTTTTAATTGTAGAGGGGAA---
TTTAAGCAGCTTCAGGCAAGAATCCTGGCTGTGGAAAGATACCTAAAGGATCAACAGCTCCTGGGGATTTGGG
GTTGCTCTGGAAAACTCATCTGCACCACTGCTGTGCCTTGGAATGCTAGTTGGAGT-----------------
---------------------------AATAAATCTTATAATGAAATTTGG------GAG------
AACATGACCTGGATGCAGTGG---GAAAAAGAAATTAGCAATTACACAGGCTTAATATAC---
TCTTTAATTGAAGAATCGCAAAACCAACAAGAA---------------------------------------
----
AAGAATGAAAAAGAATTATTGGAGTTAGATCAATGGGCAAGTTTGTGGAATTGGTTTGACATATCAAAGTGGC
TGTGGTATATAAGAATATTCATAATGATAGTAGGAGGCTTGGTAGGTTTAAGAATAGTTTTTRCT---
GTACTTTCTATAGTG---------AATAGAGTTAGGCAGGGATACTCACCA------
TTATCGTTTGCTGTTAGCTTGCTTAATGCCACAGCCATAGCAGTAGCTGAG---
GGGACAGATAGGATTATAGAAATAATTCAAGGAGCTTTTAGGGCT---------------------------
--------ATTCGCAACATA------CCTAGAAGAATAAGACAAGGCTTGGAAAGGGCTTTACTA---TAA
>JF320019
GCT---GCA--
GAGCAAGGGTGGGTCACAATCTATTATGGGGTACCTGTGTGG---AAAGAAGCAACC---
ACTACTCTCTTTTGTGCCTCAGATGCTAAAGCATATGATACAGAG---GTACATAAT---------------
--------------GTTTGGGCCACA---CATGCCTGTGTACCCACAGAC---
CCCAACCCACAAGAAGTATTATTGGAA---------AATGTGACAGAAAATTTTAAC---------------
--------------------------ATATGGAAAAATAACATGGTAGAACAGATGCAGGAGGATATA---
ATCAGCTTATGGGATCAAAGCCTA---AAGCCATGTGTAAAATTA---
ACTCCACTCTGTGTTACTTTAAATTGCTCTGACATAAGCGTCAACAAC------------------------
----
------------------------------AGTATGGGAGGGACGGAGAAAGGAGGAATGAAA---
AACTGCTCTTTCAATATC---GCCAAGTCAAAA---------------------------GATAAGGGA----
-----CGTGCAATT---TTTGATAAAGTTGATGTAGTAAAAATAAATGGT----------------------
----
AGTAGTGATAATGATAGTGCTATCTATAGGTTGATAAGTTGTAACACCTCAGTCATTACACAG---
GCCTGTCCAAAGATAACCTTTGAGCCAATTCCCATACATTATTGTGCCCCGGCAGGGTATGCGATTATAAAGT
GTAAT---GATAAAAGGTTCAAT------GGAACAGGACCATGTAAAAAT----------------------
-GTTAGC---ACAGTGCAGTGTACACATGGAATTAGGCCAGTAGTGTCAACT---
CAGTTGCTGTTAAATGGCAGTCTAGCAGAAGAAGAT-----------
ATAGTAATTAGGTCTGCCAATCTTTCAAACAAT---------GCTAAAAACATAATAGTACAGCTGAAT----
-----------GAATCTGTAGCT---------ATTAATTGTACAAGACCTAATAACAATACAAGAAGA-----
-AGTATACATGTA------------GGACCAGGGAAAGCA---TTA------TAT---ACAACAGAC------
---------ATAATAGGGGATATA------------AGACAAGCATATTGT---ACCATTAATAAACAA---
AATTGGACTAACACTTTACAACAGATAAGTACAAAATTAAGA---------------GAGCATTTTGGG----
-----------------------------------AATAAGACAATAGTCTTTAAA------
AATGCCTCAGGAGGGGACCCAGAAATTGCA---ATGCACAGTTTTAATTGTAGAGGGGAA---
TTTAAGCAGCTCCAGGCAAGAGTCCTGGCTGTGGAAAGATACCTACAGGATCAACAGCTCCTGGGAATGTGGG
GTTGCTCTGGAAAACTCATTTGCACCACTACTGTGCCTTGGAATACTAGTTGGAGT-----------------

----------------------------AATAAGTCTCTGGATCAAATTTGG------AAT------
AACATGACCTGGATGGAGTGG---GAAAAAGAAATTGACAATTACACAGGCATAATATAT---
AACTTAATTGAAGAATCGCAGACCCAACAAGAA--

AAGAATGAACTAGAATTATTGAAATTAAATCAATGGGCAAGTTTGTGGAATTGGTTTGACATATCAAACTGGC
TGTGGTATATAAAAATATTCATAATGATAGTAGGAGGCTTAGTAGGCTTAAGAATAATTTTTGCT---
GTACTTTCTATAGTG---------AATAGAGTTAGGCAGGATACTCACCG------
TTATCGTTTGCTATCAGCTTGTTTAATGCTACAGCCATAACAGTAGCTGGG---
GGAACAGATAGGGTTCTAGAATTATTACAAAGATTTGGTAGAGCT----------------------------
--------ATTCTTAACATA------CCTAGAAGAATAAGACAGGGCTTGGAAAGAATTTTGCAA---TAA
>MH078552
GCA--
GAAGATTTGTGGGTCACAGTCTATTATGGAGTGCCTGTGTGG---AGAGACGCAACC---
ACCACTCTGTTTTGTGCATCAGATGCTAAAGCATATGATACAGAG---GTACATAAT----------------
--------------GTTTGGGCCACA---CATGCCTGTGTACCCACAGAC---
CCCAACCCACAAGAAGTAAAATTAGGA---------AATGTGACAGAAAATTTTAAC----------------
--------------------------ATGTGGAACAATAGCATGGTAGAACAGATGCATGAGGATATA---
ATCAGTTTATGGGATCAAAGCCTA---AAGCCATGTGTAAAATTA---
ACCCCACTCTGTGTTACTCTAAATTGCACTGATCTTAGTAACAGCACTACC----------------------
----
----------------------------AGTAATGGGAGCGAAAGCTTGAGGGGGGAAATGAAA---
AATTGCTCTTTCAATGTCACCCCAGACGTAAGC---------------------------
GATCGGAAGAAGAGAGAATATGCACTT---CTCTATAAGCTTGATATAGTACAAATTGATAAT----------
----

AGTAACACCAATAATAGTAATACCAGCTATAGGTTGATAAGTTGTAACACCTCAGTCATTACACAA---
ACCTGCCCAAAGATATCCTTTGAACCAATTCCCATACATTATTGTACCCCGGCTGGTTTTGCGATTCTACAAT
GTAAG---GATAAGAAATTCAGT------GGAACAGGACCATGTAAAAAT-----------------------
-GTCAGC---ACAGTACAATGTACACATGGAATTAGGCCAGTGGTATCAACT---
CAACTACTGTTAAATGGTAGTCTAGCAGAAGAAGAA------------
GTAGTAATTAGGTCTGCCAATTTCTCAGACAAT---------GCCAAAACTATAATAGTACAGCTGAAC----
-----------GAATCTGTATTT---------ATTAATTGCACAAGACCCAGCAATAATACAAGAAAA-----
-GGTATACATATA------------GGACCAGGGAGAGCA---TTC------TATGCAACAGGAGAC------
---------ATAATAGGAGATATA------------AGACAAGCACATTGT---AACATTAGTGGAAGC---
CAATGGAACAATACTTTAAGACAGATAGCTAGGAAATTACGA---------------GAACAATTTGAG----
-----------------------------------AATAAAACAATAGTCTTTAAA------
CAGTCCTCAGGAGGGGACCCAGAAATTGTA---ATGCACAGTTTTAATTGTGGAGGGGAA---
TTTAAGCAGCTCCAGGCAAGAGTCCTGGCTTTGGAAAGATACCTAAGGGATCAACAGCTCCTAGGGATTTGGG
GTTGCTCTGGAAAACTCATTTGCACCACTGCCGTACCTTGGAATAAAACTTGGAGT-----------------
----------------------------AACAAAACTATGGATGCAATTTGG------GAT------
AACATGACCTGGATGCAATGG---GATAGCGAAATTAACAATTACACAAAACATATATAC---
ACCTTGCTTGAAGAATCACAAAACCAACAAGAA--

AAGAATGAACAAGATTTATTAGAATTGGATAAGTGGGCAAGTTTATGGAATTGGTTTGACATAACAAATTGGC
TGTGGTATATAAAAATATTCATAATGATAGTAGGAGGCTTAATAGGTTTAAGAATAGTCTTTGCT---
GTGCTTTCTGTAGTG---------AATAGAGTTAGGCAGGATACTCACCA------
TTGTCGTTAGCTGTTAGCTTGCTCAACGCATCAGCCATAGCAGTAGGTGAG---
GGGACAGATAGGGTTCTAGAAATATTACAAAGAATTTATAGGGCT----------------------------
--------GTTCTTCACATA------CCTAGAAGAATAAGACAAGGCTTGGAGAGGGCTCTGCTA---TAA
>MH078553
GCT---GCA--
CAAGACTTGTGGGTCACAGTCTATTATGGGGTACCTGTGTGG---AAAGAAGCAACC---
ACCACTCTATTTTGTGCATCAGATGCTAAAGCATATGATACAGAA---GCACATAAT----------------
--------------GTTTGGGCCACA---CATGCCTGTGTACCCACAGAC---
CCTAACCCACAAGAAATGAGATTGGAA---------AATGTGACAGAAGAATTTAAT----------------
--------------------------ATGTGGAAGAATAGCATGGTAGACCAGATGCATGAGGATATA---
ATCAGTTTATGGGATCAAAGCCTA---AAACCATGTGTAAAATTA---
ACTCCACTCTGTGTTACTTTAGAGTGCTCTAATGTTATCTGTACTACTAATAGTAGTAATATTACTACCTGTA
CTAGTAATAGT--
-------ACCACTAGCCCTAACATTACTAGCCCTAACATTACCACTAGCCCTACGGAAATGAAA---

AATTGCTCTTTCAATATTACCACACCCCAGAGG---------------------------
GATAAGACGAAGGAAGAATATGCATTT---TTTTATAGCCAAGATGTAGTACCAATAGAAGAAGAAAAA----
----

AGTAATAGAACATATAGAACATATAGGTTAAGAAGCTGTAACACCTCAGTCATTACGCAG---
GCCTGTCCAAAGACATCCTTTGAGCCAATTCCCATACATTACTGCACCCCGGCTGGTTTTGCGCTTCTGAAGT
GTAAG---GAGACAGACTTCAAT------GGAACAGGAACATGTACAAAT-----------------------
-GTCAGC---ACAGTACAATGCACACATGGAATTAGACCAGTAGTGTCCACT---
CAACTACTGTTAAATGGCAGTCTAGCAGAAAAAGAA------------
ATAGTACTTAGATCTGCCAATTTCACAGACAAT---------ACTAAAACCATAATTGTACAGTTAAAA----
-----------GAACCTGTAGAA---------ATAAATTGTACAAGACCCAACAATAATACAAGAAAA-----
-AGTATACATATA------------GGGCCCGGGAGGGCA---TTT------TATGCAACAGGAGAA------
---------ATAATAGGAAATATA------------AGACAAGCACATTGT---AACATTAGTGGAACA---
AAATGGAATGAAACATTAGAGCAGCTAGTTCAGAAATTAAGA---------------AGCAAATTTGGG----
-----------------------------------AATAAGAAAATAATCTTTAAT------
CAATCCTCAGGAGGGGACCCAGAGGTTGTG---ATGCACACTTTTAATTGTGGAGGAGAA---
TTTAAGCAGCTCCAGGCAAGAGTCCTGGCTGTGGAAAGATACCTACATGATCAACAGCTCCTGGGAATTTGGG
GTTGCTCTGGGAAACTCATTTGCACCACTGCTGTGCCTTGGAATGCTAGTTGGAGT-----------------
----------------------------AATAGATCTCTGGACCACATTTGG------AAC------
AACATGACATGGATGCAGTGG---GAAAAAGAAATTGACAATTACACAACACTAATCTAC---
ACCTTGCTTGAAGATTCGCAAAACCAGCAGGAA--

AAAAATGAACAAGAATTATTGGAATTGGATAAATGGGCAAGTCTGTGGAACTGGTTTAGCATAACACAATGGC
TGTGGTATATAAAAATATTCATAATGATAGTAGGAGGCTTGGTAGGTCTAAGAATAGTTTGTGCT---
GTAGTTTCTATAGTG---------AATAGAGTCAGGCAGGGATACTCACCT------
TTATCATTTGCTGTCAGCTTGCTTAACACTGTAGCTATAGTAGTAGCTGAG---
GGGACAGATAGGGTTATAGAAGTAGGACAAATACTTGGCAGAGCT----------------------------
--------ATTCTCCACATA------CCTAGAAGAATAAGACAAGGCTTAGAAAGGCTTTTGCTA---TAA
>MH078555
GCT---GAA--
GACAACTTGTGGGTTACAGTTTATTATGGGGTACCAGTGTGG---AAAGAAGCAACC---
ACCACTCTATTTTGTGCATCAGATGCTAAAGCATATGTTACAGAA---AAGCATAAT----------------
--------------ATTTGGGCTACA---CATGCCTGTGTACCCACAGAC---
CCCAACCCACAAGAATTGGCATTAATA---------AATGTGACAGAAAACTTTAAC----------------
--------------------------ATGTGGAAAAATGATATGGTAGATCAGATGCATGAGGATATA---
ATCAGTTTATGGGATGAAAGCCTA---AAGCCATGTGTAAAATTG---
ACTCCACTTTGTGTTACTTTAAATTGCACTAATGTTACTATTACTGGGAATAATACCACTGAT----------
----
-------------------GGTGGTAATGATACTAGGATTAATGTCACTAGTGACGAAATAAAA---
AATTGCTCTTTTCATACT------CATATAGGA---------------------------
GACCAAAAGCAAAGAGAATATGCAATA---TTTCATACACTTGATATGGTACCAATAAAAAATGATAATATT-
----

AGTAATAAGAATGATACTTTGTATAGGTTAATGCATTGTAACACATCAGTCATTAGGCAG---
GCATGTCCAAAAGTATCCTTTGAGCCAATTCCCATACATTATTGTGCCCCAGCTGGTTTTGCGATTCTAAAGT
GTAAG---GATAAAAAATTCAAT------GGAACAGGAACATGTCAAAAT-----------------------
-GTCAGC---ACAGTACAATGCACGCATGGAATTAAGCCAGTAGTGTCAACC---
CAACTGCTGTTAAATGGCAGCCTAGCAGAAGAAGAG------------
GTAGTAATTAGATCTGCCAACTTCACAGACAAT---------GCTAAAACCATAATAGTGCAGTTAAAG----
-----------GACCCTGTAAAA---------ATAAATTGTATAAGACCCAACAATAATACAATAAAA-----
-GAGGTACGCATA------------GGTCCAGGTGCA------TAT------TTTAGAGCAGGACAG------
---------ATAATAGGAGATATA------------AGAAAAGCATATTGT---AACATTAGTAGAACA---
GATTGGAATACCACTTTATATCAGATAACTCAAAAATTAAGA---------------GAACAATAT-------
-----------------------------------AATAAAACAATAATTTTTAAT------
CAATCCACAGGAGGGGACCCAGAGGTTGTA---ATGCACACTTTTAATTGTGGAGGAGAA---
TTTAAGCAGCTCCAGGCAAGACTCCTGGCTGTAGAAAGTTACCTAAGGGATCAACAGCTCCTAGGGATTTGGG
GTTGCTCTGGGAAACACATCTGTGCCACTTCTGTGCCTTGGAATGCTAGTTGGAGT-----------------
----------------------------AATAGATCTCTGGATTACATTTGG------GAT------
AACATGACCTGGATGGAGTGG---GAAAAAGAAATTGACAATTACACAGGACTAATATAC---
ACCTTACTTGAACAATCGCAGTACCAGCAAGAA--

AAGAATGAAAAAGAATTATTGGAATTGGATAAGTGGGCAAGTCTGTGGAATTGGTTTGCCATATCAAATTGGC
TGTGGTATATAAGGATATTCATAATAATAGTAGGAGGCTTGATAGGTTTAAGAATAATTTTTGCT---
GTAATTTCTATAGTG---------AATAGAGTTAGGCAGGGATACTCACCA------
TTGTCGTTTGCTGTTAGCTTGCTTAATGCCACAGCTATCGCAGTAGCTGAG---
GGGACAGATAGAATTATAGAAGTAGGACAAAGAATTGGTAGAGCT----------------------------
--------ATTCTCCACATA------CCTAGAAGAATAAGACAGGGCTTGGAAAGAAGTTTGCTA---TAA
>EF210726
ACT---ACA--
GAACAATTGTGGGTCACGGTATATTATGGGGTACCTGTGTGG---AAAGAAGCAACC---
ACCACTCTATTTTGTGCATCAAATGCTAAAGCATATAGTACAGAG---AAACATAAT----------------
--------------GTTTGGGCCACA---CATGCCTGTGTACCCACAGAC---
CCCAACCCACAAGAAGTAGTATTGGAA---------AATGTGACAGAAAATTTTAAC----------------
--------------------------ATGTGGAAAAATAGCATGGTAGACCAGATGCAAGAGGATATA---
ATCAGTTTATGGGATCAAAGCCTG---AAGCCATGTGTAAAACTA---
ACTCCACTCTGTGTTACCTTAAATTGTACTGATAGGCTAAATGCCACTAGAACTAAT----------------
----
----------------------------GCCACCACTGATGCCCATATTACCAATGAAATAAAA---
AACTGCTCTTTCAATGTCACCGCAAGCATAAGA---------------------------
GATAAGGTAAAGACAGAATATGCACTT---TTTTATAGCCTTGATGTAGTACCCATAGAGGAT----------
----

AATGATAATACCAGCTATAGGTTGAGAAGTTGTAACACCTCAGTCATTACACAG---
GCCTGTCCAAAGGTAACCTTTGAGCCAATTCCCATACATTATTGTACCCCGGCTGGTTTTGCACTCCTAAAGT
GTAAT---GATAAGAAGTTCAAT------GGAACAGGACCATGTACAAAT-----------------------
-GTTAGC---ACAGTACAATGTACTCATGGAATTAAGCCAGTAGTATCAACT---
CAACTGCTGTTAAATGGCAGTTTAGCAGAAGAAGAG------------
GCAGTAATTAGATCTGAAAATTTCACGAACAAT---------GCTAAAACCATAATAGTACAACTGAAA----
-----------GAACCTGTAGAG---------ATTAATTGTACAAGACCCAATAACAATACAAGAAAA-----
-AGTATACATATA------------GGGCCAGGGAGAGCA---TTT------TATGCAACAGGAGAC------
---------ATAATAGGAGATATA------------AGGCAAGCACATTGT---AACATTAGTAGAGCA---
AAATGGAATAGCACTTTAACACAGATAGTGGGAAAATTAAGA---------------GAACAATTTGGG----
-----------------------------------AATAAAACAATAATCTTTAAT------
CAATCCGCAGGAGGGGACCCAGAAATTACA---ATGCACAGTTTTAATTGTGGAGGGGAA---
TTTAAGCAGCTCCAGGCAAGAGTCCTGGCTGTGGAAAGATACCTAAGGGATCAACAGCTCCTAGGGATTTGGG
GTTGCTCTGGAAAACTCATTTGCACCACTAATGTGCCTTGGAATGCTAGTTGGAGT-----------------
----------------------------AATAAATCTCTGGATGAGGTTTGG------GGC------
AACATGACATGGTTACAGTGG---GAAAGAGAAATTGACAATTACACAAGCTTAATATAC---
ACATTACTTGAAGAATCACAGAACCAGCAAGAA--

AAGAATGAAAAAGAATTACTAGAACTAGATAAGTGGGCAAGTCTGTGGAATTGGTTTGACATAACAAAATGGC
TGTGGTATATAAAAATATTCATAATGATAGTAGGAGGCTTGGTAGGTTTAAGGATAATTTTTGCT---
GTGCTTTCTATAGTG---------AATAGGGTTAGGCAGGGATACTCACCA------
TTATCGTTGGCTGTTAGCTTGCTCAATACTACAGCTATAGTAGTAGCTGAA---
GGGACAGATAGGGTGCTAGAAGCGGGGCAAAGAATTTTTAGAGCT----------------------------
--------TTTATCCACATA------CCTAGAAGAATAAGACAAGGCTTAGAAAGAATTTTGCTA---TAA
>EF210730
GCT---GCA--
GAAGACAGGTGGGTCACAGTCTATTATGGGGTACCAGTGTGG---AAAGAAGCAACC---
ACCACTCTATTCTGTGCATCAGATGCTAAAGCATATGATACAGAG---GTACATAAT----------------
--------------GTTTGGGCCACA---CATGCCTGTGTACCCACAGAC---
CCCAACCCACAAGAGTTAAGATTGGAA---------AATGTGACAGAAGAGTTTAAC----------------
--------------------------ATATGGAAAAATGACATGGTAGAACAGATGCATGAGGATATA---
ATTAGTTTATGGGATCAAAGCCTA---AAGCCATGTGTAAAATTA---
ACTCCACTCTGTGTTACTTTAAATTGCACTAAGATTACCATGAAAAGTACTAATAATACT-------------
----
-------------------------AATACTAATACTAGTTGGGAAAGGGGGGAAGAAATGAAA---
AATTGCTCTTTCAATGTCAGCTCAAGCGTAAGA---------------------------
GACAAGATGAAGAGAGAATATGCACTT---TTTTATAAACTTGATATAGTACCAATAGATGATAGTAAT----
----

AGTAGCAGCAGTAACTATAGTAGCTATAGGTTGATAAATTGTAACACCTCAGTCATTACACAG---
GCTTGTCCAAAGGTAACCTTTGAGCCAATTCCCATACATTATTGTACCCCGGCTGGTTTTGCGCTTTTAAAGT
GTAAT---GATAAAAGGTTCAAT------GGAACAGGAAAATGTACAAAT-----------------------
-GTCAGT---ACAGTACAATGCACACATGGAATTAAGCCAGTAGTGTCAACT---
CAACTGCTGTTAAACGGCAGTCTAGCAGAAGAAGAG-----------
GTAATACTTAAATCTGCCAATATCACAGACAAT---------GCTAAGACCATAATAGTGCAGCTAAAT----
-----------GAAACTGTAGAA---------ATAAATTGTACAAGACCCAACAATAATACAAGAAAA-----
-AGTATACCTATA------------GGGCCAGGGAGAGCA---TTT------TATACAACAGGAGAT------
---------ATAATAGGAGATATA------------AGGCAAGCACATTGT---AACATTAGTAGAGCA---
AAATGGAATCTAACTTTACACCGTATAGCTACAGCATTAAAA---------------GCCAAGTTTAAT----
-----------------------------------GATAATACAATAGTCTTTAAT------
CAATCCTCAGGAGGGGACCCAGAAGTTGTG---ATGCACACTTTTAATTGTGGAGGAGAA---
TTTAAGCAGCTCCAGGCAAGAGTCCTGGCTGTGGAAAGATACCTACAGGATCAACAGCTCCTGGGAATTTGGG
GTTGCTCTGGAAAACTCATTTGCACCACTTCCGTGCCTTGGAATACTAGTTGGAGT-----------------
----------------------------AATAAATCTCAGGAACAAATTTGG------AAT------
AACATGACTTGGATGGAGTGG---GAAAAAGAAATTGACAATTATACAGGATTAATATAC---
AACTTACTTGAAAAATCGCAGAACCAGCAGGAA--

AAGAATGAACAGGAATTATTGGAATTGGATAAGTGGGCAAGTCTGTGGAATTGGTTTGACATAACACAATGGC
TGTGGTATATAAAAATATTCATAATGATAGTAGGAGGCTTGATAGGTTTAAGAATAGTTTTTGCT---
GTACTTTCTATAGTA---------AATAGAGTTAGGCAGGGATACTCACCA------
TTATCATTTGCTATTAGCTTGCTCAACGCCACAGCTATAGCAGTAGCTGAG---
GGGACAGATAGGATTATAGAAGTAGGACAAAGAGCTGGTAGAGCT----------------------------
--------ATCCTCCACATA------CCTAGAAGAATAAGACAGGGCTTAGAAAGGCTTTTGCTA---TAA
>EF210732
GCT---GCA--
GATGATTTATGGGTCACAGTCTATTATGGGGTACCTGTGTGG---AAAGAAGCAATC---
ACCACCCTATTTTGTGCATCAGATGCTAAAGCATATAAAACAGAG---GCACATAAT----------------
--------------ATTTGGGCCACA---CATGCCTGCGTACCCACAGAC---
CCCAACCCACAAGAGGTAATATTGGAA---------AATGTGACAGAAAATTTTAAC---------------
--------------------------ATGTGGAAAAATAACATGGTAGACCAAATGCATGAGGATATA---
ATCAGTTTATGGGATCAAAGCCTA---AAGCCATGTGTAAAGTTA---
ACTCCACTCTGTGTTACTTTAAAGTGCACTAATGTCAATAGTACTACTAGCACC------------------
----
-------------------------------AATAGTAGCGAGGAAGGGATAGGGGAAATGAAA---
AACTGCTCTTTCAATATCACCACAAGCGTAAAA--------------------------
GACAAAGTACAAAAAGAATATGCACTT---TTTTATAGACTTGATATAGTACCAATAGATAAT----------
----

ACCAGCTATAGGTTAGTAAAGTGTAACACCTCAGTCATTACACAG---
GCCTGTCCAAAGGTATCCTTTGAGCCAATTCCCATACATTATTGTGCCCCAGCTGGTTTTGCGATTTTAAAAT
GTAGA---GATAAGAAATTCAAT------GGAACAGGACCATGTAAAAAT-----------------------
-GTCAGT---ACAGTACAATGTACACATGGAATTAGGCCAACAGTATCAACT---
CACTTGCTGTTAAATGGCAGTCTAGCAGAAGAAGAG-----------
GTAGTAATTAGATCTGAAAATTTCACAAAAAAT---------GAGAAAACCATAATAGTACAGCTGAAT----
-----------CAATCTGTAGAA---------ATTACGTGTATAAGACCCTACAACAATACGAGAAAA-----
-AGTATACATATG------------GGACCAGGAAGAGCA---TTT------TATGCAACAGGAGAA------
---------ATAATAGGAGATATA------------AGAAAAGCACATTGT---AATGTTAATAAAACA---
AAATGGCATGAGGCTTTAAAAAAGATAGCTAAAAAATTAGAA---------------GAACAATATAAT----
--------------------------------AATAATAAAACAATAAGTTTTAAT------
CAATCCACAGGAGGGGACCCAGAAACTGCA---ATGTTTACCTTTAATTGTGGAGGGGAA---
TTTAAGCAGCTCCAGGCAAGAGTCCTGGCTGTGGAAAGATACCTACAGGATCAACAGCTCCTGGGGATTTGGG
GTTGCTCTGGAAAACTCATTTGCACCACTTCTGTGCCTTGGAATGCTAGTTGGAGT-----------------
----------------------------AATAAATCTATGAGTGAGATTTGG------GAG------
AACATGACCTGGATGGAGTGG---GAAAAAGAAATTAGCAATTACACAGGCTTAATATAT---
AACTTAATTGAAAAATCGCAAAACCAGCAAGAA--

CAGAATGAACAAGAATTATTGGCATTAGATAAATGGGCAAGTTTGTGGAATTGGTTTGACATATCAAACTGGC
TGTGGTATATAAAAATATTCATAATGATAGTAGGAGGCTTAGTAGGTTTAAGAATAGTTTTTGCT---

ATACTGTCTATAGTA---------AATAGAGTTAGGCAGGGATATTCACCA------
TTATCGTTGGCTATTAGCTTGCTTGATGCCACAGCTATAGCAGTAGCTGAG---
GGGACAGATAGGATTATAGAAGTAGCACAAAGAACTTATAGAGGC----------------------------
--------ATTCTTAACATA------CCTACCAGAATAAGACAGGGCTTGGAAAGGGCTTTGCTA---TAA
>KC596069
GCT---GCA--
GAACAATTGTGGGTCACAGTATATTATGGGGTACCTGTGTGG---AAAGAAGCAACC---
ACCACTCTATTTTGTGCATCAGATGCTAAAGCATATGACACAGAG---GTACATAAT----------------
--------------GTTTGGGCCACA---CATGCCTGTGTACCCACAGAC---
CCCAACCCACAAGAAATATTATTGGAA---------AATGTGACAGAGAATTTTAAC----------------
--------------------------ATGTGGAAAAATAACATGGTAGAACAGATGCATGAGGATATA---
ATCAGCTTATGGGATCAAAGCCTA---AAGCCATGTGTACAATTA---
ACCCCACTTTGTGTTACTTTAAATTGCACGGATGTGAAAAATACTACTAATGTCAATAATACT----------
----
----------------------GCTAGTGTCAGTAATAGTACTGTCAATAATAGTGATATGAAA---
AACTGCTCTTTCAATATCACCTCAGGCATAAGA---------------------------
GATAAGGTGCAGAAAAAGTATGCAACA---TTTTATAACCTTGATCTAGTACAAATAGAAAAT----------
----

GACAATACTAGCTATAGGATAGTAAGTTGTAACACCTCAGTCATTACACAA---
GCCTGTCCAAAAGTATCCTTTGAGCCAATTCCCATACATTATTGTGCCCCGGCTGGTTTTGCGATTCTAAAGT
GTAAG---GATAAGAATTACAAT------GGGAGCGGGCCATGTAAAAAT-----------------------
-ATTAGT---TCAGTACAATGTACACATGGAATTAGACCAGTGGTATCAACT---
CAGCTGCTGTTAAATGGCAGTCTAGCAGAAGAAGAG------------
GTAGTCATTAGATCTGAAAACTTTACAAACAAT---------GCTAAAACCATAATAGTACAGCTAAAT----
-----------GAAACTATAGAA---------ATTAATTGCACAAGACCCAACAATAATACAAGAAAA-----
-AGTATAAATATA------------GGACCAGGGAGAGCA---TTT------TATACAACAGGAGAA------
---------ATAATAGGGGATATA------------AGACAAGCACATTGT---AACATCAGTAGAGCA---
AGATGGGGAAAAGTGTTAAAACAGGTAGCTATAAAATTACGA---------------GAACAATTT-------
-----------------------------------AATAAAACAATAGCCTTTAAT------
AAATCCTCAGGAGGGGACCCAGAAATTGTA---TATCACACTTTTAATTGTGGAGGGGAA---
TTTAAGCAGCTCCAGGCAAGAGTCCTGGCTGTGGAAAGATACCTGAAGGATCAACAGCTCCTGGGAATTTGGG
GTTGCTCTGGAAAACTCATTTGCACCACTACTGTGCCTTGGAATGCTAGTTGGAGT-----------------
-------------------------AATAATAAAACTCTGGACGACATTTGG------AAT------
AACATGACCTGGATGCAGTGG---GAAAAAGAAATTGACAATTACACAGGCTTAATATAC---
ACCTTAATTGAAGAATCGCAGATCCAACAAGAA--

AAGAATGAACTAGAATTATTGAAATTGGATACATGGGCAGGTTTGTGGAATTGGTTTGACATATCAAAATGGC
TGTGGTATATAAAAATATTCATAATGATAGTAGGAGGCTTGATAGGATTAAGAATAGTTTTTACT---
GTACTGTCTATAGTG---------AATAGGATTAGGAAGGGATACTCACCA------
TTATCGTTTGCTGTTAGCTTGATCAATTTCATAGCTGTAGCAGTAGCTGAG---
GGGACAGATAGGATTATAGAAGTATCACAAAGGATTTTTAGAGCT----------------------------
--------ATTCTCCACATA------CCTACAAGAATAAGACAGGGCTTGGAAAGGGCTTTGCAG---TAA
>JN251896
GCT---ACA------AAACGTAGT---------------GCT---ACA---
GAACAATTGTGGGTCACAGTCTACTATGGGGTGCCTGTGTGG---AAAGAAGCAACC---
ACCACCCTATTTTGTGCATCAGATGCTAAAGCAGTTGACACAGAG---GTACATAAT----------------
--------------GTTTGGGCTACA---CATGCCTGTGTACCCACAGAC---
CCTAGCCCACAGGAAGTAGCATTGGAA---------AATGTGACAGAAAATTTTAAC----------------
--------------------------ATGTGGAACAATGATATGGTAGAACAGATGCATGAGGATATA---
ATCAGTTTATGGGATCAAAGCCTA---AAACCATGTGTAAAATTA---
ACCCCACTCTGTGTTACTTTAAATTGCACTGATTATTGCACTAATAGCACTAATAGCATTAGTAGCAAT----
----
-------------------AATGGGACTAAAAATTGTACTAATGAAATGAAGGGAGAAATAAAA---
AACTGCTTTTTCAATATCACCACAGGCAGAGGA---------------------------
GATAAGCTGCAAAAAGAATATGCACTT---TTTTATAAAAGTGATTTAATGCAAACAGAAAAT----------
----

GAYAACAGTAGTTATATGTTGACAAGTTGTAACACCTCAGTCATTACACAG---
GCTTGTCCAAAGGTAACCTTTGAACCAATTCCCATACATTATTGTGCCCCGGCTGGTTTTGCGATTCTAAAAT

GTAAT---AATAAGACATTTGAG------GGGAAAGGACCATGTAACAAT-----------------------
-GTTAGC---ACAGTACAATGTACACATGGAAGTACGCCWGGAGTGACGACT---CAACTGCTGTT-
AATGGCAGTCTAGCAGAAGAAGAG------------ATAGTAATTAGATCAAAAAATTTCTCGAACAAT----
-----GCTGAGACCATAATAGTGCAGTTGAAC---------------GAATCTATAGCA---------
ATTGATTGTACAAGACCCAACAACAATACAAGAAAA------GGTATACATATG------------
GGATTTGGGAGAACA---TTT------TATGCAACAGGAGAA---------------ATAATAGGAGATATA-
-----------AGACAAGCATATTGY---ATACTTAATAAAACA---
GAATGGGAAAACACTTTAAAAAAGGTAGCTAGAAAATTAGRG---------------GGACAATTTAGG----
-----------------------------------AACAGAACAATAGCCTTTAAC------
AAATCCTCAGGAGGGGACCCAGAAATTGTA---ATGCATACTTTTAATTGTGGAGGGGAA---
TTTAAGCAGCTCCAGGCAAGAGTCCTGGCTGTGGAAAGATACCTAAAGGATCAACAGCTCCTAGGGATTTGGG
GTTGCTCTGGAAAACTCATCTGCACCACTGCTGTGCCTTGGAATACTAGTTGGAGT-----------------
----------------------------AATAGATCTCAAGATTACATTTGG------AAT------
AATATGACCTGGATGCAGTGG---GAGAGAGAAATTGACAATTACACAGGATTAATATAC---
ACTTTGCTTGAAGACTCGCAAAACCAGCAGGAA--

AAGAATGAACAAGAATTATTGGAATTGGATAAGTGGGCAAGTTTGTGGAGTTGGTTTGACATAACAAATTGGC
TGTGGTACATAAGATTATTCATAATAATAGTAGGAGGATTAGTAGGTTTAAGAATAGTTTTTGCT---
GTGCTTTCTATAGTG---------AATAAAGTTAGGCAGGGATACTCACCA------
TTATCGTTCGCTGTTAGTTTGTTTAATGCAATAGCTATAGCAGTAGCTGAG---
GGAACAGATAGGATTATAGAAGTAGTACAAAGAATCTGTAGAGCT----------------------------
--------ATCCTCCACATA------CCTGTAAGAATAAGACAGGGCTTGGAAAGGGCTTTGCTA---TAA
>JN251901
GCT---GCA--
GGGCAATTGTGGGTCACAGTCTATTATGGGGTACCTGTGTGG---AAAGAAGCAACC---
ACCACTCTATTTTGTGCATCAGATGCTAAAGCATATGATACAGAG---GCACATAAT----------------
--------------GTTTGGCCACA---CATGCCTGTGTACCCACAGAC---
CCTAACCCACAAGAAATAGTATTGAAC---------AATGTGACAGAAAATTTTAAC----------------
--------------------------ATGTGGAAAAATAACATGGTAGAACAAATGCATGAGGATATA---
ATCAATTTATGGATCAAAGCCTA---AAGCCATGTGTAAAATTA---
ACCCCACTCTGTATTACTTTAAATTGCAATAACATTACTACTAATAGCACTATT-------------------
----
-------------------------------ACCTGGGGAAAGATGGAGCCAGGAGAAATAAAA---
AACTGCTCTTTCAATACCACCACAGACAGAAGG---------------------------
GATAGGGTGCGAAAAGAATATGCACTT---TTTTATAAACTTGATATAGTACCAATAAATGAA----------
----

AATGATAATAAAACTAATACCAACTATAGGTTAATAAGTTGTAATACCTCAACCATTACACAA---
GCATGTCCAAAGATATCCTGGGAACCAATTCCCATACATTATTGTGCCCCGGCTGGTTTTGCGATTCTAAAAT
GCAAT---GATAAGGAGTTCAAT------GGAACAGGACCATGCACAAAT-----------------------
-GTCAGC---ACAGTACAATGTACACATGGAATCAGGCCAGTAGTGTCAACT---
CAGTTGTTGTTAAATGGCAGTCTAGCAGAAGAAGAG------------
ATAATAATTAGATCTGAAAATATCACAAACAAT---------GCTAAAACCATAATAGTACAGCTGAAG----
-----------GACTCTGTAAAC---------ATTACGTGTGTAAGACCCAGCAACAATACAAGAAGA-----
-AGTATAAATATA------------GGACCAGGGAGAGCA---TTT------TATACAACAGGAGAA------
---------ATAATAGGAGATATA------------AGACAAGCACATTGT---AACCTTAGTAGAGGA---
GCATGGGAAAAAACTTTAAACCAGACAGCTATAAAATTAAGA---------------GAACATTTTAAG----
-----------------------------------AATAAAACAATAATCTTCAAG------
CAATCCTCAGGAGGGGACCCAGAAATTGTA---ATGCACACTTTTAATTGTGGAGGGGAA---
TTTAAGCAGCTCCAGGCAAGAGTCCTGGCTGTGGAAAGATACCTAAAGGATCAACAGCTCCTAGGGATTTGGG
GTTGCTCTGGAAAACTCATTTGCACCACTGCTGTGCCTTGGAATAATAGTTGGAGT-----------------
----------------------------AATAGATCTTTGGGACACATTTGG------AAT------
AACATGACCTGGATGCAGTGG---GAGAAGGAAATTGATAATTTCACAGGCTTAATATAC---
AATTTGCTTGAAGAATCGCAGAACCAGCAGGAA--

AAGAATGAACAAGAATTATTAGAATTGGATAAATGGGCAAATTTGTGGAATTGGTTTGACATATCAAATTGGC
TGTGGTATATAAGAATATTCATAATGATAGTAGGAGGCTTAATAGGGTTAAGAATAGTTTTTACT---
GTAATTTCTATAGTG---------AATAGAGTTAGGCAGGGATACTCACCC------
TTATCATTCGCTGTTAATTTGCTTAATACTGTAGCTATAGCAGTAGCTGAG---

GGGACAGATTGGGTTATAGAAATAGTACTAAGATTTTGTAGAGCT----------------------------
--------ATCCTTCACATA------CCTAGAAGAATAAGACAGGGCTTGGAGAGGGCTTTGCTA---TAA
>JN251906
GCT---ACA--
GGYAATCTGTGGGTCACAGTCTATTATGGGGTACCTGTGTGG---AGAGAAGCAACC---
ACCACTCTATTTTGCGCATCAGATGCTAAAGGATATGTGAAAGAG---GCACATAAT----------------
--------------ATTTGGGCCACR---CATGCCTGTGTACCCACAGAC---
CCCAACCCACAAGAAATAAAATTGGAA---------AATGTGACAGAAAATTTTAAC---------------
--------------------------ATGTGGAAAAATTACATGGTAGAACAGATGCATGAGGATATA---
ATCAGTTTATGGGATCAAAGCTTA---AAGCCATGTGTAAAATTA---
ACCCCACTTTGTGTTACTTTAAATTGTACTGATTATTGGAATAATACCAAAACTAMTAAT------------
----
-------------------------AGYACCGGGGAAATAATGATGGACAWGGGAGAAATAAAA---
AACTGCTCTTTCAATATCACCACAACAATAAGA--------------------------
GATAAAGTGCAGAAAGAATATGCACTT---TTTTATAAACTTGATGTAGTACCAATAGGGGAA----------
----

GATAATAGTAATACTAGTTATAGGTTGATAAGTTGTAACACCTCAGTCATTACACAG---
GCTTGTCCAAAGGTAACCTTTGAGCCAATTCCTATACATTATTGTGCCCCGGCTGGCTTTGCGATTCTAAAGT
GTAAC---AATAAAACATTCAAT------GGAAAAGGACCATGTACAAAT----------------------
-GTCAGC---ACAGTACAATGYACACATGGAATTAGGCCAGTAGTATCAACT---
CAACTGCTGTTAAATGGCAGTCTAGCAGAAGAAGAA------------
GTAGTAATTAGATCTGACAATTTCTCTGACAAT---------GCTAAAACCATAATAGTACAGCTGAAA----
-----------AATGCTGTACCA---------ATTAATTGTACAAGACCCAACAACAACACAAGAAAA-----
-GGTATACATATA------------GGACCAGGGAGAGCA---ATG------TTGGCAACAGGAGAA------
---------ATAATAGGAAATATA-----------AGAAAAGCATATTGT---AACATTAGCAGAGAA---
AAATGGGATAACACTTTAGAACAGGTAGCTACAAAATTAAGA---------------GAACAATTTAAG----
-----------------------------------AATAAAACAATAGTCTTTAAT------
CACTCCTCAGGAGGGGATCCAGAAATTGTA---ATGCACAGCTTTAATTGTGGAGGGGAA---
TTTAAGCAGCTCCAGGCAAGAGTCCTGGCTGTGGAAAGATACCTAAAGGATCAACAGCTCCTAGGAATTTGGG
GTTGCTCTGGAAAACTCATCTGCACCACTGCTGTGCCTTGGAACACTAGTTGGAGT----------------
----------------------------AATAAATCTATGGAATATATTTGG------CAA------
AACATGACATGGATGCAGTGG---GAAAGAGAAATTGACAATTACACAGACATAATATAC---
AATTTAATTGAAGAATCGCAGAACCAACAAGAA--

AAGAATGAACAAGATTTATTGGCATTAGACAAATGGGCAAGTCTGTGGAGTTGGTTTGACATAACAAGTTGGC
TGTGGTATATAAAAATATTCATAATGATAGTAGGAGGCTTGATAGGTTTAAGGATAGTTTTTACT---
GTATTTTCTATAGTG---------AATAGAGTTAGGCAGGGATACTCACCA------
TTATCATTCGCTGTTAGCTTGCTCAACGCCACCGCCATAGCAGTAGCTGAG---
GGGACAGACAGGGTTATAGAAGTAGCACAAAGATTTGGTAGAGCT--------------------------
--------ATTCTCCACATA------CCTAGAAGAATAAGACAGGGCCTTGAAAGGGCTTTGCTA---TAA
>AY819715
ACT---GCA--
GGACAATTGTGGGTCACAGTCTATTATGGGGTACCTGTATGG---AAAGAAGCAAAC---
ACCACTCTATTTTGTGCATCAGATGCTAAAGCATATRMTACAGAR---GTGCATAAT----------------
--------------GTTTGGGCCACA---CACGCCTGTGTACCCACAGAC---
CCCAACCCACAAGAAGTGTTTTTGATA---------AATGTAACCGAAAATTTTAAT---------------
--------------------------ATGTGGGAAAATAACATGGTAGAACAGATGCAAGAGGATATA---
ATCAGTTTATGGGATCAGAGCATA---AAACCATGTGTAAAATTA---
ACCCCACTCTGTGTTACTTTAAATTGCACTGATTGGGTGGAGAAGAATACTACCACTGCT------------
----
-------------------------ACTCCTCCCACTTTGAAGCTTGAGAAAGGAGAAGTAAAA---
AACTGCTCTTTCAATGCCACCACAAACATAAGC--------------------------
GATAAGGTGCAGAGACAATATGCACTG---TTGTATAAAGCTGATGTAGTACAAATAGAAGAA----------
----

AATAGTACTGACTATAGGTTGATAAGTTGTAACACCTCAGTCATTACACAG---
GCCTGTCCAAAGATATCCTTTGAGCCAATTCCCATACATTATTGTGCCCCGGCTGGTTTTGCGATTCTAAAAT
GTAAT---GACAAGAAGTTCAAT------GGAACAGGACCATGTAAAAAT----------------------
-GTCAGC---ACAGTACAATGTACACATGGAATTAGACCAGTAGTGTCAACT---

CAACTGCTGTTAAATGGCAGTCTAGCAGAAGAAGAG------------
ATAGTAATTAGGTCTGAAAATCTCACGGACAAT---------GTTAAAACCATAATAGTACATCTGAAT----
-----------GAATCTGTACAA---------ATTAATTGTACAAGGCCCAGCAACAATACAAGAAAA-----
-GGTATACCTATA------------GGACCAGGGAGAGCA---TGG------TATGCAACAGGAGAT------
---------ATAACAGGAGATATA------------AGAAAAGCACATTGT---AACATTAGTAGAGCA---
AAATGGGATGAAACYTTAAAGAAGGTAGTTAAAAAGTTAAGA---------------GAACAATTTAAG----
-----------------------------------AATAAGACAATAATCTTTAAG------
CAATCCTCAGGAGGGGACCCAGAAATTGTA---ATGCACACTTTTAATTGTGGAGGGGAA---
TTCAAGCAGCTCCAGGCGAGAGTCCTGGCTGTGGAGAGATACCTAAAGGATCAACAGCTCCTGGGGATTTGGG
GTTGCTCTGGAAAACTCATTTGCACCACTACTGTGCCTTGGAATACTAGTTGGAGT-----------------
---------------------------AATAAATCTCTAGATGAGATTTGG------GAT------
AACATGACCTGGATGGAGTGG---GAAAAAGAAATTAATAATTACACAGGTTTAATATAC---
AGGTTAATAGAAGAATCGCAGAACCAACAAGAA--

AAGAATGAACAAGAATTATTGGCATTAGATAAATGGGCAAGTTTGTGGAATTGGTTTAGCATATCAAACTGGC
TGTGGTATATAAAAATATTCATAATGATAGTAGGAGGCTTGGTAGGTTTAAGAATAATTTTTGCT---
GTACTTTCTATAGTG---------AATAGAGTTAGGCAGGGATATTCACCA------
TTGTCATTTGCTGTTAGCTTGTTTAATGTCATAGCTATAGCAGTAGCTGAG---
GGGACAGACAGGGTTATAGAACTAATACAAACAGCTTTTAGAGCT---------------------------
--------TTCATCCACATA------CCTAGAAGAATAAGACAGGGCTTGGAAAGGGCTTTGCTA---TAA
>HQ896488
GCT---ACA--
GACAATTGGTGGGTCACAGTCTATTATGGGGTGCCTGTGTGG---AAAGAAGCAAAC---
ACCACTCTATTTTGTGCATCAGATGCTAAAGCATATGAGACAGAA---GTACATAAT----------------
--------------GTTTGGGCCACA---CATGCGTGTGTACCCACAGAC---
CCCAACCCACAAGAAGTAAATTTGGCA---------AATGTGACAGAACCATTTAAT----------------
-------------------------ATGTGGAAAAATAACATGGTAGAACAGATGCATGAGGATATA---
ATCAGTTTATGGGATCAAAGCCTA---AAGCCATGTGTAAAATTA---
ACCCCACTCTGTGTTACTTTAAATTGCACTGATTTAAAGAATACTACTAATACCACTGAT-------------
----
---CCAAATACNAATGCCATGAAG---
AACTGCTCTTTCAATATTACCACAAACATACAA---------------------------
GATAAAAGGCAGAAAGAATATGCACTT---TTTTATAAACTTGATATAGCACCAATAGATGATAAT-------
----

AATAGCAGCAGTTATACCTTGATAAGTTGTAACACCTCAATCATTACACAG---
GCCTGTCCAAAAATATCCTTTGAGCCAATTCCCATACATTTTTGTGCCCCGGCTGGTTTTGCGATTCTAAAGT
GTAAC---CAGAAGAATTTCAAT------GGAACAGGTCCATGTACAAAT-----------------------
-GTCAGC---ACAGTACAGTGTACACATGGAATTAAGCCAGTAGTATCAACT---
CAATTGCTGCTAAATGGCAGCTTATCAGAAGAAGAG------------
GTAGTAATTAGATCTAGTAATTTCTCAGACAAT---------GCTAAAAACATAATAGTGCAACTAAAT----
-----------ACACCTGTAAAA---------ATTAATTGTACAAGACCAAATAACAATACAAGAAAA-----
-AGTATACATATA------------GGACCAGGAAGAGCA---TTT------TATGCAACAGGACAA------
---------ATAATAGGAGATATA------------AGACAAGCACATTGT---AATATTAGTCTAACA---
GATTGGAATAACACTTTACAGCAGATAAGTAAAAAATTAACA---------------GAACAATTTGGA----
--------------------------------GAAAATAAAACAATAAGATTTGCG------
CCATCCTCAGGAGGGGACCAAGAAATTACA---ATGCACAGTTTTAATTGTAGAGGGGAA---
TTTAAGCAACTCCAGGCAAGAGTCCTGGCTGTGGAAAGATACCTAAGGGATCAACAGCTCCTGGGAATTTGGG
GTTGCTCTGGAAAACTTATCTGCACCACTGCTGTGCCTTGGAATAATAGTTGGAGTGGAGGT-----------
---------------------------AATAAAAGTCTGACTGACATTTGG------AAC------
AACATGACCTGGATGGAATGG---GAAAGAGAAATTAACAATTACACAGACATAATATAC---
AATTTACTTGAAAAATCGCAAAACCAGCAAGAA--

AAGAATGAACAAGAATTGTTGGAATTGGATAAATGGGCAAATTTGTGGAATTGGTTTGACATAACAAATTGGT
TGTGGTACATAAAAATATTCATAATGATAGTAGGAGGCTTGATAGGTTTAAGAATAGTTTTTACT---
GTACTTTCTATAGTG---------AATAGAGTTAGGCAGGGATACTCACCA------
TTATCGTTGGCTGTTAGCTTGCTCAACGCCACAGCCATAGCAGTAGGTGAG---
GGGACAGATAGGGTTATAGAAATATTACAAAGAGCTGGTAGAGCT---------------------------
--------GTTCTCCACATA------CCTACAAGAATAAGACAGGGCTTGGAAAGGGCTTTGCAG---TAA
>JX500709

GCT---ACA---
GAAAGGTGGTGGGTCACAGTCTATTATGGGGTACCTGTGTGG---AAAGAAGCAACC---
ACCACTCTATTTTGTGCATCAGATGCTAAAGCATATGATACAGAA---GTACATAAT----------------
--------------GTTTGGGCCACA---CATGCTTGTGTACCCACAGAC---
CCCAACCCACAAGAAATAGTATTGGAA---------AATGTGACAGAAGAGTTTAAC----------------
--------------------------ATGTGGAAAAATGACATGGTAGAACAGATGCAGGAGGATGTC---
ATCAGTTTATGGGATCAGAGCCTA---AAGCCATGTGTAAAATTA---
ACCCCACTCTGTGTTACTTTAGACTGCACAAATGTAAACAAGAATGAG------------------------
----
----------------------------ACTTTAGGGAATGAGACTTTGACAGGAGAACTAAAA---
AACTGCTCTTTCAATATTACCACAAACATAAGA--------------------------
GATAAGGTAAAGAAAGCAAATGCAATT---TTTGATAAACTTGATATAGTACAAATAGATGAT----------
----

GATAATAATAGTGATAGTTATAGGTTGATAAATTGTAACACCTCAGTCATTACACAA---
GCCTGTCCAAAGGTATCCTTTGAGCCAATTCCCATACATTATTGTGCCCCGGCTGGTTTTGCGATTCTAAAGT
GTAAC---AACAAGACGTTTGGT------GGAAAAGGACTATGTACAAAT-----------------------
-GTCAGC---ACTGTACAATGTACACATGGAATTAAGCCAGTAGTGTCAACT---
CAACTGCTGTTAAATGGCAGTTTAGCAGAAACAGAA------------
ATAGTAATTAGATCTGACAATTTCTCGGACAAT---------GCTAAAAACATAATAGTACAGCTGAAT----
-----------GAATCTGTAGCA---------ATTACTTGTGAAAGGACCAACAATAATACTAGAACA-----
-GATATTCATATA------------GGACCAGGAAGAGCA---TTT------CATGCAATAGGAGAT------
---------ATAAAAGGAGATATA------------AGAGTAGCACACTGT---AACATTAGTAAAGCA---
AATTGGAGTAGAACTTTAGAGCAGATAGCTAACAAATTAAGA------------GGAATTGAACAATTTAAG-
-----------------------------------AATAAAACAATAGTCTTTAAT------
AGCTCCTCAGGAGGGGACCCAGAAATTGTA---ATGCATAGTTTTAATTGTGGAGGGGAA---
TTTAAGCAGCTCCAGGCAAGAGTCCTGGCTGTGGAAAGATACCTAAGAGATCAACAGCTCCTAGGGATCTGGG
GTTGCTCTGGAAAACTCATTTGCACCACTGCTGTACCTTGGAATATAAGTTGGAGT-----------------
-------------AAGAGGTGGGAT---AATGGAAGTCTGACTGATATTTGG------GAT------
AATATGACCTGGATGCAATGG---GAAAAAGAAATTGACAATTACACAGACATAATATAC---
AGCTTACTTGAAAAAGCGCAAAACCAGCAAGAA---

AAAAATGAACAGGAATTGTTGGAGTTGGACAAATGGGCAAGTTTATGGAATTGGTTTGACATAACAAACTGGC
TGTGGTACATAAAAATATTCATAATGATAGTAGGAGGCTTGATAGGTTTAAGAATAGTTTTTTCT---
GTAATTTCTATAGTG---------AATAGAGTTAGGAAGGGATACTCACCA------
TTATCATTAGCCATTAGCTTGTTCAACGCCACAGCCATAGCAGTAGCTGAG---
GGAACAGATAGGATTATAGATTTAGCACAACAAATTTGTAGAGCT---------------------------
--------ATCCGCCACATA------CCTAGAAGAGTGAGACAGGGCTTGGAAAGGGCTTTGCTG---TAA
>JX500707
GCT---ACA---
GGAAACTTGTGGGTCACAGTCTATTATGGGGTACCTGTATGG---AAAGAAGCAAAC---
ACCACTCTATTTTGTGCATCAGATGCTAAAGGATATAATACAGAG---GTACATAAT----------------
--------------GTTTGGGCCACA---CATGCATGTGTACCCACAGAC---
CCCAACCCACAAGAAATAGAATTGGGA---------AATGTAACAGAAAATTTTAAC----------------
--------------------------ATGTGGAAAAATAACATGGTAGAACAGATGCATGAGGATATA---
ATCAGCTTATGGGATCAAAGCCTA---AAGCCATGTGTAAAATTA---
ACCCCACTTTGTGTTACCTTAAATTGCACTGATTTTGAGTTGAGGAATAGTACTAATAATACC----------
----
--ATTAACAGTGGAGAAATGAAA---
AACTGCTCTTTCAAT---ACCACAAACCTAAGA--------------------------
AATAAAATACAGAAAGAATATGCACTT---TTTTATAAGCTGGATTTAGTACCAATAGATAAT----------
----

GATAATACCAGTTATATGTTGATAAGTTGTAACACCTCGGTCATTACACAA---
GCCTGTCCAAAGGTATCCTTTGAACCAATTCCCATACATTATTGTGCCCCGGCTGGATTTGCGATTCTAAAGT
GTAAA---GATAATAAGTTCAAT------GGAACAGGACCATGTAAAAAT-----------------------
-GTCAGC---ACAGTGCAGTGTACACATGGAATTAGGCCAGTAGTATCCACT---
CAATTGCTGTTAAATGGCAGTCTAGCAGAAGAAGAG------------
GTAGTAGTTAGATCTAGCAATTTCTCAGACAAT---------GCTAAAAACATAATAGTGCAACTAAAA----
-----------GAACATGTAAAA---------ATTAATTGTACAAGACCCAACAACAATACAAGAAAA-----

-AGTATAAATATA------------GGACCAGGGAGAGCA---TTT------TATGCAACAGGAGAT------
---------ATAATAGGAGATATA------------AGACAAGCACATTGT---AACCTTAGTCAAACA---
GAATGGAATAAAACTTTAGGACAGATAGTTGAAAAATTAAAA---------------GAACAATATGGT----
-----------------------------------AATAAAACAATAATCTTTAAC------
CAATCCTCAGGAGGAGACCCAGAAATTGTA---ATGCACAGTTTTAATTGTGGAGGGGAA---
TTTAAGCAGCTCCAGGCAAGAGTCCTGGCTGTGGAAAGATACCTACAGGATCAACAGCTCCTGGGGATTTGGG
GTTGCTCTGGAAAACTCATCTGCACCACTGCTGTGCCATGGAATGCTAGTTGGAGT-----------------
----------------------------AATAAATCTCATGATGATATTTGG------AAT------
AACATGACCTGGATGCAGTGG---GAAAAAGAAATTGACAATTATACAGACATAATATAC---
AACTTACTTGAAAAATCGCAAAACCAGCAAGAA--

AAAAATGAACAAGAATTATTGGAATTGGATAAATGGGCAAGTTTGTGGAATTGGTTTGACATAACACGATGGC
TGTGGTATATAAAAATATTCATAATGATAGTAGGAGGGTTAATAGGTTTAAGAATAGTTTTTGCT---
GTACTTTCTATAGTG---------AATAGTGTTAGGCAGGGGTACTCACCA------
TTATCGTTTGCTATTAGCTTGCTCAACGCCACAGCCATAGCAGTAGCTGAG---
GGGACAGATAGGATTATAGAAGTATTACAAAGAGCTGGTAGAGCT----------------------------
--------ATTCTCCATATA------CCTACAAGAATAAGACAGGGCTTGGAAAGGGCTTTGCTA---TAA
>JX500708
GCT---ACT---
CAAAATTTATGGGTCACAGTCTATTATGGGGTACCTGTGTGG---AAAGAAGCAACT---
ACCACTCTATTTTGTGCATCAGATGCTAAAGCATATAGTAAGGAA---GCACATAAT----------------
--------------GTTTGGGCTACA---TATGCCTGTGTACCCACGGAC---
CCCAGCCCACAAGAAATACCATTGAAG---------AATGTGACAGAAAATTTTAAC----------------
-------------------------ATGGGGAAAAATAACATGGTAGACCAGATGCATGAGGATATA---
ATCAGTTTATGGGATCAAAGCCTA---AAGCCATGTGTAAGGTTA---
ACCCCACTCTGTGTTATTTTAAATTGCACTAATGTTGCTAGTACCAATAATAGTAGC----------------
----
---ATAGAAGGGTCAGAAATGAAA---
AACTGCTCTTTCAACATCACCACAAGCATGAAA--------------------------
AAGGGAGTGAAAAAAGAATATGCACTC---TTTTATAATCTGGATGTAGTACAAATAGATAATAAT-------
----

GATACTAGTAGCTATAGGTTGATAAGTTGTAATACCTCAGTCGTTACACAA---
GCCTGTCCAAAGCTATCCTTTGAGCCAATTCCCATACATTATTGTGCCCCGGCTGGTTTTGCGATTATAAAGT
GCAAC---AATAAAACATTCAAT------GGAACAGGGCCATGTACAGAT----------------------
-GTCAGT---ACAGTACAATGTACACATGGAATTAAGCCAGTAGTATCAACC---
CAACTACTGTTAAATGGTAGTCTAGCAGAAGAAGAG-----------
ATAGTAATTAGATCTGTCAATCTCACAGACAAT---------ACTAAAACAATAATAGTACAGCTGAAA----
-----------GAACCTGTAGAA---------ATTAATTGTACAAGACCCAACAACAATACAAGAAAA-----
-GGTATTCATATG------------GGACCAGGGAAAGCA---TTT------TATGCAACAGGAGAC------
---------ATAATAGGAGATATA------------AGACAAGCACATTGT---AATGTTAGTAAAATA---
AAATGGAATAACACATTAATACAGGTAGTGGGCAAATTAAGA---------------GAACAATTTAAG----
-----------------------------------AATAAAACACTAAACTTTACT------
AAATCCTCAGGAGGGGACCCAGAAATTGTA---ATGCACAGTTTTAATTGTGGAGGGGAA---
TTTAAGCAGCTCCAGGCGAGAATCCTTGCTGTGGAGAGATACCTAAAGGATCAACAGCTCCTGGGGATTTGGG
GTTGCTCTGGAAAACTCATTTGCACCACTGCTGTGCCTTGGAACACTAGTTGGAGT-----------------
----------------------------AATAAATCTCTAGATGACATTTGG------AAT------
AACACGACCTGGATGGAGTGG---GAAAGAGAAATTGATAATTACACAGAGGTAATATAC---
AGTTTAATAGAACAAACGCAAAACCAACAAGAA--

AAGAATGAACAAGAATTATTGGCATTAGATAAATGGGCAAGTTTGTGGAGTTGGTTTGACATAACAAACTGGC
TGTGGTATATAAAGATATTCATAATGATAGTAGGAGGCTTGATAGGTTTAAGAATAATTTTTGCT---
GTACTTTCTGTAGTG---------AATAGAGTTAGGCAGGGATATTCACCA------
TTATCATTTGCTGTCAGCTTGTTCAACGCCACAGCTATAGCAGTAGGTGAG---
GGAACAGATAGGGTTATAGAAGTAGTACAAAGAACTTTTAGAGCT----------------------------
--------GTACTCCACATA------CCTAGAAGAATAAGACAGGGCTTCGAAAGGGCTTTGCTA---TAA
>MF373130
GCT---GCA---
GGACAATTGTGGGTCACAGTCTATTATGGGGTACCTGTGTGG---AAAGAAGCGACC---
ACCACTCTATTTTGTGCATCAGATGCTAAAGCATATGATACAGAG---GTACATAAT----------------

--------------GTTTGGGCCACA---CATGCCTGTGTACCCACAGAC---
CCCAACCCACAAGAAATGGTATTGGAA----------AATGTGACAGAAAATTTTAAC-----------------
---------------------------ATGTGGAAAAATAACATGGTAGAACAAATGCATGAGGATATA---
ATCAGTTTATGGATCAAAGCCTA---AAGCCATGTGTAAAATTA---
ACCCCTCTCTGTGTTACTTTAAATTGCACTGATTTGGAAAATGAAACTAATAGTACTTTAAAAAATGAAACTA
ATAGTAATAAT--
----------------------GCGACTATTAGTGGGAAAAGATGGAGAGAGGAGAAATAAAA---
AACTGCTCTTTCAATATC---ACAAGCCTAGGA----------------------------
AATAAGATGCAGAAAGAATATGCAACT---TTTTATAAGCTTGATATAGGACCAATAGATAAT----------
----

GATAATACTAGCTATAGGTTGATAAGTTGTAACACCTCAGTCATTACACAG---
GCCTGTCCAAAGATATCCTTTGAGCCAATTCCCATACATTATTGTGCCCCGGCTGGTTTTGCGATTCTAAAGT
GTAGA---GATACAAAGTTCAAT------GGAACAGGACCATGTAAAAAT------------------------
-GTCAGC---ACAGTACAATGTACACATGGAATTAAGCCAGTGGTATCAACT---
CAACTGCTGTTGAATGGCAGTCTAGCAGAAGAAGAT------------
ATAGTCATTAGGTCTGAAAATTTCACGGACAAT---------GCTAAAACTATAATAGTACAGCTGAAC----
-----------AAGTCTGTAGAA---------ATTCATTGTATAAGACCCAACAACAATACAAGAAAA-----
-GGTATACATATG------------GGACTAGGAGAGCA---TTT------TATACAACAGGAGAA------
---------ATAATAGGAGATATA------------AGGCAAGCACATTGT---AACCTTAGTAGCACA---
AATTGGACTAACACTTTAAGACAGATAGCTGTCAAATTAAGA---------------GAACAATTTAAG----
------------------------------------AATAAAACAATAAACTTTACT------
CGACACTCAGGAGGGGACCTAGAAGTTGTA---ATGCATAGTTTTAATTGTGGAGGGGAA---
TTTAAGCAGCTCCAGGCAAGAGTCCTGGCTGTGGAAAGATACCTAAAGGATCAACAGCTCCTGGGGATTTGGG
GTTGCTCTGGAAAACTCATTTGCACCACTACTGTGCCTTGGAATAAGACTTGGAGT-----------------
----------------------------AATAAATCTCTGGATGACATTTGG------AAA------
AACATGACCTGGATGCAGTGG---GAAAGAGAAATTGATAATTACACAGACTTAATATAC---
ACCTTACTTGCAGACTCACAGCGCCAACAAGAA---

AAGAATGAACAAGAATTATTGGAATTAGATAAATGGGCAAGTTTGTGGAATTGGTTTGACATAACAAACTGGC
TGTGGTATATAAAAATATTCATAATGATAATAGGAGGCTTGGTAGGTTTAAGAATAGTTTTTACT---
GTACTTTCTATAGTG---------AATAGAGTTAGGCAGGGATACTCACCA------
TTATCGTTTGCTGTTAGCTTGTTTAATGCCATAGCCATAGTAGTAGCTGAG---
GGAACAGATAGGGTTATAGAAGTATTACAAAGAGCTTTTAGGGCT-----------------------------
--------ATCCGCCATATA------CCTACAAGAATAAGACAGGGCGCGGAAAGGGCTTTGCTA---TAA
>MF373142
GCT---GCA---
GAACAATTATGGGTCACAATCTATTATGGGGTACCTGTGTGG---CAAGAAGCAAAC---
ACCACTCTATTTTGTGCATCAGATGCTAAAGCATATGATACAGAG---GTGCATAAT----------------
--------------GTTTGGGCCACA---CACGCCTGTGTACCCACAGAC---
CCCAACCCACAAGAGATAGTGTTAGGA----------AATGTGACCGAAAATTTTAAT-----------------
---------------------------ATGTGGAAAAATAACATGGTAGAACAAATGCAGGAGGATATA---
ATCAGTTTATGGATCAGATGTTA---AAGCCATGTGTAAAATTA---
ACCCCACTCTGTGTTACTTTACATTGCACTGATTATGTGGGAAATACTACTAATACCACTAGTACAACT----
----
----------------------------GATGCCACTAGTATGGGAATGGATAGAGAAATAAAA---
AACTGCTCTTTCAATATCACCACAAGCATGAGA----------------------------
GATAAGATGCAGAAGGAATATGCACTC---TTGTATAAATATGATATAGAACCATTAGATAGA----------
----

AGTACTACTAGTTATAGGTTGAAAAGTTGTAACACCTCAGTCATTACACAG---
GCCTGTCCAAAGATATCCTTTGAGCCAATTCCCATACATTATTGTGCCCCGGCTGGTTTTGCGATTCTAAAAT
GTAAT---AATGAGACGTTCAAT------GGAACAGGACCATGTACAAAT------------------------
-GTCAGC---ACAGTACAATGTACACATGGAATTAGGCCGGTAGTGTCAACT---
CAACTGCTGTTAAATGGCAGTCTAGCAAAAGAAGAG------------
GTAGTAATTAGATCTGAAAATTTTACAGACAAT---------GCTAAAATCATAATAGTACAGCTGAAG----
-----------GAAGCTGTAGAA---------ATTAATTGTACAAGACCCAACAATAATACAGCAAAA-----
-AGTATACCTATG------------GGACCAGGAAGAGCA---TTT------TATACAACAGGAAGA------
---------ATAATAGGAGATATA------------AGACAAGCACATTGT---AACATTAGTGGAAAA---
AAATGGAATAACACTTTAAAGCAGATAGTTACAAAATTAAGA---------------GAACAATTTGGA----

-----------------------------------AATAAGACAATAGTCTTTAAT------
CAATCCTCAGGAGGGGACCCAGAAATTGTA---ATGCACAGTTTTAATTGTGGAGGGGAA---
TTTAAGCAGCTCCAGGCTAGAGTCCTGGCTGTGGAAAGATACCTAAAGGATCAACAGCTCCTAGGAATTTGGG
GTTGCTCTGGAAAACTCATTTGCACCACTGCTGTGCCTTGGAATACTAGTTGGAGC-----------------
----------------------------AATAAATCGCTGGACAGAATTTGG------GAT------
AACATGACCTGGATGGAGTGG---GAAAAAGAAATTGACAATTATACACAAATAATATAC---
ACATTGATTGAAGAATCGCAAACTCAACAAGAA--

AAGAATGAACAAGAGTTATTGGAATTGGGAAAGTGGGACAGTTTGTGGAGTTGGTTTGACATAACAAATTGGC
TATGGTATATAAAAATATTCATAATGATAGTAGGAGGCTTAGTAGGTTTAAGAATAATTTTTACT---
GTACTGTCAATAGTG---------AATAGAGTTAGGCAGGGATACTCACCA------
TTATCATTTGCTGTTAGCTTGTTTAATGCAACAGCTGTAGCAGTAGCTGAG---
GGAACAGATAGGGTTATAGAAATAGTACAAAGAGGTTTTAGAGCT----------------------------
--------TTCCTTCACATA------CCTAGAAGAATAAGACAGGGCTTTGAAAGAGCTTTGCTA---TAG
>MF373144
ACT---GCA--
CCAAGCTTGTGGGTCACAGTCTATTATGGAGTGCCTGTGTGG---AAAGAAGCAACT---
ACCACCCTATTTTGTGCATCAGATGCTAAGGCATATGAGAAAGAG---GCACATAAT----------------
--------------ATTTGGGCCACA---CATGCCTGTGTACCCACAGAC---
CCCAATCCACAAGAAGTAGTATTGGAA---------AATGTGACAGAGGATTTTAAC----------------
--------------------------ATGTGGAAAAATAACATGGTAGAACAGATGCATGAGGATATA---
ATCAGTTTATGGGATCAAAGCTTA---AAGCCATGTGTAAAATTA---
ACCCCACTCTGTGTCACTTTAAATTGCACTGATCCTGTTTTGAGAAATATTACTAGGAATAATGATACTACA-
----
-------------GCCACTAATGAGACAATAAAGAATGAGACAATAAAGAATGGAGAAATGAGA---
AACTGCTCGTTCAATGTCAGCACAGACGTGAGA---------------------------
GATAAGATGCAGACACAATATGCACTT---CTTTATAAACTTGATTTAGTGAAAACAGAGAAT----------
----

GATAATACCAGTTATAGGCTGATAAGTTGTAACACCTCAGTCCTTACACAG---
GCTTGTCCAAAAGTATCCTTTGAGCCAATTCCCATACATTATTGTACCCCGGCTGGTTTTGCGATTCTAAAGT
GTAAA---GATAAGAATTTCAAT------GGAACAGGAAAATGTAACAAT-----------------------
-GTCAGC---ACAGTACAATGTACACATGGAATTAGGCCAGTAGTATCAACT---
CAGCTGTTGTTAAATGGCAGCCTAGCAGAAGAAGAG------------
GTAGTAATTAGATCTGAGAATTTCACAAACAAT---------GTTAAAACCATAATAGTACAGCTCAAT----
-----------GAATCTGTAGCA---------ATTAATTGTACAAGACCCAATAACAATACAAGAAAA-----
-AGTATACATATA------------GGACCAGGGAGAGCA---TTT------TATGCAACAGGAGAC------
---------ATAATAGGAGATATA------------AGACAAGCACATTGT---AACATTAGTCAAGCA---
GAATGGAATAAAACTTTAGAAAAGATAGTTACAAAATTGAGA---------------GAACAATTTAAG----
-----------------------------------AACAAGACAATAGTCTTTTCT------
CCCCCCTCAGGAGGGGATCCAGAAATTGTA---TCACACAGTTTTAATTGTGGAGGAGAG---
TTTAAGCAGCTCCAGGCAAGAGTCCTGGCTGTGGAAAGATACCTAAAGGATCAGCAGCTCCTAGGGATTTGGG
GCTGTTCTGGAAAACTCATTTGCACCACCACTGTGCCTTGGAATGCTAGTTGGAGT-----------------
----------------------------AATAAAACTCTAGAGCAGATTTGG------GAT------
AACATGACCTGGATGCAGTGG---GACAAAGAAATTCACAATTACACAGAATTAATATAC---
ACCTTAATTGAAGAATCGCAGATCCAGCAGGAA--

AAGAACGAACAAGAATTATTAGAGTTAGATAAATGGTCAAATTTGTGGAATTGGTTTGACATATCAAACTGGC
TGTGGTATATAAAAATATTCATAATGATAGTAGGAGGCTTGGTAGGTTTAAGAATAGTTTTTGTT---
GTGCTTTCTATAATA---------AATAGAGTTAGGAAGGGATACTCACCA------
TTATCGTTCGCTATTAGCTTGCTTAATGCTACAGCCATAACAGTAGCTGAG---
GGAACAGATAGGGTTATAGAAGCAGTACAAACAGCTGGTAGAGCT----------------------------
--------ATCCTCCACATA------CCTAGAAGAATAAGACAGGGCTTGGAAAGGCTTTTGCTA---TAA
>MF373161
GCT---GAA--
GACCAACTGTGGGTCACAGTCTATTATGGGGTACCTGTGTGG---AAAGAAGCAACC---
ACCACTCTATTTTGTGCATCAGATGCTAAAGCATACGATACAGAG---GCACATAAT----------------
--------------GTTTGGGCCACA---CATGCCTGTGTACCCACAGAC---
CCCAACCCACAAGAAGTAGTATTGGAA---------AATGTGACAGAAAAGTTTAAC----------------
--------------------------ATGTGGAAAAATAGCATGGTAGAACAGATGCATGAGGATATA---

ATCAGTTTATGGGATCAGAGTTTA---AAGCCATGTGTAAAATTA---
ACCCCACTCTGTGTCACTTTACATTGCACGAACTTAACTAATAGTACTACCACTAGTATT-------------
----
-------------------------------------AGCCCGACAATGAGTCCAGAAATAAAA---
AATTGCTCTTTCAATACCACCACAAGTATGAGA---------------------------
GATAAGATAGAGAAAAAATATGCATTA---TTTTATGACCTTGATGTAGTACCAATAGATAATAATAATACT-
----

AATACCAGCTATACCAGCTATAGGTTGATAAGTTGTAACACCTCAGTCATTACACAG---
GCCTGTCCAAAGGTATCCTTTGAGCCAATTCCCATACATTTTTGTACCCCTGCTGGTTTTGCGCTTCTAAAAT
GTAAT---GATAAGAAGTTCAAT------GGAACAGGACCATGTAAAAAT-----------------------
-GTCAGC---ACCGTACAATGTACACATGGAATTAGGCCAGTAGTATCAACT---
CAACTGCTGTTAAATGGCAGTCTAGCAGAAGAAGAG------------
GCGGTACTTAGATCTGAGATATCTCCAGACAAT---------GCTAAAACCATAATAGTACAGCTGAAC----
-----------GAAACTGTACAA---------ATTAATTGTACAAGACCCAATAACAATACAAGGAAA-----
-AGTATACATATA------------GGACCAGGGAGAGCA---TTT------TATGCAACAGGAGAC------
---------ATAATAGGAGATATA------------AGACAGGCATATTGT---AACGTTAGTGGAAA---
GCCTGGAATAAAACTTTAGGAAAAGTAGTTATAAAATTAAGA---------------GAACAATTTAAT----
-----------------------------------AATAAAACAATAGTCTTTAAG------
CAATCCTCAGGAGGGGACCCAGAAATTGTA---ATGCACAATTTTAATTGTGGAGGGGAA---
TTCAAGCAGCTCCAGGCAAGAGTCCTGGCTGGGGAAAAATACCTAAAGGATCAACAGCTCCTAGGAATTTGGG
GTTGCTCTGGAAAACTCATCTGCACCACAGCTGTGCCTTGGAATACTAGTTGGAGT-----------------
----------------------------AATAGATCTCTGGATGACATTTGG------AAT------
AATATGACCTGGATGCAGTGG---GAAAAAGAAATTGATAATTACACAAGTTTAATATAC---
ACCTTAATTGAAGAATCGCAGAACCAACAAGAA--

AAGAATGAACTAGACTTATTGGAATTAGATAAGTGGGCAAGTTTGTGGAATTGGTTCGATATATCGAATTGGC
TGTGGTATATAAAAATATTCATAATGATAGTAGGAGGCTTGATAGGTTTAAGGATAATTTTGCCT---
GTACTTTCTATAGTG---------AATAGAGTTAGGCAGGGATATTCACCA------
TTATCATTTGCTATTAGCTTATTCAACGCCACCGCCATAGCAGTAGCTGAG---
GGAACAGATAGGGTCATAGAACTATTACAAAGAACTGGTAGAGCT----------------------------
--------ATCCTCCACATA------CCTGTAAGAATAAGACAAGGATTGGAAAGGCTTTTGCTA---TAA
>KP411828
GCT---AAA---GAA---------------------------------
GAGAGAAAGTCATGGGTCACAGTCTATTATGGGGTACCTGTATGG---AAAGAAGCAACC---
ACCACTCTATTTTGTGCATCAGATGCTAGAGTATATAGTAAAGAA---GCACATAAT----------------
--------------GTTTGGCCACA---TATGCCTGTGTACCCACAGAC---
CCCAACCCACAAGAATTAGTATTGGAA---------AATGTGACAGAAAATTTTAAC----------------
--------------------------ATGGGAAAAATGACATGATAGAACAGATGCATGAGGATATA---
ATCAGTTTATGGGATCAAAGCCTA---AAGCCATGTGTAAAGTTA---
ACCCCACTCTGTGTTACTTTAAATTGCACTGATTGGGAAAAAAATAATACCAGCACTAATAACAATACT----
----
-------------AGTAGCGCTAGTAACAACAGCTGGAGTATAATGGAAAAAGGGGAGATAAAA---
AACTGCTCTTTCAACACCACCACAGACGTGAAA---------------------------
GATAAAATGCAAAAAGAATATGCACTC---TTTTATAAACTAGATGTTCAACAAATGGGTAAT----------
----

AGTAATAATAGTACTAGCTATAGGTTGACAAGTTGTAATACCTCAGTCATTACACAG---
GCCTGTCCAAAGATATCCTTTGAGCCAATTCCCATACATTATTGTGCCCCGGCTGGTTTTGCGATTCTAAAGT
GTAAC---GATAAAAAGTTCAAT------GGGACAGGGCCATGTAAAAAT-----------------------
-GTCAGT---ACTGTACAATGTACACATGGAATTAAGCCAGTAGTATCAACT---
CAACTGCTGTTAAATGGCAGTCTAGCAGAAGAAGAG------------
GTAGTAATTAGATCTGTCAATCTCACGGACAAT---------GCTAAAACCATAATAGTACAGCTAAAA----
-----------GAACCTGTAAAC---------ATTAGTTGTATAAGACCCAATAACAATACAAGAAAA-----
-GGTATTCATATA------------GGACCAGGGAGAGCA---TTT------TATGCAACAGGAGAC------
---------ATAATAGGAGATATA------------AGAAAAGCACATTGT---AACATTAGTTTTAAC---
CAATGGAATAACACATTAAAACAGATAGTTGACAAATTAAGA---------------GAACAATTTGGG----
-----------------------------------AACAAAACAATAGTCTTTAAT------
AAATCCTCAGGAGGGGACCCAGAAATTGTA---ATGCACAGTTTTAATTGTGGAGGGGAA---
TTTAAGCAGCTCCAGGCGAGAATCCTGGCTGTGGAAAGATACCTCAAGGATCAACAGCTCCTGGGAATTTGGG

GTTGCTCTGGAAAACTCATTTGTACCACTGCTGTGCCTTGGAATGATAGTTGGAGT-----------------
----------------------------AATAAATCTGTAGATCACATTTGG------GAG------
AACATGACCTGGATGGAGTGG---GAAAGAGAAATTGATAATTACACAGGAACAATATAC---
TTCTTACTTCATGAATCGCAACTCCAACAAGAA--

AAGAATGAACAAGAATTATTGGAATTGGATAAATGGGCAAATTTGTGGAATTGGTTTGACATATCAAATTGGC
TGTGGTATATAAAAATATTCATAATGATAGTAGGAGGCTTAATAGGTCTAAGAATAATTTTTGCT---
GTGCTTTCCATAGTG---------AATAGAGTTAGGCAGGGATACTCACCA------
TTGTCATTTGCTGTTAGCTTACTTAATGCCACAGCTATAGCAGTAGCTAAG---
GGAACAGATAGGGTTATAGAAGTAATACAAAGAACTTGTAGAGCT----------------------------
--------ATTCGCCACATA------CCTAGAAGAATAAGACAGGGCTTGGAAAGGGCTTTGCTA---TAA
>MF373204
GCT---GCA--
AACAATTTGTGGGTCACAGTTTATTATGGGGTACCTGTATGG---AGGGAAGCAAAC---
GTCACTTTATTTTGTGCATCAGATGCTAAAGCATATGATACAGAG---GTACATAAT----------------
--------------GTTTGGGCCACT---CACGCCTGTGTACCCACAGAC---
CCCAACCCACAAGAGATAGCACTGGAA---------AATGTAACAGAAAACTTTAAT----------------
--------------------------ATGTGGAAAAATCCCATGGTAGAACAGATGCATGAAGATGTA---
ATCAATTTATGGGATCAAAGCCTC---ACACCATGTGTAAAATTA---
ACCCCACTCTGTGTCACTCTAAATTGCACTGATGTAAAAGAGGGTAGTAATAGCACTGAG-------------
----
-------------------------------AACAGCACTATGACGGGAGAAGCAGTAATGACA---
AACTGCTCTTTCAATGTCACTGCAGGGATAAAG---------------------------
GATAAGATGCAGAAGGAATATGCCTTT---TTCTATAAACTTGATGGAGGACCAATAAGTACT----------
----

GATAATACCAGCTATAGGTTGAAAAGTTGTAACCCCTCAGTCATTAACCAA---
GGCTGTCCAAAGAAAACCTTTGAGCCAATTCCCATACATTATTGTGCCCCAGCTGGGTTTGCGATTCTAAAGT
GTAGA---GATAAGACATTCAAT------GGAACAGGACCATGTAAAAAT-----------------------
-GTCAGC---ACAGTACAATGTACACATGGAATTAAGCCAGTAGTGTCAACT---
CAACTGCTGTTAAATGGCAGTCTAGCAGAAGAGGAG------------
GTAGTAATTAGATATCAAAATATTAGTGACAAT---------GCTAAAACCATAATAGTACAGCTAAAA----
-----------GAACCTGTACAA---------ATTAATTGTACAAGACCAGGCAACAATACAAGAAAA-----
-AGCATACATTTT------------GGACCAGGGAAAACA---TTT------TATGCAACAGGAGAC------
---------ATAATAGGAGATATA------------AGACAAGCACATTGT---AACATTAGTAAAGAA---
ACTTGGAATAACACTTTAAAACAGATAGTTGAAAAATTAAGA---------------GAGCGTTTT-------
-----------------------------------AACAAAACAATAGTCTTTAAT------
CAATCCACAGGAGGGGACCCAGAAATTGTA---ATGCACAGTTTTAATTGTAGAGGGGAA---
TTTAAGCAGCTCCAGGCAAGAGTCCTGGCTGTGGAAAGATACCTACAGGACCAACAGCTCCTAGGAATTTGGG
GTTGCTCTGGAAGACACATCTGCACCACTGCTGTGCCTTGGAATACTAGTTGGAGC-----------------
----------------------------AATAAATCTCTGGATATGATTTGG------GGA------
AACATGACCTGGATGGAGTGG---GAAAAAGAGATTGGTAATTACACAGGCTTAATATAC---
TCCTTAATTGAAGAATCGCAAACCCAACAAGAA--

AAGAATGAAAAGGATTTATTGGAATTGGATAAGTGGGACAGTTTGTGGAATTGGTTTAGTATAACAAAATGGC
TGTGGTATATAAAAATATTTATAATGATAATAGCAGGCTTGGTAGGTTTAAAAATAGTTTTTGTT---
GTATTTTCTATAGTG---------AATAGATGTAGGAAGGGATACTCACCA------
TTATCGTTTGCTGTTAGTTTGTTCAACGCCATAGCTATAGCAGTTGCTGAA---
GGGACAGATAGGATTATAGAAGCAATACAAAGAACAGGTAGAGCT----------------------------
--------ATTCTTCATATA------CCTACAAGAATAAGACAGGGCTTAGAAAGGGCTTTGCAA---TAA
>GU204920
GCT---ACA--
GAAAAATTGTGGGTCACAGTCTATTATGGGGTACCTGTGTGG---AAAGAAGCAACC---
ACCACTCTATTTTGTGCATCAGATGCTAAAGCATATGATACAGAG---GTGCATAAT----------------
--------------GTTTGGGCCACA---CACGCCTGTGTACCCACAGAC---
CCCAACCCACAAGAAGTAGTATTGGAA---------AATGTGACAGAAAATTTTAAC----------------
--------------------------ATGTGGAAAAATAACATGGTAGACCAGATGCATGAGGATATA---
ATCAGCTTATGGGATCAAAGCCTA---AAGCCATGTGTAAAATTA---
ACCCCACTCTGTGTTACTTTAAAGTGCGAGAATGCTACTAGTATCACTGGTAGTAGCGGGAACGTTACTAATA
GCACTACT---

----AGTAACGGGAACACTGCTAGTAGCAATAATAGTAGCGGGAACATGACGGAAAAAATGAAA---
AAATGCTCTTTCAATATCACCACAGCCATAAGA---------------------------
GATAAGGTGCAGCAACAATATGCACTT---TTTTATAACCTTGATATAGTACCAATAAATAAT----------
----

ACAGATAATACCAGCTATAGGTTGATAAGTTGTAACACCTCAGTCATTACCCAG---
GCCTGTCCAAAGGTATCCTTTGAGCCAATTCCCATACATTATTGTACCCCGGCTGGTTTTGCGATTCTACAGT
GTAAT---GATAAGAGGTTCAAT------GGAAAAGGACCATGTACAAAT-----------------------
-GTCAGC---ACAGTACAGTGTACACATGGAATTAGGCCAGTAGTATCAACT---
CAACTGTTGTTAAATGGCAGCCTAGCAGAAGAAGAG------------
GTAGTAATTAGATCTGACAATTTCAGTGACAAT---------GCTAAAACCATAATAGTGCAGCTGAAC----
-----------AAATCTGTAGAA---------ATTCATTGTGCAAGACCCAACAACAATACAAGAAAA-----
-AGTATACATATA------------GCACCAGGGAGGGCA---TTT------CATACAACAGGAAGC------
---------ATAATAGGAGATATA------------AGAAAAGCATACTGT---AACATTAGTAGAGCA---
AACTGGACTAACACTCTAGAACAGATAGCTAGCAAATTAGGA---------------AAAGAACTTAAC----
-----------------------------------AAAACAATAATAGCTTTTAAG------
CCATCTTCAGGAGGGGACCCAGAAATTGTA---ATGCACAGTTTTAATTGTAGAGGGGAA---
TTTAAGCAGCTCCAGGCAAGAGTCCTGGCTGTGGAAAGATACCTAAAGGATCAACAGCTCCTGGGGATTTGGG
GTTGCTCTGAAAAACTCATCTGCACCACTGCTGTGCCTTGGAATACTAGTTGGAGT-----------------
----------------------------AATAAATCTCTGGATAAGATTTGG------AAT------
AACATGACCTGGATGGAGTGG---GAAAGAGAAATTGACAATTACACAAGCTTAATATAC---
ACCTTGATTGAAGAATCGCAGAACCAGCAGGAA--

AAGAATGAGCAAGAATTATTGGCATTGGATAACTGGGAGAGCTTGTGGAGTTGGTTTAGCATAACAAATTGGC
TGTGGTATATAAAAATATTCATAATGATAGTAGGAGGCTTGGTAGGTTTAAGAATAGTTTTTGCT---
GTAATTTCTATAGTG---------AATAGAGTTAGGCAGGGATACTCACCA------
TTATCGTTGGCTATCAGCTTGCTTAATGCCACAGCAATAACAGTAGGTGAG---
GGGACAGATAGGGTTATAAAAGCAGTACAAAGAGCTTGTAAAGCT----------------------------
--------ATTCGCCACATA------CCTAGAAGAATAAGACAGGGCCTGGAAAGGGCTTTGCTA---TAA
>JN251856
GCT---GCA--
GACAAATTGTGGGTAACAATCTATTATGGGGTACCTGTGTGG---AAAGAAGCAACT---
ACCACTCTATTTTGTGCATCAGATGCTAAAGCATATGATACAGAG---GTACATAAT----------------
--------------GTCTGGGCTACA---CATGCCTGTGTACCCACAGAC---
CCCAACCCACAAGAAGTAGAATTGAAA---------AATGTGACAGAAAATTTTAAC----------------
--------------------------ATGTGGAAAAATAACATGGTAGAACAAATGCATGAGGATATA---
ATCAGTTTATGGGATCAAAGCTTA---AAGCCATGTGTAAAATTA---
ACCCCACTCTGTGTTACTTTAAATTGCACTGGTTTGAGAAATGCTACTAATGCCACTAATAGTAGCGGGGGAA
CTAATGCCACTAAT---
-AGTGGCGGGGGAACTAATGCCACTAATAGTAGCGAGGGAACGATGGAGGGAGGAGAAATGAAA---
AACTGCTCTTTCAATATCACCACAAATATAAGA---------------------------
GATAAGACGAAGAACGAATATGCACTT---TTTTATAAACTTGATGTAATACCAATAGATGGT----------
----

GATAATACTAGCTATAGGTTGATAAATTGTAACACCTCAGTCATTACACAG---
GCCTGTCCAAAGGTATCCTTTGAACCAATTCCCATACATTATTGTACCCCGGCTGGTTTTGCGATTCTAAAGT
GTAAT---GATACAAAGTTCAAT------GGATCAGGACCATGTAAAAAT-----------------------
-GTCAGC---ACAGTACAATGTACACATGGAATTAGGCCAACAGTGTCAACT---
CAACTGTTATTAAATGGCAGTCTAGCAGAAGAAGGG------------
GTAGTACTTAGGTCTGAAAATTTCACGAATAAT---------GCTAAAATCATAATAGTGCAGCTGAAG----
-----------GAACCTGTAAAC---------ATTACTTGTACAAGACCCAACAACAATACAAGAAAA-----
-GGTATACATATA------------GGACCAGGGAGAACA---CTT------TATGCAACAGGAGGA------
---------ATAATAGGAAATATA------------AGACAGGCACATTGT---AACATTAATGGAACA---
GAATGGAATAATACTTTACAACAGATAGTTATAAAATTAAGA---------------GAACAATTTGAG----
-----------------------------------AATAAAACAATAGCCTTTAAT------
CGATCCTCAGGAGGGGACCCAGAAGTTGTA---ATGCACATGTTTAATTGTGGGGGGGAA---
TTTAAACAGCTCCAGGCAAGAGTCCTGGCTGTGGAAAGATACCTAAAGGATCAACAGCTCCTGGGAATTTGGG
GTTGCTCTGGAAAACTCATTTGCACCACTGCTGTGCCTTGGAATACTAGTTGGAGT-----------------
----------------------------AATAAATCTTTGAATATGATTTGG------GAC------
AACATGACCTGGATGGAGTGG---GAAAGAGAAATTAGCAATTACACAAGCTTAATATAC---

ACTTTAATTGAAAAATCGCAGAACCAACAAGAA---------------------------------------
----
AAGAATGAACAAGAATTATTGGGATTAGATAAATGGGCAAGTTTGTGGAATTGGTTTGACATAACAAAATGGC
TGTGGTATATAAAAATATTCATAATGATAGTAGGAGGCTTGGTAGGTTTAAGAATAGTTTTTGCT---
GTACTTTCTATAGTG---------AATAGAGTTAGGCAGGGATACTCACCA------
TTATCATTTGCTGTTAGCTTGCTCAATACCATAGCCATAGCAGTAGCTGAG---
GGGACAGATAGGATTATAGAAGTATTACAAAGAGCTTGGAAAGCT----------------------------
--------ATTATCCACATA------CCTAGAAGAATAAGACAGGGCTTGGAAAGGATTTTGCAA---TAA
>KY213741
GCT---GCA---
GACAATTTGTGGGTCACAGTCTATTATGGGGTACCTGTGTGG---AGAGATGCAAAC---
ACCACTCTATTTTGTGCATCAGATGCTAAAGCATATGAAAGAGAA---GTACATAAT----------------
--------------GTTTGGGCCACA---CATGCCTGTGTACCCACAGAC---
CCCAACCCACAAGAATTGGTATTGGAA---------AATGTGACAGAAGAGTTTAAT----------------
-------------------------ATGTGGAAAAATAACATGGTAGAACAGATGCATGAGGACATA---
ATCAGTTTATGGGATCAAAGCCTA---AAACCATGTGTAAAATTA---
ACCCCACTCTGTGTCACTTTAAATTGCACTGACGCTACCAGTGGTAATAATACCAGTGGT-------------
----
-------------------------------------AATATAACGGGGGAGGGAGGAATGAAA---
AACTGCTCTTTTAATGTCACCACAGACATAAAA--------------------------GATAAGAGA---
ATAGAAAAAGCATTG---TTTTATAAAGATGATTTAGTACAAACAGGGAAG---------------------
----
----------AATAATACTATTTATAGGTTGATGAGCTGTAACACCTCAGTCATTACCCAA---
GCCTGTCCAAAGGTATCCTTTGAGCCAATTCCCATACATTATTGTACCCCGGCTGGTTATGCGATTCTTAAGT
GTAAT---AATAAGACATTTAAT------GGATTAGGGCGATGTACAAAT-----------------------
-GTCAGC---ACAGTACTATGTACACATGGAATTAGGCCAGTAGTATCAACT---
CAACTGATGTTAAATGGCAGTTTAGCAGAAGAAGAG-----------
GTAGTAATTAGATCTGAAAATTTCACAAACAAT---------GCTAAAACCATAATAGTACAGCTGAAA----
-----------GAAGCTGTAAAA---------ATTAATTGCACAAGACCCAACAACAATACAAGAAAA-----
-AGTATACATATG------------GGGCAAGGGAAAGCA---TGG------TATACAACAGGAGAG------
---------ATAATAGGAGACATA------------AGACAAGCATATTGT---ACAATTAACAAAACA---
AAATGGAATGACACTTTAAAACAGATAGCTGAAAAATTAAGA---------------AGACAATTTGGG----
----------------------------------AATAAAACAATAGTCTTTAAT------
CAGCCCTCAGGAGGGGACCCAGAGATTGTA---ATGCACAGTTTTAATTGTGGAGGGGAA---
TTTAAGCAGCTCCAGGCAAGAGTCCTGGCTGTGGAAAGATACCTAAGGGATCAACAGCTCCTAGGAATGTGGG
GTTGCTCTGGAAAACTCATTTGCCCCACCTCTGTGCCTTGGAATGCTAGTTGGAGC-----------------
----------------------------AATAAATCTCTGGAAGATATTTGG------AAT------
AACATGACCTGGAGGCAATGG---GAGACAGAAATTGACAATTACACAGGCTTAATATAC---
ACCTTACTTACAGAATCGCAGAACCAACAGGAA---------------------------------------
----
AAGAATGAACAAGAATTATTAGAATTGGATAAATGGGCAAATTTATGGAATTGGTTTGACATATCAAACTGGC
TGTGGTATATAAGAATATTCATAATGATAGTAGGAGGCTTGATAAGTTTAAGAATAGTTTTTACT---
GTACTTTCTATAATA---------AATAGAGTTAGGCAGGGATACTCACCA------
TTGTCGTTGGCTATTAGCTTGCTCAACACCACAGCCATAGCAGTAGCTGAG---
GGGACAGATAGGCTTATAGAATTAGTGCAATTAACTTTTAGAGCT----------------------------
--------ATTCTTCACATA------CCTAGAAGAATAAGACAGGGCTTAGAAAGGACTTTGCTA---TAA
>KY213740
TCT---GCA---
GAAAAATTGTGGGTCACAGTCTATTATGGGGTACCTGTGTGG---AAAGAAACAGTC---
ACCACTCTATTTTGTGCATCAGATGCTAAAGCATATGATACAGAG---GTACATAAT----------------
--------------GTCTGGGCCACA---CATGCCTGTGTACCCACAGAC---
CCCAACCCACAAGAAGTACCATTGATA---------AATGTGACAGAAAATTTTAAC----------------
-------------------------ATGTGGAAAAATAACATGGCAGAACAGATGCATGAGGATATA---
ATCAGCTTATGGGATGAAAGCTTA---AAGCCATGTGTAGAATTA---
ACCCCACTCTGTGTTACTTTAAATTGCAGTGATGTAAATAGAACCAGGGGGAATGATACC-------------
----
----------------------AAGGGGAATGGTACCCGTATAATGGAGAAAGGAGACGTAAAA---
AACTGCTCTTTCGATATCTACACAAACATGAGA--------------------------
GATAAGAAGCAAAAACAAAATGCACTT---TTTTATAATCTTGATATAGTACCAATAAATGAG----------
----

GGTGGGAATAATACTAATACTAGCTATAGGTTGATAAGTTGTAACGCCTCAGTCACTAAACAA---
GCCTGTCCAAAAATATCATTTGAGCCAATTCCCATACATTATTGTGCCCCGGCTGGTTTTGCGATTCTATTAT
GTAAG---GATAAAAGGTTCAAT------GGAACAGGAATATGTAAAAAT-----------------------
-GTCAGC---ACAGTACAATGCACACATGGAATTAGGCCAGTAGTGTCTACA---
CAACTGCTGTTAAATGGCAGTCTAGCAGAAGAAGAG-----------
ATAATAATCAAATCTGAAAATATCAGTAACAAT---------GCTAAAAACATAATAGTACAGCTGAAG----
-----------ACTCCTGTGAAA---------ATTAATTGTACAAGACCCAGCAACAATACAAGAAAA-----
-AGTATACATATA------------GGGCCAGGGAGTGCA---TTT------TATGCAACAGGAGAC------
---------ATAATAGGAGATATA------------AGACAAGCACATTGT---AACCTTAGTAGAGCA---
GCATGGAATAACACTTTAAAACAGATAGTTGAAAAATTTACA---------------AAAATTGAAAAT----
-----------------------------------AAAACAACAATAATCTTTAAC------
AGCTCCTCAGGAGGGGACCAAGAAATTGTA---ATGCACAGTTTTAATTGTGGAGGGGAA---
TTCAAGCAGCTCCAGGCAAGAGTCCTGGCTGTGGAAAGATACCTAAGGGATCAACAGCTCCTGGGAATTTGGG
GTTGCTCTGGAAAACTCAGGTGCTACACTGATGTGCCTTGGAATGCTAGTTGGAGT----------------
----------------------------AATAAATCTCTCACTGAGATTTGG------GAT------
AACATGACTTGGAGGGAGTGG---GAAAGCGAAATTGATAATTACACAAACATAATATAT---
TCCTTGCTTGAAGCATCGCAAAACCAACAAGAC---------------------------------------
----
AATAATGAACAGGAATTATTAGAATTGGATAGTTGGGCAAATTTGTGGAATTGGTTTAACATAACAAATTGGC
TGTGGTATGTAAAAATATTCATAATGGTAATAGGAGGCTTAATAGGGTTAAGAATAGTTTTTGTT---
GTGCTTTCTATAGTG---------AATAGAGTTAGGCAGGGTTACTCACCA------
CTATCGTTCGCTGTCAGCTTGCTTAACTACACAGCTATAGCAGTAGCTGAG---
GGAACAGATAGGGTTATAGAAGCAGCAAGAAGAGCTTTTAGAGCT---------------------------
--------ATCCTCCACATA------CCTAGAAGAATAAGGCAGGGCGCGGAAAGGGCTCTGGGA---TAA
>KY213747
GCT---ACA--
GAAAACTTGTGGGTCACAGTCTATTATGGGGTACCTGTATGG---AAAGAAGCAACC---
ACCACTTTATTTTGTGCATCAGATGCCAGAGCATATGAGACAGAG---GTACATAAT----------------
--------------GTTTGGGCCACA---CATGCCTGTGTACCCACAGAC---
CCCAACCCACAAGAAGTAGTCTTGGAA---------AATGTGACAGAAAACTTTAAC---------------
--------------------------ATGTGGAAAAATGACATGGTAGAACAGATGCAAGAAGATATA---
ATCAGTTTATGGGATCAAAGCCTA---AAGCCATGTGTAAGATTA---
ACCCCACTCTGTGTTGCTTTGAATTGCACTGATTTGGGCAGTAATACTACTAATTTGGGC------------
----
----------------------AATATTTCTGCCGGTAGTATAATGGAAGGAGGAGAAATAAAG---
AACTGCTCTTTCAATATCACAACAAGCATAAAA--------------------------ACTAGGGTG---
AAAGAACATGCAATT---TTTAATAGCCTTGATGTAGTACCAATAGATAAT---------------------
----
----------------ACCAGCTATAGGATGGTGCATTGTAATACCACAGTCATTACACAG---
GTTTGTCCAAAGGTATCTTTTGAGCCAATTCCCATACATTACTGTACCCCGGCTGGTTTTGCGATCCTAAAGT
GTAAC---AATAAAACATTCGAT------GGAACAGGACCATGTACAAAT-----------------------
-GTCAGT---ACAGTACAATGTACACATGGAATTAGGCCAGTAGTATCAACT---
CAACTACTGCTAAATGGCAGTTTGGCAGAAGAAGAG-----------
GTAATAATTAGATCTAGCAACTTCACAAACAAT---------GCTAAAGTCATAATAGTACAGCTGAAT----
-----------GAATCTGTAGAA---------ATTAATTGTACAAGACCCAACAACAATACAAGAAAG-----
-AGTATACATCTA------------GGACCAGGGAAAGCA---TGG------TATGCAACA---GAC------
---------ATAATAGGAGATATA------------AGACAAGCACATTGT---AACCTTAGTTACACA---
AAATGGCAGGACACTTTAAAAAAGATAGTTGAAAAATTAAGA---------------AAACAATTTGGG----
-----------------------------------AACAAAACAATAGTATTTAAT------
CAATCCTCAGGAGGGGACCCAGAGATTGTA---ACGCACAGTTTTAATTGTGGGGGGGAA---
TTTAAGCAGCTCCAGGCAAGAGTCCTGGCTGTGGAAAGATACCTGAAGGATCAACAACTCCTAGGGATTTGGG
GCTGCTCTGGAAAACTCATTTGCACCACTGCTGTGCCTTGGAATACTAGTTGGAGT----------------
----------------------------AATAAATCTTACAATGACATTTGG------GAG------
AACATGACTTGGATGGAATGG---GAAAGAGAAATTGCCAATTACACAGGAGAAATATAC---
ACCTTAATTGAAAAATCGCAGAACCAGCAAGAA---------------------------------------
----
AAGAATGAACTAGAATTATTGGAATTGGACAAGTGGGCAAGTTTGTGGAATTGGTTTGACATAACAAAATGGC
TGTGGTATATAAAAATATTCATAATGATAGTAGGAGGCTTGGTAGGTTTAAGAATAGCTTTTGCT---
GTACTTTCTATAGTA---------AATAGGGTTAGGCAGGGATACTCACCA------

TTGTCATTAGCTGTTAGCTTGCTGAATGCCACAGCTATAGCAGTAGCTGAA---
GGGACAGATAGGGTTATAGAAGTAGCACAAAGAGCTGGGAGAGCC---------------------------
--------ATTCTCAATATA------CCTAGAAGAATCAGACAAGGTTTAGAAAGGGCTTTGCTA---TAA
>KY213751
GCT---ACA--
AACTTGTGGGTCACAGTCTATTATGGGGTACCTGTATGG---AAAGACGCAACC---
ACCACTCTATTTTGTGCATCAGATGCTAGGGCATATGATACAGAG---GTACATAAT----------------
--------------ATTTGGGCCACA---CATGCCTGTGTACCCACAGAC---
CCCAACCCACAAGAAGTAGTCTTGGAA---------AATGTGACAGAAAACTTTAAC----------------
-------------------------ATGTGGAAAAATGACATGGTAGAACAGATGCAAGAAGATATA---
ATCAGTTTATGGGATCAAAGCCTA---AAGCCATGTGTAAAATTA---
ACCCCACTCTGTGTTGCTATGAATTGCACTAATGTGAGCAGTAATACTACTAATACTACC-------------
----
------------------------------AGTGGTATAATGGAAGGAGTAGGAGAAATAAAA---
AACTGCTCTTTCAATATCACAACAAGCATAGAA--------------------------GGT---
ATGAAGAAAAAACATGCAATT---TTTAATAGCCTTGATGTAGTACCAATAGATAAT----------------
----
---------------------ACCAGCTATATACTGACGCATTGTAACACCACAGTCATTACACAA---
GCTTGTCCAAAGGTATCCTTTGAGCCAATTCCCATACATTACTGTACCCCGGCTGGTTTTGCGCTTCTAAAGT
GTAAC---AATAAAACGTACAAT------GGAACAGGACCATGTACAAAT----------------------
-GTCAGT---ACAGTACAATGTACACATGGAATTAGGCCAGTAGTATCAACT---
CAACTGCTGTTAAATGGCAGTTTAGCAGAAGAAGAA------------
GTAATAATTAGATCTAGCAACATCATGGACAAT---------GCTAAAGTCATAATAGTACAGCTGAAT----
-----------GAATCTGTAGAA---------ATTAATTGTACAAGACCCAACAACAATACAAGAAAG-----
-AGTATACATCTA------------GGACTAGGGAGAAGA---TGG------TATGCAACA---GAA------
---------ATAATAGGAGATATA-----------AGACAAGCACATTGT---AACCTTAGTTCCACA---
AAATGGCAGAAAACTTTAAAACAGATAGCTCAAAAGTTAAGA--------------GAACAATTTGGG----
-----------------------------------AACAAAACAATAGTCTTTAAT------
CAATCCTCAGGAGGGGACCCAGAGATTGTA---ATGCACAGTTTTAATTGTGGGGGGGAA---
TTTAAGCAGCTCCAGGCAAGAGTCCTGGCTGTGGAAAGATACCTGAAGGATCAACAGCTCCTAGGAATTTGGG
GCTGCTCTGGAAAACTCATTTGCACCACTGCTGTGCCTTGGAATGCTAGTTGGAGT----------------
----------------------------AATAAATCTTACACTGACATTTGG------GAT------
AATATGACTTGGATGGAGTGG---GAAAGAGAAATTGCCAATTACACAGGAGAAATATAC---
AACTTAATTGAAAAATCGCAGAACCAACAAGAA--

AAGAATGAACTAGAATTATTGGAATTGGACCAGTGGGCAAGTTTGTGGAATTGGTTTGACATAACAAAATGGC
TGTGGTATATAAAAATATTCATAATGATAGTAGGAGGCTTGGTAGGTTTAAGAATAGTTTTTGCT---
GTGCTTTCTATAGTA---------AATAGGGTTAGGCAGGGATACTCACCA------
TTGTCATTGGCTGTTAGCTTGCTGAATGCCACAGCTATAGCAGTAGCTGAA---
GGGACAGATAGGTTTATAGAAGTAGTACAAGGAGTTTATAGAGCT--------------------------
--------ATTATCCACATA------CCTACAAGAATAAGACAGGGCTTAGAAAGGGCTTTGCTA---TAA
>KY213748
GCT---ACA--
GAAAACTTGTGGGTCACAGTCTATTATGGGGTACCTGTATGG---AAAGAAGCAACC---
ACCACCTTATTTTGTGCATCAGATGCTAAAGCATATGATACAGAG---GTACATAAT----------------
--------------GTTTGGGCCACA---CATGCCTGTGTACCCACAGAC---
CCCAACCCACAAGAAGTAGTCTTGGGA---------AATGTGACAGAAAACTTTAAC----------------
-------------------------ATGTGGAAAAATGACATGGTAGAACAGATGCAAGAAGATGTA---
ATCAGTTTATGGGATCAAAGCCTA---AAGCCATGTGTAAGATTA---
ACCCCACTCTGTGTTGCTTTGAATTGCACTGATTTGAATACTACTAATTGGACTAATGCCAGT----------
----
-------------------------------------AGTATAATGGAGAAAGGAGAACTAAAA---
AACTGCTCTTTCAATATCACAACGAGCATAAAA--------------------------GATAGGGTG---
AGAAGCCATGCAATT---TTTAATAGCCTTGATGTAGTACCAATAAATAAT---------------------
----
----------------GCCAGCTATAGGTTGATGCATTGTAACACCACAGTCATTACACAA---
GCTTGTCCAAAGGTATCCTTTGAGCCAATTCCCATACATTACTGTACCCCGGCTGGTTTTGCGATTCTAAAGT
GTAAC---AATAAAATGTTCAAT------GGAACAGGACCATGTACAAAT----------------------
-GTCAGT---ACAGTACAATGTACACATGGAATTAGGCCAGTAGTATCAACT---
CAACTGCTGCTAAATGGCAGTTTAGCAGAAGAAGAG-----------

GTAATAATTAGATCTAGCAACTTCACGAACAAT---------GCTAAAGTCATAATAGTACAGCTGAAT----
-----------GAATCTATAGAA---------ATTAACTGTACGAGACCCAACAACAATACAAGAAAG-----
-AGTATACATCTA------------GGACCAGGGAAAGCA---TGG------TATGCAACA---GAC------
---------ATAATAGGAGATATA------------AGACAAGCACATTGT---AACCTTAGTTATTCA---
AAATGGCAGAACACTTTAAATCACATAACTAAAAAATTAAGA---------------GAACAATTTGGG----
-----------------------------------AACAAAACAATAATCTTTAAT------
CAATCCTCAGGAGGGGACCCAGAGATTGTA---ATGCACAATTTTAATTGTGGGGGGAA---
TTTAAGCAGCTCCAGGCAAGAGTCCTGGCTGTGGAAAGATACCTGAAGGATCAACAGCTCCTAGGGATTTGGG
GCTGCTCTGGAAAACTCATTTGCACCACTGCTGTGCCTTGGAATGCTAGTTGGAGT-----------------
----------------------------AATAAATCTTACACTGACATTTGG------GAT------
AACATGACTTGGATGGAGTGG---GAAAGAGAAATTGCCAATTATACAGGAGAAATATAC---
ACCTTAATTGAAAAATCACAGAACCAACAAGAA--

AAGAATGAATTAGAATTATTGGAATTGGACCAATGGGCAAGTTTGTGGAATTGGTTTAGCATAACAAACTGGC
TGTGGTATATAAAAATATTCATAATGATAGTAGGAGGCTTGGTAGGTTTAAGAATAGTTTTTGCT---
TTACTTTCTATAGTA---------AATAGAGTTAGGCAGGGATACTCACCA------
TTGTCATTAGCTATTAGCTTGCTGAATGCCACAGCTATAGCAGTAGCTGAA---
GGGACAGATAGGGTTATAGAACTAGTACAAAGAGCTTATAGAGCT----------------------------
--------ATTATCCACATA------CCTACAAGAATAAGACAGGGCTTAGAAAGGACTTTGCTA---TAA
>AY945710
GCT---GCA--
GAAAACTTGTGGGTCACAGTCTATTATGGGGTACCTGTATGG---AAAGAAGCAATC---
ACCACTCTATTTTGTGCATCAGATGCTAAAGCATATGATACAGAG---GCACATAAT----------------
--------------GTTTGGGCCACA---CATGCCTGTGTACCCACAGAC---
CCCAACCCACAAGAAGTAGCATTGATA---------AATGTGACAGAAAACTTTAAC----------------
--------------------------ATGTGGAAAAATGACATGGTAGAACAGATGCATGAAGATATA---
ATCGGCTTATGGATCAAAGCCTA---AAGCCATGTGTAAAATTA---
ACCCCACTCTGTGTTACTTTACATTGCACTAATTTGAATGCTACCAATAGTAGTACG----------------
----
----------------------------ACCAATAGTAGTATGATAGGGGGAGGAGAAATGAAG---
AACTGCTCTTTCAATGTCACCACAAACATAGGA---------------------------GCTAAGGTA---
ACAGAATATGCACTT---TTTTATAAAACTGATATAGTACAAATAGCTAAT----------------------
----
----------GATAATACCAGCTATAGGCTGACAAAGTGTAACACCTCAGTCATTACACAA---
GCTTGTCCAAAGATATCCTTTGAGCCAATTCCTATACGTTATTGTGCCCCGGCTGGTTTTGCGATTCTAAAGT
GTAAC---AATGCAAGGTTCAAT------GGAACAGGACCATGTACAAAT-----------------------
-GTCAGT---ACAGTACAATGTACACATGGAATTAGGCCAGTAGTATCAACT---
CAACTGCTGTTAAATGGCAGTTTAGCAGAAGAAGAG------------
GTAATGATTAGATCTAGCAATTTCTCGGACAAT---------ACTAAAGTCATAATAGTACAGCTGAAT----
-----------GAATCTGTACAA---------ATTAATTGTACAAGACCTAACAACAATACAATAAAA-----
-GGTATACATATA------------GGACCAGGGCAAGCA---TGG------TATACAACAGGACAA------
---------ATAATAGGAGATATA------------AGAAAAGCACATTGT---AACCTTAGTAGCACA---
AATTGGAATAACACTTTAAAACAGATAGTTAAAAAATTAAGA---------------GAACAATTTAGA----
-----------------------------------AATAAAACAATAGTCTTTAAG------
CAATCCTCAGGAGGGGACCCAGAGATTACA---ATGCTCAGTTTTAATTGTGGAGGGGAA---
TTTAAGCAGCTCCAGGCAAGAGTCCTGGCTGTGGAAAGATACCTAAAGGATCAACAGCTCCTAGGGATTTGGG
GTTGCTCTGGAAAACTCATTTGCACCACTGCTGTGCCTTGGAATAATAGTTGGAGT-----------------
-------------------------AATAATAAATCTTATGAGCAGATTTGG------AAT------
AATATGACTTGGATGCAGTGG---GAAACAGAAATTGACAATTACACAAAAGAAATATAC---
ACCTTAATTGAACAATCGCAGAACCAACAAGAA--

AAGAATGAACAAGAATTATTGGAATTGGATAACTGGGCAAGTTTGTGGAGTTGGTTTGACATAACAAAGTGGC
TGTGGTATATAAGGATATTCATAATAATAGTAGGAGGCTTGGTAGGTTTAAGAATAGTTTTTGCT---
GTACTTGCTGTAGTA---------AATAGAGTTAGGCAGGGGTACTCACCA------
TTGTCATTAGCTGTTAGTTTGCTCAATGCCACAGCTGTAGCAGTAGCTGAA---
AGGACAGATAGGATTATAGAAGTAGGACTAAGAGCTTATAGAGCT----------------------------
--------ATTCTCCACATA------CCTACAAGAATAAGACAGGGCTTAGAAAGGCTTTTGGTA---TAA
>KJ952535
GCT---AAA--
GAAAAATTGTGGGTCACAGTCTATTATGGGGTACCTGTATGG---AAAGAAGCAACT---

ACCACTCTATTTTGTGCATCAGATGCTAAAGCATATGATACAGAG---GTACATAAT----------------
--------------GTTTGGGCCACA---CATGCCTGTGTACCCACAGAC---
CCCAACCCACAAGAAGTAGTCTTGGGA---------AATGTGACAGAAAACTTTAAC----------------
--------------------------ATGTGGAAAAATAACATGGTAGAACAGATGCATGAAGATATA---
ATCAGTTTATGGGATCAAAGCCTA---AAGCCATGTGTAAAATTA---
ACCCCACTCTGTGTTACTTTAAATTGCACTGAATTGTATGTTACTAATACCAATAGTACGGTTAATAATACC-
----
-------------AATAGTACGGTTACTAATACCAATATTACAGTGGAGGAAGGAGTAATGAAA---
AACTGCTCTTTCAATATCACCACAAGCATAGGA--------------------------AATCAGGTG---
AAAGACTATGCATTT---TTTTATAACCTTGATATAAAACAAATAAAGGAGAAT------------------
----
----------GATAATACCAGTTATAGGGTAATAAATTGTAACACCTCAGTCATTACACAA---
GCTTGTCCAAAGGTATCCTTTGAGCCAATTCCCATACATTATTGTGCCCCGGCTGGTTTTGCGATTCTAAAGT
GTAAC---AATAAAAAGTTCAAT------GGAACAGGACCATGTACAAAT----------------------
-GTCAGT---ACAGTACAATGTACACATGGAATTAGACCAGTAGTATCAACT---
CAACTGCTGTTAAATGGCAGTTTGGCAGAAGAAGAG------------
GTAGTAATTAGATCTAGCAATTTCACAGATAAT---------GCTAAAATCATAATAGTACAGCTGAAT----
-----------GAATCTGTAGAA---------ATTAATTGTACAAGACCCAACAACAATACAAGAAAA-----
-AGTATACCTCTA------------GGACCAGGGAGAGCA---TGG------TATACAACAGGACAA------
---------ATAATAGGAGATATA------------AGACACGCACATTGT---AACCTTAGTAGCACA---
AAATGGAATAACACTTTAAGACTGATAACTAACAAATTAAAA---------------GAACAATTTGGG----
-----------------------------------AACAAAACAATAGTCTTTGAT------
CAATCCTCAGGTGGGGACCCAGAGATTGTA---ATGCACACTGTTAATTGTGGAGGGGAA---
TTTAAGCAGCTCCAGGCAAGAGTCCTGGCTGTGGAAAGATACCTAAAGGATCAACAGCTCCTAGGGATTTGGG
GCTGCTCTGGAAAGCTCATTTGCACCACTGATGTGCCTTGGAATACTAGTTGGAGT-----------------
-------------------------AATAATAGATCCCTGGATGACATTTGG------AAT------
AATATGACTTGGATGGAGTGG---GAAAGAGAAATTGACAATTACACAGAAGTAATATAC---
AACCTAATTGAAAAAATCGCAGAACCAACAAGAA---------------------------------------
----
AAGAATGAACTAGAATTATTGGAATTGGATAAGTGGGCAAGTTTGTGGAATTGGTTTAACATATCAAACTGGC
TGTGGTATATAAAAATATTTATAATGATAGTAGGAGGCTTGATAGGTTTAAGAATAGTTTTTGCT---
GTACTTTCTATAGTA---------AATAGAGTTAGGCAGGGATACTCACCA------
TTGTCATTAGCTGTTAGCTTGCTCAATACCACAGCTGTAGTAGTAGCTGAG---
GGGACAGATAGGGTCATAGAAGTAGTACAAAGAGCTTTTAGAGCT---------------------------
--------TTTATCCACATA------CCTACAAGAATAAGACAGGGCTTAGAAAGGGCTTTGCTA---TAA
>JN248321
GCT---ACA---
AACAACTTGTGGGTCACAGTCTATTATGGGGTACCTGTGTGG---AAAGAAGCGACC---
ACCACTCTATTTTGTGCATCAGACGCTAAAGCCTATATGACAGAG---GTACATAAT----------------
--------------GTCTGGGCCACA---CATGCCTGTGTACCCACAGAC---
CCCAACCCACAGGAAATATTCTTGACA---------AATGTGACAGAAAATTTTAAC----------------
--------------------------ATGTGGAAAAATGACATGGTAGAACAGATGCAGGAGGATATA---
ATCAGTTTATGGGATCAAAGCCTA---AAGCCATGTGTCAAATTA---
ACCCCACTCTGTGTTACTTTAAATTGCACTGATGTTACTAACAATAATAGTAGCACGGGAAGGAAT-------
----
----------GGTACTAATCCCACCAGTAGTAGCATGGGAAAGATGGATAAAGGAGAAATAAAA---
AACTGCTCTTTCAATGTCACCACAGGCATAAGA--------------------------
GATAAGATACAGAGAGAATATGCACTT---TTTTATAAACTTGATGTAGTACCAATAGATAAG----------
----

GATAAGGATAACACAAGCTATATATTGATGAATTGTAACACCTCAGTCATTACACAG---
GCCTGTCCAAAGGTATCCTTTGAACCAATTCCCATACATTATTGTGCCCCGGCTGGTTTTGCGATTGTAAAGT
GTAAT---GATAAGAAGTTCAAT------GGAACAGGAAAATGTAAAAAT----------------------
-GTCAGC---ACAGTACAATGTACACATGGAATTAGACCAGTAGTGTCAACT---
CAACTGCTATTAAATGGCAGTCTATCAGAAGAAGAG------------
GTAGTAATTAGATCTGAAAATTTCACGGACAAT---------GCTAAAATTATAATAGTACAGCTGAAT----
-----------GAATCTATAGTA---------ATTAATTGTACAAGGCCCAACAACAATACAAGAAAA-----
-AGTATACATATA------------GCACCGGGGAGGGCA---TTT------TATACAACAGGAGAA------
---------ATAATAGGAAATATA------------AGACAAGCACATTGT---AACATTAGTAGAACA---
AAATGGAATGACACTTTAAGACAGATAGTTACAAAATTAAGA---------------GAACAATTTGGG----

-----------------------------------AATAAAACAATAACCTTTAAT------
CACTCCTCAGGAGGAGACCCAGAAATTGTA---ATGCACAGTTTTAATTGTGGAGGGGAA---
TTTAAGCAGCTCCAGGCAAGAGTCCTGGCTGTGGAAAGATACCTAAAGGATCAACAGCTCCTGGGAATTTGGG
GTTGCTCTGGAAAACTCATCTGCACCACCTCTGTGCCTTGGAATAGCAGTTGGAGT-----------------
---------------------------AATAAATCCCTAAATGAGATTTGG------GAT------
AACATGACCTGGATGCAGTGG---GAAAGAGAAATTGACAATTACACAGGCTTAATATAC---
ACCTTACTTGAACAATCGCAGAACCAACAGGAA--

AAGAATGAACAAGAATTATTGGGATTGGATAAGTGGGCAAATTTGTGGAGTTGGTTTGACATAACAAACTGGC
TGTGGTATATAAGAATATTCATAATGATAGTAGGAGGCTTGATAGGTTTAAGAATAGTTTTTACT---
GTGCTATCTATAGTG---------AATAGAGTTAGGCAGGGATATTCACCA------
TTATCGTTTGCAATTAGCTTGCTTAACGCCGCAACCATAGCAGTAGCTGAG---
GGGACAGATAGGATTATAGAAGTAGTACAAAGAGCTGGGAGAGCT----------------------------
--------ATTCTCCACATA------CCTAGAAGAATAAGACAAGGTTTGGAAAGAGCTTTGCAA---TAA
>JN248329
GCT---GCA---
AAAAACTTGTGGGTCACAGTTTATTACGGGGTACCTGTATGG---AAAGATGCAACC---
ACAACTCTATTTTGTGCATCAGATGCTAAAGCATATGATACAGAG---GTACATAAT----------------
--------------GTTTGGGCCACA---CATGCCTGTGTACCCACAGAC---
CCCAACCCACAAGAAGTAGTATTGGAA---------AATGTGACAGAAAACTTTAAC----------------
--------------------------ATGTGGAAAAATAACATGGTAGAACAGATGCAGGAAGATATA---
ATCAGTTTATGGGATCAAAGCCTA---AAGCCATGTGTAAGATTA---
ACCCCACTCTGTGTTACTTTAAATTGCAAAAATGCTACTATTAATATCAAT---------------------
----
---ATGACGACAGAAGAAATGAAA---
AACTGCTCTTTCAATGTCACCACAAGCATACAG---------------------------ACTAAGGTG---
AAGGACTATGCACTT---TTTTATAAAGTTGATGTAGTAGAATTAAATAAA---------------------
----
-AGTAATAATAATGATACCGAATATAGGATGATACATTGTAACACCTCAGTCATTACACAA---
GCTTGTCCAAAGGTATCCTTTGAGCCAATTCCCATACATTATTGTACCCCGGCTGGTTTTGCGATTATAAAGT
GTAAC---AATAAAAAGTTCAAT------GGAACAGGACCATGTACAAAT-----------------------
-GTCAGT---ACAGTACAATGTACACATGGAATTAGGCCAGTAGTATCAACT---
CAACTGCTGTTAAATGGCAGTTTAGCAGAAGAAGAG-----------
GTAGTAATTAGATCTAGCAACTTCACGGACAAT---------GCTAGAGTCATAATAGTGCAGCTAAAT----
-----------AAATCTGTAGAA---------ATTAACTGTACAAGACCCAACAACTATACAAGAAAA-----
-AGAATAAGTATA------------GGACCAGGGCGAGCA---TGG------ATTACAACAGGACAA------
---------ATAATAGGAGATATA------------AGACAAGCACATTGT---AACCTTAGTAGAACA---
AACTGGACTAACATGTTAAAACAGGTAGCTGCAAAATTAAGG---------------GAACAATTTGGG----
---------------------------------AACAACAAAACAATAGTCTTTAAT------
AAATCCTCAGGAGGGGACCCAGAGATTACG---AGGCACAGTTTTAATTGTGGAGGAGAA---
TTTAAGCAGCTCCAGGCAAGAGTCCTGGCTATAGAAAGATACCTAAAGGATCAACAGCTCCTAGGGATTTGGG
GTTGCTCTGGAAAACTCATTTGCACCACTAATGTGCCTTGGAATACTAGTTGGAGT-----------------
---------------------------AATAAATCTCTGGGTGACATTTGG------GAT------
AATATGACTTGGATGGAGTGG---GACAGAGAAATTCACAATTACACAAAAGAAATATAT---
GCCTTAATTGAACAATCGCAGAACCAACAAGAA--

AAGAATGAACTAGAATTATTGAAATTGGATAAGTGGGCAAGCTTGTGGAGTTGGTTTGACATATCAAACTGGC
TGTGGTATATAAAAATATTCATAATGATAGTAGGAGGCTTGATAGGTTTAAGAATAGTTTTTGCT---
GTACTTTCTATAGTA---------AATAGAGTTAGGCAGGGATACTCACCA------
GTGTCTTTAGCTGTTAGCTTGCTCAATGCCACAGCCATAGCAGTAGCTGAG---
GGGACAGATAGGGTTATAGAAGTAGCACAAAGAGCTTGTAGAGCT----------------------------
--------GTTCTCCACATA------CCTAGAAGAATAAGACAGGGCTTCGAAAGGGCTTTGGTA---TAA
>JN248333
GCT---ACA---
GAAAAATTGTGGGTCACAGTCTATTATGGGGTACCTGTGTGG---AAAGAAGCAACC---
ACCACGCTATTCTGTGCATCAGATGCTAAAGCATATGATACAGAG---GTACATAAT----------------
--------------GTTTGGGCCACA---CATGCCTGTGTACCCACAGAC---
CCTGACCCACAAGAAGTAGCATTGGAA---------AATGTGACAGAAAGTTTTAAC----------------
--------------------------ATGTGGCAAAATAACATGGTAGAGCAGATGCATGAGGATATA---
ATCAGTTTATGGGATCAAAGCCTA---AAACCATGTGTAAAACTA---

ACCCCACTCTGTGTTACTCTAAATTGTACTGATTTAACTTTTAATAATACCGAAGCTACTACCAATAGT----
----
----------------------AGCGGTAATAGTAGTAACTTTGAGGGAGCGAAAGACATGAGA---
AACTGCTCTTTCAATGTAACCACAAGCATAAGA---------------------------
GATAGGATAAAGAAAGAATATGCATTC---TTTTATAAACTTGATACAGTTGCAATAGATGGT----------
----

GTTAATAACAGCTATAGGTTGATAAATTGTAACACCACAACCATCACACAG---
ACCTGTCCAAAGGTATCCTTTGAGCCAATTCCCATACATTATTGTACTCCAGCTGGTTTTGCACTTCTAAAAT
GTAAG---GATAAGAAGTTCAGT------GGAACAGGACCATGTAAAAAT-----------------------
-GTCAGC---ACAGTACAATGTACACATGGAATTAGGCCAGTAGTGTCAACT---
CAACTGCTATTAAATGGCAGTCTAGCAGAAGAAGAG------------
GTAGTAATTAGATCTGTCAATTTCTCAAACAAT---------GCTAAAAATATAATAGTTCAGCTGAAT----
-----------GAGCCTGTAGAA---------ATTAATTGTACAAGACCCAACAATAATACAAGAAAA-----
-GGTATACATTTT------------GCACCAGGGAGCACA---TTT------TATGCAACAGGAGAA------
---------ATAATAGGAGACATA------------AGACAAGCATATTGC---AACGTTAGTGGAGCG---
AAATGGAATCGCACTTTAAAACAAGTAGCTATGAAATTAAGA---------------GAACAATTTGTG----
-----------------------------------AATAAAACAATAATTTTTAAT------
AGCTCCTCAGGAGGGGACCCAGAAATTGTA---CTGCACAGTTTTACTTGTGGAGGGGAA---
TTTAAACAGCTCCAGGCAAGAGTCCTGGCTGTGGAAAGATACCTAAGGGATCAACAGCTCCTAGGGATTTGGG
GTTGCTCTGGAAAACTCATCTGCACCACTGCTGTGCCTTGGAATGCTAGTTGGAGT-----------------
----------------------------AATAAATCTCTAGATAATATTTGG------CAT------
AACATGACCTGGATGGAGTGG---GAAAGAGAAATTGACAATTACACAGGCATAATATAC---
TCCTTAATTGAAGAGTCACAGAACCAACAAGAA--

AAGAATGAACAAGACTTATTAGCATTAGATGAATGGGCAAGTTTGTGGGATTGGTTTAGTATAACAAAATGGC
TGTGGTATATAAGAATATTCATAATGATAGTAGGAGGCTTGGTAGGTTTAAAAATAGTTTTTACT---
GTATTTTCCATAATA---------AATAGAGTTAGGCAGGGATACTCACCA------
TTATCATTTGCTGTTAGCTTGCTCAACGCCACAGCTATAGCAGTAGCTGAG---
GGGACAGATAGGGTCATAGAAGTAGCACAAAGAGCTTGGAGAGCT----------------------------
--------TTTATCCACATA------CCTAGAAGAATTAGACAGGGCTTAGAAAGGTTTTTACTA---TA-
>JN248335
GCT---GCA--
GAAAACTTGTGGGTCACAGTCTATTATGGGGTACCGGTATGG---AAAGATGCATCC---
ACCACTCTATTTTGTGCATCAGATGCTAAAGCATATGATACAGAG---GTACATAAT----------------
--------------GTTTGGGCCACA---CATGCCTGTGTACCCACAGAC---
CCCAACCCACAAGAAGTAGTATTGGGA---------AATGTGACAGAAAACTTTAAC----------------
--------------------------ATGTGGAAAAATGACATGGTAGAACAGATGCAGGAAGATATA---
ATCAATTTATGGGATCAAAGCCTA---AAGCCATGTGTAAAATTA---
ACCCCACTCTGTGTCACTTTAAATTGCAATGATTTGAGAAATACTACTACTAATACCAGT-------------
----
--ATTGAAGAGAAAGTAGAAATAAAA---
AACTGCTCTTTCAATATCACCACAAGCATAAAA---------------------------ACTAAGGTA---
AGAGACTATGCATTT---TTTTATAATTCTGATATAGTACAAATAGATAAAGATAAGAATGAG----------
----
----ACTACCAACTATACCCAATATAGGCTGATAAATTGTAACACCTCAGTCATTACACAA---
GCTTGTCCAAAAGTATCCTTTGAGCCAATTCCCATACATTATTGTACCCCGGCTGGTTTTGCGCTTCTAAAGT
GTAAC---GATGAAAAGTTCAAT------GGAACAGGACCATGTACAAAT-----------------------
-GTCAGT---ACAGTACAATGTACACATGGAATTAGGCCAGTAGTATCAACT---
CAACTGCTGTTAAATGGCAGTTTAGCAGAAGAAGAT------------
GTAGTAATTAGATCCAGAAATTTCACGGACAAT---------ACTAAAATTATAATAATACAGCTGAAT----
-----------GAATCTATAGTA---------ATTAATTGTACAAGACCCAACAACAATACAAGAAAA-----
-AGTATCCCTCTA------------GGACCAGGGAAAGCA---TGG------TTTGCAACAGGAGAA------
---------ATAATAGGAGATATA------------AGACAAGCACATTGT---AACCTTAGTAGCATA---
AAATGGAATGCCACTTTAAAAAGGATAACTGCAAAATTAAGA---------------GAACAATTTGGG----
-----------------------------------AACAAAACAATAATCTTTAAT------
CAATCTGCAGGAGGGGACCCAGAGACTGTA---GTGCACAGTTTTAATTGTGGAGGGGAA---
TTTAAGCAGCTCCAGGCAAGAGTCCTGGCTGTGGAAAGATACCTAAAGGATCAACAGCTCCTAGGGATTTGGG
GTTGCTCTGGAAAACTCATTTGCACCACTACTGTGCCTTGGAATACTAGTTGGAGT-----------------
----------------------------AATAAATCTCTGAGTGAGATTTGG------GAT------

AATATGACTTGGATGCAGTGG---GATAGAGAAATTAACAACTACACAAGAGAAATATAC---
ACATTAATTGAAGAATCGCAAAACCAACAAGAA--

AAAAATGAACTAGATTTATTGAAACTGGATGAGTGGGCAAGCTTGTGGAATTGGTTTGACATATCAAAATGGC
TGTGGTATATAAAAATATTCATAATGATAGTAGGAGGCTTGGTAGGTTTAAGAATAGTTTTTGCT---
GTGCTTTCAATAGTG---------AATAGAGTTAGGCAGGGATACTCACCA------
TTGTCATTTGCTGTAAGCTTGCTCAATGCCACAGCTATAGCAGTAGCTGAG---
GGGATAGATAGGGTTATAGAAGTAGTACAAAGAGCTTATAGAGCT----------------------------
--------GTTATCCACATA------CCTACAAGAATCAGACAGGGCTTAGAAAGGGCTTTACTA---TAA
>JN248337
GCT---GAA---
AATTTGTGGGTCACAGTCTATTATGGGGTACCTGTGTGG---AAGGATGCAAAC---
ACCACTCTATTTTGTGCATCAGATGCTAAAGCATACAAAACAGAG---GTGCATAAT----------------
--------------GTCTGGCCACA---CATGCCTGTGTACCCACAGAC---
CCCAGCCCACAAGAAGTAGTAATGGAA---------AATGTGACAGAAAATTTTAAC----------------
--------------------------ATGTGGAAAAATAACATGGTAGAACAGATGCATGAGGATATA---
ATCAGTTTATGGGATCAAAGCTTA---AAGCCATGTGTTAAATTA---
ACACCACTCTGTGTTACTTTACATTGTAATGATACAGGGAATAAGACTAGGAATGGGACT-------------
----
-------------------------GATGTCAATGATAATTGGGATGCGGGAAGTGACATAAAA---
AACTGCTCTTTCAATGTCACCACAATGTTAGAA---------------------------
GGGAAGGTGAAGTCAACATATGCTTTC---TTCAATAGCCTTGATATAACACAATTGGAAAAT----------
----

GATAATAGTACATATAGATTGACAAAGTGTAATACCACAGTCATTACACAA---
GCCTGTCCTAAGGTAACCTTTGAGCCAATTCCCATACATTATTGTACCCCGGCTGGTTTTGCGATTATAAAGT
GTAAT---GATAAGAAGTTCAAT------GGAACAGGACCATGTAAAAAT-----------------------
-GTCAGC---ACAGTACAATGTACACATGGAATTAGGCCAGTAGTATCAACT---
CAATTGTTGTTAAATGGCAGTCTAGCAGAAGAAGAG------------
GTAATAATTAGATCTGAAAATTTCACAAACAAT---------GCTAAAACCATAATAGTACAGCTGAAC----
-----------CACTCTGTAGCA---------ATTAATTGTACAAGACCCAACAACAATACAAGGAAA-----
-AGTATACATCTT------------GGACCAGGGAGAGCA---TTA------TTTACAACA---GAC------
---------ATAATAGGAGATATA------------AGAAAAGCATATTGT---AACATTAGTAGAGAA---
GAGTGGAATAAAACTTTAACACAGATAGCTAAAAAGTTAAGT---------------GATCAATATGGG----
-----------------------------------AATAGAAACATAACCTTTAAG------
CCATCCTCAGGAGGGGACCCAGAAATTACA---ACTCACAGTTTTAACTGTGGAGGGGAA---
TTTAAACAGCTCCAGGCAAGAGTCCTGGCTGTGGAAAGATACCTAAGGGATCAACAGCTCCTGGGGATTTGGG
GTTGCTCTGGAAAACTCATTTGCACCACTAGTGTGCCTTGGAATAATACTTGGAGT-----------------
-------------------------AAGAATAAAACCCTGAATGACATTTGG------AAT------
AACATGACCTGGATAGAGTGG---GAAAGAGAAATTGACAATTACACAAGCTTAATATAC---
ACCTTAATTGAAGACTCGCAGAACCAGCAAGAA--

AAGAATGAACAGGAATTATTGGAATTGGATAAGTGGGACAGTTTGTGGAGTTGGTTTAGTATAACACAATGGC
TGTGGTATATAAAAATATTCATAATGATAGTAGGAGGCTTGATAGGTTTAAGAATAGTTTTTGCT---
GTAATTTCTATAGTA---------AATAGAGTTAGGCAGGGATACTCACCA------
ATATCATTAGCTGTTAGTTTGTTTAATGCAACAGCAATAGCAGTAGCTGAA---
GGGACAGATAGAATTATAGAAATAGCACAAAGAGCATTTAGAGCT----------------------------
--------TTTATCCACATA------CCTAGAAGAATAAGACAGGGCCTTGAGAGGGCTTTACTAGAATAA
>JN248343
GCT---GAA---
AACTTGTGGGTCACAGTCTATTATGGGGTACCTGTGTGG---AAAGAAGCAACC---
ACCACTCTATTTTGTGCATCAGATGCTAAATCATATGACACAGAG---GTACATAAT----------------
--------------GTTTGGCCACA---CATGCCTGTGTACCCACAGAC---
CCTAACCCACAAGAAGTAGTATTGGAA---------AATGTGACAGAAAATTTTAAC----------------
--------------------------ATGTGGAAAAATAACATGGTAGACCAGATGCATGAGGATATA---
ATCAATTTATGGGATCAAAGCCTA---AAGCCATGTGTAAAACTA---
ACCCCACTCTGTGTCACTTTAAATTGCAGTAATGTGAATTGCAGTAATGTGAATGCTACTACTTGTAGTAAT-
----
-------------------GTGAATGTTACTAATGCTACTGTCGAGGGAATGAAGGACTTAAAA---
AACTGCTCTTTCAATATCACCACAAGAATGAGA---------------------------

GAGAAGGGGCAGAAAGAGTACGCATTT---TTCTATAAATWTGATGTAGTACCAATAGATAAT----------
----

ACTAGTAACTATAGGTTGATAAGTTGTAACACCTCAGTCATTACACAA---
GCCTGTCCAAAAGTATCCTTTGAGCCAATTCCTATACATTATTGTACCCCGGCTGGTTTTGCACTTCTAAAGT
GTAAC---GACAAGAAATTCAAT------GGAACAGGACCATGTAAAAAT-----------------------
-GTCAGC---ACAGTACAATGTACACATGGAATTAGGCCAGTAGTATCAACT---
CAACTGCTGTTAAATGGCAGTCTAGCAGAA---GAG------------
ATAGTAATTAGATCTAAAAATTTCACGGACAAT---------GCTAAAACCATAATAGTACAGCTGAAT----
-----------GAATCTGTAGTA---------ATTAATTGTACAAGACCCAATAACAATACAAGAAGA-----
-AGTATACCTGTA------------GGACCAGGGAGAGTA---TTG------TATACAACAGGA---------
---------ATAATAGGAGACATA------------AGACAAGCACATTGT---AACATTAGTAATGTG---
GGATGGAATAACACCTTAAAACAGATAGTTATAAAATTAAGA---------------GAACAATTTGGA----
-----------------------------------AATAAAACAATAACCTTTAAG------
CCCTCCTCAGGAGGGGACCCAGAAATTGAA---ATGCACAGTTTTAATTGTGGAGGGGAA---
TTTAAGCAGCTCCAGGCAAGAATCCTGGCTGTGGAAAGATACCTACAGGATCAACAGCTCCTGGGGATTTGGG
GTTGCTCTGGAAAACTCATTTGCACCACTGCTGTGCCTTGGAATGCTAGTTGGAGT-----------------
----------------------------AACAAATCTCTGGAAACCATTTGG------AAC------
AACATGACCTGGATGGAGTGG---GATAGAGAAATTAACAATTACACAAGCTTAATATAC---
ACCTTAATTGAARAATCGCAGAACCAGCAAGAA--

AAGAATGAACAAGAATTATTAGCATTAGATAAGTGGGCAAGTCTGTGGAGTTGGTTTGACATAACAAAGTGGC
TGTGGTATATAAAAATATTCATAATGATAGTAGGAGGCTTGATAGGTTTAAGGATAGTTTTTACT---
GTACTTTCTATAATA---------AATAGAGTTAGGCAGGGATACTCACCA------
TTATCGTTTGCAGTTAGCTTGCTCAACGCCACAGCCATAGCAGTAGCTGAG---
GGGACAGATAGGTTTATAGAAATCTTACGAAGAGCTTATAGGGCT----------------------------
--------TTTATCCACATA------CCTAGAAGAATAAGACAGGGCTTAGAAAGGGCTTTGCAA---TAA
>JN248344
GCT---GCT---GTA------------------------------------
GACAACTTGTGGGTCACAGTCTATTATGGGGTACCTGTGTGG---AAAGAAGCGACC---
ACCACCCTATTTTGTGCATCAGAAGCTAAAGCATATGATACAGAG---GTACATAAT----------------
--------------GTTTGGGCCACA---CATGCCTGTGTACCCACAGAC---
CCCAACCCACAGGAGATATTATTGGAA---------AATGTGACAGAAAATTTTAAC----------------
--------------------------ATGTGGAAAAATGACATGGTAGAACAGATGCAAGAGGACATA---
ATCAGTTTATGGGATCAAAGCCTA---AAGCCATGTGTAAAATTA---
ACCCCACTCTGTGTTACTTTAAATTGCACTGATGCTAAAAGCACTGATAGTAGCATGGGAAAGAAT-------
----
----------GGTACTAGTAACACTACTAGTAGCCCGAGAACGATGGAAGAAGGAGAAATGAAA---
AACTGCTCTTTCAATGTCACCACAAACATAGGA---------------------------
GATAAGAGAAAGAAAGAATATGCACTT---TTTTATAGATTAGATGTAATGTCAATAGATAAG----------
----

GATAACCAAAGTTATGCATTAATAAATTGTAACACCTCAGTCATTACACAA---
GCCTGTCCAAAGGTATCCTTTGAACCAATTCCCATACATTATTGTGCCCCGGCTGGTTTTGCGATTCTAAAGT
GTAAT---GATAAGCAGTTCAAT------GGAACAGGACCATGTACAAAT-----------------------
-GTCAGC---ACAGTACAATGTACACATGGAATTAAGCCAGTGGTGTCAACT---
CAACTACTATTAAATGGCAGTCTATCAGAAGGAGAG------------
GTAGTAATTAGATCTGAAAATATCACGAACAAT---------GTTAAAAATATAATAGTACAGCTGAAT----
-----------GAATCTGTAGTA---------ATTAATTGTACAAGGCCCAACAACAATACAAGAAAA-----
-AGTATAAGCTTC------------ACACCTGGGAGTACA---TTT------TATGCAACA---GAT------
---------ATAATAGGAAATATA------------AGACAAGCACATTGT---AATATTAGTGCAGAA---
AAATGGAAGAGCACTTTAAAAAAGATAGCTACAAAATTAAGA---------------
GAACAATTTGAGGATAAG---------------------------------AATAAAACAATATCCTTTGAG-
-----CACTCCACAGGAGGGGACCCAGAAATCGCA---ATGCACAGTTTTAATTGTGGAGGGGAA---
TTTAAGCAGCTCCAGGCAAGAGTCCTGGCTGTGGAAAGATACCTAAAGGATCAACAGCTCCTAGGAATTTGGG
GTTGCTCTGGAAAACTCATCTGCACCACCGCTGTGCCTTGGAATGCCAGTTGGAGT-----------------
----------------------------AATAGATCTCTAAATGATATTTGG------GAG------
AACATGACCTGGATGGAGTGG---GAAAGAGAAATTGACAATTACACAAGCTTAATATAC---
ACCTTACTTGAACAATCACAAAACCAGCAGGAA--

AAGAATGAAAAAGACTTATTGGAATTGGATAAGTGGGAGAGTTTGTGGAGTTGGTTTGACATAACAAATTGGC
TGTGGTATATAAAAATATTCATAATGATAGTAGGAGGCATGATAGGTTTAAGAATAGTTTTTACT---
GTGCTATCTATAGTG---------AATAGAGTTAGGCAGGGATACTCACCA------
TTATCGTTGGCAACTAGCTTGCTTAACGCAACAGCCATAGCAGTAGCTGAG---
GGGACAGATAGGATTATAAAAGTAGTACAAAGGGCTTGGAGAGCT----------------------------
--------ATTCTCAACATA------CCTAGAAGAATAAGACAGGGCTTGGAAAGAGCTTTGCTA---TAA
>JN248347
GGT---GCA--
GAAAACTTGTGGGTCACAGTCTATTATGGGGTACCTGTATGG---AAAGAAGCAACC---
ACCACTCTATTTTGTGCATCAGATGCTAAAGCATATGATACAGAG---GTACATAAT----------------
--------------GTTTGGGCCACA---CATGCCTGTGTACCCACAGAC---
CCCAACCCACAAGAAATAGTATTGGAA---------AATGTGACAGAAAACTTTAAC----------------
--------------------------ATGTGGAAAAATGACATGGTAGAACAGATGCAGGAAGATATA---
ATCAGTTTATGGGATCAAAGCCTA---AAGCCATGTGTAAGATTA---
ACCCCACTTTGTGTTACTTTAAATTGCACTAATTTAAGGAATAATAATAGTACCAAT----------------
----
-------------------------------------ATCACGACAGGGGGAGGAGAAATAAAA---
AACTGCTCTTTCAACATCACCACAGGCATGCAA---------------------------ACTAAGGTG---
AAAGACTATGCATTG---TTTTCTACATTTGATATAGTACCAATAGATAAT----------------------
----
----------------ACCAGCTATAGGTTGATAAGTTGTAACACCTCAGTCCTTACACAA---
GCTTGTCCAAAGGTATCCTTTGAGCCAATTCCCATACATTATTGTGCCCCGGCTGGTTTTGCGATTATAAAGT
GTAAT---GAAAAAAATTTCAAT------GGAAAAGGATCATGTAAAAAT-----------------------
-GTCAGT---ACAGTACAATGCACACATGGAATTAAGCCAGTAGTATCAACC---
CAATTGCTGTTAAATGGCAGTTTAGCAGAAGAAGAG-----------
GTAGTAATTAAATCTAGCAATTTCACGAACAAT---------GCTAGAACCATAATAGTACAGTTGAAT----
-----------GAATCTGTAGAA---------ATTAATTGTACAAGGCCCAACAACAATACAAGAAAA-----
-AGTATACATCTA------------GGACAAGGGCGAGCA---TGG------TACGCAACAGGAGAC------
---------ATAATAGGAGATATC------------AGACAAGCACATTGT---AACCTTAGTAGCACA---
AAATGGAATAACACTTTAAGACAGATAGTTGAGAAATTAAGA---------------AAACAATTTGAG----
-----------------------------------AACAAAACAATAATCTTTAAT------
CACTCCTCAGGAGGGGACCCAGAAATTATA---ATGCACAGTTTTAATTGTGGAGGGGAA---
TTCAAGCAGCTCCAGGCAAGAGTCCTGGCTGTGGAAAGATACCTAAAGGATCAACAGCTCCTAGGAATTTGGG
GATGCTCTGGAAAACTCATTTGCACCACTGCTGTGCCTTGGAATACTAGTTGGAGT-----------------
----------------------------AATAGATCCCATGATGACATTTGG------AAT------
AACATGACCTGGATGCAGTGG---GAAAAAGAAATTGATAATTACACAAGAGAAATATAC---
AAATTAATTGAAGAATCGCAGAACCAACAAGAA--

AAGAATGAACTAGAATTATTGGAGTTGGATAAATGGGCAAGCTTGTGGAACTGGTTTGACATAACACAATGGC
TGTGGTATATAAAAATATTCATAATGATAGTAGGAGGCTTAGTAGGTTTAAGAATAGTTTTTGCT---
GTACTTTCTATAGTG---------AATAGAGTTAGGCAGGGATACTCACCA------
TTGTCATTAGCTGTTAGCTTGCTCAATGTCACAGCTATAGCAGTAGCTGAG---
GGGACAGATAGGGTTATAGAAGTAGTACAAAGAGCTTATAGAGCT----------------------------
--------ATTCTCCACATA------CCTACAAGAATAAGACAGGGCTTAGAAAGGGCTTTGGTA---TAA
>JN248353
GCT---GTA--
GGAAATTTGTGGGTCACAGTCTATTATGGGGTACCTGTGTGG---AAAGATGCAAAT---
ACCACCCTATTTTGTGCATCAGATGCTAGAGCATATTCTACAGAG---GTACATAAT----------------
--------------ATTTGGGCCACA---CATGCCTGTGTACCCACAGAC---
CCCAGCCCACAAGAAGTAGAGTTGAAA---------AATGTGACAGAGAATTTTAAC----------------
--------------------------ATGTGGAAAAATAACATGGTAGAACAGATGCATGAGGATATA---
ATCAGTTTATGGGATCAAAGCCTA---AAGCCATGTGTAAAATTA---
ACTCCACTTTGTGTTACTTTAAATTGCACTGAT--

-------------------ACCAATAGTAGTACTGTGGAAAGGACGGAGGGAGGAGAGGTAAAA---
AACTGTTCTTTCAATATCACCACAGAAATAATA---------------------------
GATAAGAAGAAAAAAGAATATGCACTG---
TTTTATAAACTTGATGTAGTTCCAATAGAAGATAGTGATAATGCC----------------------------
----
AGTAATAGTAATTTTTCCAGCTATAGATTGATAAATTGTAACACCTCAGTCGTTACACAG---

GCCTGTCCAAAGGTATCCTTTGAACCAATTCCCATACATTATTGTGCTCCAGCTGGTTTTGCGATTCTAAAGT
GTAAT---AATAAAACGTTCGAG------GGGAAAGGACCATGTACAAAT-----------------------
-GTCAGC---ACAGTCCAATGTACACATGGAATTAGGCCAGTAGTATCAACT---
CAACTGTTGTTAAATGGCAGTTTAGCAGAAAAAGAG------------
GTAGTAATTAGAGTTGACAATCACACGAACAAT---------GCTAACACCATAATAGTGCAGCTGACA----
-----------GAAGCTGTAGAA---------ATTAATTGTACAAGACCCAACAACAATACAAGAAGA-----
-GGTATACATATA------------GGACCAGGGAGAGGA---ATG------TTCTATGCAACAGAC------
---------ATAATAGGAGATATA------------AGAAAAGCACATTGT---AACATTAGTAGAATA---
AAATGGAATAACACTTTAAAAAAGGTAGCTGAAAAATTAGGG---------------GAAAAATTTAAT----
-----------------------------------CAAACTAAAATAATCTTTAAT------
TCCTCCTCAGGAGGGGACCCAGAAGTTGTA---ATGCACATTTTTAATTGTGGAGGGGAA---
TTCAAGCAGCTCCAGGCACGAGTCCTGGCTGTGGAAAGATACCTAAGGGATCAACAGCTCCTGGGAATTTGGG
GTTGCTCTGGAAAACTCATCTGCACCACTACTGTGCCTTGGAATGATACTTGGAGT-----------------
----------------------------AATAAAACTCTGAAAGATATTTGG------GAT------
AACATGACCTGGATGGAGTGG---GATAAAGAAATTAACAAATACACAAATACAATATAC---
AACTTGCTTGAAGAATCGCAGAACCAACAAGAA--

CAGAATGAACAAGAATTATTGGAATTAGGTAGATGGGCAAACTTGTGGAATTGGTTTGACATATCAAACTGGC
TGTGGTATATAAAAATATTCATAATGATAGTAGGAGGCTTGATAGGTTTAAGAATAGTTTTTACT---
GTGCTATCTATAGTG---------AATAGAGTTAGGCAGGGATACTCACCA------
TTATCGTTTGCTATTAGCCTGTTTAATGCCATAGCCATAGCAGTAGCTGAA---
GGGACAGATAGGATTATAGAAGGATTACAAAGATTTGGTAGAGGT----------------------------
--------ATTCTCCACATA------CCTAGAAGAATAAGACAGGGCTTGGAAAGGACTTTGCTT---TAA
>JN248354
ACT---GCA--
GAGAAATTGTGGGTTACAATCTATTATGGGGTACCTGTATGG---AAAGAAGCAACC---
ACCACTCTATTTTGTGCATCAGATGCTAGAGCATATGATACAGAG---GTACATAAT----------------
--------------GTTTGGGCCACA---CATGCCTGTGTACCCACAGAC---
CCCAACCCACAAGAAGTAGTATTGGGA---------AATGTGACAGAAAATTTTAAC----------------
--------------------------ATGTGGAAAAATAACATGGTAGAACAGATGCATGAGGATGTA---
ATTAGTTTATGGGATCAAAGCCTA---AAGCCATGTGTAAAATTA---
ACCCCACTCTGTGTTATTTTAAATTGCTCTGATTATTTGGGAAATAATAATACCAATAGTAATAATACCACTA
GTAATAGTGCC--
-------AGTGATAATAGTACCATTAATAATAGTAGTCGGGAAATGATGGGGGGAGAAATGAAA---
AACTGCTCTTTCAATATTACCACAAGCATAGGA---------------------------
GAGAAGGCGAAGAACGCA---GCACTT---TTTTATAGCCTTGATGTTGAACCAATAGAGAAT----------
----

AGTAATACTAGCTATAGATTGAGAAGTTGTAACACTTCAATTATTACACAG---
GCCTGTCCAAAGGTATCCTTTGAGCCAATTCCCATACATTATTGTGCCCCGGCTGGTTTTGCGATTCTAAAGT
GTAAC---AATAAGAAGTTCAAT------GGCACAGGACCATGTAAAAAT-----------------------
-GTCAGC---ACAGTACAATGTACACATGGAATTAAGCCAGTAGTGTCAACT---
CAACTCCTGTTAAATGGCAGTCTAGCAGAAGGAGAG------------
GTAGTAATTAGATCTAGCAATTTCACAGACAAT---------GCTAAAATTATACTAGTACAGCTGCAG----
-----------GAACCTGTAACA---------ATTAATTGTATAAGACCCAACAACAACACGAGAAAA-----
-GGTATACATTTA------------GGACCAGGGAGAGCA---TTA------TACACAACA---GAA------
---------ATAGTAGGAAATATA------------AGACAAGCATATTGT---AACATTAGTAGGGAA---
AAATGGAATAACACTTTAGGAGAGATAGTTAAAAAATTAAGA---------------GAAGAATTTCAG----
-----------------------------------AATAAAACAATAATCTTTAAT------
CAATCCTCAGGGGGGGACCCAGAAATAATG---ATGCACAGTTTTAATTGTGGAGGAGAA---
TTTAAGCAGCTCCAGGCAAGAGTCCTGGCTGTGGAAAGATACCTAAAGGATCAACAACTCCTAGGGATTTGGG
GTTGCTCTGGAAAACTCATTTGCACCACTGCTGTGCCTTGGAATACTAGTTGGAGT-----------------
----------------------------AATAAATCATATGATGACATTTGG------AAT------
AACATGACCTGGATGCAGTGG---GAAAAAGAAATTGACAATTACACAAGCTTTATATAC---
ACACTAATTGAAGAATCGCAGATCCAACAAGAA--

AAGAATGAACAAGAATTATTGGAATTAGATAAGTGGGCAAGTTTGTGGAATTGGTTTAGCATAACAAACTGGC
TGTGGTATATAAAATTATTCATAATGATAGTAGGAGGCTTAGTAGGTTTAAGAATAGTTTTTACT---
GTACTTTCTATAATA---------AATAGAGTTAGGAAGGGATACTCACCA------
TTATCATTCGCTGTTAGCTTACTCAATGCCACGGCTGTAGCAGTAGCTGAG---

GGGACAGACAGGGTTATAGAAGGAATACAAAGAGCTTATAGAGGT---------------------------
--------TTTATCCACATA------CCTACAAGAATAAGACAGGGTTTGGAAAGGGCTTTGCTA---TAA
>JF297222
GCT---GTA---
GAACAATCGTGGGTCACAGTCTATTATGGGGTACCTGTGTGG---AGAGAAGCAACC---
ACCACTCTATTTTGTGCATCAGATGCGAAAGCACATAAGACAGAG---GTACATAAT---------------
--------------ATTTGGGCCACA---CATGCCTGTGTACCCACAGAC---
CCCAACCCACAAGAAGTAGTATTGAAA---------AATGTGACAGAATATTTTAAC---------------
--------------------------ATGTGGAAACATGGCATGGTAGAACAGATGCAGGAGGATATA---
ATCAGTTTATGGGATGAAAGCCTA---AAGCCATGTGTAAAATTA---
ACCCCACTCTGTGTTTCCTTAAATTGCACTGATTTGAGAAATGATACTAATACCAATAATAGTAGTAGTAGTA
ATAGTAGTAGTAGT--
-AATACCACTCATAGTGCTAGTAATAGTAGTAGTGAGAATAAGATGGACCTAGGAGAAATGAAA---
AACTGCTCTTTTAATGTCACCACAGACACAGGA--------------------------GATAGGGTG---
GTGCAACGTGCACTT---TTTTATAGACTTGATGTAGAACAAATAGGTAAT---------------------
----
----------AGTACTACTAGCTATAGGTTGATAAAGTGTAACACCTCAGTCATTAAACAA---
GCCTGTCCAAAGGTATCCTTTGAACCAATTCCCATACATTATTGTACCCCGGCTGGTTTTGCGCTTCTAAAGT
GTAAA---GATGAGCATTTCAGT------GGAAAAGGAAACTGTACAAAT----------------------
-GTCAGC---ACAGTACAATGTACGCATGGAATTAAGCCAGTAGTATCAACT---
CAACTGCTGTTAAATGGCAGTCTAGCAGAAAAGGAG-----------
GTAGTAATTAGGTCTGACAATTTCACAGATAAT---------GCAAAAAGCATAATAGTGCAGCTGAGT----
-----------GAAGCTGTAGAA---------ATTAATTGTACGAGACCCAACAATAATACAAGACAA-----
-GGTATACACCTA------------GGACCAGGGAGAGCA---TTT------TATTCAACAGGAGAC------
---------ATAATAGGAGATATA------------AGACAAGCATATTGT---ACCCTTAATGAATCA---
AAATGGAATAATACTTTACAGAAGATAAGTGTAAAATTAAAA---------------GAAATATTTAAT----
-----------------------------------AATAAAACAATAGTCTTTAAT------
CACTCCTCAGGAGGGGATCCAGAAATTACA---ATGCACAGTTTTAATTGTGGAGGGGAA---
TTTAAGCAGCTCCAGGCGAGAGTCCTGGCTGTGGAAAGATACCTAAGGGATCAACAGCTCCTGGGGATTTGGG
GTTGCTCTGGAAAACTCATTTGCACCACTACTGTGCCTTGGAATACTAGTTGGAGT----------------
---------------------------AATAAATCCCTGGATTATATTTGG------GAA------
AACATGACCTGGATGCAGTGG---GACAAAGAAATTAATAATTACACAGATTTAATATAC---
ACCCTACTTGAAAGTGCGCAGAACCAGCAAGAA---------------------------------------
----
AAGAATGAACAAGAATTATTAGAATTAGATAAATGGGCAAATTTGTGGAGTTGGTTTAGCATAACAAACTGGC
TGTGGTATATAAGAATATTCATAATAATAGTAGGAGGCTTGGTAGGTTTAAGAATAGTTTTTGCT---
GTACTATCTATAGTG---------AATAGAGTTAGGAAGGGATACTCACCA------
TTATCGTTTGCTGTTAGCTTGCTCGACGCCACAGCCATAGCAGTAGCTGAG---
GGAACAGATAGGGTAATAGAAGTAGTACAAAGAGCTTGTAGAGCT---------------------------
--------ATTCTCCACATA------CCTACACGCATAAGACAGGGCTTAGAAAGAGCTTTGGTA---TAA
>JX446800
GCT---ACA---
GACCAAATGTGGGTCACAGTCTATTATGGTGTGCCTGTATGG---AAAGAAGCAACC---
ACCACTTTATTTTGTGCATCAGATGCTAAAGCACATTCCACAGAG---GCACATAAT---------------
--------------GTTTGGGCCACA---CATGCCTGTGTACCCACAGAC---
CCCAACCCACAAGAAGTAGTATTGAAA---------AATGTGACAGAAGAATTTGAT---------------
--------------------------GTGTGGAAAAATGACATGGTAGAACAAATGCAGGAGGATATA---
ATCAGTTTATGGGATCAAAGCCTA---AAGCCATGTGTAAAATTA---
ACCCCACTCTGTGTTACCTTAAACTGCTCAGATCTTAACTGCACAAATCAGAATAGTACTTCACACTGC----
----
----------------------ACAGTTCAGAATACTACTGATAGTGGGATAAGAGACATGAAA---
AACTGCTCTTTCAATGTTACCACAGAGATAAGG--------------------------
GATAGGGTGCGGCAAGATTATGCAATT---TTTAATAAACTTGATATAGTACCAATAGATAATGAT-------
----

AGTAGGAATGATAGTAGGAATGATAGCTATAGGTTGATAAATTGTAACACCTCAGTCATTACACAA---
GCCTGTCCAAAGATGTCCTTTGAACCAATTCCCATACATTATTGTACCCCAGCTGGTTTTGCGATTCTAAAGT
GTAAT---GATAAGAAGTTCAAT------GGAAAAGGATCATGTACAAAT----------------------
-GTCAGC---ACAGTACAATGTACACATGGAATTAGGCCAGTAGTGTCAACT---
CAACTGCTGTTAAATGGCAGTTTAGCAGAAGAGGAG-----------

GTAGTAATCAGATCTTACAATTTCTCGGACAAT---------GCTAAAAACATACTAGTACAGCTGAAA----
-----------AACTCTGTACAA---------ATTAATTGTACAAGACCCAACAATAATACAAGAAAA-----
-AGTATACATATA------------GGACCAGGGAGAACA---TTT------TATGCAACAGGAGAT------
---------ATAATAGGAGATATA------------AGAAAAGCATATTGT---AACATTAGTAGAAAA---
GAATGGAATGACACTTTAGAAGAGATAGTTGGGAAATTAGGA---------------AAACAATTTAAT----
-----------------------------------AAAACAACAATAGTCTTTAAC------
CACTCCTCAGGAGGGGACCCAGAAATTGTA---ATGCACAGCTTTAATTGTGGAGGGGAA---
TTCAAGCAGCTCCAGGCAAGAGTCCTCGCTGTAGAAAGATACCTAAGGGATCAACAGCTCCTGGGGATTTGGG
GTTGCTCTGGAAAACTCATTTGCACCACTACTGTGCCTTGGAATACTAGTTGGAGT-----------------
----------------------------AATAAATCCTACGAGGATATTTGG------TAT------
AACATGACCTGGATGCAGTGG---GAAAAAGAAATTGACAATTACACAGCACTAATATAT---
GATTTAATTGAAAAATCACAAAACCAGCAAGAA--

ATAAATGAAAAAGATTTATTGGAATTAGATAAGTGGGCAAGTTTGTGGAATTGGTTTGACATATCAAAATGGC
TGTGGTATATAAGAATATTCATAATGATAGTAGGAGGCTTAATAGGGTTAAGAATAATTTTTGCT---
GTACTTTCTATAGTA---------AATAGAGTTAGGCAGGGATATTCACCA------
TTATCATTTGCCATTAGCTTGCTTAACGCGACAGCTATAGCAGTAGGTGAG---
GGAACAGATAGGGTTATAGATATAGTACAAAGAACTGGTAGAGCT----------------------------
--------ATTCTCAACATA------CCTAGAAGAGTGAGACAGGGTTTGGAAAGGACTTTGCTA---TAA
>JX446818
GCT---GCA--
GAACAATCATGGGTCACAGTCTATTATGGGGTACCTGTGTGG---AAAGAAGCAACC---
ACCACTCTATTTTGTGCATCAGATGCTAAAGGATACAACACAGAG---GCACATAAT----------------
--------------ATTTGGGCCACA---CATGCCTGTGTACCCACAGAT---
CCCAACCCGCAAGAAATAGTATTAGAA---------AATGTGACAGAAAAGTTTAAT----------------
--------------------------ATGTGGAAAAATAACATGGTAGAGCAAATGCAAGAGGATATA---
ATCAGTTTATGGGATCAAAGCCTA---AAGCCATGTGTAAAATTA---
ACCCCACTCTGTGTTACTTTACATTGCGCTGATGTTACTAAGGCAAATACTACAAATACTACT----------
----
----------------------------GAGACCCTTGATAATAATATGAAAGGAGAGATAAAA---
AACTGCTCTTTCAATGTCACCACAACTATAAGA---------------------------
GATAGGATAACAAAAGAACATGCACTC---TTTTATAGACTTGATGTAGTACCAATAGAAGAT----------
----

AATAGTAATAGTAGCAATAGTACCTATAGATTGATAAATTGTAACACCTCAGTCATTACACAG---
GCCTGTCCAAAGGTATCCTTTGAGCCAATTCCCATACATTACTGTGCCCCGGCTGGTTTTGCGATTGTAAAGT
GTAAT---GATAAGAATTTCAAT------GGAACAGGACTATGTAGAAAT-----------------------
-GTCAGC---ACTGTACAATGTACACATGGAATTAAGCCAGTAGTGTCAACT---
CAATTGCTATTAAATGGTAGTCTAGCAGAAGAAGAT------------
GTGGTAATTAGATCTGAAAATTTCACAGACAAT---------GTTAAAACCATAATAGTACAGTTGAAT----
-----------GAATCGGTACAA---------ATTAATTGTACAAGACCCAACAACAATACAAGAAAA-----
-AGTATACATATA------------GGGCCAGGGAGAGCA---TTT------TATGCAGCAGGAGAT------
---------ATAATAGGAGATATA------------AGACAAGCACATTGT---GACATTAGTAGTGAA---
AAATGGAATAAAGCTTTAGGACAGATAGTTATAAAATTAAGA---------------GAACACTATCAG----
-----------------------------------AATAAAACAATAAAATTTAAT------
TCATCCACAGGAGGGGATCCAGAAATTGTA---ATGCACAGTTTTAATTGTGGAGGGGAA---
TTTAAACAGCTCCAGGCAAGAGTCCTGGCTGTGGAAAGATACCTAAGAGATCAACAGCTCCTAGGGCTTTGGG
GTTGCTCTGGAAAACTCATTTGCACCACTAATGTGCCTTGGAACACTAGCTGGAGT-----------------
----------------------------AATAAATCTATAGATGACATTTGG------AAC------
AACATGACCTGGATACAGTGG---GAAACAGAAATTGACAATTACACAGGCTTAATATAC---
AACTTACTTGAACAATCGCAGAATCAGCAAGAA--

AAAAATGAACAAGAGTTATTGGAATTGGACAAGTGGGCAAGTTTGTGGAATTGGTTTGATATAACAAACTGGC
TATGGTACATAAAAATATTCATAATGATAGTAGGAGGTTTGATAGGGTTAAGAATAGTTTTTGCT---
GTACTTTCTATAGTG---------AATAGAGTTAGGCAGGGATACTCACCA------
TTATCATTCGCTGTTAGCTTGTTTAATACCGCAGCAATAATAGTAGCTGAA---
GGGACAGATTGGATTATAGAAGTAGCACAAAGATTTCTTAGAGGA----------------------------
--------ATTCTCCACATA------CCTAGAAGAATAAGACAGGGCTTGGAAAGGGCTTTGCTA---TAA
>JX447795

GCT---GCA--
GAAAACTTGTGGGTCACAGTCTATTATGGGGTGCCTGTATGG---AAAGAAGCAACA---
ACCACTCTATTTTGTGCATCAGATGCTAAAGCATATGTTGCAGAG---GCACACAAT----------------
--------------GTTTGGGCCACA---CATGCCTGTGTACCCACAGAC---
CCCAACCCACAAGAAATAGTAATGGAA---------AATGTGACAGAAAACTTCAAC----------------
--------------------------ATGTGGAAAAATAACATGGTAGAACAGATGCATGAGGATTTA---
ATCAGCTTATGGGATCAAAGCCTA---AAGCCCTGTGTAAAATTA---
ACCCCACTCTGTGTTACTTTAAATTGCACTGATTGGAAGAATGATAATGATACTAATATCAATAGTACTACTG
CCAATAGTACCAATGGTGCTACTGCCAAT--
----------AGTACGGTGAATAGTACTAATACCCATAGTACAACGGGGGGAATAGAAATGAAA---
AACTGCTCTTTCAATATAACCACAAGCATACAA---------------------------ACTAAGGTG---
AAAAACCATGCACTT---TTTTATAAACTTGATGTAGTACCAATAAACAAT----------------------
----
----GATAGTGATACTACCAGCTATAGGTTAATAAGTTGTAACACCTCAACCATTACACAA---
GCTTGTCCAAAGGTATCCTTTGAGCCAATTCCCATACATTATTGTACCCCGGCTGGTTTTGCGATTCTACAAT
GTAAC---AATAAAAAGTTTAAT------GGAACAGGACCATGTACAAAT-----------------------
-GTCAGT---ACAGTACAATGCACACATGGAATTAGGCCAGTAGTATCAACT---
CAACTGCTGTTAAATGGCAGTTTAGCAGAAGAAGAG------------
GTAATAATTAGATCCAGCAATTTCACAGATAAT---------GCTAAAATCATAATAGTACAGTTGAAT----
-----------GAAACTGTACAA---------ATTAATTGTTCGAGACCTAGCAACAATACAAGAAAA-----
-GGTATAAATATA------------GGACCAGGGCAAGCA---TGG------TATACAACAGACCAA------
---------GTAGTAGGAAATATA------------AGACAAGCACATTGT---AACATTAGTATCACA---
AAATGGAATAACACTTTAAAATGGGTAACTGAAAAATTAAGA---------------GAACAATTTAAG----
-----------------------------------AACAAAACAATAGTCTTTCAG------
CCACCCTCAGGAGGGGACCCAGAGATTGTA---ATGCACAGTTTTAATTGTGGAGGGGAA---
TTTAAGCAGCTCCAGGCAAGAGTCCTGGCTGTGGAAAGATACCTAAAGGATCAACAGCTCCTAGGGATTTGGG
GTTGCTCTGGAAAACTCATTTGCCCCACTGCTGTGCCTTGGAATAGTAGTTGGAGT-----------------
----------------------------AATAAATCTATGGAGTATATTTGG------CAG------
AACATGACTTGGATGCAGTGG---GAAAGAGAAATTGACAATTACACAACAGAAATATAC---
ACCTTAATTGAACAATCACAGAACAAACAAGAA--

[truncated: 524,859 more chars]
