# Supplementary material for: Extending the Coding Potential of Viral Genomes with Overlapping Antisense ORFs: A Case for the De Novo Creation of the Gene Encoding the Antisense Protein ASP of HIV-1
Source: Viruses. 2022 Jan 14;14(1):146. doi: 10.3390/v14010146 (PMC8781085; doi:10.3390/v14010146)
Supplement: Supplementary file 1 [file viruses-14-00146-s001.zip › Supplementary File S1A.pdf]

Supplementary File S1A.

Sample set of 3725 *env*/*asp* overlapping regions fulfilling the following criteria: i) an *asp* anti-sense frame not interrupted by premature *stop* codons; ii) delimited by an ATG initiator at canonical nucleotide positions (triplet CAT at the end of the sequence) and a *stop* codon at canonical nucleotide positions (triplet CTA at the beginning of the sequence). Definition of canonical nucleotide positions is reported in the Materials and Methods**;** *2.1 Assembly of a sample set of 3725 env/asp overlaps*. All sequences belong to the group M of HIV-1 except for SIVcpzMB66 (NCBI acc. number DQ373063). The nucleotide sequence of each *env*/*asp* overlap is reported both in the alignment format with gaps and in the format without gaps.

>K03455
TTCTACTGTAATTCAACACAACTGTTTAATAGTACTTGGTTTAATAGTACTTGGAGTACTGAAGGGTCAAATA
ACACT--

GAAGGAAGTGACACAATCACCCTCCCATGCAGAATAAAACAAATTATAAACATGTGGCAGAAAGTAGGAAAAG
CAATGTATGCCCCTCCCATCAGTGGACAAATTAGATGTTCATCAAAT---
ATTACAGGGCTGCTATTAACAAGAGATGGTGGTAATAGCAACAAT----------------------------
----

GAGTCCGAGATCTTCAGACCTGGAGGAGGAGATATGAGGGACAATTGGAGAAGTGAATTATATAAATATAAAG
TAGTAAAAATTGAACCATTA---GGAGTAGCACCCACCAAGGCAAAGAGAAGAGTGGTG--------------
-------------------------CAGAGAGAAAAAAGAGCA---GTGGGA---ATAGGAGCT---------
TTGTTCCTTGGGTTCTTGGGAGCA---GCAGGAAGCACTATG---GGCGCAGCCTCA---
ATGACGCTGACGGTACAG---GCCAGACAA---TTATTG---TCTGGTATAGTGCAGCAGCAGAAC---
AATTTG---CTGAGG---GCTATTGAGGCGCAACAGCATCTGTTGCAACTCACAGTCTGGGGCATC
>KF725913
TTCTATTGTAATACATCAGGACTGTTTAATAGCACCTGGAGGAATGGAACCATGCAGGAGCCAAACAGCACAC
AG---

TTAAATGACACTATAACTCTCAAGTGCAGAATAAGGCAAATTATAAATATGTGGCAGAGAGCAGGAAAAGCAA
TGTATGCCCCTCCCATCCAAGGAAAAATACAGTGTAAATCAAAT---
ATTACAGGATTACTATTGACAAGAGATGGTGGTGCGGAAAATAAT----------------------------
----

GCAAGTGTACCTGAAATCTTCAGACCTGGAGGGGGAGATATGAGAGACAATTGGAGAAGTGAATTATATAGGT
ATAAAGTAGTAAAAATTGAACCACTA---GGAGTAGCACCCACCAAGGCAAAGAGAAGAGTGGTG--------
-------------------------------GAGAGAGAAAAAAGAGCACTAATTGGA---CTAGGAGCT---
------GTCTTCATTGGGTTCTTAGGAGCA---GCAGGAAGCACTATG---GGCGCGGCGTCA---
ATAACGCTGACGGTACAG---GCCAGGCAA---TTATTG---TCTGGCATAGTGCAACAGCAAGCC---
AACTTG---CTGAGG---GCTATAGAGGCGCAACAACACCTGTTGAGACTCACGGTCTGGGGCATT
>KY968401
TTCTACTGTAATACAACACAGCTGTTTAATTTTAATAGTACTTGGAATAATAATGACACTGAAGGAAATTTCA
CTGAAGAT---

AATAAAACTACTATCACACTCCCATGCAGAATAAAACAAATTATAAACAGGTGGCAGGAAGTAGGAAAAGCAA
TGTATGCCCCTCCCATCAGAGGACAAATTAGCTGTTCATCAAAT---
ATTACAGGGCTGCTATTAACAAGAGATGGTGGTAACAACACTAAC----------------------------
----

AACAATGAAACTTTCAGACCTGGAGGAGGAGATATGAGGGACAATTGGAGAAGTGAATTATATAAATATAAAG
TAGTAAGAATTGAGCCATTG---GGAATAGCACCCACCAAGGCAAAGAGAAGAGTGGTG--------------
-------------------------CAGAGAGAAAGAAGAGCA---GTGGGA---ATGGGAGCC---------
ATGTTCCTTGGGTTCTTGGGAGCA---GCAGGAAGCACTATG---GGCGCAGCGTCA---
ATAACGCTGACGGTACAG---GCCAGACAA---TTATTG---TCTGGTATAGTGCAACAGCAGAAC---
AATCTG---CTGAGA---GCTATTGATGCGCAACAGCATCTGTTGCAACTCACAGTCTGGGGCATC
>KY968403

TTTTACTGTAATACATCAAAGCTGTTTAATAACACTTGGAATGGCACTGCATGGCCAAGCACTGAA-------
----

GAAAATGAAACTATCACACTTCCATGCAGAATAAAACAAATTATAAACAGGTGGCAGGAAGTAGGAAAGGCAA
TGTATGCCCCTCCCATCAAAGGTGTAATTAGCTGTTCATCAAAT---
ATCACAGGGCTGTTATTAACAAGAGATGGGGATAACAATAACGGG----------------------------
----

TCCGAGACTTTCAGACCTGGAGGAGGAGATATGAGGGACAATTGGAGAAGTGAATTATATAAATATAAAGTAG
TAAGAATTGAGCCATTA---GGAATAGCACCCACCGAGGCAAAGAGAAGAGTGGTG-----------------
----------------------CAGAGAGAAAAAAGAGCA---GTGGGA---ATGGGAGCT---------
ATGTTCCTTGGGTTCTTGGGAGCA---GCAGGAAGCACTATG---GGCGCAGCGTCC---
ATGACGCTGACGGTACAG---GCCAGACAA---TTATTG---TCTGGTATAGTGCAACAGCAGAAC---
AACCTG---CTGAGA---GCTATTAAGGCGCAACAGCATCTGTTGCAGCTCACAGTCTGGGGCATC
>AY037269
TTCTACTGTAATACAACAAAGCTGTTTAATAGTACTTGGGTAAATGGCACTTTAAATAACACTGCAGGAAATG
AC---

AGTGACACAAGCATCATACTCCCATGCAGAATAAAACAAATTATAAACAGATGGCAGGAAGTAGGAAAAGCAA
TGTATGCCCCTCCCATTAGCGGAGTAATTAGCTGTGTATCAAAT---
ATTACAGGGCTGCTATTAGCAAGAGATGGTGGTGGGAACACGAGC----------------------------
----

AGAGACAACGAAACCTTCAGACCTGTAGGGGGAGATATGAGGGACAATTGGAGAAGTGAATTATATAAATATA
AAGTAGTAAGAATTGAGCCAGTA---GGAATAGCACCCACCAAGGCAAAGAGAAGAGTGGTG-----------
----------------------------CAGAGAGAAAAAAGAGCA---GTGGGA---TTAGGAGCT------
---ATGTTCCTTGGGTTCTTGGGAGCA---GCAGGAAGCACTATG---GGCGCAGCGTCA---
GTGACGTTGACGGTACAG---GCCAGACAA---TTGTTG---TCTGGTATAGTGCAACAGCAGAAC---
AATCTG---CTGAGA---GCTATTGAGGCGCAACAACACCTGTTGCAACTCACAGTCTGGGGCATC
>KY968394
TTCTACTGTAATTCATCACAACTGTTTAATAGCACTTGGGCTTTGAATAGTACTAACACTACTGCTGAA----
----

GGAAATGAAACTATCATACTCCCATGCAGAATAAAACAAATTATAAACATGTGGCAACAAGTAGGAAGAGCAA
TGTATGCCCCTCCTATCAAAGGACTAATTAGATGCTCATCAAAT---
ATTACAGGGATACTATTAACAAGAGATGGTGATAGTAATAGAACT----------------------------
----

AATAATGAGACCTTCAGACCTGGAGGAGGAAATATGAAGGATAATTGGAGAAGTGAATTATATAAATATAAAG
TAGTAAAGATTGAGCCATTA---GGACTAGCACCCACCAAGGCAAAAAGAAGAGTGGTG--------------
-------------------------CAGAGAGAGAAAAGAGCA---GTGGGA---TTAGGAGCT---------
ATGTTCCTTGGGTTCTTGGGAGCA---GCAGGAAGCACTATG---GGCGCAGCGTCA---
TTGACGCTGACGGTACAG---GCCAGACTA---TTATTG---TCTGGTATAGTGCAACAGCAGAAC---
AATCTG---CTGAGA---GCTATTGAGGCGCAACAACATATGTTGCAACTCACAGTCTGGGGCATT
>KY968407
TTCTACTGTAATTCATCAAAGCTGTTTAACAGTATTTGGTGGTCTAATAATACTAATACTACAGAGGGAATT-
----

GCAGGAAAAAATATCACACTCCCATGCAGAATAAAACAAATTATAAACAGATGGCAGGAAGTAGGAAAAGCAA
TGTATGCCCCTCCCATCAGTGGACTAGTTAGATGTTCATCAAAT---
ATTACAGGACTGCTATTAACAAGAGATGGGGGTAAAGATAATGAAAATAGCAAT-------------------
----

AATAGGACTGAAACCTTCAGACCTGGAGGAGGAAATATGAAGGACAATTGGAGAAGTGAATTATATAAATATA
AAGTAGTGAAGATTGAGCCATTA---GGAGTAGCACCCACCAGGGCAAAGAGAAGAGTGGTG-----------
----------------------------CAGAGAGAAAAAAGAGCA---GTAACA---ATAGGAGCT------
---GTGTTCCTTGGGTTCTTGGGAGCA---GCAGGAAGCACTATG---GGCGCAGCGTCA---
ATGACGCTGACGGTACAG---GCCAGACAA---TTACTG---TCTGGTATAGTGCAACAGCAGAAC---
AATTTG---CTGAGG---GCTATTGAGGCGCAACAGCATCTGTTGCAACTCACAGTCTGGGGCATC
>DQ383746

```
TTCTACTGTAATACAACACAACTGTTCAATAGTACTTGGAATTCAACACAACTTAATCCTAATAGCTCTTTGA
GTACC--------------------------------------------------------------------
-------------------------
AATGATACTGCTACTATCACACTCCCATGCAGAATAAAACAAATTATAAACATGTGGCAGAGAGTAGGAAAAG
CAATGTATGCCCCTCCCATTGAAGGACACATTAGGTGTTCATCAAAT---
ATTACAGGACTGCTATTAACAAGAGATGGTGGTAACGAGAGCGAG----------------------------
-------------------------------------------------------------------------
----------------
TCTGAGATCTTCAGGCCTGGAGGAGGAGATATGAGGGATAATTGGAGAAGCGAATTATATAAATATAAAGTAG
TAAAAGTTGAACCATTA---GGAATAGCACCCACCAAGGCAAAGAGAAGAGTGGTG-----------------
----------------------CAGAGAGAAAAAAGAGCA---GTGGGA---CTAGGAGCT---------
ATGTTCCTTGGGTTCTTGGGAGCA---GCAGGAAGCACTATG---GGCGCAGCGTCA---
ATGGCGCTGACGGTACAG---GCCAGACAA---TTATTG---TCTGGTATAGTGCAACAGCAAAAC---
AATTTG---CTGATG---GCTATTGAGGCGCAACAGCATATGTTGCGACTCACAGTCTGGGGCATT
>KY968396
TTCTACTGTAATACAACACAGCTATTTAATAGTACGTGGAACGAGACATGGGCAAATGACACAGAA-------
-------------------------------------------------------------------------
----------------------------
GGAAATGACACAATCACACTCCCATGCAGAATAAAACAAATTATAAACATGTGGCAGGAAGTAGGAAAAGCAA
TGTATGCCCCTCCCATCAGCGGACCAATTAACTGTACATCCAAT---
ATTACAGGGCTGCTATTAACAAGAGATGGTGGGAATAGGAATACAAGCGAG----------------------
-------------------------------------------------------------------------
-------------
CTCCTCGAGACCTTCAGACCTGGAGGAGGAGATATGAGGGACAATTGGAGAAGTGAATTATATAAATATAAAG
TAGTAAAGATTGAACCATTA---GGAGTAGCGCCCACCAAGGCAAAGAGAAGAGTGGTG--------------
-------------------------CAAAGAGAAAAAAGAGCA---GTGGGA---TTTGGAGCT---------
ATATTCCTTGGGTTCTTGGGAGCA---GCAGGAAGCACTATG---GGCGCAGCGTCA---
GTGACGCTGACGGTACAG---GCCAGACAA---TTATTG---TCTGGTATAGTGCAGCAGCAGAAC---
AATCTG---CTGAGG---GCTATTGAGGCGCAACAACACCTGTTGCAACTCACAGTCTGGGGCATC
>DQ383748
TTCTACTGTAATACAACAAAACTGTTTGATAATAGTACTTGGAATAACACTGAGGAGTCA-------------
-------------------------------------------------------------------------
----------------------------
AATAGCACTATATTCACACTCCCATGCAGAATAAAACAAATTATAAACATGTGGCAGGAAGTAGGAAAAGCAA
TGTATGCCCCTCCCATCAGAGGACAAATTAACTGTTCATCAAAT---
ATTACAGGGCTGCTATTAACAAGAGATGGTGGGACCGAGGAGAAG----------------------------
-------------------------------------------------------------------------
-------------------
GAGATCTTCAGACCTGGAGGAGGAGATATGAGGGACAATTGGAGAAGTGAATTATATAAATACAAAGTAGTAA
AAATTGAACCATTA---GGAGTAGCACCCACCAAGGCAAAGAGAAGAGTGGTG--------------------
-------------------CAAAGAGAGAGAAGAGCA---GTGGGA---ATAGGAGCC---------
CTGTTCCTTGGGTTCTTAGGAGCA---GCAGGAAGCACTATG---GGCGCAGCGTCG---
CTGACGCTGACGGTACAG---GCCAGACAA---TTATTG---TCTGGTATAGTGCAACAGCAGAAC---
AATATG---CTGAGG---GCTATTGAGGCGCAACAGCATATGTTGCAACTCACAGTCTGGGGCATC
>DQ383751
TTCTACTGTAATACAACACAACTGTTTAATAGTGCTTGGAATGATACTAATATAAACAGAACTGAAGGGTTAA
ACAAC--------------------------------------------------------------------
-------------------------
ACAGGAAGTGGCACTATCACACTCCCATGCAGAATAAAACAAATTATAAACCTGTGGCAGGAAGTAGGAAAAG
CAATGTATGCCCCTCCCATCAGTGGACAAATTAATTGTTCATCAAAT---
ATTACAGGGTTGCTATTAACAAGAGATGGGGGTAATGGTAATAACACA-------------------------
-------------------------------------------------------------------------
----------
AACAAGACAGAGATCTTCAGACCTGGAGGAGGAAATATGAAGGACAATTGGAGAAGTGAATTATATAAATATA
AAGTAGTAAAAGTTGAACCATTA---GGAATAGCACCCACCATGGCAAAGAGAAGAGTGGTG-----------
----------------------------CAAAGAGAAAAAAGAGCA---GTGGGA---ATAGGAGCG------
---TTGTTCCTTGGGTTCTTGGGAGCA---GCAGGAAGCACTATG---GGCGCAGCGTCA---
ATAACGCTGACGGTACAG---GCCAGACAA---CTATTG---TCTGGTATAGTGCAACAGCAAAAT---
AATTTG---CTGAGA---GCTATTGAAGCGCAACAGCATATGTTGCAGCTCACAGTCTGGGGCATC
>KY968399
```

TTCTACTGTAATACAACACAACTGTTTAATAGTACTTGGAGTACTTGGAATAGTACTAAAGGGGTAAATGGCA
CT---

GAAGAAAAGAACATCACACTCCCATGCAGAATAAAACAAATTATAAACATGTGGCAGGAAGTAGGAAGAGCAA
TGTATGCCCCTCCCATCAATGGACAAATTAGATGTTCATCAAAA---
ATTACAGGGCTACTATTAACAAGAGATGGTGGTACTAACAGTACC----------------------------
----

AACGAGACCTTCAGACCTGGAGGAGGAGATATGAGGGACAATTGGAGAAGTGAATTATATAAATACAAAGTAG
TAAAAATTGAACCATTA---GGAGTAGCACCCACCGAGGCAAAGAGAAGAGTGGTG-----------------
----------------------CAGAGAGAAAAAAGAGCA---CTGGGA---CTAGGAGCT---------
CTGTTCCTTGGGTTCTTGGGAGCA---GCAGGAAGCACTATG---GGCGCAGCGTCA---
CTGACGCTGACGGTACAG---GCCAGACAA---TTATTG---TCTGGTATAGTGCAACAGCAGAAC---
AATTTG---CTGAGG---GCTATTGAGGCGCAACAGCATATGTTGCAACTCACAGTCTGGGGCATC
>KY658686
TTCTACTGTAATATATCACAACTGTTTAATAGTACTTGGAATCCAAATACTACAGAATCAGACAACACAAAT-
----

AACACTGAACTTATCATACTCCCATGTAGAATAAAACAAATTATAAACTTGTGGCAGGAAGTAGGAAAAGCAA
TGTATGCTCCTCCCATCCAAGGAAACATTACCTGTTCATCAAAT---
ATTACAGGGCTAGTATTAACAAGAGATGGTGGTGATAATGCTACT----------------------------
----

CTTGAGGTCTTCAGACCTGGAGGAGGAAATATGAAGGACAATTGGAGAAGTGAATTATATAAATATAAAGTAG
TAAGAATTGAACCATTA---GGAGTAGCACCCACCAAGGCAAAGAGAAGAGTGGTG-----------------
----------------------CAGAGAGAAAGAAGAGCA---GTGGGAGCGCTAGGAGCT---------
ATGTTCCTTGGGTTCTTGGGAACA---GCAGGAAGCACTATG---GGCGCAGCGGCA---
ATGACGCTGACGGTACAG---GCCAGACAA---TTATTG---TCTGGTATAGTGCAACAGCAAAAC---
AATTTG---CTGAGA---GCTATTGAGGCGCAACAGCATCTGTTGCAACTCACAGTCTGGGGCATC
>KY658683
TTCTACTGTAATACAACACAGTTGTTTAATAGTACTTGGAATGCAAATAATACCCAAGGGTCAAATGGA----
----

ACTGGAGAAATTATCACACTCCCATGCAGAATAAAACAAATTATAAACAAATGGCAGGAAGTAGGAAAAGCAA
TGTATGCCCCTCCCATCAAAGGAAACATTAGTTGTTCCTCAAAT---
ATCACAGGGCTGCTACTAACAAGGGATGGTGGTAGCAATAGCAGC----------------------------
----

AACGAAACCTTCAGACCTGGAGGAGGAGATATGAGGGACAATTGGAGAAGTGAACTATATAAATATAAAGTGG
TAAAAATTGAACCATTA---GGAGTAGCACCTACCAGGGCAAGGAGAAGAGTGGTG-----------------
----------------------CAAAGAGAAAAAAGAGCA---CTAGGGATTGTGGGAGCT---------
GGGCTCCTTGGGTTCTTAGCAGCA---GCAGGAAGCACTATG---GGCGCAGCGTCG---
GTGACGCTGACGGTACAG---GCCAGACTA---TTATTG---TCTGGTATAGTGCAACAGCAGAAC---
AATCTG---CTGAGG---GCTATTGAGGCGCAACAGCACCTGTTGCAACTCACAGTCTGGGGCATC
>MH078530
TTCTACTGTAATACAACAAAGCTGTTTAATAGTAATAGTAATTGGCATTCCAATAAGACTTGGGAGGGG----
----

CTAAATGACACTATCACACTCCCATGCAGAATAAAACAAATTATAAACACATGGCAGGAAGTAGGAAAAGCAA
TGTATGCCCCTCCCATCAGAGGACGAATTAATTGTTCATCAAAT---
ATTACAGGGCTGCTACTAACAAGAGATGGTAGTGGCAGCAATGACACT-------------------------
----

AATAATACCGAGATCTTCAGACCTGGAGGAGGAAATATGAGGGACAATTGGAGAAGTGAATTATATAAATATA
AAGTAGTGAAAATTGAACCAATA---GGAATAGCACCCACCAAGGCAAAGAGAAGAGTGGTG-----------
----------------------------CAGAGAGAAAAAAGAGCG---ATAACG---CTGGGAGCT------
---ATGTTCCTTGGGTTCTTGGGAACA---GCAGGAAGCACTATG---GGCGCAGCGTCG---
CTGACGCTGACGGTACAA---GCCAGACAA---TTGTTG---TCTGGTATAGTGCAACAGCAGAAC---
AATCTG---CTGAGA---GCTATTGAGGCGCAACAGCATCTGTTGCAGCTCACAGTCTGGGGCATC
>KY658685

TTCTACTGTAACACAACACAACTGTTTAATAGTACTTGGAACAGTACTTGGAATTGGAACAGTACTACTGAGA
ACTCA--

ACAAACTTTAACATAACAATTCCATGCAAAATAAAACAAATTGTAAACAGGTGGCAAGAGGTAGGAAAAGCAA
TGTATGCCCCTCCCATCAGAGGAATAATCCAATGCACATCAAAC---
ATTACAGGGTTGCTATTAACAAGAGATGGTGGTAATGAGAATAAT----------------------------
----

ATTAGTGAGATTTTCAGACCTGGAGGAGGAGATATGAGGGACAATTGGAGAAGTGAATTATATAAATACAAAG
TAGTACAAATTGAACCAATA---GGAATAGCACCCACCAAGGCAAAGAGAAGAGTGGTG--------------
-------------------------CAGAGAGAGAAAAGAGCA---GTGGGA---ATAGGAGCT---------
ATGTTCCTTGGGTTCTTGAGCGCA---GCAGGAAGCACTATG---GGCGCAGCGTCA---
GTGACGCTGACGGTACAG---GCCAGACAA---TTATTG---TCTGGTATAGTGCAGCAGCAGAAC---
AATCTG---CTGAAG---GCTATTGAGGCACAACAGCATCTGTTGCAACTCACAGTCTGGGGCATC
>MH078533
TTCTACTGTAATTCAACACCATTGTTTAATAGTACTTGGAATAGTAGTACTTGGAATAATAGTAATATTGAAC
AGGTTAAT---
-------------------GGA---
ACTGAACAGGATACTATCACACTCCACTGCAGAATAAAACAAATAGTAAACATGTGGCAGGAAGTAGGAAAAG
CAATGTATGCCCCTCCTATCAGAGGACAAATTAGATGTTCATCAAAG---
ATTACGGGGCTACTGTTAGTAAGAGATGGGGGTAAAAACAACACA----------------------------
----

CAGAACGAGACCTTCAGGCCTACAGGAGGAAACATGAAGGACAATTGGAGAAGTGAATTATATAAATATAAAG
TAGTAGAAATTAAGCCATTA---GGAATAGCACCAACCAAGGCAAAGAGAAGAGTGGTG--------------
-------------------------CAGAGAGAAAAAAGAGCA---GTGGGA---GTAGGAGCT---------
ATGATCCTTGGTTTCTTGAGTACA---GCAGGAAGCACTATG---GGCGCAGCGTCA---
GTGACGCTGACGGTACAA---GCCAGACAA---TTATTG---TCTGGTATAGTGCAGCAGCAAAGC---
AATTTG---CTGAGG---GCTATTGAGGCGCAACAACATATGTTGCAACTCACAGTCTGGGGCATC
>AY037268
TTCTACTGTAATACAACACAACTGTTTAGTAGTACTTGGAATGAGACTAATATTTGGAATGAGACTAATACA-
----

GATAATAGCACAATCACACTCCAATGCAGAATAAAGCAATTTATAAACATGTGGCAGAAAGTAGGAAAAGCAA
TGTATGCCCCTCCCATCAGAGGACACATTAGATGTTCATCAAAT---
ATTACAGGGCTGCTATTAACAAGAGATGGTGGTATTATCAACACC----------------------------
----

ACCGAGACCTTCAGACCTGGAGGAGGAGATATGAGGGACAATTGGAGAAGTGAATTATATAAATACAAAGTAG
TAAGAATTGAACCATTA---GGAGTAGCACCCACCAAGGCAAAGAGAAGAGTGGTG-----------------
----------------------CAGAGAGAAAAAAGAGCA---ATAGGGGTAATAGGAGCT---------
TTGTTCCTTGGGTTCTTGGGAGCA---GCAGGAAGCACTATG---GGCGCAGCGTCA---
GTGACGTTGACGGTACAG---GCCAGACAA---TTATTG---TCTGGTATAGTGCAACAGCAGAAC---
AATCTG---CTAAGG---GCTATTGAGGCGCAACAGCATCTGTTGCAACTCACAGTCTGGGGCATT
>AY037282
TTCTACTGTGATACAACAAAACTGTTTAATAGTACTTGGACTACTGAAGGGTTAAATAGCACTGAAGGAAAT-
----

AGCACAAAAATAATCACACTCCCATGTAGAATAAGACAAATTATAAACATGTGGCAGGAAGTAGGAAAAGCAA
TGTATGCCCCTCCCATCAGTGGACAAATTAACTGTTCATCAAAT---
ATTACAGGGCTGCTATTAACAAGAGATGGTGGTAATGACACTAACAGT-------------------------
----

AGCAATACCGAGATATTCAGACCTGGAGGAGGAAATATGAAGGACAATTGGAGAAGTGAATTATATAAATACA
AAGTAGTACAAATTGAACCAATA---GGAGTAGCGCCCACCAAGGCAAAGAGAAGAGTGGTG-----------
----------------------------CAGAGAGAAAAAAGAGCA---GTGGGA---CTAGGAGCC------
---TTGTTCCTTGGGTTCTTGGGAGCA---GCAGGAAGCACTATG---GGCGCAGCGTCA---
ATAACGCTGACGGTACAG---GCCAGACAA---TTATTG---TCTGGTATAGTGCAACAGCAGAAC---
AATCTG---CTGAGA---GCTATTGAGGCGCAACAACATCTGTTGCAACTCACAGTCTGGGGCATC
>FJ952154

TTCTACTGTAACACAACAAAATTGTTTAATAGTACTTGGAATAGTAATGCTCCTTGGAATAGTACTGAATGG-
----

TTAAATGACACTATCACACTCCCATGCAGAATAAAACAAATTATAAACATGTGGCAGAAAGTAGGAAAAGCAA
TGTATGCCCCTCCCATCAGGGGACAAATTAGATGCTCATCAAAT---
ATTACAGGGCTAATATTAACAAGAGATGGTGGCAGAAATGGTAACAGTAGC----------------------
----

AACAACGGGAGCGAGACCTTCAGACCTGGAGGAGGAGATATGAGGGACAATTGGAGAAGTGAATTATATAAAT
ATAAAGTAGTAAAAATTGAGCCATTA---GGACTAGCACCCACCAAGGCAAAGAGAAGAGTGGTG--------
-------------------------------CAGAGAGAAAAAAGAGCA---GTGGGA---CTAGGAGCT---
------GTGTTCCTTGGGTTCTTGGGAGCA---GCAGGAAGCACTATG---GGCGCAGCGTCA---
GTAACGCTGACGGTACAG---GCCAGACTA---TTATTG---TCTGGTATAGTGCAACAGCAAAGC---
AATTTG---CTGAGG---GCTATTGAGGCGCAACAGCATCTGTTGCAACTCACAGTCTGGGGCATC
>DQ676886
TTCTACTGTAATTCAACACCACTGTTTAATAGTACTTGGGTGCGAGATGGTAATACTTGGAGGGAGTCTAATA
GAACTAAT---
-------------AGTAGT------
ACTTCAAGTGTTAATATCACACTCCCATGCAGAATAAAACAAATTATAAACATGTGGCAGGAAGTAGGAAAAG
CAATGTATGCCCCTCCTATCAAAGGACTAATTAGATGTTCATCAAAT---
ATTACAGGGATACTATTAACAAGAGATGGTGGTAATAACAATACC----------------------------
----

ACCAATGAAACTTTCAGACCTGGAGGAGGAGATATGAGAGACAATTGGAGAAGTGAATTATATAAATATAAAG
TAGTAAAAATTGAACCATTA---GGGATAGCACCCACCAGGGCAAAGAGAAGAGTGGTG--------------
-------------------------CAGAGAGAAAAAAGAGCA---GTAACA---TTAGGAGCT---------
ATGTTCCTTGGGTTCTTGGGAGCA---GCAGGAAGCACTATG---GGCGCAGCGTCA---
CTGACGCTGACGGTACAG---GCCAGACTA---TTATTG---TCTGGTATAGTGCAACAGCAGAAC---
AATCTG---CTGAGG---GCTATTGAGGCGCAACAACATCTGTTGCAACTCACAGTCTGGGGCATT
>AF042100
TTCTACTGTAATTCAACACAACTGTTTAATAGTACTTGGAATATTAATAGTACTTGGAATGATACTGGAGAGT
CAAATAAC---
-------------------ACT---
GTAGGCAATGTCACACTCACACTCCCATGCAGAATAAAACAAATTGTAAACCTGTGGCAGGAAGTAGGAAAAG
CAATGTATGCCCCTCCCATCAGCGGACTAATTAGATGTTCATCAAAT---
ATTACAGGGCTGCTATTAACAAGAGATGGGGGTAAGGGTAATGAC----------------------------
----

ACGGTCGAGACCTTCAGACCTGGAGGAGGAGATATGAGGGACAATTGGAGAAGTGAATTATATAAATATAAAG
TAGTAAAAATTGAACCATTA---GGAGTAGCACCCACTAAGGCTAAGAGAAGAGTGGTG--------------
-------------------------CAGAGAGAAAAAAGAGCA---GTGGGAATAGGAGCTGTG---------
TTGTTCCTTGGGTTCTTGGGAGCA---GCAGGAAGCACTATG---GGCGCAGCGTCA---
ATAACGCTGACGGTACAG---GCCAGACAA---TTATTG---TCTGGTATAGTGCAACAGCAGAAC---
AATTTG---CTGAGG---GCTACTGAGGCGCAACAGCATCTGTTGCAACTCACAGTCTGGGGCATC
>AF042101
TTTTACTGTAATTCAACACAACTGTTTAATGGTACTTGGAATGATACTGAAAGGTCATGGTCATATAACACTG
CA---

GAAAATAATGACACAATCACGCTCCCATGTAAAATAAAACAAATTATAAACAGGTGGCAGGAAGTAGGAAAAG
CAATATATGCCCCTCCCATCAGAGGACAAATTAAATGTTCATCAAAC---
ATTACAGGGCTGCTCTTAACAAGAGATGGTGGCCCTGACAATAAA----------------------------
----

GACACTGAGATCTTCAGACCTGGAGGAGGAGATATGAGGGACAATTGGAGAAGTGAATTATATAAATATAAAG
TAGTAAAAATTGAACCATTA---GGAATAGCACCCACTAAGGCAAAGAGAAGAGTGGTG--------------
-------------------------CAGAGAGAAAAAAGAGCA---GTGGGA---CTAGGAGCT---------
TTGTTCCTTGGGTTCTTGGGAGCA---GCAGGAAGCACTATG---GGCGCAGCGTCA---
ATAACGCTGACGGTACAG---GCCAGACAA---TTATTG---TCTGGTATAGTGCAGCAGCAGAAC---
AATCTG---CTGAGG---GCTATTGAGGCGCAACAGCATCTGTTGCAACTCACAGTCTGGGGCATC
>AF538304

TTCTACTGTAATACAACACAACTGTTTAATAGTACTTGGAATGTTACTGAAAGGTTAAATAACACTGAAAGA-
----

GATAATACTACAATCACACTCCCATGCAGAATAAAACAACTTATAAACATGTGGCAGGAGGTAGGAAAAGCAA
TGTATGCCCCTCCCATTAGCGGACGAATTAGATGTTCATCAAAT---
ATTACAGGGCTGATATTAACAAGAGATAGTGGTAGCGGGAACGGG----------------------------
----

ACCGAGACCTTCAGACCTGGAGGAGGAAATATGAAGGACAATTGGAGAAGTGAATTATATAAATATAAAGTAG
TAAGAATTGAACCATTA---GGAGTAGCACCCACCAAGGCAAAGAGAAGAGTGGTG-----------------
----------------------CAGAGAGAAAAAAGAGCA---TTAGGGGGACTAGGAGCT---------
ATGTTCCTTGGGTTCTTGGGAGCA---GCAGGAAGCACTATG---GGCGCAGCGTCA---
ATGGCGCTGACGGTACAG---GCCAGACAA---TTATTG---TCTGGTATAGTGCAACAGCAGAAC---
AATCTG---CTGAGA---GCTATTGAGGCGCAACAGCATCTGTTGCAACTCACAGTCTGGGGCATT
>AF538306
TTCTACTGTAATTCAACACCACTGTTTAATAGTACTTGGAATGTTACTACAGGGTCAAGTAACATGGAAGAGG
AT---

AGTCCAGAAAATATTACACTCACATGCAGAATAAAACAAATTATAAACATGTGGCAGGAAGTAGGAAAAGCAA
TGTATGCCCCTCCCATCAAAGGACTAATTAGCTGTTCATCAAAC---
ATTACAGGGCTGCTATTAACAAGAGATGGTGGTAATAATAGCGAGACC-------------------------
----

AAGACCACCGAGACCTTCAGACCTGGAGGAGGAGATATGAGGGACAATTGGAGAAGTGAATTATATAAATACA
AAGTAGTAAAAATTGAACCATTA---GGAGTAGCACCCACCAGGGCAAAGAGAAGAGTGGTG-----------
----------------------------CAGAGAGAAGAGAGAGCA---GTGGGA---ATAGGAGCT------
---TTGTTCCTTGGGTTCTTGGGAGCA---GCAGGAAGCGCTATG---GGCGCAGCATCA---
ATGACGCTGACGGTACAG---GCCAGACAA---TTATTG---TCTGGTATAGTGCAGCAGCAGAAC---
AATTTG---CTGTGG---GCTATTGAGGCGCAACAGCATCTGTTGCAACTCACAGTCTGGGGCATC
>AF042103
TTCTACTGTGATTCAACACGACTGTTTAATAGTACTTGGATTAATGGTACTCGGAATGAAACTGAAGGC----
----

AATGGAAGCACTATCACACTCCCCTGCAAAATAAAACAAATTATAAACCTGTGGCAGGAAGTAGGAAAAGCAA
TGTATGCCCCTCCCATCAGAGGACAAATTAGATGTACATCAAAT---
ATTACAGGGCTGATATTAACAAGAGATGGTGGTAATAACACGAAC----------------------------
----

GAGACCGAGATCTTCAGACCTGGAGGAGGAGATATGAGGGACAATTGGAGAAGTGAATTATATAGGTATAAAG
TAGTAAAAATTGAACCATTA---GGAGTAGCACCCACCAAGGCAAAGAGAAGAGTGGTG--------------
-------------------------CAGAGAGAAAAAAGAGCA---GTGGGA---ATAGGAGCT---------
GTGTTCCTTGGGTTCTTAGGAGCA---GCAGGAAGCACTATG---GGCGCAGCGTCA---
CTGACGCTGACGGTACAA---GCCAGACAA---TTATTG---TCTGGTATAGTGCAGCAGCAGAGC---
AATTTG---CTGAGG---GCTATTGAGGCGCAACAGCATCTGTTGCAACTCACAGTCTGGGGCATT
>AF042104
TTCTACTGTAATTCAACACAACTGTTTAATAGTATTTGGAAAAATGATACTTGGAATAATAGTACTTGGGATG
AC---

ACTGCAGGACATATCACACTTCCATGCAGAATAAAACACATTGTAAACATGTGGCAGGAAGTAGGAAAAGCCA
TGTATGCCCCTCCCATCAGAGGACAAATTAGATGTACATCAAAT---
ATTACAGGGATGCTACTAACAAGAGATGGTGGTAATAGCGATAACGCG-------------------------
----

AACGGGACCGAGACCTTCAGACCTGGAGGCGGAGATATGAGGGACAATTGGAGATGTGAACTATATAGATATA
AAGTAGTAAAAATTGAACCATTA---GGGATAGCACCCACCAAGGCAAAGAGAAGAGTGGTG-----------
----------------------------CAGAGAGAAAAAAGAGCA---GTGGGA---ATAGGAGCT------
---GTGTTCCTTGGGTTCTTGGGAGCA---GCAGGAAGCACTATG---GGCGCAGCGTCA---
ATGACGCTGACGGTACAG---GCCAGACTA---TTATTG---TCTGGTATAGTGCAACAGCAGATC---
ATTTTG---CTGAGG---GCTATTGAGGCGCAACAGCATCTGTTGCAACTCACAGTCTGGGGCATC
>AF042105

TTCTACTGTAATACAACACCACTGTTTAATAGAACATGGGTTAATGGTACCTGGGATAATACTTGGGATAATA
CTACA--

GACATTAACGGAAATATCATACTTCCATGCAGAATAAAACAAATTATAAACATGTGGCAGAAAGTAGGAAAAG
CAATGTATGCCCCTCCCATCAGAGGAATAATTAACTGTACATCAAAT---
ATTACAGGGCTGATATTAACAAGAGATGGTGGTTTGAATAGTACA----------------------------
----

AACGAGACTTTCAGACCTGGAGGAGGAGATATGAGGGACAATTGGAAAAGTAAATTATATAAGTATAAAGTAG
TAGAAATTGAACCATTA---GGAGTAGCACCCACCAAGGCAAAAAGAAGAGTGGTG-----------------
----------------------CAGAGAGAAAAAAGAGCG---GTGACA---CTAGGAGCT---------
ATGTTCCTTGGGTTCTTAGGAGCA---GCAGGAAGCACTATG---GGCGCAGCGTCA---
ATAACGCTGACGGCACAA---GCCAGACAA---TTATTG---TCTGGTATAGTGCAGCAGCAGAAC---
ACTCTG---CTGAGG---GCTATTAAGGCGCAACAGCATCTGTTGCAACTCACAGTCTGGGGCATC
>AF538302
TTCTATTGTAATTCAACAGGATTGTTTAATAGTACCTGGAATAATAGTACCTGGAATGATACTGAAAAGTCAA
ATAGC--
-------------------ACT---
GGAGGAAATGATACAATCACACTCCAATGCAGAATAAAACAAATTATAAACTTGTGGCAGGAAGTAGGAAAAG
CAATGTATGCCCCTCCCATCAGAGGAGAAATTAGATGTACATCAAAT---
ATTACAGGGCTGTTATTAACAAGAGATGGTGGTATTGTCAAGGAG----------------------------
----
----------------AACGAGACCTTCAGACCTGGAGGAGGC---GATATGAGG---
GACAATTGGAGAAGTGAATTATATAAAGTAGTACAAATTGAACCATTA---
GGAGTAGCACCCACCGAGGCAAAGAGAAGAGTGGTG-------------------------------------
--CAGAGAGAAAAAAGAGCA---ATAGGA---ATAGGAGCT---------
GTGTTCCTTGGGTTCTTGGGAGCA---GCAGGAAGCACTATG---GGCGCAGCGTCA---
ATGACGCTGACGGTACAG---GCCAGACTA---TTATTG---TCTGGTATAGTGCAACAGCAGAAC---
AATTTG---CTGAGG---GCTATTGAGGCGCAACAGCATCTGTTGCAACTCACAGTCTGGGGCATC
>AY624305
TTCTACTGTAATACATCAAAACTGTTTAATAGCACTTGGAATAGTAATGGGACTTGGAATGGTGATAAGGAA-
----

ACTAAAGGAATTATCGTACTCCCATGTAGAATAAAACAAATTATAAACATGTGGCAGGGAGTAGGAAAAGCAA
TGTATGCCCCTCCCGTCAAAGGAAAAATTGAATGTTCATCAAAT---
ATTACAGGGCTGCTATTAACAAGAGATGGTGGTATTAACAGCAGC----------------------------
----

GATAGCGAAACCTTCAGACCTGGAGGAGGAAATATGAAGGACAATTGGAGAAGTGAATTATATAAATATAAAG
TAGTAGAAATTGAACCATTA---GGAGTAGCACCCACCAAGGCAAAGAGAAGAGTGGTG--------------
-------------------------CAGAGAGAAAAAAGAGCA---GTGGGAATGATAGGAGCT---------
ATGTTCCTTGGGTTCTTGGGAGCA---GCAGGAAGCACTATG---GGCGCAGCGTCA---
ATGGCGCTGACGGTACAG---ACCAGACAA---TTATTG---TCTGGTATAGTGCAACAGCAGAAC---
AACTTG---CTGAAG---GCTATTGAGGCGCAACAGCATCTGTTGCAACTCACAGTCTGGGGCATC
>JN002002
TTCTACTGTAACACAACACAGCTGTTTAATAGTACTTGGAATTCAACACAACTGTTTAATAGTACTGGGGATG
ATACT--
-------------------GGA---
GAGTCAAATAAAATAATCACACTCCCATGTAGAATAAAACAAATTATAAACATGTGGCAGGAAGTAGGAAAAG
CAATGTATGCCCCTCCCATCAGAGGACAAATTAGATGTTTATCAAAT---
ATTACAGGGCTGATATTAACAAGAGATGGTGGTAATAACAGTGACACG-------------------------
----

AACACCACCGAGATCTTCAGACCGGGAGGAGGAGATATGAGGGACAATTGGAGAAGTGAACTATATAAATATA
AAGTAGTAAAAATTGAACCATTA---GGAGTAGCACCTACCAAGGCAAAGAGAAGAGTGGTG-----------
----------------------------CAGAGAGAAAAAAGAGCA---GTGGGAACGCTGGGAGCT------
---ATGTTCCTTGGGTTCTTGGGAGCA---GCAGGAAGCACTATG---GGCGCAGCGTCA---
ATAACGCTGACGGTACAG---GCCAGACTA---TTATTG---ACTGGTATAGTGCAACAGCAGAAC---
AACCTG---CTGAGG---GCTATTGAGGCGCAACAGCATCTGTTGCAACTCACAGTCTGGGGCATC
>JN002009

TTCTACTGTAATTCAACACAACTGTTTAATAGTACTTGGTTTAATAGTATTTGGAAAGTTAATGATACTGAAG
AGTCAAAT---
-------------AGCACT------
ACAGAAAATATTACAATCACACTCCCATGTAGAATAAAACAAATTATAAATATGTGGCAGGAAGTAGGAAAAG
CAATGTATGCCCCTCCCATCAGAGGACAAATTAGCTGTTTATCAAAT---
ATTACAGGGCTGTTATTAACAAGAGATGGTGGTAAGAATAACCAGAGC-------------------------
----

AACACCACCGAGACCTTCAGACCAGGAGGAGGAAATATGAAGGACAATTGGAGAAGTGAATTATATAAATATA
AAGTAGTAAGAATTGAACCATTA---GGAGTAGCACCCACCAAGGCAAAGAGAAGAGTGGTG-----------
----------------------------CAGAGAGAAAAAAGAGCA---GTGGGAACGATAGGAGCT------
---ATGTTCCTTGGGTTCTTGGGAGCA---GCAGGAAGCACTATG---GGCGCAGCGTCG---
CTGACGCTGACGGTACAG---GCCAGACTA---TTATTG---TCTGGTATAGTGCAGCAGCAGAAC---
AATCTG---CTGAGG---GCTATTGAGGCGCAACAGCATCTGTTGCAACTCACAGTCTGGGGCATC
>GU367406
TTCTACTGTAATTCAACACAACTGTTTGATAGTACATGGAATGATACTGAAAGTCCAAATAGCAAT-------
----

GACACCACAAATATCACACTCCCATGTAGAATAAAACAAATTATAAACATGTGGCAGAAAGTAGGAAAAGCAA
TGTATGCCCCTCCCATCAGCGGAATAATCAAATGTTCATCAAGT---
ATTACAGGGCTGCTTCTAACAAGAGATGGTGGTAGAAGTAACAGCAATGAC----------------------
----

ACTAATAACACTGAGATCTTCAGACCTGCAGGGGGAGATATGAAGGACAATTGGAGAAGTGAATTATATAAGT
ATAAAGTAGTAAAAATTGAACCATTA---GGAGTAGCACCCACCAAGGCAAAGAGAAGAGTGGTG--------
-------------------------------CAGAGAGAAAAAAGAGCA---GTGGGAATGCTAGGAGCT---
------ATGTTCCTCGGGTTCTTGGGAGCA---GCAGGAAGCACTATG---GGCGCAGCGTCA---
ATGACGCTGACGGTACAG---GCCAGACAA---TTATTG---TCTGGTATAGTGCAGCAGCAGAAC---
AACTTG---CTGAAG---GCTATTGAGGCGCAACAGCATCTGTTGCAACTCACAGTCTGGGGCATC
>GU367395
TTCTACTGTGATACATCAAAACTGTTTAATAGTACTTGGAATGGTCGGAATGAAAGTATCACAAAT-------
----

AGCACTGCAAATTTCACACTCCCATGTAGAATAAAACAGATTATAAACATGTGGCAGGGAGTAGGAAAAGCAA
TGTATGCCCCTCCCATCAGAGGACAAATTAGATGTTTATCAAAT---
ATTACAGGGATACTATTAACAAGAGATGGTGGTAATACTGGGAAC----------------------------
----

AACACCACCGAGATATTCAGACCTGGAGGAGGAGATATGAGGGACAATTGGAGAAGTGAACTATATAAATATA
AAGTAGTAGAAATTAAACCATTA---GGAGTAGCACCCACCCAGGCAAAGAGAAGAGTGGTG-----------
----------------------------CAAAGAGAAAAAAGAGCA---GTGGGAGTAATAGGAGCT------
---ATGTTCCTTGGGTTCTTGGGAGCA---GCAGGAAGCACTATG---GGCGCAGCGTCA---
ATGACACTGACGGTACAA---GCCAGACAA---TTATTG---TCTGGTATAGTGCAGCAGCAGAAC---
AATCTG---CTGAGA---GCTATTGAGGCACAACAGCATCTGTTGCAACTCACAGTCTGGGGCATC
>FJ653258
TTCTACTGTAATACAATACAATTGTTCAACAGTACTTGGAATGAAAATGATACTGAGAATGACAATAGTACTG
AA---

GGATCAAATGACACTATCACACTCCAATGCAAAATAAAGCAAATTGTAAACATGTGGCAGGAAGTAGGAAAAG
CAATGTATGCCCCTCCCATCAGCGGGAACATTACATGCTCATCACAC---
ATTACAGGGTTGCTATTAACAAGAGATGGTGGTGCGAACAGCAAC----------------------------
----

AATACCGAGATCTTCAGACCTGGAGGAGGAGATATGAGGGACAATTGGAGAAGTGAATTATATAAATATAAAG
TAGTAAGAATTGAACCATTA---GGAGTAGCACCCACCAAGGCAAAAAGAAGAGTGGTG--------------
-------------------------CAGAGAGAAAAAAGAGCA---GTGGGA---TTAGGAGTT---------
GTGTTCCTTGGGTTCTTGGGAGCA---GCAGGAAGCATTATG---GGCGCAGCGTCA---
CTAACGCTGACGGTACAA---GCCAGACAA---TTATTG---TCTGGTATAGTGCAACAGCAGGAC---
AACCTG---CTGAGA---GCTATTGAGGCGCAACAGCACCTGTTGCAACTCACAGTCTGGGGCATC
>MN485973

TTCTACTGTAATTCAACACAGCTGTTTAATAATAGTACTTGG-------------------------------
----
-------------------AAT---
GAAACTGATAACATTATCACACTCCCATGCAGAATAAAACAAATTATAAACAGATGGCAGGAAGTAGGAAAAG
CAATGTATGCCCCTCCCATTGCAGGACAAATTAGATGTTCATCAAAC---
ATTACAGGGCTGCTATTAACAAGAGATGGTGGTGAGAACGCAAGC----------------------------
----

GCCGAGGTCTTCAGACCGGGAGGAGGAGATATGAGGGATAATTGGAGAAGTGAATTATATAAATATAAGGTAG
TAAAAATCGAACCATTA---GGGGTAGCACCCACCAAGGCAAAGAGAAGAGTGGTG-----------------
----------------------CAGAGAGAAAAAAGAGCA---GTGGGA---ATAGGAGCC---------
ATGTTCCTTGGGTTCTTGGGAGCA---GCAGGGAGCACTATG---GGCGCAGCGTCA---
ATGACGCTGACGGTACAG---GCCAGACTA---TTATTG---TCTGGTATAGTGCAACAGCAGAAC---
AATCTG---CTGAGG---GCTATTGAGGCACAACAGCATATATTGCAACTCACAGTCTGGGGCATC
>MN485981
TTCTACTGTAATTCATCACAACTGTTTAATAGTACTTGGCAGTCTAATGATACTTGGCAGAGTAATAGTGAG-
----

AATGATACTATCATCACGCTTCCATGCAGAATAAAACAAATTATAAACAGATGGCAGGAAGTAGGAAAAGCAA
TATATGCCCCTCCCATTAGCGGACTCATTAACTGTACATCAAAC---
ATTACAGGGCTAATACTAACAAGAGATGGTGGTAATGTTAGCGAC----------------------------
----

AACCCTGAGACCTTCAGACCTACAGGAGGAGATATGAAGGACAATTGGAGGAGTGAACTATATAAATATAAAG
TAGTAAAAATTGAGCCATTA---GGAATAGCACCCACCAAGGCAAAGAGAAGAGTGGTG--------------
-------------------------CAGAGAGAGAAAAGA------GTGGGAGCACTAGGAGCT---------
GTGTTCCTTGGGTTCTTGGGAACA---GCAGGAAGCACTATG---GGCGCAGCGTCA---
TTGACGCTGACGGTACAG---GCCAGAACA---TTATTG---TCTGGTATAGTGCAACAGCAGAAC---
AATTTG---CTGAAG---GCTATTGAGGCGCAACAACATCTGTTGCAACTCACAGTCTGGGGCATC
>MN485993
TTCTACTGTAATACAACACAACTATTTAATAGTACTTGGAGTGGTAATGAGACTAAGACTGCAGAAAAC----
----

AATACAGAAACTATCACGCTCCCATGCAGAATAAAACAAATTGTGCGCCTGTGGCAGGAAGTAGGAACAGCAA
TGTATGCCCCTCCCATCAGTGGACTAATTCGGTGTTCATCAAAT---
ATTACAGGACTGTTATTGGTAAGAGATGGTGGTAATATCAATGGAACC-------------------------
----

AATAACACTGAGATATTCAGACCTGGGGGAGGAGATATGAGGGACAATTGGAGAAGTGAATTATATAAATATA
AAGTAGTAAAAATTGAACCAATA---GGAGTAGCACCCACCAAGGCGAAGAGAAGAGTGGTG-----------
----------------------------CAGAGAGAAAAAAGAGCA---GTGACA---CTGGGAGCT------
---ATGTTCCTTGGGTTCTTGGGAGCA---GCCGGAAGCACTATG---GGCGCAGCATCA---
GTAACGCTGACGGTACAA---GCCAGACAA---TTATTG---TCTGGTATAGTGCAACAGCAGAAC---
AATCTG---CTGAGG---GCTATTGAGGCGCAACAGCATCTGTTGCAACTCACAGTCTGGGGCATC
>MN485996
TTCTATTGTAATACAACGCAGCTGTTTAATAGTACTTGGAATAGTACTTTGGAAAATAGTACTAGGAATAAT-
----

ACAAATGCCACCATTACACTCCCATGCAGAATAAAGCAAATTGTAAACCTGTGGCAAGAAGTAGGAAAAGCAA
TGTATGCCCCTCCCATCAGAGGACAAATTAGATGTATATCAAAT---
ATTACAGGGATGCTATTAACAAGAGATGGTGGTACAGGTAATAACACT-------------------------
----

AACAATACTGAGATCTTCAGACCTGGAGGAGGAGATATGAGAGACAATTGGAGAAGTGAACTATATAAATATA
AAGTAGTAAAAATTGAACCATTA---GGAGTAGCACCCACCAAGGCAAAGAGAAGAGTGGTG-----------
----------------------------CAGAGAGAAAAAAGAGCA---GTGGGA---CTAGGAGCT------
---TTGTTCCTTGGGTTCTTGGGAGCA---GCAGGAAGCACTATG---GGCGCAGCGTCA---
GTGACGCTGACGGTACAG---GCTAGACAA---TTATTG---TCTGGTATAGTGCAGCAGCAGAAC---
AACCTG---CTGAGG---GCTATTGAGGCGCAACAACATCTGTTGCAACTCACAGTCTGGGGCATC
>MN485998

TTCTACTGTAATACATCACAGCTGTTTAATAGTACTTGGAGTAGTAGCAATTGGACTGATAATAGCAATAACA
GCACACAA--

GGGTCAAATGACACTATAACTCTCCCATGCAGAATAAAACAAATTATAAACAAATGGCAAGAAGTAGGAAGAG
CAATGTATGCCCCTCCCATTAGAGGACAAATTAGATGCGAATCAAAT---
ATTACAGGGCTGCTATTAACAAGAGATGGAGGGATCCTGAACAGT-------------------------
----

ACCAATGAGACTTTCAGACCTGAAGGAGGAGATATGAGGAACAATTGGAGGAGTGAATTATATAAATATAAAG
TAGTAGAAATTGAACCATTA---GGAATAGCACCCACCAAGGCAAAGAGAAGAGTGGTG-------------
------------------------CAGAGAGAAAAAAGAGCA---GTGGGA---ATAGGAGCT---------
CTGTTCCTTGGGTTCTTGGGAGCA---GCAGGAAGCACTATG---GGCGCAGCATCA---
ATGACGCTGACGGTACAG---GCCAGGCAA---TTACTG---TCTGGTATAGTGCAACAGCAGAAC---
AATTTG---CTGAGG---GCTATTGAGGCGCAACAGCATATGTTGCAACTCACAGTCTGGGGCATC
>MN486009
TTCTATTGTAATACATCACAACTGTTTAATAGCACTTGGGGGGTTAATAGTACCAGGAATGGTACACTTGCTT
GGTGGGTTAAT---
-GGTACCAGGAATGATACA------
CTTGTAAATGATACTATCACACTCCCATGCAGAATAAAACAATTTATAAACCTGTGGCAGGAAGTAGGAAAAG
CAATGTATGCCCCTCCCATCAAAGGACTAATTAAATGCTCATCAAAC---
ATTACAGGGTTACTATTAACAAGAGATGGTGGTAAAACCAATGAAACA----------------------
----

AATAACACCGAGATCTTCAGACCGGGAGGAGGAAATATGAAGGATAATTGGAGAAGTGAATTATATAAATATA
AAGTAGTAAAAATTGAACCATTA---GGAGTAGCACCCACCAAGGCAAAGAGAAGAGTGGTG-----------
----------------------------CAAAGAGAAAAAAGAGCA---GTGGGA---ATAGGAACA------
---TTGTTCCTTGGGTTCTTGGGAGCA---GCAGGAAGCACTATG---GGCGCAGCATCA---
ATAACGCTGACGGTACAG---GCCAGACAA---TTATTG---TCTGGTATAGTGCAGCAGCAGAAC---
AACCTG---CTGAGA---GCTATTGAGGCGCAACAACATATGTTGCAACTCACAGTCTGGGGCATC
>MN486010
TTCTACTGTAATACATCACAGCTGTTTAATAGTACTTGGGAGTTTAATGATACAAGTAAAACTTGGTACATCA
ACAATACAATT---
-------------AGCAAC------
AACACAAATAGCACAATCATACTCCCATGCAGAATAAAACAAATTATAAACAAATGGCAGGAAGTAGGAAGAG
CAATATATGCCCCTCCCATCGAAGGACAAATTAGATGCGAATCAAAT---
ATTACAGGGCTGCTATTAACAAGAGATGGAGGGATGAATAACACC-------------------------
----

AATGAGACCTTCAGACCTGAAGGAGGAGATATGAGGAACAATTGGAGGAGTGAATTATATAAATATAAAGTAG
TAGAAATTGAACCATTA---GGAGTAGCACCCACCAAGGCAAAGAGAAGAGTGGTG----------------
----------------------CAGAGAGAAAAAAGAGCA---GTGGGA---ATAGGAGCT---------
TTGTTCCTTGGGTTCTTGGGAGCA---GCAGGAAGCACTATG---GGCGCAGCGTCA---
TTAACGCTGACGGTACAG---GCCAGACAA---CTACTG---TCTGGTATAGTGCAACAGCAGAAC---
AATTTG---CTGAGA---GCTATTGAGGCGCAACAGCATATGTTGCAACTCACAGTCTGGGGCATC
>MN486013
TTCTACTGTAATACAACAAAACTGTTT---

----------ACTGAAAAC------
ATAAATAGCACAAATATCACACTCCCATGCAGAATAAAACAAATTATAAACATGTGGCAAAAAGTTGGAAAAG
CAATGTATGCCCCTCCCATCAGTGGAATAATTAGGTGTTCATCAAAT---
ATCACAGGGCTACTATTAATGAGAGATGGTGGTAATAGTAGTGGT-------------------------
----

AATGAGTCCGAGGTCTTCAGACCTGGAGGAGGAAATATGAAGGACAATTGGAGAAGTGAATTATATAAATATA
AAGTAGTAAGAGTTGAACCATTA---GGAATAGCACCCACCACGGCAAAGAGAAGAGTGGTG-----------
----------------------------CAGAGAGAAAAAAGAGCA---GTAGGA---CTAGGAGCT------
---GTGTTCCTTGGGTTCTTGGGAGCA---GCAGGAAGCACTATG---GGCGCAGCGTCA---
ATAACGCTGACGGTACAG---GCCAGACAA---TTATTG---TCTGGTATAGTGCAACAGCAAAAC---
AATATG---CTGAGA---GCTATTGAGGCGCAACAACATATGTTGCAACTCACAGTCTGGGGCATC
>MN486014

CTCTATTGTAATACATCACAGCTGTTTAATAGTACTTGGTTTAATGGTACTTGGAATGAGACTGGAGAGTCAA
AT---

AAAACAGAACTTATCATACTCCCATGCAGAATAAAACAAATCATAAACATGTGGCAGGAAGTAGGAAAAGCAA
TGTATGCCCCTCCCATCAAAGGAAACATTTCCTGTTCATCAAAT---
ATTACAGGACTGCTATTAACAAGGGATGGTGGTAACCTCAACGGGAGT-------------------------
----

AATGAGACCGAGATCTTCAGACCTGCAGGGGGAGATATGAGGGACAATTGGAGAAGTGAATTATATAAATATA
AAGTAGTAAGAATTGAACCATTA---GGAGTAGCACCCACCAGGGCAAAAAGAAGAGTGGTG-----------
----------------------------CAGAGAGAAAAAAGAGCA---TTGGGA---TTTGGAGCT------
---GTGTTCATTGGGTTCTTGGGAGCA---GCAGGAAGCACTATG---GGCGCAGCGTCA---
TTGACGCTGACGGTACAG---GCCAGAAAC---TTATTA---TCTGGTATAGTGCAGCAGCAGAAC---
AATCTG---CTGAGG---GCTATTGAGGCGCAACAGCATCTGTTGCAACTCACAGTCTGGGGCATC
>MN486022
TTCTACTGTAATACAACACAACTGTTTAATAGTACTTGGTATGCCAATAATACTTGGAATAATACTGGAGAG-
----

TCAAACATCACAATCACACTCCCCTGCAGAATAAGACAAATTGTAAACCTGTGGCAGGAAGTAGGAAAAGCAA
TGTATGCCCCTCCCATTAGAGGACCAATTACCTGTAGATCAAAT---
ATTACAGGGCTAATATTAACAAGAGATGGTGGTGAGAGTAACAATACC-------------------------
----

AAGAACAATGAGACCTTCAGACCCGGAGGAGGAGATATGAGGGACAATTGGAGAAGTGAATTATATAAATATA
AAGTAGTAAAAATTGAACCATTA---GGAGTAGCACCTACCAAGGCAAAGAGAAGAGTGGTG-----------
----------------------------CAGAGAGAAAGAAGAGCA---ATAGGA---TTAGGAGCT------
---ATGTTCCTTGGTTTCTTGGGAGCA---GCAGGAAGCACTATG---GGCGCAGCGTCA---
GTGACGCTGACGGTACAG---GCCAGACAA---TTATTG---TCTGGTATAGTGCAACAGCAGAGC---
AATCTG---CTGCAG---GCTATTGAGGCACAACAGCATCTGTTGAAACTCACAGTCTGGGGCATC
>DQ313249
TTCTACTGTAATACAACAAAACTGTTTAATAGTACTTGGAATGGTACTGAAGGGTCAAACAACACTGGAGGA-
----

GAAAATGACACGATCACACTCCCATGCAGAATAAAACAAATTGTAAACATGTGGCAGGAAGTAGGAAAAGCAA
TGTATGCACCTCCCATCAGAGGACAAATTAGATGTTCATCAAAT---
ATTACAGGGCTGATATTAACAAGAGATGGTGGTAATAATAATAAC----------------------------
----

ACGAACGAGACCTTCAGACCTGGAGGAGGAGATATGAGGGACAATTGGAGAAGTGAATTATATAAATATAAAG
TAGTAAAAATTGAACCATTA---GGAGTAGCACCCACCAGGGCAAAGAGAAGAGTGGTG--------------
-------------------------CAGAGAGAAAAAAGAGCA---ATAGCG------GGAGCT---------
GTGTTCCTTGGGTTCTTGGGAGCA---GCAGGAAGCACTATG---GGCGCAGCGTCA---
GTGGCGCTGACGGTACAG---GCCAGACTA---TTATTA---TCTGGTATAGTGCAACAGCAGAAC---
AATTTG---CTGAGG---GCTATTGAGGCGCAACAGCATCTGTTGCAACTCACAGTCTGGGGCATC
>L07421
TTCTACTGTAATTCAACAAAGCTGTTTAATAGTACTTGGAATGATACTAGTACTTGGAATGATACTACAGGAT
CA---

AATAAAGGAAATATCACACTCCTATGCAGAATAAAACAAATTATAAACAGGTGGCAAGAAGTAGGAAAAGCAA
TGTATGCCCCTCCCATCGAAGGACATATTAGATGTTCATCAAAT---
ATTACAGGGCTGTTACTAACAAGAGATGGTGGTAAGAACGAGAGC----------------------------
----

GAGACCACCGAGACCTTCAGACCTGGAGGAGGAGATATGAGGGATAATTGGAGAAGTGAATTATACAAATATA
AAGTGGTGAAGATTGAGCCATTA---GGAGTAGCACCCACCAAGGCAAAGAGAAGAGTGGTG-----------
----------------------------CAGAGAGAAAAAAGAGCA---GTGGGACTGATAGGAGCT------
---TTGTTCCTTGGATTCTTGGGAGCA---GCAGGAAGCACTATG---GGCGCGGCGTCA---
ATGACGCTGACGGTACAG---GCCAGACAA---TTATTG---TCTGGTATAGTGCAACAGCAGAGC---
AATTTG---CTGAGG---GCTATTGAGGCGCAACAGCATCTGTTGCAACTCACAGTCTGGGGCATC
>DQ313250

TTCTACTGTAATACATCACAACTGTTTAATACTACTTGGAGTGATACTACTACTTGGAATAATACTAACAAC-
----

ACAAATGGCAATATCACACTCCCATGCAGAATAAAACAAATTATAAACATGTGGCAGGGAGTAGGAAAAGCAA
TGTATGCTCCTCCCATCAGTGGACAAATTAGATGTTCATCAAAT---
ATTACAGGGCTGATATTAACAAGAGATGGTGGTCTCGCGAACAGG----------------------------
----

ACCAAAGAGACCTTCAGACCTGGAGGAGGAGATATGAGGGACAATTGGAGAAGTGAATTATATAAATATAAAG
TAGTAAAAATTGAACCATTA---GGAGTAGCACCCACCAAGGCAAAGAGAAGAGTGGTG--------------
-------------------------CAGAGAGAAAAAAGAGCA---GTGGGAATGCTAGGAGCT---------
GTGTTCCTTGGGTTCTTGGGAGCA---GCAGGAAGCACTATG---GGCGCAGCGTCA---
ATAACGCTGACGGTACAG---GCCAGACAA---TTATTG---TCTGGTATAGTGCAACAGCAGAAC---
AATCTG---CTGAAG---GCTATTGAGGCGCAACAGCATCTGTTGCAACTCACAGTCTGGGGCATC
>DQ313253
TTCTACTGTGATACAACACAACTGTTTAATAGTACTTGGCCTGTAGGGTCAAATACAAATGCCACAAATACC-
----

ACTGAAGAAATACTCACGCTCCCATGCAAAATAAAACAGATTATAAACAGGTGGCAGGAAGTAGGAAAAGCAA
TGTATGCCCCTCCCATCAGAGGAAAAATTAGATGTTCATCAAAT---
ATTACAGGGCTGCTAATAACAAGAGATGGTGGTAATAACAATAAC----------------------------
----

AATACGAACGAGACCTTCAGACCTGGAGGGGGAGATATGAGGGACAATTGGAGAAGTGAATTATATAAATATA
AAGTAGTAAAAATTGAACCATTA---GGAATAGCACCCACCAAGGCAAAGAGAAGAGTGGTG-----------
----------------------------CAGAGAGAAAAAAGGACA---GTGGGA---ATAGGAGCT------
---TTGTTCCTTGGTTTCTTGGGAGCA---GCAGGAAGCACTATG---GGCGCAGCGTCA---
CTGACGCTGACGGTACAG---GCCAGACAA---TTATTG---TCTGGTATAGTGCAACAGCAGAAC---
AATTTG---CTGAGG---GCTATTGAGGCGCAACAGCATATGTTGCAACTCACAGTCTGGGGCATC
>DQ313252
TTCTACTGTAATTCAACACCACTGTTTAATAGTACTTGGAATGATACTTGGAATGATAATGAACAGTCAAATG
AC---

ACTGAAGACACAATCACACTCCAATGCAGAATAAAACAAATTATAAACATGTGGCAGGAAGTAGGAAAAGCAA
TGTATGCCCCTCCCATCAGAGGACAAATTAGATGTTCATCAAAT---
ATTACAGGGCTACTATTAACAAGAGATGGTGGTAAAAATGATACCAATGAT----------------------
----

ACCGAGACCATCGAGGTCTTCAGACCTGGAGGAGGAGATATGAGGGACAATTGGAGAAGTGAATTATATAAAT
ATAAAGTAGTAAAAATTGAACCATTA---GGAGTAGCACCCACCAAGGCAAAGAGAAGAGTGGTG--------
-------------------------------CAGAGAGAAAAAAGAGCA---GTGGGA---ATAGGAGCT---
------GTGTTCCTTGGGTTCTTGGGAGCA---GCAGGAAGCACTATG---GGCGCAGCGTCA---
ATAACGCTGACGGTACAG---GCCAGACTA---TTATTG---TCTGGTATAGTGCAACAGCAGAAC---
AATTTG---CTGAGG---GCTATTGAGGCGCAACAACATCTGTTGCAACTCACAGTCTGGGGCATC
>EU191616
TTCTACTGTAATACAGCACAACTGTTTAATAGTACTTGGCATGCAAATGGCACTGTAGAGTCAACT-------
----

ACCACTGGAAATATCACACTCCCATGCAGAATAAAACAAATTATAAACATGTGGCAGGGAGTAGGAAAAGCAA
TGTATGCCCCTCCCATCAGAGGACAAATAAGATGTTCATCAAAT---
ATTACAGGGCTGCTCTTAACGAGAGATGGGGGGGATAATAACACG----------------------------
----

GACGAACCCGAAACCTTCAGACCTGCAGGAGGAGATATGAGGGACAATTGGAGAAGTGAACTATATAAATATA
AAGTAGTACAAATTGAACCATTA---GGAGTAGCACCCACCAAGGCAAAGAGGAGAGTGGTG-----------
----------------------------CAGAGAGAAAAAAGAGCA---ATAGGAACGATAGGAGCT------
---ATGTTCCTTGGGTTCTTGGGAGCA---GCAGGAAGCACTATG---GGCGCAGCGTCA---
ATAACGCTGACGGTACAG---GCCAGACAA---TTATTG---TCTGGTATAGTGCAACAGCAGAAC---
AACTTG---CTGAGG---GCTATTGAGGCGCAACAGCATCTGTTGCAACTCACAGTCTGGGGCATC
>MH746266

TTTTACTGTAATTCAACACAGCTATTTAATAGTACTTGGCTATTTAATAGTACTTGGAATGATACCAATTGGA
AGGGTACC--

GAAGGGAATGGTACCATCATACTCCCATGCAGAATAAAACAAATTATAAACAGATGGCAGGAAGTAGGACAAG
CAATGTATGCCCCTCCCATCAGCGGACTAATTAAATGTACATCAAAC---
ATTACAGGGCTATTACTAACAAGAGATGGTGGTAATAATGGAAGT---------------------------
----

AGTAACAATGAGACCTTCAGACCTGGAGGAGGAGACATGAGGGACAATTGGAGAAGTGAATTATATAAATATA
AGGTAGTAAAAATTGAGCCATTA---GGAGTAGCACCCACCAAGGCAAAGAGAAGAGTGGTG----------
---------------------------CAGAGAGAAAAAAGAGCG---GTGGGAGCAATAGGAGCT------
---ATGTTCCTTGGGTTCCTGGGAGCA---GCAGGAAGCACTATG---GGCGCAGCGTCG---
ATGACGCTGACGGTACAG---GCCAGACAA---TTATTG---TCTGGTATAGTGCAACAGCAGAAC---
AACCTG---CTGAGG---GCTATTGAGGCGCAACAGCATCTGTTGCAACTCACAGTCTGGGGCATC
>MH746265
TTCTACTGTAATTCATCACAACTGTTTAATAGTACTTGGGATAGGAATAGTACTTGGAATGATCCTAAT----
----

AATAGCACTACTATCACACTCCCATGCAGAATAAAACAAATTATAAACATGTGGCAGGAAGTAGGAAAAGCAA
TGTATGCTCCTCCCATCAGTGGACTAATTAAATGCTCATCAAAT---
ATTACAGGACTACTATTGACAAGAGATGGTGGTAATAATAATAACACA-----------------------
----

AATGAGACAGAGGTCTTCAGACCTGGAGGAGGAGATATGAGGGACAATTGGAGAAGTGAATTATATAAATATA
AAGTAGTACAAATTGAACCATTA---GGAGTAGCACCCACTAAGGCAAAAAGAAGAGTGGTG----------
---------------------------CAGAGAGAAAAAAGAGCT---CTGGGA---ATGGGAGCT------
---TTGTTCCTTGGGTTCTTGGGAGCA---GCAGGAAGCACTATG---GGCGCAGCGTCA---
ATAACGCTGACGGTACAA---GCCAGACAA---CTATTG---TCTGGTATAGTGCAACAGCAGAAC---
AATCTG---CTGAGG---GCTATTGAGGCGCAACAGCATATGTTGCAACTCACAGTCTGGGGCATC
>MH746264
TTCTACTGTAATTCAACACAACTGTTTAATAGCAGTTGGAGTTGGAATGGTACTAATGGTAATTGGAGTACTG
AAGAGCTAAAT--

GGCACTGAAAATATTACACTCCCATGTAGAATAAAACAAATAATAAACATGTGGCAAACAGTAGGAAAAGCAA
TGTATGCCCCTCCCATCAGAGGACAAATTAGATGTGCATCAAAT---
ATTACAGGGATGATACTAATAAGAGATGGTGGAAATGATGGAGGA---------------------------
----

GCCAACGAGACCTTCAGACCTGGAGGAGGAAATATGAGGGATAATTGGAGAAGTGAATTATATAAATATAAAG
TAGTACAAATTGAACCATTA---GGAGTAGCACCCACCAAGGCAAAGAGAAGAGTGGTG-------------
------------------------CAGAGAGAAAAAAGAGCA---GTGGGAACAATAGGAGCT---------
ATGTTCCTTGGGTTCTTGGGAGCA---GCAGGAAGCACTATG---GGCGCAGCGTCA---
ATGACACTGACGGTACAG---GCCAGACAA---TTATTG---TCTGGTATAGTGCAACAGCAGAAC---
AATCTG---CTGAGG---GCTATTGAGGCGCAACAGCATCTGTTGCAACTCACAGTCTGGGGCATT
>MH746261
TTCTATTGTAATACATCAGAGTTGTTTAATAGCACTTGGAATGAGACTACTACAAATTTAACTGGTATA----
----

AATGACTCAACTATCATACTCCCATGTAGAATAAAACAAATTATAAATATGTGGCAGGGAGTAGGAAAAGCAA
TGTATGCCCCTCCCATCAACGGAAAAATTATGTGTAACTCAACT---
ATTACAGGACTGCTCTTAACAAGAGATGGTGGTAGTAATAATGAGACC-----------------------
----

AACACCACCGAGATCTTCAGACCTGCAGGAGGAGATATGAGGGACAATTGGAGAAGTGAATTATATAAATATA
AAGTAGTAAAAATTGAGCCAATA---GGAATAGCACCCACCAAGGCAAAGAGAAGAGTGGTG----------
---------------------------CAGAGAGAAAAAAGAGCA---GTGGGAGTACTGGGAGCT------
---ATGTTCCTTGGGTTCTTGGGAGCA---GCAGGAAGCACTATG---GGCGCAGCGGCA---
ACAGCGCTGACGGTACAG---ACCAGACAA---TTATTG---TCTGGTATAGTGCGACAGCAGGAC---
AATCTG---CTGAAG---GCTATTGAGGCGCAACAGCACATGTTGCAACTCACAGTCTGGGGCATC
>MH746254

TTCTACTGTAATACAACAAAACTGTTTAATAGTACTTGGTATAGGAATAATACCAGTGACAGTAAAGAA----
----

GGAAATGGCACTATCACAATCCAATGCAGAATAAGACAAGTGATAAACATGTGGCAGGAAGTTGCAAAGCAA
TGTATGCCCCTCCTATCAGAGGAAAAATTATCTGTTCATCAAAT---
ATCACAGGAATAATATTGACAAGAGATGGTGGTGAGAATAACACA---------------------------
----

AATAACAACGAGACCTTCAGACCTGGAGGAGGAGATATGAGGGACAATTGGAGAAGTGAATTATACAAATATA
AAGTAGTAAAAATTGAACCACTA---GGAGTAGCACCCACCAAGGCAAAGAGAAGAGTGGTG-----------
---------------------------CAGAGAGAAAAAAGAGCA---ATAACA---CTAGGAGCT------
---ATGTTCCTTGGGTTCTTGGGAGCA---GCAGGAAGCACTATG---GGCGCAGCGTCA---
CTGACGCTGACGGTACAG---GCCAGACAA---TTATTG---TCTGGTATAGTGCAGCAGCAGAAC---
AATCTG---CTGAGG---GCTATTGAGGCGCAACAACATCTGTTGCAACTCACGGTCTGGGGCATC
>DQ358809
TTTTACTGTAATACAACAAAGCTGTTTAATAGTACTTGGAATGATAATAGTACTGGAGAGGCTACTAGTACCT
GGAATAGTACT---
----------GAAGAGCCA------
GAAAATAGCGCAATTATCATACTCCCATGCATAATACAACTCATCATAAACAGGTGGCAGGAAGTAGGAAAAG
CAATGTATGCCCCTCCCATCAAGGGAATAATTAGCTGCTCATCAAAT---
ATTACAGGGTTGCTATTAACAAGAGATGGTGGTAACAATAGCGAG--------------------------
----

ACCAATGAGACCTTCAGACCTGGAGGAGGAGATATGAGGGACAATTGGAGAAGTGAATTATATAAATATAAAG
TAGTAAAAATTGAGCCATTA---GGAGTAGCACCCACCAAGGCAAAGAGAAGAGTGGTG-------------
------------------------CAGAGAGAAAAAAGAGCA---GTGGGAACGCTAGGAGCT---------
ATGTTCCTTGGGTTCTTGGGAGCA---GCAGGAAGCACTATG---GGCGCAGCGTCA---
GTGACGCTGACGGTACAG---GCCAGACAA---TTATTG---TCTGGTATAGTGCAACAGCAAAAC---
AATTTG---CTGAAG---GCTATTGAGGCGCAACAACATTTGTTGCAACTCACAGTCTGGGGCATC
>DQ358810
TTCTACTGCAATACATCACGGCTGTTTAATACTACTTGGAATACTACTACTTGGAACAGTACTGAGCTGGCTA
ATATTAGT--
-------------GCTATT------
AACGATAATGAAACTATCACACTCCAGTGCAGGATAAAACAAATTATAAACATGTGGCAGGCGGTAGGAAAAG
CAATGTATGCCCCTCCCATCCAAGGACTAATTAGATGTTCATCAAAT---
ATAACAGGGCTGCTATTAACAAGGGATGGTGGTAATAATAGTATTAAGTGGAAC------------------
----
-
CAGAGCACATCCGACTCCGAGGTCTTCAGACCCCTAGGAGGAAATATGAAGGACAATTGGAGAAGTGAATTAT
ATAAATATAAAGTAGTACAAATTGAGCCATTA---GGAATAGCACCCACCAAGGCAAAGAGAAGAGTGGTG--
-------------------------------------CAGAGAGAGAAAAGAGCA---GTGGGA---
CTAGGAGCT---------GTGTTCATTGGGTTCTTGGGAGCA---GCAGGAAGCACTATG---
GGCGCAGCGTCA---ATAACGCTGACGGTACAA---GCCAGACAA---TTGTTG---
TCTGGTATAGTGCAACAGCAAAAC---AATTTG---CTGAAG---
GCTATTGAGGCGCAACAGCATCTGTTGCAACTCACAGTCTGGGGCATT
>JN692435
TTTTACTGTAATTCAACACAGCTGTTTAATAGTACTTGGCTGCATAATAACACTTGGAATGGTACTACAGGAT
CA--

AATAACAATGACACTATCACACTCCCATGCAGAATAAGACAAATTATAAACAGGTGGCAAGAAGTAGGAAAAG
CAATGTATGCCCCTCCCATCGCAGGACTAATTAGGTGTTCATCAAAT---
ATTACAGGGTTACTCTTAACAAGAGATGGTGGTCCGAACGTGACC--------------------------
----

ACCGAAATCTTTAGACCTGGAGGAGGAGATATGAGGGACAATTGGAGAAGTGAATTATATAAATATAAAGTAG
TAAGAATTGAGCCATTA---GGAGTAGCACCCACCAAGGCAAGGAGAAGAGTGGTG----------------
----------------------CAGAGAGAAAAAAGAGCG---GTGACG---CTGGGAGCT---------
ATGTTCCTTGGGTTCTTGGGAGCA---GCAGGAAGCACTATG---GGCGCAGCGTCG---
ATAACGCTGACGGTACAG---GCCAGACAA---TTATTG---TCTGGTATAGTGCAACAGCAAAAC---
AATTTG---CTGAGA---GCTATTGAGGCGCAACAGCATCTGTTGCAGCTCACAGTCTGGGGCATC

>JN692440
TTCTACTGTGATACAACAAAGCTGTTTAATAGTACTTGGAATACTACTAGGAATACAACTAAAAGTGAAGAAG
AA---

GGAGATAGCACAATCATACTCCCATGCAGAATAAAACAAATTGTAAATATGTGGCAGGAAGTAGGAAACGCAA
TGTATGCCCCTCCCATCAGCGGACTAATTAGATGTTCATCAAAT---
ATTACAGGGCTGCTATTAACAAGAGATGGTGGGGGTGGTAACAGG----------------------------
----

TCGAACGAGGAGACCTTCAGACCTATAGGAGGAGATATGAGGGACAATTGGAGAAGTGAATTATATAAATATA
AAGTAGTAAAAATTGAACCACTA---GGAATAGCACCCACCAAGGCAAAGAGAAGAGTGGTG-----------
----------------------------CAGAGAGAAAAAAGAGCA---GTGGGA---CTAGGAGCT------
---TTGTTCCTTGGGTTCTTGGGAGCA---GCAGGAAGCACTATG---GGCGCAGCGGCA---
ATGACGCTGACGGTACAG---ACCAGACAA---TTGTTG---TCTGGTATAGTGCAACAGCAGAAC---
AATTTG---CTGAGA---GCTATTGAGGCGCAACAGCATCTGTTGCAACTCACAGTCTGGGGCATC
>JN692443
TTCTACTGTAATACAACAAAACTGTTCAATAGTAGTTGGCAGATTAATGGTACTGGTAGTGGAGAGCCAGATG
AT---

GAGAATGGCACTATCACACTCCCATGCAGAATAAAACAAATTATAAACAGGTGGCAGGAAGTAGGAAAAGCAA
TATATGCTCCTCCCATCAGAGGACTAATTAGATGTTCCTCAAAT---
ATTACAGGGCTGCTACTCACAAGAGATGGTGGTAATAGTAACACG----------------------------
----

AGCAATAATGAGACATTCAGACCTGGAGGAGGAAATATGAAGGACAATTGGAGAAGTGAATTATATAAATATA
AAGTAGTACAAATTGAGCCATTA---GGAGTAGCACCCACTAAGGCAAAAAGAAGAGTGGTG-----------
----------------------------CAGAGAGAAAAAAGAGCA---GTAACG---CTGGGAGCT------
---ATGTTCCTTGGGTTCTTGGGAGCA---GCAGGAAGCACTATG---GGCGCAGCATCG---
GTGACGCTGACGGTACAG---GCCAGACTA---TTATTG---TCTGGTATAGTGCAACAGCAAAAC---
AATTTG---CTGAGG---GCTATTGAGGCGCAACAGCACCTGTTGCAACTCACAGTCTGGGGCATC
>JN692444
TTCTATTGTAATACAACACGGCTGTTTAATAGTACTTGGCCTATTAATGATACAAATATGAATAATACAAATA
ACTCA--

AATAGCACTAGAATTATCACACTCCAATGCAAAATAAAACAAATTATAAACAGGTGGCAGGAAGTAGGAAAAG
CAATGTATGCCCCTCCCATTAAAGGACCAATTAGCTGTTCATCAAAT---
ATTACAGGGCTATTATTAACAAGAGATGGTGGTAATACTAGTAGAACT-------------------------
----

AATGGTACTGAAATCTTCAGACCTGGAGGAGGAAATATGAAGGACAATTGGAGAAGTGAATTATACAAATATA
AAATAGTACAAATTAACCCATTA---GGAGTAGCACCCACCAAGGCAAAGAGAAGAGTGGTG-----------
----------------------------CAGAGAGAGAAAAGAGCA---GTGGGAGCGCTGGGAGCT------
---ATGTTCCTTGGGTTCTTGGGAGCA---GCAGGAAGCACTATG---GGCGCAGCGTCA---
CTGACGCTGACGGTACAG---GCCAGACAA---TTATTG---TCTGGTATAGTGCAACAGCAAAAC---
AATTTG---CTGAAA---GCTATTGAGGCGCAGCAGCATCTGTTGCAACTCACAGTCTGGGGCATC
>JN692445
TTCTACTGTAACACAACAAAGCTGTTTAATAGTGATTGGGACAATGGTACTTGGAATGATACTAAC-------
----

AAGGGAGAAAATATCACAATCCCATGCAGAATAAAACAAATCATAAACAGATGGCAGGAAGTAGGAAAAGCAA
TGTATGCCCCTCCCATCCAAGGACAAATTAGATGTTCATCAAAT---
ATTACAGGGCTGTTATTGACAAGAGATGGTGGCCTTAACACTAGCGAG-------------------------
----

ACTAACAGGACCGAAATCTTCAGGCCTACAGGAGGAGATATGAGGGACAATTGGAGAAGTGAATTATATAAAT
ATAAAGTAGTAAGAATTGAGCCACTA---GGAATAGCACCCACCAAGGCAAAGAGAAGAGTGGTG--------
-------------------------------CAGAGAGAAAAAAGAGCA---GTGGGAACATTAGGAGCT---
------ATGTTCCTTGGGTTCTTGGGAGCA---GCAGGAAGCACTATG---GGCGCAGCGTCA---
ATGACGCTGACGGTACAG---GCCAGACTA---TTATTG---TCTGGTATAGTGCAACAGCAGAAC---
AATTTG---CTGAAG---GCTATTGAGGCGCAACAGCATATGTTGCAACTCACAGTCTGGGGCATT

>JN692447
TTCTATTGTAACACAACACAATTGTTTAATTGGGATGATAATAGTACTTGG----------------------
----

ACAGAGGGCAAAATCATACTCCCATGCAGAATAAAACAAATAGTAAACAGGTGGCAGGAAGTAGGCAAAGCAA
TGTATGCCCCTCCCATCAGAGGATCAATTAACTGTACATCAAAC---
ATTACAGGGTTGCTATTAACAAGAGATGGTGGTAATGGAACCAAC----------------------------
----

AATGAGACCTTCAGACCTGGAGGAGGAGATATGAGGGACAGTTGGAGAAGTGAATTATATAAATATAAAGTAG
TAAAAATTGAACCTTTA---GGAGTAGCACCCACCAAGGCAAAGAGAAGGGTGGTG-----------------
----------------------CAGAGAGAAAAAAGAGCA---GTGGGAGCAATAGGAGCT---------
GTGTTCCTTGGGTTCCTGGGAACA---GCAGGAAGCACTATG---GGCGCAGCGTCA---
CTAACGCTGACGGTACAA---GCCAGACTA---TTACTG---TCTGGTATAGTGCAACAGCAGAAC---
AATCTG---CTGAGA---GCTATTGAGGCGCAACAACATCTGTTGCAACTCACGGTCTGGGGCATT
>EF637056
TTCTACTGTAATACAACAAGGCTGTTTAATGGCACTTGGGATGGTACTAAATGGAATAATACTGTATGG----
----

GGAAATAACACGCTTATACTTCCATGCAGAATAAAACAAATTATAAATAAGTGGCAGGAAGTAGGAAAAGCAA
TGTATGCCCCTCCCATTAGAGGACAAATTAGATGTTCATCAAAT---
ATTACAGGGCTGCTATTAACAAGAGATGGTGGTAACAGTAACAAT----------------------------
----

AAGTCTGAAATTTTCAGACCAGGAGGAGGATATATGAAGGACAATTGGAGAAGTGAATTATATAAATATAAAG
TAGTAAGAATCGAGCCATTA---GGAATAGCACCCAACAGGGCAAGGAGAAGAGTGGTG--------------
-------------------------CAAAGAGAAAAAAGGGCA---GTGGGG---ACCGATAGG---------
ATGTTCCTTGGGTTCTTGGGAGCA---GCAGGAAGCACTATG---GGCGCAGCGTCA---
GTGACGCTGACGGTACAG---GCCAGACAA---TTATTG---TCTGGTATAGTGCAGCAGCAAAAC---
AATTTG---CTGAGA---GCTATTGAGGCGCAACAACATATGTTGCAACTCACAGTCTGGGGCATC
>EF637054
TTCTACTGTGATACAGCGAAGCTGTTCAATAGCACGTGGATTTGGAATAGTACTCGGAATGATTCTAGCACTA
AT---

GAAACCAGCATAATCACACTCCCATGCAAAATAAAACAAATTATAAACAGGTGGCAGGAAGTAGGAAAAGCAA
TGTATGCCCCTCCTATCAGAGGACAAATTGAATGTTCCTCCAAT---
ATTACAGGGTTGCTATTAACCAGAGATGGTGGTAACAGTAGTAAC----------------------------
----

AACGAGACCTTCAGACCTGGAGGAGGAGATATGAGGGACAATTGGAGAAGTGAATTATATAAATATAAGGTAG
TAAAAATTGAACCATTA---GGATTAGCACCCACCAAGGCAAAGAGAAGAGTGGTG-----------------
----------------------CAGAGAGAAAAAAGAGCA---GTGGGAACAATGGGAGCT---------
ATGTTCCTTGGGTTCTTGGGAGCA---GCAGGAAGCACTATG---GGCGCAGCGTCA---
CTGACGCTGACGGTACAG---GCCAGACTA---TTATTG---TCTGGTATAGTGCAACAGCAGAAC---
AATTTG---CTGAGA---GCTATTGAGGCGCAACAGCATATGTTGCAGCTCACAGTCTGGGGCATC
>EF637051
TTCTACTGTAATACAACACAGCTGTTTAATAGTACTTGGGGGTTAAATAGTAATGGCACAAATAGTAATGGCA
CAAT---

AGTAATGGCACAAATATCACACTCCCATGCAGAATAAAACAAATTATAAACAGGTGGCAGGAAGTAGGAAAAG
CAATGTATGCTCCTCCCATCAGGGGAAATATTAGCTGTACATCAAAT---
ATCACAGGGATACTATTAACAAGGGATGGTGGTACCAACCAGACC----------------------------
----

AGTGAAACCGAAACCTTCAGACCTGGAGGGGGCGATATGAGGGATAATTGGAGAAATGAATTATATAAATATA
AAGTAGTAAAAATTGAGCCATTA---GGAGTAGCACCCACCACGGCAAAGAGAAGAGTGGTG-----------
----------------------------CAGAGAGAAAAAAGAGCA---GTGGGG---ATAGGAGCT------
---TTGTTCCTTGGGTTCTTGGGAGCA---GCGGGAAGCACTATG---GGCGCAGCGTCA---
ATGACGCTGACGGTACAG---GCCAGACTA---TTATTG---TCTGGTATAGTGCAACAGCAGAAC---
AATCTG---CTGAGG---GCTATTGAGGCGCAACAGCATATGTTGCAACTCACAGTCTGGGGCATC

```
>EF637050
TTCTACTGTAATACAACACAGCTGTTTAATAGTACTTGGTATTCTAATGGTACTTTGAATGATACTGGTACTT
GGAATGGT-----------------------------------------------------------------
-------------ACTGAG------
GGGTTAAATGACACTATCACACTCCCATGCAAAATAAAACAATTTATAAACAGGTGGCAAGAAGTAGGAAAAG
CAATGTATGCCCCTCCCATCAGAGGACAAATTAGATGCTCATCAAAT---
ATTACAGGGCTGCTATTAACAAGAGATGGAGGGAAAGTGAACGGGTCT-------------------------
-------------------------------------------------------------------------
----------
AACGGGACTGAGATCTTCAGACCTGGAGGAGGAAATATGAGGGACAATTGGAGAAGGGAATTATATAAATATA
AAGTAGTAAAAATTGAACCATTA---GGAGTAGGACCCACCAGAGCAAAAAGAAGAGTGGTG-----------
----------------------------CAGAGAGAGAAAAGAGCA---GTGGGA---ATAGGAGCT------
---CTGTTCCTTGGGTTCTTGGGAGCA---GCAGGAAGCACTATG---GGCGCAGCGTCA---
TTGACGCTGACGGTACAG---GCCAGACAA---TTATTG---TCTGGTATAGTGCAACAGCAAAAC---
AACTTG---CTGATG---GCTATTGAAGCGCAACAGCATCTGTTGAAGCTCACAGTCTGGGGCATC
>EF637048
TTCTACTGTAATACAACACAGTTGTTTAATAGTACTTGGGTTAATGGTGCTTGGATTAATGGTACTTGGTCC-
-------------------------------------------------------------------------
----------------------------
AATAATACTAATATCATACTCCCATGCAGAATAAAACAAATTATAAACAGGTGGCAGGAAGTAGGAAGAGCAA
TGTATGCCCCTCCCATCAGAGGACAAATTAGCTGTTCATCAAAT---
ATTACAGGGCTGCTATTAATGAGAGATGGTGGTAAGGACAACAAT----------------------------
-------------------------------------------------------------------------
-------------
ATTTCCGAAATCTTTAGACCTGCAGGAGGAGATATGAGGGACAATTGGAGAAGTGAATTATATAAATATAAAG
TAGTAAAAATTGAGCCATTA---GGAATAGCACCCACCAAGGCAAGGAGAAGGGTGGTG--------------
-------------------------CAGAGAGAAAAAAGAGCA---GTGACA---CTAGGAGCT---------
GTGTTCCTTGGGTTCTTGGGAGCA---GCAGGAAGCACTATG---GGCGCAGCGTCA---
GTGACGCTGACGGTACAG---GCCAGGCTA---TTATTG---TCTGGTATAGTGCAACAGCAAAAC---
AACTTG---CTGAGG---GCTATTGAGGCGCAACAGAGTATGTTGCAACTCACCGTCTGGGGCATC
>EF637047
TTTACTGTAATACATCAAAGCTGTTTAATAGTACTTGGACTTGGAATGGTACTGGGCCTGAAATGGTAAATA
ACAAC--------------------------------------------------------------------
-------------------ACA---
GAAAACACAACAGAAATTACACTCCCATGCAGAATAAAACAAATTATAAACAGGTGGCAGGAAGTAGGAAAAG
CAATGTATGCCCCTCCCATCAGTGGAATAATTAGATGTACATCAAAT---
ATTACAGGGCTACTGTTAACAAGAGATGGTGGTAATGTAAGTAAT----------------------------
-------------------------------------------------------------------------
-------------
ACCACTGATATCTTCAGACCTGGAGGAGGAGATATGAGGGACAATTGGAGAAGTGAACTATATAAATATAAAG
TAGTAAAAATTGAGCCATTA---GGAGTAGCACCCACCAAGGCAAGGAGAAGAGTGGTG--------------
-------------------------CAGAGAGAAAAAAGAGAA---GTGGGAACATTCGGAGCT---------
ATGTTCCTTGGGTTCTTAGGAGCA---GCAGGAAGCACTATG---GGCGCAGCGTCA---
GTGACGCTGACGGTACAG---GCCAGACAA---TTATTG---TCTGGTATAGTGCAACAGCAGAAC---
AATTTG---CTGAGG---GCTATTGAGGCGCAACAGCATATGTTGCAACTCACAGTCTGGGGCATC
>EF637046
TTCTACTGTAATACAACACCGCTGTTTAATAATAGTAAGTGGGTGAATGGTGGGTGGAATAGTACTATG----
-------------------------------------------------------------------------
----------------------------
AAGAATGGAACTATCACCCTCCCATGCAGAATAAAACAAATTGTAAACAGGTGGCAGGAAGTAGGAAAAGCAA
TGTATGCCCCTCCCATCAGAGGACAAATTAACTGTACATCACAA---
ATTACAGGGCTGCTCTTAACAAGAGATGGTGGTATTAACCAGACC----------------------------
-------------------------------------------------------------------------
-------------
AACAATGAAACCTTCAGACCAGCAGGAGGAGATATGAGGGACAATTGGAGAAGTGAATTATATAAATATAAAG
TAGTGAAAATTGAGCCATTA---GGAGTAGCACCCACCAAGGCAAGGAGAAGAGTGGTG--------------
-------------------------CAAAGAGAAAAAAGAGCA---GTGGGAACAATAGGAGCT---------
ATGTTCCTTGGGTTCTTGGGAGCA---GCAGGAAGCACTATG---GGCGCAGCGGCA---
ATGACGCTGACGGTACAG---GCCAGACTA---TTATTG---TCTGGTATAGTGCAACAGCAGAAC---
AACTTG---CTGAGA---GCTATTGAGGCGCAACAGCATATGTTGCAGCTCACAGTCTGGGGCATC
```

>JN692450
TTCTACTGCAATACAACAGATCTGTTTAATAGTGCTTGGGATTCTACAAATGATACTGACAATCTTACAGATG
ATACTGGC---
-------------ATGGGA------
GGAGATGGTTCAGTTATCATACTCCCATGCAGAATAAAACAAATTATAAACAGATGGCAGGAAGTAGGAAAAG
CAATGTATGCCCCTCCCATCAGAGGACAAATTAGCTGTTCATCAAAT---
ATTACAGGGCTGATATTAACAAGAGATGGTGGTAAAAATGGTAATAAC-------------------------
----

ACAAACGGGACTGAAACCTTCAGACCTGGAGGAGGAGATATGAGGGACAATTGGAGAAGTGAATTATATAAAT
ATAAAGTAGTAAAAATTGAGCCATTA---GGGAGTACACCCACCAAGGCAAAGAGGAGAACGGTG--------
-------------------------------CAGAGAGAAAAAAGAGCA---GTGGGAACGCTAGGAGCT---
------ATGTTCCTTGGGTTCTTGGGAACA---GCAGGAAGCACTATG---GGCGCAGCGTCA---
ATAACGCTGACGGTACAG---GCCAGACAA---TTATTG---TCTGGTATAGTGCAACAGCAGAAC---
AATTTG---CTGAGG---GCTATTGAGGCGCAACAGCATATGTTGCAACTCACAGTCTGGGGCATT
>JN692451
TTCTACTGTAATACAACACCACTGTTTAATAGCACATGGCATGATAATAATAATGTAAGCATATGGCATGTTA
ATAGAACTGTA--
-AATAGTACTGAAGGG---------
TTAAAGGGAGAAAATATCACACTCCAATGCAGAATAAAACAGATTATAAACAGGTGGCAGGAAGTAGGAAAAG
CAATGTATAACCCTCCCATTAGGGGCTTAATTAGATGTTCATCAAAT---
ATTACAGGGATACTATTAATGAGAGATGGTGGTGGTCCTAACAAC----------------------------
----

CGTACTAACGAAACCTTCAGACCAGGAGGAGGAAATATGAAGGACAATTGGAGAAGTGAATTATATAAATACA
AAGTAGTAAAAATTGAGCCATTA---GGACTAGCACCCACAAGGGCAAAGAGAAGAGTGGTG-----------
----------------------------CAGAGAGAAAAAAGAGCA---GTGGGAACAATAGGAGCT------
---ATGTTCCTTGGGTTCTTGGGAGCA---GCAGGAAGCACTATG---GGTGCAGCGGCA---
GTGACGCTGACGGTACAG---GCCAGGCTA---TTGTTG---TCTGGTATAGTGCAACAGCAGAAC---
AATCTG---CTGAAG---GCTATTGAGGCGCAACAACGTATGTTGCAACTCACAGTCTGGGGCATT
>JN692452
TTCTACTGTAATACATCACAACTGTTTAATAGTACTTGGGGGCCTAATGGTACTTGGCTGTCAAATAGTACTG
AGAATAGG---
-------------GCTAAT------
GGGTCTAATGACATTATCATACTCCCATGCAGAATAAAACAAATTATAAACATGTGGCAGGAAGTAGGAAAAG
CAATGTATGCCCCTCCCATCCAAGGACAAATTAACTGTTCATCACAT---
ATAACAGGACTGCTATTAACAAGAGATGGTGGAAATAGTAATAGAACAAAC----------------------
----

GGGTCCGAAACCGAAATCTTCAGACCTGGAGGAGGAGATATGAGGGACAATTGGAGAAGTGAATTATATAAAT
ATAAAGTAGTAAAAATTGAGCCATTA---GGAATAGCACCCACCAAGGCAAGGAGAAGAGTGGTG--------
-------------------------------CAGAGAGAAAGAAGAGCA---ATAACA---CTAGGAGCT---
------ATGTTCCTTGGGTTCTTGGGAGCA---GCAGGAAGCACTATG---GGCGCAGTGTCG---
ATGACGCTGACGGTACAG---GCCAGACAA---TTATTG---TCTGGAATAGTGCAACAGCAGAAC---
AATCTG---CTGAAG---GCTATTGAGGCGCAACAGCATATGTTGAAACTCACAGTCTGGGGCATC
>JN692453
TTCTATTGTAATTCATCACAGCTGTTTAATAGCACTTGGAATAATACCAATTGGAATGATACTAATATACAGG
CA---

AATGACACTGAAAATATCACACTCCCATGCAGGATAAAACAAATTATAAACAGATGGCAGGAAGTAGGAAAAG
CAATGTACGCCCCTCCCATCAGCGGAATAATTAAATGTACATCAAAT---
ATTACAGGGCTACTATTAACAAGAGATGGTGGTAACAAGAATGGGACG-------------------------
----

GAAAGGAATGAGACCTTCAGACCTATAGGAGGAAATATGAAGGACAATTGGAGAAGTGAATTATATAAATATA
AAGTAGTAAAAATTGAGCCATTA---GGAATAGCACCCACCAAGGCAAAGAGAAGAGTGGTG-----------
----------------------------CAGAGAGAAAAAAGAGCA---GTCACA---ATAGGAGCT------
---ATGTTCCTTGGGTTCTTGGGAGCA---GCAGGAAGCACTATG---GGCGCAGCGTCA---
ATGACGCTGACGGTACAG---GCCAGACTA---TTATTG---TCTGGTATAGTGCAACAGCAGAAC---
AATTTG---CTGAGA---GCTATTGAGGCGCAACAGCATATATTGCAACTCACAGTCTGGGGCATT

>JN692457
TTCTACTGTAATACAACAAAGCTGTTTAATAGTACTTGGCATATTAATGGTACTTGGAATGACACTAAA----
----

GGTAATGAGACTATCACACTCCCATGCAGAATAAAACAAATTATAAACAGGTGGCAGGAGGTAGGAAGAGCAA
TGTATGCCCCTCCCATTAGTGGATGGATCAACTGTTCATCAAAT---
ATTACAGGGCTGATATTAACAAGAGATGGTGGTAACCAAAGCGGG----------------------------
----

CCCGACTCCGAAACCTTCAGACCTGGAGGAGGAGATATGAGGGACAATTGGAGAAGTGAATTATATAAATATA
AAGTAGTAAAAATTGAGCCATTA---GGAATAGCACCCACCAAGGCAAAGAGAAGAGTGGTG-----------
----------------------------CAAAGAGAAAAAAGAGAA---CTAACA---CTAGGAGCG------
---TTGTTTCTTGGGTTCTTGGGAGCA---GCAGGAAGCACTATG---GGCGCAGCGTCA---
TTAACGCTGACGGTACAG---GCCAGACAA---TTATTG---TCTGGTATAGTGCAACAGCAGAAC---
AATCTG---CTGAGG---GCTATTGAGGCGCAACAGCATATGTTGCAACTCACAGTCTGGGGCATC
>FJ195090
TTCTACTGTAATACAACACAACTATTTAATTTCACTTGGAATGGTACTGAGATTGGAAATAATAGTACT----
----

AAAGAAAAAAATATCACACTCCCATGTAGAATAAAACAAATTGTAAACAGATGGCAGGAAGTAGGAAAAGCAA
TGTATGCCCCTCCCATCAGTGGATACATTAAATGTGTATCAAAT---
ATTACAGGGCTGCTATTAGCAAGAGATGGTGGTAATACTAGTGAAAAT-------------------------
----

AACAACACCGAGATCTTTAGACCTTTAGGAGGAAATATGAAGGACAATTGGAGAAATGAATTATATAAATATA
AAGTAGTAAAAATTGAGCCATTA---GGAGTAGCACCCACCTTGGCAAAGAGAAGAGTGGTG-----------
----------------------------CAAAGAGAAAAAAGAGCA---GTGGGAACAATAGGAGCT------
---ATGTTCCTTGGGTTCTTGGGAGCA---GCAGGAAGCACTATG---GGCGCAGCATCA---
CTAACGCTGACGGTACAA---GCCAGGCTA---CTACTG---TCTGGTATAGTGCAACAGCAGAAC---
AATCTG---CTGAGG---GCTATTGAGGCGCAACAACATCTGTTGCAACTCACAGTCTGGGGCATC
>JN692459
TTCTACTGTAATACAACACAGCTATTTAAGGATACTTGGAATGGCACTTGGATTGATAATATTTGGAAGGAGA
ATGATACT---
-------------GAAAAG------
ACAGATAACACTACTATCACACTCCCATGCAGAATAAAACAAATTATAAACAGGTGGCAGGACGTAGGAAAAG
CAATGTATGCCCCTCCTATCAGAGGATACATTAACTGTTCATCAAAT---
ATTACAGGGCTAATATTAACAAGAGATGGGGGTGACAGCAACTTGAATAAGACC------
TCGAATGGGACCTGCTTGAAGGGGACC--

TGCTCGAATGAGACTGAGACCTTTAGACCTGTAGGAGGAGATATGAGGGATAATTGGAGAAGTGAATTATATA
AATATAAAGTAGTAAAAATTGAGCCATTA---GGGATAGCACCCACCAAGGCAAAGAGAAGAGTGGTG-----
----------------------------------CAAAGAGAAAAAAGAGCA---
GTGGGAACGATAGGAGCT---------ATGTTCCTTGGGTTCTTGGGAGCA---GCAGGAAGCACTATG---
GGCGCAGCGTCA---CTGACGCTGACGGTACAG---GCCAGACTA---TTATTG---
TCTGGTATAGTGCAACAGCAGAAT---AATTTG---CTGAGG---
GCTATTAAGGCGCAACAACATCTGTTGCAACTCACAGTCTGGGGCATC
>JN692460
TTCTACTGTAATACATCAGGGCTGTTTAATAGTACTTGGCTAAATAATGGTACTAGGAATGATACTAACTCTA
CAAGT--
-------------------GAT---
AATAGCCCAAAGAATATCACACTCCCATGCAGAATAAAACAAATTATAAACAGGTGGCAGGAAACGGGAAGAG
CAGTATATGCCCCTCCCATCAAAGGAATAATTAGCTGTTCATCCAAT---
ATTACAGGGCTGCTATTAACAAGAGATGGTGGCAGCGGTGGGAACGACACC----------------------
----

GGAAACGCCACCGAGGTCTTCAGACCTGGGGGAGGAGATATGAGGGACAATTGGAGAAGTGAATTATATAAAT
ATAAAGTAGTAAGAATTGAACCATTA---GGAATGGCACCCACCGAGGCAAAGAGAAGAGTGGTG--------
-------------------------------CAGAGAGAAAAAAGAGCA---GTGGGA---ATAGGAGCT---
------TTGTTCCTTGGGTTCTTGGGAGCA---GCAGGAAGCACTATG---GGCGCAGCGGCA---

ATGACGCTGACGGTACAG---GCCAGACTA---TTATTG---TCTGGTATAGTGCAACAGCAAAAT---
AATTTG---CTGAGA---GCTATTGAAGCACAACAGCATATGTTGCAACTCACAGTCTGGGGCATC
>JN692463
TTCTACTGTAATACGACACAACTGTTTAATAGTACTTGGGGTATGGATGACAAGGATGTGAATGGTACTACA-
----

GGAAATGAAACTATCATACTTCCATGCAGAATAAAACAAATTGTAAACATGTGGCAGGAAGTAGGAAAAGCAA
TGTATGCCCCTCCCATCAGAGGACAAATTAGATGTTCATCAAAT---
ATCACAGGGCTGCTATTAACAAGAGATGGTGGTGGCAATAAAAAC----------------------------
----

AGAAGTAATGAGACCTTCAGACCTGGAGGAGGAGATATGAAGGACAATTGGAGAAGTGAATTATATAAATATA
AAGTAGTAAAAATTGAACCATTA---GGAGTAGCACCCACCAAGGCAAGGAGAAGAGTGGTG-----------
----------------------------CAGAGAGAAAAAAGAGCA---GTGGGAACACTAGGAGCT------
---CTGTTCCTTGGGTTCTTGGGAGCA---GCAGGAAGCACTATG---GGCGCAGCGTCA---
ATGACGCTGACGGTACAG---GCCAGACTA---TTATTG---TCTGGTATAGTGCAACAGCAGAAC---
AATTTG---CTGAGG---GCTATTGAGGCGCAACAGCATATGTTGCAACTCACAGTCTGGGGCATC
>JN692467
TTCTACTGTAATTCAACACAACTGTTTGATACTTGGAATGATACTACCCATGGA-------------------
----

GGAAATGACACAATCATACTCCCATGCAGAATAAAACAAATTATAAACAGGTGGCAGGAAGTAGGAAAAGCAA
TGTATGCCCCTCCCATCAAAGGACAAATTAATTGTGTATCAAAT---
ATCACAGGGCTGATATTAACAAGAGATGGTGGTAACACGAATGACCAG-------------------------
----

AACGGGTCCGAGATCTTCAGACCTGGAGGAGGAAACATGAAGGACAATTGGAGAAGTGAATTATATAAATATA
AAGTAGTAAAAATTGAACCATTA---GGAGTAGCACCCACCAGGGCAAAAAGAAGAGTGGTG-----------
----------------------------CAGAGAGAAAAAAGAGCA---GTGGGA---CTAGGAGCT------
---TTGTTCCTTGGGTTCTTGGGAGCA---GCAGGAAGCACTATG---GGCGCAGCGTCA---
ATGACGCTGACGGTACAG---GCCAGACAA---TTATTG---TCTGGTATAGTGCAACAGCAAAAC---
AACTTG---CTGAGG---GCTATTGAGGCGCAACAGCGTCTGTTGCAACTCACAGTCTGGGGCATC
>JN692475
TTCTACTGTAATACAACACAGCTGTTTAATAGTACTTGGAAGTTTAATGGTACCTGGAGTATGTCCAATGGGA
AT---

AGTACTGAAAAGAATATCACACTCCCATGCAGAATAAAACAAATTATAAATAAGTGGCAAGAAGTAGGAAAGG
CAATGTATGCCCCTCCCATCAGCGGAATAATTAAATGTTCATCAAAT---
ATTACAGGGCTGCTATTAACAAGAGATGGGGGTGATAAT----------------------------------
----

TCGACAGAAACCTTCAGACCGGGAGGAGGAGATATGAGGGACAATTGGAGAAGTGAATTATATAAATATAAAG
TAATAAGGATTGAGCCATTA---GGAATAGCACCCACCAAGGCAAAGAGAAGAGTGGTG--------------
-------------------------CAGAGAGAAAAAAGAGCA---ATAGGA---ATAGGAGCT---------
GTGTTCCTTGGGTTCTTGGGAGCA---GCAGGAAGCACTATG---GGCGCAGCGTCA---
GTAACGCTGACGGTACAG---GCCAGACAA---TTATTG---TCTGGTATAGTGCAACAGCAGAAC---
AATTTG---CTGAAG---GCTATTGAGGCGCAGCAGCATATGTTGCAACTCACTGTCTGGGGCATC
>FJ195091
TTCTACTGTGATACAACTAGGCTGTTTAATAATGAAACACAG-------------------------------
----

AATGACACTATCACACTCCCATGCAAAATAAGACAAATTATAAACAGATGGCAGGAAGTAGGAAAATCAATGT
ATGCCCCTCCCATCAGAGGAGAAATTAAATGTTCATCAAAT---
ATTACAGGGCTGATATTAACAAGAGATGGTGGTAACAGTACCAGC----------------------------
----

ACCATTGAGACCTTCAGACCTGGAGGAGGAAATATGAAGGACAATTGGAGGAGTGAATTATATAAATATAAAG
TAGTAAAAATTGAGCCACTA---GGAATAGCACCCACCAAGGCAAGGAGAAGAGTGGTG--------------
-------------------------CAAAGAGAAAAAAGGGCA---GTGGGAACGTTTGGAGCT---------
ATGTTCCTTGGATTCTTGGGAGCA---GCAGGAAGCACTATG---GGCGCAGCGTCA---

CTGACGCTGACGGTACAG---GCCAGACTA---TTATTG---TCTGGTATAGTGCAACAGCAAAAC---
AATTTG---CTGAGA---GCTATTGAGGCGCAACAACATATGTTGCAACTCACAGTCTGGGGCATC
>FJ195088
TTCTACTGTAATACAACACAGTTATTTAATAGTACTTGGGAGTTTAATAGTACTACGCAGTTGAATATT----
----

ACAGGTAAAACTATCACACTTCCATGCAGAATAAAACAAATTATAAACAGGTGGCAGGAAGTAGGAAAAGCAA
TGTATGCCCCTCCCATCGGAGGACAAATTAGCTGTTCATCAAAA---
ATTACAGGGCTGCTATTAACAAGAGATGGTGGAAATGATGGTAACAAT-------------------------
----

ACTGGGAACGAGACCTTCAGACCTGGAGGAGGAGATATGAGGGACAATTGGAGAAGTGAATTATATAAATATA
AAGTAGTAAAAATTGAACCATTA---GGAGTAGCACCCACCACGGCAAAGAGAAGAGTGGTG-----------
----------------------------CAGAGAGAAAAAAGAGCA---GTGGGAACGATAGGAGCT------
---ATGTTCCTTGGGTTCTTGGGAGCA---GCAGGAAGCACTATG---GGCGCAGCGTCA---
ATAACGCTGACGGTACAG---GCCAGACTA---TTATTG---TCTGGTATAGTGCAGCAGCAGAAC---
AATTTG---CTGAGG---GCTATTGAGGCGCAACAGCATATGTTGCAACTCACAGTCTGGGGCATC
>FJ195089
TTCTACTGTAACACAACAAAGCTGTTTAATAGTATTTGGAAGCATAATAGTACTACTAATATTAAAGGAAATT
CC---

ACTAGTAATGACACTATTACACTCCCATGCAGAATAAAACAAATTATAAACAAGTGGCAGGAAGTAGGAAAAG
CAATGTATGCCCCTCCCATCAGAGGACAAATTAAATGTAAATCAAAT---
ATTACAGGGCTGCTATTAACAAGAGATGGTGGTAACACCAACAATAGTGAA----------------------
----

GCCAACAGCAGTGAAACCTTCAGACCTGGAGGAGGAGATATAAGGGACAATTGGAGAAGTGAATTATATAAAT
ATAAAGTAGTAAGAATTGAGCCATTA---GGAATAGCACCCACCAAGGCAAAGAGAAGAGTGGTG--------
-------------------------------CAGAGAGAAAAAAGAGCA---GTGGGAACATTAGGAGCT---
------GTGTTCCTTGGGTTCTTGGGAACA---GCAGGAAGCACGATG---GGCGCAGCGTCA---
ATAACGCTGACGGTACAG---GCCAGACTA---TTATTG---TCTGGTATAGTGCAACAGCAGAAC---
AATTTG---CTGAGG---GCTATTGAGGCGCAACAGCATATGTTGCAACTCACAGTCTGGGGCATC
>JN692479
TTCTACTGTGATTCAACACAGTTGTTTAATAGTACTTGGCATAATACTACAGACAATAATACTACA-------
----

GACAATAGCAATATCACACTCCCATGCAGAATAAAACAAATTATAAACAGGTGGCAAAAAGTAGGAAAAGCAA
TGTATGCCCCTCCCATCGCAGGACCAATTAGCTGTACATCAAAC---
ATTACAGGGCTGCTATTAACAAGAGATGGTGGAGAAAGCAATGGG----------------------------
----

ACTGAGACCTTCAGACCTGGAGGGGGAGATATGAGGGACAATTGGAGAAGTGAATTATATAAATACAAAGTAG
TAAAAATTGAGCCATTA---GGAATAGCACCCACCAAGGCAAGGAGGAGAGTGGTG-----------------
----------------------CAAAAAGAGAAAAGAGCA---GTGACG---CTGGGAGCT---------
ATGTTCCTTGGGTTCTTGGGAGCA---GCAGGAAGCACTATG---GGCGCAGCGTCA---
CTGACGCTGACGGTACAG---GCCAGACTA---TTGTTG---TCTGGTATAGTGCAACAGCAAAAT---
AATTTG---CTGAAT---GCTATTGAGGCGCAACAACATCTGTTGCAACTCACAGTCTGGGGCATC
>HQ377443
TTCTACTGCAATACATCACAACTGTTTAATAGTACTTGGAATATTGATGGTACTTGGAATGATACTGGA----
----

GGAAAGCAAAATATCACACTCCCATGCAGAATAAGACAAATTGTAAACAGATGGCAAGAAGTAGGGAAGGCAA
TGTATGCCCCTCCCATCCCAGGACAAATTACATGTAGATCAAAT---
ATTACAGGGATACTATTAACAAGAGATGGTGGCGGCAAAAATGAT----------------------------
----

AGCCTTAATGAAACCTTCAGACCTGGAGGAGGAGATATGAGGGACAATTGGAGAAGTGAATTATATAAATATA
AAGTAGTAAGAATTGAACCATTA---GGAATAGCACCCACCAAGGCAAAGAGAAGAGTGGTG-----------
----------------------------CAGAGAGAAAAAAGAGCA---GTGACA---TTAGGAGCT------
---ATGTTCCTTGGGTTCTTGGGAGCA---GCAGGAAGCACTATG---GGCGCAGCGGCA---

ACAACGCTGACGGTACAG---GCCAGACAA---TTATTG---TCTGGTATAGTGCAACAGCAGAAC---
AATCTG---CTGATG---GCTATTGAGGCGCAACAGCATATGTTGCAACTCACAGTCTGGGGCATC
>HQ377487
TTCTACTGCAATACAACACAGCTGTTTAATAGTACCTGGACCCAGGTAAACGGTACTACAGTTAGGAATGGTA
CAAATAGC---
-------------ACTGCT------
AACACAAATGGCACTATCACACTCCCATGCAGAATAAAACAAATTGTAAACACGTGGCAGGCAGTAGGGAAAG
CAATGTATGCCCCTCCCATCAGAGGACAGATTAGCTGTAACTCAAGT---
ATTACAGGGCTGTTATTAACAAGAGATGGTGGTAGTGGTGTCAATGTC-------------------------
----

ACGAACGACACCGAAATCTTCAGACCTGCAGGAGGAGATATGAGGGACAATTGGAGAAGTGAATTATATAAAT
ATAAAGTAGTGAAAATTGAGCCACTA---GGAATAGCACCCACCAAGGCAAGGAGAAGAGTGGTG--------
-------------------------------CAAAGAGAAAAAAGAGCA---GTGGGA---ATAGGAGCT---
------GTGTTCCTTGGGTTCTTGGGAGCA---GCAGGAAGCACTATG---GGCGCAGCGTCA---
GTGACGCTGACGGTACAG---GCCAGACTA---TTATTG---TCTGGTATAGTGCAGCAGCAGAAC---
AATTTG---CTGAGA---GCTATTGAGGCGCAACAGCACATGTTGCAACTCACAGTCTGGGGCATC
>JN687739
TTCTACTGTAATACAACAAAACTGTTTAATAGTATTTGGACTTGGAATGATACACAGCTGTTGAATGATACTG
GAGAG--

CCAGACAATGAAACTATCACACTCCCATGCAGAATAAGACAAATTATAAACAGGTGGCAGGAAGTAGGAAAAG
CAATGTATGCCCCTCCCATCAGAGGACATATTAACTGTTCATCAAAT---
ATTACAGGGCTGCTATTAACAAGGGATGGTGGTAATAACACGAAC----------------------------
----

GGGACCGAGACCTTCAGACCTGGGGGAGGAGATATGAGGGACAATTGGAGAAGTGAATTATATAAATATAAAG
TAGTACAAATTGAGCCAGTA---GGAGTAGCACCCTCCAAGGCAAAGAGAAGAGTGGTG--------------
-------------------------CAGAGAGAGAAAAGAGCA---GTGGGAGCAATAGGAGCT---------
ATGTTCCTTGGGTTCTTGGGAGCA---GCAGGAAGCACTATG---GGCGCAGCGTCA---
CTCACGCTGACGGTACAG---GCCAGACAA---TTATTG---TCTGGTATAGTGCAACAGCAGAAC---
AATATG---CTGAGG---GCTATTGAGGCGCAACAACATCTGTTGCAACTCACAGTCTGGGGCATC
>JF680907
TTCTACTGTAATACAACAAAACTGTTTAATAGTAACCGGACTATAGGGTCGAATGACACTAAAGAG-------
----

AATGACACAGACATCACACTCCCATGCAAAATAAAACAAATTGTAAACATGTGGCAGGAAGTAGGAAAAGCAA
TGTATGCCCCTCCTATCAAGGGAAAAATTGAATGTTCATCAAAT---
ATTACAGGGATACTATTAACAAGAGATGGAGGTGATAATAGAACA----------------------------
----

AACGAGACCTTCAGACCAGCAGGAGGAGATATGAGGGACAATTGGAGAAGTGAATTATATAAATATAAAGTAG
TAAGAATTGAACCATTA---GGAGTAGCACCTACCAGGGCAAGGAGAAGAGTGGTG-----------------
----------------------CAGAGAGAAAAAAGAGCA---GTGGGAACAATAGGAGCT---------
ATGTTCCTTGGGTTCCTAGGAGCA---GCAGGAAGCACTATG---GGCGCAGCGTCA---
GTAACGCTGACGGTACAG---GCCAGACTA---TTATTG---TCTGGTATAGTGCAACAGCAAAAC---
AATTTG---CTGAAG---GCTATTGAGGCGCAACAGCATCTGTTGCAACTCACGGTCTGGGGCATC
>KU749389
TTCTACTGTAACACAACAAAACTGTTTAATAGTAGTTGGCCTCTTAATGGTTCAGCAAATATAACT-------
----

GCAGAAGACCCTATCAGACTCCCATGCAGAATTAGACAAATTGTAAACATGTGGCAGAAAGTAGGAAAAGCAA
TGTATGCCCCTCCCATCGAAGGACAGATTAGATGTTCATCAAAT---
ATTACAGGACTGCTATTAGTAAGAGATGGTGGTAATGGTGGTAATGAC-------------------------
----

ACAAACACGACCGAGATCTTCAGACCTGAAGGAGGAAATATGAAGGACAATTGGAGAAGTGAATTATATAAAT
ATAAAGTAGTAAGAATTGAACCCTTA---GGAATAGCACCCACCAAGGCAAAGAGAAGAGTGGTG--------
-------------------------------CAGAGAGAAAAAAGAGCA---TTGGGA---TTCGGAGCG---
------TTGTTCCTTGGGTTCTTGGGAGCA---GCAGGAAGCACTATG---GGCGCAGCGTCA---

GTGACGCTGACGGTACAG---GCCAGACAA---TTATTG---TCTGGTATAGTGCAACAGCAAAAC---
AACTTG---CTGAGG---GCTATTGAGGCGCAACAACATCTGTTGCAACTCACAGTCTGGGGCATC
>KT427690
TTCTACTGTAATACCACAACGCTATTTAATAGTACGTGGAATAAGAATGATACTAGGAATGATACTACT----
----

AGGAATGATACTATCACACTCCAATGCAGAATAAAACAAATTATAAACAGGTGGCAGGAAGTAGGAAAAGCAA
TATATGCCCCTCCCATCCCAGGACAAATTAAATGCTCATCCAAT---
ATTACAGGGCTGCTACTAACAAGAGATGGTGGTAACAGTACCGAG----------------------------
----

AGTGAAACCTTCAGACCTGGAGGAGGAGATATAAGGGACAATTGGAGAAGTGAATTATATAAATATAAAGTAG
TACAGATTGAGCCATTA---GGAGTAGCACCCACCAGGGCAAAAAGACAAGTGGTG-----------------
----------------------AGGAGAGAAAAAAGAGCA---GTGGGA---CTGGGAGCT---------
TTGTTCCTTGGGTTCTTGGGAGCA---GCAGGAAGCACTATG---GGCGCGGCGTCA---
CTAACGCTGACGGTACAG---GCCAGGCAA---TTATTG---TCTGGGATAGTGCAACAGCAGAAC---
AATCTG---CTGAGG---GCTATTGAAGCGCAACAGCACCTGTTGCAGCTCACAGTCTGGGGCATT
>KT427688
TTCTACTGTAATACATCAAAACTGTTTAATAGTACTGAGAATGGTAATGACACCAATAGTACTGGCAA----
----

TCTAACAGTACCATCACACTCCAATGCAGAATAAAACAAATTATTAATAGGTGGCAGGAAGTAGGAAAAGCAA
TGTATGCCCCTCCCATCAGAGGAAACATTAGCTGTTCATCAAAC---
ATTACAGGGCTACTATTAACAAGAGATGGGGGTAACACAACTGAC----------------------------
----

AATGAGACCTTCAGGCCAACAGGAGGAGATATGAAGGACAATTGGAGAAGTGAGTTATATAAATATAAAGTAG
TAAGAATTGAACCAATA---GGAGTAGCCCCCACCAAGGCAAAGAGAAGAGTGGTG-----------------
----------------------CAGAGAGAAAAAAGAGCA---GTAACA---CTGGGAGCT---------
GTGTTCCTTGGGTTCTTGGGAGCA---GCAGGAAGCACTATG---GGCGCAGCGTCA---
ATAACGCTGACGGCACAG---GCCAGACAA---TTGTTG---TCTGGTATAGTGCAACAGCAGAGC---
AATCTG---CTGAGA---GCTATTGAGGCGCAACAGCATCTGTTGCAACTCACAGTCTGGGGCATC
>KT427686
TTCTACTGTGATACAACACAGCTGTTTAATAGTACTTGGGATGGAAATAGTACTGAAAACAATAACACAGGA-
----

GGAAATGAAACTATCATACTCCCATGCAGAATAAAACAAATTATAAATACATGGCAGGGAGTAGGAAAAGCAA
TATATGCCCCTCCCATCAGTGGAAAGATTAATTGTTTATCAAAT---
ATTACAGGGCTGCTATTAACAAGAGATGGTGGG--

TCCAATGAGAGTGAGACCTTCAGACCCACAGGAGGAAATATGATGGATAATTGGAGAAGTGAATTATATAAAT
ATAAAGTAGTAAGAATTGAGCCCTTA---GGAGTAGCACCCACCAAGGCAAAGAGAAGAGTGGTG--------
-------------------------------CAAAGAGAAAAAAGAGCT---GTGGGAGCAATAGGAGCT---
------ATGTTCCTTGGGTTCTTGGGAGCA---GCAGGAAGCACTATG---GGCGCAGCGTCG---
CTGACGCTGACGGTACAG---GCCAGACTA---TTATTG---TCTGGTATAGTGCAACAGCAAAAC---
AACTTG---CTGAGG---GCTATTGAGGCGCAACAGCACCTGTTGCAACTCACAGTCTGGGGCATT
>KT427685
TTCTACTGTAATACAACACAACTGTTTAACAGGACCTGGAATGAGACTACCTTGAATGGTACTAACCTTAATG
TAACT--

TCCAATGAAAATATCACAATTCCATGCAGAATAAAACAAATTATAAACAGATGGCAGGAAGTGGGAAAAGCAA
TGTATGCCCCTCCCATCAGAGGACAAATTAGGTGCTCATCAAAT---
ATTACAGGACTGTTATTAACAAGAGATGGTGGCGGAGAGAACAGG----------------------------
----

TCCAATGAAACATTCAGACCTGGAGGAGGAGATATGAGGGACAATTGGAGAAGTGAATTATATAAATATAAAG
TGGTAAAAATTGAGCCATTA---GGAATAGCACCCACCAAGGCAAAGAGAATAGTGGTG--------------
-------------------------CAGAGAGAAAAAAGAGCA---GTAACA---CTAGGAGCT---------
ATGTTCCTTGGGTTCTTGGGAACA---GCAGGAAGCACTATG---GGCGCAGCGTCA---

CTAACGCTGACGGTACAG---GCCAGACTA---TTATTG---TCTGGTATAGTGCAACAGCAGAAC---
AATTTG---CTGAGA---GCTATTGAGGCGCAACAGCATATGTTGAAACTCACAGTCTGGGGCATC
>KT427683
TTCTACTGTAATACATCAGGACTGTTTAATAGTACTTGGTTGGTTAATGGCACCAGCAATACAGAT------
----

TCAAATGAAACAATCGAACTTCCATGTAGAATAAAACAAATTATAAACAGATGGCAGGAAGTAGGAAAAGCAA
TGTATGCCCCTCCCATCAGAGGACAAATTAGCTGTTCATCAAAC---
ATTACAGGGCTGCTATTAATAAGAGATGGCGGTAATGGGTCTGGG---------------------------
----

AACAATGAGACCTTCAGACCTGGTGGAGGAGATATGAGGGACAATTGGAGAAGTGAATTATATAAATATAAAG
TAGTAAAAATTGAGCCATTG---GGAGTAGCGCCTTCCAAGGCAAAGAGAAGAGTGGTG-------------
-------------------------CAGAGAGAAAAAAGAGCG---GTGGGAACACTAGGAGCC---------
ATGTTCCTTGGGTTCTTGGGAGCA---GCAGGAAGCACTATG---GGCGCAGCGTCA---
ATAACGCTGACGGTACAG---GCCAGACAA---TTACTG---TCTGGTATAGTGCAACAGCAGAAC---
AATCTG---CTGAGA---GCTATTGAGGCGCAACAACACCTGTTGCAACTCACAGTCTGGGGCATC
>KT427682
TTTACTGTAATACAACACAGCTGTTTAATAGTACTTGGAATGGGACACAGCTGTTTAATAGTACTTGGAATG
GGACGGAATCA---TCA------GAT--

AACTCAAATGACACTATCATACTCCCATGCAGAATAAAACAAATTATAAATAGGTGGCAGGAAGTAGGAAAAG
CAATGTATGCCCCTCCCATCAAAGGAATAATTAGATGTTCATCAAAT---
ATTACAGGGCTACTACTAACAAGAGATGGTGGTAGCGACAGTAGT---------------------------
----

AATGGCACCGAAACCTTCAGGCCTATAGGAGGAGATATGAGGGACAATTGGAGAAGTGAATTATATAAATATA
AAGTAGTAAGAATTGAGCCATTA---GGAATAGCACCCACCAAGGCAAGGAGAAGAGTGGTG----------
----------------------------CAGAGAGAAAAAAGAGCA---GTGGGC---ATAGGAGCT------
---GTGTTCCTTGGGTTCTTGGGAGCA---GCAGGAAGCACTATG---GGCGCAGCGTCA---
CTGACGCTGACGGTACAA---GCCAGACAA---TTACTG---TCTGGTATAGTGCAACAGCAAAAC---
AATTTG---CTGAGA---GCTATTGAGGCGCAACAACATCTGTTGCAACTCACAGTCTGGGGCATC
>KT427680
TTCTACTGTAAGACAACAAACCTATTTAATAGTAGTTGGTCAAAGAATAATAGTATTTGGTCAAAGGATAATA
CTGAGTCA--

GATAATAATGATACCATCACACTCCAGTGTAGAATAAAGCAAATTATAAACAGGTGGCAGGAAGTAGGAAAAG
CAATGTATGCCCCTCCCATCAGCGGAGTAATTAACTGTGTATCAAAC---
ATTACAGGGCTGATATTAACAAGAGATGGTGGCAACGATGATAATAACGCCACC------------------
----

AAGAACGGGCCTGAAATCTTCAGACCTGGAGGAGGAAATATGAAGGACAATTGGAGAAGTGAATTATACAAAT
ATAAAGTAGTAAAAATTGAGCCATTA---GGAATAGCACCCACCAAGGCAAAGAGAAGAGTGGTG--------
-------------------------------CAGAAAGAAAAAAGAGCA---GTAACA---CTAGGAGCT---
------GTGTTCCTTGGGTTCTTGGGAGCA---GCAGGAAGCACTATG---GGCGCAGCGTCA---
ATAACGCTGACGGCACAG---GCCAGACAA---TTACTG---TCTGGTATAGTGCAACAGCAGAAC---
AATCTG---CTGAGA---GCTATTGAGGCGCAACAGCATATGTTGCAACTCACGATCTGGGGCATC
>KT427675
TTCTATTGTAATACAACACAACTATTTAATAGTACCTGGGTCTGGAATGCTACTAGGAATGATACTGGAGGAC
TAAATAAC--

ATCACACTTCCATGCAGAATAAAACAAATTATAAACATGTGGCAGGAAGTAGGAAAAGCAATGTATGCCCCTC
CCATCAGAGGACAAATTAGATGTTCATCAAAT---
ATCACAGGGCTACTATTAACAAGAGATGGTGATAATGGGACTAAC---------------------------
----

AATGAGACCTTCAGACCTGGAGGAGGAGATATGAGGGACAATTGGAGAAGTGAATTATATAAATATAAAGTAG
TAAAAATTGAACCATTG---GGAGTAGCACCCACCAAGGCAAGGAGAAGAGTGGTG----------------
----------------------CAGAGAGAAAAAAGAGCA---GTAGGAGCGCTAGGAGCT---------
ATGTTCCTTGGGTTCTTGGGAGCA---GCAGGAAGCACTATG---GGCGCAGCGTCA---

CTGACGCTGACGGTACAG---GCCAGACGA---TTATTG---TCTGGTATAGTGCAACAGCAAAAC---
AATTTG---CTGAGA---GCTATTGAGGCGCAACAGCATATGTTGCAACTCACAGTCTGGGGCATC
>KT427671
TTCTACTGTAATACAACACCATTATTTAATAAGACTTGGAATGGTACATCGACAGGGGACTGGAAAAT-----
----

GCCACAGGAAGTATCACACTCCATTGCAGGATAAAACAAATCATAAACAGGTGGCAGGAAGTAGGAAAAGCAA
TGTATGCCCCTCCCATCAGAGGAGAGATTAGCTGTTCATCAAAT---
ATTACAGGGCTGCTATTAACAAGAGATGGTGGGTCTAATAATGGGACT-------------------------
----

AAAAATGACAATGAGACCTTCAGACCTGGAGGAGGAGATATGAGGGACAATTGGAGAAGTGAATTATATAAAT
ATAAAGTAGTAAAAATTGAGCCATTA---GGAATAGCACCCACCAGGGCAAGGAGAAGAGTGGTG--------
-------------------------------CAGAGAGAAAAAAGAGCG---GTAACG---CTGGGAGCT---
------ATGTTCCTTGGGTTCTTGGGAGCA---GCAGGAAGCACTATG---GGCGCAGCGTCA---
ATAACGCTGACGGTACAG---GCTAGACAA---TTATTG---TCTGGTATAGTGCAACAGCAGAGC---
AATCTG---CTGAAT---GCTATTGAGGCGCAACAGCGTCTGTTGCAGCTCACAGTCTGGGGCATC
>KT427745
TTCTACTGTAATACATCAAGGCTGTTTAATAGTGCTTGGCTGAATAATGGTACTAGGAATGGTACTAACTCTA
ATTCA--

ACAGATAATAAGATCACACTCCCATGCAGAATAAAACAAATTATAAACAGGTGGCAGGAAACAGGAAAAGCAA
TGTATGCCCCTCCCATCAGAGGAACAATTAGATGTTCATCCAAC---
ATTACAGGGCTGCTATTAACAAGAGATGGTGGCAGTGGTGGGAACGACACCGGG-------------------
----

AACGCCACCGAGGTCTTCAGACCTGGAGGAGGAGATATGAGGGACAATTGGAGAAGTGAATTATATAAATATA
AAGTAGTAAGAATTGAACCATTA---GGAATAGCACCCACCGAGGCAAAGAGAAGAGTGGTG-----------
----------------------------CAGAGAGAAAAAAGAGCA---GTAGGGGGACTAGGAGCT------
---TTGTTCCTTGGGTTCTTGGGAGCA---GCAGGAAGCACTATG---GGCGCAGCGGCA---
ATGACGCTGACGGTACAG---GCCAGAATA---TTATTG---TCTGGTATAGTGCAACAGCAAAAT---
AATTTG---CTGAGA---GCTATTGAAGCACAACAGCATATGTTGCAACTCACAGTCTGGGGCATC
>KJ849788
TTCTACTGTAATACAACACCACTGTTTAATAGTACTTGGCATAATAATGGTACT-------------------
----
-AGGCCTAATAGTACT---------
TGGTCAAATAACACAATCACACTCCAATGCAGAATAAAACAAATTGTAAACCTGTGGCAGCAAGTAGGAAAAG
CAATGTATGCCCCTCCCATTAGTGGATATATTAATTGTTCATCAAAT---
ATTACAGGGCTACTATTAACAAGAGATGGTAGT--

ATCAGCAGTAGCAGTAAT---
AACACTGAAGTCTTTAGGCCTAGAGGAGGAGATATGAAGGACAATTGGAGAAGTGAATTATATAAATATAAAG
TAGTACAACTTGAACCACTA---GGAGTAGCACCAACCAAGGCAAAGAGGAGAACGGTG--------------
-------------------------CAGAGAGAAAAAAGAGCA---GTGGGA---CTGGGAGCT---------
CTGTTCATTGGGTTCTTGGGAGCA---GCAGGAAGCACTATG---GGCGCAGCGTCA---
GTGACGCTGACGGTACAA---GCCAGACAA---TTATTG---TCTGGTATAGTGCAGCAGCAAAAC---
AATTTG---CTGAGG---GCTATTGAGGCGCAACAGCATCTGTTGCAACTCACAGTCTGGGGCATT
>KT427740
TTCTACTGTAATACAACACAGCTGTTTAATAGTACTTGGAATGAAACACAGCTGGATGATTGGAATAAAAGTA
AT---

GATACAAGTAATATCACACTCCCATGCAGAATAAGACAAATTGTAAATAAGTGGCAGGAAGTAGGAAAAGCAA
TGTATGCCCCTCCCATCCAAGGACAAATTAAATGTACATCAAAT---
ATTACAGGGATGCTATTGACAAGAGATGGGGGTAGTAAAAATGACACG-------------------------
----

AACGGGAGCGAAACCTTCAGACCTGCAGGAGGAGATATGAGGGACAATTGGAGAAGTGAATTATATAAATACA
AAGTGGTAAAAATTGAACCATTA---GGAATAGCACCCACCAAGGCAAGGAGAATAGTGGTG-----------
----------------------------CAAAGAGAAAAAAGAGCA---GTGGGA---ATAGGAGCT------
---GTGTTCCTTGGGTTCTTGGGAGCA---GCGGGAAGCACTATG---GGCGCAGCGTCA---

ATAGCGCTGACGGCACAG---GCCAGACAA---TTATTG---TCTGGTATAGTGCAACAGCAAAAC---
AATTTG---CTGAGG---GCTATTGAGGCGCAACAGCATATGTTGCAACTCACAGTTTGGGGCATC
>KT427739
TTCTACTGTAATACATCAGGGCTGTTTAATAGTACTTGGCTAAATAATGGTACTAGGAATGATACTAACTCTA
CAAGTGAT---

ACTAACTCTAATATCACACTCCCATGCAGAATAAAACAAATTATAAACAGGTGGCAGGAAACGGGGAAAGCAA
TATATGCCCCTCCCATCAGAGGAATGATTAGCTGTTCATCCAAC---
ATTACAGGGCTGCTATTAACAAGGGATGGTGGCAGCGGTGGGAACGCCACCGGG-------------------
----

AACGCCACCGAGATCTTCAGACCTGGAGGGGGAGATATGAGAGACAATTGGAGAAGTGAATTATACAAATATA
AAGTAGTAAGAATTGAACCATTA---GGAATAGCACCCACTGAGGCAAAGAGAAGAGTGGTG-----------
----------------------------CAAAGAGAAAAAAGAGCA---GTGGGA---ATAGGAGCT------
---TTGTTCCTTGGGTTCTTGGGAACA---GCAGGAAGCACTATG---GGCGCAGCGGCA---
ATGACGCTGACGGTACAG---GCCAGACTA---TTATTG---TCTGGTATAGTGCAACAGCAAAAT---
AATTTG---CTGAGA---GCTATTGAGGCACAACAGCATATATTGCAACTCACAGTCTGGGGCATC
>KT427738
TTCTACTGTAATACAACACAACTGTTTAATAGTACCTGGCAAAGTAATGGTACCTGGAATAATAGTAATAATA
AA---

GAGAATGACACATACACACTCCCATGCAGAATAAAACAAATTATAAACAGGTGGCAGGAAGTAGGAAGAGCAA
TATATGCCCCTCCCATCAGTGGACAAATTAACTGTTCATCAAAT---
ATTACAGGGATAATATTAACAAGAGATGGTGGTAGGAACGAAAGT----------------------------
----

AATGGGACAGAAATCTTCAGACCTGGAGGAGGAGATATGAGGGACAATTGGAGAAGTGAATTATATAAATATA
AAGTAGTAAAAATTGAGCCATTA---GGAGTAGCACCCACCAAGGCAAGGAGAAGAGTGGTG-----------
----------------------------CAGAGAGAAAAAAGAGCA---GTAACA---CTAGGAGCT------
---GTGTTCATTGGGTTCTTGGGAGCA---GCAGGAAGCACTATG---GGCGCAGCGTCA---
GTGACGCTGACGGTACAG---GCCAGACAA---TTATTG---TCTGGTATAGTGCAACAGCAGAGC---
AATTTG---CTGAGG---GCTATTGAGGCGCAACACCACATGTTGCAACTCACAGTCTGGGGCATT
>KT427735
TTCTACTGTAATACAGCACAGTTGTTTAATTCTGATTGGAATTCTACTTGGACTTGGAGTGATAATACAAATA
ATACTGGGTTT--

GAAAACAAAACTATCACACTCCTGTGTAGAATAAAACAAATCATAAACAGGTGGCAGGGAGTAGGAAAAGCAA
TGTACGCCCCTCCAATCCAAGGAAATATTAGCTGTTCATCAAAT---
ATTACAGGGCTGCTATTAACAAGAGATGGAGGCAACAATACCGGT----------------------------
----

AACGATACCGAGACCTTCAGACCTGGAGGAGGAAATATGAAGGACAATTGGAGAAGTGAATTATATAAATACA
AAGTAGTAAGAATTAAGCCATTA---GGAGTAGCACCCACCAAGGCAAAGAGAAGAGTGGTG-----------
----------------------------CAGAGAGAAAAAAGAGCA---GTGGGAACATTAGGAGCT------
---GTGTTCCTTGGGTTCTTGGGAGCA---GCAGGAAGCACTATG---GGCGCAGCGTCA---
CTGACGCTGACGGTACAG---GCCAGACAA---TTATTG---TCTGGTATAGTGCAACAGCAGAAC---
AATCTG---CTGAGG---GCTATTGAGGCGCAACAGCACCTGTTGCAACTCACAGTCTGGGGCATC
>KT427734
TTCTACTGTAATACAACACAACTGTTTAATAGTACCTGGAATAATGGTACTGAAAAG----------------
----

ACCAATGGCACAATAACACTCCCATGCAGAATAAAACAAATTGTGAACAAGTGGCAGGAAGTAGGAAAAGCAA
TGTATGCCCCTCCCATCAGGGGATACATTAACTGTTCATCAAAT---
ATTACAGGGCTGCTATTAACAAGAGATGGTGGCAACAATAATAAC----------------------------
----

AGCCAGAACGAGACCTTCAGACCAGGAGGAGGAGATATGAGGGACAATTGGAGAAGTGAATTATATAAATATA
AAGTAGTAAGGATTGAACCATTA---GGAATAGCACCCACCAGGGCAAAGAGAAGAGTGGTG-----------
----------------------------CAAAGAGAAAAAAGAGCA---GTGGGAGCACTAGGAGCT------
---ATGTTCCTTGGGTTCTTGGGAGCA---GCAGGAAGCACTATG---GGCGCAGCGTCA---

ATAACGCTGACGGTACAG---GCCAGACAA---TTATTG---TCTGGTATAGTGCAACAGCAAAAT---
AATTTG---CTGAGA---GCTATTGAGGCGCAGCAGCATCTGCTGCAACTCACGGTCTGGGGCATT
>KT427733
TTCTACTGTAATACAACAAAGCTGTTTAATAGTACTTGGGGGTCTAATGGTACTTGGAAGGGTACAAATGAC-
----

AGTAATAGTAATATCACAATACAATGCAGAATAAAACAAATTATAAACAGGTGGCAAGAAGTAGGAAAAGCAA
TGTATGCCCCTCCCGTCAAAGGACCAATCAAATGTTCATCAAAT---
ATTACAGGACTACTATTAACAAGAGATGGTAGTAGTAGTAGTAAT----------------------------
----

GATACTGAGACCTTCAGACCTGGAGGAGGAAATATGAAGGACAATTGGAGAAGTGAATTATATAAATACAAAG
TAGTAAAAATTGAGCCATTA---GGAGTAGCACCCACCAGGGCAAGGAGAAGAGTGGTG--------------
-------------------------CAGAGAGAAAGAAGAGCG---GTAACA---CTAGGAGCT---------
ATGTTCCTTGGGTTCTTGGGAGCA---GCAGGAAGCACTATG---GGCGCAGCGTCA---
ATGACGCTGACGGTACAG---GCCAGACTA---TTATTG---TCTGGTATAGTGCAGCAGCAGAGC---
AATTTG---CTGAGG---GCTATTGAGGCGCAACAGCATCTGTTGCAACTCACAGTCTGGGGCATC
>KJ849803
TTCTACTGTAATACATCAGGGCTGTTTAATAGTACCTGGTATGGGAATGAG----------------------
----
-ACTGCAGAGGCAAAT---------
AGTACAAATACTAATATCACACTCCCATGTAGAATAAAGCAAATTATAAACAGGTGGCAGGAAGTAGGAAAAG
CAATGTATGCCCCTCCCATCAGGGGACAAATAAGATGTTCATCAAAT---
ATTACAGGGATACTACTAACAAGAGATGGTGGT--

-
GATAACAATACCGAGATCGAGACCTTCAGACCTGGAGGGGGAGATATGAGGGACAATTGGAGAAGTGAATTAT
ATAAATATAAAGTAGTAGAAATTGAGCCATTA---GGAATAGCACCTACCAAGGCAAAAGAAGAGTGGTG--
-------------------------------------CAGAGAGAAAAAAGAGCA---ATAACA---
CTGGGAGCT---------ATGTTCCTTGGGTTCTTGGGAGCA---GCAGGAAGCACTATG---
GGCGCAGCGTCA---GTAACGCTGACGGTACAG---GCCAGACAA---TTATTG---
TCTGGTATAGTGCAACAGCAGAAC---AATCTG---CTGAAG---
GCTATTGAGGCGCAACAACATCTGTTGCAACTCACAGTCTGGGGCATC
>KT427730
TTCTACTGTAATACAACACAGCTGTTTAATAGTACTTGGGTGGGGAATAGTACTATAAATGATACTGCC----
----

AAGACAGAAACTATCACACTCCCATGCAGAATAAAACAAATTATAAACAGATGGCAGGAAGTAGGAAAAGCAA
TGTATGCCCCTCCCATCAGGGGACCAATTAACTGTTCATCAAAT---
ATTACAGGGATACTATTACTAAGAGATGGTGGTGTTAATACCACG----------------------------
----

AATGAGACCTTCAGACCTGGAGGAGGAGATATGAGGGACAATTGGAGAAGTGAATTATATAAATATAAAATAG
TAAAAATTGAGCCATTA---GGAGTAGCACCCACCAAGGCAAAAAGAAGAGTGGTG-----------------
----------------------CAGAGAGAAAAAAGAGCA---GTAACA---TTAGGAGCT---------
ATGTTCCTTGGGTTCTTGGGAGCA---GCAGGAAGCACTATG---GGCGCAGCGTCA---
CTGACGCTGACGGTACAG---GCCAGACAA---TTATTG---TCTGGTATAGTGCAACAGCAGAGC---
AATATG---CTGAGA---GCTATTGAGGCGCAACAGCATCTGTTGCAACTCACAGTCTGGGGCATT
>KT427728
TTCTATTGTAATACAACAAAACTGTTTAATAGTACGTGGCCGTGGCCGCATAACAGTACTATAAATAGGACTG
AAGAG--

TCAGATAATAGCACAATCACACTCCCATGCAAAATAAAACAAATTATAAACAGATGGCAGGAAATAGGAAAAG
CAATGTATGCCCCTCCCATCAGGGGACAAATTAGATGTTCATCAAAT---
ATTACAGGGCTGCTATTAACAAGAGATGGTGGAAATGTTGGTAAT----------------------------
----

AAGACCAGTGAGACCTTCAGACCTGGAGGAGGAGATATGAGGGACAATTGGAGAAGTGAGTTATATAAATATA
AAGTAGTACAAATTGAACCATTA---GGAGTAGCACCCACCAAGGCAAAGAGAAGAGTGGTG-----------
----------------------------CAGAGAGAAAAAAGAGCA---TTGGGAACGCTGGGAGCT------

---ATGTTCCTTGGGTTCTTGGGAGCA---GCAGGAAGCACTATG---GGCGCAGCGTCA---
ATGACGCTGACGGTACAG---GCCAGGCAA---TTATTG---TCTGGTATAGTGCAACAGCAGAAC---
AATCTG---CTGAGG---GCTATTGAGGCGCAACAACATATGTTGCAACTCACAGTCTGGGGCATC
>KT427727
TTCTACTGTAATACATCAAAACTGTTTAATAGTACTTGGACTAATGATACTTTGGATACCAGGTTCAATATG-
----

ACAGATGATAATATCACACTCCCATGCAGAATAAGACAAATTATAAACAGGTGGCAGGAAGTAGGAAAAGCAA
TGTATGCCCCTCCAATCAGTGGACCAATTAGATGTTCATCAAAT---
ATTACAGGGCTATTATTAACAAGAGATGGTGGTAATAGTAATAGC----------------------------
----

ACGAATGAGACCTTCAGACCTGGAGGAGGAGATATGAGGGACAATTGGAGAAGTGAATTATATAAATATAAAG
TGGTACAAATTGAGCCATTA---GGAGTAGCACCCACCAGGGCAAAGAGAAGAGTGGTG--------------
-------------------------CAGAGAGAAAAAAGAGAA---ATAACAACACTAGGAGTT---------
ATGTTCCTTGGGTTCTTGGGAGCA---GCAGGAAGCACTATG---GGCGCAGCGTCA---
ATGACGCTGACGGTACAG---GCCAGACAA---TTATTG---TCTGGTATAGTGCAACAGCAGAAC---
AATTTG---CTGAGA---GCTATTGAGGCGCAACAGCATCTGTTGCAACTCACAGTCTGGGGCATC
>KT427725
TTCTACTGCAATACATCCAGACTGTTTAATAGTACATGGAATAGCACTGAACTGGAGGAGAGGAATAAC----
----

ACTGATAAAATTATCACACTCCCATGCAAAATAAAACAAATTATAAATATGTGGCAAGAAGTAGGAAAAGCAA
TGTATGCCCCTCCTATCAGAGGACAGCTTAACTGTTCATCAAAT---
ATTACAGGGCTGATACTAACAAGAGATGGTGGCCAAGGGAACGAG----------------------------
----

ACCAATAATGAAACATTCAGACCTGGAGGAGGAGATATGAGGGACAATTGGAGAAGTGAATTATATAAATACA
AAGTAGTAAAAATTGAACCACTA---GGAATAGCACCCACCAAGGCAAAGAGAAGAGTGGTG-----------
----------------------------CAGAGAGAAAAAAGAGCC---GTGGGAACGCTAGGAGCT------
---ATGTTCCTTGGGTTCTTGGGAGCA---GCAGGAAGCACTATG---GGCGCAGCGTCA---
ATGACGCTGACGGTACAG---GCCAGGCAA---TTATTG---TCTGGTATAGTGCAGCAGCAGAGC---
AATCTG---CTGAGA---GCTATTGAGGCGCAGCAGCATATGTTGCAACTCACAGTCTGGGGCATC
>KT427724
TTCTACTGTAATACAACACAACTGTTTACAACACAACTGTTTAATAGGACATGGGAGTTTAATAGTACTGGAC
CAAT---

GCTACAGGTAATATCACACTCCCATGCAGAATAAAACAAATTATAAATAGGTGGCAAGAAGTAGGAAAAGCAA
TGTATGCCCCTCCCATCAGCGGATATATTAACTGTTCATCAAAT---
ATTACAGGACTCCTATTAATAAGAGATGGGGGCAATGAAACCGAT----------------------------
----

GGGAGCGAGACCTTTAGACCTATAGGAGGAAATATGAGGGATAATTGGAGAAGTGAATTATATAAATATAAAG
TAGTAAGAATTGAGCCATTA---GGAGTAGCACCCACCAAGGCAAGGCGAAGAGTGGTG--------------
-------------------------CAGAGAGAAAAAAGAGCC---ATAACA---ATGGGAGCT---------
ATGTTCCTTGGGTTCTTGGGAGCA---GCAGGAAGCACTATG---GGCGCAGCGTCA---
ATGGCGCTGACGGTACAG---GCCAGATTA---TTATTA---TCTGGTATAGTGCAACAGCAGAGC---
AATTTG---CTGAGG---GCTATTGAGGCGCAACAACATCTGTTGCAACTCACAGTCTGGGGCATC
>KT427723
TTCTACTGTAATACATCAGGGCTGTTTAATAGTACTTGGATACATAATGGTACTCGGCTAAATAATGGTACT-
----

AGGAATGGTACTATCACACTCCCATGCAGAATAAAACAAATTATAAACAGGTGGCAGGAAACGGGAAAAGCAA
TGTATGCCCCTCCCATCAGAGGAATAATTAGATGTTCATCCAAC---
ATTACAGGGCTGCTATTAACAAGAGATGGTGGTGGTGGGAATGACACC-------------------------
----

GACAACTCCACCGAGGTCTTCAGACCCGGAGGAGGAGATATGAGGGACAATTGGAGAAGTGAATTATATAAAT
ATAAAGTAGTAAAAATTGAACCATTA---GGAATAGCACCCAACAAGGCAAGGAGAAGAGTGGTG--------
-------------------------------CAGAGAGAAAAAAGAGCA---GTGGGAACGCTAGGAGCT---

------CTGTTCCTTGGGTTCTTGGGAACA---GCAGGAAGCACTATG---GGCGCAGCGTCA---
GTCACGCTGACGGTACAG---GCCAGACTA---TTATTG---TCTGGTATAGTGCAACAGCAGAAC---
AATATG---CTGAGA---GCTATTGAGGCGCAACAGCACATGTTGCAACTCACAGTCTGGGGCATC
>KT427720
TTCTATTGTAATACAACAAAGCTATTTAATGATACATGGTTGAATGAAAATGAAACT----------------
----

AGCACTAGCAATATCACACTCCCATGCAGAATAAAACAAATTATAAACAGATGGCAGGAAGTAGGAAGAGCAA
TGTATGCCCCTCCCATCCAAGGACAAATTAGCTGTTCATCAAAT---
ATTACAGGGCTGCTATTAACAAGAGATGGTGGTTCAGGAGCCACT----------------------------
----

AATGAAACCTTCAGACCTGTGGGAGGAGATATGAGAGACAACTGGAGAAGTGAATTATATAAATATAAAGTAG
TAAAAATTGAGCCATTA---GGACTGGCACCCACCAAGTCAAGGAGAGTAGTGGTG-----------------
----------------------CAAAGAGAAAAAAGAGCA---GTGGGA---ATGGGAGCT---------
ATGTTCCTTGGGTTCTTGGGAGCA---GCAGGAAGCACTATG---GGCGCAGCGGCA---
GTGACGCTGACGGTACAG---GCCAGACAA---TTATTG---TCTGGTATAGTGCAACAGCAAAAC---
AATTTG---CTGAGA---GCTATTGAGGCGCAACAGCACATGTTGCAACTCACAGTCTGGGGCATC
>KT427716
TTCTACTGTAATACAACAGGGCTGTTTAATAGTACGTGGAACTCTACTACAAAGATAAATACT----------
----

ACAAGGTCAAATATCATACTCCCATGCAGAATAAAACAAATTATAAACAGGTGGCAGGAAGTAAGAAAAGCAA
TGTATAACCCTCCCATCAAAGGAAGAATTAAGTGTTTATCAAAT---
ATTACAGAGATACTATTAACAAGAGATAGTAGTAGTAACAATGACACG-------------------------
----

AACAGGACCGAGATCTTCAGACCAGGAGGAGGAGATATGAGGGACAATTGGAGAAGTGAATTATATAAATATA
AAGTAGTAAGAATTAAGCCATTA---AGAATAGCACCCACCAAGGCAAAGAGAAGAGTGGTG-----------
----------------------------CAGAGAGAAAAAAGAGCA---GTAACA---CTAGGAGCT------
---ATGTTCCTTGGGTTCTTGGGAACA---GCAGGAAGCACTATG---GGCGCAGCGGCA---
CTGACGCTGACGGTACAG---GCCAGGCTA---TTATTG---TCTGGTATAGTGCAACAGCAGAAC---
AATTTG---CTGAGA---GCTATTGAGGCGCAACAGCATATGTTGCAACTCACAGTCTGGGGCATC
>KJ849767
TTCTACTGTAATACAACACAACTGTTTAATAGCAGGTGGAATATGACTGCTAAT-------------------
----
TGGACTATGAATGTTACT---------
ACAGGGACAGATAATATCACACTCCCATGCAGAATAAAACAAATTATAAATAGATGGCAGGAAGTAGGAAAAG
CAATGTATGCCCCTCCCATCGCAGGACAAATCAAATGCTTATCAAAT---
ATTACAGGGCTACTATTAACAAGAGATGGTAGT--

-
AACCAGACTAATGGGACAGAGACCTTCAGACCAGGAGGAGGAGATATGAGGGACAATTGGAGAAGTGAATTAT
ATAAATATAAAGTAGTAAAAATTGAGCCATTA---GGAGTAGCACCCACCAAGGCAAAGAGAAGAGTGGTG--
-------------------------------------CAGAGAGAAAGAAGAGCA---ATAACA---
ATGGGAGCT---------ATGTTCCTTGGGTTCTTGGGAGCA---GCAGGAAGCACTATG---
GGCGCAGCGTCA---TTGACGCTGACGGTACAG---GCCAGACAA---TTATTG---
TCTGGTATAGTGCAACAGCAGAAC---AATTTG---CTGAGG---
GCTATTGAGGCGCAACAGCACCTGTTGCAACTCACAGTCTGGGGCATC
>KT427847
TTCTACTGTAATACAACACAGCTGTTTAATAATGCTTGGAGTGAGACTGAGCTGTTTAATAGTACTGAGAATG
AT---

GCAAACAGCACACTCACACTCCCATGCAGAATAAAACAAATTATAAACAGGTGGCAGGAAGTTGGAAAAGCAA
TGTATGCCCCTCCCATCAAAGGACCAATTAGGTGTGTATCAAAC---
ATTACAGGGCTACTATTAACAAGAGATGGTGGTAACAATTCTTCT----------------------------
----

GAAACCAATTCTTCTGAAATCTTCAGACCTGGAGGAGGAGATATGAGGGACAATTGGAGAAGTGAATTATATA
AATATAAAGTAGTAAAAATTGAGCCATTA---GGAGTAGCACCCACCAAGGCAAAGAGAAGAGTGGTG-----

----------------------------------CAGAGAGAAAAAAGAGCA---
GTGGGGACAATAGGAGCT---------ATGTTCCTTGGGTTCTTGGGAGCA---GCAGGAAGCACTATG---
GGCGCAGCGTCA---ATGACGCTGACGGTACAA---GCTAGACAA---TTATTG---
TCTGGTATAGTGCAACAGCAGAAC---AATCTG---CTGAGG---
GCTATTGAGGCGCAACAGCATATGTTGCAACTCACAGTCTGGGGCATC
>KT427853
TTCTATTGTAATACAACACAGCTGTTTAATAGTACTTGGATGGGGAATGATACCATATCAAATACTACAGGA-
----

TTAAATGACACTATCACACTCCCATGCAGAATAAAACAAATTATAAACAGGTGGCAGGAAGTAGGAAAAGCAA
TATATGCCCCTCCCATCAGCGGACAGATTAACTGTTTATCAAAT---
ATTACAGGGCTGCTATTAACAAGAGATGGTGGTGATAACACCACC----------------------------
----

ATCGAGACCTTCAGACCTGGAGGAGGAAATATGAAGGACAATTGGAGAAGTGAATTATATAAATATAAAGTAG
TAAAAATTGAGCCAGTA---GGAATAGCGCCCACCAAGGCAAAGAGAAGAGTGGTG-----------------
----------------------CAGAGAGAAAGAAGAGCA---GTAACG---CTAGGAGCT---------
ATGTTCCTTGGGTTCTTGGGAGCA---GCAGGAAGCACTATG---GGCGCAGCGTCA---
ATGACGCTGACGGTACAG---GCCAGACAA---CTATTG---TCTGGTATAGTGCAACAGCAGAAC---
AATTTG---CTGAGG---GCTATTGAGGCGCAACAGCATCTGTTGCAACTCACAGTATGGGGCATC
>KT427709
TTCTATTGTAATACAACAGACCTGTTTAATAGTGCTTGGCTGTCTAACAGTACTGGGAATGAGACT-------
----

GGACAGTCAAATATTACACTCCCATGCAGAATAAAACAAATTATAAACATGTGGCAGGAGGTAGGAAAAGCAA
TGTATGCCCCCCCCATCCAAGGACAAATTAGGTGTTCATCAAAT---
GTTACAGGGCTAATATTAGTAAGAGATGGTGGTGACAATACCAAC----------------------------
----

GGGACCGAGACCTTCAGACCTGGAGGAGGAGATATGAGGGACAATTGGAGAAGTAAATTATATAAATATAAAG
TAGTAAAAATTGAGCCAGTA---GGATTAGCACCCACCAAGGCAAGGAGAAGAGTGGTG--------------
-------------------------CAGAGAAAAAAAAGAGCG---GTGGGAACGCTGGGAGCT---------
GTGTTCCTTGGGTTCTTGGGAGCA---GCAGGAAGCACTATG---GGCGCAGCGTCA---
ATGACACTGACGGTACAG---GCCAGACAA---TTATTG---TCTGGTATAGTGCAGCAGCAGAAC---
AATTTG---CTGAAG---GCTATTGAGGCGCAACACCATCTGTTGCAACTCACAGTCTGGGGCATC
>KT427702
TTCTACTGTAATTCAACACAACTGTTTAATAGTACTTGGGGGGTTAATGGTACTTGGAATGATACTAGG----
----

TCAAATGACACTATCACACTCCCATGCAGAATAAAACAAATTATAAACATGTGGCAGGAAGTAGGAAAAGCAA
TGTATGCCCCTCCCATCAGTGGACAAATTAACTGTTCATCAAAT---
ATTACAGGGTTGCTATTAACAAGAGATGGAGGTAATAACGGGACG----------------------------
----

AACGAGACCTTCAGACCTGGAGGAGGAAATATGAGGGACAATTGGAGAAGTGAATTATATAAATATAAAGTAG
TAAAAATTGAACCATTA---GGAATAGCACCCACCAAGGCAAGAAGAAGAGTGGTG-----------------
----------------------CAAAGAGAAAAAAGAGCA---ATAGGA---TTCGGAGCT---------
ATGTTCCTTGGGTTCTTGGGAGCA---GCAGGAAGCACTATG---GGCGCAGCGTCG---
GTGACGCTGACGGTACAG---GCCAGACAA---TTATTG---TCTGGTATAGTGCAACAGCAGAGC---
AATTTG---CTGAAG---GCTATTGAGGCGCAACAGCATCTGTTGCAACTCACAGTCTGGGGCATC
>KT427700
TTCTACTGTAATACAACAAAGCTGTTTAATAGTACCTGGCCTTTGAATGATACTACTTTGAATGATACTACAG
GG---

TCAGATAAAAATATCACTCTCCCATGCAGAATAAAACAAATTATAAACAGGTGGCAAGAAGTAGGAAAAGCAA
TGTATGCCCCTCCCATCAGAGGACAAATTAGGTGTTCATCAAAT---
ATTACAGGTCTATTATTAACAAGAGATGGTGGTAATGTCAACGGGACC-------------------------
----

ACCACCACCGAGACCTTCAGACCTGGAGGAGGAGATATGAGGGACAATTGGAGAAGTAAATTATATAAATATA

AAGTAGTGAAAATTGAGCCATTA---GGAGTAGCACCTACCAAGGCAAAGAGAAGAGTGGTG-----------
----------------------------CAGAGGGAAAAAAGAGCA---ATAACAGGGCTAGGAGCT------
---GTGTTCCTTGGGTTCTTGGGAGCA---GCAGGAAGCACTATG---GGCGCAGCGTCG---
GTAACGCTGACGGTACAG---GCCAGACAA---TTATTG---TCTGGCATAGTGCAGCAGCAGAAC---
AATCTG---CTGAGG---GCTATTGAGGCGCAACAGCATATGTTGCAACTCACAGTCTGGGGCATC
>KT427699
TTCTACTGTAATACAAACCGACTGTTTAACATAAATGAAACTACTCTAAATAAAACTATAAATGAAACTAGA-
----

GTCAATGAGACAATCACACTCCCATGTAGAATAAAGCAAATTATAAACATGTGGCAGACAGTAGGAAAAGCAA
TGTATGCCCCTCCCATCAACGGACAAATTAATTGTACATCAAAT---
ATTACAGGGCTACTATTAACAAGAGATGGTGGTAAAACTAATGAGACC-------------------------
----

AATGATACCGAGGTCTTCAGACCTGGAGGAGGAGATATGAGGGATAATTGGAGAAGTGAATTATATAAATATA
AAGTAGTAAAAATTGAACCATTA---GGAATAGCACCTACCAAGGCCAGGAGAAGAGTGGTG-----------
----------------------------CAGAGAGAAAAAAGAGCA---GTGGGAATGCTAGGAGCT------
---ATGTTCCTTGGGTTCTTGGGAGCA---GCAGGAAGCACTATG---GGCGCAGCGTCA---
ATGGCGCTGACGGTACAG---ACCAGACAA---TTATTG---TCTGGTATAGTGCAACAGCAGAAT---
AATCTG---CTGAGG---GCTATTGAGGCGCAACAACATATGTTGCAACTCACAGTCTGGGGCATC
>KT427692
TTCTACTGTAATACATCAGGGCTGTTTAATAGTACTTGGCTAACTAATAGTACTAGGAATAGTACTAACCAA-
----

GGCAATAGTACTATCACACTCCCATGCAGAATAAAACAAATTATAAACAGGTGGCAGGAAACGAGAAAAGCAA
TGTATGCCCCTCCCATCAGAGGAATAATTAGATGTTCATCCAAC---
ATTACAGGGCTGCTATTAACAAGAGATGGTAGTAGTAAGAACGGCACC-------------------------
----

GACAACTCCACCGAGGTCTTCAGACCTAGAAGAGGAGATATGAAGGACAATTGGAGAAGTGAATTATATAAAT
ATAAAGTAGTAAAAATTGAACCATTA---AGAATAGCACCCAACAAGGCAAGGAGAAGAGTGGTG--------
-------------------------------CAGAGAGAAAAAAGAGCA---GTGACA---CTAGGAGCT---
------TTGTTCCTTGGGTTCTTGGGAACA---GCAGGAAGCACTATG---GGCGCAGCGGCA---
CTGACGCTGACGGTACAG---GCCAGAACA---TTACTG---TCTGGTATAGTGCAACAGCAAAAT---
AATTTG---CTGAGA---GCTATTGAAGCACAACAGCATATGTTGCAACTCACAGTCTGGGGCATC
>KT427792
TTCTACTGCAGTACAACAAAGCTGTTTAATAGTACTTGGTCTGAGAATAATACCGCAGAGAATAATACCGCAG
GG---

GTAAATGAAACTATCACACTCCCATGCAGAATAAAACAAATCATAAACAGATGGCAGGGAGTAGGAAGAGCAA
TGTATGCCCCTCCCATCAGAGGATATATTAACTGTTCAGCAAAT---
ATTACAGGGATGCTATTAACAAGAGATGGTGGCAATAATAGCAGC----------------------------
----

CCCAACGAGACTTTCAGACCTGAAGGGGGAAATATGAAGGACAATTGGAGAAGTGAGTTATATAAATATAAGG
TAGTCCAAATTGAGCCATTA---GGAATAGCACCCACCAAGGCGAAGAGAAGAGTGGTG--------------
-------------------------CAGAAAGAAAAAAGAGCA---GTGACA---CTGGGAGCT---------
ATGTTCCTTGGGTTCTTGGGAGCA---GCAGGAAGCACTATG---GGCGCAGCGTCA---
CTGACGCTGACGGTACAG---GCCAGACAA---TTATTG---TCTGGTATAGTGCAACAGCAGAAC---
AATCTG---CTGAGG---GCTATTGAGGCGCAACAGCATCTGTTGCAACTCACAGTCTGGGGCATT
>KT427791
TTCTACTGTAATACAACACAGCTATTTAATAGTACCTGGTATGCTAATGGTACTACTGAAAATAGTACT----
----

GGAAACGAAACTATCACACTCCCATGCAGAATAAAACAAATTATAAACATGTGGCAAGAAGTAGGAAGAGCAA
TGTATGCCCCTCCTATCAAGGGACAAATTAACTGTACATCAAAT---
ATTACAGGGCTGATATTAACAAGAGATGGTGGTAAAAACGACACA----------------------------
----

GAGACAGAGACCTTCAGACCTGGAGGAGGAAATATGAAGGACAATTGGAGAAGTGAATTATATAAATATAAAG

TAGTAAGAATTGAACCATTA---GGAGTAGCACCCACCAAGGCAAAGAGAAGAGTGGTG--------------
-------------------------CAGAGAGAAAAAAGAGCA---GTGGGAACACTGGGAGCC---------
ATGTTCCTTGGGTTCTTGGGAGCA---GCAGGAAGCACTATG---GGCGCAGCGTCA---
ATAACGCTGACGGTACAG---GCCAGACAA---TTATTG---TCTGGTATAGTGCAACAGCAGAAC---
AATTTG---CTGAGG---GCTATTGAGGCGCAACAACATCTGTTGCAACTCACAGTCTGGGGCATC
>KT427787
TTCTACTGTGACACAGCAGAACTGTTTAATAGTACTTGGAATAATACTGAAGAGGCAGATAACATGGGA----
----

GGAAATGATACCATCATACTCCCATGTAGAATAAGACAAATTATAAACAGGTGGCAGGAAGTAGGAAAAGCAA
TGTATGCCCCTCCCATCCAAGGACATATTAATTGCTTATCAAAT---
ATTACAGGGCTACTATTAATGAGAGATGGTGGTAATAACGTGAGC----------------------------
----

GGGCCCGAGATCTTCAGACCTGGAGGAGGAGATATGAGGGACAATTGGAGAAGTGAATTATATAAATATAAAG
TAGTAAAAATTAAACCATTA---GGAATAGCACCCACTAAAGCAAAGAGAAGAGTGGTG--------------
-------------------------CAGAGAGAAAAAAGAGCA---GTGGGA---ATAGGAGCT---------
GTGTTCCTTGGGTTCTTGGGAGCA---GCAGGAAGCACTATG---GGCGCAGCGTCA---
ATGACGCTGACGGTACAA---GCCAGACTA---TTATTG---TCTGGTATAGTGCAGCAGCAAAAC---
AATTTG---CTGCAG---GCTATTGAGGCGCAACAGCGTCTGTTGCAACTCACAGTCTGGGGCATC
>KT427786
TTCTACTGTAATACAACACGACTGTTTAATAGTACTTGGCCCGCTGGGGACACTTGGAATGATACTGAA----
----

AACACAGCCAATATCACACTCCCATGTAGAATAAAACAAATTATAAACAGGTGGCAGGCAGTAGGAAAAGCAA
TGTATGCCCCTCCCATCAGAGGACAAATTAGCTGCTCATCAAAC---
ATTACAGGGTTGCTATTAACAAGAGATGGTGGTAATGATAATGCTACA-------------------------
----

AGGAACGAGACCTTCAGACCGGGAGGAGGAGATATGAGGGACAATTGGAGAAGTGAATTATATAAATATAAAG
TAGTAAAAATTGAACCATTG---GGAATAGCACCCACCAAGGCAAAGAGAAGAGTGGTG--------------
-------------------------CAGAGAGAAAAAAGAGCA---GTGGGAGTACTAGGAGCT---------
ACGTTCCTTGGGTTCTTGGGAGCA---GCAGGAAGCACTATG---GGCGCAGCGTCA---
ATGACGCTGACGGTACAG---GCCAGACTA---TTATTG---TCTGGTATAGTGCAACAGCAGAAC---
AATTTG---CTGAAG---GCTATTGAGGCGCAACAGCATCTGTTGCAACTCACAGTCTGGGGCATT
>KT427784
TTTTACTGTAATACAACAAAGCTGTTTAATAGTAGGTGGAATATTAGTGGAGAGTCAAATAACACA-------
----

GAAGGAGAAACTATCATACTCCCATGCAGAATAAAACAAATTATAAACAGATGGCAGGAAGTAGGAAAAGCAA
TGTATGCCCCTCCAATCAGCGGATATATTAGATGTTCATCAAAT---
ATTACAGGGCTACTATTAACAAGAGATGGTGGTAATAATGGAACA----------------------------
----

AATGAGACCTTCAGACCAGGAGGAGGAGACATAAAGGATAATTGGAGAAGTGAATTATATAAATATAAAGTAG
TAAAAATTGAACCATTA---GGAGTAGCACCCACCAAGGCAAAGAGAAGAGTGGTG-----------------
----------------------CAGAGAGAAAAAAGAGCA---GTGGGA---ATAGGAGCT---------
ATGTTCCTTGGGTTCTTGGGAGCA---GCAGGAAGCACTATG---GGCGCAGCGTCA---
CTAACGCTGACGGTACAG---GCCAGACTA---TTATTG---TCTGGTATAGTGCAACAGCAGAAC---
AATTTG---CTGAGA---GCTATTGAGGCGCAACAGCATCTGTTGCAACTCACAGTCTGGGGCATC
>KT427781
TTCTACTGTAATACAACACAGCTGTTTAATAGTATTTGGCCTTGGAATGATACTGATTCAAATAGTACTAAAG
GG---

TCAAATAGCAATATCACACTCCCATGCAGAATAAAACAAATCATAAACAGGTGGCAGGAAGTAGGAAAAGCAA
TGTATGCCCCTCCCATCAGAGGAGAAATCAGGTGTACATCAAAT---
ATTACAGGACTACTATTAACAAGAGATGGTGGTTCTGAGGACAACACT-------------------------
----

TCTATATCTGAGATCTTCAGACCTGGAGGAGGAGATATGAGGGATAATTGGAGAAGTGAATTATATAAATATA

AAGTAGTACAAGTTGAGCCATTA---GGAATAGCACCCACCAGGGCAAGGAGAAGAGTGGTG-----------
----------------------------CAGAGAGAAAGAAGAGCA---GTAACA---ATGGGAGCT------
---CTGTTCCTTGGGTTCTTGGGAGCA---GCAGGAAGCACTATG---GGCGCAGCGTCA---
ATGACGCTGACGGTACAG---GCCAGACAA---TTATTG---TCTGGTATAGTGCAGCAGCAGAGC---
AATTTG---CTGAAG---GCTATTGAGGCGCAACAGCACCTGTTGCAACTCACAGTCTGGGGCATC
>KT427656
TTCTACTGTAATTCATCACAGCTGTTTAATAGTACTTGGTATACTAATGGTACTTGGAATGCTAATGGCACTG
AAAAAAAT---

TTCAATGACACCATTACGCTCCCATGCAGAATAAAACAAATTATAAACAGGTGGCAGGACATAGGAAGAGCAA
TATATGCTCCTCCCATCAGAGGATACATTAATTGTTCATCAAAT---
ATTACAGGAATGATATTAACAAGAGATGGGAGTGACAATGACTCT----------------------------
----

AGTCCTGAAATCTTCAGACCTGGAGGAGGAAATATGAAGGACAATTGGAGAAGTGAATTATATAAATATAAAG
TAGTAAAAATTGAGCCATTA---GGAATAGCACCCACCAAGGCAAAGAGAAGAGTGGTG--------------
-------------------------CAGAGAGAAAAAAGAGCA---GTGGGAACGTTGGGAGCT---------
ATGTTCCTTGGGTTCTTGGGAACA---GCCGGAAGCACTATG---GGCGCAGCGGCA---
GTAACGCTGACGGTACAG---GCCAGACAA---TTATTG---TCTGGTATAGTGCAACAGCAGAAC---
AATCTG---CTGAGG---GCTATTGAGGCGCAACAGCATATGTTGCAACTCACAGTTTGGGGCATC
>KT427650
TTCTACTGTAATACATCACAGTTGTTTAACAGTACCTGGAGTCTTAACAGTACTTGGAATGGTACTAATACT-
----

AACAATGGCACTATCACACTCCCATGCAGAATAAAACAAATCATAAACAGGTGGCAGGAAGTAGGAAGATCAA
TGTATGCCCCTCCCATCAAAGGACTAATTAATTGTTTGTCAAAT---
ATTACAGGGCTCCTGTTAACAAGAGATGGTAGTAATGGGTCCGAT----------------------------
----

CCCGAAATCTTCAGACCTGGAGGAGGAGATATGAGGGACAATTGGAGAAGTGAATTATATAAATATAAAATAG
TAAAAATCGAGCCATTA---GGAATAGCACCCACCAAGGCAAAGAGAAGAGTGGTG-----------------
----------------------CAGAGAGAGAAAAGAGGA---GTAACA---TTAGGAGCT---------
GTGTTCCTTGGGTTCTTGGGAACG---GCAGGAAGCACTATG---GGCGCAGCGGCC---
ACGACGCTGACGGTACAG---GCCAGACAA---TTATTG---TCTGGTATAGTGCAACAGCAGAAC---
AATTTG---CTGAGA---GCTATTGAGGCGCAACAGCATATGTTGCAACTCACAGTCTGGGGCATT
>KJ849811
TTCTACTGTAATACAACAGAACTATTTAATTCCACTTGGAATGGTACTGCTGGT-------------------
----
GAGAACAATAGTACTAGT---------
ACTGAGGGAGGAAACATCACACTCCCATGTAGAATAAAACAAATTGTAAACAGATGGCAGGAAGTAGGAAAAT
CAATGTATGCCCCTCCCATCAGTGGATACATCAAGTGTGTATCAAAT---
ATTACAGGGCTGCTATTAACAAGAGATGGTGGT--

ATTACTAATGAAACTAAC---
CACAACGAGACTTTTAGACCTTTAGGAGGAAATATGAAGGACAATTGGAGAAATGAATTATATAAATATAAAG
TAGTAAAAATTGAGCCATTA---GGAGTAGCACCCACCAAGGCAAAGAGAAGAGTGGTG--------------
-------------------------CAAAGAGAAAAAAGAGCA---GTGGGAGCAATAGGAGCT---------
ATGTTCCTTGGGATCTTGGGAGCA---GCAGGAAGCACTATG---GGCGCAGCGTCA---
ATAACGCTGACGGTACAA---GCCAGGCTA---CTCTTG---TCTGGTATAGTGCAACAGCAGAAC---
AATCTG---CTGAGG---GCTATTGAGGCGCAACAACATCTGTTGCAACTCACAGTCTGGGGCATC
>KJ849825
TTCTATTGTAATACAACAAAACTGTTTAATAATACTTGGAATAGTACTGCTGGAAATTGGACT----------
---GGAAAT---
TGGACTGTGACTAAAGGGCCA---------------
AGTGGTACTGATATTATCATACTCCCATGCAGAATGAAACAAATCATAAACAGGTGGCAGGAAGTAGGAAAAG
CAATGTATGCTCCTCCCATCAGTGGAAGAATTAGCTGTTTATCAAAT---
ATCACAGGGCTGATATTAACAAGAGATGGTGGT--

AATAACAAGAACAGGAAC---
CGGACCGACAATTTCAGACCTGAAGGAGGAGATATGAGGAATAATTGGAGAAGTGAATTATATAAATACAAAG

TAGTAAAAATTGAGCCATTA---GGAATAGCACCCACCAAGGCAAGGAGAAGAGTGGTG---------------
-------------------------CAGAGAGAAAAAAGAGCA---ATGGGAACACTAGGAGCT----------
GTGTTCCTTGGGTTCTTGGGAACA---GCAGGAAGCACTATG---GGCGCAGCGTCA---
CTGACGCTGACGGTACAG---GCCAGACAA---CTATTG---ACTGGTATAGTGCAACAGCAGAAC---
AATTTG---CTGAGG---GCTATTGAGGCGCAACAGCATCTGTTGCAGCTCACAGTCTGGGGCATC
>KT427775
TTCTACTGTAATACAACACAGCTGTTTAATAGTACTTGGCTGAATAGTACTTGGAATGATAATGAGACAAAAT
CA--

GACAATAGCACTATCACTCTTCCATGCAGAATAAAACAAATTATAAATAGATGGCAGGAAGTAGGAAAAGCAA
TGTATGCCCCTCCCATCGCAGGAAAAATTAGATGTTCATCAAAT---
ATTACAGGGCTGCTATTAACAAGAGATGGAGGCAATACCAGTAAT-----------------------------
----

GGCCACAGTGAGATCTTCAGGCCTGCAGGAGGAGATATGAGGGACAATTGGAGAAGTGAATTATATAAATATA
AAGTAGTAAAAATTGAGCCTTTA---GGAATAGCACCCACCAAGGCAAGGAGAAGAGTGGTG------------
----------------------------CAGAGAGAAAAAAGAGCA---GTGGGA---ATAGGAGCT-------
---GTGTTCCTTGGGTTCTTGGGAGCA---GCAGGAAGCACTATG---GGCGCAGCGTCA---
GTGACGCTGACGGTACAG---GCCAGGCAA---TTATTG---TCTGGTATAGTGCAACAGCAGAAC---
AATTTG---CTGAAG---GCTATTGAGGCGCAACAGCATCTGTTGCAACTCACAGTCTGGGGCATC
>KT427769
TTTACTGTAATACAACAAAGCTGTTTAATAGGACTTGGATTAGAAATGGGGGATTTTGGAATACTACTGAAG
AG--

GAAAATAACACTGAAATCACACTCCCATGCAAAATAAAGCAAATCATAAACAGGTGGCAGGAAGTAGGAAAAT
CGATATATGCCCCTCCCATCAGAGGACAAATTAGATGTGTATCAAAT---
ATTACAGGGCTGCTATTAACAAGAGATGGTGGTATTAACAATACTACT--------------------------
----

AACGAGACCGAGATCTTTAGACCTGCAGGGGGAGATATGAGGGACAATTGGAGAAGTGAATTATATAAATATA
AAGTAGTAAAAATTGAACCATTA---GGAGTAGCACCCACCAAGGCAAAGAGAAGAGTGGTG------------
----------------------------CAGAGAGAAAAAAGGGCA---GTGGGAGTAATAGGAGCT-------
---ATGTTCCTTGGGTTCCTGGGAGCA---GCAGGAAGCACTATG---GGCGCAGCGTCA---
GTGACGCTGACGGTACAG---GCCAGACAA---TTACTG---TCTGGTATAGTGCAACAGCAGAAC---
AATCTG---CTGAGG---GCTATTGAGGCGCAACAACATCTGTTGCAACTCACAGTCTGGGGCATC
>KT427767
TTCTACTGTAATACAACACAACTGTTTAATAGTACTTGGTTTAATAGTACATGGACTACTGCAGAGTCAAATA
ACACT---

GAAGGAAGTGACTCAATCACCCTCCCATGCAGAATAAAGCAAGTAGTAAACATGTGGCAGGAAGTAGGAAAAG
CAATGTATGCCCCTCCCATCAGCGGACGAATTAACTGTTCATCAAAC---
ATTACAGGGTTGCTATTAACAAGAGATGGTGGTAATGGGAGTGAA-----------------------------
----

CCTATAGAGATCTTCAGACCTGGAGGAGGAGATATGAGGGACAATTGGAGGAGTGAATTATATAAATATAAAG
TAGTAAAAATAGAACCATTA---GGAATAGCACCTACCAAGGCAAAGAGAAGAGTGGTG---------------
-------------------------CAGAGAGAAAAGAGAGCA---GTGACA---CTAGGAGCT----------
GTGTTCCTTGGGTTCTTGGCAGCA---GCAGGAAGCACTATG---GGCGCAGCGTCA---
ATGACGCTGACGGTACAG---GCCAGACTA---TTATTG---TCTGGTATAGTGCAACAGCAGAAC---
AATTTG---CTGAGG---GCTATTGAGGCGCAACAACATCTGTTGCAACTCACAGTCTGGGGCATC
>KT427764
TTCTACTGTAATACATCACAGCTGTTTAACAGTACTTGGATTAATGATACTAATACAGAA--------------
----

AATAACACCCATATCACCCTCCCATGCAGAATAAGACAAATCATAAACAGGTGGCAAGAAGTAGGAAAAGCAA
TGTATGCCCCTCCCATTAGCGGACAGATTAAATGTTCATCAAAT---
ATTACAGGGTTGCTATTAACAAGAGATGGTGGTGTCAATGGTACC-----------------------------
----

AATGGAACTGAGACCTTCAGACCTGGAGGAGGAGATATGAGGGACAATTGGAGAAGTGAATTATATAAATATA

AAGTAGTAAAAATTGAACCATTA---GGAATAGCACCCACCAAGGCAAGGAGAAGAGTGGTG-----------
----------------------------CAAAGAGAAAAAAGAGCG---GTGGGAGCACTAGGAGCT------
---ATGTTCCTTGGATTCTTGGGCACA---GCAGGAAGCACTATG---GGCGCAGCGTCA---
ATAACGCTGACGGTACAG---GCCAGACAA---TTATTG---TCTGGTATAGTGCAACAGCAGAAC---
AATCTG---CTGAGA---GCTATTGAGGCGCAACAGCACTTGTTGCAACTCACAGTCTGGGGCATC
>KT427761
TTCTACTGTAATACAACAAAACTGTTTAATAGTACTTGGAAAAATAATAATGTTAGTTGGAATGTTGCGGCT-
----

GAAAATGACACTATCACACTCCCATGTAGAATCAAACAAATTATAAACCTGTGGCAGGAAGTGGGAAAAGCAA
TGTATGCCCCTCCCATCCAAGGACAAATTAACTGTTCATCAAAT---
ATTACAGGGCTGTTATTAATAAGAGATGGTGGTATTAACAACCAGACC-------------------------
----

AAGGACACCGAGACCTTCAGACCTGGAGGAGGAGATATGAGGGACAATTGGAGAAGTGAATTATATAAATATA
AAGTAGTAAAAATTGAACCAGTA---GGAATAGCACCCACCAAGGCAAAGAGAAGAGTGGTG-----------
----------------------------CAAAGAGAAAAAAGAGCA---GTGGGAATGGTAGGAGCT------
---ATGTTCCTTGGGTTCTTGGGAGCA---GCAGGAAGCACTATG---GGCGCAGCGTCA---
ATAACGCTGACGGTACAG---GCCAGACAA---CTATTG---TCTGGTATAGTGCAACAGCAGAAC---
AATATG---CTGAGA---GCTATTGAGGCGCAACAGCATCTGTTGCAACTCACAGTCTGGGGCATC
>KT427757
TTCTACTGCAATACAACACAGCTGTTTAATAGTACTTGGCCTGAGAATAATATTAGTACTACAGGGGTAAAT-
----

GTAAGTGATACTATCACAATTCCATGCAGAATAAAACAATTCATAAACAGGTGGCAGGCAGTAGGAAGAGCAA
TGTATGCCCCTCCCATCAGAGGATATATTAACTGTTCATCAAAT---
ATTACAGGGCTGCTATTAACAAGAGATGGTGGTAGTAACAGCTCC----------------------------
----

TTAAATGAGACTTTCAGACCTGAAGGGGGAAATATAAAGGACAATTGGAGAAGTGAGTTATATAAATATAAGG
TAGTCCAAATTGAGCCATTA---GGAGTAGCACCCACCAAGGCAAAGAGAAGAGTGGTG--------------
-------------------------CAGAGAGAAAGAAGAGCA---GTGACA---CTAGGAGCT---------
ATGTTCCTTGGGTTCTTGGGAGCA---GCAGGAAGCACTATG---GGCGCAGCGTCA---
CTGACGCTGACGGTACAG---GCCAGACAA---TTGTTG---TCTGGTATAGTGCAACAGCAGAAC---
AATCTG---CTGAGG---GCTATTGAGGCGCAACAGCATCTGTTGCAACTCACAGTCTGGGGCATT
>KT427756
TTCTACTGCAATACATCAAATCTGTTTAATAAGTCTTGGACTAATAGTTCTTGGTCTGGTAATGAAGGGTCAA
ATAAC--
-------------------AAT---
ACAGAAAATGACACAATCACACTCCAATGCAGAATAAAACAAATTATAAACATGTGGCAGAAAGTAGGAAAAG
CAATGTATGCCCCTCCTATCCGAGGAACAATTAAATGTTCATCAAAT---
ATCACAGGGCTGCTATTAACAAGAGATGGTGGCAATAACAGCACC----------------------------
----

AATGAGACCTTCAGACCTGGAGGAGGAGATATGAGAGATAATTGGAGAAGTGAATTATATAAATATAAAGTAG
TAAAAATTGAACCATTA---GGAGTAGCACCCACCAGGGCAAAAAGAAGAGTGGTG-----------------
----------------------CAGAAAGAAAAAAGAGCA---GTGGGA---ATAGGAGCT---------
TTATTCCTTGGGTTCTTGTCGGCA---GCAGGAAGCACTATG---GGCGCAGCGTCA---
ATGACGCTGACGGTACAG---GCCAGACAA---TTATTG---ACTGGTATAGTGCAACAGCAAAAC---
AATTTG---CTGAGA---GCTATTGAGGCGCAACAGCATATGTTGCAACTCACAGTCTGGGGCATA
>KT427755
TTCTACTGTAATACATCAAAACTGTTTAATAGTACGTGGAACAGTGGGAATTTAGCAGTAGATAATAGTACTG
AT---

GATAATGGGACTATCATACTCCCATGTAGAATAAAACAAATCATAAACATGTGGCAGGAAGTAGGAAAAGCAA
TGTATGCCCCTCCTATTCGAGGAATAATTAAATGTTTATCAAAT---
ATCACAGGGATAATTTTAACAAGAGATGGTGGTAATAACAATAATAGCAACGAG-------------------
----

ACCACCGAAATCTTCAGACCTGGAGGAGGAGACATGAGGGACAATTGGAGAAGTGAATTATATAAATATAAAG

TAGTAAAAAATTGAACCATTA---GGAGTAGCACCCACTGAGGCAAGGAGGAGAGTGGTG-------------
-------------------------CAGAGAGAAAAAAGAGCA---ATAACG---ATGGGAGCT---------
ATGTTCCTTGGGTTCTTGGGAGCA---GCAGGAAGCACTATG---GGCGCAGCATCA---
ATAACGCTGACGGTACAG---GCCAGACAA---TTATTG---TCTGGAAAAGGGCGACAGCAGAAC---
AATCTG---CTGAGG---GCTATTGAGGCGCAACAGCATCTGTTGCAACTCACGGTCTGGGGCATT
>KT427754
TTCTACTGTAATACATCACAGCTGTTTAACAGTACTTGGCCTTGGAACAGTGCTGGGAATGAGAGT-------
----

GATAGTACTAATATCACACTCCCATGCAGAATAAGACAAATCATAAACAGATGGCAGGAAGTAGGAAAAGCAA
TGTATGCCCCTCCCATCAAAGGACCAATTAGATGCTCATCAAAT---
ATTACAGGGATACTATTAACAAGAGATGGTGGTAATACCAACCAG----------------------------
----

ACCACGGAGATCTTTAGACCGGGAGGAGGAAATATGAAGGACAATTGGAGAAGTGAATTATATAAATATAAAG
TAGTAAAAAATTGAGCCATTA---GGAGTAGCACCCACCAAGGCAAGGAGAAGAGTGGTG-------------
-------------------------CAGAGAGACAAAAGAGCA---GTAACA---CTAGGAGCT---------
ATGTTCCTTGGGTTCTTGGGAGCA---GCAGGAAGCACTATG---GGCGCAGCGTCA---
ATGACGCTGACGGTACAG---GCCAGACTA---TTATTG---TCTGGTATAGTGCAACAGCAGAAC---
AATTTG---CTGAAG---GCTATTGAGGCGCAACAGCGTCTGTTGCAACTCACAGTCTGGGGCATC
>KT427750
TTTTACTGTAATACAACACTGCTATTTAACAGAACTTGGAAGTTTAATGGCACTTGGAATAGTGTGAAGAATA
GTACTGGA---

GAGTCAAACAATATTACACTCCCATGCAGAATAAAACAAATCATAAACAGGTGGCAGGAAGTAGGAAAAGCAA
TGTATGCCCCTCCCATCAGAGGACTAATTAACTGTTTATCAAAT---
ATTACAGGGCTACTATTAACAAGAGATGGTGGTAATGCGAATAGC----------------------------
----

AGCAATGAGACCTTCAGACCGGGAGGGGGAGATATGAGGGACAATTGGAGAAGTGAATTATATAAATATAAAG
TAGTAAAAAATTGAACCATTA---GGAGTAGCACCCACCAAGGCAAAGAGAAGAGTGGTG-------------
-------------------------CAGAGAGAAAAAAGAGCA---ATAGGAACAATAGGAGCT---------
ATGTTCCTTGGGTTCTTGGGAGCA---GCAGGAAGCACTATG---GGCGCAGCGTCA---
GTGACGCTGACGGTACAG---GCCAGACAA---TTATTG---TCTGGTATAGTGCAACAGCAGAGC---
AATCTG---CTGAGA---GCTATTGAGGCGCAACAACATATGTTGCAACTCACAGTCTGGGGCATC
>KT427830
TTCTACTGTGATACAACTAAACTGTTTAACAGTACTTGGAATAATAGCATTGATACTTGGGAGAATAGCACT-
----

AATACTGGGAATATCACACTCCCATGTAGAATAAAACAAATTATAAACATGTGGCAGGAAGTAGGAAAAGCAA
TGTACGCCCCTCCTATCAGAGGACAAATTAGATGCTCATCAAAC---
ATTACAGGGCTGCTACTAGTGAGAGATGGTGGTACAAATAACACA----------------------------
----

AACAGCTCCGAGATTTTCAGACCTGGAGGAGGAGACATGAGGGACAATTGGAGAAGTGAATTATACAGGTATA
AAGTGGTAAAAAATTGAACCATTA---GGAGTAGCACCCACCAAGGCAAAGAGAAGAGTGGTG----------
----------------------------CAGAGAGAAAAAAGAGCA---GTGGGAACGCTGGGAGCT------
---ATGTTCCTTGGGTTCTTGGGAGCA---GCAGGAAGCACTATG---GGCGCAGCGTCA---
ATAACGCTGACGGCACAG---GCCAGGCAA---TTATTG---TCTGGTATAGTGCAGCAGCAGAAC---
AATTTG---CTGAGA---GCTATTGAGGCGCAACAGCATCTGTTGCAACTCACAGTCTGGGGCATC
>KT427829
TTCTACTGTAATACAACACAACTGTTTAATAGTACTTGGCATAATAATGGTAGTACTTGGTATACTAATGATA
GTATTTGG---
TATACTAATGGTACTGAA---------
GAACTAAATGACACTATCACCCTTCCATGCAGAATAAAACAAATTGTAAACATGTGGCAGGAAGTAGGAAAAG
CAATGTATGCCCCTCCCATCAAAGGACAAATTAGATGTTCATCAAAT---
ATCACAGGGCTGCTATTAACAAGAGATGGTGGTAATACTAATGGCACA-------------------------
----

AATGGTACTGAGATTTTCAGACCTGGAGGAGGAGATATGAGGGACAATTGGAGAAGTGAATTATATAAATATA

AAGTAATAAAAATTGAACCATTA---GGAATAGCACCCACCAAGGCAAGGAGAAGAGTGGTG-----------
----------------------------CAGAGAGAAAAAAGAGCA---CTGGGAACGCTAGGAGCT------
---ATGTTCCTTGGGTTCTTGGGAACA---GCAGGAAGCACTATG---GGCGCAGCGTCA---
CTGACGCTGACGGTACAG---GCCAGACAA---TTATTG---TCTGGTATAGTGCAGCAGCAGAAC---
AATTTG---CTGAGG---GCTATTGAGGCGCAACAGCATATGTTACAACTCACAGTCTGGGGCATC
>KT427824
TTCTACTGTAACACAACACCACTGTTTAATAGTAGTTGGCAGAAAAATAATGGAACTTGGAGTACCAATATTA
CTGGAAAT---
TGGAGTACTGATACTACTGGG------------
AATAATAGCACTAATATCACACTCCAATGTAGAATAAAACAAATTATAAACTTGTGGCAGGAAGTAGGAAAAG
CAATGTATGCCCCTCCCATCAGGGGACCAATTAAATGTGAATCAAAT---
ATTACAGGACTGCTATTAACAAGAGATGGGGGAGGAGATGGGGGTAATAAA----------------------
----
AGTAATGACCATAAC---
AAGACCGAGATCTTCAGACCTGGAGGAGGAGATATGAGGGACAATTGGAGAAGTGAATTATATAAATATAAAG
TAGTAAAAATTGAACCATTA---GGAGTAGCACCCACCAAGGCAAAGAGAAGAGTGGTG--------------
-------------------------CAGAGAGAAAAAAGAGCA---ATAGGG---CTGGGAGCT---------
ATGTTCCTTGGGTTCTTGGGAACA---GCAGGAAGCACTATG---GGCGCAGCGTCA---
ATAACGCTGACGGTACAG---GCCAGACAA---TTATTG---TCTGGTATAGTGCAACAGCAAAAT---
AATTTG---CTGAAG---GCTATTGAGGCGCAACAGCATCTGTTGCAACTCACAGTCTGGGGCATC
>KJ849790
TTCTACTGCAATACAACAAAGCTGTTTAGTAATTTTAATAGTACTTGG-------------------------
----
-------AATAATACTGAA------
GGGTCAAATGACACAATCATACTCCCATGCAGAATAAAACAAATTGTAAACAGGTGGCAGGAAGTAGGAAAAG
CAATGTATGCCCCTCCCATCAAAGGACAAATTAAATGTTCATCAAAT---
ATTACAGGGCTGCTATTAACAAGAGATGGTGGTATA-------------------------------------
----
AGGAATGAGTCTGGAGGAAAT---------
GAGTCTGAGACCTTCAGACCTGCAGGAGGGGATATGAAAGACAATTGGAGAAGTGAATTATATAAATATAAAG
TAGTACAAATTGAACCATTA---GGAGTAGCACCCACCAAGGCAAGAAGAAGAGTGGTG--------------
-------------------------CAGAGAGAAAAAAGAGAA---GTAACA---CTAGGGGCT---------
TTATTCCTTGGGTTCTTGGGAGCA---GCAGGAAGCACTATG---GGCGCAGCGTCA---
GTGACGCTGACGGTACAA---GCCAGACAA---TTATTG---TCTGGTATAGTGCAACAGCAGAAC---
AATTTG---CTGAGA---GCTATTGAGGCGCAACAGCATCTGTTGCAACTCACAGTCTGGGGCATC
>KT427819
TTCTACTGTAATACATCACAACTGTTTAATAGTACTTGGCTGCCTAATGGTACTTGGCTGTCTAATAGTACTA
GGAATGAG---

ACAAATGGAAATATCACACTCTCATGCAGAATAAAACAAATTATAAACAGGTGGCAGGAAGTAGGAAAAGCAA
TGTATGCCCCTCCCATCGCAGGGAAAATTAGCTGTTCATCAAAC---
ATTACAGGGCTGATATTAACAAGAGATGGTAGCAGCACAAATAGC----------------------------
----

ACGTCTGAAACCTTCAGACCTGGAGGAGGAGACATGAGGGACAATTGGAGAAGTGAATTATATAAATATAAAG
TAGTAAGAGTTGAGCCAGTA---GGAATAGCACCCACCAAGGCAAAGAGAAGAGTGGTG--------------
-------------------------CAGAGAGAAAAAAGAGCA---ATAGGA---TTAGGAGCT---------
ATGTTCCTTGGGTTCTTGGGAGCA---GCAGGAAGCACTATG---GGCGCAGCATCA---
GTGACGCTGACGGTACAA---GCCAGACAA---TTATTG---TCTGGTATAGTGCAACAGCAGAAT---
AATCTG---CTGAGA---GCTATTGAGGCGCAACAACATATGTTGCAACTCACAGTCTGGGGCATC
>KJ849796
TTCTACTGTAATTCAACACAGCTGTTTAATAGTTCTTGGCATTTTAATGGTTCTTGG----------------
----
TATGGTAATGAACCTGAAAGA------------
AATAAAACTGTCAATATCACACTCCCATGCAGAATAAAACAAATTATAAACATGTGGCAGGAAGTAGGAAAAG
CAATGTATGCCCCTCCCATCAGGGGACAAATTATGTGCAAATCAAAT---
ATTACAGGGCTGTTACTAGCAAGAGATGGTGGT--

AATAATGAATCTGAAATCTTCAGACCTGGAGGAGGAGATATGAGGGACAATTGGAGAAGTGAATTATATAAAT

```
ATAAAGTAGTACAAATTGAGCCATTA---GGAATAGCACCCACCACGGCAAGGAGAAGAGTGGTG--------
-------------------------------CAGAGAGAAAAAAGAGCA---GTGGGG---ATAGGAGCT---
------ATGATCCTTGGGTTCTTGGGAGCA---GCCGGAAGCACTATG---GGCGCAGCGTCA---
ATAACGCTGACGGTACAG---GCCAGACTA---TTATTG---TCTGGTATAGTGCAACAGCAGAAC---
AATTTG---CTGAGG---GCTATTGAGGCGCACCAGCATATGTTGCAACTCACAGTCTGGGGCATT
>KT427815
TTCTACTGTAATACATCACGACTGTTTAATAGTACTTGGGCTACAAATGAAACTAATGAGAGA----------
-------------------------------------------------------------------------
----------------------------
AATGAATCTACAATCACGCTCCCATGCAGAATAAGACAAATTATAAACAGGTGGCAAGAAGTAGGAAAAGCGA
TGTATGCCCCTCCCATTGCAGGACAAATTAACTGTTCATCAAAT---
ATTACAGGGCTACTATTAACAAGAGATGGTGGTACTGATAATAGT----------------------------
-------------------------------------------------------------------------
----------------
ACCGAGGTCTTCAGACCTGGAGGAGGAGATATGAGGGACAATTGGAGAAGTGAATTATATAAATACAAAGTAG
TACAAATTGAACCAATA---GGAATAGCACCCACCAAGGCAAGGAGAAGAGTGGTG-----------------
----------------------CAGAGAGAAAAAAGAGCA---ATAGGA---ATAGGAGCT---------
CTGTTCGTTGGGTTCTTGTCAGCA---GCAGGAAGCACTATA---GGCGCAGCGTCA---
GTGACGCTGACGGTACAG---GCCAGACAA---TTACTG---TCTAGTATAGTGCAACAGCAAAAC---
AATTTG---CTGAGG---GCTATTGAGGCGCAACAGCATCTGTTGCAACTCACAGTCTGGGGCATC
>KJ849807
TTCTACTGTAATACAACACAACTGTTTAATAGTACTTGGAATGATACATGGGTATAT----------------
-----------------------------------------------------------------
AACATAGCTACAGGAAACATA------------
ACTACAGATAATAAAATCACACTCCCATGCAGAATAAAACAAATTATAAACAGGTGGCAGGAAGTAGGAAAAG
CAATGTATGCCCCTCCCATCGAAGGACTAATTAGATGCTCATCAAAT---
ATTACAGGACTACTATTAACAAGAGATGGGGGT----------------------------------------
--------------------------------------------------------------------
GTTAACAGCAGCAAC---
CAGTATGAGATCTTCAGACCTGGAGGAGGAGATATGAGAGATAATTGGAGAAGTGAATTATATAAATACAAAG
TAGTAAGAATTGAACCGTTA---GGAATAGCACCCACCAAGGCAAGGAGAAGAGTGGTG--------------
-------------------------CAGAGGGAAAAAAGAGCA---GTGGGA---ATAGGAGCT---------
TTGTTCCTTGGGTTCTTGGGAGCA---GCAGGAAGCACTATG---GGCGCAGCGTCA---
ATGACGCTGACGGTACAG---GCCAGACAA---TTATTG---TCTGGTATAGTGCAACAGCAAAGC---
AATCTG---CTGAGA---GCTATTGAGGCGCAACAGCATATGTTGCAGCTCACAGTCTGGGGCATC
>KT427805
TTCTACTGTGAAACAACACAGCTATTTAATAGTACTTGGACTTTTAATAGGACGTCTAATAGTACCGGG----
-------------------------------------------------------------------------
----------------------------
AATGATACTATTATCACACTCCCATGCAGGATAAAACAAATTATAAACAGGTGGCAGGAAGTAGGAAAAGCAA
TGTATGCCCCTCCCATCAGAGGATCAATTAACTGCACATCAAAC---
ATCACAGGGCTAATACTAACAAGAGATGGTGGGATTAACAGGACT----------------------------
-------------------------------------------------------------------------
-------------
GAGAATGAAACCTTCAGACCTGGAGGAGGAGATATGAGGGACAATTGGAGAAGTGAATTATATAAATATAAAG
TAGTAAAAATTGAACCATTA---GGATTAGCACCCACCAAGGCAAAGAGAAGAGTGGTA--------------
-------------------------CAGAGAGAAAAAAGAGCA---GTAACG---CTGGGAGCT---------
ATGTTCCTTGGGTTCTTGGGAACA---GCAGGAAGCACTATG---GGCGCAGCGTCA---
TTGACGCTGACGGTACAG---GCCAGACAA---TTATTG---TCTGGTATAGTGCAACAGCAGAAC---
AATATG---CTGAGG---GCTATTGAGGCGCAACAGCATATGTTGCAACTCACAGTCTGGGGCATC
>KJ849808
TTCTACTGTAACACAACACAGCTGTTTAATGGCACATGGTCCAATGGTACA----------------------
-------------------------------------------------------------------------
-------TGGACAACACCG------
CTGCTTAATGGGACTATCACACTCCCATGCAGAATAAAACAAATTATAAACAGATGGCAGGAAGTAGGAAAAG
CAATATATGCCCCTCCCATCAGAGGACAAATTAGCTGTACATCAAAT---
ATCACAGGACTGCTATTAACAAGAGATGGGGGG----------------------------------------
-------------------------------------------------------------------------
-
GTTGGCCAGAGAGAGAATGAAACCTTCAGACCTGGAGGAGGAAATATGAAAGACAATTGGAGAAGTGAACTAT
```

ATAAATATAAAGTAGTAAAAATTGAACCATTA---GGAGTAGCACCCACCCCAGCAAAGAGAAGAGTGGTG--
-------------------------------------CAGAGAGAAAAAAGAGCA---
GTGGGAACAATAGGAGCT---------ATGTTCCTTGGGTTCTTAGGAGCA---GCAGGAAGCACTATG---
GGCGCAGCGTCG---CTGACGCTGACGGTACAG---GCCAGACTA---TTATTG---
TCTGGTATAGTGCAACAGCAAAAC---AATTTG---CTGAGG---
GCTATTGAGGCGCAACAGCATCTGTTGCAACTCACAGTCTGGGGCATC
>KT427799
TTCTACTGTAATACAACACAGTTATTTAATAGTACTTGGCATGCTAATAGCACTTGGAATGAGACTACAGGAT
CA---

GGGTCAAATGACACTATCTCACTCCCATGCAGAATAAAACAAATTATAAACAGATGGCAGGAAGTAGGAAAAG
CAATGTATGCCCCTCCCATCGGAGGACAAATTAGATGTTCATCAAAT---
ATTACAGGGATACTATTAACAAGAGATGGTGGTACAGAGAACAAC----------------------------
----

ACGTCCGAGACTTTCAGACCTGGAGGAGGAAATATGAAGGACAATTGGAGAAGTGAATTATATAAATATAAAG
TAGTAAGAATTGAGCCATTA---GGAGTAGCACCCACCAAGGCAAAGAGAAGAGTGGTG--------------
-------------------------CAGAGAGAAAAAAGAGCA---GTGACG---TTTGGAGCT---------
TTTTTCCTTGGGTTCTTAGGAACA---GCAGGAAGCACTATG---GGCGCAGCATCA---
ATAACGCTGACGGTACAG---GCCAGACAA---TTATTG---TCTGGTATAGTGCAACAGCAGAGC---
AACCTG---CTGAGA---GCTATTGAGGCGCAACAACGTATGTTGCAACTCACAGTCTGGGGCATC
>KT427667
TTCTACTGTAACACAACACAACTGTTTAACAGTACCTGGACTAATGGTACCTGGGAGACGACTAGTACTGGG-
----

AATATAAGTAATATCACACTCCCATGCAGGATAAAACAAATTATAAATAGGTGGCAGGAAGTAGGAAAAGCAA
TGTATAACCCTCCCATCAGGGGAAAAATTAACTGTTTATCAAAC---
ATTACAGGAATACTATTAACAAGAGATGGAGGAGGTAACGGGTCT----------------------------
----

AGTAACGAGACCTTCAGACCTGGAGGAGGAAATATGAAGGATAATTGGAGAAGTGAACTATATAAATATAAAG
TAGTAAAAATTGAACCATTA---GGACTAGCGCCCACCAGGGCAAGGAGAAGAGTGGTG--------------
-------------------------CAAAGAGAAAAAAGAGCA---GTGGGAACACTAGGAGCT---------
ATGTTCCTTGGATTCTTGGGAGCA---GCAGGAAGCACTATG---GGCGCAGCGTCA---
ATGGCGCTGACGGTACAA---GCCAGACAA---TTATTG---GCTGGTATAGTGCAACAGCAGAAC---
AATATG---CTGAGA---GCTATTGAGGCGCAACAGCATATGTTGCAACTCACAGTCTGGGGCATC
>KT427794
TTCTACTGTAATACAACAAAGTTGTTTAATAGTACTTGGAAGTACAACGATACTAGCAACACTTGGACTGAAG
GAAACCAT---
ACTAAAGAAGGAAACCATACT------------
GAAGGAAATAACACAATCACACTCCCATGTAGAATAAAGCAAATTATAAACAGGTGGCAGGAGGTAGGAAAAG
CAATGTATGCCCCTCCCATTAGGGGACTAATTAAATGTACATCAAAT---
ATTACAGGGATGATATTAACAAGAGATGGTGGTAAGGAGAATGAGACCGAG----------------------
----

AATACCGAGACCTTCAGACCTGGAGGAGGAGATATGAGGGACAACTGGAGAAGTGAATTATATAAATATAAAA
TAGTAAAAATTGAGCCATTA---GGAATAGCACCCACCAAGGCAAGGAGAAGAGTGGTG--------------
-------------------------CAGAGAGAAAAAAGAGCA---GTGGGAACAATAGGAGCT---------
ATGTTCCTTGGGTTCTTGGGAACA---GCAGGAAGCACTATG---GGCGCAGCGTCA---
ATCACGCTGACGGTACAG---GCCAGACAA---TTATTG---TCTGGTATAGTGCAGCAGCAGAAC---
AATTTG---CTGAAG---GCTATTGAGGCGCAACAACATCTGTTGCAACTCACAGTCTGGGGCATC
>KU749390
TTCTACTGTAATACAACACAATTGTTTAACAGTACTTGGAATGCTACTGGGAATGGTACTATAGAGGGA----
----

GATAGCGATAACATCACACTCCAATGCAAAATAAAACAAATTATAAACATGTGGCAGGAAGTAGGAAAAGCAA
TGTATGCCCCTCCCATCAGAGGAGAAATTAGATGTTCATCAAAT---
ATTACAGGGCTGCTATTAACAAGAGATGGTGGTGATAATGAGTCC----------------------------
----

AGTACTGATGAGACCTTTAGACCTGGAGGAGGAGATATGAAGGATAATTGGAGAAATGAATTATATAAATATA
AAGTAGTAAAAATTGAACCATTA---GGAATAGCACCCACCAAGGCAAAAAGAAGAGTGGTG-----------
----------------------------CAAAGAGAAAAAAGAGCA---GTGGGA---ATAGGAGCT------
---TTGTTCCTTGGGTTCTTGGGAGCA---GCAGGAAGCACTATG---GGCGCAGCGTCC---
ATGACGCTGACGGTACAG---GCCAGACAA---TTACTG---TCTGGTATAGTGCAACAGCAGAAC---
AATTTG---CTGAGA---GCTATTGAGGCGCAACAGCACCTGTTGCAACTCACAGTCTGGGGCATC
>KX181912
TTCTACTGTAATACAACATCACTGTTTAATAGTACTTGGAATAATACTAAAGAGTGGCATGGTACTACAGGG-
----

AGAAACAACACTATCACACTCCCATGTAAAATAAAACAAATCATAAACAGATGGCAGGAAGTAGGACAAGCTA
TGTATGCCCCTCCCATCAGTGGAAAAATTAACTGCTTATCAAAT---
ATTACAGGGCTGTTATTAATAAGAGATGGTGGTGTTAACGTTAGC----------------------------
----

AACACCACAGAGACCTTCAGGCCTGGGGGGGGAAATATAAAGGACAATTGGAGAAGTGAATTATATAAATATA
AAGTAGTAAAAATTGAACCCTTT---GGAGTAGCCCCCCCCGGTCAAAGAGAAGAGTGGGG-----------
----------------------------CAGAGAGAAAAAAGAGCA---GTGACG---CTAGGAGCT------
---ATGTTCCTTGGGTTTTTGGGAGCA---GCAGGAAGCACTATG---GGCGCAGCGTCA---
ATGGGGCTGACGGTACAG---GCAAAACAA---TTATTG---TCTGGTATAGTGCAGCAGCAGAAT---
AATTTT---CTGAGG---GCTATTGAGGCGCAACAGCATATGTTGCAACTCACAGTCTGGGGCATT
>KX181906
TTCTACTGTAACACATCACAGCTGTTTAATAGTACTTGGTATTGGAATGGATCAAATTTCAATGAAACAAATC
TCAAT--

GAATCTGAAACTATCACAATCCCATGCAGAATAAAACAAATTATAAACAGGTGGCAGGAAGTGGGAAAAGCAA
TGTATGCCCCTCCCATCAGAGGAATGATTAACTGCACATCAAAT---
ATTACAGGGCTGCTATTAACATGGGATGGTGGTAACAATAACAGA----------------------------
----

ACGAACGAGACCTTTAGACCTGGAGGAGGAGATATGAGGGACAATTGGAGAAGTGAATTATATAAATATAAAG
TAGTAAAAATTGAGCCATTA---GGACTAGCACCCACCAAGGCAAAGAGAAGAGTGGTG--------------
-------------------------CAGAGAGAAAAACGAGCA---GTGGGAACAATAGGAGCT---------
ATGTTCCTTGGGTTTTTGGGAGCA---GCAGGAAGCACTATG---GGCGCAGCGTCA---
GTAACGCTGACGGTACAG---GCCAGACTA---TTATTG---TCTGGTATAGTGCAACAGCAAAAC---
AATTTG---CTGAGG---GCTATTGAGGCGCAACAACATATGTTGCAACTCACAGTCTGGGGCATT
>KX181900
TTCTACTGTAATACAACAAAACTGTTTAATAGTACTTGGAATAGTAGTAGTACTTGGTATAATAAAAGTGCTG
AA---

GGAAATTCCACTATTATACTCCCATGCAGAATAAAACAAATTATAAATAGGTGGCAGGAAGTAGGGAAAGCAA
TGTATGCCCCTCCCATCCCAGGACACATTAACTGTTTATCAAAT---
ATTACAGGGCTACTATTGACAAGAGATGGTGGAAACAACAACAGT----------------------------
----

AGCTCCGAAACTTTTAGACCTGGAGGAGGAGATATGAGGGACAATTGGAGAAGTGAATTATATAAGTATAAAG
TAGTAAGAATTGAACCATTA---GGAGTAGCACCCACCAAGGCAAGGAGAAGAGTGGTG--------------
-------------------------CAGAGAGAAAAAAGAGCA---GTGGGAACAATAGGAGCT---------
ATGTTCCTTGGGTTCTTGGGAGCA---GCAGGAAGCACTATG---GGCGCAGCATCA---
ATAACGCTGACGGTACAA---GCCAGACTA---TTATTG---TCTGGTATAGTGCAACAGCAGAAC---
AATCTG---CTGAGA---GCTATTGAGGCGCAACAGCATATGTTGCAACTCACAGTCTGGGGCATT
>KX181901
TTCTACTGTGATTCAACACAGCTGTTTAATAGTACTTGGCTGTTTAATGATACTCGGAATGGTACTGACATTG
AA---

GGTAATGAAACTATCACACTCCCATGTAGAATAAAACAAATTATAAACAGGTGGCAAGAAGTAGGAAAAGCAA
TATATGCCCCTCCCATCGAAGGACAAATTAGATGTACATCAAAT---
ATTACAGGGCTGTTATTAACAAGAGATGGTGGT--

AACAGTGGGACAGAAACTTTCAGACCTGGAGGAGGAGATATGAGGGACAATTGGAGAAGTGAATTATATAAAT
ATAAAGTAGTGAAAATTGAGCCATTA---GGAATAGCACCCACCAAGGCAAAGAGAAGAGTGGTG--------
-------------------------------CAGAGAGAAAAAAGAGCA---GTGGGAACGATAGAAGCT---
------ATGTTCCTTGGGTTCTTGGGAGCA---GCAGGAAGCACTATG---GGCGCAGCGTCA---
GTAACGCTGACGGTACAG---GCCAGACTA---TTATTG---TCTGGTATAGTGCAACAGCAAAAC---
AATTTG---CTGAAG---GCTATTGAGGCGCAACAGCATATGTTGCAACTCACAGTCTGGGGCATT
>KX181891
TTCTACTGTGATACAACAAAGCTGTTCAATAGTACTTGGGTGATCAATAATACTTGGCAAAGAGTAAATAACA
CCAAA--

AATGAATCAAAGATCATACTCCCATGCAAAATAAAACAAATTATAAACAGGTGGCAGGAGGTAGGAAAAGCAA
TGTATGCCCCTCCCATCAGAGGATACATTAACTGTTCATCGAAT---
ATTACAGGGCTGCTATTAACAAGAGATGGGGGAGACAATAATAAT----------------------------
----

AATAATGAGACCTTCAGACCCCAAGGGGGAAATATGAAGGATAATTGGAGAAGTGAATTATATAAATATAAAG
TAATAAAAATTGAGCCATTA---GGAGTAGCACCCACCGAGGCAAAGAGAAGAGTGGTG--------------
-------------------------CAGAGAGAAAAAAGAGCA---ATAGGAGTGCTAGGAGCT---------
GTGTTCCTTGGGTTCTTAGGAGCA---GCAGGAAGCACTATG---GGCGCAGCGTCA---
ATAACGCTGACGGTACAG---GCCAGACAA---TTATTG---TCTGGTATAGTGCAACAGCAGAGC---
AATCTG---CTGCAG---GCTATTGAGGCGCAACAGCATCTGTTGCAACTCACAGTCTGGGGCATC
>KX181907
TTCTATTGTAATACAACACAACTGTTCAATAGCACCTGGAATAATACAGAAATGGTTAATGGTACTACTAATG
GTACT--

ACTAATGATACAATTATACTCCCATGCAGAATAAAACAAATTGTAAACATGTGGCAGGAAGTAGGAAAAGCAA
TGTATGCCCCTCCCCTCAGGGGAAAAATTAACTGTGTATCAAAT---
ATTACAGGGCTAATATTAACAAGAGATGGGGGAATAAATAGGACG----------------------------
----

AAGAACGAGACCGAGACCTTCAGACCTGGGGGGGGAGATATGAGGGACAATTGGAGAAGTGAATTATATAAAT
ATAAAGTAGTAAAAATTGAACCCTTA---GGGGTAGCCCCCCCCCGGGCAAAGAGAAGAGTGGTG--------
-------------------------------CAAAGAGAAAAAAGAGCA---GTAACC---CTAGGAGCT---
------ATGTTCCTTGGGTTTTTGGGAGCA---GCAGGAAGCAATATG---GGCGCAGCGTCA---
ATAACGCTGACGGTACAG---GCCCGACAA---TTATTG---TCTGGTATAGTGCAACAGCAAAAC---
AATTTG---CTGAGA---GCTATTGAGGCGCAACAGCATCTGTTGCAACTCACGGTCTGGGGCATC
>KX181910
TTCTACTGTAATACATCACAGCTGTTTAATAGTACTTGGTTTGCGAATGGTACTAGAAAAGAAAATGGAACTG
CA---

GGAAACAACACATTCATCACACTCCCATGCAGAATAAAACAGATCATAAACAGGTGGCAGGAAGTAGGAAAAG
CAATGTATGCCCCTCCCATCAGTGGATACATTAATTGTTCATCAAAT---
ATTACAGGGCTGTTATTAACAAGAGATGGTGGTACCGGAAACAAA----------------------------
----

ACCGAGCCCGAGATCTTCAGACCTAGAAGGGGAGATATGAAGGACAATTGGAGAAGTGAATTATATAAATACA
AAGTAGTAGAAATTGAGCCATTA---GGAATAGCCCCCACCAAGGCAAAGAGAAGAGTGGTG-----------
----------------------------CAGAGAGAAAAAAGAGCA---GTAGAAATGCTAGGAGCT------
---ATGTTCCTTAGGTCCTTAGGAGCA---GCAGGAAGCACTATA---GGCGCAGCGTCG---
GTGGCGCTGACGGTACAG---GCCAGACAA---TTATTG---TCTGGTATAGTGCAACAGCAGAGC---
AATTTG---CTGAAG---GCTATTGAAGCGCAACAACATATGTTGCAACTCACAGTCTGGGGCATT
>KX181911
TTCTACTGTAATTCAACACAGCTGTTTAATAGTAGTTGGACTAATGGTAGTTGGACTGGCATTGAAGGAAATA
ATACA--

AGTAATGAAAGTATCACACTCCCATGCAGAATAAAACAAATAATAAATAGGTGGCAGGAAGTAGGAAGAGCAA
TATATGCCCCTCCCATCAGTGGACAACTTAACTGTTCATCAAAT---
ATTACAGGGCTGCTATTAACAAGAGATGGTGGTAATAGGACCAATGGG-------------------------
----

GAGGCCGAAGTCTTCAGACCTGGAGGAGGAAATATGAAAGACAATTGGAGAAGTGAATTATATAAATATAAAG
TAGTAAAAATTGAGCCATTA---GGAATAGCACCCACCAAGGCGAAGAGAAGAGTGGTG--------------
------------------------CAGAGAGAAAAAAGAGCA---GTGGGAACAATAGGAGCT---------
ATGTTCCTTGGGTTCTTGGGAGCA---GCAGGAAGCACTATG---GGCGCAGCGTCA---
ATAACGCTGACGGTACAG---GCCAGACTA---TTATTG---TCTGGTATAGTGCAACAGCAGAAC---
AATCTG---CTGAGA---GCTATTGAGGCGCAACAGCATATGTTGCAACTCACTGTCTGGGGCATC
>KX181895
TTTTACTGTAATACAACACGGCTGTTTAATAGTTTTTGGGTGAAAGGTACTTGGGGGAATAGTACTTTACAG-
----

AACAATGGTACTATCATACTTCCCTGCAGAATAAAACAGATTATAAACAGGTGGCAAGAAGTAGGAAAAACAA
AGTACGCCCCCCCCCTCAGAGGACACCTTAACTGTTTTTCAAAC---
ATTACAGGGGTGATATTAGCAAGGGATGGTGGTATTAATGTGACC---------------------------
----

AACAATACCGAGACCTTCAGACCTGGGGGAGGGGACAAGAGGGGCAATTGGGGAAGTGAATTATACAAATATA
AAATAGTAAAAAATGAGCCATTA---GGAGTAGCACCCACCAAGGCAAAGAGAAGAGTGGTG-----------
---------------------------CAGAGAGAAAAAAGAGCA---GTGGGAACTATAGGAGCT------
---ATGTTCCTTGGGTTCTTGGGAGCA---GCAGGAAGCACTATG---GGCGCAGCATCA---
GTGACGCTGACGGTACAG---GCCAGACTA---TTATTG---TCTGGTATAGTGCAACAGCAGAGC---
AATCTG---CTGAGG---GCTATTGAGGCGCAACAGCATTTGTTGCAACTCACAGTCTGGGGCATC
>KX181897
TTCTATTGTAATACAACACAGCTGTTTAATAGTACTTGGAATGGCACACATCTGTCTAATAGTATT-------
----

GGGAATGAGACTATCACACTCCCATGCAGAATAAAACAAATTATAAACAGATGGCAGGAAGTAGGAAAGGCAA
TGTATGCCCCTCCCATCAGTGGACAAATTAGCTGTTCATCAAAT---
ATAACAGGGCTGATATTAACAAGAGATGGTAGTAATGATAATAGC---------------------------
----

AGCACGAATGAAACTTTCAGACCGGGAGGAGGAAATATGAAAGACAATTGGAGAAGTGAATTATATAAATATA
AAGTAGTAGAAATTGAGCCAGTA---GGATTAGCACCCACCAAGGCAAAAAGAAGAGTGGTG-----------
---------------------------CAGAGAGAAAAAAGAGCA---GTAGGAACGCTAGGAGCT------
---ATGTTCCTTGGGTTCTTGGGAACA---GCAGGAAGCACTATG---GGCGCAGCGTCA---
TTGACGCTGACGGTACAG---GCCAGACAA---TTAATG---TCTGGTATAGTGCAACAGCAGAAC---
AATCTG---CTGAGG---GCTATTGAGGCGCAACAACATATGTTGCAACTCACAGTCTGGGGCATC
>KX181915
TTTTACTGTAATACAACAAACCTGTTTAATAATGGTACCCAGCTGCTGCCTAATGATACTTGGCAGTTTAATG
GAAGTAAAATT---

GAAAATGGGACTATCATACTCCCATGCAGAATAAAACAAATTATAAACAGGTGGCAGGAAGTAGGAAAAGCAA
TGTATGCCCCTCCCATCAGTGGACTAATTAGATGTTCATCAAAC---
ATTACAGGGCTGATATTAACAAGAGATGGTGGGTACAACAACAAGTCT------------------------
----

AACAATACTGAGACCTTTAGACCTGGAGGAGGAGATATGAGGGACAATTGGAGAAGTGAATTATATAAATATA
AAGTAGTAAAAATTAAGCCATTA---GGGGTAGCACCCACCAAGGCAAAGAGAAGAGTGGTG-----------
---------------------------CAAAGAGAAAAAAGAGCA---GTAACA---ATAGGAGCT------
---ATGTTCCTTGGGTTTTTGGGAGCA---GCAGGAAGCACTATG---GGCGCAGCATCA---
CTAACGCTGACGGTACAG---GCCAGACAA---TTATTG---ACTGGTATAGTGCAACAGCAAAAC---
AATTTG---TTGAGG---GCTATTGAGGCGCAACAGCATTTGTTGCAACTCACAGTCTGGGGCATC
>KX181916
TTTTACTGTAATTCAACACAGCTGTTCAACAGTAGTTGGAGGTTTAATAATAATACTTGGAAGGTTGTTAATA
ATAATACT--
------------------GGG---
AATTCAAATGACAACATCACACTCCCATGCAGAATAAAACAAATTATAAACAGGTGGCAGGATGTAGGAAAAG
CAATGTATGCCCCTCCCATCAGGGGACAAATTAGCTGTTCATCAAGT---
ATTACAGGGCTGCTATTAACAAGAGATGGTGGTAATAATAAAGAG---------------------------
----

TCCCCGGAGGCCTTCAGACCTGGAGGGGGGGATATGAAGGGCAATTGGGGAAGTGAATTATATAAATATAAAG
TAGTAAAAAATAAGCCATTA---GGAATAGCCCCCCCCAAGGCAAAGAGAAGAGTGGTG--------------
-------------------------CAAAGAGAAAAAAGAGCA---GTAGGAACGATAGGAGGT---------
ATGTTCCTTGGGTTTTTGGGAGCA---GCAGGAAGCAGTATG---GGCGCAGCGTCC---
TTGACGCTGACGGTACAG---GCCAGACTA---TTATTG---TCTGGTATAGTGCAACAGCAGAAT---
AATTTG---CTGAGG---GCTATTGAGGCGCAACAACATCTGTTGCAACTCACAGTCTGGGGCATC
>MK041584
TTCTACTGTAACACAACAAAGCTGTTTAATAGTACTTGGAATAGTACTGACTGGTCAAATACCACTGAT----
----

GATAATGACACCATCACACTCCCATGCAGAATAAAACAAATTATAAACAGGTGGCAGGAAGTAGGAAAAGCAA
TGTATGCCCCTCCCATTGCAGGACAGATTATGTGTTCATCAAAT---
ATTACAGGGCTAATATTAACAAGAGATGGTGGTAACAGTAACGGGTCG-------------------------
----

ACTAATGAGACCTTCAGACCTGCAGGAGGAGATATGAGGGACAATTGGAGAAGTGAATTATATAAATATAAAG
TAGTAAAAATAGAGCCATTA---GGAATAGCACCTACCAAGGCAAGGAGAAGAGTGGTG--------------
-------------------------CAAAGAGAGAAAAGAGCA---GTGGGA---CTAGGAGCT---------
CTGTTTCTTGGGTTCTTGGGAGCA---GCAGGAAGCACTATG---GGCGCAGCGTCA---
ATGACGCTGACGGTACAG---GCCAGACAA---CTATTG---TCTGGTATAGTGCAACAGCAAAAC---
AATTTG---CTGATG---GCTATTGAGGCGCAACAGCGTATGTTGCAACTCACAGTCTGGGGCATC
>MK041586
TTCTACTGCAATACAACACAGCTGTTTAATTCAACTTGGGATACTAATTCAACTTGGACTGCTGAGGAAGCAG
AT---

GGAAATATCACTATCACACTCCCATGTAGAATAAAACAATTTATAAACAGATGGCAGGAGGTAGGAAAAGCAA
TGTATGCCCCTCCCATCAGAGGAATAATTAACTGTGTATCAAAT---
ATTACAGGACTACTATTAGTGAGAGATGGTGGTGAGAACAATGCA----------------------------
----

ACCAATGAGACCTTCAGGCCTGGAGGAGGCGATATGAGGGACAATTGGAGAAGTGAACTATATAAATATAAAG
TAGTACAGATTGAGCCGTTA---GGAATAGCACCCACCAGGGCAAGGAGAAGAGTGGTG--------------
-------------------------CAGAGAGAAAAAAGAGCA---GTGGGAACAATAGGAGCT---------
ATGTTCCTTGGGTTCTTGGGAGCA---GCAGGAAGCACTATG---GGCGCAGCGTCA---
ATGACGCTGACGGTACAG---GCCAGACTA---CTACTG---TCTGGTATAGTGCAACAGCAGAAC---
AACTTG---CTGAGA---GCTATTGAGGCGCAACAACATCTGTTGCAACTCACAGTCTGGGGCATC
>MK041551
TTTTACTGTGATACAACACAACTGTTTAATAGTACTTGGTCACTTAATAAGACTTCTTGGTATAATACTACTA
CT---

GAAGGAATAGATATCACACTCCCATGCAAAATAAAACAAATCATAAACAGGTGGCAGGGAGCAGGAAAAGCAA
TGTATGCCCCTCCCATCAGAGGAAAAATTTTCTGTTCATCAAAA---
ATTACAGGAATACTATTAACAAGAGATGGTGGGTTTAACAATGCCAGT-------------------------
----

AACAATAGCGAGACCTTCAGACCTGGAGGGGGAGATATGAGGGACAATTGGAGAAGTGAATTATACAAATATA
AAGTAGTAAAAATTGAGCCATTA---GGAGTAGCACCCACCAAGGCAAAAAGAAGAGTGGTG-----------
----------------------------CAAAGAGACAAAAGAGCA---GTGGGAACGTTAGGAGCT------
---ATGTTCCTTGGGTTCTTGGGAGCA---GCAGGAAGCACTATG---GGCGCAGCGTCA---
ATGGCGCTGACGGTACAG---GCCAGACAA---TTATTG---TCTGGTATAGTGCAGCAGCAAAAC---
AATTTG---CTGAGA---GCTATTGAGGCGCAACAACATCTGTTGCAACTCACAGTCTGGGGCATC
>MK041554
TTCTACTGTAATACATCACAGCTGTTTAATGAAACTTTTTTGAAGAAGAAT----------------------
----

GATACTGAACCTATCACACTCCAATGCAGAATAAAACAAATTATAAACAGGTGGCAGGAAGTGGGAAAAGCAA
TGTATGCCCCTCCCATCAGTGGACAAATTAACTGTTCATCAAAT---
ATTACAGGGTTGATACTAACGAGAGATGGTGGCATCAATAATACC----------------------------
----

ATGAATGAGACTTTCAGACCTGGAGGAGGAGACATGAGGGACAATTGGAGAAGTGAATTATATAAATATAAAG
TAGTAAAAATTGAGCCATTA---GGAGTAGCACCCACCAAGGCAAAGAGAAGAGTGGTG--------------
-------------------------CAGAGAGAAAAAAGAGCA---GTGGGAACGATAGGAGCT---------
ATGTTCCTTGGGTTCTTGGGAGCA---GCAGGAAGCACTATG---GGCGCAGCGTCG---
CTGACGCTGACGGTACAG---GCCAGACTA---TTATTG---TCTGGTATAGTGCAACAGCAAAAC---
AATTTG---CTGAGG---GCTATTGAGGCGCAACAGCATCTGTTGCAACTCACAGTCTGGGGCATC
>MK041558
TTCTACTGTAATACAACACAGTTGTTTAATAGTACTTGGAATAGTACTGACACTAACTGGATAAATAATGCTA
GTTCG--

ACAAAGGGTAATATCACACTCCCATGCAGGATAAAACAAATTATAAACAGGTGGCAAGAAGTAGGAAAAGCAA
TGTATGCCCCTCCCATCAGCGGACAAATTAAGTGTTCATCAAAT---
ATTACAGGACTGCTATTAACAAGAGATGGTGGTCCTGAAAATGAC----------------------------
----

TCTGCGACCTTCAGACCTGGAGGAGGAGATATGAGGAATAATTGGAGAAGTGAATTATATAAATACAAAGTAG
TAAAAATTGAGCCATTA---GGAGTAGCACCCACCAAGGCAAAGAGAAGAGTGGTG-----------------
----------------------GAGAGAGAAAAAAGAGCA---GTGACA---CTAGGAGCA---------
ATGTTCCTTGGGTTCTTGGGAGCA---GCAGGAAGCACTATG---GGCGCAGCGTCA---
ATAACGCTGACGGTACAG---GCCAGACAA---TTATTG---TCTGGTATAGTGCAACAGCAGAAC---
AATCTG---CTGAAG---GCTATTGAGGCGCAACAGCACCTGTTGCAACTCACAGTCTGGGGCATC
>MK041556
TTCTACTGTAATACATCACAGCTGTTTAATAGTATGGAGTTGGAGGCTTTGAGGAATAACAATAAA-------
----

ACTAATGACACTATCACACTCCAATGCAGAATAAAACAAATTATAAACAGGTGGCAGGAAGTAGGAAAAGCAA
TGTATGCCCCTCCCATCAGTGGACAAATTAACTGTGTATCAAAT---
ATTACAGGGTTGATACTAACAAGAGATGGTGGTAACGGGACTAAC----------------------------
----

GGGACTGAGACTTTCAGACCAGGAGGAGGAGATATGAGGGACAATTGGAGAAGTGAATTATATAAATATAAAG
TAGTAAAAATTGAGCCATTA---GGAATAGCACCCACCAAGGCAAAGAGAAGAGTGGTG--------------
-------------------------CAGAGAGAAAAAAGAGCA---TTGGGAACGATAGGAGCT---------
ATGTTCCTTGGGTTCTTGGGAGCA---GCAGGAAGCACTATG---GGCGCAGCGTCA---
CTGACGCTGACGGTACAG---GCCAGACAA---TTATTG---TCTGGTATAGTGCAACAGCAAAAC---
AATTTG---CTGAGG---GCTATTGAGGCGCAACAGCATCTGTTGCAACTCACAGTCTGGGGCATC
>MK041562
TTCTATTGTGATTCAGCACAGCTGTTTAATAGTACTTGGAATGAAACTGATAGTTGGAATAATACTTCT----
----

GAAGAATCAACTATCACACTCCCATGCAGAATAAAACAAATTGTAAACCTGTGGCAGGAAGTAGGAAAAGCAA
TGTATGCCCCTCCCAGAGAAGGGGTTATTAGCTGTTTATCAAAT---
ATCACAGGGCTGCTCTTAACAAGAGATGGTGGTAATGAGAACACGAACTCT----------------------
----

AGCACTGAAACCTTCAGACCTGGAGGAGGCAATATGAAAGACAATTGGAGAAGTGAATTATACAAATATAAAG
TAGTAAGAATTGAACCATTA---GGAGTAGCACCTACCCTGGCAAAAAGAAGAGTGGTG--------------
-------------------------CAGAGAGAAAAAAGAGCA---GTGGGA---ATAGGAGCT---------
GTGTTCCTTGGGTTCTTGGGAGCA---GCAGGAAGCACTATG---GGCGCAGCGTCG---
GTGACGCTGACGGTACAA---GCCAGACAA---TTATTG---TCTGGTATAGTGCAACAGCAGAAC---
AATTTG---CTGATG---GCTATTGAGGCGCAACAGCGTCTGTTGCAACTCACAGTCTGGGGCATC
>MG571981
TTCTACTGTAATACAACACAGCTGTTTAATAGTACTTGGAATGAAACACAGCTGTTTAATAATATTAATAATA
GTACTAAA---

GAGAATGAGAATATCACACTCCCATGCAGAATAAAACAAATTGTAAACAGGTGGCAGGAAGTAGGAAAAGCAA
TGTATGCCCCTCCCATCAGAGGACAAATTAGCTGTTCATCAAAC---
ATTACAGGGCTGATACTAACAAGAGATGGTAGCGGTAACGGGTCTGCG-------------------------
----

AGCAATGAGACTTTCAGACCTGGAGGAGGAAATATGAGGGACAATTGGAGAAGTGAATTATATAAATATAAAG
TAGTAAAAATAGAGCCATTA---GGAGTAGCACCCACCAGGGCAAAGAGAAGAGTGGTG--------------
-------------------------CAGAGAGAAAAAAGAGCA---GTGGGGACGTTAGGAGCT---------
ATGTTCCTTGGGTTCTTGGGAGCA---GCAGGAAGCACTATG---GGCGCAGCGTCA---
GTGACGCTGACGGTACAG---GCCAGACAA---TTATTG---TCTGGTATAGTGCAACAGCAGAAT---
AATCTG---CTGAAG---GCTATTGAGGCGCAACAACATCTGTTGCAACTCACAGTCTGGGGCATC
>MG571996
TTCTACTGCAATACAACACAGCTGTTTAATTCAACTTGGGATACTAATTCAACTTGGACTGCTGAGGAAGCAG
AT---

GGAAATATCACTATCACACTCCCATGTAGAATAAAACAATTTATAAACAGATGGCAGGAGGTAGGAAAAGCAA
TGTATGCCCCTCCCATCAGAGGAATAATTAACTGTGTATCAAAT---
ATTACAGGACTACTATTAGTGAGAGATGGTGGTGAGAACAATGCA----------------------------
----

ACCAATGAGACCTTCAGGCCTGGAGGAGGCGATATGAGGGACAATTGGAGAAGTGAACTATATAAATATAAAG
TAGTACAGATTGAGCCGTTA---GGAATAGCACCCACCAGGGCAAGGAGAAGAGTGGTG--------------
-------------------------CAGAGAGAAAAAAGAGCA---GTGGGAACAATAGGAGCT---------
ATGTTCCTTGGGTTCTTGGGAGCA---GCAGGAAGCACTATG---GGCGCAGCGTCA---
ATGACGCTGACGGTACAG---GCCAGACTA---CTACTG---TCTGGTATAGTGCAACAGCAGAAC---
AACTTG---CTGAGA---GCTATTGAGGCGCAACAACATCTGTTGCAACTCACAGTCTGGGGCATC
>MG571999
TTCTACTGTAATACAACAAAGCTGTTTAATGGTACTTGGAATGGTACGGATTTCAATAATACTGGAGAA----
----

TCAAATGACACTATCACACTCCCCTGCAGAATAAGACAAATTATAAATAGATGGCAGGAAGTAGGAAAAGCAA
TGTATGCACCTCCCATCAGTGGACCAATTAGCTGTTCATCAAAT---
ATTACAGGGCTAATGTTAACAAGAGATGGGGGTGAGGATAACAGT----------------------------
----

TCTACTGAAACCTTCAGACCTGGAGGAGGAAATATGAAAGACAATTGGAGAAGTGAATTATATAAATATAAAA
TAGTAAAAATTGAGCCATTA---GGAGTAGCACCCACCACGGCAAAGAGAAGAGTGGTG--------------
-------------------------CAGAGAGAAAAAAGAGCA---GTGGGAACGCTAGGAGCT---------
ATGTTCCTTGGGTTCTTGGGAGCA---GCAGGAAGCACTATG---GGCGCAGCGTCA---
GTGACGCTGACGGTACAG---GCCAGACAA---TTATTG---TCTGGTATAGTGCAACAGCAGAAC---
AATTTG---CTGAGA---GCTATTGAGGCGCAACAGCACATGTTGCAACTCACAGTCTGGGGCATT
>MG572006
TTCTACTGTAATACAACACAGCTGTTTAATAGTACTTGGGGTAATAATACTTGGAATGGTACTGAAGGAAAT-
----

GGAAATGACACTATCACACTTCCATGTAGAATAAAACAAATTATAAACAGATGGCAGGAAGTAGGAAAAGCAA
TATATAACCCTCCAGTCAGAGGAATAATTAACTGTGCATCAAAT---
ATTACAGGGCTGCTATTAACAAGAGATGGTGGTAACACAAACACAACC-------------------------
----

AATAGGTCTGAAATCTTCAGACCTGGAGGAGGAAATATGAAGGACAATTGGAGAAGTGAATTATATAAATATA
AAGTAGTAAAAATAGAACCAATA---GGAATAGCACCCACCGCGGCAAAGAGAAGAGTGGTG----------
----------------------------CAGAGAGAAAAAAGAGCA---CTGCCG---CTGGGAGCT------
---ATGTTCCTTGGGTTCTTGGGAGCA---GCAGGGAGCACTATG---GGCGCAGCGTCA---
ATGACGCTGACGGTACAG---ACCAGACAA---TTATTG---TCTGGTATAGTGCAACAGCAGAGC---
AATCTG---CTGAGG---GCTATTGAGGCGCAACAACATATGTTGCAACTCACAGTCTGGGGCATC
>AY173956
TTCTACTGTAATTCAACACAACTGTTTAACAGTACTTGGAATAGTACTTGGAATGTTGATGAAAGATCAAAT-
----

GACACTGCAACACTCAAACTCCCATGCAGAATAAAACAATTTGTAAACATGTGGCAGGAAGTAGGAAAAGCAA
TGTATGCCCCTCCCATCGATGGGCAAATTAGTTGTTCATCAAAT---
ATTACAGGGTTACTATTAACAAGAGATGGTGGTAATAACACAAAC----------------------------
----

GAGACTGAGACCTTCAGACCTGGAGGAGGAGATATGAGGGACAATTGGAGAAGTGAATTATATAAATATAAAG
TAGTAAAAGTTGAACCATTA---GGAGTAGCACCCACCAGGGCAAAGAGAAGAGTGGTG--------------
-------------------------CAAAGAGAAAAAAGAGCA---GTGGGA---ATAGGAGCT---------
GTGCTCCTTGGGTTCTTGGGAGCA---GCAGGAAGCACTATG---GGCGCAGCGTCA---
CTGACGCTGACGGTACAG---GCCAGACAA---TTATTG---TCTGGTATAGTGCAACAGCAAAAC---
AATTTG---CTGAGG---GCTATTGAGGCGCAACAGCATCTGTTGCAACTCACAGTCTGGGGCATC
>AY779552
TTCTACTGTAATTCAACACCACTGTTTAATAGTACTTGGAATAGTACTACTTGGAACAGCACTAAAGGGTCAC
ATAAC--
-------------------AGT---
ACAGGAAATGAAACTATCACACTCCAATGCATAATAAAACAAATTATAAACCTGTGGCAGGAAGTAGGAAAAG
CAATGTATGCCCCTCCCATCAAAGGACAAATTAAATGCTCATCAAAT---
ATTACAGGGCTGCTATTAACAAGAGATGGTGGTAGGGGCAATGACACG-------------------------
----

GACAATACCACCGAGATCTTCAGACCTGGAGGAGGAGATATGAGGGATAATTGGAGAAGTGAATTATACAAAT
ATAAAGTAGTAAGAATTGAGCCATTA---GGGGTAGCACCTACCAAGGCAAAGAGAAGAGTGGTG--------
-------------------------------CAGAGAGAAAAAAGAGCA---GTGGGA---ATAGGAGCC---
------TTGTTCCTTGGGTTCTTGGGAGCA---GCAGGAAGCACTATG---GGCGCAGCGTCA---
ATAACGCTGACGGTACAG---GCCAGACTA---TTATTG---TCTGGTATAGTGCAGCAGCAGAAC---
AATCTG---CTGAGG---GCTATTGAGGCGCAACAGCACCTGTTGCAACTCACAGTTTGGGGCATC
>GU562135
TTCTACTGCAACTCATCAAAACTGTTTAATTGGAATTCAACACATTGGAATTCAACACATTGGAATTCAACAC
AACAGGGT---
-------------AATAGT------
ACTGGAAATGATACTATTACACTCACATGTAGGATAAAACAAATTATAAACATGTGGCAGGAAGTAGGAAAAG
CAATGTATGCCCCTCCCATCAAAGGACAAATTAGATGTTCATCAAAT---
ATTACAGGGATACTATTAACAAGAGATGGTGGAAATAACAATGACACC-------------------------
----

AACAAAACCGAGATCTTCAGACCAGGAGGAGGAGATATGAGGGACAATTGGAGAAGTGAATTATATAAATATA
AAGTAGTAAAAATTGAACCATTA---GGAGTAGCACCCACCAAGGCAAAGAGAAGAGTGGTG-----------
----------------------------CAGAGAGAAAAAAGAGCG---GTAACG---CTAGGAGCT------
---ATGTTCCTTGGGTTCTTGGGAGCA---GCAGGAAGCACTATG---GGCGCAGCGTCA---
CTAACGCTGACGGTACAG---GCCAGACTA---TTATTG---TCTGGTATAGTGCAACAGCAGAAC---
AATCTG---CTGAGG---GCTATTGAGGCGCAGCAGCATCTGTTGCAACTCACAGTCTGGGGCATC
>GU562272
TTCTACTGTAATTCAACACCACTGTTTAATAGTACTTGGCCTAGAAATTCAACAACACAACAGTCTAAT----
----

AATAGTACACCCATTGTACTCAAATGTAGAATAAAGCAAATTATAAACATGTGGCAGGAAGTAGGAAAAGCAA
TGTATGCCCCTCCCATTAAAGGAGTAATTAGGTGTTCATCAAAT---
ATCACAGGGATAATATTAACAAGAGATGGTGGTGCTAATAATACTAAC-------------------------
----

CATAATAATACCGAGACTTTCAGACCTGCAGGAGGAGATATGAGGGACAATTGGAGAAGTGAATTATATAAAT
ATAAAGTAGTAAAAATTGAACCATTA---GGAGTAGCACCCACCAGGGCAAAGAGAAGAGTGGTG--------
-------------------------------CAAAGAGAAAAAAGAGCA---GTGGGAGTAGTAGGAGCA---
------ATGTTCCTTGGGTTCTTGGGAGCA---GCAGGAAGCACTATG---GGCGCAGCGTCA---
ATAACGCTGACGGTACAG---GCCAGACAA---TTACTG---TCTGGTATAGTGCAACAGCAGAAC---
AATCTG---CTGAAG---GCTATTGAGGCGCAACAGCATCTGTTGCAACTCACAGTCTGGGGCATA
>GU562155
TTTTACTGTAATTCAACACACCTATTTAATTCAACTTGGAATAAGAATGATTCAACTTGGAATAAGAATGATT
CAACTTGGACT--
-AAGAATGATTCAACA---------
GTAAATAACGAAACTATCACCCTCCCATGCAGAATAAAACAAATTATAAACAAGTGGCAGGAAGTAGGAAAAG
CAATGTATGCCCCTCCCATCAAAGGACAAATTAGATGCTCATCAAAT---
ATTACAGGGCTAATATTAGTAAGAGATGGTGGGAACACGAACACC----------------------------
----

```
ACCGAGACCTTCAGACCTGCAGGAGGAGATATGAGGGACAATTGGAGAAGTGAATTATATAAATATAAAGTAG
TAAAAATTGAACCATTA---GGAGTAGCACCTACCAAGGCAAAGAGAAGAGTGGTG------------------
----------------------CAGAGAGAAAAAAGAGCA---GTGGGA---CTAGGAGCT---------
GTGTTCTTTGGGTTCTTGGGAGCA---GCAGGAAGCACTATG---GGCGCAGCGTCA---
GTGACGCTGACGGTACAG---GCCAGACAA---TTATTG---TCTGGTATAGTGCAACAGCAGAAT---
AATTTG---CTGAGG---GCTATTGAGGCGCAACAGCATATGTTGCAACTCACAGTCTGGGGCATC
>JF320413
TTCTACTGTGACTCAACACCACTGTTTAATAGTACTTGGAATAAGACTGAAAATGAGTGGAATAGTACTACTG
AC------------------------------------------------------------------------
----------------------------
ATACCAGAGAACATCACCCTCAGATGTAGAATAAGACAAATTATAAACATGTGGCAGGAAGTAGGAAAAGCAA
TGTATGCCCCTCCTATCAGTGGCCCAATTAACTGTACATCAAAT---
ATTACAGGGCTGCTATTAACAAGAGATGGTGGTAACCTCAGCAGC-----------------------------
--------------------------------------------------------------------------
-------------
ACTAATGAGACCTTCAGACCTGGAGGAGGAAATATGAGGGATAATTGGAGAAGTGAATTATATAAATATAAAG
TAGTAAAAATTGAACCATTA---GGAGTAGCACCCACCAAGGCAAGGAGGAGAGTGGTG---------------
-------------------------CAGAGAGAGAAAAGAGCA---GTGGGA---ATAGGAGCA----------
TTGTTCCTTGGGTTCTTGGGAGCA---GCAGGAAGCACTATG---GGCGCAGCGTCA---
ATGACGCTGACGGTACAA---GCCAGGCTA---TTATTG---TCTGGTATAGTGCAACAGCAGAAT---
AACTTG---CTGAGA---GCTATTGAGGCGCAACAGCATATGCTGCAACTCACAGTCTGGGGCATC
>GU562266
TTCTACTGTAATACATCACAATTGTTTAATGGTACTTGGAATAGTACTACATGGTCAAATTCAATTAAC----
--------------------------------------------------------------------------
----------------------------
AGCACTGAAAATATCACACTCCCATGCAGAATAAAACAATTTATAAACATGTGGCAGGAAGTAGGAAAAGCAA
TGTATGCCCCTCCCATCCAAGGACCAATTAGATGTTTATCAAAT---
ATTACAGGGCTACTATTAACAAGAGATGGTGGTAATAATAATGACATG--------------------------
--------------------------------------------------------------------------
----------
AATAACACCGAGACTTTCAGACCTGGAGGAGGAGATATGAGGGACAATTGGAGAAGTGAATTATATAAATATA
AAGTAGTACAAATTGAACCATTA---GGAGTAGCACCCACCAAGGCAAGGAGAAGAGTGGTG------------
----------------------------CAGAGAGAAAAAAGAGCA---GTAATGGGACTAGGAGCT------
---ATGTTCCTTGGGTTCTTGGGAGCA---GCAGGAAGCACTATG---GGCGCAGCATCA---
ATAACGCTGACGGTACAG---GCCAGACAA---TTATTG---TCTGGCATAGTGCAACAGCAGAGC---
AATTTG---CTGAGG---GCTATTGAGGCGCAACAGCACATGTTGCAACTCACAGTCTGGGGCATC
>AY314061
TTCTACTGTAATACAACATCACTGTTTAACAGTACTTGGAATGGTACTGAAGGGTCAATTAACACTACA----
--------------------------------------------------------------------------
----------------------------
GAAAATGACCCACTCACACTCCCATGCAGGATAAAACAAATTATAAATATGTGGCAGGAAGTAGGAAAAGCAA
TGTATGCCCCTCCCATCGCAGGACAAATTAGATGTACATCAAAT---
ATTACAGGGCTGCTACTAACAAGAGATGGTGGTAATGACACCATCAAT--------------------------
--------------------------------------------------------------------------
----------
AAGGGGACCGAGGTCTTCAGACCTGGAGGAGGAGATATGAGGGACAATTGGAGAAGTGAATTATATAAATATA
AAGTAGTAAAAATTGAACCATTA---GGAGTAGCACCCACCAGGGCAAAGAGAAGAGTGGTG------------
----------------------------CAAAGAGAAAAGAGAGCA---GCGGGA---ATAGGAGCT------
---GTGCTCCCTGGGTTCTTGGGAGCA---GCAGGAAGCACTAAG---GGCGAA---TCC---
AGCACACTGACGGTACAA---GCCAGACAA---TTATTG---TCTGGTATAGTGCAACAGCAGAGT---
AATCTG---CTGAGG---GCTATTGAGGCGCAACAGCATCTGTTGCAACTCACAGTCTGGGGCATC
>GU562033
TTCTACTGTAATTCAACACAACTGTTTAATAGTACTTGGAATGTTAGTAGTACTTGGAATAGTACTGAAGAG-
--------------------------------------------------------------------------
----------------------------
TCAAATGACACTATTATACTCCCATGTAGGATAAAACAAATTATAAACATGTGGCAGAAAGTAGGAAAAGCCA
TGTATGCCCCTCCCATCAGAGGACAAGTTAAATGTTCATCAAAA---
ATTACAGGGATACTATTAACAAGAGATGGTGGTAATAGTGGTAATAAC--------------------------
--------------------------------------------------------------------------
-------
```

ACGAACGAGACCGAGACCTTCAGGCCTGGAGGAGGAGATATGAGGGACAATTGGAGAAGTGAATTATATAAAT
ATAAAGTAGTAAAAATTAAACCATTA---GGAGTAGCACCCACCAAGGCAAAGAGAAGAGTGGTG--------
-------------------------------CAGAGAGAAAAAAGAGCA---GTGGGAACAATAGGAGCT---
------ATGTTCCTTGGGTTCTTGGGAGCA---GCAGGAAGCACTATG---GGCGCAGCGTCA---
ATGACGCTGACGGTACAG---GCCAGACAA---TTATTG---TCTGGTATAGTGCAACAGCAAAAC---
AATTTG---CTGAGG---GCTATTGAGGCGCAACAGCATCTGTTGCAACTCACAGTCTGGGGCATC
>GU562058
TTCTACTGTAATTCAACACAGCTGTTTAATAGTACTTGGTTTAAGAATAATACTTGGATAGGGTCAAAT----
----

GGCACTGAAAATATCACACTCCCATGCAGAATAAAACAAATTATAAACAGGTGGCAGGAAGTAGGAAAAGCAA
TGTATGCCCCTCCCATCAGTGGAGTAATTAGCTGTTCATCAAAT---
ATTACAGGGCTGATATTAACAAGAGATGGGGGAAGAGGTGATGTTAATAAC----------------------
----

ACCAACGAGACTGAGATCTTCAGGCCTGGAGGAGGAGATATGAGGGACAATTGGAGAAGTGAATTATATAAAT
ATAAAGTAGTAAAAATTGAGCCATTA---GGAATAGCACCCACCAAGGCAAAGAGAAGAGTGGTG--------
-------------------------------CAAAGAGAAAAAAGAGCA---GTAACG---TTTGGAGCT---
------TTGTTCCTTGGGTTCTTGGGAGCA---GCAGGAAGCACTATG---GGCGCAGCGTCA---
ATAACGCTGACGGCACAG---GCCAGACTA---TTATTG---TCTGGTATAGTGCAACAGCAGAAC---
AATTTG---CTGAGA---GCTATTGAGGCGCAACAGCATCTGTTGCAACTCACAGTCTGGGGCATC
>GU562001
TTCTACTGTACTACAACACAGATGTTTAATAGTACTTGGAAGGATGGTACTTGGACTGAAGGATCAAATAAC-
----

ACTGGCGGAAATATCATACTCCCATGCAGAATAAAACAAATTATAAACATGTGGCAGAAAGTAGGAAAAGCAA
TATATGCCCCTCCCATTAGTGGACAAATTAACTGTTCATCAAAT---
ATTACAGGGCTGCTATTAACAAGAGATGGTGGTAACGAGGGCAATGCT-------------------------
----

ACTAATAATACTGAAACCTTCAGACCTGGTGGAGGAGATATGAGGGACAATTGGAGAAGTGAATTATATAAAT
ATAAAGTAGTAAAAATTGAACCAATA---GGAATAGCACCCACCAAGGCAAAGAGAAGAGTGGTG--------
-------------------------------CAGAGAGAAAAAAGAGCA---GTGGGA---ATAGGAGCT---
------GTGTTCCTTGGGTTCTTGGGAGCA---GCAGGAAGCACTATG---GGCGCAGCGTCA---
ATAACGCTGACGGTACAG---GCCAGACAA---TTATTG---TCTGGTATAGTGCAACAGCAGAAC---
AATTTG---CTGAGG---GCTATTGAGGCGCAACAGCACCTGTTGCAACTCACAGTCTGGGGCATC
>GU562080
TTCTACTGTAATACAACACAACTATTTAATTCAACTTGGAATAATAACTCAACTTGGAGTAGTAATTCAACGG
GAGAT--

AATGAAAATATCACTATCACCCTCCCATGCAGAATAAAACAAATTATAAACATGTGGCAGGAAGTAGGAAAAG
CAATGTATGCCCCTCCCATCGGAGGGCAAATTAGATGCTCATCAAAT---
ATTACAGGGATGATTTTAACAAGAGATGGTGGTGAGAACAACACC----------------------------
----

ACTGAGATCTTCAGACCTGGAGGAGGAGATATGAGGGACAATTGGAGAAGTGAATTATATAAATATAAAGTAG
TAAAAATTGAACCAATA---GGAGTAGCACCCACCAAGGCAAAGAGAAGAGTGGTG-----------------
----------------------CAGAGAGAAAAAAGAGCA---GTGGGA---ATGGGAGCT---------
TTGTTCCTTGGGTTCTTGGGAGCA---GCAGGAAGCACTATG---GGCGCAGCATCA---
ATAACGCTGACGGTACAG---GCCAGGCAA---TTATTG---TCTGGTATAGTGCAACAGCAGAAT---
AATTTG---CTGAGA---GCTATTGAGGCGCAACAGCATCTGTTGCAACTCACAGTCTGGGGCATC
>DQ322223
TTCTACTGTAATGTAACAGAACTGTTTAATAGTACTTGGAATGGAACTGAACAGAATAGTACCCAG-------
----

AATGAGGATACTATCACACTCCCATGCAGAATAAAACAAATTATAAACATGTGGCAGGAAGTAGGAAAAGCAA
TGTATGCCCCTCCCATCAGAGGAAATATTACTTGTTCATCAAAT---
ATTACAGGGCTGCTATTGACAAGAGATGGTGGAAACAATGAGAGC----------------------------
----

```
ACGAATGAAACCTTCAGACCTGGAGGAGGAGATATGAGGGACAATTGGAGAAGTGAACTATATAAATATAAAG
TAGTAAAACTTGAACCATTA---GGAGTAGCACCCACCAAGGCAAAGAGAAGAGTGGTG--------------
-------------------------CAAAGAGAAAAAAGAGCA---GTGGGA---ATAGGAGCT---------
ATGTTCCTTGGGTTCTTGGGAGCA---GCAGGAAGCACTATG---GGCGCAGCGTCA---
ATAACGCTGACGGTACAG---GCCAGACAA---TTACTG---TCTGGTATAGTGCAACAGCAGAGC---
AATTTG---CTGAGA---GCTATTGAGGCACAACAGCATATGTTGCAACTCACAGTCTGGGGCATC
>DQ322227
TTCTATTGTAATACAACAAAACTGTTTAATAGTACTTGGCCTAGAAACAACACTGAAGGGTCTGAA-------
-------------------------------------------------------------------------
----------------------------
GGAAATGACACAATCACACTCCCATGCAGAATAAAACAAATTATAAACATGTGGCAGGAAGTAGGAAAAGCAA
TGTATGCCCCTCCCATCAGAGGACAAATTAAATGTTCATCAAAT---
ATTACAGGGCTGTTATTAACAAGAGATGGGGGTAATAATGAGACC----------------------------
-------------------------------------------------------------------------
----------
AATGGGATTGAGACCTTCAGACCTGGAGGAGGAAATATGAAGGACAATTGGAGAAGTGAATTATATAAATATA
AAGTAGTACAAATTGAACCATTA---GGAATAGCACCCACCAAGGCAAAGAGAAGAGTGGTG-----------
----------------------------CAGAGAGAAAAAAGAGAC---CTAGGA---CTAGGAGCT------
---TTGTTAATCGGGTTCTTGGGAGCA---GCAGGAAGCACTATG---GGCGCAGCGTCA---
GTGACGCTGACGGTACAG---GCCAGGCAA---TTAATG---TCTGGTATAGTGCAACAGCAGAAC---
AATCTG---CTGAGA---GCTATTGAGGCGCAACAGCACATGTTGCAACTCACAGTCTGGGGCATT
>DQ322225
TTCTACTGTAATACAACAAAACTGTTCAATAGTACTTGGAATAGTACTGAGAATGGGAATACTTGGAATAAAA
CTACATGGTTA--------------------------------------------------------------
-------------GATAAC------
CCAGAAAATGACACAATCACACTCCCATGCAGAATAAGACAAATTATAAACATGTGGCAGGAAGTAGGAAAAG
CAATGTATGCCCCTCCCATCCAAGGACAGATTAACTGTTCATCAAAT---
ATTACAGGGCTGCTATTAACAAGAGATGGTGGTGAGGATCGTAACAATAGC----------------------
-------------------------------------------------------------------------
----
GAGAATGAGGAGAATGAGATCTTCAGACCTGGAGGAGGAGATATGAGGGATAATTGGAGAAGTGAATTATATA
AATATAAAGTAGTAAAAATTGAACCATTA---GGAGTAGCACCCACCAAGGCAAAGAGAAGAGTGGTG-----
----------------------------------CAAAGAGAAAGAAGAGCA---GTGGGA---
CTAGGAGCT---------ATGTTCCTTGGGTTCTTGGGAGCA---GCAGGAAGCACTATG---
GGCGCAGCGTCA---ATAACGCTGACGGTACAG---GCCAGACAA---TTATTG---
TCTGGTATAGTGCAGCAGCAAAAC---AATTTG---CTGAGA---
GCTATTGAGGCGCAACAGCATTTGTTGCAACTCACAGTCTGGGGCATC
>DQ322239
TTTTACTGTGATACAAAACCGCTGTTTAATAGTACTTGGAATCCAAAACAACCAGTTAATAGTACTGGGAATG
GT-----------------------------------------------------------------------
----------------------------
ACTGACGGAGATATCATACTCCAATGTAGAATAAAACAAATTATAAACATGTGGCAGAAAGTAGGAAAAGCAA
TGTATGCCCCTCCCATCAGAGGACCAATTAGTTGTTTATCAAAT---
ATTACAGGGCTACTATTAACAAGAGATGGTGGTAACGTGAGTGATAGT-------------------------
-------------------------------------------------------------------------
----------
AATATCACCGAGATCTTCAGACCTGTAGGAGGAGATATGAAGGACAATTGGAGAAGTGAATTATATAAATATA
AAGTAGTAAAAATTGAACCATTA---GGAATAGCACCCACCAAGGCAAAAAGAAGAGTGGTG-----------
----------------------------CAAAGAGAAAAAAGAGCA---GTGGGAGTGATAGGAGCT------
---ATGTTCCTTGGGTTCTTGGGAGCA---GCAGGAAGCACTATG---GGCGCAGCGTCA---
TTGACGCTGACGGTACAA---GCCAGACTA---TTATTG---TCTGGTATAGTGCAACAGCAGAAC---
AATTTG---CTGAGA---GCTATTGAGGCGCAACAGCATCTGTTGCAACTCACAGTCTGGGGCATC
>JQ403022
TTCTACTGCAATACATCACAACTATTTAATAGTACCTGGAATAGTACTTGGTCTGATACTTGGAATAGTACTG
GAGGA--------------------------------------------------------------------
----------------------------
AAGAACGGCACAATCACACTCCCATGTAGAATAAAACAAATTATAAACAGGTGGCAGGAAGTAGGAACAGCAA
TGTATGCCCCTCCCATCAGAGGACTAATTAAGTGTACATCAAAT---
ATTACAGGGCTGTTATTAACAAGAGATGGTGGTAATACCACCAAC----------------------------
-------------------------------------------------------------------------
```

AACAACAGCGAGACCTTCAGACCTGGAGGAGGAGATATGAGAGACAATTGGAGAAGTGAATTATATAAATATA
AAGTAGTAAGAATTGAACCATTA---GGAGTAGCACCCACCAAGGCAAAGAGAAGAGTGGTG-----------
----------------------------CAGAGAGAAAAAAGGGCA---GCA------CTAGGAGCT------
---ATGTTCCTTGGGTTCTTGGGAGCA---GCAGGAAGCACTATG---GGCGCAGCGTCA---
GTAGCGCTGACGGTACAA---GCCAGACAA---TTATTG---TCTGGTATAGTGCAACAGCAGAAC---
AATTTG---CTGAGA---GCTATTGAGGCGCAAACAAATATGTTGCGACTCACGGTCTGGGGCATT
>JQ403023
TTCTATTGTGATTCAACAAAATTGTTTAATAGTAATTGGACTATAGGTGAGGATACTGATTGGTTCAATAATA
CTGATTGG---

TTAAATGGCACAATCACACTCCCATGCAGAATAAAACAAATTGTAAACATGTGGCAGGAAGTAGGAAAAGCAA
TGTATGCCCCTCCCATCAGCGGACAAATTAGCTGTAATTCAACT---
ATTACAGGGCTGTTATTAACAAGAGATGGTGGTGTGGATAATAACACC-------------------------
----

GATAATAACACTGAGATCTTCAGGCCAGGAGGAGGAGATATGAGGGACAATTGGAGAAGTGAATTGTATAAAT
ATAAAGTAGTACAAATTGAACCATTA---GGAATAGCACCCACTAAGGCAAAGAGAAGAGTGGTG--------
-------------------------------CAGAGAGAAAAAAGGGGC------------ATAGGAGCT---
------GTGTTCCTTGGGTTCTTGGGAGCA---GCAGGAAGCACTATG---GGCGCAGCGTCA---
GTGACGCTGACGGTACAG---GCCAGACAA---TTATTG---TCTGGTATAGTGCAACAGCAAAAC---
AATTTG---CTGAAG---GCTATTGATGCGCAACAGCATCTGTTGCAACTCACAGTCTGGGGCATC
>KC797171
TTCTACTGTAATACAACACAACTGTTTAATAGTACCTGGTCCAATAGTAGTGGTACTTGGAATTCAACACATA
ATAGTACT---
-------------GAGGAG------
TTAGATAACGACACAATCACACTCCCATGCAGAATAAAACAAATTATAAACATGTGGCAGGAAGTAGGAAAAG
CAATGTATGCCCCTCCCATCAAGGGAAAAATTAGATGTTCATCAAAC---
ATTACAGGTCTGCTATTAACAAGAGATGGTGGCAATACTAATAATACTAAT----------------------
----

AATACCATTGAAACCTTCAGACCTGGAGGAGGGGATATGAGGGACAATTGGAGAAGTGAATTATTTAAATATA
AAGTAGTACAAATTGAACCATTA---GGAGTAGCACCTTCCAGGGCAAAGAGAAGAGCGGTG-----------
----------------------------CAGAGAGAAAAAAGAGCA---GTAGGAACACTAGGAGCT------
---ATGTTCCTTGGGTTCTTGGGAGCA---GCCGGAAGCACTATG---GGCGCAGTGTCA---
ATGACGCTGACGGTACAG---GCCAGACAA---TTATTG---TCTGGTATAGTGCAACAGCAGAGC---
AATCTG---CTGAGG---GCTATTGAGGCGCAACAGCATATGTTGCAACTCACAGTCTGGGGCATC
>JQ403025
TTCTACTGTAATTCAACAAAACTGTTTAATAGTACTTGGGATACTAATACTACAGGGTTAGATAATGACACTA
AAGGA--

GATAATGACACATTCATACTCCCATGTAGAATAAAACAGATTATAAACCTGTGGCAGGAAGTAGGAAAAGCAA
TGTATGCCCCTCCCATCAAAGGACAAATTAGATGTTCCTCAAAT---
ATTACAGGACTGCTAATAACAAGAGATGGTGGTAGTAACGGGACT----------------------------
----

GATGAGATCTTCAGACCTGGAGGAGGAGATATGAGGGACAATTGGAGAAGTGAATTATATAAATATAAAGTAG
TAAAAATTGAACCATTA---GGATTAGCACCCACCAAGGCAAAGAGAAGAGTGGTG-----------------
----------------------CAAAGAGAAAAAAGAGCA---GTGGGA---TTCGGAGCT---------
CTGTTCCTTGGGTTCTTGGGAGCA---GCAGGAAGCACTATG---GGCGCAGCGTCA---
GTGACGCTGACGGTACAG---GCCAGACAA---TTATTG---TCTGGTATAGTGCAACAGCAAAAC---
AATTTG---CTGAAG---GCTATTGAGGCGCAACAGCATCTGTTGCAACTCACAGTCTGGGGCATC
>JQ403026
TTCTACTGTAATACAACACATTTGTTTAAGGGTACTTGGAATGGTACTGAATGGTCAAATAACACTGAAGGGT
CAAT---
-------------GACACT------
GCAAGAAATGACACAATCATACTCCCATGCAGAATAAGACAATTTATAAACATGTGGCAGGAAGTAGGAAAAG
CAATGTATGCCCCTCCCATCAAAGGACAAATTAGTTGTTCATCAAAT---
ATTACAGGGCTGATATTAACAAGAGATGGTGGTAATGAGACCGAAAAG-------------------------
----

AATGAGACTGAGACCTTCAGACCTGGAGGGGGAGATATGAGGGACAATTGGAGAAGTGAATTATATAAATATA
AAGTAGTAAAAATTGAACCATTA---GGAGTAGCACCCACCAAGGCAAAGAGAAGAGTGGTG----------
----------------------------CAGAGAGAAAAAAGAGCA---GTGGGA---ATAGGAGCT------
---CTGTTCCTTGGGTTCTTGGGAGCA---GCAGGAAGCACTATG---GGCGCAGCGTCA---
ATGACGCTGACGGTACAG---GCCAGACTA---TTATTG---TCTGGTATAGTGCAGCAGCAGAAC---
AATCTG---CTGAGG---GCTATTGAGGCGCAACAACATCTGTTGCAACTCACAGTCTGGGGCATC
>EU023916
TTCTACTGTAATACAACACAGCTATTTAATAGTACCTGGGTAAATGGTACTAATACTGGGAATAGTACT----
----

AATAGCACCGATATCACACTCCCGTGCAGAATAAAACAAATTGTAAACAGGTGGCAGGAAGTAGGAAAAGCAA
TGTATGCCCCTCCCATCAGAGGACAGATTAGATGTAACTCAAGT---
ATTACAGGGCTGCTATTAACAAGAGATGGTGGAGGTAACAGGACCGAG------------------------
----

AACAGGACCGAAACCTTCAGACCTGGAGGAGGAGATATGAGGGACAATTGGAGAAGTGAATTATACAAATATA
AAGTAGTAAAAATTGAGCCATTA---GGAGTAGCACCCACCAAGGCAAAGAGAAGAGTGGTG----------
----------------------------CAGAGAGAAAAAAGAGCA---GCACTA------GGAGCC------
---ATGTTCCTTGGGTTCTTGGGAGCA---GCAGGAAGCACTATG---GGCGCAGCGTCA---
GTGACGCTGACGGTACAG---GCCAGACTA---TTATTG---TCTGGTATAGTGCAACAGCAGAAC---
AATCTG---CTGAGA---GCTATTGAGGCGCAACAGCATATGTTGCAACTCACAGTCTGGGGCATC
>JQ403019
TTCTACTGTAATACAACACAACTGTTTAATAGTACCTGGTCTGATAATAGTACCGGGAATGGTACTGAA----
----

GGAGGAGAGGTCATCACAATCCCATGCAGAATAAAACAAATTGTAAACATGTGGCAGAGAGTAGGAAAAGCAA
TATATGCCCCTCCTATTAAGGGACAAATTAACTGTACTTCAAAT---
ATTACAGGGTTGCTCTTAACAAGAGATGGTGGTAATACTAGTAGG---------------------------
----

GACAACGAGACCTTCAGACCTGGAGGAGGAAATATGAGAGACAATTGGAGAAGTGAATTATATAAATACAAAG
TAGTAAGAATTGAGCCATTA---GGAGTAGCACCCACCAAGGCAAAGAGAAGAGTGGTG-------------
-------------------------CAAAGAGAAAAAAGAGCA---GTGGGAACAATAGGAGCT---------
ATGTTCCTTGGGTTCTTGGGAGCA---GCAGGAAGCACTATG---GGCGCAGCGTCG---
ATGGCGCTGACGGTACAG---GCCAGACAA---TTATTG---TCTGGTATAGTGCAACAGCAAAAC---
AATTTG---CTGAGA---GCTATTGAGGCGCAACAACATATGTTGCAACTCACAGTCTGGGGCATT
>JQ403029
TTCTACTGTAATACAGCACAACTGTTTAATAGTACTTGGGGGGTTGCTAATAGTACTTGGAAT----------
----

GTTAGTGACAATATCACACTCCCATGCAGAATAAAACAAATTATAAACATGTGGCAAGAAGTAGGAAAAGCAA
TGTATGCCCCTCCCATCCAAGGACAAATTAGATGTTCATCAAAT---
ATTACAGGGATAATATTAACAAGAGATGGTGGTAACGGAAATGGGACC------------------------
----

GACAACACCACCGAGATCTTCAGACCTGGAGGGGGAGATATGAGGGACAATTGGAGAAGTGAATTATATAAAT
ATAAAGTAGTAAAAATTAAACCATTA---GGAGTAGCACCCACCAAGGCAAAGAGAAGAGTGGTG--------
-------------------------------CAGAGAGAAAAAAGAGCA---GTAACG---TTAGGAGCT---
------ATGTTCCTTGGGTTCTTGGGAGCA---GCAGGAAGCACTATG---GGCGCAGCGTCA---
ATAACGCTGACGGTACAG---GCCAGACAA---TTGTTG---TCTGGTATAGTGCAACAGCAGAGC---
AATTTG---CTGAGA---GCTATTGAGGCGCAACAACATCTGTTGCAACTCACAGTCTGGGGCATC
>JQ403044
TTCTACTGTGATACAGCAAAACTGTTTGATAGTACTTGGCATAATAGTACTTGGAATGATACTACAGGAGTCA
ATAACACT--
-------------GAGGGA------
AATAGCACAGACCCAATCGAACTCCCATGCAGAATAAAACAAATTATAAACATGTGGCAGGGAGTAGGAAGAG
CAATGTATGCCCCTCCCATCAGTGGACGAATTAGCTGTTCATCAAAT---
ATTACAGGACTGCTATTAACAAGAGATGGTGGCACAAATGATACA---------------------------
----

AAAGATAATGAGACCTTCAGACCTGGAGGAGGAGATATGAGAGACAATTGGAGAAGTGAATTATGGAAATATA
AAGTAGTAAAAGTTGAACCATTA---GGACTAGCACCTACCAAGGCAAAGAGAAGAGTGGTG-----------
----------------------------CAGAGGGAAAAAAGAGCA---ATAACA---CTAGGAGCT------
---ATGTTCCTTGGGTTCTTGGGAGCA---GCAGGAAGCACTATG---GGCGCAGCGTCA---
ATGACGCTGACGGTACAG---GCCAGACAA---TTATTG---TCTGGTATAGTGCAACAGCAAAAT---
AATTTG---CTGAGA---GCTATTGAGGCGCAGCAGCATATGTTGCAACTCACAGTCTGGGGCATC
>KX792621
TTCTATTGTAATACAACAGGACTGTTTAACAGCACTTGGAATTCAACACAACTGTCTAATGATACTGGGAATG
AAACTGAGGGG---TCA------GGT---

AACACTACTACAGAGATCATACTCCAATGCAGAATAAAACAAATTATAAACATGTGGCTGGAAGTAGGAAAAG
CAATGTATGCCCCTCCCATCAGAGGACAAATCAGATGTTCATCTAAT---
ATTACAGGGCTATTATTAACACGGGATGGTGGTAATACTAATAATACA-------------------------
----
GAAGGGAATGGCACCGAGACT---------
TTCACTGAGATCTTCAGACCTGAGGGAGGAGACATGAGGGACAATTGGAGAAGTGAATTATATAAATATAAAG
TAGTAAAAATTGAACCATTA---GGAGTAGCACCCACCAAGGCAAAGAGAAGAGTGGTG--------------
-------------------------CAGAGAGAAAAAAGAGCG---GCA------ATAGGAGCT---------
TTGTTCCTTGGGTTCTTGGGAGCA---GCAGGAAGCACTATG---GGCGCAGCGTCA---
ATGACGCTGACGGTACAG---GCCAGACAA---TTGTTG---TCTGGTATAGTGCAACAGCAGAGC---
AACTTG---CTGAGA---GCTATTGAGGCGCAACAGCACCTGTTGCAACTCACAGTCTGGGGCATC
>JQ403043
TTCTACTGTAATTCAACAAAACTGTTCAATAGTACTTGGTATAGGAATGATACAGGAACTTGGAATATTACAG
GAACTGGA---

GAGAATGACACACTCACACTCCCATGCAGAATAAAACAAATTATAAACAGGTGGCAGGAAGTAGGAAAAGCAA
TGTATGCCCCTCCCATCAGAGGACAAATTAGATGTACATCAAAT---
ATTACTGGGATACTATTAACAAGAGATGGTGGTAATACAAATAACAATAATACC------------ATG----
----

AACGAGACTGAGATCTTCAGACCTGGAGGAGGAGATATGAGGGACAATTGGAGAAGTGAATTATATAAATATA
AAGTAGTAAAAATTGAACCATTA---GGACTAGCACCCACCAGGGCAAAGAGAAGAGTGGTG-----------
----------------------------CGGATAGAAAAAAGAGCA---GTGACA---CTAGGAGCT------
---ATGTTCCTTGGGTTCTTGGGAGCA---GCAGGAAGCACTATG---GGCGCAGCGTCA---
GTAACGCTGACGGTACAG---GCCAGACAA---TTATTG---ACTGGTATAGTGCAACAGCAGAAT---
AATTTG---CTGAAG---GCTATTGAGGCGCAACAACATCTGTTGCAACTCACAGTCTGGGGCATC
>JQ403045
TTCTACTGTAATACAACACAACTGTTTAATAGTAGCTGGGATAATAGTACTGAGAATGGAAATGATACTGAAG
GG---

TCAAATAACCTTATCATACTCCCATGCAGAATAAAACAAATTATAAACATGTGGCAGAAAGTAGGAAAAGCAA
TGTATAACCCTCCCATCCAGGGACAAATTAGCTGTTCATCAAAT---
ATTACAGGGCTGCTATTAACACGAGATGGTGGTAAGAGTGAGAACAAGACT----------------------
----

GAGAACAAGACCACTGAGATCTTCAGACCGGGAGGAGGAGATATGAGGGACAATTGGAGAAGTGAATTATATA
AATATAAAGTAGTAAAAATTGAACCATTA---GGAGTAGCACCCACCAAGGCAAAGAGAAGAGTGGTG-----
----------------------------------CAGAGAGAAAAAAGAGCA---GTGGGA---
TTAGGAGCT---------CTGTTCCTTGGGTTCTTGGGAGCA---GCAGGAAGCACTATG---
GGCGCAGCGTCA---GTGACGCTGACGGTACAG---GCCAGACAA---TTATTG---
TCTGGTATAGTGCAGCAGCAAAAC---AATTTG---CTGAGG---
GCTATTGAGGCGCAGCAGCATCTGTTGCAACTCACAGTCTGGGGCATC
>JQ403046
TTCTACTGTGATACAACACAGCTGTTTAATAGTACTTGGAACGGTACTGAAGGAAATTTC-------------
----

ACTGAAACTAACATCACACTCCCATGCAGAATAAAACAAATTGTAAACAGGTGGCAGGAAGTAGGGAGAGCGA
TGTATGCCCCTCCCATCAGCGGAATCATTAGATGTTCATCAAAT---
ATTACAGGGCTGCTACTAACAAGAGATGGTGGTATCAGCAACAATGGG-------------------------

----

TCCGAAAATGGGTCCGAAACCTTCAGACCTGGAGGAGGAGATATGAGGGACAATTGGAGAAGTGAATTATATA
AATATAAAGTAGTAAAAATTGAGCCATTA---GGATTAGCACCCACCAAGGCAAAGAGAAGAGTGGTG-----
----------------------------------CAAAGAGAAAAAAGAGCA---GTAACG---
CTGGGAGCT---------GTGTTCCTTGGGTTCTTGGGAGCA---GCAGGAAGCACTATG---
GGCGCAGCGTCA---ATAACGCTGACGGTACAA---GCCAGACTC---TTATTG---
TCTGGTATAGTGCAGCAGCAGAAC---AATCTG---CTGAAA---
GCTATTGAGGCGCAACAGCATATGTTGCAACTCACAGTCTGGGGCATC
>EU023919
TTCTACTGTAATACAACACAACTGTTTAATAGTACTTGGGAGGTTAATAATACTGAGAGTAATACTACCATA-
----

TCAAATGACACTATTACTCTCCCATGCAGAATAAAACAAATTATAAACATGTGGCAGAAGGTAGGAAAAGCAA
TGTATGCCCCTCCCATCAAAGGACTAATTAGATGTTCATCAAAT---
ATTACAGGGCTGCTATTAACAAGAGATGGTGGTAATGGCCCAAAC----------------------------
----

GGGACAAATGAGGCCTTCAGACCTGCAGGAGGAGATATGAGGGACAATTGGAGAAGTGAACTATATAAATATA
AAGTAGTACAACTTGAACCATTA---GGAATAGCACCCACCAAGGCAAAGAGGAGAGTGGTG-----------
----------------------------CAGAGAGAAAAAAGAGCA---GTGGGAATGATAGGAGCT------
---ATGTTCCTTGGGTTCTTGGGAGCA---GCAGGAAGCACTATG---GGCGCAGCGTCA---
TTGACGCTGACGGTACAG---GCCAGACAG---TTACTG---TCTGGTATAGTGCAACAGCAGAAT---
AATCTG---CTGAGG---GCTATTGAGGCGCAACAGCATCTGTTGCAACTCACAGTCTGGGGCATC
>EU023922
TTCTACTGTAATACAACACAACTGTTTAATAGTACTTGGAATGGGACTAATACTGAAGGCACTGAA-------
----

GGAAATGACACAATCACACTCCCATGCAGAATAAGACAGATTATAAACATGTGGCAGGAAGTAGGAAAAGCAA
TGTATGCCCCTCCCATCAGAGGACAAATTAGATGTTCATCAAAT---
ATTACAGGGCTGCTATTAACAAGGGATGGTGGTACGAACGAGACC----------------------------
----

AACGAGACCGAGATCTTCAGACCAGGAGGAGGAGATATGAGGGACAATTGGAGAAGTGAATTATATAAATATA
AAGTAGTAAAAATTGAACCATTA---GGAGTAGCACCCACCAAGGCAAAGAGAAGGGTGGTG-----------
----------------------------CAGAGAGAAAAAAGAGCA---GTGGGA---CTAGGAGCT------
---ATGTTCCTTGGGTTCTTGGGAGCA---GCAGGAAGCACTATG---GGCGCAGCGTCA---
GTGACGCTGACGGTACAG---GCCAGACAA---TTATTG---TCTGGTATAGTGCAACAGCAGAAT---
AATCTG---CTGAAG---GCTATTAAGGCGCAACAACATCTGTTGCAACTCACAGTCTGGGGCATC
>KX792557
TTCTACTGTAATACAACACAGCTGTTTAACAGTACGTGGACTTGGAATAATACTGATAGTGCTGGGAACGAG-
----

ACTGAAAAAATTATCACACTCCCGTGCAGGATAAGACAAATTGTAAATAGATGGCAAGAAGTAGGAAAAGCAA
TGTATGCCAATCCCATTAGTGGACCAATTAGCTGTTCATCAAAT---
ATTACAGGGCTGCTATTAACAAGAGATGGTGGTATTAACAACGAGACC-------------------------
----

AATACCACCGAGACCTTCAGACCTTGGGGGGGAGATATGAGGGACAATTGGAGAAGTAAATTATATAAATATA
AAGTAGTAAAAATTAAACCATTA---GGACTAGCACCCACCAAGGCAAAGAGAAGAGTGGTG-----------
----------------------------CAGAGAGAAAAAAGAGCA---GTAACA---TTGGGAGCT------
---ATGTTTCTTGGGTTCTTGGGAACA---GCAGGAAGCACTATG---GGCGCAGCGTCA---
CTGACGCTGACGGTACAG---GCCAGACTA---TTATTG---TCTGGTATAGTGCAACAGCAGAAC---
AACCTG---CTGAGG---GCTATTGAGGCGCAACAGCATCTGTTGCAACTCACAGTCTGGGGCATC
>KX792831
TTCTACTGTAATACAACAAAACTGTTTGATAGTACCTGGTATCCGAATGGTACTTGGTATTCGAATGGTATTA
CTTGGTATCCG---AATGGTACTACAAGGTATCCGAATGGTACTACAAAGGCAAATAGC---
ACAGGGTCAAAT------------------------
AGCACTGAGGATACGATCACACTCCCATGCAGAATAAAACAAATTATAAACAGGTGGCAAGAAGTAGGAAAAG
CAATGTATGCCCCTCCCGCCCGAGGACAAATTAAATGCTCATCAAAT---

ATTACAGGGCTGCTATTGACAAGAGATGGTGGTAAGAGCAATACCAGTGAG----------------------
----

AACGAGGAGACCTTCAGACCGGGAGGAGGAAATATGAGGGACAATTGGAGAAGTGAATTATATAAATATAAAG
TAGTAAAAATTGAACCATTA---GGAATAGCACCCACCAAGGCAAGGAGAAGAGTGGTG--------------
-------------------------CAGAAAGAAAAAAGAGCA---GTAACA---CTAGGAGCT---------
GTGTTCCTTGGGTTCTTGGGAGCA---GCAGGAAGCACTATG---GGCGCAGCGTCA---
ATGACGCTGACGGTACAA---GCCAGACAA---TTATTG---TCTGGTATAGTGCAACAGCAGAGC---
AATCTG---CTGAGG---GCTATTGAGGCGCAACAGCACCTGTTGCAACTCACAGTCTGGGGCATC
>JQ403042
TTCTACTGTAATACAGCACAACTGTTTAATAGTACTTGGAATTGGAATGATACTACCAAATCAAATAATACA-
----

GGGCCAGATAACATCACACTCCCATGCAGAATTAAACAAATTATAAACAGGTGGCAGGAAGTAGGAAAAGCAA
TGTATGCCCCTCCCATCAGCGGACTAGTTAGATGTGTATCAAAT---
ATTACAGGGCTACTATTAACAAGGGATGGTGGTACGGACAACACC----------------------------
----

ACCGAGATCTTCAGACCTGGAGGAGGAAATATGAAGGACAATTGGAGAAGTGAATTATATAAATATAAAGTAG
TAAAAATTGAGCCATTA---GGAATAGCACCCACCAAGGCAAAAAGAAGAGTGGTG-----------------
----------------------CAGAGAGAAAAAAGAGCA---ATAACG---ATAGGAGCT---------
ATGTTCCTTGGGTTCTTGGGAGCA---GCAGGAAGCACTATG---GGCGCAGCGTCA---
ATGACGCTGACGGTACAG---GCCAGACTA---TTATTG---TCTGGTATAGTGCAACAGCAGAAC---
AATCTG---CTGAGG---GCTATTGAGGCGCAACAGCATCTGTTGCAACTCACAGTCTGGGGCATC
>KX792664
TTCTACTGTAATTCATCACGACTGTTTAATTGGACAGGGAATGGGACTAATATAACATCAAATAGCAATATCA
CA---

TCAAATATCAATATCACAATCCCATGCAGGATAAAACAAATTATAAACATGTGGCAGGAAGTAGGAAAAGCAA
TGTATGCCCCTCCTATCATGGGACAAATCAAGTGTTCAACAAAT---
ATTACAGGGATAATATTAACAAGAGATGGTGGGAACAACGAAACT----------------------------
----

AACGCCACCGAGATCTTCAGACCTGGAGGAGGAAATATGAAGGACAATTGGAGAAGTGAATTATATAGATATA
AAGTAGTAAAAATTGAACCATTA---GGAGTAGCACCCACCAAGGCAAAGAGAAGAGTGGTG-----------
----------------------------CAGAGAGAAAAAAGAGCA---ATACCA---TTAGGAGCT------
---ATGTTCCTTGGGTTCTTGGGAGCA---GCAGGAAGCACTATG---GGCGCAGCGTCA---
ATGGCGCTGACGGTACAA---GCCAGACAA---TTATTG---TCTGGTATAGTGCAGCAGCAGAAC---
AATTTG---CTGAGG---GCTATTGAGGCGCAACAGCATCTGTTGCAACTCACAGTCTGGGGCATC
>KX792688
TTCTACTGTAATACATCACAACTGTTTAATAGCACTTGGTATTTTAATAGCACTGGGGATAACAATAGTACTG
GGAATACT---

ACTAACAAAAATATCACACTCCCATGTAGAATAAAACAAATTATAAACATGTGGCAGGCAGTAGGAAAAGCAA
TATATGCCCCTCCCATCAGTGGACAAATTAGTTGTTCATCAAAC---
ATTACAGGGCTAATATTAACAAGAGATGGTGGTACAAACACCACA----------------------------
----

AATGATACCTTCAGACCTGCAGGGGGGAATATGAAAGACAATTGGAGAAGTGAATTATATAAATATAAAGTAG
TAAAAATTGAACCATTA---GGAGTGGCACCCACCAAGGCAAAGAGAAGAGTGGTG-----------------
----------------------CAGAGAGAAAAAAGAGCC---GTGGGA---CTAGGAGCT---------
TTGTTCCTTGGGTTCTTGGGAGCA---GCAGGAAGCACTATG---GGCGCAGCGTCA---
ATGACGCTGACGGTACAG---GCCAGACAA---TTATTG---TCTGGTATAGTGCAGCAGCAGAAC---
AATATG---CTGAGG---GCTATTGAGGCGCAACAGCATATGTTGCAACTCACAGTCTGGGGCATC
>KX792771
TTCTACTGTGATTCAACACCACTGTTTAACAGTACTTGGAACAGTACTTGGAGTGGTAATGATACGGGAGTAA
ACAAC--

ACTGAAAAGACTATCACACTCCAATGTAGAATAAAACAAATTATAAACATGTGGCAGAGAGTAGGAAAAGCAA
TGTATAACCCTCCCATCAGTGGACAAATTAATTGCTCATCAAAT---

ATTACAGGGATGATATTATCAAGAGATGGTGGTAGTATGAACAGG----------------------------
----

AGTAACGAGACCTTCAGACCTGGAGGAGGAGATATGAGGGACAATTGGAGAAGTGAATTATATAAATATAAAG
TAGTAAAAGTTGAACCATTA---GGAGTAGCACCCACCAAGGCAAAGAGAAGAGTGGTG--------------
-------------------------CAGAGAGAAAAAAGAGCA---ATGGGA---ATAGGAGCT---------
TTGTTCCTTGGGTTCTTGGGAGCA---GCAGGAAGCACTATG---GGCGCAGCGTCA---
ATAACGCTGACGGTACAG---GCCAGACAA---TTATTG---TCTGGTATAGTGCAACAGCAGAAC---
AATTTG---CTGAAA---GCTATTGAGGCGCAACAGCATCTGTTGCAACTCACAGTCTGGGGCATC
>KX792725
TTCTACTGTAATACAACACAACTATTTAATAGTACTTGGCTGTTTAATAGTACATGGAATAGTACT-------
--GAA---
AGATTAGGGAATGATACTAAA---------------
AGGACAAATGACACTATCACACTCCCATGCAAAATAAAACAAGTTATAAACATGTGGCAGACAGTAGGAAAAG
CAATGTATGCTCCTCCCATCAGAGGACTAATTAGATGTTCATCAAAT---
ATTACAGGGCTGATATTGACAAGAGATGGTAGTGGTAACACCACC----------------------------
----

GGTAACGAGACCTTTAGGCCTGGAGGAGGAAATATGAAGGACAATTGGAGAAGTGAATTATATAAATATAAAG
TGGTAAAAATTGAACCCTTA---GGAGTAGCACCCACTAGGGCAAAGAGAAGAGTGGTG--------------
-------------------------CAGAGAGAAAAAAGAGCA---GCGGGA---CTAGGAGCT---------
TTGTTCCTTGGGTTCTTGGGAATG---GCAGGAAGCACTATG---GGCGCAGCGTCA---
CTAACGCTGACGGTACAG---GCCAGACAA---TTATTG---TCTGGTATAGTGCAACAGCAGAAC---
AATCTG---CTGAGG---GCTATTGAGGCGCAACAGCATCTGTTGCAACTCACAGTCTGGGGCATC
>KX792808
TTCTACTGTAATTCAACACAACTGTTTAATAGTAGTCGGGATAATAATAGAAGGTCAGATAACTCTGAA----
----

GGAAATAAAACAATCACACTCCCATGCAGAATAAAACAAATTATAAACATGTGGCAGAAAGTAGGAAAAGCAA
TGTATGCCCCTCCCATCAGAGGACAAATTAGATGTTCATCAAAT---
ATTACAGGGATAATATTAACAAGAGATGGTGGTAATGGTAATGAGTCCAAT----------------------
----

AACGAGACCGAGACCTTCAGACCTGGAGGAGGGGATATGAGGGACAATTGGAGAAGTGAATTATATAAATATA
AAGTAGTAAGAATTGAACCATTA---GGATTGGCACCCACCAAGGCAAAGAGAAGAGTGGTA-----------
----------------------------CAGAGAGAAAAAAGAGCA---GTGGGA---ATAGGAGCT------
---GTGTTCCTTGGGTTCTTGGGAGCA---GCAGGAAGCACTATG---GGCGCAGCCTCG---
ATGACGCTGACGGTACAA---GCCAGACTA---TTATTG---TCTGGTATAGTGCAACAGCAGAAC---
AATCTG---CTGAGA---GCTATTGAGGCGCAACAACATCTGTTGCAACTCACAGTCTGGGGCATC
>EU023929
TTCTACTGCAATACAACACAACTGTTTAATAGTACTTGGTATAGTAATAGTACTTGGAATAGTACTGAAGGGG
CAAAT--

AACACTGCAGGAAATATCACACTCCCATGCAGAATAAAACAAATTGTAAACAGGTGGCAGGGAGTAGGAAAAG
CAATGTATGCCCCTCCCATCAGAGGACAAATTAGGTGTTCATCAAAT---
ATTACAGGGCTAATATTAACAAGAGATGGGGGGCCTAACAACACG----------------------------
----

ACAGAGACCTTCAGACCTGGAGGAGGAGATATGAGGGACAATTGGAGAAGTGAATTATATAAATATAAAGTAG
TACAAATTGAACCATTA---GGAGTAGCACCCACCAAGGCAAAAAGAAGAGTGGTG-----------------
----------------------CAGAGAGAAAAAAGAGCA---GTGGGA---ATAGGAGCT---------
GTGTTCCTCGGGTTCTTGGGAGCA---GCAGGAAGCACTATG---GGCGCAGCGTCA---
CTGACGCTGACGGTACAG---GCCAGACAA---TTATTG---TCTGGTATAGTGCAACAGCAGAAC---
AATCTG---CTGAGG---GCTATTGAGGCGCAACAGCATCTGTTGCAACTCACAGTCTGGGGCATC
>EU023933
TTCTACTGTAACACATCACAGCTGTTTAATAGTACTTGGAACGGTACTGAGAGTGGTATTACAAACCTTACAA
AC---

CCAGATGAGACTATCTCACTCCCATGCAGAATAAGACAAATTGTAAACAGGTGGCAGGAAGTAGGAAAAGCAA
TGTATGCCCCTCCAATCAGTGGACAAATTAATTGTTCATCAAAT---

ATTACAGGGCTGCTATTAGTAAGAGACGGTGGTGTGTATAATAGTACA-------------------------
----

AGTGATAACGAGACTTTCAGACCTGAAGGAGGAAATATGAAAGACAATTGGAGAAGTGAATTATATAAATATA
AAGTAGTAAAAATTGAGCCATTA---GGAATAGCACCCACCAAGGCAAGGAGAAGAGTGGTG-----------
----------------------------CAAAGAGAAAAAAGAGCA---GTGGGAACGTTAGGAGCT------
---ATGTTCCTTGGTTTCTTGGGAGCA---GCAGGAAGCACTATG---GGCGCAGCGTCA---
CTGACGCTGACGGTACAA---GCCAGATTA---TTATTA---TCTGGTATAGTGCAACAGCAAAAC---
AATTTG---CTGAGA---GCTATTGAGGCGCAACAGCATCTGTTGCAGCTCACAGTCTGGGGCATT
>EU023927
TTCTACTGTAATGTAACACCACTATTTAATAGTACTTGGAAGCCTGGTAATGAGACTGTGAATGATACT----
----

AAAGAAGAAGTCATCACACTCCCATGCAGGATAAAACAAATTATAAACCTGTGGCAGGAAGTAGGAAAAGCAA
TGTATGCCCCTCCCATCAGCGGAAAAATTAGATGTTCATCAAAT---
ATTACAGGGCTATTATTAACAAGAGATGGTGGTGTAAACGTGAGC----------------------------
----

GAAAATACTGAGATCTTCAGACCTGGAGGAGGAGATATGAGGGATAATTGGAGAAGTGAATTATATAAATATA
AAGTAGTAAAAATTGAACCATTA---GGAGTAGCACCCACCAGGGCAAAGAGAAGAGTGGTG-----------
----------------------------CAGAGAGAAAAAAGAGCA---ATGGGA---ATAGGAGCT------
---CTGTTCCTTGGGTTCTTGGGAGCA---GCAGGAAGCACTATG---GGCGCAGCGTCA---
GTAACGCTGACGGTACAG---GCCAGACTA---TTATTG---TCTGGTATAGTGCAACAGCAGAAC---
AACCTG---CTGAGG---GCTATTGAGGCGCAACAGCATGTGTTGCAGCTCACAGTCTGGGGCATC
>MH714351
TTCTACTGTAATACAACACAACTGTTTAATAGTACTTGGGATAGTACTGAAGGGTTGAATAAAACTGAAAGAA
ATGAA--

AATGACACAAAGATCATACTCCCATGCAGAATAAAACAATTTATAAACATGTGGCAGGAAGTAGGAAAAGCAA
TGTATGCCCCTCCCATCAGAGGACAAATTAAATGTATATCAAAT---
ATTACAGGGCTGATATTAACAAGAGATGGTGGTAATAACAATAATGGG-------------------------
----

AATAATGAGACCTTCAGACCTGTAGGAGGAGATATGAGGGACAATTGGAGAAGTGAATTATATAAATATAAAG
TAGTAAAAATTGAACCATTA---GGATTAGCACCCACCAGGGCAAAGAGAAGAGTGGTG--------------
-------------------------CAGAGAGAAAAAAGAGCA---GTGGGA---TTAGGAGCT---------
ATGTTCCTTGGGTTCTTGGGAGCA---GCAGGAAGCACTATG---GGCGCAGCGTCA---
GTAACGCTGACGGTACAG---GCCAGACTA---TTATTG---TCTGGTATAGTGCAACAGCAAAAC---
AATTTG---CTGAGG---GCTATTGAGGCGCAACAGCATCTGTTGCAACTCACAGTCTGGGGCATC
>MH714324
TTCTACTGTAATACATCACAACTGTTTAATAGTACTTGGAATACCTGGAATAGTACTGACTGGTCAAAAAAC-
----

ACTACAGAAAATATCACACTCCCATGCAGAATAAAACAAATTATAAACATGTGGCAGGAAGTAGGAAAAGCAA
TGTATGCCCCTCCCATCAGGGGACGAATTAAATGTTCATCAAAT---
ATTACAGGGTTGTTATTAACAAGAGATGGTGGTAACGGTACCAATACT-------------------------
----

ACCACCGAGGTCTTCAGACCTATAGGAGGAAATATGAAGGACAATTGGAGAAGTGAATTGTATAAATATAAAG
TGGTACAAATTGAACCATTA---GGAATAGCACCCACCAAGGCAAAGAGAAGAGTGGTG--------------
-------------------------CAGAGAGAAAAGCGAGCA---GTGGGA---ATAGGAGCT---------
ATGTTCCTTGGGTTCTTGGGAGCA---GCAGGAAGCACTATG---GGCGCAGCATCA---
ATGATGCTGACGGTACAG---GCCAGACAA---TTATTG---TCTGGTATAGTGCAACAGCAAAAC---
AATTTG---CTGAGA---GCCATTGAGGCGCAACAACATCTGTTGCAACTCACAGTCTGGGGCATC
>MH714343
TTCTACTGTAATACATCAAAACTGTTTGATAGTATTTGGAATGGTACTGAAAGGATAAATAACACTGAAGAA-
----

AATAGCACAATAATCACACTCCCATGCAGAATAAAACAAATTATAAACATGTGGCAGGAAGTAGGAAAAGCAA
TGTATGCCCCTCCCATCAGAGGACAAATTAGCTGTTCATCAAAC---

ATTACAGGGCTGATATTAACAAGAGATGGTGGTAATAGAACGGAGAATGGGACT-------------------
----

GAGAATGATAAAGAGATCTTCAGACCTGTAGGAGGAGATATGAGGGACAATTGGAGAAGTGAATTATATAAAT
ATAAAGTAGTAAAAATTGAACCATTA---GGAGTAGCACCCACCGAGGCAAAGAGAAGAGTGGTG--------
-------------------------------CAAAGAGAAAAAAGAGCA---GTGGGA---TTAGGAGCT---
------ATGTTCCTTGGGTTCTTGGGAGCA---GCAGGAAGCACTATG---GGCGCAGCGTCA---
ATAACGCTGACGGTACAG---GCCAGACTA---TTATTG---ACTGGTATAGTGCAACAGCAGAAC---
AATCTG---CTGAGG---GCTATTGAGGCGCAACAGCATCTGTTGCAACTCACAGTCTGGGGCATC
>MH714329
TTCTACTGTAACACAACAAAACTGTTTAACAGTACTTGGGATGCTAACAGTACTTGGGATGAAAATAATACTA
CAAATGAC---

AATGATGAAAATATCACACTCCCATGTAGAATAAAACAAATTATAAACCTGTGGCAGGAAGTAGGAAAAGCAA
TGTATGCCCCTCCCATCAGTGGACCAATTATCTGTAACTCAAGT---
ATTACAGGGCTCCTAATAACAAGGGATGGTGGTAATGACACAAAC----------------------------
----

CAGAACGAGACCTTCAGACCTGGAGGAGGAGATATGAGGGATAATTGGAGAAGTGAATTATATAAATATAAAG
TAGTAAAAATTGAACCATTA---GGAATAGCACCCACCAAGGCAAAAAGAAGAGTGGTG--------------
-------------------------CAGAGAGAAAAAAGAGCA---GTGGGA---ATAGGAGCT---------
GTGTTCCTTGGGTTCTTGTCAGCA---GCAGGAAGCACTATG---GGCGCAGCGTCA---
GTGACGCTGACGGTACAG---GCCAGACTA---TTATTG---TCTGGTATAGTGCAACAGCAGAAT---
AATTTG---CTGAGG---GCTATTGAGGCGCAGCAGCGTATGTTGCAACTCACAGTCTGGGGCATC
>KU600814
TTCTACTGTAATTCAACACCACTGTTTAATAGTACTTGGTATGGTAATAATACTGGGAATGGTACTGACGAAT
TAGGGAAC---

AGTGGAAATAACACAATTACACTACAATGCAGAATAAAACAAATTATAAACATGTGGCAGGAAGTAGGAAAAG
CAATGTATGCCCCTCCCATCAGAGGACGTATTAACTGTACATCAAAT---
ATTACAGGGTTGCTATTAACAAGAGATGGGGGAAATACTAATAATGATAGT----------------------
----

AGCGAGCCCGAGATCTTCAGACCTGGAGGAGGAAATATGAAGGACAATTGGAGAAGTGAATTATATAAATATA
AAGTAGTAAAAATTGAACCATTA---GGAGTAGCACCCACCAAGGCAAAGAGAAGAGTGGTG-----------
----------------------------CAGAGAGAAAAAAGAGCA---GTGGGA---ATAGGAGCT------
---CTGTTCCTTGGGTTCTTGGGAGCA---GCAGGAAGCACTATG---GGCGCAGCGTCA---
CTGACGCTGACGGTACAG---GCCAGACAA---TTATTG---TCTGGTATAGTGCAACAGCAGAAC---
AATTTG---CTGAGG---GCTATTGAGGCGCAACAGCATCTGTTGCAACTCACAGTCTGGGGCATC
>KU600818
TTCTACTGTAATACAACACAACTGTTTAATAGTACTTGGGAAAATCCTGAAAGGTCAAATAGCACTGAA----
----

GGAAATAGCACAATCACACTCCCATGCAGAATAAAACAAATTATAAACATGTGGCAGGAAGTAGGAAAAGCAA
TGTATGCCCCTCCCATCAGAGGACAAATTAAGTGTATATCAAAT---
ATTACAGGGTTGCTACTAACAAGAGATGGTGGTAATAATGGTACC----------------------------
----

ACGAACGAGACCTTCAGACCTGGAGGAGGAGATATGAGGAACAATTGGAGAAGTGAATTATATAAATATAAAG
TAGTAAAAATTGAACCATTA---GGAGTAGCACCCACCAAGGCAAAGAGAAGAGTGGTG--------------
-------------------------CAAAGAGAAAAAAGAGCA---GTGGGA---ATAGGAGCT---------
TTGTTCCTTGGGTTCTTGGGAGCA---GCAGGAAGCACTATG---GGCGCAGCGTCA---
ATGACGCTGACGGTACAG---GCCAGACAA---TTATTG---TCTGGTATAGTGCAACAGCAGAAT---
AATCTG---CTGAGG---GCTATTGAGGCGCAACAGCATATGTTGCAACTCACAGTCTGGGGCATC
>KU600815
TTCTACTGTAATACAACACAACTGTTTAATAGTACTTGGGATAAGGAGTCAAATAATACTTCGAAT-------
----

GAGACTGAAATAATCACACTCCCATGCAGAATAAAACAAATTATAAACATGTGGCAGGAAGTAGGAAAAGCAA
TGTATGCACCTCCCATCAGAGGAAACATTAGCTGTTCATCAAAT---

ATTACAGGGCTGCTATTAACAAGAGATGGTGGGGTGGTGAATGGTAGT-------------------------
----

AGCAATGAGACAGAGACCTTCAGACCTGGAGGAGGAAATATGAAGGACAATTGGAGAAGTGAATTATATAAAT
ATAAGGTAGTACAAATTGAACCATTA---GGACTAGCACCCACCACGGCAAAAAGAAGAGTGGTG--------
-------------------------------CAAAGAGAAAAAAGAGCA---GTGGGA---TTAGGAGCT---
------ATGTTCCTTGGGTTCTTGGGAGCA---GCAGGAAGCACTATG---GGCGCAGCGTCA---
ATAACGCTGACGGTACAG---GCCAGACAA---TTATTG---TCTGGTATAGTGCAACAGCAGAGC---
AATTTG---CTGAGG---GCTATTGAGGCGCAACAGCATCTGTTGCAACTCACAGTCTGGGGCATC
>AY180905
TTCTACTGTAATACATCACGACTGTTTAATAGTACTTGGAATAGTACTAGGAATAATATA-------------
----

GGAAATGGCACTATCACGCTCCCTTGCAGAATAAAACAAATTATAAACATGTGGCAGGAAGTAGGGAAAGCAA
TGTATGCCCCTCCCATTGCAGGAAATATTAGCTGTTCATCAAAT---
ATTACAGGGTTGTTATTAACAAGAGATGGTGGTAAGAATGAGAGC----------------------------
----

AACACCACCGAGACCTTCAGACCTGCAGGAGGAGATATGAGGGACAATTGGAGAAGTGAATTATATAAATATA
AAGTAGTAAGAATTGAACCATTA---GGAGTAGCACCCACCAAGGCAAAGAGAAGAGTGGTG-----------
----------------------------CAGAGAGAAAGAAGAGCA---GTGGGAGCGATAGGAGCT------
---ATGTTCCTTGGGTTCTTGGGAGCA---GCAGGAAGCACTATG---GGCGCAGCGTCA---
ATAACGCTGACGGTACAG---ACCAGACAA---TTGTTG---TCTGGTATAGTGCAACAGCAGAGA---
AATCTG---CTGAGG---GCTATTGAGGCGCAACAGCATCTGTTGCAACTCACAGTCTGGGGCATC
>EU363827
TTCTACTGTAATACATCACGACTGTTTAATAGTACTTGGAATGAGACTAGTGCTTGGAATGATACTACA----
----

GGAAATGACACTATCACGCTCCCTTGCAGAATAAAACAAATTGTAAACATGTGGCAGGAGGTAGGGAAAGCAA
TGTATGCCCCTCCCATTAGTGGACGCATTAAATGCTCATCAAAT---
ATTACAGGGTTACTATTGACAAGAGATGGTGGTAGGAATAACGAGAGC-------------------------
----

AACACCACCGAGACCTTCAGACCTGTAGGAGGAAATATGAGAGACAATTGGAGAAGTGAATTATATAAATATA
AAGTAGTAAAAATTGAACCATTA---GGATTAGCACCCACCAGGGCAAAGAGAAGAGTGGTG-----------
----------------------------CAGAGAGAAAAAAGAGCA---GTGGGA---ATAGGAGCT------
---ATGTTCCTTGGGTTCTTGGGAGCA---GCAGGAAGCACTATG---GGCGCAGCGTCA---
ATAACGCTGACGGTACAG---GCCAGACAA---TTGCTG---TCTGGTATAGTGCAACAGCAGAGA---
AATCTG---CTGAGT---GCTATTGAGGCGCAACAACATTTGTTGCAACTCACAGTCTGGGGCATC
>AY905493
TTCTATTGTAATACATCACAACTGTTCAATAGTACTTGGAATGGTAATACTACTTGGAATAATACTGCA----
----

GGAAATAGCAATATCACGCTCCCTTGCAGAATAAAACAAATTGTAAACATGTGGCAGGAAGTAGGGAAAGCAA
TGTATGCCCCTCCCATTAGAGGACAAATTAGATGTTCATCAAAT---
ATTACAGGGTTGCTATTAACAAGAGATGGTGGTACTAATAAGAGC----------------------------
----

GAGAACACCGAGACATTCAGACCTGGAGGAGGAGATATGAAAGACAATTGGAGAAGTGAATTATATAAATATA
AAGTAGTAAAAATTGAACCATTA---GGAATAGCACCCACCAAGGCAAAGAGAAGAGTGGTG-----------
----------------------------CAGAGAGAAAAAAGAGCA---GTGGGAACAATAGGAGCT------
---ATGTTCCTTGGGTTCTTGGGAGCA---GCAGGAAGCACTATG---GGCGCAGCGTCA---
ATAACGCTGACGGTACAG---GCCAGACAA---TTGTTG---TCTGGTATAGTGCAACAGCAGAGA---
AATTTG---CTGAGG---GCTATTGAGGCGCAACAGCATATGTTGCAACTCACAGTCTGGGGCATC
>JF932492
TTCTACTGTAATACATCACAACTGTTTAATAGTATTTGGTATAATAATGATACTTGGAATGGTACTACA----
----

GGAGATGACAATATCACACTCCCTTGCAGAATAAGACAAATTGTAAACCTATGGCAGGAAGTAGGGAAAGCAA
TGTATGCCCCTCCCATTAAAGGACAAATTAGATGTTCATCAAAT---

ATTACAGGGTTGCTATTAACAAGAGATGGTGGGACGAATGAGGGC---------------------------
----

AATACCAATGAGACCTTCAGACCTGGAGGAGGAGATATGAGAGACAATTGGAGAAGTGAATTATATAAATATA
AAGTAGTAAAAATTGAACCATTA---GGAGTAGCACCCACCAAGGCAAAGAGAAGAGTGGTG----------
----------------------------CAGAGAGAAAAAAGAGCA---GTGGGAATGATGGGAGCT------
---ATGTTCCTTGGGTTCTTGGGAGCA---GCAGGAAGCACTATG---GGCGCAGCGTCA---
ATGACGCTGACGGTACAG---GCCAGACAA---TTATTG---TCTGGTATAGTGCAACAGCAAAGA---
AATTTG---CTGAGG---GCTATTGAGGCGCAACAGCATCTGTTGCAACTCACAGTCTGGGGCATT
>AY905497
TTCTACTGTAATACATCACAACTGTTTAATAGTATTAATGGTACCTGGAATGGTACTACA------------
----

AAAAATGGCAATATCACGCTCCCTTGCAGAATAAGACAAATTGTAAACAGGTGGCAGGAAGTAGGGAAAGCAA
TGTATGCTCCTCCCATTAGCGGGCAAATTAGATGTTCATCAAAT---
ATTACAGGGTTACTATTAACAAGAGATGGTGGTACTAATGAGAGC---------------------------
----

AGCAACAACGAGACCTTCAGACCTGGAGGAGGAGATATGAGGGACAATTGGAGAAGTGAATTATATAAATATA
AAGTAGTAAAAATTGAACCATTA---GGAGTAGCACCCACCAAGGCAAGGAGAAGAGTGGTG----------
----------------------------CAGAGAGAAAAAAGAGCA---GTGGGAACGATAGGAGCT------
---ATGTTCCTTGGGTTCTTGGGAGCA---GCAGGAAGCACTATG---GGCGCAGCGTCA---
ATAACGCTGACGGTACAG---GCCAGACAA---TTGTTG---TCTGGTATAGTGCAACAGCAGAGA---
AATTTG---CTGAGG---GCTATTGAGGCGCAACAGCATATGTTGCAACTCACAGTCTGGGGCATC
>AY905495
TTCTACTGTAATACAACAAAACTGTTTAATAGTATTTGGAATACTAATGGTACTTGGAATGATACTAGTACTT
GGAATAAT--
-------------------AGT---
ACAGGGAATGATACTATCACGCTCCCTTGCAGAATAAAACAAATTGTAAACATGTGGCAGGAAGTAGGGAAAG
CAATGTATGCCCCTCCCATTAGAGGACAAATTAGATGTTCATCAAAT---
ATTACAGGGCTGCTATTAACAAGAGATGGTGGTAATAATGGGACCAAC------------------------
----

ACCACCGACACAGAGATCTTCAGACCTGCAGGAGGAGATATGAGGGACAATTGGAGAAGTGAATTATATAAAT
ATAAAGTAGTAAAAATTGAACCATTA---GGAGTAGCACCCACCAGGGCAAAGAGAAGAGTGGTG--------
-------------------------------CAAAGAGAAAAAAGAGCA---GTGGGAACAATAGGAGCT---
------ATGTTCCTTGGGTTCTTGGGAGCA---GCAGGAAGCACTATG---GGCGCAGCGTCA---
GTAACGCTGACGGTACAG---GCCAGACAA---TTGTTG---TCTGGTATAGTGCAACAGCAGAGA---
AATTTG---CTGAGG---GCTATTGAGGCGCAACAGCATATGTTGCAACTCACAGTCTGGGGCATT
>AY905494
TTCTACTGTAATACGACACAACTGTTTAATAGTACTTGGAAGTTTAATAGTACTTGGAATGATACTAGTACA-
----

GAGAATGGTACTATCATACTCCCTTGCAGAATAAAACAAATTGTAAACATGTGGCAGGAAGTAGGGAAAGCAA
TGTATGCCCCTCCCATTAGTGGGCAAATTAGATGTTCATCAAAT---
ATTACAGGGTTGCTATTAACAAGAGATGGTGGTAAGGAGGAGAAC---------------------------
----

AACACCACCGAGACCTTCAGACCTGGAGGAGGAGATATGAGAGATAATTGGAGAAGTGAATTATATAAATATA
AAGTAGTAAAAATTGAACCATTA---GGAGTAGCACCCACCAAGGCAAAGAGAAGAGTGGTG----------
----------------------------CAGAGAGAAAAAAGAGCA---GTGGGAACGATAGGAGCT------
---ATGTTCCTTGGGTTTTTGGGAGCA---GCAGGAAGCACTATG---GGCGCAGCGTCA---
ATAACGCTGACGGTACAG---GCCAGACAA---TTGTTG---TCTGGTATAGTGCAACAGCAGAGA---
AATTTG---CTGAGG---GCTATTGAGGCGCAACAGCATATGTTGCAACTCACAGTCTGGGGCATC
>HM215397
TTCTACTGTAATACATCACAACTGTTTAATAGTACTTGGAATGTTACTGGTACTGTGAATGGTACTACA----
----

GGAAATGGCACTATCACGCTCCCTTGCAGCATAAAACAAATTGTAAACAGGTGGCAGGAAGTAGGGAGAGCAA
TATATGCCCCTCCCATTAGAGGACAAATTAGATGTTCATCAAAT---

ATTACAGGGTTGCTATTAACAAGAGATGGTGGTAATGAGAGCACGACC-------------------------
----

ACCGAGACCGAGACCTTCAGACCTGCAGGAGGAGATATGAGAGACAATTGGAGAAGTGAATTATATAAATATA
AAGTAGTAAAAATTGAACCATTA---GGAGTAGCACCCACCAAGGCAAAGAGAAGAGTGGTG-----------
----------------------------CAGAGAGAAAAAAGAGCA---GTGGGAACGATAGGAGCT------
---ATGTTCCTTGGGTTCTTGGGAGCA---GCAGGAAGCACTATG---GGCGCAGCGTCA---
ATCACGCTGACGGTACAG---GCCAGACAA---TTGTTG---TCTGGTATAGTGCAACAGCAGAGA---
AATTTG---CTGAGG---GCTATTGAGGCGCAACAGCATCTGTTGCAACTCACAGTCTGGGGCATC
>HQ699990
TTCTATTGTGATACATCACAACTGTTTAATAATATTTGGTTTAATAGTACTTGGAATGATAGTACTTGGAATA
AT---
-------------------ACT---
ACAGGAGATAGCAACATCACGCTCCCCTGCAGAATAAAACAAATTGTAAACATGTGGCAGGGAGTAGGGAAAG
CAATGTATGCCCCTCCCATTAAAGGACAAATTAAATGTTCATCAAAT---
ATTACAGGGTTGCTATTAACAAGAGATGGTGGTAAAAATGTGAGC----------------------------
----

AATACCAGCGAGACCTTCAGACCTGGAGGAGGAGATATGAAAGACAATTGGAGAAGTGAATTATATAAATATA
AAGTAGTAAAAATTGAACCATTA---GGAGTAGCACCCACCAAGGCAAAGAGAAGAGTGGTG-----------
----------------------------CAGAGAGAAAAAAGAGCA---GTGGGA---ATGGGAGCT------
---ATGTTCCTTGGGTTCTTGGGAGCA---GCAGGAAGCACTATG---GGCGCAGCGTCA---
ATAACGCTGACGGTACAG---GCCAGACAA---TTGTTG---TCTGGTACAGTGCAGCAGCAGAGA---
AATTTG---CTGAGG---GCTACTGAGGCGCGACAGCATCTGTTGCAACTCACGGTCTGGGGCATC
>HQ699996
TTCTACTGTAATACATCACAACTGTTTAATAGTACTTGGAGTTGGAATAATACTAATACTGAGAATAATACTA
CAGAA--

AACAGCAATATCACTATCACGCTCCCTTGCAGAATAAAACAAATTGTAAACATGTGGCAGGAAGTAGGGAAAG
CAATGTATGCCCCTCCCATTGCAGGACAAATTAGATGTTCATCAAAT---
ATTACAGGGTTGCTATTAACAAGAGATGGTGGCAACAGTACCAAC----------------------------
----

GAGACCTTCAGGCCTGAAGGAGGAAATATGAGAGACAATTGGAGAAGTGAATTATATAAATATAAAGTAGTAA
GAATTGAACCATTA---GGAGTAGCACCCACCAAGGCAAAGAGAAGAGTGGTG--------------------
-------------------CAGAGAGAAAAAAGAGCA---GTGGGAACGATAGGAGCT---------
ATGTTCCTTGGGTTCTTGGGAGCA---GCAGGAAGCACTATG---GGCGCAGCGTCA---
ATGACGCTGACGGTACAG---GCCAGACAA---TTGTTG---TCTGGTATAGTGCAACAGCAAAGA---
AATTTG---CTGAGG---GCTATTGAGGCGCAACAGCATCTGTTGCAACCCACAGTCTGGGGCATC
>EU131797
TTCTACTGTAATACATCACAACTGTTTAATAGTATTTGGTATAATAATGGTACTTGGAATGGTACTACA----
----

GGAAATGACACTATCACGCTCCCTTGCAGAATAAAACAAATTGTAAACATGTGGCAGGAAGTAGGGAAAGCAA
TGTATGCCCCTCCCATTGAAGGACAAATTAGTTGTAAATCAAAT---
ATTACAGGGTCGCTATTAACAAGAGATGGTGGTAATAATGGGAGC----------------------------
----

AGGACCAACGAGACCTTCAGACCTACAGGAGGAGATATGAGAGACAACTGGAGAAGTGAATTATATAAATATA
AAGTAGTAAAAGTTGAACCATTA---GGAGTAGCACCCACCAAGGCAAAGAGAAGAGTGGTG-----------
----------------------------CAGAGAGAAAAAAGAGCA---GTGGGAACGATAGGAGCT------
---ATGTTCCTTGGGTTCTTGGGAGCA---GCAGGAAGCACTATG---GGCGCAGCGTCA---
ATGACGCTGACGGTACAG---GCCAGACAA---TTGTTG---TCTGGTGTAGTGCAACAGCAGAGA---
AATTTG---CTGAGG---GCTATTGAGGCGCAACAGCATATGTTGCAACTCACAGTCTGGGGCATC
>EU131791
TTCTACTGTAATACATCACAACTGTTTAAGAGTACTTGGAATGTTACTACTGGTACTCCGAATGGTACTACA-
----

GGAAATGACAATATCACGCTCCCTTGCAGAATAAGACAAATTGTAAACATGTGGCAGGAAGTAGGGAAAGCAA
TGTATGCCCCTCCCATTACAGGAGTAATTAGCTGTTCATCAAAT---

ATTACAGGGTTACTATTAACAAGAGATGGTGGTAAAAATGTGACCAAT------------------------
----

AATGACACCGAGACCTTCAGACCTGGAGGAGGAGATATGAGAGGCAATTGGAGAAGTGAATTATATAAATATA
AAGTAGTAAAAATTGAACCATTA---GGAATAGCACCCACCAAGGCAAAGAGAAGAGTGGTG----------
----------------------------CAGAGAGAAAAAAGAGCA---GTGGGAGTAATAGGAGCT------
---ATGTTCCTTGGGTTCTTGGGAGCA---GCAGGAAGCACTATG---GGCGCGGCGTCA---
ATAACGCTGACGGTACAG---GCCAGACAA---ATGTTG---TCTGGTATAGTGCAACAGCAGAGA---
AATTTG---CTGAGG---GCTATTGAGGCGCAACAGCACATGTTGCAACTCACAGTCTGGGGCATC
>EU131790
TTCTACTGTAATACATCACAACTGTTTAATAGTACTTGGCATAATAATGGTACTTGGGGTGATACTACA----
----

GGAAATAGCTCTATCACGCTCCCTTGCAGAATAAAACAAATTGTAAACATGTGGCAGGAAGTAGGGAAAGCAA
TGTATGCTCCTCCCATTGAAGGGCAAATTAGATGTTCATCAAAT---
ATTACAGGGTTGATATTAACAAGAGATGGTGGTAAAAATGAGAGC---------------------------
----

GCCACCGAGACCTTCAGACCTGAGGGAGGAGATATGAGAGACAATTGGAGAAGTGAATTATATAAATATAAAG
TAGTAAAAATTGAACCATTA---GGACTAGCACCCACCAAGGCAAAGAGAAGAGTGGTG-------------
------------------------CAAAGAGAAAAAAGAGCA---GTGGGAACAATAGGAGCT---------
ATGTTCCTTGGGTTCTTGGGAGCA---GCAGGAAGCACTATG---GGCGCAGCGTCA---
ATGACGCTGACGGTACAG---GCCAGACAA---TTGTTG---TCTGGTATAGTGCAACAGCAGAGA---
AATCTG---CTGAGG---GCTATTGAGGCGCAACAGCATATGTTGCAACTCACAGTCTGGGGCATC
>EU131788
TTCTACTGTAATACATCACAACTGTTTAATAGTACTTGGAATAATAATGGTACTTGGAATGATACTACA----
----

GACAATAGCACTATCACACTCCCTTGCAGAATAAAACAAATTATAAATAGGTGGCAGGAAGTAGGGAAAGCAA
TGTATGCCCCTCCCATTAGTGGACTAATTAGATGCTCATCAAAT---
ATTACAGGGTTACTATTAACAAGAGATGGTGGTACTAATAAGAGC---------------------------
----

AGCACCACCGAGACCTTCAGACCTGCAGGAGGAAATATGAGAGACAATTGGAGAAGTGAATTATATAAATATA
AAGTAGTACAAATTGAACCATTA---GGAGTAGCACCCACCAAGGCTAAGAGAAGAGTGGTG----------
----------------------------CAAAGAGAAAAAAGAGCA---GTGGGAGCAATAGGAGCT------
---ATGTTCCTTGGGTTCTTGGGAGCA---GCAGGAAGCACAATG---GGCGCAGCGTCA---
ATAACGCTGACGGTACAG---GCCAGGCAA---TTGCTG---TCTGGTATAGTGCAACAGCAGAGA---
AATTTG---CTGAGG---GCTATTGAGGCGCAACAGCATATGTTGCAACTCACAGTCTGGGGCATA
>EU131808
TTCTACTGTAATACAACACAACTGTTTAGTAGTACTTGGTATGAGAATGGTACTTGGAATGGGACTAGTAATT
GGAATGGG---
-------------------ACT---
ACAGGAAATGACATTATCACACTCCCTTGCAGAATAAAACAAATTGTAAACATGTGGCAGGAAGTAGGGAAAG
CAATGTATGCCCCTCCTATTGAAGGACAAATTAGATGTTCATCAAAT---
ATTACAGGGCTGCTATTAACAAGAGATGGTGGCAATAATGAGAGC---------------------------
----

AGTGCCAATGAGACCTTCAGACCTGCAGGAGGAAATATGAAAGACAATTGGAGAAGTGAATTATATAAATATA
AAGTAGTAAAAATTGAACCATTA---GGAGTAGCACCCACCAAGGCAAAAAGAAGAGTGGTG----------
----------------------------CAGAGAGGAAAAAGAGCA---GTGGGAACGATAGGAGCT------
---ATGTTCCTTGGGTTCTTGGGAGCA---GCAGGAAGCACTATG---GGCGCAGCGTCA---
ATAACGCTGACGGTACAG---GCCAGACAA---ATGTTG---TCTGGTATAGTGCAACAGCAAAGA---
AATCTG---CTGAGG---GCTATTGAGGCGCAACAGCATCTGTTGCAACTCACAGTCTGGGGCATC
>EU131789
TTCTACTGTAATACATCACAACTGTTTAATAGTACTTGGGATAGAAATAGTAGTACTTGGAGTAATACTACAG
GA---

CATAACAATAGCACTATCACACTCCCTTGCAGAATAAAACAAATTGTAAACAGGTGGCAGGAAGTAGGGAAAG
CAATGTATGCCCCTCCCATTAGCGGACTAATTAGATGCTCATCAAAG---

ATTACAGGGTTATTATTAACAAGAGATGGTGGTACTAATGAGAACAACACC----------------------
----

ATCGAGAACACCACCGAGATCTTCAGACCTGGAGGAGGAGATATGAGAGACAATTGGAGAAGTGAATTATATA
AATATAAAGTAGTACAAATTGAACCATTA---GGAGTAGCACCCACCAAGGCAAAGAGAAGGGTGGTG-----
----------------------------------CAGAGAGAAAAAAGAGCA---
GTGGGAACAATAGGAGCT---------ATGTTCCTTGGGTTCTTGGGAGCA---GCAGGAAGCACTATG---
GGCGCAGCGTCA---ATAACGCTGACGGTACAG---GCCAGACAA---TTATTG---
TCTGGTATAGTGCAACAGCAGAGA---AATTTG---CTGAGG---
GCTATTGAGGCGCAACAGCATATGTTGCAACTCACAGTCTGGGGCATA
>MK053935
TTCTACTGTAATACAACACAACTGTTTAATAGTACTTGGAATGAGAATAGTACTTGGAATGAGACTAGTACT-
----

GAGAATAATATTATCACACTCCCTTGCAGAATAAAACAAATTGTAAACATGTGGCAGGAAGTAGGGAAAGCAA
TGTATGCCCCTCCCATTAGCGGACAAATTAGATGTTCATCAAAT---
ATTACAGGATTACTATTAACAAGAGATGGTGGTAATGAGACCAAC----------------------------
----

ACCACCGAGATTTTCAGACCTGGAGGAGGAGATATGAGAGACAATTGGAGAAGTGAATTATATAAATATAAAG
TAGTAAAAATTGAACCATTA---GGAGTAGCACCCACCAAGGCAAAGAGAAGAGTGGTG--------------
-------------------------CAGAGAGAAAAAAGAGCA---GTAACA---CTTGGAGCT---------
ATGTTCCTTGGGTTCTTGGGAGCA---GCAGGAAGCACTATG---GGCGCAGCGTCA---
ATAACGCTGACGGTACAG---GCCAGACAA---TTGTTG---TCTGGTATAGTGCAACAGCAGAGA---
AATTTG---CTGAGG---GCTATTGAGGCGCAACAGCATCTGTTGCAACTCACAGTCTGGGGCATC
>DQ990880
TTCTACTGTAATACCTCACGACTGTTTAATAGTACTTGGTATAATAATGGTACTTGGAATAATGCTAGTACTG
GG---

AATGATAGCACTATTATACTCCCTTGCAGAATAAAACAAATTGTAAACAGGTGGCAGGAAGTAGGGAAAGCAA
TATATGCCCCTCCCATTGCAGGACCAATTAATTGTTCATCAAAA---
ATTACAGGGTTACTATTAACAAGAGATGGTGGGATTAATAAGACT----------------------------
----

GACACCGAGACCTTCAGACCTGGAGGAGGAGATATGAGAGACAATTGGAGAAGTGAATTATATAAATATAAAG
TAGTGAAAATTGAACCATTA---GGAGTAGCACCCACCAGGGCAAAGAGAAGAGTGGTG--------------
-------------------------CAAAGAGAAAAAAGAGCA---GTGGGAGCAATAGGAGCG---------
ATGTTCCTTGGGTTCTTGGGAGCA---GCAGGAAGCACTATG---GGCGCAGCGTCA---
ATAACGCTGACGGTACAG---GCCAGACAA---TTGTTG---ACTGGTATAGTGCAACAGCAAAGA---
AATTTG---CTGAGA---GCTATTGAGGCGCAACAGCATCTGTTGCAACTCACAGTCTGGGGCATC
>HM215420
TTTTATTGTAATACATCACAACTGTTTAATGAAACTTGGACTTGGAATAATAGTACTAGGAATGATAATACA-
----

GGAAATGGCACTATCACGCTCCCTTGCAGAATAAAACAAATTGTAAACATGTGGCAGGAAGTAGGGAAAGCAA
TGTATGCCCCTCCCATTAGAGGACAAATTAGATGTTCATCAAAT---
ATTACAGGGTTACTATTAACAAGAGATGGTGGTAATAATACCGAC----------------------------
----

AAAAACACCGAGATCTTCAGACCTGGAGGAGGAAACATGAGAGACAATTGGAGAAGTGAATTATATAAATATA
AAGTAGTAAAAATTGAACCATTA---GGAGTAGCACCCACCAAGGCAAAGAGAAGAGTGGTG-----------
----------------------------CAGAGAGAAAAAAGAGCA---GTGGGA---ATAGGAGCT------
---GTGTTCCTTGGGTTCTTGGGAGCA---GCAGGAAGCACTATG---GGCGCAGCGTCA---
ATAACGCTGACGGTACAG---GCCAGACAA---TTGTTG---TCTGGTATAGTGCAGCAGCAGAGA---
AATTTG---CTGATG---GCTATTGAGGCGCAACAGCATCTGTTGCAACTCACAGTCTGGGGCATC
>HQ699995
TTCTACTGTAATACATCACAACTGTTTAATAGTACTTGGTATAATAATGGCACTTGGGATGGTACTACA----
----

GAAAATGGCAATATCACGCTCCCTTGCAGAATAAAACAATTTGTAAACATGTGGCAGGGAGTAGGGAGAGCAA

TGTATGCCCCTCCCATTACAGGAAAGATTACATGTTTATCAAAT---
ATTACAGGGTTGATATTAACAAGAGATGGTGGGAATAATAAGACC----------------------------
----

AACACCACCGAGACCTTCAGACCTGAAGGAGGAAATATGAAAGACAATTGGAGAAGTGAATTATATAAATATA
AAGTAGTAAAAATTGAACCATTA---GGAGTAGCACCCACCAAGGCAAAGAGAAGAGTGGTG-----------
----------------------------CAGAGAGAAAAAAGAGCA---GTGGGAGCACTAGGAGCT------
---ATGTTCCTTGGGTTCTTGGGAGCA---GCAGGAAGCACTATG---GGCGCAGCGTCA---
ATAACGCTGACGGTACAG---GCCAGACAA---TTGTTG---TCTGGTATAGTGCAGCAGCAAAGA---
AATTTG---CTGAGA---GCTATTGAGGCGCAACAGCATCTGTTGCAACTCACAGTCTGGGGCATC
>HM215428
TTCTACTGTAATACATCACAACTGTTTAACAGTACTTGGTATGTGAATGGTACTTGGAGTGGTACTACA----
----

GAAAATGGCACTATCACACTCCCTTGCAGAATAAAACAAATTGTAAACATGTGGCAGGCAGTAGGGAAAGCAA
TGTATGCCCCTCCCATTGCAGGAAAAATTAGATGTTCATCAAAT---
ATTACAGGGCTGCTATTAACAAGAGATGGTGGTATTAATGTGAGC----------------------------
----

AATGACACAGAGACCTTCAGACCTGGAGGAGGAAATATGAAAGACAATTGGAGAAGTGAATTATATAAATATA
AAGTAGTAAGAATTGAACCATTA---GGAGTAGCACCCACCAAGGCAAAGAGAAGAGTGGTG-----------
----------------------------CAGAGAGAAAAAAGAGCA---GTGGGAACAATAGGAGCT------
---ATGTTCCTTGGGTTCTTGGGAGCA---GCAGGAAGCACTATG---GGCGCAGCGTCA---
ATAACGCTGACGGTACAG---GCCAGACAA---TTGTTG---TCTGGTATAGTGCAACAGCAGAGA---
AATTTG---CTGATG---GCTATTGAGGCGCAACAGCATATGTTGCAACTCACAGTCTGGGGCATC
>EU363828
TTCTACTGTAATACATCACAACTGTTTAATAGTACTTGGAATGTTACTACAGAAAATAGCAATATTACA----
----

GGAAATGACACTATCACGCTCCCTTGCAGAATAAAACAAATTATAAACATGTGGCAGGAAGTAGGGAGAGCAA
TGTATGCCCCTCCCATTAGTGGACAAATTAGATGCTCATCAAAT---
ATTACAGGGTTGCTATTAACAAGGGATGGTGGTAATAAGACCAACGAG-------------------------
----

ACCGAAGACACCGAGATCTTCAGACCTGGAGGAGGAGATATGAGAGACAATTGGAGAAGTGAATTATATAAAT
ATAAAGTAGTACAAATTGAACCATTA---GGCGTAGCACCCACCAAGGCAAAGAGAAGAGTGGTG--------
-------------------------------CAGAGAGAGAAAAGAGCA---GTGGGAACGATAGGAGCT---
------ATGTTCCTTGGGTTCTTGGGAGCA---GCAGGAAGCACTATG---GGCGCAGCGTCA---
ATGACGCTGACGGTACAG---GCCAGACAA---TTGTTG---TCTGGTATAGTGCAACAGCAGAGA---
AATTTG---CTGAGG---GCTATTGAGGCGCAACAACATCTGTTGCAACTCACAGTCTGGGGCATC
>MF591612
TTCTACTGTAATACATCAAAACTGTTTAATAGTACTTGGAATGGTAATAGTACTTGGAATGATACTACA----
----

GGAGATGATCCTATCACGCTCCCTTGCAGAATAAAACAAATTGTAAACATGTGGCAGGAAGTAGGGAAAGCAA
TGTATGCCCCTCCCATTAGAGGACAAATTAGATGCTCATCAAAT---
ATTACAGGGTTGCTATTAACAAGAGATGGTGGTAATAATACGAACGAGACCACC-------------------
----

GAGGAGACCGAGACCTTCAGACCTGGAGGAGGAGATATGAGAGACAATTGGAGAAGTGAACTATATAAATATA
AAGTAGTAAAAATTGAACCATTA---GGAGTAGCACCCACCAAGGCAAAGAGAAGAGTGGTG-----------
----------------------------CAGAGAGAAAAAAGAGCA---GTGGGAACGATAGGAGCT------
---ATGTTCCTTGGGTTCTTGGGAACA---GCAGGAAGCACTATG---GGCGCAGCGTCA---
ATAACGCTGACGGTACAG---GCCAGACAG---TTGTTA---TCTGGTATAGTGCAACAGCAGAGA---
AATTTG---CTGAGG---GCTATTGAGGCGCAACAGCATCTGTTGCAACTCACAGTCTGGGGCATC
>JF932482
TTCTACTGTAATACATCACAACTGTTTAATAGTACTTGGGATTCAAATGGTACTAATACTTGGACA-------
----

GAAAATGGCACTATCACGCTCCCTTGCAGAATAAAACAGATTGTAAACATGTGGCAGGAAGTAGGGAAAGCAA

TGTATGCCCCTCCCATTAGCGGACAAATTAACTGTTCATCAAAT---
ATTACAGGGTTGCTATTAACAAGAGATGGTGGTAAAAATGAGAGC----------------------------
----

AATACCACCGAGACCTTCAGACCTGGAGGAGGAGATATGAGAGACAATTGGAGAAGTGAATTATATAAATATA
AAGTAGTAAAAATTGAACCATTA---GGAGTAGCACCCACCAAGGCAAAGAGAAGAGTGGTG-----------
----------------------------CAGAGAGAAAAAAGAGCA---GTGGGAACGATAGGAGCT------
---ATGTTCCTTGGGTTCTTGGGAGCA---GCAGGAAGCACTATG---GGCGCAGCGTCA---
ATAACGCTGACGGTACAG---GCCAGACAA---TTGTTG---TCTGGTATAGTGCAACAGCAGAGA---
AATTTG---CTGATG---GCTATTGAGGCGCAACAGCATCTGTTGCAACTCACAGTCTGGGGCATC
>HQ699949
TTCTACTGTAATACATCACAACTGTTTGATAGTACTTGGTGGAATAATGATACTAGTACTTGGAATGGTACTG
CA---

GAAAATAATACTATCACGCTCCCTTGCAGAATAAAACAAATTGTAAACATGTGGCAGGAAGTAGGAAGAGCAA
TGTATGCCCCTCCCATTGAAGGACGAATTAGATGTTCATCAAAT---
ATTACAGGGTTGCTATTAACAAGAGATGGTGGTGATGATGAGAAC----------------------------
----

ACCACTGAGATCTTCAGACCTGGAGGAGGAAATATGAGGGACAATTGGAGAAGTGAATTATATAAATATAAAG
TAGTAAAAATTGAACCATTA---GGAGTAGCACCCACCAAGGCAAAGAGAAGAGTGGTG--------------
-------------------------CAGAGAGAAAAAAGAGCA---GTGGGAACGATAGGAGCT---------
ATGTTCCTTGGGTTCTTGGGAGCA---GCAGGAAGCACTATG---GGCGCAGCGTCA---
ATAACGCTGACGGTACAG---GCCAGACAA---TTGTTG---TCTGGTATAGTGCAACAGCAGAGA---
AATCTG---CTGAGA---GCTATTGAGGCGCAACAACATATGTTGCAACTCACAGTCTGGGGCATC
>HM215423
TTCTACTGTAATACATCACAACTATTTAATAGTACTTGGCATGTTAATGGTACTTGGAATGGTACTACA----
----

GAAAATGGCACTATCACGCTCCCTTGCAGAATAAAACAAATTGTAAACATGTGGCAGGAAGTAGGAAAAGCAA
TGTATGCCCCCCCCATTAGCGGGAAAATCAGATGTTCATCAAAA---
ATTACAGGGTTGCTATTAACAAGAGATGGTGGTGCTAATGAAAGC----------------------------
----

GACCTCATCTTCAGACCTACAGGAGGAGATATGAGGGACAATTGGAGAAGTGAATTATATAAATATAAAGTAG
TAAAAATTGAACCATTA---GGAATAGCACCCACCAAGGCAAAGAGAAGAGTGGTG-----------------
----------------------CAGAGAGAAAAAAGAGCA---GTGGGAACAATAGGAGCT---------
ATGTTCCTTGGGTTCTTGGGAGCA---GCAGGAAGCACTATG---GGCGCAGCGTCG---
GTAACGCTGACGGTACAG---GCCAGACAA---TTGTTG---TCTGGTATAGTGCAACAGCAGAGA---
AATCTG---CTGATG---GCTATTGAGGCGCAACAGCATTTGTTGCAACTCACCGTCTGGGGCATC
>EU131805
TTCTACTGTAATACATCAAAACTGTTTAGTAGTATTTGGCATGACAATGGTACTAGTATTTGGAATGATACTA
TA---

AAAGATGAAAATATCACACTCCCTTGCAGAATAAAACAAATTATAAACATGTGGCAGCAAGTAGGAAAAGCAA
TGTATGCCCCTCCCATTAGAGGACAAATTAGATGTTCATCAACT---
ATTACAGGGTTGCTATTAACAAGAGATGGTGGCAATGATACCAGT----------------------------
----

AACAACGAGACCTTCAGACCAACAGGAGGAAATATGAGAGACAATTGGAGAAGTGAATTATATAAATATAAAG
TAGTAAAAATTGAACCATTA---GGAATAGCACCCGCCAGGGCAAAGAGAATAGTGGTG--------------
-------------------------CAGAGAGAAAAAAGAGCA---GTGGGAACAATAGGAGCT---------
ATGTTCCTTGGGTTCTTGGGAGCA---GCCGGAAGCACTATG---GGCGCAGCGTCA---
ATAACGCTGACGGTACAG---GCCAGACAA---ATGTTG---TCTGGTATAGTGCAACAGCAGAGA---
AATTTG---CTGATG---GCTATTGAGGCGCAACAGCATATGTTGCAACTCACAGTCTGGGGCATC
>EU131800
TTCTACTGTAATACATCACAACTGTTTAATAGTACTTGGACTTGGAATGATACTAATAATTGGACTAATACTA
CA---

GAAAAGGGCAATATCACACTCCCATGCAAAATAAAACAAATTGTAAACATGTGGCAGGAAGTAGGGAAAGCAA

TGTATGCCCCTCCCATTGCAGGACAGATTAGATGTTCATCAAAT---
ATTACAGGGTTGCTATTAACAAGAGATGGTGGTAAGAATGAGAGC----------------------------
----

AACACCACCGAGACCTTCAGACCTGGAGGAGGAGAGATGAGAGATAATTGGAGAAGTGAATTATATAAATATA
AAGTAGTAAAAATTGAACCTTTA---GGAGTAGCACCCACCAAGGCAAAGAGAAGAGTGGTG-----------
----------------------------CAGAGAGAAAAAAGAGCA---GCGGGAACGTTAGGAGCT------
---ATGTTCCTTGGGTTCTTGGGAGCA---GCAGGAAGCACTATG---GGCGCAGCGTCA---
ATGACGCTGACGGTACCG---GCCAGACAA---TTGTTG---TCTGGTATAGCGCAACAGCACAGA---
AATTTG---CTGAGA---GCTATTGAGGCCCAACAGCATATGCTGCAACTCACAGCCTGGGGCATC
>EU131794
TTCTACTGTGATACATCACAATTGTTTATTAGTACTTGGAATAATACTAGTACTTGGGAGAATACTACA----
----

GGAAATAGCGATATCACGCTCCCTTGCAGAATAAAACAAATTATAAATATGTGGCGGGAAGTAGGGAAAGCAA
TGTATGCCCCTCCCATTGAAGGACAAATTAGATGTTCATCAAAT---
ATTACAGGGTTACTATTAACAAGAGATGGTGGTAATAAGAACGAGACC-------------------------
----

ACCAAGACGGAGACCTTCAGGCCTGGAGGAGGAGATATGAGAGATAATTGGAGAAGTGAATTATATAAGTATA
AAGTAGTAAAAATTGAACCACTA---GGAGTAGCACCCACCAAGGCAAAGAGAAGAGTGGTG-----------
----------------------------CAGAGAGAAAAAAGAGCA---GTGGAAACGATAGGAGCT------
---GTGTTCCTTGGGTTCTTGGGAGCA---GCAGGAAGCACTATG---GGCGCAGCGTCA---
ATAACGCTGACGGCACAG---GCCAGACAA---TTGTTG---TCTGGTATAGTGCAACAGCAGAGA---
AATTTG---CTGAGG---GCTATTGAGGCGCAACAGCATCTGTTGCAACTCACAGTCTGGGGCATC
>EU131807
TTCTACTGCAATACATCACAACTGTTTAATAGTACTTGGAATAATAATACTAGTACTTGGACCAATACTACA-
----

GACAATAGCACTATCACACTTCCTTGCAGAATAAAACAAATTGTAAACATGTGGCAGGAAGTAGGGAAAGCAA
TGTATGCCCCTCCCATTGAAGGACAAATCAGATGTTCATCAAAT---
ATTACAGGGTTACTATTAACAAGAGATGGTGGTGATAATAAGGAC----------------------------
----

AGTACCACTGAGACCTTCAGATCTGGAGGAGGAGATATGAGAGACAATTGGAGAAGTAAATTATATAAATATA
AAATAGTAAAAATTGGACCACTA---GGAGTAGCACCCACCAAGGCAAAGAGAAGAGTGGTG-----------
----------------------------CAGAGAGAAAAAAGAGCA---GTGGGAACAATAGGAGCT------
---ATGTTCCTTGGGTTCTTGGGAGCA---GCAGGAAGCACGATG---GGCGCAGCGTCA---
ATAACGCTAACGGTACAG---GCCAGACAA---TTGTTG---TCTGGTATAGTGCAACAGCAGAGA---
AATTTG---CTGAGG---GCTATTGAGGCGCAACAGCATCTGTTGCAACTCACAGTCTGGGGCATC
>EU131809
TTCTACTGTAATACAACAAAGCTATTTAATAATACTTTGCAGGATAATAATACTTGGAATAGTCCTTGGCAAG
GG---

TCAAATGACACTATCACACTCCAATGCAGAATAAAACAAATTATAAACAGATGGCAGGAAGTAGGAAAAGCAA
TGTATGCCCCTCCCATCAGGGGACAAATTAGATGCTCATCAAAT---
ATTACAGGACTGCTACTAACAAGAGATGGTGGTAACGAAAGCGGG----------------------------
----

TCCGAAATCTTCAGACCTGGAGGAGGAAATATGAGGGACAATTGGAGAAGTGAATTATATAAATATAAAGTAG
TAAAAATTGAGCCATTA---GGACTAGCACCCACCAAGGCAAAAAGAAGAGTGGTG-----------------
----------------------CAGAGAGAAAAAAGAGCA---GTGGGAACACTAGGAGCT---------
CTGTTCCTTGGGTTCTTGGGAGCA---GCAGGAAGCACTATG---GGCGCAGCGTCA---
ATCACGCTGACGGTACAG---GCCAGACTA---TTACTG---TCTGGTATAGTGCAACAGCAGAAC---
AATTTG---CTGAGA---GCTATTGAGGCGCAACAGCATATGTTGCAACTCACAGTCTGGGGCATC
>MH479968
TTCTACTGTAATACATCAGAACTGTTT------AATTGGAATGATACTAGTCCTTGGAAGAAT----------
----

ATCACAAGAAATATCACGCTCCCTTGCAGAATAAGACAAATTGTAAACATGTGGCAGGAAGTAGGGAAAGCAA

TGTATGCCCCTCCCATTAGAGGAAAAATTAGATGTTCATCAAAT---
ATTACAGGGTTGCTATTAACAAGAGATGGTGGTGAGAATGAGAGC---------------------------
----

AATGTCACCGAGACCTTCAGACCTGGAGGAGGAAATATGAAGGACAATTGGAGAAGTGAATTATATAAATATA
AAGTAGTAAAAATTGAACCATTA---GGAGTAGCACCCACCAAGGCAAAGAGAAGAGTGGTG----------
---------------------------CAGAGAGAAAAAAGAGCA---GTGGGAACAATAGGAGCT------
---ATGTTCCTTGGGTTCTTGGGAGCA---GCAGGAAGCACTATG---GGCGCAGCGTCA---
ATAACGCTGACGGTACAG---GCCAGACAA---TTATTG---TCTGGTATAGTGCAACAGCAGAGA---
AATTTG---CTGAGG---GCTATTGAGGCGCAACAGCATCTGTTGCAACTCACAGTCTGGGGCATC
>JF932468
TTCTACTGTAATACATCACAACTGTTTAATGGTACTTGGAATAATAATGGTACTTGGAGTGGTACTGCTTGGA
ATACTACA--

GGAAATAACACTATCACGCTCCCTTGCAGAATAAAACAAATTGTAAACATGTGGCAGGAAGTAGGGAAAGCAA
TATACGCCCCTCCCATTAGAGGACAAATCAACTGTTCATCAATT---
ATTACAGGGTTACTATTAACAAGAGATGGTGGTAAGAATGTGAGC---------------------------
----

AACACCACCGAGATCTTCAGACCTGCAGGGGGAGATATGAGAGACAATTGGAGAAGTGAATTATATAAATATA
AAGTAGTAAAAATTGAACCATTA---GGAATAGCACCCACCAAGGCAAAGAGAAGAGTGGTG----------
---------------------------CAAAGAGAAAAAAGAGCA---GTGGGAACGATAGGAGCT------
---ATGTTCCTTGGGTTCTTAGGAGCA---GCAGGAAGCACTATG---GGCGCAGCGTCA---
ATAACGCTGACGGTACAG---GCCAAACAA---TTGTTG---TCTGGTATAGTGCAGCAGCAGAGA---
AATTTG---CTGAAT---GCTATTGAGGCGCAACAACGTCTGTTGCAACTCACAGTCTGGGGCATC
>JF932470
TTCTACTGTAATACAACAAAATTGTTTAATAGTACTTGGAATGATACTAGTACTGAGAATGTTACTACA----
----

GAAAATAGCACTATCGTACTCCATTGCAGAATAAGACAAATTGTAAACATGTGGCAGGAAGTAGGGAAAGCAA
TGTATGCCCCTCCCATTGAAGGACGAATTAGATGTTCATCAAAC---
ATTACAGGATTGCTATTAACAAGAGATGGTGGTAATAATAAAGGC---------------------------
----

AACACCACCGAGACCTTCAGACCTGGAGGAGGAAATATGAAAGACAATTGGAGAAGTGAATTATATAAATATA
AAGTAGTAAAAATTGAACCATTA---GGAGTAGCACCCACCAAGGCAAAGAGAAGAGTGGTG----------
---------------------------CAAAGAGAAAAAAGAGCA---GTGGGAACGATAGGAGCT------
---ATGTTCCTTGGGTTCTTGGGAGCA---GCAGGAAGCACTATG---GGCGCAGCGTCA---
ATAACGCTGACGGTACAG---GCCAGACAA---CTGTTG---TCTGGTATAGTGCAACAGCAGAGA---
AATTTG---CTGAGG---GCTATTGAGGCGCAACAGCATCTGTTGCAACTCACAGTCTGGGGCATC
>JF932471
TTCTATTGTAATACAGCAAAACTATTTAATAGTACTTGGCATGCTAATAATACTTGGACTGGTGATAGTACTA
TA--

GGAAATAGCAATATCACGCTCCCTTGCAAAATAAAACAATTTATAAACAAGTGGCAGGAAGTAGGGAAAGCAA
TGTATGCCCCTCCCATTAGCGGACTCATTAAATGCCCATCAAAG---
ATCACAGGGTTGCTATTAACAAGAGATGGTGGTAAGGAGACCAATACC------------------------
----

ACCGACATTGAGATCTTTAGACCTGGAGGAGGAGATATGAGAGACAATTGGAGAAGTGAATTATATAAATATA
AAGTAGTAAAAATTGAACCATTA---GGGGTAGCACCCACCAAGGCAAAGAGAAGAGTGGTG----------
---------------------------CAGAGAGAAAAAAGAGCA---GTGGGAACACTAGGAGCT------
---ATGTTCCTTGGGTTCTTGGGAGCA---GCAGGAAGCACTATG---GGCGCAGCGTCA---
ATGACGCTGACGGTACAG---GCCAGACAA---TTGCTG---TCTGGTATAGTGCAACAGCAGAGA---
AATCTG---CTGAGG---GCTATTGAGGCGCAACAGCGTCTGTTGCAACTCACAGTCTGGGGCATC
>JF932473
TTCTACTGTAATACATCACAACTGTTTAATAGTACTTGGAGTAATAATAGTACCAGTACTTGGAATAATACTA
CA--

GGAAATGACACTATCACGCTTCCTTGCAGAATAAAACAAATTGTAAATATGTGGCAGGAAGTAGGGAAAGCAA

TGTATGCCCCTCCCATTGCAGGACAAATTAGATGTTCATCAAAT---
ATTACAGGGTTGCTATTAACAAGAGATGGTGGTAATAATGGGAGC----------------------------
----

GACATTGAGATCTTCAGACCTGGAGGAGGAGACATGAGAGACAATTGGAGAAGTGAATTATATAAATATAAAG
TAGTAAAAATTGAACCATTA---GGAGTAGCACCCACCAAGGCAAAGAGAAGAGTGGTG--------------
-------------------------CAGAGAGAAAAAAGAGCA---GTGGGAACAATAGGAGCT---------
ATGTTCCTTGGGTTCTTGGGAGCA---GCAGGAAGCACTATG---GGCGCAGCGTCA---
ATAACGCTGACGGTACAG---GCCAGACAA---TTGTTG---TCTGGTATAGTGCAACAGCAGAGA---
AATTTG---CTGAGG---GCTATTGAGGCGCAACAGCATATGTTGCAACTCACAGTCTGGGGCATC
>KM217584
TTCTACTGCAATACAACAAAGCTGTTTAATAATACCTTGTTGGCTAATGACACTTGGAATAGAACTTGGCAAC
AG---

TCAAATGATACTATCATACTCCAATGCAGAATAAAACAAATTATAAACAGGTGGCAGGAAGTAGGAAAAGCAA
TGTATGCTCCTCCCATCAGGGGACAAATTAGCTGTTCATCAAAT---
ATTACAGGGCTGCTATTAACAAGAGATGGTGGTGACACGAATGACACG-------------------------
----

AATAGGTCTGAAATCTTCAGACCTGGAGGAGGAGATATGAGGGACAATTGGAGAAGTGAATTATATAAATATA
AAGTAGTAAAACTTGAGCCACTA---GGAATAGCACCTACCAAGGCAAAGAGAAGAGTGGTG-----------
----------------------------CAGAGAGAAAGAAGAGCA---GTGGGAACAATAGGAGCT------
---GTGTTCCTTGGGTTCTTGGGAGCA---GCAGGAAGCACTATG---GGCGCAGCGTCA---
GTGACGCTGACGGTACAG---GCCAGACTA---TTACTG---TCTGGTATAGTGCAACAGCAGAAC---
AATCTG---CTGAGA---GCTATTGAGGCGCAACAGAACATGTTGCAACTCACAGTCTGGGGCATC
>KM217662
TTCTACTGTAATACATCAAAGCTGTTTAATAATACTTGGACTAATAATACTTGGAATGGTACTTGGCCA----
----

AATAGTACCATCATAATCATCCCATGCAGAATAAAACAAATTATAAACAAGTGGCAGGAAGTAGGAAGGGCAA
TGTATGCCCCTCCCATCAGTGGACAAATTAGATGTTCATCAAAT---
ATTACAGGGTTGCTATTAACAAGGGATGGTGGTGGTGGTAATAACTCAACTAAC------TCAACTGAG---
CCA--

ACTGAGGAAATCTTCAGACCTGCAGGAGGAGATATGAGGGACAATTGGAGAAGTGAATTATATAAATATAAAG
TGGTAAAAATTGAGCCAATA---GGACTAGCACCCACCAAGGCAAAGAGAAGAGTGGTG--------------
-------------------------CAGAGAGAAAAAAGAGCA---GTGGGAACTTTAGGAGCT---------
GTGTTCCTTGGGTTCTTGGGAGCA---GCAGGAAGCACTATG---GGCGCAGCGTCA---
TTGACGCTGACGGTACAG---GCCAGACTA---TTACTG---TCTGGTATAGTGCAACAGCAGAAC---
AATCTG---CTGAGA---GCTATTGAGGCGCAACAACACATGTTGCAACTCACAGTCTGGGGCATC
>KM217802
TTTTACTGTAATACAACACAGCTGTTTAATAGTACTTGGAATGGTACTGAAGGGGTAAATACCACTGAAGGAG
AG---

ATCATACTCCCATGCAGAATAAAACAAATTATAAACAGGTGGCAAGAAGTAGGAAAAGCAATATATGCCCCTC
CCATCAGCGGACAGATCAATTGTTCATCAAAT---
ATTACAGGGCTATTATTAACAAGAGATGGTGGTATTAATAACGAGACC-------------------------
----

CAGACTACTGAGACCTTCAGACCTGGAGGAGGAGATATGAGGGACAATTGGAGAAGTGAATTATATAAATATA
AAGTAGTAAAAAATTGAACCATTA---GGAGTAGCACCCACCAAGGCAAAGAGAAGAGTGGTG----------
----------------------------CAAAGAGAAAAAAGAGCA---GTGGGA---ATAGGAGCT------
---GTGTTCCTTGGGTTCTTGGGAGCA---GCAGGAAGCACTATG---GGCGCAGCGTCG---
ATAACGCTGACGGTACAG---GCCAGACAA---TTATTG---TCTGGTATAGTGCAACAGCAGGGT---
AACTTG---CTGAGA---GCTATTGAGGCGCAACAACATCTGTTGCAACTCACAGTCTGGGGCATC
>KM217995
TTCTACTGTAATACAACAAAGTTGTTTAATAATACTTTGCTGACTAATGATACTTGGCGAGATACTTGGCAAG
AGTCA--
-------------AATAAT------
ACTGAAGGAAATGTCATTACACTCCAATGCAGAATAAAACAAATTATAAACAGATGGCAGGAAGTAGGAAAAG

CAATGTATGCCCCTCCCATCAGGGGACAAATTAGTTGTTCATCAAAT---
ATTACAGGGCTTCTATTAACAAGAGATGGTGGCAGCATGAATGAGTCC-------------------------
----

ATAAATGAAACCTTCAGACCTGGAGGAGGAAATATGAGGGACAATTGGAGGAGTGAATTATATAAATATAAAG
TAGTAAAAATTGAGCCACTA---GGAATAGCACCCACCAAGGCAAAGAGAAGAGTGGTG--------------
-------------------------CAGAGGGAAAAAAGAGCA---GTGGGAACAATAGGAGCT---------
ATGTTCCTTGGTTTCTTGGGAGCA---GCAGGAAGCACTATG---GGCGCAGCGTCA---
ATGACGCTGACGGTACAG---GCCAGACTA---TTACTG---TCTGGTATAGTGCAACAGCAGAAC---
AATCTG---CTGAAA---GCTATTGAGGCGCAACAGCACCTGTTGCAACTCACAGTCTGGGGCATC
>JF932475
TTCTACTGTAATACATCACAACTGTTTAATAGTACTTGGTATGGTAATGATACTTGGAATAATACTACA----
----

AAAGATAGCAATATCACGCTCCATTGCAGAATAAAACAAGTTGTAAACATGTGGCAGGAAGTAGGGAAAGCAA
TGTATGCCCCTCCCATTGCAGGACTAATTAGATGTACATCAAAC---
ATTACCGGGTTGCTACTAACAAGAGATGGGGGTAATAATAGGAGCGGG-------------------------
----

GACACCGAGATCTTCAGACCTGGAGGAGGAGATATGAGAGACAATTGGAGAAGTGAATTATATAAATATAAAG
TAGTGAAAATTGAACCATTA---GGAGTAGCACCCACCAGGGCACAGAGAAGAGTGGTG--------------
-------------------------CAGAGAGAAAAAAGGGCA---GTGGGAACGATAGGAGCT---------
ATGTTCCTTGGGTTCTTGGGAGCA---GCAGGAAGCACTATG---GGCGCAGCGTCA---
ATGACGCTGACGGTACAG---GCCAGACAA---CTGTTG---TCTGGTATAGTGCAACAGCAGAGA---
AATTTG---CTGATG---GCTATTGAGGCGCAACAGCATCTGTTGCAACTCACAGTCTGGGGCATC
>JF932476
TTTTACTGTAATACATCAAAACTGTTTAATAGTACTTGGGATAGGACTGATAATAATACTCGAAATGAT----
----

AATAATAATACAATCACGCTCCCTTGCAGAATAAAACAACTTATAAACATGTGGCAGGAAGTAGGGAAAGCAA
TGTATGCCCCTCCCATTAAAGGACAAATTAACTGTTCATCACAT---
ATTACGGGATTGCTATTAACAAGAGATGGTGGTACTAATGAGAGC----------------------------
----

AACACCACCGAGACCTTCAGACCTGGAGGAGGAGATATGAGAGACAATTGGAGAAGTGAATTATATAAATATA
AAGTAGTAAAAATTGAACCATTA---GGAGTAGCACCCACCAAGGCAAAGAGAAGAGTGGTG-----------
----------------------------CAGAGAGAAAAAAGAGCA---GTGGGAGCGTTAGGAGCT------
---ATGTTCCTTGGGTTCTTGGGAGCA---GCAGGAAGCACGATG---GGCGCAGCGTCA---
ATAGCGCTGACGGTACAG---GCCAGACAA---TTATTG---TCTGGTATAGTGCAGCAGCAGAGA---
AATTTG---CTGAGG---GCTATTGAGGCGCAACAGCATATGTTGCAACTCACAGTCTGGGGCATC
>JF932477
TTCTACTGTAATACATCACAACTGTTTAATAGTACTTGGGATGGTAATGATACTAGTACTTGGACTAACGCTA
CA---

GAAAATCGCACTATCACACTCCCTTGCAGAATAAAACAAATTGTAAACATGTGGCAGGAAGTAGGGAAAGCAA
TGTATGCCCCTCCCATTAGAGGACAAATTAGATGTTCATCAAAT---
ATTACAGGGCTGATATTAATAAGAGATGGTGGTAATAATGAGAGCACG-------------------------
----

ACCAACAACGAGACCTTTAGACCTGCAGGAGGAAATATGAAAGATAATTGGAGAAGTGAATTATATAAATATA
AAGTAGTAAAAATTGAACCATTA---GGAGTAGCACCCACCAAGGCAAAGAGAAGAGTGGTG-----------
----------------------------CAGAGAGAAAAAAGAGCA---GTGGGAACGATAGGAGCT------
---ATGTTCCTTGGGTTCTTGGGAGCA---GCAGGAAGCACTATG---GGCGCAGCGTCA---
ATAACGCTGACGGTACAG---GCCAGACAA---TTGATG---TCTGGTATAGTGCAACAGCAGAGA---
AATTTG---CTGAGG---GCTATTGAGGCGCAACAGCATATGTTGCAACTCACAGTCTGGGGCATC
>JF932483
TTCTACTGTAATACATCAAAACTGTTTAATGATACTATTGCTTGGAATGATACAGAACTAGAACTGTTTAACG
ATACTAGTACT--

GAGAATGATACTATCACACTCCCTTGCAGAATAAAACAAGTTGTAAATATGTGGCAGGAAGTAGGGAAAGCAA

TGTATGCCCCTCCCATTAGTGGACAAATTAGATGTTCATCAAAT---
ATTACAGGGATGATATTAACAAGAGATGGTGGTAATGTGACCAAC----------------------------
----

AACAAGACTGAGGTCTTCAGACCTGGAGGAGGAGATATGAGAGACAATTGGAGAAGTGAATTATATAAATATA
AAGTAGTAAAAATTGAACCATTA---GGAGTAGCACCCACCAAGGCAAAG-----------------------
-------------------------------AGAAAAAAAAGAGCA---GTGGGAACAAAAGGAGCT------
---ATGTTCCTTGGGTTCTTGGGAGCA---GCAGGAAGCACTATG---GGCGCAGCATCA---
ATAACGCTGACGGTACAG---GCCAGACAA---TTATTG---TCTGGTATAGTGCAACAGCAGAGA---
AATTTG---CTGAGG---GCTATTGAGGCGCAACAGCATCTGTTGCAACTCACAGTCTGGGGCATC
>JF932484
TTCTACTGTAATACCTCACAACTGTTTAATAGTACTTGGTATGCTAATAGGACTTGGAAGGGTGATACA----
----

GGAAATGACACTATCACACTCCCTTGCAGAATAAAACAAATTGTAAACATGTGGCAGGAAGTAGGAAAAGCAA
TGTATGCCCCTCCCATTAAAGGACAAATTAGATGTTCATCAAAT---
ATTACAGGGTTGTTATTAACAAGAGATGGTGGTAATGGGAACAAGACC-------------------------
----

AACAACACTGAGACCTTCAGACCGGGAGGAGGAGACATGAGAGACAATTGGAGAAGTGAATTATATAAATATA
AAGTAGTACAAATTGAACCATTA---GGAGTAGCACCCACCAAGGCAAAGAGAAGAGTGGTG-----------
----------------------------CAAAGAGAAAAAAGAGCA---GTGGGAACGATAGGAGCT------
---ATGTTCCTTGGGTTCTTGGGAGCA---GCAGGAAGCACTATG---GGCGCAGCGTCA---
ATACCGCTGACGGTACAG---GCCAGACAA---TTGTTG---TCTGGTATAGTGCAACAGCAGAGA---
AATTTG---CTGAGG---GCTATTGAGGCGCAACAGCATATGTTGCAACTCACAGTCTGGGGCATT
>JF932485
TTCTACTGTAATACATCACAACTGTTTAATAGTACTTGGCATAATAAGGGTACTTGGAATAGTACTCGGAATG
ATACTGCA---

GAAAATGACACTATCACGCTCCCTTGCAGAATAAAACAAATTGTAAACATGTGGCAGGAAGTAGGAAAAGCAA
TGTATGCCCCTCCCATTAAAGGACAAATTAGATGTTCATCAAAT---
ATTACAGGGTTGCTATTAACAAGAGATGGTGGTAATAATGAGAACAAGACCACC-------------------
----

AACGAGACCGAGACCTTCAGACCTGGAGGAGGAGATATGAGAGACAATTGGAGAAGTGAATTATATAAATATA
AAGTAGTAAAAATTGAACCATTA---GGAGTAGCACCCACCAAGGCAAAAAGAAGAGTGGTG-----------
----------------------------CAGAGAGAAAAAAGAGCA---GTGGGAACGATAGGAGCT------
---ATGTTCCTTGGGTTCTTGGGAGCA---GCAGGAAGCACTATG---GGCGCAGCGTCA---
ATAACGCTGACGGTACAG---GCCAGACAA---TTGTTG---TCTGGTATAGTGCAACAGCAGAGA---
AATTTG---CTGAGG---GCTATTGAGGCGCAACAACATCTGTTGCAACTCACAGTCTGGGGCATC
>JF932486
TTCTACTGTGACACATCACAACTGTTTAATAGTTCTTGGAATAGTAATACTAGTACTTGGAATAAGACA----
----

GGAGATAGTAATATCACGCTCCCTTGCAGAATAAAACAAATTATAAACATGTGGCAGAAAGTAGGGAAAGCAA
TGTATGCCCCTCCTATTAGAGGACAAATTAGATGTTCATCAAAT---
ATTACAGGGTTGCTATTAACAAGAGATGGTGGTGAGGGGAACAGG----------------------------
----

ACCAACGAGACCTTCAGACCTGGAGGAGGAGATATGAGAGACAATTGGAGAAGTGAATTATATAAATATAAAG
TAGTAAGAATTGAACCATTA---GGAGTAGCACCCACCAAGGCAAAGAGAAGAGTGGTG--------------
-------------------------CAGAGAGAAAAAAGAGCA---GTGGGAACGCTAGGAGCT---------
ATGTTCCTTGGGTTCTTGGGAGCA---GCAGGAAGCACTATG---GGCGCAGCGTCA---
ATAACGCTGACGGTACAG---GCCAGACAA---TTGTTG---TCTGGTATAGTGCAACAGCAGAGA---
AATTTG---CTGAGG---GCTATTGAGGCGCAACAGCATCTGTTGCAACTCACAGTCTGGGGCATC
>MH479957
TTCTACTGTAATACATCACAACTGTTTAACAGTACTTGGAATAATACCAGTACTGGGAATGATACTAATACT-
----

ACAGATAGAATTATCACGCTCCCTTGCAGGATAAAACAAATTGTAAACATGTGGCAGGAAGTAGGAAAAGCAA

TGTATGCCCCTCCCATTGGAGGACAGATTAGGTGCTCATCAAAT---
ATTACAGGGTTGCTATTAACAAGAGATGGTGGTAATACCACCGAGTCTACCGAG-------------------
----

TCTACCGAGACCTTCAGACCTGCAGGAGGAAATATGAAGGACAATTGGAGAAGTGAATTATATAAATATAAAG
TAGTAAAAATTGAACCATTA---GGAGTAGCACCCACCAGGGCAAAGAGAAGAGTGGTG--------------
-------------------------CAAAGAGAAAAAAGAGCA---GTGGGAACGATAGGAGCT---------
ATGTTCCTTGGGTTCTTGGGAGCA---GCAGGAAGCACTATG---GGCGCAGCGTCA---
ATAACGCTGACGGTACAG---GCCAGACAA---TTGCTG---TCTGGTATAGTGCAACAGCAGAGA---
AATTTG---CTGAGG---GCTATTGAGGCGCAACAACACCTGTTGCAACTCACAGTCTGGGGCATC
>JF932487
TTCTACTGTAATACATCAAAACTGTTTAACAGTACTTGGCATGCTAATGGTACTTGGACTAATAGTACA----
----

GTAGATAGCAATATCACGCTCCATTGTAGAATAAAACAAATTGTAAACATGTGGCAAGAAGTAGGGAAAGCAA
TGTATGCCCCTCCCATTGCAGGAAAAATTAGATGTTCATCAAAT---
ATTACAGGGTTGCTATTAACAAGAGATGGGGGTAATACTGGGAAC----------------------------
----

ACTGACAACGAGACCTTCAGACCTGGAGGAGGAGATATGAGAGACAATTGGAGAAGTGAATTATATAAATATA
AAGTAGTTAAAATTGAGCCAATA---GGAGTAGCACCCACCAAGGCAAAGAGAAGAGTGGTG-----------
----------------------------CAGAGAGAAAAAAGAGCA---GTGGGAACAATAGGAGCT------
---ATGTTCCTTGGGTTCTTGGGAGCA---GCAGGAAGCTCTATG---GGCGCAGCGTCA---
ATAACGCTGACGGTACAG---GCCAGACAA---TTGTTG---TCTGGTATAGTGCAACAGCAGAGA---
AATTTG---CTGAGG---GCTATTGAGGCGCAACAGCATATGTTGCAACTCACAGTCTGGGGCATC
>JF932489
TTCTACTGTAATACATCACAACTGTTTAATGGGAATGGTACTAGTACTGGGAATAAGACTACA----------
----

GGAAATGACACTATCACACTCCCTTGCAGAATAAAACAAATTGTAAACATGTGGCAGGAGGTAGGGAAAGCAA
TGTATGCCCCTCCCATTTCAGGACGAATTAGATGTGTATCAAAC---
ATTACAGGGTTGCTATTAACAAGAGATGGTGGTAATGAGACCGAT----------------------------
----

ACCACCGAGACCTTCCGACCTTGGGGAGGAGATATGAGAGACAATTGGAGAAGTGAATTATATAAATATAAAG
TAGTAAAAATTGAACCATTA---GGAGTAGCACCCACCAAGGCAAAGAGAAGAGTGGTG--------------
-------------------------CAGAGAGAAAAAAGAGCA---GTGGGA---CTAGGAGCT---------
ATGTTCCTTGGGTTCTTGGGAGCA---GCAGGAAGCACGATG---GGCGCAGCGTCA---
ATAACGCTGACGGTACAG---GCCAGACAA---TTGATG---TCTGGTATAGTGCAACAGCAGAGA---
AATTTG---CTGAGG---GCTATTGAGGCGCAACAGCATATGTTGCAACTCACAGTCTGGGGCATC
>JF932490
TTCTACTGTAATACATCACAACTGTTTAATAGTACTTGGTATGAGAATGGTACTATTACTACTAAGAATTTT-
----

ACTACAGGAACTATCACACTCCCTTGCAGAATAAAACAAATTGTAAACATGTGGCAGCAAGTAGGGAAAGCAA
TGTATGCCCCTCCCATTGCAGGAAAAATTAGATGTTCATCAAAT---
ATTACAGGATTGCTATTAACAAGAGATGGGGGTACGGGTAATGAGACCAACAAC-------------------
----

AATGACACTGAGACTTTCAGACCTGAAGGAGGAAATATGAAAGACAATTGGAGAAGTGAATTATATAAATATA
AAGTAGTAAGAATTGAACCATTA---GGACTAGCACCCACCAAGGCAAAGAGAAGAGTGGTG-----------
----------------------------CAGAGAGAAAAAAGAGCA---GTGGGAACAATAGGAGCT------
---ATGTTCCTTGGGTTCTTGGGAGCA---GCAGGAAGCACTATG---GGCGCAGCGTCA---
ATAACGCTGACGGTACAG---GCCAGACAA---TTGTTG---TCTGGTATAGTGCAACAGCAAAGA---
AATTTG---CTGAGG---GCTATTGAGGCGCAACAGCATCTGTTGCAACTCACAGTCTGGGGCATC
>JF932491
TTCTACTGTAATACAACACAACTGTTTAATAGTACTTGGCCTGGGAATAATACTAGTACTAAGACA-------
----

GAAAATGATAATATCACGCTCCCTTGCAAAATAAGACAAATTGTAAACATGTGGCAGAAAGTAGGGAAAGCAA

TGTATGCCCCTCCCATTAAAGGACAAATTAGATGTTCATCAAAT---
ATTACAGGGTTGCTGTTAACAAGAGATGGTGGTAGTAATGAGAACACG-------------------------
----

ATCAACGAGACCTTCAGACCTGGAGGAGGAGATATGAGAGACAATTGGAGAAGTGAATTATATAGATATAAAG
TAGTAAAAATTGAACCTTTA---GGAGTAGCACCCACCAAGGCAAAGAGAAGAGTGGTG--------------
-------------------------CAGAGAGAAAAAAGAGCA---GTGGGAACGCTAGGAGCT---------
ATGTTCCTTGGGTTCTTGGGAGCA---GCAGGAAGCACTATG---GGCGCAGCGTCA---
ATAACGCTGACGGTACAG---GCCAGACAA---TTGCTG---TCTGGTATAGTGCAACAGCAGAGA---
AATTTG---CTGATG---GCTATTGAGGCGCAACAACATCTGTTGCAACTCACAGTCTGGGGCATC
>JF932493
TTCTACTGTAATACAACACAACTGTTTAATAGTACTTGGAATAATACTAGTATTTGGAATAATGTTACA----
----

GAAAATGGCACTATCACGCTTCCTTGCAGAATAAAACAAATTGTAAACATGTGGCAGGGAGTAGGGAAAGCAA
TGTATGCCCCTCCCATTGCAGGACAAATTAACTGTTCATCAAAT---
ATTACAGGGTTGCTATTAACAAGAGATGGTGGTGTTAATAAGAGC----------------------------
----

GAGGGCAACACCACCGAGACCTTCAGACCTGCAGGAGGAAATATGAGAGACAATTGGAGAAGTGAATTATATA
AATATAAAGTAGTAAAAATTGAACCATTA---GGATTAGCACCCACCAAGGCAAAGAGAAGAGTGGTG-----
----------------------------------CAAAGAGAAAAAAGAGCA---
GTGGGAACGATAGGAGCT---------ATGTTCCTTGGGTTCTTGGGAGCA---GCAGGAAGCACGATG---
GGCGCAGCGTCG---ATGACGCTGACGGTACAG---GCCAGGCAA---TTGTTG---
TCTGGTATAGTGCAACAGCAAAGA---AATTTG---CTGAGG---
GCTATTGAGGTGCAACAGCATCTGTTGCAACTCACAGTCTGGGGCATC
>JF932499
TTCTACTGTAATACATCACAACTGTTTAATAGTTCTTGGAATGATACTAGTACTTGGAATAATACTACA----
----

GAAAATAGCACTATCATGCTCCCTTGCAGAATAAAACAAATTGTAAACATGTGGCAGGAAGTAGGGAAAGCAA
TGTATGCCCCTCCCATTAGTGGAATAATTAACTGCAAATCAAAT---
ATTACAGGGTTGCTATTAACGAGAGATGGTGGTGGTAATGAGACT----------------------------
----

AACACTGAGATCTTTAGACCAGGAGGAGGAAATATGAAAGACAATTGGAGAAGTAAATTATATAAATATAAAG
TAGTAAAAATTGAACCATTA---GGAGTAGCACCCACCAAGGCAAAGAGAAGAGTGGTG--------------
-------------------------CAGAGAGAAAAAAGAGCA---GTGGGAACGATAGGAGCT---------
ATGTTCCTTGGGTTCTTGGGAACA---GCAGGAAGCACTATG---GGCGCAGCGTCA---
ATAACGCTGACGGTACAG---GCCAGACAA---TTGCTG---TCTGGTATAGTGCAACAGCAGAGA---
AATTTG---CTGAGG---GCTATTGAGGCGCAACAGCATCTGTTGCAACTCACAGTCTGGGGCATC
>JF932500
TTCTACTGTAATACATCACAACTGTTTAATAGTACTTGGCCTAATAATGGTACTTGGAATGATACTGCA----
----

AAAAATGACAATATCACTCTCCCTTGCAGAATAAAACAAATTATAAACATGTGGCAGGAAGTAGGGAAAGCAA
TGTATGCCCCTCCCATTGGAGGACAAATTAGATGTTCATCAAAT---
ATTACAGGGTTGCTATTAACAAGAGATGGTGGTAGGAATGAGAGT----------------------------
----

AACACCACCGAGACCTTCAGACCTGGAGGAGGAGACATGAGAGACAATTGGAGAAGTGAATTATATAAATATA
AAGTAGTAAAAATTGAACCATTA---GGAGTAGCACCCACCAAGGCAAAGAGAAGAGTGGTG-----------
----------------------------CAGAGAGAAAAAAGAGCA---GTGGGAACGATAGGAGCT------
---ATGTTCCTTGGGTTCTTGGGAGCA---GCAGGAAGCACTATG---GGCGCAGCGTCA---
ATAACGCTGACGGTACAG---GCCAGACAA---TTGTTG---TCTGGTATAGTGCAACAGCAGAGA---
AATTTG---CTGATG---GCTATTGAGGCGCAACAGCATCTGTTGCAACTCACAGTCTGGGGCATC
>KM218168
TTCTACTGTAATACAACAAAGCTATTTAGTAATACTTGGCAGACTAATAACTCTTGGAATGGTACTTGGCAAG
GGTCAAATAAT--

AGTGATAGAAATGTCCTTATACTCCAATGCAGAATAAAACAAATTATAAACAGATGGCAGGAAGTAGGAAAAG
CAATGTATGCCCCTCCCATCAGGGGAGAAATTAAATGTTCATCAAAT---
ATTACAGGGCTTCTATTAACAAGAGATGGTGGTAACACAACTGAGTCCAGA---------------------
----

AATGGGACTGAAATCTTCAGACCTGGAGGAGGAGATATGAGGGACAATTGGAAAAGTGAATTATATAAATATA
AAGTAGTAAAAATTGAGCCACTA---GGAATAGCACCCACCAAGGCAAAGAGAAGAGTGGTG----------
----------------------------CAGAGAGACAAAAGAGCG---GTAGGAACACTAGGAGCT------
---ATGTTCCTTGGGTTCTTGGGAGCA---GCAGGAAGCACTATG---GGCGCAGCGTCG---
ATGACGCTGACGGTACAG---GCCAGACTA---TTACTG---TCTGGTATAGTGCAACAGCAGAAC---
AATATG---CTGAAA---GCTATTGAGGCGCAACAGCACCTGTTGCAACTCACAGTCTGGGGCATC
>KM218228
TTCTACTGTAATACATCAAAATTGTTTAATAATACTTTACAGCCTAATAATACTTGGAATAGTACTTGGCAAA
CAGAT---

ATTAACGGAACCATCACACTCCCATGCAGAATAAAACAAATTATAAACAGGTGGCAGGAAGTAGGAAGAGCAA
TGTATGCCCCTCCCATCAGTGGACAAATTAAATGTTTATCAAAT---
ATTACAGGGCTGCTATTAACAAGGGATGGTGGTAAAACGAATGGGTCCGAA---------------------
----

AATGAGACCGAAATCTTCAGGCCTGGAGGAGGAGATATGAGAGACAATTGGAGAAGTGAATTATATAAATATA
AAGTAGTAAAAATTGAGCCACTA---GGAATAGCACCCACCAAGGCAAAGAGAAGAGTGGTG----------
----------------------------CAGAGAGAAAAGAGAGCG---GTGGGAACACTGGGAGCT------
---GTGTTCCTCGGGTTCTTGGGAGCA---GCAGGAAGCACTATG---GGCGCAGCGTCA---
ATGACGCTGACGGTACAG---GCCAGACTA---TTGCTG---TCTGGTATAGTGCAACAGCAGAAC---
AATATG---CTGAGA---GCTATTGAGGCGCAACAGCACATGTTGCAACTCACAGTCTGGGGCATC
>JF932479
TTCTACTGTAATACATCACAACTGTTTAATAGTGTTAATGGTACTTGGAATGGTACTGGTACA---------
----

GGAAATAACACTATCACGCTCCCTTGCAGAATAAAACAAATTGTAAACATGTGGCAGGAAGTAGGGAAAGCAA
TGTATGCCCCTCCCATTAGTGGACAAATTAGATGTTTATCAAAT---
ATTACAGGGTTGCTATTAACAAGAGATGGTGGTAATAATGGGAGC---------------------------
----

AACACCACCGAGACCTTCAGACCAGGAGGAGGAGATATGAGAGACAATTGGAGAAGTGAATTATATAAATATA
AAGTAGTACAAATTGAACCATTA---GGAGTAGCACCCACCAGGGCAAAGAGAAGAGTGGTG----------
----------------------------CAGAGAGAAAAGAGAGCA---GTGGGAACAATAGGAGCT------
---ATGTTCCTTGGGTTCTTGGGAGCA---GCAGGAAGCACTATG---GGCGCAGCATCG---
ATAACGCTGACGGTACAG---GCCAGACAA---TTGTTG---TCTGGTATAGTGCAACAGCAAAGA---
AATTTG---CTGAGG---GCTATTGAGGCGCAACAGCACATGTTGCAACTCACAGTCTGGGGCATC
>JF932480
TTTTACTGTAATACATCACAACTGTTTAATAGTTCTTGGCATGCTAATAGTACTAGGAATAACACAGATAAG-
----

ATCATACTTCCTTGTAGAATAAAACAAATTGTAAACATATGGCAGGGAGTAGGGAAAGCAATATATGCCCCTC
CCATTGAAGGACAAATTAGTTGTTTATCAAAG---
ATTACAGGGTTACTATTAACAAGAGATGGTGGTGGTGGTAGAAATGCGAGT---------------------
----

GAGAACAACACCGAGACCTTCAGACCTGGAGGGGGAGATATGAGAGACAATTGGAGAAGTGAATTATATAAAT
ATAAAGTAGTAAAAATTAAACCATTA---GGAATAGCACCCACCAAGGCAAAAAGAAGAGTGGTG-------
-------------------------------CAAAGAGAAAAAAGAGCA---GTGGGAACGATAGGAGCT---
------ATGTTCCTTGGGTTCTTGGGAACA---GCAGGAAGCACTATG---GGCGCAGCGTCA---
ATGACGCTGACGGTACAG---GCCAGACAA---TTGTTG---TCTGGTATGGTGCAACAGCAGAGA---
AATTTG---CTGAGG---GCTATTGAGGCGCAACAGCATCTGTTGCAACTCACAGTCTGGGGCATC
>JF932481
TTCTACTGTGATACATCACAACTGTTTAATAGTACTTGGATTTGGAATGATACTAGTACTTGGAATGGTACTG
CA--

GAAGATAGCACTATCACGCTCCCTTGCAGAATAAGACAAATTGTAAACATGTGGCAGGAAGTAGGGAAAGCAA
TGTATGCCCCTCCCATAGCAGGACAAATTAGATGTTCATCAAAT---
ATTATAGGGTTGCTATTAACAAGAGATGGTGGTAATGAGAACAAC----------------------------
----

AAGACCACCGAGACCTTCAGACCTGGAGGAGGAGATATGAGAGACAATTGGAGGAGTGAATTATATAAATATA
AAGTAGTAAAAATTGAACCATTA---GGAGTAGCACCCACCAGGGCAAAGAGAAGAGTGGTG-----------
----------------------------CAGAGAGAAAAAAGAGCA---GTGGGA---ATAGGAGCT------
---ATGTTCCTTGGGTTCTTGGGAGCA---GCAGGAAGCACTATG---GGCGCAGCGTCA---
ATAACGCTGACGGTACAG---GCCAGACAA---TTGTTG---TCTGGTATAGTGCAGCAGCAGAGA---
AATCTG---CTGAGG---GCTATTGAGGCGCAACAGCATCTGTTGCAACTCACAGTCTGGGGCATC
>JF346919
TTCTACTGTAATACATCACTACTGTTTAATAGTACTTGGTATGAGAATGATACTAGTAATTGGAATGCT----
----

ACTAAAGAAAAAATCACGCTCCCTTGCAGAATAAGACAAATTGTAAACATGTGGCAGGAAGTAGGGAAAGCAA
TGTATGCCCCTCCCATTGCAGGACAACTTAACTGCTCATCAAAC---
ATTACAGGGTTGCTATTAACAAGAGATGGTGGTGGTGATGGTAAT----------------------------
----

AGTACGGACGAGATCTTCAGACCTGGAGGAGGAGATATGAGGGACAATTGGAGAAGTGAATTATATAAATATA
AAGTAGTAAAAATTGAACCATTA---GGAGTAGCACCCACCAAGGCAAAGAGAAGAGTGGTG-----------
----------------------------CAGAGAGAAAAAAGAGCA---GTGGGAACGATAGGAGCT------
---ATGTTCCTTGGGTTCTTGGGAGCA---GCAGGAAGCACTATG---GGCGCAGCGTCA---
ATAACGCTGACGGTACAG---GCCAGACAA---TTATTG---TCTGGTATAGTGCAACAGCAGAGA---
AATTTG---CTGAGG---GCTATTGAGGCGCAACAGCATTTGTTGCAACTCACAGTCTGGGGCATC
>JF932498
TTCTACTGTAATACATCACGCTTGTTTAACAGTATTTGGAATGGTACTAGTACTTGGAATGATACTACA----
----

GAAAATGACACTATCATACTCCCTTGCAGAATAAAACAATTTGTAAACATGTGGCAGAAAGTAGGGAAGGCAA
TGTATGCCCCTCCCATTAGCGGACCGATTAGCTGTTCATCAAAA---
ATTACAGGGTTGCTATTAACAAGAGATGGTGGTAATGAGAGCCAG----------------------------
----

ATTTCCGAGACCTTCAGACCTGAAGGAGGGGATATGAGAGATAATTGGAGAAGTGAATTATATAAATATAAAG
TAGTAAAAATTGAACCATTA---GGAATAGCACCCACCAGGGCAAAAAGAAGAGTGGTG--------------
-------------------------CAGAGAGAAAAAAGAGCA---GTGGGAACGATAGGAGCT---------
ATGTTCCTTGGGTTCTTAGGAGCA---GCGGGAAGCACTATG---GGCGCAGCGTCA---
ATAACGCTGACGGTACAG---GCCAGACAA---TTGCTG---TCTGGTATAGTGCAACAGCAGAGA---
AATTTG---CTGAGG---GCTATTGAGGCGCAACAGCATCTGTTGCAACTCACAGTCTGGGGCATT
>JX960597
TTCTACTGTAACTCAACACCACTGTTTAACAGTACTTGGGAGAATGGGACACAGGAAGAATGGACACAGCTTA
ATGAG--
-------------AATAAT------
ACTGAAGAGTCAAATATCACCCTCCAATGCAGAATAAAACAATTTATAAACATGTGGCAGGAAGTAGGAAAAG
CAATGTATGCCCCTCCCATCAGTGGACAAATCAGGTGTTCATCAAAT---
ATTACAGGACTGCTATTAACAAGAGATGGGGGAGGG-------------------------------------
----

GAGAACGAGACCTTCAGGCCTGAAGGAGGAGATATGAGGGACAATTGGAGAAGTGAATTATATAAATATAAAG
TAGTGAAAATTGAACCATTA---GGAGTAGCACCCACTAAGGCAAAGAGAAGAGTGGTG--------------
-------------------------CAGAGAGAAAAAAGAGCA---GCG------ATAGGAGCT---------
ATGTTCCTTGGGTTCTTGGGAGCA---GCAGGAAGCACTATG---GGCGCAGCGTCA---
GTAACGCTGACGGTACAG---GCCAGACAA---TTATTG---TCTGGTATAGTGCAACAGCAGAAT---
AATTTG---CTGAGA---GCTATTGAGGCGCAACAGCATCTGTTGCAACTCACAGTCTGGGGCATC
>JX960598
TTCTACTGTAATACAACACAACTGTTTAATAGTACTTGGAATGACACTCGTATTGAGAATAGTACTGGGAACG
CA---

AATGGAAGCCATATCACACTCCCATGCAGAATAAAACAAATTATAAACATGTGGCAGGAAGTAGGAAAAGCAA
TGTATGCCCCTCCCATCAAAGGACTACTTAGATGTTCATCAAAT---
ATTACAGGGCTGATATTAGTAAGAGATGGTGGTGCTGTCAATACT----------------------------
----

ACCACCGAGACCTTCAGACCTGGAGGAGGAGATATGAGGGACAATTGGAGAAGTGAATTATATAAATATAAAG
TAGTAAAAATTGAACCATTA---GGACTAGCACCCACCAAGGCAAAAAGAAGAGTGGTG--------------
-------------------------CAAAGAGAAAAAAGAGCA---GTGGGA---ATAGGAGCT---------
CTGTTCCTTGGGTTCTTGGGAGCA---GCAGGAAGCACTATG---GGCGCAGCGTCG---
ATGACGTTGACGGTACAG---GCCAGACAA---TTATTG---TCTGGTATAGTGCAACAGCAAAAC---
AACTTG---CTGAAG---GCTATTGAGGCGCAACAGCATATGTTGCAACTCACAGTATGGGGCATC
>KC899011
TTTTACTGTAATACATCAAAACTGTTTAATAGTACTTGGAATGATACTAAA----------------------
----

GGAAATGGCACTATCACGCTCGATTGCAGAATAAAACAAATTGTAAATATGTGGCAGGGAGTAGGAAAAGCAA
TATATGCCCCTCCCATTGAAGGACAAATTAGATGTTCATCAAAG---
ATTACAGGGTTACTATTAACAAGAGATGGTGGCAATAATGGGAACAACGAG----------------------
----

ACCACCGAGACCTTCAGACCTGGAGGAGGAAATATGAAAGACAATTGGAGAAGTGAATTATATAAATATAAAG
TAGTAAAACTTGAACCATTA---GGAGTGGCACCCACCAAGGCAAAGAGAAGGGTGGTG--------------
-------------------------CAGAGAGAAAAAAGAGCA---GTGGGAACGATAGGAGCT---------
ATGTTCCTTGGGTTCTTGGGAGCA---GCAGGAAGCACTATG---GGCGCAGCGTCA---
ATAACGCTGACGGTACAG---GCCAGACAA---TTGTTG---TCTGGTATAGTGCAACAGCAGAGA---
AATTTG---CTGATG---GCAATTGAGGCGCAACAGCATCTGTTGCAACTCACCGTCTGGGGCATC
>HQ215556
TTCTACTGTAATACAACAAAGCTGTTTAATAATACTTTTAATAGTGCCTGGCAAGAGTCAAAAGATAATGGC-
----

ACCGAAGGAGTCATCAAACTTCCATGCAAAATAAAACAAATAATAAATAGATGGCAGGAAGTAGGAAAAGCAA
TGTATGCTCCTCCCATCGAGGGACAAATTAGATGTTCATCAAAT---
ATTACAGGATTACTATTAACACGAGATGGTGGTAACAAGAGTGAG----------------------------
----

ACTGAGCCTGAAACCTTCAGACCTGGGGGAGGAGATATGAGGGACAATTGGAGAAGTGAATTATATAAATATA
AAGTAATAAAAATTGAGCCATTA---GGACTGGCACCCACCAGGGCAAAGAGAAGAGTGGTG-----------
----------------------------CAGAGACAAAAAAGAGCA---GTGGGAGTACTAGGAGCT------
---ATGTTCCTTGGGTTCTTGGGAGCA---GCAGGAAGCACTATG---GGCGCAGCGTCA---
GTCACGCTGACGGTACAG---GCCAGACTA---TTGCTG---TCTGGTATAGTGCAACAGCAGAAC---
AATTTG---CTGAGA---GCTGTTGAGGCGCAACAACACCTGTTGCAACTCACAGTCTGGGGCATC
>JF346916
TTCTACTGTAATACATCACAACTGTTTAATAGTACTTGGTATGCTAATGGTACTTGGGAGGGTAATTCA----
----

AGAAATGACAATATCACGCTCCCTTGCAGAATAAGACAAATTGTAAACATGTGGCAGGAAGTAGGGAAAGCAA
TGTATGCCCCTCCCATTGCAGGACAAATTAGTTGTTTATCAAAT---
ATTACAGGGTTGCTATTAACAAGAGATGGTGGTAATGAGACCAAT----------------------------
----

ACCACCGAGACCTTCAGACCTGGAGGAGGAGATATGAGAGACAATTGGAGAAGTGAATTATATAAATATAAAG
TAGTAAAAATTGAACCATTA---GGAGTAGCACCCACCAAGGCAAAGAGAAGAGTGGTG--------------
-------------------------CAGAGAGAAAAAAGAGCA---GTGGGAATGATAGGAGCT---------
ATGTTCCTTGGGTTCTTGGGAGCA---GCAGGAAGCACTATG---GGCGCAGCGTCA---
ATAACGCTGACGGTACAA---GCCAGACAA---TTGTTG---TCTGGCATAGTGCAACAGCAGAGA---
AATTTG---CTGAGG---GCTATTGAGGCGCAACAGCATCTGTTGCAACTCACAGTCTGGGGCATC
>JF932494
TTCTACTGTAATACAACAAAACTGTTTGATAGCACTTGGCATAAAAGTAATAATTGGACA-------------
----

GAAAATGGCACTATCGAGCTCCCCTGCAGAATAAAACAAATTGTAAACATGTGGCAGGAAGTAGGAAAAGCAA
TGTATGCCCCTCCCATCAGTGGACGAATTAGCTGTTTATCAAAT---
ATTACAGGGTTGCTCTTAACAAGAGATGGTGGTGGTAATGAGACCAAT-------------------------
----

ACGACCACCGAGATCTTTAGACCTGGAGGAGGAGATATGAGGGACAATTGGAGAAGTGAATTATATAAATATA
AAGTAGTAAAAATTGAACCATTA---GGAGTAGCACCCACCCAGGCAAAGAGAAGAGTGGTG-----------
----------------------------CAGAGAGAAAAAAGAGCA---GTGGGAACGATAGGAGCT------
---ATGTTCCTTGGGTTCTTGGGAGCA---GCAGGAAGCACTATG---GGCGCAGCGTCA---
ATAACGCTGACGGTACAG---GCCAGACAA---TTATTG---TCTGGTATAGTGCAACAGCAGAGA---
AATTTG---CTGAGG---GCTATTGAGGCGCAACAGCATCTGTTGCAACTCACAGTCTGGGGCATC
>JX140658
TTCTACTGTAATACATCAAAACTGTTTAATAGCACTTGGCCAAGTGATGGTACTGATGGTAATGTTACTGGAG
GG---

TCAAATGACACTATCACACTCCAATGCAGAATAAGACAAATTATAAACAGATGGCAGGAAGTAGGAAAAGCAA
TGTATGCTCCTCCCATCAGAGGAGTAATCAACTGTACATCAAAT---
ATTACAGGACTACTGTTAGTAAGAGATGGTGGGATTAACAACAGC----------------------------
----

ACTAATGAGACCTTCAGACCTGGAGGAGGAGATATGAGAGACAATTGGAGAAGTGAATTATACAAATACAAAG
TAGTAAGACTTGAGCCATTA---GGAGTAGCACCCACCGAGGCAAAGAGAATAGTGGTG--------------
-------------------------CAGAGAGAAAAAAGAGCA---GTAGGA---CTAGGAGCT---------
ATGTTCCTTGGGTTCTTGGGAGCA---GCAGGAAGCACGATG---GGCGCAGCGTCA---
ATAACGCTGATGGTACAA---GCCAGACAA---TTATTG---ACTGGTATAGTGCAACAGCAAAGC---
AATTTG---CTGAGG---GCTATTGAGGCGCAACAGCATATGTTGCAACTCACAGTCTGGGGCATC
>KX692916
TTCTACTGTAAGACATCACAACTGTTTAATAGTATTTGGTATAGTAATAGTACTTGGAATGATACTACAGGAA
AT---

AGTACTGGGAATATCACGCTCCCTTGCAGAATAAAACAAATTATAAACATGTGGCAGGAAGTAGGGAAAGCAA
TGTATGCCCCTCCCATTAGAGGACAAATTAGCTGTTTATCAAAT---
ATTACAGGGTTGCTATTAACAAGAGATGGTGGTAAGAATGAGAGC----------------------------
----

AATACCACCGAGACCTTTAGACCTGCAGGAGGAGATTTGAGAGACAATTGGAGAAGTGAATTATATAAATATA
AAGTAGTAAAAATTGAACCATTA---GGAGTAGCACCCACCAAGGCAAGGAGAAGAGTGGTG-----------
----------------------------CAAAGAGAAAAAAGAGCA---GTGGGAGCGCTAGGAGCT------
---ATGTTCCTTGGGTTCTTGGGAGCA---GCAGGAAGCACTATG---GGCGCAGCGTCA---
ATAACGCTGACGGTACAG---GCCAGACAA---TTGTTG---TCTGGTATAGTGCAACAGCAGAGA---
AATTTG---CTGAGG---GCTATTGAGGCGCAACAGCATCTGTTGCAACTCACAGTCTGGGGCATC
>KX692929
TTCTACTGTAATACATCACAACTGTTTAGTAGTACTTGGAATAATACTGGTACTCGGACTGGTACTACT----
----

GGAAATGACACTATCTCGCTCCCTTGCAGAATAAGACAAATTGTAAACATGTGGCAGGAAGTAGGGAAAGCAA
TGTATGCCCCTCCCATTGCAGGACAAATTAATTGTTCATCAAAT---
ATTACAGGGTTGCTATTAACAAGAGATGGTGGGGAGGGGAACGCGACCAGT----------------------
----

ACTAACGACACAGAGATCTTCAGACCTACAGGAGGAAATATGAGAGACAATTGGAGAAGTGAATTATATAAAT
ATAAAGTAGTAAAAATTGAACCATTA---GGAGTAGCACCCACCAGGGCAAAGAGAAGAGTGGTG--------
-------------------------------CAGAGAGAAAAAAGAGCA---GTGGGAACGATAGGAGCT---
------ATGTTCCTTGGGTTCTTGGGAGCA---GCAGGAAGCACTATG---GGCGCAGCGTCA---
ATAACGCTGACGGTACAG---GCCAGACAA---TTGTTG---TCTGGTATAGTGCAACAGCAGAGA---
AATTTG---CTGAGG---GCTATTGAGGCGCAACAGCATCTGTTGCAACTCACAGTCTGGGGCATC
>KX692941
TTCTACTGTAATACATCACAACTGTTTAATAGTAGGTGGGATGATGTGGATGAGAATGAGACTAGTACTAGTA
ATGGCACT---

ACAGGGAATGATACTATCACGCTCCCTTGCAGAATAAAACAAATTATAAACATGTGGCAGGAAGTAGGAAAAG
CAATGTATGCCCCTCCCATTGCAGGACTAATTAGATGCTCATCAAAT---
ATTACAGGGTTGCTATTAACAAGGATGGTGGTAATATTAATGCTACT-------------------------
----
GGGAACAATACCAACAAGAATAAT---------
AACACCGAGATCTTCAGACCTGCAGGGGGAAATATGAAAGACAATTGGAGAAGTGAATTATATAGATATAAAG
TAGTAAAAGTTGAACCATTA---GGAGTAGCACCCACCAGGGCACAGAGAAGAGTGGTG-------------
------------------------CAAAGAGAAAAAAGAGCA---GTGGGAATGATAGGAGCT---------
ATGTTCCTTGGGTTCTTGGGAACA---GCAGGAAGCACTATG---GGCGCAGCGTCA---
CTAACGCTGACGGTACAG---GCCAGACAA---TTGTTG---TCTGGTATAGTGCAACAGCAGAGA---
AATCTG---CTGAGG---GCTATTGAGGCGCAACAACATCTGTTGCAACTCACAGTCTGGGGCATC
>KX692973
TTCTACTGTAATACATCAAAACTATTTGATAGTACTTGGAATGCTACTAGTGATTGGAATGATACTGAG----
----

ACTACAAAAAATATCACGCTCCCTTGCAGAATAAAACAAATTGTAAACATGTGGCAGGAAGTAGGGAAAGCAA
TGTATGCCCCTCCCATTGAAGGACAAATTGGATGTTCATCAAAT---
ATTACAGGGTTGCTATTAGCAAGAGATGGTGGTAATAGTGAGAAC---------------------------
----

ACCACCGAGACCTTCAGACCTGCAGGAGGAAACATGAGAGACAATTGGAGAAGTGAATTATATAGATATAAAA
TAGTAAAAATTGAACCATTA---GGAGTAGCACCCACCAAGGCAAAGAGAAGAGTGGTG-------------
------------------------CAGAGAGAAAAAAGAGCA---GTGGGAACAATAGGAGCT---------
ATGTTCCTTGGGTTCTTGGGAGCA---GCAGGAAGCACTATG---GGCGCAGCGTCA---
ATAACGCTGACGGTACAG---GCCAGACAA---TTGTTG---TCTGGTATAGTGCAACAGCAAAGA---
AATTTG---CTGATG---GCTATTGAGGCGCAACAGCATCTGTTGCAACTCACAGTCTGGGGCATC
>KX692981
TTCTACTGTAATACATCACAACTGTTTAATAGTACTTGGTATAAAAACAGTACTTGGAATGGTGCTACA----
----

GGAAATGGCACTATCACGCTCCCTTGCAGAATAAAACAAATTGTAAACATGTGGCAGGAAGTAGGAAAAGCAA
TGTATGCCCCTCCCATTAGCGGACAAATTAGATGTTCATCAAAT---
ATTACAGGGTTGCTATTAACAAGAGATGGTGGTAATAATGGGAACAAT------------------------
----

ACCACTGAGACTGAGACCTTCAGACCTGGAGGAGGAGATATGAGAGACAATTGGAGAAGTGAATTATATAAAT
ATAAAGTAGTAAAAATTGAACCATTA---GGAGTAGCACCCACCAAGGCAAAGAGAAGAGTGGTG--------
------------------------------CAGAGAGAAAAAAGAGCA---GTGGGATTGGTAGGAGCT---
------ATGTTCCTTGGGTTCTTGGGAGCA---GCAGGAAGCACTATG---GGCGCAGCGTCA---
ATAACGCTGACGGTACAG---GCCAGACAA---TTGTTG---TCTGGTATAGTGCAACAGCAAAGA---
AATTTG---CTGAGG---GCTATTGAGGCGCAACAGCATCTGTTGCAACTCACAGTCTGGGGCATC
>KX693001
TTCTACTGTAATACATCACAACTGTTTAATAGTACTTGGAATAATAATGGCACTTGGAATGGTGCTACA----
----

GGAAACGACACTGTCACGCTCCCTTGCAGAATAAAACAAATTGTAAACATGTGGCAGGAAGTAGGGAAAGCAA
TGTATGCCCCTCCCATTAGAGGACTAATTAACTGTTCATCAAAT---
ATTACAGGGTTGCTATTAACAAGAGATGGTGGTACAAATGAGAGC---------------------------
----

AATACCACCGAGACCTTCAGACCTGGAGGAGGAGATATGAGAGACAATTGGAGAAGTGAATTATATAAATATA
AAGTAGTAAAAATTGAACCATTA---GGAGTAGCACCCACCAAGGCAAAGAGAAGAGTGGTG-----------
---------------------------CAGAGAGAAAAAAGAGCA---GTGGGAACGATAGGAGCT------
---ATGTTCCTTGGGTTCTTGGGAGCA---GCAGGAAGCACTATG---GGCGCAGCGTCA---
ATAACGCTGACGGTACAG---GCCAGACAA---TTGTTG---TCTGGTATAGTGCAACAGCAGAGA---
AATTTG---CTGAGA---GCTATTGAGGCGCAACAGCATATGTTGCAACTCACAGTCTGGGGCATC
>KX693029
TTCTACTGTAATACATCACAACTGTTTAACAGTACTTGGCCTTGGAATGATACTAGTACTGGGAATGATACTA
CA--

GGAAATAGCACTATCACGCTCCCTTGCAAAATAAGACAAATTGTAAACAGGTGGCAGGAAGTAGGGAAAGCAA
TGTATGCCCCTCCCATTGCAGGACCAATTAGCTGCTCATCAAAT---
ATTACAGGGTTGCTATTAACAAGAGATGGTGGTAATGGGAGCGCG----------------------------
----

GACAACGAGACCTTCAGACCTGGAGGAGGAGATATGAGAGACAATTGGAGAAGTGAATTATATAAATATAAAG
TAGTACAAATTGAACCATTA---GGAGTAGCACCCACCAAGGCAAAGAGAAGAGTGGTG--------------
-------------------------CAGAGAGAAAAAAGAGCA---GTGGGAACGATAGGAGCT---------
ATGTTCCTTGGGTTCTTGGGAGCA---GCAGGAAGCACTATG---GGCGCAGCGTCA---
ATAACGCTGACGGTACAG---GCCAGACAA---TTGTTG---TCTGGTATAGTGCAACAGCAGAGA---
AATTTG---CTGAGG---GCTATTGAGGCGCAACAGCATCTGTTGCAACTCACAGTCTGGGGCATC
>KX693036
TTCTACTGTAATACATCACAACTATTTAATAGTACTTGGAATAATAATAGTACTTGGAATGGTACCAGTTTT-
----

AAGAATGATACTATCACGCTCCCTTGTAGAATAAAACAAATTGTAAACATGTGGCAGGGAATAGGGAAAGCAA
TGTATGCCCCTCCCATTGCAGGACAAATTAACTGCTCATCAAAA---
ATTACAGGGTTGCTACTAACAAGAGATGGTGGTGGTAAGAATGAGAGC-------------------------
----

AATACCACCGAGACCTTCAGACCTGGAGGAGGAGATATGAAAGACAATTGGAGAAGTGAATTATATAAATATA
AAGTAGTAAAAATTGAACCATTA---GGATTAGCACCCACCAAGGCAAAGAGAAGAGTGGTG-----------
----------------------------CAGAGAGAAAAAAGAGCA---GTGGGAACGATAGGAGCT------
---ATGTTCCTTGGGTTCTTGGGAGCA---GCAGGAAGCACTATG---GGCGCAGCGTCA---
CTAACGCTGACGGTACAG---GACAGACAA---TTGTTG---TCTGGTATAGTGCAACAGCAAAGA---
AATTTG---CTGAGG---GCTATTGAGGCGCAACAGCATCTGTTGCAACTCACAGTCTGGGGCATC
>KX693575
TTCTACTGTAATACATCAAAACTGTTTAATAGTACTTGGAGTAATACTAGTGCTTGGAATGATACTACA----
----

GGAAATGACACTATCACACTCCCTTGCAGAATAAAACAAATTGTAAACATGTGGCAGGAAGTAGGGAAAGCAA
TGTATGCCCCTCCCATTAGAGGAAAAATTAGCTGTTCATCAAAC---
ATTACAGGGTTGCTATTAACAAGAGATGGTGGTAATAATGGGAGC----------------------------
----

AACACCAACGAGACCTTCAGACCTGCAGGAGGAGATATGAGAGACAATTGGAGAAGTGAATTATATAAATATA
AAATAGTAAAAATTGAACCATTA---GGAGTAGCACCCACCAAGGCAAAGAGAAGAGTGGTG-----------
----------------------------CAGAGAGAAAAAAGAGCA---GTGGGAACACTAGGAGCT------
---ATGTTCCTTGGGTTCTTGGGAGCA---GCAGGAAGCACTATG---GGCGCAGCGTCA---
ATAACGCTGACGGTACAG---GCCAGACAA---ATGTTG---TCTGGTATAGTGCAACAGCAAAGA---
AATTTG---CTGAGG---GCTATTGAGGCGCAACAGCATATGTTGCAACTCACAGTCTGGGGCATC
>KX693068
TTCTACTGTAATACATCAAAACTGTTTAATAGTACTTGGCCTAGTACTGGGAATGATACTCCTAGTACTGGGA
ATGATACT---

ACAGGAAATGGCACTATCACACTCCCTTGCAGAATAAAACAAATTGTAAACATGTGGCAGGAAGTAGGGAAAG
CAATGTATGCCCCTCCCATTAAAGGGCAAATTAGCTGTGTATCAAAT---
ATTACAGGGTTGCTATTAATGAGAGATGGTGGTAAT-------------------------------------
----

AATGAGAACGAGACCTTCAGACCTGGAGGAGGAGATATGAGAGACAATTGGAGAAGTGAATTATATAAATATA
AAGTAGTAAAAATTGAACCATTA---GGAGTAGCACCCACCAAGGCAAAGAGAAGAGTGGTG-----------
----------------------------CAGAGAGAAAAAAGAGCA---GTGGGA---ATAGGAGCT------
---ATGTTCCTTGGGTTCTTGGGAGCA---GCAGGAAGCACTATG---GGCGCAGCGTCA---
ATAACGCTGACGGTACAG---GCCAGGCAA---TTGTTG---TCTGGTATAGTGCAACAGCAGAGA---
AATTTG---CTGATG---GCTATTGAGGCGCAACAGCATCTGTTGCAACTCACAGTCTGGGGCATC
>KX693091
TTCTACTGTAATACATCACAATTGTTTAATAGTACCTGGAACAGTAATGGTACTCGGAATGGTACCACA----
----

GGAAATGACATTATCACGCTCCCTTGCAGAATAAAACAAATTGTAAACATGTGGCAGGAAGTAGGGAAAGCAA
TGTATGCCCCTCCCATTAGTGGACAAATTAGATGTTCATCAAAT---
ATTACAGGGTTGCTATTAACAAGAGATGGTGGTAAAAATGAGACC----------------------------
----

AACACCACCGAGACCTTCAGACCCGCAGGAGGAGATATGAGAGACAATTGGAGAAGTGAATTATATAAATATA
AAGTAGTAAAAATTGAACCATTA---GGAGTAGCACCCACCAAGGCAAAGAGAAGAGTGGTG-----------
----------------------------CAGAGAGAAAAAAGAGCA---GTGGGAACAATAGGAGCT------
---ATGTTCCTTGGGTTCTTGGGAGCA---GCAGGAAGCACTATG---GGCGCAGCGTCA---
ATAACGCTGACGGTACAG---GCCAGACAA---TTGTTG---TCTGGTATAGTGCAACAGCAGAGA---
AATCTG---CTGAGG---GCTATTGAGGCGCAACAGCATATATTGCAACTCACAGTCTGGGGCATT
>KX693093
TTCTACTGTAATACATCAAGACTGTTTAATGAGACTTGGGATAATATTACTACTGGGAATATTGGGAGTAACA
TTACA--

GAAGATGATATTATCACGCTCCCTTGCAGAATAAAACAAATTGTAAACAGGTGGCAGGAAGTAGGGAAAGCAA
TGTATGCCCCTCCCATTAGCGGACTAGTTAGATGTTCATCAAAT---
ATTACAGGGTTGCTATTAACAAGAGATGGTGGTACTAGTAAGGCCAATGACACC-------------------
----
AATACTGGGACCAATAAT---
AACACAGAAATTTTCAGACCTGGAGGAGGAGATATGAGAGACAATTGGAGAAGTGAATTATATAAATATAAAG
TAATAAAACTTGAACCATTA---GGAGTAGCACCCACCAAGGCAAAGAGAAGAGTGGTG--------------
-------------------------CAGAGAGAAAAAAGAGCA---GTGGGA---ATCGGAGCT---------
ATGTTCCTTGGGTTCTTGGGAGCA---GCAGGAAGCACCATG---GGCGCAGCGTCA---
ATAACGCTGACGGTACAG---GCCAGACAA---TTGTTG---TCTGGTATAGTGCAACAGCAGAGT---
AATTTG---CTGAGG---GCTATTGAGGCGCAACAACATCTGTTGCAACTCACAGTCTGGGGCATA
>KX693114
TTCTACTGTAACACATCAAAACTGTTTAATAGTACTTGGAGTAATACTAGTGCTTGGAATGATACTGCA----
----

GAAAATGACACTGTCACGCTCCCTTGCAGAATAAAACAAATTATAAACATGTGGCAGGGAGTAGGGAAAGCAA
TGTATGCCCCTCCCATTAGTGGACAAATTAGATGTTCATCAAAT---
ATTACAGGATTGCTATTAACAAGAGATGGTGGTAATGGTGGTAATAATGGGAGC-------------------
----

AATACCACCGAAACCTTCAGACCTGCAGGAGGAAATATGAAAGACAATTGGAGAAGTGAATTATATAAATATA
AAGTAGTACAAATTGAACCATTA---GGAGTAGCACCCACCAAGGCAAAGAGAAGAGTGGTG-----------
----------------------------CAAAAAGAAAAAAGAGCA---GTGGGAACAATAGGAGCT------
---ATGTTCCTTGGGTTCTTGGGAGCA---GCAGGAAGCACTATG---GGCGCAGCGTCA---
TTAACGCTGACGGTACAG---GCCAGACAA---CTGTTG---TCTGGTATAGTGCAACAGCAGAGA---
AATCTG---CTGAGG---GCTATTGAGGCGCAACAGCATCTGTTGCAACTCACAGTCTGGGGCATC
>KX693689
TTCTACTGTGATACATCACAACTGTTTAACAGTACTTGGGACAAGAATGGTACTTGGGAAGGTACTGTAGGAA
AT---

GGAAATGGCACTATCACTCTCCCCTGCAGGATAAAACAAATTGTAAACATGTGGCAGGAAGTAGGGAAAGCAA
TGTATGCCCCTCCCATTGCAGGACAAATTAAATGTTCATCAAAT---
ATTACAGGGTTACTATTAACAAGAGACGGTGGTAGTGGTAAAAAT----------------------------
----
GAGAGCAATACCACC---
GAGACCGAGACCTTCAGGCCTGCAGGAGGAGATATGAGAGACAATTGGAGAAGTGAATTATATAAATATAAAG
TAGTAAGAATTGAACCATTA---GGAGTAGCACCCACCAAGGCAAAGAGAAGAGTGGTG--------------
-------------------------CAAAGAGAAAAAAGAGCA---GTGGGAACACTAGGAGCT---------
ATGTTCCTTGGGTTCTTGGGAGCA---GCAGGAAGCACTATG---GGCGCAGCGTCA---
ATAACGCTGACGGTACAG---GCCAGACAG---TTATTG---TCTGGTATAGTGCAACAGCAGAGA---
AATTTG---CTGAGG---GCTATTGAGGCGCAACAGCATATGTTGCAACTCACAGTCTGGGGCATC
>KX693720
TTCTACTGTAATACATCACAACTGTTTAATAGTACTTGGAATGTTAATGGTAATTGGGATGGTACTACAGGAA
ATGATACT---

ACAGGAAATGGCACTATCATGCTCCCTTGCAGAATAAAACAAATTATAAACATGTGGCAGGAAGTAGGGAAAG
CAATGTATGCCCCTCCCATTAAAGGAAAGATTAAATGTTCATCAAAT---
ATTACAGGGTTGCTATTAACAAGAGATGGTGGTGAAAATGTGACC----------------------------
----

AACACCACCGAGACCTTCAGACCTGGAGGGGGAGATATGAGAGACAATTGGAGAAGTGAATTATATAAATACA
AAGTAGTAAAAATTGAACCATTA---GGAGTAGCACCCACTAAGGCAAAGAGAAGAGTGGTG-----------
----------------------------CAGAGAGAAAAAAGAGCA---GTGGGAACGATAGGAGCT------
---ATGTTCCTTGGGTTCTTGGGAGCA---GCAGGAAGCACTATG---GGCGCAGCGTCA---
ATAACGCTGACGGTACAG---GCCAGACAA---TTGTTG---TCTGGTATAGTGCAACAGCAGAGA---
AATTTG---CTGATG---GCTATTGAGGCGCAACAGCATCTGTTGCAACTCACAGTCTGGGGCATT
>KX693770
TTCTACTGTAATACATCAAAACTGTTTAGTAGTACTTGGTATAATAATGGTACTTGGACTAGTACTTGGAATG
AGACTATA---

GAAAATGACACTATCACACTCCCTTGCAGAATAAAACAAATTGTAAACATGTGGCAGGAAGTAGGGAGAGCAA
TGTATGCCCCTCCCATTGCAGGAAAAATTAGATGTTCATCAAAC---
ATTACAGGGTTGCTATTAACAAGAGATGGTGGTAATAATAATGAGACCAACGCC-------------------
----

ACCACCACCGAGGAGATCTTCAGACCTGCAGGAGGAGATATGAGAGACAATTGGAGAAGTGAATTATATAAAT
ATAAAATAGTAAAAATTGAACCATTA---GGAATAGCACCCACCAAGGCAAAGAGAAGAGTGGTG--------
-------------------------------CAGAGAGAAAAAAGAGCA---GTGGGAACGCTAGGAGCT---
------ATGTTCCTTGGGTTCTTGGGAGCA---GCAGGAAGCACTATG---GGCGCAGCGTCA---
ATAACGCTGACGGTACAG---GCCAGACAA---TTGTTG---TCTGGTATAGTGCAACAGCAAAGA---
AATTTG---CTGAGG---GCTATTGAGGCGCAACAGCATATGTTGCAACTCACAGTCTGGGGCATC
>KX693223
TTCTACTGTAATTCATCACAATTGTTTAATAGTACTTGGTATGTTAATGGTACTCGGATTGGTACTACA----
----

GGAAATGACACTATCACGCTCCCTTGCAGAATAAGACAAATTGTAAACATGTGGCAGGAAGTAGGGAAGGCAA
TGTATGCCCCTCCCATTGCAGGACCAATTAGATGTTTATCAAAT---
ATTACAGGGTTGCTATTAACAAGAGATGGTGGTAATAATACAGAT----------------------------
----

AATACCACCGAGACCTTCAGACCTGGAGGAGGAGATATGAGAGACAATTGGAGAAGTGAATTATATAAATATA
AAGTAGTAAAAATTGAGCCATTA---GGAGTAGCACCCACCAAGGCAAAGAGAAGAGTGGTG-----------
----------------------------CAGAGAGAAAAAAGAGCA---GTGGGAACAATAGGAGCT------
---ATGTTCCTTGGGTTCTTGGGAGCA---GCAGGAAGCACCATG---GGCGCAGCGTCA---
ATAACGCTGACGGTACAG---GCCAGACAA---TTGTTG---TCTGGTATAGTGCAACAGCAGAGA---
AATTTG---CTGAGG---GCTATTGAGGCGCAACAACATCTGTTGCAACTCACAGTCTGGGGCATC
>KX693238
TTCTATTGTAATACATCACAGCTGTTTAATAGTAATTGGAATGATAGTACTTTGAATGATAATAGTACTTGG-
----

AATGAAACTACTATCACGCTCCCTTGCAGAATAAAACAAATTGTAAACATGTGGCAGGAAGTAGGAAAAGCAA
TGTATGCCCCTCCCATTGCAGGACAAATTAACTGTTCATCAGAT---
ATTACAGGGTTGCTATTAACAAGAGATGGTGGTAAGAAGAATGAGAGCAAT----------------------
----

AATAACACTGAGACCTTCAGACCTGAAGGAGGAGATATGAGAGACAATTGGAGAAGTGAATTATACAAATATA
AAGTAGTAAAAATTGAACCATTA---GGAGTAGCACCCACCAAGGCAAAGAGAAGAGTGGTG-----------
----------------------------CAGAGAGAAAAAAGAGCA---GTGGGAACGATAGGAGCT------
---ATGTTCCTTGGGTTCTTGGGAGCA---GCAGGAAGCACTATG---GGCGCAGCGTCA---
ATAACGCTGACGGTACAG---GCCAGACAA---TTGTTG---TCTGGTATAGTGCAACAGCAGAGA---
AATTTG---CTGAGG---GCTATTGAGGCGCAACAGCATCTGTTGCAACTCACAGTCTGGGGCATC
>KX693247
TTCTACTGTAATACATCACAACTGTTTAATAGTACTTGGGATGATACTAATATTTGGAATGATACTAGTACTG
GGAATGGTACT--

ACAGAAAATAGCACTATCACGCTCCCTTGCAGAATAAAACAAATTGTAAACATGTGGCAGGAAGTAGGGAAAG
CAATGTATGCCCCTCCCATTGAAGGACGAATTAGATGTTCATCAAAT---
ATTACAGGGTTGCTACTAACAAGAGATGGTGGTAATGGGAGCGATACC-------------------------
----

ACCAATAACACCGAGACCTTCAGACCTGGAGGAGGAGATATGAGAGACAATTGGAGAAGTGAATTATATAAAT
ATAAAGTAGTAAAAATTGAACCATTA---GGAGTAGCACCCACCAAGGCAAAGAGAAGAGTGGTG--------
-------------------------------CAGAGAGAAAAAAGAGCA---GTGGGAACGATAGGAGCT---
------ATGTTCCTTGGGTTCTTGGGAGCA---GCAGGAAGCACTATG---GGCGCAGCGTCA---
ATAACGCTGACGGTACAG---GCCAGACAA---TTATTG---TCTGGTATAGTGCAACAGCAGAGA---
AATTTG---CTGAGG---GCTATTGAGGCGCAACAGCATCTGTTGCAACTCACAGTCTGGGGCATC
>KX693268
TTCTATTGTAATACATCACAACTGTTTAATAGTACTTGGTATACTAATAATACTTGGAATGGTACTACA----
----

GGAAATGATACTATCACGCTCCCTTGCAGAATAAAACAAATTGTAAACATGTGGCAGGAAGTAGGGAAAGCAA
TGTATGCCCCTCCTATTGCAGGAAAAATTAAATGTTCATCAAAT---
ATTACAGGGTTGCTATTAACAAGAGATGGTGGTATAAACAATACC----------------------------
----

AATACCACTGAGACCTTCAGACCTGGAGGAGGAGATATGAGAGACAATTGGAGAAGTGAATTATATAAATATA
AAGTAGTAAAAATTGAACCATTA---GGAATAGCACCCACCAAGGCAAAAAGAAGAGTGGTG-----------
----------------------------CAGAGAGAAAAAAGAGCA---GTAGGAGCACTAGGAGCT------
---ATGTTCCTTGGGTTCTTGGGAGCA---GCAGGAAGCACTATG---GGCGCAGCGTCA---
ATAACGCTGACGGTACAG---GCCAGACAA---TTGTTG---TCTGGTATAGTGCAGCAGCAGAGG---
AATCTG---CTGAGG---GCTATTGAGGCGCAACAGCATCTGTTGCAACTCACAGTCTGGGGCATA
>KX693886
TTCTACTGTAATACATCACAACTGTTTAATAGTACTTGGAACAATAGTATTTGGAATAATAATATTACTTGGA
GTGAT--
-------------------AAA---
ACAGGAAATGACACTATCTCGCTCCCTTGCAGAATAAAACAAATTATAAACACGTGGCAGAAAGTAGGAAAAG
CAATATATGCCCCTCCCATTGCAGGACAAATTAAATGTTCATCAAAT---
ATTACAGGGTTAATATTAACAAGAGATGGTGGTAATGAAAACAAGACC-------------------------
----

ACCGAGACCGAGACCTTCAGACCTGGAGGAGGAGAGATGAGAGACAATTGGAGAAGTGAATTATATAAATATA
AAGTAGTAAAAATTGAACCATTA---GGAGTAGCACCTACCAAGGCAAAGAGAAGAGTGGTG-----------
----------------------------CAAAGAGAGAAAAGAGCA---GTGGGA---ATAGGAGCT------
---ATGTTCCTTGGGTTCTTGGGAGCA---GCAGGAAGCACTATG---GGCGCAGCATCA---
ATAACGCTGACGGTACAG---GCCAGACAA---TTGTTG---TCTGGTATAGTGCAACAGCAGAGA---
AATTTG---CTGAGG---GCTATTGAGGCGCAACAACAACTGTTGCAACTCACAGTCTGGGGCATC
>KX693314
TTCTACTGTAATACAACACGGCTGTTTAATAGTACTTGGAATGAGACTAGTACTTGGAATGATACTGCA----
----

GAAAATGACAATATCACGCTCCCTTGCAGAATAAGACAAATTATAAACAGGTGGCAGGAAGTAGGGAAGGCAA
TATATGCCCCTCCCATTGCAGGACAAATTAGATGTTCATCAAAT---
ATTACAGGGTTGCTATTAACAAGAGATGGTGGTAATAATGAGAAC----------------------------
----

GAGACCACCGAGATCTTCAGACCTGCAGGAGGAGATATGAAAGACAATTGGAGAAGTGAATTGTATAAATATA
AAGTAGTAAAAATTGAACCATTA---GGAGTAGCACCCACCAAGGCAAAGAGAAGAGTGGTG-----------
----------------------------CAGAGAGAAAAAAGAGCA---GTGGGAACGATAGGAGCT------
---ATGTTCCTTGGGTTCTTGGGAGCA---GCAGGAAGCACTATG---GGCGCAGCGTCA---
ATAACGCTGACGGTACAG---GCCAGACAA---TTGTTG---TCTGGTATAGTGCAACAGCAAAGA---
AATTTG---CTGAGA---GCTATTGAGGCGCAACAGCATCTGTTGCAACTCACAGTCTGGGGCATC
>KX693326
TTCTACTGTAATACATCAAAACTGTTCAATAGTACTTGGTATGGGAATGGTAATAGTACTTTGAATGATACCA
CA---

GAAAATAGCACTATCACGCTCCCTTGCAGAATAAAACAAATTGTAAACATGTGGCAGGAGGTAGGGAAAGCAA
TGTATGCCCCTCCCATTAAAGGACCAATTAGTTGTTCATCAAAT---
ATTACAGGGCTGCTATTAACAAGAGATGGTGGTAAAAATAATGATACTGAGAGC-------------------
----

AATACCACCGAGATCTTCAGACCTGGAGGAGGAGATATGAGAGACAATTGGAGAAGTGAATTATATAAATATA
AAGTAGTAAAAATTGAACCATTA---GGAGTAGCACCCACCAAGGCAAAGAGAAGAGTGGTG-----------
----------------------------CAGAGAGAAAAAAGAGCA---GTGGGAACGATAGGAGCT------
---ATGTTCCTTGGGTTCTTGGGAGCA---GCAGGAAGCACTATG---GGCGCAGCGTCA---
ATAACGCTGACGGTACAG---GCCAGACAA---TTGTTG---TCTGGTATAGTGCAACAGCAGAGA---
AATTTG---CTGAGG---GCTATTGAGGCGCAACAGCAGATGTTGCAACTCACAGTCTGGGGCATC
>KX693339
TTCTACTGTAATACATCAAAACTGTTTACTAATATTACAGGAAAT----------------------------
----

AGCACTGACCCTATCATGCTCCCTTGCAGAATAAAACAAATTGTAAACATGTGGCAGGAAGTAGGGAGAGCAC
TGTATGCCCCTCCCATTGAAGGACCAATTAATTGTTCATCAAAG---
ATTACAGGGTTGCTATTAACAAGAGATGGTGGTAATAGTGAGAAC----------------------------
----

AAGACCGAGACCTTCAGACCTGGAGGAGGAAATATGAGAGACAATTGGAGAAGTGAATTATATAAATATAAAG
TAGTAAAAATTGAACCATTA---GGAGTAGCACCCACCAAGGCAAAGAGAAGAGTGGTG--------------
-------------------------CAGAGAGAAAAAAGAGCA---GTGGGAACGATAGGAGCT---------
ATGTTCCTTGGGTTCTTGGGAGCA---GCAGGAAGCACTATG---GGCGCAGCGTCA---
ATAACGCTGACGGTACAG---GCCAGACAA---TTGTTG---TCTGGTATAGTGCAACAGCAGAGA---
AATTTG---CTGAGG---GCTATTGAGGCGCAACAGCATCTGTTGCAACTCACAGTCTGGGGCATC
>KX693374
TTCTACTGTAATACATCACAACTGTTTGATAGTACTTGGAATGATAGTAGTACTTGGAATGATACTAGTACTT
GGAATGTTACT--

ACAAAAAATGACACTATCACGCTCCCTTGCAGAATAAAACAAATTGTAAACATGTGGCAGGAAGTAGGGAAAG
CAATGTATGCCCCTCCTATTAGAGGACTAATTAGTTGTTCATCAAAA---
ATTACAGGGTTGTTATTAACAAGAGATGGTGGTAATAAGAATGATAGCAAT----------------------
----

ACCACCGAGATCGAGATCTTCAGACCTGGAGGAGGAGATATGAGAGACAATTGGAGAAGTGAATTATATAAAT
ATAAAGTAGTAAAAATTGAACCATTA---GGAGTAGCACCCACCAGGGCAAAGAGAAGAGTGGTG--------
-------------------------------CAAAGAGAAAAAAGAGCA---GTGGGAACGATAGGAGCT---
------ATGTTCCTTGGATTCTTGGGAGCA---GCAGGAAGCACTATG---GGCGCAGCGTCA---
TTAACGCTGACGGTACAG---GCCAGACAA---TTGCTG---TCTGGTATAGTGCAACAGCAGAGA---
AATTTG---CTGATG---GCTATTGAGGCGCAACAGCATCTGTTGCAACTCACAGTCTGGGGCATC
>KX693390
TTCTACTGTGATACATCACAACTGTTTAATAGTACTTGGGATGAAAATAGTACTAAGAATGATAATAATACTT
GGAAA--
-------------GATACT------
ACAGGAAATGACACTATCACACTCCATTGCAGAATAAAACAAGTTATAAACATGTGGCAGGAAGTAGGGAGAG
CAATGTATGCCCCTCCCATTAAAGGACAAATTAGATGTTCATCAAAT---
ATTACAGGGTTGCTATTAACAAGAGATGGGGGTACGAATGTGAGC----------------------------
----

AATACCAACGAGACCTTCAGACCTGGGGGAGGAAATATGAAAGACAATTGGAGAAGTGAATTATATAAATATA
AAGTAGTAAAAATTGAACCATTA---GGAGTAGCACCCACCAAGGCAAAGAGAAGAGTGGTG-----------
----------------------------CAGAGAGAAAAAAGAGCA---GTGGGG---CTCGGAGCT------
---ATGTTCCTTGGGTTCTTGGGAGCA---GCAGGAAGCACTATG---GGCGCAGCGTCA---
ATAACGCTGACGGTACAG---GCCAGACAA---TTGTTG---TCTGGTATAGTGCAACAGCAGAGA---
AATTTG---CTGAGG---GCTATTGAGGCGCAACAGCGTATGTTGCAACTCACAGTCTGGGGCATC
>KX693437
TTCTACTGTAATACATCACAACTGTTTAACAGTACTTGGAATGATAATAGTACTTGGAAATGGAACAGGAAAA
ATACT--

ACAGATAATGACACTCTCACGCTCCCTTGCAGGATAAAACAAATTGTAAACATGTGGCAGGAAGTAGGGAAAG
CAATGTATGCCCCTCCCATTAGAGGACCAATTAAATGTAGATCAAAT---
ATTACAGGGCTGCTATTAACAAGAGATGGTGGCAATAATGGGACC----------------------------
----

AGTAACAACGAGACCTTCAGACCTGGAGGAGGAGATATGAGAGACAATTGGAGAAGTGAATTATATAAATATA
AAGTAGTACAAATTGAACCATTA---GGAGTAGCACCCACCAGGGCAAAGAGAAGAGTGGTG-----------
----------------------------CAGAGAGAAAAAAGAGCA---GTGGGAACGATAGGAGCT------
---ATGTTCCTTGGGTTCTTGGGAGCA---GCAGGAAGCACGATG---GGCGCAGCGTCA---
ATAACGCTGACGGTACAG---GCCAGACAA---TTGCTG---TCTGGTATAGTGCAACAGCAGAGA---
AATTTG---CTGAGG---GCTATTGAGGCGCAACAACATCTGTTGCAACTCACCGTCTGGGGCATC
>KX693455
TTCTACTGCAATACATCACAACTGTTTAATAGTACTTGGAATGATACTAGTACTTGGAATGAGACTACA----
----

GGAAATAGCACTATCACGCTCCCTTGCAGAATAAGACAATTTGTAAACATGTGGCAGGAAGTAGGAAAAGCAA
TGTATGCCCCTCCCATTAGAGGACAAATTAGATGTTCATCAAAT---
ATTACAGGGTTGCTATTAACAAGAGATGGTGGTATTAATGGGAGC----------------------------
----

GAGACCACCGAGACCTTCAGACCTGGAGGAGGAAATATGAGAGACAATTGGAGAAGTAAATTATATAAATATA
AAGTAGTAAAAATTGAACCATTA---GGAGTAGCACCCACCAAGGCAAAGAGAAGAGTGGTG-----------
----------------------------CAGAGAGAAAAAAGAGCA---GTGGGAACGATAGGAGCT------
---ATGTTCCTTGGGTTCTTGGGAGCA---GCAGGAAGCACTATG---GGCGCAGCATCA---
ATAACGCTGACGGTACAG---GCCAGACAA---TTGTTG---TCTGGTATAGTGCAACAGCAGAGA---
AATTTG---CTGAGA---GCTATTGAGGCGCAACAGCATCTGTTGCAGCTCACAGTCTGGGGCATC
>KX693469
TTCTACTGTGATACATCAAAACTGTTTAGTAGTACTTGGACTAATACTAGTACTTGGAATAAGACTGAGACTT
GGAATGTTTCT--

GCAGAACATGACACTATCACGCTCCATTGCAGAATAAAACAAATTGTAAACATGTGGCAGGAAGTAGGGAAAG
CAATGTATGCCCCTCCCATTGCAGGACAAATTAACTGTTCATCAAAT---
ATTACAGGGTTGCTATTAACAAGAGATGGTGGTAATAATACCAAT----------------------------
----

AACACCGAGACCTTCAGACCTGGAGGAGGAAATATGAGAGACAATTGGAGAAGTGAATTATATAAATATAAAG
TAGTAAAAATTGAACCATTA---GGAGTAGCACCCACCAAGGCAAAGAGAAGAGTGGTG--------------
-------------------------CAGAGAGAAAAAAGAGCA---GTGGGAACGCTAGGAGCT---------
ATGTTCCTTGGGTTCTTGGGAGCA---GCAGGAAGCACTATG---GGCGCAGCGTCA---
CTAACGCTGACGGTACAG---GCCAGACAA---TTGCTG---TCTGGTATAGTGCAACAGCAGAGA---
AATTTG---CTGAGG---GCTATTGAGGCGCAACAGCGTATGTTGCAACTCACAGTCTGGGGCATC
>KX693475
TTCTATTGTAATACATCAAAGCTGTTTGATAGTACTTGGAATAATACTAGTACTGAGAATAAT----------
----

AATGATACTAATATCACGCTCCCTTGCAGAATAAGACAAATTATAAACAGGTGGCAGGAAGTAGGGAAAGCAA
TGTATGCCCCTCCCATTGCAGGACAAATTAGATGTTCATCAAAT---
ATCACAGGGTTAATATTAACAAGAGATGGTGGTAATAATGGGACC----------------------------
----

AACAACACTGAGATCTTCAGACCTGCAGGAGGAGATATGAGGGACAATTGGAGAAGTGAATTATATAAATATA
AAGTAGTAAAAATTGAACCATTA---GGAGTAGCACCCACCAAGGCAAAGAGAAGAGTGGTG-----------
----------------------------CAGAGAGAAAAAAGAGCA---GTGGGA---TTAGGAGCT------
---ATGTTCCTTGGGTTCTTGGGAGCA---GCAGGAAGCACTATG---GGCGCAGCGTCA---
ATAACGCTGACGGTACAG---GCCAGACAA---TTGTTG---TCTGGTATGGTGCAACAGCAGAGA---
AATTTG---CTGAGG---GCTATTGAGGCGCAACAACATCTGTTGCAACTCACAGTCTGGGGCATC
>KX693489
TTCTACTGTAATACATCACAATTGTTTAATAGTACTTGGACTTGGAATGATACTAGTGCTTGGAATAATAGTA
CA---

GAAAATGACACTATCACGCTCCCTTGCAGAATAAAACAAATTGTAAACATGTGGCAGGAAGTAGGGAAAGCAA
TGTATGCCCCTCCTATTGCAGGACAAATTAGATGTTTATCAAAT---
ATTACAGGGTTGCTATTAACAAGAGATGGTGGTAAGAATGAGAGC----------------------------
----

AACACCACCGAGACCTTCAGACCTGCAGGAGGAGATATGAGAGACAATTGGAGGAGTGAATTATATAAATATA
AAGTAGTAGAAATTGAACCATTA---GGAGTAGCACCCACCAAGGCAAAGAGAAGAGTGGTG-----------
----------------------------CAGAGAGAAAAAAGAGCA---GTGGGAACGATAGGAGCT------
---ATGTTCCTTGGGTTCTTGGGAGCA---GCAGGAAGCACTATG---GGCGCAGCGTCA---
ATAACGCTGACGGCACAG---GCCAGACAA---TTGTTG---TCTGGTATAGTGCAACAGCAGAGA---
AATTTG---CTGAGG---GCTATTGAGGCGCAACAGCATCTGTTGCAACTCACAGTCTGGGGCATC
>KX693491
TTCTACTGTGATACATCACAACTGTTTAACAGTACTTGGCCTTGGAATGATAATAGTACTTGGAATAATTCTA
CA---

GGAAATGGCACTATCACGCTCCCTTGCAGAATAAAACAAATTATAAACATGTGGCAGGAAGTAGGGAAAGCAA
TGTATGCCCCTCCCATTGCAGGGCAAATTAACTGTTCATCAAAA---
ATTACAGGGTTGCTATTAACAAGAGATGGTGGTATTGATAATACTACGAAC----------------------
----
GATACCACCAAGAAC---
AACACCGAGACCTTCAGACCTGCAGGAGGAGATATGAGGGACAATTGGAGAAGTGAATTATATAAATATAAAG
TAGTAAAAATTGAACCATTA---GGAGTAGCACCCACCAAGGCAAAGAGAAGAGTGGTG--------------
-------------------------CAGAGAGAAAAAAGAGCA---GTAGGAACGATAGGAGCT---------
ATGTTCCTTGGGTTCTTGGGAGCA---GCAGGAAGCACTATG---GGCGCAGCGTCA---
ATAACGCTGACGGTACAG---GCCAGACAA---TTATTG---TCTGGTATAGTGCAACAGCAGAGA---
AATTTG---CTGAGG---GCTATTGAGGCGCAACAGCATATATTGCAACTCACAGTCTGGGGCATC
>KX693496
TTCTACTGTAATACATCACAATTATTTAATAGTACTTGGAATTGGAATGATACTATTCCTTGGAATGATACTA
CA---

GGAAATGACACTATCGTGCTCCCTTGCAAAATAAAACAAATTGTAAACATGTGGCAAGAGGTAGGGAAAGCAA
TGTATGCCCCTCCCATTAGTGGACAAATTAGATGTTCCTCAAAT---
ATTACAGGGTTACTATTAACAAGAGATGGTGGTATTAATGAGAGC----------------------------
----

AACACCGCCGAGACCTTCAGACCTGGAGGAGGAGATATGAGAGACAATTGGAGAAGTGAATTATATAAATATA
AAGTAGTAAAAATTGAACCATTA---GGAGTAGCACCCACCAAGGCAAAGAGAAGAGTGGTG-----------
----------------------------CAGAGAGAAAAAAGA---------------CTAGGAGCT------
---ATGTTCCTTGGGTTCTTGGGAGCA---GCAGGAAGCACTATG---GGCGCAGCGTCA---
ATAACGCTGACGGTACAG---GCCAGACAA---TTATTG---TCTGGTATAGTGCAACAGCAGAGA---
AATTTG---CTGAGG---GCTATTGAGGCGCAACAACATCTGTTGCAACTCACAGTCTGGGGCATC
>KX693539
TTCTACTGTGATACATCACGACTGTTCAATAGTACTTGGACTAATACTGGTACTTGGAATGCTACTACAGGAA
ATAATGAT---

ACTACAGGAAATATTATCAGACTCCCTTGTAGGATAAAACAAATTGTAAACATGTGGCAGGAAGTAGGGAGGG
CAATGTATGCCCCTCCCATTGAAGGACAAATTAGATGTTCATCTAAT---
ATTACAGGGTTGCTATTAACAAGAGATGGTGGTGAGAATGAGAGC----------------------------
----

AATACCACCGAAACCTTCAGACCTGAAGGAGGAGATATGAGGGACAATTGGAGAAGTGAATTATATAAATATA
AAGTAGTAAAAATTGAACCATTA---GGAGTAGCACCCACCAAGGCAAAGAGAAGAGTGGTG-----------
----------------------------CAGAGAGAAAAAAGAGCA---GTGGGAACGATAGGAGCT------
---ATGTTCCTTGGGTTCTTGGGAGCA---GCAGGAAGCACTATG---GGCGCAGCGTCA---
ATAACGCTGACGGTACAG---GCCAGACAA---TTGTTG---TCTGGTATAGTGCAACAGCAGAGA---
AATTTG---CTGAGG---GCTATTGAGGCGCAACAGCATCTGTTGCAACTCACAGTCTGGGGCATC
>KP109512
TTCTACTGTAATACAACACAACTGTTTAATAGTACTTGGAATGGTACTATGTTTAATGGTAGTAGTACTTGGA
ATAATACT---
----------GGGAATAGT------

ACTGAAAATGACACTATCACCCTCCCATGCAGAATAAAACAAATTATAAACATGTGGCAGGAAGTAGGAAAAG
CAATGTATGCCCCTCCCATCAGTGGACTAATCAGATGTTCATCAAAT---
ATTACAGGACTGATATTAACAAGAGATGGGGGGGTGGAACAGAACAGCACG---------------------
----

AACGACACCGAGACCTTCAGGCCTGGAGGAGGAGATATGAGGGACAATTGGAGAAGTGAATTATATAAATACA
AAGTAGTGAAACTTGAACCATTA---GGAGTAGCACCCACTAGGGCAAAAAGAAGAGTGGTG----------
---------------------------CAGAGAGAAAAAAGAGCA---TTGGGA---CTAGGAGCT------
---ATGTTCCTTGGGTTCTTGGGAACA---GCCGGAAGCACTATG---GGCGCAGCGTCA---
ATAACGCTGACGGTACAG---GCCAGACAA---TTATTG---TCTGGTATAGTGCAACAGCAGAAC---
AATTTG---CTGAGG---GCTATTGAGGCGCAACAGCATCTGTTGCAACTCACAGTCTGGGGCATC
>JF932495
TTCTACTGTAATACATCAAAACTGTTTAATAGTACTTGGAATGAGACTAGTGCTTGGAATGATACTGCA----
----

GAAAATGACACTATCACGCTCCCTTGCAGAATAAAACAAATTGTAAACATGTGGCAGGAAGTAGGAAGAGCAA
TGTATGCCCCTCCCATCAGAGGACAAATTAGATGTTCATCAAAC---
ATTACAGGGTTGCTATTAACAAGAGATGGTGGTAAGAATGAGAGC---------------------------
----

AATACCACCGAGACCTTCAGACCTGGAGGAGGAGATATGAAGGACAATTGGAGAAGTGAATTATATAAATATA
AAGTAGTAAAAATTGAACCATTA---GGAGTAGCACCCACCAAGGCAAAGAGAAGAGTGGTG----------
---------------------------CAGAGAGAAAAAAGAGCG---GTGGGAGCGATAGGAGCT------
---ATGTTCCTTGGGTTCTTGGGAACG---GCAGGAAGCGCTATG---GGCGCAGCGTCA---
CTGACGCTGACGGTACAG---GCCAGACAA---TTGTTG---TCTGGTATAGTGCAACAGCAAAGA---
AATTTG---CTGAGG---GCTATTGAGGCGCAACAGCATCTGTTGCAACTCACCGTCTGGGGCATC
>JF932496
TTCTACTGTAATACAACACAATTGTTTAATAGTACTTGGACTTGGAATGATATTAGTACTTGGAATAATACTA
CA--

GGAAATAGCACTATCACGCTTCCTTGCAGAATAAAACAAATTATAAACATGTGGCAGGAAGTAGGAACAGCAA
TGTATGCCCCTCCCATCGAAGGAAAAATTAGATGTTCATCAAAT---
ATTACAGGGTTACTATTAACAAGAGATGGTGGTAATAAGAACGAGACC------------------------
----

CCCGACACCGAGACCTTCAGACCTACAGGAGGAAATATGAGGGACAATTGGAGAAGTGAATTATATAAATATA
AAGTAGTAAAAATTGAACCATTA---GGAGTAGCACCCACCAGGGCAAAGAGAAGAGTGGTG----------
---------------------------CAAAGAGAAAAAAGAGCA---GTGGGAACGATAGGAGCT------
---ATGTTCCTTGGGTTCTTGGGAGCA---GCAGGAAGCACTATG---GGCGCAGCGTCA---
ATAACGCTGACGGTACAG---GCCAGACAA---TTGCTG---TCTGGTATAGTGCAACAGCAGAGA---
AATTTG---CTGAGA---GCTATTGAGGCGCAACAGCATATGTTGCAACTCACCGTCTGGGGCATC
>GU647196
TTCTACTGTAATACAGCACAACTGTTTAATAGTACTTGGAATAATACTAGTACTTGGAATGGTACTACA----
----

GGAAATAGCATTATTACGCTCCCTTGCAGGATAAGACAAATTGTAAACATGTGGCAGAAAGTAGGGAAAGCAA
TGTATGCCCCTCCCATTGAAGGACCAATTAAATGCTCATCAAAT---
ATTACAGGGTTGCTATTAACAAGAGATGGTGGACATAATGAGAGC---------------------------
----

AATACCACTGAGACCTTCAGACCTGGAGGAGGAGATATGAGAGACAATTGGAGGAGTGAATTATATAAATATA
AAGTAGTAAAAATTGAACCATTA---GGAGTAGCACCCACCAAGGCAAAGAGAAGAGTGGTG----------
---------------------------CAGAGAGAAAAAAGAGCA---GTGGGAATGATAGGAGCT------
---ATGTTCCTTGGGTTCTTGGGAGCA---GCAGGAAGCACTATG---GGCGCAGCGTCA---
ATAACGCTGACGGTACAG---GCCAGACAG---TTGTTG---TCTGGTATAGTGCAACAGCAGAGA---
AATTTG---CTGAGG---GCTATTGAGGCGCAACAGCATCTGTTGCAACTCACAGTCTGGGGCATC
>EU363830
TTCTACTGTAATACATCACAATTGTTTAACAGTACTTGGACTCCTAATAGTACTTGGAATGGTACTGGTACT-
----

TGGAATGATACTATCACACTCCCTTGCAGGATAAAACAAATTGTAAACATGTGGCAGGAAGTAGGGAAAGCAA
TGTATGCCCCTCCCATTGAAGGACCAATTAGATGTTCATCAAAT---
ATTACAGGGTTATTATTAACAAGAGATGGTGGTAAAAATGAGAACAACACT----------------------
----

GAGAACAACACCGAGACCTTCAGACCTGCAGGAGGAGATATGAGAGACAATTGGAGAAGTGAATTATATAAAT
ATAAAGTAGTAAAAATTGAACCATTA---GGAGTAGCACCCACCAGGGCAAAGAGAAGAGTGGTG--------
-------------------------------CAGAGAGAAAAAAGAGCA---GTGGGAACGATAGGAGCT---
------ATGTTCCTTGGGTTCTTGGGAGCA---GCAGGAAGCACTATG---GGCGCAGCATCA---
ATAACGCTGACGGTACAG---GCCAGACAA---TTATTG---TCTGGTATAGTGCAACAGCAGAGA---
AATTTG---CTGAGG---GCTATTGAGGCGCAACAGCATCTGTTGCAACTCACAGTCTGGGGCATC
>AY561236
TTCTACTGTAATACAACACAACTGTTTAATAGTACTTGGAATGAGAATAGTACTTGGAATGGGACTATTACTG
AAAAT--

TCAAATAACACTAATATCACACTCCCATGTAAAATAAAGCAAATTATAAATATGTGGCAGAGAGTAGGAAAAG
CAATGTATGCCCCTCCCATCAGAGGAGTAATAAGATGTGTATCAAAC---
ATCACAGGGCTACTATTAACAAGAGATGGTGGGAACAACGATACT----------------------------
----

AACAACAATGAGACCTTCAGACCTGGAGGAGGAAACATGAGGGATAATTGGAGAAGTGAATTATATAAATATA
AAGTAGTAAAAATTGAACCATTA---GGAGTAGCACCCACCAAGGCAAAGAGAAGAGTGGTG-----------
----------------------------CAGAGAGAAAAAAGAGCA---GTAACA---CTAGGAGCT------
---ATGTTCCTTGGGTTCTTGGGAGCA---GCAGGAAGCACTATG---GGCGCAGCGTCA---
ATAACGCTGACGGTACAG---GCCAGACAA---TTATTG---TCTGGTATAGTGCAACAGCAGAAC---
AATCTG---CTGAGG---GCTATTGAGGCGCAACAACATCTGTTGCAACTCACAGTCTGGGGCATT
>AY561239
TTCTACTGTAATACAGCACAACTGTTTAATAGTACTTGGAATGCTACTGATACTTGGAATACTACTACAGGGT
TAAAT--

GATACTGGTAAAAATATCACACTCCCATGCAGAATAAAACAAATTATAAACATGTGGCAGACAGTAGGAAAAG
CAATGTATGCCCCTCCCATCAGAGGACAAATTAGGTGTTCATCAAAT---
ATCACAGGGTTGTTATTAACAAGAGATGGAGGTAATAACAGCATA----------------------------
----

AACACAGAGATCTTCAGACCTGGAGGAGGAGATATGAGGGACAATTGGAGAAGTGAATTATATAAATATAAAG
TAGTAAAAATTGAACCATTA---GGAGTAGCACCCACCAAGGCAAAGAGAAGAGTGGTG--------------
-------------------------CAGAGGGAAAAAAGAGCA---GTAACA---CTAGGAGCT---------
ATGTTCCTTGGGTTCTTGGGAGCA---GCAGGAAGCACTATG---GGCGCAGCATCA---
ATAACGCTGACGGCACAG---GCCAGACAA---TTATTG---TCTGGTATAGTGCAACAGCAGAAC---
AATCTG---CTGAGG---GCTATTGAGGCGCAACAGCATATGTTGCAGCTCACAGTCTGGGGCATC
>HQ377377
TTCTACTGTAATACAACAAAATTGTTTAATAGTACTTGGTATAGTAATGGAACGCGACTTGCAAATGACAGTG
AT---

ACAAGTGAAAACATCACACTCCCATGCAGAATAAAACAAATTGTAAACATGTGGCAGGAAGTAGGAAAAGCAA
TGTATGCCCCTCCTATCAGCGGACAAATTAGATGTGTATCAAAT---
ATAACAGGGCTACTATTAACAAGGGATGGTGGGAAAGGAATGGGAACGGA-----------------------
----

ACGAACAATAACACCGAGACCTTCAGACCTGAAGGAGGAAATATGAAGGATAATTGGAGAAGTGAATTATACA
AATATAAAGTAGTAAAAGTTGAGCCATTA---GGAGTAGCACCCACCAAGGCAAAGAGAAGAGTGGTG-----
----------------------------------CAGAGAGAAAAAAGAACA---GTGGGA---
CTGGGAGCT---------TTGTTCCTTGGGTTCTTGGGAGCA---GCAGGAAGCACTATG---
GGCGCAGCGTCA---GTGACGCTGACGGTACAA---GCCAGACAA---TTATTG---
TCTGGTATAGTGCAACAGCAGAAC---AACTTG---CTGATG---
GCTATTGAGGCGCAACAAAATATGTTGCGACTCACAGTCTGGGGCATC
>AY586542
TTCTACTGTAATACAACAAAACTGTTTAATAGTACTTGGGATGTTAATAATAATATTAGCGGGCAAAATAAC-
----

ACTGAAGCTAATATTACACTCCCATGTAGAATAAAACAAATTGTAAACCTGTGGCAGGAAGTAGGAAAAGCAA
TGTATGCCCCTCCCATCAAAGGACAAATTAGCTGTTCATCAAAT---
ATTACAGGGCTGCTATTAACAAGAGATGGTGGTAATAACGGGACC----------------------------
----

AATAACACCGAGACCTTCAGACCTGGAGGAGGAGATATGAGGGACAATTGGAGAAGTGAATTATATAAATATA
AAGTAGTAAAAATTGAACCATTA---GGAGTAGCACCCACCAAGGCAAAGAGAAGAGTGGTG-----------
----------------------------CAAAGAGAAAAAAGAGCA---GTGGGAATGGTAGGAGCT------
---ATGTTCCTTGGGTTCTTGGGAGCA---GCAGGAAGCACTATG---GGCGCAGCGGCA---
ATGACGCTGACGGTACAG---ACCAGACAA---TTATTG---TCTGGTATAGTGCAGCAGCAAAAC---
AATTTG---CTGAGG---GCTATTGAGGCGCAACAACATCTGTTGCAACTCACAGTCTGGGGCATC
>EU668963
TTCTACTGTAATACAACAAGGCTGTTTAATAGTACTTGGAATGGGAATAATACTTGGAATAATACTGATGACG
CTGCTACT---
-------------------ATC---
ACTGAGAATGCGACTATCACACTCCCATGTAGAATAAAACAAATTATAAACAGGTGGCAGGAAGTAGGAAAAG
CAATGTATGCCCCTCCCATCCGAGGACATATTAGCTGTTTATCAAAC---
ATTACAGGGCTGATACTAACAAGAGATGGTGGTAAGGGCAATGACACC-------------------------
----

AACAACAACACTGAGGTCTTCAGACCTGGAGGAGGAGATATGAGGGACAATTGGAGAAGTGAATTATATAAAT
ATAAAGTAGTAAGAATTGAGCCATTA---GGAATAGCACCCACCAAGGCAAGGAGAAGAGTGGTG--------
-------------------------------CAGAGAGAAAAAAGAGCA---GTAACA---ATAGGAGCG---
------ATGTTCCTTGGGTTCTTGGGAGCA---GCAGGAAGCACTATG---GGCGCAGCGTCA---
ATAACGCTGACGGTACAG---GCCAGACTA---TTATTG---TCTGGTATAGTGCAGCAGCAGAGC---
AATATG---CTGAGA---GCTATTGAGGCGCAACAACACCTGTTGCAACTCACAGTTTGGGGCATC
>FJ388890
TTCTACTGTAATACAACAGCACTGTTTAATAGTACTTGGAATAATACTGGATGGTCAGCTGACACAAATGGCA
CTAAT--

GGCACTGAAGGAAATATCACACTCAAATGCAGAATAAAACAATTTATAAACCTGTGGCAGCAAGTAGAAAAAG
CAATGTACGCCCCTCCCATCGAAGGACTAATTAGATGCTCATCAAAC---
ATTACCGGGATACTATTAACAAGAGATGGGGGCAATAATAATGGGACC-------------------------
----

AACACGACAGAGGTATTCAGACCTGGAGGAGGAAATATGAAAGATAATTGGAGGAGTGAATTATATAAATATA
AAGTAGTACAAATTGAACCCTTA---GGAATAGCACCCAACAAGGCACAGAGAAGAGTGGTG-----------
----------------------------CAGAGAGAAAAAAGAGCA---GTAGGA---ATAGGAGCT------
---GTGTTCCTTGGGTTCTTGGGAGCA---GCAGGAAGCACTATG---GGCGCAGCATCA---
GTGATGCTGACGGTACAA---GCCAGACAA---TTATTG---TCTGGTATAGTGCAACAGCAAAAC---
AATTTG---CTGAGA---GATAGGGAGGCGCAACAACATCTGTTGCAACTCCCTGTCTGGGGCATC
>FJ388895
TTCTACTGTAACACAACACCACTGTTCAATAGTACTTGGAATCCTAATGGTATTTGGGAGGGTACTACAGGGT
CAAATGAC---
-------------------ACT---
GGGTCAAATGACACTGTCACACTCCAATGCAGAATAAAACAAATTGTAAACCTGTGGCAGGAAGTAGGAAAAG
CAATGTATGCCCCTCCCATCGAAGGACCAATTAAATGTTCATCAAAT---
ATTACAGGGATGCTATTAACAAGAGATGGTGGTAATAGTACTAAT----------------------------
----

AATGAGACCTTCAGACCTGGAGGAGGAGATATGAGGGACAATTGGAGAAGTGAACTATATAAATATAAAGTAG
TAAAAATTGAACCATTA---GGAATAGCACCCACCAAGGCAAAGAGAAGAGTGGTG-----------------
----------------------CAGAGAGACAAAAGAGCA---GTGGGAATAATGGGAGCT---------
ATGTTCCTTGGGTTCTTGGGAACA---GCAGGAAGCACTATG---GGCGCAGCGGCA---
CTGACGCTGACGGTACAG---GCCAGACAA---TTATTG---TCTGGTATAGTGCAACAGCAGAAT---
AATTTG---CTGAGG---GCTATTGAGGCGCAGCAGCATATGTTGCGACTCACTTTTTTTGGCATC
>FJ388898
TTCTACTGTAATTCAACACCACTGTTTAATAATACCTGGAATGGTACCGAATTGACAAATAATAATAATAATG
AAGAAAAT---

-------------ATCACA------
AATAATGAAGAAAATATCACACTCCAATGCAGAATAAAACAAATTATAAACAGGTGGCAGGAAGTAGGAAAAG
CAATGTATGCCCCTCCCATTGCAGGACAAATTAGATGCTCATCAAAC---
ATTACAGGGCTGCTATTGACAAGAGATGGTGGTAATGATAGTAAC----------------------------
----

ACAACCGAAATCTTCAGACCTGGAGGAGGAGATATGAGGGACAATTGGAGAAGTGAATTATATAAATATAAAG
TAGTAAAGCTTGAACCATTA---GGAATAGCACCCACCAAGGCAAAGAGAAGAGTGGTG--------------
-------------------------CAAAGAGAAAAAAGAGCA---GTGGGA---ATAGGAGCT---------
ATGTTCCTTGGGTTCTTGGGAGCA---GCAGGAAGCACTATG---GGCGCAGCGTCA---
ATAACGCTGACGGTACAG---GCCAGACAA---TTATTG---TCTGGTATAGTGCAACAGCAGAGC---
AATCTG---CTGAGG---GCTATTGAGGCGCAACAGCATATGTTGCAACTCACAGTTTGGGGCATC
>FJ388905
TTCTACTGTAATACAACATCACTGTTTAATAGTACTTTGAAACCAAAAAATAGCACTATCCAAAGATATAGAG
ATAAT--

AGAGATAATGACACTATTGTACTCCAATGCAGAATAAAACAATTTATAAACATGTGGCAGGAAGTAGGAAAGG
CAATGTATGCCCCTCCCATCAGAGGACAGCTTAACTGCTCATCAAAT---
ATTACAGGGCTGCTATTAACAAGAGATGGTGATGTTAATATCACCGAG-------------------------
----

AACGGGACTGAGACCTTCAGACCTATAGGAGGAGATATGAGGGACAATTGGAGAAGTGAATTATATAAATATA
AAGTAATAAAACTTGAACCATTA---GGAGTAGCACCCACCAAGGCACAGAGAAGAGTGGTG-----------
----------------------------CAGAGAGAAAAAAGAGCA---GCGATA------GGAGCT------
---ATGATCCTTGGGTTCTTGGGAGCA---GCAGGAAGCACTATG---GGCGCAGCGTCA---
GTGACGCTGACGGTACAG---GCCAGACTA---TTATTG---TCTGGTATAGTGCAGCAGCAGAGC---
AATCTG---CTGAGG---GCTATTGAGGCGCAACAACATCTGTTGCAACTCACAGTCTGGGGCATC
>FJ388910
TTCTACTGTAATACAACACAACTGTTTAATAGTACCTGGAATGCTGATGGTACTCGGAACGATACTGAAGGGT
CAGATAAC---
-------------------AAC---
AACAATACAGAAACAATCACACTCCCATGCAGAATAAAGCAAGTCATAAACCTGTGGCAGGAAGTAGGAAAAG
CAATGTATGCCCCTCCCATCAGAGGACAAATTAACTGTTCCTCAAGC---
ATTACAGGGCTACTATTAACAAGAGACGGTGGTGACAACTCCACT----------------------------
----

AGCAGCCCTGAGATCTTCAGACCTGGAGGAGGAGATATGAGGGACAATTGGAGAAGTGAATTATATAAATATA
AAGTAGTAAGAGTTGAACCATTA---GGAGTAGCACCCACCAAGGCAAAGAGAAGAGTGGTG-----------
----------------------------CAGAGAGAAAAAAGAGCA---GTGACA---CTAGGAGCT------
---GTGTTCCTTGGGTTCTTGGGAGCA---GCAGGAAGCACTATG---GGCGCAGCGTCA---
TTGACGCTGACGGTACAG---GCCAGACAA---TTATTG---TCTGGTATAGTGCAACAGCAAAAC---
AATTTG---CTGAGG---GCTATTGAGGCGCAACAGCACCTGTTGCAACTCACAGTCTGGGGCATC
>FJ388911
TTCTACTGTAATACAACACAGCTGTTTAATAGTACTTGGTGGGCTAATAGTACTGGGCCTGGTAATGGAAATG
GAAATATC---
-------------------ACA---
ATCACATCTGAAAGCATCATAACTCCATGCAGAATAAAACAACTTATAATCAGAAGACCGCAAGAAGTAAGAA
CAACATCTGCCCCTCCTATAGCAGCAGAAAATATTTGTTGTTCAAAA---
ATTACAAGAGTGCTATTAACAACAGAAGGTGGTGATAATACAAAC----------------------------
----

GGGAGTGAGACCTTCAGACCTGGAGGAGGAAATATGAGGGACAATTGGAGAAGTGAATTATATAAATATAAAG
TAGTAAGAATTGAACCATTA---GGAGTAGCACCCACCAGGGCAAAGAGAAGAGTGGTG--------------
-------------------------CAAAGAGAAAAAAGAGCA---GTGGGAACAATAGGAGCT---------
ATGTTCCTTGGGTTCTTGGGAGCA---GCAGGAAGCACTATG---GGCGCAGCGTCA---
ATAACGCTGACGGTACAG---GCCAGACAA---TTATTG---TCTGGTATAGTGCAGCAGCAGAAC---
AATTTG---CTGAGA---GCTATTGAGGCGCAACAGCATCTGTTGCAACTCACAGTCTGGGGCATC
>FJ388912
TTCTACTGTAATTCAACACAACTGTTTAATAGTACTTGGAATGTTAATAGTACTTGGAATGATACCGAAAGGT
CAAATATC---

-------------------ACA---
GAAGAAAATTTCAATATCACATTCCCATTCAGAATAATTCTAATTATAAGCAGGGGACTGGAAATAGCAAAAT
CAGCCCCTCCCCCCACCGGCATAAGTAAAATTTCATGTTCTTCAACG---
AGTACGGGGTTGACAAGAGCAAGAGGTGAAGGTAATGATAACAAC----------------------------
----

GGGACCGAGACCTTCAGACCTGGAGGAGGAGACATGAGGGACAATTGGAGAAGTGAATTATATAAATATAAAG
TAGTAAAAATTGAACCATTA---GGAGTAGCACCCACCAGGGCAAAAAGAAGAGTGGTG--------------
-------------------------CAAAGAGAAAAAAGAGGA---GTGGGA---CTAGGAGCT---------
TTGTTCCTTGGGTTCTTGGGAGCA---GCAGGAAGCACTATG---GGCGCAGCGTCG---
ATAACGCTGACGGTACAG---GCCAGGCAA---TTATTG---TCTGGTATAGTGCAACAACAAAAC---
AATTTG---TTGAGA---GCTATTGAGGCGCAACAGCATCTGTTGCAGCTCACAGTCTGGGGCATC
>FJ388960
TTCTACTGTAATACAACACAACTGTTTACTAGTAAGTGGAAGAATGGTACTAGATTGTCAGAGAACAAAACTA
AC---

ACAGAAGAAAATATCACACTCCCATGCAGAATAAAACAAATTATAAACATGTGGCAGGAAGTAGGAAAAGCAA
TGTATGCCCCTCCCATCAGAGGACAAATTAGATGTTCTTCAAAT---
ATTACTGGGTTGCTATTAACAAGAGATGGGGGTGATACCGGGGAG----------------------------
----

AACGAGACAGAGGTCTTCAGACCTGGAGGAGGAAATATGAGGGACAATTGGAGAGGTGAATTATATAAATATA
AAGTGGTACAAATTGAACCCTTA---GGAGTAGCACCCACCAAGGCAAAGAGAAGAGTGGTG-----------
----------------------------CAGAGAGAAAAAAGAGCA---GTAATA------GGAGCT------
---ATGTTCCTTGGGTTCTTGGGAGCA---GCAGGAAGCACTATG---GGCGCAGCATCA---
GTGGTGCTGACGGTACAG---GCCAGACAA---TTATTG---TCTGGTATAGTGCAACAGCAAAAC---
AATTTG---CTGAAG---GCTATTGAGGCGCAACAACATCTGTTGCAACTCACAGTCTGGGGCATC
>FJ388914
TTCTACTGTAATACAACACCACTGTTTAATAGTACTTGGAATAGTACTAGCTGGCCAAATAACACAAATAACA
CAAATGAC---
-------------------ACT---
AACACAAATGACACTATCACACTCAGATGCAGAATAAAACAAATTATAAACCTGTGGCAGGAAGTAGGAAAAG
CAATGTATGCCCCTCCCATCAGAGGACAAATTAATTGTACATCAAAT---
ATTACCGGGTTGCTATTAACAAGAGATGGGAGTGATGCGAACAGC----------------------------
----

ACGATCGAGGTCTTCAGACCTATAGGAGGAAATATGAAGGACAATTGGAGAAGTGAATTATATAAATATAAAG
TGGTAAAAATTGAACCATTA---GGAATAGCACCCACCACGGCAAGGAGAAGAGTGGTG--------------
-------------------------CAGAGAGAAAAAAGAGCA---GTGGGA---CTAGGAGCT---------
TTGTTCCTTGGGTTCTTGGGAGCA---GCAGGAAGCACTATG---GGCGCAGCATCA---
ATGGGGCTGACGGTACAG---GCCAGACAA---TTATTG---TCTGGTATAGTGCAACAGCAAAAC---
AATTTG---CTGAGG---GCTATTGAGGCGCAACATAATCTGTTGCAACTCACAGTCTGGGGCATC
>FJ388915
TTCTACTGTAACACAACAAAACTGTTTAACAGTACTTGGACTTGGAATAGTGGTACTTGGAATTCAACAAATA
GTAGT--
-------------------ACT---
AATGGAATTGACACTATCACTATCACATGCCCAATAAGGCTAAGTGTAAACGCGTGGCAGGAAGTAGGAATAG
CAATGTCTATCTCTGCCCTCAGAGGCCGAATCAAGAGTTTATCAACG---
TTTACAGGTACAGGATTAACAAGAACAAGTGGTGGCGCCAACGAGGAT-------------------------
----

GAGAACAGGAATGAGACCTTCAGACCTGGGGGAGGAAATATGAAGGACAATTGGAGAAGTGAATTATATAAAT
ATAAAGTAGTAGAAATTGAACCTTTA---GGAATAGCACCCACCAAGGCAAAGAGAAGAGTGGTG--------
-------------------------------CAGAGAGAAAAAAGAGCA---GCATTA------GGAGCT---
------TTGTTCCTTGGGTTCTTGGGAGCA---GCAGGAAGCACTATG---GGCGCAGCGTCA---
ATAACGCTGACGGTACAA---GCCAGACTA---TTGTTG---TCTGGTATAGTGCAACAGCAAAGT---
AACTTG---CTGAAA---GCTATTGAGGCGCAACAGCATCTGTTGCAACTCACAGTCTGGGGCATC
>FJ388918
TTCTACTGTAACACAACACAACTGTTCAATAGTACTTGGAATTTAACACAATCGTTTAATAGTAGTACTGAAG
GGTCAAGT---

-------------GGCACT------
GGGTCAAGTGGCACTATCACACTCCCATGCAGAATAAGACAAATTGTACACTTGTGGCAGGAAGTAGGAAAAG
CAATGTATGCCCCTCCCATCAGAGGACTAATCAGGTGTTCATCAAAT---
ATTACAGGGCTGCTGTTAACAAGAGATGGTGGTAATGATGGTAATGAC-------------------------
----

TCCAATACTGAGGTCTTCAGACCTGGGGGAGGAAATATGAAGGACAATTGGAGAAGTGAATTATATAAATATA
AAGTAGTAGAAATTGAACCATTA---GGAGTAGCACCCACCAGGGCAAAGAGAAGAGTGGTG-----------
----------------------------CAGAGAGAAAAAAGAGCA---GTGCTA------GGAGCT------
---TTGCTCCTTGGGTTCTTGGGAGCA---GCAGGAAGCACTATG---GGCGCAGCGTCA---
ATAGCGCTGACGGCACAA---GCCAGACAA---TTGATG---TCTGGTATAGTGCAACAGCAAAAC---
AACTTG---CTGAGA---GCTATTGAGGCGCAACAGCATATGTTGCAACTCACAGTCTGGGGCATC
>FJ388920
TTCTACTGTAGTACAACACCACTGTTTAATAGTACTTGGGATAATAATACTGAATGGTCAAATAACACAAGTA
AAAAT--

AAAAATGACAGTAATATCACACTCCCATGCAGAATAAAACAAATTATAAACCTGTGGCAGGAAGTAGGAAAAG
CAATGTATGCCCCTCCCATCAAAGGACAAATTAACTGTTCATCAAAT---
ATTACTGGACTACTATTAACAAGAGATGGGGGTGGAAACGAGAGC----------------------------
----

AATATCGAGACCTTCAGACCCGCAGGGGGAGATATGAGGGATAATTGGAGAAGTGAATTATATAAATACAAAG
TGGTACAAATTGAACCATTA---GGAGTAGCACCCACCAAGGCAAAGAGAAGAGTGGTG--------------
-------------------------CAGAGAGAAAAAAGAGCA---GTGGGA---CTAGGAGCT---------
GTGCTCTTTGGGTTCTTGGGAGCA---GCAGGAAGCACTATG---GGCGCAGCATCA---
GTGATGCTGACGGTACAG---GCCAGACAA---TTATTG---TCTGGTATAGTGCAGCAGCAAAAC---
AATTTG---CTGAGA---GCTATTGAGGCGCAACAACATCTGTTGCAACTCACAGTCTGGGGCATC
>FJ388923
TTCTACTGTAATACAACACCACTGTTTAATAGTACTTGGAATGATACTAGTCGGCCAAATATCACAGGTGAGG
AAGAT--
-------------------AAT---
GGTGAGGAAGATAATATGATACTCACATGCAGAATAAAGCAAATTATAAACCTGTGGCAGGAAGTAGGAAAAG
CAATGTATGCCCCTCCCATCAGAGGACAAATTAGATGTTCATCAAAT---
ATTACCGGGTTGCTCTTAACAAGAGATGGGGGTAATGACAGCAGC----------------------------
----

AGGACCGAGGTCTTCAGACCTATGGGAGGAAATATGAGGGACAATTGGAGAAGTGAATTATATAAATATAAAG
TGGTGCAAGTTGAACCATTA---GGAGTAGCACCTACCACAGCAAAGAGAAGAGTGGTG--------------
-------------------------CAGAGAGAAAAAAGAGCA---GTGGGA---ATAGGAGCT---------
GTGTTCCTTGGGTTCTTGGGAGCA---GCAGGAAGCACTATG---GGCGCAGCATCA---
GTGATGCTGACGGTACAA---GCCAGACAA---TTATTG---TCTGGTATAGTGCAGCAGCAAAAC---
AATTTG---CTGAGG---GCTATTGAGGCGCAACAACATCTGTTGCAACTCACAGTCTGGGGCATC
>FJ388963
TTCTACTGTAATACAACAAAACTGTTTAATAGTACTTGGAATGTTAGTACTGGGAATGGAAATAAAACTGGAG
AGGGCACAAAT--
-------------AGTAAT------
GGCACAAATAGGAATATCACACTTCCATGCAGAATAAAACAAATTATAAACATGTGGCAGGAAGTAGGAAAAG
CAATGTATGCCCCTCCCATCAAAGGACAAATTAAATGCTCATCAAAT---
ATTACAGGGCTGTTATTACCAAGAGATGGTGGTTTAAATAATAGC----------------------------
----

ACTAATGAGACCTTCAGACCTGGAGGAGGAAATATGAGGGACAATTGGAGAAGTGAATTATATAAATATAAAG
TAGTAAAAATTGAACCATTA---GGAGTAGCACCTTCCAGGGCAAAGAGAAGAGTGGTG--------------
-------------------------CAGAGAGAAAAAAGAGCA---GTAGGG---CTAGGAGCT---------
GTGTTCATTGGGTTCTTAGGAGCA---GCCGGAAGCACTATG---GGCGCAGCGTCA---
ATAACGCTGACGGTACAG---GCCAGACAA---TTATTG---TCTGGTATAGTGCAACAGCAGAGC---
AACCTG---CTGAGG---GCTATTGAGGCGCAACAGCATCTGTTGCAACTCACAGTCTGGGGCATC
>FJ388924
TTCTACTGTAATACAACACCACTGTTTAATAGTACTTGGGGTAATGGTACTGAATGGTCAAATAACACAAATA
ACCCT--

-------------------AAT---
AACCCTGACAGAGATATCACACTCACATGCAGAATAAAACAAATTATAAACCTGTGGCAGGAAGTAGGAAAAG
CAATGTATGCCCCTCCCATCAGAGGACAAATTAGATGTTCATCAAAT---
ATTACTGGGTTACTATTAACAAGAGATGGGGGTAAAAACGAGAGC----------------------------
----

GAGGCCACCGAGGTCTTCAGACCTGCAGGGGGAGATATGAGGGACAATTGGAGAAGTGAATTATATAAATATA
AAGTGGTACAAATTGAACCATTA---GGAGTAGCACCCACCAAGGCAAAGAGAAGAGTGGTG-----------
----------------------------CAAAGAGAAAAAAGAGCA---GTGGGA---CTAGGAGCT------
---ATGCTCTTTGGGTTCTTGGGAGCA---GCAGGAAGCACTATG---GGCGCAGCATCA---
GTGATGCTGACGGTACAG---GCCAGACAA---TTATTG---TCTGGTATAGTGCAACAGCAAAAC---
AATTTG---CTGAGG---GCTATTGAGGCGCAACAACATCTGTTGCAACTCACAGTCTGGGGCATC
>FJ388964
TTCTACTGTGACACAAAACCACTGTTTAATAGTACCTGGAATAGTAATAGTACTTGGAATGTTACAAATGACA
CT--

GACACTGCAGAAAATATCACACTCCAATGCAAGATAAAACAAATTATAAACTTGTGGCAGAGAGTAGGAAGAG
CAATGTATGCCCCTCCCATCAGAGGACAAATTAGATGTTCATCAAAT---
ATTACAGGGCTGCTACTAACAAGAGATGGTGGTAAGGAAAATAACACG-------------------------
----

AACTCTACTGAGATCTTCAGACCTGGAGGAGGAGACATGAGGGACAATTGGAGAAGTGAATTATATAAATATA
AAGTAGTAAAACTTGAACCATTA---GGAGTAGCACCTTCCAAGGCAAAGAGAAGAGTGGTG-----------
----------------------------CAGAGAGAAAAAAGAGCA---GTGGGA---CTAGGAGCT------
---ATGTTCCTTGGGTTCTTGGGAGCA---GCAGGAAGCACTATG---GGCGCAGCGTCA---
ATGACGCTGACGGTACAG---GCCAGGCAA---TTACTG---TCTGGTATAGTGCAGCAGCAGAGT---
AATTTG---CTGCGG---GCTATTGAGGCGCAACAGCATCTGCTGCAACTCACAGTTTGGGGCATC
>FJ388927
TTCTACTGTAATTCAACAGAACTGTTTAATAGTATTTGGTATGCAAATGGTACTAGGAATAGTACTGAAGGGG
CACATAAC--
-------------------GAA---
TCACATAACGAAACTATCACGCTCCCATGCAGAATAAAACAAATTATAAATATGTGGCAAGAAGTAGGAAAAG
CAATGTGTGCCCCTCCCATCAGAGGACAGATTAGGTGTTCATCAAAT---
ATTACAGGGCTACTATTAACAAGAGATGGTGGTAACGAGAACAAC----------------------------
----

ACCAACACAGAGACCTTCAGACCGGGAGGAGGAGATATGAGGGACAATTGGAGAAGTGAATTATATAAATATA
AAGTAGTAAAAATTGAACCGTTA---GGAGTAGCACCTACCAAGGCAAAGAGAAGAGTGGTG-----------
----------------------------CAAAGAGAAAAAAGAGCA---ATAGGA---ATGGGAGCT------
---ATGTTCCTTGGGTTCTTGGGAGCG---GCAGGAAGCACTATG---GGCGCAGCGTCA---
CTGACGCTGACGGTACAG---GTCAGACAA---TTATTG---TCTGGTATAGTGCAACAGCAGAAC---
AATCTG---CTGATG---GCTATTGAGGCGCAACAACATCTGTTGCAACTCACAGTCTGGGGCATC
>FJ388933
TTCTACTGTAATACAACAAAATTGTTTAATAGTACTTGGAATGGTACTGAAGGGTCAAGTAACGGTACTGACA
GTGAAAATAAC---
-------------ATCAAT------
AATAACAACACTGAAATCGTACTCCCATGCAAAATAAAACAAATTATAAACATGTGGCAGGAAGTAGGAAAAG
CAATGTATGCCCCTCCCATCCAAGGACAAATTAATTGTACATCAAAT---
ATTACAGGGCTGCTACTAACAAGAGATGGTGGTAATAGTAGCGGG----------------------------
----

ACTAATGAGACCTTCAGACCTGGAGGAGGAGATATGAGGGACAATTGGAGAAGTGAATTATATAAATATAAAG
TAGTAAGAATTGAACCATTA---GGAGTAGCACCCACTAGGGCAAAGAGAAGAGTGGTG--------------
-------------------------CAGAGAGAAAAAAGAGCA---GCGATA------GGAGCT---------
ATGTTCCTTGGGTTCTTGGGAGCA---GCAGGAAGCACTATG---GGCGCAGCGTCA---
ATAACGCTGACGGTACAG---GCCAGACTA---TTATTG---TCTGGTATAGTGCAACAGCAGAAC---
AATCTG---CTGAGG---GCTATTGAGGCGCAACAGCATCTGTTGCAACTCACAGTTTGGGGCATT
>FJ388935
TTTTACTGTAATACATCACAACTGTTTAATAGTAGTTGGGATGAGAATGCTAATGCTGGGAATAATACTCCTG
GGAATGAT--

-------------------ACT---
CCTGGGAATGATACTATCACGCTTCCATGCAGAATAAAACAAATTATAAACATGTGGCAGGAAGTAGGAAAAG
CAATGTATGCCCCTCCCATCAGAGGACAAATTAAATGTGTATCAAAT---
ATTACAGGGCTGTTATTGACAAGAGATGGTGGTAAGAACAACAGTAAT-------------------------
----

GATAACAATACTGAGATCTTCAGACCTGAAGGAGGAAATATGAAGGATAATTGGAGAAGTGAATTATATAAAT
ATAAAGTAGTGAAGATTGAACCATTA---GGAATAGCACCCACCAAGGCAAAGAGAAGAGTGGTG--------
-------------------------------CAAAGAGAAAAAAGAGCA---GTGGGAACAATAGGAGCT---
------ATGTTCCTAGGGTTCTTGGGAGCA---GCAGGAAGCACTATG---GGCGCAGCGTCA---
ATGACGCTGACGGTACAG---GCTAGACAA---TTATTG---TCTGGTATAGTGCAACAGCAAAAC---
AACTTG---CTGAAG---GCTATTGATGCGCAACAGCATATGTTGCAGCTCACAGTTTGGGGCATC
>FJ388936
TTCTACTGTAATACAACGCCACTGTTTAATAGTACTTGGAATTGGAATAATACTGGAGAGCCAGAAACCACAA
ATGAC--
-------------------ACT---
GACACTAATAAGAATATCACACTCAAATGCAGAATAAAACAAATTATAAACTTGTGGCAGGAAGTAGGAAAAG
CAATGTATGCCCCTCCCATCAGAGGACAAATTAGATGTTCATCAAAC---
ATTACTGGGTTACTATTAACAAGAGATGGGGGTAGTACCAAGAACGGGACC----------------------
----

CAGAACGAGACTGAGGTCTTCAGACCTGAAGGAGGAAATATGAAGGACAATTGGAGAAGTGAATTATATAAAT
ATAAAGTGGTACAAATTGAACCATTA---GGAATAGCACCCACCAAGGCAAAGAGAAGAGTGGTG--------
-------------------------------CAGAGAGAAAAAAGAGCA---GTGGGA---ATAGGAGCT---
------ATGTTCCTTGGGTTCTTGGGAGCA---GCAGGAAGCACTATG---GGCGCAGCGGCA---
GTGACGCTGACGGTACAG---GCCAGACAA---TTGTTG---TCTGGTATAGTGCAACAGCAAAAC---
AATTTG---CTGAGA---GCTATTGAGGCGCAACAACATATGTTGCAACTCACAGTCTGGGGCATC
>FJ388937
TTCTACTGTAATTCATCACAACTGTTTAATAGTACTTGGAATAGTACTAAATGGTTAAATAGCACTGAAGGAG
AAAACATC---
-------------------ACA---
ACTGAAGGAGAAAACATCACACTCCCATGCAGAATAAAACAAATTATAAACATGTGGCAGACAGTAGGAAAAG
CAATGTATGCCCCTCCCATCAGTGGACTAATTAGATGTTCATCAAAT---
ATTACAGGGCTACTATTAGTAAGGGATGGTGGTAATACCACTAGTAGC-------------------------
----

AATTCCACCGAGACCTTCAGACCGGCAGGTGGAGATATGAGGGACAATTGGAGAAGTGAATTGTATAAATACA
AAGTAGTAAAAATTGAACCAATA---GGAATAGCACCCACCAGGGCAAGGAGAAGAATGGTG-----------
----------------------------CAGAGAGAAAAAAGAGCA---GCAATAGGAGTAGGAGCT------
---GTGTTCCTTGGGTTCTTGAGTGCA---GCAGGAAGCACTATG---GGCGCAGCGTCA---
CTGACGCTGACGGTACAG---GCCAGGCAG---TTATTG---TCTGGTATAGTGCAACAGCAGAAC---
AACTTG---CTGAGG---GCTATTGAGGCGCAACAGCATCTGTTGCAACTCACAGTTTGGGGCATC
>FJ388939
TTCTACTGTAATACAACACAACTGTTTAATAGTATTTGGAATCGGGATGATATTTGGAATACTACTGGGGGGA
ATGGT--
-------------------ACT---
GAGTCAAATAACAATATTACTCTCCCATGCAGAATAAGACAAATTGTAAATAGGTGGCAGGAAGTAGGAAAAG
CAATGTATGCCCCTCCCATCGCAGGACACATTAGTTGTTTATCAAAT---
ATTACAGGGCTACTATTAACAAGAGATGGTGGTGTTAATAAAAGT----------------------------
----

GCCATCGAGACCTTCAGACCATTAGGAGGAAATATAAAGGATAATTGGAGAAGTGAATTATATAAATATAAAG
TAGTAAAAATTGAACCATTA---GGGGTAGCACCCACCAAGGCAAAGAGAAGAGTGGTG--------------
-------------------------CAGAGAGAAAAAAGAGCG---GCACTA------GGAGCG---------
CTGTTACTCGGGTTCTTGGGAGCA---GCAGGAAGCACTATG---GGCGCAGCGTCA---
CTGACGCTGACGGTACAG---GCCAGACAA---TTATTG---TCTGGTATAGTGCAACAGCAGAAC---
AACTTG---CTGAGA---GCTATTGAGGCGCAACAACATCTGTTGCAGCTCACAGTCTGGGGCATT
>FJ388955
TTCTACTGTAATACATCACAACTGTTCAATAGTACTTGGAACACCACTGAGCTTGAGTCAAATATCACAGATG
AAAAT--

ATCACAGATGAAAATATCACACTCCCATGCAGAATAAAACAAATCATAAATATGTGGCAGGAAGTAGGAAAAG
CAATGTACGCCCCTCCCATTAAAGGACAAATTAGATGTTCATCAAAT---
ATTACAGGGCTGCTATTAACAAGAGATGGTGGTAGTAATACCACA----------------------------
----

AATGGAACCGAGATTTTCAGACCTGGAGGAGGAGATATGAGGGACAATTGGAGAAGTGAATTATATAAATACA
AAGTAGTAAAAATTGAACCATTA---GGAATAGCACCCACCAAGGCAAAGAGAAGAGTGGTG-----------
----------------------------CAGAGAGAAAAAAGAGCA---GCAATA------GGAGCT------
---TTGTTCCTTGGGTTCTTGGGAGCA---GCAGGAAGCACTATG---GGCGCAGCGTCA---
ATGACGCTGACGGTACAG---GCCAGACTA---TTATTG---TCTGGTATAGTGCAACAGCAAAAC---
AATTTG---CTGAAA---GCTATTGAGGCACAACAGCATCTGTTGCAACTCACAGTCTGGGGCATT
>JF683736
TTCTACTGTAATTCAACTGCACTGTTTAATAGTACTTGGAATAATACTGCAGGGTCAAATAACACTACAGGAA
ATAAC--
-------------------ACT---
ACAGGAAATGACACAATCACACTCCAATGCAGAATAAAACAAATTGTAAACATGTGGCAGGAAGTAGGAAGAG
CAATGTATGCCCCTCCCATCAGAGGACCAATTAGATGTTCATCAAAT---
ATTACAGGGATACTATTAACAAGAGATGGTGGTAATAATAATAGCACAAAC----------------------
----

AAGTCTGAGTCTGAGACCTTCAGACCTGGAGGAGGAAATATGAAGGACAATTGGAGAAGTGAATTATATAAAT
ATAAAGTAGTACAAATTGAACCATTA---GGATTAGCACCCACCAAGGCAAAGAGAAGAGTGGTG--------
-------------------------------CAGAGAGAAAAAAGAGCA---ATAGGA---CTAGGAGCT---
------ATGTTCCTTGGGTTCTTGGGAGCA---GCAGGAAGCACTATG---GGCGCAGCATCA---
GTAACGCTGACGGTACAG---GCCAGACAA---TTATTG---TCTGGTATAGTGCAGCAGCAGAAT---
AATCTG---CTGAGA---GCTATTGAGGCGCAACAGCACATGTTGCAACTCACAGTCTGGGGCATT
>JF683741
TTCTACTGTAATTCAGCGGGACTGTTTAATAGTACTTGGGGGACTACTAATGGTACTTGGAAGGGGACTGAAG
GGTCA--

GCTAACAATAACACTCTCATACTCCCATGCAGAATAAAACAAATTATAAACATGTGGCAGAGAGTAGGAAAAG
CAATGTATGCCCCTCCTATTAGAGGAAATATTAGCTGTTCATCAAAT---
ATTACAGGGTTACTGTTGACAAGAGATGGTGGTAATAATAATAAT----------------------------
----

AGTGGGACGGAGACTTTCAGACCTGGAGGAGGAGATATGAGAGACAATTGGAGAAGTGAATTATATAAATATA
AAGTAGTAAAAATTGAACCATTA---GGAGTAGCACCCACTAAGGCAAAGAGAAGAGTGGTG-----------
----------------------------CAGAGAGAAAAAAGAGCA---GTGGGA---TTAGGAGCT------
---TTGTTCCTTGGGTTCTTGGGAGCA---GCAGGAAGCACTATG---GGCGCAGCGTCA---
GTGACGCTGACGGTACAG---GCCAGACTA---TTATTG---TCTGGTATAGTGCAACAGCAGAAC---
AATTTG---CTGATG---GCTATTGAGGCGCAACAGCATCTGTTGCAACTCACAGTCTGGGGCATC
>JF683743
TTCTACTGTAATACAACACCATTGTTTAGTAGTATTTGGAATGGTACTAAGAGGGTAAATGGAACTAACGAAA
CT---

GAAACTGGAGACAATATCACACTCACATGCAGAATAAAACAAATTATAAACATGTGGCAGGAAGTAGGAAAAG
CAATGTATGCCCCTCCCATCGCAGGACAAATTAACTGTTCATCAAAT---
ATTACAGGGCTGTTACTAACAAGAGATGGTGGTGTTAGTAATAACACT-------------------------
----

ACAGGGAATGAAACCTTCAGACCTGGAGGAGGAAATATGAAGGACAATTGGAGAAGTGAATTATATAAATATA
AAGTAGTAAGAATTGAACCATTA---GGAATAGCACCCACTAAGGCAAAGAGAAGAGTGGTG-----------
----------------------------CAGAGAGAAAAAAGAGCA---GCA------TTTGGAGCC------
---TTGTTCCTTGGGTTCTTGGGAGCA---GCAGGAAGCACTATG---GGCGCAGCGTCA---
GTAACGCTGACGGTACAG---GCCAGACAA---TTATTG---TCTGGTATAGTGCAACAGCAGAAC---
AATTTG---CTGAGA---GCTATTGAGGCGCAACAGCATCTGTTGCAACTCACAGTCTGGGGCATC
>JF683747
TTCTACTGTAATACATCACAACTGTTTAATAGTACTTGGAATAGTACTTTGGGGGTGAATAGTACTTTGGGGG
TTAATAT--

-------------GGTACT------
GACAATGACACTATCATCTCACTCCCATGTAGAATAAAACAAATTATAAACATGTGGCAGGAAGTAGGAAAAG
CAATGTATGCCCCTCCCATCAGAGGACTAATTAGATGTTCATCAAAT---
ATTACAGGGCTGATATTAACAAGAGATGGTGGGAGTAGCAAGAATAAC-------------------------
----

AGCACGAATGAGACTTTCAGACCTGGAGGAGGAGATATGAGGGACAATTGGAGAAGTGAATTATATAAATATA
AAGTAGTAAAAATTGAACCATTA---GGAATAGCACCCACCCAGGCAAAGAGAAGAGTGGTG-----------
----------------------------CAGAGAGAAAAAAGAGCA---GTGGGG---ATAGGAGCT------
---GTGTTCCTTGGGTTCTTGGGAGCA---GCAGGAAGCACTATG---GGCGCAGCGTCT---
GTGACGCTGACGGTACAG---GCCAGACTA---TTATTG---TCTGGTATAGTGCAACAGCAGAAC---
AATCTG---CTGAAG---GCTATTGAGGCGCAACAACATCTGTTGCAACTCACAGTCTGGGGCATT
>JF683751
TTCTACTGTAATTCAACACAACTGTTTAATAGTACTTGGACTTGGAATGGTACTGAAGGGTCAAATAACACTG
AA---

GGAAATGGCACAATCACACTCCCATGCAAAATAAAACAAATTGTAAACATGTGGCAGGAAGTAGGAAAAGCAA
TGTATGCCCCTCCCATCAGGGGACAAATTAAATGTTCATCAAAT---
ATTACAGGGCTGCTATTAACAAGAGATGGGGGTAATAGCACCATC----------------------------
----

AACGAGACCTTCAGACCTGGAGGAGGAGATATGAGGGATAATTGGAGAAGTGAGTTATATAAATATAAAGTAG
TAAAAATTGAACCATTA---GGGGTAGCACCCACCAAGGCAAAGAGAAGAGTGGTG-----------------
----------------------CAGAGAGAAAAAAGAGTG---GCAGGA---CTAGGAGCT---------
ATGTTCCTTGGGTTCTTGGGAGCA---GCAGGAAGCACTATG---GGCGCAGCGTCA---
ATAACGCTGACGGTACAG---GCCAGACAG---TTATTG---TCTGGTATAGTGCAACAGCAGAAC---
AATCTG---CTGAGG---GCTATTGAGGCGCAACAGCATCTGTTGCAACTCACAGTCTGGGGCATC
>JF683753
TTCTACTGTAATACAACAAAACTATTTAATAGTGCTTGGATTAATGGTACTTGGAATGGTACTGAAGGGTCA-
----

AATAAATCAACCACCATCACACTCCCATGCAGAATAAAACAAGTTATAAACCTGTGGCAGGAAGTAGGAAAAG
CAATGTATGCCCCTCCCATCAGGGGATACATTAATTGTTCATCAAAT---
ATTACAGGACTGCTATTAACAAGGGATGGTGGTAATAAT----------------------------------
----

GAGACCGAGACCTTCAGACCTGGAGGAGGAAATATGAAGGACAATTGGAGAAGTGAATTATATAAATATAAAG
TAGTAAAAATTGAACCATTA---GGAATAGCACCCACCAAGGCAAGGAGACGAGTGGTG--------------
-------------------------CAGAGAGAAAAAAGAGCA---GTCACA---CTAGGAGCT---------
ATGTTCCTTGGGTTCTTGGGGGCA---GCAGGAAGCACTATG---GGCGCAGCGTCA---
CTGACGCTGACGGTACAA---GCCAGACTA---TTATTG---TCTGGTATAGTGCAGCAGCAAAAC---
AATTTG---CTGAGG---GCTATTGGGGCGCAACAACATCTGTTGCAACTCACAGTCTGGGGCATC
>JF683754
TTCTACTGTAATACAACACCACTATTTAATAGTACCTGGAATGATACTAGCTGGTCAGTGGCCACTAAG----
----

AATGATACAAATATCACACTCGCATGCAGAATAAAACAAATTATAAACCTGGGGCAGGAAGTAGGAAGAGCAA
TGTATGCCCCTCCCATCAGAGGACTAATTAGATGTTCATCAAAT---
ATTACAGGGTTGCTATTAACAAGAGATGGGGGTAGCAACGAGAAC----------------------------
----

AATGGAACTGAGGTCTTCAGACCTGCAGGAGGAAATATGAAGGACAATTGGAGAAGTGAATTATACAAATATA
AAGTGGTACAAATTGAACCATTA---GGAGTAGCACCCACCAGGGCACGGAGAAGAGTGGTG-----------
----------------------------CAAAGAGGAAAAAGAGCA---GTGGGA---ATAGGAGCT------
---GTTCTCCTTGGGTTCTTGGGAGCA---GCAGGAAGCACTATG---GGGGCAGCTTCA---
ATTGGGCTGACGGTACAG---GCCAGACAA---TTATTG---TCTGGTATAGTGCAGCAGCAAAGC---
AATTTG---CTGAGA---GCTATTGAGGCGCAACAACATCTGTTGCAACTCACAGTCTGGGGCATC
>JF683769
TTCTACTGTAATACAACATCACTGTTTAATAGTACTTGGAATGAAACACAATTGCATAATAGCACTTGGAATG
AG---

ACTAGAAATGACAATATCACACTCCCATGCAAAATAAAACAAATTATAAACATGTGGCAGGAAGTAGGAAAAG
CAATGTATGCCCCTCCCATCAGAGGACTAATTAAATGTACATCAAAT---
ATTACAGGAATGTTACTGACAAGAGATGGGGTAGTAACCCTAACAAT--------------------------
----

ACTAACAATACTGAGATTTTCAGACCTGGAGGAGGAGATATGAGGGACAATTGGAGAAGTGAATTATATAAAT
ATAAAGTAGTACAAATTGAACCATTA---GGAATAGCACCCACCAAGGCAAAGAGAAGAGTGGTG--------
-------------------------------CAGAGAGAAAAAAGAGCA---CTGGGA---ATAGGAGCT---
------ATGTTCCTTGGGTTCTTGGGAGCA---GCAGGAAGCACTATG---GGCGCAGCGTCA---
CTGACGCTGACGGTACAG---GCCAGGCAA---TTGTTG---TCTGGTATAGTGCAACAGCAAAAC---
AATTTG---CTGAGA---GCTGTTGAGGCGCAACAGCATCTGTTGCAACTCACAGTCTGGGGCATT
>JF683775
TTCTACTGTAATACAACACAACTATTTAATAGTAATTGGACAGTAGTTAATAGTACTTGGTCAGCTAATAGTA
CTTGGAATAGT--
-------------ACTACA------
GGGTCAAATAACACTATCACACTCCAATGCAGAATAAAACAAATTATAAACATGTGGCAGGAAGTAGGAAAAG
CAATGTATGCCCCTCCCATCAGAGGAAATATTAGTTGTTCATCAAAT---
ATTACAGGGCTGCTCCTAGTAAGAGGAGGT---AATGAATCAAAA----------------------------
----

TCACAAGAGATCTTCAGACCTATAGGAGGAAACATGAAGGACAATTGGAGAAGTGAATTATACAAATATAAAG
TAGTAAAAATTGAGCCATTA---GGAGTAGCACCTACCAAGGCAAAGAGAAGAGTGGTG--------------
-------------------------CAAAGAGAAAAAAGAGCT---GTGGGA---CTAGGAGCT---------
TTGTTCCTTGGGTTCTTGGGAGCA---GCAGGAAGCACTATG---GGCGCAGCGTCA---
ATAACGCTGACGGTACAG---GCCAGACAA---TTATTG---TCTGGTATAGTGCAACAGCAGAAC---
AACCTG---CTGAGA---GCTATTGAGGCGCAACAGCATATGTTGCAACTCACAGTCTGGGGCATT
>JF683778
TTCTACTGTAATACAACACAACTGTTTAATAGTACTTGGATTTTTACTGGGAATACCACACAACTGGTTAATG
GG---

AGTGTGAATGAGAATATCACACTCCCATGCAAAATAAAACAAATTGTAAACATGTGGCAGAAAGTAGGAAAAG
CAATGTATGCCCCTCCAATCAGAGGACCAATTAGCTGTACATCAAAT---
ATTACAGGGCTGCTATTAATAAGAGATGGTGGTGTTGGCAATGAGACC-------------------------
----

AATAGCACCGAGATCTTTAGGCCTGGAGGAGGAGATATGAGGGACAATTGGAGAAGTGAATTATATAAATATA
AAGTAGTAAGAATTGAGCCAATA---GGAGTAGCACCCACTAAGGCAAAGAGAAGAGTGGTG-----------
----------------------------CAGAGAGAAAAAAGAGCA---GTGGGA---CTAGGAGCT------
---GTGCTCTTTGGGTTCTTGGGAGCA---GCAGGAAGCACTATG---GGCGCAGCGTCA---
GTGACGCTGACGGTACAG---GCCAGACAA---TTATTG---TCTGGTATAGTGCAACAGCAGAAC---
AATTTG---CTGAAG---GCTATTGAGGCGCAACAGCATTTGTTGCAACTCACAGTCTGGGGCATC
>JF683785
TTCTACTGTAATTCATCACAACTGTTTGATAGGTCTTGGCATACTAGTGGTACTCGGAAGGATAATAATACAA
GT---

ATAAATGGAACTATCATACTCCCATGTAGAATAAAACAAATTATACATATGTGGCAGGAAGTAGGGAAAGCAA
TGTACGCCCCTCCCATCAGAGGAGCAATTAACTGTACATCAAAT---
ATTACAGGGCTGCTATTAACAAGAGATGGTGGTAATGGCAGTAAT----------------------------
----

AATGAAACTGAAACTTTCAGACCTATAGGAGGGAATATGAAGGACAATTGGAGAAGTGAATTATATAAATATA
AAGTGGTAAAAATTGAACCATTA---GGAGTAGCACCCACCAAGGCAAAGAGAAGAGTGGTG-----------
----------------------------CAAAGAGAAAAAAGAGCA---GTGGGAGTAATAGGAGCT------
---ATGTTCCTCGGGTTCTTGGGAGCA---GCAGGAAGCACTATG---GGCGCAGCGTCA---
CTGACGCTGACGGTACAG---GCCAGACAA---TTATTG---TCTGGTATAGTGCAGCAGCAGAAC---
AATCTG---CTGAGG---GCTATTGAGGCGCAACAGCATCTGTTGCAACTCACAGTCTGGGGCATC
>JF683787
TTCTACTGTAATACAACACAATTGTTTAATAGTACTTGGAATACAACAGGGAATACAACAGAATGGGGTAATA
GTACTGAA---

-------------AGTAAT------
AGTACTGAAGAAACTATCACACTCCCATGCAGAATAAAACAAATTATAAACAGATGGCAGGAAGTAGGAAAAG
CAATGTATGCCCCTCCCATCAGCGGAAAAATTAAATGCTCATCAAAT---
ATTACAGGGTTACTATTAACAAGAGATGGGGGTGATAATAACGAGACC-----------------------
----

AACGGAACTGAGGTCTTCAGACCTGGAGGAGGAGATATGAGGGACAATTGGAGAAGTGAATTATATAAATATA
AAGTAGTAAAAATTGAACCATTA---GGAGTAGCACCCACCCAGGCAAGGAGAAGAGTGGTG-----------
----------------------------CAGAGAGAAAAAAGAGGA---GTAGGA---CTAGGAGCT------
---TTGTTCCTTGGGTTCTTGGGAGCA---GCAGGAAGCACTATG---GGCGCAGCGTCA---
ATGACGCTGACGGTACAG---GCCAGACAA---CTATTG---TCTGGTATAGTGCAACAGCAAAAC---
AATTTG---CTGAGA---GCTATTGAGGCGCAACAGCGTCTGTTGCAACTCACAGTCTGGGGCATC
>JF683788
TTCTACTGTAATACAACACAGCTGTTTAATAGTACCTGGTATAATATTGATAATGCTACAGGACAGAT----
----

GGTAATGAGACTATCACACTCCCATGCAGAATAAAACAAATTATAAACAGATGGCAGGAAGTAGGAAAAGCAA
TGTATGCTCCTCCCATCAGTGGACAAATTAATTGTTCTTCAAAT---
ATTACAGGGTTACTATTAACAAGAGATGGTGGTAATAGTAATAACAGT-----------------------
----

AATGAGAGTGAGACCTTCAGACCTGGAGGAGGAGATATGAAGGACAATTGGAGAAGTGAATTATATAAATATA
AAGTAGTAAGAATTGAACCATTA---GGAGTAGCACCCACCAAGGCAAGGAGAAGAGTGGTG-----------
----------------------------CAGAGAGAAAAAAGAGCA---GTGGGAACGCTAGGAGCT------
---ATGTTCCTTGGGTTCTTGGGAGCA---GCAGGAAGCACTATG---GGCGCAGCATCA---
ATAACGCTGACGGTACAG---GCCAGACAA---TTATTG---TCTGGTATAGTGCAACAGCAGAAC---
AATTTG---CTGAGA---GCTATTGAGGCGCAACAGCATCTGTTGCAACTCACAGTCTGGGGCATC
>JF683790
TTCTACTGTAATACAACAAAACTGTTTAATAGTACTTGGCCTTGGAATAGTACTGATACTGGGGGTGAA----
----

ACTAATGAAAATATCACACTCCCTTGCAGAATAAGACAAATTGTAAACAGGTGGCAGGAAGTAGGAAAGGCAA
TGTATGCCCCTCCCATCAGCGGAGTAATTAATTGTCTATCAAAT---
ATTACAGGGATACTATTAACAAGAGATGGTGGTAATAATAATGAGACT-----------------------
----

AACAACACTGAGATCTTCAGACCTGGAGGAGGAGATATGAGGGACAATTGGAGAAGTGAATTATATAAATACA
AAGTAGTAAGAATTGAACCATTA---GGAATAGCACCCACCAAGGCAAAGAGAAGAGTGGTG-----------
----------------------------CAGAGAGAAAGAAGAGCA---GTGGGA---TTTGGAGCT------
---TTTTTCCTTGGGTTCTTGGGAGCA---GCAGGAAGCACTATG---GGCGCAGCGTCA---
GTAACGCTGACGGTACAG---GCCAGACAA---TTATTG---TCTGGTATAGTGCAACAGCAGAAC---
AACTTG---CTGAGA---GCTATTGAGGCGCAACAACATCTGTTGCAACTCACAGTCTGGGGCATC
>JF683794
TTCTACTGTGATACATCAGAACTGTTTAATAGTACTTGGAATAATACTAAAAGGGTAAATAACACGAATAAC-
----

ACAAATGAAAATATCACACTTCCATGCAGAATAAAACAAATTATAAACATGTGGCAGGGAGTAGGAAAAGCAA
TGTATGCCCCTCCCATCAGTGGACCAATTAAATGTACATCAAAT---
ATTACAGGGCTGTTATTGACTAGAGATGGTGGTAAGAATAACACG--------------------------
----

AATGGGACTGAGATCTTCAGACCAGGAGGAGGAGATATGAGGGACAATTGGAGAAGTGAATTATATAAATATA
AAGTAGTACAAATTGAACCATTA---GGAATAGCACCCACCAAGGCAAAAAGAAGAGTGGTG-----------
----------------------------CAAAGAGAAAAAAGAGAA---CTAACG---ATGGGAGCT------
---CTGTTCCTTGGGTTCTTGAGCGCA---GCAGGAAGCACTATG---GGCGCAGCGTCA---
ATAACGCTGACGGTACAG---GCCAGACAA---TTATTG---TCTGGTATAGTGCAACAGCAGAAC---
AATCTG---CTGAGA---GCTATTGAGGCGCAACAGCATATGTTGCAACTCACAGTCTGGGGCATC
>JF683801
TTCTACTGTAAGACATCACAACTGTTTAATAGTACTTGGCAGTCTAATGATACTTGGCTGCCTAATGGTAACG
AG---

```
----------------------------
AGTGATGCCAAGATCACACTCCCATGCAGAATAAAACAAATTATAAATAGGTGGCAGGAAGTAGGAAAAGCAA
TGTATGCCCCTCCCATAGCAGGACAAATTAGCTGTTCATCAAAT---
ATTACAGGGCTGCTATTAACAAGAGATGGTGGCAACAACAATGAC----------------------------
-------------------------------------------------------------------------
----------
AATAACAGCGAGATCTTCAGGCCTGCAGGAGGAGATATGAGGGACAATTGGAGAAGTGAATTATATAAATATA
AAGTAGTAAAAATTGAGCCATTA---GGAGTAGCACCCACCAAGGCAAAGAGAAGAGTGGTG-----------
----------------------------CAAAGAGAAAAAAGAGCA---GTGGGAGCAATAGGAGCT------
---ATGTTCCTTGGGTTCTTGGGAACA---GCAGGAAGCACTATG---GGCGCAGCGTCA---
CTAACGCTGACGGTACAG---GCCAGACAA---TTATTG---TCTGGTATAGTGCAACAGCAGAAC---
AATCTG---CTGAGG---GCTATTGAGGCGCAACAGCATCTGTTGCAACTCACAGTCTGGGGCATC
>JF683804
TTCTACTGTAATTCAACACGACTATTTAATAGTACTTGGTATAATAATGGTACTTGGAAAGGTACTGAAGGGA
TAAATAAC-----------------------------------------------------------------
-------------------ACC---
GGGATAAATAACACCATCATACTCCCATGCAGAATAAAACAAATTATAAACATGTGGCAGGAAGTAGGAAAAG
CAATGTATGCCCCTCCCATCGAAGGACAAATTAAATGTACATCAAAT---
ATTACAGGGCTGTTATTAACAAGAGATGGTGGTACTAACGGGACGACCGATGGT------
ACTAACGGGACGACCGAT---GGTACTAAC-------------------------------------------
--------------------------
GGGACCGAGATCTTCAGACCTATAGGAGGAAATATGAGGGACAATTGGAGAAGTAAATTATATAAATATAAAG
TAATAAAAATTGAACCATTA---GGAGTAGCACCCACCAAGGCAAAGAGAAGAGTGGTG--------------
-------------------------CAGAGAGAAAAAAGAGGA---GCA------CTAGAAGTT---------
TTTTTCCTTGGGTTCTTGGGAGCA---GCAGGAAGCACTATG---GGCGCAGCGTCA---
ATGGCGCTGACGGTACAG---GCCAGACTA---TTGTTG---TCTGGTATAGTGCAGCAGCAGAAC---
AATTTG---CTGAGA---GCCATTGAGGCGCAACAGCATATGTTGCAACTCACAGTCTGGGGCATC
>JQ403048
TTCTACTGTAATACAGCACAACTGTTCAACAGTACGTGGACAAATGATACTGGGGAAGTGAATGATACTAAAG
AG-----------------------------------------------------------------------
----------------------------
TCAAATGACACTATCACACTCCCATGCAGGATAAAACAATTCATAAATATGTGGCAGGAAGTAGGAAAAGCAA
TGTATGCCCCTCCCATCCAAGGATCAATTAGGTGTTTATCAAAT---
ATTACAGGGCTGCTACTAACAAGAGATGGTGGTAATGGTAATAGGTCC-------------------------
-------------------------------------------------------------------------
-------------
GATAATGAGACCTTCAGACCTGGAGGAGGAGATATGAGGGACAATTGGAAAAGTGAATTATATAAATATAAAG
TAGTAAAAATTGAACCATTA---GGAGTAGCACCCACCAAGGCAAAGAGAAGAGTGGTG--------------
-------------------------CAGAGAGAAAAAAGAGCA---GTGGGA---GTAGGAGCT---------
TTGTTCCTTGGGTTCTTGGGAGCA---GCAGGAAGCACTATG---GGCGCAGCATCA---
GTGATGCTGACGGTACAG---GCCAGACAA---TTATTG---TCTGGTATAGTGCAACAGCAGAAC---
AATTTG---CTGAGG---GCTATTGAGGCGCAACAGCATATGTTGCAACTCACAGTCTGGGGCATC
>KT124812
TTTTACTGTAATACAACAAACCTGTTTAATAGTACTTGGTCTAAAAATGATACTAATGTGACAAACAAAACT-
-------------------------------------------------------------------------
----------------------------
GATAATGACATTATAACACTCCCATGCAGAATAAAACAAATTATAAACATGTGGCAGGAAGTAGGAAAAGCAA
TGTATGCCCCTCCCATCAGGGGACAAATTAGATGTTCCTCAAAT---
ATTACAGGGTTGCTATTAACAAGAGATGGTGGCAATAATAGCACC----------------------------
-------------------------------------------------------------------------
----------
AATGGGTCCGAGATCTTCAGACCTGGAGGAGGAGATATGAGGGACAATTGGAGAAGTGAACTATATAAATATA
AAGTAGTAAAAATTGAACCATTA---GGAATAGCACCCACCAGGGCAAAGAGAAGAGTGGTG-----------
----------------------------CAGAGAGAGAAAAGAGCA---GTGGGA---ATAGGAGCG------
---TTGTTCCTTGGGTTCTTGGGAGCA---GCAGGAAGCACTATG---GGCGCAGCGTCA---
ATGACGCTGACGGTACAG---GCCAGACTA---TTACTG---TCTGGTATAGTGCAACAGCAGAAC---
AATTTG---CTGAGA---GCTATTGAGGCGCAACAGCATATGTTGCAACTTACAGTCTGGGGCATC
>KT124752
TTCTACTGTAATACAACAAAATTGTTTAGTAGTAATTGGACTTGGGATAATAATAATACGGGTAATTGGACTG
GT---------------------------------------------------------------------
```

ACTTGGAATAGTGTTAAT---------
AACACTGAACATAATATCACACTCATATGCAGAATAAAACAAATTATAAACATGTGGCAGGGAGTAGGAAAAG
CGATGTATGCCCCTCCCATCAGAGGACAAATTAAATGTTCATCAAAT---
ATTACAGGGCTGCTGTTAACAAGAGATGGTGGTATTACTAACACTAGC-------------------------
----

AATAATACAGAGACCTTCAGACCTGGAGGAGGAGATATGAGGGACAATTGGAGAAGTGAATTATACAAATATA
AAGTAGTACAAATTGAACCATTA---GGAGTAGCACCCACCAAGGCAAAGAGAAGAGTGGTG-----------
----------------------------CAAAGAGAAAAAAGAGAT---TTGGGA---ATAGGAGCT------
---TTGTTCCTTGGGTTCTTGGGAGCA---GCAGGAAGCACTATG---GGCACAGCATCA---
ATAATGCTGACGGTACAG---GCCAGACAA---TTACTG---TCTGGTATAGTGCAGCAGCAGAAC---
AATCTG---CTGAGA---GCTATTGAGGCGCAACAGCATATGTTGCAACTCACAGTCTGGGGCATC
>KT124755
TTCTATTGTAATACAACACAACTGTTTAATAGTACTTGGAATAGTACTGAACGTTTAAATAGCACTGAA----
----

AGTAATGATAATATCACTATCCCATGCAGAATAAAACAAATTATAAACTTGTGGCAGGAAGTAGGAAAAGCAA
TGTATGCCCCTCCCATCAGAGGACAAATTAGATGCTCATCAAAT---
ATTACAGGGCTGCTATTAACAAGAGATGGTGGTAATAACGAAAAC----------------------------
----

AATACCATCGAGATCTTCAGACCTGGAGGAGGAGATATGAGGGACAATTGGAGAAATGAACTATATAAATATA
AAGTAGTAAAAATTGAACCATTA---GGAATAGCACCCACCAAGGCAAAGAGAAGAGTGGTG-----------
----------------------------CAGAGAGAAAAAAGAGCA---GTGGGA---CTAGGAGCT------
---TTGTTCCTTGGGTTCTTGGGAGCA---GCAGGAAGCACTATG---GGCGCAGCGTCA---
ATAACGCTGACGGTACAG---GCCAGACAA---TTACTG---TCTGGTATAGTGCAACAGCAGAAC---
AATCTG---CTGAGG---GCTATTGAGGCGCAACACCAGCTGTTGCAACTCACAGTCTGGGGCATT
>KT124757
TTCTACTGTAATACATCACGATTGTTTAATAGTACTTGGAATAACACTAAAAACAGAGATATAGATATGACTG
GAGAC--

ATAAATGGAACCATCACGCTCCAATGCAGAATAAAACAATTTATAAACATGTGGCAGGGAGTAGGAAAAGCAA
TGTATGCCCCTCCCATCCCGGGACTAATTAGATGTTCATCAAAT---
ATTACAGGGCTGCTATTAACAAGAGATGGGGGTAATGATACTAAG----------------------------
----

AACAATACTACTGAGATCTTCAGACCTGGAGGAGGAGATATGAGGGACAATTGGAGAAGTGAATTATATAAAT
ACAAAGTAGTAAAAATTGAACCATTA---GGACTAGCACCCACCAGGGCAAAGAGAAGAGTGGTG--------
-------------------------------CAGAGAGAAAAAAGAGCA---GTGACA---CTAGGAGCT---
------GTGTTCCTTGGGTTCTTGGGAACA---GCAGGAAGCACTATG---GGCGCAGCGTCA---
CTAACGCTGACGGTACAG---GCCAGACAA---TTACTG---TCTGGTATAGTGCAACAGCAGAGC---
AACCTG---CTGAGA---GCTATTGAGGCGCAACAGCACATGTTGCAACTCACGGTCTGGGGCATC
>KT124794
TTCTACTGTAATTCAACAAAGCTGTTTAACAGTACTTGGTATCCTAAAGGTAATAGTAGTAATAGTACTAGTG
GTACTGAG---

AATACTACTGAGATCATCACGCTCCCATGCAGAATAAAGCAAGTTATAAACATGTGGCAGACAGTAGGAAAAG
CAATGTATGCCCCTCCCATCAGAGGAATAATTAGCTGTTCATCAAAT---
ATTACAGGGATACTATTAACAAGAGATGGTGGTACAAACCAAAAT----------------------------
----

GAAAATGAGACCTTCAGACCTGCAGGAGGAAATATGAGGGACAATTGGAGAAGTGAATTATATAAATATAAAG
TAGTAAAAGTTGAACCATTA---GGAGTAGCACCCACCAAGGCAAGGAGAAGAGTGGTG--------------
-------------------------CAGAGAGAAAGAAGAGCAGTAGTGGGA---ATAGGAGCT---------
GTGTTCCTTGGGTTCTTGTCAGCA---GCAGGAAGCACTATG---GGCGCAGCGGCA---
GTGACGCTGACGGTACAG---GCCAGACAA---TTATTG---TCTGGTATAGTGCAACAGCAGAAT---
AATCTG---CTGAGA---GCTATTGAGGCGCAACAGCATCTGTTGCAACTCACAGTCTGGGGCATC
>KT124800
TTCTACTGTAATACAACACAACTGTTTAACAGTAATTGGACGACTAATAATACTGGGAGTAATGATACGGAGA
TTAATGATACT--

-------------------ATG---
GAGATTAATGATACTATCACCCTCCCATGCAGAATAAAACAAATTATAAACAGGTGGCAGGAAGTAGGAAAAG
CAATGTTTGCCCCTCCTATTAGAGGACAAATCAGCTGTTCATCAAAT---
ATTACAGGGCTGCTATTAACAAGAGATGGTGGCGACAACAACGAGACAGAA----------------------
----

ACCACCGAAATCTTTAGGCCTGGAGGAGGAGATATGAGGGACAATTGGAGAAGTGAATTATATAAATATAAAG
TAGTAAGGGTTGAACCATTA---GGAGTAGCACCCACCAAGGCAAAGAGAAGAGTGGTG--------------
-------------------------CAGAGAGAAAAAAGAGCA---GCG------ATAGGAGCT---------
GTGTTCCTTGGGTTCTTGGGAGCA---GCAGGAAGCACTATG---GGCGCAGCGTCA---
GTGGCGCTGACGGTACAG---GCCAGACAA---TTATTG---TCTGGTATAGTGCAGCAGCAGAAC---
AATCTG---TTGAGA---GCTATTGAGGCGCAACAGCACCTGTTGCAACTCACAGTCTGGGGCATT
>KT124810
TTCTACTGTGAGTCAACACCATTATTTAATAATAGTTCAGAAGGGAACTTTAGTAGTTCAGAAGGGAGCTTTA
GTAGAAGC---
-------------TCAAGT------
AACAACAACAGCACAATCACGCTGTCATGTAAAATAAGACAAATTATAAACATGTGGCAGGAAGTAGGAAAAG
CAATGTATGCCCCTCCCATCAGAGGGCAAATTAGATGCTCATCAAAT---
ATTACAGGGCTGTTATTAACAAGAGATGGTGGTAAGGAGACAGAA----------------------------
----

AATAATACTGAGATCTTCAGACCAGGAGGAGGAAATATGAAGGACAATTGGAGAAGTGAATTATATAAATATA
AAGTAGTAAAAATTGAACCATTA---GGAGTAGCACCCACCAAGGCAAAGAGAAGAGTGGTG-----------
----------------------------CAGAGAGAAAAAAGAGCA---ATAGGAACACTAGGAGCT------
---ATGTTCCTTGGTTTCTTGGGAGCA---GCAGGAAGCACTATG---GGCGCAGCGTCA---
ATAACGCTGACGGTACAG---GCCAGACAA---TTATTG---TCTGGTATAGTGCAACAGCAGAAC---
AATTTG---CTGAGG---GCTATTGAGGCGCAACAACATCTGTTGCAACTCACAGTCTGGGGCATC
>KT124749
TTCTACTGTGATACAACAAAACTGTTTAATAGTACTTGGAATAGTACTTGGAATAATACTACTAGGAATGACA
CT---

GGAGGTGAAGATAAAATTACACTCCCATGCAGAATAAAACAAATTGTAAACCTGTGGCAGGAAGTAGGAAAAG
CAATGTATGCCCCTCCCATCAGTGGACAAATTAGTTGTTCATCAAAT---
ATTACAGGGATACTATTAACAAGAGATGGAGGTAATACAAGCAGT----------------------------
----

ACTATTGAGACCTTCAGACCTGGAGGAGGAGATATGAGGGACAATTGGAGAAGTGAATTATATAAATATAAAG
TAGTAAAAATTGAACCATTA---GGAATAGCACCCACCAAGGCAAAGAGAAGAGTGGTG--------------
-------------------------CAAAGAGAAAAAAGAGCA---TTGGGA---ATAGGAGCT---------
GTGTTCCTTGGGTTCTTGGGAGCA---GCAGGAAGCACTATG---GGCGCAGCATCA---
ATAACGCTGACGGTACAG---GCCAGACTA---TTATTG---TCTGGTATAGTGCAACAGCAAAAC---
AATTTG---CTGAGA---GCTATTGAGGCGCAACAGCATCTGTTGCAACTCACAGTCTGGGGCATT
>KT124753
TTCTACTGTAATTCAACACCACTGTTCAATAGTACTTGGAATGAGACTAGTATTTGGAATGATCCTAATAATA
ATACTTGG---
-------------AATGCT------
AATGCTACTGAATTTATCATACTCCCATGCAGAATAAAACAAATTATAAACATGTGGCAGGAAGTAGGAAAAG
CAATGTATGCCCCTCCCATCAGGGGAATAATTAGATGTTCATCAAAC---
ATTACAGGACTGATATTAACAAGAGATGGTGGTAATAACAATGACACA-------------------------
----

AATGACACCGAGATCTTCAGACCTGGAGGAGGAGATATGAGGGACAATTGGAGAAGTGAATTATATAAATATA
AAGTGGTAGAAATTGAACCATTA---GGAATAGCACCCACCAAGGCAAAGAGAAGAGTGGTG-----------
----------------------------CAGAGAGAAAAAAGAGCA---ATAGGAGCACTAGGAGCT------
---GTGTTCCTTGGGTTCTTGGGAGCA---GCAGGAAGCACTATG---GGCGCAGCGTCA---
CTGACGCTGACGGTACAG---GCCAGACAA---TTATTG---TCTGGTATAGTGCAACAGCAGAAC---
AATTTG---CTGAGG---GCTATTGAGGCGCAACAACATCTGTTGCAACTCACAGTCTGGGGCATC
>KT124754
TTCTACTGTAATACAGCAGCACTGTTTAATAGTACTTGGAATAGTACTGAAGGGTTAAATAGCACTAACGAAA
AT---

```
----------------------------
GGTACTATCCCTATCACACTCCCATGCAAGATAAAACAAATTATAAACAGGTGGCAGGAAGTAGGAAAAGCAA
TGTATGCCCCTCCCATTAGTGGAAGAATTAACTGTTTATCAAAT---
ATTACAGGGCTGCTACTAACAAGAGATGGTGGTAATAATAGAGAG----------------------------
-------------------------------------------------------------------------
-------------
AGCAATGAAACCTTTAGACCTGGAGGAGGAGATATGAGGGACAATTGGAGAAGTGAATTATATAAATATAAAG
TAGTAAGAATTGAACCATTA---GGAGTAGCACCCACCAGGGCAAAGAGAAGAGTGGTG--------------
-------------------------CAGAGAGAAAAAAGAGCA---GTAGGA---CTAGGAGCT---------
TTGTTCCTTGGGTTCTTGGGAGCA---GCAGGAAGCGCTATG---GGCGCAGCGTCA---
ATAACGCTGACGGTACAG---GCCAGACAA---TTATTG---TCTGGAATAGTGCAACAGCAGAAC---
AATCTG---CTAAGA---GCTATTGAGGCGCAACAGCATCTGTTGCAACTCACAGTCTGGGGCATC
>KC247955
TTCTACTGTAATACATCAAAACTGTTTAATGTGAGCACTAGACAGACAAATGAC-------------------
-------------------------------------------------------------------------
----------------------------
ACAAATAGAAATATCACACTCCCATGCAGAATAAAACAAATTATAAACAGGTGGCAGGAGGTAGGAAAAGCAA
TGTATGCCCCTCCCATCAGAGGACAAATTAGATGTTCATCAAAC---
ATTACAGGGCTACTATTAACAAGAGATGGTGGTAACAGCAGCAAC----------------------------
-------------------------------------------------------------------------
----------------
AATGAGACCTTCAGACCTGGAGGAGGAGATATGAGAGACAATTGGAGAAGTGAATTATATAAATATAAAGTAG
TAAAAATTGAACCATTA---GGAATAGCACCCACCAAGGCAAGGAGAAGAGTGGTG-----------------
----------------------CAGAGAGAAAAAAGAGCA---GTGACA---CTGGGAGCT---------
ATGTTCCTTGGGTTCTTAGGCATG---GCAGGAAGCACTATG---GGCGCAGCGTCA---
GTGACGCTGACGGTACAG---GCCAGACAA---TTATTG---TCTGGTATAGTGCAACAGCAAAAC---
AATTTG---CTGAGA---GCTATTGAGGCGCAACAGCATCTGTTGCAACTCACGGTCTGGGGCATT
>KT124772
TTCTACTGTAATACATCAAAATTGTTCAATAGTACTTGGCATAGGAATAATAATACAGTCTCAGGAAAT----
-------------------------------------------------------------------------
----------------------------
GTCACAGGGAATATCACACTCCAATGTAGAATAAAACAATTTATAAACATGTGGCAGGAAGTAGGAAAAGCAA
TGTATGCCCCTCCCGTCAGCGGAAAAATTAGTTGTGTATCAAAT---
ATTACAGGGATACTTTTAACAAGAGATGGAGGTAATAACAACACA----------------------------
-------------------------------------------------------------------------
-------
GAGAACAGCACAGAGACCTTCAGACCTGGAGGAGGAGATATGAGGGACAATTGGAGAAGTGAATTATATAAAT
ATAAAGTAGTAAAAATTGAACCATTA---GGACTGGCACCCACCAAGGCAAAGAGAAGAGTGGTG--------
-------------------------------CAGAGAGAAAAAAGAGCA---ATAACG---CTGGGAGCT---
------ATGTTCCTTGGGTTCTTGGGAGCA---GCAGGAAGCACTATG---GGCGCAGCGTCG---
ATGACGCTGACGGTACAG---GCCAGACTA---TTATTG---TCTGGTATAGTGCAACAGCAAAAC---
AACTTG---CTGAGA---GCTATTGAGGCGCAACAACATCTGTTGCAGCTCACAGTCTGGGGCATC
>KT124797
TTCTACTGTGATACATCAAAACTGTTTAATAGTACCTGGATTAGTAATGGTACCTGGATTATAAATAGTACTG
GTAATGGGACT--------------------------------------------------------------
-------------AATGAT------
ACTGAAGGGTCAGATATCACGCTCCCATGCAGAATAAGACAAATTGTAAATAGGTGGCAGGAAGTAGGAAAAG
CAATGTATGCCCCTCCTATCAGTGGATATATTAGATGTTCATCAAAT---
ATTACAGGGATACTATTAACAAGAGATGGTGGTAATGACGAGAATACC-------------------------
-------------------------------------------------------------------------
----------
ACCGATACCGAGACCTTCAGACCTGGAGGAGGAGATATGAGGGACAATTGGAGAAGTGAATTATATAAATATA
AAGTAGTAAAACTTGAACCCTTA---GGAATAGCACCCACCAAGGCAAAGAGGAGAGTGGTG-----------
----------------------------CAGAGAGAAAAAAGAGCA---GTGGGA---CTAGGAGCT------
---ATGTTCCTTGGGTTCTTGGGAGCA---GCAGGAAGCACTATG---GGCGCAGCGTCA---
ATAACGCTGACGGTACAG---GCCAGACTA---TTATTG---TCTGGTATAGTGCAGCAGCAAAAC---
AACTTG---CTGCAG---GCTATTGAGGCGCAACAACATCTGTTGCAACTCACAGTCTGGGGCATC
>KC248026
TTCTATTGTGACACAACACAACTGTTTAATAGCACTTGGATTAATGGTACCAGGAATGATACTGAAGGG----
-------------------------------------------------------------------------
```

```
----------------------------
TCAAATAATACTATCACACTCCAATGCAGAATAAAACAGATTATAAACATGTGGCAGGAAGTAGGAAAAGCAA
TGTATGCCCCTCCCATCAAAGGACAAATTAGATGTTCATCAAAT---
ATTACAGGGTTACTATTAACAAGAGATGGTGGTACGGACAATGACACAAGT----------------------
-------------------------------------------------------------------------
----------
AATGAGACCGAGATCTTCAGACCGGGAGGAGGAAATATGAAGGACAATTGGAGAAGTGAATTATATAAATATA
AAGTTGTAAAAATTGAACCATTA---GGAGTAGCACCCACCAAGGCAAAGAGAAGAGTGGTG-----------
----------------------------CAAAGAGAAAAAAGAGCA---GTGGGA---ATAGGAGCA------
---TTGTTCCTTGGGTTCTTGGGAGCA---GCAGGAAGCACTATG---GGCGCAGCATCA---
ATAACGCTGACGGTACAG---GCCAGACAA---TTATTG---TCTGGGATAGTGCAACAGCAGAAC---
AACTTG---CTGAGA---GCTATTGAGGCGCAACAACATATGTTGCAACTCACAGTCTGGGGCATC
>KC248015
TTCTACTGTGATTCATCAAAACTGTTTAATAGTACTTGGAATGATTCTACTTCAGGGAATACCAAT-------
-------------------------------------------------------------------------
----------------------------
GCAAATGACACTATCATACTCCCATGCAGAATAAGGCAAATTATAAACATGTGGCAGGAAGTAGGAAAAGCAA
TGTATGCCCCTCCCATTAGTGGAATAATTAGATGTTCATCAAAT---
ATTACAGGGCTGCTATTAACAAGAGATGGTGGTATTAACAAGAGTGAA-------------------------
-------------------------------------------------------------------------
-------------
ACCACCGAGATCTTCAGACCTGGAGGAGGAGATATGAGGGATAATTGGAGAAGTGAATTATATAAATATAAAG
TAGTAAAAATTGAACCAGTA---GGAGTAGCACCTACCAAGGCAAAGAGAAGAGTGGTG--------------
-------------------------CAGAGAGAAAAAAGAGCA---GTGGGA---CTAGGAGCT---------
TTGTTCCTTGGGTTCTTGGGAGCA---GCAGGAAGCACTATG---GGCGCAGCGTCA---
ATAACGCTGACGGTACAG---GCCAGACAA---GTATTG---TCTGGTATAGTGCAACAGCAGAAC---
AATCTG---CTGAGA---GCTATTGAGGCGCAACAGCACCTGTTGCAACTCACAGTCTGGGGCATC
>KT124802
TTCTACTGTAATACTTCACAACTGTTTAATAGTTCCTGGAACGGGACACAGAGCGAGACAAATAACACCAAC-
-------------------------------------------------------------------------
----------------------------
AGAAATGACACACTCACACTCCAATGCAAAATAAGACAAATTGTAAACATGTGGCAGGAAGTAGGAAAAGCAA
TGTATGCCCCTCCCATCAGGGGACCAATTAGATGTTCATCAAAC---
ATTACAGGGCTACTATTAACAAGAGATGGTGGTAGTGGTAATACAACC-------------------------
-------------------------------------------------------------------------
-------
AGCAACACTACTGAGATCTTCAGACCTGGAGGAGGGAATATGAGGGACAATTGGAGAAGTGAATTATATAAAT
ATAAAGTAGTAAGAATTGAACCATTA---GGAGTAGCACCCACCAAGGCAAAGAGAAGAGTGGTG--------
-------------------------------CAGAGAGAAAAAAGAGCA---GTGGGA---CTGGGAGCT---
------CTGTTCCTTGGGTTCTTGGGAGCA---GCAGGGAGCACTATG---GGCGCAGCGTCA---
TTGACGCTGACGGTACAG---GCCAGACAA---TTATTG---TCTGGTATAGTGCAACAGCAGAAC---
AATTTG---CTGAAG---GCTATTGAGGCGCAACAGCATCTGTTGCAACTCACAGTCTGGGGCATC
>KT124765
TTTACTGTAATACAACAGAACTGTTTAATAGTATTTGGGTTAATGGTTCTTGGGTTAATGGGACTAATAATG
ATACTGAA-----------------------------------------------------------------
----------------------------
TCAAATTCCAATATCACACTTCCATGCAGAATAAAACAAATTATAAACATGTGGCAAAAAGTAGGAAAAGCAA
TATATGCCCCTCCTATCAGAGGACAAATTAGATGTTCATCAAAT---
ATTACAGGGCTGCTACTAACAAGGGATGGTGGGATAGACAATAAC----------------------------
-------------------------------------------------------------------------
----------
AAAACCAATGAGACCTTCAGGCCTGGAGGAGGAGATATGAGAGACAATTGGAGAAGTGAATTATACAAATATA
AAGTAGTAAAAATTGAACCATTA---GGAGTAGCACCCACCAGGGCAAAGAGAAGAGTGGTG-----------
----------------------------CAGAGAGAAAAAAGAGGA------------CTAGGACTT------
---GTGTTCCTTGGGTTCTTGGGAGCA---GCAGGAAGCACTATG---GGCGCAGCGTCA---
GTGACGCTGACGGTACAG---GCCAGACAA---TTATTG---TCTGGTATAGTGCAACAGCAAAAC---
AATTTG---CTGAAG---GCTATTGAGGCGCAACAGCATCTGTTGCAACTCACAGTCTGGGGCATT
>KT124811
TTCTACTGTAATACATCACAGTTGTTTAATGGTACTTGGAATAGTACTGTGGGATGGATAACTGAAGGATCAA
ATAAC--------------------------------------------------------------------
```

ACTGGAGGAATTATCACACTCCCGTGCAGAATAAAACAAATTGTAAACAGATGGCAGGAAGTAGGAAAGGCAA
TGTATGCCCCTCCTATCAGAGGAAATATTAGCTGTCTATCCAAT---
ATCACAGGGCTATTATTGACAAGAGATGGTGGTAGCACAAACAGTAGT-------------------------
----

AATGATACAGAGACCTTCAGACCTGGAGGAGGAAATATGATGGATAATTGGAGAAGTGAATTATATAAATATA
AAGTAGTAAAAATCGAACCATTA---GGAGTAGCACCCACCGAGGCAAAGAGAAGAGTGGTG-----------
----------------------------CAAAGAGACAAAAGAGCA---GTGGGAACAATAGGAGCT------
---ATGTTCCTTGGGTTCTTGGGAGCA---GCAGGAAGCACTATG---GGCGCAGCGTCA---
ATAACGCTGACGGTACAG---GCCAGACAA---TTGTTG---TCTGGTATAGTGCAACAGCAGAGC---
AATCTG---CTGAAG---GCTATTGAGGCGCAACAGCGTATGTTGCAACTCACAGTCTGGGGCATC
>KT124767
TTCTACTGTAATACAACAGAACTGTTTAATAGTGTTTGGAATAATAGTGGAAGGGTAACTGGCACTGGA----
----

AATAGTACTAACATCACACTCCCATGCAGAATAAAACAATTTATAAATATGTGGCAAGAAGTAGGAAAAGCAA
TGTATGCCCCTCCCATCAGTGGACAAATTAAATGTTCATCAAAT---
ATTACAGGGCTGCTACTAACAAGAGATGGTGGTCCTAACAAAACG----------------------------
----

GAGAATGATACCTTCAGACCTACAGGAGGAGATATGAGAAATAATTGGAGAAGTGAATTATATAAATATAAAG
TAGTACAAATTGAACCATTA---GGAATAGCACCCACCACGGCAAAGAGAAGAGTGGTG--------------
-------------------------CAGAGAGAAAAAAGAGAC---CTAGGA---CTAGGAGCT---------
TTGTTCATTGGGTTCTTGGGAGCA---GCAGGAAGCACTATG---GGCGCAGCGTCA---
GTGACGCTGACGGTACAG---GCCAGACAA---TTATTG---TCTGGTATAGTGCAACAGCAGAAC---
AATTTG---CTGAGA---GCTATTGAGGCGCAACAACATCTGTTGCAACTCACAGTCTGGGGCATC
>KY324301
TTCTACTGTAATACAACACAACTGTTTAATAGTGACTGGACGATTAATAATGGTACTGGGAATGATACTGAAG
GGTCATTA---

AATAGCAGTGACATCACACTCCCATGCAGAATAAGACAAATTATAAATATGTGGCAGAAAGTAGGAAAAGCAA
TGTATGCCCCTCCTATCAGAGGACAAATTAGATGCTCATCAAAT---
ATTACAGGGCTGCTATTAACAAGAGATGGTGGACAGGGTAGTAATGCT-------------------------
----

ACAAATGAAACCTTCAGACCTGCAGGAGGAAATATGAAAGATAATTGGAGAAGTGAATTATATAAATATAAAG
TAGTAAAAATTGAACCATTA---GGAGTAGCACCCACCAAGGCAAAGAGAAGAGTGGTG--------------
-------------------------CAGAGAGAAAAAAGAGCA---GCA------CTAGGAGCT---------
ATGTTCCTTGGGTTCTTGGGAGCA---GCAGGAAGCACTATG---GGCGCAGCGTCA---
ATAACGCTGACGGTACAG---GCCAGACTG---TTATTG---TCTGGTATAGTGCAACAGCAAAAC---
AATTTG---CTGAGG---GCTATTGAGGCGCAACAGCATCTGTTGCAACTCACAGTCTGGGGCATC
>KY324379
TTTTATTGTAACACAACACAACTGTTTAATAGTACATGGGATAATGGTACATGGCATAATGAGAATAATGAGA
ATAATGGAACA---TTT--
-------------AATGCA------
ACAAGCCATGAAAATATCACAATCCCATGCAGAATAAAACAAATTATAAACATGTGGCAGACAGTAGGAAAAG
CAATGTATGCCCCTCCCATTAGTGGACTAATCAGATGTTCATCAAAT---
ATTACAGGGCTGCTATTAACAAGAGATGGTGGTAGTAATAACAGC----------------------------
----

CTTAATGAGACCTTCAGACCTGGAGGAGGAGATATGAGGGACAATTGGAGAAGTGAATTATATAAATATAAAG
TAGTAAAAATTGAACCATTA---GGAATAGCACCCTCCAAGGCAAAGAGAAGAGTGGTG--------------
-------------------------CAGAGAGAAAAAAGAGCA---GTGGGA---CTAGGAGCC---------
ATGTTCCTTGGGTTCTTGGGAGCA---GCAGGAAGCACTATG---GGCGCAGCGTCA---
ATGACGCTGACGGTACAG---GCCAGACAA---TTATTG---TCTGGTATAGTGCAACAGCAGAGC---
AATTTG---CTGAGA---GCTATTGAGGCGCAACAGCACATGTTGCAGCTAACAGTCTGGGGCATC
>KY324630
TTTTACTGTAATACAACCCAGCTATTTAATAGCAGTTGGAATGCAACACAACTATGGAATACTGGGATGGAGA
ATAATAGCTTT--

-------GGGATGGAGAAT------
AATAGCTTAGAAATTATCACACTCCCATGTAGAATAAAACAAATTATAAACATGTGGCAGGAAGTAGGAAAAG
CAATGTATGCCCCTCCCATCAGCGGATTAATTAGATGTTCATCAAAT---
ATCACAGGGCTGCTATTAACAAGAGATGGTGGGATGCACAATAAGAGT-------------------------
----

AATGAGACTGAGACCTTCAGACCTGCAGGGGGAGATATGAAGGACAATTGGAGAAGTGAATTATATAAATATA
AAGTAGTAAAAATTGAACCTTTA---GGGATAGCACCCACCAAGGCAAAGAGAAGAGTGGTG----------
----------------------------CAAAGAGAAAAAAGAGCA---GTGACG---CTGGGAGCT------
---ATGTTCCTTGGGTTCTTGGGAGCA---GCAGGAAGCACTATG---GGCGCAGCGTCA---
ATGACGCTGACGGTACAA---GCCAGACAA---TTATTG---TCTGGTATAGTGCAACAGCAGAAC---
AATTTG---CTGAGA---GCTATTGAGGCGCAACAGCATCTGTTGCAACTCACGGTCTGGGGCATC
>KY324641
CTCTACTGTAATACAACACAACTGTTTAACAGTACTTGGTTTAACAGTACCAACATTACTGACACTAGAGAAA
AT---

AGCACAGAAATAATCACACTCCCATGTAGAATAAAACAAATTGTAAACATGTGGCAGGAGGTAGGAAAAGCAA
TGTATGCCCCTCCCATCAAAGGAAACATTAGCTGCATATCGAAT---
ATTACAGGGCTGCTGTTAATAAGAGATGGTGGTAACATCACCAAT----------------------------
----

AGTACATCCGAGACCTTCAGACCAGGAGGAGGAGATATGAGGGACAATTGGAGAAGTGAATTATACAGATATA
AAGTAGTAAAAATTGAACCATTA---GGAGTAGCCCCCACCAAGGCAAGGAGAAGAGTGGTG----------
----------------------------CAGAGAGAAAAAAGAGCA---ATAGGGACATTTGGAGCT------
---ATGTTCCTTGGTTTCTTGGGAGCA---GCAGGAAGCACTATG---GGCGCAGCGTCA---
ATGGCGCTGACGGTACAG---GCCAGACTA---TTATTG---TCTGGTATAGTGCAACAGCAGAAC---
AATTTG---CTGAGA---GCTATTGAGGCGCAACAGCACATGTTGCAACTCACAGTCTGGGGCATC
>KY324761
TTCTACTGTAATACAACAAAATTGTTTAATAGTACTTGGAATTTTAATAGTACTTGGAATACAACACAATTGC
TTAATAGT---
-AATTGGAATACAACT---------
AGTACTGAGAAGAACATCACACTCCCATGTAGAATAAAACAAATTATAAACAGGTGGCAGGACGTAGGAAAAG
CAATGTATGCCCCTCCCATCAGAGGACAAATTAGATGTTCATCAAAC---
ATTACAGGGCTACTATTAACAAGAGATGGTGGTGTTGAGAACACC----------------------------
----

AACACCACAGAAGTCTTCAGACCTGGAGGAGGAGATATGAGAGACAATTGGAGAAGTGAATTATATAAATATA
AAATAGTAAAAATTGAACCATTA---GGAATAGCACCCACCAAGGCAAAGAGAAGAGTGGTG----------
----------------------------CAAAGAGAAAAAAGAGCT---GTAACA---TTAGGAGCT------
---CTGTTCCTTGGGTTCTTGGGAGCA---GCAGGAAGCACTATG---GGCGCAGCGTCA---
ATGACGCTGACGGTACAG---GCCAGACTA---TTATTG---TCTGGTATAGTGCAGCAGCAAAAC---
AACTTG---CTGCAG---GCTATTGAGGCGCAACAGCATCTGTTGCAACTCACAGTCTGGGGCATC
>KY324808
TTCTACTGTAATACAACACCACTGTTTAATAGTACTTGGGGGACTAATACTACAAAGCCAGAAATC-------
----

GCAAATGGAACCATCACACTCCAATGCAGAATAAAACAAATTATAAACATGTGGCAGGAAGTAGGAAAAGCAA
TGTATGCCCCTCCCATCAGGGGAGAAATTAGATGTTCATCAAAT---
ATTACAGGGCTGCTATTAACAAGAGATGGTGGTGTTAACAAGACAAATGAT----------------------
----
ACAAATGACACAAAT---
AACACAGAGACCTTTAGACCTGGAGGAGGAGATATGAGGGACAATTGGAGAAATGAGTTATATAAATATAAAG
TAGTAAAAATTGAACCATTA---GGAATAGCACCCACCAAGGCAAAGAGAAGAGTGGTG--------------
-------------------------CAGAGAGAAAAAAGAGCG---GCA------CTAGGAGCT---------
CTTGTCCTTGGGTTCTTGGGAGCA---GCAGGAAGCACTATG---GGCGCAGCGTCA---
GTGACGCTGACGGTACAG---GCCAGACTA---TTATTG---TCTGGTATAGTGCAACAACAGAAC---
AATCTG---TTGAGA---GCTATTGAGGCGCAACAGCATATGTTGCAACTCACAGTCTGGGGCATC
>MK115683
TTCTACTGTGATTCAACGCAACTGTTTAATAGTACCTGGCTGCCTAATAATGAGAGTAATTGGTATAATAATG
GGAAT--

-AATACTACAGGGTCAAAC------
AACAGTACAGCCAATATCACACTCCCATGCAGAATAAAACAAATTATAAACCTGTGGCAAGAAGTAGGAAAAG
CAATGTATGCCCCTCCCATCAGAGGAGCAATCAATTGCTTGTCAAAT---
ATTACAGGGTTGTTACTAACAAGAGATGGTGGGAATAATAACGAG---------------------------
----

ACAACCGACCCCACCGAGATCTTTAGACCTGGAGGAGGAAATATGAAGGACAATTGGAGAAGTGAATTATATA
GATATAAAGTAGTAAAAATTGAACCATTA---GGAGTAGCACCCACCACGGCAAAGAGAAGAGTGGTG-----
---------------------------------CAGAGAGAAAAAAGAGCA---GTGGGT---
CTCGGAGCC---------TTGTTCCTTGGGTTCTTGGGAGCA---GCAGGAAGCACTATG---
GGCGCAGCGTCA---GTAACGCTGACGGTACAG---GCCAGACAA---TTATTG---
TCTGGTATAGTGCAGCAGCAGAAC---AATTTG---CTGAGG---
GCTATTGAGGCGCAACAGCATATGTTGCAACTCACAGTCTGGGGCATC
>MK115918
TTTTACTGTAATACAACACAACTGTTTAATAGTACTTGGAATAGTACTGGGGATAGTACTTGGAAT-------
----

AATACAGATAATATCACACTCCCATGTAGAATAAGGCAAATTGTAAACATGTGGCAGGAAGTAGGAAAGGCAA
TGTATGCTCCTCCCATCCGTGGACAAATTAAATGTTCATCAAAT---
ATTACAGGGCTGTTATTAACAAGGGATGGTGGTAACGGGAATGAGACG------------------------
----

AGAGAAACAGAAACCTTCAGACCTGGAGGAGGAGATATGAGGGACAATTGGAGAAGTGAATTATATAAATATA
AAGTAGTAAGAATTGAACCATTA---GGAATAGCACCCACTAAGGCAAAGAGAAGAGTGGTG----------
---------------------------CAAAGAGAAAAAAGAGCA---ATAGGAACACTGGGAGCT------
---GTGTTCCTTGGGTTCTTGGGAGCA---GCAGGAAGCACTATG---GGCGCAGCGTCA---
ATGACGCTGACGGTACAG---GCCAGGCAA---TTATTG---ACTGGTATAGTGCAGCAGCAGAAC---
AATCTG---CTGAAG---GCTATTGAGGCGCAACAGCATCTGTTGCAACTCACAGTCTGGGGCATT
>MK116058
TTCTATTGTAATACAACACAATTGTTTAATAGCACTTGGGATAATGGTACTGAAAAGTCAAGTAACCTTGGAG
GA--

AATGATACAACCATCATTCTCCCATGCAGAATAAAACAAATTATAAACATGTGGCAGGAAGTAGGAAAAGCAA
TGTATGCCCCTCCCATCAAAGGAATAATTAGATGTTCATCAAAT---
ATTACAGGGCTGCTATTGACAAGAGATGGAGGTAATAGTACTGACACA------------------------
----

AATGGGAGCGAGATCTTCAGACCTGGAGGAGGAGATATGAGGGACAATTGGAGAAGTGAATTATATAAGTATA
AAGTAGTAAAAATTGAACCATTA---GGAGTAGCACCTACCAAGGCAAAGAGAAGAGTGGTG----------
---------------------------CAGAGAGAAAAAAGAGCA---GTGGGA---CTAGGAGCT------
---TTGTTCCTTGGGTTCTTGGGAGCA---GCAGGAAGCACTATG---GGCGCAGCGTCT---
CTGACGCTGACGGTACAG---GCCAGACAA---TTGTTG---TCTGGTATAGTGCAACAGCAGAAC---
AATTTG---CTGAGG---GCAATTGAGGCGCAACAGCATCTGTTGCAACTCACAGTCTGGGGCATC
>MK116212
TTTTACTGTAATTCAACACAACTGTTTAATAGTACTTGGAATAATAATGGTACTAATACTTGGAAT-------
----

AGTACAGGTAATATCACACTCCCATGTAAAATAAGGCAAATTGTAAACATGTGGCAGAAAGTAGGAAAAGCAA
TGTATGCTCCTCCCATCCGTGGACAAATTAAATGTTCATCAAAT---
ATTACAGGGCTGCTATTAACAAGAGATGGTGGTAACGAGAGTGAG---------------------------
----

AGCGAAACCTTCAGACCTGGCGGAGGAGATATGAGGGACAATTGGAGAAGTGAATTATACAAATATAAAGTAG
TAAGAATTGAACCATTA---GGACTAGCACCCACTAAGGCAAAGAGAAGAGTGGTG----------------
---------------------CAGAGAGAAAAAAGAGCA---ATAGGAACACTGGGAGCT---------
GTGTTCCTTGGGTTCTTGGGAACA---GCAGGAAGCACTATG---GGCGCAGCGTCA---
ATGACGCTGACGGTACAG---GCCAGGCAA---TTATTG---TCTGGTATAGTGCAGCAGCAGAAC---
AATCTG---CTGAAG---GCTATTGAGGCGCAACAGCATCTGTTGCAACTCACAGTCTGGGGCATT
>MK086129

TTCTACTGTAATACAACAAAACTGTTTAATAGGACCTTTAATAGTACTTGGAATAGTACTGAAGGGTCA----
----

AATAGTACAACTATTACACTCCCATGCAGAATAAAACAAATTATAAACATGTGGCAGGAAATAGGAAAAGCAA
TGTATGCCCCTCCCATCAGCGGACAAATTAGATGTTCATCAAAT---
ATCACAGGGCTGATATTAACAAGAGATGGTGGTAATGACACTAAC----------------------------
----

AATGAGACCTTCAGACCTGGAGGAGGAAATATGAAAGATAATTGGAGAAGTGAATTATATAAATATAAAGTAG
TAAAAATTGAACCATTA---GGAGTAGCACCCACCAGGGCAAAGAGGAGAGTGGTG-----------------
----------------------CAGAGAGAAAAAAGAGCA---GTGGGAGTAATAGGAGCT---------
ATGTTCCTTGGGTTTCTGGGAGCA---GCAGGAAGCACTATG---GGCGCAGCATCA---
ATAACGCTGACGGTACAG---ACCAGACGA---TTAGTG---TCTGGTATAGTGCAACAGCAGAAC---
AATCTG---CTGAGA---GCTATTGAGGCGCAACACAATCTGTTGCAACTCACAGTCTGGGGCATC
>MN090868
TTTTACTGTAATTCAACACAACTGTTTAATAGTACTTGGAATAATAATGGTACTAATACTTGGAAT-------
----

AGTACAGGTAATATCACACTCCCATGTAAAATAAGGCAAATTGTAAACATGTGGCAGAAAGTAGGAAAAGCAA
TGTATGCTCCTCCCATCCGTGGACAAATTAAATGTTCATCAAAT---
ATTACAGGGCTGCTATTAACAAGAGATGGTGGTAACGAGAGTGAG----------------------------
----

AGCGAAACCTTCAGACCTGGCGGAGGAGATATGAGGGACAATTGGAGAAGTGAATTATATAAATATAAAGTAG
TAAGAATTGAACCATTA---GGACTAGCACCCACTAAGGCAAAGAGAAGAGTGGTG-----------------
----------------------CAGAGAGAAAAAAGAGCA---ATAGGAACACTGGGAGCT---------
GTGTTCCTTGGGTTCTTGGGAACA---GCAGGAAGCACTATG---GGCGCAGCGTCA---
ATGACGCTGACGGTACAG---GCCAGGCAA---TTATTG---TCTGGTATAGTGCAGCAGCAGAAC---
AATCTG---CTGAAG---GCTATTGAGGCGCAACAGCATCTGTTGCAACTCACAGTCTGGGGCATT
>AF490512
TTCTACTGTAATACAACAAAACTGTTTAATAGTACTTGGAAGTTTAATGGTACTTGGAAGGTTGTTGAAGGAT
CAAGT--

GACACTGCAGAAAATATCACACTCCCATGCAGAATAAAACAAATTATAAACATGTGGCAGGAAGTAGGAAAAG
CAATGTATGCCCCTCCCATCAGAGGACAAATTAGATGTTCATCAAAT---
ATTACAGGGTTACTATTAACAAGAGATGGTGGTAATAACACAAAC----------------------------
----

GAGACCCCCGAGACCTTCAGACCTGGAGGAGGAAATATGAAGGACAATTGGAGAAGTGAATTATATAAATATA
AAGTAGTAAAAATTGAACCATTA---GGAGTAGCACCCACCAAGGCAAAGAGAAGAGTGGTG-----------
----------------------------CAGAGAGAAAAAAGAGCA---GTGGGA---ATAGGAGCT------
---TTGTTCCTTGGGTTCTTGGGAGCA---GCAGGAAGCACTATG---GGCGCAGCGTCA---
ATAACGCTGACGGTACAG---GCCAGACAA---TTATTG---TCTGGTATAGTGCAACAGCAAAAC---
AATTTG---CTGAGG---GCTATTGAGGCGCAACAGCATCTGTTGCAACTCACAGTCTGGGGCATC
>U43096
TTCTACTGTAATTCAGCACAACTGTTTAATAGTACTTGGAATGATACTAAAGAGTCAAATAAC----------
----

ACAAATGGAACTATCACACTCCCATGCAGAATAAAACAAATTATAAACATGTGGCAGGAAGTAGGAAAAGCAA
TGTATGCCCCTCCCATCAGTGGACAAATTAGATGTTCATCAAAT---
ATTACAGGGCTGCTATTAACAAGAGATGGTGGTAAAAATAAGGAC----------------------------
----

AACGAGACCGAGACCTTCAGACCTGGAGGAGGAAATATGAGGGACAATTGGAGAAGTGAATTATATAAATATA
AAGTAGTAAAAATTGAACCATTA---GGAGTAGCACCCACCAAGGCAAAGAGAAGAGTGGTG-----------
----------------------------CAGAGAGAAAAAAGAGCA---GTGGGACTGCTGGGAGCT------
---GTGTTCCTTGGGTTCTTGGGAGCA---GCAGGAAGCACTATG---GGCGCACGGTCA---
ATGGCGCTGACGGTACAG---GCCAGACAG---TTATTG---TCTGGTATAGTGCAACAGCAGAAC---
AATTTG---CTGAGG---GCTATTGAGGCGCAACAGCATCTGTTGCAACTCACAGTCTGGGGCATC
>U43141

TTCTACTGTAATTCAACAAAACTGTTTAATAGTACTTGGAATAATACTAGTACTTGGAATGATAAT-------
----

GGAAATGACACTATCATACTGCCATGTAGAATAAAACAAATTATAAACATGTGGCAGGAAGTAGGAAAAGCAA
TGTATGCCCCTCCCATTGGAGGACTAATTAGATGTTCATCAAAT---
ATTACAGGGCTGATATTAACAAGAGATGGTGGTAATGATAACAGT----------------------------
----

AGTACCACCGAGATCTTCAGACCTGGAGGGGGAAATATGAGGGACAATTGGAGAAATGAATTATATAAATATA
AAGTAGTAAAAATTGAACCATTA---GGAGTAGCACCCACCAAGGCAAAGAGAAGAGTGGTG-----------
----------------------------CAGAGAGAAAAAAGAGCG---GTGGGAATGCTAGGAGCT------
---ATGTTCCTTGGGTTCTTGGGAGCA---GCAGGAAGCACTATG---GGCGCACGGTCA---
CTGACGCTGACGGTACAG---GCCAGACAA---TTATTG---TCTGGTATAGTGCAACAGCAAAAC---
AATTTG---CTGAGA---GCTATTGAGGCGCAACAACATCTGTTGCAACTCACAGTCTGGGGCATC
>EF514704
TTCTACTGTAGTACAACACAATTGTTTAATAGTACTTGGAGTAGTACTGGAGAGTCAAATAACACCAATGGCA
CA---

GGAACAAATGGCAATATCATACTCCCATGCAGAATAAGACAAATTGTAAACTTGTGGCAGGAAGTAGGAAAAG
CAATGTATGCCCCTCCCATCAGAGGACTAATTAGATGTTCATCAAAT---
ATTACAGGGCTGCTATTAACAAGAGATGGTGGTAGTGGTAATAACACG-------------------------
----

GACAATGCCACCGAGATCTTCAGACCTGGAGGAGGAAATATGAGGGACAATTGGAGAAGTGAATTATATAAAT
ATAAAGTAGTAAAAATTGAACCACTA---GGAGTAGCACCCACCAAGGCAAAGAGAAGAGTGGTG--------
-------------------------------CAGAGAGAAAAAAGAGCA---GTGGGAGTAATGGGAGCT---
------ATGTTCCTTGGGTTCTTGGGAGCA---GCAGGAAGCACTATG---GGCGCAGCATCA---
ATGACGCTGACGGTACAG---GCCAGACAA---TTATTG---TCTGGTATAGTGCAACAGCAAAAC---
AATTTG---CTGAAG---GCTATTGAGGCGCAGCAGAATCTGTTGCAACTCACAGTCTGGGGCATC
>EF514705
TTCTACTGTAACACTTCACAACTGTTTAATAGTACTTGGTATAGCACTTGGAATAGTACTTGGTATAGCAATA
GGAATAGTACT--
-------GGAGGGTCAACT------
AACGAGAGCGACATAATCTCAATCCCATGCAAGATAAAACAAATTATAAACATGTGGCAGGAAGTAGGAAAAG
CAATGTATGCCCCTCCCATTAGAGGACAAATTAACTGTTTCTCAAAC---
ATTACAGGGCTACTATTAACAAGAGATGGTGGTCTAAATGTTACC----------------------------
----

AATACCAGTGAGACCTTCAGACCTGGAGGAGGAGATATGAGGGACAATTGGAGAAGTGAATTATATAAATATA
AAGTAGTAAAAATTGAACCATTA---GGAATAGCACCTACCCAGGCAAAGAGAAGAGTGGTG-----------
----------------------------CAGAGAGAAAAAAGAGCA---GTGGGA---ATAGGAGCT------
---GTATTCCTTGGGTTCTTGGGAGCA---GCAGGAAGCACTATG---GGCGCAGCGTCA---
GTGGCGCTGACGGTACAA---GCCAGACAA---TTATTG---TCTGGTATAGTGCAACAGCAAAAT---
AATTTG---CTGAGG---GCTATTGAGGCGCAACAGCACCTGTTGCAACTCACAGTCTGGGGCATC
>EF514706
TTCTACTGTAATACAACACTACTGTTTAATAGTACTTGGAATGATACTACAGGAAGGTTAAAT----------
----

ACCACTGAAGACATCACACTCCCATGCAGAATAAAACAAATTATAAACATGTGGCAGGAAGTAGGAAAAGCAA
TGTATGCCCCTCCCATCAGAGGACAAATTAGCTGTTCATCAAAT---
ATTACAGGAATACTATTAACAAGAGATGGTGGTAATAATAATAAT----------------------------
----

ACCAACGAGACCTTCAGACCTGCAGGAGGAAATATGAAGGATAATTGGAGAAGTGAATTATATAAATATAAAG
TAGTAAAAATTGAACCATTA---GGAGTAGCACCCACCGAGGCAAAGAGAAGAGTGGTG--------------
-------------------------CAAAGAGAAAAAAGAGCA---GTGGGA---CTAGGAGCT---------
CTGTTCCTTGGGTTCTTGGGAGCA---GCAGGAAGCACTATG---GGCGCAGCGTCA---
ATGACGCTGACGGTACAA---GCCAGACAA---TTATTG---TCTGGTATAGTGCAACAGCAGAAC---
AATTTG---CTGAGG---GCTATTGAGGCGCAACAGCGTATGTTGCAACTCACAGTCTGGGGCATC
>EF514707

TTCTACTGTAATACAACACCACTGTTTAATAGTACGTGGAATACTAATACTACAGAGCTAAATAACACTGATG
AC---

AACAATGGCACATTCACACTCTCATGCAGAATAAAACAAATTATAAACATGTGGCAGGAAGTAGGAAAAGCAA
TGTATGCCCCTCCAATCAAAGGACGAATTAGCTGTTCATCAAAT---
ATTACAGGGCTGCTACTAACAAGAGATGGTGGTAACCCAAACAGTACC-------------------------
----

AGTACCAACGAGACCTTCAGACCTGCAGGAGGAGATATGAGAGATAATTGGAGAAGTGAATTATATAAATATA
AAGTAGTAAGAATTGAACCATTA---GGAGTAGCACCCACCAAGGCAAAGAGAAGAGTGGTG-----------
----------------------------CAGAGAGAAAAAAGAGGA------------ATAGGAGTT------
---ATGCTCCTTGGGTTCTTGGGAGCA---GCAGGAAGCACTATG---GGCGCAGCGTCA---
ATGGCGCTGACGGTACAG---GCCAGACGA---TTATTG---TCTGGTATAGTGCAACAGCAGAAC---
AATTTG---CTGAGA---GCTATTGAGGCGCAACAGCATATGTTGCAACTCACAGTCTGGGGCATC
>EF514709
TTCTACTGTAATACAACACAACTGTTTAATAGTACTTGGAATAGTACTACAGGGTCAAATAACACTGAA----
----

AGAAATGACACAATCACACTCCCTTGCAGAATAAAACAAATTGTAAACAGGTGGCAGGAAGTAGGAAAAGCAA
TGTATGCCCCTCCCATCAAAGGACAAATTAGATGTTCATCAAAT---
ATTACAGGGCTGCTATTAACAAGAGATGGTGGTAATAACGCAAGC----------------------------
----

GGGCCTGAGATCTTCAGACCTGGAGGAGGAGATATGAGGGATAATTGGAGAAGTGAGTTATATAAATATAAAG
TAGTAAAAATTGAACCATTA---GGAGTAGCACCCACCAAGGCAAAGAGAAGAGTGGTG--------------
-------------------------CAGAGAGAAAAAAGAGCG---GCAGGA---CTAGGAGCT---------
ATGTTCCTTGGGTTCTTGGGAGCA---GCAGGAAGCACTATG---GGCGCAGCGTCA---
ATAACGCTGACGGTACAG---GCCAGACAA---TTATTG---TCTGGTATAGTGCAACAGCAGAAC---
AATCTG---CTGAGG---GCTATTGAGGCGCAACAGCATCTGTTGCAACTCACAGTCTGGGGCATC
>EF514710
TTCTACTGTAATACAACACAACTGTTTAATAGTACTTGGAATGATACTAGCAACTCAAATACC----------
----

ACAAATGGCACTATCACACTCCCATGTAGAATAAGACAAATTGTAAATTTGTGGCAGGAAGTAGGAAAAGCAA
TGTATGCCCCTCCCATCAGGGGACTAATTAAATGCTCATCAAAT---
ATTACAGGGCTGATATTAACAAGAGATGGTGGTAATAACATAAGC----------------------------
----

AATACCACCGAGATCTTCAGACCTGGAGGAGGGGATATGAGGGACAATTGGAGAAGTGAATTATATAAGTATA
AAGTAGTAAAAATTGAACCATTA---GGAGTAGCACCCACCAAGGCAAAGAGAAGAGTGGTG-----------
----------------------------CAGAGAGAAAAAAGAGCA---GTGGGA---ATAGGAGCT------
---GTGTTCCTTGGGTTCTTGGGAGCG---GCAGGAAGCACTATG---GGCGCAGCGTCA---
CTGACGCTGACGGTACAG---GCCAGACAA---TTATTG---TCTGGTATAGTGCAACAGCAAAAC---
AATTTG---CTGAAG---GCTATTGAGGCGCAGCAGCATCTGTTGCAACTCACAGTCTGGGGCATC
>EF514711
TTTTACTGTAACACAACACAATTGTTTAATAGTACTTGGAATGATACTAGAGAGTCAAATAATAATGTA----
----

GGAAATGACACAATCACACTCCCATGTAGAATAAAACAAATTATAAACATGTGGCAGGAAGTAGGAAAAGCAA
TGTATGCCCCTCCCATCAGAGGACAAATTAGATGCTCATCAAAT---
ATTACAGGGCTGATATTAACAAGAGATGGTGGTAAAGAAGGGAAC----------------------------
----

GGGACGACCGAGACCTTCAGACCAGGAGGAGGAGATATGAGGGACAATTGGAGAAGTGAATTATATAAATATA
AAGTAGTAAAAATTGAACCTCTA---GGAGTAGCACCCACCAAGGCAAGGAGAAGAGTGGTG-----------
----------------------------CAGAGAGAAAAAAGAGCA---GCGGGA---CTAGGAGCT------
---TTGTTCCTTGGGTTCCTGGGAGCG---GCAGGAAGCACTATG---GGCGCAGCGTCA---
GTGACGCTGACGGTACAG---GCCAGACAA---TTACTG---TCTGGTATAGTGCAACAGCAAAAC---
AATTTG---CTGATG---GCTATTGAGGCGCAACAGCATCTGTTGAAACTCACAGTCTGGGGCATC
>EF514712

TTCTACTGTAATACAACACAACTGTTTAGTAGTACTTGGAATGAGAGTACTTGGAATGAGAGTACATGGAATA
ATACAGGGAAT--
----------ACTACCCCT------
GAAAGAAATGAAACTATCACACTCTCATGCAGAATAAAACAAATTATAAACATGTGGCAGGAAGTAGGAAAAG
CAATGTATGCCCCGCCCATAGAAGGACACATTAACTGTTCATCAAAT---
ATTACAGGGCTGCTATTAGTAAGAGATGGGGGTAACAGGAGCAAGAGCGAG----------------------
----

AATAACGGGAACGAGACCTTCAGACCTGGAGGAGGAGATATGAGGGATAATTGGAGAAGTGAATTATATAAAT
ATAAAGTAGTAAAAATTGAACCATTA---GGAATAGCACCCACCAAGGCAAAGAGAAGAGTGGTG--------
-------------------------------CAGAGAGAAAAAAGAGGA---GTA------ATAGGAGCT---
------ATGTTCCTTGGGTTCTTGGGAGCA---GCAGGAAGCACTATG---GGCGCAGCGTCA---
ATGACGCTGACGGTACAG---GCCAAACAA---TTATTG---TCTGGCATAGTGCAACAGCAGAAC---
AATTTG---CTGAAG---GCTATTGAGGCGCAACAGCATCTGTTGCAACTCACAGTCTGGGGCATC
>EF514697
TTCTACTGTAATACAGCAAAGCTGTTTAATAGTACTCTGAATAATACTGAATGGTCAAATATCACTGCAGAAA
AT---

AATGAAGACAAAATCATACTCCAATGCAGAATAAAACAAATTATAAACATGTGGCAGGAAGTAGGAAAAGCAA
TGTATGCCCCTCCCATTAGCGGACCAATTAACTGTTCATCAAGT---
ATTACAGGGCTGATATTAACAAGAGATGGTGGTAATATTAATAATAATAGC----------------------
----

ATGAGAGATAATGACACCTTCAGACCTGGAGGAGGAGATATGAGGGATAATTGGAGAAGTGAATTATATAAAT
ATAAAGTAGTAAGAATTGAACCATTA---GGAGTAGCACCCACCAAGGCAAAGAGAAGAGTGGTG--------
-------------------------------CAGAGAGAAAAAAGAGCA---GTGGGA---ATAGGAGCT---
------GTACTCCTTGGGTTCTTGGGAGCA---GCAGGAAGCACTATG---GGCGCAGCATCA---
GTGATGCTGACGGTACAA---GCCAGGCAA---TTATTG---TCTGGTATAGTGCAACAGCAGAAC---
AATCTG---CTGAGA---GCTATTGAGGCGCAACAACATCTGTTGCAACTCACAGTCTGGGGCATC
>EF514701
TTCTACTGTAATTCATCACAACTGTTTAATAGTACTTGGAACAGTAACAGTACCTTAACTTTAACTGGTCCTG
AAGAGTCA---
-------------------AAC---
AGCACTGATAATAATATCACACTCCCATGCAGAATAAAGCAGTTTATAAACCTGTGGCAGGGAGTAGGAAAAG
CAATGTATGCCCCTCCCATCCAAGGACGAATTAAATGTACATCAAAT---
ATTACAGGGCTGATATTAACAAGAGATGGTGGGAATAGCAGCAAC----------------------------
----

ACCACCACCGAAATCTTCAGACCTGGAGGAGGAGATATGAGGGACAATTGGAGAAGTGAATTATATAAATATA
AAGTAGTAAAAATTGAACCATTA---GGAGTAGCACCCACCAAGGCAAAGAGAAGAGTGGTG-----------
----------------------------CAGAGAGAAAAAAGAGCA---GTGGGG---CTAGGAGCT------
---CTGTTCCTTGGGTTCTTGGGAGCA---GCGGGAAGCACTATG---GGCGCAGCGTCA---
GTGACGCTGACGGTACAG---GCCAGACAA---TTGTTG---TCTGGTATAGTGCAACAGCAAAAC---
AACTTG---CTGATG---GCTATTGAGGCGCAACAGCATCTGTTGCAACTCACAGTCTGGGGCATT
>FJ694790
TTCTACTGTAATACGACACGACTGTTCAATAGTATGTTCAATGGTACTTTGAAGAATACTACAGGGTCAAATA
ACACA--

GGGAATAATGACACAATCATACTCCCATGCAGAATAAAACAAATTATAAACATGTGGCAGGAAGTAGGAAAAG
CAATGTATGCCCCTCCCATCGAAGGACTAATTAACTGTGTGTCAAAT---
ATTACAGGGCTGATATTAACAAGAGATGGGGGTAAGAGTAGTAAC----------------------------
----

GAAAACGAGACCTTCAGACCTATAGGAGGAAATATGAGGGACAACTGGAGAAGTGAATTATATAAATATAAAG
TAGTAAGAATTGAACCATTA---GGAGTAGCACCCACTGCGGCAAAGAGAAGAGTGGTG--------------
-------------------------CAGAGAGAAAAAAGAGCA---GTGGGA---ATAGGAGCT---------
GTGTTCCTTGGGTTCTTGGGAGCA---GCAGGAAGCACTATG---GGCGCAGCGTCA---
ATGACGCTGACGGTACAG---GCCAGACAA---TTATTG---TCTGGTATAGTGCAACAGCAGAAT---
AATCTG---CTGAGA---GCTATTGAGGCGCAACAGCATCTGTTGCAACTCACAGTCTGGGGCATC
>EU839597

TTCTACTGTAATGCAACAGAGCTGTTTAATAGTACTTGGAATGATACTACTGGGAGTGTTACTGAA-------
----

GGTAATGACACAATCACACTTCCATGCAGAATAAAACAAATTATAAACATGTGGCAAGAAGTAGGAAAAGCAA
TGTATGCCCCTCCCATCAGGGGATATATTAACTGTTCATCAAAC---
ATTACAGGGCTGCTATTAATAAGAGATGGGGGTATTAATAATACA----------------------------
----

GGCAATGAGACCTTTAGACCTGGAGGGGGAAATATGAAGGACAATTGGAGAAGTGAATTATATAAATATAAAG
TAGTAAGAATTGAACCATTA---GGAGTAGCACCCACCAAGGCAAAGAGAAGAGTGGTG--------------
-------------------------CAGAGAGAAAAAAGAGCA---GTGGGA---ATAGGAGCT---------
GTGTTCCTTGGGTTCTTGGGAGCA---GCAGGAAGCACTATG---GGCGCAGCGTCA---
ATGACGCTGACGGTACAG---GCCAGACAA---TTATTG---TCTGGTATAGTGCAACAGCAAAAC---
AATTTG---CTGAGA---GCTATTGAGGCGCAACAGCATCTGTTGCAACTCACAGTCTGGGGCATC
>EU839596
TTCTACTGTAATTCAACACAACTGTTTAATAGTACTTGGCTTGCTAATAGTACTTGGAATGATAATGATACTA
GT---

ACTGATAATAGTACTATCACACTCCCCTGTAGAATAAAACAAATTATAAACATGTGGCAGGAAGTAGGCAAAG
CAATGTATGCCCCTCCCATCAGTGGACAAATTAACTGTTCATCAAAT---
ATCACAGGGCTACTATTAACAAGAGATGGTGGTAATGAAAGTGAG----------------------------
----

TTCGAGATCTTCAGACCTGGAGGAGGAAATATGAAGGACAATTGGAGAAGTGAATTGTATAAATATAAAGTAG
TAAAAATTGAACCATTA---GGAGTAGCACCCACCAGGGCAAGGAGAAGAGTGGTG-----------------
----------------------CAGAGAGAAAAAAGAGCA---GTGGGA---ATAGGAGCT---------
GTGTTCCTTGGGTTCTTAGGAGCA---GCAGGAAGCACTATG---GGCGCAGCGTCC---
ATAACGCTGACGGTACAA---GCCAGACAA---TTATTG---TCTGGTATAGTGCAGCAGCAAAAC---
AATTTG---CTGAGG---GCTATTGAGGCGCAACAACATTTGTTGCAACTCACAGTCTGGGGCATC
>FJ817365
TTCTACTGTAATACAACACAACTGTTTAATAGTAGTTGGAATGTAAATAAAACTGTAAATGACACT-------
----

GAAAAGGAAAATATCACACTCCCATGTAGAATAAAACAAATTATAAACCTGTGGCAGGAAGTAGGAAAAGCAA
TGTATGCCCCTCCCATCAGTGGACAAATTAGATGTTCATCAAAT---
ATTACAGGGTTACTACTAATGAGAGATGGTGGTAAAGAGGAAAAC----------------------------
----

GGGACCATCATCTTCAGACCTGGAGGAGGAAATATGAGGGACAATTGGAGAAGTGAATTATACAAATATAAAG
TAGTAAAACTTGAACCATTA---GGAATAGCACCCACCAGGGCAAAGAGAAGAGCGGTG--------------
-------------------------CAGAGAGAAAAAAGAGCA---GTGGGC---ATAGGAGCT---------
CTGTTCCTTGGGTTCTTGGGAGCA---GCAGGAAGCACTATG---GGCGCAGCGTCG---
ATGACGCTGACGGTACAG---GCCAGACAA---TTATTG---TCTGGTATAGTGCAACAGCAAAAC---
AATCTG---CTGAGG---GCTATTGAGGCGCAACAACATCTGTTGCAACTCACAGTCTGGGGCATC
>KY658702
TTCTACTGTAATACAACACCATTGTTTAATAGTACTTGGAATAGTACTACTTGGAATAGTAATAATACTGCAA
ATGAA--

AATGATACAACCATCACACTGCAATGTAGAATAAAACAAATCATAAACATGTGGCAGGAAGTAGGAAAAGCAA
TGTATGCTCCTCCCATCAGGGGAAATATTACATGTATGTCAAAT---
ATTACAGGAGTATTATTAATAAGAGATGGTGGCAATACAAGTGAGGCA-------------------------
----

AACAGCACTGAGATCTTCAGACCAGGAGGAGGAGATATGAGGGACAATTGGAGAAGTGAATTATATAAATATA
AGGTAGTAAAAATTGAACCATTA---GGTATAGCACCCACCAAGGCAAGAAGAAGAGTGGTG-----------
----------------------------CAGAGAGAAAGAAGAGCA---GTGGGAACACTGGGAGCT------
---CTGTTCCTTGGGTTCTTGGGAGCA---GCAGGAAGCACTATG---GGCGCAGCGTCA---
GTGGCGCTGACGGTACAG---GCCAGACAA---TTATTG---TCTGGTATAGTGCAACAGCAGAAC---
AATTTG---CTGAGA---GCTATTGAGGCGCAACAGCATATGTTGCAGCTCACGGTCTGGGGCATC
>AY173960

TTCTACTGTGATACAACAAACCTGTTTAATAGTACTTGGAATGAAACACAACTATTTAATAGTACTTGGAATA
AT---

ACTGGGAATGAAAATATCACACTTCCATGCAGAATAAAACAAATTATAAACAGGTGGCAGGAAGTAGGAAGAG
CAATGTATGCCCCTCCCATCCAAGGACAAATAAACTGCTCCTCAAAT---
ATTACAGGGCTGCTATTGACAAGAGATGGTGATAATAGTACAAAT----------------------------
----

AATGAAACCTTCAGACCTATAGGAGGAGATATGAAGGATAATTGGAGAAGTGAATTATATAAATATAAAGTAG
TACAAATTGAGCCATTA---GGACTAGCACCCACCAAGGCAAGGAGAAGAGTGGTG-----------------
----------------------CAAAGAGAAAAAAGAGCA---GTGGGAACATTAGGAGCT---------
ATGTTCCTTGGGTTCTTGGGAGCA---GCAGGAAGCACTATG---GGCGCAGCGTCA---
ATGGCGCTGACGGTACAG---GCCAGACAA---TTACTG---TCTGGTATAGTGCAACAGCAAAAC---
AATTTG---CTGAGA---GCTATTGAGGCGCAGCAGCATATGTTGCAACTCACAGTCTGGGGCATT
>KC595150
TTCTACTGTAATTCATCACAACTGTTTAATAGTACTTGGAATAATAATACTAAAGGGTTAAATAACACTGAA-
----

GGAAATGGCACAATCACACTCCCATGCAGAATAAAACAAATTATAAACATGCGGCAGGAAGTAGGAAAAGCAA
TGTATGCCCCTCCTATCAGAGGACAAATTAGATGTTCATCAAAT---
ATTACAGGGCTAATATTAACAAGAGATGGTGGTAATAACACGAAC----------------------------
----

GGGTCCGAGATCTTCAGACCTGGAGGAGGAAATATGAGGGACAATTGGAGAAGTGAATTATATAAATATAAAG
TAGTAGAAATTGAACCATTA---GGAGTAGCACCCACCAAGGCAAAGAGAAGAGTGGTG--------------
-------------------------CAGAGAGAAAAAAGAGCA---GTGGGA---ATAGGAGCT---------
ATGTTCCTTGGGTTCTTGGGAGCA---GCAGGAAGCACTATG---GGCGCAGCGTCA---
ATGACGCTGACGGTACAG---GCCAGACTG---TTATTG---TCTGGTATAGTGCAACAGCAGAAT---
AATTTG---CTGAGG---GCTATTGAGGCGCAACAGCATCTGTTGCAACTCACAGTCTGGGGCATC
>KC595152
TTCTACTGTAATTCAACACAACTGTTTAATAGTACTTGGAATAATACTCGGAATAATACTAAAGGG-------
----

TCAAATAACACTATCACACTCCCATGTAGAATAAAACAAATTGTAAACATGTGGCAGGAGGTAGGAAAAGCAA
TGTATGCCCCTCCCATCAGAGGACAAATTAGATGTTCATCAAAT---
ATTACAGGGCTGCTATTAACAAGAGATGGTGGTAAAAACGATAAC----------------------------
----

GAGACTGAGATCTTCAGGCCTGGAGGAGGAGATATGAGGGACAATTGGAGAAGTGAATTATATAAATATAAAG
TAGTAAAAATTGAACCATTA---GGAGTAGCACCCACCAGGGCAAAGAGAAGAGTGGTG--------------
-------------------------CAGAGAGAAAAAAGAGCA---GTGGGA---ATAGGAGCT---------
GTGTTCCTTGGGTTCTTGGGAGCA---GCAGGAAGCACTATG---GGCGCAGCGTCA---
ATGACGCTGACGGTACAG---GCCAGACAA---TTATTG---TCTGGTATAGTGCAACAGCAGAAC---
AATCTG---CTGAGG---GCTATTGAGGCGCAACAGCATCTGTTGCAACTCACAGTCTGGGGCATC
>KC595154
TTCTACTGTAATACATCACAACTGTTTAATAGTATTTGGAATAATAATAATAGTACCTGGAATAGGACGGGGG
AGGGGTCA---
-------------------AAC---
AACACTGAAGAAATTATTATGCTCCCATGCAGAATAAAGCAAATTATAAACATGTGGCAGGAAGTAGGAAAAG
CAATGTATGCCCCTCCCATCCGTGGAATAATTAGATGCTCATCAAAT---
ATTACAGGGCTACTATTAACAGGAGATGGTGGTAATAACACTGACACT-------------------------
----

AACGCCAACGAGACTTTCAGACCTGGGGGAGGAGATATGAGGGACAATTGGAGAAGTGAATTATATAAATATA
AAGTAGTAAAAATTGAACCATTA---GGAGCAGCACCCACCAAGGAAAAGAGAAGAGTGGTG-----------
----------------------------CAGAGAGAAAAAAGAGCA---GGGGGG---ATAGTCTTT------
---TTGTTCCTTGGGTTCTTGGGAGCA---ACAGGAAGCACTATG---CCCGCAACGTCA---
TTGACGTTGACGGTACAG---GCCAGACTA---TTATTG---TCTGGTATAGTGCAACAGCAGAAC---
AACCTG---CTGAGG---GCTATTGAGGCGCAACAGCATCTATTGCAACTCACAGTCTGGGGCATC
>KC595157

TTCTACTGTAACTCAACACCGTTGTTTAATAGTACTTGGAGTAATACTACCGAGTCAAATAGC----------
----

ACAGCCAATAATATCACACTCCAATGCAAAATAAAACAAATTATAAACCTGTGGCAAGAAGTAGGAAAAGCAA
TGTATGCCCCTCCCATCAGAGGACAGATTAGATGTTCATCAAAT---
ATTACAGGACTGTTATTAACAAGAGATGGTGGTGACAAGAATGAC----------------------------
----

ACCGAGGTCTTCAGACCGGGAGGAGGAAATATGAAGGACAATTGGAGAAGTGAATTATATAAATATAAAGTAG
TAAAAATTGAACCATTA---GGAGTAGCACCCACCAAGGCAAAGAGAAGAACGGTG-----------------
----------------------CAGAGAGAAAAAAGAGCA---GTA------ATAGGAGCA---------
ATGTTCCTTGGGTTCTTGGGAGCA---GCAGGAAGCACTATG---GGCGCAGCGTCA---
GTGACGTTGACGGTACAG---GCCAGACTA---TTATTG---TCTGGTATAGTGCAACAGCAGAGC---
AATCTG---CTGAGG---GCTATTGAGGCGCAACAGCATCTGTTGCAACTCACAGTCTGGGGCATC
>KC595160
TTCTACTGTAATTCAACACAACTGTTTAATAGTACTTGGAATAATAATAGTACTTGGAAGACTTGGGAGATTG
GAGGG--

CCAAATGACACACTCACACTCCCATGCAGAATAAAACAAATTATAAACATGTGGCAGGAAGTAGGAAAAGCAA
TGTATGCCCCGCCCATCAGAGGACAAATTAGATGCTCATCAACT---
ATTACAGGGTTGCTATTAACAAGAGATGGAGGTAGTAACACTAAC----------------------------
----

GAGACCGAGATTTTCAGACCTGGAGGAGGAGATATGAGGGACAATTGGAGAAGTGAATTGTATAAATATAAAG
TAGTAAAAATTGAACCATTA---GGAGTAGCACCCACCAGGGCAAAGAGAAGAGTGGTG--------------
-------------------------CAGAGAGAAAAAAGAGCA---GCA------ATAGGAGCT---------
TTGTTCCTTGGGTTCTTGGGAGCA---GCAGGAAGCACTATG---GGCGCAGCGTCA---
ATGACGCTGACGGTACAG---GCCAGACTA---TTATTG---TCTGGTATAGTGCAACAGCAGAAC---
AATTTG---CTGAGG---GCTATTGAGGCGCAACAGCATCTGTTGCAACTCACAGTCTGGGGCATC
>KC595161
TTCTACTGTAATACAACACAACTGTTTAATAGTACTTGGAGTAATACTGGAGCGTCAAATGGCACTGAA----
----

GGAAATGACACAATCACACTCCCATGCAAGATAAGACAAATTATAAACATGTGGCAGGAAGTAGGAAAAGCAA
TGTATGCTCCTCCCATCAGAGGACAAATTAGATGTTCATCAAAT---
ATTACAGGGCTGCTATTAACAAGAGATGGTGGTAATAACACGAAC----------------------------
----

GGGACCGAGATCTTCAGACCTGGGGGAGGAGATATGAGGGACAATTGGAGAAGTGAATTATATAAATATAAAG
TAGTAAAAATTGAACCATTA---GGAGTAGCACCCACCAAGGCAAAGAGAAGAGTGGTG--------------
-------------------------CAGAGAGAAAAAAGAGCG------GGA---ATAGGAGCT---------
TTGTTCCTTGGGTTCTTGGGAGCA---GCAGGAAGCACTATG---GGCGCAGCGTCA---
ATAACGCTGACGGTACAG---GCCAGACTA---TTATTG---TCTGGTATAGTGCAACAGCAGAAT---
AATTTG---CTGAGG---GCTATTGAGGCGCAACAGCACCTGTTGCAACTCACAGTCTGGGGCATC
>KC595162
TTCTACTGTAATTCAACACAACTGTTTAATAGTACTTGGAATGCTAATAGTACTTGGAATGGTACTGAAGAGC
CAAGTAAC---

TTTGCAGAAAATATCACACTCCCATGCAAAATAAAACAAATTATAAACATGTGGCAGGAAGTAGGAAAAGCAA
TGTATGCCCCTCCCATCAGAGGACAAATTAGATGCTCATCAAAT---
ATTACAGGGTTGCTATTAACAAGAGATGGTGGTAATAACAAAAGC----------------------------
----

GAGGCCACCGAGACCTTCAGACCTGGAGGAGGAGATATGAGGGACAATTGGAGAAGTGAATTATATAAATATA
AAGTAGTAAAAATTGAACCATTA---GGAGTAGCACCCACCAGGGCAAAGAGAAGAGTGGTG-----------
----------------------------CAGAGAGAAAAAAGAGCA---GTGGGA---ATAGGAGCG------
---TTGTTCCTTGGGTTCTTGGGAGCA---GCAGGAAGCACTATG---GGCGCAGCGTCA---
ATGACGTTGACGGTACAG---GCCAGACTA---TTATTG---TCTGGTATAGTGCAACAGCAGAAC---
AATTTG---CTGAGG---GCTATTGAGGCGCAACAGCATCTGTTGCAACTCACAGTCTGGGGCATC
>KC595163

TTCTACTGTGATACAACACAACTGTTTAATAGTACTTGGAATGATACTACAAATGACACTGGCAAAAAT----
----

GACAATGACACAATCATTCTCCAATGCAGAATAAAACAAATTATAAACATGTGGCAGGAAGTAGGAAAAGCAA
TATATGCCCCTCCCATCCAAGGACAAATTAACTGTTCATCAAAT---
ATTACAGGGCTGCTACTAACAAGAGATGGTGGTAATACTAATAACACG-------------------------
----

AACGGGACTGAGACCTTCAGACCTGGAGGAGGAGATATGAGGGACAATTGGAGAAGTGAATTATATAAATATA
AAGTAGTAAAAATTGAACCATTA---GGAGTAGCACCCACCAAGGCAAAGAGAAGAGTGGTG-----------
----------------------------CAGAGAGAAAAAAGAGCA---GTGGGA---ATAGGAGCT------
---GTGTTCCTTGGGTTCTTGGGAGCA---GCAGGGAGCACTATG---GGCGCAGCGTCA---
ATGACGCTGACGGTACAG---GCCAGACAA---TTATTG---TCTGGTATAGTGCAACAGCAGAAT---
AATTTG---CTGAGA---GCTATTGAGGCGCAACAACATATGTTGCAACTCACAGTCTGGGGCATC
>KC595165
TTCTACTGTAATTCAACACAATTGTTTAATAGTACTTGGAACCGGACTGGTACTGAGGGGTTAAATACCACTG
AA---

GGAAATGGCACATTCACACTTCCATGTAGAATAAAACAATTTATAAACTTGTGGCAGGGAGTAGGAAAAGCAA
TGTATGCCCCTCCCATCAGGGGACAAATTAGATGTTCATCAAAC---
ATTACAGGGCTACTATTAACAAGAGATGGTGGTAATAAAATGAAC----------------------------
----

GAGACAAGCGAGACCGAGACCTTCAGACCTGGAGGAGGAAATATGAGGGACAATTGGAGAAGTGAATTATATA
AATATAAAGTAGTAAGAATTGAACCATTA---GGAATAGCACCCACCAAGGCAAAGAGAAGAGTGGTG-----
----------------------------------CAGAGAGAAAAAAGAGCA---GTGGGA---
ATAGGAGCT---------GTGTTCCTTGGGTTCTTGGGAGCA---GCAGGAAGCACTATG---
GGCGCAGCGTCA---ATGACGCTGACGGTACAG---GCTAGACTA---TTATTG---
TCTGGTATAGTGCAACAGCAGAAT---AATTTG---CTGAGA---
GCTATTGAGGCGCAACAACATCTGTTGCAACTCACAGTCTGGGGCATC
>KC595197
TTCTACTGCAATACAACACAACTGTTTAATAGTACTTGGAATAGTACTACTAGGAATGAC-------------
----

ACTGAAGGAAATATTACACTCCCATGCAGAATAAAACAAATTATAAACATGTGGCAGGAAGTAGGAAAAGCAA
TGTATGCCCCTCCCATCAGTGGACAAATTAGTTGTTCGTCAAAT---
ATTACAGGGATACTATTAACAAGAGATGGGGGTAATACGAACGGT----------------------------
----

AATAATACTGAAACCTTCAGACCTGGAGGAGGAGATATGAGGGACAATTGGAGAAGTGAATTATATAAATATA
AAGTAGTAAAAATTGAACCATTA---GGAATAGCACCCACCAGGGCAAAGAGAAGAGTGGTG-----------
----------------------------CAGAAAGAAAAAAGAGCA---GTGGGG---ATAGGAGCT------
---GTGTTCCTTGGGTTCTTGGGAGCA---GCAGGAAGCACTATG---GGCGCAGCATCA---
ATGACGCTGACGGTACAG---GCCAGACTA---TTATTG---TCTGGTATAGTGCAACAGCAAAAC---
AATTTG---CTGAGA---GCTATTGAGGCGCAACAGCATCTGTTGCAACTCACAGTCTGGGGCATC
>KC595202
TTCTACTGTAATACAACACAACTGTTTAATAGTACTTGGAATGCTACTGAAGAGACAAATAAC----------
----

ACTGAAGGCACAATCACACTTCCATGCAGAATAAAACAAATTATAAACATGTGGCAGGAAGTAGGAAAAGCAA
TGTATGCCCCTCCCATCAGAGGAAATATTAGCTGCTCATCAAAT---
ATTACAGGGGTGATATTAACAAGAGATGGTGGCACAGACAATAGC----------------------------
----

GAGACCGAGACCTTTAGACCGGGAGGAGGAGATATGAAGGACAATTGGAGAAGTGAATTGTATAAATATAAAG
TAGTAAAAATTGAGCCATTA---GGAATAGCACCCACCAAGGCAAAAAGAAGAGTGGTG--------------
-------------------------CAGAGAGAAAAAAGAGCA---GTGGGA---ATAGGAGCT---------
GTGTTCCTTGGGTTCTTGGGAGCA---GCAGGAAGCACTATG---GGCGCAGCGTCA---
ATGACGTTGACGGTACAG---GCCAGACTA---TTATTG---TCTGGTATAGTGCAACAGCAGAAC---
AATTTG---CTGAGG---GCTATTGAGGCGCAACAGCATCTGTTGAAACTCACAGTCTGGGGCATC

>KC595203
TTCTACTGTAATTCAACACAACTGTTTAATAGTACCTGGGATAATAGTAGTATTGAGAATAATACAGAGTCA-
----

GGAAATGAAACTATCACACTCCCATGCAGGATAAAACAAATTATAAACTTGTGGCAGGAAGTAGGAAAAGCAA
TGTATGCCCCTCCCATCAGAGGGCCAATTAATTGCTCATCAGAT---
ATTACAGGGCTGCTATTAACAAGAGATGGTGGTACTAATGGCCCT----------------------------
----

AACGATACTGAGACCTTCAGACCTGGAGGAGGAAACATGAAGGACAATTGGAGAAGTGAATTATATAAATATA
AAGTAGTAAGAATTGAACCACTA---GGAATAGCACCCACCAATGCAAGGAGAAGAGTGGTG-----------
----------------------------CAGAGAGAAAAAAGAGCA---GTGGGG---CTAGGAGCC------
---TTGTTCCTTGGGTTCTTGGGAGCA---GCAGGAAGCACTATG---GGCGCAACGTCA---
ATGGCGTTGACGGTACAG---GCCAGACAA---TTATTG---TCTGGTATAGTGCAACAGCAAAAC---
AATTTG---CTGAGG---GCCATTGAGGCGCAACAGCAACTGTTGCAACTCACAGTTTGGGGCATC
>KC595158
TTCTACTGTAATACAACACAACTGTTTAATAGTACTTGGGGTAATAATAGTACTTGGAGTAATACTACCGAGT
CAGGTGGC---

AATGGAACAAATATCATACTTCCATGCAGAATAAAACAAATTATAAACATGTGGCAGGAAGTAGGAAAAGCAA
TGTATGCCCCTCCCATCAGAGGACAAATTAGCTGCTCATCAAAT---
ATTACAGGGCTGCTATTAACAAGAGATGGTGGTAACAGTAACACG----------------------------
----

ACTGAGACCTTCAGACCTGGAGGAGGAAATATGAAGGACAATTGGAGAAGTGAATTATATAAATATAAAGTAG
TAAAAATTGAACCATTA---GGAGTAGCACCCACCAGGGCAAAGAGAAGAGTGGTG-----------------
----------------------CAAAGAGAAAAAAGAGCA---GTGGGA---ATAGGAGCT---------
GTGTTCCTTGGGTTCTTGGGAGCA---GCAGGAAGCACTATG---GGCGCAGCGTCC---
ATGACGTTGACGGTACAG---GCCAGACTA---TTATTG---TCTGGTATAGTGCAACAGCAGAAC---
AATTTG---CTGAGA---GCTATTGAGGCACAACAGCATCTGTTGCAACTCACAGTCTGGGGCATC
>KC595168
TTTTACTGTAATTCAACACAACTGTTTAATAGTACTTGGGAGTTTAATAGTACTTGGAATAGTACTTGGAATG
AT---

ACTGGAGAAAAGATCACACTCCCATGCAGAATAAAACAAATTATAAACATGTGGCAGGAAGTAGGAAAAGCAA
TGTATGCCCCTCCCATCAGAGGACAAATTAGATGCTCATCAAAT---
ATTACAGGACTGCTATTAACAAGAGATGGTGGCAATAACAAGAGC----------------------------
----

GACACTGAAACCTTCAGACCTGGAGGAGGAGACATGAGGGACAATTGGAGAAGTGAATTATATAAATATAAAG
TAGTAAAAATTAAACCATTA---GGAGTAGCACCCACCAAGGCAAAAAGAAGAGTGGTG--------------
-------------------------CAGAGAGAAAAAAGAGCA---GTGGGA---ATAGGAGCT---------
ATATTCCTTGGGTTCTTGGGAGCA---GCCGGAAGCACTATG---GGCGCAGCGTCA---
ATGACGTTGACGGTACCG---GCCAGACAA---TTATTG---TCTGGTATAGTGCAACAGCAGAAC---
AATTTG---CTGAGG---GCTATTGAGGCGCAACAACATCTGTTGCAACTCACAGTCTGGGGCATT
>KC595190
TTTTACTGTAATACAACGGGACTGTTTAATAGTACTTGGAGTTGGAATGATACTAACACAAATGACACAAGAG
AA---

AATAACACACATATCATACTCCCATGCAGAATAAAACAAATTATAAACATGTGGCAGGGAGTAGGAAAAGCAA
TGTATGCCCCGCCCATCAGAGGACAAATTAGATGTTCATCAAAT---
ATTACCGGGTTGCTATTAACAAGAGATGGTGGTGGTAACGGGAAC----------------------------
----

AATACCACCGAGGTCTTCAGACCTGGAGGAGGAGATATGAAGGACAATTGGAGAAGTGAATTATATAAATATA
AAGTAGTAAAAAATTGAACCATTA---GGAGTAGCACCCACCAAGGCAAAGAGAAGAGTGGTG----------
----------------------------CAGAGAGAAAAAAGAGCA---ATAGGA---ATAGGAGCT------
---ATGTTCCTTGGGTTCTTGGGAGCA---GCAGGAAGCACTATG---GGCGCAGCATCA---
ATAACGCTGACGGTACAG---GCCAGACAA---TTGTTG---TCTGGTATAGTGCAACAGCAGAAC---
AATTTG---CTGAGG---GCTATTGAGGCGCAACAACATCTGTTGCAACTCACAGTCTGGGGCATC

>KT200351
TTCTACTGTGATACATCAAAATTGTTTAATAGTACTTGGTCTAATAATGATAGTGTTATTAATAGCACTGAAG
TA---

AATAACACAGTCATCACACTCCCATGTAGAATAAAACAAATTATAAACATGTGGCAGGAAGTAGGAAAAGCAA
TGTATGCCCCGCCCATCAGAGGACAAATTAACTGTTCATCAAAT---
ATTACAGGGCTGTTATTAACAAGAGATGGTGGTAACAATGCCACC----------------------------
----

AATGAGACTTTCAGACCGGCAGGAGGAGATATGAGGGACAATTGGAGAAGTGAATTATATAAATACAAAGTAG
TAAGAATTGAACCACTA---GGAATAGCACCCACCAAGGCAAAGAGAAGAGTGGTG-----------------
----------------------CAAAGAGAAAAAAGAGCA---GTGGGA---ATAGGAGCT---------
GTGTTCCTTGGGTTTCTGGGAGCA---GCAGGAAGCACGATG---GGCGCAGCGTCA---
TTGACGCTGACGGTACAG---GCCAGACTA---TTATTG---TCTGGTATAGTGCAGCAGCAGAAC---
AATCTG---CTGAGG---GCTATTGAGGCGCAACAGCATCTGTTGCAACTCACAGTCTGGGGCATC
>EU786676
TTTTACTGTAATACATCACTACTGTTTAATAGTACCTGGAATGGTACTGGAGGGACAGATACTGAA-------
----

GGAAATGAAACAATCACACTCCCATGCAGAATAAAACAAATTATAAACATGTGGCAAGAAGTAGGAAAAGCAA
TGTATGCCCCTCCCATCAGAGGACTAATTAGATGCTCATCAAAC---
ATTACAGGGTTGCTATTAACAAGAGATGGTGGGAAAGAGAGTAAT----------------------------
----

AGCACTACAGAAACCTTCAGACCTGGAGGAGGAGATATGAGGGACAATTGGAGAAATGAATTATACAAATATA
AAGTAGTAAAAATTGAACCATTA---GGAGTAGCACCCACCAAGGCAAAGAGAAGAGTGGTG-----------
----------------------------CAGAGAGAAAAAAGAGCA---GTGGGA---ATAGGAGCT------
---TTGTTCCTTGGGTTCTTGGGAGCA---GCAGGAAGCACTATG---GGCGCAGCGTCG---
ATGACGTTGACGGTACAA---GCCAGACAA---TTATTG---TCTGGTATAGTGCAACAGCAGAAC---
AACTTG---CTGAGG---GCTATTGAGGCGCAACAGCATCTGTTGCAACTCACAGTCTGGGGCATC
>KC595196
TTCTACTGTAATTCAACACAACTGTTTAATAGTACTTGGAACGATACTGGAGGATCAAATAATACTGGAGAAT
CAAAT--

AGCACTGGAACAATCACACTCCCATGCAGAATAAGACAAGTTGTAAACATGTGGCAGGAAGTAGGAAAAGCAA
TGTACGCCCCTCCCATCAAAGGACAAATTAGATGTTCATCAAAT---
ATTACGGGGCTGCTCTTAACAAGAGATGGTGGTGATAACGGGACC----------------------------
----

ACCGAGATTTTCAGACCTGGGGGAGGAGATATGAGGGACAATTGGAGAAGTGAATTATATAAATATAAGGTAG
TAAAAATTGAGCCATTG---GGAGTGGCACCCACCAAGGCAAAGAGAAGGGTGGTG-----------------
----------------------CAGAGAGAAAAAAGAGCA---ATGGGA---ATAGGAGCT---------
CTGTTCCTTGGGTTCTTGGGAGCA---GCAGGAAGCACTATG---GGCGCAGCGTCA---
ATGACGTTGACGGTACAG---GCCAGACTA---TTGTTG---TCTGGTATAGTGCAACAGCAGAAC---
AATTTG---CTGAGG---GCTATTGAGGCGCAACAGCATCTATTGCAACTCACAGTCTGGGGCATC
>EU786678
TTCTACTGTAATACAACAGCACTGTTTAATAGTACCTGGAATACTAATGGTACTTGGAAGGGTACAACAGGA-
----

GAAAATGACACCATCACACTCCAATGCAGAATAAAACAAATCATAAACATGTGGCAGGAAGTAGGAAAAGCAA
TGTATGCTCCTCCCATCAGAGGACAAATTAGATGCACATCAAAT---
ATTACAGGAATTCTATTAACAAGAGATGGTGGTAAAAGCAATAACAAT-------------------------
----

AGCAATGGGAGCGAGATCTTCAGACCTGGAGGAGGAGATATGAGGGACAATTGGAGAAGTGAACTATATAAAT
ATAAAGTAGTAAAAATTGAACCATTA---GGAATAGCACCCTCCAAGGCAAGGAGAAGAGTGGTG--------
-------------------------------CAGAGAGAAAAAAGAGCA---GTGGGA---ATAGGAGCT---
------CTGTTCCTTGGGTTCTTGGGAGCA---GCAGGAAGCACTATG---GGCGCAGCGTCA---
ATGGCGTTGACGGTACAG---GCCAGACAA---TTATTG---TCTGGTATAGTGCAACAGCAGAAC---
AATCTG---CTGAGG---GCTATTGAGGTGCAACAGCATATGTTGCAACTCACAGTCTGGGGCATT

>EU786680
TTCTACTGTGATTCAACAAAACTGTTTAATAGTACCTGGTATTTTAATGGTACTTGGAATAATACAAATAATA
ATAAT---

GATACCCATGATCCCATCACACTCCCATGCAGAATAAAACAAATTGTAAACATGTGGCAGGAAGTAGGAAAAG
CAATGTATGCCCCTCCCATCAAGGGACAAATTAGATGCTCATCAAAT---
ATTACAGGGCTGATATTAACAAGAGATGGTGGTGTTGATAACAGC---------------------------
----

AGCACCAATGAGACCTTCAGACCTGGAGGAGGAGATATGAGGGACAATTGGAGAAGTGAATTATATAAATATA
AAGTAGTAGAACTTAAACCATTA---GGAATAGCACCCACCAGGGCAAAGAGAAGAGTGGTG----------
---------------------------CAAAGAGAGAAAAGAGCA---GTGGGA---ATAGGAGCT------
---ATGTTCCTTGGGTTCTTGGGAGCA---GCAGGAAGCACTATG---GGCGCAGCGTCA---
ATGACGCTGACGGTACAG---GCCAGACTA---TTATTG---TCTGGTATAGTGCAACAGCAGAGC---
AATTTG---CTGAGA---GCTATTGAGGCGCAACAGCATCTGTTGCAACTCACAGTCTGGGGCATC
>HQ236609
TTCTACTGTGATTCAACACAACTGTTTAATAGTACTTGGTCGTATAATAGTACTAAGAATAGTTGGAGTAGTA
CTGAA---
-------------------GGG---
ACAAATGGCACCACCATCACACTTCCATGCAGTATAAAACAATTTGTGAGAATGTGGCAAAGAGTTGGACAAG
CAATGTATGCCCCTCCCATCGCAGGAAACATATCATGCAGGTCAAAC---
ATTACAGGGTTGATATTAACAAGAGATGGTGGCACCCCAGATGAT---------------------------
----

GGCAATAACGAAACCTTCAGACCTGGAGGTGGAGACATGAAAGACAATTGGAGAAGTGAATTATATAAATATA
AAGTAGTAAAGCTTGAACCATTA---GAAATAGCACCCACCAGGGCACAGAGAAGAGTGGTG----------
---------------------------CAGAGAGAAAAAGAG------CGGGGA---CTAGGAGCT------
---CTGTTCCTTGGGTTCTTGGGAGCA---GCAGGAAGCACTATG---GGCGCAGCGTCA---
ATGACGTTGACGGTACAG---GCCAGACAA---CTACTG---TCTGGTATAGTGCAACAGCAGAAC---
AATCTG---CTGAGG---GCTATTGAGGCGCAACAGCATATGTTGCAACTCACAGTCTGGGGCATA
>FJ817366
TTCTACTGTAATTCATCACAACTGTTTAATAGTACTTGGTATAGTAATGGGACTAGTAATGGAGGGTTAAAT-
----

AACAGTGCCAACATTACACTTCCATGCAGAATAAAACAAATTATAAATCTGTGGCAGGAAGTAGGAAAAGCAA
TGTATGCCCCTCCCATCAAAGGAGTAATTAACTGTTTATCAAAT---
ATTACAGGGATAATATTAACAAGAGATGGTGGTGAGAACAATGGC---------------------------
----

ACCACCGAGACCTTCAGACCTGGAGGGGGAGATATGAGGGACAATTGGAGAAGTGAATTATATAAATATAAAG
TAGTAAAAATTGAACCATTA---GGAATAGCACCCACCAAGGCAAAAAGAAGAGTGGTG-------------
------------------------CAAAGAGAGAAAAGAGCA---GTGGGA---TTAGGAGCT---------
GTGTTCCTTGGGTTCTTGGGACTA---GCCGGAAGCACTATG---GGCGCAGCGTCA---
GTGACGTTGACGGTACAG---GCCAGGCTA---TTATTG---TCTGGTATAGTGCAACAGCAGAAC---
AATTTG---CTGAGG---GCTATTGAGGCGCAGCAGCAGCTGCTGCAACTCACAGTCTGGGGCATC
>GQ372988
TTCTACTGTAATACATCAAAGCTGTTTAATAGTACGTTGTTTAGTAATAATACTTGGTTCAATAGTACTATGA
ATGAA---
-------------------ACT---
AATACAAATGACACTATCATGATTCCATGCAGGATAAAACAAATTATAAACAGGTGGCAAGAAGTAGGAAAAG
CAATGTATGCCCCTCCCATCAGTGGACTAGTTAGATGTTCATCAAAT---
ATTACAGGGCTGTTATTAACAAGAGATGGTGGTGGAGGCCAGAACCAGACT---------------------
----

GAAAACCAGACTGAGATCTTCAGACCTGGAGGAGGAGATATGAGGGACAATTGGAGAAGTGAATTATATAAGT
ATAAAGTAGTAAGAATTGAACCATTA---GGAATAGCACCCACCAAGGCAAAGAGAAGAGTGGTG-------
------------------------------CAGAGAGAAAAAAGAGCA---GTGGGAACAATAGGAGCT---
------ATGTTCCTTGGGTTCTTGGGAGCA---GCAGGAAGCACTATG---GGCGCAGCGTCA---
GTCACGCTGACGGTACAG---GCCAGACTA---TTGTTG---TCTGGTATAGTGCAACAGCAGAAC---
AACTTG---CTGAAG---GCTATTGAGGCGCAACAACATCTGTTGCAACTCACAGTCTGGGGCATC

>FJ670531
TTCTACTGTGATTCAAAAGGACTGTTTAATAGTACTTGGAAGCTGGTTAATAGTACTTGGGAGCTGGTTAATG
GTACTTTGGAT--
----------TCAATAACA------
AATGGTACTGTCAATATCACACTCCCATGCAAAATAAAACAAATTGTAAACCGGTGGCAGGAAGTAGGGAAAG
CAATGTATGCCCCTCCCATCAAAGGGCCAATTAACTGCACATCAAAT---
ATTACAGGGCTGCTATTAACAAGAGATGGTGGTAATGACAAAAAC----------------------------
----

CAGTCCATTGAGATCTTCAGACCGGGAGGAGGAGATATGAGGGACAATTGGAGAAGTGAATTATATAAATATA
AAGTAGTAAAAATTGAACCATTA---GGAGTAGCACCCACCAAGGCAAAGAGAAGAGTGGTG-----------
----------------------------CAGAGAGAAAAAAGAGCA---GTGGGA---TTAGGAGCT------
---ATGTTCCTTGGGTTCTTGGGAGCA---GCAGGAAGCACTATG---GGCGCAGCGTCA---
GTGACGTTGACGGTACAG---GCCAGACTA---TTATTG---TCTGGTATAGTGCAACAGCAGAAC---
AATTTG---CTGAGG---GCTATTGAGGCGCAACAGCATCTGTTGCAACTCACAGTCTGGGGCATC
>FJ853620
TTCTACTGTAATACAACACAACTGTTTAATAGTACTTGGAATAGTACTATTAGGAATGACACTGAA-------
----

GCTGGAGAAAATATTACACTCACATGCAGAATAAAACAAATTATAAATCTGTGGCAGGAAGTAGGAAAAGCAA
TGTATGCCCCTCCCATCAGCGGACAAATTAGTTGTTCATCAAAT---
ATTACAGGGATACTATTAACAAGGATGGGGGTAAAAACAGTAAT----------------------------
----

GATACTGAGATCTTCAGACCTGGAGGAGGAGATATGAGGGACAATTGGAGAAGTGAATTATATAAATATAAAG
TAGTAAAAATTGAACCATTA---GGAATAGCACCCACCAAGGCAAAGAGAAGAGTGGTG--------------
-------------------------CAAAGAGAAAAAAGAGCA---GTGGGA---ATAGGAGCT---------
GTGATCCTTGGGTTCTTGGGAGCA---GCAGGAAGCACTATG---GGCGCAGCATCA---
ATAACGCTGACGGTACAG---GCCAGGCTA---TTACTG---TCTGGTATAGTGCAACAGCAAAGC---
AATTTG---CTGAGA---GCTATTGAGGTGCAACAGCATCTGTTGCAACTCACAGTCTGGGGCATC
>KT200349
TTCTACTGTAATACATCAGAACTATTTAATAGTACTTGGCAGTTTAATAATAATAATACTGGAAATAATAGT-
----

GCTGAAAGAAATATCACAATCCCATGTAGAGTAAGACAAATTATAAACATGTGGCAGAGAGTAGGAAAAGCAA
TGTATGCCCCTCCCATCGCAGGACAAATTAGATGTGTATCAAAT---
ATTACGGGGATGCTATTAACAAGAGATGGTGGTAATGATGGTGGT----------------------------
----
AATAACAGCACGAAC---
GTGAGCGAGATCTTCAGACCGGCAGGAGGAGAGATGAGGGACAATTGGAGAAGTGAATTATATAAATACAAAG
TAGTAAAAATTGAACCATTA---GGGATAGCGCCCACCAGGGCAAAGAGAAGAGTGGTG--------------
-------------------------CAGAGAGAAAAAAGAGCA---GTGGGA---ATAGGAGCT---------
GTGTTCCTTGGGTTCTTGGGAGCA---GCAGGAAGCACTATG---GGCGCAGCATCA---
ATGATGCTGACGGTACAG---GCTAGACAA---CTATTG---TCTGGTATAGTGCAACAGCAGAAC---
AATCTG---TTGAGA---GCTATTGAGGCGCAACAACATCTGTTGCAACTCACAGTCTGGGGCATC
>KT200354
TTCTACTGTAATACAACAAAACTATTTAATAGTACTTGGCAGTTTAATAGTACTGAGAGTGGTACTGGGAAAC
TA---

AATACCACTGACATCATACTTCCATGCAGGATAAAACAAATTGTAAACATGTGGCAGGAAGTAGGAAAAGCAA
TGTATGCCCCTCCAGTCACAGGAATAATTAGTTGTTTATCAAAT---
ATTACAGGACTGCTAATAACAAGAGATGGTGGTAATGATAATAAGACC-------------------------
----

AACAACACTGAGATCTTTAGACCTGGAGGAGGAAATATGAAGGACAATTGGAGAAGCGAATTATATAAATATA
AAGTAGTAAAAATTGAACCAATA---GGAATAGCACCCACCAAGGCAAAAAGAAGAGTGGTG-----------
----------------------------CAGAGAGAAAAAAGAGCAATAGTGGGA---ATAGGAGCT------
---GTGTTCCTTGGGTTCTTGGGAGCA---GCAGGAAGCACTATG---GGCGCAGCATCA---
ATAACGCTGACGGTACAA---GCCAGACAG---CTATTG---TCTGGTATAGTGCAACAGCAGAAC---
AATCTG---CTGAGG---GCTGTTGAGGCGCAACAGCATCTGTTGCAACTCACAGTCTGGGGCATC

>KT200358
TTCTACTGTAATACAACAAAGCTGTTTAATAGTACCTGGGAAACAAACAGGAGTGAAAGTGCAAAT-------
----

ACCAGTGAAACAATTATACTCCCATGTAGAATAAGACAAATTATAAACTTGTGGCAGAGAGTAGGAAAAGCAA
TGTATGCCCCTCCTATCAGAGGACAAATTAACTGCTCATCAAAT---
ATTACAGGGCTAATATTAACAAGAGATGGTGGGAATGATAATGATACT-------------------------
----

AATAATACTGAGACCTTTAGACCTGGAGGAGGAGATATGAGGGACAATTGGAGAAATGAATTATATAAATATA
AAGTGGTACAAATTGAACCAATA---GGACTGGCACCCACCAAGGCAAAGAGAAGAGTGGTG-----------
----------------------------CAGAGAGAAAAAAGAGCA---GTAACC---ATAGGAGCT------
---TTGTTCCTTGGGTTCTTAAGAGCA---GCAGGCAGCACTATA---GGCGCAGCGTCA---
ATGGCGCTGACGGTACAG---GCCAGACAA---TTATTG---TCTGGTATAGTGCAACAGCAGAGC---
AATCTG---CTAAGA---GCTATTGAGGCGCAACAGCATCTGTTGCAACTCACAGTCTGGGGCATC
>FJ853622
TTCTACTGTAATACAACAACACTGTTTAACAGTACTTGGTATAATAATGGAAGTTCAAATGACTCTGAAAAA-
----

AATGGCACCATCATCACACTCCCATGTAAAATAAAGCAAATTATAAACATGTGGCAGGAAGTAGGAAAAGCAA
TATATGCCCCTCCTATCGCAGGACAAATTAACTGTTCATCAAAT---
ATTACAGGGCTGCTATTAACAAGAGATGGTGGTAACAGAACTGATAGT-------------------------
----

AACCAGACCGAGACATTCAGACCTGAAGGGGGAAATATGAGGGACAATTGGAGAAGTGAATTATATAAATATA
AGGTAGTAAAAATTGAACCATTA---GGAGTAGCACCCACCAAGGCAAAGAGAAGAGTGGTG-----------
----------------------------CAGAGAGAAAGAAGAGCA---GTGGGAACGATGGGAGCT------
---ATGTTCCTTGGGTTCTTGGGAGCA---GCAGGAAGCACTATG---GGCGCAGCGTCA---
ATGACGCTGACGGTACAG---GCCAGGCTA---TTATTG---TCTGGTATAGTGCAACAGCAAAAC---
AACTTG---CTGAAG---GCTATTGAGGCGCAACAACATCTGTTGCAACTCACAGTCTGGGGCATC
>GU362883
TTCTACTGTAATACTACACAACTGTTTAATAGTACTTGGCTGCCTAATAGTACTTGGAATGATACTGCAAAC-
----

GACACAATCACAATCACACTCCCCTGCAGAATAAAACAAATTGTAAACATGTGGCAGGAAGTAGGAAAAGCAA
TGTATGCCCCTCCCATCAGAGGACCAATTATCTGTAAATCAAAT---
ATTACAGGGCTACTATTAACAAGAGATGGGGGTAACAAGACCAAT----------------------------
----

GACACATACGAGACCTTCAGACCTGGAGGAGGAGATATGAAGGACAATTGGAGAAGTGAATTATATAAATACA
AAGTAGTAAAAATTGAACCATTA---GGAGTAGCACCCACCAAGGCAAAGAGAAGAGTGGTG-----------
----------------------------CAGAGAGAAAAAAGAGCT---GTGGGA---ATAGGAGCT------
---GTATTCTTTGGGTTCTTGGGAGCA---GCAGGAAGCACTATG---GGCGCAGCGTCA---
CTGACGCTGACGGTACAG---GCCAGACAA---TTATTG---TCTGGTATAGTGCAACAGCAGAGC---
AATCTG---CTGAGG---GCTATTGAGGCGCAACAGCATCTGTTGCAACTCACAGTCTGGGGCATC
>GU362886
TTCTACTGTAATACAACACAACTATTTAATAGTACTTGGAATGAAACTAAGAATGAAACTGAGACAGGGAAC-
----

ACAAGTGACAGTATCACACTCCCATGCAGAATAAAACAAATTATAAACCTATGGCAGGAAGTAGGAAAAGCAA
TGTATGCCCCTCCCATCAAAGGACCAATTAGCTGTTCATCAAAT---
ATTACAGGGTTACTATTAACAAGAGATGGTGGCATAGAAAGCAAC----------------------------
----

GAGACAGAGATCTTCAGACCTGGAGGAGGAGATATGAGGGACAATTGGAGAAATGAATTATATAAATATAAAG
TAGTAAAAATTGAACCATTA---GGAATAGCACCCACCAAGGCAAGGAGAAGAGTGGTG--------------
-------------------------CAGAGAGAAAAGAGAGCA---GTAACA---TTGGGAGCT---------
CTGTTCCTTGGGTTCTTGGGAGCA---GCAGGAAGCACTATG---GGCGCAGCGTCA---
ATGACGCTGACGGTACAG---GCCAGACTA---TTATTA---ACTGGTATAGTGCAACAGCAGAAC---
AATCTG---CTGAGA---GCTATTGAGGCGCAACAGCACCTGTTGCAACTCACAGTCTGGGGCATT

```
>JX140656
TTCTACTGTAATACAACGAGACTATTTAATAGGACTTGGAATAGTAATGATAATATTACAAATAATACAAATT
CA-----------------------------------------------------------------------
----------------------------
AATGACTCAGATATCACACTCCCATGCAGAATAAAACAAATTATAAACAGATGGCAGGAAGTAGGGAAAGCAA
TGTATGCCCCTCCCATCCAAGGACAAATTAGATGTTCATCAAAT---
ATTACCGGGATGCTATTAACAAGAGATGGTGGACATGGGAATGAAACT-------------------------
-------------------------------------------------------------------------
----------
AATAATACTGAGGTATTCAGACCTGGAGGAGGAGATATGAGGGACAATTGGAGAAGTGAATTATATAAATATA
AAGTAGTAAGAATTGAACCGTTA---GGAGTAGCACCCACCAAGGCAAAGAGAAGAGTGGTG-----------
----------------------------CAGAGAGAAAAAAGAGCA---ATAGGA---ATAGGAGCT------
---GTGTTCCTTGGGTTCTTGGGAGCA---GCAGGAAGCACTATG---GGCGCAGCATCA---
ATGACGCTGACGGTACAG---GCCAGACAA---TTATTG---TCTGGTATAGTGCAGCAGCAAAAC---
AATTTG---CTGAGG---GCTATTGAGGCGCAACAACATCTGTTGCAACTCACAGTCTGGGGCATC
>GU362881
TTCTACTGTAATACATCAAGTCTATTTAACAGTACTTGGGGGGTTAACGATACTTGGAATAATACTTGGAATA
GG-----------------------------------------------------------------------
-------------------------
AATGAAAATGACAATATCACACTCCAATGCAGGATAAAACAAATTATAAACATGTGGCAGGCAGTAGGGAAAG
CAATATATGCCCCTCCCATTGCAGGATCAATTAGATGTTCATCAAAT---
ATTACAGGGCTACTATTAACAAGAGATGGTGGAAATGGTAATAATACT-------------------------
-------------------------------------------------------------------------
-------
AATAATACCATCGAGATTTTCAGACCAGGAGGAGGAGACATGAGGGACAATTGGAGAAGTGAATTATATAAAT
ATAAAGTAGTAAGAATTGAACCATTA---GGAATAGCACCCACCAAGGCAAAGAGAAGAGTGGTG--------
-------------------------------CAGAGAGAAAAAAGAGCA---GTGGGA---CTAGGAGCT---
------CTGTTCCTTGGGTTCTTGGGAGCA---GCAGGAAGCACTATG---GGCGCAGCGTCA---
GTGACGCTGACGGTACAG---GCCAGACAA---TTATTG---TCTGGTATAGTGCAGCAGCAGAAC---
AATCTG---CTGAGG---GCTATTGAGGCGCAACAACATCTGTTGCAACTCACAGTCTGGGGCATC
>GU362885
TTCTACTGTAATACAACAAAGCTGTTTAACAGTACTTGGTCGACTAATGATACTTTGAATAATACTACAAGGG
GT-----------------------------------------------------------------------
----------------------------
AATGACACTATCATAACACTCCCCTGTAGAATAAAACAAATTATAAACATGTGGCAGGAAGTGGGAAAAGCAA
TGTATGCCCCTCCTATCAAAGGACAAATTAGATGTTCATCAAAT---
ATTACAGGACTGCTATTAACAAGAGATGGTGGTAATAAAACAGAA----------------------------
-------------------------------------------------------------------------
----------
AACGAGACCGAGACCTTTAGACCTGGAGGAGGAAATATGAAGGACAATTGGAGAAGTGAATTATATAAATATA
AAGTAGTGCAAATTGAACCATTA---GGAGTAGCACCTACCAAGGCAAAGAGAAGAGTGGTG-----------
----------------------------CAAAGAGAAAAAAGAGCA---GTGGGAGCATTAGGAGCT------
---ATGGTCCTTGGGTTCTTGGGAGCA---GCAGGAAGCACTATG---GGCGCAGCATCA---
ATAACGCTGACGGTACAG---GCCAGACGA---TTAATG---TCTGGAATAGTGCAACAGCAGAAC---
AATCTG---CTGAAG---GCTATTGATGCGCAACAACAGATGTTGCAACTCACAGTCTGGGGCATC
>KC473842
TTCTACTGTAATACAACACAACTGTTTAATAATACTTGGAATGAAACTGCTTGGAATGAGACTAACAATGTAA
AT-----------------------------------------------------------------------
----------------------------
AACAGTGAAACTATCACACTCAAATGCAGAATAAAACAAATTATAAACAGGTGGCAGGGAGTAGGAAAAGCAA
TGTATGCCCCTCCCATCAGAGGATCAATTAGATGTTCATCAGAT---
ATTACAGGGCTGCTATTAACAAGAGATGGTGGGAACACGAATGAG----------------------------
-------------------------------------------------------------------------
-------------
ACCACTGAGATCTTCAGACCGGGAGGAGGAGATATGAGGGATAATTGGAGAAGTGAATTATATAAATATAAAG
TAGTACAAATTGAACCAATA---GGAATAGCACCCACCAAGGCAAAGAGAAGAGTGGTG--------------
-------------------------CAGAGAGAAAAAAGGGCA---GCG------TTCGGAGCA---------
TTGTTCCTTGGGTTCTTGGGAGCA---GCAGGAAGCACTATG---GGCGCAGCGTCA---
CTAACGCTGACGGTACAG---GCCAGACAA---TTATTG---TCTGGTATAGTGCAACAGCAGAGC---
AATCTG---CTGAGG---GCTATTGAGGCGCAACAGCATATGTTGCAACTCACAGTCTGGGGCATC
```

>KT200353
TTCTACTGTGATACATCAAAACTGTTTAATAGTACTTGGCCGCCTAATAGTACTGTTAATGGTACTGATAATG
GA---

AGTAATGAAAATATCACACTCCAATGTAGAATAAAACAAATTATAAACATGTGGCAGGAAGTAGGAAAAGCAA
TGTATGCCCCTCCCATTAAAGGACTAATTAAATGCTCATCAAAT---
ATCACAGGGCTGCTGATAGCAAGAGATGGTGGTAATAGTGGTAAT----------------------------
----

AACACTACTGAGATCTTCAGACCTTGGGGAGGAGATATGAGGGACAATTGGAGAAGTGAGTTATACAAATATA
AAGTAGTGAAAATTGAACCATTA---GGGTTAGCACCCACCAAGGCACAGAGAAGAGTGGTG-----------
----------------------------CAGAGAGAGAAAAGAGCA---GTGGGA---ATAGGAGCC------
---GTGTTCCTTGGGTTCTTGGGAGCA---GCAGGAAGCACTATG---GGCGCAGCGTCA---
TTGACGCTGACGGTACAG---GCCAGATTA---TTATTA---TCTGGTATAGTGCAACAGCAGAAC---
AATCTG---CTGAGA---GCTATTGAGGCGCAACAACATCTGTTGCAACTCACAGTCTGGGGCATC
>KT200355
TTCTATTGTAATACATCAACACTGTTTAACAGTACTTGGAATGCTAACAGTACTTGGAATGATACTGAAGGAA
CAGATGACCAC--
-------------------AAC---
ACAAATAATCTTAACATCACACTCCAGTGCAGAATAAAACAAATTGTAAACATGTGGCAGGAAGTAGGAAAAG
CAATGTATGCCCCTCCCATTAGAGGACAAATTAGATGTACATCAAAC---
ATTACAGGGGTGTTATTAACAAGAGATGGGGGGAATAATAATAACACAAGA----------------------
----

AACGGGAATGAGACTTTCAGACCTGGAGGAGGAGATATGAGGGACAATTGGAGAAGTGAATTATATAAATATA
AGGTAGTACAAATTGAACCATTA---GGAATAGCACCCACCAAGGCAAAGAGAAGAGTGGTG-----------
----------------------------CAGAGAGAAAAAAGAGCA---GTGGGA---CTAGGAGCT------
---GTGTTCCTTGGGTTCTTGGGAGCA---GCCGGAAGCACTATG---GGCGCAGCGTCA---
CTGACGCTGACGGTACAG---GCCAGACTA---TTATTG---TCTGGTATAGTGCAACAGCAAAAC---
AATTTG---CTGAGA---GCTATTGAGGCGCAACAGCATCTGTTGCAACTCACAGTCTGGGGCATC
>KT200356
TTCTACTGTAATACAACAGAACTGTTTAATAGTACATGGAATGGTAATGTAAGCAATAATACTGAA-------
----

GGAGATAAAATTATCACAATCCCATGCAGAATAAAACAAATTATAAACAGGTGGCAGGAAGTAGGAAAATCAA
TGTATGCCCCTCCTATCAGCGGAATAATTAGTTGTTCATCAAAT---
ATTACAGGGTTGCTATTAACAAGAGATGGTGGTAATACAGGGAAC----------------------------
----

AACGCCACCGAGATCTTCAGACCTGGAGGAGGAGATATGAGGGACAATTGGAGAAGTGAATTATATAAATATA
AAGTAGTAAAAATTGAACCAATA---GGAGTAGCACCCACCAAGGCAAAGAGAAGAGTGGTG-----------
----------------------------CAGAGAGAAAAAAGAGCA---ATAGGA---CTAGGAGCT------
---TTGTTCCTTGGGTTCTTGGGAGCA---GCAGGAAGCACTATG---GGCGCAGCGTCA---
GTGACGCTGACGGTACAG---GCCAGACAA---TTATTG---TCTGGTATAGTGCAACAGCAGAAC---
AATTTG---CTGAGG---GCTATTGAGGCGCAGCAGCATCTGTTGCAACTCACAGTCTGGGGCATC
>KY465967
TTCTACTGTAATTCAACACCACTGTTTAACTGGACTTTGAATGATACTAAGAAGGAGAATGACACTGAA----
----

GAAGATAAAAATATCACACTCCAATGCAGAATAAAACAATTTATAAACATGTGGCAGGGAGTAGGAAGAGCAA
TGTATGCCCCGCCCATCCAAGGACAAATTAAATGTTCATCAAAT---
ATTACAGGGCTAATATTAACAAGAGATGGTGGTAAGAGTGATAAC----------------------------
----

CCAAGTGAGATCTTCAGACCTGGAGGAGGAAATATGAAGGACAATTGGAGAAGTGAATTATATAAATATAAAG
TAGTAAAAATTGAACCATTA---GGAGTAGCACCCACCAAGGCAAGGAGAAGAGTGGTG--------------
-------------------------CAGAGAGAAAAAAGAGCA---GCA------CTAGGAGCT---------
TTGTTCCTTGGGTTCTTGGGAGCA---GCAGGAAGCACTATG---GGCGCAGCGTCG---
CTAACGCTGACGGTACAG---GCCAGACAA---TTATTG---TCTGGTATAGTGCAACAGCAAAGC---
AATTTG---CTGAGG---GCTATTGAGGCGCAACAGCATATGTTGCAACTCACAGTCTGGGGCATC

>KT276263
TTCTACTGTAATACAACACAACTGTTTAACAGTACTTGGGATAATAATACTACACAATCAAATAAT-------
----

ACCAATGGAAACATCACACTCCACTGCAGAATAAAACAAATTATAAACATGTGGCAGGAAGTAGGAAAAGCAA
TGTATGCCCCGCCCATCGCAGGACAAATTAGTTGTTCATCAAAT---
ATTACAGGGCTGCTATTAACAAGAGATGGTGGTAATAGCAGTAGT----------------------------
----

GGCAATGAGACCTTTAGACCTGCAGGAGGAGATATGAAAGACAATTGGAGAAGTGAATTATATAAATACAAAG
TAGTAAAAATTGAGCCATTA---GGAGTAGCACCCACCAGGGCAAAAAGGAGAGTGGTG--------------
-------------------------CAGAGAGAAAAAAGAGCA---GTGGGAGCGATAGGAGCA---------
ATGTTCCTTGGGTTCTTGGGGGCA---GCAGGAAGCACTATG---GGCGCAGCGTCG---
ATGGCGCTGACGGTACAG---GCCAGACAA---TTATTG---TCTGGTATAGTGCAGCAGCAAAAT---
AACTTG---CTGAGA---GCTATTGAGGCGCAACAGCATATGTTGCAACTCACAGTCTGGGGCATC
>KT276266
TTCTACTGTAATACATCAGACCTGTTTAATGAGACTAAGTTTAAT----------------------------
----

GAGACTGAGTCTATCATACTTCCATGCAGGATAAAGCAAATTATAAACATGTGGCAAGAAGCAGGAAAAGCAA
TGTATGCCCCTCCCATTAGCGGAATTATTAGCTGTTCATCAAAT---
ATTACAGGGCTGCTATTAACAAGAGATGGTGGTAATGGCACAAAC----------------------------
----

AACACGGAGATCTTCAGGCCTGCAGGAGGAAATATGAAAGATAATTGGAGAAGTGAATTATATAAATACAAAG
TAGTAAAAATTGAGCCATTA---GGAGTAGCACCCACCAAGGCAAGGAGAAGAGTGGTG--------------
-------------------------CAGAGAGAAAAAAGAGCA---ATAGGA---ATGGGAGCT---------
ATGTTCCTTGGGTTCTTGGGAGCA---GCAGGAAGCACTATG---GGCGCAGCGTCA---
ATGACGCTGACGGTACAG---GCCAGACAA---TTATTG---TCTGGTATAGTGCAACAGCAGAAC---
AACCTG---CTGAAG---GCTATTGATGCGCAACAGCATATGTTGCAGCTCACAGTCTGGGGCATC
>KT276267
TTCTACTGCAATACATCAAAACTGTTTGATAGTACTTGGCTTAATGGTACTAGGTCTAATAGTACTGGAAATA
ACAAC--

ACTGGAGAAACTATCACACTCCCATGTAGAATAAAACAAATCATAAACATGTGGCAGCAGGTAGGAAAAGCAA
TGTATGCCCCTCCTATCGAAGGACATATTAGGTGTTCATCAAAT---
ATTACAGGGCTGATCTTAACAAGAGATGGCGGTGATGACAACAAT----------------------------
----

AATAGCACAGAGATCTTCAGACCGGGAGGAGGAGATATGAGGGACAATTGGAGAAGTGAATTATATAAATACA
AAGTAGTAAAAATTGAACCATTA---GGGATAGCACCCACCAAGGCAAAGAGAAGAGTGGTG-----------
----------------------------CAGAGAGAAAAAAGGGCA---GTGGGA---ATAGGAGCT------
---GTGTTCCTTGGGTTCTTGGGAGCA---GCAGGAAGCACTATG---GGCGCAGCGTCA---
CTGACGCTGACGGTACAG---GCTAGACAA---TTACTG---TCTGGTATAGTGCAACAGCAGAAC---
AATTTG---CTGAGG---GCTATTGAGGCGCAACAACATTTGCTGCAACTCACAGTCTGGGGCATC
>KU685585
TTCTACTGCAATACATCACAACTGTTTAATAGTACTTGGAATAATAATGGTACCCGGATTAATGATACCTGGT
CTAATAGT---
-------------ACTAGC------
AACACTAACGAAACTATCACACTCCCATGTAGAATAAAACAAATTATAAACATGTGGCAGAGGGTAGGAAAAG
CAATGTATGCCCCTCCCATCAGAGGATACATTAATTGTTCATCAAAT---
ATTACAGGGCTGATCTTAACAAGAGATGGCGGTGTTAACAGCAGT----------------------------
----

GGGAGCGAGATCTTCAGACCGGGAGGAGGAGATATGAGGGACAATTGGAGAAGTGAATTGTATAAATACAAAG
TAGTACAAATTGAACCATTA---GGGATAGCACCCACCAAGGCAAAGAGAAGAGTGGTG--------------
-------------------------CAAAGAGAAAAAAGAGCA---GTGGGA---TTAGGAGCT---------
ATGTTCCTTGGGTTCTTGGGAGCA---GCAGGAAGCACTATG---GGCGCAGCGTCA---
ATAACGCTGACGGCACAG---GCTAGACAA---TTATTG---TCTGGTATAGTGCAACAGCAGAAC---
AATTTG---CTGAGG---GCTATTGAGGCGCAACAACATTTGTTGCAACTCACAGTCTGGGGCATC

>KU685589
TTCTACTGTAATACATCACAACTGTTTAACAGTACTTGGGATAACAGTACTTGGAATAATAATGGAAGCTCAA
GTAACAGCACA---TCC--

TCAAATGACATCATCACACTCAGGTGTAGAATAAAACAAATTATAAACATGTGGCAGAAAGTAGGAAGAGCAA
TGTATGCCCCTCCCATCAGCGGAAACATTAGCTGTTCATCCAAT---
ATCACAGGGCTGCTCTTAACAAGAGATGGTGGCAGTGGCAACAATACC-------------------------
----

AACAACACTGAGGTCTTTAGACCTGAAGGAGGAGATATGAGGGACAATTGGAGAAGTGAATTATACAAATACA
AAGTAGTAAAAATTGAACCATTA---GGAATAGCACCCACCAAGGCAAAAAGAAGAGTGGTG---------
----------------------------CAGCGAGAAAAAAGAGCA---ATAACA---TTTGGAGCT------
---ATGATCCTTGGTTCCTTGGGAGCA---GCCGGAAGCACTATG---GGCGCAGCGTCA---
ATAGCGCTGACGGCACAG---GCCAGACAA---TTATTG---TCTGGTATAGTGCAACAGCAGAAC---
AATCTG---CTGAGG---GCTATTGAGGCGCAACAGCATCTGTTGCAACTCACAGTCTGGGGCATC
>KY989949
TTCTATTGTAATACAACACAACTGTTTAACAGTACATGGGTTAGTAATGAAAACTTAACTAATAGTACCAATG
AA---

GACAATAGCACAATCACACTCCCATGTAGAATAAAACAAATCATAAACATGTGGCAGGAAATAGGAAAAGCAA
TGTATGCCCCTCCCATCAGTGGACAAATTAGATGTTCATCAAAT---
ATCACAGGACTGCTATTAACAAGAGATGGTGGCAACACAAGCAAC----------------------------
----

CCCAACACAGAGACCTTCAGACCTGGAGGAGGAGATATGAGGGACAATTGGAGAAGTGAATTATATAAATATA
AAGTAGTAAAAATTGAACCATTA---GGAGTAGCACCCACCAGGGCAAGGAGAAGAGTGGTG---------
----------------------------CAGAGAGAAAAAAGAGCA---GTGGAA---CTAGGAGCT------
---TTGTTTCTTGGGTTCTTGGGAGCA---GCAGGAAGCACTATG---GGCGCAGCGTCA---
GTGACGCTGACGGTACAG---GCCAGACAA---TTATTG---TCTGGTATAGTGCAACAGCAGAAC---
AATTTG---CTGATG---GCGATTGAGGCGCAACAACGCATGTTGCAACTCACAGTCTGGGGCATC
>KY989954
TTCTACTGTAATACAACACCACTGTTTAATAGTACTTGGAGTAATGGTACTTGGAATGGG-------------
----

AGTGCTGAGAATATCACACTCCCATGCAGAATAAAACAAATCATAAACATGTGGCAAGAAGTAGGAAGAGCAA
TGTATGCCCCTCCCATCAGCGGACTAATTAGGTGCTCATCAAAC---
ATTACAGGACTGATATTAACAAGAGATGGTGGCAATAGCAATGATACC-------------------------
----

AATGGCACCGAGATCTTCAGACCTGGAGGAGGAGATATGAGGGATAATTGGAGAAGTGAATTATATAAATACA
AAGTAATAGAACTTGAACCATTA---GGAATAGCACCCACCAAGGCAAAGAGACGAGTGGTG-----------
----------------------------CAGAGAGAAAAAAGAGCA---GTGGGA---CTAGGAGCC------
---ATGTTCTTTGGGTTCTTGGGTGCA---GCAGGAAGCACTATG---GGCGCAGCGTCA---
GTGACGCTGACGGTACAG---GCCAGACGC---TTGTTG---TCTGGTATAGTGCAACAGCAAAAC---
AATTTG---CTGAAG---GCTATTGAGGCGCAACAGCATCTGTTGCAACTCACAGTCTGGGGCATT
>KY989956
TTCTACTGTAATACAACAAAACTGTTTAATAGTAGTTGGGATATGAGCAACCTAAATAGCACAGAAGCAAATA
GCAGC--

AGCGAAGCAAAGATCACACTCCCATGCAGAATAAAACAAATTATAAATATGTGGCAGGGAGTAGGCAAAGCAA
TGTATGCCCCTCCCATCAGAGGACAAATTAAATGTGTATCAGAT---
ATTACAGGGCTGCTATTAACAAGAGATGGT---ACTGGAAACGAT----------------------------
----

ACCCTTGAAATCTTTAGACCTGGAGGAGGAAATATGAAGGACAATTGGAGAAGTGAATTATATAAATATAAAG
TAGTAAGAATTGAACCATTA---GGAGTAGCACCCACCAAGGCAAAGAGAAGAGTGGTG--------------
-------------------------CAGAGAGAAAAAAGAGCA---TTGGGAACACTAGGAGCT---------
GTGTTTCTTGGGTTCTTGGGAACA---GCAGGGAGCACTATG---GGCGCAGCGTCA---
CTGACGCTGACGGTACAG---GCCAGACAA---TTATTA---TCTGGTATAGTGCAACAGCAGAAC---
AATCTG---CTGAAA---GCTATTGATGCACAACAGCATCTGTTGCAACTCACGGTCTGGGGCATC

>KC595171
TTCTACTGTAATACAACACAACTGTTTAATAGTACTTGGAATGGTACTGAAGGGTCAAATAACACTGAA----
----

GGAAATGACACAATCACACTCCCATGCAGAATAAAACAAATTGTAAACAGGTGGCAGGAAGTAGGAAAAGCAA
TGTATGCCCCTCCCATCAGAGGACAGATTAGATGTTCATCCAAT---
ATTACAGGGCTGCTCTTAACAAGAGATGGGGGTGAGAACAATACT----------------------------
----

ACTGGGACCGAGACCTTCAGACCTGGAGGAGGAGATATGAGGGACAATTGGAGAAGTGAATTATATAAATATA
AAGTAGTAAAAATTGAGCCATTA---GGAATAGCACCCACCAAGGCAAAGAGAAGAGTGGTG-----------
----------------------------CAGAGAGAAAAAAGAGCA---GTGGGA---ATAGGAGCT------
---GTGTTCCTTGGGTTCTTGGGAGCA---GCAGGAAGCACTATG---GGCGCAGCGTCA---
ATGACGCTGACGGTACAG---GCCAGACTA---TTATTG---TCTGGTATAGTGCAACAGCAGAAC---
AATTTG---CTGAGG---GCTATTGAGGCGCAACAGCATCTGTTGCAACTCACAGTCTGGGGCATC
>KC595172
TTCTACTGTAATACAACAAAACTGTTTAATAGTACCTGGAGTGATACTGAAGGGTTAAATACCACTGAAGGAA
ATAAA--

ACAAATGAAATAATCACACTCCCATGCAGAATAAAACAAATTGTAAACATGTGGCAGGAAGTAGGAAAAGCAA
TGTATGCCCCTCCCATCAATGGAATAATTAGATGTTCATCAAAT---
ATTACAGGGCTGCTATTAACAAGAGATGGTGGTAATAACACGAAC----------------------------
----

GGGTCCGAGATTTTCAGACCTGGAGGAGGAAATATGAGGGACAATTGGAGAAGTGAATTATATAAATATAAAG
TAGTAAAAATTGAACCATTA---GGAGTAGCACCCAACAAGGCAAAGAGAAGAGTGGTG--------------
-------------------------CAGAGAGAAAAAAGAGCA---CTGGGAATAGGAGCTGCT---------
GTGTTCCTTGGGTTCTTGGGAGCA---GCAGGAAGCACTATG---GGCGCAGCGGCA---
CTGACGCTGACGGTACAG---GCCAGACTA---TTATTG---TCTGGTATAGTGCAGCAGCAGAAT---
AAATTG---CTGAGA---GCTATTGAGGCGCAACAGCATCTGTTGCAACTCACAGTCTGGGGCATC
>KC595174
TTCTACTGTAATACAACACAACTGTTTAATAGTACTTGGAATATTACTGAAGGGTTTAATGGTACTTGGAATG
AT---

ACTGCAAATAATATCACACTCCCATGTAGAATAAAACAAATTGTAAACATGTGGCAGGAAGTAGGAAAAGCAA
TGTATGCCCCTCCCATCAAAGGACAAATTAGATGTTCATCAAAT---
ATTACAGGGCTGATATTAACAAGAGATGGTGGTAATACTACCAAC----------------------------
----

AACGAGACCGAGACTTTCAGACCTGGAGGAGGAGATATGAGGGACAATTGGAGAAGTGAATTATATAAATATA
AAGTAGTAAAACTTGAACCATTA---GGAATAGCACCCACCAAGGCAAAGAGAAGAGTGGTG-----------
----------------------------CAAAGAGAAAAAAGAGCA---GTA------ATAGGAGCT------
---TTGTTCCTTGGGTTCTTGGGAGCA---GCAGGAAGCACTATG---GGCGCAGCGTCA---
GTGACGTTGACGGTACAG---GCCAGACAA---TTATTG---TCTGGTATAGTGCAACAGCAGAAT---
AATTTG---CTGAGG---GCTATTGAGGCGCAACAGCATCTGTTGCAACTCACAGTCTGGGGCATC
>KC595177
TTCTACTGTAATTCAACACAACTGTTTAATAGTACTTGGAATAGTACTGAAGGGTCAAATAATATTACA----
----

GCAAATGGAACAATCACACTCCCATGCAGAATAAAACAAATTATAAACATGTGGCAGGAAGTAGGAAAAGCAA
TGTATGCTCCTCCCATCAGAGGACAAATTAGATGCTCATCAAAT---
ATTACAGGGCTGCTATTAACAAGAGATGGTGGTAATGGTAACGAG----------------------------
----

ACCACCGAGATTTTCAGACCTGGGGGAGGAGATATGAGGGACAATTGGAGAAGTGAATTATATAAATATAAAG
TAGTAAAAATTGAACCATTA---GGAGTAGCACCCACCAGGGCAAAGAGAAGAGTGGTG--------------
-------------------------CAGAGAGAAAAAAGAGCA---GTGGGA---ATAGGAGCT---------
TTGTTCCTTGGGTTCTTGGGAGCA---GCAGGAAGCACTATG---GGCGCAGCGTCA---
CTGACGTTGACGGTACAG---GCCAGACAA---TTATTG---TCTGGTATAGTGCAACAACAGAAC---
AATTTG---CTGAGG---GCTATTGAGGCGCAACAGCATCTGTTGCAACTCACAGTCTGGGGCATC

>KC595178
TTCTACTGTAATTCAACACAACTGTTTAATAGTACTTGGAAGGATACTGAAGGGTCAAATGACACTACA----
----

GGAAATGACACAATCACACTCCCATGCAGAATAAAACAAATTGTAAACATGTGGCAGGAAGTAGGAAAAGCAA
TGTATGCCCCTCCCATCAGAGGACAAATTAGATGTTCATCAAAT---
ATTACAGGGCTGATATTAACAAGAGACGGTGGTACAGACAATAAC----------------------------
----

AAGACCGAGACCTTCAGACCTGGAGGAGGAGATATGAGGGACAATTGGAGAAGTGAATTATATAAATATAAAG
TAGTAAAAGTTGAACCATTA---GGAGTAGCACCCACCAGGGCAAAGAGAAGAGTGGTG--------------
-------------------------CAAAGAGAAAAAAGAGCA---GTGGGA---ATAGGAGCT---------
GTGTTCCTTGGGTTCTTGGGAGCA---GCAGGAAGCACTATG---GGCGCAGCGCCA---
ATGACGCTGACGGTACAG---GCCAGACTA---TTATTG---TCTGGTATAGTGCAACAGCAGAAC---
AATTTG---CTGAGG---GCTATTGAGGCGCAACAGCATCTGTTGCAACTCACAGTCTGGGGCATC
>KC595195
TTCTACTGTAATACAACAGAACTGTTTAATAGTACTTGGAAAGGTACTGAAAGGTTAAATAGCTCTTCTACAA
ATTCA--

AATCCTACAGAAATCATGCTCCCATGCAGAATAAAGCAAATTATAAACATGTGGCAGGAAGTAGGAAAAGCAA
TGTATGCCCCTCCCATCAAAGGACAAATTAAATGTTCATCAAAC---
ATTACAGGGTTGCTACTAACAAGGGATGGTGGTAATGATAATAACACT-------------------------
----

AACGGGACCGAGATCTTCAGACCTGGAGGAGGAGATATGAGGGATAATTGGAGAAGTGAATTATATAAATATA
AAGTAGTAAGACTTGAACCATTA---GGAGTAGCACCCACCAAGGCAAAAAGGAGAGTGGTG-----------
----------------------------CAAAGAGAAAAAAGAGCA---ATAGGA---ATAGGAGCG------
---ATGTTCCTTGGGTTCTTGGGAACA---GCAGGAAGCACTATG---GGCGCAGCGTCA---
CTGACGTTGACGGTACAG---GCCAGACTA---TTGTTG---TCTGGTATAGTACAACAGCAGAAC---
AATTTG---CTGAGA---GCTATAGAGGCGCAACAACATCTGTTGCAACTCACTGTCTGGGGCATT
>DQ854716
TTCTACTGTAATTCAACACAACTGTTTAACAGTACTTGGAATGGTACTGCATGGTCAAATAACACTGAAGGAA
AT---

GAAAATGACACAATCACACTCCCATGCAGAATAAAACAAATTATAAACATGTGGCAGGAAGTAGGAAAAGCAA
TGTATGCCCCTCCCATCAGAGGACAAATTAGATGTTCATCAAAT---
ATTACAGGGCTGATATTAACAAGAGATGGTGGTATTAACCAGACC----------------------------
----

AACACCACCGAAATCTTCAGGCCTGGAGGAGGAGATATGAGGGACAATTGGAGAAGTGAATTATATAAATATA
AAGTAGTAAAAATTGAACCATTA---GGAATAGCACCCACCAAGGCAAAGAGAAGAGTGGTG-----------
----------------------------CAGAGAGAAAAAAGAGCA---GTGGGAATAATAGGAGCT------
---ATGCTCCTTGGGTTCTTGGGAGCA---GCAGGAAGCACTATG---GGCGCAGCGTCA---
ATGACGCTGACGGTACAG---GCCAGACAA---TTATTG---TCTGGTATAGTGCAACAGCAGAAC---
AATTTG---CTGAGG---GCTATTGAGGCGCAACAGCATCTGTTGCAACTCACAGTCTGGGGCATC
>KC595182
TTCTACTGTAATTCATCACAACTGTTTAATAGTACTTGGGAGTTTAATAGTACTTGGGAGTTTAATAGTACTG
GGAATAGTACT--

GAAGAAGACCCTATCATACTCCCATGCAGAATAAAACAAATTATAAACATGTGGCAGGAAGTAGGAAAAGCAA
TGTATGCCCCTCCCATCAGAGGACAAATTAGATGCTTATCAAAT---
ATTACAGGACTGCTATTAACAAGAGATGGTGGTAAGGACGATAAC----------------------------
----

ATGACCGAGACCTTCAGACCTGGAGGAGGAGATATGAGGGACAATTGGAGAAGTGAATTATATAAATATAAAG
TAGTAAAAGTTGAACCATTA---GGAGTAGCACCCACCAAGGCAAAGAGAAGAGTGGTG--------------
-------------------------CAGAGAGAAAAAAGAGCA---GTGGGA---ATAGGAGCT---------
GTGTTCCTTGGGTTCTTGGGAGCA---GCAGGAAGCACTATG---GGCGCAGCGTCG---
ATGACGTTGACGGTACAA---GCCAGACAA---TTATTG---TCTGGTATAGTGCAACAGCAAAAC---
AATTTG---CTGAGG---GCTATTGAGGCGCAACAGCATCTGTTGCAACTCACAGTCTGGGGCATC

>KC595159
TTCTACTGTAATTCAACACAACTGTTTAATAGTACGTGGAATGGTACTGAAAGGTCAAATAACACT-------
----

GAAGGAAATATAATCACACTCCCATGCAGAATAAAACAAATTATAAATATGTGGCAGGAAGTAGGAAAAGCAA
TGTATGCCCCTCCTATCAGAGGACAAATTAGATGTTCATCAAAT---
ATTACAGGGCTGATATTAACAAGAGATGGTGGTAAGAACGAGAAC----------------------------
----

GAGACCGAGATCTTCAGACCTGGAGGAGGAGATATGAGGGATAATTGGAGAAGTGAATTATATAAATATAAAG
TAGTAAAAATTGAACCATTA---GGAGTAGCACCCACCAAGGCAAAGAGGAGAGTGGTG--------------
-------------------------CAGAGAGAGAAAAGAGCA---GTGGGA---ATAGGAGCT---------
GTGTTTCTTGGGTTCTTGGGAGCA---GCAGGAAGCACTATG---GGCGCAGCGTCA---
GTGACGCTGACGGTACAG---GCCAGACTA---TTATTG---TCTGGTATAGTGCAACAGCAGAGC---
AATTTG---CTGAGG---GCTATTGAAGCGCAACAGCATCTGTTGCAACTCACAGTCTGGGGCATT
>KC595225
TTTTATTGTAATTCAACACAACTGTTTAATAGTACTTGGTATACAAATGATACAAATGTTACTGCTGAA----
----

GGAAGTGACACAATTGTACTCCCATGCAAAATAAAACAAATTATAAACATGTGGCAGGAAGTAGGAAAAGCAA
TGTATGCCCCTCCCATCAGAGGACAAATTAACTGCTCATCAAAC---
ATTACAGGGTTGCTATTAACAAGAGATGGTGGTCCAAATAATACC----------------------------
----

AACGAGACCTTTAGACCTGGAGGAGGAAATATGAAGGACAATTGGAGAAGTGAATTATATAAATATAAAGTAG
TAAAAATTGAACCATTA---GGAGTAGCACCCACCGAGGCAAAAAGAAGAGTGGTG-----------------
----------------------CAGAGAGAAAAAAGAGCA---GTGGGA---ATGGGAGCT---------
ATGTTCCTTGGGTTCTTGGCCACA---GCAGGAGGCACTATG---GGCGCAGCGTCA---
GTGACGCTGACGGTACAG---ACCAGACAA---TTATTG---TCTGGTATAGTGCAACAGCAGAGC---
AATTTG---CTGAGG---GCTATTGAGGCGCAACAGCATATGTTGCAACTCACAGTCTGGGGCATC
>KC595222
TTCTACTGTAATTCAACACAACTGTTTAACAGTACTTGGTATGCGAATGGTACTAGAGAGCCAAATGACACTG
CA---

AGAAATGGCACAATCACACTCCCATGCAGAATAAAACAAATTATAAACATGTGGCAGAAAGTAGGAAAAGCAA
TGTATGCCCCTCCCATCAAAGGACCAATTAGATGTTCATCAAAT---
ATCACAGGGGTGATATTAACAAGGGATGGTGGCGGTAATGACAGTAGTGAG----------------------
----

GAGACCTTCAGACCTGGAGGAGGAGACATGAGGGACAATTGGAGAAGTGAATTATATAAATATAAAGTAGTAG
AACTTGAGCCATTA---GGAGTAGCACCCACCAGGGCAAAGAGAAGAGTGGTG--------------------
-------------------CAGAGGGAAAAAAGAGCA---GTGGGAATATTAGGAGCT---------
ATGTTCCTTGGGTTCTTGGGAGCA---GCAGGAAGCACTATG---GGCGCAGCGGCA---
GTGACGCTGACGGTACAG---GCCAGACAA---CTAATG---TCTGGTATAGTGCAACAGCAGAAC---
AACTTG---CTGAGG---GCTATTGAGGCGCAACAGCATCTGTTGCAACTCACAGTCTGGGGCATC
>KC595206
TTCTACTGTGACTCAACACAACTGTTCAATAGTACTTGGAATGGTACTAAAAGGATAATAGGAACTGAA----
----

GAGAATGGCACGATCACGCTCCCATGCAGAATAAAACAAATTGTAAACATGTGGCAGGAAGTAGGAAAAGCAA
TGTATGCCCCTCCCATCAGAGGAAACATTAACTGCTCATCAAAT---
ATTACAGGACTGTTATTAACAAGAGATGGTGGTAAAGACGAAAAT----------------------------
----

GAGACCGATACTGAGACCTTCAGACCTGGAGGAGGAGATATGAGGGACAATTGGAGAAGTGAATTATATAAAT
ATAAAGTAGTAAAAATTGAACCATTA---GGAGTAGCACCCACCAGGGCAAAGAGAAGAGTGGTG--------
-------------------------------CAGAGAGAAAAAAGAGCA---ATTGGAGCA---GGAGTT---
------ATGCTCTTTGGGTTCTTGGGAGCA---GCAGGAAGCACTATG---GGCGCAGCGTCA---
ATGGCGTTGACGGTACAG---GCCAGACAA---TTATTG---TCTGGTATAGTGCAACAGCAGAGA---
AATTTG---CTGAGG---GCTATTGAGGCGCAACAACATCTGTTGCAACTCACAGTCTGGGGCATC

>DQ141341
TTCTACTGTAATTCATCACAACTGTTTAATAGTTCTTGGAATGTTAATGGAGAATCAAATAACACTGAA----
----

GGAAATGACACAATCACACTCCCATGCAGAATAAAACAAATTATAAACATGTGGCAGGAAGTAGGAAAAGCAA
TGTATGCCCCTCCCATTAGAGGACAAATTAGCTGTTCATCAAAT---
ATTACAGGGCTGCTATTAACAAGAGATGGTGGTAACAATAACGAGACC-------------------------
----

ACCACCACCGAGACCTTCAGACCTGGAGGAGGAGATATGAGGGACAATTGGAGAAGTGAATTATATAAATATA
AAGTAGTAAAAATTGAACCATTA---GGAATAGCACCCACCAGGGCAAAGAGAAGAGTGGTG-----------
----------------------------CAGAGAGAAAAAAGAGCA---GTGGGA---ATAGGAGCT------
---ATGTTCCTTGGGTTCTTGGGAGCA---GCAGGAAGCACTATG---GGCGCAGCGTCA---
GTGACGTTGACGGTACAG---GCCAGACTA---TTATTG---TCTGGTATAGTGCAACAGCAGAAC---
AATTTG---CTGAGG---GCTATTGAGGCGCAACAGCGTATGTTGCAACTCACAGTCTGGGGCATC
>DQ141345
TTCTACTGTAATACAACACAACTGTTTAATAGTACTTGGGAGGCTAATGGTACTTGGAATGGTGCTAAC----
----

AAGTCAGGTGACATCATACTCCCATGCAGAATAAAACAAATTATAAACATGTGGCAGGGAGTAGGAAAAGCAA
TGTATGCCCCTCCCATCAGTGGACAAATTAGATGTTCATCAAAC---
ATTACAGGGCTGCTATTAACAAGAGATGGTGGTAGTAACGAGACC----------------------------
----

AACGGGACTGAGATCTTCAGACCTGGAGGAGGAGATATGAGGGATAATTGGAGAAGTGAATTATATAAATATA
AAGTAGTAAAAATTGAACCATTA---GGAATAGCACCCACCAAGGCAAAGAGAAGAGTGGTG-----------
----------------------------CAGAGAGAAAAAAGAGCA---GCATTT------GGAGCT------
---ATGTTCCTTGGGTTCTTGGGAGCA---GCAGGAAGCACTATG---GGCGCAGCGTCA---
CTGACGCTGACGGTACAG---GCCAGGCAA---TTATTG---TCTGGTATAGTGCAACAGCAGAAC---
AATCTG---CTGAGG---GCTATTGAGGCGCAACAGCATCTGTTGCAACTCACAGTCTGGGGCATC
>EF531330
TTCTACTGTAATACATCACAGCTATTTAATAATACTTTTAATAGTACTGGGCCTAATAATAGTACTAAGAAG-
----

TTAAATGACACTATCACACTCCCATGCAGAATAAAACAAATTATAAACATGTGGCAGGAAGTAGGAAAAGCAA
TGTATGCCCCTCCCATTAGTGGACAAATTAACTGCTCATCAATT---
ATTACAGGGTTGCTATTAACAAGAGATGGTGGTGAGGGCAATGAGACC-------------------------
----

AATGGGAACGAGACCTTCAGACCTGGAGGAGGAGATATGAGGGATAATTGGAGGAGTGAATTATATAAATATA
AAGTAGTACAAATTGAACCAGTA---GGAGTAGCACCCACCAAGGCAAAGAGAAGAGTGGTG-----------
----------------------------CAGAGAGAAAAAAGAGCA---GTATTA------GGAGCT------
---ATGTTCCTTGGGTTCTTGGGAGCA---ACAGGAAGCACTATG---GGCGCAGCGTCA---
ATGGCGTTGACGGTACAG---GCCAGACAA---TTATTG---TCTGGTATAGTGCAACAGCAGAAC---
AATTTG---CTGAAG---GCTATTGAGGCGCAACAGCATCTGTTGCAACTCACAGTCTGGGGCATC
>EF531333
TTCTACTGTAATACAACACAACTGTTTACTTGGATTGTTGATGGTAATGGGAATATTAGTAGTATTGGGAATG
GTACTAAT---
-------------ACTACT------
GATGCTAACAAGAATATCACACTCCCATGTAGAATAAAACAAATTGTAAACATGTGGCAGGAAGTAGGAAAAG
CAATGTATGCTCCTCCCATAAGGGGACAAATTAGATGCTTATCAAAT---
ATTACCGGGTTGCTAATAACAAGAGATGGAGGTAGTAATAACAGT----------------------------
----

GCTAACGAGACTTTCAGACCTGGAGGAGGAGATATGAGGGACAATTGGAGAAGTGAATTATATAAATATAAAG
TGGTAAAAATTGAACCATTA---GGAGTAGCACCCACCAAGGCAAAGAGAAGAGTGGTG--------------
-------------------------CAGAGAGAAAAAAGAGCA---GTGGGA---ATAGGAGCT---------
GTGTTCCTTGGGTTCCTGGGAGCA---GCAGGAAGCACTATG---GGCGCAGCGTCA---
ATGACGTTGACGGTACAG---GCCAGACAA---TTATTG---TCTGGTATAGTGCAACAGCAGGAC---
AATCTG---CTGAGG---GCTATTGAGGCGCAACAGCATCTGTTGCAACTCACAGTCTGGGGCATC

>JX422195
TTCTACTGTAATACAACACAACTGTTTAATACAGCACTGCTTAATAGTTCT----------------------
----

AACGAGACAAATCTCACACTCCCATGCAGAATAAAACAAATTGTAAATATGTGGCAGGAAGTAGGAAGAGCAA
TGTATGCCCCTCCCAGACAAGGAATAATTAGATGTTCATCAAAT---
ATCACAGGATTGATATTAACAAGAGATGGTGGGGTCACGAACGGGTCC-------------------------
----

GAGACCGAGACCTTCAGACCAGGAGGAGGAGATATGAGGGACAATTGGAGAAGTGAATTATACAAATATAAAG
TAGTAAAAATAGAACCATTG---GGAATAGCACCCACCAAGGCAAGGAGAAGAGTGGTG--------------
-------------------------CAGAGAGAAAAAAGAGCA---GCA------CTAGGAGCT---------
ATGTTCCTTGGGTTCTTGGGAGCA---GCAGGAAGCACTATG---GGCGCAGTGTCA---
CTAACACTGACGGTACAA---GCCAGAATG---TTATTG---TCTGGTATAGTGCAACAGCAGAAT---
AATCTG---CTGAGG---GCTATTGAGGCGCAACAGCATATGTTGCAACTCACAGTCTGGGGCATC
>AY535455
TTCTACTGTAATACAACACAACTGTTTAATAGTACTTGGAAAAATAATACTACAAGTCCGAATAAA-------
----

GGAAATGGCACAATCACACTCCCATGCAGAATAAAACAAATTATAAACATGTGGCAGGAAGTAGGAAAAGCAA
TGTATGCCCCTCCCATCGCAGGACAAATTAGATGTGTATCAAAT---
ATTACAGGGCTGCTATTAACAAGAGATGGTGGTGGTGGTGGTAATATG-------------------------
----

ACTAATGGGACCGAGATCTTCAGACCGGGAGGAGGAGATATGAGGGACAATTGGAGAAGTGAATTATATAAAT
ATAAAGTAGTAAAAATTGAGCCATTG---GGAGTAGCACCCACCAAGGCAAAAAGAAGAGTGGTG--------
-------------------------------CAGAGAGAAAAAAGAGCA---GTGGGA---ATAGGAGCT---
------TTGCTCTTTGGGTTCTTGGGAGCA---GCAGGAAGCACTATG---GGCGCAGCATCA---
ATGACGCTGACGGTACAG---GCCAGACTA---TTATTG---TCTGGTATAGTGCAACAGCAGAAT---
AATCTG---CTGAGG---GCTATTGAGGCGCAACAACATCTGTTGCAACTCACAGTCTGGGGCATC
>KC699018
TTCTACTGTAATACAACACAACTGTTTAATAGTACTTGGGATGCTACGGATGCTAATAACACTAGAGTAGAT-
----

AACACCACTGTCATCACACTCCCATGCAGAATAAAACAAATTATAAACATGTGGCAGGAGGTAGGAAAGGCAA
TGTATGCCCCTCCCATCAGCGGACAAATTAACTGCTCATCAAAT---
ATTACAGGGCTGCTATTAACAAGCGATGGGGGTAATAGTAATAAT----------------------------
----

GAGAGCGAGACCTTCAGACCTGGAGGGGGAGATATGAGGGACAATTGGAGAAGTGAATTATATAAATATAAAG
TAGTAGAAATTGAACCACTA---GGAGTAGCACCCACCAGGGCAAAAAGAAGAGTGGTG--------------
-------------------------CAGAGAGAAAAAAGAGCA---GTGACA---CTAGGAGCT---------
TTTTTCCTCGGGTTCTTGGGAGCA---GCAGGAAGCACTATG---GGCGCAGCGTCA---
ATGACGCTGACGGTACAG---GCCAGACAA---TTATTG---TCTGGTATAGTGCAACAGCAGAAC---
AATCTG---CTGAGG---GCTATTGAGGCGCAACAGCATCTGTTGCAACTCACAGTCTGGGGCATC
>KU168261
TTCTACTGTGATTCATCACCACTGTTTAACAGTACTTGGAGGAGAAATAGCACGGAAGGAAATGCCACCATTG
TAGGAAAT---
----------GCCACCATT------
GGAAAAAATAACACACTTACACTCCAATGCAGAATAAAACAAATTATAAACATGTGGCAGGGAGTAGGAAGAG
CCATGTATGCCCCTCCCATTAGCGGAATCATTAGCTGCTCATCAAAT---
ATTACGGGGCTGATACTAACAAGAGATGGTGGTGACGAGAATAGGACC-------------------------
----
ACTGACGAGAATAAT---
ACCACTGAGACCTTCAGACCTGGAGGGGGAGATATGAGGGATAATTGGAGAAGTGAATTATATAAATACAAAG
TAGTAAAGATTGAACCATTA---GGAGTAGCACCCACCGAGGCCAAGAGAAGAGTGGTG--------------
-------------------------CAGAGAGAAAAAAGAGCG---ATAGGA---CTAGGAGCT---------
ATGTTCCTCGGGTTCTTAGGAGCA---GCAGGAAGCACTATG---GGCGCAGCGTCA---
TTGACGTTGACGGTACAG---GCCAGACAA---TTATTG---TCTGGTATAGTGCAACAGCAAAAC---
AACTTG---CTGAGG---GCTATTGAGGCGCAACAGCATCTGTTGCAACTCACAGTCTGGGGCATC

>JX140652
TTCTACTGTAATTCAACGCAACTGTTTAATAGTACTTGGCTGTTTAATAGTACTGGGAATGATACAACA----
----

GAAAATGGTACCATCACACTCCAATGCAGAATAAAACAAATTATAAACATGTGGCAGGAAGTAGGAAAAGCAA
TGTATGCCCCTCCCATCAGAGGAAACATTAGGTGTTCCTCAAAT---
ATTACCGGGTTGCTATTAACAAGAGATGGTGGTAGCCATGAGAACGGGACCAAC-------------------
----

ACCACCAACACTGAGGTCTTCAGACCTGGAGGAGGAAATATGAGGGACAATTGGAGAAGTGAATTATATAAAT
ATAAAGTAATAGAAATTGAACCATTA---GGAATAGCACCCACCAAGGCAAAGAGAAGAGTGGTG--------
-------------------------------CAGAGAGAAAAAAGAGCA---GCG------CTAGGAGCT---
------GTATTCCTTGGGTTCTTGGGAGCA---GCAGGAAGCACTATG---GGCGCAGCATCA---
ATGACGCTGACGGTACAG---GCCAGACAA---TTATTG---TCTGGTATAGTGCAGCAGCAAAAC---
AACTTG---CTGAGG---GCTATTGAGGCGCAACAACATCTGTTGCAACTCACAGTCTGGGGCATC
>KC699029
TTCTACTGTAATACATCACAACTGTTTAACAGTACTTGGTCAAATGATACTAATAAG----------------
----

TCAAATGCAATAATCACACTCCCATGCAGAATAAAACAAATTATAAACATGTGGCAGGAAGTAGGAAAAGCAA
TGTATGCCCCTCCTATCAGAGGAGAAATTAAATGTTCATCAAAT---
ATTACAGGGCTGCTATTAACAAGAGATGGTGGTAATGGGAATAAGACA-------------------------
----

AACATCAGCATTGAAATCTTCAGACCTGGAGGAGGAAACATGAAGGACAATTGGAGAAGTGAATTATATAAAT
ATAAAGTAGTAAAAATTGAACCATTA---GGAGTAGCACCCACCAAGGCAAAGAGAAGAGTGGTG--------
-------------------------------CAGAGAGAAAAAAGAGCA---GTAGGA---ATAGGAGCT---
------TTGTTCCTTGGGTTCTTGGGAGCA---GCAGGGAGCACTATG---GGCGCAGCGTCA---
CTGACGCTGACGGTACAG---GCCAGACAA---TTATTG---TCTGGTATAGTGCAGCAGCAGAAC---
AATCTG---CTGAAG---GCTATTGAGGCGCAACAGCATCTGTTGCAACTCACAGTCTGGGGCATC
>KC699030
TTCTATTGTAATACAACAAGACTGTTTAGTAGTACTTGGTATAATAATGGTACTTGGTATACTAATGGTACTG
AGAATGGTATT--
-------------------AAA---
GAGTCAAATGCAACAATCATGCTGCCATGCAGGATAAAACAAATTATAAACATGTGGCAGAGTGTAGGAAAAG
CAATGTATGCCCCTCCCATCAGGGGACCACTTAGGTGTTCATCAAAT---
ATTACAGGGCTGCTATTAATGAGAGATGGTGGAGGTAATGAGAGC----------------------------
----

GAGACCACCCACAATGAGACCTTCAGACCCGGAGGGGGAGACATGAGGGACAATTGGAGAAGTGAACTATATA
AATATAAAGTAGTAGCAGTTGAACCATTA---GGAATAGCGCCCACCAAGGCAAAGAGAAGAGTGGTG-----
----------------------------------CAGAGAGAAAAAAGAGCA---GTAACA---
ATGGGAGCT---------ATGTTCCTTGGGTTCTTGGGAGCA---GCAGGAAGCACTATG---
GGCGCAGCGTCA---ATGACGCTGACGGTACAG---GCCAGACAA---TTATTG---
TCTGGTATAGTGCAACAGCAAAGC---AATTTG---CTGAAG---
GCTATTGAGGCGCAGCAACATTTGTTGCAACTCACGGTCTGGGGCATT
>KC699028
TTCTACTGTAACACAACACAGCTGTTTAATAGTACTTGGACATTCAATGATACTAGGAGTAATGATACAGAGT
CA---

GATAACGCTACGATTATACTCCCATGCAGAATAAAACAAATTATAAACAGGTGGCAGGAAGTAGGAAAAGCAA
TGTATGCCCCTCCCATCAGTGGAATAATTAGATGCTCATCAAAT---
ATTACAGGGCTGCTATTAACAAGAGATGGTGGTAATGAGAAAAAT----------------------------
----

AAGAGTACCAATGAGACCTTCAGACCTGGAGGAGGAGACATGAGAGACAATTGGAGAAGTGAATTATATAAAT
ATAAAGTAGTAAGAATTGAGCCAATA---GGAATAGCACCCACCAGGGCAAGGAGAAGAGTGGTG--------
-------------------------------CAGAGAGAAAAAAGAGCA---GTGGGAACGCTGGGAGCT---
------ATGTTCCTTGGGTTCTTGGGAGCA---GCAGGAAGCACTATG---GGCGCAGCGTCA---

ATGACGCTGACGGTACAG---GCCAGACAA---TTATTG---TCTGGTATAGTGCAACAGCAAAAC---
AATTTG---CTGAGA---GCTATTGAGGCGCAACAGCATCTGTTGCAACTCACAGTCTGGGGCATT
>KC699039
TTCTACTGTAATACAACACAACTGTTTAATGGTACTTGGAATGGTACCTGGGATGGTACTTGGAATGGTACTT
GGACTCGGATA---
----------GAGCCAAAT------
ATCAATGATACAAATATCACACTCCCATGCAGAATAAAACAAATTATAAACATGTGGCAGGGAATAGGAAGAG
CAATGTATGCCCCTCCCATCAGGGGACAAATTAAATGTATATCAAAT---
ATTACCGGGTTGCTAATAACAAGAGATGGTGGTGATGAGGAGAAC----------------------------
----

AAGACTGAGGAGGTCTTCAGACCTGGAGGAGGAGATATGAGAGACAATTGGAGAAGTGAATTATATAAATATA
AAGTAGTACAAATTGAACCAATA---GGAATAGCACCCACCAAGGCAAAGAGAAGAGTGGTG-----------
----------------------------CAGAGAGAGAAAAGAGCG------GGA---ATAGGAGCT------
---TTGTTCCTTGGGTTCTTGGGAGCA---GCAGGAAGCACTATG---GGCGCAGCATCA---
ATAACGCTGACGGTACAG---GCCAGACAA---TTGTTG---TCTGGTATAGTGCAACAGCAGAAC---
AATTTG---CTGAGA---GCTATTGAGGCGCAACACCATCTGTTGCAACTCACAGTCTGGGGCATC
>JX140654
TTCTACTGTAATACAACACCACTGTTTAATAGTGCTTGGAATAGGAATAAGAGTAAGGATACTTGGAGTATCA
AAGGGTCAAAT---
----------AATAGCACT------
GGAGGAAATAACACTATCATACTCCAATGTAAAATAAAACAAATTATAAACATGTGGCAAAGAGTAGGAAAAG
CAATGTATGCCCCTCCCATCAGAGGACAAATTAACTGTTTATCAAAT---
ATTACAGGGCTACTATTAACAAGAGATGGTGGTAATATCAGTAGC----------------------------
----

ACTAATGAAACCTTCAGACCTGGAGGAGGAGATATGAGGGACAATTGGAGAAGTGAATTGTATAAATATAAGG
TAGTACAAATTGAACCATTA---GGATTAGCACCCACCAAGGCAAAGAGAAGAGTGGTA-------------
-------------------------CAAAGAGAAAAAAGAGCA---GTGGGGACACTAGGAGCT---------
ATGTTCCTTGGGTTCTTGGGAGCA---GCAGGAAGCACTATG---GGCGCAGCGTCA---
ATAACGCTGACGGCACAG---GCCAGACAA---TTATTG---TCTGGTATAGTGCAACAGCAGAAC---
AACTTG---CTGAAG---GCTATTGATGCGCAACAGCATATGTTGCAACTCACAGTCTGGGGCATC
>KC699035
TTCTACTGTGATACAACAAAACTGTTTAATAGCACTTGGAATAGCACTTGGTATAGTAATAGTACTGGGAGTG
ATATTAAA--

GGAAATGACACAATCACACTCCCGTGCAGAATAAAACAAATTATAAACATGTGGCAGGAAGTAGGAAAAGCAA
TGTACGCCCCTCCCATCAGAGGATCAATTAAATGTTCATCAAAT---
ATTACAGGGTTGCTACTAACAAGAGATGGGGGTACAGACAATAGT----------------------------
----

AGTACTACAGAGACCTTCAGACCTGGAGGAGGAAATATGAAGGACAATTGGAGAAGTGAATTATATAAATATA
AAGTAGTACAAATTGAACCATTA---GGAGTAGCACCCACCAAGGCAAAGAGAAGAGTGGTG-----------
----------------------------CAGAGAGAAAAAAGAGCA---GTGGGA---CTAGGAGCT------
---ATGTTCCTTGGGTTCCTGGGAGCA---GCAGGAAGCACTATG---GGCGCAGCGTCA---
ATAACGCTGACGGTACAG---GCCAGACAA---TTATTG---TCTGGTATAGTGCAACAGCAAAAC---
AATTTG---CTGAGA---GCTATTGAGGCGCAACAACATCTGTTGCAACTCACAGTCTGGGGCATC
>KC699036
TTCTACTGTGAAACAGAAAAACTGTTTAATAGTACTTGGTGGAATAGTACTTGGAATAGTACTGAAGGGTCAA
ATAGT---

ACTGGGGTAAATATCACACTCCCATGCAGAATAAGACAAATTATAAACATGTGGCAGAAAGTAGGAAAAGCAA
TGTATGCCCCTCCCATCGCAGGACCAATTAGATGTGTATCAAAT---
ATCACAGGGATATTCTTAACAAGAGATGGTGGTAATGAGACCAGC----------------------------
----

AACGAGACCTTCAGACCTGGAGGAGGAGATATGAGGGACAATTGGAGAAGTGAATTATATAAATATAAAATAG
TAAAAATTGAGCCAATA---GGAGTAGCACCCACCAAGGCAAAGAGAAGAGTGGTG-----------------
---------------------CAGAGAGAAAAAAGAGCA---GTGGGA---CTAGGAGCT---------
ATGTTCCTTGGGTTCTTGGGAGCA---GCAGGAAGCACTATG---GGCGCAGCATCA---

ATAACGCTGACGGTACAG---GCCAGACAA---TTACTG---TCTGGTATAGTGCAACAGCAGAAT---
AATCTG---CTGAGG---GCTATTGAAGCGCAACAACATATGTTGCAACTCACAGTCTGGGGCATA
>KF716494
TTCTACTGTAATACAACAGAACTATTTAATAGTACTTGGAAGATTGAGAATAATACTGGAATATTACTGAAC
AGGCAAAT--
-------------AACACT------
GCAAGAAATGACACAATCATACTCCCATGCAGAATAAAGCAAATTATAAACCTGTGGCAGGAAGTAGGAAAAG
CAATGTATGCCCCTCCCATTGAGGGACTAATTAACTGTACATCAAAT---
ATCACAGGGCTAATACTAACAAGGGATGGTGGCAATAACAATAGCACT------------------------
----

AATGAGACCGAGATCTTCAGACCTGGAGGAGGAAATATGAGGGACAATTGGAGAAGTGAATTATATAAATATA
AAGTAGTACAAATTGAACCAATA---GGACTAGCACCCACCAAGGCAAAGAGAAGAGTGGTG----------
---------------------------CAGAGAGAAAAAAGAGCA---CTGGGA---ATAGGAGCT------
---GTGTTCCTTGGGTTCTTGGGAGCA---GCAGGAAGCACTATG---GGCGCAGCGTCG---
CTGACGCTGACGGTACAG---GCCAGGCAA---TTACTG---TCTGGTATAGTGCAACAGCAGAGC---
AATTTG---CTGAGA---GCTATTGAGGCGCAACAGCATATGTTGCAACTCACAGTCTGGGGCATT
>KF716495
TTCTACTGTAATACAACACAACTGTTTAATAGTACTTGGAATGCTACTAGCGAGTCAAGTGAC----------
----

CTAAATGACACAATCACACTCCCATGCAGAATAAAACAAATTATAAACATGTGGCAGAAAGTAGGAAAAGCAA
TGTATGCCCCTCCCATCAAAGGACTAATTAATTGTACATCAAAT---
ATCACAGGGCTCCTATTAACAAGAGATGGTGGTAACAAGGTTAAT---------------------------
----

GAGAGCAACCATGAGACCTTCAGACCGGGAGGAGGAAATATGAAGGACAATTGGAGAAGTGAATTATATAAAT
ACAAAATAGTAAAAATTGAACCATTA---GGAGTAGCCCCCACCAAGGCAAAGAGAAGAGTGGTG-------
------------------------------CAGAGAGAAAAAAGAGCA---GTGGGA---ATAGGAGCT---
------ATGTTCCTTGGGTTCTTGGGAGCA---GCAGGAAGCACTATG---GGCGCAGCGTCA---
GTGACGCTGACGGTACAG---GCCAGAAAA---TTATTG---TCTGGTATAGTGCAACAGCAGAAC---
AATTTG---CTGAGG---GCTGTTGAGGCGCAACAACGTATGTTGCAACTCACAGTCTGGGGCATT
>KC699031
TTCTACTGTGATACAACACAGCTATTTAATAGTACCTGGAATAGTACTACTTTGAATAATACTTGGAATAATA
CTTGG---
-------------AATAAT------
AGTGAAAATGACACTATCATACTCAGATGCAGAATAAAACAGATCATAAACAGGTGGCAGGAAGTAGGAAAAG
CAACGTATGCCCCTCCCATCAGTGGAGAAATTAGATGTACATCAAAT---
ATTACAGGGCTACTATTAACAAGAGATGGTGGTAAAAACAACAAC---------------------------
----

GACAACGAGACCTTCAGACCTGGAGGAGGAGATATGAGGGACAATTGGAGAAGTGAATTATATAAATATAAAG
TAGTAAAAATTGAGCCATTA---GGACTGGCACCCACCAAGGCAAAGAGAAGAGTGGTG-------------
------------------------CAGAGAGAAAAAAGAGCA---GTGACA---ATGGGAGCT---------
TTGTTCCTTGGGTTCTTGGGAACA---GCGGGAAGCACTATG---GGCGCAGCGTCA---
GTGACGCTGACGGTACAG---GCCAGACTA---TTGTTG---TCTGGTATAGTGCAGCAGCAAAAC---
AATTTG---CTGAGG---GCTATTGAGGCGCAACAACATTTGTTGCAACTCACAGTCTGGGGCATC
>KC699034
TTCTATTGTAATGTAACACAACTGTTTAATAGTACTTGGCATGGTAATGAGACTGTGAATAGTACTGAA----
----

GAAGGAGAAGTCATCACACTTCCATGCAGAATAAAACAAATTATAAACCTGTGGCAGGAAGTAGGAAAAGCAA
TGTATGCCCCTCCCATCAGTGGAAAAATTAGATGTTCATCAAAT---
ATTACAGGGCTGTTATTAACAAGAGATGGTGGATACAACGGGGAC---------------------------
----

AACAATACTGAGACCTTCAGACCTGGAGGAGGAGATATGAGGGATAATTGGAGAAGTGAATTATACAAATATA
AAGTAGTAAAAGTTGAACCATTA---GGAATAGCACCCACCAAGGCAAAGAGAAGAGTGGTG----------
---------------------------CAGAGAGAAAAAAGAGCA---GTGGGA---GTAGGAGCT------
---ATGATCATTGGGTTCTTGGGAGCA---GCAGGAAGCACTATG---GGCGCAGCGTCA---

GTGACGCTGACGGTACAG---GCCAGACTA---TTGTTG---TCTGGTATAGTGCAACAGCAGAGC---
AACCTG---CTGAGG---GCTATTGAGGCGCAACAGCATATGTTGCAGCTCACAGTCTGGGGCATT
>KF716496
TTCTACTGTAATACAACACCACTGTTTGACAGTACCTGGAATGGGGGAGAGCCAGTGTCAAATAACACTGGAG
GA---

AATGACACAATAATCACACTCCAATGTAGAATAAAACAAATTATAAACATGTGGCAGGAAGTTGGAAAAGCAA
TGTATGCCCCTCCCATCAGAGGACAAATTAGATGCTCATCAAAT---
ATTACAGGGCTGATATTAACAAGAGATGGTGATCTTAACAATACC----------------------------
----

CAGAACGAGACCTTCAGACCTGGAGGAGGAGATATGAGGGACAATTGGAGAAGTGAATTATATAAATATAAAG
TAGTAAAAATTGAACCATTA---GGAGTAGCACCCACCAAGGCAAAGAGAAGAGTGGTG--------------
-------------------------CAGAGAGAGAAAAGAGCA---GTGGGA---ATAGGAGCT---------
CTGTTCCTTGGGTTCTTAGGAGCA---GCAGGAAGCACTATG---GGCGCAGCGTCA---
ATGACGCTGACGGTACAG---GCCAGACAA---CTATTG---TCTGGTATAGTGCAACAGCAGAAC---
AATCTG---CTAAGG---GCTATTGAGGCGCAACAACATATGTTGCAACTCACAGTCTGGGGCATC
>MH000302
TTCTACTGTAATACAACACAACTGTTTAATAGTACGTGGGCTTCG----------------------------
----

AATACTAACACTATCACACTCCCATGCAGAATAAAACAAATTATAAACATGTGGCAACAAGTAGGAAAAGCAA
TGTATGCCCCTCCCATCAGAGGAACAATTAAATGTTCATCAAAT---
ATTACAGGGCTACTATTATCAAGAGATGGAAGT--

ATCGAGACCTTCAGACCTGGAGGAGGAGATATGAGAGATAATTGGAGAAGTGAATTATATAAATATAAAGTAG
TAAAAGTTAAGCCATTA---GGAGTAGCACCCACCAGTGCAAAAAGAAGAGTGGTG-----------------
----------------------CAGAGAGAAAAAAGAGCA---ATAGGA---TTCGGAGCT---------
ATGTTCATTGGATTCTTGGGAGCT---GCAGGAAGCACTATG---GGCGCAGCGTCA---
GTGACGCTGACGGTACAG---GCCAGACAA---TTATTG---TCTGGTATAGTGCAACAGCAGAAT---
AATCTG---CTGAGG---GCTATTGAGGCGCAACAGCATCTGTTGCAACTCACAGTCTGGGGCATC
>MH000296
TTCTACTGCAATACAACAGCACTGTTTAATAGTACTTGGGTTATG----------------------------
----

AATGATTCCACTATCACACTCCCATGCAGAATAAAACAAATTATAAACAGATGGCAGGAAGTAGGAAAAGCAA
TGTATGCCCCTCCCATTGCAGGACCAATTAGGTGTTCATCAAAT---
ATTACAGGGCTGCTATTAACAAGAGATGGGGGA--

GAGGAGATCTTCAGACCAGGAGGAGGAGATATGAGGGACAATTGGAGAAGTGAATTATATAAGTATAAAGTAG
TGAAAATTGAACCATTA---GGAGTAGCACCCACCAAGGCAAAAAGAAGAGTGGTG-----------------
----------------------CAGAGAGAAAAAAGAGCA---ATAGGA---ATAGGAGCT---------
ATGTTCATTGGGTTCTTGGGAGCA---GCAGGAAGCACTATG---GGCGCAGCGTCA---
GTGACGCTGACGGTACAG---GCCAGACAA---TTATTG---TCTGGTATAGTGCAACAGCAGAAC---
AATTTG---CTGAGG---GCTATTGAGGCGCAACAACATCTGTTGCAACTCACAGTCTGGGGCATC
>MH000299
TTCTATTGTGATACATCACAACTGTTTAATAGTACTTGGAAT-------------------------------
----

AGTACTGTTAATATCACTCTCCCATGCAGATTAAAGCAATTTATAAACATGTGGCAGCAAGTAGGAAAAGCAA
TGTATGCCCCTCCCATCAGCGGACTAATCCAGTGCTCATCAAAT---
ATTACAGGGCTGCTGTTAGTAAGGGATGGTGGT--

ACTGAAATCTTCAGACCTGGAGGGGGAGATATGAGGGACAATTGGAGAAGTGAACTATATAAATATAAAGTAG
TAAAGCTTGCACCATTA---GGAATAGCACCCACCAAGGCAAAGAGAAGAGTGGTG-----------------
----------------------CAGAGAGAAAAAAGAGCA---GTGGGA---CTAGGAGCT---------
ATGTTCCTCGGGTTCTTGGGAGCA---GCAGGAAGCACTATG---GGCGCAGCGTCA---

GTGACGCTGACGGTACAG---GCCAGACAA---TTATTG---TCTGGTATAGTGCAACAGCAGAGC---
AATCTG---CTGAGA---GCTATTGAGGCGCAACAACATATGTTGCAACTCACAGTCTGGGGCATA
>KC699002
TTCTACTGTAATTCAACACAACTGTTTAATAGTATCTGGAATGGTACTTGGAATGATACTTGGAATGGTACTA
GAGGGGTA---
-------------------AAT---
AACACTGAAGACAATATCATACTCCCATGTAGAATAAAACAAATTATAAACATGTGGCAGGAAGTAGGAAAAG
CAATGTATGCCCCTCCCATCAAAGGACGAATTATGTGTTCATCAAAT---
ATTACAGGGCTGCTATTAACAAGAGATGGTGGTAATAGTAACGCT----------------------------
----

ACCACCGAGATCTTCAGACCTGGAGGAGGAGATATGAGGGACAATTGGAGAAGTGAATTATATAAATATAAAG
TAGTAAAAATTAAACCATTA---GGAGTAGCACCTACCAAGGCAAAGAGAAGAGTGGTG--------------
-------------------------CAGAGAGAAAAAAGAGCA---GTGGGA---ATAGGAGCT---------
GTGTTCCTTGGGTTCTTGGGAGCA---GCAGGAAGCACTATG---GGCGCAGCGTCA---
ATGACGCTGACGGTACAG---GCCAGACGA---TTATTG---TCTGGTATAGTGCAACAGCAGAAC---
AATTTG---CTGAGG---GCTATTGAGGCGCAACAGCATCTGTTGCAACTCACAGTCTGGGGCATC
>KC699011
TTCTACTGTAACTCAACACAACTGTTTAATAGTACTTGGGTGTTTAATAATACTTGGAATAGAACTGAAGGGT
TAAATAAC---

ACTGCAGAAAATATCACACTCCCATGCAGAATAAAACAAATTGTAAATATGTGGCAGGAAGTAGGAAAAGCAA
TGTATGCCCCTCCCATCAGAGGACAAATTAGATGTTCATCAAAT---
ATTACAGGGCTGCTATTAACAAGAGATGGTGGTAATAACACGAGA----------------------------
----

AATAGCGAGACCTTCAGACCTGGGGGAGGAGATATGAGGGACAATTGGAGAAGTGAATTATATAAATATAAAG
TAGTACAAATTGAACCATTA---GGAGTAGCACCCACTAAGGCAAGAAGAAGAGTGGTG--------------
-------------------------CAGAGAGAAAAAAGAGCA---ATAACA---CTAGGAGCT---------
GTGTTCCTTGGGTTCTTGGGAGCA---GCAGGAAGCACTATG---GGCGCAGCGTCA---
GTGACGCTGACGGTACAG---GCCAGACAA---TTACTG---TCTGGTATAGTGCAACAGCAGAAC---
AATTTG---CTGAGG---GCTATTGAGGCGCAACAGCATCTGTTGCAACTCACAGTCTGGGGCATC
>KC699010
TTCTACTGTAATACAACACAACTGTTTAATAGCACTTGGAATAGTACTAGGATTGAAAATGATACTGAAGAGT
CAAATAGC---

ACTGAAAGAACGATCACACTCCCATGCAGAATAAGACAAATTATAAACAGGTGGCAGGAAGTAGGGAAAGCAA
TGTATGCCCCTCCCATCAGCGGACAAATTAGATGCTCATCAAAC---
ATTACAGGGCTGCTATTAACAAGAGATGGCGGTAATGACAAGAAAAACGAG----------------------
----

ACTACTGAGGTCTTCAGACCTGGAGGAGGAGATATGAAGGACAATTGGAGAAGTGAATTATATAAATATAAAG
TAGTAAAAATTGAACCATTA---GGAGTAGCACCCACTAAGGCAAAGAGAAGAGTGGTG--------------
-------------------------CAGAGAGAAAAAAGAGCA---GTGGGA---CTAGGAGCT---------
GTGTTCCTTGGGTTCTTGGGAGCA---GCAGGAAGCACTATG---GGCGCAGCGTCA---
ATGACGCTGACGGTACAG---GCCAGACTA---TTATTG---TCTGGTATAGTGCAACAGCAGAAC---
AATTTG---CTGAGG---GCTATTGAGGCGCAACAGCATCTGTTGCAACTCACAGTCTGGGGCATC
>KC699009
TTCTACTGTAATTCAACACAACTGTTTAATAGTACTTGGAATGGTACTAATAGTACTTGGAAT----------
----

GGTACTGAAAGTATCACACTCCCATGCAGAATAAAACAATTTATAAACATGTGGCAGGAAGTAGGAAAAGCAA
TGTATGCCCCTCCCATCAAAGGACAAATTAGATGTTCATCAAAT---
ATTACAGGGCTGCTATTAACAAGAGATGGTGGTAATAACAATAAC----------------------------
----

GAGAGCGAGACCTTCAGACCTGGAGGAGGAGATATGAGGGACAATTGGAGAAGTGAATTATACAAATATAAAG
TAGTAAAAATTGAACCATTA---GGAGTAGCACCCACCAAGGCAAAGAGAAGAGTGGTG--------------
-------------------------CAGAGAGAAAAAAGAGCA---GTGGGA---ATAGGAGCT---------
TTGTTCCTTGGGTTCTTGGGAGCA---GCAGGAAGCACTATG---GGCGCAGCGTCA---

ATAACGCTGACGGTACAG---GCCAGACTA---TTATTG---TCTGGTATAGTGCAACAGCAAAAC---
AATTTG---CTGAGG---GCTATTGAGGCGCAACAGCATCTGTTGCAACTCACAGTCTGGGGCATC
>KC699008
TTCTACTGTAATTCAACACCACTATTTAATAGTACTTGGCATAATAATGACACTGAAGGAAATAACATC----
----

ACTAACGGAAACATCACACTCCCATGCAGAATAAAACAAATTATAAACATGTGGCAGGAAGTAGGAAAAGCAA
TGTATGCCCCTCCCATCAAAGGACAAATTAGATGCTCATCAAAT---
ATTACAGGGCTGCTATTAACAAGAGATGGAGGTAATAATAACAATAGT-------------------------
----

ACCAATGACACTGAGATCTTCAGACCTGGAGGGGGAGATATGAGGGACAATTGGAGAAGTGAATTATATAAAT
ATAAAGTAGTAAAAATTGAACCATTA---GGAGTAGCACCCACCAAGGCAAAGAGAAGAGTGGTG--------
-------------------------------CAGAGAGAAAAAAGAGCA---GTGGGAACGTTGGGAGCC---
------ATGTTCCTTGGGTTCTTGGGAGCA---GCAGGAAGCACTATG---GGCGCAGCGTCA---
ATGACGCTGACGGTACAG---GCCAGACAA---TTATTG---TCTGGTATAGTGCAACAGCAGAAC---
AATCTG---CTGAGG---GCTATTGAGGCGCAACAGCATATGTTGCAACTCACAGTCTGGGGCATC
>KC699003
TTCTACTGTAACACAACAAAACTGTTTAATAGTACTTGGAATGTTGCTGAAGGGTCAAATAAC----------
----

ACTGAAGGAAATATCACACTCCCATGCAGAATAAAACAAATTATAAACATGTGGCAGGAAGTAGGAAAGGCAA
TGTATGCCCCTCCCATCAGAGGACAAATTAGATGTGTATCAAAT---
ATTACAGGGCTGCTATTAACAAGAGATGGTGGTAATAACAGGAATGGG-------------------------
----

ACCAACGGGACCGAGATCTTCAGACCTGGAGGAGGAGATATGAGGGACAATTGGAGAAGTGAATTATATAAAT
ATAAAGTAGTAAAAATTGAACCATTA---GGAATAGCACCCACCAAGGCAAAGAGAAGAGTGGTG--------
-------------------------------CAGAGAGAAAAAAGAGCA---GTCACA---TTAGGAGCT---
------ATGTTCCTTGGGTTCTTGGGAGCA---GCAGGAAGCACTATG---GGCGCAGCGTCA---
ATAACGCTGACGGTACAG---GCCAGACTA---TTATTG---TCTGGTATAGTGCAACAGCAGAGC---
AATTTG---CTGAGA---GCTATTGAGGCGCAACAACATTTGTTGCAACTCACAGTCTGGGGCATC
>KC699004
TTCTACTGTAATACAACAAAACTGTTTAATAGTACTTGGGCTTGGAATAATGGTACTTGGAATAGTACTGAAG
GGTCAAAT---

AACACAGCAAATATCACACTCTCATGCAGAATAAGACAAATTATAAACATGTGGCAAGAAGTAGGAAAAGCAA
TGTATGCCCCTCCCATCAGCGGACAAATTAAATGCTCATCAAAT---
ATTACAGGGCTGCTGTTAACAAGAGATGGTGGTACTAACAATAGC----------------------------
----

GAGACCGAGACTTTCAGACCTGGAGGAGGAGATATGAGGGACAATTGGAGAAGTGAATTATACAAATATAAAG
TAGTAAAAATTGAACCATTA---GGAGTAGCTCCCACCAAGGCAAAGAGAAGAGTGGTG--------------
-------------------------CAGAGAGAAAAAAGAGCA---GTGGGA---ATAGGAGCT---------
TTGTTCCTTGGGTTCTTGGGAGCA---GCAGGAAGCACTATG---GGCGCAGCATCA---
ATGACGCTGACGGTACAG---GCCAGACAA---TTACTG---TCTGGTATAGTGCAACAGCAGAAC---
AATTTG---CTGAGG---GCTATTGAGGCGCAACAGCATATGTTGCAACTCACAGTCTGGGGCATC
>KC699005
TTCTACTGTAATACAACACAACTGTTTAATAGTACTTGGAATGATACTAAAGGGCCAGATAGCACTGACAATA
GTACTTGG---

ATCATACTACCATGCAGAATAAAACAAATTATAAACATGTGGCAGGAAGTAGGAAAAGCAATGTATGCCCCTC
CTATCAGAGGACAAATTAGATGTTCATCAAAT---
ATTACAGGGCTACTATTAACAAGAGATGGTGGGGGAAACAAGAAC----------------------------
----

GAGAGCACCGAGACCTTCAGACCTGGAGGAGGAGATATGAGGGACAATTGGAGAAGTGAATTATATAAATATA
AAGTAGTACAAATTGAACCATTA---GGAGTAGCACCCACCAAGGCAAAGAGAAGAGTGGTG-----------
----------------------------CAGAGAGAAAAAAGAGCA---GTGGGA---ATAGGAGCT------
---GTGTTCCTTGGGTTCTTGAGCGCA---GCAGGAAGCACTATG---GGCGCAGCGTCG---

```
GTGACGCTGACGGTACAG---GCCAGACAA---TTATTG---TCTGGTATAGTGCAGCAGCAAAAC---
AATTTG---CTGAGG---GCTATTGAGGCGCAACAACATCTGTTGCAACTCACAGTCTGGGGCATC
>KC699001
TTCTACTGTGATTCAACACAACTGTTTAATAGTACTTGGAATAGTACTACTTGGAATAGTACTGAAGGGTTAA
ATAAC--------------------------------------------------------------------
----------------------------
ACTGAAGGCAATATCACACTCCAATGCAGAATAAAACAAATTATAAACATGTGGCAGGAAGTAGGAAAAGCAA
TGTATGCTCCTCCCATCAGAGGACATATTAAATGTTCATCAAAT---
ATTACAGGGCTGTTATTAACAAGAGATGGTGGTAATAACGAGAGTAGA-------------------------
-------------------------------------------------------------------------
-------------
ACCAACGAGACCTTTAGACCTGAAGGAGGAAATATGAAAGACAATTGGAGAAGTGAATTATATAAATATAAAG
TAGTAAAAATTGAACCATTA---GGAATAGCACCCACCAAGGCAAAGAGAAGAGTGGTG--------------
-------------------------CAGAGAGAAAAAAGAGCA---GTGGGA---TTAGGAGCT---------
GTGTTCCTTGGGTTCTTGGGAGCA---GCAGGAAGCACTATG---GGCGCAGCGTCA---
CTGACGCTGACGGTACAG---GCCAGAACA---TTATTG---TCTGGTATAGTGCAACAGCAGAAC---
AATCTG---CTGAGG---GCTATTGAGGCGCAACAGCACCTGTTGCAACTCACAGTCTGGGGCATC
>AY535425
TTCTACTGTAATACAACACAACTGTTTAATAGTACTTGGATGCTTAATAATACTTGGGATGGTACT-------
-------------------------------------------------------------------------
----------------------------
AAAGAGGAAAATATTACACTCCCATGCAGAATAAAACAAATTATAAACATGTGGCAGGAAGTAGGAAAAGCAA
TGTATGCCCCTCCCATCAAAGGACAAATTAGATGTTCATCAAAC---
ATTACAGGGCTGCTATTAACAAGAGATGGTGGTGGTAACACGAGC----------------------------
-------------------------------------------------------------------------
----------
AGCGATAATGAGACCTTCAGACCTGGAGGAGGAGATATGAGGGATAATTGGAGAAGTGAATTATATAAATATA
AAGTAGTAAAAATTGAACCATTA---GGAGTAGCACCCACCAAGGCAAGGAGAAGAGTGGTG-----------
----------------------------CAGAGAGAAAAAAGAGCA---GTGGGAATGCTAGGAGCC------
---ATGTTCCTTGGGTTCTTGGGAGCA---GCAGGAAGCACTATG---GGCGCAGCGTCA---
CTGGCGTTGACGGTACAG---GCCAGACAA---GTATTG---TCTGGTATAGTGCAACAGCGGAAC---
AATTTG---CTGAGG---GCTATTGAGGCGCAACAGCATCTGTTGCAACTCACAGTCTGGGGCATC
>AY713411
TTCTACTGTAATACAACACAACTGTTTAATAGTACTTGGAATGAAACTAACAGTGAAGGAAATATC-------
-------------------------------------------------------------------------
----------------------------
ACCAGTGGAACTATAACACTCCCATGCAGAATAAAACAAATTATAAACATGTGGCAGGAAGTAGGAAAAGCAA
TGTATGCCCCTCCCATCGGAGGACAAATTAAATGTTTGTCAAAC---
ATCACAGGGCTGTTATTAACAAGAGATGGTGGTAGTGATAACAGTAGT-------------------------
-------------------------------------------------------------------------
----------
AGTGGGAAAGAGATCTTCAGACCTGGAGGGGGAGATATGAGGGACAATTGGAGAAGTGAATTATATAAATATA
AGGTAGTAAAAATTGAACCATTA---GGAATAGCACCCACCAAGGCAAAGAGAAGAGTGGTG-----------
----------------------------CAGAGAGAAAAAAGAGCA---GTGGGA---ATAGGAGCC------
---ATGTTCCTTGGGTTCTTGGGAGCA---GCAGGAAGCACTATG---GGCGCAGCGTCA---
CTAACGCTGACGGTACAG---GCCAGACAA---TTATTG---TCTGGTATAGTGCAGCAGCAAAAC---
AATTTG---CTGAGG---GCTATTGAGGCGCAACAGCACCTGTTGCAACTCACAGTCTGGGGCATC
>AF041125
TTCTACTGTAACTCAACAAAACTGTTTAACAGTACTTGGAGTGCTAATGGTACTGTCATGCCAAATGACACTG
AA-----------------------------------------------------------------------
----------------------------
GGGAATGGCACAATCATGCTCACATGCAGAATAAAACAAATTGTAAACAGGTGGCAGGAAGTAGGAAAAGCAA
TGTATGCCCCTCCCATCAGAGGACAAATTAGATGTTCATCAAAT---
ATTACAGGGCTAATATTAACAAGAGGTGGTGGTGATAAAGAGAAC----------------------------
-------------------------------------------------------------------------
----------
GAGACTACTGAGACCTTCAGACCTGGAGGAGGAAATATGAGGGATAATTGGAGAAGTGAATTATATAAATATA
AAGTAGTAAAACTCGAACCATTA---GGGATAGCACCCACCAAGGCAAAGAGAAGAGTGGTG-----------
----------------------------CAGAGAGAAAAAAGAGCA---GTGGGA---ATAGGAGCT------
---TTGTTCCTTGGGTTCTTGGGAGCA---GCAGGAAGCACTATG---GGCGCAGCGTCA---
```

CTGACGCTGACGGTACAG---GCCAGACTA---TTATTG---TCTGGTATAGTGCAACAGCAGAAC---
AATTTG---CTGAGA---GCTATTGAGGCGCAACAGCATCTGTTGCAACTCACAGTCTGGGGCATC
>AY535471
TTTTACTGTAATACAACACAACTGTTTAATAGTACTTGGAATGCTAACCATATTAGGAATGTTACTAGAGGGT
CAAATAGA---
-------------ACAACT------
GGAGGAAATGACACACTCATACTCCCATGTAGAATAAAACAAATTATAAATATGTGGCAGGAAGTAGGAAAAG
CAATGTATGCCCCTCCCGTCAGAGGACAAATTAGATGTTCATCAAAT---
ATTACAGGGTTGCTATTAACAAGAGATGGTGGTAATAACGGGAATGAG-------------------------
----

ACCAACGGGACTGAGATCTTCAGACCTGGAGGGGGAGATATGAGGAACAATTGGAGAAGTGAATTATATAAAT
ATAAAGTAGTAAAAATTGAACCATTA---GGAGTACCACCCACCAAGGCAAGGAGAAGAGTGGTG--------
-------------------------------CAGAGAGAAAAAAGAGCA---GTGACA---CTAGGAGCT---
------ATGTTCCTTGGGTTCTTAGGAGCA---GCAGGAAGCACTATG---GGCGCAGCGTCA---
ATGACGCTGACGGTACAG---GCCAGACAA---TTATTG---TCTGGTATAGTGCAACAGCAAAGC---
AATTTG---CTGAGG---GCTATTGAGGCGCAACAGCATCTGTTGCAACTCACAGTCTGGGGCATC
>AF041130
TTCTACTGTAATACAACACAGCTGTTTAATAGTACTTGGCTGGTTAATAGTACTGGGGATGATACTAAT----
----

ACTACCACCAATATCACACTCCCATGCAGAATAAAACAAATTATAAACATGTGGCAGAAAGTAGGAAAAGCAA
TGTATGCCCCTCCCATCAGAGGACAAATTAGATGTTTATCAAAT---
ATTACAGGGATACTATTAACAAGGGATGGTGGTAATAACACAAAC----------------------------
----

GGGACCGAGATCTTCAGACCTGGAGGAGGAGATATGAGGGATAATTGGAGAAGTGAATTATATAAATATAAAG
TAGTAAAAATTGAACCATTG---GGAATAGCACCCACCAAGGCAAAGAGAAGAGTGGTG--------------
-------------------------CAAAGAGAAAAAAGAGCA---GTGGGA---TTAGGAGCT---------
ATGTTCCTTGGGTTCTTGGGAGCA---GCAGGAAGCACTATG---GGCGCAGCGTCA---
CTGACGCTGACGGTACAG---GCCAGACAA---TTATTG---TCTGGTATAGTGCAACAGCAGAAC---
AATTTG---CTGAGA---GCTATTGAGGCGCAACAGCATCTGTTGCAACTCACAGTCTGGGGCATC
>AF041131
TTCTACTGTAATACAACACGACTGTTCAATAGTACTTGGAATGCTAGTAACATTGAAGAAAATAGCACCACT-
----

GAAGGAAATATCACCACTATCCCATGCAGAATAAAACAAATTATAAACATGTGGCAGGAAGTAGGAAAAGCAA
TGTATGCCCCTCCCATCAGAGGACAAATTAGATGTTCATCAAAT---
ATTACAGGGCTGCTATTAATAAGAGATGGTGGTAATGATAACAAT----------------------------
----

AATGACACCGAGATCTTCAGACCTGGAGGGGGAGATATGAGGGACAATTGGAGAAGTGAATTATATAAATATA
AAGTAGTAAAAATTGAACCATTA---GGAATAGCACCCACCAAGGCAAAGAGAAGAGTGGTG-----------
----------------------------CAGAGAGAAAAAAGAGCA---GTGGGAACGCTAGGAGCC------
---ATGTTCCTTGGGTTCTTGGGAGCA---GCAGGAAGCACTATG---GGCGCAGCGTCA---
ATGACGCTGACGGTACAG---GCCAGACAA---TTATTG---TCTGGTATAGTGCAACAGCAGAGT---
AATCTG---CTGAGG---GCTATTGAGGCGCAACAGCATCTGTTGCAACTCACAGTCTGGGGCATC
>AF041134
TTCTACTGTAATACAACCCAACTGTTTGATAGTATTTGGAATGATACTGAAGGGTCATATAACACTGAA----
----

GGAAATGACACAATCACTCTCCCATGCAGGATAAAACAAATTATAAACTTGTGGCAGGAAGTAGGAAAAGCAA
TGTATGCCCCTCCCATCAGAGGACAAATTAGATGTTCATCAAAT---
ATTACAGGGCTGCTATTAACAAGAGATGGTGGGAACGAGAGCGAA----------------------------
----

GCCACCGAGATCTTCAGACCTGGAGGAGGAGATATGAGGGACAATTGGAGAAGTGAATTATATAAATATAAAA
TAGTAAAAATTGAACCATTA---GGAGTAGCACCCACCAAGGCAAAGAGAAGAGTGGTG--------------
-------------------------CAAAGAGAGAAAAGAGCA---GTGGGA---TTAGGAGCT---------
TTGTTCCTTGGGTTCTTGGGAGCA---GCAGGAAGCACTATG---GGCGCAGCGTCA---

ATAACGCTGACGGTACAG---GCCAGACAA---TTATTG---TCTGGTATAGTGCAACAACAGAAC---
AATTTG---TTGAGG---GCTATTGAGGCGCAACAGCATCTGTTGCAACTCACAGTCTGGGGCATC
>AF041133
TTCTACTGTGATTCAACACAACTATTTAATAGTACTTGGAGTATTAATAGTACTTGGAATAGTACTGAA----
----

ATAACAGATAATATCACACTCCCATGCAGAATAAAACAAATTATAAACATGTGGCAGGAAGTAGGGAAAGCAA
TGTATGCCCCTCCCATCAGAGGACAAATTAGATGTTCATCAAAT---
ATTACAGGGCTGCTATTAACAAGAGATGGTAGTGGCACGAATGAGACG------------------------
----

AACGGGACTGAGACCTTCAGACCGGGAGGAGGAGATATGAGGGACAATTGGAGAAGTGAATTATATAAATATA
AAGTAGTAAAAATTGAACCATTA---GGAGTAGCACCCACCAAGGCAAAGAGAAGAGTGGTG-----------
---------------------------CAGAAAGAAAAAAGAGCA---GTGGGG---ATAGGAGCT------
---GTGTTCCTTGGGTTCTTGGGAGCA---GCAGGAAGCACTATG---GGCGCAGCGTCA---
CTGACGCTGACGGTACAG---GCCAGACTA---TTATTG---TCTGGTATAGTGCAACAGCAGAAC---
AATTTG---CTGAGG---GCTATTGAGGCGCAACAGCATCTGTTGCAACTCACAGTCTGGGGCATC
>KC699014
TTCTACTGTAATACAACACCACTGTTTAATAGTACTTGGACTTTTAATGGTACTACTTGGAACACAACTAACA
CTGAA---

GGAAATGACACAATCACACTCCAATGTAAAATAAAGCAAATTATAAACATGTGGCAGGAAGTAGGAAAAGCAA
TGTATGCCCCTCCCATCAAAGGACTAATTAAATGTTCATCAAAT---
ATTACTGGGTTGCTACTAACAAGGGATGGTGGTAGTAATACTACC---------------------------
----

AATACCACCGAGACCTTCAGACCTGGAGGAGGAGATATGAAGGACAATTGGAGAAGTGAATTATATAAATATA
AAGTAGTAAAAATTGAACCATTA---GGAGTAGCACCCACCAAGGCAAAGAGAAGAGTGGTG-----------
---------------------------CAGAGAGAAAAAAGAGCA---GTAACA---CTAGGAGCT------
---ATGTTCCTTGGGTTCTTGGGAGCA---GCAGGAAGCACTATG---GGCGCAGCGTCA---
ATAACGCTGACGGTACAG---GCCAGACAA---TTATTG---TCTGGTATAGTGCAACAGCAGAAC---
AATTTG---CTGAGG---GCTATTGAGGCGCAACAACATCTGTTGCAACTCACAGTCTGGGGCATC
>KC699016
TTCTACTGTGATACAACAAAACTGTTTAGTAGTACTTGGAATAGGACTGAGACTACTTGGAATATTGCTGAAG
GGTTA---
-------------------AAT---
GAGACTGAAACCACCATCACACTCCAATGCAGAATAAAACAAATTGTAAACAAGTGGCAGGAAGTAGGAAAAG
CAATGTATGCCCCTCCCATCAAAGGACAAATTAAATGCTCATCAAAT---
ATTACAGGGCTGCTCTTAACAAGAGATGGTGGGAGCGGGAACGAGACCGAG---------------------
----

AACGGGACCGAGACTTTCAGACCTGGAGGAGGAGATATGAGGGACAATTGGAGGAGTGAATTATACAAATATA
AAGTAGTACAAATTGAACCATTA---GGAGTAGCACCCACCAAGGCAAAGAGAAGAGTGGTG-----------
---------------------------CAGAGAGAAAAAAGAGCA---GTGGGA---ATAGGAGCT------
---ATGTTCCTTGGGTTCTTGGGAGCA---GCAGGAAGCACTATG---GGCGCAGCGTCA---
ATGATGCTGACGGTACAG---GCCAGACAA---TTATTG---TCTGGTATAGTGCAACAGCAGAAC---
AATTTG---CTGAGG---GCTATTGAGGCGCAACAGCATATGTTGCAACTCACAGTCTGGGGCATT
>KC699017
TTCTACTGCAATACAACACAGCTGTTTAACAGTACTTGGTATAATAATAGTGCTTGGAATGGTACTGCACAGG
AAAAT---

AGCACTGAAATTATCACACTCCCATGCAGAATAAAACAGATTATAAACAGGTGGCAGGAAGTAGGAAGAGCAA
TGTATGCCCCTCCCATCGCAGGACAAATTAGATGTTCATCAAAC---
ATTACAGGACTGCTATTAACAAGAGACGGTGGAAACTCCAATAAT---------------------------
----

AGTGAAAATGAAACCTTTAGACCTGGAGGAGGAGATATGAGAGACAATTGGAGAAGTGAGTTATATAAATATA
AAGTAGTACAAATTAAACCATTA---GGAGTAGCACCTACCATGGCAAAGAGAAGAGTGGTA-----------
---------------------------CAGAGAGAAAAAAGAGCA---GTGGGAATGCTAGGAGCT------
---ATGTTCCTTGGGTTCTTGGGAGCA---GCAGGAAGCACTATG---GGCGCAGCGTCA---

ATGACGCTGACGGTACAG---GCCAGACAA---TTATTA---TCTGGTATAGTGCAACAGCAAAAC---
AATTTG---CTGAGG---GCTATTGAGGCGCAGCAGCATCTGTTGCAACTCACAGTCTGGGGCATC
>KC699012
TTCTACTGTAATTCAACAGAACTGTTTAATAGTACTTGGACTTGGAATGATACTAGGGGGTTAAATAACACTG
AAGGA--

AATAGCACAATAATCAAACTCCCATGCAGAATAAAACAAATTATAAACATGTGGCAGGAAGTAGGAAAAGCAA
TGTATGCCCCTCCCATCAGAGGACAAATTAACTGTTTATCCAAT---
ATTACAGGGCTGCTATTAACGAGGATGGTGGTATTATTACCACAAATGGGTCC--------------------
----

GAGAACGAGACCTTCAGACCTGGAGGAGGAGATATGAGGGACAATTGGAGAAGTGAATTATACAAATATAAAG
TAGTAAAAATTGAACCATTA---GGATTGGCACCCACCAAGGCAAAGAGAAGAGTGGTG--------------
-------------------------CAGAGAGAAAAAAGAGCA---GTGGGA---ATAGGAGCT---------
TTGTTCCTTGGGTTCTTGGGAGCA---GCAGGAAGCACTATG---GGCGCAGCGTCA---
ATAACGCTGACGGTACAG---GCCAGACAA---TTATTT---TCTGGTATAGTGCAGCAGCAGAAT---
AATCTG---CTGAGG---GCTATTGAGGCGCAACAGCATCTGTTGCAACTCACAGTCTGGGGCATC
>KC699020
TTCTACTGTGATACAACACAACTGTTTAATAGTACTTGGAATGATACATGGTCAAATAACACAAATGAT----
----

ATTGCAGAAAATATTACACTCCCATGCAGAATAAAACAAATTATAAACATGTGGCAGGAAGTAGGAAAAGCAA
TGTATGCCCCTCCCATCAGAGGACAAATTAGATGTATATCAAAT---
ATTACCGGGCTGCTATTAACAAGAGATGGTGGCAAT-------------------------------------
----

AATGAGACCGAGGTCTTCAGGCCAGGAGGAGGAGATATGAGGGACAATTGGAGAAGTGAATTATATAAATATA
AAGTGGTACAAATTGAACCATTA---GGAATAGCACCCACCAAGGCAAAGAGAAGAGTGGTG-----------
----------------------------CAGAGAGAGAAAAGAGCA---GTGGGA---ATAGGAGCT------
---GTGTTCCTTGGGTTCTTGGGAGCA---GCAGGAAGCACTATG---GGCGCAGCATCA---
GTGATGCTGACGGTACAG---GCCAGACAA---TTATTG---TCTGGTATAGTGCAACAGCAAAAC---
AATTTG---CTGAGA---GCTATTGAGGCGCAACAACATCTGTTGCAACTCACAGTCTGGGGCATT
>KC699026
TTCTACTGTAATACATCAGGACTGTTTAATAGTACTTGGATTTGGAATGATACTACTTGGAATGATACTATGG
GGTCC--
-------------------AAT---
GACACAAAGGAAAATATCACACTCATATGCAGAATAAAACAAATTGTCAACATGTGGCAGGAAGTAGGAAAAG
CAATGTATGCCCCTCCCATCAGGGGACTAATTAGATGTTCATCAAAT---
ATTACAGGGCTGCTATTAACAAGAGATGGTGGTAATAATAATGAAAGC-------------------------
----

AACAACGACACTGAGACTTTCAGACCTGGAGGAGGAGATATGAGGGACAATTGGAGAAGTGAATTATATAAAT
ATAAAGTAGTAAAGATTGAACCCTTA---GGAGTAGCACCTACCAGGGCAAAGAGAAGAGTGGTG--------
-------------------------------CAGAGAGAAAAAAGAGCA---GTAACG---CTGGGAGCT---
------TTGTTCCTTGGGTTCTTGGGAGCA---GCAGGAAGCACTATG---GGCGCAGCGTCA---
ATGACGCTGACGGTACAG---GCCAGACAA---TTGTTG---TCTGGTATAGTGCAGCAGCAGAGT---
AATCTA---CTGAGA---GCTATTGAGGCGCAACAACATCTGTTGCAACTCACAGTCTGGGGCATC
>KC699015
TTCTACTGTAATACAACAAAACTGTTTAATAGTACTTGGACTTGGAATAATACTGAAGAGGCAAATAACAATA
GTACT--
-------------GACGGG------
GCAAATGACACTATCATCACACTTCCATGCAGAATAAAACAAATTATAAACATGTGGCAGACAGTAGGAAAAG
CAATGTATGCCCCTCCCATCAAAGGAAAAATTGAATGCTCATCAAAT---
ATTACAGGGCTGCTATTAACAAGAGATGGTGGTGAAAACAACACT----------------------------
----

ACCAAAGAGATCTTCAGACCTGGAGGAGGAGATATGAGGGACAATTGGAGAAGTGAATTATATAAATATAAAG
TAGTAAAAATTGAACCATTA---GGAGTAGCACCCACCAAGGCAAAGAGAAGAGTGGTG--------------
-------------------------CAGAGAGAAAAAAGAGCA---GTGGGA---ATAGGAGCT---------
TTGTTCCTTGGGTTCTTGGGAGCA---GCAGGAAGCACTATG---GGCGCAGCGTCA---

ATGACGCTGACGGTACAG---GCCAGACAA---TTATTG---TCTGGTATAGTGCAACAGCAGAAC---
AATCTG---CTGAGA---GCTATTGAGGCGCAACAGCATATGTTGCAACTCACAGTCTGGGGCATC
>KC699013
TTCTACTGTAATACAGCAGAACTGTTTAATAGTACTTGGAATGTAAGTGGTACTGAAGAGTTAAATAAC----
----

ACTGAAAAAATAATCACACTCCCATGCAGAATAAGACAAATTATAAACAAGTGGCAGGAAGTAGGAAAAGCAA
TATATGCCCCTCCCATTAGGGGACAAATTAGATGCTCATCAAAC---
ATTACAGGGCTGCTATTAACAAGAGATGGTGGGAACGAGAACGGGACC-------------------------
----

AACGGGACCACCGAGGTCTTCAGACCTGGAGGAGGAAATATGAAGGACAATTGGAGGAGTGAATTATATAAAT
ATAAAGTAGTAAAAATTGAACCATTA---GGAGTAGCACCCACCAGGGCAAAGAGAAGAGTGGTG--------
-------------------------------CAGAGAGAAAAAAGAGCA---GCGGGA---ATAGGAGCT---
------CTGTTCCTTGGGTTCTTGGGAGCA---GCAGGAAGCACTATG---GGCGCAGCGTCA---
CTGACGCTGACGGTACAG---GCCAGACAA---TTATTG---TCTGGTATAGTGCAACAGCAGAAC---
AATTTG---CTGAGG---GCTATTGAGGCGCAACAGCATATGTTGCAACTCACAGTCTGGGGCATC
>KC699021
TTCTACTGTAATACAACACAACTGTTTAATGGTACTTGGAATTGGACTGATAATAGCTGGCCAAATGACACAA
AT---

ATTAATGACACTATCACACTCCCATGCAGAATAAGACAAATTATAAATATGTGGCAGGGAGTAGGAAAAGCAA
TGTATGCCCCTCCTATCAGCGGATACATTAACTGTTCATCAAAT---
ATTACAGGGCTGATATTAACAAGAGATGGTGGTAACACAAACACATCA-------------------------
----

AATGGGACCGAGACCTTCAGACCTGGAGGAGGAAATATGAAAGACAATTGGAGAAGTGAATTATATAAATATA
AAGTAGTAAAAATTGAACCATTA---GGAATAGCACCCACCAGGGCAAAAAGAAGAGTGGTG-----------
----------------------------CAAAGAGAAAAAAGAGCA---GTGGGA---CTAGGAGCT------
---TTGTTCCTTGGGTTCTTGGGAGCA---GCAGGAAGCACTATG---GGCGCAGCGTCA---
GTGACGCTGACGGTACAG---GCCAGACAA---TTAATG---TCTGGTATAGTGCAACAGCAGAAC---
AATCTG---CTGAAG---GCTATTGAGGCGCAACAGCGTATGTTGCAGCTCACAGTCTGGGGCATC
>KF695111
TTCTACTGTAACTCAACAAAACTGTTTAATAGAACTTGGTCTAATGGTACTTGGGATGATGCTTGGAATGATA
CT---
-------------------ACA---
GACGTTAACGGAACGATCATACTCCCATGCAGAATAAAACAAATTATAAACATGTGGCAGAAAGTAGGAAAAG
CAATGTATGCCCCTCCCATCAGAGGAATAATTAACTGTACATCAAAT---
ATTACAGGGCTGATATTAACAAGAGATGGTGGTGAATAATACA------------------------------
----

AACGAGACCTTCAGACCTGGAGGGGGAGATATGAGGGACAATTGGAGAAGTGAATTATATAGATATAAAGTAG
TAGAAATTGAACCATTA---GGAGTAGCACCCACCAAGGCAAAAAGAAGAGTGGTG-----------------
----------------------CAGAGAGAAAAAAGAGCA---GTGCCA---CTAGGAGCT---------
ATGTTCCTTGGGTTCTTAGGAGCA---GCAGGAAGCACTATG---GGCGCAGCGTCA---
ATAGCGCTGACGGCACAA---GCCAGACAA---TTATTG---TCTGGTATAGTGCAGCAGCAGAAC---
AATCTG---CTGAGG---GCTATTGAGGCGCAACAGCATCTGTTGCAACTCACAGTCTGGGGCATC
>EF033658
TTCTACTGTAATACAACAGAACTGTTTAATAGTACCTGGAATGATACTGAAAGGGTAGATGACACCGAT----
----

AGGGGTGACAATATCAAACTTCCATGCAGAATAAAACAAATTGTAAACAGGTGGCAGGAAGTAGGAATAGCAA
TGTATGCCCCTCCCATCGAAGGAAATATTAGATGTTCATCAAAT---
ACTACAGGACTGCTATTAACAAGAGATGGGGGTAATGATAACACGACA-------------------------
----

GCTAACAAGACTGAGACCTTCAGACCTGGAGGAGGAGATATGAGGGACAATTGGAGAAGTGAATTATATAAAT
ATAAAGTAGTAAAAATTGAACCATTA---GGAGTAGCACCCACCCAGGCAAAAAGAAGAGTGGTG--------
-------------------------------CAGAGAGAAAAAAGAGCA---GTGACA---CTAGGAGCT---
------ATGTTCCTTGGGTTCTTGGGAGCA---GCAGGAAGCACTATG---GGCGCAGCGTCA---

ACGGTGCTGACGGTACAG---GCCAGACAA---TTATTG---TCTGGTATAGTGCAACAGCAGAAT---
AATCTG---CTGAGA---GCTATTGAGGCGCAACAGCATCTGTTGCAACTCACAGTCTGGGGCATC
>AF321080
TTCTACTGTGATACAACACAGCTGTTTAATAGTACTTGGAATGTTACTTGGAATGAAACTGGAGAGTCAAAT-
----

AGCACTGGAAATATCACACTCCCATGCAGAATAAGACAAATTATAAACAGGTGGCAGGAAGTAGGAAAAGCAA
TGTATGCTCCTCCCATCAAAGGACAAATTAGATGTTCATCAAAC---
ATTACAGGGCTGCTATTAACAAGAGACGGTGGTGCTGAGAACGCC----------------------------
----

ACCACCGAGACCTTTAGACCTGGAGGAGGAGATATGAGAGACAATTGGAGAAGTGAGTTATATAAATATAAAG
TAGTGCAAATTAAGCCATTA---GGAGTAGCACCCACCAGGGCAAAGAGAAGAGTGGTG--------------
-------------------------CAGAGAGAAAAAAGAGCA---GTGGGAACGCTAGGAGCT---------
ATGTTCCTTGGGTTCTTGGGAGCA---GCAGGAAGCACTATG---GGCGCAGCGTCA---
ATGACGCTGACGGTACAG---GCCAGACAA---TTATTG---TCTGGTATAGTGCAACAGCAAAAC---
AATTTG---CTGAGA---GCTATTGAGGCGCAACAGCATCTGTTGCAACTCACAGTCTGGGGCATT
>M26727
TTCTACTGTAATACATCACAACTGTTTAATAGTACTTGGAATGATACTACAAGGGCAAATAGC----------
----

ACTGAAGTAACTATCACACTCCCATGTAGAATAAAACAAATTGTAAACATGTGGCAGGAAGTAGGAAAAGCAA
TGTATGCCCCTCCCATCAGTGGACAAATTAGATGTTCATCAAAG---
ATTACAGGGCTGCTATTAACAAGAGATGGTGGTAAGAACACCACG----------------------------
----

AACGGGATCGAAATCTTCAGACCTGCAGGAGGAGACATGAGGGACAATTGGAGAAGTGAATTATATAAATATA
AAGTAGTAAAAATTGAACCATTA---GGAGTAGCACCCACCAAGGCAAGGAGAAGAGTGGTG-----------
----------------------------CAGAGAGAAAAAAGAGCA---GTGGGAATGCTAGGAGCT------
---ATGTTCCTTGGGTTCTTGGGAGCA---GCAGGAAGCACTATG---GGCGCACGGTCG---
ATGACGCTGACGGTACAG---GCCAGACAA---TTATTG---TCTGGTATAGTGCAGCAGCAGAAC---
AATCTG---CTGAGG---GCTATTGAGGCGCAACAGCACCTGTTGCAACTCACAGTCTGGGGCATC
>JN034158
TTCTACTGCAATTCAACACCACTGTTTAATAGTACTTGGAATGGTACTTTGGATAGTGCTGGAGAA-------
----

TCAAGTGACAATATCACACTCATATGCAGAATAAAACAGTTTATAAACCTGTGGCAGGAAGTAGGAAAAGCAA
TGTATGCCCCTCCCATCAGAGGACAAATTAGCTGTTCATCAAAG---
ATTACAGGGCTGCTATTAACAAGAGATGGTGGTATTAAGAATGACACC-------------------------
----

AACAAGACTGAGATTTTTAGACCTGCAGGAGGAGATATGAGGGACAATTGGAGAAGTGAATTATATAAATATA
AAGTAGTAAAAATTGAACCATTA---GGAGTAGCACCCACCACGGCAAAGAGAAGAGTGGTG-----------
----------------------------CAGAGAGAAAAAAGAGCA---GTGGGAACGATAGGAGCT------
---ATGTTCCTTGGGCTCTTGGGAGCA---GCAGGAAGCACTATG---GGCGCAGCGTCA---
GTGACGCTGACGGTACAG---GCCAGACTA---TTATTG---TCTGGTATAGTGCAACAGCAGAAC---
AATTTG---CTGAAG---GCTATTGAGGCGCAACAGCATATGTTGCAACTCACAGTCTGGGGCATT
>JN034139
TTCTACTGTAATTCAACACAGCTGTTTAATAGTACTTGGATAAATGGTATTATACAGTCAAATATCACTGCT-
----

GAAAATGACAACATCACACTCCCATGCAGAATAAAACAAATTATAAACAGGTGGCAGGAAGTAGGAAAAGCAA
TGTATGCCCCTCCCATCAGAGGAATAATTAGATGCACATCAAAC---
ATTACAGGGCTGCTACTAACAAGAGATGGTGGTAATAAGAATGGGAGC-------------------------
----

AACAACAATGAGACCTTTAGACCTGGAGGAGGAGATATGAGAGACAATTGGAGAAGTGAATTATATAAATATA
AAGTAGTAAAAATTGAGCCATTA---GGAGTAGCACCCACCAAGGCAAAAAGAAGAGTGGTG-----------
----------------------------CAGAGAGAAAAAAGAGCA---GTAACG---CTAGGAGCT------
---ATGTTCCTTGGGTTCTTGGGAGCA---GCAGGAAGCACTATG---GGCGCAGCGTCC---

ATGACGCTGACGGTACAG---GCCAGACAA---TTATTG---TCTGGTATAGTGCAACAGCAGAAT---
AATTTG---CTGAGG---GCTATTGAGGCGCAACAGCATCTGTTGCAACTCACAGTCTGGGGCATC
>MG902199
TTTTACTGTAATACAACACAGCTGTTTAATAGTACTTGGAATAGTACTTGGAATTGGAATAATACTGAAGAGT
CAGCTAAC---

ACTACAGCAAACATAACACTCCCATGCAGAATAAAACAAATTATAAACATGTGGCAGGAAGTAGGAAAAGCAA
TGTATGCCCCCCCCATCTCAGGACAGATTAGATGCTCATCAAAT---
ATTACAGGGCTGATCTTAACAAGAGATGGTGGTAATGATACTAGCAAT-------------------------
----

AACGGGACCGAGATCTTCAGACCTGGAGGAGGAAATATGAAGGACAATTGGAGAAGTGAATTATATAAATATA
AAGTAGTAAAAATTGAACCATTA---GGAGTAGCCCCCACCAAGGCAAAGAGAAGAGTGGTG-----------
----------------------------CAAAGAGAAAAAAGAGAC---ATGGGA---CTAGGAGCT------
---TTGTTCCTTGGGTTCTTGGGAGCA---GCAGGAAGCACTATG---GGCGCAGCGTCA---
TTGACGTTGACGGTACAG---GCCAGACAA---TTATTG---TCTGGTATAGTGCAACAGCAGAAC---
AATTTG---CTGAGA---GCTATTGAGGCGCAACAACATTTGTTGCAGCTCACAGTCTGGGGCATC
>MG902157
TTCTACTGTAATACAACACAACTGTTTAATAGCACTTGGAATACTACTGATGGTAGTACTTGGAATAATACTG
ATAATAGTACT--
GCA---AATGACATTAGT---------
ACTGCAAATGACACTATCATACTCCCATGTAGAATAAAACAAATTATAAACATGTGGCAGGAAGTAGGAAAAG
CAATGTATGCCCCTCCCATTAAAGGACTAATTAGATGTTCATCAAAC---
ATTACAGGACTGATATTAACAAGAGATGGTGGTAATAATAGTAGC----------------------------
----

AACGAGACCTTCAGACCTGCAGGGGGAAATATGAAGGACAATTGGAGAAGTGAATTATATAAATACAAAGTAG
TAAAAATTGAACCATTA---GGAATAGCACCCACCAAGGCAAAGAGAAGAGTGGTG-----------------
----------------------CAAAGAGAAAAAAGAGCA---GTGACA---CTGGGAACC---------
ATGCTCCTTGGGTTCTTGGGAGCA---GCAGGAAGCACTATG---GGCGCAGCGTCC---
TTGACGCTGACGGTACAG---GCCAGACAA---TTATTG---TCTGGTATAGTGCAACAGCAGAAC---
AATCTG---CTGAGG---GCTATTGAGGCGCAGCAGCATCTGTTGCAGCTCACAGTCTGGGGCATC
>HM586193
TTCTACTGTAGTACAACACAACTGTTTAATAGTACTTGGAATTGGAATGAGACTGACAATGATCCTGGAATA
CA---

ACTGGTAATGGCACACTCACACTCCCATGCAGAATAAAACAATTTGTAAACATGTGGCAAGAAGTAGGAAAAG
CAATGTATGCCCCTCCTATCAGCGGACCGATTAACTGTACATCAAAT---
ATTACAGGGCTGCTATTAACCAGAGATGGTGGTAATAGCTCGAAT----------------------------
----

GATTCAGAGACCTTTAGACCTGGAGGAGGAGATATGAGGGACAATTGGAGAAGTGAATTATATAAATACAAAG
TAGTAAAAATTGAACCATTA---GGAGTAGCACCCACCAAGGCAAAGAGAAGAGTGGTG--------------
-------------------------CAAAGAGAAAAAAGAGCA---GTGGGA---CTAGGAGCT---------
ATGTTCCTTGGGTTCTTGGGAGCA---GCAGGAAGCACTATG---GGCGCAGCGTCA---
GTGACGCTGACGGTACAG---GCCAGACTG---TTATTG---TCTGGTATAGTGCAACAGCAGAAT---
AATCTG---CTGAGA---GCTATTGAGGCGCAACAACATCTGTTGCAACTCACAGTCTGGGGCATC
>HM586198
TTCTACTGCAATACAACACAACTATTTAATAGTACTTGGAATAAGAACACAACC-------------------
----

ATGAATGACACAATCATACTCCCATGTAGAATAAAACAGATTATAAACATGTGGCAGGAAGTAGGAAAAGCAA
TGTATGCCCCTCCCATCAGAGGACAAATTAATTGTTTATCAAAT---
ATTACAGGGCTGCTATTAACAAGAGACGGGGGTGATACCAATGGCACC-------------------------
----

AATGGCACAGAAATATTCAGACCTGGAGGAGGAGATATGAGGGACAATTGGAGAAGTGAATTATACAAATATA
AAGTAGTAAAAATTGAACCTTTA---GGAGTAGCACCCACTAGGGCAAAGAGAAGAGTGGTG-----------
----------------------------CAAAGAGAAAAAAGGGCA---GTGGGA---CTGGGAGCT------
---CTGTTCCTTGGGTTCTTAGGAGCA---GCAGGAAGCACTATG---GGCGCAGCGTCA---

ATGACGCTGACGGTACAG---GCCAGACAA---CTGCTG---TCTGGTATAGTGCAACAGCAGAAC---
AATCTG---CTGAGA---GCTATTGAGGCGCAACAGCATCTGTTGCAACTCACAGTCTGGGGCATC
>HM586210
TTCTACTGCAATTCAACACAGCTGTTTAATAGTACTTGGAATAGTACTGAAGGCAGTATTGAAGGGTCAAATA
GCACT--
-------------------GAA---
AAGAACATCACACTCATCACACTCCCATGCAGAATAAAGCAAATTGTAAACCTGTGGCAGGGAGTAGGAAAAG
CAATATATGCCCCTCCCATCAGGGGGCTAATTAGCTGTTCATCAAAT---
ATTACAGGGTTGATATTAACAAGAGATGTTGGTAATGGTGAGAACATA-------------------------
----

AGCAACAATACTGAAACCTTCAGACCAGGAGGAGGAGATATGAGGGACAATTGGAGAAGTGAATTATATAAAT
ATAAAGTAGTAAAAATTGAACCATTA---GGAGTAGCACCCACTAAGGCAAAGAGAAGAGTGGTG--------
-------------------------------CAGAGAGAAAAAAGAGCA---ATAGGA---CTAGGAGCT---
------GTGTTCCTTGGGTTCTTGGGAGCA---GCAGGAAGCACTATG---GGCGCAGCATCA---
GTGACGCTGACGGTACAG---GCCAGACAA---TTATTG---TCTGGTATAGTGCAACAGCAGAAC---
AATCTG---CTGAAG---GCTATTGAGGCGCAACAGCATCTGTTGCAACTCACAGTCTGGGGCATC
>FJ653437
TTCTACTGTAATTCAACGAAACTGTTTAGTAGTACTTGGATGGCTAATAGTACTTGGACTAATAGTACTTTGG
AGAAG--
-------------------AAT---
AGCACTGAAAAAAATATCACACTCCCATGTAAAATAAAACAAATTATAAACATGTGGCAGAAAGTAGGAAAAG
CAATGTATGCCCCTCCTATCAGAGGACAGATTAGATGTTCAACAAAT---
ATCACAGGTCTGTTATTAACAAGAGATGGTGGTGACAACACGACC----------------------------
----

GACAGCGAGGAGGTCTTTAGACCTGGAGGAGGAAATATGAAGGACAATTGGAGAAGTGAATTATATAAATATA
AAGTAGTAAGAATTGAACCATTA---GGAGTAGCACCCACCAAGGCAAAGAGAAGAGTGGTG-----------
----------------------------CAAAGAGAAAAAAGAGCA---GTGGGAGTTGTAGGAGCT------
---ATGTTCCTTGGGTTCTTAGGAGCA---GCAGGAAGCACTATG---GGCGCAGCGGCA---
GTGACGCTGACGGTACAG---GCCAGACAA---TTATTG---TCTGGTATAGTGCAGCAGCAGAAT---
AATCTG---CTGAAG---GCTATTGAGGCGCAACAACATCTGTTGCAACTCACAGTCTGGGGCATC
>HQ595768
TTCTACTGTAATACAACACAACTGTTTAATAGTACCTGGTATGGAAATGAGACTTGGAATAATACTGAAGGTG
CAGAC--

AACACTAATGACACCATCATACTCCCATGCAGAATAAAACAAATTATAAACATGTGGCAGGAAGTAGGAAAAG
CAATGTATGCCCCTCCCATCAGTGGACTAATTAGATGCTCATCAAAC---
ATTACAGGGCTACTATTAACAAGAGATGGGGGTACTACCAATGTCACG-------------------------
----

AACGAGACCGAGACCTTCAGACCAGGAGGAGGAGATATGAGGGACAATTGGAGAAGTGAATTATACAAATATA
AAGTAGTAAAAATTGAGCCATTA---GGAATAGCACCCACCAAGGCAAAAAGAAGAGTGGTG-----------
----------------------------CAGAGAGAAAGAAGAGCA---GTGGGG---CTAGGAGCT------
---TTGTTCGTCGGGTTCTTGGGAGCA---GCAGGAAGCACTATG---GGCACAGCATCA---
ATAATGCTGACGGCACAG---ACCAGACAA---TTACTG---TCTGGTATAGTGCAACAGCAGAAC---
AATCTG---CTGAGA---GCTATTGAGGCGCAACAACATCTGTTGCAACTCACAGTCTGGGGCATT
>HQ595771
TTTTACTGTAATACAACACAACTGTTCAAAGGGACTTGGAATGGTACTAGTGGTACTTGGGAAAGTACTAAT-
----

AGTACTGAGAATATTACACTCCCATGCAGAATAAAACAAATTGTAAACATGTGGCAGGAAGTAGGAAAAGCAA
TGTATGCCCCTCCCATCAGGGGACTAATTAGATGTGAATCAAAT---
ATTACAGGGCTGCTATTAATAAGAGATGGTGGTAATAATAGTAGC----------------------------
----

GATAATGAGACTTTCAGACCTATAGGAGGAAACATGAGGGACAATTGGAGAAGTGAATTATATAAATACAAAA
TAGTAAAAGTTGAACCAATA---GGAATAGCACCCACCATGGCAAAGAGAAGAGTGGTG--------------
-------------------------CAAAGAGAAAAAAGAGCA---GTGGGA---TTAGGAGCT---------
ATGTTCCTTGGGTTCTTGGGAGCA---GCAGGAAGCACTATG---GGCGCAGCGTCG---

ATGACGCTGACGGTACAA---GCCAGACAA---TTATTG---TCTGGTATAGTGCAGCAGCAGAGC---
AATCTG---CTGAGG---GCTATTGAGGCGCAACAGCATATGTTGCAACTCACAGTCTGGGGCATC
>HQ595772
TTCTATTGCAATACAACACAACTGTTTAATAATATTTGGATTACTGATAATGTTACTGTTAATGAAACTAATA
GT---

GACAATAGCAATATCACACTCCCATGCAGAATAAAACAAATTATAAACATGTGGCAGGAAGTAGGAAAAGCAA
TGTACGCCCCTCCCATCAGAGGACAGATTAGATGTTCATCAAAT---
ATTACAGGGCTACTATTAACAAGAGATGGTGGTAATACGAACAATACG-------------------------
----

AACGGGTCTGAGACCTTCAGACCTGGAGGAGGAAATATGAAGGACAATTGGAGAAGTGAATTATATAAATATA
AAGTAGTAAAAGTTGAACCATTA---GGATTAGCACCCACCAAGGCAAAAAGAAGAGTGGTC-----------
----------------------------CAAAGAGAAAAAAGAGCA---ATAACA---CTAGGAGCT------
---GTTTTCCTTGGGTTCTTGGGAGCA---GCAGGAAGCACTATG---GGCGCAGCGTCA---
ATAACGCTGACGGTACAG---GCCAGACAA---TTATTG---TCTGGTATAGTGCAACAGCAGAAC---
AACTTG---CTGAGG---GCTATTGAGGCGCAACAACATATGTTGCAACTCACAGTCTGGGGCATC
>HQ595774
TTCTACTGTAATACAACACAATTGTTTAATAGTACCTGGAATGATACTAATATTAATAGTACTGAAGAGTCAA
AT---

AACACTGACACTATCACACTCCAATGCAGAATAAAACAAATTATAAACATGTGGCAGGAAGTAGGAAAAGCAA
TGTATGCCCCTCCTATCCGAGGACATATTAATTGTTCATCAAAT---
ATTACAGGGCTGCTATTAACAAGAGATGGTGGTAGTAATACTACC----------------------------
----

ACCCAGGAGACCTTCAGACCTGGAGGAGGAAATATGAAGGACAATTGGAGAAGTGAATTATATAAATATAAAG
TAGTAAAAATTGAACCATTA---GGAGTAGCACCCTCCAAGGCAAAAAGAAGAGTGGTG--------------
-------------------------CAGAGAGAAAAAAGAGCA---GTAGGA---CTGGGAGCT---------
TTGTTCCTTGGGTTCTTGGGAGCA---GCAGGAAGCACTATG---GGCGCAGCATCA---
CTAACGCTGACGGTACAG---GCCAGACAA---TTATTG---TCTGGTATAGTGCAACAGCAGAAC---
AATTTG---CTGAGA---GCTATTGAGGCGCAACAGCATCTGTTGCAACTCACAGTCTGGGGCATC
>HM204626
TTCTACTGTAATACAACAAAACTGTTTAATAGCACTTGGAATAGTAATAGTACTTGGAATAGTACGCAA----
----

GGAAATGACACAATCACACTCCCGTGCAGAATAAAACAAATTATAAACCTGTGGCAGGAAGTAGGAAAAGCAA
TGTATGCCCCTCCCATCAGAGGATCAATTAAATGTTCATCAAAT---
ATTACAGGGCTGCTACTAACAAGAGATGGGGGTATAAACAATAGTAAT-------------------------
----

AATAATATAGAGACCTTCAGACCTGGAGGAGGAGATATGAGGGACAATTGGAGAAGTGAATTATATAAATATA
AAGTAGTACAAATTGAACCATTA---GGAGTAGCACCCACCAGGGCAAAGAGAAGAGTGGTG-----------
----------------------------CAAAGAGAAAAAAGAGCA---GTGGGA---CTAGGAGCT------
---TTGTTCCTTGGGTTCTTGGGAGCA---GCAGGAAGCACTATG---GGCGCAGCGTCA---
ATAACGCTGACGGTACAG---GCCAGACAA---TTATTG---TCTGGTATAGTGCAACAGCAGAAC---
AATTTG---CTGAGA---GCTATTGAGGCGCAACAACATCTGTTGCAACTCACAGTCTGGGGCATC
>HM204627
TTCTACTGTAATACAACACAACTGTTTAATAGTACTTGGACTTGGAATGATACTACTTGGAATGGTACTAATG
GCGGG--
-------------------AAT---
AACACTGAAAAAACCATCACACTCCCATGCAGAATAAAACAAATTATAAACATGTGGCATGAAGTAGGAAAAG
CAATGTATGCCCCTCCCATCAGGGGAATAATTAGCTGTTCATCAAAT---
ATTACAGGTCTGCTATTAACAAGAGATGGTGGTAGTGGTAATATGAGC-------------------------
----

AACACCACCGAGACCTTCAGACCTGAAGGAGGAAATATGAAGGACAATTGGAGAAGTGAATTATATAAATATA
AAGTAGTAAGAATTGAACCATTG---GGAATAGCACCCACCAAGGCAAAGAGAAGAGTGGTG-----------
----------------------------CAAAGAGAAAGAAGGGCA---GTGGGA---TTCGGAGCT------
---ATGTTCCTTGGGTTCTTGGGAGCA---GCAGGAAGCACTATG---GGCGCAGCGTCA---

GTGACGCTGACGGTACAG---GCCAGACAA---TTATTG---TCTGGTATAGTGCAACAGCAGAAC---
AATCTG---CTGAGG---GCTATTGAGGCGCAACAGCATCTGTTGCAACTCACAGTCTGGGGCATC
>HM204628
TTCTACTGTAATACAACACCGATATTTAGTAGTACATGGAATAGTAATAGTACTAAAGATGATATTGAGAGAT
CAAT---

AGCACTGACAGGAATATCACACTCCAATGCAGAATAAGACAAATTATAAACATGTGGCAGAAAGTAGGAAAAG
CAATGTATGCCCCTCCCATCCAAGGGCTAATTAGATGTTCATCAAAT---
ATTACAGGGCTGCTATTAACAAGGGATGGTGGTAAGGACAACAAT----------------------------
----

AACAACACCGAGACCTTCAGACCTGGGGGAGGAGATATGAGGGACAATTGGAGAAGTGAATTATATAAATATA
AAGTAGTACAAATTGAACCAGTA---GGAGTGGCACCCACTAAGGCAAAGAGAAGAGTGGTG-----------
----------------------------CAGAGAGAAAAAAGAGCA---GTGGGA---ATAGGAGCT------
---TTGTTCCTTGGGTTCTTGGGAGCA---GCAGGAAGCACTATG---GGCGCAGCGTCA---
ATGACACTGACGGTACAG---GCCAGACAA---TTGTTG---TCTGGTATAGTGCAACAGCAAAAC---
AATCTG---CTGAGG---GCTATTGAGGCGCAACAGCATCTGTTGCAACTCACAGTCTGGGGCATC
>HM204629
TTCTACTGTAATACAACACAACTGTTTAATAGTACTTGGCCATTCAATGATACTGGGAATGATAATAGTAGTA
GC---

ACTGACAATACAACAATCTCACTCCCATGTAGAATAAAACAAATTGTAAACATGTGGCAGGAAGTAGGAAAAG
CAATGTATGCCCCTCCCATTAGAGGACAAATTAGATGTTCATCAAAT---
ATTACAGGACTGATATTAACAAGAGATGGTGGCACACCCGACAACCAA-------------------------
----

AGTAGTGAGGAGGTCTTCAGACCTAGTGGAGGAAATATGAAAGACAATTGGAGAAGTGAACTATATAAATATA
AAGTAGTAAAAATTGAACCGTTA---GGAATAGCACCCACCAAGGCAAAAAGAAGAGTGGTG-----------
----------------------------CAGAGAGAGAAAAGAGCA---GCCACA---CTGGGAGCT------
---ATGTTCCTTGGATTCTTGGGAGCA---GCAGGAAGCACTATG---GGCGCAGCATCA---
ATAACGCTGACGGTACAG---GCCAGACTA---TTACTG---TCTGGTATAGTGCAGCAGCAGAGC---
AATCTG---CTGAGG---GCTATTGAGGCGCAACAGCATATGTTGCAACTCACAGTCTGGGGCATC
>HM204632
TTCTACTGTAATTCAACACAACTGTTTAATAGCACTTGGAATAGTAATAGTACTTGGAATGATACTATGGGCA
CTAAT--
-------------------GAT---
ACTATGGACACTAATATCACACTTCCATGTAGAATAAAACAAATTATAAACTTGTGGCAGGAAGTAGGAAAAG
CAATGTATGCCCCTCCCATCGCAGGACAAATTAGATGTTCATCAAAT---
ATTACAGGGTTGCTGTTAACAAGAGATGGTGGTAGTAATAGCACC----------------------------
----

AACAATGAGACCTTCAGACCTGGAGGAGGAAATATGAGGGACAATTGGAGAAATGAATTATATAAATATAAAG
TAGTACAAATTGAACCATTA---GGAATAGCACCCACCAAGGCAAAGAGAAGAGTGGTG--------------
-------------------------CAGAGAGAAAAAAGAGCA---GTGGGAACGCTAGGAGCT---------
ATGTTCCTTGGGTTCTTGGGAGCA---GCAGGAAGCACTATG---GGCGCAGCGTCA---
GTGACGCTGACGGTACAG---GCCAGGCAA---TTGCTG---TCTGGTATAGTGCAGCAGCAGAAC---
AATCTG---CTGAAG---GCTATTGAGGCACAACAGCATATGTTGCAACTCACAGTCTGGGGCATT
>HM204633
TTCTACTGTAGTACAGCAGAACTGTTTAATAGTACCTGGTTTAACAATGGTACATGGATAAGTGACAATGGAA
AA---

AATGACACAAATATCACACTCCCATGCAGAATAAAACAAATCATAAACATGTGGCAGGGAGTAGGAAAAGCAA
TGTATGCCCCTCCCATCAGTGGACTAATTAGCTGTTCATCACAA---
ATTACAGGGTTGCTATTAACAAGAGATGGTGGTAATGCGAGTGTTAAC-------------------------
----

AACTGGACCGAGACCTTCAGACCTGTGGGAGGAGATATGAGGGACAATTGGAGAAGTGAATTATATAAATATA
AAGTAGTAAAAATTGAACCATTA---GGAGTAGCACCCACCAAGGCAAGGAGAAGAGTGGTG-----------
----------------------------CAGAGAGAAAAAAGAGCA---GTGGGA---ATAGGAGCT------
---GTGTTTCTTGGGTTCTTGGGAGCA---GCAGGAAGCACTATG---GGCGCAGCATCA---

GTGATGCTGACGGTACAA---GCCAGGCAA---TTATTG---TCTGGTATAGTGCAGCAGCAAAAC---
AATTTG---CTGAGA---GCTATTGAGGCGCAACAGCATCTGTTGCAACTCACTGTCTGGGGCATC
>HM204638
TTCTACTGTAATACAACACAGCTGTTTAATAGTACTTGGCAGCTGTCTAATAGTACTCTGAAGGAAACA----
----

CAAGAGAGTAATATCACACTCCCATGCAGAATAAAACAAATTATAAACAAATGGCAGGAAGTAGGAAAAGCAA
TGTATGCCCCTCCCATCCAAGGAAAGATTAGTTGTACATCAAAT---
ATTACAGGGCTGCTATTAACAAGAGATGGTGGTGTCGAGAACAAGACT-------------------------
----

AACGAGACTAACGAAACCTTCAGACCTGGAGGAGGAGATATGAGGGATAATTGGAGAAGTGAATTATATAAAT
ATAAAGTAGTAAGAATTGAGCCATTA---GGAGTAGCACCTTCCAAGGCAAGGAGAAGAGTGGTG--------
-------------------------------CAGAGAGAAAAAAGAGCA---GTAGGA---CTGGGAGCT---
------TTGTTCCTTGGGTTCTTGGGAGCA---GCAGGAAGCACTATG---GGCGCAGCGTCA---
TTAACGCTGACGGTACAG---GCCAGACAA---CTATTG---TCTGGTATAGTGCAACAGCAGAAC---
AACCTG---ATGAAA---GCTATTGAGGCGCAACAGCATTTGTTGCAACTCACAGTCTGGGGCATC
>HM204640
TTCTACTGCAATTCATCACTGCTGTTCAATAATACTTGGAATGGCAATGATACA-------------------
----

AATAATACTATACTCACACTCCCATGCAAAATAAAACAAATTATAAACCTGTGGCAGGCAGTAGGAAAAGCAA
TGTATGCCCCGCCCATCCAAGGACTAATTAGATGTTCATCAAAT---
ATTACAGGGCTGTTATTAACAAGAGATGGTGGTAATGATAATAGCACA-------------------------
----

AACCAGACCGAGGTCTTCAGACCTGGAGGAGGAAATATGAAGGACAATTGGAGAAGTGAATTATATAGATATA
AAGTAGCAAAAATTGAACCATTA---GGAGTAGCACCCACCAAGGCAAAGAGAAGAGTGGTG-----------
----------------------------CAGAGAGAGAAAAGAGCA---GTAACG---TTAGGGGCA------
---ATGTTCCTTGGGTTCTTGGGAACA---GCAGGAAGCACTATG---GGCGCAGCGTCG---
ATGACGCTGACGGTACAG---GCCAGACTC---TTATTG---TCTGGTATAGTGCAACAGCAGAAC---
AACTTG---CTGAGA---GCTATTGAGGCGCAACAGCATCTGTTGCAACTCACAGTCTGGGGCATT
>HM204641
TTCTATTGTAATACAACAAAGCTGTTTAATTCTACTTGGAACAATACTAGTACTTGGAATAATAGTACTTGGA
ATAATACAATT--
----------TGGGATACT------
GATGGCAATGACACTATCATACTCCAATGCAGAATAAGACAAATTATAAACAGGTGGCAGGAAGTAGGGAAAG
CCATGTATGCCCCTCCCATCCAAGGACAAATTAAGTGCTCCTCAAAT---
ATTACAGGGCTGCTATTAGCAAGAGATGGAGGAAGTAATGGAAGT----------------------------
----

AACAACATAGAGACCTTCAGACCAGGAGGAGGAAATATGAAGGATAATTGGAGAAGTGAATTATATAAATATA
AAGTAGTACAAATTGAGCCATTA---GGAATAGCACCCACCAAGGCAAAGAGAAGAGTGGTG-----------
----------------------------CAGAGAGAAAAAAGAGCA---GTGGGGGCACTGGGAGCT------
---ATGTTCCTTGGGTTCCTGGGAGCA---GCAGGAAGCACTATG---GGCGCAGCGTCA---
ATAGCGCTGACGGCACAG---GCCAGACAA---TTATTG---TCTGGTATAGTGCAACAGCAAAAC---
AATTTG---CTGAGG---GCTATTGAGGCGCAGCAGCATCTGTTGCAACTCACAGTCTGGGGCATC
>HM204642
TTCTACTGTAATACATCACAACTGTTTAATAGTACCTGGGTTAATGGTACCTGGAATGGTACTGAAGGGTCA-
----

AATAGCCCAAACATCACACTCCAATGTAAAATAAAACAAATTATAAACATGTGGCAAGGAGTGGGAAAAGCAA
TGTATGCCCCTCCCATCGCAGGAAAAATTAGCTGTTCATCACAA---
ATCACAGGGCTGCTATTAGCAAGAGATGGTGGTAATGACAATTCCACT-------------------------
----

GACAATAACACTGAGATCTTCAGACCTTCAGGAGGAAATATGAAAGACAATTGGAGAAGTGAATTATATAAAT
ATAAAGTAGTAAAAATTGAACCAATA---GGAATAGCACCCACCAAGGCAAAAAGAAGAGTGGTG--------
-------------------------------CAGAGAGAAAAAAGAGCA---GTAGGAATGATGGGAGCT---
------ATGTTCCTTGGGTTCTTGGGAGCA---GCAGGAAGCACTATG---GGCGCAGCGTCA---

GTGACGCTGACGGTACAG---GCCAGGCAA---TTATTG---GCTGGTATAGTGCAACAGCAGAAC---
AATTTG---CTGAGA---GCTATTGAGGCGCAACAGCATCTGTTGCAACTCACAGTCTGGGGCATT
>HM204643
TTCTACTGTAATACAACAAAACTGTTTAATAGTACTTGGAATGTTAATAGTACTTGGAATACTAATAGTACTT
GGAAT--

ACTACTGAAGAGATAGTAAATATCCCATGCAGAATAAGACAAATTGTAAATAGGTGGCAAGAAGTAGGAAAAG
CAATGTATGCCCCTCCCATCAGAGGACAAATTAGATGTTCATCAAAC---
ATTACAGGGTTACTATTAACAAGAGATGGTGGTAATGGCAACCAG----------------------------
----

TCCAATAACGAGACCTTCAGACCTGGAGGAGGGGATATGAGGGACAAATGGAGAAGTGAATTATATAAATATA
AAGTAGTAAGAGTTGAACCATTA---GGAGTAGCACCCACCAAGGCAAAGAGAAGAGTGGTG-----------
----------------------------CAGAGAGAAAAAAGAGCA---GTGGGA---ATAGGAGCT------
---GTGTTCCTTGGGTTCTTGGGAGCA---GCAGGAAGCACTATG---GGCGCAGCCTCA---
ATAACGCTGACGGTACAA---GCCAGACTA---TTATTG---TCTGGTATAGTGCAACAGCAGAAC---
AATCTG---CTGAGG---GCTATTGAGGCGCAACAACATCTGTTGCAACTCACAGTCTGGGGCATC
>HM204646
TTTTACTGTAATTCATCACAACTGTTTACTTGGAATGAGACTAGTATTCGGAATAAGACAAATAAT-------
----

ACAAATGATACTATCACACTCCCATGTAAAATAAAACAAATTATAAACAGGTGGCAGGAAGTAGGAAGAGCAA
TGTATAACCCTCCCATCAAAGGAATAATTAGATGTGAATCAAAT---
ATTACAGGGTTACTGTTAACAAGAGATGGTGGTACTAATAATAAC----------------------------
----

ACCAACGAGACCTTCAGACCTGCAGGAGGAGACATGAGGGACAATTGGAGAAGTGAACTATATAAATATAAAG
TAGTACAAATTGAACCCTTA---GGAGTAGCACCCACCAAGGCAAAAAGAAGAGTGGTG--------------
-------------------------CAGAGAGAAAGAAGAGCA---GTGGGA---ATAGGAGCT---------
ATGTTCCTTGGGTTCTTGGGGGCA---GCAGGAAGCACTATG---GGCGCAGCGTCG---
GTGACGCTGACGGTACAG---ACCAGACAA---TTATTG---TCTGGTATAGTGCAGCAGCAGAAC---
AATCTG---CTGAGG---GCTATTGAGGCGCAACAGCACATGTTGCAACTCACAGTCTGGGGCATT
>KY658697
TTCTACTGTAATACATCACACCTGTTTAATAGTACTTGGGAACGTAATAGTAGTACTTGGAACCTTACTAAAG
GGTTAAAT---

GACACTGGAGGAAATATCACACTCCAATGCAAAATAAGACAATTCGTAAGACTGTGGCAGGAAGTAGGGAAAG
CAATGTATGCCCCTCCCATCAAAGGACTAATTAGATGTTCATCAAAT---
ATTACAGGACTGTTATTAACAAGAGATGGTGGTAATAATAATAATAATGAAACA-------------------
----

AACACGACTGAAACCTTCAGACCTGCAGGAGGAGATATGAGGGATAATTGGAGAAGTGAATTATATAGATATA
AAGTAGTGAGAATTGAACCAATA---GGAGTAGCACCCACCAGGGCAAAGAGAAGAGTGGTG-----------
----------------------------CAGAGAGAAAAAAGAGCG---GCA------CTAGGAGTT------
---TGGTTCCTTGGGTTCTTGGGAGCA---GCAGGAAGCACTATG---GGCGCAGCGTCA---
GTGACGCTGACGGCACAG---GCCAGACAA---TTATTG---TCTGGTATAGTGCAACAGCAGAGC---
AATTTG---TTGAAG---GCTATTGATGCGCAACAGCATCTGCTGCAACTCACAGTCTGGGGCATC
>HQ595776
TTCTACTGTAATACATCAAAACTGTTTGATAGTAGTTGGCTGCCCAACAATACTGGTGGGCTGCCTACCAAT-
----

TCTGCAGACCCAATCATACTCCCATGCAGAATAAGACAAATTATAAACATGTGGCAAAGGGTAGGAAAAGCAA
TGTATGCCCCTCCCATCAGTGGACAAATTAACTGTACATCCAAT---
ATTACAGGGCTACTATTAACAAGAGATGGTGGTAAGGGAAATGACACT-------------------------
----

AACCAGACCGAGATCTTTAGACCTGCAGGAGGAGATATGAAGGACAATTGGAGAAGTGAACTATATAAATATA
AAGTAGTAAGAATTGAACCATTA---GGAGTAGCACCCACCAAGGCAAAGAGAAGAGTGGTG-----------
----------------------------CAGAGAGAAAAAAGAGCA---GTGGGA---TTAGGAGCT------
---GTGTTCCTTGGGTTTTTGGGAGCA---GCAGGAAGCACTATG---GGCGCAGCGTCA---

GTGACGCTGACGGTACAG---GCCAGACAA---TTATTG---TCTGGTATAGTGCAACAGCAGAGC---
AATCTG---CTGAGA---GCTATAGATGCACAACAGCATCTGTTGCAGCTCACAGTCTGGGGCATA
>HQ595778
TTCTACTGTAATTCAACACTACTGTTTAATGGTACTTGGCATGATTCTAATAGGACTTGGCATAACCCTAAAG
GA---

AATAACAGTGACACAATCACACTCCCATGCAGAATAAAACAAATTATAAACATGTGGCAGGAAGTAGGAAAAG
CAATGTATGCCCCTCCCATCCAAGGACTAATTAGATGTAATTCAAGT---
ATTACAGGGCTGATGATAACAAGAGATGGTGGTAATAAAACTAACGAGACC----------------------
----

ATCGAGAACGTCGAGACTTTTAGACCTGGAGGAGGAAATATGAAGGACAATTGGAGAAGTGAATTATATAAAT
ATAAAGTAGTAAAAATTAAACCATTA---GGAATAGCACCCACTAAGGCAAAGAGAAGAGTGGTG--------
-------------------------------CAGAGAGAAAAAAGAGCA---GTGATA------GGAGCT---
------ATGTTCCTTGGGTTCTTGGGAGCA---GCAGGAAGCACTATG---GGCGCAGCGTCA---
CTGACGCTGACGGTACAG---GCCAGACAA---TTATTG---TCTGGTATAGTGCAACAGCAGAAT---
AATTTG---CTGAGA---GCTATTGAGGCGCAACAGCATCTGTTGCAACTCACAGTCTGGGGCATC
>HQ595779
TTCTACTGTAATTCCACACAGCTGTTTAATAGTACTTGGGGAATAGGGGCAAATGTCACAAATACC-------
----

ACTGAATACAATATCACACTCCCATGTAGAATAAAACAAATTATAAACAGGTGGCAGGAAGTAGGAAGAGCAA
TGTATGCCCCTCCCATCAGTGGACAAATTAGATGTTCATCAAAT---
ATTACAGGGCTGCTATTAACAAGAGATGGTGGTGGTGGTAATATT----------------------------
----

AGTAATACAACTGAGATCTTCAGACCTGGGGGAGGAGATATGAGGGACAATTGGAGAAGTGAATTATATAAAT
ATAAAGTAGTAAAAATTGAGCCATTA---GGAGTAGCACCCACCACGGCAAAGAGAAGGGTGGTG--------
-------------------------------CAAAGAGAAAAAAGAGCA---CTGACG---CTAGGAGCT---
------ATGTTCCTTGGGTTTTTGGGAGCA---GCAGGAAGCACTATG---GGCGCAGCGTCA---
ATAACACTGACGGTACAG---GCCAGACAA---TTATTG---TCTGGTATAGTGCAACAGCAGAAC---
AATATG---CTGAAA---GCTATTGAGGCGCAACAGCACTTGTTGCAACTCACAGTCTGGGGCATT
>HQ595788
TTCTACTGTGATACAGCAAACCTGTTTAATAGTACTTGGTATGCTAATGGGAGTGGGAATATTAATGAAAGT-
----

TCAAGTAGCAATATCACAATCACATGTAGAATAAAACAAATCATAAACATGTGGCAGAAAGTAGGAAAAGCAA
TGTATGCCCCTCCCATCAGAGGACCAATTAGCTGTTCATCAAAT---
ATTACAGGGCTGCTATTAGTAAGAGATGGAGGTAACGTGACCAGT----------------------------
----

GAGAACGAGATCTTCAGACCTGCAGGGGGAGATATGAGGGACAATTGGAGAAGTGAATTATACAAATATAAGG
TAGTAAAAATTGAACCATTA---GGAATAGCACCCACCAAGGCAAAGAGAAGAGTGGTG--------------
-------------------------CAAAGAGAAAAAAGAGCA---GTGGGAACAATAGGAGCT---------
ATGATCCTTGGGTTCTTGGGAACA---GCAGGAAGCACTATG---GGCGCAGCGTCA---
CTGACGCTGACGGTACAA---GCCAGACAA---TTATTG---TCTGGTATAGTGCAACAGCAGAAC---
AATTTG---CTGAAG---GCTATTGAGGCGCAACAACATCTGTTGCAACTCACAGTCTGGGGCATT
>HQ595790
TTCTACTGTGATACAGCAAAACTGTTTAATAGTACTTGGCTGCTGTCTAATGGTACTTGGAATAATTCAGCAG
TTAAT--
-------------------AGT---
ACTGGGAATGGAAATATCACGCTCCAATGCAGAATAAAACAAATTATAAACATGTGGCAGAGAGTAGGAAGAG
CAATGTATGCCCCTCCCATTAAAGGACAAATTAGATGTTCATCAAAT---
ATTACAGGGCTGCTATTGACGAGAGATGGTGGTAACACCAATAACACT-------------------------
----

AATGGCACCGAGACTTTCAGACCTGGAGGGGGAAATATGAAGGACAATTGGAGAAGTGAATTATATAAATATA
AGGTAGTACAAATTGAGCCCTTA---GGAATAGCACCCACCAAGGCAAAGAGAAGAGTGGTG-----------
----------------------------CAAAGAGAAAAAAGAGGA---GTGGGA---TTAGGAGCT------
---CTGTTCCTTGGGTTCTTGGGAGCA---GCAGGAAGCACTATG---GGCGCAGCGTCA---

ATAACGCTGACGGTACAG---GCCAGACAG---TTATTG---TCTGGTATAGTGCAACAGCAGAAC---
AATCTG---CTGAAG---GCTATTGAGGCGCAACAGCATCTGTTGCAACTCACAGTCTGGGGCATT
>HQ595792
TTCTACTGTAATACATCACAACTGTTTAATAGTACTTGGAATAGTACTTTGGAGGTGAATAATGGTACT----
----

GACAATGACAATATCACACTCCCATGTAAAATAAAACAAATTATAAACATGTGGCAGGAAGTAGGAAAAGCAA
TGTATGCCCCTCCCATCGAAGGACTAATTAGTTGTTCATCAAAT---
ATTACAGGGCTGATATTAACAAGAGATGGTGGGAGTAACAGCACG----------------------------
----

AATGAGACTTTCAGACCTGGAGGAGGAGATATGAGGGACAATTGGAGAAGTGAATTATATAAATATAAAGTAG
TAAAAATTGAACCATTA---GGAATAGCACCCACCCAGGCAAAGAGAAGAGTGGTG-----------------
----------------------CAAAGAGAAAAAAGAACA---GTGGGG---ATAGGAGCT---------
GTGTTCCTTGGGTTCTTGGGAGCA---GCAGGAAGCACTATG---GGCGCAGCGTCA---
GTGACGCTGACGGTACAG---GCCAGACTA---TTATTG---TCTGGTATAGTGCAACAGCAGAAC---
AATCTG---CTGAAG---GCTATTGAGGCGCAACAACATCTGTTGCAACTCACAGTCTGGGGCATT
>HQ595794
TTCTACTGTAATACATCAAAACTGTTTAATAGTACTTGGAATAGTACTGAAGGGTTAAATGACACTGAAGAA-
----

GGAAATGGCACAATTGTACTCCCATGCAGAATAAAACAGATTATAAACATGTGGCAGGAAGTAGGAAAAGCGA
TGTATGCCCCTCCCATCAGTGGACAAATTAACTGTGTATCAAAT---
ATTACAGGGATGATATTAACAAGAGATGGTGGACAGGATAATAGG----------------------------
----

AGCAGCGAGACCTTCAGACCTGGAGGGGGAGATATGAGGGACAATTGGAGGAGTGAACTATATAAATATAAAG
TAGTACAAATTGAACCACTA---GGAGTAGCACCCACCAAGGCAAAGAGAAGAGTGGTG--------------
-------------------------CAGAGGGAAAAAAGAGCA---ATAGGGGTACTAGGAGCT---------
ATGTTCATTGGGTTCTTGGGAGCA---GCAGGAAGCACTATG---GGCGCAGCGTCA---
ATGACGCTGACGGTACAA---GCCAGACTA---TTATTG---TCTGGTATAGTGCAACAGCAGAAC---
AACTTG---CTGAGG---GCTATTGAGGCGCAACAGCATCTGTTGCAACTCACAGTCTGGGGCATT
>HQ595803
TTCTACTGTAATACAACACAGCTATTTAATAGTACTTGGAGTTGGAATGGTACTGACATTAGAGATAATGGCA
CTGAC--
-------------------ATT---
AGAGATAATGGCACAATCATACTCCCATGCAGGATAAAACAAATTATAAACAGGTGGCAGGAAGTGGGAAAAG
CAATGTATGCCCCTCCCATCAAGGGACAAATTATGTGTAACTCAACC---
ATTACAGGGTTGCTATTAACAAGAGATGGTGGTGATAACAATAAT----------------------------
----

GAGACTGAGATCTTCAGACCTGGAGGAGGAAATATGAAGGACAATTGGAGAAGTGAATTATTTAAATATAAAG
TAGTAAAAATTGAGCCATTA---GGAATAGCACCCACCAAGGCAAAGAGAAGAGTGGTG--------------
-------------------------CAGAGAGAAAAAAGAGCA---GTGGAA---ATAGGAGCT---------
GTGTTCCTTGGGTTCTTGGGAGCA---GCAGGAAGCACTATG---GGCGCAGCGTCA---
GTAACGCTGACGGTACAA---GCCAGACAA---TTATTA---TCTGGTATAGTGCAACAGCAGAAT---
AATTTG---CTGAAG---GCTATTGAAGCGCAACAGCATCTGTTGCAACTCACAGTCTGGGGCATC
>JF680906
TTCTACTGTGATACAGCAAAGCTGTTTAATAGTACTTGGAAAAATGGTAATATAAGCATCGCA----------
----

AAGGACAACCCTATCATACTCCCATGCAGAATAAAACAAATTATAAACAGGTGGCAGGAGGTAGGAAAAGCAA
TGTATGCCCCTCCCATCAGAGGAAAGATTAGCTGTTCATCAAAT---
ATTACAGGGTTGCTTTTAACAAGAGATGGTGGCAGTAAAAATGTGACA-------------------------
----

GATAATAAGACAGAGATCTTCAGACCTGGAGGAGGAGATATGAGGGACAATTGGAGAAGTGAATTATATAAAT
ACAAAGTAGTAAGAATTGAGCCATTA---GGAGTAGCACCCACCAAGGCAAAGAGAAGAGTGGTG--------
-------------------------------CAGAGAGAAAAAAGAGCA---GTGGGA---ATAGGAGCT---
------TTGTTCCTTGGGTTCTTGGGAGCA---GCAGGAAGCACTATG---GGCGCAGCGTCA---

ATGACGCTGACGGTACAG---GCCAGACAA---TTATTG---TCTGGTATAGTGCAACAGCAGAAC---
AATCTG---CTGAGG---GCTATTGAGGCGCAACAGCATCTGTTGCAACTCACAGTCTGGGGCATC
>HQ595769
TTCTACTGTAATACATCACAACTGTTTAATAGTACTTGGAATTCGAGTGATACTACTTGGAATTCAAATGATA
CTAAT--
-------------------ATT---
AATGGAACGGGTACAATCACACTCCCATGTAAAATAAGACAAATTATAAATATGTGGCAGGAAGTAGGAAAAG
CAATGTATGCCCCTCCCATCAGAGGATTCATTAACTGTTCATCAAAT---
ATTACAGGGCTGCTAATAACAAGAGATGGTGGTAGTGAGAATGGC----------------------------
----

ACAAACACTGAGACCTTCAGACCTGGAGGAGGAAATATGAGGGACAATTGGAGAAGTGAATTATATAAATATA
AAACAGTAAAAGTTGAACCATTA---GGAGTAGCACCAACCAGGGCAAGGAGAAGAGTGGTG-----------
----------------------------CAGAGAGAGAAAAGAGGG---CTAACT---CTAGGAGCT------
---TTGTTCCTTGGGTTCTTGGGAGCA---GCAGGAAGCACTATG---GGCGCAGCGTCA---
GTGACGCTGACGGTACAG---GCCAGACAA---TTATTG---TCTGGTATAGTGCAACAGCAGAGC---
AATCTG---CTGAGG---GCTATTGAGGCGCAACAGCATATGTTGCAACTCACAGTCTGGGGCATC
>HQ595770
TTCTACTGTAATACAACACAACTGTTTAACAGTACTTGGAACAAGGATGGTAGTATTGGGAATACTACTAAGG
GGTCA--

ATTAACACAGACACACTCACACTCCCATGCAGAATAAAACAAATTATAAACCTGTGGCAGGAAGTAGGAAAAG
CAATGTATGCCCCTCCCATCAGAGGACAAATTAGATGTACATCAAAT---
ATTACAGGGCTGATGTTAACAAGGGATGGTGGTAATAATGGGAGC----------------------------
----

GAGACCGAGATCTTCAGACCTGCAGGAGGAAATATGAGGGACAATTGGAGAAGTGAATTATATAAATATAAAG
TAGTAAAAATTGAACCATTA---GGAGTAGCACCCACCAAGGCAAAAAGAAGAGTGGTG--------------
-------------------------CAGAGAGAAAAAAGAGCA---GTAGTGGGAATAGGAGCT---------
GTGTTCCTTGGGTTCTTGGGAGCA---GCAGGAAGCACTATG---GGCGCAGCGTCA---
ATGACGCTGACGGTACAG---GCCAGACAA---TTATTG---TCTGGTATAGTGCAACAGCAGAAC---
AATCTG---CTGAGA---GCTATTGAGGCGCAACAGCATCTGTTGCAACTCACAGTCTGGGGCATC
>JF680910
TTCTACTGTGATTCAACGCGATTGTTTAATAGTACTTGGACAAATGGAAGTTGGACAAATGGTACTGAAGAG-
----

AAAAATGACACTATCACGCTCCCATGCAGAATAAAACAAATTATAAACAGGTGGCAGGAAGTAGGAAAAGCAA
TGTATGCCCCTCCCATCAGAGGACTAATTAAATGTTCATCAAAC---
ATTACAGGGCTGTTGTTAACAAGAGATGGTGGTGAGAAGAAGAACAAC-------------------------
----

AGTAATAGTACAGAAACCTTCAGACCTGGAGGAGGGGATATGAGGGATAATTGGAGAAGTGAATTATATAAAT
ATAAAGTAGTAAAAATTGAACCATTA---GGAGTAGCACCTACCAAGGCAAAGAGAAGAGTGGTA--------
-------------------------------CAGAGAGAAAAAAGAGCA---ATAGGAACGCTAGGAGCT---
------GTGTTCCTTGGATTCTTGGGAGCA---GCAGGAAGCACTATG---GGCGCAGCGTCG---
CTGACGCTGACGGTACAG---GCCAGACAA---TTATTG---TCTGGTATAGTGCAACAGCAGAAC---
AATCTG---CTAAGG---GCTATTGAGGCGCAACAACATCTGTTGCAACTCACAGTCTGGGGCATC
>JF680911
TTCTACTGTGAAACAGCACAGCTGTTTAATAGTACTTGGTATGGGAATGAAACTGAAGAGTCACAAAAT----
----

AAGACAGACCCTATCATACTCCCATGCAGAATAAAACAAATCATAAATAGGTGGCAAGAAGTAGGAAAAGCAA
TGTATGCCCCTCCCATCAGTGGATACATTAACTGTTCATCAAAT---
ATTACAGGGCTATTATTAACAAGAGATGGTGGTAAGGATGATAAC----------------------------
----

ACCACCGAGACCTTCAGACCTGGAGGAGGAGATATGAGGGATAATTGGCGAAGTGAATTATATAAATATAAAG
TAGTAAGAATTGAACCAGTA---GGAATAGCACCCACCAGGGCAAAAAGACGAGTGGTG--------------
-------------------------CAGAGAGAAAAAAGAGCA---GTGGGAACAATAGGAGCT---------
ATGTTCCTTGGGTTCTTGGGAGCA---GCAGGAAGCACTATG---GGCGCAGCGTCA---

GTGACGCTGACGGTACAG---GCCAGACAA---TTATTG---TCTGGTATAGTGCAACAGCAAAAC---
AATTTG---CTGAAA---GCTATTGAGGCGCAACAACATCTGTTGCAACTCACAGTCTGGGGCATC
>JF680913
TTCTATTGTAATACAACAAATCTGTTTAATGCAACATTTAATATAACAAACAATACAACAAAGCTACCTAAC-
----

ACAGGTGACACTATCATACTCCCATGCAGAATAAAACAGATTATAAACAGGTGGCAGGAAGTAGGAAAAGCAA
TGTATGCCCCTCCCATCAGGGGACGAATTAGCTGTTCATCAAAT---
ATTACAGGGCTGCTATTAACAAGAGATGGTGGCACTAACATGACTGAT-------------------------
----

AATGGTACAGAGACCTTCAGACCTGCAGGAGGAGATATGAGGGACAATTGGAGAAGTGAATTATATAAATACA
AAGTAGTAAGAATTGAGCCATTA---GGAATAGCACCCACCAAGGCAAAGAGAAGAGTGGTG-----------
----------------------------CAAAGAGAAAAAAGAGCA---GTGGGA---ATAGGAGCT------
---TTGTTCATTGGGTTCTTGGGAGCA---GCAGGAAGCGCTATG---GGCGCAGCGTCA---
ATGACGCTGACGGTACAG---GCCAGACAA---TTATTG---TCTGGTATAGTGCAACAGCAGAAC---
AATTTG---CTGATG---GCTATTGAGGCGCAACAGCATATGTTGCAACTCACAGTTTGGGGCATC
>HQ595783
TTCTACTGTAATTCAACACCACTATTTAATGAGAGCTTTAGAAGATCAAATGAAAGG----------------
----

AGTGAAGGGAATATCACTCTCTCATGTAGAATAAAACAAATTATAAACTTGTGGCAGGAAGTAGGAAAAGCAA
TGTATGCCCCTCCCATCAGAGGACAAATTAGATGTTCATCAAAT---
ATTACAGGGCTGCTATTAATAAGAGATGGTGGGGAAAATGGGACAGAG-------------------------
----

GGAAATAATGAGACCTTCAGACCTGGAGGGGGAGATATGAAGGACAATTGGAGAAGTGAATTATATAAATATA
AAGTAGTAAAAATTGAACCATTG---GGAGTAGCACCCACCAAGGCAAAGAGAAGAGTGGTG-----------
----------------------------CAGAGAGAAAAAAGAGCA---GTGGGAACGCTAGGAGCT------
---ATGTTCCTTGGGTTCTTGGGAGCA---GCAGGAAGCACTATG---GGCGCAGCGTCA---
ATAACGCTGACGGTACAG---GCCAGACAA---TTATTG---TCTGGTATAGTGCAACAGCAGAAC---
AATTTG---CTGAGG---GCTATTGAGGCGCAACAACATATGTTGCAACTCACAGTCTGGGGCATC
>JF680919
TTCTACTGTAATACAACACAGCTGTTTAATAGTACTTGGACCACTGATACTTGGAATAGTACTAATGGGTCAA
GC---

AATAGCAATGACACTATTATACTCCCATGTAGAATAAAACAAATTATAAACAGGTGGCAGGAAGTAGGAAAAG
CAATGTATGCCCCTCCCATCAGCGGACAAATTATATGTCCATCAAAT---
ATTACAGGGCTACTATTAACAAGAGATGGTGGTAATGATAATAGCACC-------------------------
----

AAAAACGGGACTGAAACCTTCAGACCTGGAGGAGGAGATATGAAGGACAATTGGAGAAGTGAATTATATAAAT
ATAAAGTAGTAAGAATTGAGCCATTA---GGAGTAGCACCCACCAGGGCAAAGAGAAGAGTGGTG--------
-------------------------------CAGAGAGAAAAAAGAGCA---GCG------TTCGGAGCT---
------TTGTTCCTTGGGTTCTTGGGAGCA---GCAGGAAGCACTATG---GGCGCAGCGTCC---
CTGACGCTGACGGTACAG---GCCAGAACA---TTATTG---TCTGGTATAGTGCAACAGCAGAAC---
AATTTG---CTGAGA---GCTATTGAGGCGCAACAGCATCTGTTGCAACTCACAGTCTGGGGCATC
>JF680920
TTCTACTGTAATACAACAAAACTGTTTAATAGTACTTGGCCTGTTAATGGTAATGATACTGGGTCAAATATC-
----

ACTGAAGAAAATATTACACTCCCATGTAGAATAAAACAAATTATAAACATGTGGCAGGAAGTAGGAAAAGCAA
TGTATGCCCCTCCCATCAAAGGACTAATTAGATGTTCATCAAAC---
ATTACAGGGATACTACTAACAAGAGATGGTGGGGACGGGAGCAAT----------------------------
----

GCCAACGAGACCTTCAGACCTGGAGGAGGGAATATGAGGGACAATTGGAGAAGTGAATTATATAAATATAAAG
TAGTACAAATTGAACCATTA---GGAGTAGCACCCACCAAGGCAAAGAGAAGAGTGGTG--------------
-------------------------CAGAGAGAAAAAAGAGCA---GTGGGA---ATAGGAGCT---------
TTGTTCCTTGGGTTCTTGGGAGCA---GCAGGAAGCACTATG---GGCGCAGCATCA---

ATGACGCTGACGGTACAG---GCCAGACAA---TTATTG---TCTGGTATAGTGCAACAGCAGAAC---
AATTTG---CTGAGA---GCTATTGAGGCGCAACAGCATCTGTTGCAACTCACAGTCTGGGGCATC
>HQ595786
TTCTACTGTGATACAACACAGCTATTTAATAGTACTTGGAATATAACACAGCTGTCTAATAATAATAATACAG
GAAAT--
-------------------GAC---
ATAAATAACACAACAATCGTGCTTCCATGTAGAATAAAACAAATTATAAACAGGTGGCAGGAAGTAGGAAAAG
CAATGTATGCCCCTCCCATCAGAGGAGTAATTAAATGTTCATCAAAT---
ATTACGGGGCTGATTTTAGCAAGAGATGGTGGAATTAATGAGACT----------------------------
----

AATGACACCGAGATCTTTCGACCTGCAGGAGGAGATATGAGGGACAATTGGAGAAGTGAATTATATAAATATA
AAGTAGTAAAAATTGAACCATTA---GGAGTAGCACCCACCAAGGCAAAGAGAAGAGTGGTG-----------
----------------------------CAGAGAGAAAAAAGAGCA---GTGGGAACATTAGGAGCT------
---ATGTTCCTTGGGTTCTTGGGAGCA---GCAGGAAGCACTATG---GGCGCAGCGTCA---
ATAACGCTGACGGTACAG---GCCAGACAA---TTATTG---TCTGGTATAGTGCAACAGCAGAGC---
AATTTG---CTGAGA---GCTATTGAGGCGCAACAGCACCTGTTGCAACTCACAGTCTGGGGCATC
>JF680928
TTCTACTGTAATACAACAAAACTGTTTAATAGTACTTGGGAGAATGGTACTTGGCAGAATACTACAGAG----
----

TTGAATGGCACTATCACTCTTCCTTGTAGAATAAAACAAATTATAAACATGTGGCAGGAAGTAGGGAAAGCAA
TGTATGCCCCTCCCATCAGAGGACAAATTAAGTGTTCATCAAAT---
ATTACAGGGATACTATTAACAAGAGATGGGGGTAATAGCACCAAC----------------------------
----

AACACTGAGACCTTCAGACCTGCAGGAGGAAATATGAAGGACAATTGGAGAAGTGAATTATATAAATATAAAG
TAGTAAAAATTGAACCATTA---GGAGTAGCACCCACCAAGGCAAAGAGAAGAGTGGTG--------------
-------------------------CAGAGAGAAAAAAGAGCA---GCGGGA---CTAGGAGCT---------
GTGCTCATTGGGTTCTTGGGAGCA---GCAGGAAGCACTATG---GGCGCAGCGTCA---
CTAACGCTGACGGTACAG---GCCAGACAA---TTATTG---TCTGGTATAGTGCAACAGCAGAAT---
AATCTG---CTGAGA---GCTATTGAGGCGCAACAACATCTGTTGCAACTCACAGTCTGGGGCATT
>JF680929
TTCTACTGTAATACATCACAGCTGTTTAATAGTACTTGGGAGAATAGTGCTGGGGAGAATAATACAGAGTCAA
AC---

GCTACAGAGGTAAATATCATACTCCCATGCAGAATAAAACAAATCATAAACAGGTGGCAGGAGGTAGGAAAAG
CAATGTATGCCCCTCCCATCAGAGGAAATATCAGCTGTTCATCAAAT---
ATTACAGGGCTGATATTGACAAGAGATGGTGGTAACGAAAATCGC----------------------------
----

ACAGGCAACGAGACCTTCAGGCCGGGAGGGGGAGATATGAAAGACAATTGGAGGAGTGAATTATATAAATATA
AAGTAGTAAAAATTGAGCCATTA---GGAATAGCACCCACAAAGGCAAGGAGAAGAGTGGTG-----------
----------------------------CAGAGAGAAAAAAGAGCA---GTGACA---ATAGGAGCT------
---GTACTCCTTGGGTTCTTGGGAACA---GCAGGAAGCACTATG---GGCGCAGCGGCA---
GTGACGCTGACGGTACAG---GCCAGACAA---TTACTG---TCTGGTATAGTGCAACAGCAAAAC---
AATTTG---CTGAGA---GCTATTGAGGCGCAACAACATCTGTTGCAACTCACAGTCTGGGGCATC
>JF680934
TTCTACTGTAATTCAACACAGCTGTTTAATAGTACTTGGACGTTTAATGGTACTTGGATGTTTAATGGTACTA
CTTGGAATAAT--
----------ACTGATAAC------
AGTAACCATACCAATATTACACTCCCATGCAGAATAAAACAAATTATAAACATGTGGCAGAGAGTAGGAAAGG
CAATGTATGCCCCTCCCATCAGAGGACTAATTAGATGTAAATCGAAT---
ATTACAGGGATACTATTAACAAGGGATGGTGGTGATGGGAACAAT----------------------------
----

AGTGAAGAGATCTTCAGACCTGGAGGAGGAGATATGAAGGACAATTGGAGAAGTGAATTATATAAGTACAAAG
TAGTAAAAATTGAGCCATTA---GGAGTAGCACCCACTAAGGCAAGGAGAAGAGTGGTG--------------
-------------------------CAGAGAGAAAAAAGAGCA---GTGGGAACACTAGGAGCT---------
ATGTTCCTTGGGTTCTTGGGAGCA---GCAGGAAGCACTATG---GGCGCAGCGTCG---

GTGACGCTGACGGTACAG---GCCAGACTA---TTATTG---TCTGGTATAGTGCAACAGCAGAGC---
AATTTG---CTGAGA---GCTATTGAGGCGCAACAGCATATGTTGCAACTCACAGTCTGGGGCATC
>HQ595801
TTCTACTGTAATACAACACAACTGTTTAATAGCACTTGGAATAGTACTGAGAATGTTGATAAT----------
----

AGGACAGATAACATCACGCTCCCATGCAAAATAAAACAATTTATAAATATGTGGCAGGAAGTAGGAAAAGCAA
TGTATGCCCCTCCCATTAGAGGACAAATTAGCTGTTCATCAAAT---
ATTACAGGGCTTCTATTAACAAGAGATGGTGGTAAAGATAACAATAGCAAT----------------------
----

GACAACAATATTGAGGTCTTTAGACCTGGAGGAGGAAATATGAAAGACAATTGGAGAAGTGAATTATATAAAT
ATAAAGTAGTAAAAATTGAACCATTA---GGAATAGCACCCACCAAGGCAAAGAGAAGAGTGGTA--------
-------------------------------CAGAGAGAAAAAAGAGCA---GTGGGC---CTAGGAGCT---
------TTGTTCCTTGGGTTCTTGGGAACA---GCAGGAAGCACTATG---GGCGCAGCGTCA---
ATAACGCTGACGGTACAG---GCCAGACAA---TTATTG---TCTGGTATAGTGCAACAGCAGAAC---
AATCTG---CTGAGA---GCTATTGAGGCGCAGCAGCGTATGTTGCAACTCACAGTCTGGGGCATC
>HQ595802
TTCTACTGTAATACAACACAACTGTTTAATAGCACTTGGAAACTGGTTAATAACACTTGGAGTAAGATTGATA
ATAAGAATGAG--
ACTGGTAATAGTAATATC---------
ACACTCCCTAGTAATATCACACTCCCATGTAGAATAAAACAAATTATAAACATGTGGCAGAAAGTAGGAAAAG
CAATGTATGCCCCTCCTATTGGAGGACAAATTAAATGTTCATCAAAC---
ATTACAGGGCTAATATTAGTAAGAGATGGTGGTAATGACACAGGA----------------------------
----

GGCAACGAGACCTTCAGACCTGCAGGGGGAGATATGAGGGACAATTGGAGAAGTGAATTATATAAATACAAAG
TAGTAAAAATTGAACCATTA---GGAATAGCACCCACCAGGGCAAAGAGAAGAGTGGTG--------------
-------------------------CAAAGAGAAAGAAGAGCA---GTGACA---CTGGGAACC---------
ATGCTCCTTGGGTTCTTGGGAGCA---GCAGGAAGCACTATG---GGCGCAGCGTCC---
TTGACGCTGACGGTACAG---GCCAGACAA---TTATTG---TCTGGTATAGTGCAACAGCAGAAC---
AATCTG---CTGAGG---GCTATTGAGGCGCAACAGCATCTGTTGCAGCTCACAGTCTGGGGCATC
>HQ595804
TTCTACTGTAATACATCAGAACTGTTTAATAGTACTTGGAATGATACTGCAGGGTTAAATAACACTAACACTA
CA---

GACCAAGGCACAATCACACTCCCATGCAGAATAAAACAAATTATAAACATGTGGCAAAAAGTAGGAAAAGCAA
TGTATGCCCCTCCCATTGCAGGACAAATTAACTGCTCAACAAAT---
ATTACAGGAATACTACTAACAAGAGATGGTGGTGGTAATAGCAGT----------------------------
----

AACACCACCGAGACCTTCAGACCAGCAGGAGGAGATATGAGGGATAATTGGAGAAGTGAATTATATAAATATA
AAGTAGTAAAAATTGAACCATTA---GGAATAGCACCCACCAAGGCAAAAAGGAGAGTGGTG-----------
----------------------------CAAAGAGAGAAAAGAGCA---ATAGGGGGATTGGGAGCT------
---TTGTTCCTTGGGTTCTTGGGAGCA---GCAGGAAGCACTATG---GGCGCAGCGTCA---
CTAACGCTGACGGTACAG---GCCAGACAA---TTATTG---TCTGGTATAGTGCAGCAGCAGAAC---
AATCTG---CTGAGA---GCTATTGAGGCGCAACAACATTTGTTGCAACTCACAGTCTGGGGCATC
>HQ595806
TTCTACTGTAATACAACACAATTGTTTAATAGTACTTGGAATATAAAAGAGAATACAACAAAACGGGATAATA
GTACT--
-------------------GAA---
AGTAATAGTACTACTATCGTACTCCCATGCAGAATAAAACAAATTATAAACAGATGGCAGGAAGTAGGAAAAG
CAATGTATGCCCCTCCCATCAGCGGAACAATTAAATGCTCATCAAAT---
ATTACAGGGTTACTATTAACAAGAGATGGGGGTAATAATAACGAGACC-------------------------
----

AATGGAACTGAGGTCTTCAGACCTGGAGGAGGAGATATGAGGGACAATTGGAGAAGTGAATTATATAAATATA
AAGTAGTAAAAATTGAACCATTA---GGAGTAGCACCCACCCAGGCAAGGAGAAGAGTGGTG-----------
----------------------------CAGAGAGAAAAAAGAGGA---GTAGGA---CTAGGAGCT------
---TTGTTCCTTGGGTTCTTGGGAGCA---GCAGGGAGCACTATG---GGCGCAGCGTCA---

ATGACGCTGACGGTACAG---GCCAGACAA---CTATTG---TCTGGTATAGTGCAACAGCAAAAC---
AATTTG---CTGAGA---GCTATTGAGGCGCAACAGCGTCTGTTGCAACTCACAGTCTGGGGCATC
>JF680937
TTCTACTGTAATACAACACAACTGTTTAATAGCGATTGGAATATTACTGAAAGGGCAAATGACACTGAA----
----

GGAAATGGCACAATTACACTCCCATGCAGAATAAAACAAATTATAAACATGTGGCAGGAAGTAGGAAAAGCAA
TGTATGCCCCTCCCATCAAAGGAGAAATTGGATGTTCATCAACC---
ATCACAGGATACTCTTAACAAGAGATGGTGGAACGAACAGTACC-----------------------------
----

AATGAGACCTTCAGACCTGGAGGAGGGGATATGAGAGACAATTGGAGAAGTGAATTATTTAAATATAAAGTAG
TACAAATTGAACCATTA---GGAATAGCACCCACCAGGGCAAAGAGAAGAGTGGTG-----------------
----------------------CAGAGAGAAAAAAGAGCA---GTGGGA---CTAGGAGCC---------
TTGTTCCTTGGGTTCTTGGGAGCA---GCAGGAAGCACTATG---GGCGCAGTGTCA---
ATGACGCTGACGGTACAG---GCCAGACAA---TTATTG---TCTGGTATAGTGCAACAGCAGAAC---
AATTTG---CTGAGA---GCTATTGAGGCGCAACAGCATCTGTTGCAACTCACAGTCTGGGGCATC
>D10112
TTCTACTGTAATACAACACAGCTGTTTAATACTACTTGGCTGTTTAATGGTACTTGGAATGATACTGAAGGGT
TAAAT--

AACACTGAAAGAAATATTACACTTCCATGCAGAATAAAACAAATTATAAACAGGTGGCAGGAAGTAGGAAAAG
CAATGTATGCCCCTCCCATCACAGGAACAATTAGCTGTTCATCAAAT---
ATTACAGGGCTGCTATTAACAAGAGATGGTGGTAGAGGCGAAAAT----------------------------
----

GAGACCGAGACCTTCAGACCTGGAGGAGGAGATATGAGGGACAATTGGAGAAGTGAATTATATAAATATAAAG
TAGTAAAAATTGAACCATTA---GGAGTAGCACCCACCAAGGCAAAGAGAAGAGTGGTG--------------
-------------------------CAGAGAGAAAAAAGAGCA---GTGGGAGCGATAGGAGCT---------
CTGTTCCTTGGGTTCTTGGGAGCA---GCAGGAAGCACTATG---GGCGCAGTGGCA---
CTGACGCTGACGGTACAG---ACCAGACAA---TTATTG---TCTGGTATAGTGCAACAGCAGAAC---
AATCTG---CTGAGG---GCTATTGAGGCGCAACAGCATCTGTTGCAACTCACAGTCTGGGGCATC
>Y13716
TTCTACTGTAAGACAGCACAACTGTTTAATAGTACTTGGAATAGTACTGGAAATGGTACTATAAAGTCAAAT-
----

ACCACTGAAATTATCACACTCCCATGTAGAATAAAACAAATTGTAAACATGTGGCAGGAAGTAGGAAAAGCAA
TGTATGCCCCTCCCATCACAGGACAAATTAGATGTGCATCACAT---
ATTACAGGGCTACTATTAACAAGAGATGGAGGTAGGGAGAACAATACC-------------------------
----

AACGAGACCGAGACCTTCAGACCTGGGGGAGGAGATATGAGGGACAATTGGAGAAGTGAATTATATAAATATA
AAGTAGTAAAAATTGAACCATTA---GGAGTAGCACCCACCAAGGCAAAGAGAAGAGTGGTG-----------
----------------------------CAGAGAGAAAAAAGAGCA---GTGGGAATGATAGGAGCT------
---ATGTTCCTTGGGTTCTTGGGAGCA---GCAGGAAGCACTATG---GGCGCAGCCTCA---
CTGACGCTGACGGTACAG---GCCAGACTA---TTATTG---TCTGGTATAGTGCAGCAGCAAAAT---
AATTTG---CTGAGG---GCTATTGAGGCGCAACAGCATCTGTTGCGACTCACAGTCTGGGGCATC
>JF706393
TTCTACTGTAATACATCAAAACTGTTTAATAGTACTTGGACTAATATTACTGAAGGGTTAAATAAAACTGAA-
----

AGAAATGGCACATTCATACTCCCATGCAGAATAAAACAAATTATAAACAGGTGGCAGGAAGTAGGAAAAGCAA
TGTATGCCCCTCCCATCAGAGGACAAATTAGATGTTCATCAAAT---
ATTACAGGGCTGCTATTAATGAGAGATGGTGGTAATACTAACGAGAGC-------------------------
----

AAGACCACCGAGGTCTTCAGACCTGGAGGAGGAGACATGAGGGACAATTGGAGAAGTGAATTATATAAATATA
AAGTAGTAAGAATTGAACCATTA---GGAGTAGCACCCACCAAGGCAAAGAGAAGAGTGGTG-----------
----------------------------CAGAGGGAAAAAAGAGCA---GTGGGA---ATAGGAGCT------
---TTGTTCCTTGGGTTCTTGGGAGCA---GCAGGAAGCACTATG---GGCGCAGCGTCA---

ATGACGCTGACGGTACAG---GCCAGACAA---TTATTG---TCTGGTATAGTGCAGCAGCAGAAC---
AATCTG---CTGAGG---GCTATTGAGGCGCAACAGCATCTGTTGCAACTCACAGTCTGGGGCATC
>JN786861
TTCTACTGCAATTCAACACAACTGTTTAATAGTACTTGGAATGTTAAAGGTACTTGGAATGGTACTGAAGAGT
CAAATAAC---
-------------------AAT---
GAAGGAAATGACACAATCACACTCCAATGCAGAATAAAACAAATTATAAACATGTGGCAGGAAGTAGGAAAAG
CAATGTATGCCCCTCCCATCAGAGGACAAATTAGATGTTCATCAAAT---
ATTACAGGGCTGATATTAACAAGAGATGGTGGTAATGAGAATACC----------------------------
----

ACCACAGAGATCTTCAGACCTGGAGGAGGAAATATGAGGGACAATTGGAGAAGTGAATTATATAAATATAAAG
TAGTAAAAATTGAACCATTA---GGAGTAGCACCCACCAGGGCAAAGAGAAGAGTGGTG--------------
-------------------------CAGAGAGAAAAAAGAGCA---GTGGGA---CTAGGAGCT---------
TTGTTCCTTGGGTTCTTGGGAGCA---GCAGGAAGCACTATG---GGCGCAGCGTCA---
CTGACGCTGACGGTACAA---GCCGGACAA---TTATTG---TCTGGTATAGTGCAACAGCAGAAC---
AACTTG---CTGAGG---GCTATTGAGGCGCAACAGCACCTGTTGCAACTCACAGTCTGGGGCATC
>AJ535594
TTCTACTGTGATACAACACAACTGTTTAATGGGAGTGTTCCTAAAGGAAATAACACTGAAGGAAGTAGT----
----
----------------------------AATGACCTATCACACTCCCATGTT---
AGAATAAAACAAATTATAAACAGGTGGCAGGAAGTAGGAAAAGCAATGTATGCCCCTCCCATCAGTGGAATAG
TTACATGTTCATCAAAT---ATTACAGGGCTGCTATTAACAAGAGATGGGGGTGTGACTAACGATACT-----
----

AACGGCACTGAGATTTTCAGACCTGGAGGAGGAAATATGAGGGACAATTGGAGAAGTGAATTATATAAATATA
AAGTAGTAAGAATTGAACCATTA---GGAGTAGCACCCACTACGGCAAAGAGAAGAGTGGTG-----------
----------------------------CAAAGAGAAAAAAGAGCA---GTGGGA---CTAGGAGCT------
---ATGTTCCTTGGGTTCTTGGGAGCA---GCAGGAAGCACTATG---GGCGCAGCGTCA---
ATAACGCTGACGGTACAG---GCCAGACTA---TTATTG---TCTGGTATAGTGCAACAGCAAAAC---
AATTTG---CTGAGG---GCTATTGAGGCACAACAGCATCTGTTGCAGCTCACAGTCTGGGGCATC
>AJ535607
TTCTACTGTAATACATCACAACTGTTTAATAGTACTTGGAATGAGAATAATACTTGGACAGAGTCAAATAGC-
----

ACTGGAGAAAATATCACACTCCCATGTAGGATAAAACAAATTATAAACATGTGGCAGGATGTAGGAAGAGCAA
TGTATGCCCCTCCCATCAGTGGACAAATTAGATGTTCATCAAAT---
ATTACAGGGCTGCTATTAGTAAGAGATGGTGGTATTAACGTGACCAAC-------------------------
----

GAGGCCGGCACCGAGGTCTTCAGACCTGGGGGAGGAGATATGAAGGACAATTGGAGAAGTGAATTATATAAAT
ATAAAGTAGTAAAAATTGAACCATTA---GGAATAGCACCCACCAAGGCAAAAAGAAGAGTGGTG--------
-------------------------------CAGAGAGAAAAAAGAGCA---GCA------ATAGGAGCT---
------ATGTTCCTTGGGTTCTTGGGAGCA---GCAGGAAGCACTATG---GGCGCAGCGTCA---
CTGACGCTGACGGTACAG---GCCAGACAA---TTATTG---TCTGGTATAGTGCAACAGCAGAAC---
AATCTG---CTGAGG---GCTATTGAGGCGCAACAGCATCTGTTGCAACTCACTGTCTGGGGCATC
>AJ535612
TTCTACTGTAATTCAACAAAACTGTTTAGTAGTACTTGGAATAGTAATAGTACTTGGAATAGTGCTGAAGAGT
CA---

AATAGCACTGAAGTTATCACACTCCCATGCAGAATAAAACAAATTATAAACATGTGGCAGGAAGTAGGAAAAG
CAATGTATGCCCCTCCCATCAGAGGACAAATTAGATGCTCATCAAAT---
ATTACAGGGCTGCTATTAACAAGAGATGGTGGTAAGAATCCGAACGAG-------------------------
----

AACACCACCGAGACCTTCAGACCTGGAGGAGGAAATATGAGGGACAATTGGAGAAGTGAATTATATAAATATA
AAGTAGTAAAAAATTGAACCATTA---GGAGTAGCACCCACCAAGGCAAGGAGAAGAGTGGTG----------
----------------------------CAGAGAGAAAAAAGAGCA---ATAGGA---ATGGGAGCT------
---TTGTTCCTTGGGTTCTTGGGAGCA---GCAGGAAGCACTATG---GGCGCAGCGTCA---

CTGACGCTGACGGTACAG---GCCAGGCTA---TTATTG---TCTGGTATAGTGCAACAGCAGAAT---
AATTTG---CTGAGG---GCTATTGAGGCGCAACAGCATCTGTTGCAACTCACAGTCTGGGGCATC
>AJ418531
TTCTACTGTAATACTACTAAGCTGTTTAATAGTACTTGGAATAGTACTTGGACTGGGATACTACT-------
----

GGAGATGACAATATCACACTCCCATGTAGAATAAAACAATTTATAAACAGGTGGCAGGAAGTAGGAAAAGCAA
TGTATGCCCCTCCCATCAGTGGACAAATTAGATGTTTATCAAAT---
ATTACAGGGCTGCTATTAACAAGAGATGGTGGTAGTACCAAGAACGAGACC---------------------
----

AGTAACCAGACCGAGATTTTCAGACCTGGAGGAGGAGATATGAGGGACAATTGGAGAAGTGAATTATATAAAT
ATAAAGTAGTAAAAATTGAGCCATTA---GGAGTAGCACCTACCAGGGCAAAGAGAAGAGTGGTG-------
------------------------------CAAAGAGAAAAAAGAGCA---GTGGGAACAATAGGAGCT---
------ATGTTCCTTGGGTTCTTGGGAGCA---GCAGGAAGCACTATG---GGCGCAGCGTCA---
TTGACGCTGACGGTACAG---GCCAGACTA---TTATTG---TCTGGTATAGTGCAGCAGCAAAGC---
AATTTG---CTGAGG---GCTATTGAGGCGCAACAGCATCTGTTGCAACTCACAGTCTGGGACATC
>AJ418494
TTCTACTGTAATACAACACAACTGTTTAATAGTACCTGGAATGTTAGTAATACTGAAGGATTAAATATCAATA
TCACT---

GAAAGAAATGACACATTCACACTCCCATGCAGAATAAAACAAATTATAAACATGTGGCAGGAAGTAGGAAAAG
CAATGTATGCCCCTCCCATCAAAGGAAAAATTAGATGTTCATCAAAT---
ATTACAGGGCTGCTATTAACAAGAGATGGTGGTGGTAACGAGAGC---------------------------
----

AGCAGCAGCGAGACCTTCAGACCTGGAGGAGGAGATATGAGGGACAATTGGAGAAGTGAATTATATAAATATA
AAGTAGTAAGAATTGAACCATTA---GGAGTAGCACCCACTAAGGCAAAAAGAAGAGTGGTG----------
---------------------------CAGAGAGAAAAAAGAGCA---GCA------CTAGGAGCT------
---ATGTTCCTTGGATTCTTGGGAGCA---GCAGGAAGCACTATG---GGCGCAGCGTCA---
ATGGCGCTGACGGTACAG---GCCAGACAA---TTATTG---TCTGGTATAGTGCAACAGCAGAAT---
AATCTG---CTGAGA---GCTATTGAGGCGCAACAGCACCTGTTGCAACTCACAGTCTGGGGCATT
>AJ418495
TTCTACTGTAATACAACAAAACTGTTTAATAGTACTTGGCAAAATGAGACTAGCGGGTCCATTAACATAACTG
AC--

ATTGGAGAAAATATCACACTCCCATGCAGAATAAAACAAATTATAAACCTGTGGCAGGGAGTAGGAAAAGCTA
TGTATGCCCCTCCCATCCAAGGACAAATTAGTTGTTCATCAAAT---
ATTACAGGGCTGCTATTAACAAGAGATGGTGGTAACAACAACGAGACA------------------------
----

AACAGGACCGAGACCTTCAGACCTGGAGGAGGAAATATGAGGGACAATTGGAGAAGTGAATTATATAAATATA
AAGTAGTAAAAATTGAGCCATTA---GGAGTAGCACCCACCAAGGCAAAGAGAAGAGTGGTG----------
---------------------------CAGAGAGAAAAAAGAGCA---GTAACG---CTAGGAGCT------
---ATGTTCCTTGGGTTCTTGGGAGCA---GCAGGAAGCACAATG---GGCGCAGCGTCA---
ATGACGCTGACGGTACAG---GCCAGACTA---TTATTG---TCTGGTATAGTGCAACAGCAGAAC---
AACCTG---CTGAGG---GCTATTGAGGCGCAACAGCATCTGTTGCAACTCACAGTCTGGGGCATC
>AJ418519
TTCTACTGTGATTCATCACAGCTGTTTAATGGTACTTGGAATGATACTAATACTAATGAA------------
----

GGAAATGGCACAATCACACTCCAATGCAAAATAAAACAAATTATAAACCTGTGGCAGGAAGTAGGAAAAGCAA
TGTATGCCCCTCCCATCAGAGGACGAATTAGATGTTCATCAAAT---
ATTACAGGGCTGTTATTAACAAGAGATGGTGGTAATACTAATGAC---------------------------
----

ACAGACGAGATATTCAGACCTGGAGGAGGAGATATGAGGGACAATTGGAGAAGTGAATTATATAAATATAAAG
TAGTTAAAATTGAACCATTA---GGAGTGGCACCCACCAAGGCAAAGAGAAGAGTGGTG-------------
------------------------CAGAGAGAAAAAAGAGCA---GTGGGA---ATAGGAGCT---------
GTGTTCCTTGGGTTCTTGGGAGCA---GCAGGAAGCACTATG---GGCGCAGCGTCA---

```
ATGACGCTGACGGTACAG---GCCAGACTA---TTATTG---TCTGGTATAGTGCAACAGCAGAAC---
AATTTG---CTGAGG---GCTATTGAGGCGCAACAGCATCTGTTGCAGCTCACAGTCTGGGGCATC
>AJ418521
TTCTACTGTAATACATCACAACTATTTAATAGTATTTGGAATAGTACTGAAAAGACAAATATC----------
-------------------------------------------------------------------------
----------------------------
ACTAAAGGAATTATCACACTCCCATGCAGAATAAAACAAATTATAAACATGTGGCAGGAAGTAGGGAAAGCAA
TGTACGCCCCTCCCATCAGAGGAAATATTAGTTGTTCATCAAAT---
ATTACAGGGCTACTATTAACAAGAGATGGTAGTAACGGGAACGGGACC-------------------------
-------------------------------------------------------------------------
-------
GGGAACAGGACCGAGATCTTTAGACCTGGAGGAGGAGATATGAGGGACAATTGGAGAAGTGAATTATATAAAT
ATAAAGTAGTAAAAATCGAACCATTA---GGAGTAGCACCCTCCAAGGCAAAGAGAAGAGTGGTG--------
-------------------------------CAGAGAGAAAAAAGAGCA---GTGACA---TTAGGAGCT---
------TTGTTCCTTGGGTTCTTGGGAGCA---GCAGGAAGCACTATG---GGCGCAGCGACA---
ATGACGCTGACGGTACAG---GCCAGACTA---TTATTG---TCTGGTATAGTGCAACAGCAGAAC---
AATTTG---CTGAGG---GCTATTGAGGCGCAACAGCATCTGTTGCAACTCACAGTCTGGGGCATC
>U36869
TTCTACTGTAACACAACGCAACTGTTTAATAGTACTTGGAATGGTACTGAAGGGTCTTGGAATGATACTAAA-
-------------------------------------------------------------------------
----------------------------
GGGCCAGAAAACATCACACTCCCATGCAGAATAAAACAAATTATAAACATGTGGCAAGAAGTAGGAAAAGCAA
TGTATGCCCCTCCCATTAGTGGACAAATTAGATGCTCATCAAAT---
ATTACAGGACTGCTATTAACAAGAGATGGTGGTAAGGGCAATAATACC-------------------------
-------------------------------------------------------------------------
----------
AATACCACCGAGATCTTTAGACCGGGAGGAGGAGATATGAGGGACAATTGGAGAAGTGAATTATATAAATATA
AAGTAGTAAGAATTGAACCATTA---GGAGTAGCACCCACCAAGGCAAAGAGAAGAGTGGTG-----------
----------------------------CAGAGAGAAAGGAGATCA---ATAACA---CTAGGAGCT------
---ATGTTCCTTGGGTTCTTGGGAGCA---GCAGGAAGCACTATG---GGCGCAGCGTCA---
ATAACGCTGACGGTACAG---GCCAGACAA---TTATTG---TCTGGTATAGTGCAACAGCAGAAC---
AATTTG---CTGAGG---GCTATTGAGGCGCAACAGCATCTGTTGCAGCTCACAGTCTGGGGCATC
>U36872
TTCTACTGTAATACAACAAAATTATTCAATAGTACTTGGACTTGGAATGATACTGAAGGGTCAGATAGC----
-------------------------------------------------------------------------
----------------------------
ACTGAAGGAAGTATCACACTCCCATGCAGAATAAAACAATTTATAAACATGTGGCAGGAAGTAGGAAAAGCAA
TGTATGCCCCTCCCATCAGAGGACAAATTAGATGTTCATCAAAT---
ATTACAGGGCTGCTATTAACAAGAGATGGTGGTAATGATAACAAGACT-------------------------
-------------------------------------------------------------------------
----------
AACACCACCGAGACCTTCAGACCTGGAGGAGGAGATATGAGGGACAATTGGAGAAGTGAATTATACAAATATA
AAGTAGTAAAAATTGAACCATTA---GGAGTAGCACCCACTAAGTCAAGGAGAAGAGTGGTG-----------
----------------------------CAGAGAGAAAAAAGAGCA---GTGGGA---ATAGGAGCT------
---GTGTTCCTTGGGTTCTTGGGAGCA---GCAGGAAGCACTATG---GGCGCAGCGTCA---
CTGACACTGACGGTACAG---GCCAGACTA---TTATTG---TCTGGTATAGTGCAACAGCAGAAC---
AATTTG---CTGAGG---GCTATTGAGGCGCAACAGCATCTGTTGCAACTCACAGTCTGGGGCATC
>AJ286342
TTCTACTGTAATACAAAACAGCTGTTTAATTGGAGTAGTACTTGGAATGATACTATAAGGTCAGAT-------
-------------------------------------------------------------------------
----------------------------
GCTCCAGAAATTATCACACTCCCATGCAGAATAAAACAAATTATAAACAGGTGGCAGGAAGTAGGAAAAGCAA
TGTATGCCCCTCCAATCGAAGGACAAATTAGATGTTCATCAAAC---
ATTACAGGGCTGCTATTAACAAGAGATGGTGGTAGTAGCAACAATACC-------------------------
-------------------------------------------------------------------------
-------
AACAATACCACCGAGATCTTCAGACCTGGAGGGGGAAATATGAAAGACAATTGGAGAAGTGAATTATATAAAT
ATAAAGTAGTAAAAATTGAGCCATTA---GGAGTAGCACCCACCAAGGCAAAGAGAAGAGTGGTG--------
-------------------------------CAGAGAGAAAAAAGAGCA---ATAGGAACGCTGGGAGCT---
------ATGTTCCTTGGGTTCTTGGGAGCA---GCAGGAAGCACTATG---GGCGCAGCGGCA---
```

ATGACGCTGACGGTACAA---ACCAGACAA---TTATTG---TCTGGTATAGTGCAACAGCAAAAT---
AATTTG---CTGAGG---GCTATTGAGGCGCAACAGCATCTGTTGCAACTCACAGTCTGGGGCATC
>U36875
TTCTACTGTAATACAACAAAACTGTTTAATAGTATTTGGATGCCTAATGGTACTTGGAATAGTACTGACACGT
CA---

AATAGCACTGAGATCAGGCTCCCATGCAGAATAAAACAAATTGTAAACATGTGGCAGGAAGTAGGAAAAGCAA
TGTATGCCCCTCCCATCAGTGGACGAATTAGATGTGTATCAAAT---
ATTACAGGGCTGCTATTAACAAGAGATGGTGGTAATAACAACGAG----------------------------
----

ACCACCGAGATCTTCAGACCTGGAGGAGGAGATATGAGGGACAATTGGAGAAGTGAATTATACAAATACAAAT
TAATAAAAATTGAACCATTA---GGAATAGCACCCACCAAGGCAAAAAGAAGAGTGGTG--------------
-------------------------CAAAGAGAAAAAGAGCA---GTGGGA---ATAGGAGCT----------
GTGCTCCTTGGGTTCTTGGGAGCA---GCAGGAAGCACTATG---GGCGCAGCGTCA---
CTGACGCTGACGGTACAG---GCCAGACTA---TTACTG---TCTGGTATAGTGCAACAGCAGAAC---
AATTTG---CTGAGG---GCTATTGAGGCGCAACAGCATATGTTGCAACTCACAGTCTGGGGCATC
>U36879
TTCTACTGTAATTCAACACAATTGTTTAATAGTACTTGGAATAATGGTACTAGGAATAATACTGAAGGAGGTA
AC---

ATTACAGACACAATTACACTCCCATGTAGAATAAAACAAATTATAAACATGTGGCAGGAAGTAGGAAAAGCAA
TGTATGCCCCTCCCATCAGAGGACAAATTAGATGTTCATCAAAT---
ATTACAGGGCTGTTATTAACAAGAGATGGTGGCAGGAATGAGACC----------------------------
----

ACCACCACTGAGATCTTCAGACCTGGGGGAGGAAATATGAAGGACAATTGGAGAAGTGAATTATATAAATATA
AAGTAGTAAAAATTGAACCATTA---GGAGTAGCACCCACCAAGGCAAAGAGAAGAGTGGTG-----------
----------------------------CAGAGAGAAAAAAAAGCA---ATAGGA---ATAGGAGCT------
---ATGTTCCTTGGGTTCTTGGGAGCA---GCAGGAAGCACTATG---GGCGCAGCGTCA---
ATAACGCTGACGGTACAG---GCCAGACAA---TTATTG---TCTGGTATAGTGCAACAGCAGAAC---
AATTTG---CTGAGG---GCTATTGAGGCGCAACAGCATATGTTGCAACTCACAGTCTGGGGCATT
>U36880
TTCTACTGTAATTCAACACCATTGTTTAGTGGTACTTGGAATACTAGTACTTGGAATGATACTACAGGGTCGA
ATGAC--
-------------------ACT---
GTAGGAAATAACACCATCATACTCCAATGTAGAATAAAACAAATTATAAACCTCTGCCAGAAAGTAGGAAAAG
CAATGTATGCCCCTCCCATTAGAGGACAAATTAGATGTTCATCAAAG---
ATTACAGGGCTGCTATTAACAAGAGATGGTGGGACTGACAATAAG----------------------------
----

AATATCGAGATCTTCAGACCTGGAGGAGGAGATATGAGGGACAATTGGAGAAGTGAATTATATAAATATAAAG
TAGTAAAAATTGAACCATTA---GGACTAGCACCCACTAAGGCAAAGAGAAGAGTGGTG--------------
-------------------------CAGAGAGAAAAAGAGCA---GTGGGA---CTAGGAGCT----------
ATGTTCCTTGGGTTCTTGGGAGCA---GCAGGAAGCACTATG---GGCGCAGCGTCA---
TTAACGCTGACGGTACAG---GCCAGACAA---TTATTG---TCTGGTATAGTGCAACAGCAGAGC---
GATTTG---CTGAGG---GCTATTGAGGCGCAACAGCATATGTTGCAACTCACAGTCTGGGGCATC
>U23487
TTCTACTGTAATTCAACACAATTGTTTAATAGTACTTGGAATACTGGGAATGATACTAGAGAGTCAAATGACA
CA---

AATAATACTGGAAATATCACACTCCCATGCAGAATAAAACAAATATTAAATTTGTGGCAGGAAGTAGGAAAAG
CAATGTATGCCCCTCCCATCAGCGGACAAATTAGCTGTTCATCAAAT---
ATTACAGGGCTATTATTAACAAGAGATGGTGGTAATACTAATACTACT-------------------------
----

GGGAACACCACCGAGACCTTCAGACCTGGAGGAGGAAATATGAGGGACAATTGGAGAAGTGAATTATATAAAT
ATAAAGTAGTAAAAGTTGAACCATTA---GGAATAGCACCCACCAAGGCAAAGAGAAGAGTGGTG--------
-------------------------------CAGAGAGAAAAAAGAGCA---GTGGGAATGCTAGGAGCT---
------ATGTTCCTTGGGTTCTTGGGAGCA---GCAGGAAGCACTATG---GGCGCACGGTCA---

ATAACGCTGACGGTACAG---GCCAGACAA---TTATTG---TCTGGTATAGTGCAACAGCAGAAC---
AATCTG---CTGAGG---GCTATTGAGGCGCAACAGCATCTGTTGCAACTCACAGTCTGGGGCATC
>Y13719
TTCTACTGTAATTCAACACAACTGTTTAATAGTACTTGGAATGGTACTGGAGGGTCAAATAACACTGGA----
----

GGAAATGACACAATCACACTCCCATGCAGAATAAAACAAATTATAAACATGTGGCAGGAAGTAGGAAAAGCAA
TGTATGCCCCTCCCATCAGAGGAATTATTAGCTGTTCATCAAAT---
ATTACAGGGCTGATATTAACAAGAGATGGTGGTAACAACAGCACA----------------------------
----

AACGATACCGAGACCTTCAGACCCGGAGGAGGTGATATGAGGGATAATTGGAGAAGTGAATTATATAAATATA
AAGTAGTACAAATTGAACCATTA---GGAATAGCACCCACCAAGGCAAAGAGAAGAGTGGTG-----------
----------------------------CAGAGAGAAAAAAGAGCG---GTGACA---CTAGGAGCT------
---ATGTTCCTTGGGTTCTTGGGAGCA---GCAGGAAGCACTATG---GGCGCAGCGTCA---
ATAACGCTGACGGTACAG---GCCAGACAA---TTATTG---ACTGGTATAGTGCAACAGCAAAGC---
AATTTG---CTGAGG---GCTATTGAGGCGCAACAGCATCTGTTGCAACTCACAGTCTGGGGCATC
>AJ286330
TTCTACTGTAATACAACACAACTGTTTAATAGTACTTGGGTGCGTGATGATGCTAATAGTTCTTGGAGGCTAA
ATAGCTCT---
-------------------GAA---
GGAAATGACACACTTATCATACTCCAGTGCAGAATAAAACAAATTATAAACATGTGGCAGGAAGTAGGAAGAG
CAATGTATGCCCCTCCCATCAGAGGAAACATTAGCTGTTTATCAAAT---
ATTACAGGGCTGATATTAACAAGAGATGGTGGGATCCAGAACGAGTCCCAGTCC------------CAG----
----
-
AACGGGTCCGAGAGCGAGGAGACCTTCAGACCTGTAGGAGGAGATATGAGGGACAATTGGAGAAGTGAATTAT
ACAAATATAAAGTAGTAAAAATTGAACCATTA---GGAGTAGCACCCACCAGGGCAAAGAGAAGAGTGGTG--
-------------------------------------GCAAGAGAAAAAAGAGCG---GTGGCC---
CTAGGAGCT---------GTATTCATTGGGTTCTTGGGAGCA---GCAGGAAGCACTATG---
GGCGCAGCGTCA---ATGACGCTGACGGTACAG---GCCAGACAA---TTATTG---
TCTGGTATAGTGCAACAGCAGAAC---AATTTG---CTGAGG---
GCTATTGAGGCGCAACAACATCTGTTGCAACTCACAGTCTGGGGCATT
>AJ286332
TTCTACTGTAATACAACAAAACTGTTTAACAGTACTTGGCTTAATAATGGTACCTGGATTAATGGTACTTGGA
ATAAT--
-------------------ATT---
GATGGAAATGACACAATCACACTCCCATGCAGAATAAAACAAATTGTACACATGTGGCAGGAAGTAGGAAAAG
CAATGTATGCCCCTCCCATCAGTGGACAAATTAGATGTTCATCAAAT---
ATTACAGGGCTATTATTAACAAGAGATGGTGGTACTGACAACAGTACT-------------------------
----

GACAATAACACTGAGACCTTCAGACCTGGAGGAGGAAATATGAAGGACAATTGGAGAAGTGAATTATATAAAT
ATAAAGTAGTAAAAATTGAACCATTA---GGAATAGCACCCACGAAGGCAAAGAGAAGAGTGGTG--------
-------------------------------CAGAGAGAAAAAAGAGCA---ATACCA---CTAGGAGCT---
------TTGTTCCTTGGGTTCTTGGGAGCA---GCAGGAAGCACTATG---GGCGCAGCGTCA---
ATAGCGCTGACGGCACAG---GCCAGACTA---TTATTG---ACTGGTATAGTGCAACAGCAAAAC---
AATTTG---CTGAGG---GCTATTGAGGCGCAACAGCATCTGTTGCAACTCACAGTCTGGGGCATT
>AJ286334
TTCTACTGTAATACATCACAACTGTTTAATAGTACTTGGTATGGGAATGAGACTGAAGGGTCAAATAAC----
----

CCTGATAGAAATATCACACTCCCATGCAGAATAAGACAAATTGTAAACAGATGGCAGGAAGTAGGAAAAGCAA
TGTATGCCCCTCCCATCAAAGGACTAATTAGATGTACATCAAAT---
ATTACAGGGCTGTTATTAACAAGGGATGGTGGTAATAATAATGTCACA-------------------------
----

AACCACACAGAGATCTTCAGACCTATAGGAGGAAATATGAGGGATAATTGGAGAAGTGAATTATATAAATATA
AAGTAGTAAAAATTGAACCATTA---GGAATAGCACCCACCAGGGCAAAGAGGAGAGTGGTG-----------
----------------------------CAGAGAGAAAAAAGAGCA---GTGGGA---ATAGGAGCT------

---GTGTTCCTTGGGTTCTTGGGAGCA---GCAGGAAGCACTATG---GGCGCAGCGTCA---
ATAACGCTGACGGTACAG---GCCAGACAA---TTATTG---TCTGGTATAGTGCAGCAGCAGAGC---
AACTTG---CTGAGG---GCTATTGATGCGCAACAGCATCTGTTGCAACTCACAGTCTGGGGCATC
>AJ286336
TTCTACTGTAATACAACAAAGCTGTTTAATAGTACTTGGACTTGGAATGATACTAAAGGGTCAAATAACACTG
GG---

TCAAATGACACTATCATACTCCCATGCAGAATAAAACAAATTATAAACAGGTGGCAAGGAGTAGGAAAAGCAA
TGTATGCCCCTCCCATCAAAGGACCAATTAGATGTTCATCAAAC---
ATTACAGGGCTACTATTAACAAGAGATGGTGGTAATGATAATAGTAGT-------------------------
----

GACAATTCCTCCGAGACCTTCAGACCTGGAGGAGGAGATATGAAGGACAATTGGAGAAGTGAATTATATAAAT
ATAAAGTAGTGAGAATTGAACCATTA---GGAGTAGCACCTACCAAGGCAAAGAGAAGAGTGGTG--------
-------------------------------CAGAGAGAAAAAAGAGCA---GTGGGAATGCTAGGAGCT---
------CTGTTCCTTGGGTTCTTGGGAGCA---GCAGGAAGCACTATG---GGCGCAGCGGCA---
ATGACGCTGACGGTACAA---ACCAGACAA---TTATTG---TCTGGTATAGTGCAACAGCAAAAC---
AATCTG---CTGAGG---GCTATTGAGGCGCAACAGCATCTGTTGCAACTCACAGTCTGGGGCATC
>AJ286337
TTCTACTGTAATACAACACCACTGTTTAATAGTACTTGGAATAGTAATGGTACTAGGGTGTCAAATGACACTG
AA---

GGAAATAATACCACTATCACACTCAAATGCAGAATAAAACAATTTATAAACATGTGGCAGGAAGTAGGAAAAG
CAATGTATGCCCCTCCCATCAGAGGACAAATTAGATGTTCATCAAAT---
ATCACAGGGCTGCTATTAACAAGAGATGGTGGCCATGGTATTAATGGG-------------------------
----

ACTAACGACACTGAGACCTTCAGACCTGGAGGAGGAGATATGAGGGACAATTGGAGAAGTGAATTATATAAAT
ATAAAGTAGTAAAAATTGAGCCATTA---GGAGTAGCACCCACCAGGGCAAAGAGAAGAGTGGTG--------
-------------------------------CAGAGAGAAAAAAGAGCA---GCGGGA---ATAGGAGCT---
------GTGTTCCTTGGGTTCTTGGGAGCA---GCTGGAAGCACTATG---GGCGCAGCATCA---
ATAACGCTGACGGTACAG---GCCAGACAA---TTATTG---TCTGGTATAGTGCAACAGCAGAAC---
AATCTG---CTGAGG---GCTATTGACGCGCAGCAGCATATGTTGCAACTCACAGTCTGGGGCATT
>AF491740
TTCTACTGTGATTCATCACAACTGTTTAATAGTACTTGGATGTTTAATAGTACTTGGAATGATACTGACGGGG
CAAATAGC---
-------------------ACT---
AGGAAAAATGATACAATCACACTCCAGTGCAGAATAAAACAAATTATAAACATGTGGCAGCAAGTAGGAAAAG
CAATGTATGCCCCTCCCATCAGAGGACAGATTAGATGTACATCAAAC---
ATTACAGGGCTGATATTAACAAGAGATGGTGGTGATACCCAAAACAAG-------------------------
----

AGCGACCCCGAGATCTTTAGACCTGGAGGAGGAGATATGAGGGACAATTGGAGAAGTGAATTATATAAATATA
AAGTAGTAAAAATTGAACCACTA---GGAATAGCACCCACCAGGGCAAAGAGAAGAGTGGTG-----------
----------------------------CAGAGAGAAAAAAGAGCA---GTGGGG---ATGGGAGCT------
---ATGTTCCTTGGGTTCTTGGGAGCA---GCAGGAAGCACTATG---GGCGCAGCGTCA---
GTGACGCTGACGGTACAG---GCCAGACAA---TTATTG---TCTGGTATAGTGCAACAGCAGAGC---
AATTTG---CTGAGG---GCTATTGAGGCGCAACAGCATCTGTTGCAACGCACAGTCTGGGGCATC
>HQ122397
TTCTACTGTAATTTAACAAAACTGTTTAATAGTACTTGGAATGTTAATAGTACTTGGAATGGTACTGAAAGGT
CAAATAGC---
-------------------AAT---
GCAGAAAATGACACAATCACACTCCCATGCAGAATAAAACAAATTATAAACATGTGGCAGGAAGTAGGAAAAG
CAATGTATGCCCCTCCCATTAGAGGACAAATTAGCTGTTCATCAAAT---
ATTACAGGGCTGATATTAACAAGAGATGGTGGTAATAATGGAAGC----------------------------
----

GAGACCACCGAAATCTTCAGACCTGGAGGAGGAGACATGAGGGACAATTGGAGAAGTGAATTATATAAATATA
AAGTAGTAAAAATTGAACCATTA---GGAGTAGCACCCACCAGGGCAAAGAGAAGAGTGGTG-----------
----------------------------CAGAGAGAAAAAAGAGCA---GTGGGA---ATAGGAGCT------

```
---ATGTTCCTTGGGTTCTTGGGAGCA---GCAGGAAGCACTATG---GGCGCAGCGTCA---
CTGACGCTGACGGTACAG---GCCAGACAA---TTATTG---TCTGGTATAGTGCAACAGCAGAGC---
AATCTG---CTGAGG---GCTATTGAGGCGCAACAGCATCTGTTGCAACTCACAGTCTGGGGCATC
>JN002029
TTCTACTGTAATACAACACAACTGTTTAATAGTACTTGGAATGATACTGAAGAGTCAAAGGGCATTGAA----
-------------------------------------------------------------------------
----------------------------
GGAAATGACACAATCACACTCCCATGCAGAATAAAACAAATTGTAAACATGTGGCAGGAAGTAGGAAAAGCAA
TGTATGCCCCTCCCATCAGAGGACAAATTAGATGTTCATCAAGT---
ATTACAGGGTTAATATTAACAAGGGATGGTGGTGATACCGAGAAC----------------------------
-------------------------------------------------------------------------
-------------
GGGACAGAGATCTTCAGACCTGGAGGAGGAGATATGAGGGACAATTGGAGAAGTGAATTATATAAATATAAAG
TAGTAAAAATTGAACCATTA---GGAATAGCACCCACCAAGGCAAAGAGAAGAGTGGTG--------------
-------------------------CAGAGAGAAAAAAGAGCA---GTGGGA---ATAGGAGCT---------
GTGTTCCTTGGGTTCTTGGGAGCA---GCAGGAAGCACTATG---GGCGCAGCGTCA---
GTGACGCTGACGGTACAG---GCCAGACTA---TTATTG---CCTGGTATAGTGCAGCAACAGAAC---
AATTTG---CTGAGG---GCTATTGAGGCGCAACAGCATCTGTTGCAACTCACAGTCTGGGGCATC
>U36882
TTCTACTGTAATACAACACAGCTGTTTAATAGTACTTGGCTGTCTAATGGTGCTAGGAAGGAAGAGTCAAATA
AC-----------------------------------------------------------------------
----------------------------
AATGAAACAACCATCATACTCCCATGCAGAATAAAACAAATTATAAACAGGTGGCAGGAAGTAGGAAAAGCAA
TGTATGCCCCTCCCATCAGCGGATATATTAACTGTTCATCAAAT---
ATTACAGGGCTAATATTAACAAGAGATGGTGGTACTACTAACAATACC-------------------------
-------------------------------------------------------------------------
----------
AATGCCACCGAGATTTTCAGACCTGGAGGAGGAAATATGAAGGACAATTGGAGAAGTGAATTATATAGATATA
AAGTAGTAAAAATTGAGCCATTA---GGAGTAGCACCAACCAAAGCAAAGAGAAGAGTGGTG-----------
----------------------------CAGAGAGAGAAAAGAGCA---GTGGGAACGCTAGGAGCT------
---ATGTTCCTTGGTTTCTTGGGAGCA---GCAGGAAGCACTATG---GGCGCAGCGTCA---
CTGACGCTGACGGTACAG---GCCAGACAA---TTATTG---TCTGGTATAGTGCAACAACAGAAC---
AATCTG---CTGAGA---GCTATTGAGGCGCAACAGCATCTGTTGCAACTCACAGTCTGGGGCATC
>DQ207940
TTCTACTGTGATACAAAACAATTGTTTAATAGTACTTGGAATTCTAATGATACTTGGAATTCTAATGGTACGA
GG-----------------------------------------------------------------------
-------------------------
AATGATTCAGAAATTATCACACTCCCATGTAGAATAAGACAAATTGTAAACATGTGGCAAGAAGTAGGAAAAG
CAATGTATGCCCCTCCAATCAAAGGACAAATTAAATGTGTATCAAAT---
ATTACAGGAATAATACTAACAAGAGATGGTGGTGATAATGAGACT----------------------------
-------------------------------------------------------------------------
----------
AGAGGCAATGAGACTTTCAGACCTGGAGGAGGAGATATGAGGAACAATTGGAGAAGTGAATTATATAAATATA
AAGTAGTAAGAATTGAACCATTA---GGAATAGCACCCACCCAGGCAAGGAGAAGAGTGGTG-----------
----------------------------CAAAGAGAAAAAAGAGCA---GTGGGAACGCTAGGAGCT------
---ATGTTCCTTGGGTTCTTAGGAGCA---GCAGGAAGCACTATG---GGCGCAGCGTCA---
ATGACGCTGACGGTACAG---GCCAGACTA---TTATTG---TCTGGTATAGTGCAGCAGCAGAAC---
AATCTA---CTGAGG---GCTATTGAGGCGCAACAGCATCTGTTGCAACTCACAGTCTGGGGCATC
>HQ385456
TTCTACTGCAATTCATCACAATTGTTTAATAGTACTTGGAATGCTACTGATACTGGGAATACTAGTATTAGGA
AT-----------------------------------------------------------------------
----------------------------
GAGGCTGAGAATATCACACTCCCTTGCAGAATAAAACAAATTATAAACATGTGGCAGGAAGTAGGAAAAGCAA
TGTATGCCCCTCCCATCAGAGGACAAATTAGATGTGTATCAAAT---
ATCACAGGACTGCTATTAACAAGAGATGGTGGTGACAAGAACACT----------------------------
-------------------------------------------------------------------------
-------------
ACCACCGAGACCTTCAGACCTGGAGGAGGAAATATGAAGGATAATTGGAGAAGTGAATTATATAAATATAAAG
TAGTAAGAATTGAACCATTA---GGAGTAGCACCCACCAAGGCAAAGAGAAGAGTGGTG--------------
-------------------------CAGAGAGAAAAAAGAGCA---GTGGGA---ATAGGAGCT---------
```

ATGTTCCTTGGGTTCTTGGGAGCA---GCAGGAAGCACTATG---GGCGCAGCGTCA---
GTGACGCTGACGGTACAG---GCCAGACAA---TTATTG---TCTGGTATAGTGCAACAGCAAAAC---
AATTTG---CTGAGG---GCTATTGAAGCGCAACAGCATATGTTGCAGCTCACAGTCTGGGGCATC
>EU839603
TTCTACTGTAATACAACAAAACTGTTTAATAGTACCTGGAACAGTACTATGAATGGAACTACAGAAGGAGAT-
----

GACACGGAAGTTATCATACTCCCATGCAGAATAAAACAAATTATAAACATGTGGCAGAAAGTAGGCAAAGCAA
TGTATGCCCCTCCCATTGCAGGAGAAATTAGATGTTCATCAAAT---
ATTACAGGGCTGCTATTAACAAGAGATGGTGGTAATAATGCCACC--------------------------
----

ACCGAGACCTTCAGACCTGGAGGAGGAAATATGAAGGACAATTGGAGAAGTGAATTATATAAATATAAAGTAG
TAAAAATTAAACCATTA---GGAGTAGCACCCACCAAGGCAAAGAGAAGAGTGGTG----------------
---------------------CAGAGAGAAAAAAGAGCA---GTGGGAACACTAGGAGCT---------
GTGTTCCTTGGGTTCTTGGGAGCA---GCAGGAAGCACTATG---GGCGCAGCGTCA---
ATGACGCTGACGGTACAG---GCCAGACAA---TTATTG---TCTGGTATAGTGCAACAGCAGAAC---
AATTTG---CTGAGG---GCTATTGAGGCGCAACAGCATTTGTTGCAACTCACAGTCTGGGGCATC
>EU839601
TTCTACTGTAATACAACAAGGCTGTTTAATAGTACTTGGAATGATGCTACACTAGACAGTGCTGGGAATAGTA
CTGAA--

GGGCCAGATAAAAATATCACACTCCCATGCAGAATAAAACAAATTATAAACATGTGGCAGGGAGTAGGCAAAG
CAATGTATGCCCCTCCTATCAGCGGACAAATTGCATGTTCATCAAAA---
ATTACAGGGATGATATTAACAAGAGATGGTGGGCCTGAGAATGAA--------------------------
----

ACTGAGATCTTCAGACCTGGAGGAGGAAATATGATGGACAATTGGAGAAGTGAATTATATAAATATAAAGTAG
TAAAAATTGAACCATTA---GGAGTAGCACCCACCAAGGCAAAGAGAAGAGTGGTG----------------
---------------------CAGAGAGAAAAAAGAGCA---GTGGGAACACTAGGAGCT---------
GTGTTCCTTGGGTTCTTGGGAGCA---GCAGGAAGCACTATG---GGCGCAGCGTCA---
ATGACGCTGACGGTACAG---GCCAGACAA---TTATTG---TCTGGTATAGTGCAACAGCAGAAT---
AATCTG---CTGAGG---GCTGTTGAGGCGCAACAGCATCTGTTGCAACTCACAGTCTGGGGCATC
>MH078551
TTCTACTGTAACACAACAAAGCTGTTTAATAGTACTTGGACTGGAAATGAGACAGAGAATAGTCTAAGG----
----

TCAAATGGCACAATCACACTCCCATGCAGGATAAAACAAATTGTAAACATGTGGCAGGAAGTAGGCAAAGCAA
TGTATGCCCCTCCCAGAAGAGGAATGATCAACTGTACATCAAAC---
ATTACAGGGCTACTATTGACAAGAGATGGTGGTACTAACAGTACT--------------------------
----

AATGAGACCTTCAGACCTGGAGGAGGAGATATGAGGGACAATTGGAGAAGTGAACTATATAAATATAAAGTAG
TAAAAATTGAACCATTA---GGAGTAGCACCCACCAGGGCGAAAAGAAGAGTGGTG----------------
---------------------CAGAGAGAGAAAAGAGCA---GTGGGA---CTAGGAGCT---------
CTGTTCCTTGGGTTCTTGGGAACA---GCAGGAAGCACTATG---GGCGCAGCGTCA---
ATAACGCTGACGGTACAG---GCCAGACAA---TTATTG---TCTGGTATAGTGCAACAGCAGAGC---
AATTTG---CTGAGG---GCTATTGAGGCGCAGCAGCATATGTTGCAACTCACGGTCTGGGGCATC
>MK303352
TTCTACTGTAATACAACAAAACTGTTTAACAATGATTCAACACAACTGCCTAATGAG---------------
----

ACTAATGAGAATATCATACTTCCATGCAGAGTAAAACAAATTATAAACATGTGGCAGAGAGTAGGAAAAGCAA
TGTACGCCCCTCCCATCAGAGGACAAATTAGATGTTCATCAAAT---
ATTACAGGGCTGCTATTAACAAGAGATGGGGGTAATGAGAACAAC--------------------------
----

AACAGTAACCCTGAGATCTTCAGACCTGCAGGAGGAGATATGAGGGATAATTGGAGAAGTGAATTATATAAAT
ATAAAGTAGTGCAAATTGAACCATTA---GGAGTAGCACCCACCAAAGCAAAGAGAAGAGTGGTG--------
------------------------------CAGAGAGAAAAAAGAGCG---GTGGGA---CTAGGAGCT---

------TTGTTCCTTGGGTTCTTGGGAGCA---GCAGGAAGCACTATG---GGCGCAGCATCA---
ATAACGCTGACGGTACAG---GCCAGGCAA---TTATTG---TCTGGGATAGTGCAACAGCAGAAT---
AATCTG---CTGAGG---GCTATTGAGGCGCAGCAGCATCTGTTGCAACTCACAGTCTGGGGCATC
>MK303348
TTCTACTGTAATTCATCACAATTGTTTAATAGTACTTGGAATAGTACTAGGAATAATGATACTAGTACTAGGA
ATAATGATACA--

GAGTCAGGAGTTATCACACTCCAATGCAGAATAAAACAAATTATAAACATGTGGCAGAGAGTAGGAAAAGCAA
TGTATGCCCCTCCCATCAAAGGAAAGATTAGCTGCTCATCAAAT---
ATTACAGGGCTGCTATTAACAAGAGATGGTGGTAATGAGACCAAA----------------------------
----

AACAACACTGAGATCTTCAGACCTGCAGGAGGAAATATGAAGGATAATTGGAGAAGTGAATTATATAAATATA
AAGTAGTAAAAATTGAACCATTA---GGAATAGCACCCACCAAGGCAAAGAGACGAGTGGTG-----------
----------------------------CAGAGAGAAAAAAGAGCA---GTGGGA---CTAGGAGCT------
---TTGTTCCTTGGGTTCTTGGGAGCA---GCAGGAAGCACTATG---GGCGCAGCGTCA---
ATAACGCTGACGGTACAG---GCCAGACAA---TTATTG---TCTGGTATAGTGCAACAGCAGAAC---
AATCTG---CTGAGG---GCTATTGAGGCGCAGCAGCATCTGTTGCAACTCACAGTCTGGGGCATC
>MK303350
TTCTACTGTGATACAACAAAACTGTTTAATAGTACTTGGAATGGTACTGCTTGGAATGGTACGGAGGAT----
----

GATAACGGTACTATCACACTCCCCTGTAGAATAAAACAAGTTATAAAAATGTGGCAGAGAGTAGGAAAAGCAA
TATATGCCCCTCCCATTGCAGGACAAATTAACTGTACATCAAAT---
ATTACAGGGCTGCTATTACTAAGAGATGGTGGAAGTGGTAGTAACAATAGCATT-------------------
----
-
GTTACCAACAACAACACTGAGATCTTCAGACCTGGAGGAGGAGATATGAGAGACAATTGGAGAAGTGAATTAT
ACAAATATAAAGTAGTAGAAATTAAACCATTA---GGAGTAGCACCCACCAAGGCAAAGAGAAGAGTGGTG--
-------------------------------------CAGAGAGAAAAAAGAGCA---GTGGGG---
ATAGGAGCA---------TTGTTCCTTGGGTTCTTGGGAGCA---GCAGGAAGCACTATG---
GGCGCAGCGTCA---ATAACGCTGACGGCACAG---GCCAGACAA---TTATTG---
TCTGGTATAGTGCAACAGCAGAAC---AATCTG---CTGCAG---
GCTATTGAGGCGCAACAGCATCTGTTGCAACTCACAGTCTGGGGCATC
>MK303351
TTCTACTGTAACACAACAGGACTGTTTAATAGTAGTTCAATACAACTGCTTAATGAAACTTGGAATAGTACTG
GGAAT--

GAAAATGAAAATATCACACTCCCATGCAGAATAAAACAAATTGTAAACATGTGGCAAAGAGTAGGAAAAGCAA
TGTATGCCCCTCCCATCAGTGGACAAATTAGATGTTCGTCAAAT---
ATTACAGGGCTGCTATTAACAAGAGATGGGGGTAACAACACCAAC----------------------------
----

AACAACAACACAGAGATCTTCAGACCTGTAGGAGGAAATATGAAGGATAATTGGAGAAGTGAATTATATAAAT
ATAAAGTAGTACAAATTGAACCATTA---GGAGTAGCACCCACCAAGGCAAAGAGAAGAGTGGTG--------
-------------------------------CAGAGAGAAAAAAGAGCA---GTGGGA---CTAGGAGCT---
------TTGTTCCTTGGGTTCTTGGGAGCA---GCAGGAAGCACTATG---GGCGCAGCGTCA---
ATGACGCTGACGGTACAG---GCCAGGCAA---TTATTG---TCTGGGATAGTGCAACAGCAGAAC---
AATCTG---CTGAGG---GCTATTGAGGCGCAGCAGCATCTGTTGCAACTCACAGTCTGGGGCATC
>MK303353
TTCTACTGTAATACATCACAGCTGTTTAATAGTATTTGGTTTAATGGTACCTGGAATGGTACTAGATCAAAT-
----
-AGGACTGGGGATGATACT------
ATATCAAATAACACTATCACTCTTCCATGTAGGATAAAACAAATTATAAATAGATGGCAGGAGGTAGGGAAAG
CAATGTATGCCCCTCCCATCAGGGGACTAATTCGATGTTCATCAGAC---
ATTACAGGGCTGCTATTAACAAGAGATGGAGGTGAAGGTACAGAG----------------------------
----

AAGGACAACACGACTGAGGTCTTCAGACCAGGAGGAGGAGATATGAGGGACAATTGGAGAAGTGAATTATATA
AATATAAAGTAGTAAAAGTTGAACCAATA---GGAATAGCACCCACCAAGGCACAGAGAAGAGTGGTG-----

----------------------------------CAGAGAGAAAAAAGAGCA---GTAACG---
CTGGGAGCT---------GTGTTCCTTGGGTTCTTGGGAGCA---GCAGGAAGCACTATG---
GGCGCAGCGTCG---ATAACGCTGACGGCACAG---GCCAGACAA---TTATTG---
TCTGGTATAGTGCAACAGCAAAAC---AATTTG---CTGAGA---
GCTATTGAGGCGCAACAGCACATGTTGCAACTCACAGTCTGGGGCATC
>MK303354
TTCTACTGTAATACATCAGCACTGTTTAATAGTACCTGGAATAATACTTTGACTAATACTACAGGGTCAAAT-
----

GACACTGACACTATCACACTCCCATGCAGAATAAAACAAATTATAAACATGTGGCAGGAAGTAGGAAAAGCAA
TGTATGCCCCTCCCATCAGCGGACACATTAACTGTGTATCAAAT---
ATTACAGGGCTGATATTAACAAGAGATGGTGGTAATTCTAGCAAC----------------------------
----

GAGACCTTCAGACCCGCAGGAGGAGATATGAGGGACAATTGGAGAAGTGAATTATATAAATATAAAGTAGTAA
AAATTAAACCATTA---GGAATAGCACCCACCGAGGCAAAGAGAAGAGTGGTG--------------------
-------------------CAGAGAGAAAAAAGAGCA---GTGGGA---ATGGGAGCT---------
TTGTTCCTTGGGTTCTTGGGAGCA---GCAGGAAGCACTATG---GGCGCAGCGTCA---
ATGACGCTGACGGTACAG---GCCAGACAG---CTATTG---TCTGGTATAGTGCAACAGCAAAAC---
AATTTG---CTGAGA---GCTATTGAGGCGCAACAACATATGTTGCAACTCACAGTCTGGGGCATC
>MK303355
TTCTACTGTAATACATCAAAACTGTTTAATAATAATATTACTGCTGAT-------------------------
----

GAGAATGGGACTATCATACTCCCATGCAGAATAAGACAAATTGTAAACATGTGGCAGAGAGTAGGAAAAGCAA
TGTATGCCCCTCCCATCAGTGGACTAATTAGATGCTCATCAAAT---
ATTACGGGGCTGCTATTAACAAGAGATGGTGGTAACAGGATCAACAGCACTGAT-------------------
----
AACAGCACTATTGGGTACAGCAATGACAAC---
AACACTGAGACCTTCAGACCTGGAGGAGGAAATATGAAAGATAATTGGAGAAGTGAATTATATAAATATAAAG
TAGTAAAAATTGAACCATTA---GGAGTAGCACCCACCAAGGCAAAGAGAAGAGTGGTG--------------
-------------------------CAGAGAGAAAAAAGAGCA---GTGGGA---CTAGGAGCT---------
TTGTTCCTTGGGTTCTTGGGAGCA---GCAGGAAGCACTATG---GGCGCAGCGTCA---
ATAACGCTGACGGTACAG---GCCAGACAA---TTATTG---TCTGGTATAGTGCAACAGCAGAAC---
AATCTG---CTGAGG---GCTATTGAGGCGCAGCAACATCTGTTGCAACTCACAGTCTGGGGCATC
>GU191372
TTCTACTGTAATACAACCCCACTGTTTAATAGTACATGGAATGACACTAAACAATTCACTGTAAGTAAT----
----

AGCACCGAAGTTATCACACTCCCATGCAGAATAAAACAAATTATAAACATGTGGCAGGAAGTAGGAAAAGCAA
TGTATGCCCCTCCTATCAGAGGACAACTTAGCTGCTCATCAAAG---
ATTACAGGGCTGATACTAACAAGGGATGGTGGTGGTACCGAGTCT----------------------------
----

AAGGAAAACGAGACATTCAGACCTGCAGGAGGAGATATGAGGGACAATTGGAGAAGTGAATTATATAAATATA
AAGTAGTAAAAGTTGAACCATTA---GGAATAGCACCCACCAAGGCAAAGCGAAGAGTGGTG-----------
----------------------------CAGAGAGAAAAAAGAGCA---GTAACA---ATAGGAGCT------
---ATGTTCCTTGGGTTCTTGGGAGCA---GCAGGAAGCACTATG---GGCGCAGCGTCA---
ATGACGCTGACGGTACAG---GCCAGGCAA---TTATTG---TCTGGTATAGTGCAACAGCAGAGC---
AATTTG---CTGAGA---GCTATTGAGGCGCAACAGCATATGTTGCAACTCACAGTCTGGGGCATC
>JF680936
TTCTACTGTAATACAACAAAACTGTTTAATAGTACTTGGAATGGTACTGAATCATCAAACACCACC-------
----

ACCACTGGAAGCATCACGCTCCCATGCAAAATAAGACAAATTGTAAACATGTGGCAGGAAGTAGGAAAAGCAA
TGTATGCCCCTCCTATCAGAGGAAAGATTAGTTGTTCATCAAAT---
ATTACAGGGATACTATTAACAAGAGATGGTGGTAATGATAATGGC----------------------------
----

AGTAACCCTGAAATCTTCAGACCTGGAGGAGGAGATATGAGGGACAATTGGAGAAGTGAATTATTTAAATATA

AAGTAGTAAAAATTGAACCATTA---GGAATAGCACCATCTAAGGCAAGAAGAAGAGTGGTG-----------
----------------------------CAGAGAGAAAAAAGAGCA---GTGGGA---TTTGGAGCC------
---TTGTTCCTTGGGTTCTTGGGAGCA---GCAGGAAGCACTATG---GGCGCAGCGTCA---
ATGGCGCTGACGGTACAG---GCCAGACAA---TTATTG---TCTGGTATAGTGCAACAGCAAAAC---
AATTTG---CTGAGA---GCTATTGAGGCGCAACAGCATATGTTGCAACTCACAGTCTGGGGCATT
>KF061032
TTCTACTGTGATTCAACACAACTGTTTAATAGTACTTGGAATAGTACTTGGAATAGTACTGGAGGGTCAGAA-
----

GAAAATGACACAATCACACTCCCATGCAGAATAAAACAAATTATAAACATGTGGCAGGAAGTAGGAAAAGCAA
TGTATGCCCCTCCCATCAGAGGACAAATTAGATGCTCATCAGAT---
ATTACAGGGCTGATATTAACAAGAGATGGTGGTAATAACGAGAAC----------------------------
----

GGGTCTGAGATCTTCAGACCTGGAGGAGGAGATATGAGGGACAATTGGAGAAGTGAATTATATAAATATAAAG
TAGTAAAAATTGAACCATTA---GGAATAGCACCCACCAAGGCAAAGAGAAGAGTGGTG--------------
-------------------------CAGAGAGAAAAAAGAGCA---GCA------CTAGGAGCT---------
ATGTTCCTTGGGTTCTTGGGAGCA---GCAGGAAGCACTATG---GGCGCAGCGTCA---
GTAACGCTGACGGTACAG---GCCAGACTA---TTATTG---TCTGGTATAGTGCAACAGCAGAAC---
AATTTG---CTGAGG---GCTATTGAGGCGCAACAGCACCTGTTGCAACTCACAGTCTGGGGCATC
>KF061031
TTCTACTGCAATTCAACACAACTGTTTAATAGTACATGGAATGTTACTGAAGGGTCAAATAACACT-------
----

GCAGGAGAAAATATCACACTCCCATGCAGAATAAAACAAATTATAAACATGTGGCAGGAAGTAGGAAAAGCAA
TGTATGCCCCTCCCATCAGAGGACAAATTAAATGCTCATCAAAT---
ATTACAGGGCTGATATTAACAAGAGATGGTGGTAATAGCAGTAAC----------------------------
----

ACGACCGAGATCTTCAGACCTATAGGAGGAGATATGAGAGACAATTGGAGAAGCGAATTATATAAATATAAAG
TAGTAGAACTTGAACCATTA---GGAGTAGCACCCACCAAGGCAAAGAGAAGAGTGGTG--------------
-------------------------CAGAGAGAAAAAAGAGCA---GTGGGA---CTAGGAGCT---------
TTGTTCCTTGGGTTCTTGGGAGCA---GCAGGAAGCACTATG---GGCGCAGCGTCA---
ATAACGCTGACGGTACAG---GCCAGACTA---TTATTG---TCTGGTATAGTGCAACAGCAAAAT---
AATTTG---CTGAGA---GCTATTGAGGCGCAACAGCATATGTTGCAACTCACAGTCTGGGGCATT
>AY835445
TTCTACTGTAATACAACAAAACTGTTTAATAGTACCTGGAATGGGAATAATACTACAGAATCAGATAGC----
----

ACAGGAGAAAATATCACACTCCCATGCAGAATAAAACAGATTATAAACCTGTGGCAAGAAGTAGGAAAAGCAA
TGTATGCCCCTCCTATCAAAGGACAAATTAGTTGTTCATCAAAT---
ATTACAGGGCTACTATTAACAAGAGATGGTGGAAATAACAATAGC----------------------------
----

AGCGGGCCCGAGACCTTTAGACCTGGAGGAGGAAATATGAAGGACAATTGGAGAAGTGAATTATATAAATATA
AAGTAATAAAAATTGAACCATTA---GGAGTAGCACCCACCAGGGCAAAGAGAAGAGTGGTG-----------
----------------------------CAAAGAGAAAAAAGAGCA---GTGGGAACACTAGGAGCT------
---ATGTTCCTTGGTTTCTTGGGAGCA---GCAGGAAGCACTATG---GGCGCAGCGTCA---
GTGACGCTGACGGTACAA---GCCAGACTA---CTGTTG---TCTGGTATAGTGCAGCAGCAGAAC---
AATTTG---CTGAGA---GCTATTGAGGCGCAACAGCATATGTTGCAACTCACAGTCTGGGGCATT
>AY835444
TTCTACTGTAATACAACAAAACTGTTTAATAGTACTTGGGATGCTAATGGTAATTGCACCGGTTGTGATGAAA
GT---

GATGGAAATAACACTATCACACTTCCATGCAGAATAAAACAAATTGTAAACATGTGGCAGGAAGTAGGCAAAG
CAATGTATGCCCCTCCCATCAAGGGACTAATTAAATGTACATCAAAC---
ATTACAGGGCTGCTATTAACAAGAGATGGTGGTGCTAATAATACA----------------------------
----

AACGAGACCTTCAGACCTGGAGGAGGAGATATGAGGGACAATTGGAGAAGTGAATTATATAAATATAAAGTAG

TACAAATTGAACCATTA---GGAATAGCACCCACCAGGGCAAGGAGAAGAGTGGTG-----------------
----------------------CAGAGAGAAAAAAGAGCA---GTGGGAACACTAGGAGCT---------
ATGTTCCTTGGGTTCTTGGGGGCA---GCAGGAAGCACTATG---GGCGCAGCGTCA---
GTGACGCTGACGGTACAG---GCCAGACAA---TTGTTG---TCTGGTATAGTGCAACAGCAGAAC---
AATCTG---CTGAAA---GCTATTGAGGCGCAACAGCATATGTTGCAACTCACGGTCTGGGGCATC
>EU839605
TTCTACTGTAATACAGCACAACTGTTTAATAGTACTTTTAATGGTACTCTTAGTCCAGGGGAG----------
----

AATACTACCACTATCACACTCCCATGCAGAATAAAACAAATTATAAACAGGTGGCAGGAAGTAGGCAAAGCAA
TGTATGCCCCTCCCATCAAAGGACTAATTAGATGTTTATCAAAT---
ATTACAGGGCTGCTATTAACAAGAGATGGTGGTAATAATAGTGAT----------------------------
----

GCTAATGAGACCTTCAGACCACAAGGAGGAAATATGAAAGACAATTGGAGAAGTGAATTGTATAAATATAAAG
TAGTAAGAATTGAACCATTA---GGAATAGCACCCACCAGAGCAAGGAGAAGAGTGGTG--------------
-------------------------CAGAGAGAAAAAAGAGCA---GTGGGAGTAATAGGAGCT---------
ATGTTCCTTGGGTTCTTGGGAGCA---GCAGGAAGCACTATG---GGCGCAGCGTCG---
CTGACGCTGACGGTACAG---GCCAGACAA---TTATTG---TCTGGTATAGTGCAACAGCAGAAC---
AATCTG---CTGAAG---GCTATTGAGGCGCAACAACATCTGTTGCAACTCACGGTCTGGGGCATC
>JF680925
TTCTACTGTAACACAACAGAGCTGTTTAATAGTACTTGGAATGCTAATGGTTCCTGGACGGGTACTGAAAAGA
ATGATACT---
-------------AATACT------
AACAAAACAGGCATAATCACACTCCCATGCAAAATAAAGCAGATTATAAACATGTGGCAGGAAGTAGGAAAAG
CAATGTATGCCCCTCCCATCCGAGGACCGATTTCCTGTACATCAAAT---
ATTACAGGACTACTGTTAACAAGAGATGGTGGTAGTAATGAGAGC----------------------------
----

ACAGAGATCTTCAGACCTGGAGGAGGAGATATGAGGGACAATTGGAGAAGTGAATTATATAAGTATAAAGTAG
TAAAAGTTGAACCATTA---GGAGTAGCACCTTCCAAGGCAAGGAGGAGAGTGGTG-----------------
----------------------CAGAGAGAAAAAAGAGCA---GTGGGA---ATAGGAGCT---------
GTGTTCCTTGGGTTCTTGGGGGCA---GCAGGAAGCACTATG---GGCGCAGCGTCA---
ATGACGCTGACGGTACAA---GCCAGACTA---CTACTG---TCTGGTATAGTGCAACAGCAGAAC---
AATCTG---CTGAGA---GCTATTGAGGCGCAACAACATTTGTTGCAACTCACAGTCTGGGGCATC
>HM030564
TTCTACTGTGATACAAAAGGGCTATTCAATAGCACATTCAATAGCACGTGGAATGCTACAGAAGATACTGGAC
TGCCA--
-------------------AAT---
AGCACTAGCACTAATATTACACTCCCATGCAGAATAAAACAAATTATAAACACGTGGCAGGGAGTAGGCAAGG
CAATGTATGCCCCTCCCATCCAAGGACTAATTAGATGTAGATCAAAT---
ATTACAGGGATACTATTGACAAGAGATGGTGGGAGTGGAAATAGTAGT-------------------------
----

GATAATACAGAGATCTTCAGACCGGGAGGAGGAGATATGAGGGACAATTGGAGAAGTGAATTATATAAATATA
AAGTAGTACAAATTGAACCATTA---GGAGTAGCACCCACCAAGGCAAAGAGAAGAGTGGTG-----------
----------------------------CAGAGAGAAAAAAGAGCA---GTGACA---CTGGGAGCT------
---CTGTTCCTTGGGTTCTTGGGAGCA---GCAGGAAGCACTATG---GGCGCAGCGTCA---
ATGACGCTGACGGTACAG---GCCAGACTA---CTATTG---TCTGGTATAGTGCAACAGCAGAAC---
AATTTG---CTGAGA---GCTATTGAGGCGCAACAGCATCTGTTGCAACTCACAGTCTGGGGCATT
>HM030559
TTCTACTGCAATACAACACAACTATTTAATAGTACTTGGAATATTGGGTTAAATAACACAAATGACACTGTAG
AA---

AATGATACTAATATCATACTACCGTGCAAAATAAAACAAATAGTGAACATGTGGCAGGAAGTAGGCAAAGCAA
TGTATGCCCCTCCCATCAATGGAGTAATTAAATGTTCATCAAAT---
ATTACAGGACTGTTATTAACCAGAGATGGTGGTAACACCACCGGGATTGAGAAC-------------------
----

AATATTAGCAATACTGAGGTCTTCAGACCTGGAGGAGGAAATATGAGGGACAATTGGAGAAGTGAATTATATA

AATATAAAGTAATAAAAATTGAACCATTA---GGAATAGCACCTACCAGGGCAAAAAGGAGAGTGGTG-----
----------------------------------CAGAGAGAAAAAAGAGCA---GTAGGG---
CTAGGAGCT---------ATGTTCCTTGGGTTCTTGGGAGCA---GCAGGAAGCACTATG---
GGCGCAGCGTCA---ATAACGCTGACGGTACAG---GCCAGACAA---TTGCTG---
TCTGGTATAGTGCAACAGCAGAAC---AATCTG---CTGAGA---
GCTATTGAGGCGCAACAACATATGTTGCAACTCACGGTCTGGGGCATC
>HM030562
TTCTATTGTGACTCAACACAACTGTTTAATAGCACTTGGACTAAGAATGATAGTACTTGGACTTTGAATGACA
AAGAG--
-------------------TCA---
AATAGAACTGAGAATATCACACTCCCATGCAGAATAAAACAAATTGTAAACCTGTGGCAGGAGGTAGGAAAAG
CAATGTATGCCCCTCCCATCAAAGGACCCATTAAATGCGAATCAAAT---
ATTACAGGGATGCTCTTAACAAGAGATGGTGGTAATAACGAGAGC----------------------------
----

ACAAATGAAACCTTCAGACCTGGAGGAGGAGATATGAGGGACAATTGGAGAAGTGAATTATATAAATATAAAG
TAGTACAGATTGAGCCATTA---GGATTAGCACCCACCAAGGCAAAGAGAAGAGTGGTG--------------
-------------------------CAGAGAGAAAAAAGAGCA---GTGGGA---ATAGGAGCT---------
TTGTTCCTTGGGTTCCTGGGAGCA---GCAGGAAGCACTATG---GGCGCAGCGTCA---
CTAACGCTGACGGTACAG---GCCAGATCA---TTATTG---TCTGGTATAGTGCAGCAGCAGAAC---
AATCTG---CTGAGG---GCTATTGAGGCGCAACAGCATCTGTTGAGACTCACAGTCTGGGGCATC
>HM030565
TTCTATTGTAACACAACACCGCTGTTTAATAGTATACACAAGGAAAATGACACACTCACTAACGAGACTGAA-
----

GGAAATGACACACTCACACTCCAATGCAGAATAAAACAAATCATAAACATGTGGCAGAGAGTAGGAAAAGCAA
TGTATGCCCCTCCCATCAGCGGATGGATTAGATGTTCATCAAAT---
ATTACAGGACTGCTATTAACAAGAGATGGGGGTAATAATAATAGCACT-------------------------
----

AATGAGACCGAGACCTTCAGACCGGGAGGAGGAGATATGAGGGACAATTGGAGAAGTGAATTATATAAATATA
AAGTAGTAAAAATTGAACCATTA---GGAGTGGCACCCACCAAGGCAAAGAGAAGAGTGGTG-----------
----------------------------CAGAGAGAAAAAAGAGCA---GTGGGA---GTAGGAGCT------
---ATGGTCCTTGGGTTCTTGGGAGCA---GCGGGAAGCACTATG---GGCGCAGCGTCA---
TTGACGCTGACGGTACAG---GCCAGGCTA---TTACTG---TCTGGTATAGTGCAACAGCAAAAC---
AATTTG---CTGAGG---GCTATTGAGGCGCAACAACATCTGTTGCAACTCACAGTCTGGGGCATC
>AB428551
TTCTACTGTAATACATCATCACTGTTTAATTGGAGTACTAATGGGTCAAATATTACTAATGGG----------
----

TCAAATATTACCAAAACCATCCCATGCAGAATAAAACAAATTATAAACATGTGGCAGAAAGTAGGAAAAGCAA
TGTATGCCCCTCCCATCAGAGGACAAATTAATTGTTTATCAAAT---
ATTACAGGGCTGCTATTAACAAGAGATGGTGGTAACCAGACTGAGTTT-------------------------
----

AATAATACTGAGATCTTCAGACCTGGAGGAGGAGATATGAGGGACAATTGGAGAAGTGAATTATATAAATATA
AAGTAGTAAAAATTGAACCATTA---GGAGTAGCACCCACCAAGGCAAGGAGAAGAGTGGTG-----------
----------------------------CAAAGAGAAAAAAGAGCA---GTGGGAACAATAGGAGCT------
---ATGTTCCTTGGGTTCTTGGGAGCA---GCAGGAAGCACTATG---GGCGCAGCGTCA---
TTGACGCTGACGGTACAG---GCCAGACTA---TTATTG---TCTGGTATAGTGCAACAGCAGAAC---
AATCTG---CTGAGG---GCTATTGAGGCGCAACAACATCTGTTGCAACTCACAGTCTGGGGCATC
>AB289588
TTCTACTGTAATTCAACACCACTGTTTAATAGTACTTGGAATTTTACTAATACTTGGAATATGACTGAAGGCA
TAACT--

AACACTGAAGGAAATATCACACTCAAATGCAGAATAAAACAAATTATAAACATGTGGCAGGAAGTAGGAAAAG
CAATGTATGCCCCTCCCATCGAGGGACTAATTAGATGTACATCAAAT---
ATTACAGGGCTGCTATTAACAAGAGATGGGGGGAATAATAGGACA----------------------------
----

AATGAGACTGAGACCTTCAGACCTGGAGGAGGAGACATGAGGAACAATTGGAGAAGTGAATTATATAAATATA
AAGTAGTAAAAATTGAACCATTA---GGAGTAGCACCCACCAGGGCAAGGAGAAGAGTGGTG-----------
----------------------------CAGAGAGAGAAAAGAGCA---GTGGGAGTACTAGGAGCT------
---ATGTTCCTTGGGTTCTTGGGAGCA---GCAGGAAGCACTATG---GGCGCAGCGTCA---
TTAACGCTGACGGTACAG---GCCAGACAA---TTATTG---TCTGGTATAGTGCAACAGCAAAAC---
AATTTG---CTGAGA---GCTATTGAGGCGCAACAGCAGTTGTTGCAACTCACTGTCTGGGGCATC
>AB289590
TTTTACTGTAATACAACACAATTGTTTAATAGTACTTGGAATGAAACAAACCTGTTTAATAATACTGGGAATA
AT---

ACTGAGGGTAATACTATCACACTCCCATGCAGAATAAAACAGATTATAAACATGTGGCAGGAAGTAGGAAAAG
CAATGTATGCCCCTCCTATCAGAGGACAAATTAAATGTTCATCAAAC---
ATTACAGGGCTGCTATTAACAAGAGATGGTAGTACTAATGACACA----------------------------
----

AACAATACTGAGATATTCAGACCTACAGGAGGGAATATGAGGGACAATTGGAGAAGTGAATTATATAAATATA
AAATAGTACAAATTGAACCATTA---GGAGTAGCACCTACCAAGGCAAAGAGAAGAGTGGTG-----------
----------------------------CAGAGAGAAAAAAGAGCA---GTGGGA---CTAGGAGCT------
---ATGTTCCTTGGGTTCTTGGGAGCA---GCAGGAAGCACTATG---GGCGCAGCGTCA---
ATAACGCTGACGGTACAG---GCCAGACAA---TTATTG---TCTGGTATAGTGCAACAGCAAAAC---
AATTTG---CTGAGA---GCTATTGAGGCGCAACAGAATCTGTTGCAACTCACAGTCTGGGGCATT
>AB428558
TTCTACTGCAATACAACAGAACTGTTTAATAGTACTTGGATTAATGGTACTAGGACAACCAACACTACG----
----

TCAAATGGCACTATCACACTCCGATGTAGAATAAAACAAATTATAAACATGTGGCAGAGAGTAGGAAAAGCAA
TGTATAACCCTCCCATCGCAGGACAAATTAGATGTTCATCAAAT---
ATTACAGGGATATTATTAGTGAGAGATGGTGGTGATAACAGTACG----------------------------
----

ACCAACGAAACCTTCAGACCTGGAGGAGGGAATATAAGAGACAATTGGAGAAGTGAATTATATAAATATAAAG
TAGTAAAAATTGAACCATTA---GGAGTAGCACCCACCAAGGCAAAGAGAAGAGTGGTG--------------
-------------------------CAGAGAGAAAAAAGAGGA---GTGGGA---ATAGGAGCT---------
GTGTTCCTTGGGTTCTTGGGAGCA---GCAGGAAGCACTATG---GGCGCAGCGTCA---
ATAACGCTGACGGTACAG---GCCAGACAA---TTATTG---TCTGGTATAGTGCAACAGCAGAAC---
AATTTG---CTGAGG---GCCATTGAGGCGCAACAGCATATGTTGCAACTCACAGTCTGGGGCATC
>AB221126
TTCTACTGTAATACAACACAGCTGTTTAATAGCACTTGGACTTGGAATAGTACTACGGGCACTTGGAATAATA
CTGGGAGC---
-------------------AGG---
AGTGAGAATGACACTATCACACTCCCATGCAGAATAAAACAAATTATAAACAGGTGGCAGGAAGTAGGAAAAG
CAATGTATGCCCCTCCCATCCAAGGACAAATTAACTGTTCATCAAAT---
ATTACAGGACTGCTATTAACAAGAGATGGTGGTAACGAAACTGAG----------------------------
----

AACGGGTATGATACCTTCAGACCTGGAGGAGGAGATATGAGGGATAATTGGAGAAGTGAATTATATAAATATA
AAGTGGTACAAATTGAGCCATTA---GGAATAGCACCCACCAGGGCAAAGAGAAGAGTGGTG-----------
----------------------------CAGAGAGAAAAAAGAGCA---GTGGGAACGCTAGGAGCT------
---GTGTTCCTTGGGTTCTTGGGAGCA---GCAGGAAGCACTATG---GGCGCAGCGTCA---
CTAACGCTGACGGTACAG---GCCAGACTA---TTACTG---TCTGGTATAGTGCAACAGCAGAAC---
AATCTG---CTGAAA---GCTATTGAGGCGCAACAGCATCTGTTGCAACTCACAGTCTGGGGCATC
>AB480697
TTCTACTGTAACACATCAGCACTGTTTAATAGTACTTGGAATAATAACACTTGGTATGTAAATGGCACTGAAG
GGTCA--
-------------------AAT---
GACACTGATAAAAAATATCACCCTCCCATGCAGAATAAAACAATTTATAAACATGTGGCAGGCAGTAGGAAAAG
CAATATATGCCCCTCCTATTAAAGGACAAATTAGCTGTTCATCAAAT---
ATTACAGGGCTGCTATTAACAAGAGATGGTGGTGAAGGTAACGAAACT-------------------------
----

GACAACCAGACTGAAGTCTTCAGACCTGGAGGAGGGGATATGAGGGACAATTGGAGAAGTGAATTATACAAAT
ATAAAGTAGTAAAAATTGAACCATTA---GGAGTAGCACCCACCAAGGCAAAGAGAAGAGTGGTG--------
--------------------------------GAAAGAGAAAAAAGAGCA---ATAGGGGAATAGGAGCT---
------TTGTTCCTTGGGTTCTTGGGAGCA---GCAGGAAGCACTATG---GGCGCAGCGTCA---
CTGACGCTGACGGTACAG---GCCAGACTA---TTATTG---ACTGGTATAGTGCAACAGCAGAAC---
AATTTG---CTGAGG---GCTATTGAGGCGCAACAGCATCTGTTGCAACTCACAGTCTGGGGCATC
>AB480695
TTCTATTGTAATACATCAGGACTGTTTAATAGTACTTGGGAGTTTAATAGTACTTGGAATGATAAT-------
----

AGTACTGAAAAAATCGTACTCCCATGCAGAATAAGACAAATTGTAAGCATGTGGCAGGGAGTAGGAAAAGCAA
TGTATGCCCCTCCCATTAAAGGACAAATTAGCTGTTCATCAAAT---
ATTACAGGGCTGCTATTAACAAGAGATGGTGGTGGTAATGGTACT----------------------------
----

GAGACCTTCAGACCTGAAGGAGGAAATATGAAGGACAATTGGAGAAGTGAATTATATAAATATAAAGTAGTAA
AACTTGAGCCTTTA---GGAATAGCACCTACTCCAGCAAGGAGAAGAGTGGTG--------------------
-------------------CAGAGAGAAAAAAGAGCA---GTGGGA---TTCGGAGTT---------
TTGTTCCTAGGGTTCTTGGGAGCA---GCCGGAAGCACTATG---GGCGCAGCGTCA---
ATGGCGCTGACGGTACAG---ACCAGACAA---TTGTTG---TCTGGTATAGTGCAACAGCAGAAC---
AATCTG---CTGAGG---GCTATTGAGGCGCAACAACATCTGTTGCAACTCACAGTCTGGGGCATT
>AB287363
TTCTACTGTAATTCAACACAGCTGTTTAATAGTACTTGGCATGGAAACATATCAAATGTGTCAAATAACATTG
GA---

AAAAATAGCAACATCACACTCCCGTGTAGAATAAGACAAATTGTAAACATGTGGCAGACAGTAGGAAAAGCAA
TGTATGCCCCTCCCATCAGCGGATACATTAAATGTACATCAAAT---
ATTACAGGACTGCTATTGACAAGAGATGGTGGAAAGGGAAATAAC----------------------------
----

ACGGACACGGAGGTCTTCAGACCTGGAGGAGGAGATATGAGGGACAATTGGAGAAGTGAATTATACAAATATA
AAGTAGTAAAAATTGAACCATTA---GGAATAGCACCCACCAAGGCAAGGAGAAGAGTGGTG-----------
----------------------------CAGAGAGAAAAAAGAGCA---GTAATA------GGAGCT------
---ATGTTCCTTGGGTTCTTGGGAGCA---GCAGGAAGCACTATG---GGCGCAGCGTCA---
GTGACGCTGACGGTACAG---GCCAGGCAA---TTATTG---TCTGGTATAGTGCAACAGCAGAAC---
AATCTG---CTGAAA---GCTATTGAGGCGCAACAGCATCTGTTGCAACTCACAGTCTGGGGCATC
>AB287364
TTCTACTGCAATACAACAAAATTGTTTAATAGTACATGGATCTGGAATAAAACACTAATGGTTAATGACACAA
GA---

GAGTCAAATGACATTATCAGGTTCCAATGCAGAATAAAACAGATTATAAACATGTGGCAGGAAGTAGGAAGAG
CAATGTATGCCCCTCCCATCGAAGGACTAATTAGATGCTCATCAAAT---
ATTACAGGGCTGATATTAACAAGAGATGGTGGTAATAGCAAAAAC----------------------------
----

GATACAGAAACCTTCAGACCTGCAGGAGGAAATATGAAGGACAATTGGAGAAGTGAATTATATAAATATAAAG
TAGTACAAGTTGAACCATTA---GGAATAGCACCCACCAGGGCAAAGAGAAGAGTGGTG--------------
-------------------------CAAAGAGAGAAAAGAGCC---GTGGGA---TTAGGAGCT---------
TTGTTCCTTGGGTTCTTGGGAGCA---GCAGGAAGCACTATG---GGCGCAGCGTCA---
CTAACGCTGACGGTACAG---GCCAGACAA---TTATTG---TCTGGTATAGTGCAACAGCAAAAC---
AACTTG---CTGAGG---GCTATTGAGGCGCAACAACATCTGTTGCAACTCACAGTCTGGGGCATC
>AB588283
TTCTACTGTAATACAACAAAGCTGTTTAATAGTACTTGGCATCCTAATGGGAGTATGATTGCAGAACCAAATA
AC---

ACTAACAACATTATTACACTCCCATGCAGAATAAAACAAATTATAAACAGGTGGCAGGAGGTAGGAAAAGCAA
TATATGCCCCTCCCATCCAAGGACAAATTAACTGTTCATCAAAT---
ATTACAGGACTGCTATTAACAAGAGATGGTGGTAAGGAGGATAAGGAG-------------------------
----

AATAGGTCACAGGAGACCTTCAGACCTGGAGGAGGAGATATGAGGGACAATTGGAGAAGTGAATTATATAAGT
ATAAAGTAGTACAAATTGAGCCATTA---GGAGTAGCACCCACCAGGGCAAAGAGAAGAGTGGTG--------
-------------------------------CAAAGAGAAAGAAGAGCA---GTGGGG---ATAGGAGCT---
------ATGTTCCTTGGGTTCTTGGGAGCA---GCAGGAAGCACTATG---GGCGCAGCGTCA---
CTAACGCTGACGGTACAG---GCCAGACTA---TTATTG---TCTGGTATAGTGCAACAGCAAAAT---
AATCTG---CTGAGA---GCTATTGAGGCGCAACAGCATATGTTGCAACTCACAGTCTGGGGCATT
>AB588296
TTCTACTGTAACACAACACAACTGTTTGATAGTACTTGGAATAGTACTTTAAATATTACTGAGAGGTCAAAT-
----

GACTCTGGTATAATCACCCTCCCATGCAGAATAAAACAATATATAAATTTGTGGCAGGAAGTAGGAAGAGCAA
TGTATGCCCCTCCCATCAGTGGACAAATTAAATGTTCATCAAAT---
ATTACAGGGCTGTTATTAACAAGAGATGGTGGTAAGGGGAACGAAAAC-------------------------
----

AATATCACTGAGACCTTCAGACCTGGAGGAGGAAACATGAGGGACAATTGGAGAAGTGAATTATACAAATATA
AAGTAGTAAAAATTGAACCATTA---GGAATAGCACCCACCAGGGCAAAGAGAAGAGTGGTG-----------
----------------------------CAGAGAGAAAAAAGAGCA---GTGGGA---CTGGGAGCT------
---ATGTTCCTTGGGTTCTTGGGAGCA---GCAGGAAGCACTATG---GGCGCAGCGTCA---
GTGACGCTGACGGTACAG---GCCAGACAG---TTATTG---TCTGGTATAGTGCAACAGCAGAAC---
AATTTG---CTGAGG---GCTATTGAGGCGCAACAACATCTGTTGCAACTCACAGTCTGGGGCATC
>AB287367
TTCTACTGTAATACAACAGGGCTATTTAATGATAGTATGCTGAATGATACTACT-------------------
----

GAAGGTAACAACCTCACACTCCCGTGCAGAATAAAACAAATTATAAACAGATGGCAGGAAGTAGGAAAAGCAA
TGTATGCCCCTCCCATCCAAGGACAAATTAGATGTTCATCAAAT---
ATTACAGGGTTGCTATTAACAAGAGATGGAGGAAATAGTGGCAATAAA-------------------------
----

ACTAATAATACTGAGACTTTCAGACCTGGAGGAGGAAATATGAAGGATAATTGGAGAAGTGAATTATATAAAT
ATAAAGTAGTAAAAATTGAACCATTA---GGAGTAGCACCCACCAAGGCAAGAAGAAGAGTGGTG--------
-------------------------------CAAAGAGAAAAAAGAGCA---GTGGGAACGCTGGGAGCT---
------ATGTTCCTTGGGTTCTTGGGAGCA---GCAGGAAGCACTATG---GGCGCAGCGTCA---
ATGGCGCTGACGGTACAG---GCCAGACAA---TTATTG---TCTGGTATAGTGCAACAGCAGAAC---
AATCTG---CTGAAA---GCTATTGAGGCGCAACAGCATATGTTGCAACTCACAGTCTGGGGCATC
>AB287368
TTCTACTGTAATACAACACCACTGTTTAATAGTAGCTGGTATGAAAATGGAAGTAGTATCTGGTATAGCATAA
AT---

AACACAACTGAAAACATCACACTCCCATGCAGAATAAAACAAATTATAAACATGTGGCAGGGAGTAGGAAAAG
CAATGTATGCCCCTCCCATCGCGGACAAATTAAATGTTCATCAAAT---
ATTACAGGACTGCTATTAACAAGAGATGGGGGTGGTGATGGTAAT----------------------------
----

AATGATAGTGAGATCTTCAGACCTGAAGGAGGAAATATGAAGGACAATTGGAGAAGTGAGTTATATAAATATA
AAGTAGTAAAAATTGAACCATTA---GGAATAGCACCCACCAAGGCAAGGAGAAGAGTGGTG-----------
----------------------------CAGAGAGAAAAAAGAGCA---GTG---ACGATGGGAGCT------
---ATGTTCCTTGGGTTCTTGGGAGCA---GCAGGAAGCACTATG---GGCGCAGCATCA---
ATGACGCTGACGGTACAG---GCCAGACTA---TTATTG---TCTGGTATAGTGCAACAGCAAAAC---
AACTTG---CTGAGA---GCTATTGAGGCGCAACAGCATCTGTTGCAGCTCACGGTCTGGGGCATT
>AB588311
TTCTACTGTAATACATCAGACTTGTTTAATAATGACACTTGGAATAACCCTAACAGTACA-------------
----

AAAGGAGAGAATATCACCCTCCAATGCAGAATAAAACAAATTATAAACATGTGGCAGGAAGTAGGAAGAGCAA
TGTATGCTCCTCCCATCAGCGGACCAATTAGATGTTCATCAAAT---
ATTACAGGGCTGATACTAACAAGAGATGGTGGTACTAACCAGACC----------------------------
----

```
ACCGAGATCTTCAGACCTGGAGGAGGAGATATGAGGGACAATTGGAGAAGTGAATTATACAAGTATAAAGTAG
TAAAAATTGAACCATTA---GGACTAGCACCCACCAAGGCAAAGAGAAGAGTGGTG-------------------
----------------------CAGAGAGAAAAAAGAGCA---GTGGGA---ATAGGAGCT---------
TTGTTCCTTGGGTTCTTGGGAGCA---GCAGGAAGCACTATG---GGCGCAGCGTCA---
TTGACGCTGACGGTACAA---GCCAGACTA---TTATTG---TCTGGTATAGTGCAACAGCAAAAC---
AATTTG---CTGAGG---GCTATTGAGGCGCAACAGCACATGTTGCAACTCACAGTCTGGGGCATC
>AB731663
TTCTACTGTAATTCAACACAACTGTTTAATAGTACTTGGCTGCTTAATAGTACTTGGAATAGTAATGATACAA
AT-------------------------------------------------------------------------
-------------AGCATT------
GGAGAAAATGACACTATCATACTCCCATGCAGAATAAAACAAATTATAAACATGTGGCAGGAAGTAGGAAAAG
CAATGTATGCCCCTCCTAGAGAAGGACCAATTAGATGTTCATCAAAC---
ATTACAGGGCTGATATTAACAAGAGATGGTGGTAGTAATGGGTAT------------------------------
---------------------------------------------------------------------------
-------------
AACAATGAGACCTTCAGACCTGGAGGAGGAGATATGAGGGACAATTGGAGAAGTGAATTATATAAATATAAAG
TAATAAAAATTGAACCATTA---GGAGTAGCACCAACCAAGGCAAAAAGGCGAGTGGTG----------------
-------------------------CAGAGAGAAAAAAGAGCA---GTGGGA---CTAGGAGCC---------
TTGCTCGTTGGGTTCTTGGGAGCA---GCAGGAAGCACTATG---GGCGCAGCGTCA---
CTGACGCTGACGGTACAG---GCCAGACAA---TTACTG---TCTGGTATAGTGCAACAGCAAAAC---
AACTTG---CTGAGG---GCTATTGAGGCGCAACAACATCTGTTGCAACTCACAGTCTGGGGCATC
>AB731667
TTTTACTGTAATACAACACCATTGTTTAACAGTACTTGGAATGGAACAAATCTGACTACTTGGAAT-------
---------------------------------------------------------------------------
----------------------------
ACTGGAGCAAATATCACACTCCAATGCAAAATAAAACAGATTATAAACATGTGGCAGCAAGTAGGAAAAGCAA
TGTATGCCCCTCCCATCAGAGGACAAATTAGATGTTCATCAAAT---
ATTACAGGGCTGCTATTAACAAGAGATGGT---AATACTAATAGCACA--------------------------
---------------------------------------------------------------------------
----------
AACAATACTGAGATCTTCAGACCTACAGGAGGGAATATGAAGGACAATTGGAGAAGTGAATTATATAAATATA
AAATAGTACAAATTGAACCATTA---GGAGTAGCACCTACCAAGGCAAAGAGAAGAGTGGTG-----------
----------------------------CAGAGAGAAAAAAGAGCA---GTGGGA---CTAGGAGCT------
---ATGTTCCTTGGATTCTTGGGAGCA---GCAGGAAGCACTATG---GGCGCAGCGTCA---
ATAACGCTGACGGTACAG---GCCAGACAA---TTATTG---TCTGGTATAGTGCAACAGCAAAAC---
AACTTG---CTGAGA---GCTATTGAGGCGCAACAGAATATGTTGCAACTCACAGTCTGGGGCATC
>KF716497
TTCTACTGCGATACAACACAACTATTTAATAGTACCTGGGATAATAGTACTATGAATAGTACTAAGAAT----
---------------------------------------------------------------------------
----------------------------
TCAAATGACACTATCACGCTCCCATGTAGAATAAAACAAATTATAAACATGTGGCAGACAGTAGGAAAAGCAA
TGTATGCCCCTCCCATCGCAGGACCAATTAGATGTTCATCAAAT---
ATTACAGGGTTGTTATTAGAGAGAGATGGTGGTATTAACAAGACG------------------------------
---------------------------------------------------------------------------
-------------
ACCACAGAAACCTTCAGACCTGGGGGAGGAAATATGAAGGACAATTGGAGAAGTGAACTATATAAATATAAAG
TAGTAAAAATTGAACCATTA---GGAGTAGCACCCACCAAGGCAAAGAGAAGAGTGGTG----------------
-------------------------CAGAGAGAAAAAAGAGCA---GTGGGA---ATAGGAGCT---------
GTGTTCCTTGGGTTCTTGGGAGCA---GCAGGAAGCACTATG---GGCGCAGCGTCA---
ATGGCGCTGACGGTACAG---GCCAGACAA---TTATTG---TCTGGTATAGTGCAACAGCAGAAC---
AATTTG---CTGAGA---GCCATTGAGGCGCAACAGCATATGTTGCAACTCACAGTCTGGGGCATC
>KF716498
TTCTACTGCAATACAACAAAACTGTTTAATAGTACTTGGAATAATAGTAATTGGCCAAATGACACTATGCCA-
---------------------------------------------------------------------------
----------------------------
AATAATAACACTATCACACTCCCATGTAGAATAAAACAAATTGTAAACATGTGGCAGAAAGTAGGAAAAGCAA
TGTATAACCCTCCCATCGCAGGACTAATTAGATGTTCATCAAAT---
ATTACAGGGCTATTATTAGTGAGAGATGGTGGAACACAACGAATACA--------------------------
---------------------------------------------------------------------------
-------------
```

ACCACCGAAACCTTCAGACCTGGAGGAGGAGATATGAGGGACAATTGGAGAAGTGAATTATATAAATACAAAG
TAGTAAAAATTGAACCATTA---GGAGTAGCACCCACTAAGGCAAAGAGAAGAGTGGTG--------------
-------------------------CAGAGAGAAAAAAGAGCA---GTGGGG---ATAGGAGCT---------
GTGTTCCTTGGGTTCTTGGGAGCA---GCAGGAAGCACTATG---GGCGCAGCGTCA---
CTAATGCTGACGGTACAG---GCCAGACAA---TTATTG---TCTGGTATAGTGCAACAGCAGAAC---
AATCTG---CTGAGG---GCCATTGAGGCGCAACAGCATATGTTGCAACTCACAGTCTGGGGCATC
>AB588201
TTCTACTGTAACTTATCACAACTGTTTAATAGTTCTTGGAATGTAAATGGTACTGAAGGGTCAAAT-------
----

AACACTGGAATTATCACACTCCCATGCAGAATAAAACAAATTATAAACATGTGGCAGGAAGTAGGAAAGGCAA
TGTATGCCCCTCCCATCAGAGGACAAATTAGATGTTCATCAAAT---
ATTACAGGGCTGCTATTAACAAGAGATGGTGGTATTAACAAGAGCGAG-------------------------
----

AGCACCAACACCGAGGTCTTCAGACCTGCAGGAGGAAATATGAAGGACAATTGGAGAAGTGAATTATATAAAT
ATAAAGTAGTAAAAATTGAACCATTA---GGAGTAGCACCCACCAAGGCAAAGAGAAGAGTGGTG--------
-------------------------------CAGAGAGAAAAAAGAGCA---GTGGGA---CTAGGAGCT---
------ATGTTCCTTGGGTTCTTGGGAGCA---GCAGGAAGCACTATG---GGCGCAGCGTCG---
TTGACGCTGACGGTACAG---GCCAGACTA---TTATTG---TCTGGTATAGTGCAACAGCAGAGC---
AATTTG---CTGAGG---GCTATTGAGGCGCAACAGCATCTGTTGCAACTCACAGTCTGGGGCATC
>AB588207
TTCTACTGTGATACATCACGACTGTTTAATAGTACTTGGAATGAGACTACTAAAGGGTTAAATATTACTGGG-
----

GAAAATGACACAATCACAATCCCATGCAGAATAAAACAAATTATAAACATGTGGCAGGAAGTAGGGAAAGCAA
TGTATGCCCCTCCCATCAGTGGAGTAATTAGATGTTCATCAAAT---
ATTACAGGGCTGCTATTAACAAGAGATGGTGGTAACGGTAACGGGAGC-------------------------
----

AATACCACTGAGATCTTCAGACCTATAGGAGGAGATATGAGGGACAATTGGAGAAGTGAATTATATAAATATA
AAGTAGTAAAACTTGAACCATTA---GGAATAGCACCCACCAAGGCAAAGAGAAGAGTGGTG-----------
----------------------------CAGAGAGAAAAAAGAGCA---GTGGGA---ATGGGAGCT------
---ATGTTCCTTGGGTTCTTGGGAGCA---GCAGGAAGCACTATG---GGCGCAGCGTCA---
ATAACGCTGACGGTACAG---GCCAGACAA---TTATTG---TCTGGTATAGTGCAACAGCAGAGC---
AATTTG---CTGAGG---GCTATTGAGGCGCAACAGCATATGTTGCAACTCACAGTCTGGGGCATC
>AB588209
TTCTACTGTAATACAACACAGCTGTTTAATAGTACTTGGACTTGGACTGGGAATAATACTGAAGGAAATAAC-
----

ACTGAATACAATATCACACTCCCATGCAGAATAAAACAAATTATAAACAGGTGGCAGGAAGTAGGAAAAGCAA
TGTATGCCCCTCCCATCAAAGGACAAATTAGTTGTTCATCAAAT---
ATTACAGGGCTGCTATTAACAAGAGATGGTGGTGGTGGCAATAAC----------------------------
----

GAGTCCGAGACCTTCAGACCTGGAGGAGGAGATATGAGGGACAATTGGAGAAGTGAATTATATAAATATAAAG
TAGTACAAATTGAGCCATTA---GGAGTAGCACCCACCAAGGCAAAGAGAAGAGTGGTG--------------
-------------------------CAAAGAGAAAAAAGAGCA---GTGGGA---ATAGGAGCT---------
ATGTTCATTGGGTTCTTGGGAGCA---GCAGGAAGCACTATG---GGCGCAGCGTCA---
GTAACGCTGACGGTACAG---GCCAGACTA---TTATTG---TCTGGTATAGTGCAACAGCAGAAC---
AATCTG---CTGAAA---GCTATTGAGGCGCAACAGCATCTGTTGCAACTCACAGTCTGGGGCATC
>AB588211
TTCTACTGTAATACAACAAAGCTATTTAATAATAGTATGCTGTCTTCTAATAATACTGGAGGA----------
----

TCAAATAAGACTATCACAATCCCATGCAGAATAAAACAAATTATAAACAGGTGGCAGGAAGTAGGAAAAGCAA
TGTATGCCCCTCCCATCAAAGGACAAATTAGCTGTTCATCAAAT---
ATTACAGGACTGCTATTAACAAGAGATGGTGGTGGCGGGAACGGT----------------------------
----

TCCGAGACCGAGACCTTCAGACCTGCAGGAGGAGATATGAGGGACAATTGGAGAAGTGAATTATATAAATATA
AAGTAGTAAAAATTGAGCCATTA---GGAGTAGCACCCACCAAGGCAAAGAGAAGAGTGGTG-----------
----------------------------CAGAGAGAAAAAAGAGCA---GTGGGAACACTGGGAGCT------
---ATGTTCCTTGGGTTCTTGGGAGCA---GCAGGAAGCACTATG---GGCGCAGCGTCA---
CTAACGCTGACGGTACAG---GCCAGACTA---TTATTG---TCTGGTATAGTGCAACAGCAGAAC---
AACATG---CTGAGA---GCTATTGAGGCGCAACAGCACCTGTTGCAACTCACAGTCTGGGGCATC
>AB588233
TTCTACTGTAATTCAACACCACTGTTTAATAGTGCTTGGAATTTTACTAGTACTTGGAATATTATTGAAGGAT
CA---

GATAACACTGAAAATATCACACTCCAATGCAGAATAAAACAAATTATAAACATGTGGCAGGAAGTAGGAAAAG
CAATGTATGCCCCTCCCATCCAGGGACAAATTAACTGCACATCAAAT---
ATTACAGGACTGTTATTAACAAGAGATGGGGGTAAGGATGAGAAT----------------------------
----

AAGACGGAGGAGACCTTCAGACCTGGAGGAGGAGATATGAGGGACAATTGGAGAAGTGAATTATATAAATATA
AAGTAGTAAAAATTGAACCATTA---GGAATAGCACCCACCAAGGCGAGGAGAAGAGTGGTG-----------
----------------------------CAGAGAGAAAAAAGAGCA---GTGGGAGCAATAGGAGCT------
---ATGTTCCTTGGGTTCTTGGGAGCA---GCAGGAAGCACTATG---GGCGCAGCGTCA---
ATAACGCTGACGGTACAG---GCCAGACAA---TTATTG---TCTGGTATAGTGCAACAGCAGAAC---
AATTTG---CTGAGA---GCTATTGAGGCGCAACAGCATCTGTTGCAACTCACTGTCTGGGGCATC
>AB480698
TTCTACTGTAATACAACACAGCTGTTTGATAGTACTTGGCTGTCTAATGGTACTAGGAATGATACTACAGAGT
CA---

GATAGCACTGAGATCATACTCCAGTGCAGAATAAAACAAATTATAAATAGATGGCAGGAAGTAGGAAAAGCAA
TGTATGCCCCTCCCATCAGTGGACAAATTAGATGTACATCAAAT---
ATTACAGGATGATAATAACAAGAGATGGTGGTACCAATGACACG-----------------------------
----

CACGGAGACGAGATCTTCAGACCAGCAGGAGGAGACATGAGGGACAATTGGAGAAGTGAATTATATAAATATA
AAGTAGTAAAAATTGAACCATTA---GGAATAGCACCCACCAAGGCAAAGAGAAGAGTGGTG-----------
----------------------------CAGAGAGGAAAAAGAGCA---GTGGGAACATTAGGAGCT------
---ATGTTCCTTGGGTTCTTGGGAGCA---GCAGGAAGCACTATG---GGCGCAGCGTCG---
CTGACGCTGACGGTACAG---GCCAGACAA---TTATTG---TCTGGTATAGTGCAACAGCAAAAC---
AACATG---CTGAAA---GCTATTGAGGCGCAACAACATATGTTGCAACTCACAGTCTGGGGCATC
>AB564745
TTCTACTGTAATACAACAAAACTGTTTAATAGTACTTGGAATAATACTGAAAGGGTAAATAGCACTATG----
----

GAAAATGGCACAATCACACTCCCATGCAGAATAAAACAAATTATACACATGTGGCAAGAGGTAGGAAAAGCAA
TGTATGCCCCTCCCATCGAAGGAAACATTAGCTGCTCATCAAAT---
ATTACAGGGCTGCTATTAACAAGAGATGGGGGTATTAAGAACGAG----------------------------
----

AACAAGCCCGAGATCTTCAGACCTATAGGAGGAAATATGAAGGACAATTGGAGAAGTGAATTATATAAATATA
AAGTAGTAAAAATTGAACCATTA---GGAATAGCACCCACTAGGGCAAAGAGGAGAGTGGTG-----------
----------------------------CAGAGAGAAAAAAGAGCA---GTGGGA---CTAGGAGCT------
---TTGTTCCTTGGGTTCTTGGGAGCA---GCAGGAAGCACTATG---GGCGCAGCGTCA---
ATGACACTGACGGTACAG---GCCAGACAA---TTATTG---TCTGGTATAGTGCAACAGCAAAAT---
AATTTG---CTGAGG---GCTATTGAGGCGCAACAGCATCTGTTGCAACTCACAGTCTGGGGCATC
>AB604946
TTCTACTGTAACACAACACAACTGTTTAACAGTACTTGGCTGTCTAATGGTACTTTGAATAGTACTGAAACGT
CA---

AATGACACCATTATCACACTCCCATGCAGAATAAAACAAGTTATAAACATGTGGCAGGAAGTAGGAAAAGCCA
TGTATGCCCCTCCCATCAGTGGACAAATTAACTGTACATCAAAT---
ATTACAGGGTTGCTATTGACAAGAGATGGGGGTGAGAATAATACT----------------------------
----

GAAGAGATCTTCAGACCTGGAGGAGGAAATATGAAAGACAATTGGAGAAGTGAATTATACAAATATAAAGTAG
TAAGAATTAAACCATTA---GGAGTAGCACCCACCAAGGCAAAGAGAAGAGTGGTG-----------------
----------------------CAGAGAGAAAAAAGAGAA---CTGACA---CTAGGAGCT---------
TTGTTCCTTGGGTTCTTGGGAGCA---GCAGGAAGCACTATG---GGCGCAGCGTCA---
TTGACGCTGACGGTACAG---GCCAGACAA---TTGCTG---TCTGGTATAGTGCAACAGCAAAAC---
AATTTG---CTGAGG---GCTATTGAGGCGCAGCAGCATCTGTTGCAACTCACAGTCTGGGGCATT
>D12582
TTCTACTGTGATTCATCACAACTGTTTAATAGTACACACCTGTCTAATGGTACTTGGTGGAATGGTACT----
----

GGGCCAGAAAATATCACACTCCCCTGCAGAATAAAACAAATTGTAAACATGTGGCAGGAAGTAGGAAAAGCAA
TGTATGCCCCTCCCATCAGAGGACAAATTAGATGTTCATCAAAT---
ATTACAGGGCTGCTATTAACAAGAGATGGTGGTAACACCCAGAACAATAAC----------------------
----

ACCAATAGCAGTATCGAGATCTTCAGACCTGGAGGAGGAGATATGAGGGACAATTGGAGAAGTGAATTATATA
AATATAAAGTAGTAAAAATTGAACCATTA---GGAGTAGCACCCACCAGGGCAAAGAGAAGAGTGGTG-----
----------------------------------CAGAGAGAAAAAAGAGCA---GTGGGA---
ATAGGAGCT---------GTGTTCCTTGGGTTCTTGGGAGCA---GCAGGAAGCACTATG---
GGCGCAGCGGCA---GTGACGCTGACGGTACAG---GCCAGACAA---TTATTG---
CCTGGTATAGTGCAACAGCAGAAC---AATTTG---CTGAGG---
GCTATTGATGCGCAACAACATCTGTTGCAACTCACAGTCTGGGGCATC
>AB565497
TTCTACTGTAATACAACACAACTATTTAATAGTACTTGGCATTGGAATTGGAATAATTCTACTTGGGAGCGGA
ATGATACTGAA--
----------GGGTCAAAT------
AATACTGAGGACAAAATTACACTCCCATGCAGAATAAAACAAATTATAAACATGTGGCAGGAAGTAGGAAAAG
CAATGTATGCCCCTCCCATCAGAGGACTAATTAGATGTGCATCAAAT---
ATTACAGGGCTGATATTAACAAGAGATGGTGGTGGTGTGAACAGA----------------------------
----

ACCACCGAGACATTCAGACCTGGAGGAGGGAATATGAGGGACAATTGGAGAAGTGAATTATATAAATATAAAG
TAGTAAAAATTGAACCATTA---GGAGTAGCACCCACTAAGGCAAAGAGAAGAGTGGTG--------------
-------------------------CAGAGAGAAAAAAGAGCA---GTGGGA---ATTGGAGCT---------
ATATTCCTTGGGTTCTTGGGAGCA---GCAGGAAGCACTATG---GGCGCAGCGTCA---
GTGACGCTGACGGTACAA---GCCAGACAA---TTACTG---TCTGGTATAGTGCAACAGCAGAGC---
AATCTG---CTGCAG---GCTATTGAGGCGCAACAACAACTGTTGAAACTCACAGTCTGGGGCATC
>AB565502
TTCTACTGTAATACATCAAAACTGTTTAATAGTTCTTGGAATATTAATGGTACTTGGAATGGTACTACA----
----

GGAAATGGCACTCTCACGCTCCCTTGCAGAATAAGACAAATTGTAAACATGTGGCAGCAAGTAGGAAAAGCAA
TGTATGCCCCTCCCGTTGCAGGAATAATTAGATGTTCATCAAAT---
ATTACAGGGATGATATTAACAAGAGATGGTGGTACTAATGAGACC----------------------------
----

ATCGAGGTGGAGACCTTCAGACCTGCAGGAGGAGATATGAGGGACAATTGGCGAAGTGAATTGTATAAATATA
AAGTAGTAAGAATTGAACCATTA---GGAGTAGCACCCACCAAGGCAAGGAGAAGAGTGGTG-----------
----------------------------CAGAGAGAAAAAAGAGCA---GTGGGA---CTAGGAGCT------
---GTGTTCCTTGGGTTCTTGGGAGCA---GCAGGAAGCACTATG---GGCGCAGCATCA---
ATAACGCTGACGGTACAG---GCCAGACAA---TTGCTG---TCTGGTATAGTGCAACAGCAGAGA---
AATTTG---CTGAGA---GCTATTGAGGTGCAGCAGCATCTGTTGCAACTCACAGTCTGGGGCATC
>KT961002
TTCTATTGTAATACAACAAAACTGTTTAATAGTAGTTGGAATGAAACAACACTGTTTAATAATACTTGGAATA
GTACTGAA---

GAGTCAAATAGCACAATCACACTCCCATGCAGAATAAAACAAACTATAAACATGTGGCAGGAAGTAGGAAAAG
CAATGTATGCCCCTCCCATCCGCGGACAAATTAATTGTTCATCAAAT---
ATTACAGGGCTGCTATTAACTAGAGATGGTGGTAATAATAACACG----------------------------
----

AATAAAAACGAGACCTTCAGACCTGGAGGAGGAAATATGAAGGACAATTGGAGAAGTGAATTATATAAATATA
AAGTAGTAAAAATTGAACCATTA---GGAATAGCACCCACCAAGGCAAAAAGAAGAGTGGTG-----------
----------------------------CAGAGAGAAAAAAGAGCA---GCA------ATAGGAGCT------
---ATGTTCCTTGGGTTCTTGGGAGCA---GCAGGAAGCACTATG---GGCGCAGCGGCA---
GTGACGCTGACGGTACAG---GCCAGACAA---TTATTG---TCTGGTATAGTGCAACAGCAAAAC---
AATTTG---CTGAGA---GCTATTGAGGCGCAACAACATATGTTGCAACTCACAGTCTGGGGCATC
>AB742153
TTCTACTGTAGTACAACTAAATTGTTTAATAGTACTTGGAATAGTACTGAGAATTTGAATATTACTACTGAAA
GGTCAAAT---
-------------AGCACT------
GGAGAAAACGACACAATCATACTCCACTGTAGAATAAAACAAATTATAAATATGTGGCAAGAAGTAGGAAAAG
CAATGTATGCCCCTCCCATCAGTGGGCCAATTAACTGCTCATCAAAA---
ATTACAGGGCTGCTGTTAACAAGAGATGGTGGTAGTGGTAATGAGACT-------------------------
----

ACCAATAACACTGAAACCTTCAGACCTGGAGGAGGAGATATGAGGGACAATTGGAGAAGTGAATTATATAAAT
ATAAAGTAATAAAAATTGAACCATTA---GGAATAGCACCCACCAAGGCACAGAGAAGAGTGGTG--------
-------------------------------CAGAGAGAGAAAAGAGCA---GTGGGA---ATAGGAGCT---
------GTGTTCCTTGGGTTCTTGGGAGCA---GCAGGAAGCACTATG---GGCGCAGCGTCA---
GTGACGCTGACGGTACAG---GCCAGACTA---TTATTG---TCTGGTATAGTGCAACAGCAGAAT---
AATCTG---CTGAGA---GCTATTGAGGCGCAACAGCATCTGTTGCAACTCACAGTCTGGGGCATC
>AB641836
TTCTACTGCAATACAACAGAACTATTTAATAGTAGTTGGAATGAGAGTACCTGGAATGAGAGTACTCGGAATG
ATACT--
-------------------GAC---
GGGTCAGGAAATATCATCACACTCCCATGTAGAATAAAACAAATTATAAACATGTGGCAGGAAGTAGGAAAAG
CAATGTATGCCCCTCCCATCAGAGGACAAATTAATTGTACATCAAAC---
ATCACAGGGTTGCTATTAACAAGAGATGGTGGTAATAATAATAGCACC-------------------------
----

AATGAGACGGAGACCTTCAGACCTGGAGGAGGAGACATGAAGGACAATTGGAGAAGTGAATTATATAAATATA
AAGTAGTAAAACTTGAACCATTA---GGAATAGCACCCACCAAGGCGAAGAGAAGAGTGGTG-----------
----------------------------CAGAGAGAAAAAAGAGCA---GTGGGAACAATAGGAGCT------
---ATGTTCCTTGGGTTCTTGGGAGCA---GCAGGAAGCACTATG---GGCGCAGCGTCA---
ATGACGCTGACGGTACAG---GCCAGGCTA---TTATTG---TCTGGTATAGTGCAGCAGCAGAAT---
AATCTG---CTGAAG---GCTATTGAGGCGCAACAGCATCTGTTGCAACTCACAGTCTGGGGCATC
>KU869602
TTCTACTGTAATACAACACAACTGTTTAATAGTACTTGGGATTTTAATAGTACTTGGAATAGTACTGAAGGG-
----

TCAAATATCACACTCACACTCCCATGCAGAATAAAACAAATTATAAACAGGTGGCAGGAAGTAGGAAAAGCAA
TGTATGCCCCTCCCATCAGTGGACAAATTAGATGTTCATCAAAT---
ATTACAGGGCTGCTATTAACAAGAGATGGGGGGAAGACTGGGGAC----------------------------
----

AACGGGAGTGAGATCTTCAGACCTGGGGGAGGAGATATGAGGGACAATTGGAGAAGTGAATTATATAAATATA
AAGTAGTAAAAATTGAACCATTA---GGGATAGCACCCACCGAGGCAAGGAGAAGAGTGGTG-----------
----------------------------CAGAGAGAAAAAAGAGCA---GTGGGAACAATAGGAGCT------
---ATGTTCCTTGGGTTCTTGGGAGCA---GCAGGAAGCACTATG---GGCGCAGCGTCA---
ATGACGTTGACGGTACAG---GCCAGACTA---TTGTTG---TCTGGTATAGTGCAACAGCAGAAT---
AACTTG---CTGAGA---GCTGTTGAGGCGCAACAACATCTGTTGCAACTCACAGTCTGGGGCATC
>MN043590
TTCTACTGTAATACAACACAACTGTTTAATAGTACTTGGGATTTTAATAGTACTTGGAATAGTACTGAAGGG-
----

TCAAATATCACACTCACACTCCCATGCAGAATAAAACAAATTATAAACAGGTGGCAGGAAGTAGGAAAAGCAA
TGTATGCCCCTCCCATCAGTGGACAAATTAGATGTTCATCAAAT---
ATTACAGGGCTGCTATTAACAAGAGATGGGGGGAAGACTGGGGAC----------------------------
----

```
----------
AACGGGAGTGAGATCTTCAGACCTGGGGGAGGAGATATGAGGGACAATTGGAGAAGTGAATTATATAAATATA
AAGTAGTAAAAATTGAACCATTA---GGGATAGCACCCACCGAGGCAAGGAGAAGAGTGGTG-----------
----------------------------CAGAGAGAAAAAAGAGCA---GTGGGAACAATAGGAGCT------
---ATGTTCCTTGGGTTCTTGGGAGCA---GCAGGAAGCACTATG---GGCGCAGCGTCA---
ATGACGTTGACGGTACAG---GCCAGACTA---TTGTTG---TCTGGTATAGTGCAACAGCAGAAT---
AACTTG---CTGAGA---GCTGTTGAGGCGCAACAACATCTGTTGCAACTCACAGTCTGGGGCATC
>MN043603
TTCTACTGTAATACAACAAAACTGTTCAATAGTACTTGGAATAGTACTGGAGAGTTGCTTAATAGTACTGGGA
ATAGTACT-----------------------------------------------------------------
----------------------------
AACAATGGAACTATCATACTCCAATGCAGAATAAAACAAATTATAAACAGGTGGCAGGAAGTAGGAAAAGCAA
TGTATGCCCCTCCCATTGAAGGGATAATTAGATGTTCATCAAAT---
ATTACAGGGCTGCTATTAACAAGAGATGGGGGAATGAAAACGGGACT--------------------------
-------------------------------------------------------------------------
----------
AACAGTACTGAGACCTTCAGACCTGGGGGAGGAAATATGAAGGACAATTGGAGAAGTGAATTATATAAATATA
AAGTAGTAAGAATTGAGCCATTA---GGGATAGCACCCACCGAGGCAAGGAGAAGAGTGGTG-----------
----------------------------CAGAGAGAAAAAAGAGCA---GTGGGAACAATAGGAGCT------
---ATGTTCCTTGGGTTCTTGGGAGCA---GCAGGAAGCACTATG---GGCGCAGCGTCG---
ATGACGCTGACGGTACAG---GCCAGACTA---TTATTG---TCTGGTATAGTGCAACAGCAGAAT---
AATTTG---CTGAGA---GCTATTGAGGCGCAACAGCATCTGTTGCAACTCACAGTCTGGGGCATC
>JQ316128
TTCTACTGTAATACAACAAAACTGTTTAGTAGTACTTGGAATGATACTGAAGGCCCAAATAATGGA-------
-------------------------------------------------------------------------
----------------------------
ACCAATAGAAATATCACACTCCAATGCAGAATAAAACAAATTATCAACAGGTGGCAGGAAGTAGGAAAGGCAA
TGTATGCCCCTCCCATCGCAGGAATAATTAGATGTTCATCAAAT---
ATTACAGGACTGCTATTAACAAGAGATGGGGGAATAATGAGAATGGG--------------------------
-------------------------------------------------------------------------
-------
ACCAGTGGGAATGAGACCTTCAGACCTGGGGGAGGAAATATGAAGGACAATTGGAGAAGTGAATTATATAAAT
ATAAAGTAGTAAAAATTGAGCCATTG---GGGATAGCACCCACCGAGGCAAGGAGAAGAGTGGTG--------
-------------------------------CAGAGAGAAAAAAGAGCA---GTGGGAGCGCTAGGAGCT---
------ATGTTCCTTGGGTTCTTGGGAGCA---GCAGGAAGCACTATG---GGCGCAGCGTCA---
CTGACGCTGACGGTACAG---GCCAGACAA---TTATTG---TCTGGTATAGTGCAACAGCAGAAT---
AATTTG---CTGAGA---GCTATTGAGGCGCAACAGCATCTGTTGCAACTCACAGTCTGGGGCATC
>JQ316132
TTTTACTGTAATACAACACAACTGTTTAATAGTACTTGGAGGGTGAATGGTACTGAAGGGTTAAATGTAACTG
AA-----------------------------------------------------------------------
----------------------------
GGAAATAGACACTTTACCCTCCCATGCAGAATAAAACAAATTATAAACAGGTGGCAGGAAGTAGGAAGAGCAA
TGTATGCCCCTCCCATCGCGGGACAAATTAGATGTTCCTCAAAT---
ATTACAGGACTGCTATTAACAAGAGATGGGGGAACTAAGAACAACAGCAACAAC-------------------
-------------------------------------------------------------------------
-------
GAGACTGATACTGAGATCTTCAGACCTGGGGGAGGAGATATGAGGGACAATTGGAGAAGTGAATTATATAAAT
ATAAAGTAGTAAAAATTGAGCCATTA---GGGATAGCACCCACCGAGGCAAGGAGAAGAGTGGTG--------
-------------------------------CAGAGAGAAAAAAGAGCA---GTGGGAGTGATAGGAGCT---
------ATGTTCCTTGGGTTCTTGGGAGCA---GCAGGAAGCGCTATG---GGCGCAGCGGCA---
ATAACGCTGACGGTACAG---GCCAGACAA---TTATTG---TCTGGTATAGTGCAACAGCAAAAT---
AATTTG---CTGAGA---GCTATTGAGGCGCAACAGCATCTGTTGCAACTCACAGTCTGGGGCATC
>JQ316127
TTCTACTGTAATACAACACAACTGTTTAATAGTACTTGGGAGTTTAATAGTACTGAAGGGTCAAATACAACTG
AAGGA--------------------------------------------------------------------
----------------------------
AATAACACACTTATCACACTCCCATGCAGAATAAAACAAATTATAAACAGGTGGCAGGAAGTAGGAAAAGCAA
TGTATGCCCCTCCCATCAGAGGACAAATTAAATGTTCATCAAAT---
ATTACAGGGTTGCTATTAACAAGAGATGGGGGACTGGAACAACAAG--------------------------
-------------------------------------------------------------------------
```

ACTGACAACGGGACTGAGATCTTCAGACCTGGGGGAGGAGATATGAGGGACAATTGGAGAAGTGAATTATATA
AATATAAAGTAGTAAAAATTGAGCCATTA---GGGATAGCACCCTCCAAGGCAAGGAGAAGAGTGGTG-----
----------------------------------CAGAGAGAAAAAAGAGCA---
GTGGGAACGATAGGAGCT---------ATGTTCCTTGGGTTCTTGGGAGCA---GCAGGAAGCACTATG---
GGCGCAGCGTCA---CTGACGCTGACGGTACAG---GCCAGACTC---TTATTG---
TCTGGTATAGTGCAACAGCAGAAT---AATTTG---CTGAGA---
GCTATTGAAGCGCAACAGCATCTGTTGCAACTCACAGTCTGGGGCATC
>JQ316135
TTTTATTGTAATACAACACAACTGTTTAATAGTACTTGGAATGGTATTACTTTTAATAGAACTTGGGGTGAC-
----

CTTAATAAAAAGATTATATTCCCTTGCAAATTAAAACAAATTATAAACAGGTGCCAGGAAGTAGGAAAAGCAA
TGTATGCCCCTCCCTTCAGAGGACAAATTAGATGTTCATCAAAT---
ATTACAGGGCTGTTATTACCAAGAGATGGGGGGACCAAGAACAAAACTGAC---------------------
----

AAGGCCATTATTAAATTTTTCAGCCTTGGGGGAGGAGATATGAGGGACAATTGGAGAAGTAAATTATATAAAT
ATAAAGTAGTAAAAATTGAGCCATTA---GGGATAGCACCCACCACGGCAAGGAAAAGAGTGGTG--------
-------------------------------CAGAGAGAAAAAAGAGCA---GTGGGAACGATAGGAGCT---
------ATGTTCCTTGGGTTCTTGGGAGCA---GCAGGAAGCACTATG---GGCGCAGCGTCA---
ATGACGCTGACGGTACAG---GCCAGACTA---TTATTG---TCTGGTATAGTGCAACAGCAGAAT---
AATTTG---CTGAAA---GCTATTGAGGCGCAACAGCATCTGTTGCAACTCACAGTCTGGGGCATC
>JQ316130
TTCTACTGTAATACCACACCACTGTTTAATAGTACTTGGCGGTATAATAGTACTGGG----------------
----

GAAGAGGAAACTATCATACTCCCATCCAGGATAAAACAAATTATTATCAGCTGGCAGGAAGTAGGAACAGCAA
CGTATGCCCCTCCCATCAGCGGAATAATTAGCTGTTCATCAAAT---
ATTACAGGGGGGCTATTAACAACAGATGGGGGGAGTGAGAAAAACGGG-------------------------
----

ACTAACGATACTGAGACCTTCAGACCTGGGGGAGGAGATATGAGGGACAATTGGAGAAGTGAATTATATAAAT
ATAAAGTAGTAAAAATTGAGCCATTA---GGGATAGCACCCACCGAGGCAAGGAGAAGAGTGGTG--------
-------------------------------CAGAGAGAAAAAAGAGCA---GTAACA---ATAGGAGCT---
------CTGTTCCTTGGGTTCTTGGGAGCA---GCAGGAAGCACTATG---GGCGCAGCGTCA---
ATGACGTTGACGGTACAG---GCCAGACTA---TTGTTG---TCTGGTATAGTGCAACAGCAGAAT---
AACTTG---CTGAGA---GCTATTGAGGCGCAACAGCATATGTTGCAACTCACAGTCTGGGGCATC
>DQ295195
TTTTACTGTAATACAACACAGCTGTTTAATAGCACTTGGCCTAATAGTAGTGAGAATTCCAATAATACTGCT-
----

GATAATGGCACATTTATCCTCCCCTGCAGAATAAAACAAATTATAATCAGCTGGCAGGAAGTAGGAAAAGCAA
TATATGCCCCTCCCATCAGTGGACTAATTAAATGTTCATCAAAT---
ATTACAGGGCTAATATTAACAAGAGATGGGGGAACTAAAAACAATTGCATT---------------------
----

AACATCACCGGGGAGATCTTCAGACCTGGGGGAGGAAATATGAAGGACAATTGGAGAAGTGAATTATATAAAT
ATAAAGTAGTAAGAATTGAGCCATTA---GGGGTAGCACCCACTGGGGCAAAGAGAAGAGTGGTG--------
-------------------------------CAGAGAGAAAAAAGAGCA---GTGGGAACGATAGGAGCT---
------ATGTTCCTTGGGTTCTTGGGAGCA---GCAGGAAGCACTATG---GGCGTCGCGTCA---
ATGACGCTGACGGTACAG---GCCAGACTA---TTATTGTCTGGCTGTATAGTGCAACAGCAGAAT---
AATCTG---CTGAGA---GCTATTGAGGCGCAACAGCATCTGTTGCAACTCACAGTTTGGGGCATC
>DQ295193
TTCTACTGTAATACAACACAACTGTTTAATAGTACTTGGCTGCTTAATGATACTGGGAATGAGAGTAAACAG-
----

CCAAATGAAACTATCACACTCCCATGCAGAATAAAACAAATTATAAACAGGTGGCAGGAAGTAGGAAAAGCAA
TGTATGCCCCTCCCATCAGCGGACAAATTAGATGTTCATCAAAT---
ATTACAGGGCTGTTGTTAACAAGAGATGGGGGGAATACTAATGAGACT------------------------

----

GGGAATGGGACTGAGACCTTCAGACCTGGGGGAGGAGATATGAGGGACAATTGGAGAAGTGAATTATATAAAT
ATAAAGTAGTACAAATTGAGCCATTA---GGGATAGCACCCACTAAGGCAAGGAGAAGAGTGGTG--------
-------------------------------CAGAGAGAAAAAAGAGCA---GTGGGAGTAATAGGAGCT---
------ATGTTCCTTGGGTTCTTGGGAGCA---GCAGGAAGCACTATG---GGCGCAGCGTCA---
GTGACGTTGACGGTACAG---GCCAGACTA---TTATTG---TCTGGTATAGTGCAACAGCAAAAT---
AATTTG---CTGAGA---GCTATTGAGGCGCAACAGCATCTGTTGCAACTCACAGTCTGGGGCATC
>JQ316126
TTTTACTGTAATACAACAAAACTGTTTAATAGTACTTGGTATGGTAATGGTACTCGGAAGGAAGGGTCAAAT-
----

GAAAATGGAACTATCATACTCCCATGCAGGATAAGACAAATTGTAAACAGGTGGCAGGAAGTAGGAAAAGCAA
TGTATGCCCCTCCCATTACAGGACAAATTAGATGTTCATCAAAT---
ATTACAGGGCTGCTATTAACAAGAGATGGGGGGACTGGGAACGGGACC-------------------------
----

AACACGAATGAGACTTTCAGACCTGGGGGAGGAAATATGAAGGACAATTGGAGAAGTGAATTATATAAATATA
AAGTAGTAAGAATTGAGCCATTA---GGGATAGCACCCACCCAGGCAAGGAGAAGAGTGGTG-----------
----------------------------CAGAGAGAAAAAAGAGCA---GTAACG---CTAGGAGCT------
---ATGTTCCTTGGGTTCTTGGGAGCA---GCAGGAAGCACTATG---GGCGCAGCGTCA---
ATAACACTGACGGTACAG---GCCAGACTA---TTATTG---TCTGGTATAGTGCAACAGCAAAAT---
AATTTG---CTGAGA---GCTATTGAGGCGCAACAGCATCTGTTGCAACTCACAGTCTGGGGCATC
>DQ295196
TTCTACTGTAATACATCACAACTGTTTAATAGAGCTTGGAATGGTTCTGAAAGGTCCAATAACACTGAA----
----

GGAAATACAACTATCACACTCCCATGCAGAATAAAACAAATTATAAACAGGTGGCAAGAAGTAGGAAAAGCAA
TGTATGCCCCTCCCATCAGCGGACAAATTAGATGTTCATCAAAT---
ATTACAGGGCTGTTATTAACAAGAGATGGGGGGAATGGGAACGAGACT-------------------------
----

GAGAACGGGACTGAAATCTTCAGACCTGGGGGAGGAAATATGAGGGACAATTGGAGAAGTGAATTATATAAAT
ATAAAGTAGTAAAAATTGAGCCATTA---GGGATAGCACCCACCAGGGCAAAGAGAAGAGTGGTG--------
-------------------------------CAGAGAGAAAAAAGAGCA---GTGGGAACGATAGGAGCT---
------ATGTTCCTTGGGTTCTTGGGAGCA---GCAGGAAGCACTATG---GGCGCAGCGTCA---
GTAACGCTGACGGTACAG---GCCAGACTA---TTATTG---TCTGGTATAGTGCAACAGCAGAAT---
AATTTA---CTGAAA---GCTATTGAGGCGCAACAGCGTCTGTTGCAACTCACAGTCTGGGGCATC
>AY839827
TTCTACTGTAATACAACACAACTGTTTAATAGTACTTGGTACAGTAATAATACTGAAGGGTCAAATAATACTA
AA---

GGAAATGACACAATTACCCTCCCATGCAGAATAAGACAAATTATAAACAGATGGCAGGAAGTAGGAAAAGCAA
TGTATGCTCCTCCCATCAGTGGACAAATTAGATGTTCATCAAAT---
ATTACAGGGCTGCTGTTAACAAGAGATGGGGGAATCAACAATACA----------------------------
----

ATTAATGAGACCTTCAGACCTGAGGGAGGAGATATGAGGGACAATTGGAGAAGTGAATTATATAAATATAAAG
TAGTAAGAATTGAGCCATTA---GGGATAGCACCCACCGAGTCAAAAAGAAGAGTGGTG--------------
-------------------------CAGAGAGAAAAAAGAACA---GTGGGAACATTAGGAGCT---------
ATGTTCCTTGGGTTCTTGGGAGCA---GCAGGAAGCACTATG---GGCGCAGCGTCG---
ATGACGTTGACGGTACAG---GCCAGACTA---TTATTG---TCTGGTATAGTGCAACAGCAAAAT---
AACTTG---CTGAGA---GCTATTGAGGCGCAACAGCATCTGTTGCAACTCACAGTCTGGGGCATC
>DQ837381
TTCTACTGTAATACAACACAACTGTTTAATGGTAATTGGAGTAATGGTACCTGGAATGATCTAAACAACTCT-
----

AGTTCTATAAATATCACACTTCCATGCAGAATAAAACAAATTGTAAACATGTGGCAGGAAGTAGGAAAAGCAA
TGTATGCCCCTCCCATCAAAGGACAAATTAGCTGTTCATCAAAT---
ATTACAGGGCTGATATTAACAAGAGATGGTGGTAAGGACAATACTACCGGCAGC-------------------

----

AATGAGACAAAAGGGGAGATCTTCAGACCTGCAGGAGGAGATATGAGGGACAATTGGAGAAGTGAATTATATA
AATATAAAGTAGTAAGAATTGAACCATTA---GGAGTAGCACCCACCAAGGCAAGGAGAAGAGTGGTG-----
------------------------------------CAGAGAGAAAAAAGAGCA---GTGACA---
CTGGGAGCG---------ATGTTCCTTGGTTTCTTGGGAGCA---GCAGGAAGCACTATG---
GGCGCAGCGTCA---ATGACGCTGACGGTACAG---GCCAGACAA---TTATTA---
TCTGGTATAGTGCAACAGCAAAAC---AACTTG---CTGAGG---
GCTATTGAGGCGCAACAGCATCTGTTGCAACTCACAGTCTGGGGCATC
>JQ316134
TTCTACTGTAACACAACAAAACTGTTCAATAGTACTTGGAATAGTACTGAGAATGGTACTGGTATTGAACAT-
----

TTAAATGGGACTATCACACTCCCATGCAGAATAAAACAATTTATAAACAGGTGGCAGGAAGTAGGAAAAGCAA
TGTATGCCCCTCCCATCAGAGGAATAATTAGATGTTCATCAAAT---
ATTACAGGGCTGCTATTAACAAGAGATGGGGGGACTGAGAACAACAAAACTGAC-------------------
----

AGCACTGGGACTGACATCTTCGGACCTGGGGGAGGAGATATGAGGGACAATTGGAGAAGTGAATTATATAAAT
ATAAAGTAGTAAGAATTGAGCCATTA---GGGATAGCACCCACCAAGGCAAGGAGAAGAGTGGTG--------
--------------------------------CAGAGAGAAAAAAGAGCA---GTAACG---ATAGGAGCT---
------ATGTTCCTTGGGTTCTTGGGAGCA---GCAGGAAGCACTACG---GCCGCGACGTCG---
ATGACACTGACCGTACAA---GCCATACTA---TTATTG---TCTGGTATAGTGCAACAGCAGAAT---
AATTTG---CTGAGA---GCTATTGAGGCGCAACAACATCTGTTGCAACTCACAGTCTGGGGCATC
>MN043605
TTCTACTGTAGTACAACAAATCTGTTTAATAGTTCTTGGAATGAAACACTGCTTAATAGTACTTGGAATGAGA
AT--

GACAATAAAAATATCACACTCCGATGCAGAATAAAACAAATTATAAACAGGTGGCAGGAAGTAGGAAAAGCAA
TGTATGCCCCTCCCATCAGAGGACAAACTAGATGTTCATCAAAT---
ATTACAGGGCTGCTATTAACAAGAGATGGGGGGCCTGAGAACAAT----------------------------
----

AGCAATGAAATCTTCAGACCTGGGGGAGGAAATATGAAGGACAATTGGAGAAGTGAACTATATAAATACAAAG
TAGTAAAGATAGAGCCATTA---GGGATAGCACCCACCAAGGCAAGGAGAAGAGTGGTG--------------
-------------------------CAGAGAGAAAAAAGAGAA---GTAACA---TTAGGAGCC---------
TTGTTCCTTGGGTTCTTGGGAGCA---GCAGGAAGCACTATG---GGCGCAGCGTCA---
ATGACGCTGACGGTACAG---GCCAGACTA---TTATTG---TCTGGTATAGTGCAACAGCAAAAT---
AATTTG---CTGAGA---GCTATTGAGGCGCAACAGCATATGTTGCAACTCACAGTCTGGGGCATC
>KJ140263
TTCTACTGTGAGACAACAAAACTGTTTAATAGTACTTGGAGGTTTAATAGTACT-------------------
----
-TGGAATGGTACTGAA---------
GAGATAAATGACCCAATTACACTCCCATGCAGAATAAAACAAATTATAAACAGGTGGCAGGAAGTAGGAAAAG
CAATGTATGCCCCTCCCATCAGAGGACAAATTAAATGTTCATCAAAT---
ATTACAGGGCTGCTATTAACAAGAGATGGGGGGACT-------------------------------------
----
GAGAACAACAAGACTGGGAGCAACAGGACTGAGATCTTCAGACCAGGGGGAGGAGATATGAGGGACAATTGGA
GAAGTGAATTATATAAATATAAAGTAGTAAGAATTGAGCCATTA---
GGGATAGCACCCACCAAGGCAAGGAGGAGAGTGGTG-------------------------------------
--CAGAGAGAAAAAAGAGCA---GTGGGAACGATAGGAGCT---------
ATGTTCCTTGGGTTCTTGGGAGCA---GCAGGAAGCACTATG---GGCGCAGCGTCA---
ATGACGCTGACGGTACAG---GCCAGACTA---TTATTG---TCTGGTATAGTGCAACAGCAGAAT---
AATTTG---CTGAGA---GCTATTGAGGCGCAACAGCATCTGTTGCAACTCACAGTCTGGGGCATC
>KT878030
TTCTACTGTAATACATCACAACTGTTTAATAGTACTTGGAATGATACTTGGAGGGGTAGTAAAAGA-------
----

TCCAATGGTACTATCACACTCCCATGCAGAATAAAACAAATTATAAATAGGTGGCAGGAAGTAGGGAAAGCAA
TGTATGCCCCTCCCATCAGTGGACAAATTAGATGTACATCAAAT---

ATTACAGGGCTGATATTAACAAGAGATGGGGGGAATAACAGCGAG----------------------------
----

ACTACAGAGATCTTCAGACCTGGGGGAGGAAATATGAAGGACAATTGGAGAAGTGAATTATATAAATATAAAG
TAGTAAGAATTGAGCCATTA---GGGATAGCACCCACCAAGTCAAAGAGAATAGTGATG--------------
-------------------------CAGAGAGAAAAAAGAGCA---GTGGGAACAATAGGAGCT---------
ATGTTCCTTGGGTTCTTGGGAGCA---GCAGGAAGCACTATG---GGCGCAGCGTCA---
TTGACGCTGACGGTACAG---GCCAGACAA---TTATTG---TCTGGTATAGTGCAACAGCAGAAT---
AATTTG---CTGAGA---GCTATTGAGGCGCAACAACATATGTTGCAGCTCACAGTCTGGGGCATC
>KT878031
TTCTACTGTAATACATCACAATTGTTTAATAGTACCTGGAATGAAACACTGTTGAATAGTACCTGGAAT----
--GAA---
ACACTGTCGAATAGAACCTGG---------------
AATAGTAATGAAACTATCACACTTCCATGCAGAATAAAACAAATTGTAAACAGGTGGCAGGAAGTAGGAACAG
CAATGTATGCCCCTCCCATCAAAGGACAAATTAAATGCTCATCAAAT---
ATTACAGGGCTGTTATTAACAAGAGATGGGGGGAACAACAGCAAC----------------------------
----

AGCAATGAGACCTTCAGACCTGGGGGAGGAGATATGAGGGACAATTGGAGAAGTGAATTATATAAATATAAAG
TAGTAAAAATTGAGCCATTA---GGGATAGCACCCACCAAGGCAAGGAGAAGAGTGGTG--------------
-------------------------CAGAGAGAAAAAAGAGCA---GTGGGAACAATAGGAGCT---------
ATGTTCCTTGGGTTCTTGGGAGCA---GCAGGAAGCACTATG---GGCGCAGCGTCA---
ATGACGCTGACGGTACAG---GCCAGACAA---TTATTG---TCTGGTATAGTGCAACAGCAGAAT---
AACCTG---CTGAGA---GCTGTTGAGGCGCAACAGCATCTGTTGAAACTCACAGTCTGGGGCATC
>KT878032
TTCTACTGTAATACAACACGACTGTTTAATAGTACTTGGAAGAATGGTACACTGATAAATGGAACTGAAGACA
ATAGC--

AATAGCACCTTCATCACACTCCCATGCAGAATAAAACAAATTATAAACAGGTGGCAGGAAGTAGGAAAGGCAA
TGTATGCCCCTCCCATCAGTGGACTAATTAGATGTACATCAAAT---
ATTACAGGGCTGCTATTAACAAGAGATGGGGGGAACGACAACAGT----------------------------
----

AGTAGCAACGAGACCTTCAGACCTGGGGGAGGAAATATGATGGACAATTGGAGAAGTGAATTATATAAATATA
AAGTAGTAAGAATTGAGCCATTA---GGGATAGCACCCACCGATGCAAAGAGAAGAGTGGTG-----------
----------------------------CAGAGAGAAAAAAGAGCA---GTGGGAACACTGGGAGCT------
---ATGTTCCTTGGGTTCTTGGGAGCA---GCAGGAAGCACTATG---GGCGCAGCGTCA---
ATGACGCTGACGGTACAG---GCCAGACTA---TTATTG---GCTGGTATAGTGCAACAGCAAAAT---
AATTTG---ATGAGA---GCTATTGAGGCGCAACAGCATCTGTTGCAACTCACAGTCTGGGGCATC
>KT878033
TTCTACTGTAACACATCAAAATTGTTTAATACTACTTGGAGTAAGGATAATAATACTGAACCAATGAAAATG
GA---

ACTGAGAAAGTTATCACAATCCCATGCAGAATAAAACAAATTATAAACAGGTGGCAGGAAGTAGGAAGAGCAA
CATATGCCCCTCCCATCAAAGGAATAATTAGATGTTCATCAAAT---
ATTACAGGGCTGCTACTAACAAGAGATGGGGGAGGAGGAAACAAG----------------------------
----

ACAGAAGAAACCTTCAGACCTGGGGGAGGAGATATGAAGGACAATTGGAGAAGTGAATTATACAAATATAAAG
TAGTAAAAATTGAGCCATTA---GGGATAGCACCCACCGAGGCAAGGAGAAGAGTGGTG--------------
-------------------------CAGAGAGAAAAAAGAGCA---GTGGGAACAATAGGAGCT---------
ATGTTCCTTGGGTTCTTGGGAGCA---GCAGGAAGCACTATG---GGCGCAGCGTCA---
CTGACGCTGACGGTACAG---GCCAGACTA---TTATTG---TCTGGTATAGTGCAACAGCAGAGT---
AATTTG---CTGAGA---GCTATTGAGGCGCAACAGCATATGTTGCAACTCACAGTCTGGGGCATC
>KT878036
TTCTACTGTAACACAACACAACTGTTTAATAGTACCTGGCTTGGGAATGATACTAATAAC-------------
----

AATGAAACACCTATCACGCTCCCATGCAGAATAAAACAGTTTATAAACAGGTGGCAGGGAGTAGGAAGAGCAA
TGTATGCCCCTCCCATCGCGGGAAACATTAGCTGTTCATCAAAT---

ATTACAGGGCTACTATTAGCAAGAGATGGGGGGAAGAATGAGACC----------------------------
----

AAGTCTGAGACCTTCAGGCCTTGGGGAGGAAATATGAAGGACAATTGGAGAAGTGAATTATATAAATATAAAG
TAGTAAAAATTGAGCCATTA---GGGATAGCACCCACCGAGGCAAGAAGAAGAGTGGTG--------------
-------------------------CAGAGAGAAAGAAGAGCA---GTGGGAACGATAGGAGCT---------
ATGTTCCTTGGGTTCTTGGGAACA---GCAGGAAGCACTATG---GGCGCAGCGTCA---
CTGACGCTGACGGTACAG---GCCAGACAA---TTGTTG---TCTGGTATAGTGCAACAGCAAAGT---
AATTTG---CTAAGA---GCTATTGAGGCGCAACAGCATATGTTGCAACTCACAGTCTGGGGCATT
>KT878037
TTCTACTGTAATACATCAAAACTGTTTAATAGTACTTGGGATGATAATAATACTAGTACTTGGGAAAATAGTA
CT---

GAAAATGACATTATCGAACTCCCATGTAGAATAAAACAAATTATAAACAGGTGGCAGGAAGTAGGAAAAGCAA
TGTTTGCCCCTCCCATAGCAGGATACATTAATTGCTCATCAAAT---
ATTACAGGGCTGTTATTAGTGAGAGATGGTGGTATTGTTAACAGT----------------------------
----

ACCAACGACACCTTCAGACCTGGAGGAGGAAATATGAAGGACAATTGGAGAAGTGAATTATATAAATATAAAG
TAGTAAAAATTGAACCATTA---GGAATAGCACCCACCAAGGCAAGGAGAAGAGTGGTG--------------
-------------------------CAGAGAGAAAAAAGAGCA---GCGGGA---ATAGGAGCT---------
TTGCTCCTTGGGTTCTTGGGAGCA---GCAGGAAGCACTATG---GGCGCAGCGTCA---
ATGACGCTGACGGTACAG---GCCAGACAA---TTATTG---TCTGGTATAGTGCAACAGCAGAAC---
AATCTG---CTGAAG---GCCATTGAGGCGCAACAGCGTGTGTTGCAACTCACAGTCTGGGGCATC
>KT878038
TTCTACTGTAATACAACAAAACTGTTTAGTGGTACCTGGAATGGTACTTGGTATGGTACCTGGAATGGTACTT
GGGATGGT---
GCTACAGAAAATAGTACTAAT------------
AAAAATATTACAGATATCATAATCCCATGCAGAATAAAACAAATTATAAACAGATGGCAAGAAGTAGGAAAAG
CAATGTATGCCCCTCCTATCGCAGGACGAATCAGATGTTCATCAAAT---
ATTACAGGACTGTTACTAACAAGAGATGGGGGCACAAACACAAGCACC-------------------------
----

AGCAATGAGACGGAGACCTTCAGACCTGGGGGAGGAGATATGAGAGACAATTGGAGAAGTGAATTATATAAAT
ATAAAGTAGTAAGAATTGAGCCACTG---GGGATAGCACCCACCAAGGCAAAGAGAATAGTGGTG--------
-------------------------------CAGAGAGAAAAAAGAGCT---GTGGGAACATTAGGAGCT---
------ATGTTCCTTGGGTTCTTGGGAGCA---GCAGGAAGCACTATG---GGCGCAGCGTCA---
GTGACGCTGACGGTACAG---GCCAGACAA---TTATTG---TCTGGTATAGTGCAACAGCAGAAT---
AATTTG---CTGAGA---GCTATTGAGGCGCAACAGCATATGTTGCAACTCACAATCTGGGGCATC
>KT878022
TTCTACTGTAATACAACAAAACTGTTTAATAGTATCTGGAATGGTACTTCGAATGGTTTACA-----------
----

GGAAATGATACAATCGTACTCCCATGCAGAATAAAACAAATTATAAACAGATGGCAAGAAGTAGGAAAAGCAA
TGTATGCCCCTCCTATCGCAGGAAATATTAGCTGTTCATCAAAT---
ATTACAGGGCTGTTATTAACAAGAGATGGGGGGCAGGAGAATGGCACC-------------------------
----

AATGGGACGGAGACCTTCAGACCTGGGGGAGGAGATATGAGGGACAATTGGAGAAGTGAATTATATAAATATA
AAGTAGTAAGAATTGAGCCATTA---GGGATAGCACCCACCAAGGCAAAGAGAAGAGTGGTG-----------
----------------------------CAGAGAGAAAAAAGAGCA---GTGGGAACAATAGGAGCT------
---ATGTTCCTTGGGTTCTTGGGAGCA---GCAGGAAGCACTATG---GGCGCAGCGTCA---
GTGACGCTGACGGTACAG---GCCAGGCAA---TTATTG---TCTGGTATAGTGCAACAGCAGCAT---
AATTTG---CTAAGA---GCTATTGAGGCGCAACAGCATATGTTGCAACTCACAGTCTGGGGCATC
>KT878023
TTCTACTGTAATACAACATCATTGTTTAATAGTACTTGGACTACTAATGATACTAGGAATGATACTGAGAAT-
----

AATAATGGAACAATCACACTCCAATGCAAAATAAAACAGATTATAAACTTGTGGCAGGAAGTAGGAAAAGCAA
TGTATGCCCCTCCCATCAGTGGACAAATTAACTGTTCATCAAAC---

```
ATTACAGGGCTGATATTAACAAGAGATGGTGGTAATGACACAAAA----------------------------
-------------------------------------------------------------------------
-------------
GATAATGAGACCTTCAGACCTATAGGAGGGAATATGAAGGACAATTGGAGAAGTGAATTATATAAATATAAAA
TAGTACAACTTGAACCATTA---GGAGTAGCACCCACCAAGGCAAAGAGAAGAGTGGTG--------------
-------------------------CAGAGAGAAAAAAGAGCA---GTGGGA---CTAGGAGCT---------
ATGTTCCTTGGGTTCTTGGGAGCA---GCAGGAAGCACTATG---GGCGCAGCGTCA---
ATAACGCTGACGGTACAG---GCCAGACAA---TTATTG---TCTGGTATAGTGCAACAGCAAAAC---
AATTTG---CTGAGA---GCTATTGAGGCGCAACAGAATCTGTTGCAACTCACAGTCTGGGGCATT
>KT878024
TTCTACTGTGATACATCAAAATTGTTTAATAGTACTTGGAATAATACTGACACTTGGAATAATACTGACACTG
GGAATAGT-----------------------------------------------------------------
-------------------------
ACTGAAACAAGAAATATCACACTCCCATGCCAAATAAAACAATTTATAAACAGGTGGCAGGAAGTAGGAAGAG
CAATGTATGCCCCTCCCATCGCAGGACTAATTAGATGTTCATCAAAC---
ATTACAGGGCTGCTATTAACAAGAGATGGGGGGAATAAAAATAATACA-------------------------
-------------------------------------------------------------------------
-------------
AGTAACGAGACCTTCAGGCCTGGGGGAGGAAATATGAAGGACAATTGGAGGAGTGAATTATATAAATATAAAG
TAGTAAAAATTGAGCCATTA---GGGATAGCACCCACCAAGGCAAGGAGAAGAGTGGTG--------------
-------------------------CAGAGAGAAAAAAGAGCA---ATAGGA---CTAGGAGCT---------
GTGTTCCTTGGGTTCTTGGGAGCA---GCAGGAAGCACTATG---GGCGCAGCGTCA---
CTGACGCTGACGGCACAG---GCCAGACAA---TTATTG---TCCGGTATAGTGCAACAGCAGAAT---
AATTTG---CTGAGA---GCTATTGAGGTGCAACAGCATATGTTGCAGCTCACAGTCTGGGGCATC
>KT878025
TTCTACTGTAATACAACAAAATTGTTTAAGAGTACTTGGAATAGTACTTGGAGTGATTGGAATAGTACTTGGA
GTGATAAT-----------------------------------------------------------------
-------------------GAA---
GGAAAAGAACATAATATCATACTCCCATGCAGAATAAGACAAATTATAAACAGGTGGCAGGAAGTAGGAAAAG
CAATGTATGCCCCTCCCATCGAAGGACAAATTAGATGCTCATCAAAT---
ATTACAGGGCTGCTATTAACAAGAGATGGGGGGACAGAAGACAATAGG-------------------------
-------------------------------------------------------------------------
-------
ACTAATGGGACTGAGACCTTCAGACCTGGGGGAGGAGATATGAGGGACAATTGGAGAAGTGAATTATATAAAT
ATAAAGTAGTAAAAATTGAGCCATTA---GGGATAGCACCCACCAAGGCAAAGAGAAGAGTGGTG--------
-------------------------------CAGAGAGAAAAAAGAGCA---GTAGGAACACTAGGAGCT---
------ATGTTCCTTGGGTTCTTGGGAGCA---GCAGGAAGCACTATG---GGCGCAGCGTCA---
ATGACGCTGACGGTACAG---GCCAGACTA---TTATTG---TCTGGTATAGTGCAACAGCAGAAT---
AATTTG---CTGAGA---GCTGTTGAGGCGCAACAGCATCTGTTGCAACTCACAGTCTGGGGCATT
>KT878027
TTCTACTGTAATACAACACAACTGTTTAACAGTACTTGGAATGACACAAACCTGTATAATGCAACAAAA----
-------------------------------------------------------------------------
----------------------------
CAGAATGGCACAATTACACTCCCATGCAGAATAAAACAAATTATAAACAGATGGCAGGAAGTAGGAAAAGCAA
TGTATGCCCCTCCCATCGCAGGACAAATTAGATGCTCATCAAAT---
ATTACAGGGCTGCTATTAACAAGAGATGGGGGGATTAATACCTCT----------------------------
-------------------------------------------------------------------------
----------------
ACGGAAACCTTCAGACCTGGGGGAGGAGATATGAGGGACAATTGGAGAAGTGAATTGTACAAATATAAGGTAG
TAAGAATAGAACCATTA---GGGATAGCACCCACCAAGGCAAGGAGAAGAGTGGTG-----------------
----------------------CAGAGAGAAAAAAGAGCA---GTGGGAACAATAGGAGCT---------
ATGTTCCTTGGGCTCTTGGGAGCA---GCAGGAAGCACTATG---GGCGCAGCGTCA---
ATGACGCTGACGGTACAG---GCCAGACTG---TTATTG---TCTGGTATAGTGCAACAGCAAAAT---
AATTTG---CTGAGA---GCTATTGAGGCGCAACAGCGTCTGTTGCAACTCACAGTCTGGGGCATC
>MK871374
TTCTACTGTGATACAACACCACTGTTTAATAGTACTTGGAATATTAATGGGACTAAAAGGTCAAATGAAACTG
CA-----------------------------------------------------------------------
----------------------------
GAAAATGGCACAATTACCCTCCACTGCAGAATAAGACAAATTGTAAACAGGTGGCAGGAAGTAGGAAAAGCAA
TGTATGCCCCTCCCATCAGCGGACAAATTAACTGTACATCAAAT---
```

ATTACAGGGCTGTTATTAACAAGAGATGGGGGCGGTAATCCCGCC---------------------------
----

AACAAAACTAATGACACCTTCAGGCCTGGGGGAGGAGATATGAGGGACAATTGGAGAAGTGAATTGTATAAAT
ATAAAGTAGTAGAAATTGAACCATTA---GGGATAGCACCCACCAGGGCAAGGAGAATAGTGGTG--------
------------------------------CAGAGAGAAAAAAGACCA---GTGGGAACATTAGGAGCC---
------ATGTTCCTTGGGTTCTTGGGAGCA---GCAGGAAGCACTATG---GGCGCAGCGTCA---
CTGACGCTGACGGTACAG---GCCAAGAAA---GTATTG---TCTGGTATAGTGCGACAGCAGCAT---
AATTTG---CTGAGA---GCTATTGAGGCGCAACAGCGTCTGTTGCAACTCACAGTCTGGGGCATC
>MN043576
TTCTACTGTGATACAACACAACTGTTTAATAGTACTTGGAATAGTACTTGGAATAGTACTGAAGGGTCAAAAA
CTGAA--

AGAAATGACACACTTACCCTCCCATGCAGAATAAAACAAATTATAAACAGGTGGCAGGAAGTAGGAAAAGCAA
TGTATGCCCCTCCCATCAAAGGACAAATTAGATGTTCATCAAAT---
ATTACAGGGCTGCTATTAACAAGAGATGGGGGGACTGAGAACAAC---------------------------
----

AGCGAGACTGAGATCTTCAGACCTGGGGGAGGAAATTTGAGGGACAATTGGAGAAGTGAATTATATAAATATA
AAATAGTAAAAATTGAGCCATTA---GGGATAGCACCCACCAGGGCAAGGAGAAGAGTGGTG-----------
---------------------------CAGAGAGAAAAAAGAGCA---GTGGGAACGATAGGAGCT------
---ATGTTCCTTGGGTTCTTGGGAGCA---GCAGGAAGCACTATG---GGCGCAGCGTCA---
ATGACGCTGACGGTACAG---GCCAGACTA---TTATTG---TCTGGTATAGTGCAACAGCAGAAT---
AATTTG---CTGAGA---GCTATTGAGGCGCAACAGCATCTGTTGCAACTCACAGTCTGGGGCATC
>KU896118
TTCTACTGTGATACAGCACAACTGTTTAATAGTACTTGGAATGATACACAACTGTTTAATAGTACTGGGAATG
ATACTGAA---

GGGTTAAGTGAAAATATCACACTCCCATGCAGAATAAAACAAATTATAAACAGGTGGCAGGAAGTAGGAAAAG
CAATGTATGCCCCTCCCATCGCAGGACAAATTAGATGTTCATCAAAT---
ATTACAGGGCTGCTATTAACAAGAGATGGGGGGATTGAGAACAAC---------------------------
----
GGGACTAACAGGACT---
GAGATTGAGATCTTCAGACCTGGGGGAGGAGATATGAGGGACAATTGGAGAAGTGAATTATATAAATATAAAG
TAGTAAAAATTGATCCATTA---GGGATAGCACCCACCAGGGCAAGGAGAAGAGTGGTG-------------
-------------------------CAGAGAGAAAAAAGAGCA---GTG---ACGATAGGAGCT---------
ATGTTCCTTGGGTTCTTGGGAGCA---GCAGGAAGCACTATG---GGCGCAGCGTCA---
CTGACGCTGACGGTACAG---GCCAGACAA---TTATTG---TCTGGTATAGTGCAACAGCAGAAT---
AATTTG---CTGAGA---GCTATTGAGGCGCAACAGCATCTGTTGCAACTCACAGTCTGGGGCATC
>MN043598
TTCTACTGTGATACAGCACAACTGTTTAATAGTACTTGGAATGATACACAACTGTTTAATAGTACTGGGAATG
ATACTGAA---

GGGTTAAGTGAAAATATCACACTCCCATGCAGAATAAAACAAATTATAAACAGGTGGCAGGAAGTAGGAAAAG
CAATGTATGCCCCTCCCATCGCAGGACAAATTAGATGTTCATCAAAT---
ATTACAGGGCTGCTATTAACAAGAGATGGGGGGATTGAGAACAACGGGACTAAC------------------
----

AGGACTGAGATTGAGATCTTCAGACCTGGGGGAGGAGATATGAGGGACAATTGGAGAAGTGAATTATATAAAT
ATAAAGTAGTAAAAATTGATCCATTA---GGGATAGCACCCACCAGGGCAAGGAGAAGAGTGGTG--------
------------------------------CAGAGAGAAAAAAGAGCA---GTG---ACGATAGGAGCT---
------ATGTTCCTTGGGTTCTTGGGAGCA---GCAGGAAGCACTATG---GGCGCAGCGTCA---
CTGACGCTGACGGTACAG---GCCAGACAA---TTATTG---TCTGGTATAGTGCAACAGCAGAAT---
AATTTG---CTGAGA---GCTATTGAGGCGCAACAGCATCTGTTGCAACTCACAGTCTGGGGCATC
>MN043588
TTCTACTGTAATACAACACAATTGTTTAATAGTACTTGGAATGGGAATGATACTGTAGGGTCAAATAAA----
----

ACTGAAGGAAATATCACACTCCCATGCAGAATAAAACAAATTATAAACAGGTGGCAGGAAGTAGGAAAAGCAA
TGTATGCCCCTCCCATCAGAGGACAAATTAGATGTTCATCAAAT---

ATTACAGGGCTGCTATTAACAAGAGATGGGGGGAATGAAGGCAAC----------------------------
----

GAGACTGAGATCTTCAGACCTGGGGGAGGAGACATGAGGGACAATTGGAGAAGTGAATTATATAAATATAAAG
TAGTAAGAATTGAGCCATTA---GGGATAGCACCCACCAAGGCAAGGAGAAGAGTGGTG--------------
-------------------------CAGAGAGAAAAAAGAGCA---GTGGGAACGATAGGAGCT---------
ATGTTCCTTGGGTTCTTGGGAGCA---GCAGGAAGCACTATG---GGCGCAGCGTCA---
CTGACGCTGACGGTACAG---GCCAGACTA---TTATTG---TCTGGTATAGTGCAACAGCAGAAT---
AATTTG---CTGAGA---GCTATTGAGGCGCAACAGCGTCTGTTGCAACTCACAGTCTGGGGCATC
>KU869580
TTCTACTGTAATACAACACAACTGTTTAATAGTACTTGGAATGAGAATAGTACTGAATGGTCAAATACAACTG
AA---

GGAAATGACACAATTATCACACTCCCATGTAGAATAAAACAAATTATAAACAGGTGGCAGGAAGTAGGAAAAG
CAATGTATGCCCCTCCCATCAGAGGACAAATTAGATGTTCATCAAAT---
ATTACAGGGCTGCTATTAACAAGAGATGGAGGGACTGAGAAAAACAGG-------------------------
----
ACTGAGAACAACGGGACGACTGAGATCTTCAGGCCAACGGGAGGGAATATGAGGGACAATTGGAGAAGTGAAT
TATATAAATATAAAGTAGTAAAAATTGAGCCATTA---
GGGATAGCACCCACCAAGGCAAGGAGAAGAGTGGTG-------------------------------------
--CAGAGAGAAAAAAGAGCA---GTGGGAACGATAGGAGCT---------
ATGTTCCTTGGGTTCTTGGGAGCA---GCAGGAAGCACTATG---GGCGCAGCGTCA---
CTGACGCTGACGGTACAG---GCCAGACTA---CTATTG---TCTGGTATAGTGCACCAGCAGAAT---
AATTTG---CTGAGA---GCTATTGAGGCGCAACAGCATCTGTTGCAACTCACAGTCTGGGGCATC
>MN043589
TTCTACTGTAATACAACACAACTGTTTAATAGCACTTGGAATAGTACTGAAAGGTTCAATAACACTGAAGGA-
----

AATATCACAACTATCAGACTCCCATGCCGAATTATACACATTAGAAACGGGTGGCAAGAAGTAAGGAAAGCAA
TGTATGCCCCTCCCATCATAGGACGCACTACATGTTCTTCAGGC---
ATTACAGGGCTGCTATTAGCAAGAGATGGGGGGACTGAGAACAAC----------------------------
----

GGGACTGAAATCTTCGGACCTGGGGGAGGAGATATGAGGGATAATTGGAGAAGTGAATTATATAAATATAAAG
TAGTAAAAATTGAGCCATTA---GGGATACCACCCACCCAGGCAAAGAGAAGAGTGGTG--------------
-------------------------CAAAGAGAAAAAAGAGCA---GTGGGAACGATAGGGGCT---------
ATGTTCCTTGGGTTCTTGGGACCA---CCAGGAACCATTATG---GGCGCAGCTTCA---
ATGACGCTGACGGTACAG---GCCAGATTA---TTATTG---TCTGGTATAGTGCAACAGCAGAAT---
AATTTG---CTGAGA---GCTATTGAGGCGCAACAGCATCTGTTGCAACTCACAGTTTGGGGCATC
>KU869596
TTCTACTGTAATACAACACAACTGTTTAATAGTACTTGGACCGGGAATGGTACTGAAGGGTTAAATGAAACTG
GA---

GGAAATAACACACTTATCACACTCCCATGCAGAATAAAACAAATTATAAACAGGTGGCAGGAAGTAGGAAAAG
CAATGTATGCCCCTCCCATCAGCGGAGTAATTAGATGTTCATCAAAT---
ATTACAGGGCTACTATTAACAAGAGATGGGGGGATTGAGAACAAC----------------------------
----
GAGACTAATAGCAACAGGACTGAGATCTTCAGACCTGGGGGAGGAGATATGAGGGACAATTGGAGAAGTGAAT
TATATAAATATAAAGTAGTAAAAATTGAGCCATTA---
GGGATAGCACCCACCATGGCAAGGAGAAGAGTGGTG-------------------------------------
--CAGAGAGAAAAAAGAGCA---GTGGGACTGATAGGAGCT---------
ATGTTCCTTGGGTTCTTGGGAGCA---GCAGGAAGCACTATG---GGCGCAGCGTCA---
TTGACGCTGACGGTACAG---GCCAGACAA---TTATTG---TCTGGTATAGTGCAACAGCAGAAT---
AATTTG---CTGAGA---GCTATTGAGGCGCAACAGCATCTGTTGCAACTCACCGTCTGGGGCATC
>KX960971
TTCTACTGTGATACAACAAAACTGTTTAACAGTACTTGGAATAATACTAAAAGGGTAGAGGGAACTGAAGGA-
----

GAAAATGGCACACTTACACTCCCATGCAGAATAAAACAAATTATAAACAGATGGCAGGAAGTAGGAAAAGCAA
TGTATGCCCCTCCCATCAGAGGACAAATTAGATGTTCATCAAAT---

ATTACAGGGCTGTTATTAACAAGAGATGGGGGTGAGAGGAATGACACC-------------------------
----

AACGAGACTGAAACCTTCAGACCTGGGGGAGGAGATATGAGGGACAATTGGAGAAGTGAATTATATAAATATA
AAGTAGTAAAAATTGAGCCATTA---GGGATAGCACCCACCAAGGCAAGGAGAAGAGTGGTG-----------
----------------------------CAGAGAGAAAAAAGAGCA---GTGGGAACAATAGGAGCT------
---ATGTTCCTTGGGTTCTTGGGAGCA---GCAGGAAGCACTATG---GGCGCAGCGTCA---
CTGACGTTGACGGTACAG---GCCAGACTA---TTATTG---TCTGGTATAGTGCAACAGCAGAAT---
AATTTG---CTGAGA---GCTATTGAGGCGCAACAGCATCTGTTGCAACTCACAGTCTGGGGCATC
>KJ140267
TTCTACTGCAATACAACACAACTGTTTAATAGTACTTGGTTTAATAGTACTTGGAATGATACT----------
----

GAAGGGTCAAATATCACTGGTACTGTAGGGTCAAATATCACACTCCCATGCAGAATAAAACAAATTATAAACA
GGTGGCAGGAAGTAGGAAAAGCAATGTATGCCCCTCCCATCAGAGGACAAATTAGATGTTCATCAAAT---
ATTACAGGACTGCTATTAACAAGAGATGGGGGG--

AGTGGGGACAACGAGACTAACGGGACGACTGAGACCTTCAGACCTGGGGGAGGAGATATGAGGGACAATTGGA
GAAGTGAATTATATAAATATAAAGTAGTAAAAATTGAGCCATTA---
GGGATAGCACCCACCAAGGCAAAGAGAAGAGTGGTG-------------------------------------
--CAGAGAGAAAAAAGAGCA---GTGGGAACGATAGGAGCT---------
ATGTTCCTTGGGTTCTTGGGAGCA---GCAGGAAGCACTATG---GGCGCAGCGTCA---
CTGACGCTGACGGTACAG---GCCAGACTA---CTATTG---TCTGGTATAGTGCAACAGCAGAAT---
AATTTG---CTGAGA---GCTATTGAGGCGCAACAGCATCTGTTGCAACTCACAGTCTGGGGCATC
>KJ140248
TTCTACTGCAATACAACACGACTGTTTAATAGTACTTGGAATATTAATAGTACTTGGAATGATACTGAA----
----

AGGTCAAATAAAACTGCAAATGACACACTTACACTCCCATGCAGAATAAAACAAATTATAAACATGTGGCAGG
AAGTAGGAAAAGCAATGTATGCCCCTCCCATCAGTGGACAAATTAGATGTTCATCAAAT---
ATTACAGGGCTGCTATTAACAAGAGATGGGGGGGCTAAGAACAACGGGACT----------------------
----

GAGAACAACGGGACTGAGATCTTCAGACCTGGGGGAGGAGATATGAAGGACAATTGGAGAAGTGAATTATATA
AGTATAAAGTAGTAAAAATTGAGCCATTA---GGGATAGCACCCACCAAGGCAAGGAGAAGAGTGGTG-----
----------------------------------CAGAGAGAAAAAAGAGCA---
GTGGGAACGATAGGAGCT---------ATGTTCCTTGGGTTCTTGGGAGCA---GCAGGAAGCACTATG---
GGCGCAACGTCA---ATGACGCTAACAGTACAG---GCCGGACTA---TTATTG---
TCTGGTATAGTGCAACAGCAGAAT---AATTTG---CTGAGA---
GCTATTGAGGCGCAACAGCATCTGTTGCAACTCACAGTCTGGGGCATC
>KJ140252
TTCTACTGTAATACAACACAACTGTTTAATAGCACTTGGAATAGTACTGAAGGGTTTAATAGTAGTACTGAAG
GG---
----------------TTA------------
AATAAAACTATCACACTCCCATGCAGAATAAAACAAATTATAAACAGATGGCAGGAAGTAGGAAAAGCAATGT
ATGCCCCTCCCATCAGAGGACAAATTAGATGTTCATCAAAT---
ATTACAGGGCTGCTATTAACAAGAGATGGGGGG--

-
AATGAGAGCAACGAGGCTGAGATCTTCAGACCTGGGGGAGGAAATATGAGGGACAATTGGAGAAGTGAATTAT
ATAAATATAAAGTAGTAAAAATTGAGCCATTA---GGGATAGCACCCACCCAGGCAAGGAGAAGAGTGGTG--
-------------------------------------CAGAGAGAAAAAAGAGCA---
GTGGGAACGATAGGAGCT---------ATGTTCCTTGGGTTCTTGGGAGCA---GCAGGAAGCACTATG---
GGCGCAGCGTCG---ATGACGCTGACGGTACAG---GCCAGACTA---TTATTG---
TCTGGTATAGTGCAACAGCAGAAT---AATCTG---CTAAGA---
GCTATTGAGGCGCAACAGCATCTGTTGCAACTCACAGTCTGGGGCATC
>AJ417425
TTCTACTGTAATACAACACAACTGTTTAATAGTACTTGGAATGGGAACAGCACTTGGAATAAGACTGATAAG-
----

GCAAATGACACCATCACACTCCCATGCAGAATGAAGCAAGTCGTAAACATGTGGCAGGAGGTAGGAAAAGCAA
TGTATGCCCCTCCCATCAGAGGGCAAATTAGCTGTTCATCAAAT---
ATTACAGGGCTGCTACTAACAAGAGATGGTGGTAATACCACGAAT----------------------------
----

GATACCGAGACCTTCAGACCTGGAGGAGGGGATATGAGGGACAATTGGAGAAGTGAATTATATAAATATAAAG
TAGTAAAAATTGAACCATTA---GGAGTAGCACCCACCAAGGCAAAAAGGAGAGTGGTG--------------
-------------------------CAAAGAGAAAAAAGAGCA---GTGGGA---ATAGGAGCT---------
TTGTTCCTTGGGTTCTTGGGAGCA---GCAGGAAGCACTATG---GGCGCAGCGTCA---
GTGACGCTGACGGTACAG---GCCAGACAA---TTATTG---TCTGGTATAGTGCAACAGCGGAGT---
AATTTG---CTGATG---GCTATTGAGGCGCAACAACATCTATTGCAACTCACGGTCTGGGGCATC
>KY820529
TTCTACTGTAATACAACACAACTGTTTAATAGTACTTGGAAATGGGATAATAGTACTTGGAATAGAACTGAAG
GGTTGAAT---

ATAACTGGAAGAAATATCACACTCCCATGCAGCATAAAACAAATTATAAACAGGTGGCAGGAAGTAGGAAAAG
CAATGTATGCCCCTCCCATCAGTGGACAAATTAGATGTTCATCAAAT---
ATTACAGGGCTGCTACTAACAAGAGATGGGGGGGCTGGGAACAAC----------------------------
----

GGGACTAACGGGACTGAGATCTTCAGACCTGGGGGAGGAGATATGAGGGACAATTGGAGAAGTGAATTATATA
AATATAAAGTAGTAAAAATTGAGCCATTA---GGGATAGCACCCACCAGGGCAAAGAGAAGAGTGGTG-----
----------------------------------CAGAGAGAAAAAAGAGCA---
GTGGGAACGATAGGAGCT---------ATGTTCCTTGGGTTCTTGGGAGCA---GCAGGAAGCACTATG---
GGCGCAGCGTCA---CTGACGCTGACGGTACAG---GCCAGACTA---TTATTG---
TCTGGTATAGTGCAACAGCAGAAT---AATTTG---CTGAGA---
GCTATTGAGGCGCAACAGCATCTGTTGCAACTCACAGTCTGGGGCATC
>MK577479
TTCTACTGTAATACAACACAACTGTTTAATAGTACTTGGAATGAGAATAGTACTGAATGGTCAAATACAACTG
AAGGA--

AATGACACAATTATCACACTCCCATGTAGAATAAAACAAATTATAAACAGGTGGCAGGAAGTAGGAAAAGCAA
TGTATGCCCCTCCCATCAGAGGACAAATTAGATGTTCATCAAAT---
ATTACAGGGCTGCTATTAACAAGAGATGGAGGGACTGAGAAAAACAGGACTGAG-------------------
----

AACAACGGGACGACTGAGATCTTCAGGCCAACGGGAGGGAATATGAGGGACAATTGGAGAAGTGAATTATATA
AATATAAAGTAGTAAAAATTGAGCCATTA---GGGATAGCACCCACCAAGGCAAGGAGAAGAGTGGTG-----
----------------------------------CAGAGAGAAAAAAGAGCA---
GTGGGAACGATAGGAGCT---------ATGTTCCTTGGGTTCTTGGGAGCA---GCAGGAAGCACTATG---
GGCGCAGCGTCA---ATGACGCTGACGGTACAG---GCCAGACGA---TTATTG---
TCTGGTATAGTGCAACAGCAGAAT---AATTTG---CTGAGA---
GCTATTGAGGCGCAACAGCATCTGTTGCAACTCACAGTCTGGGGCATC
>KU869586
TTCTACTGTAATACAACACAACTGTTTAATAGCACTTGGAATAGTACTGAAAGGTTCAATAACACTGAAGGA-
----

AATATCACAACTATCAGACTCCCATGCCGAATTATACACATTAGAAACGGGTGGCAAGAAGTAAGGAAAGCAA
TGTATGCCCCTCCCATCATAGGACGCACTACATGTTCTTCAGGC---
ATTACAGGGCTGCTATTAGCAAGAGATGGGGGGACTGAGAACAAC----------------------------
----

GGGACTGAAATCTTCGGACCTGGGGGAGGAGATATGAGGGATAATTGGAGAAGTGAATTATATAAATATAAAG
TAGTAAAAATTGAGCCATTA---GGGATACCACCCACCCAGGCAAAGAGAAGAGTGGTG--------------
-------------------------CAAAGAGAAAAAAGAGCA---GTGGGAACGATAGGGGCT---------
ATGTTCCTTGGGTTCTTGGGACCA---CCAGGAACCATTATG---GGCGCAGCTTCA---
ATGACGCTGACGGTACAG---GCCAGATTA---TTATTG---TCTGGTATAGTGCAACAGCAGAAT---
AATTTG---CTGAGA---GCTATTGAGGCGCAACAGCATCTGTTGCAACTCACAGTTTGGGGCATC
>MN043595

TTCTACTGTAATACAACACAACTGTTTAATAGTACTTGGACCGGGAATGGTACTGAAGGGTTAAATGAAACTG
GAGGA---

AATAACACACTTATCACACTCCCATGCAGAATAAAACAAATTATAAACAGGTGGCAGGAAGTAGGAAAAGCAA
TGTATGCCCCTCCCATCAGCGGAGTAATTAGATGTTCATCAAAT---
ATTACAGGGCTACTATTAACAAGAGATGGGGGGATTGAGAACAACGAGACT---------------------
----

AATAGCAACAGGACTGAGATCTTCAGACCTGGGGGAGGAGATATGAGGGACAATTGGAGAAGTGAATTATATA
AATATAAAGTAGTAAAAATTGAGCCATTA---GGGATAGCACCCACCATGGCAAGGAGAAGAGTGGTG----
---------------------------------CAGAGAGAAAAAAGAGCA---
GTGGGACTGATAGGAGCT---------ATGTTCCTTGGGTTCTTGGGAGCA---GCAGGAAGCACTATG---
GGCGCAGCGTCA---TTGACGCTGACGGTACAG---GCCAGACAA---TTATTG---
TCTGGTATAGTGCAACAGCAGAAT---AATTTG---CTGAGA---
GCTATTGAGGCGCAACAGCATCTGTTGCAACTCACCGTCTGGGGCATC
>KX960978
TTCTACTGTAATACAACACAACTGTTTAATAGTACTTGGAATAGTAATAGTACTGAAGGGCCAAATAGAACTG
AA--

GGAAATAGCACACTTACCCTCCCATGCAGAATAAAACAAATTATAAACAGGTGGCAGGAAGTAGGAAAAGCAA
TGTATGCCCCTCCCATCAGTGGACAAATTAGATGCTCATCAAAT---
ATTACAGGGCTGCTATTAACAAGAGATGGGGGGAATGGGACCAAC---------------------------
----

GAGACTGAAACCTTCAGACCTGGGGGAGGAGATATGAGGGACAATTGGAGAAGTGAATTATATAAATATAAAG
TAGTAAAAATTGAGCCATTA---GGGATAGCACCCACCGGGGCAAGTAGAAGGGTGGTG-------------
------------------------CAGAGAGAAAAAAGAGCA---GTGGGAACCATAGGAGCT---------
GTGTTCCTTGGGTTCTTGGGAGCA---GCAGGAAGCACTATG---GGCGCAGCGGCA---
GTGACGCTGACGGTACAG---GCCAGACTA---TTATTG---TCTGGTATAGTGCAACAGCAGAAT---
AATTTG---CTGAGA---GCTATTGAGGCGCAACAGCATCTGTTGCAACTCACAGTCTGGGGCATC
>AJ417415
TTCTACTGTAATACAACAAACCTGTTTAATGGTACTTGGAATGGTACTGCTTGGAATGGTACTTGGAATGTTA
GT--

AAAACTGATAAAAATATCACACTCTCATGCAGAATAAAACAAATTATAAACAGGTGGCAGGAAGTAGGAAAAG
CAATGTATGCCCCTCCCATCGCAGGAAAAATTAGCTGTTCATCTAAT---
ATTACAGGGCTGTTATTAACAAGAGATGGGGGGAATGAGACCAAC---------------------------
----

GGGACCCTGGAGATCTTCAGACCTGGGGGAGGAGATATGAGGGACAATTGGAGAAGTGAATTATATAAATATA
AAGTAGTAAAAATTGAGCCATTA---GGGATAGCACCCACCAAGGCAAGGAGAAGAGTGGTG----------
---------------------------CAGAGAGGAAAAAGAGCA---GTGGGAACGATAGGAGCT------
---ATGTTCCTTGGGTTCTTGGGAGCA---GCAGGAAGCACTATG---GGCGCAGCGTCA---
TTGACGCTGACGGTACAG---GCCAGACTA---TTATCG---TCTGGTATAGTGCAACAGCAGAGT---
AATTTG---CTGAGA---GCTATTGAGGCGCAACAGCATCTGTTGCAACTCACAGTCTGGGGCATT
>AJ417431
TTCTACTGTAATACATCAAAACTGTTTAATAGTACTTGGGGTCTTAACCAGACAGCTAACCATGAAGGA----
----

AATGATACAACAATCACACTCCCATGCAGAATAAAACAAATTATAAACATGTGGCAGAAAGTAGGAAAAGCCA
TGTATGCCCCTCCCATTGCGGGACGGATTGCATGTTCATCACAT---
ATTACAGGGCTACTATTAACAAGAGATGGGGGTAATGACACTAAC---------------------------
----

AATGAGACCTTCAGACCTGGAGGAGGAAATATGAGGGACAATTGGAGGAGTGAATTATATAAATATAAAGTAG
TAAAAATTAAACCATTA---GGAATAGCACCCACCAAGGCAAAGAGAAGAGTGGTG----------------
---------------------CAGAGAGAAAAAAGAGCA---GTGGGAACACTGGGAGCT---------
ATGTTCCTTGGGTTCTTGGGAGCA---GCAGGAAGCACTATG---GGCGCAGCGTCA---
GTGACGCTGACGGTACAG---GCCAGACAA---TTATTG---TCTGGTATAGTGCAACAGCAGAAC---
AACTTG---CTGAGG---GCTATTGAGGCGCAACAGCATCTGTTGCAACTCACGGTCTGGGGCATC

```
>AJ417410
TTCTACTGTAATACAACACAACTGTTTAATAGTACTTGGAATATTACTGAAGGGTCAAATACAACTGAAAGAA
AT-----------------------------------------------------------------------
----------------------------
AATAATACACATATCACACTCCAATGCAGAATAAGACAAATTATAAACAGATGGCAGGAAGTAGGAAAAGCAA
TGTATGCCCCTCCCATCAGAGGACAAATTAGATGTTCATCAAAT---
ATTACAGGGCTGCTATTAACAAGAGATGGGGGATTGAGAACAACGAG--------------------------
-------------------------------------------------------------------------
-------
ACCAAGACTATTGAGATCTTCAGACCTGGGGGAGGAAACATGAAGGACAATTGGAGAAGTGAATTATATAAAT
ATAAAGTAGTAAAAATTGAGCCATTA---GGGATAGCACCCACCAAGGCAAGGAGAAGAGTGGTG--------
-------------------------------CAGAGAGAAAAAAGAGCA---GTGGGAACGATAGGAGCT---
------ATGTTCCTTGGATTCTTGGGAGCA---GCAGGAAGCACTATG---GGCGCAGCGTCA---
ATGACGCTGACGGTACAG---GCCAGACTA---TTATTG---TCTGGTATAGTGCAACAGCAGAAT---
AATTTG---CTGAGA---GCTATTGAGGCGCAACAGCATATGTTGCAACTCACAGTCTGGGGCATC
>AJ417413
TTCTACTGTAATACAACACAACTGTTTAATAGTACTTGGAATAGTAATAGTACTTGGAATAGTACTGAA----
-------------------------------------------------------------------------
----------------------------
AGAAATGACACAATTACCCTCCCATGCAGAATAAAACAAATTATAAACAGGTGGCAGGAAGTAGGAAAAGCAA
TGTATGCCCCTCCCATCAGAGGACAAATTAGATGCTCATCAAAT---
ATTACAGGGTTGTTATTAACAAGAGATGGGGGAACTGGGAACAACGAG--------------------------
-------------------------------------------------------------------------
-------
ACCAACGAGACTGAGATCTTCAGACCTGGGGGAGGAGACATGAGGGACAATTGGAGAAGTGAATTATATAAAT
ATAAAGTAGTAAAAATTGAACCATTA---GGGATAGCACCCACCAAGGCAAGGAGAATAGTGGTG--------
-------------------------------CAGAGAGAAAAAAGAGCA---GTGGGAACGCTAGGAGCC---
------ATGTTCCTTGGGTTCTTGGGAGCA---GCAGGAAGCACTATG---GGCGCAGCGTCA---
CTGACGCTGACGGTACAG---GCCAGACTA---TTATTG---TCTGGTATAGTGCAACAGCAGAAT---
AATTTG---CTGAGA---GCTATTGAGGCGCAACAGCATCTGTTGCAACTCACAGTCTGGGGCATC
>AJ417419
TTCTACTGTAATACAACAAAACTGTTTAATAGTACTTGGAATGAAACAAGACTTAGTAATTGGAATAGTACTG
AA-----------------------------------------------------------------------
-------------------------
AAGTTAAATGAAAATATCACACTCCAATGCAGAATAAAACAAATTATAAACAGGTGGCAGGAAGTAGGAAAAG
CAATGTATGCCCCTCCCATCGAAGGAATAATTAGATGTTCATCAAAT---
ATTACAGGGCTGCTATTAACAAGAGATGGGGGAACTGGGAACAACGAGACT----------------------
-------------------------------------------------------------------------
-------
GGCAACGAGACTGAGATCTTCAGACCTGGGGGAGGAGACATGAGGGACAATTGGAGAAGTGAATTATATAAAT
ATAAAGTAGTAAGAATTGAGCCATTA---GGGCTAGCACCCACCAAGGCAAGGAGAAGAGTGGTG--------
-------------------------------CAGAGAGAAAAAAGAGCA---GTGGGAGCAATAGGAGCT---
------ATGTTCCTTGGGCTCTTGGGAGCA---GCAGGAAGCACTATG---GGCGCAGCGTCA---
CTGACGCTGACGGTACAG---GCCAGACTA---TTATTG---TCTGGTATAGTGCAACAGCAGAAT---
AATTTG---CTGAGA---GCTATTGAGGCGCAACAGCGTTTGTTGCAACTCACAGTCTGGGGCATC
>AF224507
TTCTACTGTAATACAACACAACTGTCTAATAGTACTTGGCAGAGGTCTGATGGTACTTGGAATAGAACTGGAG
GGTTA--------------------------------------------------------------------
-------------------AAT---
GAAACTAAAGAAAATATCACACTCCCATGCAGAATAAAACAAATTATAAACAGGTGGCAGGAAGTAGGAAAAG
CAATGTATGCCCCTCCCATCAGTGGACTAATTAGATGTTCATCAAAT---
ATTACAGGGCTGCTATTAACAAGAGATGGGGGAATGAGAACAATGGG--------------------------
-------------------------------------------------------------------------
-------
ACCAATGGGACTGAGACCTTCAGACCTGAGGGAGGAAATATGAAGGACAATTGGAGAAGTAAATTATATAAAT
ATAAAGTAGTAAGAATTGAGCCATTA---GGGATAGCACCCACCAGGGCAAGGAGAAGAGTGGTG--------
-------------------------------CAGAGAGAAAAAAGAGCA---GTAACA---TTCGGAGCT---
------CTGTTCCTTGGGTTCTTGGGAGCA---GCAGGAAGCACTATG---GGCTGCACGTCA---
ATGACGCTGACGGTACAG---GCCAGACTA---TTATTG---TCTGGTATAGTGCAACAGCAGAAT---
AATTTG---CTGAGA---GCTATTGAGGCGCAACAGCATCTGTTGCAACTCACAGTCTGGGGCATC
```

>AB097870
TTCTACTGTAATACATCACAACTGTTTAATAGTACTTGGTATGGGAATAATACTAGTACTTGGAATAAT----
----

ACTGCAGAAAATATCACGCTCCCCTGCAGAATAAAACAAATTGTAAACATGTGGCAGGAAGTAGGAAGAGCAA
TGTATGCCCCTCCCATCGCAGGACAAATTAAGTGTTCATCAAAT---
ATTACAGGGTTGCTGTTAACAAGAGATGGTGGTAATAAGACCAAC----------------------------
----

CCCACCGAGATCTTCAGACCTGCAGGAGGAGATATGAGGGACAATTGGAGAAGTGAATTATATAAATATAAAG
TAGTAAGAATTGAACCATTA---GGAGTAGCACCCACCAGGGCAAAGAGAAGAGTGGTG--------------
-------------------------CAAAGAGAAAAAAGAGCA---GTGGGAACGCTAGGAGCT---------
ATGTTCCTTGGGTTCTTGGGAGCA---GCAGGAAGCACTATG---GGCGCAGCGTCA---
ATAACGCTGACGGTACAG---GCCAGACAA---TTATTG---TCTGGTATAGTGCAACAGCAAAGA---
AATTTG---CTGAGA---GCTATTGAGGCGCAACAGCATTTGTTGCAACTCACAGTCTGGGGCATC
>AY423386
TTCTACTGTAATACATCACAGCTGTTTAATAGTACTTGGGATGTGAATGCTACTGGGAATGGTACTACAGAA-
----

CCAAATAGTACTATAACACTCCCATGCAGAATAAAACAAATTATAAACAGATGGCAGGAAGTAGGAAAAGCAA
TGTATGCCCCTCCCATCGAAGGACAAATTAGCTGTTCATCAAAC---
ATCACAGGGCTGCTATTAACAAGGGATGGTGGTAGGAATAGGAACGAG-------------------------
----

AGCACCGAGATCGAGATCTTTAGACCTGGAGGAGGAGATATGAGAGACAATTGGAGAAGTGAATTATATAAAT
ATAAAGTAGTAAAAATTGAGCCATTA---GGAGTAGCACCCACCAAGGCAAAGAGAAGAGTGGTG--------
-------------------------------CAGAGAGAAAAAAGAGCA---ATAGCG---CTAGGAGCT---
------ATGTTCCTTGGGTTCTTGGGAGCA---GCAGGAAGCACTATG---GGCGCAGCGTCA---
ATGGCGCTGACGGTACAG---GCCAGACAA---TTATTG---TCTGGTATAGTGCAACAGCAAAAC---
AATTTG---CTGAGG---GCTATTGAGGCGCAACAGCATCTGTTGCAACTCACAGTCTGGGGCATT
>HQ386146
TTCTACTGTAATACAACACAACTGTTTAATAGTACTTGGGAAAAGGATACTAAAGGGTCAAATGAC-------
----

ACTGAACGCATAATCCAACTTCCATGCAGAATAAAACAAATTATAAATATGTGGCAGGAAGTAGGAAAAGCAA
TGTATGCCCCTCCCATCAAAGGACAAATTAGATGTTCATCAAAT---
ATTACAGGGCTGATATTAACAAGAGATGGTGGTAATAGTAGTAATACCACGAGC-------------------
----

AATTCTAACAAAAATGAGACCTTCAGACCTGGAGGAGGAAACATGAAGGATAATTGGAGAAGTGAACTATATA
AATATAAAGTAGTACAAATTGAACCATTA---GGAGTAGCACCCACCAAGGCAAAGAGAAGAGTGGTG-----
----------------------------------CAAAGAGAAAAAAGAGCA---GTGGGA---
TTCGGAGCT---------CTGTTCCTTGGGTTCTTGGGAGCA---GCAGGAAGCACTATG---
GGCGCAGCATCA---ATAACGCTGACGGTACAG---GCCAGACAA---TTACTG---
TCTGAAATAGTGCAACAGCAGAAC---AATCTG---CTGAAG---
GCTATTGAGGCGCAACAGCATCTGTTGCAACTCACAGTCTGGGGCATT
>HQ386218
TTCTACTGTAATACAACAAAACTGTTCAATAGTACTTGGTGGTCTAATGGTACTTCGATTGGTACAAATAAC-
----

TCTAACGAAAATATCACTCTCCCATGCAGAATAAGACAAATTATAAACAGGTGGCAGGAAGTAGGAAAAGCAA
TGTATGCCCCTCCCATTGGAGGACTAATTAGATGTTCATCAAAT---
ATTACAGGGATGATATTAACAAGAGATGGGGGTAATAATAATACAACA-------------------------
----

AATGAGACAGAGACCTTCAGACCTGGAGGAGGAGATATGAGGGATAATTGGAGAAGTGAATTATATAAATATA
AAGTAGTAAAAATTGAACCATTA---GGAGTAGCACCCACCAAGGCAAAGAGAAGAGTGGTG-----------
----------------------------CAGAGAGAAAAAAGAGCA---GTGGGA---ATAGGAGCT------
---TTGTTCCTTGGGTTCTTGGGAGCA---GCAGGAAGCACTATG---GGCGCAGCGTCA---

TTGACGCTGACGGTACAG---GCCAGACAA---TTATTG---TCTGGTATAGTGCAACAGCAGAAC---
GATCTG---CTGAGG---GCTATTGAGGCGCAACAGCATATATTGCAACTCACAGTCTGGGGCATC
>U34603
TTCTACTGTGATACAACACAACTGTTTAATAGTACTTGGAATGGTACTGAAGGAAATAACACTGAA-------
----

GGAAATAGCACAATCACACTCCCATGCAGAATAAAACAAATTATAAACATGTGGCAGGGAGTAGGAAAAGCAA
TGTATGCCCCTCCCATCAGTGGACAAATTAGATGTTCATCAAAT---
ATTACAGGGCTGCTATTAACAAGAGATGGTGGTAAGGACGAGAACAAG-------------------------
----

ACTGGGACTGAGATCTTCAGACCTGGAGGAGGAGATATGAAGGATAATTGGAGAAGTGAATTATATAAATATA
AAGTAGTAAAAATTGAACCATTA---GGAGTAGCACCCACCAGGGCAAAGAGAAGAGTGGTG-----------
----------------------------CAGAGAGAAAAAAGAGCA---GTGGGA---ATAGGAGCG------
---TTGTTCCTTGGGTTCTTGGCAGCA---GCAGGAAGCACTATG---GGCGCACGGTCA---
ATAACGCTGACGGTACAG---GCCAGACAA---TTATTG---TCTGGTATAGTGCAACAGCAAAAC---
AATTTG---CTGAGG---GCTATTGAGGCGCAACAGCATCTGTTGCAACTCACAGTCTGGGGCATC
>AY970946
TTCTACTGTAATTCAACACAACTGTTTAATAGCACTTGGAAAGATACTGGAAAGTTAAATAACCCTGAA----
----

GGAAATGGCAACATCACACTCCCATGTAAAATAAAACAAATTATAAACAGGTGGCAGGGAGTAGGAAAAGCAA
TGTATGCCCCTCCCATCAGAGGACAAATTAGATGTTCATCAAAT---
ATTACAGGGCTGCTATTAACAAGAGATGGTGGTAACAACGTGAAT----------------------------
----
----------AATACCACCGAGGTCTTCAGACCTGGAGGAGGAGATATGAGG---------------
GAATTATATAAATATAAAGTAGTAAAAATTGAACCATTA---
GGAGTAGCACCTACCAAGGCAAAGAGAAGAGTGGTG-------------------------------------
--CAGAGAGAAAAAAGAGCA---GTGGGAACGATAGGAGCT---------
ATGTTCCTTGGATTCTTGGGAGCA---GCAGGAAGCACTATG---GGCGCAGCGTCA---
GTGGCGCTGACGGTACAG---GCCAGACAA---TTATTG---TCCGGTATAGTGCAACAGCAGAAT---
AATTTG---CTGAAA---GCTATTGAGGCGCAACAACATCTGTTGCAACTCACAGTCTGGGGCATC
>GU455480
TTCTACTGTAATTCAACAAAACTGTTTAATAGTACTTGGAATTTGAATGGTACTTGGCATGGTACTGAAAGGC
CAAATAGC---
-------------------ACT---
GAAAGAAATGACACACTCACAATCCCATGCAGAATAAAACAAATTATAAACATGTGGCAGGAAGTAGGAAAAG
CAATGTATGCCCCTCCCATCAAAGGACAAATTAGCTGTGTATCAAAT---
ATTACAGGGTTGCTATTAACAAGAGATGGTGGTACCAGCAAGAACGGGACC----------------------
----

ACCAACGACACCGAGGTCTTCAGACCTGGAGGAGGAAATATGAAGGACAATTGGAGAAGTGAATTATACAAAT
ATAAAGTAGTAAAAATTGAACCATTA---GGGGTAGCACCCACCAGGGCAAAGAGAAGAGTGGTG--------
-------------------------------CAGAGAGAAAAAAGAGGA---GTGGGA---
TTAGGAGTGCTGTATGGATTAGGAGCTTTGTTCCTTGGGTTCTTGGGAGCAGCAGGAAGCACTATGGGCGCAG
CGTCAATGACGCTGACGGTACAG---GCCAGACTA---TTATTG---TCTGGTATAGTGCAACAGCAGAAC--
-AATCTG---CTGAGG---GCTATTGAGACGCAACAGCATCTGTTGCAACTCACAGTCTGGGGCATC
>GU455465
TTCTACTGCAATACAACACAGCTGTTTAATAGTACTTGGCTGCTTAATAGTACTTGGAATAGTACTGACGAGT
CATCT--

AACAAGGAAATTAATATCACACTCCCATGCAGAATAAAACAAATTATAAACAGGTGGCAGGAAGTAGGAAAAG
CAATGTATGCCCCTCCCATCCAAGGACTAATTAAATGTTCATCAAAT---
ATTACAGGGCTGTTATTAACAAGAGATGGTGGTAATGGGACCGAG----------------------------
----

AATGAGACCGAGACCTTCAGACCTGGGGGAGGAGATATGAGGGACAATTGGAGAAGTGAATTATATAAATATA
AAGTAGTTAAAATTGAGCCATTA---GGAGTAGCACCCACCAAGGCAAAGAGAAGAGTGGTG-----------
----------------------------CGAAGAGAAAAAAGAGCA---GTGGGAGTGATAGGAGCT------
---ATGTTCCTTGGGTTCTTGGGAGCA---GCAGGAAGCGCTATG---GGCGCAGCGTCA---

GTGACGCTGACGGTACAG---GCCAGACAA---TTATTG---TCTGGTATAGTGCAACAGCAGAAC---
AATCTG---CTGAGA---GCTATTGAGGCGCAACAGCATCTGTTGCAACTCACAGTCTGGGGCATC
>EU744159
TTCTACTGTAATTCAGCACAACTGTTTAATAGTACTTGGGAGGTTAATAGAACGAAAGAAAAT----------
----

GACAATGGCACAATCACACTCCCATGCAGAATAAAACAGATTATAAACATGTGGCAGGAAGTAGGAAAAGCAA
TGTATGCCCCTCCCATCAGCGGACAAATTAGATGTTCATCAAAT---
ATTACAGGGCTGCTATTAACAAGAGATGGTGGTAACGAGACTAATGGG-------------------------
----

ACTGAGACTGAGACCTTCAGACCTGGAGGAGGAAATATGAGGGACAATTGGAGAAGTGAATTATATAAATATA
AAGTAGTAAAAATTGAACCATTA---GGAGTAGCACCCACCAAGGCAAAGAGAAGAGTGGTG-----------
----------------------------CAAAGAGAAAAAAGAGCA---GTGGGA---ATAGGAGCT------
---GTGTTCCTTGGGTTCTTAGGAGCA---GCAGGAAGTACTATG---GGCGCAGCGTCA---
ATAACGCTGACGGTACAG---GCCAGACTA---TTATTG---TCTGGTATAGTACAACAGCAGAAC---
AATCTG---CTGAGG---GCTATTGAGGCGCAACAACATCTGTTGCAACTCACAGTCTGGGGCATC
>GU455505
TTCTACTGTAATACAACAAAACTGTTTAGTAGTACTTGGAATTTTACTAACGGGTCATGGTCATCTAACAATG
CGGAAAAT---
-------------------ATC---
ACACTCTCAGAAAATACCACACTCCCATGCAGAATAAAACAAATTATAAACATGTGGCAGGAAGTAGGAAAGG
CAATGTATGCCCCTCCCATCAGAGGACGAATTAGATGTTCATCAAAT---
ATTACAGGGCTGCTATTAACAAGAGATGGTGGTAGGGACAATGAAACA-------------------------
----

AACGGGACCGAGATATTCAGACCTGGAGGAGGAAATATGAAGGACAATTGGAGAAGTGAATTATATAAATATA
AAGTAGTAAAAATTGAACCATTA---GGAGTAGCACCCACCAAGGCAAAGAGAAGAGTGGTG-----------
----------------------------CAGAGAGAAAAAAGAGCA---GTGGGA---CTAGGAGCT------
---TTGTTCCTTGGGTTCTTGGGAGCA---GCAGGAAGCACTATG---GGCGCAGCGTCA---
ATGACGCTGACGGTACAG---GCCAGACTA---TTATTG---TCTGGTATAGTGCAACAGCAGAAC---
AATTTG---CTGAGG---GCCATTGAGGCGCAACAGCATTTGTTGCAACTCACAGTCTGGGGCATC
>GU455448
TTCTACTGTAATACTTCAAAACTGTTTAATAGTACTTGGAATGATACTACTTTGACTTGGAATGACACTATTT
TAAT---
-------------------GAG---
ACAATGGACACAAATATCACACTTCCATGCAGAATAAAACAAATTATAAACATGTGGCAGGAAGTAGGAAAAG
CAATGTATGCCCCTCCCATCAAAGGACAAATTAGATGTTCATCAAAC---
ATTACAGGGCTGCTATTAACAAGAGATGGTGGTACTACTGACAATAAA-------------------------
----

ACAAACATCGAGACCTTCAGACCTGGAGGAGGAGATATGAGGGACAATTGGAGAAGTGAATTATATAAATATA
AAGTAGTAAGAATTGAACCATTA---GGAGTAGCACCCACCAAGGCAAAGAGAAGAGTGGTG-----------
----------------------------CAGAGAGGAAAAAGAGCAATAGGGGGA---CTAGGAGCT------
---TTGTTCCTTGGGTTCTTGGGAACA---GCAGGAAGCACTATG---GGCGCAGCGTCA---
ATAACGCTGACGGTACAG---GCCAGACAA---TTATTG---TCTGGTATAGTGCAACAGCAAAAC---
AATTTG---CTGAGG---GCTATTGAGGCGCAACAACATCTGTTGCAACTCACAGTCTGGGGCATC
>DQ178989
TTTTACTGTAATACATCACAGCTGTTTGATAGTATTTGGAATAATAGTACTAGGAATGACACTAGAGAGTCAA
AT---

AGCACTGATCCTATCATACTCCCATGCAGAATAAAGCAAATTATAAACAGGTGGCAGGAAGTAGGAAAAGCAA
TGTATGCCCCTCCCATCAGTGGACTGATTAGGTGTTCATCAAAT---
ATTACAGGGCTACTATTAACAAGAGATGGTGGTAATAGTGTTAACACCACC----------------------
----

AAGAACACCACCGAGACCTTCAGACCTGGAGGAGGAGATATGAGGGACAATTGGAGAAGTAAATTATATAAAT
ATAAAGTAGTAAAAATTGAACCATTA---GGAGTAGCACCCACCAAGGCAAAAAGAAGAGTGGTG--------
-------------------------------CAGAGAGAAAAAAGAGCA---GTAACG---CTAGGAGCT---
------ATGTTCCTTGGGTTCTTGGGAGCA---GCAGGAAGCACTATG---GGCGCAGCGTCG---

CTGACGCTGACGGTACAG---GCCAGACTA---TTATTG---TCTGGTATAGTGCAACAGCAAAAC---
AACTTG---CTGAGA---GCTATTGAGGCGCAACAGCATCTGTTGCAACTCACAGTCTGGGGCATC
>EU744007
TTCTACTGTAATACAACAGAACTGTTTAAAGGTATTTGGAATTTAAATAATACTTGGACTCCAAATAGTACTT
GGGATTCACTT--------------------------------AATAATACTTTGGAT---
ACATCATGGAATAGTACTATA--------------
GAACCAAATAAGACTATCATACTCCCATGCAGAATAAAACAAATTATAAACATGTGGCAGGAAGTAGGAAAAG
TAATGTATGCCCCTCCCATCAAAGGAATAATTAAATGCTCATCAAAC---
ATTACAGGGCTACTATTAACAAGAGATGGTGGTAATAAAGAGAATGAGACC---------------------
----

CAGAATGGGACCGAGATCTTTAGACCTGGAGGAGGAGATATGAGGGACAATTGGAGAAGTGAATTATATAAAT
ATAAAGTAGTAAAAATTGAACCATTA---GGAGTAGCACCCACCAGGGCAAAGAGAAGAGTGGTG--------
------------------------------CAGAGAGAAAAAAGAGCA---ATGGGA---ATAGGAGCT---
------ATGTTCCTTGGGTTCTTGGGAGCA---GCAGGAAGCACTATG---GGCGCAGCGTCG---
ATAACGCTGACGGTACAG---GCCAGACAA---TTATTG---TCTGGTATAGTGCAACAGCAGAGC---
AATTTG---CTGAGG---GCTATTGAGGCGCAACAGCATCTGTTGCAACTCACAGTCTGGGGCATT
>GU455525
TTCTACTGTAATACAACAAAACTGTTTGATTGGAAGGATACAACACAACAGTTTAATTGGAATATTACTAACG
GGTCA--

TATAACAATGGCACAATTACACTCCCATGCAGAATAAAACAAATTATAAACATGTGGCAGGAAGTAGGAAAAG
CAATGTATGCCCCTCCCATCGAAGGACGAATTAGATGTTTATCAAAT---
ATTACAGGGCTGCTATTAACAAGAGATGGTGGTAATAATAAGACG----------------------------
----

AGCGACCCCGAGATCTTCAGACCTGGAGGAGGAGATATGAGGGACAATTGGAGAAGTGAATTATATAAATATA
AAGTAGTAAAAATTGAACCATTA---GGAGTAGCACCCACCAAGGCAAAGAGAAGAGTGGTG----------
---------------------------CAGAGAGAAAAAAGAGCA---GTGGGA---ATAGGAGCT------
---CTGTTCCTTGGGTTCTTGGGAGCA---GCAGGAAGCACTATG---GGCGCAGCGTCA---
CTAACGCTGACGGTACAG---GCCAGACAA---GTATTG---TCTGGTATAGTGCAACAGCAGAAC---
AATTTG---CTGAGG---GCTATTGAGGCGCAACAGCATCTGTTGCAACTCACAGTCTGGGGCATC
>EU744052
TTCTACTGTAATTCAACACCACTGTTTAATAGTACTTGGTATATTAATAGTACTGAGAATGTTACTGAAAAG-
----

GCAAATGAAACAATTACACTCCAATGCAGAATAAAACAAATTATAAACATGTGGCAGGAAGTAGGAAAAGCAA
TGTATGCCCCTCCCATCAGAGGACAAATTAAATGTTCATCAAAT---
ATTACAGGGCTGCTATTAACAAGAGATGGTGGTGAGCACGAGAAC----------------------------
----

AGCACCACCGAGACCTTCAGACCTGGAGGGGGAGATATGAGGGACAATTGGAGAAGTGAATTATATAAATATA
AAGTAGTAAGAATTGAACCATTA---GGAGTAGCACCCACCAAGGCAAAGAGAAGAGTGGTG----------
---------------------------CAGAGAGAAAAAAGAGCA---GTGGGA---ATAGGAGCT------
---TTATTCCTTGGGTTCTTGGGAGCA---GCAGGAAGCACTATG---GGCGCAGCATCA---
ATAACGCTGACGGTACAG---GCCAGACAA---TTATTG---TCTGGTATAGTGCAACAGCAGAAC---
AATCTG---CTGAGG---GCTATTGAGGCGCAACAGCACCTGTTGCAACTCACAGTCTGGGGCATC
>U15030
TTCTACTGTAATACAACAGGACTGTTTAATAGTACTTGGAATGGTACTGAAGAGATTGTTACTGAAAGGTCAA
ATAGA--
-------------------ACA---
GGAGAAAATGACACACTCATACTCCAATGCAAAATAAGACAAATTGTAAACCTGTGGCAGCAAGTAGGAAAAG
CAATGTATGCCCCTCCCATCCAAGGACAAATTAGCTGTTCATCAAAT---
ATTACAGGGCTGCTATTAACAAGAGATGGTGGTAATAATAATAGTAGT-------------------------
----

AATAACAACACTGAAATCTTCAGACCTGGAGGAGGAGATATGAGGGACAATTGGAGAAGTGAATTATATAAAT
ATAAAGTAGTAAAAATTGAACCATTA---GGACTAGCACCCACCAAGGCAAAGAGAAGAGTGGTG--------
------------------------------CAGAGAGAAAAAAGAGCA---GTGGGA---ATAGGAGTT---
------TTGTTCCTTGGGTTCTTAGGAGCA---GCAGGAAGCACTATG---GGCGCAGCGGCA---

GTGACGCTGACGGTACAG---GCCAGACAA---TTGTTG---TCTGGTATAGTGCAACAGCAGAAC---
AACCTG---CTGAGA---GCTATTGAGGCGCAACAGCATCTGTTGCAACTCACAGTCTGGGGCATC
>L08655
TTCTACTGTAATACAACACAACTGTTTAATAGTACTTGGTATGCTAATGATACTGAAGGGTCAAATGAC----
----

ACTAAAGCAAATATCACACTCCCATGTAGAATAAAACAAATTATAAACATGTGGCAGGAAGTAGGGAAAGCAA
TGTATGCCCCTCCCATCAAAGGACAAATTAGATGTTCATCAAAT---
ATTACAGGGCTACTATTAACAAGAGATGGTGGTAATAATGAGAACGGGACCGAT-------------------
----

GAGAACAGGACCGAGGAGATCTTCAGACCTGGAGGAGGAGATATGAGGGACAATTGGAGAAGTGAATTATATA
AATATAAAGTAGTAAAAATTGAGCCATTA---GGAGTAGCACCCACCAAGGCAAAGAGAAGAGTGGTG-----
----------------------------------CAGAGAGAAAAAAGAGCA---
GTGGGAATGATAGGAGCT---------ATGTTCCTTGGGTTCTTGGGAGCA---GCAGGAAGCACTATG---
GGCGCAGCGTCG---CTGACGCTGACGGTACAG---GCCAGACAA---TTATTG---
TCTGGTATAGTGCAACAGCAGAAC---AATTTG---CTGAGG---
GCTATTGAGGCGCAACAGCATCTGTTGCAACTCACAGTCTGGGGCATC
>JF320008
TTTACTGTAATACAACATCATTGTTTAATGGGACTTGGAATGGGACTTGGGATAATACCTGGCAAGGG----
----

TCAAATGGCACTATCACACTCCAATGTAGGATAAAACAAATTATAAACATGTGGCAGGAAGTAGGAAAAGCAA
TGTATGCTCCTCCCAGAGCAGGAATAATTAATTGTGTATCAAAT---
ATTACAGGGCTGATGTTAATGAGAGATGGTGGTAAGGAGGACAATAGTACC----------------------
----

GAGAATAGTACCGAGATCTTCAGACCTGGAGGAGGGAATATGAAGGACAATTGGAGAAGTGAATTATATAAAT
ATAAAGTAGTAAAAATTGAACCATTA---GGAGTAGCACCCACCAAGGCAAAGAGAAGAGTGGTG--------
-------------------------------CAGAGAGAAAAAAGAGCA---GTAACG---ATAGGAGCT---
------ATGTTCCTTGGGTTCTTGGGAGCA---GCAGGAAGCACTATG---GGCGCAGCGTCA---
ATGACGCTGACGGTACAG---GCCAGGCAA---TTATTG---TCTGGTATAGTGCAACAGCAGAAC---
AATCTG---CTGAGA---GCTATTGAGGCGCAACAGCATCTGTTGCAACTCACAGTCTGGGGCATC
>JF320215
TTCTACTGTGATACAACACAACTGTTTAATAGTACTTGGAATACTACTACAGGGTCAAATGCTACTACAGGGT
CAAATGGC---
-------------------ATT---
AGTACAAATGACACAATCACGCTCCCATGTAGAATAAAGCAAATTATAAACATGTGGCAGGAAGTAGGAAAAG
CAATGTATGCTCCTCCCATCAAAGGACTAATTAGATGTTCATCAAAC---
ATTACAGGGCTACTATTAACAAGGGATGGTGGTGATAGCCCCAAC----------------------------
----

AAGAGCCAAGAGACCTTTAGACCTGGCGGAGGAGATATGAGGGACAATTGGAGAAGTGAACTATATAAATATA
AAGTAGTAAAAATTGAACCATTA---GGAGTAGCACCTACCATGGCAAAGAGAAGAGTGGTG-----------
----------------------------CAGAGAGAAAAGAGAGCA---GTGGGA---CTAGGAGCT------
---CTGTTCCTTGGGTTCCTGGGAGCA---GCAGGAAGCACTATG---GGCGCAGCGTCA---
ATAACGCTGACGGTACAG---GCCAGACAA---TTATTA---TCTGGTATAGTGCAACAGCAGAGC---
AATTTG---CTGAGG---GCTATTGAGGCGCAACAGCATATGTTGCAACTCACAGTCTGGGGCATC
>JF320208
TTCTACTGTAATACAACAAAACTGTTTAATAGTACTTGGAATTGGAATAATACAATAGAGGGCAATAGCACTG
AA---

GATAATGAGACTATCACACTCCCATGTAGAATAAAACAAATTATAAACATGTGGCAGACAGTAGGAAAAGCAA
TGTATGCCCCTCCCATCAGAGGACCAATTAGATGTTCATCAAAC---
ATTACAGGGATACTACTGACAAGGGATGGTGGTAATGAGAACAACAAC-------------------------
----

TCCATCCCCGAGATCTTCAGACCTGGAGGAGGAGATATGAGGGACAATTGGAGAAGTGAACTATATAAATATA
AAGTAGTACAAATTGAACCATTA---GGGATAGCACCCACCAAGGCAAAGAGAAGAGTGGTG-----------
----------------------------CAAAGAGAAAAAAGAGCA---GTGGGA---CTAGGAGCG------

---TTGTTCATTGGGTTCTTGGGAGCA---GCAGGAAGCACTATG---GGCGCAGCGTCA---
GTGACGCTGACGGTACAG---GCCAGACAA---TTACTG---TCTGGTATAGTGCAACAGCAGAAC---
AATTTG---CTGAGG---GCTATTGAGGCGCAACAGCATATGTTGCAACTCACAGTCTGGGGCATC
>JF320230
TTCTACTGTAATGCATCACAACTGTTTAATAGTACTTGGAGTGTTTTTGCAAATAATGGTACTTGGACTGCAA
CTAATGGC---
-------------------ACT---
GAAGGAAATGATACTATCACTCTCCCATGTAGAATAAAACAATTTGTAAACATGTGGCAGGAAGTAGGAAAAG
CAATGTATGCCCCTCCTATCAGAGGACTAATTAGATGTTCATCAAAT---
ATTACAGGGCTGCTATTAACAAGAGATGGTGGTAAGGATAATAGCAGC-------------------------
----

AATAGCATGGAGGTCTTCAGACCTGGAGGAGGAAATATGAAGGACAATTGGAGAAGTGAATTATATAAATATA
AAGTAGTAAAAATTGAGCCATTA---GGAATAGCACCCACCAAGGCAAAGAGAAGAGTGGTG-----------
----------------------------CAGAGAGAAAAAAGAGCA---GTGGGAGTACTGGGAGCT------
---ATGTTCCTTGGGTTCTTGGGAACA---GCAGGAAGCACTATG---GGCGCAGCGTCA---
GTGACGCTGACGGTACAG---GCCAGACAA---TTATTG---TCTGGTATAGTGCAACAGCAGAAC---
AATCTG---CTGAAG---GCTATTGAGGCGCAACAGCATCTGTTGCAACTCACAGTCTGGGGCATC
>JF320013
TTCTACTGTAATACAACAAAACTGTTTAACAGTACTTGGTATAGTAATGGTACTTGGACTGGTACTAATGAAA
CA---

AATAACAATGATACTATCACACTCCCATGCAGAATAAGACAAATTGTAAACATGTGGCAGGAAGTAGGAAAAG
CAATGTATGCCCCTCCCATCAAAGGAGTAATCAGATGTACATCAAAT---
ATTACAGGGCTGCTATTAGTAAGAGATGGAGGCCAACAGAACAATACT-------------------------
----

GGGAACGAGACTGAGGTCTTCAGACCTTCAGGAGGAAATATGAAGGACAATTGGAGGAGTGAATTATATAAAT
ATAAAGTAGTAAGAATTGAACCATTA---GGAATAGCGCCCACTAGGGCAAAGAGAAGAGTGGTG--------
-------------------------------CAAAGAGAAAAGAGAGCA---GTAGGA---CTAGGAGCT---
------GTGTTCCTTGGGTTCTTGGGAGCA---GCAGGAAGCACTATG---GGCGCAGCGTCA---
ATTACGCTGACGGTACAG---GCCAGACAA---TTATTG---TCTGGTATAGTGCAACAGCAGAAC---
AATTTG---CTGAGA---GCTATTGAGGCGCAACAGTATATGTTGCAACTCACAGTCTGGGGCATT
>JF320018
TTCTACTGTGACTCTACACAGCTGTTTAATAGTACTTGGAATTGGAGTAATACTATAGGGATAAATGACACGG
AA---

GAAAATTTCAATATCACACTCCCATGCAGAATAAAACAAATTGTAAACATGTGGCAGACAGTAGGAAAAGCAA
TGTATGCCCCTCCCATCAGAGGACAAATTAGATGTTCATCAAAT---
ATTACAGGACTAATATTAACAAGAGATGGTGGAAAAAATAATGATACT-------------------------
----

AATAACACAGAAACCTTCAGACCAGGAGGAGGAAATATGAAGGATAATTGGAGAAGTGAATTATATAAATATA
AAGTAGTAAGAATTGAACCATTA---GGAGTAGCACCCACCAAGGCAAAGAGAAGAGTGGTG-----------
----------------------------CAAAGAGAAAAAAGAGCA---GTGGGA---ATGGGAGCT------
---TTGTTCATTGGGTTCTTGGGAGCA---GCAGGAAGCACTATG---GGCGCAGCGTCA---
GTGACGCTGACGGTACAG---GCCAGACAA---TTATTG---TCTGGTATAGTGCAACAGCAGAAT---
AATCTG---CTGCAA---GCTATTGAGGCGCAACAGCATCTGTTGCAACTCACAGTCTGGGGCATC
>JF320028
TTCTACTGTAATACAACAAAATTATTTAATAGTACGTGGAATGAGAATGGGAGTTGGAATGATACTGACTGG-
----

TCAAATGACACTATCATACTCCCATGCAAAATAAAACAAATTATACGCATGTGGCAGGGGGTAGGACAAGCAA
TATATGCCCCTCCCATCAGCGGACAAATCAGATGTTCATCAAAC---
ATTACAGGGCTATTACTAACAAGAGATGGTGGTATCGAGAACAAT----------------------------
----

GACACTGAAACCTTCAGACCTGTAGGAGGAGATATGAGGGACAATTGGAGAAGTGAATTATATAAATATAAAG
TAGTAAGAATTGAACCATTA---GGAGTAGCACCCACCAAGGCAAAAAGAAGAGTGGTG--------------
-------------------------CAGAGAGAAAAAAGAGCA---GTGGGA---TTCGGAGCT---------

GTGTTCCTTGGGTTCTTGGGAGCA---GCAGGAAGCACTATG---GGCGCAGCGTCA---
GTAACGCTGACGGTACAG---GCCAGACAA---TTATTG---TCTGGTATAGTGCAACAGCAGAAC---
AATTTG---CTGAGG---GCTATTGAGGCGCAACAGCATCTGTTGCAACTCACAGTCTGGGGCATC
>JF320019
TTCTACTGCAATACAACATCACTGTTTAATGGTACTTGGTTTAATAGTACTGATAATAATACTGCAGAAACCA
TT---

ACTGGGAATAATACAATCACACTTCCATGCAGAATAAAACAAATTATAAACTTGTGGCAGGAAGTAGGAAAAG
CAATGTATGCCCCTCCCATCGCAGGACAAATTAATTGTTTATCGAAT---
ATTACGGGGCTGCTATTAACAAGAGATGGTGGTAATAACAACAGCACA-------------------------
----

GACAATAATAATGAAACCTTCAGACCTGCAGGAGGAGATATGAGGGACAATTGGAGAAGTGAATTATATAAAT
ATAAAGTAGTAAGAATTGAACCATTG---GGAGTAGCACCCACCAAGGCAAAGAGAAGAGTGGTG--------
-------------------------------CAGAGAGAAAAAAGAGCA---GTAGGA---CTAGGAGCT---
------TTGTTCCTTGGGTTCTTGGGAGCA---GCAGGAAGCACTATG---GGCGCAGCATCA---
CTAACGCTGACGGTACAA---GCCAGACAA---TTATTG---TCTGGTATAGTGCAACAGCAGAAC---
AATTTG---CTGAGG---GCTATTGAGGCGCAACAGCATCTGTTGCAACTCACAGTCTGGGGCATC
>MH078552
TTCTACTGTAATACAACACAACTATTTAATAGTACTTGGAATAGTACTGCAGTGACCACTCGGAATACTAATA
ATACT--

GATGATGATAAGATTATACTCCCATGCAGAATAAAACAAATTATAAACATGTGGCAGGAAGTAGGAAAAGCAA
TGTATGCTCCTCCCAGAGCAGGAAATATTAGATGTAATTCAAAT---
ATTACAGGGCTGCTATTAATAAGAGATGGGGGTCTGAATGAGACC----------------------------
----

AACAATACCGAGATCTTCAGACCTACAGGAGGGAACATGAAGGACAATTGGAGAAGTGAATTATATAAATATA
AAGTAGTAGAAGTGGAACCAGTA---GGAGTAGCACCCACCAAGGCAAAGAGGAGAGTGGTG-----------
----------------------------CAAAGAGAAAAAAGAGCA---GTAGTGACGGCAGGAGCT------
---ATGTTCCTTGGGTTCTTGAGTGCA---GCAGGAAGCACTATG---GGCGCAGCGTCA---
CTAACGCTGACGGTACAG---GCCAGGCAA---TTATTG---TCTGGTATAGTGCAACAGCAGAAC---
AATCTG---CTGAGA---GCTATTGAGGCGCAACAGCATATGTTGCAACTCACAGTCTGGGGCATC
>MH078553
TTCTACTGTAACACAACACCACTATTTAATAGTACCTGGCATCCGAATAATACCAACATAACTAGC-------
----

ACTAGAGAAAACATCACACTCACATGTAAAATAAGACAAATTATAAACATGTGGCAGGAAGTAGGAAAAGCAA
TGTATGCCCCTCCCATCAGAGGACTAATTAGGTGTTCATCAAAT---
ATTACAGGACTATTACTAACAAGAGATGGGGGTAGTGATAAGAACAGTAAC----------------------
----

CTTAATGGGACTGAAACCTTCAGACCTGGAGGAGGAGATATGAGGGACAATTGGAGAAGTGAATTATATAAAT
ATAAAGTAGTAGAAATTGAACCACTA---GGAATAGCACCCACCAAGGCAAAGAGAAGAGTGGTG--------
-------------------------------CAAAGAGAAAAAAGAGCA---GTGGGA---CTAGGAGCT---
------CTGTTCCTTGGGTTCTTGGGAGCA---GCAGGAAGCACTATG---GGCGCAGCGTCA---
ATAACGCTGACGGTACAG---GCCAGACAA---TTATTG---TCTGGTATAGTGCAGCAGCAAAAC---
AATTTG---CTGAGA---GCTATTGAGGCGCAACAGCATATGTTGCAACTCACAGTCTGGGGCATC
>MH078555
TTCTACTGTAATACAGCACAACTATTTAATGGTACTTGGGAGGCATATAAAAATAGCTCC-------------
----

ATGAATGATACTATAACACTCCCATGTAGAATAAAGCAAATTATAAACATGTGGCAGAAAGTAGGAAAAGCAA
TGTATGCCCCTCCCATCAGTGGAATAATTAACTGCACATCAAAT---
ATAACAGGGCTGTTCTTAACAAGAGATGGAGGCATTAACAATAGT----------------------------
----

AATAATGAAACCTTCAGACCTGGAGGAGGAGACATGAAGGACAATTGGAGAAGTGAATTATATAAATATAAGG
TAATAAAAGTTGAACCTATA---GGACTGGCACCCACCAAGGCAAGAAGAAGAGTGGTA--------------
-------------------------CAGAGGGAAAAAAGAGCA---ATAACA---CTGGGAGCT---------

ATGTTCCTTGGGTTCTTGGGAACA---GCAGGAAGCACTATG---GGCGCAGCGTCA---
GTGGCGCTGACGGTACAG---GCCAGACAG---TTACTA---TCTGGTATAGTGCAACAGCAGAAC---
AATTTG---CTGAGG---GCTATTGAGGCGCAACAGCATATGTTGCAACTCACAGTCTGGGGCATC
>EF210726
TTCTACTGTAATACAACACCACTATTTAATACTACTTGGGTTAATGAGACTGGGCCTAATAATACCCAG----
----

GGGAATGATACCATCACACTCCAATGCAGAATAAAACAAATTATAAACCTGTGGCAGAAAGTAGGAAAAGCAA
TGTATGCCCCTCCTATCAGAGGACGAATTAGATGTTCATCAAAT---
ATTACAGGACTACTATTAACAAGAGATGGTGGGAATAACAATACT----------------------------
----

AATGAGACCTTCAGACCGGGAGGAGGAGATATGAGGGACAATTGGAGAAGTGAATTATATAAATATAAAGTAG
TACAAGTTGAACCATTA---GGATTAGCACCCACCAAGGCAAGGAGAAGAGTGGTG-----------------
----------------------CAGAGAGAAAAAAGAGCA---GTGGGA---CTGGGAGCT--------
GTGTTCCTTGGGTTCTTGGGAGCA---GCAGGAAGCACTATG---GGCGCAGCGTCA---
ATGGCGCTGACGGTACAG---GCCAGACAA---TTATTG---TCTGGTATAGTGCAGCAGCAGAAC---
AACATG---CTGCGG---GCTATTGAGGCGCAACAGCACCTGTTGCAACTCACAGTCTGGGGCATT
>EF210730
TTCTACTGTAATACATCACAACTGTTTAATAGTACTTGGAATGGAACAGAGCCAACTAATAGCAATAAA----
----

GAAAATGGAACTATCACACTCCCATGTAGAATAAAACAAATTGTAAACATGTGGCAGGAAGTAGGAAAAGCAA
TGTATGCCCCTCCCATCAGAGGACAAATTAGATGTTCATCAAAT---
ATTACAGGGCTACTCTTAACAAGAGATGGTGGTAATAGCAGTACC----------------------------
----

GAGACCTTCAGACCTGGAGGAGGAGATATGAGGGACAATTGGAGAAGTGAATTATATAAATATAAAGTAGTAA
AAATTGAACCATTA---GGAGTAGCCCCCACCAAGGCAAAAAGAAGAGTGGTG--------------------
------------------CAGAGAGAAAAAAGAGCA---GTGACA---CTAGGAGCT---------
ATGTTCCTTGGGTTCTTGGGAGCA---GCAGGAAGCACTATG---GGCGCAGCGTCA---
ATGACGCTGACGGTACAG---GCCAGACAA---TTATTG---TCTGGTATAGTGCAACAGCAGAAC---
AATTTG---CTGAAG---GCTATTGAGGCGCAACAGCATCTGTTGCAACTCACAGTCTGGGGCATC
>EF210732
TTCTACTGTAATTCATCACAACTGTTTAACAGTACTTCAAAACAACTGTTTAACAGTACTTGGAATAAT----
----

ACAAATGCAAATATCGAACTCCCGTGCAGAATAAGACAAATTGTAAATATGTGGCAGAGAATAGGAAAAGCAA
TGTACGCCCCTCCCATCAGTGGACAAATTAACTGTACATCAAAT---
ATTACAGGACTGCTATTAACAAGAGATGGTGATAAGAACAACAAG----------------------------
----

TCCACCGAGGTCTTCAGACCTATAGGAGGAGAAATGAGGGACAATTGGAGAAGTGAATTATATAAATATAAGG
TAGTACAAATTGAACCATTA---GGAGTAGCACCCACCAGGGCAAAAAGAAGAGTGGTG--------------
-------------------------CAGAGAGAAAAGAGAGCA---GTGGGA---ATAGGAGCT---------
GTGTTCCTTGGGTTCTTGGGAGCA---GCAGGAAGCACTATG---GGCGCAGCGGCA---
GTGACGCTGACGGTACAG---GCCAGACTA---TTATTG---TCTGGTATAGTGCAACAGCAAAGC---
AATTTG---CTGAGG---GCTATTGAGGCGCAACAGCATCTGTTGCAACTCACAGTCTGGGGCATC
>KC596069
CTCTACTGTAATACAACACAACTGTTCAATGGGACTTGGAATGGTACTGCCTGGTCAAATGGTACTGACTGGT
CAAATAGCACT--
------------------GAA---
GGAGATAATACCACACTCACACTCCCATGCAGAATAAAACAAATTATAAACATGTGGCAGAAGGTAGGAAAAG
CAATGTATGCCCCTCCCATCAGAGGAGAAATTAGATGTTCATCAAAT---
ATTACAGGTCTGCTATTAACAAGAGATGGTGGTGAGCAAAATAGC----------------------------
----

TCAGAAGAGACCTTCAGACCTGGAGGAGGAGATATGAGGGACAATTGGAGAAGTGAATTATACAAATATAAAG
TAGTAAAAATTGAACCATTA---GGAATAGCACCCACCAAGGCAAAGAGAAGAGTGGTG--------------
-------------------------CAAAGAGAAAAAAGAGCA---GTGGGA---TTCGGAGCT---------

CTGTTCCTTGGGTTCTTGGGAGCA---GCAGGAAGCACTATG---GGCGCAGCGTCA---
GTGACGCTGACGGTACAG---GCCAGACTG---TTACTG---TCTGGTATAGTGCAACAGCAAAAC---
AACTTG---CTGAGG---GCTATTGAGGCGCAACAGCATCTGTTGCAACTCACAGTCTGGGGCATC
>JN251896
TTCTACTGTAATACAACACCGCTGTTTAATAAGACTTGGAATACAACAGAGCTGTCACAGTATGATAGTAGTG
ATACT--

AATAGTAATGCTACTATCATACTCCCATGCAGAATAAAACAAATTATAAACAGATGGCAGGAAGTAGGAAAAG
CAATGTATGCCCCTCCCATCAGAGGACAAATTAGATGTAAATCAAAT---
ATTACAGGGCTGCTATTAACAAGAGATGGTAGTACCAATGATACA----------------------------
----

ACCGAGACCTTCAGACCTGGAGGAGGAAATATGAAGGACAATTGGAGAAGTGAATTATATAAATATAAAGTAG
TAAAAATTGAGCCATTA---GGAATAGCACCCACCAAGGCAAAGAGAAGAGTGGTG-----------------
----------------------CAGAGAGAAAAAAGAGCA---GTAACG---ATGGGAGCT---------
ATGTTCCTTGGGTTCTTGGGAGCA---GCAGGAAGCACTATG---GGCGCAGCGTCA---
GTGACGCTGACGGTACAG---GCCAGACTA---TTATTG---TCTGGTATAGTGCAACAGCAAAAC---
AATTTG---CTGAGA---GCTATTGAGGCGCAACAGCATCTGTTGCAACTCACAGTCTGGGGCATC
>JN251901
TTCTACTGTAATACAACAAAATTGTTTAATAGCACTTTTAATATTAATAGTACTTTAAATGGGACTGAGAGC-
----

AGTAATGAAACTATCACACTCCCATGCAGAATAAAACAAATTATAAACAGGTGGCAGGAAGTAGGAAAGGCAA
TGTATGCCCCTCCCATCAGTGGACAAATTAAGTGTGAATCAAAT---
ATTACGGGGATGCTATTAACAAGAGATGGTGACGGGAACAACAGC----------------------------
----

AGCTCTGAGACCTTCAGACCTGGAGGAGGAGATATGAGGGATAATTGGAAAAGTGAATTATATAAATATAAAG
TAGTAAAAATTGAACCACTA---GGAGTAGCACCCACCAAGGCAAAGAGAAGAGTGGTG--------------
-------------------------CAGAGAGAAAAAAGAGCA---ATAGGA---ATAGGAGCT---------
GTGTTCCTTGGGTTCTTGGGAGCA---GCAGGAAGCACTATG---GGCGCAGCGTCG---
GTGACGCTGACGGTACAG---GCCAGACAA---TTACTG---TCTGGTATAGTGCAACAGCAGAAC---
AATCTG---CTGAGG---GCTATTGAGGCGCAACAGCATATGTTGCAACTCACAGTCTGGGGCATC
>JN251906
TTCTATTGTAATACAACACAACTGTTTAATAGTACATGGAATGGTACTGAAGGGTCAAATAGCACA-------
----

AAAAATGGCACTATCATACTCCCATGCAGAATAAAACAAATTATAAACATCTGGCAGAGAGTAGGAAAAGCAA
TGTATGCCCCTCCCATCCAAGGACAAATTAGATGTTCATCACAT---
ATTACAGGGCTGCTATTAACAAGAGATGGAGGTATGCAAAATAACAAGAAA----------------------
----

ACTAGCATAACTGAGATTTTCAGACCTGGAGGAGGAGACATGAGGGACAATTGGAGAAGTGAATTATATAAAT
ATAAAGTAGTAAAAATTGAACCATTA---GGAATAGCACCCACTAAGGCAAAGAGAAGAGTGGTG--------
-------------------------------CAGAGAGAAAAAAGAGCA---GTGGGA---ATAGGAGCT---
------TTGTTCCTTGGGTTCTTGGGAGCA---GCAGGAAGCACTATG---GGCGCAGCGTCA---
TTGACGCTGACGGTACAG---GCCAGACAA---TTATTG---TCTGGTATAGTGCAACAGCAGAAC---
AATTTG---CTGAGG---GCTATTGAGGCGCAACAGCATATGTTGCAACTCACAGTCTGGGGCATC
>AY819715
TTCTACTGTAATACAGCACAACTGTTTAATAGTACTTGGAATACTAATGATACTAGGGATAGTACTAATGAC-
----

ACAAATAAAAATATCACACTCCCATGCAGAATAAAACAAATTATAAACATGTGGCAGGAAGTAGGAAAAGCAA
TATATGCCCCTCCCATCGAGGGAGCAATTAACTGTTCATCAAAT---
ATTACAGGGTTGCTATTAACAAGAGATGGTGGTGAGGGTAATGTGAGC-------------------------
----

GGGAGCAATGAAACCTTCAGACCTGGAGGAGGAAATATGAGGGACAATTGGAGAAGTGAATTATATAAATACA
AAGTAGTAAAAATCCAACCATTA---GGAGTAGCACCCACCAAGGCAAGGAGAAGAGTGGTG-----------
----------------------------CAGAGAGAAAAAAGAGCA---GTAGGA---CTGGGAGCT------

---GTGTTCCTTGGGTTCTTGGGAGCA---GCAGGAAGCACTATG---GGCGCAGCGTCA---
GTGACGCTGACGGTACAG---GCCAGACAA---TTATTG---TCTGGTATAGTGCAACAGCAGAAC---
AATTTG---CTGAGA---GCTATTGAGGCGCAACAGCACCTGTTGCAACTCACAGTCTGGGGCATC
>HQ896488
TTCTACTGTAATACATCACAATTGTTTAATAGTACTTGGAAGAATGGTACTTTTGCCAATGGCACTGAA----
----

GGAAATGGCACAATCACACTCCCATGCAGAATAAAGCAATTTATAAACATGTGGCAACAAGTAGGAAAAGCAA
TGTATGCCCCTCCCATCAGTGGACAAATTAACTGCTCATCAAAT---
ATTACAGGGCTACTATTAACAAGAGATGGTGGTAGTGGTAACAGCACAGAG----------------------
----

AACGAGACTGAGATCTTCAGACCTGCAGGGCGAGATATGAGGGACAATTGGAGAAGTGAATTATATAAATATA
AAGTAGTACAAGTTGAGCCATTA---GGATTACCACCCACCAGGGCAAAGAGAAGAGTGGTG-----------
----------------------------CAGAGAGAAAAAAGAGCA---ATAGGA---CTGGGAGCT------
---GCCTTCCTTGGGTTCTTGGGAGCA---GCAGGAAGCACTATG---GGCGCAGCGTCA---
GTGACGCTGACGGTACAG---GCCAGACAA---TTATTG---TCTGGTATAGTGCAACAGCAAAAC---
AATTTG---CTGAGG---GCTATTGAGGCGCAACAGCATATGTTGCAACTCACAGTCTGGGGCATC
>JX500709
TTCTACTGCAAGACAACAGAACTGTTTAATAGTGCTTGGCATAATAATGGTACTAGTACTGTGAATAATACTG
GGAAA--

AATACTGAAAATATTACACTCCCATGTAGAATAAAACAAATTATAAACATGTGGCAGGAAGTAGGAAAAGCAA
TGTATGCCCCTCCCATCAAAGGACTAATTAGATGTACATCAAAT---
ATTACAGGCCTGATATTAACAAGAGATGGCGGTAATAATACTAGT----------------------------
----

AGCAATGAGACCTTCAGACCTGTAGGGGGAGATATGAGGGACAATTGGAGAAGTGAATTATATAAATATAAAG
TAGTAAAAATTGAACCATTA---GGAATAGCACCCACCAAGGCAAAGAGGAGAGTGGTG--------------
-------------------------CAAAGAGAGAAAAGAGCA---ATAGGAACAATGGGAGCT---------
GTGTTCCTTGGGTTCTTGGGAACA---GCAGGAAGCACTATG---GGCGCAGCGTCA---
CTGACGCTGACGGTACAG---GCCAGACAA---TTATTG---TCTGGTATAGTGCAGCAGCAGAAC---
AATCTG---CTGAGG---GCTATTGATGCGCAACAGCATATGTTGCAACTCACAGTCTGGGGCATT
>JX500707
TTCTATTGTAATACAACAAAACTGTTTAATAGTACTTGGGAGAACAATAATACTACTAGTACT----------
----

GGGAATGGGACAATCACACTCCCATGTAGAATAAAACAAATTATAAACAGATGGCAGGAAGTAGGAAAAGCAA
TGTATGCCCCTCCCATCAAAGGAAAAATTCTATGCTCATCAAAT---
ATTACAGGGCTAATATTATCAAGAGATGGTGGTAACAGCAGCACC----------------------------
----

ACCGAGACCTTCAGACCTGCAGGAGGAGATATGAGGGACAATTGGAGAAGTGAATTATACAAATATAAAGTAG
TAAAAATTGAACCATTG---GGAGTAGCACCCACCAAGGCAAAGAGGAGAGTGGTG-----------------
----------------------CAGAGAGAAAAAAGAGCA---GTGGGA---CTAGGAGCG---------
TTATTCCTTGGGTTCTTGGGAGCA---GCAGGAAGCACTATG---GGCGCAGCGTCA---
CTGACGCTGACGGTACAG---GCCAGACAA---TTATTG---TCTGGTATAGTGCAACAGCAAAAC---
AATTTG---CTGAGG---GCTATTGAGGCGCAACAACATCTGTTGCAACTCACAGTCTGGGGCATC
>JX500708
TTCTACTGTAATACAACACAATTGTTTAATAGCACTTGGGATAAGAATATTACAGAGTTTAATAAGACTAATG
AGTTAAAT---

AACACTGAAATTATCACACTCCCATGTAGAATAAAACAAATTATAAACATGTGGCAGGCAGTAGGAAAAGCAA
TGTATGCCCCTCCCATCTCAGGACAAATTAAATGTTCATCAAAT---
ATTACAGGACTGCTATTAACAAGAGATGGTGGTAACAATGACACC----------------------------
----

GAGACCTTCAGACCTGGAGGAGGAGATATGAGGGACAATTGGAGAAGTGAATTATATAAATATAAAGTAGTAA
GAATTGAACCATTA---GGAATAGCACCCACCAAGGCAAAGAGAAGAGTGGTG--------------------
-------------------CAGAGAGAGAAAAGAGCA---GTGGGA---ATAGGAGCT---------

GTGTTCCTTGGGTTCTTGGGAGCA---GCAGGAAGCACTATG---GGCGCGGCGTCA---
ATAACGCTGACGGTACAG---GCCAGGCAA---TTATTG---GCTGGTATAGTGCAACAGCAGAAC---
AATCTG---CTGAGA---GCTATTGAGGCGCAACAACATCTGTTGCAACTCACAGTCTGGGGCATC
>MF373130
TTCTACTGTAATACAACACAACTATTTAATAGTTCTTGGGATATGAATAGAATTGTAAATAGTTCCGAAAATA
AC---

ACTGACAAGAATATCACACTCCCATGCAGAATAAAACAAATTGTAAACATGTGGCAGGAAGTAGGAAAAGCAA
TGTATGCCCCTCCCATCAGTGGACAAATTAGATGTTCATCAAAT---
ATTACAGGGCTGCTATTAACAAGAGATGGTGGTAAAAACAGCAGTAACGAG----------------------
----

ACCGAGACCGAAATCTTCAGACCTGTGGGAGGAAATATGAGGGACAATTGGAGAAGTGAATTATATAAATATA
AAGTAGTACAAATTGAACCATTA---GGAGTAGCACCCACCAAGGCAAAGAGAAGAGTGGTG-----------
----------------------------CAAAGAGAAAAAAGAGCA---GTGGGA---TTAGGAGCA------
---ATGTTCCTTGGGTTCTTGGGAGCA---GCAGGAAGCACTATG---GGCGCAGCGTCA---
ATAACGCTGACGGTACAG---GCCAGACAA---TTATTG---TCTGGTATAGTGCAACAGCAGAAT---
AATTTG---CTGAGA---GCTATTGAGGCGCAACAGCATCTGTTGCAACTCACAGTCTGGGGCATC
>MF373142
TTCTACTGTAATACTACACAACTATTTAATAGTACTTGGAATGGTACTTTGGAAAATACTAATAGTACTGGGA
AT---

GGTGATAATAAAACTATCACACTCCCATGCAGAATAAGACAAATTATAAACATGTGGCAGGAAGTAGGAAAAG
CAATGTATGCCCCTCCCATCAAAGGACAAATTAAATGCTCATCAAAG---
ATTACAGGGCTGTTATTAACAAGAGATGGGGGCAATGTACCAAATGATGGGGGC-------------------
----

AATAATAGCACAGAAATCTTCAGACCTGGAGGAGGAGATATGAGGGACAATTGGAGAAGTGAATTATATAAAT
ACAAAATAGTAAAAGTTAAACCAATA---GGAATAGCACCCACCAAGGCAAAGAGAAGAGTGGTG--------
-------------------------------CAGAGAGAAAAAAGAGCA---GTAACG---CTGGGAGCT---
------GTGTTCCTTGGGTTCTTGGGAGCA---GCAGGAAGCACTATG---GGCGCAGCGTCG---
ATGACGCTGACGGTACAG---GCCAGACAA---TTATTG---TCTGGTATAGTGCAACAGCAGAAC---
AATCTG---CTGAAG---GCTATTGAGGCGCAGCAGCATATACTGCAACTCACAGTCTGGGGCATC
>MF373144
TTCTACTGCAATTCAACACCACTGTTTAATAGCACCTGGTATACTAATGGGACTAGCACCTGGTATACTAATG
GGACTTGG---
AATGTCACTGAAGGGCAT--------
AATAGCACTACAAACATCACACTCCAATGCAGAATAAAACAAATTATAAACATGTGGCAGGAAGTAGGAAAAG
CAATGTATGCCCCTCCCATTGCAGGAATAATTAAATGCTCATCAAAT---
ATCACAGGGCTGCTATTAACAAGAGATGGTGGTAAGGGCAACGGGACTGAC----------------------
----

AACAATACTGAGATCTTCAGACCTGGAGGAGGAGATATGAGGGACAATTGGAGAAATGAATTATATAAATATA
AAGTAGTACAAATTGAACCATTA---GGAGTAGCACCCACCATGGCAAAGAGAAGAGTGGTG-----------
----------------------------CAAAGAGAGAAAAGAGAT---TTGGGA---CTAGGAGCT------
---CTGTTCCTTGGGTTCTTGGGAGCA---GCAGGAAGCACTATG---GGCGCAGCGTCA---
ATAACGCTGACGGTACAG---GCCAGACAA---TTATTG---TCTGGTATAGTGCAACAGCAGAAC---
AACTTG---CTGAGG---GCTATTGAGGCGCAACAGCATCTGTTGCAACTCACAGTCTGGGGCATT
>MF373161
TTCTACTGTAACACAACAAAACTATTTGATAGTACTTGGAATACGAATGCTACTTGGGATAGGAATTGGAATG
ATACCAGT---

GGAGAAAATGACACAATCAGACTCCCATGTAGAATAAAGCAAATTATAAACATGTGGCAGGAAGTAGGAAAAG
CAATGTATGCCCCTCCCATTAGTGGACTAATTAGCTGCTCATCAAAC---
ATTACCGGGTTGTTATTAGTAAGAGATGGTGGTGAAAACACTACC----------------------------
----

GAAGAAGAGATCTTCAGACCTGGAGGAGGAAATATGAAAGATAACTGGAGAAGTGAATTATATAAATATAAAG
TAGTAAAAATTGAACCATTA---GGAGTAGCACCCACCAAGGCAAAGAGGAGAGTGGTG--------------
-------------------------CAGAGAGAAAAAAGAGCA---GTAACG---TTCGGAGCT---------

ATGTTCCTTGGGTTCTTGGGAGCA---GCAGGAAGCACTATG---GGCGCAGCGTCA---
ATAACGCTGACGGTACAG---GCCAGACAA---TTATTG---TCTGGGATAGTGCAACAGCAGAGC---
AATTTG---CTGAAA---GCTATTGAGGCGCAACAACATCTGTTGCAACTCACAGTCTGGGGCATT
>KP411828
TTCTACTGTAATACAACACAACTGTTTAATAGCACTTGGAATATGACTAATAGGTCTAAAAATAACACTGCAG
AA---

AATAATACTACATTCATACTCCCATGCAGAATAAAACAAATTGTAAACTTGTGGCAAGAAGTAGGAAAAGCAA
TGTATGCCCCTCCCATCGCAGGACAAATTAACTGTTCATCAATT---
ATTACAGGACTGCTATTAACAAGAGATGGTGGTAGTAATAATGACACA-------------------------
----

AATGAGACCGAGATCTTTAGACCTGGAGGAGGAGATATGAGTGACAATTGTAGAAGTGAATTATATAAATATA
AAGTAGTAAAAATTGAACCATTA---GGAATAGCACCCACCAGGGCAAAGAGAAGAGTGGTG-----------
----------------------------CAGAGAGAGAAAAGAGCA---GTGACG---CTAGGAGCT------
---GTGTTCCTTGGGTTCTTGGGAGCA---GCAGGAAGCACTATG---GGCGCAGCGTCA---
GTAGCGCTGACGGCACAG---GCCAGACAA---TTGTTG---TTTGGTATAGTGCAACAGCAGAAC---
AATCTG---CTGCGA---GCTATTGAGGCGCAACAACATATGTTGCAACTCACAGTCTGGGGCATC
>MF373204
TTCTACTGTAACACAACAAGACTATTCAACAGTACTTGGAGGATCAATGGTACTCTGAATAGTACTGAAGGGC
CA---

AATAATGCAACTATCATACTCCCATGCAGAATAAAACAAGTTATAAACCTGTGGCAAGAAGTAGGAAAAGCAA
TGTATGCCCCTCCCATCAGGGGAAACATTAGTTGTTCATCAAAC---
ATTACAGGACTGCTATTAACAAGGGATGGTGGTGAT-------------------------------------
----

AATGAAACTGAGACCTTCAGACCTGGAGGAGGAAATATGAAGGACAATTGGAGAAGTGAATTATATAGATATA
AAGTAGTAAAAATTGAACCATTA---GGAATAGCACCCACCACGGCAAGGAGACGAGTGGTG-----------
----------------------------CAGAGAGAAAAAAGAGCA---GTAACA---CTAGGAGCT------
---ATGTTCCTTGGGTTCTTGGGGGCA---GCAGGAAGCACTATG---GGCGCAGCGTCA---
CTGACGCTGACGGTACAA---GCCAGACAA---TTATTG---TCTGGTATAGTGCAGCAGCAAAAC---
AATTTG---CTGAGG---GCTATTGAAGCGCAACAGCATCTGTTGCAACTCACAGTCTGGGGCATT
>GU204920
TTCTACTGTAATACAACACAACTGTTTAATAGTACTTGGAATGGTACTACTGGTACTTGGAGTAGAACTGATA
CTGAA--
-------------------GTG---
TCAAATAGCACTAATATCATACTCCCCTGCAGAATAAAACAAATTATAAACATGTGGCAGGAAGTAGGAAAAG
CAATGTATGCCCCTCCCATCAGTGGACCAATTAACTGTTTATCAAAT---
ATTACAGGGCTGTTATTAACAAGAGATGGTGGTACTAACGAGACTAAC-------------------------
----

GAAAGCAATGAGACCGTCAGACCTGGAGGAGGAGATATGAGGGACAATTGGAGAAGTGAATTATATAAATATA
AAGTAGTAAAAATTGAACCATTA---GGAGTAGCACCCACCAAGGCAAAAAGAAGAGTGGTG-----------
----------------------------CAGAGAGAAAAAAGAGCA---ATAGGA---ATAGGAGCT------
---GTGTTCCTTGGGTTCTTGGGAGCA---GCAGGAAGCACTATG---GGCGCAGCGTCA---
GTGACGCTGACGGTACAG---GCCAGACAA---TTATTG---TCTGGTATAGTGCAACAGCAGAAC---
AATTTG---CTGAGG---GCTATTGAGGCGCAACAGCATATGTTGCAACTCACAGTCTGGGGCATC
>JN251856
TTCTACTGTGATTCATCACCACTGTTTAATAGTACTTGGCCGGTTAATGGTACTAGTACTAATAGC-------
----

ATTAACGAAACTATCACACTCCCATGTAGAATAAAACAAATTATAAACATGTGGCAGGAGGTAGGAAAAGCAA
TGTATGCCCCTCCCATCAGAGGACCAATTAAGTGTTCATCAAAT---
ATTACAGGGCTGATATTAACAAGAGATGGTGGTGATAACATGAAC----------------------------
----

CAAAGCACAGAGACCTTCAGACCGGGAGGAGGAGATATGAGGGACAATTGGAGAAGTGAATTATATAAATACA
AAGTAGTAAAAATTGAACCATTA---GGGGTAGCACCCACCAAGGCAAAGAGACGAGTGGTG-----------
----------------------------CAGAGAGAAAAAAGATCA---GTGACA---CTAGGAGCT------

---ATGTTCCTTGGATTCTTGGGAGCA---GCAGGAAGCACTATG---GGCGCAGCGTCA---
ATGGCGCTGACGGTACAG---GCTAGACAA---TTATTG---TCTGGTATAGTGCAACAGCAGAAC---
AATTTG---CTGAAG---GCTATTGAGGCGCAACAACATCTGTTGCAACTCACAGTCTGGGGCATC
>KY213741
TTCTACTGTAATACATCACAACTATTTAATAGTACTTGGGATGGAAATAGTACTAGGGAGAATAGC-------
----

ACTGGACAGTTATTCACACTCCCATGCAGAATAAAACAAATTATAAACATGTGGCAGAAAGTAGGAAAAGCAA
TGTATGCCCCTCCCATCGCGGGCCCAATTAACTGTTTATCAAAC---
ATTACAGGGCTGTTATTAACAAGAGATGGTGGTAATAATACA-------------------------------
----

AATAATGAGACCTTCAGACCAGGAGGAGGAGATATGAGGGACAATTGGAGAAGTGAACTATACAAATATAAAG
TAGTAAGAGTTGAACCATTA---GGACTGGCACCCACCAGGGCAAAGAGAAGAGTGGTG--------------
-------------------------CAGAGAGAAAAAAGAGCA---GTGGGA---CTAGGAGCA---------
TTGTTCATTGGGTTCTTGGGAGCA---GCAGGAAGCACTATG---GGCGCAGCGTCA---
GTGACGCTGACGGTACAG---GCCAGACTA---TTATTG---TCTGGTATAGTGCAACAGCAGAAC---
AATCTG---CTGAGG---GCTATTGAGGCGCAACAGCATCTGTTGCAACTCACAGTCTGGGGCATC
>KY213740
TTTTACTGTAATACAAAACCACTGTTTAATAGTACTTGGACTGGGAAGAATAGTACCTGGAATAGTAATGATA
CTACA--

GGAAATGAAACAATCACACTCCAGTGTAGAATAAAACAAATTATAAACATGTGGCAGAAAGTAGGAAGAGCAA
TGTATGCTCCTCCCATCAGTGGACAAATTAACTGTTCATCAAAT---
ATTACAGGGCTAATATTAACAAGAGATTGGGAGAGAAATAACACG----------------------------
----

AGGGAGAACGAGACCCTCAGACCGGGAGGAGGAGATATGAGGGACATTTGGAGAAATGAATTATACAAATATA
AAGTAGTAAAAATTGAACCATTA---GGAATAGCACCCACCAAGGCAAAGAGAAGAGTGGTG-----------
----------------------------CAGAGAGAAAAAAGAGCA---GTGGGA---ATGGGAGCC------
---TTGTTCCTTGGGTTCTTGGGAGCA---GCAGGAAGCACTATG---GGCGCAGCATCA---
ATAACGCTGACGGTACAG---GCTAGACAA---TTATTG---TCTGGTATAGTGCAACAGCAAAAC---
AATTTG---CTGAGG---GCTATTGAGGCGCAACAACACATGTTGCAACTCACAGTCTGGGGCATC
>KY213747
TTCTACTGTAATACAACACAACTGTTTAATTCAACACACCTGTTTGATACTACTAATAGCACTGGGAAT----
----

GCTACAGGAAATATCACACTTCCTTGCAGAATAAAACAAATTGTAAACATGTGGCAGGGAGTAGGAAAAGCAA
TGTATGCCCCTCCCATTAGAGGACCAATTAGCTGTTCATCAAAG---
ATTACAGGATTGCTATTAACAAGAGATGGTGGTAGTAATAATGAGACA-------------------------
----

AACACCACCGAGACCTTCAGACCTGGGGGAGGAGATATGAAGGACAATTGGAGAAGTGAATTATATAAATATA
AAGTAGTAAAAATTGAACCATTA---GGAGTAGCACCTACCAAGGCAAAGAGAAGAGTGGTG-----------
----------------------------CAGAGAGAAAAAAGAGCA---GTGGGAACAATAGGAGCG------
---ATGTTCCTTGGGTTCTTGGGAGCA---GCAGGAAGCACTATG---GGCGCAGCGTCA---
GTGACGCTGACGGTACAG---GCCAGACAA---TTGTTG---TCTGGTATAGTGCAACAGCAGAGA---
AATTTG---CTGAGA---GCTATTGAGGCGCAACAGCATATGTTGCAACTCACAGTCTGGGGCATC
>KY213751
TTCTACTGCAATACAACACAACTGTTTAACTCTACTTGGAATAGTACTGGGAATATTGCAGAAAATAAT----
----

ACTGCAGAAAATATCACGCTTCCTTGCAGAATAAAACAAATTATAAACATGTGGCAGGGAGTAGGAAAAGCAA
TGTATGCCCCTCCCATTAGCGGACCAATTAGCTGTTCATCAAAT---
ATTACAGGGTTGCTATTAACAAGAGATGGTGGTAATAATGAGACG----------------------------
----

AACACCACCGAGACCTTCAGACCTGGGGGAGGAGATATGAGGGACAATTGGAGAAGTGAATTATATAAATATA
AAATAGTAAAAATAGAACCACTA---GGAATAGCACCTACCAAGGCAAAGAGAAGAGTGGTG-----------
----------------------------CAGAGAGAAAAAAGAGCA---GTGGGAATGATGGGAGCG------

```
---ATGTTCCTTGGGTTCTTGGGAGCA---GCAGGAAGCACTATG---GGCGCAGCGTCA---
ATGGCGCTGACGGTACAG---GCCAGACAA---TTGTTG---TCTGGTATAGTGCAACAGCAGAGA---
AATCTG---CTGAGG---GCTATTGAGGCGCAACAGCATCTGTTGCAACTCACAGTCTGGGGCATC
>KY213748
TTCTACTGTAATACAACACAACTGTTTAATTCAACACACCCGCCTAATACTACTTGGAATGAGAAT-------
-------------------------------------------------------------------------
----------------------------
AATACTGACATTATCACGCTTCCTTGCAGAATAAAACAAATTGTAAACATGTGGCAGACAGTAGGAAAAGCAA
TGTATGCCCCTCCCATTAGCGGACCAATTAGCTGTTCATCAAAA---
ATTACAGGGTTGCTATTAACAAGAGATGGTGGTAATAGTAACAAT----------------------------
-------------------------------------------------------------------------
----
GAGACAAACACCACCGAGACCTTCAGACCTGGGGGAGGAGATATGAGGGACAATTGGAGAAGCGAATTATATA
AATATAAAGTAGTAAAAATTGAACCATTA---GGAGTAGCACCTACCAAGGCAAAGAGAAGAGTGGTG-----
----------------------------------CAGAGAGAAAAAAGAGCA---
GTGGGAACAATAGGAGCG---------ATGTTCCTTGGGTTCTTGGGAGCA---GCAGGAAGCACTATG---
GGCGCAGCGTCA---GTGACGCTGACGGTACAG---GCCAGACAA---TTGTTG---
TCTGGTATAGTGCAACAGCAGAGA---AATTTG---CTGAGA---
GCTATTGAGGCGCAACAGCATCTGTTGCAACTCACAGTCTGGGGCATC
>AY945710
TTCTACTGTAATACATCAAAACTGTTTAATAGTACTTGGAATACTAATGGTACTTGGAATGATACTACA----
-------------------------------------------------------------------------
----------------------------
GGAGATGACACTATCACGCTCCCTTGCAGAATAAAGCAAATTGTAAACATGTGGCAGGAAGTAGGAAAAGCAA
TGTATGCCCCTCCCATCGCAGGAAACATCAGCTGTTCATCAAAT---
ATTACAGGGTTAATATTAACATGGGATGGTGGTAATAAGAACGGGAACAAG----------------------
-------------------------------------------------------------------------
----
ACCACCAATGATACAGAGACCTTTAGACCTGGAGGAGGAGATATGAGGGACAATTGGAGAAGTGAACTATATA
AATATAAAGTAGTGAAAATTGAACCATTA---GGAGTAGCACCCACCAAGGCAAAGAGAAGAGTGGTG-----
----------------------------------CAGAGAGAAAAAAGAGCA---GTGGGA---
ATAGGAGCT---------TTGTTCCTTGGGTTCTTGGGAGCA---GCAGGAAGCACTATG---
GGCGCAGCGTCA---ATGACGCTGACGGTACAG---GCCAGACAA---TTGTTG---
TCTGGTATAGTGCAACAGCAAAGA---AATTTG---CTGAGT---
GCTATTGAGGCGCAACAGCGTCTGTTGCAACTCACAGTCTGGGGCATC
>KJ952535
TTCTACTGTAATACATCAAAACTGTTTAATAGTACTTGGTATAATAATAGTACTTGGAATGGTGCTTGGAATA
ATACTAGA-----------------------------------------------------------------
----------------------------
GAAGATGACAATATCACGCTCCCTTGCAGAATAAAACAAATTGTAAACATGTGGCAGGAAGTAGGAAAAGCAA
TGTATGCCCCTCCCATCAGAGGACTAATTAGATGTTCATCAAGT---
ATTACAGGGTTGCTATTATTAAGAGATGGTGGTATTAAGAACCAAACC-------------------------
-------------------------------------------------------------------------
----------
AACGAGACCGAGATCTTCAGACCTGCAGGAGGAGATATGAGGGATAATTGGAGAAGTGAATTATATAAATATA
AAGTAGTACAAATTGAACCATTA---GGAGTAGCACCCACCAGGGCAAAGAGAAGAGTGGTG-----------
----------------------------CAGAGAGAAAAAAGAGCA---GTGGGAACGATAGGAGCT------
---ATGTTCCTTGGGTTCTTGGGAGCA---GCAGGAAGCACTATG---GGCGCAGCGTCA---
ATAACGCTGACGGTACAG---GCCAGACAA---TTGCTG---TCTGGTATAGTGCAACAGCAGAGA---
AATTTG---CTGAGG---GCTATTGAGGCGCAACAGCATCTGTTGCAACTCACAGTCTGGGGCATC
>JN248321
TTCTACTGCAATACATCACAGCTGTTTAATAGTACTTGGCTGTTTAATAGTACTTGGAACAGTACTTGGGGTG
ATACTAGTACT--------------------------------------------------------------
----------GAAGGGTCA------
AATGGAACAAAAGACATCGTACTCCCATGCAAAATAAAACAAATTATAAACATGTGGCAGGAAGTAGGAAAAG
CAATGTATGCCCCTCCCATCAGAGGACTAATTAAATGTTCATCAAAC---
ATTACAGGGCTGTTATTAACAAGGGATGGAGGTGAAAATAATGACACA-------------------------
-------------------------------------------------------------------------
----------
AACCAAACTGAGGTCTTCAGACCTGGAGGAGGAGATATGAGGGACAATTGGAGAAGTGAATTATATAAATATA
```

AAGTAGTAAAAATTGAACCATTA---GGAGTGGCACCCACCAAGGCAAGGAGAAGAGTGGTG-----------
----------------------------CAGAGAGAAAAAAGAGCA---GTGGGA---GTAGGAGCT------
---TTGCTTCTTGGGTTCTTAAGTGCA---GCAGGAAGCACTATG---GGCGCAGCGTCA---
GTGACGTTGACGGTACAG---GCCAGACAA---TTATTG---TCTGGTATAGTGCAACAGCAGAAC---
AATTTG---CTGAAG---GCTATTGATGCGCAACAGCATCTGTTGCAACTCACAGTCTGGGGCATC
>JN248329
TTCTACTGTAATACAGCACAACTGTTTAATAGCACTTGGAATAATAATAGTACTGAGAATGATACTATA----
----

GGAGATGGAACTATCACGCTCCCTTGCAGAATAAGACAAATTGTAAACATGTGGCAGGAAGTAGGAAAAGCAA
TGTATGCCCCTCCCATTGAAGGACAAATTAGATGTTCATCAAAT---
ATTACAGGGTTGCTATTAACAAGAGATGGTGGTAATAATGGGAGC----------------------------
----

AATACCAACGAGACCTTCAGACCTGGAGGAGGAGATATGAGGGACAATTGGAGAAGTGAATTATATAAGTATA
AAGTAGTAAAAATTGAACCATTA---GGAGTAGCACCCACCGAGGCAAGGAGAAGAGTGGTG-----------
----------------------------CAGAGAGAAAAAAGAGCA---GTGGGAGCGATAGGAGCT------
---ATGTTCCTTGGGTTCTTGGGAGCA---GCAGGAAGCACTATG---GGCGCAGCGTCA---
ATAACGCTGACGGTACAG---ACCAGACAA---TTGTTG---TCTGGTATAGTGCAACAGCAGAGA---
AATCTG---CTGAGG---GCTATTGAGGCACAACAACATCTGTTGCAACTCACAGTCTGGGGCATC
>JN248333
TTCTACTGTAATACAACACAACTGTTTAATAGTACTTGGAAGGTTAATAGTACTTGGAATGAGAATAGTACTG
GGGACAAT---
-------------AATGAT------
GAAAATAACAGCACTATCACACTCCCATGCAGAATAAAACAAATTATAAACAGGTGGCAGGAAGTAGGAAAAG
CAATGTATGCCCCTCCTATCAGCGGACTAATTAGCTGTTCATCAAAT---
ATTACAGGGCTGCTACTAACAAGAGATGGTGGGGATGATAGTAAT----------------------------
----

AATACTGAGACCTTCAGACCTGCAGGAGGAGATATGAAAGACAATTGGAGAAGTGAATTATATAAATATAAAG
TAGTAAGAATTGAACCATTA---GGAGTAGCACCCACCAAGGCAAAGAGAAGAGTGGTG--------------
-------------------------CAGAGAGAGAAAAGAGCA---GTGGGAACACTAGGAGCT---------
ATGTTCCTTGGGTTCTTGGGAGCA---GCAGGAAGCACTATG---GGCGCAGCGTCA---
ATAACGCTGACGGTACAG---GCCAGACAA---TTACTG---TCTGGTATAGTGCAACAGCAGAAC---
AATTTG---CTGAGA---GCTATTGAGGCGCAACAGCACATGTTGCAACTCACAGTCTGGGGCATC
>JN248335
TTCTACTGTAATACATCACAACTGTTTAATAGTACTTGGTATGCTAATGGTACTAGTACTGGGAATAATACTA
TA---

AAAAATGGCACTATCACGCTCCCTTGCAGAATAAGACAAATTATAAACATGTGGCAGGAAGTAGGAAAAGCAA
TGTATGCCCCTCCCATTAAAGGACTAATTAGATGTTCATCAAAT---
ATTACAGGGATACTATTAACAAGAGATGGTGGTAGTGGTAATGAGACC-------------------------
----

ACCAATACCACTGAGACCTTCAGACCTACAGGAGGAGATATGAGGGATAATTGGAGAAGTGAATTATATAAAT
ATAAAGTAGTAAAAATTGAACCATTA---GGAGTAGCACCCACCAAGGCAAAGAGAAGAGTGGTG--------
-------------------------------CAGAGAGAAAAAAGAGCA---GTGACA---ATAGGAGCT---
------ATGTTCCTTGGGTTCTTGGGAGCA---GCAGGAAGCACTATG---GGCGCAGCGTCA---
ATAACGCTGACGGTACAG---GCCAGACAA---TTGCTG---TCTGGTATAGTGCAACAGCAGAGA---
AATTTG---CTGAGG---GCGATTGAGGCGCAACAGCATCTGTTGCAGCTCACAGTCTGGGGCATC
>JN248337
TTCTACTGTAATACAACACGACTGTTTAACGGTACCTGGAGTAATGGTACCTGGGTTAATAGCACCTGGAATG
AT---

ACCACTAACGAGATCACCCTCCCATGCAGAATAAAACAAATTGTGAACATGTGGCAGGAAGTAGGAAAAGCCA
TATATGCCCCTCCCATCCAGGGACAAATTAACTGTTCATCAAAT---
ATTACAGGGCTGCTATTAACAAGAGATGGTGGTAATAATAATGATACAAAC----------------------
----

AAGACCCAGGGCGAGATCTTCAGACCTGGAGGAGGAAATATGAAGGACAATTGGAGAAGTGAATTATATAAAT

ATAAAGTAGTAAGAATTGAACCATTA---GGAATAGCACCCACCAAGGCAAGGAGAAGAGTGGTG--------
-------------------------------CAAAGAGAAAAAAGAGCA---GTGGGAACAATAGGAGCT---
------ATGTTCCTTGGGTTCTTGGGAGCA---GCAGGAAGCACTATG---GGCGCAGCGTCA---
ATAACGCTGACGGTACAG---GCCAGACTA---TTGTTG---TCTGGTATAGTGCAACAGCAGAAT---
AATTTG---CTGAGA---GCTATTGAGGCGCAGCAGCACATGTTGCAACTCACAGTCTGGGGCATC
>JN248343
TTCTATTGTAATACAACACAGCTGTTTGAGAGTACTTGGCAGGGTGATAATATTGAAGAGTCAAACAGT----
----

ACTGAACAAAATATCACACTCCCATGCAGAATAAAACAAATTATAAACATGTGGCAGGAAGTAGGAAAAGCAA
TGTATGCCCCTCCCATCGCAGGACCAATTAGATGTTCATCAAAT---
ATTACAGGGTTGCTATTAACAAGAGATGGGGGTAATGTCACTAAC----------------------------
----

GGTACTGAGATCTTCAGACCTGGAGGAGGAGACATGAAGGACAATTGGAGAAGTGAATTATATAAATATAAAG
TAGTAAAAATTGAACCATTA---GGAATAGCACCCACTAAGGCAAAGAGAAGAGTGGTG--------------
-------------------------CAAAGAGAAAAGAGAGCA---GTGGGTATAATAGGAGCT---------
ATGTTCCTTGGGTTCTTGGGAGCA---GCAGGAAGCACTATG---GGCGCAGCGTCA---
ATAACGCTGACGGTACAG---GCCAGACAA---TTATTG---TCAGGTATAGTGCAACAGCAGAAC---
AATTTG---CTGAGG---GCTATTGAGGCGCAACAGCATTTGTTGCAACTCACAGTCTGGGGCATT
>JN248344
TTCTACTGCAATACAACACAGCTGTTTAATACTACTTGGATGCCTAATGGTACTTTGAATGGTACTTGGGAA-
----

CATAACAGCACAATCATACTCCCATGCAAAATAAAACAAATTATAAACAGGTGGCAGGAAGTAGGAAAAGCAA
TGTATGCCCCTCCCATCCAAGGACTAATTAACTGTGCATCAAAT---
ATTACAGGACTGTTATTAACAAGGGATGGTGGTGAGGGTAATGGCACA-------------------------
----

AACCAGACTGCTGAGGTCTTCAGACCTGTAGGAGGAAATATGAAGGACAATTGGAGAAGTGAATTATATAAAT
ATAAAGTAGTAAAAATTGAACCACTA---GGAGTGGCACCCACCAAGGCAAGGAGAAGAGTGGTG--------
-------------------------------CAGAGAGAAAGAAGAGCA---GTGGGA---GTAGGAGCT---
------TTGCTCCTTGGGTTCTTGAGTGCA---GCAGGAAGCACTATG---GGCGCAGCGTCA---
GTGACGCTGACGGTACAG---GCCAGACAA---TTATTG---TCTGGTATAGTGCAACAGCAAAAC---
AATTTG---CTGAGG---GCTATTGATGCGCAACAGCATATGTTGCAGCTCACAGTCTGGGGCATC
>JN248347
TTCTACTGTAATACATCACAACTGTTTAATAGTACTTGGCTGAATAGTACTTGGACTAACACCAGTACTTGGA
AT---
-------------GATACT------
GCAGGAAATGACACTATCACGCTCCCCTGCAGAATAAAACAAATTGTAAACATGTGGCAGGGAGTAGGAAAAG
CAATGTATGCCCCTCCCATCGCAGGAAAAATTAGATGTTCATCAAAT---
ATTACAGGGTTGTTATTAACAAGAGATGGTGGTAATAGCACGAAC----------------------------
----

GAGACCTTCAGACCTGGAGGAGGAGAGATGAGGGACAATTGGAGAAGTGAATTATATAAATATAAAGTAGTAA
AAATTGAACCATTA---GGAGTAGCACCCACCAGGGCAAAGAGAAGAGTGGTG--------------------
-------------------CAGAGAGAAAAGAGAGCG------GGA---ATAGGAGCT---------
GTGTTCCTTGGGTTTTTGGGAGCA---GCAGGAAGCACTATG---GGCGCAGCGTCA---
ATAACGCTGACGGTACAG---GCCAGACAA---TTGTTG---TCTGGTGTAGTGCAGCAGCAGAGA---
AATTTG---CTGAAG---GCTATTGAGGCGCAACAGCATCTGTTGCAACTCACAGTCTGGGGCATC
>JN248353
TTTTACTGTAATACAACAACACTGTTTAATAGTACTTGGACTAATAGTACTTGGACTAGCAACTCAACTGACA
CTGAA--

GGCAATATCACACTCACACTCCCATGCAGAATAAAACAAATTGTAAACATGTGGCAGGAAGTAGGAAAAGCAA
TGTATGCCCCTCCCATCCAAGGAAAGATTACATGTTTATCAAAT---
ATTACAGGGCTGCTATTGACAAGAGATGGTGACAAAAACGAAACAGGG-------------------------
----

GAGACCTTCAGACCTATAGGAGGAAATATTAAGGACAATTGGAGAAGTGAATTATATAAATATAAAGTAGTAC

AGATTGAACCATTA---GGAGTAGCACCCACCCAGGCAAAGAGAAGAGTGGTG--------------------
-------------------CAGAGAGAAAAAAGAGCA---ATAGGGGAATGGGAGCT---------
ATGTTCCTTGGGTTCTTGGCAGCA---GCAGGAAGCACTATG---GGCGCAGCGTCA---
GTGACGCTGACGGTACAG---GCCAGGCAA---TTATTG---TCTGGTATAGTGCAACAGCAGAAC---
AACCTG---CTGAGG---GCTATTGAGGCGCAACAGCACCTGTTGCAACTCACAGTCTGGGGCATC
>JN248354
TTCTACTGTAATACATCACAACTGTTTAATAGTACTTGGTTTGTCAATGGTACTAATGGTACTACAGTGCCAA
ATAATGAC---

ACTGAAAAAATTATCACACTCCCATGTAAAATAAAACAAATTATAAACATGTGGCAAGAAGTAGGAAAAGCAA
TATATGCCCCTCCCATTGCAGGACCAATTAACTGTTCATCAAAT---
ATTACAGGGCTGCTATTAACAAGAGATGGTGGTAATAATAACGAGACC-------------------------
----

ACCACCAATGAAACCTTCAGACCTGGAGGAGGAGATATGAGGGACAATTGGAGAAGTGAATTATATAAATATA
AAGTAGTAAAAATTGAACCTTTA---GGAATAGCACCCACCAGGGCAAAGAGAAGAGTGGTG-----------
----------------------------CAAAGAGAAAAAAGAGCA---GTAACG---CTAGGAGCT------
---ATGTTCCTTGGGTTCTTGGGAGCA---GCAGGAAGCACTATG---GGCGCAGCGTCA---
ATGACGCTGACGGTACAG---GCCAGACTA---TTATTG---TCTGGTATAGTGCAGCAGCAAAAC---
AATTTG---CTGAGA---GCTATTGAGGCGCAACAGCATATGTTGCAACTCACAGTCTGGGGCATC
>JF297222
TTCTACTGTAATACATCAAAACTGTTTAATAGTGCTTGGCCGCCTAATAGTAATTGGAATAACAGGAATAAC-
----

ACTGGAGGAAATATCACACTCCCATGCAGAATAAAACAAATTATAAACATGTGGCAGAGAGTAGGACAAGCAA
TGTATGCCCCTCCCATCAGTGGAATCATTAACTGTGTATCAAAT---
ATTACAGGGCTAATATTAACAAGAGATGGGGGTAATACTAGTGATACTAAG----------------------
----

AATGACACAGAGATCTTCAGGCCTGCAGGAGGAGATATGAGAGACAATTGGAGAAGTGAGTTATACAGATATA
AAGTAGTAAGAATTGAACCATTA---GGAGTAGCCCCCACCAAGGCAAAAAGAAGAGTGGTG-----------
----------------------------CAGAGAGAAAAAAGAGCA---GTGGGA---ATAGGAGCT------
---GTGTTCATTGGGTTCTTGGGAGCA---GCAGGAAGCGCTATG---GGCGCAGCGTCA---
ATGACGCTGACGGTACAG---GCCAGACAA---TTATTG---TCTGGTATAGTGCAACAGCAAAGT---
AATTTG---CTGAGA---GCTATTGAGGCGCAACAACATATGTTGCAACTCACAGTCTGGGGCATT
>JX446800
TTCTACTGTAATACAACACCATTGTTTAATAGTACCTGGTTTAGTACTGAGAATAGTACCTGGAATAGTAATA
ATAGTATT---

GGGAATGATACTATTATACTCACATGCAGAATAAAACAAATTATAAACATGTGGCAGCAAGTAGGAAAAGCGA
TGTATGCCCCTCCCATCAAGGGACGAATTAACTGTACCTCAAAT---
ATTACAGGACTGATATTAACAAGAGATGGTGGCCATAATAATTCT----------------------------
----

AGCAAGAATGAGACCTTCAGACCTGGAGGAGGAGATATGAGGGACAATTGGAGAAATGAATTATATAAATATA
AAGTAGTAAAAATTGAACCATTA---GGAATAGCACCCACCAAGGCAAAGAGGAGAGTGGTG-----------
----------------------------CAGAGAGAAAAAAGAGCA---ATAGGAACACTAGGAGCT------
---GTGTTCCTTGGGTTCTTGGGAACA---GCAGGAAGCACTATG---GGCGCAGCAGCA---
GTGACGCTGACGGTACAG---GCCAGACAA---TTATTG---TCTGGTATAGTGCAACAGCAGAAC---
AATCTG---CTGATG---GCTATTGAGGCGCAACAGCGTCTGTTGCAACTCACAGTCTGGGGCATC
>JX446818
TTCTACTGTAATACATCAAAATTATTTAATAGTACTTGGAATAATACAAGTAATGACAGTACA----------
----

AGTAATGGCACAATCACACTCCCATGCAGAATAAAACAAATCATAAACATGTGGCAGCAAGTAGGAAAAGCAA
TGTATGCCCCTCCCATCCAGGGACAAATTAACTGTTCATCAAAT---
ATCACAGGGATACTATTAACAAGAGATGGTGGTAATGGTGGTAATACC-------------------------
----

AACGATACAGAGGTCTTCAGACCTGCAGGAGGGGATATGAGGAACAATTGGAGGAGTGAATTATACAAATATA

AAGTAGTACAAATTGAACCAGTA---GGAATAGCACCCACCAAGGCAAGGAGAAGAGTGGTG-----------
----------------------------CAAAGAGAAAAAAGAGCA---GTAACA---CTAGGAGCT------
---GTGTTCCTTGGGTTCTTGGGAACA---GCAGGAAGCACTATG---GGCGCAGCGTCA---
ATAGCGCTGACGGCACAG---GCCAGACAA---TTGTTG---TCTGGTATAGTGCAACAGCAGAAC---
AATCTG---CTGAGG---GCTATTGAGGCGCAACAGCATCTGTTGCAACTCACGGTCTGGGGCATT
>JX447795
TTCTACTGTAATACCTCACAACTGTTTAATAGTACTTGGCATACTAATGATACTTGGAATGGTACAAAGGGT-
----

AACAATGGCACTATCACGCTCCCTTGCAGAATAAAACAAATTATAAACATGTGGCAGGAAGTAGGAAAAGCAA
TGTATGCCCCTCCCATCAAAGGACCGATTGGATGTTTATCAAAC---
ATTACAGGGTTGCTATTAACAAGAGATGGTGGTGGGGGAACGAGACC--------------------------
----

AGGAACAACGAGACCTTCAGACCTGCAGGAGGAGATATGAGGGACAATTGGAGAAGTGAATTATATAAATATA
AAGTAGTAAGAATTGAACCATTA---GGAGTAGCACCTACCAAGGCAAAGAGAAGAGTGGTG-----------
----------------------------CAGAGAGAAAAAAGAGCA---GTGGGAATGCTAGGAGCT------
---ATGTTCCTTGGGTTCTTGGGAACA---GCAGGAAGCACTATG---GGCGCAGCGTCA---
ATAACGCTGACGGTACAG---GCCAGGCAA---TTGTTG---TCTGGTATAGTGCAACAGCAGAGA---
AATCTG---CTGAGG---GCTATTGAGGCGCAGCAGCACCTGATGCAACTCACAGTCTGGGGCATC
>JX447156
TTCTACTGTGATACATCACAACTGTTTAATAGTACTTGGGATGTTAATAGTACTTGGAATGGTACTGGGAATG
ATACCCCA---

GAAAATAGCACTATCATGCTCCCTTGCAGAATAAAACAAATTGTAAACATGTGGCAGGAAGTAGGAAAAGCAA
TGTATGCCCCTCCTATTGCAGGACAAATTAACTGTTCATCAAAT---
ATTACCGGGTTGCTATTAACAAGAGATGGTGGTGATAATAGCAAT----------------------------
----

ACCAGCGAGACCTTCAGACCTGGAGGAGGAAATATGAAGGACAATTGGAGAAGTGAATTATATAAATACAAAG
TAGTAAAAATTGAACCATTA---GGAGTAGCACCTACCAGGGCTAAGAGAAGAGTGGTG--------------
-------------------------CAGAGAGAAAAAAGAGCA---GTGGGAACGATAGGAGCT---------
ATGTTCCTTGGGTTCTTGGGAGCA---GCAGGAAGCACTATG---GGCGCAGCGTCA---
ATAACGCTGACGGTACAG---GCCAGACAA---TTGTTG---TCTGGTATAGTGCAACAGCAGAGA---
AATTTG---CTGAGA---GCTATTGAGGCGCAACAACATATGTTGCAACTCACAGTCTGGGGCATC
>JF297229
TTCTACTGTAATACATCACAACTGTTTAATAGTACTTGGAATGTTAATGATACTAAGAATTGGAATAAT----
----

ACTGCAGGAAATATCACGCTCATCTGTAGAATAAGACAAATTGTAAACATGTGGCAGGAAGTAGGAAAAGCAA
TGTATGCCCCTCCCATTGCAGGAATAATTAACTGTGTATCAAAT---
ATTACAGGGTTGATTTTAACAAGAGATGGTGGTAAAAATGGTGGTAATGAT----------------------
----

ACAAATAACTTCACTGAGATCTTCAGACCTGGAGGAGGAGATATGAAGGACAATTGGAGAAGTGAATTATATA
AATATAAAGTAGTAAAAATTGAACCATTA---GGAGTAGCACCTACCAAGGCAAAGAGAAGAGTGGTG-----
----------------------------------CAGAGAGAAAAAAGAGCA---GTG------
CTAGGAGCT---------ATGTTCCTTGGGTTCTTGGGAGCA---GCAGGAAGCACTATG---
GGCGCAGCGTCA---ATAACGCTGACGGTACAG---GCCAGACAA---TTGTTG---
TCGGGTATAGTGCAACAGCAGAGA---AATCTG---CTGAGG---
GCTATTGAGCTGCAACAGCATCTGTTGCAACTCACAGTCTGGGGCATC
>MG989620
TTCTACTGTAACACAACAAAACTGTTTAATAGTACTTGGAATAATACTGGGGAGAACATTACTAGTCCTGCAG
AT---

ACAAACGACACTATCATACTCCCATGCAGAATAAAACAAATTGTAAGAATGTGGCAGGAAGAAGGCAAAGCAA
TGTATGCCCCTCCCATCAGAGGAAATATTAGCTGTTCATCAAAT---
ATTACAGGGTTGATATTAACAAGAGATGGCGGGGACGAAGGCAAC----------------------------
----

CAAACCCAAGTAAATGAAACCTTCAGACCTGGAGGAGGAAATATGATGGACAATTGGAGAAGTGAATTATATA
GATATAAAGTAGTAAAAATTGAACCATTA---GGAATAGCACCCACCAAGGCAAAGAGAAGAGTGGTG-----
----------------------------------CAAAGAGAAAGAAGAGCA---
GTGGGAGTAATAGGAGCT---------ATGTTCCTTGGATTCCTAGGAACA---GCAGGAAGCACTATG---
GGCGCAGCGTCA---ATAACGCTGACGGTACAA---GCCAGCAAA---TTATTG---
TCTGGTATAGTGCAACAGCAGAGC---AATCTG---CTGAGA---
GCTATTGAGGCGCAACAGCATCTGTTGCAACTCACGGTCTGGGGCATT
>MG989578
TTCTACTGTAATACAACACCACTGTTTAATAGTATTTGGCATAGTAATAATACTTGGAATAGTAGGACTGGA-
----
-GAGCCAACTAACACCACT------
GGAGGAAATGGCACCATCACACTCCAATGCAGAATAAAACAAATTATAAACATGTGGCAGCAAGTAGGGAAAG
CAATGTATGCCCCTCCCATCAGTGGACCAATTAACTGCTCATCAAAT---
ATTACAGGGCTACTATTACTAAGAGACGGGGGTAACCACAAAGATAGC-------------------------
----

AATGACACCGAGATCTTCAGACCGGGAGGAGGAGATATGAGGGACAATTGGAGAAGTGAATTATATAAATATA
AAGTAGTAAAAATTGAACCATTA---GGAGTAGCACCAACCATGGCAAGAAGAAGAGTGGTG-----------
----------------------------CAGAGAGACAAAAGAGCG---GCA------CTAGGAGCG------
---GTGTTCCTTGCGTTCTTGGGAGCA---GCAGGAAGCACTATG---GGCGCAGCGTCA---
TTGACGCTGACGGTACAG---GCCAGACAA---TTGCTG---TCTGGTATAGTGCAACAGCAGAAC---
AATTTG---CTGAGA---GCTATTGAGGCGCAACAGCATATGTTGCAACTCACAGTCTGGGGCATA
>MG989587
TTCTACTGTAATACAACACCACTGTTTAATAGTACTTGGAATTTTACTAGTGCTGGGGAGCTTAATAACACTG
CTAATGAGACT--

AATGATGATAACATCACACTCCAATGCAGAATAAAACAAATTATAAACATGTGGCAGCAAACAGGAAAAGCAA
TGTATGCCCCTCCCATCAAAGGACTAATTGACTGTACATCAAAT---
ATTACAGGGCTACTATTAATAAGAGATGGTGGTAACGAGAGTAGC----------------------------
----

ACAAATGAGACCTTCAGACCAGAAGGAGGAAATATGAAGGACAATTGGAGAAGTGAACTATATAAATATAAAG
TAGTACAAATTGAACCATTA---GGAATAGCACCCACCAAGGCAAAGAGAAGAGTGGTG--------------
-------------------------CAGAGAGAAAAAAGAGCA---GTGGGA---ATGGGAGCT---------
TTGTTCCTTACGTTCTTGGGAACA---GCAGGAAGCACTATG---GGCGCAGCGTCA---
TTAACGCTGACGGTACAG---GCCAGACAA---TTATTG---TCTGGTATAGTGCAGCAGCAGAGC---
AATCTG---CTGAAG---GCTATTGAGGCGCAACAGCATATGTTGCAACTCACCGTCTGGGGCATC
>MG989506
TTTTATTGTAATACCTCACAGCTGTTTAATAGTATTTGGAATAGTAGTACTGGGAATAATAATAGTACTGGG-
----

AATAAGAGCACTATCATACTCCCTTGTAGAATAAAACAAATTATAAACATGTGGCAGAAGGTAGGAAAAGCAA
TGTACGCCCCTCCCATCGAAGGAATGATTAACTGTTTATCAAAT---
ATCACAGGGTTGCTGTTAACAAGAGATGGTGGCAGGGTGAATGGGACT-------------------------
----

GAGGAAAATAGGACTGAGATCTTCAGACCTGGAGGAGGAGATATGAGGGACAATTGGAGAAGTGAATTATATA
AATATAAAGTAGTAAAAATTGAACCATTA---GGAGTAGCACCCACCAAAGCAAAGAGAAGAGTGGTG-----
----------------------------------CAGAGAGAAAAAAGAGCA---GTAGGA---
ATAGGAGCT---------GTGTTCCTTGGGTTCCTAGGAGCA---GCAGGAAGCACTATG---
GGCGCAGCGTCA---ATAACGCTGACGGTACAG---GCCAGACAA---TTAATG---
TCTGGCATAGTGCAACAGCAAAGA---AATTTG---CTGAGG---
GCTATTGAGGCGCAACAGCATCTGTTGCAACTCACAGTCTGGGGCATC
>MG989541
TTCTACTGTAATACAACACAGCTATTTAATAGTAAGTGGAATGATAGTAATTGGAATAGTACTTGGAATAATA
CTGAAAAG---

TCAAATGATATAATCACACTCCCCTGCAGAATAAAACAGATTATAAACATGTGGCAGGAAGTAGGAAAAGCAA
TGTATGCCCCTCCCATCCAGGGACTAATTAAATGTTCATCAAAC---
ATTACAGGGATACTATTAACAAGAGATGGTGGTAAT-------------------------------------

----
GTGACTTTAGATAATGGACTTCCAGTAATGAGACTGAGATCTTCAGACCTGCAGGAGGAGATATGAGAGACA
ATTGGAGAAGTGAATTATATAAATATAAAGTAGTAAGAGTTGAACCAGTA---
GGAATAGCACCCACCAGGGCAAAGAGAAGAGTGGTG------------------------------------
--CAGAGAGAAAAAAGAGCG---GTAACA---CTGGGAGCT---------
GTGTTCCTTGGGTTCTTGGGAACA---GCAGGAAGCACTATG---GGCGCAGCGTCA---
ATAGCGCTGACGGCACAG---GCCAGACAA---TTATTG---TCTGGTATAGTGCAACAGCAAAAC---
AATTTG---CTGAGA---GCTATTGAGGCGCAACAGCACCTGTTGCAACTCACGGTCTGGGGCATC
>MG989610
TTTTACTGTAATACAACACCACTGTTTAATGGGACACTGATTAATGGGACTTGGAATGGTATTGAATGGCCAA
ATAAT---

ACGAATGGAACTATCACACTCCAGTGCAGAGTAAAACAAATTATAAACATGTGGCAAAAAGTAGGAAAAGCAA
TGTATGCCCCGCCCATCAAAGGACTAATTAAATGCTCATCAAAT---
ATTACAGGGCTGATATTAACAAGAGATGGTGGCGGGAATGGGACA---------------------------
----

AATGAGACCTTCAGACCTGCAGGAGGAGATATGAGGGACAATTGGAGAAGTGAATTATATAAGTATAAAGTAG
TAAAACTTGAACCATTA---GGAGTAGCACCCACCAAGGCAAAGAGAAGAGTGGTG----------------
----------------------CAGAGAGAAAAAAGAGCA---GTGGGA---ATAGGAGCT---------
TTGTTCATTGGGTTCTTGGGAGCA---GCAGGAAGCACTATG---GGCGCAGCGTCA---
CTAACGCTGACGGTACAG---GCCAGACAA---TTGTTG---TCTGGTATAGTGCAACAGCAGAGC---
AATCTG---CTGAGG---GCTATTGAGGCGCAACAGCATATGTTGCAACTCACAGTCTGGGGCATT
>MG989551
TTCTACTGTAATACAACACCACTGTTTAATATTACTAACACCACAAATGACAAT------------------
----

GACAATAACACAATCATACTCCAATGCAAAATAAAACAGATTATAAATATGTGGCAGAAAGTAGGGAGAGCAA
TGTATGCCCCTCCCATCAGTGGAAAAATTATGTGTTCATCAAAC---
ATTACAGGGCTACTGTTAACAAGAGATGGTGGGAATAATAGGACT---------------------------
----

AATGAGACCTTCAGACCTGCAGGGGGAGATATGAGGGACAATTGGAGAAGTGAATTATATAAATATAAGGTAG
TAAAAATTGAACCTTTA---GGAATAGCACCCACCAAGTCAAGGAGGCTAGTGGTG----------------
----------------------CAAAGAGAAAAACGAGCA---GTGGGA---ATGGGAGCT---------
ATGTTCCTTGGGTTCTTGGGAGCA---GCAGGAAGCACTATG---GGCGCGGCGTCA---
ATGACGTTGACGGTACAG---GCCAGACAA---TTATTG---TCTGGTATAGTGCAACAGCAGAAC---
AACTTG---CTGAAG---GCTATTGAGGCGCAACAGCACCTGTTGCGACTCACAGTCTGGGGCATC
>U39258
TTCTACTGTAATACATCACAACTGTTTAATGGTACTTGGAATAATACTATA---------------------
----

GAAAATAGCACTATCACGCTCCCTTGCAGAATAAGACAAATTGTAAACATGTGGCAGGAAGTAGGAAAAGCAA
TGTATGCCCCTCCCATCAGAGGACAAATTAGATGTTCATCAAAT---
ATTACAGGGCTGCTATTAACAAGAGATGGTGGTAATAATGGGAGC---------------------------
----

AATACCACCGAGACCTTCAGACCTGGAGGAGGAAATATGAGGGACAATTGGAGAAGTGAATTATATAGATATA
AAGTAGTAAAAATTGAACCATTA---GGAGTAGCACCCACCAAGGCAAAGAGAAGAGTGGTG----------
----------------------------CAGAGAGAAAAAAGAGCA---GTGGGAACAATAGGAGCT-----
---ATGTTCCTTGGGTTCTTGGGAGCA---GCAGGAAGCACTATG---GGCGCAGCGTCA---
ATAACGCTGACGGTACAG---GCCAGACAA---TTGTTG---TCTGGTATAGTGCAACAGCAGAGA---
AATTTG---CTGAGG---GCTATTGAGGCGCAACAGCATCTGTTGCAACTCACAGTCTGGGGCATC
>DQ354116
TTCTACTGTAATACAACAGGACTGTTTAATAGTACTTGGAATAATACTAGTACTGGGAATAATACTACA---
----

GGAAATGACAATATCACGCTCCCTTGCAGAATAAGACAAATTGTAAACATGTGGCAGGAAGTAGGAAGAGCAA
TGTATGCCCCTCCCATCGAGGGACAGATTAGATGTTCATCAAAT---
ATTACAGGGTTGCTATTAACAAGAGATGGTGGTAATGATAAGAAC---------------------------

```
------------------------------------------------------------------------
----------
GCGACCACCGAGACCTTCAGACCTGGAGGAGGAGATATGAGGGACAATTGGAGAAGTGAATTATATAAATATA
AAGTAGTAAAAATTGAACCATTA---GGAGTAGCACCCACCAGGGCAAAGAGAAGAGTGGTG----------
---------------------------CAAAGAGAAAAAAGAGCA---GTGGGAACGATAGGAGCT------
---ATGTTCCTTGGGTTCTTGGGAGCA---GCAGGAAGCACTATG---GGCGCAGCGTCA---
ATAACGCTGACGGTACAG---GCCAGACAA---TTGTTG---TCTGGTATAGTGCAACAGCAGAGA---
AATTTG---CTGAGA---GCTATTGAGGCGCAACAGCATCTGTTGCAACTCACAGTCTGGGGCATC
>DQ354118
TTCTACTGTAATACATCAAAACTGTTTAATAGTACTTGGCATACTAATAGTACTTGGCATACTAATAGTACT-
------------------------------------------------------------------------
----------------------------
GGGAATGATACTATCACGCTCCCTTGCAGAATAAAACAAGTTGTAAACATGTGGCAAGAAGTAGGAAAAGCAA
TGTATGCCCCTCCCATCAAAGGACCAATTAGATGCTCATCAAAT---
ATTACAGGGTTGCTATTAACAAGAGATGGTGGTAAAAAAGGGAACGAA------------------------
------------------------------------------------------------------------
-------
ACCAACGAGACCGAAACCTTCAGACCTGCAGGAGGAAATATGAAGGACAATTGGAGAAGTGAATTATATAAAT
ATAAAGTAGTAAAAATTGAACCATTA---GGAGTAGCACCCACCAGGGCAAAGAGAAGAGTGGTG--------
-------------------------------CAGAGAGAAAAAAGAGCA---GTGGGGACGATAGGAGCT---
------ATGTTCCTTGGGTTCTTGGGAGCA---GCAGGAAGCACTATG---GGCGCAGCGTCA---
ATAACGCTGACGGTACAG---GCCAGACAA---TTGCTG---TCTGGTATAGTGCAACAGCAGAGA---
AATTTG---CTGAGG---GCTATTGAGGCGCAACAGCATCTGTTGCAACTCACAGTCTGGGGCATT
>DQ354119
TTCTACTGTAATTCAACACCGCTGTTTAATGGGACGTTTAATAGTACTGGGAATGGAACTGGAGAGTCACAG-
------------------------------------------------------------------------
----------------------------
AGCAATGGAAATATCACACTCCAGTGTAGAATAAGACAAATTATAAACAGGTGGCAGGAAGTAGGAAAAGCAA
TGTATGCCCCTCCCATCAGCGGACTAATTAGATGTTCATCAAAT---
ATTACAGGGCTACTATTAACAAGAGATGGTGGAAACGGGAGTACG---------------------------
------------------------------------------------------------------------
----------------
AATGAGACCTTCAGACCTGGGGGAGGAAACATGAGGGACAATTGGAGAAGTGAATTATATAAATATAAAGTAG
TAAAAATTGAGCCATTA---GGGGTAGCACCCACCAAGGCAAAGAGAAGAGTGGTG----------------
----------------------CAGAGAGAAAAAAGAGCA---GCGATA------GGAGCT---------
ATGTTCCTTGGGTTCTTGGGAGCA---GCAGGAAGCACTATG---GGCGCAGCGTCG---
GTGACGCTGACGGTACAG---GCCAGACAA---TTATTG---TCTGGTATAGTGCAGCAGCAGAAC---
AATCTG---CTGAGG---GCTATTAACGCGCAACAGCATCTGTTGCAGCTCACAGTCTGGGGCATC
>AY945711
TTCTACTGTGATACATCAAAACTGTTTACTTGGAATAGTACTTGGAATAGTACTTGGAATAATAATACTTGGA
ATGAT-------------------------------------------------------------------
-------------------ACT---
ATAGGAGATAGCAATATCACGCTCCCTTGCAGAATAAAACAAGTTGTAAACATGTGGCAGGAAGTGGGAAAAG
CAATGTATGCCCCTCCTATTGAAGGACAAATTAGATGTTCATCAAAT---
ATTACAGGGTTGCTATTAACAAGAGATGGTGGTAATAATGGAGAC---------------------------
------------------------------------------------------------------------
----------
AATGACACCGAGATCTTCAGACCTGGAGGAGGAGATATGAGGGACAATTGGAGAAGTGAATTATATAAATATA
AAGTAGTAAAAATTGAACCATTA---GGAGTAGCACCCACCAGGGCAAAGAGAAGAGTGGTG----------
---------------------------CAGAGAGAAAAAAGAGCA---GTGGGAACAATAGGAGCT------
---ATGTTCCTTGGGTTCTTGGGAGCA---GCAGGAAGCACTATG---GGCGCAGCGTCA---
ATAACGCTGACGGTACAG---GCCAGACAG---CTGTTG---TCTGGTATAGTGCAACAGCAGAGA---
AATTTG---CTGAGG---GCTATTGAGGCGCAACAGCATCTGTTGCAACTCACAGTCTGGGGCATC
>HM215431
TTCTACTGTGACTCAACACCACTGTTTAATAGTACTTGGAAGAATATTACAGAGTTAGATGCTACAAAGTTAG
ATAAC-------------------------------------------------------------------
-------------------GCT---
ACAGAAAAAGGAAATATCACACTCAAATGTAGAATAAAACAAATTATAAACATGTGGCAGGGAGTAGGAAAAG
CAATGTATGCCCCTCCTATCAGTGGACCAATTAGCTGTTTATCAGAA---
ATTACTGGGCTACTATTAACAAGAGATGGTGGTGTCACTAAGAATAATGAGACC------
```

AATGGGACCACTGGGAAT---GAT---

AAGACCGAGGTCTTCAGACCTGGAGGAGGAGACATGAGGGACAATTGGAGAAGTGAATTATATAAATATAAAG
TAGTACAAATTAAGCCATTA---GGAATAGCACCCACCAAGGCAAAGAGGAGAGTGGTG--------------
-------------------------CAGAGAGAAAAAAGGGCA---GTGACA---TTTGGAGCT---------
ATGTTCCTTGGGTTCTTGGGAGCA---GCAGGAAGCACTATG---GGCGCAGCGTCA---
ATAGCGCTGACGGGACAG---GCCAGACAA---TTATTG---TCTGGTATAGTGCAACAGCAGAAT---
AATCTG---CTGAGG---GCTATTGAGGCGCAACAGCATCTGTTGCAACTCACTGTCTGGGGCATC
>HM215435
TTCTACTGTAATACATCACAACTGTTTAATAGCACTTGGCTTAATAACAATTCA-------------------
----

ACTAATGGAACTATCATACTCCCATGCAGAATAAAACAATTTATAAACATGTGGCAGGAAGTAGGAAAAGCAA
TGTATGCCCCTCCCATCAGCGGAAATATTAGCTGTTCATCAAAT---
ATTACAGGGCTGCTATTAACAAGAGATGGTGGTAATGGCAAAAACACC-------------------------
----

AACTCCTCTGAAACCTTCAGACCTGGAGGAGGAGATATGAGGGACAATTGGAGAAGTGAATTATATAAATACA
AAGTAGTAAGAATTGAACCACTA---GGAATAGCACCCACCAAGGCAAGGAGAAGAGTGGTG-----------
----------------------------CAGAGAGAAAAAAGAGCG---GTAATA------GGAGCT------
---ATGTTCCTTGGGTTCTTGGGAGCA---GCAGGAAGCACTATG---GGCGCAGCGTCA---
GTGACGCTGACGGTACAG---GCCAGACTA---TTATTG---TCTGGTATAGTGCAACAGCAGAAC---
AATCTG---CTGAGA---GCTATTGAGGCGCAACAGCATATGTTGCAACTCACAGTCTGGGGCATC
>EU839606
TTCTACTGTAATACAACAAAACTGTTTAATAGTACTTGGAATAGTAATGGTATTTGGAATAATACTATAGAGT
CAAATGAC---
-------------------ACA---
GAGTCAAATGATACTATCACACTCCCATGCAGAATAAAACAAATCATAAACATGTGGCAGGGAGTAGGTAAAG
CAACGTATGCCCCTCCCATTAGAGGACAAATTAGATGTTCATCAAAT---
ATTACAGGATTACTATTAACAAGAGACGGTGGTAATAACACAAGT----------------------------
----

GGCACCGAGACCTTCAGACCTGGAGGAGGAGATATGAGGGACAATTGGAGAAGTGAATTATATAAATATAAAG
TAGTAAAAATTGAACCATTA---GGAGTAGCACCCACCAAGGCAAAAAGAAGAGTGGTG--------------
-------------------------CAGAGAGAAAAAAGAGCA---GTGGGAACAATAGGAGCT---------
ATGTTCCTTGGGTTCTTGGGAGCA---GCAGGAAGCACTATG---GGCGCAGCATCA---
CTAACGCTGACGGTACAG---GCCAGACAA---TTATTG---TCTGGTATAGTGCAACAGCAGAAC---
AATCTG---CTGAGG---GCTATTGAGGCGCAACAACATCTGTTGCAACTCACGGTCTGGGGCATC
>EU839607
TTCTACTGTAACACATCACACCTGTTTAACAATACTTGGAATGGTACTGAGACTGATACTTGGAATATTACAG
GG---

GAAGATAGTAATATCACACTTCCATGTAGAATAAAACAAATTGTAAACATGTGGCAGGAAGTAGGTAAAGCAA
TGTATGCCCCTCCCATCAGAGGACAAATTAGCTGTTCATCAAAT---
ATTACAGGGATGCTATTAACAAGAGATGGTGGTAATGGTAATGACACG-------------------------
----

AACAACGAGACTGAGATCTTCAGACCTGCAGGAGGAGATATGAGGGACAATTGGAGAAGTGAATTATATAAAT
ATAAAGTAGTACAAATTGAACCATTA---GGAGTAGCACCCACCAAGGCAAAGAGAAGAGTGGTG--------
-------------------------------CAAAGAGAGAAAAGAGCA---GTGGGAGCAATAGGAGCT---
------ATGTTCCTTGGGTTCCTGGGAGCA---GCAGGAAGCACTATG---GGCGCAGCGTCG---
ATAACGCTGACGGTACAG---GCCAGACAA---CTACTG---TCTGGTATAGTGCAACAGCAGAAC---
AATCTG---CTGAAG---GCTATTGAGGCGCAACAGCATCTGTTGCAACTCACGGTCTGGGGCATC
>HQ217662
TTCTATTGTAATGCAACACAATTGTTTAATAGTACTTGGAGTATTGGGAATGATACTATAGAAAAT-------
----

AGCAATATCAATATCACACTCCCATGCAGAATAAAACAAATTGTAAACATGTGGCAGGAAGTAGGCAAGGCAA
TATATGCCCCTCCCATTAGAGGACAAATTAGATGTTCATCAAAT---
ATTACAGGAATACTATTAACAAGAGATGGTGGTGATACTAATGAC----------------------------

----

ACGACCGAGATCTTCAGACCTGGAGGAGGAGATATGAGGGAAAATTGGAGAAGTGAATTATATAAATATAAAG
TAGTAAGGATTGAACCAATA---GGAGTAGCACCCACCAAGGCAAAGAGGAGAGTGGTG--------------
-------------------------CAAAGAGAAAAAAGAGCA---GTGGGAGCAATAGGAGCT---------
ATGTTCCTTGGGTTCTTGGGAGCA---GCAGGAAGCACTATG---GGCGCAGCGTCA---
CTGACGCTGACGGTACAG---GCCAGACAA---TTATTG---TCTGGTATAGTGCAACAGCAGAAC---
AATCTG---CTGAGG---GCTATTGAGGCGCAACAACATCTGTTGCAACTCACGGTCTGGGGCATC
>EU839608
TTCTACTGTAATACATCCCAACTATTTAATAGTACTTGGAAGAATGGCACTTGGTATAGTAATAGTACTAATA
CTACAGAG---
-------------TCAAGC------
AGAAATATTACACTCATTACACTCCCATGCAGAATAAAACAAATTATAAACATGTGGCAGGCAGTAGGCAAAG
CAATGTATGCCCCTCCCATTAGAGGACCAATTACATGTGTATCAAAC---
ATCACAGGGCTGCTATTAATAAGAGATGGTGGTAGAAACGAGAATGAC-------------------------
----

AATAACACTGAGATCTTCAGACCTGGAGGAGGAAATATGAAGGACAATTGGAGAAGTGAATTATATAAATATA
AAGTAGTAAAAATTGAACCATTA---GGAATAGCACCCACCAAGGCAAAGAGAAGAGTGGTG-----------
----------------------------CAGAGAGAAAAAAGAGCA---GTGGGAACAATAGGAGCT------
---ATGTTCCTTGGGTTCTTGGGAGCA---GCAGGAAGCACTATG---GGCGCAGCGTCA---
ATGACGCTGACGGTACAG---GCCAGACTA---TTATTG---TCTGGTATAGTGCAACAGCAAAAC---
AATTTG---CTGAGG---GCTATTGAGGCGCAACAGCATCTGTTGCAACTCACGGTCTGGGGCATC
>EF593260
TTCTACTGTAATACAACACAGCTGTTTAATAGTACTTGGAATATTAGTAGTACTTGGAATGATACTGGAGGG-
----

ACTGATAGTAACATCACACTCCCATGCAGAATAAAACAAATTATAAACATGTGGCAGCAAGTAGGCAGAGCAA
TGTATGCCCCTCCTATCAGAGGACAAATTAGATGTACATCAAAT---
ATCACAGGGCTGCTATTAACAAGAGATGGTGGTAATGACACCACTAAT-------------------------
----

AATAATACAGAGATCTTCAGACCTGGAGGAGGAAATATGAAGGATAATTGGAGAAGTGAATTATATAAATATA
AAGTAGTAAAAATTGAACCATTA---GGAGTAGCACCCACCAAGGCAAAGAGAAGAGTGGTG-----------
----------------------------CAAAGAGAAAGAAGAGCA---GTGGGAACACTGGGAGCT------
---ATGTTCCTTGGGTTCTTGGGAGCA---GCAGGAAGCACTATG---GGCGCAGCGTCA---
CTGACGCTGACGGTACAG---GCCAGACTA---TTATTG---TCTGGTATAGTGCAACAGCAAAAC---
AATTTG---CTGAGA---GCTATTGAGGCGCAACAGCATATGTTGCAACTCACTGTCTGGGGCATC
>EU578580
TTCTACTGTAATACAACACAGCTGTTTGATAGTACTTGGCTGTTTAATAGTACTTGGAATGATAGTAGTACTG
AGAATGGTACT--
----------ACAAAGCAA------
AATGGCAATGACACACTCACACTTCCATGCAGAATAAAACAAATTGTAAACATGTGGCAGACAGTAGGCAAAG
CAATGTATGCCCCTCCCATCAAAGGACAAATTAGATGTTCATCAAAA---
ATTACAGGCTTGCTATTAACAAGAGATGGTGGTAATAGAGATGATGGT-------------------------
----

AATAACACCGAGATCTTCAGACCTGGAGGAGGAGATATGAGGGACAATTGGAGAAGTGAATTATATAAATATA
AAATAGTAAAAATTGAACCATTA---GGAGTAGCACCCACCAAGGCAAGGAGAAGAGTGGTG-----------
----------------------------CAAAGAGAAAAAAGAGCA---GTGGGAACAATAGGAGCT------
---ATGATCCTTGGGTTCTTGGGAACA---GCAGGAAGCACTATG---GGCGCAGCGTCA---
ATAACGCTGACGGTACAG---GCCAGACAA---TTATTG---TCTGGTATAGTGCGACAGCAGAAC---
AATCTG---CTGAGA---GCTGTTGAGGCGCAACAACATCTGTTGCAACTCACAGTCTGGGGCATC
>EU578603
TTTTACTGTAATACAACAAAGCTGTTTAGTAGTACTTGGCATACTAACCATACTAACGGCACTTGGAATAATA
ATGGG--
-------------------GAT---
AATACTGGAGAGAATATCACACTCCCATGCAGAATAAAACAAATTATAAACATGTGGCAGAGAGTAGGCAAAG
CAATGTATGCCCCTCCCATCAGAGGACAAATTAGATGTACATCAAAT---
ATCACGGGGCTGCTATTAACAAGAGATGGTGGTAATGACAAGAAC----------------------------

----

AGCACCTCCGAGACCTTCAGACCTGGAGGAGGAAATATGAAGGACAATTGGAGAAGTGAATTATATAAATATA
AAGTAGTAAAAATTGAACCATTA---GGAGTAGCACCCACCAAGGCAAAGAGAAGAGTGGTG-----------
----------------------------CAAAGAGAAAAAAGAGCA---GTGGGAGCAATAGGAGCT------
---ATGTTCCTTGGGTTCTTGGGAGCA---GCAGGAAGCACTATG---GGCGCAGCGTCA---
GTGACGCTGACGGTACAA---GCCAGACAA---TTATTG---TCTGGTATAGTGCAACAGCAGAAC---
AATTTG---CTGAGG---GCTATTGAGGCGCAACAGCATATGTTGCAACTCACGGTCTGGGGCATC
>AF277060
TTCTACTGTAATACAACACAACTGTTTAATAGTACTTGGAATATTAATGATACTCGGAATGGTACTACAGAG-
----

TCAAGTAAAACTATCACACTCCCATGCAGAATAAAACAAATTATAAACATGTGGCAGGAAGTAGGCAAAGCAA
TGTATGCCCCTCCCATTAGAGGACAGATTAGATGTTCATCAAAT---
ATTACAGGATTACTATTAACAAGAGACGGTGGTAACCAAAACACG----------------------------
----

AGCGGCACCGAGATTTTCAGACCTGGAGGAGGAAATATGAGAGACAATTGGAGAAGTGAATTATATAAATATA
AAGTAGTAAAAATTGAACCATTA---GGAATAGCACCCACCAAGGCAAAGAGAAGAGTGGTG-----------
----------------------------CAAAGAGAAAAAAGAGCA---GTGGGAACAATAGGAGCT------
---ATGTTCCTTGGGTTCTTGGGAGCA---GCAGGAAGCACTATG---GGCGCAGCATCA---
ATAACGCTGACGGTACAG---GCCAGACTA---TTATTG---TCTGGTATAGTGCAACAGCAGAAC---
AATCTG---ATGAGG---GCTATTGAGGCGCAACAGCATATGTTGCAACTCACAGTCTGGGGCATC
>AF277055
TTCTACTGTAATGCAACACCACTGTTTAATAGTAGCTGGTATGTTAATGGTACTAACACTACAGGGTCA----
----

GATGGAGAAAATATCACACTCCTATGCAGAATAAAACAAATTGTAAACATGTGGCAGGAAGTAGGCAAAGCAA
TGTATGCCCCTCCCATTAGAGGACAAATTACATGCTCATCAAAG---
ATCACAGGGTTGCTATTAACAAGAGATGGTGGTAACACGAACGGGACC-------------------------
----

AACGACACCGAGACCTTCAGACCTGGAGGAGGAGATATGAGGGACAATTGGAGAAGTGAATTATATAAATATA
AAGTAGTAAAAATTGAACCATTA---GGAGTAGCACCCACCAAGGCAAAGAGAAGAGTGGTG-----------
----------------------------CAGAGAGAAAAAAGAGCA---GTGGGAACAATAGGAGCT------
---ATGTTCCTTGGATTCTTGGGAGCA---GCAGGAAGCACTATG---GGCGCAGCGTCA---
CTGACGCTGACGGTACAG---GCCAGACTA---TTATTG---TCTGGTATAGTGCAACAGCAGAAC---
AATCTG---CTGAGG---GCTATTGAGGCGCAACAACATCTGTTGCAACTCACGGTCTGGGGCATT
>EF593245
TTCTACTGTAATTCAACACAACTGTTTAATAGTACTTGGAATAATAATGGTACTAGGAATGATACTAGGGAGT
CA---

AATGACACTATCATCACACTACCATGCAGAATCAAACAAATTATAAACATGTGGCAGCAAGTAGGCAAAGCAA
TGTATGCCCCTCCCATCAGAGGACAAATTAGATGTTCATCAAAT---
ATCACAGGGCTGCTGTTAACAAGAGATGGTGGTAACGAGAGCGAC----------------------------
----

GCTGAGACCTTCAGACCTGGAGGAGGAGATATGAGGGACAATTGGAGAAGTGAATTATACAAATATAAAGTAG
TAAAAATTGAACCATTA---GGAGTAGCACCCTCCAAGGCAAAGAGAAGAGTGGTG-----------------
----------------------CAGAGAGAAAAAAGAGCA---GTGGGAACAATAGGAGCT---------
ATGTTCCTTGGGTTCTTGGGAGCA---GCAGGAAGCACTATG---GGCGCAGCGTCA---
CTGACGCTGACGGTACAG---GCCAGACTA---TTATTG---TCTGGTATAGTGCAACAGCAGAAC---
AATCTG---CTGAGG---GCTATTGAGGCGCAACAACATATTTTGCAACTCACGGTCTGGGGCATT
>AF277070
TTCTACTGTAATACAACACAGCTGTTTAATAGTACTTGGAATGCTAACAGCACTTGGAATGATACTAGAAAG-
----

TCAAATGACACTATCACACTCCCGTGCAGAATAAAACAAATTATAAACATGTGGCAGAGAGTAGGCAGAGCAA
TGTATGCCCCTCCCATCAAAGGACAAATTAGATGTGCATCAAAT---
ATCACAGGGCTGTTATTAACAAGAGATGGTGGTAATGAGAACAGC----------------------------

----

ACCGAGAACATCTTCAGACCTGGAGGAGGAAATATGAGGGACAATTGGAGAAGTGAATTATATAAATATAAAG
TAGTAAAAATTGAACCATTA---GGAGTAGCACCCACCAAGGCAAAGAGAAGAGTGGTG--------------
-------------------------CAGAGAGAAAAAAGAGCA---GTGGGAACAATAGGAGCT---------
ATGTTCCTTGGGTTCTTGGGAGCA---GCAGGAAGCACTATG---GGCGCAGCGTCA---
CTGACGCTGACGGTACAG---GCCAGACTA---TTATTG---TCTGGTATAGTGCAACAGCAGAAC---
AATTTG---CTGAGA---GCTATTGAGGCGCAACAGCATCTGTTGCAACTCACGGTCTGGGGCATC
>AF277072
TTCTACTGTAATACAACAAAACTGTTTAACACAACACTGCTTAATGATACTGCAGAG----------------
----

TCAAATGACAATATCACAATCCCATGCAGAATAAAACAAATTATAAACATGTGGCAGGAAGTAGGTAAAGCAA
TGTATGCCCCTCCCATTAGAGGACAAATTAGATGTTCATCAAAT---
ATCACAGGATTACTATTAACAAGAGACGGTGGTAATGATAATAACACG-------------------------
----

GATAACACCACCGAGATCTTCAGACCTGGAGGAGGAGATATGAGGGACAATTGGAGAAGTGAATTATATAAAT
ATAAAGTAGTACAAATTGAACCAATA---GGAATAGCACCCACCAAGGCAAAGAGAAGAGTGGTG--------
-------------------------------CAAAGAGAAAAAAGAGCA---GTGGGAACAATAGGAGCT---
------ATGTTCCTTGGGTTCTTGGGAGCA---GCAGGAAGCACTATG---GGCGCAGCATCA---
ATAACGCTGACGGTACAG---GCCAGACAA---TTATTG---TCTGGTATAGTGCAGCAGCAGAAC---
AATCTG---CTGAGG---GCTATTGAGGCGCAACAGCATCTGTTGCAGCTCACGGTCTGGGGCATC
>AF277073
TTCTACTGTAATACAACACAGCTGTTTAATAGTACTTGGAATGTTAATGGTACTTGGAATGGTACT-------
----

GGAAGTGAAAATATCACACTCCCATGCAGAATAAAACAAATTATAAACATGTGGCAAAAAGTAGGCAAAGCAA
TGTATGCCCCCCCCATCAAAGGACAAATTAGATGTGCATCAAAT---
ATCACAGGGCTGCTGTTAACAAGAGATGGTGGAAATAGTAGTAATACA-------------------------
----

AGCAACACCGAGATCTTCAGACCTGGAGGAGGAAATATGAAGGACAATTGGCGAAGTGAATTATATAAATATA
AAGTAGTAAAAATTGAACCATTA---GGAGTAGCACCCACCAAGGCAAAGAGAAGAGTGGTG-----------
----------------------------CAGAGAGAAAAAAGAGCA---GTGGGAACAATAGGAGCT------
---ATGTTCCTTGGGTTCTTGGGAGCA---GCAGGAAGCACTATG---GGCGCAGCGTCA---
ATGACGCTGACGGTACAG---GCCAGACTA---TTATTG---TCTGGTATAGTGCAACAGCAGAAC---
AATCTG---CTGAGA---GCTATTGAGGCGCAACAGCATCTGTTGCAACTCACGGTCTGGGGCATC
>EU578429
TTCTATTGTAATACAACACCACTGTTTAATAGTACTTGGTATGAAAATGGTACTTGGAGTGGTACTCCAAAGT
CA---

GATAATGACACTATCACACTCCCATGCAGGATAAAACAAATTATAAACATGTGGCAGAGAGTAGGCAAAGCAA
TGTATGCCCCTCCCATCAGAGGACAAATTAGATGTTCATCAAAT---
ATCACAGGGCTGTTATTAACAAGAGATGGTGGTAGTAGTAATAAC----------------------------
----

AGCACCGAGACCTTCAGACCTGGAGGAGGAGATATGAGGGACAATTGGAGAAGTGAACTATATAAATATAAAG
TAGTAAAAATTGAACCATTA---GGAATAGCACCCACCAAGGCAAAGAGAAGAGTGGTG--------------
-------------------------CAAAGAGAAAAAAGAGCA---GTGGGAACAATAGGAGCT---------
ATGTTCCTTGGGTTCTTGGGAGCA---GCAGGAAGCACTATG---GGCGCAGCATCA---
CTAACGCTGACGGTACAG---GCCAGACTA---TTATTG---TCTGGTATAGTGCAACAGCAGAAC---
AATCTG---CTGAGG---GCTATTGAGGCGCAACAGCACCTGTTGCAACTCACAGTCTGGGGCATC
>EU578386
TTCTACTGTAATTCAACACAACTGTTCAATAGTACTTGGAATGGGAATAATACTTGGAATGGTACT-------
----

GGGGAGGATACTATCACACTCCCATGCAGAATAAAACAAATTGTAAATATGTGGCAGAGAGTAGGCAAAGCAA
TGTATGCCCCTCCCATCAAAGGACAAATTAGATGTTCATCAAAC---
ATTACAGGGCTGCTATTAACAAGGGATGGTGGTACTAACGGGACC----------------------------

----

ACCGAGACCTTCAGACCTGGAGGAGGAAATATGAAGGACAATTGGAGAAGTGAATTATATAAATATAAAGTAG
TAAAAATTGAACCATTA---GGAATAGCACCCACCAAGGCAAAGAGAAGAGTGGTG-----------------
---------------------CAGAGAGAAAAAAGAGCA---GTGGGAGTAATAGGAGCT---------
CTGTTCCTTGGGTTCTTGGGAGCA---GCAGGAAGCACTATG---GGCGCAGCGTCA---
CTGACGCTGACGGTACAG---GCCAGACAA---TTATTG---TCTGGTATAGTGCAACAGCAGAAC---
AATCTG---CTGAGG---GCTATTGAAGCGCAACAGCATCTGTTGCAACTCACGGTCTGGGGCATT
>EU577148
TTCTACTGTAATTCAACACAACTGTTTAACAGTACTTGGAATGCTACTGATACTTGGAAATGGAATGATAGTA
ATACT--
------------------TGG---
TATAATTCCAGCAATATCACACTTCCATGTAGAATAAAGCAAATTATAAACATGTGGCAGAGAGTAGGCAAAG
CAATGTATGCCCCTCCCATCAGAGGACAAATTAACTGTACATCCAAT---
ATCACAGGGCTGCTATTAACAAGAGATGGTGGTGATAACGAGAAC----------------------------
----

GACACCGAGACCTTCAGACCTGGAGGAGGAGATATGAGGAACAATTGGAGAAGTGAATTATATAGATATAAAG
TAGTAAAAATTGAACCATTA---GGAGTAGCACCCACCAAGGCAAAGAGAAGAGTGGTG--------------
------------------------CAGAGAGAAAAACGAGCG---GTGGGAACACTAGGAGCT---------
ATGTTCCTTGGGTTCTTGGGAGCA---GCAGGAAGCACTATG---GGCGCAGCGTCG---
CTGACGCTGACGGTACAG---GCCAGACTA---TTACTG---TCTGGTATAGTGCAGCAGCAAAAT---
AATTTG---CTGAGG---GCTATTGAGGCGCAACAGCATCTGTTGCAACTCACGGTCTGGGGCATC
>EF593264
TTCTACTGTAATACAACACAACTGTTTAATAGTACTTGGAATAATAGTACTTGGAATGGTATTGACGAGTCAG
GT---

GATGATGACAATATCACACTACCATGCAGAATAAAACAAATTATAAACATGTGGCAGCAAGTAGGCAAAGCAA
TGTATGCCCCTCCCATCAGAGGACAAATTAGCTGTTCATCAAAT---
ATCACAGGGCTGCTGTTAACAAGAGATGGTGGTGGTAACAGTAGC----------------------------
----

AACAATGAGACCTTCAGACCTGGAGGAGGAGATATGAGGGACAATTGGAGAAGTGAATTATATAAATATAAAG
TAGTAAAAATTGAACCATTA---GGAGTAGCACCCACCAAGGCAAAGAGAAGAGTGGTG--------------
------------------------CAGAGGGAAAAAAGAGCA---GTGGGAACAATAGGAGCT---------
ATGTTCCTTGGGTTCTTGGGAGCA---GCAGGAAGCACTATG---GGCGCAGCGTCA---
GTGACGCTGACGGTACAG---GCCAGACTA---CTATTG---TCTGGGATAGTGCAACAGCAGAAC---
AATCTG---CTGAGG---GCTATTGAGGCGCAACAGCACCTGTTGCAACTCACAGTCTGGGGCATC
>EF593268
TTCTACTGTAATTCAACAGCACTGTTTACTGGTACTTGGAATGGTACTACTTGGAATGGTACTACTTGGAATG
GTACTGAGCCT--
-GATACTGAAGAGTCA---------
AATAACACCACCACCATCATACTCCCATGCAGAATAAAACAAATTGTAAACATGTGGCAGGAGGTAGGCAAAG
CAATGTATGCCCCTCCCATCAGAGGACAAATTAACTGTTCATCAAGT---
ATTACAGGGCTGCTATTAACATGGGATGGTGGTAATAACACGGACCAG-------------------------
----

AACATGACCGAGACCTTCAGACCTGGAGGAGGAAATATGAGGGACAATTGGAGAAGTGAACTATATAAATATA
AAGTAGTAAAAATTGAACCATTA---GGAATAGCACCCACCAAGGCAAAGAGAAGAGTGGTG-----------
---------------------------CAAAGAGAAAAAAGAGCA---GTGGGAACAATAGGAGCT------
---ATGTTCCTTGGGTTCTTGGGAGCA---GCAGGAAGCACTATG---GGCGCAGCGTCA---
CTAACGCTGACGGTACAG---GCCAGATTA---TTATTG---TCTGGTATAGTGCAACAGCAGAAC---
AATTTG---CTGAGG---GCTATTGAGGCGCAACAGCATCTGTTGCAGCTCACGGTCTGGGGCATC
>U32396
TTCTACTGTAATTCAACACAACTGTTTAATAGTACTTGGAATGCTAATGGTAATGGGAATGGTACTGGAGGAT
CA---

AATGACAATGACACTATCACACTCCCATGCAGAATAAAACAAATTGTAAACATGTGGCAAGAAGTAGGCAAAG
CAATGTATGCCCCCCCCATCAGAGGATACATTAACTGTTCATCAAAT---
ATTACAGGGCTGTTATTAACAAGAGATGGTGGTATTGGCACGAAC----------------------------

----

GGGACCGAGATATTCAGACCTGGAGGGGGAGATATGAGGGACAATTGGAGAAGTGAATTATATAAATATAAAG
TAGTAGAAATCGAACCATTA---GGAGTAGCACCCACCAAGGCAAAGAGAAGAGTGGTG--------------
-------------------------CAGAGAGAAAAAAGAGCA---GTGGGAACAATAGGAGCT---------
ATGTTCCTTGGGTTCTTGGGAGCA---GCAGGAAGCACTATG---GGCGCAGCGTCA---
CTGACGCTGACGGTACAG---GCCAGACTA---TTATTG---TCTGGTATAGTGCAACAGCAGAAC---
AATCTG---CTGAGG---GCTATTGAGGCGCAACAGCATCTGTTGCAGCTCACGGTCTGGGGCATC
>AF086817
TTCTACTGTAATGCAACACCACTGTTTAATAGTACTTGGAATGCTACCAGTACTTTGAATGCTACTAATGAA-
----

GAAAATGAAAATATCACACTCCTATGTAGAATAAAACAAATTATAAACATGTGGCAGAGAGTAGGAAAAGCAA
TGTATGCTCCTCCCATCGAAGGACTAATTAAATGTTCATCAAAT---
ATTACAGGGCTGATGTTAACAAGAGATGGGGGTACAAATGACAGT----------------------------
----

GAGGTCGAGGTCTTCAGACCTGGAGGAGGAGATATGAAGGACATTTGGAGAAATGAATTATATAAATATAAAG
TAGTAAAAGTTGAACCATTA---GGACTAGCACCCACCAGGGCAAGGAGAAGAGTGGTG--------------
-------------------------CAGAGAGAAAAAAGAGCA---GTGGGA---ATAGGAGCT---------
TTGTTCCTTGGGTTCTTGGGAGCA---GCAGGAAGCACTATG---GGCGCAGCGTCA---
CTAACGCTGACGGTACAG---GCCAGACAA---TTATTG---TCTGGTATAGTGCAACAGCAGAAC---
AATCTG---CTGAGA---GCTATTGAGGCGCAACAGCACATGTTGCAACTCACAGTCTGGGGCATC
>DQ823362
TTCTATTGTAATACATCAAAGCTGTTCAATAGTACTTGGAATAATGGTACTTGGACGAGTAATATTGATGAGG
GTAAT--
-------------------ACT---
ACAGGGCCAGACCACATCACACTCCCATGCAGAATAAAACAAATTATAAACAGATGGCAGGAAGTAGGAAAAG
CAATATATGCCCCTCCCATCAGCGGACTAATTAGATGTTCATCAAAT---
ATTACAGGGATACTATTAGTAAGAGATGGTGGTAATAATAGTAGT----------------------------
----

AACAACGAGACTTTCAGACCTGGAGGAGGGAATATGAAGGACAATTGGAGAAGTGAATTATATAAATATAAAG
TAGTAAAAATTGAGCCATTA---GGAGTAGCACCCACCAAGGCAAAGAGAAGAGTGGTG--------------
-------------------------CAGAGAGAAAAAAGAAGT---CCAACG---CTTGGAGCT---------
CTGTTCCTTGGGTTCTTGGGAGCA---GCAGGAAGCACTATG---GGCGCAGCGTCA---
ATAACGCTGACGGTACAG---GCCAGACAA---TTATTG---TCTGGTATAGTGCAACAGCAGAAC---
AACTTG---CTGAGA---GCTATTGAGGCGCAACAGCATCTGTTGCAACTCACAGTCTGGGGCATT
>DQ823363
TTCTATTGTAATACAACACAGCTGTTCAATAGTACTTGGAATAATACTTGGAGTGGTGCTACA----------
----

GGATCAGACAACATCACACTCCCGTGCAGAATAAAACAAATTATAAACAGGTGGCAGGAAGTAGGAAAAGCAA
TGTATGCCCCTCCCATCGAAGGACAAATTAAATGTTCATCAAAT---
ATTACAGGGCTATTATTAGTAAGAGATGGTGGTATTGGTAATAAT----------------------------
----

GAAACAGAGATCTTCAGACCTGGAGGAGGAGATATGAAGGACAATTGGAGAAGTGAATTATATAAATATAAAG
TAGTAAAAATTGAACCATTA---GGAGTAGCACCCACCAGGGCAAAGAGAAGAGTGGTG--------------
-------------------------CAAAGAGAAAAAAGAGCA---GTAACG---TTTGGAGCT---------
ATGTTCCTTGGGTTCTTGGGAGCA---GCAGGAAGCACTATG---GGCGCAGCGTCA---
ATAACGCTGACGGTACAG---GCCAGACAA---TTATTG---TCTGGTATAGTGCAACAGCAGAGT---
AATTTG---CTGAGA---GCTATTGAAGCGCAACAGCATATGTTGCAACTCACAGTCTGGGGCATT
>DQ823364
TTCTATTGTAATACAACACAGCTGTTCAATAGTACTTGGCAGGCTAATAGTACTAGGAATGTTAGTACAACAT
CAGAC--
-------------------AAC---
TCAGACAACAGCACAATCACACTCCCGTGCAGAATAAAACAAATTATAAACAGGTGGCAAGAAGTAGGAAAAG
CAATGTATGCCCCTCCCATCAGAGGACAAATTAAATGTTCATCAAAT---
ATTACAGGGCTACTATTAGTAAGAGATGGTGGTAATGGTAATAATAGT-------------------------

----

AGTAACGAGACAGAGATCTTCAGACCTGGAGGAGGAAATATAAAGGACAATTGGAGAAGTGAATTATATAAAT
ATAAAGTAGTAAAAATTGAGCCGTTA---GGAGTAGCACCCACCAGGGCAAAGAGAAGAGTGGTG--------
-------------------------------CAGAGAGAAAAAAGAGCA---ATAACA---TTTGGAGCT---
------ATGTTCCTTGGGTTCTTGGGAGCA---GCAGGAAGCACTATG---GGCGCAGCGTCA---
GTGACGCTGACGGTACAG---GCCAGACAA---TTATTG---TCTGGTATAGTGCAACAGCAGAGC---
AATTTG---CTGAGA---GCTATTGAGGCGCAACAGCATATGTTGCAACTCACAGTCTGGGGCATT
>JN562768
TTCTACTGTAATACATCACAACTGTTTAACAGTACTTCTAATGTTACTTGGACTACTGGAGAGTCA-------
----

GATAACAAAACAATCACACTCCCATGCAGAATAAAGCAAATTATAAACATGTGGCAGAAAGTAGGAAAAGCAA
TGTATGCCCCTCCCATCCAAGGACTAATTAAATGTTCATCAAAT---
ATTACAGGGCTGATATTAACAAGAGATGGTGGTGTTAATATGACAAATGGTGAT------GTTAAT-------
----
GGGACAAACAACAAC---
AACACAGAGACCTTTAGACCTGCAGGAGGAGATATGAGGGACAATTGGAGAAGTGAATTATATAAGTATAAAG
TAGTAAGAATTGAACCATTA---GGAGTAGCACCCACCAAGGCAAAGAGAAGAGTGGTG--------------
-------------------------CAAAAAGAAAAAAGAGCA---GTGGGA---ATAGGAGCT---------
CTGTTCCTTGGGTTCTTGGGAACA---GCAGGAAGCACTATG---GGCGCAGCGTCA---
ATGGCGCTGACGGTACAG---GCCAGACAA---TTATTG---TCTGGTATAGTGCAACAGCAGAAC---
AACTTG---CTGAGG---GCTATTGAGGCGCAACAACATCTGTTGCAACTCACAGTCTGGGGCATC
>DQ869026
TTCTACTGTAATTCAACACAACTGTTTAATAGCACTTGGTATAGCAATAATACTTGGGAAGGGTTAAATAGC-
----

ACTGAAGCAAATATCACACTCCCATGCAGAATAAAACAAATTGTGAATATGTGGCAGCAAGTGGGAAAAGCAA
TGTATGCCCCTCCCATCAGCGGACGAATTAGCTGCACATCAAAT---
ATTACAGGGCTGTTATTAACAAGAGATGGGGGTGGTAATAGCACAAAC-------------------------
----

GAGACCACCGAGATCTTCAGACCTGGAGGAGGAGATATGAGGGACAATTGGAGAAGTGAATTATATAAATATA
AAGTAGTAGAAATTGAACCATTA---GGAGTAGCACCCACCAAGGCAAGGAGAAGAGTGGTG-----------
----------------------------CAGAGAGAAAAAAGAGGA---GTA------TTAGGAGCT------
---ATGTTCCTTGGGTTCTTGGGAGCA---GCCGGAAGCACTATG---GGCGCAGCGTCA---
CTGACGCTGACGGTACAG---GCCAGACAA---TTATTG---TCTGGTATAGTGCAACAGCAGAAC---
AATCTG---CTGAGG---GCTATTGAGGCGCAACAGCATATGTTGCAACTCACAGTCTGGGGCATT
>EF363123
TTCTACTGTAATACAACAAAACTATTTAATAGTACTTGGAACAATGGTACTTGGACTTGGAACAATGGTACTT
GGAATGGT---
-------------ACTGAA------
TGGTTAAATGACACTATCACACTCCCATGCAGAATAAAACAAATTATAAACATGTGGCAGGAAGTAGGAAAAG
CAATGTATGCCCCTCCCATCAGAGGACAAATTAGATGTTCATCAAAT---
ATTACAGGGCTGCTATTAACAAGAGATGGTGGTAATACTGAGAAC----------------------------
----

AAGACCGAGGTCTTCAGACCTGGAGGAGGAAATATGAAGGACAATTGGAGAAGTGAATTATATAAATATAAAG
TAGTAAAAATTGAACCACTA---GGAGTAGCACCCACCAAGGCAAAGAGAAGAGTGGTG--------------
-------------------------CAGAGAGAAAAAAGAGCA---GTGGGA---ATAGGAGCT---------
ATGTTCCTTGGGTTCTTGGGAGCA---GCAGGAAGCACTATG---GGCGCAGCGTCA---
ATGACGCTGACGGTACAG---GCCAGACAA---TTATTG---TCTGGTATAGTGCAACAGCAAAGC---
AATTTG---CTGAGG---GCTATTGAGGCGCAACAAAAACTGTTGCAACTCACAGTCTGGGGCATC
>EU289199
TTCTACTGTAATACAACAGGACTGTTTAATAGTACTTGGTATAATGAGACTGAAAAGATAAATAGCACTACA-
----

AGTGACAATATCACAACACTTCCATGCAGAATAAAACAAATTGTAAACATGTGGCAGGAAGTAGGAAAAGCAA
TGTATGCCCCTCCCATCAGAGGACAAATTAACTGTTCATCAAAT---
ATCACAGGGATGATATTAACAAGAGATGGTGGTAATAATAATAACAGT-------------------------

----

AGCAATGACACGGAGATCTTCAGACCTGGAGGAGGAGATATGAGGGACAATTGGAGAAGTGAATTATATAAAT
ATAAAGTAGTACAAATTGAACCATTA---GGAGTAGCCCCCACCACGGCAAAGAGAAGAGTGGTG--------
------------------------------CAGAGAGAAAAAAGAGCA---GTGGGA---CTAGGAGCT---
------ATGTTCCTTGGGTTCTTGGGAGCA---GCAGGAAGCACTATG---GGCGCAGCGTCA---
ATAACGCTGACGGTACAA---GCCAGACAA---TTATTG---TCTGGTATAGTGCAGCAGCAGAGC---
AATCTG---CTGAGG---GCTATTGAGGCGCAACAGCATCTGTTGCAACTCACAGTCTGGGGCATT
>JN944917
TTCTACTGTAATACAACAAAATTGTTTACTAGTACTTGGAATAGTACTTGGAATAGTACTTGGAATAATACTG
AAGGGTCA---

AATAGCACTGTTATCACCCTGCCATGCAGAATAAGACAAATCATAAACATGTGGCAGGAAGTAGGAAAAGCGA
TGTATGCCCCTCCCATCCAAGGACAAATTAAATGTTCATCAAAT---
ATTACAGGGCTGTTATTAACAAGAGATGGTGGTAATAACGACACC---------------------------
----

ACAAAAGAGACCTTCAGACCTGGAGGAGGAAATATGAAGGACAATTGGAGAAGTGAATTATACAAATATAAAG
TAGTAAGAATTGAGCCATTA---GGAGTAGCCCCCACCAAGGCAAAAAGAAGAGTGGTG-------------
------------------------CAGAGAGAAAAAAGAGCA---GTGGGA---ATAGGAGCT---------
ATGTTCCTTGGGTTCTTAGGAGCA---GCAGGAAGCACTATG---GGCGCAGCGTCA---
ATAACGCTGACGGTACAG---GCCAGACTA---CTATTG---TCTGGTATAGTGCAACAGCAGAGC---
AATCTG---CTGAGG---GCTATTGAGGCGCAACAGCATCTGTTGCAACTCACAGTCTGGGGCATC
>JN944930
TTCTACTGTAATACAACAAACCTGTTCAATAGTAGTTCAACAGAACTTAATAGTACTTGGAGTGGTAATAGTA
AT---
-------------GACACA------
GGAAAAAATGATACAATCACACTCCCATGCAGAATAAAACAAATTATAAACATGTGGCAGCAAGTAGGAAAAG
CAATGTATGCCCCTCCCATCAGTGGAAAAATTAACTGTTTATCAAAT---
ATTACAGGGCTGCTATTAACAAGAGATGGGGGTAGTGATGGGGGTAGT------------------------
----
AAGAACAGTAGTAAGAATGAGACC---------
GGTACTGAGATCTTCAGACCTGGAGGAGGAGATATGAGGGACAATTGGAGAAGTGAATTATATAAATATAAAG
TAGTAAGAATTGAACCATTA---GGAGTAGCACCCACCAAGGCAAAGAGGAGAGCGGTA-------------
------------------------CAGAGAGAAAAAAGAGAT---CTGGGA---CTAGGAGCT---------
TTGTTCCTTGGGTTCTTGGGAGCA---GCAGGAAGCACTATG---GGCGCAGCGTCA---
GTGACGCTGACGGTACAG---GCCAGACAG---TTATTG---TCTGGTATAGTGCAACAGCAGAAC---
AATCTG---CTGAGA---GCTATTGAGGCGCAACAGCACCTGTTGCAACTCACAGTCTGGGGCATC
>JN944938
TTCTACTGTAATTCAACACAACTGTTTAATAGTACTTGGTTTAATAATGGTACTAGTACTTGGAATAGT----
----

ACTGCAGACAATATCACACTCCCATGTAGAATAAAACAAGTTATAAACATGTGGCAGGAAGTAGGAAAAGCAA
TGTATGCCCCTCCCATCAGAGGACAAATTGATTGTTCATCAAAT---
ATTACAGGGTTGATATTAACAAGAGATGGTGGTAGTAACAGTAGC---------------------------
----

CAGAACGAAACCTTCAGACCTGGAGGAGGAAATATGAAGGACAATTGGAGAAGTGAATTATATAAATATAAAG
TAGTAAAAATTGAACCATTA---GGAATAGCACCCACCAGGGCAAAGAGAAGAGTGGTG-------------
------------------------CAGAGAGAAAAAAGAGCA---GTGACG---CTGGGAGCT---------
GTGTTCCTTGGGTTCTTGGGAGCT---GCAGGAAGCACTATG---GGCGCAGCGTCA---
CTGACGCTGACGGTACAG---GCCAGACTA---TTATTG---TCTGGTATAGTGCAACAGCAGAGC---
AATCTG---CTGAGA---GCTATTGAGGCGCAACAACATATGTTGCAACTCACAGTCTGGGGCATT
>EF593288
TTCTACTGTAATACAACACAACTGTTTAATAGTACTTGGAATTCTAATGATACTTGGAATAGTACTACAGGGT
CAAAT--

GGCACTGGAGGAAATATCACACTCCCATGCAGAATAAAACAAATTATAAACATGTGGCAGAAAGTAGGAAAAG
CAATGTATGCCCCTCCCATCAAAGGACAAATTAAATGTTTATCAAAT---
ATTACAGGGATACTATTAACAAGAGATGGGGGTAATGAGAACAGC---------------------------

----

ACTATTGAGACCTTCAGACCTGCAGGAGGAGATATGAGGGACAATTGGAGAAGTGAATTATATAAATATAAAG
TAGTACAAGTTGAACCATTA---GGAGTAGCACCCACCAAGGCAAAGAGAAGAGTGGTG--------------
-------------------------CAAAGAGAAAAAAGAGCA---ATAGTGGGAATAGGAGCT---------
GTGTTCCTTGGTTTCTTGGGAGCA---GCAGGAAGCACTATG---GGCGCAGCGTCA---
TTGACGCTGACGGTACAG---GCCAGACAA---TTATTG---TCTGGTATAGTGCAACAGCAGAAT---
AATCTG---CTGAGG---GCTATTGAGGCGCAACAACATATATTGCAACTCACAGTCTGGGGCATC
>EF593289
TTCTACTGTAATACAACACAGCTGTTTAATAGTACTTGGAATAGTACTAATTGGAATAATACTGAAAAGTCAA
AT---

AGCACTGAAGTCATCACACTCCCATGCAGAATAAAACAAATTATAAACATGTGGCAGAAGGTAGGAAAAGCAA
TGTATGCCCCTCCCATTGCAGGACAAATTAACTGTTCATCAAGT---
ATTACAGGACTGATATTAACAAGAGATGGTGGTGACAACAGCACA----------------------------
----

AACAATACAGAGATCTTCAGACCTGTAGGAGGAGATATGAGGGACAATTGGAGAAGTGAATTATATAAATATA
AAGTAGTAAGAATTGAACCATTA---GGAGTAGCGCCCACCAGGGCAAGGAGAAGAGTGGTG-----------
----------------------------CAGAGAGAAAAAAGAGCA---ATAGGA---CTAGGAGCT------
---GTGTTCATTGGGTTCTTGGGAGCA---GCAGGAAGCACTATG---GGCGCAGCGTCG---
GTGACGCTGACGGTACAA---GCCAGACAA---TTACTG---TCTGGTATAGTGCAACAGCAGAAC---
AATCTG---CTGAAG---GCTATTGAGGCGCAACAGCATATGTTGCAACTCACAGTCTGGGGCATT
>HM769944
TTCTACTGTAATACAACAGAACTGTTTAGTAGCACTTGGAATATAACAGGAGATAGCATTGGGAATATAACAG
GA---

GAGTCTTTAAATATCACACTCCCATGCCGAATAAAACAAATTATAAACATGTGGCAGGGAGTAGGAAAAGCAA
TGTATGCCCCTCCCATCAGTGGACAGATTAGATGCATATCAAAT---
ATTACAGGGCTGCTATTAACAAGAGATGGTGGTGATAATAACACCGAGAAT----------------------
----

GACAACAACACCGAAATCTTCAGACCTTGGGGAGGAGATATGAGGGATAATTGGAGAAGTGAATTATATAAAT
ATAAAGTAGTAAAACTTGAACCATTA---GGATTAGCACCCACCAAGGCAAAGAGAAGAGTGGTG--------
-------------------------------CAAAGAGAAAAAAGAGCA---ATAGGA---GTTGGAGCT---
------ATGTTCCTTGGGTTCTTGGGAGCA---GCAGGAAGCACTATG---GGCGCAGCGTCA---
CTGACGCTGACGGTACAG---GCCAGGCAA---TTATTG---TCTGGTATAGTGCAACAGCAGAAC---
AATCTG---CTGAGG---GCTATTGAGGCGCAACAGCATCTGTTGCAACTCACAGTCTGGGGCATC
>JQ609746
TTCTACTGTAATACAACACAACTGTTTAATAGCACTTGGCATGGGAATGAGACTGAAGAATCAAATACCACCA
AAGAC--

AATAGCACAATAATCACTCTCCCATGCAGAATAAGACAAATTGTAAACCGGTGGCAGGAAGTAGGGAAAGCAA
TGTATGCCCCTCCCATCAGCGGACGGATTAACTGTTCATCAAAT---
ATTACTGGGCTGCTATTAACAAGAGATGGTGGTAATATCACGACC----------------------------
----

GAGAATACTACTGAGACCTTCAGACCTGGAGGAGGAGATATGAGGGACAATTGGAGAAGTGAATTATATAAAT
ATAAAGTAGTAAAAATTGAACCATTA---GGAATAGCACCCACCAGGGCAAAGAGAAGAGTGGTG--------
-------------------------------CAAAGAGAAAAGAGAGCA---GTGGGA---ATAGGAGCT---
------GTGTTCCTTGGGTTCTTGGGAGCA---GCAGGAAGCACTATG---GGCGCAGCGTCA---
CTGACGCTGACGGTACAG---GCCAGACTC---TTATTG---TCTGGTATAGTGCAACAGCAGAAT---
AATTTG---CTGAGA---GCTATTGAGGCGCAACAACATCTGTTGCAACTCACAGTCTGGGGCATC
>DQ869017
TTCTACTGTAATACAGCACAATTGTTTAATAGTACTTGGAAGAGGGATGGTACTACTGGGACCTGGAGTGGTA
ATAATACTGAG--
-------AATGATACTGAA------
CATTCAAATGACACTATCACACTCCCATGCAGAATAAGACAAATTGTAAACATGTGGCAAGAAGTAGGAAAAG
CCATGTATGCTCCTCCCATCAGTGGACAAATTAGATGTTCATCAAAT---
ATTACAGGGTTACTATTAACAAGAGATGGTGGTGAGAGCAATGCC----------------------------

----

ACTGAGGTCTTCAGACCCGCAGGAGGAGATATGAGGGACAATTGGAGAAGTGAACTATATAAATATAAAGTAG
TAAAAATTGAACCATTA---GGAATAGCACCTTCCAAGGCAAAGAGAAGAGTGGTG-----------------
----------------------CAGAGAGAAAAAAGAGCA---GTGGGA---ATAGGAGCT---------
GTGTTCCTTGGGTTCTTAGGAGCT---GCAGGAAGCACTATG---GGCGCAGCATCC---
TTAACGCTGACGGTACAG---GCCAGACTA---TTATTG---TCTGGTATAGTGCAACAGCAAAAC---
AATTTG---CTGAGA---GCTATTGAGGCGCAACAGCATCTGTTGCAACTCACAGTCTGGGGCATT
>DQ869019
TTCTACTGTAATACAACACAATTGTTTAATGGTACTTGGAATAGTACTTGGAGTAGTACTGTA----------
----

GAAAATGGCACAATCACACTCCCATGCAAAATAAAACAGATTATAAACATGTGGCAGGAAGTAGGAAAAGCAA
TGTATGCCCCTCCCATCAGAGGACAAATTAACTGTACATCAAAT---
ATTACAGGGCTGCTATTAACAAGAGATGGTGGTAATAATAATAATACT-------------------------
----

GCTAATGAAACCGAGATCTTCAGACCTGGAGGAGGAGATATGAGGGACAATTGGAGAAGCGAATTGTATAAAT
ATAAAGTAGTAAAAATTGAACCATTA---GGACTAGCACCCACTAAGGCAAAGAGAAGAGTGGTG--------
-------------------------------CAAAGAGAAAAAAGAGCA---GTGGGA---CTAGGAGCT---
------TTGTTCCTTGGGTTCTTAGGAGCA---GCAGGAAGCACTATG---GGCGCAGCGTCA---
ATGACGCTGACGGTACAG---GCCAGACTA---TTATTG---TCTGGTATAGTGCAACAGCAAAAC---
AACTTG---CTGAGA---GCTATTGAGGCGCAACAGCAGCTGTTGCAACTCACAGTCTGGGGCATT
>DQ869014
TTCTACTGTAATACAACACAGCTGTTTAATAGTACTTGGAATACTACTCGGGAGAATGTAAAT----------
----

AACACTGAAACTATCACACTCCCATGCAGAATAAAACAGATTGTAAACAGGTGGCAGGAAGTAGGAAAAGCAA
TGTATGCCCCTCCCATCAGTGGACAAATTAGATGTTCATCAGAA---
ATTACAGGGCTGCTATTAATAAGAGATGGTGGTAATAGTAACAATAGT-------------------------
----

AACGGGACTGAGACCTTCAGACCTGGAGGAGGAAATATGAAAGACAATTGGAGAAGTGAATTATATAAATATA
AAGTAGTAAAAATTGAGCCATTA---GGAATAGCACCCACCAAGGCAAAGAGAAGAGTGGTG-----------
----------------------------CAGAGAGAAAAAAGAGCG---GTGGGAACGCTGGGAGCT------
---GTGTTCCTTGGGTTCTTGGGAACA---GCAGGAAGCACTATG---GGCGCAGCGTCA---
ATAACGCTGACGGTACAG---GCCAGACAA---TTATTG---TCTGGTATAGTGCAGCAGCAAAAC---
AATTTG---CTGAGG---GCTATTGATGCGCAACAGCATATGTTGCAACTCACTGTTTGGGGCATC
>DQ869027
TTCTACTGTAATTCAACACAACTGTTTAAAAGTACTTGGCATGTTAATGGTACTCGGAATGGTACTGAA----
----

GGAAATGACACAATCACTCTCCCATGTAAAATAAAACAAATTATAAACATGTGGCAGAAAGTAGGAAAAGCAA
TGTATGCCCCTCCCATCAAAGGAAATATTACATGTTCATCAAAT---
ATTACAGGGCTCATACTAACAAGAGATGGTGGTAGCAATGGCGAGAACACCACT-------------------
----
-
GAGATCGAGAACACCACCGAGATCTTCAGACCTGGGGGAGGAAATATGAGGGACAATTGGAGAAATGAATTAT
ATAAATATAAAATAATAAAACTTGAACCATTA---GGAATAGCACCCACTAAGGCAAAGAGAAGAGCGGTG--
-------------------------------------CAGAGAGAAAAAAGAGCT---GTGGGA---
ATAGGAGCT---------ATGTTCCTTGGGTTCTTGGGGGCA---GCAGGAAGCACTATG---
GGCGCAGCGTCA---CTGACGCTGACGGTACAG---GCCAGGCAA---TTAATG---
TCTGGTATAGTGCAACAGCAGAAC---AATTTG---CTGAGG---
GCTATTGAGGCGCAGCAGCATATGTTGCAACTCACAGTCTGGGGCATC
>EU578061
TTCTACTGTAATACATCTGGGCTGTTTAATAGTACATGGGTGTTTAATAATAGTAATAAGGTGTATGAGAGTG
ACTGGAAT---
-------------AATACT------
GAAGAGCAAAATATCACCACACTCCAGTGCAGAATAAAACAAATTGTAAACTTGTGGCAGGAAGTAGGAAAAG
CAATGTATGCCCCTCCCATCGAGGGAAAAATTAAATGTAAATCAAAT---

ATCACAGGGCTGCTATTAACAAGAGATGGTGGTACAAATAGTACA----------------------------
----

AATAAAAGTGAGACCTTCAGACCTGGAGGAGGAGATATGAGGGACAATTGGAGAAGTGAATTATATAAATATA
AAGTAGTAAGAATTGAACCATTA---GGAATAGCACCCACCAAGGCAAGGAGAAGAGTGGTG-----------
----------------------------CAGAGAGAAAAAAGAGCA---GTGGGAGCAATAGGAGCT------
---TTGTTCCTTGGGTTCTTGGGAGCA---GCAGGAAGCACTATG---GGCGCAGCGTCG---
ATGACGCTGACGGTACAG---ACCAGACAA---TTATTG---TCTGGTATAGTGCAACAGCAGAAC---
AATCTG---CTGAAG---GCTATTGAGGCGCAACAGCATATGTTGCAGCTCACGGTCTGGGGCATC
>FJ469738
TTCTACTGTAATACAACACAACTGTTTAATAGTACTTGGTATAGGAATAGGACTGAAGGGTTAAATAAAACTA
AA---

GGAAATAACACAATCACACTCCCCTGCAGAATAAAACAAATTGTAAACATGTGGCAGGAAGTAGGAAAAGCAA
TGTATGCCCCTCCTATCAGAGGACAGATTAACTGTTCATCCAAT---
ATTACAGGGCTGCTATTAACAAGAGATGGTGGTGATACTAGCGAG----------------------------
----

AACGAGAACGAGACCTTCAGACCTGGAGGAGGAGATATGAGGGACAATTGGAGAAGTGAATTATATAAATATA
AAGTAGTAAAAATAGAACCATTA---GGAGTAGCACCCACCAAGGCAAAGAGAAGAGTGGTG-----------
----------------------------CAGAGAGAAAAAAGAGCA---GTAGGA---ATAGGAGCT------
---TTGTTCCTTGGGTTCTTGGGAGCA---GCAGGAAGCACTATG---GGCGCAGCGTCA---
GTGACGCTGACGGTACAG---GCCAGACAA---TTACTG---TCTGGTATAGTGCAACAGCAGAAC---
AATTTG---CTGAGA---GCTATTGAGGCGCAACAACATCTGTTGCAACTCACAGTCTGGGGCATC
>KT283937
TTCTACTGTAATACAACACAACTGTTTAATAGTGAATGGCCTTGGAATAGTACTAATAGTACTGGAGGGTCAA
AC---
-------------AGCACT------
GAGGGAGATGACACTATCATACTCCAATGCAGAATAAAACAAATTATAAATATGTGGCAGGAAGTAGGGAAAG
CAATGTATGCCCCTCCCATTGGAGGACAAATTAAATGTGTATCAAAT---
ATTACAGGGCTGATATTAACAAGAGATGGAGGTAATGATAATAAG----------------------------
----

ACGGACAATAACACAGAGACCTTCAGACCTGGAGGAGGAGATATGAGAGAAAATTGGAGAAGTGAATTATATA
AATATAAAGTAGTAAAAATTGAACCATTA---GGAGTAGCACCCACCAAGGCAAAGAGAAGAGTGGTG-----
----------------------------------CAGAGAGAGAAAAGAGCA---
GTGGGAACACTAGGAGCT---------ATGTTCCTTGGGTTCTTGGGAGCA---GCAGGAAGCACTATG---
GGCGCAGCGTCA---ATGACGCTGACGGTACAG---GCCAGACAA---TTATTG---
TCTGGTATAGTGCAACAGCAGAAC---AACTTG---CTGAGA---
GCTATTGAGGCGCAACAGCACCTGTTGCAACTCACGGTCTGGGGCATC
>EF593254
TTCTACTGTAATACAACAAAACTGTTTAATAGTAGTTGGAATGGGAATGATAATGATACTGAAGAGGTAAAC-
----

AGCACAATAAGCATCACACTCCCATGTAGAATAAAACAAATTATAAACATGTGGCAGGAAGTAGGAAAAGCAA
TGTATGCCCCTCCCATCAAAGGACAAATTAGATGTTCATCAAAT---
ATTACAGGGCTGCTATTAACAAGAGATGGTGGTATTGGGAACAATGCCACT----------------------
----

GAGAATACCACTGAGATCTTCAGACCGGGAGGAGGAAATATGAGGGACAATTGGAGAAGTGAATTATATAAAT
ACAAAGTAGTAAAAATTGAACCATTA---GGAGTAGCACCCACCAGGGCAAAGAGAAGAACGGTG--------
-------------------------------CAGAGAGAAAAAAGAGCA---GTAACA---CTGGGAGCT---
------TTGTTCCTTGGGTTCTTGGGATCA---GCAGGGAGCACTATG---GGCGCAGCGTCA---
ATAACGCTGACGGTACAG---GCCAGACAA---TTACTG---TCTGGTATAGTGCAGCAGCAGAGC---
AATTTG---CTGCGA---GCTATTGAGGCGCAACAACACCTGTTGCAACTCACAGTCTGGGGCATC
>JN944936
TTCTACTGTAATACATCAAAACTGTTTAATGGAACCTGGAATAGTACTAAAAGAGCAAATAAC----------
----

ACGGAAGGGATTATCATACTCCAATGCAGAATAAAACAAATTATAAATAGGTGGCAAGAAGTAGGAAAAGCAA

TGTATGCCCCTCCCATCGAAGGACAAATTAAATGCTCATCAAAT---
ATTACAGGGTTGCTATTAACAAGAGATGGTGGTAAAACCACCAAC----------------------------
----

AACACCACAGAATTCTTCAGACCTGGAGGAGGAAATATGAAAGATAATTGGAGAAGTGAATTATATAAATATA
AAGTAGTAAGAATTGAACCATTA---GGAGTAGCACCCACCAAGGCAAAGAGAAGAGTGGTG-----------
----------------------------CAGAGAGAAAAAAGAGCA---ATAGGA---ATAGGAGCT------
---GTGTTCCTTGGGTTCTTAGGAGCA---GCAGGAAGCACTATG---GGCGCAGCGTCA---
ATAACGCTGACGGTACAG---GCCAGAAAA---TTATTG---TCTGGTATAGTGCAACAGCAGAAC---
AATCTG---CTGAGG---GCTATTGAGGCGCAACAGCATCTGTTGCAACTCACAGTCTGGGGCATC
>EF593291
TTCTATTGTAATACATCAAAACTGTTTAATAGTACTTGGGGTACTTGGAATAGTACTAAAGGGTCAAATGAC-
----

ACTGGAGGAAATATCACACTCCCATGCAGAATAAAACAAATCATAAACATGTGGCAGGGAGTAGGAAGAGCAA
TGTATGCCCCTCCCATCAGAGGACAAATTAAATGTTCATCAAAT---
ATTACAGGGCTGATATTAACAAGAGATGGTGGTAATGGCACAAAC----------------------------
----

AGCACCACCGAGATCTTCAGACCTGGAGGAGGAGATATGAGGGACAATTGGAGAAGTGAATTATATAAATATA
AAGTAGTGAAAATTGAACCATTA---GGAATAGCACCCACCAAGGCAAAGAGAAGAGTGGTG-----------
----------------------------CAAAGAGAAAAAAGAGCA---GTGGGA---ATAGGAGCT------
---ATGTTCCTCGGGTTCTTGGGAGCA---GCAGGAAGCACTATG---GGCGCAGCGTCA---
GTGACGCTGACGGTACAG---GCCAGACAA---TTATTG---TCTGGTATAGTGCAACAGCAAAGC---
AATTTG---CTGAGA---GCTATTGAGGCGCAGCAGCATATGTTGCAACTCACAGTCTGGGGCATC
>EF593293
TTCTACTGTAATACAGCACAACTGTTTAATAGTACTTGGAGTGGGGGACAGTCAAATGGAACTGAAGAC----
----

AATAGCACCAATATCAGACTCCCATGCAGAATAAAGCAAATTATAAACATGTGGCAGGAAGTAGGAAAAGCAA
TGTATGCCCCTCCCATCAGAGGACAAATTAGATGTTCATCAAAT---
ATTACAGGGCTGCTATTAATAAGAGATGGTGGTAATAGTAACACCACGAAC----------------------
----

AACAGCAACACGGAGACCTTTAGACCTGGAGGAGGAAATATGAGGGATAATTGGAGAAGTGAATTATATAAAT
ATAAAGTAGTAAAAATTGAACCATTA---GGAGTAGCACCCACCAAGGCAAGGAGAAGAGTGGTG--------
-------------------------------CAGAGAGAAAAAAGAGCA---GTGGGA---ATAGGAGCT---
------GTGTTCCTTGGGTTCTTGGGAGCA---GCAGGAAGCACTATG---GGCGCAGCGTCA---
GTGGCGCTGACGGTACAG---GCCAGACGA---TTATTG---TCTGGTATAGTGCAACAGCAGAAC---
AATTTG---CTGAGG---GCTATTGAGGCGCAACAGCATCTGTTGCAACTCACAGTCTGGGGCATC
>EF593294
TTCTACTGTAATACAACAAAGCTGTTTAATAGTACTTGGCCGAGTAATGGTACTGAGCCGTTGAAT-------
----

GATACTGAAAATATCATACTCCCATGCAGAATAAAACAAATTATAAACAGATGGCAGGAAGTGGGAAAAGCAA
TGTATGCCCCTCCCATCAGCGGACTAATTAGATGCTCATCAAAT---
ATTACAGGACTGCTCTTAACAAGAGATGGTGGTAATAACAGCAGC----------------------------
----

AAGAACGAGACTTTCAGACCAGGAGGAGGAGATATGAGGGACAATTGGAGAAGTGAATTATATAAATATAAAG
TAGTAAAAATTGAGCCATTA---GGGATAGCACCCACCAAGGCAAGGAGAAGAGTGGTG--------------
-------------------------CAGAGAGAAAAAAGAGCA---GTGGGA---ATAGGAGCT---------
TTGTTCCTTGGGTTCTTGGGAGCA---GCAGGAAGCACTATG---GGCGCAGCGTCA---
GTGACGCTGACGGTACAG---GCCAGACTA---TTATTG---TCTGGTATAGTGCAACAGCAAAAC---
AATTTG---CTGAGG---GCTATTGAGGCGCAACAGCATATGTTGCAACTCACAGTCTGGGGCATT
>GU330249
TTCTACTGTAATTCAACACCACTGTTTAATAGTAGTTGGACATCTAATGGTACATCTAATGGTACTTGGAAGC
CTGATGATGCT--
----------TGGAATAAT------
AATACTAAAGAAAATATCACACTCCTATGCAGAATAAAACAAATTATAAACATGTGGCAGGAAGTAGGAAAAG

CAATGTATGCCCCTCCTATTAGAGGACAAATTAGTTGTTTATCAAAT---
ATTACAGGGCTGCTATTAACAAGAGATGGTGGGGGTAATAACGGG----------------------------
----

AGCAACAATGAAACCTTCAGGCCTGGAGGAGGAAATATGAAAGACAATTGGAGAAGTGAATTATATAAATATA
AAGTAGTAAAAATTGAACCATTA---GGAGTAGCACCCACCAAGGCAAAAAGAAGAGTGGTG-----------
----------------------------CAAAGAGAAAAAAGAGCA---GTGGGAACACTAGGAGCT------
---ATGTTTCTTGGGTTCTTGGGAGCA---GCAGGAAGCACTATG---GGCGCAGCGTCA---
ATGGCGCTGACGGTACAG---GCCAGACAA---TTATTG---TCTGGTATAGTGCAGCAGCAGAAC---
AATCTG---CTGAGA---GCTATTGAGGCGCAACAGCATATGTTGCAACTCACAGTCTGGGGCATT
>GU330333
TTCTATTGTAATACAACAAAACTGTTTAATAGTACTTGGCATACTAATAGTACTGGGAATGAGACTACGGGGT
CAAATAAC---
-------------------ACG---
GGAGAAAATGACACAATCATGCTCCCATGCAGAATAAAACAAATTATAAACAGGTGGCAGGAAGTAGGAACAG
CAATGTATGCCCCTCCCATCAGAGGACAAATTAGCTGTTCATCAAAT---
ATTACAGGGCTGCTATTGACAAGAGACGGTGGTATAAATATCACT----------------------------
----

AACGAGAATGAAACCTTCAGACCTGAAGGAGGAGATATGAGGAACAATTGGAGAAGTGAATTATATAAATATA
AAGTAGTAAAAATTGAACCATTA---GGAATAGCACCCACCAAGGCAAAGAGAAGAGTGGTG-----------
----------------------------CAGAGAGAAAAAAGAGCA---GTGGGA---ATGGGAGCT------
---TTGTTCCTTGGGTTCTTAGGAGCA---GCAGGAAGCACTATG---GGCGCAGCGTCA---
GTGACGTTGACGGTACAG---GCCAGACAA---TTATTG---TCTGGTATAGTGCAACAGCAGAAC---
AACTTG---CTGAGG---GCTATTGAGGCGCAACAACATATGTTGCAACTCACAGTCTGGGGCATC
>KT124777
TTCTACTGTAACTCATCAAAATTGTTTAATAGTACTTGGTTTAATAGGACTGGGGAGGATAATAATACTACAG
CAGAT--
-------------AGCAGT------
GCAGGAAATGGTACAATCACAATCCCATGCAGAATAAAACAAATTATAAACATGTGGCAGGAAGTAGGAAAAG
CAATGTATGCTCCTCCCATCAGAGGACAGATTAGATGTATATCAAAT---
ATTACAGGGCTGCTATTAACAAGGGATGGTGGCGAGGAAGAGAAC----------------------------
----

GAGACCGAGGTCTTTAGACCCGGGGGAGGTGATATGAGGGACAATTGGAGAAGTGAATTATATAAGTATAAAG
TAGTAAGAATTGAACCATTA---GGAATAGCACCCACCAAGGCAAAGAGAAGAGTGGTG--------------
-------------------------CAGAGAGGAAAAAGAGCA---GTGGGG---ATAGGAGCT---------
CTGTTCCTTGGGTTCTTGGGAGCA---GCAGGAAGCACTATG---GGCGCAGCGTCA---
ATGACGCTGACGGTACAG---GCCAGACTA---TTATTG---TCTGGTATAGTGCAACAGCAGAAC---
AACTTG---CTGAGG---GCTATTGAGGCGCAACAGCATCTGTTGCAACTCACAGTCTGGGGCATC
>KT124795
TTCTACTGCAATTCAACACAACTGTTTAATAGTACTTGGAATTGGAATGATACTGAAAGGATAAGTAATAAC-
----

ACTGGAAAAAATATCACACTCCCATGCAGAATAAAACAAGTTATAAACCTGTGGCAGGAAGTAGGAAAAGCAA
TGTATGCCCCTCCCATCAGCGGAAACATTAGCTGTTCATCAAAT---
ATTACAGGGCTACTACTAACAAGAGATGGTGGTGGGGACAATAAC----------------------------
----

AGGACGAACGAGACCTTCAGACCTGGAGGAGGAAATATGAGGGACAATTGGAGAAGTGAATTATATAAATATA
AAGTAGTAAAAATTGAACCATTA---GGAGTAGCACCCACCAGGGCAAAGAGAAGAGTGGTG-----------
----------------------------CAAAGAGAAAAAAGAGCA---GTGGGAACAATAGGAGCT------
---ATGTTCCTTGGGTTCTTGGGAGCA---GCAGGAAGCACTATG---GGCGCAGCGTCA---
CTAACGCTGACGGTACAG---GCCAGACTA---TTACTG---TCTGGTATAGTGCAACAGCAGAAC---
AATTTG---CTGAGA---GCTATTGAGGCACAACAGCATCTGTTGCAACTCACAGTCTGGGGCATC
>DQ869020
TTCTACTGTAATACAACACTACTGTTTGATAGTACTTGGAATAATAATAGTACTTGGAATGCAAAT-------
----

GTGTCAGAAACTATCATACTCCCATGCAGAATAAAACAAATTATAAACATGTGGCAGGCAGTAGGAAAGGCAA

```
TGTATGCTCCTCCCATCAGAGGACTAATTAACTGTTCCTCAAAT---
ATCACAGGGCTGCTATTAACAAGAGATGGTGGTATTGGTAATAGTAAT-------------------------
-------------------------------------------------------------------------
----------
AGTGAAAATGAGACCTTCAGACCTGAAGGAGGAGATATGAGGGACAATTGGAGAAGTGAATTATATAAATATA
AAGTAGTAAAAATTGAACCATTA---GGAATAGCACCCACCAAGGCAAAGAGAAGAGTGGTG-----------
----------------------------CAGAGAGAGAAAAGAGCA---GTGGGA---ATAGGAGCT------
---GTGTTTCTTGGGTTCTTGGGAGCT---GCAGGAAGCACTATG---GGCGCAGCGTCA---
ATAACGCTGACGGTACAG---GCCAGACTA---TTGTTG---TCTGGTATAGTGCAACAGCAAAAT---
AATTTG---CTGAGG---GCTATTGAGGCGCAACAGCATCTGTTGCAACTCACAGTCTGGGGCATC
>DQ869021
TTCTACTGTGATTCAACACAACTGTTTAGTAGTACTTGGAATAATGGTACTGAGAAGGCAGAAGGAAATGAC-
-------------------------------------------------------------------------
----------------------------
ACAAGTGACACAATTACACTCCCATGCAAAATAAAACAAATTATAAACATGTGGCAGGAAGTGGGAAAAGCAA
TGTATGCTCCTCCCATCAGTGGATATATTAACTGTCTATCAAAT---
ATTACAGGGCTGATATTAACAAGAGATAGGGATGGAGGGGTGAACGAG-------------------------
-------------------------------------------------------------------------
-------
ACAAATATGAACGAGACCTTCAGACCTATAGGAGGAAATATGAAGGACAATTGGAGAAGTGAATTATATAAAT
ATAAAGTAGTGAAAATTGAACCATTA---GGAGTAGCACCCACTAAGGCAAAGAGAAGAGTGGTG--------
-------------------------------CAGAGAGGAAAAAGAGCA---GTGGGA---ATAGGAGCT---
------CTGTTCCTTGGGTTCTTGGGAGCA---GCAGGAAGCACTATG---GGCGCAGCGTCA---
ATGACACTGACGGTACAG---GCCAGACAA---TTATTG---TCTGGTATAGTGCAACAGCAAAAC---
AATTTG---CTGAGA---GCTATTGAGGCGCAACAACATATGTTGCAACTCACAGTCTGGGGCATC
>DQ869022
TTCTACTGTAATTCAACACAGCTGTTTAATAGTACTTGGACCAGGAATGAGACCTTGAATGGGACTGAAGGGT
CAAAT--------------------------------------------------------------------
-------------------------
AGCAATGACACATTCATCACACTCCCATGCAGAATAAAACAAATTATAAACATGTGGCAACAAGTAGGAAAAG
CAATGTATGCCCCTCCCATCAGAGGAAACATTAGTTGCTCATCAAAT---
ATTACAGGGTTGCTATTAACAAGAGATGGTAGTACCGAAAATGAG----------------------------
-------------------------------------------------------------------------
----------
AGTAACACCGAGATTTTCAGACCTGGAGGAGGAGATATGAGGGACAATTGGAGAAGTGAATTATATAAATATA
AAGTAGTAAGAATTGAACCATTA---GGAATAGCACCCACCAAGGCACAGAGAAGAACGGTG-----------
----------------------------CAGAGAGAAAAAAGAGCA---GTGGGA---CTAGGAGCT------
---TTGTTCCTTGGGTTCTTGGGAGCA---GCAGGAAGCACTATG---GGCGCAGCGTCA---
ATAACGCTGACGGTACAG---GCCAGACTA---TTATTG---TCTGGTATAGTGCAACAGCAGAAC---
AATTTG---CTGAGG---GCTATTGAGGCGCAACAGCATCTGTTGCAACTCACAGTCTGGGGCATC
>FJ469687
TTCTACTGTAGTACAACACCACTGTTTAATAGTACTTGGGAGCCTAATGATACTAGAAGGTTCAATGAC----
-------------------------------------------------------------------------
----------------------------
ACTGAAGAAATTATCACACTCATATGCAAAATAAAACAAGTTGTAAACATGTGGCAGAAAGTAGGAAAAGCAA
TGTATGCCCCTCCCATCAGAGGACAAATTAGATGTTCATCAAAT---
ATTACAGGGCTAATATTAACAAGAGATGGTGGTAGTGACGAGAAC----------------------------
-------------------------------------------------------------------------
----------
ACGACCACCGAGATCTTCAGACCTGGAGGAGGAGATATGAGGGACAATTGGAGAAGTGAATTATATAAATATA
AAGTAGTAAAAATTGAACCATTA---GGAGTAGCACCCACCAGGGCAAAGAGAAGAGTGGTG-----------
----------------------------CAGAGAGAAAAAAGAGCA---GTGGGA---CTAGGAGCT------
---ATGTTCCTTGGGTTCTTGGGAGCA---GCAGGAAGCACTATG---GGCGCAGCGTCA---
ATAACGCTGACGGTACAG---GCCAGACAA---TTATTG---TCTGGTATAGTGCAACAGCAGAAC---
AACCTG---CTGAAG---GCTATTGAGGCGCAACAGCGTATGTTGCAACTCACTGTCTGGGGCATC
>FJ469688
TTCTACTGTAATACATCAAAGCTGTTTAATAGTACTTGGAATACTAGCAGTACTGGGAATGTTACTGATGAG-
-------------------------------------------------------------------------
----------------------------
ACAAATGACACAATCACACTCCCATGCAGAATAAAACAGATTGTAAACCTGTGGCAGGAAGTAGGAAAAGCAA
```

TGTATGCCCCTCCCATCAGAGGACAAATTAGATGTTCATCAAAC---
ATTACAGGGCTACTACTAACAAGAGATGGAGGTAATGGGAATAAT----------------------------
----

AGCACAATCGAGATCTTCAGACCTGGAGGAGGAGATATGAGGGACAATTGGAGAAGTGAATTATATAAATATA
AAGTAGTAAAAATTGAACCATTA---GGAGTAGCACCCACCAAGGCAAAGAGAAGAGTGGTG-----------
----------------------------CAGAGAGAAAAAAGAGCA---GCGGGA---CTAGGAGCT-------
---TTGTTCCTTGGGTTCTTGGGAGCA---GCAGGAAGCACTATG---GGCGCAGCGTCA---
ATAACGCTGACGGTACAG---GCCAGACAA---TTATTG---TCTGGTATAGTGCAACAGCAGAAC---
AATTTG---CTGAGG---GCTATTGAGGCGCAACAGCATATGTTGCAACTCACAGTCTGGGGCATC
>FJ469693
TTCTACTGTAATACAACAGGACTGTTTAATAGTACTTGGAATAGTACTGGGAATATTGAAGGGTCAAATGGGA
CT---

GAAGGAAATGACACAATTACACTCCCATGCAGAATAAAACAAATTATAATCATGCGGCAGGAAGTAGAAAAAG
CAAAGTCTCCCCCTCCCATCAGAGGACAAATTAAATCTTCATCAATT---
ACTACAGGGCTATTATCAACAAGAGATGGTGGTAATAAGAATGGGACTAAT----------------------
----

GAGAGTAGCGACACTGAGATCTTCAGACCTGGAGGAGGAGATATGAGGGACAATTGGAGAAGTGAATTATATA
AATATAAAGTAGTAAAAGTTGAACCATTA---GGAGTAGCACCCACCAAGGCAAAGAGAAGAGTGGTG-----
----------------------------------CAGAGAGAAAAAAGAGCA---
GTAGTGGGATTAGGAGCC---------GTGTTCCTTGGGTTCTTGTCAGCA---GCAGGAAGCACTATG---
GGCGCAGCGTCA---TTAACGCTGACGGTACAG---GCCAGACAA---TTATTG---
TCTGGTATAGTGCAACAGCAGAAC---AATTTG---CTGAGA---
GCTATTGAGGCGCAACAGCATATGTTGCAACTCACAGTCTGGGGCATC
>HQ217201
TTCTACTGTAATACAACAAAACTGTTTAAGGGGACTTGGAATGGTACTACTTGGTGGAATGGTGCTAATGGGA
CAAATATCACA--
-------GATAACAATGAA------
GGGTCAAATGACACAATCATACTCCCATGCAGGATAAAACAAATTGTAAACTTGTGGCAGGAAGTAGGAAAAG
CAATGTATGCCCCTCCCATCCAAGGACAAATTAGATGTTCATCAAAT---
ATTACAGGGCTGATATTAACAAGAGATGGGGGTAACCAAAACAAAACATGCAAC-------------------
----
-
GAGACTAATCCATGCACTGAGACCTTCAGACCAGGAGGAGGAAATATGAAAGACAATTGGAGAAGTGAATTAT
ATAAATATAAAGTAGTAAAAATTGAACCATTA---GGAGTAGCACCCACCAAGGCAAAGAGAAGAGTGGTG--
-------------------------------------CAAAGAGAAAAAAGAGCA---GTGGGA---
ATAGGAGCT---------TTGTTCCTTGGGTTCTTGGGAGCA---GCAGGAAGCACTATG---
GGCGCAGCGTCA---TTGACGCTGACGGTACAG---GCCAGACAA---TTATTG---
TCTGGTATAGTGCAACAGCAAAAC---AATTTG---CTGAGA---
GCTATTGAGGCGCAACAGCATATGTTGCAACTCACAGTCTGGGGCATT
>FJ469730
TTCTACTGTAATACAACACAGCTGTTTAATGGTACTTGGAATAATGGTACTTGGAATAATGGTACTTGGAATA
GTACC--
-------------------GAA---
GGGTCAAATGACACTATCACACTTCCATGCAGAATAAAACAAATTATAAACATGTGGCAAAAAGTAGGAAAAG
CAATGTATGCCCCTCCCATTAGAGGACAAATTAGATGTTCATCAAAT---
ATTACAGGGCTGCTATTAACAAGCGATGGTGGTGGTAATGGGAAT----------------------------
----

AGTAGCGAGACGTTCAGACCTGGAGGAGGGAATATGAAGGACAATTGGAGGAGTGAATTATATAAATATAAAG
TAGTGAAAATTGAACCATTA---GGAGTAGCACCCACCAAGGCAAAGAGAAGAGTGGTG--------------
-------------------------CAGAGAGAAAAAAGAGCA---ATGGGA---CTAGGAGCT---------
CTGTTCCTTGGGTTCTTGGGAGCA---GCAGGAAGCACTATG---GGCGCAGCGTCA---
GTGACGCTGACGGTACAG---GCCAGACTA---TTATTG---TCTGGTATAGTGCAACAGCAGAAC---
AATTTG---CTGAGG---GCTATTGAGGCGCAACAGCATCTGTTGCAACTCACAGTCTGGGGCATC
>FJ469737
TTCTACTGTAATACAACAAGGCTGTTTAATAGTACTTGGATAGGTACTAATAGTACTTGGAAT----------
----

GATACTGATATCATCACACTCCCATGCAGAATAAAACAAATTGTAAACATGTGGCAGGAAGTAGGAAAAGCAA
TGTATGCCCCTCCCATCAGTGGACAAATTAGATGTTCATCAAAT---
ATTACAGGGCTACTATTAACAAGAGATGGGGGTAGCCAGAACCAAACG------------------------
----

GAGAACAACACGGAGACCTTCAGACCTGGAGGAGGAGATATGAGGGACAATTGGAGAAGTGAATTATATAAAT
ACAAAGTAGTAAGAATTGAACCATTA---GGAGTAGCACCCACCACGGCAAAAAGGAGAGTGGTG--------
------------------------------CAAAGAGAAAAAAGAGCA---GTGGGA---ATAGGAGCT---
------TTGTTCCTTGGGATCTTGGGAGCA---GCAGGAAGCACTATG---GGCGCAGCATCA---
CTGACGCTGACGGTACAG---ACCAGACTA---TTATTG---TCTGGTATAGTGCAACAGCAGAAC---
AATTTG---CTGAGG---GCTATTGAGGCGCAACAGCATATGTTGCAACTCACAGTCTGGGGCATC
>JQ403102
TTCTACTGTAATACAACACAACTATTTAATAGTAGTTGGGTTGTCAATGGGACTATAGAGTCAAATGCCACCG
AA--

GGAGATAGCAAGATCACCCTCCCATGCAGAATAAAACAAATTGTAAACATGTGGCAGGAAGTAGGAAAAGCAA
TGTATGCCCCTCCCATCAGAGGAAAAATTAGCTGTCCATCAAAT---
ATTACAGGGCTGCTATTAACAAGAGATGGTGGGCATGATGGTAATGGGAATAGC------------------
----

AGCAACAGTGAGACATTCAGACCTGGAGGAGGAGATATGAGGGACAATTGGAGAAGTGAATTATATAAATATA
AAGTAGTAGAAATTAAACCATTA---GGACTAGCACCCAACAAGGCAAAGAGAAGAGTGGTG-----------
---------------------------CAAAGAGAAAAAAGAGCA---GTGGGA---ATAGGAGCT------
---GTGTTCCTTGGGTTCTTGGGAGCA---GCAGGAAGCACTATG---GGCGCAGCGGCA---
GTGACGCTGACGGTACAG---GCCAGACAA---TTATTG---TCTGGTATAGTGCAACAGCAGAAC---
AATTTG---CTGAGA---GCTATTGAGGCGCAACAGCATCTGTTGCAACTCACAGTCTGGGGCATC
>FJ469741
TTCTACTGTAATACAACACGACTGTTTAACAGTACTTGGACTTGGGATAATGATACTGAAGAA----------
----

TCAAATGACACTATCACACTCCCATGCAGAATAAAACAAATTATAAACATGTGGCAGGAAGTAGGAAAAGCAA
TGTATGCCCCTCCCATCAGAGGACAAATTAAATGTTCATCAAAT---
ATTACAGGGCTGCTATTAATAAGAGATGGTGGTGAGGACGGGAAC---------------------------
----

AGTACCACCGAGATCTTCAGACCTGGAGGAGGAAATATGAGGGACAATTGGAGAAGTGAATTGTATAAATATA
AAGTAGTAAAAATTGAACCATTA---GGATTAGCACCCACCAAGGCAAAGAGAAGAGTGGTG-----------
---------------------------CAGAGAGAAAAAAGAGCA---GCA------ATAGGAGCT------
---ATGATCCTTGGGTTCTTGGGAGCA---GCAGGAAGCACTATG---GGCGCAGCGTCA---
CTGACGCTGACGGTACAG---GCCAGACTA---TTATTG---TCTGGTATAGTGCAACAGCAGAAC---
AATTTG---CTGAGA---GCTATTGAGGCGCAACAGCATCTGTTGCAACTCACAGTCTGGGGCATC
>FJ469748
TTCTATTGTAATACTACACAACTGTTTAATAGTACTTGGTATGGAAATGGTACTGTACAGTCAATATCTGAA-
----

GGAAATGAAACAATCACACTCCCATGCAGAATAAAACAGATTGTAAACATGTGGCAACAAGTAGGAAAAGCAA
CGTATGCCCCTCCCATCAGTGGACAAATTAACTGTACATCAAAT---
ATTACAGGGCTGCTAATAACAAGAGATGGTGGGGGTAACGAGTCC---------------------------
----

AGGAATAATGAGACCTTTAGACCTGCAGGAGGAGATATGAGGGACAATTGGAGAAGTGAATTATATAAATATA
AAGTAGTAAAAATTGAACCATTA---GGAGTAGCACCCACCAAGGCAAAAAGAAGAGTGGTG-----------
---------------------------CAGAGAGAAAAAAGAGCA---GTGACA---CTAGGAGCT------
---GTGTTCCTTGGGTTCTTGGGAGCA---GCAGGAAGCACTATG---GGCGCAGCGTCA---
ATGACGCTGACGGTACAG---GCCAGACTA---TTGATG---TCTGGTATAGTGCAACAGCAGAAC---
AACTTG---CTGAAA---GCTATTGAGGCGCAACAGCATCTGTTGCAACTCACTGTCTGGGGCATC
>FJ469760
TTCTACTGTAATTCAACAAAACTGTTTAATAGTACTTGGCCACTTAATAGTACTGTGAATGATACTACCGGGT
CA--

AATAACAATGATACCATAGTTCTCCCATGCAGAATAAAACAAATTATAAACATGTGGCAACAAGTAGGAAGAG
CAATGTATGCCCCTCCTATCAGAGGAAAAATTAGATGTTCATCAAAT---
ATTACAGGGCTGATATTAACAAGGGATGGTGGTGGTGGTGCAAACGAG-------------------------
----

ACCACCAGGAACGAGACCTTCAGACCTGCAGGAGGAGATATGAGGGACAATTGGAGAAGTGAATTATATAAAT
ATAAAGTAGTAAGAATTGAACCATTA---GGAGTAGCACCCACTAAGGCAAAGAGAAGAGTGGTG--------
-------------------------------CAGAGAGAAAAAAGAGCC---GTGGGA---CTAGGAGCC---
------ATGTTCCTTGGGTTCTTGGGAGCA---GCAGGAAGCACTATG---GGCACAGCGTCA---
CTGACGCTGACGGTACAG---GCCAGACAA---TTATTG---TCTGGTATAGTGCAACAGCAGAAC---
AATCTG---CTGAGG---GCTATTGAGGCGCAACAGCATCTGTTGCAACTCACAGTCTGGGGCATC
>FJ469762
TTCTACTGTAATACAACACAACTGTTTAATAGTACTTGGACTGATAATAGTACTACCGGGTCAAATAAC----
----

ACAAAAGGAATTATCACACTCCCATGCAGAATAAAACAAATTATAAATATGTGGCAAGAAGTAGGAAAAGCAA
TGTATGCCCCTCCCATCAGTGGATTAATTAGATGTAAATCAAAT---
ATTACAGGGCTGCTATTAACAAGAGATGGTGGTAATAAGAATAACAAC-------------------------
----

GAGACTGAGACCGAAATCTTTAGGCCTGGAGGAGGAGATATGAGGGACAATTGGAGAAGTGAATTATATAAAT
ATAAAGTAGTAAAAATTGAACCATTA---GGAATAGCACCCACCAGGGCAAAGAGAAGAGTGGTG--------
-------------------------------CAGAGAGAAAAAAGAGCA---GTGGGA---TTAGGAGCT---
------TTGTTCCTTGGGTTCTTGGGTACA---GCAGGAAGCACTATG---GGCGCAGCGTCA---
ATAACGCTGACGGTACAG---GCCAGACAA---TTATTG---TCTGGTATAGTGCAACAGCAGAAC---
AACTTG---CTGAGA---GCTATTGAGGCGCAACAACATCTGTTGCAACTCACAGTCTGGGGCATC
>EU578460
TTCTACTGTAATACAACACAGCTGTTTAATAGTACTTGGCCTAATGATACTAGTACTTGGTATGGAATGAT-
----

ACTGCAGGAGATATCACACTCCCATGCAGAATAAAACAAATTATAAACAGGTGGCAGGAAGTAGGAAAAGCAA
TGTATGCCCCTCCCATCAGCGGATAATTAGATGTTCATCAAAT---
ATTACAGGGATAATTTTAACAAGAGATGGTGGTAATAACATCACGAAC-------------------------
----

AACACCACCGAGACCTTCAGACCTGAAGGAGGAAATATGAAGGATAATTGGAGAAGTGAATTATATAAATACA
AAGTAGTAAAAATTGAACCATTA---GGAATAGCACCCACCAAGGCAAGGAGAAGAGTGGTG-----------
----------------------------CAGAGAGAAAAAAGAGCA---GTGGGAACAATAGGAGCT------
---ATGTTCCTTGGGTTCTTGGGAGCA---GCAGGAAGCACTATG---GGCGCAGCGTCG---
CTGACGCTGACGGTACAA---GCCAGACTA---TTATTG---TCTGGTATAGTGCAACAGCAGAAC---
AATCTG---CTGAGG---GCTATTGAGGCGCAACAGCATATGTTGCAACTCACAGTCTGGGGCATC
>EF593295
TTCTACTGTGATTCAGCACCACTGTTTAATAGTAGTTGGATACGGAATACAAGTACTTGGACACGGAATGATA
GCGCTTGGAAT--
-AAAACTGAAGGGACA---------
AATAGCACTGATCCAATCACACTCACATGTAGAATAAAACAATTTATAAACATGTGGCAGAAAGTAGGAAAAG
CAATGTATGCTCCTCCCATCAGTGGACAAATTAAATGTTTATCAAAT---
ATTACAGGGCTGATACTAACAAGAGATGGTGGTAATAACAAAAATACAAGT----------------------
----

GACAACGAGACTAGCGAGATCTTCAGACCTGGAGGAGGAGATATGAGGGACAATTGGAGAAGTGAATTATATA
AATATAAAGTGGTGAAAATTGAACCATTA---GGAGTAGCACCCACCAAGGCAAAGAGAAGAGTGGTG-----
----------------------------------CAGAGAGAAAAAAGAGCA---GTGACG---
CTAGGAGCT---------ATGTTCCTTGGGTTCTTGGGAGCA---GCAGGAAGCACTATG---
GGCGCAGCGTCA---ATGACGCTGACGGTACAG---GCCAGACTA---TTATTG---
TCTGGTATAGTGCAACAGCAGAAC---AATCTG---CTGAGG---
GCTATTGAGGCGCAGCAGCATCTGTTGCAACTCACAGTCTGGGGCATT
>EF593296

TTCTACTGTAATACAACAAAACTGTTTAATAGTACAGGGGGGAATAATAACACTTGGACTGAT----------
----

ACTACAGGAAATATCACACTCCAATGCAGAATAAAACAAATTATAAACATGTGGCAGGAAGTAGGAAAAGCAA
TGTATGCCCCTCCCATCAGAGGACACATTAAATGTTCATCAAAT---
ATTACAGGACTGATATTAACAAGAGATGGTGGTGGTAGTAATAAC----------------------------
----

AATACTACTGAGACCTTTAGGCCGGGAGGAGGAAATATGAAGGACAATTGGAGAAGTGAATTATATAAATATA
AAGTAGTACAAATTGAACCATTA---GGAGTAGCACCCACCAGGGCAAGGAGAAGAGTGGTG-----------
----------------------------CAGAGAGAAAAAAGAGCA---ATAGGA---CTGGGAGCT------
---CTCCTCCTTGGGTTCTTGGGAACA---GCAGGAAGCACTATG---GGCGCAGCGTCA---
CTGACGTTGACGGTACAA---GCCAGACAA---TTATTG---TCTGGTATAGTGCAACAGCAGAAC---
AATCTG---CTGAGG---GCTATTGAGGCGCAGCAGCATATGTTGCAACTCACAGTCTGGGGCATC
>EF593297
TTCTACTGTAATACAGCAGAACTGTTTAATAGTACTTGGGATACTACTACAGGGTCAAATAACACAAAT----
----

GACACTGAAATTATCACACTTCCATGTAGAATAAAACAAATTATAAACATGTGGCAGCAAGTAGGAAAAGCAA
TGTATGCCCCTCCCATCAAAGGACAAATTAGATGTTCATCAAAT---
ATTACAGGACTACTATTAACAAGGGATGGAGGTAATAACACTGAC----------------------------
----

AACGAGACCTTCAGACCTGGAGGAGGAGATATGAGGGACAATTGGAGAAGTGAATTATATAAATATAAAGTAG
TAAAAATTGAACCATTA---GGACTAGCACCCACCAAGGCAAGGAGAAGAGTGGTG-----------------
----------------------CAGAGAGAAAAAAGAGCA---GTGGGA---ATAGGAGCT---------
CTGTTCCTTGGGTTCTTGGGAGCA---GCAGGAAGCACTATG---GGCGCAGCGTCG---
ATGACGCTGACGGTACAA---GCCAGGCAA---TTATTG---TCTGGTATAGTGCAACAGCAGAAC---
AACTTG---CTGAGA---GCTATTGAGGCGCAACAGCATATGTTGCAACTCACAGTCTGGGGCATC
>GU330460
TTCTACTGTAATACAACAGGACTGTTTAATAATACTTGGGGGTTTAATAGTACTTGGGATGCTATTAATGTGC
CA---

GAAAATGACACAATCACACTCCCATGCAGAATAAAACAAATTGTAAACATGTGGCAGGAAGTAGGAAAAGCAA
TGTATGCCCCTCCCATCAGAGGACGACTTAATTGTTCATCAAAT---
ATTACAGGGCTGCTATTAACAAGAGATGGTGGTAATACCACAAAT----------------------------
----

AACACTGAGGTCTTCAGACCTGGAGGAGGAGATATGAGAGACAATTGGAGAAGTGAATTATATAAATATAAAG
TAGTAAAAATTGAACCATTA---GGAATAGCACCCACCAAGGCAAAGAGAAGAGTGGTG--------------
-------------------------CAGAGAGAAAAAAGAGCA---GTGGGA---ATAGGAGCT---------
TTGTTCCTTGGGTTCTTGGGAGCA---GCAGGAAGCACTATG---GGCGCAGCGTCG---
ATGACGCTGACGGTACAG---GCCAGACTA---TTATTG---TCTGGTATAGTGCAACAGCAGAAC---
AATTTG---CTGAGA---GCTATTGAGGCGCAACAGCATCTGTTGCAACTCACAGTCTGGGGCATC
>HM234503
TTCTATTGTAATACAACGCAACTGTTTAATAGTACTTGGAAAATTAATAGTACTTGGAATGATACTGAAGGG-
----

TCAAATGACACTATCGCACTCCCATGTAGAATAAAACAAATCATAAACATGTGGCAGAAAGTAGGAAAAGCAA
TGTATGCCCCTCCCATCCAAGGACAAATTAACTGTTCATCAAAA---
ATCACAGGGCTGCTATTAACAAGAGATGGTGGCAGCACCGATAAC----------------------------
----

AACAACACCGAGACCTTCAGACCTGGAGGAGGAGATATGAGGGACAATTGGAGAAGTGAGTTATATAAATATA
AAGTAGTAAAAGTTGAACCATTA---GGAATAGCACCCGCCAAGGCAAAGAGAAGAGTGGTG-----------
----------------------------CAGAGAGAAAAAAGAGCA---GTGGGA---ATGGGAGCT------
---CTGTTCCTTGGGTTCTTGGGAGCA---GCAGGAAGCACTATG---GGCGCAGCGTCA---
CTAACGCTGACGGTACAG---GCCAGACAA---TTATTG---TCTGGTATAGTGCAACAGCAGAAC---
AATTTG---CTGAGG---GCTATTGAGGCGCAACAGCATATGTTGCAACTCACAGTCTGGGGCATT
>KC312470

TTCTACTGTAATACAACACAACTGTTTAATAGTACTATCAATGGTACTGAAATGTAC----------------
----

AGTGCAGGAAATATCACACTCCCATGCAAAATAAAGCAAATTATAAACATGTGGCAGGAAGTAGGAAAAGCAA
TGTATGCCCCTCCCATAAGAGGACAAATTAGATGTTCATCAAAT---
ATTACAGGGCTGCTATTAGTGAGAGATGGTGGTAATGCTGAGAATGATACCAAC-------------------
----

ACTACCAACATCGAGACCTTCAGACCTGGAGGAGGAGATATGAGGGACAATTGGAGAAGTGAATTATATAAAT
ATAAAGTAGTAAAAATTGAACCATTA---GGAATAGCACCCACCAAGGCAAAAAGAAGGGTGGTG--------
-------------------------------CAGAGAGAGAAAAGAGCG---GTGGGA---CTAGGAGCT---
------TTGTTCATTGGGTTCTTAGGAGCA---GCAGGAAGCACTATG---GGCGCAGCGTCA---
ATAACGCTGACGGTACAG---GCCAGGCTA---TTATTG---TCTGGTATAGTGCAACAGCAAAAC---
AATTTG---CTGAGA---GCTATTGAGGCGCAACAGCATCTGTTGCAACTCACAGTCTGGGGCATC
>KT124807
TTTTACTGTAATACAACACAGCTGTTTAGTACTTGGAATAGTACTGGGCAGTCTAATAGTACT----------
----

GGGAATGATACCATCACACTCCCATGCAGAATAAAACAAATTATAAACAAGTGGCAGGGAGTAGGAAAAGCAA
TGTATGCCCCTCCCATCAGCGGAGTAATTAATTGTACATCAAAT---
ATTACAGGGCTGCTATTGACAAGAGATGGTGGTAAGACGGGCGAG----------------------------
----

AATGAGAGTGAGACCTTCAGACCTGGAGGGGGAGATATGAGGGACAATTGGAGAAGTGAATTATATAAATATA
AAGTAGTAAAAATTGAACCAATA---GGAGTAGCACCCACCAAAGCCAAGAGAAGAGTGGTG----------
----------------------------CAGAGAGAAAAAAGAGCA---GTAACA---CTGGGAGCT------
---ATGTTCCTTGGGTTCTTGGGAGCA---GCAGGAAGCACTATG---GGCGCAGCGTCA---
ATGACGCTGACGGTACAG---GCCAGACAA---TTATTG---TCTGGTATAGTGCAACAGCAGAAC---
AATCTG---CTGAAG---GCTATTGAGGCGCAACAGCATCTGTTGCAACTTACAGTCTGGGGCATC
>KT124813
TTCTACTGTAATACGTCACAACTATTTAATAGTACTTGGAGTAATGGGACTAATGGTAATTGGACTATAAATA
ACACC--
-ACAGGGGGAAATAAC---------
ATTGAGAATGACACTATCACACTCCCATGCAGGATAAAACAAATTATAAACTTGTGGCAGAAAGTAGGAAGGG
CAATGTATGCCCCTCCCATTAGTGGACAGATTAGCTGTTCATCAAAT---
ATTACAGGGCTAATATTAACAAGAGATGGTGGTGCTACTGATAAT----------------------------
----

AGCATGAACGAGACCTTCAGACCTGCAGGAGGAGATATGAAGGACAATTGGAGAAGTGAATTATATAAATATA
AAGTAGTAAGAATTGAACCATTA---GGAGTAGCACCCACCCCGGCAAGGAGAAGAGTGGTG----------
----------------------------CAGAGAGAAAAAAGAGAA---CTAACA---ATGGGAGCT------
---CTGTTCCTTGGGTTCTTGGGAGCA---GCAGGAAGCACTATG---GGCGCAGCGTCG---
CTGACGCTGACGGTACAA---GCCAGACTA---TTATTG---TCTGGTATAGTGCAACAGCAGAAC---
AATATG---CTGAGG---GCTATTGAGGCGCAACAACATCTGTTGCAACTCACAGTCTGGGGCATC
>DQ869016
TTCTACTGTAATACAACAAAATTGTTCAATAGTACTTGGAACAATACTGATTCAAATACCACTGAG-------
----

AACAATGACACAATCACACTCCCATGCAGGATAAAACAAATCGTAAACTTGTGGCAGCAAGTAGGAAAAGCAA
TGTATGCCCCTCCCATCAGCGGACAGATTAGATGTTCATCAAAT---
ATCACAGGGCTTTATTAACAAGAGATGGTGGTGATATTAATAGA-----------------------------
----

ACTGAGGAGATATTCAGACCTGGAGGAGGAAATATGAAGGACAATTGGAGAAGTGAATTATATAAATATAAGG
TAGTACAAATTGAACCATTA---GGAGTAGCACCCACCAAGGCAAAGAGAAGAGTGGTG--------------
-------------------------CAGAGAGAAAAAAGAGCT---TTGGGA---ATGGGAGCT---------
TTGTTCCTTGGGTTCTTGGGAGCA---GCAGGAAGCACTATG---GGCGCAGCGTCA---
TTGACGCTGACGGTACAG---GCCAGACAA---TTATTG---TCTGGTATAGTGCAACAGCAGAAC---
AATCTG---CTGAGA---GCTATTGAGGCGCAACAGCATCTGTTGCAACTCACAGTCTGGGGCATT
>DQ869024

TTCTACTGTAATACAACACAACTGTTTAATAGTACTTGGAGGAGTAATGATACAACACAACTGTCTAATAGTA
CTTGGAGGAGT--
AATGATACTGCTTCTGAA---------
GGGTCAAATGACACTATCATCCTCCCATGTAGAATAAAACAAATTATAAACTTGTGGCAGGAAGTAGGAAAAG
CAATGTATGCCCCTCCCATTAGTGGACCAATTAGCTGTTCATCAAAT---
ATTACAGGGCTGCTATTAACAAGAGATGGTGGTAACAACAGCACC----------------------------
----

AATAACACTGAGGTCTTCAGACCTGGAGGAGGAAATATGAGGGACAATTGGAGAAGTGAACTATATAAATATA
AAGTAGTAAAAATTGAACCATTA---GGAATAGCACCCACTAGGGCAAAGAGAAGAGTGGTG-----------
----------------------------CAAAGAGAAAAAAGAGCA---GTGGGA---ATCGGAGCT------
---GTGTTCCTTGGGTTCTTGGGAGCA---GCAGGAAGCACTATG---GGCGCAGCGTCA---
ATGGCGCTGACGGTACAG---GCCAGACAA---TTATTG---TCTGGTATAGTGCAACAGCAGAAT---
GATCTG---CTGAGG---GCTATTGAGGCGCAACAACATCTGTTGCAACTCACAGTCTGGGGCATC
>DQ869025
TTCTATTGTAATACAACACAACTGTTTAATAGTATTTGGAACAGTACTTGGAGTGCTAATAGTAGTGGTAATG
TA---

GAAAATAAAACGATCACACTCCCATGCAGAATAAAACAAATTATAAACATGTGGCAGGAAGTAGGAAAAGCAA
TGTATGCCCCTCCCATCGCAGGAATAATTAGCTGTTCATCAAAT---
ATTACAGGGCTGATATTAACAAGAGATGGTGGTAATGATAATGATACG-------------------------
----

AACAAGGAGGAGGAGGTCTTCAGACCTGGAGGAGGAAATATGAAGGACAATTGGAGAAGTGAATTATATAAAT
ATAAAGTAGTAAGAATTGAACCCTTA---GGAGTAGCACCTACTAAGGCAAAGAGAAGAGTGGTG--------
-------------------------------CAGAGAGAAAAAAGAGCA---GTGGGA---ATAGGAGCT---
------GTGTTCCTTGGGTTCTTGGGAGCA---GCAGGAAGCACTATG---GGCGCAGCGTCA---
GTGGCGCTGACGGTACAG---GCCAGACAA---TTATTG---TCTGGTATAGTGCAACAGCAAAAC---
AATTTG---CTGAGG---GCTATTGAGGCGCAACAGCATATCTTGCAACTCACAGTCTGGGGCATC
>DQ869029
TTCTACTGTAATACAACACAACTGTTTACTTGGAATGATACTAGTATTGAAAATTCAAATGACACTGAGGGA-
----

TCAAATAAAATTATCATTCTTCCATGCAAAATAAAACAAATTATAAAAATGTGGCAGAGAGTAGGAAAAGCAA
CGTATGCCCCTCCCATCAGAGGACAAATTAGATGTTCATCGAAT---
ATTACAGGGCTGCTATTAACAAGAGATGGTGGTAGTAACCAGAGT----------------------------
----

AATAGTGAGACCTTCAGACCTGGAGGAGGAGATATGAGGGACAATTGGAGGAGTGAATTATATAAATATAAAG
TAGTAAAAATTGAACCATTA---GGAGTAGCACCCACCAAGGCAAAGAGAAGAGTGGTG--------------
-------------------------CAAAGGGAAAGAAGAGCA---GTGGGA---ATAGGAGCT---------
ATGCTCCTTGGGTTCTTAGGAACA---GCCGGAAGCACTATG---GGCGCAGCGTCA---
GTGGCGCTGACGGTACAG---GCCAGACTA---TTATTG---TCTGGTATAGTGCAGCAGCAGAAC---
AATTTG---CTGAGA---GCTATTGAGGCGCAACAGCACCTGTTGCAACTCACAGTCTGGGGCATC
>DQ869030
TTCTATTGTAATACAACAGAGCTGTTTAATAGTACTTGGCTGGATAATAATACTAGGAATGATACTGAAGGGT
CA---

AATAGCACTGATATCACACTCCAATGCAGAATAAAACAAATTATAAACAGGTGGCAGGAAGTAGGAAAAGCAA
TGTATGCCCCTCCCATCAGAGGACCAATTAATTGTACATCAAAT---
ATTACAGGGCTACTATTAATAAGAGATGGTGGTAATGACAATAGTACT-------------------------
----

GACAATAACACTGAGATCTTCAGACCTACAGGAGGAAACATGAGGGGCAATTGGAGAAGTGAATTATATAAAT
ATAAAGTAGTAAAAATTGAGCCATTA---GGAATAGCACCCACCGAGGCAAAGAGAAGAGTGGTG--------
-------------------------------CAGAGAGAAAAAAGAGCA---GTAGGC---CTGGGAGCT---
------ATGTTCCTTGGGTTCTTGGGAGCA---GCAGGCAGCACTATG---GGCGCAGCGTCA---
CTGACGCTGACGGTACAG---GCCAGACAA---TTACTG---TCTGGGATAGTGCAACAGCAAAAC---
AATTTG---CTGAGA---GCTATTGAGGCGCAACAGCATCTGTTGCAACTCACAGTCTGGGGCATC
>DQ869031

TTCTACTGTGATTCAACACAACTGTTTAATAGTACTTGGAATACTTGGAATGCTACAGAGTCGAATGGCACTA
GA---

GAAAATGACACAATCACACTCCCATGCAGAATAAGACAAATTATAAACATGTGGCAGAAAGTAGGAAAAGCAA
TGTATGCCCCTCCCATCAGAGGACGAATTAACTGTTCATCAATA---
ATTACAGGGCTGCTATTAACAAGAGATGGT---AGTAACAGTAGC----------------------------
----

AATGAGACCTTCAGACCTGGAGGAGGAGATATGAGGGATAATTGGAGAAGTGAATTATATAAATATAAAGTAG
TAAAAGTTGAACCGTTA---GGAATAGCACCCACCACGGCAAGGAGAAGAGTGGTG-----------------
----------------------CAGAGAGAAAAAAGAGCA---CTAGGGGGACTAGGAGCT---------
ATGTTCCTTGGGTTCTTGAGTGCA---GCAGGAAGCACTATG---GGCGCAGCGTCA---
CTGACGCTGACGGTACAG---GCCAGACAA---TTATTG---TCTGGTATAGTGCAACAGCAGAGC---
AATCTG---CTGATG---GCTATTGAGGCGCAACAACATATGTTGAAACTCACAGTCTGGGGCATT
>FJ469701
TTCTACTGTAATACATCAAAACTGTTTAATAGTACTTGGAGTACTAATAGTACTTGGAATGATACTGAAAGG-
----

TTAAATGAGACTATCATACTCCCATGCAGAATAAAACAAGTTATAAACTTGTGGCAGGAAGTAGGAAAAGCAA
TGTATGCCCCTCCCATCAGAGGAGAAATTAACTGTGCATCAAAT---
ATTACAGGACTGCTATTAATAAGAGATGGTGGAAATGAGAAAAACAAG-------------------------
----

ACCGAGGAGATCGAGATCTTTAGACCTGGAGGAGGAGATATGAGGGACAATTGGAGAAGTGAATTATATAAAT
ATAAGGTAGTAAAAATTGAACCATTA---GGAATAGCACCCACCAAGGCAAAGAGAAGAGTGGTG--------
-------------------------------CAGAGAGAAAAAAGAGCA---GCAGCG---CTAGGAACT---
------ATGATCCTTGGGTTCTTGGGAGCA---GCAGGAAGCACTATG---GGCGCAGCGTCA---
CTGACGCTGACGGTACAG---GCCAGACAA---TTATTG---TCTGGTATAGTGCAACAGCAGAGC---
AATTTG---CTGAAG---GCTATTGAGGCGCAACAGCATCTGTTGCAACTCACAGTCTGGGGCATC
>KC473846
TTCTACTGCAATACAACACCACTGTTTAATAGTACTTGGGATAGTAGTACTTGGAATAGTACTAAAAATAATA
CGGGG--

TCAAATGATACTATCACAATCCAATGTAGGATAAAACAAATTATAAACATGTGGCAGACAGTGGGAAAAGCGA
TGTATGCCCCTCCCATCGCAGGACAAATTAGATGTTTATCAAAT---
ATTACAGGGCTATTATTAGAGAGAGATGGTGGTAATAATAAGACA----------------------------
----

GACCACAACGAAACCTTCAGACCTAGCGGAGGAAATATGAAGGACAATTGGAGAAGTGAATTATATAAATATA
AAGTAGTAAAAGTTGAACCAATA---GGAATAGCACCCACCAGGGCAAAGAGAAGAGTGGTG-----------
----------------------------CAGAGAGAAAAAAGAGCG------GGA---ATAGGAGCT------
---GTGTTCCTTGGGTTCTTGGGAGCA---GCAGGAAGCACTATG---GGCGCAGCGTCA---
GTAACGCTGACGGTACAG---GCCAGACAA---TTATTG---TCTGGTATAGTGCAACAGCAAAAT---
AATTTG---CTGAGG---GCCATTGAGGCGCAACAGCATATGTTGCAACTCACTGTCTGGGGCATC
>EF593222
TTCTACTGCAATACAACAAAACTGTTTAATAGTACTTGGAATAGTGGGAATAATACTAATAATACTGAAGGAT
CA---

AATGACACTATTATCACACTCCCATGCAGAATAAAACAAATTATAAACTTATGGCAGGAAGTAGGAAAAGCAA
TGTATGCCCCTCCCATCAGAGGACAAATTAACTGTTCATCAAAT---
ATTACAGGACTGTTATTAATAAGAGATGGGGGTAATACCACAAAC----------------------------
----

AACACAGAGACCTTCAGACCTTTAGGAGGAAATATGAAGGACAATTGGAGAAGTGAATTATATAAATATAAAG
TAGTACAAATTGAACCATTA---GGAGTAGCACCCACTAAGGCAAAGAGAAGAGTGGTG--------------
-------------------------CAGAGAGAAAAAAGAGCA---GTGGGA---ATAGGAGCT---------
TTGTTCCTTGGGTTCTTGGGAGCA---GCAGGAAGCACTATG---GGCGCAGCGTCA---
GTGACGCTGACGGTACAG---GCCAGACAA---TTATTG---TCTGGTATAGTGCAACAGCAGAAC---
AATTTG---CTGAGA---GCTATTGAGGCGCAACAGCATATGTTGCAACTCACAGTCTGGGGCATC
>DQ410465

TTCTACTGTAATACAACACAACTGTTTAATAGTACTTGGAATACAACACAACTGTTTAATAGTACTTGGAATG
ATACTGGG---
-------------CCAAAT------
AACGAAAATGACACACTCACACTCCCATGCAGAATAAAACAAATTATAAACAGGTGGCAGGAAGTAGGAAAAG
CAATGTATGCCCCTCCCATCAGAGGACAAATTAGATGTTCATCAAAT---
ATTACGGGGCTGCTATTAACAAGAGATGGTGGTAACGACAATAACAAGACC----------------------
----

AATAACCAGACCGAGATCTTCAGACCTGGAGGAGGAAATATGAGGGACAATTGGAGAAGTGAATTATATAAAT
ATAAAGTAGTAAGAATTGAACCATTA---GGAGTAGCACCCACCCCGGCAAAGAGAAGAGTGGTG--------
-------------------------------CAAAGAGAAAAAAGAGCA---GTGGGA---ATAGGAGCT---
------ATGTTCCTTGGGTTCTTGGGAGCA---GCAGGAAGCACTATG---GGCGCAGCATCA---
ATGACGCTGACGGTACAG---GCCAGACTA---TTATTG---TCTGGTATAGTGCAACAGCAGAAC---
AATTTG---CTGAGG---GCTATTGAGGCGCAACAGCACCTGTTGCAACTCACAGTCTGGGGCATC
>DQ410422
TTTTACTGTAATACAACACAATTATTTAATAGTACTTGGAATGATACTGATAGTGGAGGGTCAAATAACACAA
AT---

AACAATGATACTATCACACTCCCATGCAGAATAAAACAGATTATAAACATGTGGCAGGAAGTAGGAAAAGCAA
TGTATGCCCCTCCCATCATGGGACAAATTAGCTGTTCATCAAAT---
ATTACAGGGATGATATTAACAAGAGATGGTGGTACTAATAGGAGCAAGACC----------------------
----

GAGACCGAGACCGAGACCTTCAGACCTGGAGGAGGAGATATGAGGGACAATTGGAGAAGTGAATTATATAAAT
ATAAAGTAGTAAAACTTGAACCATTA---GGAGTAGCACCCACCAAGGCAAAGAGAAGAGTGGTG--------
-------------------------------CAGAGAGAAAAAAGAGCG---ATAACA---GTAGGAGCT---
------ATGCTCCTTGGGTTCTTGGGAACA---GCAGGAAGCACTATG---GGCGCAGCGGCG---
ATAACGCTGACGGTACAG---GCCAGACAA---TTATTG---TCTGGTATAGTGCAACAGCAGAAC---
AATCTG---CTGAGG---GCTATTGAGGCGCAACAGCATCTGTTGCAACTCACAGTCTGGGGCATC
>FJ469739
TTTTACTGTAATACAACAAAACTGTTTAATAGTACTTGGACTTGGAATAATACTGCAGAGTTAAATAACACTG
AA---

CAGGATGTCATAATCCCACTCCCCTGTAGAATAAAACAAATTGTAAACCTGTGGCAGGAAGTAGGAAAAGCAA
TGTATGCCCCTCCCATCCAAGGACAAATTAGATGCTCATCAAAT---
ATCACAGGGGTGCTATTAACAAGAGATGGTGGTATAAAAGGGAGCAACGAGAGC-------------------
----
-
TGCAACGAGAGCCAACCCGAGACCTTCAGACCTGGAGGAGGAGATATGAGGGACAATTGGAGAAGTGAATTAT
ATAAATATAAAGTAGTAAAAATTGAACCATTA---GGACTAGCACCCACCAGGGCAAAGAGAAGAGTGGTG--
-------------------------------------CAAAGAGAAAAAAGAGCA---GTGGGA---
CTAGGAGCT---------TTGTTCCTTGGGTTCTTGGGAGCA---GCAGGAAGCACTATG---
GGCGCAGCGTCA---CTGACGCTGACGGTACAG---GCCAGACTA---TTATTG---
TCTGGTATAGTGCAGCAGCAGAAT---AACTTG---CTGATG---
GCTATTGAGGCGCAACAGCGTATGTTGCAACTCACAGTCTGGGGCATC
>FJ469743
TTCTACTGTAATACAACACCACTGTTCAATAGTACTTGGAGGACAAATGGAACTTGGATAAATGGAACAGCAA
ATAAC--

ACTGGAGAAAACACTATCACACTCCAGTGCAAAATAAAGCAAATTGTAAACATGTGGCAGGAAGTAGGAAAAG
CAATGTATGCCCCTCCCATCCGAGGACTAATTAGATGTTTATCAAAT---
ATTACAGGGCTGATATTAACAAGAGATGGTGGTAATCCAAATAAGACTGATAAG-------------------
----

ACTGATACTACTAGTGAGATCTTCAGACCGGGAGGAGGAAATATGAAGGACAATTGGAGAAGTGAATTATATA
AATATAAAGTAGTAAAAATTGAACCATTA---GGAGTAGCACCCACCAAGGCAAAGAGAAGAGTGGTG-----
----------------------------------CAGAGAGAAAAAAGAGCA---
GTAGGAGCGATAGGAGCT---------ATGTTCCTTGGGTTCTTGGGAGCA---GCAGGAAGCACTATG---
GGCGCAGCGTCA---ATGGCGCTGACGGTACAG---GCCAGACAA---TTATTG---

TCTGGTATAGTGCAACAGCAGAAT---AATTTG---CTGAGG---
GCTATTGAGGCGCAACAGCACCTGTTGCAACTCACGGTCTGGGGCATC
>EF593228
TTCTATTGTAATACAACACAACTGTTTAATAGTACTTGGGGGCCTAATAGAACTTTGAATGAGACTGGTGAG-
----

TCAAATGAAACTATCACCCTCCCATGCAGAATAAAACAAATTATAAACTTGTGGCAGGAAGTAGGAAAAGCAA
TGTATGCCCCTCCTATCAGTGGACAAATTAGTTGTTCATCAAAT---
ATTACAGGGCTGCTATTAATAAGAGATGGTGGTGATGATAACAAT----------------------------
----

AACACCATCGAGATCTTCAGACCTGGAGGAGGAAATATGAGGGACAATTGGAGAAGTGAATTATATAAATATA
AAGTAGTAAAAATTGAACCATTA---GGAATAGCACCCACCAAGGCAAAGAGAAGAGTGGTG-----------
----------------------------CAGAGAGAAAAAAGAGCA---GTGGGA---ATAGGAGCT------
---GTGTTCCTTGGGTTCTTAGGAGCA---GCAGGAAGCACTATG---GGCGCAGCGTCA---
GTAACGCTGACGGTACAA---GCCAGACTA---TTAATG---TCTGGTATAGTGCAACAGCAAAAC---
AACTTG---CTGAGA---GCTATTGAGGCGCAACAGCATCTGTTGCAACTCACGGTCTGGGGCATC
>FJ469753
TTCTATTGTAATTCAACACAACTGTTTAATAGTACTTGGTATACTAATGGTACTTGGAGTGGTACTAAC----
----

AAAACTGGTGATATCACACTCCCATGCAAAGTAAAACAAATTGTACACATGTGGCAGAGAGTAGGAAAAGCAA
TGTATGCCCCTCCCATCGAAGGAGTAATTAGCTGTTCATCAAAT---
ATTACAGGGCTGCTATTAACAAGAGACGGTGGTAATACCGATAATACCAAT----------------------
----

AATGCCCAGATTGAGACTTTCAGACCTGGAGGAGGAAATATGAGGGACAATTGGAGAAATGAATTATATAAAT
ATAAGGTAGTAAAAATTGAACCAATA---GGAATAGCACCCACCAAGGCAAAGAGAAGAGTGGTG--------
-------------------------------CAGAGAGAAAAAAGAGCA---GTGGGA---CTAGGAGCT---
------ATGTTCCTTGGGTTCTTGGGAGCA---GCAGGAAGCACTATG---GGCGCAGCGTCA---
ATAACGCTGACGGTACAG---GCCAGATCA---TTATTG---ACTGGTATAGTGCAACAACAGAGA---
AATTTG---CTGACG---GCTATTGAGGCGCAACAGCATCTGTTGAAACTCACAGTCTGGGGCATC
>FJ469755
TTCTACTGTAGTACAACACAACTGTTTAATAGTACTTGGACAGGGAATGATACTGCAGAAGGAAAG-------
----

GGAAATGGCACACTTACACTCCCCTGCAGAATAAGACAAATTGTAAACCTGTGGCAGGAAGTAGGAAAAGCAA
TGTATGCCCCTCCCATCTCTGGACAAATTAACTGTTCATCAAAA---
ATTACAGGGATGATCTTAACAAGAGATGGTGGTACAAAACAGAAC----------------------------
----

AACGAGAGTGAGACCTTCAGGCCTGGAGGAGGAGATATGAGGGACAATTGGAGAAGTGAATTATATAAATATA
AAGTAGTAAAAATTGAACCACTA---GGAGTAGCACCCACCAGGGCAAAGAGAAGAGTGGTG-----------
----------------------------CAGAGAGAAAAAAGAGCA---GTGGGC---CTAGGAGCC------
---TTGTTCCTTGGGTTCTTGGGAGCA---GCAGGAAGCACTATG---GGCGCAGCGTCA---
GTGACGCTGACGGTACAG---GCCAGACTA---TTACTG---TCTGGTATAGTGCAGCAGCAAAAT---
AACTTG---CTGAAG---GCTATTGAGGCGCAACAGCGTATGTTGCAACTCACAGTCTGGGGCATC
>FJ469765
TTCTACTGTAATTCAACACAACTGTTTAATAGTACTTGGGATGCTAGGGAAGTTAATGAGACTAGGGAAGGGG
CAAAT--

AACACTGATAAAACTATCATACTCCCATGCAGAATAAAACAAATTGTAAATATGTGGCAGCAAGTAGGAAAGG
CAATGTATGCCCCTCCCATCAGAGGACAAATTAGATGCTCATCAAAT---
ATTACAGGACTGCTATTAACCAGAGATGGTGGTAACAACAATAAC----------------------------
----

AGTACTGAGACCTTCAGACCTGGAGGGGGAGATATGAGGGACAATTGGAGAAGTGAATTATACAAATATAAAG
TAGTAAGAATTGAACCATTA---GGAATAGCACCCACCAAGGCAAAAAGAAGAGTGGTG--------------
-------------------------CAGAGAGAAAAAAGAGCA---GTAACT---TTCGGAGCT---------
ATGTTCCTTGGGTTCTTGGGAGCA---GCAGGAAGCACTATG---GGCGCAGCGTCA---

```
CTAACGCTGACGGTACAG---GCCAGACAA---TTATTG---TCTGGTATAGTGCAACAGCAGAAC---
AATCTG---CTGAGA---GCTATTGAGGCGCAACAGCATCTGTTGCAACTCACAGTCTGGGGCATC
>FJ469770
TTCTACTGTAATTCAACACAACTGTTTAATAGTACTTGGAATGCTACTACAGGGTTACCTAACAAGGAA----
-------------------------------------------------------------------------
----------------------------
GAAAATGGCACGATCACACTCCCATGTAGAATAAAACAAATTATAAATATGTGGCAGGGAGTAGGAAGAGCAA
TATATGCCCCTCCCATCGCAGGACCAATTACCTGTTCATCAAAC---
ATTACAGGGCTAATATTAACAAGAGATGGTGGTAATGTGACGGGC----------------------------
-------------------------------------------------------------------------
-------------
CAGGAAGAAACCTTCAGACCAGGAGGAGGAAATATGAAGGACAATTGGAGAAGTGAATTATATAAATATAAAG
TAGTAAAAATTGAACCATTA---GGAGTAGCACCTACCAAGGCAAAGAGAAGAGTGGTG--------------
-------------------------CAAAGAGAAAAAAGAGAT---CTGGGA---CTAGGAGCT---------
TTGTTCCTTGGGTTCTTGGGAGCA---GCAGGAAGCACTATG---GGCGCAGCGTCA---
GTAACGCTGACGGTACAG---GCCAGACTA---TTATTG---TCTGGTATAGTGCAACAGCAGAAC---
AATTTG---CTGAGG---GCTATTGAGGCGCAACAGCATCTGTTGCAACTCACAGTCTGGGGCATC
>EF593256
TTCTACTGCAATACAACACAACTGTTTAATAGTACTTGGGATGATACTACAGCGTCAAATATCACTAAA----
-------------------------------------------------------------------------
----------------------------
GGAAATGACAACATCACACTCCCATGCAGAATAAGACAAGTTGTACACTTGTGGCAGGAAGTGGGAAAAGCAA
TGTATGCCCCTCCCATCCAAGGACAAATTAGATGTTCATCAAAT---
ATTACAGGGCTGCTATTAACAAGAGATGGTGGTAATAGTGAAAACAAC-------------------------
-------------------------------------------------------------------------
-------
ACCAACACCGAGGAGGTCTTCAGACCTGCAGGAGGAGATATGAGGGACAATTGGAGAAGTGAATTATATAAAT
ATAAAGTAGTAAAAATTGAGCCATTA---GGAATAGCACCCACCAAGGCAAAAAGAAGAGTGGTG--------
-------------------------------CAGAGAGAAAAAAGAGCA---GTAGGC---ATAGGAGCT---
------GTGTTCCTCGGGTTCTTGGGAGCA---GCAGGAAGCACTATG---GGCGCAGCGTCA---
ATAACGCTGACGGTACAA---GCCAGACTA---TTATTG---TCTGGTATAGTGCAACAGCAGAAT---
AATTTG---CTGAGA---GCTATTGAGGCGCAACAGCATCTGTTGCAACTCACAGTTTGGGGCATC
>EF593299
TTCTACTGTAATACAACAAAGCTGTTTAATAGTACTTGGTCATTTAATAGTACTGGGAATGGTAGTACTACAG
AG-----------------------------------------------------------------------
----------------------------
TCAAACGCCACTATCACACTCCCATGCAGAATAAGACAAATTATAAACAGATGGCAAGAAGTAGGAAAAGCAA
TGTATGCCCCTCCCATCAGTGGAAAAATTAGATGTAAATCAATT---
ATTACAGGGCTGCTACTAACAAGAGATGGTGGTACAAACACCACC----------------------------
-------------------------------------------------------------------------
----------
AACAAAACTGAGATCTTCAGACCTGGAGGAGGAGATATGAGGGACAATTGGAGAAGTGAATTATATAAATATA
AAGTAGTAAAAATCAAGCCATTA---GGAATAGCACCCACCAAGGCAAAGAGAAGAGTGGTG-----------
----------------------------CAGAGAGAAAAAAGAGCA---GTGGGAACACTAGGAGCT------
---ATGTTCCTTGGGTTCTTGGGAGCA---GCAGGAAGCACTATG---GGCGCAGCGTCA---
GTGACGCTGACGGTACAG---GCCAGACTA---TTATTG---TCTGGTATAGTGCAGCAGCAGAAC---
AATCTG---CTGAGG---GCTATTGAGGCGCAACAGCACCTGTTGCAACTCACAGTATGGGGCATC
>FJ469684
TTCTACTGTGATACAGCAAAATTGTTTAATAGTACTTGGAATAGTACTAAATCAAATAGC-------------
-------------------------------------------------------------------------
----------------------------
ACGGAAGTAAAGATCACACTACCATGCAAAATAAAACAAATTATAAACAGATGGCAGGAAGTAGGAAAAGCAA
TGTATGCCCCTCCCATCAGAGGACAGATTAAATGTTCATCAAAT---
ATTACAGGGCTGCTATTAACAAGAGATGGTGGTAATACCACAAAC----------------------------
-------------------------------------------------------------------------
-------------
AGGTCAGAGATCTTCAGACCTGGAGGAGGAGATATGAGGGACAATTGGAGAAGTGAATTATATAAATATAAAG
TAGTACAAATTGAACCATTA---GGAATAGCACCCACCAAGGCAAAGAGAAGAGTGGTG--------------
-------------------------CAGAGAGAAAAAAGAGCA---GTGGGA---TTAGGAGCT---------
TTGTTCCTTGGGTTCTTGGGAGCA---GCAGGAAGCACTATG---GGCGCAGCGTCA---
```

ATGACGCTGACGGTACAG---GCCAGACAA---TTATTG---TCTGTTATAGTGCAACAGCAGAAC---
AATTTG---CTGATG---GCTATTGAGGCGCAACAGCATCTGTTGCAACTCACAGTCTGGGGCATC
>HQ216370
TTCTACTGTGATACAACACCACTGTTTAGTAGTAGATGGTCACCTAATGGTAGTAGTAGTAGTACGATTAATG
AAACT--
-------------------AGC---
ACTGAAGGAAATATCACCACACTCCAATGCAGAATAAGACAAATTATAAATATGTGGCAGAGAGTAGGCAAAG
CAATATATGCCCCTCCCATCGCGGACCAATTGGATGTAATTCAAGT---
ATCACAGGGCTGTTATTAACAAGAGATGGTGGGAGAAACAACAACAGC-------------------------
----

AACAACACAGAAGTTTTCAGACCTGGAGGAGGAGATATGAGGGACAACTGGAGAAGTGAATTATACAAATATA
AAATAGTAAGGATTGAACCATTA---GGAATAGCACCCACCAAGGCAAAGAGAAGAGTGGTG-----------
----------------------------CAAAGAGAAAAAAGAGCA---GTGGGA---ATAGGAGCT------
---GTGTTCCTTGGGTTCTTGGGAGCA---GCAGGAAGCACTATG---GGCGCAGCATCA---
ATAACGCTGACGGTACAG---GCCAGACAA---TTATTG---TCTGGTATAGTGCAACAGCAGAAC---
AATTTG---CTGAGA---GCTATTGAGGCGCAACAGCACCTGTTGCAACTCACGGTCTGGGGCATC
>HQ216504
TTCTACTGTAATACAACAGCACTGTTTAATAGCACCTGGAATATTACTGAAGGGTTAAATAGCACATTCTCAA
ATAAC--
-------------------CCT---
GAAGGAAATGACACAATCACGCTTCCATGCAGAATAAAACAAATTATAAACATGTGGCAGGAAGTAGGAAAAG
CAATGTATGCCCCTCCCATCAGAGGACAAATTAGATGTTCATCAAAT---
ATTACAGGGCTGCTATTAACAAGAGATGGTGGTGATAACAATAAC----------------------------
----

AGTGACAACGAGACCTTCAGACCTGGAGGAGGAGATATGAGGGACAATTGGAGAAGTGAATTATATAAATATA
AAGTAGTAAAAATTGAACCATTA---GGAGTAGCACCAACCAAGGCAAAGAGAAGAGTGGTG-----------
----------------------------CAGAGAGAAAAAAGAGCA---ATAGGA---ATAGGAGCT------
---GTGTTCCTTGGGTTCTTGGGAGCA---GCAGGAAGCACTATG---GGCGCAGCGTCA---
ATAACGCTGACGGTACAG---GCCAGACAA---TTATTG---TCTGGTATAGTGCAACAGCAGAAC---
AACCTG---CTGAGA---GCTATTGAGGCGCAACAACATATGTTGCAACTCACAGTCTGGGGCATC
>JX863969
TTCTACTGTAATACAACACAACTGTTTAATAGTACTTGGAATAGTACTGAAGGGATTGAAGGGACAAAT----
----

AACACTGATAATATCACACTCCCATGCAGAATAAAACAAATTATAAACCTGTGGCAGAGAGTAGGAAAAGCAA
TGTATGCCCCTCCCATCAGAGGACAGATTAGATGTTCATCAAAT---
ATCACAGGGCTGCTATTAGTAAGAGATGGTGGTAACGAACAGAACGGG-------------------------
----

ACAAGCGCAACCGAGATCTTCAGACCTGGAGGAGGAGACATGAGGGACAATTGGAGAAGTGAATTATATAAAT
ATAAAGTAGTAGAAATTAAACCATTA---GGAGTAGCACCCACCAGGGCAAAGAGAAGAGTGGTG--------
-------------------------------CAGAGAGAAAAAAGAGCA---GTGACA---CTAGGAGCT---
------TTGTTCCTTGGGTTCTTGGGAGCA---GCAGGAAGCACTATG---GGCGCAGCGTCA---
ATAACGCTGACGGTACAG---GCCAGACAA---TTGTTG---TCTGGTATAGTGCAACAGCAGAAC---
AATCTG---CTGAGG---GCTATTGAGGCGCAACAGCGTCTGTTGCAACTCACAGTCTGGGGCATC
>JN562798
TTCTACTGTACTACTACAGGACTGTTTAATAGTACTTGGCATTGGAATGGTACTAGGGAGAATAATACTACGG
AG---

CCAAATAGCAATATCACACTCCCATGCAGAATAAAACAGATTATAAACATGTGGCAAGAAGTAGGAAAAGCAA
TGTATGCCCCTCCAATCAAAGGACAAATTAGATGTTCATCAAAC---
ATTACAGGGCTGCTAATAACAAGAGATGGTGGTAACAGTGACCATAGC-------------------------
----

AACGAGACCGAGATCTTCAGACCTGGAGGAGGAGATATGAGGGACAATTGGAGAAGTGAATTATATAAATATA
AAGTAGTAAAAATTGAACCATTA---GGAGTAGCACCCACTGAGGCAAGGAGAAGAGTGGTG-----------
----------------------------CAGAGAGAAAAAAGAGCG---ATAACA---CTGGGAGCT------
---TTGTTCCTTGGGTTCTTGGGAGCA---GCAGGAAGCACTATG---GGCGCAGCGTCA---

ATGACGCTGACGGTACAG---GCCAGACAA---TTATTG---TCTGGTATAGTGCAACAGCAGAAC---
AATTTG---CTGAGG---GCTATTGAGGCGCAACAGCATCTGTTGCAACTCACAGTCTGGGGCATC
>EU577811
TTCTACTGTAATACAACACAACTGTTCAATAGTACTTGGTATTGGAATAATACTCGGGATGAAGGGTCAAATA
ACACT--

GGAGGCAATGGCACAATCATACTCCCGTGTAGAATAAAACAATTTATAAACATGTGGCAGGAAGTAGGAAAAG
CAATGTATGCCCCTCCCATCAGAGGACAAATTAGCTGTTCATCAAAT---
ATTACAGGGCTGATGCTATTAAGAGATGGTGGTAATAACGTGAGC----------------------------
----

AATACCACTGAGACCTTCAGACCTGGAGGAGGAGATATGAGGGACAATTGGAGAAGTGAATTATATAAATATA
AAGTAGTAAAAATTGAACCATTA---GGAATAGCACCCACCAAGGCAAAGAGAAGAGTGGTG-----------
----------------------------CAGAGAGAAAAAAGAGCA---GTGGGA---ATAGGAGCT------
---ATGTTCCTTGGGTTCTTGGGAGCA---GCAGGAAGCACTATG---GGCGCAGCGTCA---
GTGACGCTGACGGTACAA---GCCAGGCTA---TTATTG---TCTGGTATAGTGCAACAGCAGAGC---
AATTTG---CTGAGA---GCTATTGAGGCGCAACAGCATCTGCTGCAACTCACAGTCTGGGGCATC
>EU577862
TTCTATTGTAATACATCACAACTGTATGATAGTACTTGGAATCTTACTAATACTGAAAGG-------------
----

TTAAATGACACTATCCAACTCCCATGCAGAATAAAACAAATTATAAACATGTGGCAGGAAGTAGGAAAAGCAA
TGTATGCCCCTCCCATCAGAGGACTAATTAAGTGTTCATCAAAT---
ATTACAGGGCTGTTATTAACAAGAGATGGTGGTAGTGATAATGCCACA-------------------------
----

AACGGTACTGAGACCTTCAGACCTGGAGGAGGAGATATGAGGGACAATTGGAGAAGCGAATTATACAAATATA
AAGTAATAGAAATTAAACCAGTA---GGATTAGCACCCACCAAGGCAAAGAGAAGAGTGGTG-----------
----------------------------CAGAGAGAAAAAAGAGCA---GCA------CTAGGAGCT------
---ATGCTCCTTGGGTTCTTGGGAGCA---GCAGGAAGCACTATG---GGCGCAGCGTCA---
ATAACGCTGACGGTACAG---GCCAGACAA---CTGTTG---TCTGGTATAGTGCAACAGCAAAAT---
AATTTG---CTGAGA---GCTATTGAGGCGCAACAGCATCTATTGCAACTCACAGTCTGGGGCATT
>EU577947
TTCTACTGCAATACAACACAACTGTTTAATAGTACTTGGCTGTTTAATGGTACTTGGAATGGTACTGCATTAA
ATGGC--
-------------------ACT---
GAAGGAAATGAAACAATTACACTTCCATGCAGAATAAAACAAATTATAAACATGTGGCAGGAAGTAGGAAAAG
CAATGTATGCCCCTCCCATCAAAGGACTAATTGAATGTTCATCAAAT---
ATTACAGGGCTACTGTTAACAAGAGATGGTGGTAATAATGAGAGC----------------------------
----

ACTGAGACCTTTAGGCCTAGAGGAGGAGATATGAGGGACAATTGGAGAAGTGAATTATATAAATATAAAGTAG
TACAAGTTGAACCATTA---GGAATAGCACCCACCAGGGCAAAGAGAAGAGTGGTG-----------------
----------------------CAGAGAGAAAAAAGAGCA---GGA------ATAGGAGCT---------
CTGTTCCTTGGGTTCTTGGGAGCA---GCAGGAAGCACTATG---GGCGCAGCTTCG---
ATAACGCTGACGGTACAA---GCCAGACAA---TTATTG---TCTGGTATAGTGCAACAGCAGAAC---
AATCTG---CTGAGG---GCTATTGAGGCGCAACAGCATATGTTGCAACTCACAGTCTGGGGCATC
>EU577989
TTCTACTGTAACACATCAAAACTGTTTAATAGTAGTTGGAATGCTGATGGTAGTGTTAATACTACTGGA----
----

GAATCAGGAAACATAACACTCCAATGCAGAATAAAACAAATTATAAACATGTGGCAGGAAGTAGGAAAAGCAA
TGTATGCCCCTCCCATCCAAGGACAAATTAATTGTTCATCAAAC---
ATTACAGGGATACTATTAACAAGAGATGGTGGTAGTGATAACACAACAAAC----------------------
----

AATACTGAGATCGAGATCTTCAGACCTACAGGAGGAGATATGAGGGACAATTGGAGAAGTGAATTATATAAAT
ATAAAGTAGTAAGAATTGAACCATTA---GGAATAGCACCCACCAAGGCAAAGAGAAGAGTGGTG--------
-------------------------------CAGAGAGATAAAAGAGCA---GTGGGA---CTAGGAGCT---
------TTGTTCCTTGGGTTCTTGGGAACA---GCAGGAAGCGCTATG---GGCGCAGCGTCA---

CTGACGCTGACGGTACAG---GCCAGACAA---TTATTG---TCTGGTATAGTGCAACAGCAAAAC---
AATTTG---CTGAGG---GCTATTGAGGCGCAACACCATATGTTGCAACTCACAGTCTGGGGCATC
>KX156438
TTTTACTGTAATACAACACAAATGTTTAATAGTACTTGGAATGATACTGAGAATAATATT-------------
----

GGAAATGGAACTATCACACTCCCCTGCAGAATAAAACAAATTATAAATAAGTGGCAGGAAGTAGGAAAAGCAA
TGTATGCCCCTCCCATTGAAGGACGAATTAACTGTACATCAAAT---
ATTACAGGGCTGATATTAACAAGAGATGGTGGTAGCGGGAACAAC----------------------------
----

GACACCAACACCACCGAGATCTTCAGGCCTGCAGGGGGAGATATGAGGGACAATTGGAGAAGTGAATTATATA
AATATAAGGTAGTAAAAATTGAACCATTA---GGAGTAGCACCCACCAAGGCAAAGAGAAGAGTGGTA-----
----------------------------------CAGAGAGAAAAAAGAGCA---GTGGGA---
ATAGGAGCT---------TTGTTCCTTGGGTTCTTGGGAGCA---GCAGGAAGCACTATG---
GGCGCAGCGTCA---ATAACGCTGACGGTACAG---GCCAGACAA---TTACTG---
TCTGGTATAGTGCAACAGCAAAAC---AACTTG---CTGAGA---
GCTATTGAGGCGCAACAGCATCTGTTGCAACTCACAGTCTGGGGCATC
>EU578032
TTCTACTGTAACTCAACACAACTGTTTAATAGCTCTTGGAATGGTACTGCTGGGAATGTTACTACAGGGGGGT
CA---

GATAACACCACTATCACACTCCCATGTAGAATAAAACAAATTATAAACATGTGGCAGGGAGTAGGAAAAGCAA
TGTATGCCCCTCCGATCCCAGGAAAAATTAGCTGTAAATCAATT---
ATTACAGGGCTGCTATTAACAAGAGATGGTGGCAACAATAACAGT----------------------------
----

GAGACCTTCGAAACCTTCAGACCTGGGGGAGGAAATATGAAAGACAATTGGAGAAATGAATTATATAAATATA
AAGTAGTAAAAATTGAACCAATA---GGAGTAGCACCCACCAAGGCAAAGAGAAGAGTGGTG-----------
----------------------------CAGAGAGAAAAAAGAGCA---GTGGGA---ATAGGAGCT------
---GTGCTCTTTGGGTTCTTGGGAGCA---GCAGGAAGCACTATG---GGCGCAGCGTCG---
ATAGCGCTGACGGTACAG---GCCAGACAA---TTATTG---TCTGGTATAGTGCAACAGCAGAAC---
AATCTG---CTGAGG---GCTATTGAGGCGCAACAACATATATTGCAACTCACAGTCTGGGGCATC
>KX156451
TTTTACTGTAACACAACACAACTGTTTAATAGTACTTGGAATATTACTGAAGTGTTAAATAGTACTGGAGAGT
CAAAA--

AGCAATGACTTAATCATACTCCCATGCAGGATAAAACAAATCGTAAACATGTGGCAGAGAGTAGGACAAGCAA
CGTATGCCCCTCCCATCGAAGGATTGATTAGATGTTCATCAAAA---
ATTACAGGGATACTATTAACAAGAGATGGTGGTATTAACAATAGG----------------------------
----

ACCAACGAAACCTTCAGACCTGGAGGAGGAGACATGAGAGACAATTGGAGAAGTGAATTATATAGATATAAGG
TAGTACAAATTGAACCAGTA---GGACTGGCACCCACCAAGGCGCAGCGAAGAGTGGTG--------------
-------------------------CAGAGGGAAAAAAGAGCA---GTGGGA---ATAGGAGCG---------
TTGTTCCTTGGGTTCTTGGGAACA---GCAGGAAGCACTATG---GGCGCAGCGTCA---
ATAACGCTGACGGCACAG---GCCAGACAA---TTATTG---TCTAGTATAGTGCAACAGCAGAAC---
AACTTG---CTGAGA---GCTATTGAGGCGCAACAGCATCTGTTGCAACTCACAGTCTGGGGCATC
>JN562778
TTCTACTGTAATTCAACACAACTGTTTAATAGTACTTGGATTAGGAATGATACTAATAATACTTGGCATAGTG
AAGGG--
-------------------TTA---
AATGGCACTGAAACAATCACACTCCCATGTAGAATAAAACAATTTATAAACATGTGGCAGGAAGTAGGAAAAG
CAATGTATGCCCCTCCCATCAGAGGACAAATAAAATGTTCATCAAAT---
ATTACAGGGCTATTACTAGTAAGAGATGGTGGTAATAACAATAAGAGCAAT----------------------
----

AGCACTAACAATGCTGAGATCTTCAGACCTGGAGGAGGAAATATGAAAGACAATTGGAGAAGTGAATTATATA
GATATAAAGTGGTACAAGTTGAACCATTA---GGAATAGCACCCACCAGGGCAAAGAGAAGAGTGGTG-----
----------------------------------CAGAGAGAAAAAAGAGCT---GTGGGA---

GTAGGAGCT---------TTGTTCCTTGGGTTCTTGTCAGCA---GCGGGAAGCACTATG---
GGCGCAGCGTCA---ATGACGCTGACGGTACAG---GCCAGACAA---TTATTG---
TCTGGTATAGTGCAACAGCAGAGC---AATCTG---CTGAGG---
GCTATTGAGGCGCAACAGCATATGTTGCAACTCACAGTCTGGGGCATC
>JN562811
TTCTACTGTAATTCATCAGGACTGTTTAATAGTACTTGGCATAGTAATAGTACCCAGAATAGTACTGAAGGG-
----

TCAAAGGACACTATTACACTCCCATGCAGAATAAGACAAATCATAAACATGTGGCAGGAAGTAGGAAAAGCAA
TGTATGCCCCTCCCATCCAAGGACAACTTAGATGTTCATCAAAT---
ATTACAGGGCTGCTCTTAACAAGAGATGGAGGTAATAACGGGGGC----------------------------
----

GACAACACCGAGATCTTCAGACCTGGAGGAGGAAATATGAAAGACAATTGGAGAAGTGAATTATATAAATATA
AAGTAGTAAAAATTGAACCATTA---GGATTAGCACCCACCAGGGCAAGGAGAAGAGTGGTG-----------
----------------------------CAGAGAGAAAAAAGAGCA---GTGGGA---ATGGGAGCT------
---TTGTTCCTTGGGTTCTTGGGAGCA---GCAGGAAGCACTATG---GGCGCAGCGTCA---
ATGACGCTGACGGTACAG---GCCAGAACA---CTATTG---TCTGGTATAGTGCAACAGCAAAGC---
AACTTG---CTGAGG---GCTATTGAGGCGCAACAGCATCTGTTGCAACTCACAGTCTGGGGCATC
>DQ869015
TTCTACTGTGATACAACACAACTCTTTAATAGTACTTGGAATGATACTGTTGGGAATGATACTGAAGAG----
----

TCAAATAATACTATCACACTCCCATGCAGAATAAAACAAATTGTAAACCTGTGGCAGGAAGTAGGAAAAGCAA
TGTATGCCCCTCCCATTAGAGGACAAATTAGATGTTCATCAAAT---
ATTACAGGGCTGCTATTAACAAGAGATGGTGGTAATATCAGCACC----------------------------
----

ACCACCACCGAGACCTTCAGACCTGGAGGAGGAAATATGAGGAACAATTGGAGAAGTGAATTATATAAATATA
AAGTAGTAAAAATTAAACCATTA---GGAGTAGCACCCACCAGCGCAAGGAGAAGAGTGGTG-----------
----------------------------CAGAGAGAAAAAAGAGCA---ATAGGA---ATAGGAGCT------
---ATGTTCCTCGGGTTCTTGGGAGCA---GCAGGAAGCACTATG---GGCGCAGCGTCA---
ATAACGCTGACGGTACAG---GCCAGACAA---TTATTG---TCTGGTATAGTGCAACAGCAAAGC---
AATTTG---CTGAGG---GCTATAGAAGCGCAACAGCATCTGTTGCAGCTTACAGTCTGGGGCATT
>DQ869033
TTCTACTGTAATACAACAAAACTGTTTAATAGTACTTGGAATGAGACTAGTATTGAAAATAGCACTAGAGGAA
AT---

AGCACTGGAGGAGATATCACAATTCTATGCAGAATAAAACAAGTTATAAACCTGTGGCAGGAAGTAGGAAAAG
CAATGTATGCCCCTCCCATCGAAGGACTAATCAATTGTACATCAAAT---
GTTACAGGGCTATTATTAATAAGAGATGGTGGTAACATTACCAAC----------------------------
----

AACGACACAGAGATCTTTAGACCTGGAGGAGGAGATATGAGGGACAATTGGAGAAGTGAACTGTACAAATATA
AAGTAGTAAGAATTGAACCATTA---GGAATAGCACCCACCAAGGCAAAGAGAAGAGTGGTG-----------
----------------------------CAGAGAGAAAAAAGAGCA---GTGGGA---CTAGGAGCT------
---ATGTTCCTTGGGTTCTTGGGAGCA---GCAGGAAGCACTATG---GGCGCAGCATCA---
GTGACGCTGACGGTACAG---GCCAGACAA---TTATTG---TCTGGTATAGTGCAACAGCAGAAC---
AATCTG---CTGAGG---GCTATTGAGGCGCAACAGCATCTGTTGCAACTCACAGTCTGGGGCATC
>KM259097
TTCTACTGTAATACAACAAAACTGTTTAATAGTACTTGGAATGATACTGCAGGG-------------------
----
AATGATACTGCAGGGAAT---------
GTTACTGAAGAAAATATAACACTCCCATGCAGAATAAAACAAATTATAAACATGTGGCAGGAAGTAGGAAAAG
CAATGTATGCCCCTCCCATCAGAGGACAAATTAGCTGTTCATCAAAT---
ATTACAGGGCTGCTATTAACAAGAGATGGTGGA--

AAAACCAACGAAACAAAC---
GAGACCGAGATCTTTAGACCTGGAGGAGGGAATATGAAGGACAATTGGAGAAGTGAATTATATAAATATAAAG
TAGTAAAAATTGAACCATTA---GGAGTAGCACCCACCAAGGCAAAGAGAAGAGTGGTG--------------

-------------------------CAGAGAGAAAAAAGAGCA---GTGGGAGTAATAGGAGCT---------
ATGTTCCTTGGGTTCTTGGGAACA---GCAGGAAGCACTATG---GGCGCAGCGTCA---
GTGACGCTGACGGTACAG---GCCAGACAA---TTATTG---TCTGGTATAGTGCAACAGCAGAAC---
AATTTG---CTGAGA---GCTATTGAGGCGCAACAGCATCTGTTGCAACTCACAGTCTGGGGCATC
>DQ410535
TTCTACTGTAATTCAACACCACTGTTTAATAGTAGTTGGAATGATACTACCATAGAGCAAAATGACACA----
----

ACGGAAGAGAATATCACACTCACATGCAGAATAAAACAAATTATAAATATGTGGCAGGAAGTAGGAAAAGCAA
TGTATGCCCCTCCCATCAGAGGACAAATTAGATGTATATCAAAC---
ATTACAGGGCTGCTATTAACAAGAGATGGTGGTATAAATGAGACC----------------------------
----

AACGGGACTGAGATCTTCAGACCTGGAGGAGGAGATATGAGGGACAATTGGAGAAGTGAATTATACAAATATA
AAGTAGTAAAAATTGAGCCATTA---GGAGTAGCACCCACCAAGGCAAAGAGAAGAGTGGTG-----------
----------------------------CAGAGGGAAAAAAGAGCA---GTGGGG---ATAGGAGCT------
---CTGTTCCTTGGGTTCTTGGGAGCA---GCAGGAAGCACTATG---GGCGCAGCGTCA---
GTGACGCTGACGGTACAG---GCCAGGCAA---TTATTG---TCTGGTATAGTGCAACAGCAGAAC---
AATTTG---CTGAGG---GCTATTGAGGCGCAACAGCATCTGTTGCAACTCACAGTCTGGGGCATC
>AY835452
TTTACTGTAACTCAACACAGCTGTTTAATAGTACTTGGTGGCCTAATGGTACTACGACTGAATGGTCAAATG
AA---

ACGTCAAATGGGACTATCACACTCCCATGCAGAATAAAACAAATTATAAACATGTGGCAGGAAGTAGGAAAAG
CAATGTATGCCCCTCCTATCAGCGGACCAATTAGCTGTTCATCGAAT---
ATTACAGGGCTGCTATTAGTAAGAGATGGTGGTAATGACAATGAGACT-------------------------
----

AACGGGACTGAGACCTTCAGACCTGGAGGAGGAGATATGAGAGACAATTGGAGAAGTGAATTATATAAATATA
AAGTAGTAAAAATTGAACCATTA---GGAGTAGCACCCACCAAGGCAAAGAGAAGAGTGGTG-----------
----------------------------CAAAGAGAAAAAAGAGCA---GTGGGA---CTAGGAGCT------
---ATGTTCCTTGGGTTCTTGGGAGCA---GCAGGAAGCACTATG---GGCGCAGCGTCA---
ATAACGCTGACGGTACAG---GCCAGACTA---TTATTG---TCTGGTATAGTGCAACAGCAAAAC---
AACTTG---CTGAGA---GCTATTGAGGCGCAACAGCATCTGTTGCAACTCACAGTCTGGGGCATC
>KX156493
TTCTACTGTAATTCAACACCACTGTTTAATAGTATTTGGAATGTTAGTAGTGGTTGGAATGGTACTAAAGGG-
----

TCAAATGGCACTATCACACTCTCATGCAGAATAAAACAAATTATAAACATGTGGCAGGAGGTAGGAAAAGCAA
TGTATAACCCTCCCATCAGTGGAATAATTAGATGTAACTCAACT---
ATTACAGGGCTGCTATTAACAAGAGATGGTGGTGAGAACAGGTCTGGGAGT----------------------
----

GATAACTGGACCGAGACCTTCAGACCTGGGGGAGGAGATATGAGGGACAATTGGAGAAGTGAATTATATAAAT
ATAAAGTAGTAAAAATTGAACCATTA---GGAATAGCACCCACCAGGGCAAAGAGAAGAGTGGTG--------
-------------------------------CAAAGAGAAAAAAGAGCA---GTGGGA---TTTGGAGCT---
------ATGTTCCTTGGGTTCTTGGGAGCA---GCAGGAAGCACTATG---GGCGCAGCGTCA---
GTGACGCTGACGGTACAG---GCCAGACAA---TTATTG---TCTGGTATAGTGCAACAGCAGAGC---
AACTTG---CTGAGG---GCTATTGAGGCGCAACAGCATCTGTTGCAACTCACAGTCTGGGGCATC
>KX156542
TTCTACTGTGATTCAACACAGCTATTTAATAGTACTTGGAATAATACTGAACAGCCTACCTGGAATGAT----
----

ACTGGAGAAATTATCACACTCCCGTGCAGAATAAAACAAATTATAAACAGGTGGCAAGAAGTAGGAAAAGCAA
TGTATGCCCCTCCCATCAGAGGACAAATTAGCTGCTCATCACTT---
ATTACAGGGATACTATTAACAAGAGATGGTGGTAAAAAGGAGAGC----------------------------
----

AATGAAACCGAAACCTTTAGACCTGGAGGAGGAGATATGAGGGACAATTGGAGAAATGAATTATATAAATATA
AAGTAGTAAAAATTGAGCCATTA---GGAGTAGCACCCACCAAGGCAAAGAGAAGAGTGGTG-----------

```
----------------------------CAAAGAGAAAAAAGAGCA---GTGGGAACAATAGGAGCT------
---ATGTTCCTTGGGTTCTTGGGAGCA---GCAGGAAGCACTATG---GGCGCAGCGGCA---
GTAACGCTGACGGTACAG---GCCAGACAA---TTATTG---TCTGGTATAGTGCAACAGCAAAAC---
AACTTG---CTGAGG---GCTATTGAGGCGCAACAGCATATGTTGCAACTCACAGTCTGGGGCATC
>KX156380
TTTTATTGTAATTCAACACCACTGTTTAATAGTACTTGGGCTTGGAATGGTACTTTCAATAAGGAAAATAATA
CTGAAAAT-----------------------------------------------------------------
----------------------------
TCAAATAGCACTATCACCCTCCAATGCAGAATAAGGCAAATTGTAAACATGTGGCAGGAAGTAGGAAAAGCAA
TGTATGCCCCTCCCATCAGAGGACAAATTAATTGTTCATCAAAC---
ATTACAGGGCTGCTATTACTAAGAGATGGTGGTGTTAATAATAACACA-------------------------
-------------------------------------------------------------------------
----------
AATGATACAGAGATCTTCAGACCTGGAGGAGGAAACATGAAGGACAATTGGAGAAGTGAATTATATAAGTATA
AAGTAGTACAAATTGAACCATTA---GGAGTAGCACCCACCAAGGCAAAGAGAAGAGTGGTG-----------
----------------------------CAGAGAGAAAAAAGAGCA---GTAACA---CTGGGAGCT------
---ATGTTCCTTGGGTTCTTGGGAGCA---GCAGGAAGCACTATG---GGCGCAGCGTCA---
ATGGCGCTGACGGTACAA---GCCAGACAA---TTGTTG---TCTGGTATAGTGCAACAGCAAAAC---
AACTTG---CTGAGA---GCTATTGAGGCGCAACAGCACTTGTTGCAACTCACAGTCTGGGGCATT
>FJ469697
TTCTACTGTGATACAACACAACTGTTTAATAGTACTTGGAATGATACTGAAGAGAATAGTGATAAAGGGAACA
CT-----------------------------------------------------------------------
-------------------------
GAAGGAAATGAACCAATCATACTCCCATGCAGAATAAAACAATTTATAAACATGTGGCAAGCAGTAGGAAAAG
CAATGTATGCCCCTCCCATCAGAGGACTAATTAGATGTTCATCAAAC---
ATTACAGGGCTGTTATTAACAAGAGATGGTGGTAACGAGACTAAT----------------------------
-------------------------------------------------------------------------
-------------
ACTACTGAGACCTTCAGACCGCAAGGAGGAAATATGAGGGACAATTGGAGAAGTGAATTATATAGATATAAAA
TAGTGAAAATTGAACCATTA---GGGGTAGCACCCACCAAGGCAAAAAGAAGAGTGGTG--------------
-------------------------CAGAGAGAAAAAAGAGCA---GTGGGAACATTAGGAGCT---------
ATGTTCCTTGGGTTCTTGGGAGCA---GCAGGAAGCACTATG---GGCGCAGCGTCA---
TTGACGCTGACGGTACAG---GCCAGACTA---TTACTG---TCTGGTATAGTGCAACAGCAGAAT---
AATATG---CTGAGG---GCTATTGAGGCGCAACAGCATCTGTTGCAACTCACAGTCTGGGGCATC
>FJ469698
TTCTACTGTAATTCAACACCACTGTTTAATAGTACTTGGCATAGTAATGGTACTATAGAGTCAGCT-------
-------------------------------------------------------------------------
----------------------------
AACAGAACAAATATCAGACTCATGTGCAGAATAAAACAAATTATAAACATGTGGCAGGGAGTAGGAAAAGCAA
TGTATGCCCCTCCCATCACAGGATTAATTAGGTGTTCATCAAAT---
ATTACAGGGCTGCTATTAACAAGAGATGGTGGTCTTAATAATAGT----------------------------
-------------------------------------------------------------------------
-------------
GACAATGAGACCTTCAGACCTGGAGGAGGAAATATGAAGGACAATTGGAGAAGTGAATTATATAAATATAAAG
TAGTAAGAGTTGAACCACTA---GGAGTAGCACCCACCAAGGCAAAGAGAAGAGTGGTG--------------
-------------------------CAGAGAGAAAAAAGAGCA---TTAGGGGTACTAGGAGCT---------
GTGTTCCTTGGGTTCTTAGGAGCA---GCAGGAAGCACTATG---GGCGCAGCGTCA---
ATAGCGCTGACGGCACAG---GCCAGACTA---TTATTG---TCTGGTATAGTGCAACAGCAGAAC---
AACCTG---CTGAGG---GCTATTGAGGCGCAACAACATCTGTTGCAACTCACAGTCTGGGGCATT
>HQ217185
TTCTACTGTAATACAACACAATTGTTTAATAGTACTTGGAAGAGTACCGAGTCTAATGATACTGGG-------
-------------------------------------------------------------------------
----------------------------
GGAAATGAAACTATCATACTCCCATGCAGAATAAAACAAATTGTAAACATGTGGCAGGAAGTAGGAAAAGCAA
TGTATGCCCCTCCCATCAGTGGACTAATTAACTGCTCATCAAAT---
ATTACAGGGTTATTATTAACAAGAGATGGTGATAATAGTACTAATGAC-------------------------
-------------------------------------------------------------------------
----------
ACTGACACTGAGATCTTCAGACCTATAGGAGGAAACATGAAAGACAATTGGAGAAGTGAATTATATAAATATA
AAGTAGTAAGAATTGAACCATTA---GGACTAGCACCCACCAAGGCAAAGAGAAGAGTGGTG-----------
```

----------------------------CAGAGAGAAAAAAGAGCA---GTGGGA---TTAGGAGCT------
---GTGTTCTTTGGGTTCTTGGGAGCA---GCAGGAAGCACTATG---GGCGCAGCGTCA---
ATGACGCTGACGGTACAG---GCCAGGCAA---TTACTG---TCTGGTATAGTGCAGCAGCAGAAC---
AATCTG---CTGAGG---GCTATTGAGGCGCAACAACATCTGTTGCAACTCACAGTCTGGGGCATC
>KC935957
TTCTACTGTAATTCAACACAACTATTTAATAGTACTTGGAATGGAACTGAGTCAAATGACACTAGA-------
----

GGAAATGACACAATCACACTCCCATGCAGAATAAAACAAATTATAAACATGTGGCAGGAAGTAGGAAAAGCAA
TGTATGCCCCTCCCATCAGAGGACAAATTAGATGTTCATCAAAT---
ATTACAGGGCTGCTATTAACAAGAGATGGTGGTGAAAACAAGAAC----------------------------
----

AAGACCGAGGTCTTCAGACCTGGAGGGGGAGATATGAGGGACAATTGGAGAAGTGAATTATATAAATATAAAG
TAGTAAAAATTGAACCATTA---GGAATAGCACCCACCAGGGCGAAGAGAAGAGTGGTG--------------
-------------------------CAGAGAGAAAAAAGAGCA---GTGGGA---ATAGGAGCT---------
GTGTTCCTTGGGTTCTTGGGAGCA---GCAGGAAGCACTATG---GGCGCAGCGTCA---
CTAGCGCTGACGGGACAG---GCCAGACTA---TTATTG---TCTGGTGTAGTGCAACAGCAGAAC---
AATTTG---CTGAGG---GCTATTGAGGCGCAACAGCATCTGTTGCAACTCACAGTCTGGGGCATC
>DQ410068
TTCTACTGTGATTCAACACAACTGTTTAACAGTACTTGGACTGAAGGGTCAAATAACACTGAA----------
----

GGAAATGACCCAATCACACTCCCATGCAGAATAAAACAAATTATAAACATGTGGCAAGAAGTAGGAAAAGCAA
TGTATGCCCCTCCCGTCGGAGGACAAATTAGATGTTCATCAAAT---
ATTACAGGACTGATCTTGATAAGAGATGGTGGTAATAGTAGTTCC----------------------------
----

CACAATGAGACTTTCAGACCTGGAGGAGGAGATATGAGAGACAATTGGAGGAGTGAATTATATAAATATAAAG
TAGTAAAAATTGAACCATTA---GGAGTAGCACCCACCAAGGCAAAGAGAAGAGTGGTG--------------
-------------------------CAGAGAGAAAAAAGAACA---GTGGGA---ATAGGAGCT---------
GTGTTCCTTGGGTTCTTGGGAGCA---GCAGGAAGCACTATG---GGCGCAGCGTCA---
GTGACGTTGACGGTACAG---GCCAGACTA---TTGTTA---TCTGGTATAGTGCAACAGCAGAAC---
AATCTA---CTGAGG---GCTATTGATGCGCAACAGCATCTGTTGCAACTCACAGTCTGGGGCATC
>EF363124
TTCTACTGCGATTCAACACCACTGTTTAATAGTACTTGGAATGTTACTAGTACTTGGAATGTTACTAGTACTG
GGAAT--
-------------------GAT---
ACTGAGTCAAATAACATCACACTCCCATGCAGAATAAAGCAAATTATAAACATGTGGCAGGAAGTAGGAAAAG
CAATATATGCCCCTCCCATCAGAGGACAAATTAGATGTTCATCAAAT---
ATTACAGGGCTGCTATTAACAAGAGATGGTGGTAATAACAAGAAC----------------------------
----

GAGACCGAGATCTTCAGACCTGGAGGAGGAGATATGAGGGACAATTGGAGAAGTGAATTATATAAATATAAAG
TAGTAAAAATTGAACCATTA---GGAATAGCACCCACCAAGGCAAAGAGAAGAGTGGTG--------------
-------------------------CAGAGAGAAAAAAGAGCA---GTGGGA---ATAGGAGCT---------
GTGTTCCTTGGGTTCTTGGGAGCA---GCAGGAAGCACTATG---GGCGCAGCGTCA---
ATGACGCTGGCGGTACAG---GCCAGACTA---TTATTG---TCCGGTATAGTGCAACAGCAGAAC---
AATTTG---CTGAGG---GCTATTGAGGCGCAACAGCATCTGTTGCAACTCACCGTCTGGGGCATC
>EF363126
TTCTACTGTAACACATCACAACTGTTTAATAACACTTGGAATGGTACTGAGTTAAGTAATAGTAAA-------
----

GAAAATGGTAATATCACACTCCAATGCAGAATAAAACAAATTATAAACATGTGGCAGGAAGTAGGGAAAGCAA
TGTATGCCCCTCCCATCAGAGGACAAATTAGATGTGTGTCAAAT---
ATTACAGGGCTGCTATTAACAAGAGATGGTGGTACAAACAGCACC----------------------------
----

ACCGAGACCTTCAGGCCTGCAGGAGGAAATATGAGGGACAATTGGAGAAGTGAATTATATAAATATAAAGTAG
TAAAAATTGAACCATTA---GGAATAGCACCCACCAAGGCAAAGAGAAGAGTGGTG-----------------

----------------------CAGAGAGAAAAAAGAGCA---GCGGGA---ATAGGAGCT---------
TTTTTCCTTGGGTTCTTGGGACTA---GCAGGAAGCACTATG---GGCGCAGCGTCA---
GTGACGCTGACGGTACAG---GCCAGACAA---TTATTG---TCTGGTATAGTGCAACAGCAGAAC---
AATCTG---CTGAGG---GCTATTGAGGCGCAACAGCACATGTTGCAACTCACAGTCTGGGGCATC
>DQ410498
TTCTACTGTAACTCAACAAAACTGTTTAATAGTACTTGGAATGATACTAATAGTACTTGGAATGGTACTGAGT
CACATAAC---
-------------------ACA---
AAAGAAAATGACACAATCATACTCCAATGCAGAATAAAACAAATTATAAACATGTGGCAGAAAGTAGGAAAAG
CAATGTATGCCCCTCCCATCATAGGACAAATTAGATGTTCATCAAAT---
ATTACAGGGCTGCTATTAACAAGAGATGGTGGTAATAGCGGGAACAAT-------------------------
----

ACCACCACCGAGACCTTCAGACCTGGAGGAGGAGAGATGAGGGACAATTGGAGAAATGAATTATATAAATATA
AAGTAGTAAAAATTGAACCATTA---GGAGTAGCACCCACCAAGGCAAAGAGAAGAGTGGTG-----------
----------------------------CAGAGAGAAAAAAGAGCA---GTGGGA---ATAGGAGCT------
---CTGTTCCTTGGGTTCTTGGGAGCA---GCAGGAAGCGCTATG---GGCGCAGCGTCA---
ATGGCGCTGACGGTACAG---GCCAGACCA---TTATTG---TCTGGTATAGTGCAACAGCAGAAC---
AATTTG---CTGAGG---GCTATTGAGGCGCAACAGCATCTGTTGCAACTCACAGTCTGGGGCATC
>JQ251132
TTCTACTGTAATACAACACCACTGTTTAATAGTAAGTGGACCAATGGTAATGGGAGTGGTTCTGAA-------
----

GGGAATGAGACTATCACACTCCAATGTAGAATAAAACAATTTATAAACATGTGGCAGGAAGTAGGAAAAGCAA
TGTATGCCCCTCCTATCAAAGGACAAATTAGATGTTTATCAAAT---
ATTACAGGGCTACTATTAACAAGAGATGGGGGTAATAACCAGAACAATACC----------------------
----

CCTAATGCCACTGAGACCTTCAGACCTGGAGGAGGAGATATGAGGGACAATTGGAGAAGTGAATTATACAAAT
ATAAAGTAGTAAAAATTGAACCATTA---GGAGTAGCACCCACCAAGGCAAAGAGAAGAGTGGTG--------
-------------------------------CAGAGAGAAAAAAGAGCAGCAATAGGA---ATGGGAGCC---
------TTGTTCCTTGGGTTCTTGTCAGCA---GCAGGAAGCACTATG---GGCGCAGCGTCA---
ATAACGCTGACGGTACAG---GCCAGGCAA---TTATTG---TCTGGAATAGTGCAACAGCAGAAC---
AATTTG---CTGAGG---GCTATTGAGGCGCAACAGCATCTGTTGCAACTCACAGTCTGGGGCATC
>DQ410260
TTCTACTGTGATACAGCACGACTGTTTAATAGTACTTGGAGTGTTAATGTTACTGAAGGAGCAAATAACACTG
CA---

GGAAATGACACAATCACACTCCCATGCAAAATAAAACAAATTATAAACATGTGGCAGGAAGTAGGAAAAGCAA
TGTATGCCCCTCCCATCAGAGGACTAATTAATTGTTCATCAAAT---
ATTACAGGGCTGCTATTAACAAGAGATGGTGGTCCTAACACAAAC----------------------------
----

GAGACTGAGGTCTTTAGACCTGGAGGAGGAGATATGAGGGACAATTGGAGAAGTGAATTATATAAATATAAAG
TAGTAAAAATTGAACCATTA---GGAGTAGCACCCACCAAGGCAAAGAGAAGAGTGGTG--------------
-------------------------CAAAGAGAAAAAAGGGCA---GTAGTGGGAATAGGAGCT---------
TTGTTCCTTGGGTTCTTGGGAGCA---GCAGGAAGCACTATG---GGCGCAGCGTCA---
GTGACGCTGACGGTACAG---GCCAGACAC---TTATTG---TCTGGTATAGTGCAACAGCAAAAC---
AATTTG---CTGAGA---GCTATTGAGGCGCAACAACATCTGTTGCAACTCACAGTCTGGGGCATC
>DQ410292
TTCTACTGTAATTCAACACAACTGTTTAATAGTACTTGGAATAAAACTAGGGATGGTACTTGGAACAGTACTG
GGTCA--
-------------------ACT---
ACAGGAAATGAAACAATCACACTCCCATGCAGAATAAAACAAATTATAAACATGTGGCAGGAAGTAGGAAAAG
CAATGTATGCCCCTCCCATCAGAGGACAAATTAGATGCTCATCAAAT---
ATTACAGGGCTGATATTAACAAGAGATGGTGGTAAAGACAACAATACC-------------------------
----

AATACCATCGAGATCTTCAGACCTGGAGGAGGAGAGATGAGGGACAATTGGAGAAATGAATTATATAAATATA
AAGTAGTAAGAATTGAACCATTA---GGAATAGCACCCAACAGGGCAAAGAGAAGAGTGGTG-----------

```
----------------------------CAGAGAGAAAAAAGAGCA---ATA------GCGGGAGCT------
---TTGTTCCTTGGGTTCTTGGGAACA---GCAGGAAGCACTATG---GGCGCAGCGTCA---
CTGACGCTGACGGTACAG---GCCAGACAA---TTATTG---TCTGGTATAGTGCAACAGCAGAAC---
AATTTG---CTAAGG---GCTATTGAGGCGCAACAGCATCTGTTGCAACTCACAGTCTGGGGCATC
>DQ410356
TTCTACTGTAATACAACAAAACTGTTTACCTGGAATAGTACTGAAGAGTCAAATAACACTGAG----------
-------------------------------------------------------------------------
----------------------------
GGGAATAGAACAATCACACTCCCATGCAGAATAAAACAAATTATAAACATGTGGCAGGAAGTAGGAAAAGCAA
TGTATGCCCCTCCCATCAAGGGACTAATTAACTGTACATCAAAG---
ATTACAGGACTACTATTAACAAGAGATGGTGGTGGTAATAGTACA----------------------------
-------------------------------------------------------------------------
----------
AACAGGACTGAGATCTTCAGACCTGGAGGAGGAGATATGAGGGACAATTGGAGAAGTGAATTATATAAATATA
AAGTAGTAAGAATTGAACCAGTA---GGAATAGCACCCGGCAAGGCAAAGAGAAGAGTGGTG-----------
----------------------------CAGAGAGAAAAAAGAGCG---GTAACA---CTGGGAGCT------
---ATGTTCCTTGGGTTCTTGGGAGCA---GCAGGAAGCACTATG---GGCGTAGCGTCA---
ATGACGCTGACGGTACAG---GCCAGACAA---TTACTG---TCTGGTATAGTGCAACAGCAGAAC---
AACCTG---CTGAAG---GCGATTGAGGCGCAACAGCACCTGTTGCAACTCACGGTCTGGGGCATT
>JQ403103
TTCTACTGTAATACAACAAAACTGTTTAACAGTACTTGGAGGATTAGTAATGGTACGGAGATTAATGAT----
-------------------------------------------------------------------------
----------------------------
ACTAATAGTACTATCACCCTCCCATGCAGAATAAAACAAATTATAAACAGGTGGCAGGAAGTAGGAAAAGCAA
TGTTTGCCCCTCCTATCAGAGGACAAATTAGTTGTTCATCAAAT---
ATTACAGGGCTGCTATTAACAAGAGATGGTGGGAACGTCAGCAAC----------------------------
-------------------------------------------------------------------------
-------------
ACGGCAGAAACCTTTAGGCCTGGAGGAGGAGATATGAGGGACAATTGGAGAAGTGAATTATATAAATATAAAG
TAGTAAGGGTTGAACCATTA---GGAGTAGCACCCACCAAGGCGAAGAGAAGAGTGGTG--------------
-------------------------CAGAGAGAAAAAAGAGCA---GCA------ATAGGAGCT---------
GTGTTCCTTGGGTTCTTGGGAGCA---GCAGGAAGCACTATG---GGCGCAGCGTCA---
GTGGCGCTGACGGTACAG---GCCAGGCAA---TTATTG---TCTGGTATAGTGCAGCAGCAGAAC---
AATCTG---CTGAGA---GCTATTGAGGCGCAACAGCATCTGTTGCAACTCACAGTCTGGGGCATC
>JQ403104
TTCTACTGTAATACATCACAACTGTTTAATAGCACTTTCAGTGGTAATGAGAGTATAAATACTACT-------
-------------------------------------------------------------------------
----------------------------
GAGAACGCTACAATCACACTCCCATGTAGAATAAAACAAATTATAAATATGTGGCAGGAAGTAGGAAAAGCAA
TGTATGCCCCTCCCATCAGTGGACAAATTAACTGTACATCAAAC---
ATTACAGGCCTGTTATTAACAAGAGATGGTGGAGATAACAATACC----------------------------
-------------------------------------------------------------------------
----------
ACCAACACTGAGACCTTCAGACCTACAGGGGGAAATATGAAGGACAATTGGAGAAGTGAATTATATAAATATA
AAGTAGTAAAAATTGAACCATTA---GGACTAGCACCCTCCAAGGCAAAGAGAAGAGTGGTG-----------
----------------------------CAGAGAGAAAAAAGAGCA---GTGGGA---CTAGGAGCT------
---TTGTTCATTGGGTTCTTGGGAGCA---GCAGGAAGCACTATG---GGCGCAGCGTCG---
ATGACGCTGACGGTACAG---GCCAGACAA---TTATTG---TCTGGTATAGTGCAACAGCAGAAC---
AATCTG---CTGAGA---GCTATTGAGGCGCAACAACATCTGTTGCAACTCACAGTCTGGGGCATC
>HQ217329
TTCTACTGTAATTCAACACAACTGTTTGATAGTACTTGGAATGAGACTACAACAAGGTCAAAT----------
-------------------------------------------------------------------------
----------------------------
AGAAGTGGTGACATCACACTCCCATGCAGAATAAAACAATTTATAAACATGTGGCAGGGAGTAGGAAAAGCAA
TATATGCCCCTCCCATCAGTGGACAAATTAACTGCTCATCAAAT---
ATTACTGGGCTACTATTAACAAGAGATGGTGGTAACGGAAACAAT----------------------------
-------------------------------------------------------------------------
-------------
ACCACCGAGATCTTCAGACCTGGAGGAGGAGATATGAGGGACAATTGGAGAAGTGAATTATATAAATATAAGG
TAGTAAAAATTGAGCCATTA---GGAATAGCACCCACCAAGGCAAAAAGAAGAGTGGTG--------------
```

-------------------------CAGAGAGAAAAAAGAGCA---GTGGGA---CTAGGAGTT---------
TTGTTCCTTGGGTTCTTAGGAGCA---GCAGGAAGCACTATG---GGCGCAGCGTCA---
GTGGCGCTGACGGTACAG---GCCAGACAA---TTATTG---TCTGGTATGGTGCAGCAGCAGAAC---
AATCTG---CTGAGA---GCTATTGAGGCGCAACAGCATATGTTGCAACTCACAGTCTGGGGCATC
>FJ469745
TTCTACTGTAGTACAACACAACTGTTTAGTAGTACTTGGAATAGTACTAACAGGACCGATACA----------
----

GGAAATGAAACTATCACACTCCCATGCAAAATAAAACAAATTATAAACTTGTGGCAAGAAGTAGGAAAAGCAA
TGTATGCCCCTCCCATCAGAGGACAAATTAAATGTTCATCAAAT---
ATTACAGGGCTACTATTAACAAGAGATGGTGGTAATGACAATAAT----------------------------
----

ACCACCGAGGAAATTTTCAGACCTGCAGGAGGAGACATGAGGGACAATTGGAGAAGTGAGCTATATAAATATA
AAGTAGTAAAAATTGAACCATTA---GGAGTAGCACCCACCAAGGCAAAGAGAAGAGTGGTG-----------
----------------------------CAGAGAGAAAAAAGAGCA---ATAGGA---ATAGGAGCT------
---ATGTTCCTTGGGTTCTTGGGAGCA---GCAGGAAGCACTATG---GGCGCAGCATCA---
GTGACGCTGACGGTACAG---GCCAGACAA---TTATTG---TCTGGTATAGTGCAACAGCAGAGC---
AATCTG---CTGAGG---GCTATTGAGGCGCAACAGCATCTGTTGCAACTCACAGTCTGGGGCATC
>MK147530
TTCTACTGTAATTCAACACAACTGTTTAATAGTACTTGGACATTTAATAATACTTGGAAGCCTAATATTACTG
GGAATGATACT--

GGAGGGGACAATATCATACTCCAATGCAGAATAAAACAAATTATAAACATGTGGCAGGAAGTAGGAAAAGCAA
TGTATGCCCCTCCCATCAGGGGAGAAATTAGATGTACATCAAAT---
ATTACAGGGCTGCTATTAACAAGAGATGGTGGTAAGGATAATACCACT-------------------------
----

AACGGGACTGAGACCTTCAGACCTGGAGGAGGAGATATGAGAGACAATTGGAGAAGTGAATTATATAAATATA
AAGTAGTAAGAATTGAACCATTA---GGAGTAGCACCCACCAAGGCAAAGAGAAGAGTGGTG-----------
----------------------------CAGAGAGAAAAAAGAGCA---GTGGGA---ATAGGAGCT------
---ATGTTCTTTGGGCTCTTGGGAGCA---GCAGGAAGCACTATG---GGCGCAGCGTCA---
GTGACGCTGACGGTACAA---GCCAGACTA---TTATTG---TCTGGTATAGTGCAACAGCAAAAC---
AATTTG---CTGAGG---GCTATTGAGGCGCAACAGCATTTGTTGCAACTCACAGTCTGGGGCATC
>FJ469772
TTCTACTGTAATACAACACAACTGTTTAATAGTACTTGGCTTAACAGTAATAGTACTGAAAATGGGACT----
----

ACTAACGGCACTATCACACTCCCCTGCAGAATAAAACAAATTATAAACATGTGGCAAGGAATAGGAAGAGCAA
TGTATGCCCCTCCCATCAGTGGACAAATTAGGTGCTTATCAAAT---
ATTACAGGGCTACTATTAACAAGAGATGGTGGTGGTGGGAACGAGACC-------------------------
----

GAGACCAATACCGAGATTTTCAGACCTGCAGGAGGAGATATGAAGGACAATTGGAGAAGTGAATTATATAAAT
ATAAAGTAGTAAGAATTGAACCCTTA---GGAATAGCACCCACCAAGGCAAGGAGAAGAGTGGTG--------
-------------------------------CAGAGGGAAAAAAGAGCA---GTGGGA---ATAGGAGCT---
------ATGTTCCTTGGGATCTTGGGAGCA---GCAGGAAGCACTATG---GGCGCAGTGTCA---
ATGACGCTGACGGTACAG---GCCAGACTA---TTATTG---TCTGGTATAGTGCAACAGCAGAAC---
AATCTG---CTGAGG---GCTATTGAGGCGCAACAGCATCTGTTGAAACTCACAGTCTGGGGCATC
>EF593269
TTCTACTGTAATACAACACAACTGTTTAATAGTACTTGGGGGACTAATAGTACTTGGGAGATTAATACTACT-
----

GGGAATGATACTATCACACTCCCATGCAGAATAAAACAAATTATAAACATGTGGCAGGAAGTAGGAAAAGCAA
TATATGCCCCTCCCATCAGAGGACAAATTAGATGTTCGTCAAAT---
ATTACAGGGCTGCTATTAACAAGAGATGGTGGTGAAAATAGCACA----------------------------
----

AACGGCACCGAGATCTTCAGACCTGGAGGAGGGGATATGAGGGACAATTGGAGAAGTGAATTATATAAATATA
AAGTAGTACAAGTTGAACCATTA---GGAGTAGCACCCACCAAGGCAAAGAGAAGAGTGGTG-----------

----------------------------CAGAGAGAAAAAAGAGCA---GTGGGT---ATAGGAGCT------
---CTATTCCTTGGGTTCTTAGGAGCA---GCAGGAAGCACTATG---GGCGCAGCGTCA---
ATAACGCTGACGGCACAG---GCCAGACAA---TTATTG---TCTGGTATAGTGCAGCAGCAGAGC---
AATTTG---CTGAGG---GCTATTGAGGCGCAACAGCATCTGTTGCAACTCACAGTCTGGGGCATC
>EF593271
TTCTACTGTGATACAAAAATACTGTTTAATAGTACTTGGAATTTTACTAACGGGAAAATTACTGCAGGGCCAA
ATAGC--
-------------------ACT---
GAAGGAAATGACACAATCACAATCCCATGCAGAATAAAACAAATTATAAATATGTGGCAAGAAGTAGGAAAAG
CAATGTATGCTCCTCCCATCAGAGGAATTATTAGCTGTTTATCAAAT---
ATTACAGGGCTGCTATTAACAAGAGATGGTGGTAGGAGTAATGATAGCGCG----------------------
----

ACCAATAAAACTGAAGTCTTCAGACCTGGAGGAGGAGATATGAGGGACAATTGGAGAAGTGAATTATATAAAT
ATAAGGTAGTAAAAATTGAACCATTA---GGGGTAGCACCCACCAAGGCAAAGAGAAGAGTGGTG--------
-------------------------------CAGAGAGAAAAAAGAGCA---GTGGGA---TTTGGAGCT---
------TTGTTCCTTGGGTTCTTGGGAGCA---GCAGGAAGCACTATG---GGCGCAGCGTCG---
GTGACGCTGACGGTACAG---GCCAGACAA---CTATTG---TCTGGTATAGTGCAACAGCAAAAC---
AACTTG---CTGAGA---GCTATTGAGGCGCAACAACATCTGTTGCAACTCACAGTCTGGGGCATC
>EF593273
TTCTATTGTGATTCAACAAAATTGTTTAATAGTACTTGGGGAATTAATGGTACTTGGAATACCACT-------
----

GAAGGGGCAAATATCACACTCCCATGCAGAATAAAACAGATTATAAATCTGTGGCAGCAAGTAGGAAAAGCAA
TGTATGCCCCTCCCATCAAAGGAAAAATTAGCTGCTCATCAAAT---
ATTACAGGGCTGATATTGACAAGAGATGGTGGTGATAATGGAAAT----------------------------
----

GGGACAGAGGAGATCTTCAGACCTGGAGGAGGAGATATGAGGGACAATTGGAGAAGTGAATTATATAAATATA
AGGTAGTAAAAATTGAACCATTA---GGAATAGCACCCACCAAGGCAAAGAGAAGAGTGGTG-----------
----------------------------CAGAGAGAGAAAAGAGCA---GTGGGA---ATGGGAGCT------
---CTGTTCCTTGGGTTCTTGGGAGCA---GCAGGAAGCACTATG---GGCGCAGCGTCA---
GTGACGCTGACGGTACAA---GCCAGACGA---TTATTG---TCTGGTATAGTGCAACAGCAGAAC---
AACTTG---CTGAGG---GCTATTGAGGCGCAACAGCATCTGTTGCAACTCACAGTCTGGGGCATT
>EF593274
TTCTACTGTAATACAACACAACTGTTTAATAGTACTTGGTTTCCTAATGGTACTTGGTTTCCTAATGATACTG
TGAAT--
-------------------AAT---
ACTGGAGATGTGAATATCACCCTACCATGCAGAATAAAACAAATTGTAAACATGTGGCAGGAAGTAGGAAAAG
CAATGTATGCCCCTCCCATTGCAGGACAAATTAGATGTTCATCAAAT---
ATTACAGGGTTACTATTAACAAGAGATGGTGGTATTGGTAATAGCACA-------------------------
----

AATAATGAGACCGAGACCTTCAGACCTGGAGGAGGAGATATGAGGGATAATTGGAGAAGTGAATTATATAATT
CCAAAGTAGTAAAAATTGAACCATTA---GGAATAGCACCCACCAAGGCCAAGAGAAGAGTGGTG--------
-------------------------------CAGAGAGAAAAAAGAGCA---GTGGGA---ATAGGAGCT---
------ATGTTCCTTGGATTCTTGGGAGCA---GCAGGAAGCACTATG---GGCGCAGCGTCA---
CTGACGCTGACGGTACAG---GCCAGACAA---TTATTG---TCTGGTATAGTGCAACAGCAAAAC---
AACTTG---CTGAGA---GCTATTGAGGCGCAACAACATCTGTTGCAACTCACAGTCTGGGGCATC
>EF593276
TTCTACTGTAATACAACGAAGTTGTTTGATAGTGCTTGGCCTAATGCTACTTGGAAGAATGCC----------
----

AATGATACAATTATCACACTCCCATGCAGAATAAAACAAATTATAAACATGTGGCAAGAAGTAGGAAAAGCCA
TGTATGCCCCTCCCATCAGTGGACAAATTAACTGTTCATCAAAT---
ATTACAGGGCTGCTATTAACAAGAGATGGTGGTACCGGGAACAATGAGACA----------------------
----

GACAATAATACTGAAATTTTCAGACCTGGGGGAGGAGACATGAGGGACAATTGGAGAAGTGAATTATATAAAT
ATAAAGTAGTACAAATTGAACCATTA---GGAATAGCACCCACCAAGGCAAGGAGGAGAACGGTG--------

-------------------------------CAGAGAGAAAGAAGAGCA---GCA------ATAGGAGCT---
------ATGATCCTTGGGTTCTTGGGAGTA---GCCGGAAGCACTATG---GGCGCAGCGTCA---
GTGACGCTGACGGTACAG---GCCAGACTA---TTATTG---TCTGGTATAGTGCAACAGCAGAAC---
AATCTG---CTGAGG---GCTATTGAGGCGCAACAGCATCTGTTGCAACTCACCGTCTGGGGCATT
>JN024210
TTCTACTGTAATACAACACAACTGTTTAATAGTACTTGGAATGTGACTGGGTCAAATAACACTGAA-------
----

GGAAATGACACAATCACGCTCCCATGCAGAATAAAACAAATTATAAACATGTGGCAGGAAGTAGGAAAAGCAA
TGTATGCCCCTCCCACCAGAGGACAAATTAGATGTTCATCAAAT---
ATTACAGGGCTGCTATTAACAAGAGATGGTGGTGTGAACACCACCACC-------------------------
----

ACCAACGAGACAGAGGTCTTCAGACCTGGAGGAGGAAATATGAAGGACAATTGGAGAAGTGAATTATATAAAT
ATAAAGTAGTAAAAATTGAACCATTA---GGAGTAGCACCCACCAAGGCAAAGAGAAGAGTGGTG--------
-------------------------------CAGAGAGAAAAAAGAGCA---ATAGGA---ATAGGAGCT---
------GTGTTCCTTGGGTTCTTGGGAGCA---GCAGGAAGCACTATG---GGCGCAGCGTCA---
ATAACGCTGACGGTACAG---GCCAGACAA---TTATTG---TCTGGTATAGTGCAACAGCAGAAC---
AACTTG---CTGAGA---GCTATTGAGGCGCAACAACATATGTTGCAACTCACAGTCTGGGGCATC
>FJ469683
TTCTACTGTAATACAACACAACTGTTTAATAGTACTTGGTTTAATGGTACTTGGAATGGTACTATAGAGTCAA
GT---

AACACTACTAATATCACACTCCCATGCAGAATAAAACAAATTGTAAACATGTGGCAGGAAGTAGGAAAAGCAA
TGTATGCCCCTCCCATCAGAGGACAAATTAGATGTTCATCAAAT---
ATTACAGGGCTGCTATTGACAAGAGATGGTGGTAAGAACAACAGC----------------------------
----

ACCAATGAGACCTTCAGACCTGAAGGAGGAAATATGAGGGACAATTGGAGAAGTGAATTATATAAATATAAAG
TAGTAAGAATTGAACCATTA---GGAGTAGCACCCACCAAGGCAAAGAGAAGAGTGGTG--------------
-------------------------CAAAGAGAAAAAAGAGCA---GTGGGAACGATAGGAGCT---------
ATGTTCCTTGGGTTCTTGGGAGCA---GCAGGAAGCACTATG---GGCGCAGCGTCA---
ATGACGCTGACGGTACAA---GCCAGACAA---TTATTG---TCTGGTATAGTGCAACAGCAAAAC---
AATTTG---CTGAGG---GCTATTGAGGCGCAACAACATCTGTTGCAACTCACAGTCTGGGGCATT
>HQ238279
TTCTACTGTAATACAACACAACTGTTTAATAGTACTTGGAATAATAATAGTACTGAGAATAAGAATGATACTA
AC---

AGCAATGAAACTATCATGCTCCCATGCAGAATAAAACAAATTATAAACAGATGGCAAGAAGTAGGAAAAGCAA
TGTATGCCCCTCCCATCGCTGGACAAATTAGATGTTCATCAAAT---
ATTACAGGGCTGCTATTGACAAGAGATGGTGGTAATAGCAGCAGCGAC-------------------------
----

AATACAAGTACAGAGATCTTCAGACCTGGAGGAGGAGATATGAGAGACAATTGGAGAAGTGAATTATATAAAT
ATAAAGTAGTAAAAATTGAACCATTA---GGAGTAGCACCCACTAAGGCAAAGAGAAGAGTGGTG--------
-------------------------------CAGAGAGAAAAAAGAGCA---GTAGTGGGAATAGGAGCT---
------GTGTTCCTTGGGTTCTTGGGAGCA---GCAGGAAGCACTATG---GGCGCAGCATCA---
TTAACGCTGACGGTACAG---GCCAGACAA---TTATTG---TCTGGTATAGTGCAGCAGCAGAAC---
AATCTG---CTGAGG---GCTATTGAGGCGCAACAGCATCTGTTGCAACTCACAGTCTGGGGCATC
>FJ496085
TTCTACTGTAATTTAACACAACTGTTTAATAGTACTTGGAATAGTACTGAAAGCTTTAATAGTACTGAAAGCT
CTAATAGT---
-------------------ACT---
GAAGGAAATGACACAATCAGCCTCCCATGCAGAATAAAACAAATTATAAACATGTGGCAGGAAATAGGAAAAG
CAATGTATGCCCCTCCCATTGCAGGAATAATTAGATGTTCATCAAAT---
ATTACAGGAATACTATTAACAAGAGATGGTGGTATAAATAGTACAGAT-------------------------
----

AATACCACTGAGATCTTCAGACCTGCAGGAGGAGATATGAGAGACAATTGGAGAAGTGAATTATATAAATACA
AAGTAGTAAAAATTGAACCATTA---GGAATAGCACCCACCAAGGCAAAGAGAAGAGTGGTG-----------

----------------------------CAGAGAGAAAAAAGAGCA---GTGGGA---ATAGGAGCT------
---GTGTTCCTTGGGTTCTTGGGCGCA---GCAGGAAGCACTATG---GGCGCAGCGTCA---
GTGGCGCTGACGGTACAG---GCCAGACAA---TTATTG---TCTGGTATAGTGCAACAGCAGAAC---
AATTTG---CTGAGG---GCTATTGAGGCGCAACAGCATCTGTTGCAACTCACAGTCTGGGGCATC
>JF689854
TTCTACTGTAATACAACACAACTGTTTAATAGTACTTGGAATGATACTACTGAAGGGTCAAATAAC-------
----

ACTGGAAGAACTATCACACTCCCATGCAGAATAAAACAAATTATAAATATGTGGCAGGAAGTAGGAAAAGCAA
TGTATGCCCCTCCCATCAGCGGACAAATTAGATGTTCATCAAAT---
ATTACAGGGCTTCTATTAACAAGGGATGGTGGTAATAATAACGAGACC-------------------------
----

AATAACGGGACCGAGACCTTCAGACCTGGAGGGGGAAATATAAGGGACAATTGGAGAAGTGAATTATATAAAT
ATAAAGTAGTAAAAATTGAACCATTA---GGAGTAGCACCCACCAGGGCAAAGAGAAGAGTGGTG--------
-------------------------------CAGAGAGAAAAAAGAGCA---GTGGGA---ATAGGAGCT---
------GTGTTCCTTGGGTTCTTGGGAGCA---GCAGGAAGCACTATG---GGCGCAGCGTCA---
ATAACGCTGACGGTACAG---GCCAGACAA---TTATTG---TCTGGTATAGTGCAACAGCAAAGC---
AATTTG---CTGAGG---GCTATTGAGGCGCAACAGCATATGTTGCAGCTCACAGTCTGGGGCATC
>JF689856
TTCTACTGTAATACAACAAGACTGTTTAATAGTACGTGGTTTTGGAATAATGCTACTGCTAATTGGACTTTGA
AAAATGATACT--
-AAGGGAGAATTCAAT---------
ATCTCTGGAGGGAATATCACACTCCCATGCAGAATAAAACAAATTATAAACATGTGGCAGGAAGTAGGGAAAG
CAATGTATGCCCCTCCCATCAGAGGACAAATTAGATGTTCATCAAAT---
ATTACAGGGCTGCTATTAACAAGAGATGGTGGAGAGGAGCCTAAC----------------------------
----

GATACTGAGACTTTCAGACCTGCAGGAGGAAATATGAAGGACAATTGGAGAAGTGAATTATATAAATATAAAG
TAGTAAAAATTGAACCATTA---GGAATAGCACCCACCAGGGCAAAGAGAAGAGTGGTG--------------
-------------------------CAGAGAGAAAAAAGAGCA---GTGGGA---ATAGGAGCT---------
TTGTTCCTTGGGTTCTTGGGAGCA---GCAGGAAGCACTATG---GGCGCAGCGTCA---
GTAACGCTGACGGTACAG---GCCAGAATG---CTATTG---TCTGGTATAGTGCAACAGCAGAAC---
AATCTG---CTGAGG---GCTATTGAGGCGCAACAGCATCTGTTGCAACTCACAGTCTGGGGCATC
>JF689857
TTCTACTGTAACACATCACAACTGTTTAATAATACTTGGAATGGAACTGCATGGTCAACTAACAGTTCA----
----

AATAGCACTGATATCACACTCCCATGCAGAATAAAACAAATTATAAACATGTGGCAGGAAGTAGGAAAAGCAA
TGTATGCCCCTCCCATTAGAGGACTAATTAGATGTTCATCAAAT---
ATTACAGGGCTACTATTAACAAGAGATGGTGGTAATAATAATAAT----------------------------
----

ACTAATGAGACCTTCAGGCCTGGAGGAGGAGATATGAGGGACAATTGGAGAAGTGAATTATATAAATATAAAG
TAGTAAAAATTGAACCATTA---GGAATAGCACCCACTAGGGCAAAGAGAAGAGTGGTG--------------
-------------------------CAGAGAGAAAAAAGAGCA---GTAGGA---ATAGGAGCT---------
ATGTTCCTTGGGTTCTTGGGAGCA---GCAGGAAGCACTATG---GGCGCAGCGTCA---
ATGACGCTGACGGTACAA---GCCAGACAA---TTATTG---TCTGGTATAGTGCAACAGCAGAAC---
AATCTG---CTGAGG---GCTATTGAGGCGCAACAGCATCTGTTGCAACTCACAGTCTGGGGCATC
>JF689859
TTCTACTGTGATTCAACACAACTATTTAATAGTACTTGGAATGGTACTGAAGGGTCAAATAACACTGAA----
----

GGAAATAGCACACTCACACTCCCATGCAGAATAAAACAGATTATAAACATGTGGCAGGAAGTAGGAAAAGCAA
TGTATGCCCCTCCCATCAGAGGACAAATTAGATGTTCATCAAAT---
ATTACAGGGCTGCTATTAACAAGAGATGGTGGTACTAACAATAGC----------------------------
----

GAGAACACCGAGATCTTCAGACCTGGAGGAGGAGATATGAGGGATAATTGGAGAAGTGAATTATATAAATATA
AAGTAGTAAAAATTGAACCATTA---GGAATAGCACCCACCAAGGCAAAGAGAAGAGTGGTG-----------

----------------------------CAGAGAGAAAAAAGAGCA---GTGGGA---ATAGGAGCT------
---GTGTTCCTTGGGTTCTTGGGAGCA---GCAGGAAGCACTATG---GGCGCAGCGTCA---
CTGACGCTGACGGTACAG---GCCAGACTA---TTATTG---TCTGGTATAGTGCAACAGCAGAAC---
AATTTG---CTGAGG---GCTATTGAGGCGCAACAGCATCTGTTGCAACTCACAGTCTGGGGCATC
>JF689860
TTCTACTGTAATACATCAGAGCTATTTAATTGGACTGAGGGAAAACCTGCTAAC-------------------
----

ACTGAAGACAAAATTACAATCCAATGCAGAATAAAACAAATTATAAACATGTGGCAGGAAGTAGGAAAAGCAA
TGTATGCCCCTCCCATCAGTGGACAAATTAGATGTTCCTCAACT---
ATTACAGGGTTGATATTAACAAGAGATGGTGGTAATGACACAAGG----------------------------
----

GAGAACGAGACCTTCAGACCTGGAGGAGGAGATATGAGGGACAATTGGAGAAGTGAATTATATAAATATAAAG
TAGTAAAAATTGAACCATTA---GGAATAGCACCCACCAAGGCAAAGAGAAGAGTGGTG--------------
-------------------------CAGAGAGAAAAAAGAGCA---GTGGGA---ATAGGAGCT---------
TTATTCCTTGGGTTCTTGGGAGCA---GCAGGAAGCACTATG---GGCGCAGCGTCA---
CTGACGCTGACGGTACAG---GCCAGACAA---TTATTG---TCTGGTATAGTGCAACAGCAGAGC---
AATTTG---CTGAGG---GCTATTGAGGTGCAACAGCATCTGTTGCAACTCACAGTCTGGGGCATC
>MK383403
TTCTATTGTAATACAACACAACTGTTTAATAGTACTTGGAATTTAACACAACCGTTTAATAGTACTTGGAATG
GTACTGAA---

GGGTCAAGTAATATAATCACACTCCCATGCAGAATAAAACAAGTTATAAACATGTGGCAGGAAGTAGGAAAAG
CAATGTATGCCCCTCCCATCAGAGGATACATTAGATGTTCATCAAAT---
ATTACAGGGCTACTATTAACAAGAGATGGTGGTAATGACAAAAACGGGACA----------------------
----

ACAACCGAGATCTTCAGACCTGGAGGAGGAGATATGAGGGATAATTGGAGAAGTGAATTATATAAATATAAAG
TAGTAAAAATTGAACCATTA---GGAGTAGCACCCACCAAGGCAAAGAGAAGAGTGGTG--------------
-------------------------CAGAGAGAAAAAAGAGCA---GTGGGA---ATAGGAGCC---------
TTGTTCCTTGGGTTCTTGGGAGCA---GCAGGAAGCACTATG---GGCGCAGCGTCA---
ATGACGCTGACGGTACAG---GCCAGACAA---TTATTG---TCTGGTATAGTGCAACAGCAGAAC---
AACTTG---CTGAGA---GCTATTGAGGCGCAACAACATCTGTTGCGACTCACAGTCTGGGGCATC
>HQ216575
TTCTACTGTAATACAACAAAACTGTTTAATAGTACTTGGAGTGATAATAGTACTTGGAATAATAGTAGTAAAT
GGTTC--
-------------------AAT---
GGCACTGGAAAAAATATCACACTCCTATGCAGAATAAAACAAATTGTAAATATGTGGCAGGAAGTAGGAAGAG
CAATGTACGCCCCTCCCATCAGTGGAATAATTGACTGTACATCAAAT---
ATTACAGGGATAATACTAGTAAGAGATGGTGGTAATATAAATGGGAGT-------------------------
----

AGGGAGAAAGAGACATTCAGACCTATAGGTGGAAATATGAAGGACAATTGGAGAAGTGAACTATATAAATATA
AAGTAGTAAAACTTGAACCATTA---GGAATAGCACCCACCAAGGCAAAGAGAAGAGTGGTG-----------
----------------------------CAGAGAGAAAAAAGAGCA---GTGGGA---CTAGGAGCT------
---CTGTTCTTTGGGTTCTTGGGAGCA---GCAGGAAGCACTATG---GGCGCAGCGTCA---
TTGACGCTGACGGTACAG---GCCAGACTA---TTATTG---TCTGGTATAGTGCAACAGCAAAAC---
AATTTG---CTAAAA---GCTATTGAGGCGCAACAGCATCTGTTGAAGCTCACAGTCTGGGGCATT
>HQ216584
TTCTACTGTAATACAACAAAACTGTTTAATAGTACTTGGGTTAATGGAACTAAGAATGACACAAAGGGG----
----

GGTAATGGAACTATCACACTCCAATGCAGAATAAAACAAATTATAAACATGTGGCAGCAAGTAGGAAAAGCAA
TGTATGCCCCTCCCATCAGCGGACCAATTAGTTGTTCATCAAAT---
ATTACAGGGCTGATATTAACAAGAGATGGTGGTACAAACACCACC----------------------------
----

AATGAGACCTTCAGACCTGGAGGAGGAGATATGAGGGACAATTGGAGAAGTGAATTATATAAATATAAAGTAG
TAAAAATTGAACCAATA---GGAGTAGCACCCACCAAGGCAAGGAGAAGAGTGGTG-----------------

----------------------CAGAGAGAAAAAAGGGCA---GTGGGA---ATAGGAGCT---------
TTTTTCCTTGGGTTCTTGGGAGCA---GCAGGAAGCACTATG---GGCGCAGCATCA---
ATAACGCTGACGGTACAA---GCCAGACTA---TTATTG---TCTGGTATAGTGCAACAGCAAAGC---
AACTTG---CTGAGA---GCTATTGAGGCGCAACAGCACCTGTTGCAACTCACAGTCTGGGGCATC
>HQ216686
TTCTACTGTGATTCAACACAGCTGTTTAATAGTACTTGGCCGTCTAATAGTACTTGGTCGTTTAATGATACTA
CAAAT--
-------------------AAT---
ACTGTAGGCACAAATATCACACTCCCATGCAGAATAAAACAAATTATAAACATGTGGCAGGAAGTAGGAAAAG
CAATGTATGCCCCTCCCATCAGAGGACAAATTAGATGTTCATCAAAT---
ATTACAGGCCTGCTATTAACAAGGGATGGTGGGGTTGGTAATAATAGCAGC----------------------
----

AATACAACGAACCCCGAGATCTTCAGACCTGGAGGAGGAGATATGAGGGACAATTGGAGAAGTGAATTATATA
AATATAAAGTAGTAAAAATTGAACCATTA---GGAATAGCGCCCACTAAGGCAAAGAGAAGAGTGGTG-----
----------------------------------CAAAGAGAAAAAAGAGCA---GTGGGG---
CTAGGAGCT---------ATGTTCCTTGGGTTCTTGGGAGCA---GCAGGAAGCACTATG---
GGCGCAGCGTCA---ATAACGCTGACGGTACAG---GCCAGACAA---TTATTG---
TCTGGTATAGTGCAACAGCAGAAC---AATTTG---CTGAAG---
GCTATTGAGGCGCAACAGCATCTGTTGCAACTCACAGTCTGGGGCATC
>HQ216727
TTCTACTGTAATTCAACAAAGCTGTTTAATAGTACTTGGCATTGGAATGGTACGATGTTTAATAGTACTGGGA
TTGAA--
-------------------AGG---
TCAAATGAGACTAATATCACACTCCCATGCAGAATAAAACAAATTGTAAACATGTGGCAGGAAGTAGGAAAAG
CAATGTATGCCCCTCCCATCAGAGGACAAATTAGATGTACATCAAAT---
ATTACAGGGCTGCTGTTATTTAGAGATGGTGGTAATACCACGAAGAAC-------------------------
----

AATACCGACGAGACTTTCAGACCTGGAGGAGGAGAGATGAGGGACAATTGGAGAAGTGAATTATATAAATATA
AAGTAGTAAAAATTGAACCATTA---GGAGTAGCACCCACTAAGGCAAAGAGAAGAGTGGTG-----------
----------------------------CAGAGAGAAAAAAGAGCA---GTGGGA---ATAGGAGCT------
---GTGTTCCTTGGGTTCTTGGGAGCA---GCAGGAAGCACTATG---GGCGCAGCGTCA---
ATGACGCTGACGGTACAG---GCCAGACTA---TTATTG---TCTGGTATAGTGCAACAGCAGAAC---
AATTTG---CTGAGG---GCTATTGAGGCGCAACAGCATCTGTTGCAACTCACAGTCTGGAGCATT
>HQ216762
TTCTACTGTAATACAACACAACTGTTTAATAGTACTTGGCCTACTGATAAAAATATTACTGGGAAGGATAGT-
----

GGCAATAGAAACATCACCCTCCCATGCAGAATAAAACAATTTATAAACATGTGGCAGGAAGTAGGAAAAGCAA
TGTATGCCCCTCCCATTAAAGGACAAATTAACTGCTTATCAAAT---
ATTACAGGGTTGCTATTAACAAGAGATGGTGGTAGTAACAACAAC----------------------------
----

AACAGCGAGATCTTCAGACCTGGAGGAGGAGATATGAGGGACAATTGGAGAAGTGAATTATATAAATATAAAG
TAATAGAAATTGAACCATTA---GGAATAGCACCCACCAAGGCAAAAAGAAGAGTGGTG--------------
-------------------------CAAAGAGAAAAAAGAGCA---GTGGGAGTAGTAGGAGCT---------
ATGATCCTTGGGTTCTTGGGAACA---GCAGGAAGCACTATG---GGCGCAGCGTCA---
CTGACGCTGACGGTACAG---GCCAAACAA---TTATTG---TCTGGTATAGTGCAGCAGCAGAAC---
AATCTG---CTGAGA---GCTATTGAGGCGCAACAGCACCTGTTGCAACTCACCGTCTGGGGCATC
>HQ216816
TTCTACTGTAATACAACACAGCTGTTTAATAGTACTTGGAATCTTAATGGTACTTGGAGTGGTACTACA----
----

GGAGAGGGGAACATTACACTCCCATGCAGAATAAAACAAATTGTAAATAGGTGGCAGGAAGTAGGAAAAGCAA
TGTATGCCCCTCCCATCAGCGGAAGAATTAGCTGTTCATCAAAT---
ATTACAGGGTTATTATTAACAATAGATGGTGGTAATAACAGGACA----------------------------
----

AACGAGACCTTCAGACCTGGAGGAGGAAATATGAGGGATAATTGGAGAAGTGAATTATATAAATATAAAGTAG

TACAAATTGAGCCATTA---GGAGTAGCACCCACCAAGGCAAAGAGAAGAGTGGTG-----------------
----------------------CAGAGAGAAAAAAGAGCA---GTGGGAATACTAGGAGCT---------
ATGTTCCTTGGGTTCTTGGGAGCA---GCAGGAAGCACTATG---GGCGCAGCATCG---
ATGACGCTGACGGTACAG---GCCAGACAA---CTATTG---TCTGGTATAGTGCAGCAGCAAAAC---
AATTTG---CTGAGG---GCTATTGAGGCGCAACAACATATGTTGCAACTCACAGTCTGGGGCATC
>HQ216841
TTCTACTGTAATACAACGAATCTGTTTAATAGTACTTGGAATAGTACTAGTACTGTAAATAAGACTACAGGG-
----

CCAAATGGGACTATCACACTCCCATGTAGGATAAAACAAATTATAAACATGTGGCAGGAAGTAGGAAAAGCAA
TGTATGCCCCTCCCATCCAAGGAAAAATTAGATGTACATCAAAT---
ATTACAGGGCTGTTATTAGTAAGAGATGGTGGCAACAACAATGAT----------------------------
----

ACTAATACAGAGATATTCAGACCTGGAGGAGGAAATATGAGGGACAATTGGAGAAGTGAATTATATAAATATA
AAGTAGTAAAAGTTGAACCATTA---GGAATAGCGCCCACCGAGGCAAAAAGAAGAGTGGTG-----------
----------------------------CAGAGAGAGAAAAGAGCA---GTGGGA---CTAGGAGCT------
---TTGTTCCTTGGGTTCTTGGGAGCA---GCAGGAAGCACTATG---GGCGCAGCGTCA---
ATGACGCTGACGGTACAG---GCCAGACAA---TTACTG---TCTGGTATAGTGCAACAGCAAAAC---
AACTTG---CTGAAG---GCTATTGAGGCGCAACAGCATATGTTGCAACTCACAGTCTGGGGCATC
>JX863966
TTCTACTGTAATCTAACACAACTGTTTAATAGTACTTGGAATGTAAGTGAAGGGTTAAATATCACAGAAAGTG
TC---

AATGACACAGATATCATACTCCCATGCAGGCTAAAACAAATTATAAACATGTGGCAGAAAGTAGGAAGAGCAA
TGTATGCCCCTCCCATCCCAGGACTAATTAACTGTACCTCAAAT---
ATTACAGGGTTGTTAATAACAAGAGATGGTGGTAACGAGAGCAAG----------------------------
----

AACGGGACTGAGATCTTCAGACCTTGGGGAGGAGATATGAGGGACAATTGGAGAAGTGAATTATATAAATATA
AAGTAGTAAAAATTGAACCATTA---GGAGTAGCACCCACCAAGGCAAAGAGAAGAGTGGTG-----------
----------------------------CAGAGAGAAAAAAGAGCA---TTAGGAGGATTAGGAGCT------
---ATGTTCCTTGGGTTCTTGGGAGCA---GCAGGAAGCACTATG---GGCGCAGCGTCA---
GTGACGCTGACGGTACAG---GCCAGACTA---TTATTG---TCTGGTATAGTGCAGCAGCAAAAC---
AATTTG---CTGAAG---GCTATTGAGGCGCAACAACATCTGTTGCAACTCACAGTCTGGGGCATC
>JX863971
TTCTACTGTAACACAACGCCACTGTTTACTAATTCTTGGAATGGTACCCATTGGGCTGATGAGTGGAATTGGG
AGAATAGT---

ACTAATGGAACAATCACACTCCAATGCAGAATAAAACAAATTATAAATATGTGGCAGGAAGTAGGAAAAGCAA
TGTATGCCCCTCCCATCAGGGGACAAATTAACTGTACATCAAAT---
ATTACAGGGCTGCTATTAGTAAGAGATGGTGGTAATAATGATAGC----------------------------
----

AGCACCAACGAGACCTTCAGACCTGGAGGAGGAAATATGAAGGACAATTGGAGAAGTGAATTATATAAATATA
AAGTAGTAAGAATTGAACCATTA---GGAGTAGCACCCACCAAGGCAAAGAGAAGAGTGGTG-----------
----------------------------CAAAGAGAAAAAAGAGCA---GTGGGA---ATAGGAGCT------
---CTGTTCCTTGGGTTCTTGGGAGCA---GCAGGAAGCACTATG---GGCGCAGCGTCA---
GTGACGCTGACGGTACAG---GCCAGACTA---TTATTG---TCTGGTATAGTGCAACAGCAAAAC---
AATTTG---CTGAGG---GCTATTGAGGCGCAACAGCACATGTTGCAGCTCACAGTCTGGGGCATC
>JX863972
TTCTACTGTAATACAACACCACTGTTTAATAGTAGTTGGAGGAATTCAAATAATACTTGGGATAGTACTGAAG
GATCAAAT---
-------------AACACT------
GCAGGAAATGACACAATCATACTCCAATGCAGAATAAAACAAATTATAAACATGTGGCAAGAAGTAGGAAAAG
CAATATATGCCCCTCCCATCAGAGGAAACATTACATGTTTATCAAAT---
ATTACAGGACTGCTATTAACAAGAGATGGAGGCAAAAGAGATAATAGCAGTAAC-------------------
----

AATAATGATACTGAAATCTTCAGACCTGGAGGAGGGGATATGAGGGACAATTGGAGAAGTGAATTATATAAAT

ATAAAGTAGTAAAAATTGAGCCATTA---GGAGTAGCACCCACTAAGGCAAAGAGAAGAGTGGTG-------
------------------------------CAAAGAGAAAAAAGAGCA---GTGGGA---TTAGGAGCT---
------ATGTTCCTTGGGTTCTTGGGAGCA---GCAGGAAGCACTATG---GGCGCAGCGTCA---
CTGACGCTGACGGTACAG---GCCAGACAA---TTATTG---TCTGGTATAGTGCAACAGCAGAAC---
AATTTG---CTGAGA---GCTATTGAGGCGCAACAGCATCTGTTGCAACTCACAGTCTGGGGCATC
>JX863919
TTCTACTGTAATTCATCACGACTGTTTGATAGTACTTGGTCACTGGAGGACACT------------------
----

GGAAATGACACAATCATGCTACCATGCAGAATAAGACAAATTGTAAACATGTGGCAGGGAGTAGGAAAAGCAA
TGTATGCCCCTCCCATCAGAGGACAAATTAACTGTACATCAAAT---
ATTACAGGGCTGATATTAACAAGAGATGGTGGTGCTGATATGAAT---------------------------
----

GAGACTTTCAGACCTGGAGGAGGAAATATGAAGGACAATTGGAGAAGTGAATTATATAAATATAAAGTAGTAA
GAATTGAACCAATA---GGATTAGCACCCACCAAGGCACAGAGAAGAGTGGTG-------------------
------------------CAGAGAGAAAAAAGAGCA---ATAGGA---CTAGGAGCT---------
GTGTTCCTTGGGTTTCTAGGAGCA---GCAGGAAGCACTATG---GGCGCAGCGTCA---
CTGACGCTGACGGTACAG---GCCAGACAA---TTATTG---TCTGGTATAGTGCAACAGCAGAAC---
AATTTG---CTGAGG---GCTATTGAGGCGCAACAACATATGTTGCAACTCACAGTCTGGGGCATC
>JX863983
TTCTACTGTAATACAACACCACTATTTAACAGTAGTTGGCATAGTAATGGGACTAGAAATGATACTGGAGGAT
CAAAC---

AGCACAGACCCAATCACGCTCCAATGCAGAATAAAACAAATTATAAACATGTGGCAGGAAGTAGGAAAAGCAA
TGTATGCCCCTCCCATCAGTGGCAAATTAACTGTTCATCAAAT---
ATTACAGGGCTGATATTAACAAGAGATGGTGGTACTAACAATAAC---------------------------
----

ACTACCACCGAGACCTTCAGACCTGGAGGAGGAGATATGAGGGACAATTGGAGAAGTGAACTATATAAATATA
AAGTAGTAAAAATTGAACCATTA---GGAATAGCACCCACCAGGGCAAAGAGAAGAGTGGTG----------
---------------------------CAGAGAGAAAAAAGAGCA---GCA------CTAGGAGCT------
---CTGTTCCTTGGGTTCTTGGGAGCA---GCAGGAAGCACTATG---GGCGCAGCATCA---
ATGACGCTGACGGTACAG---GCCAGACTA---CTATTG---TCTGGTATAGTGCAACAGCAGAAC---
AATTTG---CTGAGA---GCTATTGAGGCGCAACAGCATCTGTTGCAACTCACAGTCTGGGGCATC
>JX863984
TTCTACTGTAATACAACACTACTGTTTAATTGGACTGAAGAGGAAAATAACACAAATAAA------------
----

TATAATAAAACAATCAAGCTCCCATGCAGAATAAAACAAGTTATAAACATGTGGCAGGAAGTAGGAAAAGCAA
TGTATGCCCCTCCCATCAAAGGAGTAATTAACTGCTTATCAAAT---
ATTACAGGACTGTTATTAGTAAGAGATGGGGGTAATGGCAATAACACC------------------------
----

AACCGCACCGAGACCTTCAGACCTGGAGGAGGAGATATGAGGGACAATTGGAGAAGTGAGCTATATAAATATA
AAGTAGTAAAAATTGAACCAATA---GGAATAGCACCCACTAAGGCAAAGAGAAGAGTGGTG----------
---------------------------CAAAGAGAAAAAAGAGCAGTAGTGGGA---ATAGGAGCT------
---ATGTTCCTTGGGTTCTTGGGAGCT---GCAGGAAGCACTATG---GGCGCAGCGTCA---
TTGACGCTGACGGTACAG---GCCAGACTA---TTATTG---TCTGGTATAGTGCAACAGCAGAAC---
AATTTG---CTGAGG---GCTATTGAGGCGCAACAACGTATGTTGCAACTCACAGTCTGGGGCATC
>JX863985
TTCTACTGTAATTCAACACCACTGTTTACAACTGCAAATAACTCT---------------------------
----

GGAGGAGTTAATATCACACTCCAATGCAAAATAAAACAAGTTATAAACAGGTGGCAGGAAGTAGGAAAAGCAA
TGTATGCCCCTCCCATCAGAGGACAAATTAGATGTTCATCAAAT---
ATTACAGGGCTGCTATTAATAAGGGATGGTGGTAATAATAGCAAG---------------------------
----

AGTGATCCCGAGATCTTCAGACCTGGAGGAGGAGATATGAGGGACAATTGGAGAAGTGAATTATATAAATATA

```
AAGTAGTGAAAATTGAACCATTA---GGAATAGCACCCACCAAGGCAAAGAGAAGAGTGGTG-----------
----------------------------CAGAGAGAAAAAAGAGCA---GTGGGA---CTAGGAGCT------
---ATGTTCCTTGGGTTCTTGGGAGCA---GCAGGAAGCACTATG---GGCGCAGCATCA---
ATAACGCTGACGGTACAG---GCCAGACAA---TTATTG---TCTGGTATAGTGCAACAGCAGAGC---
AATCTG---CTGAGA---GCTATTGAGGCGCAACAGCATCTGTTGCAACTCACAGTCTGGGGCATC
>JX863986
TTCTACTGTAATTCAACACCACTGTTTAATAGTACTTGGTATGGGAATGGTACTAGTGCTGAGAATGATAGTA
CAAAT--------------------------------------------------------------------
----------------------------
CCAAGTGAAAATATCACACTCCAATGTAGAATAAAACAAATTATAAACATGTGGCAGGAAGTAGGAAAAGCAA
TGTATGCCCCTCCCATCAGTGGACAAATTAGCTGTACATCAAAT---
ATTACAGGGCTGTTATTAACAAGAGATGGTGGTAAGAACAATAGCACC-------------------------
-------------------------------------------------------------------------
-------
AACAACACCGATGAGATCTTTAGACCTGGAGGAGGAAATATGAAGGACAATTGGAGAAGTGAATTATATAAAT
ATAAAGTAGTACAAATTGAACCAATA---GGAATAGCACCCACCAAGGCAAAAAGAAGAGTGGTG--------
-------------------------------CAAAGAGAAAAAAGAGGA---GTGGGA---ATAGGAGCT---
------TTGTTCCTTGGGTTCTTGGGAGCA---GCAGGAAGCACTATG---GGCGCAGCATCA---
ATAACGCTGACGGTACAG---GCCAGACAA---TTATTG---TCTGGTATAGTGCAACAGCAAAAT---
AATTTG---CTGAGA---GCTATTGAGGCGCAACAGCATCTGTTGCAACTCACAGTCTGGGGCATC
>JF320059
TTCTATTGCAATACAACACAATTGTTTAATAGTATTTGGCCTACTAATGATACTAGCAAAGGGACTAAT----
-------------------------------------------------------------------------
----------------------------
ATCAAGGGAAATATCACACTCCCATGCAGAATAAGACAAATTGTAAACAGGTGGCAGGAAGTAGGAAAAGCAA
TGTATGCCCCTCCCATTAAAGGACCAATTACATGTATATCAAAT---
ATTACAGGGTTGCTATTAACAAGAGATGGTGGAGGTGCCAGTAAT----------------------------
-------------------------------------------------------------------------
----------
GGGACAAATGAGACCTTCAGACCTGGAGGAGGAGATATGAGGGACAATTGGAGAAGTGAATTATATAAATACA
AAGTGGTGAAAATTGAACCATTA---GGAATAGCACCCACCAGGGCAAGGAGAAGAGTGGTG-----------
----------------------------CAGAGAGAAAAAAGAGCA---GTGGGA---TTTGGAGCT------
---TTGTTCCTTGGGTTCTTGGGAGCA---GCAGGAAGCACTATG---GGCGCAGCTTCA---
ATGACGCTGACGGTACAG---GCCAGACAA---TTACTG---TCTGGTATAGTGCAACAGCAGAAC---
AATCTG---CTGAGG---GCTATTGAGGCGCAACAGCATCTGTTGCAACTCACAGTCTGGGGCATA
>JF320361
TTCTACTGTAACACAACACCACTATTTAATAGTACTTGGAATAATACTGAAAGGTGGAATGGTACTGAAGGG-
-------------------------------------------------------------------------
----------------------------
TTAAATGGTACCATCACACTCCAATGCAGAATAAAACAAATTATAAACCTGTGGCAGGAAGTAGGAAAAGCAA
TGTATGCCCCTCCCATCAGCGGACAAATTAGATGTTCATCAAAT---
ATTACAGGGCTGCTGATAGTAAGAGATGGTGGTATTAATACTACTAAC-------------------------
-------------------------------------------------------------------------
----------
GAGACGACCGAGATCTTCAGACCTGGAGGAGGAGACATGAGGGACAATTGGAGAAGTGAATTATATAAATATA
AAGTAGTAAAAATTGAACCATTA---GGAGTAGCACCCACCAAGGCAAAGAGAAGAGTGGTG-----------
----------------------------CAAAGAGAAAAAAGAGCA---GTGGGAACTATAGGAGCT------
---ATGTTCCTTGGGTTCTTGGGAGCA---GCAGGAAGCACTATG---GGCGCAGCGTCA---
ACAGCGCTGACGGTACAG---GCCAGACAA---TTATTG---TCTGGTATAGTGCAACAGCAGAGC---
AATCTG---CTGAGG---GCTATTGAGGCACAACAGCATGTGTTACAACTCACAGTCTGGAGCATC
>JF320054
TTCTACTGTAATACAACACAACTGTTTAATAGTTCTTGGAGGGTTAATGAGACTTGGAATAATACT-------
-------------------------------------------------------------------------
----------------------------
GAAGAGGGCAATATCATACTCCCCTGCAGAATAAAACAATTTATAAACATGTGGCAGGGAGTAGGAAAAGCTA
TGTATGCCTCTCCCATTGCAGGACGAATTAAATGTAGATCAGAT---
ATTACAGGGCTGCTATTACTAAGAGATGGTGGACAAGAAAATAACACAGAG----------------------
-------------------------------------------------------------------------
-------
ACCAATAACACAGAGACCTTCAGACCTGGAGGAGGAGATATGAAAGACAATTGGAGAAGTGAATTATATAAAT
```

ATAAAGTAGTAAAAATTGAACCATTA---GGGATAGCACCCACTAAGGCAAAGAGAAGAGTGGTG--------
-------------------------------CAGAGAGAGAAAAGAGGA---GCGGGA---ATAGGAGCT---
------CTGTTCCTTGGGTTTCTGGGAGCA---GCAGGAAGCACTATG---GGCGCAGCGTCA---
ATGACGCTGACGGTACAG---GCCAGACAA---TTATTG---TCTGGTATAGTGCAACAGCAGAAT---
AATCTG---CTGAAG---GCTATTGAGGCGCAACAGCATATGTTGCAACTCACAGTCTGGGGCATC
>EU577917
TTCTACTGTGACACAACAAAACTGTTTGATAGTATTTGGGAGTTTACTAAAGGGAATATTACGAATATTACTG
CAAGATCAAAT--
-------------AGCACT------
GAAAGAAATAACACAATCACACTCCAATGTAGAATAAAACAAATTATAAATATGTGGCAGGAAGTAGGAAAAG
CAATGTATGCCCCTCCCATTAGAGGAGATATTAGCTGTTCATCAAAA---
ATTACAGGGCTGCTATTAACAAGAGATGGTGGTAGTAACAACGATAGCGAG----------------------
----

ACCAACACCACTGAGGTCTTCAGACCTGGAGGAGGAGATATGAGGGACAATTGGAGAAGTGAATTATATAAAT
ATAAGGTAGTAAGAATTGAACCATTA---GGAGTAGCACCCACCAAGGCAAAGAGAAGAGTGGTG--------
-------------------------------CAGAGAGAAAAAAGAGCA---TTGGGA---TTCGGAGCG---
------TTGTTCCTTGGGTTCTTGGGAGCA---GCAGGAAGCACTATG---GGCGCAGCGTCG---
GTGACGCTGACGGTACAG---GCCAGACAA---TTATTG---TCTGGTATAGTGCAACAGCAAAAC---
AACTTG---CTGAGA---GCTATTGAGGCGCAACAGCATCTGTTGCAACTCACCGTCTGGGGCATC
>EU578004
TTCTACTGTAATACATCAGGACTGTTTAATAGTACTTGGAATGTTAATAAAAATGAAACTAATAGCACA----
----

CACAATACCAATATCACACTCCCATGTAGAATAAAACAAATTATAAACATGTGGCAGGAAGTAGGAAAAGCAA
TGTATGCCCCTCCCATCAGAGGACAAATTAGATGTGTATCAAAT---
ATTACAGGATTGCTATTAACAAGAGATGGTGGTAATAGTAATGGCAATAGT----------------------
----

ACTGAAAATGAGACTGAGATCTTCAGACCTGGAGGAGGAGATATGAGGGACAATTGGAGAAGTGAATTATATA
AATATAAAGTAGTAAAAATTGAACCATTA---GGAATAGCACCCACCAAGGCAAGGAGAAGAGTGGTG-----
----------------------------------CAAAGAGAAAAAAGAGTA---GTGGGA---
CTAGGAGCT---------CTGTTCCTTGGGTTCTTGGGAGCA---GCAGGAAGCACTATG---
GGCGCAGCGTCA---ATAACGCTGACGGTACAG---GCCAGAATA---TTACTG---
TCTGGTATAGTGCAACAGCAGAAC---AACCTG---CTGAAG---
GCTATTGAGGCGCAACAGCGTATGTTGCAACTCACAGTCTGGGGCATT
>KM258984
TTCTATTGTAATACAACAAAACTGTTCAAAAATAGTACTTGG-------------------------------
----
-------------------AAT---
GAGACTGAAGGGAATATCACAATCCCATGCAGAATAAAGCAAATTATAAACATGTGGCAGGAAGTAGGAAAAG
CAATGTATGCCCCTCCCATTAGAGGACAAATCAGATGTTCATCAAAT---
ATTACAGGGCTGATATTAACAAGAGATGGTGGG--

ACTAATGGGACTAAT---
GAGACAGAAACCTTCAGACCGGGAGGAGGAAATATGAAGGACAATTGGAGAAGTGAATTATATAAATATAAAG
TAGTAAAAATTGAACCATTA---GGAGTAGCACCCACCAGGGCAAAGAGAAGAGTGGTG--------------
-------------------------CAAAGAGAAAAAAGAGCA---GTGGGA---CTAGGAGCC---------
TTGTTCCTTGGGTTCTTGGGAGCA---GCAGGAAGCACTATG---GGCGCAGCGTCG---
ATAACGCTGACGGTACAG---GCCAGAATA---TTATTG---TCTGGTATAGTGCAACAGCAGAAC---
AACTTG---CTGAAA---GCTATTGAGGCGCAACAGCATATGTTGAAACTCACAGTCTGGGGCATC
>KM259000
TTCTACTGTGATACAACAAATCTGTTTAATAGTACTTGGTTTCGGAATGATACTGGAAAGTCACAGAATAATA
CTATGGAGCCA---TGGACTTGGAATAATGAGACAGGGACATGGGATGATAAAAAGTTA---
TGGACATGGACTAATATT------------------
AATGATACAGAACACATCACACTCCAATGTAGAATAAAACAAATTATAAACAGATGGCAGGAAGTAGGAAAAG
CAATGTATGCCCCTCCCATCAGCGGACTAATTAATTGTTCATCAAAT---
ATTACAGGGCTAATATTAGCAAGAGATGGT---

ATTGACAATAAAACTGAGACCTTCAGACCTATAGGAGGAAATATGAAGGACAATTGGAGAAGTGAATTATATA
AATATAAAGTAGTAAAAATCGAACCATTA---GGAATAGCACCCACCAGGGCAAAGAGAAGAGTGGTG-----
----------------------------------CAGAGAGAAAAAAGATCA---GTGACG---
CTGGGAGCT---------ATGTTCCTTGGGTTCTTGGGAGCA---GCAGGAAGCACTATG---
GGCGCAGCGTCG---CTGACGCTGACGGTACAG---GCCAGACTA---TTATTG---
TCTGGTATAGTGCAACAGCAGAAC---AATCTG---CTGAGG---
GCTATTGAGGCGCAACAGCATATGTTGCAACTCACAGTCTGGGGCATC
>KM259100
TTCTACTGTAATTCAACACAACTGTTTAATAATACTTGGGATGGTACTAAGTGGAATGAT-------------
--ACT---
GCTTGGAATGTTACTGCAGTG---------------
TCAAAAAACGAAAGCATCACACTCCAATGCAAAATAAAACAAATTATAAACATGTGGCAGAAAGTAGGAAAAG
CAATGTATGCCCCTCCCATTAGGGGAACAATTAGATGTTCATCAAAC---
ATTACAGGACTGATATTAACAAGAGATGGTGGTATTAAG----------------------------------
----
AACGAATCCGAGAATAACGGGTCC---------
AAGACCGAGATCTTCAGACCTGGAGGAGGAGATATGAGGGACAATTGGAGAAGTGAATTATATAAATATAAAG
TAGTAAAAATTGAACCATTA---GGAGTAGCACACACCAAGGCAAAGAGAAGAGTGGTG--------------
-------------------------CAGAGAGAAAAAAGAGCA---GTGGGA---ATAGGAGCT---------
GTGTTCCTTGGGTTCTTGGGAGCA---GCAGGAAGCACTATG---GGCGCAGCATCA---
ATGACGCTGACGGTACAG---GCCAGGCTA---TTATTG---TCTGGTATAGTGCAACAGCAGAAC---
AATTTG---CTGAGG---GCTATTGAGGCGCAACAGCATCTGTTGCAACTCACAGTCTGGGGCATC
>DQ410616
TTCTACTGCAGTACAACAAGACTGTTCAATAGTACTTGGTATGGGAATGGTACTGGCACAATCGAGAAT----
----

GGTACTGACACAATCATACTCCCATGCCGAATAAAACAAATTATAAACATGTGGCAGGAAGTAGGAAAAGCAA
TGTATGCCCCTCCCATCAGGGGAAAAATTATGTGTTCATCAAAT---
ATTACAGGGCTGCTATTAACAAGAGATGGCGGGAATAACAATAACGAAACG----------------------
----

AACAACACTGAGATCTTCAGACCTGGAGGAGGAGATATGAGGGACAATTGGGGGGAAAGTGAATTATATAAAT
ATAAAGTAGTAAAAATTGAACCATTA---GGAATAGCACCCACCAAGGCAAAGAGAAGAGTGGTG--------
-------------------------------CAGAGAGAAAAAAGAGCA---GTGGGA---ATAGGAGCT---
------GTGTTCCTTGGGTTCTTGGGAGCA---GCAGGAAGCACTATG---GGCGCAGCGTCA---
GTGACGCTGACGGTACAG---GCCAGACTG---TTATTG---TCTGGTATAGTGCAACAGCAAAAC---
AATTTG---CTGAGG---GCTATTGAGGCGCAACAGCATCTGTTGCAACTCACAGTCTGGGGCATC
>DQ410553
TTCTACTGTAATACATCCCAACTGTTTAATAGTACTTGGAATCCTAATGGTACTTGGGAAGGGACGAAT----
----

GTTACAGAAAATACCACACTCCCATGTAGAATAAAACAAATTATAAACATGTGGCAGGAAGTAGGAAAAGCAA
TGTACGCCCCTCCCATCAGTGGAATAATTAAATGTACATCAAAT---
ATTACAGGGCTACTGTTAACAAGAGATGGTAGTGGTGGTGCTACTAAC-------------------------
----

AACACCACCGAGATCTTCAGACCATTAGGCGGAGATATGAGGGACAATTGGAGAAGTGAATTATATAAATATA
AAGTAGTAAAAATTGAACCATTA---GGAGTAGCACCCACCAGGGCAAAGAGAAGAGTGGTG-----------
----------------------------CAGAGAGAAAAAAGAGCA---GTGGGA---CTAGGAGCT------
---GTGTTCCTTGGGTTCTTGGGAGCA---GCAGGAAGCACTATG---GGCGCAGCGTCA---
GTGGCGCTGACGGTACAG---GCCAGACAA---TTGTTG---TCTGGTATAGTGCAACAGCAGAAC---
AATCTG---CTGAGG---GCTATTGAGGCGCAACAACATCTGTTGCAACTCACAGTCTGGGGCATC
>DQ410585
TTCTACTGCAATACGACATCACTGTTTAATAGTACTTGGGATAATAGTACTTGGGGTAATACTATTGAAGGGT
CAAT---

AACACAAACACAAATATCACACTCCTATGTAGAATAAAACAGATTATAAACATGTGGCAGGGAGTAGGAAAAG
CAATGTATGCCCCTCCCGTCAGGGGACCAATTAAGTGTACATCAATT---
ATTACAGGGCTGTTGTTAACAAGGGATGGTGGTAGTAACAACCCT----------------------------
----

AATGAGACTTTCAGACCTGGAGGAGGAGAGATGAGGGACAATTGGAGAAGTGAATTTAATAAATATAAAGTAG
TAAAATTGAACCCAATGGGA---GGGGTAGCACCCCCCCCGGCAAAGAGGAGAGTGGTG--------------
-------------------------CAGAGAGAGAAAAGAGCA---GTGGGAACGCTGGGAGCT---------
ATGTTCCTTGGGTTCTTGGGAGCA---GCAGGAAGCACTATG---GGCGCAGCGTCA---
GTGACGTTGACGGTACAG---GCCAGACAG---TTATTG---TCTGGTATAGTGCAACAGCAGAAC---
AATCTG---CTGAGG---GCTATTGAGGCGCAACAACATATATTGCAACTCACAGTCTGGGGCATC
>DQ410596
TTCTACTGTAATACAACACAGCTGTTTAACAGTACTTGGCCGAGTAATAGTACTGATAATAGTACTGGTGAAG
GG---

TCAAATAGCACTATCACACTTCCATGCAGAATAAAACAAATTATAAACATGTGGCAACAAGTAGGAAAAGCAA
TATATAACCCTCCCATTCGAGGACGAATTAACTGTTTATCAAAT---
ATTACAGGGTTGCTACCCGTAAGGATGGGGGGGAGTGCCAATAGC----------------------------
----

AGTAGTGAGACCTTCAAACCTGGGGGAGGAGATATGAGGGACAATGGGAGAAGTGAATTATATAAATATAAAG
TAGTAAAAATTGAACCATTAGGG---GTAGCCCCCCCCCCCAAGGCAAAGAGAAGAGTGGTG-----------
----------------------------CAGAGAGAAAAAAGAGCAGGGGGAACAATAGGAGTTTTT------
---ATGTTCCTTGGGTTCTTGGGAGCA---GCAGGAAGCACTATG---GGCGCAGCGTCA---
GTGACGCTGACGGTACAG---GCCAGACAA---TTATTT---TTTGGTATAGTGCAACAGCAGAAC---
AATTTG---CTGAAA---GCCATTGAGGCACAACAACATATGTTGCAACTCACAGTCTGGGGCATC
>KX156472
TTCTACTGTAATACAACAGTACTGTTCAATAGTACTTGGAATGGTACTGAAGGATCAATTGCTGGA-------
----

GAAAATGACACAATCACACTCCCATGCAGAATAAAACAAATTATAAACTTGTGGCAGGAAGTAGGAAAAGCAA
TGTATGCCCCTCCCATCAAAGGACTAATTAGATGTTCATCAAAT---
ATTACAGGACTGTTATTAACAAGAGATGGTGGTGACCCAAATAGC----------------------------
----

ACTACTGAGACATTCAGACCTGGAGGAGGAGATATGAGGGACAATTGGAGAAGTGAATTATATAAATATAAAG
TAGTAAAAATTGAACCATTA---GGAATAGCACCCACCAAGGCAAAGAGAAGAGTGGTG--------------
-------------------------CAGAGAGAAAAAAGAGCA---GTGGGA---ATAGGAGCT---------
GTGTTCCTCGGGTTCTTGGGAGCA---GCAGGAAGCACTATG---GGCGCAGCGGCA---
GTGACGCTGACGGTACAG---GCCAGACAA---TTATTG---TCTGGTATAGTGCAACAGCAGAAC---
AATTTG---CTGAGA---GCTATTGAGGCGCAACAGCATATGTTGCAACTCACAGTCTGGGGCATC
>KX156483
TTCTACTGTAATTCAGCACAACTGTTTAATAGTACTTGGAATGGTACTGAAGGGATAAATACCACT-------
----

GGCAATGAAACTATCATACTCCCATGCAGAATAAAACAAATTATAAATATGTGGCAAGAAGTAGGAAAAGCAA
TGTATGCCCCTCCCATCAGAGGACAAATTAGATGTTCATCAAAT---
ATTACAGGGCTGCTATTAACAAGAGATGGTGGTAATAGCAATAGC----------------------------
----

ACAATCGAGACCTTCAGACCTGGGGGAGGAAATATGAAGGACAATTGGAGAAGTGAATTATATAAATATAAAG
TAGTAAAAATTGAGCCATTA---GGAATAGCACCCACCAAGGCAAAGAGAAGAGTGGTG--------------
-------------------------CAGAGAGAAAAAAGAGCA---ATAGCA------GGAGCG---------
TTGTTCCTTGGGTTCTTGGGAGCA---GCAGGAAGCACTATG---GGCGCAGCATCA---
GTGACGCTGACGGTACAG---GCCAGACAA---TTATTG---TCTGGTATAGTGCAACAGCAGAAC---
AATCTG---CTAAGG---GCTATTGAGGCGCAACAGCATATGTTGCAACTCACAGTCTGGGGCATC
>KX156523
TTCTACTGTAATTCAACACAACTGTTTGATAGCACTTGGAATATTACTTGGGATGGTAATAGTACTTGGAATA
GTACTGAG---

GAAGATAGAAATATCACACTCCCATGCAGAATAAAACAAATCATAAACATGTGGCAGGAAGTAGGAAAAGCGA
TGTATGCCCCTCCCATCAGTGGACAAATTAGATGTTCATCAAAT---
ATTACAGGGCTGCTAATAACAAGAGATGGTGGTAAGAGTAATGACACA-------------------------
----

AACAACGAGACTGAGATCTTTAGACCTGGAGGAGGAAATATGAAGGACAATTGGAGAAGTGAATTATATAAAT
ATAAAGTAGTAAGAATTGAACCATTA---GGAGTAGCACCCACCAAGGCAAAGAGAAGAGTGGTG--------
-------------------------------CAGAGAGAAAAAAGAGCA---GTGGGA---TTAGGAGCT---
------TTGTTCCTTGGGTTCTTGGGAGCA---GCAGGAAGCACTATG---GGCGCAGCATCA---
CTAACGCTGACGGTACAG---GCCAGACAA---TTATTG---TCTGGTATAGTGCAACAGCAGAAC---
AATTTG---CTGAGG---GCTATTGAGGCGCAACAGCATCTGTTGCAACTCACAGTCTGGGGCATC
>KX156374
TTCTACTGTAATACAACGCCATTGTTTAATAGTACTTCAACGCAGCTATTTGGGAATACTACTGAAGGG----
----

TCAAATGCCATTATCAAACTCCAATGCAGAATAAAACAAATTATAAACAGGTGGCAGGAAGTAGGAAAAGCAA
TGTATGCCCCTCCCATCAAAGGACCAATTAGCTGTTTGTCAAAT---
ATTACAGGGCTGCTACTACTAAGGGACGGTGGTACAAAGAATGAGTCCAAT----------------------
----

GGTACAGGGTCCGAAATCTTTAGACCTGGAGGAGGAGATATGAGAGACAATTGGAGAAGTGAATTATATAAAT
ACAAAGTAGTAAAAATTGAGCCATTA---GGAGTAGCACCTACCAAGGCAAAAAGAAGAGTGGTG--------
-------------------------------CAAAGAGAAAAAAGAGCA---GTGGGAACGATAGGAGCT---
------ATGTTCCTTGGATTCTTGGGAGCA---GCAGGAAGCACTATG---GGCGCAGCGTCA---
GTGACGCTGACGGTACAG---GCCAGACAA---TTATTG---TCTGGTATAGTGCAACAGCAGAAC---
AATTTG---CTGAGG---GCTATTGATGCGCAACAGCATATGTTGCAACTCACAGTCTGGGGCATC
>FJ469703
TTCTACTGTAATACAACACCACTGTTTAATAGTACTTGGACTTGGAATAGTACTAGAATGTCTAGTACTGAAA
AT---

ATTACTGAGGGAAATATCACACTCCAATGCAGGATAAAACAAATTGTAAACATGTGGCAGGAAGTAGGAAAAG
CAATGTATGCCCCTCCCATCAAAGGACAGATTAGATGTTCATCAAAT---
ATTACAGGTCTAATATTAACAAGAGATGGTGGTAACATCAGCACT----------------------------
----

AACAACGAGACCTTCAGACCTGCAGGAGGAGATATGAGGGACAATTGGAGAAGTGAATTATATAAATATAAAG
TAGTAAAAATTGAACCATTA---GGGGTAGCACCCACCAAGGCAAAGAGAAGAGTGGTG--------------
-------------------------CAAAGAGAAAAAAGAGCA---ATAGGA---ATAGGAGCT---------
GTGTTCCTTGGGTTCTTGGGAGCA---GCAGGAAGCACTATG---GGCGCAGCGTCA---
GTAACGCTGACGGTACAG---GCCAGGCAA---TTATTG---TCTGGTATAGTGCAACAGCAGAAC---
AATCTG---CTGAGA---GCTATTGAGGCGCAACAGCATCTGTTGCAACTCACAGTCTGGGGCATC
>FJ469706
TTCTACTGTAATACATCAAAACTGTTTAATAGCACCTGGGAATGGAATAATAGTACCAAAGATTGGAATAATA
CTGAAATGTCA--
----------AATAACACT------
GAAGGAAATAGAACTATCACTCTCCAATGCAGAATAAAACAAATTGTAAATATGTGGCAGGAAGTAGGAAAAG
CAATGTATGCCCCTCCCATCAGTGGACAAATTAACTGTTCATCAAAT---
ATTACCGGGTTGCTCTTAACAAGAGATGGTGGTATTAGCAACAGC----------------------------
----

AGCACCACCGAGATCTTCAGACTTGGAGGAGGAGATATGAGGGATAATTGGAGAAGTGAATTATACAGATATA
AAGTAGTAAAAATTGAACCATTA---GGAATAGCACCCACCAAGGCAAGGAGAAGAGTGGTG-----------
----------------------------CAAAGAGAAAAAAGAGCA---GTGGGA---TTAGGAGCT------
---ATGTTCCTTGGGTTCTTGGGAGCA---GCAGGAAGCACTATG---GGCGCAGCATCA---
ATGATGCTGACGGTACAG---GCCAGACAA---TTATTG---TCTGGTATAGTGCAACAGCAGAGC---
AATCTG---CTGCAG---GCTATTGAGGCACAACAGCATCTGTTGAAACTCACAGTCTGGGGCATT
>FJ469718
TTCTACTGTAATTCAACACAACTGTTTAATAGTACCTGGACTAATAGTACTTGGTATAATAGTACTTGGAATA
AAACT--
-------------------AAA---
GAGTCAAATGACACAATCATACTCCCATGCAGAATAAAACAAATTATAAACATGTGGCAGGAAGTAGGAAGAG
CAATGTATGCCAATCCCATCGCAGGACAAATTAGATGTTCATCAAAT---
ATCACAGGGTTGCTACTAACAAGAGATGGTGGTAATAACACAAAT----------------------------
----

GGGACCACCGAGATCTTCAGACCGGGAGGAGGAAATATGAAGGACAATTGGAGAAGTGAGCTATATAAATATA
AGGTAGTAGAAATTAAACCATTA---GGAGTAGCACCCACCAAGGCAAAAAGAAGAGTGGTG----------
---------------------------CAGAGAGAAAAAAGAGCA---GTAACA---CTAGGAGCT------
---TTGTTCCTTGGGTTCTTGAGTGCA---GCAGGAAGCACTATG---GGCGCAGCGTCA---
ATAACGCTGACGGTACAG---GCCAGACAA---ATGTTG---TCTGGTATAGTGCAACAGCAAACC---
AACTTG---CTGAGG---GCTATTGAGGCGCAGCAGCATCTGCTGCAACTCACAGTCTGGGGCATC
>DQ410115
TTCTACTGTAATTCAACACAACTGTTTAATAGTACTTGGAATAGTAGTACTTGGAATAGTACTAGGTCAGATA
ATACT---

ACAGAAAATGGCACAATCACACTCCCATGCAGAATAAAACAAATTATAAACATGTGGCAGAAAGTAGGAAAAG
CAATGTATGCCCCTCCCATCAGAGGACAAATTAGATGTTCATCAAAT---
ATCACAGGGCTGCTATTAACAAGAGATGGTGGTAACGAGAACGAG---------------------------
----

ACTGAGAGCTTCAGACCTGGAGGAGGAGATATGAGGGACAATTGGAGAAGTGAATTATATAAATATAAAGTAG
TAAAAATTGAACCATTA---GGAGTAGCACCCACCAGAGCAAAGAGAAGAGTGGTG----------------
---------------------CAGAGAGAAAAAAGAGCA---GTGGGA---ATAGGAGCT---------
GTGTTCCTTGGGTTCTTGGGAGCA---GCAGGAAGCACTATG---GGCGCAGCGTCA---
ATGACGCTGACGGTACAG---GCCAGACTA---TTATTG---TCTGGTATAGTGCAACAGCAGAAC---
AATTTG---CTGAGG---GCTATTGAAGCGCAACAGCATCTGTTGCAACTCACAGTCTGGGGCATC
>DQ886037
TTCTACTGTAATTCATCACAACTGTTTAATAGCACTTGGCATACGAATAAAACTAAGGGGTTAAATGACACTG
GA--

ATTAATGGCACAATCATACTCCCATGCAGAATAAAACAAATTGCTATCTTGCGGTTGGAAGTAGGTAAAGCAA
TGTATGCCCCCCCCCAATTAAACAACAAAGATGTGAGTCATAT---
ATTACAGGGCTGCTGTTAACAAGAGATGGTGGTAATAACAATAAT---------------------------
----

ACCGAAAACGAGATATTCAGACCTGCAGGAGGAAATATGAAGGACAATTGGAGAAGTGAATTATATAAATACA
AAGTAGTACAAATTGAACCATTA---GGAATAGCACCCACCAAGGCAAAGAGAAGAGTGGTG----------
---------------------------CAGAGAGAAAAAAGAGCA---GTGGGA---CTAGGAGCA------
---CTGTTCCTTGGGTTCTTGGGAGCA---GCAGGAAGCACTATG---GGCGCAGCGTCG---
GTGACGCTGACGGTACAG---GCCAGACAA---TTATTG---TCTGGTATAGTGCAACAGCAGAGC---
AATTTG---CTGAGA---GCTATTGAGGCGCAACAGCATCTGTTGCAACTCACAGTCTGGGGCATC
>FJ653236
TTCTACTGTAGTACAACAAACCTGTTTAATAGTACTTGGAATAGTACTAGGAATGATAGAGGG---------
----

TTCAATGGCACTATCACACTCCCATGCAGAATAAAACAAATTATAAACATGTGGCAGGAAGTAGGAAAAGCGA
TGTATGCCCCTCCCATCAAAGGACAAATTAGATGTTCATCAAAT---
ATTACAGGGCTGCTATTAACAAGAGATGGGGGTACTAATAATAATAATGGT---------------------
----

ACTGAGAACGGGACAGAGATCTTTAGACCTGGAGGAGGAAATATGAAGGACAATTGGAGAAGTGAATTATATA
AATATAAAGTAGTAAAAATTGAACCAGTA---GGAATAGCACCCACCAAGGCAAAGAGAAGAGTGGTG-----
---------------------------------CAGAGAGAAAAAAGAGCA---GTGGGA---
ATAGGAGCT---------GTGTTCCTTGGGTTCTTGGGAGCA---GCAGGAAGCACTATG---
GGCGCAGCGTCA---GTGACGCTGACGGTACAA---GCCAGACAA---TTATTG---
TCTGGTATAGTGCAACAGCAAAGC---AACTTG---CTGAGG---
GCTATTGAGGCGCAACAGCATCTGTTGCAACTCACAGTCTGGGGCATC
>FJ653196
TTCTACTGTAATACAACAAAACTGTTTAACAGTACTTGGAATAGTACTGAAGGGTCAGTGGATGACACAATTG
AAGGG---

ACAGGGAATGACACAATTACACTCCCATGCAGAATAAAACAAATTATAAACATGTGGCAGGAAGTAGGAAAAG
CAATGTATGCCCCTCCCATCGAAGGACCAATTAAATGCACATCAAAT---
ATTACAGGGCTGCTACTAACAAGAGATGGTGGTAATGACACCACT---------------------------

----

AATAATGAGACTTTTAGACCTGGAGGAGGAGATATGAGGGACAATTGGAGAAGTGAACTATATAAATATAAAG
TAGTAAAACTTGAACCATTA---GGAATAGCACCCACCAAGGCAAAGAGAAGAGTGGTG--------------
-------------------------CAGAGAGAAAAAAGAGCA---GTAACA---ATAGGAGCC---------
ATGTTCCTTGGGTTCTTGGGAGCA---GCAGGAAGCACTATG---GGCGCAGCGTCA---
GTGACGCTGACGGTACAG---GCCAGACTA---TTATTG---TCTGGTATAGTGCAGCAGCAGAAC---
AATCTG---CTGAGG---GCTATTGAGGCGCAACAGCATCTGTTGCAACTCACAGTCTGGGGCATC
>FJ653597
TTCTACTGTGATACAACACAGCTGTTTAACAGCACTTGGCAGTCTAATGGTACTAGGAATGATACTGAGGAGT
CA---

GGGTCAAATGACGCTATCACACTCCAATGCAGAATAAAACAAATTATAAACAGGTGGCAGGAAGTAGGAAAAG
CAATGTATGCCCCTCCCATCAGTGGACACATCAACTGCTCATCAAAT---
ATTACAGGGATACTATTAACAAGAGATGGTGGTAACGAGAGCAGT----------------------------
----

AGCACCGAGACCTTCAGACCTGCAGGAGGAGATATGAGGGACAATTGGAGAAATGAATTATATAAATATAAAG
TAGTAAAAATAGAGCCATTA---GGAATAGCACCCACCAAGGCAAAGAGAAGAGTGGTG--------------
-------------------------CAGAGAGAAAAAAGAGCA---GTGACG---CTGGGAGCT---------
ATGTTCCTTGGGTTCTTGGGAGCA---GCAGGAAGCACTATG---GGCGCAGCGTCG---
ATGACGCTGACGGTACAG---GCCAGACAA---TTATTG---TCTGGTATAGTGCAACAGCAGAGT---
AATTTG---CTGAAG---GCTATTGAGGCGCAACAACATCTGTTGCAACTCACAGTCTGGGGCATC
>FJ653474
TTCTACTGTAATACAACACCACTGTTTAATAGTACTTGGAATGGTAATGAAACTTGGAATAGCACTGAAAAGT
CA---

GATAACACCACCATCACACTCCAATGCAGAATAAAACAAATTATAAACATGTGGCAGGAAGTAGGAAAAGCAA
TGTATGCCCCTCCCATCGCGGGACCAATTAGATGTTCATCAAAT---
ATTACAGGACTGCTATTAATAAGAGATGGTGGTAGTAATGGTAATGAG-------------------------
----

ACCAATAAAACTGAGATTTTTAGACCTGGAGGAGGAAATATGAAGGATAATTGGAGAAGTGAGTTATATAAAT
ATAAAGTAGTAAAAATTGAACCATTA---GGAATAGCACCCACCAAGGCAAAGAGAAGAGTGGTG--------
-------------------------------CAGAGAGAAAAAAGAGCA---GTGGGA---CTAGGAGCT---
------TTGTTCCTTGGGTTCTTGGGAGCA---GCAGGAAGCACTATG---GGCGCAGCGTCA---
GTGACGCTGACGGTACAG---GCCAGGCGA---TTATTG---TCTGGTATAGTGCAACAGCAGAAC---
AACTTG---CTGAGG---GCTATTGAGGCGCAACAGCATCTGTTGCAACTCACGGTCTGGGGCATC
>FJ653390
TTCTACTGTAATACAACAAAGCTGTTTAGCAGTACTTGGAGTGGTAATAGTACTTTGAATGATACTGAAAGGT
CAGATACT---
-------------------GAA---
AGGTCAGATATCAATATCACACTCCCATGTAGAATAAGGCAAATTGTAAACAGATGGCAGGAAGTAGGGAAAG
CAATGTATGCCCCTCCCATCAGAGGACAAATTAGCTGTTCATCAAAT---
ATTACAGGGCTGCTATTAACAAGAGATGGTGGTGATATTAGTGACAGT-------------------------
----

AATGGGACCGAGACCTTCAGACCTGGAGGAGGAGATATGAGAGACAATTGGAGAAGTGAATTATATAAATATA
AAGTAATAAAAATTGAACCATTA---GGAGTAGCACCCACCAAGGCAAAGAGAAGAGTGGTG-----------
----------------------------CAGAGAGAAAAAAGAGCA---GTGGGA---TTGGGAGCT------
---GTGTTCATTGGGTTCTTGGGAGCA---GCAGGAAGCACTATG---GGCGCAGCGTCA---
GTGACGCTGACGGTACAG---GCTAGACAA---TTATTG---TCTGGTATAGTGCAACAGCAGAAC---
AATCTG---CTGAGA---GCTATTGAGGCGCAACAACATTTGTTGCAACTCACAGTCTGGGGCATT
>FJ653478
TTCTACTGTAATACAACAGCACTGTTTAATGGCACTTGGTATAATAGTACTTGGAATACTTGGAATACTACTG
CAAAT--
-------------------GGC---
ACCGCAGAGTCAAATATCACACTCCCCTGCAGAATAAAACAAATTATAAACATGTGGCAGGGAGTAGGAAGAG
CAATGTATGCTCCTCCTATCAGAGGACAAATTAAATGCTCATCAAAT---
ATTACAGGGCTACTATTAACAAGAGATGGTGGGGATGGTAATGATACC-------------------------

----

AATAGACCCGAGACCTTCAGACCTGGAGGAGGAGATATGAGGGACAATTGGAGAAGTGAATTATATAAATATA
AAGTAGTAAGCATTGAACCATTA---GGAGTAGCACCCACCAAGGCAAAGAGAAGAGTGGTG-----------
----------------------------CAGAGAGAAAAAAGAGCA---GTGGGA---ATGGGAGCT------
---ATGATCCTTGGGTTCTTGGGAGCA---GCAGGAAGCACTATG---GGCGCAGCGTCA---
ATAACGCTGACGGTACAG---GCCAGACAA---TTACTG---TCTGGTATAGTGCAACAGCAGAAT---
AATTTG---CTGAGG---GCTATTGATGCGCAACAGCATATGTTGCAACTCACCGTCTGGGGCATC
>FJ653547
TTCTACTGTAATACATCAGGACTGTTTAATAACAGGACCGGGAATAGTACTGAAGGA----------------
----

GTAAATACCACCATCACACTCCCATGCAAAATAAAACAAATTGTAAACATGTGGCAGGAAGTAGGAAAGGCAA
TGTATGCCCCTCCCATCAGAGGACAAATTAACTGTACATCAAAT---
ATTACAGGGATACTATTAACAAGAGATGGTGGTAACACAAATATCACC-------------------------
----

ACCACCACCGAGACCTTCAGACTTGGAGGAGGAGATATGAGGGACAATTGGAGAAATGAGTTATATAAATATA
AAGTAGTAAAAATTGAACCATTA---GGAGTAGCACCCACCAGGGCAAAGAGAAGAGTGGTG-----------
----------------------------CAAAGAGAAAAAAGAGCA---GTGGGA---CTAGGAGCT------
---GTGCTCATTGGGTTCTTGGGAGCA---GCAGGAAGCACTATG---GGCGCAGCGTCA---
ATAACGCTGACGGTACAG---GCCAGACAA---TTATTG---TCTGGTATAGTGCAACAGCAGAAC---
AATCTG---CTGAGA---GCTATTGAGGCGCAACAGCATATGTTGCAACTCACAGTCTGGGGCATC
>HQ217476
TTCTACTGTGATACAACACAGCTGTTTAATAGTACTTGGGTAAATGGTAATTGGGTTAATGGTACTAGGCTGT
CGAAT--
-------------------GAA---
ACTGGGAATGATACTATCACACTCCCATGCAAAATAAAACAAATCATAAACAGATGGCAGGAAGTAGGAAAAG
CAATGTATGCCCCTCCCATCAGAGGACAAATTAGATGTTCATCAAAT---
ATTACAGGGCTGCTCTTGACAAGAGATGGTGGTCTACACAACAATAGC-------------------------
----

AATAGCAACGAGACTTTCAGACCTGGAGGAGGAGATATGAGGGACAATTGGAGAAGTGAATTATATAGATATA
AAGTAGTAAAAATTGAACCATTA---GGAATAGCACCCACCAAGGCGAGGAGAAGAGTGGTG-----------
----------------------------CAGAGAGAAAAAAGAGCA---GCGGGA---ATAGCAGCT------
---ATGCTCATTGGGTTCTTGGGAGCA---GCAGGAAGCACTATG---GGCGCAGCGTCA---
GTGACGCTGACGGTACAA---GCCAGACAA---TTATTG---TCTGGTATAGTGCAACAGCAAAAC---
AATTTG---CTGAGA---GCTATTGAGGCGCAACAGCATATGTTGCAACTCACAGTCTGGGGCATT
>HQ217546
TTCTACTGTAATTCCACAGAACTGTTTAGTAGTACTTGGGATGGCACTAACTTGACTAATGATACTGAAAGGT
CA---

AATGACACTGTTATCACACTCCCATGCAGAATAAAACAAATAATAAACATGCGGCAGGAAGTAGGAAAAGCAA
TGTATGCCCCTCCCATCACAGGACAAATTAGATGTTCATCAAAT---
ATTACAGGGCTGCTATTAACAAGAGATGGTGGTAAAGGCACTAACGGAACT----------------------
----

GATAACCAGACTGAGATCTTTAGACCTGCAGGAGGAGATATGAAGGACAATTGGAGAAGTGAATTATACAAAT
ATAAAGTAGTAAAAGTTGAACCATTA---GGAGTAGCACCCACCAAGGCAAGGAGAAGAGTGGTG--------
-------------------------------CAAAGAGAAAAAAGAGCA---GTGGGA---ATAGGAGTG---
------TTACTCCTTGGGTTCTTGGGAGCA---GCAGGAAGCACTATG---GGCGCAGCGGCG---
ATGACGCTGACGGTACAG---ACCAGACAA---TTATTG---TCTGGTATAGTGCAACAGCAGAAC---
AATCTG---CTGAGG---GCTATTGAGGCGCAACAGCATCTGTTGCAACTCACAGTCTGGGGCATC
>HQ217818
TTCTACTGTAATACAACACCACTGTTTACTGGTACTTGGGATGGTAGTACTTGGAATAGCAGTAACAATGAG-
----

GAAAATGAAACACTCACACTCCAATGCAGAATAAAACAAATTATACGCCGGTGGCAGGAAGTAGGACAAGCAA
TGTATGCCCCTCCCATCAGAGGACAGATTAGATGTTCATCAAAT---
ATTACAGGGTTACTAATAACAAGGGATGGTGGTGACGACAACGGG----------------------------

----

ACGACCGAGGTCTTTAGACCTGGAGGAGGGGATATGAGAGACAATTGGAGAAGTGAATTATATAAATATAAAG
TAGTAAAAATTGAACCATTA---GGAGTAGCACCCACCAAGGCAAAAAGAAGAGTGGTG--------------
-------------------------CAGAGAGAAAAAAGAGCA---GTGGGA---TTCGGAGCT---------
ATGTTCCTTGGGTTCTTGGGAGCA---GCAGGAAGCACTATG---GGCGCAGCATCA---
CTAACGCTGACGGTACAG---GCCAGACAA---TTGTTG---TCTGGTATAGTGCAACAGCAAAAC---
AATCTG---CTGATG---GCTATTGAGGCGCAACAACATCTGTTGCAACTCACAGTCTGGGGCATC
>HQ217889
TTCTACTGTAATACAACAGATCTGTTTAAGAGCAATTGGACCTTTAATGAGAATGCTACTTGGCATGATAAAA
GC---

AATGGAACAAATATCATACTCCCATGCAAGATAAAACAAATTATAAACATGTGGCAGGAGGTAGGAAAAGCAA
TGTATGCCCCTCCCATCAGAGGACTAATTAGCTGTTCATCAAAT---
ATTACAGGGCTGCTATTAACAAGAGATGGGGTAACAAGAATGAGACT--------------------------
----

AACGAGACCGAAACCTTCAGACCTATAGGAGGAAATGTGAAGGACAATTGGAGAAGTGAATTATATAAATATA
AAGTAGTAAGAATTGAACCATTA---GGAGTAGCACCCACCAAGGCAAGGAGAAGAGTGGTG-----------
----------------------------CAGAGAGAAAAAAGAGCA---GTGGGAATAGTAGGAGCT------
---ATGTTCCTTGGGTTCTTGGGAGCA---GCAGGAAGCACTATG---GGCGCAGCGTCA---
TTGGCGCTGACGGTACAG---ACCAGACAA---TTATTG---TCTGGTATAGTGCAGCAGCAGAAC---
AATTTG---CTGAGG---GCTATTGAGGCGCAACAGCATCTGTTGCAACTCACAGTCTGGGGCATC
>GU330736
TTCTACTGTAACTCATCACAACTGTTTAATAGTACTTGGAATAGTACTGAGGATAATGAGACTAATAGC----
----

ACTGAAAGAAATATCACAATCCCATGCAGAATAAAACAAATTATAAACATGTGGCAGGAAGTAGGAAGAGCAA
TGTATGCCCCTCCCATCCAAGGGCTAATTAGATGTTCATCAAAT---
ATTACAGGGCTACTCTTAACAAGAGATGGTGGTAATATAACTGAGACT-------------------------
----

AATAATACAGAGATCTTCAGACCTGAAGGAGGAAATATGAAGGACAATTGGAGAAGTGAATTATATAAATATA
AAGTAGTACAAATTGAACCATTA---GGAATAGCACCCACCAAGGCAAAGAGAAGAGTGGTG-----------
----------------------------CAGAGAGAAAAAAGAGCA---GTGGGAATGCTAGGAGCT------
---ATGTTCCTTGGGTTCTTAGGAGCA---GCAGGAAGCACTATG---GGCGCAGCGTCA---
TTGACGCTGACGGTACAG---GCCAGACAA---TTATTG---TCTGGTATAGTGCAGCAGCAAAAC---
AACTTG---CTGAGA---GCTATTGAGGCGCAACAACATCTGTTGCAACTCACAGTCTGGGGCATT
>GU330809
TTCTACTGTAATTCAACACAACTGTTTAATAGTACTTGGTTTAATGGTACTTGGGATAATACTGCAGAGTCAA
ATAGC--
-------------ACTGAA------
GGAAATGGCACAATGATCACACTCCCATGCAGAATAAAACAAATTATAAACATGTGGCAGGCAGTAGGAAAAG
CAATGTATGCCCCTCCCATCAGAGGACAAATTAGATGTTCATCAAAT---
ATTACAGGACTACTGTTAACAAGAGATGGTGGTATCAATACTCCCGAT-------------------------
----

AACAATGAGACTGAGATCTTCAGACCTGCAGGGGGAAATATGAAGGACAATTGGAGAAGTGAATTATATAAAT
ATAAAGTAGTAAAAATTGAACCATTA---GGGATAGCACCTACCAAGGCAAAGAGAAGAGTGGTG--------
-------------------------------CAGAGAGAAAAGAGAGCA---ATAGGA---ATAGGAGCT---
------GTGTTCCTTGGGTTCTTGGGAGCA---GCAGGAAGCACTATG---GGCGCAGCGTCA---
ATAACGCTGACGGTACAG---GCCAGACAA---TTATTG---TCTGGTATAGTGCAGCAGCAGAGC---
AATTTG---CTGAGG---GCTATTGATGCGCAACAACATCTGTTGCAACTCACAGTCTGGGGCATC
>GU330839
TTCTACTGTAATACAACACAACTGTTTAATAGTACTTGGAATATAACACAACCGCTTACTTTGAATAGGACTA
AA---

GGGCCAAATGATACAATTACACTCCCATGCAGAATAAAACAAATTATAAATATGTGGCAGAAAGTAGGAAAAG
CAATGTATGCCCCTCCCATCAAAGGACAAATTAATTGTACATCGAAT---
ATTACAGGACTAATATTAACAAGAGATGGTGGAAATGAGACTAGG----------------------------

----

AACAACGAGACCTTCAGACCTACAGGAGGAGATATGAAGGACAATTGGAGAAGTGAATTATATAAATATAAAG
TAGTAAAAATTGAACCATTA---GGAGTAGCACCCACCAAGGCAAAGAGAAGAGTGGTG--------------
-------------------------CAGAGAGAAAAAAGAGCA---GTGGGA---CTAGGAGCT---------
CTGTTCCTTGGGTTCTTGGGAGCA---GCAGGAAGCACTATG---GGCGCAGCGTCA---
GTGACGTTGACGGTACAG---GCCAGACTA---TTATTG---TCTGGTATAGTGCAGCAGCAGAAC---
AACCTG---CTGAGA---GCTATTGAGGCGCAACAACATCTGTTGCAACTCACAGTCTGGGGCATC
>JF689862
TTCTACTGTAGTACAACACACCTGTTTAATAGTTCTTGGAATGGGACTGCATGGGTAAATGCCACAAATAAC-
----

ACTGGAGAAACTATCACACTCCCATGTAGAATTAAACAAATTATAAATATGTGGCAGGAAGTAGGAAAAGCAA
TGTATGCTCCTCCCATCAGTGGACCAATTAGATGTTTATCAAAC---
ATTACAGGCCTAATATTAACAAGAGATGGTGGTATTAACAATGAC----------------------------
----

ACCGGGGTCGAGGTCTTCAGACCTGGAGGAGGAAATATGAAGGACAATTGGAGAAGTGAATTATATAAATATA
AAGTAGTACAAATTGAACCATTA---GGAGTAGCACCTACCAAGGCAAAGAGAAGAGTGGTG-----------
----------------------------CAGAGAGAAAAAAGAGCA---GTGGGAACGATAGGAGCT------
---ATGTTCCTTGGGTTCTTGGGAGCA---GCAGGAAGCACTATG---GGCGCAGCGTCA---
GTGACGCTGACGGTACAG---GCCAGACTA---TTATTG---TCTGGTATAGTGCAACAGCAGAAC---
AATCTG---CTGAGA---GCTATTGAGGCGCAACAGCATCTGTTGCAACTCACAGTCTGGGGCATC
>JF689863
TTCTATTGTAATACAACACAACTGTTTAATAGTACATGGTATAGTAATAGTACTTGGAATGAGACTGAAGAG-
----

ACAAATGACACTATCATACTCCCATGCAGAATAAAACAAATTATAAACCTGTGGCAGGAAGTAGGAAAAGCAA
TGTATGCCCCTCCCATCAAAGGAGTAATTAACTGTTTATCAAAC---
ATTACAGGGCTGTTATTGACAAGAGATGGTGGTAATGGTAATAGCAGC-------------------------
----

AATGGGACTGAGGTCTTCAGACCTGGAGGAGGAGATATGAGGGACAATTGGAGAAGTGAATTATATAAATATA
AAGTAGTAAAAATTGAACCATTA---GGAATAGCACCCACCAAGGCAAAAAGAAGGGTAGTG-----------
----------------------------CAAAGAGAAAAAAGAGCA---GTGGGAACATTAGGAGCT------
---ATGTTCCTTGGGTTCTTGGGAGCA---GCAGGAAGCACTATG---GGCGCAGCGTCA---
ATGACGCTGACGGTACAG---GCCAGACTA---TTATTG---TCTGGTATAGTGCAGCAGCAGAGC---
AATCTG---CTGAGA---GCTATTGAGGCGCAACAGCGACTGTTGCAACTCACAGTCTGGGGCATC
>JF689866
TTCTACTGTAACACAACAAAACTGTTTAATAGTATTTGGTGGAGGAATGGTACTAATAATTGGAATGATACTA
AG---

AGTGACAATGACACAATCACACTCAAATGCAGAATAAAGCAAATTATAAACATGTGGCAGGAGGTAGGAAAAG
CAATGTATGCCCCTCCCATCAGAGGACAAATTAGATGTTCATCAAAT---
ATTACAGGGCTGATATTAACAAGGGACGGTGGGCGTACCAATGAAACA-------------------------
----

AACAATACTGAGATCTTTAGACCTGCAGGAGGAAATATGAAGGACAATTGGAGAAGTGAATTATATAAATATA
AAGTAGTACAAATTGAACCATTA---GGAATAGCACCCACCAAGGCAAAGAGAAGAGTGGTG-----------
----------------------------CAGAGAGAAAAAAGAGCA---GTGGGA---ATAGGAGCT------
---CTGTTCCTTGGGTTCTTGGGAGCA---GCAGGAAGCACTATG---GGCGTAGCGTCA---
ATAACGCTAACGGTACAG---GCCAGACAA---TTATTG---TCTGGTATAGTGCAGCAGCAGAAC---
AATCTG---CTGAGG---GCTATTGAGGCGCAACAACATATGTTGCAACTCACAGTCTGGGGCATA
>JF689867
TTCTACTGTAACACAACAGGACTGTTTAACTCAACATTTAACTCAACAAAACTGCCTGATAGTAATAAT----
----

ACTGATTCAAATTTCACGATCCCATGCAGAATAAAACAAATTATAAACATGTGGCAGAAAGTAGGAAAAGCAA
TGTATGCCCCTCCCATCAGAGGACCAATTAGATGTTCATCAAAC---
ATTACAGGGCTGATATTAACAAGAGATGGTGGTGGTAATGAGAGC----------------------------

----

GGTAGAGAGACTTTCAGACCTGGAGGAGGAAATATGAAGGACAATTGGAGAAGTGAATTATATAAATATAAAG
TAGTAAAAATTGAACCATTA---GGAGTAGCACCCAACAAGGCAAAGAGAAGAGTGGTG--------------
-------------------------CAGAGAGAAAAAAGAGCA---GTGGGA---ATAGGAGCT---------
CTGTTCCTTGGGTTCTTGGGAGCA---GCAGGAAGCACTATG---GGCGCAGCGTCC---
GTGACGCTGACGGTACAG---GCCAGAAAA---TTACTG---TCTGGTATAGTGCAACAGCAGAAC---
AATTTG---CTGAAG---GCTATTGAGGCGCAACAGCATCTGTTGCAACTCACCGTCTGGGGCATC
>JF689870
TTCTATTGTAATTCAACACAACTGTTTAATAGTACTTGGAATGATACTTGGTCAAATAGCACTAGA-------
----

GGAAATGATACAATCACACTCCCATGCAGAATAAAACAAATTATAAACATGTGGCAGAAAGTAGGAAAAGCAA
TGTATGCCCCTCCCATCAGAGGACAAATTAGATGCTCATCAAAT---
ATTACAGGGCTGCTATTAACAAGAGATGGTGGCAATACAACCGGG----------------------------
----

AGCAGGTCAGAGACCTTTAGACCTGGAGGAGGAGACATGAGGGACAATTGGAGAAGTGAATTATATAAATATA
AAGTAGTAAAAATTGAACCATTA---GGAGTAGCACCTACCAGGGCAAAGAGAAGAGTGGTG-----------
----------------------------CAGAGAGAAAAAAGAGCA---GTGGGA---ATAGGAGCT------
---ATGTTCCTTGGGTTATTGGGAGCA---GCAGGAAGCACTATG---GGCGCAGCGTCA---
ATGACGCTGACGGTACAG---GCCAGACTA---TTATTG---TCTGGTATAGTGCAACAGCAGAAC---
AACTTG---CTGAAG---GCTATTGAGGCGCAACAGCATCTGTTGCAACTCACAGTCTGGGGCATC
>JF689871
TTCTATTGTAATTCATCCCAATTGTTTAATAGTACTTGGAATAGTACTGAAGGGTCAAATAATGGTACTGAAG
TAACA--
-------------------AAT---
AATGGTACTGAAGTAATTACACTCCCATGCAGAATAAAACAAATTATAAACATGTGGCAGGAAGTAGGAAAAG
CAATGTATGCCCCTCCCATCAGAGGACAAATTAACTGCTCATCAAAT---
ATTACAGGGCTGTTATTAACAAGAGATGGTGGTCGTAACAGCACTGAG-------------------------
----

GACGATACTGAGACCTTCAGGCCTGGAGGAGGAGACATGAGGGACAATTGGAGAAGTGAATTATATAAATATA
AAGTAGTAAAAATTGAACCATTA---GGAGTAGCACCCACCAGGGCAAAGAGAAGAGTGGTG-----------
----------------------------CAGAGAGAAAGAAGAGCA---GTGGGAACAATAGGAGCT------
---ATGTTCCTTGGGTTCTTGGGAGCA---GCAGGAAGCACTATG---GGCGCAGCGTCA---
GTGACGCTGACGGTACAG---GCCAGACAA---TTATTA---TCTGGTATAGTGCAACAGCAGAAC---
AATTTG---CTGAGA---GCTATTGAGGCGCAACAGCATCTGTTGCAACTCACAGTCTGGGGCATC
>JF689872
TTCTACTGTAATTCAACACAACTGTTTAATAGTACTTGGACTTGGAATGTTACTGGAGGAAATGCCACACTCA
CATCATCAAAC--
-------------AACACT------
GAAGGGAATGACACAATCAGACTCCCATGCAGAATAAAACAAATTATAAACATGTGGCAGGAAGTAGGAAAAG
CAATGTATGCCCCTCCCATCAGAGGGCAAATTAACTGTTCATCAAAT---
ATTACAGGGCTACTATTAATAAGAGATGGTGGTAACAAGAGAGAGAGCGGGAAC------------AAG----
----
AACGAGAGCGGGAAC---
AAGACTGAGATTTTCAGACCTGGAGGAGGAGATATGAGGGACAATTGGAGAAGTGAATTATATAAATATAAAG
TAGTAAAAATTGAACCATTA---GGAGTAGCACCCACCAAGGCAAAAAGAAGAGTGGTG--------------
-------------------------CAGAGAGAAAAAAGAGCA---GTGGGA---TTAGGAGCT---------
ATGTTCCTTGGGTTCTTGGGAGCA---GCAGGAAGCACTATG---GGCGCAGCGTCA---
GTGACGCTGACGGTACAG---GCCAGACAA---TTATTG---TCTGGTATAGTGCAACAGCAGAAC---
AATCTG---CTGAGG---GCTATTGAGGCGCAACAGCATCTGTTGCAACTCACAGTCTGGGGCATC
>JF689873
TTCTACTGTAATTCAACACAACTGTTTAATAGTACTTGGAATGTTACTAGTCCTTGGAATGTTACTGAAGGGT
CAAAT--

AACACTGAAAGAACTATCACACTCCCATGCAGAATAAAACAAATTATAAACATGTGGCAGAAAGTAGGAAAAG
CAATGTATGCCCCTCCCATCAAAGGACAAATTAGATGTTCATCAAAT---
ATTACAGGGCTGCTATTAACAAGAGATGGTGGTAATAGCGAGAAC----------------------------

----

GAGACCACCGAGATCTTCAGACCTGCAGGAGGAGATATGAGGGACAATTGGAGAAGTGAATTATATAAATATA
AAGTAGTAAAAATTGAGCCATTA---GGAGTAGCACCCACCAAGGCAAAGAGAAGAGTGGTG-----------
----------------------------CAGAGAGAAAAAAGAGCA---GTGGGA---ATAGGAGCT------
---TTGTTCCTTGGGTTCTTGGGAGCA---GCAGGAAGCACTATG---GGCGCAGCGGCA---
GTGACGCTGACGGTACAG---GCCAGACTA---TTATTG---TCTGGTATAGTGCAACAGCAGAAC---
AATTTG---CTGAGG---GCTATTGAGGCGCAACAGCATCTGTTGCAACTCACAGTCTGGGGCATC
>JF689876
TTCTACTGCAATACATCACAACTGTTTAATAGTACTTGGAATGGTACTAAGTCAAATAACAATGGA-------
----

ATAAATGACACAATCATACTCCCATGTAGAATAAGACAAATTATAAACATGTGGCAGAAAGTAGGAAAAGCAA
TGTATGCCCCTCCCATCAGAGGAAAAATTGAGTGTAAGTCAAAT---
ATTACAGGGCTGCTATTAACAAGAGATGGTGGTACAAACGAGAACAATACC----------------------
----

AATACCAACACCGAGACCTTCAGGCCTGGAGGAGGGAATATGAAGGACAATTGGAGAAGTGAATTATATAAAT
ATAAAGTAGTAAAAATTGAACCATTA---GGAGTAGCACCCACCAGGGCAAAGAGAAGAGTGGTG--------
-------------------------------CAGAGAGAGAAAAGAGCA---GTGGGA---CTAGGGGCT---
------GTGTTCCTTGGGTTCTTGGGAGCA---GCAGGAAGCACTATG---GGCGCAGCGTCA---
ATAACGCTGACGGTACAG---GCCAGACTG---TTATTG---TCTGGTATAGTGCAACAGCAGAAC---
AATTTG---CTGAGG---GCTATTGAGGCGCAACAGCATCTGTTGCAACTCACAGTCTGGGGCATT
>HQ216908
TTCTACTGTAATTCAACACAACTGTTTAATAGTACTTGGGGGAATGATAATAGTACTTGGGAAAATGATACT-
----

GAAGCAACTAACATCACTCTCCCATGCAGAATAAAACAAATTGTAAACATGTGGCAGCAAGTAGGAAAAGCAA
TGTATGCCCCTCCCATCAGAGGACAAATTAGATGTTCATCAAAT---
ATTACAGGGCTGATATTAACAAGAGATGGTGGTAACAACACGAAT----------------------------
----

GGGTCCGAGACCTTCAGACCTGGAGGAGGAGATATGAGGGATAATTGGAGAAGTGAATTATATAAATATAAAG
TAGTAAAGATTGAGCCATTA---GGAGTAGCACCCACCAAGGCAAAGAGAAGAGTGGTG--------------
-------------------------CAGAGAGAAAAAAGAGCA---GTGGGA---ATGGGAGCT---------
GTGTTCCTTGGGTTCTTGGGAGCA---GCAGGGAGCACTATG---GGCGCAGCGTCA---
ATGACGCTGACGGTACAG---GCCAGACTA---TTATTG---TCTGGTATAGTGCAACAGCAAAAC---
AATTTG---CTGAGG---GCTATTGAGGCGCAACAGCATCTGTTGCAACTCACAGTCTGGGGCATC
>HQ216925
TTTTACTGTAATACAACAAAACTGTTTAATAGTACAGAACTATTTAATAGTACTAAAGTATCAAATACA----
----

ACTGGAGACACTATCACACTCCAATGCAGAATAAAACAAATTATAAACATGTGGCAGAAAGTAGGAAAAGCAA
TGTATGCCCCTCCCATCAAAGGGAGAATTAGTTGTTCATCAAAC---
ATTACAGGACTGCTACTAACAAGAGATGGTGGTAATAACACTGGTAAC-------------------------
----

AATGACAATGAGACCTTCAGACCTATAGGAGGAAATATGAAGGACAATTGGAGAAGTGAATTATATAAATATA
AAGTAGTGAAAATTGAACCATTA---GGAATAGCACCCACCAAGGCAAAGAGAAGAGTGGTG-----------
----------------------------CAGAGAGAAAAAAGAGCA---GTGGGA---TTTGGAGCG------
---GTGATCTTTGGGTTCTTGGGAGCA---GCAGGAAGCACTATG---GGCGCAGCGGCG---
GTAACGCTGACGGCACAG---GCCAGACTG---TTATTG---TCTGGTATAGTGCAACAACAGAAC---
AATCTG---TTGAGG---GCTATTGAGGCGCAACAGCATCTGTTGCAACTCACAGTCTGGGGCATC
>FJ152546
TTCTACTGTGATACAACAAAACTATTTAATAGTACTTGGAATAATAGTACTGAACTGGTAAATGGCACA----
----

GTTGACACAAATATCACACTCCCATGTAGAATAAAACAAATTATAAACATGTGGCAGGAAGTAGGAAAAGCAA
TGTATGCCCCTCCCATCAGAGGAAACATTAACTGTACATCAAAT---
ATTACAGGGCTGATATTAACTAGAGATGGTGGTAATCCAGATAACACCACA----------------------

----

GATAACACCACTGAGACCTTCAGACCTGGAGGAGGAGACATGAGGGACAATTGGAGAAGTGAATTATATAAAT
ATAAAGTAGTACAAATTGAGCCATTA---GGAATAGCACCCACCAAGGCAAAGAGAAGAGTGGTG--------
-------------------------------CAAAGAGAAAAAAGAGCA---GTGGGAACGCTGGGAGCT---
------ATGTTCCTTGGGTTCTTGGGAGCA---GCAGGAAGCACTATG---GGCGCAGCGTCA---
CTAACGCTGACGGTACAA---GCCAGACTA---TTGTTG---TCTGGTATAGTGCAACAGCAGAAC---
AATCTG---CTGAGA---GCTATTGAGGCGCAACAGCATCTGTTGCAACTCACAGTCTGGGGCATC
>FJ152547
TTCTACTGTGATACAACAAAACTGTTTAATAATTCTTGGAATGGTACATGGTCAAATAACAGTACA-------
----

GCAAATATCACAATCACACTCCCATGCAGAATAAGACAGATTGTAAACATGTGGCAGGAAGTAGGAAAAGCAA
TGTATGCCCCTCCTATCAGAGGAATAATTAACTGTACATCAAAT---
ATTACAGGGCTGCTATTAACAAGAGATGGTGGTAGTACCACGAGTAATAAT----------------------
----

ACCATAAACAAAACTGAAACCTTCAGACCTGGAGGAGGAGATATGAGAGACAATTGGAGAAGTGAATTATATA
AATATAAAGTAGTACAAATTAAACCATTA---GGAATAGCACCCACCCAGGCAAAGAGAAGAGTGGTG-----
----------------------------------CAAAGAGAA---AAAAGG---GGA------
ATAGGAGCT---------CTTTTCCTTGGGTTCTTGGGAGCA---GCAGGAAGCACTATG---
GGCGCAGCATCA---GTAACGCTGACGGTACAG---GCCAGACTA---TTATTG---
TCTGGTATAGTGCAACAGCAGAAC---AATCTG---CTGAGA---
GCTATTGAGGCGCAACAGCATCTGTTGCAACTCACAGTCTGGGGCATC
>JX863987
TTCTACTGTAATTCAACACAACTGTTCAATAGTTCTTGGAAGTGGAATAATGGTACTTGGAGTAGCAATGATT
CTAAAGGG---
-------------TTAAAT------
GGCACTGAGGACATAATCATACTCCCATGCAGAATAAAACAAATTATAAACATGTGGCAGAGAGTGGGAAAAG
CAATGTATGCCCCTCCCATCAGAGGAATAATTAACTGTACATCAAAC---
ATTACAGGGATACTGCTAACAAGAGATGGTGGTAGTAACACAAAC----------------------------
----

GATAATGAGACCTTCAGACCTGGAGGAGGAGATATGAGGGACAATTGGAGAAGTGAATTATATAAATATAAAG
TAGTAAAAATTGAACCATTA---GGAATAGCACCTACCAGGGCAAAGAGAAGAGTGGTG--------------
-------------------------CAGAGAGAAAAAAGAGCA---GTGGGA---ATAGGAGCT---------
ATGTTCCTTGGGTTCTTGGGAGCA---GCAGGGAGCACTATG---GGCGCAGCGTCA---
CTGACGCTGACGGTACAG---GCCAGACAA---TTATTG---TCTGGTATAGTGCAACAGCAGAAC---
AACCTG---CTGAGG---GCTATTGAGGCGCAACAGCATATGTTGCAACTCACAGTCTGGGGCATC
>JX863988
TTCTACTGTAGTACAACACAATTATTTAATGGTACTTGGAATGGTACTTGGGTTAATAATACTTGGAATGATA
CTGGAAAC---

AGCAATATAACTATCTCACTCCCATGCAAAATAAAACAAATTATAAACATGTGGCAGGGAGTAGGGAAGGCAA
TATATGCCCCTCCAATCAGGGGAGTAATTAAATGTACATCAAAT---
ATCACAGGGCTGATATTAACAAGAGATGGTGGTATTAATAATAGCGCC-------------------------
----

GAGGGCGAGACCTTCAGACCCCAAGGAGGAAATATGAAGGACAATTGGAGAAGTGAATTATATAAATATAAAG
TAGTAAAAATTGAACCATTA---GGAGTAGCACCCACCAAGGCAAAGAGAAGAGTGGTG--------------
-------------------------CAGAGAGAAAAAAGAGCA---GTGGGA---ATGGGAGCT---------
TTGTTCCTTGGGTTCTTGGGAGCA---GCAGGAAGCACTATG---GGCGCAGCGTCA---
ATAACGCTGACGGTACAA---GCCAGACAA---TTGTTG---TCTGGTATAGTGCAACAGCAAAAC---
AATTTG---CTGAGA---GCTATTGAGGCGCAGCAGCATCTGTTGCAACTCACAGTCTGGGGCATC
>JX863991
TTCTACTGTAATACAACAGAATTGTTTAATGGTACTTTGGAGGCTAATGATACTTGGAAGAATGGGACTATAA
AGCCA--

CAGAACGCCAACATCACACTTCCATGCAGAATAAAACAAATTATAAACCTGTGGCAAGAAGTAGGAAAAGCAA
TGTATGCCCCGCCCATCAGTGGACAAATTAGATGTTCATCAAAT---

ATTACAGAGCTACTATTAACAAGAGATGGTGGTAATGGGAACGAGACC-------------------------
----

GACAATGAGACCGAGATCTTCAGACCTGGAGGAGGAGATATGAGGGAAAATTGGAGAAGTGAATTATATAAAT
ATAAAGTAGTAAGAGTTGAACCATTA---GGATTAGCACCCACCAAGGCAAAGAGGAGAGTGGTG--------
-------------------------------CAGAGAGAAAAAAGAGCA---GTGGGA---CTAGGAGCT---
------GTGTTCCTGGGGTTCTTGGGAGCA---GCAGGAAGCACTATG---GGCGCAGCGTCG---
TTGACGCTGACGGTACAG---GCCAGAATG---TTATTG---TCTGGTATAGTGCAACAGCAGAAC---
AATCTG---CTGAGA---GCTATTGAGGCGCAACAGCGTATGTTGCAACTCACAGTCTGGGGCATC
>JX863992
TTCTACTGTAATTCAACACAACTGTTTAATAGTAATAGCACTAAAGTAAATGAC-------------------
----

ACTAGAGATAATATCACACTCCCATGCAGAATAAAACAAATTGTAAACATGTGGCAGGAAGTAGGAAAAGCAA
TGTATGCCCCTCCCATCAGAGGACAAATTAAATGCTCATCAAAT---
ATTACAGGGCTGATATTAACAAGAGATGGTGGTAATGCGACAGAC----------------------------
----

GGGAACGAGACTTTTAGACCTGGAGGAGGAAATATGAAGGACAATTGGAGAAGTGAATTATATAAATATAAAG
TAGTAAAAATTGAACCAATA---GGAATAGCACCCACCAAGGCAAAGAGAAGAGTGGTG--------------
-------------------------CAAAGAGAAAGAAGAGCA---GTGGGA---ATAGGAGCT---------
GTGTTCCTTGGGTTCTTGGGAGCA---GCAGGAAGCACTATG---GGCGCAGCGTCA---
CTAACGCTGACGGTACAG---GCCAGACTA---TTATTG---TCTGGTATAGTGCAACAGCAGAGC---
AATCTG---CTGAGG---GCTATTGAGGCGCAACAACATCTGTTGCAACTCACAGTCTGGGGCATC
>JN562781
TTCTACTGTAATACATCAAAACTGTTTAATAGTACTTGGAATAGTACTAGTACTTGGAATAATACTGAA----
----

GAAAATAGCACACTCACACTCCCATGTAAAATAAGACAAATTATAAACATGTGGCAGAAAGTAGGAAAAGCAA
TGTATGCCCCTCCCATCAGTGGACGAATTAACTGCTTATCAAAT---
ATTACTGGGCTGCTATTAATAAGAGATGGTGGTGGTGGTGGTAAT----------------------------
----

GACACGACCGAGACCTTCAGACCTGGAGGAGGAGATATGAGGGACAATTGGAGAAGTGAGTTATATAAATACA
AAGTAATCAAAATTGAACCATTA---GGAATAGCACCCACCAAGGCAAAGAGAAGAGTGGTG-----------
----------------------------CAGAGAGAAAAAAGAGCA---GTGGGA---TTAGGAGCT------
---GTGTTCCTTGGGTTCTTGGGAGCA---GCAGGAAGCACTATG---GGCGCAGCGTCA---
CTAACGCTGACGGTACAG---GCCAGACAA---TTATTG---TCTGGTATAGTGCAACAGCAAAGC---
AATTTG---CTGAGA---GCTATTGAGGCGCAACAGCATCTGTTGCAACTCACAGTCTGGGGCATA
>JF320615
TTCTATTGTAATACAGCACAGCTGTTTAATAGCACTTGGCCTAAGAATGATACTAACAGTACAGATACTGAAG
AA---

AATGGAAATGGCACAATCACACTCCCATGTAGAATAAAACAAATTATAAATATGTGGCAGGAAGTAGGAAAAG
CAATATATGCCCCTCCCATCAGAGGACAAATTAGCTGTACATCAAAT---
ATTACAGGGCTGCTATTAACAAGAGATGGTGGTAATGAGAGCAGT----------------------------
----

GCTGACGAGATCTTCAGACCTGGAGGAGGAGATATGAGGGACAATTGGAGAAGTGAATTATATAAATATAAAG
TAGTAAAAATTGAACCATTA---GGAGTAGCACCCACTAAGGCAAAGAGAAGAGTGGTG--------------
-------------------------CAGAGAGAAAAAAGAGCA---GTGGGA---ATAGGAGCT---------
GTGTTCCTTGGGTTCTTGGGAGCA---GCAGGAAGCACTATG---GGCGCAGCGTCA---
ATAACGCTGACGGTACAG---GCCAGACAA---TTATTG---TCTGGTATAGTGCAGCAGCAAAAT---
AATTTG---CTAAGG---GCTATTGATGCACAGCAGAGTCTGTTGCAACTCACAGTCTGGGGCATC
>JF320613
TTCTACTGTGATACAACAAAACTGTTTAACAGTACTTGGCAGGATAACAGTACTGTGAATGCTAGTAGT----
----

GATGAGGATCCCATCATACTCCCCTGTAAAATAAGACAAATTATAAACATGTGGCAGGAAGTAGGAAAAGCAA
TGTATACCCCTCCCATCAGAGGACTAATTAAATGTTCATCAAAT---

ATTACAGGGCTACTATTAACAAGAGATGGGGGGGAAGATAACGAC----------------------------
----

ACTGAGATATTCAGACCTGGAGGAGGAAATATGAGGGACAATTGGAGAAGTGAATTATATAAATATAAAGTAG
TAAGAATTGAACCATTA---GGAGTAGCACCCACCAGGGCAAAGAGAAGAGTGGTG-----------------
----------------------CAGAGAGAAAAAAGAGCA---GTGGGG---CTGGGAGCG---------
TTGTTCCTTGGGTTCTTGGGAGCA---GCAGGAAGCACTATG---GGCGCAGCGTCA---
GTGACGCTGACGGTACAG---GCCAGACTA---TTATTG---TCTGGTATAGTGCAACAGCAGAAC---
AACTTG---CTGAGA---GCTATTGAGGCGCAACAGCATCTGTTGCAACTCACAGTCTGGGGCATT
>JF320048
TTCTACTGTAATACAACACAACTGTTTAACAGTACTTGGAATAGTACTGGAGAGTCTTGGAATAGGACTGGAG
GGTCAAAT---
-------------AACACG------
GGAAGAAATGGCACAATCACACTCCAATGCAGAATAAAACAAATTATAAACATGTGGCAGAAAGTAGGAAAAG
CAATATATGCCCCTCCCATCAAAGGACTAATTAGATGTTCATCAAAC---
ATTACAGGGCTGATATTAACAAGAGATGGTGGTAATAATAATAGC----------------------------
----

ACCATCTCCGAGATCTTCAGACCTGGAGGAGGAGATATAAGGGACAATTGGAGAAGTGAATTATATAAATACA
AAGTAGTAAAAATTGTACCAATA---GGGATAGCACCCACTAAGGCAAAGAGAAGAGTGGTG-----------
----------------------------CAGAGAGAAAAAAGGGCA---GTGGGA---ATAGGAGCT------
---ATGTTCCTTGGGTTCTTGGGAGCA---GCAGGAAGCACTATG---GGCGCAGCGTCA---
GTGACGCTGACGGTACAG---GCCAGACAA---TTATTG---TCTGGTATAGTGCAACAGCAAAAC---
AACTTG---CTGAGG---GCTGTGGAGGCGCAACAGCATCTGTTGCAACTCACAGTCTGGGGCATC
>JF320308
TTCTACTGTAATACCACAGGACTGTTTAATAGTACTTGGAACAATGAAACTAAAGAGTCAAAT----------
----

AACACTGAAACTATCATACTCCCATGCAGAATAAAACAAATTATAAACATGTGGCAGAAAGTAGGAAAAGCAA
TGTATGCCCCTCCCATCAGTGGACTAATTAGCTGTTCATCAAAT---
ATTACAGGGCTACTATTGACAAGAGATGGTGGTAATAACAATGGTAGTGAT----------------------
----

AACAACAGAAGTGAGATCTTCAGACCTGGAGGAGGAGATATGAGGGACAATTGGAGAAGTGAATTATATAAAT
ATAAAGTAGTAAAAATTGAACCATTA---GGAATAGCACCCACCAAGGCAAAGAGAAGAGTGGTG--------
-------------------------------CAGAGAGAAAAAAGAGCA---GTGGGA---TTAGGAGCT---
------TTGTTCCTTGGGTTCTTGTCAGCA---GCAGGAAGCACTATG---GGCGCAGCGTCA---
ATGACGCTGACGGTACAG---GCCAGACAA---TTATTG---TCTGGTATAGTGCAACAGCAGAAC---
AATTTG---CTGAGA---GCTATTGAGGCGCAACAGCATCTGTTGCAACTCACAGTCTGGGGCATC
>JF320003
TTCTACTGTGATACAACACAACTGTTTAATAGTACTTGGAATACTAGTAGTGCTTGGAATAGTACTGAAGGG-
----

TCAAATGACACTATCACACTCCCATGCAGAATAAAACAAATTATAAACATGTGGCAGGAAGTAGGAAAAGCAA
TGTATGCCCCTCCCATCAGAGGAGAAATTAAATGTTCATCAAAT---
ATTACAGGGCTGCTATTAACAAGGGATGGTGGCAGTAGTAACGAGACT-------------------------
----

AACGGGACTGAGATATTCAGGCCTGGAGGAGGAGATATGAGGGACAATTGGAGAAGTGAGTTATATAAATATA
AAGTAGTAAAAATTGAACCATTA---GGAGTAGCACCCACCAAGGCAAAGAGAAGAGTGGTG-----------
----------------------------CAAAGAGAAAAAAGAGCA---GTGGGA---CTAGGAGCT------
---TTGTTCCTTGGGTTCTTGGGAACA---GCAGGAAGCACTATG---GGCGCAGCGTCA---
CTGACGCTGACGGTACAG---GCCAGACAA---TTATTG---TCTGGTATAGTGCAACAGCAGAGC---
AACTTG---CTGAGA---GCTATTGAGGCGCAACAGCATATGTTGAAACTCACAGTCTGGGGCATC
>JF320097
TTCTACTGTAACTCAACACCACTGTTTAATAGTACTTGGAATGCTAATGGTACTTGGAATGGTACCGGAAATT
TA---

ACTACTGGAAACATCACACTCACATGTAGAATAAAACAAATTATAAACATGTGGCAGGAAGTAGGAAAAGCAA
TGTACGCCCCTCCCATAAGAGGACTAATTAGTTGTTCATCAAAT---

ATTACAGGACTGTTATTAACAAGAGATGGTGGTGAAGGTAATGACACC-------------------------
----

AACAATATCACCGAGGTCTTTAGACCTGGAGGAGGAGATATGAGGGACAATTGGAGAAGTGAATTATATAAAT
ATAAAGTAGTAAAAATTGAGCCATTA---GGAGTAGCACCCACCAAGGCAAAGAGAAGAGTGGTG--------
-------------------------------CAAAGAGAAAAAAGAGCA---GTGGGA---ATAGGAGCT---
------CTGTTCCTTGGGTTCTTGGGAGCA---GCAGGAAGCACTATG---GGCGCAGCGTCG---
ATGACGCTGACGGTACAA---GCCAGACAA---CTATTG---TCTGGTATAGTGCAACAGCAGAAC---
AATCTG---CTGAGA---GCTATTGAAGCGCAACAACATCTGTTGCAACTCACAGTCTGGGGCATC
>JF320038
TTCTACTGTAATACAACACAACTGTTTAATAGCACTTGGAATAATGGTACTAATAATGCTACTACAGACTCA-
----

AATGCCACTGTCATTACACTCCCATGTAGAATAAAACAAATTGTAAACATGTGGCAAGAAGTAGGAAAAGCAA
TGTATGCCCCTCCCATCAAAGGAAAAATCAGATGTTCATCAAAT---
ATTACAGGGCTGCTATTAACAAGAGATGGTGGCAGCGATGGGAGCTCC-------------------------
----

TCGAATGAGACTGAGACCTTCAGACCTGGAGGAGGAAATATGAGGGACAATTGGAGGAGTGAATTATATAAAT
ATAAAGTAGTAAGAGTTGAACCATTA---GGAGTAGCACCCACCAAGGCAAAGAGAAGAGTGGTG--------
-------------------------------CAGAGAGAAAAAAGAGCA---GTGGGA---ATAGGAGCT---
------GTGTTCCTTGGGTTCTTGGGAGCA---GCAGGAAGCACTATG---GGCGCAGCGTCA---
ATAACGCTGACGGTACAG---GCTAGACAA---TTATTG---TCTGGTATAGTGCAACAACAGAAC---
AATCTG---TTGAGG---GCTATTGAGGCGCAACAGCATCTGTTGCAACTCACAGTCTGGGGCATC
>JF320526
TTCTACTGTGATACAACAGAACTGTTTAATAGTACTTGGAATAATACAAGGTCAAATGACACTGCA-------
----

AGCAATAAGACTATCATACTCCAATGTAGAATAAGACAAATTATAAACAGATGGCAGGAAGTAGGAAAAGCAA
TGTATGCCCCTCCCATCAGCGGACAAATTAACTGTTCATCAAAT---
ATTACAGGGCTGATATTAACAAGAGATGGTGGAAACAATAGTCCAGGA-------------------------
----

AACGGGAGTGAGATCTTCAGACCTGGAGGGGGAGATATGAGGGACAATTGGAGAAGTGAATTATATAAATATA
AAGTAGTAAAAGTTGAGCCATTA---GGAGTAGCACCCACCAAGGCAAAGAGAAGAGTGGTG-----------
----------------------------CAGAGAGAAAAAAGAGCA---GTAGGAACAATGGGAGCT------
---TTGTTCCTTGGGTTCTTGGGAACA---GCAGGAAGCACTATG---GGCGCAGCGGCA---
ATGACGCTGACGGTACAG---GCCAGGCTA---TTATTG---TCTGGTATAGTGCAACAGCAGAAC---
AATTTG---CTGAGG---GCTATTGAGGCGCAACAGCATCTGTTGCAACTCACAGTCTGGGGCATC
>JF320160
TTCTACTGTAATACATCACAACTGTTTAATAGTACTTGGCCTTGGAATGCTACTGAAGGAGTAAATAGC----
----

ACTAATGATAATATCACACTCCCATGCAGAATAAAACAAATTGTAAACATGTGGCAGGAAGTAGGAAAAGCAA
TGTATGCCCCTCCTATCAGCGGAATAATTAGGTGTTCATCAAAT---
ATTACAGGAATGATATTAACAAGAGATGGGGGAGCGGGGAACGGGAGC-------------------------
----

AATAACGAGACTGAGATCTTCAGACCTGGAGGAGGAAATATGAAGGACAATTGGAGAAGTGAATTATATAAAT
ATAAAGTAGTAAAAATTGAACCATTA---GGAATAGCACCCACCAGGGCAAAGAGAAGAGTGGTG--------
-------------------------------CAGAGAGAAAAAAGAGCA---ATAACA---CTAGGAGCT---
------TTTTTCCTTGGGTTCTTGGGAGCA---GCAGGAAGCACTATG---GGCGCAGCGTCA---
ATAACGCTGACGGTACAG---GCCAGACTA---TTACTG---TCTGGTATAGTGCAGCAGCAGAGC---
AATCTG---CTGAGG---GCTATTGAGGCGCAACAGCATATGTTGCAACTCACAGTCTGGGGCATC
>JF320169
TTCTATTGTAACACAACAGCACTGTTTAATAGTACTTGGGGGGCTAACAGTACTTGGGAGATTACA-------
----

AATGACACTGAAATCATACTCCCATGCAGAATAAGACAAATCATAAACATGTGGCAGGAAGTAGGAAAAGCAA
TGTATGCCCCTCCCATTAGTGGAAACATTAGTTGCTCATCAAAT---

ATTACAGGGTTACTATTAACAAGAGATGGGGGTAATGGAAATGGT----------------------------
----

ACCAAAAACGAGACCTTCAGACCTACAGGAGGGGATATGAGGGACAATTGGAGAAGTGAATTGTATAAATATA
AAGTAGTAAGAATTGAACCATTA---GGAGTAGCACCCACCAAGGCAAAGAGAAGAGTGGTG-----------
----------------------------CAGAGAGAAAAAAGAGCA---GTGGGA---CTAGGAGCT------
---ATGTTCCTTGGGTTCTTGGGAGCA---GCAGGAAGCACTATG---GGCGCAGCGTCG---
GTAACGCTGACGGTACAG---GCCAGACAA---TTATTG---TCTGGTATAGTGCAGCAGCAGAGC---
AATCTG---CTGAGA---GCTATTGAGGCGCAACAGCATATGTTGCAACTCACAGTCTGGGGCATC
>JF320053
TTCTACTGTAATACAGCACAACTGTTCAATAGTACCTGGATCAATGGTACTGAAAGGTCAAATACCACTGAA-
----

GGAAATACCACAATCACACTCCCATGCAGAATAAAACAAATAGTAAATATGTGGCAGGAAGTAGGAAAAGCCA
TGTATGCCCCTCCCATCAGAGGAAAAATTAACTGCTTCTCAAAT---
ATCACAGGGCTGCTATTAACAAGAGATGGTGGCTCTGATAACAACACCACT----------------------
----

GGCAACAACACCGAGATCTTCAGACCTGGAGGAGGAGATATGAGGGACAATTGGAGAAGTGAATTATATAAAT
ATAAAGTAGTAAAAATCGAACCATTA---GGAGTAGCACCTACCAGGGCAAAGAGAAGAGTGGTG--------
-------------------------------CAGAGAGAAAAAAGAGCA---GCAGGA---ATAGGAGCT---
------TTGTTCCTTGGGTTCTTGGGAGCA---GCAGGAAGCACTATG---GGCGCAGCGTCA---
GTGACGCTGACGGTACAG---GCCAGACAA---TTATTG---TCTGGTATAGTGCAGCAGCAAAAC---
AATTTG---TTGAAG---GCTATTGAGGCGCAACAGCATCTGTTGCAACTCACAGTCTGGGGCATC
>JF320356
TTCTACTGTAATACAACAATACTGTTTAATAGCACTTGGAATGATACTAAAGGGACAAATAGC----------
----

ACAAATGACACTATCACACTCCCATGCAGAATAAAACAAATTATAAACATGTGGCAGAGAGTAGGAAAAGCAA
TATATGCCCCTCCCATCAGCGGACCAATTAGGTGTTCATCAAAT---
ATTACAGGGCTGCTATTAACAAGAGATGGTAGTACTAACAACAACAGC-------------------------
----

ACTAAGAATGAGACCTTCAGACCTGGAGGAGGAGACATGAGGGACAATTGGAGAAGTGAATTATATAAATATA
AAGTAGTAAAAGTTGAACCAGTA---GGAATAGCACCCACCAAGGCAAAGAGAAGAGTGGTG-----------
----------------------------CAGAGAGAAAAAAGAGCA---GTGGGAACACTAGGAGCT------
---GTGTTCCTTGGGTTCTTGGGAGCA---GCAGGAAGCACTATG---GGCGCAGCGTCA---
ATGACGCTGACGGTACAG---GCCAGACAA---CTATTG---TCTGGTATAGTGCAACAGCAGAAC---
AATCTG---CTGAAG---GCTATTGAGGCGCAACAGCACATGTTGCAACTCACAGTCTGGGGCATT
>JF320592
TTCTACTGTAATACAACAAAGCTGTTTAATAGTACTTGGGTTAATGGTACTAGGAATGATACAGGGTCAAAT-
----

GACACTGAAACCATAATACTCCCATGCAGAATAAAACAAATTATAAACATGTGGCAGAAAGTAGGAAAAGCAA
TGTATGCCCCTCCCATCAAAGGACAAATTAGCTGTTCATCAAAC---
ATTACAGGGCTGCTATTAACAAGAGATGGATCTCTAAAGAATGAG----------------------------
----

ACCACCTTGACCTTCAGACCTGGAGGAGGAAATATGAGGGACAATTGGAGAAGTGAATTATATAAATATAAAG
TAGTAAAAATTGAACCATTA---GGGGTAGCACCCTCCAAGGCAAAGAGACGGGTGGTG--------------
-------------------------CAGAGAGAAAAAAGAGCA---GCGGGA---CTAGGAGCT---------
ATGTTCCTTGGGTTCTTGGGAGCA---GCAGGAAGCACTATG---GGCGCAGCGTCA---
GTGACGCTGACGGTACAG---GCCAGACTA---TTATTG---TCTGGTATAGTGCAACAGCAGAAC---
AATCTG---CTGAGA---GCTATTGAGGCGCAACAGCATCTGTTGCAGCTCACGGTCTGGGGCATC
>KT124791
TTCTACTGTAATACAACACAACTGTTTAATAGTACTTGGAATAGTACTAGAGAGAAAAATAGCACA-------
----

AGAAATGACACTATCACACTCCCATGCAGAATAAAACAAATTATAAATATGTGGCAAAAAGTAGGAAAAGCAA
TGTATGCCCCTCCCATCAGGGGACAAATTAGATGTTCATCAAAT---

ATTACAGGGTTGCTATTAACAAGAGATGGTGGTAGTAACCAAAAT----------------------------
----

AGGACTGAGATCTTTAGACCTGCAGGAGGAAATATGAAGGACAATTGGAGAAGTGAATTATATAAATATAAAG
TAGTAAGAATTGAACCATTA---GGAGTAGCACCCACTGGGGCAAAGAGAAGAGTGGTG--------------
-------------------------CAGAGAGAAAAAAGAGCA---GTGGGA---ATAGGAGCT---------
GTGTTCCTTGGGTTCTTGGGAGCA---GCAGGAAGCACTATG---GGCGCAGCGTCA---
CTGACGCTGACGGTACAG---GCCAGACTC---TTATTG---TCTGGTATAGTGCAACAGCAGAAC---
AATCTG---CTGAGG---GCTATTGAGGCGCAACAGCATCTGTTGCAACTCACAGTCTGGGGCATC
>KR423026
TTCTATTGTAATACAGCAAGATTGTTTAATAGCTCTTGGAATGATACCGAAGGGTCAATCAATGAGACAAAC-
----

TCAGAAAACATCATCACACTCCCATGCAGAATAAAACAAATTGTAAACATGTGGCAGGAAGTAGGAAAAGCAA
TGTATGCCCCTCCCATCAGGGGAATAATTAACTGTTCATCAACT---
ATTACAGGGCTGTTATTAACAAGAGATGGTGGTAATGGTAAGAATGGGAGC----------------------
----

AATGAGACCAATGAGATTTTCAGACCTGGAGGAGGAGATATGAGGGACAATTGGAGAAGTGAATTATATAAAT
ATAAAATAGTAAAAATTGAACCATTA---GGACTAGCACCCACCCCGGCAAAGAGAAGAGTGGTG--------
-------------------------------CAGAGAGAAAAAAGAGCA---GTGGGA---CTAGGAGCA---
------TTGTTCCTTGGGTTCTTGGGAGCA---GCAGGAAGCACTATG---GGCGCAGCGTCA---
ATGACGCTGACGGTACAG---GCCAGACAA---TTATTG---TCTGGTATAGTGCAACAGCAGAAC---
AATTTG---CTGAGA---GCTATTGAGGCGCAACAGCATATGTTGCAACTCACAGTCTGGGGCATA
>GU331039
TTCTACTGTAATACAACAGAACTGTTTAATAGTACTTGGAATAATGGTAGCAGTACTTGGACTAGCAATGATA
CTAAA--

GGGTTAAATAACACTATCACACTCCGATGCAGAATAAAACAAATTATAAACATGTGGCAGGAAGTGGGAAAAG
CAATGTATGCCCCTCCCATCAGAGGACAAATTAGATGTTCATCAAAT---
ATTACAGGGCTACTATTAACAAGAGATGGTGGTAATAATAAGACCGAA-------------------------
----

AACAAAACGGAGATCTTCAGACCTGGAGGAGGAGATATGAGGGACAATTGGAGAAGTGAATTATATAAATATA
AAGTGGTACAAATTGAACCAATA---GGAGTAGCACCCACCAAGGCAAAGAGAAGAGTGGTG-----------
----------------------------CAAAGAGAAAAAAGAGCA---GTAACA---CTGGGAGCT------
---ATGTTCCTTGGGTTCTTGGGAGCA---GCAGGAAGCACTATG---GGCGCAGCGTCA---
ATGGCGCTGACGGTACAG---GCCAGACAA---TTATTG---TCTGGTATAGTGCAACAGCAAAAC---
AATTTG---CTGAGA---GCTATTGAGGCGCAACAGCACCTGTTGCAACTCACAGTCTGGGGCATC
>GU331147
TTCTACTGTAATTCAACACAACTGTTTAATAGTATCTGGACTAAAAATGATAATGGTATCTGGACTAAAAATG
AGACTAATGGT---AAT---
TGGACTAGAAGCGGGAGCACA---------------
AATGACAATGACACAATCATACTCCCTTGCAGAATAAGACAAATTATAAACATGTGGCAGGAAGTAGGAAAAG
CAATGTACGCTCCTCCCATTAGAGGAAACATTAGCTGTTCATCAAAT---
ATTACAGGGCTGTTACTAACAAGAGATGGAGGTAACAATAATGAT----------------------------
----

AGCGCAAATGAGACGTTCAGACCTGGAGGAGGAAATATGAAGGACAATTGGAGAAGTGAATTATATAAATATA
AAGTAGTAGAAATTGAGCCATTA---GGACTAGCACCCACCAGGGCAAAAAGAAGAGTGGTG-----------
----------------------------CAGAGAGAAAAAAGAGCA---GCC------CTAGGAGCT------
---TTGTTCCTTGGGTTCTTAGGAGCT---GCAGGAAGCACTATG---GGCGCAGCGTCA---
GTGACGCTGACGGTACAA---GCCAGACTA---TTATTG---ACTGGTATAGTGCAGCAGCAGAAC---
AATCTG---CTGAAG---GCTATTGAGGCGCAACAACATCTGTTGCAACTCACAGTCTGGGGCATC
>KM353701
TTCTACTGCAATACAACAAAACTATTTAGCAGTACCTGGAGTATAAATGAAACTATCTGG-------------
----
CATCTAAATGAAACTACAAAT------------
AACACTAATGACACTATCACACTCCCATGCAAAATAAAACAATTTATAAACATGTGGCAGCAAGTAGGAAAAG
CAATGTATGCCCCTCCTATCAGAGGACAAATTAGATGTAAATCAGAT---

ATTACAGGGCTGATATTAATGAGAGATGGTGGTGAA-------------------------------------
----
ACTAACAGGGGCAACGAGACCTTC---------
AGCAATGAGACCTTCAGACCTGGGGGAGGAAATATGAGGGACAATTGGAGAAGTGAATTATATAAATATAAAG
TAGTAAAGATTGAGCCATTA---GGACTGGCACCCACCGAGGCAAAGAGAAGAGTGGTG--------------
-------------------------CAGAGAGAAAAAAGAGCA---GTGGAA---ATAGGAGCT---------
ATGTTCCTTGGGTTCTTGGGAGCA---GCAGGAAGCACTATG---GGCGCAGCATCA---
CTAACGCTGACGGTACAG---GCCAGAATG---TTATTG---TCTGGTATAGTGCAACAGCAGAGC---
AATTTG---CTGAAG---GCTATTGAGGCGCAACAACATCTGTTGCAACTCACAGTCTGGGGCATC
>JN687749
TTCTACTGTGATACAACACCACTGTTTAATAGTACGTGGCCTTGGAATAGTACTTGGAATGATACAAATACA-
----

AATAATACCAATATCACACTCCAGTGCAGAATAAAACAAATTATAAACATGTGGCAGGAAGTAGGAAAAGCAA
TGTATGCCCCTCCCATCAAAGGACAAATTAGGTGCTCATCAATT---
ATTACAGGGCTGCTGTTAACAAGAGATGGTGGTAATAGCAGCGAA----------------------------
----

AATAATACTGAGATCTTCAGACCTGGAGGGAGAGATATGAGGGACAATTGGAGAAGTGAATTATATAAATATA
AAGTTGTAAAACTTGAACCATTA---GGAGTAGCACCCACCAAGGCAAAGAGAAGAGTGGTG-----------
----------------------------CAAAGAGAAAAAAGAGCA---GTGAAA---ATAGGAGCT------
---CTGTTCCTTGGATTCTTGGGAGCA---GCAGGAAGCACTATG---GGCGCAGCGTCA---
GTGACGCTGACGGTACAG---GCCAGACTA---TTATTG---TCTGGTATAGTGCAACAGCAGAAC---
AATCTG---CTGAGG---GCTATTGAGGCGCAACAACATATGTTGCAACTCACAGTCTGGGGCATC
>JN687758
TTCTACTGTAATACAACACAACTATTTGATAGTAATTGGACTCTATCTGATATTACTAAAGGGTCAAATACCA
CTGAA--

GGCAACAGCACACACATCACACTCCCATGCAGAATAAAACAAATTATAAACATGTGGCAAGAAGTAGGAAAAG
CAATGTATGCCCCTCCTATCAGAGGAAACATTACTTGCATATCAAAT---
ATTACAGGGTTGTTACTAGTAAGAGATGGTGGTAATAATAATGAG----------------------------
----

ACAGAGATCTTTAGACCTGGAGGAGGAGATATGAGGGACAATTGGAGAAGTGAATTGTATAAATATAAAGTAA
TAAAAATTGAACCATTA---GGAATAGCACCCACCAAGGCAAAAAGAAGAGTGGTG-----------------
----------------------CAGAGAGAAAAAAGAGCA---GCA------TTAGGAGCT---------
TTGTTCCTTGGGTTCTTGGGAGCA---GCAGGAAGCACTATG---GGCGCAGCGTCA---
CTGACGCTGACGGTACAG---GCCAGGCTA---TTATTG---TCTGGTATAGTGCAACAGCAGAAC---
AATCTG---CTGAGG---GCTATTGAGGCGCAACAGCATCTGTTGCAACTCACAGTCTGGGGCATC
>HQ908116
TTCTACTGTAATACAACAGAACTGTTTAATAGTACTTGGAATGCTAATGAAGGATCAAATAAAACAGGATCA-
----

AATGACACTACAATCATACTCCCATGTAGACTAAGACAAATTGTAAACATGTGGCAGGAAGTAGGAAAAGCAA
TGTATGCTCCTCCCATTAGAGGACAGATTAGATGTTCATCAAAT---
ATTACAGGGCTGCTATTAACAAGAGATGGTGGTATTACTAATACTACCAAC----------------------
----

AAGACTAAGACTGAGACCTTCAGACCTGCAGGAGGAGATATGAGGGACAATTGGAGAAGTGAATTATATAAAT
ATAAAGTAGTAAAAATTGAACCATTA---GGAATAGCACCTTCCAGGGCAAAGAGAAGAGTGGTG--------
-------------------------------CAAAGAGAAAAAAGAGCA---GTGGGA---ATAGGAGCT---
------CTGTTCCTTGGGTTCTTGGGAGCA---GCAGGAAGCACTATG---GGCGCAGCGTCA---
ATGACGCTGACGGTACAG---GCCAGACTA---TTATTG---TCTGGTATAGTGCAACAGCAGAAC---
AACTTG---CTGAGA---GCTATTGAGGCGCAACAGCATTTGTTGCAACTCACAGTCTGGGGCATC
>JQ251004
TTCTACTGTAATACAACACAACTATTTAGTAGTACTTGGCTGCCTAATAGTACTCGGAATGATACT-------
----

GATAATAGCCCTATCACACTCCCATGCAGAATAAAACAAATTGTAAACATGTGGCAGGAAGTAGGAAAAGCAA
TGTATGCCCCTCCCATCAGAGGAAACATTAGTTGCTCATCAAAT---

ATTACAGGGCTACTATTAACAAGAGATGGGGGTAATGATAATAAT----------------------------
----

AATACCGAGATCTTCAGACCTGGAGGAGGAGATATGAGGGACAATTGGAGAAGTGAATTATACAAATATAAAG
TAGTAAAAATTGAACCATTA---GGAATAGCACCCACCAAGGCAAAGAGAAGAGTGGTG--------------
-------------------------CAGAGAGAAAAAAGAGCA---GTGGGA---ATAGGAGCT---------
GTGTTCCTTGGGTTCTTGGGAGCA---GCAGGAAGCACTATG---GGCGCAGCGTCA---
ATAACGCTGACGGTACAA---GCCAGACAA---TTATTG---TCTGGTATAGTGCAACAGCAGAAC---
AATTTG---CTGAGG---GCTATTGAGGCGCAACAGCATCTGTTGCAACTCACAGTCTGGGGCATC
>JN944897
TTCTACTGCAATACAACACAACTGTTTAATAGTACCTGGAATCTTAATAGTACCTGGAATGGAACTAAAAATT
TAAATAAC---
-------------------ACT---
ACAAGAAATGACACAATCATACTCCCATGCAGAATAAGACAAATTATAAACTTGTGGCAAAAAGTAGGAAAAG
CAATGTATGCCCCTCCCATCAGAGGACAAATTAATTGTACATCAAAT---
ATCACAGGGCTGCTATTAACAAGAGATGGTGGAAATACAAGTGAGACC-------------------------
----

AACACCACTGAGGTCTTCAGACCTGGAGGAGGAAACATGAGGGACAATTGGAGAAGTGAATTATATAAATATA
AAGTAGTAGAAATTGAACCAATA---GGGATAGCACCCACCAAGGCAAAGAGAAGAGTGGTG-----------
----------------------------CAGAGAGAAAAAAGAGCA---GTGGGA---ATAGGAGCT------
---GTGTTCCTTGGGTTCTTGGGAGCA---GCAGGAAGCACTATG---GGCGCAGCGTCG---
GTAGCGCTGACGGTACAG---GCCAGACAG---TTATTG---TCTGGTATAGTGCAACAGCAGAAC---
AACTTG---CTGAGG---GCTATTGAGGCGCAACAACATATGTTGAAACTCACGGTCTGGGGCATT
>JN944907
TTCTACTGTGATACAAAACAACTGTTTAATAGTACTTGGAATGCTACTAAGGCAAATGGCACTACA-------
----

GGAAATGACACAATCATACTCCCATGCAGAATAAAGCAAATTATAAACATGTGGCAGAAAGTAGGAAAAGCAA
TGTATGCCCCTCCCATCAAAGGAAAAATTAGTTGCTCATCAAAT---
ATCACAGGGCTGCTATTAACAAGAGATGGAGGAGGTGGGGGACAG----------------------------
----

AATGAGACCTTCAGACCTGCAGGGGGAGATATGAGGGACAATTGGAGAAGTGAATTATATAAATATAAGGTAG
TAAAAATTGAACCATTA---GGAGTAGCACCCACCAGGGCAAAGAGAAGAGTGGTG-----------------
----------------------CAGAGAGAAAAAAGAGGA---GTAGGA---ATAGGAGCT---------
ATGGTCCTTGGGTTCTTGGGAGCA---GCAGGAAGCACTATG---GGCGCAGCATCA---
ATGATGCTGACGGTACAA---GCCAGACAA---TTGTTG---TCTGGTATAGTGCAACAGCAGAAC---
AATCTG---CTGAGA---GCTATTGAGGCGCAACAACATATGTTGCAACTCACAGTCTGGGGCATC
>JN944909
TTCTACTGCGATTCAACAGCGCTGTTTAATAGTACATGGAGGAGAAATAACACTTGGACTGGT----------
----

ACTACAGGAAATATCACACTCCAATGCAGAATAAAACAAATTATAAACATGTGGCAGAAAGTAGGAAAAGCAA
TGTATGCCCCTCCCATCAGAGGATACATTAACTGTTCATCAAAT---
ATTACAGGACTGATATTAACAAGAGATGGTGGTAATAATGATAGT----------------------------
----

GAAACCGAGATCTTCAGGCCTGGAGGAGGGAATATGAAGGACAATTGGAGAAGTGAATTATATAAATATAAAG
TAATAAAAATTGAACCATTA---GGAATAGCACCCACCAAGGCAAAGAGAAGAGTGGTG--------------
-------------------------CAAAGAGAAAAAAGAGCA---ATAGGA---ATAGGAGCT---------
CTTCTCCTTGGGTTCTTAGGAGCA---GCAGGAAGCACTATG---GGCGCAGCGTCA---
GTGACGCTGACGGTACAA---GCCAGACAA---TTATTG---TCTGGTATAGTGCAACAGCAGAAC---
AATCTG---CTGAGG---GCTATTGAGGCGCAACAGCATCTGTTGCAACTCACAGTCTGGGGCATC
>FJ469691
TTCTACTGTAATACAACACAACTGTTTAATAGTACTTGGACTGGGAATAATACTACAAATAAC----------
----

ATTTCAGGATATATCACACTCCCATGCAGAATAAAACAAATTATAAACATGTGGCAAGAAGTAGGAAAAGCAA
TGTATGCCCCTCCCATCAGTGGACAAATCAGGTGTTCATCAAAT---

ATTACAGGGCTACTATTAACAAGAGATGGTGGTAATAGTAATAACACC--------------------------
----

AACGGGACGACCGAGGTTTTCAGACCTATAGGAGGAAATATGAGGGACAATTGGAGAAGTGAATTATATAAAT
ATAAAGTAGTAAAAATTGAACCATTA---GGAGTAGCACCCACCACGGCAAAGAGAAGAGTGGTG--------
--------------------------------CAAAGAGAAAAAAGAGCA---GTGGGA---ATAGGAGCT---
------TTGTTCCTTGGGTTCTTGGGAGCA---GCAGGAAGCACTATG---GGCGCAGCGTCA---
TTGACGCTGACGGTACAG---GCCAGGCTA---TTATTG---TCTGGTATAGTGCAACAGCAGAAC---
AATCTG---CTGAGA---GCTATTGAGGCGCAACAACATCTGTTGCAACTCACAGTCTGGGGCATC
>FJ469696
TTCTACTGTAATACAACACAATTGTTTAATAGTACTTGGAATAGTACTGAAGGGTCAAATAAGACTGGAGGA-
----

AATGACACAACAATCACACTCCCATGCAGAATAAAACAAATTATAAACATGTGGCAGGAAGTAGGAAAAGCAA
TATATGCCCCTCCCATCAGAGGACTAATTAGATGTTCATCAAAT---
ATTACAGGGTTGCTAATAACAAGAGATGGTGGTAATAACAATAAC-----------------------------
----

AACAAGACAGAGACCTTCAGACCTGGAGGAGGAGATATGAGGGACAATTGGAGAAGTGAATTATATAAATATA
AAGTAGTAAAAATTGAACCATTA---GGAGTAGCACCAACCAAGGCAAAGAGGAGAGTGGTG-----------
-----------------------------CAGAGAGAAAAAAGAGCA---ATGGGA---ATGGGAGCT------
---TTGTTCCTTGGGTTCTTGGCATCA---GCAGGAAGCACTATG---GGCGCAGCGTCA---
ATAACGCTGACGGTACAG---GCCAGACAA---TTATTG---TCTGGTATAGTGCAACAGCAGAAC---
AATTTG---CTGAGA---GCTATTGAGGCGCAACAGCATCTGTTGCAACTCACAGTCTGGGGCATC
>FJ469700
TTCTACTGTGACTCAACACAACTGTTTAATAGTTCCTGGAATGGTACTGGAGGGTCAAGTAAC----------
----

ACTGCAGAGAATATCACACTCCATTGTAGAATAAGACAAATTGTAAACTTGTGGCAAGAAGTGGGAAGAGCAA
TGTATGCCCCTCCTATCAGAGGACAAATTAGCTGCTCATCAAAT---
ATTACAGGACTGATATTAACAAGAGATGGAGGTAATAACACTGAAAGT--------------------------
----

AATAACACCGAGACCTTCAGACCTGGAGGAGGAAATATGAGGGATAATTGGAGAAGTGAATTATATAAATATA
AAGTAGTAAAAATTGAACCAATA---GGAGTAGCACCCACCAAGGCAAAGAGAAGAGTGGTG-----------
-----------------------------CAGAGAGAAAAAAGAGCG---GTGGGA---TTCGGAGCT------
---ATGTTCCTTGGGTTCTTGGGAGCA---GCAGGAAGCACTATG---GGCGCAGCGTCG---
ATAACGCTGACGGTACAG---GCCAGACAA---TTATTG---TCTGGTATAGTGCAACAGCAGAAC---
AATTTG---CTGAGG---GCTATTGAGGCGCAACAGCATCTGTTGCAACTCACGGTCTGGGGCATC
>FJ469708
TTCTATTGTAATACAACACAACTGTTTAATAGTACTTGGAACAGTAATGAAGAGACAAAGAATGTCACA----
----

GGAAATGGAACTATCACACTCCCATGCAGAATAAGACAAATTATAAACATGTGGCAACAAGTAGGAAAAGCAA
TGTATGCCCCTCCCATTAGCGGAAAAATTAGCTGTTCATCAAAC---
ATTACAGGGCTACTATTAACAAGAGATGGGGGTGAAAACACGAGC-----------------------------
----

GAGACCGAGATTTTTAGACCTGCAGGAGGAAATATGAAGGACAATTGGAGAAGTGAATTATACAAATATAAAG
TAGTAAAAATTGAACCATTA---GGAATAGCACCCACCAAGGCAAAGCGAAGAGTGGTG--------------
--------------------------CAAAGAGAAAAAAGAGCA---ATAGGA---ATAGGAGCT---------
ATGTTCCTTGGGTTCTTGGGAGCA---GCAGGAAGCACTATG---GGCGCAGCGTCA---
CTGACGCTGACGGTACAG---GCCAGACTA---TTATTG---TCTGGTATAGTGCAACAGCAGAAC---
AATCTG---CTGAGG---GCTATTGAGGCGCAGCAGCATCTGTTGCAACTCACAGTCTGGGGCATC
>FJ469709
TTCTACTGTGTTACAACAAAACTGTTTAATAGTACTTGGACGTTTAATAGTACTAATGGTACTTGGACGTTGA
ATAGTACTTGG--
AATAGCACTGAAGGGCTA---------
AACAACAATGACACTATCATACTCCCATGCAGAATAAAACAAATTATAAACATGTGGCAAACAGTAGGAAAAG
CAATGTATGCCCCTCCAATCAGAGGACCAATTAACTGTACATCAAAT---

ATTACAGGGTTGCTATTAACAAGAGATGGTGGTAAGGAGACCAAC----------------------------
----

ACCACCACCGAGACCTTCAGACCTGGAGGAGGAGACATGAGGGACAATTGGAGAAGTGAATTATATAAATATA
AAGTAGTAAAAATTGAGCCATTA---GGAATAGCACCCACCAAGGCAAGGAGAAGAGTGGTG-----------
----------------------------CAAAGAGAAAAAAGAGCA---GTGGGA---ATAGGAGCT------
---GTGTTCCTTGGGTTCTTGGGAGCA---GCAGGAAGCACTATG---GGCGCAGCGTCA---
CTGACGCTGACGGTACAG---GCCAGACTA---TTGTTG---TCTGGTATAGTGCAGCAGCAGAAC---
AATCTG---CTGAGG---GCTCTTGAGGCGCAACAACATCTGTTGCAACTCACAGTCTGGGGCATC
>JQ250880
TTCTACTGTAATACAACACCATTGTTTAATGGTAGTAGTTGGAATCTAAATAAGACTAAAGAAAATACAACTA
ATCTA--

GAAAATGGCACAATCACCCTCCAATGCAGAATAAAACAATTTGTAAACATGTGGCAGAAAGTAGGAAAAGCAA
TGTATGCTCCTCCCATCAGAGGACGGATTGAATGTTCATCAAAT---
ATTACAGGGCTGATATTAACAAGAGATGGGGACATGAATAGTACA----------------------------
----

GGGAACGAGACCTTCAGACCTGGAGGAGGAGATATGAGGGACAATTGGAGAAGTGAATTATACAAATACAAAG
TAGTAAAAATTGAGCCATTA---GGAGTAGCACCCACCAAGGCAAAGAGAAGAGTGGTG--------------
-------------------------CAGAGAGAAAAAAGAGCA---GTGGGAGCACTAGGAGCT---------
ATGTTCCTTGGGTTCTTGGGAGCA---GCAGGAAGCACTATG---GGCGCAGCGTCA---
GTGACGCTGACGGTACAG---GCCAGACTA---TTATTG---TCTGGTATAGTGCAACAGCAAAAT---
AATTTG---CTGAAG---GCTATTGAGGCGCAACAGCGTCTGTTGCAACTCACAGTCTGGGGCATC
>HQ217266
TTCTACTGTAACACAACACAATTGTTTAATAGTACTTGGAATAAAAATAGTACTATTGAGGATAGA-------
----

AATGATGCTAATATCACACTCCCATGCAGGATAAGGCAAATTGTAAACATGTGGCAGCGAGTAGGAAAAGCAA
TGTATGCTCCTCCCATCAGCGGAATAATTAGATGTTCATCAAAT---
ATTACTGGGCTGCTATTAACAAGAGATGGAGGTAGTAATGCTACT----------------------------
----

ACCGAGACCTTCAGACCTGGAGGAGGAGATATGAGGGATAATTGGAGAAGTGAATTATATAAATACAAAGTAG
TAAAAATTGAACCATTA---GGAATAGCACCCACCAAGGCAAAGAGAAGAGTGGTG-----------------
----------------------CAGAGAGAAAAAAGAGCG---GCAATAGGACTAGGAGCT---------
TTGTTCCTTGGGTTCTTGGGAGCA---GCAGGAAGCACTATG---GGCGCAGCGTCA---
ATAACGCTGACGGTACAG---GCCAGACTA---TTATTG---TCTGGTATAGTGCAGCAGCAGAAC---
AATTTG---CTGAGG---GCTATTGAGGCGCAACACCATCTGTTGCAACTCACAGTCTGGGGCATC
>JQ403068
TTCTACTGTGATTCATCAAAACTGTTTAATAGTACTTGGAATGTAACTAGTTGGAATGTAACTGGTTGGAATG
GTACTGAA---

GAGTCAAATGGCACTATCACACTCCCATGCAGAATAAAACAGATTATAAACATGTGGCAAGAAGTAGGAAAAG
CAATGTATGCCCCTCCCATCAGAGGACTAATTAGATGTTCAACAAAT---
ATTACAGGGCTGATATTAACAAGAGATGGTGGTAACGAGAACAAT----------------------------
----

AACAATAGTGAGGTCTTTAGACCTGGAGGAGGAGATATGAGGGACAATTGGAGAAGTGAATTATATAAATATA
AAGTAGTAAAAATTGAACCATTA---GGAGTAGCACCCACCAGGGCAAAAAGAAGAGTGGTG-----------
----------------------------CAGAGAGAAAAAAGAGCA---GTGGGA---CTAGGAGCA------
---CTGTTCCTTGGGTTCTTGGGAGCA---GCAGGAAGCACTATG---GGCGCAGCGTCA---
ATGACGCTGACGGTACAA---GCCAGACAA---TTATTG---TCTGGTATAGTGCAACAGCAGAAC---
AATTTG---CTGAGG---GCTATTGAGGCGCAACAACATTTGTTGCAACTCACAGTCTGGGGCATC
>JQ403095
TTCTACTGTAATTCAACGCAACTGTTTAATAGTACTTGGGATATTAATAATACTAGTAGGGCAAAT-------
----

AGCACTGGAAATATCACACTCCCATGCAGAATAAGACAAATTATAAACATGTGGCAGGAAGTAGGAAAAGCAA
TGTATGCCCCTCCCATCGCGGGACAAATTAGATGTACATCAAAT---

```
ATTACAGGACTGTTATTAACAAGAGATGGTGGTAATGACACTACC----------------------------
-------------------------------------------------------------------------
----------------
ACCGAGATCTTCAGACCTGGAGGAGGAAATATGAAGGACAATTGGAGAAGTGAATTATATAAATACAAAGTAG
TAAAAATTGAACCATTA---GGAATAGCACCCACCAAGGCAAAGAGAAGAGTGGTG-----------------
----------------------CAGAGAGAAAAAAGAGCA---ATAGGA---CTGGGAGCT---------
ATGTTCCTTGGGTTCTTGGGAGCA---GCAGGAAGCACCATG---GGCGCAGCGTCA---
GTGACGCTGACGGTACAG---GCCAGACAA---TTATTG---TTTGGTATAGTGCAACAGCAGAAC---
AATCTG---CTGAGG---GCTATTGAGGCGCAACAGCATATGTTGCAACTCACAGTCTGGGGCATC
>KM259364
TTCTACTGTGATACAACACCACTATTTAATAGTATTTGGAATACAACACAAATATTTAATGGT----------
--------------------------------------------------------ACT---
TGGAATGATACTGTAGGGTCT---------------
GGAGAAAATGACACAATCATACTCCAATGCAGAATAAAACAAATTATAAACATGTGGCAAGAAGTAGGAAAAG
CAATGTATGCCCCTCCCATCAGAGGACAAATTAGATGTGAATCAAAT---
ATTACAGGGCTGCTATTAACAAGAGATGGTGGTACT-------------------------------------
-----------------------------------------------------
GGTGAGAACAACACTGGTGAGAAC---------
AACACTGAGATCTTCAGACCTGGAGGAGGAGATATGAGGGACAATTGGAGAAGTGAATTATATAAATATAAAG
TAGTAAAAATTGAACCATTA---GGAGTAGCACCTACTAAGGCAAAGAGGAGAGTGGTG--------------
-------------------------CAGAGAGAAAAAAGAGCA---GTGGGA---TTAGGAGCT---------
GTGCTCTTTGGGTTCTTGGGAGCA---GCAGGAAGCACTATG---GGCGCAGCGTCA---
ATAACGCTGACGGTACAG---GCCAGACAA---TTATTG---TCTGGTATAGTGCAACAGCAAAAC---
AATTTG---CTGAAG---GCTATTGAGGCGCAACAGCATCTGTTGCAGCTCACAGTCTGGGGCATC
>KM259479
TTCTACTGTAATACAACACGGCTGTTTAATAGTGAGTGGCTGTCCAATGGTACT-------------------
-------------------------------------------------------------------------
-TGGAAAGGGTCAAAT---------
GACACTGGAGAAAATATTACACTCCCATGCAGAATAAAACAAATTATAAACATGTGGCAGGAAGTAGGAAAAG
CAATGTATGCTCCTCCCATCAGAGGACAAATCAATTGTGTATCAAAT---
ATTACAGGAATATTATTAACAAGAGATGGGGGT----------------------------------------
--------------------------------------------------------------------
AATGGCAATAACAGT---
ACCAATGAGACTTTCAGACCTGGAGGAGGAGATATGAGGGACAATTGGAGAAGTGAATTATATAAATATAAAG
TAGTAAAAATTGAACCATTA---GGAGTAGCACCCACCAAGGCAAAGAGAAGAGTGGTG--------------
-------------------------CAGAGAGAAAAAAGAGCA---GCA------CTAGGAGCT---------
ATATTCCTTGGGTTCTTGGGAGCA---GCAGGAAGCACTATG---GGCGCAGCGTCA---
TTGACGCTGACGGTACAA---GCCAGACTT---TTATTG---TCTGGTATAGTGCAACAGCAAAAC---
AATTTG---CTGAGG---GCTATTGAGGCGCAACAGCATCTGTTGCAACTCACAGTCTGGGGCATC
>EU578667
TTCTACTGCAATTCAACACAACTGTTTAATAGTACTTGGAATGATAGTACTTGGAATGATACTACAGAGCCAA
AT-----------------------------------------------------------------------
-------------------------
AACACTGACGACACAATCACACTCCCATGTAGAATAAGACAAATTGTAAACAGGTGGCAGGAAGTAGGAAGAG
CAATGTATGCCCCTCCCATCAGTGGACAAATTAACTGTTCATCACAG---
ATTACAGGGCTGATATTAACAAGAGATGGTGGTATTAACGAGAGT----------------------------
-------------------------------------------------------------------------
-------------
AATACCGAGACCTTCAGACCTGGAGGAGGAGATATGAGGGACAATTGGAGAAGTGAATTATATAAATATAAAG
TAGTAAAAATTGAACCATTA---GGATTAGCACCCACCAAGGCAAGGAGAAGAGTGGTG--------------
-------------------------CAGAGAGAAAAAAGAGCA---GCA------CTAGGAGCT---------
GTGTTCCTTGGGTTCTTGGGAGCA---GCAGGAAGCACTATG---GGCGCAGCGTCA---
ATAGCGCTGACGGTACAG---GCCAGACTA---CTATTG---TCTGGTATAGTGCAACAGCAGAGC---
AATCTG---CTGAGA---GCTATTGAGGCGCAACAACATATGTTGCAACTCACAGTCTGGGGCATC
>HQ217967
TTCTACTGTAATACAACACAATTGTTTAATAGTACATGGAAGAATGGTACTGCTGGGGTCAATGATACTACA-
-------------------------------------------------------------------------
-------------------------
GGAAATGACACCCATATCACCCTCCCATGCAGAATAAAACAAATTATAAACATGTGGCAGCAAGTAGGAAAAG
CAATGTATGCCCCGCCCATCAGAGGACAAATTAGTTGTTTATCAAAT---
```

ATTACAGGGCTACTATTAACAAGAGATGGTGGTAATAACGACAAAAACGGG---------------------
----

ACCAACATCACCGAGATCTTCAGACCTGGAGGAGGAGATATGAGGGATAATTGGAGAAATGAATTATATAGAT
ATAAAGTAGTAAAAATTGAACCATTA---GGAGTAGCACCCACCAAGGCAAAGAGAAGAGTGGTG-------
------------------------------CAAAGAGAAAAAAGAGCA---GCGGGG---TTAGGAGCT---
------TTGTTCCTCGGGTTCTTGGGAGCA---GCAGGAAGCACTATG---GGCGCAGCATCA---
GTGACGCTGACGGTACAG---GCCAGACAA---TTATTG---TCTGGTATAGTGCAACAGCAGAAC---
AATTTG---CTGAGG---GCTATTGAGGCGCAACAGCATCTGTTGCAACTCACAGTCTGGGGCATC
>HQ218020
TTCTACTGTAATACAGATAAACTGTTTAATAGTAATTGGACAACAAATACTAGCTCTGAGAATGTAACAAGTG
GCACT---

GAAGGAAATGATATAATCACACTCCCATGCAGAATAAGACAAATTATAAACCTGTGGCAGGAAGTAGGAAAAG
CAATGTATGCCCCTCCCATCCAAGGACAAATTAATTGTTCATCAAAT---
ATTACAGGGCTGTTATTAACAAGAGATGGTGGTGCTAACCAAACA---------------------------
----

AACAACACTGAGGTCTTCAGACCTGGAGGAGGAGATATGAGGGACAATTGGAGAAGTGAATTGTATAAATATA
AAGTAATAAAAATTGAGCCAGTA---GGAATAGCACCCACCAAGGCAAAGAGAAGAGTGGTG----------
---------------------------CAGAGAGAAAAAAGAGCA---GTGACG---ATAGGAGCT------
---GTGTTCCTTGGGTTCTTGGGAACA---GCAGGAAGCACTATG---GGCGTAGCGTCA---
CTGACGCTGACGGTACAG---GCCAGACAA---TTATTG---TCTGGTATAGTGCAGCAGCAGAAC---
AATTTG---CTGAGA---GCTATTGAGGCGCAACAGCATCTATTGCAACTCACAGTCTGGGGCATC
>HQ218034
TTCTACTGTAATACATCAGGGCTGTTTAATAGTACTTGGAATGTTTCTGCAGATTTGAATACCACTGAAGAGT
CA--

AATAGTAATGAAACCATCACACTCCAATGCAGAATAAAACAATTTATAAACATGTGGCAGGAAGTAGGAAGAG
CAATGTATGCTCCTCCCATCAGAGGACAAATTAAATGTTCATCAAAT---
ATTACAGGGCTACTATTAACAAGAGATGGTGGTAACGACAGCGCC---------------------------
----

CCCACAGAGACCTTCAGACCAGGAGGAGGAGATATGAGGGACAATTGGAGAAGTGAATTATATAAATATAAAG
TAGTACAGATTGAACCAATA---GGAGTAGCACCCACCAAGGCAAAGAGAAGAGTGGTG-------------
------------------------CAAAGAGAAAAAAGAGCA---GTGGGA---TTAGGAGCT---------
ATGTTCCTTGGGTTCTTGGGAGCA---GCAGGAAGCACTATG---GGCGCAGCGTCA---
GTGACGCTGACGGTACAG---GCCAGACAA---TTATTG---TCTGGTATAGTGCAACAGCAGAAC---
AATTTG---CTGATG---GCTATTGAGGCGCAACAAAAACTGTTGCAACTCACAGTCTGGGGCATC
>GU330865
TTCTATTGTAATACAACAAAACTGTTTAACAGTACTTGGATGGCTAATAGTACTTGGAATGATACTACACAGT
CA--

GATAACAATGACACTATCATACTCCAATGCAGAATAAAACAAATTATAAACAGGTGGCAGGAAGTAGGAAGAG
CTATGTATGCCCCTCCCATTAAGGGAGAAATTAAATGTTCATCAAAT---
ATTACAGGGTTGCTATTAACAAGAGATGGTGGTAATATTGATAAC---------------------------
----

GAGACTGAGACCTTTAGACCTGGGGGAGGAGACATGAGGGACAATTGGAGAAGTGAATTATATAAATATAAAG
TAGTAAAAATTGAGCCATTA---GGAGTAGCACCCACCAAGGCAAAGAGAAGAGTGGTG-------------
------------------------CAGAGAGAAAAAAGAGCA---GTAACA---CTGGGAGCT---------
ATGTTCCTTGGGTTCTTGGGAGCA---GCAGGAAGCACTATG---GGCGCAGCGTCG---
ATGACGCTGACGGTACAG---GCCAGACAA---TTATTG---TCTGGTATAGTGCAACAGCAGAGC---
AACATG---CTGAGA---GCTATTGAGGCGCAACAACATCTGTTGCAACTCACAGTCTGGGGCATC
>JF689877
TTCTACTGTAATACATCACAATTGTTTAATATGAGTTCAACACAACTGTTTAATAAGACTTGGACTAATGAC-
----

ACTAGAGAAAACATCACACTCCCATGTAGGATAAGACAAATTGTAAACCTGTGGCAGGAAGTAGGAAAAGCAA
TGTATGCCCCTCCCATCAGTGGACCAATTAGATGTGAATCAAAT---

ATTACAGGGCTGATATTAACAAGAGATGGTGGTAATGAGACCAATGGG-------------------------
----

AATGAGACGATTGAGACTTTCAGACCTGGAGGAGGTAATATGAAGGACAATTGGAGAAGTGAATTATATAAAT
ATAAAGTAGTAAGAATTGAACCAATA---GGAGTAGCACCCACCAAGGCAAAGAGAAGAGTGGTG--------
-------------------------------CAGAGAGAAAAAAGAGCA---GTGGGA---ATAGGAGCT---
------GTGTTCCTTGGGTTCTTGGGAGCA---GCAGGAAGCACTATG---GGCGCAGCGTCA---
ATGACGCTGACGGTACAG---GCCAGACAA---TTATTG---TCTGGTATAGTGCAACAGCAGAAC---
AATCTG---CTGAGG---GCTATTGAGGCGCAACAACATCTGTTGCAACTCACAGTCTGGGGCATC
>JF689879
TTCTACTGTGATACAACAAAGCTGTTTAATAGCACTTGGCAGGTTAATAGTACTTGGAATGATACTGAAAAG-
----

TCAAATGACACTCTCACAATCCCATGTAGAATAAGGCAAATTGTAAATAGGTGGCAGGAAGTAGGAAAAGCAA
TGTATGCCCCTCCCATCAGTGGACTAATTAGTTGTTCATCAAAT---
ATTACAGGAATGATATTAACAAGAGATGGTGGGTATAATAATGGGAGC-------------------------
----

AATAATACGGAGGTCTTCAGACCTGGAGGAGGAAATATGAAGGATAATTGGAGAAGTGAATTATATAAATATA
AAGTAGTAGAAATTGAGCCATTA---GGAGTAGCACCTACCAGGGCAAGGAGAAGAGTGGTG-----------
----------------------------CAAAGAGAAAAAAGAGCA---GTGGGAACATTAGGAGTT------
---ATGTTCCTTGGGTTCTTGGGAGCA---GCAGGAAGCACTATG---GGCGCAGCGTCG---
CTGACGCTGACGGTACAA---GCCAGGCAA---TTATTG---TCTGGTATAGTGCAACAGCAGAGC---
AATCTG---CTAAGG---GCTATTGAGGCGCAACAGCATATGTTGCAACTCACAGTCTGGGGCATC
>JF689883
TTCTACTGTAATTCAACACAACTGTTTAATAGCACTTGGCATGCTAATGGGAGTAATACAAAT----------
----

CTCGCTACTAACATCACACTCCCATGTAAAATAAAGCAAGTTATAAACATGTGGCAGGAAGTAGGAAAAGCAA
TGTATGCCCCTCCCATCGAAGGACTAATTAGATGCTCATCAAAT---
ATTACAGGAATACTATTAACAAGAGATGGTGGTAACAGGAACGGGACC-------------------------
----

AGTGATACTGAGATCTTCAGGCCTGCAGGAGGAGATATGAGGGACAATTGGAGAAGTGAATTATATAAATATA
AAGTAGTAAAAATTGAACCATTA---GGAGTAGCACCCACCAAGGCTAAGAGAAGAGTGGTG-----------
----------------------------CAGAGAGAAAAAAGAGCA---GTGGGA---ATCGGAGCG------
---TTGTTCCTTGGGTTCTTGGGAGCA---GCAGGAAGCACTATG---GGCGCAGCGTCA---
ATGGCGCTGACGGTACAG---GCCAGACAA---TTATTG---TCTGGTATAGTGCAACAGCAGAAC---
AATCTG---CTGAGG---GCTATTGAGGCGCAACAGCATCTGTTGCAACTCACAGTCTGGGGCATC
>JF689885
TTCTACTGTGATACAAAAGAACTGTTTAATAGTACTTGGACTAACAATGGGACTGAGAATAAGAATGAGACTG
AG---

AATAAGAATGGGACTATCACACTCCCATGTAGAATAAAACAAATTGTAAACATGTGGCAGGAAGTAGGAAAAG
CAATGTATGCCCCTCCCATCAGGGGACAAATTAGATGTGAATCAAAT---
ATTACAGGGCTGCTATTAACAAGAGATGGGGGTACGGATAATGATAATAGC----------------------
----

ACAAACACCACCGAGATCTTTAGACCTGTGGGAGGAGACATGAAGGATAATTGGAGAAGTGAATTATATAAAT
ACAAAGTAGTAAAAGTTGAACCATTA---GGAATAGCACCCACCAAGGCAAAGAGAAGAGTGGTG--------
-------------------------------CAGAGAGAAAAAAGAGCA---TTAACA---CTAGGAGCG---
------ATGTTCCTTGGGTTCTTAGGAGCA---GCAGGAAGCACTATG---GGCGCAGCGTCA---
ATAACGCTGACGGTACAT---GCCAGGCAA---TTATTG---ACTGGTATAGTGCAACAGCAGAGC---
AATCTG---CTGAAG---GCTATTGAGGCGCAACACCACCTGTTGCAACTCACAGTCTGGGGCATC
>JF689890
TTCTACTGTAATACAACAAAACTGTTTAATAGTACTTGGGAGGTTAATGGAACTTGGCCTGAAGGGTCAAATA
ACACT--

GAAGGAAATGACACAATCACACTCCCATGCAGAATAAAACAAATTATAAACATGTGGCAGGAAGTAGGAAAAG
CAATGTATGCCCCTCCCATCAGAGGACAAATTAGATGTGTATCAAAT---

ATTACAGGGCTGCTATTAACAAGAGATGGTGGTAGTGACAACCAGACT-------------------------
----

AACAACAATACTGAGACCTTCAGACCTGGAGGAGGAGATATGAAGGACAATTGGAGAAGTGAATTATATAAAT
ATAAAGTAGTACAAATTGAACCATTA---GGAATAGCACCCAACAAGGCAAAGAGAAGAGTGGTG--------
-------------------------------CAGAGAGAAAAAAGAGCA---GTGGGAGTAGTAGGAGCT---
------ATGTTCCTTGGGTTCTTGGGAGCA---GCAGGAAGCACTATG---GGCGCAGCGTCA---
GTGACGCTGACGGTACAG---GCCAGACTA---TTATTG---TCTGGTATAGTGCAACAGCAAAAC---
AATTTG---CTGAGA---GCTATTGAGGCGCAACAGCATCTGTTGCAACTCACAGTCTGGGGCATC
>JF689893
TTCTACTGTAATTCAACACCACTGTTTAATAGTACTTGGAAGGTTAATGATACTCGGAATGATACTGGAGGA-
----

GAAAATATCACACTCACACTCACATGCAGAATAAAACAAATTATAAACATGTGGCAGACAGTAGGAAAAGCAA
TGTATGCCCCTCCTATCAAAGGACAAATTAGATGTTCATCAAAT---
ATTACAGGGCTGTTATTAACCAGAGATGGTGGTAATGAGAACGAG----------------------------
----

AACAACACCGAGATCTTCAGACCTGCAGGAGGAGATATGAGGGACAATTGGAGAAGTGAATTATATAAATATA
AAGTAGTACAAATTGAACCATTA---GGAGTAGCACCCAACAGGGCAAAGAGAAGAGTGGTG-----------
----------------------------CAGAGAGAAAAAAGAACA---GTGGGA---ATAGGAGCT------
---TTGTTCCTTGGGTTCTTGGGAGCA---GCAGGAAGCACTATG---GGCGCAGCGTCA---
ATGACGCTGACGGTACAG---GCCAGACAA---TTATTG---TCTGGTATAGTGCAACAGCAGAGC---
AATCTG---CTGAGG---GCTATTGAGGCGCAACAGCATCTGTTGCAACTCACAGTCTGGGGCATT
>MK383385
TTCTACTGTAATACAACAAAACTATTTAATAGTACTTGGAATAGTACTGAAGAATTAAATATTACTGAAGGAT
CAAATACC---

ACGGGAGACCCTATCATACTCCCATGCAGGATAAAACAAGTTATAAACCTGTGGCAGGAAGTAGGAACAGCGA
TGTACGCCCCTCCCATCAGTGGACAAATTAACTGTTCATCAAAT---
ATTACAGGGCTGCTATTAACAAGAGATGGTGGTGTTGACAGTAAC----------------------------
----

ACGACCGAGATTTTCAGACCTGGAGGAGGAGATATGAGGGACAATTGGAGAAGTGAATTATATAAATATAAAG
TAGTAAAAATTGAACCATTA---GGAGTAGCACCCACCAGGGCAAAGAGAAGAGTGGTG--------------
-------------------------CAAAGAGAAAAAAGAGCA---GTGGGA---ATAGGAGTT---------
TTGTTCCTTGGGTTCTTGGGAGCA---GCAGGAAGCACTATG---GGCGCAGCATCA---
CTGACGCTGACGGTACAG---GCCAGACAG---TTATTG---TCTGGTATAGTGCAACAGCAGAAC---
AATCTG---CTGAGG---GCTATTGAGGCGCAACAGCACCTGTTGCAACTCACAGTCTGGGGCATC
>JX863994
TTCTACTGTAATACAACACAACTGTTTAATAGCACTTGGTTTGGGAATGATACTCGGAATGGTAGTGAAGAG-
----

GCAAATGACACAATTACACTCCCATGCAGAATAAAACAGATTATAAACATGTGGCAGGAAGTAGGAAAAGCAA
TGTATGCCCCTCCCATCAGAGGACAAATTAGCTGTTCTTCAAAC---
ATTACAGGGCTGTTATTAACAAGAGATGGTGGTATAAGAAATAAC----------------------------
----

AATAGCGAGACCTTCAGACCTGGGGGAGGAGACATGAGGGATAATTGGAGAAGTGAATTATATAAATATAAAG
TAGTAAAAATTGAACCATTA---GGAATAGCACCCACCAAGGCAAAGAGAAGAGTGGTG--------------
-------------------------CAGAGAGAAAAAAGAGCA---GTGGGA---TTGGGAGCT---------
TTGTTCCTTGGTGTCTTGGGAGCA---GCAGGAAGCACTATG---GGCGCAGCGTCG---
ATAACGCTGACGGTACAA---GCCAGAACT---TTATTG---TCTGGTATAGTGCAACAGCAGAAC---
AATCTG---CTGAGG---GCTATTGAGGCGCAACAGCATCTGTTGCAACTCACAGTCTGGGGCATC
>JX863995
TTCTACTGTAGTACATCACAACTCTTTAATAGTACTTGGTATGAGAATGGAACTAGGAATATTAGTGGA----
----

GGGACAGAAATTATCACACTCCCATGCAGAATAAAACAAATTATAAACATGTGGCAGCAGGTAGGAAAAGCAA
TGTATGCCCCTCCCATCAGGGGATTAATTAACTGTTCATCAAAT---

```
ATTACAGGGCTGCTATTAACAAGAGACGGTGGTGGGGAGAATAGA----------------------------
-------------------------------------------------------------------------
-------------
ACTAATGAAACATTCAGACCTGGAGGAGGAGACATGAGGGACAATTGGAGAAGTGAATTATATAAATATAAAG
TAGTAAAAATTGAACCCTTA---GGATTAGCACCCACCAAGGCAAAGAGAAGAGTGGTG--------------
-------------------------CAGAGAGAAAAAAGAGCA---GTGGGA---ATAGGAGCT---------
CTGTTCCTTGGGTTCTTGGGAGCA---GCAGGAAGCACTATG---GGCGCAGCGTCA---
ATAACGCTGACGGTACAG---GCCAGGCTA---TTATTG---TCTGGTATAGTGCAGCAGCAGAAC---
AATCTG---CTGAGG---GCTATTGAGGCGCAACAGCACCTGTTGCAACTCACAGTCTGGGGCATC
>JX863921
TTCTACTGTAATACAGCAGAACTGTTTAATAGTACTTGGGATAATGACAGGTCAAATAGCACAGAA-------
-------------------------------------------------------------------------
----------------------------------------
ATCACACTCCAATGTAGAATAAAACAAATTATAAACATGTGGCAGGAAGTAGGAAGAGCAATGTATGCCCCTC
CCATTGCAGGACAAATTAACTGCACATCAAAT---
ATTACAGGGCTGCTATTGACAAGAGATGGTGGTACAGACAGTAAT----------------------------
-------------------------------------------------------------------------
-------------
AATACTGAGATCTTCAGACCTGGAGGAGGAGATATGAGGGATAATTGGAGAAGTGAATTATATAAATATAAGG
TAGTAAAAATTGAACCATTA---GGAATAGCACCCACCAGGGCAAGGAGAAGAGTGGTG--------------
-------------------------CAGAGAGAGAAAAGAGCA---GTGGGA---CTGGGAGCT---------
TTGTTCCTTGGGTTCTTGGGAACA---GCAGGAAGCACTATG---GGCGCAGCGTCA---
ATAGCGCTGACGGTACAG---GCCAGACAA---TTATTG---TCTGGTATAGTGCAACAGCAGAAC---
AATTTG---CTGAGG---GCTATTGAGGCGCAACAGCGTATGTTGCAACTCACAGTCTGGGGCATC
>JX864007
TTCTACTGTAATACAGCACAGCTGTTTAATAGTACTTGGAGTACTTGGAATGGTACTGAAGGAGCAAATAACA
CTGAA--------------------------------------------------------------------
----------------------------
GGAAATGATACAATCACACTCCCATGCAGAATAAAACAAATTATAAACATGTGGCAGGAAGTAGGAAAAGCAA
TGTATGCCCCTCCCATCAGTGGAATAATTAACTGTTCATCAAAT---
ATTACAGGGTTGCTATTAACAAGAGATGGTAGTAATAACACAAAG----------------------------
-------------------------------------------------------------------------
----------------
AACGAGACCTTCAGACCTGAAGGAGGGAATATGAGGGACAATTGGAGAAGTGAATTATATAAATACAAAGTAG
TAAAAATTGAACCATTA---GGAGTAGCACCTACCAAGGCAAGGAGAAGAGTGGTG-----------------
----------------------CAAAGAGAAAAAAGAGCA---GTGGGA---TTAGGAGCT---------
ATGTTCCTTGGGTTCTTGTCAGCA---GCAGGAAGCACTATG---GGCGCAGCGTCA---
GTGACGCTGACGGTACAG---GCCAGACAA---TTATTG---TCTGGTATAGTGCAACAGCAAAAC---
AACTTG---CTGAGG---GCTATTGAGGCGCAACAGCATCTGTTGCAACTCACAGTCTGGGGCATC
>JX864008
TTCTACTGTAATACAACAAGGCTGTTTAATAGTACCTGGCATCCGAATGGTGCTAAAATATCAAATGGC----
-------------------------------------------------------------------------
----------------------------
ACTGAAGAAACTATCACACTCCCATGTAGAATAAAACAAATTATAAACAGATGGCAGGAAGTGGGAAAAGCAA
TGTATGCCCCTCCCATCCAAGGAAAAATTAGCTGTGTATCAAAT---
ATTACAGGGCTACTATTAACAAGAGACGGTGGTAACAAGAGCCAT----------------------------
-------------------------------------------------------------------------
----------
AACAGTACCGAGACCTTCAGACCTGGAGGAGGAGATATGAGGGACAATTGGAGAAGTGAATTATATAAATATA
AAGTAGTAAAAATTGAGCCATTA---GGAGTAGCACCGACCAAGGCAAAGAGAAGAGTGGTG-----------
----------------------------CAGAGAGAAAAAAGAGCA---GTAACG---CTGGGAGCT------
---ATGTTCCTTGGTTTCTTGGGAGCA---GCAGGAAGCACTATG---GGCGCAGCGTCG---
ATGACGCTGACGGTACAG---GCCAGACTA---TTATTG---TCTGGTATAGTGCAACAGCAGAAC---
AACTTG---CTGAGA---GCTATTGAGGCGCAACAACATCTGTTGCAGCTCACAGTCTGGGGCATC
>JX864020
TTCTACTGTAATACAACAATGCTGTTTAATAGTACTTGGTGGGCTAATGATACTTGGAATAGCACTTGGAATA
ATAATATC-----------------------------------------------------------------
-------------------AGT---
AAAGAAAATGACACAATCACACTCCCATGCAGAATAAGACAAATTGTAAACATGTGGCAACAAGTAGGAAAAG
CAATGTATGCACCTCCCATCAGAGGAGAAATTAGATGTTCATCAAAT---
```

ATTACAGGGCTGCTAATAACAAGAGATGGGGGGAATAACAACACTGGGAGAGGG-------------------
----

GAGAATAAGACTGAGATCTTCAGACCTGTAGGAGGAGATATGAAGGACAATTGGAGAAGTGAATTATATAAAT
ATAAAGTAGTAAGAATTGAACCATTA---GGAATAGCACCCACCAAGGCAAAGAGAAGAGTGGTG--------
-------------------------------CAGAGAGAAAGAAGAGCA---GTGGGA---CTCGGAGTT---
------TTTTTTCTTGGGTTCTTGGCAACA---GCAGGAAGCACTATG---GGCGCAGCATCA---
ATAACGCTGACGGTACAG---GCCAGACAA---TTATTG---TCTGGTATAGTGCAGCAGCAGAAC---
AATCTG---CTGAGA---GCTATTGAGGCGCAACAGCATCTGTTGCAACTCACAGTCTGGGGCATT
>JF320375
TTCTACTGTAATACAACAAAGCTGTTTAATAGTACTTGGAATGGTACTGATACTTGGGATACTACTACAGAGG
CA---

GATAACAATGACACTATCACACTCCCATGCAGAATAAAACAAATTATAAACAGGTGGCAGGAAGTAGGAAAAG
CAATGTATGCCCCTCCCATTGCAGGACAGATCAGATGCTCATCAAAT---
ATTACAGGGATACTCTTAACAAGAGATGGTGGTAGTAATGACACA----------------------------
----

GACAGTGAGACCTTCAGACCAGGAGGAGGAGATATGAGGGACAATTGGAGAAGTGAATTATATAAGTATAAAG
TAGTAAGAATTGAGCCATTA---GGAATAGCACCCACCCAGGCAAAAAGAAGAGTGGTG--------------
-------------------------CAGAGAGAAAAAAGAGCA---GTGGGAACAATAGGAGCT---------
ATGTTCCTTGGGTTCTTGGGAGCA---GCAGGAAGCACTATG---GGCGCAGCGTCA---
TTGACGCTGACGGTACAG---GCCAGACAA---TTATTG---TCTGGTATAGTGCAGCAGCAGAAC---
AATCTG---CTGAGA---GCTATTGAGGCGCAACAGCATATGTTGCAACTCACAGTCTGGGGCATC
>JF320563
TTCTACTGTAATTCAACAAAACTGTTTAATAGTACTTGGAATGCTAATGGTACTATTAATGATACTAACGGGT
CA---

AATACAACAGAGGTGATCACGCTCCCATGCAGAATAAAACAATTTATAAACATGTGGCAGGAAGTAGGAAAAG
CAATGTATGCCCCTCCCATCAGAGGAGCAATTAACTGCTCATCAAAT---
ATTACAGGAATACTGTTAACAAGAGATGGTGGAAGTAACCAAAGT----------------------------
----

AATAACATCGAGGTCTTCAGACCTGGAGGAGGAGATATGAGGGACAATTGGAGAAGTGAATTATATAAATATA
AAGTAGTAAGAATTGAACCATTA---GGACTAGCACCTACTAAGGCAAGGAGAAGAGTGGTG-----------
----------------------------CAGAGAGAAAAAAGAGCA---GCGGGC---CTGGGAGCT------
---GTGTTCCTTGGGTTCTTGGCAGCA---GCAGGAAGCACTATG---GGCGCAGCGTCA---
ATAACGCTGACGGTACAG---GCCAGACAA---TTATTG---TCTGGTATAGTGCAACAGCAGAAC---
AATTTG---CTGAGG---GCTATTGAGGCGCAACAACGCATGTTGCAACTCGCAGTCTGGGGCATC
>JF320530
TTCTACTGTGATACAACACCGCTGTTTAATTCTACTTGGAACAATACTACCAATGAAAATAACATTACT----
----

AGAGGAGAAAACATCACACTCCCATGCAGAATAAAACAAATTATAAATAGGTGGCAAGAAGTAGGAAAAGCAA
TGTACGCCCCTCCCATCGCAGGACAAATTAGATGCTCATCAAAC---
ATTACAGGGCTGTTATTAACAAGAGATGGTGGTAATAATAATAACACC-------------------------
----

ACCACCACCGAGACCTTCAGACCAGGAGGAGGGGATATGAGAGATAATTGGAGAAGTGAATTATATAAATATA
AAGTAGTAAAAATTGAGCCATTA---GGAATAGCACCCACCAAGGCAAGGAGAAGAGTGGTG-----------
----------------------------CAAAGAGAAAAGAGAGCA---GTGGGAACAATAGGAGCT------
---ATGTTCCTTGGGTTCTTGGGAGCA---GCAGGAAGCACTATG---GGCGCAGCGTCA---
ATAACGCTGACGGTACAA---GCCAGACTA---TTATTG---TCTGGTATAGTGCAGCAGCAGAAC---
AATCTG---CTGCAA---GCTATTGAGGCGCAACAGCATCTGTTGCAGCTCACAGTCTGGGGCATC
>JF320631
TTCTACTGTGACTCAACACAACTGTTTAATAGTACTTGGACATTTACTGACGGGAGGTTAAATGAAACTGGGT
TAAAT--
-------------------GAA---
ACTGAAGCTGGAAATATCACACTCCCATGCAGAATAAAACAATTTATAAATATGTGGCAGGGAGTAGGAAGAG
CAATGTATGCCCCTCCCATCAGAGGACACATTAACTGTACATCAAAT---

ATTACAGGGTTGCTATTAACAAGAGATAGTGGTAGTAATGAGACTAGT-------------------------
----

AAGAACGGGTCAGAGACCTTCAGACCTGGAGGAGAAAATATGAAGGACAATTGGAGAAGTGAATTATATAAAT
ACAAAGTAGTAAGAATTGAACCATTA---GGAATAGCACCCACCAGGGCAAGGAGAAGAGTGGTA--------
-------------------------------CAAAGAGAAAAAAGAGCA---GTGGGA---ATAGGAGCT---
------ATGTTCCTTGGGTTCTTGGGAGCA---GCAGGAAGCACTATG---GGCGCAGCGTCA---
CTGACGCTGACGGTACAG---GCCAGACAA---TTATTG---ACTGGTATAGTGCAACAGCAAAGA---
AATTTG---CTGAGA---GCTATTGAGGCGCAACAGCATATGTTGCAACTCACAGTCTGGGGCATT
>JF320045
TTCTACTGTAATACATCAAAACTGTTTAACAGTACTTGGAGTATGAATAGTACTGAAGGGAGTATGAATGGTA
CTGAAGGG---
-------------GCAAAT------
AACACTGATGAGAAAATCGTACTCCAATGCAGAATAAAACAAATTATAAACATGTGGCAGGAAGTAGGAAAAG
CAATGTATGCCCCTCCCATCAGCGGACTAATTAGATGTTCATCAAAT---
ATTACAGGGTTGTTATTAACAAGAGATGGCGGTAAAAAGGATAACATGACA----------------------
----

GAGAAGGATGAGACAGAGACTTTCAGACCTTGGGGAGGAGATATGAGGGACAATTGGAGAAGTGAATTATATA
AATATAAAGTAGTACAAATTGAACCATTA---GGAATAGCACCCACCAGGGCAAAGAGAAGAGTGGTG-----
----------------------------------CAGAGAGAAAAAAGAGCA---GTGGGA---
ATAGGAGCT---------CTATTCCTTGGGTTCTTGGGAGCA---GCAGGAAGCACTATG---
GGCGCAGCGGCA---GTGACGCTGACGGTACAG---GCCAGACAA---TTATTG---
TCTGGTATAGTGCAACAGCAGAAC---AATCTG---CTGAGA---
GCTATTGAGGCGCAACAGCATCTGTTGCAACTCACAGTCTGGGGCATC
>JF320173
TTCTATTGTAATACCACACAACTGTTTAATAATACTTGGTCTAACGGTACTTGGAATAGTACTTGGAATAGT-
----

AGTATCACACATATCACACTCCCATGCAAAATAAGACAAATTATAAACATGTGGCAGGAGGTAGGAAAAGCAA
TATATGCCCCTCCCATCAGAGGGCTAATTAGCTGTTCATCAAAT---
ATTACAGGGCTGCTGTTAGTAAGAGATGGTGGTACTAATACTACTGAA-------------------------
----

GACAATACTGAGATCTTCAGACCTGGAGGAGGAGATATGAGGGACAATTGGAGAAGTGAATTATATAAATATA
AAGTAGTAAAAATTGAACCATTA---GGAGTAGCACCCACCAGGGCAAAGAGAAGAGTGGTG-----------
----------------------------CAAAGAGAAAAAAGAGCA---GTGGGAACAATAGGAGCT------
---ATGTTCCTTGGGTTCTTGGGAGCA---GCAGGAAGCACTATG---GGCGCAGCGTCA---
ATGGCGCTGACGGTACAG---GCCAGACAA---TTATTG---TCTGGTATAGTGCAACAGCAAAAT---
AATTTG---CTGAGG---GCTATTGAGGCGCAACAACATTTGTTGCAACTCACAGTCTGGGGCATT
>JF320387
TTCTACTGTAATTTATCACAGCTGTTTAATAGTACTTGGAATTTTACTGAAGGGTCAAATAGCACAGGAAAT-
----

TTGTCAGACACTATCACAATCCCATGCAGAATAAAGCAAATTATAAACATGTGGCAGGAAGTAGGAAAAGCAA
TATATGCTCCTCCTATCAGGGGACTAATTAGATGTTCATCAAAT---
ATTACAGGGCTGTTAATAACAAGAGATGGTGGTATTAATAATAGTGATAGT----------------------
----

GATGATAACACCGAGATCTTTAGACCTGCAGGGGGAAATATGAAGAACAATTGGAGAAGTGAATTATATAAAT
ATAAAGTAGTAAAACTTGAACCATTA---GGAGTGGCACCCACCAAGGCAAAGAGAAGAGTGGTG--------
-------------------------------CAAAGAGACAAAAGAGCA---GTGGGAATGATAGGAGCT---
------ATGTTCCTTGGGTTCTTGGGAGCA---GCAGGAAGCACTATG---GGCGCAGCGTCA---
CTAACGCTGACGGTACAG---GCCAGACAA---TTATTG---ACTGGTATAGTGCAACAGCAGAGC---
AATTTG---CTGAGA---GCTATTGAGGCGCAACAGCACCTGTTGCAACTCACAGTCTGGGGCATC
>JF320117
TTCTACTGTAATTCAACACAATTATTTAACAGTACTTGGAATGATACTACAGGGACAGGGAACATT-------
----

ACAGGAAATATTATTACACTCCCATGCAGAATAAGACAAATTATAAATATGTGGCAGGAAGTAGGAAAAGCAA

TGTATGCCCCTCCCATCAGAGGAATAATTAGCTGTTCATCAAAT---
ATTACAGGGCTGATATTAACAAGAGATGGGGGTAATAATGAGACC----------------------------
----

AATGGACCTGAGATCTTCAGACCTCTAGGAGGAAATATGAGGGATAATTGGAGAAGTGAACTATATAAATATA
AAGTAGTAAAGATAGAACCATTA---GGAGTAGCACCCACCAAGGCAAAGAGAAGAGTGGTG-----------
----------------------------CAGAGAGAAAAAAGAGCA---ATAGGA---CTAGGAGCT------
---GTGTTCATTGGGTTCTTGGGAGCA---GCAGGAAGCACTATG---GGCGCAGCGTCA---
GTGACGCTGACGGTACAG---GCCAGACAA---TTGTTG---TCTGGTATAGTGCAACAGCAGAAC---
AACTTG---CTGAAG---GCTATTGAGGCGCAACAGCATCTGTTGCAACTCACAGTCTGGGGCATC
>JF320182
TTCTACTGTAATACATCACAGCTGTTTAATAGCACGTGGCCAATTAATGGTGCATGGAATGGAACT-------
----

GAAGGGTCAGATATCACACTTCCATGTAGAATAAGACAAATTGTAAACAGATGGCAGGAAGTAGGAAAAGCAA
TGTATGCCCCTCCCATCAGTGGACTAATTAGATGTTCATCAGAT---
ATTACAGGGATACTATTAACAAGAGATGGTGGGGGCAATGAGACA----------------------------
----

GAGACAGAGACCTTCAGACCTGGAGGAGGAAATATGAGGGACAATTGGAGAAGTGAATTATATAAATATAAAG
TAGTAAAAATTGAGCCATTA---GGGATAGCACCCACCAAGGCAAGGAGAAGAGTGGTG--------------
-------------------------CAGAGAGAAAAAAGAGCA---GTGGGAACAATAGGAGCT---------
ATGTTCCTTGGGTTCTTGGGAGCA---GCAGGAAGCACTATG---GGCGCAGCGTCA---
GTGACGCTGACGGTACAG---GCCAGACTA---TTATTG---TCTGGTATAGTGCAACAGCAGAAT---
AATCTG---CTGAGG---GCTATTGAGGCGCAGCAGCATCTGTTGCAGCTCACAGTCTGGGGCATC
>JF320577
TTCTACTGTAATACAACATCACTGTTTAATAGTACTTGGAATAGTACTGGGAGGCTTAATGATACTGAAGGG-
----

TCAAATGACACCATTACACTCCAATGCAGAATAAAACAAGTTATAAACTTGTGGCAGGAAGTAGGAAAAGCAA
TGTATGCTCCTCCCATCAAAGGACCAATTAGGTGTTCATCAAAT---
ATTACAGGACTGATATTAACAAGAGATGGTGGTAACAGTAACGAG----------------------------
----

AGCGAAAGCGAGACCTTCAGACCTGGAGGAGGAGATATGAGGGATAATTGGAGAAGTGAATTATATAAATACA
AAGTAGTAAAAATTGAACCATTA---GGAGTAGCACCCACCAAGGCGAAGAGAAGAGTGGTG-----------
----------------------------CAGAGAGAAAAAAGAGCA---ATTGGA---CTAGGAGCT------
---ATGTTCCTTGGGTTCTTGGGAGCA---GCAGGAAGCACTATG---GGCGCAGCGTCA---
ATAACGCTGACGGTACAG---GCCAGACAA---TTATTG---TCTGGTATAGTGCAACAGCAGAAC---
AATTTG---CTGAGA---GCTATTGAGGCGCAACAACATCTGTTGCAACTCACAGTCTGGGGCATC
>KX156431
TTCTACTGTAATACAACACGACTGTTTAATAGTACTTGGGATGTTAATAATACTTGGGTTGATGGTAATTGGA
GTGATACC---
----------AACAGCGGA------
GTAAATATCACAAATATCACCCTCCCATGCAGAATAAAACAAATTGTAAACATGTGGCAGAAAGTAGGAAAAG
CAATGTATGCCCCTCCCATTAGAGGACAAATTAGATGCTCATCAAAT---
ATTACAGGGCTGCTATTAACAAGAGATGGTGGTGATATCAATAGC----------------------------
----

ACGACCGAGACCTTCAGACCTGGAGGAGGAGATATGAAGGACAATTGGAGAAGTGAATTATATAAATATAAAG
TAGTAAGAATTGAACCATTA---GGAATAGCACCCACCAAGGCAAAGAGAAGAGTGGTG--------------
-------------------------CAGAGAGAAAAAAGAGCA---GTGGGA---ATAGGAGCT---------
CTGTTCCTTGGGTTCTTGGGAGCA---GCAGGAAGCACTATG---GGCGCAGTGTCA---
ATGACGCTGACGGTACAG---GCCAGACTA---TTATTG---TCTGGTATAGTGCAACAGCAGAAC---
AATTTG---CTGAGG---GCTATTGAGGCGCAACAGCATCTGTTGCAACTCACAGTCTGGGGCATC
>HQ216971
TTCTACTGTAATACAACAAGGCTGTTTAATAGTACTTGGAATGGTACTGAGGTAACTAACAATGGAGAA----
----

AATAGCACAATTATCACACTCCAATGCAAAATAAAACAATTTATAAACATGTGGCAGAGAGTAGGAATAGCAA

TGTATGCCCCTCCCATCACAGGACAAATCAGTTGTTCATCAAAT---
ATTACAGGGCTGCTATTAACAAGAGACGGTGGTAATAATAACAGT----------------------------
----

ACAGAGACCTTCAGACCTCTAGGAGGAAATATGAGGGACAATTGGAGAAGTGAATTATATAAATATAAAGTAG
TAAAGGTTGAACCATTA---GGAATAGCACCCTCCAGGGCAAGAAGAAGAGTGGTG-----------------
----------------------CAGAGAGAAAAAAGAGCG---GTGGGAGCACTAGGAGCT---------
TTGTTCCTTGGGTTCTTGGGAGCA---GCAGGAAGCACTATG---GGCGCAGCGTCT---
CTGACACTGACGGTACAG---GCCAGACAA---TTATTG---TCTGGTATAGTGCAACAGCAGAAC---
AATCTG---CTGAGA---GCTGTTGAGGCGCAACAACATCTGTTGCAACTCACAGTCTGGGGCATC
>HQ908150
TTCTACTGTAATACAACAGGACTATTTAATAGTACTTGGAATAGTACAGAAGGGTCAAATAAT----------
----

GATGGCAAAAACATAACACTCCAGTGCAGAATAAAACAAATTATAAACCTGTGGCAGGAAGTAGGAAAAGCAA
TGTATGCCCCTCCCATCCGAGGAGAAATTAGATGTTCATCAAAC---
ATTACAGGGTTGCTACTAACAAGAGATGGTGGTAATGACACAGAA----------------------------
----

AACATCGAAACCTTCAGACCTGCAGGAGGGGATATGAGGGACAATTGGAGAAGTGAATTATATAAATATAAAG
TAGTAAAAATTGAACCATTA---GGAGTAGCACCCACCAAGGCAAAGAGAAGAGTGGTG--------------
-------------------------CAGAGAGAAAAAAGAGCA---GTGGGA---ATAGGAGCT---------
ATATTCCTTGGGTTCTTGGGAGCA---GCAGGAAGCACTATG---GGCGCAGCGTCA---
GTGACGTTGACGGTACAG---GCCAGACAA---TTATTG---TCTGGTATAGTGCAACAGCAAAAC---
AATTTG---CTGAGG---GCTATTGAGGCGCAACAAAGTCTGTTGCAACTCACAGTCTGGGGCATC
>HQ217045
TTCTACTGTGATACAGCAAAACTGTTTAATGGTACTTTAAACAATACTATAAATAATACTACATGGAAGTTTG
AA---

GGGTCAAATGGCACTATCATACTCCCATGCAGAATAAGACAAATTGTAAACATGTGGCAGGAAGTAGGAAAAG
CAATGTATGCCCCTCCCATCAAAGGAAACATTAGTTGTACATCAAAT---
ATTACAGGGCTGCTATTAGTAAGAGATGGTGGTGTTAAAAACAGC----------------------------
----

ACGAACGAGACCTTCAGACCTGGAGGAGGAAATATGAAAGACAATTGGAGGAGTGAATTATACAAATATAAAG
TAGTAAAAATTGAGCCATTA---GGAATAGCACCCACCAAGGCAAAGAGAAGAGTGGTG--------------
-------------------------CAGAGAGAAAAAAGAGCA---GTGGGA---ATAGGAGCT---------
TTGTTCCTTGGGTTCTTGGGAGCA---GCAGGAAGCACTATG---GGCGCAGCGTCA---
ATGACGCTGACGGTACAG---GCCAGACTA---TTATTG---TCTGGTATAGTGCAACAGCAGAAC---
AATCTG---CTGAGG---GCTATTGAGGCGCAACAGCATCTGTTGCAACTCACAGTCTGGGGCATT
>HQ908184
TTCTACTGTAATACAACAAATCTGTTTACTAACGAGCTAAATGAC----------------------------
----

ACTGATGGAAATATCATACTCCCATGTAGAATAAAACAAATTATAAACATGTGGCAGGAAGTAGGAAAAGCAA
TGTATGCTCCGCCCATTAGTGGACCAATTAATTGTTTATCAAAT---
ATCACAGGGCTGCTATTAACAAGAGATGGTGGTATAGGCAACAAC----------------------------
----

ACCATCAACGAAACCTTCAGACCTGGAGGAGGAGATATGAGGGACAATTGGAGAAGTGAACTATATAAATACA
AAGTAGTAAAAATTAAACCATTA---GGATTAGCACCCACTAAGGCAAAGAGAAGAGTGGTG-----------
----------------------------CAAAGAGAAAAAAGAGCA---GTGGGA---ATAGGAGCT------
---TTGTTCCTTGGGTTCTTGGGAGCA---GCAGGAAGCACTATG---GGCGCAGCGTCA---
TTGACGTTGACGGTACAG---GCCAGATCC---TTATTG---TCTGGTATAGTGCAACAGCAGAAC---
AATCTG---CTGAGG---GCTATTGAGGCGCAACAGCATCTGTTGCAACTCACAGTCTGGGGCATC
>KM353733
TTCTACTGTAATACAACACAACTGTTTAATAGTACTTGGCCGCTTAATAGTACCTGG----------------
----
GAGGAGACTGAAAGGTCA---------
AATGGCACTGGTGTATACACACTCCAATGCAAAATAAAACAAATTATAAACAGGTGGCAGGAAGTAGGAAAAG

```
CAATGTATGCTCCTCCCATCAAGGGAATCATCAGTTGTGCATCAAAT---
ATTACAGGTCTGCTATTAACAAGAGATGGTGGTAAT-------------------------------------
-----------------------------------------------------
AAGGCAAACACAAACGGAACTGAT---------
GAGACTGAGATCTTCAGACCTGGAGGAGGAGATATGAGGGACAATTGGAGAAGTGAATTATATAAATATAAAG
TAGTAAGGATTGAGCCATTG---GGAGTAGCACCCACTAAGGCAAAAAGAAGAGTGGTG--------------
-------------------------CAGAGAGAAAAAAGAGCA---GTAGGA---CTAGGAGCT---------
ATGTTCCTTGGGTTCTTGAGCACA---GCAGGAAGCACTATG---GGCGCAGCGTCA---
GTGACGCTGACGGTACAA---GCCAGACAA---TTATTG---TCTGGTATAGTGCAACAGCAGAAC---
AATTTG---CTGAGG---GCTATTGAGGCGCAACAGCATCTGTTGCAACTCACAGTCTGGGGCATC
>KM354114
TTCTACTGTAATACAACAAAACTGTTTAATGGTACTTGGTTTAATAGT-------------------------
-------------------------------------------------------------------------
----------ACTGTGAAT------
GATACTGAAGAAGAAATCATACTCCCATGCAGGATAAAACAAATTATAAATAGATGGCAGGAAGTAGGAAAGG
CAATGTATGCCCCTCCCATCAGAGGATACATTAATTGTTCATCAAAT---
ATTACAGGAATGATATTGACAAGAGATGGTGGT----------------------------------------
-------------------------------------------------------------------------
----
AATAACACCAGCAATGAGACCTTCAGACCTGGAGGAGGAGATATGAGGGACAATTGGAGAAGTGAATTATATA
AATATAAAGTAGTAAGAATTGAACCATTA---GGAATAGCACCCACTAAGGCAAGAAGAAGAGTGGTG-----
----------------------------------CAGAGAGAAAAAAGAGCA---GTGGGA---
ATAGGAGCT---------ATATTCCTTGGGTTCTTGGGAGCA---GCAGGAAGCACTATG---
GGCGCAGCATCA---ATGACGCTGACGGTACAG---GCCAGACAA---TTATTG---
TCTGGTATAGTGCAACAGCAGAAC---AACCTG---CTGAGG---
GCTATTGAGGCGCAACAGCATCTGTTGCAACTCACAGTCTGGGGCATT
>HQ217096
TTCTACTGTAATTCAACACAATTGTTTAATAGTACTTGGGATAAGGCTAATGACACTTTTTCTAGGAATGGGA
CT-----------------------------------------------------------------------
-------------------------
AGGTTAAATGGAACTATCACACTCCCATGTAGAATAAAACAAATTATAAACATGTGGCAGGGAGTAGGAAAAG
CAATGTATGCCCCTCCTATCAAAGGACAAATTAGCTGTTCATCAAAT---
ATTACAGGGCTGCTATTAACAAGAGATGGTGATAAGAATAAGAAC----------------------------
-------------------------------------------------------------------------
-------------
GAGACCGAGATCTTCAGACCGATAGGAGGAGATATGAGGGGCAATTGGAGAAATGAATTATATAAATATAAAG
TAGTAAAAATTGAACCATTA---GGAGTAGCACCCACCAAGGCAAAAAGAAGAGTGGTG--------------
-------------------------CAGAGAGAAAAAAGAGCA---GTGGGA---ATAGGAGCA---------
TTGTTCCTTGGGTTCTTGGGAGCA---GCAGGAAGCACTATG---GGCGCAGCGTCA---
CTAACGCTGACGGTACAG---GCCAGACAA---TTATTG---TCTGGTATAGTGCAGCAGCAAAGC---
AATTTG---CTGAGG---GCTATTGAGGCGCAGCAGCAGCTGTTGCAACTCACAGTCTGGGGCATC
>HQ217102
TTCTACTGTAATACAACACCACTGTTTAGTAGTACTTGGAATGAGACTACTTGGTCTAATAGTACTGCAAATA
CCACT--------------------------------------------------------------------
-------------------GAA---
AAGGAAAGTGAAAATATTACACTCCAATGTAGACTAAAACAAATTGTAAACCTGTGGCAGGAGGTAGGAAAAG
CAATGTATGCCTCTCCCATCAGTGGACCAATTAGCTGTAAATCAAAT---
ATTACAGGGCTGATATTAACAAGAGATGGTGGTACAAATAATAATACG-------------------------
-------------------------------------------------------------------------
-------
AACGGACCACCGAGACCTTCAGACCTGGAGGAGGAGATATGAGGGACAATTGGAGAAGTGAATTATATAAAT
ACAAAGTAGTAAAAATTGAACCATTA---GGAATAGCACCCACCAGGGCAAAGAGAAGAGTGGTG--------
-------------------------------CAGAGAGAAAAAAGAGCA---GTGGGA---ATAGGAGCT---
------TTGTTCCTTGGGTTCTTGGGAGCA---GCAGGAAGCACTATG---GGCGCAGCGTCA---
ATGACGCTGACGGTACAG---GCCAGACTA---TTATTG---TCTGGTATAGTGCAACAGCAGAAC---
AATTTG---CTGAGG---GCTATTGAGGCGCAACAGCATCTGCTGCAACTTACAGTCTGGGGCATC
>JN687759
TTCTACTGTAATACATCATCACTGTTTAATAGTACTTGGAATGAAAATACAACTACAGATCTAAAT-------
-------------------------------------------------------------------------
----------------------------
```

AATAATGGAAATATCACACTCAAATGCAGGATAAAACAAATTATAAGAATGTGGCAGGAAGTAGGAAAAGCAA
TGTATGCTCCTCCCATCGAAGGACAAATTAGCTGTTCATCAAAT---
ATTACAGGGCTGCTATTAACAAGAGATGGTGGAAATGAGAACGAGACC-------------------------
----

AACGCGACCACCGAGACCTTCAGACCTGGGGGAGGAGATATGAGGGACAACTGGAGAAGTGAATTATATAAAT
ATAAAGTAGTACAACTTGAACCATTA---GGAGTGGCACCCACCAAGGCAAAGAGAAGAGTGGTG--------
-------------------------------CAGAGAGAAAAAAGAGCA---GTGGGA---ATAGGAGCT---
------GTGTTTCTTGGGTTCTTGGGAGCA---GCAGGAAGCACTATG---GGCGCAGCGTCA---
CTGACGCTGACGGTACAG---GCCAGACAA---TTATTG---TCTGGTATAGTGCAACAGCAGAAC---
AATCTG---CTGAGG---GCTATTGAGGCGCAACAGAGTCTGTTGCAACTCACAGTCTGGGGCATT
>JN687761
TTCTACTGTAATTCAACACAACTGTTTAATAGCACTTGGGATTCAAATAGCACTTGGGATGATTCAAATAATA
CTATT--

GAGGAGAATGGGACTATCACACTCCCATGTAGAATAAAACAAATTGTAAACATGTGGCAAAAAGTAGGGAGAG
CAATGTATGCCCCTCCCATCAGGGGACAAATTAGATGCTCATCAAAT---
ATTACAGGACTGCTATTAGCAAGAGATGGGAGTGACAACAGCATT----------------------------
----

AACGAGACCTTCCGACCTGGAGGAGGAGATATGAGGGACAATTGGAGAAGTGAATTATACAAATATAAAGTAG
TACAAATTGAACCAGTA---GGAATAGCACCCACCAAGGCAAAGAGAAGAGTGGTG-----------------
----------------------CAGAGAGAAAAAAGAGCA---GTGGGA---CTAGGAGCT---------
ATGTTCCTTGGGTTCTTGGGAGCA---GCAGGAAGCACTATG---GGCGCAGCGTCA---
ATAACGCTGACGGTACAA---GCCAGACAA---TTATTG---TCTGGTATAGTGCAACAGCAGAAC---
AACCTG---CTGAGA---GCTATTGAGGCGCAACAGCATCTGTTGCAACTCACAGTCTGGGGCATC
>JN687762
TTCTACTGCAATTCAACACAGCTGTTTAATAGTAGTTGGAATAGTACTGCTAATAGTACTGAAGGGAATGATG
AAACT--

ATCAATAATGAAATTATCACACTCCCATGCAGAATAAAACAAATTATAAACATGTGGCAGGAAGTAGGAAAAG
CAATGTATGCCCCTCCCATCGCAGGACCAATTAGATGTTCATCAAAT---
ATTACAGGGCTGTTATTAACAAGAGATGGTGGGAATAATGGGACT----------------------------
----

AATGAGACCGAGACCTTCAGACCTGCAGGAGGAAATATGAAGGACAATTGGAGAAGTGAATTATATAAATATA
AAGTAGTAAAAATTGAACCATTA---GGAGTAGCACCCACCAAGGCAAGGAGAAGGGTGGTG-----------
----------------------------CAGAGAGAAAAAAGGGCA---GCA------CTAGGAGCT------
---GTGTTCCTTGGGTTCTTGGGAGCA---GCAGGAAGCACTATG---GGCGCAGCATCA---
GTAATGCTGACGGTACAG---GCCAGACAA---TTATTG---TCTGGTATAGTGCAACAGCAGAAC---
AATCTG---CTGAGA---GCTATTGAGGCGCAACAGCACCTGTTGCAACTCACAGTCTGGGGCATC
>HQ217115
TTCTACTGTAATTCAACAAAACTGTTTAATAGTACTTGGGAGACTAATGGTACTTCGAATTTCAATGAGACTA
GA---

GGGTCAAATGACCATATCACACTCCCATGCAGAATAAAACAAATTATAAACATGTGGCAGGAAGTAGGAAAAG
CAATGTATGCCCCTCCTATCAGTGGACCAATTAGATGTTCATCAAAT---
ATTACAGGGCTGCTATTAACAAGAGATGGTGGTAATGAGAGCAGC----------------------------
----

ACCAACGAGACCTTCAGACCTGGGGGAGGAAACATGAAGGACAATTGGAGAAGTGAATTATATAAATATAAAG
TAGTAAAAGTTGAACCATTA---GGATTAGCACCCACCAAGGCAAAAAGAAGAGTGGTG--------------
-------------------------CAGAGAGAAAAAAGAGCA---ATAATGGGAATGGGAGCT---------
TTGTTCCTTGGGTTCTTGGGAGCA---GCAGGAAGCACTATG---GGCGCAGCGTCA---
ATAACGCTGACGGCACAG---GCCAGACAA---TTATTG---TCTGGTATAGTGCAGCAGCAAAAC---
AACTTG---CTGAGA---GCTATTGAGGCGCAACAGCATATGTTGCAACTCACAGTCTGGGGCATC
>KM259082
TTCTACTGTAATACAACAAAACTGTTTAATAGTACTTGGACTTGGAATAGTACTATT----------------
----
AATAGTACCGAGAATAGT---------

```
AGAAATGACACTAATATCACACTCCCATGCAGAATAAAACAAATTATAAACATGTGGCAGAAAGTAGGAAGAG
CAATGTATGCCCCTCCCATCGCAGGAATAATTAAATGCTCATCAAAT---
ATTACAGGGCTGCTATTAACAAGAGATGGTGGTAAAAAG----------------------------------
-----------------------------------------------------
ACTAAGGATAACGGGACTGAGAGC---------
GACACTGAGATCTTCAGGCCTGGAGGAGGAGATATGAGGGACAATTGGAGAAGTGAATTATATAAATATAAAG
TAGTAAAAATTGAACCATTA---GGAGTAGCACCCACCAAGGCAAAAAGAAGAGTGGTG--------------
-------------------------CAAAGAGAAAAAAGAGCA---GTGGGA---ATAGGAGCT---------
GTGTTCCTTGGGTTCTTGGGAGCA---GCAGGAAGCACTATG---GGCGCAGCGGCA---
TTAACGCTGACGGTACAG---GCCAGACAA---TTATTG---TCTGGTATAGTGCGACAGCAGAAC---
AATCTG---CTGAGA---GCTATTGAGGCGCAGCAACATCTGCTGCAACTCACAGTCTGGGGCATC
>HQ908139
TTCTACTGTAATACAACAAAACTGTTTAATAGTACTTGGTATAGTAATACAACTGATACAGATAAT-------
-------------------------------------------------------------------------
----------------------------
ACTACAGGGAATATCACACTCCCATGCAGAATAAAACAAGTTATAAACATGTGGCAGGAAGTAGGAAAAGCAA
TGTATGCCCCTCCCATCAGAGGACAAATCAGATGTTCATCAAAT---
ATTACAGGGCTGTTATTAACAAGAGACGGTGGTAAGAACAATACC----------------------------
-------------------------------------------------------------------------
----------
AACGGTACCGAGACTTTCAGACCTGGAGGAGGAGATATGAGGGACAATTGGAGAAGTGAATTATATAAATATA
AAGTAGTAAAAATTGAACCATTA---GGAGTAGCACCTACCAAGGCAAGGAGAAGAGTGGTG-----------
----------------------------CAGAGAGAAAAAAGAGCA---GTGGGA---ATAGGAGCT------
---GTACTCCTTGGGTTCTTGGGAGCA---GCAGGAAGCACTATG---GGCGCAGCATCA---
ATAACGCTGACGGTACAG---GCCAGACAA---TTATTG---TCTGGTATAGTGCAACAGCAGACC---
AATTTG---CTGAGG---GCTATTGAGGCGCAACAGCACCTGTTGCAACTCACAGTCTGGGGCATC
>HQ217160
TTCTACTGTAACACAACACAATTGTTTAATAGTACTTGGAATAATACTGAACTTAGTAGTACTTGGGAGACAG
AGCAA--------------------------------------------------------------------
-------------------------
AATGACAATGACAATATCACACTCCCATGCAGAATAAAACAAATTATAAACACATGGCAGGAAGTAGGAAAAG
CAATGTATGCCCCTCCCATCAAAGGAGTAATTAGATGTTCATCAAAT---
ATTACAGGGTTGTTATTAACAAGAGATGGTGGTAAGAACACGAAC----------------------------
-------------------------------------------------------------------------
-------------
ACCACCGAGACCTTCAGGCCTGGAGGAGGAGATATGAGGGACAATTGGAGGAGTGAATTATATAAATATAAAG
TAATACAAATTGAACCATTA---GGGGTAGCACCCACCAAGGCAAAGAGAAGAGCGGTG--------------
-------------------------CAGAGAGAAAAAAGAGCA---ATAACA---ATAGGAGCT---------
ATGTTCCTTGGGTTCTTGGGAGCA---GCAGGAAGCACTATG---GGCGCAGCGTCA---
TTGACGCTGACGGTACAG---GCCAGACAG---TTGTTG---TCCGGTATAGTGCAACAGCAGAAC---
AATCTG---CTGAGA---GCTATTGAGGCGCAACAGCACCTGTTGCAACTCACAGTCTGGGGCATC
>FJ469689
TTCTACTGTAATACAACGAAATTGTTTAATAGTACTTGGAATAAGACTGATAGTGTTAATAGTACTTGGAATT
TTATTGAAGGG--------------------------------------------------------------
-------TTAAATAACACT------
GGAGAAAATGATATAATCACACTCCCATGCAGAATAAAACAAATTATAAACATGTGGCAGGAAGTAGGAAAAG
CAATGTATGCCCCTCCCATCAAAGGAATAATTAGCTGTAGATCAAAT---
ATTACAGGGCTGATATTAACAAGAGATGGTGGTAGTAATAACACA----------------------------
-------------------------------------------------------------------------
----------------
AACGAGACCTTCAGACCTGGAGGAGGAGATATGAGGGACAATTGGAGAAGTGAATTATATAAATATAAAGTAG
TAAAAATTGAACCATTA---GGAGTAGCACCCACCAGGGCAAAGAGAAGAGTGGTG-----------------
----------------------CAGAGAGAAAAAAGAGCA---GTGGGA---CTAGGAGCT---------
CTGTTCCTTGGGTTCTTGGGAGCA---GCAGGAAGCACTATG---GGCGCAGCGTCA---
GTGACGCTGACGGTACAG---GCCAGACAA---TTACTG---TCTGGTATAGTGCAACAGCAGAAC---
AATTTG---CTGAGG---GCTATTGAGGCGCAACAGCATCTGTTGCAACTCACAGTCTGGGGCATC
>FJ469707
TTCTACTGTAACACAACACAACTGTTTAATAGTACTTGGAATGTTACTGAAGGAGAAGGGTCAAATGACACTG
AA-----------------------------------------------------------------------
----------------------------
```

```
ACAAATGACACAATCACACTCCCGTGCAGAATAAGACAAATTATAAATATGTGGCAGGAAGTAGGAAAAGCAA
TGTATGCCCCTCCCATCAGAGGACCAATTAGCTGTTCATCAAAT---
ATTACAGGGTTACTGTTAACAAGAGATGGTGGTAATACCACGGGG----------------------------
-------------------------------------------------------------------------
-------------
GAAAGTGAGACCTTCAGACCTGGAGGAGGAAATATGAAGGACAATTGGAGAAGTGAATTATATAAATATAAAG
TAGTAAAAATTGAACCATTA---GGAATAGCACCCACCACGGCAAAGAGAAGAGTGGTG--------------
-------------------------CAGAGAGAGAAAAGAGCA---GTGGGA---ATAGGAGCT---------
TTGTTCCTTGGGTTCTTGGGAGCA---GCAGGAAGCACTATG---GGCGCAGCGTCA---
ATGACGCTGACGGTACAA---GCCAGACTA---CTATTG---TCTGGTATAGTGCAACAGCAGAAC---
AATTTG---CTGAGG---GCTATTGAGGCGCAACAGCATCTGTTGCAACTCACAGTCTGGGGCATC
>FJ469716
TTCTACTGTGATACAACAAAACTGTTTAATAGTATCTGGCATAGTAATGATACCTGGACTGGGGCAAATAAC-
-------------------------------------------------------------------------
----------------------------
ACTGAAAAGAATATCACACTCCCATGCAGAATAAAACAAATTGTAAACATGTGGCAGGAAGTAGGAAAAGCAA
TGTATGCCCCTCCTATCGAGGGACAAATTAAATGTACATCAAAT---
ATTACAGGGCTGCTATTAACAAGAGATGGTGGTAATGAGACGAAG----------------------------
-------------------------------------------------------------------------
-------------------
GAAACTTTCAGACCTGGAGGAGGAGATATGAGGGACAATTGGAGAAGTGAATTATATAAATATAAAGTAGTAA
AAATTGAACCATTA---GGAATAGCACCCACCGAGGCAAAGAGAAGAGTGGTG--------------------
-------------------CAGAGAGAAAAAAGAGCA---GTGGGAGCACTAGGAGTT---------
ATGTTCCTTGGGTTCTTGGGAGCA---GCAGGAAGCACTATG---GGCGCAGCGTCA---
ATGGCGCTGACGGTACAG---ACCAGACAA---TTATTG---TCTGGTATAGTGCAACAGCAAAAC---
AATCTG---CTGAAG---GCTATTGAGGCGCAACAGCATCTGTTGCAACTCACGGTCTGGGGCATC
>FJ469721
TTCTACTGTAATTCAACACAACTGTTTAATAGTACTTGGAATAGTACTACTTGGAATAATACTAATGTGAATG
ATACTATT-----------------------------------------------------------------
-------------------GGG---
AATGAGACTGACCCAATCATACTCCCATGCAGAATAAAACAAATTATAAACATGTGGCAGGGAGTAGGAAGAG
CAATGTATGCCCCTCCCATCAGTGGACTAATTAGATGTTCATCAAAT---
ATTACAGGACTGCTATTAGTAAGGGATGGTGGTGTTAACAACAGC----------------------------
-------------------------------------------------------------------------
----------
CGCACAAATGAGACCTTTAGACCTTTAGGAGGAAATATGAAGGATAATTGGAGAAGTGAATTATACAAATATA
AAGTAGTAAGAATTGAACCATTA---GGAGTAGCACCCACCAAGGCAAGGAGAAGAGTGGTG-----------
----------------------------CAGAGAGAAAAAAGAGCA---CTGGGA---ATAGGAGCT------
---GTGTTCATTGGGTTCTTGGGAGCA---GCAGGAAGCACTATG---GGCGCAGCGTCA---
GTGACGCTGACGGTACAG---GCCAGACTA---TTGCTG---TCTGGTATAGTGCAACAGCAGAAC---
AACTTG---CTGAGA---GCTATTGAGGCGCAACAACATCTGTTGCAACTCACAGTCTGGGGCATT
>MN516423
TTCTACTGTAATACAACACAACTATTTAATAGTACATGGACATGGAATACTACCGGGGATGGTACCTGGAAG-
-------------------------------------------------------------------------
-GTTACTGAAGGGTCAAAT------
AACACTGCAGAAAACATCACACTCCCATGTAGAATAAAACAAATTATAAATATGTGGCAGGAAGTAGGAAAAG
CAATGTATGCCCCTCCCATCAGAGGAAAAATTAGATGCTCATCAAAT---
ATTACAGGGCTACTATTAACAAGAGATGGGGGTGATACAAACGAA----------------------------
-------------------------------------------------------------------------
----------
GGCAACAATGAGACCTTCAGACCTGCAGGAGGGGATATGAAGGACAATTGGAGAAGTGAACTATATAAATATA
AAGTAGTACAAATTGAACCATTA---GGAGTAGCACCCACCAAGGCAAAGAGAAGAGTGGTG-----------
----------------------------CAGAGAGAAAAAAGAGCT---GTGGGA---CTAGGAGCG------
---TTGTTCCTTGGGTTCCTGGGAGCA---GCAGGAAGCACTATG---GGCGCAGCGTCG---
CTGACGCTGACGGTACAG---GCCAGACAA---TTATTG---TCTGGTATAGTGCAACAGCAAAAC---
AATTTG---CTGCAG---GCTATTGAGGCGCAACAGCGTCTGTTGCAACTCACAGTCTGGGGCATC
>HQ217308
TTCTACTGTAATACAACAAAACTATTTAGTAGTACTTGGAATAATACTTGGAATAGTACTTGGAATAGTACTA
AT-----------------------------------------------------------------------
-------------------------
```

GAATCAAATGACACAATTACACTCCCATGCAGAATAAAACAAATTATAAACAGATGGCAAGAAGTAGGAAGAG
CAATGTATGCCCCTCCCATCGAGGGACAAATCAGATGTTCATCAAAT---
ATTACAGGGTTGCTATTAACAAGAGATGGTGGCAATGGAGAGAATGAG-------------------------
----

TCTGATACTGAGATCTTCAGACCTGGGGGAGGAGATATGAGGGACAATTGGAGAAGTGAATTGTACAAATATA
AAGTAGTAAAAATTGAGCCATTA---GGAGTAGCACCCACCAAGGCAAAGAGAAGAGTGGTG-----------
----------------------------CAAAGAGAAAAAAGAGCA---ATAGGAACGATAGGAGCT------
---ATGTTCCTTGGGTTCTTGGGAGCA---GCAGGAAGCACTATG---GGCGCAGCGTCA---
ATAACGCTGACGGTACAA---GCCAGACAA---TTATTG---TCTGGTATAGTGCAACAGCAGAAC---
AATCTG---CTGAGG---GCTATTGATGCGCAACAGCATATGTTGCAGCTCACAGTCTGGGGCATC
>JQ403058
TTCTACTGTGATTCAACACAACTGTTTAATAGTACTTGGTCGTCCAATGGTACTTGGACTAATGATACTACAG
GGTTAAAT---
-------------ACAACT------
GATAAATATGATAACTTCACACTCCCATGCAAAATAAAACAAATTATAAACATGTGGCAGAAAGTAGGAAAAG
CAATGTATGCCCCTCCCATTAGCGGACTAATTAGCTGTGAATCAAAT---
ATCACAGGACTGATATTAGTAAGAGATGGTGGTAATAATGCAAACGAGACC----------------------
----

GACAACACCACCGAGATCTTCAGACCTGGAGGAGGAAATATGAAGGACAATTGGAGAAGTGAATTATATAAAT
ATAAAGTAGTAAGAATTGAACCATTA---GGAGTAGCACCCACCAGGGCAAAGAGAAGAGTGGTG--------
-------------------------------CAAAGAGAAAAAAGAGCA---GTGGGAACATTAGGAGCT---
------ATGTTCCTTGGGTTCTTGGGAGCA---GCAGGAAGCACTATG---GGCGCAGCATCA---
ATGACGCTGACGGTACAG---GCCAGACAA---TTATTG---TCTGGTATAGTGCAACAGCAGAAC---
AATTTG---CTGAGG---GCTATTGAGGCGCAACAGCATTTGTTGCAACTCACAGTCTGGGGCATC
>JQ403059
TTCTACTGTAATACAACACAGCTGTTTAATAGTACTTGGAATGTTACTGAAGAGACAAATAGCACTAGA----
----

GGAAATGACACAATCACACTCCAATGCAGAATAAAACAAATTATAAACATGTGGCAGGAAGTAGGAAAAGCAA
TGTATGCCCCTCCCATCAGGGGACAGATTAGATGTTCATCAAAT---
ATTACAGGGTTGCTACTAACAAGAGATGGTGGTGGTAACAATAAC----------------------------
----

AACAGCGAGGAGGTCTTCAGACCTGGAGGAGGAGATATGAGGGACAATTGGAGAAGTGAATTATATAAATATA
AAGTAGTAAAAGTTGAACCATTA---GGAGTAGCACCCACCAAGGCAAAGAGAAGAGCGGTG-----------
----------------------------CAGAGAGAAAAAAGAGCA---GTGGGG---ATAGGAGCT------
---ATGTTCCTTGGGTTCTTGGGAGCA---GCAGGAAGCACTATG---GGCGCAGCGTCA---
CTAACGCTGACGGTACAG---GCCAGACTA---TTATTG---TCTGGTATAGTGCAACAGCAGAAC---
AATTTG---CTGAAG---GCTATTGAGGCGCAACAGCATATGTTGCAACTCACAGTCTGGGGCATC
>JQ403060
TTCTACTGTAATTCAACACCACTGTTTAATAGTAATTGGTCAGCTAGTAGTCCTTGGAATAGTAGTACT----
----

GAACGAGGAAATATCACACTCCAATGCAGAATAAAACAAATTGTAAACATGTGGCAGGAAGTAGGAAGAGCAA
TGTATGCACCTCCCATCAAAGGAGTAATTAAATGTACATCAAAT---
ATTACAGGACTGATATTAACAAGAGATGGTAGTAATAACACTAGT----------------------------
----

GAAACTGAAACCTTCAGACCTGGAGGAGGAAATATGAAGGACAATTGGAGAAGTGAATTATATAAATATAAAG
TAGTAAAAATTGAACCATTA---GGAGTAGCACCCACTACAGCCAAGAGAAGAGTGGTG--------------
-------------------------CAGAGAGAAAAAAGAGCA---GTGGGA---ATAGGAGCT---------
GTGTTCCTTGGGTTCTTGGGAGCA---GCAGGAAGCACTATG---GGCGCAGCGTCA---
ATAACGCTGACGGTACAG---GCCAGACTA---TTATTG---TCTGGTATAGTGCAACAGCAGAAC---
AATCTG---CTGAGG---GCTATTGAGGCGCAACAGCACCTGTTGCAACTCACAGTCTGGGGCATT
>JQ403064
TTCTACTGTAATACAACACAACTGTTTAACACTAATTGGACTAGGACTAATGCTAATTGGACTGTTACTAATT
GGACTGAT---

GCTGGAGTAAATATTACACTCCCATGCAGAATAAAACAAATTATAAATATGTGGCAGGAAGTAGGAAAAGCAA
TGTATGCCCCTCCCATCAGAGGACAAATTAGATGCTCATCAAAT---
ATTACAGGGTTGCTATTAACAAGAGATGGTGGTAATATCACGAAC----------------------------
----

GAGACCACCGAGATCTTCAGGCCTGGAGGAGGAAATATGAGGGACAATTGGAGAAGTGAATTATATAAATATA
AAGTAGTAAAAATTGAACCATTA---GGAATAGCACCCACCAAGGCAAGGAGAAGAGTGGTG-----------
----------------------------CAAAGAGAAAAACGAACA---GTGGGA---CTAGGAGCT------
---TTGTTCATTGGGTTCTTGGGAGCA---GCAGGAAGCACTATG---GGCGCAGCGTCA---
CTGACGCTGACGGTACAA---GCCAGACTA---TTATTG---TCTGGTATAGTGCAACAGCAGAAC---
AACCTG---CTGAAG---GCTATTGAGGCGCAACAGCATCTGTTGCAACTCACAGTCTGGGGCATC
>JQ403077
TTCTACTGTGATACAACAGGACTGTTTAACAGCACTTGGAACTCAACAGAATGGGATAATAGCACAGATAAAA
AAGTCACA---
-------------ATCAAT------
AGCACAGATAAAAAAGTCACAATCAAATGTAGAATAAAACAAATTATAAACTTGTGGCAGGAAGTAGGAAAAG
CAATGTATGCCCCTCCCATCAAAGGACTAATTAGATGTAGTTCAAAT---
ATTACAGGGCTGCTGTTAACAAGAGATGGTGGTAACAGTAACAATAACAGTAAC-------------------
----

AACGGGACCGAGGTCTTTAGACCTGGAGGAGGAAATATGAAAGACAATTGGAGAAGTGAATTATATAAATATA
AAGTAGTGAAAATTGAGCCATTA---GGAATAGCACCCACCAAGGCAAAGAGACGAGTGGTG-----------
----------------------------CAGAGAGAAAAAAGAGCA---GTAACG---TTAGGAGCT------
---ATGTTCCTTGGGTTCTTGGGAACA---GCAGGAAGCACTATG---GGCGCAGCGTCA---
TTGACACTGACGGTACAG---GCCAGACTA---TTATTG---TCTGGTATAGTGCAACAGCAGAAT---
AATCTG---CTGAGA---GCTATTGAGGCGCAACAGCATATGTTGCAACTCACAGTCTGGGGCATT
>JQ403092
TTCTACTGTAATACAACACAGTTATTTAATAGTACTTGGGGTAATGGTACTTGGCAAGGGTCAAAT-------
----

GGGAATGATACTATCACACTCCCGTGCAGAATAAAACAGATTATAAACAGGTGGCAAGAAGTAGGAAAAGCAA
TGTATGCCCCTCCCATCAAAGGAATAATTAGATGTTCATCAAAA---
ATTACAGGGTTAATATTAACAAGAGATGGTGGTAACGTTAACAGT----------------------------
----

AGTGACCCCGAGATCTTCAGACCTGGAGGAGGAGATATGAGGGATAATTGGAGAAGTGAATTATATAAATATA
AAGTAGTAAGAATTGAGCCATTA---GGAATAGCACCCACCAAGGCAAAGAGAAGAGTGGTG-----------
----------------------------CGGAGAGAAAAAAGAGCA---GTAACA---CTGGGAGCT------
---ATGTTCCTTGGGTTCTTGGGAGCA---GCAGGAAGCACTATG---GGCGCAGCGTCA---
ATAACGCTGACGGTACAG---GCCAGACAA---TTATTG---TCTGGTATAGTGCAACAGCAGAAC---
AATTTG---CTGAGG---GCTATTGAGGCGCAACAACATATATTGCAACTCACAGTCTGGGGCATC
>JQ403093
TTCTACTGTAATACAACAGAATTGTTTAATGGTACTTTGAATAACACTGGGTGGAGTAATAGTAGCACTGGG-
----

GTAAATGGAACTATCATACTCCCATGCAGAATAAAACAAATTATAAACCTGTGGCAGGAAGTAGGAAAAGCAA
TGTATGCCCCTCCCATCAGAGGACAAATTAACTGTTCATCAAAG---
ATTACAGGGCTGTTATTAACAAGAGATGGTGGTACTAATAACGAGACC-------------------------
----

ACGAACGCCACCACCGAGATCTTTAGACCTGGAGGAGGAGATATGAGGGACAATTGGAGAAGCGAATTATATA
AATATAAAGTAGTAAAAATTGAACCATTA---GGAGTAGCACCCACCAAGGCAAAGAGAAGAGTGGTG-----
----------------------------------CAGAGAGAAAAAAGAGAA---
GTGGGAACACTGGGAGCT---------ATGTTCCTTGGGTTCTTGGGAGCA---GCAGGAAGCACTATG---
GGCGCAGCGTCA---CTGACGCTGACGGTACAG---GCCAGACTA---TTATTG---
TCTGGTATAGTGCAACAGCAGAAT---AATTTG---CTGAGG---
GCTATTGAGGCGCAACAGCATCTGTTGCAACTCACGGTCTGGGGCATC
>JQ403096
TTCTACTGTGATACAACACGGCTGTTTAATACTACCTGGAATGATACAAATAACAGTGGACTT----------
----

AACAATGAAACTATCACCCTCCCATGCAGAATAAGACAAATTATAAACAGGTGGCAGGAAGTAGGAAAAGCAA
TGTATGCCCCTCCCATCAGCGGACAAATTAATTGTTCATCAAAA---
ATTACAGGGCTGCTGTTAACAAGAGATGGTGGTACTAATGACACAAATAAC----------------------
----

ACAAATAAGACCGAGACCTTCAGACCAGGGGGAGGAGATATGAGGGACAATTGGAGAAGTGAATTATATAAAT
ATAAAGTAGTAAAAATTGAGCCATTA---GGAGTAGCACCCACCAAGGCAAAGAGAAGAGTGGTG--------
-------------------------------CAAAGAGAAAAAAGAGCA---GTGGGAGTGATAGGAGCT---
------ATGTTCCTTGGGTTCTTGGGAGCA---GCAGGAAGCACTATG---GGCGCAGCGTCA---
ATGACGCTGACGGTACAG---GCCAGACAA---TTATTG---TCTGGTATAGTGCAACAGCAGAAT---
AACTTG---CTGAGG---GCTATTGAGGCGCAACAGCACCTGTTGCAGCTCACAGTCTGGGGCATC
>HQ217347
TTCTACTGTAATTCAACAGTACTATTTAATAGTACTTGGAATTCAACAACTTGGAATTCAACACTGTTTAATA
GTACTGGG---
-------------AATGAG------
ACTAGTGGAGAAAATATCACCCTCAAATGCAGAATAAAACAGATTATAAACATGTGGCAAGAAGTAGGAAAAG
CAATGTATGCCCCTCCCATCAAAGGAAAAATTAAATGTAAATCAAAT---
ATTACAGGGCTGCTTTTAACAAGAGATGGGGGTAATGGGGGTAATGAT-------------------------
----

ACGAACAAGGAGATCTTCAGACCTGGAGGAGGAGATATGAGGGACAATTGGAGAAGTGAATTATATAAATATA
AAGTAGTAAGAATTGAACCATTA---GGAATAGCGCCCACCAAGGCAAAGAGAAGAGTGGTG-----------
----------------------------CAAAGAGAAAAAAGAGAT---TTGGGA---ATAGGAGCA------
---TTGTTCCTTGGGTTCTTGGGAGCA---GCAGGAAGCACTATG---GGCGCAGCGTCA---
ATGACGCTGACGGTACAG---GCCAGACAA---TTATTG---TCTGGTATAGTGCAACAGCAAAAC---
AATCTG---CTGAGG---GCTATTGAGGCGCAACAACATCTGTTGCAACTCACAGTCTGGGGCATC
>HQ217386
TTCTACTGTAACTCATCAAAACTATTTAATAGTACTTGGAATGTTAATAATACTTGGAATAATACTGGAGAAA
AA---

GAAAATGACACAATAATACTCCCATGCAGAATAAAACAGATTATAAACATGTGGCAGGAAGTAGGAAAAGCAA
TGTATGCCCCTCCCATCAGAGGACAAATTAAATGTTCATCAAAT---
ATTACAGGGTTGCTGTTAACAAGAGATGGAAGAAATAGTGAGGAC----------------------------
----

AATGACACTGAGACCTTCAGACCTGGAGGAGGGGATATGAGGGACAATTGGAGAAGTGAATTATATAAATATA
AAGTAGTAAAACTTGAACCATTA---GGAATAGCACCCACCAGGGCAAAGAGAAGAGTGGTG-----------
----------------------------CAGAGAGAAAAAAGAGCA---ATAGGA---ATAGGGGCT------
---ATGTTCCTCGGGTTCTTGGGAGCA---GCCGGAAGCACTATG---GGCGCAGCGTCA---
ATGACACTGACGGTACAG---GCCAGACAA---TTATTG---TCTGGTATAGTGCAACAGCAGAAC---
AATCTG---CTGAAG---GCTATTGAGGCGCAACAACATCTGTTGCAACTCACTGTCTGGGGCATT
>KC312583
TTCTACTGTAATACAACAAAGCTGTTTAATAGCACTTGGTTGTCTAATGATACTACTACTTGGAATGAGACTA
AT---

GACAACAGCACAATCATACTCCCGTGCAGAATAAGACAAATTATAAATAGATGGCAGGAAGTAGGAAAAGCAA
TGTATGCCCCTCCCATCAGCGGTCTAATTAAATGTACATCAAAT---
ATTACAGGGCTACTATTAACAAGAGATGGAGGTAATAATAATAATAATAGT----------------------
----

AATACCTCAGAAGAGATCTTCAGACCTGGAGGAGGGGATATGAGGGACAATTGGAGAAGTGAATTATATAAAT
ACAAAGTAGTCAAAATTGAGCCATTA---GGATTAGCACCCACCAAGGCAAAGAGAAGAGTGGTG--------
-------------------------------CAGAGAGAAAAAAGAGCA---ATAACG---ATGGGAGCT---
------TTGTTCCTTGGGTTCCTGGGAGCA---GCAGGAAGCACTATG---GGCGCAGCGTCA---
TTGACGCTGACGGTACAG---GCCAGACTA---TTATTG---TCTGGTATAGTGCAACAGCAGAAC---
AATCTG---CTGAGA---GCTATTGAGGCGCAACAGCATCTGTTGCAACTCACAGTCTGGGGCATC
>HQ217430
TTCTACTGTAATTCAACACAGCTGTTTAATAGTACTTGGACTTTTAATAGTAATAATACTACCTCTAGCAATG
GA---

AATAGCAATGGAAATATCATACTCCCGTGCAGAATAAAACAAATTATAAATAGGTGGCAGGAAGTAGGAAAAG
CAATGTATGCCCCTCCCATCAGTGGATTAATTAATTGCTCATCAAAT---
ATTACAGGGTTACTATTAACAAGAGATGGTGGTGATAATGAGACCAAC-------------------------
----

AACACCACCGAGACCTTCAGACCTGGGGGAGGAGATATGAGGGACAATTGGAGAAGTGAATTATATAAATATA
AAGTAGTAAAAATTGAGCCATTA---GGGATAGCACCCTCCAGGGCAAAGAGAAGAGTGGTG-----------
----------------------------CAGAGAGAAAAAAGAGCA---GTGGGAGTAATAGGAGCT------
---ATGTTCCTTGGGTTCTTGGGAGCA---GCAGGAAGCACTATG---GGCGCAGCGTCG---
ATGACGCTGACGGTACAG---GCCAGACAA---TTATTG---TCTGGTATAGTGCAACAGCAGAAC---
AATTTG---CTGAAA---GCTATTGAGGCGCAACAACATTTGTTGCAACTCACAGTCTGGGGCATC
>KM259264
TTCTACTGTAATACAACAGAATTGTTTAATAGTACTTGGAATGGTACTGTG----------------------
----
-------AACGAGTCAAAC------
AACACTGGAGAAACTATCACACTCCCATGCAGAATAAAACAAATTATAAACATGTGGCAGGAAGTAGGAAAAG
CAATGTACGCCCCTCCCATCAAAGGACAAATTAAATGTTCATCAAAT---
ATTACAGGGCTGCTGTTAACACGAGATGGTGG---

ACTAATAAGACTAAT---
GATACTGAGACCTTTAGACCTGGAGGGGGAAATATGAAGGACAATTGGAGAAGTGAATTATATAAATATAAAA
TAGTAAAAATTGAACCATTA---GGAATAGCACCCACCAAGGCAAAGAGAAGAGTGGTG--------------
-------------------------CAGAGAGAAAAAAGAGCA---GTGGGAACATTAGGAGCT---------
ATGTTCCTTGGGTTCTTGGGAGCA---GCAGGAAGCACTATG---GGCGCAGCGTCA---
CTGACGCTGACGGTACAG---GCCAGACTA---TTATTG---TCTGGTATAGTGCAACAGCAAAAC---
AATTTG---CTGAGA---GCTATTGAGGCGCAACAACATATGTTGCAACTCACAGTCTGGGGCATC
>KM259566
TTCTACTGTAACTCAACACCACTGTTTAACAGTACTTGGTTGTCTAATGGTACT-------------------
----
-TTGAATGAAACTGAA---------
GGCAACGGGGGCAACATCACCCTCCTATGCAGAATAAAACAAATTATAAATGTATGGCAGGAAGTAGGAAAAG
CAATGTATGCCCCTCCTATCAGAGGACAAATTAAATGTACATCAAAT---
ATTACAGGACTGCTATTAACAAGAGATGGTGGG--

AACAATGGGTCTGAA---
CCCACCGAGACCTTCAGACCTGGAGGGGGAAATATGAAGGACAATTGGAGAAGTGAATTATATAAATATAAGG
TAGTAAAAGTTGAACCATTA---GGATTGGCACCCACCAAGGCAAAGAGAAGAGTGGTG--------------
-------------------------CAGAGAGAAAAAAGAGCAATAGGGGGA---CTAGGAGCT---------
TTGTTCCTTGGGCTCTTGGGAGCA---GCAGGAAGCACTATG---GGCGCAGCGTCA---
CTAACGCTGACGGTACAG---GCTAGACAA---TTATTG---TCTGGTATAGTGCAACAGCAAAAC---
AACTTG---CTGAGG---GCTATTGAGGCGCAACAGCATCTGTTGCAACTCACAGTCTGGGGCATC
>HQ217509
TTCTACTGTAATACAACACAACTGTTTAATAGTACTTGGGGGTCAAATAACACTGAAGAGCCAAATAACACTG
GA---

GGAAATGACACAATCACACTCCCATGTAGACTAAAACAAATTATAAACATGTGGCAGGAAGTAGGAAAAGCAA
TGTATGCCCCTCCCATCAGAGGACAAATTAGATGTTCATCAAAT---
ATTACAGGACTGTTATTAACAAGAGATGGGGGGAACAGGAATGAGACTAAC----------------------
----

GGGAACGGGACTGAGATCTTCAGGCCTGGAGGAGGAGATATGAGGGACAATTGGAGAAGTGAATTATACAAAT
ATAAAGTAGTAAAAATTGAACCATTA---GGAGTAGCACCCACTAAGGCAAAAAGAAGAGTGGTG--------
-------------------------------CAAAGAGAAAAAAGAGCA---GTGGGA---TTAGGAGCT---
------ATGTTCCTTGGGTTCTTGGGAGCA---GCAGGAAGCACTATG---GGCGCAGCGTCA---
ATGACGTTGACGGTACAG---GCCAGACTG---TTATTG---TCTGGTATAGTGCAACAGCAGAAC---
AATCTG---CTGAGG---GCTATTGAGGCGCAACAACATCTGTTGCAGCTCACAGTCTGGGGCATC
>HQ217601
TTCTACTGTAATACAACAAAACTGTTTAATAGTACTTGGAATAGGACTACTAGGGAAAGCAATGTTACTGAAG
GGTCA--

GATAACAATGAAACTATCACACTCCAATGCAGGATAAAACAGATTATAAACATGTGGCAGGAAGTAGGAAAAG
CCATATATGCCCCTCCTATCAGCGGACCGATTAGATGTTCATCAAAT---
ATCACAGGGCTGCTATTAACAAGAGATGGAGGTAATACAAATAAGAGCAAT----------------------
----

GAGAACGAGACTGAGACCTTCAGACCTGGAGGAGGAGATATGAGGGACAATTGGAGAAGTGAATTATATAAAT
ATAAAGTAGTACAAATTGAACCATTA---GGAGTAGCACCCACCAAGGCAAAGAGAAGAGTGGTG--------
-------------------------------CAGAGAGAAAAAAGAGCA---GTGGGA---ATAGGAGCT---
------CTGTTCCTTGGGTTCTTGGGAGCA---GCAGGAAGCACTATG---GGCGCAGCGTCA---
ATAACGCTGACGGTACAG---GCCAGACAA---TTATTG---TCTGGTATAGTGCAACAGCAGAAC---
AACTTG---CTGAGG---GCTATTGAGGCGCAACAGCATATGTTGCAACTCACAGTCTGGGGCATC
>GQ256646
TTCTATTGTAATACAGCACAACTGTTTAATAGTACTTGGCCTGAGAATAGTACTTGGCCTGGGAATAATGGTA
CTACAGAGTCA--
-------AATAACAACACT------
GGAGAAAATGACACAATCACACTCCCATGCAAAATAAAACAAATTATAAACATGTGGCAGAAAGTAGGAAAAG
CAATGTATGCCCCTCCCATCAAAGGGATAATTAAATGTTCATCAAAT---
ATTACAGGATTGCTATTAGTAAGAGATGGTGGTAACACCAGCATG----------------------------
----

ACCAACGAGACTTTCAGACCTGGAGGAGGAGATATGAGAGACAATTGGAGAAGTGAATTATATAAATATAAAG
TAGTAAAAATTGAACCGTTA---GGAGTAGCACCCACCAAGGCAAAGAGAAGAGTGGTG--------------
-------------------------CAGAGAGAAAAAAGAGCA---GTGGGA---TTAGGAGCT---------
ATGTTCCTTGGGTTCTTGGGAGCA---GCAGGAAGCACTATG---GGCGCAGCGTCA---
CTATCGCTGACGGTACAG---GCCAGAAAC---TTATTG---TCTGGTATAGTGCAACAGCAAAAC---
AATTTG---CTGAGG---GCTATTGAGGCGCAACAGCATATGTTGCAACTCACAGTCTGGGGCATC
>JF689895
TTCTACTGTAATACAACACAACTGTTTAATAGTACCTGGAATAATAATAATACCTGGACTGGTAACACTGAA-
----

GGAAATGACACACTCATACTCCCATGCAGAATAAAACAAATTATAAACAGGTGGCAAGAAGTAGGAAAAGCAA
TGTATGCCCCTCCCATCAGAGGACCAATTAACTGTTTATCAAAT---
ATTACAGGGTTGCTATTAACAAGAGATGGAGGTGATAATAACAAC----------------------------
----

ATGAGTGAGATTTTCAGACCTGCAGGAGGAGATATGAGGGACAATTGGAGAAGTGAATTATATAAGTATAAAG
TAGTAAAAATTGAACCACTA---GGAATAGCACCTACCAAGGCAAAGAGAAGAGTGGTG--------------
-------------------------CAGAGAGAAAAAAGAGCA---GTGGGA---ATAGGAGCT---------
GTGCTCCTTGGGTTCTTAGGAGCA---GCAGGAAGCACTATG---GGCGCAGCGTCA---
ATGACGCTGACGGTACAA---GCCAGGCTA---TTATTG---TCTGGTATAGTGCAACAGCAGAGC---
AATTTG---CTGAGG---GCTATTGAGGCGCAACAGCATCTGTTGCAACTCACAGTCTGGGGCATT
>JF689896
TTCTACTGTAATTCAACACAACTGTTTAATAGTACTTGGAGGTTAAATAACACTGAAGGAAATAGCACTGGGT
CAGGTAAC---
-------------------ACT---
GAAGGAAATAACACAATCACACTCCCATGCAAAATAAAACAAATTGTAAACATGTGGCAGAAAGTAGGAAAAG
CAATGTATGCCCCTCCCATCAAAGGACAAATTAGATGTTCATCAAAT---
ATTACAGGGCTGCTATTAACAAGAGATGGGGGTAAAAACAACAGT----------------------------
----

GGGACCGAGACCTTCAGACCTGGAGGAGGAGATATGAGGGACAATTGGAGAAGTGAATTATATAAATATAAAG
TAGTAAAGCTTGAACCATTA---GGAGTAGCACCCACCAGGGCAAAGAGAAGAGTGGTG--------------
-------------------------CAGAGAGAAAAGAGAGCA---ATAGGA---ATAGGAGCT---------
ATGTTCCTTGGGTTCTTGGGAGCA---GCAGGAAGCACTATG---GGCGCAGCGTCG---
ATGACGCTGGCGGTACAG---GCCAGACAA---TTATTG---TCTGGTATAGTGCAACAGCAGAAC---
AATTTG---CTGAAG---GCTATTGAGGCGCAACAGCATCTGTTGCAACTCACAGTCTGGGGCATC
>JX864023
TTCTATTGTAATACAACAAAGCTGTTTAATAGTACTTGGGTGTTTAATGGTACTTGGACTATTAATGGGAGTG
TT---

ACTGAAGGAAATATCACACTCCCGTGCAGAATAAAACAAATTATAAACAGATGGCAGGAAGTAGGAAAAGCAA
TGTATGCCCCTCCCATCAGAGGACAAATTAGATGTATATCAAAT---
ATTACAGGGTTACTATTAACAAGAGATGGGGGTACTAAAAATAATAGTAATAAC-------------------
----

ACCAGTGAACCCGAGATCTTCAGACCAGGAGGAGGGGATATGAGAGACAATTGGAGAAGTGAATTATATAAAT
ATAAAGTAGTAAGAATTGAGCCATTA---GGAGTAGCACCCACCAAGGCAAAGAGAAGAGTGGTG--------
-------------------------------CAAAGAGAAAAAAGAGCAGCAGTT------ATGGGAGCT---
------ATGTTCCTTGGGTTCTTGGGAGCA---GCAGGAAGCACTATG---GGCGCAGCGGCA---
ATAACGCTGACGGTACAG---GCCAGACAA---TTATTG---TCTGGTATAGTGCAACAGCAAAAT---
AATTTG---CTGAGA---GCTATTGAGGCGCAGCAGCATATGCTGCAACTCACAGTCTGGGGCATT
>JX864024
TTCTACTGCAATACAACACAACTGTTTAATAGTACTTGGAACTCTAATAGTACTTTGAAC-------------
----

AGTACTGGGAATATCACACTCCCATGTAGAATAAAACAAATTGTAAACATGTGGCAGGAAGTAGGAAAAGCAA
TGTATGCCCCTCCCATCCAAGGACTAATTAGATGCTCATCAAAT---
ATTACAGGGCTGTTATTAGTAAGAGATGGTGGTAACAGTAACAGA----------------------------
----

ACCAATGAAACCTTCAGACCTGGAGGAGGAAATATGAAGGACAATTGGAGAAATGAATTATATAAATATAAAG
TAGTAAAAATTGAACCATTA---GGAGTAGCACCCACCAGGGCAAAGAGAAGAGTGGTG--------------
-------------------------CAGAGAGAAAAAAGAGCA---GTGGGA---ATAGGAGCT---------
GTGTTCCTTGGGTTCTTAGGAGCA---GCAGGAAGCACTATG---GGCGCAGCGTCA---
GTGACGCTGACGGTACAG---GCCAGACAA---TTATTG---TCTGGTATAGTGCAGCAGCAGAAC---
AATCTG---CTGAGG---GCCATTGAGGCGCAACAGCATCTGTTGCAACTCACAGTCTGGGGCATT
>JX864025
TTCTACTGCAATACAACAAAGCTGTTTAACAGTACGTGGACTAATCGGACTACAACTAACGAGACAACTAGT-
----

AGTAATGAAACTATTACAATCCCATGCAGAATAAAACAAATTATAAACATGTGGCAGGAAGTAGGAAAAGCAA
TGTATGCCCCTCCTATCAGGGGACCAATTAGATGTTCATCAAAT---
ATTACAGGACTAATATTACTAAGAGATGGTGGTAATAGCAATAACACAAACAAT------------AAC----
----

ACAAACAAGACTGAGATCTTCAGACCTGGAGGAGGAGACATGAGGGACAATTGGAGAAGTGAATTATATAAAT
ATAAAGTAGTACAACTTGAACCATTA---GGAATAGCACCCACCAGGGCAAAGAGAAGAGTGGTG--------
-------------------------------CAGAGAGAAAAAAGAGCA---ATAGGA---ATAGGAGCC---
------TTGTTCCTTGGGTTCTTGGGAACA---GCAGGAAGCACTATG---GGCGCAGCGTCA---
ATAACGCTGACGGTACAG---GCCAGACAA---TTATTG---TCTGGTATAGTGCAACAGCAGAAC---
AATCTG---CTGAGG---GCTATTGAGGCGCAACAACATCTGTTGCAACTCACAGTCTGGGGCATC
>HM638517
TTCTACTGTAATTCAACAAAACTGTTTAATAGTACCTGGAATGGGACTAGTACTGAAGAGAGCAAGGGA----
----

GAAAATGACACAATCACACTCCCATGCAGAATAAAACAATTTATAAACATGTGGCAGAGAGTAGGAAAAGCAA
TGTATGCCCCTCCTATCAGCGGAAAAATTAGATGTTCATCAAAT---
ATTACAGGGCTGTTATTAACAAGAGATGGTGGCATTAACACCACCAACGAGACCTTCAGACCTGGAGGAGGAA
ATATGAGGGACAATTGGAGAAGTGAATTATATAAATATAAAGTAGTAAGAGTTGAACCATTAGGAGTAGCACC
TACCAAGGACACCACCAACGAGACCTTCAGACCTGGAGGAGGAAATATGAGGGACAATTGGAGAAGTGAATTA
TATAAATATAAAGTAGTAAGAGTTGAACCATTA---GGAGTAGCACCTACCAAGGCAAAGAGAAGAGTGGTG-
--------------------------------------CAGAGAGAAAAAAGAGCA---GTGACA---
TTTGGAGCT---------GTGTTCCTTGGGTTCTTGGGAGCA---GCAGGAAGCACTATG---
GGCGCAGCGTCA---GTGACGCTGACGGTACAG---GCCAGACAA---TTATTG---
TCTGGTATAGTGCAACAGCAAAGT---AATTTG---CTGAGG---
GCTATTGAGGCGCAACAGCATCTGTTGCAACTCACAGTCTGGGGCATC
>MF499166
TTCTACTGTAATACAACAAAACTGTTCAACACGAATTTG----------------------------------
----

----ACACAACTGTCTAAT------
AACACTAAAGAAAACATCACACTCCCATGTAGGATAAAACAAATTATAAACATGTGGCAGGAAGTAGGAAAAG
CAATGTATGCCCCTCCTATCAGTGGACTAATTAGATGCTCATCAAAT---
ATTACAGGGCTGCTGTTAACAAGAGATGGTGGTAATAACAACAAC----------------------------
----

AGCCAAAATGAGACCTTCAGACCTATAGGGGGAAATATGAGGGACAATTGGAGAAGTGAATTATATAAATATA
AAGTAGTAAGAATTGAACCAGTA---GGAATAGCACCCACCAAGGCAAAGAGAAGAGTGGTG-----------
----------------------------CAGAGAGAAAAAAGAGCA---GTGGGA---ATAGGAGCT------
---ATGTTCCTTGGGTTCTTGGGAGCA---GCAGGAAGCACTATG---GGCGCAGCGTCA---
GTGACGCTGACGGTACAG---GCCAGACAA---TTATTG---TCTGGTATAGTGCAACAGCAGAAC---
AATCTG---CTGAGG---GCGATTGAGGTGCAACAGCATATGTTGCAACTCACAGTCTGGGGCATC
>KR423340
TTCTACTGTAACTTAACAAAACTATTTAATAGTACTTGGCCTAATAATGATACTTCAAAGTTTAATAGTACTG
GG---

GGTAATGATACTATCAGACTCCCATGTAGAATAAAACAAATTATAAACATGTGGCAGGAAGTAGGAAAAGCAA
TGTATGCCCCTCCCATCGAAGGAGAAATTACATGTAATTCAAGT---
ATTACAGGGCTGCTATTAACAAGAGATGGTGGTAATACTAATGACACT-------------------------
----

AAGGTGAATGAGACCTTCAGGCCTGGAGGAGGAGACATGAGGGACAATTGGAGAAGTGAATTATATAAATATA
AAGTAGTAAGAATTGAACCATTA---GGAATAGCACCCACCAAGGCAAGGAGAAGAGTGGTG-----------
----------------------------CAGAGAGAAAAAAGAGCA---GTGGGA---ATAGGAGCT------
---GTGTTCATTGGGTTCTTGGGAGCA---GCAGGAAGCACTATG---GGCGCAGCGTCA---
GTGGCGCTGACGGTACAG---ACCAAACAG---TTGCTG---TTTGGGATAGTGCAACAGCAAAAC---
AACTTG---CTGAGG---GCTATTGAGGCGCAACAGCATATGTTGCAACTCACAGTCTGGGGCATC
>HM638584
TTCTACTGCAATACAACACAGCTGTTTAATAGTACTTGGTATAGTAATGAGACTGAAGAATTAAATAACACAG
GAGTG--

AATAGCACAGAAACTATCACACTCCCATGCAAGATAAAGCAAATTATAAACATGTGGCAAGGAGTAGGAAGAG
CAATGTATGCCCCTCCCATCAGTGGACAAATTAATTGTTCATCAAAT---
ATTACAGGGCTGCTACTACTAAGAGATGGTGGTAATATTAATGACACG-------------------------
----

AGAGACAATGAAACCTTCAGACCTGGAGGAGGAAATATGAGGGACAATTGGAGAAGTGAATTATATAAATATA
AAGTAGTACAAATTGAACCATTA---GGAGTAGCACCCACCAAAGCAAGGAGAAGAGTGGTG-----------
----------------------------CAGAGAGAAAAAAGAGCA---GCAGTGGGAATAGGAGCT------
---TTGCTCATTGGGTTCTTGGGAACA---GCAGGAAGCACTATG---GGCGCAGCGTCA---
ATAACGCTGACGGTACAA---GCCAGACAA---TTATTG---TCTGGTATAGTGCAGCAGCAGAAC---
AATCTG---CTGAGA---GCTATTGATGCGCAACAACATCTGTTGCAGCTCACGGTCTGGGGCATC
>KM354155
TTCTACTGTAATTTAACACAACTGTTTAATAAAACATTTAATAACACCGGGGAT-------------------
----
-GATACTGACAACAGT---------
GAAGGTAATGACACAATAATACTCCCGTGCAGAATAAAACAAATTATAAACATGTGGCAGGAAGTAGGAAAAG
CAATGTATGCCCCTCCCATCAGAGGACAAATTAAATGTTCATCAAAT---
ATTACAGGGCTACTATTAACAAGAGATGGTGGT--

GTTGTGGAGGACAAT---
AACACTGAGACCTTCAGACCTGCAGGAGGAGATATGAGGGACAATTGGAGAAGTGAATTATATAAATATAAAG
TAGTAAAAATTGAACCATTA---GGAGTAGCACCCACCAGGGGAAAGAGACGGGTGGTG--------------
-------------------------CAAAGAGAAAAAAGAGCAGTAGTGGGA---ATAGGAGCT---------
GTGCTCTTTGGGTTCTTGGGAGCA---GCAGGAAGCACTATG---GGCGCAGCGGCA---
GTAACGCTGACGGTACAA---GCCAGACAA---TTATTG---TCTGGTATAGTGCAACAGCAGAAC---
AATCTG---CTGAGG---GCTATTGAGGCGCAACAGCATATGTTGCAACTCACAGTCTGGGGCATT
>KM354232
TTCTACTGTGATACAACAGCATTGTTTAATAATACTTGGAATAATAATGATACT-------------------
----

-TGGAAGGGTACTAGA---------
GCACCAAATGACACCATCACACTCCCATGTAGAATAAAACAAATTATAAACATGTGGCAGGAAGTAGGAAAAG
CAATGTATGCCCCTCCCATTAGAGGACGAATTAGATGTGTATCAAAT---
ATTACAGGACTACTATTAATAAGGGATGGTGGT--

AATACTAACACAACAAAC---
AAGTCTGAAACCTTCAGACCTGGAGGAGGAAATATGAAAGACAATTGGAGAAGTGAATTATATAAATATAAAG
TAGTAAAAATTGAACCATTA---GGAGTAGCACCCACCAAGGCAAAGAGAAGAGTGGTG--------------
-------------------------CAAAGAGAAAAAAGAGCA---GTGGGAACAATAGGAGCT---------
ATGTTCCTTGGGTTCTTGGGAGCA---GCAGGAAGCACTATG---GGCGCAGCGTCA---
CTGACGCTGACGGTACAG---GCCAGACTA---TTGTTG---TCTGGTATAGTGCAACAGCAGAAC---
AATCTG---CTGAGA---GCTATTGAGGCGCAACAGCACATGTTGCAACTCACAGTCTGGGGCATC
>KM354355
TTCTATTGTAATACAACACAACTGTTTAATAGTACTTGGAATGAGACT-------------------------
----
-------GGAGAGCCAGAT------
AACATTGTAAAAGAGATCATACTCCCATGCAGAATAAAACAAATTATAAACATGTGGCAGGGAGTAGGAAAGG
CAATGTATGCCCCTCCCATCAGAGGACGAATTAGCTGTGTATCAAAT---
ATTACAGGGCTGCTACTAGTAAGAGACGGAGGT--

-
AACGAGACTAACGGGAATGAGACTTTCAGACCTGGAGGAGGAAATATGAAGGACAATTGGAGAAGTGAATTAT
ATAAATATAAAGTAGTAGAAGTTGAACCATTA---GGAATAGCACCCACTAGGGCAAGGAGAAGAGTGGTG--
-------------------------------------CAGAGAGAAAAAAGAGCA---GTGACA---
TTAGGAGCT---------CTGTTCCTTGGGTTCTTGGGAGCA---GCAGGAAGCACTATG---
GGCGCAGCGTCA---GTGACGCTGACGGTACAG---GCCAGACAA---TTGTTG---
TCTGGTATAGTGCAACAGCAAAAC---AATTTG---CTGAGA---
GCTATTGATGCGCAACAACATATGTTGCAACTCACAGTCTGGGGCATC
>JN687773
TTCTACTGTAATACATCACAGCTGTTTAATACTACTTGGAATGATACTAATAGCACCAGGTATGAG-------
----

AATAACAGCAATATCACACTCCCATGCAGAATAAAACAAATTATAAACAGATGGCAGGAAGTAGGAAAAGCAA
TGTACGCCCCTCCCATCGCAGGACAAATTAGCTGTACATCAAAT---
ATTACAGGGGTGCTATTAACAAGAGATGGTGGTATCAATGGGAGC----------------------------
----

AACGAGACCGAGGTCTTCAGACCTGGAGGGGGAGATATGAGGGACAATTGGAGAAGTGAGTTATATAAATATA
AAGTAGTAAAAATTGAACCATTA---GGAATAGCACCCACCAAGGCAAAGAGAAGAGCGGTG-----------
----------------------------CAGAGAGAAAAAAGAGCA---GTGACA---TTCGGAGCT------
---ATGTTCCTTGGGTTCTTGGGAGCA---GCAGGAAGCACTATG---GGCGCAGCATCA---
ATGACGCTGACGGTACAG---GCCAGACTA---TTATTG---TCTGGTATAGTGCAACAGCAGAAC---
AATCTG---CTGAGA---GCTATTGAGGCGCAACAACATCTGTTGCAACTCACAGTCTGGGGCATC
>HQ908219
TTCTATTGTAACACAACACGGCTGTTTAATAATTGGACTTCTAATAATACTTGGAATGATACTACAGGGTCA-
----

AATAACAGCACAATCACACTCCCATGCAGAATAAAACAAATTATAAATAGGTGGCAGGAAATAGGAAAAGCAA
TGTATGCCCCTCCCATCGCAGGACAAATTAACTGTTCATCAAAT---
ATTACAGGGTTGCTATTAACAAGAGATGGTGGTAAGACCAACAACAGC-------------------------
----

AACAGCAGTGAGACCTTCAGACCAGGGGGAGGAAATATGAAGGACAATTGGAGAAGTGAATTATATAAATATA
AAGTAGTAAAAATTGAACCATTA---GGAATAGCACCCACCGAGGCAAGGAGAAGAGTGGTG-----------
----------------------------CAGAGAGAAAAAAGAACA---GTAACA---TTAGGAGCT------
---ATGTTCCTTGGGTTCTTGGGAGCA---GCAGGAAGCACTATG---GGCGCAGCATCA---
ATGACGCTGACGGTACAG---GCCAGACAA---TTATTG---TCTGGTATAGTGCAACAGCAGAGC---
AATCTG---CTGAGG---GCTATTGAGGCGCAACAGCATATGTTGCAACTCACAGTCTGGGGCATC
>HM204596

TTCTACTGTAATACATCACAATTGTTCAATAGTACTTGGGAGTTTAATAGTACTTGGAATGGGAATGAAACTG
AA---

GGAAATGACACCATCATTCTCCCATGTAGAATAAAACAGATTATAAACCTGTGGCAGAGAGTAGGAAAAGCAA
TGTATGCCCCTCCCATCAAAGGAATAATTAGATGTTCGTCAAAT---
ATTACAGGGCTGCTATTAACAAGAGATGGTGGTAATAGTAATACCTCCAAC----------------------
----

TCCAGCAATATTGAAACCTTTAGACCTGGAGGAGGAAATATGAAGGACAATTGGAGAAGTGAATTATATAAAT
ATAAAGTAGTAAGACTTGAACCATTA---GGAGTAGCACCCACCAAGGCAAGGAGAAGAGTGGTG--------
-------------------------------CAGAGAGAAAAAAGAGCA---GTGGGA---CTAGGAGCT---
------TTGTTCCTTGGGTTCTTGGGAGCA---GCAGGAAGCACTATG---GGCGCAGCGTCA---
GTGACGCTGACGGTACAG---GCCAGACAA---TTGTTG---TCTGGTATAGTGCAACAGCAGAAC---
AACCTG---CTGAGA---GCTATTGAGGCGCAACAGCATCTGTTGCAACTCACAGTCTGGGGCATC
>KY112149
TTCTACTGTAATACATCACGACTGTTTAATAGTACTTGGAATACTACACAATTGTTTAATAGTACTGAGAGTG
CTAATGATACT--
------------GGAGGG------
TTGAATAACACAGTTATTACCCTCCCATGCAGAATAAAACAAATTATAAACATGTGGCAGGAAGTAGGAAAAG
CAATGTATGCCCCTCCCATCGAAGGATTACTTAATTGTTCATCAAAC---
ATTACAGGGCTGCTATTAACAAGAGATGGTGGTAGTGATCTTAACAGTACTGAC-------------------
----

AACAATACCAATATAGAGGTCTTCAGACCGCAAGGAGGAGATATGAGGGATAATTGGAGAAGTGAATTATATA
AATATAAAGTAGTAAAAATTGAACCATTA---GGAGTAGCACCCACCAAGGCAAAGAGAAGAGTGGTG-----
----------------------------------CAGAGAGAAAAAAGAGCA---GTGGGA---
ATAGGAGCT---------CTGTTCCTTGGGTTCTTGGGAGCA---GCAGGAAGCACTATG---
GGCGCAGCGTCC---ATGACGCTGACGGTACAG---GCCAGGCTA---TTATTG---
TCTGGTATAGTGCAGCAGCAAAAC---AATTTG---CTGAAG---
GCTATTGAGGCGCAACAGCGTCTGTTGCAACTCACAGTCTGGGGCATC
>KY112490
TTCTACTGTGATTCATCACAACTGTTTAATAGCACTTGGAATAATGAGACTTGGAGTGATAATAGTACTAAAG
GGTCA--

AATAGCACAGAAATTATTACACTCCCATGCAGAATAAAACAATTTATAAACATGTGGCAGGAAGTAGGGAAAG
CAATGTATGCCCCTCCTATCAAAGGACAACTTAATTGTTCATCAAAC---
ATTACAGGGCTGCTGTTAACAAGAGATGGCGGTAGTGGTCTTAACAGGACTGAC-------------------
----
AACAGGACTGAGAAC---
AATACTGAGGTCTTCAGACCGCAAGGAGGAGATATGAGGGATAATTGGAGAAGTGAATTATATAAATATAAAG
TAGTAAGAATTGAACCATTA---GGAATAGCACCCACCAAGGCAAAGAGAAGAGTGGTG--------------
-------------------------CAGAGAGAAAAAAGAGCA---GTGGGA---GTAGGAGCT---------
CTGTTCCTTGGGTTCTTGGGAGCA---GCAGGAAGCACTATG---GGCGCAGCGTCA---
ATGACGCTGACGGTACAG---GCCAGGCTA---TTATTG---TCTGGTATAGTGCAACAGCAGAAC---
AATTTG---CTGAAG---GCTATTGAGGCGCAACAGCGTCTGTTGCAACTCACAGTCTGGGGCATC
>JQ403067
TTCTACTGTAATACAACAAAGCTGTTTAATAGTACTTGGAATGGTACTGAGAATGGTACTCTTACAGGGTCA-
----

AATGACACTAGCATCACACTCCCATGTAGAATAAAACAAATTATAAACAGGTGGCAAGAAGTAGGAAAAGCAA
TGTATGCCCCTCCCATCAGTGGGCAAATTACATGTTCATCAAAT---
ATTACAGGGCTACTATTAACAAGAGATGGAGGTCCGAATGAGAAT----------------------------
----

GAGACTGAGACCTTCAGACCTGGAGGAGGAAATATGAGGGATAATTGGAGAAGTGAATTATATAGATATAAAG
TAGTAAGAATTGAGCCATTA---GGAGTAGCACCCACCAAGGCAAAGAGAAGAGTGGTG--------------
-------------------------CAGAGAGAAAAAAGAGCA---GTGACG---CTAGGAGCT---------
GTGTTCCTTGGGTTCTTGGGAACA---GCAGGAAGCACTATG---GGCGCAGCGTCA---
GTGGCGCTGACGGCACAG---GCCAGACAA---TTATTG---TCTGGTATAGTGCAACAGCAGAAC---
AATTTG---CTGAGG---GCTATTGAGGCGCAACAGCATCTGTTGCAACTCACAGTCTGGGGCATC

```
>JQ403069
TTCTACTGCAATACAACACCACTATTTAATAGTATTTGGAATGATACACAACTGTCTAATAGTACTTGGAATG
AGACTGACTGG--------------------------------------------------------------
----------TCAAATAAA------
ACTACAAGAGAAAACATCACACTCCAATGCAGAATAAAACAAATTGTAAACATGTGGCAGGAAGTAGGAAGAG
CAATGTACGCCCCTCCCATTAGGGGAATAATTAAATGTTCATCAAAT---
ATCACAGGGCTACTATTAACAAGAGATGGAGGGGATTCTAATGAC----------------------------
-------------------------------------------------------------------------
----------------
AATGAAACCTTCAGACCTGGAGGGGGAGATATGAGGGACAATTGGAGAAGTGAATTATATAAATATAAAGTTG
TAAAAATTGAGCCATTA---GGAGTAGCACCCACCAAGGCAAAGAGAAGAGTGGTG-----------------
----------------------CAGAGAGAAAAAAGAGCA---GTGGGA---TTAGGAGCT---------
ATGTTCCTGGGGTTTCTGGGAGCA---GCAGGAAGCACTATG---GGCGCAGCGTCA---
ATAACGCTGACGGTACAG---GCCAGACAA---CTATTG---TCTGGTATAGTGCAACAGCAGAGC---
AATTTG---CTGAGA---GCTATTGAGGCGCAACAGCATCTGTTGCAACTCACAGTCTGGGGCATC
>JQ403082
TTCTACTGTAATACATCACAGCTATTTAATACATCACTTCTATTTGATAAGAGTCCTGAAAAGCCA-------
-------------------------------------------------------------------------
----------------------------
AATAATGGCACTATCACACTCCCATGTAGAATAAAACAAATTATAAACCTGTGGCAGGAAGTAGGGAAAGCAA
TGTATGCCCCTCCCATCAGTGGACCAATTAACTGTTCATCAGAA---
ATTACAGGGCTGCTATTAACAAGAGATGGTGGTAATAATAATGACACCAAT----------------------
-------------------------------------------------------------------------
----------
AGCACCCACGAGGTCTTCAGACCTGGAGGAGGAGATATGAGGGACAATTGGAGAAGTGAATTATATAAATATA
AAGTAGTGAAAATTGAACCATTA---GGAGTAGCACCCACCAAGGCAAAGAGAAGAGTGGTG-----------
----------------------------CAGAGAGAAAGAAGAGCA---GTGGGA---ATAGGAGCT------
---ATATTCCTTGGGTTCTTGGGAGCA---GCAGGAAGCACTATG---GGCGCAGCGTCA---
ATAACGCTGACGGTACAG---GCCAGGCAA---TTATTG---TCTGGTATAGTGCAACAGCAGAGC---
AATTTG---CTGAGA---GCTATCGAGGCGCAACAGCATCTGTTGCAGCTCACAGTCTGGGGCATT
>JQ403035
TTCTACTGTAATACATCAAGACTGTTTAATAGTACTTGGCATAGTAATGGTACTGTAGAGTCAAGTAACAAT-
-------------------------------------------------------------------------
----------------------------
AACAGTACAAATATCACACTCCAATGCAGAATAAAACAAATTGTAAACATGTGGCAGGAAGTAGGAAGAGCAA
TATATGCCCCTCCCATCAGAGGACTAATTAGATGTTCATCAAAC---
ATTACTGGGCTGCTATTAACAAGAGATGGTGGTAGTAACACCACC----------------------------
-------------------------------------------------------------------------
----------
AATGGGACCGAGATCTTCAGGCCTGGAGGAGGAGATATGAGGGACAACTGGAGAAGTGAATTATACAAATATA
AAGTAGTAAAAATTGAACCATTA---GGAATAGCACCCACCAAGGCAAAGAGAAGAGTGGTG-----------
----------------------------CAGAGAGAAAAAAGAGCA---GTGGGA---CTAGGAGCT------
---ATGTTCCTTGGGTTCTTGGGAGCA---GCAGGAAGCACTATG---GGCGCAGCGTCA---
GTGACGCTGACGGTACAA---GCCAGACAA---TTATTG---TCTGGTATAGTGCAACAGCAAAAC---
AATTTG---CTGAGG---GCCATTGAGGCGCAACAACATCTGTTGCAACTCACAGTCTGGGGCATT
>JQ403083
TTCTATTGTGATTCAACAAAACTGTTTAATAGTACTTGGTATACTAATGGGAATACTACTGGAGAGCCAATTA
ACACTGAC-----------------------------------------------------------------
----------------------------
GGAAATGGTACAATCACACTCCCATGCAGAATAAAACAAGTGATAAACATGTGGCAGGAAGTAGGAAAAGCAA
TGTATGCCCCTCCCATCAGCGGAATAATTAACTGTTCATCAAGT---
ATTACAGGGCTAATATTAACAAGGGATGGTGGTAACGAGAACGGGACC-------------------------
-------------------------------------------------------------------------
----------
AACGAGACCGAGATCTTCAGACCTGGAGGAGGAGATATGAGGGATAATTGGAGAAGTGAATTATATAAATATA
AAGTAGTGAAACTTGAACCATTA---GGAATAGCACCCACCAAGGCAAGGAGAAGAGTGGTG-----------
----------------------------CAAAGAGAAAAAAGAACA---GTGGGA---CTAGGAGCT------
---ATGTTCCTTGGGTTCTTGGGAGCA---GCAGGAAGCACTATG---GGCGCAGCGTCA---
GTGACGCTGACGGTACAG---GCCAGACTA---CTATTG---TCTGGTATAGTGCAACAGCAGAGC---
AATTTG---CTGAGG---GCTATTGAGGCGCAACAGCATATGTTGCAACTCACAGTCTGGGGCATC
```

>JQ403084
TTCTACTGTAATACAACACAGCTGTTTAATGATAGTATTTGGAATGGGACTAGGACTAAAGAGCAAAAT----
----

GAAAATGAAACTATCACACTCCCATGCAGAATAAAACAAATTATAAACATGTGGCAGAAAGTGGGCAAAGCAA
TGTATGCCCCTCCCATCAGAGGACAGATTAAATGTTCATCAAAT---
ATTACAGGGCTGCTATTAACAAGAGATGGTGGTACCCAGAGCAACGGT-------------------------
----
-
ACCCAGGGCAATGAGACCGAGATCTTCAGACCTGCAGGAGGAGATATGAGGGACAATTGGAGAAGTGAATTAT
ATAAATATAAAGTAGTACAAATTGAGCCAGTA---GGAGTGGCACCCACCAAGGCAAAGAGAAGAGTGGTG--
-------------------------------------CAAAGAGAAAAAAGAGCA---
GTGGGAACAATAGGAGCT---------ATGTTCCTTGGGTTCTTGGGAGCA---GCAGGAAGCACTATG---
GGCGCAGCGTCA---ATAACGCTGACGGCACAG---GCCAGACAA---TTATTG---
TCTGGTATAGTGCAACAGCAGAAC---AACTTG---CTGAGA---
GCTATTGAGGCGCAACAGCATTTGTTGCAACTCACAGTCTGGGGCATT
>JQ403086
TTCTACTGTAATACAACACAACTGTTTAATAGTGCTTGGAATAGTACTAGGGATAATAGTACTGCAGAGTCAA
GT---

AACAGTACACATATCACACTCCCATGCAGAATAAAACAAATTATAAACATGTGGCAGGAAGTAGGAAGAGCAA
TATATGCCCCTCCCATCAGAGGACTGATTAGATGTTCATCAAAT---
ATTACAGGGCTACTATTAACAAGAGATGGTGGTAGTAACAACACC----------------------------
----

AATGAGATCGAGACCTTCAGGCCAGCAGGAGGAGATATGAGGGACAATTGGAGAAGTGAATTATATAAATACA
AAGTAGTAAAAATTGAACCATTA---GGAATAGCACCCACCAAGGCAAAGAGAAGAGTGGTG-----------
----------------------------CAAAGAGAAAAAAGAGCA---GTGGGAGTAATAGGAGCT------
---ATGTTCCTTGGGTTCTTGGGAACA---GCAGGAAGCACTATG---GGCGCAGCGTCA---
GTGACGCTGACGGTACAA---GCCAGACAA---TTATTG---TCTGGTATAGTGCAACAGCAAAAC---
AACTTG---CTGAGG---GCTATTGAGGCGCAACAACATCTGTTGCAACTCACAGTCTGGGGCATC
>JQ403087
TTCTATTGTAATACAACACAACTGTTTAATAGTACTTGGATAAATAATGGTACTTGGAAGGGTACTAACGTGT
CAGAT--

TCAAATGACACTGTCACACTCCCATGCAGAATAAAACAAATCATAAACAGGTGGCAAGAAGTAGGAAAAGCAA
TATATGCCCCCCCCATCGCAGGACGACTTGAGTGCATATCAAAT---
ATTACAGGGCTGCTATTAACAAGAGATGGTGGTATTATTAACACCACC-------------------------
----

AAGGACAATGAGACCTTCAGACCTGGAGGAGGAGACATGAGGGACAATTGGAGAAATGAGTTATATAAATATA
AAGTAGTAAAAATTGAACCATTA---GGAATAGCGCCCTCCAAGGCAAGGAGAAGAGTGGTG-----------
----------------------------CAAAGAGAAAAAAGAGCA---GTGGGG---TTCGGAGCC------
---TTGTTCCTTGGGTTCTTGGGAGCA---GCAGGAAGCACTATG---GGCGCAGCATCA---
TTGATGCTGACGGTACAG---GCCAGACAA---TTATTG---TCTGGTATAGTGCAACAGCAGAAC---
AATCTG---CTGAGA---GCTATTGAGGCGCAACAACATATGTTGCAACTCACAGTCTGGGGCATC
>JQ403088
TTCTACTGTAATACAACACAACTGTTTAATAGTAATTGGACTAATACAACTAGAGAGGGA-------------
----

GAGAATGGCACAATCACACTCCCATGCAGAATAAAACAAATTATAAACATGTGGCAGGAAGTAGGAAAGGCAA
TGTATGCCCCTCCCATCAGAGGACATATTAACTGTTCATCAAAT---
ATTACAGGGTTGCTATTAACAAGAGATGGTGGTGAAAACAATGGGAGT-------------------------
----

AATGACAGCGAGACCTTCAGACCTGGAGGAGGAGATATGAGAGACAATTGGAGAAGTGAATTATATAAATATA
AAGTAGTACAAATTGAACCATTA---GGAGTAGCACCCACCAAGGCAAAGAGAAGAGTGGTG-----------
----------------------------CAGAGAGAAAAAAGAGCA---GTGACA---ATAGGAGCT------
---ATGTTCCTTGGGTTCTTGGGAGCA---GCAGGAAGCACTATG---GGCGCAGCGTCA---

ATAACGCTGACGGTACAG---GCCAGACAA---TTATTG---TCTGGTATAGTGCAACAGCAGAGC---
AATTTG---CTGAGG---GCTATTGAGGCGCAACAGCATCTGTTGCAACTCACAGTCTGGGGCATC
>JQ403089
TTCTACTGCAATACAACAAAACTGTTTAATAGTACTTGGACTGAGAATAGAACTGTAGGGACTGAA-------
----

GGAAATGACACAATCACAATCCAATGCAGAATAAAACAAATTATAAACATGTGGCAGCAAGTAGGAAAAGCAA
TGTATGCCCCTCCCATCAGCGGACCCATTAGATGCTCATCAAAT---
ATTACAGGACTGCTATTAATGAGAGATGGTGGTACAAACCAGACC----------------------------
----

AACAATACCGAGATCTTCAGACCTGGAGGAGGAGATATGAGGGACAATTGGAGAAGTGAACTATATAAATATA
AAGTAGTGCAAATTGAACCATTA---GGAATAGCACCCACCAAGGCAAGGAGAAGAGTGGTG-----------
----------------------------CAGAGAGAAAAAAGAGCA---GTGGGA---ATAGGAGCT------
---CTGTTCCTTGGGTTCTTGGGAGCA---GCAGGAAGCACTATG---GGCGCAGCGTCA---
ATAACGCTGACGGTACAG---GCCAGACAA---TTATTG---TCTGGTATAGTGCAACAGCAGAAC---
AATTTG---CTGAAA---GCTATTGAGGCGCAACAGCATCTGTTGCAACTCACAGTCTGGGGCATC
>JQ403091
TTCTACTGTAATACAACACAACTGTTTAATAGTAGTTGGGTTAATAGTAGTTGGGTTAGTGGGAGTAATGAAT
CTAGCGAT---

GCCAATGGCACAATAACACTCCCATGCAGAATAAAACAAATTATAAACATGTGGCAGGAGGTAGGAAAAGCAA
TGTATGCCCCTCCCATCAAGGGACAAATTAGATGCACATCAAAT---
ATCACAGGGCTGATATTAACAAGAGATGGTGGTAAAAATACAAGT----------------------------
----

AACAACAGCGAGACCTTCAGACCTGGAGGAGGAGATATGAGGGACAATTGGAGAAGTGAGCTATATAAATATA
AGGTAGTAAAAATTGAACCTTTA---GGAATAGCACCCACCAAGGCAAAGAGAAGGGTGGTG-----------
----------------------------CAGAGAGAAAAAAGAGCA---GTGGGA---GTAGGAGCT------
---TTGTTCCTCGGGTTCTTGGGAGCA---GCAGGAAGCACTATG---GGCGCAGCGTCA---
ATAACGCTGACGGTACAG---GCCAGACTA---TTGTTG---TCTGGTATAGTGCAACAGCAGAAC---
AATTTG---CTGAGA---GCTATTGAGGCGCAACAGCATATGTTGCAACTCACAGTCTGGGGCATT
>JQ610123
TTCTATTGTAACACAACACAACTATTTGATAGTACTTGGGATAGTAATAGTACTTGGAATAGTACTGGGAATA
CAAATAAA---
-------------------ACT---
GGAGGAAAGGACACAATCACACTCCCATGCAGAATAAAACAAATTATAAACAGGTGGCAGGAAGTAGGAAAAG
CAATGTATGCCCCTCCCATCAGCGGACAAATTAGATGTTCATCAAAT---
ATTACAGGACTGCTATTAACAAGAGATGGTGGTAAGAATAATAGTACG-------------------------
----

AACAACACTGAGACTTTCAGACCTGCAGGAGGAGATATGAAAGACAATTGGAGAAGTGAATTATATAAATACA
AAGTGGTAAGAATTGAGCCATTA---GGAGTAGCACCCACCAAGGCAAAAAGAAGAGTGGTG-----------
----------------------------CAGAGAGAAAAAAGAGCA---GCA------CTAGGAGCT------
---ATGTTCCTTGGGTTCTTGGGAGCA---GCAGGAAGCACTATG---GGCGCAGCGTCA---
GTGACGCTGACGGTACAG---GCCAGACTA---TTATTG---TCTGGTATAGTGCAACAGCAGAAT---
AATTTG---CTGAGG---GCTATTGAGGCGCAACAGCATATGTTGCAACTCACAGTCTGGGGCATC
>KM081848
TTCTACTGTAATTCAACACCACTGTTTGGTAGTACTTGGCTGTTAAATAATACTGGGAATGGTACTTGGAATA
GGAATGATACT--
----------GAAAAGCCA------
AATAACACTGACCCAATCACACTCCAATGCAGAATAAAACAATTTATAAACATGTGGCAGGAAGTAGGAAAAG
CAATGTATGCCCCTCCCATCCAAGGACATATTAGATGTTCATCAAAT---
ATTACAGGGCTGCTATTGACAAGAGATGGTGGTAGTAATGACACCACG-------------------------
----

AACGGCACAGAGACCTTCAGACCTGGAGGAGGAGATATGAGGGACAATTGGAGAAGTGAATTATATAAATATA
AAGTAGTAAAAATTGAACCATTA---GGAGTAGCACCCACTAGGGCAAAGAGGAGAGTGGTG-----------
----------------------------CAGAGAGAAAAAAGAGCA---GTGACA---TTAGGAGCT------
---ATGTTCCTTGGGTTCTTGGGAGCA---GCAGGAAGCACTATG---GGCGCAGCGTCA---

ATAACGCTGACGGTACAG---GCCAGACAA---TTATTG---TCTGGTATAGTGCAACAGCAGAAC---
AATTTG---CTGAGA---GCCATTGAGGCGCAACAACATATGTTGCAACTCACAGTCTGGGGCATC
>KM081861
TTCTACTGTGACTCAACAAAACTGTTTAATAGTACTTGGAATAATAACACTGGGAATGTAAGTGATACTGAAG
GG--

TCAAATAACACTATCACGCTCCCATGCAGGATAAAACAAATTATAAACATGTGGCAGACAGTGGGAAAAGCAA
TGTACGCCCCTCCTATCCGAGGACAAATTAACTGTTTATCAAAT---
ATTACAGGGCTACTCTTAACAAGAGATGGTGGTAACGGGAACAACACT--------------------------
----

AATACTAATGAGACCTTCAGACCTGGAGGAGGAGATATGAGGGACAATTGGAGAAGTGAATTATATAAATATA
AAGTAGTAAAAATTGAACCATTA---GGAGTAGCACCCACCAAGGCAAAGAGAAGAGTGGTG-----------
----------------------------CAAAGAGAAAAAAGAGCA---GTGGGA---ATAGGAGCT------
---GTGTTCCTTGGGTTCTTGGGAGCA---GCAGGAAGCACTATG---GGCGCAGCGTCA---
CTGACGCTGACGGTACAG---GCCAGACAA---TTATTG---TCTGGTATAGTGCAACAGCAGAAC---
AATTTG---CTGAGG---GCTATCGAGATGCAACAGCATCTGTTGCAACTCACAGTCTGGGGCATC
>KM081893
TTCTACTGTAATACAACACAACTATTTAATAGTACTTGGACTTGGAATAGTACTGGGGAGGACAATAAC----
----

ACTGCAGAAAATATCACACTCCCCTGTAGAATAAAACAAATTATAAACATGTGGCAGGAAGTAGGAAAAGCAA
TGTATGCCCCTCCCATCAGAGGACAAATTAGATGTTCATCTAAT---
ATTACAGGGTTGCTATTAACAAGAGATGGTGGTAGAGGTAGCGGGAAC--------------------------
----
AATTCCAAGACCAAC---
GATTCCGAGATCTTCAGACCTGGAGGAGGAGATATGAGGGACAATTGGAGGAGTGAATTATATAAATATAAAG
TAGTAAAAATTGAACCATTA---GGAGTAGCACCCACCAAGGCAAAGAGAAGAGTGGTG--------------
-------------------------CAGAGAGAAAAAAGAGCA---GTGGGAACACTGGGAGCT---------
ATGTTCCTTGGGTTCTTGGGAGCA---GCAGGAAGCACTATG---GGCGCAGCGGCA---
GTGACGCTGACGGCACAG---GCCAGACAA---TTATTG---TCTGGTATAGTGCAGCAGCAGAAC---
AATCTG---CTGAGG---GCTATTGAGGCGCAACAGCATCTGTTGCAACTCACAGTCTGGGGCATC
>KM081913
TTCTACTGTAACTCAACACCACTGTTTAATAGCACTTGGTATCCTAATAATAGCACTTGGTATCCTAATGAGA
CTGTGGCAGAT---

ACTGAAGGAAATATCACACTCCAATGCCGAATAAAACAAGTCATAAACCTGTGGCAGGAAGTAGGAAAGGCAA
TGTATGCCCCTCCCATCAGCGGACCAATTAAATGTTCATCAAAT---
ATTACAGGGCTGCTATTAACAAGAGATGGTGGTAATGGCAATACCACA--------------------------
----

AACACCATCGAGGTCTTTAGACCTGGAGGAGGAGATATGAGGGATAATTGGAGAAGTGAGTTATACAAATACA
AAGTAGTAAGAATTGAACCATTA---GGAGTAGCACCCACCAGGGCAAAGAGAAGAGTGGTG-----------
----------------------------CAGAGAGAAAAAAGAGCA---ATAGGA---ATAGGAGCT------
---GTGTTCCTTGGGTTCTTGGGAGCA---GCAGGAAGCACTATG---GGCGCAGCGTCA---
ATAACGCTGACGGTACAA---GCCAGACAA---TTATTG---TCTGGTATAGTGCAACAGCAGAAC---
AACTTG---CTGATG---GCTATTGAGGCGCAACAGCATCTGTTGCAACTCACAGTCTGGGGCATC
>KM081963
TTCTACTGTAATACATCACAGCTGTTTAATGGTACTTTACAGCTTAATGGTACTTGGATTTGGAATGGTACTT
GGAATGGTACT---
-------------TGGAAT------
GGTACTATAGGTACCATCACACTTCCATGCAGAATAAAACAAATTATAAACAGGTGGCAGGAAGTAGGAAAAG
CAATGTATGCCCCTCCCATCAGTGGACAAATTAGATGCTCATCCAAT---
ATTACAGGGCTACTATTAACAAGAGATGGAGGAGATAAAAGTAGTAACAGC-----------------------
----

ACAAACAATACTGAGACCTTCAGACCTGGAGGAGGAAATATGAGGGACAATTGGAGAAGTGAATTATATAAAT
ATAAAGTAGTAAGAATTGAGCCATTA---GGAATAGCACCCACCAAGGCAAAGAGAAGAGTGGTG--------
-------------------------------CAGAGAGAAAAAAGAGCA---GTGGGAACACTAGGAGCT---
------ATGTTCCTCGGGTTCTTGGGAGCA---GCAGGAAGCACTATG---GGCGCAGCGTCA---

ATGACGCTGACGGTACAA---GCCAGACAA---TTATTG---TCTGGTATAGTGCAACAGCAGAGC---
AATTTG---CTGAGA---GCTATTGAGGCGCAACAGCATATGTTGCAACTCACAGTCTGGGGCATC
>JX864027
TTCTACTGCAATACAACACCACTATTTAATAGCACTTGGAAAAGGAATGGTAATTGGAGTAGAAATGATACTG
AAGAA--
-------------------TCA---
AACAGCACAGAACCAATCACACTCCAATGCAGAATAAAACAAATTGTAAACATGTGGCAGGAAGTAGGAAGAG
CAATGTATGCCCCTCCCATCAGTGGACGAATTAACTGTTCATCAAAT---
ATTACAGGGCTGATACTAACAAGAGATGGTGGTAATAACAGTGCC----------------------------
----

GCCAACGAGACCTTCAGACCAGCAGGAGGAGATATGAGGGACAATTGGAGAAGTGAACTATATAAATATAAAG
TAGTAAAAATTGAACCATTA---GGAATAGCACCCACCAAGGCAAAGAGAAGAGTGGTG--------------
-------------------------CAGAGAGAAAAAAGAGCA---GCA------GTAGGAGCT---------
CTGTTCCTTGGGTTCTTGGGAGCA---GCAGGAAGCACTATG---GGCGCAGCGTCA---
GTGACGCTGACGGTACAG---GCCAGACTA---TTATTG---TCTGGTATAGTGCAACAGCAGAAC---
AATTTG---CTGAAG---GCTATTGAGGCGCAACAGCATCTGTTGCAACTCACAGTCTGGGGCATC
>MH666175
TTCTACTGTAATACGACAATACTGTTTAATAGTACTTGGAATTCAACACAACTGCAGAATAATGACACTGAAC
AG---

TCAAATGACACTATCACACTCCCATGCAGAATAAAACAGTTTATAAACATGTGGCAGGAAGTAGGAAAAGCAA
TGTATGCCCCTCCCATCAGAGGACAAATTAAATGTTCATCAAAT---
ATTACAGGGCTGATATTAACAAGAGATGGTAATAAGGAAGAAAACAATACCGAA-------------------
----

GGGACCGAGATCTTCAGACCTGGAGGAGGAGATATGAGAGACAATTGGAGAAGTGAATTATATAAATATAAAG
TAGTAAGAATTGAACCATTA---GGAGTAGCACCCACCAAGGCAAAGAAAAGAGTGGTG--------------
-------------------------CAGAGAGAAAAAAGAGCA---GTGGGA---ATAGGAGCT---------
TTGTTCCTTGGTTTCTTGGGAGCA---GCAGGAAGCACTATG---GGCGCAGCGTCA---
GTGACGCTGACGGTACAA---GCCAGACAA---TTATTG---TCTGGTATAGTGCAGCAGCAAAAT---
AATTTG---CTGAGG---GCTATTGAGGCGCAACAACATATGTTGCAACTCACAGTCTGGGGCATC
>MH666186
TTCTACTGTAATACAACAAAACTATTTAATAGCATCTGGAATGGCACCAGGAGTGAGAATAATAATACTGGAG
GG---

TCAAATGAAAACATCACACTCCCATGCAGAATAAAACAAATTATAAACATGTGGCAGAGAGTAGGAAAAGCAA
TGTATGCCCCTCCCATCAGAGGACAAATTAGATGTACATCAAAT---
ATTACAGGGCTGATATTAACAAGAGATGGAGGCCTAAACAACAAC----------------------------
----

ACCAACGAGACCTTCAGACCGGGAGGAGGAAATATGAAGGATAAGTGGAGAAGTGAATTATATAAATATAAAG
TAGTAAGAATTGAACCATTA---GGAGTAGCACCCACCAGGGCAAAAAGGAGAGTGGTG--------------
-------------------------CAGAGAGAAAAAAGAGCA---GTAGGA---ATAGGAGCT---------
TTGTTCCTTGGATTCTTGGGAGCA---GCAGGAAGCACTATG---GGCGCAGCGTCA---
CTGACGCTGACGGTACAG---GCCAGACAA---TTATTG---TCTGGTATAGTGCAACAGCAGAAC---
AACTTG---CTGAGA---GCTATAGAGGCGCAACAGCATATGTTGCAACTCACAGTCTGGGGCATC
>MH666216
TTCTACTGTAATACATCAAAGCTGTTTAGTAGTACTTGGAATAGTACTACTAATTGGAATAGTACTAATTGGA
ATAGTACTACT--

GATGGGAATAATACTATCATACTCCCATGCAGAATAAAACAAATTATAAACATGTGGCAGGCAGTAGGAAAAG
CAATGTATGCCCCTCCCATTAAAGGAGTAATTAAGTGTTTATCAAAT---
ATTACAGGGTTGCTATTAGTAAGAGATGGTGGTATTAACCAGACCAACACCACC-------------------
----

GAGATCGAGACCTTCAGACCTGGAGGAGGAGATATGAGGGACAATTGGAGAAGTGAATTATATAAATATAAAG
TAGTAAGAATTGAACCATTA---GGAGTAGCACCCACCAAGGCAAAAAGGAGAGTGGTG--------------
-------------------------CAGAGAGAAAGAAGAGCG---GTGGGA---ATAGGAGCT---------
GTGTTCCTTGGGTTCTTGGGAGCA---GCAGGAAGCACTATG---GGCGCAGCGTCA---

ATAACGCTGACGGTACAG---GCCAGACAA---TTATTG---TCTGGTATAGTGCAACAGCAAAAC---
AATTTG---CTGAGG---GCTATTGATGCGCAACAGCATCTGTTGCAACTCACAGTCTGGGGCATA
>MH666227
TTCTACTGTAATACAACAAAATTATTTAGTAGTACTTGGCATAATGAAACCAATAGCACTTTGAATGATCCTG
AAAATAGC---

AATAACGACACAATCACACTCCCATGTAGAATAAAACAATTTATAAACATGTGGCAGGAAGTAGGAAAAGCAA
TGTATGCCCCTCCCATCCAAGGACTAATTAACTGTTCATCAAAC---
ATTACAGGGCTATTATTAGTAAGAGATGGTGGTGGTAATGATACA----------------------------
----

AGGGACAATGAGACCTTCAGACCTGGAGGAGGAAATATGAAAGACAATTGGAGAAGTGAATTATATAAATATA
AAGTAGTAAAAATTGAACCATTA---GGGGTAGCACCCACTAAGGCAAAGAGAAGAGTGGTG-----------
----------------------------CAGAGAGAAAAAAGAGCA---GTGGGA---ATAGGAGCT------
---ATGTTCCTTGGGTTCTTGGGAGCA---GCAGGAAGCACTATG---GGCGCAGCGTCA---
ATAACGCTGACGGTACAG---GCCAGACAA---CTATTG---TCTGGTATAGTGCAACAGCAGAAC---
AATTTG---CTGAGG---GCTATTGAGGCGCAACAGCATCTGTTGCAACTCACAGTCTGGGGCATT
>MH666240
TTCTACTGTAATACAACACAGCTGTTTAATAGCACTTGGAAGGGGGGAGAGTTAAATAGCACTGAAGAAAATA
ATAGCACT---

AAAGAAAGTAATATCACACTCCCATGCAGGATAAAACAAATTATAAATATGTGGCAGGAAGTAGGAAAAGCAA
TGTATGCCCCTCCCATCAAAGGAGAAATTAGATGTTCATCAAAT---
ATTACAGGACTGCTAATAACAAGAGATGGTGGTAACAAGAATGATACT-------------------------
----

AATGATACTGATACTGAGATCTTCAGACCTGGAGGAGGAAATATGAAGGACAATTGGAGAAGTGAATTATATA
AATATAAAGTAGTAAAAATTGAGCCATTA---GGAGTAGCACCCACCAAGGCAAAGAGAAGAGTGGTG-----
----------------------------------CAGAGAGAAAAAAGAGCA---GTGGGA---
ATAGGAGCT---------TTGTTCCTTGGGTTCTTGTCAGCA---GCAGGAAGCACTATG---
GGCGCAGCGTCA---ATAACGCTGACGGTACAG---GCCAGACAA---TTACTG---
TCTGGTATAGTGCAACAGCAGAAC---AATTTG---CTGAGG---
GCTATTGAGGCGCAACAACATCTGTTGCAACTCACAGTCTGGGGCATC
>MH666253
TTCTATTGTAATACAACACCACTGTTTAATAGTACCTGGAATAAGAATGATAGTTCCGGGAACTGGACTGGG-
----
-AATGATACTGGAGTGGCA------
AATAACAATGGCACAATCACCCTCCAATGCAGACTAAAACAAATTATAAACATGTGGCAGGAAGTAGGAAAGG
CAATGTATGCCCCTCCCATCCGGGGAAACATTAGCTGTTCATCAAAC---
ATTACAGGGCTGCTATTAACAAGAGATGGTGGTGTTAATGCCACA----------------------------
----

AGCAACGAAACCTTTAGACCTGCAGGAGGAGATATGAGGGACAATTGGAGAAGTGAATTATATAAATATAAAG
TAGTACAAATTGAACCATTA---GGAATAGCACCCACCAAGGCAAAGAGAAGAGTGGTG--------------
-------------------------CAAAGAGAAAAAAGAGCA---GTGGGA---TTTGGAGCT---------
ATGTTCCTTGGGTTCTTGGGAGCA---GCAGGAAGCACTATG---GGCGCAGCGTCA---
GTAACGCTGACGGTACAG---GCCAGACAA---TTATTG---TCTGGTATAGTGCAACAGCAGAGC---
AACCTG---CTGAGA---GCTATTGAGGCGCAACAACATATGTTGCAACTCACGGTCTGGGGCATT
>MH666262
TTTTACTGTGATACAACACTACTGTTTAACAGTACTTGGAACAGTAACAGTACTGGGAACAGTACTGGGAATG
GTACTGAA---

GAGGGTGATCCAATCACACTCCAATGCAGAATAAAACAAATTATAAACATGTGGCAGGAAGTAGGAAAAGCAA
TGTATGCCCCTCCCATCAGAGGACAAATTAGATGTTCATCAAAC---
ATTACAGGGTTGCTATTAACAAGAGATGGTGGCAACAGTGATAGCACT-------------------------
----

AACGGAACTGAGATCTTCAGACCTGCAGGAGGAGATATGAAGGACAATTGGAGAAGTGAATTATACAAATATA
AAGTAGTAAAAGTTGAACCATTA---GGAGTAGCACCCACCATGGCAAGGAGAAGAGTGGTG-----------
----------------------------CAGAGAGAGAAAAGAGCA---GTGGGA---CTAGGAGCT------

---TTGTTCCTTGGGTTCTTGGGAGCA---GCAGGAAGCACTATG---GGCGCAGCGTCA---
ATGACACTGACGGTACAG---GCCAGACAA---CTATTG---TCTGGTATAGTGCAGCAGCAGAAC---
AATCTG---CTGAGG---GCTATTGAGGCGCAACAGCATATGTTGCAACTCACAGTCTGGGGCATC
>KM354431
TTCTACTGTAATACATCACAACTGTTTAATAATACTTGGAATAGTACTGATAAA-------------------
----
-ACGTTAAATAACACT---------
GAAGGAAATGACACAATTACCCTCCCATGCAGAATAAGACAGATTATAAACAGGTGGCAGGAAGTAGGAAAAG
CAATGTATGCCCCTCCAATCGCAGGAATAATTACATGTTCATCAAAT---
ATTACAGGGTTAATATTAACAAGAGATGGGGGG--

ACAGAGAATGGGACTAACACC---------
ACCACTGAGACCTTCAGACCTGGGGGAGGAAACATGAAAGACAATTGGAGAAGTGAATTATATAAATATAAAG
TAGTAAGAATTGAACCATTA---GGGATAGCACCCACCAGGGCAAGGAGAATAGTGGTG--------------
-------------------------CAGAGAGAAAAAAGAGCA---GTGGGAACGATAGGAGCC---------
ATGTTCCTTGGGTTCTTGGGAGCA---GCAGGAAGCACTATG---GGCGCAGCGTCA---
GTGACGCTGACGGTACAG---GCCAGACTA---TTATTG---TCTGGTATAGTGCAACAGCAAAAT---
AATTTG---CTGAGA---GCTATTGAGGCGCAACAGCGTATGTTGCAACTCACAGTCTGGGGCATC
>KM354585
TTCTACTGTAATACATCAGGACTGTTTAATAGCACTTGGACTCGGAACAATACTACTGGCACT----------
--TGG---
AACAGTATCAATACCACCACC---------------
ATAGGAAATGACACCATCACACTCCAATGCAGAATAAGACAAATTATAAACAGGTGGCAGGAAGTAGGAAAAG
CAATGTATGCCCCTCCCATCGCAGGAATAATTAGATGTTCATCAAAT---
ATTACAGGGCTGCTAATAACAAGAGATGGTGGG--

-
AACGAAACAGTGCACAATGAGACCTTCAGACCTGCAGGAGGGGATATGAGGGACAATTGGAGAAGTGAATTAT
ATAAATATAAAGTAGTAAAAGTTGAACCATTA---GGAATAGCACCCACCAAGGCAAGGAGAAGAGTGGTG--
-------------------------------------CAGAGAGAAAAAAGAGCA---GTGGGA---
ATAGGAGCT---------GTATTCCTTGGGTTCTTGGGAGCA---GCAGGAAGCACTATG---
GGCGCAGCGTCA---CTGACGCTGACGGTACAG---GCCAGACTA---TTATTG---
TCTGGTATAGTGCAACAGCAAAAC---AATTTG---CTGAAG---
GCTATTGAGGCGCAGCAGCATATGTTGCAACTCACAGTCTGGGGCATT
>KM354670
TTCTACTGTAATACAACACGACTATTTAATAGTATTTGGTATGCTAATGGTAGTAGTAAAGAG----------
--ACA---
GAGCCTGTTAATAGTACTGAA---------------
GGGACAAATGCCACTATCAGACTCCCATGCAGAATAAAACAAATTATAAACCTGTGGCAGGAAGTAGGAAAGG
CAATGTATGCCCCTCCCATCAAAGGACTAATTAGATGTTCATCAAAT---
ATTACAGGGATACTATTAACAAGAGATGGTGGTAGT-------------------------------------
----
ATAAGGAATGACACAGATAAC---------
ACCACAGAGGTCTTCAGACCTGGAGGTGGAGATATGAGGGACAATTGGAGAAGTGAATTATATAAATATAAAG
TAGTAAAAATTGAACCATTA---GGAGTAGCACCCACCAAGGCAAAGAGAAGAGTGGTG--------------
-------------------------CAGAGAGAAAAAAGAGCA---GTGGGA---ATAGGAGCT---------
GTGTTCCTTGGGTTCTTGGGAGCA---GCCGGAAGCACTATG---GGCGCAGCGTCA---
ATAACGCTGACGGTACAG---GCCAGACAA---TTATTG---TCTGGTATAGTGCAACAGCAAAGC---
AATTTG---CTGAGG---GCTATTGAGGCGCAACAACATCTGTTGCAACTCACAGTCTGGGGCATC
>KX587204
TTCTATTGTAATACATCAAAACTGTTTAATAGTACTTGGAATAGTACTAGGAATGATAGTGTTACTGGAGAG-
----

TCAAATAACACTATCACACTCCAATGCAGGATAAAACAAATTATAAACATGTGGCAGGAAGTTGGAAAAGCAA
TGTATGCCCCTCCCATCAGCGGACCAATTAGATGCTCATCAAAT---
ATTACAGGGTTGCTATTAACAAGAGATGGTGGGAAGGAGTCCAAT----------------------------
----

ACGACTGAGACCTTCAGACCAGGGGGAGGAAATATGAAGGATAATTGGAGAAATGAATTATATAAATATAAGG
TAGTAAAAATTGAACCATTA---GGAGTAGCACCCACCAAGGCAAGGAGAAGAGTGGTG--------------

-------------------------CAGAGAGAAAAAAGAGCA---GTGGGA---CTAGGAGCT---------
ATGTTCCTTGGGTTCTTGGGAGCA---GCAGGAAGCACTATG---GGCGCAGCGTCA---
GTGACGCTGACGGTACAG---GCCAGACAA---TTATTG---TCTGGTATAGTGCAACAGCAAAAC---
AATTTG---CTGAGA---GCTATTGAGGCGCAACAGCATCTGTTGCAGCTCACAGTCTGGGGCATC
>KC473824
TTCTACTGTGATACAACACAACTGTTTAATAGTACTTGGAATAATACTAATATTAATTCAAATGACACTGAGA
GA---

TCAAATAAAACTATCATTCTTCCATGCAAAATAAAACAAATTGTAAAAATGTGGCAGAGAGTAGGAAAAGCAA
CGTATGCCCCTCCCATCAGAGGACAAATTAGATGCTCATCAAAT---
ATTACAGGGCTGTTATTAACAAGAGATGGTGGTAGTAACCATAAT----------------------------
----

AATAGTGAGACCTTCAGACCTGGAGGAGGAGATATGAGGGACAATTGGAGGAGTGAATTATATAAATATAAAG
TAGTAAAAATTGAACCATTA---GGAGTAGCACCCACCAGGGCAAAGAGAAGAGTGGTG--------------
-------------------------CAAAGGGAGAGAAGAGCA---GTGGGA---ATAGGAGCT---------
ATGCTCCTTGGGTTCTTAGGAACA---GCCGGAAGCACTATG---GGCGCAGCGTCA---
GTGGCGCTGACGGTACAG---GCCAGACTA---TTATTG---TCTGGTATAGTGCAGCAGCAGAAC---
AATTTG---CTGAGA---GCTATTGAGGCGCAACAGCACCTGTTGCAACTCACAGTCTGGGGCATC
>MK164663
TTCTACTGTAATACAACAGAACTGTTTGAAAGAAGTTGGAGTAATAGTACTTGGAATGTTACTACCAATAATA
CTGAAGGG---

GAAGATAAAAATATCACTCTCCCATGCAGAATCAAACAGATTATAAACATGTGGCAGGAAGTAGGAAAAGCAA
TGTATGCCCCTCCCATCAAAGGACAAATTAGCTGTTCATCAAAT---
ATTACAGGGCTAATATTAACAAGAGATGGTGGTAACAACAACGGCAGTAATAAC-------------------
----
AACAACGGCAGTAATAACAACAAC------
AGTAGTGAGATCTTCAGACCTACAGGAGGAAATATGAGGGACAATTGGAGAAGTGAATTATATAAATATAAAG
TAGTAAAAATTGAACCATTA---GGAATAGCCCCCACCAAGGCAAAGAGAAGAGTGGTG--------------
-------------------------CAGAGAGAAAAAAGAGCA---GTGGGAGTACTAGGAGCT---------
ATGTTCCTTGGGTTCTTGGGAGCA---GCAGGAAGCACTATG---GGCGCAGCGTCA---
CTGACGCTGACGGTACAG---GCCAGACAA---CTATTG---TCTGGTATAGTGCAACAGCAGAAC---
AACCTG---CTGAGG---GCTATTGAGGCGCAACAGCATCTGTTGCAACTCACAGTATGGGGCATC
>MK164670
TTCTACTGTAATACAACAAAACTGTTTAATAATACTTGGGTTAATGGTACTTGGAGTAATATGCCTAACAATG
ATACTGAA---

GGGGCAGATGGAAAGATCACTCTCCCATGCAGAATCAAACAGATTATAAACATGTGGCAGGAAGTAGGAAAAG
CAATGTATGCCCCTCCCATCAAAGGACAAATTAGCTGTTCATCAAAT---
ATTACAGGGCTAATATTAACAAGAGATGGTGGCGACAACAACAGTCGGACCAAC-------------------
----

GACAGTAGTGAGATCTTCAGACCTAATGGAGGAAATATGAGGGACAATTGGAGAAGTGAATTATATAAATATA
AAGTAGTAAAAATTGAACCATTA---GGAATAGCCCCCACCAAGGCAAAGAGAAGAGTGGTG-----------
----------------------------CAGAGAGAAAAAAGAGCA---GTGGGAGTACTAGGAGCT------
---ATGTTCCTTGGGTTCTTGGGAGCA---GCAGGAAGCACTATG---GGCGCAGCGTCA---
TTGACGCTGACGGTACAG---GCCAGACAA---CTATTG---TCTGGTATAGTGCAACAGCAGAAC---
AACCTG---CTGAGG---GCTATTGAGGCGCAACAGCATCTGTTGCAACTCACAGTCTGGGGCATC
>KC935958
TTCTACTGTAATACAACAAAACTGTTTAATAGTACTTGGAATGGTACTGAGTCAGCTAACAATACA-------
----

GAAAATGGGACAATCACACTCCCATGCAGAATAAAACAAATCATAAATATGTGGCAGACAGTAGGAAAAGCAA
TGTATGCCCCTCCCATTAGGGGACAAATTACATGTATATCAAAT---
ATTACAGGGCTGCTATTAACAAGAGATGGTGGTGAGGAAAATAATACAACC----------------------
----

AATGCAACCATCGAGACCTTTAGACCGGGAGGAGGAGATATGAGGGACAATTGGAGAAGTGAATTATATAAAT
ATAAAGTAGTAGAAATTGAACCATTA---GGAATAGCACCCACCAAGGCAAAGAGAAGAGTGGTG--------

```
-------------------------------CAGAGAGAAAAAAGAGCA---GTGACA---CTAGGAGCT---
------CTGTTCCTTGGGTTCTTGGGAGCA---GCAGGAAGCACTATG---GGCGCAGCATCA---
ATGACGCTGACGGTACAA---GCCAGACAA---TTATTG---TCTGGTATAGTGCAACAGCAGAAT---
AATCTG---CTGAGG---GCTATTGAGGCGCAACAGCACCTGTTGCAGCTCACAGTCTGGGGCATC
>JF680914
TTCTACTGTAATACAACAAAACTGTTTAATAGTACTTGGTATTCGAATGATACTAGCAAGGGAGATAACAATG
AA-----------------------------------------------------------------------
----------------------------
GGAAATGATACAATCACACTCCCATGCAGAATAAAACAAATTGTAAACATGTGGCAGGAAGTAGGAAAAGCAA
TGTATGCCCCTCCCATCAGAGGACAAATTAGATGTTCATCAAAT---
ATTACAGGGCTGCTATTAACAAGAGATGGTGGTAACCATACTGAA----------------------------
-------------------------------------------------------------------------
-------------
AATAACGAGACTTTCAGACCTGGAGGAGGAGATATGAGGGACAATTGGAGAAGTGAATTATATAGGTATAAGG
TAGTAAAAATTGAACCATTA---GGAGTAGCACCCACCAAGGCAAAGAGAAGAGTGGTG--------------
-------------------------CAGAGAGAAAAAAGAGCA---GTAGTGGGAATGGGAGCT---------
CTGTTCCTTGGGTTCTTGGGAGCA---GCAGGAAGCACTATG---GGCGCAGCGTCA---
GTGACGCTGACGGTACAG---GCCAGACTA---TTATTG---TCTGGTATAGTGCAACAGCAGAAC---
AACTTG---CTGAGG---GCTATTGAGGCGCAACAACATCTGTTGCAACTCACAGTCTGGGGCATC
>JF680916
TTTTACTGTAATACAACAAGGCTGTTTAATAGTAATAGTACTATCTTGGATGCTTGGAATGAAACTACTGCTG
AT-----------------------------------------------------------------------
----------------------------
GAGAACAAGACTATCACACTCCCATGTAGGATAAAACAAATTATAAACATGTGGCAGGAAGTAGGAAAAGCAA
TGTATGCCCCTCCCATCAGTGGACAAATTAAGTGTTCATCAAAT---
ATTACAGGACTAATATTAACAAGAGATGGGGGTAATAATGATACA----------------------------
-------------------------------------------------------------------------
----------
AGCGGGACTGAGACCTTCAGACCTGGAGGAGGAGATATGAGAGACAATTGGAGAAGTGAATTATATAAATACA
AAGTAGTAAAAGTTGAACCATTA---GGAGTAGCACCCAATAAGGCAAAGAGAAGAGTGGTG-----------
----------------------------CAGAGAGAAAAAAGAGCA---GTGGGA---ATAGGAGCT------
---ATGTTCCTTGGGTTCTTGGGAGCA---GCAGGAAGCACTATG---GGCGCAGCGTCA---
CTGACGCTGACGGTACAG---GCCAGACAA---TTATTG---TCTGGTATAGTGCAACAGCAGAAC---
AATATG---CTGAGA---GCTATTGAGGCGCAACAACATCTGTTGCAACTCACAGTCTGGGGCATC
>MK383384
TTCTACTGTAATACAACAAAACTGTTTAATAGTACCTGGATCTGGGATAATGGTGCTGAAAACTGGAATGGTA
CTGAAAGGCCA--------------------------------------------------------------
----------------------------
GAGAATGACACTATCACACTCAAATGCAGAATAAAACAAATTATAAACATGTGGCAGGAAGTGGGAAAAGCAA
TGTATGCCCCTCCCATCAGTGGACAACTTAATTGTTCATCAAAT---
ATTACAGGTCTGCTTTTAACAAGAGATGGTGGTAAAAACAATAGT----------------------------
-------------------------------------------------------------------------
-------------
ACTAATGAGACCTTCAGACCTGGAGGAGGGGATATGAGGGACAATTGGAGAAGTGAATTATATAAATATAAAG
TAGTAAAAATTGAACCATTA---GGAATCGCACCCACCAAGGCAAAGAGAAGAGTGGTG--------------
-------------------------CAAAGAGAAAAAAGAGCA---GTGACA---TTTGGAGCT---------
ATGTTCCTTGGGTTCTTGGGAGCA---GCAGGAAGCACTATG---GGCGCAGCGTCA---
ATGACGCTGACGGTACAG---GCCAGACAA---TTATTG---TCTGGTATAGTGCAACAGCAGAGC---
AATTTG---CTGAGA---GCTATTGAGGCGCAACAGCATCTGTTGCAACTTACGGTCTGGGGCATC
>KF526141
TTCTATTGTAATTCAACACCACTATTTAATAGTACTTGGAATGTTAATGATACTAGTACTGGGAATGATACT-
-------------------------------------------------------------------------
----------------------------
GAAGAAGGAGATATCACACTCCAATGCAGAATAAAACAAATTGTAAACATGTGGCAAGAAGTAGGAAAAGCAA
TGTATGCCCCTCCCATCAGAGGACCAATTAGTTGTTCATCAAAT---
ATTACAGGGCTGCTATTAACAAGAGATGGCGGTGTTAATAGGAGC----------------------------
-------------------------------------------------------------------------
----------
AATGGAACCGAGGTCTTCAGACCTTCAGGGGGAGATATGAGGGACAATTGGAGAAGTGAATTATATAAATATA
AAGTAGTACAAATTGAACCATTA---GGAGTAGCACCCAGCAAGGCAAAAAGAAGAGTGGTG-----------
```

----------------------------CAGAGAGAAAAAAGAGCA---GCGGGA---ATGGGAGCT------
---CTGTTCCTTGGGTTCTTGGCAGCA---GCAGGAAGCACTATG---GGCGCAGCATCA---
ATGACGCTGACGGTACAG---GCCAGACAG---TTACTG---TCTGGTATAGTGCAACAGCAGAGC---
AATTTG---CTGAGG---GCTATTGAGGCGCAACAGCATCTGTTGCAACTCACAGTCTGGGGCATT
>KM081852
TTCTACTGTAATACAACACAGCTGTTTAATAGTACTTGGCTTAGCAATGGAACTTGGACTAATGCTAGA----
----

AGCAATGAGACTATCATACTCCAATGCAGAATAAAACAAATTATAAACAAGTGGCAGGGAGTAGGAAGAGTAA
TGTATGCCCCTCCCATCAGTGGAAATATTAGCTGTTTATCAAAT---
ATTACAGGGCTACTATTAACAAGAGATGGGGGTACAAAAAATGACATA-------------------------
----

AATGAGGAGATCTTCAGACCTGGAGGAGGAGATATGAGGGACAATTGGAGAAGTGAATTATATAAATATAAAG
TAGTAAAAATTGAACCAATA---GGAGTAGCACCCACCAAGGCAAAGAGAAGAGTGGTG--------------
-------------------------CAGAGAGAAAGAAGAGCA---GCA------TTAGGAGCT---------
ATGTTCCTTGGGTTCTTGGGAGCA---GCAGGAAGCACTATG---GGCGCAGCGTCG---
GTGACGCTGACGGTACAG---GCCAGACAA---TTATTG---TCTGGTATAGTGCAGCAGCAGAGC---
AATCTG---CTGAGG---GCTATTGAGGCGCAACAACATCTGTTGCAACTCACAGTCTGGGGCATT
>KM082074
TTCTACTGTAATACATCACAACTGTTTAATAGTACTTGGACTTGGAATGGTACTGAAGGGTCCAATAACACAA
AT---

AGCACTGATAATATCACACTCCAATGTAGAATAAAACAAATTGTAAATTTGTGGCAAGAAGTAGGGAAAGCAA
TGTATGCCCCTCCCATTAGAGGACAGATTAGATGTTCATCAAAT---
ATTACAGGGCTAATATTAACAAGAGATGGTGGACATGGAAATGAT----------------------------
----

AACACCACCGAGATCTTCAGACCTGGAGGGGGAGATATGAGGGACAATTGGAGAAGTGAATTATATAAGTATA
AAGTAGTACAAATTGAACCATTG---GGAATAGCACCCACCAAGGCAAAGAGAAGAGTGGTG-----------
----------------------------CAGAGAGAAAAAAGAGCA---GCA------ATAGGAGCT------
---GTGTTCCTTGGGTTCTTGGGAGCA---GCAGGAAGCACTATG---GGCGCAGCGTCG---
GTAGCGCTGACGGTACAG---GCCAGACAA---TTATTG---TCTGGTATAGTGCAACAGCAGAAC---
AATCTG---CTGAGA---GCTATTGATGCGCAACAGCATATGTTGCAGCTCACAGTCTGGGGCATC
>MG196787
TTCTACTGTAATACAACACAACTGTTTAATAGTACTTGGCATTGGAATAGCACTGAAGAATCAAATACT----
----

ACTGAAAGAAATATCACACTTCCATGCAGAATAAAACAAATCATAAACATGTGGCAGGAAGTAGGAAAAGCAA
TGTATGCCCCTCCCATCAGAGGACTAATTAAATGTTCATCAAAT---
ATTACAGGGCTGCTATTGACAAAAGATGGGGGTACTGGAAATAAG----------------------------
----

ACTGAGGAGATCTTCAGACCTGGGGGAGGAAATATGAAGGACAATTGGAGAAGTGAATTGTATAAATATAAAG
TAGTAAAAATTGAACCATTA---GGAATAGCACCCACCAGGGCAAAGAGAAGAGTGGTG--------------
-------------------------CAGAGAGAAAAAAGAGCA---GTGGGA---CTAGGAGCT---------
GTGTTCCTTGGGTTCTTGGGAGCA---GCAGGAAGCACTATG---GGCGCAGCGTCA---
GTGACGCTGACGGTACAG---GCCAGACAA---TTATTG---TCTGGCATAGTGCAACAGCAAAAC---
AATTTG---CTGAGG---GCTATTGATGCGCAACAGCATCTGTTGCAACTCACAGTCTGGGGCATC
>MG196997
TTCTATTGTAATTCAACACAACTGTTCAACAGTACTTGGAATAGTACTTTGACTAATAACTGGAATGATACTT
GGAATGCTAAT--
-------------AGCACT------
GATGTCAATGACACTATCACAATCCCATGCAGAATAAAACAAATTATAAACATGTGGCAGGAAGTAGGAAAAG
CCATGTATGCCCCTCCCATCAGAGGACAAATTAGATGTGAATCAAAT---
ATTACAGGGCTGCTATTGACAAGAGATGGTGGTGATAGCGATGGC----------------------------
----

ACAGACGAGATCTTCAGACCTGGAGGAGGAGATATGAGGGACAATTGGAGAAGTGAATTATATAAATATAAAG
TAGTAAAAATTGAGCCATTA---GGAATAGCACCCACCAGGGCAAGGAGAAGAGTGGTG--------------

-------------------------CAGAGAGAAAAAAGAGCA---GTGGGA---CTAGGAGCT---------
TTGTTCCTTGGGTTCTTGGGAGCA---GCAGGAAGCACTATG---GGCGCAGCGTCA---
ATAACGCTGACGGTACAG---GCCAGGCAA---TTATTG---TCTGGTATAGTGCAGCAGCAAAGC---
AACTTG---CTGAGG---GCTATTGAGGCGCAACAACATCTGTTGCAACTCACAGTCTGGGGCATC
>KM354703
TTCTACTGTAATTCAACGCCACTATTTAACAGTAATTGGACTCGGAACAATGGGACT----------------
----
TGGAAGCTAAATAACACTTCA------------
AATAGCACTGAGCCAATCACACTCCAATGCAGAATAAAACAAATTGTAAACAGGTGGCAAGAAGTAGGAAAAG
CAATGTATGCCCCTCCCATCGGAGGACAAATTAACTGTACATCAAAT---
ATTACAGGGCTACTAATAACAAGGGATGGTGGT--

-
GACCCGAACAGCACGACTGAGACCTTCAGACCAGGAGGAGGAGATATGAGGGACAATTGGAGAAGTGAATTAT
ATAAATATAAAGTAGTAAAAATTGAACCATTG---GGAATAGCACCCACCAAGGCAAAGAGAAGAGTGGTG--
-------------------------------------CAGAGAGAAAAAAGAGCA---GTGACA---
CTGGGAGCT---------ATGTTCCTTGGGTTCTTGGGAGCA---GCAGGAAGCACTATG---
GGCGCAGCGTCA---ATAACGCTGACGGTACAG---GCCAGACAA---TTACTG---
TCTGGTATAGTGCAACAGCAGAAC---AATCTG---CTGAAG---
GCTATTGATGCGCAACAGCATATGTTGCAACTCACCGTCTGGGGCATC
>KM354781
TTCTACTGTAATACAACAAAACTGTTTAATAGTACTTGGAATAGTACTTGGAATAGTGAG-------------
----
GAACAGTCAGGTGTTGATAAC------------
ACATCAAATAAGATTATCACAATCCAATGCAGAATAAAACAAATTGTACACATGTGGCAGAGAGTAGGAAGGG
CAATGTATGCCCCTCCCATCAGTGGACCAATTAGATGTTCATCAAAT---
ATTACAGGGTTGCTATTAGTAAGAGATGGTGCC--

AACGATACCAAGGAGACTTTCAGACCAGGAGGAGGAAATATGAAGGACAATTGGAGAAGTGAATTATATAAAT
ACAAAGTAGTAAAAATTGAACCATTA---GGAATAGCACCCACTAGGGCAAGGAGAAGAGTGGTG--------
-------------------------------CAGAGAGAAAAAAGAGCA---GTGGGA---CTAGGAGCT---
------ATGTTCCTTGGGTTCTTAGGAGCA---GCAGGAAGCACTATG---GGCGCAGCGTCG---
ATGGCGCTGACGGTACAA---GCCAGACAA---TTATTG---TCTGGCATAGTGCAGCAGCAGAAC---
AATCTG---CTGAGG---GCTATTGAGGCGCAACAGCACATGTTGCAACTCACAGTCTGGGGCATC
>JN397365
TTCTACTGTAACACATCAGGACTGTTTAATAGTACTTGGACTGTTATCGGTGAGTGGGAGGATCCTACACGG-
----

AATGATACCAACATCACACTCCCATGTAGAATAAAACAAATTATAAACATGTGGCAGGAAGTAGGGAAAGCAA
TGTATGCCCCTCCCATCAAAGGACTAATCAACTGTACATCAAAT---
ATCACAGGGCTGCTATTAACAAGAGATGGTGGTATTAATAGAACT----------------------------
----

AATAACGAGACCTTCAGACCTGCAGGAGGAAATATGAAGGACAATTGGAGAAGTGAATTATATAAATATAAAG
TAGTACAAATTGAACCATTA---GGATTAGCACCCACCAAGGCAAAGAGGAGAGTGGTG--------------
-------------------------CAGAGAGAAAGAAGAGCA---GTGACA---CTCGGAGCT---------
ATGCTCCTTGGGTTCTTGAGCGCA---GCAGGAAGCACTATG---GGCGCAGCGTCG---
CTGACGCTGACGGTACAG---GCCAGACTA---TTATTG---TCTGGTATAGTGCAACAGCAGAAC---
AATCTG---CTGAGA---GCTATTGAGGTGCAACAGCATCTGTTGCAACTCACAGTCTGGGGCATT
>HQ846901
TTCTACTGTAATTCAACACCACTGTTTAATAGTACTTGGAATGTTACTGGAGAGTCTTGGAATGATACAAAA-
----

GGGGGAGTAAACATCACACTTCCATGCAGAATAAGACAAATTATAAACATGTGGCAGAAAGTAGGAAAAGCAA
TGTATGCCCCTCCCATCAGAGGACAAATTAGATGTACATCAAAT---
ATTACAGGGTTGATATTAACAAGAGATGGTGGTAATAACAAGAGTGAG-------------------------
----

AACGAGACTGAGACCTTCAGACCTGGAGGAGGAGATATGAGGGACAATTGGAGAAGTGAACTATATAAATATA

AAGTAGTAAAAATTGAACCATTA---GGAATAGCACCCACCAAGGCAAAGAGAAGAGTGGTG-----------
----------------------------CAGAGAGAAAAAAGAGCA---GTGGGA---ATAGGAGCT------
---GTGTTCCTTGGGTTCTTGGGAGCA---GCAGGAAGCACTATG---GGCGCAGCGTCA---
CTGACGCTGACGGTACAG---GCCAGACTA---TTATTG---TCTGGTATAGTGCAGCAGCAGAAC---
AATCTG---CTGAGG---GCTATTGAGGCGCAACAGCATCTGTTGCAGCTCACAGTCTGGGGCATC
>KC473825
TTCTACTGTAATACAACAAGACTGTTTGCTTGGAATAGTGCTTGGAATAGTACTGGGAATGAAACT-------
----

GCAGATAGAAATATTACACTCCCATGCAGAATAAAACAAATTATAAACATGTGGCAGGCAGTAGGAAAAGCAA
TGTATGCCCCTCCCATCAGAGGAGCAATTAATTGTGCATCAAAT---
ATTACAGGGCTGTTATTATTAAGAGATGGTGGTAATAGCAGCGAA----------------------------
----

GAGATCTTCAGACCTGGAGGAGGAGATATGAGGGACAATTGGAGAAGTGAATTATATAAATATAAAGTAGTAA
AAATTGAACCAATA---GGAGTAGCACCCACCAAGGCAAAGAGAAGAGTGGTG--------------------
-------------------CAGAGAGAAAAAAGAGCA---GTAGGA---ATAGGAGCT---------
CTGTTCCTTGGGTTCTTGGGAGCA---GCAGGAAGCACTATG---GGCGCAGCGTCA---
ATAACGCTGACGGTACAA---GCCAGACAA---TTATTG---ACTGGTATAGTGCAACAGCAGAAC---
AATCTG---CTAAGG---GCTATTGAGGCGCAACAGCACCTGTTGCAACTCACAGTCTGGGGCATT
>KC473827
TTCTACTGTAATACAACAGCACTGTTTAATAGCACTTGGAATAGGAATGAGACTTGGAATTCTACTGGAGAG-
----

TCAAATGAAACTATCACACTCCAATGTAGAATAAAACAAATTATAAACATGTGGCAGGAAGTAGGAAAAGCAA
TGTATGCCCCTCCCATCAGAGGACAAATTAGATGTTCATCCAAT---
ATTACAGGGCTGTTATTAACAAGAGATGGGGGTAACACAAACGAG----------------------------
----

ACTACCTCTGAGACTTTTAGACCCGGAGGAGGAAATATGAGGGACAATTGGAGAAGTGAATTATATAAATATA
AAGTAGTAAAGATTGAACCATTA---GGAATAGCACCCACCAAGGCAAAGAGAAGAGTGGTG-----------
----------------------------CAGAGAGAAAAAAGAGCA---GTGGGA---ATAGGAGCT------
---ATGTTCCTTGGGTTCTTGGGAGCA---GCAGGAAGCACTATG---GGCGCAGCGTCA---
ATGACGCTGACGGTACAG---GCCAGACTA---TTATTG---TCTGGTATAGTGCAACAGCAGAAC---
AATCTG---CTGAGG---GCTATTGAGGCGCAACAACATCTGTTGCAACTCACAGTCTGGGGCATC
>KU678074
TTCTACTGTAATACAACACAACTGTTTAATAGTACTTGGTATCCTAATGATACAAGTACTTGGAATGATACTA
AT---

GGAATTAATGACACACTCACACTACCATGTAAAATAAAACAATTTGTAAACTTGTGGCAGGAGGTAGGAAAGG
CAATGTATGCCCCTCCCATCCAGGGACAAATTAATTGTGCATCAAAT---
ATTACAGGGCTGCTATTAACAAGAGATGGAGGTGTTAACACTACC----------------------------
----

AACACCACTGAGGTCTTCAGACCTGCAGGAGGAGATATGAGGGACAATTGGAGAAGTGAACTATATAAATATA
AAGTAGTAAAAATTGAACCATTA---GGAATAGCACCCACTAAGGCAAAGAGAAGAGTGGTA-----------
----------------------------CAAAGAGAAAAAAGAGCA---GTGGGA---ATAGGAGCT------
---ATGTTCCTTGGGTTCTTGGGAGCA---GCAGGAAGCACTATG---GGCGCAGCGTCA---
GTGACGCTGACGGTACAG---GCCAGACAA---TTAATG---TCTGGTATAGTGCAACAGCAGAGA---
AATTTG---CTGAGG---GCTATTGAGGCGCAGCAGCATCTGTTGCAACTCACAGTCTGGGGCATT
>KM082156
TTCTACTGCAATACAACACAACTGTTTAATAGTACTTGGGCTAATAGTACTTGGAATGACACTGAAGGGTTTA
GTGACACT---

GGAAATGATACAATCACACTCCCATGCAGAATAAAACAAATTATAAATCTGTGGCAGGAAGTAGGAAAAGCAA
TGTACGCCCCTCCCATCAGAGGACAAATTAGATGTTCATCAAAT---
ATTACAGGGCTGCTATTAACAAGAGATGGTGGTAATATTACCAAT----------------------------
----
-
AATAATACAAACAATAATGAGACCTTCAGACCTGGAGGAGGAGATATGAGGGACAATTGGAGAAGTGAATTAT

ATAAATATAAAGTAGTAAAAATTGAACCATTA---GGAATAGCCCCCACCAAGGCAAAGAGGAGAGTGGTG--
-------------------------------------CAGAGAGAAAAAAGAGCA---GTGGGA---
ATAGGAGCT---------GTGTTCCTTGGGTTCTTGGGAGCA---GCAGGAAGCACTATG---
GGCGCAGCGTCA---ATAACGCTGACGGTACAG---GCCAGACTG---TTATTG---
TCTGGTATAGTGCAACAGCAGAAC---AATTTG---CTGAGA---
GCTATTGAGGCGCAACAACATCTGTTGCAACTCACAGTCTGGGGCATC
>KF526228
TTCTACTGTAATACAACACAACTGTTTAACAGTACTTGGAATTCACCACAACCTAATAGGAATTGGACA----
----

GGAAATGACACAATCACACTCCCATGCAGAATAAAACAATTTATAAACATGTGGCAGGAAGTAGGAAAAGCAA
TGTATGCCCCTCCCATCAAAGGAATAATTAGATGTGTATCAAAT---
ATTACAGGAATGATATTAACAAGAGATGGTGGTATTGGTAACACCACCGGG----------------------
----

GAGAACGGAACAGAGATCTTTAGACCAGGAGGAGGAGATATGAGGGACAATTGGAGAAGTGAACTATATAAAT
ATAAAGTAGTAAGAATTGAACCATTA---GGAATAGCACCCACCAAGGCAAAGAGAAGAGCGGTG--------
-------------------------------CAGAGAGAAAAAAGAGCA---GTGGGA---ATAGGAGCT---
------GTGTTCCTTGGGTTCTTGGGAGCA---GCAGGAAGCACTATG---GGCGCAGCGTCA---
CTAACGCTGACGGTACAA---GCCAGACTA---TTATTG---TCTGGTATAGTGCAACAGCAGAAC---
AATCTG---CTGAGG---GCTATTGAGGCGCAACAGCATCTGTTGCAACTCACTGTCTGGGGCATC
>KF526265
TTCTACTGCAATTCATCACAATTGTTTAATAGTACTTGGAATGGTACTGAGTCTTGGAATAGTACTGGG----
----

CATGACACTAACATCACACTCCAATGCAGAATTAAACAAATTATAAACATGTGGCAGGAAGTAGGAAAAGCAA
TGTATGCCCCTCCCATCCAAGGACCAATTAGCTGTTCATCAAAT---
ATTACAGGGCTGCTATTAACAAGAGATGGTGGTGGGAACAAGAGC----------------------------
----

GAGACTGAGACTGAGACTTTCAGACCTGTAGGAGGAAATATGAGGGACAATTGGAGAAGTGAATTATATAAAT
ATAAAGTAGTAAAAATTGAACCATTA---GGAATAGCACCCACCAAGGCAAAGAGAAGAGTGGTG--------
-------------------------------CAGAGAGAAAAAAGAGCA---ATGGGA---CTAGGAGCT---
------TTGTTCATTGGGTTCTTGGGAGCA---GCAGGAAGCACTATG---GGCGCAGCGTCA---
ATAACGCTGACGGCACAG---GCCAGACTA---CTATTG---TCTGGTATAGTGCAACAGCAGAAC---
AATTTG---CTGAGA---GCTATTGAGGTGCACCAACATATGTTGCAACTCACAGTCTGGGGCATC
>KF526312
TTCTACTGTAATGCAACAAAACTGTTTAATTCTACGTGGAATAGTAGTACTTGGAATGATAATAATCCTGGG-
----

GAAAATGTTACCATCACACTCCCATGCAGGATAAAACAAATTATAAACATGTGGCAGAAAACAGGAAAAGCAA
TGTATGCCCCTCCCATCAGTGGACGAATTAGCTGTTTATCAAAT---
ATTACAGGGCTGATATTAACAAGAGATGGGGGTATTAATAACACG----------------------------
----

AACGAGACCTTCAGACCTGCAGGAGGAGATATGAGGGACAATTGGAGAAGTGAATTATATAAATATAAAGTAG
TAAAAATTGAACCATTA---GGAATAGCACCCAACAAGGCAAGGAGAAGAGTGGTG-----------------
----------------------CAGAGAGAAAAAAGAGCA---GTGGGA---ATAGGAGCT---------
GTGTTCCTTGGGTTCTTGGGAGCA---GCAGGAAGCACTATG---GGCGCAGCGTCA---
GTAACGCTGACGGTACAG---GCCAGACTA---TTGCTG---TCTGGTATAGTGCAACAGCAGAAC---
AATCTG---CTGAGG---GCTATTGAGGCGCAACAGCATCTGTTGCAACTCACAGTCTGGGGCATC
>KF526323
TTCTATTGTAATACATCAAAATTATTTAATAGTACCTGGAATGAGACTAGTACTTGGAATGAGATGGA----
----

AAAGATGATAATATCATACTCCCATGTAGAATAAAACAAATTATAAACAGATGGCAGGAACCAGGAAAAGCAA
TGTATGCCCCTCCCATCGCAGGACTAATTAGATGTTCATCAAAT---
ATTACAGGGCTGCTATTAATAAGAGATGGTGGTAACAAGGGCAGTAATGGT----------------------
----

AGCAACACCGAGATCTTCAGACCTGGAGGAGGAGACATGAGGGACAATTGGAGAAGTGAATTATATAAATATA
AAGTAGTAAAAATTGAACCATTA---GGAATAGCACCCACCAAGGCAAAGAGAAGAGTGGTG----------
----------------------------CAGAGAGAAAAAAGAGCA---GTGGGAGTACTAGGAGCT------
---ATGTTCCTTGGGTTCTTGGGAACA---GCAGGAAGCACTATG---GGCGCAGCGTCA---
GTGACGCTGACGGTACAG---GCCAGGCAA---TTAATG---GCTGGTATAGTGCAACAGCAGAAC---
AATCTG---CTGAGA---GCTATTGATGCGCAACAGCAACTGTTGCAACTCACGGTCTGGGGCATC
>KT124766
TTCTACTGTAACACAACAGAACTGTTTAACAGTACTTGGAATGTTACTAGTGATGAGTCAAATAACACA----
----

AATAACACTACTATCACGCTCCAATGCAGAATAAAACAAATTGTAAACATGTGGCAGACAGTAGGAAAAGCAA
TGTATGCCCCTCCCATCCAAGGAATAATTAGATGTTCATCAAAT---
ATTACAGGGCTGCTATTAACAAGAGATGGTGGGTATAATAATGGCACCAAT----------------------
----

AATGATACCGAGATCTTCAGACCTGGAGGAGGAGATATGAGGGACAATTGGAGAAGTGAATTATATAAATATA
AAATAGTAAGAATTGAACCATTA---GGAATAGCACCCAACAAGGCAAAGAGAAGAGTGGTG----------
----------------------------CAAAGAGAAAAAAGAGCA---GTGGGGATGATAGGAGCA------
---ATGTTCCTTGGGTTCTTGGGAGCA---GCAGGAAGCACTATG---GGCGCAGCGTCA---
CTAACGCTGACGGTACAT---GCCAGACAA---GTATTG---GCTGGTATAGTGCAACAGCAGAAC---
AATCTG---CTGAGG---GCTATTGAGGCGCAACAACATATATTGCAACTCACAGTCTGGGGCATC
>KT124773
TTCTATTGTAATACAACACAACTGTTTAATAGTACCTGGGTTAATAGTACATGGAATAGTACTGAAGGAATG-
----

AACAACACCAACATCACACTCCCATGCAGAATAAAACAGATTGTAAACATGTGGCAGGAAGTAGGAAAAGCAA
TGTATGCCCCTCCCATCAGAGGAAATATTACATGTGCATCAAAT---
ATTACAGGGGTGCTATTAACAAGAGATGGTGGACATAATGGATCA----------------------------
----

AATGAGACCTTCAGACCTGCAGGAGGAGATATGAGGGATAATTGGAGAAGTGAATTATATAAATATAAAATAG
TAAAAATTGAACCATTA---GGAATAGCACCCACCAAGGCAAAAAGGAGAGTGGTG-----------------
----------------------CAGAGAGAAAAAAGATCA---CCAACA---CTGGGAGCT---------
TTGTTCATTGGGTTCTTGGGAGCA---GCAGGAAGCACTATG---GGCGCAGCGTCA---
CTGACGCTGACGGTACAA---GCCAGGCAA---TTATTG---TCTGGTATAGTGCAACAGCAGAGC---
AATCTG---CTGAGG---GCTATTGAGGCGCAACAACACATATTGCAACTCACGGTCTGGGGCATC
>KT124775
TTCTACTGTAACACCACACAGCTGTTTAATGAAACCTGGATTAACAGTAACAGTACCGGG------------
----

AGTAATAGCAGTATCACACTCCCATGCAGAATAAAACAAATTATAAACAGATGGCAGGAAGTAGGAAAAGCAA
TGTATGCCCCTCCCATGCAAGGACAAATTAGTTGCTCATCAAAT---
ATTACAGGACTACTATTAACAAGAGATGGAGGAGGAGGTAATGGCACA-------------------------
----

AACAATACTGAGACCTTCAGACCTGGAGGAGGAGATATGAGAGATAATTGGAGAAGTGAATTATATAAATATA
AGGTAGTAAGAATTGAGCCATTA---GGAGTAGCACCCACCAAGGCAAAGAGAAGAGTGGTG----------
----------------------------CAGAGAGAAAAAAGAGCA---GTGGGAACAATAGGAGCT------
---ATGTTCCTTGGGTTCTTGGGAGCA---GCTGGAAGCACTATG---GGCGCAGCATCA---
GTGATGCTGACGGTACAG---GCCAGACAA---TTATTG---TCTGGTATAGTGCAACAGCAGAAT---
AATCTG---CTGAGA---GCTATTGAGGCGCAACAGCATATGTTGCAACTCACGGTCTGGGGCATC
>MG196689
TTCTATTGTAACTCAACAAAACTGTTCAACAGTATTTGGAATAGTAGTTTGATTAATGAGAATATTACTTGGA
ATGCTAAT--
-------------AGCACT------
GATGCCAATGACACTATCACACTCACATGCAGAATAAAACAAATTGTAAACATGTGGCAGGAAGTAGGAAAAG
CCATGTATGCCCCTCCCATCAGAGGACAAATTAGATGTGAATCAAAT---
ATTACAGGGCTGCTATTAACAAGAGATGGTGGTGATACCAGTAGC----------------------------
----

ACAAATGAGACCTTCAGACCTGGAGGAGGAGATATGAGGGACAATTGGAGAAGTGAATTATATAAATACAAGG
TAGTAAAAATTGAGCCATTA---GGAATAGCACCCACCAAGGCAAGGAGAAGAGTGGTG--------------
-------------------------CAGAGAGAAAAAAGAGCA---GTGGGA---CTAGGAGCT---------
TTTTTCCTTGGGTTCTTGGGAGCA---GCAGGAAGCACTATG---GGCGCAGCGTCA---
ATAACGCTGACGGTACAG---GCCAGGCAA---TTATTG---TCTGGTATAGTGCAGCAGCAAAAC---
AACTTG---CTGAGG---GCTATTGAGGCGCAACAACATCTGTTGCAACTCACAGTCTGGGGCATC
>MG196815
TTCTACTGTAATTCAACACAATTGTTTAATAGTACTTGGCATAGTAATAGTACTTTGAATAGTACTGAGAAT-
----

GTAAATAAAACTATCACACTCCCATGCAAAATAAAACAAATTGTAAACATGTGGCAGGAAGTAGGAAGAGCAA
TATATGCCCCTCCCATCAGAGGAGTAATCAGATGTTCATCAAAT---
ATTACAGGGCTGCTACTAACAAGAGATGGTGGTAAGAGTAGCAACACCAGCAAC-------------------
----

ACCACCGAGGAGGTCTTCAGACCTGAGGGAGGAAATATGAGGGACAATTGGAGAAGTGAATTATATAAATATA
AAGTAGTAAGAATTGAACCATTA---GGAGTAGCACCCACCAAGGCAAAGAGAAGAGTGGTG-----------
----------------------------CAGAGAGAAAAGAGAGCA---GTGGGAACACTAGGAGCT------
---ATGTTCCTTGGATTCTTGGGAACA---GCAGGAAGCACTATG---GGCGCAGCGTCA---
CTGGCGCTGACGGTACAG---GCCAGACAA---TTATTG---TCTGGAATAGTGCAACAGCAAAAC---
AATTTG---CTGAGA---GCTATTGAGGCGCAACAGCATCTGTTGCAACTCACAGTCTGGGGCATT
>MG196840
TTCTACTGTAATTCAACACCGTTGTTTAATAGTACTTGGTCTAATAATACTTGGAACGATACTGAAGAATTAA
GTGCC--
-------------------ACT---
GAAGGAAATGAAACAATCACACTCCCATGCAGAATAAAACAAATTATAAACATGTGGCAAGAAGTAGGAAAAG
CAATGTATGCCCCTCCCATCAGAGGACTGATTAGATGCTCATCAAAC---
ATTACAGGGCTGCTATTAACAAGGGATGGTGGTCCTAATAACACC----------------------------
----

AACGAAACCTTCAGACCTACAGGAGGAGATATGAGGGATAATTGGAGAAGTGAATTGTATAAATATAAAGTAG
TAGAACTTGAACCATTA---GGAGTAGCACCCACCAAGGCAAAAAGAAGAGTGGTG-----------------
----------------------CAGAGAGAAAAAAGAGCA---GTGGGA---CTAGGAACT---------
TTGTTCCTTGGGTTCTTGGGAGCA---GCAGGAAGCACTATG---GGCGCAGCATCA---
ATGGTGCTGACGGTACAG---GCCAGGCAA---TTATTG---TCTGGTATAGTGCAACAGCAGAAC---
AATTTG---CTGAGA---GCTATTGAGGCGCAACAGCATCTGTTGCAACTCACAGTCTGGGGCATC
>MG196846
TTCTATTGTGATACAGCAAGCCTGTTTAATAGTACTTGGCAGGCAAAGGAGACTGAAAGAATGACACTGGA-
----

GGAAATGACACAATCACACTCCCCTGTAGAATAAAACAAATTATAAACATGTGGCAGGCAGTAGGGAAAGCAA
TATATGCCCCTCCTATTGCAGGGTTAATTAGATGTTCATCAAAT---
ATAACAGGGCTGCTGTTAACAAGAGATGGTGGTAATGAGACCATGACC-------------------------
----

AAGGATAACGAGACCTTCAGACCTGGAGAAGGAAATATGAGGGATAATTGGAGGAGTGAATTATATAAATATA
AAGTAGTAAAAGTTGAACCATTA---GGAATAGCACCCACCAAGGCAAGGAGAAGAGTGGTG-----------
----------------------------CAAAGAGAAAAAAGAGCA---GTGACA---ATAGGAGCT------
---GTGTTCCTTGGGTTCTTGGGAGCA---GCAGGAAGCACTATG---GGCGCAGCATCA---
GTGACGCTGACGGTACAG---GCCAGACAA---TTATTG---TCTGGTATAGTGCAGCAGCAAAAC---
AACTTG---CTGAAA---GCTATTGAGGCGCAACAGCATCTGTTGCAACTCACAGTCTGGGGCATC
>MG196942
TTCTACTGTAACACAACAGCACTGTTTAATAGCAGTTGGAATGAGAATAATACTTGGGTTAATAATACTGAAA
CA---

GGAGAGAGAAACATCACACTCCAATGTAGAATAAAACAAGTTATAAACTTGTGGCAGCAAGTAGGAAAAGCAA
TGTATGCCCCTCCCATCAGAGGGCAAATTAGATGTTCATCAAAT---
ATTACAGGGATACTATTAACAAGAGATGGTGGTGAGCCTAACAATACC-------------------------
----

CAAAATGAGACCTTTAGACCTGGGGGAGGAAATATGAAGGACAATTGGAGAAGTGAATTATATAAATATAAAG
TAGTAAGGATTGAACCATTA---GGAGTAGCACCCACCAAGGCAAAAAGAAGAGTGGTG--------------
-------------------------CAGAGAGAAAAAAGAGCA---GTGGGA---ATAGGAGCT---------
GTGTTCCTTGGATTCTTGGGAGCA---GCAGGAAGCACTATG---GGCGCAGCGTCA---
CTGACGCTGACGGTACAG---GCCAGACAA---TTATTG---TCTGGTATAGTGCAACAGCAGAGC---
AATTTG---CTGAGA---GCTATTGAGGCGCAACAGCATATGTTGCAACTCACAGTCTGGGGCATC
>MG197084
TTCTATTGTAATACAACAAAACTATTTGATAGTACTTGGAATGTTACTTGGAAAGAGGCAAATAACACTGGA-
----

GAGAATGACACAATCATCCTCCAGTGCAGGATAAAACAAATTATAAACATGTGGCAGGAAGTAGGAAAAGCAA
TGTATGCCCCTCCCATCAGAGGAAATATTAGTTGCTCATCAAAT---
ATTACTGGATTGCTATTAACAAGAGATGGTGGTGACGATAGGAAC----------------------------
----

ACCACCGAGATCTTTCGACCTGGAGGAGGAGATATGAGGGACAATTGGAGAAGTGAATTATATAAATATAAAG
TAGTACAAGTTGAACCATTA---GGAATAGCACCTACCAAGGCAAAAAGAAGAGTGGTG--------------
-------------------------CAGAGAGAAAAAAGAGCA---GTGGGA---ATAGGAGCT---------
CTGTTCCTTGGGTTCTTGGGAGCA---GCAGGAAGCACTATG---GGCGCAGCATCA---
ATGACGCTGACGGTACAG---GCCAGACAA---TTATTG---TCTGGTATAGTGCAACAGCAGAGC---
AATCTG---CTGAGA---GCTATTGAGGCGCAACAGCATCTGTTGCAACTCACAGTCTGGGGCATC
>MG197146
TTCTACTGTAATTCAATACCACTGTTTAATAGTACTTGGGATAGTAATAGTACTTGGGGTAATGATACCATAG
GG---

TTAAATGAAACTATTACACTCCAATGCAGAATAAAACAAATTATAAACATGTGGCAGGAAGTAGGAAAAGCAA
TGTATGCCCCTCCCATCAAAGGACAGATTGGATGTTCCTCAAAT---
ATTACAGGGCTATTGTTAACAAGAGATGGTGGTAATGCTAAGAGG----------------------------
----

GACAAAAACGACACAGAGATCTTCAGACCTGCAGGAGGAGATATGAGGGACAATTGGAGAAGTGAATTATATA
GATATAAAGTAGTAAGAATTGAACCATTA---GGAGTAGCACCCACCAAGGCAAGGAGAAGAGTGGTG-----
----------------------------------CAAAAAGAAAAAAGAGCA---GTGGGA---
ATAGGAGCT---------TTGTTCATTGGGTTCCTGGGAGCA---GCTGGGAGCACTATG---
GGCGCAGCGTCA---CTGACGCTGACGGTACAG---GCCAGACAA---TTGTTG---
TCTGGTATAGTGCAGCAGCAAAAC---AATTTG---CTGATG---
GCTATTGAGGCGCAACAGCATCTGTTGCAACTCACAGTCTGGGGCATT
>KM354470
TTCTACTGTAATACAACACAACTGTTTAATAGTACTTGGAATGATACAGAGTTA-------------------
----
AATAGTACTACAGGGAAT---------
AGTACTACAGACACAATCACACTCCCATGCAGAATAAAACAAATTATAAACATGTGGCAGGAGGTAGGAAAAG
CAATGTATGCCCCTCCCATAAGGGGATACATTAACTGTTCATCAAAT---
ATTACAGGGCTGCTATTAACAAGAGATGGAGGA--

-
GAGGGCAACAACAGGAGTGAGACCTTTAGACCTGGAGGAGGAAATATGAGGGACAATTGGAGAAGTGAATTAT
ATAAATATAAAGTAGTAAAAATTGAACCATTA---GGAATAGCACCCACCAAGGCAAAGAGAAGAGTGGTG--
-------------------------------------CAAAGAGAAAAAAGAGCA---
GTGGGAACAATAGGAGCT---------ATGTTCCTTGGGTTCTTGGGAGCA---GCAGGAAGCACTATG---
GGCGCAGCGTCA---ATAACGCTGACGGTACAG---GCCAGACAA---TTATTG---
TCTGGTATAGTGCAACAGCAGAAC---AATCTG---CTGAGA---
GCTATTGAGGCGCAACAACATCTGTTGCAACTCACAGTCTGGGGCATC
>KM354904
TTCTACTGTAACACAACACAACTGTTTAATAGTACTTGGACTAATAGTGGTGATGGT----------------
----
AATTGGACTAGTAGTGAT---------
AAGTTAAATGGCACTATCACACTCCCATGTAGAATAAGACAAATTATAAACATGTGGCAGGAAGTAGGAAAAG
CAATGTATGCCCCTCCCATCAGAGGATATATTAACTGCTCATCAAAT---
ATTACAGGAATACTATTAACAAGAGATGGTGGT--

ATTGAGAACAATACCAAC---
AGTACTGAGATCTTCAGACCTGGAGGAGGAGATATGAGGGACAATTGGAGAAGTGAGCTATATAAATATAAAG
TAGTACAAATTAAACCATTA---GGAGTAGCACCCACCGAGGCAAAAAGAAGAGTGGTA--------------
-------------------------CAGAGAGAAAAAAGAGCA---GCA------CTAGGAGCT---------
CTGTTCCTTGGGTTCTTGGGAGCA---GCCGGAAGCACTATG---GGCGCAGCGTCA---
ATGACGCTGACGGTACAG---GCCAGACAA---TTATTG---TCTGGTATAGTGCAACAGCAGAGC---
AATCTG---CTGAGG---GCTATTGAGGCGCAACAGCATCTGTTGCAACTCACAGTCTGGGGCATA
>KM355003
TTCTACTGTAATACATCACGACTGTTTAATAGTACTTGGAATAGTACTGGA----------------------
----
-TCAAATAACACTGAG---------
GGAAATGACACAACAATCACAATCCCATGTAAAATAAAACAAGTTATAAACCTGTGGCAGGAAGTAGGAAAAG
CAATGTATGCCCCTCCCATCAGAGGACAGATTAAATGCTCATCAAAT---
ATTACAGGGTTATTACTAACAAGAGATGGGGGT--

ATTAATGCCACAAACGGG---
ACCATGGAGATCTTCAGACCTGGAGGAGGAGATATGAGGGACAATTGGAGAAGTGAATTATACAAATATAAAG
TAGTACAAATTGAACCATTA---GGAATAGCACCCACCAGGGCAAAGAGAAGAGTGGTG--------------
-------------------------CAGAGAGAAAAAAGAGCA---GTAGGA---TTAGGAGCT---------
CTGTTCCTTGGGTTCTTGGGAGCA---GCAGGAAGCACTATG---GGCGCAGCGTCA---
GTGACGCTGACGGTACAG---GCCAGACAA---TTATTG---TCTGGTATAGTGCAGCAGCAGAAC---
AATCTG---CTGAGG---GCTATTGAGGCGCAACAGCATCTGTTGCAACTCACAGTCTGGGGCATC
>KM355044
TTCTACTGTAATTCAACAAAATTGTTTAATAGTACTTGGACGAATAGTAGTTTG-------------------
----
-GAAAATAATACTACA---------
GAGTCAAATATCAATATCACACTCCCATGCAGAATAAAACAGATTATAAACATGTGGCAGGGAGTAGGAAAAG
CGATGTATGCCCCTCCCATCAGTGGACCAATTAGATGTTCAACAAAT---
ATCACAGGGCTACTATTAACAAGAGATGGTGGG--

-
AATATCACTAATAACACTGAGGTCTTCAGACCTGGAGGAGGAGATATGAGAGACAATTGGAGAAGTGAATTAT
ATAAATATAAAGTAGTGAAAATTGAACCATTA---GGAGTAGCACCCACTAAGGCAAAGAGAAGAGTGGTG--
-------------------------------------CAGAGAGAAAAAAGAGCA---GTGGGA---
TTAGGAGCT---------ATGTTCCTTGGGTTCTTGGGAGCA---GCCGGAAGCACTATG---
GGCGCAGCGTCA---ATAACGCTGACGGTACAG---GCCAGACAA---TTATTG---
TCTGGTATAGTGCAACAGCAGAAC---AATCTG---CTGAGG---
GCTATTGAGGCGCAACAGCATCTGTTGCAACTCACAGTCTGGGGCATC
>KU901727
TTCTACTGCGATACAACACAACTGTTTAATAGTACTTGGAATTGGAATGATACTCGGACAGGGTCAAAT----
----

GGCAATGACAAGATCATACTCCCATGTAGAATAAGACAAATTGTAAATAGGTGGCAGGAGGTAGGAAAGGCAA
TGTATGCTCCTCCCATTAGAGGACAAATTAAATGCTCATCAAAC---
ATTACAGGGCTACTATTAACAAGAGATGGGGGCAATAACAATACA----------------------------
----

AATGAAAATGAGACCTTCAGACCTGGAGGAGGAGATATGAGGGACAATTGGAGAAGTGAATTATATAAATATA
AGGTGGTACAGGTTGAACCAATA---GGAATAGCACCCACCACGGCAAGGAGAAGAGTGGTG-----------
----------------------------CGAAGAGAAAAAAGAGCG---GTAACA---ATGGGAGCT------
---GTGTTCCTTGGGTTCTTGGGAACA---GCAGGAAGCACTATG---GGCGCAGCGTCA---
CTGACGCTGACGGTACAG---GTCAGACAA---TTATTG---TCTGGCATAGTGCAGCAGCAAAGC---
AATTTG---CTGAGG---GCTATTGAGGCGCAACAGCATCTGTTGCAACTCACAGTCTGGGGCATC
>KF384799
TTCTACTGTAATACAGCACAACTGTTTAATAGTATTTGGAATGGTACTGAGCCTAATGCTGAATGGGCAACTA
AC---

AATAAAGCAAATATCACACTCCCATGCAGAATAAAACAAATTATAAACATGTGGCAGGAAGTAGGAAAAGCAA
TGTATGCCCCTCCCATCGAAGGACTAATTAGATGTACATCAAAT---

ATTACAGGACTATTACTAACAAGAGATGGTGGTAATAGCGGGAAAAATGATAGT-------------------
----

AATAATAACACTGAGATCTTCAGACCTGGAGGGGGAGATATGAGAGACAATTGGAGAAGTGAATTATATAAAT
ATAAAGTAGTAAAAGTTGAACCATTA---GGAGTAGCACCCACCAGGGCAAAGAGAAGAGTGGTG--------
-------------------------------CAGAGAGAAAAAAGAGCA---ATAGGC---CTAGGAGCT---
------GTGTTCATTGGGTTCTTGGGAGCA---GCAGGAAGCACTATG---GGCGCAGCGTCA---
GTGACGCTGACGGTACAA---GCCAGACAA---TTATTG---TCTGGTATAGTGCAACAGCAGAAT---
AATCTG---CTGAGG---GCTATTGAGGCGCAGCAGCACCTGTTACAACTCACAGTCTGGGGCATC
>KF384805
TTCTACTGTAATACAACACAACTGTTTAATAGTACTTGGTTGTCAAATAGTACTTGGATGTCAAATAACACTG
GAGGA--

AATGACACAACAATCATACTACCATGCAGAATAAAACAAATTATAAACATGTGGCAGGAAGTAGGAAAAGCAA
TGTATGCCCCTCCCATTCCAGGACAAATTAGCTGTTTATCAAAC---
ATTACAGGGCTACTATTAACAAGAGATGGTGGGAATGTGAGCAACAGC-------------------------
----

AACACCACCGAGATCTTCAGACCTGTAGGAGGAGATATGAGGGACAATTGGAGAAGTGAATTATATAAATATA
AAGTAGTAAAAATTGAACCATTA---GGAGTAGCACCCTCCAAGGCAAAAAGAAGAGTGGTG-----------
----------------------------CAGAGAGAAAAAAGAGGA---GCG---------GGACTG------
---GTGTTCCTTGGGTTCTTGGCAACA---GCAGGAAGCACTATG---GGCGCGGCGTCA---
CTGACGCTGACGGTACAG---GCCAGACAA---TTATTG---TCTGGTATAGTGCAACAGCAGAAC---
AATCTG---CTGAGA---GCTATTGAGGCGCAACAACATCTGTTGCAACTCACAGTCTGGGGCATT
>KF384806
TTCTACTGTAATACATCACAATTGTTTAATAGTACTTGGTTTGGGAATACTACTAAACAGTCAAATGAC----
----

ACTACAGAATATATCAGACTCCCATGCAGAATAAAACAAATTGTAAACCTGTGGCAGGAAGTAGGAAAAGCAA
TGTATGCCCCTCCCATCAGCGGAGAAATTAACTGTACATCAAAT---
ATTACAGGGCTGCTACTAACAAGAGATGGTGGTAATGATAATAACACA-------------------------
----

AATGGGACTGAGACCTTTAGACCTGGAGGAGGAGATATGAGGGACAATTGGAGAAGTGAATTATACAAGTATA
AGGTAGTACAAATTGAACCCTTA---GGGGTAGCGCCCACCAAGGCAAAGAGAAGAGTGGTG-----------
----------------------------CAGAGAGAAAAAAGAGCA---ATAGGA---CTAGGAGCT------
---TTATTCCTTGGGTTCTTGGGAGCA---GCAGGAAGCACAATG---GGCGCAGCGTCA---
ATGACGCTGACGGTACAG---GCCAGACTA---TTATTG---ACTGGTATAGTGCAACAGCAAAAC---
AACTTG---CTGAGG---GCTATTGAGGCGCAACAGCATCTGTTGCAACTCACAGTCTGGGGCATC
>KC473831
TTCTACTGTAATTCAACACAACTGTTTAATAGTAAATGGGAGGGTAATAGTACTTGGAATAGTACTGGAGAGT
CAAATAAC---

ACTGCAGAAAACATCATACTCCCATGCAGAATAAAGCAAATTGTAAACATGTGGCAGGAAGTAGGAAAAGCCA
TATATGCCCCTCCCATCAGCGGACCAATTAGATGCTCAACAAAT---
ATTACAGGGCTGCTATTAACAAGAGATGGGGGAAAGGATGAGAAC----------------------------
----

CAAACAGAGATCTTTAGACCTGGAGGAGGAGATATGAGGGACAATTGGAGAAGTGAATTATACAAATATAAAG
TAGTAAAAATTGAACCATTA---GGAGTAGCACCCACCAAGGCAAAGAGAAGAGTGGTG--------------
-------------------------CAGAGAGAAAAAAGAGCA---GTGGGA---ATGGGAGCT---------
CTGTTCCTTGGTTTCTTGGGAGCA---GCAGGAAGCACTATG---GGCGCAGCGTCA---
ATAACGCTGACGGCACAG---GCTAGACAA---TTATTG---TCTGGTATAGTGCAACAGCAGAGC---
AATTTG---CTGAGG---GCTATTGAGGCGCAGCAACATCTGCTGCAACTCACAGTCTGGGGCATC
>KC473833
TTCTACTGCAATACATCAGCACTGTTTAATAGTACTTGGAATAGTACTATAAATACAAGT-------------
----

GAAAATGACACAATCATACTCCCATGCAGAATAAAACAAATTATAAATCTGTGGCAGGAAGTAGGAAGAGCAA
TGTATGCTCCTCCCATCAGGGGAAACATTAGCTGTACATCAAAT---

ATTACGGGGGTGCTATTAACAAGAGATGGTGGCGATGACCCTAACGGG-------------------------
----

ACCAACGACACCGAGACCTTCAGACCTGGAGGAGGAGATATGAGGGACAATTGGAGAAATGAATTGTATAAAT
ACAAAGTAGTAAAAATTGAACCATTG---GGAATAGCACCCACCAGGGCAAAGAGAAGAGTGGTG--------
-------------------------------CAAAGAGAAAAAAGAGCA---GTGGGA---ATAGGAGCA---
------TTGTTCCTTGGGTTCTTGGGAGCA---GCAGGAAGCACTATG---GGCGCAGCGTCA---
GTGACGCTGACGGTACAA---GCCAGGCTA---TTATTG---TCTGGTATAGTGCAACAGCAGAAC---
AATTTG---CTGAGA---GCTATTGAGGCGCAACAGCATCTGTTGCAACTCACAGTCTGGGGCATC
>KC473834
TTCTACTGTAATACAACAAAACTGTTTAATGGTAATTTGACTAATACTTGGAATGGAAATTGGTCTGAT----
----

ACTAATGGTACTATCATACTCCAGTGCAGAATAAAACAGATTGTAAACATGTGGCAGGAAGTAGGAAAAGCAA
TGTATGCTCCTCCCATCAAAGGACAGATTAACTGCACATCAAAT---
ATTACAGGATTGCTATTAACAAGAGATGGTGGCACAGGCAATGACAGT-------------------------
----

GACAGCAAGGAGACCTTCAGACCTGGAGGAGGAGATATGAGAGACAATTGGAGAAGTGAATTATATAAATATA
AAGTAGTAAAAGTTGAACCATTA---GGAATAGCACCCACCAAGGCAAAGAGAAGAGTGGTG-----------
----------------------------CAGAGAGAAAAAAGAGCA---GTGGGA---ATAGGAGCT------
---GTGTTCCTTGGGTTCTTGGGAGCA---GCAGGAAGCACTATG---GGCGCAGCGTCA---
ATAACGCTGACGGTACAG---GCCAGGCTA---TTATTG---TCTGGTATAGTGCAGCAGCAGAAC---
AATCTG---CTGAGG---GCTATTGAGGCGCAACAGCATCTGTTGCAACTCACAGTCTGGGGCATT
>JN397362
TTCTACTGTAATACAACAAAACTGTTTAACAGTACTTGGGAAAAAACAAATGATACTTGGAGTGGTAATGAAA
CGTTA--
-------------------AAT---
GGCACAATCACACCAATCACACTCCCATGCAGAATAAAACAAATAATAAACATGTGGCAGAAAATAGGAAAAG
CAATGTATGCCCCTCCTATTCCAGGACCAATTAACTGTTCATCAAAT---
ATTACAGGGCTGATATTAACAAGAGATGGTGGAGGTGATAGGAGTAAT-------------------------
----

GCCACCAACGAGACCTTCAGACCTGGAGGAGGAGATATGAGGGACAATTGGAGAAGTGAATTATATAAATATA
AAGTAGTAAAAAATTGAACCATTA---GGGTTAGCACCCACCAAGGCACAGAGAAGAGTGGTG----------
----------------------------CAGAGAGAAAAAAGAGCT---GTGGGA---TTTGGAGCG------
---CTGTTCCTTGGGTTCTTGGGAGCA---GCAGGAAGCACTATG---GGCGCAGCGTCA---
GTGACGCTGACGGTACAG---GCCAGACAA---TTATTG---TCTGGTATAGTGCAACAGCAGAAC---
AATTTG---CTGAGA---GCTATTGAGGCGCAACAGCACCTGTTGCAACTCACAGTCTGGGGCATC
>KF384810
TTCTACTGTAATACAACACAGCTGTTTAATAGTACTTGGGAATGGAATAGTACCAATTGGAGTAATAACACT-
----

AGCAATGAAAATATCATACTCCCGTGCAGAATAAGACAAATTGTAAACAGGTGGCAGGAAGTAGGAAAAGCAA
TGTATGCCCCTCCCATCAGTGGACGGATTAGTTGTTCCTCAAAT---
ATTACAGGACTGCTACTAACAAGAGATGGTGGTGATAGTAATGATACA-------------------------
----

AATAATACTGAGATCTTCAGACCAGGAGGAGGAGATATGAGGGACAATTGGAGAAGTGAATTATATAAATATA
AAGTAGTAGAGATTGAACCATTA---GGAATAGCACCCACCAAGGCAAAAAGGAGAGTGGTG-----------
----------------------------CAGAGAGAAAAAAGAGCA---ATAACG---CTGGGAGCT------
---TTATTCCTCGGGTTCTTGGGAACA---GCAGGAAGCACTATG---GGCGCAGCGTCA---
ATGACGCTGACGGTACAG---GCCAGGCAA---TTATTG---TCTGGTATAGTGCAACAGCAGAAT---
AATCTG---CTGAGA---GCTGTTGAGGCGCAACAGCATATGTTGCAACTCACAGTCTGGGGCATC
>KM516891
TTCTACTGTAATACAACACAACTGTTTGATAATACTTGGAACAGCACTAAG----------------------
----
-GATGCGACTGGTACT---------
GAAAGAAATGATACTATTACACTTCCATGTAGAATAAAACAAATTATCAATATGTGGCAAAGAGTAGGAAAAG
CAATATATGCCCCTCCCATCAGAGGACAAATTAATTGTACATCAAAT---

ATTACAGGGCTGTTATTAACAAGAGATGGTGGT--

GTGAACGTGACAACCAAC---
AACACTGAGACCTTCAGACCAGGAGGAGGAGATATGAGGGATAATTGGAGAAGTGAATTATATAAATATAAAG
TAGTAAGAATTGAACCATTA---GGAATAGCGCCCACCAAGGCAAAGAGAAGAGTGGTG--------------
-------------------------CAAAGAGAGAGAAGAGCA---GTA------CTGGGAGCT---------
ATGTTCCTTGGGTTCTTGGGAGCA---GCAGGAAGCACTATG---GGCGCAGCGGCA---
GTGACGCTGACGGTACAG---GCCAGACAA---TTATTG---TCTGGTATAGTGCAACAGCAGAGC---
AATTTG---CTGAGG---GCTATTGAGGCGCAACAGCATCTGTTGCAACTCACAGTCTGGGGCATT
>KU901848
TTCTACTGTAATACAACAAAACTGTTTAATAGTACTTGGAATAGTACTGAAGAGTCAAATAACACTACTGCA-
----

GGAAATGATATAATCAAACTCCCATGCAGAATAAAACAAATTGTAAACATGTGGCAGGAGGTAGGAAAAGCAA
TGTATGCCAATCCCAACAGAGAACTAATTAGATGTTCATCAAAT---
ATTACGGGGCTGCTATTAACAAGAGATGGTGGTGTCACCAACACCACCAATGAG-------------------
----

GCCAAAATTGAGACCTTCAGACCAGGAGGAGGAGATATGAGGGACAATTGGAGAAGTGAATTATATAAATATA
AAGTAGTACAAATTGAACCATTA---GGAGTAGCACCCACCAGGGCAAAGAGAAGAGTGGTG-----------
----------------------------CAAAGAGAAAAAAGAGCA---GTGGGA---ATAGGAGCT------
---TTGTTCCTTGGGTTCTTGGGAGCA---GCAGGAAGCACTATG---GGCGCAGCGGCA---
GTGACGCTGACGGTACAG---GCCAGACAA---TTATTG---TCTGGTATAGTGCAACAGCAAAAC---
AACTTG---CTGAGG---GCTATTGAGGCGCAACAGCATCTGTTGCAACTCACAGTCTGGGGCATC
>KF384812
TTCTATTGTAATACAACAGAACTGTTTAATAGTACTTGGAATGATACTGAAGGGTCAAATAACACTGGGGGGT
CAGAAAAC---

ACTGACAACACAATCACACTCCCATGTAGAATAAAACAATTTATAAACATGTGGCAGGAAGTAGGAAAAGCAA
TGTATGCCCCTCCCATCAGAGGACAGATTAGTTGTGTATCAAAC---
ATCACAGGGCTGCTATTACTAAGAGATGGTGGTAAAAAGAATGAGACTGAC----------------------
----

GGGAACGAGACTGAGACCTTCAGACCTGGAGGAGGAGATATGAGGGACAATTGGAGAAGTGAGTTATATAAAT
ATAAAGTAGTGAAAATTGAACCATTA---GGAGTAGCACCCACCAAGGCAAAGAGAAGAGTGGTG--------
-------------------------------CAGAGAGAAAAAAGAGCA---GTGGGA---ATAGGAGCT---
------GTGTTCCTTGGATTCTTGGGAGCA---GCAGGAAGCACTATG---GGCGCAGCGTCA---
ATGACGCTGACGGTACAG---GCCAGACTA---TTATTG---TCTGGTATAGTGCAACAGCAGAAC---
AATCTG---CTGAGA---GCTATTGAGGCGCAACAGCATCTGTTGCAACTCACAGTCTGGGGCATT
>KF384813
TTCTACTGTAATTCAACACAACTGTTTAATAGTACTTGGACTGGTAATGAGTCAGATAGTACTAAC-------
----

AAAAGTGTCACAATCACACTCCCATGCAGAATAAAACAAATTATAAACATGTGGCAGGAAGTAGGAAAAGCAA
TGTATGCCCCTCCCATTAGAGGACAAATTAGATGTTCATCAAAC---
ATTACAGGGCTGCTCTTAACAAGAGATGGTGGTATAGACAACAAGACAGAC----------------------
----

AAGAACGAGACCGAGATCTTTAGGCCTGCAGGAGGAGATATGAGGGACAATTGGAGAAGTGAATTATATAAAT
ATAAAGTAGTAAAAATTGAACCGTTA---GGAGTAGCACCCACCAGGGCAAAGAGAAGAGTGGTG--------
-------------------------------CAGAGAGAAAAAAGAGCA---GTGGGA---ATAGGAGCT---
------ATGCTCCTTGGGTTCTTGGGAGCA---GCAGGAAGCACTATG---GGCGCAGCGTCA---
ATGACGCTGACGGTACAG---GCCAGACTA---TTATTG---TCTGGTATAGTGCAACAGCAGAAC---
AATTTG---CTGAGG---GCTATTGAGGCGCAACAGCATCTGTTGCAACTCACAGTCTGGGGCATC
>MK383400
TTCTACTGTAATTCAACACCACTGTTTAATAGTACCTGGAATTGGAATGATACTAAAGGGTCAAATAGCACTG
GAGAA--

AATAGCACACTCATCACACTCCAATGCAGAATAAAACAAATTATAAACATGTGGCAGGAAGTAGGAAGAGCAA
TGTATGCCCCTCCCATTAGAGGAAACATTAGCTGTTCATCAAAT---

ATTACAGGGCTGCTATTAACAAGAGATGGTGGTAACCAGACTAAC----------------------------
----

AACACCGAGATCTTCAGGCCTGAAGGAGGAAATATGAAGGATAATTGGAGAAGTGAATTATATAAATATAAAG
TAGTAAGAATTGAACCATTA---GGAGTAGCACCCACCAAGGCAAGGAGAAGAGTGGTG--------------
-------------------------CAGAGAGAAAAGAGAGCA---GTGGGA---ATAGGAGCT---------
TTGTTCCTTGGGTTCTTGGGAGCA---GCAGGAAGCACTATG---GGCGCAGCGTCG---
ATGACGCTGACGGTACAG---GCCAGACTA---TTACTG---TCTGGTATAGTGCAACAGCAGAAC---
AATTTG---CTGAGG---GCTATTGAGGCGCAACAGCGTCTGTTGCAACTCACAGTCTGGGGCATC
>KY778473
TTCTACTGTAATTTAACACAGTTGTTTAATAGTACTTGGGGGCATAATAATTCAATACAATTGGATAATAGTA
CTTGGGAT---

ACTAATAATACTATCACACTCCCATGTAAGATAAAACAAATTATAAACATGTGGCAAGAAGTAGGAAAAGCAA
TGTATGCCCCTCCCATCAGAGGACAAATTAAATGTATATCAAAT---
ATTACAGGGCTGTTATTAACAAGAGATGGTGGTAGCAATACCACC----------------------------
----

AACGAAACCTTCAGACCTGGAGGAGGAGATATGAGGGACAATTGGAGAAGTGAATTATATAAATATAAAGTAG
TAAAAATTGAGCCATTA---GGAATAGCACCCACCAAGGCAAAGAGAAGAGTGGTG-----------------
----------------------CAGAGAGAAAAAAGAGCA---GTGGGA---GTGGGAGCT---------
CTGCTCATTGGGTTCTTGGGAGCA---GCAGGAAGCACTATG---GGCGCAGCGTCA---
ATAACGCTGACGGTACAA---GCCAGACAA---TTGTTG---TCTGGTATAGTGCAACAGCAGAGC---
AATCTG---CTGAAA---GCTATTGAGGCGCAACAGCATCTGTTGCAACTCACAGTCTGGGGCATT
>KT124756
TTCTACTGCAATACAACACAACTGTTTAATAGCAGTTGGACAGATGGGCAAGAGCTAAATAACACTGGGCAAG
AG---

CTAAATAGCACTATCACGCTCCCGTGCAGAATAAAACAAATTATAAACAGGTGGCAGGAAGTAGGAAAAGCAA
TGTATGCTCCTCCCATTAGTGGACCAATTAAATGTACATCCAAT---
ATTACAGGGCTGTTATTAACAAGAGATGGTGGTAACTCTAACTCTAGTGGGAAG-------------------
----

AACGACACTGAGACTTTCAGACCTGGAGGAGGAGATATGAGGGACAATTGGAGAAGTGAATTATATAAATATA
AGGTAGTAAAAATTGAACCATTA---GGAGTAGCACCCACCAAGGCAAGGAGAAGAGTGGTG-----------
----------------------------CAGAGAGAGAAAAGAGCA---GTGGGA---ATAGGAGCT------
---GTGTTCCTTGGATTCTTGGGAGCA---GCAGGAAGCACTATG---GGCGCAGCGTCA---
TTAACGCTGACGGTACAG---GCCAGACAA---TTATTG---TCTGGTATAGTGCAACAGCAGAAC---
AATTTG---CTGAGG---GCTATTGAGGCGCAACAGCATCTGTTGCAACTCACAGTCTGGGGCATC
>MH897911
TTCTACTGTAATACATCAAAACTATTTAATAGTACTTGGCAGACTGATAATAGTACTTGGCAGCCTGAAGCT-
----

GAGAATAACACTATCACACTCCAATGCAGAATAAGACAAATTATAAACATGTGGCAAGAAGTAGGAAAAGCAA
TGTATGCCCCTCCCATCAGTGGACAAATTAGATGTTCATCAAAT---
ATTACAGGACTGCTACTAACAAGAGATGGTGACAATGAGAACAAC----------------------------
----

AGCACTGAGGTCTTCAGACCTGCAGGAGGAGATATGAGGGACAATTGGAGAAGTGAATTATACAAATATAAAG
TAGTAAAAATTGAACCATTA---GGAATAGCACCCACCAAGGCAAAGAGAAGAGTGGTG--------------
-------------------------CAAAGAGAGAAAAGGGCT---GTGACG---CTGGGAGCC---------
TTGTTCCTTGGGTTCTTGGGAGCA---GCAGGAAGCACTATG---GGCGCAACGTCA---
ATGACGTTGACGGTACAG---GCCAGACAG---TTATTG---TCTGGTATAGTGCAACAGCAGAGC---
AATCTG---CTGAGA---GCTATTGAGGCGCAACAGCATCTGTTGCAACTCACAGTCTGGGGCATC
>MH897913
TTCTACTGTAATACAACAAAACTATTTAATAGTACTTGGACAAATGGTACTGAAGAGGCAAATAACACTGGAG
GA---

AATGACACACATATCACACTCCCATGCAGAATAAAACAAATTATAAACATGTGGCAGGGAGTAGGAAAAGCAA
TGTATGCCCCTCCCATCAAAGGACAAATCAGATGTTCATCAAAT---

ATTACAGGGCTGCTATTAACAAGAGATGGTGGTAATAACAATAACGGG----------------------
----

ACGACCGAGATCTTCAGACCTGGGGGAGGAGATATGAGGGACAATTGGAGAAGTGAATTATATAAATATAAAG
TAGTAAAAATAGAACCATTA---GGAGTAGCTCCCACCAAGGCAAAGAGAAGAGTGGTG-----------
------------------------CAAAGAGAAAAAAGAGCA---GTGGGAACAATAGGAGCT--------
ATGTTCCTTGGGTTCTTAGGAGCA---GCAGGAAGCACTATG---GGCGCAGCGTCA---
ATGACACTGACGGTACAG---GCCAGACAA---TTATTG---TCTGGTATAGTGCAACAGCAAAAC---
AATTTG---CTGAGG---GCTGTTGAGGCGCAACAGCATCTGTTGCAACTCACAGTCTGGGGCATC
>MH897916
TTCTACTGTAATACAACACAACTATTTAATAGTACTTTTAATAGTCCTTGGAATGATACTAAAATGTCAAATG
GCACTGAA--

GGATATAACACAACCATCACACTCCAATGCAGAATAAAACAAATTATAAACAGGTGGCAGGAAGTGGGAAAAG
CAATATATGCCCCTCCCATCCAAGGACAAATTAATTGTTCATCAAAT---
ATTACAGGGCTGCTCTTAGTGAGAGATGGTGGTATTGATAATGCCACA----------------------
----

AACAATATTGAAACCTTCAGACCTGGAGGAGGAGATATGAGGGACAATTGGAGAAGTGAATTATATAGATATA
AAGTAGTAAAAATTGAACCATTA---GGAGTAGCACCCACTAAGGCAAGGAGAAGAGTGGTG--------
---------------------------CAGAGAGAAAAAAGAGCA---ATTGGA---CTAGGAGCT-----
---ATGTTCCTTGGGTTCTTGGGAGCA---GCAGGAAGCACTATG---GGCGCAGCATCA---
ATAACGCTGACGGTACAG---GCCAGACAA---TTATTG---TCTGGTATAGTGCAACAGCAAAAC---
AACTTG---CTGAGG---GCTATTGAGGCGCAACAACATCTGTTGCAACTCACAGTCTGGGGCATC
>MH897917
TTCTACTGCAATACAACACCACTGTTTGATAATGTTTGGAATGGCACTAAGTTTACAAAGAATTTTACAAAGA
ATAAA---

GGAGAGAATATCACTACTCTCTCATGCAGAATAAAACAATTTATAAACCTGTGGCAGGAAGTAGGGAAAGCAA
TGTATGCCCCTCCCATCAGAGGAGAAATTAACTGTTCATCCAAT---
ATTACAGGACTGATACTAACAAGGGAGGGTGGTGTTAACAGGACT-------------------------
----

GACAATGACACCTTCAGACCTATAGGAGGAAATATGAAGGACAATTGGAGAAGTGAATTGTACAAATATAAAG
TAGTAAGAATTGAACCATTA---GGAGTAGCACCCGACAAGGCAAAGAGAAGAGTGGTG-----------
------------------------CAGAGAGAAAAAAGAGCA---GTGGGA---CTAGGAGCG--------
TTGATCCTTGGGTTCTTAGGAACA---GCAGGAAGCACTATG---GGCGCAGCGTCA---
CTGACGCTGACGGTACAA---GCCAGACAA---TTATTG---TCTGGTATAGTGCAACAGCAGAGC---
AATTTG---CTGAGG---GCTATTGAGGCGCAACAACATCTGTTGCAACTCACAGTCTGGGGCATC
>MG196702
TTCTACTGTAATACAACACAACTGTTTAATAGTACTTGGCCTTTGAATGATACTGAAGGGACAAAT-------
----

AGAACTGAAAATATCACGCTCCCATGCAGAATAAGACAAATTGTAAACATGTGGCAGGAAGTAGGAAAAGCAA
TGTATGCCCCTCCCATCAAAGGACAAATTAATTGTGTATCAAAT---
ATTACAGGGCTGTTATTAACAAGAGATGGTGGTATTAACTCTACA-------------------------
----

AAGAATGAGACCTTTAGACCTGGAGGAGGAGATATGAGGGACAATTGGAGAAGTGAATTATATAAATATAAAG
TAGTAAGAATTGAACCATTA---GGAGTAGCACCCACCAAAGCAAGGAGAAGAGTGGTG-----------
------------------------CAGAGAGAAAAAAGAGCA---GTGGGA---CTAGGAGCT--------
TTGTTCCTTGGGTTCTTGGGAGCA---GCAGGAAGCACTATG---GGCGCAGCGTCA---
ATAACGCTGACGGCACAA---GCCAGGCAA---TTATTG---TCTGGTATAGTGCAACAGCAGAAC---
AATCTG---CTGAGG---GCTATTGAGGCGCAACAGCATCTGTTGCAACTCACAGTCTGGGGCATC
>MG196732
TTCTACTGTAATACAACACAATTGTTTGAAGAGTCAAATAACACTGAAAACAACACTAAAAGAGAAAATATCA
CACTT---

GAAAGAGAAAATATCACACTCCCATGCAGAATAAGACAAATTGTAAACCTGTGGCAGAAAGTAGGCAAAGCAA
TGTATGCCCCTCCCATCAGAGGACAAATTAGCTGTTCATCAAAC---

ATTACAGGACTGTTATTAACAAGAGATGGTGGCAATAATAATAATACT-------------------------
----

GATGATCCGGAGATCTTCAGACCTGGAGGAGGAGATATGAGGGACAATTGGAGAAGTGAATTATACAAATATA
AAGTAGTAAAAATTGAACCATTA---GGAATAGCACCCACCAAGGCAAAGAGAAGAGTGGTG-----------
----------------------------CAGAGAGAAAAAAGAGCA---ATAGGGGGACTAGGAGCT------
---TTGTTCCTTGGATTCTTGGGGGCA---GCAGGAAGCACTATG---GGCGCAGCGTCA---
GTGACGCTGACGGTACAG---GCCAGACAA---TTACTG---TCTGGTATAGTGCAACAGCAAAAC---
AATTTG---CTGAGA---GCTATTGAGGCGCAGCAACATATGTTGCAACTCACAGTCTGGGGCATC
>MG196767
TTCTATTGTAATATAACACAGTTATTTAATAGTACTTGGCTGGGTAATGGTACTGGGAATAGT----------
----

ACTACAGGCAATATCACACTCCCATGCAGAATAAAACAAATTATAAATAGGTGGCAGGAAGTAGGAAAAGCAA
TGTATGCCCCTCCCATCAGCGGAGAGATCAGCTGTAGTTCAAAT---
ATTACAGGGCTATTATTAACAAGAGATGGTGGTAAGCCCGGAAACGCCGCC----------------------
----

GAGAGCATCAATGAGACCTTCAGACCTGGAGGAGGAAATATGAAGGACAATTGGAGAAGTGAATTATATAAAT
ATAAAGTAGTAAAGATTGAACCATTA---GGAGTAGCACCCACCAAGGCAAAGAGAAGAGTGGTG--------
-------------------------------CAAGGAGAAAAAAGAGCA---GTGGGAACATTTGGAGCT---
------CTGTTCCTTGGGTTCTTGAGTGCA---GCGGGAAGCACTATG---GGCGCAGCGTCA---
ATGACGCTGACGGTACAG---GCCAGACAA---TTATTG---TCCGGTATAGTGCAACAGCAGAAC---
AACTTG---CTGAGA---GCTATTGAGGCGCAACAGCATATGTTGCAACTCACAGTCTGGGGCATC
>MG196819
TTCTACTGTGATTCAACAAAACTGTTTAATAGTACTTGGAATGCTAATAGTACTTGGAATAATACTGAA----
----

GAGCCAGAGAACATCACCCTCCCATGTAGAATAAAACAATTTATCAACATGTGGCAGGAAGTAGGAAAAGCAA
TGTATGCCCCTCCCATCAGAGGACCAATTAAATGTTCATCAAAT---
ATTACAGGGATACTATTGACTAGAGATGGTGGTAATGGTAGCAGCACCGAG----------------------
----

AACAATACCGAGACCTTCAGACCTGGAGGAGGAGACATGAGGGATAATTGGAGAAGTGAATTATATAAATACA
AAGTGGTAAAAATTGAACCATTA---GGAGTAGCACCCACCAGGGCAAAGAGAAGAGTGGTG-----------
----------------------------CAGAGAGAAAAGAGAGCA---GTGACA---CTGGGAGCT------
---TTGTTCCTTGGGTTCTTGGGAGCA---GCAGGAAGCACTATG---GGCGCAGCGTCA---
ATGACGCTGACGGTACAA---GCCAGACAA---TTATTG---TCTGGTATAGTGCAACAGCAAAAC---
AATTTG---CTGAGG---GCTATTGAGGCGCAGCAGCATCTGTTGCAACTCACAGTCTGGGGCATC
>MG196866
TTCTACTGTAATTCAACACCACTGTTTAATAGTAGTTGGCATAGTACTACTAAGTATAGTACTAACAGGACAA
CTGAA--

GGAAATGACACAATCACACTCCAATGTAGAATAAAACAAATTATAAACATGTGGCAGGAAGTAGGAAAAGCAA
TGTACGCCCCTCCCATCAGCGGACCAATTGGATGTTTATCAAAT---
ATTACAGGGCTGCTATTAACAAGAGATGGTGGTACTAACATGAGC----------------------------
----

GATAACGAAACTTTCAGACCTGGAGGAGGAGATATGAGGGACAATTGGAGAAGTGAATTATATAAATATAAAG
TAGTGAAAATTGAACCATTA---GGAGTAGCACCCACTCGGGCAAGGAGAAGAGTGGTG--------------
-------------------------CAGAGAGAAAAAAGAGCA------GGA---CTAGGAGCT---------
CTGCTCCTTGGGTTCTTGGGAGCA---GCAGGAAGCACTATG---GGCGCAGCGTCA---
ATAACGCTGACGGCACAG---GCCAGAAAA---TTATTG---TCTGGTATAGTGCAACAGCAGAAC---
AATCTG---CTGAGG---GCTATTGAGGCGCAACAGCATCTGTTGCAACTCACAGTCTGGGGCATC
>MG196902
TTCTACTGTAATACAACACAGCTGTTTAATAGTACTTGGCTGTTTAATGATACTATGAATAATGCTACAGGAT
CA---

AACAGCAGTGATATCATACTCCCATGCAGAATAAAACAAATTATAAACAGGTGGCAGGAAGTAGGAAAAGCAA
TGTATGCCCCTCCCATAGCAGGAATAATTAAATGTTCATCAAAT---

ATTACAGGGCTACTATTAACAAGAGATGGTGGTAATAGTAACAAC---------------------------
----

ACCAAGGAGACCTTCAGACCTGGAGGAGGAGATATGAGGGACAATTGGAGAAGTGAATTATATAAATATAAAG
TAGTAAAAATTAAGCCATTA---GGAGTAGCACCCACCAAGGCCAAGAGAAGAGTGGTG-------------
-------------------------CAGAGAGAAAAAAGAGCA---GTAACA---ATAGGAGCC--------
TTTTTCCTTGGGTTCTTGGGAGCA---GCAGGAAGCACTATG---GGCGCAGCGTCG---
GTGACGCTGACGGTACAG---GCCAGACAA---CTATTG---TCTGGTATAGTGCAACAGCAGAGC---
AATCTG---CTGAGA---GCTATTGAGGCGCAACAGCATATGTTGCAACTCACAGTCTGGGGCATA
>MG196921
TTCTACTGTAACTCAACACCACTGTTTGATAGTACTTGGAATAGTAATAGTACAGGGTTAAATAGCACCAAA-
----

ACCAATAACACAATCACACTCCAATGCAGAATAAAACAAATTATAAACATGTGGCAGGAAGTAGGAAGAGCAA
TGTACGCCCCTCCCATCAGTGGACACATTAACTGTTCATCAAAT---
ATCACAGGGCTGCTGTTAACAAGAGATGGTGGTGTTGACAAGAAT---------------------------
----

GGCAGTGAGACCTTTAGACCTGGAGGAGGAGATATGAGGGACAATTGGAGAAGTGAATTGTATAAATATAAAG
TAGTAAAAATTGAACCATTA---GGAGTAGCACCCACCAAGGCAAAGAGAAGAGTGGTG-------------
-------------------------CAGAGAGAAAAAAGAGCA---GTGGGA---TTAGGAGCT--------
ATGTTCCTTGGGTTCTTGGGAGCA---GCAGGAAGCACTATG---GGCGCAGCGTCA---
CTGACGCTGACGGTACAG---GCCAGACAA---TTACTG---TCTGGTATAGTGCAACAGCAGAAC---
AATTTG---CTGAGG---GCCATTGAGGCGCAACAGCATCTGTTGCAACTCACAGTCTGGGGCATC
>MG196922
TTCTACTGTAATACAACAAAACTGTTTAATGGGACTGCAGAACAGATAGAAAATAACACTAAAGCTGAA----
----

AATATCACAACAATCATACTCCCATGCAGAATAAAACAAATTGTAAACAGGTGGCAGGAAGTAGGAAAAGCAA
TGTATGCCCCTCCCATCCAAGGACGAATTAGCTGTTCATCAAAT---
ATTACAGGGCTGCTATTAACAAGAGATGGTGGGAACAATACTAGT---------------------------
----

AACAACGAGACCTTCAGACCTGCAGGAGGAGATATGAGGGACAATTGGAGAAGTGAATTATATAAATATAAAG
TAGTGAGAATTGAGCCATTA---GGAGTAGCACCCACCAAGGCAAAAAGAAGAGTGGTG-------------
-------------------------CAGAGAGAAAAAAGAGCA---GTGGGAGCACTAGGAGCT--------
ATGTTCCTTGGGTTTCTGGGAACA---GCAGGAAGCACTATG---GGCGCAGCGGCA---
GTGACGCTGACGGTACAG---GCCAGGCAA---TTATTG---TCTGGTATAGTGCAACAGCAGAAC---
AACCTG---CTGAGA---GCTATTGAGGCGCAACAACACCTGTTGCAACTCACAGTCTGGGGCATT
>MG196952
TTCTACTGCAATACAACACCACTGTTTAATACATTACTGTTCAATGAAACAAGTGGT---------------
----

GATAATAAGACTATTACACTCCAATGCAGAATAAAACAAATTATAAACATGTGGCAGCAAGTAGGCAAAGCAA
TGTATGCCCCTCCCATCAGGGGACAAATTAACTGTTCATCAACT---
ATCACAGGGCTGCTATTAACAAGAGATGGTGGTAACCCAAACGCCAGC------------------------
----

AACAGCAACGAGACCTTCAGACCTGCAGGAGGAAATATGAGGGACAATTGGAGAAGTGAACTATATAAATATA
AAGTAGTAAAAATTGAACCATTA---GGGATAGCACCCACCAAGGCAAAGAGAAGAGTGGTG----------
----------------------------CAAAGAGAAAAAAGAGCA---GTGGGAGTAATCGGAGCT-----
---ATGTTCCTTGGGTTCTTGGGAACA---GCAGGAAGCACTATG---GGCGCAGCGTCA---
ATAGCGCTGACGGTACAG---ACCAGACAA---TTATTG---TCTGGTATAGTGCAACAGCAGAAC---
AATTTG---CTGAGA---GCTATTGAGGCGCAACAACATCTGTTGCAACTCACGGTCTGGGGCATC
>MG196979
TTCTACTGTAATACAACACCATTGTTTAATAGTAATTGGACTTGGAATAATAATACAGGGTTAAAT------
----

AACAGTGGTACTATCACACTCCAATGTAGAATAAAACAAATTATAAACCTGTGGCAGGAAGTAGGAAAAGCAA
TGTATGCCCCTCCTATCGCAGGACAAATTAACTGTTTATCAAAT---

ATTACAGGGCTAATATTAACAAGAGATGGTGGTAACAGTAACCCA----------------------------
----

AATAATACTGAGATCTTCAGACCTGGAGGAGGAGATATGAGGGACAATTGGAAAAGTGAATTATATAAATATA
AAGTAGTAAAAATTGAACCATTA---GGAGTAGCACCCACTAAGGCAAAGAGAAGAGTGGTG-----------
----------------------------CAGAGAGAAAAAAGAGCA---GTGGGA---ATAGGAGCT------
---GTGTTCCTTGGGTTCTTGGGAGCA---GCAGGAAGCACTATG---GGCGCAGCGTCA---
ATAACGCTGACGGCACAG---GCTAGACAA---TTATTG---TCTGGTATAGTGCAACAGCAGAGT---
AATCTG---CTAAGG---GCTATCGAGGCGCAACAACATCTGTTGCAACTCACAGTCTGGGGCATC
>MG197083
TTCTACTGTAATACAACACCCCTGTTTAATAGTAGCTGGAGTAATAGTAATAGTACCAATGCAACAAGTGGTC
CTGAA--

AAAGATGACACAATCATACTCCACTGCAGAATAAAGCAAATTATAAACATGTGGCAAAAAGTAGGGAAAGCAA
TGTATGCCCCTCCCATTAGAGGACAAATTAGATGCTCATCAAAC---
ATCACAGGTCTGCTATTAACAAGAGACGGTGGTAACACAACTGGG----------------------------
----

ACCGAGACCTTCAGACCTGGAGGAGGAGATATGAGGAACAATTGGAGAAGTGAATTATATAAATATAAAGTAG
TAAAAATTGAACCATTA---GGAGTAGCACCCACCAAGGCAAAGAGAAGAGTGGTG-----------------
----------------------CAGAGAGAAAAAAGAGCA---CTAGGA---TTCGGAGCA---------
ATGTTCCTTGGGTTCTTGGCAGCA---GCAGGAAGCACTATG---GGCGCAGCGTCA---
ATGACGCTGACGGTACAG---GCCAGGCAA---TTGTTG---TCTGGTATAGTGCAACAGCAGAAC---
AATCTG---CTGAGG---GCTATTGAGGCGCAACAACATCTGTTGCAACTCACAGTCTGGGGCATC
>MG197164
TTTACTGTAATACAACACAACTGTTTAATAGTACTTGGCCTTTAAATAGTAGTACTTGGTGGAATAATGATA
CTGTG--

AATAATGGAAGCATCACACTCCCATGCAGAATAAAACAAATTATAAACAGGTGGCAGGAAGTAGGAAAAGCAA
TGTATGCCCCTCCCATCAGGGGACAAATTAAATGTACATCAAAT---
ATTACAGGGATACTACTAACAAGAGATGGTGGTGCTAACAATACA----------------------------
----

CAGAACGAGACCTTCAGACCTACAGGAGGAAATATGAAGGACAATTGGAGAAGTGAATTATATAAATATAAAG
TAGTAGAAATTGAGCCATTA---GGAATAGCACCCACCAGGGCAAAAAGAAGAGTGGTG--------------
-------------------------CAGAGAGAAAAAAGAGCA---GTGGGA---CTGGGAGCT---------
GTCTTCCTTGGGTTCTTGGGAGCA---GCAGGCAGCACTATG---GGCGCAGCGTCA---
ATAACGCTGACGGCACAA---GCCAGACAA---TTAATG---TCTGGTATAGTGCAACAGCAGAAC---
AATTTG---CTGAGG---GCTATTGAGGCGCAACAGCATCTGTTGCAACTCACAGTCTGGGGCATC
>MG197187
TTCTACTGTAATACATCACAACTGTTTAATAGTACTTGGGGTTCAACACAGTTGTCTAATAGTAGTTGGGATA
AGACT--
-------------------AAA---
GGACCAAATGACACACTCACACTCCTATGCAGAATAAAACAAGTCGTCAACATGTGGCAGGGAGTAGGAAAAG
CAATGTATGCTAATCCCATCAAAGGACAAATTCTATGTAGATCAAAT---
ATTACAGGGATAATATTAACAAGAGATGGTGGTGAGAGTAATAACGGAAGTGAG-------------------
----

AACAACGAGACCTTCAGACCTGGAGGAGGAGATATGAGGGATAATTGGAGAAGTGAATTATATAAATATAAAG
TAGTAAAAATTGAGCCATTA---GGAGTAGCACCCACCAAGGCAAAGAGAAGAGTGGTG--------------
-------------------------CAGAGAGAAAAAAGAGCA---GTGGGAACACTAGGAGCT---------
ATGTTCCTTGGGTTCTTGGGAGCA---GCAGGAAGCACTATG---GGCGCAGCGTCA---
ATGACGCTGACGGTACAG---GCCAGACTA---TTATTG---TCTGGTATAGTGCAGCAGCAAAAC---
AATTTG---CTGAGG---GCTATTGAGGCGCAACAGCATATGTTGCAACTCACAGTCTGGGGCATC
>KT124785
TTCTATTGTAATACAACAAAACTGTTTAATAGTACTTGGAATGAAACTGAAATAGTGAATGGCACTGGA----
----

ACAAATGAAACAATCACACTCCCATGTAGAATAAAACAAATTATAAACAGATGGCAGGAAGTAGGAAAAGCAA
TATATGCCCCTCCCATTGCGGGGCCAATTAACTGTTCATCAAAT---

ATTACAGGACTGTTATTAACAAGGGATGGTGGTAATAATAAAACC----------------------------
----

ACTGAGACCTTCAGACCTGGAGGAGGAAACATGAAGGACAATTGGAGAAGTGAATTATATAAATACAAAGTAG
TAAAAATTGAACCATTA---GGAATAGCACCCACCAGGGCAAAGAGAAGGGTGGTG-----------------
----------------------CAGAAAGAAAAAAGAGCA---GTGGGA---CTAGGAGCT---------
ATGTTCCTTGGGTTCTTGGGAGCA---GCAGGAAGCACTATG---GGCGCAGCGTCG---
ATAACGCTGACGGTACAG---GCCAGACAA---TTATTG---TCTGGTATTGTGCAGCAGCAGAAC---
AATCTG---CTGAGG---GCTATTGAGGCGCAACAGCATCTGTTGCAACTCACGGTCTGGGGCATC
>KT124790
TTCTATTGTAATACAACACAACTGTTTAATAGTATTTGGAATGGGACAAATGTTACTGGAGAG----------
----

TTAAATGGTACAATTACACTCCCATGTAGAATAAAACAAATTATAAACATGTGGCAGGAAGTAGGAAAAGCAA
TGTATGCCCCTCCCATCAAAGGACCAATTACGTGCTTATCAAAT---
ATTACAGGGATACTGTTAGTAAGAGATGGAGGTAATAATAATAAT----------------------------
----

ACTGCGAACGAGACCTTCAGACCTGGAGGGGGAGATATGAGGGACAATTGGAGAAGTGAATTATATAAATATA
AGGTAGTAAAAATTGAACCATTA---GGAATAGCACCCACCAAGGCAAAGCGAAGGGTGGTG-----------
----------------------------CAGAGAGAAAAAAGAGCA---GTGGGA---ATAGGAGCT------
---GTGTTCCTTGGGTTCTTATCAGCA---GCAGGAAGCACTATG---GGCGCAGCGTCA---
GTGACGCTGACGGTACAG---GCCAGGCTA---TTATTG---TCTGGTATAGTGCAACAGCAAAAC---
AATTTG---CTGAGG---GCTATTGAGGCGCAACAGCATCTGTTGCAACTCACAGTCTGGGGCATC
>KM355085
TTCTACTGTAATACAACACAACTGTTTAATAGTACTTGGGAAAATGGTACT----------------------
----
-------TCAACTAATACT------
ACAGGGAATGAAACTATCACACTCCCATGCAGAATAAAGCAAATTATAAACATGTGGCAGGAAGTAGGGAAAG
CAATGTATGCCCCTCCCATCAGAGGACAAATTAGATGTTCATCAAAT---
ATTACAGGGTTGCTATTAACAAGAGATGGGGGG--

-
AACAACTCCAGCAATAATGACACCTTCAGACCTGGAGGAGGGAATATGAAGGACAATTGGAGAAGTGAATTAT
ACAAATATAAAGTAGTAAGCATAGAACCAATA---GGAGTGGCACCCACCAAGGCAAGGAGAAGAGTGGTG--
-------------------------------------CAGAGAGAAAAAAGAGCA---
GTGGGAACAATAGGAGCT---------ATGTTCCTTGGGTTCTTGGGAGCA---GCAGGAAGCACTATG---
GGCGCAGCGTCA---GTGACGCTGACGGTACAG---GCCAGACAA---TTATTG---
TCTGGTATAGTGCAACAGCAGAAC---AATCTG---CTGAGA---
GCTATTGATGTGCAACAGCATATGTTGCAGCTCACAGTCTGGGGCATC
>KX587079
TTTTACTGTAATTCATCAATATTATTTAATAGTACTTGGTATACTAATAGCACTTGGAATAGTACTTGGAATG
AAACTGGA---

AATAAAAATGACACACTCACACTCCAGTGCAGAATAAAACAAATTATAAACATGTGGCAGGGAGTAGGAAAAG
CAATATATGCCCCTCCCATCCAAGGACAAATTAATTGCTCATCAAAT---
ATTACAGGACTACTATTAGTAAGAGATGGTGGTAATAGTAATGACACA-------------------------
----

AAAAACAATGAGACTTTCAGACCTCTAGGAGGAAATATGATGGACAATTGGAGAAGTGAATTATATAAATACA
AAGTAGTACAAATTGAACCATTA---GGAGTAGCACCCACCAAGGCAAAAAGGAGAGTGGTG-----------
----------------------------CAGAGAGAAAAAAGAGCA---CTAGGAGGAATGGGAGCT------
---ATGTTCCTTGGGTTCTTAAGTGCA---GCAGGAAGCACTATG---GGCGCAGCGTCA---
CTAACGCTGACGGTACAG---GCCAGACAA---TTGTTG---TCTGGTATAGTGCAACAGCAGAGC---
AATCTG---CTGAGG---GCTATTGAGGTGCAACAGCATCTGCTGCAACTCACAGTCTGGGGCATC
>KU901792
TTCTACTGTAATACAACAAAGCTGTTTAATAGTACCTGGGATAGTAATAGTACCTGGAATAGTAATAGTACCA
CAGGGGTA---

AATAGCACTGTTATCACACTGCCATGCAGAATAAAACAAATTATAAACATGTGGCAGGAAGTAGGAAAAGCAA

TGTATGCCCCTCCCATCAGAGGAGAAATTAAATGTACATCAAAT---
ATTACAGGGCTGTTACTGGTAAGAGATGGTGGTAATAACAGCAGC---------------------------
----

CAAAATGAGACCTTCAGACCTGAAGGAGGAGATATGAGGGACAATTGGAGAAGTGAATTATATAAATATAAAG
TAGTAAAAATTGAACCATTA---GGAGTAGCACCCACCAAGGCAAAGAGAAGGGTGGTG-------------
-------------------------CAGAGAGAAAAAAGAGCA---GCA------TTCGGAGCT---------
ATGTTCCTTGGGTTCTTGGGAGCA---GCAGGAAGCACTATG---GGCGCAGCGTCA---
ATAACGCTGACGGTACAA---GCCAGACAA---TTATTG---TCTGGTATAGTGCAACAGCAGAAC---
AATTTG---CTGAAA---GCTATTGATGCGCAACAACATATGTTGCAACTCACAGTCTGGGGCATC
>KU901816
TTCTACTGTAATACAACAAAGCTGTTTAATAGTACTTGGTATACTAATAGTACTGGAAATGGTACTGAAGGG-
----

TCAAATGACACAATCACACTCCCATGCCGAATAAGACAAATTATAAACAAATGGCAGAGAGTAGGAAAAGCAA
TGTATGCCCCTCCCATTAAAGGACACATTAACTGTACATCAAAT---
ATTACAGGGCTGCTATTAACAAGAGATGGTGGTGATAACAGCAGT---------------------------
----

ACGAACGAGACCTTCAGACCTGGAGGAGGAGATATGAGGGACAATTGGAGAAGTGAATTATATAAATATAAAG
TAGTAAGAATTGAGCCAGTA---GGAATAGCTCCCACCAAGGCAAGAAGAAGAGTGGTA-------------
-------------------------CAGAGAGAAAAAAGAGCA---GCC------TTAGGAGCT---------
GTGTTCCTTGGGTTCTTGGGAGCA---GCAGGAAGCACTATG---GGCGCAGCGTCA---
CTAACGCTGACGGTACAA---GCCAGACTA---TTATTG---TCTGGTATAGTGCAACAGCAAAGC---
AATTTG---CTGAAG---GCTATTGAGGCGCAACAGCGTCTGTTGCAACTCACAGTCTGGGGCATC
>KM986883
TTCTACTGTAACTCATCAAAACTGTTTAATAGTACTTGGAATAGTACTGGGAGTGAAAATAATAACACTGAAG
GG--

TCAACTGGCACTATCACACTCCAATGCAGGATAAAACAAATTATAAATATGTGGCAGGAAGTAGGAAAAGCAA
TGTATGCCCCTCCCATCAGAGGACAAATTAGCTGTAATTCAACT---
ATTACAGGGCTGCTACTAACAAGAGATGGGGGTGATACGGACAAT---------------------------
----

ACCTCCGAGGTCTTCAGACCTGGAGGAGGAGATATGAGGGACAATTGGAGAAATGAATTATACAAATATAAAG
TAGTAAAAGTAGAACCATTA---GGAATAGCACCCACTAAAGCAAAAAGAAGAGTGGTG-------------
-------------------------CAGAGAGAAAAAAGAGCA---GTAGGA---CTAGGAGCT---------
GTGTTCCTTGGGTTTTTGTCAGCA---GCAGGAAGCACTATG---GGCGCAGCGTCG---
GTGACGCTGACGGTACAG---GCCAGACAA---TTACTG---TCTGGTATAGTGCAACAGCAGAAC---
AATCTG---CTGAGG---GCTGTTGAGGCGCAACAACACCTGTTGCAACTCACAGTCTGGGGCATC
>KU901976
TTCTACTGTAGTACAACACAACTGTTTGATAGTACTTGGGCAACTAATAGTACTGGGAATATT---------
----

ACTAACGGAACTATCACACTCCCATGTAGAATAAAACAAATTATAAACATGTGGCAGGGAGTAGGAAAAGCAA
TGTATGCCCCTCCCATTAAAGGACAAATTAGATGTTCATCAAAT---
ATTACAGGGCTGCTATTAACAAGGGATGGTGGTAATAATGACACG---------------------------
----

AACGAGAACGAGACCTTCAGACCTGGAGGAGGAGATATGAGGGACAACTGGAGAAGTGAATTATATAAATATA
AAGTAGTAAAAATTGAACCATTA---GGAGTAGCACCCACCAAGGCAAAGAGAAGAGTGGTG----------
----------------------------CAGAGAGAAAAAAGAGCA---GTGGGA---ATAGGAGCT------
---TTGTTCCTTGGGTTCTTGGGAGCA---GCAGGAAGCACTATG---GGCGCAGCGTCA---
ATGACGCTGACGGTACAA---GCCAGGCAA---TTATTG---TCTGGTATAGTGCAACAGCAGAAC---
AACTTG---CTGAGA---GCTATTGAGGCGCAACAGCATCTGTTGCAACTCACAGTCTGGGGCATC
>KY766175
TTCTACTGTAATACAATACAGCTGTTTAATAGTACTTGGGATAATAATACTTGGAATAGGTATAATAGTACTA
CTGGG---

TCAAATGAAATTATCAGGCTCCCATGCAGAATAAGACAAATTGTAAACAGGTGGCAGGAAGTAGGAAAAGCAA

TGTATGCCCCTCCCATCGAAGGACAAATTAGATGTTCATCAAAT---
ATTACAGGGCTGATATTAACAAGAGATGGTGGTGGTAATGGCACG-------------------------
----

AAAGGAAACGAGACCTTCAGACCTGGAGGAGGAGATATGAAGGACAATTGGAGAAGTGAATTATATAAATATA
AAGTAGTAAAAATTGAGCCATTA---GGAATAGCACCCACCGCGGCAAAGAGAAGAGTGGTG----------
---------------------------CAGAGAGAAAAAAGAGCA---ATAGGAATGCTAGGAGCT------
---ATGTTCCTTGGGTTCTTGGGAGCA---GCAGGAAGCACTATG---GGCGCAGCGTCA---
ATGACGCTGACGGTACAG---GCCAGACAA---TTATTG---TCTGGTATAGTGCAACAGCAGAAC---
AATTTG---CTGAGG---GCTATTGAGGCGCAACAGCATCTGTTGCAACTCACAGTCTGGGGCATT
>KY778395
TTCTACTGTAATACAACACAACTGTTTAATGGTACTTGGGAGAATAGTACTAGGAATGATAATAACACTGCA-
----

AAAAATGGCACAATCACACTCCCATGCAGAATAAAACAAATTATAAACATGTGGCAAAAAGTAGGAAAAGCAA
TATATGCCCCTCCCATCAGAGGAGAAATTAGGTGTTCATCTAAC---
ATTACAGGGCTGCTACTAACTAGAGATGGTGGTAATGGGAACAGCACC----------------------
----

AAGGACAACGAGACCTTCAGACCTGGGGGAGGAGATATGAGGGACAATTGGAGAAGTGAATTATATAAATACA
AAGTAGTAAAAATTGAACCATTA---GGAATAGCACCCACTAAGGCAAAGAGAAGAGTGGTG----------
---------------------------CAGAGAGAAAAAAGAGCA---GTGGGA---CTAGGAGCT------
---GTGTTCCTTGGGTTCTTGGGAGCA---GCAGGAAGCACTATG---GGCGCAGCGTCA---
ATGACGCTGACGGTACAG---GCCAGACTA---TTATTG---TCTGGTATAGTGCAACAGCAAAAC---
AATCTG---CTGAGA---GCTATTGAGGCGCAACAGCATCTGTTGCAACTCACAGTCTGGGGCATC
>MH897918
TTCTACTGTGATTCATCACAACTGTTTAATAGTACTTGGAGTAATGATACTAATACTGGGAAT---------
----

GATAATGGGAATATCACACTCCTATGCAGAATAAAACAATTTATAAACATGTGGCAGCAAGTAGGAAAAGCGA
TGTATGCCCCTCCCATCAGAGGACAAATTAAATGTTCATCAAAT---
ATTACAGGGCTACTGTTAACAAGAGATGGTGGTAATAATAACAATACTGGA-------------------
----

AACCGTACTGAGACCTTCAGACCTGGAGGAGGAGATATGAGGGACAATTGGAGAAGTGAATTATATAAATATA
AAGTAGTAAGAATTGAACCATTA---GGATTAGCACCCACCAAGGCAAAGAGAAGAGTGGTG----------
---------------------------CAGAGAGAAAAAAGAGCA---GTGGGA---ATAGGAGCT------
---ATGTTCCTTGGGTTCTTGGGAGCA---GCAGGAAGCACTATG---GGCGCAGCGTCA---
CTGACGCTGACGGTACAG---GCCAGACAA---TTATTG---TCTGGTATAGTGCAACAGCAGAAC---
AATTTG---CTGAGG---GCTATTGAGGCGCAACAGCATTTGTTGCAACTCACAGTCTGGGGCATT
>MG196711
TTCTACTGTAATACAACACAGTTGTTTAATAGTACTTGGCAGCCGGTTAATAGTACTACTTGGAGTAATTTCG
GTCCGGGG--

CCAAATGAAAATATCACACTCCCATGTAGAATAAAACAAATTATAAACAGATGGCAGGAAGTAGGAAAATCAA
TGTATGCCCCTCCCATTAGAGGAGTCATTAGCTGTTCATCAAAT---
ATTACAGGGTTGATATTAACAAGAGATGGTGGTGGGACCAATAAT-------------------------
----

ACTAACGAAACCTTCAGACCTGGGGGAGGAGATATGAGGGACAATTGGAGAAGTGAATTGTATAAATACAAAG
TAGTAAAAATTGAGCCATTA---GGAATAGCACCCACCAGGGCAAAGAGAAGAGTGGTG-------------
------------------------CAGAGAGAAAAAAGAGCA---GTAACA---ATAGGAGCT---------
ATGTTCCTTGGGTTCTTGGGAGCA---GCAGGAAGCACTATG---GGCGCAGCATCA---
CTGACGCTGACGGTACAG---GCCAGACTA---TTATTG---TCTGGTATAGTGCAACAGCAAAAC---
AATTTG---CTGAGA---GCTATTGAGGCGCAACAGCATCTGTTGCAGCTCACAGTCTGGGGCATC
>MG197127
TTCTACTGTAATACAACACCACTGTTTAATAGTACTTGGAATAAAAACAATAGTACTTGGACTTGGAATAGCA
CTAGAAAA--
-------------AACTTT------
ACAGAAAATGGCAATATCACACTACAGTGTAGAATCAGACAAATTGTAAACAGGTGGCAAGAAGTAGGAAAAG

CAATGTATGCTCCTCCCATCAGGGGACTAATTAGATGTTCATCAAAT---
ATTACTGGGCTATTGTTAACAAGAGATGGTGGTAATAATAAAACT----------------------------
----

AATGGGAGTGAGATCTTCAGACCTGGAGGAGGAAATATGAGGGACAATTGGAGAAGTGAATTATATAAATATA
AAGTGGTAAAAATTGAACCATTA---GGAGTAGCACCCACCAGGGCAAAGAGAAGAGTGGTG-----------
----------------------------CAGAGAGAAAAAAGAGCA---GTAGGA---TTGGGAGCT------
---TTGTTCCTCGGGTTCTTGGGAGCA---GCAGGAAGCACTATG---GGCGCAGCATCA---
CTAACGCTGACGGTACAG---GCCAGACAA---TTATTG---TCTGGTATAGTGCAACAGCAAAAC---
AATTTG---CTGAGG---GCTATTGAGGCGCAACAACATATGTTGCAACTCACAGTCTGGGGCATC
>KT124796
TTCTACTGTAATTTAACAAAACTGTTTAATAGTACTTGGAATGAAACAGGGTCAAATAACACT----------
----

TTAAATGGCACAGTCATACTCCCATGCAGAATAAAACAAATTGTAAATATGTGGCAGGAAGTAGGAAAAGCAA
TGTATGCCCCTCCCATCGCAGGGCTAATTAACTGTGTATCAAAT---
ATTACAGGGCTGATATTAACAAGAGATGGTGGT--

ACTAATGACACTGAGACCTTCAGACCTGGAGGAGGGGATATGAGGGACAATTGGAGAAGTGAATTATATAAAT
ATAAAGTAGTAAAAGTTGAACCATTA---GGAGTAGCACCCACCAGGGCAAAGAGGAGAACGGTG--------
-------------------------------CAAAGAGAAAAAAGAGCA---GTGGGA---CTAGGAGCT---
------GTGTTCCTTGGGTTCTTGGGAGCA---GCAGGAAGCACTATG---GGCGCAGCATCA---
ATGACGCTGACGGTACAG---GCCAGACAA---TTATTG---TCTGGTATAGTGCAACAGCAGAAC---
AATCTG---CTGAGG---GCTATTGAGGCGCAACAGCACCTGTTGCAACTCACAGTCTGGGGCATC
>KX505648
TTCTACTGTAATACAGCACAACTGTTTAATAGTACTTGGAATGGTACTGAAGGGTCAAATAACATTGAAGGAA
ATAACAAT---

GAAGAAAATGACACAATCACACTCCAATGCAGAATAAAACAAATTATAAACATGTGGCAGGAAGTAGGAAAAG
CAATGTATGCCCCTCCCATCAATGGGCAAATTAGATGTACATCAAAT---
ATTACAGGGCTGCTATTAGTAAGAGATGGAGGGGTAAAAAATGGTACTGAGGAT-------------------
----

GAAAACAAGACCGAGATTTTCAGACCTGGAGGAGGAGATATGAGGGACAATTGGAGAAGTGAATTATATAAAT
ATAAAGTAGTAAAAATTGAACCGTTA---GGAATAGCACCCACCAGGGCAAAGAGAAGAGTGGTG--------
-------------------------------CAAAGAGAGAAAAGAGCA---GTGGGATTAGTAGGAGCT---
------ATGTTCCTTGGGTTCTTGGGAGCA---GCAGGAAGCACTATG---GGCGCAGCGGCA---
ATAACGCTGACGGTACAG---GTCAGACAA---ATGTTG---TCTGGTATAGTGCAACAGCAGAAC---
AATCTG---CTGAGG---GCTATTGAGGCGCAACAGCATCTGTTGCAACTCACAGTCTGGGGCATC
>KX505652
TTCTACTGTAATACAACACAACTGTTTAATAGTACTTGGAATGATACCGAAGGGTTAAGTAACACTGAA----
----

GGAGATGACACACTCACACTCCCATGCAGAATAAAACAAATTGTAAACATGTGGCAGGAAGTAGGAAAAGCAA
TGTATGCCCCTCCCATCAAAGGACAAATTAATTGTACATCAAAT---
ATTACAGGGCTGCTATTAACAAGAGATGGTGGGAATAATGAGAGC----------------------------
----

AGCACCACCGAGACCTTCAGACCTGGAGGAGGAGATATGAGGGACAATTGGAGAAATGAATTATATAAATATA
AAGTAGTAAAGGTTGAACCATTA---GGAGTAGCACCCACCAAGGCAAGGAGAAGAGTGGTG-----------
----------------------------CAGAGAGAAAAAAGAGCA---GTGGGA---ATAGGAGCT------
---ATGTTCCTTGGGTTCTTGGGAGCA---GCCGGAAGCACTATG---GGCGCAGCGTCA---
ATGACGCTGACGGTACAG---GCCAGACAA---TTATTG---TCTGGTATAGTGCAACAGCAAAAC---
AATTTG---CTGAGG---GCTATTGAGGCGCAACAGCATATGTTGCAACTCACAGTCTGGGGCATC
>KX505686
TTCTACTGTAATTCAACACAACTGTTTAATAAGACATGGAATGATACTGAACTATCTGATAAAACTTGGAAAG
TTGATAAA---
-------------------AAT---
AATGCTAGTGACATCATCACACTCCCATGTAAAATAAAACAAATCATAAACCTGTGGCAGGAAGTAGGAAAAG

CAATGTATGCCCCTCCTATCAGAGGATCAATTACATGTTCATCAAAT---
ATTACAGGGCTGGTATTAACAAGAGATGGTGGTACGAACAATACC----------------------------
----

ATGAATGAAACCTTCAGACCTGGAGGAGGAGATATGAGGGATAATTGGAGAAGTGAATTATATAAATATAAAG
TAGTAAGAATTGAACCATTA---GGAGTAGCACCCACCAAGGCAAGGAGAAGAGTGGTG--------------
-------------------------CAGAGAGAAAAAAGAGCA---GTGGGA---CTAGGAGCT---------
CTGTTCTTTGGGTTCTTATCAGCA---GCAGGAACCACTATG---GGCGCAGCGTCA---
ATGACGTTGACGGTACAG---GCCAGACAG---TTACTG---TCTGGTATAGTGCAACAGCAGAAC---
AATATG---CTGAGA---GCTATTGAGGCGCAACAGCATCTGTTGCAACTCACAGTCTGGGGCATT
>KX505707
TTTTACTGTAATACATCAAAACTGTTTAATAGTATTTGGAATGATAATAGTACTTGGAATAATACTAGAGAGT
TTAATAAC---

ACTGACAGAAATATCACACTCCCATGCAGAATAAAACAAATTGTAAACAGGTGGCAGGAAGTAGGAAAAGCAA
TGTATGCCCCTCCCATCAGTGGAAAAATTAGTTGTTCATCAAAT---
ATTACAGGACTGCTATTAACAAGAGATGGTGGTAAGGGTAACAAGACAAAT----------------------
----

AACACCACCGAGATCTTCAGACCAGGGGGAGGAAATATGAGGGACAATTGGAGAAGTGAATTATACAAATATA
AAGTAGTAAAAATTGAACCATTA---GGAATAGCACCCACCAAGGCAAAGAGAAGAGTGGTG-----------
----------------------------CAGAGAGAAAAAAGAGCA---GTGGGA---ATAGGAGCT------
---ATGTTCCTTGGGTTCTTGGGAGCA---GCAGGAAGCACTATG---GGCGCAGCGTCA---
GTGACGCTGACGGTACAG---GCCAGACAA---TTATTG---TCTGGTATAGTGCAACAGCAGAAC---
AATCTG---CTGAAG---GCTATTGAGGCGCAACAGCGTATGTTGCAACTCACAGTCTGGGGCATT
>KY057587
TTCTACTGTAATACAACACCACTGTTTAATAGTATTTGGAATACAAGTGATACTTGGAATAGTACTAATGAAG
AATCAAAT---
-------------AACACT------
GCAGGAAATGACACAATCATACTTCGATGCAGAATAAAACAAATTATAAACATGTGGCAAGAAGTAGGAAAAG
CAATGTATGCCCCTCCCATCAGAGGAAATATTACATGTTTATCAAAT---
ATTACAGGACTGCTGTTAACAAGAGATGGAGGCAATAGCAGTAAT----------------------------
----

AATGGGACTGAGATCTTCAGACCTGGAGGAGGGGATATGAGGGACAATTGGAGAAGTGAATTATATAAATATA
AAGTAGTAAAAATTGAACCATTA---GGAGTAGCACCCACTAAGGCAAAGAGAAGAGTGGTG-----------
----------------------------CAGAGAGAAAAAAGAGCA---GTGGGG---TTAGGAGCT------
---ATGTTCCTTGGGTTCTTGGGAGCA---GCAGGAAGCACTATG---GGCGCAGCGTCA---
CTGACGCTGACGGTACAG---GCCAGACAA---TTATTG---TCTGGTATAGTGCAACAGCAGAAT---
AATTTG---CTGAGA---GCTATTGAGGCGCAACAGCATCTGTTGCAACTCACAGTCTGGGGCATC
>KY748513
TTCTACTGTAACACAACAAGGCTGTTTAATAGTACTTGGAATGGTACGGATACAATAAATAACACTGCA----
----

GAAAACAGTACACTTACACTCCCATGCAGAATAAAACAAATTATAAATAGGTGGCAGGAAGTAGGAAAAGCAA
TATATGCCCCTCCCATCAGGGGACAAATTAGATGTAAATCAAAT---
ATTACAGGGTTGCTATTAACAAGAGATGGTGGTCCCGAAGGGAATAAGACC----------------------
----

ACCTTCAGACCTGGAGGAGGAGATATGAGGGACAATTGGAGAAGTGAATTATATAAATATAAAGTAGTAAAAA
TTGAGCCATTA---GGGATAGCACCCACCAAGGCAAAGAGAAGAGTGGTG-----------------------
----------------CAGAGAGAAAAAAGAGCA---GTGGGC---ATAGGAGCT---------
ATGTTCCTTGGGTTCTTGGGAGCA---GCAGGAAGCACTATG---GGCGCAGCGTCA---
ATGACGCTGACGGTACAG---GCCAGACAA---TTATTG---TCTGGTATAGTGCAACAGCAGAAC---
AATTTG---CTGAGG---GCTATTGAGGCGCAACAGCATCTGTTGCAACTCACAGTCTGGGGCATC
>KY748576
TTCTACTGTGATTCAACAAAACTGTTTAATAGCACTTGGAATGATACTTGGGATGTGAATATCACTGAAAGG-
----

ACAAATGACACTATCATACTCCCATGCAGAATAAAACAAATTGTAAACATGTGGCAGGAAGTAGGAAAAGCAA

TGTATGCCCCTCCCATCAAAGGACCAATTAGCTGTTCATCAAAC---
ATTACAGGACTACTATTAACAAGAGATGGTGGTAGTGGCAACGAGACCACT----------------------
----

AACACCACCGAGATCTTCAGACCTGGAGGAGGAGATATGAGGGACAAGTGGAGAAGTGAATTATACAAATATA
AAGTAGTAAAAATTGAACCATTA---GGAGTAGCACCCACCAAGGCAAAGAGAAGAGTGGTG-----------
----------------------------CAGAGAGAAAAAAGAGCA---GTGGGA---ATAGGAGCT------
---TTGTTCCTTGGGTTCTTGGGAACA---GCAGGAAGCACTATG---GGCGCAGCGGCA---
GTGACGCTGACGGTACAG---GCCAGACAA---TTATTG---TCTGGTATAGTGCAACAGCAGAAC---
AATCTG---CTGAGG---GCTATTGAGGCGCAACAACATCTGTTGCAACTCACAGTCTGGGGCATC
>KT284371
TTCTACTGTAATACAACAAAACTGTTTAATAGTACATGGTATGTAAATAATACTGAAGAGTTAAATAAG----
----

GAAGGAGACACGATCACACTCCCATGCAGAATAAAACAAATTGTAAACATGTGGCAGGAAGTAGGAAAAGCAA
TGTATGCCCCTCCCATCAGAGGACAAATTAGCTGCTCATCAAAT---
ATTACAGGGCTACTATTAACGAGAGATGGTGGTACCGACAACAGCACG-------------------------
----

AACAATACCGAGACCTTCAGACCTGGAGGGGGAGATATGAGGGACAATTGGAGAAGTGAATTATATAAATATA
AAGTAGTAAAAATTGAACCATTA---GGAGTAGCACCCACCAAGGCAAAGAGAGTAGTGGTG-----------
----------------------------CGAAGAGAAAAAAGAGCA---GCGGGA---ATAGGAGCT------
---TTGTTCCTTGGGTTCTTGGGAGCA---GCAGGAGGCACTATG---GGCGCAGCGTCA---
CTGACGCTGACGGTACAG---GCCAGACAA---TTATTG---TCTAGTATAGTGCAACAGCAGAAT---
AACTTG---CTGAGA---GCTATAGAGGCGCAACAGCATCTGTTGCAGCTCACAGTCTGGGGCATC
>KR182173
TTCTACTGTAATACAACAAAACTGTTTAATAGTACTTGGAATAAGACGGGTGAGATTTGGAATAGTACTATGG
AGAATAATAAT--

GACACTGAACCAATCACACTCCCATGCAGAATAAAACAAATTGTAAACATGTGGCAGGAAGTAGGAAAAGCAA
TGTATGCCCCTCCCATCAGTGGACCAATTAGATGTGTATCAAAT---
ATCACAGGGCTGCTATTAACAAGAGATGGTGGAAATGATGGTAATACC-------------------------
----

AACACCAGGAATGAGACTTTCAGACCTGGAGGAGGAGATATGAGGGACAATTGGAGAAGTGAATTATATAAAT
ATAAAGTAGTAAGAATTGAACCAATA---GGACTAGCACCTACCAGGGCAAAGAGAAGAGTGGTG--------
-------------------------------CAGAGAGAAAAAAGAGCA---ATAGGA---ATAGGAGCT---
------GTGTTCCTTGGGTTCTTGGGAGCA---GCAGGAAGCACTATG---GGCGCAGCGTCA---
CTGACGCTGACGGTACAA---GCCAGACAA---TTATTG---TCTGGTATAGTGCAACAGCAGAGC---
AACTTG---CTGAGG---GCTATTGAGGCGCAACAACATCTGTTGCAACTCACAGTCTGGGGCATC
>KR182183
TTCTACTGTAATACAACACAGCTGTTTGATAGTATTTGGGAGCGTAATAGAACTTTGAATGATACAGAAAATA
CA---

AATGACCGTAACATCACACTCCCATGCAGAATAAAACAAATTATAAACAGATGGCAGGGAGTAGGAAAAGCAA
TGTATGCCCCTCCCATAAAAGGAGAAATTAGATGCACATCAAAT---
ATTACAGGGCTAATATTAACAAGAGATGGTGGTAGTGATACTAACAGG-------------------------
----
GATAATAACACGACA---
GGGAATGAGACCTTCAGACCTGGAGGAGGAGATATGAGGGACAATTGGAGAAGTGAATTATATAAATATAAAG
TAGTAAAAATTGAGCCATTA---GGAGTAGCACCCACCAAGGCAAAGAGAAGAGTGGTG--------------
-------------------------CAGAGAGAAAAAAGAGCA---GTGACA---ATGGGAGCT---------
ATGCTCCTTGGGTTCTTGGGAGCA---GCAGGAAGCACTATG---GGCGCAGCATCG---
ATAACGCTGACGGTACAA---GCCAGACAA---TTATTG---TCTGGTATAGTGCAACAGCAGAGC---
AATTTG---CTGAAG---GCTATTGACGCGCAACAGCACCTGTTGCAACTCACAGTCTGGGGCATT
>KR182187
TTCTACTGTAATACAACACAGCTGTTTAATAGTACTTGGTCGCTGTTTAATAGTACTTGGTCGCATAATGAAA
CTAGG--
-------------AATGCT------
ACAAATGACCGTAACATCACACTTCCATGCAGAATAAAACAAATTATAAACAGATGGCAGGGAGTAGGAAAAG

CAATGTATGCCCCTCCCATCAAGGGAGAAATTAGATGCACATCAAAT---
ATTACAGGGCTAATATTAACAAGAGATGGTGGTAGTGATACTAACAAT-------------------------
----
GATACTAACAAGACA---
GGGAATGAGACCTTCAGACCTGGAGGAGGAGATATGAGGGACAATTGGAGAAGTGAATTATATAAATATAAAG
TAGTAAAAATTGAGCCATTA---GGGGTAGCACCCACCAAGGCAAAGAGAAGAGTGGTG--------------
-------------------------CAGAGAGAAAAAAGAGCA---GCGGGAACGATGGGAGCT---------
ATGTTCCTTGGGTTCTTGGGAGCA---GCAGGAAGCACTATG---GGCGCAGCATCG---
ATAACGCTGACGGTACAA---GCCAGACAA---TTATTG---TCTGGTATAGTGCAACAGCAGAGC---
AATTTG---CTGAAG---GCTATTGACGCGCAACAGCACCTGTTGCAACTCACAGTCTGGGGCATT
>KR182191
TTCTACTGTAATACAACACAGCTGTTTAATAGTACTTGGAATAGTACTTGGCCGCTTAATGAAACTTGGGATG
GTATCGAAGGA--

AATAACACTAACATCACACTTCCATGCAGAATAAAACAAATTATAAACAGATGGCAGGGAGTAGGAAAAGCAA
TGTATGCCCCTCCCATCAAGGGACAAATTAGCTGTTCATCAAAT---
ATTACAGGGCTAATATTAACAAGAGATGGTGGTAATAACACGAGT----------------------------
----

GGGAGTGAGACCTTCAGACCGGGAGGAGGAGATATGAGGGACAATTGGAGAAGTGAATTATATAAATATAAAG
TAGTAAAAATTGAGCCATTA---GGGGTAGCACCCACCAGGGCAAAGAGAAGAGTGGTG--------------
-------------------------CAGAGAGAAAAAAGAGCA---GTGACG---ATGGGAGCT---------
ATGCTCCTTGGGTTCTTGGGAGCA---GCAGGAAGCACTATG---GGCGCAGCGTCG---
ATAACGCTGACGGTACAG---GCCAGACTA---TTGTTG---TCTGGTATAGTGCAACAGCAGAGC---
AATTTG---CTGAAG---GCTATTGAGGCGCAACAGCACCTGTTGCAACTCACAGTCTGGGGCATC
>KR182196
TTCTACTGTAACACAACACAACTGTTTAATAGTACTTGGAATGCTAATAGTACTTGGAATGATACTAGTTCTA
ATATTGGA---

GAGTCAAATGACACAATCACACTCCCATGCAAAATAAGACAGATTGTAAATATGTGGCAGGAAGTAGGAAAAG
CAATGTATGCTCCTCCCATTGCAGGACAACTTAATTGCTCATCAAAT---
ATTACAGGAATATTACTAACAAGAGATGGTGGTGAGAGTAATACCACG-------------------------
----

AATAGTAACGAGACTTTCAGACCTGGAGGAGGAAATATGAGGGACAATTGGAGGAGTAAATTATATAAATATA
AAGTAATAAAAATTGAACCATTA---GGACTGGCACCCACCAAGGCAAAGAGAAGAGTGGTG-----------
----------------------------CAGAGAAAAGCAAGAGCA---GCAGGA---TTAGGAGCT------
---GTGATCTTTGGGTTCTTGGGAGCA---GCAGGAAGCACTATG---GGCGCAGCGTCA---
GTAGCGCTGACGGTACAG---GCCAGACAA---TTATTG---TCTGGTATAGTGCAACAGCAAAAC---
AATTTG---CTGAGG---GCTATTGAGGCGCAACAGCATATGTTGCAACTCACAGTCTGGGGCATC
>KR182341
TTCTATTGTAATACAACACAACTGTTTAATAGTACTTGGTTTAATGGTACTTGGAATAGTACTAATGACAATA
GT---

ACAAATGGCACTATCACGCTCCCATGCAAAATAAAACAAATTATAAACATGTGGCAGAAAGTAGGAAAAGCAA
TGTATGCCCCTCCCATCAGAGGAAACATTAGCTGTTCATCAAAT---
ATAACGGGGTTGCTATTAATAAGAGATGGTGGTATTAATAGTACA----------------------------
----

GAGAATAGCACAGAAACCTTCAGACCGGGAGGAGGAGATATGAGGGACAATTGGAGAAGTGAATTATATAAAT
ATAAAGTAGTACAAATTGAACCAATA---GGACTAGCACCCACAAAGGCAAAGAGAAGAGTGGTG--------
-------------------------------CAGAGAGAAAAAAGAGCA---GTGGGAACATTAGGAGCT---
------GTGTTCCTTGGGTTCTTGGGAGCA---GCAGGAAGCACTATG---GGCGCAGCGTCA---
ATAACGCTGACGGTACAG---GCCAGACAA---TTATTG---TCTGGTATAGTGCAACAGCAGAAT---
AATCTG---CTGAAG---GCTATTGATGCGCAACAACATCTGTTGCAGCTCACAGTCTGGGGCATC
>KR182409
TTCTACTGTGAGTCAACACCACTGTTTAATAGCACTTGGAATAGTAATGACTTGACTCCTACTATAGAGTCAA
ATAGC--

AATGGAGAAAACATTACACTAACATGCAGAATAAAACAAATTATAAACATGTGGCAGGAAGTAGGAAAAGCAA

TGTATGCCCCTCCCATCAGTGGACCAATTGAATGTACATCAAAT---
ATTACAGGGCTACTATTAACAAGAGATGGTGGTGATAACAATAATATG-------------------------
----

AGCAGCGAGACTTTCAGACCTGGAGGAGGAAATATGAAAGATAATTGGAGAAATGAATTATATAAATATAAAG
TAGTAAAAATTAAACCATTA---GGAATAGCACCCACCAAGGCGAGAAGAAGAGTGGTG--------------
-------------------------CAAAGAGAAAAAAGAGCA---GTGGGA---ATAGGAGCA---------
TTATTCCTTGGGTTCTTGGGAGCA---GCAGGAAGCACTATG---GGCGCAGCGTCA---
TTGACGCTGACGGTACAG---GCCAGACAA---TTATTG---ACTGGTATAGTGCAACAGCAGAAC---
AATCTG---CTGAGG---GCTATTGAAGCGCAACAGCATCTGTTGCAACTCACAGTCTGGGGCATC
>KR182465
TTCTACTGTAATACAACACAACTGTTTAATAGTACCTGGATCAATGGTACTAAAAAGTCAAATACC-------
----

ACTGAAGACACAATCATACTCCCATGCAGAATAAGACAAATTATAAACATGTGGCAGGAAACAGGAAAAGCAA
TGTATGCCCCTCCCATAAGAGGACAACTTAGATGTTCATCAAAT---
ATTACAGGATTACTATTAACAAGAGATGGTGGGAATGATAATAAT----------------------------
----

GAGACTGAGACCTTCAGACCTGGGGGAGGAAATATGAAAGACAATTGGAGAAGTGAACTATATAAATACAAAG
TAGTAAAAGTTAAACCATTA---GGAATAGCACCCACCAGGGCAAAGAGAAGAGTGGTG--------------
-------------------------CAGAGAGAAAAAAGAGCA---GTGGGA---ATAGGAGCT---------
GTGTTCCTTGGGTTCTTGGGAGCA---GCAGGAAGCACTATG---GGCGCAGCGTCA---
CTGACGCTGACGGTACAG---GCCAGACAA---TTATTG---TCTGGTATAGTGCAACAGCAGAAC---
AATCTG---CTGAGA---GCTATTGAGGCGCAACAGCATATGTTGCAACTCACAGTCTGGGGCATC
>MG171201
TTCTACTGTAATTCAACAAAACTGTTTAATAGTACTTGGAGTGTTAATAGTACTCGGAGTGGTAATAGTACAG
GGTCAAAT---
-------------------AAC---
ACTGGAAACTCAACAATCACACTCCCATGCAGAATAAAACAAGTTATAAACATGTGGCAGGCAGTAGGAAAAG
CAATGTATGCCCCTCCCATCAGCGGACAAATTAGATGTTCATCAAAT---
ATTACAGGGCTGATATTAACAAGAGATGGTGGTGATAACGCTAGC----------------------------
----

ACTACTGAGACTTTCAGACCTGGAGGAGGGGATATGAGGGACAATTGGAGAAGTGAATTATATAAATATAAAA
TAGTAAAAATTGAACCATTA---GGAGTAGCACCCACCAAGGCAAAGAGAAGAGTGGTG--------------
-------------------------CAGAGAGAAAAAAGAGCA---GTGGGA---ATAGGAGCT---------
GTGTTCCTTGGGTTCTTGGGAGCA---GCAGGAAGCACTATG---GGCGCAGCGTCA---
ATGACGCTGACGGTACAG---GCTAGACTA---TTATTG---TCTGGTATAGTGCAACAGCAGAAC---
AATTTG---CTGAGG---GCTATTGAGGCGCAACAGCATCTGTTGCAACTCACAGTCTGGGGCATC
>KY778299
TTCTACTGTAATACAACACAACTGTTTAATAGTACTTGGCAGGGTAATACTTGGAATGGT-------------
----

ACTAAAGAAAATATCACACTCCCATGCAAAATAAGACAAATTGTAAACATGTGGCAAGAAGTAGGAAAAGCAA
TGTATGCCCCTCCTATCAGAGGACAAATTACATGCTCATCAAAT---
ATTACAGGGCTGTTATTAACAAGAGATGGTGGTAATACCACAAAC----------------------------
----

AATACCGAGGTCTTCAGACCTGGAGGAGGAGATATGAAGGACAATTGGAGAAGTGAATTATACAAATATAAAG
TAATAAAAATTGAACCATTG---GGAGTAGCACCCACTAAGGCAAAAAGAAGAGTGGTG--------------
-------------------------CAGAGAGAAAAAAGAGCA---GTGGGA---TTAGGAGCT---------
GTGTTCCTTGGGTTCTTGGGAGCA---GCAGGAAGCACTATG---GGCGCAGCGTCA---
ATCACGCTGACGGTACAG---GCCAGACAA---TTATTG---TCTGGTATAGTGCAACAGCAGAAC---
AACTTG---CTGAGA---GCTATTGAGGCGCAACAGCATCTGTTGCAACTCACAGTCTGGGGCATC
>MK384347
TTCTACTGTAATACAACAAAACTGTTTAATAGTACCTGGACCTGGAATAGTACTGAAGGGTCAGGGAATGACA
CTGAGAGGAAT--

ATCACTGAGAGGAATATCACACTCCCATGCAGAATAAAACAAATTGTAAACATGTGGCAGGAAGTGGGAAAAG

CAATGTATGCCCCTCCCATCAGTGGACAAATTAAATGTTCATCAAAT---
ATTACAGGTCTGCTTTTAACAAGAGATGGTGGTGGAAAAAAATAAC---------------------------
----

AGTGACAACGAGACCTTCAGACCTGGAGGAGGAGATATGAGGGACAATTGGAGAAGCGAATTATATAAATATA
AAGTAGTAAAAATTGAACCATTA---GGAGTCGCACCCACCAAGGCAAAGAGAAGAGTGGTG-----------
----------------------------CAGAGAGAGAAAAGAGCA---GTGACA---TTTGGAGCT------
---ATGTTCCTTGGGTTCTTGGGAGCA---GCAGGAAGCACTATG---GGCGCAGCGTCA---
ATAACGCTGACGGTACAG---GCCAGACAA---TTATTG---TCTGGTATAGTGCAACAGCAGAGC---
AATTTG---CTGAGA---GCTATTGAGGCGCAACAGCATCTGTTGCAACTCACGGTCTGGGGCATC
>KX505396
TTCTACTGTGATACAACAAAACTATTTAATAGTACTTGGAATGAAACTAACACTGTAGTAAACCAA-------
----

ACTACAGAAAATATCACACTCTCATGCAGAATAAAACAAATTATAAACAGGTGGCAGGAAGTAGGAAAGGCAA
TGTATGCCCCTCCCATCGAAGGACAAATTAGGTGTTCATCAAAC---
ATTACAGGGCTGTTATTAACAAGAGATGGTGGTATTAATAATGAGACT-------------------------
----

AACACGACCGAGATCTTCAGACCTGGAGGAGGAGATATGAGGGACAATTGGAGAAGTGAATTATATAAATATA
AAGTAGTAAGAATTGAACCATTA---GGAGTAGCACCCACCAAGGCAAAGAGAAGAGTGGTG-----------
----------------------------CAGAGAGAAAAAAGAGCA---GTGGGA---TTAGGAGCT------
---GTGTTCCTTGGGTTCTTAGGAGCA---GCAGGAAGCACTATG---GGCGCAGCGTCA---
ATAACGCTGACGGTACAG---GCCAGGCAA---TTATTG---TCTGGTATAGTGCAACAGCAGAAC---
AACTTG---CTGAAA---GCTATTGAGGCGCAACAGCATCTGTTGCAGCTCACAGTCTGGGGCATC
>KX505419
TTCTACTGTAATACAACAAACCTGTTTAATAGTACTTGGAATGTTAGTGATACTACTGGTAGGGGA-------
----

GTGTCAGACAACATCACACTCCCATGCAGAATAAAACAAGTTATAAACATGTGGCAGGAAGTAGGAAAAGCAA
TGTATGCTCCTCCCATCCCAGGACAAATTAGATGCTCATCAAAT---
ATTACAGGGTTACTATTAACAAGAGATGGTGGTAACACCTCTGAG----------------------------
----

TCTGAGACCTTCAGACCTGGAGGAGGAGATATGAGAGACAATTGGAGAAGTGAACTATATAAATATAAAGTAG
TAAAGATTGAACCATTA---GGAGTAGCACCCACTAAGGCAAAGAGAAGAGTGGTG-----------------
----------------------CAGAGAGAAAAAAGAGCA---GTGGGA---ATAGGAGCT---------
GTGTTCCTTGGGTTCTTGGGAGCA---GCAGGAAGCACTATG---GGCGCAGCGTCA---
ATAACGCTGACGGTACAG---GCCAGACAA---TTATTG---TCTGGTATAGTGCAACAGCAGAAC---
AATCTG---CTGAGA---GCTATTGAGACGCAACAGCATCTGTTGCAGCTCACAGTCTGGGGCATC
>KX505435
TTCTACTGTAATACATCAAAGCTGTTTAACAGTACTTGGAATGATACAAGTACTGGGGAGTGGGAGTCAAAT-
----

GATAATGAAACAATCATACTCCCGTGCAAAATAAGACAAATTATAAACAGATGGCAGAAAGTAGGAAGAGCCA
TGTATGCCCCTCCCATCAGAGGACAAATTAGATGTTCATCAAAT---
ATTACAGGGTTACTATTAACAAGAGATGGTGGTATAAATAGGACC----------------------------
----

GAGAATGAGACCGAGATCTTCAGACCTGGAGGAGGAGATATGAGGGACAATTGGAGAAGTGAATTGTATAAAT
ATAAAGTAGTAAAAATTGAGCCATTA---GGAATAGCACCCACCAGGGCAAGGAGAAGAGTGGTG--------
-------------------------------CAGAGAGAAAAAAGAGCA---GTGGGAACGATAGGAGCT---
------ATGTTCCTTGGGTTCTTGGGAGCA---GCAGGAAGCACTATG---GGCGCAGCGTCA---
GTGACGCTGACGGTACAG---GCCAGACTA---TTATTG---TCTGGTATAGTGCAACAGCAGAAC---
AATCTG---CTAAGA---GCTATTGAGGCGCAACAGCATCTGTTGCAACTCACAGTCTGGGGCATC
>KX505475
TTCTACTGTAATACAACAAAACTGTTTAATAGTACTTGGAATAGTACTTGGAATAGTACTTGGAATAGTGAGG
AACAGTCA---

GGTGTTGATAACATTATCACAATCCAATGCAGAATAAAACAAATTGTACACATGTGGCAGAGAGTAGGAAGGG

CAATGTATGCCCCTCCCATCAGTGGACCAATTAGATGTTCATCAAAT---
ATTACAGGGTTGCTATTAGTAAGAGATGGTGGTAGTTCCAACGATACC------------------------
----

AAGGAGACTTTCAAGGAGACTTTCAGACCAGGAGGAGGAAATATGAAGGACAATTGGAGAAGTGAATTATATA
AATACAAAGTAGTAAAAATTGAACCATTA---GGAATAGCACCCACTAAGGCAAAGAGAAGAGTGGTG-----
----------------------------------CAGAGAGAAAAAAGAGCA---GTGGGA---
CTAGGAGCT---------ATGTTCCTTGGGTTCTTAGGAGCA---GCAGGAAGCACTATG---
GGCGCAGCGTCG---ATGGCGCTGACGGTACAA---GCCAGACAA---TTATTG---
TCTGGCATAGTGCAGCAGCAGAAC---AATCTG---CTGAGG---
GCTATTGAGGCGCAACAGCACATGTTGCAACTCACAGTCTGGGGCATC
>KX028091
TTCTACTGTAATACAACAAAACTATTTAATAGTTCTTGGAATAGTACAGAAAGGTCACAATACAAAAACATAA
CA--

CAAAACGGAACCATAACACTCCAGTGCAGAATAAAACAAATTATAAACATGTGGCAGGAAGTAGGAAAAGCAA
TGTATGCCCCTCCCATCCGAGGAGAAATTAGATGTTCATCAAAC---
ATTACAGGGTTGCTACTAACAAGAGATGGTGGTGGTGGTAATAGCACAGGGGAC------------------
----

AACGAAACCGAAACCTTCAGACCTGCAGGAGGAGATATGAGGGACAATTGGAGAAGTGAATTATATAAATATA
AAGTAGTAAAAATTGAACCATTA---GGAGTAGCACCCACCCCGGCAAGGAGAAGAGTGGTG----------
-----------------------------CAGAGAGAAAAAAGAGCA---GTGGGA---ATAGGAGCT------
---CTGTTCCTTGGGTTCTTGGGAGCA---GCAGGAAGCACTATG---GGCGCAGCGTCA---
GTGACGTTGACGGTACAG---GCCAGACAA---TTATTG---TCTGGTATAGTGCAACAGCAAAAC---
AACTTG---CTGAGG---GCTATTGAGATGCAACAAAATCTGTTGCAACTCACAGTCTGGGGCATC
>KX028321
TTCTACTGTAATACAACAGCACTGTTTAATAGTACTTGGAATAGTACTTGGAATGGTACTACAGAGACAAGTG
GCACTGAA--

GAAAATGGAAACATCACACTCCCATGCAGAATAAAACAAATCATAAACATGTGGCAGACAGTAGGAAAAGCAA
TGTATGCCCCTCCCATCAGAGGACAAATTAGATGTTCATCAAAT---
ATTACAGGGCTACTATTAACAAGAGATGGTGGGAGTAGCACTAAT---------------------------
----

GGGACGGAAATCTTCAGACCTGGAGGAGGAGATATGAGGGATAATTGGAGAAGTGAATTGTATAAATATAAAG
TAGTGAAGATTGAACCATTA---GGAGTAGCACCCACTAAGGCAAAGAGAAGAGTGGTG-------------
--------------------------CAGAGAGAAAAAAGAGCA---GTGGGA---TTAGGAGCT---------
TTGTTCCTTGGGTTCTTGGGAGCA---GCAGGAAGCACTATG---GGCGCAGCGTCA---
CTGACGCTGACGGTACAG---GCCAGACAA---TTGTTG---TCTGGTATAGTGCAACAGCAGAAC---
AACTTG---CTGAGA---GCTATTGAGGCGCAACAACATCTGTTGCAACTCACAGTCTGGGGCATC
>KX028508
TTCTACTGTAATACAACACAACTGTTCAATAGTACTTGGAATAATACTGGAAATAATTGGACTGATACTGGAG
AGTCA---

GACAATATCACACTTATACTCCCATGCAGAATAAAACAAATTGTAAACCTGTGGCAGAGAGTAGGAAAAGCAA
TGTATGCCCCTCCCAGCAGGGGACCAATTAGATGCTCATCAAAT---
ATTACAGGGATATTATTAACAAGAGATGGTGGTAGTGGTGTGAAC---------------------------
----

GTGAGCAGCACAACTGAAACTTTTAGACCCGGAGGAGGAAATATGAGGGATAATTGGAGAAGTGAATTATATA
AATATAAAATAGTACAAATTGAACCAATA---GGAATAGCACCCACCAGGGCAAAGAGAAGAGTGGTG-----
----------------------------------CAGAGAGAAAAAAGAGCA---ATAGGA---
CTAGGAGCT---------ATGTTCCTTGGGTTCTTGGGAGCA---GCAGGAAGCACTATG---
GGCGCAGCGTCA---ATGACGCTGACGGTACAG---GCCAGGCAA---CTATTG---
TCTGGTATAGTGCAACAGCAGAAC---AATTTG---CTGAGG---
GCTATTGAGGCGCAACAGCATATGTTGCAACTCACAGTCTGGGGCATC
>KX505739
TTCTACTGTAATTCAACAAAACTGTTTAATAGTACTTGGGAGGCAGGTATTACTGAAAAGTCAGAA-------
----

TCAAATGACACTATTACACTCCCATGCAGGATAAAACAAATTATAAACATGTGGCAGAAAGTAGGAAAAGCAA
TGTATGCTCCTCCCATCAGTGGAATAATTAAATGTAAATCAAAT---
ATTACAGGGCTACTATTAACAAGAGATGGTGGTAATAGTAGCGAG----------------------------
----

AATAAGAGTGAGATCTTCAGACCTGCAGGAGGAGATATGAGGGACAATTGGAGAAGTGAATTATATAAATATA
AAGTAGTAAAAATTGAACCATTA---GGAGTAGCACCCACCAAGGCAAAGAGAAGAGTGGTG-----------
----------------------------CAGAGAGAAAAAAGAGCA---GTGGGA---ATAGGAGCT------
---GTGTTCCTTGGGTTCTTGGGAGCA---GCAGGAAGCACTATG---GGCGCAGCGTCA---
ATGACGCTGACGGTACAG---GCCAGGCAA---TTATTG---TCTGGTATAGTGCAACAGCAGAAC---
AATCTG---CTGAGA---GCCATTGAGGCGCAACAGCATCTGTTGCAACTCACAGTCTGGGGCATC
>KU678060
TTCTACTGTAATACATCACAACTGTTTAACAGTACTTGGTATGGTAATTCAACACAGGATAATACTACTGGG-
----

GATAGTACTGTTATCACACTCCCATGTAGAATAAAACAAATTATAAACATGTGGCAGGGAGTAGGAAAAGCAA
TGTATGCCCCTCCCATCAGTGGATACATTAATTGTTCATCAAAC---
ATTACAGGGCTACTATTAACAAGAGATGGTATTGTGGCTAACAATACCGAT----------------------
----

AACGATACCGAGACATTCAGACCTGAAGGAGGAAACATGAAGGACAATTGGAGAAGTGAATTATATAAATATA
AAGTAGTACAAATTGAACCATTA---GGAATAGCACCCACCAAGGCAAGGAGAAGAGTGGTG-----------
----------------------------CAGAGAGAAAAAAGAGCA---GTGGGAGTGCTGGGAGCT------
---ATGTTCCTTGGGTTCTTGGGAGCA---GCAGGAAGCACTATG---GGCGCAGCGTCA---
ATAACGCTGACGGTACAG---GCCAGACTA---TTATTG---TCTGGTATAGTGCAGCAGCAGAAC---
AATCTG---CTGCGG---GCTATTGAGGCGCAACAGCATCTGCTGCAACTCACAGTCTGGGGCATC
>KX595124
TTCTACTGTAATACAACACCACTGTTTAATAGTACTTGGCCTTGGAATGGTAACAAGACACATGATACA----
----

AATAGAAACAATATCACACTCTCATGCAGAATAAAACAAATTATAAACAGATGGCAGGAAGTAGGAAAAGCAA
TGTATGCTCCTCCCATTGCAGGACGAATTAGCTGTTTATCAAAT---
ATTACAGGGATGATATTAACAAGAGATGGTGGTAACAATATCGGA----------------------------
----

GGGGAAAATAACACTGAGATCTTCAGACCTGCAGGAGGAGATATGAGGGACAATTGGAGAAGTGAATTATATA
AATATAAAGTAGTAAAAGTTGAACCATTA---GGAGTAGCACCCACCAGGGCAAGGAGAAGAGTGGTG-----
----------------------------------CAAAGAGAGAAAAGAGCA---GCGGGA---
ATAGGAGCG---------TTACTCCTTGGGTTCTTGGGAGCG---GCAGGAAGCACTATG---
GGCGCAGCGTCA---CTGACGCTGACGGTACAA---GCCAGACAA---TTATTG---
TCTGGTATAGTGCAACAGCAGAAC---AACCTG---CTGAGG---
GCTATTGAGGCGCAACAGCATATGTTGCAACTCACAGTCTGGGGCATC
>KY323732
TTCTATTGTAATTCAACACGACTGTTTAATAGTACCTGGGAGAGCCCTGATGGCCCTAGGAATGATAGTGGGG
AG---

CTCAATGACAATATCACACTCCCATGCAGAATAAAACAAATTATAAACATGTGGCAGGAAGTAGGAAAAGCAA
TGTATGCCCCTCCCATCAAAGGTATAATTAAATGCTCATCAAAC---
ATTACAGGGCTGCTATTAACAAGAGATGGGGGGAATGAGAATGTCACAGGCAAC-------------------
----

AATAAGACTGAGACCTTCAGACCTGGGGGAGGAGATATGAGGGACAATTGGAGAAGTGAATTATACAAATATA
AAGTAGTGCAAGTTGAACCACTA---GGAGTAGCGCCCACCAGGGCAAAAAGAAGAGTGGTG-----------
----------------------------CAGAGAGAAAAAAGAGCA---GTGACA---ATGGGAGCT------
---ATGTTCCTTGGGTTCTTGGGAGCA---GCAGGAAGCACTATG---GGCGCAGCATCA---
ATGATGCTGACGGTACAA---GCCAGACAA---TTGTTG---TCTGGTATAGTGCAGCAGCAGAGC---
AATTTG---CTGAAG---GCTATTGAGGCGCAACAACATCTGTTGCAACTAACAGTCTGGGGCATC
>KY323909

TTCTACTGTAATACAACACGACTGTTCAATAGTACTTGGAATAATAATAATGGTACTTGGAGTGATAATAGTA
CTGGGAAT---
-------------AATACT------
GGAGAAAATGACACACTCACACTCCCATGCAAAATAAAACAAATTATAAACATGTGGCAGAAAGTAGGAAAAG
CAATGTATGCCCCTCCTATCAGAGGGCAAATTAGATGTTCATCAAAC---
ATTACAGGGTTATTATTGACAAGAGACGGGGGAGTTAATAATACAACC-------------------------
----

AACAACACTGAGACCTTCAGACCTGGAGGGGGAGATATGAGGGACAATTGGAGAAGTGAATTATATAAATATA
AGGTAGTAAAAATTGAACCATTA---GGAGTAGCACCCACCAAGGCAAAGAGAAGAGTGGTG-----------
----------------------------CAGAGAGGAAAAAGAGCA---GTAGGC---ATAGGAGCT------
---GTGTTCCTTGGTTTCTTGGGAGCA---GCAGGAAGCACGATG---GGCGCAGCGTCA---
CTGACGCTGACGGTACAG---GCCAGACTA---TTATTG---TCTGGTATAGTGCAACAGCAGAAC---
AATTTG---CTGAGA---GCTATTGAGGCGCAACAGCATCTGTTGCAACTCACAGTCTGGGGCATC
>KY324122
TTCTACTGTAATACATCCCAACTGTTTAATAGTACTTGGAATAGTACTTGGGATAATACTGGAGAGTCAAATA
GCACTGAA---

GGAAATGACACAATCACACTCCCATGTAGAATAAAACAATTTATAAATTTATGGCAGCAAGTAGGAAAAGCAA
TGTATGCCCCTCCCATCAGGGGAAACATTACCTGTTCATCAAAT---
ATTACAGGGCTGATATTAACAAGAGATGGTGGTAATGATAACGGG----------------------------
----

AGTAACGAGACCTTCAGACCTGCAGGGGGAGATATGAGGGACAATTGGAGAAGTGAATTATATAAATATAAAG
TAGTAAAAATTGAACCATTA---GGAGTAGCACCCACCAAGGCAAAGAGAAGAGTGGTG--------------
-------------------------CAGAGAGAAAAAAGAGCA---GTGGGC---ATAGGAGCT---------
TTGTTCCTTGGGTTCTTGGGAGCA---GCAGGAAGCACTATG---GGCGCAGCGTCA---
ATGACGCTGACGGTACAG---GCCAGACAA---TTGTTG---TCTGGTATAGTGCAACAGCAAAAC---
AACCTG---CTGAGA---GCTATTGAGGCGCAACAGCATATGTTGCAACTCACAGTCTGGGGCATC
>KX028240
TTCTATTGTAATACAACACAATTGTTTAATAGTACTTGGGATGCTAATACTACTGTACAAAATACTGGAGAAA
ATGACACT---

GGAGAAAATGACACTATTATACTCCAATGCAAAATAAAACAAATTATAAACATGTGGCAGCGAACAGGAAAAG
CAATGTATGCCCCTCCCATCAGAGGACAAATTAGATGTTCATCAAAT---
ATTACAGGGCTGCTTTTAGCAAGAGATGGTGGCAACATGACCGAG----------------------------
----

AGCACGGAGGTCTTCAGACCTACAGGAGGAAATATGAAGGACAATTGGAGAAGTGAATTATATAAATACAAAG
TAGTTGAACTTGAACCATTA---GGAATAGCACCCACCAAGGCAAAGAGAAGAGTGGTG--------------
-------------------------CAGAGAGAGAAAAGAGGA------------ATAGGAGCT---------
GTGTTCCTTGGGTTCTTGGGAGCA---GCAGGAAGCACTATG---GGCGCAGCGTCA---
GTGACGCTGACGGTACAG---GCCAGACAA---TTATTG---TCTGGTATAGTGCAACAGCAGAGC---
AATTTG---CTGAAG---GCTATTGAGGCGCAACAACATTTGTTGCAACTCACAGTCTGGGGCATC
>KX505555
TTCTACTGTAACACTGACAAACTGTTTAATAGTACTTGGAATAGTAATGGTAGTGGTACCTGGAATAGGGATG
ATACTGAA---

GAGTCAAATGACACAATCACACTCCAATGCAGAATAAAACAAATCATAAACATGTGGCAGGAAGTAGGAAAAG
CAATGTATGCCCCTCCCATCGAGGGACAAATTAAATGTTCATCAAAT---
ATTACAGGGCTGCTATTAACAAGAGATGGTGGTAATTCTAGCAGCACG-------------------------
----

AACGACGAGATATTTAGACCTGGAGGAGGAGATATGAAGGACAATTGGAGAAGTGAATTATACAAATATAAAG
TAGTAAAAATTGAACCAATA---GGGATAGCACCTGAAAAGGCACAGAGAAGAGTGGTG--------------
-------------------------CAGAGAGAAAAAAGAGCA---GTGGGAGCATTAGGAGCT---------
TTGTTCCTTGGGTTCTTAGGAGCA---GCAGGAAGCACTATG---GGCGCAGCGTCA---
GTGACGCTGACGGTACAG---GCCAGACAA---TTATTG---TCTGGTATAGTGCAACAGCAAAAC---
AATTTG---CTGAGA---GCTATTGAGGCGCAACAGCATCTGTTGCAACTCACGGTCTGGGGCATC
>MH897920

TTCTACTGTAATACATCAGAACTGTTTAAAGGTACTTGGAATGGTACTGAAAGGCCAAATGACACTGAAAAG-
----

TTAAATGATACAATTACACTCCAATGCAGAATAAAACAATTTATAAACATGTGGCAGGAAGTAGGAAAAGCAA
TGTATGCCCCTCCAATCAGAGGACAAATTAACTGTTCATCAAAT---
ATTACAGGGCTGCTACTAACAAGAGATGGTGGAAATGGAAATGAGACG-------------------------
----

AAAGAGACTGAGGTCTTCAGACCTGGAGGAGGAGATATGAGGGACAATTGGAGAAGTGAATTATATAAATATA
AAGTAGTAAAAATTGAACCATTA---GGAATAGCACCCACCAAGGCAAGGAGAAGAGTGGTG-----------
----------------------------CAGAGAGAAAAAAGAGCA---GTGGGA---ATAGGAGCT------
---CTGTTCCTTGGGTTCTTGGGAGCA---GCAGGAAGCACTATG---GGCGCAGCGTCA---
GTGACGCTGACGGTACAG---GCCAGACAA---TTATTG---TCTGGTATAGTGCAACAGCAGAGC---
AATTTG---CTGAGA---GCTATTGAGGCGCAGCAGCATATGTTGCAACTCACAGTCTGGGGCATC
>KX587137
TTCTACTGTAATACATCAAAACTGTTTAATAGTACTTGGTTTAATAATGGGACTAATAGTGAAGAGTCG----
----

TCAAATGACACTATCACACTTCCATGCAGGATAAAACAAATTGTAAATATGTGGCAGGAGGTAGGAAAAGCAA
TGTATGCCCCTCCCATTAGAGGACAAATTAGATGTAACTCAAGT---
ATTACAGGGATACTATTAGTAAGAGATGGTGGTAAGACCAACAGTACCAATGAG------------AGC----
----
AGTACCAATGAGAGC---
GAGATCGAAATCTTCAGACCTGGAGGAGGAGACATGAGGGACAATTGGAGAAGTGAATTATATAAATATAAAG
TAGTACAAATTGAACCATTA---GGAGTAGCACCCACCAAGGCAAAAAGAAGAGTGGTG--------------
-------------------------CAGAGAGAAAAAAGAGCA---ATAGGA---ATGGGAGCT---------
TTGTTCCTTGGGTTCTTAAGCGCA---GCAGGAAGCACTATG---GGCGCAGCGTCA---
GTGACGCTGACGGTACAA---GCCAGACAA---TTACTG---TCTGGTATAGTGCAACAGCAGAAC---
AACCTG---CTGAGG---GCTATTGAGGCGCAGCAGCATCTGCTGCAACTCACAGTCTGGGGCATC
>KX587284
TTCTACTGCAATACAACAAAACTGTTTAATAGCACTTGGAATTCAACACATAGTTGGAACGATACAGGG----
----

CCAAATGACAATGTCACACTTCCATGCAGGATAAAACAAATCATAAACATGTGGCAGGAAGTAGGAAAAGCAA
TGTATGCCCCTCCTATTAAGGGAGAAATTAAATGTTCATCAAAT---
ATTACAGGGCTGATATTAACTAGAGATGGTGGGACAGGTAATGAGACT-------------------------
----

GACAACAACACTGAGACCTTCAGACCAGGAGGAGGAGATATGAGAAACAATTGGAGAAGTGAATTATATAAAT
ATAAAGTAATAAGAATTGAACCAATA---GGATTAGCACCCACCAAGGCAAAGAGAAGAGTGGTG--------
-------------------------------CAGAGAGAAAAAAGAGCA---ATACCA---ATAGGAGCT---
------GTGTTCTTTGGGTTCTTGGGAGCA---GCAGGAAGCACTATG---GGCGCAGCGTCA---
ATAGCGCTGACGGCACAG---GCCAGACAA---TTACTG---TCTGGTATAGTGCAACAGCAAAAC---
AATTTG---CTGAGG---GCTATTGAGGCGCAACAACATCTGTTGCAACTCACAGTCTGGGGCATC
>KY113738
TTCTACTGTAATTCAACACAACTGTTTAATAGTACTTGGAATAGTAATAGTACTCGGAATGGTACTGAAGGGT
CAAATATCACT--

GTAGAAAATGACACAATCATACTTACTTGCAGAATAAAACAAATTATAAACATGTGGCAGGAAGTAGGAAAAG
CAATGTATGCCCCTCCCATCAGTGGAATAATTAGATGTTCATCAAAT---
ATTACAGGGCTGCTATTAACAAGAGATGGTGGTAACAGTAGCAAC----------------------------
----

GGGACAGAGGTCTTCAGACCTGGAGGAGGAAATATGAAAGACAATTGGAGGAGTGAATTATATAAATATAAAG
TAGTAAAAATTGAACCATTA---GGAATAGCACCCACCAAGGCGAAGAGAAGAGTGGTG--------------
-------------------------CAGAAAGAAAAAAGAGCA---GCA------CTAGGAGCC---------
TTGTTCCTTGGGTTCTTGGGAGCA---GCAGGAAGCACTATG---GGCGCAGCGTCA---
ATGACGCTGACGGTACAG---GCCAGACTA---TTATTG---TCTGGTATAGTGCAACAGCAAAAT---
AATTTG---CTGAGG---GCTATTGAGGCGCAACAGCATCTGTTGCAACTCACAGTCTGGGGCATT
>KY113566

TTCTACTGTAATACATCAAAACTGTTTAATAGTACTTGGAATAATAATAGTACTTGGAATAGTACTACTACTG
AAAGAAAT---

GAGACAGAAGGAAATATCACACTCCCATGTAGAATAAAACAAATTATAAACATGTGGCAGGAAGTAGGAAAAG
CAATGTATGCCCCTCCCATTAGGGGAATAATTAAATGTTCATCAAAT---
ATTACAGGATTGCTATTGACAAGAGATGGTGGTAAAGACAATACC----------------------------
----
ACAAATAATAATGGG---
ACTGAGGAGGTCTTCAGACCAGGAGGAGGAGATATGAGGGACAATTGGAGAAGTGAATTGTATAAATATAAAG
TAGTAAGAATTGAACCATTA---GGAGTAGCACCCACCAAGGCAAAGAGAAGAGTGGTG--------------
-------------------------CAGAGAGAAAAAAGAGCA---GTGACA---CTAGGAGCT---------
ATGTTCCTTGGGTTCTTGGGAGCA---GCAGGAAGCACTATG---GGCGCAGCGTCA---
ATAACGCTGACGGCACAG---GCCAGACAA---TTATTG---TCTGGTATAGTGCAACAGCAGAAT---
AATCTG---CTGAGA---GCTATTGAGGCGCAACAGCATCTGTTGCAACTCACAGTCTGGGGCATC
>KY113379
TTCTATTGTAATTCAACACAACTGTTTAATAGTACTTGGAATAATACTGATTGGAATAATACTGAAGAGGCAA
ATAAA--
-------------ACTAGA------
GGAGGAAATGACACAATTATACTCCCATGTAGAATAAAACAAATTATAAACATGTGGCAGGAAGTAGGAAAAG
CAATGTATGCCCCTCCCATCAGAGGAAACATTAGTTGTTTTTCAAAG---
ATTACAGGGCTGTTACTCACAAGAGATGGTGGTAATAGTAATATTACT-------------------------
----

AGTGAGAGCGAGACCTTCAGACCTGCAGGAGGAGATATGAGGGACAATTGGAGAAGTGAGTTATATAAATATA
AAGTAGTAAAAATTGAACCCTTA---GGAGTAGCACCCACCAAGGCAAAGAGAAGAGTGGTG-----------
----------------------------CAGAGGGAAAAAAGAGCA---GTTGGA---ATTGGAGCT------
---TTGTTCCTTGGGTTCTTGGGAGCA---GCAGGAAGCACTATG---GGCGCAGCATCA---
TTAACGCTGACGGTACAG---GCCAGACAA---TTATTG---TCTGGTATAGTGCAACAGCAGAAC---
AATTTG---CTGAGA---GCTATTGAGGCGCAACAGCATCTGTTGCAACTCACAGTCTGGGGCATC
>KY057390
TTCTACTGTAACACATCAAAACTATTTAATGGAACTTGGAATGGTACTGATGGAACTTGGAATGATATT----
----

ACTGATGAAATTATCACAATCCCATGCAGAATAAAACAATTTATAAACCTGTGGCAAGAAGTAGGAAAAGCAA
TGTATGCCCCTCCCATCGCAGGACCAATTAGCTGTTCATCAAAC---
ATTACAGGACTGATATTAACAAGAGATGGTGGTAATACTAATAGCACT-------------------------
----

AAGGGGAAGGAGACCTTCAGACCGGGAGGAGGAGATATGAGGGACAATTGGAGAAGTGAATTATATAAATATA
AAGTAGTAAAAATTGAACCATTA---GGAATAGCACCCACCAGGGCAAGGAGAAGAGTGGTG-----------
----------------------------CAGAGAGAAAAAAGAGCA---GCA------ATAGGAGCT------
---CTGTTCCTTGGGTTCTTGGGAGCA---GCAGGAAGCACGATG---GGCGCAGCATCA---
GTGACGCTGACGGTACAG---GCCAGACTA---CTATTG---TCTGGTATAGTGCAACAGCAGAAC---
AACCTG---CTGAGA---GCTATTGAGGCGCAACAGCATATGTTGCAACTCACAGTCTGGGGCATC
>KY748402
TTCTACTGTAATTCAACACAACTGTTTAATAGTACTTGGAATAGTACTTTGAATACTACTTGGGAGGTTAAT-
----

GAGACTGACATTATCACACTCCCATGCAGAATAAAACAAATTATAAACATGTGGCAGGAAGTAGGAAAAGCAA
TGTATGCCCCTCCCATCCAAGGAAGAATTAAATGCTCATCGAAT---
ATTACAGGGCTGTTACTAATAAGAGATGGTGGTAATAACAATGGTAGCGAA----------------------
----

ACAACAGAAATCTTCAGACCTGGAGGAGGAGACATGAGGGACAATTGGAGAAGTGAATTATATAAATATAAGG
TAGTAAAAATTGAGCCATTA---GGAATAGCACCCACCAAGGCAAAGAGAAGAGTGGTG--------------
-------------------------CAAAGAGAAAAAAGAGCA---GTGGGAGTATTTGGAGCT---------
ATGTTCCTCGGGTTCTTGGGAACA---GCAGGAAGCACTATG---GGCGCAGCGTCA---
ATAACGCTGACGGTACAG---GCCAGACAA---TTATTA---TCTGGTATAGTGCAACAGCAAAAC---
AATTTG---CTGAGA---GCTATTGAGGCGCAACAGCATCTGTTGCAACTCACAGTCTGGGGCATT
>KY057518

TTCTACTGTAATACAACACAACTGTTTAATAGTGCTTGGAATAATACAGGGTCAAATGGAGCT----------
----

ACCAGTGACAACATCACGCTCCCATGCAGAATAAAGCAAATTATAAACATGTGGCAGGAAGTAGGAAAAGCAA
TGTATGCCCCGCCCATCAGTGGACTAATTAACTGTACATCAAAT---
ATTACAGGGCTACTATTAACAAGAGATGGGGGAAATGGAAATGACACAAAT----------------------
----

AAAACCGATGAGATCTTCAGACCTGGAGGAGGAAATATGAGGGACAATTGGAGAAATAAATTATATAAATATA
AAGTAGTGAAAATTGAACCATTA---GGAGTAGCACCCACCAAGGCAAAGAGAAGAGTAGTG-----------
----------------------------CAGAGAAAAAAAAAGAGCA---TTGGGA---ATAGGAGCT------
---GTGTTCCTTGGGTTCTTGGGAGCA---GCAGGAAGCACTATG---GGCGCAGCGTCA---
ATGACGCTGACGGTACAG---GCCAGACAA---TTATTG---TCTGGTATAGTGCAGCAGCAGAAC---
AATTTG---CTGAGA---GCTATTGAGGCGCAACAGCATGTGTTGCAACTCACAGTCTGGGGCATC
>KY324010
TTCTACTGTGATACAACAAAATTGTTTAATAGTACTTGGAAGCTTAATAGTACTTGGAATAGTATGGAAGAAG
TGACAAAT---
-------------AGCACT------
AAAGGAGATGCCAACATCACACTCCCCTGCAGAATAAAACAAATTGTAAACATGTGGCAGAGAGTAGGAAGAG
CAATGTATGCCCCTCCCATCCAAGGACGAATTAGCTGTTCATCAGAT---
ATTACAGGGCTGCTATTACTAAGAGATGGTGGTAGTGGTAATAATACCACG----------------------
----

AATGATACTGAGATCTTCAGACCTGGAGGAGGAAATATGAAGGATAATTGGAGAAGTGAATTATATAAATATA
AAGTAGTAAAAATTGAACCATTA---GGAGTAGCACCCACCAAGGCAAAGAGAAGAGTGGTG-----------
----------------------------CAGAGAGAAAAAAGAGCA---GCGGGA---ATAGGAGCT------
---GTGCTCCTTGGGTTCTTGGGAGCA---GCAGGAAGCACTATG---GGCGCAGCGGCA---
GTGACGCTGACGGTACAG---GCCAGACAA---TTATTG---TCTGGTATAGTGCAGCAGCAGAAT---
AATTTG---CTGAAG---GCTATTGAGGCGCAACAACATCTGTTGCAACTCACAGTCTGGGGCATC
>KY324473
TTCTACTGTAATACAACATCATTGTTTAATAGTACTTGGTATGCTAATAATACTACTGAAGGGACAAATAGC-
----

ACTGGAAGAAATATCACACTCCAATGCAGAATAAGACAAATTATAAACATGTGGCAGAAAGTAGGAAAAGCAA
TGTATGCCCCTCCCATCAGTGGAGTAATTAACTGTAAATCAAAT---
ATTACAGGGCTGATATTAACGAGAGATGGTGGTAACAATAATGGTAACAAT----------------------
----
-
AATAATACCAACGAAACCGAGACCTTCAGACCAGGAGGAGGAGATATGAGGGACAATTGGAGAAGTGAATTAT
ATAAATATAAAGTAGTAAAAATTGAACCATTA---GGAATAGCACCCACCAGGGCACAAAGAAGAGTGGTG--
-------------------------------------CAGAGAGAAAAAAGAGCA---GTGGGA---
TTAGGAGCT---------TTGTTCCTTGGGTTCTTGGGAGCA---GCAGGAAGCACTATG---
GGCGCAGCATCA---ATGACGCTGACGGTACAG---GCCAGACAA---TTATTG---
TCTGGTATAGTGCAGCAGCAAAGC---AATTTG---CTGAGA---
GCTATTGAGACGCAACAGCATCTGTTGCAACTCACAGTCTGGGGCATT
>MK148554
TTCTACTGTAATACAACACAACTGTTTAATAGTACTTGGAATGGTACTACTGAAGGGTTAAATAACACTGCA-
----

GGAAATGGCACAATCACACTTCCATGCAGAATAAAACAAATTATAAACATGTGGCAGGAAGTAGGAAAAGCAA
TGTATGCCCCTCCCATCAGAGGACTAATTAGATGTTCATCAAAT---
ATTACAGGGCTGCTATTAACAAGGGATGGTGGTGATGGTAATAGC----------------------------
----

AACGAGACCGAGGTTTTCAGACCTGGAGGAGGAGATATGAGGGACAATTGGAGAAGTGAATTATATAAATATA
AAGTAGTAAAAATTGAACCATTA---GGAGTAGCACCCACCAAGGCAAAGAGAAGAGTGGTG-----------
----------------------------CAAAGAGAAAAAAGAGCA---GTGGGA---ATAGGAGCT------
---ATGTTCCTTGGGTTCTTGGGAGCA---GCAGGAAGCACTATG---GGCGCAGCGTCA---
ATGACGCTGACGGTACAG---GCCAGACTA---TTATTG---TCTGGTATAGTGCAACAGCAGAAC---
AATTTG---CTGAGG---GCTATTGAGGCGCAACAACATCTGTTGCAACTCACAGTCTGGGGCATC

>MK114705
TTCTACTGTAATACAACAAAACTATTTAATAGTACTTGGCCACGTAATAGTACTTGGAATAATACTGAAGGGT
CAAAT--

GACACTGAAATAATCACACTCCCGTGCAGAATAAAACAAATTGTAAACAGGTGGCAGGAAGTAGGCAAAGCAA
TGTATGCCCCTCCCATCCAAGGACAAATTAGTTGTTCATCAAAT---
ATTACAGGGCTGCTACTAGTTAGAGATGGTGGAATTAACACCAGT----------------------------
----

GAGAGCAACGAGACCTTCAGACCTGGAGGAGGAGATATGAGGGACAATTGGAGAAGTGAATTATATAAATATA
AGGTAGTAAAAATTGAACCATTA---GGAGTAGCACCCACCAAGGCAAAGAGAAGAGTGGTG----------
----------------------------CAGAGAGAAAAAAGAGCA---GTGGGAATGCTGGGAGCT------
---ATGTTCCTTGGGCTCTTGGGAGCA---GCAGGAAGCACTATG---GGCGCAGCGTCA---
GTGACGTTGACGGTACAG---ACCAGACTA---TTATTG---TCTGGTATAGTGCAACAGCAGAAC---
AATCTG---CTGAGG---GCTATTGAGGCGCAGCAGCATCTGTTGCAACTCACAGTCTGGGGCATC
>MK114983
TTCTACTGTAACACAACACGACTGTTTAATAGTACTTGGAGTGTTAATGGAACTAGCATAAACGAAACT----
----

AACAATAAAAATATCACACTCCCGTGCAGAATAAAACAAATTATAAACAGGTGGCAGGAAGTAGGAAAAGCAA
TGTATGCCCCTCCTATCAGTGGGATAATTAGGTGCTCATCAAAT---
ATTACAGGACTAATATTAACAAGAGATGGTGGTACAACTGATGGTGGTACA----------------------
----

ACTAATAGTACAAAGGAGACCTTCAGACCTGGAGGGGGAAATATGAAGGACAATTGGAGAAGTGAATTATATA
AATACAAAGTAGTAAAAATTGAGCCATTA---GGAGTAGCACCCACCAAGGCACAGAGAAGAGTGGTG-----
----------------------------------CAAAGAGAAAAAAGAGCA---
ATAGGAACGTTAGGAGCT---------ATGTTCCTCGGGTTCTTGGGAGCA---GCAGGAAGCACTATG---
GGCGCAGCGTCA---CTGACGCTGACGGTACAA---GCCAGACAA---TTATTG---
TCTGGTATAGTGCAACAGCAGAAC---AATCTG---CTGAGG---
GCTATTGAGGCGCAACAGCATATGTTGCAACTCACAGTCTGGGGCATC
>MK115065
TTCTACTGTAATACAACACAGCTGTTTAATAGTACTTGGAATAATAGTACTTGGAATGGTACTGACAATTGGA
AT---
-------------GGTACT------
GAATCAAATAGCACTATCACACTCCCATGCAGAATAAAACAAATTATAAACTTGTGGCAGGAAGTAGGAAGAG
CAATGTATGCCCCTCCCATCCAAGGGCAAATTAGATGTTCATCAAAT---
ATTACAGGGCTGCTGCTAGTAAGAGATGGTGGGAGCAACAATAGTAGT-------------------------
----

AATGATACAGAGACCTTCAGGCCTGGAGGAGGAGATATGAGGGACAATTGGAGAAGTGAATTATATAAATATA
AAGTAGTCAAAATTGAACCATTA---GGAATAGCACCCACCCATGCAAAGAGAAGAGTGGTG-----------
----------------------------CAAAGGGAAAAAAGAGCA---ATAGGA---CTTGGAGCT------
---TTCTTCCTTGGGTTCTTGGGAGCA---GCAGGAAGCACTATG---GGCGCAGCGTCA---
GTGACGCTGACGGTACAG---GCCAGACAA---TTATTG---TCTGGTATAGTGCAGCAGCAAAAC---
AATTTG---CTGAGA---GCTATTGAGGCGCAACAGCATCTGTTGCAACTCACAGTCTGGGGCATC
>MK115195
TTCTACTGTAATACAACACAACTGTTTAATAGTACTTGGTATAGTACTACAGGAGTAAATGGTACAGGAGCAA
ATAACACA---

GAAGTAAATATTATCACACTCCCATGCAGGATAAGGCAAATTGTAAACATGTGGCAGGAAGTAGGAAAAGCAA
TGTATGCCCCTCCCATCAGTGGACAAATTAGTTGTTCATCAAAT---
ATTACAGGGCTACTACTACTAAGAGATGGTGGTAATAGCAATAAC----------------------------
----

ACGAATAACATGACTGAAATCTTCAGACCTGGAGGAGGAGATATGAGAGACAATTGGAGAAGTGAATTATATA
AATATAAAGTAGTACAAATTGAACCATTA---GGAATAGCACCCACCAAGGCAAGGAGAAGAGTGGTG-----
----------------------------------CAGAGAGAAAAAAGAGCA---GTGGGA---
ATAGGAGCT---------CTGTTCCTTGGGTTCTTGGGAGCA---GCAGGAAGCACTATG---
GGCGCAGCATCA---ATAACGCTGACGGTACAA---GCCAGACAC---TTATTG---

TCTGGTATAGTGCAACAGCAGAAC---AATTTG---CTGAGA---
GCTATTGAGGCGCAACAGCATATGTTGCAGCTCACAGTCTGGGGCATC
>MK115360
TTCTACTGTAATACAACACAACTGTTCAATAGTACCTGGCCCAATAATAGTACTGGGAATGATACGGGAGGA-
----

CCAAATGGTACTATCACACTCCCATGCAAAATAAAACAGATTGTAAACTTATGGCAGGAAGTAGGAAAAGCAA
TGTATGCCCCTCCCATCAAAGGACAAATTAGATGTTCATCAAAT---
ATTACAGGGATGATATTAATAAGAGATGGTGGATCAGGTAATGAAAGTAATACC-------------------
----

AATAGTGAGACCTTCAGACCTGGAGGAGGAAATATGAAGGACAATTGGAGAAGTGAATTATACAAATATAAAG
TAGTAAAAATTGAACCATTA---GGAATAGCACCCACCAGGGCAAAGAGAAGAGTGGTG---------------
-------------------------CAGAGAGAAAGAAGAGCA---GTGGGA---ATAGGAGCT---------
TTGTTCCTTGGGTTCTTGGGAGCG---GCAGGAAGCACTATG---GGCGCAGCGTCG---
ATGACGCTGACGGTACAG---GCCAGACAA---TTATTG---TCTGGTATAGTGCAGCAGCAGAAC---
AACCTG---CTGAGG---GCTATTGAGGCACAACAGCATCTGTTGCAACTCACAGTCTGGGGCATT
>MK115488
TTCTATTGTAATACAACACAGCTGTTTAATAGTACTTGGAATAGTACTTGGACTGGGAATGCTACCGAA----
----

TTAGATGGAAACATCACACTCCCATGCAGAATAAAACAGATTATAAACAGGTGGCAAGAAGTAGGAAAAGCAA
TGTATGCCCCTCCCATTAGAGGAAAAATTAGATGTTCATCAAAT---
ATTACAGGGTTACTATTAGTAAGAGATGGTGGTAACACGAGCGGG----------------------------
----

ACTGAGACCTTCAGACCTGGAGGAGGAGATATGAAGGACAATTGGAGGAGTGAATTATATAAATATAAAGTAG
TAAAAATTGAGCCATTA---GGAGTAGCACCCACCAGGGCAAAGAGAAGAGTGGTG-----------------
----------------------CAGAGAGAAAAAAGAGCA---GTGGGA---ATAGGAGCT---------
GTGTTCCTTGGGTTCTTGGGAGCA---GCAGGAAGCACTATG---GGCGCAGCGTCA---
GTGACGCTGACGGTACAA---GCCAGGCTG---TTATTG---TCTGGTATAGTGCAACAGCAAAAC---
AATTTG---CTAAGA---GCTATTGAGGCGCAACAACATCTGTTGCAACTCACAGTCTGGGGCATC
>MK115745
TTCTACTGTAATACAACAAAACTGTTT------ACTTGGAATGCTACAGAT----------------------
----

AGCAATGGAACCATCATACTCCCATGTAGAATAAAACAAATTATAAACATGTGGCAAGAGGTAGGAAAAGCAA
TGTATGCCCCTCCCATTCGTGGACAAATTAGATGTTCATCAAAT---
ATTACAGGACTGCTATTAACAAGAGATGGTGGGACTAACAGGACA----------------------------
----

GAGAACAGGACTGAGATCTTCAGACCTGGAGGGGGAAATATGAAGGACAATTGGAGAAGTGAATTATATAAAT
ACAAAGTAGTAGAAATTAAACCATTA---GGAGTAGCACCCACCAAGGCAAAGAGAAGAGTGGTG--------
-------------------------------CAGAGAGAAAAAAGAGCA---GTGACC---ATAGGAGCT---
------ATGTTCCTTGGGTTCCTGGGAGCA---GCAGGAAGCACTATG---GGCGCAGCATCA---
CTGACGCTGACGGTACAG---GCCAGACAA---TTATTG---TCTGGTATAGTGCAACAGCAGAAC---
AATCTG---CTGAGG---GCGATTGAGGCGCAGCAGCATCTGTTGCAACTCACAGTCTGGGGCATA
>KX587450
TTCTACTGTAATACAACACCACTGTTTAGCAGCTCTTGGAATGTTACTACAGGGGCAAATATCACTGGAGGAA
ATAGC--

ACACAAAATAATATCACACTCCGATGCAGAATAAGACAATTTATAAACATGTGGCAGGAAGTAGGAAAAGCAA
TGTATGCCCCTCCCATCAAAGGACCCATTAAATGCTCATCGAAT---
ATTACAGGGCTGCTATTAGTAAGAGATGGTGGTATAAATGTGACA----------------------------
----
-
GACACAGACAACAACACAGAGACCTTCAGACCTGGAGGAGGAAATATGAAAGACAATTGGAGAAGTGAATTAT
ATAAATATAAAGTAGTAAAAATTGAACCATTA---GGAGTAGCACCTACCAAGGCAAAGAGAAGAGTGGTG--
-------------------------------------CAGAGAGAAAAAAGAGCA---GTGGGA---
CTAGGAGCT---------ATGTTCCTTGGGTTCTTAGGAGCA---GCAGGAAGCACTATG---

GGCGCAGCGTCG---CTGACGCTGACGGTACAG---GCCAGACAG---TTACTG---
TCTGGTATAGTGCAGCAGCAGAAC---AATCTG---CTGAGG---
GCTATTGAGGCGCAACAACATCTGTTGCAACTCACAGTCTGGGGCATC
>MK499378
TTCTACTGTGATACAACACAACTGTTTAATAGTACTTGGTCAAATGATACCTGGTTGAATAGTACTTGGAAT-
----
-GGAAATGAAGGGTCAAAT------
AGCAATGGAGAACCTATCACACTCCAATGCAGAATAAAACAGATTATAAACATGTGGCAGGAAGTAGGAAAAG
CAATGTATGCCCCTCCCATCAGAGGAGAAATTAACTGTTCATCAAAT---
ATTACAGGGCTGCTCTTAACAAGAGATGGTGGTAATAATAATACC----------------------------
----

ATGAACGAGACCTTCAGACCAGGAGGAGGAGATATGAGGGACAATTGGAGAAGTGAATTATATAAATATAAAG
TAGTAAAAGTTGAACCATTA---GGAGTAGCACCCACCAAGGCAAAGAGAAGAGTGGTG--------------
-------------------------CAAAGAGAAAAAAGAGCA---GTGGGA---ATAGGAGCT---------
GTGTTCCTTGGGTTCTTGGGAGCA---GCAGGGAGCACTATG---GGCGCAGCGTCA---
ATGACGCTGACGGTACAA---GCCAGACTA---TTATTG---TCTGGTATAGTGCAACAGCAGAAC---
AATTTG---CTGAGG---GCTATTGAGGCGCAACAGCATATGTTGCAACTCACAGTCTGGGGCATC
>MK169493
TTCTACTGTAACACAACAAAACTGTTTAATAGCTCTTGGCCTGAGAATAATACTAAAAATAATACTAGTAATA
AC---

ACTGGAGAAATAATAACACTCCCATGTAAAATAAAACAAATTATAAACATGTGGCAGGAAGTAGGAAGAGCAA
TGTATGCCTCTCCCATCAGTGGACAAATTAACTGTAACTCAAGT---
ATTACAGGGCTGTTGTTAACAAGAGATGGTGGTAAAAAGACTGAT----------------------------
----

AATGAGACCTTCAGACCTGCAGGGGGAGATATGAGGGACAATTGGAGAAGTGAATTATATAAATATAAAGTAG
TAAAAGTTGAACCATTA---GGAATAGCACCCACCAAGGCAAAGAGAAGAGTGGTG-----------------
----------------------CAGAGAGAAAAAAGAGCA---GTGGGA---ATAGGAGCT---------
GTGTTCCTTGGGTTCTTGGGAGCA---GCAGGAAGCACTATG---GGCGCAGCGTCA---
ATAACGCTGACGGTACAG---GCCAGACAG---TTATTG---TCTGGTATAGTGCAACAGCAGAGC---
AATCTG---CTGAGA---GCTATTGAGGCGCAACAGCATATGTTGCAACTCACAGTCTGGGGCATT
>MK169546
TTCTACTGTAATACAACAGCATTGTTTAATAGTACTTGGTTTAATGGTACTTGGTCTAATAGCACTGTGGAAT
GG---

CCAAATGATACTATCACACTCCCATGCAGAATAAAACAAATTATAAACATGTGGCAGGAAGTAGGAAAAGCAA
TGTACGCCCCTCCCATCAGAGGACAAATTAATTGTTCATCAAAT---
ATTACAGGGCTATTATTACACCGAGATGGTGGTAATAATGTTAGT----------------------------
----

AATAATGAGACCTTTAGACCTATAGGAGGAGATATGAAAAACAATTGGAGAAGTGAATTATACAAATATAAAG
TAGTAAGAATTGAACCAATA---GGAGTAGCACCCACCAAGGCAAAGAGAAGGGTGGTG--------------
-------------------------CAGAGAGAAAAAAGAGCA---ATAACA---CTAGGAGCT---------
ATGTTCCTTGGGTTCTTGGGAGCA---GCAGGAAGCACTATG---GGCGCAGCGTCA---
CTGACGCTGACGGTACAG---GCCAGACAA---TTATTG---TCTGGTATAGTGCAACAGCAAAAC---
AATTTG---CTGAGA---GCTATTGAGGCGCAACAACATCTGTTGCAACTCACAGTCTGGGGCATC
>MK169551
TTCTACTGTAATACAACAGCATTGTTTAATAGTACTTGGTGGTCTAATAGTACTTGGCCTAATAGTACTGTGG
AAGAG--

TCAAATAGAACTATCACACTCCCATGCAGAATAAAACAAATTATAAACATGTGGCAGGAAGTAGGAAAAGCAA
TGTATGCCCCTCCCATCAGCGGACAAATTAATTGTGTATCAAAT---
ATTACAGGGCTGTTATTACACCGAGATGGTGGTGAAAATAGTAATAATAATACT-------------------
----
-
GAGGTTAGTAATAGTACTGAGACCTTTAGACCCATAGGAGGAGATATGAAGAACAATTGGAGAAGTGAATTAT
ACAAATATAAAGTAGTAAGAATTGAACCAATA---GGAGTAGCACCCACCAAGGCAAAGAGAAGGGTGGTG--
-------------------------------------CAGAGAGAAAAAAGAGCA---GTAACA---

CTAGGAGCT---------ATGTTCCTTGGGTTCTTGGGAGCA---GCAGGAAGCACTATG---
GGCGCAGCGTCA---CTGACGCTGACGGTACAG---GCCAGACAA---TTATTG---
TCTGGTATAGTGCAACAGCAAAAC---AATTTG---CTGAGA---
GCTATTGAGGCGCAACAACATCTGTTGCAACTCACAGTCTGGGGCATC
>MK169676
TTCTACTGTAATACAACACTATTGTTTAATAGTACTTGGTCTAATAGTACTGTGGAAGGGTCAAATAACACTG
AA---

GAAAATAGCACACTCACACTCCCATGCAGAATAAAACAAATTATAAACATGTGGCAGGGAGTAGGAAAAGCAA
TGTATGCCCCTCCCATCAGAGGACAAATTAGATGTTCATCAAAT---
ATTACAGGACTGTTATTGACACGAGATGGTGGTAATAATAGTGCT----------------------------
----

AATAATGAGACCTTTAGACCTGGAGGAGGAAATATGAGGGACAATTGGAGAAGTGAATTATATAAATATAAAG
TAGTAAGAATTGAACCATTA---GGAGTAGCACCCACCAAGGCAAAGAGAAGAGTGGTG--------------
-------------------------CAGAGAGAAAAAAGAGCA---GTAACA---CTAGGAGCT---------
ATGTTCCTTGGGTTCTTGGGAGCA---GCAGGAAGCACTATG---GGCGCAGCGTCA---
CTGACGCTGACGGTACAG---GCCAGACAA---TTGTTG---TCTGGTATAGTGCAACAGCAAAAC---
AATTTG---CTGAGA---GCTATTGAGGCGCAACAACATCTGTTGCAACTCACAGTCTGGGGCATC
>MK169581
TTCTACTGTAATACAACAGCATTGTTTAATAGTACTTGGTTTAATGGTACTTGGTCTAATAGTACTGTGAAAG
GG---

TCAAATAATACTATCACACTCCCATGCAGAATAAAACAAATTATAAACATGTGGCAGGAAGTAGGAAAAGCAA
TGTATGCCCCTCCCATCAGAGGACAAATTAATTGTGTATCAAAT---
ATTACAGGGCTGTTATTACACCGAGATGGTGGTAAAAAAGTTAGT----------------------------
----

AATGATACTGAGACCTTTAGACCTATAGGAGGAAATATGAAGGACAATTGGAGAAGTGAATTATACAAATATA
AAGTAGTAAGAATTGAACCAATA---GGAGTAGCACCCACCAAGGCAAAGAGAAGGGTGGTG-----------
----------------------------CAGAGAGAAAAAAGAGCA---GTAACA---CTAGGAGCT------
---ATGTTCCTTGGGTTCTTGGGAGCA---GCAGGAAGCACTATG---GGCGCAGCGTCA---
CTGACGCTGACGGTACAG---GCCAGACAA---TTATTG---TCTGGTATAGTGCAACAGCAAAAC---
AATTTG---CTGAGA---GCTATTGAGGCGCAACAACATCTGTTGCAACTCACAGTCTGGGGCATC
>MK169545
TTCTACTGTAATACAACACTATTGTTTAATAGTACTTGGTCTAATAGTACTGTGGAAGGGTCAAATAAC----
----

ACTGAAGAAAATATCACACTCCCATGCAGAATAAAACAAATTATAAACATGTGGCAGGGAGTAGGAAAAGCAA
TGTATGCCCCTCCCATCAGAGGACAAATTAGATGTTCATCAAAT---
ATTACAGGGCTGTTATTAACACGAGATGGTGGTAATAATAGTGCT----------------------------
----

GATACTGAGATCTTTAGACCTGGAGGAGGAGATATGAGGGACAATTGGAGAAGTGAATTATATAAATATAAAG
TAGTAAAAATTGAACCATTA---GGAGTAGCACCCACCAAGGCAAAGAGAAGAGTGGTG--------------
-------------------------CAGAGAGAAAAAAGAGCA---GTGGGAACACTAGGAGCT---------
ATGTTCCTTGGGTTCTTGGGAGCA---GCAGGAAGCACTATG---GGCGCAGCGTCA---
CTGACGCTGACGGTACAG---GCCAGACTA---TTATTG---TCTGGTATAGTGCAACAGCAAAAC---
AATTTG---CTGAGG---GCTATTGAGGCGCAACAACATCTGTTGCAACTCACAGTCTGGGGCATC
>MK169677
TTCTACTGTAATACAACATCACTGTTTAATAGTACTTGGGATAATAGTACAAATAGTACTGGA----------
----

GAGGGAGAAGATATCACACTCCCATGCAGACTAAAACAGTTTATAAACATGTGGCAGAAAGTAGGAAAAGCAA
TGTATGCCCCTCCCATCAGAGGACAAATTAAATGTTCATCAAAT---
ATTACAGGGCTGCTATTAACAAGAGATGGTGGTGGAGGTAATAGCACT-------------------------
----

ACCACAAACGAGACCTTCAGACCAGGAGGGGGAGATATGAGAGATAATTGGAGAAGTGAATTATATAAATATA
AAGTAGTAAAAATTGAACCATTA---GGAATAGCACCCACCAAGGCAAAGAGGAGAGTGGTG-----------

----------------------------CAGAGAGAAAAAAGAGCA---GTGGGA---ATAGGAGCT------
---TTGTTCCTTGGGTTCTTGGGAGCA---GCAGGAAGCACTATG---GGCGCAGCGTCA---
GTAACGCTGACGGTACAG---GCCAGACTA---TTATTG---TCTGGTATAGTGCAACAGCAGAAC---
AATTTG---CTGAGG---GCTATTGAGGCGCAACAGCATCTGTTGCAACTCACAGTCTGGGGCATC
>MN090376
TTCTACTGTAATACAACACAACTGTTTAATAGTACTTGGAATAGTACTACAGGAGTAAATGGTACAGGAGCAA
ATAACACA---

GAAGTAAATATTATCACACTCCCATGCAAGATAAGGCAAATTGTAAACATGTGGCAGGAAGTAGGAAAAGCAA
TGTATGCCCCTCCCATCAGTGGACAAATTAGTTGTTCATCAAAT---
ATTACAGGGCTACTACTACTAAGAGATGGTGGTAATAGCAATGAC----------------------------
----

ACGGATAACACGACTGAAATCTTCAGACCTGGAGGAGGAGATATGAGGGACAATTGGAGAAGTGAATTATATA
AATATAAAGTAGTACAAATTGAACCATTA---GGAGTAGCACCCACCAAGGCAAGGAGAAGAGTGGTG-----
----------------------------------CAGAGAGAAAAAAGAGCA---GTGGGA---
ATAGGAGCT---------CTGTTCCTTGGGTTCTTGGGAGCA---GCAGGAAGCACTATG---
GGCGCAGCATCA---ATAACGCTGACGGTACAA---GCCAGACAT---TTATTG---
TCTGGTATAGTGCAACAGCAGAAC---AATTTG---CTGAGA---
GCTATTGAGGCGCAACAGCATATGTTGCAGCTCACAGTCTGGGGCATC
>MN090399
TTCTACTGTAATACAACACAACTGTTTAATAGTACTTGGAATAGTACTACAGGAGTAAATGGTACAGGAGCAA
ATAACACA---

GAAGTAAATATTATCACACTCCCATGCAAGATAAGGCAAATTGTAAACATGTGGCAGGAAGTAGGAAAAGCAA
TGTATGCCCCTCCCATCAGTGGACAGATTAGTTGTTCATCAAAT---
ATTACAGGGCTACTACTACTAAGAGATGGTGGTAATAGCAATGAC----------------------------
----

ACGAATGACACGGCTGAAATCTTCAGACCTGGAGGAGGAGATATGAGGGACAATTGGAGAAGTGAATTATATA
AATATAAAGTAGTACAAATTGAACCATTA---GGAGTAGCACCCACCAAGGCAAGGAGAAGAGTGGTG-----
----------------------------------CAGAGAGAAAAAAGAGCA---GTGGGA---
ATAGGAGCT---------CTGTTCCTTGGGTTCTTGGGAGCA---GCAGGAAGCACTATG---
GGCGCAGCATCA---ATAACGCTGACGGTACAA---GCCAGACAC---TTATTG---
TCTGGTATAGTGCAACAGCAGAAC---AATTTG---CTGAGA---
GCTATTGAGGCGCAACAGCATATGTTGCAGCTCACAGTCTGGGGCATC
>AY835781
TTCTACTGTAATTCATCACAACTGTTTAATAGTACTTGGAATGATAGTACTTGGAATGATACTAAAGGGTTAA
AT---

AACACTGAAGGAATTATCACACTCCCATGCAGAATAAAACAATTTATAAACATGTGGCAGGAAGTAGGAAAAG
CAATGTATGCCCCTCCCATCAGAGGACAAATTAGATGTTCATCAAAT---
ATTACAGGGCTGCTCTTAACAAGAGATGGTGGTAATAGCGAGAAC----------------------------
----

GATACCACCGAGATCTTCAGACCTGGAGGAGGAGATATGAGGGACAATTGGAGAAGTGAATTATATAAATATA
AAGTAATAAAAATTGAACCATTA---GGAGTAGCACCCACCAAGGCAAAGAGAAGAGTGGTG-----------
----------------------------CAGAGAGAAAAAAGAGCA---GTGGGAACGCTAGGAGCT------
---GTGTTCCTTGGGTTCTTGGGAGCA---GCAGGAAGCACCATG---GGCGCAGCGTCA---
ATGACGCTGACGGTACAG---GCCAGACAA---TTATTG---TCTGGTATAGTGCAACAGCAGAAC---
AATTTG---CTGAGG---GCTATTGAGGCGCAACAGCATCTGTTGCAACTCACAGTCTGGGGCATC
>M17451
TTCTACTGTAATACAACACAACTGTTTAATAGTACTTGGAATAGTACTGAAGGGTCAAATAACACTGGA----
----

GGAAATGACACAATCACACTCCCATGCAGAATAAAACAAATTGTAAACATGTGGCAGGAAGTAGGAAAAGCAA
TGTATGCCCCTCCCATCAGTGGACAAATTAAATGTATATCAAAT---
ATTACAGGGCTACTATTAACAAGAGATGGGGGTGAAGATACAACT----------------------------
----

AATACTACAGAGATCTTCAGACTTGGAGGAGGAAATATGAGGGACAATTGGAGAAGTGAATTATATAAATATA
AAGTGGTAAGAATTGAGCCATTA---GGAGTGGCACCCACTAGGGCAAAGAGAAGAGTGGTG-----------
----------------------------CAAAGAGAAAAAAGAGCA---GTGGGAACAATAGGAGCT------
---ATGTTCCTTGGGTTCTTGGGAGCA---GCAGGAAGCACTATG---GGCGCAGGCTCA---
ATAACGCTAACGGTACAG---GCCAGACAC---TTATTG---TCTGGTATAGTGCAACAGCAAAAC---
AATTTG---CTGAGG---GCTATTGAGGCGCAACAGCATCTGTTGCAACTCACGGTCTGGGGCATC
>AY835779
TTCTACTGTAATTCAACACAACTGTTTAATAGTACTTGGAATGTTACTGGAGGGACAAATGGCACTGAAGGA-
----

AGTAACCCAAATATCACACTCCCATGCAGAATAAAACAAATTATAAACATGTGGCAGGAAGTAGGAAAAGCAA
TGTATGCCCCTCCCATCAGTGGACAAATTAGATGTTCATCAAAT---
ATTACAGGACTGCTATTAACAAGAGATGGTGGTAGTAACAAGAGC----------------------------
----

GATACCGAGATCTTCAGACCTGGAGGAGGAGATATGAGGGACAATTGGAGAAGTGAATTATATAAATATAAAG
TAGTAAAAATTGAACCATTA---GGAGTAGCACCCACCAAGGCAAAGAGAAGAGTGGTG--------------
-------------------------CAGAGAGAAAAAAGAGCA---GCGGGA---CTAGGAGCT---------
TTGTTCCTTGGGTTCTTGGGAGCA---GCAGGAAGCACTATG---GGCGCAGCGGCA---
GTAACGCTGACGGTACAG---GCCAGACTA---TTATTG---TCTGGTATAGTGCAACAGCAGAAC---
AATTTG---CTGAGG---GCTATTGAGGCGCAACAGCATCTGTTGCAACTAACAGTCTGGGGCATC
>M38431
TTCTACTGTAAGACAACACAGCTGTTTAATAGTACTTGGCTGTTTAATAGTACTTGGAATGATACTGAAAGGT
CA---

GATAACAATGAAACTATCATACTCCCATGCAGAATAAAACAAATTATAAACAGGTGGCAGGAAGTAGGAAAAG
CAATGTATGCCCCTCCCATCAGTGGACAAATTAGATGCTCATCAAAT---
ATTACAGGGCTGCTATTAACAAGAGATGGTGGTGATAAAGAGAAC----------------------------
----

AGTACCACCGAGATCTTCAGACCTGGAGGAGGAGATATGAGGGACAATTGGAGAAGTGAATTATATAAATATA
AAGTAGTAAAAATTGAGCCATTA---GGAGTAGCACCCACCAAGGCAAAGAGAAGAGTGGTG-----------
----------------------------CAGAGAGAAAAAAGAGCA---GTGGGAGCGCTAGGAGCT------
---CTGTTCCTTGGGTTCTTGGGAGCA---GCAGGAAGCACTATG---GGCGCAGCGTCA---
ATGGCGCTGACGGTACAG---ACCAGACAA---TTAATG---TCTGGTATAGTGCAACAGCAGAAC---
AATTTG---CTGAAG---GCTATTGAGGCGCAACAGCATCTGTTGCAACTCACAGTCTGGGGCATC
>U90935
TTCTACTGTAATACAACAAAACTGTTTAATAGTACTTGGATGTTTAATAATACTTGGAATGATACTGAAGACA
CT---

GAAGGAAATGGCGCAATCACACTCCCATGCAGAATAAAACAGATTATAAACAGGTGGCAGGAAGTAGAAAAAG
CAATGTATGCCCCTCCCATCAGAGGACAAATTAGATGTTCATCAAAC---
ATTACAGGGATACTATTAACAAGAGATGGTGGTACTAACAATAGT----------------------------
----

ACCAACGAGACCTTCAGACCTGGAGGAGGAGATATGAGGGACAATTGGAGAAGTGAATTATATAAATATAAAG
TAGTAAAAATTGAACCATTA---GGAGTAGCACCCACCAAGGCAAAGAGAAGAGTGGTG--------------
-------------------------CAGAGAGAAAAAAGAGCA---GTGGGA---ATAGGAGCT---------
TTGTTCCTTGGGTTCTTGGGAGCA---GCAGGAAGCACTATG---GGCGCAGCGGCA---
GTGACGCTGACGGTACAG---GCCAGACAA---TTATTG---TCTGGTATAGTGCAACAGCAGAAC---
AACTTG---CTGAGG---GCTATTGAGGCGCAACAGCATCTGTTGCAACTCACAGTCTGGGGCATC
>M17450
TTCTACTGTAATTCAACACAACTGTTTAGTAGTACTTGGAATGGTACTGAAGGGTCAAATAACACTGGA----
----

GGAAATGACACAATCACCCTCCCATGCAGAATAAAAGAAATTATAAACATGTGGCAGGAAGTAGGAAAAGCAA
TGTATGCCCCTCCCATCAAAGGACAAGTTAAATGTTCATCAAAT---
ATTACAGGGCTGCTATTAACAAGAGATGGTGGTAATAGCAAGAATGGTAGC----------------------
----

AAGAATGAGAACACAGAGATCTTTAGACCGGGAGGAGGAGATATGAGGGACAATTGGAGAAGTGAATTATATA
AATATAAAGTAGTAAAAATTGAACCATTA---GGAGTAGCACCCACCAAGGCAAAGAGAAGAGTGGTG-----
----------------------------------CAGAGAGAAAAAAGAGCA---
GTGGGAACGATAGGAGCT---------ATGTTCCTTGGGTTCTTGGGAGCA---GCAGGAAGCACTATG---
GGCGCTACGTCA---ATGACGCTGACGGTACAG---GCCAGACTA---TTATTG---
TCTGGTATAGTGCAACAGCAGAAC---AATTTG---CTGAGG---
GCTATTGAGGCGCAACAGCATCTGTTGCAACTCACAGTCTGGGGCATC
>AY352275
TTCTACTGTAATACAACAAAACTGTTTAATAGTACTTGGAATGAAAATAGTACTTGGAATGCTACT-------
----

GGAAATGACACTATCACACTCCCATGTAGAATAAAACAAATTATAAACATGTGGCAGGAAGTAGGAAAAGCAA
TGTATGCCCCTCCCATCGAAGGACAAATTAGATGTTCATCAAAT---
ATTACAGGGCTGCTATTAACAAGAGATGGTGGTGGTGACAAGAAC----------------------------
----

AGTACCACCGAGATCTTTAGACCTGCAGGAGGAAATATGAAGGACAATTGGAGAAGTGAATTATATAAATATA
AAGTAGTAAAAATTGAACCATTA---GGAGTAGCACCCACCAAGGCAAAGAGAAGAGTGGTG-----------
----------------------------CAAAGAGAAAAAAGAGCA---GTGGGAGTGATAGGAGCT------
---ATGTTCCTTGGGTTCTTGGGAGCA---GCAGGAAGCACTATG---GGCGCAGCGTCA---
ATAACGCTGACGGTACAG---GCCAGAAAA---CTATTG---TCTGGTATAGTGCAACAGCAGAAC---
AATCTG---CTGAGA---GCTATTGAGGCGCAACAGCATCTGTTGCAACTCACAGTCTGGGGCATC
>AY835769
TTCTACTGTGATTCAACACAACTGTTTAACAGTACTTGGCATGGTAATGGTACTTGGAATGGTACTGAA----
----

GGAAATGGCACACTCACACTCCCATGCAGAATAAAACAAATTATAAACATGTGGCAGGAAGTAGGAAAAGCAA
TGTATGCCCCTCCCATCAGAGGACAAATTAGATGTTCATCAAAT---
ATTACAGGGCTGCTATTAACAAGAGATGGTGGTAGGAACGAGAGC----------------------------
----

AATATCACCCTCAGACCTGGAGGAGGGGATATGAGGGACAATTGGAGAAGTGAATTATATAAATATAAAGTAG
TAAAAATTGAACCATTA---GGAGTAGCACCCACTAAGGCAAAGAGAAGAGTGGTG-----------------
----------------------CAGAGAGAAAAAAGAGCA---GCGGGA---TTAGGAGCT---------
ATGTTCCTTGGGTTCTTGGGAGCA---GCAGGAAGCACTATG---GGCGCAGCGTCA---
ATAACGCTGACGGTACAG---GCCAGACTA---TTATTG---TCTGGTATAGTGCAACAGCAGAAT---
AATTTG---CTGAGG---GCTATTGAGGCGCAACAGCATATGTTGAAACTCACAGTCTGGGGCATC
>M38430
TTCTACTGTAATTCAACACAACTATTTAATAGTACTTGGAATAGTACTGAAGGGCCTAAAGGGTCAAATAAC-
----

ACTGAAGAAAATATCACACTCCCATGCAGAATAAAACAAATTGTAAACATGTGGCAGGAAGTAGGAAAAGCAA
TGTATGCCCCTCCCATCGAAGGACAAATTAGATGCTCATCAAAT---
ATTACAGGGCTGCTATTAACAAGAGATGGTGGTAATAGCAGCAAC----------------------------
----

AACACCGAGATCTTCAGACCTGGAGGAGGAGATATGAGGGACAATTGGAGAAGTGAATTATATAAATATAAAG
TAGTAAAAATTGAACCATTA---GGAGTAGCACCCACCAGGGCAAAGAGAAGAGTGGTG--------------
-------------------------CAGAGAGAAAAAAGAGCA---GTGGGA---CTAGGAGCT---------
TTGTTCCTTGGGTTCTTGGGAGCA---GCAGGAAGCACTATG---GGCGCACGGTCA---
ATGACGCTGACGGTACAG---GCCAGACAA---TTATTG---TCTGGTATAGTGCAACAGCAGAAC---
AATTTG---CTGAGG---GCTATTGAGGCGCAACAGCATCTGTTGCAACTCACAGTCTGGGGCATC
>AB221005
TTCTACTGTAATTCAACACAACTGTTTAATAGTACTTGGAATGTTACTGAAGAGTCAAATAACACTGTA----
----

GAAAATAACACAATCACACTCCCATGCAGAATAAAACAAATTATAAACATGTGGCAGGAAGTAGGAAGAGCAA
TGTATGCCCCTCCCATCAGAGGACAAATTAGATGTTCATCAAAT---
ATTACAGGGCTGCTATTAACAAGAGATGGTGGTCCTGAGGACAAC----------------------------
----

AAGACCGAGGTCTTCAGACCTGGAGGAGGAGATATGAGGGATAATTGGAGAAGTGAATTATATAAATATAAAG
TAGTAAAAATTGAACCATTA---GGAGTAGCACCCACCAAGGCAAAGAGAAGAGTGGTG--------------
-------------------------CAGAGAGAAAAAAGAGCA---GTGGGA---ATAGGAGCT---------
GTGTTCCTTGGGTTCTTGGGAGCA---GCAGGAAGCACTATG---GGCGCAGCGTCA---
ATGACGCTGACGGTACAG---GCCAGACTA---TTATTG---TCTGGTATAGTGCAACAGCAGAAC---
AATCTG---CTGAGA---GCTATTGAGGCGCAACAGCATCTGTTGCAACTCACAGTCTGGGGCATC
>AY357406
TTCTACTGTAAGTCAACAAAACTGTTTAATAGTACTTGGACTAGGAATAATGGTACTTGGACTCGGAATGATA
CTGAAAGG---
-------------TCAAAT------
AGCACTGAAGAACATATCACACTCCCATGCAGAATAAAACAAATTATAAACATGTGGCAGGAAGTAGGAAAAG
CAATGTATGCCCCTCCCATCAGAGGACAAATTAGATGTTCATCAAAT---
ATTACAGGGCTGCTATTAACAAGAGATGGTGGTAATGACACGAGC----------------------------
----

GGGACTGAGACCTTCAGACCTGGAGGAGGAGATATGAGGGACAATTGGAGAAGTGAATTATATAAATATAAAG
TAGTAAGAATTGTACCATTA---GGAGTAGCACCCACCGAGGCAAAGAGAAGAGTGGTG--------------
-------------------------CAGAGAGAAAAAAGAGCA---GTGGGA---ATAGGAGCT---------
GTGTTCCTTGGGTTCTTGGGAGCA---GCAGGAAGCACTATG---GGCGCAGCGGCA---
GTGACGCTGACGGTACAG---GCCAGACTA---TTATTG---TCTGGTATAGTGCAACAGCAGAAC---
AATTTG---CTGAGG---GCTATTGAGGCGCAACAGCATCTGTTGCAACTCACAGTCTGGGGCATC
>AF025758
CTCTACTGTAATACAACACAACTGTTTAATAGTACTTGGAATGCTAATGGGACTTGGAATGATACAGGAGGGT
CAAAT--

AGCACTGAAGGAATTATCTCACTCCCATGCAGAATAAAACAAATTATAAACATGTGGCAGGAAGTAGGAAAAG
CAATGTATGCCCCTCCCATCGAAGGACAAATTAGATGTTCATCAAAT---
ATTACAGGGCTATTATTAACAAGAGATGGTGGTAGTAGCCAAAACAAG-------------------------
----

AGCGAGACCGAGATCTTCAGACCTGGAGGAGGAGATATGAGGGACAATTGGAGAAGTGCATTATATAAATACA
AAGTAGTAAAAATTGAGCCATTA---GGAGTAGCACCCACTAAGGCAAAGAGAAGAGTGGTG-----------
----------------------------CAGAGAGAAAAAAGAGCA---GTGGGA---ATAGGAGCT------
---GTACTCCTTGGGTTCTTGGGAGCA---GCAGGAAGCACTATG---GGCGCAGCGTCA---
TTGACGCTGACGGTACAG---GCCAGACTA---TTATTG---TCTGGTATAGTGCAACAGCAGAAC---
AATTTG---CTGAGG---GCTATTGAGGCGCAACAGCATCTGTTGCAACTCACAGTCTGGGGCATC
>AF025764
TTCTACTGTAATTCAACACAACTGTTTAATAGTACTTGGCCATTTAATAGTACTTTGAATGATACTAGCACTG
AA---

GGAAATGCCACAATCACACTCCCATGCCGAATAAAACAAGTTGTAAACACGTGGCAGGAAGTAGGAAAAGCAA
TGTATGCCCCTCCCATCAGAGGACAAATTAGATGTTCATCAAAT---
ATTACAGGGCTGCTATTAACAAGAGATGGAGGGACTAGCGAGAACGGG-------------------------
----

ACGAACGAGATTGAGATCTTCAGACCTGGAGGAGGAGATATGAGGGACAATTGGAGAAGTGAATTATATAAAT
ATAAAGTAGTAAAAATTGAACCATTA---GGAGTAGCACCCACCAAGGCAAAGAGAAGAGTGGTC--------
-------------------------------CAAAGAGAAAAAAGAGCA---GTGGGAACAATAGGAGCT---
------ATGTTCCTTGGGTTCTTGGGAGCA---GCAGGAAGCACTATG---GGCGCAGCGTCA---
ATAACGCTGACGGTACAG---GCCAGACTA---TTATTG---TCTGGTATAGTGCAGCAGCAGAAC---
AATCTG---CTGAGG---GCTATTGAGGCGCAACAGCATCTGTTGCAACTCACAGTCTGGGGCATC
>AF025751
TTCTACTGTAATTCAACACAACTGTTTAATAGTACTTGGAATGTTACTAGAGAGTCAAATAACAGTACTGAA-
----

GGAAATGGGACTATCACACTCCCATGCAGAATAAAACAAATTATAAACATGTGGCAGGGAGTAGGAAAAGCAA
TGTACGCCCCTCCCATCAGAGGACAAATTAGATGTTCATCAAAT---
ATTACAGGGCTGCTATTAACAAGGGATGGTGGTAATAACGAGAGC----------------------------
----

AATGAGACCGAGACCTTCAGACCTGGAGGAGGAGATATGAGGGACAATTGGAGAAGTGAATTATATAAATATA
AAGTAGTAAAAATTGAACCATTA---GGAGTAGCACCCACCAAGGCAAAAAGAAGAGTGGTG-----------
----------------------------CAGAGAGAAAAAAGAGCA---GTGGGA---ATAGGAGCT------
---GTGTTCCTTGGGTTCTTGGGAGCA---GCAGGAAGCACTATG---GGCGCAGCGTCA---
ATGACGCTGACGGTACAG---GCCAGACTA---TTATTG---TCTGGTATAGTGCAACAGCAGAAC---
AATTTG---CTGAGG---GCTATTGAGGCGCAACAGCATCTGTTGCAACTCACAGTCTGGGGCATC
>U69584
TTCTACTGTAATACATCACAACTGTTTAATAGTACTTGGAATAGTACTGGGAATAATATTAGCGAGTCAGATA
AC---

ACTGAAAGAAATATCACACTCCCATGCAGAATAAAACAAATTATAAACCTGTGGCAGGAAGTAGGAAAAGCAA
TGTATGCCCCTCCCATCAGAGGACAAATTAGATGTTCATCAAAT---
ATTACAGGGCTGCTATTAACAAGAGATGGTGGTAGTAACACGGACGAG-------------------------
----

AACAGGACCGAGATCTTCAGACCTGGAGGAGGAGATATGAGGGACAATTGGAGAAGTGAATTATATAAATATA
AAGTAGTAAAAATTGAACCATTA---GGAGTAGCACCCACCAAGGCAAAGAGAAGAGTGGTG-----------
----------------------------CAGAGAGAAAAAAGAGCA---GTGGGAACAATAGGAGCT------
---ATGTTCCTTGGGTTCTTGGGAGCA---GCAGGAAGCACTATG---GGCGCAGCGTCG---
ATGACGCTGACGGTACAG---GCCAGACTA---TTATTG---TCTGGTATAGTGCAACAGCAGAAC---
AATTTG---CTGAGG---GCTATTGAGGCGCAACAGCATCTGTTGCAACTCACAGTCTGGGGCATC
>AY835778
TTCTACTGTAATTCAAGAGAGCTGTTTAATAGTACTTGGAATGATACTGAAGGGTGGAATAATACTGACAGG-
----

TCAAATAAAACTATCACACTCCCATGCAAAATAAAACAAATTATAAACAGATGGCAGGAAGTAGGAAAAGCAA
TGTATGCCCCTCCCATCAGAGGACAAATTAGATGTACATCAAAT---
ATTACAGGGCTGCTACTAACAAGAGATGGTGGTAGGAACAATAGC----------------------------
----

GAAAACGAGACCTTCAGACCTGGAGGAGACGATATGAGGGACAATTGGAGAAGTGAATTATATAAATATAAAG
TAGTAAAAATTGGACCATTG---GGAGTAGCACCCACCAAGGCAAAGAGAAGAGTGGTG--------------
-------------------------CAGAGAGAAAAAAGAGCA---GTGGGAGTAATAGGAGCT---------
ATGTTCCTTGGGTTCTTGGGAGCA---GCAGGAAGCACTATG---GGCGCAGCGTCA---
ATGACGCTGACGGTACAA---GCCAGACAA---TTATTG---TCTGGTATAGTGCAACAGCAGAAC---
AATCTG---CTGAGG---GCTATTGAGGCGCAACAGCATCTGTTGCGACTCACAGTCTGGGGCATC
>AY835775
TTCTACTGTAATTCAACACAACTATTTAATAGTACTTGGAATGGTACTGAGAGGTCATATAACACTTCA----
----

GAAAATATCAATATCACACTCCCATGCAGAATAAAACAAATTGTAAACATGTGGCAGGAAGTAGGAAAAGCAA
TGTATGCTCCTCCCATCAGTGGACAAATTAGATGTTCATCAAAT---
ATTACAGGGCTGCTATTAACAAGAGATGGTGGTAAGAAACAGAACGAGACC----------------------
----

AAGAACGAGACCGAGATCTTTAGACCTGGAGGAGGAGATATGAGGGACAATTGGAGAAGTGAATTATATAGAT
ATAAAGTAGTAAAAATTGAACCATTA---GGAGTAGCACCCATCAAGGCAAAGAGAAGAGTGGTG--------
-------------------------------CAGAGAGAAAAAAGAGCA---GCGGGA---ATAGGAGCT---
------TTGTTCCTTGGGTTCTTGGGAGCA---GCAGGAAGCACTATG---GGCGCAGCGTCA---
ATAACGCTGACGGTACAG---GCCAGACTA---TTATTG---TCTGGTATAGTGCAACAGCAGAAC---
AATTTG---CTGAGG---GCTATTGAGGCGCAACAGCATCTGTTGCAACTCACAGTCTGGGGCATC
>AY835749
TTCTACTGTAATACAACACAACTATTTAATAGTACTTGGAATGTTAATAGTACTTGGAGTAGTACTGAAGGGT
CAAATAAC---
-------------------ACT---
GAAAGAAATGACACACTCACACTCCCATGCAGAATAAAACAAATTATAAACATGTGGCAGGAAGTAGGAAAAG
CAATGTATGCCCCTCCCATCAGCGGACAAATTAGTTGTTCATCAAAT---
ATTACAGGGCTGATATTAACAAGAGATGGTGATAAAAACAACAAT----------------------------
----

```
-------------
GAGACCGAGATCTTCAGACCTGTAGGAGGAGATATGAGGGACAATTGGAGAAGTGAATTATATAAATATAAAG
TAGTAAAGATTGAACCATTA---GGAGTAGCACCCACTAGGGCAAAGAGAAGAGTGGTG--------------
-------------------------CAGAGAGAAAAAAGAGTAGCAGTGGGA---CTAGGAGCT---------
GTGTTCCTTGGGTTCTTGGGAGCA---GCAGGAAGCACTATG---GGCGCAGCGGCA---
GTGACGCTGACGGTACAG---GCCAGACTA---TTATTG---TCTGGTATAGTGCAACAGCAGAAC---
AATTTG---CTGAGA---GCTATTGAGGCGCAACAGCATCTGTTGCAACTCACAGTCTGGGGCATC
>AY426119
TTCTACTGTAATTCAACACAACTGTTTAATAGTACTTGGAATTTTAATGGTACTTGGAATTTAACACAATCGA
ATGGT--------------------------------------------------------------------
-------------------ACT---
GAAGGAAATGACACTATCACACTCCCATGTAGAATAAAACAAATTATAAATATGTGGCAGGAAGTAGGAAAAG
CAATGTATGCCCCTCCCATCAGAGGACAAATTAGATGCTCATCAAAT---
ATTACAGGGCTAATATTAACAAGAGATGGTGGAACTAACAGTAGT----------------------------
-------------------------------------------------------------------------
-------------
GGGTCCGAGATCTTCAGACCTGGGGGAGGAGATATGAGGGACAATTGGAGAAGTGAATTATATAAATATAAAG
TAGTAAAAATTGAACCATTA---GGAGTAGCACCCACCAAGGCAAAAAGAAGAGTGGTG--------------
-------------------------CAGAGAGAAAAAAGAGCA---GTGGGAACGATAGGAGCT---------
ATGTTCCTTGGGTTCTTGGGAGCA---GCAGGAAGCACTATG---GGCGCAGCGTCA---
ATAACGCTGACGGTACAG---GCCAGACTA---TTATTG---TCTGGTATAGTGCAACAGCAGAAC---
AATTTG---CTGAGG---GCTATTGAGGCGCAACAGCATCTGTTGCAACTCACAGTCTGGGGCATC
>AY426125
TTCTACTGTAATTCAACACAACTGTTTAATAGTACTTGGAATGATACTGAAAAGTCAAGTGGCACTGAA----
-------------------------------------------------------------------------
----------------------------
GGAAATGACACCATCATACTCCCATGCAGAATAAAACAAATTATAAACATGTGGCAGGAAGTGGGAAAAGCAA
TGTATGCTCCTCCCATTAAAGGACAAATTAGATGTTCATCAAAT---
ATTACAGGGCTGCTATTAACAAGAGATGGTGGTAAAAATGAGAGT----------------------------
-------------------------------------------------------------------------
-------------
GAGATCGAGATCTTCAGACCTGGAGGAGGAGACATGAGGGACAATTGGAGAAGTGAATTATATAAATATAAAG
TAGTAAAAATTGAACCATTA---GGAGTAGCACCCACCAAGGCAAAGAGAAGAGTGGTG--------------
-------------------------CAAAGAGAAAAAAGAGCA---GTGGGA---ATAGGAGCT---------
TTGTTCCTTGGGTTCTTGGGAGCA---GCAGGAAGCACTATG---GGCGCAGCGTCA---
ATGACACTGACGGTACAG---GCCAGACAA---TTATTG---TCTGGTATAGTGCAACAGCAAAAC---
AATTTG---CTGAGG---GCTATTGAGGCGCAACAGCATATGTTGCAACTCACAGTCTGGGGCATC
>AF025759
TTCTACTGTAATTCAACACAGCTATTTAATAGTACTTGGCTGTTTAATAGTACTGGGAATGATACTACAGGGT
CA-----------------------------------------------------------------------
-------------------------
AATAACACGGAACCTATCACACTCCCATGCAGAATAAAACAAATTATAAATATGTGGCAAAAAGTAGGAAAAG
CAATGTATGCCCCTCCCATCAGAGGACAAATTAGATGTTCATCAAAT---
ATTACAGGGCTGCTATTAACAAGAGATGGTGGTCTATTAACAAGAAATGACATC-------------------
-------------------------------------------------------------------------
----
AAAGAGAACGAGACTGAGATCTTCAGACCTGGAGGAGGAGATATGAGGGACAATTGGAGAAGTGAATTATATA
AATATAAAGTAGTAAAAATTGAGCCATTA---GGAGTAGCACCCACCAAGGCAATGAGAAGAGTGGTG-----
----------------------------------CAGAGAGAAAAAAGAGCA---
GTGGGAACGATAGGAGCT---------ATGTTCCTTGGGTTCTTGGGAGCA---GCAGGAAGCACTATG---
GGCGCAGCGTCG---ATGACGCTGACGGTACAG---GCCAGACTA---TTATTG---
TCTGGTATAGTGCAACAGCAGAAC---AATTTG---CTGAGG---
GCTATTGAGGCGCAACAGCATCTGTTGCAACTCACAGTCTGGGGCATC
>AF025761
TTCTACTGTAATACAACACAACTGTTTAATAGTACTTGGAATAGTACTGGGGATGAAGGGTCAAATAACACTG
AAGGA--------------------------------------------------------------------
-------------------------
AATAACACACTCCCAATCATACTTCCATGCAGAATAAAACAAATTATAAACATGTGGCAGGGAGTAGGAAAAG
CAATGTATGCCCCTCCCATCGGAGGACAAATTAGATGTTCATCAAAT---
ATTACAGGGCTGCTATTAACAAGAGATGGTGGTAAGAGTCCAAAC----------------------------
```


GATACCGAGACCTTCAGACCTGCAGGAGGAGATATGAGGGACAATTGGAGAAGTGAATTATATAAATATAAAG
TAGTAAAAATTGAACCATTA---GAAGTAGCACCCACTAGGGCAAAGAGAAGAGTGGTG--------------
-------------------------CAAAGAGAAAAAAGAGCA---GTAGGA---ATAGGAGCT---------
ATGTTCCTTGGGTTCTTGGGAGCA---GCAGGAAGCACTATG---GGCGCAGCGTCG---
ATAACGCTGACGGTACAG---GCCAGACTA---TTATTG---TCTGGTATAGTGCAACAGCAGAAC---
AATCTG---CTGAGG---GCTATTGAGGCGCAACAGCATCTGTTGCAACTCACAGTCTGGGGCATC
>AF025749
TTCTACTGTAATACAGCAAAACTATTTAATAGTACTTGGAATAGTACTTGGAGTTCAAATAATACTGAAGGAA
AT---

AGCACTGAAGAAATTATCATACTCCCATGTAGAATAAAACAAATTATAAACAGGTGGCAGGAAGTAGGAAAAG
CAATGTATGCCCCTCCCATCAGAGGACAAATCAGATGTTCATCAAAT---
ATTACAGGGCTGCTATTAACAAGAGATGGTGGTACTAATGGGAAGAAC-------------------------
----

GAGACCCCCGAGATCTTCAGACCTGGAGGAGGAGATATGAGGGACAATTGGAGAAGTGAATTATATAAATATA
AAGTAGTAGAAATTGAACCATTA---GGAGTAGCACCCACCAGGGCAAGGAGAAGAGTGGTG-----------
----------------------------CAGAGAGAAAAAAGAGCA---GTGGGAACAATAGGAGCT------
---ATGTTCCTTGGGTTCTTGGGAGCA---GCAGGAAGCACTATG---GGCGCAGCGTCA---
ATGACGCTGACGGTACAG---GCCAGACTA---TTATTG---TCTGGTATAGTGCAACAGCAGAAC---
AATTTG---CTGAGG---GCTATTGAGGCGCAACAGCATCTGTTGCAACTCACAGTCTGGGGCATC
>AF025750
TTCTACTGTAATTCAGCACAACTGTTTAACAGTACTTGGAATGATACTGAAAGGTCAAATAACACTAGAGAA-
----

AATGACACAACAATCACACTCCCATGCAGAATAAAACAAATTATAAACATGTGGCAGGAAGTAGGAAAAGCAA
TGTATGCCCCTCCTATCAGAGGACAAATTAAATGTGCATCAAAT---
ATCACAGGGCTGCTATTAACAAGAGATGGTGGTAATAACAATACT----------------------------
----

GGGACCACCGAGACCTTCAGACCTGGAGGAGGAGATATGAGGGACAATTGGAGAAGTGAATTATATAAATATA
AAGTAGTAAAAATTGAACCATTA---GGAATAGCACCCACTAAGGCAAAGAGAAGAGTGGTG-----------
----------------------------CAGAGAGAAAAAAGAGCA---GTGGGA---ATAGGAGCT------
---GTGTTCCTTGGGTTCTTGGGAGCA---GCAGGAAGCACTATG---GGCGCAGCGTCA---
GTGACGCTGACGGTACAA---GCCAGACTA---TTATTG---TCTGGTATAGTGCAACAGCAAAAC---
AATCTG---CTGAGG---GCTATTGAGGCGCAACAGCATCTGTTGCAACTCACAGTCTGGGGCATC
>AF025752
CTCTACTGTAATTCAACACAATTGTTTAATAGTACTTGGAAGTTTAATAGTACTTGGAATGATACTGAAGGGT
CAAT---

AACACTGAAGGAAATATCACACTCCCATGCAGAATAAAACAAATTATAAACATGTGGCAGGAAGTAGGAAAAG
CAATGTATGCCCCTCCCATCAGAGGACAAATTAGATGTTCATCAAAG---
ATTACAGGGCTGTTATTAACAAGAGATGGTGGTACTACCAAAAACGGGAAC----------------------
----

AACAACGATACTGAGATCTTTGGACCTGGAGGAGAAGATATGAAGGACAATTGGAGAAGTGAATTATATAAAT
ATAAAGTAGTAAAAATTGAACCATTA---GGAGTAGCACCCACTAAGGCAAAGAGAAGAGTGGTG--------
-------------------------------CAGAGAGAAAAAAGAGCA---GTGGGA---CTAGGAGCT---
------ATGTTCCTTGGGTTCTTGGGAGCA---GCAGGAAGCACTATG---GGCGCAGCGTCA---
ATGACGCTGACGGTACAG---GCCAGACAA---TTATTG---TCTGGTATAGTGCAACAGCAAAAC---
AATTTG---CTGAGG---GCTATTGATGCGCAACAGCATATGTTGCAACTCACAGTCTGGGGCATC
>AF025756
TTCTACTGTAATTCAACAAAACTGTTTAATAGAACTTGGAGTGTTAATAGTACTTGGAATGATACTGAAGGGG
TAAAT--

AACACTGGAGAAAATATCACACTCCCATGCAGAATAAAACAAATTATAAACATGTGGCAGGAAGTAGGAAAAG
CAATGTATGCCCCTCCCATCAAAGGACAAATTAGATGTTCATCAAAT---
ATTACAGGGCTGCTATTAACAAGAGATGGTGGTAATAGCAACAAC----------------------------

----

GAGACCGAGACTTTCAGACCTGGAGGAGGAGATATGAGGGACAATTGGAGAAGTGAATTATATAAATATAAAG
TAGTAAAAATTGAACCATTA---GGAGTAGCACCTACTAAGGCAAAGAGAAGAGTGGTG--------------
-------------------------CAAAGAGAAAAAAGAGCA---GTAGGA---ATAGGAGCT---------
GTGTTCCTTGGGTTCTTGGGAGCA---GCAGGAAGCACTATG---GGCGCAGCGTCA---
ATGACGCTGACGGTACAG---GCCAGACTA---TTATTG---TCTGGTATAGTGCAACAGCAGAAC---
AATTTG---CTGAGG---GCTATTGAGGCGCAACAGCATCTGTTGCAACTCACAGTCTGGGGCATC
>M93258
TTCTACTGTAATTCAACACAACTGTTTACTTGGAATGATACTAGAAAGTTAAATAAC----------------
----

ACTGGAAGAAATATCACACTCCCATGTAGAATAAAACAAATTATAAATATGTGGCAGGAAGTAGGAAAAGCAA
TGTATGCCCCTCCCATCAGAGGACAAATTAGATGTTCATCAAAT---
ATTACAGGGCTGCTATTAACAAGAGATGGTGGTAAGGACACGAAC----------------------------
----

GGGACTGAGATCTTCAGACCTGGAGGAGGAGATATGAGGGACAATTGGAGAAGTGAATTATATAAATATAAAG
TAGTAAAAATTGAACCATTA---GGAGTAGCACCCACCAAGGCAAAGAGAAGAGTGGTG--------------
-------------------------CAGAGAGAAAAAAGAGCA---GTGGGA---CTAGGAGCT---------
TTGTTCCTTGGGTTCTTGGGAGCA---GCAGGAAGCACTATG---GGCGCAGCGTCA---
ATAACGCTGACGGTACAG---GCCAGACAA---TTATTG---TCTGGTATAGTGCAACAGCAGAAC---
AATCTG---CTGAGG---GCTATTGAGGCGCAACAGCACCTGTTGCAACTCACAGTCTGGGGCATC
>AY835758
TTCTACTGTAACACAACAAAACTGTTTAATAGTACTTGGAATAATAGTACTTGGAATGCTACTGCAGGCCAAG
AATTA--
-------------------AAT---
AGCACTGAAGACACCATCACACTCCCATGCAAAATAAAACAAATTATAAACATGTGGCAGGAAGTAGGAAAAG
CAATGTATGCCCCTCCCATCAAAGGACTACTTAGATGTTCATCAAAT---
ATTACAGGGCTGCTATTAACAAGAGATGGTGGTAATAGCAATAACACG-------------------------
----

AACACGACCGAGATCTTCAGACCTGGGGGAGGAGATATGAGGGACAATTGGAGAAGTGAATTATATAAATATA
AAGTAGTAAAAATTGAACCACTA---GGAATAGCACCCACTAAGGCAAAGAGAAGAGTGGTG-----------
----------------------------CAGAGAGAAAAAAGAGCA---GTGGGA---ATAGGAGCT------
---TTGTTCCTTGGGTTCTTGGGAGCA---GCAGGAAGCACTATG---GGCGCAGCGTCA---
ATGACGCTGACGGTACAG---GCCAGACTA---TTATTG---TCTGGTATAGTGCAACAGCAGAAC---
AATTTG---CTGAGG---GCTATTGAGGCGCAACAGCACCTGTTGCAACTCACAGTCTGGGGCATC
>L02317
TTCTACTGTAAGTCAACACAACTGTTTAATAGTACTTGGGCTGGGAATAATACTTGGAATAGTAGTGCTGAAA
GGTCA--
-------------------GAT---
GACACTGGAGGAAATATCACACTCCCATGCAGAATAAAACAAATTATAAACATGTGGCAGGAAGTAGGAAAAG
CAATGTATGCCCCTCCCATCAGCGGACAAATTAGATGTACATCAAAT---
ATTACAGGGCTGCTATTAACAAGAGATGGTGGTACGAGCGATACC----------------------------
----

AATACCACCGAGACCTTCAGACCTGGAGGAGGAGATATGAGGGACAATTGGAGAAGTGAATTATATAAATATA
AAGTAGTAAAAATTGAACCATTA---GGAGTAGCACCCACCACGGCAAAGAGAAGAGTGGTG-----------
----------------------------CAAAGAGAAAAAAGAGCA---GTGGGA---ATAGGAGCT------
---CTGTTCCTTGGGTTCTTGGGAGCA---GCAGGAAGCACTATG---GGCGCAGCGTCA---
ATGACGTTGACGGTACAG---GCCAGACAA---TTATTG---TCTGGTATAGTGCAACAGCAGAAC---
AATTTG---CTGAGG---GCTATTGAGGCGCAACAGCATCTGTTGCAACTCACAGTCTGGGGCATC
>AF025755
TTCTACTGTAATTCAACAAAACTGTTTAATAGTACTTGGAAGTTTAATAATACTTGGAATATTACTGAAAGGT
CA---

AATAACACTGACACAATCACACTCCCATGTAGAATAAAACAAATTATAAACATGTGGCAGGGAGTAGGAAAAG
CAATGTATGCCCCTCCCATCAGTGGACTAATTAGCTGTTCATCAAAT---
ATTACAGGGCTGCTATTAACAAGAGATGGGGGTAATAACAATGGGACCGAGGGT-------------------

----
-
GATAATAACAACGAGACTGAGATTTTCAGACCTGGAGGAGGGAATATGAAGGACAATTGGAGAAGTGAATTAT
ATAAATATAAAGTAGTAAAAGTTGAACCATTA---GGAATAGCACCCACTAAGGCAAAGAGAAGAGTGGTG--
-------------------------------------CAAAGAGAAAAAGGAGCA---GTGGGA---
ATAGGAGCT---------GTGTTCCTTGGGTTCTTGGGAGCA---GCAGGAAGCACTATG---
GGCGCAGCGTCA---ATAACGCTGACGGTACAG---GCCAGACAA---TTATTG---
TCTGGTATAGTGCAACAGCAGAAC---AATTTG---CTGAGG---
GCTATTGAAGCGCAACAGCATCTCTTGCAACTCACAGTCTGGGGCATC
>AF025757
TTCTACTGTAATTCAACACCGCTGTTTAATAGTACTTGGAATAATAATAATTGGAGTGATCCTAAT-------
----

AACGATGTAAATATCACACTCCCATGCAGAATAAAACAAATTATAAACAGGTGGCAGGAAGTAGGAAAAGCAA
TGTATGCCCCTCCCATCAGTGGCCAAATTAGATGTTCATCAAAT---
ATTACAGGGCTGCTATTAACAAGAGATGGTGGTAATACCACGGAG----------------------------
----

GAGATCTTCAGACCTGGAGGAGGAGATATGAGGGACAATTGGAGAAGTGAATTATGTAAATATAAAGTAGTAA
AAATTGAGCCATTA---GGAGTAGCACCCACCAAGGCAAAGAGAAGAGTGGTG--------------------
-------------------CAGAGAGAAAAAAGAGCA---GTGGGAACGATAGGAGCT---------
ATGTTCCTTGGGTTCTTGGGAGCA---GCAGGAAGCACTATG---GGCGCAGCGTCA---
ATAACGCTGACGGTACAG---GCCAGACTA---TTATTG---TCCGGTATAGTGCAACAGCAGAAC---
AATTTG---CTGAGG---GCTATTGAGGCGCAACAGCATCTGTTGCAACTCACAGTCTGGGGCATC
>AY835763
TTCTACTGTAATTCAACAAAACTGTTTAATAGTACTTGGAAGTTTAATGGTACTACTTGGAATTGGACTACAG
GGTTA--
-------------------AAT---
AAAACTGAAGTAAATGTCACACTCCCATGCAGAATAAAACAAATTATAAACATGTGGCAGGAAGTAGGAAAAG
CAATGTATGCCCCTCCCATCAAAGGAGTAATTAGATGTTCATCAAAT---
ATTACAGGGCTGCTATTAACAAGAGATGGTGGTAGTAACAATGGTAGTAACAAT------------GGG----
----
ACCGGGATTAACAAT---
GAGACCGAGATCTTCAGACCTGGAGGAGGAAATATGAAGGACAATTGGAGAAGTGAATTATATAAATATAAAG
TAGTAAGAATTGAACCATTA---GGAATAGCACCCACCAAGGCAAAGAGAAGAGTGGTG--------------
-------------------------CAGAGAGAGAAAAGAACA---GTGGGA---TTAGGAGCC---------
TTGTTCCTTGGGTTCTTGGGAGCA---GCAGGAAGCACTATG---GGCGCAGCGTCA---
CTGACGCTGACGGTACAG---GCCAGACTA---TTATTG---TCTGGTATAGTGCAACAGCAGAAC---
AATTTG---CTGAGG---GCTATTGAGGCGCAACAGCGTATGTTGCAACTTACAGTCTGGGGCATC
>AF025762
TTCTACTGTAATTCAACACAACTGTTTAATAGTACTTGGACTGAATGGTCAAATAACACTGAAGGAAATATC-
----

ACTAAAGAAAATATTACACTCCCATGCAGAATAAAACAAATTATGAACATGTGCCAGGAAGTAGGAAGAGCAA
TGTATGCCCCTCCCATCAAAGGACAAATTAGATGTTCATCAAAA---
ATTACAGGGCTGCTATTAACAAGAGATGGTGGTACTGGCGGGAAC----------------------------
----

GGGTCCGAGTTCTTCAGACCTGGAGGAGGAGATATAAGGGACAATTGGAGAAGTGAATTATATAAATATAAAG
TAGTAAAAATTGAACCATTA---GGAGTAGCACCCACCAGGGCAAAGAGAAGAGTGGTG--------------
-------------------------CAAAGAGAAAAAAGAGCA---GTGGGA---ATAGGAGCT---------
GTGTTCCTTGGGTTCTTGGGAGCA---GCAGGAAGCACTATG---GGCGCAGCGTCA---
CTGACGCTGACGGTACAG---GCCAGACTA---TTATTG---TCTGGTATAGTGCAACAGCAGAAC---
AATTTG---CTGAGG---GCTATTGAGGCGCAACAGCATCTGTTGCAACTCACAGTCTGGGGCATC
>AF025754
TTCTACTGTAATTCAACACAACTGTTTAGTAGTACTTGGAATAATACTGAATCTGAAGGGGTAAATAACACTG
AA---

GGAAATGACACAATCATACTCCTATGCAGAATAAAACAAATTGTAAACATGTGGCAGGAAGTAGGAAAAGCAA
TGTATGCCCCTCCCATCAGAGGACAAATTAGATGCTCATCAAAT---

ATTACAGGGCTGCTATTAACAAGAGATGGTGGTAATAGTAATGGCAGC-------------------------
----

AACAAGGCCGAGGTCTTCAGACCTGGAGGAGGAGATATGAGGGACAATTGGAGAAGTGAATTATATAAATATA
AAATAGTAAAAATTGAACCATTA---GAAGTAGCACCCACTAAGGCAAAGAGAAGAGTGGTG-----------
----------------------------CAGAGAGAAAAAAGAGCA---GCGGGA---CTAGGAGTT------
---ATGTTCCTTGGGTTCTTGGGAGCA---GCAGGAAGCACTATG---GGCGCAGCGTCA---
ATGACGCTGACGGTACAG---GCCAGACAA---TTATTG---TCTGGTATAGTGCAACAGCAGAAC---
AATTTG---CTGAGG---GCTATTGAGGCGCAACAGCATCTGTTGCAACTCACAGTCTGGGGCATC
>AF286365
TTCTACTGTAATTCAACACAACTGTTTAATAGTACTTGGAATAGTACTGAAGGAAATAGTACTTGG-------
----

TCAGATAAAATTATCAGACTCCCATGCAGAATAAAACAAATTATAAACATGTGGCAGGAAGTAGGAAAGGCAA
TGTATGCCCCTCCCATCGATGGACAAATTAGATGTTCATCAAAT---
ATTACAGGGCTGCTATTAACAAGAGATGGTGGGAATAGCAACGAG----------------------------
----

ACCACCGAGATCTTCAGACCTGGGGGAGGAGATATGAGGGACAATTGGAGAAGTGAATTATACAAATATAAAG
TAGTAAAAATTGAACCATTA---GGAGTAGCACCCACCAAGGCAAAGAGAAGAGTGGTG--------------
-------------------------CAAAGAGAAAAAAGAGCA---GTGGGAGTAATAGGAGCT---------
ATGTTCCTTGGGTTCTTGGGAGCA---GCAGGAAGCACTATG---GGCGCAGCGTCA---
ATGGCGCTGACGGTACAA---GCCAGACAA---TTATTG---TCTGGTATAGTGCAGCAGCAGAAC---
AATCTG---CTGAGA---GCTATTGAGGCGCAACAACATATGTTGCAGCTCACAGTCTGGGGCATC
>U39362
TTCTACTGTAATACAGCACAACTGTTTAATAGTACTTGGAATGTTACTGGAGGGACAAATGGCACTGAA----
----

GGAAATGACATAATCACACTCCAATGCAGAATAAAACAAATTATAAATATGTGGCAGAAAGTAGGAAAAGCAA
TGTATGCCCCTCCCATCACAGGACAAATTAGATGTTCATCAAAT---
ATTACAGGGCTGCTACTAACAAGAGATGGAGGTAATAGTACTGAG----------------------------
----

ACTGAGACTGAGATCTTCAGACCTGGAGGAGGAGATATGAGGGACAATTGGAGAAGTGAATTATATAAATATA
AAGTAGTAAGAATTGAACCAATA---GGAGTAGCACCCACCAGGGCAAAGAGAAGAACAGTG-----------
----------------------------CAAAGAGAAAAAAGAGCA---GTGGGA---ATAGGAGCT------
---GTGTTCCTTGGGTTCTTGGGAGCA---GCAGGAAGCACTATG---GGCGCAGCGTCA---
GTGACGCTGACGGTACAG---GCCAGGCTA---TTATTG---TCTGGTATAGTGCAGCAGCAGAAC---
AATCTG---CTGAGG---GCTATTGAGGCGCAACAGCATATGTTGCAACTCACAGTCTGGGGCATC
>AF128126
TTCTACTGTAATACAACACAACTGTTTGATAGTATTTGGAATAGTGAGAATGGTACTTGGAATATTACTAGGG
GGTTAAAT---
-------------AACACT------
GGAAGAAATGACACAATCACACTCCCATGCAGGATAAAACAAATTATAAACAGGTGGCAGGAAGTAGGAAAAG
CAATGTATGCCCCTCCCATCAAAGGAAACATTAGCTGTTCATCAAAT---
ATTACAGGGCTGCTATTAACAAGAGATGGTGGTAAGGATGATAATAGC-------------------------
----

AGGGACGGGAACGAGACCTTCAGACCTGGAGGAGGAGATATGAGGGACAATTGGAGAAGTGAATTATATAAAT
ATAAAGTAGTAAAAATTGAACCATTA---GGAGTAGCACCCACCAAGGCAAAGAGAAGAGTGGTG--------
-------------------------------CAAAGAGAAGAAAGAGCA---GTGGGA---CTAGGAGCT---
------ATGTTCATTGGGTTCTTGGGAGCA---GCAGGAAGCACTATG---GGCGCAGCGTCA---
GTGACGCTGACGGTACAG---GCCAGGCAA---TTATTG---TCTGGTATAGTGCAACAGCAGAGC---
AATTTG---CTGAGA---GCTATTGAGGCGCAACAGCATCTGTTGCAACTCACAGTCTGGGGCATC
>AF025763
TTCTACTGTAATACAACACAACTATTTAATAGTACTTGGAATGTTGCTGAAGGGTCAAATAGCACTGAAGGA-
----

AATAGCACACTAATCGAACTCCCATGCAGAATAAAACAAATTATAAATCTGTGGCAGGAAGTAGGAAAAGCAA
TGTATGCCCCTCCCATCAGAGGACAAATTAGATGTTCGTCAAAT---

ATTACGGGGCTGCTATTAACAAGAGATGGTGGTAATAATAGCGAG----------------------------
----

AGCGCCCCCGAGATCTTCAGACCTGGAGGAGGAGATATGAGGGACAATTGGAGAAGTGAATTATATAAATATA
AAGTAGTACAAATTGAACCATTA---GGAGTAGCACCCACTAGGGCGAAGAGAAGAGTGGTG-----------
----------------------------CAAAGAGAAAAAAGAGCG------GCA---ATAGGAGCT------
---CTGTTCCTTGGGTTCTTGGGAGCA---GCAGGAAGCACTATG---GGCGCAGCGTCA---
ATAACGCTGACGGTACAG---GCCAGACTA---TTATTG---TCTGGTATAGTGCAACAGCAGAAC---
AATTTG---CTGAGA---GCTATTGAGGCGCAACAGCATCTGTTGCAACTCACAGTCTGGGGCATC
>MH012793
TTCTACTGCAATACAACACAACTGTTTAATAGTACTTGGGGGAATGGTACTGAGAGGTCAAATGAA-------
----

ACGGAAGATACTATCACACTCCCATGCAGAATAAAACAAATTGTAAACATGTGGCAGCAAGTAGGAAAAGCAA
TGTATGCCCCTCCCATCAGAGGACAAATTAGATGTTCATCAAAT---
ATTACAGGGCTGCTATTAACAAGGGATGGTGGTGATAAATTAACAAGGGATGGT-------------------
----
-
GGTGATAATGAGACCACCGAGATCTTCAGACCTGGAGGAGGAGATATGAAGGACAATTGGAGAAGTGAATTAT
ATAAATATAAAGTAGTAAAAATTGAACCATTA---GGAGTAGCACCCACCAGGGCAAAAAGAAGAGTGGTG--
-------------------------------------CAGAGAGAAAAAAGAGCA---
GTGGGAACGATAGGAGCT---------ATGTTCCTTGGGTTCTTGGGAGCA---GCAGGAAGCACTATG---
GGCGCAGCGTCA---CTGACGCTGACGGTACAG---GCCAGACTA---TTGTTG---
TCTGGTATAGTGCAACAGCAGAAC---AATTTG---CTGAGA---
GCTATTGAGGCGCAACAGCATCTGTTGCAACTCACAGTCTGGGGCATC
>EU576292
TTCTACTGTAATTCAACACAACTGTTTAATAGTACTTGGAGTTTTAATAGTACTTGGAATAAGAATTTTAATA
GTACTTGGAAT--
-------ATTACTGAAGGG------
ACAAATAACACTGAAATCACACTCCCATGTAGAATAAAACAAATTATAAACATGTGGCAGGAAGTAGGAAAAG
CAATGTATGCCCCTCCCATCAGAGGACAAATTAAATGTTCATCTAAC---
ATTACAGGGCTGCTATTAACAAGAGATGGTGGGGGTAATGGGAGC----------------------------
----

GGGACCACTGAGGTCTTCAGACCTGGAGGGGGAGATATGAGGGACAATTGGAGAAGTGAATTATACAAATATA
AAGTAGTAAAAATTGAACCATTA---GGAGTAGCACCTACCAAGGCAAAGAGAAGAGTGGTG-----------
----------------------------CAAGGAGAAAAAAGAGCA---GTGGGAGCAATAGGAGCT------
---ATGTTCCTTGGGTTCTTGGGAGCA---GCAGGAAGCACTATG---GGCGCAGCGTCA---
GTGGCGCTGACGGTACAG---GCCAGACAA---TTATTG---TCTGGTATAGTGCAACAGCAGAAC---
AATTTG---CTGAGG---GCTATTGAGGCGCAACAGCATCTGTTGCAACTCACAGTCTGGGGCATC
>KT283697
TTCTACTGTGATTCAACACAATTGTTTAATAGTACTTGGAATAGTACTTGGCTGAATGATACCGAAGGGTTAA
ATAAAACT---

GAAGGAGACCCTATTATACTCCAATGCAGAATAAAACAATTTATAAACATGTGGCAGGAAGTAGGAAAAGCAA
TGTATGCCCCTCCCATTGCAGGACCAATTAATTGTTCATCAAAT---
ATTACAGGACTGCTATTAACAAGAGATGGTGGCAATAACAATGACACT-------------------------
----

AGCAACACCGAGATTTTTAGACCTGGAGGAGGAGATATGAGGGACAATTGGAGAAGTGAATTATATAAATATA
AAGTAGTAAAAATTGAACCATTA---GGACTAGCACCTACCAAGGCAAAGAGAAGAGTGGTG-----------
----------------------------CAGAGAGAAAAAAGAGCA---GTGGGA---ATAGGAGCT------
---ATGTTCCTTGGGTTCTTGGGAGCA---GCAGGAAGCACTATG---GGCGCAGCGTCA---
GTGACGCTGACGGTACAG---GCCAGACTA---TTATTG---TCTGGTATAGTGCAACAGCAGAAC---
AATTTG---CTGAGG---GCTATTGAGGCGCAACAGCATCTGTTGCAACTCACAGTCTGGGGCATC
>AY173952
TTCTACTGTAATTCAACACAACTGTTTAATAGTACTTGGGGGGCTAATGATACTTGGACTGAGTCAAAT----
----

AACAATGAAACAATCACACTCCCATGCAGAATAAAACAAATTATAAACATGTGGCAGGAAGTAGGAAAAGCAA

TGTATGCCCCTCCCATCGCAGGACAAATTAGATGTTCATCAAAT---
ATTACAGGGCTGCTACTAACAAGAGATGGTGGTAATAAAAACGATAGC-------------------------
----

AACGGGACCGAGATCTTCAGACCTGGAGGAGGAGATATGAGGGACAATTGGAGAAGTGAATTATATAAATATA
AAGTAGTAAAAATTGAACCATTA---GGAATAGCACCCACCAAGGCAAAGAGAAGAGTGGTG-----------
----------------------------CAGAGAGAAAAAAGAGCA---GTAGGC---CTAGGAGCT------
---ATGTTCCTTGGGTTCTTGGGAGCA---GCAGGAAGCACTATG---GGCGCAGCGTCA---
ATAACGCTGACGGTACAG---GCCAGACAA---TTATTG---TCTGGTATAGTGCAACAGCAGAAC---
AATTTG---CTGAGG---GCTATTGAGGCGCAACAGCATCTGTTGCAACTCACAGTCTGGGGCATC
>AY173953
TTCTACTGTAATACAACAAATCTGTTTAATAGTACTTGGAATGGTACTGAAGAGTTTGATAGTACTTTGAGT-
----

GATGCTGAAAACATCACACTCCAATGCAGAATAAAACAAATTATAAACATGTGGCAGGAAGTAGGAAAAGCAA
TGTATGCCCCTCCCATCAGAGGACACATTAGATGTGTATCAAAC---
ATTACAGGGCTGTTATTAACAAGAGATGGGGGTATGAACAACGGGACCAAG----------------------
----

GACAACGAGACCGAGGTCTTCAGACCTGGAGGAGGAGATATGAGGGACAATTGGAGAAGTGAATTATATAAAT
ATAAAGTAGTAAAAATTGAACCATTA---GGAGTAGCACCCACCAAGGCAAAGAGAAGAGTGGTG--------
-------------------------------CAGAGAGAAAAAAGAGCA---GTGGGA---ATAGGAGCT---
------ATGTTCCTTGGGTTCTTGGGAGCA---GCAGGAAGCACTATG---GGCGCAGCGTCA---
CTGACGCTGACGGTACAG---GCCAGACAA---TTATTG---TCTGGTATAGTGCAACAGCAGAAC---
AATTTG---CTGAGG---GCTATTGAGGCGCAACAGCATCTGTTGCAACTCACAGTCTGGGGCATC
>AY173954
TTCTACTGTAATTCAACACAGCTGTTTAATAGTACTTGGATTAATAATAATGGTACTTGGAATAGTACTTGGA
GTAGTAATAAT--
-------------AATACT------
GATAACAATGGAACTATCACACTCCCATGCAGAATAAGACAAATTATAAACAGGTGGCAGGAAGTAGGAAAAG
CAATGTATGCCCCTCCCATCAGAGGACAAATTAGATGTTCATCAAAT---
ATTACAGGGCTGCTATTAACAAGAGATGGTGGTAATAACAATAACACG-------------------------
----

AACAGGACCGAGACCTTCAGACCTGGAGGAGGAGATATGAGGGACAATTGGAGAAGTGAATTATATAAGTATA
AAGTAGTACAAATTGAGCCATTA---GGAGTAGCACCCACCAGGGCAAAGAGAAGAGTGGTG-----------
----------------------------CAGAGAGAAAAAAGAGCA---GTGGGAACGATAGGAGCT------
---ATGTTCCTTGGGTTCTTGGGAGCA---GCAGGAAGCACTATG---GGCGCAGCGTCA---
ATGACGCTGACGGTACAG---GCCAGGCTA---TTATTG---TCTGGTATAGTGCAGCAGCAGAAC---
AATTTG---CTGAGG---GCTATTGAGGCACAACAGCATATGTTGCAACTCACAGTCTGGGGCATC
>U21135
TTCTACTGTAATTCAACACAGCTGTTTAATAGTACTTGGCATGCTAATGGTACTTGGAAGAATACTGAAGGG-
----

GCAGATAACAATATCACACTCCCATGCAGAATAAAACAAATTATAAACAGGTGGCAGGAAGTAGGAAAAGCAA
TGTATGCCCCACCCATCGAAGGACAAATTAGATGTTTATCAAAT---
ATTACAGGGTTACTATTAACAAGAGATGGTGGTAGTAGTGAAGAG----------------------------
----

AACCAGACCGAGATCTTCAGACCTGGAGGAGGAAATATGAAGGATAATTGGAGAAGTGAATTATATAAATATA
AAGTAGTAAAAATTGAACCATTA---GGAGTAGCACCCACCAAGGCAAAGAGAAGAGTGGTG-----------
----------------------------CAGAGAGAAAAAAGAGCA---GTGGGAATGCTAGGAGCT------
---ATGTTCCTTGGGTTCTTGGGAGCA---GCAGGAAGCACTATG---GGCGCAGCGTCA---
ATGACGCTGACGGTACAG---GCCAGACTA---TTATTG---TCTGGTATAGTGCAACAGCAGAAC---
AATCTG---CTGAGG---GCTATTGAGGCGCAACAGCATCTGTTCGAACTCACAGTCTGGGGCATC
>MH012589
TTCTACTGTAATACATCAAAACTGTTTAATAGTACTTGGAATGGTACTACAGAGTCAAATAAC----------
----

ATTGGAGAAAATATCACACTCCCATGCAGAATAAAACAAATTATAAACATGTGGCAGGAAGTAGGAAAAGCAA

TGTATGCTCCTCCCATCAGTGGACAAATTAGATGTTCATCAAAT---
ATTACAGGGCTGCTATTAACAAGAGATGGTGGTAATAACACAAAT----------------------------
----

AATATCGAGACCTTCAGACCTGGAGGAGGAGATATGAGGGACAATTGGAGAAGTGAATTATATAAATACAAAG
TAGTAAGAGTTGAACCATTA---GGACTAGCACCCACCAAGGCAAGGAGAAGAGTGGTG--------------
-------------------------CAGAGAGAAAAAAGAGCA---GTGGGA---ATAGGAGCT---------
GTGTTCCTGGGGTTCTTGGGAGCA---GCAGGAAGCACTATG---GGCGCAGCGTCA---
GTGACGCTGACGGTACAG---GCCAGACAA---TTATTG---TCTGGTATAGTGCAACAGCAGAAT---
AATCTG---CTGAGG---GCTATTGAGGCGCAACAGCATATGTTGCAACTCACAGTCTGGGGCATC
>MH012650
TTCTACTGTAATACAACACAACTGTTTAATAGTACTTGGAATAGTACTGAAACGTCAAATATCACT-------
----

GGAGAAGATATCATCACACTCCCATGCAGAATAAAACAAATTATAAACATGTGGCAGGAAGTAGGAAAAGCAA
TGTATGCCCCTCCCATCGCAGGACCAATTAGATGTACATCAAAT---
ATTACAGGGATGATACTAACAAGAGATGGAGGTAAAAACAACGGG----------------------------
----

AGCGATGAGGTCCTCAGACCTGGAGGAGGAGATATGAGGGACAATTGGAGAAGTGAATTATATAAATATAAAG
TAGTAAGAATTGAACCATTA---GGAGTAGCACCCTCCAGGGCAAAGAGAAGAGTGGTG--------------
-------------------------CAAAGAGAAAAAAGAGCA---GTGGGAGCGATAGGAGCT---------
ATGTTCCTTGGGTTCTTAGGAGCA---GCAGGAAGCACTATG---GGCGCAGCGGCA---
GTGACGCTGACGGTACAG---GCCAGACAG---CTACTG---TCTGGTATAGTGCAACAGCAGAAC---
AATTTG---CTGAGG---GCTATTGAGGCGCAACAGCATCTGTTGCAACTCACAGTCTGGGGCATC
>MH012866
TTCTACTGTAATTCAACACAACTGTTTAATAGTACTTGGGTTAATGGTACTTTGAATAGTACT----------
----

GGAGGAGAAAATATCACAATCCCATGCAGAATAAAACAAATTATAAACATGTGGCAGACAGTAGGAAAAGCAA
TGTATGCCCCTCCCATCAGAGGACAAATTAAATGTTCATCAAAT---
ATTACAGGGCTACTATTAACAAGAGATGGTGGTGAAAACGGGAGC----------------------------
----

AATACTGAGATCTTCAGACCTGGAGGAGGAGATATGAGGGACAATTGGAGAAGTGAATTATATAAATATAAAG
TAGTAAAAATTGAACCATTA---GGAGTAGCACCCACCAAGGCAAAGAGAAGAGTGGTG--------------
-------------------------CAAAGAGAAAAAAGAGCA---GTGGGA---ATAGGAGCT---------
TTGTTCCTTGGGTTCTTGGGAGCA---GCAGGAAGCACTATG---GGCGCAGCGTCA---
ATGACGCTGACGGTACAG---GCCAGACAA---TTATTG---TCTGGAATAGTGCAACAGCAAAAC---
AATTTG---CTGAGG---GCTATTGAGGCGCAACAGCATATATTGCAACTCACAGTCTGGGGCATC
>MH012925
TTCTACTGTAATACAACAAAACTGTTTAATAGTACTTGGACTTGGAATGGTACTGCTTGGACTTGGAATAGTA
CTGAAGGGTCA--

CCTGACAATGACACAATCATACTCCCATGCAGAATAAAACAAATTATAAACATGTGGCAGGAAGTAGGAAAAG
CAATGTATGCCCCTCCCATCAGGGGACAAATTAAATGTTCATCAAAT---
ATTACAGGGTTGCTATTAACAAGAGATGGTGGTGATAACAGGAAC----------------------------
----

AACACCACTGAGATCTTCAGACCAGGAGGAGGAGATATGAGGGACAATTGGAGAAGTGAATTATATAAATATA
AAGTAGTAAAAATTGAACCATTA---GGAGTAGCACCCACCAAGGCAAAGAGAAGAGTGGTG-----------
----------------------------CAGAGAGAAAAAAGAGCA---GTGGGA---ATAGGAGCT------
---TTGTTCCTTGGGTTCTTGGGAGCA---GCAGGAAGCACTATG---GGCGCAGCGTCA---
ATGACGCTGACGGTACAG---GCCAGACAA---TTATTG---TCTGGTATAGTGCAACAGCAGAAC---
AATCTG---CTGAGG---GCTATTGAGGCGCAACAACATCTGTTGCAACTCACAGTCTGGGGCATC
>AY835761
TTCTACTGTAATACAACAGCACTGTTTAATAGGACATTTAATAGGACTGAGAATGATAATGGTACCTGGAAT-
----

AGTACTGACCCTATCATACTCCAATGCAGGATAAAACAAATTGTAAACATGTGGCAGGAAGTAGGAAAAGCAA

TGTATGCCCCTCCCATCAAAGGACAAATTAGATGCTCATCAAAT---
ATTACAGGGCTGCTATTAACAAGAGATGGTGGTAATGGCAATGATACT-------------------------
----

AAAAATGATACCGAGATCTTCAGACCTGGAGGAGGAGATATGAGGGACAATTGGAGAAGTGAATTATATAAAT
ATAAAGTAGTAAGAATTGAACCATTA---GGAATAGCACCCACCAAGGCAAAGAGAAGAGTGGTG--------
-------------------------------CAGAGAGAAAAAAGAGCA---GTGGGA---ATAGGAGCT---
------GTGTTCCTTGGGTTCTTGGGAGCA---GCAGGAAGCACTATG---GGCGCAGCGTCA---
ATAACGCTGACGGTACAA---GCCAGACTA---TTATTG---TCTGGTATAGTGCAACAGCAGAAC---
AATTTG---CTGAGG---GCTATTGAGGCGCAACAGCATCTGTTGCAACTCACAGTCTGGGGCATC
>AF069140
TTCTACTGTAATACAAAAAAACTGTTTAATAGTACTTGGAATGGTACTGAAGGGTCATATAACATTGAA----
----

GGAAATGACACTATCACACTCCCATGCAGAATAAAACAAATTATAAACATGTGGCAGGAAGTAGGAAAAGCAA
TGTATGCCCCTCCCATCAGTGGACAAATTTGGTGCTCATCAAAT---
ATTACAGGGCTGCTACTAACAAGAGATGGTGGTAAGAACAGCAGC----------------------------
----

ACCGAAATCTTCAGACCTGGAGGAGGAGATATGAGGGACAATTGGAGAAGTGAATTATATAAATATAAAGTAG
TAAGAGTTGAACCATTA---GGAATAGCACCCACCAAGGCAAAGAGAAGAGTGGTG-----------------
----------------------CAGAGAGAAAAAAGAGCA---GTGGGA---ATAGGAGCT---------
GTGTTCCTTGGGTTCTTGGGAGCA---GCAGGAAGCACTATG---GGCGCAGCGTCA---
ATAACGCTGACGGTACAG---GCCAGACAA---TTATTG---TCTGGTATAGTGCAACAGCAGAAC---
AATTTG---CTGAGG---GCTATTGAAGCGCAACAGCATATGTTGCAACTCACAGTCTGGGGCATC
>GU331649
TTCTACTGTAATACAACACAGCTGTTTAATAGTACTTGGCCTTTTAATAGTACTTGGAATGATACTAACACTG
AA---

GGAAATGACACAATCACACTCCCATGTAGAATAAAACAATTTATAAACATGTGGCAGGAAGTAGGAAAAGCAA
TGTATGCCCCTCCCATCAGTGGACAAATTAGATGTTCATCAAAT---
ATTACAGGGCTGTTATTAACAAGAGATGGTGGTATAAACCAGAGC----------------------------
----

AGGACGAACGAGACCTTCAGACCTGGAGGAGGAAATATGAAGGACAATTGGAGAAGTGAATTGTACAAATATA
AAGTAGTAAAAATTAAACCATTA---GGAATAGCACCCACTAAGGCAAAGAGAAGAGTGGTG-----------
----------------------------CAGAGAGAAAAAAGAGCA---GTGGGA---ATAGGAGCT------
---CTGTTCCTTGGGTTCTTGGGAGCA---GCAGGAAGCACTATG---GGCGCAGCGTCA---
GTGACGCTGACGGTACAG---GCCAGACTA---TTATTG---TCTGGTATAGTGCAACAGCAGAAC---
AATTTG---CTGAGA---GCTATTGAGGCGCAACAGCATCTGTTGCAACTCACAGTCTGGGGCATC
>AY608577
TTCTACTGCAATTCAACACAACTGTTTAATGGTACTTGGAATGGTACTTGGTTAGATGTCAAACAA-------
----

GGAAATGGCACAATCACACTCCCATGCAGAATAAAACAAATTATAAACCTGTGGCAGGAAGTAGGAAAAGCAA
TGTATGCCCCTCCCATCAGCGGACAAATTAGATGTTCATCAAAT---
ATTACAGGGCTGTTATTAACAAGAGATGGTGGTACAAGCAATGAGACC-------------------------
----

ACCACCACCGAGACCTTCAGACCTGGAGGAGGAAATATGAAGGACAATTGGAGAAGTGAATTATATAGATATA
AAGTAATAAAAATTGAACCATTA---GGAGTAGCACCCACCAAGGCAAGGAGAAGAGTGGTG-----------
----------------------------CAGAGAGAAAAAAGAGCA---GTGGGA---ATAGGAGCT------
---GTGTTCCTTGGGTTCTTGGGAGCA---GCAGGAAGCACTATG---GGCGCAGCGTCA---
ATGACGCTGACGGTACAG---GCCAGACAA---TTATTG---TCTGGTATAGTGCAACAGCAGAAC---
AATTTG---CTGAGG---GCTATTGAGGCGCAACAGCAACTGTTGAAACTCACAGTCTGGGGCATC
>JN944928
TTCTATTGTAATACAACACAACTGTTTAATAGTACTTGGTATATTAATAATACTGGGAATGGTGCTAAAGGGT
CAGAT--

AACACTGACACTATCAAACTCCCATGCAGAATAAAACAAATTATAAACATGTGGCAGGAGGTAGGAAAAGCAA

TGTATGCCCCTCCCATCAGAGGACAAATTAGATGTTCCTCAAAT---
ATTACAGGACTGATATTAACAAGAGATGGGGTAATAACAATGATACC--------------------------
----

AACAATGATACCGAGGTCTTCAGACCTGGAGGAGGAGATATGAGAGACAATTGGAGAAGTGAATTATATAAAT
ATAAAGTAGTAAAAATTGAACCATTA---GGAGTAGCACCCACTAAGGCAAAGAGAAGAGTGGTG--------
-------------------------------CAGAGAGAAAAAAGAGCA---GTGGGA---ATAGGAGCC---
------TTGTTCCTTGGGTTCTTGGGAGCA---GCAGGAAGCACTATG---GGCGCAGCGTCA---
GTGACGCTGACGGTACAG---GCCAGACAA---TTGTTG---TCTGGTATAGTGCAACAGCAGAAC---
AATTTG---CTGAAG---GCTATTGAGGCGCAACAGCATCTGTTGCAACTCACAGTCTGGGGCATC
>MH013129
TTCTACTGTAATTCAACACAACTATTTAATAGTACTTGGGAGAGGGAAGAGTCAAATGACACTCGA-------
----

GGGAATGACACAATCACACTCCCATGCAGAATAAAACAAATTATAAACATGTGGCAGGAAGTAGGAAAAGCAA
TGTATGCCCCTCCCATCAGAGGACAAATTAGCTGTTCATCAAAT---
ATTACAGGGCTGCTATTAACAAGAGATGGTGGTAATGTGAGCGAC----------------------------
----

CCCGAGACCTTCAGACCTGGAGGAGGAGATATGAGGGACAATTGGAGAAGTGAATTATATAAATATAAAGTAG
TAAAAATTGAACCATTA---GGAGTAGCACCCACCAAGGCAAAGAGAAGAGTGGTG-----------------
----------------------CAGAGAGAGAGAAGAGCA---GTGGGC---CTAGGAGCT---------
ATGTTCCTTGGGTTCTTGGGAGCA---GCAGGAAGCACTATG---GGCGCAGCGTCA---
ATAACGCTGACGGTACAG---GCCAGACAA---TTATTG---TCTGGTATAGTGCAACAGCAGAAC---
AATCTG---CTGAGG---GCTATTGAGGCGCAACAGCATCTGTTGCAACTCACAGTCTGGGGCATC
>U04908
TTCTACTGTAATACAACACAGTTATTCAATAGTACTTGGTATCGGAATGGTACTTGGTATTGGAATGGTACTA
GGATTGAGAAT--
-------GGTACTGAAGGG------
TTAAATGACACAATTATCACCCTCCCATGCAGAATAAAACAAATTATAAACATGTGGCAGGAAGTAGGAAAAG
CAATGTATGCTCCTCCCATCAAAGGACAAATTAGCTGTTCATCAAAT---
ATTACAGGGCTGCTATTAACAAGAGATGGTGGTAATACTAATAAGACCGGG----------------------
----

ACCGAGAACGAGACCGAGATCTTTAGACCTGGAGGAGGAGATATGAGGGACAATTGGAGAAGTGAATTATATA
AATATAAAGTAGTAAAAATTGAACCATTA---GGAGTAGCACCCACCAGGGCAAAGAGAAGAGTGGTG-----
----------------------------------CAGAGAGAAAAAAGAGCA---GTGGGA---
ATAGGAGCT---------ATGCTCTTTGGGTTCTTGGGAGCA---GCAGGAAGCACTATG---
GGCGCAGCGTCA---ATAACGCTGACGGTACAA---GCCAGACAA---TTGTTG---
TCTGGTATAGTGCAACAGCAGAGT---AATCTG---CTGAGG---
GCTATTGAGGCGCAACAACATCTGTTGCAACTCACAGTCTGGGGCATC
>KT283706
TTCTACTGTAATACAACACAACTGTTTAATAGTACTTGGAATAGTACTGGAGGACTAAATAACACTGGA----
----

GGAAATGACACAATCACACTCCCATGCAGAATAAAACAAATTATAAACATGTGGCAGGAAGTAGGAAAAGCAA
TGTATGCCCCTCCCATCAGAGGACAAATTAGATGTTCATCAAAT---
ATTACAGGGCTGCTATTAACAAGAGATGGTGGTAATAATGGGACC----------------------------
----

AGCGGGGCCGAGGTCTTCAGACCTGGAGGAGGAGATATGAGGGACAATTGGAGAAGTGAATTATATAAATATA
AAGTAGTACAAATTGAACCATTA---GGAGTAGCACCCACCAAGGCAAAGAGAAGAGTGGTG-----------
----------------------------CAGAGAGAAAAAAGAGCA---GTGGGA---ATAGGAGCT------
---GTGTTCCTTGGGTTCTTGGGAGCA---GCAGGAAGCACTATG---GGCGCAGCGTCA---
ATGACGCTGACGGTACAG---GCCAGACTA---TTATTG---TCTGGTATAGTGCAACAGCAGAAC---
AATTTG---CTGAGA---GCTATTGAGGCGCAACAGCATCTGTTGCAACTCACAGTCTGGGGCATC
>MH012332
TTCTACTGTAATTCAACACAACTGTTTAATAGTACTTGGAATGGTACGGAAGGGTCAAATAACCCTGTA----
----

GACAATGGCACAATCACGCTCCCATGCAGAATAAAACAAATTGTAAACATGTGGCAGGAAGTAGGAAAAGCAA
TGTATGCCCCTCCCATTAGAGGACAAATTAACTGTTTATCAAAT---
ATTACAGGGCTGCTATTAACAAGAGATGGTGGTGATAACGGGAGC----------------------------
----

AATACCACCGAGACCTTCAGACCTGGAGGAGGAGATATGAGGGACAATTGGAGAAGTGAATTATATAAATATA
AAGTAGTAAAAATTGAACCATTA---GGAGTAGCACCTACCAAGGCAAAGAGAAGAGTGGTA-----------
----------------------------CAGAGAGAAAAAAGAGCA---GTGGGA---ATAGGAGCT------
---CTGTTCCTTGGGTTCTTGGGAGCA---GCAGGAAGCACTATG---GGCGCAGCGTCA---
TTGACGCTGACGGTACAG---GCCAGACTA---TTATTG---TCTGGTATAGTGCAACAGCAGAAC---
AATTTG---CTGAGG---GCTATTGAGGCGCAACAGCGTGTGTTGCAACTCACAGTCTGGGGCATC
>MH012400
TTCTATTGCAATACAACACAACTGTTT------
ACTTGGAATAGTAGTAGTCCTTGGAATATTACTGAAGGGGTAAATAGCACT----------------------
----
ATGGGAAATGACACACTCACACTCCCCTGCAAAATAAAACAAATTATAAACATGTGGCAGGAAGTAGGAAAAG
CAATGTATGCCCCTCCCATCAGAGGACAAATTAGATGTTCATCAAAT---
ATTACAGGGCTGCTATTAACAAGAGATGGTGGTAATGGAAACGAGAGC-------------------------
----

CAGGTCAATAATGAGACCTTCAGACCTGCAGGAGGAGATATGAGAGACAATTGGAGAAGTGAATTATATAAAT
ATAAAGTAGTAAAAATTGAACCATTA---GGAGTAGCACCCACCAGGGCAAAGAGAAGAGTGGTG--------
-------------------------------CAGAGAGAAAGAAGAGCA---GTGGGA---GTAGGAGCT---
------ATGTTCCTTGGGTTCTTGGGAGCA---GCAGGAAGCACTATG---GGCGCAGCGTCA---
CTGACGCTGACGGTACAA---GCCAGACTA---TTATTG---TCTGGTATAGTGCAACAGCAGAGC---
AATCTG---CTGAGG---GCTATTGAGGCGCAACAGCATCTGTTGCGACTCACAGTCTGGGGCATC
>MH012257
TTCTACTGTAATTCAACACAACTGTTTAATAGTACTTGGAATGGTACTGAAGGGTCAAATAACACTGAA----
----

GGAAATGATACAATCATACTCCCATGCAGAATAAAACAAATTATAAACATGTGGCAGGAAGTAGGAAAAGCAA
TGTATGCCCCTCCCATCAAAGGACAAATTAGATGTTCATCAAAT---
ATCACAGGGCTGCTATTAACAAGAGATGGTGGTAATAGCAGCGGG----------------------------
----

ACCACCGAGTTCTTCAGACCTGGGGGAGGAGATATGAGGGACAATTGGAGAAGTGAATTATATAAATATAAGG
TAGTAAAAATTGAACCATTA---GGAGTAGCACCCACCAGGGCAAAAAGAAGAGTGGTG--------------
-------------------------CAGAGAGAAAGGAGAGCA---GTGGGA---CTAGGAGCT---------
GTGTTCCTTGGGTTCTTAGGAGCA---GCAGGAAGCACTATG---GGCGCAGCGTCA---
GTGACGCTGACGGTACAG---GCCAGACAA---TTATTG---TCTGGTATAGTGCAGCAGCAGAAC---
AATTTG---CTGAAG---GCTATTGAGGCGCAACACCATCTGTTGCAACTCACAGTCTGGGGCATC
>MH012478
TTCTACTGTAATACAACAAAACTGTTTAATAGTACTTGGTATGGGAATGGTACTAAAGTGTCAACTAACACGG
AA---

GGAAATGGCACAATCATACTCCCATGCAGAATAAAACAACTTATAAACATGTGGCAGGAAACAGGAAAAGCAA
TGTATGCCCCTCCCATTAGAGGACAAATTAGATGTTCATCAAAT---
ATTACAGGGGTGCTATTAACAAGAGATGGGGGTAACAATGATGGGACT-------------------------
----

GGGACTGAAACCTTCAGACCTGGAGGAGGAGATATGAGGGATAATTGGAGAAGTGAATTATATAAATATAAAG
TAGTAAAAATTGAACCATTA---GGAGTAGCACCCACCAAGGCAAAGAGGAGAGTGGTG--------------
-------------------------CAGAGAGAAAAAAGAGCA---GTGGGA---ATAGGAGCT---------
GTGTTCCTTGGGTTCTTGGGAGCA---GCAGGAAGCACTATG---GGCGCAGCGTCA---
GTGACGCTGACGGTACAG---GCCAGACTA---TTATTG---TCTGGTATAGTGCAGCAGCAGAAT---
AATTTG---CTGAGG---GCTATTGAGGCGCAACAGCATCTGTTGCAACTCACAGTCTGGGGCATC
>MH013027
TTCTACTGTAATACAACAGACCTGTTTAGTAGTACTTGGAATGACACACAACTGAATAATACTGGGAGTGAAG
GGTCAAAT---
-------------AGTACT------

GGAAAAAATGACACAATCACACTCCGATGCAGAATAAAACAAATTATAAACATGTGGCAAGAAGTAGGAAAAG
CAATGTATGCCCCTCCCATCAAAGGACAAATTAGATGTGTATCAAAT---
ATTACAGGGCTGCTATTAACAAGAGATGGTGGTAGTAGTAACGAGACT-------------------------
----

AACGGGACCGAGATCTTCAGACCTGGAGGAGGAGATATGAGAGACAATTGGAGAAGTGAATTATATAAATATA
AAGTAGTACAAATTGAACCAGTA---GGAATAGCACCCACCAAGGCAAGGAGAAGAGTGGTG-----------
----------------------------CAGAGAGAAAAAAGAGCA---GTGGGA---ATAGGAGCT------
---CTGTTCCTTGGGTTCTTGGGAGCA---GCAGGAAGCACTATG---GGCGCAGCGTCA---
GTGACGCTGACGGTACAA---GCCAGACAA---TTATTG---TCTGGTATAGTGCAACAGCAGAAC---
AATCTG---CTAAGG---GCTATTGAGGCGCAACAGCATCTGTTGCAACTCACAGTCTGGGGCATC
>MH013075
TTCTACTGTAATACAACAAAACTGTTTAATAGTACTTGGAATTCAACACAACTTAGTAGTACTGAGAATAGTA
CCGAA--

GGGTCAGAGAACATCACACTCCCATGCAGCATAAAACAAATTATAAACATGTGGCAGAAAGTAGGAAAAGCAA
TGTATGCCCCGCCCATCGAAGGACAAATTAGATGTTCATCAAAT---
ATTACAGGGCTGCTATTAACAAGAGATGGTGGTGGTGGGAATGCGAGCAATGAG-------------------
----

ACCACCGAGACTTTCAGGCCTGGAGGAGGAGATATGAGAGAAAATTGGAGAAGTGAATTATATAAATATAAAG
TAGTAAGAATTGAACCATTA---GGAGTAGCACCCTCCACGGCAAAGAGAAGAGTGGTG--------------
-------------------------CAGAGAGAAAAAAGAGCA---GTGGGA---ATAGGAGCA------
TTGTTGTTCCTTGGGTTCTTGGGAGCA---GCAGGAAGCACTATG---GGCGCAGCGTCA---
ATGATGCTGACGGTACAG---GCCAGACTA---TTATTG---TCTGGTATAGTGCAACAGCAGAAC---
AATATG---CTGAGG---GCTATTGAGGCGCAACAGCATCTGTTGCAACTCACAGTCTGGGGCATC
>DQ487188
TTCTACTGTGATACAACACGACTGTTTAATAGTACTTGGAATAATACTTGGACTGGGAATGATACTTGGTCAA
ATAAC--
-------------------ACT---
AGAAGAAATGATACAATCACACTCCCATGCAGAATAAAACAAATTATAAACATGTGGCAGGAAGTAGGAAAAG
CAATGTATGCCCCTCCCATCAGAGGAAACATTACCTGTTCATCAAAT---
ATTACAGGGCTAATATTAACAAGAGATGGTGGTGATAACAATAGCACG-------------------------
----

AACGGGACTGAGACCTTCAGACCTGGAGGAGGAGATATGAGGGACAATTGGAGAAGTGAATTATATAAATATA
AAGTAGTACAAATTGAACCATTA---GGAGTAGCACCCACCAAGGCAAAGAGAAGAGTGGTG-----------
----------------------------CAAAGAGAAAAAAGAGCA---GTGGGC---ATAGGAGCT------
---GTGTTCCTTGGGTTCTTGGGAGCA---GCAGGAAGCACTATG---GGCGCAGCGTCA---
ATAACGCTGACGGTACAG---GCCAGACAA---TTATTG---TCTGGTATAGTGCAACAGCAGAAC---
AATTTG---CTGAGG---GCTATTGAGGCGCAACAACATCTGTTGCAACTCACAGTCTGGGGCATC
>DQ487190
TTCTACTGTGATGCAACACAACTGTTTAATAGTACTTGGAATAGTACTGAAGATTGGAATAGTACTGAAGATT
CAACTAAC---
-------------------GCT---
GATGGAAATAACACAATCATACTCCCATGCAGAATAAAACAGATTGTAAACATGTGGCAGGAAGTAGGAAAAG
CAATGTATGCCCCTCCCATCAGAGGACAGATTAGATGTTCATCAAAT---
ATTACAGGGCTGCTATTAACAAGAGATGGTGGTAACGGCACCAAT----------------------------
----

AACGACAATGAGACCTTCAGACCTGGGGGAGGAGATATGAGGGACAATTGGAGAAGTGAATTATATAAATATA
AAGTAGTAAAAATTGAACCATTA---GGAGTAGCACCCACCAAGGCAAAGAGAAGAGTGGTG-----------
----------------------------CAGAGAGAGAAAAGAGCG------GGC---ATAGGAGCT------
---TTGTTCCTTGGGTTCTTGGGAGCA---GCAGGAAGCACTATG---GGCGCAGCGTCC---
ATGACGCTGACGGTACAG---GCCAGACAA---TTATTG---TCTGGTATAGTGCAACAGCAGAAT---
AATTTG---CTGAGG---GCTATTGAGGCGCAACAGCATCTGTTGCAACTCACAGTCTGGGGCATC
>AY612855
TTCTACTGTAATACAACACAGCTGTTTAATAGTACTTGGAATGAGAATGATACTACCTGGAATAATACAGCAG
GGTCA--

```
AATAACAATGAAACTATCACACTCCCATGTAGAATAAAACAAATTATAAACAGGTGGCAGGAAGTAGGAAAAG
CAATGTATGCCCCTCCCATCAGTGGACCAATTAATTGTTTATCAAAT---
ATCACAGGGCTATTATTAACAAGAGATGGTGGTGACAACAATAAT----------------------------
-------------------------------------------------------------------------
-------------
ACAATAGAGACCTTCAGACCTGGAGGAGGAGATATGAGGGACAATTGGAGAAGTGAATTATATAAATATAAAG
TAGTAAGAATTGAGCCATTA---GGAATAGCACCCACCAAGGCAAAGAGAAGAGTGGTG--------------
-------------------------CAAAGAGAAAAAAGAGCA---GTGGGA---ATAGGAGCT---------
ATGTTCCTTGGGTTCTTGGGAGCA---GCAGGAAGCACTATG---GGCGCAGCGTCA---
GTGACGCTGACGGTACAG---GCCAGACTA---TTATTG---TCTGGTATAGTGCAACAGCAAAAC---
AATTTG---CTGAGA---GCTATCGAGGCGCAACAGCATCTGTTGCAACTCACAGTCTGGGGCATC
>AY835437
TTCTACTGTAATTCAACACAGCTGTTTAATAGTACTTGGACGCGGCCGCCTAATGGTACTTGGCGGTCTAATG
GTACTTGGAAT--------------------------------------------------------------
----------AGTACTGAA------
GGGACAAATGACACTATCACACTCCCATGTAGAATAAAACAAATTATAAACATGTGGCAGGGAGTAGGAAAAG
CAATGTATGCCCCTCCCATCAGTGGACAAATTAGATGTACATCAAAT---
ATCACAGGGCTGCTATTAACAAGAGATGGTGGTGAGAACAGGACAGAT-------------------------
-------------------------------------------------------------------------
----------
AACGGACAGAGATATTCAGACCTGGAGGAGGAAATATGAGGGACAACTGGAGAAGTGAATTATATAAATATA
AAGTAGTAAAAATTGAGCCATTA---GGAGTAGCACCCACCAAGGCAAAGAGAAGAGTGGTG-----------
----------------------------CAAAGAGAAAAAAGAGCA---GTGGGAACGATAGGAGCT------
---ATGTTCCTTGGGTTCTTGGGAGCA---GCAGGAAGCACTATG---GGCGCAGCGTCA---
ATAACGCTGACGGTACAG---GCCAGACTA---TTATTG---TCTGGTATAGTGCAACAGCAGAAC---
AATTTG---CTGAGG---GCTATTGAGGCGCAACAGCATATGTTGCAACTCACAGTCTGGGGCATC
>KT283729
TTCTACTGTAATACAACACAACTGTTTAATAGTACTTGGGCTTGGAATAGTACTGAAGGGTCAAATAGCACTG
GA-----------------------------------------------------------------------
----------------------------
GAAAATGGCACTATCACACTCCAATGCAGAATAAAACAAATTATAAATATGTGGCAGGAAGTAGGGAAAGCAA
TGTATGCCCCTCCCATCAGAGGACAAATTAAATGTTCATCAAAT---
ATTACAGGGCTGATATTAACAAGAGATGGAGGTAATAATAATGGT----------------------------
-------------------------------------------------------------------------
----
ACGAACAATACCATAGAGATCTTCAGACCTGGAGGAGGAGATATGAGAGAAAATTGGAGAAGTGAATTATATA
AATATAAAGTAGTAAAAATTGAACCATTA---GGAGTAGCACCCACCAGGGCAAAGAGAAGAGTGGTG-----
----------------------------------CAGAGAGAAAAAAGAGCA---
GTGGGAACAATAGGAGCT---------ATGTTCCTTGGGTTCTTGGGAGCA---GCAGGAAGCACTATG---
GGCGCAGCGTCA---ATGACGCTGACGGTACAG---GCCAGACAA---TTATTG---
TCTGGTATAGTGCAACAGCAGAAC---AACTTG---CTGAAA---
GCTATTGAGGCGCAACAGCACCTGTTGCAACTCACGGTCTGGGGCATC
>DQ313246
TTCTACTGTAATTCATCACAACTGTTTAATAGTACTTGGAATACTAATAGTACTTGGAGTGATACTAGAGGG-
-------------------------------------------------------------------------
----------------------------
TCAAATGACACTATCACACTCCCATGTAGAATAAAACAAATTATAAACATGTGGCAGGAAGTGGGAAAAGCAA
TGTATGCCCCTCCCATCAGAGGACAAATTAGCTGTTCATCAAAT---
ATTACAGGGCTGCTATTAACAAGAGATGGTGGGAACGAGAGCAAG----------------------------
-------------------------------------------------------------------------
----------
CCCGACACCGAGACCTTCAGACCTGGAGGAGGAGATATGAGGGACAATTGGAGAAGTGAATTATATAAATATA
AAGTAGTAAAGGTTGAACCATTA---GGAGTAGCACCCACCAGGGCAAGGAGAAGAGTGGTG-----------
----------------------------CAAAGAGAAAAAAGAGCA---GTGGGAACGTTAGGAGCT------
---GTGTTCCTTGGGTTCTTGGGAGCA---GCAGGAAGCACTATG---GGCGCAGCGTCA---
ATGACGCTGACGGTACAG---GCCAGACAA---TTGTTG---TCTGGTATAGTGCAACAGCAGAAC---
AATTTG---CTGAGA---GCTATTGAGGCGCAACAGCATATGTTGCAACTCACAGTCTGGGGCATC
>EU289197
TTCTACTGTGATTCATCACCACTGTTTAATAGTACTTGGGAGAATGATACAGAAGTGTTAAATAACACTGTG-
-------------------------------------------------------------------------
```

GGAAATGAAACAATCACGCTCCCATGCAAAATAAAACAAATTATAAACTTGTGGCAGGAAGTAGGGAAAGCAA
TGTATGCCCCTCCCATCAGAGGACAAATTAAATGTTCATCAAAT---
ATTACAGGGCTACTATTAACAAGAGATGGTGGTACTAACGGGGAC----------------------------
----

AATACCACTGAGACCTTCAGACCTGGAGGAGGAGATATGAGGGACAATTGGAGAAGTGAATTATATAAATATA
AAGTAGTAAAGATTGAACCATTA---GGAGTAGCACCCACCAAGGCAAAGAGAAGAGTGGTG-----------
----------------------------CAAAGAGAAAAAAGAGCA---GTGGGA---TTAGGAGCT------
---CTGTTCCTTGGGTTCTTGGGAGCA---GCAGGAAGCACTATG---GGCGCAGCGTCA---
GTGACGCTGACGGTACAG---GCCAGACAA---TTACTG---TCTGGTATAGTGCAACAGCAGAAC---
AATCTG---CTGAGG---GCTATTGAAGCGCAACAGCATCTGTTGCAACTCACAGTCTGGGGCATC
>JQ609870
TTCTACTGCAATACATCACAACTGTTTAATAGTACTTGGCTTAATAAAACTTGGACTGGGTCAAAT-------
----

GACACTGAAAATATCACACTCCCATGCAGAATAAAACAAATTATAAACATGTGGCAGGAAGTAGGAAAAGCAA
TGTATGCCCCTCCCATCAGCGGACAAATTAGATGTTCATCAAAT---
ATTACAGGGCTGCTATTAATAAGAGATGGTGGTAAGACAAACCAGAGT-------------------------
----

AACACGACCGAGACCTTCAGACCTGGAGGAGGAGATATGAGGGACAACTGGAGAAGTGAATTATATAAATATA
AAGTAGTAAGAATTGAACCAATA---GGAGTAGCACCCACCAAGGCAAAGAGAAGAGTGGTG-----------
----------------------------CAGAGAGAAAAAAGA------GTAGCA---CTAGGAGCT------
---ATGTTCCTTGGGTTCTTGGGAGCA---GCAGGAAGCACTATG---GGCGCAGCGTCA---
GTGACGCTGACGGTACAG---GCCAGACTA---TTATTG---TCTGGTATAGTGCAACAGCAGAAC---
AATTTG---CTGAGG---GCTATTGAGGCGCAACAGCGTCTGTTGCAACTCACAGTCTGGGGCATC
>AY835436
TTCTACTGTAATTCAACACAGCTGTTTAATAGTACTTGGAGTGTTAATAGTACTGGGAATGATACTACT----
----

GAAGAGCCGAATATCACATTCCCATGCAGAATAAAACAAATTATAAACATGTGGCAGGAAGTAGGAAAAGCAA
TGTATAACCCTCCCATTAAAGGAATAATTAGTTGTAGATCAAAT---
ATTACAGGGCTGTTATTAACAAGAGATGGTGGTAATAACAACAACACC-------------------------
----

AACACCACCGAAACCTTCAGACCTGAAGGAGGAAATATGAAGGACAATTGGAGAAGTGAATTATATAAATATA
AAGTAGTAAAAATTGAACCATTA---GGAGTAGCACCCACCAAGGCAAAGAGAAGAGTGGTG-----------
----------------------------CAAAGAGAGAAAAGAGCA---GTAGGA---ATAGGAGCT------
---GTGTTCCTTGGGTTCTTGGGAGCA---GCAGGAAGCACTATG---GGCGCAGCGTCA---
GTGACGCTGACGGTACAA---GCCAGACAA---TTATTG---TCTGGTATAGTGCAACAGCAGAAC---
AATCTG---CTGAGG---GCTATTGAGGCGCAACAGCATATGTTGCAACTCACAGTCTGGGGCATC
>AY835768
TTCTACTGTAATACAACAAAACTGTTTAATAGTACTTGGATGAATAATGGTACTTGGATTGGTACTAACGAG-
----

TCAGATAGCAACATCACGCTCCCATGCAGAATAAAACAAATTATAAACATGTGGCAGGAAGTAGGAAAAGCAA
TGTATGCCCCTCCCATCGAAGAACAAATTAGTTGTTTATCAAAC---
ATTACAGGGCTGCTATTAACAAGGGATGGTGGTGGGAACGAGAGCAAG-------------------------
----

AACGGGAGCGAGACCTTCAGACCTGGAGGAGGAGATATGAGGGACAATTGGAGAAGTGAATTATATAAATATA
AAGTAGTAAAAATTGAACCATTA---GGAGTAGCACCCACTGAGGCAAAGAGAAGAGTGGTG-----------
----------------------------CAGAGAGAAAAAAGAGCA---GTGGGA---ATAGGAGCT------
---TTGTTCCTTGGGTTCTTGGGAGCA---GCAGGAAGCACTATG---GGCGCAGCGGCA---
ATGACGCTGACGGTACAG---GCCAGACAA---TTATTG---ACTGGTATAGTGCAACAGCAGAAC---
AATTTG---CTGAGG---GCTATTGAGGCGCAACAGCATCTGTTGCAACTCACAGTCTGGGGCATC
>EU289190
TTCTACTGTGATACAACACAGCTGTTTAATAGTACTTGGTCGTCTAATGATACTTGGAATAGTACTGGAGTG-
----

CAAGATAACAATATCACACTCCCATGCAGAATAAAACAAATTATAAACATGTGGCAGGAAGTAGGAAAAGCAA
TGTATGCCCCTCCCATCCAAGGACTAATTAGCTGTTCATCAAAT---
ATTACAGGGCTGCTATTAACAAGAGATGGTGGTACTAACAATACC----------------------------
----

AATGCCACCGAGATCTTCAGACCAGGAGGAGGAGATATGAGGGACAATTGGAGAAGTGAATTATATAAATATA
AAGTAGTAAAAATTGAACCATTA---GGAATAGCACCCACCAAGGCAAAGAGAAGAGTGGTG-----------
----------------------------CAAAGAGAAAAAAGAGCA---GTGGGA---CTAGGAGCT------
---GTGTTCATTGGGTTCTTGGGAGCA---GCCGGAAGCACTATG---GGCGCAGCGTCC---
GTGACGCTGACGGTACAG---GCCAGACAA---TTACTG---TCTGGTATAGTGCAACAGCAGAAC---
AATCTG---CTGAGA---GCTATTGAGGCGCAACAACATATGTTGCAACTCACAGTCTGGGGCATC
>AY835438
TTTTACTGTGATACATCACAACTGTTTAATAGTACTTGGAATTCTAATAGTACTTGGAATGATACTAACAATA
AT---

AATAGCACTGAAAAGATTATACTCTCATGTAGAATAAGACAAATTATAAACAGGTGGCAGGAAGTAGGAAAAG
CAATGTATGCCCCTCCCATCAGCGGACCAATTAAGTGTTCATCAAAT---
ATTACAGGGCTGCTATTAGCAAGAGATGGTGGAAATGAGACTAAT----------------------------
----

GTGACCGAGACCTTCAGACCTGCAGGAGGAGATATGAGGGACAATTGGAGAAGTGAATTATATAAATATAAAG
TAGTACAAATTGAACCATTA---GGAATAGCACCCACCAAGGCAAAGAGAAGAGTGGTG--------------
-------------------------CAGAGAGAAAAAAGAGCA---GTGGGAATGCTAGGAGCT---------
ATGTTCCTTGGGTTCTTGGGAGCA---GCAGGAAGCACTATA---GGCGCAGCGTCA---
ATGACGCTGACGGTACAG---GCCAGACAA---TTATTG---TCTGGCATAGTGCAACAGCAAAAC---
AATTTG---CTGAGG---GCTATTGAGGCGCAACAACATATGTTGCAACTCACAGTCTGGGGCATC
>KT283753
TTCTACTGTAATACAACACAACTGTTTAACAGTACTTGGAATGTTACTGATACTGAAGTGTCAAATAACACTA
AT---

GGAAATGACACAATCACACTCCCATGCAGAATAAAACAAATTATAAACATGTGGCAGGAAGTAGGAAAAGCAA
TGTATGCCCCTCCCAGAGAAGGGCAAATTAAATGTTCATCAAAT---
ATTACAGGGCTACTATTAACAAGAGATGGTGGTAATAGTAATAATACT-------------------------
----

GACAATGGAACCGAGGTCTTCAGACCTGGAGGAGGAAACATGAAGGACAATTGGAGAAGTGAATTATATAAAT
ATAAAGTAGTAAGAATTGAACCATTA---GGGATAGCACCCACCAAGGCAAAGAGAAGAGTGGTG--------
-------------------------------CAGAGAGAAAAAAGAGCA---GTGGGA---TTCGGAGCT---
------GTGTTCCTTGGGTTCTTGGGAGCA---GCAGGAAGCACTATG---GGCGCAGCGTCA---
ATAACGCTGACGGTACAG---GCCAGACAA---TTATTG---TCTGGTATAGTGCAACAGCAAAAC---
AATTTG---CTGAGG---GCTATTGAGGCGCAACAGCATCTGTTGCAACTCACAGTCTGGGGCATC
>EU289198
TTCTACTGTAATACAACACAACTGTTTAATAGTACTTGGCCTTGGAATGTTACTGAAGAGTCAAAT-------
----

AGCACTGACAATATCACACTCCCATGCAGAATAAAACAAATTGTAAACATGTGGCAGGAAGTAGGAAAAGCAA
TGTATGCCCCTCCCATCAGAGGACCAATTAGATGTTCATCAAAT---
ATTACAGGTCTGCTATTAACAAGGGATGGTGGGAATACCATAAAC----------------------------
----

GGAACTGAGATCTTCAGACCTGGAGGAGGAGATATGAGGGACAATTGGAGAAGTGAATTATATAAATATAAAG
TAGTAAAAATTGAACCATTA---GGAGTAGCACCCACCAAGGCAAAGAGAAGAGTGGTG--------------
-------------------------CAGAGAGAAAAAAGAGCA---GTGGGA---ATAGGAGCT---------
ATGTTCCTTGGGTTCTTGGGAGCA---GCAGGAAGCACTATG---GGCGCAGCGTCA---
ATAACGCTGACGGTACAG---GCCAGACAA---TTATTG---TCTGGTATAGTGCAACAGCAGAAC---
AATTTG---CTGAAG---GCTATTGAGGCGCAACAGCATCTGTTGCAACTCACAGTCTGGGGCATC
>KU678164
TTCTATTGTAATACAACACAACTGTTTAATAGTACTTGGTATGCTAATAGTACTTGGAATAGCACTGAAGGAA
ATAAC--

```
----------------------------
ACTGACATACCTATCACACTTCCATGCAGAATAAAGCAAATTATAAACATGTGGCAAGAAGTAGGAAAAGCAA
TGTATGCCCCTCCAATCTCAGGAAAGATTAGATGTTCATCAAAT---
ATTACAGGGATGCTATTAACAAGAGATGGTGGTAATAATAGGAACAGT-------------------------
-------------------------------------------------------------------------
----------
AGTAATCCCGAGATCTTCAGACCTGGAGGAGGAGATATGAGGGACAATTGGAGAAGTGAATTATATAAATATA
AAGTAGTAAAAATTGAACCATTA---GGAGTAGCACCCACCAAGGCAAAAAGAAGGGTGGTG-----------
----------------------------CAGAGAGAAAAAAGAGCA---GTGGGA---ATAGGAGCT------
---TTGTTCCTTGGGTTCTTGGGAGCA---GCAGGAAGCACTATG---GGCGCAGCGTCA---
ATGACGCTGACGGTACAG---GCCAGACAA---TTATTG---TCTGGTATAGTGCAACAGCAGAAC---
AATTTG---CTGAGA---GCTATTGAGGTGCAACAGCACCTGTTGCAACTCACAGTCTGGGGCATC
>JN024303
TTCTACTGTAATACAACAAGGCTGTTTAATTGGAGTGATACTGAAAGTGGGAATGGTACTGAAGGG-------
-------------------------------------------------------------------------
----------------------------
AAAAATGACACAATTATACTCCCATGCAAAATAAAACAAATTATAAACCTGTGGCAAGAAGTAGGAAAAGCAA
TGTATGCCCCTCCCATCAGCGGACCAATTAGATGCTCATCAAAT---
ATTACAGGGCTACTATTAACAAGAGATGGTGGTAGTAGTAATACG----------------------------
-------------------------------------------------------------------------
-------------
ACCACCGAGATCTTCAGACCTGGAGGAGGAGATATGAGGGACAATTGGAGAAGTGAATTATATAAATATAAAG
TAGTAAAAATTGAACCATTA---GGAGTAGCACCCACCGAGGCAAAGAGAAGAGTGGTG--------------
-------------------------CAGAGAGAAAAAAGAGCA---GTGGGA---TTTGGAGCT---------
TTCTTCCTTGGGTTCTTGGGAGCA---GCAGGAAGCACTATG---GGCGCAGCGTCA---
ATAACGCTGACGGCACAG---GCCAGACTA---TTATTG---ACTGGTATAGTGCAACAGCAGAAC---
AATCTG---CTGAGG---GCTATTGAGGCGCAACAGCATCTGTTGCAACTCACAGTCTGGGGCATC
>AY332237
TTCTATTGTAATACAACAAAACTGTTTAATAGTACTTGGAATCTGACTAGTGCTGAAGGGTTAAATGACACTG
AA-----------------------------------------------------------------------
----------------------------
GGAAATGACACACTCACACTCCCATGCAGAATAAAACAAATTATAAACATGTGGCAGGAAGTAGGAAAAGCAA
TGTATGCCCCTCCCATCAGAGGACAAATTAGATGTTCATCAAAT---
ATTACAGGGCTGCTATTAACAAGAGATGGTGGTAGAGAGAATGGG----------------------------
-------------------------------------------------------------------------
-------------
GACACTGAGACCTTCAGACCTGGAGGAGGAGATATGAGGGACAATTGGAGAAGTGAATTATATAAATATAAAG
TAGTAAAAATTAAACCATTA---GGAATAGCACCCACCAGGGCAAAGAGAAGAGTGGTG--------------
-------------------------CAGAGAGAAAAAAGAACA---GTGGGA---CTAGGAGCT---------
TTGTTCCTTGGGTTCTTGGGAGCA---GCAGGAAGCACTATG---GGCGCAGCGTCG---
TTGACGCTGACGGTACAG---GCCAGACAA---TTACTG---TCTGGTATAGTGCAGCAGCAGAAC---
AATTTG---CTGAGA---GCTATTGAGGCGCAACACCAGCTGTTGCAACTCACAGTCTGGGGCATC
>AY308762
TTCTACTGTAATACAACTAAACTGTTTAATAGTACTTGGGGGGTTAATGGTACTTGGAATGGGACTGAAGGGA
AT-----------------------------------------------------------------------
-------------------------
AATGGGAATAACACTATCATACTCCCATGCAGAATAAAACAAATTATAAACATGTGGCAGCAAGTAGGAAAAG
CAATGTATGCCCCTCCCATCAGCGGATTAATTAGATGTTCATCAAAT---
ATTACAGGGTTGATGTTAACAAGAGATGGTGGTGGTGGTAATAGC----------------------------
-------------------------------------------------------------------------
----------
AGCACAAACGAGACCTTCAGACCTGGAGGAGGAGATATGAGGGACAATTGGAGAAGTGAATTATATAAATATA
AAGTAGTACAAATTAAACCATTA---GGAGTAGCACCCACCAAGGCAAAGAGAAGAGTGGTG-----------
----------------------------CAGAGAGAAAAAAGAGCA---GTGGGA---ATAGGAGCT------
---GTGTTCCTTGGGTTCTTGGGAGCA---GCAGGAAGCACTATG---GGCGCAGCGTCA---
ATAACGCTGACGGTACAG---GCCAGACAA---TTATTG---TCTGGTATAGTGCAACAGCAGAAC---
AATTTG---CTGAGG---GCTATTGAGGCGCAACAGCATCTGTTGCAACTCACAGTCTGGGGCATT
>AY835753
TTCTACTGTGAGACAACACAACTGTTTAATAGCACTTGGATTAGGGATAATGGTAATTGGACTAGGGCTGAGA
ATATT--------------------------------------------------------------------
```

ACTGAAGGGAATAATATCACACTCCCATGCAGAATAAGACAAATTGTAAACCTGTGGCAGCAAGTAGGAAAAG
CAATGTATGCCCCTCCCATCAGTGGACCAATTAACTGTTTATCAAAT---
ATTACAGGACTGCTATTAACATGGGATGGTGGTAATAATAGCACTAGT-------------------------
----

AATAACAACACCGAGATCTTCAGACCTGGAGGAGGAGATATGAGGGACAACTGGAGAAGTGAATTATATAAAT
ATAAAGTAGTAAAAATTGAACCATTA---GGAGTAGCACCCACCAAGGCAAAGAGAAGAGTGGTG--------
-------------------------------CAGAGGGAAAAAAGAGCA---GTGGGA---CTAGGAGCT---
------TTGTTCCTTGGGTTCTTGGGAACA---GCAGGAAGCACTATG---GGCGCAGCATCA---
TTGACGCTGACGGTACAG---GCCAGACAA---TTATTG---TCTGGTATAGTGCAACAGCAGAAC---
AATTTG---CTGAGG---GCTATTGAGGCGCAACAACATCTGTTGCAGCTTACAGTCTGGGGCATC
>EU575474
TTCTACTGTAGTACAACACAACTGTTTAATAGTACTTGGATTGCAAATAAGACTGGGAATGATACTGGAGGA-
----

TCAAATGGAACTATTACACTTCCATGCAGAATAAAACAAATTGTAAACATGTGGCAGGAAGTAGGAAAAGCAA
TGTATGCCCCTCCCATCGAAGGACAAATTAGATGTTCATCAAAC---
ATTACAGGACTGCTATTATTAAGAGATGGTGGTAAGAATAACGGGACA-------------------------
----

GGAAACATGACAGAAATCTTCAGACCTGGAGGAGGAGATATGAGGGACAATTGGAGAAGTGAATTATATAAAT
ATAAAGTAGTAAAAATTGAACCATTA---GGAGTAGCACCCACCAAGGCAAAGAGAAGAGTGGTG--------
-------------------------------CAGAGAGAAAAAAGAGCG---GTGGGAACACTAGGAGCC---
------TTGTTCCTTGGGTTCTTGGGAACA---GCAGGAAGCACTATG---GGCGCAGCATCA---
CTAACGCTGACGGTACAG---GCCAGACTA---TTATTG---TCTGGTATAGTGCAACAGCAAAAC---
AATTTG---CTGAGA---GCTATTGAGGCGCAACAGCATTTGTTGCAACTCACAGTCTGGGGCATC
>JN024428
TTCTACTGTAATACAACACAGCTGTTTAATAGTATTTGGTATAATACTACAAATAGTTCTTGGAATAATAAGA
CATGG--

TCAAATAACACGATCACACTCCCATGTAGAATAAAACAAATTATAAACATGTGGCAGCAAGTAGGAAAAGCAA
TATATGCCCCTTCCATCAAAGGAAAAATTAAATGTATATCAAAT---
ATTACAGGGCTGCTATTAACAAGAGATGGTGGTGATACCAATAGT----------------------------
----

GGGACAGAGATCTTCAGACCTGGAGGAGGAGATATGAGGGATAATTGGAGAAGTGAATTATATAAATATAAAG
TAGTACAAATTGAACCATTA---GGAGTAGCACCCACCAAGGCAAAGAGAAGAGTGGTG--------------
-------------------------CAAAGAGAAAAAAGAGCA---GTGGGA---TTAGGAGCT---------
ATGTTCCTTGGGTTCTTGGGAGCA---GCAGGAAGCACTGTG---GGCGCAGCGTCA---
ATAACGCTGACGGTACAG---GCCAGACAA---TTGTTG---TCTGGTATAGTGCAACAGCAGAAC---
AATCTG---CTGAGA---GCTATTGAGGCGCAACAGCGTCTGTTGCAACTCACAGTCTGGGGCATC
>AY331284
TTCTACTGTAATACAACACAACTGTTTAATAGTACTTGGAATGCTAATGTTACTGCAAATAGCACGGAA----
----

GGAAATGACACCCTCATACTCCCATGCAAAATAAAACAAATTATAAACATGTGGCAAGAGGTAGGAAAAGCAA
TGTATGCCCCTCCCATCCAAGGGATAATTAGCTGTTCATCAAAT---
ATCACAGGGCTATTATTAACAAGAGATGGTGGAAGTGAAAACAGG----------------------------
----

CCTAACGAGACCTTCAGACCTGGAGGAGGAGATATGAGGGACAATTGGAGAAGTGAATTATATAAATATAAAG
TAGTACAAATTGAACCACTA---GGAGTAGCACCCACCAAGGCAAAAAGAAGAGTGGTG--------------
-------------------------CAGAGAGAAAAAAGAGCA---ATAGGA---ATGGGAGCG---------
TTGTTCCTTGGGTTCTTGGGAGCA---GCAGGAAGCACTATG---GGCGCAGCGTCA---
GTGACGCTGACGGTACAG---GCCAGACAA---TTATTG---TCTGGTATAGTGCAACAGCAAAAC---
AATTTG---CTGAAG---GCTATTGAGGCGCAACAGCATCTGTTGCAACTCACAGTCTGGGGCATC
>AY331285
TTCTACTGTAATACAACAAAACTGTTTAATAGTACTTGGATTAGGGAGGGTAATAATGGTACTTGGAATGGTA
CTATAGGGTTA--

----------AATGACACT------
GCAGGAAATGACACAATCATACTCCCATGTAAAATAAAACAAATTATAAACATGTGGCAGGAAGTGGGAAAAG
CAATGTATGCCCCTCCCATCAGAGGACAAATTAGATGTTCATCAAAT---
ATTACAGGGCTGATATTAACAAGAGATGGCGGTAAGGACGATAGTAATGGG----------------------
----

AGTGAGATCCTCGAGATCTTCAGACCTGGAGGAGGAGATATGAGGGACAATTGGAGAAGTGAATTATATAAAT
ATAAAGTAGTAAGAATTGAACCATTA---GGAGTAGCACCCACCAGGGCAAGGAGGAGAGTGGTG--------
-------------------------------CAAAAAGAAAAAAGAGCA---GTGGGA---TTAGGAGCT---
------ATGTTCCTTGGGTTCTTAGGAGCA---GCAGGAAGCGCTATG---GGCGCAGCGTCA---
ATGACGCTGACGGTACAG---GCCAGACAA---TTACTG---TCTGGTATAGTGCAACAGCAGAAT---
AATTTG---CTGAGG---GCTATTGAGGCGCAACAGCATATGTTGCAACTCACAGTCTGGGGCATC
>KT124745
TTCTACTGTAATACAACACAACTGTTTAATAGTACTTGGGCTTGGAATAATACTGAAGAGTCAAATAACACTG
AA---

GGAAATAGCACAACAATCACACTGCAATGCAGAATAAAACAAATTGTAAACATGTGGCAGGAAGTAGGAAAAG
CAATGTATGCCCCTCCCATTAGAGGACAAATTAAATGTTTATCAAAT---
GTTACAGGGCTGCTATTAACAAGAGATGGGGGTAATAACAATGAC----------------------------
----

ACTGAAACCTTCAGACCTGGAGGAGGAAATATAAAGGACAATTGGAGAAGTGAATTATATAAATATAAAGTAA
TAAAAATTAAACCATTA---GGAATAGCACCCACCAAGGCAAAGAGAAGAGTGGTG-----------------
----------------------CAAAGAGAAAAAAGAGCA---GTGGGAACATTAGGAGCT---------
ATGTTCCTTGGGTTCTTGGGAGCA---GCAGGAAGCACTATG---GGCGCAGCGTCA---
TTGACACTGACGGTACAG---GCCAGACTA---TTGTTG---TCTGGTATAGTGCAACAGCAAAAC---
AATTTG---CTGAGG---GCTATTGAGGCGCAACAACATCTGTTGCAACTCACGGTCTGGGGCATC
>JN562755
TTCTACTGTGATACAACACAATTGTTTAATAGTACTTGGAATGGTACTGAAGAGTCAAATGACACTGAA----
----

AGTAATATCACACTCACACTCCCATGCAGAATAAAACAAATTGTAAACATGTGGCAGGAAGTAGGAAAAGCAA
TGTATGCCCCTCCCATCAGAGGAATAATTAGCTGCTCATCAAAT---
ATTACAGGGCTGCTATTAGTAAGAGATGGTGGTAAGCCCAATAATGAGACT----------------------
----

AATAATGGTACTGAGGTCTTCAGACCTGGAGGAGGAAATATGAAGGACAATTGGAGAAGTGAATTATATAAAT
ATAAAGTAGTAAAAATTGAACCATTA---GGAGTAGCACCCACTAAGGCAAAAAGAAGAGTGGTG--------
-------------------------------CAGAGAGAGAAAAGAGCA---GTGGGA---TTAGGAGCT---
------ATGTTCCTTGGGTTCTTGGGAGCA---GCAGGAAGCACTATG---GGCGCAGCGTCA---
ATAACGCTGACGGTACAG---GCCAGACAC---TTATTG---TCTGGTATAGTGCAACAGCAAAAC---
AATTTG---CTGAAG---GCTATTGAGGCGCAACAGCATCTGTTGCAACTCACAGTCTGGGGCATC
>JN562794
TTCTACTGTAATACAACACTATTGTTTAATAGTAATTGGACTTGGAATGATACTGGAGGATATTTTACTAAAG
GGTCAAATAAC--
ACAAATGATACTGAAGGG---------
TCAAATAGCACACATATCACACTCCCATGCAGAATAAAACAAATTATAAACATGTGGCAGGGAGTAGGAAAAG
CAATGTATGCCCCTCCTATCAGTGGACTAATTAGATGTTCATCAAAT---
ATCACAGGGCTGCTACTAACAAGGGATGGTGGTATGACGAATGATACC-------------------------
----

AATACCACCGAGACCTTCAGACCTCTAGGAGGAAATATGAAGGACAATTGGAGAAGTGAATTATATAAATATA
AAGTAGTAGAAATTGAACCATTA---GGAGTAGCACCCACCAAGGCAAAGAGAAGAGTGGTG-----------
----------------------------CAGAGAGAAAAAAGAGCA---GTGGGA---ATAGGAGCT------
---TTGTTCCTTGGGTTCTTGGGAGCA---GCAGGAAGCACTATG---GGCGCAGCGTCA---
CTAACGCTGACGGTACAG---GCCAGACAA---TTATTG---TCTGGTATAGTGCAACAGCAGAAT---
AATCTG---CTGAGG---GCTATTGAGGCGCAGCAGCATCTGTTGCAACTCACAGTCTGGGGCATC
>JN562786
TTCTACTGTAATACAACAAAATTGTTTAATAGTACTTGGAATATTAATGGTACTTGGAAGGGTACA-------
----

GGAGAGCCAAATATCACACTCCCATGCAGAATAAAACAAATTGTACACATGTGGCAGGAAGTAGGAAAAGCAA
TGTATGCCCCTCCCATCAGAGGACAAATTAACTGCTCATCACAT---
ATTACAGGGCTGCTATTAACAAGAGATGGTGGGCATACTAACAATAATGGG----------------------
----

AGCAATGAGACAGAGATCTTCAGACCTGGAGGAGGAGATATGAGGGACAATTGGAGAAGTGAATTATACAAAT
ATAAAGTAGTAAGAATTGAACCATTA---GGAGTAGCACCCACCAAGGCAAAGAGGAGAGTGGTG--------
-------------------------------CAGAGAGAAAAAAGAGCA---GTGGGA---ATAGGAGCT---
------GTGTTCCTTGGGTTCTTGGGAGCA---GCAGGAAGCACTATG---GGCGCAGCATCA---
ATAACGCTGACGGTACAG---GCCAGACAA---TTATTG---TCTGGCATAGTGCAACAGCAGAAC---
AATTTG---CTGAAG---GCTATTGAGGCGCAACAGCATCTGTTGCAACTCACAGTCTGGGGCATC
>JN562773
TTCTACTGTAATTTAACACAACTGTTTAATAGTACTTGGAATGGTACAGAGTCAAATAACACTGAA-------
----

GAAGATAACACAATCACACTCCCATGCAGAATAAAACAAATCATAAACATGTGGCAGGAAGTAGGGAAAGCAA
TGTATGCCCCTCCCATCAGAGGAAATATTAGCTGTTCATCAAAT---
ATTACAGGGCTACTATTAACAAGAGATGGTGGTATTAACCAGACC----------------------------
----

AAAGACACTGAGATCTTCAGACCTGGAGGAGGAGATATGAGGGACAATTGGAGAAGTGAACTATATAAATATA
AAGTAGTAAAGGTTGAACCATTA---GGAATAGCACCCACCAAGGCAAAGAGAAGAGTGGTG-----------
----------------------------CAGAGAGAAAAAAGAGCA---GTAGGA---ATAGGAGCT------
---GTGTTCCTTGGGTTCTTGGGAGCA---GCAGGAAGCACTATG---GGCGCAGCGTCA---
GTGACGCTGACGGTACAG---GCCAGACTA---TTGTTG---TCTGGTATAGTGCAACAGCAGAAC---
AATCTG---CTGAGG---GCTATTGAGGCGCAGCAGCATCTGTTGCAACTCACAGTCTGGGGCATC
>EU289189
TTCTACTGTAATACAACAAAACTGTTTAATAATACTTGGAATAGTAATGATACTTGGACTAATACTGAAGAGT
CA---

AATGAAACTGAAATCAGGCTCCCATGCAGAATAAAACAAATTATAAACATGTGGCAGGAAGTAGGAAAGGCAA
TGTATGCCCCTCCCATCCAAGGACAAATAAGATGTTCATCAAAT---
ATTACAGGGCTGCTACTAACAAGAGATGGGGGTAACAACACGAAC----------------------------
----

AATACCGAGGTCTTCAGACCTGGAGGAGGAGATATGAGAGATAATTGGAGAAGTGAATTATATAAATATAAGG
TAGTAAAAATTGAACCATTA---GGAGTAGCACCCACTAAGGCTAAGAGAAGAGTGGTG--------------
-------------------------CAGAGAGAAAAAAGAGCA---GTGGGA---CTAGGAGCT---------
TTGTTCCTTGGGTTCTTGGGAGCA---GCAGGAAGCACTATG---GGCGCAGCGTCA---
GTGACGCTGACGGTACAG---GCCAGACAA---TTATTG---TCTGGTATAGTGCAACAGCAGAGC---
AATTTG---CTGAGG---GCTATTGAGGCGCAACAGCATCTGTTGCAACTCACAGTCTGGGGCATC
>EF593200
TTCTACTGTAATACAACACAACTGTTTAATAGTACTTGGGAGAATTCAACACAACTGTTTAATAGTACTGGG-
----

GAGAATGATACTATCACACTCCCATGCAGAATAAAACAAATTATAAACATGTGGCAGGCAGTGGGAAAAGCAA
TGTATGCCCCTCCTATCAGAGGACCAATTACATGTAAATCAAAT---
ATCACAGGGCTGATACTAACAAGAGATGGTGGGAACAGCAGCAGC----------------------------
----

AACAACGAGACCTTCAGACCTGGAGGAGGAGATATGAGGGACAATTGGAGAAGTGAATTATATAAATATAAAG
TAGTAAAAATTGAACCATTA---GGAGTAGCACCCACCAAGGCAAAGAGAAGAGTGGTG--------------
-------------------------CAGAGAGAAAAAAGAGCA---GTGGGAGTGATAGGAGCT---------
ATGTTCCTTGGGTTCTTGGGAGCA---GCAGGAAGCACTATG---GGCGCAGCGTCA---
GTGGCGCTGACGGTACAG---GCCAGACAA---TTATTG---TCTGGTATAGTGCAGCAGCAGAAC---
AATTTG---CTGAGG---GCTATTGAGGCGCAACAACATCTGTTGCAACTCACAGTCTGGGGCATC
>EF593201
TTCTACTGTAATACAACAAGACTGTTTAATAGTACGTGGAATGCCACTAGGGAGGAGAATGGTACTAAAGAGA
CAAGT--

GACAATACCACACTTATCACACTCCCATGCAGAATAAAACAATTTATAAACATGTGGCAGGAAGTAGGAAAAG
CAATGTATGCCCCTCCAATCAGTGGACAAATTAGTTGTTCATCAAAT---
ATTACAGGGCTGCTATTAACAAGAGATGGTGGTAATGACAATGGTTCT-------------------------
----

AACAACACCGAGATCTTCAGACCTGGAGGAGGAAATATGAGGGACAATTGGAGAAGTGAATTATATAGATATA
AAGTAGTAAAAATTGAACCATTA---GGAGTAGCACCCACCAAGGCAAAGAGAAGAGTGGTG-----------
----------------------------CAGAGGGAAAAAAGAGCA---ATAGGA---CTAGGAGCT------
---TTGTTTCTTGGGTTCTTGGGAGCA---GCAGGAAGCACTATG---GGCGCAGCGTCA---
CTGATGCTGACGGTACAG---GCCAGACAC---TTATTG---TCTGGTATAGTGCAACAGCAGAAC---
AATCTG---CTGAGG---GCTATTGAGGCGCAACAGCATATGTTGCAACTCACAGTCTGGGGCATT
>EU289192
TTCTACTGTAATACAACACAATTGTTTAATAGTACTTGGAATACAACACAACTTAATAGTACTTGGATGTTA
CAGGAGGG---
-------------------TCA---
AATACTACAGAGAATATCACACTCCAATGTAGAATAAAACAAATTATAAACATGTGGCAGGAAGTAGGCAAAG
CAATGTATGCCCCTCCCATCAGTGGAATAATTAAATGTACATCAAAT---
ATTACAGGGCTACTATTAACAAGAGATGGTGGTACAAACAATACA----------------------------
----

AACGGTAGCGAGATTTTCAGACCTGGAGGAGGAGATATGAAGGACAATTGGAGAAGTGAATTATATAAATACA
AAGTAGTAAAAATTGAACCATTA---GGAGTAGCACCCACCAAGGCAAAGAGAAGAGTGGTG-----------
----------------------------CAAAGAGAAAAAAGAGCA---GTAACA---CTAGGAGCT------
---ATGTTCCTTGGGTTCTTGGGAGCA---GCAGGAAGCACGATG---GGCGCAGCGTCA---
ATGACGCTGACGGTACAG---GCCAGGCAA---TTATTG---TCTGGTATAGTGCAACAGCAGAAC---
AATCTG---CTGCGG---GCTATTGAGGCGCAACAACATCTGTTGCAACTCACAGTCTGGGGCATC
>EF593204
TTCTACTGTAATACAACAAAACTGTTTAATAGTACTTGGTCGCTTAATAGTACTGGGCTTAATAGTACTGGG-
----

AATGGTACTGAGATCATACTCCCATGTAGAATAAAACAAATTATAAACATGTGGCAGGAAGTAGGAAAAGCAA
TGTATGCCCCTCCCATCAGAGGACAAATTAGATGCTCATCAAAT---
ATTACAGGACTACTATTAACAAGAGATGGAGGGAGCAATGACACC----------------------------
----

CGCCAGGAGACTTTCAGACCTGGAGGAGGAGATATGAGAGACAATTGGAGAAGTGAATTATATAAATATAAAG
TAGTACAAATTGAACCATTA---GGATTAGCACCCACCAAGGCAAGGAGAAGAGTGGTG--------------
-------------------------CAGAGAGAAAGAAGAGCA---ATAGGA---CTAGGAGCT---------
ATGTTCCTTGGGTTCTTGGGAGCA---GCAGGAAGCACTATG---GGCGCAGCGTCA---
CTGACGCTGACGGTACAG---GCCAAACAA---TTATTG---TCTGGTATAGTGCAACAGCAGAAC---
AATCTG---CTGAGG---GCTATTGAGGCGCAACAGCATCTGTTGCAACTCACAGTCTGGGGCATC
>EU289195
TTCTACTGTAATACAACAAAGCTGTTTAATAGTACTTGGAATAATACTAATGGTGATTGGAATCTTGCTAGTG
AAGGATCAAAT--
-------------AACACT------
GCAGTAAATGGCAGTATCATACTTCCATGCAGAATTAAACAAATTATAAACATGTGGCAGGAAGTAGGAAAAG
CAATGTATGCCCCTCCCATCAAAGGACAAATTAGATGTTCATCAAAT---
ATTACAGGGCTGATATTAACAAGAGATGGTGGTGTTAATGCGACC----------------------------
----

AATGCCACTGAGATCTTCAGACCAGGAGGAGGAGATATGAGGGACAATTGGAGAAGTGAATTATACAAATATA
AAGTAGTACAAATTGAACCATTA---GGAGTAGCACCTACCAAGGCAAAGAGAAGAGTGGTG-----------
----------------------------CAGAGAGAAAAAAGAGCA---GCATTGGAATAGGAGCT------
---CTGTTCCTTGGGTTCTTGGGAGCA---GCAGGAAGCACTATG---GGCGCAGCGTCG---
ATAACGCTGACGGTACAG---GCCAGACAA---TTATTG---TCTGGTATAGTGCAACAGCAGAAC---
AACTTG---CTGAGA---GCTATTGAGGCGCAACAGCATCTGTTGCAGCTCACAGTCTGGGGCATC
>EU575815
TTCTACTGTAATTCAACACCACTGTTTAATAGTACCTGGGCTATGAATAGAACCTGGAATGATACTGAAGGGT
CA---

AATAACAATAAAACTATCACACTCCAATGCAAAATAAAACAAATTATAAACATGTGGCAGGAAGTAGGAAAAG
CAATGTATGCCCCTCCCATCAGAGGACAAATTAGATGTTCATCAAAT---
ATTACAGGGCTGCTATTAACAAGAGATGGTGGTAATAGTGAAAATGCG-------------------------
----

AACAAGACCGAGATCTTCAGACCTGGAGGAGGAGATATGAGGGATAATTGGAGAAGTGAATTATATAAATATA
AAGTAGTAAAAATAGAACCATTA---GGAGTAGCACCCACCAAGGCAAAGAGAAGAGTGGTG----------
----------------------------CAGAGAGAAAAAAGAGCA---GTGGGA---CTAGGAGCT------
---TTGTTCCTTGGGTTCTTGGGAACA---GCAGGAAGCACTATG---GGCGCAGCATCA---
ATAACGCTGACGGTACAG---GCCAGGCAA---TTATTG---TCTGGTATAGTGCAACAGCAGAGC---
AATCTG---CTGAGG---GCTATTGAGGCGCAACAGCATCTGTTGCAACTCACAGTCTGGGGCATC
>EU575850
TTCTACTGCAATACAACAGCACTGTTTAATAGTACTTGGAATAATACTGAATGGTCAAATGACACTAGGTCAA
ATGAC--
-------------------ACT---
AGGTCAAATGCCACTATCACACTCCCATGCAGAATAAAACAAATTATAAACATGTGGCAGGAAGTAGGAAAAG
CAATGTATGCCCCTCCCATCAAAGGACAAATTAGCTGTTCATCAAAT---
ATTACAGGGCTGTTATTAACAAGAGATGGTGGCAGTAGCAATGATAGCACG----------------------
----

AACGGGACCACCGAGATCTTCAGACCTGGAGGAGGAGATATGAGGGACAATTGGAGAAGTGAATTATATAAAT
ATAAAGTAGTACAAATTGAACCATTA---GGAGTAGCACCCAGCAGGGCAAAGAGAAGAGTGGTG--------
-------------------------------CAGAGAGAAAAAAGAGCA---ATAGGA---CTAGGAGCT---
------ATGTTCCTTGGGTTCTTGGGAGCA---GCAGGAAGCACTATG---GGCGCAGCGTCA---
CTAACGCTGACGGTACAG---GCCAGACTA---TTATTG---TCTGGTATAGTGCAACAGCAGAGC---
AATCTG---CTGAGG---GCTATTGAGGCGCAACAGCATCTGTTGCAACTCACAGTCTGGGGCATT
>EF593232
TTCTACTGTAAGTCAACAGAACTGTTCAATAGTACTTGGAATACTAATAGTACTTGGAATACTACTAGTACTT
GGAATACTACT--
-------------AGAGGG------
CCTAACAATAGAACAATCACACTCCCATGCAGAATAAAACAAATTATAAACATGTGGCAGGAAGTAGGAAAAG
CAATGTATGCCCCTCCCATCAGAGGACAAATTAGGTGTTCATCAAAT---
ATTACAGGGCTGCTATTAACAAGAGATGGTGGGAACAATGGGACC----------------------------
----

ACCGAGACCTTCAGACCTGGAGGAGGAAATATGAAAGACAATTGGAGAAGTGAATTATATAAATATAAAGTAG
TAAAAATTGAACCATTA---GGAGTAGCACCCACCAAGGCAAGGAGAAGAGTGGTG-----------------
----------------------CAAAGAGAAAAAAGAGCA---GTGGGAGCAATAGGAGCT---------
ATGTTCCTTGGGTTCTTGGGAGCA---GCAGGAAGCACTATG---GGCGCAGCGTCA---
GTGACGCTGACGGTACAG---GCCAGACTA---CTATTG---TCTGGTATAGTGCAACAGCAGAAC---
AATCTA---CTGAGG---GCTATTGAGGCGCAACAGCATATGTTGCAACTCACAGTCTGGGGCATC
>JN400469
TTCTACTGTAATACAACAAAACTGTTTAATAGTACTTGGAACATTACTGAAGGGTCAAATAACACTGAAGGGT
CAAAT--

AACACTGAAGAACTTATCACACTCCCATGCAGAATAAAACAAATTATAAACATGTGGCAGAGAGTAGGAAAAG
CAATGTATGCCCCTCCCATCCGTGGACAAATTAAATGTTCATCAAAT---
ATTACAGGGCTGTTATTAACAAGAGATGGTGGTAATAGTAGCAGC----------------------------
----

AACGGGTCAGAAATCTTCAGACCTGGAGGAGGAGATATGAGGGACAATTGGAGAAGTGAATTATATAAATATA
AAGTAGTAAAAATTGAACCATTA---GGAGTAGCGCCCACCAAGGCAAAAAGAAGAGTGGTG-----------
----------------------------CAAAGAGAAAAAAGAGCA---GTGGGA---TTAGGAGCT------
---TTGTTCCTTGGGTTCTTGGGAGCA---GCAGGAAGCACTATG---GGCGCAGCGTCA---
ATGACGCTGACGGTACAG---GCCAGACAA---TTATTG---TCTGGTATAGTGCAACAGCAGAAC---
AATTTG---CTGAGA---GCTATTGAGGCGCAACAGCATATGTTGCAACTCACAGTCTGGGGCATC
>EF593304
TTCTACTGCAATACAACAATACTGTTTAATAGTACTTGGAATGAAACTAACCTGTTTAATGCCACTGAAGGAA
AT---

AACACAGAAGTTATTACACTCCAATGCAGAATAAAACAAATTATAAACATGTGGCAGGAAGTAGGAAAAGCAA
TGTATGCCCCTCCCATCAGTGGACAAATTAACTGTTCATCAAAC---
ATTACAGGGCTGCTATTAACAAGAGATGGCGGTATTGGGAACAATATC-------------------------
----

ACCAATACCTCCGAGATCTTCAGACCTATAGGAGGAAATATGAGGGACAATTGGAGAAGTGAATTATATAAAT
ATAAAGTAGTCAAAATTGAACCAATA---GGAATAGCACCCACCAAGGCAAAGAGAAGAGTGGTG--------
-------------------------------CAGAGAGAAAAAAGAGCA---GTGGGA---ATAGGAGCT---
------ATGTTCCTTGGGTTCTTGGGAGCA---GCAGGAAGCACTATG---GGCGCAGCGTCA---
GTGACGCTGACGGTACAG---GCCAGACTA---TTATTG---TCTGGTATAGTGCAACAGCAGAAC---
AATCTG---CTGAGA---GCTATTGAGGCGCAACAACATCTGTTGCAACTCACAGTCTGGGGCATC
>AY331294
TTCTATTGCAACTCAACCAAACTATTTAATAGCATTTGGGATAATAATAAAGATAGCACTAAAACAAATGAAC
CA---

AATGATGGAAAAAATATCACACTCCCATGCAGAATAAAACAAATTATAAACATGTGGCAGGGAGTAGGAAAAG
CAATGTATGCCCCTCCCATCAGAGGACAAATTAGATGTACATCAAAT---
ATTACAGGGCTACTATTAACAAGAGATGGTGGCAAGAACAATGGCACT-------------------------
----

AATGGGACCGAGGTCTTCAGACCTGGAGGAGAAAATATGAAGGACAATTGGAAAAGTGAATTATATAAATATA
AAGTAGTAAAAATTGAACCATTA---GGAGTAGCACCCACCACGGCAAAGAGACGAGTGGTG-----------
----------------------------CAGAGAGAAAAAAGAGCA---GTGACA---CTAGGAGCT------
---CTGTTCCTTGGGTTCTTGGGAGCA---GCAGGAAGCACTATG---GGCGCAGCGTCA---
ATGACGCTGACGGTACAG---GCCAGACTA---TTATTG---TCTGGTATAGTGCAACAGCAGAAC---
AACTTG---CTGAGG---GCTATTGAGGCGCAACAACATCTGTTGCAACTCACGGTCTGGGGCATC
>KT124746
TTCTACTGTAATACAACACAACTGTTTAATAGTACTTGGAATGGAAATGATACTGGAGAGTTTAATAACACTG
GAAAA--

AATATCACATATATCACACTCCCATGCAGGATAAAACAAATTATAAACATGTGGCAGGAAGTAGGAAAAGCAA
TGTATGCCCCTCCCATCGCAGGACAAATTAGATGTTCATCAAAT---
ATTACAGGGATACTATTAACAAGAGATGGTGGTAATAGTAGCGAG----------------------------
----

GACAAGGAGATCTTCAGACCTGAAGGAGGAAATATGAGGGATAATTGGAGGAGTGAATTATACAAATATAAAG
TAGTAAAAATTGAACCATTA---GGAGTAGCACCCACCAAGGCAAAGAGAAGAGTGGTG--------------
-------------------------CAGAGAGAAAAAAGAGCA---GTGGGA---ATAGGAGCT---------
GTGTTCCTTGGGTTCTTGGGAGCA---GCAGGAAGCACTATG---GGCGCAGCGTCA---
ATGACGCTGACGGTACAG---GCCAGACTA---TTATTG---TCTGGTATAGTGCAACAGCAGAAC---
AATTTG---CTGAGG---GCTATTGAGGCGCAACAGCATCTGTTGCAACTCACAGTCTGGGGCATC
>KT124747
TTCTACTGTAATACAACACAGCTGTTTAATAGTACCTGGCCATGGCCGGATAATGATACTAATGATAACGGTA
CA---

GGCAATAGCACAATCACACTTCCATGCAAAATAAAACAAATTATAAACAGGTGGCAAGAAGTAGGAAAAGCAA
TGTATGCCCCTCCCATCAGTGGACAAATTAGATGTACATCAAAT---
ATTACAGGACTGATATTAACAAGAGATGGTGGTCCCACAAATGATACC-------------------------
----

AACAACACCGAGGTCTTCAGACCTGGAGGAGGAGATATGAGGGACAATTGGAGAAGTGAATTATATAAATATA
AAGTAGTAAAAATTGACCCATTA---GGAGTAGCACCCACCAAGGCAAAGAGAAGAGTGGTG-----------
----------------------------CAAAGAGGAAAAAGAGCA---GTGGGG---ATAGGAGCT------
---ATGTTCCTTGGGTTCTTGGGAGCA---GCAGGAAGCACTATG---GGCGCAGCGTCA---
CTAACGCTGACGGTACAA---GCCAGACTA---CTATTG---TCTGGTATAGTGCAACAGCAGAAC---
AATCTG---CTGAGG---GCTATTGAGGCGCAACAGCATATGTTGCAACTCACAGTCTGGGGCATT
>EF593208
TTCTACTGTAACTTAACAAGGCTGTTTAATAATACTTGGTATAATGAGAGCAGTAGTACTTGGAAT-------
----

GTAAATGGCACCATCACACTCCCATGCAGGATAAAACAAATTATAAACATGTGGCAGGAAGTAGGAAAAGCAA
TGTATGCCCCTCCCATCATGGGATCAATTAACTGTTCATCAAAT---
ATTACAGGGCTACTATTAACAAGAGATGGTGGTAACCATAACAAT----------------------------
----

GACAGCAACGAGACCTTCAGACCTGGAGGAGGAGATATGAGGGACAATTGGAGAAGTGAATTATATAAATATA
AAGTAGTAAAAATTGAACCATTA---GGAGTAGCACCCACCAAGGCAAAGAGAAGAGTGGTG-----------
----------------------------CAAAGAGAAAAAAGAGGA---GTGACA---CTAGGAGCT------
---ATGTTCCTTGGGTTCTTGGGAGCA---GCAGGAAGCACTATG---GGCGCAGCGTCA---
ATGGCGCTGACGGTACAG---GCCAGACAA---TTATTG---TCTGGTATAGTGCAACAGCAGGAC---
AATTTG---CTGAGG---GCTATTGAGGCGCAACAGCATATGTTGCAACTCACAGTCTGGGGCATC
>AY560107
TTCTACTGTAATACAGCAAAACTGTTTAATAGCATTTGGAATAATAGCAGTTGGAGTGGTACTGAAAGGTCAA
GT---

AACGAGACAAGGAACATCATACTCCCATGTAAAATAAGACAAATTGTAAACCTGTGGCAGGAAGTAGGAAAAG
CAATGTATGCCCCTCCCATCAGAGGACAAATTAGATGTTCATCAAAT---
ATTACAGGGATACTATTAACAAGAGATGGTGGTAATAACACAGAA----------------------------
----

GGGAACGAGACCTTCAGACCTGGAGGAGGAGATATGAGGGACAATTGGAGAAGTGAATTATATAAATATAAAG
TAGTAAAAATTGAACCATTA---GGAGTAGCACCCACCAAGGCAAAGAGAAGAGTGGTG--------------
-------------------------CAGAGAGAAAAAAGAGCA---GTGGGA---TTAGGAGCT---------
TTGTTCCTTGGGTTCTTGGGAGCA---GCAGGAAGCACTATG---GGCGCAGCGTCA---
GTAACGCTGACGGTACAG---GCCAGACAA---TTATTG---TCTGGTATAGTGCAGCAGCAAAAC---
AATTTG---CTGAGG---GCTATTGAGGCGCAACAGCACCTGTTGCAACTCACAGTCTGGGGCATC
>AY560108
TTTTACTGTAATACAACACCACTGTTTAATAGTACTTGGGTTAATGGTACTAGGAATGATACTGCAAGGCCAA
AT---

GACACTGACACAATTAAACTTCCATGCAGAATAAAACAAATTATAAACCTGTGGCAGGAAGTAGGAAAAGCAA
TGTATGCTCCTCCCATCGAAGGACAAATTAGCTGTTCATCAAAT---
ATTACAGGGCTACTATTAACAAGAGATGGTGGTATTAACAGCAGCAAT-------------------------
----

ACCAATACCACCGAGACCTTCAGACCTGGAGGAGGAGATATGAGGGACAATTGGAGAAATGAATTATATAAAT
ATAAAGTAGTAAGAATTGAGCCATTA---GGAGTAGCACCCACCAAGGCAAAGAGAAGAGTGGTG--------
-------------------------------CAGAGAGAAAAAAGAGCA---GTGGGA---ATAGGAGCT---
------ATGTTCCTTGGGATCTTGGGAACA---GCAGGAAGCGCTATG---GGCGCAGCGTCA---
ATGACGCTGACGGTACAG---GCCAGACAA---TTATTG---TCTGGTATAGTGCAACAGCAGAAC---
AATATG---CTGAGG---GCTATTGGGGCGCAACAGCACCTGTTGCAACTCACAGTCTGGGGCATC
>AY560109
TTCTACTGTAATACAACACAACTGTTTAATAGCACTTGGTATAGTAATAGTACTGGGAATAGTACTGGAGGG-
----

TCAAATGACACTATCACACTCCCATGTAGAATAAAACAAATTATAAACATGTGGCAGGAAGTAGGAAGAGCAA
TGTACGCCCCTCCCATCAGTGGAAAAATTAGTTGTTCATCAAAT---
ATTACAGGGCTACTGTTAACAAGAGATGGTGGTATTAACATGACC----------------------------
----

AATACCACCGAGACCTTCAGACCTGGAGGAGGAGATATGAGGGACAACTGGAGAAGTGAATTATATAAATATA
AAGTAGTACAAATTGAACCATTA---GGAATAGCACCCACCAAGGCAAAGAGAAGAGTGGTG-----------
----------------------------CAGAGAGAAAAAAGAGCA---GTGGGA---ATAGGAGCT------
---CTGTTCCTTGGGTTCTTAGGAGCA---GCAGGAAGCACTATG---GGCGCAGCGTCA---
GTGACGCTGACGGTACAG---GCCAGGCAC---TTATTG---CCTGGTATAGTGCAGCAGCAGAAC---
AATCTG---CTGAAG---GCTATTGAGGCGCAACAGCATCTGTTGCAACTCACAGTCTGGGGCATT
>AY560110
TTCTACTGTGATTCAACACCACTGTTTAATAGTACTTGGAATAGTACTACTTGGAATGATACTACTGACACTG
AA---

GGAAATGAAACAATCACACTCAAATGTAGAATAAAACAAATTATAAACATGTGGCAGGAAGTAGGAAAAGCAA
TGTATGCCCCTCCTATCGAGGGAGTAATTAAATGTTTATCAAAT---
ATTACAGGGCTGCTATTAGTAAGAGATGGT---ATAAGCAACAGT----------------------------
----

AGTGACGAGACCTTCAGACCTGGAGGAGGAAATATGAAGGACAATTGGAGAAGTGAATTATATAAATATAAAG
TAGTAAAAATTGAACCATTA---GGAGTAGCACCCACCAAGGCAAGGAGAAGAGTGGTG--------------
-------------------------CAAAGAGAAAAAAGAGCA---GTGGGAACACTGGGAGCT---------
ATGTTCCTTGGGTTCTTGGGAGCA---GCAGGAAGCGCTATG---GGCGCAGCGTCA---
GCGACGCTGACGGTACAG---GCCAGACAA---TTATTG---TCTGGTATAGTGCAACAGCAAAAC---
AACTTG---CTGAGA---GCTATTGAGGCGCAACAGCATATGTTGCAACTCACAGTCTGGGGCATC
>EF175212
TTCTACTGTAATACAACAAAACTGTTTAATAGTAATTGGACTTTGGAGGGTAATGTGACATATACCACAAATA
AC---

ACAAATGGCACAAATATCATACTCCCATGCAGAATAAAACAAATTATAAACATGTGGCAGGGAGTAGGAAAAG
CAATGTATGCCAATCCCATCAGTGGACTAATTAACTGTACATCAAAG---
ATTACAGGGCTGCTATTAACAAGAGATGGGGGTACAGAGGGTAATGGCAGCAACGGGACCGGGACAGAGATCG
AGAAC--

AGGACCGAGATCTTCAGACCTGGGGGAGGAAATATGAAGGACAATTGGAGAAGTGAATTATATAAATATAAAG
TAGTAAGAATTGAACCAATA---GGGATAGCACCCACCAAGGCAAAGAGAAGAGTGGTG--------------
-------------------------CAGAGAGAAAAAAGAGCA---GTGGGGGTGCTGGGAGCT---------
ATGTTCCTTGGGTTCTTGGGAACA---GCAGGAAGCACTATG---GGCGCAGCATCA---
GTAACGCTGACGGTACAG---GCCAGACAA---TTATTG---TCTGGTATAGTGCAACAGCAGAAC---
AATCTG---CTGCAG---GCTATTGAGGCGCAACAGCATCTGTTGCAGCTCACAGTCTGGGGCATC
>EU577426
TTCTACTGTAATTCAACAGAACTGTTTAATAGTACTTGGAATAATACTAATACTTGGAATGATACTGGAGAG-
----

TCAAATGAAACTATCATACTTCCATGTAGAATAAAACAATTTATAAACATGTGGCAGGGAGTAGGAAAAGCAA
TGTATGCCCCTCCCATCAGAGGAGAAATTAACTGCTCATCACAA---
ATTACAGGGCTGCTATTAACAAGAGATGGTGGTAATGATAATACCACT-------------------------
----

AACATGATTGAGGTCTTCAGACCTGGAGGAGGAAATATGAAGGACAATTGGAGAAGTGAATTATCTAAATATA
AAGTAGTAAAAATTGAGCCATTA---GGAGTAGCACCCACTAAGGCAAAGAGAAGAGTGGTG-----------
----------------------------CAGAGAGAAAAAAGGGGA------------ATAGGAGCT------
---GTGTTCCTTGGGTTCTTGGGAGCA---GCAGGAAGCACTATG---GGCGCAGCGTCA---
GTGGCGCTGACGGTACAG---GCCAGACAA---TTATTG---TCTGGTATAGTGCAACAGCAGAAC---
AATCTG---CTGAAG---GCTATTGAGGCGCAACAGCATCTGTTGCAACTCACAGTCTGGGGCATC
>EF593284
TTCTACTGTAATTCAACACAATTGTTTAATAGTACTTGGTATGGTAATGAGACTATTGAAGGGTTAAATGAC-
----

ACTACAGAAAATATCACACTCCCATGCAAAATAAAACAAATTATAAACATGTGGCAGGAAGTAGGAAAAGCAA
TGTATGCCCCTCCCATCAGTGGACAAATTAGATGTTCATCAAAT---
ATTACAGGGCTGATATTAACAAGAGATGGTGGTTTTAGCAACAATACCCAG----------------------
----

GAGAAGAACAAGACCGAGATCTTCAGACCTGGGGGAGGAGATATGAGGGACAATTGGAGAAGTGAATTATATA
AATATAAAGTAGTGCAAGTTGAACCATTA---GGACTAGCACCCACCAAGGCAAAGAGAAGAGTGGTG-----
----------------------------------CAGAGAGAAAAAAGAGCA---GTGGGT---
TTAGGAGCT---------TTGTTCCTTGGGTTCTTGGGAGCA---GCAGGAAGCACTATG---
GGCGCAGCGTCA---GTGACGCTGACGGTACAA---GCCAGACAA---TTATTG---
TCTGGTATAGTGCAACAGCAGAAC---AACTTG---CTGAGA---
GCTATTGAGGCGCAACAGCATCTGTTGCAACTCACAGTCTGGGGCATC
>EF593310

TTCTACTGTAATTCAACACCACTGTTTGATAGTATTTGGAATAGTACTGGGAATGATACTGAAGGGTCAAATA
GT---

ACTGAAAAAGATATCATACTCCAATGCAGAATAAAACAAGTTATAAATATGTGGCAGCAAGTAGGAAAAGCAA
TGTATGCCCCTCCCATCAAAGGACAAATTAACTGTACATCAAAT---
ATTACAGGGCTGCTATTAACAAGAGATGGTGGTAACAGGGGCGAG----------------------------
----

TCTGAGACCTTCAGACCTGGAGGAGGAGATATGAAGGACAATTGGAGAAGTGAATTATATAAATATAAAGTAG
TAAAAATTAAACCATTA---GGAATAGCACCCACCAAGGCAAAGAGAAGAGTGGTG-----------------
----------------------CAGAGAGAAAAGAGATCA---GTGACA---TTAGGAGCT---------
ATGTTCCTTGGGTTCTTGGGAGCA---GCAGGAAGCACTATG---GGCGCAGCGTCA---
ATGGCGCTGACGGTACAA---GCCAGACAA---TTATTG---TCTGGTATAGTGCAACAGCAGAAC---
AATTTG---CTGAAA---GCTATTGAGGCGCAACAGCATCTGTTGCAACTCACAGTCTGGGGCATC
>EF593312
TTCTACTGTAATTCAACACAACTGTTTAATAGTACTTGGTATGGTAATGAGACTGAGACTGGAGGGACAAATG
AC---

ACTATAGGAAATATCACACTCCCATGCAGAATAAGACAAATTATAAACATGTGGCAGGAAGTAGGAAAAGCAA
TGTATGCCCCTCCCATCAGAGGACAAATTAGTTGTTCATCAAAA---
ATTACAGGGCTGCTATTAACAAGGGATGGTGGTAACAACAATGAGACC-------------------------
----

AATACCACCGAGACATTCAGACCAGGAGGAGGAGATATGAGGGATAATTGGAGAAGTGAATTATATAAATATA
AAGTAGTCAAAATTGAGCCACTA---GGAATAGCACCCACCAAGGCAAAGAGAAGAGTGGTG-----------
----------------------------CAAAGAGAAAAAAGAGCA---GTGGGAACAATAGGAGCT------
---ATGTTCCTTGGGTTCTTGGGAGCA---GCAGGAAGCACTATG---GGCGCAGCGTCA---
ATGACGCTGACGGTACAG---GCCAGACTA---TTATTG---TCTGGTATAGTGCAACAGCAGAAC---
AATTTG---CTGAGA---GCTATTGAGGCGCAACAGCATATGTTGCAACTCACAGTCTGGGGCATC
>EF593314
TTCTACTGTGATTCAACACAACTATTTAATAGTACTTGGACTGAAGGGGCGACTAACAATACA----------
----

GGAAATGACACAATCATACTCCCATGCAGAATAAGACAAATTATAAACATGTGGCAGGAGGTAGGAAAAGCAA
TGTATGCCCCTCCCATCAGAGGACAAATTAGATGTTCATCAAAT---
ATTACAGGGCTGCTATTAACAAGAGATGGTGGTAATAGCAGCAAA----------------------------
----

ACAACCCCTGAGATCTTCAGACCTGGAGGAGGAGATATGAGGGACAATTGGAGAAGTGAATTATATAAATATA
AAGTAGTAAAGATTGAACCATTA---GGAATAGCACCCACCAAGGCAAAGAGAAGAGTGGTG-----------
----------------------------CAAAGAGAAAAAAGAGCA---GTGGGG---ATAGGAGCT------
---GTGTTCCTTGGGTTCTTGGGAACA---GCAGGAAGCACTATG---GGCGCAGCGGCA---
GTAACGCTGACGGTACAA---GCCAGACTA---TTATTG---TCTGGTATAGTGCAACAGCAAAAC---
AACTTG---CTGAGG---GCTATTGAGGCGCAACAGCATCTGTTGCAACTCACAGTCTGGGGCATC
>EF593316
TTCTATTGTAATTCAACACAACTGTTTAATAGTACTTGGCCTACTAATAGTACTTGGAATGCTACAGAAGGG-
----

TCAAATAGCACTATCACACTCCCATGTAAAATAAAACAAATTATAAACATGTGGCATGAAGTAGGAAAAGCAA
TGTATGCCCCTCCCATCAGAGGACAAATTAGATGTTCATCAAAT---
ATTACAGGGATATTTTTAACTAGAGATGGTGGTAAAGAGAAGAAC----------------------------
----

AACACCGAGATCTTCAGACCTGGAGGAGGAGATATGAGGGACAATTGGAGAAGTGAATTATATAAATATAAAG
TAGTAAAAATTGAACCATTA---GGAATAGCACCCACCAAGGCAAAGAGAAGAGTGGTG--------------
-------------------------CAGAGAGAAAAAAGAGCG---GGA------ATAGGAGCA---------
CTGTTCCTTGGGTTCTTGGGAGCA---GCAGGAAGCACTATG---GGCGCAGCGTCA---
ATGACGCTGACGGTACAG---GCCAGACTC---TTATTG---TCTGGTATAGTGCAACAGCAGAAC---
AATCTG---CTGAGA---GCTATTGATGCGCAACAGCATCTGTTGCAACTCACAGTCTGGGGCATC
>EF593318

TTCTACTGTGATACAACAAAACTGTTTAATAGTACTTGGCCTTGGAATGGTACTGAAGGGCCAAATATCACTG
GAGGAGAT---
-------------------ACT---
AACATTAACATTAACATTACACTCCCATGCAGAATAAGACAAATTGTAAACATGTGGCAGGAAGTAGGAAAAG
CAATGTATGCCCCTCCCATCAGAGGACAAATTAGATGTTCATCAAAT---
ATCACAGGGTTGTTGTTAACAAGAGATGGAGGTAACAATAGTACA----------------------------
----

AACGAGACCTTCAGACCTGGAGGAGGAAATATGAAGGACAATTGGAGAAGTGAATTATATAAATATAAAGTAG
TAAGAATTGAACCATTA---GGAGTAGCACCCACCGAGGCAAGGAGAAGAGTGGTG-----------------
----------------------CAGAGAGAAAAAAGAGCA---GTAACA---CTAGGAGCT---------
ATGTTCCTTGGGTTCTTGGGAGCA---GCAGGAAGCACTATG---GGCGCAGCGTCA---
ATGGCGCTGACGGTACAG---GCCAGACAA---TTATTG---TCTGGTATAGTGCAACAGCAGAAC---
AATTTG---CTGAGA---GCTATTGAGGCGCAACAACATCTGTTGCAACTCACAGTCTGGGGCATC
>JN786786
TTCTACTGTAATACAACAGAACTGTTTAATAGCACTTGGTATACTAATGGGTCAAGTAATAATATTGAAGGA-
----

AATGACACAATAATCACACTCCCATGCAGAATAAAACAAATTGTACGCATGTGGCAGGGAGTAGGAAAAGCAA
TGTATGCCCCTCCCATCGATGGAATAATTAGATGTACCTCAAAT---
ATTACAGGGCTGCTATTAACAAGGGATGGTGGTATTGATAATAACACG-------------------------
----

ACCGATCCCGAGATCTTCAGACCTGAAGGAGGAGATATGAAAAACAATTGGAGAAGTGAATTATATAGATATA
AAGTAATAAAAATTGAACCATTA---GGAGTAGCACCCACCAAGGCAAGGAGAAGAGTGGTG-----------
----------------------------CAGAGAGAAAAAAGAGCA---GCA------CTAGGAGCT------
---GTGTTCCTTGGGTTCTTGGGAGCA---GCAGGAAGCACTATG---GGCGCAGCGTCA---
ATAACGCTGACGGTACAG---GCCAGACTA---TTATTG---TCTGGTATAGTGCAACAGCAGAGT---
AATTTG---CTGAGA---GCTATTGAGGCGCAACAGCATCTGTTGAAACTCCCAGTCTGGGGCATT
>JN599165
TTCTACTGTAATACAGCAGGACTGTTTAATAGTACTTGGAATAGTACTTGGAGTTTAAATGACACTAAAAGAA
AT---

GGAAATGAAAATATCACACTCCCATGCAGAATAAAACAAATTATAAACATGTGGCAGGGAGTAGGAAAAGCAA
TGTATGCCCCTCCCATCAGTGGACAAATTAGATGCTCATCAAAT---
ATTACAGGGTTGCTATTAACAAGAGATGGTGGGAACGGGAACATCACC-------------------------
----

AACACCACCGAGACCTTCAGACCTGGAGGAGGAGATATGAGGGACAATTGGAGAAGTGAATTATATAAATATA
AAGTAGTAAAAATTGAACCATTA---GGAATAGCACCCACCAAGGCAAAGAGAAGAGTGGTG-----------
----------------------------CAGAGAGAAAAAAGAGCA---GTGGGA---ATAGGAGCT------
---GTGCTCCTTGGGTTCTTGGGAGCA---GCAGGAAGCACTATG---GGCGCAGCGTCA---
GTGACGCTGACGGTACAG---GCCAGACTA---TTATTG---TCTGGTATAGTGCAACAGCAGAAC---
AATTTG---CTGAGA---GCTATTGAGGCGCAACAGCATTTGTTGCAGCTCACAGTCTGGGGCATC
>KT124759
TTCTACTGTAATACAACAGACCTGTTTAATAGAACTGTGGAAAAAAATATTACTTGGCCTAGGGCAAACGACA
CAGAAGGA---

AATGAAAATGAAACAATCACACTCCAATGCAGAATAAAACAAATTATAAACATGTGGCAGGAAGTAGGAAAAG
CAATGTATGCCCCTCCTATCAGAGGACAAATTAAATGTACATCAAAC---
ATTACAGGACTACTATTAACAAGAGATGGTGGGAATAGTAATGATACT-------------------------
----

AATGATACCGAGACCTTTAGACCTGGAGGAGGAGATATGAGGGACAATTGGAGAAGTGAATTATATAAATATA
AAGTAGTAAAAATTGAACCATTA---GGAATAGCACCCACCAAGGCAAAGAGAAGAGTGGTG-----------
----------------------------CAGAGAGAAAAAAGAGCA---GTGGGA---CTAGGAGCA------
---CTGTTCCTTGGGTTCTTGGGAGCA---GCAGGAAGCACTATG---GGCGCAGCGTCA---
TTGACGTTGACGGTACAG---GCCAGACAA---TTATTG---TCTGGTATAGTGCAACAGCAGAAC---
AATCTG---CTGAGG---GCTATTGAGGCGCAACAGCATATGTTGCAACTCACAGTCTGGGGCATT
>JN562762

TTCTACTGTAATTCAACACAACTGTTTAGTAGCAGTTGGAATAGTACTAGTACTTGGAATAGTACTACAGTGT
CAAAT--

GATACTGACAAAAATATTACACTCCCATGTAGAATAAAACAATTTATAAACATGTGGCAGGGAGTAGGAAAAG
CAATGTATGCCCCTCCCATCAGCGGAATAATCAGCTGTTCATCAAAT---
ATTACAGGGCTGCTATTAACAAGAGATGGGGGTAATAGCAAGAATGGGAGTACT------AACAGTAGC---
AGT--
GATGGGAGTAATAACAGTAAC---------
AAGAGCGAGATCTTCAGACCTGGAGGAGGAGATATGAGGGACAATTGGAGAAGTGAATTATATAAATATAAAG
TAGTACAAATTGAACCATTA---GGAGTAGCACCCACCAGGGCAAAGAGAAGAGTGGTG--------------
-------------------------CAAAGAGAAAAAAGAGCA---GTGGGA---ATAGGAGCT---------
TTGTTCCTTGGGTTCTTGGGAGCA---GCAGGAAGCACTATG---GGCGCAGCATCA---
ATAACGCTGACGGTACAG---GCCAGACAG---TTATTG---TCTGGTATAGTGCAACAGCAGAAC---
AATTTG---CTGAGG---GCTATTGAGGCGCAACAACATATGTTGCAACTCACAGTCTGGGGCATC
>KT124776
TTCTACTGTAATACAACACAACTGTTTAATAGTATCTGGAGTAGTAATGGTACCAGGAATAGTACAAATAGCA
CTAGG--

ACAAATAGTACTATCATCACACTCCCATGCAAAATAAAACAAATTATAAACATGTGGCAGGAAGTAGGAAAAA
CAATGTATGCCCCTCCCATCAGAGGACAAATTAGCTGTGTATCAAAT---
ATTACAGGGCTATTGTTAACAAGAGATGGTGGTACAAATATCACC----------------------------
----

AACGAAACCTTCAGACCTGGAGGAGGAGACATGAGGGACAATTGGAGAAGTGAATTATATAAATATAAAGTAG
TAAAAATTGAACCATTA---GGAGTAGCACCTACCAAGGCAAAGAGAAGGGTGGTG-----------------
----------------------CAAAGAGAAAAAAGAGCA---GCGGGA---CTAGGAGCT---------
CTGTTCCTTGGGTTCTTGGGAGCA---GCAGGAAGCACTATG---GGCGCAGCGTCA---
ATAACGCTGACGGTACAG---GCCAGACAA---TTACTG---TCTGGTATAGTGCAACAGCAAAAT---
AATTTG---CTGAGG---GCTATTGAGGCGCAACAACATATGTTGCAACTCACAGTCTGGGGCATC
>KT124798
TTCTACTGTAATACAACACCACTGTTTAATAGTACTTGGAAGCGTACTTGGAATAATGTTAAAGGAACAATG
AT---

ACTAATAAAAATATCACACTCCGATGTAGGATAAAACAAATTATAAACATGTGGCAGAAAGTAGGAAAAGCAA
TGTATGCCCCTCCCATCCAAGGAGAAATTAGATGTTCATCAAAT---
ATTACAGGGCTGCTATTAACAAGGGATGGTGGTATTAGCAACAGCACGAAT----------------------
----

GACACCACCGAGATCTTCAGACCTGAAGGAGGAGATATGAGGGATAATTGGAGAAGTGAATTATATAAATATA
AAGTAGTAAGAATTGAACCAATA---GGAGTAGCACCCACCAAGGCAAAGAGAAGAGTGGTG-----------
----------------------------CAGAGAGAAAAAAGAGCA---GTGGGC---TTCGGAGCT------
---ATGTTCCTTGGGTTCTTGGGAGCA---GCAGGAAGCACTATG---GGCGCAGCATCA---
TTAACGCTGACGGTACAG---GCCAGACAA---TTATTG---TCTGGTATAGTGCAACAGCAGAAC---
AATTTG---CTGAGA---GCTATTGAGGCGCAACAGCATCTGTTGCAGCTCACAGTCTGGGGCATC
>GU331247
TTCTACTGTAATACAACAGAACTGTTTAATAGTACTTGGAATGGTACTGAAGTGGTAAATATCAATGAA----
----

GGAAATGACAATATCACTCTTCCATGCAGAATAAAACAAATTATAAACATGTGGCAGGAAGTAGGAAAAGCAA
TGTATGCCCCTCCCATCAGAGGACCAATTAAATGTTCATCGAAT---
ATTACAGGGCTAATATTAACAAGAGATGGTGGTACTAATGACACC----------------------------
----

ACCGAGGTCTTCAGACCTGCAGGAGGAGATATGATGGACAATTGGAGAAGTGAACTATATAAATATAAAGTGG
TGAAAATTGAACCATTA---GGAGTAGCACCCACCAAGGCAAAGAGAAGAGTGGTG-----------------
----------------------CAGAGAGAAAAAAGAGCA---ATAACA---ATAGGAGCG---------
GTGTTCCTTGGGTTCTTGGGAACA---GCAGGAAGCACTATG---GGCGCAGCGTCC---
GTGGCGCTGACGGTACAG---GCCAGACAA---TTATTG---TCTGGTATAGTGCAACAGCAAAGC---
AACTTG---TTGAGA---GCTATTGAGGCGCAACAGCATATGTTGCAACTCACAGTCTGGGGCATC
>JN786831

TTCTACTGCAATACTTCACCACTGTTTAATAGTATTTGGAATAGTAGTACTTGGAATGACACAAATAATACTG
AA---
-------------------GGG---
GCAAATGACACTGTTATCACACTTCGATGCAGGATAAAACAAATTATAAACATGTGGCAGGCAGTGGGAAAAG
CAATGTATGCCCCTCCCATTGAAGGACAAATTAGGTGTTCATCAAAT---
ATTACAGGGCTGATATTAACAAGAGATGGTGGTAAAGAAAACGCTACC-------------------------
----

AGGAACAACGAGACCTTCAGACCTGCAGGAGGAGATATGAGGGACAATTGGAGAAGTGAATTATATAAATATA
AAATAGTACAAATTGAACCATTA---GGAGTAGCACCCACCAAGGCAAAGAGAAGAGTGGTG-----------
----------------------------CAGAGAGAAAAAAGAGCA---GTGGGA---ATGGGAGCT------
---TTGTTCCTTGGGTTCTTGGGAGCA---GCAGGAAGCACTATG---GGCGCAGCGTCA---
CTGACGCTGACGGTACAG---GCCAGACAA---TTATTG---TCTGGTATAGTGCAACAGCAGAAC---
AATTTG---CTGAGG---GCTATTGAGGCGCAACAACATCTGTTGCAACTCACAGTCTGGGGCATC
>JQ403100
TTCTACTGTAAGACAGCACAACTGTTTAATAGTACTTGGAATGTGTTTAATAGTACTTGGAATGGTATTAAAA
GGGTAAAT---
-------------AGCACT------
GAAGGAAATGACACTATCACACTCCAATGCAGAATAAAACAAATTATAAACATGTGGCAGGAAGTAGGAAAAG
CAATGTATGCCCCTCCCATCAGAGGACAGATTAGATGCTCATCAAAT---
ATTACAGGGCTGCTGTTAACAAGAGATGGTGGCAATAACGAGAACGGA-------------------------
----

ACCCCCGAGGTCTTCAGACCTGGAGGAGGAGATATGAGGGACAATTGGAGAAGTGAATTATATAAATATAAAG
TAGTAAAAATTGAACCATTA---GGAATAGCACCCACCAAGGCAAAGAGAAGAGTGGTG--------------
-------------------------CAGAGAGAAAAAAGAGCA---GTGGGA---CTGGGAGCT---------
ATGTTCCTTGGGTTCTTGGGAGCA---GCAGGAAGCACTATG---GGCGCAGCGTCA---
GTAACGCTGACGGTACAG---GCCAGACAA---TTATTG---TCTGGTATAGTGCAACAGCAGAAC---
AATTTG---CTGAGA---GCTATTGAGGCGCAACAGCATATGTTGCAACTCACAGTCTGGGGCATT
>EU576617
TTCTACTGTAATACATCACCACTGTTTAATAGTACTTGGAATAGTACTACTAATGGGAATGAGACTGAAGGGT
CA---

AATAACGGAACTATCACACTCCAATGCAAAATAAAACAAATTATAAACATGTGGCAGGAAGTAGGGAAAGCAA
TGTATGCCCCTCCCATCAGAGGACAAATTAGATGTATATCAAAT---
ATTACAGGGCTGCTAATAACAAGAGATGGTGGTAATAACGAGACC----------------------------
----

GACAGCACAGAGAGCTTCAGACCTGGAGGAGGAGATATGAGGGACAATTGGAGAAGTGAATTATATAAATATA
AAGTAGTAAAAATTGAACCATTA---GGAGTAGCACCCACCAAGGCAAAGAGGAGAGTGGTG-----------
----------------------------CAGAGAGAAAAAAGAGCA---GTGGGA---TTAGGAGCT------
---CTGTTCCTTGGGTTCTTGGGAGCA---GCAGGAAGCACTATG---GGCGCAGCGTCA---
ATGACGCTGACGGTACAG---GCCAGACAA---TTGCTG---TCTGGTATAGTGCAACAGCAGAAC---
AATCTG---CTGAGG---GCTATTGAGGCGCAACAGCACCTGTTGCAACTCACAGTCTGGGGCATC
>AY331296
TTCTACTGTAATACAACACAACTGTTTAATAGTACTTGGAATGGTACCGAAGAGTTAAATAGTACA-------
----

AATGACAAAATTATCACACTCCCATGCAGAATAAAACAAATTATAAACATGTGGCAGGAAGTAGGAAAAGCAA
TGTATGCCCCTCCTATCAGAGGACAAATTAGATGTTCATCAAAT---
ATTACAGGGCTGCTATTAACAAGAGATGGTGGTAATAATAACAACAGC-------------------------
----

GATACTCCTGAGACCTTCAGACCTGGAGGAGGAGATATGAGGGATAATTGGAGAAGTGAATTATATAAATATA
AAGTAGTAAAAGTTGAACCATTA---GGAATAGCACCCACCAAGGCAAAGAGAAGAACGGTG-----------
----------------------------CAGAGAGAAAAAAGAGCA---GTGGGA---ATAGGAGCT------
---GTGTTCCTTGGGTTCTTGGGAGCA---GCAGGAAGCACTATG---GGCGCAGCGTCA---
ATAACGCTGACGGTACAG---GCCAGACTA---TTATTG---TCTGGTATAGTGCAACAGCAGAAC---
AATCTG---CTGAGG---GCTATTGAGGCGCAACAGCACATGTTGCAACTCACAGTCTGGGGCATC
>EF593286

TTCTACTGTAATACAACAGAACTGTTTAATAGTACTTGGTATGGTAATGATACT-------------------
----

AGGAATGATACTATCACACTCTCATGCAGAATAAAACAAATAATAAACATGTGGCAGGAAGTAGGAAAAGCAA
TGTATGCCCCTCCCATCAAAGGACAAATTAGATGCTCATCAAAT---
ATTACAGGGCTACTATTAACAAGGGATGGTGGTAATGACACGGAC----------------------------
----

GGGAAAGAGACCTTCAGACCTGTAGGAGGAAATATGAAGGACAATTGGAGAAGTGAATTATATAAATATAAAG
TAGTAAGAATTGAACCATTA---GGGGTAGCACCCACCAAGGCAAAGAGAAGAGTGGTG--------------
-------------------------CAGAGAGAAAAAAGAGCA---GTGGGA---TTCGGAGCT---------
TTGTTCCTTGGGTTCTTGGGAGCA---GCAGGAAGCACTATG---GGCGCAGCGTCA---
CTAACGCTGACGGTACAA---GCCAGACAG---TTACTG---TCTGGTATAGTGCAACAGCAAAAC---
AATCTG---CTGAGG---GCTATTGAGGCGCAACAGCATCTGTTGCAACTCACAGTCTGGGGCATT
>EU577525
TTTTACTGTAATACAACAAACCTGTTTAGTAGTACTTGGTATGGGAATGAAACTGAAGAGCCAAATAATACTA
CA---

GGAAATAGCACAACAATCACACTCCCATGCAGAATAAAACAAATTATAAACATGTGGCAGCAAGTAGGAAAAG
CAATGTATGCCCCTCCCATCAGTGGCCAATTAGCTGTTCATCAAAT---
ATTACAGGGCTGATATTAACAAGAGATGGTGGTAACACGAACGAG----------------------------
----

ACCACCGAAACCTTCAGACCTGGAGGAGGAAATATGAGAGATAATTGGAGAAGTGAATTATATAAATATAAGG
TGGTAAAAATTGAACCACTA---GGAGTAGCGCCCACCAGAGCAAGGAGAAGAGTGGTG--------------
-------------------------CAAAGAGAAAAAAGAGCA---GTGGGA---ATAGGAGCT---------
GTGCTCCTTGGGTTCTTGGGAGCA---GCAGGAAGCACTATG---GGCGCAGCGTCA---
GTGACGCTGACGGTACAA---GCCAGACAG---CTATTG---TCTGGTATAGTGCAACAGCAGAGC---
AATCTG---CTGAGG---GCTATTGATGCACAACAGCATATGTTGCAGCTCACAGTCTGGGGCATC
>JQ085297
TTCTACTGCGATTCAACAAAACTGTTTAATAGTACTTGGAATGGTACTGAAGATGATTCTGGA----------
----

AAAAATAGAACAATCACACTCCCATGCAGAATAAAGCAATTTATAAACATGTGGCAGGAAGTAGGAAAAGCAA
TGTATGCCCCTCCTATCAAGGGACAAATTAGCTGTTTATCAAAT---
ATTACAGGGCTGCTATTAACAAGAGATGGTGGTAACAATGTGAGC----------------------------
----

AACACCACCGAGGTCTTCAGACCTGGAGGAGGAAATATGAGGGACAATTGGAGAAGTGAATTATATAAATATA
AAGTAGTAGAAATTGAACCATTA---GGATTAGCACCCACCAAGGCAAAGAGGAGAGTGGTG-----------
----------------------------CAGAGAGAAAAAAGAGCA---GTGGGA---ATAGGAGCT------
---TTGTTCCTTGGATTCTTGGGAGCA---GCAGGAAGCACTATG---GGCGCAGCGTCA---
ATGACGCTGACGGTACAG---GCCAGACAA---TTATTG---TCTGGTATAGTGCAACAGCAGAAC---
AATCTG---CTGAGA---GCTATTGAGGCGCAACAGCACCTGTTGCAACTCACAGTCTGGGGCATC
>JQ085295
TTCTACTGTAATACAACACCACTATTCAATAGTATTTGGAAGGATGGACAGTTAAATAACACATACACT----
----

AAAGAGAAAAATATCACACTCCAATGTAAAATAAAACAAATTGTAAACAGGTGGCAGGAAGTAGGAAAAGCAA
TGTATGCCCCTCCCATCAGAGGACAAATTACATGTATATCAAAT---
ATTACAGGGCTACTATTAACAAGAGATGGTGGTATTGGCAATAATGAAACTGGC-------------------
----

AATAACAATACTGAGATCTTCAGACCTGGAGGAGGAGATATAAGGGACAATTGGAGAAGTGAATTATATAAAT
ATAAAGTAGTAAAAATTGAACCATTA---GGAGTAGCACCCACCAAGGCAAAAAGAAGAGTGGTG--------
-------------------------------CAGAGAGACAAAAGAGCA---ATAGGAACACTAGGGGCT---
------ATGTTCCTTGGGTTCTTGGGAGCA---GCAGGAAGCACTATG---GGCGCAGCGTCA---
TTGACGCTGACGGTACAA---GCCAGATTA---CTATTA---TCTGGTATAGTGCAACAGCAGAAC---
AATCTG---CTGAGA---GCTATTGAGGCGCAACAGCATATGTTGCAACTCACAGTCTGGGGCATT
>FJ798397

TTCTATTGTAATACAACAAAACTGTTTAACAGTACTTGGAATGGTACTGAAGGACTAAACAACTCTACAGGAA
AT---

AGCACAGGAAGTATCACACTCCCATGCAAAATAAAACAAATTATAAACATGTGGCAAGAAGTAGGAAAAGCAA
TGTATGCCCCTCCCATCAGAGGACAAATTAGCTGTTCATCAATT---
ATTACAGGGCTGATATTAACAAGAGATGGTGGTATTGACAATAAAAATGCCACT-------------------
----

AACACAAACAACACAGAGACCTTTAGACCAGGAGGAGGAAATATGAAGGATAATTGGAGAAGTGAATTATATA
AATATAAAGTAGTAAAAATTGAACCATTA---GGAATAGCACCCACCAAGGCAAAGGAGAGTGGTG-----
----------------------------------CAGAGAGAAAAAAGAGCA---GTGGGA---
CTAGGAGCT---------GTGTTCCTTGGGTTCTTGGCAACA---GCAGGGAGCACTATG---
GGCGCAGCGTCA---GTGACGCTGACGGTACAG---GCCAGACAA---TTATTG---
TCTGGTATAGTGCAACAGCAAAAC---AATCTG---CTGAAG---
GCTATTGAGGCGCAACAACGTATGTTGCAACTCACAGTCTGGGGCATC
>FJ469685
TTCTACTGTAATACAACACAACTGTTTAATAGTACTTGGAACATTACAGGAAATAACACCATCGCACCA----
----

GGAAATGACACAATCACACTCCCATGCAGAATAAAACAAATTATAAACATGTGGCAGGAAGTAGGAAAAGCAA
TGTATGCCCCTCCCATCAGAGGACCAATTAGATGTTCATCAAAT---
ATTACAGGGCTGCTATTAACAAGAGATGGTGGTAGTGACAAAAAT----------------------------
----

GATACTGAGGTCTTCAGACCTGCAGGAGGAAATATGAAGGACAATTGGAGAAGTGAATTATATAAATATAAAG
TAGTAAGAATTGAACCATTA---GGAATAGCACCCACCAAGGCAAAGAGAAGAGTGGTG--------------
-------------------------CAAAGAGAAAAAAGAGCA---GTGGGA---ATAGGAGCT---------
ATGTTCCTTGGGTTCTTGGGAGCA---GCAGGAAGCACTATG---GGCGCAGCGTCA---
GTGGCGCTGACGGTACAG---GCCAGACTA---TTATTG---TCTGGTATAGTGCAACAGCAGAAC---
AATCTG---CTGAGG---GCTATTGAGGCGCAACAGCATATGTTGCAACTCACAGTCTGGGGCATC
>FJ798416
TTCTACTGTAATACAACAGGACTGTTTAAAAGTACTTGGTATATGAATACAACAAGGAATGAT----------
----

AGTGCTGAAAATATAACACTCCCATGCAGGATAAAACAAATTATAAACATGTGGCAGGAAGTAGGAAAAGCAA
TGTATGCCCCTCCCATCAGTGGACCAATTAGTTGTTCATCAAAT---
ATTACAGGGTTGATATTAACAAGGGATGGTGGTGATAGGAACGAT----------------------------
----

ACTGAGACCTTCAGACCTGGAGGAGGAGATATGAGGGACAATTGGAGAAGTGAGTTATATAAATATAAAGTAG
TAACAATTGAACCATTA---GGAATAGCACCCACCAAGGCGAGGAGAAGAGTGGTG-----------------
----------------------CAGAGAGAAAAAAGAGCA---ATAGGC---CTGGGAGCT---------
TTGTTCATTGGGTTCTTGGGAGCA---GCAGGAAGCACTATG---GGCGCAGCGTCA---
ATAACGCTGACGGTACAA---GCCAGACAA---TTATTG---TCTGGTATAGTGCAACAGCAGAAT---
AATCTG---CTGAGG---GCTATTGAGGCGCAACAACATCTGTTGCAACTCACAGTCTGGGGCATC
>JQ085289
TTCTACTGTAATACAACATCACTGTTTAATAGTACCTGGAATGGGAATGAGACCATAGAGTCAAATAAC----
----

AGCACTGAAAATATTATACTCCAATGCAGAATAAAACAAACTATAAACATGTGGCAGGAAGTAGGAAAAGCAA
TGTATGCCCCTCCCATCAGTGGACAAATTAGATGCTCATCAAAT---
ATTACAGGGCTAATATTAACAAGAGATGGTGGTAATAATGCTAGC----------------------------
----

CAGACAGAGATCTTCAGACCTGGAGGAGGAGATATGAGGGACAATTGGAGAAGTGAATTATATAAATATAAAG
TAGTAGAGATTGAACCATTA---GGAGTAGCACCCACCAAGGCAAAGAGAAGAGTGGTG--------------
-------------------------CAGAGAGAAAAAAGAGCA---GTGGGAACACTAGGAGTT---------
ATGTTCCTTGGGTTCTTGGGAGCA---GCAGGAAGCACTATG---GGCGCAGCGTCG---
ATGGCGCTGACGGTACAG---GCCAGACAA---TTATTG---TCTGGTATAGTGCAACAGCAGAAC---
AATCTG---CTGAAG---GCTATTGAGGCGCAACAGCATCTGTTGCAACTCACGGTCTGGGGCATC

>JQ085293
TTCTACTGTGATACATCACTACTGTTTAATAGTACTTGGAATGAAACTACTTGGAACAAAACTACTGGGAATA
GTACTAAC---
-------------------GAT---
GCAAATAACACAGTAATTACACTCCCATGCAGAATAAAACAAATTATAAACATGTGGCAGGGAGTAGGAAGAG
CAATGTATGCCCCTCCCATCAGCGGAAATATTAGCTGTTCATCAAAT---
ATTACAGGGCTGATATTGACAAGAGATGGTGGTAATGAAAGTAAGAAT-------------------------
----

AACAGCGCTGAGATTTTCAGACCTGCAGGAGGAGATATGAGGGACAATTGGAGAAGTGAATTATATAAATATA
AAGTAGTAAAAATTGAACCATTA---GGAATAGCCCCCACCAGGGCAAAAAGAAGAGTGGTG-----------
----------------------------CAGAGAGAAAAAAGAGCA---GTGGGA---TTCGGAGCT------
---CTGTTCCTTGGGTTCTTGGGAGCA---GCAGGAAGCACTATG---GGCGCAGCGTCA---
CTGACGTTGACGGTACAG---GCCAGACAA---TTATTG---TCTGGTATAGTGCAACAGCAGAGC---
AATATG---CTGAGA---GCTATTGAGGCGCAACAGCATATGTTGCAACTCACAGTCTGGGGCATC
>AF217150
TTCTACTGTAATTCAACACCATTGTTTAATAGTATTTGGAATATTACTGGGAATATTACTGAAGAGTCAAATA
ACACT--

AAAGGAAATGACACAATCATACTCACATGCAGAATAAAACAAATTATAAACATGTGGCAGGAAGTAGGAAAAG
CAATGTATGCCCCTCCCATCAGAGGACAAATTAAATGTGTATCAAAT---
ATTACAGGGCTACTATTAACAAGAGATGGTGGTAAGGATGAAGGTAAA-------------------------
----

GAGAACGATACCGAGACCTTCAGACCACAAGGAGGAAATATGAGGGATAATTGGAGAAGTGAATTGTATAAAT
ATAAAGTAGTAAAAATTGAACCATTA---GGAGTAGCACCCACCAAGGCAAAGAGAAGAGTGGTG--------
-------------------------------CAGAGAGAAAAAAGAGCA---GTGGGA---ATAGGAGCT---
------ATGTTCCTTGGGTTCTTAGGAGCA---GCAGGAAGCACTATG---GGCGCAGCGTCA---
ATAACGCTGACGGTACAG---GCCAGACAA---TTATTG---TCTGGTATAGTGCAACAGCAGAGC---
AATTTG---CTGAGG---GCTATTGAGGCGCAACAGCATCTGTTGCAACTCACAGTCTGGGGCATC
>MH262735
TTCTACTGTAATACAACACAGCTGTTTAACAGTACTTGGAGGGTGGTCAATGGTACTTGGAATGATACTATAG
AA---

TCAAATAAAACTATCACACTCCCATGCAGAATAAAACAAATTATAAATAGGTGGCAGGAAGTAGGAAAAGCAA
TGTATGCCCCTCCCATTGAAGGACAAATTAACTGTTCATCACAG---
ATTACAGGGCTACTATTAACAAGAGATGGTGGTAATGACACAAACGGG-------------------------
----

ACGACAGAGACCTTCAGACCTGGAGGAGGAAATATGAAAGACAATTGGAGAAATGAATTATATAAATATAAAG
TAGTAAAAATTGAGCCATTA---GGAATAGCACCCACCAAGGCAAAGAGAAGAGTGGTG--------------
-------------------------CAGAGAGAAAAAAGAGCA---ATA------ATAGGAGCT---------
GTGTTCCTTGGGTTCTTGGGGGCA---GCAGGAATCACTATG---GGCGCAGCGTCG---
ATGACGCTGACGGTACAG---GCCAGACAA---TTATTG---TCTGGTATAGTGCAACAGCAAAAC---
AATTTG---CTGAGG---GCTATTGAGGCGCAACAGCATCTGTTGCAACTCACAGTCTGGGGCATT
>MH263144
TTCTACTGTAATACAACACCACTGTTTAATAGCTCATGGAATGGTACTGAAGGATTCAAAAAGTTCAATGAGA
CT---

GAAGACAAAAACATTACACTCCAATGTAGAATAAAACAAATTATAAACATGTGGCAGGGAGTAGGAAAAGCAA
TGTATGCCCCTCCCATCAGCGGAATAATTAAATGTTCATCAAAT---
ATTACAGGGCTGATACTAACAAGAGATGGTGGTAGAGATGATGGTACTAATACC-------------------
----

ACAACAAACGATACCGAGATCTTCAGACCTATAGGAGGAGATATGAAGGATAATTGGAGAAGTGAATTATATA
AATATAAAGTAGTAAGAATTGAACCAGTA---GGAATAGCACCCACCAGGGCAAAGAGAAGAGTGGTG-----
----------------------------------CAGAGAGAAAAAAGAGCA---GCA------
CTAGGAGCA---------GTGTTCCTTGGGTTCTTGGGAGCA---GCAGGAAGCACTATG---
GGCGCAGCGTCA---ATAACGCTGACGGTACAG---GCCAGGCTA---TTATTG---

TCTGGTATAGTGCAGCAGCAGAAC---AATTTG---CTGAGG---
GCTATTGACGCGCAACAACACCTGTTGCAACTCACAGTCTGGGGCATC
>MH263362
TTTTACTGTAATTCAACACCACTGTTTAATAGTACTTGGAAGAGGAATGATACTAATAGTACTTGGAATGTAA
CACAATGGAAT---
-------------------GAT---
AGTACTAATGGTACAATCACACTCCAATGCAGAATAAAACAAATTATAAACATGTGGCAAGAAGTAGGAAAAG
CAATGTATGCCCCTCCTATTAGAGGAAACATTAGCTGCTCATCAAAT---
ATTACAGGGCTCATATTAACAAGAGATGGTGGTGAGAAAAATGAG---------------------------
----

ACCGATGAGATCTTCAGACCTGCAGGAGGAGATATGAGGGACAATTGGAGAAGTGAACTATATAAGTATAAAG
TAGTACAACTTGAGCCATTA---GGAGTAGCCCCCACCAAGGCAAAGAGAAGAGTGGTG-------------
-------------------------CAGAGAGAAAAAAGAGCA---GTGGGAATGATAGGAGCT---------
ATGTTCCTTGGGTTCTTGGGAACA---GCAGGAAGCACTATG---GGCGCAGCGTCA---
ATAGCGCTGACGGCACAG---GCCAGACAA---TTATTG---TCTGGTATAGTGCAACAGCAGAAT---
AATCTG---CTGAGG---GCTATTGAGGCGCAACAGCATCTGTTGCAACTCACAGTCTGGGGCATC
>MH263630
TTCTACTGTAATACAACGAAACTGTTT------ACTTGGAATAGTACTCGGGATGGGAATAGCACTAAAGAG-
----

GCAAATGACACACTCATTCTCCCATGTAGAATAAAACAAATTATAAACATGTGGCAGGAAGTAGGAAAAGCAA
TGTATGCCCCTCCCATCAGAGGACCCATTAATTGTGTATCAAAC---
ATTACAGGGTTGATATTAACAAGAGATGGTGGTACTGGAAAAAATACC------------------------
----

ACAGACAACAACACTGAGGTCTTCAGACCTACAGGAGGAAATATGAAGGACAATTGGAGAAGTGAATTATATA
AATATAAAGTAGTAAGAATTGAACCATTA---GGAGTAGCACCCACCAAGGCAAAGAGAAGAGTGGTG-----
----------------------------------CAGAGAGAAAAAAGAGCA---GTGGGA---
ATAGGAGCT---------GTGTTCCTTGGGTTCTTGGGAGCA---GCAGGAAGCACTATG---
GGCGCAGCGTCA---GTGACGCTGACGGTACAG---GCCAGACAA---TTATTG---
TCTGGTATAGTGCAACAGCAGAAC---AACTTG---CTGAGG---
GCCATTGAGGCGCAACAACATCTGTTGCAACTCACGGTCTGGGGCATC
>KX984368
TTCTACTGTAATACAACACAACTGTTTAATAGTACTTGGGAGAATGAGACTACAGGGATAAATACCACTGAA-
----

GGAAATGGCACATTCACACTCCAATGCAGAATAAAACAAATTATAAACATGTGGCAGGAAGTAGGAAAAGCAA
TGTATGCCCCTCCCATTAGAGGACAGATTAGATGTTCATCAAAT---
ATTACAGGGCTGCTATTAACAAGAGATGGTGGTAATAGAGATGGT---------------------------
----
GGTAATGACACAAAC---
AGGAATGAGACCTTTAGACCTGGAGGAGGAGATATGAGGGACAATTGGAGAAGTGAATTATATAAATATAAAG
TAGTAAAAATTGAACCATTA---GGAGTAGCACCCACCAAGGCAAAGAGAAGAGTGGTG-------------
-------------------------CAGAGAGAAAAAAGAGCA---GTGGGA---ATAGGAGCT---------
CTGTTCCTTGGGTTCTTGGGAGCA---GCAGGAAGCACTATG---GGCGCAGCGTCA---
CTGACGCTGACGGTACAG---GCCAGACTA---TTATTG---TCTGGTATAGTGCAACAGCAGAAC---
AATTTG---CTGAGA---GCTATTGAGGCGCAACAGCATCTGTTGCAACTCACAGTCTGGGGCATT
>KX984537
TTCTACTGTAATACAACACAACTGTTTAATAGTACCTGGGATAATAGTACCTGGGATACTAACTGGACTAGTC
CTGAA---
-------------------AAT---
TCTACCAATACCAATATCACACTCCCATGCAGAATAAAACAAATTATAAACATGTGGCAGGAAGTAGGAAAAG
CAATGTATGCCCCTCCCATCAGAGGACAAATTAAGTGTTCATCAAAT---
ATTACAGGACTGCTGTTAACAAGAGATGGTGGTAATAGCACAGATGGA------------------------
----

AACAACTCTGAGATCTTCAGACCTGGAGGAGGAGATATGAGGGACAATTGGAGAAGTGAATTATACAAATATA
AAGTAGTGCAAATTGAACCATTA---GGAGTAGCACCTACCAAGGCAAAGAGAAGAGTGGTG----------
----------------------------CAGAGAGAAAAAAGAGCA---GTGGGA---ATAGGAGCT------

---TTATTCCTTGGGTTCTTGGGAGCA---GCAGGAAGCACTATG---GGCGCAGCATCA---
ATAACGCTGACGGTACAG---GCCAGACAA---TTATTA---TCTGGTATAGTGCAACAGCAGAAC---
AATCTG---CTGAGG---GCTATTGAGGCGCAACAGCATCTGTTGCAACTCACAGTCTGGGGCATC
>KX984588
TTCTACTGTAACACAACACAACTGTTTAATAGTACTTGGTATGCTAATGGTTCTGGGAATAATACTAAGTGG-
----

ACAAATGACACCATTACACTCCCATGTAGAATAAAACAAATTATAAACATGTGGCAGGAAGTAGGAAAAGCAA
TGTATGCCCCTCCCATTGCAGGAGTGATTAACTGTGTATCAAGT---
ATTACAGGGCTACTATTAACAAGAGATGGTGGTAGTGGAAATAACACC-------------------------
----

AACACCACTGAGGTCTTCAGACCTGGAGGAGGAGATATGAGGGACAAGTGGAGAAGTGAATTATATAAATATA
AAGTAGTACAAATTGAACCATTA---GGAATAGCTCCCACCAAGGCAAAGAGAAGAGTGGTG-----------
----------------------------CAGAGAGAAAAAAGAGCA---GTAACA---TTAGGAGCT------
---ATGTTCCTTGGGTTCTTGGGAGCA---GCAGGAAGCACTATG---GGCGCAGCGTCA---
GTGACGCTGACGGTACAG---GCCAGACAA---TTATTG---TCTGGTATAGTGCAACAGCAGAAC---
AATCTG---CTGAGA---GCTATTGAGGCGCAACAACATATGTTGCAACTCACAGTCTGGGGCATC
>AY842786
TTCTACTGTAATTCAACACAACTGTTTAATAGTACTTGGGTTAATGGTACCAGGAATGATACTAAAGGGTCAA
AT---

AGCACTGAAATCATCACACTCCCATGCAGAATAAAACAAATTATAAACAGGTGGCAGGGAGTAGGAAAAGCAA
TGTATGCCCCTCCCATCCAAGGACTAATTAAATGTTCATCAAAT---
ATTACAGGGTTGTTATTAACAAGAGATGGGGGTAACGAGGATAAC----------------------------
----

AAGACCGAGACCTTCAGACCTGCAGGAGGAGAGATGAGGGACAATTGGAGAAGTGAATTATATAAATATAAAG
TAGTAAAAATTGAACCATTA---GGAGTAGCACCCACCAAGGCAAAGAGAAGGGTGGTG--------------
-------------------------CAGAGAGAAAAAAGAGCA---GTGGGA---ATAGGAGCT---------
GTGTTCCTTGGGTTCTTGGGAGCA---GCAGGAAGCACTATG---GGCGCAGCATCA---
ATGACGCTGACGGTACAG---GCCAGACGA---TTATTG---TCTGGTATAGTGCAACAGCAGAAC---
AATTTG---CTGAGG---GCTATTGAGGCGCAACAGCATCTGTTGCAACTCACAGTCTGGGGCATC
>AY842808
TTCTACTGTAACACAACACAACTGTTTAATAGTACTTGGAACGATACTACTATTAATAGAACTGAAGGGTCA-
----

AATACAAGAAATATCACACTCCCATGCAGAATAAAACAAATTATAAACAAGTGGCAGGAAGTAGGGAAAGCAA
TGTATGCCCCTCCCATCAGAGGACAAATTAGATGTTCATCAAAT---
ATTACAGGGCTGTTATTAACAAGAGATGGTGGTGCTGATACTAAC----------------------------
----

ACCACCGAAATCTTCAGACCTGGAGGAGGAGATATGAGGGACAATTGGAGAAGTGAATTATATAAATATAAAG
TAGTAAGAATTGAACCAATA---GGAGTAGCACCCACTAAGGCAAAGAGAAGAGTGGTG--------------
-------------------------CAGAGAGAAAAAAGAGCA---GTGGGA---ATAGGAGCT---------
TTGCTCCTTGGGTTCTTGGGAGCA---GCAGGAAGCACTATG---GGCGCAGCGTCA---
TTGACGCTGACGGTACAG---GCCAGACAA---TTATTG---TCTGGTATAGTGCAACAGCAGAAC---
AACCTG---CTGAGG---GCTATTGAGGCGCAACAGCACCTGTTGCAACTCACAGTCTGGGGCATC
>KX984877
TTCTACTGTAATACAACAAAATTGTTTGATAATAATATTGCTAACAGGTCA----------------------
----

GCTAATAGCACAATCATACTCTCATGCAGAATAAAACAAATTATAAACATGTGGCAGGAAGTAGGGAAAGCAA
TGTATGCCCCTCCCATCGAAGGACAGATTAGCTGTTCATCAAAT---
ATTACAGGGCTGTTATTAACAAGAGATGGTGGTAGTAGTAATGAGAAT-------------------------
----

AATGGGAGTGAGATTTTCAGACCTGGAGGAGGAGATATGAGGGACAATTGGAGAAGTGAATTATATAAGTACA
AAGTAATAAAAATTGAACCATTA---GGAGTAGCACCCACCAAGGCAAGGAGAAGAGTGGTG-----------
----------------------------CAGAGAGAAAAAAGAGCA---GTGGGA---ATGGCAGCA------

---GTGTTCCTTGGGTTCTTGGGAGCA---GCAGGAAGCGCTATG---GGCGCAGCGTCA---
ATGACGCTGACGGTACAG---GCCAGACAA---TTATTG---TCTGGTATAGTGCAACAGCAAAAC---
AATTTG---CTGAGA---GCTATTGAGGCGCAGCAGCATCTGCTGCAACTCACAGTCTGGGGCATC
>AY842824
TTCTACTGTAATACAGACCAGCTGTTTAATAGTACTTGGCAGTTTAATAGTACTTGGCCGCCTAATAGTACTG
AGTATAATACT--
----------ACAGGGTCA------
GATAACAATGGAACGATCACACTCCAGTGTAGAATAAAACAAATTATAAATAGATGGCAGGAAGTAGGAAAAG
CAATGTATGCCCCTCCCATCGAAGGACCAATTAACTGTTCATCAAAT---
ATTACAGGGCTGCTATTAACAAGAGATGGTGGTGAGAACAGGAACAAT-------------------------
----

AGCAGTACAGAGATCTTCAGACCTGGAGGAGGAAATATGAGGGACAATTGGAGAAGTGAATTATATAAATATA
AAGTAGTAAAAATTGAACCACTA---GGAGTAGCACCCACCAAGGCAAAGAGAAGAGTGGTG-----------
----------------------------CATAAAGAAAAAAGAGCA---GTGGGA---CTTGGAGCT------
---ATGTTCCTTGGGTTCTTGGGAGCA---GCAGGAAGCACTATG---GGCGCAGCGTCA---
ATAACGCTGACGGTACAG---GCCAGACAA---TTATTG---TCTGGTATAGTGCAACAGCAGAAC---
AATCTG---CTGAGA---GCTATTGAGGCGCAACAACATATGTTGCAACTCACAGTCTGGGGCATC
>MH632949
TTCTACTGTAATACAACACAACTGTTTAATAGTAGTAATATTTGGATAAATGGACGACA--------------
----

AATAACACTAATATTACACTCCCATGCAGAATAAAACAAATCATAAATAGGTGGCAGGAAGTAGGAAGAGCAA
TGTATGCCCCTCCCATCGAGGGACGAATTAATTGTGTGTCCAAT---
ATAACAGGACTGATATTAACAAGAGATGGTGGTAACAAGACCAACACC-------------------------
----

CCTGAGACCTTCGAGACCTTCAGACCTATAGGAGGAGATATGAGGGACAATTGGAGAAGTGAATTATATAAAT
ATAAAGTAGTAAAAATTGAGCCGTTA---GGAGTAGCACCCACCAAGGCAAAAAGAAGAGTGGTG--------
-------------------------------CAAAGAGACAAAAGAGCA---GTGGGA---ATAGGAGCT---
------GTGTTCCTCGGGTTCTTGGGAGCA---GCAGGAAGCACTATG---GGCGCAGCGTCA---
ATAACGCTGACGGTACAA---GCCAGGCAA---TTACTG---TCTGGGATAGTGCAACAGCAGAAC---
AATCTG---CTGAGA---GCTATTGAGGCGCAACAGCATATATTGCAACTCACAGTCTGGGGCATC
>MH632956
TTCTACTGTAACACAACACAGTTGTTTAATAGTACTTGGCCTCCTGAGAATGGGACAACAAAT----------
----

GATACAGGAAATATAATACTCCCATGTAGAATAAAACAAATTATAAACAGATGGCAGGAAGTAGGAAAAGCAA
TGTATGCCCCTCCCATCAGAGGACAAATTAGCTGTGAATCAAAT---
ATTACAGGGTTGCTATTGACAAGAGATGGTGGTACTCCTGTGAAC----------------------------
----

CAGACCGAGACTTTCAGACCTGGAGGAGGAGATATGAGAGACAATTGGAGAAGTGAATTATACAAATATAAAG
TAGTAAAAATTGAACCATTA---GGAATAGCACCCACCAGGGCAAAGAGAAGAGTGGTG--------------
-------------------------CAGAGAGAAAAAAGAGCA---GTGGGAACTATGGGAGCT---------
CTGTTCCTTGGGTTCTTGGGAACA---GCAGGAAGCACTATG---GGCGCAGCATCA---
TTAACGCTGACGGTACAG---GCCAGACTA---CTATTG---TCTGGTATAGTGCAACAGCAAAGC---
AATTTG---CTGAAG---GCTATTGAGGCGCAACAACATCTGTTGCAACTCACAGTCTGGGGCATC
>MH632822
TTCTACTGTAATACATCACAACTGTTTAATACTACTTGGAGGACTAATAATACTGATAACAGGTCAAAT----
----

AGAACTAATACAATCACCCTCCCATGTAGAATAAAACAAATTGTAAACATGTGGCAGGAAGTAGGAAGAGCAA
TGTACGCCCCTCCCATCAGTGGACCACTTAGTTGCTTATCAAAT---
ATTACAGGGCTGCTATTAACAAGAGATGGTGGCAACGCCAAAAATGACAGCGAC-------------------
----

AACAACACAGAGACCTTCAGACCTGGAGGAGGAGATATGAGGGACAATTGGAGAAGTGAATTATATAAATATA
AAGTAGTAAAAATTGAACCATTA---GGAATAGCACCCACCAAGGCAAAGAGAAGAGTGGTG-----------
----------------------------CAGAGAGAAAAAAGAGCA---GTGGGAGTAATGGGAGCT------

```
---ATGTTCCTTGGGTTCTTGGGAGCA---GCAGGAAGCACTATG---GGCGCAGCGTCA---
GTGACGCTGACGGTACAG---GCCAGACAA---TTATTG---TCTGGTATAGTGCAACAGCAGAAC---
AATCTG---CTGAAG---GCTATTGAGGCGCAGCAGCATATGTTGCAACTCACAGTCTGGGGCATT
>MH575991
TTCTACTGTAATACAACAAAACTATTTAATAGTACTTGGCTACTTAATAGTACTTGGAATAATACTGAAAGGA
CAAAT--------------------------------------------------------------------
----------------------------
GACACTGAAATAATCACACTCCCATGCAGAATAAAACAAATTGTAAACAGGTGGCAGGAAGTAGGCAAAGCAA
TGTATGCCCCTCCCATCCAAGGACAAATTAGTTGCTCATCAGAT---
ATTACAGGGCTGCTACTAGTTAGAGATGGTGGAATTAATACCAGCAACAACAGC-------------------
-------------------------------------------------------------------------
-------
AGCAACGAGACCGAGACCTTCAGACCTGGAGGAGGAGATATGAGGGACAATTGGAGAAGTGAATTATATAAAT
ATAAGGTGGTAAAAATTGAACCATTA---GGAGTAGCACCCACCAAGGCAAAGAGAAGAGTGGTG--------
-------------------------------CAGAGAGAAAAAAGAGCA---GTGGGAATGCTGGGAGCT---
------ATGTTCCTTGGGCTCTTGGGAGCA---GCAGGAAGCACTATG---GGCGCAGCGTCA---
ATGACGTTGACGGTACAG---ACCAGACAA---TTATTG---TCTGGTATAGTGCAACAGCAGAAC---
AATCTG---CTGAGG---GCTATTGAGGCGCAGCAGCATCTGTTGCAACTCACAGTCTGGGGCATC
>MH576349
TTCTACTGTAACACAACACGACTGTTTAATAGTACTTGGAGTGTTAATGGAACTAGCATAAACGAAACT----
-------------------------------------------------------------------------
----------------------------
AACAATAAAAATATCACACTCCCGTGCAGAATAAAACAAATTATAAACAGGTGGCAGGAAGTAGGAAAAGCAA
TGTATGCCCCTCCTATCAGTGGGATAATTAGGTGCTCATCAAAT---
ATTACAGGACTAATATTAACAAGAGATGGTGGTACAACTGATGGTGGTACAACT-------------------
-------------------------------------------------------------------------
-------
AATAGTACAAAGGAGACCTTCAGACCTGGAGGGGGAAATATGAAGGACAATTGGAGAAGTGAATTATATAAAT
ACAAAGTAGTAAAAATTGAGCCATTA---GGAGTAGCACCCACCAAGGCACAGAGAAGAGTGGTG--------
-------------------------------CAAAGAGAAAAAAGAGCA---ATAGGAACGTTAGGAGCT---
------ATGTTCCTCGGGTTCTTGGGAGCA---GCAGGAAGCACTATG---GGCGCAGCGTCA---
CTGACGCTGACGGTACAA---GCCAGACAA---TTATTG---TCTGGTATAGTGCAACAGCAGAAC---
AATCTG---CTGAGG---GCTATTGAGGCGCAACAGCATATGTTGCAACTCACAGTCTGGGGCATC
>MH576262
TTCTACTGTAATACAACACAGCTGTTTAATAGTACTTGGAATAATAGTACTTGGAATGGTACTGATAATTGGG
AT-----------------------------------------------------------------------
-------------GGTACT------
GAATCAAATAGCACTATCACACTCCCATGCAGAATAAAACAAATTATAAACTTGTGGCAGGAAGTAGGAAGAG
CAATGTATGCCCCTCCCATCCAAGGGCAAATTAGATGTTCATCAAAT---
ATTACAGGGCTGCTGCTAGTAAGAGATGGTGGGAGCAACAATAGTAGT-------------------------
-------------------------------------------------------------------------
----------
AATGATACAGAGACCTTCAGGCCTGGAGGAGGAGATATGAGGGACAATTGGAGAAGTGAATTATATAAATATA
AAGTAGTCAAAATTGAACCATTA---GGAATAGCACCCACCCATGCAAAGAGAAGAGTGGTG-----------
----------------------------CAAAGGGAAAAAAGAGCA---ATAGGA---CTTGGAGCT------
---TTCTTCCTTGGGTTCTTGGGAGCA---GCAGGAAGCACTATG---GGCGCAGCGTCA---
GTGACGCTGACGGTACAG---GCCAGACAA---TTATTG---TCTGGTATAGTGCAGCAGCAAAAC---
AATTTG---CTGAGA---GCTATTGAGGCGCAACAGCATCTGTTGCAACTCACAGTCTGGGGCATC
>MH576152
TTCTACTGTAATACAACACAACTGTTTAATAGTACTTGGAATGGTACTACAGGAGTAAATGGTACAGGAGCAA
ATAACACA-----------------------------------------------------------------
----------------------------
GAAGTAAATATTATCACACTCCCATGCAAGATAAGGCAAATTGTAAACATGTGGCAGGAAGTAGGAAAAGCAA
TGTATGCCCCTCCCATCAGTGGACAAATTAGTTGTTCATCAAAT---
ATTACAGGGCTACTACTACTAAGAGATGGTGGTAATAGCAATGACACGGAT----------------------
-------------------------------------------------------------------------
----------
AACACGACTGAAATCTTCAGACCTGGAGGAGGAGATATGAGGGACAATTGGAGAAGTGAATTATATAAATATA
AAGTAGTACAAATTGAACCATTA---GGAATAGCACCCACCAAGGCAAGGAGAAGAGTGGTG-----------
----------------------------CAGAGAGAAAAAAGAGCA---GTGGGA---ATAGGAGCT------
```

---CTGTTCCTTGGGTTCTTGGGAGCA---GCAGGAAGCACTATG---GGCGCAGCATCA---
ATAACGCTGACGGTACAA---GCCAGACAC---TTATTG---TCTGGTATAGTGCAACAGCAGAAC---
AATTTG---CTGAGA---GCTATTGAGGCGCAACAGCATATGTTGCAGCTCACAGTCTGGGGCATC
>MH576039
TTCTATTGTAATACAACACAGCTGTTTAATAGTACTTGGAATAGTACTTGGACTGGGAATGCTACCGAA----
----

TTAGATGGAAACATCACACTCCCATGCAGAATAAAACAGATTATAAACAGGTGGCAAGAAGTAGGAAAAGCAA
TGTATGCCCCTCCCATTAGAGGAAAAATTAGATGTTCATCAAAT---
ATTACAGGGTTACTATTAGTAAGAGATGGTGGTAACACGAGCGGG----------------------------
----

ACTGAGACCTTCAGACCTGGAGGAGGAGATATGAAGGACAATTGGAGGAGTGAATTATATAAATATAAAGTAG
TAAAAATTGAGCCATTA---GGAGTAGCACCCACCAGGGCAAAGAGAAGAGTGGTG-----------------
----------------------CAAAGAGAAAAAAGAGCA---GTGGGA---ATAGGAGCT---------
GTGTTCCTTGGGTTCTTGGGAGCA---GCAGGAAGCACTATG---GGCGCAGCGTCA---
GTGACGCTGACGGTACAA---GCCAGGCTG---TTATTG---TCTGGTATAGTGCAACAGCAAAAC---
AATTTG---CTGAGA---GCTATTGAGGCGCAACAACATCTATTGCAACTCACAGTCTGGGGCATC
>MH575857
TTCTACTGTAATACAACAAAACTGTTTAATAGTACTTGGAACAAAAATAATACTAATTGGACTATTACTGAAG
GGTCAGAT---
-------------AACACT------
GCAGAGAATGACACACTCATGCTCCCATGCAGAATAAAACAAATTATAAACATGTGGCAGGAAGTAGGAAAAG
CAATGTATGCTCCTCCCATTAGAGGAGTAATCAAATGTTCATCAAAT---
ATTACAGGGTTGCTATTAACAAGGGATGGTGGTAGAAACCAGAGTAATAAC----------------------
----

CAGAATGAGACAGAGATCTTCAGACCTGGAGGAGGAGATATGAGGGACAATTGGAGAAGTGAATTATATAAAT
ATAAAGTAGTAAGAATTGAACCATTA---GGAGTAGCACCCACCAAGGCAAAGAGAAGAGTGGTG--------
-------------------------------CAAAGAGAAAAAAGAGCA---GTGGGA---ATAGGAGCT---
------GTGTTCCTTGGGTTCTTAGGAGCA---GCAGGAAGCACTATG---GGCGCAGCGTCG---
GTGACGCTGACGGTACAG---GCCAGACAA---TTATTG---TCTGGTATAGTGCAGCAGCAGAAC---
AACTTG---CTGAGA---GCTATTGAGGCGCAGCACCATCTGTTGCAACTCACGGTCTGGGGCATC
>MH575796
TTCTACTGTGATTCAACGCAACTGTTTAATAGTACCTGGCTGCCTAATAATGAGAGTATTTGGTATAATAATA
GGAATAATACT--
----ACAGGGTCAAAC---------
AGCAGTGCAGTCAATATCACACTCCCATGTAGAATAAAACAAATTATAAACCTATGGCAAGAAGTAGGAAAAG
CAATGTATGCCCCTCCCATCAGAGGAGCAATCAACTGCACGTCAAAT---
ATTACAGGGTTGATATTAACAAGAGATGGTGGGAATAATCCTAAC----------------------------
----

GGGACTAACACCTCTGAGACTTTCAGACCTGGAGGAGGAAATATGAGGGACAATTGGAGAAGTGAATTATATA
GATATAAAGTAGTAAAAATTGAACCATTA---GGAGTAGCACCCACCACGGCAAAGAGAAGAGTGGTG-----
----------------------------------CAGAGAGAAAAAAGAGCA---GTGGGT---
CTCGGAGCC---------TTGTTCCTTGGGTTCTTGGGAGCA---GCAGGAAGCACTATG---
GGCGCAGCGTCA---GTAACGCTGACGGTACAG---GCCAGACAA---CTATTG---
TCTGGTATAGTGCAGCAGCAGAAC---AATCTG---CTGAGG---
GCTATTGAGGCGCAACAGCATCTGTTGCAACTCACAGTCTGGGGCATC
>MH575728
TTCTACTGTAATTCAACAAAACTGTTTAATAGTACTTGGAGTAGTAATGGGAGTAGTACTTGGAATAGTACTG
AG---

ACGAATGAAATTATCACACTCCCATGCAGAATAAAACAAATTATAAACATGTGGCAGGAAGTAGGAAAAGCAA
TGTATGCCCCTCCTATCAGAGGACAAATTAGATGTTCATCAAAC---
ATTACAGGGCTGCTGTTAACAAGAGATGGTGGTAACAATAGCAACGACACTAGC-------------------
----

AACGACACTGAGATCTTTAGACCTGGAGGAGGAAATATGAAGGATAATTGGAGAAGTGAATTATATAAATACA
AAGTAGTAAAAATTGAACCAATA---GGAGTAGCACCCACCAAGGCAAAGAGAAGGGTGGTG-----------

----------------------------CAGAGAGAGAAAAGAGCA---GTAGGA---CTAGGAGCT------
---GTGTTCCTTGGGTTCTTGGGAGCA---GCCGGAAGCACTATG---GGCGCAGCGTCA---
ATGACGCTGACGGTACAG---GCCAGACAA---TTGTTG---TCTGGTATAGTGCAACAGCAGAAC---
AACCTG---CTGAAG---GCTATTGAGGCGCAGCAGCATCTGTTGCAACTCACAGTCTGGGGCATC
>MH575694
TTCTACTGTAACACATCAAATCTGTTTAATAGTACTTGGAATAGTACTACTTGGAATAGTTGGAATAGTAGTA
CTCGGGGTAAT--

ACTACAGGAAATATCACACTCCAATGCAGAATAAAACAAATTGTAAACATGTGGCAGGAAGTAGGAAAAGCAA
TGTATGCCCCTCCTATCAAAGGACCAATTAAATGTTCATCAAAT---
ATCACAGGGCTGCTACTAACAAGAGATGGGGGTGAGGAGAGTGAGAAT-------------------------
----

AACAACGATTCCGAGATCTTCAGACCTGGAGGGGGAGATATGAGGGATAATTGGAGAAGTGAATTATATAAAT
ATAAAGTAGTAAAAATTGAACCATTA---GGTCTAGCACCCACCAAGGCAAAGAGAAGAGTGGTG--------
-------------------------------CAGAGAGAAAAAAGAGCA---GTGGGAACATTAGGAGCT---
------TTGTTTCTTGGGTTCTTGGGAACA---GCGGGAAGCACTATG---GGCGCAGCGTCA---
ATGACGCTGACGGTACAG---GCCAGGCTA---TTATTG---TCTGGTATAGTGCAACAGCAGAAC---
AATTTG---CTGAGA---GCTATTGAGGCGCAACAGCATATGTTGCAACTCACAGTATGGGGCATC
>MH575680
TTCTACTGTAATACAACAAAACTGTTT------
ACTTGGAATGCTACAACAAAACTGTTTACGTGGAATGCTACAAAT----------------------------
----
AGCAATGGAACCATCATACTCCCATGTAGAATAAAACAAATTATAAACATGTGGCAAGAGGTAGGAAAAGCAA
TGTATGCCCCTCCCATTCGTGGACAAATTAGATGTTCATCAAAT---
ATTACAGGACTGCTATTAACAAGAGATGGTGGGACTAACAAGACA----------------------------
----

GGGAACAGGACTGAGATCTTCAGACCTGGAGGGGGAAATATGAAGGACAATTGGAGAAGTGAATTATATAAAT
ACAAAGTAGTAGAAATTAAACCATTA---GGAGTAGCACCCACCAAGGCAAAGAGAAGAGTGGTG--------
-------------------------------CAGAGAGAAAAAAGAGCA---GTGACC---ATAGGAGCT---
------ATGTTCCTTGGGTTCCTGGGAGCA---GCAGGAAGCACTATG---GGCGCAGCATCA---
CTGACGCTGACGGTACAG---GCCAGACAA---TTATTG---TCTGGTATAGTGCAACAGCAGAAC---
AATCTG---CTGAGG---GCGATTGAGGCGCAGCAGCATCTGTTGCAACTCACAGTCTGGGGCATA
>MH575478
TTCTATTGTAATACAACACAATTGTTTAATAGCACTTGGGATAATGGTACTGAAAAGTCAAGTAACCTTGGAG
GA---

AATGATACAACCATCATTCTCCCATGCAGAATAAAACAAATTATAAACATGTGGCAGGAAGTAGGAAAAGCAA
TGTATGCCCCTCCCATCAAAGGAATAATTAGATGTTCATCAAAT---
ATTACAGGGCTGCTATTGACAAGAGATGGAGGTAATAGTACTGACACA-------------------------
----

AATGGGAGCGAGATCTTCAGACCTGGAGGAGGAGATATGAGGGACAATTGGAGAAGTGAATTATATAAGTATA
AAGTAGTAAAAATTGAACCATTA---GGAGTAGCACCTACCAAGGCAAAGAGAAGAGTGGTG-----------
----------------------------CAGAGAGAAAAAAGAGCA---GTGGGA---CTAGGAGCT------
---TTGTTCCTTGGGTTCTTGGGAGCA---GCAGGAAGCACTATG---GGCGCAGCGTCT---
CTGACGCTGACGGTACAG---GCCAGACAA---TTGTTG---TCTGGTATAGTGCAACAGCAGAAC---
AATTTG---CTGAGG---GCAATTGAGGCGCAACAGCATCTGTTGCAACTCACAGTCTGGGGCATC
>MH575379
TTTTACTGTAATTCAACACAACTGTTTAATAGTACTTGGAATAGTGATGGTACTCTGAATAGTACTTGGAAT-
----

AGTCCAGGTAACATCACACTCCCATGTAAAATAAGGCAAATTGTAAACATGTGGCAGAAAGTAGGAAAAGCAA
TGTATGCTCCTCCCATCCGTGGACAAATTAAATGTTCATCAAAT---
ATTACAGGGCTGCTATTAACAAGAGATGGTGGTAACGAGAGTGGG----------------------------
----

AACAGCGAAACCTTCAGACCTGGCGGAGGAGATATGAGGGACAATTGGAGAAGTGAATTATACAAATATAAAG
TAGTAAGAATTGAACCATTA---GGACTAGCACCCACTAAGGCAAAGAGAAGAGTGGTG--------------

-------------------------CAGAGAGAAAAAAGAGCA---ATAGGAACACTGGGAGCT---------
GTGTTCCTTGGGTTCTTGGGAACA---GCAGGAAGCACTATG---GGCGCAGCGTCA---
ATGACGCTGACGGTACAG---GCCAGGCAA---TTATTG---TCTGGTATAGTGCAGCAGCAGAAC---
AATCTG---CTGAAG---GCTATTGAGGCGCAACAGCATCTGTTGCAACTCACAGTCTGGGGCATT
>MH633031
TTCTACTGTAATACAACAGTACTGTTCAATAGTACTTGGAATTTTAATGATAGTGATACTGGTATTGGG----
----

AATAATACTAATATCACACTCCCATGCAGAATAAGACAAGTTATAAACATGTGGCAGGAAGTAGGAAAGGCAA
TGTATGCCCCTCCCATTAGAGGACAGATTAGATGTTCATCAAAT---
ATTACAGGGCTGATATTAACAAGAGATGGTGGTGATACTAATGTTACCAAT----------------------
----

AACAAGACTGAAACCTTCAGACCTGCAGGAGGAAATATGAAGGATAATTGGAGAAGTGAACTATATAAATATA
AAGTAGTACAAATTGAACCATTA---GGAGTAGCACCCACCAAGGCAAAGAGAAGAGTGGTG-----------
----------------------------CAAAGAGAAAAAAGAGCT---GTGGGA---TTTGGAGCT------
---ATGTTCCTTGGGTTCTTGGGAGCA---GCAGGAAGCACTATG---GGCGCAGCGTCA---
GTGGCGCTGACGGTACAG---GCCAGACAA---TTATTG---TCTGGTATAGTGCAACAGCAAAAC---
AATTTG---CTGAGG---GCTATTGAGGCGCAACAGCATCTGTTGCAACTCACAGTCTGGGGCATT
>MH633155
TTCTATTGCAATACAACAGAATTATTTGACAGTACTTGGATTAATGGTACTGACATTACTACTAATGCTACTG
GAGGGAACAAT--

AGCACAAAAAAATATCACCCTCACATGCAGAATAAAACAAATTATAAATATGTGGCAGAAAGTAGGAAAAGCAA
TGTATGCCCCTCCCATCAGAGGACAAATTAGCTGTTCATCAAAT---
ATTACAGGGCTGCTATTAACTAGAGATGGGGGTGGTAATAGCACA----------------------------
----

AACAACAATGAAACCTTCAGACCTACTGGAGGAGATATGAGGGACAATTGGAGGAGTGAATTATATAAATATA
AAGTAGTAAGAATTGAACCATTA---GGGGTAGCACCCAACAAGGCAAAGAGAAGAGTGGTG-----------
----------------------------CAGAGAGAAAAAAGAGCA---GTGGGAGTAATAGGAGCT------
---ATGTTCCTTGGGTTCTTGGGAGCA---GCAGGAAGCACTATG---GGCGCAGCGTCA---
ATAACGCTGACGGTACAG---GCCAGACAA---TTATTG---TCTGGTATAGTGCAACAGCAGAAT---
AACTTG---CTGAGG---GCTATTGAGGCGCAACAGCATCTGTTGCAACTCACAGTCTGGGGCATT
>DQ127534
TTCTACTGTAATACAACAAACCTGTTTAATAGTACCTGGAATGCAACAAACCTGGTTAATGGTACCGGGAATA
GTACT--

GAAGGGAATGACACAATCAAACTCCCATGCAGAATAAGACAATTTATAAACATGTGGCAGACAGTGGGAAAAG
CAATGTATGCCCCTCCCATCGAAGGACAAATTAAATGTACATCAAAT---
ATTACAGGGCTGCTATTAACAAGAGATGGTGGTAATTCGACGGAA----------------------------
----

CAGTCCGAGACCTTCAGACCGGGAGGAGGAGATATGAGGGACAATTGGAGAAGTGAATTATATAAATATAAAG
TAGTAGGAATTGAACCATTA---GGAATAGCACCCACCACGGCAAAGAGAAGAGTGGTG--------------
-------------------------CAGAGAGAAAAAAGAGCA---GTGGGAGCACTAGGAGCT---------
ATGTTCCTTGGGTTCTTGGGAGCA---GCAGGAAGCACTATG---GGCGCAGCGTCA---
CTGACGCTGACGGTACAG---GCCAGACTA---TTATTG---TCTGGTATAGTGCAACAGCAGAAC---
AATTTG---CTGAGG---GCTATTGAGGCGCAGCAGCATATGTTGCAACTCACGGTCTGGGGCATC
>DQ127537
TTCTACTGTGACTCAACACAACTGTTTAATAGTACTTGGAATGAGACTGATTGGAATAATAAAACTGAT----
----

GGAGATGAAACTATCACACTCCCATGCAGAATAAAACAAGTTATAAACAGGTGGCAGGAAGTAGGAAAAGCAA
TGTATGCCCCTCCTATCAGAGGAAACATTAGCTGTTCATCAAAT---
ATTACAGGGCTGATACTAACAAGAGATGGTGGTAATAGTAATAACACT-------------------------
----

AACAACATCGAGATCTTTAGACCTGGGGGAGGAAATATGAAGGACAATTGGAGAAGTGAATTATATAAATATA
AAGTAGTACAAATTGAACCATTA---GGAGTAGCACCCACCAGGGCAAAGAGAAGAGTGGTG-----------

----------------------------CAGAGAGAAAAAAGAGCA---GTGGGAATGATAGGAGCT------
---ATGTTCCTTGGGTTCTTGGGAGCA---GCAGGAAGCACTATG---GGCGCAGCGTCA---
ATAACGCTGACGGTACAG---GCCAGACAA---TTATTG---TCTGGTATAGTGCAGCAGCAGAAC---
AATCTG---CTGAGG---GCTATTGAGGCGCAACAGCATCTGTTGCAACTCACAGTCTGGGGCATT
>DQ127548
TTCTACTGTAATTCAACACAATTGTTTAATAGTATTTGGAAGTATAATAATGGCACTTGGAAGTATAATGATA
GTATTTGGAAT---
-AGTACTGAAAGTTCA---------
AATAACACTGAAAACATCATGCTCCCGTGCAGAATAAGACAAATTATAAACATGTGGCAGGAAGTAGGAAAAG
CAATGTATGCCCCTCCCATCAGAGGACCAATTAGATGTACATCAAAT---
ATTACAGGGCTAATATTAACAAGAGATGGTGGCACAAACAACAAC----------------------------
----

ACGAAGGAGGTCTTCAGACCTGGAGGAGGAGATATGAGGGACAATTGGAGAAGTGAACTATATAAATATAAAG
TAGTACAAATTGAACCATTA---GGAATAGCACCCACCAGGGCAAAAAGAAGAGTGGTG--------------
-------------------------CAGAGAGAAAAAAGAGCA---GTGGGA---ATAGGAGCT---------
TTGTTCCTTGGGTTCTTGGGAGCA---GCAGGAAGCACTATG---GGCGCAGTGTCA---
ATGACGCTGACGGTACAG---GCCAGACAA---TTGTTG---TCTGGTATAGTGCAACAGCAGAAC---
AATCTG---CTGCGG---GCTATTGAGGCGCAACAGCATATGTTGCAACTCACAGTCTGGGGCATC
>AF219627
TTTTACTGTAATACAACACAACTGTTTAATAGTACTTGGATTCGGAATGGTACTGATTGGACTCGAAATGATA
CTGAAGGA---
-------------TCAGAC------
ATCACTAACGAAAATATCACGCTCCCATGTAGAATAAAACAAATTATAAACATGTGGCAGAAAGTAGGGAAAG
CAATGTATGCCCCTCCCATCAGTGGACAAATTAGCTGTTCCTCAAAT---
ATTACAGGGCTGCTATTAACACACGATGGTGTTGTTGGTCTGTACACG-------------------------
----

GACGCGAACAACGTGACCTTCAGACCGGGAGGAGGAAATATGAGGGACAATTGGAGAAGTGAATTATATAAAT
ATAAAGTAATAAAAGTTGAACCAATA---GGAATAGCACCCACCAAGGCAAAGAGAAGAGTGGTG--------
-------------------------------CAGAGAGAAAAAAGAGCA---GTGGGA---ATAGGAGCT---
------ATGTTCCTTGGGTTCTTGGGAACA---GCAGGAAGCGCTATG---GGCGCAGCGTCA---
GTGACGCTGACGGTACAA---GCCAGACAA---TTATTG---TCTGGTATAGTGCAACAGCAGAAC---
AATCTG---CTGAGG---GCTATTGAGGCGCAACAGCATATGTTGCAACTCACAGTCTGGGGCATC
>GU728244
TTCTATTGTAATACAACATCACTGTTTAATAGTACTAATAGTACTTGGAATATTGAAAGGTCAAATAACTCTA
AA---

GTTAATGACACACTCACACTCCCATGCAGAATAAAACAAATTATAAACATGTGGCAGGAAGTAGGAAAAGCAA
TGTATGCTCCTCCCATCAAAGGGAACATTAGCTGTTCATCAAAT---
ATTACAGGGCTACTATTAGTAAGAGATGGTGGTGAAAACCAGAAC----------------------------
----

AGCAGCACCGAGACTTTCAGACCTGGAGGAGGAGATATGAGAGACAATTGGAGAAGTGAATTATATAAATATA
AAGTAGTAAAACTTGAACCGTTA---GGAATAGCACCCACCAAGGCAAAGAGAAGAGTGGTG-----------
----------------------------CAGAGAGAAAAAAGAGCA---TTGGGA---TTCGGAGCT------
---ATGTTCCTTGGGTTCTTGGGAGCA---GCAGGAAGCACTATG---GGCGCAGCGTCA---
ATAGCGCTGACGGTACAG---GCCAGACAA---TTATTG---TCTGGTATAGTGCAACAGCAGAAC---
AATCTG---CTGAGA---GCTATTGAAGCGCAACAGCATCTGTTGCAGCTCACAGTCTGGGGCATC
>GU728216
TTCTACTGTAATACATCAGAGCTGTTTAATAGTGCTTGGCATGCTAATGATACTGGGAATGGCACAAAT----
----

GGGACAGAATATATCACACTCCAATGTAGAATAAAACAAATTATAAACATGTGGCAGAAAGTAGGAAAAGCAA
TGTATGCCCCTCCCATCAGAGGACCAATTAAATGTTCATCAAAT---
ATTACAGGGCTGCTATTAGTAAGAGATGGTGGCAATAATAACGAG----------------------------
----

AGCAACAATGAGACCTTCAGACCTGGAGGAGGAGATATGAGGGACAATTGGAGAAGTGAATTATATAAATATA
AAGTAGTAAAAATTGAACCATTA---GGACTGGCACCCACCAAGGCAAAGAGAAGAGTGGTG-----------

---------------------------CAGAGAGAAAAAGAGCA---GTGGGA---CTAGGAGCA------
---TTTTTCCTTGGGTTCTTGGGAGCA---GCAGGAAGCACTATG---GGCGCAGCGTCA---
ATGGCGCTGACGGTACAG---GCCAGACAA---TTATTG---TCTGGTATAGTGCAGCAGCAGAAC---
AACCTG---CTGAGG---GCTATTGAGGCGCAACAGCATCTGTTGCAACTCACAGTCTGGGGCATC
>GU728376
TTCTACTGTAACTCAACACCACTGTTTAATAGTACTTGGAATATTCGGAATAGTACTGAAGAG----------
----

TTAAATGATACTATCATACTCAAATGCAGAATAAAACAAATTATAAACATGTGGCAGGAAGTAGGAAAGGCAA
TGTACGCCCCTCCCATCAGAGGACAAATTAGATGTTCATCAAAT---
ATTACAGGGCTGCTATTAACAAGAGATGGTGGTGACACTAAAAAGAATGGG---------------------
----

ACCGATGATACAGAGACCTTCAGACCTGGAGGAGGAAATATGAGGGACAATTGGAGAAGTGAATTATATAAAT
ATAAAGTAGTAAAAATTAAACCATTA---GGAATAGCACCCACCAAGGCAAAGAGAAGAGTGGTG--------
------------------------------CAGAGAGAAAAAGAGCA---GTGGGA---TTAGGAGCT---
------ATGTTCCTTGGGTTCTTGGGAGCA---GCAGGAAGCACTATG---GGCGCAGCGTCA---
GTGACGCTGACGGTACAG---GCCAGACAA---TTATTG---TCTGGTATAGTGCAACAGCAAAAC---
AATTTG---CTGAGG---GCTATTGAGGCGCAACAGCATATGTTGCAACTCACAGTCTGGGGCATC
>FJ469690
TTCTACTGTAATTTAACACAACTGTTTAATAGTACTTGGAATGAAACAGGGTCAAATAACACTGAG-------
----

GGAAATGGCACCTTCATACTCCCATGCAGAATAAAACAAATTATAAATATGTGGCAGGAAGTAGGAAAAGCAA
TGTATGCCCCTCCCATCGAAGGGCTAATTAACTGTTTATCAAAT---
ATTACAGGGCTGATATTAACAAGAGATGGTGGTAGTAGTAATAAC---------------------------
----

ACCACCGAGATCTTCAGACCTGGAGGAGGGGATATGAGGGACAATTGGAGAAGTGAATTATATAAATATAAAG
TAGTAAAAGTTGAACCATTA---GGAGTAGCACCCACCAGGGCAAAGAGGAGAACGGTG--------------
------------------------CAAAGAGAAAAAAGAGCA---GTGGGA---ATAGGAGCT---------
TTGTTCCTTGGGTTCTTGGGAGCA---GCAGGAAGCACTATG---GGCGCAGCGTCA---
ATGACGCTGACGGTACAG---GCCAGACAA---TTATTG---TCTGGTATAGTGCAACAGCAGAAC---
AATCTG---CTGAGG---GCTATTGAGGCGCAACAGCACCTGTTGCAACTCACAGTCTGGGGCATC
>FJ469692
TTCTACTGTACTACAACACCACTGTTTAATAGTACTTGGGATAATAATAGTACTGGGAATATTAACAGT----
----

ACTAACAAAAATATCACACTCCAATGCAGAATAAAACAAATTATAAACATGTGGCAGGAAGTAGGAAAAGCAA
TGTATGCCCCTCCTATCGAGGGAACAATTAGGTGTTCATCAAAT---
ATTACAGGGTTGATATTAACAAGAGATGGCGGTATTAACAATACT---------------------------
----

GACAACAAGGAGGTCTTCAGACCAGGAGGAGGAGACATGAGGGACAATTGGAGAAGTGAATTATACAAATATA
AAGTAGTAAAAATTGAACCATTA---GGATTAGCACCCACCAAGGCAAAGAGAAGAGTGGTG-----------
---------------------------CAAAGAGAAAAAAGAGCA---GTGACA---CTGGGAGCT------
---ATGTTCCTTGGGTTCTTAGGAGCA---GCAGGAAGCACTATG---GGCGCAGCATCA---
ATGACGCTGACGGTACAG---GCCAGACTA---TTATTG---TCTGGTATAGTGCAACAGCAGAAC---
AATCTG---CTGAGG---GCTATTGAGGCGCAACAGCATCTGTTGCAACTCACAGTCTGGGGCATC
>FJ469694
TTCTACTGTAATTCAACACAACTGTTTAATAGTACTTGGAAGTATAATGATACTAATGACACTGAAAGACACG
AA--

AAAGGCAATGACAATATTACACTCCCATGCAGAATAAGACAAATTGTAAACCTGTGGCAAGAAGTAGGGAAAG
CAATGTATGCCCCTCCCATCCAAGGACTAATTAAATGTTCATCAAAT---
ATTACAGGGCTGCTATTAACAAGGGATGGTGGTAATAATGACACC---------------------------
----

TCCCAGACCGAGACCTTCAGACCTGCAGGAGGAGATATGAGGGACAATTGGAGAAGTGAATTATATAAATATA
AAGTAGTACGAATTGAACCATTA---GGAATAGCACCCACCAAGGCAAAGAGAAGAGTGGTGCAG---AGA--

----------------------GAGGTGCAGAGAGAAAAAAGAGCA---ATAGGAATGGGAGCAGTT------
---TTGTTCCTTGGGTTCTTGGGAGCA---GCAGGAAGCACTATG---GGCGCAGCGTCA---
ATGGCGCTGACGGTACAG---GCCAGACAA---TTATTG---TCTGGTATAGTGCAACAGCAAAAC---
AATTTG---CTGAGG---GCTATTGAGGCGCAACAGCATATGTTGCAACTCACAGTCTGGGGCATC
>FJ469699
TTCTACTGTAATACAACACAACTGTTTAAAGGTACTTGGAATGGTACTACTTTGATAAATACAACAGGA----
----

GGAAATGAAACAATCACACTCCCATGCAGAATAAAACAATTTATAAACATGTGGCAGAGAGTAGGAAAAGCAA
TATATGCCCCTCCTATCAGAGGACATATTAACTGTACATCAAAT---
ATTACAGGGCTGCTATTAACAAGAGATGGTGGTAATAATAACGGGACC-------------------------
----

AACACCACCGAAATCTTTAGACCTGGAGGGGGAGATATGAGGGACAATTGGAGAAGTGAATTATATAAATATA
AAGTAGTAAAAATTGAACCATTA---GGAATAGCACCCACCAAGGCAAAGAGAAGAGTGGTG-----------
----------------------------CAGAGAGAAAAAAGAGCA---GTGGGA---ATAGGAGCT------
---CTGTTCCTTGGGTTCTTGGGAGCA---GCAGGAAGCACTATG---GGCGCAGCGTCA---
TTAACGCTGACGGTACAG---GCCAGACAA---TTATTG---TCTGGTATAGTGCAACAGCAGAAC---
AATCTG---TTGAAG---GCTATTGAGGCGCAACAGCATCTGTTGCAACTCACAGTCTGGGGCATC
>FJ469702
TTCTACTGTGACACAACACAACTGTTTAATAGTACCTGGAATGATACTACCTTGAATGATACTAGTAATTGGA
GTAAT--

GATACTACTGAAGTTATCACACTCCCATGCAGAATAAAACAGATTATAAACTTGTGGCAGGAAGTAGGAAAAG
CAATGTATGCCCCTCCCATCAGTGGACAAATTAATTGTTCATCTAAT---
ATTACAGGGCTGCTATTAACAAGAGATGGTGGTAATAATAATAATAGC-------------------------
----

AACAACAGCGAGACCTTCAGACCTGCAGGAGGAAATATGAAGGACAATTGGAGAAGTGAATTATATAAATACA
AAGTAGTAAAAATTGAACCATTA---GGAATAGCACCCACCAAGGCAAGGAGAAGAGTGGTG-----------
----------------------------CAAAGAGAAAAAAGAGCA---GTGGGA---CTAGGAGCT------
---ATGTTCCTTGGGTTCTTGGGAGCA---GCAGGAAGCACTATG---GGCGCAGCGTCA---
ATAACGCTGACGGTACAG---GCCAGACAA---TTATTG---TCTGGTATAGTGCAACAGCAAAAC---
AATTTG---CTGAGA---GCTATTGAGGCGCAACACCATTTGTTGCAACTCACAGTCTGGGGCATC
>FJ469705
TTCTACTGTAATACAACAAAACTGTTTAATAGTACTTGGAATAGTACTTGGAATAATAGTACTTGGAATGGTA
CTGGA--

ACTGAAAATGACACAATCACACTCCCATGCAGAATAAAACAATTTATAAACCTGTGGCAGCAAGTAGGAAAAG
CAATGTATGCCCCTCCTATCCCAGGAAATATTAGCTGTTCATCAAAT---
ATTACAGGGCTGCTATTAACAAGAGATGGTGGTAATAGTAACAAC----------------------------
----

AGCAACAACGAGACCTTCAGACCTGGAGGAGGAGATATGAGGGACAATTGGAGAAGTGAATTATACAAATATA
AAGTAGTAAAAGTTGAACCATTA---GGAGTAGCACCCACTAAGGCAAAGAGAAGAGTGGTG-----------
----------------------------CAGAGAGAAAAAAGAGCA---GCAGGA---ATAGGAGCT------
---CTGCTCCTTGGGTTCTTGTCAGCA---GCAGGAAGCACTATG---GGCGCAGCGTCA---
GTGACGCTGACGGTACAG---GCCAGACAA---TTATTG---TCTGGTATAGTGCAACAGCAAAAC---
AATTTG---CTGAGA---GCTATTGAGGCGCAACAGCATCTGTTGCAACTCACGGTCTGGGGCATT
>FJ469711
TTCTACTGTAATTCAACACAACTGTTTAATAGTACTTGGCATAAGAATGGTACTAATAGTACTGGGGGG----
----

GCAAATGACACTATCACACTCCCATGTAAAATAAAACAAATTATAAACATGTGGCAGGAAGTAGGAAAAGCAA
TGTATGCCCCTCCCATCGAAGGACAAATTAACTGTTCATCAAAA---
ATTACAGGGCTGCTATTAACAAGAGATGGTGGTAATAACAGCACTGCC-------------------------
----

AATGACACCGAGACCTTCAGACCTGGAGGAGGAGATATGAGGGACAATTGGAGAAGTGAATTGTATAAATATA
AAGTAGTAAAAATTGAACCACTA---GGATTAGCACCCACCAGGGCAAGGAGAAGAGTGGTG-----------

---------------------------CAGAGAGAAAAAAGAGCA---GTGGGA---CTAGGAGCT------
---GTGTTCCTTGGGTTCTTGGGAGCA---GCAGGAAGCACTATG---GGCGCAGCGTCA---
ATAACGCTGACGGTACAG---GCCAGGCGA---TTAATG---TCTGGTATAGTGCAACAGCAGAAC---
AATTTG---CTGAGA---GCTATTGAGGCGCAACAGCATCTGTTGCAACTCACAGTCTGGGGCATT
>FJ469712
TTCTATTGTAATTCAACACAACTGTTTAATAGTACCTGGTATGCTAATGATACTAACAGTATTAGGAATAGTA
GCAGG---

GAAGGAAACGATACAATTATACTCCAATGTAGAATAAAACAAATTATAAACATGTGGCATGAAGTAGGAAAAG
CAATGTATGCCCCTCCCATCAAAGGAAACATTAGCTGTTCATCAAAC---
ATTACAGGGATACTATTAACAAGAGATGGTGGTAATAAGAATAACGAG------------------------
----

ACTAACAACACTGAGATCTTCAGACCTGGAGGAGGAGATATGAGGGACAATTGGAGAAGTGAACTATATAAAT
ATAAAGTAGTAAAAATTGAACCATTA---GGAATAGCACCCACCACGGCAAGGAGAAGAGTGGTG-------
------------------------------CAGAGAGAAAAAAGAGCA---GTGGGA---CTAGGAGCA---
------CTGTTCCTTGGGTTCTTGGGAGCA---GCAGGAAGCACTATG---GGCGCAGCGTCA---
TTGACGCTGACGGTACAG---GCCAGACTC---TTATTG---TCTGGTATAGTGCAGCAGCAGAAT---
AATCTG---CTGAGA---GCTATTGATGCGCAACAACATCTGTTGCAACTCACAGTCTGGGGCATT
>FJ469719
TTCTACTGTAATACAACACAACTGTTTAATACTACTTGGTATAACAATGAAACTGTGGATAATAAAACTTGGA
CTGAAGATGAT---
-------ACTAGAAACTTA------
ACTGACAAAGACATTATCATACTCCCATGCAGAATAAAACAAATTATAAACATGTGGCAGAAAGTAGGAAAAG
CAATGTATGCCCCTCCTATCCAAGGAATGATTAGCTGTTCATCAAAT---
ATTACAGGGCTACTCTTAACAAGAGATGGTGGTAATGATACAGATACAGAT---------------------
----

CCGAATGGGACAGAGACCTTCAGACCTATAGGAGGAGATATGAGGGACAATTGGAGAAGTGAATTATATAAAT
ATAAAGTAGTAAGAATTGAACCATTA---GGGATAGCACCCTCCAAGGCAAAGAGAAGAGTGGTG-------
------------------------------CAGAGAGAAAAAAGAGCA---GCA------CTAGGAGCT---
------GTGTTCCTTGGGTTCTTGGGAGCA---GCAGGAAGCACTATG---GGCGCAGCGTCA---
ATAACGCTGACGGTACAG---GCCAGACAA---TTATTG---TCTGGTATAGTGCAACAGCAGAGC---
AGATTG---CTGAGA---GCTATTGAGGCGCAACAACATATGTTGCAACTCACAGTCTGGGGCATC
>FJ469726
TTCTACTGTAATTCAACACCACTATTTAATAGTATTTGGCATGCTAATGGTACCTGGAATGGTACTGAAAAGT
CAAAT---

AACACTGAAAGAAATATCACACTCCAATGCAGAATAAAACAAATTATAAATATGTGGCAGGGAGTAGGAAAAG
CAATGTATGCCCCTCCCATCAGAGGACAAATTAGATGTTCATCAAAC---
ATTACAGGGCTGTTATTAACAAGAGATGGGGGTCCAGAGAACGAG---------------------------
----

ACTAAGCCTGAGATCTTCAGACCTGGAGGAGGAGATATGAGGAATAATTGGAGAAGTGAATTATACAAATATA
AAGTAGTAAAAATTGAACCATTA---GGAGTAGCACCCACCAAGGCAAAGAAAAGAGTGGTG----------
---------------------------CAGAGAGAAAAAAGAGCA---ATAGGGGGATTAGGAGCT------
---TTGTTCCTTGGGTTCTTAAGAGCA---GCAGGAAGCACTATG---GGCGCAGCGTCA---
ATAACGCTGACGGTACAA---GCCAGACAA---TTATTG---ACTGGTATAGTGCAACAGCAGAAC---
AATTTG---CTGAGA---GCTATTGAGGCGCAACAACACCTGTTGCAACTCACAGTCTGGGGCATC
>GU728225
TTCTACTGTAATTCATCACAACTGTTTAATAGCACTTGGAGCAGGAATGGGACGTGGAGTAGTACTTGGAACA
CC--

ACGGGAGAAAATATCACACTCCCATGCAAAATAAAACAAATTATAAACATGTGGCAAGAAGTAGGAAAAGCGA
TGTATGCCCCTCCCATTGAAGGACTAATTAGATGTGTATCAAAT---
ATCACAGGGCTGCTATTAACAAGAGATGGTGGGAACACCACCGAC---------------------------
----

AACGACACCGAGACCTTCAGACCTTTAGGAGGAAATATGAAGGACAATTGGAGAAGTGAATTATATAAATATA
AAGTAGTAAGAATTGAACCATTA---GGGTTAGCACCCACCAAGGCAAAGAGAAGAGTGGTG----------

----------------------------CAGAGGGAAAAAAGAGCA---GTGGGA---ATGGGAGCT------
---CTGTTCCTTGGGTTCTTGGGAGCA---GCAGGGAGCACTATG---GGCGCAGCGGCA---
ATGACGCTGACGGTACAA---GCCAGACAA---TTATTG---TCTGGTATAGTGCAGCAGCAGAAC---
AATCTG---CTGAGA---GCTATTGAGGCGCAACAGAGTATGTTGCAACTCACAGTCTGGGGCATT
>MN515912
TTCTACTGTAATTCAACACAACTGTTTAATAGTACTTGGAGTGTTAATGAAGGGTCAAATAACACTGAAGGA-
----

AACAATGGCACAATCACACTCCCATGTAGAATAAAACAAATTATAAACATGTGGCAGGAAGTAGGAAAAGCAA
TGTATGCCCCTCCCATCAGAGGACAGATTAAATGTTCATCAAAT---
ATTACAGGGCTGTTGTTAACAAGAGATGGTGGCAATAACAACAGC----------------------------
----

AATACAGAGATCTTCAGACCTACAGGAGGAAATATGAAGGACAATTGGAGGAGTGAATTATATAAATATAAAG
TAGTAAAAATTGAACCATTA---GGAGTAGCACCCACCCCGGCAAAGAGAAGAGTGGTG--------------
-------------------------CAGAGAGAAAAAAGAGCA---GTGGGA---TTAGGAGCT---------
TTGTTCCTTGGGTTCTTGGGAGCA---GCAGGAAGCACTATG---GGCGCAGCGTCA---
GTGACGCTGACGGTACAG---GCCAGACAA---TTATTG---TCTGGTATAGTGCAACAGCAGAAC---
AATCTG---CTGAGG---GCTATTGAGGCGCAACAGCATCTGTTGCAACTCACAGTCTGGGGCATA
>MN515943
TTCTACTGTAATTCAACACAACTGTTTAATAGTACTTGGGCTGTTAATACTACTTGGAATAATACTGAAAACA
ATGAA--

GGAAATGACACAATCACCCTTCCATGCAGAATAAAACAAATTATAAACATGTGGCAGGAAGTAGGAAAAGCAA
TGTATGCCCCTCCCATTAGAGGACAAATAAGATGTTCATCAAAT---
ATTACAGGGCTGCTATTAACAAGAGATGGTGGTAATAGTAATGACACT-------------------------
----

ACCAGGCCCGAGACCTTCAGACCTGGAGGAGGAGATATGAGGGACAATTGGAGAAGTGAATTATATAAATATA
AAGTAGTAAAAATTGAACCATTA---GGAATAGCACCCACCAAGGCAAAGAGAAGAGTGGTG-----------
----------------------------CAGAGAGAAAAAAGAGCA---GTGGGA---ATAGGAGCT------
---ATGTTCCTTGGGTTCTTGGGAGCA---GCAGGAAGCACTATG---GGCGCAGCGTCA---
ATAACGCTGACGGTACAG---GCCAGACTA---TTATTA---TCTGGTATAGTGCAACAGCAGAAC---
AATTTG---CTGAGG---GCTATTGAGGCGCAACAGCATCTGTTGCAACTCACAGTCTGGGGCATC
>MN515956
TTCTACTGTAATACAACAAAACTGTTTAGTAGCACTTGGAATGCTACTGAAAAGATCACTTGGAATAATACTG
AAAGG--

TCAAATGACACTATCATACTCCCATGCAGAATAAAACAAATTATAAACATGTGGCAGGAAGTAGGAAAAGCAA
TGTATGCCCCTCCCATCCAGGGACTAATTAGATGTTCATCAAAT---
ATTACAGGGCTGCTATTAACAAGAGATGGTGGTAATGAAAGTGACACA-------------------------
----

AATAGGTCTGAGACCTTCAGACCTGGAGGAGGAGATATGAAGGACAATTGGAGAAGTGAATTATATAAATATA
AAGTAATAAAACTTGAACCATTA---GGACTAGCACCCACTAAGGCAAAGAGAAGAGTGGTG-----------
----------------------------CAGAGAGAAAAAAGAGCA---CTGGGA---TTCGGAGCT------
---TTGTTCCTTGGGTTCTTGGGAGCA---GCAGGAAGCACTATG---GGCGCAGCGTCA---
ATAACGCTGACGGTACAG---GCCAGACAG---TTACTG---TCTGGTATAGTGCAACAGCAGAAC---
AATCTG---CTGATG---GCTATTGAGGCGCAACAGCATATGTTGCAACTCACAGTCTGGGGCATT
>MN515787
TTCTATTGTAATACAACAAAGCTGTTTAATAGCACCTTTCCCTTTAATAGTACTTCAAATAATACTAATGGGG
CAAAT--

AACACTGAAATAATCACACTCCCATGTAAAATAAAACAAATTATAAACAGGTGGCAGGAAGTAGGAAAAGCAA
TGTATGCCCCTCCCATCAGAGGAGAAATTAAATGTTTATCAAAA---
ATTACAGGGCTGCTATTAACAAGAGATGGTGGTAATAACACAAAT----------------------------
----

AACACATATGAGACCTTCAGACCTGGAGGAGGAAATATGAAGGACAATTGGAGAAGTGAATTATATAAATATA
AAGTAGTACAAATTGAGCCATTA---GGAATAGCACCCACCACGGCAAAGAGAAGAGTGGTG-----------

---------------------------CAGAGAGAAGAAAGAGCA---GTGGGG---TTTGGAGCT------
---ATGTTCCTTGGGTTCTTGGGAGCA---GCAGGAAGCACTATG---GGCGCAGCGTCA---
GTGACGCTGACGGTACAG---GCCAGACAA---TTATTG---TCTGGTATAGTGCAACAGCAGAAC---
AATTTG---CTGAGA---GCTATTGAGGCGCAACAGCATCTGTTGCAGCTCACAGTCTGGGGCATC
>EU850430
TTCTACTGTAATTCTACACAACTGTTTAATAGTACTTGGAATAGTACTGAAGGATCAAATAGCACTGAA----
----

AGAAATACCACACTCACACTCCCATGCAGAATAAAACAAATTGTAAACATGTGGCAGGAAGTAGGAAAAGCAA
TGTATGCCCCTCCCATCAGAGGACAAATTAGATGTTCATCAAAT---
ATTACAGGGCTGATATTAACAAGAGATGGT---AATAGTAACGAG----------------------------
----

ACCGAGATCTTCAGACCTGGAGGAGGAGATATGAGGGACAATTGGAGAAGTGAATTATATAAATATAAAGTAG
TAAAAATTGAACCATTA---GGAGTAGCACCCACCAAGGCAAAGAGAAGAGTGGTG-----------------
----------------------CAGAGAGAAAAAAGAGCA---GTGGGA---ATAGGAGCT---------
TTGTTCCTTGGGTTCTTGGGAGCA---GCAGGAAGCACTATG---GGCGCAGCGTCG---
ATGACGCTGACGGTACAG---GCCAGACTA---TTATTG---TCTGGTATAGTGCAACAGCAGAAC---
AATTTG---CTGAGG---GCTATTGAGGCGCAACAACATATGTTGCAACTCACAGTCTGGGGCATC
>GU728304
TTCTATTGTAATACAACACAACTGTTTAATAGTACTTGGAATGAGACTAACATAAATAGCACTGGA-------
----

GGAAATGGCACAATCACACTCCCATGCAGAATAAAACAGATTATAAATATGTGGCAGCAAGTAGGAAAAGCAA
TGTACGCCCCTCCCATCAGGGGACAAATTAACTGTACATCAAAT---
ATTACAGGGCTGCTATTAACAAGAGATGGTGGGATAAACACGACC----------------------------
----

AACACCACTGAGATCTTTAGACCTGCAGGAGGAGATATGAGGGACAATTGGAGAAGTGAATTATATAAATATA
AAGTAGTAAAAATTGAACCATTA---GGAATAGCACCCACCAAGGCAAGGAGAAGAGTGGTG-----------
---------------------------CAGAGAGAAAAAAGAGCA---GTGGGG---ATAGGAGCT------
---CTGTTCCTTGGGTTCTTGGGAGCA---GCAGGAAGCACTATG---GGCGCAGCATCA---
ATAACGCTGACGGTACAG---GCCAGAAAC---TTATTG---TCTGGTATAGTGCAACAGCAGAAC---
AATATG---CTGAGG---GCTATTGAGGCGCAACAACATATATTGCAACTCACGGTCTGGGGCATC
>GU728410
TTCTACTGTAACACATCACGGCTGTTTAATAGCACATGGTATGTAAATGGAAGTAGTACTGGAGAG-------
----

TCAAATAGCACTATCACCCTCCCATGTAGGATAAAACAAATTATAAACATGTGGCAGAAAGTAGGAAAGGCAA
TGTATGCCCCTCCTATCAAAGGACAAATTAAATGTACATCAAAC---
ATTACAGGGCTGCTATTAACAAGAGATGGTGGTAATAATAGCCAG----------------------------
----

CCCGAAGAAATCTTCAGACCTGCAGGGGGAGATATGAGGGACAATTGGAGAAGTGAATTATATAAATATAAAG
TAGTAAAAATTGAACCATTA---GGAATAGCACCCACCAAGGCACAGAGAAGAGTGGTG--------------
-------------------------CAGAGAGCAAGAAGAGCA---GTGGGA---CTAGGAGCT---------
TTGTTCCTTGGGTTCTTGGGAGCA---GCAGGAAGCACTATG---GGCGCAGCGTCA---
GTGACGCTGACGGTACAA---GCCAGACAA---TTATTG---ACTGGTATAGTGCAACAGCAGAGC---
AATTTG---CTGAGA---GCTATTGAGGCGCAACAGCATCTGTTGCAACTCACAGTCTGGGGCATC
>GU728295
TTCTACTGTAATACAACAAAACTGTTTAATAGTACATGGAATGAGACTGATAGTAACAATACTAAA-------
----

GGAGAAGACACTATCACACTCCCATGCAGAATAAAACAAATTATAAACCTGTGGCAGGAAGTAGGAAAAGCAA
TATATGCCCCTCCCATCAGAGGACAAATTAACTGTTCATCAAAT---
ATTACAGGACTGTTATTAACAAGTGATGGGGGTGGGGGTGGCAAC----------------------------
----

AGGACGGACGAGACCTTCAGACCTATAGGAGGAGATATGAGGGACAATTGGAGAAGTGAATTATATAAATATA
AAGTAGTAAGAATTGAACCATTA---GGAGTAGCACCCACCAAGGCAAAGAGAAGAGCGGTG----------

----------------------------CAGAGAGAAAAAAGAGCA---ATAGGGGACTAGGAGCT-------
---TTTTTCCTTGGGTTCTTGGGAGCA---GCAGGAAGCACTATG---GGCGCAGCGTCA---
CTGACGCTGACGGTACAG---GCCAGACTA---TTATTG---TCTGGTATAGTGCAACAGCAGAGC---
AACTTG---CTGAGA---GCTATTGAGGCGCAACAGCATCTGTTGCAACTCACTGTCTGGGGCATC
>GU728383
TTCTATTGTGATACAACATCACTGTTTAACAGTACTTGGAATAACAGTACTTGGGAGAATAATACTAAAGGAA
TC---

CTCCCAAACGCAACCATCACACTCCCATGCAGAATAAAGCAAATTATAAACATGTGGCAAGGAGTAGGAAGAG
CAATGTATGCCCCTCCTATTAGAGGACAAATTAGATGTTCATCAAAT---
ATTACAGGGCTACTGTTAACAAGAGATGGTGGTATTGGTAATAATATC-------------------------
----

ACCAATGGGTCCGAGACCTTCAGACCTGGAGGAGGGAATATGAAGGACAATTGGAGAAGTGAATTATATAAAT
ATAAAGTAGTACAACTTGAACCAGTA---GGAATAGCACCCACCAAGGCAAGGAGAAGAGTGGTG--------
-------------------------------CAGAGAGAAAAAAGAGCA---ATAGGA---CTAGGAGCT---
------ATGTTCCTTGGGTTCTTGGGAGCA---GCAGGAAGCACTATG---GGCGCAGCGTCA---
CTAACGCTGACGGTACAG---GCCAGACAA---TTATTG---TCTGGTATAGTGCAACAGCAGAAC---
AATTTG---CTGAGA---GCTATTGAGGCGCAACAGCATATGTTGCAACTCACAGTCTGGGGCATC
>FJ469729
TTCTACTGTGATTCATCAGGACTGTTTAATAGTACTTGGAATGGAACACACCTGGATACTTGGAATGGT----
----

ACTGGAGAAAATATCACACTCCAATGCAGGATAAGACAAATTATAAACATGTGGCAGGAGGTAGGAAAAGCAA
TGTATGCCCCTCCCATCAAGGGAATAATTAAATGTTCATCAAAC---
ATTACAGGATTGCTATTAACAAGAGATGGTGGTCCTGGTAATAGC----------------------------
----

ACCAATGAGACCTTCAGACCTGAAGGAGGAGATATGAGGAACAATTGGAGAAGTGAATTATATAAATACAAAG
TAGTACAAATTGAGCCATTA---GGAGTAGCACCCACCAAGGCAAGGAGAAGAGTGGTG--------------
-------------------------CAGAGAGAAAAAAGAGCA---GTGGGA---ATAGGAGCT---------
GTGCTCCTTGGGTTCTTGGGAGCA---GCAGGAAGCACTATG---GGCGCAGCGTCA---
ATGGCGCTGACGGTACAA---GCCAGACAA---TTATTG---TCTGGTATAGTGCAACAGCAGAAC---
AATTTG---CTGAGA---GCTATTGAGGCGCAACAACATCTGTTGCAACTCACAGTCTGGGGCATT
>DQ886033
TTCTACTGTAATACAACAAAACTTTTTAATAGTACTTGGATGTGGAATACTACTACTAGTAATTGGACTCTAA
ATAGCACTACA--
-------------GGGGCA------
GATGACAATGACATAATCATACTCCCATGCAGAATAAAACAAATTATAAACATGTGGCAGGAAGTAGGAAAAG
CAATGTATGCCCCTCCCATCCAAGGACAAATTAGCTGTTCATCAAAT---
ATCACAGGGTTGCTGTTAACAAGAGATGGTGGTATTAACAATGACACG-------------------------
----

AATGGGACTGAGATCTTCAGACCTGGAGGGGGAGATATGAGGGACAATTGGAGAAGTGAATTATATAAATATA
AAGTAGTAAAAATTAAACCATTA---GGAATAGCACCCACCAGGGCAAAAAGAAGAGTGGTG-----------
----------------------------CAGAGAGAGAAAAGAGCA---GTGGGA---CTAGGAGCT------
---TTGTTCCTTGGGTTCTTGGGAGCA---GCAGGAAGCACTATG---GGCGCAGCGTCA---
GTGACGCTGACGGTACAG---GCCAGACAA---TTATTG---TCTGGTATAGTGCAACAGCAGAAC---
AATTTG---CTGAGA---GCTATTGAGGCGCAACAGCATATGTTGCAACTCACAGTCTGGGGCATC
>GU728280
TTCTACTGTAATACAACAAAACTGTTTACTTGGAATAGTACTTGGGATAATATTGAAAAAGCAAATAAC----
----

ACTGACTCAAATATCACACTCCCATGCAGAATAAAACAAATTATAAACATGTGGCAGGAAGTAGGAAAAGCAA
TGTATGCCCCTCCCATCAAAGGAAGCATTATGTGTTCATCAAAC---
ATTACAGGGTTACTATTAACAAGAGATGGTGGTAACAACGGGACA----------------------------
----

AACGATACTGAAATCTTCAGACCTGGAGGAGGAGATATGAGGGACAATTGGAGAAGTGAATTATATAAATATA
AAATAGTACAAATTGAACCATTA---GGAATAGCACCCACTAGGGCAAGGAGAAGAGTGGTG-----------

----------------------------CAGAGAGAAAAAAGAGCA---ATAGGA---ATAGGAGCT------
---TTGTTCCTTGGGTTCTTGGGAGCA---GCAGGAAGCACTATG---GGCGCAGCGTCA---
ATGACGCTGACGGTACAG---GCCAGACAA---GTATTG---TCTGGTATAGTGCAGCAGCAGAAC---
AATCTG---CTGAAG---GCTATTGAGGCGCAACAGCATCTGTTGCAACTCACAGTTTGGGGCATC
>GU728339
TTTTACTGTAATACAACACAACTGTTTAATAGTACTTGGAATGGTACTGAGAATGGTACTGAGAATGATACTA
CA---

GGAGAAGAAACTATCATACTCCCATGCAGGATAAAACAAATTATAAACAGGTGGCAGGAAGTAGGAAAGGCAA
TGTATGCCCCTCCCATCAGTGGACAAATTAAATGTTCATCAAAT---
ATTACAGGGCTGCTATTAACAAGGGATGGC---AATACCAACGGT----------------------------
----

ACTGAGATCTTCAGACCTGGAGGAGGAGATATGAGGGACAATTGGAGAAGTGAATTATACAAATATAAAGTAG
TAAAAGTTGAACCAGTA---GGAGTAGCACCCACCCAGGCAAAGAGAAGAGTGGTG-----------------
----------------------CAAAGAGAGAAAAGAGCA---GTGGGA---ATAGGAGCT---------
TTGTTCCTTGGGTTCTTGGGAGCA---GCAGGAAGCACTATG---GGCGCAGCGTCA---
ATGACGCTGACGGTACAG---GCCAGACAA---TTATTG---TCTGGTATAGTGCAACAGCAGAAT---
AATCTG---CTGAGG---GCTATTGAGGCGCAGCAGCATATGTTGCAACTCACAGTCTGGGGCATC
>DQ222211
TTCTACTGTAATTCAACACAACTGTTTAATAGTACTTGGAGAGGGTTTAAGAGCTCTAATAGTACTACTTGGA
ATAAAACCGGA--
-------------GGGTCA------
AATAACACCGGACTAATCACACTCCCATGCAGAATAAAACAAATTATAAACATGTGGCAGGAAGTAGGAAAAG
CAATGTATGCCCCTCCCATCAGAGGACAAATTAGATGTTCGTCAAAT---
ATTACAGGGCTGCTATTAACAAGAGATGGTGGTAATGATAATGACCCG-------------------------
----

AACAGGACTGAGATCTTCAGACCTGGAGGAGGAGATATGAGGGACAATTGGAGAAGTGAATTATATAAATATA
AAGTAGTAAAAATTGAGCCATTA---GGAGTAGCACCCACCAAGGCAAAAAGAAGAGTGGTG-----------
----------------------------CAGAGAGAAAAAAGAGCA---GTGGGA---CTAGGAGCT------
---ATGTTCCTTGGGTTCTTGGGGGCA---GCAGGAAGCACTATG---GGCGCAGCGTCA---
GTGACGCTGACGGTACAG---GCCAGACAA---TTATTG---TCTGGTATAGTGCAACAGCAGAAC---
AATTTG---CTGAGG---GCTATTGAGGCGCAACAGCATCTGTTGCAACTCACAGTCTGGGGCATC
>GU367407
TTCTACTGTGATACAACACAACTGTTTAATAGTACTTGGGTTAATAGTACATGGAATAATACTGAAGAG----
----

GTGAATGGCACAATCACACTCCCATGCAAAATAAAACAGATTATAAACATGTGGCAGGGAGTAGGAAAAGCAA
TGTATGCCCCTCCCATCAGTGGAAAAATTAGATGTTCATCAAAT---
ATTACAGGGCTGCTATTAACAAGAGATGGTGGTGACAGTAGTAACAGT-------------------------
----

AATAGCAATGAAACCTTCAGACCTATAGGAGGAGATATGAAGGACAATTGGAGAAGTGAATTATATCCATATA
AAGTAGTAAGAATTGAACCATTA---GGAATAGCACCCACCAAGGCAAAGAGAAGAGTGGTG-----------
----------------------------CAGAAAGAAAAAAGAGCA---TTGGGTGGTTTAGGAGCT------
---TTGTTCCTTGGGTTCTTGGGAACA---GCAGGAAGCACTATG---GGCGCAGCGTCA---
CTGACGCTGACGGTACAG---GCCAGACAA---TTATTG---TCTGGTATAGTGCAACAGCAGAAT---
AATTTG---CTGAGG---GCTATTGAGGCGCAACAGCATCTGTTGCAACTCACAGTCTGGGGCATC
>GU367408
TTCTACTGTAATACAACACAACTGTTTAATAGTACTTGGAATAGTACTACTTGGAATGGAAATAATACAGATA
AC---

ACTGACAAAAACATCACACTCCAATGCAAAATAAAACAAGTTATAAACATGTGGCAGACAGTGGGAAAAGCAA
TGTATGCCCCTCCCATCCCAGGACAAATTAGATGTTCATCAAAT---
ATTACAGGGCTGCTATTAACAAGAGATGGTGGTAACGAGACCTGG----------------------------
----

GAGACCTTCAGACCTGGGGGAGGAAATATGAGGGACAATTGGAGAAGTGAATTATATAAATATAAAGTGGTGA
AAATTGAACCATTA---GGAATAGCACCCACCAAGGCAAAGAGAAGAGTGGTG--------------------

-------------------CAGAGAGAAAAAAGAGCA---GTGGGA---ATAGGAGCT---------
GTGTTCCTTGGGTTCTTGGGAGCA---GCAGGAAGCACTATG---GGCGCAGCGTCA---
TTGACGCTGACGGTACAG---GCCAGACTA---TTATTG---TCTGGTATAGTGCAACAGCAGAAT---
AATCTG---CTGAGG---GCTATTGAGGCGCAACAGCATATGTTGCAGCTCACAGTCTGGGGCATC
>KP754463
TTCTACTGTGATACAACACAATTGTTTAATAGTACTTGGAATGGGACTAATACTGAGAATAAAACTAAAGAGT
CA---

GATGACACTACTATCACACTCCCATGTAGGATAAAACAAATTATAAACATGTGGCAGGAAGTAGGAAGAGCAA
TGTATGCCCCTCCCATCAGCGGGCAAATTAACTGTTCATCAAAT---
ATTACAGGGCTGATATTAATAAGAGATGGTGGAGACAAGAATACT----------------------------
----

ACCGAGACCTTCAGACCTGGAGGAGGAGATATGAGGGACAATTGGAGAAGTGAATTATATAAATACAAAGTAG
TGAGCCTTGAGCCAATA---GGAATAGCACCAACAAAGGCAAAGAGAAGAGTGGTG-----------------
----------------------CAGAGAGAAAAAAGAGCA---GTGACC---CTGGGAGCT---------
ATGTTCCTTGGATTCTTGGGAGCA---GCAGGAAGCACTATG---GGCGCAGCATCA---
ATAACGCTGACGGTACAG---GCCAGACAA---TTATTG---TCTGGTATAGTGCAACAGCAGAAT---
AATCTG---CTGAGA---GCTATTGAGGCGCAACAGCATATGTTGCAACTCACAGTCTGGGGCATC
>KP754465
TTCTACTGTAATACAACAAAACTGTTTGAGGGTACTTGGAATGGTAATAATTGGACTACAGAGTCAAATAAAA
CTGAAGAC---

AATAACACAACAATCACAATTCCATGCAGAATAAAACAAGTTATAAACCTGTGGCAGACAGTAGGAAAAGCAA
TGTATGCCCCTCCTATTGCAGGACTAATTAACTGTTCATCAAAT---
ATTACAGGACTGTTATTAATAAGAGATGGGGGTAAAAATAGCACT----------------------------
----

AATGAAACCTTCAGACCTGGAGGAGGAGATATGAGAGACAATTGGAGAAGTGAATTATACAAATATAAAGTAG
TAAAAATTGAACCATTA---GGAATAGCACCCACTAAGGCAAAGAGAAGAGTGGTG-----------------
----------------------CAGAGAGAAAAAAGAGCA---GTGGGA---ATAGGAGCT---------
GTGTTCCTTGGGTTCTTAGGAGCA---GCAGGAAGCACTATG---GGCGCAGCGTCA---
ATGACGCTGACGGTACAG---GCCAGACAA---TTATTG---TCTGGTATAGTGCAGCAGCAGAAC---
AATCTG---CTGAGG---GCTATTGAGGCGCAACAACATCTGTTGCAACTCACAGTCTGGGGCATC
>KP754466
TTCTACTGTAATACATCACAACTGTTTAATAGTACTTGGAATAGTACTACTGACCATACTAACCTTACA----
----

GGAGATGCAATTATCACGCTCCCATGCAGAATAAAACAAATTATAAACATGTGGCAAGGAGTAGGAAGAGCAA
TGTATGCCCCTCCCATCAGAGGACAAATTAACTGTTCATCAAAT---
ATTACAGGGCTGCTATTAACAAGAGATGGTGGTGTTAACAATACCACCGAT----------------------
----

AATAATACCGAGATCTTCAGACCAGGAGGAGGAGATATGAGGGACAATTGGAGAAGTGAATTATATAAATATA
AAGTGGTAAAAATTGAACCATTA---GGAATAGCACCCACCAAGGCAAAGAGAAGAGTGGTG-----------
----------------------------CATAGAGAAAGAAGAGCG---GCA------CTAGGAGCT------
---ATGTTCCTTGGGTTCTTGGGAGCA---GCAGGAAGCACTATG---GGCGCAGCGTCA---
GTGACGCTGACGGTACAG---GCCAGACTA---TTATTG---TCTGGTATAGTGCAGCAGCAGAAC---
AATTTG---CTGAGG---GCTATTGAGGCGCAACAGCATATGTTGCAACTCACAGTCTGGGGCATC
>KP754468
TTCTACTGTAATACAGCGAAACTGTTTAACAGTACTTGGTTCAATAACACTGAAGGCATAAATACCACTGAAG
GAAATGGCAAT--

ATCACACGCAATATCACACTCCAATGCAGAATAAAACAATTTATAAACATGTGGCAGGAAGTAGGAAAAGCAA
TGTATGCCCCTCCCATCAGTGGACCGATTAGCTGTAAATCAAAT---
ATTACAGGGTTGATATTAACAAGAGATGGTGGTGAGGATAACAAC----------------------------
----

ACCGAGACCGAGATTTTTAGACCTGGAGGAGGAGATATGAGGGACAATTGGAGAAGTGAATTATATAAATATA
AAGTAGTAAGAATTGAACCATTA---GGAGTAGCACCCACCAAGGCAAAGAGAAGAGTGGTG-----------

----------------------------CAGAGAGAAAAAAGAGCA---GTGGGG---CTGGGAGCT------
---TTGTTCCTTGGGTTCTTGGGAGCA---GCAGGAAGCACTATG---GGCGCAGCGTCA---
TTGACGCTGACGGTACAA---GCCAGACAA---TTATTG---TCTGGTATAGTGCAGCAGCAGAAC---
AATCTG---CTGAGG---GCTATTGAGGCGCAACAGCATCTGTTGCAACTCACTGTCTGGGGCATC
>KP754470
TTCTACTGTAACACAGCGAGACTGTTTAATAGTACTTGGGGGGTTAATAGCACGGGAAAATCAAATAAC----
----

AATGAAACACATATCATACTCCCATGCAGAATAAAACAAATTATAAACATGTGGCAAAGAGTAGGAAAAGCAA
TGTATAACCCTCCCATCAGTGGACCAATTAGTTGTACATCAAAT---
ATTACAGGAATAATATTAACAAGAGATGGTGGACAGACCAATGAC----------------------------
----

AGTAGCACTGAGATCTTCAGACCTGGAGGAGGAGACATGAGGGATAATTGGAGAAGTGAATTATACAAATATA
AAGTAGTAAAAATTGAACCATTA---GGAGTAGCACCAACCAAGGCAAGGAGAAGAGTGGTG-----------
----------------------------CAGAGAGAAAAAAGAGCA---ATAGGA---CTAGGAGCT------
---CTGTTCCTTGGGTTCTTGGGAGCA---GCAGGAAGCACTATG---GGCGCAGCGTCA---
TTGACGCTGACGGTACAG---GCCAGACAA---TTATTG---TCTGGTATAGTGCAACAGCAGAAC---
AATTTG---CTGAGA---GCTATTGAGGCGCAACAGCATCTGTTGCAACTCACAGTCTGGGGCATT
>KP754472
TTCTACTGTAATTCAACACAACTGTTTAATAGTACTTGGATAAATGGTACTTGGACAAATGGTACTACAAATA
CCACT--

GGAGAAGATAAGATTATACTCCCATGCAGGATAAAACAAATTATAAACTTGTGGCAGGAAGTAGGAAAGGCAA
TGTACGCCCCTCCCATCAGAGGACAAATTACCTGTTCATCAAAT---
ATTACAGGGTTACTATTAACAAGAGATGGGGGTAATAAGAACGGGAGTAATGAG-------------------
----

GCCGTTGAGACCTTCAGACCTGCAGGAGGAGATATGAGGGACAATTGGAGAAGTGAATTATACAAATATAAAG
TAGTAAAAATTGAACCATTA---GGAGTAGCACCCACCAAGGCAAAGAGAAGAGTGGTG--------------
-------------------------CAGAGAGAAAAAAGAGCA---GTGGGA---TTAGGAGCT---------
TTGTTCATTGGGTTCTTGGGAGCC---GCAGGAAGCACTATG---GGCGCAGCGTCA---
GTGACGCTGACGGTACAG---GCCAGACAA---TTATTG---TCTGGTATAGTGCAACAGCAGAGC---
AATCTG---CTGAGG---GCTATTGAGGCGCAACAGCATCTGTTGCAACTCACAGTCTGGGGCATC
>KP754473
TTCTACTGTAATACTACACCACTGTTTAATAAAATTTGGTTTAATAGGACTGACTGGTCTAATAATACTGCAG
GGTCA--
-------------------AAT---
ACCACTAACGGCATAATTACACTCCAATGCAGGATAAAACAAATTATAAACATGTGGCAGGGAATAGGAAAAG
CAATGTATGCCCCTCCCATCAGAGGACAGATTAAATGTACATCAAAC---
ATTACAGGGCTGCTATTAACAAGAGATGGTGGTAATCCTTCAAACGCA-------------------------
----

ACCGATGAGATCTTCAGACCCATAGGGGGAAATATGAAGGACAATTGGAGAAGTGAATTATATAAATATAAAG
TAGTAAAACTTGAACCAATA---GGACTGGCACCCACCAAGGCGCAGAGAAGAGTGGTG--------------
-------------------------CAGAGAGAAAAAAGAGCA---GTGGGA---CTAGGAGCT---------
GTGTTCCTTGGGTTCTTGGGAGCA---GCAGGAAGCACTATG---GGCGCAGTGTCA---
ATGACGCTGACGGTACAG---GCCAGACAA---TTATTG---TCTGGTATAGTGCAACAGCAAAGC---
AATTTG---CTGAGG---GCTATTGAGGCGCAACAACATATGTTGCAACTCACAGTCTGGGGCATC
>DQ444258
TTCTACTGTAATACAACAAAACTGTTTAACAGTACTTGGAATGATAACAGTACTTGGAATGTTAACGGTACTG
GG---

AATGATAATGACACCATCATACTCCCATGCAGAATTAAACAAATTGTAAACATGTGGCAGGAAGTAGGAAAAG
CAATGTATGCCCCTCCCATCAGAGGACTAATTAAATGTTCATCAAAT---
ATCACAGGGCTGATATTAACAAGAGATGGTGGTAATAACATCACT----------------------------
----

AACGAGACCGAGATCTTCAGACCTGCAGGAGGAGATATGAAGGACAATTGGAGAAGTGAATTATATAAATATA
AAGTAGTACAAATTGAACCATTA---GGAGTAGCACCCACTAAGGCAAAGAGAAGAGTGGTG-----------

----------------------------CAGAGAGAAAAAAGAGCA---GTGGGA---ATAGGAGCT------
---TTGTTCCTTGGGTTCTTGGGAGCA---GCAGGAAGCACTATG---GGCGCAGCGTCA---
ATAACGCTGACGGTACAG---GCCAGGCAA---TTATTG---TCTGGTATAGTGCAACAGCAAAAC---
AACTTG---CTGAGG---GCTATTGAGGCGCAACAGCACCTGTTGCAACTCACAGTCTGGGGCATC
>GU728371
TTCTACTGTAACTCAACAAAACTGTTTAATAGTACTTGGTCTAATAATGATACTTGGAATAGTAGTGATGCTA
CA---

AGGTCAAATGAAACTATCACACTCCCATGCAGAATAAAACAAATTATAAACATGTGGCAGAAAGTAGGAAAAG
CGATGTACGCCCCTCCCATCAGAGGACAAATTAAATGTTCATCAAAT---
ATTACAGGGCTGCTGTTAGTAAGAGATGGTGGTACGAATAAGAGC----------------------------
----

GAGTCCGAGACATTCAGACCTGGAGGAGGAGATATGAGGGACAATTGGAGAAGTGAATTATACAAATATAAAG
TAGTAAAAATTGAACCATTA---GGAGTAGCACCCACCAAGGCAAAGAGAAGAGTGGTG--------------
-------------------------CAGAGAGAAAAAAGAGCA---CTGGGA---CTAGGAGCT---------
ATGTTCATTGGGTTCTTGGGAGCA---GCAGGAAGCACTATG---GGCGCAGCGTCA---
GTAACGCTGACGGTACAA---GCCAGACAA---TTATTG---TCTGGCATAGTGCAACAGCAGAAC---
AATCTG---CTGAGA---GCTATTGAGGCGCAACAGCATTTGTTGCAACTCACAGTCTGGGGCATC
>GU728322
TTCTACTGTAATACAACAAAACTGTTTAATAGTACTTGGCTTAATGGTACTTGGAATAGTAATGATACTGGA-
----

TTGTCAGAAAATATCACACTCCCATGCAGAATAAAACAAATTGTAAACATGTGGCAGGAAGTAGGAAAAGCAA
TGTATGCCCCACCCATCAGGGGACAAATTAGGTGTTCATCAAAT---
ATTACAGGGCTACTATTAGAAAGAGATGGTGGTGAGAACAACAGG----------------------------
----

TCCAATGAGACCTTCAGACCTATAGGAGGAAATATGAAGGACAATTGGAGAAGTGAATTATATAAATATAAAG
TAATAAAAATTGAACCATTA---GGAATAGCACCCACCAAGGCAAGGAGAAGAGTGGTG--------------
-------------------------CAAAGAGAAAAAAGAGCA---GTGGGA---ATAGGAGCT---------
ATGCTCTTTGGGTTCTTGGGAGCA---GCAGGAAGCACTATG---GGCGCAGCGTCA---
ATGACGCTGACGGTACAG---GCCAGGCTA---TTATTG---ACTGGTATAGTGCAACAGCAGAAC---
AATCTG---CTGAGG---GCTATTGAGGCGCAACAGCATCTGTTGCAGCTCACAGTCTGGGGCATC
>EF440778
TTCTACTGTAATTCAACAAAACTGTTTAATAGTACTTGGAATAGTGATGGTACTAGAAATAGTAGTGAAACGT
CA---

TCTGAAACAGTTATCACACTCCCATGTAGAATAAAACAAATTGTAAACATGTGGCAGGAAGTAGGAAAAGCAA
TGTATGCCCCTCCCATCAGTGGACAGATTAGGTGCTCATCAAAT---
ATTACAGGGCTAATATTAACAAGAGATGGTGGCAAAGGTGACAACATC-------------------------
----

ACCAACAACACCGAGATTTTCAGACCTGGAGGAGGAAATATGCAGGACAATTGGAGAAGTGAATTATATAAAT
ATAAGGTAGTACAAATTGAACCATTA---GGAATAGCACCCACCAAGGCAAAGAGAAGAGTGGTG--------
-------------------------------CAAAGAGAAAAAAGAGCA---GTGGGG---ATAGGAGCT---
------GTGTTCCTTGGGTTCTTGGGAGCA---GCAGGAAGCACTATG---GGCGCAGCGTCA---
ATAGCGCTGACGGCACAG---GCCAGACAA---TTGTTG---TCTGGTATAGTGCAACAGCAGAAC---
AATCTG---CTGAGG---GCTATTGAGGCGCAACAGCATCTGTTGCAACTCACAGTCTGGGGCATC
>GU728392
TTCTACTGCAACACATCACAACTGTTTAATAGTACTTGGAATGGTACTGAAGGGACAAATAACAATAAA----
----

GGAAATGAAACAATCACACTGCCATGCAGAATAAAACAGATTATAAACATGTGGCAGGAAGTAGGAAAAGCAA
TGTATGCTCCTCCCATCAGAGGACAAATTAGATGTTCATCAAAT---
ATTACAGGGTTGCTATTGACAAGAGATGGTGGTAATAGGACAAAC----------------------------
----

AATACAGAGACTTTCAGACCTGGAGGAGGAAATATGAAGGACAATTGGAGAAGTGAGCTATATAAATATAAAG
TAGTAAAAATTGAACCAATA---GGAATAGCACCCACCAAGGCAAAGAGAAGAGTGGTG--------------

-------------------------CAGAGAGAAAAAAGAGCA---GTGGGA---GTAGGAGCG---------
TTTATCATTGGATTCTTGGGAGCA---GCAGGAAGCACTATG---GGCGCAGCATCA---
ATGACGCTGACGGTACAG---GCCAGACTA---TTGTTG---TCTGGTATAGTGCAACAGCAAAGC---
AATTTG---CTGAAG---GCTATTGAGGCGCAACAACGTCTGTTGCAACTCACAGTCTGGGGCATC
>DQ886034
TTCTACTGTAACACAACAAAACTGTTCAATAGTACTTGGACTTGGAATAGTACTGAAGGGTCAGAA-------
----

GGAAATGAAACAATCACACTCCCATGCAGAATAAAACAAATTATAAACATGTGGCAGGAAGTAGGAAAAGCAA
TGTATGCCCCTCCCATCAGAGGACAAATTAAATGTTCATCAAAT---
ATTACAGGACTGCTATTAACAAGAGATGGTGGTGACCAAAATACC----------------------------
----

ACCACCGAGGTCTTCAGACCTGGAGGAGGAGATATGAGGGACAATTGGAGAAGTGAATTATATAAATATAAAG
TAGTGAAAATTGAGCCATTA---GGCATAGCACCCACCAAGGCAAAGAGAAGAGTGGTG--------------
-------------------------CAGAGAGAAAAAAGAGCA---GTGGGA---CTAGGAGCT---------
ATGTTCCTTGGGTTCTTGGGAGCA---GCAGGAAGCACTATG---GGCGCAGCGTCC---
ATAACGCTGACGGTACAG---GCCAGACTA---TTATTG---TCTGGTATAGTGCAACAGCAGAAC---
AATTTG---CTGAGG---GCTATTGAGGCGCAACAGCATCTGTTGCAACTCACAGTCTGGGGCATC
>DQ886035
TTCTACTGTGATTCAACACAACTGTTCAATAATACTTGGGATGGTACTAATATGTCAAATGCAAGA-------
----

AATGACACAAACATTACACTCCCATGCAGAATAAAACAATTTATAAACATGTGGCAGAGAGTAGGAAAAGCAA
TGTATGCCCCTCCCATCAGAGGAAACATTAGCTGTTCATCAAAT---
ATTACAGGGCTGATATTAACAAGAGATGGTGGTCCCAACGCGACC----------------------------
----

AACGAGACTTTCAGACCTGGAGGAGGAGATATGAGGGACAATTGGAGAAGTGAATTATATAAATATAAAGTAG
TAAGAATTGAACCATTA---GGAGTAGCACCCACCAAGGCAAAGAGAAGAGTGGTG-----------------
----------------------CAGAGAGAGAAAAGAGCA---GTGGGA---ATAGGAGCT---------
ATGTTCCTTGGGATCTTGGGAGCA---GCAGGAAGCACTATG---GGCGCAGCGTCA---
ATAACGCTGACGGTACAG---GCCAGACAA---TTATTG---TCTGGTATAGTGCAACAGCAGAGC---
AATTTG---CTGAGG---GCTATTGAGGCGCAACAGCATCTGTTGCAACTCACAGTCTGGGGCATC
>DQ886036
TTCTACTGTAATACAACACAACTGTTTAATAGTACTTGGTATGAGAATGAGACTATTGAATGGTCAAATAACA
CT---

GTAGGAAATGACATTATCACACTCCCATGCAGAATAAAACAAATTGTAAACATGTGGCAAGAGGTAGGAAAAG
CAATGTATGCCCCTCCCATCAGCGGACAAATTAGATGCTCATCAAAT---
ATTACAGGGTTACTATTAACAAGAGATGGTGGTAATAACACAAAT----------------------------
----

GAGACCGAGATTTTCAGACCTGTAGGAGGAAACATGAGGGACAATTGGAGAAGTGAATTATATAAATATAAAG
TAGTAAGAATTGAACCATTA---GGAGTAGCACCCACCAAGGCAAAGAGAAGAGTGGTG--------------
-------------------------CAGAGAGAAAAAAGAGCA---GTGGGA---CTAGGAGCT---------
ATGTTCCTTGGGTTCTTGGGAGCA---GCAGGAAGCACTATG---GGCGCAGCGTCA---
GTGACGCTGACGGTACAG---GCCAGACAA---TTATTG---TCTGGTATAGTGCAACAGCAGAAC---
AATCTG---CTGAGG---GCTATTGAGGCGCAACAGCATCTGTTGCAACTCACAGTCTGGGGCATC
>FJ469750
TTCTACTGCGATTCAACACCACTGTTTAATAGTACTTGGAATACTACTGAAGGAAATGACACAATCACACGC-
----

GGAAATGAAACAATCACACTCCCATGCAGAATAAAACAAATTATAAACATGTGGCAAGAAGTAGGAAAAGCAA
TGTATGCCCCTCCCATCAAAGGACAAATTAGCTGTTCATCAAAT---
ATTACAGGGCTGATATTAACAAGAGATGGTGGTAATAATAATGGGACC-------------------------
----

AATAACAATACTGAGATCTTCAGACCTGCAGGAGGAGATATGAGGGACAATTGGAGAAGTGAATTATATAAAT
ATAAAGTAGTAAAAATTGAACCATTA---GGAATAGCACCCACCACGGCAAAAAGAAGAGTGGTG--------

-------------------------------CAGAGAGAAAAAAGAGCA---GTAGGA---CTAGGAGCT---
------ATGTTCCTTGGGTTCTTGGGAGCA---GCAGGAAGCACTATG---GGCGCAGCGTCA---
CTGACACTGACGGTACAG---GCCAGACTA---TTATTG---TCTGGTATAGTGCAACAGCAGAAC---
AATTTG---CTGAGG---GCTATTGAGGCGCAACAGCATCTGTTGCAACTCACAGTCTGGGGCATC
>GU728413
TTCTACTGTAATACAACAAAACTATTTAATAGTACTTGGAATGATACTAAGGGGTCAAATGACACTGTA----
----

GGAAATGACACAATCATACTCCCATGCAGAATAAAACAAATTATAAACCTGTGGCAGGAAGTAGGAAAAGCAA
TGTACGCCCCTCCCATCAGAGGACAAATTAGGTGTTCATCAAAT---
ATTACAGGGCTGCTATTAACAAGAGATGGTGGTAACGAGAGCAGC----------------------------
----

ACCACCGAGACCTTTAGACCTGGAGGAGGAAATATGAAGGATAATTGGAGAAGTGAATTATATAAATATAAAG
TAGTAAGAGTTGAACCATTA---GGAATAGCACCCACCATGGCAAGGAGAAGAGTGGTG--------------
-------------------------CAGAGAGAAAAAAGAGCA---GTGGGA---ATGGGAGCT---------
CTGTTCCTAGGGTTCTTAGGAGCA---GCAGGAAGCACTATG---GGCGCAGCGTCC---
GTAACGCTGACGGTACAG---GCCAGACAA---TTATTG---TCTGGTATAGTGCAACAGCAGAAC---
AATCTG---CTGAGA---GCTATTGATGCGCAACAGCATCTGTTGCAGCTCACTGTCTGGGGCATC
>GU728313
TTCTACTGTAATACAACACGACTGTTTAATAGTACCTGGAATAATACTACCTGGAGTAATTCTAATGTAGCAA
AT---

GGCACTGAAAGAATTATCACACTTCCATGCAGAATAAAACAAATTATAAACATGTGGCAGGAAGTAGGAAAAG
CAATGTATGCCCCTCCCATTAGAGGACAAATTAGTTGCTCATCAAAT---
ATTACAGGGCTACTGATAACAAGAGATGGTGGGAAGAATGTGACT----------------------------
----

AGGGGGACTGAGATCTTCAGACCTGGAGGAGGAGATATGAGGGACAATTGGAGAAGTGTATTATATAAATATA
AAGTAGTACAAATTGAACCATTA---GGAGTAGCACCCACCAAGGCAAAGAGAAGAGTGGTG-----------
----------------------------CAAAGAGAAAAAAGAGCA---GTGGGA---ATAGGAGCT------
---GTGTTCCTTGGGTTCTTGGGAGCA---GCAGGAAGCACTATG---GGCGCAGCGTCA---
ATGACGCTGACGGTACAG---GCCAGACAA---TTATTG---TCTGGTATAGTGCAACAGCAGAAC---
AATCTG---CTGAGA---GCTATTGAGGCGCAACAGCATCTGTTGCAACTCACAGTCTGGGGCATC
>GU728179
TTCTACTGTAATACAACACAACTGTTTAATAGTACTTGGCTTAACAGTACTTGGAATAATAGTGGAAATGATA
CTGCA--

GAATCAAATGACACTATCACACTCCCATGTAGAATAAGACAAATTATAAACATGTGGCAGCGAGTAGGAAAAG
CAATGTATGCCCCTCCCATCAGCGGAAAGATAAAGTGTATATCAAAT---
ATTACAGGGCTACTATTAACAAGAGATGGTGCTAGGAACAACAGC----------------------------
----

GAGAATGAGACCTTCAGACCCGAAGGAGGGAATATGAGGGACAATTGGAGAAGTGAATTATATAAATATAAAG
TAGTACAAATTGAACCATTA---GGAGTAGCACCCACTAAGGCAAAGAGGAGAGTGGTG--------------
-------------------------CAGAGAGAAAAAAGAGCA---GTGGGA---CTAGGAGCT---------
GTGTTCCTTGGGTTCTTGGGAGCA---GCAGGAAGCACTATG---GGCGCAGCGTCA---
CTGACGCTGACGGTACAG---GCCAGACAA---TTATTG---TCTGGTATAGTGCAACAGCAGAAC---
AATCTG---CTGAGA---GCTATTGAGGCGCAACAACATATGTTGCAACTCACAGTCTGGGGCATC
>GU728409
TTCTATTGCAGTACAACAGAACTATTTAATAGTACTTGGAATGTTACTGAAAAGTTAAATAAAACTAAAGAAA
AT---

GGAAGTGACACAATCACACTCCCATGCAGAATAAAACAAATTATAAACCTGTGGCAGGAAGTAGGAAAAGCAA
TGTATGCCCCTCCCATCAGCGGACAAATTAGATGTTCCTCAAAT---
ATTACAGGGCTGATATTAACAAGAGATGGTGGTAATGAAAACAAG----------------------------
----

ACCACCGAGACCTTCAGACCTGAAGGAGGAAATATGAGGGACAATTGGAGAAGTGAATTATATAAATATAAAG
TAGTACAAATTGAACCATTA---GGAGTAGCACCCACCAGGGCAAAAAGAAGAGTGGTG--------------

-------------------------CAGAGAGAAAAAAGAGCG---GGAATA------GGAGCT---------
CTGCTCCTTGGGTTCTTGGGAGCA---GCAGGAAGCACTATG---GGCGCAGCGTCA---
ATGGCGCTGACGGTACAG---GCCAGGCAA---TTATTG---TCTGGTATAGTGCAACAGCAGAAC---
AATCTG---CTGAGG---GCTATTGAGGCGCAACAGCATCTGTTGCAACTCACAGTCTGGGGCATT
>AF491737
TTCTACTGTGATACAACAAAACTGTTTAATAGTACTTGGAGTAGTAATAGTACTTGGAATGATACTACAGGGT
TAAATAAC---
-------------------ACT---
GGGGAGGATGATACAATCACACTCCCATGCAGAATAAAACAAATTATAGATATGTGGCAGGAAGTAGGAAAAG
CAATGTATGCCCCTCCCAGCAGTGGACAAATTAGATGTTCATCAAAT---
ATTACAGGGCTGTTATTAACAAGAGATGGTGGTATTAACGGGAGC----------------------------
----

GAAGGGAATGAGACCTTCAGACCTGGAGGAGGAGATATGAGGGACAATTGGAGAAGTGAATTATACAAATATA
AAGTAGTAAGAATTGAACCATTA---GGAGTAGCACCCACCAAGGCAAAGAGAAGAGTGGTG-----------
----------------------------CGGAGAGAAAAAAGAGCA---GTGGGA---TTAGGAGCT------
---ATGTTCCTTGGGTTCTTGGGAGCA---GCAGGAAGCACTATG---GGCGCAGCGTCA---
ATAGCGCTGACGGTACAG---GCCAGACAA---TTGTTG---TCTGGTATAGTGCAACAGCAGAAC---
AATTTG---CTGAGG---GCTATTGAGGCGCAACAACATTTGTTGCAACTCACAATCTGGGGCATC
>JN002043
TTCTACTGTGATTCATCACAACTGTTTAATAGTACTTGGATGTTTAATAGTACTTGGAATGATACTGACGGGG
CAAATAGC---
-------------------ACT---
AGGAAAAAATGATACAATCACACTCCAATGCAGAATAAAACAAATTATAAACATGTGGCAGCAAGTAGGAAAAG
CAATGTATGCCCCTCCCATCAGAGGACAGATTAGATGTACATCAAAC---
ATTACAGGGCTGATATTAACAAGAGATGGTGGTGATACCCAAAACAAG-------------------------
----

AGCGACCCCGAGATCTTTAGACCTGGAGGAGGAGATATGAGGGACAATTGGAGAAGTGAATTATATAAATATA
AAGTAGTAAAAAATTGAACCACTA---GGAATAGCACCCACCAGGGCAAAGAGAAGAGTGGTG----------
----------------------------CAGAGAGAAAAAAGAGCA---GTGGGAATGCTAGGAGCT------
---ATGTTCCTTGGGTTCTTGGGAGCA---GCAGGAAGCACTATG---GGCGCAGCGTCA---
CTGACGCTGACGGTACAG---GCCAGACAA---TTATTG---TCTGGTATAGTGCAACAGCAAAAC---
AATTTG---CTGAGA---GCTATTGAGGCGCAACAGCATCTGTTGCAACTCACAGTCTGGGGCATT
>AF049495
TTCTACTGTAATACAACAAAACTGTTTAATAGTACTTGGAATGAGACTACAGAGTCAAATGGC----------
----

AAGGGAGAAAATATCACACTCCCATGCAGAATAAGACAATTTGTAAACATGTGGCAGAAAGTAGGAAAAGCAA
TGTATGCCCCTCCCAGCGATGGACAAATTAGGTGTACATCAAAT---
ATTACTGGGCTACTATTAACAAGAGATGGGGGTGGTCCTAGTGATAAC-------------------------
----

AAAACCGACAAGACCTTCAGACCAGGAGGAGGAGATATGAGGGACAATTGGAGAAGTGAATTATATAAATATA
AAGTAATAAAAAATTGAACCATTA---GGAGTAGCACCCACCAAGGCAAAGAGAAGAGTGGTG----------
----------------------------CAAAGAGAAAAAAGAGCA---GTGGGAATGGTAGGAGCT------
---ATGTTCCTTGGGTTCTTGGGAGCA---GCAGGAAGCACTATG---GGCGCAGCGTCA---
TTGACGCTGACGGTACAG---GCCAGACAA---TTATTG---TCTGGTATAGTGCAGCAGCAGAAC---
AATCTG---CTGAGA---GCTATTGAGGCGCAACAACATCTGTTGCAACTCACAGTCTGGGGCATC
>FJ469752
TTCTACTGTAATACAACAAAACTGTTTAATAGCACTTGGAATGACACTTGGGTTGATACTAGAAATACC----
----

ACTGAAGAAAATATCACACTCACATGCAGAATAAAACAAATTATAAACATGTGGCAGGAAGTAGGAAGAGCAA
TGTATGCCCCTCCCATCAGTGGATGGATTAACTGTACATCAAAT---
ATTACAGGGCTGCTATTGACAAGGGATGGTGGGAATAAAACAGGC----------------------------
----

GAAAACGAGACCTTCAGACCTGGAGGAGGAAATATGATGGACAATTGGAGAAGTGAACTATACAAATATAAAG
TAGTAAAAATTGAACCATTA---GGAATAGCACCCACCAGGGCAAAGAGAAGAGTGGTG--------------

-------------------------CAGAGAGAAAAAAGAGCA---GTGGGAGCACTAGGAGCT---------
ATGTTCCTTGGGTTCTTGGGAACA---GCAGGAAGCACTATG---GGCGCAGCGTCA---
ATGGCGCTGACGGTACAA---GCCAGACAA---GTATTG---TCTGGTATAGTGCAGCAGCAGAAT---
AATCTG---CTGAGG---GCTATTGAGGCGCAACAGCATATGTTGCAACTCACAGTCTGGGGCATT
>GU728189
TTCTACTGTAACACAACACAGCTGTTTAATAGTACTTGGCTGTTTAATAGTACTTGGATTGATAATACTACA-
----

GGGTCAGAGAGACTCATACTCCCCTGCAAAATAAAACAAATTATAAACAGGTGGCAGGGAGTAGGGAAAGCAA
TGTATGCCCCTCCCATTAGAGGACTAATTAAATGCACATCAAAT---
ATCACAGGGCTAATATTAACAAGAGATGGCGGTCCCAACAACGAG----------------------------
----

AGCGAGACCTTCAGACCTGGAGGAGGAGATATGAGGGACAATTGGAGAAGTGAATTATATAAATATAAAGTAG
TAAAAATTGAGCCATTA---GGAGTAGCACCCACCAGGGCAAAGAGAAGAGTGGTG-----------------
----------------------CAAAGAGAAAAAAGAGCA---GTGGGAACAATAGGAGCT---------
ATGTTCCTTGGGTTCTTGGGAACA---GCAGGAAGCACTATG---GGCGCAGCGTCA---
ATGGCGCTGACGGTACAG---GTCAGACAA---TTATTG---TCTGGTATAGTGCAACAGCAGAAC---
AATTTG---CTGAGG---GCTATTGAGGCGCAACAGCATCTGTTGCAACTCACAGTCTGGGGCATC
>KU252650
TTCTACTGTAATACAACACAACTGTTTAAGAGTACTTGGTATGCTAATGGTTCTAGGAAAGGGTTAGATGAC-
----

CCTGGAGAAAATATCACACTCCCATGCAGGATAAAACAAATTATAAACATGTGGCAGGAAGTAGGAAAAGCAA
TGTATGCCCCTCCCATTAGTGGACCAATTAACTGTTTATCAAAT---
ATTACAGGGCTACTATTAACAAGAGATGGTGGGACAAACAATGAGACC-------------------------
----

AACAATACCGAGGTCTTCAGACCTGTAGGAGGGGATATGAGGGACAATTGGAGAAGTGAATTATATAAATATA
AAGTAGTAAGAATTGAACCATTA---GGAATAGCACCCACCAAGGCAAAAAGAAGAGTGGTG-----------
----------------------------CAGAGAGAAAGAAGAGCA---ATAGGA---ATAGGAGCT------
---GTGTTCCTTGGGTTCTTGGGAGCA---GCAGGAAGCACTATG---GGCGCAGCGTCA---
GTGGCGCTGACGGTACAA---GCCAGACAA---TTATTG---TCTGGTATAGTGCAACAGCAGAAC---
AATTTG---CTGAGA---GCTATTGAGGCGCAACAGCATCTGTTGCAACTCACAGTCTGGGGCATT
>GU728294
TTCTACTGTAACTCAACACAACTATTTAATAGTACTTGGCATAGAAATGGCACTGTAGTAGGGTTAAATACC-
----

ACTGAAGAAAATATCATACTCCCATGCAGGATAAAACAAATTATAAACATGTGGCAGAAAGTAGGAAAAGCAA
TGTATGCCCCTCCCATCAAGGGACCAATTAACTGTACATCAAAT---
ATCACAGGGCTGATATTAACAAGAGATGGTGGTAGTGAGGATATCACA-------------------------
----

AATAACACCGAGACCTTCAGACCTGGAGGAGGAAATATGGAGGACAATTGGAGAAGTGAATTATATAAATATA
AAGTAGTAAAAATTGAACCATTA---GGAGTAGCACCCACCAAGGCAAAGAGGAGAGTGGTG-----------
----------------------------CAAAGAGAAAAAAGAGCG---GGAATA------GGAGCT------
---GTGTTCCTTGGGTTCTTGGGAGCA---GCAGGAAGCACTATG---GGCGCAGCGTCA---
ATGACGCTGACGGTACAG---GCCAGACTA---TTATTG---TCTGGTATAGTGCAACAGCAGAGC---
AATCTG---CTGAGG---GCTATTGAGGCGCAGCAGCATCTGTTGCAACTCACAGTCTGGGGCATC
>FJ469756
TTCTACTGTGAGTCATTAGCATTGTTTAATAGTACCTGGCATGCTAATGGTACTATGGAAAGGACAAAT----
----

AGCAGTGAAAATATCACACTCCCATGCAGAATAAAACAAATTATAAACATGTGGCAGGGAGTAGGAAAAGCAA
TGTATGCCCCTCCCATCAGAGGACCAATTAAATGTTCATCAAAT---
ATTACAGGGCTAATATTAACAAGAGATGGTGGTAATATTAATGCCACAAATGGA-------------------
----

AATGGGAATGAGACCTTCAGACCTGGAGGAGGAAATATGAGGGACAATTGGAGAAGTGAACTATATAAAATAC
AAAGTAGTACAAAATTGAACCAATTA---GGAATAGCACCCACCAAGGCAAGGAGAAGAGTGGTG--------

-------------------------------CAGAGAGAAAAAAGAGCA---GTGGGA---CTAGGAGCT---
------TTGTTCCTTGGGTTCTTGGGAGCA---GCAGGAAGCACTATG---GGCGCAGTGTCA---
ATGACGCTGACGGTACAG---GCCAGGCAA---TTATTG---ACTGGTATAGTGCAACAGCAGAAC---
AATTTG---CTGAAG---GCTATTGAGGCGCAACAGCATCTGTTGCAACTCACAGTCTGGGGCATC
>FJ469761
TTCTACTGTAACTCAACAAAACTGTTTAATAGTACTTGGAGTGCTAATGGTACTGGGGAAGAGTCAAATAGCA
CT---

GGAGATAATGATAATATCACACTCCCATGCAGAATAAAACAAATTATAAACATGTGGCAGGAAGTAGGAAGAG
CAATGTATGCCCCTCCCATCGGAGGACAAATTAGATGTTCATCAAAT---
ATTACAGGGCTGATATTAACAAGAGATGGTGGTAATAACACGAATGAG-------------------------
----

ACTGAGCCCGAGATCTTCAGACCTGGAGGAGGAAATATGAAGGACAATTGGAGAAGTGAATTATATAAATACA
AAGTAGTAAAGATTGAACCGTTA---GGAGTAGCACCCACCAAGGCAAAGAGAAGAGTGGTG-----------
----------------------------CAGAGAGAAAAAAGAGCA---GTGGGATTAGTAGGAGCT------
---ATGTTCCTTGGGTTCTTGGGAGCA---GCAGGAAGCACTATG---GGCGCAGCGGCA---
ATGACGCTGACGGTACAA---GCCAGACAA---TTATTG---TCTGGTATAGTGCAACAGCAGAAC---
AATTTG---CTGAGG---GCTATTGAGGCGCAACAACATTTGTTGCAACTCACAGTCTGGGGCATC
>FJ469763
TTCTACTGTAATACAACATCACTGTTCAATAGTACTTGGGATAGTAATGGTACTAGACAACTGTTCAATGGTA
CTGGG--

GATAATAACATCACAATCACACTCCCATGCAGAATAAAACAAATTATAAACTTGTGGCAGGAAGTAGGAAAAG
CAATGTATGCCCCTCCCATCAGCGGACCAATTAAATGTTCATCAAAT---
ATTACAGGACTTCTATTAACAAGAGATGGTGGTAACACGAACGAGACC-------------------------
----

ACCAACAAGACCGAGGTCTTCAGACCTGCAGGAGGAGATATGAGGGATAATTGGAGAAGTGAATTATATAAAT
ATAAAGTAGTAAAAATTGAACCATTA---GGAGTAGCACCCACCAAGGCAAAGAGAAGAGTGGTG--------
-------------------------------CAGAGAGAAAAAAGAGCA---ACA------ATAGGAGCT---
------TTGTTCCTTGGGTTCTTGGGAGCA---GCAGGAAGCACTATG---GGCGCAGCGTCA---
ATAACGCTGACGGTACAG---GCCAGACTA---TTATTG---TCTGGTATAGTGCAACAGCAGAAC---
AATCTG---CTGAGG---GCTATTGAGGCGCAACAACATCTGTTGCAACTCACAGTCTGGGGCATC
>FJ469766
TTCTACTGTAATTCAACACAACTGTTCAATAGTACTTGGCTTAGGAATAATACTGCAGAGTCAAATAGCACTG
AA---

AGAAATAACACAATCACACTCCCATGCAGAATAAAACAAATTATAAACATGTGGCAGGAGGTAGGAAAAGCAA
TGTATGCCCCTCCCATCAGAGGACAAATTAGATGTTCATCAAAG---
ATTACAGGGCTGCTATTAACAAGAGATGGTGGCAATAACGAGACT----------------------------
----

ACTGAGGAGGTCTTCAGACCTGGAGGAGGAGATATGAGGGATAATTGGAGAAGTGAATTATATAAATACAAAG
TGGTAAAAATTGAACCATTA---GGAGTAGCACCCACCAAGGCAAAGAGAAGAGTGGTG--------------
-------------------------CAGAGAGAAAAAAGAGCA---GCGGGA---CTAGGAGCT---------
ATGTTCCTTGGGTTCTTGGGAGCA---GCAGGAAGCACTATG---GGCGCAGCATCA---
ATGGTGCTGACGGTACAG---GCCAGACAA---TTATTG---TCTGGTATAGTGCAACAGCAGAAC---
AATTTG---CTGAGG---GCTATTGAGGCGCAACAGCATCTGTTGCAACTCACAGTCTGGGGCATC
>GU728073
TTCTACTGCAATTCAACACAACTGTTTAATAGTACTTGGATTACGAATGGTACTGAATGGTCACCTAACACTA
CTTTA--

GGAGATACAGAAAATATCACACTCATATGCAGGATAAAACAAATTATAAACATGTGGCAGGAAGTAGGAAAAG
CCATGTATGCCCCTCCCATCGAAGGACAAATTAAATGCTCATCAAAT---
ATTACAGGGCTACTATTAACAAGAGATGGTGGTAATGGGAATGAGACC-------------------------
----

AGGAACAATGAGACCTTCAGACCTACAGGAGGAAACATGAGGGACAATTGGAGAAGTGAATTATATAAATATA
AAGTAGTAAAAATTGAGCCATTA---GGACTAGCACCCACCAGGGCAAAGAGAAGAGTGGTG-----------

```
----------------------------CAGAGAGAAAAAAGAGCA---GTGGGA---ATAGGAGCT------
---ATGTTCCTTGGGTTCTTGGGAGCA---GCAGGAAGCACTATG---GGCGCAGCGTCA---
ATAACGCTGACGGCACAA---GCCAGACAA---TTACTG---TCTGGTATAGTGCAGCAGCAGAAC---
AATTTG---CTGAGG---GCTATTGAGGCGCAACAGCATCTGTTGCAACTCACAGTCTGGGGCATC
>GU727875
TTCTACTGTAATACAACACAGCTGTTTAATAGTACTTGGAATTCTGATAGTACTCGGAATTGGACTGTACAGG
CAAAC--------------------------------------------------------------------
-------------------------
AACAGTGGAGACAATATCACACTCCCATGCAGAATAAAACAAATTGTAAACATGTGGCAGGAAGTGGGAAAAG
CAATGTATGCCCCTCCCATTAAAGGACCAATTGGATGTTTATCAAAT---
ATTACAGGGCTGCTACTAACAAGAGATGGTGGGAATGGGAATGACACA-------------------------
-------------------------------------------------------------------------
----------
AACACCACTGAGATCTTCAGACCTGGAGGAGGAAATATGAGGGACAATTGGAGAAGTGAATTATATAAATATA
AAGTAGTAAAAATTGAGCCATTA---GGAGTAGCACCCACCAAGGCAAAGAGAAGAGTGGTG-----------
----------------------------CAGAGAGAAAAAAGAGCA---ATAGGAACACTGGGAGCT------
---GTGTTCCTTGGGTTCTTGGGAGCA---GCAGGAAGCACTATG---GGCGCAGCGTCA---
ATAACGCTGACGGTACAA---GCCAGACAA---TTATTG---TCTGGTATAGTGCAACAGCAGAAC---
AATCTG---CTGAAA---GCTATTGATGCGCAACAGCGTATGTTGCAACTCACAGTCTGGGGCATC
>GU728234
TTCTACTGTAATTCAACACAACTGTTTAATAGTACTTGGAATTCAACACAAATATATAATAGTACTTGGGATA
CAAAG--------------------------------------------------------------------
-------------------------
AACACAACAGAAAGAATCATACTTCCATGCAGAATAAAACAAATTATAAACATGTGGCAGGAAGTAGGAAAAG
CAATGTATGCCCCTCCCATCCAAGGAATAATTAGCTGTGTATCAAAT---
ATTACAGGGCTGATATTAACAAGAGATGGTGGTGTGAACAGTAGC----------------------------
-------------------------------------------------------------------------
----------
AATACCACCGAGACCTTCAGACCTGCAGGAGGAGATATGAGGGACAATTGGAGAAGTGAATTATATAAATATA
AAGTAGTAAAAATTGAGCCATTA---GGAGTAGCACCCACCAAGGCAAGGAGAAGAGTGGTG-----------
----------------------------CAGAGAGAAAAAAGAACA---ATA------GCGGGAGCT------
---GTGTTCCTTGCGTTCTTGGGAGCA---GCAGGAAGCACTATG---GGCGCAGCGTCA---
ATAACGCTGACGGTACAG---GCCAGACAA---TTATTG---TCTGGTATAGTGCAACAGCAGAAC---
AATTTG---CTGAGA---GCTATTGAGGCGCAACAGCATATGTTGCAACTCACAGTCTGGGGCATC
>M95292
TTCTACTGTAATACAACAAAACTGTTTAATAGTACTTGGAATGATACTAGACTGTTTAATAGTACTTGGAATA
ATACTATAGGG--------------------------------------------------------------
-------------TCAAAT------
AACACTGAAGGAAATATCACACTCCCATGTAGAATAAAACAAATTATAAACATGTGGCAGGAAGTAGGAAAAG
CAATGTATGCCCCTCCCATCAGAGGACAAATTAGATGTTCATCAAAT---
ATTACAGGGCTGCTATTAACAAGAGATGGTGGTGAAACTAATGAG----------------------------
-------------------------------------------------------------------------
-------------
ACCACCGAGGTCTTCAGACCTGGAGGAGGAGATATGAGGGACAATTGGAGAAGTGAATTATATAAATATAAAG
TAGTAAAAATTGAACCATTA---GGAGTAGCACCCACCAGGGCAAAGAGAAGAGTGGTG--------------
-------------------------CAGAGAGAAAAAAGAGCA---GTGGGA---ATAGGAGCT---------
GTGTTCCTTGGGTTCTTGGGAGCA---GCAGGAAGCACTATG---GGCGCAGCGTCA---
GTGGCGCTGACGGTACAG---GCCAGACAA---TTATTG---TCTGGTATAGTGCAACAGCAGAAC---
AATTTG---CTGAGG---GCTATTGAGGCGCAACAGCACCTGTTGCAACTCACAGTCTGGGGCATC
>FJ469767
TTCTACTGTAATACAACACAGCTGTTTAATAGTACTTGGAATACAACTACTCTGGATAATTGGAATGAAACTA
CTTGG--------------------------------------------------------------------
-------------------------
AATACTACTAAAATTATCACACTCCCATGCAGAATAAAACAAATTATAAACAGGTGGCAGGAAGTAGGAAAAG
CAATGTATGCCCCTCCCATCAGTGGACCAATTAGATGTGCATCACAT---
ATTACAGGGCTGCTATTAACAAGAGATGGGGGTAATAATGATAATTCT-------------------------
-------------------------------------------------------------------------
-------
ACAAACGGGACTGAGGTCTTCAGACCTGGAGGAGGAGATATGAGGGACAATTGGAGAAGTGAATTATATAAAT
ACAAAGTAGTAAAAATTGAACCATTA---GGAATAGCACCCACCAAGGCAAAGAGAAGAGTGGTG--------
```

-------------------------------CAGAGAGAAAAAAGAGCA---GTGGGA---ATAGGAGCT---
------GTGTTCCTTGGGTTCTTGGGAGCA---GCAGGAAGCACTATG---GGCGCAGCGTCA---
ATGACGCTGACGGTACAG---GCCAGACTA---TTATTG---TCTGGTATAGTGCAACAGCAGAAC---
AATTTG---CTGAGA---GCTATTGAGGCGCAACAGCATATGTTGCAACTCACAGTCTGGGGCATT
>FJ469768
TTCTACTGTAATTCAACACAACTGTTTAATAGTACTTGGGATTCAGCACAACTATTTAATAGTACTTGGAATG
GCACG--
-------------------AAA---
GAAAATGACACGACAATCGTACTCCCATGCAGAATAAAACAAATTATAAACATGTGGCAGGAAGTAGGAAAAG
CAATGTATGCCCCTCCCATTAGAGGACAAATTAGATGTTTATCAAAT---
ATTACAGGGCTGATATTAACAAGAGATGGTGGTAATAACACTAGC----------------------------
----

AGGATTGAGACCTTCAGACCTGGAGGAGGAGATATGAGGGACAATTGGAGAAGTGAATTATATAAATATAAAG
TAGTTAAAATTGAACCATTA---GGAATAGCACCCACCAAGGCAAAGAGAAGAGTGGTG--------------
-------------------------CAGAGAGAAAAAAGAGCA---GTGGGA---CTAGGAGCT---------
ATGTTCCTTGGGTTCTTGGGAGCA---GCAGGAAGCACTATG---GGCGCAGCGTCA---
ATGACGCTGACGGTACAG---GCCAGACAA---CTGTTG---TCTGGTATAGTGCAACAGCAGAAC---
AATTTG---CTGAAG---GCTATTGAGGCGCAACAGCGTATGTTGCAACTCACAGTCTGGGGCATC
>FJ469769
TTCTACTGTAATACAACACAATTGTTTAACAGTACGTGGAATGCTACTGATATGTGGAACATTACTGAAGGGT
CAGGA--

AATACCACAACCAAAATCACACTCCCATGCAGAATAAAACAAGTTATAAACTTGTGGCAGGAAGTAGGGAAAG
CAATGTATGCCCCTCCCATCCAAGGACGAATTAGATGCTCATCAAAT---
ATTACAGGGCTGTTATTAACAAGAGATGGTGGTAAGGGCATTGAAACT-------------------------
----

AAAAATGACACTGAGACCTTCAGACCAGGAGGAGGAGACATGAGGGACAATTGGAGAAGTGAATTATATAAAT
ATAAAGTAGTAAAAATTGAACCATTA---GGAGTAGCACCCACCAGGGCAAAGAGAAGAGTGGTG--------
-------------------------------CAGAGAGACAAAAGAGCA---GTGGGA---ATAGGAGCT---
------GTACTCCTTGGGTTCTTGGGAGCA---GCAGGAAGCACTATG---GGCGCAGCGTCA---
ATAGCGCTGACGGCACAG---GCCAGACAA---TTACTG---TCTGGTATAGTGCAACAGCAGAAC---
AACTTG---CTGAGG---GCTATTGAGGCGCAACAGCATCTGTTGCAACTCACAGTCTGGGGCATC
>FJ469771
TTCTACTGTAATACAACACAATTGTTTAATAGTACTTGGAATAAAAATAGTACTTGGAATAATACTGAAAAGA
CAAT---

AGCACTGAAAACATTATCACACTCCCATGCAGGATAAAACAAATTATAAACATGTGGCAGAAAGTAGGAAAAG
CAATGTATGCCCCTCCCATCCAAGGACAAATTAAATGTTCATCAAAT---
ATTACAGGGATGATATTAACAAGAGATGGGGGTAATGAAAACAAC----------------------------
----

AACACCGAGATCTTCAGACCTGGAGGAGGAGATATGAGGGACAATTGGAGAAGTGAATTATATAAATATAAAG
TAGTAAAAATTGAACCATTA---GGAGTAGCACCCACCAAGGCAAAAAGGAGAGTGGTG--------------
-------------------------CAAAGAGAAAAAAGAGCA---GTGGGA---CTAGGAGCT---------
GTGTTCCTTGGGTTCTTGGGAACA---GCAGGAAGCACTATG---GGCGCAGCGGCA---
GTGACGCTGACGGTACAG---GCCAGACTA---TTATTG---TCTGGAATAGTGCAGCAGCAGAAC---
AATTTG---CTGAGA---GCTATTGAGGCACAACAGCATCTGTTGCAACTCACAGTCTGGGGCATC
>GU728204
TTCTACTGCAATACAACACAACTGTTTAATAGTACTTGGAATAGTAGTACTTGGAATAGTAATGATACTGAAG
GG---

TCAAATATCACAATCACACTCCCATGCAGAATAAAACAAATTATAAACATGTGGCAGGAAGTAGGAAAAGCAA
TGTATGCCCCTCCCATCAGGGGAAACATTAGCTGTTCATCAAAT---
ATTACAGGGCTGATATTAACAAGAGATGGTGGGAACAATACCACCAATAGTAAT-------------------
----
-
ACCACCAATACGACCACCGAGATCTTCAGACCTGGAGGGGGAGATATGAGGGATAATTGGAGAAGTGAATTAT
ATAAATATAAAGTAGTAAAAATTGAGCCATTA---GGAGTAGCACCCACCAAGGCAAAGAGAAGAGTGGTG--

-------------------------------------CAGAGAGAAAAAAGAGCA---TTGGGA---
ATGGGAGCT---------TTGTTCCTTGGGTTCTTGGGAGCA---GCAGGAAGCACTATG---
GGCGCAGCGTCA---GTGACGCTGACGGTACAG---GCCAGACAA---TTATTG---
TCTGGTATAGTGCAACAGCAGAAC---AATCTG---CTGAGA---
GCTATTGAGGCGCAACAGCATTTGTTGCAACTCACAGTCTGGGGCATC
>GU728223
TTCTACTGTAATTCAACAAATCTATTTAATAGTACTTGGACTTGGAATGGTACTTGGATTAATGGTACTGAAG
GA---

TCAAATGACACAATCACACTCCCATGCAGAATAAAGCAAATTATAAACATGTGGCAGGGAGTAGGACAAGCAA
TGTATGCCCCTCCCATTAAAGGACAAATTAGATGTTCATCGAAT---
ATTACAGGGTTGATATTAACAAGAGATGGTGGTAGTGATACCAATGAT-------------------------
----

ACCAGTAATGAGGTCTTCAGACCTGCAGGAGGAAATATGAAAGACAATTGGAGAAGTGAATTATATAAATATA
AAGTAGTAAGAATTGAACCATTA---GGAGTAGCACCCACCAGGGCAAGGAGAAGAGTGGTG-----------
----------------------------CAAAGAGAAAAAAGAGCA---GTGGGA---TTAGGAACT-------
---GTGCTCTTTGGGTTCTTGAGTGCA---GCAGGAAGCACTATG---GGCGCAGCGTCA---
ATGACGCTGACGGTACAG---GCCAGACAG---TTATTG---TCTGGTATAGTGCAACAGCAGAAC---
AACCTG---CTGAGA---GCTATTGAGGCGCAACAGCATATGTTGCAACTCACAGTCTGGGGCATT
>GU728030
TTCTACTGTAATACAACAAAGCTGTTTAATAGTACTTGGCCGTCTAATGATACTAAAGGGTCAGAT-------
----

GGCAATGAAACTATCATACTCCCGTGCAGAATAAAACAAATTATAAACAGATGGCAGGAAGTAGGAAAAGCAA
TGTATGCCCCTCCCATCAGCGGAATAATTAGTTGTTCATCAAAT---
ATTACAGGGCTAATATTAACAAGAGATGGTGGTGACAATAACCAC----------------------------
----

AGCGAGACCTTTAGACCTGGGGGAGGAAATATGAGGGACAATTGGAGAAGTGAATTATATAAATATAAAATAG
TAAGGATTGAACCATTA---GGAGTAGCGCCCACCAGGTCAAGGAGACTAGTGGTG-----------------
----------------------CAGAGAGAAAAAAGAGCA---GTGGGAACGATAGGAGCT---------
ATGTTCCTTGGGTTCTTGGGAGCA---GCAGGAAGCACTATG---GGCGCAGCGTCG---
GTGACGCTGACGGTACAG---GCCAGACAA---TTATTG---TCTGGTATAGTGCAACAGCAGAAC---
AATTTG---CTGAAG---GCTATTGAGGCGCAACAGCATATGTTGCAACTCACAGTCTGGGGCATC
>GU728229
TTCTACTGTAATTCAACACCACTGTTTAATAGTACATGGGAAAGGAAGGGGGATGGTACTTGGAACAAGAGTG
AAACAAATAGC--
ACTAAAGGAAGGTCAAAC---------
AGCACTGAAGAAAAGATCATACTCCGATGCAGAATAAAACAAATTATGAACATGTGGCAGGAAGTAGGAATAG
CAATGTATGCCCCTCCCATTAACGGACTAATTAGATGTTCATCAAAT---
ATTACAGGGTTACTATTAACAAGGGATGGTGGTGAGACTAACGAGACT-------------------------
----

ACCAAAACTGAGACCTTCAGACCGGGAGGAGGAGATATGAGGGACAATTGGAGAAGTGAATTATATAAATATA
AAGTAGTAAAAATTGAACCATTA---GGTGTAGCACCCACCAAGGCAAAGAGAAGAGTGGTA-----------
----------------------------CAGAGAGAAAAAAGAGCA---ATAGGA---ATAGGAGCG------
---TTGTTCCTTGGGTTCTTGGGAGCA---GCAGGAAGCACTATG---GGCGCAGCGTCA---
CTAACGCTGACGGTACAA---GCCAGACAA---TTGTTG---TCTGGTATAGTGCAACAGCAGAAC---
AATTTG---CTGAGA---GCTATTGAGGCGCAACAACATCTGTTGCAACTCACAGTCTGGGGCATC
>AY189526
TTCTACTGTAATACATCACAAATGTTTAATGTTACTGAAGTGTCAAGGAATGACCCTAGA-------------
----

GCAAATGACACTATCACACTCCAATGCAGAATAAAACAAATTGTAAACATGTGGCAGAGAGTAGGAAGAGCAA
TGTATGCCCCTCCCATCAGTGGACTAATTAGATGTTCATCAAAT---
ATTACTGGGATAATGTTAACAAGAGATGGAGGTAAAATTGATAACGGA-------------------------
----

ACCAACAACACTGAGATCTTCAGACCTGGAGGAGGGGATATGAAGGACAATTGGAGAAGTGAATTATATAAAT

ATAAAGTAGTAAAAATTGAACCATTA---GGACTAGCACCCACCAAGGCAAAGAGAAGAGTGGTG--------
-------------------------------CAGAGAGAAAAAAGAGCA---GTGGGA---TTCGGAGCT---
------ATGTTCCTTGGGTTCTTGGGAGCA---GCAGGAAGCACTATG---GGCGCAGCATCA---
ATAACGCTGACGGTACAG---GCCAGAAAA---TTATTG---TCTGGTATAGTGCAACAGCAGAGC---
AATTTG---CTGAGG---GCTATTGAGGCGCAACAACGTATGTTGCAACTCACAGTCTGGGGCATC
>GU728370
TTCTACTGTAATACAACACAACTGTTTAATAGTACTTGGAATTCAACACAAACAGCTAATAGTAATAGTACTG
AC---

AATGGTACTGTAATCACACTCCCATGTAAAATAAAACAAATTATAAACATGTGGCAAGGAGTAGGAAAAGCAA
TGTATGCCCCTCCTATCAGAGGACAAATTAACTGCTCATCAAAT---
ATTACAGGGCTAATATTAACAAGAGATGGTGGTAATAACAATGAGAGC-------------------------
----

ACCGAGATCGAGATCTTCAGACCTGCAGGAGGAAATATGGAGGACAATTGGAGAAGTGAATTATACAAATACA
AAGTAGTAGAGATTAAACCATTA---GGAATAGCACCCACCAAGGCAAAAAGAAGAGTGGTG-----------
----------------------------CAGAGAGAAAAAAGAGCA---GTAGGAACGATAGGAGCT------
---ATGTTCCTTGGGTTCTTGGGAGCA---GCAGGAAGCACTATG---GGCGCAGCGTCA---
GTGACGCTGACGGTACAG---GCCAGACTA---TTATTG---TCTGGTATAGTGCAACAGCAAAAC---
AATTTG---CTGAAG---GCTATTGATGCGCAACAGCATCTGTTGCAACTCACAGTCTGGGGCATC
>GU728321
TTCTACTGCAATACAACAAAACTGTTTAATGGCACTTTGCATACAAATGGCACTTGGTATTTTAATGGCACTT
GGAATTTTACT--
----------GGAGAGTCA------
AATAGCACTGAAAATATCATACTCACATGCAGAATAAAACAAATTATAAACATGTGGCAGGAAGTAGGAAAAG
CAATGTATGCCCCTCCCATCAGTGGACAAATTAGATGTTCATCAAAT---
ATTACAGGGCTGCTATTAACAAGAGATGGTGGTAATAACAGTACCACA-------------------------
----

AACGAAAGTGAGACTTTCAGACCTGAAGGAGGAAATATGAAGGACAATTGGAGAAGTGAATTATATAAATATA
AAGTAGTGCAAATTGAACCATTA---GGAGTAGCACCCACCAAGGCAAAGAGAAGAGTGGTG-----------
----------------------------CAGAGAGAAAAAAGAGCA---ATAGGA---ATAGGAGCT------
---ATGTTCCTTGGGTTCTTGGGAGCA---GCAGGAAGCACTATG---GGCGCAGCGTCA---
ATGACACTGACGGTACAG---GCCAGACAA---TTATTG---TCTGGTATAGTGCAACAGCAAAAC---
AATTTG---CTGAGG---GCTATTGAGGCGCAACAGCATCTGTTGCAACTCACAGTCTGGGGCATC
>GU727980
TTCTACTGTAATTCAACACCACTGTTTAATAATATTGTGCGTACGGATGGGACTATAGAGAAAATTAAAAATG
ACACG--

GAAACAAATGACACAATCACACTCCAATGTAGAATAAAACAAATTATAAACATGTGGCAGGAAGTAGGAAAAG
CAATGTATGCCCCTCCCATCAGTGGACAAATTAGATGTTCATCAAAT---
ATAACAGGACTATTATTAACAAGAGATGGTGGTAATGGTGTTAACTTAACAGGA------
AATGGGGGTAACGTAACA---GGA---
AATAGTAGTACACACGGAAAT---------
GGGACCGAGATCTTCAGACCTGGAGGAGGAGATATGAGGGACAATTGGAGAAGTGAATTATATAAATATAAAG
TAGTAAGAATTGAACCATTA---GGAGTAGCACCCACCAGGGCAAAGAGAAGAGTGGTG--------------
-------------------------CAGAGAGAAAAAAGAGCA---GTAACA---CTAGGAGCT---------
ATGTTCCTTGGGTTCTTGGGAGCA---GCAGGAAGCACTATG---GGCGCAGCGTCA---
ATAACGCTGACGGTACAG---GCCAGACTA---TTATTG---TCTGGTATAGTGCAACAGCAGAAC---
AATTTG---CTGAGG---GCTATTGAGGCGCAACAACATCTGTTGCAACTCACAGTCTGGGGCATC
>GU728054
TTCTACTGTAATACAACACAACTGTTTAATAGTACTTGGACTTGGAATGATACTAACTGGAATGATACTAGAG
GGTCA--

AACAACTCTGAAAATATCACACTGCCATGCAGAATAAAACAAATCATAAACATGTGGCAGAGAGTAGGAAAAG
CAATGTATGCCCCTCCCATTAAAGGACAGATTAGCTGTTTATCAAAT---
ATTACAGGGCTGCTATTAACAAGAGATGGTGGTAATACTACCGAG----------------------------
----

ACCGAGACCTTCAGGCCTGCAGGAGGAGATATGAGGGACAATTGGAGAAGTGAATTATATAAATACAAAGTAG

TAAAAATTGAACCATTA---GGAATAGCGCCCACCAAGGCAAAAAGAAGAGTGGTG-----------------
----------------------CAGAGAGAAAAAAGAGCA---GTGGGA---CTAGGAGCT---------
ATGTTCCTTGGGTTCTTGGGAGCA---GCAGGAAGCACTATG---GGCGCAGCGTCC---
GTGACGCTGACGGTACAG---GCCAGACAA---TTGCTG---TCTGGTATAGTGCAACAGCAGAAC---
AATCTG---CTGCAA---GCTATTGAGGCGCAACAGCATCTGTTGCAACTCACAGTCTGGGGCATC
>GU728181
TTCTACTGTGATTCAACACCACTGTTTAATAGCACTTGGCATACTAATAATACTTTGGAGAGTACTAACGGG-
----

ACAAATGAAAATATCACACTCACATGCAGAATAAAACAAATTATAAACATGTGGCAGGAAGTAGGAAAAGCAA
TGTATGCCCCTCCCATCAGAGGACAAATTAGATGTATATCAGAT---
ATTACAGGGATGCTACTAACAAGAGATGGTGGTATTAACAACACCAATGGG----------------------
----

GAGAACAACACCGAGATATTCAGACCTGGAGGAGGAAATATGAGAGACAATTGGAGAAATGAATTATATAAAT
ATAAAGTAGTAAAACTTGAACCAATA---GGGTTAGCACCTACCAAGGCAAAGAGAAGAGTGGTA--------
-------------------------------CAGAGAGAAAAAAGAGCA---GTAACA---CTGGGAGCT---
------ATGTTCCTTGGGTTCTTGGGAGCA---GCAGGAAGCACTATG---GGCGCAGCGTCA---
CTGACGCTGACGGTACAG---GCCAGACTA---TTGTTG---TCTGGTATAGTGCAACAGCAGAGC---
AATCTG---CTGAGG---GCTATTGAGGCGCAACAGCATATGTTGCAACTCACAGTCTGGGGCATC
>EU604556
TTCTACTGTAATTCAACACAACTGTTTAATAGTACTTGGGATTCAACACAACTGTTTAATAGTACTTGGTATA
ACGAT--

ACAGGAAATGACACAATCATACTCCAATGCAGAATAAAACAATTTATAAACATGTGGCAGGAAGTAGGAAAAG
CAATGTATGCCCCTCCTATCAAAGGACAAATTAGCTGTTTGTCAAAT---
ATTACAGGGATACTATTAACAAGAGATGGTGGTAGTAACACCAGC----------------------------
----

AACACAACTGAGACCTTCAGACCTGGAGGAGGAGATATGAAGGATAATTGGAGAAGTGAATTATATAAATATA
AAGTAGTACAAATTGAACCATTA---GGAATAGCACCCACCAAGGCAAAGAGAAGAGTGGTG-----------
----------------------------CAGAGAGAAAAAAGAGCA---GTGGGA---ATAGGAGCT------
---TTATTCCTTGGGTTCTTGGGAGCA---GCAGGAAGCACTATG---GGCGCAGCGTCA---
ATGACGCTGACGGTACAG---GCCAGACAA---TTATTG---TCTGGTATAGTGCAACAGCAGAAC---
AATTTG---CTGAGA---GCTATTGAGATGCAACAGCATCTGTTGCAACTCACAGTCTGGGGCATC
>JX503075
TTCTATTGTAATACAACACAACTGTTTAATAGTACCTGGGATGTTAATAGTACCTGGAATGGAGGGTCAGAGA
ACACT--
-------------------ACA---
GGAGGAAATGATACAATCACACTCCCATGTAGAATAAAACAAATTATAAATATGTGGCAGGAAGTAGGCAAAG
CAATGTATGCCCCTCCCATCAAAGGACAAATTAGATGTTCATCAAAT---
ATTACAGGGCTACTACTAGTAAGAGATGGTGGAAATGATAATGAAACT-------------------------
----

AACGACACCGAGATCTTCAGACCTGGAGGAGGAGATATGAGGGACAATTGGAGAAGTGAATTGTATAAATATA
AAGTAGTAAGAATTGAACCATTA---GGAGTAGCACCCACCAAGGCAAAGAGAAGAGTGGTG-----------
----------------------------CAGAAGGAAAAAAGAGCA---ATAACA---TTAGGAGCT------
---ATGTTCCTTGGGTTCTTGGGAACA---GCAGGAAGCACGATG---GGCGCAGCGTCA---
ATAACGCTGACGGTACAG---GCCAGACAA---TTATTG---TCTGGTATAGTGCAGCAGCAGAAC---
AATCTG---CTGAGG---GCTATTGAGGCGCAACAACATTTGTTGCAACTCACAGTCTGGGGCATT
>AY781127
TTCTACTGTAATACAACAGAATTGTTTAATAGTACTTGGCCGTTTAATAGTACTGAAAATAAGGCTAACGAGT
CAGGG--

ACAAGTGAAAGAAATATCACACTCCCATGCAGAATAAAACAAATTATAAACAGGTGGCAGGAAGTAGGAAAAG
CAATGTATGCCCCTCCCATCCAAGGACGAATTAGCTGTTCATCAAAA---
ATTACAGGGATGCTATTAACAAGAGATGGTGGTAAAGGGAACGAGACA-------------------------
----

AGGAACGATACCGAGACCTTCAGACCTGCAGGAGGAGATATGAAGGACAATTGGAGAAGTGAATTATATAAAT

ATAAAGTAGTAAAAATTGAGCCATTA---GGAATAGCACCCACCAGGGCACAGAGAAGAGTGGTG--------
-------------------------------CAGAGAGAAAAAAGAGCA---GTGGGAATGATAGGAGCT---
------ATGTTCCTTGGGTTCTTGGGAGCA---GCAGGAAGCACTATG---GGCGCAGCGTCA---
GTGACGCTGACGGTACAG---GCCAGACAA---TTATTG---TCTGGTATAGTGCAGCAGCAGAAC---
AATCTG---CTGAGG---GCTATTGAGGCGCAACAGCATCTGTTGCAACTCACAGTCTGGGGCATC
>JN235958
TTCTACTGTAATACAACACAGCTGTTTAATAGTACCTGGAATGATAGTACCTGGAATGGTACTGCAGGA----
----

CGGAATGATACTATTACACTCCCATGCAGAATAAGACAAATTATAAACAGGTGGCAGGAAGTAGGAAGAGCAA
TGTATGCCCCTCCCATCAGCGGAATGATTAGCTGTTCATCAAAT---
ATCACAGGCCTGCTATTAACAAGAGATGGGGGTAACAATAGTAAC----------------------------
----

AGCAACAACGAGACCTTCAGACCAGCAGGAGGAGATATGAGGGACAATTGGAGAAGTGAATTATATAAATATA
AAGTAGTAAAAATTGAGCCATTA---GGAATAGCACCCACCAAGGCAAAGAGAAGAGTGGTG-----------
----------------------------CAGAGAGAAAAAAGAGCA---GTAACG---CTAGGAGCT------
---ATGTTCCTTGGGTTCTTGGGAGCA---GCAGGAAGCACTATG---GGCGCAGCGTCA---
CTGACGCTGACGGTACAG---GCCAGACAA---TTATTG---TCTGGTATAGTGCAACAGCAGAAC---
AATTTG---CTGAGG---GCTATTGAGGCGCAACAGCATATGTTGCAACTCACAGTCTGGGGCATC
>AY795905
TTCTACTGTGAAACAGGAAGACTGTTTAATAGTACTTGGAATTTAACACAACTGACTTGGAATGATACTAAAG
AG---

TTAAATAACACGATCACACTCCCATGCAAAATAAAACAAATTATAAACATGTGGCAGGAAGTAGGAAAAGCAA
TGTATGCCCCTCCCATCGCAGGACAAATTAACTGTTTATCAAAT---
ATTACAGGGCTGATTTTAACAAGGGATGGTGGTAATAAGGGTAATGGGACC----------------------
----

GATGACACCACCGAGATCTTCAGACCTGGAGGAGGAGATATGAGGGACAATTGGAGAAGTGAATTATATAAGT
ATAAAGTAGTAAAAATTGAACCATTA---GGAATAGCACCCACCAAGGCAAAGAGAAGAGTGGTG--------
-------------------------------CAGAGAGAAAGAAGAGCA---GTAGGA---TTGGGAGCT---
------TTGCTCTTTGGGTTCTTGGGAGCA---GCAGGAAGCACTATG---GGCGCAGCGTCA---
ATAACGCTGACGGTACAG---GCCAGACAA---TTATTG---TCTGGTATAGTGCAGCAGCAAAAT---
AATTTG---CTGAGA---GCTGTGGAGGCGCAACAACATCTGTTGCAACTCACAGTCTGGGGCATC
>KJ948657
TTCTACTGTAATACAACACAACTGTTTAATAGTACTTGGCATGTTAATATGACTGGGAAT-------------
--GAT---
ACTGAGGCTGCTAAAAATATT---------------
ACAGGAAATGACACAATCACACTCCCATGCAGAATAAAACAAATTATAAACATGTGGCAGGAAGTAGGAAAAG
CAATGTATGCCCCTCCCATCAGAGGAATAATTAGATGTTCATCAAAT---
ATTACAGGGCTGCTATTAACAAGAGATGGGGGT--

-
AAAGAGAACAATGAGACTGAGATCTTCAGACCTGGAGGAGGAGATATGAGGGATAATTGGAGAAGTGAATTAT
ATAAATATAAAGTAGTACAAATTGAACCATTA---GGAGTAGCACCCACCAAGGCAAAGAGAAGAGTGGTG--
-------------------------------------CAAAGAGAAAAAAGAGCAATAACGGGA---
TTAGGAGTT---------ATGTTCCTTGGGTTCTTGGGAGCA---GCAGGAAGCACTATG---
GGCGCAGCGTCA---ATAACGCTGACGGTACAG---GCCAGACAA---TTATTG---
TCTGGTATAGTGCAACAGCAGAAC---AATCTG---CTGAGG---
GCTATTGAGGCGCAACAGCATCTGTTGCAACTCACAGTCTGGGGCATC
>KJ948660
TTCTACTGTAATACAACACAACTGTTTAATAATACTGGTATGGCGGGTGATAATTGG----------------
----
ACTACTAGTTGGAATAATACT------------
ACACGAAATGTCAATATCACACTCCCATGCAGAATAAAACAAATTATAAACATGTGGCAGGAAGTAGGAAAAG
CAATGTATGCCCCTCCCATCAATGGACACATTATATGTGCATCAAAT---
ATTACAGGGCTGCTATTAACAAGAGATGGTGGTATC-------------------------------------
----
GAGGATAATGGGACTACTATT---------

AGGAATGAGACCTTCAGACCTGGAGGAGGAGATATGAAGGATAACTGGAGAAGTGAATTATATAAATATAAAG
TAGTAAAAATTGAACCATTA---GGAATAGCACCCACCAAGGCAAAGAGAAGAGTGGTG--------------
-------------------------CAGAGAGAAAAAAGAGCA---TTGGGA---ATAGGAGCT---------
TTGTTCCTTGGGTTCTTGGGAGCA---GCAGGAAGCACTATG---GGCGCAGCGGCA---
CTGACGCTGACGGTACAG---GCCAGACCA---TTATTG---TCTGGTATAGTGGAACAGCAAAAC---
AATTTG---CTGAGG---GCTATTGAGGCGCAACAGCATCTGTTGCAACTCACAGTCTGGGGCATC
>HQ595755
TTCTACTGTAATACAACACAACTGTTCAATAATACTTGGAATGGTACTACCTGGTCAAATAACAGGGCAACTA
AC---

AGCAGCAAAAATATCATCACACTCCCATGCAAAATAAAACAAATTATAAACATGTGGCAGCAGGTAGGAAAAG
CAATGTATGCCCCTCCCATCAGAGGACAAATCAGATGTTCATCAAAT---
ATTACAGGGCTGCTATTAACAAGAGATGGTGGTAATGATAATAATAAT-------------------------
----

ACAAATGGGTCCGAGATCTTCAGACCTGGGGGAGGAGATATGAGGGACAATTGGAGAAGTGAATTATATAAAT
ATAAAGTAGTAACAATTGAACCAGTA---GGAATAGCACCCACCAAGGCAAAGAGAAGAGTGGTG--------
-------------------------------CAGAGAGAAAAAAGAGCA---ATAGGA---ATAGGAGCT---
------GTGTTCCTTGGGTTCTTGGGAGCA---GCAGGAAGCACTATG---GGCGCAGCATCA---
ATAACGCTGACGGTACAG---GCCAGACAA---TTATTG---TCTGGTATAGTGCAACAGCAGAAC---
AATTTG---CTGAGG---GCTATTGAGGCGCAACAGCATATGTTGCAACTCACAGTCTGGGGCATC
>KP109515
TTCTACTGTAATACAACACAACTGTTCAATAATACTTGGAATGGTACTACCTGGTCAAATAACAGGGCAACTA
ACAGC--

AGCAAAAATATCATCACACTCCCATGCAAAATAAAACAAATTATAAACATGTGGCAGCAGGTAGGAAAAGCAA
TGTATGCCCCTCCCATCAGAGGACAAATCAGATGTTCATCAAAT---
ATTACAGGGCTGCTATTAACAAGAGATGGTGGTAATGATAATAATAAT-------------------------
----

ACAAATGGGTCCGAGATCTTCAGACCTGGGGGAGGAGATATGAGGGACAATTGGAGAAGTGAATTATATAAAT
ATAAAGTAGTAACAATTGAACCAGTA---GGAATAGCACCCACCAAGGCAAAGAGAAGAGTGGTG--------
-------------------------------CAGAGAGAAAAAAGAGCA---ATAGGA---ATAGGAGCT---
------GTGTTCCTTGGGTTCTTGGGAGCA---GCAGGAAGCACTATG---GGCGCAGCATCA---
ATAACGCTGACGGTACAG---GCCAGACAA---TTATTG---TCTGGTATAGTGCAACAGCAGAAC---
AATTTG---CTGAGG---GCTATTGAGGCGCAACAGCATATGTTGCAACTCACAGTCTGGGGCATC
>KF725915
TTCTACTGTAATTTAACACAGCTGTTTAATAGTACTTGGCGGAAAAGTAATAGTACTAGGCAGATGAATAACA
CT---
-------------GGAACA------
AGGTTAAATGGAACCATCATACTCCCATGCAGAATAAAACAAATTATAAACAGGTGGCAAGGAGTAGGAAGAG
CAATGTATGCCCCTCCCATCAGAGGACCAATTAATTGTTCATCAAAG---
ATTACAGGGCTACTATTAATAAGAGATGGTGGTAAAACCAATAAT----------------------------
----

TCTACAGAGATCTTCAGGCCTGGAGGGGGAAATATGAAGGACAATTGGAGAAGTGAATTATATAAATATAAAG
TAGTAAAAATTGAGCCATTA---GGAATAGCACCCACCAAGGCAAAGAGAAGAGTGGTG--------------
-------------------------CAGAGAGAAAAAAGAGCA---GTTGGAGTAATAGGAGCT---------
ATGTTCCTTGGGTTCTTGGGAGCA---GCAGGAAGCGCTATG---GGCGCAGCGTCA---
CTGACGCTGACGGTACAG---GCCAGACAA---TTATTG---TCTGGTATAGTGCAACAGCAGAAC---
AATCTG---CTGAGA---GCTATTGAGGCGCAACAGCATATGTTGCAACTCACAGTCTGGGGCATC
>KF725918
TTCTACTGTGATTCATCAAAACTGTTTAATAGTACTTGGCATGTTAATAGCACTGAAGGAAATAGC-------
----

ACTAATGAAGAAATCATACTCCCATGTAAAATAAAGCAAATTATAAACATGTGGCAGAAAGTAGGAAAAGCAA
TGTATGCTCCTCCCATCAAAGGGAAAATTAACTGTTTATCAAAC---
ATTACAGGACTGCTATTAACAAGGGATGGTGGTAATAATAGGACC----------------------------
----

AACGAGACCTTTAGACCTGTGGGAGGAGATATGAGGGACAATTGGAGAAGTGAATTATATAAATATAAAGTAG
TAAAAATTGAACCATTA---GGAGTAGCACCCACCAAGGCAAAGAGAAGAGTGGTG-----------------
----------------------CAGAGAGAAAAAAGAGCA---GTGGGAACGATGGAGCT---------
ATGTTCCTTGGGTTCTTGGGAGCA---GCAGGAAGCACTATG---GGCGCAGCGTCA---
ATAACGCTGACGGTACAG---GCCAGACAA---TTATTG---ACTGGTATAGTGCAACAGCAGAAC---
AATTTG---CTGAAG---GCTATTGAGGCGCAACAGCATCTGTTGCAACTCACAGTCTGGGGCATC
>KF725946
TTCTACTGTAACACAACAAAACTGTTTAATAGTATGTGGAATAGTAGTACTTGGAGTAACAATAGTATTGAAG
ACTCA--
-------------------AAT---
AACACTGAGAACAATATCACACTCCCATGCAGGATAAAACAAATTATAAACATGTGGCAGAAGGTAGGAAAGG
CAATGTATGCCCCTCCCATCAGTGGACAAATTAGGTGTTCATCAAAT---
ATTACAGGGCTACTATTAACAAGAGATGGTGGTAATGATAGTAACACC-------------------------
----

AATAGCAAGGAGACCTTCAGACCTGGAGGAGGAGATATGAGGGACAATTGGAGAAGTGAGTTATATAAATATA
AAGTAGTAAAAATTGAACCATTA---GGAATAGCACCCACCAAGGCAAAGAGAAGAGTGGTG-----------
----------------------------CAGAGAGAAAAGAGAGCA---GTGGGA---ATAGGAGCG------
---TTGTTCCTTGGGTTCTTGGGAGCA---GCAGGAAGCACTATG---GGCGCAGCGTCA---
ATAACGCTGACGGTACAG---GCCAGACTA---TTATTG---TCTGGTATAGTGCAGCAGCAAAAC---
AATTTG---CTGAGG---GCTATTGAGGCGCAACAACACTTGTTGCAACTCACAGTCTGGGGCATC
>KF725977
TTCTACTGTAATACAACACAACTATTTAAGGGGTCTTGGAATGGTACTGAGTCTTGGAATGATACTGAGTCTT
GGAATGGT---

AATGACACCATTCTCGAAATCCCATGCAGAATAAAACAAATTGTAAACATGTGGCAGGAAGTAGGAAAAGCAA
TGTATGCCCCTCCCATCAGAGGACAAATTAGCTGTTCCTCAAAT---
ATTACAGGATTACTATTAACAAGAGATGGTGGTAACACCAATAGA----------------------------
----

ACCGACGAGACTTTTAGACCTGGAGGAGGAGATATGAGGGACAATTGGAGGAGTGAATTATATAAATATAAAG
TAGTAAAAATTGAACCATTA---GGAATAGCACCCACCAAGGCAAAGAGAAGAGTGGTG--------------
-------------------------CAGAGAGAAAAAAGAGCA---GTGGGA---CTAGGAGCT---------
ATGTTCCTTGGGTTCTTGGGAGCA---GCAGGAAGCACTATG---GGCGCAGCGTCA---
CTGACGCTGACGGTACAG---GCCAGACTA---TTATTG---TCTGGTATAGTGCAACAGCAGAAC---
AATTTG---CTGAGA---GCTATTGAGGCGCAACAACATTTGTTGCAACTCACAGTCTGGGGCATC
>KF726002
TTCTACTGTAATACAGCACAACTGTTTAATAGTACTTGGAATACAACACAACTTAATAATAATAATACTGGAG
GGGGC--

GACAATGAAACTATCACACTCCAATGTAAAATAAAACAAATTATCAACATGTGGCAGGGAGTAGGAAAAGCAA
TGTATGCCCCTCCCATTAAAGGACAAATTAGATGCACATCAAAT---
ATTACAGGGCTACTATTAACAAGAGATGGTGGTGGGAACGAGAGC----------------------------
----

ACGAACGAGACCTTCAGACCTGGAGGAGGAGATATGAGGGATAATTGGAGAAGTGAATTATACAAATATAAAG
TAGTGAAAATTGAACCATTA---GGAGTAGCACCCACCAAGGCAAGGAGAAGAGTGGTG--------------
-------------------------CAGAGAGAGAAAAGAACA---GTGGGA---CTAGGAGCT---------
CTGTTCCTTGGGTTCTTGGGAGCA---GCAGGAAGCACTATG---GGCGCAGCGTCA---
GTAACGCTGACGGTACAG---GCCAGACTA---TTATTG---ACTGGTATAGTGCAACAGCAGAAT---
AATTTG---CTGAGG---GCTATTGAGGCGCAACAACATCTGTTGCAACTCACAGTCTGGGGCATC
>KF726004
TTCTACTGTAATACAACAGGACTGTTTGATAGTACTTGGAATAATACTGAAAAAGTAAATGGCACT-------
----

AACAATGACACCATCACACTCCCATGCAGAATAAGGCAATTTATAAACATGTGGCAGAAAGTAGGAAAAGCAA
TGTATGCCCCTCCCATTAAAGGACAAATTAAATGTTCATCAAAT---
ATTACAGGACTGATATTAACAAGAGATGGTGGTAATAATAACAGTACC-------------------------
----

AACGAGACCGAGACCTTCAGACCTGCAGGAGGAAATATGAGGGACAATTGGAGAAGTGAATTATATAAATACA
AAGTAGTAAAAATTGAACCATTA---GGATTAGCACCCACCAAGGCAAAGAGAAGAGTGGTG-----------
----------------------------CAGAGAGAAAAAAGAGCA---GTGGGA---TTAGGAGCT------
---ATGTTCCTTGGGTTCTTGGGAGCA---GCAGGAAGCACTATG---GGCGCAGCGTCA---
CTGACGCTGACGGTACAA---GCCAGACAA---TTATTG---TCTGGTATAGTGCAGCAGCAAAAC---
AATTTG---CTGAGA---GCTATTGAGGCGCAACAACATATGTTGCAACTCACAGTCTGGGGCATC
>KF726016
TTCTACTGTAATACAACACAACTGTTTAATAGTACTTGGATTAATGATACTAGGAATGATACA----------
----

GGAAATGAAACAATCACACTCCCATGCAGAATAAAACAAATTATAAACATGTGGCAGGAGGTAGGAAAAGCAA
TGTATGCCCCTCCCATCAGTGGACAAATTAGATGTTCATCAAAT---
ATTACAGGGCTGATATTAACAAGAGATGGTGGGCAGAATAACACT----------------------------
----

AATGACACTGAGATCTTCAGACCTGGAGGAGGAAATATGAAAGACAACTGGAGAAGTGAATTATATAAATATA
AAGTAGTAAGAATTGAACCATTA---GGAGTAGCACCCACCAAGGCAAAGAGAAGAGTGGTG-----------
----------------------------CAAAGAGAAAAAAGAGCA---GTGGGA---ATAGGAGCT------
---TTGTTCCTTGGGTTCTTGGGAACA---GCAGGAAGCGCTATG---GGCGCAGCGTCA---
CTGACGCTGACGGTACAG---GCCAGGCAA---TTATTG---TCTGGTATAGTGCAACAGCAAAAC---
AATTTG---CTGAGG---GCTATTGAGGCGCAACAGCATCTGTTGCAACTCACAGTCTGGGGCATC
>KF726018
TTCTACTGTAATACAACACAACTGTTTAATAGTACTTGGAATAATACAAATAACAATGAG-------------
----

GGAAATGGCACAATCACACTCCAATGTAGAATAAAACAAATTATAAACATGTGGCAGGAAGTAGGAAAAGCAA
TGTATGCCCCTCCCATCAGTGGACAAATTAACTGTTCATCAAAT---
ATTACAGGACTTCTATTAACAAGGGATGGTGGTAGTACTAATGAGACG-------------------------
----

ACCACCGAGACCTTCAGACCTGGAGGGGGAAATATGAAGGACAATTGGAGAAGTGAATTATATAAATACAAAG
TAGTAAAAATTGAACCATTA---GGAGTAGCACCCACCAAGGCAAAGAGAAGAGTGGTG--------------
-------------------------CAGAGAGAAAAAAGAGCA---GTGGGAGCACTAGGAGCT---------
ATGTTCCTTGGGTTCTTGGGAGCA---GCAGGAAGCACAATG---GGCGCGGCGTCA---
CTGACGCTGACGGTACAG---GCCAGACAA---TTATTG---TCTGGTATAGTGCAACAGCAGAAC---
AATCTG---CTGCAA---GCTATTGAGGCGCAACAGCATCTGTTGCAACTCACAGTCTGGGGCATT
>KF726021
TTCTACTGTAATTCATCAGCACTGTTTAATAGTACTTGGGATGTTAATAGCACTGGATTAAATAGC-------
----

ACTGGGGGAAATATCACACTCCCATGTAGAATAAAACAAATTATAAACATGTGGCAGACAGTAGGAAAAGCAA
TGTATGCCCCTCCCATCAGCGGATAATTAACTGTACATCAAAC---
ATTACAGGACTGCTATTAACGAGAGATGGTGGTGGTAATAATAGCACC-------------------------
----

AACGAGAACGAGACCTTCAGACCTGCAGGAGGAGATATGAGGGACAATTGGAGAAGTGAATTATATAAATATA
AAGTAGTAAAAATTGAACCATTA---GGAGTAGCACCCACCAAGGCAAAGAGAAGAGTGGTG-----------
----------------------------CAGAGAGAAAAAAGAGCA---GTGGGAACACTAGGAGCT------
---ATGTTCCTTGGGTTCTTGGGAGCA---GCAGGAAGCACTATG---GGCGCAGCGTCA---
ATGACGCTGACGGTACAG---GCCAGACAA---TTATTG---ACTGGTATAGTGCAACAGCAGAAC---
AATTTG---CTGAAG---GCTATTGAGGCGCAACAGCATCTGTTGCAACTCACAGTCTGGGGCATC
>MH234643
TTCTACTGTAATTCAACAAAACTGTTTAATAGTACTTGGAATGGGAATGATACTGAAGGGTCAAATAACACTG
AA---

GGAAATGACACACTCACACTCCCATGCAGAATAAAACAAATTATAAACATGTGGCAGGAAGTAGGAAAAGCAA
TGTATGCCCCTCCCATCCAAGGACAAATTAGATGTTCATCAAAT---
ATTACAGGGCTGCTATTAACAAGAGATGGTGGTAATGGCAGCGAG----------------------------
----

ACCACCGAGATCTTCAGACCTGGAGGAGGAGATATGAGGGATAATTGGAGAAGTGAATTATATAAATATAAAG
TAGTAAAAATTGAACCATTA---GGAGTAGCACCCACCAAGGCAAAAAGAAGAGTGGTG--------------
-------------------------CAGAGAGAGAAAAGAGCA---GTGGGA---ATAGGAGCT---------
TTGTTCCTTGGGTTCTTGGGAGCA---GCAGGAAGCACTATG---GGCGCAGCGTCA---
ATGACGCTGACGGTACAG---GCCAGACAA---TTATTG---TCTGGTATAGTGCAACAGCAGAAC---
AATTTG---CTGAGG---GCTATTGAGGCGCAACAGCATCTGTTGCAACTCACAGTCTGGGGCATT
>FJ647145
TTCTACTGTAACTTAACACAACTGTTTAATAGTACTTGGAATGTAAATGGTACTGAAGGGTCAAATAAC----
----

ACTGACAAAAATATCACACTCCCATGCAGGATAAAACAAATTATAAACATGTGGCAGACAGTAGGAAAAGCAA
TGTATGCCCCTCCCATCAGAGGACAAATTAGATGTTCATCAAAT---
ATTACAGGGCTGCTATTAACAAGAGATGGTGGTAATAACGAGAAC----------------------------
----
-------------GATCCCGAAAAT---
TCACCCGGAGGAGGAGATATGAGGGACAATTGGAGAAGTGAATTATATAAATATAAAGTATTAAAAATTAAAG
CATTA---GGAGTAGCACCCCCCAAGGCCAAGAGAAGAGTGGTG-----------------------------
----------CAAAGAGTAAAAAGAGCA---GTGGGA---ATAGGAGCT---------
GTGTTCTTTGGGTTCTTGGGAGCA---GCAGGAAGCACTATG---GGCGCAGCGTCA---
ATGACGCTGACGGTACAG---GCCAGACTA---TTATTG---TCTGGTATAGTGCAACAGCAAAAC---
AATTTG---CTGAAG---GCTATTGAGGCGCAACAGCACCTGTTGCAACTCACAGTCTGGGGCATC
>MH234640
TTCTACTGTAATACAACACAACTGTTTAATAGTACTTGGGATAATAGTACTTTGAGTACTGGGAATGATGATA
AT---

AACACTGGGATTATCACACTCCCATGTAAAATAAAACAAATTATAAACATGTGGCAGGAAGTAGGAAAAGCAA
TGTATGCCCCTCCCATCAGAGGACAAATTAACTGCTTATCAAAC---
ATTACAGGGCTGCTATTAACAAGAGATGGTGGTAATAACACAAACGGG-------------------------
----

ACCACCGAGATCTTCAGACCTGGAGGAGGAAATATGAGGGACAATTGGAGAAGTGAATTATATAAATATAAAG
TAGTAAAAATTGAACCATTA---GGAGTAGCACCCACCAGGGCAAAGAGAAGAGTGGTG--------------
-------------------------CAGAGAGAAAAAAGAGCA---GTGGGA---ATAGGAGCT---------
GTGTTCCTTGGGTTCTTGGGAGCA---GCAGGAAGCACTATG---GGCGCAGCGTCA---
ATAACGCTGACGGTACAG---GCCAGACTA---TTATTG---TCTGGTATAGTGCAACAGCAGAAC---
AATCTG---CTGAGG---GCTATTGAGGCGCAACAGCATCTGTTGCAACTCACAGTCTGGGGCATC
>AY505010
TTCTACTGTAATACAACACAACTGTTTAATAGTACTTGGAATAACACTGAAGAGTCAGATAACACGGAA----
----

GGAAATGGCACAATCACACTCCCATGTAGAATAAAACAAATTATAAACATGTGGCAGGAAGTAGGAAAAGCAA
TGTATGCCCCTCCCATCGCAGGACAAATTAGATGTTCATCAAAT---
ATTACAGGGCTTCTATTAACAAGAGATGGTGGTAATAGTACTAAT----------------------------
----

GGGAGTAGGGAGGTCTTCAGACCTGGAGGAGGAGATATGAGGGACAATTGGAGAAGTGAATTATATAAATACA
AAGTAGTAAAAATTGAGCCATTA---GGAGTAGCACCCACCAAGGCAAAGAGAAGAGTGGTG-----------
----------------------------CAGAGAGAAAAAAGAGCA---GCA------ATAGGAGCT------
---ATGTTCCTTGGGTTCTTGGGAGCA---GCAGGAAGCACTATG---GGCGCAGCGGCA---
GTGACGCTGACGGTACAG---GCCAGGCTA---TTATTG---TCTGGTATAGTGCAACAGCAAAAC---
AATTTG---CTGAGG---GCTATTGAGGCGCAACAGCATATGTTGCAACTCACAGTCTGGGGCATC
>KU168257
TTCTACTGTAATACATCAGGGCTGTTTAATAGTACTTGGTATAAGAATGATACTACTTGGAATGGTACAGAAA
AA---

TCAAATGGCACTATCACACTCCCATGCAGAATAAAACAAATTATAAACAGATGGCAGGAAGTAGGAAAAGCAA
TGTATGCCCCTCCTATCAGTGGATCAATTAATTGTTCATCACAT---
ATTACAGGGCTGCTATTAGTAAGAGATGGCGGCAATACCAGTAGT----------------------------
----

AATAACGAGACTTTCAGACCTGGAGGAGGAGATATGAGGGACAATTGGAGAAGTGAATTATATAAATATAAAG
TAGTAAGAATTGAGCCATTA---GGAGTAGCACCCACCAAGGCAAAGAGAAGAGTGGTG--------------
-------------------------CAGAGAGAAAAAAGAGCA---GTGGGAACGCTAGGAGCT---------
ATGTTCCTTGGGTTCTTGGGAGCA---GCAGGAAGCACTATG---GGCGCAGCATCA---
CTAACGCTGACGGTACAG---GCCAGACTA---TTATTG---TCTGGTATAGTGCAACAGCAGAAC---
AATCTG---CTAAGA---GCTATTGAGGCGCAACAGCATCTGTTGCAACTCACAGTCTGGGGCATC
>KU168258
TTCTACTGTAATACAACACAACTGTTTAATAGTACTTGGGTTAATGGTACTTGGAATGATACTGAAGCA----
----

CGAAAAGAAAATATCACACTCCCATGCAGAATAAAACAAATTATAAACATGTGGCAGGAAGTAGGAAAAGCAA
TGTATGCCCCTCCCATTGCAGGAAAAATTAGCTGTTCATCAAAT---
ATTACAGGGCTGCTATTAACAAGAGATGGTGGTATTGATAATAAC----------------------------
----

AACAAGAATGAGACCTTCAGACCTGGAGGAGGAGATATGAGGGACAATTGGAGAAGTGAATTATATAAATATA
AAGTAGTAAAAATTGAACCATTA---GGAGTAGCACCCACCAAGGCAAAGAGAAGAGTGGTG-----------
----------------------------CAGAGAGAAAAAAGAGCA---CCAGTA---CTAGGAGCT------
---TTGTTCCTTGGGTTCTTAGGAGCA---GCAGGAAGCGCTATG---GGCGCAGCGGCA---
TTAACGCTGACGGTACAG---GCCAGACTA---TTACTG---TCTGGTATAGTGCAACAGCAGAAT---
AATCTG---CTGAGG---GCTATTGAGGCGCAACAGCACCTGTTGCAACTCACAGTCTGGGGCATC
>KU168259
TTCTATTGTAATTCAACACAACTGTTTAGTAGCACTTGGAATACGACTAAAATTTGGAATGATACTGACACTA
AAGGA--
-------------AATAGC------
ACCACCAATGACACCATCATACTCCCATGTAAAATAAGACAAATTATAAACATGTGGCAGGAAGTAGGAAAAG
CAATGTATGCCCCTCCCATCGAAGGACTAATTAGTTGTTCATCAAAT---
ATTACAGGGCTGTTATTAACAAGAGATGGAGGTAATAATAGCACT----------------------------
----

AATGAGTCTGAGATCTTCAGACCTGGAGGGGGAGATATGAGGGACAATTGGAGAAGTGAATTATATAAATATA
AAGTAGTAACAATTGAACCATTA---GGAGTAGCACCCACCAAGGCAAAGAGAAGAGTGGTG-----------
----------------------------CAGAGAGAAAAAAGAGCA---GTGGGAATGTTAGGAGCC------
---ATGTTCCTTGGGTTCTTGGGAGCA---GCAGGAAGCACTATG---GGCGCAGCGTCA---
ATAACGCTGACGGTACAG---GCCAGGCAA---TTATTG---ACTGGTATAGTGCAACAGCAGAAC---
AATCTG---CTGAGG---GCTATTGAGGCGCAACAGCACATGTTGCAACTCACGGTCTGGGGCATT
>JF680931
TTCTACTGTAACACAACAGGATTGTTTAATAGTACTTGGCCAGTTAATAGTAATTGGACAGAAACTAATGGTA
CT---

AACAGGTCAGAGGTCATCACTCTCCCATGCAGGATAAAACAAATTATAAACATGTGGCAGGAAGTAGGAAAAG
CAATGTATGCTCCTCCCATCCAAGGGCCAATTAGCTGTTCCTCAAAT---
ATTACAGGGCTACTATTAACAAGAGATGGTGGTGGGGGTAACGAAACC-------------------------
----

AACACCACCACTGAGATCTTCAGACCTGGAGGAGGAGACATGAGGGACAATTGGAGAAATGAATTATATAAAT
ATAAAGTAGTACAAATTGAACCATTA---GGAGTAGCACCCACCATGGCAAAGAGAAGAGTGGTG--------
-------------------------------CAGAGAGAAAAAAGAGCA---GTGGGA---ATAGGAGCT---
------CTGTTCCTTGGGTTCTTGGGAGCA---GCAGGAAGCACTATG---GGCGCAGCGTCA---
TTGACGCTGACGGTACAG---GCCAGACAA---TTGCTG---TCTGGTATAGTGCAACAGCAGAAC---
AACTTG---CTGAGG---GCTATTGAGGCGCAACAGCACCTGTTGCAACTCACAGTCTGGGGCATT
>KC834604
TTCTACTGTAATACAACACAACTGTTTAACAGTACTTGGAATTTTTCTAATTCAACACACCTGTTTAATTGGA
CTGGGTCAAT---

AACACTGAGCCTATCACACTCCCATGCAGAATAAAACAAATTATAAACATGTGGCAGGAAGTAGGAAAAGCAA
TGTATGCCCCGCCCATCAGAGGAGAAATTAACTGTTCATCAAAT---
ATTACAGGGGTGCTGCTAGTAAGAGATGGTGGTAAAGAAAACAGC----------------------------
----

```
ACTAGCGAGATCTTCAGACCTGGAGGAGGAGATATGAGGGACAATTGGAGAAGTGAATTATATAAATATAAAG
TAGTACAAATTGAACCATTA---GGAGTAGCACCCACCAAGGCAAAGCGAAGAGTGGTG--------------
-------------------------CAAAGAGAAAAAAGAGCA---GTGGGA---ATAGGAGCT---------
GTGCTCCTTGGGTTCTTGGGAGCA---GCAGGAAGCACTATG---GGCGCAGCGTCA---
CTGACGCTGACGGTACAG---GCCAGACTA---TTATTG---TCTGGTATAGCGCAACAGCAGAGC---
AATTTG---CTGAGA---GCTATTGAGGCGCAACAACATCTGTTGCAACTCACAGTCTGGGGCATT
>KC834602
TTCTACTGTAATACAACACAGCTGTTTAATAGTACTTGGCTGTCTAATGGCACTAGGAATGATACTGAAAAA-
-------------------------------------------------------------------------
----------------------------
TTAAATGACATTATCACACTCCCATGCAGAATAAAACAAATTGTAAACAGGTGGCAAGAAGTGGGAAAAGCAA
TGTATGCCCCTCCCATCAGCGGACAAATTAGGTGTTCATCAAAT---
ATTACAGGGTTGCTATTAACAAGAGATGGTGGTAGCAATGTCACC----------------------------
-------------------------------------------------------------------------
----------------
ACCGAGACCTTCAGACCTGAAGGAGGAAATATGAAGGACAATTGGAGAAGTGAATTATATAAATATAAAGTAG
TAAAACTTGAGCCATTA---GGAATAGCACCCACCAAGGCAAAGAGGCGAGTGGTG-----------------
----------------------CAGAGAGAAAAAAGA------GTGGCA---CTAGGAGCT---------
TTGTTCCTTGGGTTCTTGGGAGCA---GCAGGAAGCACTATG---GGCGCAGCGTCA---
ATAACGCTGACGGTACAA---GCCAGACTA---CTATTG---GCTGGTATAGAGCAACAGCAGAAC---
AATCTG---CTGAGA---GCTATTGAGGCGCAACAACATCTGTTGCAACTCACTGTCTGGGGCATC
>KT452273
TTCTACTGTAATACAACAAAACTGTTTAATAGTACTTGGCCAGTTAATAGTACTGGGAATAGTACTGAA----
-------------------------------------------------------------------------
----------------------------
GAAAAAGAAAATATCACACTCCCATGCAGAATAAAACAAATTATAAACATGTGGCAGGAAGTAGGAAAAGCAA
TGTATGCCCCTCCCATCAAAGGACAAATTAGATGTTCATCAAAC---
ATTACAGGGCTGCTATTAACAAGAGACGGTGGTGCTACCAACAAT----------------------------
-------------------------------------------------------------------------
-------------
ACCACCGAGATCTTCAGACCTGGAGGAGGAAATATGAAAGACAATTGGAGAAGTGAATTATATAAATATAAAG
TAGTGAAAATTGAACCATTA---GGGGTAGCACCCACCAAGGCAAAGAGGAGAGTGGTG--------------
-------------------------CAAAGAGAAAAAAGAGCA---GTGGGA---TTTGGAGCT---------
CTGTTCCTTGGGTTCTTGGGAGCA---GCAGGAAGCACTATG---GGCGCAGCGTCA---
GTGACGCTGACGGTACAA---GCCAGACAA---TTATTG---TCTGGTATAGTGCAGCAGCAAAAC---
GATTTG---CTGAGA---GCTATTGAGGCGCAACAACATCTGTTGCAACTCACAGTCTGGGGCATC
>KT452314
TTCTACTGTAATACAACAAAACTGTTTAATAGTACTTGGAGTAATAGTTCTGAACTGATTAATGGTACTGAAC
TG-----------------------------------------------------------------------
-ACGATGAATGGTACT---------
GGGGAACATGAAAATATCACCCTTCCATGCAGGATAAAACAAATTGTAAACATGTGGCAGGAAGTAGGAAAAG
CAATGTATGCCCCTCCCATTAGAGGACAAATTTTATGTACATCAAAC---
ATTACAGGGCTGATATTAACAAGAGATGGTGGAAATGTTAGTGAGAGC-------------------------
-------------------------------------------------------------------------
----------
AATGGCACGGAGATCTTCAGACCTGGAGGAGGAGACATGAGGGACAATTGGAGAAGTGAATTATATAAATATA
AAGTAATAAGAATTGAACCATTA---GGAATAGCACCCACCAAGGCAAAGAGAAGAGTGGTG-----------
----------------------------CAGAGAGAAAAAAGAGCA---GTGGGT---TTTGGAGCT------
---ATGTTCCTTGGGTTCTTGGGAGCA---GCCGGAAGCACTATG---GGCGCAGCGTCA---
GTGACGCTGACGGTACAG---GCCAGACTA---TTATTG---TCTGGTATAGTGCAACAGCAGAAC---
AATTTG---CTGAGG---GCTATTGAGGCGCAACAGCATCTGTTGCAACTCACGGTCTGGGGCATC
>KT452341
TTCTACTGTAATACAACAAGGTTGTTTAATAGTATTTGGAATAAAACTGAGATTGACAGTAATGCTACAGAAT
CAAT---------------------------------------------------------------------
----------------------------
AGCAATGACACTATCACACTCCCATGCAGAATAAAACAAATTATAAACAGATGGCAAGAAGTAGGAAAAGCAA
TGTATGCCCCTCCCATCAGTGGACAGATTAGGTGTTTATCAAAT---
ATTACAGGGCTGATATTAACAAGAGATGGAGGTAATAAAACGAAC----------------------------
-------------------------------------------------------------------------
-------
```

GAGACCAACATCGAAGTCTTCAGACCTGAAGGAGGAGATATGAGACACAATTGGAGAAGTGAATTATATAAGT
ATAAAGTAGTAAGAATTGAGCCATTA---GGGATAGCACCCACCAGGGCAAAGAGAAGAGTGGTG--------
-------------------------------GGAAGAGAAAAAAGAGCG---GTCACA---CTAGGAGCT---
------ATGTTCCTTGGGTTCTTAGGAGCA---GCAGGAAGCACTATG---GGCGCAGCGTCA---
ATGACGCTGACGGTACAG---GCCAGACAA---TTATTG---TCTGGTATAGTGCAGCAGCAAAAT---
AATTTG---CTGAGA---GCTATTGAGGCGCAACAGCATATGTTGCAACTCACAGTCTGGGGCATC
>KT452551
TTTTACTGTAATTCAACACCATTGTTTAATAGTATTTGGTATGATAATGGAACAAGCATCGCTACAGGAAATG
GGACA--

GTAAATAATACTATCATACTCCAATGCAGAATAAAACAAATTATAAACATGTGGCAAAAAGTAGGAAAAGCAA
TGTATGCTCCTCCCATTAAAGGACTAATTAAATGTTCATCAAAT---
ATTACGGGACTGCTATTAACAAGAGATGGTGGTAAAAACCAAACC----------------------------
----

AATGACAGTGAGACCTTCAGGCCGGGAGGAGGAGATATGAGGGACAATTGGAGAAGTGAACTATACAAATATA
AAGTAGTAAAAATTGAACCATTA---GGAGTAGCACCCACCAAGGCAAAAAGAAGAGTGGTG-----------
----------------------------CAGAGAGAAAAAAGAGCA---ATAACA---CTGGGAGCT------
---ATGTTCCTTGGGTTCTTGGGAGCA---GCAGGAAGCACTATG---GGCGCAGCGTCA---
ATGACGCTGACGGTACAG---GCCAGACAA---TTATTG---TCTGGTATAGTGCAACAGCAAAAC---
AATTTG---CTGAGA---GCTATTGAGGCGCAACAGCATCTGTTGCAACTCACAGTCTGGGGCATC
>KT452612
TTCTACTGTAATACAACACCGCTGTTTAATAGTACTTGGAAGTGGAATAATGGTACTGGAGGGTCAAGTATCA
CTGAAGGAAAT--

CTCACAGAACCTATCACACTCCAATGCAAAATAAAACAAATTATAAACATGTGGCAGGAAGTAGGAAAAGCAA
TGTATGCCCCCCCCATCCAGGGACAAATTAACTGTTCCTCAAAT---
ATTACAGGGCTGATATTGACAAGAGATGGAGGTGACGATAATACG----------------------------
----

CAAAATGACACCTTCAGACCTGCAGGAGGAGATATGAGGGACAATTGGAGAAGTGAATTATATAAATATAAAG
TAGTAAGAATTGAACCATTA---GGAATAGCACCTTCCAGGGCAAAGAGAAGAGTGGTG--------------
-------------------------CAGAGAGAAAAAAGAGCA---GTGGGA---TTTGGAGCT---------
TTGTTCCTTGGGTTCTTGGGAGCA---GCAGGAAGCACTATG---GGCGCAGCGTCA---
GTGGCGCTGACGGTACAG---GCCAGATCA---TTATTG---TCTGGTATAATGCAGCAGCAAAAC---
AACTTG---CTGAGG---GCTGTTGAGGCGCAACAGCATCTGTTGCAACTCACAGTCTGGGGCATC
>KT452359
TTCTACTGTAATTCAACACAACTGTTTAATAGTACTTGGAATACTACTGAAGGGTTAAATAGCACTGAAGGA-
----

GATGACACAACAATCACACTCCCATGCAGAATAAAACAAATTATAAACATGTGGCACAAAGTAGGCAGAGCAA
TGTATGCCCCTCCCATCCAAGGACAAATTAGATGTTCATCAAAT---
ATTACAGGGCTGCTATTAACAAGAGATGGTGGTAAGAATGTGAGC----------------------------
----

AATATCAGCGAGACCTTCAGACCTGGAGGAGGAAATATGAAGGACAATTGGAGAAGTGAATTATATAAATATA
AAGTAGTAAGAATTGAACCTTTA---GGAATAGCACCCACCAAGGCAAAGAGAAGAGTGGTG-----------
----------------------------CAAAGAGAGAAAAGAGCA---GTGGGA---ATGGGAGCT------
---CTGTTCCTTGGGTTCTTGGGAACA---GCAGGAAGCACTATG---GGCGCAGCGTCA---
TTGACGCTGACGGTACAG---GCCAGACAA---TTATTG---TCTGGTATAGTGCAGCAGCAGAAT---
GATATG---CTGAGA---GCTATTGAGGCGCAACAGCACCTGTTGCAACTCACAGTCTGGGGCATC
>KT452109
TTCTACTGTAATACAACACAACTGTTCACTAGTACTTGGAAAAATTCTAGTGATGGGAACGTTACAAGAAGT-
----

GAAAATGACACAATCACACTCCCATGCAGAATAAAACAAATTATAAACATGTGGCAGGAAGTAGGAAAAGCAA
TGTATGCCCCTCCCATCAGAGGACACATTAACTGTTCATCAAAT---
ATTACAGGGCTGCTATTAACAAGAGATGGTGGGGGCAACGAGAGCAGA-------------------------
----

GAAAATGAAACCTTCAGACCTGGAGGGGGAGATATGAAGGATAATTGGAGAAGTGAACTATATAAATATAAAG
TAGTAAAAATTGAACCATTA---GGAATAGCACCCACCGAGGCACAGAGAAGAGTGGTG--------------
-------------------------CAGAGAGAAAAAAGAGCA---GTGGGA---CTAGGAGCT---------
ATGCTCTTTGGGTTCTTGGGAGCA---GCAGGAAGCACTATG---GGCGCAGCGTCA---
TTGACGTTGACGGTACAA---GCCAAACTA---TTATTG---TCTGGTATAGTGCAACAGCAGAAT---
AATTTG---CTGAGA---GCTATTGAGGCGCAACAGCATATGTTGCAACTCACAGTCTGGGGCATC
>KT452090
TTCTACTGTAACTCATCACAATTGTTTAATAGTACTTGGAATCTTAATGATACTTGGAATGGTACTAGTGAC-
----

ACCAATGAAAATATTACACTCCCATGCAGAATAAAACAAATCATAAACCTGTGGCAGAAAGTAGGAAAAGCAA
TGTATGCCCCTCCCATCAGTGGATGGATTAACTGTTCATCAAAT---
ATTACAGGGCTGCTATTAACAAGAGATGGTGGTAATAATGAG-------------------------------
----

AATACTAGTGAGACCTTCAGACCTGGAGGAGGAAATATGAAGGACAATTGGAGAAGTGAATTGTACAAATACA
AAGTAGTAAAAATTGAACCATTA---GGAGTAGCACCCACCAGGGCAAAGAGAAGAGTGGTG-----------
----------------------------CAGAGAGAAAAAAGAGCA---GTGGGAACACTGGGAGCT------
---ATATTCCTTGGGTTCTTGGGAACA---GCAGGAAGCACTATG---GGCGCAGCGTCA---
GTGACGCTGACGGTACAA---GCCAGACAA---TTATTG---TCTGGTATAGTGCAGCAGCAAAAC---
AATTTG---CTGAGG---GCTATTGAGGCGCAGCAGCATCTGTTGCAACTCACAGTCTGGGGCATC
>KT452407
TTCTACTGTGACACAACACAACTGTTTAATAGCACTTGGCATCCTAATGGCACCAATAGTACTTGGCCTACTA
ATAGTACT---
-------------AATGGA------
AGAGAAAATGATACAATCATACTCCCATGCAGAATAAAACAAATTATAAACATGTGGCAGGAAGTAGGAAAAG
CAATGTATGCCCCTCCCATCAGTGGAGTAATTAAATGTTCATCAAAT---
ATTACAGGGCTGTTATTAACAAGAGATGGTGGTAGGATAATGGCACG--------------------------
----

AATGGTACCGAGACCTTCAGACCTGGAGGGGGAGATATGAGGGACAATTGGAGAAGTGAATTATACAAATATA
AAGTAGTAAGAATTGAACCATTA---GGAATAGCACCCACTAAGGCAAAGAGAAGAGTGGTG-----------
----------------------------CCGAGAGAAAAAAGAGCA---ATCGGA---ATAGGAGCT------
---ATGTTCATTGGGTTCTTGGGAGCA---GCAGGAAGCACTATG---GGCGCAGCGTCA---
GTGACGCTGACGGTACAG---GCCAGACAA---TTAATG---TCTGAGATAGTGCAACAGCAAAAC---
AATTTG---CTGAGA---GCTATTGAGGCGCAACAGCATATGTTGCAACTCACAGTCTGGGGCATC
>KT452194
TTCTACTGTGATACAACAAGTCTGTTTAATAGTAATTGGACGAAAGATAATAATGAGTCTTGGAGTAATACTA
CTGACTGG---

TCAAATGGAACTATCACACTCCAATGCAGAATAAAACAAATTATAAACCTGTGGCAGCAAGTAGGAAAGGCAA
TGTATGCCCCTCCCATCAGCGGAAACATTAGCTGTTCATCAAAT---
ATTACAGGGGTGATATTAACAAGAGATGGTGGGGGGGTAATAACACGGAA-----------------------
----

AACAATACTGAGATCTTCAGACCAGGAGGAGGAGATATGAAGGACAATTGGAGAAGTGAATTATATAAATATA
AAGTAGTACAAATTGAGCCACTA---GGAATAGCACCCACCAGGGCAAAGAGAAGAGTGGTG-----------
----------------------------CAGAGAGAAAAAAGAGCA---GTTGGC---ATAGGAGCG------
---GTGTTCCTTGGGTTCTTGTCTGCA---GCAGGAAGCACTATG---GGCGCAGCGGCA---
GTAACGCTGACGGTACAG---GCCAGACAA---TTATTG---TCTGGTATAGTGCAACAGCAGACC---
GCTCTG---CTGAGG---GCTATTGAGGCGCAACAGCATATGTTGCAACTCACAGTCTGGGGCATC
>KT452218
TTCTACTGTAATTCAACACAACTGTTTAATAGTACTTGGAATATTACTGGAGAACCAAATAGCACTGCA----
----

GGAAGTGACACAATCATACTGCCATGCAGAATAAAACAAATTATAAACATGTGGCAAGAAGTAGGAAAGGCAA
CGTATGCCCCTCCCATTAAAGGACAAATTAGATGCTCATCAAAT---
ATTACAGGGCTGCTATTAACAAGAGACGGTGGTACTAACAGTAGC----------------------------
----

AACAGTAGTGAGATCTTCAGACCTGGAGGAGGAAATATGAAGGACAATTGGAGAAGTGAACTATATAAATATA
AAGTAGTAAAAATTGAACCATTA---GGAATAGCACCCACCAAGGCAAAGAGAAGAGTGGTG-----------
----------------------------CAGAGAGAAAAAAGAGCA---GTGGGAGTAATAGGAGCT------
---ATGTTCCTTGGGTTCTTGGGAGCA---GCAGGAAGCACTATG---GGCGCAGCGTCA---
GTGACGCTGACGGTACAG---GCTAGACAA---TTATTG---TCTAGTATAGTGCAACAGCAGAAC---
GATTTG---CTGAGA---GCTATTGAGGCGCAACAGCATCTGTTGCAGCTCACAGTCTGGGGCATC
>KT452245
TTCTACTGTAATACATCACAACTGTTTAATAGTACTTGGAATGTTACTGAAGGGACAACTAACACTGAAGGGT
CAAATAACACT--

GGAGGAAGTGACCCTATCACACTCCCCTGCAGAATAAAACAAATTATAAACCTATGGCAGGAAGTAGGAAAAG
CAATGTATGCCCCTCCCATCAGAGGACAACTTAGTTGCTCATCAAAT---
ATTACAGGGCTGCTATTAACAAGAGATGGTGGTAATAACAGCGGA----------------------------
----

AGCGAGGTCGAGACCTTTAGACCAGGAGGAGGAGATATGAGGGACAATTGGAGAAATGAATTATATAAATATA
AAGTAGTAAAAGTTGAACCATTA---GGAATAGCACCCACCAGGGCAAAGAGAAGAGTGGTG-----------
----------------------------CAGAGAGAAAAAAGAGCG---GTAACG---CTAGGAGCT------
---GTGTTCCTTGGGTTCTTGGGAGCA---GCAGGAAGCACTATG---GGCGCAGCATCA---
GTAACGCTGACGGTACAG---GCCAGACAA---TTATTG---TCTGGTATAGTGCAACAGCAGAAC---
AATCTG---CTGAGG---GCTATTGAGGCGCAACAACATATGTTGCAACTCACAGTCTGGGGCATT
>MN486011
TTCTATTGCAATACATCACGCTTGTTTAATAGTACACATAATGTTACAGAAAATGCATATAATATTACAGGAA
ATGCT--

TCAGATTCAACCATCATCCTCCCATGCAGAATAAAACAAATTATAAATATGTGGCAGGAGGTAGGACGAGCAA
TGTATGCCCCTCCCATTGCAGGAAACATAACATGCCACTCAAAT---
ATCACAGGACTATTATTGACACGTGATGGAGGTGAAAATAACAGT----------------------------
----

ACATCAGAAACATTTAGACCTGCAGGAGGAGATATGAGGGACAATTGGAGAAGTGAATTATATAAATATAAAG
TGGTAGAAATTAAACCATTA---GGAATAGCACCCACTGGCGCAAAAAGGAGAGTGGTG--------------
-------------------------GAGAGAGAAAAAAGAGCA---GTGGGA---ATAGGAGCT---------
GTGCTCCTTGGGTTCTTGGGAGCA---GCAGGAAGCACTATG---GGCGCAGCGTCA---
ATAACGCTGACGGTACAG---GCCAGACAA---CTGTTG---TCTGGTATAGTGCAACAGCAAAGC---
AATTTG---CTGAGA---GCTATAGAGGCGCAACAGCATATGTTGCAACTCACGGTCTGGGGCATT
>HQ912708
TTCTATTGCAACACATCAAAACTGTTTAATGAGACAGAACTGCTTAAAAGT-----------------------
----

ACAGACAGTACTTTAACCATCCCATGCAGAATAAAACAAATTATAAACATGTGGCAGGGGGTAGGACGAGCAA
TATATGCCCCTCCCATTAAAGGAAAAATAACATGTAAATCAAAT---
ATTACAGGACTACTACTAACACGTGATGGAGGAGTAAATAACGAT----------------------------
----

AGCACAGAAACATTCAGACCTGTAGGAGGAAACATGAAGGACAATTGGAGAAGTGAATTATATAAATATAAAG
TGGTAGAAATTAAACCATTA---GGAGTAGCACCCACTAAGGCAAAAAGGAGAGTGGTG--------------
-------------------------GAGAGAGAAAAAAGAGCA---GTGGGA---ATAGGAGCT---------
GTGTTCCTTGGGTTCTTGGGAGCA---GCAGGAAGCACTATG---GGCGCAGCATCA---
ATAACGCTGACGGTACAG---GCCAGACAA---TTATTG---TCGGGCATAGTGCAACAGCAAAGC---
AATTTG---CTGAGG---GCCATAGAAGCGCAACAGCATATGTTGCAACTCACGGTCTGGGGCATT
>EU191613
TTCTATTGCAATACATCAGGATTGTTTAATGGTACATACATGAATAGTACATACATGAATAGT----------
----

ACAGATAAAATCATCAGACTCCCATGCAGAATAAAACAAATTATAAACATGTGGCAGGAAGTAGGACGAGCAA
TGTATGCCTCTCCCATTAAAGGACAAATAAAATGTGAATCAAAT---
ATCACAGGATTATTATTGACACGTGATGGAGGAAATGAAAATAAT----------------------------
----

GTGACAGAGATCTTCAGACCTGGAGGAGGAAATATGAAGGACAATTGGAGAAGTGAATTATATAAATATAAAG
TGGTGGAAATTAAACCATTA---GGAATAGCACCCACTACAGCAAAAAGGAGAGTGGTG--------------
-------------------------GAGAGAGAAAAAAGAGCA---GCGGGA---CTAGGAGCT---------
GTGTTCTTTGGGTTCTTGGGAGCA---GCAGGAAGCACTATG---GGCGCAGCATCA---
ATAACGCTGACGGTACAG---GCCAGACAA---TTATTG---TCTGGTATAGTGCAACAGCAAAGC---
AATTTG---CTGAGA---GCCATAGAGGCGCAACAGCACATGTTGCAACTCACGGTCTGGGGCATT
>MH746230
TTTTATTGTAACACATCAAATCTGTTTAATAGTACACACAGGGCAGATGATACAAATTCAACCATCACAAATG
ATACAGAT---

TCAAATAGGACCATCACACTCCAATGCAGAATAAAACAAATCATAAACATGTGGCAGAGGGTAGGACGAGCAA
TGTATGCCCCTCCCATTAAAGGAAACATAACATGTATATCAAAT---
ATCACAGGACTACTACTAACAAGGGATGGAGGAGTAAAGAATGAAACAGGGAAG------
AATGAAACAGGGAAGGACTCAGAT---GGG---

AACAACACAGAGATATTCAGACCTGGAGGAGGAGATATGAGGGACAATTGGAGAAGTGAATTATATAAATATA
AAGTGGTAGAAATTAAGCCATTA---GGAGTAGCACCCACTGAGGCAAAAAGGAGAGTGGTG-----------
----------------------------GAGAGAGAAAAAAGAGCA---GTGGGA---ATAGGAGCT------
---ATGTTCCTTGGGTTCTTGGGAGCA---GCAGGAAGCACTATG---GGCGCAGCATCA---
ATAACGCTGACGGTACAG---GCCAGACAA---TTGTTG---TCTGGTATAGTGCAACAGCAAAGC---
AATTTG---CTGAAA---GCTATAGAGGCGCAACAGCATATGTTGCAACTCACGGTCTGGGGCATT
>MH746248
TTCTATTGCAATACATCAAGCCTGTTTAATAGTAATAGTACAAGCCTAGGTAATAGTAGTAATAGTACAGAAA
CA---

GAAAATGCAAACATCACACTCCCATGCAGAATAAAACAAATCATAAACATGTGGCAGGGGGTAGGACAAGCAA
TGTATGCTCCTCCCATTAAAGGAATAATAACATGTAGATCAAAT---
ATCACAGGACTACTACTAACACGTGATGGAGGAGACAACAACAAA----------------------------
----

ACAGAGACATTCAGGCCTGGAGGAGGAGATATGAGGGACAATTGGAGAAGTGAATTATATAAATATAAAGTGG
TAGAAATTAAACCATTA---GGAATAGCACCCACTAAGGCAAAAAGGAGAGTGGTG-----------------
----------------------GAGAGAGAAAAAAGAGCA---GTGGGA---ATAGGAGCT---------
GTGTTCCTTGGGTTCTTGGGAGCA---GCAGGAAGCACTATG---GGCGCAGCATCA---
ATAACGCTGACGGTACAG---GCCAGACAA---TTATTG---TCTGGGATAGTGCAACAGCAAAGC---
AATTTG---CTGAGG---GCTATAGAGGCGCAACAGCATATGTTGCAACTCACGGTCTGGGGCATT
>U39233
TTCTATTGTAATACATCAAAACTGTTTAATAGTACAGACAGGTCTAGCAGTACA-------------------
----

GAAAGTGCAAACATAACCATCCCATGCAGAATAAAACAAATTATAAACATGTGGCAGGGGGTAGGACGAGCAA
TGTATGCCCCTCCCATTAAAGGAAAAATAACATGTAAATCAAAT---
ATCACAGGACTACTACTGACACGTGATGGAGGAACAACAAATGAT----------------------------
----

AGCACAGAGGCGTTCAGGCCTGCAGGAGGAGATATGAAGGACAATTGGAGAAGTGAATTATATAAGTATAAAG
TAGTAGAAATTAAACCATTG---GGAGTAGCACCCACCAAGGCAAAGAGGAGAGTGGTG--------------
-------------------------GAGAGAGAAAAAAGAGCA---GTGGGA---ATAGGAGCT---------
GTGTTCCTTAGGTTCTTGGGAGCA---GCAGGAAGCACTATG---GGCGCAGCATCG---
ATAACGCTGACGGTACAA---GCCAGACAA---TTATTG---TCGGGTATAGTGCAACAGCAAAGC---
AATTTG---CTGAGG---GCTATAGAGACGCAACAGCATATGTTGCAACTCACGGTCTGGGGCATT
>U39237
TTCTATTGCAATACATCAGGCCTGTTTAATAGTACATACAATAGTAATAGTACAGACAATGCTAATAGCACA-
----

GACAATTCAACCATCACGCTTCCATGTAGAATAAAACAAATTATAAACATGTGGCAGGGGGTAGGACAAGCAA
TATATGCCCCTCCCATTAGAGGAAACATAACATGTAAATCAAAT---
ATCACAGGAATATTACTGACACGTGATGGAGGATCAGATGCAAAT----------------------------
----

GAAACAGAGACATTCAGGCCTGGAGGAGGAGATATGAGGGACAATTGGAGAAGTGAATTATATAAATATAAAG
TGGTAGAAGTTAAACCATTA---GGATTAGCACCCACTAAGGCAAAAAGAAGAGTGGTG-------------
-------------------------GAGAGAGAAAAAAGAGCA---GTGGGA---CTAGGAGCT---------
GTGTTCCTTGGGTTCTTGGGAGCA---GCAGGAAGCACTATG---GGCGCAGCGTCA---
ATAACGCTGATGGTACAG---GCCAGACAA---TTGTTG---TCCGGTATAGTGCAACAGCAAAGC---
AATTTG---CTGAGG---GCTATAGAGGCGCAACAGCATATGTTGCAACTCACGGTCTGGGGCATT
>U39239
TTCTATTGCAATACATCAGGACTGTTTAATAGTACATACTGGCCTAACAGTACAAGAGAGGAGATTACAACA-
----

GACAATTCAAACATCACACTCCAATGCAGAATAAAACAAATTGTAAACATGTGGCAGGGGGTAGGACGAGCAA
CATATGCCCCTCCCATTGCAGGAAAAATAACATGTAACTCAAAC---
ATCACAGGACTGTTACTGACACGTGATGGAGGGATAAATGGGACA----------------------------
----

AATAGCACAGAGACATTCAGGCCTGGAGGAGGAAATATGAAGGACAATTGGAGAAGTGAATTATATAAATATA
AAGTGGTAGAAGTTAATCCATTA---GGAGTAGCACCCACTGGGGCGAAAAGGAGAGTGGTG-----------
---------------------------GAGAGAGAAAAAAGAGCA---GTGGGA---ATAGGAGCT------
---GTGTTCCTTGGGTTCTTGGGAGCA---GCAGGAAGCACTATG---GGCGCAGCGTCA---
ATAACGCTGACGGTACAG---GCCAGACAA---TTATTG---TCAGGTATAGTGCAACAGCAAAGC---
AATTTG---CTGAGG---GCTATCGAGGCGCAACAGCATATGTTGCAACTCACGGTCTGGGGCATT
>U39246
TTCTATTGTAATACATCAAAACTGTTTAATGGTGCATACAATAATGGTACATACAATAGTACAGAGAATATA-
----

GAAAATTCAACTATCACACTCCCATGCAGAATAAAACAAATTATAAACATGTGGCAGGGGGTAGGGCAAGCAA
TGTATGCCCCTCCCATCAAAGGAAACATAACATGTAAATCAAAT---
ATCACAGGACTACTACTGACACGTGATGGAGGAAGCAGCGAGGCAAAT-------------------------
----

AAAACAGGGGAGACATTCAGGCCTGGAGGAGGAGATATGAAGGACAATTGGAGAAGTGAATTATATAAATATA
AAGTGGTAGAAATTAAACCATTA---GGAATAGCACCCACTAAGGCAAAAAGGAGAGTGGTG-----------
---------------------------GAGAGAGAAAAAAGAGCA---GCA------ATAGGAGCT------
---ATGTTCCTTGGGTTCTTGGGAGCA---GCTGGAAGCACTATG---GGCGCAGCATCA---
ATAACGCTGACGGTACAG---GCCAGACAA---TTATTG---TCGGGCATAGTGCAACAGCAAAGC---
AATTTG---CTGAGG---GCTATAGAGGCGCAACAGCATATGTTGCAACTCACGGTCTGGGGCATT
>U39245
TTCTATTGCAATACATCAAAATTGTTTAAGACATCAGTTAAC-------------------------------
----

CCAAACATCACACTCACACTCCCATGCAGAATAAAACAAATTATAAACATGTGGCAGGAGGTAGGGCGAGCAA
TGTATGCCCCTCCCATTTCAGGAATAATAACATGTAAATCAAAT---
ATCACAGGACTACTACTAACACGTGATGGAGGGGGGCAGAATAAT----------------------------
----

GGACCAGAGACATTCAGACCTGGAGGAGGAAATATGAGAGACAATTGGAGAAGTGAATTATATAAATATAAAG
TGGTAGAAATTAAGCCATTA---GGAATAGCACCCTCTAAGGCAAAAAGGAGAGTGGTG-------------
-------------------------GAGAGAGAAAAAAGAGCA---GTGGGA---ATAGGAGCT---------
GTGTTCCTTGGGTTCTTGGGAGCA---GCAGGAAGCACTATG---GGCGCAGCATCA---
ATAACGCTGACGGTACAG---GCCAGACAA---TTATTG---TCTGGTATAGTGCAACAGCAAAGC---
AATTTG---CTGAGA---GCTATAGAGGCGCAACAGCACATGTTGCAACTCACAGTCTGGGGCATT
>U39251
TTCTATTGCAATACATCACAACTGTTTAATAGTACATACTGGTTTAATAGTACACACTGGTTTAACAGTACAG
AAAGCGCT---
-------------AATGGA------
ACAGAAAATGACACTATCACACTCCCATGCAGAATAAAACAAATTATAAACATGTGGCAGGGGGTAGGACAAG
CAATGTATGCCCCTCCCATTAAAGGAAACATAACATGTAAATCAAAT---
ATCACAGGAATACTCCTAACACGTGATGGAGGAAATTTAACTAAT----------------------------
----

GGCACAGAGACATTCAGGCCTGGAGGAGGAGATATGAGGGACAATTGGAGAAATGAATTATATAAATATAAAG
TGGTAGAAATTAAACCATTA---GGAGTAGCACCCACTGCGGCAAAAAGGAGAGTGGTG-------------
------------------------GAGAGAGAAAAAAGAGCA---GCA------CTAGGAGCT---------
GTGTTCCTTGGGTTCTTGGGAGCA---GCAGGAAGCACTATG---GGCGCAGCGTCA---
ATAACGCTGACGGTACAG---GCCAGACAA---TTATTG---TCCGGCATAGTGCAACAGCAAAGC---
AATTTG---CTGAGG---GCCATAGAGGCGCAACAGCATATGTTGCAACTCACGGTCTGGGGCATT
>JN692434
TTCTATTGCAATACAGCAAGCCTGTTTAATAGTACATACAATAGTACAGGAAACATTACTGAGACAGAAAAGT
CA--

AACAGCACAGTCATCACAATCCCATGCAGAATAAAACAAATTATAAACATGTGGCAGGAGGTAGGACGAGCAA
TGTATGCCTCCCCTATAAAAGGAGAAATAACATGTAACTCAAGT---
ATCACAGGATTACTACTGACACGGGATGGAGGCCTGAATAGAACA---------------------------
----

AATGAGACATTCAGGCCTGGAGGAGGAGATATGAGAGACAATTGGAGAAGTGAATTATATAAATATAAAGTGG
TAGAAATTAAACCATTA---GGAATAGCACCCACTAGAGCAAAAAGGAGAGTGGTG----------------
---------------------GGGAGAGAAAAAAGAGCA---GTGGGA---ATAGGAGCT---------
GTGTTCCTTGGGTTCTTGGGAGCA---GCAGGAAGCACTATG---GGCGCAGCATCA---
ATAACGCTGACGGTACAG---GCCAGACAA---TTGTTG---TCTGGGATAGTGCAACAGCAAAGC---
AATTTG---CTGAGA---GCTATAGAGGCGCAACAGCATATGTTGCAACTCACGGTCTGGGGCATT
>AY727522
TTCTATTGCAATACATCAGGCTTGTTTAATGGTACATACAATGGTACAGAAAGCAATAATGAAACAGAAAAT-
----

GCAAACGCAAACATCACACTCCCATGCAAAATAAAACAAATTGTACGCATGTGGCAGGAAGTAGGACAAGCAA
TGTATGCCCCTCCCGTTAAGGGAATGATAACATGTAGATCAAAT---
ATCACAGGATTACTATTGACACGGGATGGAGGCTCTAACACCACG---------------------------
----

AATAACACAGAAACATTCAGGCCTGGAGGAGGAGATATGAGGGACAATTGGAGAAGTGAACTATATAAATATA
AAGTGGTAGAAATTAAACCATTA---GGAATAGCACCCACTGAGGCAAAAAGGAGAGTGGTG----------
---------------------------CAGAGAGAAAAAAGAGCA---GTGGGA---ATAGGAGCT------
---GTGTTCCTTGGGTTCTTGGGAGCA---GCAGGAAGCACTATG---GGCGCAGCATCA---
ATAACGCTGACGGTACAG---GCCAGACAA---TTATTG---TCTGGAATAGTGCAACAGCAAAGC---
AATTTG---CTGAGG---GCTATAGAGGCGCAACAGCATATGTTGCAACTCACGGTCTGGGGCATT
>AY727523
TTCTATTGTAATACAACAGGCCTGTTTAATGGTACATACATAAAAAATTCTAATGCAACAGGAAAC-------
----

ATTACTAGAAACATCACAATCCCATGCAGAATAAAACAAATTATAAACATGTGGCAGAAGGTAGGACGAGCAA
TGTATGCCCCTCCCATTCGAGGAATAATAACCTGTAGGTCAAAT---
ATCACAGGACTACTACTGACACGTGATGGAGGAGAGAATGATACA---------------------------
----

GAGACAGTCAGGCCTGGAGGAGGAGATATGAGGGACAATTGGAGAAGTGAATTATATAAATATAAAGTGGTAG
AAATTAAACCATTA---GGAATAGCACCCACTAAGGCAAAAAGGAGAGTGGTG-------------------
------------------GAGAGAGAAAAAAGAGCA---GTGGGA---ATAGGAGCT---------
GTGTTCCTTGGGTTCTTGGGAGCA---GCAGGAAGCACTATG---GGCGCAGCATCA---
ATAACGCTGACGGTACAG---GCCAGACAA---TTGTTG---TCTGGGATAGTGCAACAGCAAAGC---
AATTTG---CTGAGG---GCTATAGAGGCGCAACAGCATATGTTGCAACTCACAGTCTGGGGCATT
>AY727524
TTCTATTGCAATACATCAAGCCTGTTCAATGGTACATACAATGGTACATACAATAGTACAGGAAACATTAATG
GA--

ACAGACACAAACATCACAATACCATGCAGAATAAAACAATTTATAAACATGTGGCAGGAGGTAGGACGAGCAA
TGTATGCCCCTCCCATTAGAGGAATAATAATGTGTAGATCAAAT---
ATCACAGGACTACTACTGACACGTGATGGGGGAATTAGCAGAGATAACAAC---------------------
----

ACAGATAACACCACAGAGATATTCAGACCTGGAGGAGGAGATATGAGGGACAATTGGAGAAGTGAATTATATA
AATATAAAGTGGTAGAAATTAAACCATTA---GGACTAGCACCCACTAAGGCAAAAGGAGAGTGGTG-----
-----------------------------------GAGAGAGAAAAAAGAGCA---GCA------
CTAGGAGCT---------ATGTTCCTTGGGTTCTTGGGAGCA---GCCGGAAGCACTATG---
GGCGCAGCATCA---ATAACGCTGACGGTACAG---GCCAGACAA---TTATTG---
TCTGGGATAGTGCAACAGCAAAGC---AATTTG---CTGAGG---
GCTATAGAGGCGCAACAGCATATGTTGCAACTCACGGTCTGGGGCATT
>AY727525
TTCTATTGCAATACAGCAAACCTGTTTAATGGTACATACAATGATACAGGAAACATTAAT------------
----

GAAACAGAAACCATTACAATCCCATGCAGAATAAAACAAATTATAAACATGTGGCAGAAGGTAGGACGAGCAA
TGTATACCCCTCCCATTAACGGAATAATAACATGTAGATCAAAT---
ATCACAGGGTTACTACTGACACGGGATGGAGGCAATAATAATGGC---------------------------
----

ACGAATGAGACATTCAGGCCTGAAGGAGGAGATATGAAGGACAATTGGAGAAGTGAATTATATAAATATAAAG
TGGTGGAAATTAAACCATTA---GGAATAGCACCCACTAAGGCAAAAGGAGAGTGGTG-------------
-------------------------GAGAGAGAAAAAAGAGCA---GCAGGA---ATAGGAGCT---------
GTGTTCCTTGGGTTCTTGGGAGCA---GCCGGAAGCACTATG---GGCGCAGCATCA---
ATAACGCTGACGGTACAG---GCCAGACAA---TTATTG---TCTGGTATAGTGCAACAGCAAAGT---
AATTTG---CTGAGG---GCTATAGAGGCGCAACAGCATATGTTGCAACTCACGGTTTGGGGCATT
>JX140663
TTCTATTGCAATACATCAAGCCTGTTTAATAGTGAATTAGACAGTAATGGTACATTCAAAATTAATGGGACAG
AAAATGGAACT---
-------------------GGA---
ACAGAAAATTCAAACATCACACTCCAATGCAGAATAAAACAAATTATAAACATGTGGCAGGAGGTAGGACGAG
CAATGTATGCCCCTCCCATTGCAGGAGAAATAACATGTAGATCAAAT---
ATCACAGGATTACTACTAACAAGGGATGGAGGAGACACGAGTGAC---------------------------
----

GAGATATTCAGGCCTGGAGGAGGAGATATGAGGGACAATTGGAGAAGTGAATTATATAAATATAAAGTGGTAG
AAGTTAAACCATTA---GGAGTAGCACCCACCGAGGCAAAAGGAGAGTGGTG-------------------
-------------------GAGAGAGAAAAAAGAGCA---GTGGGA---ATAGGAGCT---------
GTGTTCCTTGGGATCTTGGGAGCA---GCAGGAAGCACTATG---GGCGCAGCATCA---
ATGACGCTGACGGTACAG---GCCAGACAA---TTATTG---TCTGGGATAGTGCAACAGCAAAGT---
AATTTG---CTGAGG---GCTATAGAGGCGCAACAGCATATGTTGCAACTCACGGTCTGGGGCATT
>KX181929
TTCTACTGCAATACATCAGGCCTGTTTAATAGTACATACTGGCCTAATGCTACATATAATGCAACAGAAAATT
CA--

GAAAATTCAACCATCACAATCCCATGCAGAATAAAACAATTTGTAAACATGTGGCAGGAGGTAGGACGAGCAA
TGTATGCCCCTCCCATTAGAGGACCAATAAACTGTACATCAAAT---
ATAACAGGACTATTGATAACACGTGATGGAGGAATCAACTCAAGT---------------------------
----

GACACAGAGATATTCAGACCTGGAGGAGGAGATATGAGGGACAATTGGAGAAGTGAATTATATAAATACAAAG
TGGTAGAAATTAAACCATTA---GGAATAGCACCCACCGAGGCAAAAGGAGAGTGGTG-------------
-------------------------GAGAGAGAAAAAAGAGCA---GTGGGA---ATAGGAGCT---------
GTGTTCCTTGGGTTCTTGGGAGCC---GCAGGAAGCATTATG---GGCGCAGCGTCA---
CTAACGCTGACGGTACAG---GCCCGGCAA---TTATTG---TTTGGGATAGTGCAACAGCAAACC---
AATTTG---TTGAGG---GCTATAGAGGCGCAACAGCATATGTTGCAACTCACAGTCTGGGGCATT
>KX181930
TTCTATTGTAATACATCAAGCCTGTTTAATAGGAATATTACAGAAAAAATTACTGAT---------------
----

AATAATTCAAGCATCACTATCACATGCAGAATAAAACAAATTGTAAACATGTGGCAGGGGGTAGGACGAGCAA
TATATGCCCCTCCCATTGAAGGAGTAATAACTTGTAGATCAAAT---
ATCACAGGATTGCTACTGACACGGGATGGAGGCAACACAAATAAC---------------------------
----

ACAACAGAGACATTCAGGCCTGGAGGAGGAGATATGAGGGACAATTGGAGAAGTGAATTATATAAATATAAAG
TGGTAGAAATTAAACCATTA---GGAATAGCACCCACTAAGGCAAAAAGGAGAGTGGTG--------------
-------------------------GAGAGAGAAAAAAGAGCA---GTGGGA---ATAGGAGCT---------
GTGTTCCTTGGGTTCTTGGGAGCA---GCAGGAAGCACTATG---GGCGCAGCATCA---
ATAACGCTGACGGTACAG---GCCAGACAA---TTGTTG---TCTGGGATAGTGCAACAGCAAAGC---
AATTTG---CTGAGA---GCTATAGAGGCGCAACAGCATATGTTGCAACTCACGGTCTGGGGCATT
>KX181931
TTTTATTGCAATACATCAAACCTGTTTAATAATATATACCCGAAATGGAACATGTTTGACTGGAGCAATGCAA
ACAAA--
-------------------ACA---
AACACTACAACAAACATCACACTCCGATGCAGAATAAGACAATTTATAAACATGTGGCAGGAGGTAGGACGAG
CAATGTATGCCCCTCCCATTGAAGGAATAATAACATGTAGATCAAAT---
ATCACAGGATTACTACTGACCCGGGATGGAGGCAGGGGAGACACAAATGGCACA-------------------
----

GAGACAAATAAAACAGAGACATTCAGGCCCGGAGGGGGAGATATGAGGGACAATTGGAGAAGTGAATTATATA
AATATAAAGTGGTAGAAATTAAACCATTA---GGAGTAGCACCAACTAAGGCAAAAAGGAGAGTGGTG-----
----------------------------------GAGAGAGAAAAAAGAGCA---GTGGGA---
ATAGGAGCT---------GTGTTCCTTGGGTTCTTGGGAGCA---GCAGGAAGCACTATG---
GGCGCAGCATCA---ATAACGCTGACGGTACAG---GCCAGACAA---TTATTG---
TCTGGAATAGTGCAACAGCAAAGC---AATTTG---CTGAGG---
GCTATAGAGGCGCAACAGCATATGTTGCAACTCACGGTCTGGGGCATT
>KX181932
TTCTATTGCAATACATCAATCCTGTTTAATGATACATACTGGGTTAATGGGACAGGAAGCACTAAT-------
----

GGAACAGAAAACATCACACTCCCATGCAGAATAAAACAAATTATAAACATGTGGCAGGGAGTAGGACAAGCAA
TATATGCCCCTCCCATTAAAGGAAGAATAACATGTAAATCAAAT---
ATCACAGGAATACTACTGACACGTGATGGAGGAGAAACTAATGAAACT-------------------------
----

AATGCCACAGAGGTATTTAGGCCTGGAGGAGGAGACATGAGGGACAACTGGAGAAGTGAACTATATAAATATA
AAGTGGTAGAAATTAAACCATTA---GGAATAGCACCCACTAGGGCAAAAAGGAGAGTGGTG-----------
----------------------------GAGAGAGAAAAAAGAGCA---GCA------CTAGGAGCT------
---ATGTTCCTTGGGTTCTTGGGAGCA---GCAGGAAGCACTATG---GGCGCAGCGTCA---
ATAGCGCTGACGGTACAG---GCCAGACAA---TTATTG---TCTGGGATAGTGCAACAGCAAAGC---
AATTTG---CTGAGG---GCTATAGAGGCGCAACAGCATATGTTGCAACTCACGGTCTGGGGCATT
>KX181933
TTCTATTGCGATACAACAGGACTGTTTACAGAAAACAGTACTGAGATAGACAACGGTACA-------------
----

GAAAATTCAACCATCATAATCCCATGCAGAATAAGACAATTTGTAAACATGTGGCAAGAGGTAGGACGAGCAA
TATATGCCCCTCCCATTGCAGGAAACATAACATGTAGATCAAAT---
ATCACAGGACTACTACTGACACGTGATGGAGGAGAAGGAAATGAGACA-------------------------
----

CAAAATGATACAGAAACATTCAGGCCTGGAGGGGGAAATATGAAGGACAATTGGAGAAGTGAATTATATAAAT
ATAAGGTGGTAGAAATTAAGCCCTTA---GGAATAGCCCCCCCCAAGGCAAAAAGGAGAGTGGTG--------
-------------------------------GAGAGAGAAAAAAGAGCG---GCA------TTAGGAGGT---
------GTGTTCCTTGGGTTTTTGGGAGCA---GCAGGAAGCACTATG---GGCGCAGCATCA---
ATGATGCTGACGGCACAG---GCCCGACAA---TTATTG---TTTGGGATAGTGCAACAGCAAAGC---
AATTTG---TTGAGA---GCTATAGGGGCGCAACAGCACATGTTGCAACCCCCGTTTTGGGGCATT
>KX181934
TTCTATTGCAATACATCAAACCTGTTTAATAGTACATACAATAGTAACTGCACATCTAATAGGACA-------
----

GGAACTGAAAACATAACAATCCCATGCAGAATAAAACAAATTATAAACATGTGGCAGGGGGTGGGACGAGCAA
TGTATGCCCCTCCCATTAAAGGACAAATAACATGTAGATCAAAT---
ATCACAGGATTAGTACTAGAAAGGGATGGAGGCACAAATAAGACA----------------------------

----

GAAGAGACATTCTGGCCTACAGGAGGAGATATGAGGGACAATTGGAGAAGTGAATTATATAAATATAAAGTGG
TAGAAGTTAGACCATTA---GGAATAGCACCCACTAGAGCAAAAAGGAGAGTGGTG-----------------
----------------------GAGAGAGAAAAAAGAGAT---TTGGGA---CTAGGAGCT---------
GTGTTCCTTGGGTTCTTGGGAGCA---GCAGGAAGCACTATG---GGCGCAGCATCA---
ATAACGCTGACGGTACAG---GCCAGACAA---TTGTTG---TCTGGGATAGTGCAACAGCAAAGC---
AATTTG---CTGAGG---GCTATAGAGGCGCAACAGCATATGTTGCAACTCACTGTCTGGGGCATT
>KX181935
TTCTATTGCAATACATCAAAACTGTTTAATAGTACATACTGGCCTAATGGTACAGAAAAC-------------
----

ACAAACATCACCATCACAATTCCATGCAGAATAAAACAAATTATAAACATGTGGCAGGAGGTAGGACGAGCAA
TGTATGCCCCTCCCATTAAAGGAATAATAACATGTAGATCAAAT---
ATCACAGGGCTACTACTGACACGTGATGGAGGAAATGGAGATTTCACAGAT----------------------
----

AGCCCAGAAACATTCAGGCCTGGAGGAGGAGATATGAGGGACAATTGGAGAAGTGAATTATATAAATATAAAG
TGGTAGAAATTAAACCATTA---GGAATAGCACCCACTAAGGCAAAAAGGAGAGTGGTG--------------
-------------------------GAGAGAGAAAAAAGAGCA---GTGGGA---ATAGGAGCT---------
GTGTTCCTTGGGTTCTTGGGAGCA---GCAGGAAGCACTATG---GGCGCAGCATCA---
ATAACGCTGACGGTACAG---GCCAGACAA---TTATTG---TCTGGGATAGTGCAACAGCAAAGC---
AATTTG---CTGAGG---GCTATAGAGGCGCAACAGCATATGTTGCAACTCACGGTCTGGGGCATT
>KX181938
TTCTATTGCAATACATCAGGCCTGTTTAATGGTACATATACACCTAATAGTAATAGTACTGAAACA-------
----

GGAAATTCAACAATCACCATCCCATGCAGAATAAAACAAATTATAAATATGTGGCAAGGGGTAGGGCGAGCAA
TGTATGCCCCTCCCATTAGTGGAATAATAACATGTAGATCAAAT---
ATCACAGGGATACTACTGACACGGGATGGAGGCGATAGCAACAAT----------------------------
----

GATACAGAGATATTCAGGCCTACAGGAGGAGATATGAGGGACAATTGGAGAAGTGAATTATATAAATATAAAG
TGGTAGAGATTAAACCCGTA---GGGATAGCCCCCCCCAAGGCAAAGAGAAGAGTGGTG--------------
-------------------------GAGAGAGAAAAAAGGGGA---ATAGGA---ATAGGAGCT---------
GTGTTCCTTGGGTTTTTGGGAGCA---GCAGGAAGCACTATG---GGCGCAGCATCA---
ATAACGCTGACGGTACAG---GCCCGACAA---TTATTG---TTTGGAATAGTGCAACAGCAAAGC---
AATTTG---CTGAGA---GCTATAGAGGCGCAACAGCATATGTTGCAACTCACTGTCTGGGGCATC
>KX181939
TTCTATTGCAATACATCAGGACTGTTTAATAGTACATATTTTAATGGTACATATAATAGTACAGGA-------
----

AACAGTCCAAACATCACACTCCCATGCAGAATAAAACAAATTGTAAGAATGTGGCAGAGGGTAGGACAAGCAA
TGTATGCCCCTCCCATTCCAGGAATAATAACATGTAGATCAAAT---
ATCACAGGATTGTTACTGACACGGGATGGAGGCCCGGATAATAAT----------------------------
----

CGGACAGAGACATTCAGGCCTGGAGGAGGAGATATGAGGGACAATTGGAGAAGTGAATTATATAAATATAAAG
TGGTAGAAATTAAACCATTA---GGAATAGCACCCACTAAGGCAAAAAGGAGAGTGGTG--------------
-------------------------GAGAGAGAAAAAAGAGCA---GTAGGA---CTAGGAGCC---------
ATGTTCCTTGGGTTCTTGGGAGCA---GCAGGAAGCACTATG---GGCGCAGCATCA---
ATAACGCTGACGGTACAT---GCCAGACAA---TTACTG---TCTGGGATAGTGCAACAGCAAAGC---
AATTTG---CTGAGG---GCTATAGAGGCGCAACAGCATATGTTGCAACTCACGGTCTGGGGCATT
>KX181940
TTCTATTGTAATACATCAAGCCTGTTTAATAGGACATACCCAGCTAATAGTACAGGATACAATAATGAAACA-
----

GGAAATTCAACCATTACAATCCCATGCAGAATAAAACAAATTATAAATAGATGGCAGGAGGTGGGACAAGCAA
TGTATGCCCCTCCCATTGCAGGAATAATAACATGTAGATCAAAT---
ATCACAGGATTACTACTGACACGGGATGGAGGCAACACAACAAAT----------------------------

----

AAAACTGAGATATTCAGGCCTGAAGGAGGGAATATGAGGGACAATTGGAGAAGTGAATTATATAAATATAAAG
TGGTAGAAATTAAACCATTA---GGAATAGCACCCACTAAGGCAAAAAGGAGAGTGGTG--------------
-------------------------GAGAGAGAAAAAAGAGCA---GTGGGA---ATAGGAGCT---------
GTGTTCCTTGGGTTCTTGGGAGCA---GCAGGAAGCACTATG---GGCGCAGCATCA---
ATAACGCTGACGGTACAA---GCCAGACAA---TTATTG---TCTGGGATAGTGCAACAGCAAAGC---
AATTTG---CTGAGG---GCTATAGAGGCGCAACAGCATATGTTGCAACTCACGGTCTGGGGCATT
>KU749391
TTCTATTGCAATACATCAAGCCTGTTTAACCATACATACCACAACCTGAATAGTAGTAGTACAAACACA----
----

ACAGCAAATATCACAAGAATACCATGCAGAATAAAACAAATTATAAATATGTGGCAGGGGGTAGGACGAGCAA
TGTATGCCCCTCCCATTAAAGGAAAAATAACATGTAGATCAAAC---
ATCACAGGATTAGTACTGACACGGGATGGAGGTAATACAGGAGGG----------------------------
----

ACAGAGATATTCAGGCCTGGAGGAGGAGATATGAGGGACAATTGGAGAAGTGAACTATATAAATATAAGGTGG
TAGAAATTAAACCATTA---GGAATAGCACCCACTAAGGCAAAAAGGAGAGTGGTG-----------------
----------------------GAGAGAGAAAAAAGAGCA---GTGGGA---ATAGGAGCT---------
GTGTTCCTTGGGTTCTTGGGAGCA---GCAGGAAGCACTATG---GGCGCAGCATCA---
CTAACGCTGACGGTACAG---GCCAGACAA---TTATTG---TCTGGGATAGTGCAACAGCAAAGC---
AATTTG---CTGAGG---GCTATAGAGGCGCAACAGCATATGTTGCAACTCACGGTCTGGGGCATT
>KT427678
TTCTATTGCAATACGTCAGGGCTGTTTAATAATACATGGCTTAACGTGACTAATAAAACA-------------
----

GAAAATCCAACCATCACAATCCCATGTAGAATAAAACAAATTATAAACATGTGGCAGGAAGTAGGAAAAGCAA
TGTATGCCCCTCCCATTGCAGGAAAGATAACATGTAGATCAAAT---
ATCACAGGACTACTACTGACACGTGATGGAGGTGACAATATCACA----------------------------
----

GAAGAAATATTCAGGCCTGCAGGAGGAGACATGAGGGACAATTGGAGAAGTGAATTATATAAATATAAAGTGG
TAGAAATTAAACCATTA---GGAATAGCACCCACCAAGGCAAAAAGGAGAGTGGTG-----------------
----------------------GAGAGAGAAAAAAGAGCAGTAGTGGGA---ATAGGAGCT---------
GTGTTCCTTGGGTTCTTGGGAGCA---GCAGGAAGCACTATG---GGCGCAGCATCA---
ATAACGCTGACGGTACAG---GCCAGACAA---TTATTG---TCTGGAATAGTGCAACAGCAAAGC---
AATTTG---CTGAGA---GCTATAGAGGCGCAACAGCATATGTTGCAACTCACGGTCTGGGGCATT
>KT427736
TTCTATTGCGATACATCAGGCCTGTTTAATAGTACATTTAATAAGACAGGAAACAGTAATAGTACA-------
----

GTAAACAGCACAATCACAATCCAATGCAGAATAAAACAAATTATAAACATGTGGCAGGAGGTAGGACGAGCAA
TGTATGCCCCTCCCATTAAAGGAAACATAACATGTAGAACAAAT---
ATCACAGGAATACTACTGACACGTGATGGAGGAAACGAAACTAAC----------------------------
----

ACAGAAGAGATATTCAGACCTGGAGGAGGAGATATGAGGGACAATTGGAGAAGTGAATTATATAAATATAAAG
TGGTAGAAATTAAACCATTA---GGAATAGCACCCACTGAGGCAAGAAGGAGAGTGGTG--------------
-------------------------GAGAGAGAAAAAAGAGCA---ATAGGGGGAATAGGAGCT---------
GTGTTCCTTGGGTTCTTGGGAGCA---GCAGGAAGCACTATG---GGCGCAGCATCA---
ATAACGCTGACGGTACAG---GCCAGACAA---TTATTG---TCTGGGATAGTGCAAGAGCAAAGC---
AATTTG---CTGAGG---GCTATAGAGGCGCAACAGCATATGTTGCAACTCACGGTCTGGGGCATT
>KT427800
TTCTATTGCAATACATCAGGGCTGTTTAATGCTACATTTAATGCTACACACTGGTCTAATATGACAGAAAGTA
CTAATGAA---

ACAGACAATACAACCATCATAATCCCATGTAAAATAAAACAAATTGTAAACCTGTGGCAGGAGGTAGGAAAAG
CAATGTATGCCCCTCCCATTGCAGGAATGATAACATGTAGGTCAAAT---
ATCACAGGACTACTACTGACACGTGATGGAGGTAATACAGATAAC----------------------------

----

AGTAGCACAGAAATATTCAGGCCTGGAGGAGGAGACATGAGGGACAATTGGAGAAGTGAATTATATAAATATA
AAGTGGTAGAAATTAAACCATTA---GGAATAGCACCCACCGAGGCAAAAAGGAGAGTGGTG-----------
----------------------------GAGAGAGAAAAAAGAGCA---GTGGGA---ATAGGAGCT------
---TTGTTCCTGGGGTTCTTGGGAGCA---GCAGGAAGCACTATG---GGCGCAGCATCA---
ATAACGCTGACGGTACAG---GCCAGACAA---TTATTG---TCTGGAATAGTGCAACAGCAAAGC---
AATTTG---CTGAGA---GCTATAGAGGCGCAACAGCATATGTTGCAACTCACGGTCTGGGGCATT
>KU749392
TTCTATTGCAATACATCAAGCCTGTTTAATAAGACATACCTGTTTAATAATACATACCTGGTTAAGAATGGCA
CG---
GTTACAGAAAACAATAAT---------
GGAACAAACATCACAGTCACAATCCCATGCAGAATAAAACAATTTATAAACATGTGGCAGGAGGTAGGACGAG
CAATGTATGCCCCTCCCATTGCAGGACCAATAACATGTATATCAAAT---
ATCACAGGGTTACTACTGACACGGGATGGAGGCCTTAACACAACG----------------------------
----

AACGAGACATTCAGGCCTGGAGGAGGAGATATGAGGGACAATTGGAGAAATGAATTATACAAATATAAAGTGG
TAGAAATTAAACCATTA---GGAATAGCGCCCACTAAGGCAAAAAGAAGAGTGGTG-----------------
----------------------GAGAGAGAAAAAAGAGCA---GTGGGA---ATAGGAGCT---------
GTGTTCCTTGGGTTCTTGGGAGCA---GCAGGAAGCACTATG---GGCGCAGCGTCA---
ATAACGCTGACGGTACAG---GCCAGACAA---TTACTG---TTTGGGATAGTGCAACAGCAAAGT---
AATTTG---CTGAGA---GCTATAGAGGCGCAACAACATATGTTGCAACTCACGGTCTGGGGCATT
>KU749393
TTCTATTGCAATACATCAGATCTGTTTAATGATATAGACACATCTAAT-------------------------
----

AACACTGAGAACATCACAATCCCATGCAGGATAAAACAGATTATAAACATGTGGCAGGAAGTAGGACGAGCAA
TGTATGCCTCTCCCATTAAAGGACCAATAACATGTAGATCAAAT---
ATCACAGGATTATTACTGACACGGGATGGAGGCACGGACGGGGGA----------------------------
----
GGCGAAAAGGACAAC---
AACACGGAGACATTCAGGCCTGGAGGAGGAGATATGAGGGACAATTGGAGAAGTGAATTATATAAATATAAAG
TGGTAGAAATTAAACCATTA---GGAATAGCACCAACTAGGGCAAAAAGGAGAGTGGTG--------------
-------------------------GAGAGAGAAAAAAGAGCG---GCA------ATAGGAGCT---------
ATGTTCCTTGGGTTCTTAGGAGCA---GCAGGAAGCACTATG---GGCGCAGCATCA---
ATAACGCTGACGGTACAG---GCCAGACAA---TTGTTG---TCTGGGATAGTGCAACAGCAAAGC---
AATTTG---CTGAGA---GCTATAGAGGCGCAACAACATATGTTGCAACTCACGGTCTGGGGCATC
>MK041570
TTCTATTGCAATACAACAAAGCTATTTAAT---AATTGCACAGGAAACAGTAGTTGCAACACAGAAAACAGT-
----

GATGGAGAAGAGATCACAATCCCATGCAGAATAAGGCAAATTGTAAGAATGTGGCAGGGGGTAGGACAAGCAA
TGTATGCCCCTCCTATAGCGGGATACGTAACATGTAACTCAAAT---
ATCACAGGATTATTACTGACAAGGGATGGAGGGGATACAGGGAAT----------------------------
----

GAGAGTGAGACATTCAGGCCTGGAGGAGGAGATATGAGGGACAATTGGAGAAGTGAATTATATAAATATAAAG
TGGTAGAAATTAAACCATTA---GGAATAGCACCCACTGAGGCAAAAAGGAGAGTGGTG--------------
-------------------------GAGAGAGAAAAAAGAGCA---GTGGGA---ATAGGAGCT---------
ATGTTCCTTGGGTTCTTGGGAATA---GCAGGAAGCACTATG---GGCGCAGCGTCA---
ATAACGCTGGCGGTACAG---GCCAGACAA---TTATTG---TCTGGAATAGTGCAACAGCAAAGT---
AATTTG---CTGAGG---GCTATAGAGGCGCAACAGCATATGTTGCAACTCACGGTCTGGGGCATT
>MK041576
TTCTATTGCAATACATCAAGGCTGTTTAATAGTACATATGCACCTAATGGTACAGAAAACAGTACTGAAACA-
----

AACAATTCAACCATCACAATCCCATGCAGAATAAAACAAATTGTAAGAATGTGGCAGGGGGTAGGACAAGCAA
TGTATGCCCCTCCCATTAAAGGAAACATAACATGTAGATCAAAT---
ATCACAGGACTACTACTAACACGTGATGGAGGTGATGGAGGAAAT----------------------------

----

AATACAAATAATACAGAAACATTCAGGCCTGAAGGAGGAGATATGAGGGACAATTGGAGAAGTGAATTATATA
AATATAAAGTGGTAGAAATCAAACCATTA---GGAATAGCACCCACTGAGGCAAAAAGGAGAGTGGTG-----
----------------------------------GGGAGAGAAAAAAGAGCA---GTGGGA---
ATAGGAGCT---------GTGTTCCTTGGGTTCTTGGGAGCA---GCAGGAAGCACTATG---
GGCGCAGCATCA---ATAACGCTGACGGTACAG---GCCAGACAA---TTATTG---
TCTGGGATAGTGCAACAGCAAAGC---AATTTG---CTGAGG---
GCTATAGAGGCGCAACAGCATATGTTGCAACTCACGGTCTGGGGCATT
>MK041550
TTCTATTGCAACACATCAACACTATTTAATAGTACACACAATGGTACATACAATGGTACAGAAAACATTACAA
TCCCATACAAT--

GGTACAGAAAACATTACAATCCCATGCAAAATAAAACAAATTATAAACATGTGGCAGAAAGTGGGAAGCGCAA
TGTATGCCCCTCCCATTAAAGGAGTAATAACATGTCTATCAAAT---
ATCACAGGATTACTACTGACACGTGATGGAGGGGACACAGAGGAA----------------------------
----

AATGAGACAGAGACATTCAGGCCTGGAGGAGGAGATATGAGGGACAATTGGAGAAGTGAGTTATATAAATATA
AAGTGGTAGAAATTAAACCATTG---GGAGTAGCACCCACTAGGGCAAAAAGGAGAGTGGTG-----------
----------------------------GAGAGAGAAAAAAGAGCA---GTGGGA---TTAGGAGCT------
---GTGTTCCTTGGGTTCTTGGGAGCA---GCAGGAAGCACTATG---GGCGCAGCATCA---
ATAACGCTGACGGTACAG---GCCAGACAA---TTATTG---TCTGGGATAGTGCAACAGCAGAGC---
AATTTG---CTGAGG---GCTATAGAGGCGCAACAGCACATGTTGCAACTCACGGTCTGGGGCATT
>MK041553
TTTATTGCAATACATCAGGCCTGTTTAATGGTACATATCCACCTAATAGTACAGGAAATGGTAATGGAACA-
----

GATGAGGAAAACATTACCCTCCCATGCAGAATAAAACAAATTATAAATATGTGGCAGGGGGTAGGACGAGCAA
TGTATGCCCCTCCCATTAAAGGACCGATAACATGTAGATCAAAC---
ATCACAGGATTACTGTTGACACGGGATGGAGGGGTAAACAGCAAC----------------------------
----

AACAGCACCGAGACATTCAGGCCTGAAGGAGGAGATATGAGGGACAATTGGAGAAGTGAATTATACAAATATA
AAGTGGTAGAAATCAAACCATTA---GGAATAGCACCCACTAAGGCAAAAAGAAGAGTGGTG-----------
----------------------------GAGAGAGAAAAAAGAGCA---GTGGGA---ATAGGAGCT------
---GTGTTCCTTGGGTTCTTGGGAGCA---GCCGGAAGCACTATG---GGCGCAGCATCA---
ATAACGCTGACGGTACAG---GCCAGACAA---TTATTG---TCTGGGATAGTGCAGCAGCAAAGC---
AATTTG---CTGAGG---GCTATAGAGGCGCAACAGCATATGTTGCAACTCACGGTCTGGGGCATT
>MK041561
TTCTATTGCAATACATCAAGATTGTTTAATGGTACATATAATACTACAGGAAACAGTAATGAAACA-------
----

AACAATGCAAACATCACAATCCCATGTAGAATAAAACAAATTGTAAACATGTGGCAGGAGGTAGGACGAGCAA
TGTATGCCCCTCCCATTGCAGGAGCAATAACATGTGAATCAAAT---
ATCACAGGATTGCTACTGACATGGGATGGCGGCAAGGGGAATGACACG-------------------------
----

AACAACACAGAGACATTCAGGCCTGGAGGAGGAGATATGAAGGACAATTGGAGAAGTGAATTATATAAATATA
AAGTGGTAGAGATTAAACCATTA---GGAATAGCACCCACTGGAGCAAAAAGGAGAGTGGTG-----------
----------------------------GAGAGAGAAAAAAGAGCA---GTGGGA---ATAGGAGCT------
---GTATTCCTTGGGTTCTTGGGAGCA---GCCGGAAGCACTATG---GGCGCAGCATCA---
ATAACGCTGACGGTACAG---GCCAGACAA---CTATTG---TCTGGTATAGTGCAACAGCAAAGC---
AATTTG---CTGAGA---GCTATAGAGGCGCAACAGCATATATTGCAACTCACGGTCTGGGGCATT
>MK041563
TTCTATTGCAATACATCAAGACTATTTAATGGTACATACAACTCTACTACTACAGGAAACAGTACT-------
----

GAAAATGAAATCATCACAATCCAATGCAGAATAAAACAAATTATAAACATGTGGCAGGGGGTAGGACGAGCAA
TGTATGCCCCTCCCATTGCAGGAAACATAACATGTAGATCAAAT---

ATCACAGGATTACTACTGACACGGGATGGAGGGAATAGCAGCAGC----------------------------
----

CCAGAGGAGATATTCAGGCTTGGAGGAGGAGATATGAAGGACAATTGGAGAAGTGAATTGTATAAATATAAAG
TGGTAGAAATTAAACCATTA---GGAATAGCACCCACTAAGGCAAAGAGAAGAGTGGTG--------------
-------------------------GAGAGAGAAAAAAGAGCA---GTGGGA---ATAGGAGCT---------
GTGTTCTTTGGGTTCTTGGGAGCC---GCAGGAAGCACTATG---GGCGCAGCATCA---
ATAACGCTGACGGTACAG---GCCAGACAA---TTATTG---TCTGGGATAGTGCAACAGCAAAGC---
AATTTG---CTGAGG---GCTATAGAGGCGCAACAGCATATGTTGCAACTCACAGTCTGGGGCATT
>MK041564
TTCTATTGCAATACAACACAACTGTTTAATAGAACATATTGGTTTAATAGTACAACTAATGACACAGGCACTA
ATGACACA---

GAAGATAAAAACATCACAATCCCATGCAGAATAAAGCAAATTATAAACCTGTGGCAGGAGGTAGGACGAGCAA
TGTATGCCCCTCCCATTGAGGGAGCCATAACATGTAACTCAAAT---
ATCACAGGATTACTACTGACACGTGATGGAGGCACAGGAAATGATACCACA----------------------
----

AATGCCACAGAGACATTCAGGCCTGGAGGAGGAGATATGAGGGACAATTGGAGAAGTGAATTATATAAATATA
AAGTGGTAGAAATTAAGCCATTA---GGAATAGCACCCACTAAGGCAAAGAGAAGAGTGGTG-----------
----------------------------GAGAGAGAAAAAAGAGCA---GCGGGA---CTAGGAGCT------
---GTGTTCCTTGGGTTCTTGGGAATA---GCAGGAAGCACTATG---GGCGCAGCGTCC---
ATAACGCTGACGGCACAG---GCCAGACAA---TTACTG---TCTGGGATAGTGCAACAGCAAAGC---
AATTTG---CTGAGG---GCTATAGAGGCGCAACAGCATATGTTGCAACTCACGGTCTGGGGCATC
>U52953
TTCTATTGCAATACATCAAGCCTGTTTAATAGTACATACACACCTAATAGTACAGAAAACATTACTGGAACA-
----

GAAAATTCAATCATCACAATCCCATGCAGAATAAAACAAATTATAAATATGTGGCAGGGGGTCGGACGAGCAA
TGTATGCCCCTCCCATTGAAGGAATATTAACATGTAGATCAAAT---
ATCACAGGATTACTACTGACACGGGATGGAGGTACAGGTATGCAT----------------------------
----

GACACAGAGATATTCAGGCCTGAAGGAGGAGATATGAGGGACAATTGGAGAAGTGAATTATATAAATATAAAG
TGGTAGAAATTAAACCATTA---GGAATAGCACCCACTAAGGCGAAAAGGAGAGTGGTG--------------
-------------------------GAGAGAGAAAAAAGAGCA---GTGGGA---ATAGGAGCT---------
GTGTTCCTTGGGTTCCTGGGAGCA---GCAGGAAGCACTATG---GGCGCAGCATCA---
ATAACGCTGACGGTACAG---GTCAGACAA---TTATTG---TCTGGGATAGTGCAACAGCAAAGC---
AATTTG---CTGAGA---GCTATAGAGGCGCAACAGCATATGTTGCAACTCACGGTCTGGGGCATT
>AF443088
TTCTATTGCAATACAACAAGACTGTTTAATGGTACATATAATAGTACAGGAAATGATACAAATTCA-------
----

ACAAATTCAACCATCACACTCCAATGCAGAATAAAACAAATTATAAACATGTGGCAGGGGGTAGGAAAAGCAA
TGTATGCCCCGCCCATTGCAGGAAACATAATATGTAAATCAAAC---
ATTACAGGACTACTATTGACACGTGATGGAGGTGAAGGTGAAAAT----------------------------
----

ACAACAGAGACATTCAGGCCTGCAGGAGGAGATATGAGGGACAATTGGAGAAGTGAATTATATAAATATAAAG
TGGTAGAAATTAAGCCATTA---GGAATAGCACCCACTAGCGCAAAAAGGAGAGTGGTG--------------
-------------------------GAGAGAGAAAAAAGAGCA---GTAGTGGGAATAGGAGCT---------
GTGTTCCTTGGATTCTTGGGAGCA---GCAGGAAGCACTATG---GGCGCAGCATCA---
ATAACGCTGATGGTACAG---GCCAGACAA---CTGTTG---TCTGGTATAGTGCAACAGCAAAAC---
AATTTG---CTGAGA---GCTATAGAGGCGCAACAGCATATGTTGCAACTCACGGTCTGGGGCATT
>AF443089
TTCTATTGCGATACATCAAACCTGTTTAATAAGACACGCAGGGATAAT-------------------------
----

GCTAATGAAACTATCACACTCCCATGCAGAATAAAACAAATTATAAACATGTGGCAGGAGGTAGGACGAGCAA
TGTATGCCCCTCCCATTGAAGGAAACATAACATGTAAATCAAAT---

ATCACAGGACTACTCTTGGTACGTGATGGAGGAAAAACAGAGGAT----------------------------
----

AACAAAAGTGAGATATTCAGACCTGGAGGAGGAGATATGAGGGACAATTGGAGAAGTGAATTATATAAATATA
AAGTGGTAGAAATAAAGCCATTG---GGAGTAGCACCCACTGAGGCAAAAAGGAGAGTGGTG-----------
----------------------------GAGAGAGAAAAAAGAGCA---GTGGGA---ATAGGAGCT------
---GTGCTCCTTGGGTTCTTAGGAGCA---GCAGGAAGCACTATG---GGCGCAGCGTCA---
ATAACGCTGACGGTACAG---GCCAGACAA---GTGCTG---TCTGGAATAGTGCAACAGCAAAGT---
AATTTG---CTGAGG---GCTATAGAGGCGCAACAGCATATGTTGCAACTCACGGTCTGGGGCATT
>AF443092
TTCTATTGCAATACATCAAAACTGTTTAATGGTACATATAATAATACAAATTCTAATAAT-------------
----

AATACAGCAGACATCACACTCCAATGCAGAATAAAACAAATTATAAACTTGTGGCAGGGGGTAGGACGAGCAA
TGTATGCCCCTCCCATTGCAGGAAACATAACATGTAAATCAAAT---
ATCACAGGACTACTATTGACACGTGATGGAGGAGGAGGAGAGAAC----------------------------
----

AACTCAACAGAGACATTCAGACCTGCAGGAGGAGAAATGAGGGACAATTGGAGGAGTGAATTATATAAATATA
AAGTGGTAGAAGTTAAGCCATTA---GGAATAGCACCCACTGAGGCAAAAAGAAGGGTGGTG-----------
----------------------------CAGAGAGAAAAAAGAGCA---GTAGGA---ATAGGAGCT------
---GTGTTCCTTGGGTTCTTGGGAGCA---GCAGGAAGCACTATG---GGCGCGGCGTCC---
ATAACGCTGACGGTACAG---GCCAGACAA---TTATTG---TCTGGTATAGTGCAACAGCAGAAC---
AACTTG---CTGAGA---GCTATAGAGGCGCAACAGCATATGTTGCAACTCACGGTCTGGGGCATT
>AF443093
TTCTATTGCAATACATCAAATCTGTCTAATGAGACATACCTGGCAAACCTGACACGGATGTCTAATGTAACA-
----

AAAAACGCCACAATCACACTCCCATGCAGAATAAAACAAATTATAAACATGTGGCAGGAGGTAGGACGGGCAA
TATATGCCCCTCCTATTGCAGGAAAAATAACATGTATATCAAAT---
ATCACAGGAACACTATTGACGCGTGATGGAGGAGTGTCAAATACAACA-------------------------
----

GAGGGCAATGAGACATTCAGACCTGGAGGAGGAGATATGAGGAACAATTGGAGAAGTGAATTATACAAATACA
AAGTGGTAGAAATCAGGCCATTG---GGAGTAGCACCCACTGAGGCAAGAAGGCGAGTGGTG-----------
----------------------------GAGAGAGAAAAAAGAGCA---GTAGGAGCAATAGGAGCT------
---GTGCTCCTTGGGTTCTTGGGAGCA---GCCGGAAGCACTATG---GGCGCAGCATCA---
ATGACGCTGACGGTACAG---GCCAGACAA---TTGCTG---TCTGGTATAGTGCAACAGCAAAGT---
AATTTG---CTGAGG---GCTATAGAGGCGCAACAGCATATGTTGCAACTCACGGTCTGGGGCATT
>AF443094
TTCTATTGCAATACATCAAACTTGTTTAATAATACATACAGGGCTGATAGTACATCCAATAATATAACAAAT-
----

GATAATTCAAATATCACACTCCAATGCAGGATAAAACAAATTATAAACATGTGGCAGGAAGTAGGACGAGCAA
TGTATGCCCCTCCCATTGAAGGAAACATAACATGTAACTCAAGT---
ATCACAGGACTACTATTGACACGTGATGGAGGAAAAGAAGAGAACAATAGC----------------------
----

ACAAACAATGGCACAGAGATATTCAGACCTGGAGGAGGAGATATGAGGGACAATTGGAGAAGTGAATTATATA
AATATAAAGTGGTAAAAATTAAGCCATTA---GGAATAGCACCCACCAAGGCACAAAGGAGAGTGGTG-----
----------------------------------AAAAGAGAAAAAAGAGCA---GTGGGA---
ATAGGAGCT---------GTGTTCCTTGGGTTCTTGGGAGCA---GCCGGAAGCACTATG---
GGCGCAGCATCA---ATAACGCTGACGGTACAG---GCCAGACAA---TTGCTG---
TCTGGTATAGTGCAACAGCAAAGC---AATTTG---CTGAGG---
GCTATAGAGGCGCAACAGCATATGTTGCAACTCACGGTCTGGGGCATT
>AF443095
TTCTATTGCAACACATCAGCACTGTTTAATAGTACATATAATAGTACATACAATACAAGCGGGCATAAT----
----

GATACAAGAATCATCACACTCCCATGCAGAATAAAACAAATTATAAACATGTGGCAGAAGGTAGGACGAGCAA

TGTATGCCCCTCCCATTGCAGGAAACATAACATGTAAATCCAAT---
ATCACAGGACTACTCTTGACACGTGATGGAGGAAATACAAGTAGC----------------------------
----

ACAGAAGAGATATTCAGACCTGAAGGAGGGGATATGAGGGATAACTGGAGGAGTGAATTATATAAATATAAAG
TGGTAGAAATTAAACCATTG---GGAGTAGCACCCACTAAGGCAAAAAGGAGAGTGGTG--------------
-------------------------GAGAGAGAAAAAAGAGCA---GTGGGA---ATAGGAGCT---------
ATGTTCCTTGGGTTCTTGGGAGCA---GCAGGAAGCACTATG---GGCGCGGCGTCA---
ATAACGCTGACGGTACAG---GCCAGACAA---TTGCTG---TCTGGTATAGTGCAGCAGCAAAGC---
AATTTG---CTGAGG---GCTATAGAGGCGCAACAGCATCTGTTGCAACTCACGGTCTGGGGCATT
>AF443096
TTCTATTGTAATACATCAAAACTGTTTAATGGTACATACAATGGTACATCCATCAGTACGGAC----------
----

AGTAACTCAAGCATCACACTCCAATGCAGAATAAAACAAATTATAAACATGTGGCAGGGGGTAGGACAAGCAA
TATATGCCCCTCCCATTGCAGGAAACATAACATGTAAATCAAAT---
ATCACAGGACTACTATTGACACGTGATGGAGGGAATAACACAGAG----------------------------
----

AACACAGAGACATTCAGACCTGGAGGAGGAGACATGAGGGATAATTGGAGAAATGAATTATATAAATATAAAG
TAGTAGAAATTAAGCCATTA---GGAATAGCACCCACTAGTGCAAAAAGGAGAGTGGTG--------------
-------------------------GAGAGAGAAAAAAGAGCA---GTAGGG---ATAGGAGCT---------
GTGTTCCTTGGGTTCTTGGGGGCA---GCAGGAAGCACTATG---GGCGCGGCGTCA---
ATAACGCTGACGGTACAG---GTCAGACAA---TTGTTG---TCTGGTATAGTGCAACAGCAAAAC---
AATTTG---CTGAGG---GCTATAGAGGCGCAACAGCATATGTTGCAACTCACAGTCTGGGGCATT
>AF443098
TTCTACTGCAATACATCACAACTGTTTAATGGTACATACATGCCTAATAGTACATACATGTCTAATAGTTCA-
----

GACAATAGAAACATCACAATCCCATGCAGAATAAAACAAATTATAAACCTATGGCAGGAGGTAGGACGAGCAA
TGTATGCCCCTCCCATTGCAGGAAACATAACATGTAAATCAAAT---
ATCACAGGGCTACTATTGACACGTGATGGAGGAGGAGAAAGTAACACA-------------------------
----

ACAAATGCAACAGAGACATTCAGACCTGGAGGAGGGGACATGAGGGACAATTGGAGAAGTGAATTATACAAAT
ATAAAGTGGTAGAAGTTAAACCATTG---GGATTAGCACCCACTGAGGCAAAAAGGAGAGTGGTG--------
-------------------------------GAGAGAGAAAAAAGAGCA---GTGGGA---ATAGGAGCT---
------GTGTTCCTTGGGTTCTTGGGAGCA---GCAGGAAGCACTATG---GGCGCGGCGTCA---
ATGACGCTGACGGTACAA---GCCAGACAA---CTATTG---TCTGGTATAGTGCAACAGCAAAGC---
AATTTG---CTGAAG---GCTATAGAGGCGCAACAGCATCTGTTGCAACTCACGGTCTGGGGCATT
>AF443101
TTCTATTGCAATACATCACAACTGTTCAATGGTACATACAATGATACATACAATGGTACAGAAAGTAATTCA-
----

GGTAATTCAACTATCACACTCCCATGCAGAATAAAACAAATTATAAACATGTGGCAGGGGGTAGGACGAGCAA
TATATGCCCCTCCCATTGAAGGAAACATAACATGTAAATCAAAT---
ATCACAGGACTACTATTGACGCGGGATGGAGGGAAAGGAATGAT-----------------------------
----

ACCGCAGAGATATTCAGACCTGAAGGAGGAGATATGAAGAACAATTGGAGAAGTGAATTATATAAATATAAAG
TAGTAGAAATTAGGCCATTG---GGAGTAGCACCCACTAAAGCAAAAAGGAGAGTGGTG--------------
-------------------------GAGAGAGAAAAAAGAGCA---GCA------CTAGGAGCT---------
GTGCTCCTTGGGTTCTTGGGAGCA---GCAGGAAGCACTATG---GGCGCAGCATCA---
ATAACGCTGACGGTACAG---GCCAGACAA---TTGCTG---TCTGGAATAGTGCAACAGCAAAGC---
AATTTG---CTGAAG---GCTATAGAGGCGCAACAGCATCTGTTGCAACTCACGGTCTGGGGCATT
>AF443104
TTCTATTGCAATACATCAGGCCTGTTTAATGGAACGTTTAATGGAACACACCTAAACATATCT----------
----

ACTAATACTAACATCACACTCCCATGCAGAATAAAACAAATTATAAACATGTGGCAAGAGGTAGGACGAGCAA

TGTATGCCCCTCCCATTGCAGGAAACATAACATGTAAATCAAAT---
ATCACAGGAATACTATTGACACGAGATGGAGGACCAGAAGATACAAAG-------------------------
----

AATAAGACAGAGACATTCAGACCTGGAGGAGGAGATATGAGGGACAATTGGAGAAGTGAATTATATAAATATA
AAGTGGTAGAAATTAAGCCATTG---GGAGTAGCACCCACTAAAGCAAAAAGGAGAGTGGTG-----------
----------------------------GAGAGAGAAAAAAGAGCA---GTGGGA---ATAGGAGCT------
---GTGTTCCTTGGGTTCTTGGGAGCA---GCAGGAAGCACTATG---GGCGCGGCGTCA---
ATAACGCTGACGGTACAG---ACCAAACAA---TTGTTG---TCTGGTATAGTGCAACAGCAAAGC---
AATTTG---CTGAAG---GCTATAGAGGCGCAACAGCATCTGTTGCAACTCACAGTCTGGGGCATT
>AF443110
TTCTATTGTAATACATCAATTCTGTTTAATGATACATATTGGTTTAATGGGACAGACATGGCTAATGATACA-
----

GGAAGTAATAACATCACAATCCCATGCAGGATAAAACAAATTATAAACATGTGGCAGGAGGTAGGACGAGCAA
TATATGCCCCTCCCATTAGAGGAATCATAACATGTACATCAAAT---
ATCACAGGACTGTTATTGACACGTGATGGAGGAAACACAGGAGGA----------------------------
----

AACACAACAGAGATATTCAGACCTGAAGGAGGAGATATGAGGAACAATTGGAGAAATGAATTATATAAATATA
AAGTGGTAGAAATTAAGCCATTG---GGAATAGCACCCACTGGGGCAAAAAGGAGAGTGGTG-----------
----------------------------GAGAGAGAAAAAAGAGCA---GTGGGA---ATAGGAGCT------
---GTGTTCCTTGGGTTCTTGGGAGCA---GCAGGAAGCACTATG---GGCGCAGCGTCA---
ATAACGCTGACGGCACAG---GCCAGACAA---TTGCTG---TCTGGAATAGTGCAACAGCAAAGC---
AATTTG---CTGAGG---GCTATAGAGGCGCAACAACATATGTTGCAACTCACGGTCTGGGGCATT
>AF443111
TTCTATTGTAATACATCAGGCCTGTTTAATAATAACCTGATTAATAATGGG----------------------
----

GCAGAAGATACTATCAGACTCCCATGCCGAATAAAACAAATTATAAACATGTGGCAGGAGGTAGGACGAGCAA
TGTATGCCCCTCCCATTGCGGGAAACATAACATGCACATCAAAT---
ATCACAGGACTGTTGCTGACACGGGATGGGGGAAACGGAGGAAAT----------------------------
----

AATACAGAGACATTCAGACCTGGAGGAGGAAATATGAAGGACAATTGGAGAAGTGAATTATATAAATATAAAG
TAGTAGAAATTAAGCCATTG---GGAGTAGCACCCACTGAGGCAAAAAGGAGAGTGGTG--------------
-------------------------GAGAGAGAAAAAAGAGCA---GTGGAA---ATAGGAGCT---------
GTGTTCCTTGGGTTCTTGGGAGCA---GCAGGAAGCACTATG---GGCGCGGCGTCA---
ATAACGCTGACGGTACAG---GCCAGACAA---TTGCTG---TCTGGTATAGTGCAACAGCAAAGC---
AATTTG---CTGAGG---GCTATAGAGGCGCAACAACATCTGTTGCAACTCACGGTCTGGGGCATT
>AF443112
TTCTATTGTAATACATCAAAACTGTTTAATAGTACAAATAATAATACAGAAAGTGAG----------------
----

TCAAATGCAACCATCACACTCCCATGCAGAATAAAACAATTTATAAGAATGTGGCAGAGGGTAGGACAAGCAA
TGTATGCCCCTCCCATTGCAGGAAACATAACATGTAGATCAAAT---
ATCACAGGGCTACTATTGACAAGGGATGGA---AGCAAAAATGAT----------------------------
----

ACAGAGACATTCAGACCTGGAGGAGGAAATATGAGAGATAATTGGAGAAATGAATTATATAAATATAAAGTGG
TAGAAATTAAGCCATTG---GGAATAGCACCCACTGAGGCAAAAAGGAGAGCGGTG-----------------
----------------------GAGAGAGAAAAAAGAGCA---GTGGGA---ATAGGAGCT---------
GTGATCCTTGGGTTCTTGGGAGCT---GCAGGAAGCACTATG---GGCGCAGCGTCA---
ATAACGCTGACGGCACAG---GCCAGACAA---CTGCTG---TCTGGAATAGTGCAACAGCAAAGC---
AATTTG---CTGAGA---GCTATAGAGGCGCAACAACACCTGTTGCAACTCACAGTCTGGGGCATT
>AF443113
TTTTATTGCAATATATCAAGATTGTTTAATAGGCCAAACATGACTAAAGAGACAAACATGACTAGTGATATAA
AA---

AATAATTCAACAATCACACTCCCATGCAGAATAAAACAAATTATAAACATGTGGCAGGGGGTAGGACGAGCAA

TGTATGCCCCTCCCATTGCAGGAAGAATAATATGTAAATCAAAT---
ATCACAGGACTATTATTGGTACGTGATGGAGGACAGGGAGATAATGTC-------------------------
----

ATGAATGCCACCGAGACATTCAGACCTGGAGGAGGAGATATGAGAGATAATTGGAGAAGTGAATTATACAAAT
ATAAAGTGGTAGAAATTAAGCCATTG---GGAGTAGCACCCACTAGTGCAAAAAGGAGAGTGGTG--------
-------------------------------GAGAGAGAAAAAAGAGCA---GCA------ATAGGAGCT---
------ATGTTCCTTGGGTTCTTGGGAGCA---GCAGGAAGCACTATG---GGCGCGGCGTCA---
ATAACGCTGACGGTACAG---GCCAGAAAA---TTGTTG---TCTGGTATAGTGCAACAGCAAAGC---
AATTTG---CTGAGG---GCTATAGAGGCGCAACAGCATATGTTGCAACTCACGGTCTGGGGCATT
>AF443115
TTCTATTGCAATACATCACAACTGTTTAATAGTACATACAGGGCCAATAGTACAAATAGTACT----------
----

TCAAATTCGAACATCACACTCCCATGCAGAATAAAACAAATTATAAACATGTGGCAGGGGGTAGGACGAGCAA
TGTATGCCCCCCCCATTGCAGGAAACATAATATGTAAATCAAAT---
ATCACAGGAGTACTATTGACATATGATGGAGGAGAAGGGGTAAAG----------------------------
----

GAAAATGAGACATTCAGACCTGCAGGAGGAAATATGAAGGACAATTGGAGAAGTGAATTATATAAATATAAAG
TGGTAGAAATTAAACCATTA---GGAATAGCACCCACTAAGGCAAAAAGGAGAGTGGTG--------------
-------------------------GAGAGAGAAAAAAGAGCA---GCA------CTAGGAGCT---------
GTGTTCCTTGGGTTCTTGGGAGCA---GCAGGAAGCACTATG---GGCGCAGCATCA---
ATAACGCTGACGGTACAG---GCCAGACAA---TTGCTG---TCTGGTATAGTGCAACAGCAAAGC---
AATTTG---CTGAGA---GCTATCGAGGCGCAACAGCATATGTTACAACTCACAGTCTGGGGCATT
>KY658704
TTTTATTGCAATACAACACAACTGTTTAATGGTACATACAATGGCACATACATTAATGATACAGAA-------
----

AGTAATTCAACCTTCACACTCCCATGCAGAATAAAACAAATTATAAACATGTGGCAGGAAGTAGGACGAGCAA
TGTATGCACCTCCAATTGCAGGAAACATAACATGTAAATCAAGT---
ATCACAGGACTACTATTGACACGGGATGGAGGTGACAAAAACGAC----------------------------
----

AACAACAGTGAAATATTCAGACCTGGAGGAGGAGATATGAGGAACAATTGGAGAAGTGAATTATATAAATATA
AAGTGGTAGAAATTAAGCCATTG---GGACTAGCACCCACTGGGGCAAAAAGGAGAGTGGTG-----------
----------------------------GAGAGAGAAAAAAGAGCA---GTGGGA---ATAGGAGCT------
---GTGTTCCTTGGGTTCTTGGGAGCA---GCTGGAAGCACTATG---GGCGCAGCGTCA---
ATAACGCTGACGGCACAG---GCCAGACAA---GTATTG---TCTGGTATAGTGCAACAGCAAAGC---
AATTTG---CTGAGG---GCTATAGAGGCGCAACAGCATATGTTGCAACTCACAGTCTGGGGCATT
>KY658705
TTCTATTGCAATACATCAGGCCTGTTTAATGGTACATATAATGGTACATACCTTGATGAGACAAATTCA----
----

ACAAATTCAACCATCACACTCCAATGCAGAATAAAACAAATTATAAACATGTGGCAGGGGGTAGGACGAGCAA
TGTATGCCCCCCCTATTGCAGGAAACATAACATGTAAATCAAAT---
ATCACAGGAATACTGTTGACACGTGATGGAGGAAAAGGAGAAAAT----------------------------
----

AATAGTACAGAGATATTCAGACCTGGAGGAGGAGATATGAGGGACAATTGGAGAAGTGAGTTATATAAATATA
AAGTGGTTGAAATTAAACCATTA---GGAATAGCACCCACTAAGGCAAGAAGGAGAGTGGTG-----------
----------------------------GAGAGAGAGAAGAGAGCA---GTGGGA---ATAGGAGCT------
---GTGTTCCTTGGGTTCTTGGGAGCA---GCAGGAAGCACTATG---GGCGCGGCATCG---
ATAACGCTGACGGTACAG---GCCAGACAA---TTATTG---TCTGGTATAGTGCAACAGCAAAGC---
AATTTG---CTGAGA---GCTATAGAGACGCAACAACGTCTGTTGCAACTCACAGTCTGGGGCATT
>KY658706
TTCTATTGCAATACATCAGGCCTGTTTAATGGTACATATAATGGTACACATAATAATGCGACAGACTCA----
----

AAAAATCCAACCATCACCCTCCAATGCAGAATAAAACAAATTATAAACATGTGGCAGAAGGTAGGACAAGCAA

TGTATGCCCCCCCCATTGCAGGAAACATAACATGTACATCAAAT---
ATCACAGGACTACTATTGACACGTGATGGAGGTAATGAAAATGAAAATGAAACC------------------
----

AACAAAAATGAGACATTCAGACCTGCAGGAGGAGATATGAGGGACAATTGGAGAAGTGAATTATATAAATATA
AAGTGGTGGAGATTAAGCCATTA---GGAATAGCACCCACTGAGGCAAAAAGGAGAGTGGTG----------
---------------------------GAGAGAGAGAAAAGAGCA---GTAGGA---ATAGGAGCT------
---GTGTTCCTTGGGTTCTTGGGAGCA---GCAGGAAGCACTATG---GGCGCGGCGTCA---
ATAACGCTGACGGTACAG---GCCAGACAA---TTATTG---TCTGGTATAGTGCAACAGCAAAGC---
AATTTG---CTGAGA---GCTATAGAGGCGCAACAACATATGTTGCAACTCACAGTCTGGGGCATT
>KC154013
TTCTATTGCAATACATCAAAACTGTTTAATGAAACATACCTTAATGATACA---------------------
----

AAGAGTGAAACAATCATACTCCCATGCAGAATAAAACAGATTATAAACATGTGGCAGGAGGTAGGACGAGCAA
TGTATGCCCCTCCCATTGCAGGAAACATAACATGTAACTCAAGT---
ATCACAGGACTACTGTTGACACGGGATGGAGGAGAGACAAATGGCACA------------------------
----

AATGGCACAGAAACGTTCAGACCTGGAGGAGGAGATATGAGGGACAATTGGAGAAGTGAATTATATAAATATA
AAGTGGTAGAAATTAATCCATTG---GGAATAGCACCCACTAAGGCAAAAAGGAGAGTGGTG----------
---------------------------GAGAGAGAAAAAAGAGCA---GTGGGA---ATGGGAGCT------
---GTGTTCCTTGGGTTCTTGGGAGCA---GCAGGAAGCACTATG---GGCGCAGCGTCA---
CTAACGCTGACGGCACAG---GCCAGACAA---TTGTTG---TCTGGAATAGTGCAACAGCAAAGC---
AATTTG---CTGAGG---GCTATAGAGGCGCAACAGCATATGTTGCAACTCACGGTCTGGGGCATA
>KC154015
TTTTATTGTAATACATCAGGACTGTTTAATAGTACATACTGGGGTAGGACTAATAGTACTGATATTCTT----
----

TCAAATTCAACTATCACACTCCAATGCAGAATAAAACAAATTATAAACATGTGGCAGGAGGTAGGACGAGCAA
TGTATGCCCCTCCCATTGCAGGAAACATAACATGTAATTCAAGT---
ATCACAGGATTACTATTGACGCGTGATGGAGGAACATGGGAAAAC---------------------------
----

AAGACAGAAGAGATATTCAGACCTGGAGGAGGAGATATGAGGGACAATTGGAGAAGTGAATTATACAAATATA
AAGTGGTAGAAATTAAGCCATTA---GGAGTAGCACCCACTAAGGCAAAAAGGAGAGTGGTGGAG-------
------------------------AAACAAATAGAAAAAAGAGCA---GTGGGA---CTAGGAGCT------
---GTGTTCTTTGGGTTCTTGGGAGCA---GCAGGAAGCACTATG---GGCGCAGCGTCA---
ATAACGCTGACGGTACAG---GCCAGAAAC---TTATTG---TCTGGCATAGTGCAACAGCAAAGC---
AATTTG---CTGAGA---GCTATAGAGGCGCAACAGCATATGTTGCAACTCACAGTCTGGGGCATT
>KF114882
TTCTATTGCAATACATCAAAGCTGTTTAATGAAATAGACACAAATAAAACAGGAAAC---------------
----

GAAAGTGAAAACATCACACTCCCATGCAGAATAAAACAAATTATAAACATGTGGCAGGAGGTAGGACGAGCAA
TGTATGCCCCTCCCATTGCAGGAAGCATAACATGTAAATCAAAT---
ATCACAGGACTACTATTGCTACGTGATGGAGGCACAAATACAAGC---------------------------
----

GACAACAACAGTGAGATATTCAGACCTGGAGGAGGAAATATGAAGGATAATTGGAGAAGTGAATTATATAAAT
ATAAAGTGGTAGAAATTAAGCCATTG---GGAATAGCACCAACTGAGGCAAAAAGGAGAGTGGTG-------
------------------------------GAAAGAGAAAAAAGAGCA---GTGGGA---ATAGGAGCT---
------GTACTCCTTGGGTTCTTGGGAGCA---GCAGGAAGCACTATG---GGCGCAGCGTCA---
ATAACGCTGACGGTACAG---GCCAGACAG---CTGTTG---TCTGGTATAGTGCAGCAGCAAAGC---
AATTTG---CTGAGA---GCTATAGAGGCGCAACAGCATATGTTGCAACTCACAGTCTGGGGCATT
>KR861313
TTCTATTGCGATACATCAAAACTGTTTAATGACTCGTCTAATAGTACA------------------------
----

AGCAAGGAGCCAATCACACTCCCATGCAGAATAAAACAAATTATAAACATGTGGCAGGAGGTAGGACGAGCAA

TATATGCCCCTCCCATTGCAGGAAACATAACATGCAGATCTAAT---
ATCACAGGACTACTATTGACACGTGATGGAGGGAGGGTCAATGACACA-------------------------
----

ACAGAGGAGATATTCAGACCTGGAGGAGGAGATATGAGGGACAATTGGAGAAGTGAATTATATAAATATAAAG
TAGTAGAAATTACACCATTG---GGAGTAGCACCCACTGGGGCAAAAAGGAGAGTGGTG--------------
-------------------------GAGAGAGAAAAAAGAGCA---GTTGGA---ATAGGAGCT---------
GTACTCCTTGGGTTCTTGGGAGCA---GCAGGAAGCACTATG---GGCGCAGCGTCG---
ATGACGCTGACGGTACAG---GCCAGACAA---TTGTTG---TCTGGAATAGTGCAACAGCAAAGC---
AATTTG---CTGAGA---GCTATAGAGGCGCAACAGCATATGTTGCAACTCACGGTCTGGGGCATT
>KR861315
TTCTATTGCAATACAACAAAATTGTTTAACAGTACATACAATTCTAATGGTACATACAGTAAT----------
----

AGTACTTCAAATATCACACTCCAATGCAGAATAAAACAAATTATAAACATGCGGCAGGAGGTAGGACGAGCAA
TGTATGCCCCTCCCATTGCAGGAAACATAACATGCGAATCAAAT---
ATCACAGGACTACTATTGACACGTGATGGAGGAAATGGGTCCAGT----------------------------
----

GATGCCCCAGAGATATTCAGACCAGGAGGAGGAAATATGAAGGACAATTGGAGAAGTGAACTATATAAATATA
AAGTGGTAGAAATTAAACCATTA---GGAGTAGCACCCACTACGGCAAAAAGAAGAGTGGTG-----------
----------------------------GAGAGAGAAAAAAGAGCA---GTGGGA---ATAGGAGCT------
---GTGTTCCTTGGGTTCTTGGGAGCA---GCAGGAAGCACTATG---GGCGCAGCATCA---
ATAACGCTGACGGTACAG---GCCAGACAA---TTGTTG---TCTGGGATAGTGCAACAGCAAAGC---
AATTTG---CTGAAG---GCTATAGAGGCGCAACAGCATCTGCTGCAACTCACGGTCTGGGGCATT
>KR861316
TTCTATTGCAATACATCACAACTGTTTAATAAGACAATGAACAATATTAATGGTACAGAAAACAGTACAGACA
CA---

GAAAACAGGACCATCACAATCCCATGCAGAATAAAACAAATTATAAACATGTGGCAGAGGGTGGGACGAGCAA
TGTATGCTCCTCCCATTGCAGGAAACATAACATGTGAATCAAAT---
ATCACAGGAATACTATTGACACGTGATGGAGGAGAATCAAATAAC----------------------------
----

ACCAATGAGACATTCAGACCTGGAGGAGGAGATATGAGGGATAACTGGAGAAGTGAATTATATAAATATAAAG
TGGTAGAAATTAAGCCATTG---GGAGTAGCACCCACTGAGGCAAGAAGGAGAGTGGTG--------------
-------------------------GAGAGAGAAAAAAGAGCA---GTGGGA---ATGGGAGCT---------
GTGTTACTTGGGTTCTTGGCAACA---GCAGGAAGCACTATG---GGCGCGGCGTCA---
GTGACGCTGACGGTACAG---GCCAGACAA---TTGTTG---TCTGGTATAGTGCAACAGCAAAGC---
AATTTG---CTGAAG---GCTATAGAGGCGCAACAGCATCTGTTGCAACTCACAGTCTGGGGCATT
>KR861325
TTCTATTGCAATACATCAAGTCTGTTTAATGAGACATACCTATATAATGGTACAGCAGAC-------------
----

AATACAGTAAACATTACACTCCCATGCAAAATAAAACAAATTATAAACATGTGGCAGGGGGTAGGACGAGCGA
TGTATGCCCCCCCCATTGCAGGAAACATAACATGTAAATCAAAT---
ATCACAGGATTACTATTGGTACGTGATGGGGGGAAAGGAAATAAT----------------------------
----

GACACTGAGACATTCAGACCTGGAGGAGGAAATATGAAGGACAATTGGAGAAGTGAATTATATAAATATAAGG
TAGTAGAAATTAAACCATTA---GGAGTAGCACCCACCAAGGCAAAAAGGAGAGTGGTG--------------
-------------------------GAGAGAGAAAAACGAGCA---GCGGGA---CTAGGAGCT---------
GTGTTCCTTGGGTTCTTGGGAGCG---GCAGGAAGCACTATG---GGCGCAGCATCA---
ATAGTGCTGACGGTACAG---GCCAGACAA---TTGTTG---TCTGGTATAGTGCAACAGCAAAAC---
AATTTG---CTGAGA---GCTATAGAGGCGCAACAACATCTGTTGCAACTCACAGTCTGGGGCATT
>KR861326
TTCTATTGCGATACATCACAACTGTTTAATGAGTCCCTGTTTAATAGTACA----------------------
----

AACAATGATACAATCATAGTTCCATGCAAAATAAAACAAATTATAAACATGTGGCAGGGGGTAGGACGAGCAA

TGTATGCCCCTCCCATTGCAGGGAACATTACATGTAACTCTAGT---
ATCACAGGACTACTATTGACATGGGATGGAGGGATAAATAATAGG----------------------------
----

ACTACAGAGATATTCAGACCTGGAGGAGGAAATATGAAGGATAATTGGAGAAATGAATTATATAAATATAAAG
TGGTAGAAATTAGGCCATTG---GGAATAGCACCCACTGGAGCAAAAAGGAGAGTGGTG--------------
-------------------------GAGAGAGAAAAAAGAGCAGTAATGGGA---TTAGGAGCT---------
GTGTTCCTTGGGATCTTGGGAGCA---GCAGGAAGCACTATG---GGCGCAGCGTCA---
ATAACGCTGACGGTACAG---GCCAGGCAA---TTGTTG---TCTGGAATAGTGCAGCAGCAAAAC---
AATTTG---CTGAGA---GCTATAGAGGCGCAACAGCATATGTTGCAACTCACAGTCTGGGGCATT
>KR861337
TTCTATTGTAATACATCAGACCTGTTTAATGGTACATACAATGGTACATACAATGATATAGATCTG-------
----

AATAATGATACACTCACACTCACATGCAGAATAAAACAAATTATAAACATGTGGCAGGAGGTAGGACGAGCAA
TGTATGCCCCTCCCATTGAAGGAAACATAACATGCACATCAAAT---
ATCACAGGACTACTGCTACTGCGGGATGGAGGAATTGGAAATGAG----------------------------
----

ACAGAAGAGATATTCAGACCTGGAGGAGGAGATATGAGAGACAATTGGAGAAGTGAATTATATAAATATAAAG
TAGTAGAAATTAAGCCATTG---GGAGTAGCACCCACTAAGGCAAAAAGGAGAGTGGTG--------------
-------------------------GAGAGAGAAAAAAGAGCA---GTGGGA---ATTGGAGCT---------
GTACTCCTTGGGTTCTTGGGAGCA---GCAGGAAGCACTATG---GGCGCGGCGTCA---
ATAACGCTGACGGTACAA---GCCAGACAA---TTGTTG---TCTGGTATAGTGCAACAGCAAAGC---
AATTTG---CTGAGG---GCTATAGAGGCGCAACAGCATCTGTTGCAACTCACGGTCTGGGGCATT
>KR861345
TTCTATTGCAATACAACAAAACTGTTTGACACAGCAACACTGGTTAATGGTACATACATGAACAGTACAGAA-
----

AGTAATTCAACCATCACAATCAAATGCAGAATAAAACAATTTATAAACATGTGGCAGGGGGTAGGACGAGCAA
TGTATGCCCCTCCCATTGCAGGAAATATAACATGTATATCAAAT---
ATCACAGGATTAATATTGACACGTGATGGAGGACCAAATAGTAGTGATAAT----------------------
----
GTGACTACAACTGGAAATAATACTAAT---------
ATTACTGAGATATTCAGACCTGCAGGAGGAGACATGAGGGACAATTGGAGAAGTGAACTATATAAATATAAAG
TAGTAGAAATTAAGCCATTA---GGACTAGCACCCACCAAGGCAAAAAGGAGAGTGGTG--------------
-------------------------GAGAGAGAAAAAAGAGCG---GTGGGA---ATAGGAGCT---------
GTGTTCCTTGGGTTCTTGGGAGCA---GCAGGAAGCACTATG---GGCGCGGCGTCA---
ATAACGCTGACGGTACAG---ACCAGACAA---CTGTTG---TCTGGTATAGTGCAACAGCAAAGT---
AATTTG---CTGAGA---GCTATAGAGGCGCAACAGCATATGTTGCAACTCACGGTCTGGGGCATT
>KR861312
TTCTATTGCAATACATCAAAACTGTTTAATGGTACATACCCGCAGCTTAATAGGACAGAAGAT----------
----

GTAAATCGAACCATCACACTTAAATGCAGAATAAAACAAATTATAAACATGTGGCAGGGGGTAGGACGAGCAA
TGTATGCCCCTCCCATTGCAGGAAATATAACATGTATATCAAAT---
ATCACAGGATTAATATTGACACGTGATGGAAAATATGAAAGTAGT----------------------------
----
AAGAATGAAAGTAGT---
AAGAATGAGACATTCAGACCTGCAGGAGGAGATATGAGGGACAATTGGAGGAGTGAATTATATAAATATAAAG
TAGTAGAAATTAAGCCATTA---GGACTAGCACCCACCAAGGCAAAAAGGAGAGTGGTG--------------
-------------------------GAGAGAGAAAAAAGAGCA---GTGGGA---ATAGGAGCT---------
GTGTTCCTTGGGTTCTTGGGAGCA---GCAGGAAGCACTATG---GGCGCAGCGTCA---
ATAACGCTGACGGTACAG---GCCAGACAA---CTGTTG---TCTGGTATAGTGCAACAGCAAAGC---
AATTTG---CTGAGG---GCTATAGAGGCGCAACAGCATATGTTGCAACTCACAGTCTGGGGCATT
>KR861322
TTTTATTGTAATACATCAAAATTGTTTAATGAGAGCCATGTGTATAATAAT----------------------
----

ACAAATGGAACCATCACACTCCCATGCAGAATAAAACAAATTATAAACATGTGGCAAGGGGTAGGAAGAGCAA

TGTATGCCCCTCCCATTGCAGGAAACATAACATGTATATCAAAT---
ATCACAGGACTGCTATTGACACGTGATGGAGGAGAGAATAATGGG----------------------------
----

TCAGAAGAAGTATTCAGACCTGGAGGAGGAGATATGAGAGACAATTGGAGAAGTGAATTATATAAATATAAAG
TGGTAGAAGTTAAACCATTA---GGAATAGCACCCACTGAGGCAAGAAGGAGAGTGGTG--------------
-------------------------GAGAGAGAAAAAAGAGCA---GTGGGA---ATAGGAGCT---------
GTGTTCCTTGGGTTCTTGGGAGCA---GCAGGAAGCACTATG---GGCGCGGCGTCA---
ATAACGCTGACGGTACAG---GCCAGACAG---TTATTG---TCTGGTATAGTGCAACAGCAAAGC---
AATTTG---CTGAGG---GCTATAGAGGCGCAACAGCATATGTTGCAACTCACGGTCTGGGGCATT
>KR861324
TTCTATTGTAACACATCAGGCCTGTTTAATAGCACCATCCGGCCTAATGGCACTATCCAGTTTAATAGCACTA
ATGCA--

ACAGCCCCAAACATCACACTTCCATGCAGAATAAAACAATTTATAAACATGTGGCAAGGGGTAGGACGAGCAA
TGTATGCCCCCCCCATTGCAGGAAATATAATGTGTAGATCAAAT---
ATCACAGGACTACTATTGACACGTGATGGAGGTGGCAATGAGACT----------------------------
----

GATACTGAGATATTCAGACCTGAGGGAGGAGATATGAGGAACAATTGGAGAAGTGAATTATATAAATATAAAG
TGGTGGAGATCCAGCCATTA---GGAGTAGCACCCACTGAGGCAAAAAGGAGAGTGGTG--------------
-------------------------GAGAGAGAGAAAAGAGCA---GTGGGA---ATAGGAGCT---------
GTGTTCCTTGGGTTCTTAGGAGCA---GCAGGAAGCACTATG---GGCGCAGCATCA---
ATAACGCTGACGGTACAA---GCCAGACAA---TTATTG---TCTGGTATAGTGCAACAGCAAAGC---
AATTTG---CTGAGG---GCTATAGAGGCGCAACAACATATGTTGCAACTCACAGTCTGGGGCATT
>KR861330
TTCTATTGCAATACATCAAATCTGTTTAATAAGACATACTGTCCTAATGATACAAAGAGTAATGAGACAAAG-
----

AGTAATGAAACCATCACACTGCAATGCAGAATAAAACAAATTATAAACAGGTGGCAGGAGGTAGGACGAGCAA
TGTATGCCCCTCCTATTGAAGGAAACATAACATGTAAATCAAAT---
ATCACAGGACTACTGTTGGTACGTGATGGAGTTAACACAACTGAG----------------------------
----

GAGATATTCAGACCTGGAGGAGGAAATATGAAAGACAATTGGAGAAGTGAATTATATAAATACAAAGTAGTAG
AAATTAAGCCATTG---GGAATAGCACCCACTGCAGCAAAAAGGAGAGTGGTG--------------------
-------------------GAGAGAGAAAAAAGAGCA---GCA------CTGGGAGCT---------
ATGTTCCTTGGGTTCTTGGGATTA---GCAGGAAGCACTATG---GGCGCAGCGTCA---
GTGACGCTGACGGTACAG---GCCAGACAA---CTGTTG---TCTGGTATAGTGCAACAGCAAAAC---
AATTTG---CTGAGA---GCTATAGAGGCGCAACAGCATATGTTGCAACTCACGGTCTGGGGCATT
>KR861335
TTCTATTGCAATACAACAAAACTGTTCAATACAACAATACCGCCTAATGAGACATACAGGAGTACAAAC----
----

AATTCAAGTACCATCACACTCAAATGCAGAATAAAACAAATTATAAATATGTGGCAGGGGGTAGGACGAGCAA
TGTATGCCCCTCCCATTGCAGGAAACATAACATGTATATCAAAT---
ATCACAGGATTAATATTGACACGTGATGGAGGAAACAATAATAAT----------------------------
----

GAGACTGAGACATTCAGACCTGCAGGAGGAAATATGAAGGACAATTGGAGAAGTGAATTATATAAATATAAAG
TAGTAGAAATTAAGCCATTA---GGACTAGCACCCAACAAGGCAAAAAGGAGAGTGGTG--------------
-------------------------GAGAGAGAAAAAAGAGCA---GTGGGA---ATAGGAGCT---------
GTGTTCCTTGGGTTCTTGGGAGCA---GCAGGAAGCACTATG---GGCGCGGCGTCA---
ATAACGCTGACGGTACAG---GCCAGACAA---CTGTTG---TCTGGTATAGTGCAACAGCAAAGT---
AATTTG---CTGAGA---GCTATAGAGGCGCAACAGCATATGTTGCAACTCACGGTCTGGGGCATT
>KR861336
TTTTATTGCGATACATCAAGATTGTTTAATGATACACACTGGAATGACACAGAAGATAATATA----------
----

GCTAATAGTAACACCATCATACCATGCAAAATAAGACAAATTATAAACATGTGGCAGGAAGTAGGACGAGCAA

TATATGCCCCTCCCATTGAAGGAAACATAACATGTAAATCACGT---
ATCACAGGATTACTATTGGAACGTGATGGAGGAAAAAAAAATGAC----------------------------
----

CCAAAAAAAATAGAAATATTCAGACCTGGAGGAGGAGATATGAGGAACAATTGGAGAAATGAATTATATAAAT
ATAAAGTGGTAGAAATTAAGCCATTG---GGAGTAGCACCCACTGAGGCAAAAAGGAGAGTGGTG--------
-------------------------------GAGAGAGAAAAAAGAGCA---GTGGGC---ATAGGAGCT---
------GTGCTCCTTGGGTTCTTGGGAGCA---GCAGGAAGCACTATG---GGCGCAGCATCA---
ATAACGCTGACGGTACAG---GCCAGACAA---TTGTTG---TCTGGAATAGTGCAACAGCAAAAC---
AATTTG---CTGAGG---GCTATAGAGGCGCAACAGCATATGTTGCAACTCACAGTCTGGGGCATT
>KR861343
TTCTATTGCAATACATCAAACCTATTTCCAGGAAATGAGACGCAAGGCACAGGG-------------------
----

ACAAACAGCACAATAACAATACAATGCAGAATAAAACAAATTATAAACATGTGGCAGGAGGTAGGACGAGCAA
TATATGCCCCTCCCATTGAAGGAAACATAACATGTATATCAAAG---
ATCACAGGACTAATACTGACACGTGATGGAGGAAACAGTGGAAACAAT-------------------------
----

ACAAAGCAGCCAGAGATATTCAGACCTGGAGGAGGAGATATGAGAGACAATTGGAGAAGTGAACTATATAAAT
ATAAAGTGGTAGAAATTAAGCCATTG---GGAGTAGCACCCACTGGGGCAAAAAGGAGAGTGGTG--------
-------------------------------GAGAGAGAAAAAAGAGCA---GTGGGA---ATAGGAGCT---
------GTGCTCCTTGGGTTCTTGGGAGCA---GCAGGAAGCACTATG---GGCGCAGCATCA---
ATAACGCTGACGGTACAG---GCCAGACAA---TTGTTG---TCTGGAATAGTGCAACAGCAAAGC---
AATTTG---CTGAGG---GCTATAGAGGCGCAACAACATATGTTGCAACTCACGGTCTGGGGCATT
>KR861311
TTCTATTGTAATACATCAAAACTGTTTAATAGGACATATGAGGTTAATGATACAGGTAGGACTAATGATACTA
GT---

GATAATTCAACCATCATACTCCCATGCAGAATAAAACAAATTATAAACTTGTGGCAGGGAGTAGGACGAGCAA
TATATGCCCCGCCCATTGCAGGAAACATAACATGTACTTCAAAT---
ATCACAGGAATGCTATTGACACGGGATGGAGGAAACAAAAACAATACA-------------------------
----

GAGCCAGAGACATTCAGACCTGGAGGAGGAGATATGAGGGACAATTGGAGAAATGAATTATATAAATATAAGG
TGGTAGAAATTAAGCCATTA---GGAATAGCACCCACTAGGGCAAAAAGAAGAGTGGTG--------------
-------------------------GAGAGAGAAAAAAGAGGA------------ATAGGAGCT---------
GTGTTCCTTGGGTTCTTGGGAGCA---GCAGGAAGCACTATG---GGCGCGGCATCA---
GTGATGCTGACGGTACAA---GCCAGACAA---TTATTG---TCTGGTATAGTGCAACAGCAAAGC---
AATTTG---CTGAGG---GCTATAGAGGCGCAACAGCATATGTTGCAACTCACAGTCTGGGGCATT
>KR861327
TTCTATTGCAATACAACAAAGCTGTTTAATAGTACTGATAAAAGT----------------------------
----

GATAATGGTACCATCACACTCCCATGCAAAATAAAACAAATTATAAACATGTGGCAGGGGGTAGGAAGAGCAA
TGTATGCCCCTCCCATTGCAGGAAACATAACATGTAACTCAAGT---
ATCACAGGACTAATATTGACACGGGATGGAGGAAACGATACAAACACT-------------------------
----

AATAACACAGAGATATTCAGACCTGGAGGAGGAAATATGAAGGACAATTGGAGAAATGAATTATATAAATATA
AAGTGGTAGAAATTAAGCCATTG---GGAATAGCACCCACCCAGGCAAAAAGGAGAGTGGTG-----------
----------------------------GAGAGAGAGAAAAGAGCA---GTGGGA---ATAGGAGCT------
---GTGTTCCTTGGGTTCTTGAGTGCA---GCAGGAAGCACTATG---GGCGCGGCGTCA---
ATAACGCTGACGGTACAG---GCCAGACAA---CTGTTG---TCTGGTATAGTGCAACAGCAAAGT---
AATTTG---CTGAGG---GCTATAGAGGCGCAACAGCATATGTTGCAACTCACGGTCTGGGGCATT
>KR861344
TTCTATTGCAATACAACAAAACTATTTAGTGGGGTTAATGATACA----------------------------
----

GACAATAGGACAATAATACTCCCATGCAGAATAAAACAAATTATAAACATGTGGCAGGGGGTAGGACGAGCAA

TATATGCCCCTCCCATTGCAGGAAACATAACATGTAAATCTAAT---
ATCACAGGACTACTATTGGTACGAGATGGAGGTGATACCAATGCAACA-------------------------
----

GAGATAGAGATATTCAGGCCTGGAGGAGGAGATATGAGAGACAATTGGAGAAGTGAATTATATAAATATAAGG
TGGTAGAAATCAAGCCATTG---GGAATAGCACCCACTGAGTCAAAAAGGAGAGTGGTG--------------
-------------------------GAGAGAGAAAAGAGAGCAGTATTGGGAGGAATAGGAGCT---------
GTGTTCCTTGGGTTCTTGGGAGCA---GCAGGAAGCACTATG---GGCGCGGCGTCA---
CTGACGCTGACGGTACAG---GCCAGGCAA---CTGTTG---TCTGGTATAGTGCAGCAGCAAAGC---
AATTTG---CTGAAG---GCTATAGAGGCGCAACAGCATCTGTTGCAACTCACAGTCTGGGGCATT
>KR861279
TTCTATTGCAATACATCAAATCTGTTTAATGGAAAGCTATTTAATGATACATACAACAAGACAGGA-------
----

GATACTTCAAACATCACACTCTCATGCAGAATAAAACAAATTATAAACATGTGGCAGGAGGTAGGACGAGCAA
TGTATGCCCCTCCCATTGCAGGAAACATAACATGTATATCAAAT---
ATCACAGGAATAATATTGACACGTGATGGAGGAATTAATAACACTACTAAC----------------------
----
AACACTGAGAAAAAT---
GGCGCAGAGACATTCAGACCTGGAGGAGGAAATATGAAGGACAATTGGAGAAGTGAATTATATAAATATAAAG
TGGTAGAAATTAAGCCATTA---GGACTAGCACCCACTGCGGCAAAAAGGAGAGTGGTG--------------
-------------------------GAGAGAGAAAAAAGAGCA---GTGGGA---ATAGGAGCT---------
GTTTTCCTTGGGTTCTTGGGAGCA---GCAGGAAGCACTATG---GGCGCGGCGTCA---
ATAACGCTGACGGTACAG---GCCAGACAA---CTGTTG---TCTGGTATAGTGCAACAGCAAAGT---
AATTTG---CTGAGA---GCTATAGAGGCGCAACAGCATATGTTGCAACTCACGGTCTGGGGCATT
>KR861271
TTCTATTGCAATACAACAAACCTGTTTAATGGAACATATGGGGATAAAGATACAAAC----------------
----

AGTACTTCAAACATCACACTCTCATGCAAAATAAAACAAATTATAAACACATGGCAGGAGGTAGGAAAGGCAA
TATACGCCCCTCCCATTGCAGGAAGCATAAACTGCACATCACAG---
ATCACAGGACTACTCTTGGTACGTGATGGAGGGGGAAATAGCACA----------------------------
----

GATAATGACACATTCAGACCTGAAGGGGGAGATATGAGAGACAATTGGAGAAGTGAATTATATAAATATAAAG
TGGTAGAAATTAAACCATTG---GGAATAGCACCCACCAAGGCAAAAAGGAGAGTGGTG--------------
-------------------------GAGAAAGAAAAAAGAGCA---GTCGGA---ATGGGAGCT---------
GTGATTCTTGGGTTCTTGGGAGCA---GCTGGAAGCACTATG---GGCGCGGCGTCA---
ATAACGCTGACGGCACAG---GCCAGACAA---GTATTG---TCTGGTATAGTGCAACAGCAAAGC---
AATTTG---CTGAAG---GCTATAGAGGCGCAACAGCGTATGTTGCAACTCACGGTCTGGGGCATT
>KR861282
TTCTATTGCGACACATCAAAACTGTTTAATAGTACATACAACGATCTATTTAATAAAACATACAATGATACAG
CT---

AATAGATTAAACATCACAATTCCATGCAGAATAAAACAAATTATAAACATGTGGCAGAAGGTAGGACGAGCAA
TGTATGCTCCTCCCATTGCAGGAAACATAACATGTGAATCCAAT---
ATCACAGGACTGCTACTGGTACGTGATGGAGGTAGAAGTAACAAT----------------------------
----

AACAATGGCACAGAGACATTTAGGCCTGGAGGAGGAAATATGAAGGACAATTGGAGAAGTGAATTATATAAAT
ATAAAGTGGTAGAAATTAAACCATTA---GGAATAGCACCCACTGAAGCAAAAAGGAGAGTGGTG--------
-------------------------------GAGAGAGAAAAAAGAGCA---GTGGGA---ATGGGAGCT---
------GTGTTCCTTGGGTTCTTGGGAGCA---GCAGGAAGCACTATG---GGCGCGGCGTCA---
GTGGCGCTGACGGTACAG---GTCAGACAA---TTGTTG---TCTGGTATAGTGCAACAGCAAAAC---
AATTTG---CTGAGG---GCTATAGAGGCGCAACAGCATATGTTGCAACTCACAGTCTGGGGCATT
>KR861304
TTCTATTGCAATACATCATGCCTGTTTAATGATAGTTGTACAAAAAATGAATCAGTC----------------
----

ATCACACTCCCATGCAGAATAAAACAAATTACAAACATGTGGCAGGAAGTAGGACGAGCAATATATGCCCCTC

```
CCATTGCAGGAAACATAACATGTAATTCAAGT---
ATCACAGGACTACTCTTAACACGTGATGGAGGACGGTCAAGTGAA----------------------------
--------------------------------------------------------------------
CCAAATAACACAAAT---
AACAATGAGACATTCAGACCTGCAGGAGGTGATATGAGGGACAATTGGAGAAGTGAATTATATAAATATAAAG
TGGTAGAAGTTAAGCCATTG---GGAATAGCACCCACTAACGCAAAAAGGAGAGTGGTG--------------
-------------------------GAGAGAGAAAAAAGAGCA---GTGGGA---ATAGGAGCT---------
GTGTTCCTTGGGTTCTTGGGAGCA---GCAGGAAGCACTATG---GGCGCAGCATCA---
ATAACGCTGACGGTACAA---GCCAGACAA---CTGTTG---TCTGGTATAGTGCAACAGCAAAGC---
AATTTG---CTGAGA---GCTATAGAGGCGCAACAACATATGTTGCAACTCACAGTCTGGGGCATT
>AF110959
TTTTATTGCGATACATCAGAACTGTTTAATAGTACATACATGTCTAATGGTACAGAAGGTAATATAAGC----
-------------------------------------------------------------------------
----------------------------
CCAAGTACAATCATCATGCTCCCATGCAGAATAAAACAAATTATAAACATGTGGCAGGGGGTGGGACGAGCAA
TGTATGCCCCTCCCATTAAAGGAAGTATAACATGTAGATCAAAT---
ATTACAGGGTTACTATTGACACGTGATGGAGGGCTAAATAGGAGCACA-------------------------
-------------------------------------------------------------------------
----------
GAGGAGCCAGAGATATTCAGACCTGGAGGAGGAGATATGAGGAACAATTGGAGAAGTGAATTATATAAATATA
AAGTGGTAGGAATTAAGCCATTA---GGAGTAGCACCCACTGGGGCAAAAAGGAGAGTGGTG-----------
----------------------------GAGAGAGAAAAAAGAGCA---GTGGGA---ATAGGAGCT------
---GTGTTCCTTGGGTCCTTGGGAGCA---GCAGGAAGCACTATG---GGCGCGGCGTCA---
ATCACGCTGACGGTACAG---GCCAGACAA---TTATTG---TCTGGTATAGTGCAACAGCAAAGC---
AATTTG---CTGAGG---GCCATAGAGGCGCAACAGCACATGTTGCAACTCACGGTCTGGGGCATT
>AF290028
TTTTATTGCAATACATCAAGACTGTTTGATGAGACATACCTGTCTAATGGGACAGACGAG-------------
-------------------------------------------------------------------------
----------------------------
GATAATGGTACAATCACACTCCCATGCAAAATAAAACAAATTATAAACATGTGGCAAGAGGTAGGACGAGCAA
TATATGCCCCTCCCATTGCAGGAAACATAACATGTAAATCAAAT---
ATCACAGGACTATTATTGACACGTGATGGAGGGTTAAGCCCTAAT----------------------------
-------------------------------------------------------------------------
----------
AATGACAGTGAGACATTCAGACCTATAGGAGGAGAGATGAGGAACAATTGGAGAAGTGAATTATATAAATATA
AAGTGGTAGAAATTAAGCCATTG---GGAATAGCACCCACTAAGGCAAAAAGGAGAGTGGTG-----------
----------------------------GAGAGAGAAAAAAGAGCA---GTGGGA---ATAGGAGCT------
---GTGTTCCTTGGGTTCTTGGGAGCA---GCAGGAAGCACTATG---GGCGCAGCATCA---
ATAACGCTGACGGTACAG---GCCAAACAA---TTGTTG---TCTGGAATAGTGCAACAGCAAAGC---
AATTTG---CTGAAA---GCTATAGAGGCGCAACAGCATATGTTGCAACTCACGGTCTGGGGCATT
>AF443075
TTCTATTGCAATACATCAGAACTGTTTAATGGAACATATAATGGAACAGACAACAAT----------------
-------------------------------------------------------------------------
----------------------------
AGCAATAAAACCATCACACTCCTATGCAGGATAAAACAAATTATAAACACATGGCAGGAGGTAGGACGGGCAA
TATACGCCCCTCCCATTGCAGGAAACATAATATGTATATCAAAC---
ATCACAGGACTACTCTTGACACGTGATGGAGGAAAAACAAATGACACA-------------------------
-------------------------------------------------------------------------
----------
AATGACACAGAAATATTCAGACCTGGAGGAGGAAATATGAAGGACAATTGGAGAAGTGAATTATATAAATATA
AAGTGGTAGAAATTAAACCATTG---GGAGTAGCACCCACCAAGGCAAAAAGGCGAGTGGTG-----------
----------------------------GAGAGAGAAAAAAGAGCA---GTCGGA---ATAGGAGCT------
---GTGCTCCTTGGGTTCTTGGGAACA---GCAGGAAGCACTATG---GGCGCGGCGTCA---
ATAACGCTGACGGTACAG---GCCAGACAA---GTATTG---TCTGGTATAGTGCAACAGCAAAGC---
AATTTG---CTGAGG---GCTATAGAGGCGCAACAGCATATGTTGCAACTCACGGTCTGGGGCATT
>AF443074
ATCTATTGTAATACATCAAAGCTGTTTAATGGTACATACAACAGTACAGGAGGTACT----------------
-------------------------------------------------------------------------
----------------------------
TCAAATTCAACAATCACACTCTCATGCAGAATAAAACAAATTATAAACATGTGGCAGGGGGTAGGACGAGCAA
```

TGTATGCCTCTCCCATTGCAGGAAACATAACATGTAAATCAAAT---
ATCACAGGACTACTATTGACACGTGATGGAGGAAATGAGACAAGT----------------------------
----

GGGATTGAGATATTCAGACCTGCAGGAGGAGATATGAGGGACAATTGGAGAAGTGAATTATATAAATATAAAG
TGGTAGAAATTAAGCCATTA---GGATTAGCACCCACTAAGTCAAAACGGAGAGTGGTG--------------
-------------------------GGGAGAGAAAAAAGAGCA---GTGACA---TTCGGAGCT---------
ATGTTCCTTGGGTTCTTGGGAGCA---GCAGGAAGCACTATG---GGCGCGGCGTCC---
ATGACGCTGACGGTACAG---GCCAGACAA---CTGTTG---TCTGGTATAGTGCAACAGCAAAGC---
AATTTG---CTGAGG---GCTATAGAGGCGCAACAGCATATGTTGCAACTCACGATCTGGGGCATT
>KY658709
TTCTATTGCAATACATCAAAACTGTTTAATGGTACATATAACAGTACAGGAAGTACT----------------
----

TCAAATTCAACCATCACACTCCCATGCAGAATAAAACAATTTATAAACTTGTGGCAGGAGGTAGGACGAGCAA
TGTATGCCCCTCCCATTGCAGGAAACATAACATGTAGATCAAAT---
ATCACAGGACTAATATTGACACGTGATGGAGGAGTTGGTAATGAC----------------------------
----

TCAGAGACATTCAGACCTGAAGGAGGAGATATGAGGAACAATTGGAGAAGTGAATTATATAAATACAAAGTGG
TAGAAATTAAGCCATTA---GGACTAGCACCCACTAAGGCAAAAAGGAGAGTGGTG-----------------
----------------------GAGAGAGAAAAAAGAGCA---GTGGGA---ATAGGAGCT---------
GTGTTCCTTGGGTTCTTGGGAGCA---GCAGGAAGCACTATG---GGCGCGGCGTCA---
ATAACGCTGACGGTACAG---GCTAGACAA---CTGTTG---TCTGGTATAGTGCAACAGCAAAGC---
AATTTG---CTGAGG---GCTATAGAGGCGCAACAACATATGTTGCAACTCACGGTCTGGGGCATT
>KY658711
TTCTATTGCAATACATCAAAACTGTTTAATAGTACATACAATAGTACAGACAGGTCTAAT-------------
----

AATACAGACAACATCATAATCCAATGCAGAATAAAACAAATTATAAACATGTGGCAGGGGGTAGGACGAGCAA
TGTATGCCCCTCCCATTGAAGGAAACATAACATGTAAATCAAAT---
ATCACAGGACTACTATTGGTACGTGATGGAGGGAAAGAAGAGAATAACACA----------------------
----

GGGACAGAGATATTCAGACCTGAAGGAGGAGATATGAGGGATAACTGGAGAAGTGAATTATATAAATATAAAG
TGGTAGAAATTAAGCCATTG---GGAATAGCACCCACTAAGGCAAAAAGGAGAGTGGTG--------------
-------------------------GAGAGAGAAAAAAGAGCA---GTGGGA---ATAGGAGCT---------
GTGTTCCTTGGGTTCTTGGGAGCA---GCAGGAAGCACTATG---GGCGCGGCGTCA---
ATAACGCTGACGGTACAG---GCCAGACAA---TTGTTG---TCTGGTATAGTGCAACAGCAAAGC---
AATTTG---CTGAAA---GCTATAGAGGCGCAACAACATCTGTTGCAACTCACGGTCTGGGGCATT
>AF443076
TTCTATTGCAATACATCAGGCTTGTTTAATAGTACATACAATCCTAATAGTACATACAATCTTAATAGTACAG
AAAGT--

AAGGCAAATTCAAACATCACACTCCACTGCAGAATAAAACAAATTATAAACATGTGGCAGGAGGTAGGACGAG
CAATGTATGCCCCTCCCATTGCAGGAAACATAACATGTAGATCAAAT---
ATCACAGGATTACTATTGACACGTGATGGAGGAAATACAACAGAG----------------------------
----

ACAAAAGAGACATTCAGACCTGGAGGAGGGGATATGAGGGACAATTGGAGAAGTGAATTATATAAATATAAAG
TGGTAGAAATTAAACCATTG---GGAGTAGCACCCACTGAGGCGAAAAGGAGAGTGGTG--------------
-------------------------GAGAGAGGAAAAAGAGCA---GCGGGA---CTAGGAGCT---------
GTGCTCTTTGGGTTCTTGGGAGCA---GCAGGAAGCACTATG---GGCGCAGCGTCA---
ATAACGCTGACGGTACAG---GCCAGACAA---CTGTTG---TCTGGTATAGTGCAACAGCAAAGC---
AATTTG---CTGAGG---GCCATAGAGGCGCAACAGCATATGTTGCAACTCACGGTCTGGGGCATT
>AF443078
TTCTATTGCAATACATCACAACTGTTTAATAGTACATACAATGGTACAGAAAGAAATTCAACA----------
----

ACAAATGCAACCATTACACTCCCATGCAGAATAAAACAAATCATAAACATGTGGCAGGAGGTAGGACGAGCAA

TATATGCCCCTCCCATTAAAGGAAACATAACATGTGAATCAAAT---
ATCACAGGACTACTATTGACACGTGATGGAGGGTCGAATGATACA----------------------------
----

ACAGAAACATTTAGACCTGAGGGAGGAGATATGAGGAACAATTGGAGAAGTGAATTGTATAAATATAAAGTAG
TAGAAATTAGGCCATTA---GGGATAGCACCCACTGGGGCAAAAAGGAGAGTGGTG-----------------
----------------------AATAGAGAAAAAAGAGCA---GTGGGA---GTAGCAGCT---------
GTGTTCCTTGGGTTCTTATCAGCA---GCAGGAAGCACTATG---GGCGCAGCATCA---
ATAACGCTGACGGTACAG---GCCAGACAA---TCGTTG---TCTGGTATAGTGCAACAGCAAAGC---
AATTTG---CTGAGA---GCTATAGAGGCGCAACAGCATCTGTTGCAACTCACGGTCTGGGGCATT
>AF443079
TTCTATTGCAATACATCAAAACTGTTTAATAGTACATACAATGCTACATACAATAGTACATACAATGATACA-
----

TCAAATTCAACCATCACAATCCCATGCAGAATAAAACAAATTATAAACATGTGGCAGGGGGTAGGACAAGCAA
TGTATGCCCCTCCCATTGCAGGAAACATAACATGTAAATCAAAT---
ATCACAGGAATACTGTTGACACGTGATGGAGGCATAAATAACACA----------------------------
----

AATGGCACAGAGACATTCAGACCTGGAGGAGGAGATATGAGGGACAATTGGAGAAGTGAATTATATAAATATA
AAGTGGTAGAAGTTAAACCATTA---GGGATAGCACCCACCAAGGCACAAAGGAGAGTGGTG-----------
----------------------------GAGAGAGAAAAAAGAGCA---GTGGGA---ATAGGAGCT------
---GTGTTCCTTGGGTTCTTGGGAGCA---GCAGGAAGCACTATG---GGCGCAGCGTCA---
ATAACGCTGACGGTACAG---GCCAGACAA---TTGTTG---TCTGGTATAGTGCAACAGCAAAGC---
AATTTG---CTGAGA---GCTATAGAGGCGCAACAGCATATGTTGCAACTCACAGTCTGGGGCATA
>AF443080
TTCTATTGTAATACATCAGGCCTGTTTAATAGTGCATTTAATGATAAT-------------------------
----

AGTGGTGGGACCATCACCCTCCAATGCAGAATAGAACAAATTATAAACATGTGGCAGAAGGTAGGACGAGCAA
TATATGCACCTCCCATTGCAGGAAACATAACATGTAGTTCAAGG---
ATCACAGGACTACTATTGACACGGGATGGTGGAAAAAAATGATACA---------------------------
----

CATGAGACATTCAGACCTGCAGGAGGAGATATGAGGGACAATTGGAGAAGTGAATTATATAAATATAAAGTGG
TGGAGATTAAGCCATTA---GGAGTAGCACCCAGTGAGGCAAAAAGAAGAGTGGTG-----------------
----------------------GAGAGAGAGAAAAGAGCG---GCA------CTGGGAGCT---------
GTGTTCCTTGGGTTCTTGGGAGCA---GCAGGAAGCACTATG---GGCGCGGCATCA---
ATAACGCTGACGGTACAG---ACCAGAAAA---TTATTG---TCTGGTATAGTGCAACAGCAAAGT---
AATTTG---CTGAAA---GCTATAGAGGCGCAACAACATATGTTGCAACTCACAGTCTGGGGCATT
>AF443082
TTCTATTGCAATACATCAAAGCTGTTTAATACATCATGGCTGGATTCATACATATCTAATAATACAGGA----
----

AATAATTCAATCATCACACTTCCATGCAGAATAAAACAAATTATAAACATGTGGCAGAAGGTAGGACGAGCAA
TGTATGCCAACCCCATTGAAGGAAATATAACATGTAGGTCAAAT---
ATCACAGGATTACTATTAGAAAATGATGGA---AACATG----------------------------------
----

ACATTCAGACCTGGAGGAGGAGATATGAAGGACAATTGGAGAAGTGAATTATATAAATATAAAGTAGTAGAAA
TTAAGCCATTG---GGAATAGCACCCACTGAAGCAAAAAGGAGAGTGGTG-----------------------
----------------GAGAGAGAAAAAAGAGCA---GTGGGA---ATAGGAGCT---------
GTGTTCCTTGGGTTCTTGGGAGCA---GCAGGAAGCACTATG---GGCGCGGCGTCA---
ATAACGCTGATGGTACAG---GCCAGACAA---TTGTTG---TCTGGTATAGTGCAACAGCAAAGC---
AATTTG---CTGAGA---GCTATAGAGGCGCAACAGCATCTGTTGCAACTCACAGTCTGGGGCATT
>AF443083
TTCTATTGCAATACATCACGCCTGTTTAATAGTACATACAATCCTAATAGTACAAAAAGCAAT----------
----

ACAGGCTCATGGATCATACTCCCATGCAGAATAAAACAAATTATAAACATGTGGCAGAAGGTAGGACGAGCAA

TGTATGCTCCTCCCATTGCAGGAAACATAACATGTAAATCAAAT---
ATCACAGGATTACTATTGGTGCGCGATGGAGGAACAACAAATGCC----------------------------
----

ACGGACGAGATATTCAGACCTGGAGGAGGAGATATGAGGGACAATTGGAGAAGTGAACTATATAAATATAAAG
TGGTAGAAATTAAGCCATTG---GGAATAGCACCCACTGAGGCAAAAAGGAGAGTGGTG--------------
-------------------------GAGAGAGAAAAAAGAGCA---GTAGGA---CTAGGAGCT---------
GTGTTCCTTGGGTTCTTGGGAGCA---GCAGGAAGCACTATG---GGCGCGGCGTCA---
ATAACGCTGACGGTACAG---GCCAGACAA---CTGCTG---TCTGGTATAGTGCAACAGCAAAAT---
AATTTG---CTGAGA---GCTATAGAGGCGCAACAACATCTGTTGCAACTCACGGTCTGGGGCATT
>AF443085
TTCTATTGCAATACATCAGAACTGTTTAACAGTACGTACAATGCAAATATGACATATAATACAAGCATGACTA
ATGCTACA---
-------------------GGA---
AATAACTCAACAACAATCATACTCCCATGCAGAATAAAACAAATTATAAACATGTGGCAGGAGGTAGGACGAG
CAATGTATGCCCCGCCCATTGAAGGGAACATAACATGTAAATCAAAT---
ATCACAGGACTACTATTGGTACGTGATGGAGGAGGAGGAAAGAATGCC-------------------------
----

ACAAATGACACAGAGATATTCAGACCTGAAGGAGGAGACATGAGAAACAATTGGAGAAGTGAATTATATAAAT
ATAAAGTGGTAGAAATTAAGCCATTG---GGAGTAGCACCCACTAAGGCAAAGAGAAGAGTGGTG--------
-------------------------------GACAAAGAAAAAAGAGCA---ATA------GCAGGAGCT---
------GTGTTCCTTGGGTTCTTGGGAGTA---GCAGGAAGCACTATG---GGCGCGGCGTCA---
GTGGCGCTGACGGTACAG---GCCAGACAA---TTGTTG---TCTGGTATAGTGCAACAGCAAAGC---
AATTTG---CTGAGA---GCTATAGAGGCGCAACAGCATATGTTGCAACTCACGGTCTGGGGCATT
>AF443086
TTCTATTGCAATACATCAAAACTGTTTAACAGTACGTTTAATAGCAATGGTTCATACATATGGCATGACAGTA
CA---

GGAAATGATCCACTAACCATCCCATGTAGAATAAAACAAATTATAAACATGTGGCAGGAGGTAGGACGAGCAA
TGTATGCCCCTCCCATTGCAGGAAGAATAATATGTAACTCAACT---
ATCACAGGGTTAATATTGACACGTGATGGAGGAAATAATACTAAT----------------------------
----

AATACAGAGATATTCAGACCTATAGGAGGAAATATGAGGGACAATTGGAGAAGTGAATTATATAAATATAAAG
TGGTAGAAATTAAGCCATTA---GGAATAGCACCCACTAAGGCAAAAAGGAGAGTGGTG--------------
-------------------------GAGAGAGAAAAAAGAGCA---GTGGGA---ATAGGAGCT---------
GTGTTCCTTGGGTTCTTGGGAGCA---GCAGGAAGCACTATG---GGCGCAGCATCA---
ATAACGCTGACGGTACAG---GCCAGACAA---CTGTTG---TCTGGTATAGTGCAACAGCAAAGC---
AATTTG---CTGAGG---GCTATAGAGGCGCAACAGCATATGTTGCAACTCACGGTCTGGGGCATT
>HM215421
TTCTATTGTAATACATCAAGCCTGTTTAATAGCACATACATGTCTAATGGCACATACATGTTTAATGACATGT
CTAATGGTACA--
-------------GAAAGG------
AATTCAAGCTCAATCATCGCAATTCCATGCAGAATAAAGCAAGTTATAAACATGTGGCAGGAGGTAGGACGAG
CGATGTATGCCCCTCCCATTGCAGGAAAATTAACATGTAGATCAAAT---
ATCACAGGACTACTATTGGTACGTGATGGAGGAACAAATAATGCT----------------------------
----

ACAACAGAGACATTCAGACCTGGAGGAGGAGATATGAGGAACAATTGGAGAAGTGAATTATACAAATATAAAG
TGGTAGAAATTAAGCCATTG---GGAGTAGCACCCACTGAGGCAAAAAGGAGAGTGGTG--------------
-------------------------GAGAGAGAAAAAAGAGCA---GTGGGA---ATAGGAGCT---------
GTGTTCCTTGGGTTCTTGGGTGCA---GCCGGAAGCACTATG---GGCGCGGCGTCA---
ATAACGCTGACGGTACAG---GCCAGACAA---TTGCTG---TCTGGTATAGTGCAACAGCAAAGC---
AATTTG---CTGAGG---GCTATAGAGGCGCAACAGCATATGTTGCAACTCACGGTCTGGGGCATT
>HQ699992
TTCTATTGTAATACATCAGGCCTGTTTAATAGTACATACATGACTAATGGTACATACATGTTTAATGGTACAT
ACAGGCTTAAT--
-------------AATATA------
ACAAGTAATTCAAACATCACAATTCCATGCAGAATAAAGCAAATTATAAACACGTGGCAGGAGGTAGGGCGAG

CAATGTATGCCAATCCCATTGCAGGAAACATAACATGTAAATCAAAT---
ATCACAGGACTACTATTGGTACGTGATGGAGGAACAAATAATGCT----------------------------
----

ACAACAGAGACATTCAGACCTGGAGGAGGAAATATGAGGGACAATTGGAGAAGTGAATTATATAAATATAAGG
TGGTAGAAATTAAGCCATTG---GGAATAGCACCCACTGAAGCAAAAAGGCGAGTGGTG--------------
-------------------------GAGAGAGAAAAAAGAGCA---GTGGGA---ATAGGAGCT---------
GTGTTCCTTGGGTTCTTGGGTGCA---GCCGGAAGCACTATG---GGCGCAGCGTCA---
ATAACGCTGACGGTACAG---GCCAGACAA---TTGCTG---TCTGGTATAGTGCAACAGCAAAGC---
AATTTG---CTGAGG---GCTATAGAGGCGCAACAGCATATGTTGCAACTCACGGTCTGGGGCATT
>KF835515
TTCTATTGTGATACATCAGGCCTGTTTAATAGTACATACAATAGTACATACGTGCCTAATGGTACAGAA----
----

AGTAAGCCAAACATCACAATCCAATGCAGAATAAAGCAAATTATAAACATGTGGCAGGAGGTAGGACGAGCAA
TGTACGCCCCTCCCATTAAAGGAAGCATAACATGTAAATCAAAT---
ATCACAGGACTACTATTGGTACGTGATGGAGGAGCAAATACTACA----------------------------
----

GAAGAGATATTCAGACCTGGAGGAGGAGATATGAGGGACAATTGGAGAAGTGAATTATATAAATATAAAGTGG
TAGAAATCAAGCCATTG---GGAATAGCACCCACTGAAGCAAAAAGGAGAGTGGTG-----------------
----------------------GAGAGAGAAAAAAGAGCA---GTGGGA---CTAGGAGCT---------
GTGTTCCTTGGGTTCTTGGGCGCA---GCAGGAAGCACTATG---GGCGCGGCGTCA---
ATAACGCTGACGGTACAG---GCCAGACAA---TTGCTG---TCTGGTATAGTGCAACAGCAAAAC---
AATTTG---CTGAAG---GCTATAGAGGCGCAACAGCATATGTTGCAACTCACGGTCTGGGGCATT
>HQ699985
TTCTATTGTAATACATCAAAACTGTTTAATGGTACATACATGCCTAATGGTACAAACAGTAATTCAAGCAGTA
AT---

CCAAACTCAACCATCACAATCCCATGCAGTATAAAGCAAATTATAAATATGTGGCAGGAGGTAGGACGAGCAA
TGTATGCCCCTCCTATTGCAGGAAACATAACATGTAAATCAAAC---
ATCACGGGACTACTATTGGTACGTGATGGAGGAAATGTTACAGGGAGA-------------------------
----

AATGGTACAGAGATATTCAGACCTGAAGGAGGAAATATGAGGGACAATTGGAGAAGTGAATTATATAAATATA
AAGTGGTAGAAATTAAGCCATTG---GGAGTAGCACCCACTGGAGCAAAAAGGAGAGTGGTG-----------
----------------------------GAGAGAGAAAAAAGAGCA---GTGGGA---ATAGGAGCT------
---ATGTTCCTTGGGTTCTTGGGAGCA---GCAGGAAGCACTATG---GGCGCGGCGTCA---
ATAACGCTGACGGTACAG---GCCAGACAA---TTGCTG---TCTGGTATAGTGCAACAGCAAAAC---
AATTTG---CTGAGA---GCTATAGAGGCGCAACAACATCTGTTGCAACTCACGGTCTGGGGCATT
>HM215411
TTCTATTGTAACACATCAGACCTGTTTAATTCTACATACATGTCTAATGGTACATTCAGGTTTAATGGTACAG
TGTTTAATGGT--
----------ACAGGAGGT------
GATTCAAGCATAAACATCACAATCCCATGCAGAATAAAGCAAATTATAAACATGTGGCAGGAAGTAGGACGAG
CAATGTATGCCCCTCCCATTGCAGGAAATATAACATGTAAATCAAAT---
ATCACAGGACTACTATTGGTACGTGATGGAGGAACAACAAATAATACA-------------------------
----

AATAATACAGAGATATTCAGACCTGGAGGAGGAGATATGAGGGACAATTGGAGAAGTGAATTATATAAATATA
AAGTGGTAGAAGTTAAACCATTG---GGAATAGCACCCACTACAGCAAAAAGGAGAGTGGTA-----------
----------------------------GAGAGAGAAAAAAGAGCA---GTGGGA---CTAGGAGCT------
---GTGTTCCTTGGGTTCTTGGGAGCA---GCAGGAAGCACTATG---GGCGCAGCGTCA---
ATAACGCTGACGGTACAG---GCCAGACAA---TTGCTG---TCTGGTATAGTGCAACAGCAAAGC---
AATTTG---CTGAAA---GCTATAGAGGCGCAACAGCATCTGTTGCAACTCACGGTCTGGGGCATT
>KC898995
TTCTATTGTAATACATCAAGACTGTTTAATAGTACATATATGGATAATAGTACAGAAAGTAAT----------
----

TCAAACTCAACCATCACAATCCCATGTAGAATAAAGCAATTTATAAACATGTGGCAGCAGGTAGGACGAGCAA

```
TGTATGCCCCTCCCATTGAAGGAAATATAACATGTAAATCAAAT---
ATCACAGGACTACTATTGGTACGTGATGGAGGAAAAACAAATGAT---------------------------
------------------------------------------------------------------------
-------------
ACAACAGAGACATTCAGACCTGGAGGAGGAGATATGAGGGACAATTGGAGAAGTGAATTATATAAATATAAAG
TGGTAGAAATTAAACCATTG---GGAATAGCACCCACTGAAGCAAAAAGGAGAGTGGTG-------------
------------------------GAGAGAGAAAAAAGAGCA---GTGGGA---ATAGGAGCT---------
GTGTTCCTTGGGTTCTTGGGAGCA---GCAGGAAGCACTATG---GGCGCGGCGTCA---
ATAACGCTGACGGTACAG---GCCAGACAA---TTATTG---TCTGGTATAGTGCAACAGCAAAGC---
AATTTG---CTGAGG---GCTATAGAGGCGCAACAGCATATGCTGCAACTCACGGTCTGGGGCATT
>KC898996
TTCTATTGTAATACATCAGGCCTGTTTAATAGTACATACACGTCTAAGGGTACATACAGGTTTAATGGTACAG
AAAGTAATGGT-------------------------------------------------------------
----------ACAGAAAGT------
AATTCAAGCTCAATCATCACAATCCCATGCAGAATAAAGCAAATTATAAACATGTGGCAGGAGGTAGGACGAG
CAATGTATGCCCCTCCCATTGAAGGAAACATAACATGTAAATCAAAT---
ATCACAGGACTATTATTGGTACGTGATGGAGGAAAAGGAGACACAGGG------------------------
------------------------------------------------------------------------
-------
CTAAATGATACAGAGACATTCAGACCTGGAGGAGGAGATATGAGAGACAATTGGAGAAATGAATTATATAAAT
ATAAAGTGGTAGAAATTAAGCCATTG---GGAATAGCACCCACTGCAGCAAAAAGGAGAGTGGTG-------
------------------------------GAGAGAGGAAAAAGAGCA---GTGGGA---ATAGGAGCT---
------GTGTTCCTTGGGTTCTTGGGAGCA---GCAGGAAGCACTATG---GGCGCGGCGTCA---
ATAACGCTGACGGTACAG---GCCAGACAA---TTATTG---TCTGGTATAGTGCAACAGCAAAGC---
AATTTG---CTGAAG---GCTATAGAGGCGCAACAGCATCTGTTGCAACTCACGGTCTGGGGCATT
>KF250403
TTCTATTGCAATACATCAGGCCTGTTTAATAGTACATACACGCCTTTTAATAGTACATACATGCCTAATGGTA
CAGAAAGTAAT-------------------------------------------------------------
----------------------------
TCAAGCTTAACCATCACACTCCCATGCAGAATAAAGCAAATTATAAACATGTGGCAGGAGGTAGGACGAGCAA
TGTATGCCCCTCCCGTTGCAGGAAACATAACATGTAAATCAAAT---
ATCACAGGACTACTATTGGTACGTGATGGAGGCATAAATAATACA---------------------------
------------------------------------------------------------------------
-------------
AGAGAGGAGACATTCAGACCTGGAGGAGGAGAAATGAGGGACAATTGGAGAAGTGAATTATATAAATATAAAG
TGGTAGAAATTAAGCCATTG---GGAATAGCACCCACTGCAGCAAAAAGGAGAGTAGTG-------------
------------------------GAGAGAGAAAAAAGAGCA---GTGGGA---ATAGGAGCT---------
GTGTTCCTTGGGTTCTTGGGAGTA---GCCGGAAGCACTATG---GGCGCGGCGTCA---
ATGACGCTGACGGTACAG---GCCAGACAA---TTATTG---TCTGGTATAGTGCAACAGCAAAGC---
AATTTG---CTGAAG---GCTATAGAGGCGCAACAGCATCTGTTGCAACTCACAGTCTGGGGCATT
>KF250404
TTCTATTGTAATACATCAGATCTGTTTAATAGTACATACATGCCTAATAGTACAGAAAGTAATTCAAACTCCT
CC----------------------------------------------------------------------
----------------------------
TCAAATTTAACCATCACACTCCAATGCAGAATAAAGCAAATTATAAACATGTGGCAGGAGGTAGGACGAGCAA
TGTATGCCCCTCCCATTGCAGGAAACATAACATGTAGATCAAAT---
ATTACAGGACTACTATTGGTACGTGATGGAGGT---------------------------------------
------------------------------------------------------------------------
-------
TCGAATGATACAGAGACATTCAGACCTGGAGGAGGAGATATGAGGGACAATTGGAGAAGTGAATTATATAAAT
ATAAAGTGGTAGAAATTAAGCCATTG---GGAATAGCACCAACTGGAGCAAAAAGGAGAGTGGTG-------
------------------------------GAGAGAGAAAAAAGAGCA---GTGGGA---ATAGGAGCT---
------GTGTTCCTTGGGTTCTTGGGAGTA---GCAGGAAGCACTATG---GGCGCGGCGTCA---
ATAACGCTGACGGCACAG---GCCAGAAAA---TTATTG---TCTGGTATAGTGCAACAGCAAAGC---
AATTTG---CTGAGA---GCTATAGAGGCGCAACAGCATATGTTGCAACTCACGGTCTGGGGCATT
>KC807921
TTCTATTGTAATACATCAAAACTGTTTAATAGTACATACATACATAATGGTACATACATGAGTGGTACAAAAA
AT----------------------------------------------------------------------
----------------------------
AATTCAAGCTTCATCAGAATACCATGCAAAATAAAGCAAATTATAAACATGTGGCAGGAGGTAGGACGAGCAA
```

TGTATGCCCCTCCCATTGAAGGAAACATAACATGTAACTCAAGT---
ATCACAGGACTACTATTGGTACGTGATGGAGTATCAGACACAAAT----------------------------
----

GAGACATTCAGACCTGGAGGAGGAGATATGAGAGACAATTGGAGAAATGAATTATATAAATATAAAGTGGTAG
AAATTAAGCCATTA---GGACTAGCACCCACTGCAGCAAAAAGGAGAGTGGTG--------------------
-------------------GAGAGAGAAAAAAGAGCA---GTGGGA---CTAGGAGCT---------
GTGTTCCTTGGGTTCTTGGGAATG---GCAGGAAGCACTATG---GGCGCGGCGTCA---
ATAACGCTGACGGTACAG---GCCAGACAA---TTATTG---TCTGGTATAGTGCAACAGCAAAAC---
AATTTG---CTGAGA---GCTATAGAGGCGCAACAGCATATGTTGCAACTCACGGTCTGGGGCATT
>KC870038
TTCTATTGTAATACATCACAACTGTTTAATGGTACATACAATGGTACATAC----------------------
----
-ATGTCTAATGGTACA---------
GAAAGTAATGCAACCATCACAATTCCATGCAGAATAAAGCAAATTATAAACATGTGGCAGGAGGTAGGACGAG
CAATGTATGCCAATCCCATTGCAGGAAACATAACATGTAAATCAAAT---
ATTACAGGACTACTATTGGTACGTGATGGAGGA--

GAAGGTACAAATGCT---
ACAAATGAGACATTCAGACCTGGAGGAGGAGATATGAGGGACAATTGGAGAAGTGAATTATATAAATATAAAG
TGGTAGAAATTAAGCCATTG---GGGTTAGCACCCACTGAAGCAAAAAGGAGAGTGGTG--------------
-------------------------GAGAGAGAAAAAAGAGCA---GTGGGA---ATAGGAGCT---------
GTGTTCCTTGGGTTCTTGGGAGCA---GCCGGAAGCACTATG---GGCGCGGCGTCA---
ATAACGCTGACGGTACAG---GCCAGACAG---TTATTG---TCTGGTATAGTGCAACAGCAAAGC---
AATTTG---CTGAGG---GCTATAGAGGCGCAACAGCATCTGTTGCAACTCACGGTCTGGGGCATT
>FJ388913
TTCTATTGCAATACATCAAACCTGTTTAATAGTACATACATGACTAATGGTACATTCATACTTAATGGTACAG
GCATG--
-------------------AAT---
ACAGGTAATGAAACCATCATACTCCCATCCAGGATAAGACAATTTGTAATCCTGTGGCAGGAGGTAGGGCGAG
CAACGTATGCTCCTCCCATCGCAGGAAACATCACATGTAAATCAAAT---
ATTACAGGAGTACTAGTGGTACGTGATGGAGGCACAGGCAACGGGACA-------------------------
----

GAAAATAACACAGAGATATTCAGACCTGGAGGAGGGAATATGAAGGACAATTGGAGAAGTGAATTATATAAAT
ATAAAGTGGTAGAAATTAAGCCATTG---GGAATAGCACCCACTGGAGCAAAGAGGAGAGTGGTG--------
-------------------------------GAGAGAGAGAAAAGAGCA---GTGGGA---CTGGGAGCT---
------GTGATCTTTGGGTTCTTGGGAGCA---GCAGGAAGCACTATG---GGCGCGGCGTCA---
ATGGCGCTGACGGTACAG---GCCAGACAA---GTATTG---GCTGGTATAGTGCAACAGCAAAAC---
AATTTG---CTGAGA---GCCATAGAGGCGCAACAGCATATGTTGCAACTCACGGTCTGGGGCATT
>FJ388948
TTCTATTGCAATACATCAAAACTGTTTGACAGTACACTATTGGATAATAGTACAAATTTAAACTTGACTGGCA
ATATC--

AATTTAACTGGCAATATCACGCTTCCATGCAGAATAAAACAAATTATAAACATGTGGCAGGAGGTAGGACGAG
CAATGTATGCCTCTCCCATAGCAGGAAACATAACATGTAAATCAAAT---
ATCACAGGACTACTGTTAACACGTGATGGAGGAAAAGACAACAAT----------------------------
----

GGCACAGAAACATTCAGACCTGGAGGAGGAAATATGAAAGACAATTGGAGAAGTGAATTATATAAATATAAAG
TAGTAGAAATTAAGCCATTA---GGAATAGCACCCACTACAGCAAAAAGGAGAGTGGTG--------------
-------------------------GAGAGAGAAAAAAGAGCA---GTAGGA---ATAGGAGCT---------
GTGTTCCTTGGGTTCTTGGGAGCA---GCAGGAAGCACTATG---GGCGCGGCGTCT---
GTAACGCTGACGGTACAG---GCCAGACAA---CTGTTG---TCTGGTATAGTGCAGCAACAAAGC---
AATTTG---CTGAAG---GCTATAGAGGCGCAACAACATCTGTTGCAACTCACGGTCTGGGGCATA
>FJ388952
TTCTATTGCAATACATCAAAGCTGTTTAATAGTACGTACATGTCTAATGGTACATACATGGTTAATGGTTCTA
ATGGT--
-------------------GCA---
ACTCCAAATTCAACCATCACACTCCAATGTAGAATAAAACAAATTATAAACATGTGGCAGGGGGTAGGACAAG

CAATGTATGCCCCTCCCATTGCAGGAAACATAACCTGTAACTCAAGT---
ATCACAGGAATACTATTGACACGTGATGGAGGAATTAATAATGACACA-------------------------
----

AATAACATAGAGACTTTCAGACCTGGAGGAGGAGACATGAGGGACAATTGGAGAAGTGAATTGTATAAATATA
AAGTGGTAGAAATTAAACCATTA---GGAATAGCACCCACTAAGGCAAAAAGGAGAGTGGTG-----------
----------------------------GAGAGAGAAAAAAGAGCA---GCAACA---TTAGGAGCT------
---GTGTTCCTTGGGTTCTTGGGAGCA---GCTGGAAGCACTATG---GGCGCAGCATCA---
ATAACGCTGACGGTACAG---GCCAGACAA---TTATTG---TCGGGTATAGTGCAACAGCAAAGC---
AATTTG---CTGAGG---GCTATAGAGGCGCAACAGCATATGTTGCAACTCACGGTTTGGGGCATT
>JF683740
TTCTATTGCAATACATCAAAACTGTTTAATAGGAAATACACATTTAATGGCACAAATGGCACAAAC-------
----

ACTAATATCACTATCACATTCCCGAGCAAGATAAAAAAAAACATAAACCTGGGGCAGGGGGGAGCAAGATCAA
CCTCTGCCCCTGCCGGAACCGTAACCATAACA------TCAAGT---
TTAACAGGAACAGGATTGTTACGGGAAGGAGAAGGGAGAAAGAGCAAA-------------------------
----

CACACTAATGAGACATTCAGACCTGGAGGAGGAAATATGAAGGATAATTGGAGGAGTGAATTATATAAATATA
AAGTAGTAGAAATTAAACCATTA---GGAATAGCTCCCACTGAAGCAGAAAGGAGAGCGGTG-----------
----------------------------AAGAGAGAAAAAAGAGCA---GTGGGA---ATAGGAGCT------
---GTGTTCCTTGGGTTCTTGGGAGCA---GCAGGAAGCACTATG---GGCGCGGCGTCA---
ATAACGCTGACGGCACAG---GCCAGACAA---CTGTTG---TCTGGTATAGTGCAACAGCAAAGC---
AATTTG---CTGAGA---GCTATAGAAGCACAACAGCATCTGTTGCAGCTCACGGTCTGGGGCATT
>JF683755
TTCTATTGCAATACATCAGGACTGTTTAATGGCACAAATAGCACAGATAGCACA-------------------
----

AACAGCACAGTCATCACACTCCCATGCAGAATAAAACAAATTATAAGGATGTGGCAGGGAGTAGGACAAGCAA
TGTATGCTCCTCCCATTAGAGCAAACATAACATGTAGATCAAAT---
ATCACAGGACTACTATTAACACGTGATGGAGGACATAATTTTACT----------------------------
----

AATGAGACCTTCAGACCTGGAGGAGGAGATATGAGGGACAATTGGAGAAGTGAATTATATAAATATAAAGTGG
TAGAAATTAAACCATTA---GGAATAGCCCCCACTACAGCAAAAAGGAGAGTGGTGGAT---GGA--------
-------------GAAAAAATACCAAGAGAAAAAAGAGCA---ATAGGGGGACTAGGAGCT---------
GTGTTCCTTGGGTTCTTAGGAGTA---GCAGGAAGCACTATG---GGCGCGGCGTCA---
ATAACGCTGACGGTACAG---GCCAGAGAA---TTATTG---TCTGGTATAGTGCAACAGCAAAGC---
AATTTG---CTGAAG---GCTATAGAGGCGCAACAGCATATGTTGCAACTCACGGTCTGGGGCATT
>JF683768
TTCTATTGCAATACATCGAGCCTGTTTAATGGTACATACAATAGTACAGACAATAGTAGTTCATCTGAAACCA
TC---

AGTTCATCTGAAACCATCACACTTCCATGCAGAATAAAACAAATTATAAACATGTGGCAGGAGGTAGGACGAG
CAATGTATGCCCCTCCCATTGCAGGAAATATAACATGTAGATCAAAT---
ATCACAGGAATACTATTAACACGTGATGGAGGTAATGGAGGAAACAACACAAAC-------------------
----
-
GGAACTGAAGAGACATCAGAGATATTCAGACCTGGAGGAGGAGATATGAGGGACAATTGGAGAAGTGAATTAT
ATAAATATAAAGTGGTAGAAATTAAACCATTA---GGAGTAGCACCCGAGGAAGCAAAAAGAAGAGTGGTA--
-------------------------------------GAGAGAGAAAAAAGGGCA---GTGGGA---
CTAGGAGTT---------TTGTTCTTTGGGTTCTTGGGAGCA---GCAGGAAGCACTATG---
GGCGCGGCGTCA---TTAACGCTGACGGTACAG---GCCAGACAA---TTGTTG---
TCTGGTATAGTGCAGCAGCAAAGC---AATTTG---CTGAGA---
GCTATAGAGGCGCAACAGCATATGTTGCAACTCACAGTCTGGGGCATT
>KT124786
TTCTATTGTAATACATCAAAACTATTTAACAGTACATTTAATAGTACACACTTAGAAGATAATGCTACAAATA
ATAAT--

GATACAACATCCATTACACTTGCATGCAGAATAAAACAATTTGTGAATATGTGGCAGGAAGTAGGAAGAGCAA
TGTATGCCCCTCCCATTGCAGGAAACATAACATGTAAATCAAAT---
ATCACAGGAATACTGTTAGTACGCGATGGAGTC---------------------------------------
----

GGCAATGAGACAGAGATATTCAGACCTGGAGGAGGAAATATGAAGGACAATTGGAGAAGTGAATTATATAAAT
ATAAAGTGGTAGAAATTAAACCATTG---GGAATAGCACCCACTAAAGCAAAAAGGAGAGTGGTG-------
------------------------------GAGAGAGAAAAAAGAGCA---GCA------CTAGGAGCT---
------GTGTTCCTTGGGTTCTTGGGAGCA---GCCGGAAGCACTATG---GGCGCGGCGTCA---
CTAACGCTGACGGTACAG---GTCAGACAA---CTGTTG---TCTGGTATAGTGCAACAGCAAAGC---
AATTTG---CTGAGG---GCTATAGAGGCGCAACAGCATATGTTGCAACTCACGGTCTGGGGCATT
>KC473844
TTCTATTGCAATACATCAAAACTATTTAATAGTACAATTAATAGTACAGTCATGGTTAATGATACA------
----

GACAACAGCACAATCACACTCCCATGCAGGATAAAACAAATTATAAACATGTGGCAAGGGGTAGGACGAGCAA
TGTATGCCCCTCCCATTGCAGGAAATATAACATGTAACTCAAGT---
ATCACAGGACTAATATTGACACGTGATGGAGGAATAGGAAATGGAACT------------------------
----

GATAAGACAGAGATATTTAGACCAGCAGGAGGAGATATGAGGGACAATTGGAGAAGTGAATTATATAAATATA
AAGTGGTAGAAATTAAACCATTA---GGGATAGCACCCACTGAGGCAAAAAGGAGAGTGGTG----------
---------------------------GAGAGAGAGAAAAGAGCA---GTGGGA---ATAGGAGCT------
---GTGTTCCTTGGGTTCTTGGGAGTA---GCAGGAAGCACTATG---GGCGCGGCGTCA---
ATGGCGCTGACGGTACAA---GCCAGACAA---TTATTG---TCTGGTATAGTGCAACAGCAAAGC---
AATTTG---CTGAGG---GCTATAGAGGCGCAACAGCATATGTTGCAACTCACAGTCTGGGGCATT
>HQ236594
TTTATTGCAATACATCACCCCTGTTTAATAGTACATACAATAAGACAGAAAGCATAATTGAGGAA-------
----

GACAATTCAACCATCACCATCCAATGCAGAATAAAACAAATTATAAACATGTGGCAGGAGGTAGGACAAGCAA
TGTATGCCCCCCCCATAAAAGGAATAATAAAATGTAGGTCAAAC---
ATCACAGGATTACTACTAACACGGGATGGAGGTACGGGTAACGAAACA------------------------
----

GATAATGGAACAGAGACATTCAGGCCTGGAGGAGGAGATATGAGAGACAATTGGAGAAGTGAATTATATAAAT
ATAAAGTGGTAGAAATTAAACCATTA---GGAATAGCACCCACTAAGGCAAAAAGGAGAGTGGTG-------
------------------------------GAGAGAGAAAAAAGAGCA---GTGGGA---ATTGGAGCT---
------GTGCTCCTTGGGTTCTTGGGAGCA---GCAGGAAGCACTATG---GGCGCAGCATCA---
ATAATGCTGACGGTACAG---GCCAGACAA---TTATTG---TCTGGGATAGTGCAACAGCAAAGC---
AATTTG---CTGAGA---GCTATAGAGGCGCAACAGCATATGTTGCAACTCACGGTCTGGGGCATT
>HQ236601
TTCTATTGCAATACATCAAAACTGTTTAATAGTACATACAATAGTGCATACAATAGTACATACAACAGAACTG
AAAAA---

GGAGATGCAAACATCACAACCATCCAGTGCAGAATAAGGCAAATTATAAACATGTGGCAAGGGGTAGGACGAG
CAATGTATGCCCCTCCCATTAAAGGAAACATAACATGTAGATCAAAT---
ATCACAGGACTACTATTGACACGTGATGGAGGAGTAAATAATAGT---------------------------
----

GAAACAACAGAGACATTTAGGCCTGGAGGAGGAGATATGAGGGACAATTGGAGAAGTGAATTATATAAATATA
AAGTGGTAGAAATTAAACCATTA---GGAATAGCACCTACTAGAGCAAAAAGGAGAGTGGTG----------
---------------------------CAGAGAGAAAAAAGAGCA---GTAACT---CTGGGAGCT------
---ATGTTCCTTGGTTTCTTGGGAGCA---GCAGGAAGCACTATG---GGCGCAGCATCA---
ATAACGCTGACGGTACAG---GCCAGACAA---TTATTG---TCTGGGATAGTGCAACAGCAAAGC---
AATTTG---CTGAGG---GCTATAGAGGCGCAACAGCATATGTTGCAACTCACAGTCTGGGGCATT
>KY496624
TTCTATTGCAATACAACAAGCCTGTTTAATGGTACATACAATAATAGTACAGGAGATCTTACA---------
----

GAAGGAGGAACCATCACGATCCCATGCAAAATAAAACAATTTATAAACATGTGGCAGGAGGTAGGGCGAGCAA
TGTATGCCCCTCCCATTGAAGGAATGATAACATGTAACTCAAAT---
ATTACAGGATTACTACTGACACGGGATGGAGGCAGCGAAGGCAAC----------------------------
----

AATACAGAGATATTCAGGCCTGGAGGAGGAGATATGAGGGACAATTGGAGAAGTGAATTATATAAATATAAAG
TAGTAGAAATTAAACCATTA---GGAATAGCACCCACCAAAGCAAAAAGGAGAGTGGTG--------------
-------------------------GAGAGAGAAAAAAGAGCA---GTGGGA---ATAGGAGCT---------
GTGTTCCTTGGGTTCTTGGGAGTA---GCAGGAAGCACTATG---GGCGCGGCGTCG---
CTGACGTTGACGGTACAG---GCCAGACAA---TTATTG---TCTGGTATAGTGCAACAGCAAAGC---
AATTTG---CTGAGA---GCTATAGAGGCGCAACAACATATGTTGCAACTCACAGTCTGGGGCATT
>AY713417
TTCTATTGCAATACATCAAAACTGTTTAATACAACAGGACTGTCT----------------------------
----

GATAATGCAACTTTAACCATCCCATGCAGAATAAAGCAAATTATAAACATGTGGCAGGAGGTAGGACGAGCAA
TGTATGCCCCTCCCATTAAAGGAATAATAACATGTAGATCAAAT---
ATCACAGGATTATTACTGACACGTGATGGGGGGATGAACAACACA----------------------------
----

AACAACACAGAGACATTCAGACCTGGAGGAGGAGATATGAGGGACAATTGGAGAAGTGAATTATATAAGTATA
AAGTGGTAGAAATTAAGCCATTA---GGAGTAGCACCCACTGGGGCAAAAAGGGCAGTGGTG-----------
----------------------------GGGAGAGAAAAAAGAGCA---GCA------CTAGGAGCT------
---TTGTTCCTTGGGTTCTTGGGAGCG---GCAGGAAGCACTATG---GGCGCAGCGTCA---
ATAACGCTGACGGTACAG---GCCAGACAA---TTATTG---TCGGGTATAGTGCAGCAGCAAAGC---
AATTTG---CTGAGA---GCTATAGAGGCGCAACAGCATATGTTGCAACTCACGGTCTGGGGCATT
>KU319531
TTCTACTGCAATCCATCAGGACTGTTTAATAGGACATACATGTTTAATAGTACAGACGTGTTTAATAGTAATA
GTACAGACAGG---TCT------AAT---
-------------------AGT---
ACTAATAGTACTTCCATCACACTCCAATGCAGAATAAAGCAAATTATAAACCTGTGGCAGGGGGTAGGAAAAG
CAATATATGCCCCTCCCATTGCAGGGAAAATAACATGTAAATCAAAC---
ATTACAGGAATACTACTGACACGAGATGGAGGAAGTAATAACAGT----------------------------
----

AGCACAGAGACATTCAGACCTGGAGGAGGAGATATGAGGGACAATTGGAGAAGTGAACTATATAAATATAAAG
TGGTAGAAATTAAACCATTA---GGAATAGTACCCACCGAGGCACAAAGAAGAGTGGTG--------------
-------------------------GGGAGAGAAAAAAGAGCA---ATAGGA---ATAGGAGCT---------
ATGTTCCTTGGGTTCTTGGGAGCA---GCAGGAAGCACTATG---GGCGCAGCGTCA---
ATAGCGCTGACGGTACAG---GCCAGACAA---TTATTG---TCGGGTATAGTGCAACAGCAAAAT---
AACTTG---CTGAGG---GCTATAGAGGCGCAACAGCATATGTTGCAACTCACAGTCTGGGGCATC
>KU319532
TTTTACTGTGATACAACAAACCTGTTTAATAGTACATACAATGGTACTAGTACTAATAGTACT----------
----
-------------TCCAGT------
GGTACCATCACCCTCTCATGCAGTATAAAACAAATTATACAGATGTGGCAAGGATTACGAAAAGTATTGAATG
CCCCTGTCTGCGCTCCCCTTGACGGAAACATAAAATGTACATCAAAT---
TTCACAGGACTACTATTGCCACGTGCTGGAGGAAGTGGACTTAAT----------------------------
----

GATACTGACGCAGAAGTATTCAGACCAGCAGGAGGAGACATGAGGGACAATTGGAGAAGTGAATTATATAAAT
ATAAAGTGGTAGAAATTAAACCATTA---GGAGTAGCACCAACTACAGCAAAAAGGAGAGTGGTG--------
-------------------------------GAGAGAGAAAAAAGAGCA---GTGGGA---ATAGGAGCT---
------GTGTTCCTTGGGTTCTTGGGAGCA---GCAGGAAGCACTATG---GGCGCAGCATCA---
ATAACGCTGACGGTACAG---GCCAGACAA---TTATTG---TCTGGTATAGTGCAACAGCAAAGC---
AATTTG---CTGAGG---GCTATAGAGGCGCAACAGCATCTGTTGCAACTCACAGTCTGGGGCATT
>KU319534
TTCTATTGGGATACAACAAAACTGTTTAATGAGACATATAATAATACAGAT----------------------
----

GCAAATGGCACCATCATAATGCCATGCAGAATAAAACAAATTATAAACATGTGGCAGAAGGTAGGAAGAGCAA
TGTATGCCCCTCCCATTGAAGGACCAATAACATGTATATCAAAC---
ATCACAGGACTACTACTAACACATGATGGAGAGGAA-------------------------------------
----

AATGACACAGAGACATTCAGACCTTGGGGAGGAGATATGAGGGACAATTGGAGAAGTGAATTATATAAATACA
AAGTGGTAGAAATTAAGCCATTA---GGAATAGCACCCACTAAGGCAAAAAGGAGAGTGGTG-----------
----------------------------CAGAGAGAAAAAAGAGCA---GCA------CTAGGAGCT------
---ATGTTCCTTGGGTTCTTGGGAGCA---GCAGGAAGCACTATG---GGCGCAGCATCA---
ATAACGCTGACGGTACAG---GCCAGACAA---TTATTG---TCTGGTATAGTGCAACAGCAAAGC---
AATTTG---CTGAGA---GCTATAGAGGCGCAACAGCATCTGTTGCAACTCACGGTCTGGGGCATT
>KU319537
TTTTATTGCAATACATCACACTTGTTTAAGGATAATATTAGT-------------------------------
----

AGCACGGGAAACATCACAATCCCATGCAGAATAAGACAAATTATAAACTTGTGGCAGGAAGTAGGACGAGCAA
TGTATGCTCCTCCCATTGAAGGACTAATAAAATGTAGATCAAAT---
ATCACAGGACTATTGCTGACACGTGATGGAGGCAATAGACAAAAT----------------------------
----

GGCACAGAGATATTCAGACCAGGAGGAGGAGATATGAGGGACAATTGGAGAAGTGAATTATATAAATATAAAG
TAGTAGAAATTAAACCATTA---GGAATAGCACCCACTAAAGCAAAAAGGAAAGTGGTG--------------
-------------------------AAGAGAGAAAAAAGAGCA---GCA------ATAGGAGCT---------
GTGTTCCTTGGGTTCTTGGGAGCA---GCTGGAAGCACTATG---GGCGCAGCATCA---
ATAACGCTGACGGTACAG---GCCAGACAA---TTGTTG---TCTGGTATAGTGCAACAGCAAAGC---
AATTTG---CTGAGG---GCTATAGAGGCGCAACAGCATATGTTGCAACTCACAGTCTGGGGCATT
>KU319541
TTCTATTGCAATACATCAGGCCTGTTTAATAATAGTATG----------------------------------
----
-ACAAATGGCACCCTC---------
AGACTCCCATGCAGAATAAAACAAATTATAAACATGTGGCAGGGAGTAGGACGAGCAATGTATGCTCCTCCCA
TTAGCGGAGCAATAAGTAGGAAGGCCATAGTATCATGCTTATCAAAT---
ATCACAGGGATACTCTTAACATGTGATGGAGGCATTAATAGGCACAGA-------------------------
----

CATAACACAGAGACATTCAGACCTGTAGGAGGAGACATGAAGGACAATTGTAGAAGTGAATTATATAAATATA
AGGTGGTAGAAGTTAAAGCCATA---GGAATAGCACCCACACAGGCAAAAAGGAGAGTGGTG-----------
----------------------------CAGAGAGAAAAAAGAGCA---GCA------TTAGGAGTT------
---TTATTCTTTGGGTTCTTGGGAGCA---GCCGGAAGCATTATG---GGCGCAGCATCA---
ATATTTTTTTTGGTACAG---GCCAGACAA---TTATTG---TCTGGTATAGTGCAACAGCAGAGC---
AATTTG---CTGAGG---GCTATAGAGGCGCAGCAGCATCTGTTGCAACTCACGGTCTGGGGCATT
>KU319544
TTCTATTGCAATACAACAGGACTGTTTAACAGTACGTACAATAGTACACACATATTTAATAATACT-------
----

TCAAATGCAACCATCACACTCCCCTGCAGAATAAAACAAATTATCAACATGTGGCAGGGGGTAGGACGAGCAA
TGTATGCCCCTCCCACTGCAGGAAACATAACATGTAGATCAAAT---
ATCACAGGGCTACTATTAACACGTGATGGAGGAAGCGACAGCGACACG-------------------------
----

ACTGAGGAGACATTCAGACCTGGAGGAGGAAATATGAGAGACAATTGGAGAAGTGAATTATATAAATATAAAG
TGGTAGAAATTAAACCATTA---GGAGTAGTACCCACTACTGCAAAAAGGAGAGTGGTG--------------
-------------------------GAAAGAGAAAAAAGAGCA---GTGGGA---ATAGGAGCT---------
GTGTTCCTTGGGTTCTTGGGAGCA---GCAGGAAGCACTATG---GGCGCAGCATCA---
ATAACGCTGACGGTACAG---GCCAGACAA---TTGCTG---TCTGGTATAGTGCAACAGCAAAGC---
AATTTG---CTGAGG---GCTATAGAGGCGCAACAGCATATGTTGCAACTCACGGTTTGGGGCATT
>KU319546
TTCTATTGCAATACATCAAAACTGTTTAATGGTACATACAACTCTAATAGTACTAATAGTACT----------
----

TCAAATTCAAGCATCACAATCCAATGCAGAATAAAACAAATTATAAACATGTGGCAGGAGGTAGGACGAGCAA
TGTATGCCCCTCCCATTGCAGGAAACATAACATGTAGATCAAAT---
ATCACAGGAATACTATTGACATGGGATGGAGGAAACCAAAACGGAACC-------------------------
----

AACAATACAGAGACATTCAGACCTGGAGGAGGAGATATGAGGGACAATTGGAGAAGTGAATTATATAAATATA
AAGTGGTAGAAATTAAACCATTA---GGAGTAGCACCCACTAAGGCAAAGAGGACAGTGGTG-----------
----------------------------GAAAGAGAAAAAAGAGCA---GTGGGA---ATAGGAGCT------
---GTGTTCCTTGGGTTCTTGGGAGCA---GCAGGAAGCACTATG---GGCGCAGCATCA---
ATAACGCTGACGGTACAG---GCCAAACTA---TTGTTG---GCTGGTATAGTGCAGCAGCAAAAT---
AATTTG---CTGAGG---GCTATAGAGGCGCAACAACATATGTTGCAACTCACGGTCTGGGGCATT
>KU319551
TTCTACTGTAGTACTACACCCTTGTTTACTAGTACTAGTACTACTGGGGTACTACTGATTCAAATTCA-----
----

TACATCACAGTCATCACTCCCCCATGCAGTATACTACTATGTCGTAACATGTGGCAGGAGGTAGGACGAGCCA
TATATGCTCCTCCTATTAAAGGAGTAATAACATGTAGATCAAAT---
ATCACAGGACTACTGCTGACACGGGATGGCGGCCATAGCACAAAT----------------------------
----

GACACAGAGATATTCAGACCTATAGGAGGAAATATGAAGGACAATTGGAGAAGTGAATTATATAAATATAAAG
TGGTAGAAGTTAAGCCATTA---GGAGTAGCACCCACTGAGGCAAGAAGGAGAGTGGTG--------------
-------------------------GAGAGAGAAAAAAGAGCA---GCA------CTAGGAGCT---------
TTGTTCCTTGGGTTCTTGGGAGCA---GCAGGAAGCACTATG---GGCGCAGCGTCA---
ATAACGCTGACGGTACAG---GCCAGACAA---TTACTG---TCGGGTATAGTGCAACAGCAAAGC---
AATTTG---CTGAGA---GCTATTGAGGCGCAACAACATATGTTGCAGCTCACGGTCTGGGGCATT
>U46016
TTCTATTGCAATACATCAAACCTGTTTAATAGCACAAAACTAGAACTGTTTAATAGCAGT-------------
----

ACAAATTTAAACATCACACTCCAATGCAGAATAAAACAAATTATAAACATGTGGCAGGGAGTAGGACGAGCAA
TGTATGCCCCTCCCATTGAAGGAATAATAATGTGTAGATCAAAT---
ATCACAGGACTACTACTGACACGTGATGGAGCCAAAGAGCCACAT----------------------------
----

AGCACAAAAGAGATATTCAGACCTGAAGGAGGAGATATGAGGGACAATTGGAGAAGTGAATTATATAAATATA
AAGTGGTAGAAATTAAGCCACTA---GGAGTAGCACCCACTAAGCCAAAAAGGAGAGTGGTG-----------
----------------------------GAGAGAGAAAAAAGAGCA---GCA------CTAGGAGCT------
---TTGTTCCTTGGGTTCTTGGGAGCA---GCAGGAAGCACTATG---GGCGCAGCATCA---
ATAACGCTGACGGTACAG---GCCAGACAA---TTATTG---TCGGGTATAGTGCAACAGCAAAGC---
AATTTG---CTGAAA---GCTATAGAGGCGCAACAGCATATGTTGCAGCTCACGGTCTGGGGCATT
>AF219262
TTCTATTGCAATACAACAGACCTATTTAATAGCACAAAACTGTTTAATAGCAGTAGT----------------
----

ACAAATTCAACCATCACACTTCCATGCAGAATAAAACAAATTATAAACATGTGGCAGGGGGTAGGACGAGCAA
TGTATGCCCCTCCCATTAAAGGAATAATAACATGTAGATCAAAT---
ATCACAGGAATACTACTGACACGTGATGGAGGGAATGACAATAGCACA-------------------------
----

AATGGCACAGAGACATTCAGACCTGGAGGAGGAGATATGAGAGACAATTGGAGAAGTGAATTATATAAATATA
AAGTGGTAGAGATTAAGCCATTA---GGAATAGCACCCACTGGGGCAAAAAGGCGAGTGGTG-----------
----------------------------GAGAGAGAAAAAAGAGCA---GCA------CTAGGAGCT------
---ATGTTCCTTGGGTTCTTGGGAGCA---GCTGGAAGCACTATG---GGCGCAGCATCA---
ATAACGCTGACGGTACAG---GCCAGACAA---TTATTG---TCGGGTATAGTGCAACAGCAAAAT---
AATTTG---CTGAGG---GCTATAGAGGCGCAACAGCATATGTTGCAACTCACGGTCTGGGGCATT
>AF219263
TTCTATTGCAATACATCAAAATTGTCTAATAGGACATACAATGCATCAGACCTGGTTAATAGTACA-------
----

AGTAATTCAACCATCACACTCCCATGCAGAATAAAGCAAATTGTAAACATGTGGCAGGAAGTGGGACGAGCAA
TGTATGCCCCTCCCATTGCAGGAAACATAACATGTAACTCAAAT---
ATCACAGGACTACTATTGACACGTGATGGAGGAAATAATGCAAATAAT-------------------------
----

GCAAGTACCAACGAGACATTCAGACCTGGAGGAGGAGATATGAGGGACAATTGGAGAAGTGAATTGTATAAAT
ATAAAGTGGTAGAAATTAAGCCATTG---GGAGTAGCACCCACCAAGGCAAAAAGGAGAGTGGTG--------
-------------------------------GGGAGAGAGAAAAGAGCA---GTGGGA---CTAGGAGCT---
------GCGTTCCTTGGGTTCTTAGGAGCA---GCAGGAAGCACTATG---GGCGCGGCGTCA---
ATAACGCTGACGGCACAG---GCCAGGCAA---GTGTTG---TCTGGTACAGTGCAACAGCAAAGC---
AATTTG---CTGAGG---GCTATAGAGGCGCAACAGCATATGTTGCAACTCACAGTCTGGGGCATT
>AF219269
TTCTATTGCAATACATCAAAACTGTTTAAGGGTACATACATGTATAATGATACAGAT----------------
----

ACAAACATCACACTCACACTCCCATGCAGAATAAAACAGATTATAAACATGTGGCAGGGGGTAGGACGAGCAA
TATATGCCCCTCCCATTGAAGGAAGCATAAAGTGTACATCGAAT---
ATCACAGGACTACTATTGACACGTGATGGA---GACAAAAATGAT----------------------------
----

ACAGAGACCTTCAGACCTGGAGGAGGAGATATGAGGGACAATTGGAGAAGTGAATTATATAAATATAAAGTGG
TAGAAATTAAGCCATTA---GGAATAGCACCCGCTAAGGCAAAAAGGAGAGTGGTG-----------------
----------------------GAGAGAGAAAAAAGAGCA---GTAGGA---ATGGGAGCT---------
GTGCTCCTTGGGTTCTTGGGAGCA---GCAGGAAGCACTATG---GGCGCAGCGTCA---
ATAACGCTGACGGTACAG---GCCAGGCAA---TTGTTG---TCTGGTATAGTGCAACAGCAAAGC---
AATTTG---CTGAGA---GCTATAGAGGCGCAACAGCATATGTTGCAACTCATGGTCTGGGGCATT
>AF219274
TTCTATTGCAATACATCAAAACTGTTTAATAGTACATATATGTTTAATAGTACATATACGTCTAATAATACAG
GAAGT--

AATTCCAACTCAACCATCACACTCCCATGCAGAATAAGACAATTTATAAACATGTGGCAGGAGGTAGGACGAG
CAATGTATGCCCCTCCCATTGAAGGAAACATAACATGTAAATCAAAT---
ATCACAGGACTACTATTGACACGGATGGAGGCGTGGAGACACAAGATACCACA--------------------
----
-
GAGACACAAAATAACACAGAGACATTCAGACCTGGGGGAGGAGATATGAGGAACAATTGGAGAAGTGAATTAT
ATAAATATAAAGTGGTAGAAATTAAGCCATTG---GGAGTAGCACCTACTACGGCAAAAAGGAGAGTGGTG--
-------------------------------------GAGAGAGAAAAAAGAGCA---GTGGGA---
ATAGGAGCT---------GTGACCCTTGGGTTCTTGGGAGCA---GCAGGAAGCACTATG---
GGCGCGGCGTCA---GCAGCGCTGACGGTACAG---GCCAGACAA---TTGTTG---
TCTGGTATAGTGCAACAGCAAAGC---AATTTG---CTGAGG---
GCTATAGAGGCGCAACAGCATATGTTGCAACTCACAGTCTGGGGCATT
>FJ977094
TTCTATTGCAATACATCAGGCCTGTTTAATGGTACATTCAAGTTTAATGATACAGACATGACTAAT-------
----

GATACAGGAAACATCACACTTCCATGCAGAATAAAACAAATTATAAACATGTGGCAGGAGGTAGGACGAGCAA
TGTATGCCCCTCCCATTGCAGGAAACATAACATGTAAATCAAAT---
ATCACAGGACTACTATTGCTACGGATGGAGGGACAAATGTAACA-----------------------------
----

AATGAAACAGAAATATTCAGACCTGGAGGAGGAGATATGAGGGACAATTGGAGAAGTGAATTATATAAATATA
AAGTGGTTGAAATTAAGCCATTG---GGAATAGCACCCACTAAGGCAAAAAGGAGAGTGGTG-----------
----------------------------GAAAGACAAAAAAGAGCA---GCA------ATAGGAGCT------
---ATGATCATTGGGTTCTTGGGAGCA---GCAGGAAGCACTATG---GGCGCAGCGTCA---
ATAGCGCTGACGGCACAG---GCCAGACAA---GTATTG---TCTGGTATAGTGCAACAGCAAAGC---
AATTTG---CTGAGA---GCTATAGAGGCGCAACAGCATATGTTGCAACTCACAGTCTGGGGCATT
>HQ595789
TTCTATTGCAATACATCAGGCCTGTTTAACAGTAAATACATGCATAACATGACAATCAATGGGACAGACATGA
CAAGCAATGGG--

-------------ACAGAC------
AGTAATGCAACAATCATCACACTTCCATGCAGAATAAAACAAATTATAAACATGTGGCAGGAGGTAGGACGAG
CAATGTATGCCCCTCCCATTGCAGGAAACATAACATGTAACTCAAGC---
ATTACAGGACTACTATTAGTACGTGATGGAGGCACTGGAGACAGCAGC-------------------------
----

AGCAATGACACAGAGACTTTCAGACCTGGAGGAGGAAATATGAAGGACAATTGGAGAAGTGAATTATATAAAT
ATAAAGTGGTAGAAATCAAGCCATTA---GGAATAGCACCCACTGGAGCAAAAAGGAGAGTGGTG--------
-------------------------------GAGAGAGAAAAAAGAGCA---GTGGGA---ATGGGAGCT---
------TTGTTCCTTGGGTTCTTGGGAGCA---GCAGGAAGCACTATG---GGCGCAGCGTCA---
CTAACGCTGACGGTACAG---GCCAGACAA---GTATTG---TCTGGTATAGTGCAACAGCAAAGC---
AATTTG---CTGAAG---GCTATAGAGGCGCAACAGCATATGTTGCAACTCACGGTCTGGGGCATT
>JF680927
TTCTATTGCAATACATCAGGGATATTTAATAGTACACAGCTGTTTAATGATACATTAAATAATAGTACAGGA-
----

AATAATGAGACCATCACACTCCCATGCAGAATAAAACAAATTGTAAACATGTGGCAGCAGGTAGGACGAGCAA
TGTATGCCCCGCCCATTGCAGGAAACCTAACATGTAACTCAAAC---
ATCACAGGACTGCTATTGACACGTGATGGAGGAAAAGATAACACAACA-------------------------
----

GATAACAATACAGAGATATTCAGACCTGCAGGAGGAGATATGAGGGATAATTGGAGAAGTGAATTATATAAAT
ATAAAGTGGTAGAAATTAAGCCATTG---GGAATAGCTCCCACTGGGGCAAAAAGGAGAGTGGTG--------
-------------------------------GAGAGACCAAAAAGAGCA---GTGGGA---ATGGGAGCT---
------GTGTTCCTTGGGTTCTTGTCAGCT---GCAGGAAGCACTATG---GGCGCAGCGTCG---
ATGACGCTGACGGTACAA---GCCAGACAA---TTGCTG---TCTGGTATAGTGCAACAGCAAAGC---
AATTTG---CTGAGA---GCTATAGAGGCGCAACAACATTTGTTGCAACTCACAGTCTGGGGCATA
>HQ385458
TTCTATTGCAATACATCAAAACTGTTTAATAATAGTACAAATAGTACAGAA----------------------
----

GGAAATTCAACCATCACACTCCCATGCAGAATAAAACAAATTATAAACCTGTGGCAGGAGGTAGGAAAAGCAA
TATATGCCCCTCCCATTGCAGGAAACATAACATGTGAATCAAAT---
ATCACAGGACTACTCTTGGTACGAGATGGAGGCAATAGTACAAATAAT-------------------------
----

GATACAACAGAGATATTCAGACCAGGAGGAGGAAATATGAAGGACAATTGGAGAAGTGAACTATATAAATATA
AAGTAGTAGAAATTAGGCCATTG---GGGGTAGCACCCACCAAGGCAAAAAGGAGAGTGGTG-----------
----------------------------GAGAGAGAAAAAAGAGCA---GTGGGA---ATAGGAGCT------
---GTGTTCCTTGGGTTCTTGGGAGCA---GCAGGAAGCACTATG---GGCGCAGCGTCA---
ATAACGCTGACGGTACAG---GCCAGACAA---TTGTTG---TCTGGTATAGTGCAACAGCAAAGC---
AATTTG---CTGAGA---GCTATAGAGGCGCAACAGCATATGTTGCAACTCACGGTCTGGGGCATT
>AF286233
TTCTATTGCAATACATCAAAACTGTTTAATAGTACATACCTGTCTAATAGTACATCATATAATAGTACT----
----

TCAAATTCAACCATCATACTCCAATGCAGAATAAAACAAATTATAAACATGTGGCAGGAGGTAGGACGAGCAA
TGTATGCCCCTCCCATTGAGGGAAACATAACATGTAAATCAGAT---
ATCACAGGACTGCTACTGATACGTGATGGAGGCATAAATAATGACACA-------------------------
----

AACGAGACAGAGACATTCAGACCGGGGGGAGGAGATATGAGGGACAATTGGAGAAGTGAATTATATAAATACA
AAGTGGTAGAGATTAAGCCATTA---GGAATAGCACCCACTAAGGCAAAAAGGAGAGTGGTG-----------
----------------------------GAGAGAGAAAAAAGAGCA---GCA------CTAGGAGCT------
---ATGTTCCTTGGGTTCTTGGGAGCA---GCCGGAAGCACTATG---GGCGCAGCGTCA---
ATAACGCTGACGGTACAG---GCCAGGCAA---TTGTTG---TCTGGTATAGTGCAACAGCAAAAC---
AATTTG---CTGAGG---GCTATAGAGGCGCAACAGCATATGTTGCAACTCACGGTCTGGGGCATT
>AY255824
TTCTACTGCAATACATCAAACCTGTTTAATAGTACATACAGGTCTAATAGTACTAATAGTACT----------
----

TCAGATACATCCATCATACTCCCATGCAGAATAAAACAAATTATAAACATGTGGCAGGGGGTAGGACGAGCAA
TGTATGCCCCTCCCATTGAAGGAAACATAACATGTAAATCAAAT---
ATTACAGGACTACTATTGACACGTGATGGAGGAACGGACAATGAA----------------------------
----

ACAATAGAGACATTCAGACCTGGAGGAGGAGATATGAGGGATAATTGGAGAAGTGAATTATACAAATATAAAA
TAGTAGAAATTAAGCCATTA---GGAGTAGCTCCAACTGCGGCAAAAAGGAGAGTGGTG--------------
-------------------------GAGAGAGAAAAAAGAGCA---GCGGGA---ATAGGAGCT---------
TTGTTCCTTGGGCTCTTGGGAGCA---GCAGGAAGCACTATG---GGCGCAGCGTCA---
ATAACGCTGACGGTACAG---GCCAGGCAA---TTGTTG---TCTGGTATAGTGCAACAGCAAAGC---
AATTTG---CTGAGG---GCTATAGAGGCGCAACAGCATATGTTGCAACTCACAGTCTGGGGCATT
>KP109481
TTCTATTGCAATACATCAGGCCTGTTTAATAGTACATATATGCCTAATGGTACATATATGCCTAATGGTACAA
ATAAT--

TCAAACTCAACCATCATCCTCCCATGCAGAATAAAACAAATTATAAACATGTGGCAGGAGGTAGGACGAGCAA
TGTATGCCCCTCCCATTGCAGGAAACATAACATGTAACTCAAAT---
ATCACAGGACTACTATTGGTACGAGATGGAGGAAAGAACAATAAT----------------------------
----

ACAGAGATATTCAGACCTGGAGGAGGAGATATGAGGGACAATTGGAGAAGTGAATTATATAAATATAAAGTAG
TGGAGATTAAGCCATTG---GGAGTAGCACCCACTAGAGCAAAAAGGAGAGTGGTG-----------------
----------------------GAGAGAGAAAAAAGAGCA---GTGGGA---CTGGGAGCT---------
GTGTTCCTTGGGTTCTTGGGAGCA---GCAGGAAGCACTATG---GGCGCGGCGTCA---
ATAACGCTGACGGTACAG---GCCAGACAA---TTGTTG---TCTGGTATAGTGCAACAGCAAAGC---
AATTTG---CTGCAG---GCTATAGAGGCGCAACAGCATCTGTTGCAACTCACGGTCTGGGGCATT
>KP109482
TTCTATTGTAATACATCAGGCCTGTTTAATGGTACATATATGCCTACATACATGCCTAATAGTACAAACAGTA
AT---

TCAAGCTCAAACATCACAATCCCATGCAGAATAAAGCAAGTTATAAACATGTGGCAGGAGGTAGGAAGAGCAA
TGTATGCCCCTCCCATTGAAGGAGAAATAACATGTAAATCAAAT---
ATCACAGGACTACTATTGGTACGTGATGGAGGAAATGGAAATGATACA-------------------------
----

AATAAAACAGAGATATTCAGACCTGAAGGAGGAGATATGAGGGACAATTGGAGAAGTGAATTATATAAATATA
AAGTGGTAGAAATTAAGCCATTG---GGAATAGCACCCACTGAAGCAAAAAGGAGAGTGGTG-----------
----------------------------GAGAGAGAAAAAAGAGCA---GTGGGA---ATAGGAGCT------
---GTGATCCTTGGGTTCTTGGGAGCA---GCAGGAAGCACTATG---GGCGCGGCGTCA---
ATAACGCTGACGGCACAG---GCCAGACAA---TTGTTG---TCTGGTATAGTGCAACAGCAAAGC---
AATTTG---CTGAAG---GCTATAGATGCGCAACAGCATCTGTTGCAACTCACAGTCTGGGGCATT
>KP109484
TTCTATTGCAATACATCAAAACTGTTTAATGGTATATACAATGGTACACAAAGTAATTCAAGTAAT-------
----

TCAAACTCAACCATCATAATCCCATGCAAAATAAAACAAATTGTAAACATGTGGCAGAAGGTAGGACGGGCAA
TGTATGCCCCTCCCATTGCAGGAAACATAACATGTACATCAAAT---
ATCACAGGACTACTACTGGTACGTGATGGAGGACCAGATAATGTT----------------------------
----

ACAGAGATATTCAGACCTGGAGGAGGAGATATGAGGGACAATTGGAGAAGTGAATTATATAAATATAAAGTGG
TAGAAATTAAGCCATTG---GGAATAGCACCCACTGGGGCAAAAAGGAGAGTGGTG-----------------
----------------------GAGAGAGAAAAAAGAGCA---GTGGGA---TTAGGAGCT---------
GTGTTCCTTGGGTTCTTGGGAGCA---GCCGGAAGCACTATG---GGCGCAGCGTCA---
ATAACGCTGACGGTACAG---GCCAGACAA---TTGTTG---TCTGGTATAGTGCAACAGCAAAGC---
AATTTG---CTGAGG---GCTATCGAGGCGCAACAGCATATGTTGCAACTCACGGTCTGGGGCATT
>EU521729
TTCTATTGTAATACATCACGCTTGTTTAAGGGTACATACACGCCTAATGGTACAGGAAGTATATCAGAAAGTA
ATTCAAAT---

-------------------TCA---
ACCTCAAACATCAGCATCACAATCCCATGCAGAATAAAGCAAATTATAAACATGTGGCAGGAGGTAGGACGAG
CAATGTATGCCCCTCCCATTGAAGGAAGCATAAAATGTAACTCAACT---
ATCACAGGACTACTATTGGTACGTGATGGAGGACTAGGTAATGAGACA-------------------------
----

AAGACAGAAGAGAGATTCAGACCTGGAGGAGGAGATATGAGGGACAATTGGAGAAGTGAATTATATAAATATA
AAGTGGTAGAGATTAGGCCATTG---GGATTAGCACCCACTAAAGCAAAAAGGAGAGTGGTG-----------
----------------------------GAGAGAGAAAAAAGAGCA---GTAGTGGGAATAGGAGCT------
---ATGTTCCTTGGGCTCTTGAGTGCA---GCTGGAAGCACTATG---GGCGCGGCGGCA---
ATAACGCTGACGGTACAG---ACCAGACAA---TTGTTG---TCTGGTATAGTGCAACAGCAAAGC---
AATTTG---CTGATG---GCTATAGAGGCGCAACAGCATTTGTTGCAACTCACGGTCTGGGGCATT
>EF469243
TTCTATTGCAATACATCAGTACTGTTTAATAGTACATACAACCATACAACAAAGCAGTTTAGTAGTCCATACA
AT---

GATACAAACTCAACCATCACAATCCACTGCAAAATAAAGCAAATTATAAACATGTGGCAGGAGGTGGGGCGAG
CAATATATGCCCCTCCCATTGCAGGAAACATAACATGTAAATCCAAT---
ATCACAGGATTACTATTGGTACGAGATGGAGGAAATACTGAGTCA----------------------------
----

AATAATACAGAAATATTCAGACCTGGAGGAGGAGATATGAGGGACAATTGGAGAAGTGAATTGTACAAATATA
AAGTGGTAGAAGTTAAGCCATTG---GGAATAGCACCAACTGCAGCAAAAAGGAGAGTGGTG-----------
----------------------------GAAAGAGAAAAAAGAGCAGTATTGGGTGGAATAGGAGCT------
---GTGTTCCTTGGGTTCTTGGGAGCG---GCAGGAAGCACTATG---GGCGCGGCGTCA---
ATAACGCTGACGGTACAG---GCCAGACAA---TTATTG---TCTGGTATAGTGCAACAGCAGAGC---
AATCTG---CTGAGA---GCTATAGAGGCGCAACAACATATATTGCAACTCACGGTCTGGGGCATT
>KF766537
TTCTATTGTAATACATCAGGCCTGTTTAATAGTACATACAATGTGACAGACAATGGTACAGAAAGTACTTCA-
----

AACTCAACCACCATCACAATCACATGCAGAATAAAGCAAATTATAAATATGTGGCAGGGGGTAGGACGAGCAA
TATATGCCCCTCCCATTGCAGGAAACATAACATGTAATTCAAGC---
ATCACAGGATTACTATTGGAACGTGATGGAGGAGACGAGACAAAA----------------------------
----

AACGGTACAGAGATATTCAGACCTGGAGGAGGAGATATGAAGCACAATTGGAGAAGTGAATTATATAAATATA
AAGTAGTAGAAATTAAGCCATTG---GGAATAGCACCCACTGCAGCAAAAAGGAGAGTGGTG-----------
----------------------------GAGAGAGAAAAAAGAGCA---GTGGGA---CTAGGAGCT------
---GTGTTCCTTGGGTTCTTGGGAGCA---GCAGGAAGCACTATG---GGCGCAGCGTCA---
ATAACGCTGACGGTACAG---GCCAGACAA---TTGTTG---TCTGGTATAGTGCAACAGCAAAGC---
AATTTG---CTGAAG---GCTATAGAGGCGCAACAGCATCTGTTGCAACTCACGGTCTGGGGCATT
>KF766540
TTCTATTGCAATACAACACAACTGTTTAATAGTACATACATACCTAACATGTCTGATAATACAACAAGTAAT-
----

GAAAGCTCAGACATCACAATCCCATGTAGCATAAGACAATTTATAAACATGTGGCAGAGGGTAGGACGAGCAA
TGTATGCCCCTCCCATTTCAGGAAACATAACATGTAAATCAAAT---
ATAACAGGGCTACTATTGGAACGTGATGGAGGGGATGATAATAAG----------------------------
----

ACAGAGACATTCAGACCTGGAGGAGGAGATATGAGGGACAATTGGAGAAGTGAATTATATAAATGTAAAGTGG
TAGAAATTAAGCCATTA---GGAGTTGCACCCACTGGAGCAAAGAGGAGAGTGGTG-----------------
----------------------GAGAGAGAAAAAAGAGCA---GTGGGA---ATAGGAGCC---------
ATGTTCCTTGGGTTCTTGGGAGCA---GCAGGAAGCACTATG---GGCGCAGCGTCA---
ATAACGCTGACGGTACAG---GCCAGACAA---TTGTTG---TCTGGTATAGTGCAACAGCAAAGC---
AATTTG---CTGAGG---GCTATAGAGGCGCAACAACATCTGTTGCAACTCACAGTCTGGGGCATT
>EU908218
TTCTATTGTAATACGTCAGGCCTGTTTAATAGTACATACATGACTAATGGTACATTTACATACAAGCCT----
----

```
---------------------------
AATGGTACAAACATCACAATCCCATGCAGAATAAAGCAAATTATAAACATGTGGCAGGAGGTAGGACGAGCAA
TGTATGCCCCTCCCATTGCAGGAAACATAACATGTAAATCAAAT---
ATCACAGGAGTGATATTGGTACGTGATGGAGGGGAAAATGAAAAT-------------------------
----------------------------------------------------------------------
----------
ATTACAGAAGAGACATTTAGACCTGGAGGAGGAAACATGAGGGACAATTGGAGAAGTGAATTATATAAATATA
AAGTAGTAGAAATTAAGCCATTG---GGAGTAGCACCCACTAAAGCAAAAAGGAGAGTAGTG----------
---------------------------GAGAGAGAAAAAAGAGCA---GTAGGA---CTAGGAGCT------
---ATGTTCCTTGGGTTCTTGGGAGCA---GCAGGAAGCACTATG---GGCGCGGCGTCA---
ATAACGCTGACGGTACAA---GCCAGACAA---TTGTTG---TCTGGTATAGTGCAGCAGCAAAGA---
ATTTTG---CTGCAG---GCTATAGAGGCGCAACAGCATCTGTTGCAACTCACGGTCTGGGGCATT
>EU521727
TTCTATTGCAACACATCAATCCTGTTTAATGGTACATACAACCCTAATGGTACAAAAAGTAAT---------
----------------------------------------------------------------------
---------------------------
TCAAGTAATCCCATCACAGTCCCATGCCGCATAAAACAAATTATAAACATGTGGCAGGAGGTAGGACGAGCAA
TGTATGCTCCTCCCATTGAAGGAAACATAACATGCAAATCAAAT---
ATCACAGGACTGCTATTGGTACGTGATGGAGGAGAAGAGTCAAATAAA----------------------
----------------------------------------------------------------------
----------
ACTAATACAGAGACATTCAGACCTGGAGGAGGAGATATGAGGGACAATTGGAGAAGTGAATTATATAAATACA
AAGTAGTAGAAATTAAGCCATTA---GGAATAGCACCCACTGAAGCAAAAAGGAGAGTGGTG----------
---------------------------GAGAGAGAAAAAAGAGCA---GTGGGA---ATAGGAGCT------
---GTGTTCCTTGGATTCTTGGGAGCA---GCAGGAAGCACTATG---GGCGCGGCGTCA---
ATAACGCTGACGGTACAG---GCCAGACAA---TTGCTG---TCTGGTATAGTGCAACAGCAAAGC---
AATTTG---CTGAGG---GCTATAGAGGCGCAACAGCATATGTTGCAACTCACGGTCTGGGGCATT
>KC156210
TTCTATTGTAATACATCAGGCCTGTTTAATAGGACATATACAACAGACCTGTTTAATAAGACATATAATGATA
ATGATACA--------------------------------------------------------------
-------------GAAGAT------
AATTCAGGCCCAGTCATCATAATCCCATGTAGAATAAAGCAAATTATAAACATGTGGCAGGAGGTAGGACGAG
CAATGTATGCCCCTCCCATTGCAGGAAACATAACATGTAACTCAAGC---
ATCACAGGTCTGCTATTGACACGGGATGGAGGCAATGACAATAAAACGTCA-------------------
----------------------------------------------------------------------
-------------
ACGAATGAGACATTCAGACCTCTAGGAGGAAATATGAGGGACAATTGGAGAAATGAATTATATAAGTATAAAG
TGGTAGAAATTAAGCCACTA---GGAATAGCACCCACTACAGCAAAAAGGAGAGTGGTG-------------
------------------------GAGAGAGAAAAAAGAGCA---GTGGGAATGATGGGAGCT---------
ATGTTCCTTGGGTTCTTGGGAGCT---GCAGGAAGCACTATG---GGCGCGGCGTCA---
ATAACGCTGACGGTACAG---GCCAGGCAA---TTGTTG---TCTGGTATAGTGCAACAGCAAAGC---
AATTTG---CTGAGG---GCTATAGAGGCGCAACAGCATCTGTTGCAACTCACGGTCTGGGGCATT
>KY929361
TTCTATTGCAATACATCAAGTCTATTTAATAGCACATACATTAATGGCACATACATTAATGGCACAGTAAGTA
ATTCAGGC--------------------------------------------------------------
---------------------------
TCAGACTCAAACATCACAATCCCATGTAGAATAAAACAAATTGTAAGAATGTGGCAGCAGGTAGGACAAGCAA
TATATGCCCCTCCTATTGAAGGAAACATAACATGTAAATCAAAT---
ATCACAGGACTACTCTTAGTACGTGATGGAACAATTACAGGTGAGGCA----------------------
----------------------------------------------------------------------
----------
AATGATACAGAGACATTCAGACCTGGAGGAGGAGAGATGAGGGATAATTGGAGAAGTGAATTATATAAATATA
AAGTGGTAGAAATTAAACCATTA---GGAATAGCACCCACTGGATCAAAAAGGAGAGTGGTG----------
---------------------------GAGAGAGAGAAAAGAGCA---GCA------GTGTCAGCT------
---ATGCTCTTTGGGTTCTTGGGATTG---GCAGGAAGCACTATG---GGCGCGGCGTCA---
ATATCGCTGACGGTACAG---GCCAGACAA---TTGTTG---TCTGGTATAGTGCAACAGCAAAGC---
AATTTG---CTGAGA---GCTATAGAGGCGCAACAGCATCTGTTGCAACTCACAGTCTGGGGCATT
>KY929362
TTCTATTGCAATACATCAAAACTGTTTAATAGAAAATACCTGCCTGATGGTACAGACAACACTACGGATACAG
ATGCA-----------------------------------------------------------------
```

AACAATACAGACATTACAATCCCATGTAGGCTAAAACAATTTATAAACATGTGGCAGGGGGTAGGGAGAGCGA
TGTATGCCCCTCCCATTGCAGGAAACATAACGTGTATATCAAAT---
ATCACAGGGATACTATTGACACGTGATGGAGGAAATAATAATAAT----------------------------
----

AGTAATAATGAGACATTCAGACCGGGAGGAGGAGATATGAGGGACAATTGGAGAAGTGAATTATATAAATATA
AAGTGGTAGAAATTAAACCATTG---GGAATAGCACCCACTACAGCAAAAAGGAGAGTGGTG-----------
----------------------------GAGAGAGAAAAAAGAGCA---GTGGGA---CTAGGAGCT------
---ATGTTCCTTGGGTTCTTGGGAGCA---GCAGGAAGCACTATG---GGCGCGGCGTCA---
ATAACGCTGACGGTACAG---GCCAGACAA---TTGTTG---TCTGGTATAGTGCAACAGCAAAGC---
AATTTG---CTGAGA---GCTATAGAGGCGCAACAGCATCTGTTGCAACTCACAGTCTGGGGCATT
>KY929366
TTCTATTGCAATACATCAAGCCTGTTTAATGCTACATACAAAAATGGTACATTCATTAATAATACCACC----
----

CTCAAATCAAACATGACCATCCCCTGCAGAATAAGACAATTTATAAACCTGTGGCAGGAGGTAGGAAAAGCAA
TGTATGCCCCTCCCATTGCAGGGAACATAACATGTAGATCAAAT---
ATCACAGGAATACTATTGACAAGTGATGGAGGAATAAGAAATGACACA-------------------------
----

AATAATAATGAGACATTCAGACCTGCAGGAGGAGATATGAGGGACAATTGGAGAAGTGAATTATACAAATATA
AAGTGGTAGAAATTCGGCCATTG---GGAGTAGCACCCACTACAGCAAAAAGGAGAGTGGTG-----------
----------------------------GAGAGAGAAAAAAGAGCA---GTGGGA---CTAGGAGCT------
---ATGTTCCTTGGGTTCTTGGGAACA---GCCGGAAGCACTATG---GGCGCAGCGTCA---
ATGGCGCTGACGGTACAG---GCCAGACAA---TTGTTG---TCTGGTATAGTGCAACAGCAAAGC---
AATTTG---CTGAGG---GCTATAGAGGCGCAACAACACATGTTGCAACTCACGGTCTGGGGCATT
>KY929368
TTCTATTGTAATACATCAGGCCTGTTTAATAGTACATACATTACATACATTAATGGTAACATGTCTATATACA
ATGGTAGT---
-------------GGAAAC------
TCCAATAACACAAACATCACACTCCCATGCCGCCTAAAGCAAATTATAAACATGTGGCAGAGGGTAGGACGAG
CAATGTATGCCCCTCCCATTCCAGGAAACATAACATGTAACTCAACT---
ATCACAGGACTACTATTGGTACGTGATGGAGGAAACGATAATAGAACA-------------------------
----

AATGGGACAGAGATATTTAGACCTGGAGGAGGAGATATGAGGGACAATTGGAGAAGTGAACTATATAAATATA
AAGTGGTAGAAATTAAACCATTG---GGACTAGCACCCACTAAAGCAAAAAGGAGAGTGGTG-----------
----------------------------GAGAGAGAAAAAAGAGCA---GTGGGA---ATAGGAGCT------
---TTGCTCTTTGGGTTCTTGGGAATG---GCAGGAAGCACTATG---GGCGCGGCGTCA---
ATAACGCTGACGGTACAG---GCCAGAGAC---ATATTG---TCTGGTATAGTGCAACAGCAAAGT---
AATTTG---CTGAGA---GCTATAGAGGCGCAACAGCATCTGTTGCAACTCACGGTCTGGGGCATT
>KX069227
TTCTATTGCAATACATCAGGTCTATTTAATGGTACATACAATGGTACAGAAAGTGAT----------------
----

TTAAACTCAACCATCACAATCCCATGCAAAATAAAACAAATTATAAACATGTGGCAGGAGGTAGGAAGAGCAA
TGTATGCCCCTCCTATTGAAGGAAACATAACATGTAAATCAAAT---
ATCACAGGACTACTATTAGAACGTGATGGAGGACAGGGAAATGGGGCAGAA----------------------
----

AATGATACAGAGATATTCAGACCTGGAGGAGGAGACATGAGGGATAATTGGAGAAGTGAATTATATAAATATA
AAGTGGTAGAAATTAAACCATTA---GGAGTAGCACCCACTGGATCAAAAAGGAGAGTGGTG-----------
----------------------------GAGAGAGAAAAAAGAGCA---GCA------GTGTCAGCT------
---ATGCTCTTTGGGTTCTTGGGATTG---GCAGGAAGCACTATG---GGCGCGGCGTCA---
ATATCGCTGACGGTACAG---GCCAGACAA---TTGTTG---TCTGGTATAGTGCAACAGCAAAGC---
AATTTG---CTGAGA---GCTATAGAGGCGCAACAGCATCTGTTGCAACTCACAGTCTGGGGCATT
>KX069228
TTCTATTGCAATACAACAAAGTTGTTTAATAGTACATACAGTACATACAATGATACAAAAAATTCA------
----

ACAAACTCAACCATCACAATCCCATGCAGAATAAAACAAATTATAAACATGTGGCAGGAGGTAGGACGAGCAA
TGTATGCCCCTCCCATTGCAGGATACTTAACATGTAAGTCAAAT---
ATCACAGGACTACTATTGACACGTGATGGAGGAATAGAGGATAAGAATACT----------------------
----
AGTACAGGTAGTAATGACACA---------
AAGACAGAGACATTCAGACCTATAGGAGGAGATATGAGGGACAATTGGAGAAGTGAATTATATAAATACAAAG
TAGTAGAAGTTAAGCCATTG---GGAATAGCACCCACTGAAGCAAAAAGGAGAGTGGTG--------------
-------------------------GAGAGAGGAAAAAGAGCA---GTAACA---ATGGGAGCT---------
ATGTTCCTTGGGTTCTTGGGAGCA---GCAGGAAGCACTATG---GGCGTGGCGTCA---
ATGGCGCTGACGGTACAG---GCCAGACAA---TTGTTG---TCTGGTATAGTGCAACAGCAAAGC---
AATTTG---CTGAGA---GCTATAGAGGCGCAACAGCATCTGTTGCAACTCACAGTCTGGGGCATT
>KX069222
TTCTATTGTAATACATCAAAACTGTTTAATAGTACATACCTGTCTAATGGCACATACATGCCTAATGAGGCAC
TA---

AATAGTACTTCCATCACGATCCCATGCAGAATAAAGCAAATTATAAACATGTGGCAGGAGATAGGACGAGCAA
TGTATGCCCCTCCCATTGCAGGAAACATAACATGTACATCAAAT---
ATCACAGGACTACTATTGGTACGTGATGGGGGAAAAGAGAAAAAT----------------------------
----

GAAACAGAAATATTCAGACCGGGAGGAGGAAACATGAAGGACAATTGGAGAAGTGAATTATATAAATATAAAG
TGGTAGAGATTAAACCATTG---GGAGTAGCACCCACTGGAGCAACAAGGAGAGTGGTG--------------
-------------------------AAGAGAGAGAAAAGAGCA---GCC------ATAGGAGCT---------
GTGTTCCTTGGGTTCTTGGGAGCA---GCAGGAAGCACTATG---GGCGCGGCGTCA---
ATGGCGCTGACGGTACAG---GCCAGACAA---TTGTTG---TCTGGTATAGTGCAACAGCAAAGC---
AATTTG---CTGAGG---GCTATAGAGGCGCAACAGCATCTGTTGCAACTCACGGTCTGGGGCATT
>KX756601
TTCTATTGTAATACATCAAGCCTGTTTAATAGGACATACATATTTAATGAGACAACAAGTAATTCAAGCTCA-
----

AATAGTTCACACATCACAATCCAATGCAGAATAAAGCAAATTATAAACATGTGGCAGGAGGTAGGACGAGCAA
TGTATGCCCCTCCCATTGAAGGAACCATAACATGTAAATCAAAC---
ATCACAGGGATACTATTGACAAGGGATGGTGGAACACATAATGGA----------------------------
----

ACAGAGACATTCAGACCGGGAGGAGGAAATATGAAGGACAATTGGAGAAATGAATTATATAAGTATAAAGTGG
TAGAAATCAAGCCACTG---GGAGTAGCACCCACTGGAGCAAAAAGGAGAGTGGTG-----------------
----------------------GAGAGAGAAAAAAGAGCA---GTGGGA---CTAGGAGCT---------
ATGTTCCTTGGGATCTTAGGAGCA---GCAGGAAGCACTATG---GGCGCGGCGTCA---
ATAACGCTGACGGTACAG---GCCAGACAA---TTGTTG---TCTGGTATAGTGCAACAGCAAAGC---
AATTTG---CTGAGA---GCCATAGAGGCGCAACAGCATATGTTGCAACTCACAGTCTGGGGCATT
>KX069219
TTCTATTGCAATACATCAAGCCTATTTAATAGTACATACATGTCCAATGGTACATACATGTTTAATGGGACAA
TAAGTAAT---
-------------GGGACA------
ATAAGTAATGATAGCATCACAATCCCATGCAGGATAAAACAAATTGTAAACATGTGGCAGCAGGTAGGACGAG
CAATGTATGCCCCTCCCATTGCAGGAAACATAACGTGTAACTCAAAC---
ATTACAGGAATATTATTGGAACGTGATGGAGGGTTAAGTAATAGG----------------------------
----

TCAGAGGAGACATTCAGACCTGTAGGGGGAGATATGAAGAACAATTGGAGAAGTGAATTATATAAATATAAAG
TGGTAGAAATTAAACCATTG---GGAGTAGCACCCACTGCAGCAAAAAGGAGAGTGGTG--------------
-------------------------GAGAGAGAAAAAAGAGCA---GTGGGC---CTAGGAGTT---------
ATGTTCCTTGGGTTCTTGGGAGCA---GCAGGAAGCACTATG---GGCGCGGCGTCA---
ATAACGCTGACGGTACAG---GCCAGACAA---TTGCTG---TCTGGTATAGTGCAGCAGCAAAGC---
AATTTG---CTGAGG---GCTATAGAGGCGCAACAGCATTTGTTGCAACTCACAGTCTGGGGCATT
>KX069221
TTCTATTGTAATACATCAAAACTGTTTAAGGGTAATGGTACAAGCACAGACACAGGA----------------
----

AATAATTCAAGCATCACAATCCCATGCAGAATAAAGCAAATTATAAATATGTGGCAGGGGGTAGGACGAGCAA
TGTATGCCCCTCCCATTGAAGGACACATAACATGTGAATCAAAT---
ATCACAGGACTACTATTGGTACGTGATGGGGGAGCATCAAATGAGACAAGT---------------------
----

AATGATACAGAGACATTCAGACCTGGAGGAGGAAATATGAAGGACAATTGGAGAAGTGAATTATATAAATATA
AAGTGGTAGAAATTAAACCATTG---GGAGTAGCACCCACTGAAGCAAAAAGGAGAGTGGTG----------
---------------------------GAGAGAGAAAAAAGAGCA---GTG------TTGGGAGCT------
---GCGATCCTTGGGTTCTTGGGAGCA---GCAGGAAGCACTATG---GGCGCGGCGTCA---
ATAACGCTGACGGTACAG---GCCAGACAA---TTGCTG---TCTGGTATAGTGCAACAGCAAAGC---
AATTTG---CTGAGA---GCTATAGAGGCGCAACAGCATCTGTTGCAACTCACAGTCTGGGGCATT
>KX069223
TTCTATTGCAATACATCAAGACTGTTTAATGGAACATATGATGGAACATATAATGGTACAGCAAGTAAC----
----

AATACAGGAAACATCACCCTCCCATGCAGAATAAAACAAATTATAAACATGTGGCAGGGGGTAGGACGAGCAA
TATATGCCCCTCCCATTGCAGGAAACATAACATGTGTATCAAAT---
ATCACAGGACTATTATTGGTACGTGATGGAGGAACAGAGAATGGCACA------------------------
----

AATGGCACAGAAACATTCAGACCTACAGGAGGAAATATGAGGGACAATTGGAGAAGTGAATTATATAAATATA
AAGTGGTAGAAATTAAGCCATTG---GGAATAGCACCCACTGCAGCAAGAAGGAGAGTGGTG----------
---------------------------GAGAGAGGAAAAAAGAGCA---GTGGGA---ATAGGAGCT------
---GTGTTCCTTGGGTTCTTGGGAGCA---GCAGGAAGCACTATG---GGCGCGGCGTCA---
ATAACGCTGACGGTACAG---GCCAGACAA---TTGTTG---TCTGGTATAGTGCAGCAGCAAAGC---
AATTTG---CTGAGA---GCTATAGAGGCGCAACAGCATCTGTTGCAACTCACGGTCTGGGGCATT
>KX069224
TTCTATTGCAATACATCAAAACTGTTTAATAGTACATATAATGGTACAGACAATGGTAACTCAAGCAGCTCAA
AC--

TCAAGCTCAATCATCACAATCGCATGCAGACTAAAACAAATTGTAAAACTGTGGCAGGAGGTAGGACGAGCAA
TGTATGCCCCCCCCATTGCAGGAAACATAACGTGCAAATCAAAT---
ATCACAGGAATACTATTGGTGCGTGATGGAGGGACAAATGGGACA---------------------------
----

GAGGAGACCGAGACATTTAGACCTGGAGGAGGAAATATGAGGGATAATTGGAGAAGTGAATTGTATAAATATA
AAGTGGTAGAAATTAAGCCATTG---GGAATAGCACCCACTACAGCAAAAAGGAGAGTGGTG----------
---------------------------GAGAGAGAAAAAAGAGCA---GTGGGA---ATAGGAGCT------
---GTGTTCCTTGGGTTCTTGGGAGCA---GCAGGAAGCACTATG---GGCGCGGCGTCA---
ATGGCGCTGACGGTACAG---GCCAGACAA---TTGTTG---TCTGGTATAGTGCAACAGCAAAGC---
AATTTG---CTGAGG---GCTATAGAGGCGCAACAGCATCTGTTGCAACTCACGGTCTGGGGCATT
>KX069226
TTCTACTGCAACACAACAAACCTGTTTAATAGTACATACCTAGCGAATAGTACATTA---------------
----

AGTAATTCAACCATCACAATCCCATGCAGCATAAAACAAGTGATAAACATGTGGCAGGAGGTAGGACGAGCAA
TGTATGCCCCTCCCATTGCAGGAAACATAACCTGTAAATCAAAT---
ATCACAGGGCTACTATTGGTACGTGATGGGGGAACAAACAATAGC---------------------------
----

ACTACAGAGGTATTCAGACCTGGAGGAGGAGATATGAGGGACAATTGGAGAAGTGAATTATATAAATATAAAG
TGGTAGAAATTAAGCCATTA---GGAATAGCACCCACTGAAGCAAAAAGGAGAGTGGTG-------------
------------------------GAGAGAGAAAAAAGAGCA---GTGGGA---ATAGGAGCT---------
GTGTTCCTTGGGTTCTTGGGAGCA---GCAGGAAGCACTATG---GGCGCGGCGTCA---
ATAACGCTGACGGTACAG---GCCAGACAA---TTGTTG---TCTGGTATAGTGCAACAGCAAAGC---
AATTTG---CTGAGG---GCTATAGAGGCGCAACAGCATCTGTTGCAACTCACGGTCTGGGGCATT
>KY713230
TTCTATTGCAATACATCAGGCCTGTTTAATGGTACATATAATAGTACATACAATGGTACATCCATG------
----

TCTAATGATACCATCACACTCCCATGCAGAATAAAACAAATTATAAACATGTGGCAGGAGGTAGGAAGAGCAA
TGTATGCCCCTCCCATTGCAGGAAACATAACATGTAAATCAAAT---
ATCACAGGAATACTATTGGTACGTGATGGAGGACCAAATAGGACA----------------------------
----

AATGAGACATTCAGACCTGGAGGAGGAGATATGAGAGACAATTGGAGAAGTGAATTATATAAATACAAAGTGG
TAGAAGTTAAGCCATTG---GGAATAGCACCCACTACAGCAAAAAGGAGAGTGGTG-----------------
----------------------GAGAGAGAAAAAAGAGCA---GTGGGA---CTAGGAGCT---------
GTGTTCCTTGGGTTCTTGGGCGCA---GCAGGAAGCACTATG---GGCGCGGCGTCA---
ATAACGCTGACGGTACAG---GCCAGACAA---TTGTTG---TCTGGTATAGTGCAACAGCAAAAC---
AATTTG---CTGAAA---GCTATAGAGGCGCAACAACATCTGTTGCAACTCACGGTCTGGGGCATT
>KY713236
TTCTATTGTAATACATCAGACCTGTTTATTGGTACATATAAGTTTATTAATGGTACATATGAGTTTAATGATA
CAACAAGTAAT--

TCAAGCAGATCAGACATCACAATCCAATGCAGAATAAAGCAATTTATAAACATGTGGCAGGAGGTAGGAAAAG
CAATGTATGCCCCTCCCATTGAAGGAAACATAACATGTAAATCAAAC---
ATCACAGGACTAATATTGGTACGTGATGGAGGGCTAAATACCAAT----------------------------
----

GAGACAAATAATACAGAGATATTCAGACCTGGAGGAGGAGATATGAGGGACAATTGGAGAAGTGAATTATATA
AATATAAAGTGGTAGAAATTAAGCCATTA---GGAATAGCACCCACTGCAGCAAAAAGGAGAGTGGTG-----
----------------------------------GAGAGACCAAGAAGAGCA---GTGGGA---
CTAGGAGCT---------GTGTTCCTTGGGTTCTTGGGAGCA---GCCGGAAGCACTATG---
GGCGCGGCGTCA---ATAACGCTGACGGTACAG---GCCAGACAA---TTGTTG---
TCTGGTATAGTGCAACAGCAAAGC---AATTTG---CTGAGG---
GCTATAGAGGCGCAACACCATCTGTTGCAACTCACAGTCTGGGGCATT
>MN703367
TTCTATTGCAATACATCAAAACTGTTTAGTGGTGTATATAATGGTACATATAATGTTACAACAGGTAATTCAA
CAGGA--

GATAATTCAACTATCACAATCCCATGCAGAATAAGGCAAGTTATAAACCTGTGGCAGGAGGTAGGAAGAGCAA
TATATGCCCCTCCCATTGCAGGAAACATAACATGTAAATCAAAT---
ATCACAGGAATACTGTTAACGCGTGATGGAGGGCAAGGGGCAAAC----------------------------
----

ATTACAAATGATACAGAGACATTTAGACCTGCAGGAGGAGATATGAGGGACAATTGGAGAAGTGAACTATATA
AATATAAAGTGGTAGAAATTAGGCCATTA---GGAATAGCACCCACTCCAGCAAAAAGGAGAGTAGTG-----
----------------------------------GAGAGAGAGAAAAGAGCA---GCGGGA---
TTAGGAGCT---------GTGCTCCTTGGGTTCTTGGGAGCA---GCCGGAAGCACTATG---
GGCGCAGCGTCA---ATAACGCTGACGGTACAG---GCCAGACAA---TTGTTG---
TCTGGTATAGTGCAACAGCAAAGC---AATTTG---CTGAAG---
GCTATAGAGGCGCAACAACACATGTTGCAACTCACTGTCTGGGGCATC
>MN703351
TTCTATTGTAATACATCAAAACTGTTTAATGGTACATATAAAAGTGATTCAAACTCAACC-------------
----

TCAGACAAAAACATCACAATCCCATGCAGAATAAAGCAAATTATAAACATGTGGCAGGAGGTAGGACGAGCAA
TGTATGCCCCTCCCATTGCAGGAAGCATAATATGTAAATCACAA---
ATCACAGGACTCCTATTGGTACGTGATGGAGGAGTAACAAATGACACAGAAAAT-------------------
----

AATGATACAGAGATATTCAGACCTGGAGGAGGAGATATGAAGAACAATTGGAGAAGTGAATTATATAAATATA
AAGTAGTAGAGATTAAGCCATTG---GGAGTAGCACCCACTGGAGCAAAAGGGAGAGTAGTG-----------
----------------------------GAGAGAGAAAAAAGAGCA---GTGGGA---CTGGGAGCT------
---GTGTTCCTCGGGTTTCTGGGAGCA---GCAGGAAGCACTATG---GGCGCGGCGTCA---
ATGACGCCGACGGTACAG---GCCAGACAA---TTGTTA---TCTGGTATAGTGCAACAGCAAAAC---
AATTTG---CTAAAA---GCTATAGAGGCGCAACAGCATCTGTTGCAACTCACAGTCTGGGGCATT
>MN703360

TTCTATTGTAATACATCAGGCCTGTTTAATAGTATATATAATACAACAAGTAATTCAAGCTCCAAT-------
----
----------------------------TCAAATGAA---
ACCAGAATCCCATGCAGAATAAAGCAAATTATAAACATGTGGCAGGGGGTAGGACGAGCAATGTATGCCCCTC
CCATTGCAGGAAACATAACATGTAAATCAAAT---
ATCACAGGACTACTATTGGCACGAGATGGAGGAAAAGAGACAAAT----------------------------
----

GAGACAAGCGCGACAGAGATATTCAGACCTGGGGGAGGAAATATGAAGGACAACTGGAGAAGTGAATTATATA
AATATAAAGTGGTAGAAATTAAACCATTA---GGAATAGCACCCACTAAAGCAAAAAGGAGAGTGGTG-----
----------------------------------GAGAAAGGAAAAAGAGCA---GCGGGA---
ATGGCAGCT---------GTGTTCCTTGGGTTCTTGGGAGCA---GCCGGAAGCACTATG---
GGCGCGGCGTCA---ATAGCGCTGACGGTACAG---GCCAGACAA---TTGTTG---
TCTGGTATAGTGCAACAGCAAAGC---AATTTG---CTGAGG---
GCTATAGAGGCGCAACAGCATCTGTTGCAACTCACGGTCTGGGGCATT
>MN703391
TTCTATTGTAATACATCAGGCCTGTTTAATGATACATATGATACAACAGTTAATTCAAGCCCCACT-------
----

TCAAACGAAACCATCACAATCCTATGCAGAATAAAGCAAATTATAAACATGTGGCAGAAGGTAGGACGAGCAA
TGTATGCCCCTCCCATTGCAGGAAACATAACATGTAGATCAAAT---
ATCACAGGGCTACTATTGGTACGTGATGGAGGGATAAATGTGACAAGCAACACA-------------------
----

ACAGACAACATGACAGAGACATTCAGACCTGGAGGAGGAGATATGAGGGACAATTGGAGAAGTGAATTATACA
AATATAAAGTGGTAGAAATTAAGCCATTG---GGAATAGCACCCACTAAAGCAAAAAGGAGAGTGGTG-----
----------------------------------GAGAGAGAAAAAAGAGCA---GTGGGA---
ATGGCAGCT---------GTGTTCCTTGGGTTCTTGGGAGCA---GCCGGAAGCACTATG---
GGCGCGGCGTCA---ATAGCGCTGACGGTACAG---GCCAGACAA---TTGTTG---
TCTGGTATAGTGCAACAGCAAAAC---AATTTG---CTGAGA---
GCTATAGAGGCGCAACAGCATTTGTTGCAACTCACAGTCTGGGGCATC
>AF067154
TTCTATTGCAATACATCAAGCCTGTTTGATAGCCTGTTTAATCCTAATGGTACAAGAAATGAT----------
----

ACAAACTTAACCATTACAATCCCATGCAGAATAAAACAAATTATAAACATGTGGCAGGAGGTAGGACGAGCAA
TGTATGCCCCTCCCATTGCAGGAAACATAACATGTAAATCAAAC---
ATCACAGGACTACTATTGGTGCGTGATGGAGGAAGAGGTAATGATACA-------------------------
----

GAGAATAATACAGAGATATTCAGACCTGGAGGAGGAGATATGAGGAACAATTGGAGAAGTGAATTATATAAAT
ATAAAGTGGTAGAAATTAAGCCATTG---GGAGTAGCACCCACTAAAGCAAAAAGGAGAGTGGTG--------
-------------------------------GAGAGAGAAAAAAGAGCA---GTAGTGGGACTAGGAGCT---
------GTGTTCCTTGGGTTCTTGGGAGCA---GCAGGAAGCACTATG---GGCGCGGCGTCA---
ATAACGCTGACGGTACAG---GCCAGACAA---TTGTTG---TCTGGTATAGTGCAACAGCAAAGC---
AATTTG---CTGAGG---GCTATAGAGGCGCAACAGCATCTGTTGCAACTCACGGTCTGGGGCATT
>JQ715368
TTCTATTGTAATACATCAGGCCTGTTTAATGGTACATACATGTCTAATGGTACAGAAAGTAAT----------
----

TCAAGCTCAATCATCACAATCCCATGCAGAATAAAGCAAATTATAAACATGTGGCAGGAGGTAGGACGAACCA
TGTATGCCCCTCCCATTAAAGGAAACATAGCATGTAAATCAAAT---
ATCACCGGACTACTATTGGTACGTGATGGAGGAACAGAGCCAAAT----------------------------
----

GATACAGAGACATTCAGACCTGGAGGAGGAGATATGAGGAACAATTGGAGAAGTGAATTATATAAATATAAAG
TGGTAGAAATTAAGCCATTG---GGAATAGCACCCACTGCAGCAAAAAGAAGAGTGGTG--------------
-------------------------GAGAGAGAAAAAAGAGCA---GTGGGA---CTGGGAGCT---------
GTGTTCCTTGGGTTCTTGGGAGCA---GCAGGAAGCACTATG---GGCGCGGCGTCA---

ATAACGCTGACGGCACAG---GCCAGACAA---TTGTTG---TCTGGTATAGTGCAACAGCAAAGC---
AATTTG---CTGAGG---GCTATAGAGGCGCAACAGCATCTGTTGCAACTCACGGTCTGGGGCATT
>AF286223
TTTTATTGTAATACATCAAGACTGTTTAACGGTACATACAATGGTACAGACATGCCTACATACAATGGTACAA
AT---

TCCAGTTCAGACATCATCATGCTTCCATGCAGAATAAGGCAATTTATAAACATTTGGCAGAAGGTAGGACGAG
CAATGTATGCCCCTCCCATTGAAGGAAACATAACATGTGAATCAAAT---
ATCACAGGACTACTATTAGTACGTGATGGAGGCGACACAAATAGT----------------------------
----

AGCACAGAGATATTCAGACCTGGAGGAGGAGATATGAGGGACAATTGGAGAAGTGAATTATATAAATATAAAG
TGGTAGAAATTAAGCCATTA---GGAATAGCACCTACTGAAGCAAAAAGGAGAGTGGTG--------------
-------------------------GAGAGAGAAAAAAGAGCA---GTGGGA---ATAGGAGCT---------
GTGTTCCTTGGGTTCTTGGGAGTA---GCAGGAAGCACTATG---GGCGCGGCGTCA---
ATGACGCTGACGGTACAG---GCCAGACAA---TTGTTG---TCTGGTATAGTGCAACAGCAAAGC---
AATTTG---CTGAAG---GCTATAGAGGCGCAACAGCATATGTTGCAACTCACAGTCTGGGGCATT
>AF067155
TTCTATTGCAATACATCAGGCCTGTTTAATAGGACATACATGCCTAATGATACAAAGAGTAATTCAAGCAGTA
AT---

CCAAACGCAAACATCACAATCCCATGCAGAATAAAGCAAATTATAAACATGTGGCAGGAGGTAGGACGAGCAA
TGTATGCCCCTCCCATTGAAGGAAAAATAACATGTAGATCAAAT---
ATCACAGGACTACTATTGGTACGTGATGGAGGAGAAGACAAAAATAATACA----------------------
----

GAGACAAATAAGACGGAGACATTCAGACCTGGAGGAGGAGATATGAGGGACAATTGGAGAAGTGAATTATATA
AATACAAAGTGGTAGAAGTTAAGCCATTG---GGAGTAGCACCCACTACAGCAAAAAGGAGAGTGGTG-----
----------------------------------GAGAGAGAAAAAAGAGCA---GTGGGA---
ATAGGAGCT---------GTGTTCCTTGGGTTCTTGGGAGCA---GCAGGAAGCACTATG---
GGCGCGGCGTCA---ATAACGCTGACGGTACAG---GCCAGACAA---TTGTTG---
TCTGGTATAGTGCAACAGCAAAGC---AATTTG---CTGAGG---
GCTATAGAGGCGCAACAGCATCTGTTGCAACTCACGGTCTGGGGCATT
>AF286231
TTCTATTGCAGCACATCAAGACTGTTTAATAGTACATACATGCCTAATGATACAAAAAGTAAG----------
----

TCAAACAAAACCATCACAATCCCATGCAGCATAAAACAAATTGTAAACATGTGGCAGGAGGTAGGACGAGCAA
TGTATGCCCCTCCCATTGAAGGAAACATAACCTGTAGATCAAAT---
ATCACAGGAATACTATTGGTACGTGATGGAGGAGTAGATTCAGAAGATCCA----------------------
----

GAAAATAATAAGACAGAGACATTCCGACCTGGAGGAGGAGATATGAGGAACAATTGGAGAAGTGAATTATATA
AATATAAAGCGGCAGAAATTAAGCCATTG---GGAGTAGCACCCACTCCAGCAAAAAGGAGAGTGGTG-----
----------------------------------GAGAGAGAAAAAAGAGCA---GTAGGA---
TTAGGAGCT---------GTGTTCCTTGGATTCTTGGGAGCA---GCAGGAAGCACTATG---
GGCGCAGCGTCA---ATAACGCTGACGGTACAG---GCCAGACAA---TTGTTG---
TCTGGTATAGTGCAACAGCAAAGC---AATTTG---CTGAGG---
GCTATCGAGGCGCAACAGCATCTGTTGCAACTCACGGTCTGGGGCATT
>AF286232
TTCTATTGTAACACATCAGGTCTGTTTAATGGTACATACAATCCTAATGTTACAGAAAGTAATTCAAACTCA-
----

ACAAATGCAACCATCACACTCCCATGCAGAATAAAGCAAATTATAAATTTGTGGCAGGAGGTGGGACGAGCAA
TGTATGCCCCTCCCATTGCAGGAAACATAACATGTAAATCAAAT---
ATCACAGGACTATTATTGACACGTGATGGAGGAACAAATGATACA----------------------------
----

AGTAAGACAGAGATATTCAGACCTGGAGGAGGAGATATGAGGGACAATTGGAGAAGTGAATTATACAAATATA
AAGTGGTAGAAGTTAAGCCATTG---GGAGTAGCACCCACTGGAGCAAAAAGGAGAGTGGTG-----------

----------------------------GAGAGAGAAAAAAGAGCA---GTGGGA---ATAGGAGCT------
---GTGTTCCTTGGGATCTTGGGAGCA---GCAGGAAGCACTATG---GGCGCGGCGTCA---
ATAACGCTGACGGTACAG---GCCAGACAA---TTGTTG---TCTGGTATAGTGCAACAGCAAAGC---
AATTTG---CTGAGA---GCTATAGAGGCGCAACAGCATCTGTTGCAACTCACAGTCTGGGGCATT
>KP109486
TTCTATTGCAATACATCAAGCCTGTTTAACAGTACATACAATCCTAATGATACAAACAGTAATTCAAGCTCAA
GTAAT--

TCAAGCTTAGACATCACAATCCCATGCAGAATAAAACAAATTATAAACATGTGGCAGGAGGTAGGACGAGCAA
TGTATGCCCCTCCTATTGAAGGAAACATAACATGTAAATCAAAT---
ATCACAGGACTACTATTGGTACGTAATGGAGGAATAGAGTCAAAT----------------------------
----

GAAACAGAGATATTCAGACCTGGAGGGGGAGATATGAGGAACAATTGGAGAAGTGAATTATATAAATATAAGG
TGGTGGAAATTAAGCCATTG---GGAATAGCACCCACTGCAGCAAAAAGGAGAGTGGTG--------------
-------------------------GAGAGAGAAAAAAGAGCA---GTGGGA---CTGGGAGCT---------
GTGATCTTTGGGTTCTTGGGAGCG---GCAGGAAGCACTATG---GGCGCGGCGTCA---
ATAACGCTGACGGTACAG---GCCAGACAA---TTGTTG---TCTGGTATAGTGCAACAGCAAAGC---
AATTTG---CTGAAG---GCTATAGAAGCGCAACAACATTTGTTGCAGCTCACGGTCTGGGGCATT
>KP109487
TTCTATTGTAATACATCAGGCCTGTTTAATGGTACATTTAATGGTACATACGTGTCACCTAATAGTACAGACA
GTAAT--

TCAAGTTCAATCATCACGATCCCCTGCAGAATAAAGCAAATTATAAACATGTGGCAGGAGGTAGGACGAGCAA
TGTATGCCCCTCCCATTGCAGGAAACATAACATGCAAATCAAAT---
ATCACAGGACTACTATTGGTACGCGATGGGGGAACAGGGTCAGAGTCA-------------------------
----

AATAAGACAGAGATATTCAGACCTGGAGGAGGAGATATGAGGGACAATTGGAGAAGTGAATTATATAAATATA
AAGTGGTAGAAATTAAGCCATTG---GGAGTAGCACCCACTAAAGCAAAAAGGAGAGTGGTG-----------
----------------------------GAGAGAGAAAAAAGAGCA---GTGGGA---ATAGGAGCT------
---GTGTTCCTTGGGTTCTTGGGAGCA---GCAGGAAGCACTATG---GGCGCGGCGTCA---
ATAACGCTGACGGTACAG---GCCAGACAA---TTGTTG---TCTGGTATAGTGCAACAGCAAAGC---
AATTTG---CTGAGG---GCTATAGAAGCGCAACAGCATCTGTTGCAACTCACGGTCTGGGGCATT
>KP109488
TTCTATTGTAATACATCAGGCTTGTTTAATGGTACATACATGAATAATGATACAAAAAGTAATGATACAAAAA
GTAAT--

TCAAGCTCCATCATCACAATCCCATGCAGAATAAAGCAAATTATAAACATGTGGCAAGAGGTAGGACGAGCAA
TGTATGCCCCTCCCATTGCAGGAAACATAACATGTAAATCAAAT---
ATCACAGGAATACTATTGACACGAGATGGAGGACGAGGAGAAGAG----------------------------
----

GTAAAAAATGATACAGAGACATTCAGACCTGGAGGAGGAAATATGAAGGACAATTGGAGAAGTGAATTATATA
AATACAAAGTAGTAGAAATTAAGCCATTA---GGAGTAGCACCAACTGCAGCAAAAAGGAGAGTGGTG-----
----------------------------------GAGAGAGAAAAAAGAGCA---GTGGGA---
CTAGGAGCT---------GTACTCCTTGGGTTCTTGGGAGCA---GCAGGAAGCACTATG---
GGCGCGGCGTCA---ATAACGCTGACGGTACAG---GCCAGACAA---TTGTTG---
TCTGGAATAGTGCAACAGCAAAGC---AATTTG---CTGAGA---
GCTATAGAGGCGCAACAGCATATGTTGCAACTCACAGTCTGGGGCATT
>KP109489
TTCTATTGTAATACATCAAAACTGTTTAATAGTACATACATACCTACATACAGGCCTAATAATACACAAGGTA
AT---

TCAAGCTCAACCATCACAATCCCATGTAGAATAAAGCAAATTATAAACATGTGGCAGGAGGTAGGACGAGCAA
TGTATGCCCCTCCCATTGCAGGAAACATAACATGTAAATCACAT---
ATCACAGGACTACTATTGGTACGGGATGGAGGAACAGGGCTAAAT----------------------------
----

AGTAGTACAGAGACATTTAGACCTGGAGGAGGGGATATGAGGGACAATTGGAGAAGTGAATTATATAAATATA

AAGTAGTAGAAATTAAGCCATTG---GGAGTAGCACCAACTGCAGCAAAAAGAAGAGTGGTG-----------
----------------------------CAGAGAGAAAAACGAGCA---GTGGGA---TTAGGAGCT------
---GTGTTCCTTGGGTTCTTGGGAACA---GCAGGAAGCACTATG---GGCGCAGCGTCA---
ATAACGCTGACGGTACAG---GCCAGACAA---TTGTTG---TCTGGTATAGTGCAACAGCAGAGC---
AATCTG---CTGAGG---GCTATAGAGGCGCAACAGCATATGTTGCAACTCACAGTCTGGGGCATT
>AJ276221
TTCTATTGTAATACAACAGGCCTGTTTAATGGTACATTTAATGGTACATACATGAATAATACAGGA-------
----

AGTAATTCAAGCATCACAATCCCATGCAGAATAAAGCAAATTATAAACATGTGGCAGAAGGTAGGACAAGCAA
TGTATGCCCCTCCCATTGCAGGAAACATAACCTGTAGGTCAAAT---
ATTACAGGACTACTATTGGTACGCGATGGAGGATTGGGGACAAAT----------------------------
----

GACACAGAGACATTTAGACCTGGAGGAGGAGATATGAGGGACGATTGGAGAAGCGAATTATATAAATATAAAG
TGGTAGAAATTAAGCCATTG---GGAGTAGCACCCACAGGAGCAAAAAGGAGAGTGGTG--------------
-------------------------GAGAGAGGAAAAAGAGCA---GTGGGA---CTAGGAGCT---------
GTGTTCCAAGGGTTCTTGGGAGCA---GCAGGAAGCACTATG---GGCGCGGCGTCA---
ATAACGCTGACGGTACAG---GCCAGACAA---TTGTTGTCTGGTATAGTGCAACAACAAAGCAGC---
AATTTG---CTGAGG---GCTATAGAGGCGCAACAGCATCTGTTGCAACTCACGGTCTGGGGCATT
>EU521728
TTCTATTGCAACACATCAACACTGTTTAATAGTACGTACATGTACATGAATAGTACGTACATGTTTAATGGTA
CAGAAAGTAAT--
-------GGTACAGAAAGT------
AATTCAAACTCAACCATCACAATCCAATGCAGAATAAAACAAATTATAAACATGTGGCAGAAGGTAGGAAGAG
CAATGTATGCCCCTCCCATTGCAGGAAACATAACATGTAAATCAGGT---
ATCACAGGACTATTATTGGTACGTGATGGAGGAAATCCAAATAATACA-------------------------
----

AATAATACAGAGATCTTCAGACCTGGAGGAGGAGATATGAGGGACAATTGGAGAAGTGAATTATATAAATATA
AAGTGGTAGAAATTAAGCCATTG---GGAATAGCACCCACTGCAGCAAAAAGGAGAGTGGTG-----------
----------------------------GAGAGAGAGAAAAGAGCA---GTGGGA---CTAGGAGCT------
---ATGTTCTTTGGGTTCTTAGGAGCA---GCAGGAAGCACTATG---GGCGCGGCGTCA---
GTGACGCTGACGGTACAG---GCCAGACAA---TTGTTG---TCTGGTATAGTGCAACAGCAAAGC---
AATTTG---CTGAGG---GCTATAGAGGCGCAACAGCAGTTGTTGCAACTCACAGTCTGGGGCATT
>EF694033
TTCTATTGCAATACATCACAACTGTTTAATAGTACATATGATCCTGCATACAGGCCTAATAATACAGAA----
----

AACAATTCAACCATCATACTCCCATGCAGAATAAGACAAGTTGTAAACCTGTGGCAGGAGGTAGGACGAGCAA
TGTATGCCCCTCCCATTGAAGGAAACATAACGTGTAAATCAAAT---
ATCACAGGAATACTATTGGTACGTGATGGAGGGACAGAATTAAATAATACA----------------------
----

GAATTTAATAATACAGAAATATTCAGACCGGGAGGAGGAGATATGAGAGACAATTGGAGAAGTGAATTATATA
AATATAAAGTGGTAGAAATTAAGCCATTG---GGAGTAGCACCCACTACGGCAAAAAGGAGAGTGGTG-----
----------------------------------GAGAGAGAAAAAAGAGCA---
GTAGTGGGAATAGGAGCT---------GTGTTCCTTGGGATCTTGGGAGCA---GCAGGAAGCACTATG---
GGCGCGGCATCA---ATAACGCTGACGGTACAG---GCCAGACAA---CTGCTG---
TCTGGTGTAGTGCAACAGCAAAGC---AATTTG---CTGAGG---
GCTATAGAGGCGCAACAGCATATGTTGCAACTCACGGTCTGGGGCATT
>AF457054
TTCTATTGTAATACATCAGGCCTGTTTAATGAAACTCTAACTCGGTTGAATGATACATACAGTTCT-------
----

AATGGGACAAACATCACACTCCCATGTAGAATAAAACAAATTATAAACATGTGGCAGAAGGTAGGACGAGCAA
TGTATGCCCCTCCCATTGCAGGAAACATAACATGTACATCAAAA---
ATCACAGGACTATTATTGCAACGTGATGGAGGGAACAGTACAAAT----------------------------
----

AAAACTGAGACATTCAGGCCTGGAGGAGGAAATATGAAGGACAACTGGAGAAGTGAATTATACAAATATAAAG
TGGTGGAAATTAAGCCATTG---GGAATAGCACCCACTGAGGCAAAAAGGAGAGTGGTG-------------
------------------------GAGAGAGAAAAAAGAGCA---GTGGGA---ATAGGAGCT---------
GTACTCCTTGGGTTCTTGGGAGCA---GCAGGAAGCACTATG---GGCGCGGCGTCA---
ATAACGCTGACGGTACAG---GCCAGACAA---CTGTTG---TCTGGTATAGTGCAACAGCAAAGC---
AATTTG---CTGAGG---GCTATAGAAGCGCAACAGCATATGTTGCAGCTCACGGTCTGGGGCATT
>FJ396028
TTCTATTGCAATACATCAGATTTGTTTAACTTTCCTAATGGTAGTAAGGCTATTAAT---------------
----

GGTAATTCAACCATTACACTCCAATGCAGAATAAAACAAATTATAAACATGTGGCAGGGGGTAGGACAAGCAA
TGTATGCTCCTCCCATTAGAGGGAACATAACATGTAGATCAAAT---
ATCACAGGACTATTACTGACACGCGATGGAGGAGAAGGACGAAATGGCACA---------------------
----

GAGAATGTCACAGAGATATTCAGACCTGGAGGAGGAGATATGAGGGACAATTGGAGAAGTGAATTATACAAAT
ATAAAGTGGTAGAAATTAAGCCATTA---GGAGTAGCACCCACTAAGGCAAAAAGGAGAGTGGTG-------
------------------------------GAGAGAGAAAAAAGAGCA---GTGGGA---ATAGGAGCT---
------GTGTTCCTTGGGTTCTTGGGAGCA---GCAGGAAGCACTATG---GGCGCAGCGTCA---
ATAACGCTGACGGTACAG---GCCAGACAA---TTATTG---TCAGGCATAGTGCAACAGCAAAAC---
AATTTG---CTGAGA---GCTATAGAGGCGCAACAACATCTGTTGCAACTCACGGTCTGGGGCATT
>KT022366
TTCTATTGCAATACAACAATCCTGTTTAATGAGACATACAGGGAATACATGGCTAATAAAACACAA------
----

GATAATACGACACTCACACTCCAATGCAGAATAAAACAGATTATAAGGATGTGGCAGGGGGTAGGACAAGCGA
TGTATGCCCCTCCCATTGCAGGCAACATAACATGTAGATCAAAT---
ATCACAGGACTACTATTGATACGTGATGGAGGAACAAACAATAGT---------------------------
----

GAGACATTCAGACCTGGAGGAGGAGATATGAGGGACAATTGGAGAAGTGAATTATATAAATATAAAGTGGTAG
AAATTAAACCATTA---GGAATAGCACCCACCAAGGCAAAAAGGAGAGTGGTG-------------------
------------------GAGAGAGAAAAAAGAGCA---GCA------CTAGGAGCT---------
TTGTTCCTTGGGTTCTTGGGAGTA---GCAGGAAGCACTATG---GGCGCGGCGTCA---
ATGACGCTGACGGTACAA---GCCAGACAA---TTGCTG---TCTGGTATAGTGCTACAGCAAAGC---
AATTTG---CTGAGA---GCCATAGAGGCGCAACAGCATATGTTGCAACTCACGGTCTGGGGCATT
>KT022371
TTCTATTGCAATACATCAAAACTGTTTAAGGATATTGTTATAAATGATACTGAT------------------
----

ACAAATACAACCATCATGCTCCCATGCAGAATAAAACAAATTATAAACATGTGGCAGCAGGTAGGACGAGCAA
TGTATGCCCCTCCCATCCGAGGGCAAATAACATGTAACTCAACT---
ATCACAGGATTAATACTGACACGTGATGGAGGAAATACTAATAACAATAACACC------------------
----

AATGACACAGAGATAGAGATATTCAGACCTGCAGGAGGAGATATGAGGGACAATTGGAGAAGTGAATTATATA
AATATAAAGTGGTAGAAGTTAAGCCATTA---GGAATAGCACCCACTGAGGCAAAAAGGAGAGTGGTG----
----
GAGAGAGAAAAAAGAGCAGTAGTAGGGGGACTAGGAGTT---------GTGTTCCTTGGGTTCTTGGGAGCA-
--GCCGGAAGCACTATG---GGCGCAGCGTCA---ATGGCGCTGACGGTACAG---GCCAGACAA---
TTACTG---TCGGGTATAGTGCAACAGCAAAAC---AATTTG---CTGAGG---
GCTATAGAGGCGCAACAGCATATGTTGCAACTCACGGTCTGGGGCATT
>KU921723
TTCTATTGCAATACAACAATCCTGTTTAATAGTAGTAGGATTTTTAAAAGAAAT------------------
----

TTTACAGAAAATATCACACTCCCATGCAGAATAAAACAATTTATAAACATGTGGCAGAGAGTAGGACAAGCAA
TGTATGCTCCTCCCATTGCAGGAAACATAATATGTAACTCAAAC---
ATCACAGGACTAATATTGACACGTGATGGAGGTAATGATAATGATACA------------------------
----

```
-------
TCAAACAACAATGAGACATTCAGACCTGCAGGAGGAGATATGAGGGACAATTGGAGAAGTGAATTATATAAAT
ATAAAGTGGTAGAAATTAAACCATTA---GGAATAGCACCCACAAAGGCAAAAAGGAGAGTGGTG--------
-------------------------------GAGAGAGAAAAAAGAGCA---GTGGGA---ATAGGAGCT---
------ATGATCCTTGGGTTCTTGGGAACA---GCAGGAAGCACTATG---GGCGCGGCGTCA---
ATAACGCTGACGGTACAA---GCCAGACAA---TTGTTG---TCAGGCATAGTGCAACAGCAAAGT---
AATTTG---CTGAGA---GCTATAGAGGCGCAACAGCACATGTTGCAACTCACAGTCTGGGGCATT
>KU921743
TTCTATTGCAATACATCAAGCCTGTTTAATAGTATATACAATCCTAATATTACATACAATGCTAGTGATGAGT
CAAGCTCA-----------------------------------------------------------------
-------------------------
AACATCACACGAAACATCACACTCCCATGCAGAATAAGACAAATTATAAACATGTGGCAGACGGTAGGACGAG
CAGTATATGCTCCTCCCATTAGAGGAAACATAACATGTATATCAAAC---
ATCACAGGACTATTATTGGTACGTGATGGAGGGGACAATAATAACACA-------------------------
-------------------------------------------------------------------------
----------
AATAATACAGAGACATTCAGACCTGGAGGAGGAGATATGAGGGACAATTGGAGAAGTGAATTATATAAATATA
AGGTGGTAGAAATTAAGCCATTG---GGAGTAGCACCAACTGGAGCAAAAAGGAGAGTGGTG-----------
----------------------------GACAGAGGAAAAAGAGCA---GTGGGA---CTAGGAGCT------
---ATGTTCCTTGGGTTCTTGGGAGCA---GCAGGAAGCACTATG---GGCGCGGCGTCA---
ATAACGCTGACGGTACAG---GCCAGACAA---CTGTTG---TCTGGTATAGTGCAACAGCAAAAC---
AATTTG---CTGAGG---GCTATAGAAGCGCAACAGCATATGTTGCAACTCACGGTCTGGGGCATT
>KU921790
TTCTATTGCGAGACAGACAACCTGTTTAATAGTACATACATGGAGGCATACAATAGTACAGATAATAGT----
-------------------------------------------------------------------------
----------------------------
ACCAATTCAACCATCACACTCCCATGCAGAATAAAACAAATTATAAACATGTGGCAGGAAGTAGGACGAGCAA
TGTATGCCCCTCCCATTGCAGGAAACATAACATGTATATCAAAT---
ATCACAGGACTATTATTGACACGTGATGGAGGACAGGGAACTGAT----------------------------
-------------------------------------------------------------------------
----------
AATGAAACAGAGATATTCAGACCAGCAGGAGGAGATATGAGGGACAATTGGAGAAGTGAATTATATAAATATA
AGGTGGTAGAAATTAAACCATTA---GGTATAGCACCCACTTCAGCAAAAAGGAACGTGGTG-----------
----------------------------AAGAGAGAAAAAAGAGCA---GTGGGA---CTAGGAGCT------
---GTGTTCCTTGGGTTCTTGGGAGCA---GCAGGAAGCACTATG---GGCGCAGCGTCA---
GTGACGCTGACGGTACAG---GCCAGGCAA---TTGCTG---TCTGGTATAGTGCAACAGCAAAAC---
AATTTG---CTGAGG---GCTATAGAGGCGCAACAGCATCTGTTGCAACTCACAGTCTGGGGCATT
>KU921807
TTCTATTGCAATACATCCAAACTGTTTAATAGTACATACATGGAGAAATACAATAGTACAGGCAATAGT----
-------------------------------------------------------------------------
----------------------------
ACCAATTCAACCATCACACTCCCATGTAGAATAAAACAAATTATAAACATGTGGCAGGGGGTAGGACGAGCAA
TGTATGCCCCTCCCATTGAAGGAAACATAACATGTAAATCAAAT---
ATCACAGGAATAATATTGACACGTGATGGAGGAGGGGTAGATAAT----------------------------
-------------------------------------------------------------------------
-------------
ACAACAGAGATATTCAGACCAGGAGGAGGAGATATGAGGGACAATTGGAGAAGTGAATTATATAAATATAAGG
TAGTAGAAATTAAACCATTA---GGAATAGCACCCACTAAGGCAAAAAGGAACGTGGTG--------------
-------------------------CAGAGAGAAAAAAGAGCAGTAATGGGA---ATAGGAGCT----------
GTGTTCCTTGGGTTCTTGGGAGCA---GCAGGAAGCACTATG---GGCGCAGCATCA---
ATAACGCTGACGGTACAG---GCCAGGCAA---TTGCTG---TCTGGTATAGTGCAACAGCAAAGC---
AATTTG---CTGAGG---GCTATAGAGGCGCAACAGCATCTGTTGCAACTCACGGTCTGGGGCATT
>FJ866132
TTCTATTGTAATACATCAGGCCTGTTTAATAGGGTAATTAATGATACATACATGACTAATGATACT-------
-------------------------------------------------------------------------
----------------------------
TCAAATTCAACCATCACACTCCGCTGCAGAATAAAACAAATTATAAACACGTGGCAGGGGGTAGGACGAGCAA
TGTATGCCCCACCCATTGAAGGAAACATAACATGTATATCAAAC---
ATCACAGGACTACTATTGACACGTGATGGAGGACAAGAAAATAGA----------------------------
-------------------------------------------------------------------------
```

ACAGAGGTATTTAGGCCTGGAGGAGGAGATATGAGGGACAATTGGAGAAGTGAATTATATAAATATAAGGTGG
TAGAAATTAAACCATTA---GGAATAGCACCCACTACTGCAAAAAGGAGAGTGGTG-----------------
----------------------GAGAGAGAAAAAAGAGCA---GTGGGA---CTAGGAGCT---------
GTGTTCCTTGGGTTCTTGGGAGCA---GCGGGAAGCACTATG---GGCGCAGCGTCA---
ATAACGCTGACGGTACAG---GCCAGACAA---TTATTG---TCTGGTATAGTGCAACAGCAAAGC---
AATTTG---CTGAGA---GCTATAGAGGCGCAACAGCATATGTTGCAACTCACGGTCTGGGGCATT
>JN983803
TTCTATTGCAATACATCAGGTCTGTTTAATGGTACATATGGATCTAATAATACAAAAGGTGAT----------
----

TCAAATGCAACTATCACACTCCCATGCAGAATAAAACAAATTATAAACATGTGGCAGGGGGTAGGACGAGCAA
TATATGCCCCTCCCATTGCAGGAAACATAACATGTACATCAAAT---
ATCACAGGATTAATATTGACACGTGATGGAGGGTCAGAGGATAAT----------------------------
----

ACCACAGAGATATTCAGACCTGCAGGAGGAGATATGAGGGACAATTGGAGAAGTGAAATATACAAATATAAAG
TGGTAGAAATTAAACCATTG---GGAATAGCACCCACTGGGGCAAAAAGGAGAGTGGTG--------------
-------------------------GAGAGAGAGAAAAGAGCA---GTGGGA---ATAGGAGCT---------
GTGTTCCTTGGGTTCTTGGGAGCA---GCAGGAAGCACTATG---GGCGCGGCGTCA---
ATAACGCTGACGGTACAG---GCCAGACAA---CTGTTG---TCTGGTATAGTGCAACAGCAAAGC---
AATTTG---CTGAGA---GCTATAGAGGCGCAACAACATTTGTTGCAACTCACAGTCTGGGGCATT
>JN983804
TTCTATTGTGATACATCAAAACTGTTTAATAGAACATACATGGCTAATAGTACAGAA----------------
----

AATAATTCATCCATCACGCTGCCCTGCAAAATAAAACAAATTATAAACCTGTGGCAGGGAGTAGGACGAGCAA
TGTATGCCCCTCCCATTAAAGGAAAAATAACATGTAACTCAACG---
ATCACAGGACTACTATTGACACGTGATGGAGGGAATGAGAGTGAA----------------------------
----

GAGACGTTCAGACCTGGAGGAGGAGATATGAGAGACAATTGGAGAAGTGAATTATATAAATATAAAGTGGTAG
AAATTAAGCCATTA---GGAATAGCACCCACTGAGGCAAAAAGGAGAGTGGTG--------------------
-------------------GAGAGAGAGAAAAGAGCA---GTGGGA---ATAGGAGCT---------
GTGTTCCTTGGGTTCTTGGGAGCA---GCAGGAAGCACTATG---GGCGCGGCGTCA---
ATAACGCTGACGGTACAG---GCCAGACAA---TTGCTG---TCTGGTATAGTGCAACAGCAAAGC---
AATTTG---CTGAGG---GCTATAGAGGCGCAACAGCATATGTTGCAACTCACAGTCTGGGGCATT
>JN983805
TTCTATTGCAATACATCAGAATTGTTTACAGAAAAAATTTCA-------------------------------
----

AATGAGACAAACATCACGCTTTCATGCAGAATAAAACAGATTATAAATATGTGGCAGGGGGTAGGACGGGCAA
TGTATGCCCCTCCCATTGAAGGAAACATAACATGTACATCAAAT---
ATCACAGGACTACTCATGACATATGATGGAGGACAACACACAGAA----------------------------
----

AATAAAACAGAGACCTTCAGACCTGGAGGAGGAGATATGAGGGACAATTGGAGAAGTGAATTATATAAATATA
AAGTGGTAGAAATTAGGCCATTA---GGAATAGCACCCACTAAGGCAAAAAGGAGAGTGGTG-----------
----------------------------GAGAGAGAAAAAAGAGCA---GTGGGA---ATGGGAGCT------
---GTGCTCCTTGGGTTCTTGGGAGCA---GCAGGAAGCACTATG---GGCGCAGCATCA---
ATAACGCTGACGGTACAG---GCCAGACAA---CTGTTG---TCTGGTATAGTGCAACAGCAAAGC---
AATTTG---CTGAGA---GCTATAGATGCGCAACAGCATATGTTGCAACTCACGGTCTGGGGCATT
>HM215360
TTCTATTGCAATACATCAAGATTGTTTACATACACAGAAGAG-------------------------------
----

ACAGGAAATATTACCCTCCCATGCAGAATAAAACAAATTATAAACATGTGGCAGGAGGTAGGACGAGCAATGT
ACGCCCCTCCCATTGCAGGAAACATAATATGTAGATCAAAT---
ATCACAGGATTACTATTGACACGTGATGGAGGAAAAAATAATAGT----------------------------
----

GGACCTGAGACATTCAGACCTGGAGGAGGAGATATGAGGGACAATTGGAGAAGTGAATTATATAAATATAAAG
TAGTAGAAATTAAACCATTA---GGAATAGCACCCACTAAGGCAAAAAGGAGAGTGGTG--------------
-------------------------GAGAGAGAGAAAAGAGCA---GTGGGA---CTAGGAGCT---------
GTGTTCCTTGGGTTCTTGGGAGCA---GCAGGAAGCACTATG---GGCGCGGCGTCA---
ATCACGCTGACGGTACAG---GCCAGACAA---TTATTG---TCTGGTATAGTGCAACAGCAAAGC---
AATTTG---CTGAGA---GCCATAGAGGCGCAACAGCATATGTTGCAACTCACGGTCTGGGGCATT
>KC894075
TTCTATTGCAATACATCAAAACTGTTCAATGGTACATACAATAGTACATACAAGACTAATACTACAGAA----
----

AGTAATGACACCATCACCATCCCATGCAGAATAAAACAAATTATAAACATGTGGCAGGGGGTAGGACGAGCAA
TGTATGCCCCTCCCATTGCAGGAAACATAACATGTAGATCAAAT---
ATCACAGGACTACTATTGACACGTGATGGAGGAAAAGTAAATAGC----------------------------
----

ACAAATGAGACATTCAGACCTGAAGGAGGAAATATGAAAGACAATTGGAGAAGTGAATTGTATAAATATAAAG
TGGTAGAAATTAAGCCATTA---GGAGTAGCACCCACGGAGGCAAAAAGGACAGCGGTG--------------
-------------------------GGGAGAGGAAAAAGAGCA---GTGGGA---ATAGGAGCT---------
GTGCTCCTTGGGTTCTTGGGAGCA---GCAGGAAGCACTATG---GGCGCGGCGTCA---
ATAACGCTGACGGCACAG---GCCAGACAA---GTATTG---TCTGGTATAGTGCAACAGCAAAGC---
AATTTG---CTGAGA---GCCATAGAGGCGCAACAGCATATGTTGCAACTCACGGTCTGGGGCATT
>KC894077
TTCTATTGCGATACATCAAAATTATTTAATAGTACACACAGG-------------------------------
----

GCTGATGATTCACCCATCACACTCCAATGCAGAATAAAACAAATTATAAACATGTGGCAGGGGGTAGGAAGAG
CAATGTATGCCCCTCCCATTGCAGGAAACATAGTATGTAACTCAAGT---
ATCACAGGACTACTATTAACACGTGATGGAGGAGGGAATGGTACA----------------------------
----

GAGACATTCAGACCTGGAGGAGGAGATATGAGGGACAATTGGAGAAGTGAGTTATATAAATATAAAGTGGTAG
AAATTAAGCCATTA---GGAATAGCACCCACTAAGGCAAAAAGGAGAGTGGTG--------------------
-------------------GAGAGAGAAAAAAGAGCA---GCT------CTGGGAGCT---------
ATGTTCCTTGGGTTCTTGGGAATG---GCAGGAAGCACTATG---GGCGCAGCATCA---
ATAACGCTGACGGTACAG---GCCAGGCAA---TTGTTG---TCTGGTATAGTGCAACAGCAGAAC---
AATTTG---CTGAGA---GCTATAGAGGCGCAACAGCATATGTTGCAACTCACAGTCTGGGGCATT
>FJ444184
TTCTATTGCAATACAACAAAACTGTTTAACAGTACACACCAGGTTAATAGTACCCGGGATAATAGTACAGAA-
----

AGTAATTCAACCATCACACTCCAATGCAGAATAAAACAAATTATAAACATGTGGCAGGGGGTAGGACGAGCAA
TGTATGCCCCTCCCATTGCAGGAGATATAAGATGTGAATCAAAT---
ATCACAGGACTACTATTGACACGTGATGGAGGAATAGGTAACAATGAC-------------------------
----

ACAGAGAAAACAGAGATATTCAGACCTGGAGGAGGAAATATGAGGGACAATTGGAGAAGTGAATTATATAAAT
ATAAAGTGATAGAAATTAAACCATTA---GGAATAGCACCCACTAAGGCAAAAAGGAGAGTGGTG--------
-------------------------------GAGAGAGAAAAAAGAGCA---GTGGGA---ATAGGAGCT---
------GTGTTCCTTGGGTTCTTGGGAGCA---GCAGGAAGCACTATG---GGCGCAGCATCA---
ATAACGCTGACGGTACAG---GCCAGACAA---TTGTTG---TCTGGTATAGTGCAACAGCAAAAC---
AATTTG---CTGAGG---GCTATAGAAGCGCAACAGCATATGTTGCAGCTCACGGTCTGGGGCATT
>FJ444251
TTCTATTGCAATACATCAGGTCTGTTTAATGGTACATACCCGTCTAATGATAATAGTACATACATG-------
----

TCTAATGATACAATAACCCTCCCATGCAGAATAAAACAAATTATAAACATGTGGCAGGGGGTAGGACGAGCAA
TGTATGCTCCTCCCATTAAAGGAAATATAACATGTGAATCAAAC---
ATCACAGGATTACTATTGACGCGTGATGGAGGAGAAACTAATACCACT-------------------------
----

AATAACACAGAGATATTCAGACCTGGAGGAGGAGATATGAGGGACAATTGGAGAAATGAACTATATAAGTATA
AAGTGGTAGAAATTAAGCCATTG---GGAATAGCACCCACTAAGGCAAAAAGGAGAGTGGTG----------
-----------------------------GAGAGAGAAAAAAGAGCA---GTGGGA---ATAGGAGCT------
---GTGTTCCTTGGGTTCTTGGGAGCA---GCAGGAAGCACTATG---GGCGCGGCATCA---
ATAACGCTGACGGTACAG---GCCAGACAA---TTGTTG---TCTGGTATAGTGCAACAGCAAAGC---
AATTTG---CTGAGG---GCTATAGAGGCGCAACAGCATATGTTGCAACTCACGGTCTGGGGCATT
>FJ444277
TTCTATTGCAATACATCAGGACTGTTTAATGAGACATACACATACAAATTTAATGAAAGTAATTCGACAAATG
GA---

ACTAACTCAACAATCAAACTCCTATGCAGAATAAAGCAAATTATAAACATGTGGCAGGGGGTAGGACGAGCAA
TGTATGCCCCTCCCATTGAAGGAAACATAACATGCAAATCAAGT---
ATCACAGGATTACTATTGACACGTGATGGAGGAAATACTAATGAG----------------------------
----

ACAGAGATATTCAGACCTGCAGGAGGAGATATGAGGGACAATTGGAGAAGTGAATTATACAAATATAAAGTGG
TAGAAATTAAGCCATTA---GGAATAGCACCCACTGAAGCAAAAAGGAGAGTGGTG-----------------
----------------------GAGAGGGAGAAAAGAGCA---GTGGGA---ATAGGAGCT---------
GTGTTCCTTGGGTTCTTGGGAGCA---GCAGGAAGCACTATG---GGCGCGGCGTCA---
ATAACGCTGACGGTACAG---GCCAGACAA---TTGTTG---TCTGGTATAGTGCAACAGCAAAGC---
AATTTG---CTGAGG---GCTATAGAGGCGCAACAGCATATGTTGCAACTCACGGTCTGGGGCATT
>FJ444476
TTCTATTGCGATACATCAAAACTGTTTAATGGTAATGGTACCAACAGTAATGGTACTGGTACCAACAGTACT-
----

ACAAACTCAACAATCATGATCCCATGCAGAATAAAACAATTTATAAACATGTGGCAGGAGGTAGGACAAGCAA
TGTATGCCCCTCCCATTGAAGGAAAGATAACCTGTAAATCAAAT---
ATCACAGGACTGCTATTGACACGTGATGGAGGAAATAGTACTGGAAGTAAT----------------------
----

AAAAGTGACACAGAAACATTCAGACCTATAGGAGGAGATATGAGGGACAATTGGAGAAGTGAATTATATAAAT
ATAAAGTGGTAGAAATTAAGCCATTA---GGAATAGCACCCACTAAGGCAAAAAGGAGAGTGGTG--------
--------------------------------GAGAGAGAGAAAAGAGCA---GTGGGA---ATAGGAGCT---
------ATGTTCCTTGGGTTCTTGGGGGCA---GCAGGAAGCACTATG---GGCGCAGCGTCA---
ATAACGCTGACGGTACAT---GCCAAACAA---TTATTG---TCTGGTATAGTGCAACAGCAAAGC---
AATTTG---CTGAGG---GCTATAGAGGCGCAACAGCATATGTTGCAACTCACGGTCTGGGGCATT
>KC894083
TTCTATTGCAATACATCAAAACTGTTTAATAGTACATATCCTAATAATAGTACAGACAAC-------------
----

ACTTCAGATACCATCACACTCCAATGCAGAATAAAACAAATTATAAACATGTGGCAGGAGGTAGGACGAGCAA
TGTATGCCCCTCCCATTGCAGGAAACATAACATGTAGATCAAAT---
ATCACAGGACTGCTATTGACACGTGATGGAGGAGGAAAAACTAAC----------------------------
----

AGCACAAATGAGACATTCAGACCTTTGGGAGGAGATATGAGGGACAATTGGAGAAGTGAATTATATAAATACA
AAGTGGTAGAAATTAAGCCATTA---GGATTAGCACCTACTAAGGCAAAAAGGAGAGTAGTG----------
-----------------------------GACAGAGAAAAAAGAGCA---GTAGGA---ATGGGAGCT------
---TTGTTCCTTGGGTTCTTGGGAGTA---GCCGGAAGCACTATG---GGCGCAGCATCA---
ATGACGCTGACGGTACAA---ACCAGACAA---TTGCTG---TCTGGTATAGTGCAACAGCAAAGC---
AATTTG---CTGAGG---GCCATAGAGGCGCAACAACATATGTTGCAACTCACGGTCTGGGGCATT
>KC894085
TTTTATTGCAATACAACAAAACTGTTTAATAGTACAGATAATAGCACAGGTACAAATAATAGCACAGAA----
----

ATCATACTCCCATGCAGAATAAAACAGATTATAAACATGTGGCAAGAGGTAGGGCGAGCAATGTATGCCCCTC
CCATTGCAGGAAACATAACATGTAAATCAAAT---
ATCACAGGGCTAATATTGACACGCGATGGAGGAGATAACAATAAC----------------------------
----

AATGACATAGAGACATTCAGACCTGGAGGAGGAGATATGAGGGATAATTGGAGAAGTGAATTATATAAATATA
AAGTGGTAGAAATTAAGCCATTG---GGAATAGCACCCACTGAGGCAAAAAGGAGAGTGGTG-----------
----------------------------CAGAGAGAAAAAAGAGCA---GTGGGA---ATAGGAGCT------
---ATGTTCCTTGGGTTCTTGGGATTC---GCAGGAAGCACTATG---GGCGCGGCGTCA---
ATAACGCTGACGGTACAG---GCCAGACTA---CTGTTG---TCTGGTATAGTGCAACAGCAAAAC---
AATTTG---CTGAGA---GCTATAGAGGCGCAACAGCATATGTTGCAACTCACAGTCTGGGGCATT
>KC894087
TTCTACTGCAATACATCAAAACTGTTTAATGGTATATATAATAGTACATATAATAGTACAGGCAACATTACA-
----

GAGGGTAAGAACATCACACTCCAATGCAGAATAAAACAAATTATAAATATGTGGCAGGGGGTGGACAGGCAA
TGTATGCCCCGCCCATTGCAGGAAACATAACATGTAAGTCAAAT---
ATCACAGGAATACTATTGACACGTGATGGAGGAAATAATAGTACAAGA-------------------------
----

ACAGAAGAGATATTCAGACCTGGAGGAGGAAACATGAAGGACAATTGGAGAAGTGAATTATATAAATATAAAG
TAGTAGAAATTAAGCCATTA---GGAGTAGCACCCACTGAGGCAAAAAGGAGAGTGGTG--------------
-------------------------GAGAGAGAAAAAAGAGCA---GTGGGA---CTGGGAGCG---------
TTGTTCCTTGGGTTCTTGGGAGCA---GCAGGAAGCACTATG---GGCGCAGCATCA---
ATAACGCTGACGGTACAG---GCCAGACAA---TTGTTG---TCTGGTATAGTGCAACAGCAAAGC---
AATTTG---CTGAGA---GCTATAGAGGCGCAACAGCATATGTTGCAACTCACAGTCTGGGGCATT
>KC894088
TTCTATTGCAATACATCAAGCCTGTTTAATGATGCATTTAATGATACAAACAGTACAGAA-------------
----

TCAAATTCAACCATCACACTCCCATGCAGAATAAAACAAATTGTAAACATGTGGCAGAGGGTAGGACGAGCAA
TGTATGCCTCTCCCATTAAAGGAAACATTACATGTAAATCAAAT---
ATCACAGGACTAATATTGACACGTGATGGAGGAAACGAAAATAAT----------------------------
----

GGAACAGACATATTCAGACCTATAGGAGGAAATATGAAGGACAATTGGAGAAGTGAATTATATAAATATAAAG
TAGTAGAAATTAAGCCATTA---GGAGTAGCACCCACTACTGCAAAAAGGAGAGTGGTG--------------
-------------------------GAGAGAGAAAAAAGAGCA---GTGACA---CTAGGAGCT---------
GTGTTCGTTGGGTTCTTGGGAGCA---GCAGGAAGCACCATG---GGCGCAGCATCC---
GTAATGCTGACGGTACAG---GCCAGACAA---TTATTG---TCTGGTATAGTGCAACAGCAAAGC---
AATTTG---CTGAGG---GCTATAGAGGCGCAACAGCATATGTTGCAACTCACGGTCTGGGGCATT
>KC894090
TTCTATTGTAACACATCAAGACTGTTTAATAATACATATAAGTTTAATGATACAAACATAGTTAATAGTACAA
ATAGTACA---

ACAAATTCAACCATCACACTCCAATGCAGAATAAAACAAATTATAAATATGTGGCAGGGGGTAGGACGGGCAA
TGTATGCCCCTCCTATTGCAGGAAACATAACATGTAAATCAAAT---
ATCACAGGACTACTATTGACACGTGATGGAGGTGGAAATAATAAAACA-------------------------
----

GAGACAGAGACATTCAGACCTGGAGGAGGAGATATGAGAGACAATTGGAGAAGTGAATTATATAAATATAAAG
TGATAGAAATTAAACCATTA---GGAATAGCACCCACTAGGGCAAAAAGGAGAGTGGTG--------------
-------------------------GAGAGAGAAAAAAGAGCA---GTGGGA---CTAGGAGCT---------
ATGATCCTTGGGTTCTTGGGAGCA---GCTGGAAGCACTATG---GGCGCAGCATCA---
ATAACGCTGACGGTACAG---GCCAGACAA---TTGTTG---TTTGGTATAGTGCAACAGCAAAAC---
AATTTG---CTGAGG---GCTATAGAGGCGCAACAGCATATGTTGCAACTCACAGTCTGGGGCATT
>KC894091
TTCTATTGCAATACAACACAACTGTTTAATGAAACAGATATA-------------------------------
----

GAAAGTAATTCAACTATAACAATCCCATGCAGATTAAAACAAATCATAAACATGTGGCAGGAGGTAGGACGAG
CAATGTATGCCCCTCCCATTGCAGGAAACATAACATGTAAATCAAAT---
ATCACAGGATTACTATTGACACGTGATGGAGGAAGAAATACAACA----------------------------
----

AATGACACAGAGATATTCAGACCTGCAGGAGGAGATATGAGGGACAATTGGAGAAGTGAATTATATAAATATA
AAGTGGTAGAAATTAAACCATTG---GGAATAGCACCCACTAAGGCAAAAAGGAGAGTGGTG----------
----------------------------GAGAGAGAAAAAAGAGCA---GCA------ATAGGAGCT------
---ATGTTCCTTGGGTTCTTGGGAGCA---GCAGGAAGCACTATG---GGCGCAGCATCA---
ATAACGCTGACGGTACAG---GCCAGACAA---TTGCTG---TCTGGTATAGTGCACCAGCAAAGC---
AATTTG---CTGAGG---GCTATAGAGGCACAACAACATATGTTGCAACTCACAGTCTGGGGCATT
>KC894093
TTCTATTGCAATACATCAAATCTGTTTAATAGTACACCAGAACTGTTTAATAGTGAGGGTACAGAAAGTAATT
CAACC---

AGCAACAAAAACATCACCCTCCCATGCAGAATAAAACAGATTATAAACATGTGGCAGGAGGTAGGACGAGCAA
TGTATGCCCCTCCCATTGAAGGAAACATAACATGTATATCAAAT---
ATCACAGGACTACTATTGACACGTGATGGAGGAATTGACCCCAAT---------------------------
----

AGCACAGATGAGACATTCAGGCCTGGAGGAGGAGAAATGAGAGACAATTGGAGGAGTGAATTATATAAATATA
AGGTGGTAGAAATTAAGCCATTG---GGAATAGCACCCACTGAGGCAAAAAGGAGAGTGGTG----------
----------------------------GAGAGAGAAAAAAGAGCA---GTGGGA---ATAGGAGCT------
---GTGTTCCTTGGGTTCTTGGGAGCA---GCAGGAAGCACTATG---GGCGCGGCGTCA---
ATAACGCTGACGGTACAG---GCCAGACAA---CTGTTG---TCTGGTATAGTGCAACAGCAAAGC---
AATTTG---CTGAAG---GCTATAGAGGCGCAACAGCATCTGTTGCAACTCACGGTCTGGGGCATA
>KC894094
TTCTATTGCGATACATCAGGCCTGTTTAATAGCACATACAATAGCACATACAATGATACAGATAACAGTACA-
----

GAAAACGACACGATCACGCTCCAATGTAGAATAAAACAAATTATAAGAATGTGGCAGGGGGTAGGACAAGCAA
TGTATGCCCCTCCCATTGCAGGAAACATAACATGTATATCAAAG---
ATCACAGGACTACTATTGACACGTGATGGAGGTATAAATAATGCT---------------------------
----

ACTAATGAGACATTCAGGCCTGCAGGAGGAGATATGAGGGACAATTGGAGAAGTGAATTATATAAATACAAAG
TGATAGAAATTAAGCCATTA---GGAATAGCACCCACTAAGGCAAAAAGGAGAGTGGTG-------------
-------------------------GAGAGAGAAAAAAGAGCA---GCA------CTAGGAGCT---------
ATGTTCCTTGGGTTCTTGGGAGCA---GCAGGAAGCACTATG---GGCGCAGCATCA---
ATAACGCTGACGGTACAG---GCCAGACAA---TTGTTG---GCTGGTATAGTGCAACAGCAAAAC---
AATTTG---CTGAGG---GCTATAGAGGCGCAACAGCATCTGTTGCAACTCACGGTCTGGGGCATT
>KC894095
TTCTACTGCAATACAACAAACTTGTTTAATAAAACATATGATAGTACG------------------------
----

TCTAACAAGACAATCATACTCACATGCAAAATAAAACAAATTATAAACATGTGGCAGGAGGTAGGACGAGCAA
TGTATGCCCCTCCTATTGCAGGAAACATAACATGTGAATCAGAT---
ATCACAGGAATACTATTGGTACGTGATGGAGGAAGTGAAAATGGCACA------------------------
----

GAAAGTAACACTGAGATATTCAGACCTGAAGGAGGAAATATGAAGGACAATTGGAGAAGTGAATTATACAAAT
ATAAAGTGGTAGAAATTAAGCCATTG---GGAGTAGCACCCACTGAGGCAAAAAGGAGAGTGGTG-------
-------------------------------GAGAGAGAAAAAAGAGCA---GTGGGA---ATAGGAGCT---
------GTGTTCCTTGGGTTCTTGGGAGCA---GCAGGAAGCACTATG---GGCGCGGCGTCA---
ATAACGCTGACGGTACAG---GCCAGACAA---GTGCTG---TCTGGTATAGTGCAACAGCAAAGC---
AATTTG---CTGAGG---GCTATAGAGGCGCAACAGCACTTGTTGCAACTCACGGTCTGGGGCATT
>KC862818
TTCTATTGCAATACATCAAACCTGTTTAATAAGACATATAACTTTAATGAGACT------------------
----

AAGAATGGAGCAATCACACTCCCATGCAAAATAAAACAAATTATAAGAATGTGGCAAAAAGTAGGACAAGCAA
TGTATGCTCCTCCCATTGCAGGAAACATAACATGTAAGTCTAAT---
ATCACAGGGTTACTATTGACGCGTGATGGAGGGAACACAACTGGGAATGAGACA------------TTC----
----

GAGACATTCAGACCTGGAGGAGGAGATATGAGGGACAATTGGAGAAATGAATTATATAAATATAAAGTGGTAG
AAATTAAGCCATTA---GGAGTAGCACCCACCAAGGCAAAAAGGAGAGTGGTG-------------------
-------------------GAGAGAGAAAAAAGAGCA---GTGGGA---ATAGGAGCT--------
GTGTTCCTTGGGTTCTTGGGAGCA---GCAGGAAGCACTATG---GGCGCAGCATCA---
ATAACGCTGACGGTACAG---GCCAGACAA---TTGCTG---TCTGGTATAGTGCAACAGCAGAGC---
AATTTG---CTGAGA---GCTATAGAGGCGCAACAGCATATGTTGCAACTCACAGTCTGGGGCATT
>KC894098
TTCTATTGCAATACATCAAAACTGTTTAATAGTACATACAATGGTACACACATGATTAATAGTACT------
----

TCAAACTCAACCATCACACTCCCATGCAGAATAAAACAAATTATAAACATGTGGCAGGAGGTAGGACGAGCAA
TGTATGCCCCTCCCATTAAGGGAAACATAACATGTACATCAAAT---
ATCACAGGACTACTGTTGACACGGGATGGGGGAGTTAATTTGACAGGGGGTGAG------------------
----

GGAGTTAATGACACAGAAACATTCAGACCTGAAGGAGGAAATATGAAGGACAATTGGAGAAGTGAATTATATA
AATATAAAGTGGTAGAAATTAAGCCATTA---GGAGTAGCACCCACTAAGGCAAAAAGGAGAGTGGTG-----
---------------------------------GAGAGAGAAAAAAGAGCA---GCA------
ATAGGAGCT---------ATGTTCCTTGGGTTCTTGGGAGCA---GCAGGAAGCACTATG---
GGCGCAGCATCA---ATAACGCTGACGGTACAA---GCCAGACAA---TTGCTG---
TCTGGTATAGTGCAACAGCAAAGC---AATTTG---CTGAGA---
GCTATAGAGGCGCAACAGCATCTGTTGCAACTCACAGTCTGGGGCATT
>KC862876
TTCTATTGCAATACATCAAACCTGTTTAATTTTACATACCTGCCTAATGGAACTAGTATAAACAGCGCTAACA
TT--

TTAAATGGGACCATCACACTCCCATGCAGAATAAAACAAATTATAAACATGTGGCAGGAGGTAGGAAAAGCAA
TGTATGCCCCTCCAGTTGCAGGAAAAATAACATGTAAATCAAAT---
ATCACAGGAATACTATTGACACGTGATGGAGGCAAAGATAACAAT---------------------------
----

GAGACAGAGACATTCAGACCTGGAGGAGGAGATATGAGGGACAATTGGAGAAGTGAATTATATAAATATAAAG
TGGTAGAAATTAAGCCATTA---GGAATAGCACCCACTGATGCAAAAAGGAGAGTGGTG-------------
-------------------------GAGAGAGAAAAAAGAGCA---GTGGGA---ATAGGAGCT--------
GTGTTCCTTGGGTTCTTGGGAGCA---GCAGGAAGCACTATG---GGCGCGGCGTCT---
ATAACGCTGACGGTACAG---GCCAGACAA---TTATTG---TCTGGTATAGTGCAACAGCAAAGC---
AATTTG---CTGAGG---GCTATAGAGGCGCAGCAGCATATGTTGCAACTCACGGTCTGGGGCATT
>KC894100
TTCTATTGCAATACATCAGGCCTGTTTAAAGGCCTGTTTAATGGTAGCAGAGAATGGGTTAATGGTACAGACA
ATAGT---
----------ACAAATAGT------
ACTCTAGATTCAGTCATCACACTCCCATGCAGAATAAAACAAATTATAAGAATGTGGCAGGGGGTAGGACAAG
CAATGTATGCCCCTCCCATTGCAGGAAACATAACATGTAAATCAAAT---
ATCACAGGAATACTATTGACACGTGATGGAGGAATACGTGAGGGA---GGAAAT------
ACAAGTGAGGGAGGAAAT--

GACACAGAGGTATTCAGACCTGGAGGAGGAGACATGAGGGACAATTGGAGAAGTGAATTATATAAATATAAAG
TGGTAGAAATTAAGCCATTA---GGAGTAGCACCCACTCCTGCAAAAAGGAGAGTGGTG-------------
-------------------------GAGAAACAGAAAAGAGCA---GTGGGA---ATGGGAGCT--------
GTGATCTTTGGGTTCTTGGGAGCA---GCAGGAAGCACTATG---GGCGCGGCGTCA---
ATAGCGCTGACGGCACAG---GCCAGACAA---TTATTG---TCTGGTATAGTGCAACAGCAAAGC---
AATTTG---CTGAGG---GCTATAGAGGCGCAACAGCATATGTTGCAACTCACAGTCTGGGGCATT
>KC862949
TTCTATTGCAATACATCAAAACTGTTTAATATTACAAACTTGAATAAAACAGAC------------------
----

AGTAATTCAACAATCACACTCCCATGCAGAATAAAACAAATCATAAACATGTGGCAGGGGGTAGGACGAGCAA
TATATGCTCCTCCCATTGCAGGAAACATAACATGTAACTCTAGC---
ATCACAGGATTACTATTGACACGTGATGGAGGATCCAATGGTAAC---------------------------

----

GACACTGAGGTATTCAGACCTGGAGGAGGAGATATGAGGGACAATTGGAGAAGTGAATTATATAAATATAAAG
TGGTAGAAATTAAGCCATTG---GGAATAGCACCCACGGCGTCAAAAAGGAGAGTGGTG--------------
-------------------------GAGAGAGAAAAAAGAGCA---GTGGGA---ATAGGAGCT---------
GTGTTCCTTGGGTTCTTGGGAGCA---GCAGGAAGCACTATG---GGCGCAGCGTCA---
ATAACGCTGACGGTACAG---GCCAGACAA---TTGCTG---TCTGGTATAGTGCAGCAGCAAAAC---
AACTTG---CTGAGG---GCTATAGAGGCGCAACAGCATCTGTTGCAACTCACGGTCTGGGGCATT
>KC863009
TTCTATTGCAATACATCACGACTGTTCAATGGTACATACAGTACTAATAATACAAATAGTACT----------
----

TCAAATTCAACCATCACAATTCCATGCAGAATAAAACAAATTATAAACATGTGGCAGGGGGTAGGACAAGCAA
TGTATGCCCCTCCCATTGAAGGAAACATAACATGCAGGTCAAAT---
ATCACAGGACTATTACTGACACGTGATGGAGGAAAAGGAAATGACACA-------------------------
----

AATGACACACAGACATTCAGACCTGGAGGAGGAGATATGAGGGACAATTGGAGAAGTGAATTATATAAATATA
AAGTAGTAGAAATTAAGCCATTA---GGAATAGCACCCACTAAGGCAAAAAGGAGAGTGGTG-----------
----------------------------GAGAGAGAAAAAAGAGCA---GCG------CTAGGAGCT------
---GTGTTCCTTGGGTTCTTGGGAGCG---GCAGGAAGCACTATG---GGCGCAGCGTCA---
ATAACGCTGACGGTACAA---GCCAGACAA---CTGCTG---TCTGGTATAGTGCAACAGCAAAGC---
AATTTG---CTGAGG---GCTATAGAGGCGCAACAGCATATGTTGCAACTCACGGTCTGGGGCATT
>KC894385
TTTTATTGCAATACATCAAACCTGTTTAATAGGATATATAATATTACAGACAATAGTACAGGAAGTGAT----
----

GACAATTCAACCATCACACTCCAATGCAGAATAAAACAAATTATAAACATGTGGCAGAAGGTAGGAAAAGCAA
TATATGCCCCTCCCATTGCAGGGAACATAACATGTGAATCAAAT---
ATCACAGGACTACTATTGACACGTGATGGAGGAAACAATGATAGT----------------------------
----

ATCACCACAGAGACATTCAGACCTGGAGGGGGAGATATGAAGGACAATTGGAGAAGTGAATTATATAAATATA
AAGTGGTAGAAATTAAGCCATTA---GGAATAGCACCCACCAGGGCAAAAAGGAGAGTGGTG-----------
----------------------------GAGAGAGAAAAAAGAGCA---GTGGGA---ATAGGAGCT------
---GTGTTCATTGGGTTCTTGAGTGCA---GCAGGAAGCACTATG---GGCGCAGCGTCA---
ATAACGCTGACGGTACAG---GCCAGACAA---CTGTTG---TCTGGTATAGTGCAACAGCAAAGC---
AATTTG---CTGAGG---GCTATAGAGGCGCAACAGCATATGTTGAAACTCACGGTCTGGGGCATT
>KC156212
TTCTATTGCAATACATCAAGATTGTTTAGTAGTAATAGTACAGAAATTAATAGTACAGAAAGT----------
----

TCCAATTCAACAATCATACTCCCATGCAGAATAAAACAAATTATAAATATGTGGCAGGGGGTGGGACGGGCAA
TATATGCAGCTCCTATTGCAGGAAAAATAACATGTAAATCAAAT---
ATCACAGGACTAATATTGACACGTGATGGAGGAGAGGGCAATAATACA-------------------------
----

GAAAACAGAAGTGAGATATTCAGACCTACAGGAGGAGATATGAGGGACAATTGGAGAAGTGAATTATACAAAT
ACAAAGTAGTAGAAATTAAGCCATTA---GGAGTAGCACCCACTAATGCAAAAAGGAGAGTGGTG--------
-------------------------------GAGAGAGAAAAAAGAGCA---GTGGGA---TTAGGAGCT---
------ATGTTCCTTGGGTTCTTGGGAATG---GCCGGAAGCACTATG---GGCGCAGCATCA---
ATAACGCTGACGGTACAG---GCCAGACAA---GTATTG---TCTGGTATAGTGCAACAGCAAAGC---
AATTTG---CTGAGG---GCTATAGAGGCGCAACAGCATATGTTGCAACTCACGGTCTGGGGCATT
>KC156211
TTCTATTGCAATACATCAGGTCTATTTAATGGGACATACAATAGTACAAGAACTAATTCAACTAAT-------
----

TCAACTTCAATCATCACACTCCAATGCAGAATAAAACAAATTGTAAACCTATGGCAGGGGGTAGGAAAAGCAA
TGTATGCCCCTCCCATTGCAGGAAACATAACATGCAGATCAGAT---
ATCACAGGACTACTATTGACACGTGATGGAGGAAACGGAAATAATAGT-------------------------

----

ACAGAGACAGAGACATTCAGGCCTGGAGGAGGAGATATGAGGGACAATTGGAGAAGTGAATTATACAAATACA
AAGTGGTAGAAATTAAGCCATTA---GGATTAGCACCCACTGAGGCAAAAAGGAGAGTGGTG-----------
----------------------------GAGAGAGAAAAAAGAGCA---GTGGGA---CTGGGAGCG------
---ATGTTCCTTGGGTTCTTAGGAGCA---GCAGGAAGCACTATG---GGCGCAGCATCA---
ATAACGCTGACGGTACAG---GCCAGACAA---TTATTG---TCTGGTATAGTGCAACAGCAAAGC---
AATTTG---CTGAGA---GCTATAGAGGCGCAACAGCATATGTTGCAACTCACAGTCTGGGGCATT
>KC156213
TTCTATTGTAATACATCAGGACTGTTCAATAGTACATACAATAGTACATACAATGGTCCATACAAT-------
----

GGTACAGCAATAATCACACTCCCATGCAGGATAAAACAAATTATAAACATGTGGCAGGAGGTAGGCAGAGCAA
TGTACGCCCCTCCCATTGAAGGAAACATAACATGTAACTCAAGC---
ATCACGGGACTACTCTTGGTACGTGATGGAGGCGAAACAAATAGTTCC-------------------------
----

CAAGAGGAGATATTCAGACCTGGAGGAGGAGATATGAGAGACAATTGGAGAAGTGAATTATATAAATATAAAG
TGGTAGAAATTAAACCATTA---GGAATAGCACCTACTGAGGCAAAAAGGAGAGTGGTG--------------
-------------------------GAGAGAGAAAAAAGAGCA---GTGGGA---ATAGGAGCT---------
GTGTTCCTTGGGTTCTTGGGAGCA---GCAGGAAGCACTATG---GGCGCAGCATCA---
ATAACGCTGACGGTACAG---GCCAGACAA---TTGTTG---TCTGGTATAGTGCAGCAGCAAAGT---
AATTTG---CTGAGA---GCCATAGAGGCGCAACAGCATATGTTGCAACTCACAGTCTGGGGCATT
>KC156119
TTCTATTGCAATACATCAAAACTGTTTAATAGTACATATAATTTTAATTTTAATGGTACAGAAGATAAC----
----

TCAAGCTCAGTCATCACTCTCTCATGCAGAATAAAACAAATTATAAACATGTGGCAAGAGGTAGGACGAGCAA
TGTATGCCCCTCCCATTGAAGGAAAAATAACATGTAGGTCAGAT---
ATCACAGGACTACTATTGACACGTGATGGAGGAAACAATAATACA----------------------------
----

GAGGAAATATTTAGACCTGGGGGGGGAAATATGAAAGACAATTGGAGAAGTGAGTTATATAAATACAAGGTGG
TAGAAATAAAGCCATTA---GGAGTAGCACCCACTAAGGCAAAAAGAAGAGTGGTG-----------------
----------------------GAGAGAGAGAAAAGAGCA---GTAGGA---ATAGGAGCT---------
GTGTTCCTTGGGTTCTTGGGAGCA---GCAGGAAGCACTATG---GGCGCGGCGTCA---
ATAACGCTGACGGTACAG---GCCAGACAA---TTATTG---TCTGGTATAGTGCAACAGCAAAGT---
AATTTG---CTGAAG---GCTATAGAGGCGCAACAGCATATGTTGCGACTCACAGTCTGGGGCATT
>KC894109
TTCTATTGCGATACATCAGAACTGTTTAATGGTACATACAATAATAGTACATACAAT----------------
----

AGTAGTAATAATATCACACTCCAATGCAAAATAAAACAAATCATAAACATGTGGCAGGGAGTAGGACGAGCAA
TGTATGCCCCTCCCATTGCAGGAAACATAACATGTGAATCAAAT---
ATCACAGGACTATTATTGACACGGGATGGAGGTAATAATAAGAGC----------------------------
----

ACTCCTGAAACATTCAGACCTGGAGGAGGAGATATGAGGGACAATTGGAGAAGTGAATTATATAAATATAAAG
TGGTAGAAATTAAGCCATTA---GGAATAGCACCCACTAAGGCAAAAAGGAGAGTGGTG--------------
-------------------------GAGAGAGAAAAAAGAGCA---GTGGGA---ATGGGAGCT---------
GTGTTCCTTGGGTTCTTGGGAGCA---GCCGGAAGCACTATG---GGCGCAGCATCA---
TTAACGCTGACGGTACAG---GCCAGACAA---TTGCTG---TCTGGTATAGTGCAACAGCAAAAC---
AATTTG---CTGAGA---GCTATAGAGGCGCAACAGCATATGTTGCAACTCACGGTCTGGGGCATT
>KC148599
TTCTATTGCAATACATCAGGACTGTTTAATGCTACAAGCAATTTTACAAGAGAT-------------------
----

TCAAATTCAACTATCACAATCCCATGCAGAATAAAGCAAATTATAAACATGTGGCAGGAGGTAGGAAAAGCAA
TGTATGCCCCTCCCATAGAAGGAAACATAACATGTGTATCAAAT---
ATCACAGGACTACTATTGGTACGTGATGGAGGTGGGACAAATAACTCAACCTCA-------------------

----

ACAAATGAGACATTCAGACCTGGAGGAGGAGATATGAGGGACAATTGGAGAAGTGAACTATATAAATATAAAG
TGGTAGAAATTAAGCCATTG---GGAATAGCACCCACTAAGGCAAAAAGGAGAGTGGTG--------------
-------------------------GAGAGAGAAAAAAGAGCA---GTAACA---ATAGGAGCT---------
GTATTCCTTGGGTTCTTGGGAGCA---GCAGGAAGCACTATG---GGCGCGGCGTCA---
ATGACGCTGACGGTACAG---GCCAGACAA---CTGTTG---TCTGGTATAGTGCAACAGCAAAGC---
AATTTG---CTGCGG---GCTATAGAGGCGCAACAACATATGTTGCAACTCACAGTCTGGGGCATT
>KC148663
TTCTATTGCAATACAACAAGTCTGTTTAACGGTCTGTTTAACGGTACATATATGTCTAATAGTACAGATAGTA
CT---

GAAAATGGAACCATCACACTCCCATGCAGAATAAAACAAATCATAAACATGTGGCAGAAGGTAGGACGAGCCA
TATATGCCCCTCCCATTGCAGGAAACATAACATGTATATCAGAT---
ATCACAGGATTACTATTGACACGTGATGGAGGAATAAATAACACA----------------------------
----

GAGGATGAGACGTTCAGACCTGGCGGAGGAGATATGAGGGACAATTGGAGAAGTGAATTATACAAATATAAAG
TGATAGAAATTAAGCCACTA---GGAATAGCACCCACTACTGCAAAAAGGAGAGTGGTG--------------
-------------------------GAAAAAGAAAAAAGAGCA---TTGGGA---GTAGGAGCT---------
GTGCTCCTTGGGTTCTTGGGAGCA---GCAGGAAGCACTATG---GGCGCAGCATCA---
ATAACGCTGACGGTACAG---GCCAGACAA---TTGCTG---TCTGGTATAGTGCAACAGCAAAAC---
AATTTG---CTGAAA---GCTATAGAGGCGCAACAGCATTTGTTGCAACTCACGGTCTGGGGCATT
>HM204583
TTCTATTGTAATACATCAGGCCTATTTAATAGAACAGCAAACATGACTAGAACGGCAAACATGACT-------
----

ACAAATTCAACCATTATACTCCGATGCAGAATAAAACAAATTGTAAACATGTGGCAAGGGGTAGGACGAGCAA
TGTATGCTCCTCCCATTGCAGGAAACATAACATGTGCATCTAAT---
ATCACAGGACTACTGTTGACACGTGATGGAGGAGAAAATAACACC----------------------------
----

GAAAATAAGGAGATATTCAGACCAGGAGGAGGAGACATGAGGGACAATTGGAGAAGTGAATTATATAAATATA
AAGTAGTAGAAATTAAGCCATTA---GGAATAGCACCCACTACGGCAAAAAGGAGAGTGGTG-----------
----------------------------GAGAGAGAAAAAAGAGCA---GTGGGA---ATAGGAGCT------
---GTGCTCCTTGGGTTCTTGGGAGCA---GCAGGAAGCACTATG---GGCGCGGCGTCA---
CTGACGCTGACGGTACAG---GCCAGACAA---TTGTTG---TCTGGTATAGTGCAACAGCAAAGC---
AATTTG---CTGAGA---GCTGTAGAGGCGCAACAGCACATGTTGCAACTCACGGTCTGGGGCATT
>HM204591
TTCTATTGCAATACATCAAACCTGTTCGATAAATTAAATAGTACT----------------------------
----

AATGGTACAAACATCACACTCCCATGCAGAATAAAACAAATTATAAACACGTGGCAGGGGGTAGGACGGGCAA
TATATGCAGCTCCTATTGCAGGAAAAATACAATGTATATCAAAT---
ATCACAGGACTACTATTGACACGTGATGGAGGAAAGGACAATAATAATAATACA-------------------
----

AATAACAGTATTACTGAGACAATCAGACCTGGAGGAGGAGATATGAGGGACAATTGGAGAAGTGAACTATATA
AATATAAAGTAGTAGAAATTAAGCCATTA---GGAATAGCACCCACTAATGCAAAAAGGAGAGTGGTG-----
----------------------------------GAGAGAGAAAAAAGAGCA---GTGGGA---
TTAGGAGCT---------ATGTTCCTTGGGTTCTTGGGAATG---GCCGGAAGCACTATG---
GGCGCAGCATCA---ATAACGCTGACGGTACAG---GCCAGACAA---GTATTG---
TCTGGTATAGTGCAACAGCAAAGC---AATTTG---CTGAGG---
GCTATAGAGGCGCAACAGCATATGTTGCAACTCACGGTCTGGGGCATT
>FJ443615
TTCTACTGCAATACATCACCATTGTTTAATAGTACATACGATCTTAATGGT----------------------
----

ACTGCTTCAACCATCACACTCCAATGTAAAATAAAACAATCATAAACATGTGGCAAGGGGTAGGAAAAGCAA
TGTATGCTCCTCCCATTGAAGGGAACATAACATGTAACTCAAGT---

```
ATTACAGGATTACTATTGACACGTGATGGAGGAACACGAGATCGA----------------------------
-------------------------------------------------------------------------
----------
AATGACACAGAAACATTTAGACCTGGAGGAGGAGATATGAGGGACAATTGGAGAAGTGAGTTATACAAATATA
AAGTGGTGGAAATTAAGCCATTG---GGAATAGCACCCACTGAGGCAAAAAGGAGAGTGGTG-----------
----------------------------GAAAGAGAAAAAAGAGCA---GTAGGA---ATAGGAGCT------
---GTGTTCCTTGGGATCTTGGGAGCA---GCAGGAAGCACTATG---GGCGCGGCGTCA---
ATAACGCTGACGGTACAG---GCCAGACAA---CTGTTG---TCTGGTATAGTGCAACAGCAAAAC---
AATTTG---CTGAGA---GCTATAGAGGCGCAACAGCATATGTTGCAACTCACGGTCTGGGGCATT
>KP109520
TTCTATTGCAATACATCAAACCTATTTAATAGTACGTTGTCTAATAGTACTAACAAT----------------
-------------------------------------------------------------------------
----------------------------
AGTACTTCAACCATCACACTCCAATGTAAAATAAAACAAATAATAAACATGTGGCAGGGGGTAGGACGGCCA
TGTATGCCCCTCCCATTGCAGGAAACATAACATGTAAATCAAAT---
ATCACAGGAATACTTTTGTCACGTGATGGAGGCAAGGCTGAAAATGGT-------------------------
-------------------------------------------------------------------------
----------
ACAAAGATAGAGATATTCAGACCTGGAGGAGGAGATATGAGAGACAATTGGAGAAGTGAACTATACAAATATA
AGGTGATAGAAATAAAACCATTA---GGAATAGCACCCACTAAGGCAAAGAGGAGAGTGGTG-----------
----------------------------GAGAGAAAAAAAAGAGCA---GTAGGA---ATAGGAGCT------
---GTGTTCCTTGGGTTCTTGGGAGCA---GCCGGAAGCACTATG---GGCGCAGCATCA---
ATAACGCTGACGGTACAA---GCCAGACAA---TTGTTG---TCTGGTATAGTGCAACAGCAAAGC---
AATTTG---CTGAAG---GCTATAGAAGCGCAACAACATATGTTGCAACTCACAGTCTGGGGCATT
>HM070539
TTCTACTGCAATACCTCAGCACTGTTTAATAGTACATGGAGGTGTGATAATACTTCATGTTCACGTGTGTCTA
ATAAT--------------------------------------------------------------------
-------------------ACA---
GATAATTCTCCAAACATCACACTCCAATGCAGAATAAAACAAATTATAAACATGTGGCAGGGGGTAGGAAGAG
CAATGTATGCCTCTCCCATTGCAGGAAACATAACATGTAGATCAAAC---
ATTACAGGACTACTATTGACATGGGATGGAGATAATAGTAGTAAT----------------------------
-------------------------------------------------------------------------
-------------
ACAACAGAGACATTCAGACCTGGAGGAGGAAATATGAAAGACAATTGGAGAAGTGAATTATATAAATATAAAG
TGGTAGAAATTAAGCCATTA---GGAATAGCACCTACTGGGGCAAAAAGGAGAGTGGTG--------------
-------------------------GAGAGAGGGAAAAGAGCA---GTGACA---CTAGGAGCT---------
GTGTTCCTTGGGTTCTTGGGAGCA---GCAGGAAGCACTATG---GGCGCGGCGTCA---
ATAACGCTGACGGTACAG---GCCAGACAA---TTATTG---TCTGGTATAGTGCAACAGCAGAGC---
AATCTG---CTGAGA---GCTATAGAGGCGCAGCAGCATCTGTTGCAACTCACGGTCTGGGGCATT
>HQ595984
TTCTATTGCGATACATCAACATTGTTTAATAGTACATACATGCCTAATATTACTTCAAATTCAAGTACTTCAA
AT-----------------------------------------------------------------------
----------------------------
TCAAGTTCACTCATCACACTCCAATGCAAAATAAAACAAATTATAAATATGTGGCAGGAGGTAGGACGAGCAA
TGTATGCCCCTCCCATTAAAGGGAACATAACATGCAACTCAACT---
ATCACAGGATTAATATTGACACGTGATGGAGGAACTGGAAATAACACT-------------------------
-------------------------------------------------------------------------
----------
AACGATACAGAGATATTCAGACCTGGAGGAGGAGATATGAGAGACAATTGGAGAAGTGAATTATATAAATATA
AAGTGGTAGAAATTAAGCCATTA---GGAGTGGCACCCACTAAGGCAAAAAGGAGAGTGGTG-----------
----------------------------GAGAGAGAAAAAAGAGCA---GTGGGA---ATAGGAGCT------
---GTGCTCCTTGGGTTCTTGGGAGCA---GCAGGAAGCACTATG---GGCGCAGCATCA---
ATAACGCTGACGGTACAG---GCCAGACAA---TTATTG---TCTGGTATAGTGCAACAGCAAAGC---
AATTTG---CTGAGA---GCTATAGAGGCGCAACAGCACCTGTTGCAACTCACAGTCTGGGGCATT
>HQ596030
TTCTATTGCAATACATCAGGCCTGTTTAATGGGACATATGATCCTAGATATAATAGTACATATAATAGTACAG
AAAAT--------------------------------------------------------------------
-------------------AAT---
AGTACAACCCATAACATCACACTCCAATGCAGAATAAAGCAAATTATAAACATGTGGCAGGAGGTAGGAAAAG
CAATATATGCCCCTCCTATTGCAGGAAGTCTAACATGTACATCAAAC---
```

ATCACAGGACTACTATTGGTACGTGATGGAGGTAAAAGAAACAATACC------------------------
----

AATGACCCAGAGATATTCAGACCTGGAGGAGGAAATATGAAGGACAATTGGAGAAGTGAATTATATAAATATA
AAGTAGTAGAAATTAAGCCCTTA---GGAGTAGCACCCACTGAGGCAAAAAGGAGAGTGGTG----------
----------------------------GAGAGAGAAAAAAGAGCA-----------ATAGGAGCT------
---GTGTTCCTTGGGTTCTTGGGAGCA---GCAGGAAGCACTATG---GGCGCGGCGTCA---
GTGGCGCTGACGGTACAC---ACCAGAAAA---CTGTTG---TCTGGTATAGTGCAACAGCAAAGC---
AATTTG---CTGAGA---GCTATAGAGGCGCAACAGCATATGTTGCAACTCACGGTCTGGGGCATT
>HM070639
TTCTATTGCAATACATCAAACCTGTTTAATAAGACATATTGGATTAATAGTACAGAAGGTAATACAGAAGATG
ATACA---

AGCTCAAACTCAACGATCACACTCCCATGCAGAATAAAACAAATTATAAATATGTGGCAGGGGGTAGGACGAG
CAATATATGCCCCTCCCATTGCAGGAAACATAACATGTAAATCAAAT---
ATCACAGGACTACTATTGACACGTGATGGAGGAAATGATAATAGA---------------------------
----

AGTACAAATGAAACATTCAGACCTGGAGGAGGAGATATGAGGGACAATTGGAGAAGTGAATTATACAAATATA
AAGTGGTAGAAATTAAGCCATTA---GGAATAGCACCAACTGCTGCCAAGAGAAGAGTGGTG----------
----------------------------GAGAGAGAAAAAAGAGCA---GTGGGA---ATAGGAGCT-----
---GTGTTCCTTGGGTTCTTGGGAGCA---GCAGGAAGCACTATG---GGCGCAGCGTCA---
ATAACGCTGACGGTACAG---GCCAGACAA---TTATTG---TTTGGTATAGTGCAACAGCAAAGC---
AATTTG---CTGAGG---GCCATAGAGGCGCAACAGCATATGTTGCAACTCACGGTCTGGGGCATT
>KF527172
TTCTATTGCGATACATCAGGATTGTTTAATGGCACATACAATCCTAATGATACAACAAGTAATTCAAGCTCAC
TT--

AACAACATCACACTCACACTCCCATGCAAAATAAAGCAAGTTATAAACATGTGGCAGGGGGTAGGAAAAGCGA
TATATGCCCCTCCCATTGCAGGAAACATAACATGTAGATCAAAT---
ATCACAGGACTACTATTGCTACGTGATGGAGGAACTACAAATGAC---------------------------
----

ACAGAAGAGATATTCAGACCTGGAGGAGGAGATATGAGGGACAATTGGAGAAGTGAATTATATAAATACAAAG
TGGTAGAAATTAAGCCATTG---GGAGTAGCACCCACTAAGGCAAAAAGGAGAGTGGTG-------------
-------------------------GAGAGAGAAAAAAGAGCA---GTGGGA---ATAGGAGCT--------
GTACTCCTTGGGTTCTTAGGAGCG---GCAGGAAGCACTATG---GGCGCGGCGTCA---
ATAACGCTGACGGTACAG---GCCAGACAA---CTGTTG---TCTGGTATAGTGCAACAGCAAAGC---
AATTTG---CTGAGG---GCTATAGAGGCGCAACAGCATATGTTGCAACTCACGGTCTGGGGCATT
>HM070807
TTCTATTGCAATACATCACGCCTGTTTAATGGTACATATAATAGTACAAGATATAATACAACAGAA------
----

GCAAATTCAACCATCACACTCCAATGCAGAATAAAACAAATTATAAACATGTGGCAGGAGGTAGGACGGGCGA
TGTATGCCCCTCCCATTGCAGGAAACATAACATGTAAATCAAAT---
ATCACAGGACTACTATTGACACGTGATGGAGGACACGGAAATGGGACA------------------------
----

GACAATAAGACAGAAACATTTAGACCAGGAGGAGGAGATATGAGGGACAATTGGAGGAGTGAATTATATAAAT
ATAAAGTGGTAGAAGTTAAGCCATTA---GGAATAGCACCCACTAAGGCAAAAAGAAGAGTGGTG-------
------------------------------GAGAGAGAGAAAAGAGCA---GTGGGA---GTAGGAGCT---
------ATGTTCCTTGGGTTCTTGGGAGCG---GCCGGAAGCACTATG---GGCGCAGCATCA---
ATAACGCTGACGGTACAG---GCCAGACAA---TTATTG---TCTGGTATAGTGCAACAGCAAAGC---
AATTTG---CTGAGG---GCTATAGAGGCGCAACAGCATATGTTGCAACTCACGGTCTGGGGCATC
>HQ596147
TTCTATTGTAATACATCAAAACTGTTTAATAGTACATACATGCCTAATAGTACTTCAAATTCAACACACATG-
----

TCTAATGGTACCATCACACTCCAATGCAGAATAAAACAAATTATAAACATGTGGCAGGAGGTAGGACGAGCGA
TGTATGCCCCTCCTATCGAAGGAAACATAATATGTAAATCAAAT---

ATCACAGGAATACTATTGACACGTGATGGAGGTAAGAATGAGACT----------------------------
----

ACAGAGACATTCAGACCTGGAGGAGGAGATATGAGGGACAATTGGAGAAGTGAGTTATATAAATATAAAGTGG
TAGAAGTTAAGCCATTA---GGGATAGCACCTACTACTGCAAAAAGGAGAGTGGTG-----------------
----------------------GAGAGAGAAAAAAGAGCA---GTGGGA---ATAGGAGCT---------
GTGTTCCTTGGGTTCTTGGGTGCA---GCAGGAAGCACTATG---GGCGCGGCGTCA---
ATAACGCTGACGGTACAG---GCCAGACAA---TTATTG---TCTGGTATAGTGCAACAACAAAGC---
AATTTG---TTGAGG---GCCATAGAGGCGCAACAACACATGTTGCAACTCACGGTCTGGGGCATT
>KC156218
TTCTATTGTGATACATCAAAACTGTTTAATAGGACATACACGGTTAATGACACACACGAGACTAATAGTACTG
AT---

ACAAACAGTACCATCACACTCCCATGCAGAATAAAACAAATTATAAACATGTGGCAGAGAGTAGGACAAGCAA
TGTATGCTTCTCCCATTGCAGGAAACATAACATGTAACTCAAGT---
ATCACAGGATTGCTACTGACGAGTGATGAAGGAGGAAATAACAAC----------------------------
----

GAAAACGAGACATTCAGGCCTGGAGGAGGAGATATGAGGGACAATTGGAGAAGTGAATTATATAAATATAAAG
TGGTAGAAGTTAAGCCATTG---GGAATAGCACCCACCAAGGCAAAAAGGAGAGTGGTG--------------
-------------------------GAGAGAGAGAAAAGAGCA---GTAGGA---ATAGGAGCT---------
TTGTTCCTTGGGTTCTTGGGAGCA---GCAGGAAGCACTATG---GGCGCGGCGTCA---
ATGACGCTGACGGTACAG---GCCAGACAA---TTGTTG---TCTGGTATAGTGCAACAGCAAAGC---
AATTTG---CTGAGG---GCTATAGAGGCGCAACAACATATGTTGCAACTCACAGTCTGGGGCATT
>KC156215
TTCTATTGTAATACATCACGCCTGTTTAATAGTACATACAATGGTACAGACGGGTCTAAT-------------
----

AGCACAACAAACATCACACTCCCATGCAGAATAAAACAAATTATAAACATGTGGCAGGAGGTAGGACGAGCAA
TGTATGCCCCTCCCATTGCAGGAAACATAACATGTAAATCAAAT---
ATCACAGGAATACTATTGACACGTGATGGAGGAGGGAACAGCACA----------------------------
----

GACCCAGAGATATTCAGACCTGGAGGAGGAGATATGAGGGACAATTGGAGAAGTGAATTATACAAATATAAAG
TGGTAGAAATTAAGCCATTA---GGAATAGCACCCACTACTGCAAAAAGGAGAGTGGTG--------------
-------------------------GAGAGAGAGAAAAGAGCA---GTGGGA---ATAGGAGCT---------
GTGTTCCTTGGGTTCTTGGGAGCA---GCAGGAAGCACTATG---GGCGCGGCGTCA---
ATAACGCTGACGGTACAG---GCCAGACAA---TTATTG---TCTGGTATAGTGCAACAGCAAAAC---
AATTTG---CTGAGA---GCTATAGAGGCGCAACAGCATATGTTGCAACTCACAGTCTGGGGCATT
>HQ615962
TTCTATTGCGATACATCAAAACTGTTTAATAGGACATACACCAATGACACACACTTCAAAGAGACAGATCCAA
GT---

ATAAATGGCACAATCACAATCACATGCAAAATAAAACAATTTATAAACATGTGGCAGGAGGTAGGAAAAGCAA
TGTATGCCCCTCCCATTGCAGGAAACATAACATGTAAGTCAAAT---
ATCACAGGAATACTATTGGTACGCGATGGGGGTAAAACCAATAGT----------------------------
----

ACAAATGAGACATTCAGACCTGGAGGGGGAGACATGAGGGACAATTGGAGAAGTGAATTATATAAATATAAAG
TGGTAAGAATTGAGCCATTG---GGAGTAGCACCCACTACGGCAAAAAGGAGAGTGGTG--------------
-------------------------GAGAGAGAAAAAAGAGCA---GTGGGA---CTAGGAGCT---------
GTGTTCCTTGGGTTCTTGGGGGCA---GCCGGAAGCACTATG---GGCGCGGCGTCA---
ATAACGCTGACGGTACAG---GCCAGACAA---CTGTTG---TCTGGCATAGTGCAGCAGCAAAGC---
AATTTG---CTGATG---GCTATAGAGGCGCAACAACATATGTTGCAACTCACAGTCTGGGGCATT
>KY112213
TTCTATTGTAATACATCAGGTCTGTTTAATAGGACATACTCGCTTAATAATGATACAGAACAGAATAATAGT-
----

ACAAACAAAACCATCACACTCCCATGCAGAATAAAACAAATTATAAACATGTGGCAGGAGGTAGGACGAGCCA
TGTATGCCCCTCCCATTGCAGGAAACATAACATGTAACTCAAAT---

ATTACAGGACTACTATTGACACGTGATGGAGGAGAAGGAAATGAT----------------------------
----

AGTGATGAGATTTTCAGACCTGGAGGAGGAGATATGAGAGACAATTGGAGAAGTGAATTATATAAATACAAAG
TAGTAGAAATAAAACCATTA---GGAATAGCACCCACTAAGGCAAGAAGGAGAGTGGTG--------------
-------------------------GAGAGAGAGAAAAGAGCA---GTGGGA---ATAGGAGCT---------
GTGTTCCTTGGGTTCTTGGGAGCA---GCAGGAAGCACTATG---GGCGCAGCGTCA---
ATAACGCTGACGGTACAG---GCCAGACAA---TTATTG---TCTGGAATAGTGCAACAGCAAAGC---
AATTTG---CTGAGG---GCAATCGAGGCGCAACAGCATATGTTGCAACTCACGGTCTGGGGCATT
>KP109521
TTCTATTGCAATACATCAAAATTGTTTAATAGTACATTTAATAGTACACACATGGGTAATGACACACACAGGA
ATAATGAT---

ACAGACAGTACCATCACGATCCCATGCAGAATAAAACAAATTATAAACATGTGGCAGAGGGTAGGACAAGCAA
TGTATGCTTCTCCCATTGCAGGAAACATAACATGTAACTCAAGT---
ATCACAGGACTGCTATTAACACGTGATGGAGGTAACGAGACAGGC----------------------------
----

GAGGAAGAGACATTCAGGCCTGGAGGAGGAGATATGAGGGACAATTGGAGAAGTGAATTATATAAATATAAAG
TGGTAGAAGTTAAGCCATTG---GGAATAGCACCCACCAAGGCAAAAAGGAGAGTGGTG--------------
-------------------------GAGAGAGAGAAAAGAGCA---GTGGGA---ATAGGAGCG---------
TTGTTCCTTGGGTTCTTGGGAGCA---GCAGGAAGCACTATG---GGCGCGGCGTCA---
ATGACGCTGACGGTACAG---GCCAGACAA---TTGTTG---TCTGGTATAGTGCAACAGCAAAGC---
AATTTG---CTGAGG---GCTATAGAGGCGCAACAACATATGTTGCAACTCACGGTCTGGGGCATT
>KP109522
TTCTATTGTAATACATCAAGATTGTTTACATACAATGGTACAGAAAGTAAGAATTCAACAAACAAAAGTAAG-
----

AATTCAACAGACATCATACTCCCATGCAGAATAAAGCAGATTATAAACATGTGGCAAGAGGTAGGACGAGCAA
TGTATGCTCCTCCCATTGCAGGAAACATAACATGTACGTCAAAT---
ATCACAGGACTACTATTGACACGAGATGGAGGAGGTAAAAATAACACA-------------------------
----

AAAGAAGAGATATTCAGACCTGGAGGAGGAGATATGAGGGACAATTGGAGAAGTGAATTATACAAGTATAAAG
TGATAGAAATTAAGCCATTA---GGAATAGCTCCAACTACGGCAAAAAGGAGAGTGGTG--------------
-------------------------GAGAGAGAAAAAAGAGCA---GTGGGA---ATAGGAGCT---------
GTGCTCCTTGGGTTCTTGGGAGCA---GCCGGAAGCACTATG---GGCGCAGCATCA---
ATAACGCTGACGGTACAG---GCCAGACAA---TTGCTG---TCTGGTATAGTGCAACAGCAAAGC---
AATTTG---TTGAGG---GCTATAGAGGCGCAACAGCATATGTTGCAACTCACAGTCTGGGGCATT
>KC634164
TTCTATTGCAATACATCAAACTTGTTTAATAGTAGTATACCA-------------------------------
----

AATTCAACTATCACACTCCAGTGCAGAATAAAACAGATTATAAACATGTGGCAGGAGGTAGGACGAGCAATGT
ATGCCCCTCCCATTGCAGGAAACATAACATGTAAGTCAAAT---
ATCACAGGACTATTATTGACACGTGATGGAGGGACAGATAATAACAATACAAAT-------------------
----

ACTAACAATACAGAGACATTCAGACCTGGAGGAGGAGATATGAGAGACAATTGGAGAAGTGAACTATATAAAT
ATAAAGTAGTAGAAATTAAGCCCTTA---GGAATAGCACCTACTAAGGCAAAAAGGAGAGTGGTG--------
-------------------------------GAGAGAGAAAAAAGAGCA---GTGGGA---ATAGGAGCT---
------GTACTCCTTGGGTTCTTGGGAGCA---GCAGGAAGCACTATG---GGCGCGGCGTCA---
ATAACGCTGACGGTACAG---GCCAGACAA---TTATTG---TCTGGTATAGTGCAACAGCAAAAC---
AATTTG---CTGAGG---GCTATAGAGGCGCAACAGCATATGTTGCAACTCACGGTCTGGGGCATT
>KC187352
TTCTATTGTAACACATCAAACCTGTTTAATAGGACATACTCGTCTAATAGTACAAATAGTGCCTCAAATGCTA
CC---

TCAAATGCAACCATCACACTCCCATGCAGAATAAAGCAAATTATAAATATGTGGCAGGGAGTAGGACAAGCAA
TATATGCCCCTCCCGTTGCAGGAAACATAACATGTAACTCAAGC---

ATCACAGGACTACTATTGACACGTGATGGAGGAAATACTAATGGAGAAAAT---------------------
----

AATGATACAGAGACATTCAGACCTGGAGGAGGAGATATGAGGGACAATTGGAGAAGTGAATTATATAAATATA
AAGTGGTAGAAATTAAACCATTA---GGAATAGCACCCACTAAGGCAAAAAGGAGAGTGGTG--------
---------------------------GAGAAAGAAAAAAGAGCA---GTGGGA---ATAGGAGCT------
---GTGTTCCTTGGGTTCTTGGGAGCA---GCAGGAAGCACTATG---GGCGCAGCATCG---
ATAACGCTGACGGTACAG---GCCAGACAA---TTGTTG---TCTGGTATAGTGCAACAGCAAAGT---
AATTTG---CTGAGG---GCTATAGAGGCGCAACAGCATATGTTGCAACTCACAGTCTGGGGCATT
>KC186723
TTCTATTGCAATACATCACAATTGTTTAATATTACAAACACAAGTAATATTACAAACACA------------
----

AGTACTGGGAATATAATTCTCCAATGTAGAATAAAACAAATTATAAACATGTGGCAGGAGGTAGGAAGAGCAA
TGTATGCCCCTCCCATTGAAGGAAACATAACATGTAAATCAAAT---
ATCACAGGACTACTATTGACACGGGATGGAGGAAACACAGATAAT---------------------------
----

ACAACTGAGAAATTTAGGCCTGGAGGAGGAGATATGAGGGACAATTGGAGAAGTGAACTATATAAATACAAAG
TGGTAGAAATTAAGCCATTA---GGAGTAGCACCCACTGAGGCAAAAAGGAGAGTGGTG-------------
------------------------GAGAGAGAAAAAAGAGGA-----------ATAGGAGCT--------
GTGTTCCTTGGGTTCTTGGGAGCA---GCAGGAAGCACTATG---GGCGCAGCGTCA---
ATAACGCTGACGGTACAG---GTCAGACAA---TTATTG---TCTGGCATAGTGCAACAGCAAAGC---
AATTTG---CTGAGG---GCTATAGAGGCGCAACAGCATATGTTGCAACTCACAGTCTGGGGCATT
>KC186899
TTCTATTGCAACACATCAAGGCTGTTCAATAGCACATTTAATAGTACATTTAATAGTACACTGGATAACAGTA
CAAAT---

AGTACAGGAAACATCACACTCCAATGCAGAATAAAACAAATTATAAACATGTGGCAGGGGGTAGGAAAAGCAA
TATATGCCCCTCCCGTTGCAGGAGACATAATGTGTGAATCAAAT---
ATCACAGGATTACTATTAACAAGGGATGGAGGAAATGAAAATAAT---------------------------
----

AAAACAGAGACATTCAGACCTGCAGGAGGAAATATGAGGGACAATTGGAGAAGTGAATTATATAAATATAAAG
TGGTAGAAATTAAGCCATTA---GGAATAGCACCCACTAAGGCAAAAAGGAGAGTGGTG-------------
------------------------GAGAGAGAAAAAAGAGCA---GTGGGA---ATAGGAGCT--------
GTGTTCCTTGGGTTCTTGGGAGCA---GCAGGAAGCACTATG---GGCGCAGCATCG---
ATAACGCTGACGGTACAG---GCCAGACAA---TTGCTG---TCTGGTATAGTGCAACAGCAAAGT---
AACTTG---CTGAGG---GCTATAGAGGCGCAACAGCAAATGTTGCAACTCACGGTCTGGGGCATT
>KC186242
TTCTATTGCAATACATCAGGTCTGTTTAGAGATACAAACATATCT---------------------------
----

ACTACGCTCCAATGCAGGATAAAACAAATTATAAATATGTGGCAGGGGGTAGGGCGAGCAATGTACGCCCCTC
CCATTGCAGGAATCATAAAGTGTAAATCAAAG---
ATCACAGGAATACTATTGACAAGGGATGGAGGGGCAGATAATACC---------------------------
----

ACAGAGACATTCAGACCTGCAGGAGGAGATATGAGGGACAATTGGAGAAGTGAATTATATAAATATAAAGTAG
TAGAAATTAAGCCATTA---GGAATAGCACCAACTGAGTCAAAAAGGAGAGTGGTG----------------
---------------------GAGAGAGAAAAAAGAGCA---GTGGGAGTAGTAGGAGCT---------
ATGTTCCTTGGGTTCTTGGGAGCA---GCCGGAAGCACTATG---GGCGCAGCGGCA---
GTGACGCTGACGGCACAG---GCCAGACAA---TTATTG---TCTGGTATAGTGCAACAGCAAAGC---
AATTTG---CTGAGG---GCTATAGAGGCGCAACAGCATATGTTGCAACTCACAGTCTGGGGCATT
>KC187207
TTCTACTGCAATACATCAAAACTGTTTAATAGTACAATGTTTAGTACTACAGAGCATTCAAGTGAT-------
----

TCAAGCGAAAACATCACACTCCCATGCAGATTAAAGCAAATTATAAACATGTGGCAGGAGGTAGGAAGAGCAA
TATATGCCCCTCCCATTGCAGGAAACATAACATGTAAATCAAAT---

ATCACAGGGCTACTATTGCTACGTGATGGAGGTAATGATGGTAAAAAT-------------------------
----

AACACAAATGAAACATTCAGACCTGGAGGAGGAGATATGAGGGACAATTGGAGAAGTGAATTATACAAATACA
AAGTGGTAGAAATCAAGCCATTA---GGAGTGGCACCCACTAATGCAAAAAGGAGAGTGGTG-----------
----------------------------GAGAGAGAAAAAAGAGCA---GTGGGA---CTAGGAGCT------
---GTGTTCCTTGGGTTCTTGGGAGCA---GCAGGAAGCACTATG---GGCGCAGCGTCA---
ATAACGCTGACGGTACAA---GCCAGACAA---CTGTTG---ACTGGTATAGTGCAACAGCAAAGC---
AATTTG---CTGAGG---GCTATAGAGGCGCAACAGCATATGTTGCAACTCACGGTCTGGGGCATT
>KC186306
TTCTATTGCAATACATCAGGGCTGTTTAATAGCACATACATGTCTGGTGACACCCCCATGAATGATACAGACA
GTAATAAT---
-------------------ACA---
GACAGTAATAGTACAATCACACTCCAATGCAGAATAAAACAAATTATAAACATGTGGCAACAGGTAGGACGAG
CAATGTATGCTTCTCCCATTGAAGGAAACATAACATGTAAATCAAAT---
ATCACAGGGTTACTATTGACACGTGATGGAGGAAAAAATAACCAA----------------------------
----

AGTGAAAATGAGACATTCAGACCTGGAGGAGGAGATATGAGGGACAATTGGAGAAGTGAATTATACAAATATA
AAGTGGTAGAAATTAAGCCATTA---GGAATAGCACCCACTAAGGCAAAAAGGAGAGTGGTG-----------
----------------------------GAGAGAGAAAAAAGAGCA---ATAACAGGAATAGGAGCT------
---GTGTTCCTTGGGTTCTTGGGAGCA---GCAGGAAGCACTATG---GGCGCGGCGTCA---
ATAACGCTGACGGTACAG---GCCAGACAA---TTATTG---TCTGGTATAGTGCAACAGCAAAGC---
AATTTG---CTGAGG---GCTATAGAGGCGCAACAGCATATGTTGCAACTCACAGTCTGGGGCATT
>KC187437
TTCTATTGCAATACATCAGGACTGTTTAATGGTACAAACACA-------------------------------
----

AGGAATGGCTCCATCACACTCCCATGCAGAATAAAACAAATTATAAACATGTGGCAGGGGGTAGGACGAGCAA
TATATGCCCCTCCCATTGAAGGAAACATAACATGTAAATCAAAT---
ATCACAGGACTACTATTGTCACGTGATGGAGGAAATGGAAATAACACA-------------------------
----

AGTACCACAGAGACATTCAGACCTGGAGGAGGAGATATGAGGGACAACTGGAGAAGTGAATTATACAAATATA
AAGTGGTAGAAATTAAGCCATTA---GGAGTAGCACCCACTGGGGCAAAAAGGAAAGTGGTG-----------
----------------------------GAGAGAGAAAAAAGAGCA---GTGGGA---ATAGGAGCT------
---GTGTTCCTTGGGTTCTTGGGAGCA---GCAGGAAGCACTATG---GGCGCGGCGTCA---
ATAACGCTGACGGTACAG---GCCAGACAA---CTGTTG---ACTGGTATAGTGCAACAGCAAAGC---
AATTTG---CTGAGG---GCTATAGAGGTGCAACAGCATATGTTGCAACTCACGGTCTGGGGCATT
>KC186647
TTCTATTGCAACACATCAATATTGTTTAATGGTACATACCTGACTAATAATACAAACAGTACTTCAAATACT-
----

TCAAATTCAACCATTACACTCCAATGCAAAATAAAACAAATTATAAACATGTGGCAGGGGGTAGGACAAGCAA
TGTATGCCCCTCCCATTTCAGGAAACATAACCTGTGAATCAAAT---
ATCACAGGACTATTATTGACACGTGATGGAGGAAATAACACAGAG----------------------------
----

AACACTGAGATATTCAGACCTGGAGGAGGGGACATGAGGGACAATTGGAGAAGTGAATTATATAAATATAAAG
TGGTAGAGATCAAGCCATTA---GGAGTAGCACCCAATAAGGCAAAAAGACGAGTGGTG--------------
-------------------------GAGAGAGAAAAAAGAGCA---GTAGGA---ATAGGAGCT---------
GTACTCCTTGGGTTCTTGGGAGCA---GCAGGAAGCACTATG---GGCGCGGCGTCA---
ATGACGCTGACGGTACAG---GCCAGACAA---TTATTG---TCTGGTATAGTGCAACAGCAAAAC---
AATTTG---CTGAGA---GCTATAGAGGCGCAACAGCATATGTTGCAACTCACGGTCTGGGGCATT
>KC187550
TTCTATTGCAATACATCAAAACTGTTTAATAGTACAAAACTGTTTAATAGTACTAATAAT-------------
----

AATAGTGCAATAATCACACTCCCATGTAAAATAAAACAAATTATAAATATGTGGCAGAAGGTAGGACGAGCAA
TGTATGCTCCTCCCATTGAAGGAAACATAACATGTAACTCAAGT---

ATCACAGGATTACTATTGACGCGTGATGGAGCAGGAGGGGGGGGC----------------------------
----

AATGAGACATTCAGACCTGGAGGAGGAGATATGAGGGACAATTGGAGAAGTGAATTATATAAATATAAAGTGG
TAGAAATTAAGCCATTA---GGAATAGCACCAACTGGAGCAAAAAGGAGAGTGGTG-----------------
----------------------GAGAGAGGAAAACGAGCA---GTGGGA---CTAGGAGCT---------
GTGTTCCTTGGGTTCTTGGGAGCA---GCAGGAAGCACTATG---GGCGCGGCGGCA---
GTAACGCTGACGGCACAG---ACCAGACAA---TTGTTG---TCTGGTATAGTGCAACAGCAAAGT---
AATTTG---CTGAAG---GCTATAGAGGCGCAACAACATATGTTGCAACTCACGGTCTGGGGCATT
>KC187376
TTCTATTGCAATACATCAGGCCTGTTTAGTGGAACATACAATAGTACAGGCAATACTAAT-------------
----

AGTACAACAAACATCACACTCCCATGCAGAATAAGACAAATTATAAGAATGTGGCAGGGAGTAGGACAAGCAA
TGTATGCCCCTCCCATTGCAGGAAACATAATATGTATTTCAAAT---
ATCACAGGGCTACTATTGACACGTGATGGAGGACAAAATAATGGGACAAAT----------------------
----

AATAATACAGAGACATTCAGACCTGGAGGGGGAGACATGAGGGACAATTGGAGAAGTGAATTATATAAATATA
AAGTGGTAGAAATTAAGCCATTG---GGAATAGCACCCACTAAGGCAAAAAGGAGAGTGGTG-----------
----------------------------GAGAGAGAAAAAAGAGCA---GTGGGA---ATAGGAGCT------
---GTGTTCCTTGGGTTCTTGGGAGCA---GCAGGAAGCACTATG---GGCGCAGCGTCA---
ATAACGCTGACGGTACAG---GCCAGACAA---TTGTTG---TCTGGTATAGTGCAACAGCAAAGC---
AATTTG---CTGAAG---GCTATAGAGGCGCAACAGCATATGTTGCAACTCACGGTCTGGGGCATT
>KC187723
TTCTATTGCAATACATCAGCATTGTTTAACACAACACTGGGTAACACAACACTGGGTAACATAACCATACAAA
ATGAT--

TCAAACGCAACCATCACACTCCCATGCAGAATAAAGCAGATTATAAACATGTGGCAGGAGGTAGGACGAGCAA
TGTATGCTCCTCCCATTAAAGGAAATATAACCTGTAAATCAAAC---
ATCACAGGACTACTATTGTTACGTGATGGAGGAGTGAACACAACT----------------------------
----

AACGAGACATTCAGACCTGGAGGAGGAGATATGAGGGACAATTGGAGAAGTGAATTATATAAATATAAAGTGG
TAGAAATTAAGCCATTG---GGAGTAGCACCCACTAAAGCAAAAAGGAGAGTGGTG-----------------
----------------------GAGAGAGAAAAAAGAGCA---GTGGGA---ATAGGAGCT---------
GTGTTCCTTGGGTTCTTGGGAGCA---GCAGGAAGCACTATG---GGCGCGGCGTCA---
ATAACGCTGACGGTACAG---GCCAGACAA---CTGTTG---TCTGGTATAGTGCAACAGCAAAGC---
AATTTG---CTGAGG---GCTATTGAGGCGCAACAGCATATGTTGCAACTCACGGTCTGGGGCATT
>KC186458
TTCTATTGCAATACATCAGGACTGTTTAATAACTCACTACTGACAGAAAATGGTACAGAA-------------
----

AGTAACGAAACAATAACAATCCCATGCAAAATAAAACAATTTTTAAACATGTGGCAGCAGGTAGGACGAGCAA
TGTATGCCCCTCCCATTGCAGGAAACATAACATGTAAATCAAAC---
ATCACAGGACTACTATTGACACGGGATGGAGGAACTACTAATGGGACA-------------------------
----

GGAAATGAGACATTCAGACCTGGAGGAGGAAATATGAGGGACAATTGGAGAAGTGAATTATATAAATATAAAG
TGGTAGAAATTAAGCCATTA---GGAATAGCACCCACTGGAGCAAAAAGGAGAGTGGTG--------------
-------------------------GAGAGAGAAAAAAGAGCA---GTGGGA---ATAGGAGCT---------
TTGTTCCTTGGGTTCTTGGGAGCA---GCAGGAAGCACTATG---GGCGCGGCGTCA---
ATAACGCTGACGGTACAG---GCCAGACAA---TTATTG---TCTGGTATAGTGCAACAGCAAAGC---
AATTTG---CTGAGA---GCTATAGAGGTGCAACAGCACATGTTGCAACTCACAGTCTGGGGCATT
>KC187049
TTCTATTGCAATACATCAAAGCTGTTCAATGGTACATATGTGAATAGTACATACGTGAATAGT----------
----

ACAGATTCAAACATCAACATCACATGCAGAATAAAACAAATTATTAACATGTGGCAGGGAGTAGGACGAGCAA
TGTATGCCCCTCCCATTGAAGGAGTCATAACATGTAAATCAAAT---

ATCACGGGACTACTATTGACAAGAGATGGAGGAAAAAATAATACT----------------------------
----

AATACAGAGACATTCAGACCTGGAGGGGGAGATATGAGGGACAATTGGAGAAGTGAATTATATAAATATAAAG
TGGTAGAAATTAAGCCATTA---GGAATAGCACCAACTGCGGCAAAAAGACGAGTGGTG--------------
-------------------------GAGAGAGAAAAAAGAGCA---GTGGGA---CTAGGAGCT---------
GTGTTCCTTGGGTTCTTGGGAGCA---GCAGGAAGCACTATG---GGCGCGGCATCA---
ATAACGCTGACGGTACAA---GCCAGACAA---TTGTTG---TCTGGTATAGTGCAACAGCAAAGC---
AATTTG---CTGAGG---GCTATAGAGGCGCAACAGCATATGTTGCAACTCACAGTCTGGGGCATT
>KC186320
TTCTATTGTAATACATCAGGACTGTTTAATGGTACATATTGGCCTAATGGTACAAATACATGGGGGAATGCAA
GCAAT--
-------------GAAAAC------
ATCACACTCCAAAACATCACACTCCCATGCAAAATAAAACAAATTATAAACATGTGGCAGGGAGTAGGACGAG
CAATATATGCCCCTCCCATTGCAGGAAACATAACATGTACATCAAAT---
ATCACAGGACTACTATTGGTACGTGATGGAGGGACAAATGAAAGTGCT-------------------------
----

ACAAATGAGACATTCAGACCTGGAGGAGGAGATATGAGGGACAACTGGAGAAGTGAATTATATAAATATAAAG
TGGTAGAAATTAAACCATTA---GGAGTAGCACCCACTGCTGCAAAAAGAAGAGTGGTG--------------
-------------------------GAGAGAGAAAAAAGAGCA---GTAACA---ATGGGAGCT---------
TTGTTCCTTGGGTTCTTGGGAGCA---GCAGGAAGCACTATG---GGCGCGGCGTCA---
ATAACGCTGACGGTACAG---GCCAGACAA---CTGTTG---TCTGGTATAGTGCAACAGCAAAGC---
AATTTG---CTGAGA---GCTATAGAGGCGCAACAGCATATGTTGCAACTCACGGTCTGGGGCATT
>KC187482
TTTTATTGCAATACATCAGATCTGTTCAATGATACATTT----------------------------------
----

ACAAACTCAACCATCACGCTTCCATGTAGGATAAAACAAATTATAAACATGTGGCAGGGGGTAGGACGAGCAA
TGTACGCCCCTCCCATTGAAGGAAACATAACATGTAAATCAAAT---
ATTACAGGACTACTATTGACACGTGATGGAGGAAACAGTAATAAT----------------------------
----

GGAACAGAGATATTCAGACCTGGAGGAGGAGATATGAGAGACAATTGGAGAAGTGAATTATATAAATATAAAG
TGGTAGAAATTAAGCCATTG---GGAATAGCACCCACTGAGGCAAAAAGGAGAGTGGTG--------------
-------------------------GAGAGAGAAAAAAGAGCA---GTGGGG---ATAGGAGCT---------
GTGTTCCTTGGGTTCTTGGGAGCA---GCAGGAAGCACTATG---GGCGCGGCGTCA---
ATAACGCTGACGGTACAG---GCCAGACAA---CTGTTG---TCTGGTATAGTGCAACAGCAAAGC---
AATTTG---CTGAGA---GCTATAGAGGCGCAACAACATCTGTTGCAACTCACAGTCTGGGGCATT
>KC186143
TTCTATTGCAATACATCAGGACTGTTTAATACATCAGGACAGTCTACAAT-----------------------
----

TCAAGTTCAAACATCACACTCCCATGCAAAATAAAACAAATTATAAACATGTGGCAGAAAGTAGGACGAGCAA
TATATGCCCCTCCCACTAAAGGAATCATAACATGTAGCTCAAAT---
ATCACAGGACTACTATTAACACGTGATGGAGGAAGCAACAAAAACAAT-------------------------
----

AACACTACAGAGATATTCAGACCTGGAGGAGGAGATATGAGGGACAATTGGAGAAGTGAATTATATAAATATA
AAGTGGTAGAAATTAAACCATTA---GGAATAGCACCCACTGAGGCAAAAAGGAGAGTGGTG-----------
----------------------------GAGAGAGAGAAAAGAGCA---GTGGGA---ATAGGAGCT------
---GTGTTCCTTGGGTTCTTGGGAGCG---GCAGGAAGCACTATG---GGCGCGGCGTCA---
ATAACGCTGACGGTACAG---GCCAGACAA---TTATTG---TCTGGTATAGTGCAACAGCAAAGC---
AATTTG---CTGAGA---GCTATAGAGGCGCAACAGCATATGTTGCAACTCACGGTCTGGGGCATT
>KC156214
TTCTATTGCAATACATCAAAAATATTTGATAGTGACAATGCAAACAAA-------------------------
----

ACTTCAGGAAATATCACACTCCCATGCAAAATAAAACAAATCATAAACATGTGGCAGGGGGTAGGAAGAGCAA
TGTATGCCCCTCCCATTGCAGGAAACATAACATGTACATCAAAT---

ATCACAGGACTACTATTGACACGTGATGGAGGCAATGTTACAAAT---------------------------
----

GACACAGAGATATTCAGACCTGGAGGAGGAAATATGAGGGACAATTGGAGAAGTGAATTATACAATATAAAG
TGGTAGAAATTAAGCCATTA---GGGATAGCACCCACCAAGGCAAAAAGGCGAGTGGTG-------------
-------------------------GAGAGAGAAAAAAGAGCA---GTGGGA---ATAGGAGCT--------
GTATTCCTTGGGTTCTTGGGAGCA---GCAGGAAGCACTATG---GGCGCAGCGTCA---
ATAGCGCTGACGGTACAG---GCCAGACAA---TTGTTG---TCTGGTATAGTGCAACAGCAAAAC---
AATTTG---CTGAGA---GCTATAGAGGCGCAACAGCATATGTTGCAACTCACGGTCTGGGGCATT
>KP109523
TTCTACTGCAATACATCAAGTCTGTTTAATAGTAATAGT---------------------------------
----

ACAGAAGATCCCATCACACTCAAATGCAGAATAAAACAAATCATAAACATGTGGCAGGAGGTAGGAAGAGCAA
TGTATGCCCCTCCCATTGCAGGAGAAATCACGTGTAACTCAAGT---
ATCACAGGACTATTACTGCTACGTGATGGAGGTAACTCAAATGAC---------------------------
----

TCCAAAGAGAAAGAGACATTCAGACCTGGAGGAGGAAATATGAAGGACAATTGGAGGAGTGAATTATATAAAT
ATAAGGTGGTAGAAATTAAGCCATTA---GGAGTAGCACCCACTGAGGCAAAAAGGAGAGTGGTG-------
------------------------------GAGAGAGAAAAAAGAGCA---GTGGGA---ATAGGAGCT---
------GTGATCTTTGGGTTCTTGGGAGCA---GCAGGAAGCACTATG---GGCGCGGCGTCA---
ATAACGCTGACGGTACAA---GCCAGACAA---CTGCTG---TCTGGTATAGTGCAACAGCAAAAC---
AATTTG---CTGAGG---GCTATAGAGGCGCAACAGCATATGTTGCAACTCACAGTCTGGGGCATT
>KP109524
TTTTATTGCAATACAACAAACCTGTTTAATGGTATTAATGAGACAGAAAAT---------------------
----

ACCAATTCAACCATCATACTTCGTTGCAGAATAAAACAAATAATAAACATGTGGCAGGGGGTAGGACGAGCAA
TGTATGCCCCTCCCATTGAAGGAAACATAACATGTAGATCAAAT---
ATCACAGGGCTACTATTGACACGTGATGGAGGAGTAAATGCGACA---------------------------
----

AAAGAGACATTCAGACCTGGAGGAGGAGATATGAGGGACAATTGGAGAAGTGAACTATATAAATATAAAGTGG
TAGAAATTAAGCCATTA---GGAGTAGCACCCACTAGTGCAAAAAGGAGAGTGGTA----------------
-------------------------AGAGAGAAAAGAGCA---GTGGGA---CTAGGAGCT--------
GTGTTCCTTGGGTTCTTGGGAGCA---GCAGGAAGCACTATG---GGCGCGGCATCA---
GTAATGCTGACGGTACAG---GCCAGACAA---TTATTG---TCTGGTATAGTGCAACAGCAAAGC---
AATTTG---CTGAAG---GCCATCGAGGCGCAACAGCATATGTTGCAACTCACAGTCTGGGGCATT
>KP109527
TTCTATTGCGATACATCAGGTCTGTTTAATGGTACATACAACGCTACAGACATGTCTAATAGT---------
----
----------------------------------ACAAACGAAACCATCATA--------
AAACAGATTATAAACATGTGGCAGGGGGTAGGACGAGCAATGTATGCCCCTCCCATTGCAGGAAACATAACAT
GTAAATCCAAT---ATCACAGGACTATTATTGACACGTGATGGAGGTAATGATAAAAAT-------------
----

AACACAGAAATATTCAGACCTGGAGGAGGAGATATGAGGGACAATTGGAGAAGTGAATTATATAAATACAAAG
TGGTAGAAATTAAGCCATTG---GGAGTAGCACCCACTGAGGCAAAAAGGAGAGTGGTG-------------
-------------------------AAGAAAGAAAAAAGAGCA---GTGGGA---CTAGGAGCT--------
ATGTTCCTTGGGTTCTTGACAGCA---GCAGGAAGCACTATG---GGCGCGGCGTCA---
ATAACGCTGACGGTACAG---GCCAGACAA---TTGTTG---TCTGGTATAGTGCAACAGCAAAAC---
AATTTG---CTGAGA---GCTATAGAGGCGCAACAGCATATGTTGCAACTCACGGTCTGGGGCATT
>KC634109
TTCTATTGTAATACATCAGGGCTGTTTAATGGTACATACCCGGCTAATGATACAGAAAGTAAC---------
----

TCAAGCTCACCAATCACACTCCCATGCAGAATAAAACAAATTATAAACATGTGGCAGGAAGTAGGACGAGCAA
TGTATGCTCCTCCCATTGCAGGAAATATAACATGTACATCAAAT---
ATCACGGGATTACTCTTGACACGTGATGGAGGCACAGGAAATGGCACAGAATCA------------------

----

AATAGCACAGAAACATTCAGACCTGGAGGAGGAGATATGAGGGACAATTGGAGAAGTGAATTATATAAATACA
AAGTGGTAGAAATTAAGCCATTG---GGAATAGCACCCACTACGGCAAAAAGGAGAGTGGTG-----------
----------------------------GAGAGAGAAAAAAGAGCA---GTAGGA---ATAGGAGCT------
---GTGTTCCTTGGGTTCTTGGGAGCA---GCAGGAAGCACTATG---GGCGCGGCGTCA---
ATCACGCTGACGGTACAT---GCCAGACAA---TTGTTG---TCTGGTATAGTGCAACAGCAAAGC---
AATTTG---CTGAAG---GCTATAGAGGCGCAACAACATTTGTTGCAACTCACAGTCTGGGGCATT
>KC187500
TTCTATTGCAATACATCAGAACTGTTTAATAGTACACTGTTTAATAGTACACACATGTCTAGTACAAATGCAA
CT---

AAAAACAAAACCATCACACTTTCATGCAGAATAAAACAAATCATAAACATGTGGCAGGGGGTAGGACGAGCAA
TGTATGCCCCTCCCATTGCAGGAAACATAACATGTAGGTCAAAC---
ATCACAGGACTAATAATAACACGTGATGGGGGAACCGGAGAGAATACAACAGGG-------------------
----

GATAGAACAGAGGAAACATTCAGACCTGCAGGAGGAGATATGAGGGACAATTGGAGAAGTGAATTATATAAAT
ATAAAGTGGTAGAAATTAAGCCATTA---GGAATAGCACCCACTAGAGCAAAAAGGAGAGTGGTG--------
-------------------------------GAGAGAGAAAAAAGAGCA---GTGGGA---ATAGGAGCT---
------GTGTTCCTTGGGTTCTTGGGAGCA---GCAGGAAGCACTATG---GGCGCGGCGTCA---
ATAACGCTGACGGTACAG---GCCAGACAA---TTGCTG---TCTGGTATAGTGCAACAGCAAAGC---
AATTTG---CTGAGG---GCTATAGAGGCGCAACAGCATATGTTGCAACTCACAGTCTGGGGCATT
>KC187630
TTCTATTGCAATACATCAAACCTGTTTAATATGACATACATATGGAAAAATGGGTCATACGTGCTTAATAATA
CAGACAAT---

AATGATACAACAATCACACTCCAATGCAGAATAAAACAATTTATAAACATGTGGCAGAGGGTAGGAAGAGCCA
TGTATGCTCCTCCCATTGCAGGAAACATAACATGTGTATCAAAT---
ATCACAGGATTACTATTGACACATGATGGGATAAAGACAAATAGATCAAGTGAC-------------------
----

TCAAATTGGACATGGACATTCAGACCTGGGGGAGGAAATATGAAGGACAATTGGAGTAATGAATTATACAAAT
ATAAAGTGGTAGAAATTAAACCATTG---GGAATAGCACCCACTAAGGCAAAAAGGAGAGTGGTG--------
-------------------------------GAGAGAGAAAAAAGAGCA---GTGGGA---ATAGGAGCT---
------GTGATCTTTGGGTTCTTGGGAGCA---GCAGGAAGCACTATG---GGCGCAGCGTCA---
ATAACGCTGACGGTACAG---GCCAGACAA---TTGCTG---TCTGGTATAGTGCAACAGCAAAGC---
AATTTG---CTGAAA---GCTATAGAGGCGCAACAACATATGTTGCAACTCACGGTCTGGGGCATT
>KC186203
TTCTATTGCAATACATCAGGACTATTTAATAGTACATTTAATAAGGCATACCAGCCTAATAGTACAGAAGAT-
----

GACTCAAAAAACATCACACTCCAATGCAGAATAAAGCAAATTATAAACATGTGGCAGGGGGTAGGACAAGCAA
TGTATGCTCCTCCCATAGCAGGAAACATAACATGTAGATCAAAT---
ATAACAGGACTGCTATTAACACGTGATGGAGGAGATAACAATAGCACA-------------------------
----

AATGGCACAGAGATATTCAGACCTGGAGGAGGAGATATGAAGGACAATTGGAGAAGTGAATTATATAAATATA
AAGTGGTAGAAGTTATGCCATTG---GGATTAGCACCCACTAAGGCCAAAAGGAGAGTGGTGGAGAGA-----
-------GAGGCCAAAACGAGAGTGGTGGAGAGAAAGAAAAGAGCA---GTGGGA---ATAGGAGCT------
---GTGTTCCTTGGGTTCTTAGGAGCA---GCAGGAAGCACTATG---GGCGCAGCGTCA---
GTGACGCTGACGGTACAG---GCTAGACAA---TTATTG---TCTGGTATAGTGCAACAGCAAAGC---
AATCTG---CTGAAG---GCTATAGAGGCGCAACAGCATATGTTGCAACTCACAGTCTGGGGCATT
>KC186594
TTCTATTGCAATACATCAGGCCTGTTCAATGGCACATACAATGGCACATACAATGGCACATACAATGGCATAT
ACAAT--

AGTACAGACAATATCACACTCCCATGTAGAATAAAACAAATTATAAACATGTGGCAGAGGGTAGGACGAGCAA
TATATGCCCCTCCCATTGCAGGAAATATAACATGTAAATCAAAT---
ATCACAGGACTATTATTGACACGTGATGGAGGAAAAAATAATACA----------------------------

----

ACAGATGAAGTATTCAGACCTGGAGGAGGAGATATGAGGGACAATTGGAGAAGTGAATTATATAAATATAAAG
TGGTAGAAATTAAGCCATTA---GGAATAGCACCCACTAATGCAAAAAGGAGAGTGGTG--------------
-------------------------GAGAAAGAAAAAAGAGCA---GTGGGA---CTGGGAGCT---------
GTGTTCCTTGGGTTCTTGGGAGCA---GCAGGAAGCACTATG---GGCGCGGCGTCG---
ATAACGCTGACGGTACAG---GCCAGACAA---TTGTTG---TCTGGTATAGTGCAACAGCAAAGC---
AATTTG---CTGAGG---GCTATAGAGGCGCAACAGCATATGTTGCAACTCACGGTCTGGGGCATT
>KC186169
TTCTATTGCAATACATCAAAACTGTTTAATACTACAGGCAATAGTACA-------------------------
----

GCAAATACAACCATCACACTCCCATGCCAAATAAAACAAATTATAAACATGTGGCAGGGGGTAGGACGAGCGA
TGTATGCCCCTCCCATTGCAGGAAACATAACATGTGAATCAAAT---
ATCACAGGGCTACTATTGACACGGGATGGAGGAAAAGGTAAAGAT----------------------------
----

AATGGAACAGAGACATTCAGACCTGAAGGAGGAGATATGAGGGACAATTGGAGGAGTGAATTATATAAATATA
AAGTGGTAGAAATTAGGCCATTG---GGAATAGCACCCACTACGGCAAAAAGGAGAGTGGTG-----------
----------------------------GAGAGAGAAAAAAGAGCA---GTGGGA---CTAGGAGCT------
---GTGTTCCTTGGGTTCTTAGGAGCA---GCAGGAAGCACTATG---GGCGCAGCGTCA---
ATAACGCTGACGGTACAG---GCCAGACAA---CTGTTG---TCTGGTATAGTGCAACAGCAAAGC---
AATTTG---CTGAGA---GCTATAGAGGCGCAACAGCATATGTTGCAACTCACGGTCTGGGGCATT
>KC186446
TTCTATTGCAATACAACAGACCTGTTTAATGGGACAGGAAATGGTACAAAG----------------------
----

GCAAATGACACAATCAAAATCCCATGCAAAATAAAACAAATTATAAACATGTGGCAGGGAGTAGGAAGAGCAA
TATATGCTCCTCCCATTAAAGGAAACATAACATGTAAATCAAAT---
ATCACAGGAATACTATTGACACGTGATGGAGGAAGAGATGGAGGAGAAGAT----------------------
----

AACGAGACAGAGATATTCAGACCTGGAGGAGGAGATATGAGGGACAATTGGAGGAGTGAATTATATAAATATA
AAGTGGTAGAAATCAAGCCATTG---GGAATAGCACCCACTGCGGCAAAACGGAGAGTGGTG-----------
----------------------------GAGAAAGAAAAAAGAGCA---GTGGGA---ATAGGAGCT------
---GTGTTCCTTGGGTTCTTGGGAGCA---GCAGGAAGCACTATG---GGCGCGGCATCA---
ATAACGCTGACGGTACAG---GCCAGACAA---TTATTG---TTTGGTATAGTGCAACAGCAAAGC---
AATTTG---CTGAGA---GCTATAGAGGCGCAGCAGCATATGTTGCAACTCACAGTCTGGGGCATT
>KT896551
TTCTATTGTAATACATCAAAACTGTTTAATGGTGCATATAATAATTCAAATTCAAGTACAGAAGATACT----
----

TCAAACTCAACAATCACACTCCCATGCAAAATAAAACAAATTATAAACATGTGGCAGGAGGTAGGACGAGCAA
TGTATGCTCCTCCCATTAGAGGAAACATAACATGTGAATCGAAT---
ATCACAGGATTACTATTGGTGCGTGATGGAGGAAATACTACGAATAATGAGACA-------------------
----

GAGACAGAGACATTCAGACCTGGAGGAGGAGACATGAGGGACAATTGGAGAAGTGAATTATACAAGTATAAAG
TGGTAGAAATTAAGCCATTG---GGAATAGCACCCACTAAGGCAAGAAGAATAGTGGTG--------------
-------------------------GAGAGAGAAAAAAGAGCA---GTAGGA---CTAGGAGCT---------
GTGATCTTTGGGTTCTTGGGAGCA---GCAGGAAGCACTATG---GGCGCGGCGTCA---
ATAGCGCTGACGGTACAG---GCCAGGCAA---GTGTTG---TCTGGTATAGTGCAACAGCAAAGC---
AATTTG---CTGAAA---GCCATAGAGGCGCAACAGCATCTGTTGCAACTCACAGTCTGGGGCATT
>MG898649
TTCTACTGCAATACATCAGGCCTATTTAATAGGACATACGATGCAAATGAAACAGCAAAGTATGGTAACAGT-
----

ACAAATGGTAACATCACACTCCAATGCAGAATAAAACAATTTATAAACATGTGGCAGAGGGTAGGACGAGCAA
TGTATGCCCCTCCCATTGCAGGAATCATAACATGTAGATCAAAT---
ATCACAGGACTACTATTGACACGAGATGGAGGAGATGGAGGAGAAAATGGAACA-------------------

----

GAGACAGAGACATTCAGACCTGGAGGAGGAGATATGAGGGACAATTGGAGGAGTGAATTATATAAATATAAAG
TGGTAGAAATTAAGCCATTA---GGAATAGCACCCACCGAGGCAAAAAGGAGAGTGGTG--------------
-------------------------GAGAGAGAAAAAAGAGCA---GTGGGG---CTAGGAGCT---------
GTGCTCCTTGGGTTCTTGGGAGCA---GCAGGAAGCACTATG---GGCGCGGCGTCA---
ATAACGCTGACGGTACAG---GCCAGACAA---TTGTTG---TCTGGTATAGTGCAACAGCAAAGC---
AATTTG---CTGAAG---GCTATAGAGGCGCAACACCATCTGTTGCAACTCACGGTCTGGGGCATT
>MG899863
TTTTATTGCAATACATCACAACTGTTTAATAGGACATACAACCCTACAGAAAATAGTACAGAA----------
----

AGTAATAAAACCATCACAATCCCATGCAGAATAAAACAGATTATAAATATGTGGCAGAAAGTAGGAAAAGCAA
TGTATGCCCCTCCCATTGCAGGATACATAACATGTAGATCAAAC---
ATCACAGGACTACTATTGTCACGTGATGGAGGCAACGATAACACAACAGAA----------------------
----

AGAACAGAAACCTTCAGACCTGCAGGAGGAGATATGAGGGACAATTGGAGAAGTGAATTATATAAATATAAAG
TGGTAGAAATTAAGCCATTG---GGAATAGCACCCACCGAGGCAAAAAGGAGAGTGGTG--------------
-------------------------GAGAGACAAAAAAGAGCA---GTGGGA---ATAGGAGCT---------
GTGCTCCTTGGGTTCTTGGTAGCA---GCAGGAAGCACTATG---GGCGCGGCGTCA---
ATAACGCTGACGGTACAG---GCCAGACAA---CTGTTG---TCTGGTATAGTGCAACAGCAAAGC---
AATTTG---CTGAGA---GCTATAGAGGCGCAACAGCATATGTTGCAACTCACGGTCTGGGGCATT
>AY713413
TTCTATTGTAATACATCAAACCTGTTTACATCAAACCTGTTTACTAACAATCTAACA----------------
----

AATACTACAAACATCACACTCCCATGCAGAATAAAACAAATTATAAACATGTGGCAGGGGGTAGGACGAGCAA
TGTATGCCCCTCCCATTGCAGGAAACATAACATGTAAATCGAAT---
ATCACAGGACTACTACTGACACGTGATGGAGGAGAAAATAATAGG----------------------------
----

ACTGAGACATTCAGACCTGGAGGAGGAGATATGAAGGACAATTGGAGAAGTGAATTATATAAATATAAGGTGG
TAGAAATTAAGCCATTA---GGAGTAGCACCCACTGGTGCAAAAAGGAGAGTGGTG-----------------
----------------------GAGAGAGAGAAAAGAGCA---GTGGGA---ATAGGAGCT---------
GTGTTCCTTGGGTTCTTAGGAGCA---GCAGGAAGCACTATG---GGCGCGGCGTCA---
ATAACGCTGACGGTACAG---GCCAGACAA---TTATTG---TCTGGTATAGTGCAACAGCAAAGC---
AATTTG---CTGAGG---GCTATAGAGGCGCAACAGCATATGTTGCAACTCACGGTCTGGGGCATT
>HM638662
TTCTATTGTAATACATCAAAACTGTTTAATAGTACATACATGCATAATGGTACAACAGGTAAC----------
----

TCAAGCGGAGTCATCACACTCCCATGCAAAATAAAACAAATTATAAACATGTGGCAGGAGGTAGGACGAGCAA
TGTATGCTCCTCCCATTGCAGGAAACATAACATGCAAATCAAAT---
ATCACAGGACTACTATTGACACGTGATGGAGGGTCTGACCCAAACACA-------------------------
----

AATAATGACACAGAGACATTCAGACCTGGAGGAGGAGATATGAGGGACAATTGGAGAAGTGAATTATATAAAT
ATAAAGTGGTAGAAATTAAGCCATTA---GGAGTAGCACCCACTGGAGCAAAAAGGAGAGTGGTG--------
-------------------------------GAGAGAGAAAAAAGAGCA---GTTGGA---ATAGGAGCT---
------GTGCTCCTTGGGTTCTTGGGAACA---GCAGGAAGCACTATG---GGCGCGGCGTCA---
ATAACGCTGACGGTACAG---GCCAGACAA---GTATTG---TCTGGTATAGTGCAACAGCAAAGC---
AATTTG---CTGAGG---GCTATAGAGGCGCAACAGCATATGTTGCAACTCACGGTCTGGGGCATT
>HM638713
TTCTATTGCAATACATCAAACCTGTTTAATAGTACATACATGACAAACATGACTAATAGTACTTCAGATTCTA
AT---

AGTACTTCAAACATTACACTCCAATGCAGAATAAAACAAATTGTAAACATGTGGCAGGAGGTAGGACGAGCAA
TGTATGCCCCTCCCATTGCAGGAAACATAACATGTGAATCAAAT---
ATCACAGGACTACTATTGACACGTGATGGAGGAACAACTAATAACACAGGAACA------------AAT----

----
GGAACAGAAAATAAT---
AACACAGAAACATTCAGACCTGGAGGAGGAGATATGAGGGACAATTGGAGAAATGAATTATACAAATATAAAG
TAGTAGAAATTAAGCCATTG---GGAATAGCACCCACTAAGGCAAAAAGGAGAGTGGTG--------------
-------------------------GAGAGAGAAAAAAGAGCA---GTGGGA---CTAGGAGCT---------
GCGTTCCTTGGGTTCTTGGGAGCA---GCCGGAAGCACTATG---GGCGCAGCATCA---
ATAACGCTGACGGTACAG---GCCAGACAA---TTGTTG---TCTGGTATAGTGCAACAGCAAAGC---
AATTTG---CTGAGG---GCTATAGAGGCGCAACAGCATATGTTGCAACTCACGGTCTGGGGCATT
>HM638770
TTCTATTGCAATACATCAAAACTGTTTAATGGGACATACAATGCGACAGACATGCCTAATGGGACATACAATT
CT---

AATAGTACAAAAGTCATCACACTCCCATGCAGAATAAAGCAAATTATAAACATGTGGCAGGAGGTAGGACGAG
CAATGTATGCAAATCCCATTGAAGGAAACATAACATGTAAATCAAAT---
ATCACAGGAATACTATTGGTACGTGATGGAGGCAAAGAGGACAATAAA-------------------------
----

ACAGACCCAAAAGAGACATTCAGACCTGGAGGAGGAGATATGAGGGACAATTGGAGAAGTGAATTATATAAAT
ATAAAGTGGTAGAAATTAAACCATTA---GGAATAGCACCCACTAAGGCAAAAAGGAGAGTGGTG--------
-------------------------------GAGAGAGAAAAAAGAGCA---GTGGGA---ATAGGAGCT---
------GTGTTCCTTGGGTTCTTGGGAGCA---GCAGGAAGCACTATG---GGCGCGGCGTCA---
ATAACGCTGACGGTACAG---GCCAGACAA---CTGTTG---TCTGGTATAGTGCAACAGCAGAGT---
AATTTG---CTGAGG---GCTATAGAGGCGCAACAGCATATGTTGCAACTCACGGTCTGGGGCATT
>HM638848
TTCTATTGCAATACATCAGGACTGTTTAATAGTACATACAATAGTACATACATGCCTAATGGTACAGAG----
----

AGTAATTCAACAATCACACTCCCATGCAGAATAAAACAAATTATAAACATGTGGCAGGAGGTAGGACGAGCAG
TGTATGCTCCTCCCATTGAAGGACACATAACATGTAACTCAACA---
ATCACAGGATTACTATTGACACGCGATGGAGGCAATAACACAAAT----------------------------
----

GGAACTGAGACATTCAGACCTGGAGGAGGAGATATGAGGGACAATTGGAGAAATGAATTATACAAATATAAAG
TAGTAGAAATTAAGCCATTA---GGAATAGCACCCACTGGAGCAAAAAGGAGAGTGGTG--------------
-------------------------GAGAGAGAAAAAAGAGCA---GTGGGA---ATAGGAGCC---------
TTGTTCCTTGGGTTCTTAGGAGCA---GCAGGAAGCACTATG---GGCGCAGCGTCA---
ATAACGCTGACGGTACAG---GCCAGACAA---CTGCTG---TCTGGTATAGTGCAACAGCAAAGC---
AATTTG---CTGAGA---GCTATAGAGGCGCAACAGCATATGTTGCAACTCACGGTCTGGGGCATT
>HM638911
TTCTATTGCAATACATCAGGCCTGTTTAATAGTACATACAATATAACAAATTCTTCAGATTCAGAAAATTCT-
----

TCAAATTCAACCATCACACTCCAATGCAGAATAAAACAAATTATAAACATGTGGCAGGAGGTAGGACGAGCAA
TGTATGCTCCTCCCATTGAAGGAAGCATAACCTGTAACTCAAGT---
ATCACAGGATTACTATTGACCCGTGATGGAGGCACATCTCCAGGAGAT-------------------------
----

GCTAATAACACAGAGACATTCAGACCTGGAGGAGGAGATATGAGGAACAATTGGAGAAGTGAATTATATAAAT
ATAAAGTGGTAGAAATTAAGCCATTA---GGAATAGCACCCACTAAGGCAAAAAGGAGAGTGGTG--------
-------------------------------GAGAGAGAGAAAAGAGCA---GTGGGA---ATAGGAGCT---
------GTACTCCTTGGGTTCTTGGGAGCA---GCAGGAAGCACTATG---GGCGCGGCGTCA---
ATAACGCTGACGGTACAG---GCCAGACAA---TTGTTG---TCTGGTATAGTGCAACAGCAAAGC---
AATTTG---CTGAGA---GCTATAGAGGCGCAACAGCATATGTTGCAACTCACGGTCTGGGGCATT
>KC894126
TTCTATTGCAATACATCAAAACTGTTTAATGGCACATACATGTCTAATAGTACGTCTAATAGTACAGGA----
----

AACATCACAATCATCACAATCCCATGCAGAATAAAACAAATTATAAACATGTGGCAGGAGGTAGGACGAGCAA
TGTATGCCCCTCCCATTGCAGGAAACATAACATGTAAATCAAAT---
ATCACAGGACTACTATTGACTCGTGATGGAGGAGTCGATAATGGAAATGGA----------------------

----

ACAGAGGAGATATTCAGACCTGGAGGAGGAGATATGAGGGACAATTGGAGAAGTGAATTATATAAATATAAAG
TGGTAGAAATTAAGCCATTA---GGAATAGCACCCACTACTGCAAAAAGGAGAGTGGTG--------------
-------------------------GAGAGAGAGAAAAGAGCA---GTGGGA---ATAGGAGCT---------
GTGTTCCTTGGGTTCTTGGGAGCA---GCAGGAAGCACTATG---GGCGCAGCGTCA---
ATAACGCTGACGGTACAG---GCCAGACAA---TTATTG---TCTGGTATAGTGCAACAGCAAAGC---
AATTTG---CTGAGG---GCTATAGAGGCGCAACAGCATATGTTGCAACTCACGGTCTGGGGCATT
>HM638986
TTCTATTGTAATACATCAAACCTATTTAATAGTACATACATGATTAATGGTACATACATGGCTAATGGTACAG
CAAAT--

ATTCCAAATTCAACCATCACACTCCAATGCAGAATAAAACAAATTGTAAACATGTGGCAAGGGGTAGGACGAG
CAATGTATGCCCCTCCCATTGCAGGAAACATAACATGTGTATCAAAT---
ATCACAGGACTACTATTGACACGTGATGGAGGATATGGAAATAACACC-------------------------
----

AATAACACAGAAGAAATATTCAGACCTGGAGGAGGAAACATGAAGGACAATTGGAGAAGTGAATTATATAAAT
ATAAAGTAGTAGAAATCCAGCCATTA---GGAATAGCACCCACTGAGGCAAAAAGGAGAGTGGTG--------
-------------------------------GAGAGAGAAAAAAGAGCA---GTGGGA---CTAGGAGCT---
------GTGTTCCTTGGGTTCTTGGGAGCA---GCAGGAAGCACTATG---GGCGCAGCATCA---
ATAACGCTGACGGTACAG---GCCAGACAA---TTGTTG---TCTGGTATAGTGCAACAGCAAAGC---
AATTTG---CTGAGG---GCTATAGAGGCGCAACAGCATATGTTGCAACTCACGGTCTGGGGCATT
>KC894128
TTCTATTGTAATACATCAAACCTATTTAATAGTACATATAGTACAAACATGACTAATGATACAGAAAATACT-
----

TTAAATTCAACCATCATACTCCAATGCAGAATAAAACAAATTGTAAACATGTGGCAAGGAGTAGGACGAGCAA
TGTATGCCCCTCCCATTGCAGGAAGCATAACATGTATATCAAAT---
ATCACAGGACTACTATTGACACGTGATGGAGGAAATGGAAATAACACCAAT----------------------
----

AACACAGAAGAAATATTCAGGCCTGGAGGAGGAAACATGAAGGACAATTGGAGAAGTGAATTATATAAATATA
AAGTAGTAGAAATTAAGCCATTA---GGAATAGCACCCACTGAGGCAAAAAGGAGAGTGGTG-----------
----------------------------GAGAGAGAAAAAAGAGCA---GTGGGA---CTAGGAGCT------
---GTGTTCCTTGGGTTCTTGGGAGCA---GCAGGAAGCACTATG---GGCGCAGCATCA---
ATAACGCTGACGGTACAG---GCCAGACAA---TTGTTG---TCTGGTATAGTGCAACAGCAAAGC---
AATTTG---CTGAGG---GCTATAGAGGCGCAACAGCACATGTTGCAACTCACGGTCTGGGGCATT
>KC863127
TTCTATTGCAATACATCAAACTTGTTTAATAAAACAAACCTGTTTAATAGTACAGATAATAGTACAGAAGGGG
AAAAAGGAGTT--

TCAAATGCAACCATCACACTCCAATGCAGAATAAAACAAATTATAAATATGTGGCAGGGGGTAGGACGAGCAA
TGTATGCCCCTCCCATTGCAGGAAACATAACATGTAAATCAAAT---
ATTACAGGACTACTGTTAACACGGGATGGAGGAGATGATAATAAA----------------------------
----

ACAGAGACATTCAGACCTGGAGGAGGAGATATGAGGGACAATTGGAGAAGTGAATTATATAAATATAAAGTGG
TAGAAATTAAGCCATTA---GGAGTAGCACCCACTAAGGCAAAAAGGAGAGTGGTG-----------------
----------------------GAGAGAGAAAAAAGAGCA---GTGGGA---ATAGGAGCT---------
GTGTTCCTTGGGTTCTTGGGAGCA---GCAGGAAGCACTATG---GGCGCAGCATCA---
ATAACGCTGACGGTACAG---GCCAGACAA---TTGTTG---TCTGGTATAGTGCAACAGCAAAGC---
AATTTG---CTGAGG---GCTATAGAGGCGCAACAGCATATGTTGCAACTCACGGTCTGGGGCATT
>KC894129
TTCTATTGCAATACATCAGGCCTGTTTAATGGTACCCTTAATGGTACATATATGCCTAATAATACAGGCAGTA
AT---

TCAAGTTCAACCATCACACTCCAATGCAGGATAAAACAAATTATAAACATGTGGCAGGAGGTAGGACGAGCAA
TGTATGCCCCTCCCATTGCAGGAAACATAACATGTAGATCAAAT---
ATCACAGGACTACTATTAACACGCGATGGAGGAAATACTACTAAT----------------------------

----

AATACAGAAATATTCAGGCCTGGAGGAGGAAATATGAAGGACAATTGGAGAAGTGAATTATATAAATATAAAG
TGGTAGAAATTAAGCCATTA---GGAATAGCACCCACTGAGGCAAAAAGGAGAGTGGTG--------------
-------------------------GAGAGAGAGAAAAGAGCA---GTGGGA---ATAGGAGCT---------
GTGTTCCTTGGGTTCTTGGAACA---GCAGGAAGCACTATG---GGCGCGGCGTCA---
ATAACGCTGACGGTACAG---GCCAGACAA---TTGTTG---TCTGGTATAGTGCAACAGCAAAGC---
AATTTG---CTGAGG---GCTATAGAGGCGCAACAGCATATGTTGCAACTCACGGTCTGGGGCATT
>HM639015
TTCTATTGCAATACATCAGGACTGTTTAATAGTATATACACATTTAATAGTACATACAATAGTAATAGTACAT
ACAATAGTAAT--
TCAAGTTCATACAATAGT---------
AATTCAAGTTCAATTATCACACTCCAATGCAGAATCAAACAAATTATAAACATGTGGCAGGGGGTAGGGCGAG
CAATGTATGCTCCTCCCATTGCAGGAAACATAACATGTATATCAAAT---
ATCACAGGACTACTATTGGTGCGTGATGGAGGCAATGTGACAGAC----------------------------
----

ACAGAGATATTCAGACCTGGAGGAGGAGATATGAAGGACAATTGGAGAAGTGAATTATATAAATATAAAGTGG
TAGAAATTAAGCCATTA---GGAATAGCACCCACTGGGGCAAAAAGGAGAGTGGTG-----------------
----------------------AAGATAGAAAAAAGAGCA---GTGGGA---ATAGGAGCT---------
GTGCTCCTTGGGTTCTTGGGAGCA---GCAGGAAGCACTATG---GGCGCGGCGTCC---
ATAACGCTGACGGTACAG---GCCAGACAA---TTGTTG---TCTGGTATAGTGCAACAGCAAAGC---
AATTTG---CTGAGG---GCTATAGAGGCGCAACAACATATGTTGCAACTCACGGTCTGGGGCATT
>HM639196
TTCTATTGCAATACATCAAGACTGTTTAATAGGACATACAATTTTAATGGTACAGAGAATCCTAATGGTACAG
AG---

AGTAATTCAAGCTTCATCGAACTCCCATGCAGAATAAAACAAATTATAAACATGTGGCAGGAGGTAGGACGAG
CAATGTATGCCCCTCCCATTGAAGGAAACATAACATGTAAATCAAAT---
ATCACAGGACTACTATTGCTACGTGATGGAGGAACAGGAAGTGAGGTA-------------------------
----

CCAAATGACACAGAGATATTCAGACCTGGAGGAGGAAATATGAGGGACAACTGGAGAAGTGAATTATATAAAT
ATAAAGTGGTAGAAATTAAACCATTG---GGAGTAGCACCCACTAAGGCAAAAAGGAGAGTGGTG--------
-------------------------------GAGAGAGAAAAAAGAGCA---GTG------GGAGCTGCT---
------ATGTTCTTTGGGTTCTTGGGAGCA---GCAGGAAGCACTATG---GGCGCGGCGTCA---
ATAACGCTGACGGTACAG---GCCAGACAA---CTGCTG---TCTGGTATAGTGCAACAGCAAAGT---
AATTTG---CTGAGG---GCTATAGAGGCGCAACAGCATATGTTGCAACTCACGGTCTGGGGCATT
>KC894132
TTCTATTGCAATACATCAGGACTGTTTAATAGTACATACAATCCTGATCTTAATAATGCAAAGAATGAT----
----

TCAAATGCAACCATCACGCTCCCATGCAGAATAAAACAAATTATAAACATGTGGCAGGAGGTAGGACGAGCAA
TGTATGCCCCTCCCATTGCAGGAAACATAACATGTAGATCAAAT---
ATCACAGGACTACTGTTGCTACGTGATGGAGGAAAAGATCCAAAC----------------------------
----

AACACAGAGATATTCAGGCCTGGAGGAGGAAATATGAGGGACAACTGGAGAAGTGAATTATATAAATATAAAG
TGGTAGAAATTAAGCCATTG---GGAGTAGCACCCACTAAGGCAAAAAGGAGAGTGGTG--------------
-------------------------GAGAGAGAAAAAAGAGCA---GTAGGA------GCTGCT---------
ATGTTCTTTGGGTTCTTGGGAGCA---GCAGGAAGCACTATG---GGCGCGGCGTCA---
ATAACGCTGACGGTACAG---GCCAGACAA---CTGCTG---TCTGGTATAGTGCAACAGCAAAGT---
AATTTG---CTGAGA---GCTATAGAGGCGCAACAGCATATGTTGCAACTCACAGTCTGGGGCATT
>HM215361
TTCTATTGCAATACATCAGGCCTGTTTAATAATAATCTTACAGAAAATAAT----------------------
----

CTTACAGGCATCATCACACTCCCATGCAGAATAAAACAAATTATAAACATGTGGCAGGGGGTAGGAAGAGCAA
TATATGCCCCTCCCATTGCAGGAAACATAACATGTAAATCAAAT---
ATCACAGGACTACTATTGACACGTGATGGAGGCGATAACACAACT----------------------------

----

GAGACATTCAGACCTGGAGGAGGAGATATGAGGGACAATTGGAGAAGTGAATTATATAAATATAAGGTGGTAG
AAATTAAGCCATTA---GGAATAGCACCCACTGAGGCAAAAAGAAGAGTGGCG--------------------
-------------------GAGAGAGAAAGAAGAGCA---GTGGGA---ATAGGAGCT---------
GTGTTCCTTGGGTTCTTGGGAGCA---GCAGGAAGCACTATG---GGCGCGGCGTCA---
ATAACGCTGACGGTACAG---GCCAGACAA---CTGTTG---TCTGGTATAGTGCAACAGCAAGAC---
AATTTG---CTGAGG---GCTATAGAGGCGCAACAGCATCTGTTGCAACTCACGGTCTGGGGCATT
>HM215362
TTCTATTGCAATACATCAGGACTGTTTAATAGTACATACAATGATACATACATTCCTAATGGTACAAGAAATG
GA---

AATGATTCAATCATCAGACTCCCATGCAGAATAAAACAAATTATAAACATGTGGCAGGGGGTAGGACGAGCAA
TGTATGCCCCTCCCATTGCAGGAAACATAACATGTAGATCAAAT---
ATCACAGGAATACTATTGACACGTGATGGAGGAGAAAATAATGGT----------------------------
----

ACAGAGATATTCAGACCTGGAGGAGGAAATATGAAGGACAATTGGAGAAGTGAGTTATATAAATATAAAGTGG
TAGAAATCAAGCCATTG---GGAATAGCACCCACTGGGGCAAAAAGGAGAGTGGTG-----------------
----------------------GAGAGAGAAAAAAGAGCA---GTGGGA---ATAGGAGCT---------
GTGTTCCTTGGGTTCTTGGGAGCA---GCAGGAAGCACTATG---GGCGCGGCGTCA---
ATAACGCTGACGGTACAG---GCCAGACAA---CTGTTG---TCTGGTATAGTGCAACAGCAAAGC---
AATTTG---CTGAGA---GCTATAGAGGCGCAACAGCATATGTTGCAACTCACGGTCTGGGGCATT
>KU341731
TTCTATTGTAATACATCAGGACTGTTTAATAGTACATACATGCCTACATACATGCCTAATGGTACAGAAAGTA
ATGAT--

TCAAACACAACTATCATAATCCCATGCAGAATAAAGCAAATTGTAAACATGTGGCAGGAGGTAGGACGAGCAA
TGTATGCCCCTCCCATTGCAGGGAACATAACATGTACATCAAAT---
ATCACAGGATTACTATTGGTACGTGATGGAGGAATAAAGGAAAAT----------------------------
----
-
GATACAGAGAATAAGACAGAGACATTCAGACCTGAAGGAGGAAATATGAGGGACAATTGGAGAAGTGAATTAT
ACAAATATAAAGTAGTAGAAATCAAGCCATTG---GGAATAGCACCCACTACAGCAAAAAGGAGAGTGGTG--
-------------------------------------GAGAGAGAAAAAAGAGCA---GTGGGA---
TTAGGAGCT---------GTGTTCCTTGGGTTCTTGGGAGCA---GCCGGAAGCACTATG---
GGCGCAGCGTCA---ATAACGCTGACGGTACAG---GCCAGACAA---TTGTTG---
TCTGGTATAGTGCAACAGCAAAGC---AATTTG---CTGAGA---
GCTATTGAGGCACAACAGCATCTGTTGAAACTCACGGTCTGGGGCATT
>KU341730
TTCTATTGTAATACATCAGGCCTGTTTAATGGTACATACATGCCTACATACATGCCTAATGGTACAGAAAGTA
AT---

TCAAACTCCACCATCACAATCCCATGCCGCATAAAACAAATTATAAACATGTGGCAAGAGGTAGGACGAGCAA
TGTATGCCCCTCCCATTGCAGGAAACATCACATGTACATCAAAT---
ATTACAGGACTATTATTGGTACGTGATGGAGGAATAAAGGAAAAT----------------------------
----
-
GATACAGAGAATAAGACAGAGATATTCAGACCTGAAGGAGGAGATATGAGGGACAATTGGAGAAGTGAATTAT
ATAAATACAAAGTAGTAGAAATTAAGCCATTG---GGAGTAGCACCCACTGAAGCGAAAAGGAGAGTGGTG--
-------------------------------------GAGAGAGAAAAAAGAGCA---GTGGGA---
ATAGGAGCC---------GTGTTCCTTGGGTTCTTGGGAGCA---GCAGGAAGCACTATG---
GGCGCGGCGTCA---ATGACGCTGACGGTACAG---GCCAGACAA---TTATTG---
TCTGGTATAGTGCAACAGCAAAGC---AATTTG---CTGAGA---
GCTATAGAGGCGCAACAGCATCTGTTGCAACTCACGGTCTGGGGCATT
>KU341726
TTCTATTGCAACACATCAGGACTGTTTAATGGTACATACATGCCTACATACATGCCTAATGGTACAGAAAGTA
AT---

TCAAACTCAACTATCACAATCCCATGCAGAATAAAACAATTTGTAAACATGTGGCAGAAGGTAGGACGAGCAA

TGTATGCCCCTCCCATTGAAGGAAACATAACATGTAGATCAAAC---
ATCACAGGAATACTCTTGGTACGTGATGGAGGAATAAAGGAAAAT----------------------------
----
-
GATACAGAGAATAAGACAGAAATATTCAGACCTGAAGGAGGAGATATGAGGGATAATTGGAGAAGTGAATTAT
ATAAATATAAAGTGGTAGAAATTAAGCCATTG---GGAGTAGCACCCACTGGAGCAAGAAGGAGAGTGGTG--
-------------------------------------GAGAGAGAAAAAAGAGCA---ATAGGA---
ATGGGAGCT---------GTGTTCCTTGGGTTCTTGGGAGCA---GCAGGAAGCACTATG---
GGCGCGGCGTCA---ATAACGCTGACGGTACAG---GCCAGACAA---CTGTTG---
TCTGGTATAGTGCAACAGCAAAGC---AATTTG---CTGAGG---
GCTATAGAGGCGCAACAACATATGTTGCAGCTCACGGTTTGGGGCATT
>KU341725
TTCTATTGTAATACAGCAGGCATGTTTAATAGGACATACATGCCTACATACATGCCTAATGGTACAGAAAGTA
AT---

TCAAACTCAACTATCACAATCCCATGCAGAATAAAGCAAATTATTAATATGTGGCAGGAGATAGGAAGGGCAA
TGTATGCCCCTCCCATTGCAGGAGAAATAACATGTAAATCAAAC---
ATTACAGGAATACTATTGGTACGTGATGGAGGAATACAGGCAAAT----------------------------
----
-
GATACAGAGAATAAGACAGAGATATTCAGACCTGGAGGAGGAGATATGAGAGATAATTGGAGAAGTGAATTAT
ATAAATATAAAGTGGTAGAAATTAAGCCATTG---GGAATAGCACCCACTGCAGCAAAAAGGAGAGTGGTG--
-------------------------------------GAGAGAGAAAAAAGAGCA---GTGGGA---
CTAGGAGCT---------GTGTTCCTTGGGTTCTTAGGAGCA---GCAGGAAGCACTATG---
GGCGCGGCGTCA---ATAGCGCTGACGGTACAG---GCCAGACAA---TTGTTG---
TCTGGTATAGTGCAACAGCAAAGT---AATTTG---CTGAGG---
GCTATAGAGGCGCAACAGCATATGTTGCAACTCACGGTCTGGGGCATT
>KU341723
TTCTATTGTAATACATCAGGCCTGTTTAATGGTACATACATGCCTACATACATGCCTAATGGTACAGAAAGTA
AT---

TCAAACTCAACTATCACAATTCCATGTAGAATAAAGCAAATTATAAACATGTGGCAGGAGGTAGGACGAGCAA
TGTATGCCCCTCCCATTGCAGGAGAAATAACATGTATATCAAAT---
ATCACAGGATTGCTATTGGTACATGATGGAGGAATAAAGGAAAAT----------------------------
----
-
GATACAGAGAATAAGCCAGAGATATTCAGACCTGGAGGAGGAGATATGAGGGACAATTGGAGAAGTGAATTAT
ACAAATATAAAGTGGTAGAAATTAGGCCATTA---GGAATAGCACCCACTGCTGCAAAAAGAAGAGTGGTG--
-------------------------------------GAAAGACAAAAAAGAGCA---GTGGGA---
ATGGGAGCT---------GTGATCTTTGGGTTCTTGGGAGCA---GCAGGAAGCACTATG---
GGCGCAGCGTCG---ATAGCGCTGACGGCACAG---GCCAGACAA---TTGTTG---
TCTGGTATAGTGCAACAGCAAAAC---AATTTG---CTGAGG---
GCTATAGAGGCGCAACAGCATCTGTTGCAACTCACGGTCTGGGGCATT
>KU341724
TTCTATTGTAATACAACAGGCTTGTTTAATGGTACATACATGCCTACAGAAAGGCCTAATGGTACAGAAAGTA
AT---

ACAAACTCAACTATCACAATCCCATGCAGAATAAAACAAATTGTAAACATGTGGCAGGAGATAGGACGAGCAA
TGTATGCCCCTCCCATTGCAGGAAACATAACATGTAGATCAAAT---
ATCACAGGATTACTATTGGTACGTGATGGAGGAAAAAAGGCAAATAAT-------------------------
----

ACAAATGATACAGAGACATTCAGACCTGGAGGAGGGGATATGAGGGACAATTGGAGAAGTGAATTATATAAAT
ACAAAGTGGTAGAAATTAAGCCATTG---GGAGTAGCACCCACTGCAGCAAAAAGGAGAGTGGTG--------
-------------------------------GAGAGAGAAAAAAGAGCA---GTGGGA---CTGGGAGCT---
------GTGTTCCTTGGGTTCTTGGGAGCA---GCAGGAAGCACTATG---GGCGCGGCGTCA---
ATAACGCTGACGGTACAG---GCCAGACAA---TTGTTG---TCTGGTATAGTGCAACAGCAAAGC---
AATTTG---CTGATG---GCTATTGAGGCGCAACAGCATCTGTTGCAACTCACGGTCTGGGGCATT
>KJ158430

TTCTATTGTGATACAAAAAAACTGTTTAATGCTACATACATGAATGATGATACA-------------------
----
GCAAATAATTCAAGAAGC---------
AACGGCAATGACACCGTCACTCTCCCATGCAAGATAAAGCAAATTATAAACATGTGGCAGGAGGTAGGGCGAG
CAATGTATGCCCCTCCCATTGCAGGAAACATAACATGTAAATCAAAT---
ATCACAGGACTACTATTGGTACGTGATGGAGGAAAA-------------------------------------
----
ATGAACCAAAATATAGAGACAAAT---------
AAGACGGAGACATTCAGACCTGAAGGAGGAAATATGAAGGACAATTGGAGAAGTGAATTGTATAAATATAAAG
TGGTAGAAATTAAACCATTG---GGAATAGCACCCACTAAAGCAAAAAGGAGAGTGGTG--------------
-------------------------GAGAGAGAAAAAAGAGCA---GTGGGA---CTAGGAGCT---------
GTGTTCCTTGGATTCTTGGGAGTA---GCAGGGAGCACTATG---GGCGCGGCGTCA---
ATAACGCTGACGGCACAG---GCCAGACAA---TTGCTG---TCTGGTATAGTGCAACAGCAAAGC---
AATTTG---CTGAAG---GCTATAGAGGCGCAACAGCATCTGTTGCAACTCACGGTCTGGGGCATT
>KJ158433
TTCTATTGTGGGTCCTTTGCCTGTTTAATAGGACATACATGATTCCGCCCAAAAG------------------
----
AGTAATTCAAGCAGTAATCCA------------
AAATACAATAAATCAATCACAATGCACTGCAGAATAATACTAATTATCATCATGAGGGGGAGGTGGAATTAG
CAACGTATTTTCCTCCCATCGCAGGATGCATTACATCAGAATCAAAT---
ATCACAGGAATAATGTTAACCCGAAATGGGGAC--

AAAAAAAGGAATAAGGCAACA---------
GGGACAGAGACATTCAGACCTGGAGGAGGAGATATGAGGAACAATTGGAGAAGTGAATTATATAAATATAAAG
TGGTAGAAATTAAGCCATTG---GGGGTAGCACCCACTACAGCAAAAAGGAGAGTGGTG--------------
-------------------------GAGAGAGAAAAAAGAGCA---ATGGGA---CTAGGAGCT---------
TTGTTCCTTGGGTTCTTGGGAACA---GCAGGAAGCACTATG---GGCGCGGCGTCA---
ATAACGCTGACGGTACAG---GCCAGACAA---TTGTTG---TCTGGTATAGTGCAACAGCAAAGT---
AATTTG---CTGAAA---GCTATAGAGGCGCAACAGCATCTGTTGCAACTCACGGTCTGGGGCATT
>KJ541840
TTCTATTGTAATACATCAGGCCTGTTTATTGATGCTACCAATGGTAATTCCAGG-------------------
----
GAAAATGGTACAAACAGT---------
AATTCAAGTTTAAACATCACAATCCCATGCAGAATAAAGCAAATTATAAATATGTGGCAGGAGGTAGGACGAG
CAATGTATGCTCCTCCCATTAAAGGAAACATAACATGTAAATCAAAT---
ATCACAGGAATACTATTGGCACGAGATGGTGGACAA-------------------------------------
----
GAAGCAAATAGCACCGAGGGGGGG---------
GACACAGATACATTCAGACCTGGAGGAGGAAATATGAAGGACAATTGGAGAAGTGAATTATATAAATATAAAG
TAGTAGAGATTAAGCCATTG---GGAATAGCACCCACTAAAGCAAAAAGGAGAGTGGTG--------------
-------------------------GAGAGAGAAAAAAGAGCA---GTGGGA---ATAGGAGCT---------
GTGTTCCTTGGGTTCTTGGGAGCA---GCAGGAAGCACTATG---GGCGCGGCGTCA---
ATAACGCTGACGGTACAG---GCCAGACAA---TTGTTG---TCTGGTATAGTGCAACAGCAAAGC---
AATTTG---CTGCAG---GCTATAGAGGCGCAACAGCATCTGTTGCAACTCACAGTCTGGGGCATT
>KJ158435
TTCTATTGCAATACATCAAAACTGTTTAATAGCACATACATGAGTAATGGTACA-------------------
----
AATAGGGGTTTAATTATT---------
ACTAATGATAAAAACATCACAATCCCATGCAGAATAAAACAAATTATAAACATGTGGCAGGAGGTAGGAAGAG
CAATGTATGCCCCTCCCATTGCAGGAAACATAACATGCAGATCAAAT---
ATCACAGGAATACTATTGGTACGTGATGGAGGAGAA-------------------------------------
----
GACAAAAATGGAGACACAAATGAT---------
ACGACCGAGACATTTAGACCTGGAGGAGGAAATATGAGGGACAATTGGAGAAGTGAGTTATATAAATATAAAG
TGGTAGAAATTAAGCCATTG---GGAATAGCGCCCACTGGAGCAAAAAGGAGAGTGGTG--------------
-------------------------GAGAGAGAAAAAAGAGCA---GTGGGA---ATAGGAGCT---------
GTGTTCCTTGGGTTCTTGGGAGCA---GCAGGAAGCACTATG---GGCGCGGCGTCA---
ATAACGCTGACGGTACAG---GCCAGACAA---TTGTTG---TCTGGTATAGTGCAACAGCAAAGC---
AATTTG---CTGAGG---GCTATAGAGGCGCAACAGCATATGTTGCAACTCACAGTCTGGGGCATT
>KJ541849

TTCTATTGTAATACATCAAGCCTGTTTAATGGGTCTAATGATACTGCCAATATT-------------------
----
GGTACCGACAATTTTACA---------
AACAGTAATGATAACATCACAATCCCATGCAGAATAAAGCAAATTATAAACATGTGGCAGGAGGTAGGACGAG
CAATGTATGCCCCTCCCATTGAAGGAAGTATAACATGTAACTCAAAT---
ATCACAGGACTACTATTGGTGCGTGATGGAGGAGGA-------------------------------------
----
GACAATAATGATACTAATACACAC---------
AAACCAGAGATATTCAGACCTGGAGGAGGAAATATGAGGGACAATTGGAGAAGTGAATTATATAAATATAAAG
TGGTAGAAATTAAGCCATTA---GGAGTAGCACCCACTGGGGCAAAAAGGAGAGTAGTG--------------
-------------------------GAGAGAGGAAAAAGAGCA---GTGGGA---ATAGGAGCT---------
GTGTTCCTTGGGTTCTTGGGAGCA---GCAGGAAGCACTATG---GGCGCGGCGTCA---
ATAACGCTGACGGCACAG---GCCAGACAA---TTGTTG---TCTGGTATAGTGCAACAGCAAAAT---
AATTTG---CTGAGG---GCTATAGAGGCGCAACAGCATCTGTTGCAACTCACGGTCTGGGGCATT
>MF373126
TTCTACTGCAATACATCAAACCTGATTAATAATAATATAAACATTGCTTCAGCG-------------------
----

AGCACAATACAACGCAGAATAAAACAAATTATAAACATGTGGCAGGGGGTAGGACGAGCAATGTATGCCCCTC
CCATTGCAGGAAACATAACATGTAAATCGAAT---
ATCACAGGACTACTACTGACACGTGATGGAGGAGAAAATAATAGG----------------------------
----

ACTGAGACATTCAGACCTGGAGGAGGAGATATGAAGGACAATTGGAGAAGTGAATTATATAAATATAAGGTGG
TAGAAAGTAAGCCATTA---GGAATAGCACCCACAAAGGCAAAAAGGAGAGTGGTG-----------------
----------------------GAGAGAGAGAAACGAGCA---GTGGGA---CTAGGAGCT---------
GTGTTCCTTGGGTTCTTAGGAGCA---GCAGGAAGCACTATG---GGCGCAGCATCA---
ATAACGCTGACGGTACAG---GCCAGACAA---TTATTG---TCTGGTATAGTGCAACAGCAAAGT---
AATTTG---CTGAAG---GCCATAGAGGCGCAACAGCATATGTTGCAACTCACAGTCTGGGGCATT
>MF373131
TTCTATTGCAATACATCACAACTGTTTAATGCATCAGATATTTTTAATGATACAACT----------------
----

AATAATACTACCAGCACAATTCCCTGCACTATATACAGGATAATGGACATAAGGCAAGAGATGTGGCCAGCAC
CGTATGCACCTATGATTGCCCGCACGATTACAAGAACGTCAACA---
ATCACTTCAATTCTCCCAAGACTTCTTCTAAGGAATGAGACAGGTAATGAGAAT------
GAGACACACCTGTATGAG---ACATATGAACAA--

CCTGGCAGAGACAATAGGAGAAATAATTGGAGGAAAGATTTACAGGCATATGAAGTGGCATCACTTGGAGCAA
TA---ACAATAGCGCCG---AAGAGAGATAAGAGAACAGTG--------------------------------
-------GAGAAAGCAGAAGGAGCA---CTAGGC---ATTCGAGCT---------
GTTCTCCGTGCATTCGTGAGACTT---CCAGGGAGCACTATG---GGCGCAGCATCA---
ATAACGCTGACGGTACAG---GCCAGACAA---TTACTG---TCTGGTATAGTGCAACAGCAAAAC---
AATTTG---GTGAGA---GCTATAGAGGCGCAACAGCACATGTTGCAACTCACAGTCTGGGGCATT
>MF373136
TTCTATTGTAATACATCAGGCCTGTTTAATGGTACATACATGCCTACATACATGCCTAATGGTACAGAAAGTA
AT---

TCAAACTCAACTATCACAATCCCATGCAGAATAAAGCAAATTATAAACATGTGGCAGGAGGTAGGACGAGCAA
TGTATGCCCCTCCCATTGCAGGAAACATAACATGTATATCAAAT---
ATCACAGGACTACTACTAGTACGTGATGGAGGACATGATGGAGAAAAT-------------------------
----

AATGACACGGAGACATTCAGACCTGGAGGAGGAAATATGAAGGACAATTGGAGAAGTGAATTATATAAATATA
AAGTGGTAGAAGTTAAACCATTG---GGGATAGCACCCACTAAAGCAAAAAGGAGAGTGGTG-----------
----------------------------GAGAGAGAAAAAAGAGCA---GTGGGA---ATAGGAGCT------
---GTGTTCCTTGGTTTCTTGGGAGCT---GCAGGAAGCACTATG---GGCGCGGCGTCA---
ATGACGCTGACGGTACAG---GCCAGACAA---TTGTTG---TCTGGTATAGTGCAACAGCAAAGC---
AATTTG---CTGAAG---GCTATAGAGGCGCAACAGCATCTGTTGCAACTCACGGTCTGGGGCATT
>KP411832

TTCTATTGCGACACATCACAACTGTTTAATGGTACATACAAGGCTACTAGTACTACTACTACT----------
----

TCACATTCCACCATCACACTCCAATGCAGAAGAAAACAAATTATAAATCTGTGGCAGGAGGTAGGACGAGCAA
TGTATGCCCCTTCCCTTGCAGGAAATATAACATGCGAATCAGGT---
ATCTCAGGATTACTAGTGACACGTGATGGAGGTAATGGTAATGAAACTGAT----------------------
----

AATAAGACAGAGACATTCAGACCTACAGGAGGAGATATGAGGGACAATTGGAGAAGTGAATTATATAAATATA
AAGTGGTAGAAATTAAACCATTA---GGAGTAGCACCAACTGGGGCAAAAAGGAGAGTGGTG-----------
----------------------------GAGAGAGAAAAAAGAGCA---GTGGGA---ATGGGAGCT------
---GTGATCCTTGGGTTCTTAGGAGCA---GCAGGAAGCACTATG---GGCGCAGCATCA---
ATAACGCTGACGGTACAG---GCCAGACAA---TTGTTG---TCTGGTATAGTGCAACAGCAAAGC---
AATTTG---CTGAGG---GCTATAGAGGCGCAACAGCATATGTTGCAACTCACAGTCTGGGGCATA
>KP411833
TTCTATTGCGATACATCAAAATTGTTTAATATTACATTTAATGGTAGCCTGTCTAATAATGTT----------
----

CCAAATTCAACCATCACACTCCAATGCAAAATAAAACAATTTATAAACATGTGGCAGGAGGTAGGACAAGCAA
TGTATGCCCCTCCTATTGCAGGAAACATAACATGTACATCAAAT---
ATCACAGGACTACTATTGACACGTGATGGAGGAACAGGGAAAAATAGT-------------------------
----

AAAAATGACACAGAGACATTCACACCTGGAGGAGGAGATATGAGGGACAATTGGAGAAGTGAATTATATAAAT
ATAAAGTGGTAGAAATTAAACCATTA---GGAATAGCACCCACTACGGCAAAAAGGAGAGTGGTG--------
-------------------------------GAGAAAGAAAAAAGAGCA---GTAACA---CTAGGAGCC---
------TTGTTCCTTGGGTTCTTGGGAGCA---GCCGGAAGCACTATG---GGCGCAGCATCA---
ATAACGCTGACGGTACAG---GCCAGACAA---TTATTG---TCTGGTATAGTGCAACAGCAGAAC---
AACCTG---CTGAGG---GCTATAGAGGCGCAACAGCATATGTTGCAACTCACGGTCTGGGGCATT
>MF373145
TTTTATTGCAATACAACAAACCTGTTTAATGGCAGTTTAGAAACACCAGTTATTACATGCAGACAAAATGTTC
CAATGCACCAT---CTA------CTT---
----------------------------------AATGCCAAT------------------
AAACAATTTATAAACATGTGGCAGGAGGTAGGACGAGCAATGTATGCCCCTCCTATTAAAGGAAACATAACAT
GTAGATCAAAT---ATCACAGGACTGCTACTGACAAGGGATGGGGGCAACAACAGCAAC--------------
----

GATAGCGAGACATTCAGACCTGCAGGAGGAGATATGAGGGACAATTGGAGAAGTGAATTATATAAATATAAAG
TGGTAGAAATTAAGCCATTA---GGAATAGCACCCACTTCTGCAAAAAGGAGAGTGGTG--------------
-------------------------GAGAGAGAAAAAAGAGCA---GCA------CTAGGAGCT---------
ATGTTCCTTGGGTTCTTGGGAGCA---GCTGGAAGCACTATG---GGCGCAGCATCA---
ATAATGCTGACGGTACAG---GCCAGACAA---TTATTG---TCTGGTATAGTGCAACAGCAAAGC---
AATTTG---CTGAAA---GCTATAGAGGCGCAACAGCATATGTTGCAACTCACGGTCTGGGGCATT
>MF373146
TTTTATTGTAATACATCAGCCCTGTTCAATACATCAATCCCGTTCAATAGTACTATTACTAATAGTAGT----
----

ACAGATTCAAACATCACACTCCCATGCAGGATAAAACAAATTATAAACATGTGGCAAGGGGTAGGACAAGCAA
TGTATGCCCCTCCCATTGAAGGAAAAATAACATGTAGCTCAAAT---
ATCACAGGACTACTACTGACACGAGATGGAGGAGGAAATGAAACT----------------------------
----

GAAACTGAGACATTCAGGCCTGTAGGAGGAGATATGAGGAACAATTGGAGGAGTGAATTATATAAATATAAAG
TGGTAGAAATCAAGCCATTA---GGAATAGCACCCACTGGAGCAAAAAGGAGAGTGGTG--------------
-------------------------GAGAGAGAAAAAAGAGCG---GCA------CTAGGAGCT---------
CTGTTCCTTGGGTTCTTGGGAGCA---GCTGGAAGCACTATG---GGCGCAGCATCA---
CTAACGCTGACGGTACAG---GCCAGACAA---TTGTTG---TCGGGTATAGTGCAACAGCAAAAT---
AATTTG---CTGAGG---GCTATAGAGGCGCAACAGCATATGTTGCAACTCACGGTCTGGGGCATT
>MF373154

```
TTCTATTGCAATCCAACAAAATTGTTTAATGGTAATAGTACTTCA---------------------------
------------------------------------------------------------------------
---------------------------
ACTTCAGACCCCATCATACTCCCATGCAGAATAAAACAAATTATAAACATGTGGCAGGGGGTAGGCCGAGCAA
TGTATGCCCCTCCCATTGCAGGAAAGATAACATGTAAATCAAAC---
ATCACAGGACTACTATTGACACGTGATGGAGGACACAACAATAAC---------------------------
------------------------------------------------------------------------
------------
GAGACAGAAACATTCAGACCTGAAGGAGGAAATATGAGGGACAATTGGAGAAGTGAATTATACAAATATAAAG
TGGTGGAAATTAATCCATTA---GGAATAGCACCCACTAAGGCAAAAAGGAGAGTGGTG-------------
------------------------GAGAGAGAAAAAAGAGCA---GTGGGA---ATAGGAGCT---------
GTGTTCCTTGGGTTCTTGGGAGTA---GCAGGAAGCACTATG---GGCGCAGCGTCA---
TTGACGCTGACGGTACAG---GCCAGACAA---TTGCTG---TCTGGTATAGTGCAACAGCAAAAC---
AATTTG---CTGAGG---GCTATAGAGGCGCAACAGCATATGTTGCAACTCACAGTCTGGGGCATT
>KP411837
TTCTATTGCAACACATCAAACCTATTTAATAGAAATTTTTCAGAACTGGTTAATAACAGTACTAATAGTACTA
AT----------------------------------------------------------------------
---------------------------
AGTACTTCAATCATCACACTCCGATGTAGAATAAAACAAATCATAAACATGTGGCAGGGGGTAGGACGAGCAA
TGTATGCCCCTCCCATTGAAGGAGACATAACATGTAAGTCAAAT---
ATCACAGGAATACTACTGACACGTGAGGGAGGAAAAAGACAGCAC---------------------------
------------------------------------------------------------------------
------------
CAGACAGAGATATTCAGACCTGGAGGAGGAGATATGAGGGACAATTGGAGAAGTGAATTATATAAATACAAAG
TGGTAGAGATTAAGCCACTA---GGAATAGCACCCACTAGAGCAAAAAGGAGAGTGGTG-------------
------------------------GAGAGAGAAAAAAGAGCA---GTGGGA---ATAGGAGCT---------
GTGATCTTTGGGTTCTTGGGAGCA---GCCGGAAGCACTATG---GGCGCAGCATCA---
ATAGTGCTGACGGCACAG---GCCAGACAA---TTATTG---TCTGGTATAGTGCAACAGCAAAAC---
AATTTG---CTGAGG---GCCATAGAGGCGCAACAGCATATGTTGCAACTCACGGTCTGGGGCATT
>MF373156
TTCTATTGTGATACAACAAAATTGTTTAATGGTACATATGAGCATGAGAGTAATGCTACTTCAAATTCAAGT-
------------------------------------------------------------------------
---------------------------
TCAAATTCAACCATCACACTCCAATGCAGAATAAAACAAATTATAAATATGTGGCAGGGGGTAGGACGAGCAA
TGTATGCCCCTCCCATTGAAGGAATCATACAATGTGCATCAAAT---
ATCACAGGACTATTATTGACACGTGATGGAGGAGAGAATAATAAT---------------------------
------------------------------------------------------------------------
---------
AATGACACAGAGACATTCAGACCTGGAGGAGGAGATATGAGGGACAATTGGAGAAGTGAATTATATAAATATA
AAGTGGTAGAAATTAAACCATTA---GGAATTGCACCCACTACTGCAAAAAGGAGAGTGGTG----------
---------------------------GAGAGAGAAAAAAGAGCA---GTGGGA---ATAGGAGCT------
---GTGTTCCTTGGGTTCTTGGGGGCA---GCAGGAAGCACTATG---GGCGCAGCGTCA---
ATAACGCTGACGGTACAG---GCCAGACAA---TTGTTG---TCTGGCATAGTGCAACAGCAAAGC---
AATTTG---CTGAGG---GCTATAGAGGCGCAACAGCATATGTTGCAACTCACGGTCTGGGGCATT
>MF373157
TTCTATTGCAATACATCACAGCTGTTTGATTCAACACAGCTGGTTAATAGTAGTGGT---------------
------------------------------------------------------------------------
---------------------------
AGTAATGCACACATCACAATCCCATGCAGAATAAAACAAATTATAAACCTGTGGCAAGAGGTAGGACGAGCAA
TGTATGCCCCTCCCATTGCAGGAAACATAACATGTAAATCAAAT---
ATCACAGGAATACTATTGACACGTGATGGAGGAAATACCAATGAAACA------------------------
------------------------------------------------------------------------
---------
AATAGCACAGAGACATTCAGACCTGGAGGAGGAGATATGAGGGACAATTGGAGAAGTGAATTATACAAATATA
AAGTGGTAGAAGTTAAACCATTA---GGAGTAGCACCCACTAAGGCAAAAAGGAGAGTGGTG----------
---------------------------GAGAAACAAAAAAGAGCA---GTGGGA---CTAGGAGCT------
---ATGTTCCTTGGGATCTTGGGAGCA---GCAGGAAGCACTATG---GGCGCAGCATCA---
ATAACGCTGACGGTACAG---GCCAGGCAA---TTGTTG---TCTGGTATAGTGCAACAGCAAAGC---
AATTTG---CTGAGG---GCTATAGAGGCGCAACAGCATATGTTGCAACTCACAGTCTGGGGCATT
>MF373170
```

TTCTACTGCAATACATCAGGCCTGTTTAATGGTACATACATGGGTAATATGACTAATAGTACTACAAAT----
----

GAAAATGACACCATCACCCTCCAATGCAGAATAAAACAAATGATAAACATGTGGCAGGAGGTAGGACGAGCAA
TGTATGCTCCTCCCATTGCAGGAAACATAGCATGTAGATCAGAA---
ATCACAGGACTACTATTGACACGTGATGGGGGAAATGGAAATGAAACAGAT----------------------
----

AATAACACAGAGATATTTAGACCTGGAGGAGGAGATATGAGAAACAACTGGAGAAGTGAATTATATAAATATA
AAGTAGTAGAAATCAAGCCATTA---GGATTAGCACCCACTAGAGCAAGAAGGCAAGTGGTG-----------
----------------------------CCGAGACAAAAAAGAGCA---GCA------ATAGGAGCT------
---ATATTCCTTGGGTTCTTGGGAGCA---GCTGGAAGCACTATG---GGCGCAGCGTCA---
ATGACGCTGACGGTACAG---GCCAGACAA---TTATTG---TCGGGTATAGTGCAACAGCAGAGC---
AATCTG---CTGAGA---GCTATAGAGGCGCAACAACATATGTTGCAACTCACAGTCTGGGGCATT
>MF373171
TTCTACTGCAATACATCAGGTCTGTTTAATGGTACATACATGGGTAATATGACTAATGGTAATATAAATGGA-
----

ACAAATGAAACCATCATCCTCCCATGCAGAATAAAACAAATGATAAACATGTGGCAGGAGGTAGGACGAGCAA
TGTATGCTCCTCCCATTGCAGGAAACATAGCATGTAACTCAACA---
ATCACAGGATTACTATTGACACGTGATGGAGGATCTGGAAATGAT----------------------------
----

AGTACCACAGAGATATTCAGACCTGGAGGAGGAGATATGAGAAACAACTGGAGAAGTGAATTATATAAATATA
AAGTAGTAGAAATCAAGCCATTA---GGATTAGCACCCACTACAGCAAGAAGGAGAGTGGTG-----------
----------------------------CAGAGAGAAAAAAGAGCA---GCA------ATAGGAGCT------
---ATGCTCCTTGGGTTCTTGGGAGCA---GCTGGAAGCACTATG---GGCGCAGCGTCA---
ATAACGCTGACGGTACAG---GCCAGACAA---TTATTG---TCGGGTATAGTGCAACAGCAGAGC---
AATCTG---CTGAGG---GCTATAGAGGCGCAACAACATATGTTGCAACTCACAGTCTGGGGCATT
>MF373177
TTCTATTGCGATACATCCAACCTGTTTAATGAAACAGCATTTAATAATAGT----------------------
----

GATGGTACAAACATCACACTCCCATGCAGAATAAAACAAATTATAAACATGTGGCAGAAGGTAGGACGGGCAA
TGTATGCCCCTCCCATTAAAGGACCAATAAAGTGTGACTCAAAT---
ATCACAGGATTACTACTAACACGAGATGGAGGCAATAGAGGCAAT----------------------------
----

GGGGGAAATGACACAGAAATATTCAGACCTGCAGGAGGAGATATGAGGGACAATTGGAGAAGTGAATTATATA
AATATAAGGTGGTAGAAATTAAGCCATTA---GGAATAGCACCCACTGCTGCAAAAAGGAGAGTGGTG-----
----------------------------------GGGAGAGAAAAAAGAGCA---GCA------
CTAGGAGCT---------ATGTTCCTTGGGTTCTTGGGAGCA---GCAGGAAGCACTATG---
GGCGCAGCATCA---ATAACGCTGACGGTACAG---GCCAGACAA---TTATTG---
TCTGGTATAGTGCAACAGCAAAGC---AATTTG---CTGAGA---
GCTATAGAGGCGCAACAACATATGTTGCAGCTCACAGTCTGGGGCATT
>MF373187
TTCTATTGCAATACATCAGGCCTGTTCAATGATACATACTGGAACAATAATACGAAAAATAATACAAAA----
----

GGAAATTCAAACTTCACAATCCAATGCAGAATAAAACAAATTATAAACATGTGGCAGGGAGTAGGAAGAGCAA
TGTATGCTCCTCCCATTGCAGGAGAAATAACATGTAGATCAAAT---
ATCACAGGACTACTACTGACACGGGATGGAGGAAATAAGACAGGAAATGGT----------------------
----

AATGAGACAGAGACATTCAGGCCGGGAGGAGGAGATATGAAGGACAATTGGAGAAGTGAATTATATAAATATA
AAGTGGTAGAAATTAGACCATTA---GGAATAGCACCCACCATGGCAAAAAGGAGAGTGGTG-----------
----------------------------GAGAGAGAAAAAAGAGCA---GTGGGA---ATAGGAGCT------
---GTGCTCTTTGGGTTCTTGGGAGCA---GCAGGAAGCACTATG---GGCGCAGCGTCA---
ATAGCGCTGACGGTACAG---GCCAGACAA---TTATTG---TCGGGCATAGTGCAACAGCAAAGC---
AATTTG---CTGAGG---GCTATAGAGGCGCAACAGCATATGTTGCAACTCACGGTCTGGGGCATT

```
>MF373193
TTCTATTGCAATACATCAGGCCTGTTTAATAGTATATACTGGCCTAATGGTACATTCGCATTCAATGGTAGTA
CAAACAGT-----------------------------------------------------------------
-------------------AAC---
TCAACCAACAGCACAATCACAATCCCATGCAGAATAAAACAAATTATAAATATGTGGCAGGAGGTAGGACGAG
CAATGTATGCCCCTCCCATTAGGGGAAACATAACATGTACCTCAAAT---
ATCACAGGACTACTAATAATACGTGATGGAGGAAACAACGAGACA----------------------------
-------------------------------------------------------------------------
----------
AATGAGACAGAGATATTCAGACCAGGGGGAGGAAATATGAAGGACAACTGGAGAAGTGAATTATACAAATATA
AGGTGGTAGAAATTAAGCCATTA---GGAGTAGCACCCACTGAAGCAAAAAGGAGAGTGGTG-----------
----------------------------GGGAGAGAAAAAAGAGCA---GTGGGA---ATAGGAGCT------
---GTGTTCCTTGGGTTCTTGGGATTA---GCAGGAAGCACTATG---GGCGCGGCGTCA---
ATGACGCTGACGGTACAA---GCCAGACAA---TTGCTG---TCTGGTATAGTGCAACAGCAAAGC---
AATTTG---CTGAGG---GCTATAGAGGCGCAACAGCATATGTTGCAACTCACGGTCTGGGGCATT
>MF373199
TTCTATTGCAATACGTCTAGACTGATTAATAGTACATACAGGTCTAATAGTACTAATAGTACT----------
-------------------------------------------------------------------------
----------------------------
TCAGATACATCCATCATACTCCCATGCAGAATAAAACAAATTATAAACATGTGGCAGGGGGTAGGACGAGCAA
TGTATGCCCCTCCCATTGAAGGAAACATAACATGTAAATCAAAT---
ATTACAGGACTACTATTGACACGTGATGGAGGAACGGACAATGAA----------------------------
-------------------------------------------------------------------------
-------------
ACAATAGAGACATTCAGACCTGGAGGAGGAGATATGAGGGACAATTGGAGAAGTGAATTATATAAATATAAAG
TGGTAGAAATTAAACCATTA---GGAATAGCACCCACTAAGGCAAAAAGGAGAGTGGTG--------------
-------------------------GAGAGAGAAAAAAGAGCA---GTGGGA---ATAGGAGCT---------
GTGTTCCTTGGGTTCTTGGGAGCA---GCAGGAAGCACTATG---GGCGCAGCATCA---
CTGACGCTGACGGTACAG---GCCAGGCAA---TTGCTG---TCTGGTATAGTGCAACAGCAAAGC---
AATTTG---CTGAGG---GCTATAGAGGCGCAACAGCATATGTTGCAACTCACGGTCTGGGGCATT
>MF373202
TTCTATTGCAATACATCAAACCTGTTTAATAGATCAATCCTGAATGTAACTGAAACAAATGTA----------
-------------------------------------------------------------------------
----------------------------
AATGAAGCAAACATCACCCTCCCATGCAGAATAAACCAATTTTTAAACATGTGGCAAGGGGTAGGACGAGCAA
TGTATGCCCCTCCCATTGAAGGAGAAATAACATGTAAATCAAAT---
ATCACAGGACTACTCCTGACACGTGATGGGGGTAATGGGAGAGAT----------------------------
-------------------------------------------------------------------------
----------
AAGCAAAATGAAACATTTAGACCTGCAGGAGGAGATATGAGGGACAATTGGAGAAGTGAGTTATACAAATATA
AAGTGGTAGAACTTAAGCCATTA---GGACTAGCACCCACTACGGCAAAAAGGAGAGTGGTG-----------
----------------------------GAGAGAGAAAAAAGAGCA---GTGGGA---TTTGGAGCT------
---CTGTTCTTTGGGTTCTTAGGAGCA---GCAGGAAGCACTATG---GGCGCAGCATCA---
ATAACGCTGACGGTACAG---GCCAGACAA---TTACTG---TTTGGTATAGTGCAACAGCAAAAT---
AATTTG---CTGAAA---GCTATAGAGGCGCAACAGCATATGTTGCAACTCACAGTCTGGGGCATT
>MH705137
TTTTATTGCAATACATCACAACTGTTTAATGGTACAATTACA-------------------------------
-------------------------------------------------------------------------
-------------------------
ACAGATGGTTTAAACATCACAATCCCATGCAGAATAAAACAAATTATAAACATGTGGCAGGAGGTAGGACGAG
CAATGTATGCTCCTCCCATTGCGGGAAATATAATATGTAACTCAAGT---
ATCACAGGAATACTACTGACACGCGATGGAGGA----------------------------------------
-------------------------------------------------------------------------
-------
GAGAATACTACAGAGACATTCAGACCTGGAGGAGGAGATATGAGGGACAATTGGAGAAGTGAATTATATAAAT
ATAAAGTAGTAGAAATTAAGCCATTA---GGAATAGCACCCACCAAGGCAAAAAGGAGAGTGGTG--------
-------------------------------GAGAGAGAAAAAAGAGCA---GTGACA---CTAGGAGCT---
------TTGTTCCTTGGGTTCTTGGGAGCA---GCAGGAAGCACTATG---GGCGCAGCGTCA---
ATAACGCTGACGGTACAG---GCCAGACAA---TTGTTG---TCTGGTATAGTGCAACAGCAAAGC---
AATTTG---CTGAGA---GCTATAGAGGCGCAACAGCATATGTTGCAACTCACAGTCTGGGGCATT
```

>MG989567
TTCTATTGTAACACAGCAGGACTGTTCAATGCAACATACAATAGTACAGAAGGAGATACA-------------
----

AACATTACAAACATCACACTCCCATGCAGAATAAGACAAATTATAAATATGTGGCAGGAAGTAGGAAGAGCAA
TATATGCCTCTCCCATTGAAGGAAATATAGCTTGTAACTCAAGT---
ATCACAGGACTACTATTGGTACGAGATGGAGGAAAT-------------------------------------
----

AGCTCAAATGAGACATTTAGACCTATAGGAGGAGATATGAAGAACAATTGGAGAAATGAATTATATAAATATA
AAGTGGTAGAAATTAAGCCATTG---GGAGTAGCCCCCACTGAAGCAAGAAGGAGAGTGGTG-----------
----------------------------GAGAGAGAAAAAAGAGCA---GTAGGA---CTGGGAGCT------
---GTGTTCCTTGGGTTCCTGGGAGCA---GCAGGAAGCACTATG---GGCGCGGCGTCA---
ATAACGCTGACGGTACAG---GCCAGACAA---TTACTG---TCTGGTATAGTGCAACAGCAAAGT---
AATTTG---CTGAGA---GCTATAGAGGCGCAACAGCATTTGTTGCAACTCACAGTCTGGGGCATT
>HM215433
TTCTATTGCAATACATCAAAACTGTTTAATAGTACATACAATCCTAATGGAACAAAAAATGATACAAAC----
----

TCAAGTGCATACATCATCCTTCCATGCAGAATAAAACAGATTATAAACATGTGGCAGGAGGTAGGACGAGCAA
TGTATGCCAATCCCATTGAAGGATACATAACATGTAGATCAAAT---
ATCACAGGACTACTATTAGTACGTGATGGAAGCACAAATAGTAGT----------------------------
----

AACGAGACATTCAGACCTGGAGGAGGAAATATGAAGGATAATTGGAGAAGTGAATTATATAAATATAAAGTGG
TAGAAATTAAGCCATTG---GGAATAGCACCCACTGAAGCAAAAAGGAGAGTGGTG-----------------
----------------------GAGAGAGAAAAAAGAGCA---GTGGGA---ATAGGAGCT---------
GTGTTCCTTGGGTTCTTGGGAGCA---GCAGGAAGCACTATG---GGCGCGGCGTCA---
ATGACGCTGACGGTACAG---GCCAGACAA---TTGTTG---TCTGGTATAGTGCAACAGCAAAGC---
AATTTG---CTGAGG---GCTATAGAGGCGCAACAGCATTTGTTGCAACTCACGGTCTGGGGCATT
>HQ659585
TTCTATTGCAATACATCAGGGCTGTTTGGTAGTACATACAGGAATACA-------------------------
----

GAAAATGGTACAATCATACTCCAATGCAGAATAAAGCAAATTATAAACATGTGGCAGGAGGTAGGAAGAGCAA
TGTATGCTCCTCCCATTGCAGGAAACATAACATGTAAGTCAAAT---
ATCACAGGATTACTATTGACGCGTGATGGAGGAACAAAT----------------------------------
----

GAAACAGAAATATTCAGACCTGGAGGAGGAGATATGAGGGACAATTGGAGAAGTGAATTATACAAATATAAAG
TGGTAGAAATTAAGCCATTA---GGAATAGCACCCACTGGGGCAAAAAGGAGAGTGGTG--------------
-------------------------GAGAGAGGAAAAAGAGCA---GTGGGA---ATAGGAGCT---------
GTGTTCCTTGGGTTCTTGGGAGCA---GCAGGAAGCACTATG---GGCGCGGCGTCA---
ATAACGCTGACGGTACAG---GCCAGACAA---TTATTG---TCTGGCATAGTGCAACAGCAAAAC---
AATTTG---CTGAGG---GCTATAGAGGCGCAACAGCATATGTTGCAACTCACGGTCTGGGGCATT
>HQ697934
TTCTATTGCAATACATCAAAACTGTTTAATGGTATATACAACTCTAATAATACAAGAAATGAT----------
----

TCAAATTCAACCATCATACTCCCATGCAGAATAAAACAAATTGTAAACATGTGGCAGGAGGTAGGACGAGCAA
TGTATGCCCCTCCCATTGCAGGAAACATAACATGTAAATCACAA---
ATCACAGGGCTACTATTGACATGGGATGGAGGCAAAGACAATAACTCAACA----------------------
----

GAGGATAACAATACAGAGATATTCAGACCTGGAGGAGGAGATATGAGGGACAATTGGAGAAGTGAATTATATA
AATATAAAGTGGTAGAAATTAAGCCATTA---GGAGTAGCACCCACTGGGGCAAAAAGGAGAGTGGTG-----
----------------------------------GAGAGAGAAAAAAGAGCA---GTGGGA---
ATAGGAGCT---------GTGTTCCTTGGGTTCTTGGGAGCA---GCAGGAAGCACTATG---
GGCGCGGCGTCA---ATCACGCTGACGGTACAG---ACCAGACAA---TTATTG---

TCTGGTATAGTGCAACAGCAAAGC---AATTTG---CTGAAG---
GCTATAGAGGCGCAACAGCATATGTTGCAGCTCACAGTCTGGGGCATT
>HQ697983
TTCTATTGCAATACATCAAACCTGTTTAATAATAACATAACT-------------------------------
----

TTAAACGGCACACTCACACTCCCATGCAGGATAAAACAAATTGTAAACATGTGGCAGGGGGTAGGACAAGCAA
TGTATGCCCCTCCCATTGAAGGAAACATAACATGTAAATCAAAC---
ATCACAGGAATATTATTGACACGGGATGGAGGGACAAATAGGACTGAA-------------------------
----

GGGAATAATAATGAGACATTCAGACCTGGAGGAGGAGACATGAGGGACAATTGGAGAAGTGAATTATATAAAT
ATAAAGTAGTAGAAATTAAGCCACTA---GGATTAGCACCCACCGAGGCAAAAAGGAGAGTGGTG--------
-------------------------------GAGAGAGAAAAAAGAGCA---GTGGGA---ATAGGAGCT---
------GTGTTCCTTGGGTTCTTGGGAGCA---GCAGGAAGCACTATG---GGCGCGGCGTCA---
ATAACGCTGACGGTACAG---GCCAGACAA---TTGTTG---TCTGGTATAGTGCAACAGCAAAGC---
AATTTG---CTGAGG---GCTATAGAGGCGCAACAGCATATGTTGCAACTCACGGTCTGGGGCATT
>HQ698009
TTCTATTGCAATACATCAGGACTGTTTCTGTTTAACAAATCAAGACCATTTACA-------------------
----

AAAAATGGGACAATCATACTCCCATGCAGAATAAAACAAATTATAAACATGTGGCAGGGGGTAGGACGAGCAA
TGTATGCCCCTCCCATTGCAGGAAACATAACATGTAACTCAAAT---
ATCACAGGACTATTATTGACACGTGATGGGGGAGGAAAAGGTAAT----------------------------
----

AACACAGAAACATTTAGACCTGGAGGAGGAGATATGAGGGACAATTGGAGAAGTGAATTATATAAATATAAAG
TAGTAGAAATTAAACCATTA---GGAATAGCACCCACTAAGGCAAAAAGGAGAGTGGTG--------------
-------------------------GAGAGAGAAAAAAGAGCA---GCA------ATAGGAGCT---------
GTGTTCCTTGGGTTCTTGGGAGCA---GCAGGAAGCACTATG---GGCGCGGCGTCA---
ATAACGCTGACGGTACAG---GCCAGACAA---CTGTTG---TCTGGTATAGTGCAACAGCAAAGC---
AATTTG---CTGAGA---GCTATAGAAGCGCAACAGCATCTGTTGCAGCTCACGGTCTGGGGCATT
>HM215286
TTCTATTGCAATACATTAGACCTGTTTAATAGTACATATGATAGTACATACGGGTCTAATAGTACA-------
----

GATAATTCAACCATCACACTCCACTGCAGAATAAAACAAATTATAAACATGTGGCAGGAGGTAGGACGAGCAA
TGTATGCCCCTCCCATTGAAGGAAACATAACATGTATATCAAAT---
ATTACAGGACTACTATTAACACGTGATGGAGGAGATAGAAATGATACA-------------------------
----

AATGACACAGAGACATTCAGACCTGGAGGAGGAGACATGAGGGATAATTGGAGAAGTGAATTATATAAATATA
AAGTAGTAGAAATTAAACCATTA---GGAATAGCACCCACACAGGCAAGAAGGAGAGTGGTG-----------
----------------------------GAGAGAGAAAAAAGAGCA---GTGGGA---ATAGGAGCT------
---GTGTTCCTTGGGTTCTTGGGAGCA---GCAGGAAGCACTATG---GGCGCGGCGTCA---
ATAACGCTGACGGTACAG---GCCAGACAA---TTGTTG---TCTGGTATAGTGCAACAGCAAAGC---
AATTTG---CTGAGG---GCCATAGAGGCGCAACAGCATATGTTGCAACTCACGGTCTGGGGCATT
>HM215302
TTCTATTGCAATACATCAAACCTGTTTAATGAAAGTTTATTTAATAGTACAGAAAATAGTAGT----------
----

AACAACACCATCATCACCCTCCCATGCAGAATAAAACAAATTATAAACATGTGGCAGGAGGTAGGACGAGCCA
TATATGCCCCTCCCATCGCAGGAAACATAACATGTAAATCAAAT---
ATTACAGGACTAATTTTGACACGTGATGGAGGAAACTCAAGTGAAACC-------------------------
----

AACCACACAGAGACATTCAGACCTGGAGGAGGAAATATGAGGGACAATTGGAGAAGTGAATTATATAAATATA
AAGTGGTAGAAATTAAGCCATTG---GGAATAGCACCTACCAAGGCAAAAAGGAGAGTGGTG-----------
----------------------------GAGAGAGAAAAAAGAGCA---GTGGGA---ATGGGAGCT------
---ATGTTCCTTGGTCTCTTGAGTGCA---GCAGGAAGCACTATG---GGCGCGGCGTCA---

ATAACGCTGACGGTACAG---GCCAGACAA---CTGTTG---TCTGGTATAGTGCAGCAGCAAAGC---
AATTTG---CTGCGG---GCTATAGAGGCGCAACAGCATATGTTGCAACTCACAGTCTGGGGCATT
>AY253304
TTCTATTGCAATACATCAAAACTGTTTACAGGTGAATATAATGGTACTGAGTATAATGATACAGAATCAAATT
CA---

ACTTCATCAATCATCACACTCCCATGTAGAATAAAACAAATTATAAACATGTGGCAGAAGGTAGGACGAGCAA
TGTATGCCCCTCCTATTGAAGGAAACATAACGTGTAACTCAAGT---
ATCACAGGACTACTATTGACACGTGATGGAGGAAACAATACTAAT----------------------------
----

AACACAGAGACATTCAGACCTGGAGGAGGAGATATGAGGGATAATTGGAGAAGTGAATTATATAAATATAAAG
TGGTAGAAATTAAACCATTA---GGAATAGCACCCACTAGTGCAAAAAGGAGAGTGGTG--------------
-------------------------GAGAGAGAGAAAAGAGCA---GTGGGA---ATAGGAGCT---------
TTGTTCCTTGGGTTCTTAGGAGCA---GCAGGAAGCACTATG---GGCGCAGCATCA---
ATAACGCTGACGGTACAG---GCCAGACAA---TTATTG---TCTGGTATAGTGCAACAGCAGAGC---
AATTTG---CTGAGG---GCCATAGAGGCGCAACAGCATATGTTGCAACTCACAGTCTGGGGCATT
>AY253308
TTCTATTGCGATACATCAGACCTGTTCAATAGAACATACGTGATGCCTAATGATACAGAAAATACAGAAGGTA
CG---

GCAAATACAACCATCAGACTCCAATGCAGAATAAAACAAATTGTAAATATGTGGCAGGGGGTAGGACGAGCAA
TGTATGCCCCTCCCATTGAAGGAAATATAGAATGTATATCAAAC---
ATCACAGGACTACTATTGACATGGGATGGAGGAAATGAAGCTAAT----------------------------
----

GACACAGAAGAGACATTCAGACCTGGAGGAGGAGACATGAGGGATAATTGGAGAAGTGAATTATATAAATATA
AAGTGGTAGAAATTAAGCCATTA---GGAATAGCACCCAGTATGGCAAAAAGGAGAGTGGTG-----------
----------------------------GAGAGAGAAAAAAGAGCA---GTGGGA---ATAGGAGCT------
---GTGCTCCTTGGGTTCTTGGGAGCA---GCAGGAAGCACTATG---GGCGTGGCGTCA---
ATGACGCTGACGGTACAG---GCCAGACAA---TTGTTG---TCTGGTATAGTGCAACAGCAAAGC---
AATTTG---CTGAGG---GCTATAGAGGCGCAACAGCATATGTTGCAACTCACAGTCTGGGGCATT
>AY253310
TTCTATTGCAATACATCACAACTATTTAATAGTATATATAATAATAGTACATTTAATAGTAATAGTACATCTA
ATAGT--
-------------------ACA---
AATAATACTGAATACATCACACTCCAATGTAGAATAAAACAAATTATAAACATGTGGCAGGAGGTAGGACGAG
CAATGTATGCCCCTCCCGTTGCAGGAAACATAACATGTATATCAAAT---
ATTACAGGACTACTATTGACACGTGATGGAGGAAGCAGTGGAGGAAACAAT----------------------
----

AGCGAAGAGACAGAGGAGACATTCAGACCTGGAGGAGGAGATATGAGGGACAATTGGAGAAGTGAATTATACA
AGTATAAAGTGGTAGAAATTAAGCCATTG---GGAATAGCGCCCACTACAGCAAAAAGGAGAGTGGTG-----
----------------------------------GAGAGAGAAAAAAGAGCA---GTTGGA---
ATAGGAGCT---------GTGCTCTTTGGGTTCTTGGGAGCA---GCAGGAAGCACTATG---
GGCGCGGCGTCA---ATAACGCTGACGGTACAG---GCCAGACAA---GTGTTG---
TCTGGTATAGTGCAACAACAAAGC---AATCTG---CTGAGG---
GCTATAGAGGCGCAACAGCATATGTTGCAACTCACGGTCTGGGGCATT
>AY253312
TTCTATTGCAATACATCAGGGCTGTTCAATGGTACATACAATGGCACATACAATGGTACA-------------
----

ACAAATTCAACCATCACACTCCAATGCAGAATAAAACAAATTATAAACATGTGGCAGAAGGTAGGACGAGCAA
TGTATGCCCCTCCCATTGCAGGAAACATAACATGTAACTCAACT---
ATCACAGGACTAATATTGACACGGGATGGAGGAAACGGAAACGTT----------------------------
----

ACAGGAAACGAAACATTCAGACCTGCAGGAGGAGATATGAAAGACAATTGGAGAAGTGAATTATATAAATATA
AAGTGGTAGAAGTTAAGCCATTA---GGAATAGCACCCACTAAGGCACAAAGAAAAGTGGTG-----------
----------------------------CAGAGAGAGAAAAGAGCA---GTGGGA---ATAGGAGCT------

---GTGTTCCTTGGGTTCTTGGGAGCA---GCAGGAAGCACTATG---GGCGCGGCATCA---
ATAACGCTGACGGTACAG---GCCAGACAA---TTATTG---TCTGGTATAGTGCAACAGCAAAGC---
AATTTG---CTGAGA---GCTATAGAGGCGCAACAGCATATGTTGCAACTCACGGTCTGGGGCATT
>AY253303
TTCTATTGCAATACATCAAACTTGTTTAATAAGACAATTTTGGCTAATATAACGGGTAATGGT---------
----

ACAAATGCAAACATCACACTCCCATGCAGGATAAAACAAATTATAAACATGTGGCAGGGGGTAGGACGAGCAA
TGTATGCCCCTCCCATCGCAGGAGAAATAACATGTAAATCAAAT---
ATCACAGGATTACTATTGACACGTGATGGAGGAAATAATAAGACA---------------------------
----

AACGAATCAGAGATATTTAGACCTATAGGAGGAGATATGAGGGACAATTGGAGAAATGAATTATATAAATATA
AAGTGGTAGAAATTAAACCATTA---GGAGTAGCACCCACTGGTGCAAAAAGGAGAGTGGTG----------
----------------------------GAGAGAGAAAAAAGAGCA------------GCGGGAGTT-----
---GTGCTCCTTGGGTTCTTGGGAGCA---GCAGGAAGCACTATG---GGCGCAGCATCA---
ATGTGGCTGACGGTACAG---GCCAGACAA---TTACTG---TCTGGTATAGTGCAACAGCAAAGT---
AATTTG---CTGAGG---GCTATAGAGGCGCAACAGCATCTGTTGCAACTCACAGTCTGGGGCATT
>AY253313
TTCTATTGCAATACATCAATACTGTTTAATAATAGCATGCTTAATGGTACAGAAGGTACTCCAAAT------
----

TCAACTGAAGATATCACAATCCCATGTAGAATAAAACAAATTATAAACATGTGGCAGGGGGTAGGACGAGCAA
TGTATGCCCCTCCCATTGCAGGAAGCATAACATGTAAGTCAAAT---
ATCACAGGACTATTATTGACACGTGATGGAGGAATAAATGAGGAT---------------------------
----

AATACAACAGAGATATTCAGGCCTGGAGGAGGAGATATGAGGGATAATTGGAGAAGTGAATTATATAAATATA
AAGTGGTAGAAATTAAGCCATTA---GGAGTAGTACCCACTAATGCAAAAAGGAGAGTGGTG----------
----------------------------GAGAGAGAAAAAAGAGCA---GTGGGA---ATAGGAGCT-----
---GTGTTCCTTGGGTTCTTAGGAGCA---GCAGGAAGCACTATG---GGCGCAGCATCA---
ATAACGCTGACGGTACAG---GCCAGACAA---TTATTG---TCTGGTATAGTGCAACAGCAAAGC---
AATTTG---CTGAGG---GCTATAGAGGCGCAACAGCATATGTTGCAACTCACAGTCTGGGGCATT
>AY253322
TTCTATTGCAACACATCAGGTCTGTTTAATAGTACATACAACTCTAATGGTACCAATAGTACA---------
----

ACAAACTCAACCATCACACTTCCATGCAGAATAAAACAAATTATAAACATGTGGCAGGGGGTAGGACGAGCAA
TGTATGCCAATCCCATTGCAGGAAACATAACATGTAAATCAAAT---
ATCACAGGACTATTATTGGTACGTGATGGAGGTACAGATAATGAA---------------------------
----

ACAGAAACATTCAGACCTGGAGGAGGAAATATGAGGGACAATTGGAGAAGTGAATTATATAAATATAAAGTGG
TAGAAATTAGGCCATTG---GGAATAGCACCCACTAAGGCAAAAAGGAGAGTGGTG----------------
----------------------GAGAGAGAAAAAAGAGCA---GTGGGA---ATAGGAGCT--------
GTGCTCCTTGGGTTCTTGGGAGCA---GCAGGAAGCACTATG---GGCGCGGCGTCA---
ATGACGCTGACGGTACAG---GCCAGACAA---CTGTTG---TCTGGTATAGTGCAACAGCAAAGC---
AATTTG---CTGAGA---GCTATAGAGGCGCAACAGCATCTGTTGCAACTCACGGTCTGGGGCATT
>MK501601
TTCTATTGCAACACATCAGGTCTGTTTAATAGTACATACAACTCTGCATACAAGTCTAATGATACA------
----

ACAAACTCAACCATCACACTTCCATGCAGAATAAAACAAATTGTAAACATGTGGCAGGAGGTAGGACGAGCAA
TGTATGCCCCTCCCATTGCAGGAAACATAACATGTAAATCAAAT---
ATCACAGGATTATTATTAGTGCGTGATGGAGGAGGTGAAAGTAAT---------------------------
----

GAAACAGAAATATTCAGACCTGGAGGAGGAGATATGAGGGACAATTGGAGAAGTGAATTATATAAATACAAAG
TGGTAGAAGTTAAACCATTG---GGAATAGCACCCACTGAAGCAAAAAGGAGAGTGGTG-------------
-------------------------AAGAGAGATAAAAGAGCA---GTAGGA---ATAGGAGCT--------

ATGTTCCTTGGGTTCTTGGGAGCA---GCAGGAAGCACTATG---GGCGCGGCGTCA---
ATGACGCTGACGGTACAG---GCCAGACAA---GTGCTG---TCTGGTATAGTGCAACAGCAAAGC---
AATTTG---CTGAGG---GCTATAGAGGCGCAACAGCATATGTTGCAACTCACAGTCTGGGGCATT
>AY734550
TTCTATTGCAATACATCAAGCCTGTTTAATGATAGCCTGGTTAATGATACAGAAAGTGAGAATAACACAAAAA
GT---

GATAATGGTACAATCACACTCCCATGCAGAATAAAACAAATTATAAACCTGTGGCAGGAAGTAGGACGAGCAA
TGTATGCCCCTCCCATTGCAGGAAACATAACATGTAAATCAAAT---
ATCACAGGATTACTCTTGACACGTGATGGAGGAGACAAAAATAACACA-------------------------
----

AACAATACCGCAGAGATATTCAGACCTGGAGGAGGAAATATGAGGGACAACTGGAGAAGTGAATTATATAAAT
ATAAAGTGGTAGAAATTAAACCATTG---GGAATAGCACCCACTGCGGCAAAAAGGAGAGTGGTG--------
-------------------------------GAGAGAGAAAAAAGAGCA---GTAGGA---ATAGGAGCT---
------GTGTTCCTTGGGTTCTTGGGAGCA---GCTGGAAGCACTATG---GGCGCAGCGTCG---
ATAACGCTGACGGTACAG---GCCAGACAA---CTGTTG---TCTGGTATAGTGCAGCAGCAAAGC---
AATTTG---CTGAGG---GCTATAGAGGCGCAACAGCATATGTTGCAACTCACGGTCTGGGGCATT
>AY734551
TTCTATTGCAATACAACAATACTGTTTAATAGGACATACAGCCCTAATGGCACAGACAATAATGGCACATACA
AT---

AATAGTGATTCGACCATCACAATCCCATGCAGAATAAAGCAAATTATAAACATGTGGCAGGAGGTAGGACGAG
CAATGTATGCCCCTCCCATTGCAGGAAACATAACATGTAATTCAAGT---
ATCACAGGACTAATATTGACACGTGATGGTGGTAATAAAAATGATACA-------------------------
----

AATGATACAGAGATATTTAGACCTGAAGGAGGAAATATGAAAGACAATTGGAGAAGTGAATTGTATAAATATA
AGGTGGTAGAAATTAAGCCATTA---GGAATAGCACCCACTAATGCAAAAAGGAGAGTGGTG-----------
----------------------------GAGAGAGAGAAAAGAGCA---GTGGGA---ATAGGAGCT------
---GTGTTCCTTGGGTTCTTGGGAGCA---GCAGGAAGCACTATG---GGCGCAGCATCA---
ATAACGCTGACGGTACAG---GCCAGACAA---TTATTG---TCTGGTATAGTGCAACAGCAAAGC---
AATTTG---CTGAGG---GCTATAGAGGCGCAACAACATATGTTGCAACTCACGGTCTGGGGCATT
>AY734558
TTCTACTGCAATACATCAAAGCTGTTTAATAGTCTTAATGATACAGAC-------------------------
----

AATAGTACAGACATCACACTCCCATGCAGAATAAAACAAATTATAAACATGTGGCAGGGGGTAGGACGAGCAA
TGTATGCCCCTCCCATTGCAGGAAACATAACATGTAAATCAAAT---
ATCACAGGACTACTATTGACACGAGATGGAGGAACAAACAATACA----------------------------
----

GATACAGAGATATTCAGGCCTGGAGGAGGAGATATGAGGGACAATTGGAGAAGTGAATTATATAAATATAAAG
TGGTAGAAATTAAGCCATTA---GGAATAGCACCAACCACGGCAAAAAGGAGAGTGGTG--------------
-------------------------GAGAGAGAAAAAAGAGCA---GTGGGA---GTAGGAGCT---------
GTGTTCCTTGGGTTTTTAGGAGCA---GCCGGAAGCACTATG---GGCGCAGCATCA---
ATAACGCTGACGGTACAG---GCCAGACAA---TTATTG---TCTGGTATAGTGCAACAGCAAAGC---
AATTTG---CTGAGG---GCTATAGAGGCGCAACAGCATATGTTGCAACTCACAGTCTGGGGCATT
>AY734559
TTCTATTGCAATACATCAGGCCTGTTCAATAGTACGTTTAATGAGGGATATAATAATACAAGAAATACTTCAG
AAAAAACA---
-------------------AAT---
AATACTTCAGAAACCATCACACTCCAATGCAGAATAAAACAATTTATAAACATGTGGCAGGGGGTAGGACGAG
CAATGTATGCCCCTCCCATTGCAGGAAACATAACATGTAAATCAAAT---
ATCACAGGATTATTATTAACACGTGATGGAGGAGAAGGAAACGGTACAGAT----------------------
----

ACAAATACAACAGAGATATTCAGACCACAAGGAGGAAATATGAAGGACAATTGGAGAAGTGAATTATATAAAT
ATAAAGTAGTAGAAATTAGACCATTA---GGAATAGCACCTACTAATTCAAAAAGGAGAGTGGTG--------
-------------------------------CAGAGAGAGAAAAGAGCA---GTGGGAGTAATGGGAGCT---

------ATGTTCCTTGGGTTCTTGGGAGCT---GCAGGAAGCACTATG---GGCGCGGCATCA---
ATAACGCTGACGGTACAG---GCCAGACAA---TTATTG---TCTGGTATAGTGCAACAGCAAAAT---
AATTTG---CTGAAA---GCTATAGAAGCGCAACAGCATATGTTGCAACTCACGGTCTGGGGCATT
>AY734560
TTCTATTGTAATACATCAAACCTGTTTAATAATTCACTGTTTAATGATACTTCA------------------
----

AATGATACAGTCATCACACTCCCCTGCAGAATAAAACAAATTATAAACATGTGGCAGGAGGTAGGACGAGCCA
TATATGCCCCTCCCATTGCTGGAAATATAACATGTAGATCAAAT---
ATCACAGGACTATTGTTGACACGTGATGGAGGAGTTAATGCCAGT---------------------------
----

AAAGAAACATTCAGACCTGGAGGAGGAGATATGAGGGATAATTGGAGAAGTGAATTATATAAATATAAAGTAG
TAGAAATTAATCCATTA---GGAGTAGCACCAACTAAGGCAAAAAGGAGAGTGGTG-----------------
---------------------GAGAGAGAGAAAAGAGCA---GCGGGA---ATAGGAGCT---------
TTGATCCTTGGGTTCTTAGGAGCA---GCAGGAAGCACTATG---GGCGCAGCATCA---
ATAACGCTGACGGTACAG---GCCAGACAA---TTGTTG---TCTGGTATAGTGCAACAGCAGAGC---
AATTTG---CTGAGG---GCTATAGAGGCGCAACAGCATATGTTGCAACTCACAGTCTGGGGCATT
>KX907356
TTCTATTGTAATACAACACAACTATTTAATAATATTAGTATAGGAAGATTCAATACTAGTATAGAA-------
----

AATGATACAAACATCATACTCCCATGCAGAATAAAACAAATTATAAATATGTGGCAGGAGGTAGGACGAGCAA
TGTATGCCCCTCCTATTGAAGGGAACATAACATGCAGATCAAAC---
ATCACAGGAATACTATTAACACGTGATGGAGGCAATGGAGGCGCT---------------------------
----

GACAATTCAACAGAGACATTCAGACCTGGAGGAGGAAATATGAAGGACAATTGGAGAAGTGAATTATATAAAT
ATAAAGTGGTAGAAATCAAGCCATTA---GGAATAGCACCCACTAGGGCAAAAAGGAGAGTGGTG--------
------------------------------GAGAGAGAAAAAAGAGCA---GTGGGA---ATAGGAGCT---
------GTGTTCCTTGGGTTCTTGGGAGCA---GCTGGAAGCACTATG---GGCGCGGCGTCA---
ATAACGCTGACGGTACAG---GCCAGACAA---CTGTTG---TCTGGTATAGTGCAACAGCAAAGC---
AATTTG---CTGAGG---GCTATAGAGGCACAGCAACATATGTTGCAGCTCACTGTCTGGGGCATT
>KX907388
TTCTATTGCAATACATCAAAACTGTTTAATGATACATACAATGGGACAGAA---------------------
----

AGTAACTCAACCATCACACTCCCATGCAGAATAAAACAAATTATAAACATGTGGCAGGGGGTAGGACGAGCAA
TGTATGCCCCTCCCATTGCAGGAAACATAACATGTAAATCAATG---
ATCACAGGACTACTATTGGTACGGGATGGGGGCACGGGTAAAAAT--------------------------
----
-
GACACAGAAAATGCCACAGAAACATTCAGACCTGAAGGAGGAGATATGAGGGATAATTGGAGAAGTGAATTAT
ATAAATATAAAGTGGTAGAAATTAAGCCATTG---GGAATAGCACCCACTGAGGCAAAAAGGAGAGTGGTG--
-------------------------------------GAGAGAGAAAAAAGAGCA---GTGGGA---
ATAGGAGCT---------GTGTTCCTTGGGTTCTTGGGAGCA---GCAGGAAGCACTATG---
GGCGCAGCGTCA---ATAACGCTGACGGTACAG---GCCAGACAA---CTGTTG---
TCTGGTATAGTGCAACAGCAAAAC---AATTTG---CTGAGG---
GCTATAGAGGCGCAACAGCATATGTTGCAACTCACAGTCTGGGGCATT
>KX907407
TTCTATTGCAACACATCAAAACTGTTTAATAGTACATACATGCCTGGGTACTGGCCTAATGCTACAGAAAGAA
AT---

TCAACCTCAAATATCACACTCCATTGCAGAATAAAACAAATTATAAACATGTGGCAGGGAGTAGGACGGGCAA
TGTATGCCCCTCCCATTAGAGGAAACATAACATGTATATCAAAT---
ATCACAGGACTACTATTGACATGGGATGGAGATAAAACAAGCAAT---------------------------
----

GACCCAGACACAGACGTATTCAGACCTGGAGGAGGAAATATGAAGGATAATTGGAGAAGTGAATTATATAAAT
ATAAAGTGGTAGAAATTAAGCCATTA---GGAATAGCACCCACCAGGGCAAAAAGGAGAGTGGTG--------

-------------------------------GAGAGAGAAAAAAGAGCA---GTGGGA---ATAGGAGCT---
------GTGCTCCTTGGGTTCTTGGGAGCA---GCCGGAAGCACTATG---GGCGTCGCGTCA---
ATAACGCTGACGGTACAG---GCCAGACAA---TTATTG---TCCGGTATAGTGCAACAGCAAAGT---
AATTTG---CTGAGG---GCTATAGAGGCGCAACAGCATATGTTGCAACTCACAGTCTGGGGCATT
>KX907424
TTCTATTGCAATACATCAAAATTGTTTAATAATAATGATACAGAA----------------------------
----

AATAATTTAACCATCACACTCCCATGCAGAATAAAGCAAATTGTAAACATGTGGCAGGGGGTAGGACGAGCAA
TGTATGCCCCTCCCATTGAAGGAAACATAACATGTAAATCAAAT---
ATCACAGGATTATTATTGACACGTGATGGAGGCAATGGCACGGAG----------------------------
----

AGGAATGAGACATTCAGACCTGGAGGAGGAGATATGAGGAACAATTGGAGAAGTGAATTATATAAATATAAAG
TGGTAGAAATTAAACCATTG---GGAATAGCACCCACTGGGGCAAAAAGGAGAGTGGTG--------------
-------------------------GAGAGAGAAAAGAGAGCA---GTGGGC---ATGGGAGCT---------
GTGTTCCTTGGGTTCTTGGGAGCA---GCAGGAAGCACTATG---GGCGCGGCGTCA---
CTAACGCTGACGGTACAA---GCCAGACAA---GTGTTG---TCTGGTATAGTGCAGCAGCAAAGC---
AATTTG---CTGAGA---GCTATAGAGGCGCAACAACATATGTTGCAACTCACAGTTTGGGGCATT
>KX907426
TTTTATTGCAATACATCAAGCCTGTTTAAGGGTACATTTAATGGTACGGTTACAGATACCTCAAATTTAACAG
AA---

ATCACACTCCACTGTAGAATAAAACAAATTGTAAACATGTGGCAAGGAGTAGGACGAGCAATGTATGCCCCTC
CCATTGCAGGAAATATAACTTGTAAATCAAAT---
ATCACAGGATTACTATTGACACGTGATGGAGGATGTGATAACAAC----------------------------
----

GGTACAGAGATATTCAGGCCTGGAGGAGGAGATATGAGGGATAATTGGAGAAGTGAGTTATACAAATATAAAG
TAGTAGAAATTAAGCCATTA---GGAATAGCACCCACTAAGGCAAAAAGGAGAGTGGTG--------------
-------------------------GAGAGAGAAAAAAGAGCA---GTGGGA---ATAGGAGCT---------
GTGTTCCTTGGGTTCTTAGGAGCA---GCAGGAAGCACTATG---GGCGCAGCATCA---
ATAACGCTGACGGTACAG---GCCAGACAA---TTATTG---TCTGGTATAGTGCAACAGCAAAAC---
AATTTG---CTGAGG---GCTATAGAGGCGCAACAGCATATGTTGCAACTCACAGTCTGGGGCATT
>KX907345
TTCTATTGCAATACATCAAAACTGGTTAATGAGAATGAGACAGACCCTAATAGAGCTAAT-------------
----

AGTAGTGAGCCAATCACACTCCAATGCAGAATAAAACAAATTATAAACATGTGGCAGGGGGTAGGACGAGCAA
TGTATGCCCCTCCCATTGAAGGAAACATAACATGTAGGTCAAAT---
ATCACAGGACTGCTATTGACACGCGACGGAGGTGCCAATAGTACC----------------------------
----

ACAAATGAAACATTCAGATTTGAAGGAGGAAATATGAAGGACAATTGGAGAAGTGAATTATATAAATATAAGG
TGGTAGAGATTAAGCCATTA---GGAATAGCACCCACTAAGGCAAAAAGGAGAGTGGTG--------------
-------------------------GAGAGAGAAAAAAGAGCA---GTGGGA---ATAGGAGCT---------
GTGTTCCTTGGGTTCTTGGGAGCA---GCAGGAAGCACTATG---GGCGCAGCATCA---
ATAACGCTGACGGTACAG---GCCAGACAA---TTGTTG---TCTGGTATAGTGCAACAGCAAAGC---
AATTTG---CTGAGG---GCCATAGAGGCGCAACAGCACATGTTGCAACTCACGGTCTGGGGCATT
>KX907384
TTCTATTGTAACACATCACAACTATTTAATGGTACCAGTACAGGATTTAGTAATAAGAGTACA----------
----

GGAAATGAAACCTTCACACTCCCATGCAGAATAAAACAAATTATAAACATGTGGCAGGAGGTAGGACGAGCAA
TGTATGCCCCTCCCATTGCAGGAAACATAACATGTGTATCAAAT---
ATCACAGGACTACTATTGACACGCGATGGAGGAGATAATAATACT----------------------------
----

AGAACTGAGACATTCAGACCTGGAGGAGGAGATATGAGGGACAATTGGAGAAGTGAATTATATAAATATAAAG
TAGTAGAAGTTAAACCACTA---GGAGTAGCACCCTCTGAGGCAAGAAGGAGAGTGGTG--------------

------------------------GAGAGAGAAAAAAGAGCA---GTGGGA---CTAGGAGCT---------
GTGTTCCTTGGGTTCTTGGGAGCA---GCCGGAAGCACTATG---GGCGCGGCGTCG---
ATAACGCTGACGGTACAG---GCCAGACAA---CTGTTG---TCTGGTATAGTGCAACAGCAAAGC---
AATTTG---CTGAGA---GCTATAGAAGCACAGCAGCATATGTTGCAGCTCACGGTCTGGGGCATT
>KX907392
TTCTATTGCAATACATCAAAACTGTTTAATGATACAAACTTCACCACGTCT---------------------
----

GATGATGAAAACATCACACTCCCATGCAGAATAAAACAAATTATAAACATGTGGCAGGAGGTAGGACGAGCAA
TGTATGCCCCTCCCATTGCAGGAAACATAACATGCACATCAAAA---
ATCACAGGACTACTATTGACACGTGATGGGGGAACCACTGGCCCC---------------------------
----

AATAATACCGAGACATTCAGACCTATAGGAGGAAATATGAAGGACAATTGGAGAAGTGAATTATACAAATATA
AGGTGGTGGAAATTAAGCCATTA---GGAGTAGCACCCACTAAGGCAAAAAGGAGAGTGGTG----------
---------------------------GAGAGAGAAAAAAGAGCA---GTGGGA---ATAGGAGCT------
---ATGTTCCTTGGGTTCTTGGGGGCA---GCAGGAAGCACTATG---GGCGCGGCGTCA---
ATAACGCTGACGGTACAG---GCCAGACAA---GTGTTG---TCTGGTATAGTGCAACAGCAAAGC---
AATTTG---CTGAGG---GCTATAGAGGCGCAACAGAATATGTTGCAACTCACGGTCTGGGGCATT
>KX907394
TTCTATTGCAATACATCAAAACTGTTTAATGGTACATACAGGTCTAATAGTACAGAAAAGAAT---------
----

TCAAATGCAAACATCACACTCCCATGCAGAATAAAACAAATTGTAAACATGTGGCAGGAGGTAGGGCGAGCAA
TGTATGCCCCTCCCATTGCAGGAAACATAACATGTAAATCAAAT---
ATCACAGGACTACTATTGACACGTGATGGGGGAAAGGATAATAATACT------------------------
----

AATACCACAGAGACATTCAGACCAGGAGGAGGAGATATGAGGGACAATTGGAGAAGTGAATTATATAAATATA
AAGTAGTAGAAGTTAATCCACTA---GGAATAGCACCCACTACTGCAAAAAGGAGAGTGGTG----------
---------------------------GAGAGAGAGAAAAGAGCA---GTGGGA---ATAGGAGCT------
---TTGTTCCTTGGGTTCTTGGGAGCA---GCAGGAAGCACTATG---GGCGCGGCGTCA---
ATGGCGCTGACGGTACAG---GCCAGACAA---TTATTG---TCTGGTATAGTGCAACAGCAAAGC---
AATTTG---CTGAGA---GCTATAGAGGCGCAACAACATATGTTGCAACTCACAGTCTGGGGCATT
>KX907395
TTCTATTGCAATACATCAAAACTGTTTAATGGTACAGAAAAG------------------------------
----

AATTCAAATGCAAACATCACACTCCCATGCAGAATAAAACAAATTGTAAACATGTGGCAGGGGGTAGGGCGAG
CAATGTATGCCCCTCCCATTGCAGGAAACCTAACATGTAAATCAAAA---
ATCACAGGACTACTATTGACACGTGATGGGGGACCAAATGAGAAT---------------------------
----

GACACAGAGATATTCAGACCTGGAGGAGGAGACATGAGGGACAATTGGAGAAGTGAATTATATAAATATAAAG
TAGTAGAAATTAATCCATTA---GGAGTAGCACCCACTACTGCAAAAAGGAGAGTGGTG-------------
------------------------GAGAGAGAGAAAAGAGCA---GTGGGA---GTAGGAGCT---------
ATGTTCCTTGGGTTCTTGGGAGCA---GCAGGAAGCACTATG---GGCGCGGCGTCA---
CTAACGCTGACGGTACAG---GCCAGACAA---TTATTG---TCTGGTATAGTGCAACAGCAAAGC---
AATTTG---CTGAGG---GCCATAGAGGCGCAACAACATATGTTGCAACTCACAGTCTGGGGCATT
>KX907405
TTCTATTGCAATACATCACAACTGTTTAATGAGACACACACTAATGGT------------------------
----

ACCAATAGTACCATCATACTCCCATGCAGAATAAAACAAATTATAAACATGTGGCAGGAGGTAGGACGAGCAA
TGTATGCCCCTCCCATTGCAGGGAACATAACATGTACATCAAAT---
ATTACAGGACTATTATTGGAACGGGATGGAGGCAAGGACAATAAT---------------------------
----

ATGACTGAGATATTCAGACCTGGAGGAGGAGATATGAGGGATAATTGGAGAAGTGAACTATATAAATATAAAG
TGGTAGAAATTAAACCATTG---GGAGTAGCACCCACTAAGGCAAAAAGGAGAGTGGTG-------------

-------------------------GAGAGAGAAAAAAGAGCA---GCA------CTAGGAGCT---------
GTGTTCCTTGGGTTCTTGGGGGCA---GCAGGAAGCACTATG---GGCGCGGCGTCA---
ATAACGCTGACGGTACAG---GCCAGACAG---TTGTTG---TCTGGTATAGTGCAGCAGCAAAGC---
AATTTG---CTAAGA---GCTATAGAGGCGCAACAACATCTGTTGCAACTCACGGTTTGGGGCATT
>KX907413
TTCTATTGCAATACATCAGCACTGTTTAATAGTACATATAATGAAACATATAGATATAATGCTACAAACGAT-
----

ACAAATCCAACCATCACACTCAAATGCAGAATAAAGCAAATTATAAACATGTGGCAGGAGGTAGGACGAGCAA
TGTATGCCCCTCCCATTGCAGGAAACATAACGTGTAGATCAAAT---
ATCACAGGGATACTATTGACACGTGATGGAGGACCTAACAGTACT----------------------------
----

AATGAAACATTCAGACCTGAAGGAGGAGATATGAGGAATAATTGGAGAAGTGAACTATATAAATATAAAGTGG
TAGAAATTAAACCATTG---GGAATAGCACCCACCAAGGCAAAAAGGAGAGTGGTG-----------------
----------------------GAGAGAGAAAAAAGAGCA---GTCGGA---ATAGGAGCC---------
TTGTTCCTTGGTTTCTTGGGAGCA---GCAGGAAGCACTATG---GGCGCAGCGTCA---
ATAACGCTGACGGTACAG---GCCAGACAA---TTGTTG---TCTGGTATAGTGCAACAGCAAAGC---
AATTTG---CTGAGG---GCTATAGAGGCGCAACAGCATATGTTGCAACTCACTGTCTGGGGCATT
>KX907422
TTCTATTGCAACACATCACAACTGTTTAATAAACTGTTTAATGGTACTAATGATACAGAAAGTACTAAT----
----

TCAACTAAGAATATCACACTCCAATGTAAAATAAAACAAATTATAAATATGTGGCAGGGGGTAGGACGAGCAA
TATATGCTCCTCCCATTGAAGGAAACATCACATGTAAATCAAAC---
ATCACAGGACTAATATTAACACGAGATGGAGGAAGAAATAATAACACT-------------------------
----

AACATAACAGAGATATTCAGACCTGGAGGAGGAGATATGAGGGACAATTGGAGAAGTGAATTATATAAATATA
AAGTAGTAGAAATTAAGCCATTA---GGAATAGCACCCACTGGAGCAAAAAGGAGAGTGGTG-----------
----------------------------GAGAGAGAAAAAAGAGCA---GTGGGA---ATGGGAGCT------
---GTGCTCCTTGGGTTCTTAGGAGCA---GCAGGAAGCACTATG---GGCGCGGCGTCA---
ATAACGCTGACGGTACAG---GCCAGACAA---CTGCTG---TCTGGTATAGTGCAACAGCAAGAC---
AACTTG---CTGAGG---GCTATAGAGGCGCAACAGCATATGTTGCAACTCACAGTCTGGGGCATT
>KX907338
TTCTATTGCAATACATCAGGTCTGTTTAATGGTACAAATTCAACTAAT-------------------------
----

CCAAATGCAACTATCATACTCCCATGCAGAATAAAACAAATTATTAATATGTGGCAGGAAGTAGGACGAGCAA
TATATGCCCCTCCCATTGCAGGAAGCATATCATGCACATCAAAT---
ATTACAGGACTACTATTGACACGTGATGGAGGAAATAACACAAAC----------------------------
----

AATGAGACATTCAGACCCGGAGGAGGAGATATGAGAAATAATTGGAGAAGTGAATTATATAAATATAAAGTGG
TAGAAATTAAACCATTA---GGACTAGCACCCACTAAGGCAAAAAGGAGAGTGGTG-----------------
----------------------GAGAGAGAAAAAAGAGGA------------ATAGGAGCT---------
GTACTCCTTGGGTTCTTGGGAGCT---GCAGGAAGCACTATG---GGCGCGGCGTCA---
ATAGCGCTGACGGTACAG---GCCAGACAG---CTATTG---TCTGGTATAGTGCAACAGCAAAGC---
AATTTG---CTGAGG---GCTATAGAGGCGCAACAGCATATGTTGCAACTCACAGTCTGGGGCATT
>KX907363
TTCTATTGCAATACAACAAAACTGTTTACTGGTATTAGATACATTAATGGTACATACATAACTAATGATACAG
AAATAGCT---
----------AATGATACA------
GAGAATAATTCAACCATCACACTCCAATGCAGAATAAAACAGATTATAAACATGTGGCAGGGGGTAGGAAAAG
CAATGTATGCCCCTCCCATTGCAGGAAACATAATATGTAAATCAAAG---
ATCACAGGACTACTATTGACACGTGATGGAGAAGGGGAAAATGGAACA-------------------------
----

AATGAGACTGAGACATTCAGACCTGGAGGAGGAGATATGAGGGACAATTGGAGAAGTGAATTGTATAAATATA
AAGTGGTAGAAATTAAGCCATTA---GGAATAGCACCCGCTGAGGCAAAAAGGAGAGTGGTG-----------

----------------------------GAGAGAGAAAAAAGAGCA---GTGGGA---ATGGGAGCT------
---GTGCTCTTTGGGTTCTTGGGAGCA---GCAGGAAGCACTATG---GGCGCAGCATCA---
GTGATGCTGACGGTACAG---GCCAGACAA---TTGTTG---TCTGGTATAGTGCAACAGCAAAGC---
AATTTG---CTGAGA---GCTATAGAGGCGCAACAGCATATGTTGCAACTCACAGTCTGGGGCATT
>KX907374
TTCTATTGCAACACATCAGCACTGTTTAATAAACTGTTTAATAATACTAATGATACAGAAAGTAATACT----
----

TCAGATACAACTATCACACTCCAGTGCAAAATAAAACAAATTATAAACATGTGGCAGGGGGTAGGACGAGCAA
TATATGCTCCTCCCATTGAAGGAATCATCAATTGTACCTCAAAC---
ATCACAGGACTACTGTTGACACGTGATGGTGGGAATAACAGTGATGGGAATAAC-------------------
----

AATAACACAGAAACATTCAGACCTGGAGGAGGAGATATGAGGGACAATTGGAGAAGTGAATTATATAAATATA
AAGTGGTAGAAATTAAACCATTA---GGAATAGCACCCACTAAGGCAAAAAGGAGAGTGGTG-----------
----------------------------GAGAGAGAAAAAAGAGCA---GTGAGC---CTAGGAGCT------
---GTGTTCCTTGGGTTCTTGGGAGCA---GCAGGAAGCACTATG---GGCGCGGCGTCA---
GTGACGCTGACGGTACAG---GCCAGACAA---TTGTTG---TCTGGTATAGTGCAACAGCAAAGC---
AATTTG---CTAAGA---GCTATAGAGGCGCAACAGCATCTGTTGCAACTCACGGTCTGGGGCATT
>KX907380
TTTTATTGCAATACATCAGGCCTGTTTAATAATACACTGCTTAATGATACATTGACAATAGAC----------
----

AATAATGAAACCATCACACTCCCATGCAGAATAAAACAAATTATAAACATGTGGCAGGAGGTAGGACGAGCAA
TGTATGCCCCTCCCATTGCAGGAAACATAACATGTAAATCAAAT---
ATCACAGGACTATTATTGGTACGGGATGGAGGCATATCAAATGGGACAGAT----------------------
----

AACAAAAATGAGACATTCAGACCTGGAGGAGGAGATATGAGGAATAATTGGAGAAGTGAATTATACAAATATA
AAGTGGTAGAAATTAAACCATTG---GGAGTGGCACCCACCAGGGCAAGAAGGAGAGTGGTG-----------
----------------------------GAGAGAGAAAAAAGAGCA---GTGGGA---ATAGGAGCT------
---GTGATCTTTGGGTTCTTAGGAGCA---GCAGGAAGCACTATG---GGCGCAGCGTCA---
ATAGCGCTGACGGTACAG---GCCAGACAA---CTGTTG---TCTGGTATAGTGCAACAGCAGGGC---
AACCTG---CTGAGG---GCTATAGAGGCGCAACAGCATCTGTTGCAACTCACGGTCTGGGGCATT
>KX907416
TTCTATTGCGATACATCAAATCTGTTTAATAGTACATACAGCCCTAACAGTACAGATGATGGAGCGCAAGATG
TA---

AATGACACAATAATCACACTCACATGCAAAATAAAACAAATTGTAAACATGTGGCAGGGAGTAGGAAAAGCAA
TGTATGCCCCTCCCATTAGAGGAAACATAACATGCAGATCAAAT---
ATTACAGGACTACTACTGACAAGGGATGGAGGAGACTCAGAGGGG----------------------------
----

ACAGAAACATTTAGGCCTGGAGGGGGAGATATGAAGGACAATTGGAGAAGTGAATTATATAAATATAAAGTGG
TAGAAGTTAAACCATTA---GGAGTAGCACCAACTACTGCAAAAAGGAGAGTGGTG-----------------
----------------------GAGAGAGAAAAAAGAGCA---GTGGGA---ATAGGAGCT---------
GTGTTCCTTGGGTTCTTGGGAGCA---GCAGGAAGCACTATG---GGCGCAGCGTCA---
ATAACGCTGACGGTACAG---GCCAGACAA---TTATTG---TCTGGTATAGTGCAACAACAAAAC---
AATTTG---CTAAGA---GCTATAGAGGCGCAACAGCATATGTTGCAACTCACGGTCTGGGGCATT
>KX907375
TTCTATTGCAATACAACAAAACTGTTTAATGATAGCATGTCAAATGAAAGCTCAAACATC-------------
----

ACAAACAGCACACTCACACTCCCATGCAGAATAAAACAAATTATAAATATGTGGCAGGAGGTAGGAAGAGCAA
TGTATGCCCCTCCCGTTGAAGGAAATATAACATGTAAATCAAAT---
ATTACAGGACTACTATTGGTACGGGATGGGGGT--

GTGTCAAATGATACAAAT---
GGGACAGAGACATTCAGACCTGGAGGAGGAGATATGAGGGATAATTGGAGAAGTGAATTATATAAATATAAAG
TAGTAGAAATTAAGCCATTG---GGAGTAGCACCAACTGGGGCAAAAAGGAGAGTGGTG--------------

-------------------------GAGAGAGAAAAAAGAGCA---GTGGGA---ATAGGAGCT---------
GTGTTCCTTGGGTTCTTGGGAGCG---GCAGGAAGCACTATG---GGCGCGGCGTCA---
ATAGCGCTGACGGCACAG---GCCAGACAA---GTGTTG---TCTGGTATAGTGCAACAGCAAAAC---
AATTTG---CTGAGA---GCTATAGAGGCGCAACAGCATCTGTTGCAACTCACAGTCTGGGGCATT
>KX907390
TTCTATTGCAATACATCAAAACTGTTTAATACTACATACCTGTATAATGATACAGAAGGTAAC----------
----

TCAACCATCACACTCACACTCCCATGCAGAATAAAACAAATTATAAACTTGTGGCAGGAGGTAGGACGAGCAA
TGTATGCCCCTCCCATTAAAGGAGACATAATATGTAACTCAAGT---
ATCACAGGGCTATTATTGACACGTGATGGAGGAGACAATAGAACA----------------------------
----

AAGGAGACATTCAGACCTGGAGGAGGAGATATGAGGGACAATTGGAGAAGTGAATTATACAAATATAAGGTAA
TACAAATTAAGCCACTA---GGAATAGCACCCACTGCGGCAAGAAGGAGAGTGGTG-----------------
----------------------GAGAGAGAAAAAAGAGCA---ATTGGA---CTAGGAGCC---------
ATGTTCCTTGGGTTCTTGGGAGCA---GCAGGAAGCACTATG---GGCGCGGCGTCA---
ATAACGCTGACGGGACAG---GCCAGACAA---TTGTTG---TCTGGTATAGTGCAACAGCAAAGC---
AATTTG---CTGAGA---GCTATAGAGGCGCAACAGCATATGTTGCAACTCACAGTCTGGGGCATT
>KX907391
TTCTATTGCAATACATCAAACCTATTCAATGGTACCAATTCT-------------------------------
----

TCAGGCAGTAACATCACACTCCCATGTAGAATAAAACAAATTATAAACATGTGGCAGGGAGTAGGAAGAGCAA
TGTATGCTCCTCCCATTGCAGGAGAAATAAAATGTGCATCAAAT---
ATCACAGGGCTAATATTAACATGGGATGGAGGAAAGAACAATAAT----------------------------
----

ACCACAGAGACATTCAGACCTGGAGGAGGGGATATGAGGGATAATTGGAGAAGTGAATTATATAAATATAAAG
TGGTAGAAATTAAGCCATTA---GGAATAGCACCCACTGAGGCAAAAAGGAGAGTGGTG--------------
-------------------------GAGAGAGGAAAAAGAGCA---GTGGGA---ATAGGAGCC---------
GTGTTCCTTGGGTTCTTGGGAGCA---GCAGGAAGCACTATG---GGCGCAGCGTCA---
ATAACGCTGACGGTACAG---GCCAGACAA---CTGTTG---TCTGGTATAGTGCAACAGCAAAGC---
AATTTG---CTGAGG---GCTATAGAGGCGCAACAGCATATGTTGCAACTCACAATCTGGGGCATT
>KX907399
TTCTATTGCGATACAACAAAACTGTTTAATAGTACTAATGGTACTAATAGTTTTAAC----------------
----

TCAAATGAAACTATCATACTCCAATGTAGAATAAAACAAATTATAAATATGTGGCAGGGGGTAGGACGAGCAA
TATATGCTCCTCCCATTGAAGGAAACGTCACATGTACATCAAAC---
ATCACAGGACTACTATTGACACGTGATGGAGGAAATGTAACTGAC----------------------------
----

ACAGAAAAAATTCAGACCTGGAGGAGGAGATATGAGGGACAATTGGAGAAGTGAATTATACAAATATAAAGTGG
TAGAAATTAAGCCATTA---GGAATAGCACCCACTGGGGCAAAAAGGAGAGTGGTG-----------------
----------------------GAGAGAGAAAAAAGAGCA---GTGGGA---ATAGGAGCT---------
GTGTTCCTTGGGTTCTTGGGAGCA---GCAGGAAGCACTATG---GGCGCGGCGTCA---
ATAACGCTGACGGTACAG---GCCAGACAA---CTATTG---TCTGGCATAGTGCAACAGCAAAGT---
AATATG---CTGAGA---GCTATAGAGGCGCAACAGCATATGTTGCAACTTACAGTCTGGGGCATA
>KC156220
TTCTATTGTAATACATCAAAACTGTTTAATAATAATATAACAAGCGAT-------------------------
----

AATTCAAGCAACATCACAATCCCATGCAAAATAAAACAAATTATAAACATGTGGCAGAAGGTAGGAAAAGCAA
TGTATGCCCCTCCCATTGCAGGGAACATAACATGTACCTCGAAT---
ATCACAGGAATACTATTAACACGTGATGGAGGAAATAATAGTGAC----------------------------
----

AGTGATCCAGAGACATTCAGACCTACAGGAGGAGATATGAGGGACAATTGGAGAAGTGAATTATATAAATATA
AAGTAGTAGAAGTAGAACCATTA---GGAATAGCACCCACTGGAGCAAAAAGGAGAGTGGTG-----------

----------------------------GAGAGACAGAAAAGAGCA---GTGGGA---ATGGGAGCT------
---ATGTTCCTTGGGTTCTTGGGAGCA---GCAGGAAGCACTATG---GGCGCAGCGTCA---
ATAACGCTGACGGTACAG---GCCAGACAA---TTATTG---TCTGGTATAGTGCAACAGCAAAGC---
AATTTG---CTGAGG---GCTATAGAGGCGCAACAGCATATATTGCAACTCACAGTCTGGGGCATT
>AF361874
TTCTATTGCGATACATCAAGGCTATTTAATGAAAGTCTTAATGAAAGACTGTTTAATGAAAGTAGTGAG----
----

TCAAATGACACCATCACACTCCAATGTAGAATAAAACAAATTATAAACATGTGGCAGAAGGTAGGACAAGCAA
TATATGCCCCTCCCATAAAAGGAAACATATCATGTACATCAAAC---
ATCACAGGACTACTATTGACACGTGATGGAGGAAATGATGATGGAGGAAATGAT-------------------
----

ACAGGGTCAAGGCCAGAGACATTCAGACCTGGAGGAGGAGATATGAGGGACAATTGGAGAAGTGAATTATATA
AATATAAAGTGGTAGAAATTAAGCCATTA---GGAATAGCACCCACTGCGGCAAAAAGAAGAGTGGTG-----
----------------------------------GAGAGAGAAAAAAGAGCA---GTGGGA---
ATAGGAGCT---------GTGTTCCTTGGGTTCTTGGGAGCA---GCAGGAAGCACTATG---
GGCGCGGCGTCA---ATAACGCTGACGGTACAG---GCCAGACAA---CTATTG---
TCTGGTATAGTGCAACAGCAAAGC---AATTTG---CTGAGA---
GCTATAGAGGCGCAACAGCATATGTTGCAACTCACGGTCTGGGGCATT
>AF286234
TTCTATTGCAATACATCAAATCTGTTTACATCAAAACTGTTTAATAGTACTAATACTACAGAGAATGCCACAC
AA---

AGAAACGAAACCCTCACACTCCAATGCAGAATAAAACAGATTATAAACATGTGGCAGGAGGTAGGACGAGCAA
TATATGCCCCTCCCATTGCAGGGAGCATAACATGTACATCAAAT---
ATCACAGGAATACTATTGACACGAGATGGAGAAAATGCAGGAAATAAC-------------------------
----

ACAGGGAATGAGACATTCAGACCTGGAGGAAGAAATATGAGGGATAATTGGAGAAGTGAATTATATAAATATA
AAGTGGTAGAAATTAAGCCATTG---GGAATAGCACCCACTGAGGCAAAAAGGAGAGTGGTG-----------
----------------------------GAGAGAGAAAAAAGAGCA---GTGGGA---CTAGGAGCT------
---GTGTTCCTTGGGTTCTTGGGAGCA---GCCGGAAGCACTATG---GGCGCGGCGTCA---
ATAACGCTGACGGTACAG---GCCAGACAA---TTATTG---TCTGGTATAGTGCAACAGCAAAGC---
AATCTG---CTGAGG---GCTATAGAGGCGCAACAGCATCTGTTGCAACTCACGGTCTGGGGCATT
>AF286235
TTCTATTGCAATACATCAGGCCTGTTTAATGAAAGTCTGGTTAAAAGTAATAAT-------------------
----

TCAAATGGAACTATCGAACTCCAATGTAGAATAAAACAAATTATAAACATGTGGCAGGAGGTAGGACGAGCAA
TATATGCCCCTCCCATTAGAGGAAACATAACATGTATATCAAAC---
ATCACAGGACTATTATTGACACGTGATGGAGGAAATAATGGCACT----------------------------
----

AACGCAACGGAGATATTCAGACCTGGAGGAGGGGATATGAGGGACAATTGGAGAAGTGAATTATACAAATATA
AAGTGGTAGAAATTAAACCATTA---GGAATAGCACCCACTGGGGCAAAAAGAAGAGTGGTG-----------
----------------------------GAGAGAGAAAAAAGAGCA---GTGGGA---ATGGGAGCT------
---GTGCTCCTTGGGTTCTTGGGAGCG---GCAGGAAGCACTATG---GGCGCGGCGTCG---
ATGGCGCTGACGGTACAG---GCCAGACAA---CTATTG---TCTGGTATAGTGCAACAGCAAAGC---
AATTTG---CTGAGG---GCTATAGAGGCGCAACAGCATATGTTGCAACTCACGGTCTGGGGCATT
>L22948
TTCTATTGCAATACATCAAGCCTGTTTAATAGTAGTGATAATAAT----------------------------
----

AATAGTACCATCATCACACTCCCATGCAGAATAAAACAAATTATAAACATGTGGCAGGGGGTAGGACGAGCAA
TGTATGCCCCTCCCATTAAAGGAAAAATAACATGTAGATCAAAT---
ATCACAGGACTACTACTGACACGTGATGGAGGGGAGACAAGTGAGACA-------------------------
----

AATAGCACAGAGACATTCAGACCTGGAGGAGGAGATATGAGGGACAATTGGAGAAGTGAATTATATAAATATA

AAGTGGTAGAAGTTAAGCCACTA---GGAGTAGCACCCACTAAGGCAAAAAGGAGAGTGGTG-----------
----------------------------GAGAGAGAAAAAAGAGCA---GTGGGA---ATAGGAGCT------
---GTGTTCCTTGGGTTCTTGGGAGCC---GCAGGAAGCACTATG---GGCGCAGCATCA---
ATAACGCTGACGGTACAG---GCCAGACAA---TTATTG---TCTGGTATAGTGCAACAGCAAAAC---
AATTTG---CTGAGG---GCTATAGAGGCGCAACAGCATATGTTGCAACTCACGGTCTGGGGCATT
>EU663619
TTCTATTGCGATACATCAGGACTGTTCAATAGGACATACTTGCTAGCAAATGTTACAGGAAATATTTCAGACA
CAGACCGC---
-------------------AAC---
GTCACAGACCGCAACATCACACTTTCATGCAGAATAAAACAAATTATAAACACGTGGCAGGAGGTAGGAAAAG
CAATGTATGCTCCTCCCATTGCAGGAAACATAACATGTACATCAAAC---
ATCACAGGACTACTATTGACACGTGATGGAGGTATAAGTAATGACACC-------------------------
----

AATAACACAGAGACATTCAGACCTGGAGGAGGAGATATGAAGAACAATTGGAGAAGTGAATTATATAAATATA
AAGTGGTAGAAATTAAGCCATTA---GGATTAGCACCCACTGCAGCAAAAAGGAGAGTGGTG-----------
----------------------------GAGAGAGAAAAAAGAGCA---GTAGGA---ATAGGAGCT------
---GTACTCCTTGGGTTCTTGGGAGCA---GCAGGAAGCACTATG---GGCGCGGCGTCA---
ATAACGCTGACGGTACAA---GCCAGACAA---GTATTG---TCTGATATAGTGCAACAGCAAAGC---
AATTTG---CTGAAG---GCTATAGATGCGCAACAGCATATGTTGCAACTCACAGTCTGGGGCATT
>AY444800
TTCTATTGCAATACATCAGGGCTGTTTAATGGTACGTATAATGATACAAGGGGTGAG----------------
----

CCAAATGGAACCATCACACTCCCATGCAGAATAAAACAAATTATAGACATGTGGCAGAAGGTAGGACGAGCAA
TGTATGCTCCTCCCATTGAAGGAAATATAACATGTAAATCAAAT---
ATCACAGGACTATTATTGACACGTGATGGAGGCCTGTTAGATAAT----------------------------
----

AGTAGCACAGAGACATTCAGACCTGCAGGAGGAGATATGAGGGACAATTGGAGAAGTGAATTATATAAATATA
AAGTGGTGGAAATTAAGCCATTA---GGAGTAGCACCCACTGCTGCAAAAAGGAGAGTGGTG-----------
----------------------------GAGAGAGAAAAAAGAGCA---GTGGGA---ATAGGAGCT------
---GTGTTCCTTGGGTTCTTGGGAGCA---GCAGGAAGCACTATG---GGCGCGGCGTCA---
ATAACGCTGACGGTACAG---GCCAAACAA---TTGTTG---TCTGGTATAGTGCAGCAGCAAAGC---
AATTTG---CTGAGA---GCTATAGAGGCGCAACAGCATATGTTGCAACTCACGGTCTGGGGCATT
>AY444801
TTCTATTGCAATACATCAGTCCTGTTTAATAGGTCATATAATCTTACTGATACAAATAGTAATAATATAAATA
GC---

ACTTCAACCGACACACTCACACTCCCATGCAGAATAAAACAAATTATAAACATGTGGCAGGAGGTAGGACGGG
CAATGTATGCCCCTCCCATTGCAGGAAACATAACATGTAACTCAAGC---
ATCACAGGACTACTATTGACATGGGATGGAGGCGAAGAGAAAAACAACACA----------------------
----

GAGAAAAACAACACAGAGATATTCAGACCTGGAGGAGGAGATATGAGGGACAATTGGAGAAGTGAACTATATA
AATATAAAGTGGTAGAAATTAAGCCATTA---GGAATAGCACCCACCAAGGCAAAAAGGAGAGTGGTG-----
----------------------------------GAGAGAGAAAAAAGAGCA---GTGGGA---
ATAGGAGCT---------GTGCTCCTTGGGTTCTTGGGAGCA---GCAGGAAGCACTATG---
GGCGCGGCGTCA---ATAACGCTGACGGCACAG---GCCAGACAA---GTGTTG---
TCTGGTATAGTGCAACAGCAAAGT---AATTTG---CTGAGA---
GCTATAGAGGCGCAACAGCAAATGTTGCAACTCACAGTCTGGGGCATT
>AY563169
TTCTATTGCAATACATCAAGCCTGTTTAATAGTACATACAATACAACAGGGACAGAAAGCAGTAATGGACAG
AAAGC--
-------------------AGT---
AATGAAACAGGAAGCATTACAATCCCATGCAGAATAAAACAAGTTATAAACATGTGGCAAGGGGTAGGACGAG
CAATGTATGCCCCTCCCATTGAAGGAAACATAACATGTAGGTCAAAT---
ATCACAGGATTACTACTGACACGGGATGGAGGAGACGGGAATGGC----------------------------
----

ACAACAGAGACATTCAGACCTGGAGGAGGAGATATGAGGGACAATTGGAGAAGTGAATTATACAAATATAAAG
TGGTAGAAATTAAACCATTA---GGAATAGCACCCACTACTGCAAAAAGGAGAGTGGTG--------------
-------------------------GAGAGAGAAAAAAGAGCA---GCG------CTAGGAGCT---------
ATGTTCCTTGGGTTCTTGGGAGCA---GCAGGAAGCACTATG---GGCGCAGCGTCA---
ATAACGCTGACGGTACAG---GCCAGACAA---TTATTG---TCTGGGATAGTGCAACAGCAAAGC---
AATTTG---CTGAAG---GCTATAGAGGCGCAACAGCATATGTTGCAACTCACGGTCTGGGGCATT
>AY795906
TTCTATTGCAACACATCAAAATTGTTTAATAGGACATACATGTTTAATAGTACATACATGCCTAATAGTACTA
ATAGT--

ACTTCAAATTCAACCATCACACTCCCTTGCAGAATAAAACAAATTATAAACATGTGGCAGGGGGTAGGACGAG
CAATGTATGCCCCTCCTATTGCAGGAAACATAACATGTACATCAAAC---
ATCACAGGACTACTATTGACACGTGATGGAGGAGGAAAAGACAAC----------------------------
----

GAGTCAGAGACATTCAGACCTGGAGGAGGAGATATGAGGGATAATTGGAGAAGTGAATTATATAAATATAAAG
TGGTAGAAATTAAACCATTA---GGAATAGCACCCACTGCAGCAAAGAGGAGAGTGGTG--------------
-------------------------GAGAGAGAAAAAAGAGCA---GCAGGA---ATGGGAGCT---------
TTGTTCCTTGGGTTCTTGTCAGCA---GCAGGAAGCACTATG---GGCGCAGCATCA---
ATAACGCTGACGGTACAG---GCCAGGCAA---TTGTTG---TCTGGTATAGTGCAACAGCAAAGC---
AATTTG---CTGAGG---GCTATAGAGGCGCAACAGCATCTGTTGCAACTCACGGTCTGGGGCATT
>AY529667
TTCTATTGCAATACATCAAACCTGTTTAATGAGAGTAATATAGAAAGG-------------------------
----

AATGATTCAATCATCACACTCCCATGCAGAATAAAACAAATTATAAACATGTGGCAGGAGGTAGGACGAGCAA
TATATGCTCCTCCCATTGCAGGAAACATAACATGTAGATCAAAT---
ATCACAGGACTACTATTGACACGTGATGGAGGGTCAAATAATGGGGTG-------------------------
----

CCAAATGACACAGAGACATTCAGACCTGGAGGAGGCGATATGAGGAACAATTGGAGAAGTGAATTATATAAAT
ATAAAGTGGTAGAAGTTAAACCATTG---GGAGTAGCACCCACTGAGGCAAAAAGGCGAGTGGTG--------
-------------------------------GAGAGAGAAAAAAGAGCA---GTAGGA---ATAGGAGCT---
------GTGTTCCTTGGGATCTTGGGAGCA---GCCGGAAGCACTATG---GGCGCAGCATCA---
ATAACGCTGACGGTACAG---GCCAGACAA---TTGCTG---TCTGGTATAGTGCAACAGCAAAGC---
AATTTG---CTGAGG---GCTATAGAGGCGCAACAGCATATGTTGCAACTCACAGTCTGGGGCATT
>AY463217
TTCTATTGCAATACAACACAACTGTTTGAAAATATTACTAAT-------------------------------
----

AATGTAAGTAACATCATACTCCCATGCAGAATAAAACAATTTATAAACATGTGGCAGGAGGTAGGACGAGCAA
TGTATGCCCCTCCCATTGCAGGAAACATAACATGTACCTCAAAT---
ATCACAGGACTGCTATTGGTACGTGATGGTGGAAACACAAATGGCACA-------------------------
----

AATAACTCAGAAATATTCAGACCTGCAGGAGGAAATATGAAGGACAATTGGAGAAGTGAATTATATAAATATA
AAGTGATAGAACTTAAGCCATTG---GGAGTAGCACCCACTGAGGCAAGAAGGAGAGTGGTG-----------
----------------------------GAGAGAGAAAAAAGAGCA---GTGGGA---ATAGGAGCT------
---GTGTTCCTTGGGTTCTTGGGAGCA---GCAGGAAGCACTATG---GGCGCGGCGTCA---
ATAACGCTGACGGTACAG---GCCAGACAA---CTGTTG---TCTGGTATAGTGCAACAGCAAAGC---
AATTTG---CTGAGA---GCTATAGAGGCGCAACAGCATATGTTGCAACTCACGGTCTGGGGCATT
>AY463230
TTCTATTGCAATACATCACAACTGTTTAATGGCACATACAATGGCACATATAGTAAT----------------
----

ATCACAGACCTCATCACCCTCCCATGCAAGATAAAACAAATTATAAACCTGTGGCAGGGGGTAGGACGAGCAA
TGTATGCCCCTCCCATTGCAGGAAACATAACATGTAACTCAAAT---
ATCACAGGAATACTATTGACACGTGATGGAGGACACGAAAGGAATAACACA----------------------
----

```
AAGAATGACACAGAGATATTCAGACCTGGAGGAGGAAATATGAAGGACAATTGGAGAAGTGCATTATATAAAT
ATAAAGTGGTAGAAGTTAAGCCATTA---GGAATAGCACCAACTGAGGCAAAAAGGAGAGTGGTG--------
-------------------------------GAGAGAGAAAAAAGAGCA---GTGGGA---ATGGGAGCT---
------GTACTCCTTGGGTTCTTGGGAGCA---GCAGGAAGCACTATG---GGCGCAGCGTCA---
ATAACGCTGACGGTACAG---GCCAGGCAA---CTGTTG---TCTGGTATAGTGCAACAGCAAAAT---
AATTTG---CTGAGA---GCTATAGAGGCGCAACAGCATATGTTGCAACTCACGGTCTGGGGCATT
>AY463231
TTCTATTGTAATACATCAAACCTGTTTAATGATAATGGTACAGAAAGT-------------------------
-------------------------------------------------------------------------
----------------------------
AATTCAACACACACATTACACTTCCATGCAGAATAAAACAAATTATAAACATGTGGCAGGGGGTAGGGCGAGCAA
TGTATGCCCCTCCCATTGAAGGAAATATAACATGTAACTCAAGT---
ATCACAGGACTACTACTGACACGTGATGGAGGAGACATAATAAATGGCACA----------------------
-------------------------------------------------------------------------
-------
GACAATGAAACAGAGACATTCAGACCTGGAGGAGGAAATATGAAGGACAATTGGAGAAGTGAATTATATAAAT
ATAAAGTGGTAGAAATTAAGCCATTA---GGGATAGCACCCACTAAGGCAAAAAGGAGAGTGGTG--------
-------------------------------GAGAGAGAAAAAAGAGCA---GTAGGA---TTAGGAGCT---
------GTGATCTTTGGGTTCTTGGGAGCA---GCAGGAAGCACTATG---GGCGCGGCGTCA---
ATAACGCTGACGGTACAG---GCCAGACAA---CTGTTG---TCTGGTATAGTGCAACAGCAAAGC---
AATTTG---CTGAGA---GCTATAGAGGCGCAACAGCATATGTTGCAACTCACGGTCTGGGGCATT
>AY463225
TTCTATTGCAATACGTCAGACTTGTTTAATAGTAATAATAAGACAGAA-------------------------
-------------------------------------------------------------------------
----------------------------
AGCAATTCAACTATCACACTTCCATGCAGAATAAAACAAATTATAAACATGTGGCAGGGGGTAGGACGAACAA
TGTATGCCCCTCCCATTGCAGGAAACATAACATGTAACTCAAAT---
ATCACAGGACTACTATTGACACGTGATGGAGGGAAAAATGACACA----------------------------
-------------------------------------------------------------------------
-------------
GGGGAGGAGATATTCAGACCTGGAGGAGGAAATATGAAGGACAATTGGAGAAATGAATTATATAAATATAAAG
TGGTAGAAATTAAGCCATTG---GGAGTAGCACCCACTGAGGCAAAAAGGAGAGTGGTG--------------
-------------------------GAGAGAGAAAAAAGAGCA---GTGGGA---ATAGGAGCT---------
GTGATCCTTGGGTTCTTGGGAGCA---GCAGGAAGCACTATG---GGCGCGGCGTCA---
ATAACGCTGACGGTACAG---GCCAGACAA---CTGTTG---TCTGGTATAGTGCAACAGCAAAAT---
AATTTG---CTGAGA---GCTATAGAGGCGCAACAGCATATGTTGCAACTCACGGTCTGGGGCATT
>AY463232
TTCTATTGCAATACATCAAACCTGTTTAATAGTACATTGCTTAATAGTACATTGTCTAATAGTACAAATAAT-
-------------------------------------------------------------------------
----------------------------
ACTAATGGTATCATCATACTCCCATGCAGAATAAAACAAATTATAAATATGTGGCAGGGGGTAGGACGGGCAA
TGTATGCCCCTCCCATTGCAGGAAATATAACATGTAAATCAAAT---
ATCACAGGACTAATATTGACACGTGATGGAGGAGAACCTAATATA----------------------------
-------------------------------------------------------------------------
-------------
ACGGAGGAGACATTCAGACCTGGAGGAGGAGATATGAGAGACAATTGGAGAAGTGAATTATATAAATACAAAG
TGGTAGAAATTAATCCATTA---GGAGTAGCTCCCACTAGAGCAAAAAGGAGAGTGGTG--------------
-------------------------GAGAGAGAGAAAAGAGCA---GTGGGA---ATAGGAGCT---------
GTATTCCTTGGGTTCTTAGGAGCA---GCAGGAAGCACTATG---GGCGCAGCATCA---
ATAACGCTGACGGTACAG---GCCAGACAA---TTATTG---TCTGGTATAGTGCAACAGCAAAGC---
AATTTG---CTGAGG---GCTATAGAGGCGCAACAGCATATGTTGCAACTCACAGTCTGGGGCATT
>AY463218
TTCTATTGCAATACAACAAAACTGTTCACTAGTACTAATATG-------------------------------
-------------------------------------------------------------------------
----------------------------
ACAAACACAACCATCATGATCCCATGCAGAATAAGGCAAATCGTACGCCTGTGGCAGGAGGTAGGACGAGCAA
TGTATGCCCCTCCCATTGAAGGAAACATAACATGTAGCTCAAAT---
ATCACGGGACTACTATTGGTACGTGATGGAGGAAAAGGAAACGAAACA-------------------------
-------------------------------------------------------------------------
-------
```

CAGAATAACACAGAAATATTCAGACCTGGAGGAGGAAATATGAAGGACAATTGGAGAAGTGAATTATATAAAT
ATAAGGTGGTAGAGCTTAAGCCATTG---GGAATAGCACCCACTGATGCAAAAAGGAGAGTGGTG--------
-------------------------------GAGAGAGAAAAAAGAGCA---GTGGGAGTAATAGGAGCT---
------GTGCTCCTTGGGTTCTTGGGAGCA---GCAGGAAGCACTATG---GGCGCGGCGTCA---
ATAACGCTGACGGTACAG---GCCAGACAA---CTGTTG---TCTGGTATAGTGCAACAGCAAAGC---
AATTTG---CTGAGA---GCTATTGAGGCGCAACAGCATTTGTTGCAACTCACGGTCTGGGGCATT
>AY463233
TTCTATTGCAATACATCAAAACTGTTTAATAGTACATACAATAGTACATACAATAGTACATACAATAGTAGTA
CA---

GGAAATTCAACCATTACACTCCAATGCAGAATAAAACAAATTATAAATATGTGGCAGGGGGTAGGACGAGCAA
TGTATGCCCCTCCCATTGCAGGAAATATAACATGTAAATCAAAT---
ATCACAGGACTACTATTGACAAGCGATGGAGGGGGGAATAATGAA----------------------------
----

ACAGAGACATTTAGACCTGGAGGAGGAAATATGAGGGACAATTGGAGAAGTGAATTGTATAAATATAAAGTGG
TAGAAATTAAGCCATTA---GGAGTAGCACCCACTGAGGCAAAAAGGAGAGTGGTG-----------------
----------------------GAGAGAGAAAAAAGAGCA---GTGGGA---ATAGGAGCT---------
GTACTCCTTGGATTCTTGGGAGCC---GCAGGAAGCACTATG---GGCGCAGCATCA---
ATAACGCTGACGGTACAG---GCCAGACAA---TTGTTG---TCTGGTATAGTGCAACAGCAAAGC---
AATTTG---CTGAGG---GCTATAGAGGCGCAACAGCATATGTTGCAACTCACGGTCTGGGGCATT
>AY463219
TTCTATTGCAATACAACAGGACTGTTTAATAACAATGCAGCT-------------------------------
----

AACACAACAACCATCAACATCCCATGCAGAATAAAACAAATTGTAAACATGTGGCAAGAGGTAGGACGAGCAA
TGTATGCCCCTCCTATTGCAGGAAACATAACATGTAAATCAAAC---
ATCACAGGATTACTATTGCTACGTGATGGAGGAAACAATAGTAAC----------------------------
----

ACAGAGATATTTAGACCTGGAGGAGGAAATACGAAGGACAATTGGAGAAGTGAATTATATAAATACAAAGTAG
TAGAAATTAAGCCATTG---GGAGTAGCACCCACTAAGGCAAAAAGGAGAGTGGTG-----------------
----------------------GAGAGAGAAAAAAGAGCA---GTGGGA---ATAGGAGCT---------
GTGCTCCTTGGGTTCTTGGGAGCA---GCAGGAAGCACTATG---GGCGCGGCGTCA---
ATAACGCTGACGGTACAG---GCCAGACAA---TTGTTG---TCTGGTATAGTGCAACAGCAAAGC---
AATTTG---CTGAGA---GCTATAGAGGCGCAACAGCATATGTTGCAACTCACTGTCTGGGGCATT
>AY463220
TTCTATTGCAATACATCAAAGCTGTTTAATATATCTACTAGTGAAAATGTAACT-------------------
----

AGTGAAAATGTAACTACACTCCCATGCAGAATAAAACAAATTATAAGGATGTGGCAGGGAGTAGGACAAGCAA
TGTATGCCCCTCCCATTGAAGGAAACATAACATGTAACTCAAGT---
ATCACAGGACTACTACTGACACGGGATGGAGGAAACAACACGACT----------------------------
----

AATGGCACGGAGATATTCAGACCTGGAGGAGGAAATATGAAAGACAATTGGAGAAGTGAATTATATAAATATA
AAGTGGTAGAAATTAAGCCATTG---GGAGTAGCACCCACAAAGGCAAAAAGGAGAGTGGTG-----------
----------------------------GAGAAAGAAAAAAGAGCA---GTGGGA---ATAGGAGCT------
---GTGTTCCTCGGGTTCTTGGGAGCA---GCAGGAAGCACTATG---GGCGCGGCGTCA---
ATAACGCTGACGGTACAG---GCCAGACAA---CTGTTG---TTTGGTATAGTGCAACAGCAAAGC---
AATTTG---CTGAGA---GCTATAGAGGCGCAACAGCATATGTTGCAACTCACAGTCTGGGGCATT
>AY463234
TTCTATTGCAATACAACAGAACTGTTTAATGATAATAACAGTGAT----------------------------
----

GACAACGCAACCATCACACTCCCATGCAGAATAAAACAAATCATAAACATGTGGCAAGGGGTAGGACGAGCAA
TGTATGCCCCTCCCATTGAAGGAAGCATAACATGTAACTCAAAT---
ATCACAGGACTACTATTGACACGTGATGGAGGAACAAACAACACA----------------------------
----

GATGAGACATTCAGACCTGGAGGAGGAAATATGAAGGACAATTGGAGAAGTGAATTATATAAATATAAAGTGG
TAGAAATTAAACCATTG---GGAATAGCACCCACAGGGGCAAAAAGGAGAGTGGTG-----------------
----------------------GAGAGAGAAAAAAGAGCA---GTGGGA---CTAGGAGCT---------
GTGTTCCTTGGGTTCTTGGGAGCA---GCAGGAAGCACTATG---GGCGCGGCGTCA---
ATAACGCTGACGGTACAG---GCCAGACAA---CTGTTG---TCTGGTATAGTGCAACAGCAAAGC---
AATTTG---CTGAGA---GCTATAGAGGCGCAACAGCATATGTTGCAACTCACGGTCTGGGGCATT
>AY463221
TTCTATTGCAATACAACAAACCTGTTTAATGAAAGTCTGGTTAATACA-------------------------
----

AGCACAGGAGACATCACGCTCCCATGCAAAATAAAACAAATTATAAACATGTGGCAGGAAGTAGGGCGAGCAA
TGTATGCCCCTCCCATTGAAGGAAGCATAACATGTAAATCAAAT---
ATCACAGGACTACTATTGCTACGTGATGGAGGAAAGAATAACACA----------------------------
----

AATGAGACATTCAGGCCTGGAGGAGGAAATATGAAAGACAATTGGAGAAGTGAATTATATAAATATAAAGTGG
TAGGACTTAAGCCATTG---GGAATAGCACCCACTGATTCAAAAAGGAGAGTGGTG-----------------
----------------------GAGAGAGAAAAAAGAGCA---GTGGGA---ATAGGAGCT---------
ATGTTCCTTGGGTTCTTGGGAGCA---GCAGGAAGCACTATG---GGCGCGGCGTCG---
ATAACGCTGACGGTACAG---GCCAGACAA---CTGCTG---TCTGGTATAGTGCAACAGCAAAGC---
AATTTG---CTGAGA---GCTATAGAGGCGCAACAGCATTTGTTGCAACTCACGGTCTGGGGCATT
>AY463236
TTCTATTGCGATACAGCAGCCCTGTTTAATGGCACATATAATGGCACATATGGTAAT----------------
----

GAGACTTCAAACATCACACTCCAATGCAGAATAAAACAGATGATAAACATGTGGCAGGGGGTAGGACGAGCAA
TGTATGCTCCTCCCATTGCAGGAAACATAACATGTAGATCGAAT---
ATCACAGGACTACTATTGACACGTGATGGAAGTAACGATACTGGG----------------------------
----

CCAGAGATATTCAGACCTGGAGGAGGAAATATGAAGGACAATTGGAGAAGTGAATTATATAAATACAAAGTGG
TAGAAATTAAGCCATTA---GGAATAGCACCCACTAAAGCAAAAAGGAGAGTGGTG-----------------
----------------------GAGAGAGAGAAAAGAGCA---GTGGGA---ATAGGAGCT---------
GTGTTCCTTGGGTTCTTAGGAGCA---GCAGGAAGCACTATG---GGCGCAGCATCA---
ATAACGCTGACGGTACAG---GCCAGACAA---TTATTG---TCTGGTATAGTGCAACAGCAAAGC---
AATTTG---CTGAGA---GCTATAGAGGCGCAACAGCATATGTTGCAACTCACAGTCTGGGGCATT
>AY463226
TTCTATTGCAATACCACAAACCTGTTTGCCACAGAACTGGGTTCTAATAGAAGTGAGTCAAACATCACA----
----

AAGGAGTCAAACATCACACTCCCATGTAGGATAAAACAAATTATAAACATGTGGCAGGGGGTAGGACGAGCAA
TGTATGCCCCTCCCATTGAAGGAAACATAATGTGTAACTCAAGT---
ATCACAGGAATACTATTAACACGTGATGGAGGAGGAGGAGGAGAC----------------------------
----

AATCTCACAGAGATATTCAGACCTGGAGGAGGAAATATGAAGGACAATTGGAGAAATGAATTATATAAATATA
AAGTGGTAGAAATTAAGCCATTG---GGAGTGGCACCCACTGAATCAAAAAGGAGAGTGGTG-----------
----------------------------GAGAGAGAAAAAAGAGCA---GTGGGA---ATAGGAGCT------
---GTACTCCTTGGGTTCTTGGGAGCA---GCAGGAAGCACTATG---GGCGCGGCGTCA---
ATAACGCTGACGGTACAG---GCCAGACAA---TTGTTG---TCTGGTATAGTGCAACAGCAAAGC---
AATTTG---CTGAGA---GTCATAGAGGCGCAACAGCATATGTTGCAACTCACGGTCTGGGGCATT
>AY463222
TTCTATTGCAATACAACAATACTGTTTAATGAAACAAAACCT-------------------------------
----

TGTGAAAACACAACCATCCCATGCAGAATAAAACAAATTATAAACATATGGCAGGAGGTAGGAAGAGCAATGT
ATGCCCCTCCCATTGCAGGACACATAACATGTAACTCAACT---
ATCACAGGACTACTATTGTCCCGCGATGGAGGAGCCACAAACGAT----------------------------
----

ACCGAGATATTCAGGCCTGGAGGAGGAAATATGAAGGATAATTGGAGAAGTGAATTATATAAGTATAAAGTGG
TAGAAATTAAGCCATTA---GGAATAGCACCCACTACGGCAAAGAGGAGAGTGGTG-----------------
----------------------GAGAGAGAAAAAAGAGCA---GTGGGA---ATAGGAGCT---------
GTGTTCCTTGGGTTCTTGGGAGCA---GCAGGAAGCACTATG---GGCGCGGCGTCA---
ATAACGCTGACGGTACAG---GCCAGACAA---CTGCTG---TCTGGTATAGTGCAACAGCAAAGC---
AATTTG---CTGAGG---GCTATAGAGGCGCAACAGCATATGTTGCAACTCACGGTCTGGGGCATT
>JX845606
TTCTATTGCAATACAACAAACCTGTTTAACGAAAATATAACACAACTGTTTAATAAAAGTGAA----------
----

GCAAACGGAACCATCACACTCCCATGCAGGATAAAACAAATTATAAACATGTGGCAGAAGGTAGGACGAGCAA
TGTATGCCCCTCCTATTGCAGGAAATATAACATGTAAATCAAT---
ATCACAGGACTACTATTGACACGCGATGGAGAAGGATACAACACA----------------------------
----

CCGAATGAGACATTCAGACCTACAGGAGGAAATATGAAAGATAATTGGAGAAGTGAATTATATAAATATAAAG
TGGTAGAAATTAAGCCATTA---GGAATAGCACCCACTGAGTCAAAAAGGAGAGTGGTG--------------
-------------------------GAGAGAGAAAAAAGAGCA---GTGGGA---CTAGGAGCT---------
GTACTCCTTGGGTTCTTGGGAGCA---GCAGGAAGCACTATG---GGCGCGGCGTCA---
ATAACGCTGACGGTACAG---GCCAGACAA---CTGTTG---TCTGGTATAGTGCAACAGCAAAGC---
AATTTG---CTGAGA---GCTATAGAGGCGCAACAGCATATGTTGCAACTCACAGTCTGGGGCATT
>KF146947
TTCTATTGCAATACATCAAAACTGTTTAATAGTACATTGAATAGTACATATATGCCTAATGGTACAGAA----
----

AGTAATTCAAACATCACACTCCCATGCAGAATAAGACAATTTGTAAACATGTGGCAGGAAGTAGGACGAGCAA
TGTATGCCCCTCCCATTGAAGGAAAGATAACATGTAACTCAAAT---
ATTACAGGACTACTATTGTTACGTGATGGAGGAATAAATAATGACAGC-------------------------
----

AGTGAGAATGAGACATTCAGACCGGGAGGAGGAGATATGAGGGACAATTGGAGAAGTGAATTATATAAATATA
AAGTGATAGAAATTAAGCCATTG---GGAATAGCACCCACTGGAGCAAAAAGGAGAGTGGTG-----------
----------------------------GAGAAAGAAAAAAGAGCA---GTGGGA---ATAGCAGCT------
---GTGTTCCTTGGGTTCTTGGGAGCA---GCAGGAAGCACTATG---GGCGCGGCGTCA---
TTAACGCTGACGGTACAG---GCCAGACAA---TTGTTG---TCTGGTATAGTGCAGCAGCAAAGC---
AATTTG---CTGAGG---GCTATAGAGGCGCAACAGCATATGTTGCAACTCACGGTCTGGGGCATT
>AY838568
TTCTATTGCAATACATCAAAACTATTTAAATTTAATAGTTCGGAACCATTTAATAGTACAGAAAGTAATAGTA
CA---

GAAAGTAATAAAAATATCACAATTCCATGCAGAATAAAACAAATTATAAACATGTGGCAGGAGGTAGGACGAG
CAATGTATGCCCCTCCCATTGCAGGGAACATAACATGTACATCAAAC---
ATCACAGGACTACTATTAGTACGTGATGGGGGCAAAGAAAATGACCAA-------------------------
----

AATAACACAGAAATATTCAGACCTGGAGGAGGAAATATGAAGGACAATTGGAGAAGTGAATTATATAAATATA
AAGTGGTAGAAATTAAGCCATTA---GGAATAGCACCCACTGGAGCAAAAAGGAGAGTGGTG-----------
----------------------------CAGAGAGAGAAAAGAGCA---GTGGGA---ATAGGAGCT------
---ATACTCCTTGGGTTCTTGGGAGCA---GCAGGAAGCACTATG---GGCGCAGCGTCA---
ATAGCGCTGACGGCACAG---GCCAGACAA---GTGTTG---TCTGGTATAGTGCAACAGCAAAAC---
AATCTG---CTGAGA---GCTATAGAGGCGCAACAGCATATGTTGCAACTCACGGTCTGGGGCATT
>AY463228
TTCTATTGCAATACAACAAAATTGTTCCTGTTTAATGAAAGTGATGGAAGTGCT-------------------
----

CTAAACAAAACCATCACACTCCCATGCAGAATAAAACAGATTATAAACATGTGGCAGGAGGTAGGACGAGCAA
TGTATGCCCCTCCCATTGCAGGAAACATAACATGTAACTCAAGT---
ACCACAGGATTACTATTGCTACGTGATGGCGGAAACCAAAACAAT----------------------------
----

GAGACAGAGATATTCAGACCTGGAGGAGGAAATATGAAGGACAATTGGAGAAGTGAATTATATAAATATAAAG
TGGTAGAACTTAAGCCATTA---GGAGTAGCACCCACTGAGGCAAAAAGGAGAGTGGTG--------------
-------------------------GAGAGAGAAAAAAGAGCA---GTGGGA---ATAGGAGCT---------
GTGTTCCTTGGGTTCTTGGGAGCA---GCAGGAAGCACTATG---GGCGCGGCGTCA---
ATAACGCTGACGGTACAG---GCCAGACAA---CTGCTG---TCTGGTATAGTGCAACAGCAAAGC---
AATTTG---CTGAGA---GCTATAGAGGCGCAACAGCATATGTTGCAACTCACGGTCTGGGGCATT
>EU161645
TTCTATTGCAATACATCAGGACTGTTTAATAGCATTTCAAATGAAACTACCACAAATGGAACC----------
----

ACAAATGGAACCATCACAATTCCATGCAGAATAAAACAAATTATAAACATGTGGCAGGAGGTAGGACGAGCAA
TGTATGCCCCTCCCATTGCAGGAAACATAACATGTAAATCAAAT---
ATCACAGGACTACTATTATCAAGTGATGGAGGCACAGATAATAGCACA-------------------------
----

AAGCCAGAGACAGAGACCTTCAGACCTGGAGGAGGAGATATGAGGGATAACTGGAGAAGTGAATTATATAAAT
ATAAAGTGGTAGAAATTAAACCATTA---GGAATAGCACCCACTGAGGCAAAAAGGAGAGTGGTG--------
-------------------------------GAGAGAGAAAAAAGAGCA---GTGGGA---ATAGGAGCT---
------GTACTCCTTGGGTTCTTGGGAGCA---GCCGGAAGCACTATG---GGCGCGGCGTCA---
ATAACGCTGACGGTACAG---GCCAGACAA---TTGTTG---TCTGGTATAGTGCAACAGCAAAGC---
AATTTG---CTGAAG---GCTATAGAGGCGCAACAGCATATGTTGCAACTCACAGTCTGGGGCATT
>FJ846632
TTCTATTGCAATACAACAAATCTGTTTAATATAAGCATGACCAATGAC-------------------------
----

ACAGACACATTCATCACACTCCCATGTAGAATAAAACAAATTATAAATATGTGGCAGAGGGTAGGACGAGCAA
TGTATGCCCCTCCCATTACAGGAAACATAACATGTAACTCAAGT---
ATCACAGGACTACTATTGACACGTGATGGAGGAACAACAAATGGCACAGAG----------------------
----

AATAATAACACAGAGATATTCAGACCTGCAGGAGGAAATATGAAGGATAATTGGAGAAGTGAATTATATAAAT
ATAAAGTGGTAGAGATTAAGCCATTA---GGAGTAGCACCCACTGGAGCAAAAAGGAGAGTGGTG--------
-------------------------------GAGAGAGAAAAAAGAGCA---GTGGGA---ATAGGAGCT---
------GTGTTCCTTGGGTTCTTGGGAGCA---GCAGGAAGCACTATG---GGCGCGGCGTCA---
ATCACGCTGACGGTACAG---GCCAGACAA---CTGTTG---TCTGGTATAGTGCAACAGCAAAGC---
AATTTG---CTGAGA---GCTATAGAGGCGCAACAGCATATGTTGCAACTCACGGTCTGGGGCATT
>AY529672
TTCTATTGTGATACATCAAGATTATTTAATAGAACATATAATGGTACAGAAGATAACTCAGGA----------
----

GATAACACAACTATTACAATTCCATGCAGAATAAGACAAATTATAAACATGTGGCAGGAGGTAGGACGAGCAA
TGTATGCCCCTCCCATTGCAGGAAACATAACATGTAAATCAAAT---
ATCACAGGACTACTATTGGTGAGTGATGGAGGCAACAGAAGCAGC----------------------------
----

TCAAATGAAACATTTAGACCTGGAGGGGGAAACATGAAGGACAATTGGAGAAGTGAATTATATAAATATAAAG
TGGTAGAAATTAGGCCATTG---GGAATAGCACCCACTAAAGCAAAAAGGAGAGTGGTG--------------
-------------------------GAGAAAGAAAAAAGAGCA---GTGGGAATAATAGGAGCT---------
GTGTTCCTTGGGATCTTGGGAATA---GCAGGAAGCACTATG---GGCGCAGCGTCA---
ATAACGCTGACGGCACAG---GCCAGACAA---GTGATG---TCTGGTATAGTGCAACAGCAAAGC---
AATTTG---CTGAGG---GCTATAGAGGCGCAACAGCATATGTTGCAACTCACAGTCTGGGGCATT
>AY463237
TTCTATTGCAATACATCAAAACTGTTTAATAGTACAAGTAATGATACACAA----------------------
----

AGTAACACAACCATCACACTCCCATGCAGAATAAAACAAATTATAAACATATGGCAGGAGGTAGGACGAGCAA
TGTATGCCCCTCCCATTGCAGGAAACATAACATGTAACTCAAGC---
ATCACAGGACTGCTGTTGCTACGTGATGGAGGAGAGGGGAACAAC----------------------------
----

AGCACAGAGATATTCAGACCTGGAGGAGGGAATATGAAGGACAATTGGAGAAGTGAATTATATAAATATAAAG
TGGTAGAAATTGAGCCATTG---GGAATAGCACCCACTACGGCAAAAGGAGAGTGGTG---------------
-------------------------GAGAGAGAAAAAAGAGCA---GTGGGA---ATAGGAGCT---------
GTGCTCCTTGGGTTCTTGGGAGCA---GCAGGAAGCACTATG---GGCGCGGCGTCA---
ATAACGCTGACGGTACAG---GCCAGACAA---CTGTTG---TCTGGTATAGTGCAACAGCGAAGC---
AATTTG---CTGAGA---GCTATAGAGGCGCAACAGCATATGTTGCAACTCACGGTCTGGGGCATT
>KC863203
TTCTATTGCAATACATCAAAACTATTTAATAATACATACAGTCCTACATACAGTCCTAATGGTACAGGA----
----

GGAAATTCAACCATCAAAATTCCATGCAGAATAAAACAAATTATAAACATGTGGCAGGGGGTAGGACGAGCAA
TGTATGCCAATCCCATTGCAGGGAACATAACATGTAAATCAAAT---
ATCACAGGACTACTATTAGTACGTGATGGAGGCACAAACAACAGC----------------------------
----

ATGGAAATATTCAGACCTGGAGGAGGAAATATGAAGGACAATTGGAGAAGTGAATTATATAAATATAAAGTGG
TAGAAATTAAGCCATTG---GGAATAGCACCCACTGGAGCAAAAAGGAGAGTGGTG-----------------
----------------------GAGAGAGAAAAAAGAACA---GCGGGA---ATAGGAGCT---------
GTGTTCCTTGGGTTCTTGGGAGCA---GCAGGAAGCACTATG---GGCGCGGCGTCA---
TTGACGCTGACGGTACAG---GCTAGACAA---ATATTG---TCTGGTATAGTGCAACAGCAAAGC---
AATTTG---CTGAGG---GCTATAGAGGCGCAACAGCATATGTTGCAACTCACGGTCTGGGGCATT
>AY463223
TTCTACTGCAATACAACAGAACTGTTTAATTCAACAAAACTGTTCAATGTAACTAAGGTAACTAAT-------
----

GCAACTGAAAATATCACTCTCCCATGCAGAATAAAACAAATTGTAAACATGTGGCAGGAGGTGGGACGAGCAA
TGTATGCCCCTCCCATTGCAGGAAAAATAACATGTAGGTCAAAT---
ATCACAGGACTACTATTGTCACGTGATGGAGGCATGGATAATAACACGAAT----------------------
----

AGCACAAAAGAGGAGATATTCAGACCTGGAGGAGGAAATATGAAGGACAATTGGAGAAGTGAATTATATAAAT
ATAAAGTGGTAGAAGTTAAGCCATTG---GGAGTAGCACCCACGGAGGCAAAAAGGCGAGTGGTG--------
-------------------------------GAGAGAGAAAAAAGAGCA---GTGGGA---ATAGGAGCT---
------GTGTTCCTTGGGTTCTTGGGAGCA---GCAGGAAGCACTATG---GGCGCAGCGTCA---
ATAACGCTGACGGCACAG---GCCAGGCAA---CTGTTG---TCTGGTATAGTGCAACAGCAAAGC---
AATTTG---CTGAGA---GCTATAGAGGCGCAACAGCATATGTTGCAACTCACGGTCTGGGGCATT
>DQ351235
TTCTATTGCAATACATCAGGCCTGTTTAATGGTACGTACAATGGTACAGACATGTCTAATAGT----------
----

ACAGAAAGTACCATCACACTCCCATGCAGAATAAAACAAATTATAAACATGTGGCAGGGGGTAGGACGAGCAA
TGTATGCCCCTCCCATTGCAGGAAACATAACCTGTAAATCAAAC---
ATTACAGGATTACTATTGACACATGATGGAGGAAGAGAGGGTAAT----------------------------
----

AGCACTGAAACATTCAGACCTGGAGGAGGAAATATGAGGGATAATTGGAGAAGTGAATTATATAAATATAAAG
TAGTAGAAATCAAACCACTA---GGAATAGCACCCACAGAGGCAAAAAGGAGAGTGGTG--------------
-------------------------GAGAGAGAAAAAAGAGCA---GTGGGA---CTGGGAGCT---------
GTGTTCCTTGGGTTCTTGGGAGCA---GCAGGAAGCACTATG---GGCGCAGCGTCA---
ATAACGCTGACGGTACAG---GCCAGACAG---TTATTG---TCTGGTATAGTGCAACAGCAAAGC---
AATTTG---CTGAAG---GCTATAGAGGCGCAACAGCATATGTTGCAACTCACGGTCTGGGGCATT
>DQ351222
TTCTATTGCGATACAACAAGACTGTTTAATAGCAGTACATTTGGT----------------------------
----

GAAGGTGAAATCATCACACTCCCGTGCAAAATAAAACAAATTATAAACATGTGGCAGGGGGTAGGACGAGCAA
TGTATGCCCCTCCCGTTGAAGGAAATATAACATGTAACTCAAGT---
ACCACAGGAATACTATTGACACGTGATGGAGGAGACCAGGACAAT----------------------------
----

```
ACCACAGAGAAATTCAGACCTGGAGGAGGGAATATGAAGGACAATTGGAGAAGTGAATTATATAAATATAAAG
TGGTAGAAATTAATCCAGTG---GGAGTAGCACCCACGGAGGCAAAGAGGAGCGTGGTG--------------
-------------------------GCAAGAGAAAAAAGAACA---GTA------ATAGGAGCT---------
ATGATCCTTGGGTTCTTGGGAGCA---GCAGGAAGCACTATG---GGCGCGGCGTCA---
ATAACGCTGACGGTACAG---GCCAGACAA---CTGCTG---TCTGGTATAGTGCAACAGCAAAGC---
AATTTG---CTGAGA---GCTATAGAGGCGCAACAGCATCTGTTGCAACCCACGGTCTGGGGCATT
>DQ351218
TTCTATTGCAATACAACAAACCTGTTCAGTACAACAATCCTGGTCAACAACTCTACT----------------
-------------------------------------------------------------------------
----------------------------
GAAAAGGAAAACATCACACTCCCATGCAGAATAAAACAAATTATAAACATGTGGCAGGAGGTAGGACGAGCAA
TGTATGCCCCTCCCATTGAAGGAAACATAACATGTACATCAAAG---
ATCACAGGAATACTACTGACACGTGATGGAGGAGACAACACAACAACA-------------------------
-------------------------------------------------------------------------
----------
AGAAATAACGGGACATTCAGACCTGGAGGAGGAAATATGAAGGACAATTGGAGAAATGAATTATATAAATATA
AAGTAGTAGAAATTAAACCATTG---GGAGTAGCACCCACTAAGGCAAAAAGGAGAGTGGTG-----------
----------------------------GAGAGAGAAAAAAGAGCA---GTGGGA---ATAGGAGCT------
---GTACTCCTTGGGTTCTTGGGAGCA---GCAGGAAGCACTATG---GGCGCAGCGTCA---
CTAACGCTGACGGTACAG---GCCAGACAA---CTGTTG---TCTGGTATAGTGCAACAGCAAAGC---
AATTTG---CTGAGG---GCTATAGAGGCGCAACAGCATATGTTGCAACTCACGGTCTGGGGCATT
>DQ447269
TTCTATTGCGATACCTCAAACTTGTTTAATAGGACATACCCATTGAATAATAGTAGTGCAGGAAATGACAATA
GT-----------------------------------------------------------------------
-------------------------
AGCAGTGATTCAACCATCACACTCCAATGCAGGATAAAACAAATTATAAACATGTGGCAGGGGGTAGGACGAG
CAATGTATGCCCCTCCCATTGAAGGAAACATAACATGTAAGTCAAAT---
ATCACAGGACTACTATTGACACGTGATGGAGGACCAAGTAATGAA----------------------------
-------------------------------------------------------------------------
----------------
AGTGAGACATTCAGACCTGCAGGAGGGGATATGAGGGACAATTGGAGAAGTGAATTATATAAATACAAAGTGG
TGGAAGTGAATCCATTA---GGAATAGCACCCACCAAGGCAAAAAGGAGAGTGGTG-----------------
----------------------GAGAGAGAGAAAAGAGCA---GTGGGA---ATAGGAGCT---------
GTGTTCCTTGGGTTCTTGGGAGCA---GCAGGAAGCACTATG---GGCGCAGCATCA---
ATAACGCTGACGGTACAG---GCCAGACAA---TTATTG---TCTGGTATAGTGCAACAGCAAAGC---
AATTTG---CTGAGG---GCTATAGAGGCGCAACAGCATATGTTGCAACTCACAGTCTGGGGCATT
>DQ369978
TTCTATTGCAATACAACACCACTGTTTAATATGTCTGCTGACAATAACAGCACC-------------------
-------------------------------------------------------------------------
----------------------------
AATAATGGCACCATCACACTCCAATGCAAGATAAAACAAATTATAAACATGTGGCAGGGGGTAGGACGAGCAA
TGTATGCCCCTCCCATTGCAGGAAACATAACATGTGCGTCAAAT---
ATCACAGGACTACTATTGACACATGACGGAGGCATAAAAAATGAC----------------------------
-------------------------------------------------------------------------
----------
ACAGTCCCAGAGGTATTTAGACCTGGAGGAGGAAATATGAAGGACAATTGGAGAAGTGAATTATATGAATATA
AAGTGGTAGAAATTAAGCCATTG---GGAATAGCCCCCACTGAGTCAAGAAGGATAGTGGTG-----------
----------------------------GAGAGAGAAAAAAGAGCA---GTGGGA---ATGGGAGCT------
---GTGATCTTTGGGTTCTTAGGAGCA---GCAGGAAGCACTATG---GGCGCGGCGTCA---
ACGGCGCTGACGGTACAG---GCCAGACAA---CTGTTG---TCTGGTATAGTGCAACAGCAAAGC---
AATTTG---CTGAGA---GCTATAGAGGCGCAACAGCATATGTTGCAACTCACAGTCTGGGGCATT
>DQ351225
TTCTATTGCAATACATCAAAACTGTTTAACGGTACATACAGCCATAATGTCAGTACAAATAAC----------
-------------------------------------------------------------------------
----------------------------
AGTACAGAACCCATCACAATTCCATGCAGGATAAAACAAATTATAAACATGTGGCAAGAGGTAGGACGAGCAA
TGTATGCCCCTCCCATTGCAGGAAACATAACATGTAAATCAAAT---
ATCACAGGACTACTATTAACACGTGATGGAGGGAACACAAACACAAGC-------------------------
-------------------------------------------------------------------------
----------
```

AATAACACAGAGACATTCAGACCTGGAGGAGGAAATATGAAGGACAATTGGAGAAATGAATTATATAAATATA
AAGTGGTAGAAATTAAGCCATTG---GGAGTAGCACCCACTAAGGCAAAAAGGAGAGTGGTG----------
----------------------------GAGAGAGAAAAAAGAGCA---GTGGGA---ATAGGAGCT------
---GTGCTCCTTGGGTTCTTGGGAGCA---GCAGGAAGCACTATG---GGCGCGGCGTCA---
ATAACGCTGACGGTACAG---GCCAGACAA---CTGCTG---TCTGGTATAGTGCAACAGCAAAGC---
AATTTG---CTGAGA---GCCATAGAGGCGCAACAGCATATGTTGCAACTCACGGTCTGGGGCATT
>DQ396367
TTCTATTGCAATACAACATACCTGTTTTCTAATAAGACAGGCAACACAACC---------------------
----

GACAACGCAACCATCACGCTCCCATGCAGGATAAAACAATTTATAAACATGTGGCAGGAGGTAGGACGAGCAA
TATATGCCCCTCCCATTGCAGGAAAAATAACATGTAACTCAAAT---
ATCACAGGACTACTATTGACACGTGATGGAGGTGTGAATACTACGAATAATTCG------------AGT----
----
AATACAAGTAAAACA---
GAGACAGAGATATTCAGACCTGGAGGAGGAAATATGAAAGACAATTGGAGAAGTGAATTATATAAATACAAAG
TGGTAGAAATTAATCCATTG---GGAGTAGCACCCACTAATGCAAAAAGGAGAGTGGTG-------------
------------------------GAGAGAGAAAAAAGAGCA---GTGGGG---ATAGGAGCT---------
GTACTCCTTGGGTTCTTGGGAGCC---GCAGGAAGCACTATG---GGCGCGGCGTCA---
ATAACGCTGACGGTACAG---GCCAGACAA---TTGTTG---TCTGGTATAGTGCAACAGCAAAGC---
AATTTG---CTGAGA---GCTATAGAGGCGCAACAGCATATGTTGCAACTCACAGTCTGGGGCATT
>DQ351226
TTCTATTGTAATACAACAAACCTGTTTAACATAAACCTGACTAACTGGAGGTCTGATAGTAAGGGA-------
----

AATTCCACAATTATCACACTCCCATGTAGAATAAGACAATTTGTAAACCTGTGGCAGGGGGTAGGAAGGGCAA
TGTATGCCCCTCCCATTGAAGGTAATATAACATGTAAGTCAAAT---
ATCACAGGACTACTATTGACACGTGATGGAGGCAATGTCACAAAT---------------------------
----

AATGGCACAGAGATATTCAGACCTGGAGGAGGAGATATGAGGGACAATTGGAGAAGTGAATTATATAAATATA
AAGTGGTAGAAATTAAGCCATTG---GGAGTAGCACCCACAGAGGCAAAAAGGAGAGTGGTG----------
----------------------------GAGAGAGGAAAAAGAGCA---GTGGGA---ATGGGAGCT------
---GTGATCCTTGGTTTCTTGGGAGCA---GCAGGAAGCACTATG---GGCGCGGCGTCA---
ATAACGCTGACGGTACAG---GCCAGACAA---CTGTTG---TCTGGTATAGTGCAACAGCAAAGC---
AATTTG---CTGAGA---GCTATAGAGGCGCAACAGCACATGTTGAAACTCACAGTCTGGGGCATT
>DQ369985
TTCTATTGCAATACATCAAACCTGTTTAATAGTACAGTTAATGGTAATAGTACAGTTAATGGTACACACCTGG
GTAATAGT--
-------------------AAT---
AGTACAAATAACAACATCACACTCCAATGCAGAATAAAACAAATTATAAACATGTGGCAGGGGGTAGGACGAG
CAATGTATGCCCCTCCTATTGCAGGAAACATAACCTGTGTATCAAAT---
ATCACAGGACTACTATTGACACGTGATGGTGGCAGGGAGAATGGCACA------------------------
----

AATAACACAGAGATATTCAGACCTGCAGGAGGAAACATGAAGGACAATTGGAGAAGTGAATTATATAAATATA
AAGTGGTAGAAATTAATCCATTG---GGAGTAGCACCCACTGAGGCAAAAAGGAGAGTGGTG----------
----------------------------GAGAGAGAAAGAAGAGCA---GTGGGA---ATAGGAGCT------
---GTGTTCCTTGGGTTCTTGGGAGCA---GCAGGAAGCACTATG---GGCGCAGCGTCA---
ATAACGCTGACGGTACAG---GCCAGACAA---CTGTTG---TCTGGTATAGTGCAACAGCAAAGC---
AATTTG---CTGAGG---GCTATAGAGGCGCAACAGCATATGTTGCAACCCACGGTCTGGGGCATT
>DQ369996
TTCTATTGCAATACATCAAAACTGTTTAATAGTACATACAGTAGTACATACAATAATACATCTGATAATACT-
----

TCACATCCAAACATCACACTCCCATGCAGAATAAAACAAATTATAAACCTGTGGCGGGAGGTAGGACGGGCAA
TGTATGCCCCTCCCATTGCAGGAAACATAACATGTAACTCAAGT---
ATCACAGGACTACTATCGACACGTGATGGGGGAGAAAATAACTCA---------------------------
----

ACAACAGAGACATTCAGACCTGGAGGAGGAGATATGAGGGACAATTGGAGAAGTGAATTATATAAATATAAAG
TGGTAGGAATTGAGCCATTA---GGAGTAGCACCCACCAAGGCAAAAAGGAGAGTGGTGGAG-----------
----------------------AGGGAGAGAGAAAAAAGAGCA---ATGGGA---ATAGGAGCT---------
GTGTTCCTTGGGTTCTTGGGAGCA---GCAGGAAGCACTATG---GGCGCGGCGTCA---
ATAACGCTGACGGTACAG---GCCAGACAA---CTGTTG---TCTGGTATAGTGCAACAGCAAAGC---
AATTTG---CTGAAG---GCTATAGAGGCGCAACAGCATCTGTTGCAACTCACAGTCTGGGGCATT
>DQ445633
TTCTATTGCAATACAACAAACTTGTTTAATAATATAAAAAATGATGAGGATACAAAAAAT-------------
----

GGTAGTGATCCAATCATACTCCCATGCAGAATAAAACAAATTATAAACATGTGGCAGGGGGTAGGACGAGCAA
TGTATGCCCCTCCCATTGCAGGAAACATAACATGTAACTCAAGT---
ATCACAGGACTACTATTGACACGTGATGGAGGGAATGGTACTAAT----------------------------
----

AATACTGAGATATTCAGACCTGGAGGAGGAAATATGAAGGACAACTGGAGAAATGAATTATACAAATATAAAG
TGGTAGAAATTAAACCATTA---GGAATAGCACCCACCAAGGCAAAAAGGAGAGTGGTG--------------
-------------------------GAGAGAGAAAAAAGAGCA---GTGGGA---ATAGGAGCT---------
GTGTTCCTTGGGTTCTTGGGAGCA---GCAGGAAGCACTATG---GGCGCGGCGTCT---
ATCACGCTGACGGTACAG---GCCAGACAA---CAGTTG---TCTGGTATAGTGCAACAGCAAAGC---
AATTTG---CTGAGA---GCTATAGAGGCGCAACAGCATATGTTGCAACTCACGGTCTGGGGCATT
>DQ369979
TTCTATTGCAATACAACAAGACTGTTCAACACAACTAGCACAAGAGAG-------------------------
----

TCAAACAAAACCATCACACTCCCATGCAGAATAAAACAAATTATAAACATGTGGCAGGGGGTAGGACGAGCAA
TGTATGCCCCTCCCATTGCAGGAAACATAACATGTAACTCAAGT---
ATCACAGGAATACTATTGCAACGTGATGGAGGAAACACGAGTGAG----------------------------
----

CCCACAGAGATATTCAGACCTGGAGGAGGAAATATGAAGGACAATTGGAGAAGCGAATTATATAAATATAAAG
TGGTAGAAATTAAGCCATTG---GGAATAGCACCCACGAAGGCAAAAAGGAGAGTGGTG--------------
-------------------------GAGAGAGAAAAAAGAGCA---GTGGGA---ATAGGAGCT---------
GTGTTCCTTGGGTTCTTGGGAGCA---GCAGGAAGCACTATG---GGCGCGGCGTCA---
ATAACGCTGACGGTACAG---GCCAGACAA---CTGTTG---TCTGGTATAGTGCAACAGCAAAGC---
AATTTG---CTGAGA---GCTATAGAGGCGCAACAGCATCTGTTGCAACTCACGGTCTGGGGCATT
>DQ369984
TTCTACTGCAATACAACAAAACTGTTTAATACAACAAGACTGTTAAAAGAAAGTGATTCA-------------
----

AACACAAACACCACCACACTCCCATGCAGGATAAAACAGATTGTAAACATGTGGCAGGAGGTAGGACGAGCAA
TGTATGCCCCTCCCATTGCAGGAAACATAACATGTAAATCAAAT---
ATCACAGGACTACTATTGACACGTGATGGAGGGAATAACACTGACACAGAT----------------------
----

GGCGATAACACAGAAACATTCAGACCTGGAGGAGGAAATATGAAGGACAATTGGAGAAGTGAATTATATAAGT
ATAAGGTGATAAAAATTAAGCCATTA---GGAATAGCACCCACTGAGGCAAAAAGAAGAGTGGTG--------
-------------------------------AAGAGAGAAAAAAGAGCA---GTGGGA---ATAGGAGCT---
------ATGATCTTTGGGTTCTTGGGAGCA---GCAGGAAGCACTATG---GGCGCGGCGTCA---
ATAACGCTGACGGTACAG---GCCAGACAA---CTGTTG---TCTGGTATAGTGCAACAGCAAAGC---
AATTTG---CTGAGA---GCTATAGAGGCGCAACAGCATATGTTGCAACTCACGGTCTGGGGCATT
>DQ396364
TTCTATTGCGATACAACAGGACTGTTTAATAGTACATACATGCCTGAATACTGGCCTAATAGTACAGAAAGTA
AT---

TCAAGCTCAGTAATCACACTCCCATGCAGAATAAAACAAATTATAAACATGTGGCAGGAGGTAGGAAGAGCAA
TGTATGCTCCTCCCATTGCAGGAAACATAACATGTAGATCAAAT---
ATCACAGGATTACTATTGGTGCGTGATGGAGGAACAAATAACACA----------------------------
----

AATAACACAGAGACCTTCAGACCTGGAGGAGGAGATATGAGGGACAATTGGAGAAGTGAATTATACAAATATA
AAGTGGTAGAAATTAAGCCATTG---GGAATAGCACCTACTGGGGCAAAAAGGAGAGTGGTG-----------
----------------------------GAGAGAGAAAAAAGAGCA---GTTGGA---ATAGGAGCT------
---GTGTTCCTTGGGTTCTTGGGAGCA---GCAGGAAGCACTATG---GGCGCAGCGTCA---
ATAACGCTGACGGTACAG---GCCAGACAA---TTGTTG---TCTGGTATAGTGCAACAGCAAAGT---
AATTTG---CTGAGG---GCCATAGAGGCGCAACAGCATATGTTGCAACTCACGGTCTGGGGCATC
>DQ369986
TTCTATTGCAACACATCAGGACTATTTAATGGTACATACACGAGTAACATGACAGGAAATGGAAATGAT----
----

TCAAACTCAAACATCACACTTCCATGCAGAATAAAACAAATTATAAACATGTGGCAAGAGGTAGGACGAGCAA
TGTATGCCCCTCCCATTAAAGGAAACATAACATGTAGATCAAAT---
ATCACAGGACTACTATTAGTACGTGATGGAGGCAACCACAACAACAGC-------------------------
----

AACAACACAGAGACATTCAGACCTGGAGGAGGAAATATGAAGGACAATTGGAGAAGTGAATTGTATAAATATA
AAGTGGTAGAAATTAAACCATTA---GGAATAGCACCCACTACTGCAAAAAGGAGAGTGGTG-----------
----------------------------GAGAGAGAAAAAAGAGCA---GTGGGA---ATAGGAGCT------
---GTATTCCTTGGGTTCTTGGGAACA---GCAGGAAGCACTATG---GGCGCAGCGTCA---
ATAACGCTGACGGTACAG---GCCAGACAA---CTGTTG---TCTGGTATAGTGCAACAGCAAAGC---
AATTTG---CTGAGA---GCTATAGAGGCGCAACAGCATCTGTTGCAACTCACGGTCTGGGGCATT
>DQ369980
TTCTATTGCAATACATCAAACCTGTTTAATAGTACATACAAGCCTAATGGTACAAGAAGTACT----------
----

TCAAATTTAACCATCACACTCCAGTGCAGAATAAAACAAATTATAAACATGTGGCAGGAGGTGGGACGAGCAA
TGTATGCCCCTCCCATTGCAGGAAACATAACATGTAAATCAAAT---
ATCACAGGGCTACTATTGACACGTGACGGAGGAAAAGGAAATGAT----------------------------
----

ACGTCTGAAACATTCAGACCTGGAGGAGGAGATATGAGGGACAATTGGAGAAGTGAATTATATAAATATAAGG
TGGTAGAAATTAAGCCATTA---GGATTAGCACCCACTAAGGCAAAAAGGAGAGTGGTG--------------
-------------------------CAGAGAGAGAAAAGAGCA---GTGGGA---ATAGGAGCT---------
GTGTTCCTTGGGTTCTTGGGGGCA---GCAGGAAGCACTATG---GGCGCGGCGTCA---
ATAACGCTGACGGTACAG---GCCAGACAA---TTATTG---TCTGGCATAGTGCAACAGCAAAGC---
AATTTG---CTGAAG---GCTATAGAGGCGCAACAACATCTGTTGCAACTCACAGTCTGGGGCATT
>DQ396385
TTCTATTGTAATACAACAAAGCTGTTTAATGGTACATACAGGCCTGCATACAATAATGGTACAGGA-------
----

AGTAATTCAAGCATCACACTCCAATGCAGGATAAAACAAATTATAAACATGTGGCAGCAGGTAGGACGAGCAA
TGTATGCTCCTCCCATTGAAGGAAACATAACATGTAAATCAACT---
ATCACAGGACTACTATTGACACGTGATGGAGGGGAAAATGGCACA----------------------------
----

GAAAATGAGACATTCAGACCTGGAGGAGGAAATATGAGGGACAATTGGAGAAGTGAATTATACAAATATAAAG
TGGTAGAAGTTAAACCATTG---GGAATAGCACCTACTGGGGCAAAAAGGAGAGTGGTG--------------
-------------------------GAGAGAGAAAAAAGAGCA---GTGGGA---ATAGGAGCT---------
GTGTTCCTTGGGTTCTTGGGGGCA---GCAGGAAGCACTATG---GGCGCGGCGTCA---
CTGACGCTGACGGTACAG---GCCAGACAA---CTATTG---TCTGGTATAGTGCAACAGCAAAGC---
AATTTG---CTGAGG---GCCATAGAGGCGCAACAGCATATGTTGCAACTCACGGTCTGGGGCATT
>DQ369987
TTCTATTGCAATACATCAAGACTGTTTAATAGCACATTTAATAGTACATACATGCCTAATGCTACAGAAAGTA
AT---

TCAAGTTCAGTCATCACGATTCCCTGCAGAATAAAACAAATTATAAACATGTGGCAGGAGATAGGACGGGCAA
TGTATGCCCCTCCTATTGCAGGAAACATAACATGTAAATCAAAT---
ATCACAGGACTACTATTAGTACGTGATGGAGGCAACAGTAACAAT----------------------------
----

GACACAGAGATATTCAGACCTGGAGGAGGAAATATGAAGGACAATTGGAGAAGTGAATTATACAAATATAAAG
TGGTAGAAATTAAGCCATTG---GGAATAGCACCCACGAAGGCAAAAAGGAGAGTGGTG--------------
-------------------------GAGAGAGAAAAAAGAGCA---GTAGGA---ATAGGAGCT---------
GTGTTCCTTGGGTTCTTGGGAGCA---GCAGGAAGCACTATG---GGCGCGGCGTCA---
ATAACGCTGACGGCACAG---GCCAGACAA---TTGTTG---TCTGGTATAGTGCAACAGCAAAGC---
AATTTG---CTGAGG---GCTATAGAGGCGCAACAGCATATGTTGCAACTCACGGTCTGGGGCATT
>DQ369988
TTCTATTGCGAAACAACAAGACTGTTTAATACAACACAACTTAATACAACA----------------------
----

CAAAATGGAACAATCATACTCCCATGTAGAATAAAGCAAATTATAAACATGTGGCAGGGGGTAGGACGAGCAA
TGTATGCCCCTCCCATTGCAGGAATGATAACATGTAACTCAAGT---
ATCACAGGACTACTATTGGTACGCGATGGAGGCACGAATGACACA----------------------------
----

GAGATATTCAGACCTGGAGGAGGAAATATGAAGGACAATTGGAGAAGTGAATTATATAAATATAAAGTGGTAG
AAATTAAGCCATTG---GGAATAGCACCCACTGGGGCAAAAAGGAGAGTGGTG--------------------
-------------------GAGAGAGAAAAAAGAGCA---GTGGGA---ATAGGAGCT---------
GTACTCCTTGGGTTCTTGGGAGCA---GCAGGAAGCACTATG---GGCGCAGCATCA---
ATAACGCTGACGGTACAG---GCCAGACAA---CTGTTG---TCTGGTATAGTGCAACAGCAAAGC---
AATTTG---CTGAGG---GCTATAGAGGCGCAACAGCATATGTTGCAACTCACGGTCTGGGGCATT
>DQ396373
TTCTATTGCAATACATCACAACTGTTTAATAGTACATACAACAACCATACATACACGAATAATGGGACA----
----

GGAAATTCAAACATCACACTTCCATGCAAAATAAAACAGATTATAAATATGTGGCAGGGGGTAGGACGAGCAA
TGTATGCCCCTCCCATTGCAGGAAACATAACATGTATCTCAAAT---
ATCACAGGACTAATATTGACACGTGATGGAGGAGAAGAAGGAAACAAC-------------------------
----

AACAAAACAGAGATATTCAGACCTGGAGGAGGAAATATGAAGGATAATTGGAGAAATGAATTATATAAATATA
AAGTGGTAGAAATTAAGCCATTG---GGAATAGCACCCACTAAGGCAAAAAGAAGAGTGGTG-----------
----------------------------GAGAGAGAAAAAAGAGCA---GTGGGA---CTAGGAGCT------
---GTGTTCCTTGGGTTCTTGGGAGCA---GCAGGAAGCACTATG---GGCGCGGCGTCA---
ATAACGCTGACGGTACAA---GCCAGACAA---CTGTTA---GCTGGTATAGTGCAACAGCAAAAT---
AATTTG---CTGAGG---GCTATAGAGGCGCAACAGCATATGTTGCAACTCACGGTCTGGGGCATT
>DQ396374
TTCTATTGCAATACATCAAAACTGTTTAATACAACAGTTAATGGAACAGAGTTAGAGTTTAACACAACAGGAA
GG---

TCTAATAAAACCATCACTCTCCAATGCAAGATAAAACAAATTATAAACATGTGGCAAGAGGTAGGACGAGCAA
TGTATGCCCCTCCCATTGCAGGAAACATAACATGTAGATCAAAT---
ATCACAGGATTACTATTGACATGGGATGGACCAACAAACAATGGC----------------------------
----

ACAGACATATTTAGACCTGGAGGAGGAAATATGAAAGACAATTGGAGAAGTGAATTATATAAATATAAAGTGG
TAGAAATTAAGCCATTA---GGAATAGCACCCACTGAATCAAAAAGGAGAGTGGTG-----------------
----------------------GAAAGAGAAAAAAGAGCA---GTGGGA---CTAGGAGCT---------
TTGATCCTTGGGTTCTTGGGAACA---GCAGGAAGCACTATG---GGCGCGGCGTCA---
ATAGCGCTGACGGTACAG---GCCAGACAA---CTGTTG---TCTGGTATAGTGCAGCAGCAAAAC---
AATTTG---CTGAGA---GCTATAGAGGCGCAACAGCATATGTTGCAACTCACAGTCTGGGGCATT
>DQ369989
TTCTATTGCAATACATCAGAACTATTTAACAGTACATACAATAAGGATACAAAGAGTAATGGTACAAAG----
----

AGTAATTTAACCATCACAATTCCATGCAGGATAAAACAAATTATAAACATGTGGCAGCAGGTGGGACGAGCAA
TGTATGCTCCTCCCATTGCAGGAAACATAACATGTAAATCAAAT---
ATCACAGGACTACTATTGGTGCGTGATGGAGGAACAAATAACATA----------------------------
----

AATAACACAGAGACATTCAGACCTGGAGGAGGAGATATGAGGGATAATTGGAGAAGTGAATTATACAAATATA
AAGTGGTAGAAATTAAACCATTG---GGAATGGCACCAACTAAGGCAAAACGGAGAGTGGTG----------
---------------------------GAGAGAGAAAAAAGAGCA---GTGGGA---ATAGGAGCT------
---GTGCTCTTTGGGTTCTTGGGAGCA---GCAGGAAGCACTATG---GGCGTGGCGTCA---
ATAACGCTGACGGTACAG---GCCAGACAA---TTGTTG---TCTGGTATAGTGCAACAGCAAAGC---
AATTTG---CTGAGA---GCCATAGAGGCGCAACAGCGTATGTTGCAACTCACGGTCTGGGGCATT
>DQ396375
TTCTATTGCAATACAACAGAACTGTTTAATAATAGTGAAGTAAACATAACTGCAGCTACTGCA---------
----

ACAAATGAAACCTTCACACTCCCATGCAAAATAAAACAAATTATAAACATGTGGCAGGGGGTAGGACGAGCAA
TGTATGCCCCTCCCATTAAAGGAAACATAACATGTAAATCAAAC---
ATCACAGGAATACTATTGACATGGGATGGAGGAAATGAAACTAGGACAGATAAT------------------
----
-
AGGACAAATAATAAAACAGAGATATTCAGACCTGGAGGAGGAAATATGAAGGACAATTGGAGAAATGAATTAT
ATAAATATAAAGTGGTAGAAATTAAGCCATTG---GGAGTAGCACCCACTACGGCAAAAAGGAGAGTGGTG--
------------------------------------GAGAGAGAAAAAAGAGCA---GTAGGA---
ATAGGAGCT---------GTGCTCCTTGGGTTCTTGGGAGCA---GCAGGAAGCACTATG---
GGCGCGGCGTCA---ATAACGCTGACGGTACAG---GCCAGACAA---CTGTTG---
TCTGGTATAGTGCAACAGCAAAGC---AATTTG---CTGAGA---
GCTATAGAGGCGCAACAGCATATGTTGCAACTCACAGTCTGGGGCATT
>DQ396389
TTCTATTGCAATACATCAAGACTGTTTAATGGTACATACAACAGCACAGAAAACAGCACAGAAAAT------
----

GCAAATAGTACCATCACACTCCCATGTAGAATAAAACAAATTGTAAACATGTGGCAGGGAGTAGGACAAGCAA
TGTATGCCCCTCCCATTGCAGGAAACATAACATGTAACTCAAAT---
ATCACAGGACTACTATTGACACGTGATGGGGGAACAACAATAAC----------------------------
----

GGGACAGAGACATTCAGACCTGGAGGAGGAGATATGAGGGACAATTGGAGAAGTGAATTATATAAATATAAAG
TGGTAGAAATTAAGCCATTA---GGAATAGCACCCACTAAGGCACAAAGGAGAGTGGTG-------------
------------------------CAGAGAGAAAAAAGAGCA---GTAGGA---ATAGGAGCT---------
GTACTCCTTGGGTTCTTGTCAGCA---GCAGGAAGCACTATG---GGCGCGGCGTCG---
ATAACGCTGACGGTACAG---GCCAGACAA---GTGCTG---TCTGGTATAGTGCAACAGCAGAGC---
AATCTG---CTGAGG---GCTATAGAGGCGCAACAGCATATGTTGCAACTCACGGTCTGGGGCATT
>DQ396376
TTCTATTGCAATACAACAAAACTATTTAATGACAATGAGACA------------------------------
----

AACGAATACATCATACTCCCATGCAGAATAAAACAAATTATAAACATGTGGCAGGGGGTAGGACGAGCCATGT
ATGCCCCTCCCATTGAAGGAAACATAACATGTAAATCAAAT---
ATCACAGGACTATTATTGACACGTGATGGAGGAGGCGAAAATGAA---------------------------
----

ACCAAAATCGAGATATTCAGACCTGGAGGAGGAAATATGAAGGACAATTGGAGAAGTGAATTATATAAATATA
AAGTGGTAGAACTTAAGCCATTG---GGCATAGCACCCACTGAGGCAAAAAGGAGAGTGGTG----------
---------------------------GAGAGAGAAAAAAGAGCA---GTGGGA---ATGGGAGCT------
---GTGCTCCTTGGGTTCTTGGGAGCA---GCGGGAAGCACTATG---GGCGCGGCGTCA---
ATAACGCTGACGGTACAG---GCCAGACAA---CTGTTG---TCTGGTATAGTGCAACAGCAAAGC---
AATTTG---CTGAGG---GCTATAGAGGCGCAACAACATATGTTGCAACTCACGGTCTGGGGCATT
>DQ093591
TTCTACTGCAATACAAGCGGACTGTTCAATGCAACTTTCAATACAACTAATACA------------------
----

ACTGATGCAAACATCACACTCCCATGCAGAATAAAACAAATTATAAACATGTGGCAGGAGGTAGGACGAGCAA
TGTATGCCCCTCCCATTGAAGGAAACATAATATGTAGATCAAAC---
ATCACAGGACTGCTATTGCAACGTGATGGAGGCAAGGGCAATGGCACT------------------------
----

GACAACGAAACAGAGATATTCAGACCTGGAGGAGGAAATATGAAAGACAATTGGAGAAGTGAATTATATAAAT
ATAAGGTGGTAGCAATTAAGCCATTA---GGAGTAGCACCCACGGATGCACAAAGGAGAGTGGTG--------
-------------------------------GAGAGAGAAAAAAGAGCA---GTGGGA---ATAGGAGCT---
------ATGCTCTTTGGGTTCTTAGGAGCA---GCAGGAAGCACTATG---GGCGCAGCGTCA---
ATAACGCTGACGGTACAG---GCCAGACAA---CTGTTG---TCTGGTATAGTGCAACAGCAAAGC---
AATTTG---CTGAGA---GCTATAGAGGCGCAACAGCATCTGTTGCAACTCACAGTCTGGGGCATT
>DQ351219
TTCTATTGTAATACAACAGGACTGTTTTCTAATAATGGAAGTGCAGGA-------------------------
----

AACGCAACCACCATCACACTCCCATGCAGAATAAAACAAATTATAAACATGTGGCAGGGGGTAGGACGAGCGA
TGTATGCCCCTCCCATTGCAGGAAACATAACATGTACCTCAAAT---
ATCACAGGACTACTATTGACACGGGATGGAGGAATAAAGGATAACACA-------------------------
----

ACAGACATAACAGAGACATTCAGACCTGGAGGAGGAAATATGAAGGACAATTGGAGAAATGAATTATATAAAT
ATAAAGTGGTAGAAATTAAACCATTG---GGAGTAGCACCCACTGAGGCAAAAAGGAGAGTGGTG--------
-------------------------------GATAGAGAAAAAAGAGCA---GTA------CTAGGAGCT---
------ATGTTCCTTGGGTTCTTGGGAGTA---GCAGGAAGCACTATG---GGCGCAGCGTCA---
GTAACGCTGACGGTACAG---GCTAGACAA---TTATTG---TCTGGTATAGTGCAACAGCAAAGC---
AATTTG---CTGAGG---GCTATAGAGGCGCAACAGCATATGCCGCAACTCACAGTCTGGGGCATT
>DQ351229
TTCTATTGCAATACATCAAAACTGTTTAATAGTACATTTAATAGTACATATATGCATAATGATACAGGAAGTA
AT---

ACAAGCTCAACAATCACAATCCCATGCAAAATAAAACAAATTATAAACATGTGGCAGGAGGTAGGAAGAGCAA
TGTATGCTCCTCCCATTGCAGGAAACATAACATGTAAATCAAAT---
ATCACAGGACTACTATTGACACGTGATGGGGGAAATTCAACTGAT----------------------------
----

AATGAAACAGAAACATTCAGACCTGAAGGAGGAGACATGAGGGACAATTGGAGAAATGAATTATACAAATATA
AAGTGGTAGAAATTAAGCCATTG---GGAATAGCACCCACTGAGGCAAAGAGGAGAGTGGTG-----------
----------------------------GAGAGAGAAAAAAGAGCA---GTGGGA---ATAGGAGCT------
---GTGCTCCTTGGGTTCTTGGGAGCA---GCAGGAAGCACTATG---GGCGCGGCGTCA---
ATAACGCTGACGGTACAA---GCCAGACAA---CTGTTG---TCTGGTATAGTGCAACAGCAAAGC---
AATTTG---CTGAGA---GCTATAGAGGCGCAACAGCATATGTTGCAACTCACGGTCTGGGGCATT
>DQ275654
TTCTATTGCAATACATCACAACTATTTAATGATAGTAATACAGAATTTAATATTAGTAGTACAGAA-------
----

AATAATAAAACCATCACAATTCTATGCAGAATAAAACAATTTATAAACATGTGGCAGAAGGTAGGACAAGCAA
TGTATGCCCCTCCCATAGCAGGAAACATAACATGTAACTCAAGC---
ATCACAGGATTATTACTGACACGTGATGGTGGAAATGAAAGTATCACA-------------------------
----

AATAACACAGAGATATTCAGACCTGGAGGAGGAAATATGAAGGACAATTGGAGAAGTGAATTATATAAATATA
AAGTGGTAGAAATTATGCCATTG---GGAATAGCACCCACTACTGCAAAAAGGAGAGTGGTG-----------
----------------------------GAGAGAGAAAAAAGAGCA---GTGGGA---ATAGGAGCT------
---GTGATCTTTGGGTTCTTGGGAGCA---GCAGGAAGCACTATG---GGCGCGGCGTCA---
ATAACGCTGACGGTACAG---GCCAGACAA---CTGCTG---TCTGGTATAGTGCAACAGCAAAGT---
AATTTG---CTGAGA---GCTATAGAGGCGCAACAACATATGTTGCAACTCACGGTCTGGGGCATT
>DQ351216
TTTTATTGCGATACATCGCGGCTGTTTACTAATGTAAGCAATGAAAATGACACAATCACCAATGTAAGCAAT-
----

AAAAATGACACAATCACACTCCCATGCAGAATAAAACAAATTGTAAACATGTGGCAGGGGGTAGGACGAGCAA
TGTATGCCCCTCCCATTGCAGGACAAATAACATGTAGCTCAAAT---
ATCACAGGACTACTATTGACACGTGATGGAGGAAACACAACTGTT----------------------------
----

ACTAACACAGAGGTATTCAGACCTGAAGGAGGAAATATGAAGGACAATTGGAGAAGTGAACTATATAAATATA
AAGTGGTAGAAATTAAACCATTG---GGAGTAGCACCCACTAAGGCAAAAAGGAGAGTGGTG-----------
----------------------------GAGAGAGAAAAAAGAGCA---GTGGGA---ATAGGAGCT------
---GTACTCCTTGGGTTCTTGGGAGCA---GCAGGAAGCACTATG---GGCGCGGCGTCT---
ATAACGCTGACGGTACAG---GCCAGACAA---CTGTTG---TCTGGTATAGTGCAACAGCAAAAC---
AATTTG---CTGAGA---GCTATAGAGGCGCAACAGCATATGTTGCAACTCACGGTCTGGGGCATT
>DQ275645
TTCTACTGCAATACAACAGAACTGTTTAATGTAAACAATGATCCA----------------------------
----

AACGATTCAAACATCACGCTCCCATGCAGAATAAAGCAAATTATAAACATGTGGCAGGGGGTAGGACGAGCAA
TGTATGCCCCTCCCATTGAAGGAGAGATAACATGTAAATCAAAC---
ATTACAGGACTACTATTGGAGCGTGATGGAGGAAAAACAAATGAC----------------------------
----

ACGAAAGAGACATTTAGACCTGGGGGAGGAAATATGAAGGACAATTGGAGAAGTGAATTATATAAATATAAAG
TGGTAGAAATTAAACCATTG---GGAGTAGCACCCACAGAGGCAAAAAGGAGAGTGGTG--------------
-------------------------GAGAGAGAAAAAAGAGCA---GTAACA---ATAGGAGCT---------
ATGTTCCTTGGGTTCTTGGGAGCA---GCAGGAAGCACTATG---GGCGCGGCGTCA---
ATAACGCTGACGGTACAG---GCCAGACAA---CTGTTG---TCTGGTATAGTGCAACAGCAAAGC---
AATTTG---CTGAGA---GCTATAGAGGCGCAACAGCATATGTTGCAACTCACAGTCTGGGGCATT
>DQ351230
TTCTATTGCAATACAACAAGACTGTTTAATAATGATACAGGCGAACTGAATGATAATAAT-------------
----

AAAACAACAAACATCACTCTCCCATGCAGAATAAAACAAATTATAAACATGTGGCAAGAGGTAGGACGAGCAA
TGTATGCCCCTCCCATTGAAGGAAACATAACATGTATATCAAAT---
ATCACAGGACTACTATTGCTACGAGATGGAGGAACAACAAATAACACAATTAAT-------------------
----
-
GGCACAGGGAAAAAGAATGATACAGTCAGACCTGGAGGAGGAAATATGAAAGACAATTGGAGAAGTGAATTAT
ATAAATATAAAGTGATAGAACTTAAACCATTG---GGAATAGCACCCACTGAGGCAAGAAGGAGAGTGGTG--
-------------------------------------GAGAGAGAAAAAAGAGCA---GTGGGA---
ATAGGAGCT---------GTGTTCCTTGGGTTCTTGGGAGCA---GCAGGAAGCACTATG---
GGCACGGCGTCA---ATAACGCTGACGGTACAG---GCCAGACAA---CTGCTG---
TCTGGTGTAGTGCAACAGCAAAGC---AATTTG---CTGAGA---
GCTATAGAGGCGCAACAACATCTGTTGCAACTCACAGTCTGGGGCATA
>DQ275656
TTCTATTGCAATACATCACAATTGTTTAATAGTACATACACACATAATAGTACATATAATAGTACAGAA----
----

ACTAATGGAACCATCACACTTTCATGCAGAATAAAGCAATTTATAAACATGTGGCAGAGGGTAGGACGAGCAA
TGTATGCCCCTCCCATTGCAGGAAACATAACATGTAACTCAAGT---
ATCACAGGACTACTATTAGTACGTGATGGAGGCGATACAAATAAA----------------------------
----

AGTAACACAGAGACATTCAGACCTGAAGGAGGAAATATGAAGGACAATTGGAGAAGTGAATTATATAAATATA
AAGTAGTAGAAATTAAGCCATTG---GGAATAGCACCCACTAATGCAAAAAGGAGAGTGGTG-----------
----------------------------GAGAGAGAAAAAAGAGCA---GTGGGA---ATAGGAGCT------
---GTGTTCCTTGGGTTCTTGGGAGCA---GCAGGAAGCACTATG---GGCGCAGCGTCA---
ATAACGCTGACGGTACAG---GCCAGACAA---TTGTTG---TCCGGTATAGTGCAACAGCAAAGC---
AATTTG---CTGAGA---GCTATAGAGGCGCAACAACATCTGTTGCAACTCATGGTCTGGGGCATT
>DQ445632
TTCTATTGCAATACATCAAGTCTGTTTAATGGTACATACAAGGCTAATAGTAATAGTACAAACAGTACTTCAA
AA---

GTAAATGAAACCATCACACTCCCATGCAGAATAAAACAAATTATAAACATGTGGCAGGGGGTAGGACGCGCAA
TGTATGCCCCTCCAATTGCAGGAACCATAACATGTAAATCAAAT---
ATCACAGGAATACTATTGACACGTGATGGAGGCAGTGGCGATAAT----------------------------

----

GAAACAGAAGAGATATTCAGACCTGCAGGAGGAGATATGAGGGACAATTGGAGAAGTGAATTATATAAATATA
AAGTGGTAGAAATTAAGCCATTG---GGACCAGCTCCTACTGAGGCAAAAAGGAGAGTAGTG-----------
----------------------------GAGAGAGAAAAAAGAGCA---GTGGGA---ATAGGAGCT------
---GTGTTCCTTGGGTTCTTGGGAGCA---GCAGGAAGCACTATG---GGCGCAGCATCA---
ATAACGCTGACGGTACAG---ACCAGACAA---TTGTTG---TCTGGTATAGTGCAACAGCAAAGC---
AATTTG---CTGAAG---GCTATAGAGGCGCAACAACATCTGTTGCAGCTCACGGTCTGGGGCATT
>DQ396378
TTCTATTGCAATACAACAAACCTGTTCAGCACAAGTGTTAACATAACAAATAGT-------------------
----

ACAAATAGTAGCATCACACTCCCATGCAGAATAAAACAAGTTATAAACATGTGGCAGGGGGTAGGTCGAGCAA
TGTATGCCCCTCCCATTGCAGGAAACATAACATGTAACTCTACT---
ATCACAGGACTACTACTGACACGAGATGGAGGACAGGGAACAGAAACC-------------------------
----

AATGACACAGAGATATTCAGGCCTGGAGGAGGAGATATGAGGGACAATTGGAGAAGTGAATTATATAAATACA
AAGTGGTAGAAATTAAGCCATTG---GGACTAGCACCCACTGAGGCAAAAAGGAGAGTGGTG-----------
----------------------------GAGAGAGAAAAAAGAGCA---GTAGGA---ATAGGAGCT------
---GTACTCCTTGGGTTCTTGGGAGCA---GCAGGAAGCACTATG---GGCGCGGCGTCA---
ATAACGCTGACGGTACAG---GCCAGACAA---CTGTTG---TCTGGTATAGTGCAACAGCAAAGC---
AATTTG---CTGAGA---GCTATAGAGGCGCAACAGCATATGTTGCAACTCACGGTCTGGGGCATT
>DQ396386
TTCTATTGCAACACATCATCCCTGTTTAATAGAACATACCCGCTCAATAGTGCAGGAAACAGTTCAACAGGG-
----

AATAATTCAATCATCACACTCCAATGCAGAATAAAACAATTTATAAACATGTGGCAGGAGGTAGGACGAGCAA
TGTATGCCCCTCCCATTGCAGGAAACATAACATGTAAATCAAAT---
ATCACAGGACTACTATTGGTACGTGATGGAGGAACCACAAATGACTCA-------------------------
----

AATAACAAAGAGACATTCAGACCTGGAGGAGGAGATATGAGGGACAATTGGAGAAGTGAATTATATAAATATA
AAGTGGTAGAAATTAAGCCATTA---GGAATAGCACCCACTAAGGCAAAAAGGAGAGTGGTG-----------
----------------------------GAGAGAGAAAAAAGAGCA---GTGGGA---ATAGGAGCT------
---GTGTTCCTTGGGTTCTTGGGAGCA---GCAGGAAGCACTATG---GGCGCAGCATCA---
ATAACGCTGACGGTACAG---GCCAGACAA---TTGCTG---TCTGGAATAGTGCAACAGCAAAGC---
AATTTG---CTGAGG---GCTATAGAGGCGCAACAGCATATGTTGCAACTCACGGTCTGGGGCATT
>DQ396365
TTCTATTGTGATACATCACAACTGTTTAATGATACATTTAATAATACAGGA----------------------
----

TCTAATGCTACACTTACACTCCCCTGTAGAATAAAACAAATTATAAACATGTGGCAGGGGGTAGGACGAGCAA
TGTATGCCCCTCCCATTGCAGGAAATATAACATGTAAATCGAAG---
ATCACAGGACTACTATTGACAAGTGATGGAGGAAAAGACTCAAAT----------------------------
----

GCCACAGAGACGTTCAGACCTGGGGGAGGAAATATGAGAGACAATTGGAGAAGTGAATTATATAAATATAAAG
TGGTAGAAATTAATCCATTA---GGAGTAGCTCCCACTGGAGCAAAAAGGAGAGTGGTG--------------
-------------------------GAGAGAGAAAGAAGAGCA---GTGGGA---ATAGGAGCT---------
GTGTTCCTTGGGTTCTTAGGAGCA---GCAGGAAGCACTATG---GGCGCAGCATCA---
ATAACGCTGACGGTACAG---GCCAGACAA---TTATTG---TCTGGTATAGTGCAACAGCAAAGC---
AATTTG---CTGAGG---GCTATAGAGGCGCAACAGCATATGTTGCAACTCACAGTCTGGGGCATT
>DQ445635
TTCTATTGCAATACAACAAAACTATTTTCTAATGAGAGTGAG-------------------------------
----

TCAAACAAAACTATCACACTCCCATGCAGGGTAAAACAAATTATAAACATGTGGCAGGGAGTAGGACGAGCAA
TGTATGCCCCTCCTATTGCAGGAAATATAACATGTAAATCAAAT---
ATCACAGGACTACTATTGACACGAGATGGAGGAAACACAGGAGAA----------------------------

----

AACATAACAGAGATATTCAGACCGGGGGGAGGAAACATGAAAGACAATTGGAGAAATGAGTTATATAAATACA
AAGTGGTAGAAATTAAGCCATTG---GGAATAGCACCCACTGAAGCAAAAAGGAGAGTGGTG-----------
----------------------------GAGAGAGAAAAAAGAGCA---GTGGGA---ATAGGAGCT------
---GTGTTTCTTGGGTTCTTGGGAGCA---GCAGGAAGCACTATG---GGCGCAGCGTCA---
GTGGCGCTGACGGTACAG---GCCAGACAA---CTGCTG---TCTGGTATAGTGCAACAGCAAAGC---
AATTTG---CTGAGA---GCTATAGAGGCGCAACAGCATATGTTGCAACTCACAGTCTGGGGCATT
>DQ396368
TTCTATTGCAATACAACAAATCTGTTCAATGAACTGGATAATAAT----------------------------
----

AGTAGTGGAAACATCACACTCCCATGCAAGATAAAGCAATTTATAAACATGTGGCAACAAGTGGGACGAGCAA
TGTATGCCCCTCCCATTGAAGGAAAAATTACATGTAACTCAAAT---
ATCACAGGAATTCTATTGACAAGGGATGGAGGGATTAATGGTACA----------------------------
----

GAAACATTCAGACCTACAGGAGGAAATATGAAGGACAATTGGAGAAATGAATTATATAAATATAAAGTAGTAG
AAATTAAGCCATTG---GGAATAGCACCCACTGAAGCAAAAAGGAGAGTGGTG--------------------
-------------------GAGAGAGAAAAAAGAGCA---GTGGGA---ATAGGAGCT---------
GTATTCTTTGGGTTCTTGGGAGCA---GCAGGAAGCACTATG---GGCGCGGCGTCA---
ATAACGCTGACGGTACAG---GCCAGACAA---CTGTTG---TCTGGTATAGTGCAACAGCAAAGC---
AATTTG---CTGAGA---GCTATAGAGGCGCAACAGCATCTGTTGCAACTCACGGTCTGGGGCATT
>DQ396370
TTCTATTGCAATACGACAAGACTGTTTAATGAAAGTAATTCAGATATA-------------------------
----

ACAAATACCACCATCACACTCCCATGCAGAATAAAACAAATTATAAATATGTGGCAGGGAGTAGGACGAGCAA
TGTATGCCCCTCCCATTGAAGGAAACATAACATGTAACTCAAGT---
ATCACAGGATTACTATTGCTACGTGATGGAGGAAACAAGAATGATACA-------------------------
----

AATGATACAAACGAGATATTCAGACCTGGAGGAGGAAATATGAAGGACAATTGGAGAAGTGAATTATATAAAT
ATAAAGTGATAGAAATTAAGCCATTG---GGGGTAGCCCCCACCGAGGCAAAAAGAAGAGTGGTG--------
-------------------------------GAGAGAGAAAAAAGAGCA---GTGGGA---ATAGGAGCT---
------GTACTCCTTGGGTTCTTGGGAGCA---GCAGGAAGCACTATG---GGCGCGGCGTCA---
ATAACGCTGACGGTACAG---GCCAGACAA---CTGTTG---TCTGGTATAGTGCAACAGCAAAGC---
AATTTG---CTGAGA---GCTATAGAGGCGCAACAGCATATGTTGCAACTCACGGTCTGGGGCATT
>DQ396369
TTCTATTGTAATACAACAAAACTGTTTAATAACACACAGTGGTTGAACCTGACA-------------------
----

AACAATGACACAATCACACTCCAATGCAGAATAAAACAAATTATAAACATGTGGCAGGAGGTAGGACGAGCAA
TGTATGCCCCTCCCATTGCAGGTAACATAACGTGTAACTCAAGT---
ATCACAGGATTACTATTGACACGTGATGGAGGCAATAACGACACA----------------------------
----

AAGAATGAGACATTCAGGCCTGCAGGAGGAAATATGAAGGACAATTGGAGAAGTGAATTATATAAATATAAAG
TGGTAGAAATTAAACCATTG---GGAGTAGCACCCACGGAGGCAAAAAGGAGAGTGGTG--------------
-------------------------GAGAGAGAAAAAAGAGCA---GTGGGA---ATGGGAGCT---------
GTGATCCTTGGGTTCTTGGGAGCA---GCAGGAAGCACTATG---GGCGCGGCGTCA---
ATAACGCTGACGGTACAG---GCCAGACAA---CTGTTG---TCTGGTATAGTGCAACAGCAAAGC---
AATTTG---CTGAAA---GCTATAGAAGCGCAACAGCATATGTTGCAACTCACGGTCTGGGGCATT
>DQ369976
TTTTATTGCAGTACATCACGACTGTTTAATAGTACAGAAAGTGCAAATAGC----------------------
----

ACTAATGGTACCATCACACTTCCATGCAGAATAAAGCAAATTATAAACATGTGGCAGAAGGTAGGGCGAGCAA
TATATGCCCCTCCCATTGCAGGAAACATAACATGTAATTCAAGT---
ATCACAGGACTAATATTGGTACGTGATGGAGGAACAAATACAACAAAT-------------------------

----

AATAATAATGAGATATTCAGACCTGAAGGAGGAGACATGAGAGATAATTGGAGAAGTGAATTATATAAATATA
AAGTGGTAGAAATTGAGCCATTA---GGAATAGCACCCACTGGGGCAAAAAGGAGAGTGGTG-----------
----------------------------GAGAGAGAAAAAAGAGCA---GTGGGA---ATAGGAGCT------
---ATGTTCCTTGGGTTCTTGGGAGCA---GCAGGAAGCACTATG---GGCGCGGCGTCA---
ATGACGCTGACGGTACAG---GCCAGACAA---CTGTTG---TCTGGTATAGTGCAGCAGCAAAGC---
AATTTG---CTGAGG---GCTATAGAGGCGCAACAGCATATGTTGCAACTCACAGTCTGGGGCATT
>DQ396390
TTCTATTGCAATACAACAAAACTATTTAATAGTACAGAACTATTTAATAGTACAATCAATAACACAAACAGA-
----

AACATCACAAACATCACAATTCCATGCAGAATAAAACAAATCATAAACATGTGGCAAAAGGTAGGGCGAGCAA
TGTATGCCAATCCCATTGCAGGAAACATAATATGTAGATCAAAT---
ATCACAGGACTACTATTAGTACGTGATGGAGGCAGTAACAGTAAC----------------------------
----

ACAACAGAAATATTCAGACCTGGAGGAGGAAATATGAAGGACAATTGGAGAAGTGAATTATATAAATATAAAG
TGGTAGAAATTAAGCCATTG---GGAATAGCACCCACTGGAGCAAAAAGGAGAGTGGTG--------------
-------------------------GAGAGAGAAAAAAGAGCA---GTGGGA---ATAGGAGCT---------
GTACTCCTTGGGTTCTTGGGAGCA---GCAGGAAGCACTATG---GGCGCAGCGTCA---
ATAGCGCTGACGGCACAG---GCCAGACAA---GTGCTG---TCTGGTATAGTGCAACAGCAAAGC---
AATTTG---CTGAGG---GCTATAGAGGCGCAACAGCATCTGTTGCAACTCACGGTCTGGGGCATT
>DQ275657
TTCTATTGCAATACAACAAACCTGTTCAATACAACAAACCTGTTCAATACAACAAACCTGTTCAATGCCACTA
CT---

AATGAAAATGATAACATCACACTCCAATGCAAAATAAAACAAATTATAAACATGTGGCAGGGAGTAGGACGAG
CAATGTATGCCCCTCCCATTGAAGGAAACATAACATGTAACTCTAAT---
ATCACAGGACTACTATTGACACGTGATGGAGGAACAAACGCAACC----------------------------
----

AATAACACAGAGATATTCAGACCTGGAGGAGGAAATATGAAGGACAATTGGAGAAATGAATTATATAAATATA
AAGTAGTAGAAGTTAAGCCATTG---GGAGTAGCACCCACCAAGGCAAAAAGGAGAGTGGTG-----------
----------------------------GAGAGAGAAAAAAGAGCA---GTGGGA---CTAGGAGCT------
---GTGTTCCTTGGGTTCTTGGGAGCA---GCAGGAAGCACTATG---GGCGCGGCGTCA---
ATAACGCTGACGGTACAG---GCCAGACAA---CTATTG---TCTGGTATAGTGCAACAGCAAAAT---
AATTTG---CTGAGG---GCTATAGAGGAGCAACAGCATATGTTGCAACTCACGGTCTGGGGCATT
>DQ275643
TTCTATTGCAATACAACAAATCTGTTCAATACAACAAATCTGTTTGGAAATACTTCAACCGCAAACATCACA-
----

AATACAACAAAAATCACACTCCCATGCAGGATAAAACAAATTATAAACATGTGGCAGGGGGTAGGACGAGCAA
TGTATGCCCCTCCCGTTGAAGGAAACATAACATGTGAGTCAAAT---
ATCACAGGACTACTATTGACACGTGATGGAGGAAAAAACACAACA----------------------------
----

GAGGAAACAGAGACTTTTAGACCTGGAGGAGGAAATATGAAAGACAATTGGAGAAGTGAATTATATAAATATA
AAGTGGTAGAAATTAAACCATTG---GGAATAGCACCCACTGAGGCAAAAAGGATAGTGGTG-----------
----------------------------GAGAGAGAAAAAAGAGCA---GTGGGA---ATAGGAGCT------
---GTGCTCCTTGGGTTCTTGGGAGCA---GCAGGAAGCACTATG---GGCGCAGCGTCA---
ATAACGCTGACGGTACAG---GCCAGACAA---CTGTTG---TCTGGTATAGTGCAGCAGCAAAGC---
AATTTG---CTGAGA---GCTATAGAGGCGCAACAGCATATGTTGCAACTCACAGTCTGGGGCATT
>DQ396380
TTCTATTGCAATACATCAGGCCTGTTTAATAGTTCCAACAATACAGCAGTTACTGAT----------------
----

AATAATGGCACTATTACACTCCAATGCAGAATAAAACAAATTATAAACATGTGGCAGAAGGTAGGACGAGCAA
TGTATGCCCCTCCCATTGCAGGAAACATAACATGTAGATCAAAC---
ATCACAGGACTACTATTGACACGTGATGGAGGAAATAATAGTGAA----------------------------

----

AATGCAGAGATATTCAGACCTGGAGGAGGAGATATGAGGGACAATTGGAGAAGTGAATTATATAAGTATAAAG
TGGTAGAAATTAAGCCATTA---GGAGTAGCACCCACTACTGCAAAGAGGAGAGTGGTG--------------
-------------------------GAGAGACAGAAAAGAGCA---GTGGGA---CTAGGAGCT---------
GCGTTCTTCGGGTTCTTGGGAGCA---GCAGGAAGCACTATG---GGCGCGGCATCG---
ATAACGCTGACGGTACAG---GCCAGACGA---TTATTG---TCTGGTATAGTGCAACAGCAAAGC---
AATTTG---CTGAAA---GCCATAGAGGCGCAACAGCATATGTTGCAACTCACGGTCTGGGGCATT
>DQ275646
TTCTATTGTAATACAACAAACCTGTTTACAGAACTGTCTAATACAACAAAGGGGGAAGCTACAAATAAA----
----

AGTGATGAAAACATCACACTCCAGTGCAGAATAAAACAAATTATAAACATGTGGCAGGGGGTAGGACGAGCAA
TGTATGCCCCTCCCATTGAAGGAAACATAACATGTAACTCGAGT---
ATCACAGGACTGCTATTGACACGTGATGGAGGAAGAAATGACACA----------------------------
----

GAGACATTCAGACCTGGAGGAGGAAATATGAAAGACAATTGGAGAAGTGAATTATATAAATATAAAGTGGTAG
AAATTAAGCCATTG---GGAATAGCACCCACTACGGCAAAAAGGAGAGTGGTG--------------------
-------------------GAGAGAGAAAAAAGAGCA---GTAGTAGGAATAGGAGCT---------
GTACTCCTTGGGTTCTTGGGAGCA---GCAGGAAGCACTATG---GGCGCGGCGTCA---
ATAACGCTGACGGTACAG---GCCAGACAA---CTGTTG---TCTGGTATAGTGCAACAGCAAAGT---
AATTTG---CTGAGG---GCTATAGAGGCGCAACAGCATATGTTGCAACTCACGGTCTGGGGCATT
>DQ351231
TTCTATTGCAATACATCACAACTGTTTAAGGGTACATACAATGGTACATACAATGGTACAGAA----------
----

AGTAATTCAACCATCACACTTCCATGCAGAATAAAACAAATTATAAACATGTGGCAGGAGGTAGGACGAGCAA
TGTATGCCCCTCCCATTGAAGGAAATATAACATGTAACTCAAGT---
ATCACAGGACTACTATTAGTACGTGATGGAGGCAACAATAACACA----------------------------
----

AACGAGACATTCAGACCTATAGGAGGAGATATGAAGGACAATTGGAGAAGTGAATTATATAAATACAAAGTGG
TAGAAATTAAGCCATTG---GGACTAGCACCCACTGGAGCAAAAAGGAGAGTGGTG-----------------
----------------------GAGAGAGAAAAAAGAGCA---GTAGGA---ATAGGAGCT---------
GTGTTCCTTGGGTTCTTGGGAGCA---GCAGGAAGCACTATG---GGCGCGGCATCA---
ATAACGCTGACGGTACAG---GCCAGACAA---CTGTTG---TCTGGTATAGTGCAACAGCAAAAT---
AATTTG---CTGAGG---GCTATAGAGGCGCAACAGCATATGTTGCAACTCACGGTCTGGGGCATT
>DQ369981
TTCTATTGCAACACATCAGGACTGTTCAATAGTATATACAGTCCTACATACAGGCCTAATGGGACA-------
----

AGTGATTCAACCATCACACTCCCATGCAGAATAAAACAGATTATAAACATGCGGCAGGGGGTAGGACGAGCAA
TGTATGCCCCTCCCATTGAAGGAAGCATAACATGTAACTCAACC---
ATCACAGGACTACTATTGACACGTGATGGAGGAAAAGCAATGAAT----------------------------
----

AACACAGAGATATTCAGACCTGGAGGAGGAAATATGAAGGACAATTGGAGAAGTGAATTATACAAATATAAAG
TGGTAGAAATTAAGCCATTG---GGGGTAGCACCCACTGGGGCAAAAGGGAGAGTGGTG--------------
-------------------------GAGAAAGAAAAAAGAGCA---GTGGGA---ATAGGAGCT---------
GTGCTCCTTGGGTTCTTGGGAGCA---GCAGGAAGCACTATG---GGCGCGGCGTCA---
ATAACGCTGACGGTACAG---GCCAGACAA---CTGTTG---TCTGGTATAGTGCAACAGCAAAAT---
AATTTG---CTGAGA---GCTATAGAGGCGCAACAGCATATGTTGCAACTCACGGTCTGGGGCATT
>DQ396392
TTCTATTGCAATACATCAGGCCTGTTTAATGGTACATATAATAGTACAAATACATATAATAGTACAAATAGTA
CT---

GAAAATTCAAACATCACACTCCCTTGCAGAATAAAACAAATTGTAAACATGTGGCAGGAGGTAGGACAAGCAA
TGTATGCCCCTCCCATTGCAGGAAACATAACATGTAAATCAAAT---
ATCACAGGATTACTCTTGACACGTGATGGCGGAAACAATACAAAT----------------------------

----

GGGACAGAGACATTCAGACCTGGAGGAGGAGATATGAAGAACAATTGGAGAAGTGAATTATATAAATATAAAG
TGGTAGAAATTAAGCCATTA---GGAGTAGCACCCACTAAGGCAAGAAGGATGGTGGTG--------------
-------------------------GAGAGAGAGAAAAGAGCA---GTAGGA---ATAGGAGCT---------
GTGTTCCTTGGGTTCTTGGGAGCA---GCAGGAAGCACTATG---GGCGCGGCGTCA---
ATAACGCTGACGGTACAA---GCCAGACAA---TTATTG---TCTGGTATAGTGCAACAGCAAAGC---
AATTTG---CTGAGG---GCTATAGAGGCGCAGCAGCATATGTTGCAACTCACGGTCTGGGGCATT
>DQ396399
TTTTATTGCAATACATCAGGCCTGTTTAATAGTACACTGTTTAATAGTACTTCAAACAGTACTTCA-------
----

AACAGTACTGATATCATACTCCCATGCAGAATAAAACAAATTATAAACATGTGGCAGGAGGTAGGACGAGCAA
TGTATGCCCCTCCCATTGCAGGAAACATAACATGTAAATCAAAT---
ATCACAGGACTATTATTGACACGAGATGGAGGAAAAAACATAAGC----------------------------
----

AAAACCGAGACATTCAGACCTGGAGGAGGAAATATGAAGGACAATTGGAGAAGTGAATTATATAAATATAAAG
TAGTAGAAATTAAGCCATTA---GGAATAGCACCCACATCGGCAAAAAGGAGAGTGGTG--------------
-------------------------GAGAGAGAGAAAAGAGCA---GCA------ATAGGAGCT---------
ATGCTCCTTGGGTTCTTGGGAGCA---GCCGGAAGCACTATG---GGCGCAGCGTCA---
ATAGCGCTGACGGTACAG---GCCAGACAA---CTGCTG---TCTGGTATAGTGCAACAGCAAAGC---
AATTTG---CTGAGA---GCTATAGAGGCGCAACAGCATATGTTGCAACTCACGGTCTGGGGCATT
>AY901965
TTCTATTGCAATACATCAAAGCTATTTAATAATACATACAATGGTACATACTGGATATTTAATAATAATACAT
ACTGGAAT---
-------------------AGC---
AGCACAAACGAGAACATTACAATTCCATGCAGAATAAAACAAATTATAAACATGTGGCAGGAGGTAGGACGAG
CAATGTATGCCCCTCCCATTGCAGGAAACATAACATGTAACTCAAGT---
ATCACAGGATTACTATTGACACGTGATGGAGGAGACGAAACAAAC----------------------------
----

AATACAGAAACATTCAGACCTGGAGGAGGAAATATGAAGGACAATTGGAGAAGTGAATTATATAAATATAAAG
TGGTAGAAATTAAGCCATTG---GGAATAGCACCCACTGGAGCAAAAAGGAGAGTGGTG--------------
-------------------------GAGAGAGAAAAAAGAGCA---GTGGGA---ATAGGAGCT---------
GTACTCCTTGGGTTCTTGGGAGCA---GCAGGAAGCACTATG---GGCGCGGCGTCA---
ATAACGCTGACGGTACAG---GCCAGACAA---CTGTTG---TCTGGTATAGTGCAACAGCAAAGC---
AATTTG---CTGAGA---GCTATAGAGGCGCAACAGCATATGTTGCAACTCACAGTCTGGGGCATT
>DQ275660
TTCTATTGCAATACAACAGGCCTGTTTAACACAACACTGATTACTAATAGTAGTGAGGAG-------------
----

TCAAACGAAACCATCACACTCCCATGCAGAATAAAACAAATTGTAAACATGTGGCAGGAGGTAGGACGAGCAA
TGTATGCCCCGCCCATTGCAGGAAACATAACATGTATATCAAAT---
ATCACAGGAATACTATTGACATGGGATGGAGGAAATCTTAGTGAAACA-------------------------
----

AGCAATAGGAGTGAAATATTCAGACCTGCAGGAGGAAATATGAAGGATAATTGGAGAAGTGAATTATATAAAT
ATAAAGTAGTAGAAATTAAACCATTG---GGAGTAGCACCCACTGGGGCAAAAAGGAGAGTGGTG--------
-------------------------------GAGAGAGAAAAAAGAGCA---GTGGGA---CTAGGAGCT---
------GTGTTCCTTGGGTTCTTGGGAGCA---GCAGGAAGCACTATG---GGCGCGGCGTCA---
ATCACGCTGACGGTACAG---GCCAGACAA---CTGCTG---TCTGGTATAGTGCAACAGCAAAGC---
AATTTG---CTGAGA---GCTATAGAGGCGCAACAGCATATGTTGCAACTCACGGTCTGGGGCATT
>DQ351233
TTCTATTGTAATACATCACAACTGTTTAATAATACACACTGGTTTAATAATACACACGGGATTAATAGCACAA
AA---

GGTAATGAAAACATCACACTCCCATGTAGAATAAAACAAATTATAAACATGTGGCAGAAGGTAGGACAAGCAA
TGTATGCCCCTCCCATTGCAGGAAACATAACATGTGCCTCAAAT---
ATCACAGGACTACTATTGGTACATGATGGAGGAAATGAAACAAAA----------------------------

----

GAGATATTCAGACCTGGAGGAGGAGATATGAGAGACAATTGGAGAAGTGAACTATATAAATATAAGGTGGTAG
AAGTTAAGCCATTG---GGAGTAGCACCAACCAAGGCAAGAAGGAGAGTGGTG--------------------
-------------------GAGAGAGAAAAAAGAGCAGTAGCAGGAGGACTGGGAGCT---------
TTGTTCCTTGGGTTCTTGGGAGCA---GCAGGAAGCACTATG---GGCGCAGCATCA---
ATAACGCTGACGGTACAG---GCCAGACAA---TTGTTG---TCCGGCATAGTGCAACAGCAAAGC---
AATTTG---TTGAAG---GCTATAGAGGCGCAACAGCATTTGTTGCAACTCACGGTCTGGGGCATT
>AY878063
TTCTATTGCAATACATCAAAACTGTTTAATATAACAAGTGAGTCAATCAATATAAGTAATGAC----------
----

AGTAATGTAAACATCACACTCCCATGCAGAATAAAACAAATTATAAACATGTGGCAAGGGGTAGGAAAAGCAA
TGTATGCCCCTCCCATTAAAGGAAACATAACATGTAAATCAAAT---
ATCACAGGACTACTATTGACATGGGATGGAGGACACAAAGGGAATGACACA----------------------
----

GATAAGAACACAGAAACATTCAGACCTGGAGGAGGAAATATGAAGGACAATTGGAGAAGTGAATTATATAAAT
ATAAAGTAGTAGAAATTAAGCCATTG---GGGGTAGCACCCACCAAGGCAAAAAGGAGAGTGGTG--------
-------------------------------GAGAGAGAAAAAAGAGCA---GTGGGA---ATAGGAGCT---
------GTACTCCTTGGGTTCTTGGGAGCA---GCCGGAAGCACTATG---GGCGCGGCGTCA---
ATAACGCTGACGGTACAG---GCCAGACAA---CTGTTG---TCTGGTATAGTGCAACAGCAAAGC---
AATTTG---CTGAGA---GCTATAGAGGCGCAACAGCATATGTTGCAACTCACAGTCTGGGGCATT
>AY878064
TTCTACTGCAATACAACAAATCTGTTTAATACAACAGATTTGGTTAGTGCAACAAAGAAGGCTGGT-------
----

CCGAATGATAACATCACACTTCCATGCAGAATAAAACAAATTATAAACATGTGGCAGGAGGTAGGACGAGCAA
TGTATGCCCCTCCCATTGAAGGAAACATAACATGTAAATCAAAT---
ATCACAGGACTATTATTGACACGTGATGGAGGAATCACAAATGAT----------------------------
----

AATAAAACAGAGATATTCAGACCTGGAGGAGGAAATATGAAAGACAATTGGGGAAATGAATTATATAAATATA
AAGTGGTAGAGATTAAGCCATTG---GGAGTAGCACCCACTAAGGCAAAAAGGAGAGTGGTG-----------
----------------------------GAGAGAGAAAAAAGAGCA---GTGGGA---ATAGGAGCT------
---GTGCTCCTTGGGTTCTTGGGAGCA---GCAGGAAGCACTATG---GGCGCGGCGTCA---
ATGGCGCTGACGGTACAG---GCCAGACAG---TTGTTG---TCTGGTATAGTGCAACAGCAAAGC---
AATTTG---CTGAGG---GCTATAGAGGCGCAACAGCATATGTTGCAACTCACGGTCTGGGGCATT
>AY878065
TTCTATTGCAATACAACAAACTTGTTTAATAGGACATACACGCCTGAATACGTGTTTAATGGTACAAATAGC-
----

AATTCAAGCGTAATCACCCTCCCATGCAGGATAAAACAAATTATAAATATGTGGCAGCAGGTAGGACGAGCAA
TGTATGCTCCTCCCATTGCAGGAAACATAACATGTAACTCAACA---
ATCACAGGATTACTATTGATGCGTGATGGAGGAATAAGTAATAACACA-------------------------
----

AATAAAACAGAGACATTCAGACCTGGAGGAGGAGATATGAGGGACAATTGGAGAAGTGAATTATACAAATATA
AAGTGGTAGAAATTAAGCCATTA---GGAATAGCACCTACTGGGGCAAAAAGGAGAGTGGTG-----------
----------------------------GAGAGAGAAAAAAGAGCA---GTGGGA---ATAGGAGCT------
---GTGTTCCTTGGGTTCTTAGGAGCA---GCAGGAAGCACTATG---GGCGCGGCATCA---
ATAACGCTGACGGTACAG---GCCAGACAA---TTGTTG---TCTGGTATAGTGCAACAGCAAAGC---
AATTTG---CTGAGG---GCCATAGAGGCGCAACAGCATATGTTGCAACTCACGGTCTGGGGCATT
>AY878068
TTCTATTGCAATACATCAGGACTGTTTAATAGTACATACCGTCCTACATACAGGCAGAATGGGACAGAC----
----

AATAATTCAACCATCACACTGCCATGCAGAATAAAACAAATTATAAACATGTGGCAGGAGGTAGGACGAGCAA
TGTATGCCCCTCCTATTGCAGGAAAAATAACATGTAACTCAAGT---
ATCACAGGACTACTATTGACACGTGATGGAGGAACCAATAACACAGAG-------------------------

----

AATAACACAGAGATATTCAGACCTGGAGGAGGAAATATGAAGGACAATTGGAGAAGTGAATTATATAAATATA
AAGTGGTAGAAATTAAGCCACTA---GGAATAGCACCCACTACGGCAAAAAGGAGAGTGGTG-----------
---------------------------GAGAGAGAAAAAAGAGCA---GTGGGA---CTAGGAGCT------
---TTGTTCCTTGGGTTCTTGGGAGTA---GCAGGAAGCACTATG---GGCGCGGCGTCA---
ATAACGCTGACGGTACAG---GCCAGACAA---CTGTTG---TCTGGTATAGTGCAACAGCAAAAT---
AATTTG---CTGAGA---GCTATAGAGGCGCAACAGCATATGTTGCAACTCACGGTCGGGGGCATT
>AY878069
TTCTATTGTAATACATCAAAGCTGTTTAATAGTACAGAGCTGTTTAATAGTACAGAA---------------
----

AATAATTCAACAATCACACTCCCATGCAGGATAAAACAGATTATAAACATGTGGCAACAGGTAGGACGAGCAA
TGTATGCTCCTCCCATTGCAGGAAACATAACATGTAACTCAAGT---
ATCACAGGATTACTATTAGTGCGTGATGGAGGAACAAATGCCACA---------------------------
----

AATGAGACATTCAGACCTGGAGGAGGAGATATGAGGGACAATTGGAGAAGTGAATTATACAAGTATAAAGTGG
TAGAAATTAAACCATTG---GGAATAGCTCCAACTGATGCAAGAAGGAGAGTGGTG----------------
---------------------GAGAGAGAAAAAAGAGCA---GTGGGC---TTTGGAGCT---------
TTGATCATTGGGTTCTTGGGAGCA---GCAGGAAGCACTATG---GGCGCGGCGTCA---
ATAACGCTGACGGTACAG---GCCAGACAA---CTGTTG---TCTGGTATAGTGCAACAGCAAAGC---
AATTTG---CTGAGG---GCCATAGAGGCGCAACAGCATATGTTGCAACTCACGGTCTGGGGCATT
>AY901967
TTCTATTGCGATACAACAAACCTGTTTAATCTGACTAAAAAAGACAGAACCATCAAGGATGGA----------
----

AATGAAACCATCATCACACTCCCATGCAGAATAAAACAAATTGTAAGGATGTGGCAGGGGGTAGGACAAGCAA
TGTATGCCCCTCCCATTGCAGGAAACATAACATGTAGATCAAAT---
ATCACAGGACTACTATTGACACGTGATGGAGGAAAAACAAATGGCACAGGGAAC------------------
----

AATAAAACAGAGACAGAGATATTCAGACCTGCAGGAGGGAATATGAAGGACAATTGGAGAAGTGAATTATACA
AATATAAAGTGGTAGAAATTAAGCCATTG---GGAATAGCACCCGCCAAGGCAAAAAGGAGAGTGGTG-----
---------------------------------GAGAGAGAAAAAAGAGCA---GTGGGA---
ATGGGAGCT---------GTACTCCTTGGGTTCTTGGGAGCA---GCTGGAAGCACTATG---
GGCGCGGCGTCA---ATCACGCTGACGGTACAG---GCCAGACAA---GTGCTG---
TCTGGTATAGTGCAACAGCAAAGT---AATTTG---CTGAGA---
GCTATAGAGGCGCAACAGCATATGTTGCAACTCACGGTCTGGGGCATT
>AY901968
TTCTATTGCAATACAACACAACTGTTTAATGATAGTGTGGCAAAAGACAAC---------------------
----

AACACTAACATCACTACTATCCCATGCAAGATAAAACAAATTGTAAACATGTGGCAGGGGGTAGGACAAGCAA
TGTATGCCCCTCCCATTGCAGGAAACATAACATGTGAGTCAAAT---
ATCACAGGACTATTATTGACACGTGATGGAGGAAATGCAGGAAAAAATACT---------------------
----

ACAGATAATACAGAGATATTCAGACCTGGAGGAGGAAATATGAAGGACAATTGGAGAAGTGAATTATATAAAT
ATAAAGTGGTAGAAATTAATCCATTG---GGAGTAGCACCCACTGAGGCAAAAAGGAGAGTGGTG--------
------------------------------GAGAGAGAAAAAAGAGCA---GTGGGA---CTAGGAGCT---
------GTGTTCCTTGGGTTCTTGGGAGCA---GCAGGAAGCACTATG---GGCGCGGCGTCA---
ATAACGCTGACGGTACAG---GCCAGACAA---CTGTTG---TCTGGTATAGTGCAACAGCAAAGC---
AATTTG---CTGAGA---GCTATAGAGGCGCAACAGCATATGTTGCAACTCACAGTCTGGGGCATT
>DQ164113
TTCTATTGCAATACAACATACCTGTTTAATAATACAAGTGAT------------------------------
----

CTAAATAACACCATCACACTCCCATGCAGAATAAAACAAATTATAAACATGTGGCGGGAGGTAGGGAGAGCAA
TGTATGCCCCTCCCATTGCAGGAAACATAACATGTAACTCAAGT---

ATCACAGGACTACTATTGACACGTGATGGAGGAAGCACAAACGACACGAAT---------------------
----

AATACAAACCCAGAGATATTCAGACCTGGAGGAGGAAACATGAAAGACAATTGGAGAAGTGAATTATATAAAT
ATAAAGTGGTAGAAATTAAGCCATTG---GGAGTAGCACCCACGAAAGCAAAAAGGAGAGTGGTG--------
------------------------------CAGAGAGAAAAAAGAGCA---GTGGGA---ATGGGAGCT---
------GTGCTCCTTGGGTTCTTGGGAGCA---GCAGGAAGCACTATG---GGCGCGGCGTCA---
ATAACGCTGACGGTACAG---GCCAGACAA---CTGTTG---TCTGGTATAGTGCAACAGCAAAGC---
AATTTG---CTGAGA---GCTATAGAGGCGCAACAACATATGTTGCAACTCACGGTCTGGGGCATT
>AY901969
TTCTATTGCAATACATCAAAACTGTTTAATAGTACATATAATAGTACATATAATAGTAACAGTACAAATAGTA
AT--

TCAAACTCAACCATCACACTCCAATGCGGAATAAAACAAATTATAAACATGTGGCAGGGGGTAGGACAAGCAA
TGTACGCCCCTCCTATTGCAGGAAACATAACCTGTATATCAAAT---
ATCACAGGACTACTATTGACACGTGATGGAGGAGAAAACAGGTCA---------------------------
----

GAGGAGACATTCAGACCTGGAGGAGGAGATATGAGGGACAATTGGAGAAGTGAATTATATAAATATAAAGTGG
TGGAAATTAAACCATTA---GGAATAGCACCCACTAAGGCAAAAAGAAGAGTGGTG-----------------
---------------------GAGAGAGAAAAAAGAGCA---GTGGGA---ATAGGAGCT---------
GTGTTCCTTGGGTTCTTGGGAGCA---GCAGGAAGCACTATG---GGCGCGGCATCA---
CTGACGCTGACGGTACAG---GCCAGACAA---TTATTG---TCTGGTATAGTGCAACAGCAAAGC---
AATTTG---CTGAGA---GCTATAGAGGCGCAACAGCATATGTTGCAGCTCACAGTCTGGGGCATT
>DQ093593
TTCTATTGCAATACATCACAACTGTTTAACAGTACAGATAACAGTACACACATTGAAACAGGTAAGGCAAACA
TCACA---

GAAAGTGATGCAAACATCACACTGCCATGCAGAATAAAGCAAATTATAAACATGTGGCAGGAGGTAGGACGAG
CAATGTACGCCCCTCCCATTGCAGGAAACATAATATGTAACTCAAGT---
ATCACAGGACTCCTATTGACACGAGATGGAGGAAAGGGAGAAAACACA------------------------
----

ACTAACATGACAGAGACATTCAGACCTGGAGGAGGAAATATGAAGGACAATTGGAGAAATGAATTATATAAAT
ATAAAGTGGTAGAAATTAAACCATTG---GGAATAGCACCCACTAAAGCAAAAAGGAGAGTGGTG--------
------------------------------GAGAGAGAAAAAAGAGCA---GTGGGA---ATAGGAGCT---
------GTGCTCTTTGGGTTCTTGGGAGCA---GCAGGAAGCACTATG---GGCGCGGCGTCA---
ATAACGCTGACGGTACAG---GCCAGACAA---CTGTTG---TCTGGTATAGTGCAACAGCAAAGT---
AATTTG---CTGAGG---GCTATAGAGGCGCAACAGCATATGTTGCAACTCACGGTCTGGGGCATT
>AY901970
TTCTATTGTGATACAACAAAACTATTTAATGAAACATTATTTAATGATACAACAGAACTAGTTAATGGCACAA
CA--

AGTAATTCAAGCAACATCACACTCCCCTGCAGGATAAAACAAATTATAAACATGTGGCAGCAGGTAGGACGAG
CAATGTATGCTCCTCCCATTGCAGGAAACATAACATGTAACTCAAAT---
ATCACAGGATTACTATTGGTGCGTGATGGAGGAACACATAACAAAACAGAGACA------------AAT----
----
-
ACCACAGAAAATGAAACAGAGACATTCAGACCTGGAGGAGGAGATATGAGGGACAATTGGAGAAGTGAATTAT
ACAAATATAAAGTGGTAGAAATTAAACCATTA---GGAATAGCACCTACCAAGGCAAAACGGAGAGTGGTG--
-------------------------------------GAGAGAGAAAAAAGA---------GGA---
CTAGGAGCT---------GTGATCTTTGGGTTCTTGGGAGCA---GCAGGAAGCACTATG---
GGCGCAGCGTCA---ATAGCGCTGACGGCACAG---GCCAGACAA---GTGTTG---
TCTGGTATAGTGCAACAGCAAAGC---AATTTG---CTGAGG---
GCCATAGAGGCGCAACAGCATATGTTGCAACTCACGGTCTGGGGCATT
>AY901981
TTCTATTGTAATACAACAAAACTGTTTGATGATAGTGAAAGGATAAACACG---------------------
----

ACAACCACAACCATCATACTCCCATGCAGAATAAAACAAATTGTAAACATGTGGCAGGGGGTAGGACGAGCAA

TGTATGCCCCGCCCATTGAAGGAAACATAACATGTAAATCAAAT---
ATCACAGGATTACTATTGACACGTGATGGAGGAAACAATGGAACA----------------------------
----

AATGAGACATTCAGACCTGAAGGAGGAAATATGAAGGACAATTGGAGAAGTGAATTATATAAATATAAAGTAG
TGGAAATTAAGCCATTG---GGAGTAGCACCCACTGAGTCAAAAAGAAGAGTGGTG-----------------
----------------------GAGAGAGAAAAAAGAGCA---TTGGGA---ATAGGAGCC---------
GTGTTCCTTGGGTTCTTAGGAGCA---GCAGGAAGCACTATG---GGCGCGGCGTCA---
ATCACGCTGACGGTACAG---GCCAGACAA---GTGTTG---TCTGGTATAGTGCAACAGCAAAGT---
AATTTG---CTGAGA---GCTATAGAGGCGCAACAGCATATGTTGCAACTCACGGTCTGGGGCATT
>AY878057
TTCTATTGCAATACAACAAACCTGTTTAACATTAATGGAAGTGGAAGT-------------------------
----

AGTGGTAACAGCACAACACTCCCATGCAGGATAAAACAAATTATAAACATGTGGCAGGGGGTAGGACGAGCAA
TGTATGCCCCTCCCATAGAAGGAAACATAACATGTATATCAAAT---
ATCACAGGACTACTATTGACACGTGATGGAGGATACAATGTAACAGGAGAG----------------------
----

AATAATAAATCAACAGAGATATTCAGACCAGGAGGAGGAAATATGAAGGACAATTGGAGAAATGAATTATATA
AATATAAAGTGGTAGAAATTAAGCCATTG---GGAGTAGCACCCACTGAGGCAAAAAGGAGAGTGGTG-----
----------------------------------GAGAGAGAAAAAAGAGCA---GTGGGA---
ATAGGAGCT---------GTGTTCCTTGGGTTCTTGGGAGCG---GCAGGAAGCACTATG---
GGCGCGGCGTCA---ATAACGCTGACGGTACAG---GCCAGACAA---CTATTG---
TCTGGTATAGTGCAACAGCAAAGC---AATTTG---CTAAGA---
GCTATAGAGGCGCAACAGCACATGTTGCAACTCACGGTCTGGGGCATT
>AY878060
TTCTATTGCAATACACCAGGACTGTTTAATGGGACATACAATGAGAGTAGTGAAATA----------------
----

AGTAATGATACCATCAGACTCCCATGCAGAATAAAACAAATTATAAACATGTGGCAAGGGGTAGGACGAGCAA
TGTATGCCCCTCCCATTGCAGGGAACATAACTTGTAGCTCAAGT---
ATCACAGGACTACTATTGACACGGGATGGAGGAAAGGATAATGATAAT-------------------------
----

AATGGGAATGAGACATTCAGACCTGGAGGAGGAAATATGAAGGACAATTGGAGAAGTGAATTATATAAATATA
AAGTGGTAGAAATTAAGCCATTG---GGAATAGCACCCACTGGGGCAAAAAGGAGAGTGGTG-----------
----------------------------GAAAGAGAAAAAAGAGCA---GTGGGA---ATAGGAGCT------
---GTGTTCCTTGGGTTCTTGGGAGCT---GCAGGAAGCACTATG---GGCGCGGCGTCA---
ATAACGCTGACGGTACAG---GCCAGACAA---CTGTTG---TCTGGTATAGTGCAACAGCAAAGC---
AATTTG---CTGAGA---GCTATAGAGGCGCAACAGCATATGTTGCAACTCACGGTCTGGGGCATT
>AY878061
TTCTATTGCAATACATCAGAGCTGTTTAATAATAGTACAAGTAATGCAACTACA-------------------
----

AGTAATGCAACTATTACACTCCCATGCAGAATAAAACAAATTATAAACATGTGGCAGGGGGTAGGACGAGCAA
TGTATGCCCCTCCCATTGCAGGAAACATAACATGTAACTCAAGT---
ATCACAGGACTACTATTGACACGTGATGGAGCAATTAACAATACA----------------------------
----

GAGACATTCAGACCTGGAGGAGGAGATATGAGGGACAATTGGAGAAGTGAATTATATAAATATAAAGTGGTAG
AAATTAAACCATTA---GGAGTAGCACCCACTGATGCAAAAAGGAGAGTGGTG--------------------
-------------------GAGAGAGGAAAAAGAACA---GTGGGA---ATAGGAGCT---------
GTGTTCCTTGGGTTCTTGGGAGCA---GCAGGAAGCACTATG---GGCGCGGCGGCA---
GTAACGCTGACGGTACAG---GCCAGACAA---CTATTG---TCTGGTATAGTGCAACAGCAAAGC---
AATTTG---CTGAGA---GCTATAGAGGCGCAACAGCACATGTTGCAACTCACGGTCTGGGGCATT
>DQ396395
TTCTATTGCAATACAACAAAACTGTTTTCTTCTGAGTGGTTAAACAACACCAAGGAA----------------
----

AACATCACAACCATCACACTCCAATGCAGGATAAAACAATTTATAAACATGTGGCAGAAGGTAGGAAGAGCAA
TGTATAACCCTCCTATTGAAGGAAACATAACATGTAAATCAAAT---
ATCACAGGGCTATTATTGACAAGGGATGGAGGCGAAGGAGACAATACA-----------------------
----

AGAACAACAGAGATATTCAGACCTACAGGAGGAAATATGAAGGATAATTGGAGAAGTGAATTATATAAATATA
AGGTGGTAGAAATTAAGCCATTG---GGAGTAGCTCCCACTGAATCAAAAAGGAGAGTGGTG-----------
---------------------------GAGAGAGAAAAAAGAGCA---GTGGGA---ATAGGAGCT------
---GTACTCCTTGGGTTCTTGGGAGCA---GCAGGAAGCACTATG---GGCGCGGCGTCA---
ATAACGCTGACGGTACAG---GCCAGACAA---CTGTTG---TCTGGTATAGTGCAACAGCAAAGC---
AATTTG---CTGAGA---GCCATAGAGGCGCAACAGCATATGTTGCAACTCACAGTCTGGGGCATT
>DQ056410
TTCTATTGCAATACATCAGGACTATTTAATGGTACATACAATAGTACAGAAAGTAATTCA------------
----

ACAGATAAAAACACCACAATGCAATGCAGAATAAAACAAATTATAAACATGTGGCAGGGGGTAGGACGAGCAA
TGTATGCCCCTCCCATTGCAGGAAACATAACATGTAAATCAAAC---
ATCACAGGACTACTATTAGTACGTGATGGAGGCAACGACAACAAAACT-----------------------
----

AATGACACAGAAACATTCAGACCTGGAGGAGGAGATATGAGGGACAATTGGAGAAGTGAATTATATAAATATA
AAGTGGTAGAAATTAAGCCATTG---GGAATAGCTCCAACTGAAGCAAGAAGGAGAGTGGTG-----------
---------------------------GAGAGAGAGAAAAGAGCA---GTAGGA---ATAGGAGCG------
---TTGATCCTTGGGTTCTTGGGAGCA---GCAGGAAGCACTATG---GGCGCGGCGTCA---
ATAGCGCTGACGGCACAG---GCCAGACAA---GTGCTG---TCTGGTATAGTGCAACAGCAAAAC---
AATTTG---CTGAGA---GCTATAGAGGCACAACAGCATCTGTTGCAACTCACAGTCTGGGGCATT
>DQ056411
TTCTACTGTAATACAACAGAACTGTTTACAAACAATATCACAACAAATGGCACCAACACA------------
----

ACAAATGGCAACATCACCATCCCATGCAGGATAAAACAAATTGTAAACATGTGGCAGCAGGTAGGACGAGCAA
TGTATGCCCCTCCCATTGCAGGAAACATAACATGTAGATCAAAT---
ATTACAGGACTACTATTGACACGTGATGGTGGTATCACAAATGGC--------------------------
----

ACAGAGACATTCAGACCTGAAGGAGGAAATATGAAGGACAATTGGAGAAGTGAATTATATAAATATAAAGTGG
TAGAACTTAAGCCATTG---GGAATAGCACCCACCAAGGCAAAAAGGAGAGTGGTG----------------
----------------------GAGAGAGGAAAGAGAGCA---GTGGGA---ATAGGAGCC---------
TTGTTCCTTGGGCTCTTGGGAGTA---GCAGGAAGCACTATG---GGCGCGGCGTCA---
GTGACGCTGACGGCACAG---GCCAGACAA---CTGTTG---TCTGGTATAGTGCAACAGCAAAAC---
AATTTG---CTGAGA---GCTATAGAGGCGCAACAACATATGTTGCAACTCACGGTCTGGGGCATT
>DQ056404
TTCTATTGCAATGCATCACAACTGTTTAACGAGACACTGCTTAACGAGACTATGACTAATGGTACA-------
----

ACCAATGCAACCATCACACTCCCATGCAGAATAAAACAAATTATAAACATGTGGCAGGAGGTAGGACGAGCAA
TATATGCCCCTCCCATTGAAGGAAACATAACATGTACATCAAAT---
ATCACAGGACTACTATTGGTACGTGATGGAGGAAATGATGGAGGAAAT-----------------------
----

AACACACAGACCGAGACATTCAGACCTGGAGGAGGAGATATGAGGAACAATTGGAGAAGTGAATTATATAAAT
ATAAAGTGGTAGAAATTAAGCCACTG---GGAGTAGCACCCACTGAGGCAAAAAGGAGAGTGGTG--------
------------------------------GAGAGAGAAAAAAGAGCA---GCA------CTAGGAGCT---
------ATGCTCTTTGGGTTCTTGGGAGCA---GCCGGAAGCACTATG---GGCGCAGCATCA---
ATAACGCTGACGGTACAG---GCCAGACAA---TTGTTG---TCTGGAATAGTGCAACAGCAAAGC---
AATTTG---CTGAGG---GCTATAGAGGCGCAACAGCATATGTTGCAACTGACGGTCTGGGGCATT
>DQ351237
TTCTATTGCAATACATCAGGCCTGTTTAATGTTAATAGTAGTACACTTAAAAACAGTACAGGT----------
----

GCTAATGGTACAATCACACTCCAATGCAGAATAAAACAAATTATAAACATGTGGCAGAAGGTAGGACGAGCAA
TGTATGCCCCTCCCATTGCGGGAAATATAACATGTAACTCAACT---
ATCACAGGACTCCTATTGACACGCGATGGAGGAACAGGAGAAGAA----------------------------
----

AATACTACAGAGACATTCAGACCTGGAGGAGGAGATATGAGGGACAATTGGAGAAGTGAATTATATAAATATA
AAGTGGTAGAAGTTAAGCCATTA---GGAATAGCACCCACTACTGCAAAAAGGAGAGTGGTG-----------
----------------------------GAGAGAGAGAAAAGAGCA---GTGGGA---ATAGGAGCT------
---ATGTTCCTTGGGATCTTGGGAGCA---GCAGGAAGCACTATG---GGCGCAGCATCA---
ATAACGCTGACGGTACAG---GCCAGACAA---TTATTG---TCTGGTATAGTGCAACAGCAAAGC---
AATTTG---CTGAGA---GCTATAGAGGCGCAACAGCATATGTTGCAACTCACGGTCTGGGGCATA
>DQ056408
TTCTATTGTAATACAACAAAGCTGTTTAATAGTACATACATGCCTAATGGTACATACATGTACATGCCTAATG
ATACAGAA---
-------------------AGT---
AATTCAAGTGAAGTAATCACACTCCCATGCAGGATAAAACAAATTATAAACATGTGGCAGCAGGTAGGACGAG
CAATGTATGCTCCTCCCATTGCAGGGAACATAACATGCATATCAAAT---
ATCACAGGATTACTATTGGTGAGAGATGGAGGAAAAGGAAATAGCACAGATAAT-------------------
----
-
AAAGAGGCAGAAAAACCAGAGATATTCAGACCTGAAGGAGGAGATATGAGAAACAATTGGAGAAGTGAATTAT
ACAAATATAAAGTAGTAGAAGTTAAACCATTG---GGAATAGCACCTACTAAGGCAAAAAGAAGAGTGGTG--
-------------------------------------GAGAGAGAAAAAAGAGCA---GTGGGA---
CTAGGAGCT---------GTGTTCCTTGGGTTCTTGGGAGCA---GCAGGAAGCACTATG---
GGCGCGGCGTCA---ATAACGCTGACGGTACAG---GCCAGACAA---TTGTTG---
TCTGGTATAGTGCAACAGCAAAGC---AATTTG---CTGAGG---
GCCATAGAGGCGCAACAGCATATGTTGCAACTCGCGGTCTGGGGCATT
>DQ011169
TTCTATTGCAATACAACAGGTCTGTTTAGTAATAATGGAAGTACC----------------------------
----

CAAAATGAGACCATCACACTCCCATGCAGAATAAAACAAATTGTAAACATGTGGCAGGGGGTAGGACGAGCAA
TGTATGCCCCTCCCATTGCAGGAACCATAACATGTAAATCAAAT---
ATCACAGGATTATTATTGCTACGTGATGGAGGAGACACCAGCACG----------------------------
----

AATGGCACAGAGATATTCAGACCTGGAGGAGGAAATATGAAAGACAATTGGAGAAGTGAATTATATAAATATA
AAGTGGTAGAAATTAAGCCATTA---GGAGTAGCACCCACGACGGCAAAAAGGAGAGTGGTG-----------
----------------------------GAGAGAGAAAAAAGAGCA---GTGGGA---ATAGGAGCT------
---GTGCTCTTTGGGTTCTTGGGAGCA---GCAGGAAGCACTATG---GGCGCGGCGTCA---
ATAACGCTGACGGTACAG---GCCAGACAA---CTGTTG---TCTGGTATAGTGCAACAGCAAAGC---
AATTTG---CTGAGA---GCTATAGAGGCGCAACAGCATATGTTGCAACTCACGGTCTGGGGCATT
>DQ011176
TTCTATTGCAATACAACAAAACTGTTTAATGCAACAGACAAAGACAATATAACACAATGGGCCAACACAACAA
AC---

AGTACTGAAACCATCACAATCCCATGCAGAATAAAACAAATTGTAAACATGTGGCAGGGGGTAGGACGAGCAA
TGTATGCCCCTCCCATTGCAGGAAACATAACATGTAAATCAAAT---
ATCACAGGACTACTACTGACATGGGATGGTCCGTATAACAAAAGC----------------------------
----

AGTACGAATGAGACATTCAGACCTGGAGGAGGAAATATGAAGGACAATTGGAGAAGTGAATTATATAAATATA
AAGTGGTAGAAATTAAGCCATTG---GGAGTAGCACCCACTGAGGCAAAAAGGAGAGTGGTG-----------
----------------------------GGGAGAGAAAAAAGAGCA---GCA------CTAGGAGCT------
---TTGCTCCTTGGGTTCTTGGGAGCA---GCAGGAAGCACTATG---GGCGCGGCGTCA---
ATAACGCTGACGGTACAT---GCCAAACAA---CTGATG---GCTGGTATAGTGCAACAGCAAAGC---
AATTTG---CTGAGA---GCTATAGAGGCGCAACAGCATATGTTGCAACTCACAGTTTGGGGCATT
>DQ093601
TTCTATTGCAATACAACAAAACTGTTTTCTAATTCTAATGACAGTGGTTCAAAC-------------------
----

ACAACTACCATAACCACCATCCCATGTAGGATAAAACAAATTGTAAACTTGTGGCAGGGGGTAGGAAGAGCAA
TGTATGCCCCTCCCATTGAAGGGAACATAACATGTAACTCAAGT---
ATCACAGGATTACTATTGACACGTGATGGTGGCTTCAACAACACA---------------------------
----

ACAGAGATATTCAGACCTGAAGGAGGAAATATGAAGGACAATTGGAGAAGTGAATTATATAAATATAAAGTGG
TAGAAATTAAGCCATTG---GGAGTAGCACCCACTAAGGCAAAAAGGAGAGTGGTG----------------
----------------------GAGAGAGAAAAGAGAGCA---GTGGGG---ATAGGAGCT---------
TTGTTCCTTGGGTTCTTGGGAGCA---GCAGGAAGCACTATG---GGCGCGGCGTCA---
ATGACGCTGACGGTACAG---GCCAGACAA---CTGTTG---TCCGGTATAGTGCAACAGCAAAAC---
CATTTG---CTGAGA---GCCATAGAGGCGCAACAGCATATGTTGCAACTCACGGTCTGGGGCATT
>DQ093596
TTCTATTGCAATACATCAAACCTGTTTAGTGACATGTATAAT------------------------------
----

GATACCATCACACTCCCATGCAAAATAAAGCAAATCATAAACATGTGGCAGGAGGTAGGACGAGCAATGTATG
CCCCTCCCATTGCAGGAAACATAACATGTAATTCAAGT---
ATCACAGGATTACTACTGACACGTGATGGAGGCACGAATGGAGGCACG------------------------
----

AATGACACCACAGAAACATTCAGACTTGGAGGAGGAAATATGAAGGACAACTGGAGAAGTGAATTATATAAGT
ACAAGGTGGTAGAAATTAAGCCATTA---GGAGTAGCACCCACTGGAGCAAAAAGGAGAGTGGTG--------
------------------------------GAGAGAGAAAAAAGAGCA---GTGGGA---ATGGGAGCT---
------GTGCTCCTTGGGTTCTTGGGAGCA---GCAGGAAGCACTATG---GGCGCGGCGTCA---
ATAACGCTGACGGTACAG---GCCAGACAA---CTGTTG---TCTGGTATAGTGCAACAGCAAAGC---
AATTTG---CTGAGA---GCTATAGAGGCGCAACAGCATCTGTTGCAACTCACGGTCTGGGGCATT
>DQ093607
TTCTATTGTGACACATTAAAACTGTTTAATAATAGTCTGGTTAATGATAGTGCT------------------
----

ACAAACGACTCAATCACCATCCCATGCAGAATAAAGCAAATTATAAATATGTGGCAAAAGGTAGGACGAGCAA
TGTATGCCCCTCCCATTGCAGGAAACATAACATGTAGATCAAAT---
ATCACAGGACTACTATTGACACGTGATGGAGGAACAATTAATGCAACT------------------------
----

GAAAATAATATAGAGACATTCAGACCTGGAGGAGGAGATGTGAGGGACAATTGGAGAAGTGAATTATATAAAT
ATAAAGTGGTAGAAATTAAGCCATTA---GGAATAGCACCCACTGAAGCAAAAAGGAGAGTGGTG--------
------------------------------GAGAGAGAAAAAAGAGCA---GTGGGA---ATAGGAGCC---
------GTGTTCCTTGGGTTCTTGGGAGCA---GCAGGAAGCACTATG---GGCGCAGCGTCA---
ATAACGCTGACGGTACAG---GCCAGACAA---CTATTG---TCTGGTATAGTGCAACAGCAAAGC---
AATTTG---CTGAGG---GCTATAGAGGCGCAACAGCATATGTTGCAACTCACGGTCTGGGACATT
>DQ164108
TTCTATTGCAATACATCAGCACTGTTTAATGGTACATACAATGGGACAGAAAGTAATTCA------------
----

ACTGAGTCAAACATCACACTTCCATGCAGAATAAAACAAATTGTAAACATGTGGCAGGAGGTAGGACGAGCAA
TGTATGCCCCTCCCATTGAAGGAATAATAACATGTAACTCAAGT---
ATCACAGGACTACTATTGACACGTGATGGAGGACAAACAAACAAAACA------------------------
----

AATGGCACAGAGATATTCAGACCTGGAGGAGGAAATATGAAGGACAATTGGAGAAATGAATTGTATAAATATA
AAGTGGTAGAAATTAAGCCATTG---GGAATAGCACCCTCTAAGGCAAAAAGGAGAGTGGTG----------
----------------------------GAGAGAGAAAAAAGAGCA---GCAGGA---ATGGGAGCT------
---GTGCTCCTTGGGTTCTTGGGAGCA---GCAGGAAGCACTATG---GGCGCGGCGTCA---
ATAACGCTGACGGTACAG---GCCAGACAA---CTGTTG---TCTGGTATAGTGCAACAGCAAAAT---
AATTTG---CTGAGG---GCTATTGAAGCGCAACAGCATATGTTGCAACTCACGGTCTGGGGCATT
>DQ351234
TTCTATTGCAACACATCAAAACTGTTTAATAGTACCATGTCTAATGGTACCATGTTTAATAGTACAGGA----
----

AATAATTCAGTCATCACACTCCAATGTAGAATAAAACAAATTATAAATATGTGGCAGGAGGTAGGACGAGCAA
TGTATGCCCCTCCCATTGCAGGAACCATCACATGTAACTCTAAT---
ATCACAGGACTACTATTGACACGTGATGGTGGAAATAATGAGACA----------------------------
----

GAAACGTTCAGACCTGAAGGAGGAGATATGAGGGATAATTGGAGAAGTGAATTATATAAATATAAAGTGGTAG
AAATTAAGCCATTA---GGAGTAGCACCCACTGAGGCAAAAAGGAGAGTGGTG--------------------
-------------------GAGAGAGAAAAAAGAGCA---GTGGGA---ATGGGAGCT---------
GTGTTCCTTGGGTTCTTGGGAGCA---GCAGGAAGCACTATG---GGCGCAGCGGCA---
GTGACGCTGACGGTACAG---GCCAGACAA---TTGCTG---TCTGGAATAGTGCAACAGCAAAGC---
AATTTG---CTGAGA---GCTATAGAGGCGCAACAGCATATGTTGAAACTCACGGTCTGGGGCATT
>DQ275650
TTTTATTGCAATACATCAGCACTATTTAATAGTTCGTTTAATGGTACATACATGAACAGTACAGAA-------
----

GGTAATTCAAACATCACAATCCCACGCAGAATAAAACAAATTATAAACATGTGGCAGGAGGTGGGAAAAGCAA
TGTATGCCCCTCCCATTGCAGGAAACATAACATGTAAATCAAAT---
ATCACAGGACTACTATTGATACGTGATGGAGGAACAGGCAATGAAATA-------------------------
----

GACAATACAACAGAGACATTCAGACCTGGAGGAGGAGATATGAGGGACAATTGGAGAAGTGAATTATATAAAT
ATAAAGTGGTAGAAATTAAGCCATTA---GGAATAGCACCCACTGAGGCAAAAAGGAGAGTGGTG--------
-------------------------------CAGAGGGAGAAAAGAGCA---GTGGGA---ATAGGAGCT---
------GTGCTCCTTGGGTTCTTGGGAGCA---GCAGGAAGCACTATG---GGCGCAGCATCA---
ATAACGCTGACGGTACAG---GCCAGACAA---TTATTG---TCTGGTATAGTGCAACAGCAAAGC---
AATTTG---CTGAGA---GCTATAGAGGCGCAACAACATATGTTGCAACTCACAGTCTGGGGCATT
>KT183301
TTCTATTGTAATACATCAAGACTATTTAATGGTAATGGTACACACACACAAGATAATAGTACAAAAGAG----
----

ACCAACAAGACCATCACAATTACATGCAAAATAAAACAAATAATAAATATGTGGCAAGAGGTAGGACGAGCAA
TGTATGCCCCTCCCATTGCAGGAAACATAACATGTAAATCAAAT---
ATCACAGGACTACTGTTGACACGTGATGGAGGAGGAAATAGTACT----------------------------
----

GATGAGATCTTCAGACCTGGAGGAGGAGATATGAGAGACAATTGGAGAAGTGAATTATATAAATATAAAGTGG
TAGAAATTAAACCATTA---GGAATAGCACCCACTAGTGCAAAAAGGAGAGTGGTG-----------------
----------------------GAGAGAGAAAAAAGAGCA---GTGGGA---ATAGGAGCT---------
GTGTTCCTTGGGTTCTTGGGAACA---GCAGGAAGCACTATG---GGCGCGGCGTCA---
ATAACGCTGACGGTACAG---GCCAGACAA---TTATTG---TCTGGTATAGTGCAGCAGCAAAGC---
AATTTG---CTGAGA---GCCATAGAGGCGCAACAGCATCTGTTGCAACTCACGGTCTGGGGCATT
>KC863229
TTCTATTGCAATACATCAAAACTGTTTAATAAGAATGGTACA-------------------------------
----

GAAAAGAATACAACCATCACACTCCCATGCAGAATAAAACAAATTATAAACATGTGGCAGGAGGTAGGACGAG
CAATATATGCCCCTCCCATTAAAGGAAACATAACATGTAGCTCAAAC---
ATCACAGGACTACTATTGACACGGGATGGAGGAGAAACAAACATAAAAAACACA-------------------
----
-
GAGACAGACAATACCACAGAGACATTCAGACCTGAAGGAGGAGATATGAGAAATAATTGGAGAAGTGAATTGT
ATAAATATAAAGTGGTAGAAGTTAAGCCACTA---GGAGTAGCACCTACTGAGGCAAAAAGGAGAGTGGTG--
-------------------------------------GAGAGAGAAAAAAGAGCA---GTGGGA---
ATAGGAGCT---------GTGTTCCTTGGGTTCTTGGGAGCA---GCAGGAAGCACTATG---
GGCGCGGCGTCA---ATAACGCTGACGGTACAG---GCCAGACAA---TTGTTG---
TCTGGAATAGTGCAACAGCAAAGC---AATTTG---CTGAAG---
GCTATAGAGGCGCAACAGCATATGTTGCAACTCACGGTCTGGGGCATT
>AY772690

TTCTATTGTGATACAACAAAACTATTTAATGAAAGTGAGTTT-------------------------------
----

AATGAGGACGCAATCACGCTTCCATGCAAGATAAAACAATTTATAAACATGTGGCAGGGGGTGGGACGGGCAA
TGTACGCCCCTCCCATTGAAGGAAACATAACATGTAACTCAAGT---
ATCACAGGATTACTATTGACACGTGATGGAGGCAATACCAGTAGC----------------------------
----

CGAGGAGAGATATTCAGACCTGGAGGAGGAAATATGAAGGACAATTGGAGAAATGAATTATATAAATATAAAG
TGGTAGAAATTAAGCCATTG---GGAGTAGCACCCACTGATGCAAAAAGGAGAGTGGTG--------------
-------------------------GAGAGAGAAAAAAGAGCA---GTGGGA---ATAGGAGCT---------
GTGTTCCTTGGGTTCTTGGGAGCA---GCAGGAAGCACTATG---GGCGCGGCGTCA---
ATAACGCTGACGGTACAG---GCCAGACAA---CTGTTG---TCTGGTATAGTGCAACAGCAAAGC---
AATTTG---CTGAGA---GCTATAGAGGCGCAACAGCATATGTTGCAACTCACAGTCTGGGGCATT
>AY772691
TTCTATTGCAATACATCAGGCCTGTTTAATGGTACATATGATGTTAATAATACAAACAGTACTTCAAATACAA
GC---

AGTAATTCATCCATCATACTTAAATGCCGAATAAAACAAATTATAAACATGTGGCAGGGGGTAGGACGAGCGA
TGTATGCCCCTCCCATTGCAGGAAACATAACATGTAAATCAAAT---
ATTACAGGATTACTATTGACACGTGATGGAGGAACGGGAAATGACACT-------------------------
----

GACACAGAAACAGAAACATTCAGACCTGGAGGAGGAAATATGAGGGACAATTGGAGAAGTGAATTATATAAAT
ATAAGGTAGTAGAAATTAAACCACTA---GGAATAGCACCCACTGAGGCAAAAAGGAGAGTGGTG--------
-------------------------------GAGAGAGAAAAAAGAGCA---GTGGGA---CTAGGAGCT---
------GTGTTCCTTGGGTTCTTAGGAGCA---GCAGGAAGCACTATG---GGCGCAGCATCA---
ATAACGCTGACGGTACAG---GCCAGACAA---CTATTA---TCTGGTATAGTGCAACAGCAAAAC---
AATCTG---CTGAGA---GCTATAGAGGCGCAACAACATATGTTGCAACTCACAGTCTGGGGCATT
>AY703908
TTCTATTGCAATACAGCAGACCTGTTTACTAATGTAAGTAGA-------------------------------
----

AATGGGACCATCACACTCCCATGCAGAATAAAACAAATTGTAAACATGTGGCAGGGGGTAGGACGAGCAATGT
ATGCCCCTCCCATTGAAGGAAACATAACATGTAAATCAAAT---
ATCACAGGATTATTATTGCTACGTGATGGAGGAAACATGGGAGGAGACACCAGT-------------------
----
-
ACGAATGGCATAAAGAACGAGACATTCAGACCTGGGGGAGGAAATATGAAAGACAATTGGAGAAGTGAACTAT
ATAAATATAAAGTGGTAGAAATTAAGCCATTG---GGAGTGGCACCCACAGGGGCAAAAAGGACAGTGGTG--
-------------------------------------GAGAGAGAAAAAAGAGCA---GTGGGG---
ATAGGAGCT---------GTGCTCTTTGGGTTCTTGGGAGCA---GCAGGAAGCACTATG---
GGCGCGGCGTCA---ATAACGCTGACGGTACAG---GCCAGACAA---TTGTTG---
TCTGGTATAGTGCAACAGCAAAGC---AATTTG---CTGAGA---
GCTATAGAGGCGCAACAGCATATGTTGCAACTCACAGTCTGGGGCATT
>AY772693
TTCTATTGCGATACAACAAACCTGTTTAATGAAAGTATAGTTTCTAGTAAAAGTGAG----------------
----

TTAAACGAAACCATCATACTCCCATGCAGGATAAAACAAATTATAAACATGTGGCAGGGGGTAGGACGAGCAA
TGTATGCCCCTCCCATTGCAGGAAACATAACATGTAAATCAAAT---
ATCACAGGACTACTATTGACACGTGATGGAGGAAAAAACACAACA----------------------------
----

AAAGAGATATTCAGACCTGGAGGAGGAAATATGAAAGACAATTGGAGAAGTGAATTATATAAATATAAAGTAG
TAGAAATTAAGCCATTG---GGAATAGCACCCACTGGGGCAAAAAGGAGAGTGGTG-----------------
----------------------GAGAGAGAAAAAAGAGCA---GTGGGA---ATAGGAGCT---------
GTGTTCCTTGGGTTCTTGGGAGCG---GCAGGAAGCACTATG---GGCGCGGCGTCG---
ATAACGCTGACGGTACAG---GCCAGACAA---CTGTTG---TCTGGTATAGTGCAACAGCAAAGT---
AATTTG---CTGAGA---GCTATAGAGGCGCAACAACATATGTTGCAACTCACAGTCTGGGGCATT

>AY772700
TTCTATTGTAATACAACAAACCTGTTTAATCCTAATGGTACAGAGGGAAGT----------------------
----

CCAAACTCAACCATCACACTCCAATGCAGAATAAAACAAATTATAAACATGTGGCAGGAGGTAGGACGAGCAA
TGTATGCTCCGCCCATTGCAGGAAACATAACATGTAAATCAAAC---
ATCACAGGAATACTATTGACACGTGATGGAGGAAACAATGATAATAATACA----------------------
----

GATAATAATACAGAGACATTCAGACCTGGAGGAGGAAATATGAAGGATAATTGGAGAAGTGAATTATATAAAT
ATAAAGTGGTAGAAATTAAACCATTG---GGAGTAGCACCAACTGAAGCAAAAAGGAGAGTGGTG--------
-------------------------------GAGAGAGAAAAAAGAGCA---GTGGGA---ATAGGAGCT---
------GTGTTCCTTGGGTTCTTGGGAGCA---GCAGGAAGCACTATG---GGCGCGGCGTCA---
ATAACGCTGACGGTACAG---GCCAGACAA---CTGTTG---TCTGGTATAGTGCAACAGCAAAGC---
AATTTG---CTGAGA---GCTATAGAGGCGCAACAACATATGTTGCAACTCACGGTCTGGGGCATT
>AY772694
TTCTATTGCAATACATCAGGACTGTTTAATAGTACACACAATTCTACATTCTGGAATGAGACACATTGGCCAT
ACTGGGAG---
-------------AATACA------
ACAGATAATTTAAACATCACACTCCCATGCAGAATAAAACAAATTATAAACATGTGGCAGGAGGTAGGACGAG
CAATGTATGCTCCTCCCATTGCAGGAAACATAACATGTAAATCAAAT---
ATCACAGGACTACTATTAGTACGTGATGGAGGCAAACACAACAACACA-------------------------
----

AACGAGACAGAGATATTCAGACCTGCAGGAGGAGATATGAAGGACAATTGGAGAAGTGAATTATATAAATATA
AAGTGGTAGAAGTCAAGCCATTG---GGGATAGCACCCACTGGAGCAAAAAGGAGAGTGGTA-----------
----------------------------AAGAGAGAAAAAAGAGCA---GTGGGA---ATAGGAGCT------
---GTGCTCCTTGGTTTCTTGGGAGCA---GCCGGAAGCACTATG---GGCGCGGCGTCA---
ATAACGCTGACGGTACAG---GCCAGACAG---CTGCTG---TCTGGTATAGTGCAACAGCAAAAT---
AATTTG---CTGAGG---GCTATAGAGGCGCAACAGCATATGTTGCAACTCACAGTCTGGGGCATT
>AY772701
TTCTATTGTAATACAACAGAACTATTTGATACAACAAAAATATTTAATGGCACAGAA----------------
----

GGTAATTCAAACATCACACTCCCCTGCAGGATAAAACAAGTTATAAACATGTGGCAGCAGGTAGGACGAGCAA
TGTATGCTCCTCCCATTGCAGGAAATATAACATGTAACTCAAGT---
ATCACAGGATTACTATTGGTGCGTGATGGAGGAACAAGTAACACAAATAAC----------------------
----

ACAGAGAATAACACAGAGACATTCAGACCTGGAGGAGGAGATATGAGGGACAATTGGAGAAGTGAGTTATACA
AATATAAAGTGGTAGAAATTAAACCATTA---GGAATAGCACCTACCAAGGCAAAAAGGAGAGTGGTG-----
----------------------------------GAGAGAGAAAAAAGAGCA---GCGGGA---
ATAGGGGCT---------GTGATCCTTGGGTTCTTGGGAGCA---GCAGGAAGCACTATG---
GGCGCAGCGTCA---ATAGCGCTGACGGCACAG---GCCAGACAA---TTGTTG---
TCTGGTATAGTGCAACAGCAAAGC---AATTTG---CTGAGG---
GCCATAGAGGCGCAACAGCATATGTTGCAACTCACGGTTTGGGGCATC
>AY772695
TTCTATTGTAATACAACAAACCTGTTCAGTAAGAATGTAAGT-------------------------------
----

AATGATAACATCATACTCCCATGCAGAATAAAACAAATTATAAATATGTGGCAGAAGGTGGGACGAGCAATGT
ATGCCCCTCCCATTGCAGGAAACATAACATGTAACTCAAAT---
ATCACAGGACTACTACTGACACGTGATGGAGGCACAAATAACACA----------------------------
----

GAGATATTCAGACCTGGAGGAGGAAATATGAAGGACAATTGGAGAAATGAATTATATAAATATAAAGTGGTAG
AAATTAAGCCATTG---GGAGTAGCACCCACTGAGGCAAAAAGAATAGTGGTG--------------------
-------------------GAGAGAGAAAAAAGAGCA---GTAGGA---ATAGGAGCT---------
GTACTCCTTGGGTTCTTGGGAGCA---GCAGGAAGCACTATG---GGCGCGGCGTCA---

GTGACGCTGACGGTACAG---GCCAGACAA---CTGTTG---TCTGGTATAGTGCAACAGCAAAGC---
AATTTG---CTGAGA---GCTATAGAGGCGCAACAGCATATGTTGCAACTCACAGTCTGGGGCATT
>AY772696
TTTTATTGCGATACAACAAACCTGTTCAATCGTTCTAAGGATAAT----------------------------
----

GAAAATGATACCATCATACTCCCATGCAGAATAAAACAATTTATAAACATGTGGCAGGGGGTAGGACGAGCAA
TGTATGCCCCTCCCATTGAAGGATACATAACATGTAACTCAAGT---
ATCACAGGACTACTACTGACACGTGATGGAGGTGGTAATGAGACA----------------------------
----

AGGAAAAATGAGACATTCAGACCTGGAGGAGGAAATATGAAGGACAATTGGAGAAGTGAATTATATAAATATA
AAGTGGTAGAAATTAAGCCATTG---GGAATAGCACCCACTGAGGCAAAAAGGAGAGTGGTG-----------
----------------------------GAGAGAGAAAAAAGAGCA---GTTGGA---ATAGGAGCT------
---GTGCTCCTTGGGTTCTTGGGAGCA---GCAGGGAGCACTATG---GGCGCAGCGTCA---
GTAACGCTGACGGTACAT---GCCAGAAAA---CTGTTG---GCTGGTATAGTGCAACAGCAAAGC---
AATTTG---CTGAGA---GCTATAGAGGCGCAACAGCACATGTTGCAACTCACAGCCTGGGGCATT
>DQ164115
TTCTATTGCAATACATCAAATCTGTTCAATACAACCACTAAT-------------------------------
----

AACATTACAAACATCACACTCCCATGCAGAATAAAACAATTTATAAACATGTGGCAGGAGGTAGGACGAGCAA
TGTATGCCCCGCCCATTGAAGGAAACATAACATGTAGGTCAAAT---
ATCACAGGACTACTATTGGTACGTGATGGAGGAAAAAGGAATAACACGAAT----------------------
----

GACAATGCCACAGAGATATTCAGACCTGGAGGAGGAAATATGAAGGACAATTGGAGAAGTGAATTATATAAAT
ATAAAGTAGTAGAAATTAAACCATTG---GGAATAGCACCCACAAAAGCAAAAAGGAGAGTGGTG--------
-------------------------------GAGAGAGAAAAAAGAGCA---GTGGGA---ATAGGAGCT---
------GTACTCCTTGGGTTCTTGGGAGCA---GCAGGAAGCACTATG---GGCGCGGCGTCA---
ATAACGCTGACGGTACAG---GCCAGACAA---CTGTTG---TCTGGTATAGTGCAACAGCAAAGC---
AATTTG---CTGAGA---GCTATAGAGGCGCAACAGCATATGTTGCAACTCACGGTCTGGGGCATT
>DQ351221
TTCTATTGTGATACAGCACAACTGTTTAACGAAAGTGAGGCAAACATAACCAGT-------------------
----

GAGGCAAACATAACCACTCTCCAATGCAGGATAAAACAAATTATAAACATGTGGCAGGGGGTAGGACGAGCAA
TGTATGCCCCTCCCATTGCAGGAAACATAACATGTAAATCAAAT---
ATCACAGGACTATTGTTGACACGTGATGGAGGAAACGCAGAAAAC----------------------------
----

ACAACGGAGACATTCAGACCTGGAGGAGGAAATATGAAGGACAATTGGAGAAGTGAATTATATAAATATAAAG
TGGTAGAAATTAAGCCATTG---GGAGTAGCACCCACTGAGGCAAAAAGGAGAGTGGTG--------------
-------------------------GAGAGAGAAAAAAGAGCA---GTGGGA---CTAGGAGCT---------
GTGTTCCTTGGGTTCTTGGGAGCA---GCAGGAAGCACTATG---GGCGCGGCGTCA---
ATAACGCTGACGGTACAG---GCCAGACAG---CTGTTG---TCTGGTATAGTGCAACAGCAAAGC---
AATTTG---CTGAGA---GCTATAGAGGCGCAACAGCATATGTTGCAACTCACGGTCTGGGGCATT
>DQ164121
TTCTATTGCAATACGTCAAATCTGTTTAATAGTACATATACTAGCAACATTACA-------------------
----

GGAAATTCAAGCATCACACTCCCATGCAGAATAAAACAAATTATAAACATGTGGCAGGAGGTAGGACAAGCAA
TGTATGCCCCTCCCATTAGAGGAAACATAACATGTATCTCAAAG---
ATCACAGGACTACTATTGACACGTGATGGAGGAAACAATAGGACT----------------------------
----

AATGAGACCTTCAGACCTGGAGGAGGAGATATGAGGGACAATTGGAGAAATGAATTATATAAATATAAAGTGG
TAGAAATTAAGCCATTG---GGAATAGCACCCACTGAGGCAAAAAGGACAGTGGTG-----------------
----------------------GAGAGAGAAAAAAGAGCA---GTGGGA---ATAGGAGCT---------
GTGTTCCTTGGGTTCTTAGGAGCA---GCAGGAAGCACTATG---GGCGCGGCGTCA---

ATAACGCTGACGGTACAG---GCCAGACAA---CTGTTG---TCTGGTATAGTGCAACAGCAAAGC---
AATTTG---CTGAGA---GCTATAGAGGCGCAACAGCATATGTTGCAACTCACAGTCTGGGGCATT
>DQ396393
TTCTATTGCAATACATCACTACTGTTTAATAGTATAGCAAGTAATAGTACAGAAAGTAATAGTACAAAAAGCA
GT---

AGTACAGAAAAGAAGATCACACTTCTATGCAGAATAAAACAAATTATAAACATGTGGCAGGAGGTGGGACGAG
CAATGTATGCCCCTCCCATTGCCGGAAACATAACATGTATATCAAAT---
ATAACAGGGATAATATTAACACGTGATGGAGGAGAACAGACAAAT----------------------------
----

GACACAGAGACATTCAGACCTGGAGGAGGAGATATGAGGGACAATTGGAGAAGTGAATTATATAAATATAAAG
TGGTAGAAATTAAGCCATTG---GGAGTAGCACCAACTGCGGCAAAAAGGAGAGTGGTG--------------
-------------------------AAGAGAGAAAAAAGAGCA---GTGGGA---ATAGGAGCT---------
GTGTTCCTTGGGTTCTTGGGAGCA---GCAGGAAGCACTATG---GGCGCGGCGTCA---
ATAACGCTGACGGTACAG---GCCAGACAA---CTGTTG---TCTGGTATAGTGCAACAGCAAAGC---
AATTTG---CTGAGG---GCTATAGAGGCGCAACAGCATATGTTGCAACTCACTGTCTGGGGCATT
>DQ396394
TTCTATTGCAATACATCAGGCTTGTTTAATAATGCTACAGGAAATGCATCAGGCTTGGTTAATAATGCTACAG
GA---

GATGGGTCAGTCATCACACTGCCATGCAAAATAAAACAAATAATAAACATGTGGCAGGAGGTAGGACGAGCAA
TGTATGCCCCTCCCATTGCAGGAAACATAACATGTAACTCAAGT---
ATCACAGGACTACTATTGACACGGGATGGAGGAAATACCAATGGC----------------------------
----

ACTGAAATATTCAGACCTGGAGGAGGAAATATGAAGGACAATTGGAGAAGTGAATTATATAAATATAAAGTAG
TAGAAATCAAGCCATTA---GGAGTAGCACCCACCGAGGCAAAAAGGAGAGTGGTG-----------------
----------------------GAGAGAGAAAAAAGAGCA---GTGGGA---ATAGGAGCT---------
GTTCTCCTTGGGTTCTTGGGAGCA---GCAGGAAGCACTATG---GGCGCGGCGTCA---
ATAACGCTGACGGTACAG---GCCAGACAA---CTGTTG---TCTGGTATAGTGCAACAGCAAAGC---
AATTTG---CTGAGA---GCTATAGAGGCGCAACAGCATATGTTGCAACTCACAGTCTGGGGCATT
>DQ164122
TTCTATTGCAGTACATCAAAGCTGTTTAATGCTACAATGGGTAATGATAAGTTGCTTAATGATACTTCA----
----

GATAACTCAACCATCACACTCCCATGCAGAATAAAACAATTTATAAACATGTGGCAGGGGGTTGGACGAGCAA
TGTATGCCCCGCCCATTGCAGGAAACATAACATGTAGATCAAAT---
ATCACAGGACTACTATTGACACGTGATGGAGGAGTAGGTAATGGGACA-------------------------
----

GAGAATAATACAGAGATATTCAGACCTGGAGGAGGAGATATGAAGGACAATTGGAGAAGTGAATTATATAAAT
ACAAAGTGGTAGAAGTGATGCCATTG---GGAATAGCACCCACTGGAGCAAAAAGGAGAGTGGTG--------
-------------------------------GAGAGAGAAAAAAGAGCA---GTGGGA---ATAGGAGCT---
------GTGTTCCTTGGGTTCTTGGGAGCA---GCAGGAAGCACTATG---GGCGCAGCGTCA---
ATAACGCTGACGGTACAG---GCCAGACAA---TTGTTG---TCTGGTATAGTGCAACAGCAAAAC---
AATTTG---CTGAGG---GCTATAGAGGCGCAACAGCATATGTTGCAACTCACGGTCTGGGGCATT
>DQ164117
TTCTATTGCAATACATCAGGACTATTTAATGGGACATTTGATGGTACAGAAAGTAATTCAACA----------
----

AGTAATGCAACCATCACAATTCCATGCAGAATAAAACAAATTATAAACATGTGGCAGGGGGTAGGACGAGCAA
TGTATGCCCCTCCCATTGCAGGAAACATAACATGTAAATCAAAT---
ATCACAGGACTACTATTAGTACGTGATGGAGGCAACACAGACAACACA-------------------------
----

AATGGCACAGAAATATTCAGACCTGGAGGAGGAGATATGAAGGACAATTGGAGAAGTGAATTATATAAATATA
AAGTGGTAGAAATTAAGCCATTG---GGAATAGCACCCACTGGAGCAAAAAGGAGAGTGGTG-----------
----------------------------AAGAGAGAAAAAAGAGCA---GTGGGA---ATAGGAGCT------
---GTGCTCTTTGGGTTCTTGGGAGCA---GCAGGAAGCACTATG---GGCGCAGCGTCA---

ATAGCGCTGACGGCACAA---GCCAGACAA---GTATTG---TCTGGTATAGTGCAACAGCAAAAC---
AATTTG---CTGAGG---GCTATAGAGGCGCAACAGCATATGTTGCAACTCACGGTCTGGGGCATT
>DQ164118
TTCTATTGTAATACATCAGACCTGTTTAATAGGCCTATAAATGAGTCTAATGGTACAGAG-------------
----

GATAATTCAACCATCACACTTCCATGCAGAATAAAACAAATTATAAACCTGTGGCAGGAGGTAGGACGAGCAA
TGTATGCCCCTCCCATTGAAGGAAACATAACATGTAAATCAAAT---
ATCACAGGACTACTATTGGTACGTGATGGAGGCAACACAACTGAA----------------------------
----

AACACACCTGAGATATTCAGACCTGGAGGAGGAGATATGAGGGACAATTGGAGAAATGAATTATACAAATATA
AAGTAGTAGAAATTGAGCCATTG---GGAATAGCACCCACTAAAGCAAAAAGGAGAGTGGTG-----------
----------------------------GAGAGAGAAAAAAGAGCA---GTGGGA---ATAGGAGCT------
---GTGTTCCTTGGGTTCTTGGGAGCA---GCAGGAAGCACTATG---GGCGCGGCGTCA---
CTAACGCTGACGGTACAG---GCCAGACAA---CTGTTG---TCTGGTATAGTGCAACAGCAAAGC---
AATTTG---CTGAGA---GCCATAGAGGCGCAACAGCATATGTTGCAACTCACGGTATGGGGCATT
>DQ445637
TTCTACTGCAATACGACAGAACTGTTCAACATCACTAGCAAT-------------------------------
----

ACAACTACCATCACACTCCCATGCAGAATAAAACAAATTATAAACATGTGGCAGGAGGTAGGACGAGCAATGT
ATGCCCCTCCCATTGCAGGAAACATAACATGTAACTCAAGT---
ATCACAGGATTACTATTAACACGTGATGGAGGCGGCACCCCAAATAACAAA----------------------
----

TCAGAGGGTAACGAAACATTCAGACCTGGAGGAGGAAATATGAAGGATAATTGGAGAAGTGAATTATATAAAT
ATAAAGTGGTAGAAATTAAGCCATTG---GGGATAGCACCCACTAATGCAAAAAGGAGAGTGGTG--------
-------------------------------GCGAGAGAAAAAAGAGCA---GTGGGA---GTAGGAGTT---
------ATGTTCCTTGGGTTCTTGGGAGCA---GCAGGAAGCACTATG---GGCGCAGCGGCA---
GTGACGCTGACGGTACAG---GCCAGACAA---CTGTTG---TCTGGTATAGTGCAACAGCAAAAC---
AATTTG---CTGAGA---GCTATAGAGGCGCAACAGCATCTGTTGCAGCTCACGGTCTGGGGCATT
>DQ164110
TTCTATTGCAATACAACAGAACTGTTTTCTAATAAAAGTAATTCAGGCAACACAACA----------------
----

GACAAAGCAACCATCACACTCCCATGCAGAATAAAACAAATTGTAAACATGTGGCAGGAGGTAGGACGAGCAA
TGTATGCCCCTCCCATTGAAGGAAAAATAACATGCAATTCAAGT---
ATCACAGGACTACTATTGACACGTGATGGGGGAGAAGGAGGAAACACA-------------------------
----

ACCAACGAAACAGAGATATTTAGACCTGCAGGAGGAAATATGAAGGACAATTGGGGAAGTGAATTATATAAAT
ATAAAGTGGTAGAAATCAAGCCATTG---GGAATAGCTCCCACTGAGGCAAGAAGGAGAGTAGTG--------
-------------------------------GAGAGAGAAAAAAGAGCA---GCA------CTAGGAGCT---
------GTGTTCCTTGGGTTCTTGGGAGCA---GCAGGAAGCACTATG---GGCGCGGCGTCA---
ATAACGCTGACGGTACAG---GCCAGACAA---CTGTTG---TCTGGTATAGTGCGACAGCAAAGC---
AATTTG---CTGAGA---GCTATAGAGGCGCAACAGCACCTGTTGCAACTCACGGTCTGGGGCATT
>DQ093598
TTTTATTGCAATACATCAGGCCTGTTTAATACATCAGGTCTGTCTAGGAATGGTACAGAA-------------
----

AATAATGCAAGCATCACACTCCCATGTAAAATAAAACAAATTATAAACATGTGGCAGGAGGTAGGACGAGCAA
TGTATGCCCCTCCCATTGCAGGGAACATAACATGCTCCTCAAAT---
ATCACAGGACTACTATTGGTACGTGATGGAGGCGGAGATAATAAC----------------------------
----

ACAGAAACATTCAGACCTGGAGGAGGGAATATGAAGGACAATTGGAGAAGTGAATTATATAAATATAAAGTGG
TAGAAATTAGGCCATTG---GGAGTAGCACCCACCAAGGCAAAAAGGAGAGTGGTG-----------------
----------------------GAGAGAGAAAAAAGAGCA---GTGGGA---CTGGGAGCT---------
GTGCTCTTTGGGTTCTTGGGAGCA---GCAGGAAGCACTATG---GGCGCGGCGTCA---

ATGACGTTGACGGTACAG---GCCAGACAA---TTGTTG---TCTGGTATAGTGCAACAGCAAAGC---
AATTTG---CTGAAG---GCTATAGAGGCGCAACAGCATCTGTTGCAACTCACGGTCTGGGGCATT
>DQ093599
TTCTATTGTGATACAACAAAGCTATTTAATAGTACATATAATCCTAATGTTACAACAAATGCAGCA-------
----

AATGGGTCAGTAATCACACTCCCATGCAGGATAAAACAATTTATAAATATGTGGCAGCAGGTAGGACGAGCAA
TGTATGCTCCTCCCATTGCAGGAAACATAACATGTAAATCAAAC---
ATCACAGGATTACTATTGGTGCGTGATGGAGGAAATCGCACAGAAACA-------------------------
----

GATAACACAGAGATATTCAGACCTGGAGGAGGAGATATGAGGGACAATTGGAGAAGTGAATTATACAAATATA
AAGTGGTAGAACTTAAACCATTG---GGAATAGCACCTACTAAGGCAAAAAGGAGAGTGGTG-----------
----------------------------GAGAGAGAAAAAAGAGCA---GTGGGA---ATAGGAGCT------
---GTGTTCCTTGGGTTCTTGGGATTA---GCAGGAAGCACTATG---GGCGCGGCGTCA---
ATAACGCTGACGGTACAG---GCCAGACAA---TTGTTG---TCTGGTATAGTGCAACAGCAAAGC---
AATTTG---CTGAGG---GCCATAGAGGCGCAACAGCATATGTTGCAACTCACGGTCTGGGGCATT
>DQ164126
TTCTATTGCAATACATCAGCACTGTTTAATAGTACATACAATGGTACATACATTAATGAGACAAAA-------
----

AGTAATGGGACCATCACACTTCCATGCAGAATAAAGCAAATTATAAACATGTGGCAGGGGGTAGGACGAGCAA
TGTATGCTAATCCCATTGAAGGAAATATAACATGTAAATCAAAT---
ATCACAGGAATACTATTAGTACGTGATGGAGGAAATGAAGACAGT----------------------------
----

ACAAACGAGACATTCAGACCTATAGGAGGAAATATGAAGGACAATTGGAGAAGTGAATTATATAAATATAAAG
TGGTAGAAATTAAGCCATTG---GGAATAGCACCCACTAGAGCAAAAAGGAGAGTGGTG--------------
-------------------------GACAGAGCAAAAAGAGCA---GTGGGA---CTAGGAGCT---------
GTGATCGTTGGGTTCTTGGGAGCA---GCAGGAAGCACTATG---GGCGCGGCGTCA---
ATAACGCTGACGGCACAG---GCCAGACAA---CTGTTG---TCAGGTATAGTGCAACAGCAGAAT---
AATCTG---CTGAGA---GCTATAGAGGCGCAACAACATCTGCTGCAACTCACGGTCTGGGGCATT
>DQ093588
TTCTATTGCAATACAACAGATCTGTTTAATGAAAGGTTTAATGAA----------------------------
----

AGTAATCCAAACATCACACTCCTATGCAGAATAAAACAGATTATAAACATGTGGCAGGGGGTAGGACGAGCAA
TGTATGCCCCTCCCATTAAAGGAAACATAACATGTACCTCAAAT---
ATCACAGGACTACTATTGACACATGATAGAGAAAGAAATGGCACA----------------------------
----

GAGACATTCAGACCTGGAGGAGGAAATATGAAGGACAATTGGAGAAATGAATTATATAAATATAAGGTGGTAG
AAGTTAAGCCATTG---GGAGTAGCACCCACTGAGGCAAAAAGGAGAGTGGTG--------------------
-------------------GAGAGAGAAAAAAGAGCA---GTGGGA---ATAGGAGCT---------
GTACTCCTTGGGTTCTTGGGAGCA---GCAGGAAGCACTATG---GGCGCGGCGTCA---
ATAACGCTGACGGTACAG---GCCAGACAA---CTGTTG---TCTGGTATAGTGCAACAGCAAAGC---
AATTTG---CTGAGA---GCTATAGAGGCGCAACAGCATATGTTGCAACTCACGGTCTGGGGCATT
>DQ164119
TTCTATTGTGATACAACAGAACTGTTTAATTCAACAGAACAGTCTAGTACAACAGAAGAGGAGAATAAG----
----

AATAATAATACAATCACACTCCAATGCAGAATAAAACAAATTGTAAACATGTGGCAGGGGGTAGGAAGAGCAA
TGTATGCCCCTCCCATTGCAGGAAATATAACATGTAACTCAACC---
ATCACAGGATTACTATTGACACGTGATGGTGGAATAATCAATAACACAGAA----------------------
----

GGTAACAAAACAGAGACATTCAGACCAGGAGGAGGAGATATGAGGGACAATTGGAGAGGTGAATTATATAAAT
ATAAAGTGGTAGAAATTAGACCATTG---GGAATAGCACCCACTGAGGCAAAAAGGAGAGTGGTG--------
-------------------------------GAGAGAGGAAAAAGAGCA---GTGGGA---ATAGGAGCT---
------GTGCTCTTTGGGTTCTTGGGAGCA---GCAGGAAGCACTATG---GGCGCGGCGTCA---

GTGGCGCTGACGGTACAG---GCCAGACAA---CTGTTG---ACTGGCATAGTGCAACAGCAAAGT---
AATTTG---CTGAGG---GCTATAGAGGCGCAACAGCATATGTTGCAACTCACGGTCTGGGGCATT
>DQ093585
TTCTATTGTAACACATCAACACTGTTTAATAATACAAATATC-------------------------------
----

ACAGGCATAGCCACAATCCCATGCAGAATAAAACAAATTATAAACATGTGGCAGGAGGTAGGACGAGCAACAT
ATGCCCCTCCCATTGCAGGAAACATAACATGTATCTCAAAT---
ATCACAGGACTACTATTGCAACGTGATGGAGGAAACATCACAGATGAAAAG----------------------
----

AACAGGTCCATAGAGACATTCAGACCTATAGGAGGAAATATGAAGGACAATTGGAGAAGTGAATTATATAAAT
ATAAAGTAGTAGAAATTAAGCCATTG---GGAATAGCACCCACGGGGTCAAAAAGGAGAGTGGTG--------
-------------------------------GAGAGAGAAAAAAGAGCA---GTGGGA---GTAGGAGCT---
------GTACTCCTTGGGTTCTTGGGAGCA---GCAGGAAGCACTATG---GGCGCGGCGGCA---
GTGACGCTGACGGTACAG---GCCAGACAA---CTGCTG---TCTGGTATAGTGCAACAGCAAAGC---
AATTTG---CTGAGA---GCTATAGAGGCGCAACAGCATATGTTGCAGCTCACGGTCTGGGGCATT
>DQ056412
TTCTATTGCAATACATCAAACCTGTTCCAGGTCAATAACTCTACA----------------------------
----

ACTAATGATACCATCACACTTCCATGCAGAATAAAACAAATTATCAACATGTGGCAGGGGGTAGGACGAGCAA
TGTATGCCCCTCCCATTGCAGGAAACATAACATGTAACTCAAGC---
ATCACAGGACTACTACTGACACGTGATGGAGGAGACAAAAATAGC----------------------------
----

ACAGACCCAGAGACATTCAGACCTGGAGGAGGAAATATGAAGGACAATTGGAGAAGTGAATTATATAAATATA
AAGTGGTAGAAATTAAGCCATTA---GGAATAGCCCCCACTAAGGCAAAAAGGAGAGTGGTG-----------
----------------------------GAGAGAGAAAAAAGAGCA---GTGGGA---CTAGGAGCT------
---GTACTCCTTGGGTTCTTGGGAGCG---GCAGGAAGCACTATG---GGCGCAGCGTCA---
ATAACGCTGACGGTACAG---GCCAGACAA---TTGCTG---TCTGGTATAGTGCAACAGCAAAGC---
AATTTG---CTGAGA---GCTATAGAGGCGCAACAGCATATGTTGCAACTCACAGTCTGGGGCATT
>AY878072
TTCTATTGCAATACAACACAACTGTTTAATGAAAGTCTGTTCAAT----------------------------
----

AACACAACAAACATCACACTCCCATGCAGAATAAAACAAATTATAAACATGTGGCAGGAAGTAGGACGAGCAA
TGTATGCCCCTCCCATTGCAGGAAACATAACATGTAACTCAAGT---
ATCACAGGACTACTATTGCTACGTGATGGAGGAACAGAGAATAAT----------------------------
----

GACACAGAGACATTCAGGCCTGGAGGAGGAAATATGAAAGACAATTGGAGAAGTGAATTGTATAAATATAAAG
TGGTAGGACTTAAGCCATTG---GGAGTAGCACCCACTGAGGCAAAAAGGAGAGTGGTG--------------
-------------------------GAGAGAGAAAAAAGAGCA---GTGGGA---CCGGGAGCT---------
GTGCTCTTTGGGTTCTTGGGAGCA---GCAGGAAGCACTATG---GGCGCGGCGTCA---
ATATCGCTGACGGTACAG---GCCAGACAA---TTGCTG---TCTGGTATAGTGCAGCAGCAAAGC---
AATTTG---CTGAGA---GCTATAGAGGCGCAACAGCATATGTTGCAGCTCACGGTCTGGGGCATT
>AY772699
TTCTATTGCAATACATCAAATCTGTTTAATATAACTGCAAGTAATGCAAGTGATGCA----------------
----

AACAACAACACCATCACACTCCCATGCAAAATAAAACAAATTATAAACATGTGGCAGGAGGTAGGACGAGCAA
TGTATGCCCCTCCCATTGCAGGAAACATAACATGTAACTCAAGT---
ATCACAGGACTACTATTGACACGTGATGGAGGCAATAACAATGACACAGGC----------------------
----

AATAACAATGACACAGAGATATTCAGACCTGGAGGAGGAAATATGAAGGACAATTGGAGAAGTGAGTTATATA
AATATAAAGTGGTAGAAATTAAGCCATTA---GGAATAGCACCCACTAAGGCAAAAAGGAGAGTGGTG-----
----------------------------------GAGAGAGAGAAAAGAGCA---GTGGGA---
CTAGGAGCT---------GTACTCCTTGGGTTCTTAGGAACA---GCAGGAAGCACTATG---

GGCGCGGCGTCA---ATAACGCTGACGGTACAG---GCCAGGCAA---CTGTTG---
TCTGGTATAGTGCAACAGCAAAGC---AATTTG---CTGAGG---
GCTATAGAGGCGCAACAGCACATGTTGCAACTCACGGTCTGGGGCATT
>DQ164114
TTTTATTGCAATACAACACAACTGTTTAATGAAGATGTTTTAAACACCACACTGTTTAATAAA----------
----

ACAGACACCAACATCACACTCCCATGCCGAATAAAACAAATTATAAACATGTGGCAGGGAGTAGGAAAAGCAA
TGTATGCCCCCCCCATTGAAGGACAAATAATATGTAACTCAAGT---
ATCACAGGAATACTACTGACACGGGATGGAGGAGCAGTAACGAATGGC-------------------------
----

ACGAATGTCACAGAGACATTCAGACCTGGGGGAGGAAATATGAAGGACAATTGGAGAAGTGAATTATATAAAT
ATAAAGTGGTAGAAATTAAGCCATTA---GGGGTAGCACCCACTGGGGCAAAAAGGAGAGTGGTG--------
-------------------------------GAGAGAGAAAAAAGAGCA---GTAGGA---ATAGGAGCT---
------GTGTTCCTTGGGTTCTTGGGAGCC---GCAGGAAGCACTATG---GGCGCGGCGTCA---
ATGACGCTGACGGTACAG---GCCAGACAA---CTGTTG---TCCGGTATAGTGCAACAGCAAAGC---
AATTTG---CTGAGA---GCCATAGAGGCGCAACAACATATGTTGCAACTCACGGTCTGGGGCATT
>AY901977
TTCTATTGTAATACAACAAACCTGTTTAATGAAAGTACGTCA-------------------------------
----

AACACAACCATCGGATTCCCATGCAGAATAAAACAAATTATAAACATGTGGCAGGGGGTAGGGCGAGCAATGT
ATGCCCCTCCCATTGAAGGAAACATAACATGTAAATCAAGT---
ATCACAGGGTTACTATTGGTACGTGATGGAGGAATAAATGACACA----------------------------
----

AAAGAGATATTCAGACCTGGAGGAGGAAATATGAAGGACAATTGGCGAAGTGAATTATATAAATATAAAGTGG
TAGAAATTAAGCCATTG---GGAGTAGCACCCACTGAGGCAAAAAGGAGAGTGGTG-----------------
----------------------GAGAGAGAAAAAAGAGCA---GTGGGA---ATAGGAGCT---------
GTGATCCTTGGGTTCTTGGGAGCA---GCAGGAAGCACTATG---GGCGCAGCGTCA---
ATAACGCTGACGGTACAG---GCCAGACAA---CTGTTG---TCTGGTATAGTGCAACAGCAAAGC---
AATTTG---CTGAGA---GCTGTAGAGACGCAACAGCATATGCTGCAACTCACGGTCTGGGGCATT
>AY901978
TTCTATTGCAATACAACAAAACTGTTTTCTAATGAAAGTGATGCT----------------------------
----

GACAACGGAACAATCACAATCCCATGCAGGATAAAACAAATCATAAGAATGTGGCAGGGGGTAGGACAAGCAA
TGTATGCCCCTCCCATTGTAGGAAACATAACATGTAACTCAAAT---
ATCACAGGACTACTCTTGGTACGTGATGGAGGAAAAAGCAATAAT----------------------------
----

AACACAGAGATATTTAGACCTGGAGGAGGAAATATGAAGGACAATTGGAGAAGTGAATTATATAAATATAAAG
TGGTAGAAGTTAAGCCATTG---GGAATAGCACCCACTAGAGCAAAAAGGAGAGTGGTG--------------
-------------------------GAGAGAGAAAAAAGAGCA---GTGGGA---ATAGGAGCT---------
GTGTTCCTTGGGTTCTTGGGAGCA---GCAGGAAGCACTATG---GGCGCGGCGTCA---
ATAACGCTGACGGTACAG---GCCAGACAA---CTGTTG---TCTGGTATAGTGCAACAGCAAAGC---
AATTTG---CTGAGA---GCCATAGAGGCGCAACAGCATATGTTGCAACTCACAGTCTGGGGCATT
>DQ011171
TTCTATTGCAATACATCAAACCTGTTTAATAAAACTATAACAGCAAATTCAACAAATTCAAATTCAACAAAT-
----

TCAAGTATGCCCATCACACTCCAATGTAGAATAAAACAGATTATAAACATGTGGCAGGGGGTAGGACAAGCAA
TGTATGCCCCTCCCATTAAAGGAAACATAACATGTAACTCAAGC---
ATCACAGGACTACTATTGACACGTGATGGAGGAGCAAACAACGGT----------------------------
----

AGCGAAACATTCAGACCTGAAGGAGGAAATATGAAGGACAATTGGAGAAGTGAATTATATAAATATAAAGTGG
TAGAAATTAGGCCATTA---GGAATAGCACCCACTAAGGCAAAAAGGAGAGTGGTG-----------------
----------------------GAGAGAGAAAAAAGAGCA---GTGGGA---ATGGGAGCT---------

GTGCTCTTTGGGTTCTTAGGAGCA---GCAGGAAGCACTATG---GGCGCGGCGTCA---
ATAACGCTGACGGTACAG---GCCAGACAA---TTGTTG---TCTGGTATAGTGCAACAGCAAAGT---
AATTTG---CTGAGA---GCTATAGAGGCGCAACAGCATTTGTTGCAACTTACAGTCTGGGGCATT
>DQ011179
TTCTATTGCAATACATCACAACTGTTTAACGAAAGTAGGTGGTCAAACAACACA-------------------
----

AGCGACACAAACATCACAATCCCATGCAGGATAAAACAAATTATAAACATGTGGCAGGGGGTAGGAAGAGCAA
TGTATGCCCCTCCCATTGCAGGAAACATAACATGTAAATCAAAT---
ATCACAGGACTACTATTGACAAGGGATGGAGGAGTAAGCAATGGCACA-------------------------
----

AACAATAACGAGACATTCAGACCTGCAGGAGGAGATATGAAGGACAATTGGAGAAGTAAATTATATAAATATA
AAGTGGTAGAAATTAAGCCATTG---GGAATAGCACCCACTGGAGCAAAAAGGAGAGTGGTG-----------
----------------------------GGAAGAGAAAAAAGAGCA---GCA------ATAGGAGCT------
---ATGCTCCTTGGGTTCTTGGGAGCA---GCAGGAAGCACTATG---GGCGCGGCGTTA---
ATAACGCTGACGGTACAG---GCCAGACAA---CTGTTG---TCTGGTATAGTGCAACAGCAAAGC---
AATTTG---CTGAGA---GCTATAGAGGCGCAACAACATATGTTGCAACTCACGGTCTGGGGCATT
>DQ011173
TTCTATTGTAATACATCAGGACTATTTAATGGTACATACACAAAC----------------------------
----

GAGGATTCAACCATCACAATTCCATGCAGAATAAAACAAATTATAAACATGTGGCAGGGGGTAGGACGAGCAA
TGTATGCCCCTCCCATTGCAGGAAACATAATATGTAACTCAAAT---
ATCACAGGATTACTGTTAACACGTGATGGTGGTGTTGGTAATGAATCA-------------------------
----

TTAAATAACACAGAGATATTTAGACCTGGAGGAGGAAATATGAAGGACAATTGGAGAAGTGAATTATATAAAT
ATAAAGTGGTAGAAATTAAACCATTG---GGAATAGCACCCACTGAGGCAAAAAGGAGAGTGGTG--------
-------------------------------GAGAGAGAAAAAAGAGCA---GCGGGA---ATAGGAGCT---
------TTGTTCCTTGGGTTCTTGGGAACA---GCAGGAAGCACTATG---GGCGCGGCGTCA---
ATAACGCTGACGGTACAG---GCCAGACAA---CTGTTG---TCTGGTATAGTGCAACAGCAAAGC---
AATTTG---CTGAAA---GCTATAGAGGCACAACAGCATATGTTGCAACTCACGGTCTGGGGCATT
>DQ011170
TTCTATTGCAATACAACAAAACTGTTTAATGAAAGCGGGTTAAACGAAACAAATATCAATGAAAGCGGGTCA-
----

AACAAAACAAACATCACACTCCCGTGCAGGATAAAACAAATTATAAACATGTGGCAGGAGGTAGGAAGAGCAA
TGTACGCCCCTCCTATTGCAGGAAACATAACATGTAGATCAAAT---
ATCACAGGACTACTATTGACACGTGATGGGGGAACAAACAACACA----------------------------
----

GAGATATTCAGACCTGGAGGAGGAAATATGAAGGACAATTGGAGAAGTGAATTATATAAGTACAAAGTGGTAG
AAATTAAGCCATTG---GGAGTAGCTCCCACTAAATCAAAAAGGAGAGTGGTG--------------------
-------------------GAGAGAGAAAAAAGAGCA---GTGGGA---ATGGGAGCT---------
GTACTCCTTGGGTTCTTGGGAGCA---GCAGGAAGCACTATG---GGCGCGGCGTCA---
ATAACGCTGACGGTACAG---GCCAGACAA---CTGTTG---TCTGGTATAGTGCAACAGCAAAGC---
AATTTG---CTGAGA---GCTATAGAGGCGCAACAGCATATGTTGCAACTCACGGTCTGGGGCATT
>AY901979
TTCTATTGTAATACAACAAATCTGTTTAATAGTACATACATGCCTGAATACCCGCATAATGGTACAGAAAGTA
AT---

TCAAGCACAGAAATCACACTCCCATGCAGGATAAAGCAAATTATAAACATGTGGCAGCAGGTAGGACGAGCAA
TGTATGCTCCTCCCATTGCAGGGAGCATAACATGTAACTCAAAT---
ATCACAGGAATACTATTGATGCGTGATGGAGGAAATAACTCAAATAAC-------------------------
----

ACAACTAAGATAGAGATATTCAGACCTGGAGGAGGAGATATGAGGGACAATTGGAGAAGTGAATTATACAAAT
ATAAAGTGGTAGAAATTAAGCCATTG---GGAATAGCACCTACCAAGGCAAAAAGGAGAGTGGTG--------
-------------------------------GAGAGAGAAAAGAGAGCA---GTGGGA---ATAGGAGCT---

------GTGTTCCTTGGGTTCTTGGGAGCA---GCCGGAAGCACTATG---GGCGCGGCGTCA---
ATAACGCTGACGGTACAG---GCCAGACAA---TTGTTG---TCTGGTATAGTGCAACAGCAAAGC---
AATTTG---CTGAGG---GCCATAGAGGCGCAACAGCATATGTTGCAACTCACGGTCTGGGGCATT
>DQ396387
TTCTATTGCAATACATCAGAGCTGTTTAATACATCAAAGCTGTTAGATGCTAATGAAACAGAA----------
----

AGTAATTCAACAATCATACTCCCATGCAGAATAAAACAAATTATAAACATGTGGCAAGAGGTAGGACGAGCAA
TATATGCCCCTCCCATTGCAGGAAACATAACATGTAGATCAAAT---
ATCACAGGACTATTATTGACACGTGATGGAGGGACAAATGGGACA----------------------------
----

GATAAGACAGAGATATTCAGACCTGGAGGAGGAGATATGAGGGACAATTGGAGAAGTGAATTATATAAATATA
AAGTGGTAGAAATTAAACCACTG---GGAGTAGCACCCACTGAGGCAAAAAGGAGAGTGGTG-----------
----------------------------GAGAGAGAAAAAAGAGCA---GTGGGA---ATAGGAGCT------
---GTGTTCCTTGGGTTCTTGGGAGCA---GCAGGAAGCACTATG---GGCGCAGCATCA---
ATAACGCTGACGGTACAG---GCCAGACAA---TTGTTG---TCTGGAATAGTGCAACAGCAAAGC---
AATTTG---CTGAGG---GCTATAGAGGCGCAACAGCATCTGTTGCAACTCACGGTCTGGGGCATT
>DQ164127
TTCTATTGTGATACATCAAACCTGTTTAATAGAACATACCCGTCTAACCACACAGATAATAGTACAAAA----
----

GGGAATGAAACCATTACACTTCCATGCAGAATAAAACAAATTGTAAACATGTGGCAGGGGGTAGGACAAGCAA
TATATGCCCCTCCCATTGCAGGAAACATAACATGTAACTCAAGC---
ATCACAGGACTACTATTAGTACGTGATGGAGGGAATGTGAGTGCC----------------------------
----

ACAAACGAGACATTCAGGCCTGGAGGAGGAAATATGAAGGACAATTGGAGAAGTGAATTATATAAATATAAAG
TGGTAGAAATTAAGCCATTG---GGAGTAGCACCCACTGAGGCAAAAAGGAGAGTGGTG--------------
-------------------------GAGAGAGAAAAAAGAGCA---GTGGGC---ATAGGAGCT---------
GTACTCCTTGGGTTCTTGGGCGCA---GCAGGAAGCACTATG---GGCGCGGCGTCA---
ATAACGCTGACGGTACAG---GCCAGACAA---CTGTTG---TCTGGTATAGTGCAACAGCAAAGC---
AATTTG---CTGAGA---GCTATAGAGGCGCAACAACACATGTTGCAACTCACAGTCTGGGGCATT
>AY878058
TTCTATTGTAATACAACAGCATTGTTTGATGAAAGTATTTTTAATAAAAGTGAT-------------------
----

TCAAACAGCACCATCACCCTCCCATGCAGAATAAAGCAAATTATAAACATGTGGCGGAAAGTAGGACGAGCAA
TGTATGCCCCTCCCATTGAAGGAAACATAACATGTAAATCAAAT---
ATCACGGGACTATTATTGGAACGTGATGGAGGAAAGGTAAATAAT----------------------------
----

AAAACCGAGATATTCAGACCTGGAGGAGGAAATATGAAGGACAATTGGAGAAGTGAATTATATAAATATAAAG
TGGTAGAACTTAAGCCATTG---GGAGTAGCACCCACTAAGGCAAAAAGGATAGTGGTG--------------
-------------------------GAGAGAGAAAAAAGAGCA---GTGGGA---CTAGGAGCT---------
GTGTTCTTTGGGTTCTTGGGAGCA---GCAGGAAGCACTATG---GGCGCGGCGTCA---
ATAACGCTGACGGTACAG---GCCAGACAA---TTGTTG---TCTGGTATAGTGCAGCAGCAAAGC---
AATTTG---CTGAGA---GCTATAGAGGCGCAACAGCATATGTTGCAACTCACGGTCTGGGGCATT
>DQ396381
TTCTATTGCAATACATCACGACTATTTAATAGTACATACACACAGAATAATACAGAA----------------
----

GATAATTCAAACATCGCACTTTCATGCAAAATAAAACAAATTATAAACATGTGGCAGAGGGTAGGGCGAGCAA
TGTATGCCCCTCCCATTGCAGGAAACATAACATGTAGATCAAAT---
ATCACAGGACTACTATTAGTACGTGATGGAGGCACAGAAGACGAA----------------------------
----

AATAAGACAGAAACATTCAGACCTGGAGGAGGAAATATGAAGGACAATTGGAGAAGTGAATTATATAAATATA
AAGTGGTAGAAATTAAGCCATTG---GGAATAGCACCCACTGAAGCAAAAAGGAGAGTGGTG-----------
----------------------------GAGAGAGAAAAAAGAGCA---GTGGGA---ATAGGAGCT------

---GTGTTCCTTGGGTTCTTGGGAGCA---GCAGGAAGCACTATG---GGCGTGGCGTCA---
ATGACGCTGACGGTACGA---GCCAGACAG---TTGTTG---TCTGGTATAGTGCAACAGCAAAGC---
AATTTG---CTGAGG---GCTATAGAGGCGCAACAGCATATGTTGCAACTCACGGTCTGGGGCATT
>DQ093595
TTCTATTGTAATACATCACATCTATTTAATACATCACAGCTATTTAATAGTACATTGGCTAATAGTACAGGAG
AT---

TCTAATGGCAACATCACACTCCAATGCAGAATAAAACAAATTATAAACATGTGGCAGGGGGTAGGACGAGCAA
TGTATGCCCCTCCCATTGCAGGAAATATAACATGCAGATCAAAT---
ATCACAGGGCTACTATTGACACGTGATGGAGGAAAAACTAACACAAGC-------------------------
----

AATGAGACAGAGACATTCAGACCTGGAGGAGGAGACATGAGAGACAATTGGAGAAGTGAATTATATAAATATA
AAGTGGTAGAAATTAATCCATTA---GGAGTAGCTCCCACTGGAGCAAAAAGGAGAGTGGTG-----------
----------------------------GAGAGAGAGAAAAGAGCA---GTGGGA---ATAGGAGCT------
---GTGTTCCTTGGGTTCTTAGGAACA---GCAGGAAGCACTATG---GGCGCAGCATCA---
ATAACGCTGACGGTACAG---GCCAGACAA---TTATTG---TCTGGTATAGTGCAACAGCAAAGC---
AATTTG---CTGAGA---GCTATAGAGGCGCAACAGCATATGTTGCAGCTCACAGTCTGGGGCATT
>DQ164129
TTTTATTGTGATACATCAAAGCTGTTTAAGAGGACATACATGGATAATGGTACAGAAAATAATTCAACCACA-
----

AACAGTAATTCAACCATCATTCCATGCAGAATAAAACAAATTATAAACATGTGGCAGGAGGTAGGACGAGCAA
TGTATGCACCTCCCATTGCAGGAAAAATAACATGTAAATCAAAT---
ATCACAGGACTACTATTGACACGTGATGGAGGGTTAGATGATTCAAATAAG----------------------
----

CCAAATGATAGTGAGATATTCAGACCTGGAGGAGGAGATATGAGGAACAATTGGAGAAGTGAATTATATAAAT
ATAAAGTGGTAGAAATTAAGCCATTG---GGAGTAGCACCCACCAAGGCACAAAGGGAAGTGGTG--------
-------------------------------GGGAGAGAAAAAAGAGCA---CTGGGAATAGTAGGAGCT---
------ATGTTCCTTGGGTTCTTGGGAGCA---GCAGGAAGCACTATG---GGCGCAGCGTCA---
ATAACGCTGACGGCACAG---GCCAGACAA---GTGTTG---TCTGGTATAGTGCAACAGCAAAGC---
AATTTG---CTGAGG---GCTATAGAGGCGCAACAGCATATGTTGCAACTCACGATCTGGGGCATT
>DQ056416
TTCTATTGCAATACATCAGGATTGTTTAATGGTATATACAACGGTACAAAAAGTAATTCAAGTACAAAA----
----

AGTAATTTAACCATCACACTCCCATGCAGAATAAAACAAATTGTAAACATGTGGCAGAAGGTAGGACAAGCAA
TATATGCCCCTCCTATTGCAGGAAACATAACATGTACATCAAAG---
ATCACAGGACTACTATTGACACGTGATGGAGGCATACATAACCCA----------------------------
----

ACTAATGAGACATTCAGACCTGGAGGAGGAGATATGAGGGATAATTGGAGAAGTGAATTATATAAATATAAAG
TGGTAGAAATTAAGCCATTA---GGAATAGCACCCACTAATGCAAAAAGAAGAGTGGTG--------------
-------------------------GAGAGAGAAAAAAGAGCA---GTGGGA---ATAGGAGCT---------
GTGTTCCTTGGGTTCTTGGGAGCA---GCAGGAAGCACTATG---GGCGCGGCGTCA---
ATATCGCTGACGGTACAG---GCCAGACAA---TTGTTG---TCTGGTATAGTGCAACAGCAAAGC---
AATTTG---CTGAGG---GCTATAGAGGCGCAACAGCATATGTTGCAACTCACAGTCTGGGGCATT
>AY878062
TTCTATTGTAATACATCAGACCTGTTTAATGAATCCATAACTAATAGGACAGGAAATAATAGTACAGGA----
----

AATAGTTCAGTCATCACACTCCCATGCAGAATAAAACAAATTATAAACATGTGGCAGAAGGTAGGACGAGCAA
TGTATGCCCCTCCCATTGCAGGAAACATAACATGTAAATCAAAT---
ATCACAGGACTACTATTGACACGTGATGGAGGAATAAATGGGACT----------------------------
----

AATAACATAGGGACATTCAGACCTGGAGGAGGAGATATGAGGGACAATTGGAGAAGTGAATTATATAAATATA
AAGTGGTAGAGATTAGGCCATTG---GGAATAGCACCCACTGGAGCAAAGAGGAGAGTGGTG-----------
----------------------------GAGAGAGAAAAAAGAGCA---GTGGGA---ATAGGAGCT------

---GTGTTCCTTGGGTTCTTGGGAGCA---GCAGGAAGCACTATG---GGCGCAGCATCA---
ATAACGCTGACGGTACAA---GCCAGACAA---TTGTTG---TCTGGGATAGTGCAACAGCAAAGC---
AATTTG---CTGAGA---GCGATAGAGGCGCAACAGCATATGTTGCAACTCACGGTCTGGGGCATT
>DQ056409
TTCTATTGCAATACATCAAAACTGTTTAATGGTACATACAATGGTAATGGTACATACAATGGTACAGGTACAG
AC---

ACTAATTCAACCATCACCATCCCATGCAAGATAAAACAAATAGTAAACATGTGGCAGGGGGTAGGACGAGCAA
TGTATGCCCCTCCCATTGCAGGAAACATAACATGTAAATCAAT---
ATCACAGGACTACTATTGACACGTGATGGAGGAGAAAATAGTACA----------------------------
----

GAGACATTCAGACCTGGAGGAGGAAATATGAAGGACAATTGGAGAAGTGAATTATATAAATATAAAGTAGTAG
AAATTAAGCCATTG---GGAATAGCACCCACTGGAGCAAAAAGGAGAGTGGTG--------------------
-------------------GAGAGAGAAAAAAGAGCA---GTGGGA---ATAGGAGCT---------
GTGTTCCTTGGGTTCTTGGGAGCA---GCAGGAAGCACTATG---GGCGCGGCGTCG---
ATAACGCTGACGGTACAA---GCCAGACAA---CTGTTG---TCTGGTATAGTGCAACAGCAAAGC---
AATTTG---CTGAGA---GCTATAGAGGCGCAACAGCATATGTTGCAACTCACAGTCTGGGGCATT
>DQ369993
TTTTATTGCAACACATCAAACCTGTTTAATGGTACATACAATAGTACATACAATAGTACATACAATAGT----
----

ACATCCAGAAACATCACACTCCAATGCAGAATAAAACAAATTATAAACATGTGGCAGGGAGTAGGGCAAGCAA
TGTATGCCCCTCCCATTAAAGGAAACATAACATGTAAATCAAT---
ATCACAGGAATACTATTGACACGTGATGGAGGACATGAAAATGAC----------------------------
----

AACAACACAGAGACATTCAGACCTGGAAGAGGAAATATGAAGGACAATTGGAGAAGTGAATTATATAAATATA
AAGTGGTAGAAATTAAGCCATTG---GGAATTGCCCCCACTGAAGCAAAAAGGAGAGTGGTG-----------
----------------------------GAGAGAGAAAAAAGAGCA---GTGGGA---ATAGGAGCT------
---GTGTTCCTGGGGTTCTTGGGAGCA---GCAGGAAGCACTATG---GGCGCGGCGTCA---
ATAACGCTGACGGTACAG---GCCAGACAA---CTGTTG---TCTGGTATAGTGCAACAGCAAAAT---
AATTTG---CTGAGA---GCTATAGAGGCGCAACAACATATGTTGCAACTCACAGTCTGGGGCATT
>DQ396382
TTCTATTGCAACACAACAGGACTGTTTAATATCAGTAACAGTAAA----------------------------
----

GATGATTCAAACATCACACTCCCATGCAGAATAAAACAAATTATAAACATGTGGCAGGGGGTAGGACGAGCAA
TGTATGCTCCTCCCATTGCAGGAAAGATAACATGTACCTCAAAT---
ATCACAGGAATACTATTGACACGTGATGGAGGCCCAGAGAATAAC----------------------------
----

ACAAAGATATTCAGACCTGGAGGAGGAGATACGAGGGACAATTGGAGAAGTGAATTATATAAATATAAAGTGG
TAGAAGTTAAGCCATTG---GGAGTAGCACCCACTGAGGCAAGAAGGAGAGTGGTG-----------------
----------------------GAGAGAGAAAAAAGAGCA---GTGGGA---ATAGGAGCT---------
GTGTTCCTTGGGTTCTTGGGAGCA---GCAGGAAGCACTATG---GGCGCAGCGTCA---
ATAACGCTGACGGTACAG---GCCAGACAA---CTATTG---TCTGGTATAGTGCAACAGCAAAGT---
AATTTG---CTGAGA---GCTATAGAGGCGCAACAACATATGTTGCAACTCACGGTCTGGGGCATT
>DQ396396
TTCTATTGCAATACAACAAATCTGTTCAATACAACAGATCTGTTTAATAATAGCAATGATTTA----------
----

AACACAAGTACCATCACACTCCCATGCAGAATAAAGCAAATTATAAACATGTGGCAGGGGGTAGGACGAGCCA
TGTACGCCCCTCCCGTAGCAGGAAACATAACATGTAACTCAAGT---
ATCACAGGACTACTATTGGTACGTGATGGAGGAAGGAATAATAAT----------------------------
----

ACAAATGAGACATTCAGACCTGGGGGAGGAAATATGAAGGACAATTGGAGAAGTGAATTATATAAATATAAAG
TGGTAGAAATTAAGCCATTG---GGAGTGGCACCCACTGAGGCAAAAAGAAGAGTGGTG--------------
-------------------------GAGAGAGAAAAAAGAGCA---GTGGGA---ATAGGAGCT---------

GTATTCCTCGGGTTCTTGGGAGCA---GCCGGAAGCACTATG---GGCGCAGCGTCA---
ATAACGCTGACGGTACAG---GCCAGACAA---CTGTTG---TCTGGTATAGTGCAACAGCAAAGC---
AATTTG---CTGAGA---GCTATAGAGGCGCAACAGCACATGTTGCAACTCACGGTCTGGGGCATT
>DQ056413
TTCTATTGCAATACATCAAAACTATTTAATAGCACATATGTTAATAGGACAGACATGAATGATGATACAGGA-
----

AATAATTCAACCATCACACTTCCATGCAGAATAAAACAAATTATAAACATGTGGCAGGAGGTAGGACGAGCAA
TGTATGCCCCTCCCATTGAAGGAAACATAACATGTAAATCAAAT---
ATCACAGGAATACTATTAGTACGTGATGGAGGAATAAATAAGACA----------------------------
----

AATAACACAGAGACATTCAGACCAGGAGGAGGAAATATGAAGGACAATTGGAGAAGTGAATTATATAAATATA
AAGTGGTAGAAATTAAGCCATTA---GGAATAGCACCCACTGAAGCAAAAAGGAGAGTGGTG-----------
----------------------------GAGAGAGAAAAAAGAGCA---GTAGGA---ATGGGAGCT------
---GTGTTCCTTGGGTTCTTGGGAGTA---GCAGGAAGCACTATG---GGCGCAGCGTCA---
ATAGCGCTGACGGCACAG---GCCAGACAA---GTGTTG---TCTGGTATAGTGCAACAGCAAAGC---
AATTTG---CTGAGG---GCTATAGAGGCGCAACAGCATATGTTGCAACTCACGGTCTGGGGCATT
>DQ396383
TTCTATTGCAATACAACAAAACTGTTTGATAACAGAACGTTTAAT----------------------------
----

GATGGAAGCACAATCACAATCCCATGTAGAATAAAACAAATTATAAATATGTGGCAGGGGGTAGGACGAGCAA
TGTATGCCCCTCCCATTGAGGGCAGCATAACATGTAACTCAAAT---
ATCACAGGGCTACTATTGCTACGTGATGGAGGAAACGTGGATAGC----------------------------
----

TCAGAGGAGGTATTCAGACCTGGAGGAGGAAATATGAAGGACAATTGGAGAAGTGAATTATATAAATATAAAG
TGGTAGCAATTAAGCCCTTG---GGAATAGCACCCACGACGGCAAAAAGAAGGGTGGTG--------------
-------------------------GAGAGAGAAAAAAGAGCG---GGA------ATAGGAGCT---------
GTGTTCCTTGGGTTCTTGGGAGCA---GCAGGAAGCACTATG---GGCGCGGCGTCA---
ATAACGCTGACGGTACAG---GCCAGAAAA---CTGTTG---TCTGGTATAGTGCAACAGCAAAGC---
AATTTG---CTGAGA---GCTATAGAGGCGCAACAGCATATGTTGCAACTCACGGTCTGGGGCATT
>DQ396397
TTCTATTGTAATACATCAGAACTGTTTAAGAGTACATTAGATAATTTAACAGCAAATAGTTCAATAGAA----
----

GATAATACAATCATCACAATTCCATGCAGAATAAAACAAATTATAAACATGTGGCAGGGGGTAGGAAGAGCAA
TGTATGCCCCTCCCATTGCAGGAAACATAACATGTAATTCAAGC---
ATCACAGGACTACTATTAGTACGTGATGGTGGCGATACAAATGAC----------------------------
----

ACAGAAGAAATATTCAGACCTGAAGGAGGAAATATGAAGGACAATTGGAGAAGTGAATTATATAAATATAAAG
TGGTAGAAATTAAGCCATTA---GGAATAGCACCCACTAAAGCAAAAAGGAGAGTGGTG--------------
-------------------------GAGAGAGAAAAAAGAGCA---GTGGGA---TTAGGAGCT---------
TTGTTCCTTGGGTTCTTGGGAGCA---GCAGGAAGCACTATG---GGCGCGGCGTCA---
ATAACGCTGACGGTACAA---GCCAGACAA---GTATTG---TCTGGTATAGTGCAACAGCAAAAC---
AATTTG---CTGAGG---GCTATAGAGGCGCAACAGCATATGTTGCAACTCACAGTCTGGGGCATT
>DQ011180
TTCTATTGTAATACATCAGGCCTGTTTAATAGTACATACAATACATCAGACCTGTTTAATGATACATACAACA
CA---

GAAAGTAATTCCATTATCACACTCCCATGCAGAATAAAACAAATCATAAACATGTGGCAGGAGGTAGGACGAG
CAATGTATGCTCCTCCCATTGCAGGCACCATAACATGTAAATCAAAC---
ATCACAGGATTACTATTGGAGCGTGATGGAGGAAGGGAAAGTAAT----------------------------
----

GGCAGCGAGATATTCAGACCTGGAGGAGGAGATATGAAGAACAATTGGAGAAGTGAATTATATAAATATAAAG
TGGTAGAAATTAAGCCATTG---GGAGTTGCACCCACTGAGGCAAAAAGGGCAGTGGTG--------------
-------------------------AAGAGAGAAAAAAGAGCA---GTAGTGGGACTAGGAGCT---------

ATGATCTTTGGGTTCTTGGGAGCA---GCAGGAAGCACTATG---GGCGCGGCATCA---
ATAACGCTGACGGTACAG---GCCAGACAA---CTACTG---TCTGGTATAGTGCAACAGCAAAGC---
AATTTG---CTGAGG---GCTATAGAGGCGCAACAGCATATGTTGCAACTCACGGTCTGGGGCATT
>DQ056414
TTCTATTGCAATACATCAAGCCTGTTTAATGGTATATATAATGGTACATATAATGATACG-------------
----

TCTGATAAAACCATCACATTCTCATGCAGAATAAAACAAATTATAAACATGTGGCAGAAGGTAGGACGAGCAA
TATATGCCCCTCCCATTGCAGGAAACATAACATGTAAATCAAAT---
ATCACAGGACTACTATTGACACGTGATGGAGGAGGTAGAAATAAT----------------------------
----

AATATAACAGAGACATTCAGACCTGGAGGAGGAGAGATGAGGGATAATTGGAGAAGTGAATTATATAAATACA
AGGTGGTAGAAATTAAGCCATTA---GGAGTAGCACCCACCAAGGCAAAAAGGAGAGTGGTG-----------
----------------------------GAGAGAGGAAAAAGAGCA---GTAGGA---ATAGGAGCT------
---ATGATCTTTGGGTTCTTGGGAGCA---GCAGGAAGCACTATG---GGCGCAGCATCA---
ATAACGCTGACGGTACAG---GCCAGACAA---TTGTTG---TCTGGTATAGTGCAACAGCAAAGC---
AATTTG---CTGAGG---GCTATAGAGGCGCAACAGCATATGTTGCAACTCACGGTCTGGGGCATT
>DQ056415
TTCTATTGCAATACATCAAGACTGTTTAATAGTACATACAATCCTAATGGTACATACAACTCTACATACACAT
CATACAATTTT--
-------------AGTGGT------
ACATCTAATGATACCATCACACTCCCATGCAGAATAAAACAAATTATAAACATGTGGCAGGAAGTAGGGAGAG
CAATGTATGCCCCTCCCATTACAGGAAACATAACATGTAAATCAAAT---
ATCACAGGACTACTATTGTTACGTGATGGAGGAAACAGCTCTAAT----------------------------
----

GAGACATTCAGACCTGGAGGAGGAGATATGAGGGATAATTGGAGAAGTGAACTATATAAATATAAGGTAGTAG
AAATTAAGCCATTA---GGAATAGCACCCACTACAGTAAAAAGGAGAGTGGTG--------------------
-------------------GAGAAAGAAAAAAGAGCA---GTGGGA---ATAGGAGCT---------
GTGTTCCTTGGGTTCTTGGGAGCA---GCAGGAAGCACTATG---GGCGCGGCGTCA---
ATAACGCTGACGGTACAG---GCCAGACAA---TTGTTG---TCTGGTATAGTGCAACAGCAAAGC---
AATTTG---CTGAGG---GCTATAGAGGCGCAACAGCATATGTTGCAACTCACAGTCTGGGGCATT
>DQ056406
TTCTATTGCAATACAACAGAACTGTTTAATAATACAAATAACAGTACA-------------------------
----

AATATAACAGACATCATACTCCCATGCAGAATAAAACAGATTATAAACATGTGGCAGGGGGTAGGACGAGCAA
TGTATGCCCCTCCCATTGCAGGAAACATAACATGTACCTCAAAG---
ATCACAGGACTACTATTGACACGTGATGGAGGAGGAAACGAAACA----------------------------
----

AATAACACAGAAATATTCAGACCTGGAGGAGGAAATATGAAGGACAATTGGAGAAGTGAATTATATAAATATA
AAGTGGTAGAAATTAAGCCATTG---GGAATAGCACCCACTGAGGCAAAAAGGAGAACGGTG-----------
----------------------------AAGAAAGAAAAAAGAGCA---GTGGGA---ATAGGAGCT------
---GTACTCCTTGGGTTCTTGGGAGCA---GCAGGAAGCACTATG---GGCGCGGCGTCA---
ATAACGCTGACGGTACAG---GCCAGACAA---CTATTG---TCTGGTATAGTGCAACAGCAAAAC---
AATTTG---CTGAGA---GCTATAGAGGCGCAACAGAGTATGTTGCAACTCACGGTCTGGGGCATT
>DQ056417
TTCTATTGCAATACATCAGGCCTATTTAATGGTACATACAATCCAGCATACACGTATAATGCGACA-------
----

AATAACTCAACCATCACACTCCAATGCAGAATAAGACAAATTATAAACATGTGGCAGAAGGTGGGAAGAGCAA
TGTATGCCCCTCCCATTGCAGGAAACATAACATGTAATTCGAGC---
ATCACAGGACTACTGTTGGTACGTGATGGAGGCAAAAACACAAGT----------------------------
----

GGAATAGAAACATTCAGACCCGGAGGAGGAAACATGAAGGACAATTGGAGAAGTGAATTATATAAATATAAAG
TGGTAGAAATTAAGCCATTG---GGAGTAGCACCCACGAGTGCAAAAAGGAGAGTGGTG--------------
-------------------------GAGAGAGAAAAAAGAGCA---GTGGGA---CTAGGAGCT---------

GTGTTCCTTGGGTTCTTGGGAGCA---GCAGGAAGCACTATG---GGCGCGGCGTCA---
ATAACGCTGACGGTACAG---GCCAGACAA---TTATTG---TCTGGTATAGTGCAGCAGCAAAGT---
AATTTG---CTGAGA---GCTATAGAGGCGCAACAACATATGTTGCAACTCACAGTCTGGGGCATT
>DQ093605
TTCTATTGTAATACATCAGGACTGTTTAATGGTACAGTAGGTAACTCAACTTCAAATAATTCAACT-------
----

TTAAGCGGCACAATCACACTCCCATGCAGAATAAAACAGATTATAAACATGTGGCAGACGGTAGGGCGAGCAA
TGTATAACCCTCCCATTGCAGGAAACATAACATGTAAATCAAAT---
ATCACAGGACTACTGTTGACACGGGATGGAGGGAACGACAATGAAACA-------------------------
----

AAAGAAACAGAGACATTCAGACCTGGAGGAGGAGATATGAGAGACAATTGGAGAAGTGAATTATACAAATATA
AAGTGGTAGAAATTAAGCCATTA---GGAATAGCACCCACTAAGGCACGAAGGAGAGTGGTG-----------
----------------------------GAGAGAGAGAAAAGAGCA---GTAGGA---ATAGGAGCT------
---GTGTTCCTTGGGTTCTTGGGAGCA---GCTGGAAGCACTATG---GGCGCAGCATCA---
ATAACGCTGACGGTACAG---GCCAGACAA---TTGTTG---TCTGGTATAGTGCAACAGCAAAGT---
AATTTG---CTGAGG---GCTATAGAGGCGCAACAGCATTTGTTGCAACTCACGGTCTGGGGCATT
>GQ999973
TTCTATTGCAATACAACAAAACTGTTTAACAGTACAGATAATGAGACAAAAAGTACAGAAAGTAATAATGAGA
TA---

GGAAATAATTCAATCATCACACTTCCATGCAGAATAAAACAAATTATAAACATGTGGCAGGAGGTAGGACGAG
CAATGTATGCCCCTCCCATTGCAGGAAACATAACATGTAGATCAGAG---
ATCACAGGATTATTATTGACACGTGATGGAGGGAGAAACACTACA----------------------------
----

GAGGAGACATTCAGACCTGGAGGAGGAAATATGAAGGACAATTGGAGAAGTGAATTATATAAATATAAAGTGG
TAGAAATTAAACCATTG---GGAATAGCACCCACCAAGGCAAAAAGGAGAGTGGTG-----------------
----------------------GAGAGAGAAAAAAGAGCA---GTGGGA---ATAGGAGCT---------
TTGTTCCTTGGGTTCTTGGGAGCA---GCAGGAAGCACTATG---GGCGCGGCGTCA---
ATAACGCTGACGGTACAG---GCCAGACAA---TTGTTG---TCTGGTATAGTGCAACAGCAAAAT---
AATTTG---CTGAGA---GCTATAGAGGCGCAACAGCATATGTTGCAACTCACGGTCTGGGGCATT
>GQ999975
TTTTATTGCAATACATCAGGCCTGTTTAATGGTACATTTAATGGTACAGACATGTCTAATGAG----------
----

ACAAATTCAACCATCACACTTCCATGCAAAATAAAACAAATTATAAACATGTGGCAAAAGGTAGGACGGGCAA
TGTATGCCCCTCCCATTGCAGGAAATATAACATGTACAGCAAAT---
ATCACAGGACTGCTATTGACACGTGATGGAGGGGACAGTAGCAGT----------------------------
----

ACAAACGAGACATTCAGACCTGGAGGAGGAAATATGAAGGACAATTGGAGAAGTGAATTATATAAATATAAAG
TGGTAGAAATCAAGCCATTG---GGGATAGCACCAACTGAAGCAAAAAGGAGAGTGGTG--------------
-------------------------GAGAGAGAAAAAAGAGCA---GTGGGA---ATAGGAGCT---------
TTGTTCCTTGGGTTCTTGGGAGCA---GCAGGAAGCACTATG---GGCGCGGCGTCA---
ATAACGCTGACGGCACAG---GCCAGACAA---CTGTTG---TCTGGTATAGTGCAACAGCAAAAC---
AATTTG---CTGAGA---GCTATAGAGGCGCAACAGCATATGTTGCAACTCACGGTCTGGGGCATT
>AY703909
TTCTATTGCAATACATCAAAACTGTTTAATAATAATAAGACAGAA----------------------------
----

TGGAATTCAACCATCATACTGCCATGCAGAATAAAACAAATTATAAACATGTGGCAGGAGGTAGGAAGAGCAA
TATATGCCCCTCCCATTGCAGGAAACATAACATGTATATCAAAT---
ATCACAGGACTACTATTGGAACGGGATGGAGGAGATAATTCAACAGAG-------------------------
----

AATGGCACAGAGATATTCAGACCTATAGGAGGAGATATGAGGAACAATTGGAGAAGTGAATTATATAAATATA
AAGTGGTAGAAATTAAGCCACTG---GGAGTAGCACCCACTAAGGCAAAAAGGAGAGTGGTG-----------
----------------------------GAGAGAGAAAAAAGAGCA---GTGGGA---CTAGGAGCT------

---TTGTTCCTTGGGTTCTTGGGAGCA---GCAGGAAGCACTATG---GGCGCAGCATCA---
ATAACGCTGACGGTACAG---GCCAGACAA---GTGCTG---TCTGGAATAGTGCAACAGCAAAGC---
AATTTG---CTGAGA---GCTGTAGAGGCGCAACAGCATATGTTGCAACTCACTGTCTGGGGCATT
>AY703910
TTCTATTGTAATACATCAAAACTGTTTAATAATAATAGCACAAACAGCACT----------------------
----

TCAAATGGAACCATCACACTCCCATGCAGAATAAGACAAATTATAAACATGTGGCAGGGAGTAGGACGGGCAA
TGTATGCCCCGCCCATTGCAGGAAACATAACATGTACGTCAAAT---
ATTACAGGACTACTACTGACACGTGATGGAGGAAATAACGAAACA----------------------------
----

GAGACAGAAACATTCAGACCTGGAGGAGGAGATATGAGGGACAATTGGAGAAGTGAGTTATATAAATACAAAG
TGGTAGAAATTAAGCCATTA---GGAATAGCACCTACTAGGGCAAAAAGGAGAGTGGTG--------------
-------------------------GAGAGAGAAAAAAGAGCA---GCA------ATAGGAGCT---------
GTGTTCCTTGGGTTCTTGGGAGCA---GCAGGAAGCACTATG---GGCGCAGCATCA---
ATAACGCTGACGGTACAG---GCCAGACAA---TTGTTG---TCTGGTATAGTGCAACAGCAAAGC---
AATTTG---CTGAGG---GCTATAGAGGCGCAACAGCATATGTTGCAACTCACGGTCTGGGGCATT
>AY703911
TTCTATTGCAATACATCAGATCTGTTTACAGAATTTAATGGTACAACAGAATATGATAATGCAACCATAGAT-
----

GATAATGCAACCATCACACTCCAATGTAGAATAAAACAGATTATAAACATGTGGCAGGGGGTAGGACAAGCAA
TGTATGCCCCTCCCATTGAAGGAAACATAACATGTAACTCAAAT---
ATCACAGGACTACTATTGACACGTGATGGAGGACCAGATAATAGA----------------------------
----

ACGGAGACATTCAGACCTGGAGGAGGAAATATGAAGGACAATTGGAGAAGTGAATTATATAAATATAAAGTGG
TAGAAATTAAGCCATTA---GGAATAGCACCCACTAAGGCACAAAGGAGAGTGGTG-----------------
----------------------GAAAGAGAAAAAAGAGGA---GCAGCA---CTGGGAGCT---------
ATGTTCCTTGGGTTCTTGGGAGCA---GCAGGAAGCACTATG---GGCGCGGCATCA---
ATAACGCTGACGGTACAG---GCCAGACGA---TTGTTG---TCTGGTATAGTGCAACAGCAAAGC---
AATTTG---CTGAGA---GCTATAGAGGCGCAACAGCATATGTTGCAACTCACAGTCTGGGGCATT
>DQ382369
TTCTATTGTAACACAACAGAACTGTTTAAAAATGATTCAAGCACAAAA-------------------------
----

AATGATACATACATCACACTCCCATGCAGAATAAAACAAATTATAAACATGTGGCAAGAGGTAGGACGAGCAA
TGTATGCTCCTCCCATTGCAGGAAACATAACATGTAGATCAAAT---
ATCACAGGATTGCTATTGGTACGTGATGGAGGAAACGAGAATAAC----------------------------
----

ATCACAGAGATATTCAGACCTGGAGGAGGAAATATGAAGGACAATTGGAGAAGTGAACTATATAAATACAAAG
TGGTAGAAATTAAACCATTA---GGAATAGCACCCACAACGGCAAAAAGGAGAGTGGTG--------------
-------------------------CAGAGAGAAAAAAGAGCA---GTAGGA---ATGGGAGCT---------
GTACTCCTTGGGTTCTTGGGAGCA---GCAGGAAGCACTATG---GGCGCGGCGTCA---
ATGACGCTGACGGTACAG---GCCAGACAA---CTGTTG---TCTGGTATAGTGCAACAGCAAAGC---
AATTTG---CTGAGA---GCTATAGAGGCGCAACAGCATCTGTTGCAACTCACAGTCTGGGGCATT
>DQ382370
TTCTATTGCAATACAACAAAACTGTTTAAGGAAAATAGTACAAAT----------------------------
----

GAAAATGATACAATCACACTCCCATGCAGGATAAAACAAATTGTAAACATGTGGCAGACAGTAGGACAAGCAA
TGTATGCCCCTCCCATTGCAGGAAACATAACATGTAACTCAAGT---
ATCACAGGGTTACTATTGACACGTGATGGAGGAAATGGGAATAGT----------------------------
----

ACTAATGAGACATTCAGACCTACAGGAGGAGATATGAAAGACAATTGGAGAAGTGAATTATATAAATATAAAG
TGGTAGAAATTAAGCCATTG---GGAGTAGCACCCACAGAGTCAAAAAGGAGAGTGGTG--------------
-------------------------GAGAGAGAAAAAAGAGCA---GCGGGA---ATAGGAGCT---------

GTACTCCTTGGGTTCTTGGGAGCA---GCAGGAAGCACTATG---GGCGCAGCGTCA---
ATAACGCTGACGGTACAG---GCCAGACAA---CTGTTG---TCTGGTATAGTGCAACAGCAAAGC---
AATTTG---CTGAGA---GCTATAGAGGCGCAACAGCATATGTTGCAACTCACGGTCTGGGGCATT
>DQ382371
TTCTATTGCAATACATCAAAACTGTTTAATAATAGTGAAGACAAGGATAGTCCTTTTAATTCAACC-------
----

AGAAATGCAACCATTATAATCCAATGCAGAATAAGACAATTTATAAACATGTGGCAGGAGGTAGGACGAGCAA
TGTATGCCCCTCCCATTGCAGGAAACATAACATGTAAATCAAAC---
ATCACAGGACTACTATTGACACGTGATGGAGGAGAGAATGACACA----------------------------
----

AACCCAGAGATATTCAGACCTGGAGGAGGAGATATGAGGGACAATTGGAGAAGTGAATTATATAAATACAAAG
TGGTAGAAATTAAGCCATTA---GGAATAGCACCCACTGGAGCAAAAAGGAGAGTGGTG--------------
-------------------------GAGAGAGAAAAAAGAGCA---GTGGGA---ATAGGAGCT---------
GTGCTCCTTGGGTTCTTGGGAACA---GCAGGAAGCACTATG---GGCGCGGCGTCA---
TTAGCGCTGACGGTACAG---GCCAGACAA---CTGTTG---TCTGGTATAGTGCAACAGCAAAGC---
AATTTG---CTGAGG---GCTATAGAGGCGCAACAGCATATGTTGCAACTCACGGTCTGGGGCATT
>DQ382372
TTCTATTGCAACACATCAAACCTGTTTAATAGGACATACAATAGTACAGATAAGTCAGATAGGTTTAATAGTA
CA---

GATAATTCAACCATCACAATCCAATGCAGAATAAGACAATTTATAAACATGTGGCAGAGGGTAGGACGAGCAA
TGTATGCCCCTCCCATTGAAGGAAACATAACATGTAAATCAAAA---
ATCACAGGACTACTATTGACACGTGATGGAGGCGAGGGAATAAGAATGAC-----------------------
----

ACAAATGACACAGAGATATTCAGACCTGGAGGAGGAGATATGAGGGACAATTGGAGAAGTGAATTATATAAAT
ATAAAGTGGTAGAAATTAAGCCATTA---GGACTAGCACCCACTAAAGCAAAAAGGAGAGTGGTG--------
-------------------------------GAGAGAGAAAAAAGAGCA---GTGGGA---ATAGGAGCG---
------TTGTTCCTTGGGGTCTTGGGAGTA---GCAGGAAGCACTATG---GGCGCAGCATCA---
ATAACGTTGACGGTACAG---GCCAGACAA---TTGTTG---TCTGGTATAGTGCAACAGCAAAAC---
AATTTG---CTGAGG---GCTATAGAGGCGCAACAGCATATGTTGCAACTCACGGTCTGGGGCATT
>DQ382375
TTCTATTGCAATACAGCAGGCCTGTTTAATACAAGTCTGATTAATAGTACTAAGAGTACAAGTACAAAGAAT-
----

ACACAAGACCCCATCACACTCACATGCAGAATAAAGCAATTTATAAACATGTGGCAGCAGGTAGGACGAGCAA
TGTATGCTCCTCCCATTGCAGGAAACATAACATGTAAATCAAAT---
ATCACAGGATTGCTATTGATGTATGATGGAGGAAGGAATGAGACA----------------------------
----

AATGCGACCTTCAGACCTGGAGGAGGAGATATGAGAGACAATTGGAGAAGTGAATTATACAAATATAAAGTGG
TAGAAATTAAGCCATTG---GGAATAGCACCCACCAAGGCAAAAAGGAGAGTGGTG-----------------
----------------------GAGAGAGAAAAAAGAGCA---GTGGGA---ATAGGAGCT---------
GTGCTCTTTGGGTTCTTGGGAGCA---GCAGGAAGCACTATG---GGCGCGGCGTCA---
ATAACGCTGACGGTACAG---GCCAGACAA---TTGTTG---TCTGGTATAGTGCAACAGCAAAGC---
AATTTG---CTGAGA---GCTATAGAGGCGCAACAGCATATGTTGCAACTCACGGTCTGGGGCATT
>DQ382377
TTCTATTGTAATACATCAAAACTGTTTAATGGTACATACAATGGTACATACAATGCTACAGAAAGAAACATT-
----

GGTCCCGGAACCCTCTGCCTCCAATGCCGAATAAAACAAATTATAAACATGTGGCAACAGGTGGGACGAGCAA
TGTATGCTCCTCCCATTGCAGGAAACATAACATGTACATCAAAT---
ATCACAGGACTACTATTGGTACGTGATGGAGGAAACTCAAGTAAC----------------------------
----

CTAAAGATAGAGATATTCAGACCTAACAGAGGAGAGATGAGGGACAACTGGAGAAGTAAATTATATGAGTATA
AGGTGGTAAAAATTAAACCATTA---GGAGTAGCTCCCACTGAAGCAAAAAGGAGAGTGGTG-----------
----------------------------GAGAGACAAAAAAGAGCA---GCAGGA---ATAGGAGCT------

---GTGTTCCTTGGGTTCTTGGGAGTA---GCAGGAAGCACTATG---GGCGCGGCGTCA---
ATGACGCTGACGGTACAG---GCCAGACAA---CTGTTG---TCTGGTATAGTGCAACAGCAAAGC---
AATTTG---CTGAGG---GCTATAGAGGCGCAACAGCATATGTTGCAACTCACGGTCTGGGGCATT
>DQ382378
TTCTATTGCGATACAACAAACCTGTTTAATATTTCTAATATTAAC----------------------------
----

AGTACAAGCAACATTACACTCCAATGCAAGATAAAACAAATTATAAACATGTGGCAAAAGGTAGGACGAGCAA
TGTATGCCCCTCCCATTGAAGGAACCATAACATGTATATCAAAC---
ATCACAGGACTATTATTGACACGTGATGGAGGTAGTAGTAATGAGACTAGT----------------------
----

AGTATTAATAAGACAGAGATTTTTAGACCTGGAGGAGGAAATATGAAAGACAATTGGAGAAGTGAATTGTATA
AATATAAGGTGGTAGAAATTAAGCCATTG---GGAATAGCACCCACTGGAGCAAAAAGGAGAGTGGTG-----
----------------------------------GAGAGAGAAAAAAGAGCA---GTGGGA---
ATAGGAGCT---------GTGTTCCTTGGGTTCTTGGGAGCA---GCAGGAAGCACTATG---
GGCGCGGCGTCA---ATAACGCTGACGGTACAG---GCCAGACAA---CTGCTG---
TCTGGTATAGTGCAACAGCAAAGC---AATTTG---CTGAGA---
GCTATAGAGGCGCAACAGCATATGTTGCAACTCACAGTCTGGGGCATT
>DQ382361
TTCTACTGCAATACATCAAGACTGTTTAATAGTACATGGCTTAATAGTACAGAGCCTACAGACCAGGCTAAT-
----

GATACAAGTGTAATAACACTCCCATGCAGAATAAAACAAATTATAAACATGTGGCAGGAGGTAGGACGGGCAA
TGTATGCTCCTCCCATTGCAGGAAACATAACATGTAGGTCAAAT---
ATCACAGGATTACTATTGGTGAGGGATGGAGGAAAGGGCAATAGCACA-------------------------
----

GACAATAAATCTGAGATATTCAGACCTGGAGGAGGAGATATGAGGGACAATTGGAGAAGTGAATTATACAAAT
ATAAAGTGGTAGAAGTTAAGCCATTG---GGAATAGCACCCACCCAGGCAAAAAGGAGAGTGGTG--------
-------------------------------GAGAGAGAAAAAAGAGCA---GTGGGA---ATAGGAGCT---
------GTGTTCCTTGGGTTCTTGGGAGCC---GCAGGAAGCACTATG---GGCGTGGCGTCA---
ATGGCGCTGACGGTACAG---GCCAGAAAT---TTATTG---TCTGGTATAGTGCAACAGCAAAGT---
AATTTG---CTGAGG---GCTATAGAGGCGCAACAGCATATGTTGCAACTCACGGTCTGGGGCATC
>DQ382362
TTCTATTGCAATACATCAGACCTGTTTAAGGATAATATTACTATAACTAATAGTACT----------------
----

AACAATACAGTTATTACACTCCAATGCAGAATAAAACAAATTATAAACATGTGGCAAAGGGCAGGACAGGCAA
TATATGCCCCTCCCATTAGAGGAAACATAACATGTAATTCAAAT---
ATCACAGGACTACTATTGACACGTGATGGAGGAAAAGATAATAAAACAAAT----------------------
----

AATGAGAATAAAACAGAGATATTCAGACCTGGAGGAGGAGATATGAGGGACAATTGGAGAAGTGAATTATATA
AATACAAAGTAGTAGAAATTAAGCCATTA---GGAATAGCACCCACTACTGCAAAAAGGAGAGTGGTG-----
----------------------------------GAGAGAGAGAAAAGAGCA---GTGGGA---
ATAGGAGCT---------GTGCTCCTTGGGTTCTTGGGAGCA---GCAGGAAGCACTATG---
GGCGCAGCGTCA---ATAACGCTGACGGCACAG---GCCAGACAA---GTATTG---
TCTGGTATAGTGCAACAGCAAAGT---AATTTG---CTGAGA---
GCTATAGAGGCGCAACAGCATATGTTGCAGCTCACAGTCTGGGGCATT
>DQ382364
TTCTATTGCAATACATCAAAACTGTTTAATGGTACATACAATGGTACAGACATGTCTAATAATACAGAA----
----

AGTAATTCAACCATCACACTCCAATGCAGAATAAAACAAATTATAAACATGTGGCAGGAGGTAGGACGAGCAA
TGTATGCCCCTCCCATTGCAGGAAACATAACATGTAGATCAAAT---
ATCACAGGGCTACTATTGCTACGTGATGGAGGTTTCTGCCAAAAT----------------------------
----

AATACCACAGAGACATTCAGGCCGGGAGGGGGAGATATGAGGAACAATTGGAGAAGTGAATTATATAAGTATA

AAGTGGTAGAAATTAAGCCATTG---GGAGTAGCACCCACTAAGGCAAAAAGGAGAGTGGTG-----------
----------------------------GAGAGAGAAAAAAGAGCA---GTGGGA---ATAGGAGCT------
---GTGTTCCTTGGGTTCTTGGGAGCA---GCAGGAAGCACTATG---GGCGCGGCGTCA---
ATAACGCTGACGGTACAG---GCCAGACAA---CTGTTG---TCTGGCATAGTGCAACAGCAGAGC---
AATTTG---CTGAGA---GCTATAGAGGCGCAACAGCATCTGTTGCAACTCACAGTCTGGGGCATT
>DQ382365
TTCTATTGCAATACATCAAGCCTGTTTAATGGGACATACAATAGTACACGCAATAGT----------------
----

ACAGGAAGGACTATCACACTCCAATGCAGAATAAAACAAATTATAAACATGTGGCAGGAGGTGGGACGAGCAA
TGTATGCCCCTCCCATTGCAGGGAACATAACATGTAATTCAAAT---
ATCACAGGACTAATATTGACACGTGATGGAGGATATGATAATAATAATGCCACT------
AAAGAAAATAAGACTACTAATAATACT--

AATAGTAATAAAACAGAGAAATTCAGACCTGTAGGAGGAGAGATGAGGGATAATTGGAGAAGTGAATTATATA
AATATAAAGTGGTAGAAATTAAGCCATTA---GGAATAGCACCCACTAAGGCAAAAAGGAGAGTGGTG-----
----------------------------------GAAAGGGAGAAAAGAGCA---GTGGGA---
CTAGGAGCT---------GTGTTCCTTGGGTTCTTGGGAGCA---GCAGGAAGCACTATG---
GGCGCGGCGTCA---ATAACGCTGACGGTACAG---GCCAGACAA---TTATTG---
TCTGGTATAGTGCAACAGCAAAGC---AATTTG---CTGAGA---
GCTATAGAGGCGCAACAGCATCTGTTGCAACTCACGGTCTGGGGCATT
>DQ382366
TTCTATTGCGATACATCAAAATTGTTTAATAGGACATTTAATAGTACTAATACAAAAGGTAATAGTACAGATG
CTAGA--
-------------------GGT---
AATAGTAATGCAACAATCACACTCCCATGCAGGATAAAACAATTTATACACATGTGGCAGCCAGTAGGAAGGG
CAATGTATGCTCCGCCCATTGCAGGAAAAATAACATGTAAATCAAAT---
ATCACAGGATTACTATTGGCGAGAGATGGAGGAAACACAAATAAC----------------------------
----

TCAGAGACATTCAGACCTGGAGGAGGAAATATGAAGGACAATTGGAGAAGTGAATTATACAAATATAAAGTGG
TAGAAGTTAAGCCATTG---GGAATAGCACCCACTGGAGCAAAAAGGAGAGTGGTG-----------------
----------------------GAGAGAGAAAAAAGAGCA---GTGGGA---ATAGGAGCT---------
GTGTTCCTTGGGTTCTTGGGAGCA---GCAGGAAGCACTATG---GGCGCGGCGTCA---
ATAACGCTGACGGTACAG---GCCAGACAA---CTGTTG---TCTGGTATAGTGCAACAGCAAGGC---
AATTTG---CTGAGA---GCTATAGAGGCGCAACAGCATATGTTGCAACTCACGGTCTGGGGCATT
>DQ382367
TTCTATTGTAATACATCACAACTGTTCAATAGCACATACAATAGCAATGAGACAACAAGTGAT----------
----

TCAAATTCAACCATCACACTCCCATGTACAATAAAACAAATTATAAATATGTGGCAGGAGGTGGGACGAGCAA
TGTATGCCCCTCCCATTGCAGGAAACATAACATGTGTATCAAAC---
ATCACAGGAATACTATTGACACGTGATGGAGGACAAAATGGAGGAAACAAC----------------------
----

ACAAATGTCCCAGAGATATTCAGACCTGCAGGAGGAGATATGAGGCTCAATTGGAGAAGTGAATTATTTAAAT
ATAAAGTAGTAGAAATTAAGCCATTG---GGAATAGCACCCACTGAGGCAAAAAGGAGAGTGGTG--------
-------------------------------GAGAGGGAAAAAAGAGCA---GTGGGA---ATAGGAGCT---
------GTGTTCCTTGGGTTCTTGGGAGCA---GCAGGAAGCACTATG---GGCGCGGCGTCA---
GTGACGCTGACGGTACAG---GCCAGACAA---TTGTTG---TCTGGTATAGTGCAACAGCAAAGC---
AATTTG---CTGAGG---GCTATAGAGGCGCAACAGCACATGTTGCAACTCACGGTCTGGGGCATT
>DQ396372
TTCTATTGCAATACAACAAAACTGTTTAATGAAACGCTTTTTTCGAAGGAA----------------------
----

ACTAATGAAACCATAACCATCCAATGCAGGATAAAACAAATTATAAACATGTGGCAGGGGGTAGGACGAGCAA
TGTATGCCCCTCCCGTTGCAGGAAACATAACATGTAACTCAAGT---
ATCACAGGAATACTATTAACACGTGATGGCGGTTTAAAAAACGACACC-------------------------
----

AATGACACAGAGACATTCAGACCTGGAGGAGGAAATATGAAGGACAATTGGAGAAGTGAATTATATAAATATA
AAGTGGTAGAAATTAAGCCTTTG---GGAATGGCACCCACTAGGGCAAAAAGGAGAGTGGTG-----------
----------------------------GAGAGAGAAAAAAGAGCA---GTGGGA---ATAGGAGCT------
---GTGTTCCTTGGGTTCTTGGGAGCA---GCAGGAAGCACTATG---GGCGCAGCGTCA---
ATAACGCTGACGGTACAG---GCCAGACAA---CTGTTG---TCTGGTATAGTGCAACAGCAGAGC---
AATCTG---CTGAGA---GCTATAGAGGCGCAACAGCATATGTTGCAACTCACAGTCTGGGGCATT
>DQ369992
TTCTATTGCAATACATCAAAGCTGTTTACTGAATCGCTTAATAGTACA-------------------------
----

GACAATGAAACAATCACAATCCCATGTAAACTAAAACAGATTATAAACCTGTGGCAGGAAGTAGGGCGAGCAA
TGTATGCCCCTCCCATTGAAGGAAACATAACATGTAACTCAAGT---
ATCACAGGACTACTATTGACACGTGATGGAGGAAACACGAATGACACA-------------------------
----

AATGGCCCAGAGATATTCAGACCTGGAGGAGGGAATATGAAGGACAATTGGAGAAGTGAATTATATAAATATA
AAGTGGTAGAAATTAAGCCATTG---GGAATAGCACCCACTGGAGCAAAAAGGAGAGTGGTG-----------
----------------------------GAGAGAGAAAAAAGAGCA---GTGGGA---ATAGGAGCT------
---ATGATCCTTGGGTTCTTGGGAGCA---GCAGGAAGCACTATG---GGCGCGGCGTCA---
ATAACGCTGACGGTACAG---GCCAGACAA---TTGTTG---TCTGGTATAGTGCAACAGCAAAAT---
AATTTG---CTGAGG---GCTATAGAGGCGCAACAGCATATGTTGCAACTAACGGTCTGGGGCATT
>DQ369982
TTCTATTGTAATACATCAGGACTATTTAATGGTACATATAATAGTACAGGATCTAATAGTACAGGA-------
----

AATGGTTCAGTCATTACACTTCCATGCAGAATAAAACAAATTATAAACATGTGGCAGGAAGTAGGACGAGCAA
TGTATGCCCCTCCCATTGCAGGAAACATAACATGTGAGTCAAAT---
ATCACAGGACTACTATTAGTACGTGATGGTGGCAACACAAGTACA----------------------------
----

GAAGAAATATTCAGACCTGGGGGAGGAAATATGAAGGACAATTGGAGAAGTGAATTATATAAATATAAAGTGG
TAGAGGTTAAGCCATTG---GGAATAGCACCCACTAGAGCAAAAAGGAGAGTGGTG-----------------
----------------------GAGAGAGAGAAAAGAGCA---GTGGGA---ATAGGAGCT---------
GTGTTCCTTGGGTTCTTGGGAGCA---GCAGGAAGCACTATG---GGCGCAGCGTCA---
ATAGCGCTGACGGCACAG---GCCAGACAA---GTGCTG---TCTGGTATAGTGCAACAGCAAAGC---
AATTTG---CTGAGG---GCTATAGAGGCGCAACAGCATATGTTGCAACTCACAGTCTGGGGCATT
>DQ369983
TTCTACTGCAATACAACACAACTGTTCAATACAACAAAACTGTTCAATAGCACA-------------------
----

ACTAATGAAACTATCATACTCCCATGCAGAATAAAACAAATTATAAATATGTGGCAGGAGGTAGGACGAGCAA
TGTACGCCCCTCCCATTAGAGGAAACATAACATGTAACTCAAGT---
ATCACAGGGCTGCTATTGGAACGTGATGGAGGAAATGTAAACAGCAGC-------------------------
----

AATAGCACAGAGACATTCAGACCTGGAGGAGGAAATATGAAGGACAATTGGCGTAGTGAATTATATAAATATA
AAGTGGTAGAACTTAAGCCATTG---GGAATAGCACCCACGGATGCAAAAAGGAGAGCGGTG-----------
----------------------------GAGAGAGAAAAAAGAGCA---GTGGGA---ATAGGAGCT------
---GTGCTCCTTGGGTTCTTGGGAGCA---GCAGGAAGCACTATG---GGCGCGGCGTCA---
ATAACGCTGACGGTACAG---GCCAGGCAA---TTATTG---TCTGGTATAGTGCAACAGCAAAGC---
AGTTTG---CTGAGA---GCTATAGAGGCGCAACAGCATATGTCGCAACTCACGGTCTGGGGCATT
>DQ369994
TTCTATTGCAATACATCAAGACTGTTTACATCAAACCTGTCTAGTAAT-------------------------
----

ACAGAAGACATCATCACAATCCCATGCAGAATAAAACAGGTTATAAACATGTGGCAGAAGGTAGGACGAGCAA
TGTATGCCCCTCCCATTGCAGGAAACATAACATGTAGATCCAAC---
ATCACAGGACTACTATTGACACGTGATGGAGGATCAGATAACCATACC-------------------------
----

AATAACACAGAGACTTTCAGACCTGGAGGAGGAGACATGAGGGATAATTGGAGAAGTGAATTATATAAATATA
AAGTAGTAGAAATTAAGCCATTG---GGAATAGCACCCACCAAGGCAAAAAGGAGAGTGGTG----------
----------------------------GAGAGAGAAAAAAGAGCA---GCGGGA---ATAGGAGCT-----
---GTGTTCCTTGGGTTCTTGGGAGCA---GCAGGAAGCACTATG---GGCGCGGCGTCA---
ATAACGCTGACGGTACAG---GCCAGACAA---TTGTTG---TCTGGTATAGTGCAACAGCAAAGC---
AATTTG---CTGAAG---GCTATAGAGGCGCAACAGCATCTGTTGCAACTCACGGTCTGGGGCATT
>HQ615948
TTCTATTGCAATACATCAGGCCTATTTAATAGAAAATACTCCTTTAATGATACATACAATAGTACAGAA---
----

AATAGTTCAAACATCACACTCCAATGCAGAATAAAACAAATTATAAACATGTGGCAGGGGGTAGGACGAGCAA
TGTATGCCCCTCCCATTGAAGGAAATATAACATGTAAATCAAAG---
ATCACAGGACTACTATTAGTACGTGATGGAGGCAACCCTGACAATAGC------------------------
----

AGTGGCACAGAGACATTCAGACCTGGAGGAGGAAACATGAAGGACAATTGGAGAAGTGAATTATATAAATATA
AAGTGGTAGAAATTAAACCATTA---GGAATAGCACCAACTAATGCAAAAAGGAGAGTGGTG----------
----------------------------GAGAGAGAAAAAAGAGCA---GTGGGA---ATAGGAGCT-----
---GTGTTCCTTGGGTTCTTGGGAGCA---GCAGGAAGCACTATG---GGCGCGGCGTCA---
GTGACGCTGACGGTACAG---GCCAGACAA---TTACTG---TCTGGTATAGTGCAACAGCAAAGC---
AATTTG---CTAAGG---GCTATAGAGGCGCAACAACATATGTTGCAACTCACGGTCTGGGGCATT
>JQ754193
TTCTATTGCAATACATCAAAACTGTTCAATAGTACATACATGGCTAATAATACAGACGTGTCTAATAGTACTT
TAAATAGTACT---

TCAAATGCAACCATCACACTCCCATGCAGAATAAAACAAATTATAAACATGTGGCAGGGGGTAGGACGGGCAA
TGTATGCCCCTCCCATTGCAGGAAACATAACATGTAAGTCAAAT---
ATCACAGGAGTACTATTGACACGTGATGGGGGAGAAAGCAATGGG---------------------------
----

ACAGAAGAAGTCTTCAGACCTGGAGGAGGAGATATGAGGGACAATTGGAGAAGTGAATTATATAAGTATAAAG
TGGTAGAAATTAAGCCATTA---GGAATAGCACCCACTGGGGCAAAAAGGAGAGTGGTG-------------
-------------------------GAGAGAGAAAAAAGAGCA---GTAGGA---ATAGGAGCT--------
GTGTTCCTTGGGTTCTTGGGAGCA---GCAGGAAGCACTATG---GGCGCAGCATCA---
ATAACGCTGACGGTACAG---GCCAGACAA---TTGTTG---TCTGGTATAGTGCAACAGCAAAGC---
AATTTG---CTGAGG---GCCATAGAGGCGCAACAACATTTGTTGCAACTCACGGTCTGGGGCATT
>GQ999981
TTCTATTGTAATACAGCAAAACTATTTAACACAACACAGTTTAATAACAAT---------------------
----

GATGCAAACACAACCACCATCCCATGCAGGATAAAACAAATTATAAACATGTGGCAGGGGGTAGGACGAGCAA
TGTATGCCCCTCCCATTGAAGGAAACATAACATGTAACTCAAGT---
ATCGCAGGACTACTATTGACACGTGATGGCGGCCAGCAGAATACA---------------------------
----

AATGAGACATTCAGACCTGGAGGAGGAAATATGAAGGACAATTGGAGAGGTGAATTATATAAATATAAAGTGG
TAGAAATTAAGCCATTG---GGGGTAGCACCCACTGAGGCAAAAAGGAGAGTGGTG----------------
----------------------GAGAGAGAAAAAAGAGCA---GTGGGA---ATAGGAGCT---------
GTGCTCCTTGGGTTCTTGGGAGCA---GCAGGAAGCACTATG---GGCGCGGCGTCA---
ATAACGCTGACGGTACAG---GCCAGACAA---CTGTTG---TCTGGTATAGTACAACAGCAAAGC---
AATTTG---CTGAGA---GCTATAGAGGCGCAACAGCATATGTTGCAACTCACAGTCTGGGGCATC
>KC894135
TTCTATTGCGACACAACAACACTGTTTAATGACACACTGTTTAATAACACACTGTCTGATAAT---------
----

GAAAATGAGACAATCACACTCCCATGCAGAATAAAACAAATTATAAACATGTGGCAGGGGGTAGGACGAGCAA
TATATGCCCCTCCCATTGAAGGGAAAATAACATGTAACTCAACT---
ATCACAGGACTACTATTGACACGTGATGGAGGAGTAAATGCTAGT---------------------------
----

AACAAAGCAGAGACATTCAGACCTGGAGGAGGAAATATGAAGGACAATTGGAGAAGTGAATTATATAAGTATA
AAGTGGTAGAAATTAAGCCATTG---GGAGTAGCACCCACTGGGGCAAAAAGGAGAGTGGTG-----------
----------------------------GAGAGAGAAAAAAGAGCA---GTGGGA---ATGGGAGCT------
---GTGCTCCTTGGGTTCTTGGGAGCA---GCAGGAAGCACTATG---GGCGCAGCGTCA---
ATAACGCTGACGGTACAG---GCCAGACAA---CTGTTG---TCTGGTATAGTGCAACAGCAAAGC---
AATTTG---CTGAGA---GCTATAGAGGCGCAACAGCATATGTTGCAACTCACGGTCTGGGGCATT
>GQ999983
TTCTATTGCAATACCACAAAACTGTTTAATAGTACACATAATAGTACTGATAGTACAGTAAATAGTACTGATA
GTACAGCAGAA--
----------ACAGGAAAT------
TCAACAAATACAAACATCACGCTCCCATGCAGAATAAGGCAAATTATAAACATGTGGCAGGAGGTAGGACGAG
CAATGTATGCTCCTCCCAGTAAAGGGAACATAACATGTATATCAAAC---
ATCACAGGACTATTACTGACGCGTGATGGAGGAGAAAACAAAACAGAG-------------------------
----

AATAATGACACAGAGATATTCAGGCCTGGAGGAGGAGATATGAAGGACAATTGGAGAAGTGAATTATATAAAT
ATAAAGTAGTAGAAATTAAACCATTA---GGAGTAGCACCCACTAGAGCAAAAAGGAGAGTGGTG--------
-------------------------------GAGAGAGAAAAAAGAGCA---GTGGGA---ATAGGAGCT---
------GTGTTCCTTGGGTTCTTGGGAGCA---GCAGGAAGCACTATG---GGCGCAGCATCC---
ATAACGCTGACGGTACAA---GCCAGACAA---TTATTG---TCTGGTATAGTGCAGCAGCAAAGT---
AATTTG---CTGAGA---GCTATAGAGGCGCAACAACATATGTTGCAACTCACAGTCTGGGGCATT
>GQ999984
TTCTATTGCAATACAACAAAACTGTTTAATGAAAATGATACAAAATTGTTTAATGAAAGTGCTACAATTAAAA
ATGAA--
-------------------AGT---
AATACAAATGAAAACATCACAATCCCATGCAGAATAAAACAAATTATAAACATGTGGCAGGGGGTAGGACGAG
CAATGTATGCCCCTCCCATTGCAGGAAACATAACATGTAAATCAAAT---
ATCACAGGACTACTATTGCTACGTGATGGAGGAAACAGCAGTGAGGGAGGAAAC-------------------
----

AGCAGTGATAAAAATGAGATATTCAGACCTGGAGGAGGAAATATGAAGAACAATTGGAGAAGTGAATTATATA
GATATAAAGTGATAGAACTTAAACCATTG---GGAATAGCACCCACCGAGGCAAGAAGGAGAGTGGTG-----
----------------------------------GAGAGAGAAAAAAGAGCA---GTGGGA---
CTAGGAGCT---------GCACTCATTGGGTTCTTGGGAGCA---GCAGGAAGCACTATG---
GGCGCGGCGTCA---ATAACGCTGACGGTACAG---GCCAGACAA---CTGTTG---
TCTGGTATAGTGCAACAGCAAAGC---AACTTG---CTGAGA---
GCTATAGAGGCGCAACAGCATATGTTGCAACTCACGGTCTGGGGCATT
>GQ999985
TTCTATTGCAATACATCAAAACTGTTTCTAATAATTTTACTAGT-----------------------------
----

TCAACCAACATAACCACCATCCCATGTAGGATAAAACAAATTATAAACATGTGGCAGGGGGTAGGACGAGCAA
TGTATGCCCCTCCCATTGCAGGAAACATAACATGTAACTCAAGT---
ATCACAGGACTACTATTGGTACGTGATGGTGGTAATGATAATAATAAA-------------------------
----

ACGAATGTCATAGAGACATTCAGACCTGAAGGAGGAAATATGAAGGACAATTGGAGAAGTGAATTATATAAAT
ATAAAGTAGTAGAAATTAAGCCATTG---GGAATAGCACCCACTACGGCAAAAAGGAGAGTGGTG--------
-------------------------------GAGAGAGAAAAGAGGGCA---GTGGGA---ATAGGAGCT---
------GTGTTCCTTGGGTTCTTGGGAGCA---GCAGGAAGCACTATG---GGCGCGGCGTCA---
GTGGCGCTGACGGTACAG---GCCAGACAA---TTGTTG---TCTGGAATAGTGCAACAGCAAAGC---
AATTTG---CTAAAA---GCTATAGAGGCGCAGCAGCATATGTTGCAGCTCACGGTCTGGGGCATT
>GQ999989
TTCTATTGCAATACGTCAGACCTGTTTAATGGTACATACAATAACATTAGTGTCACTAGTACTTCAGATTCA-
----

AGTACACCACACATCACACTCCAATGCAGAATAAAACAAATTATAGACATGTGGCAGGAGGTGGGACGAGCAA
TGTATGCCCCTCCCATTGCAGGAAATATAACATGTAACTCAAAT---
ATCACAGGACTACTATTGACACGTGATGGAGGAAGAGAAAGTAAT----------------------------
----

GAGACAGAGACATTCAGACCTGGAGGAGGAGATATGAGGAACAATTGGAGAAGTGAATTATATAAATATAAAG
TAGTAGAAATTAAACCATTA---GGAATAGCACCCACTGAAGCAAAAAGGAGAGTGGTG--------------
-------------------------GAGAGAGAGAAACGAGCA---GTAGGA---ATGGGAGCT---------
GTGATCTTTGGGTTCTTGGGAGCA---GCAGGAAGCACTATG---GGCGCGGCGTCA---
ATGGCGCTGACGGTACAG---GCCAGACAA---CTGCTG---TCTGGTATAGTGCAACAGCAAAGC---
AATTTG---CTGAGA---GCTATAGAGGCGCAACAGCATATGTTGCAACTCACAGTCTGGGGCATC
>GQ999990
TTCTATTGCAATACAACAAACCTGTTTAATACAAATAATAGTGATTCA-------------------------
----

GGCAACACAACCATCACACTTCCATGCAGAATAAAACAAATTGTAAACCTGTGGCAGGGAGTAGGACGAGCAA
TGTATGCCCCTCCTATTGCAGGAAACATAACATGTAACTCAACT---
ATCACAGGACTACTATTGACACGTGATGGAGGAATAAATGCATCA----------------------------
----

AAGACAGAGACATTCAGACCTGGAGGAGGAGATATGAGGGACAATTGGAGAAGTGAATTATATAAGTATAAAG
TGGTAGAAATTAAGCCATTG---GGAATAGCTCCCACTACGTCAAAAGGGAGAGTGGTG--------------
-------------------------GAGAGAGAAAAAAGAGCA---GTAGGG---ATAGGAGCT---------
GTGTTCCTTGGGTTCTTGGGAGCA---GCAGGAAGCACTATG---GGCGCAGCGTCA---
CTAACGCTGACGGTACAG---GCCAGACAA---CTGTTG---TCTGGTATAGTGCAACAGCAAAGC---
AATTTG---CTGAGA---GCTATAGAGGCGCAACAACATATGTTGCAACTCACGGTCTGGGGCATT
>JN681227
TTCTATTGCAATACAACAAATCTGTTTGAAGTTAACAGCACA-------------------------------
----

AACATTACACTCCCATGTAAGATAAAACAAATTGTAAACATGTGGCAAGAGGTAGGACGAGCAATGTATGCCC
CTCCCATTGCAGGAAACATAACATGTAAATCAAAT---
ATCACAGGACTACTATTGACAAGGGATGGAGGAGTCAACAACGGAACA-------------------------
----

AAGAATAATGAAACATTTAGACCTACAGGAGGAAATATGAAAGACAATTGGAGAAGTGAATTATATAAGTATA
AAGTGGTAGAAATTAAGCCATTA---GGAATAGCACCCACTGGATCAAAAAGGAGAGTGGTG-----------
----------------------------GAGAGAGAGAAAAGAGCA---GTGGGA---ATAGGAGCT------
---GTGTTCCTTGGGTTCTTGGGAGCA---GCAGGAAGCACTATG---GGCGCGGCGTCA---
ATAACGCTGACGGTACAG---GCCAGACAA---CTGCTG---TCTGGTATAGTGCAACAGCAAAGC---
AATTTG---CTGAGA---GCTATAGAGGCGCAACAGCATATGTTGCAACTCACAGTCTGGGGCATT
>GQ999974
TTCTATTGCAATACAACACGACTGTTTAAGTGGTCTAGTAATGTC----------------------------
----

ACAAACGACACAATCACAATCCCATGCAGAATAAAACAATTTATAAACATGTGGCAAGGGGTAGGACGAGCAA
TGTATGCCCCTCCCATTGAAGGAAACATAACACGTAACTCAAGT---
ATCACAGGACTCCTATTGACACGTGATGGAGGGAAAACAGACAGG----------------------------
----

AATGACACAGAGATATTCAGACCTGGAGGAGGAAATATGAAGGACAATTGGAGAAATGAATTATATAAATATA
AAGTGGTAGAAATTAAGCCATTG---GGAGTAGCACCCACTGAGGCAAGAAGGAGAGTGGTG-----------
----------------------------GAGAGAGAAAAAAGAGCA---GTGGGA---ATAGGAGCT------
---GTACTCCTTGGGTTCTTGGGAGCA---GCAGGAAGCACTATG---GGCGCGGCGTCA---
ATAACGCTGACGGTACAG---GCCAGGCAA---CTGTTG---TCTGGTATAGTGCAACAGCAAAGC---
AATTTG---CTGAGA---GCTATAGAGGCGCAACAGCACATGTTGCAACTCACGGTCTGGGGCATT
>GQ999976
TTCTATTGCAATACATCAGGACTGTTTAACAGTACATACATGTCTAATGGTATACACATACCAAATGGT----
----

GCAAGCGAAGTCATCACACTCCCATGCAGAATAAAACAAATTATAAACATGTGGCAGGAGGTAGGACGAGCAA
TGTACGCCCCTCCCATTGAAGGAAACATAACATGTAGCTCAAAT---
ATCACAGGACTACTATTGACACGTGATGGAGGAAACAATAGCACA----------------------------
----

AACGGAACAGAGACATTCAGACCTGGAGGAGGAAATATGAAGGACAATTGGAGAAGTGAATTATATAAATATA
AAGTGGTAGAAATTAAGCCATTA---GGAATAGCACCCACCAAGGCAAAAAGGAGAGTGGTG----------
----------------------------GGGAGAGAAAAAAGAGCA---GTGGGA---CTGGGAGCT------
---GTGTTCCTTGGGTTCTTGGGAGCA---GCAGGAAGCACTATG---GGCGCGGCGTCA---
CTAACGCTGACGGCACAG---GCCAGACAA---TTGTTG---TCTGGTATAGTGCAGCAGCAAAAC---
AATTTG---CTGAGA---GCTATAGAGGCGCAACAGCATATGTTGCAACTCACAGTCTGGGGCATT
>GQ999978
TTCTATTGCAATACAACAAATCTGTTCAATCTGAACAGTACTGATGGAAGT---------------------
----

AACATCACTAACATCACAATCCCATGCAGAATAAAACAAATTATAAACATGTGGCAGAAGGTAGGACGAGCAA
TGTATGCCCCTCCTATTGAAGGAATAATAACATGTAACTCAAGT---
ATCACAGGACTACTGCTGACACGTGATGGAGGTGGAAATGAAAGT---------------------------
----

GAAACAGAGACATTCAGACCTGCAGGAGGAGATATGAAGGACAATTGGAGAAGTGAATTATATAAATATAAAG
TGGTACAAATTAAGCCATTA---GGAATAGCACCCACTACGGCAAAAAGGAGAGTGGTG-------------
-------------------------GAGAGAGAAAAAAGAGCA---GTGGGA---ATAGGAGCT---------
GTATTCCTTGGGTTCTTGGGAGCA---GCAGGAAGCACTATG---GGCGCGGCGTCA---
ATAACGCTGACGGTACAG---GCCAGACAA---CTGTTG---TCTGGTATAGTGCAACAGCAAAGC---
AATTTG---CTGAGA---GCTATAGAGGTGCAACAGCATATGTTGCAACTCACGGTCTGGGGCATT
>GQ999979
TTCTACTGCAATACATCACAACTGTTTAATAGTACAGTGAATGGTACAGAAGGAAGTTCAACCACCACAAAC-
----

GGGAGTTCACCCATCACACTCCCATGCAGAATAAAACAAATTATAAACATGTGGCAGGGGGTAGGAAGAGCAG
TGTATGCCAACCCCATTGCAGGAAACATAACATGTATATCAAAC---
ATCACAGGACTACTATTGACACGTGATGGAGGCATGGCAGCTAACATG------------------------
----

ACAAATAACACAGAGATATTCAGACCTGGAGGAGGAAATATGAAGAATAATTGGAGAAGTGAATTATATAAGT
ATAAAGTGGTAGAAATTAAGCCATTG---GGAATAGCACCCAATAAGGCAAAAAGGAGAGTGGTG--------
-------------------------------GAGAGAGAAAAAAGAGCA---GTGGGA---ATAGGAGCT---
------GTGTTCCTTGGGTTCTTGGGAGCA---GCAGGAAGCACTATG---GGCGCAGCGTCA---
ATAACGCTGACGGCACAG---GCCAGACAA---TTGTTG---TCTGGTATAGTGCAACAGCAAAGC---
AATCTG---CTGAAG---GCTATCGAGGCGCAACAGCATCTGTTGCAACTCACGGTCTGGGGCATT
>GQ999980
TTCTATTGCAATACGTCGAAACTGTTTAATGAGAATGAGACAGAA---------------------------
----

ACAAACACAACCATCACACTCCCATGCAGAATAAAACAAATTATAAACATGTGGCAGGAGGTAGGACGAGCAA
TGTATGCCCCTCCCATTGCAGGGAACATAACATGTAACTCAAGT---
ATCACAGGACTACTATTACTACGTGATGGAGGAAACGAAAACATAACA------------------------
----

AATGGCACAGAGATATTTAGACCTGGAGGAGGAAATATGAAGGACAATTGGAGAAGTGAATTATATAAATATA
AAGTAGTAGAAATTAGACCATTG---GGAGTAGCACCCACCGAGGCAAAAAGGAGAGTGGTG----------
----------------------------GAGAGAGAAAAAAGAGCA---GCGGGA---ATAGGAGCT------
---GTGCTCCTTGGGTTCTTGGGAGCA---GCAGGAAGCACTATG---GGCGCGGCGTCA---
ATAACGCTGACGGTACAG---GCCAGACAA---CTGTTG---TCTGGTATAGTGCAACAGCAAAAC---
AATTTG---CTGAGA---GCTATAGAGGCGCAACAGCATATGTTGCAACGCACGGTCTGGGGCATT
>FJ443982
TTCTATTGCAATACCACAAAACTATTTAATAATAATGAGACAGGAAGT------------------------
----

AATTCAACCAACATCACAATTCCATGTAGAATAAAACAAATTATAAACATGTGGCAGGGGGTAGGACGAGCAA
TGTATGCCCCTCCCATTGCAGGAAACATAACATGTAACTCAAAT---
ATTACAGGACTACTATTGACACGTGATGGAGGATATGATAACAAAACC------------------------
----

AATAACACAGAGACATTCAGGCCTGGAGGAGGAGATATGAGGGACAATTGGAGAAGTGAATTATATAAATATA
AAGTGGTAGAAATCAAGCCATTG---GGAATAGCACCCACGAAGGCAAAAAGGAGAGTGGTG-----------
----------------------------GAGAGAGAAAAAAGAGCA---GTGGGC---ATGGGAGCT------
---GTGATCTTTGGGTTCTTGGGAGCA---GCAGGAAGCACTATG---GGCGCAGCGTCA---
ATAGCGCTGACGGCACAG---GCCAGACAA---CTGCTG---TCTGGTATAGTGCAACAGCAAAAC---
AATTTG---CTGAGA---GCTATAGAGGCGCAACAGCATATGTTGCAACTCACGGTCTGGGGCATT
>HQ595744
TTCTATTGCAATACATCAAAACTGTTTTCTAATGATTCAATTAAT----------------------------
----

TCAGACGACAACATCACAATCCCATGCAGGATAAAACAAATTATAAACATGTGGCAGGGGGTAGGACGAGCAA
TGTATGCCCCTCCCATTGAAGGAATAATAACATGTAACTCAAAT---
ATCACAGGACTACTATTGACACGCGATGGAGGAGACTCAAATGGC----------------------------
----

ACAGAGATATTCAGACCTGCAGGAGGAAATATGAAGGACAATTGGAGAAGTGAATTATATAAATATAAAGTGG
TAGAAATTAAGCCATTA---GGAGTAGCACCCACTGGATCAAAAAGGAGAGTGGTG-----------------
----------------------GAGAGAGAAAAAAGAGCA---GTGGGA---ATAGGAGCT---------
GTTCTCCTTGGGTTCTTGGGAGCA---GCAGGAAGCACTATG---GGCGCGGCGTCA---
ATAACGCTGACGGTACAG---GCCAGACAA---CTGTTG---TCTGGTATAGTGCAACAGCAAAAT---
AATTTG---CTGAGA---GCAATAGAGGCGCAACAACATATGTTGCAACTCACAGTCTGGGGCATT
>HQ595745
TTTTATTGCAATACATCAAAGCTGTTTACCTTCAACAGTACT-------------------------------
----

TCAAATGAAACCATTACAATCCCATGCAGAATAAAACAAATTATAAACATGTGGCAGGAGGTAGGACGAGCAA
TATATGCCCCTCCCATTGCAGGAAACATAACATGTACATCAAAT---
ATCACAGGACTACTATTGACACGTGATGGAGGAACTAATGGAACT----------------------------
----

AATAACACAGAGATATTCAGACCTGCAGGAGGAGATATGAGGGACAATTGGAGAAGTGAATTATATAAATATA
AAGTGGTAGAAGTTAAGCCATTA---GGAATAGCACCCACCATGGCAAGAAGGAGAGTGGTG-----------
----------------------------GAGAGAGAAAAAAGAGCA---GTGGGA---ATAGGAGCT------
---GTGTTCCTTGGGTTCTTGAGTGCA---GCAGGAAGCACTATG---GGCGCAGCATCA---
ATAACGCTGACGGTACAG---GCCAGACAA---TTGTTG---TCTGGTATAGTGCAACAGCAAAGC---
AATTTG---CTGAGG---GCTATAGAGGCGCAACAGCATATGTTGCAACTCACGGTCTGGGGCATT
>HQ615959
TTCTATTGCAATACAACAAAACTGTTTAAAAAAGAAAATGAAACAGAGGGAAAA-------------------
----

AATGAAACAATCATCACACTCCCATGCAAAATAAAACAAATTATAAACATGTGGCAGGAGGTAGGACGAGCAA
TGTATGCCCCTCCCATTAGAGGAAACATAACATGTAACTCAAGT---
ATCACAGGACTACTATTAACACGTGATGGAGGTGATGGAGGAAATACAACA----------------------
----

GATAATAACACAGAGACATTCAGACCTGGAGGAGGAAATATGAAAGACAATTGGAGAAGTGAATTATATAAAT
ATAAAGTGGTAGAAATTAAGCCATTG---GGAGTAGCACCCACTGAGGCAAAAAGGAGAGTGGTG--------
-------------------------------GAGAGAGAAAAAAGAGCA---ATGGGA---ATAGGAGCT---
------GTACTCCTTGGGTTCTTGGGAGCA---GCAGGAAGCACTATG---GGCGCGGCGTCG---
ATAACGCTGACGGTACAG---GCCAGACAA---TTGTTG---TCTGGTATAGTGCAACAGCAAAGC---
AATTTG---CTGAGA---GCTATAGAGGCGCAACAGCATATGTTGCAACTCACAGTCTGGGGCATT
>HQ595757
TTCTATTGCAATACATCAGGCCTGTTCAATAGTACATACCATATTAATGGTACTTTAAATTCAAGTACTGCA-
----

AATGATTCCTCCATCATACTCCAGTGCAAAATAAAACAAATTATAAACATGTGGCAGGGGGTAGGGCGAGCAA
TGTATGCCCCTCCCATTGAAGGAAACATAACATGTAAATCAAAT---
ATCACAGGGCTACTATTGCTACGTGATGGAGGTACAAATGATACA----------------------------
----

GGGACAACAGAGACATTTAGACCTGGAGGAGGAGATATGAGGGACAATTGGAGAAGTGAATTGTATAAATATA
AAGTGGTAGAAATTAAGCCACTA---GGAGTAGCACCCACTAAGGCAAGAAGGAGAGTGGTG-----------
----------------------------GAGAGAGAAAAAAGAGCA---GTGGGA---ATAGGAGCT------
---GTGTTCCTTGGGTTCTTGGGAGCA---GCAGGAAGCACTATG---GGCGCAGCATCA---
ATAACGCTGACGGTACAG---GCCAGACAA---TTGTTG---TCTGGTATAGTGCAACAGCAAAGC---
AATTTG---CTGAGG---GCTATAGAGGCGCAACAGCATATGTTGCAACTCACGGTCTGGGGCATT
>KC894111
TTCTATTGCAACACATCAGAACTGTTTAATAGTCCTAATGATACAAGCAGTACAAATGCAACCAGTCCT----
----

TCAAATGCAACCATCACACTCCAATGCAAAATAAAACAAATTATAAACATGTGGCAAGGGGTAGGACGGGCAA
TGTACGCCCCTCCCATTGCAGGAAAAATAACATGTAGGTCAAAC---
ATCACAGGACTACTATTGACACGTGATGGGGGAAATAGCGAAAAT----------------------------
----

AATACAAAGGAAACCTTCAGACCTGGAGGAGGAGATATGAGGGACAATTGGAGGAGTGAATTATATAAATACA
AAGTAGTAGAAATTAAGCCATTA---GGAATAGCACCCACTAAGGCAAAAAGGAGAGTGGTG-----------
----------------------------GAGAGAGAAAAAAGAGCA---GTAGGA---CTAGGAGCT------
---GTGCTCCTTGGGTTCTTGGGAGCA---GCCGGAAGCACTATG---GGCGCAGCATCA---
ATAACGCTGACGGTACAG---GCCAGACAA---TTGTTG---TCTGGTATAGTGCAACAGCAAAGC---
AATTTG---CTGAGG---GCTATAGAGGCGCAACAGCGTATGTTGCAACTCACGGTCTGGGGCATT
>KC863324
TTCTATTGCAATACAACAAATCTGTTTACAATTAGTAATGCAACAAAACCGGATATC----------------
----

ACTAATGGTTCCATCATACTCCCGTGCAGAATAAAACAAATTATAAACATGTGGCAGGGGTTAGGACGAGCAA
TGTATGCCCCTCCCATTGCAGGAAACATAACATGTAAATCAAAT---
ATCACAGGACTACTATTGACACGTGATGGAGGGAATAACATAAAT----------------------------
----

AATACAGAGACATTCAGACCTGGAGGAGGAAATATGAAGGACAATTGGAGAAGTGAATTATATAAATATAAAG
TGGTAGAAATTAAGCCATTG---GGAGTAGCACCCACCGCATCAAAAAGGAGAATAGTG--------------
-------------------------GAGAGAGAAAAAAGAGCA---GTGGGA---ATAGGAGCT---------
GTGTTCCTTGGGTTCTTGGGAGCA---GCAGGAAGCACTATG---GGCGCGGCGTCC---
ATAACGCTGACGGTACAG---GCCAGACAA---CTGTTG---TCTGGTATAGTGCAACAGCAAAGC---
AATTTA---CTGAGA---GCTATAGAGGCGCAACAGAATCTGTTGCAACTCACGGTTTGGGGCATT
>KC863361
TTCTATTGCGATACAACAAACCTGTTTAATAACAACAGTAGT-------------------------------
----

GCAGACAACATCACACTCACACTCCCGTGCAGGATAAAACAAATTATAAACATGTGGCAGGGGGTAGGAAAAG
CAATGTATGCCCCTCCCATTGAAGGAAAAATAACATGTAACTCAAGT---
ATCACAGGACTACTATTGATACATGATGGAGTACACAACAACACA----------------------------
----

AACAACATGACATTCAGACCTGGAGGAGGAAATATGAAGGACAATTGGAGAAGTGAATTATATAAGTATAAAG
TGGTAGAAATTAAGCCATTG---GGAGTAGCTCCCACTGAGGCAAAAAGGAGAGTGGTG--------------
-------------------------GAGAGAGAAAAAAGAGCA---GTGGGA---ATAGGAGCT---------
GTACTCCTTGGGTTCTTGGGAGCA---GCAGGAAGCACTATG---GGCGCGGCGTCA---
ATAACGCTGACGGTACAG---GCCAGACAA---CTGTTG---TCTGGTATAGTGCAACAGCAAAGC---
AATTTG---CTGAGA---GCTATAGAGGCACAACAACATATGTTGCAACTCACGGTCTGGGGCATT
>KC894133
TTCTATTGCAATACAACAAAACTATTCACAAACCAAAAC----------------------------------
----

AGTACTGAGAACATTACACTCCCATGCAGAATAAAACAAATTATAAACATGTGGCAGGAGGTAGGACGGGCAA
TGTATGCCCCTCCCATTGAAGGAAGCATAACATGTGACTCAAAT---
ATCACAGGACTACTACTGACACGTGATGGAGGAGACATGAATGGCACA-------------------------
----

AATGGCACAGAAACATTCAGACCTGGAGGAGGAAATATGAAAGACAATTGGAGAAGTGAATTATATAAATATA
AAGTGGTAGAAATTAGGCCATTG---GGAGTAGCACCCACTAAGGCAAAAAGGAGAGTGGTG-----------
----------------------------GAGAGAGGAAAAAGAGCA---GTGGGA---ATAGGAGCT------
---GTATTCCTTGGGTTCTTGGGAGCA---GCAGGAAGCACTATG---GGCGCGGCGTCA---
ATAACGCTGACGGTACAG---GCCAGACAA---CTGTTG---TCTGGTATAGTGCAACAGCAAAGC---
AATTTG---CTGAGA---GCTATAGAGGCGCAACAGCATATGTTGCAACTCACAGTCTGGGGCATA
>FJ443300
TTCTATTGTAATACATCAGAACTGTTTAATAGTACATACAATAGTACATACAATGGTACATCCAGT-------
----

GAAAACAGCACACTCACACTCCCATGCAAAATAAAACAAATTATAAGAATGTGGCAGGGGGTAGGACAGGCAA
TGTATGCCCCTCCCATTGCAGGAAACATAACATGTAAATCAAAT---
ATCACAGGACTGCTATTGACATGGGATGGCGGAGATAACAAGGAAAAT-------------------------
----

AACAATACAGAGATATTCAGACCTGGAGGAGGAGACATGAGGGACAATTGGAGAAGTGAGTTATATAAATACA
AAGTGGTAGAAATTAAGCCACTA---GGAATAGCACCCACTGAGGCAAAAAGGAGAGTGGTG-----------
----------------------------GAGAGAGAAAAAAGAGCA---GCAGGA---CTAGGAGCT------
---GTGTTCCTTGGGTTCTTGGGAGCA---GCAGGAAGCACTATG---GGCGCAGCGTCG---
ATAACGCTGACGGTACAG---GCCAGACAA---TTATTG---TCTGGTATAGTGCAACAGCAAAGC---
AATTTG---CTGAAG---GCTATAGAGGCGCAACAGAATATGTTGCGACTCACGGTCTGGGGCATT
>FJ443417
TTCTATTGCAATACATCACAACTATTTAATAGTACATACAACAATACATATAACTCTAGTGGTATAGAA----
----

AGTAGTGCAACCATCACACTTCCATGCAGAATAAAACAAATCATAAACATGTGGCAGGAGGTAGGAAGAGCAA
TGTATGCTCCTCCCATTGCAGGAAACATAGCATGCAACTCAAAT---
ATCACAGGACTACTATTGGTACGTGATGGAGGTAAAATTAACAAT----------------------------
----

ACCGAACCAGAAATATTCAGACCTGGAGGAGGAAATGTTAAGGACAATTGGAGAAGTGAATTATATAAATATA
AAGTGGTAGAAATCAAGCCATTG---GGAATAGCACCCACTGGAGCAAAAAGGAGAGTGGTG-----------
----------------------------GAGAGAGAAAAAAGAGCA---GTGGGA---ATAGGAGCT------
---TTGTTCCTTGGGTTCTTGGGAGCA---GCAGGAAGCACTATG---GGCGCGGCGTCA---
ATAACGCTGACGGTACAA---GCCAGACAA---TTGTTG---TCTGGTATAGTGCAACAGCAAAAT---
AATTTG---CTGAGG---GCTATAGAGGCGCAACAACATATGTTGCAACTCACAGTCTGGGGCATT
>FJ443426
TTCTATTGCAATACAACAAAACTGTTTAGTAGTGATTCAAAC-------------------------------
----

AATGGCACAATTACACTCCCATGCAGAATAAAACAAATTATAAACATGTGGCAGGGGGTAGGACGAGCAATGT
ATGCCCCTCCCATTGAAGGAAGCATAACATGTAACTCAAAT---
ATCACAGGACTACTATTGACACGGGATGGAGGTATGAACAACACA----------------------------
----

GAAAAAGAGATATTCAGGCCTGGAGGAGGAAATATGAAGGACAATTGGAGAAGTGAGTTATATAAATATAAAG
TGGTAGAAATTAAACCATTG---GGAATAGCACCCACAGAGGCAAAGAGGAGAGTGGTG--------------
-------------------------GAGAGAGAAAAAAGAGCA---GTGGGA---ATAGGAGCT---------
GTGCTCCTTGGGTTCTTGGGAGCA---GCAGGAAGCACTATG---GGCGCAGCGTCA---
ATAACGCTGACGGTACAG---GCCAGGCAA---TTGCTG---TCTGGTATAGTGCAACAGCAAAAC---
AATTTG---CTGAGA---GCTATAGAGGCGCAACAGCATATGTTGCAACTCACGGTCTGGGGCATT
>FJ443473
TTCTATTGCAATACATCAGGCCTGTTTAAAAACAACCTTACTGCTAGTAATACAGAA----------------
----

AGTAACCAGACCATCACACTCCCATGCAGAATAAAACAAATTGTAAACATGTGGCAGGAGGTAGGAAGAGCAA
TGTATGCCCCTCCCATTGCAGGAAACATAACATGTACATCAAAT---
ATCACGGGACTATTATTATTACGTGATGGTGGTAACCTAAATAACTCAAGTAGC------------TCA----
----

-
AATAGCACAAATATCACAGAGATATTTAGGCCTGGAGGAGGAGACATGAGGGACAATTGGAGAAGTGAATTAT
ATAAGTACAAAGTGGTAGAAATCAAGCCATTG---GGAATAGCACCCACTGAGGCAAAAAGGAGAGTGGTG--
-------------------------------------GAGAGAGAA---AAAAGA---GGA------
ATAGGAGCT---------TTGCTCGTTGGATTCTTGGGAGCA---GCAGGAAGCACTATG---
GGCGCGGCGTCA---ATAACGCTGACGGTACAG---GCCAGACAA---TTGTTG---
TCTGGTATAGTGCAACAGCAAAGC---AATTTG---CTGAGA---
GCTATAGAGGCGCAACAGCATCTGTTGCAACTCACGGTCTGGGGCATT
>FJ443488
TTCTATTGCAATACAACACCACTGTTTAATACAACACTGTTTAATACAACACATCCA----------------
----

CATAATGATACCATCACACTCCAATGTAGGATAAAACAGATTATAAACATGTGGCAAGGGGTAGGACGAGCAA
TGTATGCCCCTCCAATTGCAGGAAACATAACATGTAACTCAAGT---
ATCACAGGAATACTATTGACACGTGATGGAGGCCAAACAAATGGCACA-------------------------
----

AATGGCACAGAGACATTCAGACCTGGAGGAGGAAATATGAAGGACAATTGGAGAAGTGAATTATACAAATATA
AAGTAGTAGAAATTAAGCCATTG---GGTTTAGCACCCACTGGAGCAAAAAGGAGAGTGGTG-----------
----------------------------GAGAGAGAAAAAAGAGCA---GTGGGA---ATAGGAGCT------
---GTACTCCTTGGGTTCTTGGGAGCA---GCAGGAAGCACTATG---GGCGCGGCGTCA---
ATAACGCTGACGGCACAG---GCCAGACAA---CTGTTG---TCTGGTATAGTGCAACAGCAAAGC---
AATTTG---CTGAGA---GCTATAGAGGCGCAACAGCATATGTTGCAACTCACAGTCTGGGGCATT
>FJ443498
TTCTATTGCGATACATCAGAACTATTTAATAAAACATTTGGTAATCTAACAGAAAGC----------------
----

AATTCACCAAACATCACACTTCCATGCAGAATAAAACAAATTATAAACATGTGGCAGGGGGTAGGACGAGCAA
TGTATGCCCCTCCCATTGCAGGAAACATAACATGTAACTCAAAT---
ATCACAGGGCTACTATTAACACGTGATGGAGGCACCAACCAAACA----------------------------
----

GGAGAAAAAGAAATATTCAGACCTGGAGGAGGAGATATGAAGGACAATTGGAGAAGTGAATTATATAAATATA
AAGTGGTAGAAATTAAGCCATTG---GGAATAGCACCCACTGAAGCAAAAAGGAGAGCGGTG-----------
----------------------------GAGAGAGAAAAAAGAGCA---GTGGGA---ATAGGAGCT------
---TTGTTCCTTGGGTTCTTGGGAGCA---GCAGGAAGCACTATG---GGCGCAGCGTCA---
ATAACGCTGACGGTACAG---ACCAGACAA---CTGTTG---TCTGGTATAGTGCAACAGCAAAGC---
AATTTG---CTGAGG---GCTATAGAGGCGCAGCAGCATATGTTGCAACTCACAGTCTGGGGCATT
>FJ443515
TTCTATTGTAATACATCAAAACTGTTTAATAGTAATAGTAGTGAACTATTTAATAGTAGT-------------
----

GAAACAGAAAACATCACACTTCCATGCAAAATAAAACAAATTATAAACATGTGGCAGGGGGTAGGACGAGCAA
TGTACGCCCCTCCCATTGAAGGAAACATAACATGTAGCTCAAAT---
ATCACAGGATTGCTATTGACACGTGATGGAGAAAAAAATAGAACA----------------------------
----

AAAGAGACATTTAGGCCTGGAGGAGGAAATATGAAAGACAATTGGAGAAGTGAATTATATAAATATAAAGTGG
TAGAAATTAAGCCATTG---GGAATAGCACCAACTGGGGCAAAAAGGAGAGTGGTG-----------------
----------------------GAGAAAGAAAAAAGAGCA---GTGGGA---ATAGGAGCT---------
GTGATCCTTGGGTTCTTGGGAGCA---GCAGGAAGCACTATG---GGCGCGGCGTCA---
ATAACGCTGACGGTACAG---GCCAGACAA---CTGTTG---TCTGGTATAGTGCAACAGCAAAAT---
AATTTG---CTGAGG---GCTATAGAGGCGCAACAACATATGTTGCAACTCACAGTCTGGGGCATT
>HQ615950
TTCTATTGCAATACATCACAACTGTTTAATGGGACATACTCTAATAGTACATACTCTAATAGTACA-------
----

GGAAATGAAACCATCACAATTCCATGTAGAATAAAACAAATTATAAACATGTGGCAGGAGGTAGGACGAGCAA
TGTATGCCCCTCCCATTGCAGGAAACATAACATGTAGATCAAGT---
ATCACAGGACTACTATTGACATGGGATGGTAATGGTGATGATGGGAAT-------------------------

```
-------------------------------------------------------------------------
-------
GATACTGAAACTGAAACATTCAGACCTGGAGGAGGAGATATGAGGGACAATTGGAGAAGTGAATTATATAAGT
ATAAGGTAGTAGAAATTAAGCCATTA---GGAATAGCACCAACCCAGGCAAAAAGGATAGTGGTG--------
-------------------------------GAGAGAGAAAAAAGAGCA---GTA------ATAGGAGCT---
------GTGCTCCTTGGGTTCTTGGGAGCA---GCAGGAAGCACTATG---GGCGCGGCGTCA---
ATAACGCTGACGGTACAG---GCCAGACAA---CTGTTG---TCTGGTATAGTGCAACAGCAAAGC---
AATTTG---CTGAGA---GCTATAGAGGCGCAACAGCATATGTTGCAACTCACAGTCTGGGGCATT
>HQ615944
TTCTATTGCAATACATCAAACTTGTTTAATGGTACGTTTAATGGTACATACACTAATAGTACAGGCATGCCT-
-------------------------------------------------------------------------
----------------------------
GAAAATGTTAGCATCACACTCCAATGCAGAATAAAGCAAATTATAAATATGTGGCAGGGGGTAGGACGAGCAA
TATATGCCCCTCCCATTGCAGGAAACATAACATGTAACTCAAGT---
ATCACAGGACTACTATTGACACGTGATGGAGGAATCACTAATGAG----------------------------
-------------------------------------------------------------------------
-------------
ACTATAGAGAGATTCAGACCTGGAGGAGGAAATATGAGGGACAATTGGAGAAGTGAATTATATAAATATAAAG
TGGTAGAAATTAAGCCCTTA---GGGGTAGCACCAACTGATGCAAGGAGGAGAGTGGTG--------------
-------------------------CAGAAAGAGAAAAGAACA---GTA------ATAGGAGCC---------
ATGATCCTTGGGTTCTTGGGAGCA---GCAGGAAGCACTATG---GGCGCGGCATCA---
GTGGTGCTGACGGTACAG---GCCAGACAG---TTATTG---TCTGGTATAGTGCAACAGCAAAAC---
AATTTG---CTAAGG---GCTATAGAGGCGCAACAGCATATGTTGCAACTCACGGTCTGGGGCATT
>HQ595746
TTCTATTGCAATACATCAAAGCTGTTTAATAGTACAGAAAGTTTTAATATTACAGAA----------------
-------------------------------------------------------------------------
----------------------------
AGTAACTCAACCATCACACTGCCATGCAGGATAAAACAAATTATAAACATGTGGCAGGAGGTAGGACGAGCAA
TGTATGCCCCTCCCATTGCAGGAAACATAACATGTAACTCAAAT---
ATCACAGGATTACTATTGGTACGGGATGGAGCACATAATGAAACA----------------------------
-------------------------------------------------------------------------
-------------------
GAGACATTTAGACCTGAAGGAGGAGATATGAGGAACAATTGGAGAAGTGAATTATATAAATATAAAGTGGTAG
AAATTAAGCCATTA---GGAGTAGCACCCACTGAGGCAAAAAGGAGAGTGGTG--------------------
-------------------GAGAGAGAAAAAAGAGCA---GTGGGA---ATAGGAGCT---------
GTGTTCCTTGGGTTCTTGGGAGCA---GCAGGAAGCACTATG---GGCGCGGCGTCA---
GTAACGCTGACGGTACAA---GCCAGACTT---CTACTG---TCTGGTATAGTGCAACAGCAAAAC---
AATTTG---CTGAGG---GCTATAGAGGCGCAACAGCATCTGTTGCAACTCACGGTCTGGGGCATT
>HQ615955
TTCTATTGCAATACATCAGGACTGTTTAATGGTACATACAATGAGACATCAGAA-------------------
-------------------------------------------------------------------------
----------------------------
ACTAATGCAACCATCACACTTCCATGCAGACTAAAACAAATTATAAACATGTGGCAGGAGGTAGGACGAGCAA
TGTATGCTCCTCCCATTGAAGGCAACATAACATGTAAATCAAAT---
ATCACAGGACTAATATTAGAACGTGATGGAGGCAACACAGATAACACA-------------------------
-------------------------------------------------------------------------
----------
ACAAGGACAGAGACATTCAGACCTGCAGGAGGAAATATGAAGGACAATTGGAGAAGTGAATTATATAAATATA
AAGTGGTAGAAATTAAGCCATTG---GGAATAGCACCCACTAGAGCAAAAAGGAGAGTGGTG-----------
----------------------------GAGAGACAAAAAAGAGCA---GTGGGA---ATAGGAGCT------
---GTATTCCTTGGGTTCTTGGGAGCA---GCAGGAAGCACTATG---GGCGCGGCGTCA---
ATAACGCTGACGGTACAG---GCCAGACAA---CTGTTG---TCTGGTATAGTGCAACAGCAAAAT---
AATTTG---CTGAGG---GCTATAGAGGCGCAACAGTATATGTTGCAACTCACGGTCTGGGGCATT
>HQ595766
TTCTACTGCAATACCACACATCTGTTTTCAAATGTAACACAACTCAATGATGGTATG----------------
-------------------------------------------------------------------------
----------------------------
GCTAATGAGACCATCACACTCCCATGCAAAATAAAACAAATTATAAACATGTGGCAAGAGGTAGGACGAGCAA
TGTATGCCCCTCCCATTGCAGGAAACATAACATGTAACTCAAAT---
ATCACAGGAATACTATTGACACGTGATGGAGGAATAGTAAACAACAGC-------------------------
```


ACAGACACAGAGACATTCAGACCTGCAGGAGGAAATATGAAAGACAATTGGAGAAGTGAATTATATAAATATA
AAGTGGTAGAGATTAAACCATTG---GGAATAGCACCCACTGGATCAAAAAGGAGAGTGGTG-----------
----------------------------GAGAGAGAGAAAAGAGCA---GTAGGA---ATAGGAGCT------
---GTGTTCCTTGGGTTCTTGGGAGCA---GCAGGAAGCACTATG---GGCGCGGCGTCA---
ATAACGCTGACGGTACAG---GCCAGACAA---CTGTTG---TCTGGTATAGTGCAACAGCAAAGC---
AATTTG---CTGAGA---GCTATAGAGGCGCAACAACACATGTTGCAACTCACAGTCTGGGGCATT
>KC894386
TTCTATTGCAATACAACAGAACTGTTTAATAGTACATACAATAGTACATACATGTCTAATAGTACAGGAAATA
AT---

TCAAGCTCAAATATCACACTCCCATGCAGAATAAAACAATTTGTAAACATGTGGCAGAAGGTAGGACGAGCAA
TATATGCTCCTCCCATTGCAGGAAACATAACATGTACATCAAAC---
ATCACAGGATTAATATTGGTGCGTGATGGAGGAACAAATAATGACACA-------------------------
----

AATGACACAGAGATCTTCAGACCTGGAGGAGGAGATATGAGGGACAATTGGAGAAGTGAATTATACAAATATA
AAGTGGTAGAAATTAAGCCATTA---GGAATAGCACCCACTGGAGCAAAAAGGAGAGTGGTG-----------
----------------------------GAGAGAGAAAAAAGAGCA---GTAGGA---ATAGGAGCT------
---GTGTTCCTTGGGTTCTTGGGAGCA---GCAGGAAGCACTATG---GGCGCGGCGTCA---
ATAACGCTGACGGTACAG---GCCAGACAA---TTGTTG---TCTGGTATAGTGCAACAGCAAAGC---
AATTTG---CTGAGG---GCCATAGAGGCGCAGCAGCATATGTTGCAACTCACTGTCTGGGGCATT
>HQ595759
TTCTATTGCAATACATCAAAACTGTTTAATAGCACAGAAAATAAT----------------------------
----

GCAAGTGAAATCATCACACTCAGATGTAGAATAAAACAAATTATAAATATGTGGCAGGAGGTAGGACGAGCAA
TGTATGCTCCTCCCATTGAAGGAAACATAACATGTAAATCAAAT---
ATCACAGGGCTACTATTGACACGTGATGGAGGCAATGGCACTGGG----------------------------
----

AACAGCACAGAGACATTCAGGCCTGGAGGAGGAGATATGAGGGACAATTGGAGAAATGAATTATATAAATATA
AAGTAGTAGAAATCAAGCCATTG---GGAATAGCACCCACTGAGGCAAAAAGGAGAGTGGTGGAG--------
----------------------------AGGAGAGAAAAAAGAGCA---GTGGGA---ATAGGAGCT------
---GTGTTCCTTGGGTTTTTGGGAGCA---GCAGGAAGCACTATG---GGCGCGGCGTCA---
ATAGCGCTGACGGTACAG---GCCAGACAA---CTGTTG---TCTGGTATAGTGCAACAGCAAAGC---
AATTTG---CTGAGA---GCTATAGAGGCGCAACAGCATATGTTGCAACTCACGGTCTGGGGCATT
>HQ615942
TTCTATTGCAATACAACAAACCTGTTTCCTAGCAATACAACAAACCTAAATGTTACC----------------
----

CTAAATGATACCATCACACTCCCATGCAGGATAAAACAAATTGTAAACATGTGGCAGGGGGTAGGACGAGCAA
TGTATGCCCCTCCCATTGAAGGAAGCATAACATGTAATTCAAGT---
ATCACAGGACTACTATTGACACGGGATGGAGGACACACGAATGACACGAAT----------------------
----

AACACAGAACCAGAGACATTCAGACCTGGAGGAGGAAATATGAAGGACAATTGGAGAAGTGAATTATACAAAT
ATAAAGTGGTAGAAATTAAGCCATTG---GGAGTAGCACCCACTGCATCAAAAAGGAGAGTGGTG--------
-------------------------------GAGAGAGAAAAAAGAGCA---GTGGGA---ATAGGAGCT---
------GTACTCCTTGGGTTCTTGGGAGCA---GCAGGAAGCACTATG---GGCGCAGCGTCA---
ATAACGCTGACGGTACAG---GCCAGACAA---CTGTTG---TCTGGTATAGTGCAACAGCAAAGC---
AATTTG---CTGAGA---GCTATAGAGGCGCAACAGCATATGTTGCAACTCACAGTCTGGGGCATT
>KT183087
TTCTATTGCAACACATCAGGCCTGTTTAATGGAACATACAACAATGGAACATACAATGATGGAACAGACAAT-
----

TCCAATGACACCATTACACTCTTATGCAGAATAAAACAAATTATAAACATGTGGCAGGGGGTAGGAAAAGCAA
TATATGCCCCTCCTATTAAAGGAAACATAACATGCAACTCAAGT---
ATCACAGGACTACTATTGACACGAGATGGAGGAAAAGGAGATAATAAC-------------------------

----

ACTAATAACACAGAGACATTTAGACCTGGAGGAGGAGATATGAGGGACAATTGGAGAAATGAATTATATAAAT
ATAAAGTGGTAGAAATCAAGCCATTA---GGAGTAGCACCCACTACTGCAAAAAGGAGAGTGGTG--------
-------------------------------GAGAGAGAAAAAAGAGCA---GTAGGA---ATAGGAGCT---
------GTGTTCCTTGGGTTCTTGGGAGCA---GCAGGAAGCACTATG---GGCGCGGCGTCA---
ATAACGCTGACGGTACAG---GCCAGACAA---CTGTTG---TCTGGTATAGTGCAACAGCAAAGC---
AATTTG---CTGAGG---GCTATAGAGGCGCAACAGCACATGTTGCAACTCACGGTCTGGGGCATT
>KT183090
TTCTATTGCGATACAACAAATCTGTTTAATAGGACATATCCTAATGGTACAGAGGGTAATTCAACCCAGAAT-
----

AGTTCAAGCTCAATCACCCTCCCATGCAGAATAAGACAGATTATAAACATGTGGCAGGGGGTAGGACGAGCAA
TGTATGCCCCTCCCATTGCAGGAAACATAACATGTAAATCAAAT---
ATCACAGGACTGCTATTGACACGTGATGGTGGAAAAAATGCCACC----------------------------
----

AATGAAACATTCAGACCTGGAGGAGGAGATATGAGGGACAATTGGAGAAGTGAACTATATAAATATAAAGTGG
TAGAAATTAAACCATTG---GGAGTAGCACCCACTGAGGCAAAAAGAAGAGTGGTG-----------------
----------------------GAGAGAGAGAAAAGAGCA---GTGGGA---ATAGGAGCT---------
GTGTTCCTTGGGTTCTTGGGAGCA---GCAGGAAGCACTATG---GGCGCAGCATCA---
ATAACGCTGACGGTACAG---GCCAGACAA---CTATTG---TCTGGTATAGTGCAACAGCAAAGC---
AATTTG---CTAAGG---GCTATAGAGGCGCAACAGCATATGTTGCAACTCACGGTCTGGGGCATT
>KT183211
TTCTATTGCAATACAACAATTCTGTTCAATAAAAGTAGTTGGGACAACACAACCAATGAAAACAACACA----
----

ACCAGTGGAAACTTCACACTCCCATGCAGGATAAAACAAATTGTAAACATGTGGCAGGGGGTAGGACGAGCAA
TGTATGCCCCTCCCATTGCAGGAAATATAAACTGTAGCTCAAAT---
ATCACAGGACTATTATTGACAAGGGACGGAGGAATAAAAAACGAAACA-------------------------
----

AATGACACAGAAATATTCAGACCTGGAGGGGGAAATATGAAGGACAATTGGAGAAGTGAATTATATAAATATA
AAGTGGTAGAAATTAAACCATTG---GGAATAGCACCTACTGCTGCAAAAAGGAGAGTGGTG-----------
----------------------------GAGAGAGAAAAAAGAGCA---GTGGGA---CTAGGAGCT------
---TTACTCCTTGGGTTCTTGGGAGCA---GCAGGAAGCACTATG---GGCGCGGCGTCA---
ATAACGCTGACGGTACAT---GCCAGACAA---CTGTTG---TCTGGTATAGTGCAACAGCAAAGC---
AATTTG---CTGAGA---GCTATTGAGGCGCAACAGCATATGTTGCAACTCACGGTCTGGGGCATT
>KT183216
TTCTATTGCAACACATCAAGACTGTTTAATGGTACATATGATAGTACATATAATAATACAGGA----------
----

AATAATACCATACTCACAATCCCATGTAGAATAAAACAAATTATAAACATGTGGCAAAAGGTAGGACGAGCAA
TGTATGCTCCTCCCATTGCAGGAAACATAACTTGTAACTCAAGT---
ATCACAGGACTACTACTAACACGTGATGGAGGAACAGGAACAAAT----------------------------
----

GGAACTGAGATATTCAGACCTGGAGGAGGAGATATGAGAGACAATTGGAGAAGTGAATTATATAAATATAAAG
TGGTAGAAATTAAGCCATTA---GGAATAGCACCCACTGCCGCAAGAAGAAGAGTGGTG--------------
-------------------------GAGAGAGAGAAAAGAGCA---GTAACA---CTAGGAGCT---------
ATGTTCCTTGGGTTCTTGGGAGCA---GCAGGAAGCACTATG---GGCGCGGCGTCA---
ATAACGCTGACGGCACAA---GCCAGACAA---TTGTTG---TCTGGTATAGTGCAACAGCAAAGC---
AATTTG---CTGAGG---GCTATAGAGGCGCAACAGCATATGTTGCAACTCACGGTCTGGGGCATT
>KT183229
TTCTATTGCAATACAACAAATCTGTTTAATGAAACAATAACTAATGGAAGTGATTCA----------------
----

GACAACACAAACATCACACTCAAATGCAGAATAAAACAAATTATAAACATGTGGCAGAAGGTAGGACAAGCAA
TATATGCCCCTCCCATTAGAGGAAACATAACATGTAACTCAAAC---
ATCACGGGGCTACTATTGACACGTGATGGAGGAAGAGAAAAGAAT----------------------------

----

GAGACAGAGACATTCAGACCTGGAGGAGGAGATATGAGGGACAATTGGAGAAGTGAATTATATAAATATAAGG
TGATAGAAATTAAACCATTA---GGAATAGCACCCACTGGAGCAAAAAGGAGAGTGGTG--------------
-------------------------GAGAGAGAAAAAAGAGCA---ATAGGAGGAATGGGAGCT---------
TTGTTCCTTGGGTTCTTGGGGGCA---GCAGGAAGCACTATG---GGCGCAGCATCA---
ATAACGCTGACGGTACAG---GCCAGACAA---TTATTG---TCTGGTATAGTGCAACAGCAAAGC---
AATTTG---CTGAGG---GCTATAGAGGCGCAACAGCATATGTTGCAACTCACAGTCTGGGGCATT
>KT183253
TTTTATTGCAATACATCAGGTCTGTTTAATAGAACATACTCTCGTAATAATGATACAAATAGTGCAGACAGTA
CT---

TCAAATTCAACCATTACAATCCCATGCAAAATAAAACAAATTGTAAACATGTGGCAGGAGGTAGGACGGGCGA
TGTATGCTCCTCCCATTAAAGGAAGTATCACATGTAATTCAACT---
ATCACAGGACTACTATTGACAAGGGATGGTGGTAATACAACAGGTAAT-------------------------
----

ACAACAGAGACAGAGACATTCAGGCCTGGAGGAGGAAATATGAGGGACAATTGGAGAAGTGAATTATACAAAT
ATAAAGTGGTAGAAATTAAGCCATTG---GGAATAGCACCCACCGAGGCAAAAAGGAGAGTGGTG--------
-------------------------------GAGAGAGAAAAAAGAGCA---GTGGGG---CTAGGAGCT---
------GTGTTCCTTGGGTTCTTGGGAGCA---GCAGGAAGCACTATG---GGCGCAGCATCA---
ATAACGCTGACGGTACAA---GCCAGACAA---TTGTTG---TCTGGTATAGTGCAACAGCAAAGC---
AATTTG---CTGAGG---GCTATAGAGGCGCAACAGCATATGTTGCAACTCACAGTCTGGGGCATT
>KT183271
TTCTATTGCAATACATCAGGCCTGTTTAATAGAGACATATCTAATAGCACAGGAAGTAAT-------------
----

TCAAGTGCACCCATCATACTCCCATGCAGAATAAAACAAATTATAAACATGTGGCAGGAAGTGGGAAGAGCAA
TGTATGCCCCTCCCATTGCAGGGAACATAACATGTAAATCAAAT---
ATCACAGGACTACTACTAACACGTGATGGAGGAAGTGGAGCAGTG----------------------------
----

AACGAGACATTCAGACCTGGAGGAGGAAATATGAAGGACAATTGGAGAAGTGAATTATATAAATATAAAGTGG
TAGAAATTAAGCCATTA---GGAGTAGCACCCACTGAGGCAAAAAGGAGAGTGGTG-----------------
----------------------GAGAGAGAAAAAAGAGCA---GTAGGA---ATAGGAGCT---------
GTGCTCCTTGGGTTCTTGGGAGCA---GCAGGAAGCACTATG---GGCGCAGCGTCA---
ATAACGCTGACGGTACAG---GCCAGACAA---CTGTTG---TCTGGTATAGTGCAACAGCAAAGC---
AATTTG---CTGAGA---GCCATAGAGGCGCAACAGCATATGTTGCAACTCACGGTCTGGGGCATT
>KT183279
TTCTATTGCAATACAACACTACTGTTTGATAGTACACAAGGGGATAAT-------------------------
----

AGTACAACAATCACCCTTCCCTGTAAAATAAAACAAATTATAAACATGTGGCAGAAGGTAGGAAAAGCAATGT
ATGCCCCTCCCATTGAAGGGAATATAACATGTGAATCAAAC---
ATCACAGGACTACTATTGACACGTGATGGAGGAACAAATAATGAAACAAATGTC-------------------
----

ACAGAAGAGATATTCAGGCCTGGAGGAGGAAATATGAAGGACAATTGGAGAAGTGAATTATATAAATATAAAG
TCGTAGAAATTAAGCCACTG---GGAGTAGCACCCACTAAATCAAAAAGGAGAGTGGTG--------------
-------------------------GAGAGAGAAAAAAGAGCA---GTGGGA---ATAGGAGCT---------
GTACTCCTTGGGTTCTTGGGAGCA---GCAGGAAGCACTATG---GGCGCGGCGTCA---
ATAACGCTGACGGTACAG---GCCAGACAA---GTGTTG---TCTGGTATAGTGCAACAGCAAAGC---
AATTTG---CTGAGA---GCTATAGAGGCGCAACAGCATCTGTTGCAACTCACAGTCTGGGGCATT
>HQ595761
TTCTATTGCAATACAAATATCCTGTTTAATGAAAGTACTTCAGAC----------------------------
----

ACAAACATCACAATCACAATCCCATGCAAAATAAAACAGATTATAAACATGTGGCAAGAGGTAGGAAGAGCAA
TGTATGCCCCTCCCATTGCAGGAAACATAACATGTAGCTCAAAC---
ATCACAGGTCTACTATTGACACGTGATGGAGGAATAAGAAATAAT----------------------------

----

ACAGAAGAAATATTCAGACCTGGAGGAGGAGATATGAGAGACAATTGGAGAAGTGAATTATATAAATATAAAG
TGGTAGAAGTGAAGCCATTA---GGAGTAGCTCCCACTGAGGCAAAAAGAAGAGTGGTG--------------
-------------------------GAGAGAGAAAAAAGAGCA---GTAGGA---GTAGGAGCT---------
ATGTTCCTTGGGTTCTTGGCAGCA---GCAGGAAGCACTATG---GGCGCAGCGTCA---
ATAACGCTGACGGTACAG---GCCAGACAA---CTGTTG---TCTGGTATAGTGCAACAGCAAAGC---
AATTTG---CTGAGA---GCTATAGAGGCGCAACAGCATATGTTGCAACTCACAGTCTGGGGCATT
>KC894116
TTCTATTGTAATACAACAGAACTGTTTAATCCTAATGTAGCAAAATGGAATGGAAGTAGT-------------
----

CCAAATGAAACCATCACCATCCTATGCAGAATAAAACAAATTATAAACATGTGGCAGGGAGTAGGACAAGCGA
TGTATGCCCCTCCCATTGCAGGCAACATAACATGTGTCTCAAAT---
ATCACAGGATTACTATTGACACGTGATGGAGGAAACATCAATAATACT-------------------------
----

AATGGTACTGAGACATTCAGACCTGGAGGAGGAAATATGAAGGACAATTGGAGAAGTGAATTATATAAATATA
AAGTGGTAGAAATTAAGCCATTG---GGAATAGCCCCCACTAAGGCAAAAAGGAGAGTGGTG-----------
----------------------------GAGAGAGAAAAAAGAGCA---GTGGGA---ATAGGAGCT------
---GTGTTCCTTGGGTTCTTGGGAGCA---GCAGGAAGCACTATG---GGCGCGGCGTCA---
CTGACGCTGACGGTACAG---GCCAGACAA---CTGTTG---TCTGGTATAGTGCAACAGCAAAGC---
AATTTG---CTGAGA---GCTATAGAGGCGCAACAGCACATGTTGCAACTCACGGTCTGGGGCATT
>KC894119
TTCTATTGCAACACATCACAATTGTTTAATAGTACATACAATGGTACACACCATGGAACCATC----------
----

AAAAATGGAACCATCACACTTCCATGCAGAATAAAACAAATCATAAACATGTGGCAGGAGGTAGGACGAGCAA
TGTATGCCCCTCCCGTTGCAGGAGCCATAACATGTAACTCAACT---
ATCACAGGACTACTATTAACACGTGATGGAGACAATGAGACTAAG----------------------------
----

GAGACATTCAGACCTGGAGGAGGAAATATGAAGGACAATTGGAGAAGTGAATTGTATAAATATAAAGTGGTAG
AAGTTAAGCCATTG---GGATTAGCACCCACTAGAGCAAAAAGGAGAGTGGTG--------------------
-------------------GAAAGAGAAAAAAGAGCA---GTGGGA---ATAGGAGCC---------
GTGTTCCTTGGGTTCTTGGGAGCA---GCAGGAAGCACTATG---GGCGCAGCGTCA---
ATAACGCTGACGGTACAG---GCCAGACAA---CTGTTG---TCTGGTATAGTGCAACAGCAAGGC---
AACTTG---CTAAGA---GCTATAGAGGCGCAACAGCATATGTTGCAACTCACAGTCTGGGGCATT
>KC156130
TTCTATTGTAATACATCAAAACTGTTTAATGGGAATGATAAT-------------------------------
----

TCAAGCGAAGTAATCACACTCCCATGTAGAATAAAACAGATTATAAACATGTGGCAGAAGGTAGGACGAGCAA
TGTATGCCCCTCCCATTGAAGGAAACATAACATGTAGGTCAAAT---
ATCACAGGACTACTATTGACACGTGATGGAGGGACAGGGAATGAC----------------------------
----

ACAGAGATATTCAGACCTGGAGGAGGAAATATGAAGGACAACTGGAGAAGTGAACTATATAAATATAAAGTGG
TAGAAGTTAAGCCATTA---GGAATAGCACCCACTGGAGCAAAAAGGAGAGTGGTG-----------------
----------------------GAGAGAGAAAAAAGAGCA---GTGGGA---CTAGGAGCT---------
GTGTTCCTTGGGTTCTTAGGAGCA---GCAGGAAGCACTATG---GGCGCGGCGTCA---
ATAACGCTGACGGTACAG---GCCAGACAA---CTGTTG---TCTGGTATAGTGCAGCAGCAAAGC---
AATTTG---CTGAGA---GCTATAGAAGCGCAACAGCATATGTTGCAACTCACAGTCTGGGGCATT
>KC894115
TTCTATTGTGATACAGCTAAGCTATTTAATAAAAGTGAGTCA-------------------------------
----

AACGAAACCATCACACTCACACTCCCATGCAGGATAAAACAAATTATAAATATGTGGCAGGGGGTAGGACGAG
CAATGTATGCCCCTCCCATTGAAGGAAACATAACATGTAACTCAAGT---
ATTACAGGACTAATATTAACACGTGATGGAGGACCAGATAACCAA----------------------------

----

ACAGAGACATTCAGACCTGGAGGAGGAGATATGAAGGACAATTGGAGAAGTGAATTATATAAATATAAGGTGG
TAGAAATTAAACCATTG---GGAATAGCACCCACTGGGGCAAAAAGGAGAGTGGTG-----------------
----------------------GAGAGAGAAAAAAGAGCA---GCG------ATAGGAGCT---------
GTGTTCCTTGGGTTCTTGGGAGCA---GCAGGAAGCACTATG---GGCGCGGCGTCA---
ATAACGCTGACGGTACAG---GCCAGACAA---CTGTTG---TCTGGTATAGTGCAACAGCAAAGC---
AATTTG---CTGAGA---GCTATAGAGGCGCAACAGCATATGTTGCAACTCACGGTCTGGGGCATT
>JN687704
TTCTATTGCAATACAACAAACCTGTTTAATAGTACATACAATCCTAATGGTACAAGGGATGGC----------
----

TCAGACTCAATCATCACACTCCCATGCAGAATAAAACAAATTATAAATATGTGGCAGGGGGTAGGACGAGCAA
TGTATGCCCCTCCCATTGCAGGAATCATAACATGTAGATCAAAT---
ATCACAGGACTACTATTGACACGTGATGGAGGAAGCGATGAGGGA----------------------------
----

AACAGTACAGAGATATTCAGACCTGGAGGAGGAGAGATGAGGGACAATTGGAGAAATGAATTATATAAATATA
AAGTGGTAGAAATTAAGCCATTA---GGAGTAGCACCCACTGAGGCAAAAAGAAGAGTGGTG-----------
----------------------------GAGAGAGAAAAAAGAGCA---GTGGGG---ATAGGAGCT------
---GTGTTCCTCGGGTTCTTGGGAGCA---GCAGGAAGCACTATG---GGCGCAGCGTCA---
ATGACGCTGACGGTACAG---GCCAGACAA---CTGTTG---TCTGGTATAGTGCAACAGCAAAGC---
AATTTG---CTGAAG---GCTATAGAGGCGCAACAACATATGTTGCAACTCACGGTTTGGGGCATT
>JN687706
TTCTATTGCAATACATCAAAATTGTTTAATAGTACACACAGTCCTAATGGTAATGGTAATGATACAGAAAGTG
AT---

ACAAACTCAACAATAATCACACTTCCATGCAGAATAAAACAAATTATAAATATGTGGCAGGGGGTGGGACGAG
CAATGTATGCCCCTCCCATTGCAGGAAACATAACATGCAAATCAAAT---
ATCACAGGACTACTATTGACACGTGATGGAGGAACAAGTGAAGAAACA-------------------------
----

AACGGGACAGAGATATTCAGACCTGGAGGAGGAAATATGAGGGACAATTGGAGAAGTGAATTATATAAATATA
AAGTGGTAGAAATTAAGCCATTA---GGAATAGCACCCACTGAGGCAAAAAGGAGAGTGGTG-----------
----------------------------GAGAGAGAGAAAAGAGCA---GTGGGA---ATAGGAGCT------
---GTGTTCCTTGGGTTCTTGGGAGCA---GCAGGAAGCACTATG---GGCGCAGCATCA---
ATAACGCTGACGGTACAG---GCCAGACAA---TTGTTG---TCTGGTATAGTGCAACAGCAAAGC---
AATTTG---CTGAGG---GCTATAGAGGCGCAACAACATATGTTGCAACTCACGGTCTGGGGCATT
>JN687718
TTCTATTGCAATACATCAAGCCTGTTTAATGGTACATACAATAATGGTACATACAATACAACAGGA-------
----

ACAAACGAAACCATCACACTCCCATGCAGAATAAAACAAATTATAAACATGTGGCAGAGTGTAGGACGGGCTA
TATATGCCTCGCCCGTTGCAGGAAACATAACATGTAAATCAAAT---
ATCACAGGACTAATATTAGTACGTGATGGAGGCAACACAAATGACACA-------------------------
----

AATGGCACAGAGATATTCAGACCTGGAGGAGGAAATATGAAGGACAATTGGAGAAGTGAATTATATAAATATA
AAGTGGTAGAGATTAAGCCATTG---GGAATAGCACCCACTGAAGCAAAAAGGCGAGTGGTG-----------
----------------------------GAGAGAGAAAAAAGAGCA---GTAGGA---ATAGGAGCT------
---GTGTTCCTTGGGTTCTTGGGAGCA---GCAGGAAGCACTATG---GGCGCAGCATCA---
ATAACGCTGACGGTACAG---GCCAGACAG---TTGTTG---TCTGGTATAGTGCAACAGCAAAGT---
AATTTG---CTAAGG---GCTATAGAGGCGCAACAGCATATGTTGCAACTCACAGTCTGGGGCATT
>JN687735
TTCTATTGCGATACAACAAAGCTGTTTAATGACAGTGAGTCAAGTAATTCAACCCAAAGTAATTCA-------
----

GCCCACGCAAACATCACACTCCCATGCAAGATAAAACAAATTATAAACATGTGGCAGGGGGTAGGAAGAGCGA
TGTATGCCCCTCCTATTAAAGGAAACATAGTATGTAACTCAAAT---
ATCACAGGACTACTATTGACACGTGATGGAGGCAGAAATAAAACA----------------------------

----

GAACCAGAGATATTCAGACCAATAGGAGGAAATATGAAGGACAATTGGAGAAGTGAATTATATAAATATAAGG
TGGTAGAAATTAAGCCATTG---GGAGTAGCACCCACTGCATCAAAAAGGCGAGTGGTG--------------
-------------------------GAGAGAGAAAAAAGAGCA---GTGGGA---ATAGGAGCT---------
GTGCTCCTTGGGTTCTTGGGAGCG---GCAGGAAGCACTATG---GGCGCGGCGTCA---
GTGACGCTGACGGTACAG---GCCAGACAA---CTGTTG---TCTGGTATAGTGCAACAGCAAAGC---
AATTTG---CTGAGA---GCTATAGAGGCGCAACAGCATATGTTGCAACTCACAGTCTGGGGCATT
>JN687736
TTCTATTGCAATACATCAGGACTGTTTTCTAAGGGTACAAATAATACT-------------------------
----

TCTAAAAGGAACATCACACTCCCATGCAGAATAAAACAAATAATAAACATGTGGCAGGGGGTAGGACGAGCAA
TGTATGCCCCTCCCATTGAAGGAGACATAACATGTAAATCAAAT---
ATCACAGGACTACTATTGCTACGTGATGGAGGTTTCTTAAATATAACT-------------------------
----

AATGGCACAGAGACATTTAGACCTGGAGGAGGAGATATGAGGGACAATTGGAGAAGTGAATTGTATAAATATA
AAGTGGTAGAAATTAAGCCATTA---GGAGTAGCACCCACTAAGGCAAAAAGGAGAGTGGTG-----------
----------------------------GAGAGAGAAAAAAGAGCA---GTGGGA---CTGGGAGCT------
---GTGTCCCTTGGGTTCTTGGGAGCC---GCAGGAAGCACTATG---GGCGCAGCATCA---
ATAACGCTGACGGTACAG---GCCAGACAA---TTGTTG---TCTGGTATAGTGCAACAGCAAAGC---
AATTTG---CTGAGA---GCTATAGAGGCGCAACAGCATATGTTGCAACTCACGGTCTGGGGCATT
>JX976681
TTCTATTGCAATACATCAGGCCTGTTTAATGGTACATTTAATGGTACATTTAATGGTACAGATACAAATAGTA
CAAATAGTACT--

TCAAATGCAACCATCACACTCCAATGCAGAATAAAACAAATTATAAACATGTGGCAGGAGGTAGGAAGAGCAA
TGTATGCCCCTCCCATTGCAGGAAACATAACATGTAAATCAAAT---
ATCACAGGATTGCTCTTGACAAGGGATGGTGGAGACAGTAATGTGACA-------------------------
----

AGGCATAATGAGACATTCAGACCTGGAGGAGGAGATATGAGAGATAATTGGAGAAGTGAATTATATAAATATA
AAGTGGTAGAAATTAAGCCATTA---GGAGTAGCACCCACTGATGCAAAAAGGAGAGTGGTG-----------
----------------------------GAGAGAGAGAAAAGAGCA---GTGGGA---CTAGGAGCT------
---GTGTTCCTTGGGTTCTTGGGAGCG---GCAGGAAGCACTATG---GGCGCGGCGTCA---
ATAACGCTGACGGTACAG---GCCAGACAA---TTATTG---TCTGGTATAGTGCAACAGCAAAGC---
AATTTG---CTGAGG---GCTATAGAGGCGCAACAGCATATGTTGCAACTCACAGTCTGGGGCATT
>KC863573
TTCTATTGTAATACAACAGACTTGTTTAATAAGAGTATAATT-------------------------------
----

GAGAGAAACGCGACCATCACACTCCCATGCAGAATAAAACAAATTATAAACATGTGGCAGGAGGTGGGACGAG
CTATGTATGCCCCTCCCATTGCAGGAAACATAACATGTAATTCAAGT---
ATCACAGGACTACTATTGACTCGTGATGGAGGAAATGGGGAAAATAACACA----------------------
[truncated: 3,492,443 more chars]
