# Supplementary material for: Extending the Coding Potential of Viral Genomes with Overlapping Antisense ORFs: A Case for the De Novo Creation of the Gene Encoding the Antisense Protein ASP of HIV-1
Source: Viruses. 2022 Jan 14;14(1):146. doi: 10.3390/v14010146 (PMC8781085; doi:10.3390/v14010146)
Supplement: Supplementary file 1 [file viruses-14-00146-s001.zip › Supplementary File S2A.pdf]

Supplementary File S2A.

Supplementary file S2A contains a control set of 1753 *env/asp* overlapping regions. They are delimited, in the reverse frame, by a *start* codon (ATG) at canonical nucleotide positions (triplet CAT at the end of the sequence) and a *stop* codon at canonical nucleotide positions (triplet CTA at the beginning of the sequence). All overlaps, however, have lost the ability to encode a full-length overlapping ASP protein, because of the presence of internal stop codons. The number of internal stop codons is added at the end of to the accession number after two dots. In detail, 1231 *env* sequences have an *asp* ORF interrupted by 1 internal stop codon (e.g. MH705158..1), 403 env sequences have an *asp* ORF interrupted by 2 internal stop codons (e.g. FM165645..2), 102 env sequences have an *asp* ORF interrupted by 3 internal stop codons (e.g. AF457086..3), 11 env sequences have an *asp* ORF interrupted by 4 internal stop codons (e.g. KT022381..4), and 6 env sequences have an *asp* ORF interrupted by 5 internal stop codons (e.g. KP411836..5). All sequences belong to the group M of HIV-1. Each nucleotide sequence is reported both in the alignment format with gaps and in the format without gaps.

```
>MH705158..1
TTCTATTGCAATACATCAGGCCTGTTTAATAGTAGTTGGAACGAAAGTGACACT-------------------
-------------------------------------------------------------------------
----------------------------
GCAAATGACACTATAACGCTCCCGTGCAAAATAAAACAAATTGTAAGGATGTGGCAGAGAGTAGGACAAGCAA
TGTATGCCCCTCCCATCCAAGGGGTAATGAGGTGTGAATCTAAC---
ATTACAGGACTACTATTAACAAGAGATGGTGGGAATGAGGGCAAT----------------------------
-------------------------------------------------------------------------
----------
GAAAGTAGTGAGACGTGCAGACCTGGAGGAGGAGATATGAGGGATAATTGGAGAAGTGAACTATATAAATATA
AAGTAGTAAAAATTGAACCACTA---GGAGTAGCACCCACCAGGGCAAAGAGAAGAGTGGTG-----------
----------------------------GAGAGAGAAAAAAGAGCA---GTTGGA---CTGGGAGCT------
---GTCTTCATTGGGTTCTTAGGAGCA---GCAGGAAGCACTATG---GGCGCGGCGTCA---
GTGACGCTGACGGTACAG---GCCAGACAA---TTATTG---TCTGGCATAGTGCAACAGCAAAAC---
AATTTG---CTGAGG---GCTATAGAGGCTCAACAGCATCTGTTGAAACTCACGGTCTGGGGCATT
>MH705151..1
TTCTATTGCAATACATCAGACCTGTTTAATAGCACTTGGCGGAAACTGGCAAATGGCACTTGGAGCAACATAA
ATAATGCAACG--------------------------------------------------------------
----------------------------
TCAAATGACACTATAACTCTCCAATGCAAAATAAAGCAAATTGTAAACATGTGGCAGAGAGTAGGACAAGCAA
TGTATGCCCCTCCCATCCAAGGAGTAATACAGTGTGTATCAAAC---
ATTACAGGACTACTCTTAACAAGAGATGGTGGGAGTAGTAACAGG----------------------------
-------------------------------------------------------------------------
-------------
GCAAATGAGACCTTCAGACCTGGAGGAGGAGATATGAGGGACAATTGGAGAAGTGAATTATATAAGTATAAAG
TAGTAAAAATTGAACCACTA---GGAGTAGCACCCACCAGGGCAAGGAGAAGAGTGGTG--------------
-------------------------GAGAGAGAAAAAAGAGCA---ATTGGA---ATGGGAGCT---------
CTTTTCCTTGGGTTCTTAGGAACA---GCAGGAAGTACTATG---GGCGCGGCGTCA---
ATAACGCTGACGGTACAG---GCCAGACAA---TTATTG---TCTGGTATAGTACAACAGCAGAGC---
AATCTG---CTGAGG---GCTATAGAGGCTCAACAGCATCTGTTGAAACTCACAGTCTGGGGCATT
>MH705157..1
TTCTATTGTAATACATCAAGCCTGTTTAATAGCACTTGGAAAGAGGAGAATGGCACTGTCAGCATGCAGAATG
ACACGGAG-----------------------------------------------------------------
----------------------------
TCAACTGACCCTATAACTCTCCAATGTAGAATAAAGCAAGTTATAAACATGTGGCAGAGAGTAGGACAAGCAA
TATATGCCCCTCCCATCCAAGGAGTAATAAGATGTGTATCAAAC---
ATTACAGGACTACTATTAACAAGAGATGGTGGGAGTAATAATAATACT-------------------------
-------------------------------------------------------------------------
-------------
TCAAATGAAACCTTCAGACCTGGAGGAGGAGATATGAGGGACAATTGGAGAAGTGAATTATATAAGTATAAAG
TAGTAAAAATTGAACCACTA---GGAGTAGCACCCACCAAGGCAAAGAGAAGAGTGGTG--------------
-------------------------GAGAGAGAAAAAAGAGCA---GTTGGA---CTGGGAGCT---------
```

GTCTTCCTTGGGTTCTTAGGAGCA---GCAGGAAGTACGATG---GGCGCGGCGTCA---
ATAACGCTGACGGTACAG---GCCAGACAA---TTATTG---TCTGGCATAGTGCAACAGCAGAGC---
AATCTG---CTGAGG---GCTATAGAGGCTCAACAGCAACTGTTGAGACTCACGGTCTGGGGCATT
>MH705153..2
TTCTATTGCGATACATCAGGCCTGTTTAATGGCACTTGGAGCAATACATCAGGCCTGATTAATAGCACTTGGA
GC---

GAGAATGACACTATAACTCTCCCATGTAGAATAAAGCAAATTGTAAATATGTGGCAGAGAGTAGGACAAGCAA
TGTATGCCCCTCCCATCCAAGGAATAATAAATTGTACATCAAAC---
ATTACAGGACTACTGTTAACAAGAGATGGTGGGAATAATAACAGTATA-------------------------
----

AATGAGCCTGAGACCTTCAGACCTGGAGGAGGAGATATGAGGGACAATTGGAGAAGTGAACTATATAAGTATA
AAGTAGTAAAAATTCAACCACTA---GGAGTAGCACCCACCAGGGCAAAGAGAAGAGTGGTG-----------
----------------------------GAGAGAGAAAAAAGAGCA---GTTGGC---ATGGGAGCT------
---CTTCTCCTTGGGTTCTTAGGAGCA---GCAGGAAGCACGATG---GGCGCGGCGTCA---
ATAACGCTGACGGTACAG---GCCAGACAA---TTATTG---TCTGGTATAGTGCAACAGCAGAGC---
AATCTG---CTGAGG---GCTATAGAGGCTCAACAGCATCTGTTGAAACTCACGGTCTGGGGCATT
>MH705133..1
TTCTATTGCAATACGTCAGGCCTGTTTAATAGCACTTGGAAGAATGGCACTAAAGAGGGAAATGACACTGCAA
AG---

TTAAGTGACACTATAACTCTCCCATGCAGAATAAAGCAAATTGTAAGGATGTGGCAGAGAACAGGACAAGCAA
TGTATGCCCCTCCCATCCCAGGAGTAATAAATTGTGTCTCTAAC---
ATTACAGGGATACTATTAACAAGGGATGGTGGGAATAGTAGTAAT----------------------------
----

GCCAATGAAACCTTCAGACCTGGAGGAGGAAATATGAAGGACAATTGGAGAAGTGAACTATATAAGTATAAAG
TAGTAAAAATTGAACCAATA---GGAGTAGCACCCACCAGGGCAAAGAAAAGAGTGGTG--------------
-------------------------GAGAGAGAAAAAAGAGCA---GTTGGA---CTGGGAGCT---------
GTCTTCCTTGGGTTCTTAGGAGCA---GCAGGAAGTACTATG---GGCGCGGCGTCA---
ATAACGCTGACGGTACAG---GCCAGACAA---TTATTG---TCTGGCATAGTACAACAGCAAAGC---
AATTTG---CTGAGG---GCTATAGAGGCTCAACAGCATATGTTGAGACTCACAGTCTGGGGCATT
>JQ403028..1
TTCTACTGCAATACAACAGAACTGTTTAATAGCACGTGGAATGACACGAGCAACACATCGCAGGGGACAAAT-
----

AACAATAGTACTATTACTCTCCCATGCAGAATAAAGCAATTTGTAAATATGTGGCAGAGAGTAGGACAAGCAA
TGTATGCCCCTCCCATCAAAGGAAAAATACAGTGCATATCAAAT---
ATTACAGGTCTACTATTAACAAGAGATGGGGGTAAGGGTAAAAGC----------------------------
----

AATATTACAGAGATCTTCAGACCTGAAGGAGGAAATATGAAGGACAACTGGAGAAGTGAATTATATAAATATA
AAGTAGTAAAAATTGAACCACTA---GGAGTAGCACCCACCAAGGCAAAGAGAAGAGTGGTG-----------
----------------------------GAGAGAGAAAAAAGAGCA---GTTGGA---CTGGGAGCT------
---GTGTTCCTTGGGTTCTTAGGAGCA---GCCGGAAGTACTATG---GGCGCGGCGTCA---
ATAACGCTGACGGCACAG---GCCAGGCAA---TTATTG---TCTGGTATAGTACAACAGCAGAGC---
AATCTG---CTGAGG---GCTATAGAGGCTCAACAGCATCTGTTGAAACTCACGGTCTGGGGCATT
>KX389622..1
TTCTATTGCAATACATCAGACTTGTTTAATAGCAATTGGACAAATGGGAATTGGACAAATGGGATAGGCAGCA
CGGAG--

ACTAATGACACTATAACTCTTCCATGCAGAATAAAGCAGATTATAAGAATGTGGCAGAGAGTAGGACAAGCAA
TGTATGCCCCTCCCATCAAAGGGGTAATAGAGTGTAAATCAAAT---
ATTACAGGAATAATATTAGTAAGAGATGGTGGTAAGAATAACACA----------------------------
----

GACAAGGAGACATTTAGACCTGGAGGAGGAGATATGAGGGACAATTGGAGAAGTGAACTATACAAATATAAAG
TAGTAAAAATTGAACCATTA---GGAGTAGCACCCACCAGGGCAAAAAGAAGAGTGGTG--------------
-------------------------GAGAGAGAAAAAAGAGCA---GTTGGA---ATAGGAGCT---------

```
GTCTTCCTTGGGTTCTTAGGAGCG---GCAGGAAGCACTATG---GGCGCAGCGTCA---
ATAACGCTGACGGTACAG---GCCAGACAA---TTATTG---TCTGGCATAGTGCAACAGCAGAGC---
AATCTG---CTGAGG---GCTATAGAGGCTCAACAACATCTGTTGAAACTCACAGTCTGGGGCATT
>DQ396400..1
TTCTATTGCAATACATCAGGCCTGTTTAACAGAACTTGGAATGACACCGCTTGGAATGAAACAACTTGGAATA
ACATA--------------------------------------------------------------------
-------------------------
AATTCAAATGGAAGTATAACTCTCCAATGTAGAATAAAGCAAATTTTAAATATGTGGCAGAGAGTAGGACAGG
CGATGTATGCCCCTCCCATCCCAGGAGAAATAAGGTGTGAATCAAAC---
ATTACAGGACTAATCTTAACAAGAGACGGGGGGCTTGAGCTTAACAAT-------------------------
-------------------------------------------------------------------------
----------
ACGAATAATGAGACCTTCAGGCCTGGAGGAGGAGATATGAGGGACAATTGGAGAAGTGAATTGTATAAATACA
AAGTAATAAAAATTGAACCACTA---GGAGTAGCACCCACCAGGGCAAAAAGAAGAGTGGTG-----------
----------------------------GAGAGAGAAAAAAGAGCA---GTTGGA---CTGGGAGCT------
---GTTTCCTTGGGTTCTTAGGGGCA---GCAGGAAGCACTATG---GGCGCAGCGTCA---
ATAACGCTGACGGTACAG---GCTAGGCAA---TTATTG---TCTGGCATAGTGCAACAGCAAAGC---
AATTTG---CTGAGG---GCTATAGAGGCTCAACAACATTTGTTGAAACTCACAGTCTGGGGCATT
>FM165645..2
TTCTACTGTAACACATCAGGCCTGTTTAATAGCAGTTGGGAAAATGACACTGGCAGCACGCAGGAGTCAAATA
GAACA--------------------------------------------------------------------
-------------------------
AAGTCAAATGACACTATAACTCTCCCATGCAGAATAAAGCAAATTATAAATATGTGGCAGAGGGCAGGACAAG
CAATATATGCCCCTCCCATCCCAGGGGTAATACAGTGTCAGTCAAAC---
ATTACAGGACTACTATTAACAAGAGATGGTGGGAATAACAATACA----------------------------
-------------------------------------------------------------------------
-------------
ACTAATGAAACCTTCAGACCTGGAGGAGGAAATATGAGGGATAATTGGAGAAGTGAACTATATAAATATAAAG
TAGTAAAAATTGAACCACTA---GGAGTAGCACCCACCACGGCAAGGAGAAGAGTGGTG--------------
-------------------------GAGAGAGAAAAAAGAGCA---GTGGGA---CTGGGAGCT---------
CTGTTCATTGGGTTCTTAGGAGCC---GCAGGAAGCACTATG---GGCGCGGCGTCA---
GTGACGCTGACGGTACAG---GCCAGACAA---TTATTG---TCTGGCATAGTGCAACAGCAAAAC---
AATTTG---CTGAGG---GCTATTGAGGCTCAACAGCATCTGTTGAAACTCACGGTCTGGGGCATT
>FM165628..2
TTCTACTGCAACACATCAAGACTGTTTAATAGCAGTTGGGAACCATTAGGCCAGTATGATGACAGTCAGAGC-
-------------------------------------------------------------------------
----------------------------
AATGACACAAATATAACTCTCCCATGCAGAATAAAGCAAATTATAAGGATGTGGCAGAGGACAGGACAAGCAA
TATATGCCCCTCCCATCCAAGGGGAAATAACGTGTAGATCAAAT---
ATTACAGGACTACTATTAACAAGAGATGGTGGAAATAAAACTAAT----------------------------
-------------------------------------------------------------------------
----------
GAAACCTCCGAAACCTTCAGACCTGGAGGAGGAGATATGAGAGATAATTGGAGAAGTGAACTATATAAATATA
AAGTAGTAAAAATTGAACCACTA---GGAGTAGCACCCACCAAGGCAAAAAGAAGAGTGGTG-----------
----------------------------GAGAGAGAAAAAAGAGCA---GTGGGA---CTGGGAGCT------
---GTTCTCATTGGGTTCTTAGGAACA---GCAGGAAGCACTATG---GGCGCGGCGTCA---
GTGACGCTGACGGTACAG---GCCAGACAA---TTATTG---TCTGGCATAGTGCAACAGCAAAAC---
AATTTG---CTGAGG---GCTATAGAGGCTCAACAGCATCTGTTGAAACTCACGGTCTGGGGCATT
>EU191615..1
TTCTACTGCAACACATCAACACTGTTTAATAGCAGTTGGCCATTAAACCAGTCTAACAGTTGGAGCAATGGCG
GA-----------------------------------------------------------------------
----------------------------
AATGACACAAATATAACTCTCCCATGCAGAATAAAGCAAATTATAAGGATGTGGCAGAGGACAGGACAAGCAA
TATATGCCCCTCCCATCGAAGGGGTAATAATGTGTAGGTCAAAT---
ATTACAGGACTCCTATTAACAAGAGATGGTGGAAATGAAGCTAATAAAACT----------------------
-------------------------------------------------------------------------
-------
AATGAAACTGCTGAAATCTTCAGACCTGGAGGAGGGGATATGAGAGATAATTGGAGAAGTGAACTATATAAAT
ATAAAGTAGTAAAAATTGAACCACTA---GGAGTAGCACCCACCAGGGCAAAAAGAAGAGTGGTG--------
-------------------------------GAGAGAGAAAAAAGAGCA---GTGGGA---CTGGGAGCT---
```

------GTTCTCATTGGGTTCTTAGGAACA---GCAGGAAGCACTATG---GGCGCGGCGTCA---
GTGACGCTGACGGTACAG---GCCAGACAA---TTATTG---TCTGGCATAGTGCAACAGCAAAAC---
AATTTG---CTGAGG---GCTATAGAGGCTCAACAGCATCTGTTGAAACTCACGGTCTGGGGCATT
>MH746258..1
TTCTATTGTAACACATCAGGCCTGTTTAATAGCACATGGTCTAAAAATGGCACACAGCAGGTAGACAAC----
----

ATAACCAATCCTATAATTCTCCAATGCAGAATAAAGCAGATTATAAATATGTGGCAGAGAGCAGGACAAGCAA
TATATGCCCCTCCCATCCAAGGACTAATAAGGTGTGAATCAAAC---
ATTACAGGACTAATATTAACAAGAGATGGTGGAAATGGGAATACA---------------------------
----

ACAAACAATGAAACCGAAACCTTCAGACCTGGAGGAGGAGATATGAGGGACAATTGGAGAAGTGAATTATATA
AGTATAAGGTAGTACAAGTTGAACCACTA---GGAGTAGCACCCACCAAGGCAAAAAGAAGAGTGGTG-----
---------------------------------GAGAGAGAAAAAAGAGCA---GTTGGA---
CTAGGAGCT---------GTATTCATTGGATTCTTAGGAGCA---GCAGGAAGCACTATG---
GGCGCGGCGTCT---GTGACGCTGACGGTACAG---GCCAGGCAA---TTATTG---
TCTGGTATAGTGCAACAGCAAAGC---AATTTG---CTGAGG---
GCTATAGAGGCTCAACAGCATTTGTTGAAACTCACAGTCTGGGGCATT
>MH746253..1
TTCTATTGTAATACATCAGCCTTATTTAATAGCACTTGGAATGATACCAATAATACGGGATCAAATACTGACA
ATAACACCACG---

GGGTCAAATGAAGTTATAACTCTCCCTTGCAGAATAAAACAAATTATACGTATGTGGCAGAGAACAGGACAAG
CAATGTATGCCCCTCCCATTTCAGGAATAATAAATTGTACATCAAAC---
ATTACAGGACTAATATTGACAAGAGATGGTAGTAATGGTGATAAT----------------------------
----

AACAGTGAAACCTTCAGACCTGGAGGAGGAGATATGAGGGACAATTGGAGAAGTGAATTATATAAGTATAAAG
TAGTAAAAATTGAACCATTA---GGGGTAGCACCCACCAGGGCAAGGAGAAGAGTGGTG-------------
-------------------------CAGAGAGAAAAAAGAGCA---GTTGGA---ATAGGAGCT---------
GTCTTCCTTGGGTTCTTAGGAGCA---GCAGGAAGCACTATG---GGCGCAGCGTCA---
ATAACGCTGACGGTACAG---GCCAGACAA---TTATTG---TCTGGCATAGTGCAACAGCAAAAC---
AATTTG---CTGAGG---GCTATAGAGGCTCAACAACAGCTGTTGAAACTCACAGTCTGGGGCATT
>MH746251..1
TTCTATTGCAATACATCAGCCCTGTTCAATAGTACTTGGAATGGCACTGTCACAAATGGCACACAA-------
----

GCAAATGGAACTATAATTCTACCATGCAGAATAAAGCAAATTATAAGAATGTGGCAGAGAGTAGGACAAGCAA
TGTATGCCCCTCCTATCAGAGGAGTGATAAGGTGTGAATCAAAC---
ATTACAGGACTAATACTAACAAGAGATGGTGGAGGTGGAACTAAT---------------------------
----

AGCAGCAATGAGACCTTCAGGCCTACAGGAGGAGATATGAGGGACAATTGGAGAAGTGAACTATATAAGTATA
AAGTAGTAAAAATTGAACCAATA---GGATTAGCACCTACCGAGGCAAGGAGGAGAGTGGTG----------
----------------------------GAGAGAGAAAAAAGAGCA---ATTGGA---CTGGGAGTT------
---GCCTTCCTTGGGTTCTTAGGAGCA---GCAGGAAGTACTATG---GGCGCGGCGTCA---
TTGACGCTGACGGCACAA---GCCAGACAA---TTATTG---TCTGGTATAGTGCAACAGCAAAGC---
AATTTG---CTGAGG---GCTATAGAGGCTCAGCAGCATCTGCTGAAACTCACAGTCTGGGGCATT
>KU168256..1
TTCTATTGCAATACATCAGGCCTGTTTAATAGCACTTGGCATGTCAATAATACATCAAGCCTGGTTAAT----
----

AGCACTGGGAACATAACTCTCCCATGCAGAATAAAGCAAATTATAAATATGTGGCAGAGAGTAGGACAAGCAA
TGTATGCCCCTCCTATTCCAGGAATAATAAAGTGTGAGTCAAAC---
ATTACAGGACTACTATTGATAAGAGATGGTGGAATTAATAATAAC---------------------------
----

AGTATGAATGAGACCTTCAGACCTGGAGGAGGAGATATGAGAGACAATTGGAGAAGTGAATTATATAAATATA
AAGTAGTACAAATTGAACCACTA---GGAGTAGCACCTACCAAGGCAAAGAGAAGAGTGGTG----------

```
----------------------------GAGAGAGAAAAAAGAGCA---GTTGGA---TTTGGAGCT------
---GTCTTCCTTGGGTTCTTAGGAGCA---GCAGGAAGCACTATG---GGCGCGGCGTCA---
ATAACGCTGACGGCACAG---GCCAGACAA---TTATTG---TTTGGCATAGTGCAACAGCAAAGC---
AATTTG---CTGAGG---GCTATAGAGGCTCAACAGCATCTGTTGAAACTCACAGTCTGGGGCATT
>KU168305..1
TTCTATTGTAATACATCAGGCCTGTTTAATAGCACTTGGAAGACTAATGGCACTAGCACTTGGAAGGAGACTA
ATGGCACT-----------------------------------------------------------------
-------GGGAGCCAGGAA------
AATAGCAATGACAATATAACTCTCCCATGCAGAATAAAGCAAATTATAAATATGTGGCAGAGAGCAGGACAAG
CAATGTATGCCCCTCCCATCGCAGGAGTAATAAAGTGTAACTCAAAC---
ATTACAGGACTACTATTAACAAGAGATGGGGGAAGTAATAACAAT----------------------------
-------------------------------------------------------------------------
----------
AGCCAAGAGGAGACTTTCAGACCTGGAGGAGGAAATATGAAGGATAACTGGAGAAGTGAATTATATAGGTACA
AAGTAGTACAAATTGAACCCCTA---GGAATAGCACCCACCAGGGCAAGGAGAAGAGTGGTG-----------
----------------------------GGGAGAGAAAAAAGAGCA---GTTGGA---CTGGGAGCT------
---GTCTTCATTGGGTTCTTAGGAGCA---GCTGGAAGCACTATG---GGCGCAGCGTCA---
ATAACGCTGACGGCACAG---GCCAGACAA---TTATTG---TCTGGCATAGTGCAACAGCAAAGC---
AATTTG---CTGAGG---GCTATAGAGGCTCAACAGCATATGTTGAAACTCACGGTCTGGGGCATT
>KP718918..2
TTCTATTGTAATACATCAGGCCTGTTTAATAGCACTTGGTTTAGTAATGGGAGCAGGGGCAATGAGAGCATGG
AAGATGACACC--------------------------------------------------------------
----------------------------
AATATAACTCATATAACTCTCCCATGCAGAATAAGGCAAATTGTGAATATGTGGCAGAGAGCAGGACAAGCAA
TGTATGCCCCTCCCATCCAAGGAGTAATAAAGTGTACATCAAAT---
ATTACAGGAATACTATTAACAAGAGATGGTGGATATAATATTAGCAAT-------------------------
-------------------------------------------------------------------------
----------
AACGACACCGAGATTTTCAGACCTGGAGGAGGAAATATGAAGGACAATTGGAGAAGTGAATTATATAAATATA
AAGTAGTAAAAGTTGAACCACTA---GGAATAGCACCCACTAGGGCAAAGAGAAGAGTGGTG-----------
----------------------------GAGAGAGAAAAAAGAGCA---GTTGGA---ATAGGAGCT------
---GTGTTCATTGGGTTCCTAGGAGCA---GCAGGAAGCACTATG---GGCGCAGCGTCA---
ATAACGCTGACGGCACAG---GCCAGACAG---TTATTG---TCTGGAATAGTGCAACAGCAAAGC---
AATTTG---CTGAGG---GCTATAGAGGCTCAACAGCATTTGTTGAAACTCACGGTCTGGGGCATT
>KP718928..1
TTCTATTGTAATACATCAGGCCTGTTTAATAGCACTTGGCATAGTAATGGCACTGCAAATGGCACT-------
-------------------------------------------------------------------------
----------------------------
GAAAATGCCACTATAACTCTCCCATGCAGGATAAAGCAGATTATAAATCTATGGCAGAGAGTAGGGCAAGCAA
TGTATGCCCCTCCTATCCCAGGAATAATAAATTGTACATCAAAC---
ATTACAGGACTACTATTAACAAGAGATGGTGGGAATGCTAGTGAG----------------------------
-------------------------------------------------------------------------
-------------
GAAACTGAGACCTTCAGACCTGGAGGAGGAAATATGAAGGACAATTGGAGGAGTGAATTATATAAATATAAAG
TAGTAAAGATTGAACCACTA---GGAGTAGCACCCACCAGGGCAAAGAGAAGAGTGGTG--------------
-------------------------GAGAGAGAAAAAAGAGCA---GTCGGA---ATAGGAGCT---------
GTGTTCATTGGGTTCTTAGGAGCA---GCAGGAAGCACTATG---GGCGCAGCGTCA---
ATAACGCTGACGGCACAG---GCCAGACAG---TTACTG---TCTGGTATAGTGCAACAGCAAAGC---
AATTTG---CTGAGG---GCTATAGAGGCTCAACAGCACATGTTGAAACTCACAGTCTGGGGCATT
>AM279343..1
TTCTATTGTAATACATCAGGCCTGTTTAATAGCACTTGGTTTAATAGCACTTGGAATGACACTATCAGCAATA
ATAAC--------------------------------------------------------------------
-------------------ACA---
GGGTCAAATGACACCATATATCTCCCATGCAGAATAAAGCAAATTATAAATATGTGGCAAAGGACAGGACAAG
CAATGTATGCCCCTCCCATCCGAGGAATAATACAGTGTAATTCAACC---
ATTACAGGACTACTATTAACAAGAGATGGTGGGATTAACAACAAC----------------------------
-------------------------------------------------------------------------
----------
AGTACAAATGAAACCTTCAGACCTGGAGGAGGAAATATGAGGGATAATTGGAGAAGTGAACTATATAAATATA
AAGTAGTAAAAATTGAACCACTA---GGAGTAGCACCCACCAAGGCAAGGAGAAGAGTGGTG-----------
```

----------------------------GAGAGAGAAAAAAGAGCA---GTTGGA---CTGGGAGCT------
---GTATTCATTGGGTTCTTAGGAGCA---GCAGGAAGCACTATG---GGCGCGGCGTCA---
ATAACGCTGACGGTACAG---GCCAGACAA---TTATTG---TCTGGCATAGTGCAACAGCAAAAC---
AATTTG---CTGAGG---GCTATAGAGGCTCAACAGCATCTGTTGAAACTCACGGTCTGGGGCATT
>AM279348..2
TTCTATTGCAATACATCAGGCCTGTTTGCTCAGGAGTTAAAAAATGACACAAAT-------------------
----

GACAATAACACAATAATTCTCCTATGCAAAATAAAGCAAATTATAAATATGTGGCAGAGAGTAGGACAAGCAA
TGTATGCCCCTCCCAGAGCAGGAGTAATAAGGTGTGTATCAAAC---
ATTACAGGACTACTGTTAACAAGAGATGGTGGAAAAAATGACAATGGA-------------------------
----

ACTAATGGAACTGAGATATTCAGGCCTGGAGGAGGAGATATGAGGGACAATTGGAGAAGTGAATTATATAAGT
ATAAAGTAGTAAGAATTGAACCAGTA---GGGGTAGCACCCACCAGGGCAAAGAGAAGAGTGGTG--------
-------------------------------GAGAGAGAGAAAAGAGCA---GTTGGA---CTGGGAGCT---
------GTCTTCCTTGGGTTCTTAGGAGCA---GCAGGAAGCACTATG---GGCGCGGCGTCA---
CTGACGCTGACGGTACAG---GCCAGGCAA---TTATTG---TCTGGCATAGTGCAACAGCAGAGC---
AATTTG---CTGAGG---GCTATAGAGGCTCAACAACATATGTTGAGACTCACAGTCTGGGGCATC
>FJ388894..1
TTCTATTGTAACACATCAAGCCTGTTTAATAGCACCTGGAATATTAATACTATTTGGAATAACACTATGTCAA
ACAGC--
-------------------ACT---
GAGTCAAACGGCACTATAGTGCTCCCATGCAGAATAAAGCAAATTGTAAATGTGTGGCAGAGAGCAGGACAAG
CAATATACGCCCCTCCCATCCAAGGAATAATACAGTGTGTCTCAAAC---
ATTACAGGACTAATATTAACAAGAGATGGTGGGAATAATAATAACACA-------------------------
----

AATAATACTGAAATCTTCAGACCTGGAGGAGGAGATATGAGGGACAATTGGAGAAGTGAATTATATAAATATA
AGGTAGTAAAAATTGAACCATTA---GGAGTAGCACCCACCAAGGCAAGGAGAAGAGTGGTG-----------
----------------------------GGGAGAGAAAAAAGAGCA---GTTGGA---CTGGGAGCT------
---GTCTTCCTTGGGTTCTTAGGAACA---GCAGGAAGCACTATG---GGCGCGGCGTCA---
ATAACGCTGACGGTACAG---GCCAGGCAA---TTATTG---TCTGGCATAGTGCAACAGCAAAGC---
AATTTG---CTGAGG---GCTATAGAGGCTCAACAACATCTGTTGAAACTCACTGTCTGGGGCATT
>FJ388903..3
TTCTATTGTAACACATCGGGCCTGTTTAATAGCCAATATAACACATCAGGCCTGGTTAATAACACGCAGGAGT
CAGGAAGCACA--
GAGTCAAATGACACTGAGTCA------------
ATTGTCACAGACAATATAACTCTCCAAATCAGAATAAAGCAAATTATAAATCAGTGGCAAAGAGTAGCTCAAG
CAACATATGCCCCTCCCATCCCAGGAGTAATAAAATGTGTATCAAAC---
ATTACAGGACTAATATTAACTAGAGATGGTGGGAGTAGTAGCGGA----------------------------
----

AATGAAACCTTCAGACCTGGAGGAGGAGATATGAGGGACAATTGGAGAAGTGAATTATATAAGTATAAAGTAG
TAAAAATTGAACCACTA---GGAGTAGCACCCACCAGGGCAAAGAGAAGAGTGGTG-----------------
----------------------GAGAGAGAAAAAAGAGCA---GTTGGA---ATAGGAGCT---------
GTCTTCATTGGGTTCTTAGGAGCA---GCAGGAAGCACTATG---GGCGCGGCGTCA---
ATGACGCTGACGGTACAG---GCCAGGCAA---TTATTG---TCTGGCATAGTGCAACAGCAAAGT---
AATTTG---CTGAGG---GCTATAGAGGCTCAACAGCATCTGTTGAAACTCACGGTTTGGGGCATT
>FJ388909..1
TTCTATTGTAATACATCAGGCCTGTTTAATAGCACTTGGAATAATGACACCTGGAATGGCAATGACACACAAG
AGTCAAAT---
-------------GAAACC------
TCGTCAAATGGCACCATAATTCCACCATGCAAAATAAAACAAATTGTAAATATGTGGCAGAGAGTACAACAAG
CAATGTATCCCCCTCTCGTCGAAGGAGTAATAAGGTGTGAATCCATC---
ATTACAGGACTACTATCAACAAGAGATGGTGGGATTAACAGCAGG----------------------------
----

ACAAATGAAACCTTCAGACCTGGGGGAGGAGATATGAGGGACAATTGGAGAAGTGAACTATATAAATATAAGG
TAGTAAAAATTGAACCACTA---GGAGTAGCACCCACCACGGCAAAGAGAAGAGTGGTG--------------

-------------------------GAGAGAGAAAAAAGAGCA---GCTGGA---CTGGGAGTT---------
ATGTTCCTTGGGTTCTTAGGAGCA---GCCGGAAGCACTATG---GGCGCGGCGTCA---
ATAACGCTGACGGTACAG---GCCAGACAG---TTATTA---TCTGGTATAGTGCAACAGCAAAGC---
AATTTG---CTGAAG---GCTATAGAGGCTCAACAGCACCTGTTGAGACTCACGGTCTGGGGCATT
>FJ388925..1
TTCTATTGTAACACATCAGGCTTGTTTAATAGCACTTGGAATGCCACTGCCAGCATGCAGGAGTCAAATAGCA
CAGAG--

TCAAACGACACTACTATAACTCTCCCATGCAGAATAAAGCAAATTATAAATATGTGGCAGAGAGCAGGACAAG
CAATATATGCCCCTCCCATCCGAGGAGTAATAAGGTGTGAATCAAAC---
ATTACAGGACTAATATTAACAAGAGATGGAGGGAATAGTAACAGC----------------------------
----

ACAAATGAAACCTTCAGACCTGGAGGAGGAGATATGAGGGATAATTGGAGAAGTGAATTATATAAGTATAAAG
TAGTAAAAATTGAACCACTA---GGAGTAGCACCCACCAGGGCAAGGAGAAGAGTGGTG--------------
-------------------------GAGAGAGAAAAAAGAGCA---GTTGGA---ATAGGAGCT---------
GTCTTCATTGGGTTCTTAGGAGCA---GCAGGAAGCACTATG---GGCGCGGCGTCA---
ATAGCGCTGACGGTACAG---GCCAGGCAA---TTATTG---TCTGGCATAGTGCAACAGCAAAGC---
AATTTG---CTGAGG---GCTATAGAGGCTCAACAGCATATGTTGAAACTCACGGTCTGGGGCATT
>FJ388932..1
CTCTATTGTAACACATCAGGCCTGTTTAATAGCACTTGGCCTGCCAATAACAGTACGCAGGGGTCAAATAGCA
CA---

GAGTCAAACGACACCGTAATTCTCCAATGCAGAATAAAGCAAATTATAAATATGTGGCAAAGAGTAGGACAAG
CAATATATGCCCCTCCCATACCAGGAGAAATAAAGTGTGTATCAAAC---
ATTACAGGACTAATATTAACAAGAGATGGTGGGAGTAGCAATAAT----------------------------
----

ACAGAAAATGAAACCTTCAGACCAGGAGGAGGAGATATGAGGGACAATTGGAGAAGTGAATTATATAAGTATA
AAGTAGTAAAAATTGAACCACTA---GGAGTAGCACCCACCAGGGCAAAGAGAAGAGTGGTG-----------
----------------------------CAGAGAGAAAAAAGAGCAGTATTAGGTGGACTGGGAGCT------
---GTCTTCGTTGGGTTCTTGGGAGCA---GCAGGAAGCACTATG---GGCGCGGCGTCA---
GTGACGCTGACGGTACAG---GCCAGGAAA---TTATTG---ACTGGCATAGTGCAACAGCAAAGT---
AATTTG---CTGAGG---GCTATCGAGGCTCAACAGCATCTGTTGAAACTCACGGTCTGGGGCATT
>FJ388938..1
TTCTATTGTAACACATCAGGCCTGTTTAATAGCACTTGGAATGGCACTACCATGCAGGAGTCAAATAACACAG
AGTCAAAC---
-------------GACACT------
GAGTCAAACGACATTATAACTCTCCGATGCAGAATAAGGCAAATTATAAGAATGTGGCAGAGAGCAGGACAAG
CAATGTATGCCCCACCCATCCAAGGAATAATAAAGTGTGAATCAAAC---
ATTACAGGACTAATATTAGCAAGAGATGGTGGGAATAGAAATGAA----------------------------
----

ACTGAAACCTTCAGACCTGGAGGAGGAGACATGAGGGACAATTGGAGAAGTGAATTATATAAGTATAAAGTAG
TAAAAATTGAACCATTA---GGAGTAGCACCCACCAAGGCAAGGAGAAGAGTGGTG-----------------
----------------------CAGAGAGAAAAAAGAGCA---GTTGGA---ATAGGAGCT---------
GTCTTCATTGGGTTCTTAGGAGCA---GCAGGAAGCACTATG---GGCGCGGCGTCA---
ATAACGCTGACGGTACAG---GCCAGGCAA---CTATTG---TCTGGCATAGTGCAACAGCAAAGT---
AATTTG---CTGAAG---GCTATAGAGGCTCAACAACATCTGTTGAAACTCACGGTTTGGGGCATT
>FJ388942..1
TTCTATTGTAACACATCAGGCCTGTTTAATAGCTCTTGGCTGAAGTCAAATAGAACAGGGCCTAGCACAGAGC
CAAACAAC---
-------------ACTGAG------
CCAAACAACACTATTATAACTCTCCCATGCAAAATAAAGCAAATTATAAATATGTGGCAGAGAACAGGACAAG
CAATGTATGCCCCTCCCATCCCAGGAGTAATAAGGTGTGAATCAAAC---
ATTACAGGACTAATATTAACAAGAGATGGTGGGAATAGTAGCAAT----------------------------
----

AGCAATGAAACCTTCAGACCTGGAGGAGGAAATATGAGGGACAATTGGAGAAGTGAATTATATAAGTATAAAG
TAGTAAAAATTGAACCACTA---GGAGTAGCACCCACCAGGGCAAGGAGAAGAGTGGTG--------------

```
-------------------------GAGAGAGAAAAAAGAGCA---GTTGGA---ATAGGAGCT---------
GTCTTCATTGGGTTCTTAGGAGCA---GCAGGAAGCACTATG---GGCGCGGCGTCA---
ATGACGCTGACGGTACAG---GCCAGGCAA---TTATTG---TCTGGCATAGTGCAACAGCAAAGC---
AATTTG---CTGAGG---GCTATAGAGGCTCAACAGCATATGTTGAAACTCACGGTCTGGGGCATT
>FJ388946..2
TTTTATTGTAATACATCAGGCCTGTTTAATAGAACTTGGTCTACCAATACCAGCATGCAGGTGTCAAATAGCA
CA-----------------------------------------------------------------------
-------------------------
GAGTTAAATGGCCCTATAATTCTCCAATGCAGAATAAAGCAAATTATAAATATGTGGCAAAGAGTAGGACAAG
CAATATATGCCCCTCCCATCCCAGGAGTAATAAGGTGTGAATCAAAC---
ATTACAGGACTAATATTAACAAGAGATGGTGGAAATAATAATACC----------------------------
-------------------------------------------------------------------------
-------------
GGAAATCAAACCTTCAGACCTGTAGGAGGAGATATGAAGGACAATTGGAGAAGTGAATTATATAAGTATAAAG
TAGTAAAAATTGAACCACTA---GGAGTAGCACCCTCCAAGGCAAAGAGAAGAGTGGTG--------------
-------------------------GAGAGAGAAAAAAGAGCA---ATTGGA---ATAGGAGCT---------
GTCTTCATTGGGTTCTTAGGAGCA---GCAGGAAGCACTATG---GGCGCGGCGTCA---
GTGACGCTGACGGTACAG---GCCAGGCAA---TTATTG---TCTGGCATAGTGCAACAGCAAAGT---
AATTTG---CTGAGG---GCTATAGAGGCTCAACAGCATCTGTTGAACCTCACGGTCTGGGGCATT
>JF683737..1
TTCTATTGTAACACATCAGGCCTGTTTAATAGCACGTGGAATGCTAGTGACATCACGCAGGGG----------
-------------------------------------------------------------------------
----------------------------
TTAAATGACACAATAACTCTCCAATGCAGAATAAAGCAAATTATAAATATGTGGCAGAGAGTAGGGCAGGCAA
TGTATGCCCCTCCCATCCAGGGAGAAATAAGGTGTGAATCAAAC---
ATTACAGGACTAATATTAACAAGAGATGGTGGGAATGGGAATGGA----------------------------
-------------------------------------------------------------------------
-------------
ACAAATGAAACCTTCAGACCTGGAGGAGGAGATATGAGGGACAATTGGAGAAGTGAATTATATAAGTATAAAG
TAGTAAAAATTGAACCACTA---GGAGTAGCACCCACCAGGGCAAAGAGAAGAGTGGTG--------------
-------------------------GAGAGAGAAAAAAGAGCA---GTTGGA---CTGGGAGCT---------
GTATTCATTGGGTTCTTAGGAGCA---GCAGGAAGCACTATG---GGCGCGGCGTCA---
GTGACGCTGACGGTACAG---GCCAGGCAA---TTATTG---TCTGGCATAGTGCAACAGCAAAGC---
AATTTG---CTGAGG---GCTATAGAGGCTCAACAGCATTTGTTGAAACTCACAGTCTGGGGCATT
>JF683759..1
TTCTATTGTGACACATCAGGCCTGTTTAATAGCACTTGGAATACCACTGACACCATGCACGAGGAGACTAATA
CA-----------------------------------------------------------------------
-------------------------
GAGGCAAATAGTACTATTACTCTCCCATGCAGAATAAAGCAAATTATAAATATGTGGCAGAGAGCAGGACAAG
CAATATATGCCCCTCCCATCCCAGGAGTAATACAGTGTAAATCAAAC---
ATTACAGGACTAATATTAACAAGAGATGGTGGGAGTAATAGCAGC----------------------------
-------------------------------------------------------------------------
-------------
ACAAATGAAACCTTCAGACCTGGAGGAGGAGATATGAGGGACAATTGGAGAAGTGAATTATATAAGTATAAAG
TAGTAAAAATTGAACCACTA---GGAGTAGCACCCACCAAGGCAAGGAGAAGAGTGGTG--------------
-------------------------CAGAGAGAAAAAAGAGCA---GTAGGA---CTGGGAGCG---------
GTATTCATTGGGTTCTTAGGAGCA---GCTGGAAGCACCATG---GGCGCGGCGTCA---
ATAACGCTGACGGTACAG---GCCAGGCAA---TTATTG---TCTGGTATAGTGCAACAGCAAAGC---
AATTTG---CTGAGG---GCTATAGAGGCTCAACAGCATCTGTTGAAACTCACAGTCTGGGGCATT
>JF683760..1
TTCTATTGTAACACCTCAAAGTTGTTTAATAGCACTTGGGATCCTAATAATACCAGCACGCAGGAGTCAAATA
GC-----------------------------------------------------------------------
----------------------------
ACAAATGATAATATAATTCTCCCATGCAGAATAAGGCAAATTATAAATATGTGGCAGAGAGCAGGACAAGCAA
TATATGCCCCTCCCATCCAAGGAATAATAAACTGTGTATCAAAC---
ATTACAGGATTAATATTAACAAGAGATGGGGGTGTAGGTAACAAC----------------------------
-------------------------------------------------------------------------
-------------
GCAAAGGAAGTCTTCAGACCTGGAGGAGGAGATATGAGGGACAATTGGAGAAGTGAATTATATAAGTATAAAG
TAGTAAAAATTGAGCCACTA---GGAGTAGCACCCACTGGGGCAAGGAGAAGAGTGGTG--------------
```

-------------------------CAGAGAGAAAAAAGAGCA---GTTGGA---CTGGGAGCT---------
GTTTTCATTGGGTTCTTGGGAGCA---GCAGGAAGCACTATG---GGCGCGGCGTCA---
ATGACGCTGACGGTACAG---GCCAGACAA---TTATTG---TCTGGCATAGTGCAACAGCAAAGC---
AATTTG---CTGAGG---GCTATAGAGGCTCAACAGCATATGTTGAGACTCACGGTCTGGGGCATT
>JF683761..1
TTCTATTGTAACACCTCAAAGCTGTTTAATAGCACTTGGAATAATACCAGCACGCAGGAGTCAAATAGCACTA
AT---

CCAAATGATAATATAACTCTCCCATGCAGAATAAAGCAAATTATAAATATGTGGCAAAGAGCAGGACAAGCAA
TATATGCCCCTCCCATCCAAGGAAAAATAAACTGTGTATCAAAC---
ATTACAGGATTAATATTAACAAGAGATGGGGGGGGTAACAGCACA----------------------------
----

GAGGAAGTCTTCAGACCTGGAGGAGGAGATATGAGGGACAATTGGAGAAGTGAATTATATAAGTATAAAGTAG
TAAAAATTGAGCCAATA---GGAGTAGCACCCACTGAGGCAAGGAGAAGAGTGGTG-----------------
----------------------CAGAGAGAAAAAAGAGCG---GTTGGA---CTGGGAGCT---------
GTTTTCATTGGGTTCTTGGGAGCA---GCAGGAAGCACTATG---GGCGGGGCGTCA---
ATGACGCGGACGGTACAG---GCCAGACAA---TTATTG---TCTGGCATAGTGCAACAGCAAAGC---
AATTTG---CTGAGG---GCTATAGAGGCTCAACAGCATATGTTGAGACTCACGGTCTGGGGCATT
>JF683767..1
TTCTATTGTAATACATCAGGTCTGTTCAATAGCTCTTGGAATATCAATGGCAGCAGGTCAAATAGCACAAGG-
----

TCAAATGGCAATATAACTCTCCCATGCAGAATAAAGCAAATTATAAATATGTGGCAAAGAACAGGACAGGCAA
TGTATGCCCCTCCCATCCAAGGAGTAATAAGGTGTGTATCAAAC---
ATTACAGGACTACTGTTAACAAGAGATGGAGGGGGTAACAATACA----------------------------
----

AATGAAACTGAAATTTTCAGACCTGGAGGAGGAGATATGAGGGGCAATTGGAGAAGTGAATTATATAAGTATA
AAGTAGTAAAAATTGACCCACCA---GGAGTAGCACCCAACACAGCAAGGAGAAGAGTGGTG-----------
----------------------------GGGAGAGAAAAAAGAGCA---GCTGGAATAATAGGAGCT------
---GTCTTCCTTGGGTTCTTAGGAGCA---GCAGGAAGCACTATG---GGCGCGGCGTCA---
GTGACGCTGACGGTACAG---GCCAGACAA---TTATTG---TCTGGTATAGTGCAACAGCAAAGC---
AATTTG---CTGAGG---GCTATAGAGGCTCAACAACACCTGTTGAAACTCACGGTCTGGGGCATT
>JF683779..2
TTCTACTGTAACACATCAGGCCTGTTTAATAGCACTTGGTATACCAATAACAGCATGCAGCCAAAT-------
----

TACACAAAAACTATAACTTTGCCATGCAGAATAAAGCAAGTTATAAATATGTGGCAAAGAGTAGGACAAGCAA
TGTATGCCCCTCCCATCCCAGGAATAATACAATGTGTATCAAAC---
ATTACAGGACTAATATTAACAAGAGATGGTGGGCATAATAATAGT----------------------------
----

GCAAATGAGACCTTCAGACCTGGAGGAGGAGATATGAGGGACAATTGGAGAAGTGAATTATATAAGTATAAAG
TAGTGAAAGTTGAACCATTA---GGAGTAGCACCCACCAGGGCAAGGAGAAGAGTGGTG--------------
-------------------------GAGAGAGAAAAAAGAGCA---GTTGGA---ATAGGAGCT---------
GTCTTCCTTGGGTTCTTAGGAGCA---GCAGGAAGCACTATG---GGCGCGGCGTCA---
GTGACGCTGACGGTACAG---GCCAGGCAA---TTATTG---TCTGGCATAGTGCAACAGCAAAGC---
AATTTG---CTGAGG---GCTATAGAGGCTCAACAACATATGTTGAAACTCACGGTCTGGGGCATT
>JF683782..1
TTCTATTGTAATACATCAAAATTGTTTAATACAACTTGGAATGCCAATGGCAGCGTACAGGAGTCAGTCAACA
CT---

GCAGGCAACACTACTATATTTCTCCCATGCAGAATAAAGCAAATTATAAGGATGTGGCAGAGAGCAGGACAAG
CAATATATGCCCCTCCCATCCAGGGAAGAATAAGGTGTGAATCAAAC---
ATTACAGGACTACTGTTAACAAGAGATGGTGGAGGTAATGCAAAC----------------------------
----

AACACAGAAATCTTTAGACCTGGAGGAGGAGATATGAGGGACAATTGGAGAAGTGAATTATATAAGTACAAGG
TAGTAAAAATTGAACCACTA---GGAGTAGCACCCACCAAGGCAAGGAGAAGAGTGGTG--------------

-------------------------CAGAGAGAAAAAAGAGCA---GTTGGA---CTAGGAGCT---------
GTCTTCCTTGGGTTCTTAGGAGCA---GCAGGAAGCACTATG---GGCGCGGCGTCA---
ATAACGCTGACGGTACAG---GCCAGGCAA---TTATTG---TCTGGCATAGTGCAACAGCAAAGC---
AATTTG---CTGAGG---GCTATTGAGGCTCAACAGCAGATGTTGAGACTCACGGTCTGGGGCATT
>JF683783..1
TTCTATTGTAATACATCAAAATTGTTTAATACAACTTGGAATGTCACTGACAGCATAGAAGAGTCA-------
----

GGCAACACTATTATAGTCCTCCCATGCAAAATAAAGCAAATTATAAATATGTGGCAGAGAGCAGGACAAGCAA
TATATCCCCCTCCCATCCGGGGAATAATAAGGTGTGAATCCAAC---
ATTACAGGACTATTATTAACAAGAGATGGTGGAGATACTGAAAAC----------------------------
----

ACCACAGAAATCTTTAGACCTGGAGGAGGAGATATGAGGGACAATTGGAGAAGTGAATTATATAAGTATAAAG
TAGTAAAAATTGAACCACTA---GGAGTAGCACCCACCGAGGCAAGGAGAAGAGTGGTG--------------
-------------------------CAGAGAGAAAAAAGAGCA---GTTGGA---ATAGGAGCT---------
GTCTTCCTTGGGTTCTTAGGAGCA---GCAGGAAGCACTATG---GGCGCGGCGTCA---
ATAACGCTGACGGTACAG---GCCAGGCAA---TTATTG---TCTGGCATAGTGCAACAGCAAAGC---
AATTTG---CTGAGG---GCTATAGAGGCTCAACAGCAGATGTTGAGACTCACGGTCTGGGGCATT
>JF683789..1
TTCTATTGTAACACCTCAAAGCTGTTTAATAGCATTTGGCATCCTAATGATACCATGCAGGAGGAGACTAATA
CA---

AATCCAAATGCTAATATAACTCTCCCATGCAGAATAAAGCAAATTATAAATCTGTGGCAAAGAGTAGGGAAAG
CAATATATGCCCCTCCCATCCGAGGAAGAATAGAGTGTGTATCAAAC---
ATTACAGGATTAATAATAACAAGAGATGGTGGAAATAACACAAAT----------------------------
----

AACACTGAAATCTTCAGACCTGCAGGAGGAGACATGAGGGACAATTGGAGAAGTGAATTATATAAATATAAAG
TAGTACAAATTGAGCCACTA---GGAATAGCACCCACTGAGGCAAGGAGAAGAGTGGTG--------------
-------------------------CAGAGAGAAAAAAGAGCA---ATTGGA---CTAGGAGCT---------
ATGTTCATTGGGTTCTTAGGAGCA---GCAGGAAGCACTATG---GGCGCGGCGTCA---
GTGACGCTGACGGTACAG---GCCAGGCAA---TTATTG---TCTGGCATAGTGCAACAGCAAAGC---
AATTTG---CTGATG---GCTATAGAGGCTCAACAGCATCTGTTGAAACTCACAGTCTGGGGCATT
>FJ670523..1
TTCTATTGTAACACATCAGGCCTGTTTAATAGCACTTGGCCTAATAGCACTTGGAATGCCAATACCAGCATAC
AGGAG--
-------------------TCA---
AATAGCACAGACAATATAACTCTCCAATGCAGAATAAAGCAAATTATAAATCTGTGGCAGAGAGCAGGAAAAG
CAATATATGCCCCTCCCATCCAAGGAGCAATACAGTGTATATCAAAC---
ATTACAGGACTAATATTAACAAGAGATGGAGGTAATAATGGAACT----------------------------
----

AATAACACTGAGATCTTCAGACCTGGAGGAGGAGATATGAGAGATAATTGGAGAAGTGAATTATATAAGTATA
AGGTAGTAAAAATTGAACCATTA---GGAGTAGCACCCTCCAGGGCAAAGAGAAGAGTAGTG-----------
----------------------------GGGAGAGAAAAAAGAGCA---GTTACA---ATAGGAGCT------
---GTCTTCCTTGGGTTCTTAGGAGCA---GCAGGAAGCACTATG---GGCGCGGCGTCA---
CTGACGCTGACGGTACAG---GCCAGGCAA---TTATTG---TCTGGCATAGTGCAACAGCAAAGC---
AATTTG---CTGCAG---GCTATAGAGGCTCAACAGCATCTGTTGAAACTCACGGTCTGGGGCATT
>AF219261..2
TTCTATTGTAATACATCAGGCCTGTTTAATAGCACTTGGGTGTCAAACAACACTGGGAAATATTATAGTAATA
GCACT--

AGGGAAAATCCAAATATAACTCTCCCATGCAGAATAAAACAAATTATAAATATGTGGCAGAGAGCAGGACAAG
CAATATATGCCCCTCCCATCGAAGGAGTAATAAGGTGTGAATCAAAC---
ATTACAGGACTACTATTAACAAGAGATGGTGGGGATAAGAACAAC----------------------------
----

ACAAGTGAAATCTTCAGACCTGGAGGAGGAAATATGAAGGACAACTGGAGAAGTGAATTATATAAGTATAAAG
TAGTAAAAATTGAACCATTA---GGAGTAGCACCCACCCCGGCAAGGAGAAGAGTGGTG--------------

-------------------------CAGAGAGAAAAAAGAGCA---GTATTTGGGCTGGGAGCT---------
GTCTTTCTTGGGTTCTTAGGAGCA---GCAGGAAGCACTATG---GGCGCGGCGTCG---
ATAACGCTGACGGTACAG---GCCAGACAA---TTATTG---TCTGGCATAGTGCAACAGCAAAGT---
AATTTG---CTGAGG---GCTATAGAGGCTCAACAGCATTTGTTGAAACTCACGGTCTGGGGCATT
>AF219265..2
TTCTATTGTAATACATCAGGCCTGTTTAATAGCACTTGGAGTAATAGCACTTGGAGTTCCAATGCCAGCACGC
AGGGG--

TCAGAAAATGACACTATAACTCTCCCATGCAGAATAAAACAAATTATAAATATGTGGCAGAGAGCAGGACAAG
CAATGTATGCCCCTCCCATTCAAGGAGTAATAAGGTGTGATTCAAAT---
ATTACAGGACTAATATTAACAAGAGATGGTGGGGTTACAAATGAA----------------------------
----

ACCGAAACCTCCAGACCTGGAGGAGGAGATATGGGGGACAATTGGAGAAGTGAATTATATAAATATAAAGTAG
TGAAAATTGAACCACTA---GGAGTAGCACCCACCAGGGCAAAAAGAGTAGTGGTG-----------------
----------------------GGGAGAGAAAAAAGAGCA---ATTGGA---ATAGGAGCT---------
GTTTTCATTGGGTTCCTAGGAGCA---GCAGGAAGCACTATG---GGCGCGGCGTCA---
ATGACGCTGACGGTACAG---GCCAGACAA---TTATTG---TCTGGCATAGTGCAACAGCAAAGC---
AATTTG---CTGAGG---GCTATAGAGGCTCAACAACATCTGTTGAAACTCACGGTCTGGGGCATT
>HM204635..1
TTCTATTGTAATACATCACACCTGTTTAATAGCGCTTGGTCTGTCAATGACACCGTGCAGGAGTCAAATAGCA
CG---

GAGTCAAATGACACTATAACTCTCCAATGCAGAATAAAGCAAATTATAAATATGTGGCAGAGAACAGGACAAG
CAATGTATGCCCCTCCCATCCCAGGAGTAATAAGATGTAACTCAAAC---
ATTACAGGACTAATATTAACAAGAGATGGTGGGAATAATAATAGTTCA-------------------------
----

AATGAAAATGAAACCTTCAGACCTGGAGGAGGAGATATGAGAGACAATTGGAGAAGTGAATTATATAAGTATA
AAGTAGTAAAAATTGAACCACTA---GGAGTAGCACCCACCAAGGCAAGGAGAAGAGTGGTG-----------
----------------------------CAGAGAGAAAAAAGAGCA---GTTGGA---CTGGGAGCT------
---GTCTTCATTGGGTTCTTAGGAGCA---GCAGGAAGCACTATG---GGCGCGGCGTCA---
GTGACGCTGACGGTACAG---GCCAGACAA---TTATTG---TCTGGCATAGTGCAACAGCAAAGC---
AATTTG---CTGAGG---GCTATAGAGGCTCAACAACATCTGTTGAAACTCACAGTCTGGGGCATT
>HM204648..2
TTTATTGTAACACATCAGGCCTGTTTACTGGCACTTGGAATCAGACTAATAGTACTTGGAATAGTGGCACTT
GGACT--

AACACTACTGGCAATATAACTCTCCAATGCAGAATAAAGCAGGTCATAAGAATGTGGCAGAGAGTAGGACAAG
CAATGTATGCCCCTCCCATCCCAGGAGTAATAAGGTGTGAATCAAAC---
ATTACAGGACTAATATTAACAAGAGATGGGGAAGGTAACAGTAGC----------------------------
----

ATAAAAGATGAAACCTTCAGACCTGGAGGAGGGAATATGAAGGACAATTGGAGAAGTGAATTATATAAATATA
AAGTAGTAAAAATTGAACCAATA---GGAGTAGCACCCACCAGGGCAAAGAGAAGAGTGGTG-----------
----------------------------GAGAGAGAAAAAAGAGCA---GTTGGA---CTAGGAGCT------
---GTCTTCATTGGGTTCTTAGGAGCA---GCAGGAAGCACTATG---GGCGCAGCGTCA---
ATAACGCTGACGGTACAG---GCCAGGCAA---TTGTTG---TCTGGCATAGTGCAGCAGCAAAGC---
AATTTG---CTGAAG---GCTATTGAGGCTCAACAACAGTTGTTGAGACTCACGGTCTGGGGCATT
>HQ595782..1
TTCTATTGCAATACGTCTAAACTGTTTAATAGCACGTGGAAAAATGAAAGCAGGCAGGGG-------------
----

TCAAATGACATTATAACTCTCCCATGCAGAATAAAGCAAATTATAAGGATGTGGCAAAGAGTAGGGCAAGCAA
TGTATGCCCCTCCCATCCCAGGAGTAATAGAGTGTAATTCAAAC---
ATTACAGGGCTACTATTGACAAGAGATGGTGGTAATAGAACCGAA----------------------------
----

AATGAGACCTTCAGACCTGAGGGAGGAGATATGAGAAACAATTGGAGAAGTGAATTATATAAGTATAAAGTAG
TAAGAATTGAACCATTG---GGAGTAGCACCCACCAGGGCAAAAAGAAGAGTGGTG-----------------

----------------------GAGAGAGAAAAAAGAGCA---GCTGGA---TTGGGAGCT---------
GTCTTCCTTGGGTTCTTAGCAGCA---GCAGGAAGTACTATG---GGCGCAGCGTCA---
ATAACGCTGACGGCACAG---GCCAGACAA---TTATTG---TCTGGCATAGTACAACAGCAAAGC---
AATTTG---CTGAGG---GCTATAGAGGCTCAACAGCAACTGTTGAGGCTCACGGTCTGGGGCATT
>HQ595784..2
TTCTATTGCAACACAGCAGGTCTGTTTAATAGCAGCTGGGGGAACAATGACACTGACAGCAACCAGACA----
----

GCAAAGATAAATATAACTCTCCAATGCAGAATAAAGCAAATTGTAAGGATGTGGCAGAGAGTAGGACAAGCAA
TGTATGCCCCTCCCATCGCAGGAACGATAAACTGTTTATCAAAT---
ATTACAGGACTACTATTAACAAGAGATGGGGGGACAAATAAGACGTGG-------------------------
----

TCTCACAATGAGACCTTCAGACCTGAAGGAGGAAATATGAAGGACAATTGGAGAAGTGAATTATATAGGTATA
AAGTAGTAAAAATTGAACCACTA---GGAGTAGCACCCACCGAGGCAAGGAGAAGAGTGGTG-----------
----------------------------GAGAGAGGAAAAAGAGCA---GTTGGA---ATAGGAGCT------
---GTTCTCTTTGGGTTCTTAGGAGCA---GCAGGAAGCACTATG---GGCGCGGCGTCA---
CTAACGCTGACGGTACAG---GCCAGACAA---TTACTG---TCTGGCATAGTGCAACAGCAGAGC---
AATCTG---CTGAGG---GCTATAGAGGCTCAACAGCATCTGTTGAAGCTCACAGTCTGGGGCATT
>Y13718..1
TTCTATTGTAACACATCAAGACTGTTTAATAGCACTTGGAATGACACTACCAGTATGCTAAATGACACGAAG-
----

CCAAACGACACTATAACTCTCCCATGCAGAATAAAGCAAATTATAAATATGTGGCAAAGAGCAGGACAAGCAA
TATATGCCCCTCCCATCCAAGGAGTAATAAGGTGTGAATCAAAC---
ATTACAGGACTAATATTAACAAGAGATGGTGGGGGTAATAGCAGT----------------------------
----

GTAAGTAGTGAAACTTTCAGACCTGGAGGAGGAGATATGAGAGACAATTGGAGAAGTGAATTATATAAGTATA
AAGTAGTAAAAATTGAACCACTA---GGAGTAGCACCCACCAAGGCAAAGAGAAGAGTGGTG-----------
----------------------------GAGAGAGAAAAAAGAGCA---GTTGGA---CTAGGAGCT------
---GTTTTCATTGGGTTCTTAGGAGCA---GCAGGAAGCACTATG---GGCGCGGCGTCA---
ATAACGCTGACGGTACAG---GCCAGACAA---TTATTG---TCTGGCATAGTGCAACAGCAAAGC---
AATTTG---CTGAGG---GCTATAGAGGCTCAACAGCATCTGTTGAAACTCACGGTCTGGGGCATT
>Y13717..1
TTCTATTGTAACACATCAGGCCTGTTTAATAGCACTTGGGATAGCAGTGCCAGCATGCAAGAGTCAAAT----
----

AGCACGGCCAATATAACTCTCCCATGCAGAATAAGGCAAATTATAAATCTGTGGCAGAGAACAGGACAAGCAA
TATATGCCCCTCCCATCCAAGGAGTAATAAGGTGTGTATCAGAC---
ATCACAGGACTAATATTAACAAGAGATGGTGGGGGTAATGGTACTGAA-------------------------
----

AGTAAAAAAGAAACTTTCAGACCTGGAGGAGGAGATATGAGGGACAATTGGAGAAGTGAATTATATAAGTATA
AAGTAGTAAAAATTGAACCACTA---GGAGTAGCACCCACCAAGGCAAAGAGAAGAGTGGTG-----------
----------------------------GAGAGAGAAAAAAGAGCA---GTTGGA---ATGGGAGCT------
---GTCCTCCTGGGGTTCTTAGGAGCA---GCAGGAAGCACTATG---GGCGCGGCGTCA---
ATAACGCTGACGGTACAA---GCCAGACAA---TTATTG---TCTGGCATAGTGCAACAGCAAAGC---
AATTTG---CTGAGG---GCTATAGAGGCTCAACAGCAGGTGTTGAGACTCACGGTCTGGGGCATT
>HQ385459..2
TTCTATTGCGATACATCAGGCCTGTTTAATAGTATTTGGAATAGCACTGCCATCAGCCAGAATGTCGAGGAG-
----

TCAAATGATACTATAACTCTCCCATGCAAAATAAGACAAATTGTAAATATGTGGCAGAGAGTAGGACAAGCAA
TATATGCCCGTCCCATTCAAGGAATAATAAACTGTACATCAAAC---
ATTACAGGGCTACTGCTAACAAGAGATGGTGGGGATAATGATAAT----------------------------
----

AAAACTGAGACTTTCAGACCTGGAGGAGGAAATATGAAGGACAATTGGAGAAGTGAGTTATATAGATATAAAG
TAGTAAAAATTGAACCACTA---GGAGTAGCACCCACCAAGGCAAAGAGAAGAGTGGTG--------------

-------------------------GAGAGAGAAAAAAGAGCA---GTTGGA---CTAGGAGCT---------
GTCATCTTTGGGTTCCTGGGAGCA---GCCGGAAGTACTATG---GGCGCGGCGTCA---
ATAACGCTGACGGCACAG---GCCACACAA---TTATTG---TCTGGTATAGTACAACAGCAAAGC---
AATCTG---CTGAGG---GCTATAGAGGCTCAACAGCACCTGTTGAAACTCACGGTCTGGGGCATT
>HQ385452..1
TTCTATTGCAATACATCAGGCCTGTTTTCTGGCTCTTGGTATAATGGCACTTGGGCTAATGACTCTTGGAGTG
CTGGT--
-------------------CCT---
GGGACCAATGATAATATAACTCTCCCATGCAAAATAAAGCAAATCATAAGAATGTGGCAGAGAGTGGGACAAG
CAATGTATGCCCCTCCCATCCAAGGAGAAATAAATTGTGTATCAAAC---
ATCACAGGACTATTACTAACAAGAGATGGTGGTAATAGTAGTGAA----------------------------
----

GAGATCTTCAGACCTGGAGGAGGAGATATAAGAGACAATTGGAGGAGTGAATTATATAAATATAAAGTGGTAA
AAATTGAACCATTA---GGAGTAGCGCCCACCAGGGCAAGGAGAAGAGTGGTG--------------------
-------------------GGAAGAGAAAAAAGAGCA---GTTGGA---CTGGGAGCT---------
GTCTTCCTTGGGTTCTTAGGAGCA---GCAGGAAGCACTATG---GGCGCGGCGTCA---
ATCACGCTGACGGTACAG---GCCAGGCAA---TTATTG---TCTGGCATAGTGCAACAGCAGAGC---
AATCTG---CTGAGG---GCTATAGAGGCTCAACACCATCTGTTGAAACTCACGGTCTGGGGCATT
>HQ385453..1
TTCTACTGCGACACAACAGGCCTGTTTAATAACACAGAGAAA-------------------------------
----

AATGACACTATAATGCTCCCATGCAAAATAAGACAAATTGTACGTATGTGGCAGAGAGTAGGACAAGCAATGT
ATGCCCCTCCCATTCCAGGAGTAATAAACTGTCTATCAAAC---
ATTACAGGGCTACTGCTGACAAGAGATGGTGGGGATAGTACTAAT----------------------------
----

AATACTGAGAAATTCAGACCAGGAGGAGGAGATATAAGGGACAATTGGAGAAGTGAATTATATAAATATAAAG
TAGTAAAAATTGAACCACTA---GGAGTAGCACCTACCAAGGCAAAGAGAAGAGTGGTG--------------
-------------------------GAGAGAAGAAAAAGAGCA---GGA------CTAGCAGCT---------
GTCTTCCTTGGGTTCTTAGGAGCA---GCAGGAAGCACTATG---GGCGCAGCGTCA---
ATAACGCTGACGGTACAG---GCCAGACAA---TTATTG---TCTGGTATAGTGCAACAGCAAAAC---
AATTTG---CTGAAG---GCTATAGAGGCTCAACAACAGATGTTGAGACTCACGGTCTGGGGCATT
>KT152846..2
TTCTATTGTAATACATCAAACCTGTTTAATAGCACTTGGACGAAAAATGACAGCATGCAGGGGTTAAATCACA
CGGAG--

TCAAATGACAATATAACTCTCCCATGCAGACTAAGGCAAATTATAAAAATGTGGCAGAGAGTAGGACAAGCAA
TGTATGCCCCTCCCATCCAAGGAATAATAAAGTGTGAATCAAAC---
ATTACAGGGCTAATATTAACAAGAGATGGTGGGAATAACAACAGT----------------------------
----

GCAGAGGAAACCTTCAGACCTGGAGGAGGAGATATGAGGGACAATTGGAGAAGTGAATTATATAAGTATAAAG
TAGTACAAATTGAACCACTA---GGGGTAGCACCCACCAAGGCAAGGAGAAGAACGGTG--------------
-------------------------GGGAGAGAAAAAAGAGCA---GTTGGA---ATGGGAGCT---------
GTTTTCCTTGGGTTCTTAGGAGCA---GCAGGAAGCACTATG---GGCGCAGCGTCA---
ATAACGCTGACGGTACAG---GCCTGGCAA---TTATTG---TCTGGCATAGTGCAACAGCAAAGC---
AATTTG---CTGAGG---GCTATAGAGGCTCAACAACATCTGTTGAAACTCACGGTCTGGGGCATT
>KT152841..1
TTCTATTGTGATACATCAGCCCTGTTTAATGGCACTTGGAATAATACCAGCATGAAGGAGTCAAATAGCACGG
AA---

TCAAATGACACTATAACTCTCCCATGCAGAATAAAGCAAATTATAAATATGTGGCAGAGAGTAGGACAAGCAA
TGTATGCCCCTCCCATCCAAGGAATAATAAAGTGTGAATCAAAC---
ATTACAGGACTACTATTAACAAGAGATGGTGGTGGGGATAACAGTACA-------------------------
----

AATGAGGAAACCTTCAGACCTGGAGGAGGAGATATGAGGGACAATTGGAGAAGTGAATTATATAGGTATAAAG
TAGTAAAAATTGAACCACTA---GGAGTAGCACCCACCAGGGCAAGGAGAAGAACGGTG--------------

-------------------------GGGAGAGAAAAAAGAGCA---GTGGAA---ATAGGAGCT---------
GTCTTCCTTGGGTTCTTAGGAGCA---GCAGGAAGCACTATG---GGCGCAGCGTCA---
ATAACGCTGACGGTACAG---GCCAGACAA---TTATTG---TCTGGCATAGTGCAACAGCAAAAC---
AATTTG---CTGAGG---GAA---GAGGCTCAACAACATTTGTTGAAACTCACGGTCTGGGGCATA
>KT152842..1
TTCTATTGTAATACATCAGCTCTGTTTAATGGCACTTGGGGGCAAAATGCCAGCATGGAGTCAAATGGCACT-
----

ATAAATGACACTATAATTCTCCCATGCAGACTAAAGCAAATTGTAAGGATGTGGCAGAGAGTAGGACAAGCAA
TGTATGCCCCTCCCATCCAAGGAGAAATAAATTGTGTATCAAAC---
ATTACAGGACTAATATTAACAAGAGATGGTGGGAATAATACAGAG----------------------------
----

GAAATCTTCAGACCTGGAGGAGGAGATATGAGGGACAATTGGAGAAGTGAATTATATAAGTATAAAGTAGTAA
AAATTGAACCACTA---GGAGTAGCACCCACCAGTGCAAGGAGAAGAACGGTG--------------------
-------------------GGGAGAGAAAAAAGAGCA---GTTGGA---ATAGGAGCT---------
GTCTTCCTTGGGTTCTTAGGAGCA---GCAGGAAGCACTATG---GGCGCGGCGTCA---
ATAACGCTGACGGTACAG---GCCAGACAA---TTATTG---TCTGGCATAGTGCAACAGCAAAGC---
AATTTG---CTGAGG---GCTATAGAGGCTCAACAACATCTGTTGAAACTCACAGTCTGGGGCATT
>KT152844..1
TTCTACTGTAATACATCAGCCCTGCTTAATGGCACTTGGCAAAATGCCAGCATGCAGGAGTCAAATAGCACAG
AG---

TCAAATGACACCATAACTCTCCCATGCAGAATAAAGCAAGTTATAAATATGTGGCAGAGAGTAGGACAAGCAA
TGTATGCCCCTCCCATCCAAGGAATAATAAAGTGCGTATCAAAC---
ATCACAGGACTAATATTAACAAGAGATGGTGGGAATAGTAACAGT----------------------------
----

AGTACAGTTGAAACCTTCAGGCCTGGAGGAGGAGATATGAGGGACAATTGGAGAAGTGAATTATATAAGTATA
AAGTAGTAAAAATTGAACCACTA---GGAGTAGCACCCACCAGGGCAAGGAGAAGAACGGTG-----------
----------------------------GAGAGAGAAAAAAGAGCA---GTTGGA---ATAGGAGCT------
---GTCTTCCTTGGGTTCTTAGGAGCA---GCAGGAAGCACTATG---GGCGCAGCGTCA---
ATAACGCTGACGGTACAG---GCCAGACAA---TTATTG---TCTGGCATAGTGCAACAGCAAAGT---
AATTTG---CTGAGG---GAAATAGAGGCTCAACAACATCTGTTGAAACTCACGGTCTGGGGCATT
>AY736809..2
TTCTATTGTAATACATCAGGCCTGTTCAATAGCACTTGGCCTGTCAATGCCAGTGAGCAGGAGTCAAATAGCA
CA---

GAGTCAGATGGCATTATAACTCTCCGATGCAGAATAAAGCAAATCATAAATATGTGGCAGAGAACAGGACAAG
CAATGTATGCCCCTCCCATTCAAGGAATAATAAAGTGTGTATCAAAC---
ATTACAGGACTACTGTTAACAAGAGATGGTGGGAATAGTACTGTC----------------------------
----

AATGAAACTTTCAGACCTGGAGGAGGAGATATGAGGGACAATTGGAGAAGTGAATTATATAAGTATAAAGTAG
TAAAAATTGAACCACTA---GGAGTAGCACCCACCAAGGCAAGGAGAAGAGTGGTG-----------------
----------------------GGGAGAGAAAAAAGAGCA---GTTGGA---ATAGGAGCT---------
GTTTTCCTTGGGTTCTTAGGAGCA---GCTGGAAGCACTATG---GGCGCGGCGTCA---
ATAACGCTGACGGTACAG---GCCAGACAA---TTACTG---TCTGGCATAGTGCAACAGCAAAAC---
AATTTG---CTGAGG---GCTATAGAGGCTCAACAACAACTGTTGAAACTCACGGTCTGGGGCATT
>AY736810..2
TTTTATTGTAATACATCAGACCTGTTTAATAGTACTTGGAACATTGAAGGCACTGCCAGCATAAATGGCACGG
AG---

TCAAATGACAATATAACTCTCCCATGCAGAATAAAGCAAATTATAAATATGTGGCAGAGAGTAGGACAAGCAA
TGTATGCCCCTCCCATCCAAGGAGTAATAAGGTGTCAATCAAAC---
ATTACAGGAATACTATTAACAAGAGATGGTGGGAATACTGGGAATAAT-------------------------
----

AGCAGGACAAATGAAACCTTTAGACCTGGAGGAGGAGATATGAGGGACAATTGGAGAAGTGAATTATATAAGT
ATAAAGTAGTAAAAATTGAACCACTG---GGAGTAGCACCCACCAAGGCAAAGAGAAGAGTGGTG--------

-------------------------------GAGAGAGAAAAAAGAGCA---GTTGGA---CTGGGAGCT---
------GTATTCATTGGGTTCTTAGGAGCA---GCCGGAAGCACTATG---GGCGCGGCGTCA---
ATAACGCTGACGGTACAG---GCCAGACAA---TTATTG---TCTGGCATAGTGCAACAGCAAAGC---
AATTTG---CTGAGG---GCTATAGAGGCTCAACAGCATTTGTTGAAACTCACGGTCTGGGGCATT
>AY736812..1
TTCTATTGTAATACATCAGGTCTGTTCAATAGCACCTGGAAGGCCAACAATGGCACCTGGAAGGCCAACATAT
CAGAG--

TCAAATAACACGGAGATAACTCTCCAATGCAGAATAAAGCAAATTATAAATATGTGGCAGAGAACAGGACAAG
CAATATATGCCCCTCCCATCCAGGGAGTGATAAGGTGTGAATCAAAC---
ATCACAGGACTACTGTTAACAAGAGATGGTGGGGAGGGGAACAAT----------------------------
----

GAAAGTGAGATCTTCAGACCTGGAGGAGGAGATATGAGGGACAACTGGAGAAGTGAATTATATAAGTATAAAG
TAGTAAAAATTGAACCACTA---GGAGTAGCACCCACCAGGGCAAGGAGAAGAGTGGTG--------------
-------------------------GGAAGAGAAAAAAGAGCA---GTTGGA---ATAGGAGCT---------
GTTTTCCTTGGGTTCTTAGGAGCA---GCAGGAAGCACTATG---GGCGCGGCGTCA---
ATAACGCTGACGGTACAG---GCCAGGCAA---TTATTG---TCTGGCATAGTGCAACAGCAAAGC---
AATTTG---CTGAGG---GCTATAGAGGCTCAACAACATATGTTGAAACTCACGGTCTGGGGCATT
>AY736815..1
TTCTATTGTAATACATCAGGTCTGTTTAATAGCACCTGGGACAACAACACCGACCCAACCAACACAACG----
----

TCAAATGACACTATCACTCTCCAATGCAGAATAAAGCAATTTATAAATATGTGGCAGAGAACAGGGCAAGCAA
TGTATGCCCCTCCCATCCAAGGGGTAATAAGGTGTGATTCCAAC---
ATTACAGGACTACTATTAACAAGAGATGGTGGAGTAGGTAACAAT----------------------------
----

ACAACTGAAACCTTCAGACCTGGAGGAGGAGATATGAGGGACAATTGGAGAAGTGAATTATATAAGTATAAAG
TAGTAAAAATTGAACCACTA---GGAGTAGCACCCACCGGGGCAAAGAGAAGAGTGGTG--------------
-------------------------GAGAGAGAAAAAAGAGCA---GTTGGA---ATAGGAGCT---------
GTTTTCATTGGGTTCTTAGGAGCA---GCAGGAAGCACTATG---GGCGCGGCGTCA---
GTAACGCTGACGGTACAG---GCCAGACAA---TTATTG---TCTGGCATAGTGCAACAGCAAAGC---
AATTTG---CTGAGG---GCTATAGAGGCTCAACAGCATCTGTTGAAACTCACGGTCTGGGGCATT
>AY736813..1
TTCTATTGTGATACATCAGGCCTGTTTAATAGCAGTTGGAATAATAACACTTGGAAGGAGTCAAATAGCACGG
GG---

TCAAATGACACTATAACTCTCCTATGCAGAATAAAACAAATTATAAATATGTGGCAGAGAACAGGACAAGCAA
TGTATGCCCCTCCCATCCCAGGGTTAATAAGCTGTAAATCAAAC---
ATTACAGGTATAATATTGACAAGAGATGGTGGGAATGGTAACAGT----------------------------
----

ACAAATGAAACCTTCAGACCTGGAGGAGGAGATATGAGGGACAATTGGAGAAGTGAACTATATAGGTATAAAG
TAGTACAAATTGAACCACTA---GGAGTAGCACCCACCAGGGCAAGGAGAAGAGTGGTG--------------
-------------------------CAGAGAGAAAAAAGAGCA---GTAGGA---ATAGGAGCT---------
GTCTTCCTTGGGTTCTTAGGAGCA---GCAGGAAGCACTATG---GGCGCAGCGTCA---
ATAACGCTGACGGTACAG---GCCAGACAA---TTATTG---TCTGGCATAGTGCAACAGCAAAGC---
AATTTG---CTGAGG---GCTATAGAGGCTCAACAACATCTGTTGAAACTCACGGTCTGGGGCATT
>AY736816..1
TTCTATTGTGACACATCAGGCCTGTTTACTTGGAATAGCACTTGGAATGGCACTGAGTCAAACAACACAAAG-
----

TCAAACGACACTATAACTCTCCAATGCAGAATAAAGCAAATTATAAATATGTGGCAGAGAGTAGGACAAGCAA
TATATGCCCCTCCCATCAAAGGAGTAATAAGGTGTGAATCAGAC---
ATTACAGGACTTATACTAACAAGAGATGGTGGGAATACTAACAGT----------------------------
----

ACAGAGGAAACCTTCAGACCTGGAGGAGGAGATATGAGGGACAATTGGAGAAGTGAATTATATAAGTATAAAG
TAGTAAAAATTGAACCACTA---GGAGTAGCACCCACCAAGGCAAAGAGAAGAGTGGTG--------------

-------------------------GGGAGAGGAAAAAGAGCA---GTTGGA---ATAGGAGCT---------
GTCTTCCTTGGGTTCTTAGGAGCA---GCCGGAAGCACTATG---GGCGCGGCGTCA---
ATAACGCTGACGGTACAG---GCCAGACAA---TTATTG---TCTGGCATAGTGCAACAGCAAAGC---
AATTTG---CTAAGG---GCTATAGAGGCTCAACAGCATCTGTTGAAACTCACGGTCTGGGGCATT
>AF457053..2
TTCTATTGTAATACATCAGGCCTGTTTAACAGCACCTGGCACTATAACAGCAATGGTACTTGGCACAATAACA
GCACAAAT---
-------------AGCATG------
GACTCAAATGAAACTATAACTCTCACATGCAGAATAAAGCAAATTATAAATATGTGGCAGAGAGTAGGAAAAG
CAATGTATGCTAATCCCATCCAAGGAATAATAAAGTGTGAATCAAAC---
ATTACAGGACTACTCTTAACAAGAGATGGTGGGAATACTAGCAGT----------------------------
----

ACAAATGAAACCTTCAGACCTGGAGGAGGAGATATGAGGGACAATTGGAGAAGTGAATTATACAAATATAAAG
TAGTAAAGATTGAGCCACTA---GGAGTAGCACCCACCAGGGCACAGAGAAGAGTGGTG--------------
-------------------------GGGAGAGAAAAAAGAGCA---ATTGGA---ATAGGAGCT---------
GTCTTCCTTGGGTTCTTAGGAGCA---GCAGGAAGCACTATG---GGCGCGGCGTCA---
ATGACGCTGACGGTACAG---GCCAGACAA---TTATTG---TCTGGCATAGTGCAACAGCAAAGC---
AATTTG---CTGAGG---GCTATAGAGGCTCAACAACAACTGTTGAAACTCACGGTCTGGGGCATT
>AF457055..1
TTCTATTGTAATACAACAAACCTGTTTAATAGCACCTGGAGAGGAAATAAAACTGTCAGCAATAGCACAGAGT
TA---

GATGAAAATGGCACTATAACTCTCCCATGCAGAATAAAGCAAATTATAAATATGTGGCAGAGAACAGGACAAG
CAATGTATGCCCCTCCCATCCAAGGAGTAATAAAGTGTGTATCAAAC---
ATTACAGGACTACTATTAACAAGAGATGGTGGAAGTGATAACAAT----------------------------
----

ATAAGTAGTGAAACCTTCAGACCAGGAGGAGGAGATATGAGGGACAATTGGAGAAGTGAATTATATAAGTATA
AAGTAGTGAAACTTGAACCACTA---GGAGTAGCACCCAACAAGGCAAGGAGAAGAGTGGTG-----------
----------------------------GAGAGAGAAAAAAGAGCA---GTTGGA---CTGGGAGCT------
---GTATTCATTGGGTTCTTAGGAGCA---GCAGGAAGCACTATG---GGCGCGGCGTCA---
ATAACGCTGACGGCACAG---GCCAGACAA---TTATTG---TCTGGCATAGTGCAACAGCAAAGC---
AATTTG---CTGAAG---GCTATAGAGGCTCAACAGCATCTGTTGAAACTCACGGTCTGGGGCATT
>AF457070..1
TTCTATTGTAATACATCAGGCCTGTTTAATAGCACTTGGAATAGCACTGGGAATGACACTTGGAGTGGCAATG
CCAGCACG---
-------------------CAG---
GAGTCAAATGACACTATAACTCTCCCATGCAGAATAAAGCAAATTGTAAATATGTGGCAGAGAATAGGACAAG
CAATGTATGCCCCTCCCATCCAAGGAGTAATAAGGTGTGAATCAAAC---
ATTACAGGACTACTATTAACAAGAGATGGTGGGAACAATGAAAGT----------------------------
----

GAAACCTTCAGACCTGGAGGAGGAAATATGAGGGACAATTGGAGAAGTGAATTATATAGGTACAAAGTAGTGA
AAATTGAACCACTA---GGAGTAGCACCCACCAGGGCAAAAAGAAGAGTGGTG--------------------
-------------------GGGAGAGAAAAAAGAGCA---GTTGGA---ATAGGAGCT---------
GTGTTCCTTGGGTTCTTAGGAGCA---GCAGGAAGCACTATG---GGCGCGGCGTCA---
GTAACGCTGACGGTACAG---GCCAGACAA---TTATTG---TCTGGCATAGTGCAACAGCAAAGC---
AACTTG---CTGAGG---GCTATAGAGGCTCAACAACATCTGTTGAAACTCACGGTCTGGGGCATT
>AF457077..1
TTCTATTGTAATACATCAGGCCTGTTTAATAGCACTTGGTCTTGGAATGACACTGCAAATGGCACTGCAATGG
AG---

CCAAATAGCACTATAACTCTCCCATGCAGAATAAAGCAAATTATAAATATGTGGCAGAGAGTAGGACAAGCAA
TGTATGCCCCTCCCATCCCAGGAGTAATAAGTTGTGTATCAAAC---
ATTACAGGACTAATATTAGTAAGAGATGGAGGGAATAATGTTACA----------------------------
----

AATGAAACCTTCAGACCTGGAGGAGGAGATATGAGGGACAATTGGAGAAGTGAATTATATAAGTATAAGGTAG
TAAAAATTGAACCACTA---GGAGTAGCACCCACCAAGGCAAAGAGAAGAGTGGTG-----------------

```
----------------------GAGAGAGAAAAAAGAGCA---GTTGGA---ATAGGAGCT---------
GTCTTCCTTGGGTTCTTAGGAGCA---GCAGGAAGCACTATG---GGCGCGGCGTCA---
ATAACGCTGACGGTACAG---GCCAGACAA---TTATTG---TCTGGTATAGTGCAACAGCAAAGC---
AATTTG---CTGAGG---GCTATAGAGGCTCAACAACATCTGTTGAAACTCACAGTCTGGGGCATT
>AF457080..2
TTCTATTGTAATACATCGGGCCTGTTTAATAGCACCTACAATGGCACTGACATCGCTGAAAATAGCACGGAG-
-------------------------------------------------------------------------
----------------------------
TCAAATGACACTATAGTTCTCCCATGCAGAATAAAGCAAATTATAAGGATGTGGCAGCGAGTAGGACAAGCAA
TGTATGCCCCTCCCATCCCAGGAATAATAAGATGTGACTCAAAC---
ATTACAGGATTACTATTAACAAGAGATGGTGGGGATGATAATGAA----------------------------
-------------------------------------------------------------------------
----------------
ACTGAAACCTTTAGACCTGGAGGAGGAGATATGAGGGATAATTGGAGAAGTGAATTATATAAGTATAAAGTAG
TAAAAATTGAACCACTA---GGAGTAGCACCCACCAGGGCAAAAAGAAGAGTGGTG-----------------
----------------------GAGAGAGAAAAAAGAGCA---GTTGGA---ATAGGGGCT---------
TTTGTCCTTGGGTTCTTAGGAGCA---GCAGGAAGCACTATG---GGCGCAGCATCA---
ATAACGCTGACGGTACAG---GCCAGACAA---TTATTG---TCTGGCATAGTGCAACAGCAAAGC---
AATTTG---CTGAGG---GCTATAGAGGCTCAACAACAAATATTGAGACTCACGGTCTGGGGCATT
>AF457081..1
TTCTATTGTAATACATCAGGCCTGTTTAATAGCACTTGGACAAATGACAGTATCAGCACAAATAGCACAAAG-
-------------------------------------------------------------------------
----------------------------
TCAAATGACACTATAACTCTCCCATGCAGAATAAAGCAAATTATAAATATATGGCAGAGAGTGGGACAAGCAA
TGTATGCCCCTCCCATCCAAGGAGTAATACAGTGTGTATCAAAC---
ATTACAGGACTACTATTAACAAGAGATGGTGGGGATAACAGTAGT----------------------------
-------------------------------------------------------------------------
-------------
ACGAATGAAACCTTCAGACCTGGAGGAGGAGATATGAGGGACAATTGGAGAAGTGAATTATATAAGTATAAAG
TAGTAAAAATTGAACCACTA---GGAGTAGCACCCACCAGGGCAAGGAGAAGAGTGGTG--------------
-------------------------GAGAGAGAGAAAAGAGCA---GTTGGA---CTGGGAGCT------------
GTTTTCATTGGGTTCTTGGGAGCA---GCAGGGAGCACTATG---GGCGCAGCGTCA---
ATAACGCTGACGGTACAG---GCCAGACAA---TTATTG---TCTGGCATAGTGCAACAGCAAAGC---
AATTTG---CTGAGG---GCTATAGAGGCTCAACAACATCTGTTGAAACTCACGGTCTGGGGCATT
>AF457083..1
TTCTATTGTAATACATCAAGTCTGTTTAATAGTACTTGGAATAACAGTACTTGGAATACCAGCAGGCAGGAGG
CAAATAAT-----------------------------------------------------------------
-------------AGCCCG------
GGGTCAAACGACACTATAACTCTCAAATGCAGAATAAAGCAAATTATAAATATGTGGCAGAGAGCGGGACAAG
CAATATATGCCCCTCCCATCCAAGGAGTAATAAGGTGTAACTCAAAC---
ATTACAGGACTAATATTAACAAGAGATGGTGGGGGTAATAACAAT----------------------------
-------------------------------------------------------------------------
-------------
ACAAATGAAACCTTCAGACCTGGAGGAGGAGATATGAGGGACAATTGGAGAAGTGAATTATATAAGTACAAAG
TAGTAAAAATTGAACCACTA---GGAGTAGCACCCACCAGGGCAAAGAGAAGAGTGGTG--------------
-------------------------GAGAGAGGAAAAAGAGCA---GTTGGA---ATAGGAGCT------------
GTCCTCATTGGGTTCTTAGGAGCA---GCAGGAAGCACTATG---GGCGCGGCGTCA---
ATAACGCTGACGGCACAG---GCCAGACAA---TTATTG---TCTGGCATAGTGCAACAGCAAAAC---
AATTTG---CTGAGG---GCTATAGAGGCTCAACAACATCTGTTGAGACTCACGGTCTGGGGCATT
>AF457084..1
TTCTATTGTGACACATCAGGCCTGTTTAATAGCAGTTGGACTGCTAGCAATGATAGCATGCAGGAGGCACATA
GC-----------------------------------------------------------------------
----------------------------
ACAGAGTCAAATATAACTCTCCAATGCAGAATAAAGCAAATTATAAATATGTGGCAGAGAGCAGGACAAGCAA
TGTATGCCCCTCCCATTCCAGGAATAATAAGGTGTGAATCAAAC---
ATTACAGGACTAATATTAACAAGAGATGGTGGTGAGGGTAATAAC----------------------------
-------------------------------------------------------------------------
----------
AGTACAAATGAAACCTTCAGACCAGTAGGAGGAAATATGAGGGACAATTGGAGAAGTGAATTATATAAGTATA
AAGTAGTAAAAGTTGAACCACTA---GGAGTAGCACCCACCAAGTCAAGGAGGAGAGTGGTG-----------
```

----------------------------GAGAGAGAAAAAAGAGCA---GTTGGA---CTAGGAGCT------
---GTGTTCATTGGGTTCTTAGGAGCA---GCAGGAAGCACTATG---GGCGCGGCGTCA---
ATGACGCTGACGGTACAG---GCCAGACAA---TTATTG---TCTGGCATAGTGCAACAGCAAAGC---
AATTTG---CTGAGG---GCTATAGAGGCTCAACAGCATCTGTTGAAACTCACGGTTTGGGGCATT
>AF457086..3
TTCTATTGTAATACAACAAAACTGTTTAATAGCACTTGGAGTCATAATGACAGCACGCAAGAGTCAAATAGCA
CA---

AAGTCAAATGACACTATAACTCTCCCATGCAGAATAAAGCAAATTATAAATATGTGGCAGAGAGTAGGACAAG
CAATATATGCCCCTCCCATACAAGGAATAATAAGGTGTGAATCAAAT---
ATTACAGGACTAATATTAACAAGAGATGGAGGTACCAATAGTACA----------------------------
----

AATGAAACCTTCAGACCTGGAGGAGGAGATATGAGGGACAATTGGAGAAGTGAATTATATCAGTATAAAGTAG
TAAAAATTGAACCACTA---GGAGTAGCACCTACCAGGGCAAAGAGAAGAGTGGTG-----------------
----------------------GAGAGAGCAAAAAGAGCA---GTTGGA---ATAGGAGCT---------
GTCTTCCTTGGGTTCTTAGGAGCA---GCAGGAAGCACTATG---GGCGCGGCGTCA---
ATAACGCTGACGGTACAG---GCCAGACAA---TTATTG---TCTGGCATAGTGCAACAGCAAAGT---
AATTTG---CTGAGG---GCTATAGAGGCTCAACAGCATCTGTTGAAACTCACGGTCTGGGGCATT
>AF457089..1
TTTTATTGCAATACATCAGATCTGTTTAATAGTACTTGGCCTGTCAATGCCAGCATGGAGTCAAATAGCACGG
AG---

TCAAATGACAATATAACACTCCAATGCAGAATAAAGCAAATTATAAATATGTGGCAGAAAGTAGGACAAGCAA
TGTATGCCCCTCCCATACAAGGAGTAATAAGGTGTGAATCAAAC---
ATTACAGGACTACTATTAACAAGAGATGGTGGATCTAACAGTACA----------------------------
----

GAAGAAACCTTCAGACCTGGAGGAGGAGATATGAGGGACAATTGGAGAAGTGAATTATATAAGTATAAAGTAG
TGAAAATTGAGCCATTA---GGGGTAGCACCCACCAGGGCAAAGAGAAGAGTGGTG-----------------
----------------------GAGAGAGAAAAAAGAGCG---GTTACA---CTAGGAGCT---------
GTATTCATTGGGTTCTTAGGAGCA---GCAGGAAGCACTATG---GGCGCAGCGTCA---
GTAACGCTGACGGTACAG---GCCAGACAA---TTACTG---TCTGGCATAGTGCAACAGCAAAGC---
AATTTG---CTGAGG---GCTATAGAGGCTCAACAGCATATGTTGAAACTCACGGTCTGGGGCATT
>EU110088..2
TTCTATTGTAACACATCACGCCTGTTTAATAGCACCTGGTGGGCCAATGGCACCAGGACGGGCTACAACAGC-
----

ACTGATGGCAATATAACACTCCCATGCAGAATAAAGCAAATTATAAATATGTGGCAGAGAGTAGGACAAGCAA
TGTATGCCCCTCCCATCCCAGGAAGAATAAGTTGTGAATCAAAC---
ATTACAGGACTAATATTAACAAGAGATGGTGGGAGTGATAATACA----------------------------
----

ACTGAAACCTTCAGACCTGGAGGAGGAGATATGAGGGACAATTGGAGAAGTGAATTATATAAGTATAAAGTAG
TAGAAGTTGAACCACTA---GGAGTAGCACCCACAGAGGCAAGGAGAAGAGTGGTG-----------------
----------------------CAGAGAGAAAAAAGAGCA---GTCGGA---CTGGGAGCT---------
GTCTTCATTGGGTTCTTGGGAGCA---GCAGGAAGCACTATG---GGCGCGGCGTCA---
GTGACGCTGACGGTACAG---GCCAGACAA---TTATTG---TCTGGTATAGTGCAACAGCAAAGC---
AATTTG---CTGAGG---GCTATAGAGGCTCAACAACATCTGTTGAAACTCACGGTCTGGGGCATT
>EU110092..1
TTCTATTGTGATACATCAGCTCTGTTCAATAGCACTTGGAATGCCACTGAGTCAAATGACACAAACTCCACAG
AATCA--

AACTCAAATGACACTATAACTCTCCCATGCAGAATAAAACAAATTATAAATATGTGGCAGAGAATAGGACAAG
CAATGTATAACCCTCCCATCCAAGGAAAAATAAAGTGTGAATCAAAC---
ATTACAGGACTATTGTTAACAAGAGATGGTGGAGAAAATAGAGAAAAT-------------------------
----

AATGACAGTAGTGAAATCTTCAGACCAGGAGGAGGAGATATGAAGGACAATTGGAGAAGTGAATTGTATAAGT
ATAAAGTAGTAAAAATTGAACCACTA---GGAGTAGCACCCACCAGGGCAAGGAGAAGAGTGGTG--------

------------------------------GGGAGAGAAAAAAGAGCA---GTTGGA---ATAGGAGCT---
------GTCTTCCTTGGGTTCTTAGGAGCA---GCAGGAAGCACTATG---GGCGCGGCGTCA---
ATAACGCTGACGGTACAG---GCCAGACAA---TTATTG---TCTGGCATAGTGCAACAGCAAAGC---
AATTTG---CTGAGG---GCTATAGAGGCTCAACAACATCTGTTGAAACTCACAGTCTGGGGCATT
>EU110094..1
TTCTATTGTAATACATCAGGCCTGTTTAGTAGCACTTGGAGTTGGAATGACACTACCAAAACAATGGAATCAA
ATAGC---
------------------ACG---
GTGTCAGATGGCACTATAAATCTCCCATGCAGAATAAAGCAAATTATAAATATGTGGCAGAGAGTAGGGCAAG
CAATGTATGCCCCTCCCATCCAAGGAGCAATAAGGTGTACATCAAAC---
ATTACAGGACTAATATTAACAAGAGATGGTGGGAAAGATGACAGG---------------------------
----

AATAATGAAACCTTCAGACCTGGAGGAGGAGATATGAGGGACAATTGGAGAAGTGAATTATATAAATATAAAG
TAGTAAAAAATTGAACCACTA---GGAGTAGCACCCACCAGGGCAAAGAGAAGAGTGGTG------------
------------------------GAGAGAGAAAAAAGAGCA---GTTGGA---ATAGGAGCT---------
GTCTTCCTTGGGTTCTTAGGAGCA---GCAGGAAGCACTATG---GGCGCGGCGTCA---
ATAACGCTGACGGTACAG---GCCAGACAA---TTATTG---GCTGGCATAGTGCAACAGCAAAGC---
AATTTG---CTGAGG---GCTATAGAGGCTCAACAACATCTGTTGAAACTCACGGTCTGGGGCATT
>KT022360..2
TTCTATTGTAACACATCAGGCTTGTTTAATAACACTTGGGTGTTTAATAGCATTTGGGATAATAGCACTCAGG
AGTCCAAT--
------------AGCACA------
GAGTCAAATAAAACTATAATTCTCCAATGCAGAATAAAGCAAATTATAAATATGTGGCAGAGAGCAGGACAAG
CAATATATGCTCCTCCCATTCCAGGAGTAATAAGGTGTGAATCAAAC---
ATTACAGGACTAATATTAACAAGAGATGGTGGAAATAATAACACG---------------------------
----

ATAAATGAAACATTTAGACCTGGAGGAGGAGACATGAGGGACAATTGGAGAAGTGAATTATATAAGTACAAAG
TAGTAAAAAATTGAACCACTA---GGAGTAGCACCTACCAAGGCACGGAGAAGAGTGGTG------------
------------------------GAGAGAGAAAAAAGAGCA---GTTACA---ATAGGAGCT---------
ATGTTCATTGGGTTCTTAGGAGCA---GCAGGAAGCACTATG---GGCGCGGCGTCA---
ATGACGCTGACGGTACAG---GCCAGACAA---TTATTG---TCTGGCATAGTGCAACAGCAAAGC---
AATTTG---CTGAGG---GCTATAGAGGCTCAACAGCATCTGTTGAAACTCACGGTCTGGGGCATC
>KT022361..3
TTCTATTGTAATACATCAGGCCTGTTTAATTACAGCTACAGCATGCAGGAGTCAAATGATACGGAG-------
----

GCAAATGGCACTATAACTCTCCAATGCAAAATAAAACAAATTATAAATATGTGGCAGAGAGTAGGACAAGCAA
TGTACGCCCCTCCCATCCAAGGAGTAATACAGTGTAAATCAAAC---
ATTACAGGACTAATATTAACAAGAGATGGTGGAAATAGTACTAAT---------------------------
----

GGTACAGAAATCTTCAGACCTGGAGGAGGAGATATGAGAGACAATTGGAGAAGTGAGTTATATAAGTATAAAG
TAGTACAACTTGAACCACTA---GGAGTAGCACCCACCAAGGCAAAGAGAAGAGTGGTG-------------
------------------------GAAAGAGAAAAAAGAGCA---GTTGGA---ATAGGAGCT---------
GTCTTCCTTGGGTTCTTAGGAACA---GCAGGAAGCACTATG---GGCGCGGCGTCA---
CTGACGCTGACGGTACAA---GCCAGACTA---TTATTG---TCTGGCATAGTGCAACAGCAAAGC---
AATTTG---CTGAGG---GCTATAGAGGCTCAACAATATCTGTTGAAACTCACGGTCTGGGGCATT
>KT022363..1
TTCTATTGTGACACCTCGAAGCTGTTTAATAGCTCTTGGAATGAGAGCACTTGGGATGAAAATAGCACGAAGC
CTAAC---

AACAATGAAACTATAATTCTCCCATGCAGAATAAAGCAAATCATAAATATGTGGCAGAGAACAGGACAAGCAA
TATATGCCCCTCCCATTGAAGGAATAATAAAGTGTGACTCAAAC---
ATTACAGGACTAATATTAACAAGAGATGGTGGGAGGATTAACACA---------------------------
----

ACTAATGAAACTTTCAGACCTGGAGGAGGAGATATAAGGGACAATTGGAGAAGTGAATTATATAAATATAAAG
TAGTAAAAAATTGAGCCACTA---GGAGTAGCACCCTCCAGGGCAAGGAGAAGAGTGGTG------------

-------------------------GGGAGAGAAAAAAGAGCA---GTTGGA---ATAGGAGCT---------
GTCTTCCTTGGGTTCTTAGGAGCA---GCAGGAAGCACTATG---GGCGCGGCGTCA---
CTAACGCTGACGGTACAG---GCCAGGCAA---TTATTG---TCTGGCATAGTGCAACAGCAAAGC---
AATTTG---CTGAGG---GCTATAGAGGCTCAACAGCATATGTTGAAACTCACGGTCTGGGGCATT
>KT022364..1
TTTTATTGTGATACCTCAGGCTTGTTTAATAGTACTTGGGAAATTGAAAACAATACTGCCAGAATAAAGCCAA
ATAAC--

ACGGGGTTAAATATAACGCTCCCGTGCAGAATAAAGCAAATTATAAATATGTGGCAAAGGGTAGGACAAGCAA
TGTATGCCCCTCCCATCAAAGGAGTAATAAGGTGTGTATCAAAC---
ATCACAGGACTACTGCTAACAAGAGATGGTGGGAGTAAAAATAGA----------------------------
----

AGTAATGAAACCTTCAGACCTGGAGGAGGAGATATGAGGGACAATTGGAGAAGTGAATTATATAAATATAAAG
TAGTAAAAATTGAACCACTA---GGAGTAGCACCCTCCAGGGCAAAGAGGAGAGTGGTG--------------
-------------------------GAGAGAGAGAAAAGAGCA---GTTGGA---ATAGGAGCT---------
GTATTCCTTGGGTTCTTAGGAGCA---GCAGGAAGCACTATG---GGCGCGGCGTCA---
CTGACGCTGACGGTACAG---GCCAGACAA---TTATTG---TCTGGCATAGTGCAACAGCAAAGC---
AATTTG---CTGAGG---GCTATAGAAGCTCAACAGCATCTGTTGAAGCTTACAGTCTGGGGCATT
>KT022365..1
TTCTATTGTGATACGTCAGGCTTATTCAGTAGCACCTGGCAATTCAATGGGAGTACCTGGAACAACACAGATT
CAAACAGCACA--

GGGTCAGATGACACTATAGAACTCCCATGCAGAATAAAGCAAATTATAAATATGTGGCAGAGAGTAGGACGAG
CAATGTATGCCCCTCCCATCCAAGGAGTAATAAGTTGTGTATCAAAC---
ATCACAGGACTAATATTAACAAGAGATAGT---AATAACAGAACT----------------------------
----

AATGAAACCTTCAGACCTGGAGGAGGAGACATGAGAGACAATTGGAGAAGTGAATTATATAAGTATAAAGTAG
TAAAAATTGAACCACTA---GGAGTAGCACCCACCAAGGCAAAAAGAAGAGTGGTG-----------------
----------------------GAGAGAGAAAAAAGAGCA---GTTGGA---ATAGGAGCT---------
GTCTTCCTTGGGTTCTTAGGAGCA---GCAGGAAGCACTATG---GGCGCGGCGTCA---
ATAACGCTGACGGTACAG---GCCAGACAA---TTATTG---TCTGGCATAGTGCAACAGCAAAGC---
AATTTG---CTGAGG---GCTATAGAGGCTCAACAACATCTGTTGAAACTCACAGTCTGGGGCATT
>KT022367..1
TTCTATTGTAATACATCAGGTCTATTTAATAGCAGTTGGAATGATACTGAAATCGGGCGGGAGATAAATAGGA
CGGAG--

TCAAATGACACTATAATTCTCCCATGCAGAATAAAGCAAATTATAAATATGTGGCAGAGAGTAGGACAAGCAA
TATATGCCCCTCCCATCCAAGGAGTAATAAGGTGTGAATCAAAC---
ATTACAGGACTAATATTAACAAGGGATGGTGGGGGTAATAACAGA----------------------------
----

AAAAATGAAACCTTCAGACCTGGAGGAGGAGATATGAGGGACAATTGGAGAAGTGAATTATATAAGTATAAAG
TAGTAAAAATTGAACCAATA---GGAATAGCACCCACCAGGGCAAAGAGAAGAGTGGTG--------------
-------------------------GAGAGAGAAAAAAGAGCA---ATTGGA---ATGGGAGCT---------
GTATTCATTGGGTTCTTAGGAGCA---GCAGGAAGCACTATG---GGCGCGGCGTCA---
ATAACGCTGACGGTACAG---GCCAGACAA---TTGTTG---TCTGGCATAGTGCAACAGCAAAAC---
AATTTG---CTGAGG---GCTATAGAGGCTCAACAACATCTGTTGAAGCTCACGGTCTGGGGCATT
>KT022368..2
TTCTATTGTAATACAACAGACCTGTTTGATAGCATTTGGCTTTACAATGCCAGCATGCATAAT----------
----

GCAAATAGCACGATAACTCTCCAATGCAGAATAAAGCAAATTATCAATATGTGGCAGAGAGTAGGACAAGCAA
TGTATGCCCCTCCCGTCGAAGGAAAAATACAGTGCAAATCAAAC---
ATTACAGGACTAATATTAACAAGAGATGGTGGGGTTAACGATACA----------------------------
----

AATGGAACCGAAATCTTCAGACCTGGAGGAGGGGATATGAGGGACAATTGGAGAAGTGAATTATATAAGTATA
AAGTAGTAAAAATTGAACCACTA---GGAGTAGCACCCACCAGGGCAAGGAGAAGAGTGGTG-----------

----------------------------CAGAGAGAAAAAAGAGCA---GTTGGA---ATAGGAGCT------
---GTATTCCTTGGGTTCTTAGGAGCA---GCAGGAAGCACTATG---GGCGCAGCGTCA---
GTGACGCTGACGGTACAG---GCCAGAAGA---TTATTG---TCTGGCATAGTGCAACAGCAAAGC---
AATTTG---CTGAAG---GCTATAGAAGCTCAACAGCATCTGTTGAAGCTCACGGTCTGGGGCATT
>KT022369..2
TTCTATTGTAATACATCAGGCCTGTTTAATAGCACTTGGAATTATACTAATAGCATGCAGCAGTCAAATAGCA
CAGAG--

TCAAACGAAACTATAACTCTCCAATGCAGAATAAAGCAAATTATAAATATGTGGCAGAGAGTGGGGCAAGCAA
TATATGCCCCTCCCATCCCAGGGGTAATAAGGTGTGAATCAAAC---
ATTACAGGACTAATATTGACAAGAGATGGTGGGAGTAATAACAGT----------------------------
----

ACAAATGAAACCTTCAGACCTGGAGGAGGGAATGTGAGGGACAATTGGAGAAGTGAATTATATAAGTATAAAG
TAGTAAAAATTGAACCACTA---GGAGTAGCACCCACCAGGGCAAAGAGAAGAGTGGTG--------------
-------------------------GAGAGAGAAAAAAGAGCA---GTTGGA---CTAGGAGCT---------
GTCTTCATTGGGTTCTTGGGAACA---GCAGGAAGCACTATG---GGCGCGGCGTCA---
ATAACGCTGACGGTACAG---GCCAGACAA---TTATTG---TCTGGTATAGTGCAACAGCAAGGC---
AATTTG---CTGAGA---GCTATAGAAGCTCAACAACAACTGTTGAGACTCACGGTCTGGGGCATT
>FJ866117..1
TTTTATTGTAATACATCAGGCCTGTTTAATAGTACTTGGTCACCTGACAATAGTACTGGGACTAGTACTGGGA
ATAATGAC---
-------------------ACT---
GCCAGCAATGGCACTATAACTCTCCCATGTAGAATAAAGCAGATTATAAATATGTGGCAGAGAGTAGGACAAG
CAATGTATGCCCCTCCCATCCAAGGAGTAATAAGGTGTGACTCAAAC---
ATTACAGGACTACTATTAACAAGAGATGGTGGGAGTGGGAATAAT----------------------------
----

ACCACAGAGATCTTCAGACCTGGAGGAGGAGATATGAGGGACAATTGGAGAAGTGAATTATATAAGTATAAAG
TAGTAAGAATCGAACCTCTA---GGAGTAGCACCCACCAGGGCAAAGAGAAGAGTGGTG--------------
-------------------------GAGAGAGAAAAAAGAGCA---GTTGGA---ATAGGAGCT---------
GTATTCATTGGGTTCTTAGGAGCA---GCAGGAAGCACTATG---GGCGCAGCGTCA---
ATAACGCTGACGGTACAG---GCCAGACAA---TTATTG---TCTGGCATAGTGCAACAGCAAAGC---
AATTTG---CTGAGG---GCTATAGAGGCTCAACAGCATTTGTTGAAGCTCACAGTCTGGGGCATT
>KT022370..3
TTCTATTGTAATACATCAGGCCTGTTTAATAGAGCTTACACTAACAGCACTTACAATAACAGCACGCAGGACA
ATGACAAT---
----------AACAGCACG------
CAGGACAATGCAACTATAACTCTCCCATGCAGAATAAGGCAAATTATAAATATGTGGCAGAGAGCAGGGAAAG
CAATGTATGCCCCTCCCATCCAAGGAGTAATAAACTGTGTATCAAAC---
ATTACAGGACTAATTTTAACAAGAGATGGTGGAAAGAGTAACAGTACG-------------------------
----

GAGGGGGAAATCTTCAGACCTGGGGGAGGAGATATGAGGGATAATTGGAGAAGTGAATTATATAAGTATAAAG
TAGTAAAAATTGAACCACTA---GGAGTAGCACCTACCAAGGCAAGGAGAAGAGTGGTG--------------
-------------------------GGGAGAGAGAAAAGAGCA---GTTGGA---CTAGGAGCT---------
GTCTTCATTGGGTTCTTAGGAGCA---GCCGGAAGCACTATG---GGCGCGGCGTCA---
GTGACGCTGACGGTACAG---GCCAGGCAA---TTATTG---TCTGGCATAGTGCAACAGCAAAGC---
AATTTG---CTGAGG---GCTATAGAGGCTCAACAACATCTGTTGAAACTCACGGTCTGGGGCATT
>KT022372..2
TTCTATTGTGATACAGCAGGCCTGTTTAATAGCACTTGGAGTCACAATGACAGCATACAGGGGACAAATAGCG
CAGAG--

TCAAATGAAACTATAACTCTCCCATGCAGAATAAAGCAAATTATAAATATGTGGCAGAGAGCAGGACAAGCAA
TGTATGCCCCTCCCATCCCAGGAGTAATAAAGTGTGTATCAAAC---
ATTACAGGACTAATATTAACAAGAGATGGTGGGAAGAACAATACA----------------------------
----

GAGGAAGTCTTCAGACCTGGAGGAGGAGATATGAGGGATAATTGGAGAAGTGAATTATATAAGTATAAAGTAG
TAAAAATTGAACCACTA---GGAGTAGCACCCACCAAGGCAAGGAGAAGAGTGGTG-----------------

----------------------GGGAGAGAGAAAAGAGCA---GTTGGA---GTAGGAGCT---------
GTCTTCATTGGGTTCTTAGGAGCA---GCCGGAAGCACTATG---GGCGCGGCGTCA---
GTGACGCTGACGGTACAG---GCCAGACGA---TTATTG---TCTGGCATAGTGCAACAGCAAAGC---
AATTTG---CTGAAG---GCTATAGAGGCTCAACAACACCTGTTGAAACTCACGGTCTGGGGCATT
>KT022373..1
TTCTATTGTAACACATCAAGCTTGTTTAGGGGCGCTTGGAATAGTACAACTGCAACACTTAATAGCACTGGGA
AT---

AGCAATGACACTATAACTCTTCCATGCAGAATAAAGCAAATTATAAATATGTGGCAGAGAGCAGGACAAGCAA
TATATGCCCCTCCCATAAAAGGAGTAATAAGGTGTAACTCAAAT---
ATTACAGGACTAATATTAACAAGAGATGGTGGGGGTAGTGATAAT----------------------------
----

AGAACTGAAACTTTTAGACCTGGAGGAGGAGATATGAGAGACAATTGGAGAAGTGAATTATATAAGTATAAAG
TAGTTAAAATTGAACCACTA---GGAGTAGCACCCACCAGGGCAAAGAGAAGAGTGGTG--------------
-------------------------GAGAGAGAAAAAAGAGCA---GTTGGA---ATAGGAGCT---------
GTCTTCATTGGGTTCTTAGGAGCA---GCAGGAAGCACTATG---GGCGCGGCGTCG---
ATAACGCTGACGGTACAG---GCCAGACAA---TTATTG---TCTGGCATAGTGCAACAGCAAAGA---
AATTTG---CTGAGG---GCTATAGAGGCTCAACAACATCTGTTGAAACTCACGGTCTGGGGCATT
>KT022374..1
TTCTATTGTAATACAACAAAGCTGTTCAATAGCTCTTGGTCTGCCAATGCCAGTGAGTCAAATAGTACAGATG
GCAATAGT---

ACAGATGGCACTATAACTCTCCCATGCAAAATAAAGCAAATTATAAATATGTGGCAGAGAGTAGGACGAGCAA
TGTATGCCAATCCCATCCAAGGAAAAATAGAGTGTGCATCAAAC---
ATTACAGGACTAATATTAACAAGAGATGGTGGGAATGTGAGCAGG----------------------------
----

GAAAATGAAACCTTCAGACCTGGAGGAGGAGATATGAGGGACAATTGGAGAAGTGAATTATATAAGTATAAAG
TAGTAAAGATTGAACCACTA---GGAGTAGCACCCCATACGGCAAGGAGAAGAGTGGTG--------------
-------------------------GGGAGAGAAAAAAGAGCA---GTTGGA---ATGGGAGCT---------
GTCTTCCTTGGGTTCTTAGGAGCA---GCAGGGAGCACTATG---GGCGCAGCGTCA---
CTAACGCTGACGGTACAG---GCCAGACAA---TTATTG---TCTGGCATAGTGCAACAGCAAAGC---
AATTTG---CTGAGG---GCTATAGAGGCTCAACAACATCTGTTGAAACTCACAGTCTGGGGCATT
>KT022375..1
TTCTATTGTAATACATCAAAACTGTTTAATAGCACTTGGCCACAGTCAAACCACACGGAA-------------
----

GACAATGGAAATCTAACTCTCCCATGCAGAATAAAACAACTTATAAACCTGTGGCAAAGAGCAGGACAAGCAA
TGTATGCCCCTCCCATACCAGGAAATATAAGTTGTGTATCAAAC---
ATTACAGGACTACTATTGACAAGAGATGGTGGGAACACTGGGAAC----------------------------
----
AATACAGAGGGAAAT---
AAAACTGAAATCTTCAGACCTGGAGGAGGAAATATGAGGGACAATTGGAGAAGTGAATTATATAAGTATAAAG
TAGTGAAAATTGAACCAATA---GGAATAGCACCCACCAAGGCAAAGAGAAGAGTGGTG--------------
-------------------------GAGAGAGAAAAAAGAGCAGCACTTGCT------GGAGCT---------
TTCTTTCTTGGGTTCTTAGGAACA---GCAGGAAGCACTATG---GGCGCGGCGTCA---
ATAGCGCTGACGGTACAG---GCCAGGCAA---TTATTG---TCTGGCATAGTGCAACAGCAAAGC---
AATTTG---CTGAGG---GCTATAGAGGCTCAACAGCAATTGTTGAGACTCACAGTCTGGGGCATT
>KT022376..1
TTCTATTGTAATACATCAGGCCTGTTTGATAGCACTTGGTCGTTTAATAGCACACAGGAGTCAAATAACACGG
GG---

GAAAATGATACTATAATTCTCCCATGCAGAATAAAGCAAATTATAAACATGTGGCAGAGAGTAGGACAAGCAA
TGTATGCCCCTCCCATCCCAGGAGTAATACAGTGTAACTCAACC---
ATTACAGGGCTAATATTAACAAGAGATGGTGGGGAGGATAATAAT----------------------------
----

AACACAGAAACCTTCAGACCTGCAGGAGGAGATATGAGGGACAATTGGAGAAGTGAATTATATAAATATAAAG
TAGTAAAACTTGAACCACTA---GGAGTAGCGCCCACCAAGGCAAAGAGAAGAGTGGTG--------------

```
-------------------------GAGAGAGAAAAAAGAGCA---GTTGGA---CTAGGAGCT---------
GTCTTCCTTGGGTTCTTAGGAGCA---GCAGGAAGCACTATG---GGCGCGGCGTCA---
ATAACGCTGACGGTACAG---GCCAGGCAA---TTATTG---TCTGGCATAGTGCAACAGCAAAGC---
AATTTG---CTGAGG---GCTATAGAGGCTCAACAACATATGTTGAAACTCACAGTCTGGGGCATT
>KT022377..1
TTCTATTGTAATACATCAGGGCTGTTCAATAGTACTTGGAATACCACGAAGGTAAATAGCACAGAG-------
-------------------------------------------------------------------------
----------------------------
TCAAATGGCACTATAACTCTCCCATGCAGAATAAAGCAAATTATAAATATGTGGCAAAGAACAGGACAAGCAA
TGTATGCCCCTCCCATCAAAGGAATAATAAATTGTGCATCAAAT---
ATTACAGGACTACTATTAACAAGAGATGGTGGGGATAATAACAGT----------------------------
-------------------------------------------------------------------------
-------------
ACAAATGAAACCTTCAGACCTGGAGGAGGAGATATGAGGGACAATTGGAGAAGTGAATTATATAAGTATAAAG
TAGTAAAAATTGAACCACTA---GGGGTAGCACCCACCGAGGCAAGGAGAAGAGTGGTG--------------
-------------------------GAGAGAGAAAAAAGAGCATTTGTTGGA---ATAGGAGCT---------
GTCTTCCTTGGGTTCTTAGGAGCA---GCCGGCAGCACTATG---GGCGCGGCGTCA---
TTAACGCTGACGGTACAG---GCCAGACAA---TTATTG---TCTGGTATAGTGCAACAGCAAAAC---
AATTTG---CTGAGG---GCTATAGAGGCTCAACAACATCTGTTGAAACTCACGGTCTGGGGCATT
>FJ866116..2
TTCTACTGTGATACATCAGGACTGTTTAATAGCTCTTGGAATAATACATCGGGCCAGTTTAATAGCACTTGGA
CTGATGGC-----------------------------------------------------------------
-------------TACAAT------
AGAAAGAGCGAGAATATAACTCTCCAATGCAGAATAAAGCAATTTATAAATATGTGGCAGAGAACAGGGCAAG
CAATGTATGCCCCTCCCATCCCAGGAATAATAAAATGTGAATCAAAC---
ATTACAGGACTAATACTAACAAGAGATGGTGGGCAGGCTAACAAA----------------------------
-------------------------------------------------------------------------
-------------
ACAGAGGAAACCTTCAGACCTGGAGGAGGAGATATGAGGGACAATTGGAGAAGTGAATTATTTAGGTATAAAG
TAGTAAAAATTGAACCACTA---GGAGTAGCACCCACCAAGGCAAAGAGAAGAGTGGTG--------------
-------------------------GAGAGAGAGAAAAGAGCA---GTAATTGGCATGGGAGCT---------
GTTTTCCTTGGGTTCTTAGGAGCA---GCAGGAAGCACTATG---GGCGCGGCGTCA---
CTGACGCTGACGGTACAG---GCCAGACAA---TTATTG---TCTGGCATAGTGCAACAGCAAGGC---
AATTTG---CTGAGG---GCTATAGAGGCTCAACAACATCTGTTGAGACTCACGGTCTGGGGCATT
>FJ866118..1
TTCTATTGTAATACAACAGGACTGTTCAATCTCACTTGGAATGGCACTGACAGCACAAATAGAAAT-------
-------------------------------------------------------------------------
----------------------------
GAGTCAGGCAATATAACTCTCCCATGCAGAATAAAGCAAATTATAAATATGTGGCAAAGAGTAGGACGAGCAA
TATATGCCCCTCCCATCCCAGGAGTAGTAAAGTGTAAATCAAAT---
ATTACAGGATTAATATTAACAAGGGATGGTGGAGACGACGAGAAT----------------------------
-------------------------------------------------------------------------
-------------
AACACTGAAACCTTCAGACCTGGAGGAGGAAATATGAGGGACAATTGGAGAAGTGAATTATATAAATATAAAG
TAGTAAAAATTGAACCACTA---GGGGTAGCACCCACCAAGGCAAGGAGGAGAACGGTG--------------
-------------------------GAGAGAGAAAAAAGAGCA---GTTGGA---ATAGGAGCT---------
GTCTTCCTTGGGTTCTTAGGAGCA---GCAGGAAGCACTATG---GGCGCGGCGTCA---
ATAACGCTGACGGTACAG---GCCAGACAA---TTATTG---GCTGGCATAGTGCAACAGCAAAGC---
AATTTG---CTGAGG---GCTATAGAGGCTCAACAACAGATGTTGAGACTCACAGTCTGGGGCATT
>FJ866120..2
TTCTATTGTAATACATCACTCCTGTTTGAAAGCATTTGGTATGGCAATGAAAGCATAAATGCCACAGAAGAGG
CA-----------------------------------------------------------------------
----------------------------
AATGACACTATAATAACTCTCCGATGCAGAATAAAGCAATTTATAAAAATGTGGCAGAGAGTAGGACAAGCAA
TGTATGCCCCTCCCATCCCAGGAGCAATCAGGTGTAATTCAAAC---
ATTACAGGACTAATATTAACAAGAGATGGAGGGCTTAATGACAATGGAGCT----------------------
-------------------------------------------------------------------------
-------
GATGGAAGAAATGAAACCTTCAGACCTGGAGGAGGAGATATGAGGGACAATTGGAGAAGTGAATTATATAAGT
ATAAAGTGGTAGAAATTGAACCACTA---GGAGTAGCCCCCACTCAGGCAAGAAGAAGAGTGGTG--------
```

----------------------------CAGAGAGAAAAAAGAGCA---GTTGGA---ATAGGAGCT---
------GTCTTCATTGGGTTCTTAGGAGCA---GCAGGAAGCACTATG---GGCGCAGCGTCA---
ATAGCGCTGACGGCACAG---GCCAGACAA---TTATTG---TCTGGCATAGTGCAACAGCAAAGC---
AATTTG---CTGAGG---GCTATAGAGGTTCAACAACAGTTGTTGAAACTCACGGTCTGGGGCATT
>FJ866121..2
TTCTACTGTAACACATCAAAACTGTTTAATAGTACTTGGCATTACAATAACAGCACTGGGTATAATAATGGCA
GCCTACAG--
------------------GAG---
GAAAATAACACAGAGATAGTTCTCCTATGCAGAATAAAGCAAATTATAAATCTGTGGCAGAGGGTAGGAAAGG
CAATGTATGCCCCTCCCATCCAAGGAAGCATAAAGTGTAACTCAAAC---
ATTACAGGACTACTATTAATCAGGGATGGTGGGGTTAATACTACT-------------------------
----

AATGAAACCTTCAGACCTGGAGGAGGAGATATGAGGGACAATTGGAGAAGTGAATTATATAAATATAAAGTAA
TAAAAATTGAACCACTA---GGAATAGCACCCACCAGGGCAAAGAGAAGAGTGGTG--------------
---------------------GAGAGAGAAAAAAGAGCA---GTTGGA---CTAGGAGCT---------
GTTTTCGTTGGGTTCTTAGGAGCA---GCAGGAAGCACTATG---GGCGCAGCGTCA---
GTGACGCTGACGGTACAG---GCCAGACAG---TTATTG---TCTGGTATAGTGCAACAGCAAAGC---
AATTTG---CTGAGG---GCTATAGAGGCTCAACAACATATGTTGAAACTCACGGTCTGGGGCATT
>KT022380..1
TTCTATTGTAATACATCAGGCTTGTTTAATAGGACTTGGAAAAAGGGGATAATCAAGGGCACTGCCAATAACA
CG--

GAGTCAGACCCTATAGTGCTCCAATGCAGAATAAAGCAAATTATAAATATGTGGCAGAGAGTAGGACGAGCAA
TGTATGCCCCTCCCATCCCAGGAATAATAAAATGTGTATCAAAC---
ATTACAGGACTAATATTAACAAGAGATGGTGGGAATAGTAATGGA-------------------------
----

GATAATGAAACCTTCAGACCTGGAGGAGGAGATATGAGGGACAATTGGAGAAGTGAATTATATAAGTATAAAG
TAGTAAAAGTTGAGCCATTA---GGAGTAGCACCCACCAGGGCAAGGAGGAGAGTGGTG-----------
------------------------GCGAGAGAAAAAAGAGCAGTAGTTGGA---CTGGGAGCT---------
GTTTTCATTGGGTTCTTAGGAGCA---GCAGGAAGCACTATG---GGCGCGGCGTCA---
ATAACGCTGACGGTACAG---GCCAGACAA---TTATTG---TCTGGCATAGTGCAACAGCAAAGC---
AATTTG---CTGAGG---GCTATAGAGGCTCAACAACATCTGTTGAAACTCACGGTCTGGGGCATT
>KT022381..4
TTCTATTGTAATACATCAGGCCTGTTTAATTACACCTATCCATCAAATCACAGTGATAAGTCAAATAACACT-
----

ATAAATGAGACTATAACTCTCCCATGCAGAATAAAACAAATTATAAGAATGTGGCAGAGAACAGGACAAGCAA
TGTATGCCCCTCCCATCCCAGGAATAATTAAGTGTGAATCAAAC---
ATTACAGGACTAATATTAACAAGAGATGGT---TTTAATGGCACG-------------------------
----

AATGAAACCTTCAGGCCTGTAGGAGGAGATATGAGGGACAATTGGAGAAGTGAATTATATAAGTATAAAGTAG
TAAGAATTGAACCACTA---GGAGTAGCACCCACCAGGGCAAAGAGAAGAGTGGTG--------------
---------------------GGAAGAGAGAAAAGAGCA---GTTGGA---ATGGGAGCT---------
GTATTCCTTGGGTTCTTAGGAGCA---GCAGGAAGCACTATG---GGCGCGGCGTCA---
ATAACGCTGACGGTACAG---GCCAGACAA---TTATTG---TCTGGCATAGTGCAACAGCAAAGC---
AATTTG---CTGAAG---GCAATAGAGGCTCAACAACATCTGCTGAGACTCACGGTCTGGGGCATC
>KT022382..2
TTCTATTGTAATACATCAGGCCTGTTCAATAGTAGCTGGGGTCCAAATGACACGAAATCAGATAAC-------
----

TCAACTACAAATTACACTCTCCCATGCAGAATAAAGCAAATTGTAAGGATGTGGCAGAAAATAGGACAAGCAA
TGTATGCCCCTCCAATCGAGGGAGAAATAAATTGTGTATCAAAC---
ATTACAGGACTATTATTAACAAGAGATGGTGGGCATAACAGTATT-------------------------
----

AACAATGAAACTGAAACATTCAGACCTGGAGGAGGAGATATGAAGGACAATTGGAGAAGTGAATTATATAAGT
ATAAAGTAGTAAAAATTGAACCACTA---GGAGTAGCACCCACCAGGGCAAAGAGAAGAGTGGTG--------

-------------------------------GGGAGAGAAAAAAGAGCA---GTTGGA---ATAGGAGCT---
------GTTTTCCTTGGGTTCTTGGGAGCA---GCAGGAAGCACTATG---GGCGCGGCGTCA---
ATAACGCTGACGGTACAG---GCCAGACAA---TTATTG---TCTGGCATAGTGCAGCAGCAAAAC---
AATTTG---CTGAGG---GCTATAGAGGCTCAACAACATCTGTTGAAACTCACGGTCTGGGGCATT
>KT022383..1
TTCTATTGTAATACATCAGGCCTGTTTAATAGCACTTGGAACAGCCCTTGGAATAACACTGGGGAA-------
----

CAAGAAGGCAATATAACTCTCCCATGCAGAATAAAGCAAATTATAAATATGTGGCAGAGAGTAGGACAAGCAA
TATATGCCCCTCCCATCCCAGGAGTAATAAAGTGTGAATCGAAC---
ATTACAGGGCTACTATTAACAAGAGATGGTGGGAACACTAACAGT----------------------------
----

AGTACTGAAACCTTCAGACCTGGAGGAGGAGATATGAGGGACAATTGGAGAAGTGAATTATATAAATATAAAG
TAGTAAAAGTTGAACCACTA---GGAGTAGCACCCACCAGGGCAAGGAGAAGAGTGGTG--------------
-------------------------GGGAGAGAAAAAAGAGCA---GTTGGA---ATAGGAGCT---------
GTCTTCCTTGGGTTCTTAGGAGCA---GCAGGAAGCACTATG---GGCGCGGCGTCA---
ATAACGCTGACGGTACAG---GCCAGACAA---TTATTG---TCTGGCATAGTGCAACAGCAAAGC---
AATTTG---CTGAGG---GCTATAGAGGCTCAACAACATATGTTGAAACTCACGGTCTGGGGCATT
>FJ623487..1
TTCTATTGTAATACATCAAGACTGTTTAATAGCACTTGGAACAATGGCACTTGGAACAATACCAACATGCAGG
AGTCAAATAGC--
-------------ACGAAG------
TCAGAAAATGGCACTATAACTCTCCCATGCAGAATAAAGCAAATTATAAATATGTGGCAGAGAACAGGACAAG
CAATGTATGCCCCTCCCATCCGAGGAGTAATAAGATGTGAATCAAAC---
ATTACAGGACTAATATTAACAAGAGATAGTGTGAATAATAGCAAA----------------------------
----

GACAATGAAACCTTCAGACCAGGAGGAGGAGATATGAGGGACAATTGGAGAAGTGAATTATATAAGTATAAAG
TAGTAAAAGTTGAACCACTA---GGAGTAGCACCCACCACGGCAAGGAGAAGAGTGGTG--------------
-------------------------GGAAGAGAAAAAAGAGCA---GTGGGA---ATAGGAGCT---------
GTATTCCTTGGGTTCTTGGGAGCA---GCAGGAAGCACGATG---GGCGCGGCGTCA---
ATGACGCTGACGGTACAG---GCCAGACAA---TTATTG---TCTGGTATAGTGCAACAGCAAAGC---
AATTTG---CTGAGG---GCTATAGAGGCTCAACAACACCTGTTGAAACTCACGGTCTGGGGCATT
>FJ623481..2
TTTTATTGTAATACATCAGGCCTGTTTAATAGCACTTGGAATCACACTGACATAAAGGGGTCAAATAGCACGG
AG---

TCAAGTGAAACTATAACTCTCACATGCAGAATAAAGCAAATTATAAATATGTGGCAGAGAGTAGGGCAAGCAA
TGTATGCCCCTCCCATACCAGGAGCAATAAGGTGTGAATCAAAC---
ATTACAGGAATACTCTTAACAAGAGATGGTGGGAATAGCAAGAAC----------------------------
----

AGCACAACAGAAATCTTCAGACCTGGAGGAGGAAACATGCGGGACAATTGGAGAAGTGAATTATATAAGTATA
AAGTAGTACAAATTGAACCACTA---GGAGTAGCACCTACCAGGGCAAAGAGAAGAGTGGTG-----------
----------------------------GAGAGAGAAAAAAGAGCA---ATTGGA---ATAGGAGCT------
---GTATTCATTGGGTTCTTAGGAGCA---GCAGGAAGCACTATG---GGCGCGGCGTCA---
ATAACGCTGACGGTACAG---GCCAGACAA---TTATTG---ACTGGCATAGTGCGACAGCAAAGC---
AATTTG---CTGAGG---GCTATAGAGGCTCAACAACATCTGTTGAAACTCACGGTCTGGGGCATT
>FJ623475..1
TTTTACTGTGATACATCAGGCCTGTTTAATAGCACTTGGGGAGCCACTGACAGCATGCATGGGGCAAATGACA
CG---

GAGTCAAATGACACTATAACTCTCCCATGTAGGATAAAACAAATTATAAATAGGTGGCAGAGAGTAGGACAAG
CAATGTATGCCCCTCCCATCCAAGGAGTAATAAAGTGCAAATCAGAC---
ATTACAGGAATAATATTAACAAGAGATGGTGGGAATAATAATGAC----------------------------
----

AGTAGAAATGAAACCTTCAGACCTGGAGGAGGAGATATGAGGGACAATTGGAGAAGTGAATTGTATAAGTATA
AGGTGGTAAAGATTGAACCACTA---GGAGTAGCACCCACCAGGGCAAAGAGAAGAGTGGTG-----------

----------------------------GAGAGAGGAAAAAGAGCA---GTTGGG---ATAGGAGCT------
---GTTTTCCTTGGATTCTTAGGAGCA---GCCGGAAGCACTATG---GGCGCGGCGTCA---
ATAACGCTGACGGTACAG---GCCAGGCAA---TTATTG---TCTGGCATAGTGCAACAGCAAAGC---
AATTTG---CTGAGG---GCTATAGAGGCTCAACAACATATGTTGAAACTCACGGTCTGGGGCATA
>FJ623476..2
TTCTATTGTAATACATCAGGCTTGTTTAATAGCACTTGGAATATCACTGACAATCTCAACATAACTCAG----
----

TCAAATGAGAACATAACTCTCCCATGCAAAATAAAGCAAATTATAAATATGTGGCAGAGAGTAGGACAAGCAA
TATATGCCCCTCCCATCCCAGGAAGGGTAAGTTGTGTCTCAAAC---
ATTACAGGACTAATATTAACAAGAGATGGTGGGAACAATAACAGT----------------------------
----

ACAAATGAAACCTTCAGGCCTGGAGGAGGAGATATGAGGGACAATTGGAGAAGTGAATTATATAAGTATAAAG
TAGTAAGAATTGAACCACTA---GGAGTAGCACCTACCCAGGCAAAGAGAAGAGTGGTG--------------
-------------------------GGAAGAGAAAAAAGAGCA---ATTGGA---ATAGGAGCT---------
GTCTTCCTTGGGTTCTTAGGAGCA---GCAGGAAGCACTATG---GGCGCGGCGTCA---
ATAACGCTGACGGTACAG---GCCAGACAG---TTATTG---TCTGGCATAGTGCAACAGCAAAAC---
AATTTG---CTGAGG---GCTATAGAGGCTCAACAACAACTGTTGAAACTCACGGTCTGGGGCATT
>FJ623485..3
TTTTATTGTAATACATCAGGCCTGTTTAATAGCACTTGGTATCACAATAGCACTTGGAATGATCAAACTGACG
AG---

AGGTCAAATGACACTATAACTCTCCAATGCAGAATAAAGCAAATTATAAATATGTGGCAGAGAGTAGGACAAG
CAATATATGCCCCTCCTATCCAAGGAACAATAAGGTGTGAATCAAAC---
ATTACAGGACTAATATTAACAAGAGATGGTGGGGAGGATAGCAAT----------------------------
----

ACAACAACTGAAACCTTCAGACCTAGCGGAGGAGATATGAAGGACAATTGGAGAAGTGAATTATATAAATATA
AAGTAGTAAAAATTGAACCACTA---GGAGTAGCACCCTCCAGGGCAAGGAGAAGAGTGGTG-----------
----------------------------GGAAGAGAAAAAAGAGCA---GTTGGA---ATAGGAGCT------
---GTCTTCCTTGGGTTCTTAGGAGCA---GCAGGAAGCACTATG---GGCGCAGCGTCA---
ATAACGCTGACGGTACAG---GCCAGACAA---TTACTG---TCTGGCATAGTGCAACAGCAAAGC---
AATTTG---CTGAGG---GCTATAGAGGCTCAACAACATATGTTGAGACTCACGGTCTGGGGCATT
>FJ623488..1
TTCTATTGTAACACATCAGGTCTGTTTAATAGCACTTGGGATAACAATACTGGCAATTGGTCTAGCAATAGCA
GCAGCATGCAG--
----------GGGTCAAAT------
AGCACAGATGATACTATAACTCTCCCATGCAGAATAAAGCAAATTATAAATATGTGGCAGAGAACAGGACAAG
CAATGTATGCCCCCCCCATCCAAGGGATAATAAGGTGTGAATCAAAC---
ATCACAGGGATAATATTAACAAGAGATGGTGGGGGTACTGAGGAT----------------------------
----

AATACTACAGAAGTCTTCAGACCTGGAGGAGGAGATATGAGGGACAATTGGAGAAGTGAATTATATAAATATA
AAGTAGTACAAGTTGAACCAGTA---GGAATAGCACCCTCCAAGGCAAGGAGAAGAGTGGTG-----------
----------------------------CAGAGAGAAAAAAGAGCA---ATTGGA---ATGGGAGCT------
---GTCTTCCTTGGGTTCTTAGGAGCA---GCAGGAAGCACTATG---GGCGCGGCGTCA---
ATGACGCTGACGGTACAG---GCCAGAAAT---TTATTG---TCTGGCATAGTGCAACAGCAGAGT---
AATTTG---CTGAGG---GCTGTAGAGGCTCAACAACAACTGTTGAGACTCACGGTCTGGGGCATT
>FJ623478..4
TTCTATTGTAATACATCAGGCCTGTTTAATCACACTTACCTGCCTAAGAGCACTTACCTGCCTAATAGCACTT
GGAATAAC---
-------------AGTACG------
GGATCAAATGACAGTATAACTCTTCCATGTAGAATAAAGCAAATTATAAATATGTGGCAGAGAGTAGGACAAG
CAATGTATGCCCCTCCTATCCAAGGAGTAATAAAGTGTAAATCAGAT---
ATTACAGGACTAATATTGACAAGAGATGGTGGGAATAGTAACAGT----------------------------
----

GATAATGAAACCTTCAGACCTGGAGGAGGAGATATGAGGGACAATTGGAGAAGTGAATTATATAAGTATAAAG
TAGTAAAAATTGAACCACTA---GGAGTAGCACCCACCAGGGCAAAGAGAAGAGTGGTG--------------

-------------------------GAGAGAGAAAAAAGAGCA---GTTGGA---ATAGGAGCT---------
GTTTTCATTGGGTTCTTAGGAGCA---GCAGGAAGCACTATG---GGCGCGGCGTCA---
ATAACGCTGACGGTACAG---GCTAGACAA---TTATTG---TCTGGCATAGTGCAACAGCAAAGC---
AATTTG---CTGAGG---GCTATAGAGGCTCAACAACATATGTTGAAACTCACGGTCTGGGGCATT
>FJ623482..1
TTCTATTGTAATACATCAGACCTGTTTAATGGCAATAGCACTAGGAATGGCACTGGGAATAGCAACAGCACGC
CG---

GTAACTGGCAATATAACTCTCCCATGCAGAATAAAGCAAATTATAAATATGTGGCAGAGAGTAGGACAAGCAA
TGTATGCCCCGCCCATCCAAGGGGTAATAAGGTGTGTATCAAAC---
ATTACAGGATTAATATTGACAAGAGATGGTGGGAAACAGAATAATAATAGTAAT-------------------
----
-
AGTAATAGTAGTGATATTGAAGTCTTCAGACCTGGAGGAGGAGATATGAGGGACAATTGGAGAAGTGAATTAT
ATAAGTATAAAGTAGTAAAAATTGAACCACTA---GGAATAGCACCTACCAAGGCAAAAAGAAGAGTGGTG--
-------------------------------------GAGAGAGAAAAAAGAGCA---ATTGGA---
ATAGGAGCT---------GTCCTCCTTGGGTTCTTAGGAACA---GCAGGAAGCACTATG---
GGCGCGGCGTCA---ATAGCGCTGACGGTACAG---GCCAGACAA---TTAATG---
TCTGGCATAGTGCAACAGCAAAGC---AATTTG---CTGAGG---
GCTATAGAGGCTCAACAGCATATGTTGAAACTCACAGTCTGGGGCATT
>FJ623477..1
TTCTATTGTGATACATCAGGCCTGTTTAATAGCACCTGGAATAATAGCACCTGGAATAATAATGACACTGCCC
AG---

TCAAATGACACAATAACTCTCCCATGCAGAATAAAACAAATTGTAAATATGTGGCAGAGAACAGAACAAGCAA
TGTATGCCCCTCCCATCCAAGGAGTAATAAGGTGTGAATCAAAC---
ATCACAGGACTACTATTAACAAGAGATGGTGGGATTAATAACACA----------------------------
----

AATGAAACCTTCAGACCTGGAGGAGGAGATATGAGGGACAATTGGAGAAGTGAACTATATAAGTATAAAGTAG
TAAAAATTGAACCACTG---GGAGTAGCACCCTCCAGGGCAAGGAGAAGAGTGGTG-----------------
----------------------GGGAGAGAAAAAAGAGCA---GTTGGA---ATAGGAGCT---------
TTATTCCTTGGATTCTTGGGCGCA---GCAGGAAGCACTATG---GGCGCGGCGTCA---
ATGACGCTGACGGTACAG---GCCAGACAA---TTATTG---TCTGGCATAGTGCAACAGCAAAGC---
AATTTG---CTGAAG---GCTATAGAGGCTCAACAACATCTGTTGAGACTCACGGTCTGGGGCATT
>FJ623486..3
TTCTATTGTAATACATCAGAACTATTTAATAGCACTTGGAATCTTAATAGCACTTGGAATCACACTGGCAGCA
TACCGGAGTCA--
----------AATAGCACG------
GAGTCAAATGGCACTATAACCCTCAAATGCAGAATAAAGCAAATTATAAATATGTGGCAGAGAGTAGGGCAAG
CAATGTATGCCCCTCCCATCAAAGGGACAATAAGGTGTAAATCAAAC---
ATCACAGGACTAATATTAACAAGAGATGGTGGGGATAATAACAGT----------------------------
----

ACAAAGGAAACCTTCAGGCCTGGAGGAGGAGATATGAGGGACAATTGGAGAAGTGAATTATATCAGTATAAAG
TAGTAAAACTTGAACCACTA---GGAGTAGCACCCACCAGGGCAAGGAGAAGAGTGGTG--------------
-------------------------GGGAGAAAAAAAAGAGCA---GTTGGA---CTAGGAGCT---------
GTTTTCATTGGGTTCTTGGGAGCA---GCAGGAAGCACTATG---GGCGCGGCGTCA---
ATGACGCTGACGGTACAA---GCTAGACAA---TTATTG---TCTGGCATAGTGCAACAGCAAAGC---
AATTTG---CTGAGG---GCTATAGAGGCTCAACAACATCTGTTGAAACTCACGGTCTGGGGCATT
>FJ623479..1
TTCTATTGTAATACATCAGACCTGTTTAATGGTACTTGGAATAATAGCACTTTGAATGGCACTGCCAAGCAGG
AG---

GTAAATGACAGCACTATAACGATCTCATGCAGGATAAAGCAATTTATAAATATGTGGCAGAGAACAGGACGAG
CAATGTATGCCCCTCCCATTCCAGGAGTAATAAGTTGTAACTCAAGC---
ATTACAGGATTAATATTAACAAGAGATGGTGGGGATGATAACAAT----------------------------
----

AATAATGAAACATTCAGACCTGGAGGAGGAGATATGAGGGACAATTGGAGAAGTGAATTATATAAGTATAAAG

TAGTAAAAATTGAACCACTA---GGAGTAGCACCCACCAAGGCAAGGAGAAGAGTGGTG--------------
-------------------------GAGAGAGAAAAAAGAGCA---GTTGGA---ATAGGAGCT---------
GTACTCCTTGGGTTCTTAGGAGCA---GCAGGAAGCACTATG---GGCGCGGCGTCA---
ATAACGCTGACGGTACAG---GCCAGACAA---TTATTG---TCTGGCATAGTGCAACAGCAAAGC---
AATTTG---CTGAAG---GCTATAGAGGCTCAACAACAACTGTTGAAACTCACAGTCTGGGGCATT
>HM215275..1
TTCTATTGTAATACATCAGGTCTGTTCAATAGCACTTGGGAGGCCAATAGTACTGCCAATACC----------
----

AGCACGACAAATATAACTCTCCCATGCAAAATAAAGCAACTTGTAAGAATGTGGCAGAGGACAGGACAAGCAT
TGTATGCCCCTCCCATCGAAGGAGTAATAAAATGTGTATCAAAC---
ATTACAGGACTAATATTAACAAGAGATGGTGGGGGTAATAACAGT----------------------------
----

GCAACAAATGAAACCTTCAGACCTGGAGGAGGAGATATGAGGGACAATTGGAGAAGTGAATTATATAAGTATA
AAGTAGTACAAATTGAACCACTA---GGAGTTGCACCCACCGAGGCAAGGAGAAGAGTGGTG-----------
----------------------------AAGAGAGAAAAAAGAGCA---GTTCCT------GGACTG------
---TTTTTCCTTGGGTTCTTAGCAGCA---GCAGGAAGCACTATG---GGCGCGGCGTCA---
ATAACGCTGACGGTACAG---GCCAGACAA---TTATTG---TCTGGCATAGTGCAACAGCAAAGC---
AATTTG---CTGAGG---GCTATAGAGGCTCAACAGCATCTGTTGAAACTCACGGTCTGGGGCATT
>KT252544..1
TTCTATTGTAACACATCAGGCCTGTTTAATAGTACTTGGGATGGGAATAGCACTGAGGCAAACATTACGCAG-
----

TCAAATGAGACTATAACTCTCCCATGCAAAATAAAGCAAATTGTAAATATGTGGCAGAGAGTGGGACGAGCAA
TGTATGCCCCTCCTATCCCAGGAGTAATAAAGTGTGAATCAAAC---
ATTACAGGGCTAATACTGACAAGAGATGGTGGGAATGAAACAAAT----------------------------
----

GGAACAGAAATCTTCAGACCTGTAGGAGGAGACATGAGGGACAATTGGAGAAGTGAATTATATAAGTATAAAG
TAGTACAAATTAAACCACTA---GGAGTAGCACCCACCAAGGCAAAGAGAAGAGTGGTG--------------
-------------------------CAGAGAGAAAAAAGAGCA---GTTGGA---ATGGGAGCT---------
GTTTTCTTTGGGTTCTTAGGAGCA---GCTGGAAGCACTATG---GGCGCGGCGTCA---
ATAACGCTGACGGTACAG---GCCAGACAA---TTATTG---ACTGGCATAGTGCAACAGCAAAGC---
AATTTG---CTGAGG---GCTATAGAGGCTCAACAGCATCTGTTGAAACTCACTGTCTGGGGCATT
>HQ540689..1
TTCTACTGTAATACAACAAACCTGTTTAATAGTAGCATTCCCTTCAACGCCAGCGAGAGGGCAAATAACACCA
AT---

AGCACAGATGACATTATAACTCTCCAATGCAGAATAAAGCAAATTGTAAGGATGTGGCAAAGAGTAGGACAAG
CAATGTATGCCCCTCCCATCCCAGGAGTAATAAGGTGTGAATCAAAC---
ATTACAGGACTTATGTTAACATGGGATGGTGGGAGTAAAAATATTACTGAG----------------------
----

GGCAATAGAACTGAAACCTTTAGACCTGGAGGAGGAGACATGAGGGACAATTGGAGAAGTGAATTATATAAAT
ATAAAGTAGTAAAAATTGAACCACTA---GGAGTTGCACCCACCAAGGCAAGGAGAAGAGTGGTG--------
-------------------------------GGAAGAGAAAAAAGAGCA---GTTGGA---ATAGGAGCT---
------GTTTTCCTTGGGTTCTTAGGAGCA---GCAGGAAGCACTATG---GGCGCGGCGTCA---
GTGACGCTGACGGTACAG---GCCAGACAA---TTATTG---TCTGGTATAGTGCAACAGCAAAGC---
AATTTG---CTGAGA---GCTATAGAGGCTCAACAACAGTTGTTGAAACTCACGGTCTGGGGCATT
>KU921708..2
TTCTATTGTAATACAGTAAATCTGTTCAATAGCTCTTGGGGTGCCAACGCCACTGAGTCAAATAGCACAGGGT
TA---

AATAATCAAACTATACATCTCCCATGCAGAATAAAGCAAATTATAAATATGTGGCAGAGAACAGGACAAGCAA
TGTATGCCCCTCCCGTCGAAGGAATAATAAGTTGTGTATCAGAC---
ATTACAGGACTACTGTTAACAAGAGACGGAGGAGAAAATACAACA----------------------------
----

CATGAAATTTTCAGACCTGGAGGAGGAGATATGAGGGACAATTGGAGAAGTGAATTATATAAGTATAAAGTAG

TAAAAATTGAACCCCTA---GGAGTAGCACCCACCAAGGCAAGGAGAAGAGTGGTG-----------------
----------------------CAGAGAGAAAAAAGAGCA---GTTGGA---ATGGGAGCT---------
GTCTTCCTTGGGTTCTTAGGAGCA---GCAGGAAGCACTATG---GGCGCAGCGTCA---
ATAACGCTGACGGTACAG---GCCAGACAA---TTATTG---TCTGGTATAGTGCAACAGCAAAGC---
AATTTG---CTGAGG---GCTATAGAGGCTCAACAGCATCTGTTGAAACTCACTGTCTGGGGCATT
>KU921712..2
TTCTATTGTAATACATCAAAGCTGTCTAACAGCACATTTCCACTTAATAACACTCAGAATAACAGCACGCAG-
----

ACAAACAGTACTATAACTCTCCCATGCAGAATAAAGCAAATTATAAATATGTGGCAGAGAACAGGAAAAGCAA
TATATGCCCCTCCCATCTCAGGACCAATAAAGTGTACATCAAAC---
ATTACAGGACTAATATTAACAAGAGATGGTGGAAATGATAATAATAATGAT----------------------
----

ACCAATGGAAATGAAACCTTCAGACCTGGAGGAGGAGATATGAGGGACAATTGGAGAAGTGAATTATATAAGT
ATAAAGTAGTAAAACTTGAACCACTA---GGAGTAGCACCCACCAGGGCAAGAAGAAGAGTGGTG--------
-------------------------------CAGAGAGAGAAAAGAGCA---ATTGGA---CTGGGAGCT---
------GTATTCATTGGGTTCTTAGGAGCA---GCAGGGAGCACTATG---GGCGCGGCGTCA---
GTGACGCTGACGGTACAG---GCCAGAGCC---TTATTG---TCTGGCATAGTGCAACAGCAAAGC---
AATTTG---CTGAGG---GCTATAGAAGCTCAACAACATCTGTTGAAGCTCACGGTCTGGGGCATT
>KU921727..2
TTCTATTGTGACACATCAAGCCTGTTTAATAGCACTTGGAATGAGAATGACAATGTCAGCAGAACAGAGTCAA
AT---

AGTACAGAAGATATAACTCTCCAATGCAGAATAAAACAAATTGTAAATATGTGGCAGAGAGTAGGACAAGCGA
TGTATGCCCCTCCCATCCAAGGAATAATAAAGTGTGAATCAAAC---
ATAACAGGACTAATATTAACAAGAGATGGTGGGAATCATAATAGT----------------------------
----
ACAAATGAAACCTCC---
AGAAATGAAACCTTCAGACCTGGAGGAGGAGATATGAGGGACAATTGGAGAAGTGAATTATATAAGTATAAGG
TAGTAAAAATTGAACCTCTA---GGAGTAGCACCCACCAAGGCAAAGAGAAGAGTGGTG--------------
-------------------------GAGAGAGAAAAAAGAGCA---GTTGGA---ATAGGAGCT---------
GTCTTCCTTGGATTCTTGGGAGCA---GCAGGAAGCACTATG---GGCGCGGCGTCA---
ATAACGCTGACGGCACAG---GCCAGACAA---TTATTG---TCTGGTATAGTGCAACAGCAAAGC---
AATTTG---CTGAGG---GCTATAGAGGCTCAACAGCATCTGTTGAGACTCACAGTCTGGGGCATT
>KU921732..1
TTCTATTGTAATACATCAGGCCTTTTTGATGACACTTGGAATGGCACGAAG----------------------
----

TCAAATGACACTATAACACTCCCATGCAGAATAAAGCAAGTTATAAATATGTGGCAGAGGACAGGACAAGCAA
TGTATGCCCCTCCCATCCAAGGAGTAATAAGGTGCGAATCAAAC---
ATCACAGGACTAATATTAACAAGAGATGGTGGGCATAACATTACA----------------------------
----

AATGAAACCTTCAGACCTACAGGAGGAAATATGAGGGACAATTGGAGAAGTGAATTATATAAGTATAAAGTAG
TAAGAATTGAACCACTA---GGAGTAGCACCCACCTCGGCAAAGAGAAGAGTGGTG-----------------
----------------------GAGAGAGAAAAAAGAGCAGTAGTTGGA---ATGGGAGCT---------
GTCTTCATTGGGTTCTTGGGAGCA---GCAGGAAGCACTATG---GGCGCGGCGTCA---
ATGACGCTGACGGTACAG---GCCAGACAA---TTATTG---TCTGGCATAGTGCAACAGCAAAGC---
AATTTG---CTGAGG---GCTATAGAGGCTCAACAGCATTTGTTGAAGCTCACGGTCTGGGGCATT
>KU921734..1
TTCTATTGTAACACATCAGGTCTGTTTAATAGCACGTGGAATAAGGATAATAACACCTTGAATGGCGCTGTAA
ATAAC--

ATAACAGACAATATAACCCTCCAATGCAGAATAAAACAAATTATAAATATGTGGCAGAGAGCAGGACAAGCAA
TATATGCTCCTCCCATCCCAGGAGTAATAAGGTGCACCTCAAAT---
ATTACAGGATTAATATTAACAAGAGATGGTGGGAAAGATAGCAATGAA-------------------------
----

AGTGCTGAAACCTTCAGACCTGGAGGAGGAGATATGGGGGACAACTGGAGAAGTGAATTATATAAGTATAAGG

TAGTAAAAATTGAACCACTA---GGAATAGCACCCAACAGGGCAAAGAGAAGAGTGGTG--------------
-------------------------GAGAGAGAAAAAAGAGCAATCTTGGGA---CTGGGAGCT---------
GTTTTCGTTGGGTTCTTAGGAGCA---GCAGGAAGCACTATG---GGCGCGGCGTCA---
CTGACGCTGACGGTACAG---GCCAGAAAC---TTATTG---TCTGGCATAGTGCAACAGCAAAAC---
AATTTG---CTGAAG---GCTATAGAGGCTCAACAGCATCTGTTGAAACTCACGGTCTGGGGCATT
>KU921738..2
TTCTATTGTAACACATCAGAGCTGTTTAATAGCACTTGGAATAGGACTGACCCTGGCACTTGGAATAACACC-
----
AGCACGCAGGAGTCAAAT---------
AGCACAGATCAAAAAATAACTCTCCAATGCAGCATAAAGCAAATTATAAATATGTGGCAAAGGGTAGGACGAG
CAATGTATGCCCCTCCCATCCCAGGACCAATACAGTGTAGATCAAAC---
ATTACAGGACTAATATTAGTGAGAGATGGTGGGAATACGAATACT----------------------------
----

AGCAAGGAAACCTTTAGACCTGCAGGAGGGGATATGAGGGACAATTGGAGAAGTGAATTATATAAATATAAAG
TAGTAAAAATTGAACCACTA---GGAGTAGCACCCACCAAGGCAAAGAGAAGAGTGGTG--------------
-------------------------GAGAGAGGAAAAAGAGCA---GTTGGA---CTAGGAGCT---------
GTCTTCATTGGGTTCTTAGGAGCA---GCAGGAAGCACTATG---GGCGCAGCGTCA---
ATAACGCTGACGGTACAG---GTCAGACAA---TTATTG---TCTGGCATAGTGCGACAGCAAAGC---
AATTTG---CTGAGG---GCTATAGAAGCTCAACAACATCTGTTGAAACTCACGGTCTGGGGCATT
>KU921746..1
TTCTATTGTAACACATCAGGTCTGTTCAACAGCACTTGGGATGATACCGCCATACAGGAAAATATCACGGAGA
TCACGGAG---

TCAAATGACACAATAACTCTCCAATGCAGAATAAGGCAATTTATAAGGATGTGGCAGAGGACAGGACAGGCAA
TGTATGCCCCTCCCATACCAGGAGTAATAAGGTGTGAATCAAAC---
ATTACAGGACTAATATTAACAAGAGATGGTGGGCATAGTAATAGT----------------------------
----

ACTAGTAATGAAACCTTCAGACCTGGAGGAGGAGATATGAGGGACAATTGGAGAAGTGAATTATATAAGTATA
AAGTAGTAAAAATTGAACCACTA---GGAGTAGCACCCTCCAGGGCAAGGAGAAGAGTGGTG-----------
----------------------------GGAAGAGAAAAAAGAGCA---GTTACA---ATAGGAGCC------
---GTCTTCCTTGGGTTCTTAGGAGCA---GCCGGAAGCACTATG---GGCGCGGCGTCA---
ATAACGCTGACGGTACAG---GCCAGGCAA---TTATTG---TCTGGTATAGTGCAACAGCAAAGC---
AATTTG---CTGAAG---GCTATAGAGGCTCAACAACATCTGTTGAGACTCACGGTCTGGGGCATT
>KU921752..1
TTCTATTGTAACACATCAGGCCTGTTTAATAGCACTTGGAATGACACCAGCAATGGGAATGACACCAACATAT
CAAATAGC---
-------------------ACG---
GAGAAGAATGAGAATATAACTCTCCAATGCAGAATAAAGCAAATTATAAATATGTGGCAAAGAGTAGGAAAAG
CAATGTATGCTCCTCCCATCCCAGGAATAATAAAATGTGAATCAAAC---
ATTACAGGACTAATATTAACAAGAGATGGGGGAAAGGAGGAAAAC----------------------------
----

GAAAGTGAAACCTTCAGACCTGGAGGAGGAGATATGAGGGACAATTGGAGAAGTGAATTATATAAGTATAAAG
TAGTAAAAATTGAACCACTA---GGAGTAGCACCCACCAGGGCAAGGAGAAGAGTGGTG--------------
-------------------------GAGAGAGAAAAAAGAGCA---GTTGGA---ATGGGAGCT---------
TTGTTCATTGGGTTCTTAGGAGCA---GCTGGAAGCACTATG---GGCGCGGCGTCG---
ATGACGCTGACGGTACAG---GCTAGACAA---CTATTG---TCTGGCATAGTGCAACAGCAAAGC---
AATTTG---CTGAGG---GCTATAGAGGCTCAACAGCATCTGTTGAAACTCACAGTCTGGGGCATT
>KU921756..1
TTCTATTGTAACACATCAAACCTGTTTAATAGTACTTGGCTTAACAATGGCACATTAGGTAGTGACAATGGCA
TATCA--

GATAGTAAAAACATAACTCTCCCATGCAAAATAAGGCAAATTATCAGAATGTGGCAGAGAGTAGGACAAGCAA
TATATGCCCCTCCCATCCAAGGAGTAATAAGGTGTAAATCAGAC---
ATTACAGGACTAATATTAACAAGAGATGGTGGAGTTAATAACAAT----------------------------
----

ATAACTGAAACCTTCAGACCTGGAGGAGGAGATATGAGGGACAATTGGAGAAGTGAGTTATATAAATATAAAG

```
TAGTAAAAATTGAACCACTA---GGAGTAGCACCCACCAGGGCAAGGAGAAGAGTGGTG--------------
-------------------------GGGAGAGAAAAAAGAGCA---GTTGGA---GTAGGAGCT---------
GTATTCCTTGGGTTCTTAGGAGCA---GCAGGAAGCACTATG---GGCGCGGCGTCA---
ATAACGCTGACGGTACAG---GCCAGACAA---TTATTG---TCTGGCATAGTGCAACAGCAAAGC---
AATTTG---CTAAGG---GCTATAGAGGCTCAACAGCATCTGTTGAAACTCACAGTCTGGGGCATT
>KU921759..1
TTCTATTGCAGTACAAAAGGCCTGTTTAATAGCACTTGGAATGCTAATGGCACCAGGAATGACTTAGGGAGCT
CG-----------------------------------------------------------------------
-------------------------
GAATCAGATGGCACTATAACTCTCCAGTGCAGAATAAAACAAATTATAAATATGTGGCAGAGAACAGGACAAG
CAATGTATGCCCCTCCCATCGCAGGAGTAATAAGTTGTAATTCAACC---
ATTACAGGACTACTATTAACAAGAGATGGTGGGAACAATAGTACA----------------------------
-------------------------------------------------------------------------
----------
AGTGAAACTGAAACCTTTAGACCTGGAGGAGGAGATATGAGGGACAATTGGAGAAGTGAATTATATAAGTATA
AAGTAGTAAAAATTGAACCATTA---GGAGTAGCACCCACCAGGGCGCGGAGAAGAGCGGTG-----------
----------------------------GGGAGAGAAAAAAGAGCA---GTTGGA---CTGGGAGCT------
---GTTTTTCTTGGGTTCTTAGGAGCA---GCAGGAAGCACTATG---GGCGCGGCGGCA---
GTGACGCTGACGGTACAG---GCCAGACAA---TTATTG---TCTGGCATAGTGCAACAGCAAAGC---
AATTTG---CTGAGA---GCTATAGAGGCTCAACAACATCTGTTGAAACTCACTGTCTGGGGCATT
>KU921772..1
TTCTATTGTAACACATCAGGCTTGTTTAATAGCAGTTGGAATAGCACCAGCAAGGAGCTAAATAGCACCAACA
GGGAGCTA-----------------------------------------------------------------
----------------------------
AATAACATGACTGTAACTCTCCAATGTAGAATAAAACAAATTATAAATATGTGGCAGAGAGTGGGACAAGCAA
TGTATGCCCCTCCCATCCCAGGAGTAATAAAGTGTGTATCAAAC---
ATTACAGGATTAATATTAACAAGGGATGGTGGGCTTAATCCTACA----------------------------
-------------------------------------------------------------------------
-------
GAGAACGAAACCGAAACCTTCAGACCTGGAGGAGGAGATATGAGAGACAATTGGAGAAGTGAATTATATATGT
ATAAAGTAGTGAAAATTGAACCACTA---GGAGTAGCACCCACCAGGGCAAAGAGAAGAGTGGTG--------
-------------------------------GAGAGAGAAAAAAGAGCA---GTTGGA---ATAGGAGCT---
------GTTTTCCTTGGGTTCTTGGGAGCA---GCAGGAAGCACTATG---GGCGCGGCGTCA---
ATAACGCTGACGGTACAA---GCCAGACAA---TTATTG---TCTGGCATAGTGCAACAGCAAAGC---
AATTTG---CTGAGG---GCTATAGAGGCTCAACAGCATCTGTTGAAACTCACAGTCTGGGGCATT
>KU921780..1
TTCTATTGTAATACATCAGGCTTATTTAATGGCACTTGGACCAATGCTAGCATACAAGAAAATAGC-------
-------------------------------------------------------------------------
----------------------------
ACGGAGTCAAATATAACTCTCCAATGCAGAATAAAACAAATTATAAATATGTGGCAGACAGTAGGACGAGCAA
TGTATGCTCCTCCCATTGAAGGAGCAATAAGGTGTGTATCAAAC---
ATTACAGGACTAATATTAACCAGAGATGGTGGGGATAACACAACT----------------------------
-------------------------------------------------------------------------
-------------
ACAACTGAAATCTTCAGACCTGGAGGAGGAAATATGAAGGACAATTGGAGAAGTGAATTATATAAGTATAAAG
TAGTAAAAATTGAACCATTA---GGAGTAGCACCCACCAAGGCAAAGAGAAGAGTGGTG--------------
-------------------------GAGAGAGAAAAAAGAGCA---GTTGGA---CTGGGAGCT---------
GTCTTCCTTGGGTTCTTAGGAGCA---GCAGGAAGCACTATG---GGCGCGGCGTCA---
GTAACGCTGACGGTACAG---GCCAGACAA---TTATTG---TCTGGCATAGTGCAACAGCAAAAC---
AATTTG---CTGAGG---GCTATCGAGGCTCAACAACATCTGTTGAAACTCACGGTCTGGGGCATT
>KU921783..1
TTCTATTGTAATACAACAGGCCTGTTTAATAGCACTTGGGGGAATGGCACTTGGAATGCCAGCAGCCAGGGGG
AG-----------------------------------------------------------------------
----------------------------
TCAAATGACACTATACTCCTCCAATGCAAAATAAAGCAAATTATAAATATGTGGCAGAGAGTAGGACAAGCAA
TGTATGCTCCTCCCATCCCAGGAGTAATAAGGTGTGAATCAAAC---
ATTACAGGACTAATATTAACAAGAGATGGTGGGGGGGATGACAAT----------------------------
-------------------------------------------------------------------------
-------------
GGAACCGAAACCTTCAGACCTGGAGGAGGAGATATGAGGGATAATTGGAGAAGTGAATTATATAAGTATAAAG
```

TAGTAAAAATTGAACCACTA---GGAGTAGCACCCACCAGGGCAAGGAGAAGAGTGGTG--------------
-------------------------GAGAGAGAGAAAAGAGCA---GTTACA---ATAGGAGCT---------
GTTTTTCTTGGGTTCTTAGGAACA---GCAGGAAGCACTATG---GGCGCGGCGTCA---
ATAGCGCTGACGGCACAG---GCTAGACAA---TTATTG---TCTGGCATAGTGCAACAGCAAAGC---
AATTTG---CTGAGG---GCTATAGAGGCTCAACAACATATGTTGAGACTCACGGTCTGGGGCATT
>KU921785..1
TTCTATTGTAACACATCAGACCTGTTTAATAGCACTTGGTATGGCAATAACACCATGCAGGGATTAAATGGC-
----

ACAAATGACACTATAATTCTCAAATGCAGAATAAAGCAAATTATAAATATGTGGCAGAGAACAGGACAAGCAA
TATATGCCCCTCCCATCCCAGGAGTAATAGAGTGTGTATCAAAC---
ATAACAGGACTAATATTGACAAGAGATGGTGGGGGTAGTGAGAGC----------------------------
----

AGGACAGAGGAAACATTTAGACCTGGAGGAGGAGATACGAGGGACAATTGGAGAAGTGAATTATATAAGTATA
AAGTAGTAAAAATTGAACCACTA---GGAGTAGCACCCACCAGGGCAAAAAGAAGAGTGGTG-----------
----------------------------GAGAGAGAAAAAAGAGCA---ATTGGA---ATAGGAGCT------
---GTCTTCATTGGGTTCTTAGGAGCA---GCAGGAAGCACTATG---GGCGCGGCGTCA---
ATAACGCTGACGGTACAG---GCCAGACAA---TTATTG---TCTGGTATAGTGCAACAGCAAAGC---
AATTTG---CTGAGG---GCTATAGAGGCTCAACAGCATCTGTTGAAACTCACAGTCTGGGGCATT
>KU921788..1
TTCTATTGTGATACATCAAGCCTGTTTAACAGCACTTGGGAACCAAACAATGTTAGTAGCAAT----------
----

TTAAATGGCACTATAACTCTCCAATGCAGAATAAAGCAAGTCATAAATATGTGGCAGAGAGTAGGACAAGCAA
TGTATGCCCCTCCCATCCCAGGAAACATAACATGTAGATCAAAC---
ATTACAGGAATAATATTAACAAAAGATAAGGATGGTATAAATAAT----------------------------
----

TCAGATGAAACCTTGAGGCCAGGAGGAGGAGATATGAGGGACAATTGGAGAAGTGAATTATATAAGTATAAAG
TGGTAAAAATTGAACCACTA---GGAGTAGCACCCACCAAGGCAAGGAGAAGAGTGGTG--------------
-------------------------GAGAGAGAAAAAAGAGCA---GTTGGA---CTAGGAGCT---------
GTTTTCCTTGGGTTCTTAGGAGCA---GCAGGAAGCACTATG---GGCGCAGCGCCA---
ATAACGCTGACGGTACAG---GCCAGACAA---TTGTTG---TCTGGCATAGTGCAACAGCAAAGC---
AATTTG---CTGAGG---GCTATAGAGGCTCAACAACAGCTGTTGAAGCTCACGGTCTGGGGCATT
>KU921804..1
TTCTATTGTGATACATCAAGCCTGTTTACTGGTACTTGGAATGGTACTACTTGGATGAATAGCACT-------
----

GACACTGAAAATATAACTCTTCCATGCAGAATAAAGCAAATTATAAATATGTGGCAGAGGGTAGGGAGAGCAA
TGTATGCCCCTCCCATCCAAGGAAAAATAAATTGTACATCAAAC---
ATTACAGGACTACTATTAACAAGAGATGGTGGGAAAACTAATAAT----------------------------
----

TCATCTGAAACCTTCAGACCAGGAGGAGGAGATATGAGGGACAATTGGAGAAGTGAATTATATAAGTATAAAG
TAGTAAAGATTGAACCACTA---GGAGTAGCACCCACCAGGGCAAGAAGGAGAGTGGTG--------------
-------------------------GAAAGAGAAAAAAGAGCA---GTTGGA---CTGGGAGCT---------
GTATTCATTGGGTTCTTGGGAGCA---GCAGGGAGCACTATG---GGCGCGGCGTCA---
GTGACGCTGACGGTACAG---GCCAGACGA---TTACTA---TCTGGCATAGTGCAGCAGCAAAGC---
AATTTG---CTGAGG---GCTATAGAGGCTCAACAGCATCTGTTGAAACTCACGGTCTGGGGCATC
>KU921831..3
TTCTATTGTAATACATCACGCCTGTTTAGTTACAGGTGCACGTTCGAAAATGAGACTAATACTTACACCTGCA
CTAAC--
-ACCATGCATGAGTTC---------
AATAGCACGGCAAATATAACTCTCCAATGCAGACTAAAGCAGATCATAAGGATGTGGCAGAGGACAGGACAAG
CAATGTATGCCCCTCCCATCCCAGGGCAAATAATGTGTAGATCAAAC---
ATTACAGGGTTAATATTAACAAGAGATGGTGGGGTTGATAACAATACA-------------------------
----

GATGCTACCGAAATCTTCAGACCTGGAGGAGGAGATATGAAGGACAATTGGAGAAGTGAATTATATAAGTATA

AAGTAGTAAAAATTGAACCACTA---GGAGTAGCACCCACCAGGGCAAGGAGAAGAGTGGTG-----------
----------------------------GGGAGAGAAAAAAGAGCA---GTTGGA---CTGGGAGCT------
---GTTTTCATTGGGTTCTTAGGAGCA---GCAGGAAGCACTATG---GGCGCGGCGTCA---
GTGACGCTGACGGTACAG---GCCAGACAA---TTATTG---TCTGGTATAGTGCAACAGCAAAGC---
AATTTG---CTGAAG---GCTATTGAGGCTCAACAACATCTGTTGAGGCTCACGGTCTGGGGCATT
>KU921835..2
TTCTATTGTAACACATCAGAACTGTTTAATAGCACTTGGAGTTGGAATAACACTTGGAATAACACTCATGAG-
----

CCAAATGGCACTATAACTCTCCAATGCAGAATAAAGCAAATTATAAATATGTGGCAGAGAGTAGGACAAGCAA
TGTATGCCCCTCCCATACAAGGAGAAATAAAGTGTGAATCAAAC---
ATCACAGGACTAATATTAACAAGAGATGGT---ATGGGTAACAATGTA-------------------------
----

TCAAATGAAACCTTCAGACCTGGAGGAGGAGATATGAGGGACAACTGGAGAAGTGAGTTATATAAATATAAAG
TAGTAAAAATTGAACCATTA---GGAGTAGCACCCACTGGGGCAAGGAGAAGGGTGGTG--------------
-------------------------CAGAGAGAAAAGAGAGCG---GTTGGA---ATAGGAGCT---------
GTCTTCCTTGGGTTCTTAGGAGCA---GCAGGAAGCACTATG---GGCGCAGCGTCA---
TTAACGCTGACGGTACAG---GCCAGACAA---TTACTG---TCTGGCATAGTGCAGCAGCAAAGC---
AATTTG---CTGAGG---GCTATAGAGGCTCAACAACATATGTTGAAACTCACGGTCTGGGGCATT
>KU921841..2
TTCTATTGCAACACATCAGGCCTGTTTAATAGCACTGACATGAGTTCAAATAACACAGAG-------------
----

TTAAATGACACTATAAGACTCCCATGCAGAATAAAACAAATTATACGTATGTGGCAGAAAGTAGGACAAGCAA
TGTATGCCCCTCCCATTCAAGGAGTGATAAGATGTAGCTCAAAC---
ATTACAGGAATACTATTAACAAGAGATGGTGGGATAGAAAATGAA----------------------------
----

ACAGAAATCTTCAGACCTGGAGGAGGAAACATGAAGGACAATTGGAGAAGTGAATTATATAAGTATAAAGTAG
TAAAAATTGAACCACTA---GGAGTAGCACCCACCTCGGCAAGGAGAAGAGTGGTG-----------------
----------------------GAGAGAGAAAAAAGAGCA---GTTGGA---CTGGGAGCT---------
GTATTCATTGGGTTCTTAGGAGCA---GCAGGAAGCACTATG---GGCGCGGCGTCA---
ATGACGCTGACGGTACAA---GCCAGACAA---TTATTG---TCTGGCATAGTGCAACAGCAAAGC---
AATTTG---CTGAGG---GCTATAGAGGCTCAACAGCATCTGTTGAAACTCACGGTCTGGGGCATC
>KU921852..1
TTCTATTGTAATACATCAGGCTTGTTTAATAGCACTTGGAATGACACTGTCATGTTTAATAGCACTGGGAATG
ACACTGTC---
-------------GGCAGG------
GAGTCAAATGAAACTATAACACTCCAATGCAGGATAAAACAAATTATAAATATGTGGCAGAGAACAGGACAAG
CAATATATGCCCCTCCTATCCCAGGAGTAATAAGGTGTGTATCAAAC---
ATTACAGGACTAATATTAACAAGAGATGGTGGGAATACGGCAGGGACA-------------------------
----

GGTTCTGAAACCTTCAGACCTGGAGGAGGAGATATGAGGGACAATTGGAGAAGTGAATTATATAAGTATAAAG
TAGTAAAAGTTGAACCACTA---GGAGTAGCACCCACCAGGGCACAGAGAAGAGTGGTG--------------
-------------------------CAGAGGGAAAAAAGAGCA---GTTGGA---ATAGGAGCT---------
GTCTTCATTGGGTTCTTAGGAGCA---GCAGGAAGCACTATG---GGCGCGGCGTCA---
ATAACGCTGACGGCACAG---GCCAGACAA---TTATTG---TCTGGTATAGTGCAACAGCAAAGC---
AATTTG---CTGAGG---GCTATAGAGGCTCAACAGCATCTGTTGAAACTCACAGTCTGGGGCATC
>KF716474..1
TTCTATTGTGACACATCAGGCCTGTTTAATAGCACTTGGAATGGTACGGCCAGCAATGTCAGCATACCTGACA
CGGTG--

ACAAATGGCACTATAACACTCCCATGCAAAATAAAGCAAATTATAAATATGTGGCAGAGAGTAGGACAAGCAA
TGTATGCCCCTCCCATTGCAGGAGTAATAAGGTGTGATTCAAAC---
ATTACAGGGATGATATTAACAAGAGATGGTGGTGAT-------------------------------------
----

AACAGGACAGAAACCTTCAGACCTGGAGGAGGAGATATGAGGGACAATTGGAGAAGTGAATTATATAAATATA

AAGTAGTAAAAGTTGAACCACTA---GGAGTAGCACCTACCAGGGCAAGAAGAAGAGTGGTG-----------
----------------------------GAGAGAGAAAAAAGAGCA---GTAGGA---ATAGGAGCT------
---GTTTTCCTTGGGTTCTTAGGAGCA---GCAGGAAGCACTATG---GGCGCAGCGTCA---
ATAACGCTGACGGTACAG---GCCAGACAA---TTATTG---TCTGGTATAGTGCAACAGCAAAGT---
AATTTG---CTGAGG---GCTATAGAGGCTCAACAACAGCTGTTGAAACTCACAGTCTGGGGCATT
>KF716475..1
TTCTACTGTAATACATCAGGCCTGTTTAATAGCACTTGGTCCCTTAACAACACTTGGTCCCTTAACAGCACGC
AGTCAAAT---
-------------GACACA------
GGGATAAATGATACCATAACTCTCCCATGCAGAATAAAGCAAATTATAAATATGTGGCAGAGAGTAGGACAAG
CAATGTATGCCCCTCCCATCCAAGGAGTAATACAGTGTGTATCAAAC---
ATTACAGGACTACTGTTAACAAGAGATGGGGGGAATGTGAATAGCAGT-------------------------
----

AATGGCAATGAAATCTTCAGACCTGGAGGAGGAGATATGAGGGACAATTGGAGAAGTGAATTATATAAGTATA
AAGTAGTAAAAATTGAACCACTA---GGAGTAGCACCCACCAGGGCAAAGAGAAGAGTGGTG-----------
----------------------------GGGAGAGAAAAAAGAGCA---ATTGGTGGGCTAGGAGCT------
---GTTTTCCTTGGGTTCTTAGGAATG---GCAGGAAGCACTATG---GGCGCGGCGTCA---
ATGACGCTGACGGTACAG---GCCAGAAAC---TTATTG---TCTGGCATAGTGCAACAGCAAAGC---
AATTTG---CTGAGG---GCTATAGAGGCTCAACAACATCTGTTGAAACTCACAGTCTGGGGCATT
>AY322184..1
TTCTATTGTAACACATCAGATCTGTTTAATGAGTCAAATAGTACGGAG-------------------------
----

TTAAACGACACTATAACTCTCCAATGCAGAATAAAGCAAATTATAAATATGTGGCAGAGAGCAGGACAAGCAA
TATATGCCCCTCCCATCCCAGGAGTAATAAGGTGTGTATCAAAC---
ATTACAGGACTATTGTTAACAAGGGATGGTGGGATTAATACCAGT----------------------------
----

ACAAGTGAAACCTTCAGACCTGGAGGAGGAGATATGAGGGACAATTGGAGAAGTGAATTATATAGGTATAAAG
TAGTAAAAATTGAACCACTA---GGAGTAGCACCCACCAGGGCAAAGAGAAGAGTGGTG--------------
-------------------------GAGAGAGAAAAAAGAGCA---GTTGGA---ATAGGAGCT---------
GTTTTCATTGGGTTCTTAGGAGCA---GCAGGAAGCACTATG---GGCGCGGCGTCA---
ATAACGCTGACGGTACAG---GCCAGACAA---TTATTG---TCTGGCATAGTGCAACAGCAAAGC---
AATTTG---CTGAGG---GCTATAGAGGCTCAACAACATCTGTTGAAACTCACGGTCTGGGGCATT
>AF539405..1
TTCTATTGTAACACATCAGGCCTGTTTAATAGCACTTGGAATGGCAGTGAGGCAAATAGCACG----------
----

CCACACGAGAATATAACTCTCCAATGCAGAATAAAGCAAATTATAAATATGTGGCAGAGAGCAGGACAAGCAA
TATATGCCCCTCCCATCCCAGGAGTAATAAAATGTAATTCAAAC---
ATTACAGGACTAATATTAACAAGAGATGGTGGGGTTAATAGCAGT----------------------------
----

GCAAATGAAACCTTCAGACCTGGAGGAGGAGATATGAGGGACAATTGGAGAAGTGAATTATATAAGTATAAAG
TAGTAAAAATTGAACCACTA---GGAGTAGCACCCACCAGGGCAAAGAGAAGAGTGGTG--------------
-------------------------GAGAGAGAAAAAAGAGCA---GTTGGA---ATAGGAGCT---------
GTCTTCATTGGGTTCTTAGGAGCA---GCAGGAAGCACTATG---GGCGCGGCGTCA---
ATAACGCTGACGGTACAG---GCCAGACAA---TTATTG---TCTGGCATAGTGCAACAGCAAAGC---
AATTTG---CTGAGG---GCTATAGAGGCTCAACAACATCTGTTGAAACTCACGGTCTGGGGCATT
>L22943..1
TTCTATTGTAATTCATCAGGCCTGTTTAATAGCACTTGGACCAACATGCAGGAGTCAAATAGCACGGAG----
----
----------------------------TCAAATGACACTATAACTCTCCCATGCAGAATA---
AAAATTATAAACATGTGGCAGAGAGCAGGACAAGCAATGTATGCCCCTCCCATCCAAGGAATAATAAAGTGTG
TATCAAAC---ATTACAGGACTAATATTAACAAGAGATGGTGGGATAATAACAGT------------------
----

GAAAGTGAAACCTTCAGACCTGGAGGAGGAGATATGAGGGACAATTGGAGAAGTGAATTATATAAGTATAAAG
TAGTAAAAATTGAACCACTA---GGAGTAGCACCCACCAAGGCAAAGAGAAGAGTGGTG--------------

-------------------------GAGAGAGAAAAAAGAGCA---GTTGGA---ATAGGAGCT---------
GTCTTCCTTGGGTTCTTAGGAGCA---GCAGGAAGCACTATG---GGCGCGGCGTCA---
ATAACGCTGACGGTACAG---GCCAGACAA---TTATTG---TCTGGCATAGTGCAACAGCAAAGC---
AATTTG---CTGAGG---GCTATAGAGGCTCAACAACATCTGTTGAAACTCACGGTCTGGGGCATT
>KX168075..1
TTCTATTGTAACACATCAGGCCTGTTTAATAGCACTTGGGAGTTTAATAGCACTTGGGAGTTTAATAGCACTT
GGGAGTTT---
AATAGCACTTGGAATAATTCA------------
AATAGCACAGAGAATATAACTCTCCAATGCAGAATAAAGCAAATTGTAAATATGTGGCAAAGAGCAGGACAAG
CAATATATGCCCCTCCCATACCAGGAGTAATAAGGTGTGAATCAAAT---
ATTACAGGACTAATATTAACAAGAGATGGTGGGGATAATAATAGTACAAAT----------------------
----

AATACAAGTGAAACCTTCAGACCTGGAGGAGGAGATATGAGGGACAATTGGAGAAGTGAATTATATAAGTATA
AAGTAGTAAAAATTGAACCAATA---GGAGTAGCACCCACCAGGGCAAAAAGAAGAGTGGTG-----------
----------------------------GAGAGAGAAAAAAGAGCA---GTTGGA---ATAGGAGCT------
---GTCTTCATTGGGTTCTTAGGAGCA---GCAGGAAGCACTATG---GGCGCAGCGTCA---
GTAACGCTGACGGTACAG---GCCAGACAG---TTATTG---TCTGGTATAGTGCAACAGCAAAGC---
AATTTG---CTGAGG---GCTATAGAGGCTCAACAGCATCTGTTGAAACTCACGGTCTGGGGCATT
>AF004885..2
TTCTATTGTAATACATCAGGCTTGTTTAATAGCACTTGGTATGTTAATAGCACTTGGAATGACACTGACAGCA
CTCAG--

GAGTCAAATGACACTATAACTCTCCCATGCAGAATAAAGCAAATTATAAATATGTGGCAGAGAGCAGGACAAG
CAATGTATGCCCCTCCCATCCCAGGAGTAATAAAGTGTGAATCAAAC---
ATTACAGGACTACTATTAACAAGGGATGGTGGAAAGGATAACAAT----------------------------
----

GTAAATGAAACCTTCAGACCTGGAGGAGGAGATATGAGGGACAATTGGAGAAGTGAATTATATAAGTATAAAG
TAGTAGAAATTGAACCACTA---GGAGTAGCACCTACCAGGGCAAAGAGAAGAGTGGTG--------------
-------------------------GAGAGAGAAAAAAGAGCA---GTTGGA---ATAGGAGCT---------
GTCTTCCTTGGGTTCTTAGGAGCA---GCAGGAAGCACTATG---GGCGCAACGTCA---
ATAACGCTGACGGTACAG---GCCAGACAA---TTATTG---TCTGGCATAGTGCAACAGCAAAAC---
AATTTG---CTGAGG---GCTATAGAGGCTCAACAACATCTGTTGAAACTCACGGTCTGGGGCATT
>AF407151..2
TTCTATTGTAACACATCAGGCCTGTTTAACAGAGCTTATGGCAATGTCAGCAAGCAAGAGCCAAAT-------
----

AGCTCCGGCAATATAACACTCCAATGCAGAATAAAGCAAGTTATAAATATGTGGCAGAGAGCAGGACAAGCAA
TATATGCCCCTCCCATACAAGGAGTAATAAGATGTGACTCAAAT---
ATTACAGGACTAATATTAACAAGAGATGGTGGGAAAGATAGTAAT----------------------------
----

ATAACTGAAACATTCAGACCTGGAGGAGGAGATATGAGGGACAATTGGAGAAGTGAATTATATAAGTATAAAG
TAGTAAAAATTGAACCACTA---GGAGTAGCACCCACCAGGGCAAAGAGAAGAGTGGTG--------------
-------------------------GAGAGAGAAAAAAGAGCA---GTTGGA---ATAGGAGCT---------
GTCTTCATTGGGTTCTTAGGAGCA---GCAGGAAGCACTATG---GGCGCGGCGTCA---
ATAACGCTGACGGCACAG---GCCAGAAAA---TTATTG---TCTGGCATAGTGCAACAGCAAAGC---
AATTTG---CTGAGG---GCTATAGAGGCTCAACAACATCTGTTGAAACTCACGGTCTGGGGCATT
>AF407162..2
TTCTATTGCAATACAACAAACCTGTTTAATAGCACTTGGAATCACACTGCCAGCATGAATAGCACAGAG----
----

TCAAATGACACTATAATTCTCCCATGCAGAATAAAACAAATTATAAATATGTGGCAGAGAGTAGGACAAGCAA
TGTATGCCCCTCCCATTCGAGGAGTAATAAGGTGTGAATCAAAC---
ATTACAGGACTAATATTAACAAGAGATGGTGGGAATACTACCGGT----------------------------
----

ACAAGTGAAATCTTCAGACCTGGAGGTGGAGATATGAGGGACAATTGGAGAAGTGAATTATATAAGTATAAAG
TAGTAAAAATTGAACCACTA---GGAGTAGCACCCACCAGGGCAAAGAGAAGAGTGGTG--------------

-------------------------GAGAGAGAAAAAAGAGCA---GTTGGA---ATAGGAGCT---------
GTCTTCATTGGGTTCTTAGGAGCA---GCAGGAAGCACTATG---GGCGCGGCGTCA---
ATAACGCTGACGGTACAG---GCCAGACAA---TTATTG---TCTGGCATAGTGCAACAGCAAAGC---
AATTTG---CTGAGG---GCTATAGAGGCTCAACAGCATCTGTTGAAACTCACGGTCTGGGGCATT
>AF407156..1
TTCTATTGCAATACATCAAAACTGTTTAATAGCAGGTGGGAGAACAATGGCACCGCCAACATGTTGAAGAATG
ACACA--
-------------------GGG---
TCAAATGAGACTACTCTGATTCTCCGATGCAGAATAAAACAAATTATAAATATGTGGCAGAGAGTAGGACAAG
CGATGTATGCCCCTCCCATCCAAGGAGTAATAAATTGTACATCAAAC---
ATCACAGGACTAATATTAACAAGAGATGGTGGTGAAAACGAT----------------------------
----

ACAGAGACCTTCAGACCTGGAGGAGGAGATATGAGGGACAATTGGAGAAGTGAATTATATAAGTATAAAGTAG
TAAAACTTGAACCACTA---GGAGTAGCACCCACCATGGCAAAGAGAAGAGTGGTG-----------------
----------------------GAGAGGGAAAAAAGAGCA---GTTGGA---ATGGCAGCT---------
GTCTTCCTTGGGTTCTTGGGAACA---GCAGGGAGCACTATG---GGCGCGGCGTCA---
CTAACGCTGACGGTACAG---GCCAGACAA---TTATTG---TCTGGCATAGTGCAACAGCAAAGC---
AATTTG---CTGAAG---GCTATAGAGGCTCAACAGCATCTGTTGAGACTCACGGTCTGGGGCATT
>KT008652..1
TTCTATTGTAACACATCAGGCCTGTTTAATAGCACTTGGGGGCACAATGCCAGCAGGCAGGAGAATAGCACGG
AG---

TCAAGTGACACTATAACTCTCCCATGCAAAATAAAGCAAATTATAAATATGTGGCAGAGAGCAGGGCAAGCAA
TATATGCCCCTCCCATCCCAGGAGTAATAAGGTGTGAATCAAAC---
ATTACAGGACTAATATTAACAAGAGATGGTGGGAACAACAACAGT----------------------------
----

ACAAGTGAAACCTTCAGACCTGGAGGAGGAAATATGAGGGACAATTGGAGAAGCGAATTATATAAGTATAAAG
TAGTAAAAATTGAACCACTA---GGAGTAGCACCCACCAGGGCAAAGAGAAGAGTGGTG--------------
-------------------------GGGAGAGAAAAAAGAGCA---GTTGGA---ATAGGAGCT---------
GTTTTCCTTGGGTTCTTAGGCGCA---GCAGGAAGCACTATG---GGCGCAGCGTCA---
GTAACGCTGACGGTACAG---GCCAGACAA---TTATTG---TCTGGCATAGTGCAACAGCAAAGC---
AATTTG---CTGAGG---GCTATAGAGGCTCAACAACATCTGTTGAAACTCACGGTCTGGGGCATT
>AF407157..2
TTCTATTGTAACACATCAGGCCTGTTTAATGGCATTTGGAATGGCACGCAGGAGCCAAATAGAACGGAG----
----

TCAAACGATACTATAACACTCCAATGCAGAATAAAGCAAATTATAAATATGTGGCAAAGAGTAGGACAAGCAA
TATATGCCCCTCCCATTCAAGGAGAAATAAGGTGTGAGTCAAAC---
ATTACAGGACTAATATTAACAAGAGATGGTGGGATAATTAACGGT----------------------------
----

ACAGAGGAGACCTTCAGACCTGGAGGAGGAGATATGAGGGACAATTGGAGAAGTGAATTATATAAGTATAAAG
TAGTAAAGATTGAACCATTA---GGAGTAGCACCCACCAAGGCAAAGAGAAGAGTGGTG--------------
-------------------------GAGAGGGAAAAAAGAGCA---GTTGGA---TTCGGAGCT---------
TTCTTCCTTGGGTTCTTAGGAGCA---GCAGGAAGCACTATG---GGCGCGGCGTCA---
ATAACGCTGACGGTACAG---GCTAGACAA---TTATTG---TCTGGCATAGTGCAACAGCAAAAC---
AATTTG---CTGAGG---GCTATAGAGGCTCAACAGCATCTGTTGAAACTCACGGTCTGGGGCATT
>FJ866112..1
TTCTATTGTAATACATCAGAACTATTTAATAGCACTTGGACTTGGAAAGGCAATAATGACAGCATGCAGGGGT
TAAATAGC---
-------------------ACG---
GGGTTAAACAACACTATAACTCTCCACTGCAGCATAAAGCAAATTATAAATATGTGGCAGAGAGTAGGACAAG
CAATGTATGCCCCTCCCATCCAAGGAGTAATAAAGTGTGAATCAAAC---
ATTACTGGACTACTATTAACAAGAGATGGTGGGAATAATAACATA----------------------------
----

AACGAGACCCTCAGACCTGGAGGAGGAGATATGAGGGACAATTGGAGAAGTGAATTATATAAGTATAAAGTAG
TAAAAATTGAACCACTA---GGAGTAGCACCCACCAAGGCAAAGAGAAGAGTGGTG-----------------

----------------------GAGAGAGAAAAAAGAGCA---GTTGGA---ATAGGAGCT---------
GTCTTCCTTGGGTTCTTAGGAGCA---GCAGGAAGCACTATG---GGCGCAGCGTCA---
ATAACGCTGACGGCACAG---GCCAGAATA---TTATTG---TCTGGCATAGTGCAGCAGCAAAGC---
AATTTG---CTGAAG---GCTATAGAGGCTCAACAGCATCTGTTGAGGCTCACGGTCTGGGGCATT
>AY322190..2
TTCTATTGTAATACATCAGGCCTGTTTAATAGCATTTGGAATCACACTAATGGCACTTGGAATAGCACGGAGT
TAAAT--
-------------------GGC---
AATATAACTCTCAATATAACTCTCCCATGCAGAATAAACCAAATTATAAATATGTGGCAGAGAGTAGGACAAG
CAATATTTGCCCCTCCCATCCAAGGAGTAATACAGTGTGTATCAAAC---
ATTACAGGACTAATATTAACAAGAGATGGAGGGAATAATAACACT----------------------------
----

ACAAATGAAACCTTCAGACCTGGAGGAGGAGATATGAGGGACAATTGGAGAAGTGAATTATATAAGTATAAAG
TAGTAAAAATTGAACCACTA---GGAGTAGCACCCACCAGGGCACAGAGAAGAGTGGTG--------------
-------------------------CAGAGAGAAAAAAGAGCA---ATTGGA---ATGGGAGCT---------
GTCTTCATTGGGTTCTTAGGAGCA---GCAGGAAGCACTATG---GGCGCGGCGTCA---
ATAACGCTGACGGTACAG---GCCAGACAA---TTATTG---TCTGGTATAGTGCAACAGCAAAGC---
AATTTG---CTGAGG---GCTATAGAGGCTCAACAGCATCTGTTGAAACTCACGGTCTGGGGCATT
>AY322193..1
TTCTATTGTAACACATCAAGCCTATTTAATGGCACTTGGACGGCAATGACAGCAGGCAGGAGTCAAAT----
----

AGCAGCGACACTATAACTCTTCCATGCAGAATAAAACAAATTATAAATATGTGGCAGAGGGTAGGACAAGCAA
TATATGCCCCTCCCCTACAAGGAGTAATCAGATGTGATTCAAAT---
ATTACAGGACTAATCTTAACAAGAGATGGTGGGAGTCCGGATAAT----------------------------
----

GAAACCTTCAGACCTGTTGGAGGAGATATGAGGGACAATTGGAGAAGTGAATTATATAAGTATAAAGTAGTAA
AAATTGAACCATTA---GGAGTAGCACCCACCAGGGCAAAGAGAAGAGTGGTG--------------------
-------------------GAGAGAGAAAAAAGAGCA---GTTGGA---ATAGGAGCT---------
GTCCTCATTGGGTTCTTAGGAGCA---GCAGGAAGCACTATG---GGCGCGGCGTCA---
ATAACGCTGACGGTACAG---GCCAGACAA---TTATTG---TCTGGCATAGTGCAACAGCAAAGC---
AATTTG---CTGAGG---GCTATAGAGGCTCAACAGCATCTGTTGAAACTCACGGTCTGGGGCATT
>FJ396018..1
TTTTATTGTAATACATCAGGCCTGTTTAATAGTACTTGGGATGACAATAATACTAGGAATAATGAGACTGCC-
----

AGAAATAATACTATAACTCTCCCATGTAGAATAAAGCAAATTATAAATATGTGGCAGAGGGTAGGACAAGCAA
TGTATGCCCCTCCCATCAAAGGAGTAATAAGGTGTGAATCAAAC---
ATTACAGGACTAATATTAACAAGAGATGGTGGACTTAATAACAAT----------------------------
----

ACAGAGACCTTCAGACCTGGAGGAGGAGATATGAGGGACAATTGGAGAAGTGAATTATATAAGTATAAAGTAG
TAAAAATTGAACCACTA---GGAGTAGCACCCACCAGGGCAAGGAGAAGAGTGGTG-----------------
----------------------GGAAGAGAAAAAAGAGCA---GTTGGA---ATAGGAGCT---------
GTCTTCCTTGGGTTCTTAGGAGCA---GCAGGAAGCACTATG---GGCGCGGCGTCA---
ATAACGCTGACGGTACAG---GCCAGACAA---TTATTG---TCTGGCATAGTGCAACAGCAAAGC---
AATTTG---CTGAGG---GCTATAGAGGCTCAACGACATCTGTTGAAACTCACGGTCTGGGGCATT
>AY736811..1
TTCTATTGTAATACATCAGGCCTGTTTAATAGCACATATAGCGGGAACGAAACTGTCAGCAATAGC-------
----

ACAGAGTACAACATAACTCTCCCATGCAAAATAAAGCAAATTATAAATATGTGGCAGAGAACAGGACAAGCAA
TGTATGCCCCTCCCATCCAAGGAGTAATAAGCTGTGTGTCAAAC---
ATTACAGGACTACTATTAACAAGAGATGGTGGTAATGGTGGTAATAAC-------------------------
----

AATACAACTGAGACCTTCAGACCTGGAGGAGGAAATATGAAGGACAATTGGAGAAGTGAATTATATAAGTATA
AAGTAGTAAAAATTGAACCACTA---GGAGTAGCACCCACCAGGGCAAAGAGAAGAGTGGTG-----------

----------------------------GAGAGAGAAAAAAGAGCA---GTTGGA---CTGGGAGCT------
---GTATTCCTTGGGTTCTTAGGAGCA---GCAGGAAGCACTATG---GGCGCGGCGTCC---
ATAACGCTGACGGTACAG---GCCAGACAA---TTACTG---TCTGGCATAGTGCAACAGCAAAGC---
AATTTG---CTGAGG---GCTATAGAGGCTCAACAGCATCTGTTGAAACTCACGGTCTGGGGCATT
>AY736814..1
TTCTATTGCAACACATCAGGCCTGTTTAATAGCACTTGGGATGATAACATTAAGAATGATACTAACAGCACAC
AGGGG--

TCAAATAGCACGGAGATAATTCTCCCATGCAAAATAAAGCAAATTGTAAGGATGTGGCAGAGAACAGGACAAG
CAATGTATGCTCCTCCAATCCCAGGAGTAATAAGGTGTGTATCAAAC---
ATTACAGGACTAATACTAACAAGAGATGGTGGGAATACTAGCAGC----------------------------
----

ATAAATGAAACCTTTAGACCTGGAGGAGGAGATATGAGGGACAACTGGAGAAGTGAATTATATAAGTATAAAG
TAGTAAAAATTGAACCACTA---GGAGTGGCACCCACCAAGGCAAGGAGAAGAGTGGTG--------------
-------------------------GAGAGAGAAAAAAGAGCA---GTTGGA---ATAGGAGCT---------
GTCCTCTTTGGTTTCTTAGGAGCA---GCCGGAAGCACTATG---GGCGCGGCGTCA---
ATAACGCTGACGGTACAG---GCCAGACAA---TTATTG---GCTGGCATAGTGCAACAGCAAAGC---
AATTTG---CTGAGG---GCTATAGAGGCTCAACAGCAAATGTTGAGACTCACGGTCTGGGGCATT
>AF457075..1
TTCTATTGTGATACATCAAGCCTGTTTAATGGCACTTTAGAGGCCAATGCCACCGTGCAAAACTTCAAT----
----

AGCACAGAGCATATAATTCTCCCATGCAAAATAAAGCAAATTATAAATATGTGGCAGAGAGCAGGACAAGCAA
TGTATGCCCCTCCCATCCAAGGAGTAATAAATTGTGTGTCAAAC---
ATTACAGGACTAATATTAACAAGAGATGGTGGGGATAATAATAAT----------------------------
----

AGTACAAATGAAACATTCAGACCTGGAGGAGGAGATATGAGGGACAATTGGAGAAGTGAATTATATAAGTATA
AAGTAGTAAAAATTGAACCACTA---GGAGTAGCACCAACCAAGGCAAAAAGAAGAGTGGTG-----------
----------------------------GAGAGAGAAAAAAGAGCA---GTAATTGGACTGGGAGCT------
---GTTTTCATTGGGTTCTTAGGAGCA---GCAGGAAGCACTATG---GGCGCGGCGTCA---
ATAACGCTGACGGTACAG---GCCAGACAA---TTATTG---TCTGGTATAGTGCAACAGCAAAGC---
AATTTG---CTGAGG---GCTATAGAGGCTCAACAACATCTGTTGAAACTCACGGTCTGGGGCATT
>DQ208458..1
TTCTATTGTAACACATCAGGCCTGTTCAATAGCACTTGGATTAGCAATACCAGCGTGCAGGGGTCAAATAGCA
CG---

GGGTCAAATGACAGTATAACTCTCCCATGCAGAATAAAGCAAATTATAAATATGTGGCAGAGAATAGGACAAG
CAATGTATGCCCCTCCCATCCAAGGAGTAATAAGATGTGTATCAAAC---
ATTACAGGGCTAATATTAACAAGAGATGGTGGGAGTACTAATAGT----------------------------
----

ACAACTGAAACCTTCAGACCTGGAGGAGGAGATATGAGGGACAATTGGAGAAGTGAATTATATAAGTATAAAG
TAGTAAAAATTGAACCACTA---GGAGTAGCACCCACCAGGGCAAAGAGAAGAGTGGTG--------------
-------------------------GGGAGAGAAAAAAGAGCA---GTTGGA---ATAGGAGCT---------
GTCTTCCTTGGGTTCTTAGGAGCA---GCAGGAAGCACTATG---GGCGCGGCGTCA---
ATGACGCTGACGGTACAG---GCCAGAAAT---TTATTA---TCTGGCATAGTGCAACAGCAAAGC---
AATTTG---CTGAGG---GCTATAGAGGCTCAACAACATCTGTTGAAACTCACGGTCTGGGGCATT
>DQ208467..2
TTCTATTGTAATACATCAGGCCTGTTTAATAGCAATTGGAATTACACTAACAGCACGCAGGAGTCAAATAGCA
CG---

GAGTCAAATGGCACTATAACTCTCCAATGCAGAATAAAGCAAATTATAAATATGTGGCAGAGAGTAGGACAAG
CAATGTATGCCCCTCCTATCCCAGGAGTAATAAGGTGTGTATCAAAC---
ATTACAGGACTAATATTAACAAGAGATGGCGGGGACAACAACAGT----------------------------
----

ATAACTGAAACCTTCAGACCTGGAGGAGGAGATATGAGGGACAATTGGAGAAGTGAATTATATAAGTATAAAG
TAGTAAAAATTGAACCACTA---GGAGTAGCACCCACCAGGGCAAGGAGAAGAGTGGTG--------------

-------------------------GGAAGAGAAAAAAGAGCA---GTTGGA---ATAGGAGCT---------
GTCTTCATTGGGTTCTTAGGAGCA---GCCGGAAGCACGATG---GGCGCGGCGTCA---
ATAACGCTGACGGTACAG---GCCAGACAA---TTATTG---TCTGGCATAGTGCAACAGCAAAGC---
AATTTG---CTGAGG---GCTATAGAGGCTCAACAACATCTGTTGAAACTCACGGTCTGGGGCATT
>DQ208448..1
TTCTATTGTAACACATCAGACTTGTTTAATAGCACTTGGTATGGGAATGGGACCAGCATGCGGGAGTCAAATA
GCACG--

GAGTCAAACGACACTATAATTCTCCGATGCAGAATAAAGCAAATTATAAATATGTGGCAGAGAGTAGGACAAG
CAATATATGCCCCTCCCATCCCAGGAGTAATAAACTGTGTATCAAAC---
ATCACAGGACTAATATTAACAAGAGATGGTGGGAATAATAATAAT----------------------------
----

GAAACTGAAACCTTCAGACCTGGGGGAGGAGATATGAGGGACAATTGGAGAAGTGAATTATATAAGTATAAAG
TAGTAAAAATTGAACCACTA---GGAGTAGCACCCACCAGGGCAAGGAGAAGAGTGGTG--------------
-------------------------GAGAGAGAAAAAAGAGCA---GTAGTTGGAATAGGAGCT---------
GTCTTCCTTGGGTTCTTAGGAGCA---GCAGGAAGCACTATG---GGCGCGGCGTCA---
ATAACGCTGACGGTACAG---GCCAGACAA---TTATTG---TCTGGCATAGTGCAACAGCAAAGC---
AATTTG---CTGAGG---GCTATAGAGGCTCAACAACATCTGTTGAAACTCACAGTCTGGGGCATT
>KU749411..1
TTCTATTGTAATACAACAGGCCTATTTAATAGTACTTGGAATACAACAGGCCCATTTAATAATACTTGGCCT-
----

GATGATGTCAATATAGTACTCCCATGCAAAATAAAGCAAATTATAAATGTATGGCAGAGAGTAGGACAAGCAA
TATATGCCCCTCCCATCCAAGGACTAATACAATGTAACTCAAAT---
ATTACGGGGCTACTATTAACAAGAGATGGTGGGAATAATGGCACA----------------------------
----

CTTAATGAAACCTTCAGACCTGGAGGAGGAAATATGAAGGACAATTGGAGAAGTGAACTGTATAAATATAAAG
TAGTAAAAATTGAACCACTA---GGAGTAGCACCCACCAGGGCAAAGAGAAGAGTGGTG--------------
-------------------------GAGAGAGGAAAAAGAGCA---ATTGGA---ATAGGAGCT---------
GTGCTCATTGGGTTCTTGGGGGCA---GCCGGAAGCACTATG---GGCGCGGCGTCT---
GTAACGCTGACGGTACAG---GCCAGACAA---TTATTG---TCTGGCATAGTGCAACAGCAAAGC---
AATTTG---CTGAGG---GCTATAGAGGCTCAGCAACAGATGCTGAAACTCACAGTCTGGGGCATT
>KX232594..1
TTCTATTGTAATACAACAGGACTGTTTAATAGTACTTGGTATAAAAATGGAACGACTGAGGGAACAAATAGCA
CGGGG--

TCAAATGACACTATAACACTCCCATGCAGAATAAAGCAAGTTATAAATGTATGGCAGAGAGTAGGACAAGCAA
TATATGCCCCTCCCATCCCAGGGATAATAAGGTGTGAATCAAAT---
ATTACGGGGCTACTATTAACAAGAGATGGTGGAAATAATACAAGT----------------------------
----

AATGAAACCTTCAGACCTGGAGGAGGAAATATGAAGGACAATTGGAGAAGTGAACTGTATAAGTATAAAGTAG
TAAAACTTGAACCACTA---GGAGTAGCACCCACCAAAGCAAAGAGAAGAGTGGTG-----------------
----------------------GAGAGAGAAAAAAGAGCA---ATTGGA---TTTGGAGCT---------
TTCTTCCTTGGGTTCTTGGGAGCA---GCTGGAAGCACTATG---GGCGCGGCGTCC---
GTAACGCTGACGGTACAG---GCCAGACAA---TTATTG---TCTGGCATAGTGCAACAGCAAAGC---
AATTTG---CTGAGG---GCTATAGAGGCTCAGCAGCATATGCTGAAACTCACGGTCTGGGGCATT
>KX232597..1
TTCTATTGTAATACAACAGGTCTGTTTAATAGTACTTGGCCTAGTAATGAGACTAAGGAAGTAAATAACACGG
AG---

TCAAATGACATTATCACACTCCCATGCAGAATAAAGCAAATTGTAAATGTATGGCAAAGAGTAGGACAAGCAA
TATACGCCCCTCCCATCAAAGGACTAGTACAGTGTAATTCAAGT---
ATTACGGGGCTACTATTAAACAGAGATGGTGGGAATAATAACACA----------------------------
----

GATGAAACCTTCAGACCTGGAGGAGGAAATATGAAGGACAATTGGAGAAGTGAACTATATAAGTATAAAGTAG
TAAAAATTGAACCACTA---GGAGTAGCACCCACCAGGGCAAGGAGAAGAGTGGTG-----------------

----------------------GAGAGAGAAAAAAGAGCA---GCTGGA---CTAGGAGCT---------
TTATTCATTGGGTTCCTGGGAGCA---GCCGGAAGCACTATG---GGCGCAGCGTCT---
GTAACGCTGACGGTACAG---GCCAGACAA---TTATTG---TCTGGCATAGTGCAACAGCAAAGC---
AATTTG---CTGAGG---GCTATAGAGGCTCAGCAGCAGATGCTGAGACTCACAGTCTGGGGCATT
>KX232609..1
TTCTATTGTGATACAACAAACCTGTTTAATAGTACTTGGTTTGATAATGGGACTACTGAAGCAAATAGCACGG
GG---

TCAAGTGACATTATAACACTCCCATGCAGAATAAAACAAATTGTAAATGTATGGCAGAGAGTAGGACAAGCAA
TATATGCCCCTCCCATCAGAGGACTAATAAAGTGTGAATCAAAT---
ATTACGGGGCTACTATTAACCAGAGATGGTGGGCATAATAGCACAAATGCA----------------------
----

AGTAGTGAAACCTTCAGACCTGGAGGAGGAAATATGAAGGACAATTGGAGAAGTGAACTATATAAGTATAAAG
TAGTAAAAATTGAACCACTA---GGAGTAGCACCCACCAGAGCAAGGAGAAGAGTGGTG--------------
-------------------------GAGAGAGAAAAAAGAGCA---ATTGGA---ATAGGAGCT---------
GTACTCTTTGGGTTCTTGGGAGCA---GCCGGAAGCACTATG---GGCGCGGCGTCC---
ATAACGCTGACGGTACAG---GCCAGACAA---TTATTG---TCTGGCATAGTGCAACAGCAAAGC---
AATTTG---CTGAGG---GCTATAGAGGCTCAGCAGCATCTGCTGAAACTCACGGTCTGGGGCATT
>KX232613..1
TTCTATTGTAATACATCAGCCCTGTTTAATAGCACTTGGAGAGACAACCAGACAGATAACCAAACACATACAA
AT---

GACACTGACATTATAACTCTCCCATGCAGAATAAAGCAAGTTATAAACATGTGGCAGAGGACAGGACAAGCAA
TGTATGCCCCTCCTATCCAAGGAGTAATAAAGTGTACATCAAAT---
ATTACAGGACTATTATTAACAAGAGATGGGGGTGCTAACACAACA----------------------------
----

AATGAAACTTTCAGACCTACAGGAGGAGATATGAAAGACAATTGGAGAAGTGAATTATACAAGTATAAAGTAG
TAAAAATTGAACCACTA---GGAGTAGCACCCACCAGGGCAAAGAGAAGAGTGGTG-----------------
----------------------GAGAGAGAAAAAAGAGCA---GTTGGA---CTGGGAGCT---------
GTATTCATTGGGTTCTTGGGAGCA---GCAGGAAGCACTATG---GGCGCGGCGTCA---
ATCACGCTGACGGTACAG---GCTAGACAA---TTATTG---TCTGGCATAGTGCAACAGCAAAGC---
AATTTG---CTGAGG---GCTATAGAGGCTCAACAACATATGTTGAAGCTCACAGTCTGGGGCATT
>KX232614..1
TTCTATTGTAATACAACAAACCTGTTTAATAGTACTTGGCCTGAGAATAAAAATAGCACAGGGTCAAATGACA
CAGGG--

TCAAATGGCACTATAACTCTCCAGTGCAAAATAAAGCAAATTATAAATGTATGGCAGAGAGTAGGACAAGCAA
TATATGCCCCTCCCATCCCAGGAGCAATAAGATGTAACTCAAAT---
ATTACGGGGCTACTATTAACAAGAGATGGTGGGAATAATAACACA----------------------------
----

AATGAAACCTTCAGACCTGGAGGGGGAAATATGAAGGACAATTGGAGAAGTGAACTGTATAAATATAAAGTAG
TAAAACTTGAACCACTA---GGAGTAGCACCCACCAAGGCAAAGAGAAGAGTGGTG-----------------
----------------------GAGAGAGAAAAAAGAGCA---TTCGGA---CTAGGAGCT---------
GTATTCATTGGGTTCTTGGGAGCA---GCCGGAAGCACTATG---GGCGCGGCGTCT---
GTAACGCTGACGGTACAG---GCCAGACAA---TTATTG---TTTGGCATAGTGCAACAGCAAAGC---
AATTTG---CTGAGG---GCTATAGAGGCTCAGCAACACCTGCTGAAACTCACGGTCTGGGGCATT
>KX232618..2
TTCTATTGTAATACATCAGGCCTGTTTAATAGTACTTGGTCTAATAATGAGACTGAGGGAGCGGGG-------
----

TCAAATGACACTATAACACTCCCATGCAGAATAAAGCAAATTGTAAATGTATGGCAGAGAGTAGGACAAGCAA
TATATGCCCCTCCCATCGAAGGACTAATAAAATGTAACTCAAAT---
ATTACGGGGCTACTATTGACAAGAGATGGTGGGGATTATAACACATCTACA----------------------
----

AATAATGAAACCTTCAGACCTGGAGGAGGAAATATGAAGGACAATTGGAGAAGTGAACTGTATAAGTATAAAG
TAGTAAGACTTGAACCACTA---GGAGTAGCACCCACCAAAGCGAAGAGAAGAGTGGTG--------------

-------------------------GAGAGACAAAAAAGAGCA---ATTGGA---ATAGGAGCT---------
GTGTTCATTGGGTTCTTGGGAGCA---GCTGGAAGCACTATG---GGCGCGGCGTCC---
GTAACGCTGACGGTACAG---GCCAGACAA---TTATTG---TCTGGCATAGTGCAACAGCAAAGC---
AATTTG---CTGCAG---GCTATAGAGGCTCAGCAACATCTGCTGAAACTCACAGTCTGGGGCATT
>KX232620..2
TTCTATTGTAATACAACAAGCCTGTTTAATAGTACTTGGCCTCATAATGAGACGAAAATAAATAGCACGGAAT
CAAAT--

GACAATGGCACTATAACACTCCCATGCAGAATAAAGCAAATTGTAAATATATGGCAGAGAGTAGGACAAGCAA
TGTATGCCCCTCCCATCAAAGGACTAATAAGGTGTAATTCAACT---
ATTACGGGGCTACTATTAACAAGAGATGGGGGTGAGACTAATGGC----------------------------
----

ACAAATGAAACCTTTAGACCTGGAGGAGGAGATATGAGGGATAATTGGAGAAGTGAACTGTATAAGTATAAAG
TAGTAAAAATTGAACCACTA---GGAATAGCACCCACCAAAGCAAGGAGAAGAGTGGTG--------------
-------------------------GAGAGAGAAAAAAGAGCA---ATTGGA---CTAGGAGCT---------
GTATTCATTGGGTTCTTGGGAGCA---GCCGGAAGCACTATG---GGCGCGGCGTCT---
GTAACGCTGACGGTACAG---GCCAGACAA---TTATTG---TCTGGCATAGTGCAACAGCAAAGC---
AATTTG---CTGAAG---GCTATAGAGGCTCAGCAGCAACTGCTGAAACTCACGGTCTGGGGCATT
>KX232624..1
TTCTATTGTAATACAACAGGCCTGTTTAATAGTACTTGGTATAATAATGGAACTGAGGAAGCAAATAACACGG
GG---

TCAAATGACACTATAACACTCCCATGCAGAATAAAACAAATTATAAATGTATGGCAAAGAGTAGGACAAGCAA
TATATGCCCCTCCCATCGAAGGACTAATAAGGTGTAATTCAACT---
ATTACGGGGCTACTATTAACAAGAGATGGGGGTAATAACACAAAT----------------------------
----

GACACAAGTGAAACCTTCAGACCTGGAGGAGGAGATATGAGGGATAATTGGAGAAGTGAACTGTATAAGTATA
AAGTAGTAAAAATTGAACCACTA---GGAATAGCACCCACCAAAGCAAGGAGAAGAGTGGTG-----------
----------------------------GAGAGGGAAAAAAGAGCA---GTTGGA---CTAGGAGCT------
---GTATTCATTGGGTTCTTGGGAGCA---GCCGGAAGCACTATG---GGCGCGGCGTCC---
GTGACGCTGACGGTACAG---GCCAGGCAA---TTATTG---TCTGGCATAGTGCAACAGCAAAGC---
AATTTG---CTGAGG---GCTATAGAGGCTCAGCAACATCTGCTGAAACTCACAGTCTGGGGCATT
>KX232626..1
TTCTATTGTGATACAACAGGCCTGTTTAATAGTACTTGGCCTAACAATGAAACTGGGGAAGTAAATAGCACGG
GA---

TCAAACGATATTATAACACTCCCATGCAGAATAAAGCAAGTTGTAAATGTATGGCAGAGAGTGGGACAAGCAA
TATATGCCCCTCCCATCCAAGGACTAATAAAATGTAACTCAAAT---
ATTACGGGGATAATATTAACAAGAGATGGTGGGAATCTTAATTCT----------------------------
----

ACAAATGAAACTTTCAGACCTGGAGGAGGAAATATGAAGGACAATTGGAGAAGTGAACTGTATAAGTATAAAG
TAGTAAAACTTGAACCACTA---GGAGTAGCACCCACCAGAGCAAAGAGAAGAGTGGTG--------------
-------------------------GAGAGAGAAAAAAGAGCA---ATTACC---ATAGGAGCT---------
GTGCTCATTGGGTTCTTGGGAGCA---GCCGGAAGCACTATG---GGCGCGGCGTCT---
GTAACGCTGACGGTACAG---GCCAGACAA---TTATTG---TCTGGCATAGTGCAACAGCAAAGC---
AATTTG---CTAAGG---GCTATAGAGGCTCAGCAACATCTGCTGAAACTCACAGTCTGGGGCATT
>KU749423..1
TTCTATTGTAATACATCAGGCCTGTTCAATAGCACCTGGTATCCCAATGGCACCAACATAACTAACCCA----
----

GGACCAGAGAATATAACCCTCCCATGCAGAATAAAGCAAATTGTAAATATGTGGCAGAGAGTAGGGAGAGCAA
TGTATGCTCCTCCCATCAAAGGAGTAATACAATGTGTATCAAAC---
ATTACAGGACTACTATTAACAAGAGATGGTGGGAATAATAATAAT----------------------------
----

ACAACTGAAACCTTCAGACCAGGAGGAGGAGATATGAGGGACAATTGGAGAAGTGAATTATATAAGTATAAAG
TAGTACAAGTTGAACCACTA---GGAATAGCACCCACTAGGGCAAGGAGAAGAGTGGTG--------------

-------------------------GAGAGAGAAAAAAGAGCA---GTTGGA---ATGGGAGCT---------
CTTTTCATTGGGTTCCTAGGAGCA---GCCGGAAGCACTATG---GGCGCGGCATCA---
ATGATGCTGACGGTACAG---GCCAGACAA---TTATTG---TCTGGCATAGTGCAACAGCAAAGC---
AATTTG---CTGAGG---GCTATTGAAGCTCAACAGCATATGTTGAAGCTCACGGTCTGGGGCATT
>KX983643..1
TTCTATTGTAATACATCAGAACTGTTTAATAGCACTTGGGGAGAGGAAGGGCGAAAGGACAATATCACGAAG-
----

TCAAATGCCACTATAACTCTCCCATGTAGAATAAAGCAAGTCATCAATATGTGGCAGAGAGCAGGACGAGCAA
TGTATGCCCCTCCCATCCAAGGAGTAATAAATTGTGCATCAAAC---
ATTACAGGACTACTACTAACAAGAGACGGTGGGAATACTAATACTAGC-------------------------
----

GATCTACCTGAAATCTTCAGACCAGGAGGAGGAGATATGAGGGACAATTGGAGAAGTGAATTATATAAGTATA
AGGTAGTAAAAATTGAACCACTA---GGAGTAGCACCCACCAAGGCAAGGAGAAGAGTGGTG-----------
----------------------------GAGAGAGAAAAAAGAGCA---GTTGGA---CTGGGAGTT------
---GTCTTCCTTGGGTTCTTAGGAGCA---GCAGGAAGCACTATG---GGCGCGGCGTCA---
ATAACGCTGACGGTACAG---GCCAGACAA---TTATTG---TCTGGCATAGTGCAGCAGCAAAGC---
AATTTG---CTGAAG---GCTATAGAGGCTCAACAGCATCTGTTGAAGCTCACAGTCTGGGGCATT
>KX983542..2
TTCTATTGTGATACAACAGGCCTGTTTAATAGCACTTGGAAGAATGGCACTCTCAACATGCAGGAATTAACTA
TCACAGTC---
ACAAACTACACAAATCTC---------
ACAGACAGTATAAATATAACTCTTCCATGTAGAATAAAGCAAATTATAAATATGTGGCAGAGAGTAGGACAGG
CAATGTATGCTCCTCCCATCCAAGGAGTAATAAAATGTGTGTCAAAC---
ATTACGGGACTACTATTAACGAGAGATGGGGGAAT--------------------------------------
----

AATGAGACAGAAACCTTCAGACCTGGAGGAGGAAATATGAAGGACAATTGGAGAAGTGAATTATATAAGTATA
AAGTAGTAAAAGTTGAGCCTATA---GGAGTAGCACCCACCAGGGCAAGGAGAAGAGTGGTG-----------
----------------------------GAGAGAGAAAAAAGAGCA---GTTGGA---ATAGGAGCT------
---GTTTTCATTGGGTTCTTAGGAGCA---GCAGGAAGCACTATG---GGCGCGGCGTCA---
ATAACGCTGACGGTACAG---GCCAAACAA---TTATTG---TCTGGCATAGTGCAACAGCAAAGC---
AATTTG---CTGAGG---GCTATAGAGGCTCAACAGCATCTGTTGAAACTCACGGTCTGGGGCATT
>KX983566..2
TTCTATTGTAATACATCAAGCCTGTTTAATAGCATTTGGGTTAATGGCACTAGGAATGACACTGTCAGC----
----

AATGGAACTTATATAACTCTCCCATGCAGAATAAAGCAAATTATAAATATGTGGCAGAGAGTAGGGCAAGCAA
TGTATGCCCCTCCCATTAGAGGAGTAATAAAGTGTGAATCAAAC---
ATTACAGGACTACTGTTAACAAGAGATGGTGGGGAGAATAATAGT----------------------------
----

ACCAATGAAACCTTCAGACCTGGAGGAGGAGATATGAGGGACAATTGGAGAAGTGAATTATATAAGTACAAAG
TAGTAAAAATTAAACCACTA---GGAGTAGCACCCACCAAGGCAAAGAGAAGAGTGGTG--------------
-------------------------GAGAGAGAAAAAAGAGCAGTAGTTGGA---CTGGGAGCT---------
GTCTTCCTTGGGTTCTTAGGAGCA---GCAGGAAGCACTATG---GGCGCAGCGTCA---
ATAACGCTGACGGTACAG---GCCAGACAA---TTATTG---TCTGGCATAGTGCAACAGCAAAGC---
AATTTG---CTGAGG---GCTATAGAGGCTCAACAACATCTGTTGAAACTCACGGTCTGGGGCATT
>JX236677..1
TTCTATTGTAATACATCAGCCCTGTTTGATAGCACTTGGCGGTTTAATGGCATTGGCAGTGGAAATGACACTG
TCAATGCCACT--
-------------------AGA---
AGCGAGAATGATACTATAAATCTCCCATGCAGGATAAAGCAAATTATAAATATGTGGCAGAGAGCAGGACAGG
CAATGTATGCCCCTCCCATCCAAGGAGTAATAAGGTGTGACTCAAAC---
ATTACAGGTCTATTATTAACAAGAGATGGTGGGAGGGGGAATGAT----------------------------
----

AATAACACTGAGGTCTTCAGACCTGGAGGAGGAAATATGAAGGACAATTGGAGGAGTGAATTATATAAGTACA
AAGTAGTAAAAATTGAACCATTA---GGAGTAGCACCCACCAGGGCAAGGAGAAGAGTGGTG-----------

----------------------------GAGAGAGGAAAAAGAGCA---GTTGGA---ATAGGAGCT------
---GTTTTCATTGGGTTCTTAGGAGCA---GCAGGAAGCACTATG---GGCGCAGCGTCA---
ATAACGCTGACGGTACAG---GCCAGGCAA---TTATTG---TCTGGCATAGTGCAACAGCAAAGC---
AATTTG---CTGAGG---GCTATAGAGGCTCAACAGCATCTGTTGAGACTCACAGTCTGGGGCATT
>JX236678..2
TTCTATTGTAACACATCAACCCTGTTTAGTAGCACTTGGGAGAACGGTACGGAACTAAACAGCACGAAG----
----

TCAAATGACACTATAACTCTCCCATGCAGAATAAAGCAAATTATAAATATGTGGCAGAGAGTAGGACAAGCAA
TATATGCTCCTCCAATCGAAGGAGAAATAAGGTGTCAATCAAAT---
ATTACAGGACTAATATTAACAAGAGATGGTGGTGAGGATATTAAC----------------------------
----

AGTACAAGTGAAACCTTCAGACCTGGAGGAGGAGATATGAGGGACAATTGGAGAAGTGAATTATATAAGTATA
AGGTAGTAAAAATTGAACCACTA---GGAGTAGCACCCACCAGGGCAAGAAGAAGAGTGGTG-----------
----------------------------GAGAGAGAAAAAAGAGCA---GTTGGA---ATAGGAGCT------
---GTCTTCATTGGGTTCTTAGGAGCA---GCAGGAAGCACTATG---GGCGCGGCGTCA---
ATAACGCTGACGGTACAG---GCCAGACAA---TTATTG---TCTGGCATAGTGCAACAGCAAAGC---
AATTTG---CTGAGG---GCTATAGAGGCTCAACAACATCTGTTGAAGCTCACGGTCTGGGGCATT
>KU749424..1
TTTTATTGTAATACATCAGAACTGTTTAATAGCACTTGGGTATACAATGCATCAGAACTTAATGGCACTTGGG
TAAACAATGCC--
AGCATGCCAGGATCAAAT---------
AGCACAAGTGAAAATATAACTCTCCACTGCAGAATAAAGCAAATTATAAATATGTGGCAGAGAGCAGGAAAAG
CAATGTACGCCCCTCCCATCTCAGGAAACATAACGTGTGTTTCAAAC---
ATTACAGGACTCATATTAACAAGAGATGGTGGGAATACCAATACA----------------------------
----

ACTGAAATCTTCAGACCTGAAGGAGGAGATATGAGAGACAATTGGAGAAGTGAATTATATAAGTATAAAGTAG
TAAAAATTGAACCACTA---GGAGTAGCACCCACCAAGGCAAGGAGGAGAGTGGTG-----------------
----------------------GAGAGAGAAAAAAGAGCA---ATTGGA---ATAGGAGCT---------
GTCCTCTTTGGGTTCTTAGGAGCA---GCAGGAAGCACTATG---GGCGCGGCGTCA---
ATAACGCTGACGGTACAG---GCCAGACAA---TTATTG---TCTGGCATAGTGCAACAGCAAAGC---
AATTTG---CTGAGG---GCTATAGAGGCTCAACAACATCTGTTGAAACTCACAGTCTGGGGCATT
>MF565934..1
TTCTATTGCAATACATCAAGCCTGTTTAATAGTACTTGGGATGGGAATAGCACTACTAACAGCACGCAGAAG-
----

CCAAATGGAACTATAACTCTCCCATGCAGGATAAAGCAAATTATAAATATGTGGCAGAGAACAGGGCAAGCAA
TGTATGCCCCTCCCATCCCAGGAAAAATAAGGTGTGACTCAAAC---
ATCACAGGACTAATATTAACAAGAGATGGTGAAAATAATAACACT----------------------------
----

GAAAGTGAAACCTTTAGACCTGAAGGAGGAGACATGAGGAACAATTGGAGAAGTGAGTTATATAAGTATAAAG
TAGTAAAGATTGACCCACTA---GGAGTAGCACCCACCGGGGCAAAGAGAAGAGTGGTG--------------
-------------------------GAGAGAGAAAAAAGAGCA---GTTGGA---ATAGGAGCT---------
GTCCTCTTTGGGTTCTTAGGAGCA---GCAGGAAGCACTATG---GGCGCAGCGTCT---
CTAACGCTGACGGTACAG---GCCAGACAA---TTATTG---TCTGGCATAGTGCAACAGCAAAGC---
AATTTG---CTGAGG---GCTATAGAGGCTCAACAACATCTGTTGAGACTCACGGTCTGGGGCATT
>KX983624..1
TTCTACTGTAATACATCAGGCCTGTTTAATAGCCTCTGGATAAATGGCACCATGCTGAATGGTACCCTGAAGA
ATTCACCC---

AGCACAAATGACACCATAACTCTCCCATGCAGAATAAAGCAAATCATAAACATGTGGCAGAAAGTAGGACAAG
CAATGTATGCCCCTCCCATCCCAGGAAGCATAACGTGTGTATCAAAC---
ATTACAGGACTAATATTAACAAGAGATGGTGGGAATACTAACAGG----------------------------
----

ACAGATGAAATCTTCAGACCTGAAGGGGGAAATATGAGGGACAATTGGAGAAGTGAATTATATAAGTATAAAG
TAGTAAAAATTGAACCACTA---GGAGTAGCACCCACTAGGGCAAAGAGAAGAGTGGTG--------------

-------------------------GAGAGAGAAAAAAGAGCA---GTTGGA---CTGGGAGCT---------
GTCTTCATTGGGTTCTTAGGAGCA---GCAGGAAGCACTATG---GGCGCGGCGTCA---
ATAACGCTGACGGTACAG---GCCAGAAAC---TTATTG---TCTGGCATAGTGCAACAGCAAAGC---
AATTTG---CTGAGG---GCTATTGAGGCTCAACAGCATCTGTTGAAACTCACAGTCTGGGGCATT
>KF716472..1
TTCTATTGTAACACAACAGGTCTGTTTAATAGCACTTGGAGTCGGAACACAACAGATAATAACACTTGGTTTA
GGAACAATGCT--
-------AGCAGTAGCACA------
GGGTTAAATGGCACAATAACTCTCCAATGCAAAATAAAGCAAATTATAAATATGTGGCAGAGAACAGGACGAG
CAATGTATGCCCCTCCCATACCAGGAACAATAATGTGTAACTCAAGC---
ATCACAGGACTGCTATTAACAAGAGATGGTGGGAATAGTGGGAAC----------------------------
----

AACAGTAATGAAACCTTCAGGCCTGGAGGAGGAGATATGAAAGACAATTGGAGAAGTGAATTATATAAGTATA
AGGTAGTAAAAATTGAACCACTA---GGAGTAGCACCCACCAGGGCAAAGAGAAGAGTGGTG-----------
----------------------------GAGAGAGAAAAAAGAGCA---GTTGGA---ATGGGAGCT------
---CTTTTCATTGGGTTCTTGGGAGCA---GCAGGAAGCACTATG---GGCGCGGCGTCA---
GTGACGCTGACGGTACAG---GCCAGACAA---TTATTG---TCTGGTATAGTGCAACAGCAAAGC---
AATTTG---CTGAAG---GCTATAGAGGCTCAACAACATCTGTTGAGACTCACAGTCTGGGGCATT
>AB253421..1
TTTATTGTGATTCATCAGGCCTGTTTAATAGCACTTGGGATAACACTAACATCACGCAGCCAAATAGCACAG
GG---

TCAAATGACACCATAACTCTCCAATGCAGAATAAAGCAAATTATAAATATGTGGCAGAGAGCAGGACAGGCAA
TGTATGCCCCTCCCATCCCAGGAGTAATAAGTTGTGTATCAAAC---
ATTACAGGATTACTATTAACAAGAGATGGTGGGATTACCAGTGCA----------------------------
----

AATGAAACCTTCAGACCTGGAGGAGGAGATATGAGGGACAATTGGAGAAGTGAATTATATAAGTATAAGGTAG
TAAAACTTGAACCACTA---GGAGTAGCACCCACCAGGGCAAGGAGAAGAGTGGTG-----------------
----------------------GAGAGAGAAAAAAGAGCA---GTTGGA---ATAGGAGCT---------
GTATTCATTGGTTTCTTAGGAGCA---GCAGGAAGCACTATG---GGCGCGGCGTCA---
ATGACGCTGACGGTACAG---GCCAGACAA---TTATTG---TCTGGCATAGTGCAACAGCAAAGC---
AATTTG---CTGAGG---GCTATAGAGGCTCAACAACATCTGTTGAAACTCACGGTCTGGGGCATT
>AB287377..1
TTCTATTGTAATACATCAGGCCTGTTTAATAGCACCTGGAATAATAATAATACCAGGGAGAAGATAAATGGTA
CA---

GAGTCAAATAGCACTATAACTCTCCATTGCAGAATAAAGCAAATTATAAATAGGTGGCAGGAAGTAGGACAAG
CAATGTATGCCCCTCCCATCCCAGGAGTAATAAATTGTAGATCAAAC---
ATTACAGGACTAATATTAACAAGAGATGGTGGGGATGGGGATAAC----------------------------
----

AATACGGAAATCTTCAGACCTGGAGGAGGAAATATGAAGGACAATTGGAGAAGTGAATTATATAAGTATAAAG
TAGTAAAAATTGAACCACTG---GGAGTAGCACCCACCAGGGCTAAGAGAAGAGTGGTG--------------
-------------------------GAGAGAGCAAAAAGAGCA---GTTGGA---ATAGGAGCT---------
GTTTTCCTTGGGTTCTTAGGAGCA---GCAGGAAGCACTATG---GGCGCGGCGTCA---
ATAACGCTGACGGTACAG---GCCAGACAA---TTATTG---TCTGGCATAGTGCAACAGCAAAGC---
AATTTG---CTGAGG---GCTATAGAGGCTCAACAACATCTGTTGAAACTCACGGTCTGGGGCATT
>AB287379..1
TTCTATTGTAATACATCAGGCCTGTTTAATAGCACTTGGACCAATGAAAGCATGCAAGTAAATAGCACAGAA-
----

TCAAATGACACTATAACTCTCCCATGCAGAATAAAGCAAATTATAAATATGTGGCAGAGGGCAGGACAAGCAA
TATATGCCCCTCCCATCGAAGGAGTAATAAGGTGTAGCTCAAAC---
ATTACAGGACTACTATTAACAAGAGATGGTGGGGTTAACAATACA----------------------------
----

GTCAATGAAACCTTCAGACCTGGAGGAGGAAATATGAGGGATAATTGGAGAAGTGAACTATATAAATATAAAG
TAGTAAAAATTGAACCACTA---GGAGTAGCACCCACCAGGGCAAAAAGAAGAGTGGTG--------------

-------------------------GAGAGAGAAAAAAGAGCA---GTTGGA---CTGGGAGCT---------
GTCTTCATTGGGTTCTTAGGAGCA---GCAGGAAGCACTATG---GGCGCGGCGTCA---
ATAACGCTGACGGTACAG---GCCAGACAA---TTATTG---TCTGGCATAGTGCAACAGCAAAAC---
AATTTG---CTGAGG---GCTATAGAGGCTCAACAACATCTGTTGAAACTCACGGTCTGGGGCATT
>AY713406..1
TTCTATTGTAATACATCAAGCCTGTTTAATAGCACTTGGAATAACACTGCCAGCATGCAGAAGTCAAATAGCA
CG---

GGGTCAAATGACACTATAACTCTCCAATGCAGAATAAAGCAAATTATAAGAATGTGGCAAAGAACAGGACAGG
CAATGTATGCCCCTCCCATCAAAGGAGATATAAGGTGTGAATCAAAC---
ATTACAGGACTAATATTAACAAGAGATGGTGGGAATGATAGCAGT----------------------------
----

CCAGATGAAATCTTCAGACCTGGAGGAGGAGATATGAGGGACAATTGGAGAAGTGAATTATATAAGTATAAAG
TAGTACAAATTGAACCTCTA---GGAGTAGCACCCACCAAGGCAAGAAGAAGAGTGGTG--------------
-------------------------CAGAGAGAGAAAAGAGCA---ATTGGA---ATAGGAGCT---------
GTCTTCCTTGGGTTCTTAGGAGCA---GCAGGAAGCACTATG---GGCGCGGCGTCA---
ATAACGCTGACGGTACAG---GCCAGACAA---TTATTG---TCTGGCATAGTGCAACAGCAAAGC---
AATTTG---CTGAGG---GCTATAGAGGCTCAACAACATCTGTTGAAACTCACGGTCTGGGGCATT
>L07082..1
TTCTATTGTAATACAACAGGCCTGTTTAATAGCACCTGGAAAAATGTAAATGGCACCATGCAGGAGTCAAGCA
GCACA--

AAGTTAAATGACACTATAACACTCACATGCAGAATAAAGCAAATTATAAATATGTGGCAGAGAGCAGGACAGG
CAATGTATGCCCCTCCCATCGAAGGAATAATAAGGTGTGACTCAAAC---
ATTACAGGACTACTATTAACAAGAGATGGTGGGGTTGGGGATAACAGT-------------------------
----

ACAAATGGAACTGAAACATTCAGACCTGGAGGAGGAAATATGAGGGACAATTGGAGAAGTGAATTATATAAGT
ATAAAGTAGTAAAAATTGAACCACTA---GGAGTAGCACCCACCACGGCAAAAAGAAAAGTGGTG--------
-------------------------------GAGAGAGAAAAAAGAGCA---ATTGGA---ATAGGAGCT---
------GTCTTCCTTGGGTTCTTAGGAGCA---GCAGGAAGCACTATG---GGCGCGGCGTCA---
ATAACGCTGACGGTACAG---GCCAGACAA---TTATTG---TCTGGCATAGTGCAACAGCAAAGC---
AATTTG---CTGAGG---GCTATAGAGGCTCAACAGCATCTGTTGAAACTCACAGTCTGGGGCATT
>MF373167..1
TTCTATTGTAATACATCAGGCCTGTTTAATAGCACTTGGAATGAAACTACCATGCAGGAGTCAAATGGAACAG
AG---

TCAAACGACAGCATAACTCTCCCATGCAGAATAAAGCAGATTGTAAATATGTGGCAGAGAACAGGACAAGCAA
TGTATGCCCCTCCCATCCAAGGAGTAATACAGTGTGTATCAAAT---
ATTACAGGACTAATATTAATGAGAGATGGTGGGAATAGTAGTAGG----------------------------
----

GAAAATGAAACCTTCAGACCTGGAGGAGGAGATATGAGGGACAATTGGAGAAGTGAATTATATAAGTATAAAG
TAGTAAAAATTGAACCACTA---GGAGTAGCACCCACCAGGGCAAGGAGAAGAGTGGTG--------------
-------------------------CAGAGAGAAAAAAGAGCG---GTTGGA---CTGGGAGCT---------
GTTTTCATTGGGTTCTTAGGAGCA---GCAGGAAGCACTATG---GGCGCAGCGTCA---
GTGACGCTGACGGTACAG---GCCAGGCAA---TTATTG---TCTGGCATAGTGCAACAGCAAAGC---
AATTTG---CTGAGG---GCTATAGAGGCTCAACAGCATCTGTTGAGACTCACGGTCTGGGGCATT
>MF373168..1
TTCTATTGTGATACATCAGACCTGTTTAATAGCATTTGGCCGTTTAATAGCACTCGGGGACACAATGAGACTG
AAAGATCA---
-------AATAGCACAGAG------
ACAAATAACACTATTATAAAACTTCCATGTAGAATAAAACAAATTATAAATATGTGGCAGAGAGTAGGACAAG
CAATATTTGCCCCTCCCATCCAAGGAGTAATACAGTGTGTATCAAAC---
ATTACAGGACTAATATTAACAAGAGATGGAGGGAATAATAACACT----------------------------
----

ACAAATGAAACCTTCAGACCTGGAGGAGGAGATATGAGGGACAATTGGAGAAGTGAATTATATAAGTATAAAG
TAGTAAAAATTGAACCACTA---GGAGTAGCACCCACCAGGGCACAGAGAAGAGTGGTG--------------

-------------------------CAGAGAGAAAAAAGAGCA---ATTGGA---ATGGGAGCT---------
GTCTTCATTGGGTTCTTAGGAGCA---GCAGGAAGCACTATG---GGCGCGGCGTCA---
ATAACGCTGACGGTACAG---GCCAGACAA---TTATTG---TCTGGTATAGTGCAACAGCAAAGC---
AATTTG---CTGAGG---GCTATAGAGGCTCAACAGCATCTGTTGAAACTCACGGTCTGGGGCATT
>MF373181..2
TTCTACTGTAATACATCAGGCCTGTTTAACAGTAGTTGGGGAAATGGCACTAACAACAGCAGCACT-------
----

GGGGGGCAAACTATAACTCTCCCATGCAAAATAAAGCAAATTGTAAATATGTGGCAGAGGACAGGACAAGCAA
TGTATGCCCCTCCTATCGAAGGAGTAATAAGGTGTGTATCAAAC---
ATTACAGGAATACTATTAACTAGAGATGGTGGGCCTAATAATAGT----------------------------
----

ACAAATGAAACCTTCAGACCGGGAGGAGGAAATATGAAGGACAATTGGAGAAGTGAATTATATAACTATAAAG
TAGTACAAATTAACCCATTA---GGAATAGCACCCACCAAGGCAAAGAGAAGAGTGGTG--------------
-------------------------GAGAGAGAAAAAAGAGCAGTAGTTGGA---CTGGGAGCT---------
TTGTTCATTGGGTTCCTAGGAGCT---GCAGGAAGCACTATG---GGCGCGGCGTCA---
ATGACGCTGACGGTACAG---GCCAGACAA---TTATTG---TCTGGCATAGTGCAACAGCAAAGC---
AATTTG---CTGAGA---GCTATAGAGGCTCAACAGCATGTGTTGAGACTCACGGTCTGGGGCATT
>MF373182..1
TTCTATTGTAATACATCAGGCCTGTTTAATAGCAGTTGGGGAGTAAACAACACTGATGGCAATATA-------
----

ACTAATGGCACGATAACTCTCCCCTGCAAAATAAAACAATTTATAAATATGTGGCAGAGAGTAGGACAAGCAA
TGTATGCCCCTCCCATCGAAGGGGCAATAAGTTGTAATTCAACC---
ATCACAGGACTACTATTAACAAGAGATGGTGGGTACAACAACAGCACT-------------------------
----

AACCAAATCGACAAAATCTTCAGACCTGCAGGAGGAGATATGCAGGACAACTGGAGAAGTGAATTATATAAGT
ATAAAGTAGTAAAAATTGAACCATTA---GGAGTAGCACCCACCTGGGCAAGGAGAAGAGTGGTG--------
-------------------------------CAGAGAGAAAAAAGAGCA---GTTGGA---CTGGGAGCT---
------GTCTTTCTTGGGTTCTTAGGAGCA---GCAGGAAGCACTATG---GGCGCGGCGTCG---
ATAACGCTGACGGTACAG---GCCAGACAA---TTATTG---TCTGGCATAGTGCAACAGCAAAGC---
AATTTG---CTGATG---GCTATAGAGGCTCAACAGCATTTGTTGAAACTCACAGTCTGGGGCATT
>AF069670..1
TTCTATTGTAATACATCAGGCCTGTTTAATAGCACTTGGAGCCAAAATGACACTGGGGTATCAAATAGCACGG
AG---

TCAAATGACACTATAATTCTTCCATGCAGAATAAAACAAATTATAAATATGTGGCAGAGAGCAGGACAAGCAA
TATATGCCCCTCCCATCCCAGGAATAATAAGGTGTGAATCAAAC---
ATTACAGGGCTACTGTTAACAAGAGATGGTGGGGTTGTTAACAGT----------------------------
----

ACAAATGAAACCTTCAGACCTGGAGGAGGAAATATGAAGGACAATTGGAGAAGTGAATTATATAAGTATAAAG
TAGTAAAAATTGAACCACTA---GGAGTAGCACCCACCAGGGCAAGGAGAAGAGTGGTG--------------
-------------------------CAGAGAGAAAAAAGAGCA---GTTGGA---CTGGGAGCT---------
TTATTCATTGGGTTCTTAGGAGCA---GCAGGAAGCACTATG---GGCGCGGCGTCA---
ATAACGCTGACGGTACAG---GCCAGACAA---TTATTA---TCTGGCATAGTGCAACAGCAAAGT---
AATTTG---CTGAGG---GCTATAGAGGCTCAACAGCATCTGTTGAAACTCACTGTCTGGGGCATT
>AF069671..1
TTCTATTGTAATACATCAGGCCTGTTTAATAGCACTATTTTGAATAGCACTAAGATGAATGACAATGCCAGCA
GGGAG--

TCGTATGATGACACTATAACCCTCCAGTGCAGAATAAAGCAAATTATAAATATGTGGCAGAGAGTAGGACAAG
CAATGTATGCCCCTCCCATCCGAGGAGCAATAAGATGTAAATCAAAC---
ATTACAGGACTATTATTAACAAGAGATGGTGGAAATAGTAACAGT----------------------------
----

AGTACAAATGAAACCTTCAGACCTGGAGGAGGAGATATGAGAGATAATTGGAGAAGTGAATTATATAAATATA
AAGTAGTAAAAATTGAACCACTA---GGAGTAGCACCCACCAAGGCAAAGAGAAGAGTGGTG-----------

----------------------------GAGAGAGAAAAAAGAGCA---GTTGGA---ATAGGAGCT------
---GTCTTCCTTGGGTTCTTAGGAGCA---GCAGGAAGCACTATG---GGCGCGGCGTCA---
ATAACGCTGACGGTACAG---GCCAGACAA---TTATTG---TCTGGCATAGTGCAACAGCAAAGC---
AATTTG---CTGAGG---GCTATAGAGGTTCAACAACATCTGTTGAAACTCACGGTCTGGGGCATT
>AF069673..1
TTCTATTGTAATACATCAGGCCTGTTTAATAGTACTTGGAATGGCACTGACAGCATGCAGAAGTTAAAT----
----

AGCACGGGCAATATAACTCTCCCATGCAGAATAAAACAAATTATAAATATGTGGCAGAGAGCAGGACAAGCAA
TATATGCCCCTCCCATCCAAGGAGTAATAAGGTGTGAATCAAAC---
ATTACAGGACTAATATTAACAAGAGATGGTGGGAATGATAACAAT----------------------------
----

GAAAGTGAAATCTTCAGGCCTGGAGGAGGAGACATGAGGGACAATTGGAGAAGTGAATTATATAAGTATAAGG
TAGTAAAAATTGAACCACTA---GGAGTAGCACCCACCAAGGCAAAAAGAAGAGTGGTG--------------
-------------------------GAGAGAGAAAAAAGAGCA---ATTGGA---ATAGGAGCT---------
GTCTTCATTGGGTTCTTGGGAGCA---GCAGGAAGCACTATG---GGCGCAGCGTCA---
ATAACGCTGACGGTACAG---GCCAGGCAA---TTATTG---TCTGGCATAGTGCAACAGCAAAGC---
AATTTG---CTGATG---GCTATAGAGGCTCAACAGCATCTGTTGAAACTCACGGTCTGGGGCATT
>AF107771..2
TTCTATTGTAATACATCAGGTCTGTTTAATAGTAGTTGGAATGAAAATGACACCAAAGTAAATTACAACACTG
AG---

TCAAATGACACTATAACTCTCCAATGCAGAATAAAGCAAATTATAAATATGTGGCAGAGAACAGGACAAGCAA
CGTATGCCCCTCCCATCCCAGGGGTAATACAGTGTAGATCAAAC---
ATTACAGGACTACTATTAACAAGGGATGGTGGGGTAACTAACAAT----------------------------
----

ACAAATAATGAAACCTTCAGACCTGGAGGAGGAGATATGAGGGACAATTGGAGAAGTGAATTATACAAGTATA
AAGTAGTAAAACTTGAACCACTA---GGAGTAGCACCCACCAAGGCACAGAGAAGAGTGGTG-----------
----------------------------AAGAGAGAAAAAAGAGCA---GTTGGA---CTGGCAGCT------
---GTATTCTTTGGGTTCTTAGGAGCA---GCAGGAAGCACTATG---GGCGCGGCGTCA---
ATAACGCTGACGGTACAG---GCCAGACAA---TTATTG---TCTGGTATAGTGCAACAGCAAAAC---
AATTTG---CTGAGG---GCTATAGAGGCTCAACAGCATATGTTGAGACTCACGGTCTGGGGCATT
>HQ659604..1
TTTTATTGTGATACATCAGGCCTGTTTAATAGCACATGGGGGGGAAATAGCACTGACAGCATACAGGAGTCAA
ATAGC--
-------------------ACA---
GAGTCAAATGACACTATAATTCTCCCATGCAGAATAAAGCAAATTATAAATATGTGGCAGAGAGTAGGACAAG
CAATGTATGCCCCTCCCATCCAAGGAGTAATAAAGTGTATATCAAAT---
ATTACAGGACTTCTATTAACAAGAGATGGTGGGGATAATAACAGT----------------------------
----

GCAAATGAAACCTTCAGACCTACAGGAGGAAATATGAGGGACAATTGGAGAAGTGAATTATATAAGTATAAAG
TAGTAAAAATTGAACCACTA---GGAGTAGCACCCACCCCGGCAAAGAGAAGAGTGGTG--------------
-------------------------CAGAGAGAAAAAAGAGCA---GTTGGA---ATAGGAGCT---------
GTATTCATTGGGTTCTTAGGAGCA---GCCGGAAGCACTATG---GGCGCGGCGTCA---
ATAACGCTGACGGTACAG---GCTAGACAA---TTATTG---TCTGGCATAGTGCAACAGCAAAGC---
AATTTG---CTGAGG---GCTATAGAGGCTCAACAACATCTGTTGAAACTCACGGTCTGGGGCATT
>HQ659597..1
TTCTATTGCAACACATCAGAATTGTTTAATAGCACTTGGGATAATAGCACTTGGGATAATAGCACTTGGGATA
ATAACATG---
----------CATGAGTCG------
AATAGCACGGAGAATATAACTCTCCAATGTAGGATAAAGCAAATTATAAATATGTGGCAGAGAGCAGGACAAG
CAATATATGCCCCTCCCATCAAAGGAGTAATAGGGTGTAAATCAAAC---
ATTACAGGACTAATATTAACAAGAGATGGTGGGGAGAAGGGTAATAAC-------------------------
----

AGTATCAATGAAACCTTCAGACCTGGAGGAGGAGATATGAGGGACAATTGGAGAAGTGAATTATACAAGTATA
AAGTAGTAAAAATTGAACCACTA---GGAGTAGCACCCACCAAGGCAAAGAGAAGAGTGGTG-----------

---------------------------GAGAGAGAAAAAAGAGCA---GTTGGA---ATAGGAGCT------
---GTCTTCCTTGGATTCTTAGGAACA---GCAGGAAGCACTATG---GGCGCGGCGTCA---
GTAACGCTGACGGTACAG---GCCAGACAA---TTATTG---TCTGGCATAGTGCAACAGCAAAGC---
AATTTG---CTGAGG---GCTATAGAGGCTCAACAGCATCTGTTGAAACTCACAGTCTGGGGCATT
>HQ697994..3
TTCTATTGCTACACATCAGGCCTGTTTAATTATACCTTTAATGATACCAGCACAAATAGCACGGAGTCGAAG-
----

TCAAATGACACTATAACTCTCCAATGCAGAATAAAGCAAATTATAAATATGTGGCAGAGAGCAGGACAAGCAG
TATATGCCCCTCCCATACCAGGGATAATAAGGTGTGAATCAAAC---
ATTACAGGACTAATATTAACAAGAGATGGTGGGAATAATAATAGT---------------------------
----

AATACAAATGAAACCTTCAGACCTGGAGGAGGAGATATGAGGGACAATTGGAGAAGTGAATTATATCGGTATA
AAGTAGTAAAAATTGAACCAATA---GGAGTAGCACCTACCACGGCAAAGAGAAGAGTGGTG----------
---------------------------GAGAGAGAAAAAAGAGCA---GTTGGA---ATAGGAGCT------
---GTCTTCCTTGGGTTCTTAGGAGCA---GCAGGAAGCACTATG---GGCGCGGCGTCA---
ATAACGCTGACGGTACAG---GCCAGACAA---TTATTG---TCTGGCATAGTGCAACAGCAAAGC---
AATTTG---CTGAGG---GCTATAGAGGCTCAACAGCATCTGTTGAAACTCACGGTCTGGGGCATT
>HQ698029..1
TTCTATTGCAACACGGCAGGCCTGTTTGATAGCATTTGGAATGGTAGCATTGGGAATAACACTGACAATAGTA
GCATGGAG--
-------------TTAAAT------
GACGATAATGACACTATAACTCTCCCATGCAGAATAAAGCAAATTATAAATATGTGGCAGAGAGTAGGACAAG
CAATATATGCCCCTCCCATCCAAGGAGTAATAAGGTGTGACTCAAAT---
ATTACAGGGTTAATATTAACAAGAGATGGTGGGGATAATAATAGT---------------------------
----

GCAAATGAAACCTTCAGACCTGTAGGAGGAGATATGAGGGACAATTGGAGAAGTGAATTATATAAGTATAAAG
TAGTAAAAATTGAGCCACTA---GGAGTAGCACCCACCAGGGCAAAGAGAAGAGTGGTG-------------
------------------------GGGAGAGAAAAAAGAGCA---GTTGGA---CTAGGAGCT---------
GTCTTCATTGGGTTCTTAGGAGCA---GCAGGAAGCACTATG---GGCGCAGCGTCA---
GTAACGCTGACGGTACAG---GCCAGACAA---TTATTG---TCTGGCATAGTGCAACAGCAAAGC---
AATTTG---CTGAAG---GCTATAGAGGCTCAACAGCATCTGTTGAGACTCACAGTCTGGGGCATT
>AY253305..2
TTCTATTGTAACACATCAGGCCTGTTTAATGGCCTGTTTAATGGCACCTGGAATGGCAATCACACAAACAGCA
CG--

GAGTTAAATAGCACTATAATTCTCCAATGCAAAATAAAGCAAATTATAAATATGTGGCAGAGAGCAGGACAAG
CAATATATGCCCCTCCCATCCAAGGAGTAATAAAGTGTGTATCAAAT---
ATTACAGGACTAATATTAACAAGAGATGGTGAGAGGAACAATAGT---------------------------
----

CTAAATGAAACCTTCAGACCTGGAGGAGGAGATATGAGGGACAATTGGAGAAGTGAATTATATAAGTATAAAG
TAGTAAAAATTGAACCACTA---GGAGTAGCACCTACCAGGGCAAAGAGAAGAGTGGTG-------------
------------------------GAGAGAGAAAAAAGAGCA---GTCGGA---CTGGGAGCT---------
GTCTTCATTGGGTTCTTAGGAGCA---GCAGGAAGCACTATG---GGCGCGGCGTCA---
GTAACGCTGACGGTACAG---GCCAGACAA---TTATTG---TCTGGCATAGTGCAACAGCAAAGC---
AATTTG---CTGAGG---GCTATAGAGGCTCAACAGCATCTGTTGAAACTCACGGTCTGGGGCATT
>AY253314..1
TTCTATTGTAATACATCAGGCCTGTTTAATAGCACTTGGACTACCAACAACATGCAGGAGTCAAATGACACT-
----

AGCAATGGCACTATAACTCTCCCATGCAGAATAAAGCAAATTATAAGGATGTGGCAGAGAGTAGGACAAGCAA
TGTATGCCCCTCCCATTGAAGGAGTAATAAGATGTGAATCAAAC---
ATTACAGGACTAATACTAACAAGAGATGGCGGGAATAATAACAGT---------------------------
----

GCAAATGAAACCTTTAGACCTGGAGGAGGAGATATAAGGGACAATTGGAGAAGTGAATTATATAAGTATAAAG
TAGTAAAAATTGAACCACTA---GGAGTAGCACCCACCAGGGCAAAGAGAAGAGTGGTG-------------

-------------------------GAGAGAGAGAAAAGAGCA---GTTGGA---ATAGGAGCT---------
GTCTTCCTTGGGTTCTTAGGAGCA---GCAGGAAGCACTATG---GGCGCGGCGTCA---
ATAACGCTGACGGTACAA---GCCAGACAA---TTATTG---TCTGGCATAGTGCAGCAGCAAAGC---
AATTTG---CTGAGG---GCTATAGAGGCTCAACAACAGCTGTTGAAACTCACGGTCTGGGGCATT
>KX907352..1
TTCTATTGTAATACATCAGGCCTGTTTAATAGCACTTGGCTATTTAACAAGTCAAATGAGAATAAGTCAAATG
AG---

TCAGATGTCAATATAACACTCCCATGCAGAATAAAGCAAATTATAAATATGTGGCAGAGAGCAGGACAAGCAA
TGTATGCCCCTCCCATCCCAGGAGTAATAAAGTGTGAATCAAAC---
ATTACAGGACTACTATTAACAAGAGATGGTGGGAATAACAACAAT----------------------------
----

TCAAATGAGACCTTCAGACCTGGAGGAGGAGATATGAGGGACAATTGGAGAAGTGAATTATATAAGTATAAAG
TAGTCAAAATTGAACCACTA---GGAGTAGCACCCACCAGGGCAAGGAGAAGAGTGGTG--------------
-------------------------GGAAGAGAAAAAAGAGCA---GTTGGA---ATAGGAGCT---------
GTTTTCCTTGGGTTCTTAGGAGCA---GCAGGAAGCACTATG---GGCGCGGCGTCA---
ATAACGCTGACGGTACAG---GCCAGACAA---TTATTG---TCTGGCATAGTGCAACAGCAAAGC---
AATTTG---CTGAGG---GCTATAGAGGCTCAACAACATCTGTTGAAACTCACAGTCTGGGGCATT
>KX907348..1
TTCTATTGTAACACATCAGGTCTGTTTAATAGCACTTGGTATGGCAATGGCAGCAAGCTGGAGTCAAATAGCA
CGGGG--

TCAGACGACAATATAACTCTCCACTGTAGAATAAAGCAAATTATAAATATGTGGCAAAGAGCAGGACAAGCAA
TATATGCCCCTCCCATCCCAGGAGTAATAAACTGTCTATCAAAC---
ATTACAGGACTAATATTAACAAGAGATGGTGGGGACAATAACAGT----------------------------
----

ACAAATGAAACCTTCAGACCTGGAGGAGGAGATATGAGGGACAATTGGAGAAGTGAATTATATAAGTATAAAG
TAGTAAAAATTGAACCATTA---GGAGTAGCACCCACCAGGGCAAAGAGAAGAGTGGTG--------------
-------------------------GAAAGAGAAAAAAGAGCA---GTTGGA---CTGGGAACT---------
GTTCTCTTTGGGTTCTTAGGAGCA---GCAGGAAGCACTATG---GGCGCGGCGTCA---
GTAACGCTGACGGTACAG---GCCAGAAAG---TTACTA---TCTGGCATAGTGCAACAGCAAAGC---
AATTTG---CTGAGG---GCTATAGAGGCTCAACAGCATCTGTTGAAACTCACAGTCTGGGGCATT
>KX907336..1
TTCTATTGTAATACATCAGGCCTGTTTAATAGCACTTGGAACAGCACTGGGTCAAGAAAT-------------
----

GACAGTGGCAATATAACTCTCCCATGCAGAATAAAGCAAATTGTAAATATGTGGCAGAGGACAGGACAAGCAA
TATACGCCCCTCCCATCCAAGGAAGAATACAGTGTGTGTCAAAT---
ATTACAGGACTACTATTGACAAGAGATGGAGGGATTAACAACAGT----------------------------
----

GAGGAAACCTTCAGACCTGGAGGAGGAAATATGAGAGATAATTGGAGAAGTGAACTATATAAATATAAAGTAG
TAAAAATTGAACCACTA---GGAGTAGCACCCACCAGGGCAAAGAGAAGAGTGGTG-----------------
----------------------GAGAGAGAAAAAAGAGCAGTAGTTGGA---CTGGGAGCT---------
GTCTTCCTTGGGTTCTTAGGAGCA---GCAGGAAGCACTATG---GGCGCGGCGTCA---
CTGACGCTGACGGTACAG---GCCAGACAT---TTATTG---TCTGGCATAGTGCAACAGCAAAAC---
AATTTG---CTGAGG---GCTATAGAGGCTCAACAGCATCTGTTGAAACTCACGGTCTGGGGCATT
>KX907372..1
TTCTATTGTAATACATCAGGCCTGTTTAATAGCAGTTGGAACATTAATGGCAGTCGGGAGTCAAATAAAACAC
AG---

TCAAATGGCACTATAACTCTCCCATGCAGAATAAAGCAAATTATAAATATGTGGCAGAGAGCAGGACAAGCAA
TATATGCTCCTCCCATCCAAGGAGTAATAAAGTGTGTATCAAAC---
ATTACAGGACTAATATTAACAAGAGATGGTGGGAATAATAATAAC----------------------------
----

AGTTCAAATGAAACTTTCAGACCTGGAGGAGGAGATATGAGGGACAATTGGAGAAGTGAATTGTATAAGTATA
AGGTAGTAAAAATTGAACCACTA---GGGGTAGCACCCACCAGGGCAAAGAGAAGAGTGGTG-----------

----------------------------GAGAGAGAAAAAAGAGCA---GTTGGA---ATAGGAGCT------
---GTTTTCCTTGGGTTCTTAGGAGCA---GCAGGAAGCACTATG---GGCGCGGCGTCA---
ATAACGCTGACGGTACAG---GCCAGACAA---TTACTG---TCTGGCATAGTGCAACAGCAGAGC---
AATTTG---CTGAGG---GCTATAGAGGCTCAACAACAGTTGTTGAAACTCACGGTCTGGGGCATT
>KX907383..1
TTCTATTGTGACACATCAAGCCTGTTTAATATCACTTGGGATAGAAGTAACGTCACGCAGGAGTTAAATAACA
CAAAG--

TTAAATGACACTATAATTCTCCAATGCAGGATAAAGCAAATTATAAATATGTGGCAGAGAGCAGGACAAGCAA
TGTATGCCCCTCCCATCCCAGGAATAATAAAGTGTGAATCAAAC---
ATTACAGGACTAATATTAACAAGAGATGGGAGT--

GAGAATGAAACTGAAACCTTCAGACCTGGAGGAGGAGACATGAGGGACAATTGGAGAAGTGAGTTATATAAGT
ATAAAGTGGTAAAAAATTGAACCACTA---GGAGTAGCACCCACCAAGGCAAGGAGAAGAGTGGTG-------
-------------------------------GAGAGAGAGAAAAGAGCA---GTCGGA---CTAGGAGCT---
------GTCTTCCTTGGGTTCTTAGGAGCA---GCAGGAAGCACTATG---GGCGCAGCGTCA---
ATAACGCTGACGGTACAG---GTCAGACAA---TTGTTG---TCTGGCATAGTGCAACAGCAAAAC---
AATTTG---CTGAGG---GCTATAGAAGCTCAACAGCATCTGTTGAAGCTCACGGTCTGGGGCATT
>KX907389..2
TTCTATTGTAACACATCAATCCTGTTTAATAGCACTTGGAATAGCACTAGCACTTGGAATATCTCTGAGAGTG
TAAATAGC---
----------AGCAGTCAG------
GAGTCAAATGACACTATAACTCTCCAATGCAGAATAAAGCAAATTATAAATATGTGGCAGAGAACAGGACAAG
CAATGTATGCCCCTCCCATCCAAGGAGTAATAAAGTGCATATCAAAT---
ATTACAGGACTAATATTAACAAGAGATGGTGGGGGTGATGACAAT----------------------------
----

ACAACTGAAACCTTCAGACCTGGAGGAGGAGATATGAGGGACAATTGGAGAAGTGAATTATATAAGTATAAAG
TAGTAAAAGTTGAACCACTA---GGAATAGCACCCACCAGGGCAAGGAGAAGAGTGGTG--------------
-------------------------GAGAGAGAAAAAAGAGCA---GTTGGA---ATAGGAGCT---------
GTTTTCATTGGGTTCTTAGGAGCA---GCAGGAAGCACTATG---GGCGCGGCGTCA---
ATAACGCTGACGGCACAG---GCCAGACAA---CTATTG---GCTGGCATAGTGCAACAGCAAAGC---
AATTTG---CTGAGG---GCTATAGAGGCTCAACAGCATCTGTTGAAACTCACAGTCTGGGGCATT
>KX907341..2
TTCTATTGTAATACATCAGGCCTGTTTAATAGTACTTGGGATGACACCTCGAATCATAATACAAGTGGCACGG
AG---

TCAAATGCCAATATAACTCTCCAGTGTAGAATAAAGCAAATTATAAATATGTGGCAGAGAGCAGGACAAGCAA
TATATGCCCCTCCCATCCAAGGAATAATAAAGTGTGAATCAAAC---
ATTACAGGACTGATATTAACAAGAGATGGTGGAGATAACAATAAC----------------------------
----

ACTACCACCGAAACCTTCAGACCTGGAGGAGGAGATATGAGGGACAATTGGAGAAGTGAATTATATAAGTATA
AAGTAGTAAAAAATTGAACCACTA---GGAGTAGCACCCACCAAGGCAAGGAGACGAGTGGTG----------
----------------------------CAGAGAGAAAAAAGAGCA---GTTGGA---ATGGCAGCT------
---TTATTCTTTGGGTTCTTAGGAGCA---GCAGGAAGCACTATG---GGCGCGGCGTCC---
ATAACGCTGACGGTACAG---GCCAGACAA---TTATTG---TCTGGCATAGTGCAACAGCAAAGC---
AATTTG---CTGAGG---GCTATAGAGGCTCAACAGCATATGTTGAGACTCACGGTCTGGGGCATT
>KX907347..1
TTCTATTGTAATACATCAGGCCTGTTTAATAGCACTTGGCTGTCTAATAGTACTAACAGCACGGATAACACGG
GG---

ACAAATGACACTATAACTCTCCAATGCAGAATAAAGCAAATTATAAATATGTGGCAAAGAGTAGGACAAGCAA
TGTATGCCCCTCCCATCCCAGGAGTAATAAGGTGTGAATCAAAC---
ATTACAGGACTAATGTTAACAAGAGATGGTGGGAATAATGCTACC----------------------------
----

AATGAAACCTTTAGACCTGGAGGAGGAGATATGAGGGACAATTGGAGAAGTGAATTATATAAGTATAAAGTAG
TAAAAATTGAACCACTA---GGAGTAGCACCCACCAAGGCAAAGAGAAGAGTGGTG-----------------

----------------------GAGAGAGAAAAAAGAGCA---GTTGGA---ATAGGAGCT---------
GTCTTCCTTGGGTTCTTAGGAGCA---GCAGGAAGCACTATG---GGCGCGGCGTCA---
ATAACGCTGACGGTACAG---GCCAGACAA---TTATTG---TCTGGCATAGTGCAACAGCAAGGC---
AACTTG---CTGAGG---GCTATAGAGGCTCAACAACATCTGTTGAAACTCACAGTCTGGGGCATT
>KX907364..2
TTCTATTGTAACACATCAGCCCTGTTCAATAGCAGTTGGAGTAGTGTAAATGACAGCGGGAACATTAGCCCAG
AG---

CCAAACAAAAATATAACCCTCCGATGCAGAATAAAGCAAATTATAAATATGTGGCAGAGAGTAGGACAAGCAA
TATATGCCCCTCCCATCCAAGGAGTAATAAGGTGTGACTCAAAC---
ATTACGGGATTAATATTAACAAGCGATTATGGAGGGACTAACAGT----------------------------
----

ACACATGAAATCTTCAGACCTGCAGGAGGAAATATGAGGGACAATTGGAGAAGTGAATTATATAAGTATAAAG
TAGTACAAATTGAACCAATA---GGAGTAGCACCCATCAGGGCAAAGAGAAGAGTGGTG--------------
-------------------------GAGAGAGAAAAAAGAGCA---GTCGGA---CTGGGAGCT---------
GTCTTCCTTGGTTTCTTAGGAGCA---GCAGGAAGCACTATG---GGCGCGGCGTCA---
ATGACGCTGACGGTACAG---GCCAGACAA---TTATTG---TCTGGTATAGTGCAACAGCAAAGC---
AATTTG---CTGAGG---GCTATAGAGGCTCAACAGCATCTGTTGAAACTCACGGTCTGGGGCATT
>KX907412..3
TTCTATTGTAACACATCAGGCCTGTTTCATAGCACTTGGTTTAATAGCACTTGGTTTAATAGCACTTGGGAAG
ACAATCAGGCA---AAT--

AGCACAAATGACACTATAGTACTCCCATGCAGAATAAAGCAAATTATTAATATGTGGCAGAGAGCAGGACAAG
CAATATATGCCCCTCCCATCCCAGGGGAAATAAGGTGTGAATCAAAC---
ATTACAGGGCTAATGTTAACAAGAGATGGTGGGAATAATAACAATAACAGGACA-------------------
----

AATGAAACCAAAAATGAAACCTTCAGACCTGGAGGAGGAGATATGAGGGACAATTGGAGAAGTGAATTATATA
AGTATAAAGTAGTAAAAATTGAACCACTA---GGACTAGCACCCACCGGGGCAAAGAGAAGAGTGGTG-----
----------------------------------GAGAGAGAAAAAAGAGCA---GTTGGA---
ATAGGAGCT---------GTCTTCCTTGGGTTCTTAGGAGCA---GCAGGAAGCACTATG---
GGCGCGGCGTCA---ATAACGCTGACGGTACAG---GCCAGACAA---TTATTG---
TCTGGCATAGTGCAACAGCAAAGC---AATTTG---CTGAAG---
GCTATAGAGGCTCAACAACATCTGTTGAAACTCACGGTCTGGGGCATT
>KX907414..1
TTCTATTGTAACACATCAGGCCTGTTTAATAGCACTTGGAGTAACACTACCACTTGGGAAAACATTACCGAAA
GGCAGGAACCA--
----------AGCAGCAAC------
AAGACAAACGCAACTATAACTCTCCAATGCAGAATAAAGCAAATTATAAATATGTGGCAGAGAACAGGACGAG
CAATGTATGCCCCTCCCATCCCAGGAATAATAAGTTGTAACTCAAAT---
ATTACAGGACTAATATTAACAAGAGATGGTGGAAATAACAGTACA----------------------------
----

AATGAAACCTTCAGACCTGGAGGAGGAGACATGAGGGACAATTGGAGAAGTGAATTATATAGGTATAAAGTAG
TAAAAATTGAACCACTA---GGAGTAGCACCCACTGAGGCAAAGAGAAGAGTGGTG-----------------
----------------------GAGAGAGGAAAAAGAGCA---ATTGGA---ATAGGAGCT---------
GTTTTCATTGGGTTCTTAGGAGCA---GCAGGAAGCACTATG---GGCGCGGCGTCA---
ATAACGCTGACGGTACAG---GCCAGACAA---TTATTG---TCTGGCATAGTGCAACAGCAAAAC---
AATTTG---CTGAGG---GCTATAGAGGCTCAACAGCATCTGTTGAAACTCACGGTCTGGGGCATT
>KX907423..2
TTCTATTGTAACACATCAGGCCTGTTTAATAGCATTTGGGTTAAGGGCCAGGAAAGCAATCAGTCAAATAGC-
----

GCAAATGACACTATAGTACTCCCATGCAGAATAAAGCAAATTATAAATATGTGGCAGAGAGCAGGACAAGCAA
TATATGCCCCTCCCATCCCAGGGGAAATAAAGTGTGTATCAAAC---
ATTACAGGGCTAATATTAACAAGAGATGGTGGGAATAACGGGATA----------------------------
----

AATGAAACCTTCAGACCTGGAGGAGGAGATATGAGGGACAATTGGAGAAGTGAATTATATAAGTATAAAGTAG

TAAAAATTGAACCACTA---GGAGTAGCACCCACCAGGGCAAAGAGAAGAGTGGTG-----------------
----------------------GAGAGAGAAAAAAGAGCA---GTTGGA---ATAGGAGCT---------
GTCTTCCTTGGGTTCTTAGGAGCA---GCAGGAAGCACTATG---GGCGCGGCGTCA---
ATAACGCTGACGGTACAG---GCCAGACAA---TTATTG---TCTGGCATAGTGCAACAGCAAAGC---
AATTTG---CTGAAG---GCTATAGAGGCTCAACAACATCTGTTGAGACTCACGGTCTGGGGCATT
>KX907431..1
TTCTATTGTAACACAACAGACCTGTTTAATAGCACCTGGAATGAGACCAGCATACAGGAAAATAGCACAGAGA
CA---

AACAACGAGACTATAACTCTCCAATGCAGAATAAAGCAAATTATAAATATGTGGCAGAGAGCAGGACAAGCAA
TATATGCCCCTCCCGTACCAGGAGTAATAAGGTGTGACTCAAAT---
ATTACAGGGCTAATATTAACAAGAGATGGTGGGACTAATAATACA----------------------------
----

AATGAAACATTCAGGCCTGTAGGAGGAGATATGAAGGACAATTGGAGAAGTGAATTATATAAGTATAAAGTAG
TAAAAATTGAACCACTA---GGGGTAGCACCCACCAGGGCAAAGAGAAGAGTGGTG-----------------
----------------------GAGAGAGAAAAAAGAGCA---GTTGGA---ATAGGAGCT---------
GTCTTCCTTGGGTTCTTAGGAGCA---GCAGGAAGCACTATG---GGCGCAGCATCA---
ATAACGCTGACGGTACAG---GCCAGACAA---TTATTG---TCTGGCATAGTGCAACAGCAAAGC---
AATTTG---CTGAGG---GCTATAGAGGCTCAACAACAACTGTTGAAACTCACAGTCTGGGGCATT
>HQ615968..1
TTCTATTGCAACACATCAGACCTATTTAATAGCACTTGGGAAAATAACCCTGTCAGCCAGGAGTCAAATAGCA
CA---

AGGTCAAACGACACTATAACTCTCCAATGCAAAATAAGGCAAATTATAAGAATGTGGCAGAGAGTAGGACAAG
CAATGTATGCCCCTCCCATCCAAGGAGTAATAAACTGTACATCAAAT---
ATTACAGGACTAATATTAACAAGAGATGGTGGGAATAATAATAAC----------------------------
----

AGTGGAAATGAAACCTTCAGACCTGCAGGAGGAGATATGAGGGACAATTGGAGAAGTGAATTATATAAGTATA
AAGTAGTAAAAATTGAACCACTA---GGAGTAGCACCCACCGGGGCAAAGAGAAGAGTGGTG-----------
----------------------------GAGAGAGAAAAAAGAGCA---GTTGGA---ATAGGAGCT------
---GTCTTCATTGGGTTCTTAGGAGCA---GCAGGAAGCACTATG---GGCGCAGCGTCA---
ATAACGCTGACGGTACAG---GCCAGACAA---TTATTG---TCTGGCATAGTGCGGCAGCAAAGC---
AATTTG---CTGCAG---GCTATAGAGGCTCAACAGCATCTGTTGAAACTCACGGTCTGGGGCATT
>HQ615970..2
TTCTATTGTAATACATCAAGCCTGTTTAATAGTACTTGGAATTGGAATCACACAAACAGCTTGCAGGATTCAA
ATAGC--
-------------------ACA---
GAGACAAATGGCACTATAATTCTCAAATGCAGAATAAAACAAATTATAAGGATGTGGCAGAGAGTAGGACAAG
CAATGTATGCCCCTCCTATCCCAGGAGTAATAAGGTGTGTATCAAAC---
ATTACAGGATTAATATTAACAAGCGATGGCGGGAATAAGACCAGT----------------------------
----

ACAACAGAGACCTTCAGACCTGGGGGAGGAGATATGAGGGACAATTGGAGAAGTGAATTATATAAGTATAAAG
TAGTCAAAATTGAACCACTA---GGAGTAGCACCCACCAGGGCAAAGAGAAGAGTGGTG--------------
-------------------------GAGAGAGAAAAAAGAGCA---GTTGGA---ATAGGAGCT---------
GTCTTCCTTGGGTTCTTAGGAGCA---GCAGGAAGCACTATG---GGCGTGGCGTCA---
ATGACGCTGACGGTACAG---GCCAGGCAA---TTATTG---TCTGGCATAGTGCAACAGCAAAGC---
AATTTG---CTGAGG---GCTATAGAGGCTCAACAGCATCTGTTGAAACTCACAGTCTGGGGCATT
>HQ615971..2
TTTTATTGTAATACATCAAAACTGTTTAATAGCAGTATATCCTACAATGGCAGTACAAAGTCAGTAGAGTCAA
TA---

AACAGTAACGAGACAATAAGTCTCCCATGCAGAATAAAGCAAATTATAAATATGTGGCAGAGAGCAGGACAAG
CAATATATGCCCCTCCCATCCGAGGAGTAATAAAGTGTAAGTCAGAC---
ATTACAGGACTACTATTAACAAGAGATGGTGGGGTTACCAACACA----------------------------
----

AATGAGACCTTTAGACCTGGAGGAGGAAATATGAGGGATAATTGGAAAAGTGAACTATATAAATATAAAGTAG

TAAAAATTGAACCATTA---GGAGTGGCACCCTCTCCTGCAAAGAGAAGAGTGGTG----------------
----------------------CAGAGAGAAAAAAGAGCA---GTAGGA---CTGGGAGCT---------
GTTTTCATTGGGTTCTTAGGAGCA---GCAGGAACCACTATG---GGCGCGGCGTCA---
ATGACGCTGACGGTACAG---GCCAGACAA---TTATTG---TCTGGCATAGTGCAACAGCAAGGC---
AATTTG---CTGAGG---GCTATAGAGGCTCAACAGCATCTGTTGAAACTCACGGTCTGGGGCATT
>HM204620..2
TTCTATTGCAACACATCAGGATTGTTTAATAGCAGATGGGAAAATGACACGCAACCAAATAACACGCAG----
----

CCAAACGGCACTATAACTCTCCCATGCAGAATAAAGCAAATTATAAATATGTGGCAGAGAACAGGACAAGCAA
TATATGCCCCTCCCATCAGAGGAGTAATAAGTTGTCAATCAAAC---
ATTACAGGACTACTACTAACAAGAGATGGGGGGGGGAAGAATACT----------------------------
----

AGTGATAATGAAACCTTCAGACCTGGAGGAGGAGATATGAGGGACAATTGGAGAAGTGAATTATATAAGTATA
AAGTAGTAAAAATTGAACCATTA---GGAGTAGCACCCAACAGGGCAAGGAGAAGAGTGGTG----------
----------------------------GAGAGAGAAAAAAGAGCA---GTTGGA---ATAGGAGCT-----
---GTGTTCCTTGGGTTCTTGGGAGCA---GCAGGAAGCACTATG---GGCGCAGCGTCA---
ATGACGCTGACGGTACAG---GCTAGACAA---TTATTG---TCTGGTATAGTGCAACAGCAAAGC---
AATTTG---CTGAGG---GCTATAGAGGCTCAACAGCATCTGTTGAAACTCACAGTCTGGGGCATT
>HM204621..2
TTCTATTGTAATACATCAAACCTGTTTAATAGCACTTGGGGTAATGGCAGTTACAGCACCAATGACACAGGGG
AC--

GCAAATTCAAATATAACTATCCAATGCAGAATAAAGCAAATTGTAAGGATGTGGCAGAGGACAGGACAAGCAA
TGTATGCCCCTCCCATCAAAGGAATAATAAGATGTATGTCAAAT---
ATTACAGGCCTACTATTAACAAGAGATGGTGGGATTAATAGGACG---------------------------
----

AATGAAACCTTCAGACCTATAGGAGGAGATATGATGGATAATTGGAGAAGTGAGCTATATAAATATAAAGTAG
TAAGAATTGAACCAATA---GGAGTAGCACCCAACAGGGCAAAGAGAAGAGTGGTG----------------
----------------------GAGAGAGAAAAAAGAGCA---GTCTTTGGAATGGGAGCT---------
GTCTTCCTTGGGTTCTTAGGAGCA---GCCGGAAGCACTATG---GGCGCAGCGTCG---
ATAACGCTGACGTTACAG---GCCAGACAA---TTGTTG---TCTGGTATAGTGCAACAGCAAAGC---
AATTTG---CTGAGG---GCTATAGAGGCTCAACAGCATCTGTTGAAACTCACGGTCTGGGGCATT
>KY658695..2
TTCTATTGTAACACATCAAGTTTGTTTAAAGGCACTTGGAATAATGGCACTTGGAATGATAGCATGTCAGAGG
TAAAT---
-------------AGCACA------
GAGTCAAATGACAACATAACTCTCCCATGCAGAATAAAGCAAATTATAAATATGTGGCAGAAAGTAGGGCAAG
CAATGTATGCCCCACCCATCCAGGGAGTAATAAGGTGTGAATCAAAC---
ATTACTGGACTAATATTAACAAGAGATGGTGGGAATAGTAGCGGT---------------------------
----

CAGAATGAAACCTTTAGACCTGGAGGAGGAGATATGAGGGACAATTGGAGAAGTGAATTATATAAGTATAAAG
TAGTAAAAATTGAACCACTA---GGAGTAGCACCCACCAAGGCAAGAAGAAGAGTGGTG-------------
-------------------------CAGAGAGAAAAAAGAGCA---GTTGGA---ATGGGAGCA--------
CTTTTCATTGGGTTCTTAGGAGCA---GCAGGAAGCACTATG---GGCGCGGCGTCA---
ATAACGCTGACGGTACAG---GCCAGACAA---TTATTG---TCTGGCATAGTGCAACAGCAAAGC---
AATTTG---CTGAAG---GCTATAGAGGCTCAACAGCATCTGTTGAGACTCACTGTCTGGGGCATT
>KY658696..1
TTCTACTGTAATACATCAGGCCTGTTTAATAGCAATAGCACAGCAAATAGCACAGCAAAGTTTAATAGCAATA
GCACAGCA--

AATAGCACAGCAAATATAACTCTCCCATGCAGAATAAAACAAATTGTAAATATGTGGCAGAGGCCAGGACAAG
CAATGTATGCCCCTCCAATCCAAGGGCCAATAAGTTGTAGGTCAAAC---
ATTACAGGATTACTAATAACAAGAGATGGTGGAGAAAACAATGGA---------------------------
----

ACGAATGAAACCTTCAGACCTGGAGGAGGAGAGATGAGGGATATTTGGAGAAGTGAACTATATAAATATAAAG

TAGTGAAAATTGAACCATTA---GGAGTAGCACCCACCAGGGCAAGGAGAAGAGTGGTG--------------
-------------------------GAGAGAGAAAAAAGAGCA---GTTGGA---CTGGGAGCT---------
GTCTTCATTGGGTTCTTAGGAGCA---GCCGGAAGCACTATG---GGCGTGGCGTCA---
ATAACGCTGACGGTACAG---GCCAGACAA---TTATTG---TCTGGTATAGTGCAGCAGCAAAAC---
AATTTG---CTGAGG---GCTATAGAGGCTCAACAGCATATGTTGAAACTCACAGTCTGGGGCATT
>AF361872..2
TTCTATTGTAATACAACAAGCCTGTTTAATAGCACCTGGTATCACAAGAACAGCACGCAGGGGTCAAATGACA
CG---

GGGTCAGATGACACTATAACTCTCCCATGCAAAATAAAACAAATTGTAAGGATGTGGCAGAGAGCAGGACAAG
CAATGTATGCCCCTCCCATTCCAGGAGTAATAAGGTGTGAATCAAAC---
ATTACAGGACTAATATTAACAAGAGATGGGGGGAGTGAGGACAAT----------------------------
----

ACCACTGAAATCTTCAGACCTGGAGGAGGAAATATGAGGGACAATTGGAGAAGTGAATTATATAAGTATAAAG
TAGTAAAAATTGAACCACTA---GGAGTAGCACCCACCAAGGCAAAGAGAAGAGTGGTG--------------
-------------------------GGAAGAGAAAAAAGAGCA---GTTGGA---CTGGGAGCT---------
GTCTTCCTTGGGTTCTTAGGAGCA---GCCGGAAGCACTATG---GGCGCGGCGTCA---
GTAACGCTGACGGTACAG---GCCAGACAA---TTATTG---TCTGGCATAGTGCAACAGCAGAAC---
AATTTG---CTGAGG---GCTATAGAGGCTCAACAACATCTGTTGAAACTCACAGTCTGGGGCATC
>AF361873..1
TTCTATTGTAACACATCAGGCCTGTTTAACAGCACTTGGCATAGCAATGCCAGCATACAAGAGCCAAACAGCA
CAGAG--

TCAAATGACACTATTATAACTCTCCAATGTAGAATAAAACAAATTATAAATATGTGGCAGAGAACAGGAAAAG
CAATATATGCTCCTCCCATCCAAGGAATAATAAGGTGTGAATCAAAC---
ATTACAGGACTAATATTAACAAGAGATGGTGGGAGTAATAATAGC----------------------------
----

ACCAATGAAACCTTCAGACCTGGAGGAGGAGATATGAGGGACAATTGGAGAAGTGAATTATATAAGTATAAAG
TAGTAAAAATTGAACCACTA---GGAGTAGCACCCTCCTGGGCAAGGAGAAGAGTGGTG--------------
-------------------------CAGAGAAAAAAAAGAGCA---GTTGGA---ATAGGAGCT---------
GTCTTCCTTGGGTTCTTAGGAGCA---GCTGGAAGCACTATG---GGCGCGGCGTCA---
ATAACGCTGACGGTACAG---GCCAGACAA---TTATTG---TCTGGCATAGTGCAACAGCAAAGC---
AATTTG---CTGAGG---GCTATAGAGGCTCAACAGCATCTGTTGAAACTCACGGTCTGGGGCATT
>HM027846..2
TTCTATTGTAACACATCAGGTCTGTTTAATAGCACGTGGAATACTACCAACATGTGGGAGCAAAATAGCACGG
AG---

TCAAACAATAATACTATATATCTTCAATGCAGAATAAAGCAAATTATCAATATGTGGCAGAGAGTAGGACAAG
CAATATATGCCCCTCCCATCCCAGGAATGATAAGGTGTGTCTCAAAT---
ATTACAGGACTAATATTAACAAGGGATGGTGGGAATATTAGTGAGAAT-------------------------
----

AATGCCAGTGAAATCTTCAGACCAGGAGGAGGAGATATGAGGGACAATTGGAGAAGTGAATTATACAAGTATA
AAGTAGTAAAAATTGAACCACTA---GGAGTAGCACCAACCCCGGCAAGGAGAAGAGTGGTG-----------
----------------------------GAGAGAGAAAAAAGAGCA---GTCGGA---ATAGGAGCT------
---GTCTTCCTTGGGTTCTTAGGAGCA---GCAGGAAGCACTATG---GGCGCGGCGTCA---
ATAACGCTGACGGCACAG---GCCAGACAA---TTATTG---TCTGGTATAGTGCAACAGCAAAGC---
AATTTG---CTGAGG---GCTATAGAGGCTCAACAGCATCTGTTGAAACTCACGGTCTGGGGCATT
>JX877522..1
TTCTATTGTGACACATCAGAACTATTTAATAGCACTTGGAATGACGCTGCCATTATGAGGGAATTAAATAGAA
CGGAG--

TCAAACGACACCATAACTCTCCAATGCAAAATAAAGCAAATTATAAATATGTGGCAGAGAGCAGGACGAGCAA
TGTATGCCCCTCCCATCCGAGGAGTAATAAGATGTGAGTCAAAT---
ATTACAGGACTAATATTAACAAGAGATGGTGGGAGGGATGATAAC----------------------------
----

AATACAACTGAAACCTTCAGACCTGGAGGAGGAGATATGAGGGACAATTGGAGAAGTGAATTATATAAGTATA

AAGTAGTAAAACTTGAGCCACTA---GGAATAGCACCCACCAAGGCAAAGAGAAGAGTGGTG-----------
----------------------------GAGAGAGAAAAAAGAGCA---GTTGGA---CTGGGAGCT------
---GTTTTCATTGGGTTCTTAGGAGCA---GCAGGAAGCACTATG---GGCGCGGCGTCA---
ATAACGCTGACGGTACAG---GCCAGACAA---TTATTG---TTTGGCATAGTGCAACAGCAAAGC---
AATTTG---CTGAGG---GCTATAGAGGCTCAACAGCATATGTTGAAACTCACAGTCTGGGGCATT
>HM215272..1
TTCTATTGTAATACATCAAGCCTATTTAATAGTACTTGGTGGAGCAACAACAGCAACAACACTGACAGTGAGG
AGTCAAAT---
-------------AGCATG------
GGGTTAAATGACACTATAACTCTCCAATGCAGAATAAAGCAAATTATAAATATGTGGCAGAGAGTAGGACAAG
CAATGTATGCCCCTCCCATCCAAGGAGTAATAAGGTGTGAGTCAAAC---
ATCACAGGAATACTATTAACAAGAGATGGTGGGGATAATAACAAT----------------------------
----

GAAAGTGAAACCTTCAGACCTGTAGGAGGAGATATGAGGGACAATTGGAGAAGTGAATTATATAAATATAAAG
TAGTAAAAATTGAACCATTA---GGAGTAGCACCCACAGAGGCAAGGAGAAGAGTGGTG-------------
-------------------------GGAAGAGGAAAAAGAGCAGTTGTTGGA---ATGGGAGCT---------
ATGTTCCTTGGGTTTTTGGGAGCA---GCTGGGAGCACTATG---GGCGCGGCGTCA---
ATAACGCTGACGGTACAG---GCCAGACAA---TTATTG---TCTGGCATAGTGCAACAGCAAAGC---
AATTTG---CTGAGG---GCTATAGAGGCTCAACAGCATCTGTTGAAACTCACTGTCTGGGGCATT
>HM027824..1
TTCTATTGTAACACATCAGGTCTGTTTAATAGCACGTGGAATGGTACCCAAATGATGGAGCCAAATAGCACAG
AA---

AACATTAATGATACTATAACTCTTAAATGCAGAATAAAGCAAATTATACATATGTGGCAGAGAGTAGGACAAG
CAATATATGCCCCTCCCATCAAAGGAGTGATAAGATGTAACTCAAAC---
ATTACAGGACTAATATTAACAAGAGATGGTGGGACTGAAGGGAAT----------------------------
----

AGCAGTGAAACCTTCAGACCGGGAGGAGGTGATATGAGGGACAATTGGAGAAGTGAATTATATAAGTATAAAG
TAGTAAAAATTGAACCACTA---GGAGTAGCACCTACCAAGGCAAGGAGAAGAGTGGTG-------------
-------------------------GAGAGAGAAAAAAGAGCA---ATCGGA---CTGGGAGCT---------
GTCTTCCTTGGGTTCTTAGGAGCA---GCAGGAAGCACTATG---GGCGCGGCGTCA---
ATAACGCTGACGGCACAG---GCCAGACAA---TTATTG---TCTGGCATAGTGCAACAGCAAAGC---
AATTTG---CTGCAG---GCTATAGAGGCTCAACAGCATCTGTTGAAACTCACAGTCTGGGGCATT
>JX236669..1
TTCTACTGTAATACATCAGGCCTGTTTAATAGCACTTGGAACATTGCTGGGAACAGGACAAATGACACGAAG-
----

TCAAATGAAACTATAACTCTCCCATGCAGAATAAAGCAAATTGTAAATGTGTGGCAGAGAGTAGGACAAGCAA
TATATGCCCCTCCCATCGCAGGAGTAATAAGATGTAACTCAAAC---
ATTACAGGATTACTATTAGTAAGAGATGGTGGAGCTACTAACAAT----------------------------
----

ACTGATGAAACCTTCAGGCCTGGAGGAGGAAATATGAGGGACAATTGGAGAAGTGAATTATATAAGTATAAAG
TAGTAAAAATTGAACCACTA---GGAGTAGCACCCACCAGGGCAAGGAGAAGAGTGGTG-------------
-------------------------GAGAGAGAAAAAAGAGCA---GTTGGA---CTGGGAGCT---------
GTATTCATTGGGTTCTTAGGAGCA---GCAGGAAGCACTATG---GGCGCGGCGTCA---
GTGACGCTGACGGTACAG---GCCAGACAA---TTATTG---TCTGGCATAGTGCAACAGCAAAGC---
AATTTG---CTGAGG---GCTATAGAGGCTCAACAGCATCTGTTGAAGCTCACGGTCTGGGGCATT
>JX236671..2
TTCTATTGTAATACAGCAGGTCTGTTCAATAGAAATTGGACTTGGAGTAAGGAGAATGGGACCAGAATGCAGA
ATAAAACGGAT--

TCAAATGGCACTATAACTCTCCAATGCAGAATAAAGCAAATTATAAATATGTGGCAGAGAACAGGACAGGCAA
TGTATGCCCCTCCCATCCAAGGAGTAATAAAATGTGCATCAAAC---
ATTACAGGACTACTGTTAACAAGGGATGGTGGAGATAACAAAAACAATAGTACC------
AATAGTACCAATCAAAAT---

AACACTGAAATCTTTAGACCTATAGGAGGAGATATGAGGAATAATTGGAGAAGTGAATTATATAAGTATAAAG

TAGTAAAAATTGAACCACTA---GGAGTAGCACCTACCACGGCAAGGAGAAGAGTGGTG--------------
-------------------------GGAAGAGAAAAAAGAGCAGTACTAGGTGGACTAGGAGCT---------
GTATTCCTTGGGTTCTTAGGAGCA---GCCGGAAGCACTATG---GGCGCAGCGTCA---
ATAACGCTGACGGTACAG---GCCAGGCAA---TTATTG---TCTGGCATAGTGCAACAGCAAAGC---
AATTTG---CTGAGG---GCTATAGAGGCTCAACAACATCTGTTGAAACTCACGGTCTGGGGCATT
>JX236676..1
TTCTATTGTAACACATCAAGCCTATTTAATAGCATTTGGAATGGCAGTAAATCAAATAACACGGAG-------
----

CCAGATAGCACCATAACTCTCCAATGCAGAATAAAGCAAATTATAAATATGTGGCAGAGAGTAGGACAAGCAA
TGTATGCCCCTCCCATCCAAGGAGTAATAAGGTGTGAGTCAAAC---
ATTACAGGGCTAATATTAACAAGAGATGGTGGGAGTAATAGCAGT----------------------------
----

AGTACAAATGAAACCTTCAGACCTGGAGGAGGAGATATGAGAGACAATTGGAGAAGTGAATTATATAAGTACA
AAGTAGTAAAAATTGAACCACTA---GGAGTAGCACCCACCAAGGCAAAGAGAAGAGTGGTG-----------
----------------------------GAAAGAGAAAAAAGAGCA---GTTGGA---ATAGGAGCT------
---GTTTTCCTTGGGTTCTTAGGAGCA---GCAGGAAGCACTATG---GGCGCAGCGTCA---
ATAACGCTGACGGTACAG---GCCAGACAA---TTATTG---TTTGGCATAGTGCAACAGCAAAGT---
AATTTG---CTGAGG---GCTATAGAGGCTCAACAGCATCTGTTGAAACTCACGGTCTGGGGCATT
>KF716478..2
TTCTATTGTAATACATCAAGCCTGTTTAATAGTACTTGGGTATATGGTAATGGAAGCTGGGGGTCAAATAGCA
CGGGGTTAAAT--
AGTACAATGATAAATAATACT------------
ATTTTCAGTGATACCATAATTCTTCAATGCAGAATAAAGCAAATTATAAATATGTGGCAGAGAGCAGGACAAG
CAATATATGCCCCTCCCATCAAAGGAGTAATAAGCTGTAGATCAAAC---
ATCACAGGACTAATATTAACAAGAGATGGTGGGAGTAACAGCAATGCAAGT----------------------
----

ACAAATGGAAGTGAAACCTTCAGACCTGGAGGAGGAGATATGAGGGACAATTGGAGAAGTGAATTATATAAGT
ATAAAGTAGTAAAAGTTGAACCACTA---GGAATAGCACCCTCCAAGGCAAGGAGAAGAGTGGTG--------
-------------------------------GCGAGAGAAAAAAGAGCA---GTTGGA---ATAGGAGCT---
------GTACTCCTTGGGTTCTTAGGAACA---GCAGGAAGCACTATG---GGCGCAGCGTCA---
ATAACGCTGACGGTACAG---GCCAGAAAA---TTATTG---TCTGGCATAGTGCAACAGCAAAGC---
AATTTG---CTGAGG---GCTATAGAGGCTCAACAGCATCTGTTGAAACTCACTGTCTGGGGCATT
>KP109490..1
TTCTACTGTAATACATCAGGCCTGTTCAATAGCACTTGGAATAACACCTCGCAGGCAAATAGCTCGGAG----
----

TTAAAGGGCAATATAACTCTCCCATGCAAAATAAAACAAATTATCAGGATGTGGCAAAGAACAGGACAAGCAA
TGTATGCCCCTCCCATCCAAGGAGTAATAAATTGTGTATCAAAC---
ATTACAGGACTACTATTAACAAGAGATGGTGGGGAGGAGGACAAT----------------------------
----

AGTACTGAAACCTTCAGACCTGGAGGAGGAGATATGAGGGATAATTGGAGAAGTGAATTATATAAGTATAAAG
TAGTAAAAATTGAACCAGTA---GGAGTAGCACCCACCAAGGCAAGGAGAAGGGTGGTG--------------
-------------------------GAGAGAGAAAAAAGAGCA---GTTGGA---ATAGGAGCT---------
GTCCTCCTTGGATTCTTAGGAGCA---GCAGGAAGCACTATG---GGCGCGGCGTCA---
ATAACGCTGACGGTACAG---GCCAGACAG---TTATTG---TCTGGTATAGTGCAGCAGCAAAGC---
AATTTG---CTGAGG---GCTATAGAGGCTCAACAACAACTGTTGAAACTCACGGTCTGGGGCATT
>JF680921..1
TTCTATTGTAACACATCAGGCCTGTTTAATAGCACTTGGACTAAGAATAGCAGCACTAACACTTGGAATAGTA
CTGACATCATG--
-------TGGGGGGTAAAT------
AACAGTGACCATAATATAACTCTCCAATGCAAAATAAAGCAAATTATAACCATGTGGCAGAGGACAGGGCAGG
CAATGTACGCCCCTCCCATCCCAGGAATAATAAACTGTGTGTCAAAC---
ATTACAGGACTACTATTAACAAGAGATGGTGGTGAGGAGAGTAACAGT-------------------------
----

AGAGATAATGAAACCTTCAGACCTATAGGAGGAAATATGAAGGATAATTGGAGAAGTGAACTATATAAATATA

AAGTAGTAAAAATTGAACCACTA---GGAGTAGCACCCACCAAGGCAAAGAGAAGAGTGGTG-----------
----------------------------CAGAGAGAAAAAAGAGCA---GTTGGA---CTGGGAGCT------
---GTCTTCATTGGGTTCTTAGGAGCA---GCCGGAAGCACTATG---GGCGCGGCGTCA---
ATAACGCTGACGGTACAG---GCCAGACAA---TTATTG---TCTGGCATAGTGCAACAGCAAAAC---
AATTTG---CTGAGG---GCTATAGACGCTCAACAGCATCTGTTGAAGCTCACGGTCTGGGGCATT
>KF859745..2
TTCTATTGTAACACATCAGGCTTGTTTAATAGCACTTGGAACAATAGCACTCAAGAGTCTAAT----------
----

AGCACGGAGACTATAACTCTCCCATGCCGAATAAAGCAAATTGTAAATATGTGGCAGAGAGCAGGACAAGCCA
TATATGCCCCTCCCATCAAAGGAATAATAAATTGTAGATCAAAC---
ATTACAGGACTAATATTAACAAGAGATGGTGGACATAATAATACA----------------------------
----

AATGAAACCTTCAGGCCTGGAGGAGGAGATATGAGGGACAATTGGAGAAGTGAATTATATAAGTATAAAGTAG
TAAAAGTTGAACCACTA---GGAGTAGCACCCACCACGGCAAGGAGAAGAGTGGTG-----------------
----------------------CAGAGAGAAAAAAGAGCAGTATTTGGA---CTGGGAGCT---------
GTCTTCATTGGGTTCTTAGGAGCA---GCAGGAAGCACTATG---GGCGCGGCGTCA---
ATAACGCTGACGGTACAG---GCCAGACAA---TTATTG---TCTGGCATAGTGCAACAGCAAAGC---
AATTTG---CTGAGG---GCTATAGAGGCTCAACAGCATCTGTTGAAACTCACGGTCTGGGGCATT
>KF716486..2
TTCTATTGTAATACATCAAGTCTGTTTAATAGTACTTGGGACCTTAATGCTACTAGGGAAGAT----------
----

AGCACGGAGAACATAACTCTCCCATGCAGAATAAAGCAAATCATAAATATGTGGCAAAGAGTAGGACAAGCAA
TGTATGCCCCTCCCATCGAAGGAATAATAAGATGTGACTCAAAC---
ATTACAGGACTACTCTTAACAAGAGATGGTGGGAATAGTAGTACAAATGAA----------------------
----

ACTCAGGAAATCTTCAGACCTATAGGAGGGGATATGAAAGATAATTGGAGAAATGAATTATACAAGTATAAAG
TAGTAAAAATTGAACCTCTG---GGAGTAGCACCCTCCTGGGCAAGGAGAAGAGTGGTG--------------
-------------------------GAGAGAGGAAAAAGAGCA---GTTGGA---ATAGGAGCT---------
GTATTCCTTGGGTTCTTAGGAGCA---GCAGGGAGCACTATG---GGCGCAGCGTCA---
ATAACGCTGACGGTACAG---GCCAGGCAA---TTATTG---TCTGGCATAGTGCAACAGCAAAGC---
AATTTG---CTGAAG---GCTATAGAAGCTCAACAACAACTGTTGAGACTCACGGTCTGGGGCATT
>M62320..1
TTCTATTGCAACACATCAGGCCTGTTTAATAGCATTTGGAATGGCAGCATGTCAAATGACATGGGG-------
----

CCAAATGGCACTATAACTCTCCAATGTAGAATAAAGCAAATTATAAATATGTGGCAGAGAGTAGGACAAGCAA
TGTATGCCCCTCCCATCCAAGGAGTAATAAGGTGTGAATCAAAC---
ATTACAGGACTACTATTAACAAGAGATGGGGGTACTAACAATACA----------------------------
----

AAAAATGAGACCTTCAGACCTGGAGGAGGAGATATGAGGGACAATTGGAAAAGTGAATTATATAAGTATAAAG
TAGTAAAAATTGAACCACTA---GGAGTAGCACCCACCAGGGCAAAGAGAAGAGTGGTG--------------
-------------------------GAGAGAGAAAAAAGAGCA---GTTGGA---CTAGGAGCT---------
ATCTTCCTTGGGTTCTTAGGAGCA---GCTGGAAGCACAATG---GGCGCGGCGTCA---
ATAACGCTGACGGTACAG---GCCAGACAA---TTATTG---TCTGGTATAGTGCAACAGCAGAGC---
AATCTG---CTGAGG---GCTATAGAGGCTCAACAGCATCTGTTGAAACTCACTGTCTGGGGCATT
>L22957..2
TTCTATTGTAATACAGCAGGCCTGTTTAATAGTACTTGGAATAAAAATGACAGCATAAAGTCAAATGACACAA
AG---

TCAAATGACATTATAACTCTTCAATGCAGAATAAAGCAAATTATAAATATGTGGCAGAGAGTAGGACGAGCAA
TGTATGCCCCTCCCATCAAAGGAGTAATAAGGTGTGAATCAAAC---
ATTACAGGACTACTATTAACAAGAGATGGTGGAAATGGGAGCAAT----------------------------
----

ACAAATGAAACCTTCAGACCAGGAGGAGGAGATATGAGGGACAATTGGAGAAATGAATTATATAAGTATAAAG

TAGTACAAATTGAACCATTA---GGAGTAGCACCTACCCCAGCAAAGAGAAGAGTGGTG--------------
-------------------------CAGAGAGAAAAAAGAGCA---GTTGGA---CTAGGAGCT---------
GTATTCATTGGGTTCTTAGGAGCA---GCAGGAAGCACTATG---GGCGCGGCGTCA---
ATAACGCTGACGGTACAG---GCCAGACAA---TTATTG---TCTGGCATAGTGCAACAGCAAAGC---
AATTTG---CTGAGA---GCTATAGAGGCTCAACAGCATCTGTTGAAACTCACAGTCTGGGGCATT
>L22951..1
TTCTATTGTGACACATCAGGCCTGTTTAATAGCACTTGGAATGGCAATACCAGCAAGCAGGAGTCAAGTAGCA
CA---

GAGTCAAATGGCAATATAACTCTCCAATGCAGAATAAAGCAAATTATAAATATGTGGCAGAGAGCAGGAAAAG
CAATATATGCCCCTCCTATCAAAGGAGTAATAAGGTGTGTATCAAAC---
ATTACAGGACTAATATTAACAAGAGATGGTGGGGGTAATAACAGT----------------------------
----

GAAAATGAAACCTTCAGACCTGGAGGAAGAGATATGAGGGACAATTGGAGAAGTGAATTATATAAGTATAAAG
TAGTAAAAATTGAACCACTA---GGAGTAGCACCCACCAGGGCAAGGAGGAGAGTGGTG--------------
-------------------------GAGAGAGAAAAAAGAGCA---GTTGGA---GTAGGAGCT---------
GTCTTCCTTGGGTTCTTAGGAGCA---GCAGGAAGCACTATG---GGCGCGGCGTCA---
ATAACGCTGACGGTACAG---GCCAGACAA---TTATTG---TCTGGCATAGTGCAACAGCAAAGC---
AATTTG---CTGAAG---GCTATAGAGGCTCAACAGCATCTGTTGAAACTCACGGTCTGGGGCATT
>AB253429..1
TTCTATTGTAATACATCAGGCCTGTTTAATAGTACTTGGGTAAATGGCACTACCAGCAGCATG----------
----

TCAAATGGCACTATAACTCTCCCATGCAGAATAAAGCAAATTATAAATATGTGGCAGAGAGTAGGACAAGCAA
TGTATGCCCCTCCCATCCAAGGAGTAATAAAGTGTGAATCAAAC---
ATTACAGGACTAATATTAACAAGAGATGGTGGGGTTAATAGCAGT----------------------------
----

GACAGTGAAACCTTCAGACCTGGAGGAGGAGATATGAGGGATAATTGGAGAAGTGAATTATATAAGTATAAGG
TAGTAAAAATTGAACCACTA---GGAGTAGCACCCACCAAGGCAAGGAGAAGAGTGGTG--------------
-------------------------GAGAGAGAAAAAAGAGCA---GTTACA---CTGGGAGCT---------
GTATTCATTGGGTTCTTAGGAACA---GCTGGAAGCACAATG---GGCGCGGCGTCA---
ATAACGCTGACGGTACAG---GCCAGAAAA---TTATTG---TCTGGCATAGTGCAACAGCAAAGC---
AATTTG---CTGAGG---GCTATAGAGGCTCAACAGCATCTGTTGAAACTCACTGTCTGGGGCATT
>AY494973..1
TTCTATTGTAATACATCAGGCCTGTTTAATAGCACTTGGCCTTGGAATGAGACAAATAGCGAGACAAATAAC-
----

ACGAACGCTACTATAGTGCTCCCATGCAGAATAAAACAAATTGTAAGAATGTGGCAGAGAGTAGGACAAGCAA
TGTATGCCCCTCCCATCCAAGGAGTAATAAAGTGTACATCAAAC---
ATTACAGGACTACTATTAATAAGAGATGGTGGGGTTGGTAACAATACA-------------------------
----

AATGGAACTGAAACCTTCAGACCTGGAGGAGGAAATATGAGGGACAATTGGAGAAGTGAATTATATAAGTATA
AAGTAGTAAAAATTGAACCACTA---GGAGTAGCACCCACCGGGGCAAAAAGAAGAGTGGTG-----------
----------------------------GAGAGAGAAAAAAGAGCA---GTTGGA---ATAGGAGCT------
---GTGTTCATTGGGTTCTTAGGAGCA---GCTGGAAGCACTATG---GGCGCAGCATCA---
GTAACGCTGACGGTACAG---GCCAGACAA---TTGTTG---TCTGGCATAGTGCAACAGCAAAGC---
AATTTG---CTGAGG---GCTATAGAGGCTCAACAGCATATGTTGAAACTCACGGTCTGGGGCATT
>EU853062..2
TTCTATTGTAATACATCAGACCTGTTTAATAGCACTTGGTACTTAAATGGCACTAGCACTTGGTACAATGGCA
CTGCAAAT---
-------------AACGCA------
CCGTTAAATGGTAATATAACTCTCCCATGCAGAATAAAGCAAATCATAAGGATGTGGCAGAGAACAGGACAAG
CAATGTATGCCCCTCCCATTCAAGGAAAAATAGAATGTGTATCAAAC---
ATTACAGGACTCCTATTAGTAAGAGATGGTGGGAATGATGAGAATAACAACACA------------AGA----
----
-
AATAACACCCTCAAAGATGAAAAATTCAGACCTGGAGGAGGAGATATGAAGGACAATTGGAGAAATGAATTAT

ATAGGTATAAAGTAGTAAGAGTTGAACCACTA---GGAGTAGCACCCACCAAGGCAAGGAGAAGAGTGGTG--
-------------------------------------GGGAGAGAAAAAAGAGCA---GTTGGA---
ATAGGAGCT---------GTGTTCCTTGGGTTCTTAGGAGCA---GCAGGAAGCACTATG---
GGCGCGGCGTCA---ATAACGCTGACGGAACAG---GCCAGACAA---TTATTG---
TCTGGTATAGTGCAACAGCAAAGC---AATTTG---CTGAGG---
GCTATAGAGGCTCAACAGCAACTGTTGAAACTCACAGTCTGGGGCATT
>EU852958..1
TTCTATTGTAATACATCAGGCCTGTTTAATAGCACCTGGGATAAAAATACCACCGTGCAGGAGTCAAATAGCA
CG---

GGGTCAAATGACACTATAACTCTCCAATGTAGAATAAAGCAAATTATAAATATGTGGCAGGAAGTAGGACGAG
CAATGTATGCTCCTCCCATCAAAGGAGTAATAAGGTGTGAATCAAAC---
ATTACAGGAATGATATTAACAAGAGATGGGGGTAATACTACCGGG----------------------------
----

AAAAATGAGATCTTTAGGCCTGGAGGAGGAGATATGAGGGATAATTGGAGAAGTGAATTGTATAAGTATAAAG
TAGTAAAAATTGAACCACTA---GGAGTAGCACCCACCGAGGCAAAGAGAAGAGTGGTG--------------
-------------------------GAGAGAGAAAAAAGAGCA---ATTGGTGGAATAGGAGCT---------
GTCTTCCTTGGGTTCTTAGGAGCA---GCAGGAAGCACTATG---GGCGCAGCGTCA---
ATAACGCTGACGGCACAG---GCCAGACAA---TTATTG---TCTGGTATAGTGCAACAGCAAAGC---
AATTTG---CTGAGG---GCTATAGAGGCTCAACAGCATCTGTTGAAACTCACGGTCTGGGGCATT
>EU852954..1
TTCTATTGTAACACATCAGGCCTGTTTAATAGCACCTGGTATACAAATAATACCAGCGTGCAGGAGACAAATA
GCACG--

GGGACAAATGACACTATAATTCTCCAATGTAGAATAAAGCAAATTATAAATATGTGGCAGGAAGTAGGACGAG
CCATGTATGCTCCTCCCATCAGGGGAGTAATAAGGTGTGAATCAAAC---
ATTACAGGAATGATATTAACAAGAGATGGGGGTGAGTGTTAACAAG---------------------------
----

ACAACTGAGACTGAGGTCTTCAGACCTGGAGGAGGAGATATGAGGGATAATTGGAGAAGTGAATTGTATAAGT
ATAAAGTAGTAAAAATTGAACCACTA---GGAGTAGCACCTACCAAGGCAAAGAGAAGAGTGGTG--------
-------------------------------GAGAGAGAAAAAAGAGCA---ATTACTGGAATAGGAGCT---
------GTCTTCCTTGGGCTCTTAGGAGCA---GCAGGAAGCACTATG---GGCGCAGCGTCA---
ATAACGCTGACGGCACAG---GCCAGACAA---TTATTG---TCTGGTATAGTGCAACAGCAAAGC---
AATTTG---CTGAGG---GCTATAGAGGCTCAACAACATCTGTTGAAACTCACGGTCTGGGGCATT
>AF484507..1
TTCTATTGTAATACATCACAATTGTTTAATAGCATTTGGAATGTCACTAACAGCACAGAGTCAGAGAATAGCA
CG---

GGGTCAGATGACACTATAACTCTTCCATGCAGAATAAAGCAAATTATAAAGATGTGGCAGAGAACAGGACAAG
CAATATATGCCCCCCCCATCCAAGGAGTAATAAGGTGTGAATCAAAC---
ATTACAGGACTATTATTAACAAGAGATGGTGGGAATAACAATACA----------------------------
----

GGTAATGAAACCTTCAGACCTGCAGGAGGAGATATGAAGGACAATTGGAGAAGTGAATTATATAAGTACAAAG
TAGTAAAAATTGAACCACTA---GGAGTAGCACCCACCAAGGCAAGAAGGAGAGTGGTG--------------
-------------------------GAGAGAGAAAAAAGAGCA---GTTGGA---ATAGGAGCT---------
GTATTCCTTGGGTTCTTAGGAACA---GCAGGAAGCACTATG---GGCGCGGCGTCA---
ATAACGCTGACGGTACAG---GTCAGACAA---TTATTG---TCTGGCATAGTGCAACAGCAAGGC---
AATTTG---CTGAGG---GCTATAGAGGCTCAACAACAGCTGTTGGAACTCACGGTCTGGGGCATT
>AF484508..2
TTCTACTGTAACACATCAAGCCTGTTTAATGGCACTTGGAATGGCACTGAGAACATAACGGAGTCAAATAGC-
----

ATGAACGACTCTATAATACTCCAATGCAGAATAAAGCAAATTATAAATATGTGGCAGAGAGCAGGACAAGCAA
TATATGCCCCTCCCATCCCAGGAGTAATAAGGTGTCAATCAAAC---
ATTACAGGACTAATATTAACAAGAGATGGTGGAAATAACAGTACA----------------------------
----

AATGAAACCTCCAGACCTGGAGGGGGAGATATGAGGGACAACTGGAGAAGTGAATTATATAAGTATAAAGTAG
TAAAACTTGAACCACTA---GGAGTAGCACCCACCAGGGCAAAGAGAAGAGTGGTG-----------------
----------------------GAGAGAGAGAAAAGAGCA---GTTGGA---CTAGGAGCT---------
GTTTTCTTTGGGTTCTTAGGAGCA---GCAGGAAGCACTATG---GGCGCGGCGTCA---
ATAACGCTGACGGTACAG---GCCAGACAA---TTATTG---TCTGGTATAGTGCAACAGCAAAGC---
AATTTG---CTGAGG---GCTATAGAGGCTCAACAGCATCTGTTGAAACTCACGGTCTGGGGCATT
>AF484509..1
TTCTATTGTGACACATCAGGCCTGTTTAATAGCACTTGGTATAATACTTGGAATAGCACTGAATCAAAAAGCA
CG---

GAGTCAAACGACATTACTCTCCAATGCAGAATAAAGCAAATTATAAATATGTGGCAGAGAGCAGGACAAGCAA
TATATGCCCCTCCCATCCAAGGAGTAATAATGTGTAAATCAAAT---
ATTACAGGACTAATATTAACAAGAGATGGTGGAGGTAATAGCAGT----------------------------
----

GAACCTGAAACCTTCAGACCTGGAGGAGGAGACATGAGGGACAATTGGAGAAGTGAATTGTATAAGTATAAAG
TAGTAAAAATTGAACCACTA---GGAGTAGCACCCACCAAGGCAAGGAGAAGAGTGGTG--------------
-------------------------CAGAGAGAAAAAAGAACA---GTTGGA---ATAGGAGCT---------
GTGTTCCTTGGGTTCTTAGGAGCA---GCAGGAAGCACTATG---GGCGCGGCGTCA---
ATAACGCTGACGGCACAG---GCCAGAGCA---TTACTG---TCTGGCATAGTGCAACAGCAAAGC---
AATTTG---CTGAGG---GCTATAGAGGCTCAACAACATCTGTTGAGACTCACGGTCTGGGGCATT
>AF484512..2
TTCTATTGTGATACAACAGGACTGTTTAATAGCAATTGGACTTGGAATGACACTGCCAGCATAATGGAGTCAA
ATAGC--
-------------------ATG---
GAGTCAACTCAAAATATAACACTCCAATGCAGAATAAAGCAGATTATAAATATGTGGCAGAGAGCAGGTAAAG
CAATGTATGCCCCTCCCATCCAAGGAACAATAAGGTGTGAATCAAAC---
ATTACAGGACTACTGTTAACAAGAGATGGTGGGGATAATAGAGAT----------------------------
----

AATAGAACTGAAACCTTCAGACCTGGAGGAGGAGATATGAGGGATAATTGGAGAAGTGAATTATATAAGTATA
AAGTAGTCAAAATTGAACCACTA---GGAGTAGCACGCACCAGGGCAAAGAGAAGAGTGGTG-----------
----------------------------GAGAGAGAAAAAAGAGCA---GTTGGA---ATAGGAGCT------
---GTCTTCCTTGGGTTCTTAGGAGCA---GCAGGAAGCACTATG---GGCGCGGCGTCA---
ATAACGCTGACGGTACAG---GCCAGACTA---TTATTG---TCTGGTATAGTGCAACAGCAAAGC---
AATTTG---CTGAGG---GCTATAGAGGCTCAACAACATTTGTTGAAGCTCACGGTCTGGGGCATT
>EU853030..1
CTCTATTGTAATACATCAGGCCTGTTTAATAGCACTTGGAATAACACTGTCAGCACGGAGTTAAATAGCAAGG
AG---

GCAAATAACAATATAACTCTCCCATGCAAAATAAAGCAAATTATAAACATGTGGCAAAGAACAGGACAAGCAA
TGTATGCCCCTCCCATCCGAGGAGTAATAAATTGTGTGTCAAAC---
ATTACAGGACTAATATTAACAAGAGATGGTGGGCCTAACGATACA----------------------------
----

CATGAAAATGAAACCTTCAGACCTGGAGGAGGAAATATGAGGGACAATTGGAGAAGTGAATTATATAAATATA
AAGTAGTAAAAATTGAACCATTA---GGAGTAGCACCCACCAGGGCAAAGAGAAGAGTGGTG-----------
----------------------------GAAAGAGAAAAAGGAGCA---GTTGGA---ATAGGAGCT------
---GTCTTCCTTGGGTTCTTAGGAGCA---GCAGGAAGCACTATG---GGCGCGGCGTCA---
ATAACGCTGACGGTACAG---GCCAGACAA---TTGTTG---TCTGGCATAGTGCAACAGCAAAGC---
AATTTG---CTGAGG---GCTATAGAGGCTCAACAACATCTGTTGAAACTCACGGTCTGGGGCATT
>AF484478..1
TTCTATTGTAACACAACAAACCTGTTTAATAACATTTGGAGTAATGCCAGCATGCAGAAGGCAAATAGC----
----

ATAAACGACACTATAACTCTCCAATGCAGAATAAAGCAAATTATAAATATGTGGCAGAGAGTAGGACAAGCAA
TATATGCCCCTCCCATCCAAGGAGTGATAAAGTGTGAATCAAAC---
ATTACAGGACTAATATTAACAAGAGATGGTGGGGGTAATAACAAT----------------------------
----

ACAAATGAAACCTTCAGACCTGGAGGAGGAGATATGAGGGACAATTGGAGAAGTGAATTATATAAGTATAAAG
TAGTAAAAATTGAACCACTA---GGAGTAGCACCTACCAGGGCAAAGAGAAGAGTGGTG--------------
-------------------------GAGAGAGAAAAAAGAGCA---GTTGGA---ATGGGAGCT---------
GTCCTCATTGGGTTCTTAGGAGCA---GCAGGAAGCACTATG---GGCGCGGCGTCA---
ATAACGCTGACGGTACAG---GCCAGACAA---TTATTG---TCTGGCATAGTGCAACAGCAAAGC---
AATTTG---CTGAGG---GCTATAGAGGCTCAACAGCATCTGTTGAAACTCACGGTCTGGGGCATT
>AF484493..2
TTCTATTGTAATACATCAGGCCTGTTTAATAATACTTACACTACTGACAGCATAGAGTCAAATGACACGGAG-
----

CCAGATGCAAATATAACTCTTCCATGCAGAATAAAGCAAATTATAAATATGTGGCAGAGAGTAGGACAAGCAA
TATATGCCCCTCCCATCCAAGGAGTAATAAAGTGTGAGTCAAAC---
ATCACAGGACTACTATTAACAAGAGATGGTGGTACTAACAGTACT----------------------------
----

AAAAGTACTGAAACCTTCAGACCTGGAGGAGGAGATATGAGGGACAATTGGAGAAGTGAATTATATAAGTATA
AAGTAGTACAAGTTGAACCAATA---GGAGTAGCACCCACCAGGGCAAAGAGAAGAGTGGTG-----------
----------------------------GAGAGAGAAAAAAGAGCA---GTTGGA---ATAGGAGCT------
---GTACTCCTTGGGTTCTTAGGAGCA---GCTGGAAGCACAATG---GGCGCGGCGTCA---
ATAACGCTGACGGTACAG---GCCAGACAG---TTATTG---TCTGGCATAGTGCAGCAGCAAAGC---
AATTTG---CTGAGG---GCTATAGAGGCTCAACAGCAACTGTTGAGACTCACTGTCTGGGGCATT
>AB098330..2
TTCTATTGTAATACATCAGGCCTGTTCAATGGCACCTGGAAGCCAAACAGCACAGAGTCAAACAACACAACG-
----

CCAAATGACACTATAACTCTTACATGCAGAATAAAGCAAATTATAAATATGTGGCAGAAAGTAGGACAAGCAA
TGTATGCCCCTCCCATTCAAGGAGTAATAAGGTGTGAATCAAAC---
ATTACAGGACTACTATTAACAAGAGATGGAGGGATTAACAGTATA----------------------------
----

AATGAAACCTTCAGACCTGGAGGAGGAAATATGAGGGACAATTGGAGAAGTGAATTATATAAGTATAAAGTAG
TAAAAATTGAACCACTA---GGAGTAGCACCCTCCAGGGCAAAGAGAAGAGTGGTG-----------------
----------------------GAGAGAGAAAAAAGAGCA---GTTGGA---ATAGGAGCT---------
GTCTTCCTTGGGTTCTTAGGAGCA---GCTGGAAGCACTATG---GGCGCGGCGTCA---
ATAACGCTGACGGCACAG---GCCAGACAA---TTATTG---TCTGGTATAGTGCAACAGCAAAGC---
AATTTG---CTGAGG---GCTATAAAGGCTCAACAGCATATGTTGAAGCTCACGGTCTGGGGCATT
>GU367412..5
TTCTATTGTGATACATCAGGCCTGTTTAATAGTACTTGGAATGACACTGCCACCAATCAGACTTTTGAT----
----

GCAAATAGCAATATAACTCTTCAATGTAAAATAAGACAAATTGTACGTATGTGGCAGAGAATAGGACAAGCAT
TGTATGCCCCTCCCATTCAAGGAGTAATAAGCTGTAAATCAAAC---
ATTACAGGGCTGCTACTAACAAGAGATGGT---ATGAATGAGAGT----------------------------
----

AGTGAGACGTTCAGACCTCAAGGAGGAAATATGAAGGACAATTGGAGAAGTGAATTATATAAATATAAAGTAG
TAAAAATTGAACCACTA---GGAATAGCACCCACCAGGGCAAAGAGAAGAGTGGTG-----------------
----------------------GAGAGAGAAAAAAGAGCA---GTTGGA---CTGGGAGCT---------
GTCTTCTTTGGGTTCTTGGGAGCA---GCAGGAAGTACTATG---GGCGCGGCGTCA---
ATAACGCTGACGGCACAG---GCCAGACAA---TTATTG---TCTGGTATAGTACAACAGCAGAGC---
AATCTG---CTGAGG---GCTATAGAGGCTCAACAGCAACTGTTGAAACTCACAGTCTGGGGCATT
>FJ647148..1
TTCTATTGTAATACATCAGGTCTGTTTAGTAGCAGGTGGTTCACTAATGGCACTAACAGCACGGAGTCAAAT-
----

GGCACAGGCAATATAACTCTCCAATGCAGGATAAAGCAAATTATAAATATGTGGCAGAGAGTAGGACAAGCAA
CGTACACCCCTCCCATCCAAGGAGAAATAAGGTGTAGATCAAAC---
ATTACAGGACTACTATTAACAAGAGATGGTGGGATTAACACAACA----------------------------
----

GAGGAAATCTTCAGACCTGGAGGGGGAAATATGAAGGACAATTGGAGAAGTGAATTATATAAGTATAAAGTAG
TAAAAATTGAACCACTA---GGAGTAGCACCATCCAAGGCAAAGAGAAGAGTGGTG----------------
---------------------GGAAGAGAAAAAAGAGCA---GTTGGA---CTGGGAGCT---------
GTATTCATTGGGTTCTTGGGAGCA---GCAGGAAGCACTATG---GGCGCGGCGTCA---
GTGACGCTGACGGTACAG---GCCAGACAA---TTATTG---TCTGGCATAGTGCAGCAGCAAAGC---
AATTTG---CTGAGG---GCTATAGAGGCTCAACAGCATCTGTTGAAACTCACAGTCTGGGGCATT
>KT183312..1
TTCTATTGTGACACATCAGGTCTGTTTAATGGCAGTTGGAGTGAAAATAGCACTTGGAATAGCACTATGGAG-
----

GCAAATAGCACAATTATACTCCCATGTAGAATAAAGCAAATTATAAATATGTGGCAGAGAACAGGACGAGCAA
TGTATGCCCCTCCCATCCAAGGAACAATACAGTGTACATCAAAC---
ATCACAGGATTAATATTAACAAGAGATGGTGGTGAGAAAAACAAT---------------------------
----

AGCACTGAAGAAATCTTCAGACCTGGAGGAGGAGATATGAGGGACAATTGGAGGAGTGAATTATATAAATATA
AAGTAGTAAAAATTGAACCACTA---GGAGTAGCACCCACCAGGGCAAAGAGAAGAGTGGTG----------
---------------------------GAGAGAGAAAAAAGAGCA---GTTACA---CTAGGAGCT------
---GTATTCATTGGGTTCTTAGGAGCA---GCAGGAAGCACTATG---GGCGCGGCGTCA---
GTGACGCTGACGGTACAG---GCCAGACAA---TTATTG---TCTGGCATAGTGCAACAGCAAAGC---
AATTTG---CTGAGG---GCTATAGAGGCTCAACAGCATATGTTGAAACTCACGGTCTGGGGCATT
>KC863311..1
TTCTATTGTAATACAACAGACCTGTTTAAGGGTACTTGGCATCCTGATAATAAAACTTGGAGTGATCCTTGGA
ATGCCAGC--
-ATGAAGTCAAATAAC---------
ACATCAAATGTCAATATAACTATCCTATGCAAGATAAAGCAAATTGTAAGGATGTGGCAGAGAGTAGAACAAG
CAATGTATGCCCCTCCCATCCAAGGAGTAATAAGCTGTTCATCAAAC---
ATTACAGGACTACTATTAACAAGCGATGGTGGTAGGGATAATAGCAATAATACTGGTAAGGATAATAGCAATA
AAAAT---

AATACTGAAACCTTCAGACCTGGAGGAGGAAATATGAGGGACAATTGGAGAAGTGAATTATATAAGTATAAAG
TAGTAAAAATTGAACCACTA---GGAGTAGCACCTACCCCGGCAAGGAGAAGAGTGGTG-------------
------------------------CAGAGAGAAAAAAGAGCA---GTTGGC---CTAGGAGCT---------
GTCTTCATTGGGTTCTTAGGAGCA---GCAGGAAGCACTATG---GGCGCGGCGTCA---
GTGACGCTGACGGTACAG---GCCAGACAA---TTATTG---ACTGGTATAGTGCGACAGCAAAGC---
AATTTG---CTGAAG---GCTATAGAGGCTCAACAACATCTGTTGAGACTCACAGTCTGGGGCATT
>HM623589..3
TTCTACTGCAACACATCAAGCTTGTTTAATAGGACATTTCCGGCCAACTACACGTATAATAGGACTGCTGACA
GCACA---

CAGGGGAATACAACTATAACTATCCCATGCAGAATAAAACAAATTATAAACATGTGGCAGAGAGTAGGACAAG
CAATGTATGCCCCTCCCATTGAAGGAGTAATAAGATGTGAATCAAAC---
ATTACAGGAATACTATTGACAAGAGATGGTGGGAATGAGACAAAT---------------------------
----

GTAGAAGTCTTCAGACCAGAGGGAGGAAATATGAGGGACAATTGGAGAAGTGAATTATATAATTACAAGGTAG
TAAGAATTGAACCACTA---GGAGTAGCACCCACCAGGGCAAGGAGGAGAGTGGTG----------------
---------------------GAGAGAGAAAAAAGAGCA---GTTGGA---CTGGGAGCT---------
GTCTTCCTTGGGTTCTTAGGAGCA---GCAGGAAGCACTATG---GGCGCGGCGTCA---
ATAACGCTGACGGTACAG---GCCAGGCAA---TTATTG---GCTGGCATAGTGCAACAGCAAAGC---
AATTTG---CTGCAA---GCTATAGAGGCTCAACAGCATCTGTTGAAACTCACTGTCTGGGGCATT
>MH705163..1
TTCTATTGCAATACAACAGCCCTGTTTAATAGCACGTGGGAGAATGAGAATGACACGGCCAACATACAGAAGA
AT--
-------------GGCACG------
GAGTCAAATGACACTATAACACTCCCATGCAGAATAAAACAAATTATAAACATGTGGCAGAGAGTAGGACGAG
CAATGTATGCCCCTCCCATTGCAGGAGTAATAAAGTGTACATCAACC---
ATTACAGGAATAATATTGACAAGAGATGGTGGGGATAGTGGGAACAAT------------------------
----

```
ACCAGGGAGGAGATCTTCAGACCTGGAGGAGGAGATATGAGGGACAATTGGAGAAGTGAATTATATAAGTATA
AAGTAGTAAAACTTGAACCACTA---GGAGTAGCACCCACCAAGGCAAGGAGAAGAGTGGTG-----------
----------------------------GAGAGAGAAAAAAGAGCA---GTTGGA---CTGGGAGCT------
---GTCTTCCTTGGGTTCTTGGGAGCA---GCAGGAAGCACTATG---GGCGCAGCGTCA---
ATAACGCTGACGGTACAA---GCCAGACAA---TTATTG---TCTGGCATAGTGCAACAGCAAGGC---
AATTTG---CTGAAG---GCTATAGAGGCTCAACAGCATCTGTTGAAACTCACGGTCTGGGGCATT
>AF286241..1
TTCTATTGTAATACAACAAGCCTGTTTAATAGCACATGGAAGAATGGCGCCACCATACAGGAGAATAGCACGG
AG-----------------------------------------------------------------------
----------------------------
ACAAATGGAATTATGACTCTCCCATGCAGAATAAAACAAATTGTAGACATGTGGCAGGAAGTAGGACAAGCAA
TGTATGCCCCTCCCATTGCAGGAGTAATATATTGTACATCAAAC---
ATTACAGGAATAATATTGACAAGAGATGGTGGGAGCAGTAACACC----------------------------
-------------------------------------------------------------------------
-------------
AATAGTGAGATCTTTAGGCCTGGAGGAGGAGATATGAGGGACAATTGGAGAAGTGAATTATATAAGTATAAAG
TAGTAAAAATTGAACCACTA---GGAGTAGCACCCTCCAGGGCAAAGAGAAGAGTGGTG--------------
-------------------------GAGAGAGAAAAAAGAGCA---GTGGGA---ATAGGAGCT---------
GTTTTCCTTGGGTTCTTGGGAGCT---GCAGGAAGCACTATG---GGCGCGGCGTCA---
ATAACGCTGACGGTACAG---GCCAGACAG---TTATTA---TCTGGCATAGTGCAACAGCAAAGC---
AATTTG---CTGAAG---GCTATAGAGGCTCAACAGCATCTGTTGAAACTCACAGTCTGGGGCATT
>AF286238..2
TTCTATTGCAATACAACAGGCCTGTTTAATAGCACATGGGAAAATGGCACTAACAAACAGAATTACACGGAG-
-------------------------------------------------------------------------
----------------------------
TCAAATGACACTATAACGCTCCAATGCAGAATAAAACAAATTATAAACATGTGGCAGAGAGTAGGCCGAGCAA
TGTATGCCCCTCCCATTGCAGGAGTAATAAAGTGTACATCAAAC---
ATTACAGGAATGATATTGACAAGAGACGGTGGGAAGAACAGTATT----------------------------
-------------------------------------------------------------------------
----------------
AATGAGACCTTCAGACCTGGAGGAGGAGATATGAGGGACAATTGGAGAAGTGAATTATATAAGTATAAAGTAG
TAAAAATTGAACCACTA---GGAATAGCACCCACAGAGGCAAGGAGAAGAGTGGTG-----------------
----------------------CAGAGAGAAAAAAGAGCA---GTTGGA---TTGGGAGCT---------
GTTTTCCTTGGGTTCTTGGGAGCA---GCAGGAAGCACTATG---GGCGCGGCGTCA---
ATAACGCTGACGGTACAG---GCCAGACAA---TTATTG---ACTGGCATAGTGCAACAGCAAAGC---
AATTTG---CTGAAG---GCTATAGAGGCTCAACAACAGATGTTGAGGCTCACAGTCTGGGGCATT
>AF286237..2
TTCTATTGCAATACAACAGGCCTGTTTAATGGTACGTGGTGGAACAATGGTACGTGGAACGGGCCCTACACAC
CTAAT--------------------------------------------------------------------
------------------------
AACACCAATGGAAGTATAATCCTCCCATGCAGAATAAAACAAATTATAAACATGTGGCAGAGAGTAGGAAGAG
CAATGTATGCCCCTCCCATTGCAGGAATAATAAAGTGTACATCAAAC---
ATTACAGGAATAATATTGACAAGAGATGGTGGTAACAATGGGACT----------------------------
-------------------------------------------------------------------------
----------------
AATGAGACCTTCAGACCTGGAGGAGGAGATATGAGGGACAATTGGAGAAGTGAATTATATAAATATAAAGTAG
TAAAACTTGAACCACTA---GGAGTAGCACCTACCAGGGCAAAAAGAAGAGTGGTG-----------------
----------------------GAGAGAGAAAAAAGAGCA---GTTGGA---CTGGGAGCT---------
GTCTTCCTTGGGTTCTTGGGAGCA---GCAGGAAGCACTATG---GGCGCGGCGTCA---
CTAACGCTGACGGTACAG---GCCAGACAA---TTATTG---TCTGGTATAGTGCAACAGCAAAGC---
AATTTG---CTGCAG---GCTATAGAAGCTCAACAGCATCTGTTGAAACTCACAGTCTGGGGCATT
>AY521629..3
TTCTATTGCAATACATCAGGTCTGTTTAATAGTACTTGGAATGACACGGCCATCAATGACACTATCAACGTGC
AG-----------------------------------------------------------------------
------------------------
AATTCAACTTACAATATAACTCTCCCATGCAGAATAAGACAAATTGTACATATGTGGCAGAGAGTAGGACAAG
CAATGTATGCCCCTCCCATTCAAGGGGTAATAAACTGTACATCAAAC---
ATTACAGGGCTACTGTTAACAAGGGATGGTGGGGATAATAGTAGT----------------------------
-------------------------------------------------------------------------
-------------
```

ACAAATGAGACATTCAGACCTGGAGGAGGAAATATGAAGGACAATTGGAGAAGTGAATTATATAAATATAAAG
TAGTAAAAATTGAACCACTA---GGAGTAGCACCCACCAAGGCAAAGAGGAGAGTGGTG-------------
------------------------GAGAGAGAAAAAAGAGCA---GTTGGA---CTAGGAGCT---------
GTCTTCCTTGGGTTCTTAGGAGCA---GCAGGAAGTACTATG---GGCGCGGCGTCA---
ATGACGCTGACGGTACAG---GCCAGACAA---TTATTG---TCTGGCATAGTACAACAGCAGAGC---
AATCTG---CTGAGG---GCTATAGAGGCTCAACAGCACATGTTGAAACTCACAGTCTGGGGCATT
>AY521631..2
TTCTATTGCAATACATCAGGCCTGTTTAATAGTACTTGGTATAATAGTACTTGGAATAACACTGACAGCACAC
AGAAA---
-------------------TCA---
AATGGCACGGAGAAAATAACTCTCCCATGCAGAATAAAACAAGTTATAAAAATGTGGCAAAGAGTAGGACAAG
CAATGTATGCCCCTCCCATTCAAGGAGTAATAAAATGTGTATCAAAC---
ATTACAGGGATACTGCTAACAAGAGATGGGGGGAACCTTAATAGC---------------------------
----

TCATTTGAGACGTTCAGACCTGGAGGAGGAAATATGAAGGACAATTGGAGAAGTGAATTATATAAATATAAAG
TAGTAAAAATTGAACCACTA---GGAGTAGCACCCACCAAGGCAAAGAGAAGAGTGGTG-------------
------------------------GAGAGAGAAAAAAGAGCG---GTCGGA---CTGGGAGCT---------
GTCTTCCTTGGGTTCTTAGGAGCA---GCAGGAAGTACTATG---GGCGCGGCGTCA---
ATAACGCTGACGGCACAG---GCCAGACAA---TTATTG---TCTGGTATAGTACAACAGCAGAGC---
AATCTG---CTGAGG---GCTATAGAGGCTCAACAGCAAATGTTGAAACTCACGGTCTGGGGCATT
>AY521630..1
TTCTATTGCAATACATCAGGTCTGTTTAATAGTACTTGGAATAGCACTACCATCAAGCAGAATTTAACTAACA
AT--

GACACGGGGGTAATAAATCTCCCATGCAGAATAAGACAAATTGTACATATGTGGCAGAGAGTAGGACAAGCAA
TGTATGCCCCTCCCATTAAAGGGGTAATAAACTGTACATCAAAC---
ATTACAGGGCTACTGCTAACAAGGGATGGTGGGGATAATAATAGT---------------------------
----

ACAGATGAGAAGTTCAGACCTGGAGGAGGAGATATGAGGGACAATTGGAGAAGTGAATTATATAAATATAAAG
TAGTAAAAATTGAACCACTA---GGAGTAGCACCCACCAGGGCAAAGAGAAGAGTGGTG-------------
------------------------CAGAGAGAAAAAAGAGCA---GTTGGA---CTAGGAGCT---------
GTCTTCTTTGGGTTCTTAGGAGCA---GCAGGAAGTACTATG---GGCGCGGCGTCA---
ATAACGCTGACGGCACAG---GCCAGACAA---TTATTG---TCTGGCATAGTACAACAGCAGAGC---
AATCTG---CTGAGG---GCTATAGAGGCTCAACAGCACTTGTTGAAACTCACAGTCTGGGGCATT
>AM000053..1
TTCTATTGCAATACATCAGGCCTATTTAATAGCAGTTGGGCGCCCAACGGCAGTTGGATCAACAACATTGGCA
GT--

AGGGAAAATGACACTATGACTCTGCCATGCAGAATAAAACAAATTATAAATATGTGGCAGAGAGTAGAACAAG
CAATGTATGCCCCTCCTATCAAAGGAAACATAACTTGTGTATCAAAC---
ATTACAGGACTACTGTTAACAAGAGATGGTGGAACTAATGAAACT---------------------------
----

AGGGAGAATGAGACCTTCAGACCTATAGGAGGAGAGATGAGAGACAATTGGAGAAGTGAATTATATAAGTATA
AAGTAGTAAAAATTGAACCACTA---GGAGTAGCACCCACCAAGGCAAAGAGAAGAGTGGTGGAG-------
---------------------------GGGAGAGAAAAAAGAGCA---GTTGGA---ATGGGAGCT------
---TTCTTCCTTGGGTTCTTAGGAGCA---GCAGGAAGCACTATG---GGCGCGGCGTCA---
ATAACGCTGACGGTACAG---GCCAGACAA---TTATTG---GCTGGTATAGTGCAACAGCAAAGC---
AATTTG---CTGAAG---GCTATAGAGGCTCAACAACATCTGTTGAGACTCACAGTCTGGGGCATT
>AM000054..2
TTCTATTGTAATACATCAAGCCTGTTTAATAGCTCTTGGCCCATTAACAGCACTACCAGCAATAACAGCACTG
CC--

AGTAATGAGACCATAATTCTGCCATGTAGAATAATACAAATTATAAATATGTGGCAGAGACTAGGACAGGCAA
TGTATGCCCCTCCCATCAAAGGATTAATAAATTGTGTATCAAAC---
ATTACAGGACTACTGTTGACAAGGGATGGTGGAGATAGGAACAGT---------------------------
----

AGTTATGAGACCTTCAGACCTATAGGAGGAAATATGAAGGACAATTGGAGAAGTGAACTGTATAAGTATAAAG
TAGTAAAAATTGAACCACTA---GGAGTAGCACCTACCAAGGCAAGGAGGAGAGTGGTG--------------
-------------------------GAGAGAGAAAAAAGAGCA---GTTGGA---ATAGGAGCT---------
TTCTTCCTTGGGATCTTAGGAGCA---GCAGGAAGCACTATG---GGCGCGGCGTCA---
ATAACGCTGACGGTACAA---GCCAGGCAA---GTATTG---TCTGGTATAGTGCAACAGCAGAGC---
AATTTG---CTGAAG---GCTATAGAGGCTCAACAACATTTGTTGAAACTCACAGTCTGGGGCATC
>FJ388892..2
TTCTATTGCAATACAACAGACCTGTTTAATAGCACTTGGTACACTAATGACACTGGCACAAATAGCACAGGGG
CCAATGAA---
-------------------AAT---
GGGGCCAATGAAAATATAACTTTACCATGCAGAATAAAGCAAATTATAAATATGTGGCAGAGAGTAGGGCAAG
CAATGTATGCCCCTCCTATCAAAGGAAGTATAAGGTGTGAATCAAAC---
ATTACAGGACTACTACTAACAAGAGATGGTGGAAGTGGAAATGAT----------------------------
----

AGCTACAATGAGACCTTCAGACCTATAGGAGGAAATATGAGGGATAATTGGAGAAGTGAACTATATAAGTATA
AAGTAGTAGAAATTGAACCAATA---GGAGTAGCACCTACCAAGGCAAGGAGAAGAGTGGTG-----------
----------------------------GGGAGAGAAAAAAGAGCA---ATTGGA---CTGGGAGCT------
---GCCTTCCTTGGGTTCTTAGGAGCA---GCAGGAAGCACTATG---GGCGCGGCGTCA---
ATAACGCTGACGGTACAG---GCCAGACAA---TTATTG---TCTGGCATAGTGCAACAGCAAAGC---
AATTTG---CTGAGG---GCTATAGAGGCTCAGCAGCATCTGCTGAAACTCACAGTCTGGGGCATT
>JF683763..1
TTCTATTGCAATACAACAGACCTGTTCAGTAGCACTTGGGACGAAGATGACACTGACATCACTCTCGCCCAG-
----

GACAATGGAACTATAACTCTGCCATGCAGAATAAAGCAAATTATAAATATGTGGCAGAGAGTAGGACAAGCAA
TGTATGCCCCTCCTATCAAAGGAAGTATAAGGTGTGAATCAAAC---
ATTACAGGACTACTACTAACAAGAGATGGTGGAGCTAATAGCAGT----------------------------
----

AATGAGACCTTCAGACCTATAGGAGGAGATATGAGAGACAATTGGAGAAGTGAACTATATAAATATAAAGTGG
TACAAATTGAACCAATA---GGAATAGCACCTACCAGGGCAAGGAGAAGAGTGGTG-----------------
----------------------GAGAGAGAAAAAAGAGCA---ATTGGA---CTGGGAGCT---------
GCCTTCCTTGGGTTCTTAGGAGCA---GCAGGAAGTACTATG---GGCGCGGCGTCA---
CTGACGCTGACGGTACAG---GCCAGAAAA---TTATTG---TCTGGCATAGTGCAACAGCAAAAC---
AATTTG---CTGAGG---GCTATAGAGGCTCAGCAGCATCTGCTGAAACTCACGGTCTGGGGCATT
>JF683798..1
TTCTATTGCGATACAACAAACCTGTTCAATAGCACTTGGGACGGAAATAGCACTGTCACAAATATCACACAG-
----

ACCAATGAAAATATAACTTTACCATGCAAAATAAGGCAAATTATAAGGATGTGGCAGAGAGTAGGACAAGCAA
TGTATGCCCCTCCTATCAAAGGAATTATAAGGTGTGAATCAAAC---
ATTACAGGACTACTGCTAACAAGAGATGGTGGAAATAAAACCAAT----------------------------
----

AGCAACAATGAGACCTTCAGACCTATAGGAGGAGATATGAGGGACAATTGGAGAAGTGAACTATATAAGTATA
AAGTAGTAAAAGTGGAACCAATA---GGAGTAGCACCTACCAGAGCAAGGAGAAGAGTGGTG-----------
----------------------------GAGAGAGAAAAAAGAGCA---ATAATTGGACTGGGAGCT------
---GCCTTCATTGGGTTCTTAGGAGCA---GCAGGAAGCACTATG---GGCGCGGCGTCA---
ATAACGCTGACGGTACAG---GCCAGACAA---TTATTG---TCTGGCATAGTGCAACAGCAAAGC---
AATTTG---CTGAGG---GCTATAGAGGCTCAGCAGCATCTGCTGAAACTCACGGTCTGGGGCATT
>EF545108..1
TTCTATTGCAATACAACAGGCCTGTTCAATAATGCCAGCAACAATGGTACAAAC-------------------
----

CGCACAGACCCTATAATTCTCCCATGCAGAATAAAGCAGATTATAAATATGTGGCAAAGAGTAGGGCAGGCAA
TGTATGCTCCTCCTATCAAAGGAATCATAAGGTGTGAATCAAAC---
ATTACAGGACTACTATTAACAAGAGATGGTGGGAATAAAAATGGAAGC-------------------------
----

AACGGGACCGAGACCTTCAGACCTATAGGAGGAGATATGAGGGACAATTGGAGAAGTGAATTATATAAGTATA
AAGTAGTAAAAATTGAACCAATA---GGAGTAGCACCTACCAGGGCAAAGAGAAGAGTGGTG-----------
----------------------------GAGAGAGAAAAGAGAGCA---ATTGGA---CTGGGAGCT------
---GCCTTCCTTGGGTTCTTAGGAGCA---GCAGGAAGTACTATG---GGCGCGGCGTCA---
ATAACGCTGACGGTACAG---GCCAGACAA---TTACTG---TCTGGCATAGTACAACAGCAAAGC---
AATTTG---CTGAGG---GCTATAGAGGCTCAGCAGCATCTGCTGAAGCTCACGGTCTGGGGCATT
>GU481566..1
TTCTATTGCAATACAACAGCCCTGTTCAATAGCACTTGGGATGAAAATAGCACTGTCACAAATAGCACAACG-
----

GCCAATGAAAATATAACTCTGCCATGCAGAATAAAGCAAATTATAAATATGTGGCAGAGAGTAGGACAAGCAA
TGTATGCCCCTCCTATCGCAGGAATTATAAGGTGTGAATCAAAC---
ATTACAGGACTACTAATAACAAGAGATGGTGGAACTAATAGCAGC----------------------------
----

AATGAGACTGAGACCTTCAGACCTACAGGAGGAAATATGAGGGACAATTGGAGAAGTGAACTATATAAGTATA
AAGTAGTACAAATTGAACCAATA---GGAGTAGCACCTACCAAGGCAAAGAGAAGAGTGGTG-----------
----------------------------GAGAGAGAAAAAAGAGCA---ATTGGA---CTGGGAGCC------
---GCCTTCCTTGGGTTCTTAGGAGCA---GCAGGAAGTACTATG---GGCGCGGCGTCA---
CTGACGCTGACGGTACAG---GCCAGACAA---TTATTG---TCTGGCATAGTACAACAGCAAAGC---
AATTTG---CTGAGG---GCTATAGAGGCTCAGCAGCATCTGCTGAAACTCACGGTCTGGGGCATT
>GU481615..1
TTCTATTGCAATACAACAGAACTGTTCAATAGCACTTGGAACACAAGCAGCACTGTCACAAATAGCACACAG-
----

GCCAATGAAACTATAAGTCTACCATGCAAAATAAAGCAAATTATAAGGATGTGGCAGAGAGTAGGACAAGCAA
TGTATGCCCCTCCTATCAACGGAACTATAAGGTGTGAATCAAAC---
ATTACAGGACTACTACTAACAAGAGATGGTGGAGGTGAAAATAAT----------------------------
----

AGCAGCAATGAGACCTTCAGACCTGCAGGAGGAGATATGAAGGACAATTGGAGAAGTGAACTATATAAGTATA
AAGTAGTAGAAATTGAACCAATA---GGATTAGCACCTACCAAGGCAAGGAGAAGAGTGGTG-----------
----------------------------GAGAGAGAAAAAAGAGCA---ATTGGA---CTGGGAGCT------
---GCCTTCCTTGGGTTCTTAGGAGCA---GCAGGAAGTACTATG---GGCGCGGCGTCA---
TTGACGCTGACGGTACAG---GCCAGACAA---TTATTG---TCTGGCATAGTGCAACAGCAAAGC---
AATTTG---CTGAGG---GCTATAGAGGCTCAGCAGCATCTGCTGAAACTCACAGTCTGGGGCATT
>HQ896489..1
TTCTATTGCAATACAACAGACCTGTTCACAAATAGCACTCTCACAGATAACACAGAG----------------
----

ACCAATGAAACTATAACTCTGCCATGCAGAATAAAGCAAATCATAAATATGTGGCAGAGAGTAGGACAAGCAA
TGTATGCCCCTCCTATCAGAGGAAGTATAAGGTGTAATTCAAAC---
ATTACAGGACTACTGCTAATAAGAGATGGTGGAGCTAATAGCAGC----------------------------
----

AATGAGACCTTCAGACCTATAGGAGGAAATATGAGGGACAATTGGAGAAGTGAACTATATAAGTATAAAGTAG
TAAAAATTGAACCAATA---GGAGTAGCACCTACCAAGGCAAAGAGAAGAGTGGTG-----------------
----------------------GAGAGAGAAAAAAGAGCA---ATTGGA---CTGGGAGCC---------
GCCTTCCTTGGGTTCTTAGGAACA---GCAGGAAGCACTATG---GGCGCGGCGTCA---
CTGACGCTGACGGTACAA---GCCAGACAA---TTATTG---TCTGGCATAGTGCAACAGCAAAGC---
AATTTG---CTGAGG---GCTATAGAGGCTCAGCAACATCTGCTGAAACTCACGGTCTGGGGCATT
>JQ292897..1
TTCTATTGCGATACAACAGCCCTGTTCAATAGCACTTGGGAAAATGGCACTGTTATAAATAGCACACAG----
----

GCCAATGAGACTATAACTCTACCATGCAGAATAAAGCAAATTATAAATATGTGGCAGAGAGTAGGACGAGCAA
TGTATGCCCCTCCTATCAGAGGAATTATAAGGTGTGAATCAAAC---
ATTACAGGACTACTACTAACAAGAGATGGTGGAGGTGGAAATAAT----------------------------
----

AGCAGCAATGAGACCTTCAGACCTATAGGAGGAAATATGGAGGACAATTGGAGAAGTGAACTATATAAGTATA
AAGTAGTAGAAATTGAACCAATA---GGAGTAGCACCTACCAAGGCAAAGAGAAGAGTGGTG-----------
----------------------------GAGAGGGAAAAAAGAGCA---ATTGGA---CTGGGAGCT------
---GCCTTCCTTGGGTTCTTGGGAGCA---GCAGGAAGCACTATG---GGCGCGGCGTCA---
CTAACGCTGACGGTACAG---GCCAGACAA---TTATTG---TCTGGCATAGTGCAACAGCAAAGC---
AATTTG---CTGAGG---GCTATAGAGGCTCAGCAGCATCTGCTGAAACTCACGGTCTGGGGCATT
>JQ292899..1
TTCTATTGCAATACAACAGACCTGTTCAATTGGGACGGAAATGGCAATGTTACAAACAGCACACAG-------
----

GCCGATGGAAATATAACTCTACCATGCAGAATAAAGCAAATTATAAATATGTGGCAGAGAGTAGGACAAGCAA
TGTATGCCCCTCCTATCAAAGGAAATATAACGTGTGTATCAAAC---
ATTACAGGACTACTATTAACAAGAGATGGTGGAGGTGGAATTAAT----------------------------
----

AGTAGCAATGAGACCTTCAGACCTATAGGAGGAGATATGAGGGACAATTGGAGAAGTGAACTATATAAGTATA
AAGTAGTAAAAATTGAACCAATA---GGAGTAGCACCTACCAAGGCAAGGAGAAGAGTGGTG-----------
----------------------------GAGAGAGAAAAAAGAGCA---ATTGGA---CTGGGAGCT------
---GCCTTCCTTGGGTTCTTGGGAGCA---GCAGGAAGTACTATG---GGCGCGGCGTCA---
ATAACGCTGACGGTACAG---GCCAGACAA---TTATTG---TCTGGCATAGTGCAACAGCAAAGC---
AATTTG---CTGAGG---GCTATAGAGGCTCAGCAGCATATGCTGAAACTCACGGTCTGGGGCATT
>HQ385841..2
TTCTATTGCTATACAACAAAACTGTTCAATAGCACTTGGAACGGAAATGACACTGTTACAAATAGCACACAG-
----

GCCAATGAAAATATAACTATACCATGCAGAATAAAGCAAATTATAAATATGTGGCAGAGAGTAGGACAAGCAA
TGTATGCCCCTCCTATCAAAGGAAATATAAGCTGTACATCAAAC---
ATTACAGGACTATTACTAACAAGAGATGGTGGAACTAATAGCAGT----------------------------
----

AATGAGACCTTCAGACCTATAGGAGGAGATATGAGGGACAATTGGAGAAGTGAACTATATAAGTATAAAGTAG
TACAAATTGAACCAATA---GGAGTAGCACCTACCAGGGCAAAGAGAAGAGTGGTG-----------------
----------------------GAGAGAGAAAAAAGAGCA---ATTGGA---CTGGGAGCT---------
GCCTTCCTTGGGTTCTTGGGAGCA---GCAGGAAGTACTATG---GGCGCGGCGTCA---
ATAACGCTGACGGTACAG---GCCAGACAA---TTATTG---TCTGGCATAGTACAACAGCAAAGC---
AATTTG---CTGAGG---GCTATAGAGGCTCAGCAGCATCTGCTGAAACTCACGGTCTGGGGCATT
>HQ385843..1
TTCTATTGCAATACAACAGCCCTGTTCAATAGTACTTGGCACGAAAATAGCACTGATACAAATAGCACACAG-
----

GACAATGAAACTATAACTCTACCATGCAGAATAAAGCAAATTATAAATATGTGGCAGAGAGTAGGACAAGCAA
TGTATGCCCCTCCTATCGAAGGAAATATAAGCTGTACATCAAAC---
ATTACAGGACTACTACTAACAAGGGATGGTGGAGCTAATAGCAGC----------------------------
----

AATGAGACCTTCAGACCTATAGGAGGAGATATGAGGGACAATTGGAGAAGTGAACTATATAAGTATAAAGTAG
TACAAATTGAACCAATA---GGAGTAGCACCTACCAAGGCAAAGAGAAGAGTGGTG-----------------
----------------------GAGAGAGAAAAAAGAGCA---ATTGGA---CTGGGAGCT---------
GCCTTCCTTGGGTTCTTGGGAGCA---GCAGGAAGCACTATG---GGCGCGGCGTCA---
ATAACGCTGACGGTACAG---GCCAGACAA---TTATTG---TCTGGCATAGTGCAACAGCAAAGC---
AATTTG---CTGAGG---GCTATAGAGGCTCAGCAGCATCTGCTGAAACTCACGGTCTGGGGCATT
>HQ385846..1
TTCTATTGCAATACAACAGAACTGTTCAATAGCACTTGGGGCAATGGCACTGTCACAAATAGCACAGGG----
----

GACAATGAAAATATAATTCTACCATGCAGAATAAAGCAAATCATAAGGATGTGGCAGAGAGTAGGACAAGCAA
TGTATGCCCCTCCTATCAAAGGAATTATAAAGTGTAAATCAAAC---
ATTACAGGACTACTACTAACAAGAGATGGTGGAGGTGGAAATAGCAGC-------------------------
----

AATGAGACCAGTGAAACCTTCAGGCCTATAGGAGGAGATATGAGGGACAATTGGAGAAGTGAACTATACAAGT
ATAAAGTAGTACAAATTGAACCAATA---GGAGTAGCACCTACCAGGGCAAAGAGAAGAGTGGTG--------
-------------------------------GAGAGAGAAAAAAGAGCA---ATTGGA---CTGGGAGCT---
------GCCTTCCTTGGGTTCTTAGGAGCA---GCAGGAAGCACTATG---GGCGCGGCGTCA---
ATAACGCTGACGGTACAG---GCCAGACAA---TTATTG---TCTGGCATAGTGCAACAGCAAAGC---
AATTTG---CTGAGA---GCTATAGAGGCTCAGCAGCATCTGCTGAAACTCACGGTCTGGGGCATT
>GU481434..2
TTCTATTGCTATACAACAAACCTGTTCAATAGCACTTGGGGCGAAAATAGCACTGATATAAATAGCACACAG-
----

GCCAATGAAAATATAACTCTACCATGCAGAATAAAGCAAATTATAAATATGTGGCAGAGAGTAGGACAAGCAA
TGTATGCCCCTCCTATCAAAGGAAGTATAATGTGTACATCAAAC---
ATTACAGGGCTATTACTAACAAGAGATGGTGGAGGTGGAGCTAAT----------------------------
----

AGCAGCAATGAGACCTTCAGACCTATAGGAGGAAATATGAGGGACAATTGGAGAAGTGAACTATATAAGTATA
AAGTAGTAAAAATTGAACCAATA---GGAGTAGCACCTACCACGGCAAGGAGAAGAGTGGTG-----------
----------------------------GAGAGAGAAAAAAGGGCA---ATTGGA---CTGGGAGCT------
---GCCTTCCTTGGGTTCTTGGGAGCA---GCAGGAAGCACTATG---GGCGCGGCGTCA---
CTAACGCTGACGGTACAG---GCCAGACAA---TTATTG---TCTGGCATAGTGCAGCAGCAAAGC---
AATTTG---CTGAAG---GCTATAGAGGCTCAGCAGCATCTGCTGAAACTCACGGTCTGGGGCATT
>GU481486..1
TTCTATTGCAATACAACAGACCTGTTCAATAGCACTTGGAACGAAACTGACATTGATGCACAGGCCAATGAA-
----

GTCAATGAAACTATAACTCTACCATGCAGAATAAAGCAAATTATAAATATGTGGCAGAGAGTAGGGCAAGCAA
TGTATGCCCCTCCTATCAAAGGAAGTATAAGGTGTGAATCAAAC---
ATTACAGGACTACTACTAACAAGAGATGGTGGAAGACTTAATGGC----------------------------
----

AGCAATGAGACCTTTAGACCTATAGGAGGAGATATGAGGGACAATTGGAGAAGTGAACTATATAAGTATAAAG
TAGTACAAATTGAACCAATA---GGAGTAGCACCTACCAAGGCAAAGAGAAGAGTGGTG--------------
-------------------------GAGAGAGAAAAAAGAGCA---ATTGGA---CTGGGAGCT---------
GCCTTCCTTGGGTTCTTGGGAGCA---GCAGGAAGTACTATG---GGCGCGGCGTCA---
ATAACGCTGACGGTACAG---GCCAGACAA---TTATTG---TCTGGCATAGTGCAACAGCAAAGC---
AATTTG---CTGAGG---GCTATAGAGGCTCAGCAGCATCTGCTGAAACTCACGGTCTGGGGCATT
>GU481644..1
TTCTATTGCAATACAACAAACCTGTTCAATAGCACTTGGAATGAAACAGACATTGTTACAAATATCACACAG-
----

GGCAATGGAACTATAACTCTACCATGCAGAATAAAGCAAATTATAAATATGTGGCAGAGAGTAGGACAAGCAA
TGTATGCCCCTCCTATCAAAGGAAATATAACGTGTACATCAAAC---
ATTACAGGACTACTACTAACAAGAGATGGTGGAACTAATAGCAGC----------------------------
----

AATGAGACCTTCAGACCTATAGGAGGAGATATGAGGGACAATTGGAGAAGTGAACTATATAAATATAAAGTAG
TACAAATTGAACCAATA---GGAGTAGCACCTACCAGGGCAAGGAGAAGAGTGGTG-----------------
----------------------GAGAGAGAAAAAAGAGCA---ATTGGA---CTGGGAGCT---------
GCCTTCCTTGGGTTCTTGGGAGCA---GCAGGAAGCACTATG---GGCGCGGCGTCA---
CTAACGCTGACGGTACAG---GCCAGACAA---TTACTG---TCTGGCATAGTGCAACAGCAAAGC---
AATTTG---CTGAGG---GCTATAGAGGCTCAGCAGCATCTGCTGAAACTCACGGTCTGGGGCATT
>JQ292891..1
TTCTATTGCAATACAACAGACCTGTTCAATAGCACTTGGGACAATAGCACTGTCACAAATAGCACAGGG----
----

ACCAATGAAACTATAACTCTGCCATGTAGAATAAAGCAAATTATAAATATGCGGCAGAGAGTAGGACAAGCAA
TGTATGCCCCTCCTATTAGAGGAAGTATAAGGTGTGAATCAAAC---
ATTACAGGACTACTACTAATAAGAGATGGTGGAGCTAATAGCAGC----------------------------
----

AATGAGACCTTCAGACCTATAGGAGGAAATATGAGGGACAATTGGAGAAGTGAACTATATAAGTATAAAGTAG
TAAAAATTGAACCAATA---GGAGTAGCACCTACCAAGGCAAGGAGAAGAGTGGTG----------------
----------------------GAAAGAGAAAAAAGAGCA---ATTGGA---CTGGGAGCC---------
GCCTTCCTTGGGTTCTTAGGAGCA---GCAGGAAGCACTATG---GGCGCGGCGTCA---
ATGACGCTGACGGTACAG---GCCAGACAA---TTATTG---TCTGGCATAGTGCAACAGCAAAGC---
AATTTG---CTGAGG---GCTATAGAGGCTCAGCAGCATCTGCTGAAACTCACGGTCTGGGGCATT
>HQ385820..1
TTCTATTGCAATACAACAAACCTGTTCAATAGCACTTGGGACACAAATATCACAGAGGCCAATATCACAGAG-
----

GCCAATGAAACTATAATTCTACCATGCAAAATAAAGCAAATTATAAATATGTGGCAGAGAGTAGGAAAAGCAA
TGTATGCCCCTCCTATCAAAGGAAGTATAAGGTGTGAATCAAAC---
ATTACAGGACTACTACTAACAAGAGATGGTGGAACTAATAGCAGC---------------------------
----

AATAGCAATGAGACCTTCAGACCTATAGGAGGAAATATGAGGGACAATTGGAGAAGTGAACTATATAAATATA
AAGTAGTAAAAATTGAACCAATA---GGAGTAGCACCTACCAAGGCAAAGAGAAGAGTGGTG----------
----------------------------GAGAGAGAAAAAAGAGCA---ATTGGG---TTGGGAGCT-----
---GCCTTCCTTGGGTTCTTAGGAGCA---GCAGGAAGCACTATG---GCCGGCGGGTCA---
ATAACGCTGACGGTACAG---GCCAGACAA---TTATTG---TCTGGCATAGTGCAACAGCAAAGC---
AATTTG---CTGAGG---GCCATAGAGGCTCAGCAACATATGCTGAAACTCACGGTCTGGGGCATT
>JF952018..1
TTTTATTGCAATACAACAGCCCTGTTCAATAGCACTTGGGACAATAGTAGCATTGCCGAAAATAGCACAGAG-
----

GACAATGCAACTATAACTCTACCATGCAGAATAAAGCAAATTATAAATATGTGGCAGAGAGTAGGGCAAGCAA
TGTATGCCCCTCCTATCAAAGGAAGTATAAGGTGTGAATCAAAT---
ATTACAGGACTATTACTAACAAGAGATGGTGGAGATGCCGATGGAAATGGAACT------------------
----

AATAGCAACAATGAAACCTTCAGACCTACAGGAGGAGATATGAGGAACAATTGGAGAAGTGAACTATATAAAT
ATAAAGTAGTACAAATTGAACCAATA---GGGGTAGCACCTACCACGGCAAAGAGAAGAGTGGTG-------
------------------------------GAGAGAGAAAAAAGAGCA---ATTGGA---CTGGGAGCT---
------GCCTTCCTTGGGTTCTTAGGAGCA---GCAGGAAGCACTATG---GGCGCGGCGTCA---
ATAACGCTGACGGTACAG---GCCAGACAA---TTATTG---TCTGGCATAGTGCAACAGCAAAGC---
AATTTG---CTGAGG---GCTATAGAGGCTCAGCAGCATCTGCTGAAACTCACGGTCTGGGGCATT
>HQ338112..1
TTCTATTGCAATACAACAGAACTGTTCAATAGCACTTGGGACAATAGCACTTGGGACAAAAATGACACTAGCA
CA--

AATATCACACAGGTCATAACTCTGCCATGCAGAATAAAACAAATTATAAATATGTGGCAGAGAGTAGGACAAG
CAATGTATGCCCCTCCTATTAAAGGAGTTATGAGGTGTGAATCAAAC---
ATTACAGGACTACTACTAACAAGAGATGGTGGAGCTAATAGCAGC---------------------------
----

AATGAGACCTTCAGACCTATAGGAGGAGATATGAGGGACAATTGGAGAAGTGAACTATATAAGTATAAAGTAG
TAGAAATTGAACCAATA---GGAGTAGCACCTACCAGGGCAAAGAGAAGAGTGGTG----------------
----------------------GAGAGAGAAAAAAGAGCA---ATTACA---CTGGGAGCT---------
GCCTTCCTTGGGTTCTTAGGAGCA---GCAGGAAGCACTATG---GGCGCGGCGTCA---
TTGACGCTGACGGTACAG---GCCAGACAA---TTATTG---TCTGGCATAGTGCAACAGCAAAGC---
AATTTG---CTGAGG---GCTATAGAGGCTCAGCAGCATCTGCTGAAACTCACGGTCTGGGGCATT
>GU481277..1
TTCTATTGCAATACAGCAGACCTGTTCAATAGCACTTGGGACACAAATAGCACACAGACCAATAGAAATATAA
CT--

GCCAGTGAAAATATAACTCTACCATGCAGAATAAAGCAAATTATAAATATGTGGCAGAGAGTAGGACAAGCAA
TGTATGCCCCTCCTATCAAAGGAATTATAATGTGTACATCAAAC---
ATTACAGGACTACTCCTAACAAGAGATGGTGGAGGTGAAACTAAT---------------------------
----

```
AGCAGCAATGAGACCTTCAGACCTATAGGAGGAGACATGAGGGACAATTGGAGAAGTGAACTATATAAATATA
AAGTAGTAAAAATTGAACCAATA---GGGGTAGCACCTACCAGGGCAAAGAGAAGAGTGGTG-----------
----------------------------GAGAGAGAAAAAAGAGCA---ATTGGA---CTGGGAGCT------
---GCCTTCCTTGGGTTCTTGGGAGCG---GCAGGAAGTACTATG---GGCGCGGCGTCA---
ATAACGCTGACGGTACAG---GCCAGACAA---TTATTG---TCTGGCATAGTACAACAGCAAAGC---
AATTTG---CTGAGG---GCTATAGAGGCTCAGCAGCATCTGTTGAAGCTCACGGTCTGGGGCATT
>GU481544..1
TTCTATTGCAATACAACAAAACTGTTCAATAGCTCTTGGGGCGAAAATGGCACAAATAGCATCGAG-------
-------------------------------------------------------------------------
----------------------------
ACCAATGAAACTATAATTCTACCATGCAGAATAAAGCAAATTATAAATATGTGGCAGAGAGTAGGACAAGCAA
TGTATGCCCCTCCTATCAAAGGAATTATAAAGTGTGAATCAAAC---
ATTACAGGACTACTACTAATCAGAGATGGTGGAACTAACAGCAGT----------------------------
-------------------------------------------------------------------------
----------------
AATGAGACCTTCAGACCTATAGGAGGAGATATGAGGGACAATTGGAGAAGTGAACTATATAAGTATAAAGTAG
TAAAAATTGAACCAATA---GGAGTAGCACCTACCAAGGCAAGGAGAAGAGTGGTG-----------------
----------------------GAGAGAGAAAAAAGAGCA---ATTGGA---CTGGGAGCT---------
GCCTTCCTTGGGTTCTTAGGAGCA---GCAGGAAGCACTATG---GGCGCGGCATCA---
ATAACGCTGACGGTACAG---GCCAGACAA---TTATTG---TCTGGCATAGTGCAACAGCAAAGC---
AATTTG---CTGAGG---GCTATAGAGGCTCAGCAACATCTGCTGAAACTCACGGTCTGGGGCATT
>GU481661..1
TTCTATTGCGATACAACAAACCTGTTCAATAGCACTTGGGAAGAAAAAAGCACTGTTACAAATAGC-------
-------------------------------------------------------------------------
----------------------------
ACCAATGACACTATAACTCTACCATGCAGAATAAAGCAAATTATAAATATGTGGCAGAGAGTAGGACAAGCAA
TGTATGCCCCTCCTATCCAAGGAAATATAAGTTGTGAATCAAAC---
ATTACAGGACTACTACTAACAAGAGATGGTGGAAGTGAAAATAAT----------------------------
-------------------------------------------------------------------------
-------------------
GAGACCTTCAGACCTATAGGAGGAGATATGAGGGACAATTGGAGAAGTGAATTATACAAGTATAAAGTAGTAA
AAATTGAACCAATA---GGAGTAGCACCTACCAGGGCAAGGAGAATAGTGGTG--------------------
-------------------AAGAGAGAAAAAAGAGCA---ATTGGA---CTGGGAGTT---------
GCCTTCCTTGGGTTCTTAGGAGCA---GCAGGAAGCACTATG---GGCGCAGCGTCA---
CTAACGCTGACGGTACAG---GCCAGACAA---TTATTG---TCTGGCATAGTGCAACAGCAAAGC---
AATTTG---CTGAGG---GCTATAGAGGCTCAGCAACATCTGCTGAAACTCACGGTCTGGGGCATT
>KF716491..1
TTCTATTGCAATACAACAAACCTGTTCGATAGCACTTGGAATGACACTGACACAAATAGCACTGACACAAATA
GCACAGGG-----------------------------------------------------------------
----------------------------
GCCAATGAAATTATAACTCTACCATGCAGAATAAGGCAAATTATAAATATGTGGCAGAGAGTAGGACAAGCAA
TGTATGCCCCTCCTATCAAAGGAGTTATAAGGTGTGAATCAAAC---
ATTACAGGACTACTACTAACAAGAGATGGTGGAACTAATAGCAGC----------------------------
-------------------------------------------------------------------------
----------
AATGAGAATGAGATCTTCAGACCTATAGGAGGAGATATGAGGGACAATTGGAGAAGTGAACTATATAAGTATA
AAGTAGTAGAAGTTGAACCAATA---GGAGTAGCGCCTACCAAGGCAAGGAGAAGAGTGGTG-----------
----------------------------GAGAGAGAAAAAAGAGCA---ATTGGA---CTGGGAGCT------
---GCCTTCCTTGGGTTCTTAGGAGCA---GCAGGAAGTACTATG---GGCGCGGCGTCA---
ATAACGCTGACGGTACAG---GCCAGACAA---TTATTG---TCTGGCATAGTGCAACAGCAAAGC---
AATTTG---CTGAAA---GCTATAGAGGCTCAGCAGCATCTGCTGAAACTCACGGTCTGGGGCATT
>KF716492..1
TTCTATTGCAATACAACAGAACTGTTCAATAGCACTTGGAAAGAAAATAGCACTATCACAAATAACACAACA-
-------------------------------------------------------------------------
----------------------------
GCCAATGACACTATAACTCTACCATGCAGAATAAAGCAAATTATAAATATGTGGCAGAGAGTAGGACAAGCAA
TGTATGCCCCTCCTATCAAAGGAAGTATAAGGTGTAAATCAAAC---
ATTACAGGACTACTACTAACAAGAGATGGTGGAAGTGAACCTAAT----------------------------
-------------------------------------------------------------------------
----------
```

AGCAGTGAGGAAATCTTCAGACCTATAGGAGGAAATATGAGGGACAATTGGAGAAGTGAACTATATAAGTATA
AAGTAGTAAAAATTGAACCAATA---GGAGTAGCACCTACCAAGGCAAAGAGAAGAGTGGTG-----------
----------------------------GAGAGAGAAAAAAGAGCAATAATTGGA---CTGGGAGCT------
---GCCTTCCTTGGGTTCTTAGGAGCA---GCAGGAAGCACTATG---GGCGCGGCGTCA---
CTGACGCTGACGGTACAG---GCCAGACAA---TTATTG---TCTGGCATAGTGCAACAGCAAAGC---
AATTTG---CTGAGG---GCTATAGAGGCTCAGCAGCATCTGCTGAAACTCACGGTCTGGGGCATT
>FJ864679..1
TTCTATTGTAATACAACAGAACTGTTCAATAGCACTTGGTACAAAAATGGCACTAACACAACTAGCACACAG-
----

ACCAGTGAAAACATAACTCTGCCATGCAGAATAAAGCAAATTGTAAGGATGTGGCAGAGAGTAGGACAAGCAA
TGTATGCCCCTCCTATCAGTGGAATTATAAAGTGTGAATCAAAC---
ATTACAGGATTAATACTAACAAGAGATGGTGGAGATAATAGTAGC----------------------------
----

AGCAATGAGACCTTCAGACCTGAAGGAGGAGACATAAGGGACAATTGGAGAAATGAACTATATAAGTATAAAG
TAGTAGAAATTGAACCAATA---GGAGTAGCACCTACTGAGGCAAGGAGAAGAGTGGTG--------------
-------------------------CAGAGAGAAAAAAGAGCA---ATTGGA---CTGGGAGCT---------
GCCTTCATTGGGTTCTTAGGAGCA---GCAGGAAGCACTATG---GGCGCAGCGTCA---
CTGACGCTGACGGTACAG---GCCAGAAAA---TTATTG---TCTGGCATAGTGCAACAGCAAAGC---
AATTTG---CTGAGG---GCTATAGAGGCTCAGCAGCATCTGCTGAAACTCACGGTCTGGGGCATT
>JQ292893..1
TTCTATTGCAATACCACAGACCTGTTCAATAGCACTTGGGACAAAAATGACACTATCACAAATACCCCAGAGG
TC---

AATGGAACTACTATAACTCTGCCATGCAGAATAAAGCAAATTGTAAATATGTGGCAGAGAGTAGGACAAACAA
TGTATGCCCCTCCTATCAAAGGAAGTATAAGGTGTGAATCAAAC---
ATTACAGGACTACTGCTAATAAGAGATGGTGGAGAAACTAATAGC----------------------------
----

AGCAGTGAGACCTTCAGACCTGTAGGAGGAGATATGAGGGATAATTGGAGAAGTGAACTATATAAGTATAAAG
TAGTAAAAATTGAACCAATA---GGAGTAGCACCTACCAGGGCAAAGAGAAGAGTGGTG--------------
-------------------------GAGAGAGAAAAAAGAGCA---ATTGGA---CTGGGAGCC---------
GCCTTCCTTGGGTTCTTAGGAGCA---GCAGGAAGTACTATG---GGCGCGGCGTCA---
CTGACGCTGACGGTACAG---GCCAGACAA---TTATTG---TCTGGCATAGTGCAACAGCAAAGC---
AATTTG---CTGAGG---GCTATAGAGGCTCAGCAGCATCTGCTGAAACTCACGGTCTGGGGCATT
>JF430898..1
TTCTATTGCAATACAACAGACCTGTTCAATAGCACTTGGAGGAATGACACTGTCGCAAATATCACG-------
----

GCCAATGGAACTATAACTCTGCCATGCAGAATAAAGCAAATTATAAATATGTGGCAGAGAGTAGGACAAGCAA
TGTATGCCCCTCCTATCGAAGGAAATATAAGTTGTGAATCAAAC---
ATTACAGGGCTACTACTAATAAGAGATGGTGGAACTGATGACAGC----------------------------
----

AATGAGACTGAGTCCTTCAGACCCATAGGAGGAGATATGAGGGACAATTGGAGAAGTGAACTATATAAATATA
AAGTGGTACAAATTGAACCAATA---GGAGTAGCACCTACCAAGGCAAGGAGAAGAGTGGTG-----------
----------------------------GAGAGAGAAAAAAGAGCA---ATTGGA---CTGGGAGCT------
---GCCTTCCTTGGGTTCTTAGGAGCA---GCAGGAAGCACTATG---GGCGCGGCGTCA---
TTGACGCTGACGGTACAG---GCCAGACAA---TTATTG---TCTGGCATAGTGCAACAGCAAAAC---
AATTTG---CTGAGG---GCTATAGAGGCTCAGCAGCATCTGCTGAAACTCACGGTCTGGGGCATT
>HQ834967..1
TTTTATTGCAATACAACAGGCCTGTTCAATAGCACTTGGGGCAATAGCACTGACGCAAAT-------------
----

AGCAATGAAAATATAACTCTACCATGCAGAATAAAGCAAATTATAAATATGTGGCAGAGAGTAGGACAAGCAA
TGTATGCCCCTCCTATCAGCGGAAATATAAGTTGTGTATCAAAC---
GTTACAGGACTACTACTAGTAAGAGATGGTGGAGGTGGAAATAGT----------------------------
----

AGCAACAATGAGACCTTCAGACCTATAGGAGGAGATATGAGGGACAATTGGAAAAGTGAACTATATAAGTATA
AAGTAGTAGAAATTGAACCAATA---GGAGTAGCACCTACCAAGGCAAAGAGAAGGGTGGTG-----------
----------------------------CAGAGAGAAAAAAGAGCA---ATTGGAGGACTGGGAGCT------
---GCCCTCCTTGGATTCTTAGGAGCA---GCAGGAAGCACTATG---GGCGCGGCGTCA---
CTGACGCTGACGGTACAG---GCCAGACAA---TTATTG---TCTGGCATAGTGCAACAGCAAAGC---
AATTTG---CTGAGG---GCTATAGAGGCTCAGCAGCATCTGCTGAAACTCACCGTCTGGGGCATT
>JX500696..1
TTCTATTGTAATACAACAAAACTGTTCAATAGCACTTGGGACAAAAATAGTACTTGGAATGAAAATAGCACT-
----

GCCAATGAAAATATAACTCTGCCATGCAGGATAAAGCAAATTATAAATATGTGGCAGAGAGTAGGACAAGCAA
TGTATGCCCCTCCTATCAAAGGAGTTATAATGTGTAAATCAAAC---
ATTACAGGTCTACTCCTAACAAGAGATGGTGGAGGTGGAGCTAAT----------------------------
----

AGCAGCAATGAGACTTTCAGACCTATAGGAGGAAATATGAGGGACAATTGGAGAAGTGAACTATATAAGTATA
AAGTAGTACAAATTGAACCAATA---GGAGTAGCACCTACCAGGGCAAAGAGAAGAGTGGTG-----------
----------------------------GAGAGAGAAAAAAGAGCA---ATTGGA---CTGGGAGCC------
---GCTTTCCTTGGGTTCTTAGGAGCA---GCAGGAAGCACTATG---GGCGCGGCGTCA---
CTGACGCTGACGGTACAG---GCCAGACAA---TTATTG---TCTGGCATAGTGCAACAGCAAAGC---
AATTTG---CTGAGG---GCTATAGAGGCTCAGCAGCAGCTGCTGAAACTCACGGTCTGGGGCATT
>JX500695..1
TTCTATTGCAATACAACAGGCCTGTTCAATAGCACTTGGGACACAAATGGCACTGTCACAAGTAGCACAAATG
GA---

ACAAATAAAACTATAACTCTGCCATGCAGAATAAAACAAATTATAAATATGTGGCAGAGAGTAGGACAAGCAA
TGTACGCCCCTCCTATCAAAGGAAGTATAAGGTGTGAATCAAAC---
ATTACAGGTCTACTACTAATAAGAGATGGTGAAAGGAATAGCAGC----------------------------
----

AATGAGACCTTTAGACCTATAGGAGGAAATATGAGGGACAATTGGAGGAGTGAACTATATAAGTATAAAGTAG
TACAAATTGAACCAATA---GGACTAGCACCTACCAGGGCAAAGAGAAGAGTGGTG-----------------
----------------------GAGAGAGAAAAAAGAGCA---ATTGGA---CTGGGAGCC---------
GCCCTCCTTGGGTTCTTAGGAGCA---GCAGGAAGCACTATG---GGCGCGGCGTCA---
ATGACGCTGACGGTACAG---GCCAGACAA---TTATTG---TCTGGCATAGTGCAACAGCAAAGC---
AATTTG---CTGAGG---GCTATAGAGGCTCAGCAGCATCTGCTGAAACTCACGGTCTGGGGCATT
>JX500694..1
TTCTATTGCAATACAACAGAACTGTTCAATAGCACTTGGGACGGAAATGGCACTGCCACAAATAAC-------
----

ACAGAGGACAATGTAACTCTGCCATGCAGAATAAAGCAAATTATAAATATGTGGCAGAGAGTAGGACAAGCAA
TGTATGCCCCTCCTATCAGAGGAAGTATAAAGTGTGTATCAAAC---
ATTACAGGACTACTACTAATAAGAGATGGTGGAGGTGGAAATAAT----------------------------
----

AGCAACAATGAGACCTTCAGACCTATAGGAGGAAATATGAGGGACAATTGGAGAAGTGAACTATATAAGTATA
AGGTAGTACAAATTGAACCAATA---GGAATAGCACCTACCACAGCAAAAAGAAGAGTGGTG-----------
----------------------------GAGAGAGAAAAAAGAGCA---ATTGGA---CTGGGAGCT------
---GCCTTCCTTGGGTTCTTAGGAGCA---GCAGGAAGTACTATG---GGCGCGGCGTCA---
ATAACGCTGACGGTACAG---GCCAGACAA---TTATTG---TCTGGTATAGTACAACAGCAAAGC---
AATTTG---CTGAGG---GCTATAGAGGCTCAGCAGCATCTGCTGAAACTCACGGTCTGGGGCATT
>MH603967..1
TTCTATTGCAATACAACACAACTGTTCGATAGCACGTGGGAAGAGAATGCCAATATCACAAATACAAATAATA
CACAG--

AATAATGAAAATATAACTCTACCATGCAGAATAAAGCAAATTATAAGGATGTGGCAGGGGGTAGGACAAGCAA
TGTATGCCCCTCCTATAAGAGGAGAAATAAGGTGTGAATCAAAC---
ATTACAGGATTACTACTAACAAGAGATGGTGGAGGTACAAATAGC----------------------------
----

AGCAACAATGAGACCTTCAGACCTATAGGAGGAGACATGAGGGACAATTGGAGAAGTGAACTATATAAGTATA
AAGTAGTACAAATTGAACCCATA---GGAGTAGCACCTACCAAGGCAAAGAGAAGAGTGGTG-----------
----------------------------GAGAGAGAAAAAAGAGCA---ATTGGA---CTGGGAGCT------
---GCCTTCCTTGGGTTCTTAGGAGCA---GCAGGAAGCACTATG---GGCGCGGCGTCA---
ATAACGCTGACGGTACAG---GCCAGACAA---TTATTG---TCTGGCATAGTGCAACAGCAAAGC---
AATTTG---CTGAGG---GCTATAGAGGCTCAGCAGCATCTGCTGAAACTCACGGTCTGGGGCATT
>MH604120..1
TTTTATTGTAATACAACAGACCTGTTCAATAGCGCTTGGGAAAATGGCAATGCTACAAATATCACACAGGCCA
AT---

GACAATGAAACTATAATTCTACCATGCAGAATAAGGCAAATTATAAATATGTGGCAAAGAGTAGGACAGGCAA
TGTATGCCCCTCCTATCAGAGGAAATATAACGTGTATATCAAAT---
ATTACAGGACTACTACTAACAAGAGATGGTGGAGGTGGAAATAAT----------------------------
----

AGCAACAATGAGACCTTCAGACCTATAGGAGGAGATATGAGGGACAATTGGAGAAGTGAACTATATAAATATA
AAGTAGTACAAATTGAACCAATA---GGAATAGCACCTAACAGGGCAAAGAGAAGAGTGGTG-----------
----------------------------GAGAGAGAAAAAAGAGCA---ATTGGA---CTGGGAGCT------
---GCCTTCCTTGGGTTCTTGGGAGCA---GCAGGAAGTACTATG---GGCGCGGCGTCG---
CTAACGCTGACGGTACAG---GCCAGAAAA---TTATTG---TCTGGCATAGTGCAACAGCAAAAC---
AATTTG---CTGAAG---GCTATAGAGGCTCAGCAGCATCTGCTGAGACTCACGGTCTGGGGCATT
>MH604308..1
TTCTATTGCAATACAACAGACCTGTTCAATAGCACTTGGGACGGAAATAGCACTAGAGATGAAAATGGCAATG
ATACAGAT---
-------------AGCACA------
CAGAACAATAGACCTATAACTCTGCCATGCAGAATAAAGCAAATTATAAATATGTGGCAGAGAGTAGGACAAG
CAATGTATGCCCCTCCTATAAAAGGAATTATAAGGTGTACATCAAAC---
ATTACGGGACTACTACTAATAAGAGATGGTGGAGGCGGAGCTAAT----------------------------
----

AGCAGCAATGAGACCTTCAGACCTATAGGAGGAGATATAAGGGACAATTGGAGAAGTGAACTATTTAAGTATA
AAGTAGTACAAATTGAACCAATA---GGAGTAGCACCTACCAGGGCAAAGAGAAGAGTGGTG-----------
----------------------------GAGAGAGAAAAAAGAGCA---ATTGGA---CTGGGAGCT------
---GCCTTCCTTGGGTTCTTGGGAGCA---GCAGGAAGTACTATG---GGCGCGGCGTCA---
ATAACGCTGACGGTACAG---GCCAGACAA---TTATTG---TCTGGCATAGTACAACAGCAAAGC---
AATTTG---CTGAGG---GCTATAGAGGCTCAGCAGCATCTGCTGAAACTCACGGTCTGGGGCATT
>MH604056..1
TTCTATTGCAATACAACAGCCCTGTTCAATTGGAACGGCAATGAAACTGTTACAAATAGCACACAG-------
----

GCCAATGAAACTATAACTTTACCATGCAGAATAAAGCAAATTATAAATATGTGGCAGAGAGTAGGACAAGCAA
TGTATGCCCCTCCTATCAAAGGAAGTATAAGGTGTACATCAAAC---
ATTACAGGACTGCTGCTAACAAGAGATGGTGGAACTAACAGCAGC----------------------------
----

AACAGCACTGAGATCTTCAGACCTATAGGAGGAAATATGAAGGACAATTGGAGAAGTGAACTATATAAATATA
AAGTAGTAAAAATTGAACCAATA---GGAGTAGCACCTACCAAGGCAAGGAGAAGAGTGGTG-----------
----------------------------GAGAGAGAAAAAAGAGCA---ATTGGA---CTGGGAGCT------
---GCCTTCCTTGGGTTCTTGGGAGCA---GCAGGAAGCACTATG---GGCGCGGCGTCA---
ATAACGCTGACGGTACAG---GCCAGACAA---TTATTG---TCCGGCATAGTGCAACAGCAAAGC---
AATTTG---CTGAGG---GCTATAGAGGCTCAGCAGCATCTGCTGAAACTCACGGTCTGGGGCATT
>MH604408..1
TTCTATTGCAATACAACAAACCTGTTCAATAGCACGTGGAACGAAACTGACAGTGTTACAAATAGCACACAGG
CC---

AATAATGAAACTATAATTTTACCATGCAGGATAAAGCAAATTATAAGGACGTGGCAGAGAGTAGGACAAGCAA
TGTATGCCCCTCCTATCAAAGGAAGGATAAGGTGTGAATCAAAC---
ATTACAGGACTACTACTAACAAGAGATGGTGGAGGTAAAAGTAATAATGAGACA-------------------
----

AGTAATAATGAGACCTTCAGACCTATAGGAGGAGATATGAGGGACAATTGGAGAAGTGAACTATATAAATATA
AAGTAGTAGAAATTGAACCAATA---GGAGTAGCACCTACCAAGGCAAAGAGAAGAGTGGTG-----------
----------------------------GAGAGACAAAAAAGAGCA---ATTGGA---CTGGGAGCT------
---GCCTTCCTTGGGTTCTTAGGAGCA---GCAGGAAGCACTATG---GGCGCGGCGTCA---
CTAACGCTGACGGTACAG---GCCAGACAA---TTATTG---TCTGGCATAGTGCAACAGCAAAGC---
AATTTG---CTGAGG---GCTATAGAGGCTCAGCAGCATCTGCTGAAACTCACGGTCTGGGGCATT
>MH604109..1
TTCTACTGCAGTACAGCAGCCCTGTTCAATAGCACTTGGGACGGAAATACCACTATTGCAAATAACACACAG-
----

AACAATGAAAATATAACTATACCATGCAGAATAAAGCAAATTATCAATATGTGGCAGAGAGTAGGACAAGCAA
TGTATGCCCCTCCTATACAAGGAGTTATAACTTGTGAATCAAAC---
ATTACAGGACTACTACTAACAAGAGATGGTGGAGTTAATAGCAGC----------------------------
----

AATGAAACCTTCAGGCCTATAGGAGGAGATATGAGGGACAATTGGAGAAGTGAATTATATAAGTATAAAGTAG
TAAAAGTTGAACCAATA---GGAGTAGCACCTACCACGGCAAGGAGAAGAGTGGTG-----------------
----------------------GGGAGAGAAAAAAGAGCA---ATTGGA---CTGGGAGCT---------
GCCCTCCTTGGGTTCTTGGGAGCA---GCAGGAAGCACTATG---GGCGCGGCGTCA---
ATAACGCTGACGGTACAG---GCCAGACAA---TTATTG---TCTGGCATAGTGCAACAGCAAAGC---
AATTTG---CTAAGG---GCCATAGAGGCTCAGCAGCATCTGCTGAAACTCACGGTCTGGGGCATT
>MH604508..2
TTCTATTGCTATACAACAGGGCTGTTCAATAGCACTTGGGACGAAAATAGCACTAGCACAAATATCACACAG-
----

AAAAATGAAAATATAACTATACCATGCAGAATAAAGCAAATTATAAATATGTGGCAGAGAGTAGGACAAGCAA
TGTATGCCCCTCCTATCAGAGGAAATATAAGATGTGTATCAAAC---
ATTACAGGACTACTACTAACAAGAGATGGTGGAGATGGAGATAAT----------------------------
----

AGCAGTGAGACCTTCAGACCTATAGGAGGAGATATGAGGGACAATTGGAGAAGTGAACTATATAAATATAAAG
TAGTACAAATTGAACCCATA---GGAGTAGCACCTACCAAGGCAAGGAGAAGAGTGGTG--------------
-------------------------GAGAGAGAAAAAAGAGCA---ATTGGA---CTGGGAGCT---------
GCCTTCCTTGGGTTCTTAGGAGCG---GCAGGAAGCACTATG---GGCGCGGCGTCA---
ATAACGCTGACGGTACAG---GCCAGACAA---TTATTG---TCTGGCATAGTGCAACAGCAAAGC---
AATTTG---CTGAGG---GCTATAGAGGCTCAGCAGCATCTGCTGAAACTCACGGTCTGGGGCATT
>MH604524..1
TTCTATTGCAATACAACAAACCTGTTCAATAGTGCTTGGAAAAATGGCAGTGCTACAAATAGCACACAAGCC-
----

AATGGAACAACTATAACTCTACCATGCAGAATAAAGCAAATTATAAATATGTGGCAGAGAGTAGGACAAGCAA
TGTATGCCCCTCCTATCAAAGGAAGTATAAGGTGTGAATCAAAC---
ATTACAGGACTACTACTAACAAGAGATGGTGGAGGTGAAACTAATAGC-------------------------
----

AGCGATGAAACCGAAACCTTCAGACCTATAGGAGGAGATATGAGGAATAATTGGAGAAGTGAATTATATAAGT
ATAAAGTAGTAAAAATTGAACCAATA---GGAGTAGCACCTACCAGGGCAAGGAGAAGAGTGGTG--------
-------------------------------GAGAGAGAAAAAAGAGCA---GTTGGA---CTGGGAGCT---
------GCCTTCCTTGGGTTCTTGGGAGCA---GCAGGAAGCACTATG---GGCGCGGCGTCA---
ATGACGCTGACGGTACAG---GCCAGAAAA---TTATTG---TCTGGCATAGTGCAACAGCAAAGC---
AATTTG---CTGAGG---GCTATAGAGGCTCAGCAGCATCTGCTGAAACTCACGGTCTGGGGCATT
>MH604541..1
TTCTATTGCAATACAACAAAGCTGTTCAATAGCACTTGGGAAAATAGCACTTGGGAAAATGGCACTGTTGCAA
AT---
-------------AGCACA------
GGGGACAATGACACTATAACTATACCATGCAGAATAAAGCAAATTATAAATATGTGGCAAAGAGTAGGACAAG
CAATGTATGCCCCTCCTATCAAAGGAATTATAAGGTGTGAATCAAAC---
ATTACAGGACTACTACTAACAAGAGATGGTGGAGGTGGAACTAAT----------------------------
----

AGCAGCAATGAGACCTTCAGACCTATAGGAGGAGACATGAGGGACAATTGGAGAAGTGAACTATATAAGTATA
AAGTAGTAAAAATTGAACCAATA---GGAGTAGCACCTACCAGGGCAAAGAGAAGAGTGGTG-----------
----------------------------GAGAGAGAAAAAAGGGCA---TTTGGA---CTGGGAGCT------
---GCCTTCCTTACGTTCTTGGGAGCA---GCAGGAAGCACTATG---GGCGCGGCGTCA---
ATAACGCTGACGGTACAG---GCCAGACAA---TTATTG---TCTGGCATAGTGCAACAGCAAAGC---
AATTTG---CTGAGG---GCTATAGAGGCTCAGCAGCATCTGCTGAAACTCACGGTCTGGGGCATT
>MH330347..1
TTCTATTGCAATACCACATACCTGTTCAATAGCACTTGGGATGGAAATAGCACTGTAAATAGCACAGGG----
----

GGCGATAGAAATATAACTTTACCATGCAGAATAAAGCAAATTATAAATATGTGGCAGAGAGTAGGACAAGCAA
TGTATGCCCCTCCTATCCGAGGAAACATAAGTTGTGAATCAAAC---
ATCACAGGACTACTCCTAATAAGAGATGGTGGAGGTGGAAATAAT----------------------------
----

AAGACTAATGAGACCTTCAGACCTATAGGAGGAGATATGAGGGACAATTGGAGAAGTGAACTATATAAGTATA
AAGTAGTAAAAATTGAACCAATA---GGGGTAGCACCTACCCTGGCCAGGAGAAGAGTGGTG-----------
----------------------------GAGAGAGAAAAAAGAGCAATAATTGGA---CTGGGAGCT------
---GCCTTCATTGGGTTCTTAGGAGCA---GCAGGAAGCACTATG---GGCGCGGCGTCA---
CTGACGCTGACGGTACAG---GCCAGACAA---TTATTG---TCTGGCATAGTGCAACAGCAAAGC---
AATTTG---CTGAGG---GCTATAGAGGCTCAGCAGCATCTGCTGAAACTCACGGTCTGGGGCATT
>MH330348..1
TTCTATTGCAATACAACAAACCTGTTCAACATAAATAGCACTGACACAAATATCACACAG-------------
----

GACAATGAAACTATAACTCTACCATGCAGAATAAAGCAAATTATAAATATGTGGCAGAGAGTAGGACAAGCAA
TGTATGCCCCTCCTATCAAAGGAAGTATAATGTGTGAATCAAAC---
ATTACAGGACTACTACTAACAAGAGATGGTGGAGGTGACAGAAAT----------------------------
----

GATACTAATAGTACTGAGACCTTCAGACCTATAGGAGGAGATATGAGGGACAATTGGAGAAGTGAACTATATA
AGTATAAAGTAGTAAAAATTGAACCAATA---GGAGTAGCACCTACCAGGGCAAAGAGAAGAGTGGTG-----
----------------------------------GGGAGAGAAAAAAGAGCA---ATTGGA---
CTGGGAGCC---------GCCTTCCTTGGGTTCTTAGGAGCA---GCAGGAAGCACTATG---
GGCGCGGCGTCA---CTGACGCTGACGGTACAG---GCCAGACAA---TTATTG---
TCTGGCATAGTGCAACAGCAAAGC---AATTTG---CTGAGG---
GCTATAGAGGCTCAGCAGTATATGCTGAAACTCACGGTCTGGGGCATT
>MH330349..1
TTCTATTGCAATACAACAGCCCTGTTCAATAGCACTTGGAGTGAAAATGGCACTGCCATAAATAGCACAGAG-
----

GCCAATGGAACTATAACTCTGCCATGCAGAATAAGGCAAATTATAAATATGTGGCAGAGAGTAGGACAAGCAA
TGTATGCCCCTCCTATCAGAGGAATTATAAGGTGTGAATCAAAC---
ATTACAGGACTACTGCTAACACGAGATGGTGTAGCTAATAGCAGC----------------------------
----

AATGAGACCTTCAGACCTATAGGAGGAGATATGAGGAACAATTGGAGAAGTGAACTATATAAGTATAAAGTAG
TAAAAATTGAACCAATA---GGAGTAGCACCTACCAGGGCAAAGAGAAGAGTGGTG-----------------
----------------------GAGAGAGAAAAAAGAGCA---ATTGGA---CTGGGAGCC---------
GCCTTCCTTGGGTTCTTAGGAGCA---GCAGGAAGCACTATG---GGCGCGGCGTCA---
CTGACGCTGACGGTACAG---GCCAGACAA---CTCTTG---TCTGGCATAGTGCAACAGCAAAGC---
AATTTG---CTGAGG---GCTATAGAGGCTCAGCAGCATCTGCTGAAACTCACGGTCTGGGGCATT
>MH330350..1
TTTTATTGTAATACAACAGAACTGTTCAATAGTACTTGGGACGGAAATAACACTGTTACAAATAGCACACAG-
----

GACAATGGCACTATAACTCTACCATGCAGAATAAGGCAAATTATAAATATGTGGCAGAGAGTAGGACAAGCAA
TGTATGCCCCTCCTATCAGAGGAAATATAAGTTGTGAATCAAAC---
ATTACAGGACTACTACTAACAAGAGATGGTGGAGTTAATACCAGC----------------------------
----

AATGAGACCTTCAGACCTACAGGAGGAGATATGAGGAACAATTGGAGAAGTGAACTATATAAGTATAAAGTAG
TACAAATTGAACCAATA---GGAGTAGCACCTACCAAGGCAAAGAGAAGAGTGGTG-----------------
----------------------GAGAGAGGAAAAAGGGCA---ATTGGA---CTGGGAGCT---------
GCCTTCCTTGGGTTCTTGGGAGCA---GCAGGAAGTACTATG---GGCGCGGCGTCA---
ATAACGCTGACGGTACAG---GCCAGACAA---TTATTG---TCTGGCATAGTACAACAGCAAAGC---
AATTTG---CTGATG---GCTATAGAGGCTCAGCAGCATCTGCTGAAACTCACGGTCTGGGGCATT
>MH330351..1
TTCTATTGCAATACAACAAACCTGTTCAATAGCACTTGGGACAAAAATAGTACTGTCACAAATAGCACAAAC-
----

AGCACAGAAACTATAACTCTACCATGCAGAATAAAGCAAATTATAAATATGTGGCAGAGAGTAGGACAAGCAA
TGTATGCCCCTCCTATCAAAGGAAGTATAAGGTGTGAATCAAAC---
ATTACAGGACTAATATTAATAAGAGATGGTGGAAATAATAGCAGC----------------------------
----

AATGAGACCTTCAGACCTATAGGAGGAAATATGAGGGACAATTGGAGAAGTGAACTATATAAATATAAAGTAG
TAGAGATTGAACCAATA---GGAATAGCACCTACCAAGGCAAGAAGGAGAGTGGTG-----------------
----------------------GAGAGAGAAAAAAGAGCA---ATTGGA---CTGGGAGCT---------
GCCTTCCTTGGGTTCTTAGGAGCA---GCAGGAAGCACTATG---GGCGCGGCGTCA---
ATAACGCTGACGGTACAG---GCCAGACAA---TTATTG---TCTGGCATAGTGCAACAGCAAAGC---
AATTTG---CTGAGG---GCTATAGAAGCTCAGCAGCATCTGCTGAAGCTCACGGTCTGGGGCATT
>MH330352..1
TTCTATTGCAATACAACACAACTGTTCAATAGCACTTGGCCTTGGAATGACACTGTCACAAATAGCACAGAG-
----

ACCAGTGAAAATATAACTCTACCATGCAGAATAAAGCAAATTATAAATATGTGGCAGAGAGTAGGACAAGCAA
TGTATGCCCCTCCTATCAGAGGAAGTATAAGGTGTGAATCAAAC---
ATTACAGGACTACTACTAATAAGAGATGGTGGAACTAATAGCACC----------------------------
----

AATGAGACCTTCAGACCTATAGGAGGAGATATGAGGGACAATTGGAGAAGTGAACTATATAAGTATAAAGTAG
TACAAATTGAACCAATA---GGAGTAGCACCTACCAAGGCAAAGAGAAGAGTGGTG-----------------
----------------------GAGAGAGAAAAAAGAGCA---ATTGGA---CTGGGAGCT---------
GCCTTCCTTGGGTTCTTAGGAGCA---GCAGGAAGCACTATG---GGCGCGGCGTCA---
ATAACGCTGACGGTACAG---GCCAGACAA---TTATTG---TCTGGCATAGTGCAACAGCAAAGC---
AATTTG---CTGAGG---GCTATAGAGGCTCAGCAGCATCTGCTGAAACTCACGGTCTGGGGCATT
>MH330353..1
TTCTATTGCAATACAACAGACCTGTTCGATAGCACTTGGGATGGAAATAACACAGATAACACTGTCACAAATA
TCACG--

GGCAATGGAACTATGACTCTGCCATGCAGAATAAGGCAAATTGTAAGGATGTGGCAGAGAGTAGGACAAGCAA
TGTATGCCCCTCCTATTGAAGGGAAAATAAGGTGTGAATCAAAC---
ATTACAGGACTACTACTACTAAGAGATGGTGGAATTAATAGCAGC----------------------------
----

AATGAGACCTTCAGACCTATAGGAGGAGATATGAGGGACAATTGGAGAAGTGAGCTATATAAGTATAAAGTAG
TAAAAATTGAACCAATA---GGAGTAGCACCTACCCAGGCAAGGAGAAGAGTGGTG-----------------
----------------------GCGAGAGAAAAAAGAGCA---ATTGGA---TTAGGAGCT---------
GCCTTCCTTGGGTTCTTAGGAGCA---GCAGGAAGCACTATG---GGCGCGGCGTCA---
ATAACGCTGACGGTACAG---GCCAGACAA---TTATTG---TCTGGCATAGTGCAACAGCAAAGC---
AATTTG---CTGAAG---GCTATAGAGGCTCAGCAGCATCTGCTGAAACTCACAGTCTGGGGCATT
>MH330354..1
TTCTATTGCAATACAACAAGCCTGTTCAATAGCACTTGGGATGAAAATAGCACTGTCACAAACACAGAG----
----

GCCAATGGAACTATAATTCTGCCATGCAGAATAAAGCAAATTATAAATATGTGGCAAAGAGTAGGACAAGCAA
TGTATGCCCCTCCTATCAGAGGAAGGATAAGGTGTGAATCAAAC---
ATTACAGGACTACTAATAACAAGAGATGGTGGAGGTGGAAATAAT----------------------------
----

AGCACCAATGAAACCTTCAGACCTATAGGAGGAGATATGAGGGACAATTGGAGAAGTGAACTATATAAGTATA
AAGTAGTAAAAATTGAACCAATA---GGAGTAGCACCTACCAGGGCAAAGAGAAGAGTGGTG-----------
----------------------------GAGAGAGAAAAAAGAGCA---ATTGGA---CTGGGAGCC------
---GCCTTCCTTGGGTTCTTAGGAGCA---GCAGGAAGCACTATG---GGCGCAGCGTCA---
ATGACGCTGACGGTACAG---GCCAGACAA---TTATTG---TCTGGCATAGTGCAACAGCAAAGC---
AATTTG---CTGAGG---GCTATAGAGGCTCAGCAGCATCTGCTGAAACTCACGGTCTGGGGCATT
>MH330355..1
TTCTATTGCAATACAACAAGGCTGTTCAATAGCTCTTGGGGCGAAAATAGCACTGTCACAAATAGCACAGAT-
----

GTCAATGGAACTATAATTCTGCCATGCAGAATAAAACAAATTATAAATATGTGGCAGAGAGTAGGACAAGCAA
TGTATGCCCCTCCTATCCCAGGAAATATAACATGTATATCAAAC---
ATTACAGGACTACTACTAGTAAGAGATGGTGGACAGAACAGTAGT----------------------------
----

AACACCAGTGAAACCTTCAGACCCATAGGAGGAGATATGAAGCACAATTGGAGAAGTGAACTATATAAGTATA
AAGTAGTAGAAATTGAACCAATA---GGAGTAGCACCTACCAAGGCAAAGAGAAGAGTGGTG-----------
----------------------------GAGAGAGAAAAAAGAGCAATTATTGGA---CTGGGAGCT------
---GCCTTCATTGGGTTCTTAGGAGCA---GCAGGAAGCACTATG---GGCGCGGCGTCA---
ATAACGCTGACGGTACAG---GCCAGACAA---TTACTG---TCTGGCATAGTGCAACAGCAAAGC---
AATTTG---CTGAGG---GCTATAGAGGCTCAGCAGCATCTGCTGAAACTCACGGTCTGGGGCATT
>MH330356..1
TTCTATTGCAATACAACAGACCTGTTTAATAGCACTTGGGAGGAAAATAGCACTGTTACAAATAACACAAAT-
----

GCCAATGAAAATATAACTCTGCCATGCAGGATAAAGCAAATTATAAATTTGTGGCAGAGAGTAGGGCGAGCAA
TGTATGCCCCTCCTATCAGAGGAATTATAAGGTGTAACTCAACT---
ATTACAGGACTACTACTAACAAGAGATGGTGGAAATGGAAATAGTAGC-------------------------
----

AACAATGAGACTGAGACCTTCAGACCTATAGGAGGAAATATGAGGGACAATTGGAGAAGTGAACTATATAAGT
ATAAAGTAGTAGAAATTGAACCAATA---GGAGTAGCACCTACCAGGGCAAGGAGAAGAGTGGTG--------
-------------------------------CAGAGAGAAAAAAGAGCA---GTTGGA---CTGGGAGCC---
------GCCTTCCTTGGGTTCTTAGGAGCA---GCAGGAAGCACTATG---GGCGCGGCGTCA---
CTGACGCTGACGGTACAG---GCCAGACAA---TTATTG---TCTGGCATAGTGCAACAGCAAAGT---
AATTTG---CTGAGG---GCTATAGAGGCTCAGCAGCATCTGCTGAAACTCACGGTCTGGGGCATT
>MG902950..1
TTCTATTGCAATACAACAAACCTGTTCAGTAGCACTTGGAACGACACTATCACAAATTTCACAGAG-------
----

ACCAATGAAACTATAATTCTGCCATGCAGAATAAGGCAATTTATAAAGATGTGGCAGAGAGTAGGACAAGCAA
TGTATGCCCCTCCTATCAAAGGGAGCATAAGGTGTGAATCAAAC---
ATTACAGGACTACTACTAACAAGAGATGGTGGAAATGGAACTACT----------------------------
----

GACAGCAATGAGACCTTCAGACCTATAGGAGGAGATATGAGGGACAATTGGAGAAGTGAACTATATAAGTATA
AAGTAGTACAAATTGAACCAATA---GGAATAGCACCTACCAAGGCAAAGAGAAGAGTGGTG-----------
----------------------------GAGAGAGAAAAAAGAGCA---ATTGGA---CTGGGAGCC------
---GCTTTCCTTGGGTTCTTAGGAGCA---GCAGGAAGTACTATG---GGCGCGGCGTCA---
ATAACGCTGACGGCACAG---GCCAGACAA---TTATTG---TCTGGTATAGTACAACAGCAAAGC---
AATTTG---CTGAGG---GCTATAGAGGCTCAGCAGCATCTGCTGAAACTCACGGTCTGGGGCATT
>MG902951..1
TTCTATTGCAATACAACAGACCTGTTCAATAGCACTTGGGGAAATAACACAAATAGCACAGGG----------
----

ACCAATGAAACTATAACTCTGCCATGCAGAATAAAGCAAATTATAAATATGTGGCAGAGAGTAGGACAAGCAA
TGTATGCCCCTCCTATCCGAGGAATTATAAAGTGTGAATCAAAC---
ATTACAGGACTACTACTAACAAGAGATGGTGGAGGTGAAGCTAATAAT-------------------------
----

AGCGAGAGCGAGACCTTCAGACCTATAGGAGGAGATATGAGGGACAATTGGAGAAGTGAACTATATAAGTATA
AAGTAGTAGAAATTGAACCAATA---GGAGTAGCACCTACCAGGGCAAAGAGAAGAGTGGTG----------
----------------------------CAGAGAGAAAAAAGAGCA---ATTGGA---CTGGGAGCC------
---GCCCTCCTTGGGTTCTTAGGAGCA---GCAGGAAGCACTATG---GGCGCGGCGTCA---
ATAACGCTGACGGTACAG---GCCAGACAA---TTATTG---TCTGGTATAGTGCAACAGCAAAGC---
AATTTG---CTGGAT---GCTATAGAGGCTCAGCAGCATCTGCTGAGACTCACGGTCTGGGGCATT
>MH330337..1
TTCTATTGCAATACAACAGACCTGTTCAATAGCACTTGGGACGAAAATGACACTGTTACAAATAGCACACAG-
----

GCCAATGAAACTATAATTCTACCATGCAGGATAAAGCAAATTATAAATATGTGGCAGAGAGTAGGACAAGCAA
TGTATGCCCCTCCTATCAAAGGAAGTATAAGGTGTGAATCAAAC---
ATTACAGGACTAATACTAACAAGAGATGGTGGAACTAATAGCAGC---------------------------
----

AATGAGACCTTCAGACCTATAGGAGGAAATATGAGGGACAATTGGAGAAGTGAACTATATAAGTATAAAGTAG
TAAAAATTGAACCAATA---GGAGTAGCACCTACCAAGGCAAAGAGAAGAGTGGTG----------------
----------------------GAAAGAGAAAAAAGAGCA---ATTGGA---CTGGGAGCT---------
GCCTTCCTTGGGTTCTTGGGAGCA---GCAGGAAGTACTATG---GGCGCGGCGTCA---
ATAACGCTGACGGTACAG---GTCAGACAA---TTATTG---TCTGGCATAGTGCAACAGCAAAGC---
AATTTG---CTGAGG---GCTATAGAGGCTCAGCAGCATCTGCTGAAACTCACGGTCTGGGGCATT
>MH330338..1
TTCTATTGCGATACAACAAAACTGTTCAATAGCACTTGGAACGAAAATGGCACTAGGACAGATACAGATACCA
CA--

CAGGATGAAAATATAACTATACCATGCAGAATAAAGCAAATTATAAATATGTGGCAGAGAGTAGGACAAGCAA
TGTATGCCCCTCCCATCAGAGGAACTATAATGTGTATATCAAAC---
ATTACAGGGCTACTCCTAACAAGAGATGGTGGAGCTAATAACAGC---------------------------
----

AATGAAACCTTCAGACCTATAGGAGGAGATATGAGAGACAATTGGAGAAGTGAACTATATAAGTATAAAGTAG
TAGAAATTGAACCAATA---GGGGTAGCACCTACCAAGGCAAAGAGAAGAGTGGTG----------------
----------------------GAGAGAGAAAAAAGAGCA---GTTGGA---CTGGGAGTT---------
GCCTTCCTCGGGTTCTTAGGAGCA---GCAGGAAGTACTATG---GGCGCGGCGTCA---
CTGACGCTGACGGTACAG---GCCAGACAA---TTATTG---TCTGGCATAGTGGAACAGCAAAGC---
AATTTG---CTGAGG---GCTATAGAGGCTCAGCAGCATCTGCTGAAACTCACGGTCTGGGGCATT
>MH330339..1
TTCTATTGCAATACAACAGCACTGTTCAATAGCACTTGGGGAGGAAATAACACACAG---------------
----

GGCAATGAAAGTATAACTCTACCATGCAGAATAAAGCAAATTATAAGGATGTGGCAGAGAGTAGGACAAGCAA
TGTATGCCCCTCCTATCCAAGGAAGGATAAGGTGTGAATCAAAT---
ATTACAGGACTACTATTAACAAGAGATGGTGGAGTAACTAATAGCAGC------------------------
----

AATGGGACTGAGACCTTCAGACCTACAGGAGGAGATATGAGGGACAATTGGAGAAGTGAACTATATAAGTATA
AAGTGGTAGAAATTGAACCAATA---GGAATAGCACCTACCAGGGCAAGGAGAAGAGTGGTG----------
----------------------------CAGAGAGAAAAAAGAGCA---ATTACA---CTGGGAGCT------
---GCCTTCCTTGGGTTCTTGGGAGCA---GCAGGAAGTACTATG---GGCGCGGCGTCA---
ATAACGCTGACGGTACAG---GCCAGGCAA---TTATTG---TCTGGTATAGTACAACAGCAAAGC---
AATTTG---CTAAAG---GCTATAGAGGCTCAGCAGCATCTGCTGAAACTCACGGTCTGGGGCATT
>MH330340..1
TTCTATTGCAATACAACAGACCTGTTCAATAGCATTTGGGAAAACGGCAATGTCACAAATAGCACAGAG----
----

ACCAATGAAACTATAACTCTACAATGCAGAATAAAGCAAATTATAAATATGTGGCAAAGAGTAGGACAAGCAA
TGTATGCCCCTCCTATCAGTGGAAGCATAAGGTGTGAATCAAAC---
ATTACAGGACTAATACTAATAAGAGATGGTGGATATAATAGCAGC---------------------------
----

AATGAGACCTTCAGACCTATAGGAGGAAATATGAAGGACAATTGGAGAAGTGAACTATATAAGTATAAAGTAG
TAAAAATTGAACCAATA---GGAGTAGCACCTACCAGGGCAAAGAGAAGAGTGGTG-----------------
----------------------GAGAGAGAAAAAAGAGCA---ATCGGA---CTGGGAGCT---------
GCCTTCCTTGGGTTCTTAGGAGCA---GCAGGAAGTACTATG---GGCGCGGCGTCA---
CTGACGCTGACGGTACAA---GCCAGACAA---TTATTG---TCTGGCATAGTGCAACAGCAAAGC---
AATTTG---CTGAGG---GCTATAGAGGCTCAGCAACATCTGCTGAAACTCACGGTCTGGGGCATT
>MH330341..1
TTCTATTGCAATACAACAGCCCTGTTCAATAGCACTTGGCACGAAAATGACACTGTTACAAATAGCACACAG-
----

AACAATGAAACTATAATTCTACCATGCAGAATAAAGCAAATTATAAATATGTGGCAGAGAGTAGGACAAGCAA
TGTATGCCCCTCCGATCAGAGGAAGTATAAGGTGTGAATCAAAC---
ATTACAGGACTACTACTAACAAGAGATGGTGGGGCTAATAGCAGC----------------------------
----

AATGAGACCTTCAGACCTATAGGAGGAGATATGAGGGACAATTGGAGAAGTGAACTATATAAGTATAAAGTAG
TAAAAATTGAACCAATA---GGAGTAGCACCTACCAGGGCAAAGAGAAGAGTGGTG-----------------
----------------------GAAAGAGAAAAAAGAGCA---ATTGGA---CTGGGAGTT---------
GCCTTCCTTGGGTTCTTGGGAGCA---GCAGGAAGCACTATG---GGCGCGGCGTCA---
ATAACGCTGACGGTACAG---GCCAGACAA---TTATTG---TCTGGCATAGTGCAACAGCAAAGC---
AATTTG---CTGAGG---GCTATAGAGGCTCAGCAGCATCTGCTGAAACTCACGGTCTGGGGCATT
>MH330342..1
TTCTATTGCAATACAACAGCCCTGTTCAATAACACTGACACAAATAGCACAGAG-------------------
----

ACCAGTGGAAATATAACTATACCATGTAGAATAAAGCAAATTATAAGAATGTGGCAGAGAGTAGGACAAGCAA
TGTATGCCCCTCCTATCGAAGGAAATATAACGTGTGTATCAAAC---
ATTACAGGTCTACTACTAACAAGAGATGGTGGGGTAAATAACACC----------------------------
----

AATGAGACCTTCAGACCCATAGGAGGAGATATGAGGGACAATTGGAGAAGTGAACTATATAAGTATAAAGTAG
TAGAAATTGAACCAATA---GGAGTAGCACCTACCAGGGCAAAGAGAAGAGTGGTG-----------------
----------------------GAGAGAGAAAAAAGAGCA---ATTGGA---CTGGGAGCT---------
GCCTTCCTTGGGTTCTTAGGAGCA---GCAGGAAGCACTATG---GGCGCGGCGTCA---
ATGACGCTGACGGTACAG---GCCAGACAA---TTATTG---TCTGGCATAGTGCAGCAGCAAAGC---
AATTTG---CTGCAG---GCTATAGAGGCTCAGCAGCATCTGCTGAGACTCACGGTCTGGGGCATT
>MH330344..1
TTCTATTGTAATACAACAAAACTGTTCAATAGCACTTGGGACAAAAATAGCACAAGTACAAATATCACACAG-
----

ACAAATGGAAATATAACTCTACCATGCAGAATAAAGCAAATTATAAATATGTGGCAGAGAGTAGGACAAGCAA
TATATGCCCCTCCTATCAAAGGAAATATAATGTGTGTATCAAAC---
ATTACAGGACTACTACTAATAAGAGATGGGGGAGGTGGAAATAAT----------------------------
----

AGCATCAATGAAACTTTCAGACCTATAGGAGGAGATATGAGGGACAATTGGAGAAGTGAACTATATAAGTATA
AAGTAGTACAAATTGAACCAATA---GGGGTAGCACCTACCAGGGCAAGGAGAAGAGTGGTG-----------
----------------------------CAGAGAGAAAAAAGAGCA---ATTGGA---CTGGGAGCC------
---GCCTTCCTCGGGTTCTTAGGAGCA---GCAGGAAGCACTATG---GGCGCAGCGTCA---
CTGACGCTGACGGTACAG---GCCAGACAA---TTATTG---TCTGGCATAGTGCAACAGCAAAGC---
AATTTG---CTGAGG---GCTATAGAGGCTCAGCAGCATCTGCTGAAACTCACGGTCTGGGGCATT
>MH330345..1
TTCTATTGCAATACAACAGCCCTGTTCAATAGCATTTGGAACGGAAATGACAATGGCACAAATAGTACAGAG-
----

GACACTGACCCTATAATTCTACCATGCAGAATAAAGCAAATTGTAAATATGTGGCAGAGAGTAGGACAAGCAA
TGTATGCCCCTCCTATCCCAGGAAATATAAGTTGTACATCAAAC---
ATTACAGGACTACTACTAACAAGAGATGGA---ATTAATGGCAGC----------------------------
----

AATGAGACCTTCCGACCTATAGGAGGAGACATGAGGGACAATTGGAGAAGTGAACTATATAAGTATAAAGTAG
TAAAAATTGAACCAATA---GGAGTAGCACCTACCAAGGCAAAGAGAAGAGTGGTG-----------------
----------------------GAGAGAGAAAAAAGAGCA---ATTGGA---CTGGGAGCT---------
GCCTTCCTTGGGTTCTTAGGAGCA---GCAGGAAGCACTATG---GGCGCGGCGTCA---
ATAACGCTGACGGTACAG---GCCAGACAA---TTATTG---TCTGGCATAGTGCAACAGCAAAGC---
AATTTG---CTGAGG---GCTATAGAGGCTCAGCAGCATCTGCTGAAACTCACGGTCTGGGGCATT
>MH330346..1
TTCTATTGCAATACATCAGACCTGTTC------ACACATAATATCACAGGGGCCAATGAAACAGGG-------
----

GCCAATGGAACTATAACTATACCATGCAAAATAAAACAAATTATAAATATGTGGCAGAGAGTAGGACAAGCAA
TGTATGCCCCTCCTATCAGAGGAATTATAAGGTGTGAATCAAAC---
ATTACAGGACTATTACTAACAAGAGATGGTGGAAAG-------------------------------------
----

AGCGAGAATGAGACCTTCAGACCTATAGGGGGAGATATGAGGGACAATTGGAGAAGTGAACTATATAAGTATA
AAGTAGTAGAAATTGGACCAATA---GGAGTAGCACCTACCAGGGCACAGAGGAAAGTGGTG-----------
----------------------------AAGAGAGAAAAAAGAGCAATTATTGGA---CTGGGAGCT------
---GCCTTCCTTGGGTTCTTAGGAGCA---GCAGGAAGCACTATG---GGCGCGGCGTCA---
TTGACGCTGACGGTACAG---GCCAGACAA---TTATTG---TCTGGCATAGTGCAACAGCAAAGC---
AATTTG---CTGAGG---GCTATAGAGGCTCAGCAGCATCTGCTGAAACTCACGGTCTGGGGCATT
>HQ616082..1
TTCTATTGCAATACAACAAACCTGTTCAATGGCACTTGGTCAAAAAGCACAACGGCCAATGCAACT-------
----

GCCAATGAAACTATAACTCTACCGTGCAGAATAAAGCAAATTATAAATATGTGGCAGAGAGTAGGACAAGCAA
TGTATGCCCCTCCTATCATAGGCAATATAAGCTGTGAATCAAAC---
ATTACAGGACTGCTACTGACAAGAGATGGTGGAACAACTAATAGC----------------------------
----

AGCGATGAGACCTTCAGACCTATAGGAGGAAATATGAGGGACAATTGGAGAAGTGAACTATATAAGTATAAAG
TAGTAATAATTGATCCAATA---GGAGTAGCACCTACCAGGGCAAAGAGAAGAGTGGTG--------------
-------------------------GAGAGGGAAAAAAGAGCA---ATTGGA---CTGGGAGTT---------
GCCTTCCTTGGGTTCTTAGGAGCA---GCAGGAAGCACTATG---GGCGCGGCGTCA---
CTGACGCTGACGGTACAG---GCCAGACAA---TTATTG---TCTGGTATAGTGCAACAGCAAAGC---
AATTTG---CTGAGG---GCTATAGAGGCTCAGCAGCATCTGCTGAAACTCACGGTCTGGGGCATT
>DQ823357..1
TTCTATTGCAATACAACAGAACTGTTCAATAGCACTTGGGGCGAAAATGGCACTATCACAAATAGCACAGAG-
----

ACCAGTGAAAATATAACTCTACCATGCAGAATAAAGCAAATTATAAATATGTGGCAGAGAGTAGGACAAGCAA
TGTATGCCCCTCCTATCAGAGGAAATATAAGTTGTGTATCAAAC---
ATTACAGGACTACTACTAACAAGAGATGGTGGAACTAATAGCAGC----------------------------
----

ACTGAGACCTTCAGACCTACAGGAGGAGATATGAGGGACAATTGGAGAAGTGAACTATATAAGTATAAAGTAG
TAGAAATTGAACCAATA---GGAGTAGCACCTACCAAGGCAAAGAGAAGAGTGGTG-----------------
----------------------GAGAGAGAAAAAAGAGCA---ATAATTGGACTGGGAGCT---------
GCCTTCCTTGGGTTCTTAAGTGCA---GCAGGAAGCACTATG---GGCGCAGCGTCA---
CTGACGCTGACGGTACAG---GCCAGACAA---TTATTG---TCTGGCATAGTGCAACAGCAAAGC---
AATTTG---CTGAGG---GCTATAGAGGCTCAGCAGCATTTGCTGAGACTCACGGTCTGGGGCATT
>KU749399..1
TTCTATTGCAATACAACAGGCCTGTTCAATAGTACTTGGGACAATAGTACTTGGGACAAAAATAGCACTGTCA
CAAAT--

GGCACCGAGGACAATATAACTCTACAATGCAAAATAAAGCAAATTATAAGGATGTGGCAGAGAGTAGGACAAG
CAATGTATGCCCCTCCTATCAGAGGAAATATAAAGTGTACATCAAAC---
ATTACAGGACTACTACTAATAAGAGATGGTGGAAATGGAAATAAT----------------------------
----

AGCAACAATGAGACCTTCAGACCGACAGGAGGAGATATGAGAAATAATTGGAGAAGTGAACTATATAAGTATA
AAGTAGTAGAAGTTGAACCAATA---GGAATAGCACCTACCAAGGCAAAGAGAAGAGTGGTG-----------
----------------------------GAGAGAGAAAAAAGAGCAATAGTTGGA---CTGGGAGCT------
---GCCTTCCTTGGGTTCTTAGGAGCA---GCAGGAAGTACTATG---GGCGCGGCGTCA---
ATAACGCTGACGGTACAG---GCCAGACAA---TTACTA---TCTGGCATAGTACAACAGCAGAGC---
AATCTG---CTGAGG---GCTATAGAGGCTCAGCAGCATCTGCTGAAACTCACGGTCTGGGGCATT
>KU749401..1
TTCTATTGCAATACAACAGACCTGTTCAATAGCACTTGGAACGGCAGCAATAGCACTGCCATGAATAGCTCA-
----

GGCAATGAAAATATAACCATACACTGCAGAATAAAGCAAATTATAAATATGTGGCAGAGAGTAGGACAAGCAA
TGTATGCCCCTCCTATCAGAGGAAATATAACATGTGTATCAAAC---
ATTACAGGGCTACTACTAATAAGAGATGGGGGAAATGGAAATAAT----------------------------
----

AGCAACAATGAGACCTTCAGACCTATAGGAGGAGATATGAGGGATAATTGGAGAAGTGAACTATATAAATATA
AAGTAGTAAAAATTGAACCAATA---GGAGTAGCACCTACCAAGGCAAGGAGAAGAGTGGTG-----------
----------------------------GGGAGAGAAAAAAGAGCA---ATTGGA---CTGGGAGCT------
---GCCTTCCTTGGGTTCTTAGGAGCA---GCAGGAAGCACTATG---GGCGCGGCGTCA---
CTGACGCTGACGGTACAG---GCCAGAAAA---TTGCTA---TCTGGCATAGTGCAACAGCAAAGC---
AATTTG---CTGAGG---GCTATAGAGGCTCAGCAGCAACTGCTGAGACTCACAGTCTGGGGCATT
>KY658681..1
TTCTATTGCAATACAACAGCCCTGTTCAATAGCACTTGGAACACCACTTGGAACGAAAATAGCACTGTTGAAA
GTAGTACA---

GGGGACAATGAAAATATAACTATACCATGCAGAATAAAGCAAATTATAAATATGTGGCAGAGAGTAGGACAAG
CAATGTATGCCCCTCCTATCAAAGGAATTATAAAGTGTGAATCAAAC---
ATTACAGGACTACTACTGGTAAGAGATGGTGGAAACACTAATAGC----------------------------
----

AGCAATGAGACCTTCAGACCTATAGGAGGAGATATGAGGGACAATTGGAGAAGTGAACTATATAAGTATAAGG
TAGTAAAAATTGAACCAATA---GGAGTAGCACCAACCAAGGCACAGAGAAGAGTGGTG--------------
-------------------------GGGAGAGAAAAAAGAGCA---ATTGGA---CTGGGAGTT---------
GCCTTCCTTGGGTTCTTAGGAGCA---GCAGGAAGCACTATG---GGCGCAGCGTCA---
ATAACGCTGACGGTACAG---GCCAGACAA---TTACTG---TCTGGCATAGTGCAACAGCAAAGC---
AATTTG---CTGAAG---GCTATAGAGGCTCAGCAGCATCTGCTGAAACTCACAGTCTGGGGCATT
>KU749402..1
TTCTATTGCAATACATCAAAACTGTTCAATAGCACTTGGGACGAAAATAGCACTGCCATAAATAGTACAGAG-
----

GACAGTGGAATTATAACTATACCATGCAGAATAAGGCAAGTTATAAATATGTGGCAGAGAGTAGGACAAGCAA
TGTATGCCCCTCCTATCAGAGGAAGTATAAGGTGTGAATCAAAC---
ATTACAGGATTAATACTAACAAGAGATGGTGGAAATATTAGTAGC----------------------------
----

ACCAATGAGACCTTCAGACCTACAGGAGGAGATATGAGGGACAATTGGAGAAGTGAACTATACAAGTATAAAG
TAGTAAGCATTGAACCAATA---GGAGTAGCACCTACCAAGGCAAGGAGAAGAGTGGTG--------------
-------------------------CAGAGAGAAAAAAGAGCA---GTTGGA---CTGGGAGCT---------
GCCTTCCTTGGGTTCTTAGGAGCA---GCAGGAAGCACTATG---GGCGCGGCGTCA---
TTGACGCTGACGGTACAG---GCCAGAAAA---TTATTG---TCTGGCATAGTGCAACAGCAAAAC---
AATTTG---CTGAAG---GCTATAGAGGCTCAGCAGCATCTGCTGAAACTCACAGTCTGGGGCATT
>KU749403..1
TTCTATTGTAATACAACAGACCTGTTCAATAGCACTTGGGACAGAAATGACACTGATGCAAATAGTACACAGG
TTGATAGTACA---CAG--

GTTGATGTAACTATAACTCTACCATGCAGAATAAAGCAAATTGTAAGGATGTGGCAGAGAGTAGGACAAGCAA
TGTATGCCCCTCCTATCGGAGGAGTTATAAGGTGTGAATCAAAC---
ATTACAGGACTACTACTAACAAGGGATGGTGGAGCTAATAATAGC----------------------------
----

ACTAATGAGACCTTTAGACCTCTAGGAGGAGATATGAGGGACAATTGGAGAAGTGAACTATATAAGTATAAAG
TAATACAAGTTGAACCAATA---GGAGTAGCACCTACCCCAGCAAAGAGAAGAGTGGTG--------------
-------------------------GAGAGACAAAAAAGAGCA---ATTGGA---CTGGGAGCT---------
GCCTTCCTTGGGTTCTTAGGAGCA---GCAGGAAGTACTATG---GGCGCGGCGTCA---
ATAACGCTGACGGTACAG---GCCAGACAA---TTATTG---TCTGGCATAGTACAACAGCAAAGT---
AATTTG---CTGAGA---GCTATAGAGGCTCAGCAGCATCTGCTGAAACTCACAGTCTGGGGCATT
>KU749404..1
TTCTATTGCAATACAACAGAGCTGTTCAATAGCACTGAGGACAAAAATGGCACTGTCACAAAT----------
----

AGCAATGAAAATATAACTCTACCATGCAGGATAAAGCAAATTATAAATATGTGGCAGAGAGTAGGACAAGCAA
TGTATGCCCCTCCTATCAGAGGAAATATAACTTGTGAATCAAAC---
ATTACAGGACTACTACTAACAAGAGATGGAGATGATAATAGCAGT----------------------------
----

GAGACCTTCAGACCTATAGGAGGAAATATGAGGGACAATTGGAGAAGTGAACTATATAAATATAAAGTAGTCC
AAATTGAACCAATA---GGAGTAGCACCTACCAAGGCAAGGAGAAGAGTGGTG--------------------
-------------------GGGAGAGAAAAAAGAGCA---ATTGGA---CTGGGAGCT---------
GCCTTCCTTGGGTTCTTAGGAGCA---GCAGGAAGCACTATG---GGCGCGGCGTCA---
CTGACGCTGACGGTACAG---GCCAGACAA---TTATTG---TCTGGTATAGTGCAACAGCAAAGC---
AATTTG---TTGAGG---GCTATAGAGGCTCAGCAGCATCTGCTGAAACTCACAGTCTGGGGCATT
>KU749405..1
TTCTATTGCAATACAACAGACTTGTTCAATTGGGATAGAAATAGCACTGTCCCAAATAAC-------------
----

ACAAGTAGCAATATAACTCTGCCATGCAGAATAAAGCAAATTATAAATATGTGGCAGAGAGTAGGACAATCAA
TGTATGCCCCTCCTATCAGCGGAAAGATAAGGTGTGAATCAAAC---
ATTACAGGACTACTACTAGTAAGAGATGGTGGAGATAATAATAGC----------------------------
----

ACCAATGAGACCTTCAGACCTATAGGAGGAGAAATGAGGCACAATTGGAGAAGTGAACTATATAAGTATAAAG
TAGTAGAAATTGAACCAATA---GGAGTAGCACCTACCAAGGCAAGGAGAAGAGTGGTG--------------
-------------------------GGAAGAGAAAAAAGAGCAGCCTTTGGA---CTGGGAGCT---------
GTCTTCCTTGGGTTCTTAGGAGCA---GCAGGAAGCACTATG---GGCGCGGCGTCA---
CTGACGCTGACGGTACAG---GCCAGGCAA---TTATTG---GCTGGCATAGTGCAACAGCAAAGC---
AATTTG---CTGAGG---GCTATAGAGGCTCAGCAGCATCTGCTGAAACTCACAGTCTGGGGCATT
>KY658682..1
TTCTATTGCAATACAACAGAACTGTTCAATAGCACTTGGAATGGCACTGACACAAACAGCACAGAGGAC----
----

AATAATGTAAATATAACTATACCATGCAGAATAAAGCAAATTATAAATATGTGGCAGAGAGTAGGACGAGCAA
TGTATGCCCCTCCTATCAGAGGAAATATAACTTGTGAATCAAAC---
ATTACAGGACTACTACTAACAAGAGATGGTGGTAAAGAGAAAAAT----------------------------
----

GACAACGAAACCTTTAGACCTATAGGAGGAAATATGAAGGACAATTGGAGAAGTGAACTATATAAGTATAAAG
TAGTAGAAATTAACCCAATA---GGAGTAGCACCTACCCCGGCAAAGAGAAGAGTGGTG--------------
-------------------------GAGAGAGAAAAAAGAGCA---GTTAGC---CTAGGAGTT---------
GCCTTCCTTGGGTTCTTAGGAGCA---GCAGGAAGTACTATG---GGCGCGGCGTCA---
CTGACGCTGACGGTACAG---GCCAGACAA---CTATTG---TCTGGCATAGTACAACAGCAAAGC---
AATTTG---CTGAGG---GCTATAGAGGCTCAGCAGCAATTGCTGAAACTCACGGTCTGGGGCATT
>KU749407..1
TTCTATTGCAATACAACAGAACTGTTCAATAGCACTTGGGACGACAATAGCACTGTTGCAAATATCACAAAG-
----

ACCAATGAAAATATAACTCTACCATGCAGACTAAGGCAAATCATAAAGATGTGGCAGAGAGTAGGACAAGCAA
TGTATGCCCCTCCTATCCAAGGAGTTATAAAGTGTGAATCAAAC---
ATTACAGGACTACTACTAACAAGAGATGGTGGAGATGGAACTAAT----------------------------
----

AACAGCAATAATGAGATTTTCAGACCTATAGGAGGAAATATGAGGGACAATTGGAGAAGTGAACTATATAAGT
ATAAAGTAGTAGAAATTGAACCAATA---GGAGTAGCACCTACCAAGGCAAAGAGAAGAGTGGTG-------
------------------------------CAGAGAGAAAAAAGAGCAGTTATTGGA---CTGGGAGCT---
------GCCTTCCTTGGGTTCTTAGGAGCA---GCAGGAAGCACTATG---GGCGCGGCGTCA---
ATAACGCTGACGGTACAG---GCCAGACAA---TTACTG---TCTGGCATAGTGCAACAGCAAAGC---
AATTTG---CTGAGG---GCTATAGAGGCTCAGCAGCATCTGCTGAAACTCACGGTCTGGGGCATT
>AF082486..1
TTCTATTGCAATACAACAGACCTGTTCAATAGCACTTGGGACGAACATGGCACTGTCACAAATAGCACAATG-
----

GCCAATGGAACTATAACTCTACCATGCAGAATAAAGCAAATTATAAATATGTGGCAGAGAGTAGGACAAGCAA
TGTATGCCCCTCCTATTAAAGGAAGTATAAGGTGTGAATCAAAC---
ATTACAGGACTACTATTAACAAGAGATGGTGGAAGTGGAACTAAT---------------------------
----

AGCAGCAATGAGACCTTCAGACCTATAGGAGGAAATATGAGGGATAATTGGAGAAGTGAACTATATAAGTATA
AAGTAGTAAAAATTGAACCCATA---GGATTAGCACGTACCAGGGCAAAGAGAAGAGTGGTG----------
---------------------------GAGAGAGAAAAAAGAGCC---ATTGGA---CTGGGAGCT------
---GCCTTCCTTGGGTTCTTAGGAGCA---GCAGGAAGTACTATG---GGCGCGGCGTCA---
ATGACGCTGACGGTACAG---GCCAGACAA---TTATTG---TCTGGCATAGTGCAACAGCAAAGC---
AATTTG---CTGAGG---GCTATAAAGGCTCAGCAGCATCTGCTGAAACTCACGGTCTGGGGCATT
>KY968402..1
TTCTACTGTAACACAACAAAACTGTTTAATAGTACTTGGTCTAATAATGATACTGGGGAGGGTACTGCAGATT
CA--

AATAACGAAACTATCACAATCCCATGCAGAATAAAACAAATTATAAACATGTGGCAACAAGTAGGAAAAGCAA
TGTATGCCCCTCCCATCAGTGGAAACATTAGTTGCTCATCAAAT---
ATTACAGGGTTACTGTTAACAAGAGATGGTGGAGATAATGATGAC---------------------------
----

CTAAACGGGACCGAGACCTTCAGACCTCAAGGAGGAAACATGAAGGACAATTGGAGAAGTGAATTATATAAAT
ATAAAGTAGTAAAAATTGAACCATTA---GGAGTAGCACCCACCAAGGCAAAGAGAAGAGTGTTG-------
------------------------------CAGAGAGACAAAAGAGCA---GTGGGA---CTAGGAGCT---
------ATGTTCCTTGGGTTCTTGGGAGCA---GCAGGAAGCACTATG---GGCGCAGCGTCA---
ATAGCGCTGACGGTACAG---ACCAGACAA---TTATTG---TCTGGTATAGTGCAACAGCAGAAC---
AATATG---CTGAGG---GCTATTGAGGCGCAACAGCATATGTTGCAACTCACTGTCTGGGGCATC
>KY968404..2
TTCTACTGTAATACAACACAACTGTTTTATAGTACTTGGTTGTCTAATGGTAGTAGTTTGACAAATAACACTG
AA--

GGAAATGACACTATCACACTCCCATGCAGAATAAAACAGATTATAAATATGTGGCAGCAAGTAGGAAAAGCAA
TGTATGCCCCTCCCATCAAAGGACTAATTAGATGCTCATCGAAT---
ATTACAGGACTGCTACTAACAAGAGATGGTGGTAGTCATAACGAGAGC------------------------
----

AATGAGACTGAGACATTCAGACCTGGGGGAGGAGATATGAGGGACAATTGGAGAAGTGAATTATACAAATATA
AAGTAGTACAAATTGAACCATTA---GGAATAGCACCCACCAAGGCAAAGAGAAGAGTGGTG----------
---------------------------CAGAGAGAAAAAAGAGCA---GTGGGA---CTGGGAGCT------
---TTGTTCCTTGGGTTCTTGGGAACA---GCAGGAAGCACTATG---GGCGCAGCGTCA---
TTGACGCTGACGGTACAG---GCCAGACAA---TTATTG---TCTGGTATAGTGCAACAGCAGAAT---
AATCTG---CTGAGA---GCTATTGAGGCGCAACAACATCTGTTGCAGCTCACAGTCTGGGGCATC
>KY968408..1
TTCTACTGTAATACAACAAAACTGTTTAATAAGACTTGGAATGGTACTTGGCTTAATAATGAGAAT------
----

ACTACACAGAATATCACACTCCCATGCAGAATAAAACAAATTATAAACAGGTGGCAGGAAGTAGGAAAAGCAA
TGTATGCCCCTCCCATCAAAGGACAAATTAGCTGCTCATCAAAT---
ATTACAGGGCTGCTATTAACAAGAGATGGTGATAACGAGACTGGG---------------------------
----

TCGGAGACCTTCAGACCTGGAGGAGGAGATATGAGGGACAATTGGAGAAGTGAATTATATAAATATAAAGTAG
TAAAAATTCAGCCATTA---GGAGTAGCACCCACCAAGGCAAAGAGAAGAGTGGTG-----------------
----------------------CAAAGAGAAAAAAGAGCA---GTGGGA---ATAGGAGCT---------
GTGTTCCTTGGGTTCTTGGGAGCA---GCAGGAAGCACTATG---GGCGCAGCGTCA---
GTAACGCTGACGGTACAG---GCCAGACAA---TTATTG---TCTGGTATAGTGCAGCAGCAGAAC---
AATTTG---CTGAGG---GCTATTGAGGCGCAACAGCATCTGTTGCAACTCACAGTCTGGGGCATC
>DQ383749..1
TTCTACTGTAATACATCACAACTGTTTAATAGTACTTGGCCTTGGAATGGGAATGCGACTACAGATAATACT-
----

ACAGGAGTAAATATCACACTCCCATGCAGAATAAAACAAATTATAAACATGTGGCAGGAAGTAGGAAAAGCAA
TGTATGCCCCTCCTATTAAAGGACAAATTAGATGTTCATCAAAT---
ATTACAGGGCTGCTATTAACAAGAGATGGTGGTCATAACGAGACC----------------------------
----

AACGACACTGAGACCTTCAGACCTGGAGGAGGAAATATGAAGGATAATTGGAGAAGTGAATTATATAAATATA
AAGTAGTAAAAATTGAACCATTA---GGAATAGCACCCACCACGGCAAAGAGAAGAGTGGTG-----------
----------------------------CAGAGAGAGAAAAGAGCA---ATAACA---ATAGGAGCT------
---ATGTTACTTGGTTTCTTGGGAACA---GCAGGAAGCACTATG---GGCGCAGCGTCA---
ATCACGCTGACGGTACAG---GCCAGACAA---TTATTG---TCTGGTATAGTGCAACAGCAGAAC---
AATCTG---CTGAGG---GCTATTGAGGCGCAACAGCATCTGTTGCAACTCACAGTCTGGGGCATC
>MH078532..1
TTCTACTGTAACACATCAAAGCTGTTTAACAGTACTTGGCATAGGAATGGTACTTGGAATGGTACTAGTACT-
----

GAGAATAATACAATCACAATCCCATGCAGAATAAAACAAATTATAAACAGGTGGCAGGAAGTAGGAAAAGCAA
TGTATGCCCCTCCCATCAGAGGAAATATTACATGTACATCTAAT---
ATTACAGGACTGTTATTAATAAGAGATGGTGGTAACATTAACACCACG-------------------------
----

AATGAGACCGAGATCTTCAGACCTGGAGGAGGAGACATGAGGGATAATTGGAGAAGTGAATTATATAAATATA
AAGTAGTAAGAATTCAGCCATTA---GGAATAGCACCCACCGAGGCAAAGAGAAGAGTGGTG-----------
----------------------------CAAAGAGAAAAGAGAGCG---GTGGGAACACTAGGAGCT------
---GTGTTCCTTGGGTTCTTGGGAGCA---GCAGGAAGCACTATG---GGCGCAGCGGCA---
ACAACGCTGACGGTACAG---GCCAGACAG---TTACTG---TCTGGCATAGTGCAACAGCAAAAC---
AATTTG---CTGAGA---GCTATTGAGGCGCAACAGCATACGTTGCAACTCACAGTCTGGGGCATT
>AF538307..1
TTCTACTGTGATTCAACACAACTGTTTAATAGTACTTGGCATAATAGTACTTGGAATAATAATAAAGAGTCAA
AT---

AGCACTGATCCAATCACACTTCCATGCAGAATAAAACAAAATATAAACATGTGGCAGGAAGTAGGAAAAGCAA
TGTATGCCCCTCCCATCAGAGGACAAATTAAATGTTCATCAAAC---
ATTACAGGGCTAATATTAACAAGAGATGGTGGTAATAACACAGACAAT-------------------------
----

AACAACACCGAAACCTTCAGACCTGGAGGAGGAAATATGAAGGACAATTGGAGAAGTGAATTATATAAATATA
AAGTAGTAAAAATTGAACCATTA---GGAGTAGCACCCACCAAGGCAAGGAGAAGAGTGGTG-----------
----------------------------CAGAGAGAGAAAAGAGAA---GTAGGA---CTAGGAGTT------
---ATGTTCCTTGGGTTCTTGGGAGCA---GCAGGAAGCACTATG---GGCGCAGCGTCA---
CTGAGGCTGACGGTACAG---GCCAGACAA---TTATTG---TATGATATAGTGCAACAGCAAAAC---
AATTTG---CTGAGA---GCTATTGAGGCGCAACATCATCTGTTGCAACTCACAGTCTGGGGCATC
>GU367404..1
TTCTATTGTAATACAACACAACTGTTTAACAGTACTTGGAGAAAGGAGTCAAATAACACTGGAGGAAAT----
----

ATCACACGAAATATCACGCTCCCATGTAGAATAAGACAGATTGTAAACATGTGGCAGGAAGTAGGAAAAGCAA
TATATGCCCCTCCCATTGAAGGACTAATTAGATGTTTATCAAAT---
ATTACAGGGCTGCTATTAACAAGAGACGGTGGTAATCAGAATGGGACT-------------------------
----

AATGACACTGAAATCTTCAGACCTGGAGGGGGAGATATGAGGGACAATTGGAGAAGTGAATTATATAAATATA
AAGTAGTAAAAATTGAACCCTTA---GGAATAGCACCCACCAAGGCAAAGAGAAGAGTGGTG-----------
----------------------------CAAAGAGAAAAAAGAGCA---GTGGGAGCATTAGGAGCT------
---ATGTTCCTTGGGTTCTTGGGAGCA---GCAGGAAGCACTATG---GGCGCAGCGTCA---
GTAACGCTGACGGTACAG---GCCAGACAA---TTATTG---TCTGGTATAGTGCAACAGCAGAAT---
AATCTG---CTGAAG---GCTATTGAGGCGCAACAGCATCTGTTGCAACTCACAGTCTGGGGCATC
>AF146728..1
TTCTACTGTAATTCAACACAACTGTTTAATAGTACTTGGTTTAATAGTACTGGGAATGATACTGAAAGGGCAA
CT---

AACAATACAGAAAATATCACACTCCCATGTAGAATAAAACAAATTATAAACATGTGGCAGAAAGTAGGAAAAG
CAATGTATGCCCCTCCCATCAACGGACAGATTAGATGTTCATCAAAT---
ATTACAGGGCTGATACTAACAAGAGATGGTGGTAATCAAGAGAAC----------------------------
----

AAGACCGAGATCTTCAGACCTGGAGGAGGAGATATGAGGGACAATTGGAGAAGTGAATTATATAAATATAAAG
TAGTAAGAATTGAACCATTA---GGAGTAGCACCCACCAAGGCAAAGAGAAGAGTGGTG--------------
-------------------------CAGAGAGAAAAAAGAGCA---GTGGGAATGATAGGGGCA---------
ATGATCCTTGGGTTCTTGGGAGCA---GCAGGAAGCACTATG---GGCGCAGCGTCA---
CTGGCGCTGACGGTACAG---ACCAGGCAA---TTGTTG---TCTGGTATAGTGCAACAGCAGAAC---
AATCTG---CTGAGG---GCTATTGAGGCGCAACAGCATCTGTTGCAACTCACAGTCTGGGGCATC
>MN485974..1
TTCTATTGTAATACAACAAAACTGTTTAATGGTACTTGGAATGGTACTTGGCTATCGGGGAACTGG-------
----

CCAAATGAAAATATCACACTCCCATGTAGAATAAAACAAATTGTAAACATGTGGCAAGAAGTAGGAAAAGCAA
TGTATGCCCCTCCCATCCAAGGACAGATTAGATGTGACTCAAAT---
ATTACAGGGCTGCTATTAACAAGAGATGGTGGTTCCGGGAATGGAACTGAG----------------------
----

AATAACACTGAGATCTTCAGACCTGGAGGAGGAAATATGAGAGACAATTGGAGAAGTGAATTATATAAATATA
AAGTAGTAAAAATTGCACCATTA---GGAATAGCACCCACCAAGGCAAAGAGAAGAGTGGTG-----------
----------------------------CAGAGAGAAAAAAGA------ATAGGAACGCTAGGAGCT------
---GTGTTCCTTGGGTTCTTGGGAACA---GCAGGAAGCACTATG---GGCGCAGCGTCA---
CTGACGCTGACGGTACAG---GCCAGACAA---TTATTG---TCTGGTATAGTGCAACAGCAGAAC---
AATTTG---CTGAGG---GCTATTGAGGCGCAACATCATCTGTTGCAACTCACAGTCTGGGGCATC
>MN485990..1
TTCTACTGTGATTCAACAGAACTATTTAATAGTACTTGGAATTTTAATAATAATACTTGGTATGAGAACTCAA
ATAACACAAGT--

GACAAAGAAAACATCACACTCCCATGTAGAATAAAACAAATTAGAAACTTGTGGCAGGAAGTAGGAAAAGCAA
TGTATGCCCCTCCCATCCAAGGGCTAATTACATGTCAATCAAAT---
ATTACTGGGGTGCTACTAACAAGAGATGGTGGTATTAATAACACC----------------------------
----

AGGAACGAGACCTTTAGACCTGGAGGAGGAAATATGAAAGACAATTGGAGAAGTGAACTATACAAATATAAAG
TAATAAAAATTGAACCATTA---GGAGTGGCACCCACCAAGGCAAAGAGAAGAGTGGTG--------------
-------------------------CAGAGAGAAAAAAGAGCA---GTGGGAACAATAGGAGCT---------
GTGTTCCTTGGTTTCTTGGGAACA---GCAGGAAGCACTATG---GGCGCAGCGTCA---
ATAGCGCTGACGGCACAG---GCCAGACAA---TTATTG---TCTGGTATAGTGCAACAGCAAAAC---
AATTTG---CTGAGA---GCTATTGAGGCGCAACAACATCTGTTGCAACTCACAGTCTGGGGCATC
>MN485995..1
TTCTACTGTAATACAACACAACTGTTTAATAGTACTTGGTGGTCTAATAGTACTGGGAATATTACTGGA----
----

CAGCCAGAAAATATCACTCTCCCATGCAGAATAAAACAAATCATAAACTTGTGGCAAGAGGTAGGAAAAGCAA
TGTATGCCCCTCCCATCCAAGGACTAATTAGATGTTCATCAAAT---
ATTACAGGGTTACTATTAACAAGAGATGGTGATAATCAAACTAAA----------------------------
----

GAGACTGAGATCTTTAGACCGGGAGGAGGAGATATGAGGGACAATTGGAGAAGTGAATTATATAAATATAAAA
TAGTAAAAATTGAGCCATTG---GGAATAGCACCCACCAGGGCAAAGAGAAGAGTGGTG--------------
-------------------------CAAAGAGAAAAAAGAGCA---GTGGGA---ATAGGAGCT---------
CTGTTCCTTGGGTTCTTGGGAGCT---GCAGGAAGCACTATG---GGCGCAGCGTCG---
GTGACGCTGACGGTACAG---GCCAGACAA---TTATTG---TCTGGTATAGTGCAACAGCAAAAC---
AACTTG---CTGAGA---GCTGTTGAGGCGCAACAACATATGTTGCAACTCACAGTCTGGGGCATC
>MN486034..1
TTCTACTGTAATACAACAGAGCTATTTAATAGGAATATTACTGAAGAGTCAAACACTAATGGAAATAGCAGT-
----

AGCGCAGGAAATATCACACTCCAATGCAGAATAAAACAGATTATAAACATGTGGCAGGAAGTAGGAAAAGCAA
TGTATGCCCCTCCCATCAGAGGACAAATTCAATGTATATCAAAT---
ATTACAGGACTAATATTAACAAGAGATGGTGGTAAGACAAATAAT----------------------------
----

GAGACAAATGGGACTGAGATTTTCAGACCTGGAGGAGGAAATATGAGAGACAATTGGAGAAGTGAATTATATA
AATATAAGGTAGTAAAAATTGAACCATTA---GGAGTAGCACCCACCAGGGCAAAGAGAAGAGTGGTG-----
----------------------------------CAGAGAGAAAAAAGAGCA---GTGGGA---
ATAGGAGCT---------GTGTTCCTTGGGTTCTTGGGAGCA---GCAGGAAGCACTATG---
GGCGCAGCGTCA---ATAACGCTGACGGTACAG---GCCAGACAA---TTATTG---
TCTGGTATAGTGCAACAGCAAAAC---AATTTG---CTGAGA---
GCTATTGAGGCGCAACAGCATCTGTTGCAACTCACAGTCTGGGGCATC
>DQ358805..1
TTCTACTGTAATACAACGAAGCTGTTTACTGGTACTTTGAATAGCACTGGGACTTGGCATACAGAAAAC----
----

CAGACTGATACTATCACACTCCCATGCAAAATAAAACAAATTATAAACAGGTGGCAGGAAGTAGGAAAAGCAA
TGTATGCCCCTCCCATTAAAGGACAAATTAGCTGTTCATCAAAT---
ATTACAGGGATACTATTAACACGAGATGGTGGTGGGAATGGGTCC----------------------------
----

GACACCACCGCCTCCTTAACACCAGGAGGAGGAGATATGAGGAACAGTTGGAGAAATGAATTATATAATTATG
AGGTGGTAAAAATTCCACCATTA---GGAGTACCACCCACCGAGGCGAGGAGAAGAGTGGTG-----------
----------------------------CAGAGAGAAAAAAGAGCA---GTGGGGGCACTAGGAGCT------
---ATGTTCCTTGGGTTCTTGGGAACA---GCAGGAAGCACTATG---GGCGCAGCGTCA---
CTGACGCTGACGGTACAG---GCCAGACAA---TTATTG---TCTGGTATAGTGCAACAGCAGAAT---
AATTTG---CTGAGA---GCTATTAAGGCGCAACAGCATATGTTGCAACTCACAGTCTGGGGCATC
>JN692439..3
TTCTACTGTGATACCTCACAAATATTTAATAGTAATTGGAATAGTTCTTCAAATTTTAGTCATCATGAAAAT-
----

TCAAATGAAAATATCACACTCCAATGCAGAATAAAACAAATTATAAACAGGTGGCAGGAAGTAGGAAGAGCAA
TGTATGCCCCTCCCATCCGAGGACTAATTAGATGTTCATCAAAT---
ATTACAGGGCTGCTATTAACAAGAGATGGTGGTAAGAATGATAACAATGGG----------------------
----

ACTGAGAATGGGACTGAGACCTTCAGACCTGGAGGGGGAGATATGAGGGACAATTGGAGAAGTGAATTATATA
AATATAAAGTAGTTCAAATTGAGCCAGTA---GGAATAGCACCCACCAAGGCAAAGAGAAGAGTGGTG-----
----------------------------------CAAAGAGAAAAAAGAGCA---GTAGGA---
GTAGGAGCT---------GTGTTCCTTGGGTTCTTGGGAACA---GCAGGAAGCACTATG---
GGCGCAGCGTCA---CTCACGCTGACGGTACAG---GCCAGACAA---TTGTTG---
TCTGGTATAGTGCAACAGCAGAAC---AATCTG---CTGAAG---
GCTATTGATGCGCAACAGCATCTGTTGCAGCTCACAGTCTGGGGCATC
>JN692462..1
TTCTACTGTAATACATCACCACTGTTTAATAGTACTTGGAAAATTGGAACTAATAAGTCTATA----------
----

GAAAATGGAACTATCACACTCCCATGCAGAATAAAACAAATTATAAACAGGTGGCAGGAAGTAGGAAAAGCAA
TGTATGCCCCTCCCATCGAAGGACCAATTAGATGTTCATCAAAT---

ATTACAGGGCTGCTATTAACAAGAGATGGTGGTGAAGACAACAATGGGTCC----------------------
----
-
GAGACCTCCAATGAGACCTACACCTCTGGACCAGGAGATATGAATATGAATTGCAATTGTGAATTATATTTAT
ATAAAGATATGGAAATGGAGCCTGTACCATTA---GCACCCACCCCGACCAAGGCAAAGAGAAGAGTGGTG--
-------------------------------------CAGAGAGAAAAAAGAGCA---GTGGGA---
GTAGGAGCT---------GTGTTCCTTGGGTTCTTGGGAGCA---GCAGGAAGCACTATG---
GGCGCAGCGTCA---GTGGCGCTGACGGCACAA---GCCAGACAA---TTATTG---
TCTGGTATAGTGCAACAGCAGGAC---AATCTG---CTGAGG---
GCTATTGAGGCGCAACAGCATCTGTTGCAACTCACAGTCTGGGGCATC
>JN692465..1
TTCTATTGTAATACAACAGAGCTGTTTAAGGGTACTTGGAATGGTACTTGGAATGGTACTACTTGGGCTAATA
GTACTTGGGCT--
-------------AATGGT------
ACTGGGAATATGAACATCACACTCCCATGCAGAATAAGACAAATTATAAACATGTGGCAGGAGGTAGGAAAAG
CAATGTATGCCCCTCCCATCAGCGGACCCATTAACTGTACATCAAAT---
ATTACAGGGATGATATTAACAAGAGATGGTGGTGATGGTAACGGG----------------------------
----

AGTCAGGAGATCTTCAGACCTGGAGGAGGAGATATGAGGGACAATTGGAGAAGTGAATTATATAAATATAAAG
TAATAAAAATTGAACCATTA---GGAGTAGCACCCACCAGGGCAAAGAGAAGAGTGGTG--------------
-------------------------CAGAGAGAAAAAAGAGCA---GCT------ATAGGAGCT---------
GTGCTCATTGGGTTCTTGGGAGCA---GCAGGAAGCACTATG---GGCGCAGCGTCA---
ATAACGCTGACGGTACAA---GCCAGGCAA---TTATTG---TCTGGTATAGTGCAACAGCAGAGC---
AATCTG---CTGAGA---GCTATTGAGGCGCAACAACACCTGTTGCAACTCACTGTCTGGGGCATC
>JN692473..1
TTCTACTGTAATACATCAAAGCTGTTTAATAGGAATTGGACTGATAATAGTAATAATACTGGAAATGATACT-
----

GGGAATAATACTATCACACTCCCATGCAGAATAAAACAAATTATAAACAGATGGCAGGAAGTAGGAAAGGCAA
TGTATGCCCCTCCCATTCGAGGACTAATTAGATGTTCATCAAAT---
ATTACAGGGCTGATATTAACAAGAGATGGTGGTAATCATAACAATAGT-------------------------
----

AACGGTAGTGAAATCTTCAGACCTGGGGGAGGAGATATGAGGGACAATTGGAGAAGTGAATTATATAAATATA
AAGTAATAAAAATTGAGCCATTA---GGAATAGCACCCACCAAGGCAAAGAGAAGAGTGGTG-----------
----------------------------CAGAGAGAAAAAAGAGCA---GTGGGAACGATAGGAGCT------
---ATGTTCCTTGGGTTCTTGGGAGCA---GCAGGAAGCACTATG---GGCGCAGCGGCA---
GTGACGCTGACGGTACAG---GCCAGACAA---TTATTG---TCTGGTATAGTGCAACAGCAGAAC---
AATCTG---CTGAGA---GCTATTGAGGCGCAACAGCATATGTTGCAACTCACAGTCTGGGGCATC
>JN692474..2
TTTTACTGTAATACAACACAGCTGTTTAATAGCACTTGGCTGTTTAACAGCACTTGGAATCAAAGTGCTAAT-
----

GATGCAGACAATATCACACTCCCATGCAGAATAAAACAAATAGTAAACAGATGGCAGGAAGTAGGAAAAGCAA
TGTATGCCCCTCCCATCAAAGGACAAATTAGATGTTCATCAAAT---
ATTACAGGGCTGCTCTTAACAAGAGATGGTGATAATAACACCAAC----------------------------
----

AACAACGAGACCTTTAGACCTGGAGGAGGAGATATGAGGGACAATTGGAGAAGTGAATTATATAAATATAAAG
TAGTAAAAATTGAGCCATTA---GGAATAGCACCCACCAAGGCAAAAAGAAGAGTGGTG--------------
-------------------------CAGAGAGAAAAAAGAGCA---GTGGGAACAATAGGAGCT---------
GTGTTCCTTGGGTTCTTGGGAGCA---GCAGGAAGCACTATG---GGCGCAGCGTCA---
GTGACGCTGACGGTACAG---GCCAGACTA---TTATTG---TCTGGTATAGTGCAACAGCAGAAC---
AATCTG---CTGAAG---GCTATTGAAGCGCAACATCATCTGTTGCAGCTCACAGTCTGGGGCATC
>JN692480..1
TTCTACTGTAACACAGCACAGCTGTTTAATAATACTTGGCTGTCCAATAATACCTGGCTACAGAATAATACTT
GG---

ATTAATGGCACTATCACACTCCCATGCAGAATAAGACAAATTGTAAACAGGTGGCAGGAAGTAGGGAGAGCGA

TGTATGCTCCTCCCATCGCAGGAATAATCAACTGTGTATCAAAC---
ATTACAGGGCTGATATTAACAAGAGATGGAAACATAACAACAAATAGTACCTAC-------------------
----

TCTGAAAACAATACTGAAATCTTCAGACCTGGAGGAGGAGATATGAGGGACAATTGGAGAAGTGAATTATATA
AATATAAAGTGGTAACAATTGAGCCAGTG---GGAATAGCACCCACCGAGGCAAGGAGAAGAGTGGTG-----
----------------------------------CAGAGAGAAAAAAGAGCA---
GTGGGAACGATAGGAGCT---------ATGTTCCTTGGGTTCTTGGGAGCA---GCAGGAAGCACTATG---
GGCGCAGCGTCA---CTAACGCTGACGGTACAG---GCCAGAATA---TTATTG---
TCTGGTATAGTGCAACAGCAAAAC---AATTTG---CTGAGA---
GCTATTGAGGCGCAACAACATATGTTGCAACTCACAGTCTGGGGCATC
>HQ377423..2
TTCTACTGTGATACAAAACCACTGTTCAATAGTACTTGGAATGATACTACAGGGTCAATTCAC----------
----

ATTAATGGAACTATCACACTCCGATGCAGAATAAAACAAATTATAAACATGTGGCAGGGAGTAGGAAAAGCAA
TGTATGCCCCTCCCATCAGAGGACTAATTAACTGTACATCAAAT---
ATTACAGGGCTGCTATTAACAAGAGATGGTGGTAAAGAGAACAGG----------------------------
----

ACCACCGAGACCTTTAGACCTCAAGGAGGAAATATGAAAGACAATTGGAGAAGTGAACTATATAAATATAAAG
TAGTAAAAATTGAACCATTA---GGAATAGCACCCACCAAGGCAAAGCGAAGAGTGGTA--------------
-------------------------CAAAGAGAAAAAAGAGCA---ATAGGGGTAATAGGAGCT---------
ATGTTCCTTGGGTTCTTGGGAACA---GCAGGAAGCACTATG---GGCGCAGCGTCA---
CTGACGCTGACGGTACAG---GCCAGACAA---TTATTG---TCTGGTATAGTGCAACAGCAGAAC---
AATTTG---CTGAGA---GCTATTGAGGCGCAACAACATCTGTTGCAACTCACAGTCTGGGGCATC
>KT427681..1
TTCTACTGTAATACATCAACGCTGTTTAATTGGACTGAAAATTCAAATAACAAT-------------------
----

GCAAGTGACAATATGACAATCCCATGCAGAATAAGACAAATTGTAAACAGGTGGCAGGAAGTAGGAAAAGCAA
TGTATGCCCCTCCCATCAGCGGACAGATTAGCTGTTTATCAAAT---
ATCACAGGGCTGATATTAACAAGAGATGGTGGTATCAACAGCAGCAGCAGC----------------------
----

AATGAGACCTATGAGACCTTCAGACCTGCAGGAGGAAATATGAAAGATAATTGGAGAAGTGAATTATATAAAT
ATAAAGTAGTAAAAGTTGAGCCATTA---GGAGTAGCACCCACCAAGGCAAAGAGAAGAGTGGTG--------
-------------------------------CAGAGAGAAAAAAGAGCA---ATAACA---CTAGGAGCC---
------ATGTTCCTTGGGTTCTTGGGAGCA---GCAGGAAGCACTATG---GGCGCAGCGTCG---
ATGACGCTGACGGTACAG---GCCAGACTA---TTATTG---TCTGGTATAGTGCAACAGCAGAAC---
AACCTG---CTGAGA---GCTATTGAGGCGCAACAACATCTGTTGCAACTCACAGTCTGGGGCATT
>KT427732..1
TTCTACTGTAAGACAACACAGCTGTTTAATAGCACTTGGTTAAATACTAGTATTGAGTATAATAATACTGAAG
GGGCT--

GGAGTACCAAATATCACACTCCAATGCAGAATAAAACAAATTATAAACAGATGGCAGGGAGTAGAAAGAGCAA
TATATGCCCCTCCCATCCCGGGACAAATTAGATGTACATCAAAT---
ATTACAGGGCTGATATTGACACGGGATGGT---AATGGTAATCAAACAGAT----------------------
----

AGCACTAACGAAACCTTCAGACCAATAGGAGGAAATATGCAGGACAATTGGAGAAGTGAATTATATAAATATA
AAGTGGTAAAAATTGAGCCAGTA---GGAATAGCACCCACCAAGGCAAAGAGAAGAGTGGTG-----------
----------------------------CAGAGAGAAAAAAGAGCA---GTGGGAACACTAGGAGCT------
---ATGTTCCTTGGGTTCTTGGGAGCA---GCAGGAAGCACTATG---GGCGCAGCGTCA---
GTGACGCTGACGGTACAG---GCCAGACTA---TTATTG---TCTGGTATAGTGCAACAGCAGAAC---
AACCTG---CTGAAG---GCTATTGAGGCGCAACAGCATTTGTTGCAACTCACAGTCTGGGGCATC
>KT427713..1
TTCTATTGTAATACAACAAACCTGTTCAACAGTACTTGGAATGACACTACTGAAGGAAATAACACAGGA----
----

AATATAGGAAATATCACACTCCCGTGCAGAATAAAACAAATTATAAACATGTGGCAGCAAGTAGGAAAAGCAA
TGTATGCCCCTCCCATCGCAGGACAAATTAGCTGTTCATCAAAT---
ATTACAGGGTTGTTACTAACAAGAGATGGTGGTAATAATGATTCT----------------------------
----

AATAATTATGAAATCTTCAGGCCTGGAGGAGGAGATATGAGGGACAATTGGAGAAGTGAATTATACAAATATA
AAGTAGTAAAAATTGAACCATTA---GGAGTAGCACCCACTAAGGCAAAGAGAAGAGTGGTG-----------
----------------------------CAGAGAGAAAAAAGAGCG---GCA------CTAGGAGCT------
---TTGTTCCTTGGGTTCTTGGGAGCA---GCAGGGAGCACTATG---GGCGCAGCGGCA---
GTAACGCTGACGGTACAG---GCCAGAACA---TTATTG---TCTGGTATAGTGCAGCAGCAAAGG---
AATTTG---CTGAGA---GCTATTGAGGCGCAACAACATCTGTTGCAACTCACAGTCTGGGGCATC
>KT427870..2
TTCTACTGTAATACATCAGGACTGTTTAATAGTACTTGGAATAGGGATGGTAATTGGTCTCAGGATGATACTA
AT---

GGGTCAAATGAAACAATCACACTCCCATGCAGAATAAAACAAGTTATAAACAGGTGGCAGGAAGTAGGAAAAG
CAATGTATGCCCCTCCCATCAGCGGAAGGATTTTCTGTTCATCAAAC---
ATTACAGGGCTACTCTTAGTAAGAGATGGTGGTAACCAGAGCCAC----------------------------
----

GAGAGTGAGACCTTCCGACCTGGAGGAGGAGATATGAGGGACAATTGGAGAAGTGAATTATATAAATATAAAG
TAGTAAAAATTGAGCCATTA---GGAATAGCACCCACCAAGGCAAAGAGAAGAGTGGTG--------------
-------------------------CAGAGAGAAAAAAGAGCA---GTGGGAACGCTAGGAGCT---------
ATGTTCCTTGGGTTCTTGGGAACA---GCAGGAAGCACTATG---GGCGCAGCGTCA---
CTGACGCTGACGGTACAG---GCCAGACAA---TTATTG---TCTGGTATAGTGCAACAGCAGAAC---
AATCTG---CTGAAG---GCTATTGAGGCGCAACATCATCTGTTGCGACTCACAGTCTGGGGCATA
>KT427707..1
TTCTACTGTAATACAACAGGGCTGTTTAATAGTAATAATACTTGGAATAATACTAATAATAATAGTAGG----
----

AATAATGAAACATATACCATACCATGCAGAATAAAGCAAATTATAAACAGGTGGCAAGAAGTAGGAAAAGCAA
TGTACGCCCCTCCCATCAAAGGAATAATTCACTGTACATCAAAT---
ATTACAGGGCTGCTATTAACAAGAGATGGTGGGAATAGTTCAGAA----------------------------
----

AATGAAACTTTCAGACCTGCAGGAGGAGATATGAGGGACAATTGGAGAAGCGAATTATATAAATATAAAGTAG
TAAAAATTGAGCCAATA---GGACTAGCACCTACCAAGGCACAGAGAAGAGTGGTG-----------------
----------------------CAGAGAGAAAAAAGAGCA---GTGACA---CTGGGAGCT---------
ATGTTCCTTGGGTTCTTAGGAGCA---GCAGGAAGCACTATG---GGCGCAGCGTCA---
ATAACGCTGACGGTACAG---GCCAGACAA---TTGCTG---TCTGGTATAGTGCAACAGCAGAGT---
AATCTG---CTGAGG---GCTATTGAGGCGCAACAGCATATGTTGCAACTCACAGTCTGGGGCATC
>KT427873..1
TTCTACTGTAATTCAACACAACTGTTTAATAGTACCTGGGATACTAATGCAACCTTGAATGCTACAGACAGCC
AAGATGAA---

CTTAACAAAACTATCACACTCCCATGTAGAATAAAACAAATTATAAACATGTGGCAGGAAGTAGGAAAAGCAA
TGTATGCCCCTCCCATTAGAGGGCTAATTAGATGTACAACAAAT---
ATTACAGGGCTACTATTAACAAGAGATGGTGGCACAGGCAATAAGACT-------------------------
----

CAAAATAACACTGAGGTCTTCAGACCTGGAGGAGGAGATATGAAGGATAATTGGAGGAGTGAGTTATATAAAT
ATAAAGTAGTAAAAATTGAACCATTA---GGAGTAGCACCTACCAAGGCCAAGAGAAGAGTGGTG--------
-------------------------------CAGAGAGAAAAAAGAGCA---GTGGGAACAATAGGAGCT---
------ATGTTCCTTGGGTTCTTGGGAGCA---GCAGGAAGCACTATG---GGCGCAGCGTCA---
ATAACGCTGACGGCACAG---GCCAGACAA---TTATTG---TCTGGTATAGTGCAGCAGCAGAAT---
AATCTG---CTGAGG---GCTATTGAGGCGCAACAACATCTGTTGCAACTCACAGTCTGGGGCATC
>KT427695..1
TTCTACTGCAATACATCGGTGCTGTTTAATGATACTTGGCTGAATAATAATAAGACT----------------
----

GAACACCAAAATATCACGCTCCCATGCAAAATAAAACAGATTATAAACATGTGGCAGGAAGTAGGAAAAGCAA
TGTATGCCCCTCCCATCAGTGGACCAATTAGGTGTTCATCAAAT---
ATCACAGGGCTGCTATTAACAAGAGATGGTGGTAATAGCACAAAT----------------------------
----

AATAACGAGACCTTCAGACCTGGAGGAGGAGATATGAGGGACAATTGGAGAAGTGAATTATATAAATATAAAG
TAGTAAAACTTGAGCCATTA---GGATTAGCACCCACCAAGGCAAAAAGGAGAGTGGTG--------------
-------------------------CAGAGAGAAAAAAGAGCA---GTGGGAACGCTAGGAGCT----------
ATGTTCCTTGGGTTCTTGGGAACA---GCAGGAAGCACTATG---GGCGCAGCATCA---
CTAACGCTGACGGTACAG---GCCAGACTA---TTATTG---TCTGGCATAGTGCAACAGCAGAGC---
AATTTG---CTGAGG---GCTATTGAGGCGCATCAGCATATGTTGCAACTCACAGTCTGGGGCATC
>KT427785..1
TTTTACTGTAATACAACACAACTATTTAATAGTACTTGGGTTAATGATACTTTTAGTACTTGGGTTAATAATA
CTTGGGTT---AATGGT---
ACTGTAAAGTCAATTAGTAAT---------------
AAAACAAATATTAATATTACACTTCCATGCAGAATAAAGCAAATTATAAACATGTGGCAGGAAGTAGGAAAAG
CAATGTATGCCCCTCCCATCAAGGGACCAATTAGCTGTTCATCAAAT---
ATTACAGGACTGCTATTAACAAGAGATGGGGGAAATAGTGATAATAGTAGC----------------------
----
AAAAATGAGACAAAT---
GAGACCGAGACTTTTAGACCTGGAGGAGGAAATATGAAAGACAATTGGAGAAGTGAATTATATAAATATAAAG
TAGTGCAAATTGAACCATTA---GGAATAGCACCCACCAGGGCAAAAAGAAGAGTGGTG--------------
-------------------------CAAAGAGAGAAAAGAGCA---GTGGGA---ATAGGAGCA---------
TTGTTCATTGGGTTCTTGGGAGCA---GCAGGAAGCACTATG---GGCGCAGCGTCA---
ATGACGCTGACGGTACAG---GCCAGACAA---TTATTG---TCTGGTATAGTGCAACAGCAGAAC---
AACCTG---CTGAGG---GCTATTCAGGCGCAACAGCATTTGTTGCAGCTCACAGTCTGGGGCATC
>KJ849814..2
TTCTACTGCAATACAACACAGCTATTTAATTGGACAATTGGCAAGTCA-------------------------
----
-------AATATAACCCAA------
AATGATAGTGAAATTATTACAATCCAATGCAGAATAAAACAGATTATAAATAAGTGGCAGGAAGTAGGAAAAT
CAATATATGCCCCTCCTATCAGAGGAAATATTAGCTGTTCATCAAAT---
ATTACAGGACTGATATTAACAAGAGATGGTGGTAAT-------------------------------------
----
CACAATGAAACTAAAAACAAGACT---------
AAGACTGAGACTTTCAGACCTGGAGGAGGAGATATGAGGGACAATTGGAGAAGTGAATTATATAAATATAAGG
TAGTGAAAATTCAGCCATTA---GGAATAGCACCCACCAGGGCAAGGCGAAGAGTGGTG--------------
-------------------------CAGAGAGAAAAAAGAGCA---GTGGGAACACTAGGAGCT----------
ATGTTCCTTGGGTTCTTGGGAGCA---GCAGGAAGCACTATG---GGCGCAGCGTCA---
ATAACGCTGACGGTACAG---GCCAGACAA---TTGTTG---TCTGGTATAGTGCAGCAGCAGAAC---
AATCTG---CTGAAA---GCTATTGAGGCGCAACAGCATCTGTTGCAACTCACAGTCTGGGGCATC
>KT427771..1
TTCTACTGTAATACAACACAACTGTTTAATAGTACTTGGCAAAGTAATAGTACTGGAAATAGTACTGAGAAT-
----

AATAGCACAATAATCACACTCCCCTGTAGGATAAAACAAGTTATAAACATGTGGCAGGAGGTAGGAAAAGCAA
TGTATGCCCCTCCCATCAGTGGACTAATTCAATGTACATCAAAT---
ATTACAGGGCTGCTGTTAACAAGAGATGGTGGGTCTGATGGTGAATCTAATAAA-------------------
----

ACAGAGAGTGAGATCTTCAGACCTGGAGGAGGAGATATGAGGGACAATTGGAGAAGTGAATTATATAAATATA
AAATAGTAAAAATTGAACCATTA---GGAGTAGCGCCCACCAAGGCAAAGAGAAGAGTGGTG-----------
----------------------------CAGAGAGAAAAGAGAGCACTAGGGGGA---CTAGGAGCT------
---GTATTCCTTGGGTTCTTGGGAGCA---GCAGGAAGCACTATG---GGCGCAGCGTCA---
ATAACGCTGACGGTACAG---GCCAGACAA---TTATTG---TCTGGTATAGTGCAGCAGCAGAAC---
AATCTG---CTGAGG---GCTATTGATGCGCAACGGCACATGTTGCAACTCACAGTCTGGGGCATC
>KT427753..1
TTCTACTGTAATACAACAAAACTGTTCAATAGTACTTGGAAGTGGAATAATGGTACTTGGAATAGGAATGATA
CT---
-------GAAGGACAAGAT------

AACACTGGAGAAACTATTACACTTCCATGCAGAATAAAACAAATCATAAACATGTGGCAGGAAGTAGGAAAAG
CAATGTATGCCCCTCCCATCAGAGGACAAATTAATTGCACATCAAAT---
ATTACAGGACTGCTATTAACAAGGGATGGTGGTAATAATAATAATAGC------------------------
----

AAGAATAACGAGACTTTCAGACCTGGAGGAGGAAATATGAAGGACAATTGGAGAAGTGAATTATATAAATATA
AAGTAGTACAAATTGAACCATTA---GGAATAGCACCCACCAAGGCAAAAAGGAGAACGGTG----------
----------------------------CAGAGAGAAAAAAGAGCA---GTGGGA---ATAGGAGCT------
---GTGCTCTTTGGGTTCTTGGGAGCA---GCAGGAAGCACTATG---GGCGCAGCGTCA---
GTAACGCTGACGGTACAG---GCCAGACAA---TTGTTG---TCTGGTATAGTGCAACAGCAGAGC---
AATCTG---CTGAAG---GCTATTCAGGCGCAACAGCATCTGTTGCAGCTCACAGTCTGGGGCATC
>KT427749..3
TTCTACTGTAATACAACAGAACTGTTCTATAGTAATTGGACGTGGAATGGTACTTGGAATAGGAATGATACTG
AAGGATTA--

CAAAATAAAACTATTATACTTCCATGCAGAATAAAACAAATCATAAACATGTGGCAGGAAGTAGGAAAAGCAA
TGTATGCCCCTCCCATCAGAGGACAAATTAATTGCACATCAAAT---
ATTACAGGACTGCTATTAATAAGAGATGGTGGTAATGATACTGGAACT------------------------
----

AGTGGGACTGAAATTTTCAGACCTCAAGGAGGAAACATGAAAGACAATTGGAGAAGTGAATTATATAAATATA
AAGTAGTACAAATTGAACCATTA---GGAATAGCACCCACCAAGGCAAAGAGAAGAACGGTG----------
----------------------------CAGAGAGAAAAAAGAGCA---GTGGGA---TTTGGAGCT------
---GTGATCTTTGGGTTCTTGGGAGCA---GCAGGAAGCACTATG---GGCGCAGCGTCA---
ATAACGCTGACGGTACAG---GCCAGACAA---TTGTTG---TCTGGTATAGTGCAACAGCAGAGC---
AATTTG---CTGAAG---GCTATTCAAGCGCAACAGCATCTGTTGCAGCTCACAGTCTGGGGCATC
>KT427826..1
TTTTACTGTAATACAACAAAACTGTTTAATACTACGTGGAATATTACTGGAGAAGGAAACAACAATCTC----
----

ACTAATGAAACTATCACACTCCCATGCAGAATAAAACAGTTCATAAACATGTGGCAGGAAGTAGGAAAAGCAA
TGTATGCCCCTCCTATCAGGGGACAAATTCACTGCTCATCAAAT---
ATTACAGGGCTACTATTAACAAGAGATGGTGGTAATAGTAATAAT---------------------------
----

GAGTCGGAGACCTTCAGACCGGGAGGAGGAGATATGAGGGACAATTGGAGGAGTGAATTATATAAATATAAAG
TAGTAAAAATTGAACCATTA---GGAATAGCACCCACCAAGGCAAGAAGGAGAGTGGTG-------------
-------------------------CAAAGAGAAAAAAGAGCA---ATAGGAGGAATAGGAGTT---------
GTGATCCTTGGGTTCTTGGGAGCA---GCAGGAAGCACTATG---GGCGCAGCGTCG---
CTAACGCTGACGGTACAG---GCCAGACAA---TTATTG---TCTGGTATAGTGCAACAGCAAAGC---
AATTTG---CTGAGA---GCTATTGAGGCGCAACAGCGTTTGTTGCAACTCACTGTCTGGGGCATC
>KJ849821..1
TTCTACTGTGATACAGCAAAGCTGTTTAACAGTACTTGGGAGAGGCGTAATGACACGAAC------------
----
ACTTGGGAGTCGAACAATACA------------
ATACCAAATGAGAATATCACACTCCCATGCAGAATAAAACAAATTATAAACAGGTGGCAGGAAGTAGGAAAAG
CAATGTATGCCCCTCCCATTGCAGGACGAATTGAATGTACATCAAAT---
ATTACAGGGCTACTATTAACAAGAGATGGTGGT---------------------------------------
----
-
TCTAACAATAGTAGTAACGATACCTTCAGACCAGGAGGAGGAGATATGAAGGACAATTGGAGAAGTGAATTAT
ATAAATATAAAGTAGTACAAATTCAGCCATTA---GGAGTAGCACCCACCAGGTCAAGGAGAAGAGTGGTG--
-------------------------------------CAGAGAGAAAAAAGAGCA---GTAACA---
CTGGGAGCT---------ATGTTCCTTGGGTTCTTGGGAGCA---GCAGGAAGCACTATG---
GGCGCAGCGTCA---GTGACGCTGACGGTACAG---GCCAGACAA---TTATTG---
TCTGGTATAGTGCAGCAGCAAAGC---AATTTG---CTGAGG---
GCTATTGATGCGCAACAGCACATGTTGCAACTCACAGTCTGGGGCATC
>KT427811..1
TTTTACTGTAATACATCAAGACTGTTTAACAGTATTTGGAACAGTACCTGGCAGGATAATACAGCTGAAGAGT
CA--

AATAATACTGACATCATACTCCATTGCAGAATAAAACAAATTATAAACAGATGGCAGGAAGTAGGAAAAGCAA
TGTATGCCCCTCCCATCAAGGGAAAAATTCAATGTTCATCAAAT---
ATTACAGGGCTGCTATTAGTAAGAGATGGTGGTAACGAGAACACT---------------------------
----

AGTAACACCGAGACCTTCAGACCTGGAGGAGGAGACATGAGGGACAATTGGAGAAGTGAATTATATAAATATA
AAGTAGTGCAAATTGAGCCAGTA---GGAATAGCACCCACCAAGGCAAGAAGAAGAGTGGTG----------
---------------------------CAGAGAGAAAAAAGAGCA---GTAACA---ATAGGAGCT------
---ATGTTCCTTGGGTTCTTGGGAGCA---GCAGGAAGCACTATG---GGCGCAGCGTCA---
ATCGCGCTGACGGGACAG---GCCAGACAA---TTATTG---TCTGGTATAGTGCAACAGCAGAAT---
AATCTG---CTGAAG---GCTATTGAGGCGCAACAACATATGTTGCAACTCACAGTCTGGGGCATC
>KT427796..1
TTCTATTGTAATACATCACAGCTGTTCAATAGTACTTGGAATTTCACTAGTGCTAGGAATGAGACTGAAGAG-
----

TCAAATGACACTATCACACTCCCATGTAGGATAAAACAAATTATAAATAAGTGGCAAGAAGTAGGAAAAGCAA
TATATGCTCCTCCTATCCAAGGACGAATTTACTGTTCATCAAAC---
ATTACAGGGTTGCTATTAACAAGAGATGGGGGTGAAAACAACCTG---------------------------
----

ACCAATGAGACTTTTAGACCTGGAGGAGGAGATATGAGGGACAATTGGAGAAGTGAACTGTATAAATATAAAG
TAGTAAAAATTGAGCCATTA---GGACTAGCACCCACCAAGGCAAAGAGAAGAGTGGTG-------------
------------------------CAGAGAGAAAAAAGAGCA---GTGGGAACGCTGGGAGCA---------
ATGTTCCTTGGGTTCTTGGGAACA---GCAGGAAGCACTATG---GGCGCAGCGTCG---
CTGACGCTGACGGTACAG---GCCAGACAA---TTATTG---TCTGGTATAGTGCAACAGCAAAAC---
AATTTG---CTGAAG---GCTATTGAGGCGCAACAGCATCTGTTGCAACTCACAGTCTGGGGCATT
>KX181909..1
TTCTACTGTAATACAGCAGAGCTGTTTAGTAATACTTGGTTAAATAAGAGTGGCAAT---------------
----

AACGATGGAACTATCACACTTCCATGCAGAATAAAACAAATTATAAACAGATGGCAGGAAGTAGGAAAAGCAA
TGTATGCCCCTCCCATCAGGGGATTAATTGAATGTTCATCAAAT---
ATTACAGGGCTGATACTAACAAGAGATGGTGGTAATAATAGTGCA---------------------------
----

AACAATGCTAGTGAAACCTTCAGACCCGGAGGGGGAAATATGAAGGACAATTGGGGAAGTGAATTATATAAAT
ATAAAGTAGTAAAAATTCAGCCCTTA---GGAATAGCACCCACCAGGGCAAAAAGAAGAGTGGTG--------
------------------------------CAAAGAGAAAAAAAAGCA---GTAACG---CTAGGAGCT---
------ATGTTCCTTGGGTTCTTGGGAACA---GCAGGAAGCACTATG---GGCGCAGCGTCA---
GTGACGCTGACGGTACAG---GCCAGACAG---TTACTG---TCTGGTATAGTGCAACAGCAGAGC---
AATCTG---CTGAAG---GCTATTGAGGCGCAACAGCATCTGTTGCAACTCACAGTCTGGGGCATC
>KX181908..2
TTTTACTGTGATACAACAAAGCTGTTTAATAGTACTTGGAATAATACTGGAGAGTCAAATTACACGGAG----
----

GGAAATGCCACTATCATACTTCCATGCAGAATAAAACAAATTATAAACAGATGGCAGGAAGTAGGAAAAGCAA
TGTATGCCCCTCCCCTCAGTGGAAATATTAGCTGTTTATCAAAT---
ATTACAGGGGTACTATTAACAAGAGATGGGGGTAATGAAACAAAT---------------------------
----

GCCCCTGAGACCTTCAGACCTCAAGGGGGAAATATAAAGGATAATTGGGGAAGTGAATTATATAAATATAAAG
TAGTAAAAATTGAACCCTTA---GGAGTAGCCCCCCCCAAGGCAAAGAGAAGAGTGGGG-------------
------------------------CAAAGAGAAAAAAGAGCA---GTGGGA---ATAGGAGCT---------
ATGTTCCTTGGGTTCTTGGGAGCA---GCAGGAAGCAATATG---GGCGCAGCATCA---
CTAACGCTGACGGTACAG---GCCAGACTA---TTATTG---TCTGGTATAGTGCAACAGCAAAAC---
AATTTG---CTGAGA---GCTATTGAGGCGCAACAGCATCTGTTGCAGCTCACAGTCTGGGGCATC
>KX181898..2
TTCTACTGTAATACATCACAGCTGTTTAATTGGACTGAAAGAAATAGAAATAGCTCAGATGGGACAGGGTCAA
AT--

GAGACTGAGACCATTACACTCCCATGCAGAATAAAACAAATTATAAACAGATGGCAGGAAGTAGGAAAAGCAA
TGTATGCCCCTCCCATCAAAGGAAATATTAGCTGTTCATCAAAT---
ATTACTGGGCTGCTATTAACAAGAGATGGTGGTACAAACCTGACT----------------------------
----

GGGTCTAACGGGACCGAGACCTTCAGACCTCAAGGAGGAAATATGAAGGATAATTGGAGAAGTGAATTATATA
AATATAAAGTAGTAAGAATTGAGCCAGTA---GGAATAGCACCCACCGAGGCAAAGAGAAGAGTGGTG-----
----------------------------------CAAAGAGAAAAAAGAGCA---
GTGGGAACACTAGGAGCT---------ATGTTCCTTGGGTTCTTGGGAGCA---GCAGGAAGCACTATG---
GGCGCAGCGTCA---ATGACGCTGACGGTACAG---GCCAGACAA---TTATTG---
TCTGGTATAGTGCAACAGCAAAAC---AATCTG---CTGAAA---
GCTATTGAGGCGCAACATCATCTGTTGCAACTCACAGTCTGGGGCATC
>MK041573..1
TTCTACTGTAATACAACAAAACTATTTAATAGTACTTGGGATGGGTCAAACATCATAGGAAATGATACA----
----

AATGACACAGTGATCACACTCCCATGCAGAATAAAACAAATTATAAACATGTGGCAGCGAGTAGGAAAAGCAA
TGTATGCCCCTCCCATCAGAGGACAAATTAGATGTTCATCAAAT---
ATTACAGGGCTGCTGTTAACAAGAGATGGTGGAAATCATACAAGT----------------------------
----

GGGAATGAGACCTTCAGACCAGGAGGAGGAGATATGAGGGACAATTGGAGAAGTGAATTATATAAATATAAAG
TAGTAAAAATTGAACCATTG---GGAGTAGCACCCACCAGGGCAAAGAGAAGGGTGGTG--------------
-------------------------CAGAGAGAAAAAAGAGCA---GTAGGA---CTAGGAGCT---------
TTGTTCCTTGGGTTCTTGGGAGCA---GCAGGAAGCACTATG---GGCGCAGCGTCA---
CTAACGCTGACGGTACAG---GCCAGACAG---TTATTG---TCTGGTATAGTGCAACAGCAGAAC---
AACTTG---CTGAGG---GCTATTGAGGCGCAACAGCACATGTTGCAACTCACAGTCTGGGGCATC
>MK041575..1
TTCTACTGTAATACAACACAGCTGTTTAATAATACTTGGAATGATACTGCTTGGAATGATACTAGCTGG----
----

TCAAATGGCACTATCACACTCCCATGCAGAATAAAACAAATTATAAACAGGTGGCAGGAAGTAGGAAAAGCAA
TGTATGCCCCTCCCATCAGCGGACAAATTAATTGTTCATCAAAT---
ATTACAGGACTGTTATTAACAAGAGATGGTGGTAATGACAATGACACGAATACG-------------------
----

AATGAGACCGAGATCTTCAGACCTGCAGGAGGAGATATGAGGGACAATTGGAGAAGTGAATTATATAAATATA
AAGTAGTAAAAATTGAACCATTA---GGAATAGCACCCACCAGGGCAAAGAGAAGAGTGGTG-----------
----------------------------CAGAGAGAAAAAAGAGCA---GTGACA---TTAGGAGCT------
---GTGTTCCTTGGGTTCTTGGGAGCA---GCAGGAAGCACTATG---GGCGCAGCGTCA---
GTGACGCTGACGGTACAG---GCCAGACTA---TTATTG---TCTGGTATAGTGCAACAGCAGAGC---
AATTTG---CTGAGA---GCCATTCAGGCGCAACAGCATCTGCTGCAACTCACAGTCTGGGGCATC
>MG571983..1
TTCTACGGTAATACATCAAACCTGTTTAATAATAATATTACAGGGATTATA----------------------
----

AAGAATGGGACTATCATACTCCCATGCAGAATAAAACAAATTATAAACAGGTGGCAGGAAGTAGGAAGAGCAA
TGTATGCCCCTCCCATCAGAGGACTAATCAACTGTACATCAAAT---
ATTACAGGGCTGCTATTAACAAGAGATGGTGGTGGTGGTAATGAG----------------------------
----

AGTCATAACGAGACCTTCAGACCTGGAGGAGGAGATATGAGGGACAATTGGAGAAGTGAATTATATAAATATA
AAGTAGTAAGAATTGAGCCATTA---GGAATAGCACCCACCAAGGCACAGAGAAGAGTGGTG-----------
----------------------------CAAAGAGAAAAAAGAGCA---GTGGGAGCACTAGGAGCT------
---ATGTTCCTTGGGTTCTTGGGAGCA---GCAGGAAGCACTATG---GGCGCAGCGTCA---
ATAACGCTGACGGCACAG---GCCAGACAA---GTATTG---TCTGGTATAGTGCAACAGCAAAAC---
AATTTG---CTGAGG---GCTATTGAGGCGCAACAGCATTTGTTGCAACTCACAGTCTGGGGCATC
>MG571987..3

TTCTACTGTAATACAACATCACTGTTTAATAGTACTTGGCTGCTTAATGATACTGGAAATGTTACTGTGCCAA
ACAAA---

ACGGACGAAATTATCATACTCCCATGCAGAATAAAACAAATTATAAATATGTGGCAAAAAGTAGGAAGAGCAA
TGTATGCCCCTCCCATCAGAGGTTATATTAACTGTTCATCAAAT---
ATTACAGGGCTGCTATTAGTAAGGATGGTGGTAATAATAATCAGTCT-------------------------
----

AATCAGGCTGAAACCTTCAGACCTGGAGGAGGAGATATGAGGGACAATTGGAGAAGTGAATTATATAAATATA
AAGTAGTAAAAATTGAGCCATTA---GGAATAGCACCCACCAAGGCAAAGAGAAGAGTGGTG-----------
----------------------------CAGAGAGAAAAAAGAGCA---GTGGGGACACTAGGAGCT------
---GTGTTCCTTGGGTTCTTGGGAGCA---GCAGGGAGCACTATG---GGCGCAGCGTCA---
CTAACGCTGACGGTACAG---GCTAGACTA---TTATTG---TCTGGTATAGTGCAACAGCAGAAC---
AATTTG---CTGAGA---GCTATTGAGGCGCAACAGCATATATTGCAACTCACAGTCTGGGGCATC
>MG571990..1
TTCTACTGTAATACAACAAAACTATTTAATAGTACTTGGGATGGGTCAAACATCATAGGAAATGATACA----
----

AATGACACAGTGATCACACTCCCATGCAGAATAAAACAAATTATAAACATGTGGCAGCGAGTAGGAAAAGCAA
TGTATGCCCCTCCCATCAGAGGACAAATTAGATGTTCATCAAAT---
ATTACAGGGCTGCTGTTAACAAGAGATGGTGGAAATCATACAAGT----------------------------
----

GGGAATGAGACCTTCAGACCAGGAGGAGGAGATATGAGGGACAATTGGAGAAGTGAATTATATAAATATAAAG
TAGTAAAAATTGAACCATTG---GGAGTAGCACCCACCAGGGCAAAGAGAAGGGTGGTG--------------
-------------------------CAGAGAGAAAAAAGAGCA---GTAGGA---CTAGGAGCT---------
TTGTTCCTTGGGTTCTTGGGAGCA---GCAGGAAGCACTATG---GGCGCAGCGTCA---
CTAACGCTGACGGTACAG---GCCAGACAG---TTATTG---TCTGGTATAGTGCAACAGCAGAAC---
AACTTG---CTGAGG---GCTATTGAGGCGCAACAGCACATGTTGCAACTCACAGTCTGGGGCATC
>MG572002..1
TTCTATTGTAATTCATCACAGCTGTTTAATACAACTTGGATAAATGGCACTACAGGAAATAGAACTAATGAA-
----

AATGACACACTTATCACACTCCCATGCAGAATAAAACAAATTATAAACAGATGGCAGGAAGTAGGAACAGCAA
TATATGCCCCTCCCATTGAAGGCATGATTAAATGTTCATCAAAT---
ATTACGGGGGTGCTATTAACAAGAGATGGTGGTTATGATAACAATAGCACG----------------------
----

CGCCAGAACGAGACCTTCAGACCTGGAGGGGGAAATATGAAGGACAATTGGAGAAGTGAATTATATAAATATA
AAGTAGTAAAAGTTGAGCCATTA---GGACTAGCACCCACCAAGGCAAAGAGAAGAGTGGTG-----------
----------------------------CAAAGAGAAAAAAGAGCG---GTAACA---TTAGGAGCT------
---ATGTTCCTTGGGTTCTTGGGAGCA---GCAGGAAGCACTATG---GGCGCAGCGTCA---
ATGACGCTGACGGTACAG---GCCAGACAA---TTGTTG---TCTGGTATAGTGCAACAGCAGAAC---
AATCTG---CTGAGA---GCTATTGAGGCGCAACAGCATATGTTGCAACTCACAGTCTGGGGCATA
>JF320427..1
TTCTACTGCAATACAACAGCACTGTTTAATAGTACATGGAACGGTACAGAGGGGATAAGTAAT----------
----

GCTACAGGAAATATCACACTCCCATGCAGAATAAAACAAGTTATAAACAGATGGCAGGAAGTAGGAAAAGCAA
TGTATGCCCCTCCCATTAAAGGAGTAATTAGATGTGTATCAAAT---
ATCACAGGGATACTATTAACGAGAGATGGGGGTAATAATAATAATACT-------------------------
----

AATCACACCGAGATATTCAGACCTGGAGGAGGAGATATGAGAGACAATTGGAGAGGTGAATTATATAAATATA
AGGTAGTAAAAATTGAACCATTA---GGAGTAGCACCCACCAAGGCAAAGAGAAGAGTGGTG-----------
----------------------------CAGAGAGAAAAAAGAGCA---GTGGGA---ATAGGAGCT------
---TTGTTCCTTGGGTTCTTGGGAGCA---GCAGGAAGCACGATG---GGCGCAGCGTCA---
GTGACGCTGACGGTACAG---GCCAGACAA---TTATTG---TCTGGTATAGTGCAACAGCAGAGC---
AATTTG---CTGAGG---GCTATTGAGGCGCAACAACATATGTTGCAACTCACAGTCTGGGGCATC
>GU562236..3

TTCTACTGCAATACAACGCAGCTGTTTAATAGTACTTGGTATAGTAATGGTACTTGGTCTTGGAATGGTACTA
CTTGGAATGGT---ACT---
AGAGAGACAAATAGTACGGAA---------------
GGAAACGACACAATGATCACACTCCCATGCAGAATAAAACAATTTATAAACATGTGGCAGGCAGTAGGAAAAG
CAATATATGCCCCTCCCATCAGTGGACTAATCAAATGTACATCAAAT---
ATTACAGGGCTGTTAATAACAAGAGATGGGGGTCATGAAAGTAATAAAAGTAAC------TATGGT-------
----
TATGAAAGTAGTAAAAGT---
GAGAATGAGACCTTCAGACCTGGAGGAGGAAATATGAGGGATAATTGGAGAAGTGAATTGTATAAATATAAAA
TAGTAAGAATTGAACCATTA---GGACTAGCACCCACCAAGGCAAAGAGAAGAGTGGTG--------------
-------------------------CAGAGAGAAAAGAGAGCC---GCA------CTAGGAGCC---------
ATGTTCCTTGGGTTCTTGGGAGCA---GCAGGAAGCACTATG---GGCGCAGCGTCA---
CTGACGCTGACGGTACAG---GCCAGACAA---TTATTG---TCTGGTATAGTGCAACAGCAGAGC---
AATTTG---CTGAGG---GCTATTGAGGCGCAACAGCATATGTTGCAACTCACGGTCTGGGGCATC
>JQ403024..1
TTCTACTGTAATACAACACAACTGTTTAATAGAGATTGGAATGATACTAGTACTATTAACAGTACT-------
----

GAAAATAGAAATATCACACTCCCATGTAGAATAAAACAAATTATAAACATGTGGCAGGAAGTAGGAAAAGCAA
TGTATGCCCCTCCCATCCAAGGACAAATTAGATGTTCATCAAAT---
ATTACAGGGTTGCTATTAACAAGAGATGGTGGTAATGACAATACCACT-------------------------
----

AACACCACTGAGATCTTCAGACCTGTAGGAGGAAATATGAGGGACAATTGGAGAAGTGAATTATACAAATATA
AAGTAGTACAAATTGAACCATTA---GGATTAGCACCCACCAAGGCAAAGAGAAGAGTGGTG-----------
----------------------------CAGAGAGAAAAAAGAGCA---GTAACG---TTAGGAGCT------
---ATGTTCCTTGGGTTCTTGGGAGCA---GCAGGAAGCACTATG---GGCGCAGCGTCA---
ATGACGCTGACGGTACAG---GCCAGACAA---TTATTG---TCTGGTATAGTGCAGCAGCAAAAT---
AATTTG---CTGAAG---GCTATTGAGGCGCAACATCATCTGTTGCAACTCACAGTCTGGGGCATC
>JQ403041..2
TTCTACTGTAATACAACACGACTGTTTAATAGTACTTGGAATAGTACCGAATCAAATAATACTATA-------
----

GGAAATGGCACAATCACACTCCCATGCAGAATAAAACAAATTGTAAACATGTGGCAGGAAGTAGGAAAAGCAA
TGTATGCCCCTCCCATCAGAGGACAAATTAGATGTTCATCAAAT---
ATTACAGGGCTGATATTAACAAGAGATGGTGGTCATGGTGGTCATAATAAGAAT-------------------
----

ACCACCAACACCACAGAGATCTTCAGACCTGGAGGAGGAGATATGAGGGACAATTGGAGAAGTGAATTATATA
AATATAAAGTAGTAAAACTTGAACCATTA---GGAATAGCCCCCACCTCGGCAAGGAGAAGAGTGGTG-----
----------------------------------CAGAGAGAAAAAAGAGCA---GTGGGA---
ATAGGAGCT---------ATGTTCCTTGGGTTCTTGGGAGCA---GCAGGAAGCACTATG---
GGCGCAGCGTCA---GTGACGCTGACGGTACAA---GCCAGACTA---TTATTG---
TCTGGTATAGTGCAACAGCAGAAC---AATTTG---CTGAAG---
GCTATTGAGGCGCAACAGCATATGTTGCAACTCACAGTCTGGGGCATC
>KC797225..3
TTCTACTGTAATACATCACAATTGTTTAATAGTACTTGGAATGCTACTGGTACTCAGAATACTACTGGTACTC
AGAATGAGACT--

AAAATAGGCAATATCACACTCCCATGTAAAATAAAACAAATTATAAACAGGTGGCAAGAAGTAGGAAAAGCAA
TGTATGCCCCTCCCATCAAAGGACTAATTAACTGTGTATCAAAT---
ATTACAGGGATACTATTAACAAGAGATGGTGGTCATTCTAAGAATACAAATAGT-------------------
----

ACAGATAATGATACCGAGGTCTTCAGACCTGGAGGAGGAGATATGAGGGACAATTGGAGAAGTGAATTATATA
AATATAAAGTGGTAAAAATTGAGCCATTA---GGAGTAGCACCCACCAAGGCAAAGAGAAGAGTGGTG-----
----------------------------------CAGAGAGAAAAAAGAGCA---GTGACG---
CTAGGAGCC---------ATGTTCCTTGGGTTCTTGGGAGCA---GCAGGAAGCACTATG---
GGCGCAGCGTCG---CTGACGCTGACGGTACAG---GCCAGACAA---TTATTG---

TCTGGTATAGTGCAACAGCAGAAC---AACCTG---CTGAGG---
GCTATTGAGGCGCAACAGCATATGTTGCAACTCACAGTCTGGGGCATC
>HQ699978..2
TTCTACTGTAATACATCACAACTGTTTAATAGTACTTATAATGGTACTTGGAGTGGTACTACA----------
----

GGAAATGATACTATCACGCTCCCTTGCAGAATAAAACAAATTGTAAACAGGTGGCAGGAAGTAGGGAAAGCAA
TGTATGCCCCTCCCATTCAAGGAATAATTAGATGTTCATCAAAT---
ATTACAGGGTTGCTATTAACAAGAGATGGTGGTAAGAATGAGAGCGAT-------------------------
----

AGCAATACCACCGAGACCTTCAGACCTGGAGGAGGAAATATGAGAGACAATTGGAGGAGTGAATTATATAAAT
ATAAAGTAGTAAAAATTGAACCATTA---GGAGTAGCACCCACCAAGGCAAAAAGAAGAGTGGTG--------
-------------------------------CAGAGAGAAAAAAGAGCA---GTGGGAACGATAGGAGCT---
------ATGTTCCTTGGGTTCTTGGGAGCA---GCAGGAAGCACTATG---GGCGCAGCGTCA---
ATAACGCTGACGGTACAG---GCCAGACAA---TTGTTG---TCTGGTATAGTGCAACAGCAGAGA---
AATTTG---CTGAGG---GCTATTGAGGCGCAACAGCATCTGTTGCAACTCACAGTCTGGGGCATC
>EU131792..1
TTCTACTGTAATACATCAGAGCTGTTTAAAAGTACTTGGAATAATACTAGTACT-------------------
----

GGGAATGATCCTATCACGCTCCCTTGCAGAATAAAGCAAATTGTAAACATGTGGCAAGAAGTAGGGAAAGCAA
TGTATGCTCCTCCCATTGAAGGACAAATTAGATGTTTATCAAAT---
ATTACAGGATTGCTATTAACAAGGGATGGTGGTCATAATGGGAGC----------------------------
----

GAGACCAACGAGACCTTCAGACCTGCAGGAGGAAATATGAGAGACAATTGGAGAAGTGAATTATATAAATATA
AAGTAGTAAAAATTGAACCATTA---GGAGTAGCACCCACCAAGGCAAAGAGAAGAGTGGTG-----------
----------------------------CAGAGAGAAAAAAGAGCA---GTGGGAACAATAGGAGCT------
---ATGTTCCTTGGGTTCTTGGGAGCA---GCAGGAAGCACTATG---GGCGCAGCGTCA---
ATGACGCTGACGGTACAG---GCCAGACAA---ATGTTG---TCTGGTACAGTGCAACAGCAAAGA---
AATTTG---CTGAGG---GCTATTGAGGCGCAACAGCATCTGTTGCAACTCACAGTCTGGGGCATC
>EU131803..1
TTCTACTGTAATACATCAAAATTATTTAATAGTACTTGGTATAATAATGGTACTTGGAATAATGCTAGTACT-
----

GGGAATGATACTATCACGCTCCCTTGCAGAATAAAACAAATTGTAAACATGTGGCAGGAAGTAGGGAAAGCAA
TGTATGCCCCTCCCATTGCAGGACAAATTAGATGTTCATCAAAG---
ATTACAGGGTTGCTATTAACAAGAGATGGTGGTAATGAGAACAAGACTAAGGAC-------------------
----

AACAACACCGAGACTGAGACCTTTAGACCTGGGGGAGGAGATATGAGAGACTATTGGAGAAGTGAATTATATA
AATATAAAGTAGTAAAAATTGAACCATTA---GGAGTAGCACCCACCAAGGCAAAGAGAAGAGTGGTG-----
----------------------------------CAGAGAGAAAAAAGAGCA---
GTGGGAACAATAGGGGCT---------ATGTTCCTTGGGTTCTTGGGAGCA---GCAGGAAGCACTATG---
GGCGCAGCGTCA---ATAACGCTGACGGTACAG---GCCAGACAA---TTGTTG---
TCTGGTATAGTGCAACAGCAGAGA---AATTTG---CTGATG---
GCTATTGAGGCGCAACAACATTTGTTGCAACTCACAGTCTGGGGCATC
>EU363829..1
TTCTACTGTAATACAACAAAACTGTTTAATAGTATTTGGTATAATAATGGTACTTCGAGTGGTACTACA----
----

GGAAATGAAACTATCATACTCCCTTGCAGAATAAAACAAATTGTAAACATGTGGCAGAAAGTAGGGAGAGCAA
TGTATGCCCCTCCCATTGAAGGAAAAATTAGCTGTGTATCAAAT---
ATCACAGGGTTGCTATTAACAAGAGATGGTGGTCAGAATGAGAAC----------------------------
----

AATACCGAAACCTTCAGACCTGAAGGAGGAAATATGAAAGACAATTGGAGAAGTGAATTATATAAATATAAAG
TAGTAAAAATTGAACCATTA---GGAGTAGCACCCACCAAGGCAAAGAGAAGAGTGGTG--------------
-------------------------CAAAGAGAAAAAAGAGCA---GTGGGAGCAATAGGAGCT---------

ATGTTCCTTGGGTTCTTAGGAGCA---GCAGGAAGCACTATG---GGCGCAGCGTCA---
ATAACGCTGACGGTACAG---GCCAGACAA---TTGTTG---TCTGGCATTGTGCAACAGCAGAGA---
AATTTG---CTAAGG---GCTATTGAGGCGCAACAGCATCTGTTGCAACTCACAGTCTGGGGCATT
>HM215413..1
TTCTACTGTAATACAGCACAACTGTTTAATAGTACTTGGAATACTACTAAGATTTGGGGGAATACTACAGAAG
GT---

AACAATAGCACTATCATGCTCCCTTGCAAAATAAAGCAAATTATAAACCTGTGGCAGGAAGTAGGAAAAGCAA
TATATGCCCCTCCCATCGCAGGGCGAATTAGATGTGTATCAAAT---
ATTACAGGGTTGCTATTAACAAGAGATGGTGGTCATAATGGGAACGGG-------------------------
----

TCCAACGAGACCGAGACCTTCAGACCTGCAGGAGGAGATATGAGGGACAATTGGAGAAGTGAATTATATAAAT
ATAAAGTAGTAAAAATTGAACCAATA---GGATTAGCACCCACCAAGGCAAAGAGAAGAGTGGTG--------
-------------------------------CAGAGAGAAAAAAGAGCA---ATGGGA---ATAGGAGCT---
------ATGTTCCTTGGGTTCTTGGGAGCA---GCAGGAAGCACTATG---GGCGCAGCGTCA---
ATAACGCTGACGGTGCAG---GCCAGACGA---TTATTG---TCTGGTATAGTGCAACAGCAAAGA---
AATTTG---CTGAGG---GCTATTGAGGCGCAACAACATCTGTTGCAACTCACCGTCTGGGGCATC
>EU131802..1
TTCTACTGTAATACATCACAACTGTTTAATAGTACTTGGAATGATACTAGTACTTGGAATGGTAAT-------
----

ACAGGAACTACTGTCACGCTCCCTTGCAGAATAAAACAAATTGTAAACATGTGGCAGAAAGTAGGGAAAGCCA
TGTACGCCCCTCCCATTCAAGGACAAATTAGATGTTCATCAAAT---
ATTACAGGGTTGCTATTAACAAGAGATGGTGGTAATAATGAGAGC----------------------------
----

GAGAATACCGAGACCTTCAGACCTGCAGGAGGAAATATGAGAGATAATTGGAGAAGTGAATTATATAAATATA
AAGTAGTAAAAATTGAACCATTA---GGAGTAGCCCCCACCAAGGCAAAGAGAAGAGTGGTG----------
----------------------------CAGAGAGAAAAAAGAGCA---GTGGGATTGATAGGAGCT------
---ATGTTCCTTGGGTTCTTGGGAGCA---GCAGGAAGCACTATG---GGCGCAGCGTCA---
ATAACGCTGACGGTACAG---GCCAGGCAA---TTATTG---TCTGGTATAGTGCAACAGCAGAGA---
AATTTG---CTGATG---GCTATTGAGGCGCAACAGAATATGTTGCGACTCACAGTCTGGGGCATC
>EU131804..1
TTCTACTGTAACACATCACAACTGTTTAATAGTACTTGGTATAGTAATGGTACTAGGAAGGATCCTACA----
----

GGAAATGGCACTATCACGCTCCCTTGCAGAATAAAACAAATTATAAACATGTGGCAGCAAGTAGGGAAAGCAA
TGTATGCACCTCCCATTAAAGGACAAATTAGATGTTCATCAAAT---
ATTACAGGGTCGCTATTAACAAGAGATGGTGGTTATAATGAGAGC----------------------------
----

AACACCACCGAGACCTTCAGACCTGGAGGAGGAGATATGAGAGACAATTGGAGAAGTGAATTATATAAATATA
AAGTAGTAAAAATTGAACCATTA---GGAGTAGCACCCACCAAGGCAAAGAGAAGAGTGGTG----------
----------------------------CAGAGAGAAAAAAGAGCA---GTAGGA---CTAGGAGCT------
---ATGTTCCTTGGGTTCTTGGGAGCA---GCAGGAGGCACCATG---GGCGCAGCGTCA---
ATAACGCTGACGGTACAG---GCCAGACAA---TTGTTG---TCTGGTATAGTGCAACAGCAGAGA---
AATTTG---CTGAGT---GCTATTGAGGCGCAACAACGTTTGTTGCAACTCACAGTCTGGGGCATC
>JF932472..1
TTCTACTGTAATACAGCACAACTGTTTAATAGTACTTGGACAGGAAATGATACTAGCATTTGGAATGATACTA
CA---

GGAAATGACACTATCACACTCCCTTGCAGAATAAAACAAATTATAAACATGTGGCAGGAAGTAGGGAAAGCAA
TGTATGCCCCTCCCATTGCAGGACAAATTAGATGTTCATCAAAT---
ATTACAGGGTTGCTATTAACAAGAGATGGTGGTAATCATGAGAAC----------------------------
----

AACACTGAGACCTTCAGACCTGGAGGAGGAGATATGAGAGACAATTGGAGAAGTGAATTATATAGATATAAAG
TAGTAAAAATTGAACCATTA---GGAGTAGCACCCACCAAGGCAAAGAGAAGAGTGGTG--------------
-------------------------CAGAGAGAAAAAAGAGCA---GTGGGAACGCTAGGAGCT---------

ATGTTCCTTGGGTTCTTGGGAGCA---GCAGGAAGCACTATG---GGCGCAGCGTCA---
ATAACGCTGACGGTACAG---GCCAGACAA---TTGTTG---TCTGGTATAGTGCAACAGCAGAGA---
AATTTG---CTGAGG---GCTATTGAGGCGCAACAGCATCTGTTGCAACTCACAGTCTGGGGCATC
>JF932474..3
TTCTACTGTAATACATCACAACTGTTTAATAGTACTTGGCCTTGGAATAATGATACTAGTGTTTGGGATGATA
CTACA---

GAAAATGACATTATCACGCTCCCTTGCAGAATAAAACAAATTATAAACATGTGGCAGGAAGTAGGGAAAGCAA
TGTATGCCCCTCCCATTCAAGGACAACTTAACTGTTCATCAAAT---
ATTACAGGGTTGCTATTAACAAGAGATGGTGGTTATCATAATGAGAGC------------------------
----

AATAACACCGAGACCTTCAGACCTGGAGGAGGAGATATGAGAGACAATTGGAGAAGTGAATTATATAAATATA
AAGTAGTAAAAATTGAACCATTA---GGAGTAGCACCCACCAAGGCAAAGAGAAGAGTGGTG----------
---------------------------CAAAGAGAAAAAAGAGCA---GTGGGAGCGATAGGAGCT------
---ATGTTCCTTGGGTTCTTGGGAGCA---GCAGGAAGCACTATG---GGCGCAGCGTCA---
ATAACGCTGACGGTACAG---GCCAGACAA---CTGTTG---TCTGGTATAGTGCAACAGCAGAGA---
AATTTG---CTGAGG---GCTATTGAGGCGCAACAGCATCTGTTGCAACTCACAGTCTGGGGCATT
>HQ215554..1
TTCTACTGTAATACAACAGGGCTGTTTAATAGTACTTTGACTAATGATACCTGGAATGATAATCGGTCATATA
AC--

AATGGAACAGGTAACATCACACTCCACTGCAGAATAAAACAAATTATAAACAGGTGGCAGGAAGTAGGAAAAG
CAATGTATGCCCCTCCCATCAGTGGACCAATTAGCTGTTTATCAAAT---
ATTACAGGACTGATATTAACAAGAGATGGAGGTAACAATACCGGGAAT------------------------
----

CAATCTGATACTGAAACCTTCAGACCTGGGGGAGGGGATATGAAGGACAATTGGAGAAGTGAATTATATAAAT
ATAAAGTAGTACAAATTGAGCCAATA---GGATTAGCACCCACCAGGGCAAGGAGAAGAGTGGTG-------
------------------------------CAGAGAGAAAAAAGAGCA---GTGGGAACATTAGGAGCT---
------ATGTTCCTTGGGTTCTTGGGAGCA---GCAGGAAGCACTATG---GGCGCAGCGTCA---
GTCACGCTGACGGTACAG---GCCAGACAA---TTACTG---TCTGGTATAGTGCAACAGCAGAAC---
AATTTG---CTGAAA---GCTATTGAGGCGCAACAACATATGTTGCAACTCACAGTCTGGGGCATC
>JF346917..1
TTCTACTGTAATACATCACAACTGTTTAATAGTACTTGGGATATTAATAGTAATGGGACTAATAATACAAAT-
----

AGCAATATCAATATCACACTCCCTTGCAAAATAAAACAAATTGTAAACATGTGGCAGGAAGTAGGGAAAGCAA
TGTACGCCCCTCCCATTAGAGGACAAATTAGATGTTCATCACAA---
ATTACAGGGTTGTTATTAACAAGAGATGGTGGTACTAATAATAAT---------------------------
----

CATACCAACGAAACCTTCAGACCTGCAGGGGGAGACATGAGAGACAATTGGAGAAGTGAATTATATAAATATA
AAGTAGTAAAAATTGAACCATTA---GGAATAGCACCCACCAAGGCAAAGAGAAGAGTGGTG----------
---------------------------CAGAGAGAAAAAAGAGCA---GTGGGAACAATAGGAGCT------
---ATGTTCCTTGGGTTCTTAGGAGCA---GCAGGAAGCACTATG---GGCGCAGCGTCA---
GTAACGCTGACGGTACAG---GCCAGACAA---TTATTG---TCTGGTATAGTGCAACAGCAGAGA---
AATCTG---CTGAGG---GCTATTGAGGCGCAACAGCGTGTGTTGCAACTCACAGTCTGGGGCATC
>JX960599..1
TTCTACTGCAATACAACACAACTGTTTAATAGTACTTGGAATACTTGGGATAATATTACTGGGAATAGTAATG
AGAATAAT--

ACACAGAACAATATCACACTTCCATGTAGAATAAAACAAATTATAAACATGTGGCAGGGAGTAGGAAAAGCAA
TGTACGCCCCTCCCATCGCAGGACGAATTAAATGTTCATCAGAT---
ATTACAGGACTATTATTAGTGAGAGATGGT---AATAACAATAACAAT------------------------
----

ACAGATAACAAGACAGAAACTTTCAGACCTCAGGGAGGAAACATGAAGGACAATTGGAGAAGTGAATTATATA
AATATAAAGTAGTAAAAATTGAACCAATA---GGAATAGCACCCACTACGGCAAGGAGAAGAGTGGTG----
---------------------------------CAGAGAGAAAAAAGAGCA---GTGACA---

CTAGGAGCT---------ATGTTCCTTGGGTTCTTAGGAGCA---GCAGGAAGCACAATG---
GGCGCAGCGTCA---ATAACGCTGACGGTACAG---GCCAGACAA---TTACTG---
TCTGGTATAGTGCAACAGCAGAAC---AATTTG---CTGAGG---
GCCATTGAGGCGCAACAGCATATGTTGCAACTCACAGTCTGGGGCATC
>JF932497..2
TTCTACTGTAATACATCACGACTGTTTAGTAGTACATGGTATACTAATGATACTCAGAATGGTACTACA----
----

GGAAATGACACTATCACACTCCCTTGCAGAATAAAACAAATTGTAAACATGTGGCAGGAAGTAGGAAAAGCAA
TGTATGCCCCTCCCGTTGAAGGACTAATTAGATGTTCATCAAAT---
ATTACAGGGTTGCTATTAACAAGAGATGGTGGTTATAATGTAAGC----------------------------
----

AACAACACCGAGATCTTCAGACCTGGAGGAGGAGATATGAGGGACAATTGGAGAAGTGAATTATATAAATATA
AAGTAATAAAAATTGAACCATTA---GGAGTAGCACCCACCAAGGCAAAGAGAAGAGTGGTG-----------
----------------------------CAAAGAGAAAAAAGAGCA---GTGGGA---ATAGGAGCT------
---ATGTTCCTTGGGTTCTTGGGAGCA---GCAGGAACCACTATG---GGCGCAGCGTCA---
ATGACGCTGACGGTACAG---GCCAGACAA---TTGTTG---TCTGGTATAGTGCAACAGCAGGGA---
AATCTG---CTGAGG---GCTATTGAGGCGCAACAGCATATGTTGCAACTCACAGTCTGGGGCATT
>KX693642..1
TTCTACTGTAATACATCACAACTGTTTAATAGTACTTGGGATGTTAATGACACTAGTAATGATACTACA----
----

GGAAATGAAACTATCACGCTCCCTTGCAGAATAAAACAAATTATAAACATGTGGCAGGGAGTAGGGAAAGCAA
TGTATGCCCCTCCCATTAAAGGAAAAATTAGATGTTCATCAAAT---
ATTACAGGGTTGATATTAACAAGAGATGGTGGTGTAAATGAGAGC----------------------------
----

GAGTTCAATGAGACCTTCAGACCTCAAGGAGGAAATATGAAAGACAATTGGAGAAGTGAATTGTATAAATATA
AAGTAGTAAAAATTGAACCATTA---GGAGTAGCACCCACCAAGGCAAAGAGAAGAGTGGTG-----------
----------------------------CAGAGAGAAAAAAGAGCA------GGA---ATAGGAGCT------
---CTGTTCCTTGGGTTCTTGGGAGCA---GCAGGAAGCACTATG---GGCGCAGCGTCA---
ATAACGCTGACGGTACAG---GCCAGACAA---TTGATG---TCTGGTATAGTGCAACAGCAGAGA---
AATTTG---CTGAGA---GCTATTGAGGCGCAACAGCATCTGTTGCAACTCACAGTCTGGGGCATC
>KX693171..1
TTTTACTGTAATACATCACAACTGTTTGACAGTATTTGGAATGCTACTACTGGTACTTGGAATGGTACTACA-
----

GGAGGTGACAATATCACGCTCCCTTGCAGGATAAAACAAATTGTAAACATGTGGCAGGAAGTAGGGAAAGCAA
TGTATGCCCCTCCCATTAGAGGACAAATTTATTGTTCATCAAGT---
ATTACAGGGTTGCTATTAACAAGAGATGGTGGTACGAGTAAGAAT----------------------------
----

GAGAGCAATACCACCGAGACCTTCAGACCTGGAGGAGGAGATATGAGGGACAATTGGAGAAGTGAATTATATA
AATACAAAGTAGTAAAACTTGAACCACTA---GGAGTAGCACCCACCGAGGCAAAGAGAAGAGTGGTG-----
----------------------------------CAGAGAGAAAAAAGAGCA---
GTGGGAGCGATAGGAGCT---------ATGTTCCTTGGGTTCTTGGGAGCA---GCAGGAAGCACTATG---
GGCGCAGCGTCA---ATAACGCTGACGGTACAG---GCCAGACAA---TTATTG---
TCTGGTATAGTGCAACAGCAAAGA---AATTTG---CTGAGG---
GCTATTGAGGCGCAACAGCATATGTTGCAACTCACAGTCTGGGGCATC
>KX693309..1
TTCTACTGTAATACATCAAAACTGTTTAATAGTACTTGGAGTAAGACTAGTACTTGGAATGATACTACA----
----

GGAAATGACACTATCACGCTCCCTTGCAGAATAAAACAAATTGTAAACATGTGGCAGGAAGTAGGGAAAGCAA
TGTATGCCCCTCCCATTCAAGGACTAATTAACTGTTCATCAAAT---
ATTACCGGGTTGCTATTAACAAGAGATGGTGGTAATAATACTGAGAGC-------------------------
----

AACACCACCGAGACCTTCAGACCTACAGGAGGAAATATGAGAGACAATTGGAGAAGTGAATTATATAAATATA

AAGTAGTACAAATTGAACCATTA---GGAGTAGCACCCACCAAGGCAAAGAGAAGAGTGGTG-----------
----------------------------CAGAGAGAAAAAAGAGCA---GTGGGAACAATAGGAGCT------
---ATGTTCCTTGGGTTCTTGGGAGCA---GCAGGAAGCACTATG---GGCGCAGCGTCG---
GTAACGCTGACGGTACAG---GCCAGACAA---TTGTTG---TCTGGTATAGTGCAACAGCAGAGA---
AATTTG---CTGAGG---GCTATTGAGGCGCAACAGCATCTGTTGCAACTCACAGTCTGGGGCATT
>KX693362..1
TTCTACTGTAATACATCAAAACTGTTTAATAGTACTTGGAATGATAATAGTACTTGGAATGATAATGATAAGA
CA---

GGAGATGACAATCTCACGCTCCCTTGTAGAATAAAACAAATTGTAAACATGTGGCAGGAAGTAGGGAAAGCAA
TGTATGCCCCTCCCATTAAAGGACAAATTAGATGTTCATCAAAT---
ATTACAGGGTTGCTATTAACAAGAGATGGTGGTCAGAATGTGAGC----------------------------
----

AATACCACCGAGACCTTCAGACCTGCAGGAGGAGATATGAGAGACAATTGGAGAAGTGAATTATATAAATATA
AAGTAGTAAAAATTGAACCATTA---GGAGTAGCACCTACCAAGGCAAAGAGAAGAGTGGTG-----------
----------------------------CAGAGAGAAAAAAGAGCA---GTGGGAACGATAGGAGCT------
---ATGTTCCTTGGGTTCTTGGGAGCA---GCAGGAAGCACGATG---GGCGCAGCGTCA---
ATAACGCTGACGGTACAG---GCCAGACAA---TTGTTG---TCTGGTATAGTGCAACAGCAGAGA---
AATTTG---CTGAGG---GCTATTGAGGCGCAACAGCATCTGTTGCAACTCACAGTCTGGGGCATC
>KX693411..2
TTTTACTGTAATACATCAAAACTGTTTAATAGTACTTGGAGTAATAATAATCCTTGGAATACTACTAGTATT-
----

CAAAATGATACTATCACGCTCCCTTGCAGAATAAAACAAATTGTAAACATGTGGCAGGAAGTAGGGAAAGCAA
TGTATGCCCCTCCCATTGCAGGACCAATTAATTGTTCATCAAAT---
ATTACGGGGTTGCTATTAACAAGAGATGGTGGTAAAGGTGGGGAT----------------------------
----

GTGAGCAACTACACCGAGACCTTCAGACCTGCAGGGGGAAATATGAGAGACAATTGGAGAAGTGAATTATATA
GATATAAAGTAGTAAAAATTGAACCATTA---GGAGTAGCACCCACCAAGGCAAAGAGAAGAGTGGTG-----
----------------------------------CAGAGAGAAAAAAGAGCA---
GTGGGAGCAATAGGAGCT---------ATGTTCCTTGGGTTCTTGGGAGCA---GCAGGAAGCACTATG---
GGCGCAGCGTCA---ATAACGCTGACGGTACAG---GCCAGACAA---TTGTTG---
TCTGGTATAGTGCAACAGCAGAGA---AATTTG---CTGAGG---
GCTATTGAGGCGCAACAGCATCTGTTGCAACTCACAGTCTGGGGCATC
>KR914676..3
TTCTACTGTAACACAACACCACTGTTTAATAGTAGTTGGTACAAGTCAAATTGGAAAAGTCAAAGGACAAAGG
AGATTGGA---

GGAAGGGAGACAATCACACTCCAATGTAGAATAAAACAAATTGTAAACATGTGGCAGGAAGTAGGACGAGCAA
TGTATGCCCCTCCCATCAAAGGACAAATTAGATGTTCATCAAAT---
ATTACAGGGGTGCTATTAACAAGAGATGGTGGTAATACCACCAAT----------------------------
----

CACTCCGAGATCTTCAGACCTGGAGGAGGAAATATGAAGGACAATTGGAGAAGTGAATTATATAAATATAAAG
TAGTACAAATTGAACCATTA---GGAATAGCACCCACCAAGGCAAAGAGAAGAGTGGTG--------------
-------------------------CAGAGAGAAAAAAGAGCA---ATAGGA---TTCGGAGCT---------
GTGTTCCTTGGGTTCTTGGGAGCA---GCAGGAAGCACTATG---GGCGCAGCGTCA---
GTAACGCTGACGGTACAA---GCCAGACAA---TTATTG---TCTGGTATAGTGCAACAGCAGAGC---
AATTTG---CTGAAT---GCTATTCAGGCGCAACAGCATCTGTTGCAACTCACAGTCTGGGGCATT
>FJ388899..1
TTCTACTGCAATACAACAGAACTGTTTAATAGTACTTGGAATAGTACTACTTGGAATAGTAATAGTACTACAA
ATGCAAAT---
-------------------GCA---
ACAAATACAGCTAACCTAGTACTCCCATGCAGAATAAAACAGATTGTAAACATGTGGCAGAAAGTAGGAAAAG
CAATGTATGCCCCTCCCATCAAAGGAATAATTAGATGTTCATCAAAT---
ATTACAGGGATAATATTAACAAGAGATGGTGGTAATAGTAATCAGACC-------------------------
----

AACACCACAGAGATTTTCAGACCTGTAGGAGGAAATATGAAGGACAATTGGAGAAGTGAATTATATAAATATA
AAGTAGTACAAATTGAACCATTA---GGAGTAGCACCCACCAAGGCAAAAAGAAGAGTGGTG-----------
----------------------------CAGAGAGAAAAAAGAGCA---GTGACA---TTAGGAGCT------
---ATGTTCCTTGGGTTCTTGGGAGCA---GCAGGAAGCACTATG---GGCGCAGCGTCA---
ATAACGCTGACGGTACAG---GCCAGACAA---TTACTG---TCTGGTATAGTGCAACAGCAGAGC---
AATCTG---CTGAGG---GCTATTGAGGCGCAACAGCACCTGTTGCAACTCACAGTCTGGGGCATC
>FJ388916..3
TTCTACTGTAATACAACAGGGCTGTATAATAGTACTTGGCAGTTTAATAGTACTTGGAATACTACTTGGAATC
ATACTGAC---
-------------------CCT---
AATCATACTGACCCTATCACACTACCATGCAGAATAAGACAAATTGTAAACAGGTGGCAGGAAGTAGGAAAAG
CAATGTATGCCCCTCCCATCAGCGGACAAATTAGCTGTTCATCAAAC---
ATTACAGGGCTACTATTAACAAGAGATGGTGGTAGTAATGAGAGC----------------------------
----

AGAACCAACGAGACCTTTAGACCTGGAGGAGGAGATATGAGGGACAATTGGAGAAGTGAATTATATAAATATA
AAGTAGTAAGAATTCAACCATTA---GGAATAGCACCCACCAAGGCAAAAAGAAGAGTGGTG-----------
----------------------------CAAAGAGAAAAAAGAGCA---GTGGGAACGCTGGGAGCT------
---ATGTTCCTTGGGTTCTTGGGAGCA---GCAGGAAGCACTATG---GGCGCAGCGTCA---
CTGACGCTGACGGTACAG---GCCAGACAA---TTATTG---ACTGGTATAGTGCAACAGCAGAAC---
AATCTG---CTGAGA---GCTATTGAGGCGCAACAGCATATGTTGCAACTCACAGTCTGGGGCATC
>JF683738..2
TTCTACTGTGATACTACAAAGCTGTTTAATAGTACTTGGACTGGGAATGAAACTTGGACTGGGAATGAAACTG
AAGAG--

TCAAATAATAGTACTATCACACTCCCATGCAGAATAAAACAAATTATAAACAGATGGCAGGAAGTAGGAAAAG
CAATGTATGCCCCTCCCATCAGAGGAGAAATTAAATGTACATCAAAT---
ATTACAGGGCTACTATTAACAAGAGATGGTGGTAATGGTAATGGGACT-------------------------
----

CAGAATCAGACCGAGATCTTCAGACCTGGAGGAGGAGACATGAAGGACAATTGGAGAAGTGAATTATATAAAT
ATAAAGTAGTAAAAATTGAGCCATTA---GGAATAGCACCTACCAAGGCAAAAAGAAGAGTGGTG--------
-------------------------------CAGAGAGAAAAAAGAGCA---ATAGGA---CTGGGAGCT---
------GTGTTCATTGGGTTCTTGGGAGCA---GCAGGAAGCACTATG---GGCGCAGCGTCG---
GTGACGCTGACGGTACAG---GCCAGACAG---TTATTG---TCTGGTATAGTGCAACAGCAAAAC---
AATTTG---CTAAAA---GCTATTGAGGCGCAACAGCATCTGTTGCAACTCACAGTCTGGGGCATC
>JF683756..1
TTCTACTGTAATACATCAAAGCTGTTTAATAGTACTTGGACTAATAGTAGTAGTACTTGGAATAATACTGAAG
AG---

TCAAATGACACTATCACACTCCCATGTAAAATAAAACAAATTATAAACATGTGGCAGGAAGTAGGAAGAGCAA
TGTATGCCCCTCCCATCAGTGGGCAAATTAGGTGCTCATCAAAT---
ATTACAGGGCTACTATTAACAAGAGATGGTGGTCATAACGGAACCAAT-------------------------
----

AACGAGACCGAGATCTTCAGACCTGCAGGAGGAAATATGAAGGACAATTGGAGAAGTGAATTATATAAATATA
AAGTAGTGAAAATTGAGCCATTA---GGAGTAGCACCCACCAGGGCAAAAAGAAGAGTGGTG-----------
----------------------------CAGAGAGAAAAAAGAGCA---GTGGGA---ATGGGAGCT------
---TTGCTCCTTGGGTTCTTGGGAGCA---GCAGGAAGCACTATG---GGCGCAGCATCG---
GTGATGCTGACGGTACAG---GCCAGACAA---TTATTG---TCTGGTATAGTGCAACAGCAGAAC---
AATCTG---CTGAGA---GCTATTGAGGCGCAACAACATATGTTGCAACTCACAGTCTGGGGCATT
>JF683764..1
TTCTACTGTGATACAACACAACTGTTTAATAGTACTTGGTTTATGAATGGGACTAATACAACTACC-------
----

CTTACCGCAAATGCCACAATCCCAAGCCGAATAAAACAAATTATACACAGGAGGCAGGAAGTAGGAAGAGCAA
TGTATGTCCCTGCCCTCAGAGGACCAATTACATGTATATCAAAT---
ATGACAGGGACGTTATTAACAAGAGATGGTGGTCATAACAATGGCATT-------------------------
----

AATGACATTGAGACCTTCAGACCAGGAGGAGGAGATATGAGGGACAATTGGAGAAGTGAATTATATAAATATA
AAGTAGTAAGAGTTGAACCATTA---GGAATAGCACCCACCAGGGCAAAGAGAAGAGTGGTG-----------
----------------------------CAGAGAGAAAAAAGAGCA---GCA------CTAGGAGCT------
---ATGTTCCTTGGGTTCTTGGGAGCA---GCAGGAAGCACTATG---GGCGCAGCGTCA---
ATAACGCTGACGGTACAG---GCCAGACAA---TTGTTG---TCTGGTATAGTGCAACAGCAAAAC---
AATTTG---CTGAGA---GCTATTGAGGCGCAACAACACATGTTGCAACTCACAGTCTGGGGCATC
>JF683773..1
TTCTACTGTAATACATCACAACTGTTTAATAGTACTTGGACACCTAATGGTACTTGGACATTTAATGCTGCTA
GTGGGGCT---
-------------------TCA---
GGATTCAAGGGCACTATCACACTCCCATGTAGAATAAAACAAATTATAAACATGTGGCAGGAAGTAGGAAAAG
CAATGTATGCCCCTCCCATCAGTGGACTAATTAGATGCTCATCAAAT---
ATTACAGGACTGCTATTAACAAGAGATGGTGGTATAAACAATACC----------------------------
----

AATACCACCGAGATCTTCAGACCTGTAGGAGGAAATATGAAGGACAATTGGAGAAGTGAATTATATAAATATA
AAGTAGTACAAGTTGAACCAATA---GGAATTGCACCCACCAAGGCAAAGAGAAGAGTGGTG-----------
----------------------------CAGAGAGAAAAAAGAGCA---GTTACA---CTAGGAGCT------
---GTGTTCCTTGGGTTCTTGGGAGCA---GCAGGAAGCACTATG---GGCGCAGCGTCA---
ATGACGCTGACGGTACAG---GCCAGGCAA---TTATTG---TCTGGTATAGTGCAACAGCAGAAC---
AATCTG---CTGAGA---GCTATTCAGGCGCAACAGCATCTGTTGCAACTCACAGTCTGGGGCATC
>JQ416158..1
TTCTACTGTAATACATCACAACTGTTTAATAGTATTTGGCCTTGGAATAGTACTGCAAACAACACTGGA----
----

GGAAATGACACAATCACACTCCCATGCAGGATAAGACAAATTGTAAATAGGTGGCAGGAAGTAGGAAAAGCAA
TGTATGCCCCTCCCATCAGTGGACAAATTAGATGTTCATCAAAT---
ATTACAGGGCTGCTATTAACAAGAGATGGAGGTCAGACTAACAACCAG-------------------------
----

ACTGGGAACCAGACCGAGATTTTCAGACCTGGAGGAGGAGATATAAAAGATAATTGGAGAAGTGAATTATACA
AATATAAAGTAGTACAAATTGAACCATTA---GGAGTAGCTCCCACTAAGGCAAGGAGAAGAGTGGTG-----
----------------------------------CAGAGAGAAAAAAGAGCA---CTAACA---
CTGGGAGCT---------GTGTTCCTTGGGTTCTTGGGAACA---GCAGGAAGCACTATG---
GGCGCAGCGTCA---CTAACGCTGACGGTACAG---GCCAGACAA---TTATTG---
TCTGGTATAGTGCAGCAGCAGAAC---AATCTG---CTGAGA---
GCTATTGAGGCGCAACAACATTTGTTGCAACTCACAGTCTGGGGCATC
>KT124803..1
TTCTACTGTAATACAACAAGCCTGTTTAATAGTACTTGGTATGCAAATGGTACAAATAGCACTTGGTCTGAT-
----

GAGTCAGATGTTATCACACTTCCATGCAGAATAAGACAAATTGTAAACATGTGGCAGGGGGTAGGAAAAGCAA
TGTATGCCCCTCCCATCAGAGGACAGATTAATTGTACATCAAAT---
ATTACAGGGCTAATATTAACAAGAGATGGTGGGAACAAGAATGGCACTAATGAG-------------------
----

ACCTATGAGACCTTCAGACCTGCAGGAGGAAATATGAAGGACAATTGGAGAAGTGAATTATATAAATATAAAG
TAGTAAAGATTGAACCATTA---GGAATAGCACCCACCAAGGCAAAGAGAAGAGTGGTG--------------
-------------------------CAAAGAGAAAAAAGAGCA---ATAGGA---ATGGGAGCC---------
TTGTTCCTTGGGTTCTTGGGAGCA---GCAGGAAGCACTATG---GGCGCAGCGTCA---
ATGACGCTGACGGTACAG---GCCAGACAG---TTATTG---TCTGGTATAGTGCAACAGCAAAAC---
AATTTG---CTGAGA---GCTATTGAGGCGCAACAACACCTGTTGCAACTCACAGTCTGGGGCATC
>KT124789..1
TTCTACTGTAATACAACAGAGCTGTTTAACGGTACTTGGAATGGTACTTTCTTGAATGTTACTACA-------
----

GGGCGAGAGAATATCACACTCCCATGCAGAATAAAACAGATTATAAACAGGTGGCAGGAAGTAGGAAAAGCAA
TGTACGCCCCTCCCATCAGAGGACCAATTAGATGCTCATCAAAT---
ATTACAGGGCTGATATTAACAAGAGATGGTGGTCAGAATGCGAGTGCG-------------------------
----

ACCAACGAAACCTTCAGACCGGGAGGAGGAGATATGAGGGACAATTGGAGAAGTGAATTATATAAATATAAAG
TAGTAAAAATTGAGCCATTA---GGAATAGCACCCACCAAGGCAAGGAGGAGAGTGGTG----------------
--------------------------CAGAGAGAAAAAAGAGCA---GTGACA---CTAGGAGCT----------
ATGTTCCTTGGGTTCTTGGGAGCA---GCAGGAAGCACTATG---GGCGCAGCTTCA---
TTGACGCTGACGGTACAG---GCCAGACTA---TTATTG---TCTGGTATAGTGCAACAGCAAAAC---
AACTTG---CTGAGG---GCTATTGAGGCGCAGCAGCATCTGCTGCAACTCACAGTCTGGGGCATC
>KT124805..1
TTTTACTGTGATACAACACAACTGTTCAACAGTACGTGGGATACAAATAATACTGGGGGAGTGAATGAT----
----

ACTGACGAGCCAATTACACTCCCATGCAGGATAAAACAATTTATAAACATGTGGCAGGAAGTAGGGAAAGCAA
TGTATGCCCCTCCCATCCAAGGAATAATTCACTGTTCATCAAAT---
ATTACAGGACTACTATTAACAAGAGATGGTGGTAAGACAGAGAACGGGACAGAG---------------------
----

AATGTTACTGAGATCTTTAGACCTGGAGGAGGAGACATGAGGGACAATTGGAGAAGTGAATTATATAAATATA
AAGTAGTAAAAATTGAACCATTA---GGAGTAGCACCCACCAGGGCAAAGAGAAGAGTGGTG-------------
-----------------------------CAGAGAGAAAAAAGAGCA---GTGACA---CTGGGAGCT-------
---GTGTTCCTTGGGTTCTTGGGAGCA---GCCGGAAGCACTATG---GGCGCAGCGTCA---
CTGACGCTGACGGTACAG---GCCAGACAA---TTATTG---TCTGGTATAGTGCAACAGCAGAAC---
AATTTG---CTGAGA---GCTATTGAGGCGCAACACCATCTGTTGCAACTCACAGTCTGGGGCATT
>KT124763..1
TTCTACTGTAATTCATCGACACTGTTTAATAGTACTTGGAATGAAACTATTGCAGGGATTACCAAT-------
----

GCAAATGAAACTATCACACTCCCATGCAGAATAAGGCAAATTATAAACATGTGGCAGGAAGTAGGAAAAGCAA
TGTATGCCCCTCCCATTAGTGGAATAATTAGATGTTCATCAAAT---
ATTACAGGGCTGCTATTAACAAGAGATGGTGGTAATAATGGTAAT------------------------------
----
CATAGCGAGAATAAT---
ACCATTGAGATCTTCAGACCTGGAGGAGGAGATATGAGGGATAATTGGAGAAGTGAATTATATAAATATAAAG
TAGTACAAATTGAACCAGTA---GGAGTAGCACCTACCAAGGCAAAGAGAAGAGTGGTG----------------
--------------------------CAGAGAGAAAAAAGAGCA---GTGGGA---CTAGGAGCT----------
TTGTTCCTTGGGTTCTTGGGAGCA---GCAGGAAGCACTATG---GGCGCAGCGTCA---
ATAACGCTGACGGTACAG---GCCAGACAA---TTATTG---TTTGGTATAGTGCAACAGCAGAAC---
AATCTG---CTGAGA---GCTATTGAGGCGCAACAGCACCTGTTGCAACTCACAGTCTGGGGCATC
>KT124779..1
TTCTACTGTGATACAACACAACTGTTTAATAGTACTTGGAGTAATGCTACTTGGAATGAGACTACAGGT----
----

TCAAATGACACGATTACACTCCAATGTAAAATAAAACAAATTATAAACAGGTGGCAGGAAGTAGGAAAAGCAA
TGTATGCCCCTCCCATCCGAGGACAAATAAAGTGTTCATCAAAT---
ATTACAGGGTTGCTATTAACAAGAGATGGTGGTAACGAGACTAAC------------------------------
----

GGGACCGAGGTCTTCAGACCTATAGGAGGAGATATGAAAGACAATTGGAGAAGTGAATTATATAAATATAAAG
TAGTTCAAATTGAACCATTA---GGAATAGCACCCACCAAGGCAAAGAGAAGAGTGGTG----------------
--------------------------CAGAGAGAAAAAAGAGCG---GTGACA---CTGGGAGCT----------
ATGTTCCTTGGGTTCTTGGGAGCA---GCGGGAAGCACTATG---GGCGCAGCGTCA---
GTGGCGCTGACGGTACAG---GCCAGACAA---TTATTG---TCTGGTATAGTGCAACAGCAGAAC---
AATATG---CTGAGG---GCTATTGAGGCACAACAGCATATGTTGCAACTCACAGTCTGGGGCATC
>KT124787..2
TTCTACTGTGATACAACACAGCTGTTTAATAGCAATAGCACTTGGCTGTTTAATAACACTTGGAATAGTACTG
AGAATAATAAT---ACT--

GAGAATAATAATATCACACTCCAGTGCAGAATAAGACAAATTGTAAACATGTGGCAGAAAGTAGGAAAAGCAA
TGTATGCCCCTCCCATCAGAGGACAAATTTACTGTGCATCACAA---
ATAACAGGGCTGCTATTAACAAGAGATGGTGGTAGTAGTGATCAG------------------------------
----

```
----------
AACAACACCGAGATCTTCAGACCTGCAGGAGGAAATATGAGGGACAATTGGAGAAGTGAATTATATAAATATA
AAGTAGTAAAAATTAAACCATTA---GGAGTAGCACCCACCAAGGCAAAGAGAAGAGTGGTG-----------
----------------------------CAGAGAGAAAAAAGAGCA---GTAACG---CTAGGAGCT------
---ATGTTCCTTGGGTTCTTGGGAGCA---GCGGGAAGCACTATG---GGCGCAGCGGCA---
ATGACGCTGACGGTACAG---GCCAGACAA---TTATTG---TCTGGTATAGTGCAACAGCAGAAT---
AATCTG---CTGAGA---GCTATTGAGGCGCAACAGCATCTGTTGCAACTCACAGTCTGGGGCATC
>KC596067..1
TTCTACTGTAATACAACACAATTGTTTAACAGTACTTGGAATACTACTGAAGATTTAAATAAAACTAGAACTG
AA-----------------------------------------------------------------------
----------------------------
GTAAATGACACAATCACACTCCCATGTAGAATAAGACAAATTATAAACAGATGGCAGGAAGTGGGAAAAGCAA
TGTATGCCCCTCCCATCAGCGGAAACATTAGTTGTTCATCAAAC---
ATCACAGGGCTGCTATTAACAAGAGATGGTGGTCAGAATAATGTGAGC-------------------------
-------------------------------------------------------------------------
----------
AACACCACAGAGATCTTCAGACCTGGAGGAGGAGATATGAGGGACAATTGGAGAAGTGAATTATATAAATATA
AAATAATAAAAATCGAACCATTA---GGAATAGCACCCACCAAGGCAAGGAGAAGAGTGGTG-----------
----------------------------CAGAGAGAGAAAAGAGCA---GCA------CTAGGAGCT------
---ATGTTCCTTGGGTTCTTAGGAGCA---GCAGGAAGCACTATG---GGCGCAGCGTCA---
GTGACGCTGACGGTACAG---GCCAGACAA---TTATTG---TCTGGTATAGTGCAACAGCAAAGT---
AACTTG---CTGAAA---GCTATTGAGGCGCAACAGCATCTGTTGCAACTCACAGTCTGGGGCATC
>EF514700..2
TTTTACTGTAATACGTCAAAACTGTTTGATAGTACCTGGAATGAAACTAGTATTTGGAATTATACCACTACTA
ATGAT--------------------------------------------------------------------
-------------------ACC---
ACAAAAAATGACACACTCACACTCCCATGTAAAATAAAACAAATTATAAACATGTGGCAGGAAGTAGGAAAAG
CAATGTATGCCCCTCCCATCAGAGGACAAATCAGATGCTCATCAAAT---
ATTACAGGGCTGTTATTAACAAGAGATGGTGGTCAGGGTAACGGGACC-------------------------
-------------------------------------------------------------------------
-------
GATAACACCACCGAGATCTTCAGACCTGCAGGAGGAGATATGAGGGACAATTGGAGAAGTGAATTATATAAAT
ATAAAGTAGTAAGAATTGAACCATTA---GGAGTAGCACCCACCAAGGCAAAGAGAAGAGTGGTG--------
-------------------------------CAGAGAGAAAAAAGAGCA---GTGGGA---CTGGGAGCT---
------TTGTTCCTTGGGTTCTTGGGAACA---GCAGGAAGCACTATG---GGCGCAGCATCA---
CTAACGCTGACGGTACAG---GCCAGACAA---TTGTTG---TCTGGTATAGTGCAACAGCAGAAC---
AATCTG---CTGAGA---GCTATTGAGGCGCAACAACATCTGTTGCAACTCACAGTCTGGGGCATT
>EU839598..1
TTCTACTGTAATACAACAAAACTGTTTAACAATACTGGACAATTAGATAAAGAAGAAGATGAAGGA-------
-------------------------------------------------------------------------
----------------------------
GAAGAAGGCAATATCACACTTCCATGCAGAATAAGGCAATTCGTAAACAGGTGGCAAGAAGTAGGAAAAGCAA
TGTATGCCCCTCCCATCAGTGGACAAATTAGATGTTCATCAAAT---
ATTACAGGGCTACTATTAACAAGAGATGGTGGTGGTAGTGGTAAT----------------------------
-------------------------------------------------------------------------
----------
AGTGAGAATGAGACCTTCAGACCTGGGGGAGGAGATATGAAAGACAATTGGAGAAGTGAGTTATATAAATATA
AAGTAGTAAAAATTCAGCCATTA---GGAATAGCACCCAGCAAGGCAAAGAGAAGAGTGGTG-----------
----------------------------CAAAGAGAAAAAAGAGCA---GTGACA---CTAGGAGCT------
---ATGTTCCTTGGGTTCTTAGGAGCA---GCAGGAAGCACTATG---GGCGCAGCGTCA---
GTAACGCTGACGGTACAG---GCCAGACTA---TTATTG---TCTGGTATAGTGCAACAGCAGAAC---
AATCTG---CTGAGG---GCTATTGAGGCGCAACAACATCTGTTGCAACTCACGGTCTGGGGCATC
>KC595194..1
TTTTACTGTAATTCAACACAACTGTTTAACAGTGTTTGGAAGGCTAACAGTACTTGGAATAGTACTGAAGGGT
CAAATAAC-----------------------------------------------------------------
----------------------------
AATGCAGGAAATATCACACTCCCATGCAGAATAAAACAAATTATAAACATGTGGCAGGAAGTAGGAAAAGCAA
TGTATGCCCCTCCCATCAGAGGCCAAATTAGCTGTTCATCAAAT---
ATTACAGGGCTACTATTAACAAGAGATGGTGGTAACATCACTAAC----------------------------
-------------------------------------------------------------------------
```

GGGACCGAGATCTTCAGACCTGGAGGAGGAGATATGAGGGACAATTGGAGAAGTGAATTATATAAATATAAAG
TAGTAAAAATTGAACCATTA---GGAATAGCACCCACCAAGGCAAAGAGAAGAGTGGTG--------------
-------------------------CAGAGAGAGAAAAGAGCA---GTGGGA---ATAGGAGCT---------
ATGTTCCTTGGGTTCTTGGGAGCA---GCAGGAAGCACTATG---GGCGCGGCGTCA---
ATGACGTTGACGGTACAG---GCCAGACTA---TTATTG---TCTGGTATAGTGCAACAGCAGAAC---
AATTTG---CTAAGG---GCTATTGAGGCGCAACATCATCTGTTGCAACTCACAGTCTGGGGCATC
>KC595151..1
TTCTACTGTGATACAACAAAATTGTTTAATAGTACTTGGGATATTAATGATACTTGGAATGGTACTAACGGG-
----

CCAGATAACACTATCATACTCCCATGCAGAATAAAACAAATTATAAACATGTGGCAGGAAGTAGGAAAAGCAA
TGTATGCCCCTCCCATCAGAGGACGAATTAGGTGTGAATCAAAT---
ATTACAGGGCTGCTATTAACAAGAGATGGTGGTGATGATAGCAGT----------------------------
----

AACCAGACCGAGATCTTCAGACCTGGAGGAGGAGATATGAGGGACAATTGGAGAAGTGAATTATATAAATATA
AAGTAGTAAAAATTCAACCATTA---GGAATAGCACCCAACAAGGCAAAGAGAAGAGTGGTG-----------
----------------------------CAGAGAGAAAAAAGAGCA---GTGGGA---ATAGGAGCT------
---TTGTTCCTTGGGTTCTTGGGAGCA---GCAGGAAGCACTATG---GGCGCAGCGTCA---
ATGACGTTGACGGTACAG---GCCAGACAA---TTATTG---TCTGGTATAGTGCAACAGCAGAAC---
AATCTG---CTGAGG---GCTATTGAGGCGCAACAGCATCTGTTGCAACTCACAGTCTGGGGCATC
>EU786679..1
TTCTACTGTAATACAACACAGCTGTTTAATAGTACTTGGGGTATGAATACGACTAAAGGGACAAATACC----
----

ACTGAAGGAGATATCATACTCCCATGCAGAATAAAACAAATTATAAACATGTGGCAGGAAGTAGGAAAAGCAA
TGTATGCCCCTCCCATCAGCGGCCAATTACATGTACATCAAAT---
ATTACAGGGCTGCTACTAACAAGAGATGGGGGTCAGAGCAAGAACGAA-------------------------
----

AGTAACGAGACAGAGATCTTCAGACCAGGAGGAGGAGACATGAAGGACAATTGGAGAAGTGAATTATATAAAT
ATAAAGTAGTAAAAATTGAACCATTA---GGAATAGCACCCACCAAGGCAAAAAGAAGAGTGGTG--------
-------------------------------CAAAGAGAAAAAAGAGCA---GTGGGA---CTAGGAGCT---
------GTGTTCCTTGGGTTCTTGGGAGCA---GCAGGAAGCACTATG---GGCGCAGCGTCA---
ATAACGCTGACGGTACAG---GCCAGACAA---TTATTG---TCTGGTATAGTGCAACAGCAGAAC---
AACTTG---CTGAGG---GCTATTGATGCGCAACAGCATATGTTGCAGCTCACAGTCTGGGGCATC
>KC473841..1
TTCTACTGTAATACATCACAACTGTTTAGTAGTACTTGGAATAATACTGTAGGGTTAAATACTACA-------
----

GGAAATGATACTATCACACTCCACTGCAGAATAAGACAAATTATAAACCTGTGGCAGAGAGTAGGAAAAGCAA
TGTATGCCCCTCCCATTAGAGGACAAATTAGTTGTTCATCAAAC---
ATTACAGGGTTGCTATTAACAAGAGATGGTGGTAACAACAATCAGAGC-------------------------
----

AATAACACCGAAATCTTCAGACCTGGAGGAGGAGATATGAGGGACAATTGGAGAAGTGAATTATATAAATATA
AAGTAGTAAGAATTGAACCACTA---GGAGTAGCACCCACCAAGGCAAAGAGAAGAGTGGTG-----------
----------------------------CAGAGAGAAAAAAGAGCA---GTGGGA---CTAGGAGCT------
---GTGCTCTTTGGGTTCTTGGGAGCA---GCAGGAAGCACTATG---GGCGCAGCGTCA---
GTGACGCTGACGGTACAG---GCCAGACAA---TTATTG---TCTGGTATAGTGCAACAGCAAAAT---
AATTTG---CTGAAA---GCTATTGAGGCGCAACAGCATCTGTTGCAACTCACAGTCTGGGGCATC
>KT200357..1
TTCTACTGTAATACAACACAGCTGTTCAATAGTACTTGGGAGCCAGGGAAGAATACTACTACAGAG-------
----

GGAAATGACACTATCACGCTCCCATGCAGAATAAAACAGATTATAAACAGGTGGCAGGAAGTAGGAAAAGCAA
TGTATGCCCCTCCCATCAGAGGACGAATTAATTGTACATCAAAT---
ATTACAGGGCTGTTATTAACAAGAGATGGTGGTAACAATAATAAC----------------------------
----

CAGACAAATAGGACAGAGACCTTTAGACCTCAAGGAGGGAATATGAAGGACAATTGGAGAAGTGAATTATATA
AATATAAAGTAGTACAAGTTGAGCCATTA---GGAATAGCACCCACCAAGGCAAGGAGAAGAGTGGTG-----
----------------------------------CAGAGAGAAAAAAGAGCA---GTGGGA---
CTAGGAGCT---------GTGTTCCTTGGGTTCTTAGGAGCA---GCAGGAAGCACTATG---
GGCGCAGCGTCA---ATAACGCTGACGGTACAG---GCCAGACAA---TTGTTG---
TCTGGTATAGTGCAACAGCAGAAC---AACCTG---CTGAGG---
GCTATTGAAGCGCAACAACATATGTTGCAGCTCACAGTCTGGGGCATC
>KP109518..1
TTCTACTGTAATACAACACAACTGTTTAATAGTACCTGGAATGATACTAGTATTAGTGAAAGGTCAAT----
----

ACTACTAAAAACATCACGCTTCCATGCAGAATAAAACAAATTATAAACATGTGGCAAGAAGTAGGGAAAGCAA
TGTATGCCCCTCCCATCCCAGGACCAATTCAATGTAGATCAAAT---
ATTACAGGGATATTATTAACAAGAGATGGTGGTAATACTAGTGAT---------------------------
----

AGCCAGAATGAAACCTTCAGACCTGGAGGAGGAAATATGAAGGACAATTGGAGAAGTGAATTATATAAATATA
AAGTAGTAGAAATTGAACCATTA---GGAGTGGCACCCACCAGGGCAAGGAGAAGAGTGGTG----------
----------------------------CAGAGAGAAAAAAGAGCA---GTGGGAGTAGTAGGAGCT------
---ATGTTCCTTGGGTTCTTGGGAGCA---GCAGGAAGCACTATG---GGCGCAGCGTCA---
ATGACGTTGACGGTACAG---GCCAGACTA---TTACTG---TCTGGTATAGTGCAGCAGCAGAAC---
AATCTG---CTGAGA---GCTATTGAAGCGCAACAGCATCTGTTGCAGCTCACAGTCTGGGGCATT
>KY465969..2
TTCTACTGTAATACAACACAGCTGTTTAATAGCACTTGGAATAAAACTGTAGTTGAGAATAATAATTATGGTT
CT--

GAGAATGGTACTATCACACTCCAGTGCAGAATAAGACAAATTGTAAACAGGTGGCAGGAAGTAGGAAAAGCAA
TGTATGCCCCTCCCATCAGAGGAGTAATTAACTGTTCATCAAGT---
ATTACAGGGCTGTTATTAACAAGGGATGGTGGTAATAGTAAGAACACC------------------------
----

AACAGGTCATCTGAGATCTTCAGACCTGGAGGAGGAGATATGAGGGACAATTGGAGAAGTGAATTATACAAAT
ATAAAGTAGTAAAGATTGAACCATTA---GGAGTAGCACCCACCAAGGCAAAGAGGAGAGTGGTG-------
-------------------------------CAGAGAGAAAAAAGAGCA---GTGGGAGCATTAGGAGCT---
------ATGTTCCTTGGGTTCTTGGGAACA---GCAGGAAGCACTATG---GGCGCAGCGTCA---
CTGACGCTGACGGTACAA---GCCAGACAA---CTATTG---TCTGGTATAGTGCAACAGCAGAAC---
AATCTG---CTGCAG---GCTATCGAGGCGCAACATCATCTGTTGCAACTCACCGTCTGGGGCATC
>KC595204..2
TTCTACTGTAACTCAACACAGCTGTTTAATAGTACTTGGAATAGTACTGGGAATCAGAATAATATTGAAGGG-
----

TTAAATGACACAATCACACTCCCATGCAGAATAAAACAAATTATAAACATGTGGCAGGAAGTAGGAAGAGCAA
TGTATGCCCCTCCCATTAGAGGACAAATTAGATGTTCATCAAAT---
ATTACAGGGCTGTTATTAACAAGAGATGGTGGTCATAACACGACA---------------------------
----

GAGACCGAGATCTTCAGACCTGGAGGAGGAGATATGAGGGACAATTGGAGAAGTGAATTATATAAATATAAAG
TAGTAAAAATTGAACCATTA---GGAGTAGCACCCACCAGAGCAAAGAGAAGAGTGGTG-------------
-------------------------CAGAGAGAAAAAAGAGCA---GTGGGA---ATAGGAGCT---------
GTGTTCCTTGGGTTCTTGGGAGCA---GCAGGAAGCACTATG---GGCGCAGCGTCA---
GTGACGCTGACGGTACAG---GCCAGACTA---TTATTG---TCTGGTATAGTGCAACAGCAGAAT---
AATTTG---CTGAAG---GCTATTGAGGCGCAACAGCATCTGTTGCAACTCACAGTCTGGGGCATC
>DQ141342..1
TTCTACTGTAATACAACACAACTGTTTAATAGTACTTGGCCTAAGAATGGTACCAGCGAGTTAAAT------
----

ACCACTGACCCATTCATACTCCCATGTAAAATAAAACAAATTATAAACATGTGGCAGAAAGTAGGAAAAGCAA
TGTATGCCCCTCCCATCCGTGGACAGATTAGATGCTCATCAAAT---
ATTACAGGGCTGCTATTACCAAGAGATGGTGGTAACAACAACAATGGTAGT---------------------

----

AACAACCGCAGTCCTGAGATCTTCAGACCTGGAGGAGGAGATATGAGGGATAATTGGAGAAGTGAATTATACA
AATATAAAGTAGTAAAAATTCAACCATTA---GGAGTAGCACCCACCAAGGCAAAGAGAAGAGCGGTG-----
---------------------------------CATAGAGACAAAAGAGCA---GTGGGA---
CTCGGAGCT---------GTGTTCCTTGGATTCTTGGGAGCA---GCAGGAAGCACTATG---
GGCGCAGCGTCA---CTGACGTTGACGGTACAG---GCCAGACAA---TTATTG---
TCTGGTATAGTGCAACAGCAAAAC---AACTTG---CTGAGG---
GCTATTGAGGCGCAACAGCATCTGTTGCAACTCACAGTCTGGGGCATC
>DQ141343..2
TTCTACTGTGATACATCACAACTATTTAATAGTACTTGGAATAGTAGTACTTTGAATAATTTTACTAACGGGT
CAAAT---

ATAACTAATGAAACTTTCACACTCCCATGCAGAATAAAACAATTTATAAACATGTGGCAGGAAGTAGGAAAAG
CAATGTATGCCCCTCCTATCAGAGGAGTAATTAACTGTTTATCAAAT---
ATTACAGGGCTGCTATTAACAAGAGATGGTGGTAATAATCATAGTGAC------------------------
----

ACCAATCAGGAGATCTTCAGGCCTGCAGGAGGAGATATGAGGGACAATTGGAGAGGTGAATTATATAAGTATA
AAGTAGTAAGAGTAGAACCATTA---GGGATAGCACCCACCAAGGCAAAGAGAAGAGTGGTG-----------
---------------------------CAGAGAGAAAAAAGAGCA---GTGGGA---CTAGGAGCT------
---TTGTTCCTTGGGTTCTTGGGAGCA---GCAGGAAGCACTATG---GGCGCAGCATCA---
ATGACGCTGACGGTACAG---GCCAGACAA---TTATTG---TCTGGTATAGTGCAACAGCAGAAC---
AACCTG---CTGAGG---GCTATTGAGGCGCAACAGCATATGTTGCAACTCACAGTCTGGGGCATC
>DQ448819..1
TTCTACTGTAATACAACACAACTGTTTAATAGTACTTGGTATGCTAGTAATAATACTTGGAAGGGTACTAAC-
----

ATTACAGGCAATATCACACTCCCATGCAAAATAAAACAAATTATAAACATGTGGCAGGAAGTAGGAAAAGCAA
TGTATGCCCCTCCCATCAGAGGACAAATTAGATGCTCATCAAAT---
ATTACAGGGTTACTATTAACAAGAGATGGGGGTCAGGGAAACAACACC------------------------
----

GACAACGACACTGAGATCTTCAGACCTGGAGGAGGAAATATGAGGGACAATTGGAGAAGTGAATTATATAAAT
ATAAAGTAGTAAGAATTGAACCATTA---GGACTAGCACCCACCAGGGCAAAGAGAAGAGTGGTG--------
------------------------------CAGAGAGAGAAAAGAGCA---GTGGGA---CTAGGAGCG---
------TTGTTCATTGGGTTCTTGGGAGCA---GCAGGAAGCACTATG---GGCGCAGCGTCA---
GTGACGTTGACGGTACAG---GCCAGACAA---TTACTG---TCTGGTATAGTGCAACAGCAGAAT---
AATTTG---CTGATG---GCTATTGAGGCGCAACAGCATATGTTGAAACTCACGGTCTGGGGCATT
>EF531331..1
TTTTACTGTAATACAACACAACTATTTAATAGTACTTGGAATATAACAAATCTGTTTAATAGTACTTGGAATA
GTACTGAAGGG---
-------TCAAATAACACT------
AAAAGAAATGGCACAATCATACACCCATGCAGAATAAAACAGATTATAAACATGTGGCAGAAAGTAGGAAAAG
CAATGTATGCCCCTCCCATCGAAGGAATAATTAGATGTGAATCAAAA---
ATTACAGGGATGATACTAACAAGAGATGGTGGTCTTGGTCGTAACATT------------------------
----

AGCAACGAGACCGAGATCTTCAGACCTGGAGGAGGAGATATGAGGGACAATTGGAGAAGTGAATTGTATAAAT
ATAAAGTAGTAAAAATAGAACCCTTA---GGAATAGCACCCTCCAAGGCAAAGAGAAGAGTGGTG--------
------------------------------CAGAGAGAAAAAAGAGCA---ATAGGA---TTCGGAGCT---
------ATGTTCCTTGGGTTCTTGGGAGCA---GCAGGAAGCACTATG---GGCGCAGCGTCA---
CTAACGCTGACGGTACAG---GCCAGACAA---TTACTG---TCTGGTATAGTGCAACAGCAGAAC---
AATTTG---CTGAGG---GCTATTGAGGCGCAACATCATCTGTTGCAACTCACAGTCTGGGGCATC
>MH000288..1
TTCTATTGTAATACAACACAACTGTTTGATAGTACTTGGAATGAT---------------------------
----

ACTACAGACACAATCACACTCCCATGCAAAATAAGACAAATTGTAAACTTGTGGCAGGAAGTAGGAAAAGCAA
TGTATGCCCCTCCCATCAGTGGAATAATTCAGTGTACATCAAAT---

ATTATAGGGTTGCTATTAACAAGAGACGGTGGT--

ACCGAAATCTTCAGACCAGGAGGAGGAGATATGAGGGACAATTGGAGAAGTGAATTATATAAATATAAAGTAG
TACAAGTTGAACCATTA---GGAATAGCACCCACCAAGGCAAAGAGAAGAGTGGTG-----------------
----------------------CAGAGAGAAAAAAGAGCA---GTGGGAACGCTAGGAGCT---------
GTATTCCTCGGGTTCTTGGGAGCA---GCAGGAAGCACTATG---GGCGCAGCGTCA---
ACAGCGCTGACGGTACAG---GCCAGACAA---TTATTG---TCTGGTATAGTGCAACAGCAGAAC---
AATCTG---CTGAAG---GCTATTGAGGCGCAACAGCATTTGTTGCAGCTCACAGTCTGGGGCATC
>KC699040..1
TTCTATTGTGATACAGCACAACTGTTTAATAGTACTTGGCCTAAGAATAGCACCAGTAGCACCAATAGCACCG
GTAAAGGG---

GGGCCAACAACTATCACACTCCCATGCAGAATAAGACAAATTGTAAACATGTGGCAGGAAGTAGGAAAAGCAA
TGTATGCTCCTCCCATCAGCGGAAACATCACATGTTCATCAAAT---
ATTACAGGGCTGCTATTAACAAGAGATGGGGGTAATAAGAATGAG----------------------------
----

ACGACCAACAGGACTCAGACCTTCAGACCTATAGGAGGAGATATGAGGGACAATTGGAGAAGTGAATTATATA
AATATAAAGTAGTAAAAATTGAACCATTA---GGAATAGCACCCACCAAGGCAAAGAGAAGAGTGGTG-----
----------------------------------CAGAGAGAAAAAAGAGCA---GTGGGA---
GTAGGAGCT---------TTGTTCCTTGGGTTCTTGGGAGCA---GCAGGAAGCACTATG---
GGCGCAGCGTCA---GTGACGTTGACGGTACAA---GCCAGACAA---TTATTG---
TCTGGTATAGTGCAACAGCAGAAC---AATCTG---CTGAGA---
GCTATTGAGGCGCAACAGCATATGTTGCAACTCACAGTCTGGGGCATC
>KC699032..1
TTTTACTGTAATACAACACAGCTGTTTAATAGTACTTGGAATGCTAATGGTACTAGTCGGGATGGTACTACA-
----

GGGAATGATACTATCACAATCCAATGCAGAATAAAGCAAATTATAAACAGGTGGCAGGAGGTAGGAAAAGCAA
TGTATGCCCCTCCCATCCAAGGACAAATTTACTGTTCATCAAAT---
ATTACAGGGCTTCTATTAACAAGAGATGGGGGTAATGACAATAACACT-------------------------
----

AGCACTAATGAGACCTTCAGACCTGCAGGAGGAAATATGAAGGACAATTGGAGAAGTGAATTATATAAATATA
AAGTAGTAAAAATTGAGCCATTA---GGACTGGCACCCACCAGGGCAAGGAGAAGAGTGGTG-----------
----------------------------CAGAGAGAAAAAAGAGCA---GTGGGA---CTAGGAGCT------
---ATGTTCCTTGGGTTCTTGGGAGCA---GCAGGAAGCACTATG---GGCGCAGCGTCA---
TTGACGCTGACGGTACAG---GCCAGGCAA---TTATTG---TCTGGTATAGTGCAACAGCAAAAC---
AATTTG---CTAAGA---GCTATTGAGGCGCAACAGCATATGTTGCAACTCACAGTCTGGGGCATC
>KC699037..2
TTTTATTGTAATACATCACAACTGTTTAATAGTAGTTGGACCAATTACACTAATGGTACTTGGGACAATGACA
CCATAATA---
----------GATAACACT------
ACCACTGGAGTAAATATCACACTCCCATGCAGAATAAAACAAATTATAAACATGTGGCAGAAAGTAGGAAAAG
CCATGTATGCCCCTCCCATCAGAGGACTAATTCAATGTACATCAAAT---
ATTACAGGAATACTATTAACAAGAGATGGAGGTAATAGGAATGAGAGCAAT----------------------
----

GGCAAAAGCAATGAGACCTTCAGACCTGTAGGGGGAGATATGAGGGACAATTGGAGAAGTGAATTATATAAAT
ATAAAGTAGTAAAAATTAAACCATTA---GGAATAGCACCCACCAGGGCAAAGAGAAGAGTGGTA--------
-------------------------------CAGAGAGAAAAAAGAGCA---GTGGGAACGTTGGGAGCC---
------ATGTTCCTTGGGTTCTTGGGAGCA---GCAGGAAGCACTATG---GGCGCAGCGTCA---
CTAACGCTGACGGTACAA---GCCAGACAA---TTATTG---TCTGGTATAGTGCAACAGCAAAGC---
AATTTG---CTGAGG---GCTATTGAGGCGCAGCAGCACATGTTGCAACTCACAGTCTGGGGCATT
>MH000292..1
TTCTATTGTAACACAACACAGCTATTTAATAGTACTTGGATGCCT----------------------------
----

AATGGTGGCACTATCACACTCCCATGCAGAATAAAACAAATTATAAACAGGTGGCAGGAAGTAGGAAAAGCAA

TGTATGCCCCTCCCATTAGTGGACAAATTAACTGCTCATCAAAC---
ATTACAGGGTTACTATTAATAAGAGATGGTGGT--

AACGAGACCTTCAGACCTGGAGGAGGAAATATGAAAGACAATTGGAGAAGTGAATTATATAAATATAAAGTAG
TACAAATTCAGCCATTA---GGAGTAGCACCCACCAAGGCAAAGAGAAGAGTGGTG-----------------
----------------------CAGAGAGAAAAAAGAGCA---GTGGGAACGCTAGGAGCT---------
ATGTTCCTTGGGTTCTTGGGAGCA---GCAGGAAGCACTATG---GGCGCAGCGTCA---
GTGACGCTGACGGTACAG---GCCAGACAA---TTATTG---TCTGGTATAGTGCAACAGCAAAAC---
AACTTG---CTGAAA---GCTATTGAGGCGCAACACCATCTGTTGCAACTCACAGTCTGGGGCATT
>KC699006..1
TTCTACTGTAATACAACACAACTGTTTAATAGTACTTGGAATAGTACTTGGGATATTAATAGTACTATAGAGT
CAAATAAC---

ACTGGAGAATATATCACACTCCCATGTAGAATAAAACAAATTATAAACATGTGGCAGGAAGTAGGAAAAGCAA
TGTATGCCCCTCCCATCAAAGGACAAATTAGATGTTCATCAAAC---
ATTACAGGGCTGCTATTAACAAGAGATGGTGGTAATAACAATAGTAGC-------------------------
----

AACACCACCGAGACCTTCAGACCTGGAGGAGGAGATATGAGGGACAATTGGAGAAGTGAATTATATAAATATA
AAGTAGTAAAAATTGAACCATTA---GGAGTAGCACCCACCAAGGCAAAGAGAAGAGTGGTG-----------
----------------------------CAGAGAGAAAAAAGAGCA---GTGGGA---ATAGGAGCC------
---GTGTTCCTTGGGTTCTTGGGAGCA---GCAGGAAGCACTATG---GGCGCAGCGTCA---
ATAACGCTGACGGTACAA---GCCAGACTA---TTATTG---TCTGGTATAGTGCAACAGCAGAAC---
AATCTG---CTGAGG---GCCATTGAGGCGCAACATCATCTGTTGCAACTCACAGTCTGGGGCATC
>KC699007..1
TTCTACTGTAATTCAACACAACTGTTTAATAGTACTTGGAGTGAAGGGTCAAATAACACTGAAGGAAATAACA
CTGAA--

GGAAATGACACAATCACACTCCCATGCAGAATAAAACAAATTATAAACATGTGGCAGAACGTAGGAAAAGCAA
TGTATGCCCCTCCCATCAGCGGACAAATTAGATGTTCATCAAAT---
ATTACAGGGCTGATATTAACAAGAGATGGTGGTAATAACCCAAAC----------------------------
----

GGGACCGAGATCTTCAGACCTGGAGGAGGAGATATGAGGGACAATTGGAGAAGTGAATTATATAAATATAAAG
TAGTAAAAATTGAACCATTA---GGAGTAGCACCTACCAAGGCAAAGAGAAGAGTGGTG--------------
-------------------------CAGAGAGAAAAAAGAGCA---GCGGGA---CTAGGAGCT---------
GTGTTCCTTGGGTTCTTGGGAGCA---GCAGGAAGCACTATG---GGCGCAGCGTCA---
ATAACGCTGACGGTACAG---GCCAGACGA---TTATTG---TCTGGTATAGTGCAACAGCAGAAC---
AATTTG---CTGAGG---GCTATTGAGGCGCAACATCATCTATTGCAACTCACAGTCTGGGGCATC
>KC699025..1
TTCTACTGTAATACAACAAAACTGTTTAATAGTACTTGGAAGGAAAATGGCACCATTGAAGACGATGGTACA-
----

AAGGATACTAACATCACACTCCCATGCAAAATAAAACAAATTATAAACAGGTGGCAGGCAGTAGGAAAAGCAA
TGTATGCCCCTCCCATCAGAGGACAAATTAAATGTACATCAAAT---
ATTACAGGGTTGCTGTTAGTAAGAGATGGTGGTCATAACACTGAC----------------------------
----

AACGAGACCTTCAGACCTGGAGGGGGAGACATGAAGGACAATTGGAGAAGTGAACTATATAAATATAAAGTAG
TAAAAGTTGAACCATTA---GGAATAGCACCCACCAAGGCAAAGAGAAGAGTGGTG-----------------
----------------------CAGAGAGACAAAAGAGCA---GTGGGAACACTAGGAGCA---------
ATGTTCCTTGGGTTCTTGGGAACA---GCAGGAAGCACTATG---GGCGCAGCGTCA---
GTGGCGCTGACGGTACAG---GCCAGACAA---TTACTG---TCTGGTATAGTGCAACAGCAAAAC---
AATTTG---CTGAAG---GCTATTGAGGCGCAACAGCATCTGTTGCAACTCACAGTCTGGGGCATC
>JF706474..1
TTCTACTGTAATTCAGCACCACTGTTTAATAGTACTTGGAATAGTATTGAGGGTAGTACTGAGGGTAAGAATG
AA---

AGCAGTGGAGGAAATATCACACTCCTATGTAGAATAAAACAAATTATAAACATGTGGCAGGAAGTAGGAAAAG

CAATGTATGCCCCTCCTATCAGAGGAGTAATTCAATGCTCATCAAAT---
ATTACAGGGCTGCTATTAACAAGAGATGGTGGTAGTGGTAATAGTAGCAAT----------------------
----

GAGAACGAGACCGAGACCTTCAGACCTGGGGGGGGAAATATGAAGGACAATTGGAGAAGTGAATTATATAAAT
ATAAAGTAGTACAAATTGAACCATTA---GGAATAGCACCCACCAAGGCAAAGAGAAGAGTGGTG--------
-------------------------------CAGAGAGAAAAAAGAGCG---GTGGGAACACTAGGAGCT---
------GTGTTCCTTGGGTTCTTGGGAGCA---GCAGGAAGCACTATG---GGCGCAGCGTCG---
GTAACGCTGACGGTACAG---GCCAGACAA---TTATTG---TCTGGTATAGTGCAACAGCAGAAC---
AATTTG---CTGAAG---GCTATTGAGGCGCAACAGCATCTGTTGCAACTCACAGTCTGGGGCATC
>HM204630..1
TTCTACTGTAATACAACAAAACTGTTTAATAGTACTTGGGGGAATAATACTGCAGGACAA-------------
----

GGTAATAACACTATCACACTCCCATGCAGAATAAAACAAATTATAAACTTGTGGCAGGAAGTAGGAAAAGCAA
TGTATGCCCCTCCCATTAGTGGACACATTTACTGTTCATCAAAT---
ATTACAGGGCTACTATTAACAAGAGATGGTGGTACTACCAACATAACC-------------------------
----

AATGAAACTGAGATCTTCAGACCTGGAGGGGGAGATATGAGGGACAATTGGAGAAGTGAATTATATAAATATA
AAGTAGTAAAAGTTGAACCATTA---GGAATAGCACCCACCAAGGCGAAGAGAAGAGTGGTG-----------
----------------------------CAGAGAGAAAAAAGAGCA---GTGGGA---ATAGGAGCT------
---GTGTTCCTTGGGTTCTTGGGAGCA---GCAGGAAGCACTATG---GGCGCAGCGTCA---
CTGACGCTGACGGTACAG---GCCAGACAA---TTATTG---TCTGGTATAGTGCAACAGCAGAAC---
AATTTG---CTGAGA---GCTATTGAGGCGCAACAGCATCTGTTGCAACTCACAGTCTGGGGCATC
>HM204631..2
TTTTACTGTAATTCATCACAACTGTTTAATAGTACTTGGAATGCCACTAGTACTGGGAATAACAGTATAGGG-
----

TTAAATGGCACTATCACACTCCCATGTAAAATAAAACAGATTATAAACAGGTGGCAGGAAGTAGGAAGAGCAA
TGTATGCCCCTCCCATTCAAGGAATAATTAGATGTGAATCAAAT---
ATTACAGGGCTACTGTTAACAAGAGATGGTGGTAATATAACTAGT----------------------------
----

AATGGGAACGAGACCTTCAGACCCATAGGAGGAAATATGAAGGACAATTGGAGAAGTGAACTCTATAAATATA
AAGTAGTACAAATTGAACCCTTA---GGAATAGCACCCACCAAGGCAAAGAGAAGAGTGGTG-----------
----------------------------CAGAGAGAAAAAAGAGCA---GTGGGAACAATAGGAGCT------
---ATGTTCCTTGGGTTCTTGGGAGCA---GCAGGAAGCACTATG---GGCGCAGCGTCG---
GTGGCGCTGACGGTACAG---GCCAGACAA---TTATTG---TCTGGTATAGTGCAGCAGCAGAAC---
AACCTG---CTGAGG---GCTATTGAGGCGCAACAGCACATGTTGCAACTCACAGTCTGGGGCATT
>HM204636..1
TTCTATTGTAAAACAACAGAGCTGTTTAATAGTACTTGGCCAGGGAATAGTACTGTGAATAATGCTACAGAAT
CA---

AATGACAATGACACTATCACACTCCCATGCAGAATAAAACAAATTATAAACAGGTGGCAGGAAGTAGGAAAAG
CAATGTATGCCCCTCCCATCCAAGGAAACATTAGCTGTTCATCAAAC---
ATTACAGGACTGCTATTAACAAGAGATGGTGGTAAAGATCATAAC----------------------------
----

TCCACCGAGACCTTTAGACCTGGAGGAGGAGATATGAGAGACAATTGGAGAAGTGAATTATACAAATATAAAG
TAGTAAGAATTGAGCCATTA---GGAGTAGCACCCACCAAGGCAAGGAGAAGAGTGGTG--------------
-------------------------CAGAGAGAAAAAAGAGCA---GCACTG------GGAGCT---------
GTGTTCCTTGGATTCTTGGGAGCA---GCAGGAAGCACTATG---GGCGCAGCGTCA---
GTGGCGCTGACGGTACAG---GCCAGACAA---TTATTG---TCTGGTATAGTGCAACAGCAAAAC---
AATTTG---CTGAGG---GCTATTGAGGCGCAACAACATCTGTTGCAACTCACAGTCTGGGGCATC
>HM204637..1
TTCTACTGTAATACAACACAGTTGTTTAATAATACACAGTGGGTTAATAGTAATAGTACAAAT----------
----

AGCAATGACACCATCACACTCCCATGCAGAATAAAACAAATTATAAACAGATGGCAGGAAGTAGGAAAAGCAA

TGTATGCCCCTCCCATCGCGGGACAAATTCACTGTTCATCAAAT---
ATTACAGGGCTAATATTAACAAGAGATGGTGGTAATAAAAATGACACC-------------------------
----

AACGGGACTGAGACTTTCAGGCCTGGAGGAGGAGATATGAGGGACAATTGGAGAAGTGAATTATATAAATATA
AAGTAGTAAAAATTGAACCATTA---GGAATAGCACCCACCAAGGCAAGGAGAAGAGTGGTG-----------
----------------------------CAGAGAGAAAAAAGAGCA---GTAACT---TTAGGAGCT------
---ATGTTCCTTGGGTTCTTGGGAGCA---GCAGGAAGCACTATG---GGCGCAGCGTCA---
ATGACGCTGACGGTACAA---GCCAGACAA---TTATTG---TCTGGTATAGTGCAACAGCAAAAC---
AATTTG---CTGAGA---GCTATTGAGGCGCAACAGCACCTGTTGCAACTCACAGTCTGGGGCATC
>HM204644..2
TTCTACTGCAACACATCAAAACTGTTTAATAGTAGTTGGAGTACTAATGGAACATTGTTAAATGGAACGTGGG
TAATGAG--
-------------------ACT---
AGAATAAATGACACAATCATACTCCCATGCAAAATAAAACAAATTATAAACATGTGGCAGAAAGTAGGAAAAG
CAATGTATGCTCCCCCCATCCAAGGAATAATTAACTGCTCATCAAAT---
ATTACAGGACTGCTATTAACAAGAGATGGTGGTCATAATCAAAATAGC-------------------------
----

ACCAATGACACAGAGACCTTCAGACCAGGAGGAGGAGATATGAGGGACAATTGGAGAAGTGAATTATATAAAT
ATAAAGTAGTAAGAATTGAACCATTA---GGAATAGCACCCACCAAGGCAAAGAGAAGAGTGGTG--------
-------------------------------CAGAGAGAAAAAAGAGCA---GTGGGA---TTCGGAGCT---
------ATGTTCCTTGGGTTCTTGGGAGCA---GCAGGAAGCACTATG---GGCGCAGCGTCA---
ATAACGCTGACGGTACAG---GCCAGACAA---TTGCTG---TCTGGTATAGTGCAACAGCAAAAC---
AATTTG---CTGAGG---GCTATTGAGGCGCAACAGCATATGTTGCAACTCACAGTCTGGGGCATC
>JF680923..1
TTCTACTGTAACACAACAAAACTGTTTAATAGTACCTGGGAGAATAATAGTACTTGGGGGAATAAT-------
----

ACTACAGGTACTATCACACTCCCATGCAGAATAAAACAAATTGTAAACATGTGGCAAGAAGTAGGAAAAGCAA
TGTATGCCCCTCCCATCCAAGGACAAATTAGATGCTCATCAAAT---
ATTACAGGACTGTTATTAACAAGAGATGGTGGTAATAATAATAGCACA-------------------------
----

AATCAGAGCGAGACCTTCAGACCTGGAGGAGGAAATATGAAGGACAATTGGAGAAGTGAATTATATAAATATA
AAGTAGTAAAAATTGAGCCATTA---GGAATAGCACCCACCAGGGCAAAAAGAAGAGTGGTG-----------
----------------------------CAGAGAGAAAAAAGAGCA---GCC------ATAGGAGCT------
---GTGTTCCTTGGGTTCTTGGGAGCA---GCAGGAAGCACTATG---GGCGCAGCGTCA---
GTGACGCTGACGGTACAG---GCCAGACAA---TTATTG---TCTGGTATAGTGCAACAGCAGAAC---
AATTTG---CTGAAG---GCTATTGATGCGCAACAGCATCTGTTGCAGCTCACAGTCTGGGGCATC
>JF680924..1
TTCTACTGCAATACAACATTACTGTTCAATAGTGATTGGTATCCTAATGATACTAGCACAGGTACTCCAGGGT
CA---

GATGGGAATGACTCACTCATACTCCCATGCAGAATAAGACAAATTGTAAACATGTGGCAGGAAGTAGGAAAAG
CAATGTATGCTCCTCCCATCAAAGGACAAATTAGGTGTCATTCAAAT---
ATTACAGGGATACTACTAACAAGAGATGGTGGGATTGACAACAAC----------------------------
----

AGGACTAATGAGACCTTTAGACCTGGAGGAGGAGATATGAGGGACAATTGGAGAAGTGAATTATATAAATATA
AAGTAGTAAAACTTGAACCATTA---GGATTAGCACCCACCAAGGCAAAGAGAAGAGTGGTG-----------
----------------------------CAGAGAGAAAAAAGAGCA---GTGGGA---CTAGGAGCT------
---ATGTTCCTTGGGTTCTTGGGAGCA---GCAGGAAGCACTATG---GGCGCAGCGTCA---
ATGACGCTGACGGTACAG---GCCAGACAA---TTATTG---TCTGGTATAGTGCAACAGCAGAAC---
AATCTG---CTGAAG---GCTATTGAGGCGCAACAGCATATGTTGCAACTCACAGTCTGGGGCATC
>JF680933..1
TTCTATTGCAATACAACACAACTGTTTAATACTACTTGGAATATTACTAATAATATTAATGTTACTAGGAATG
AT---

AGTGACAACAGCATTATCACACTCCCATGCAGAATAAAACAAATTATAAACATGTGGCAGGAAGTAGGAAAAG

CAATGTACGCCCCTCCCATCAGAGGACAGATTAGATGTTCATCAAAA---
ATTACAGGGCTACTATTAACAAGAGATGGTGGTAATACAAGCATTGCG-------------------------
----

AACGGGTCTGAGATCTTCAGACCTGGAGGAGGAAATATGAAGGACAATTGGAGAAGTGAATTATACAAATATA
AAGTAGTAAAAGTTCAACCATTA---GGAATAGCACCCACCAAGGCAAAAAGAAGAGTGGTC-----------
----------------------------CAGAGAGAAAAAAGAGCA---ATAACA---CTAGGAGCT------
---GTTTTCCTTGGGTTCTTGGGAGCA---GCAGGAAGCACTATG---GGCGCAGCGTCA---
ATAACGCTGACGGTACAG---GCCAGACAA---TTATTG---TCTGGTATAGTGCAGCAGCAGAGC---
AACTTG---CTGAGG---GCTATTGAGGCGCAACAACATATGTTGCAACTCACAGTCTGGGGCATC
>HQ595797..1
TTCTACTGTAATTCAACACAACTGTTTAGTAGTACTTGGATGGCTAATGGTACTTCAAATGGTACCGTA----
----

GGGAATGACACTATCATACTCCCATGTAAAATAAAACAATTTGTAAACATGTGGCAGCGAATAGGACAAGCAG
TGTATGCCCCTCCCATTAGAGGATACATTAATTGTTCATCAAAT---
ATTACAGGACTGCTATTAACAATAGATGGTGACAGGAATGACACC----------------------------
----

CAGAGTGAGACCTTCAGACCTGGAGGAGGAGATATGAGGGACAATTGGAGAAGTGAATTATATAAATATAAAG
TAGTAAAAATTGAACCATTA---GGAATAGCACCCACTAAGGCAAAGAGAAGAGTGGTG--------------
-------------------------CAGAGAGAAAAAAGAGCA---GTAACG---CTGGGAGCT---------
ATGTTCCTTGGATTCTTGGGAGCA---GCAGGAAGCACTATG---GGCGCAGCGTCA---
ATGACGCTGACGGTACAG---GCCAGACTA---TTACTG---TCTGGTATAGTGCAACAGCAGAGC---
AATCTG---CTGAGG---GCTATTCAGGCGCAACAGCATCTGTTGCAACTTACAGTCTGGGGCATC
>AJ535611..1
TTCTACTGTAATACAACACAGCTGTTTAATAGTACTTGGAATATTACACAGCTGTTGAATAGTACTGAGTCAA
AT---

AACACTGGCAATATCACACTCCCATGCAGAATAAGACAAATTGTAAACAGGTGGCAGGAAGTAGGAAAAGCAA
TGTATGCCCCTCCCATCAGCGGACAAATTAGTTGTTCATCAAAT---
ATTACAGGGTTGCTATTAACAAGAGATGGAGGTAACAGTAGCTAC----------------------------
----

AATGAGACCGAGACCTTCAGACCAGGAGGAGGAAATATGAGGGACAATTGGAGAAGTGAATTATATAAATATA
AAGTAGTAAGAATTGAACCAATA---GGAGTAGCACCCACCAGGGCAAAGAGAAGAGTGGTA-----------
----------------------------CAGAGAGAAAAAAGAGCA---ATAACG---CTGGGAGCT------
---GTGCTCCTTGGGTTCTTGGGAGCA---GCAGGAAGCACTATG---GGCGCAGCGTCA---
ATAACGCTGACGGTACAG---GCCAGACAG---TTATTG---TCTGGTATAGTGCAGCAGCAGAAC---
AATCTG---CTGAGG---GCTATTGAGGCGCAACAGCACCTGTTGCAACTCACAGTCTGGGGCATC
>AJ286339..1
TTCTACTGTAGTACAACAGAACTGTTTAATGACACTTGGGATAAGACTAGGAATAATACTGGAGAGGCA----
----

GATAACAGCACAATCACACTCCCCTGCAGAATAAAACAAATTATAAACAAGTGGCAAGAAGTAGGAAAAGCAA
TGTATGCCCCTCCCATCAGAGGACAAATTAAGTGTACATCAAAT---
ATTACAGGGCTAGTCTTAACAAGAGATGGTGGTAATGAGACTCAT----------------------------
----

AATGAGACTGAGATCTTCAGACCTGGAGGAGGAGATATGAGGGACAATTGGAGAAGTGAATTATACAAATATA
AAGTAGTAAAAATTGAGCCATTA---GGAGTAGCACCCACCAAGGCAAGGAGAAGAGTGGTG-----------
----------------------------CAGAGAGAAAAAAGAGCA---GTGGGAACAATAGGAGCT------
---ATGTTCCTTGGGTTCTTGGGAGCA---GCAGGAAGCACTATG---GGCGCAGCGCCA---
ATGACGCTGACGGTACAG---GCCAGACAA---TTATTG---TCTGGTATAGTGCAGCAGCAGAAC---
AATCTG---CTGAAG---GCTATTGAGGCGCAACAGCATCTGTTGCAACTCACAGTCTGGGGCATC
>DQ207942..1
TTCTATTGTAATACAACACAGCTGTTCAATAATACTTGGCTGAATAATGATACTGGGAATAGCACTAAG----
----

AATAATGCTACAATCACACTCCCGTGCAGAATAAAACAAATTATAAACAGATGGCAGGAAGTAGGAAAAGCAA

TGTATGCCCCTCCCATCAGAGGACTAATTAGATGTTCCTCAAAT---
ATTACAGGGCTACTATTAGTAAGAGATGGTGGTAATAGTAGTGCT----------------------------
----

GATCAGGAGATCTTCAGACCTGGGGGAGGAAATATAAAGGACAATTGGAGAAGTGAACTATATAAATATAAAG
TAGTAAAACTTGAGCCATTA---GGAGTAGCACCCACCAAGGCAAAGAGAAGAGTGGTG--------------
-------------------------CAGAGAGAAAAAAGAGCA---GTGGGAGTGTTTGGAGCC---------
ATGTTCCTTGGGTTCTTGGGAGCA---GCAGGAAGCACTATG---GGCGCAGCGTCA---
ATAACGCTGACGGTACAG---GCCAGACAA---TTATTG---TCTGGTATAGTGCAACAGCAGAGC---
AATTTG---CTGAGA---GCTATTGAGGCGCAACAGCATCTGTTGCAACTCACAGTCTGGGGCATT
>MH078546..1
TTCTACTGTGATTCAACACAACTGTTCAATAGTACTTGGTGGACTAATGGTACTGAGAACGGTACTAGAGGG-
----

ACAATTGGCACCATCACACTCCAATGCAGAATAAAACAAATTATAAACATGTGGCAGAAAATAGGAAAAGCAA
TGTATGCCCCTCCCATCAAAGGACAAATTTATTGTTCATCAAAT---
ATTACAGGGATAATATTAACAAGAGATGGTGGTAGGGACAATAATACT-------------------------
----

AATGGCACTGAGACCTTCAGACCTGGAGGAGGAAATATGAAAGACAATTGGAGAAATGAATTATATAAATATA
AAGTAGTAAAACTTAAGCCATTA---GGAATAGCACCCACCAAGGCACGGAGAAGAGTGGTG-----------
----------------------------CAAAGAGAAAAAAGAGCA---GTGGGA---ATAGGAGCT------
---CTGTTCCTTGGGTTCTTGGGAGCA---GCAGGAAGCACTATG---GGCGCAGCGTCA---
GTGACGTTGACGGTACAG---GCCAGACAA---TTGTTG---TCTGGTATAGTGCAACAGCAACAC---
AACTTG---CTGAAT---GCTATTGAGGCGCAACAACACCTGTTGCAACTCACAGTCTGGGGCATC
>EU839600..1
TTCTACTGTAATACATCAAAGCTATTTAACAATACTTGGAATACTACTAATACTAAGAAT-------------
----

TGGACTGGTTTTATCATACTCCCATGCAGAATAAAACAAATTATAAATATGTGGCAAAAAGTAGGCAAAGCAA
TGTATGCCCCTCCCATCAGGGGACAAATCAAATGTATATCAGCT---
ATTACAGGGATGCTATTAACAAGAGATGGTGGGAACAACAACGAGAATAAGACT-------------------
----

GTAGGGAATAGGACCGAGATCTTCAGACCTACAGGAGGAAATATGAAAGATAATTGGAGAAGTGAATTATACA
GCTATAAAGTAGTAAAAATTGAACCATTA---GGATTAGCACCCACCAGGGCGAAAAGAAGAGTGGTG-----
----------------------------------CAGAGAGAAAAAAGAGCA---
GTGGGAGCACTGGGAGCT---------ATGTTCCTTGGGTTCTTGGGAGCA---GCAGGAAGCACTATG---
GGCGCAGCGTCA---TTGACGCTGACGGTACAG---GCCAGACAA---TTACTG---
TCTGGTATAGTGCAACAGCAAAAT---AATTTG---CTAAGG---
GCTATTGAGGCGCAACAGCATCTGTTGCAACTCACAGTCTGGGGCATC
>MK303349..1
TTCTACTGTGATACAACAGAACTGTTTAATAGTACTTGGAATAATAGTGTTGGAGGGTTAAATAACAGT----
----

AACAGTAATACTATCACACTCCCATGTAGAATAAAACAAATGGTAAACATGTGGCAGAGAGTAGGAAGAGCAA
TGTATGCCCCTCCCATCAAAGGACTAATTAGCTGTTCATCAGAT---
ATTACAGGGCTGCTATTAACAAGAGATGGTGGTAACTATAGTGATAACAGT----------------------
----

AATAACACTGAGACCTTCAGACCAGGAGGAGGAGATATGAAAGACAATTGGAGAAGTGAATTATATAAATATA
AAGTAGTAGAAATTAAACCACTA---GGAGTAGCGCCCACCAAGGCAAAAAGAAGAGTGGTG-----------
----------------------------CAAAGAGAAAAAAGAGCA---GTGGGA---ATAGGAGCC------
---TTGTTCCTTGGGTTCTTGGGAGCA---GCAGGAAGCACTATG---GGCGCAGCGTCA---
ATAACGCTGACGGCACAG---GCCAGACAA---TTATTG---TCTGGTATAGTGCAACAGCAGAAC---
AATCTG---CTGAGG---GCTATTGAGGCGCAACAGCAGCTATTGCAACTCACAGTCTGGGGCATC
>EF694037..1
TTCTACTGTATTACAACACAACTGTTTAATAGTACTTGGTGGAATGATACTGAAAGGATAAATAACATTGAAG
AA---

AACACAGAAAATATCACACTCCCATGCAGAATAAGGCAGATTGTAAACCTGTGGCAGACAGTAGGAAAAGCAA
TGTATGCCCCTCCCATCAGAGGACAAATTAGATGTTCATCAAAT---
ATTACGGGGCTGCTATTAACAAGAGATGGTGGTAATCCCAATACC----------------------------
----

ACGGAAGAGAGCTTCAGACCTGGAGGAGGAGATATGAGGGACTATTGGAGAAGTGAATTATATAAATATAAAG
TAGTAAAAATTGAACCATTA---GGAGTAGCACCCACCAAGGCAAAGAGAAGAGTGGTG--------------
-------------------------CAAAGAGAAAAAAGAGCA---GTAGGA---ATAGGAGCT---------
GTGTTCCTTGGGTTCTTGGGAGTA---GCAGGAAGCACTATG---GGCGCAGCGTCA---
ATGACGCTGACGGTACAG---GCCAGACTA---TTATTG---TCTGGTATAGTGCAACAGCAGAAT---
AACCTG---CTGAGG---GCTATTGAGGCGCAACAGCATCTGTTGCAACTCACAGCCTGGGGCATC
>JF680930..2
TTCTATTGTAATACAACGAAACTGTTTAGCAGTAGTTGGAATGAAACTACAAATAGCACTGAAAATGGCACT-
----

GAAAATGACACAATCACACTTCAATGCAGAATAAAACAAATTATAAACATGTGGCAGGAAGTAGGAAAAGCAA
TGTATGCCCCTCCCATCAAAGGTCCAATTAGATGCTCATCAAAT---
ATTACAGGGCTAATATTAACAAGAGATGGTGGTCATAACAATAACACG-------------------------
----

AACGAGACTGAGATATTCAGACCTGGAGGAGGAGATATGAGGGACAATTGGAGAAGTGAATTATACAAATATA
AAGTAGTAAGAATTGAACCCTTG---GGAGTAGCACCCACCAAGGCAAAGAGAAGAGTGGTG-----------
----------------------------CAGAGAGATAAAAGAGCA---GTGGGAGTATTCGGAGCT------
---ATGTTCCTTGGGTTCTTGGGAGCA---GCAGGAAGCACTATG---GGCGCAGCGTCA---
GTGGCGTTGACGGTACAG---GCCAGACAA---TTATTG---TCTGGTATAGTGCAACAGCAGAAT---
AATCTG---CTGAGA---GCTATTGAGGCGCAACAACATCTGTTGCAACTCACAGTCTGGGGCATC
>AB287372..1
TTCTACTGTAATTCAACACCACTGTTTAGTAGTAATTGGACTAAGAATACTACTACCGGGTCTTGGATTGGCA
GATATGGA---
-------------TCAAAT------
GAGACTGATGCAAATATTACACTCATATGCAGAATAAAACAAATTATAAACATGTGGCAGGGGGTAGGAAAAG
CAATGTATGCCCCTCCCATCAGTGGACCAATCAGATGTTCATCAAAT---
ATTACAGGGCTGCTATTAACAAGAGATGGTGGTGAGGATAAGAAC----------------------------
----

AGCACTAAAGAGACCTTCAGACCCCTAGGAGGAAATATGAAAGACAATTGGAGAAGTGAATTATATCAATATA
AAGTAGTAGGAATTGAACCATTA---GGAGTAGCACCCACCAAGGCAAGGAGAAGAGTGGTG-----------
----------------------------CAGAGAGAAAAAAGAGCA---GTGGGAACGCTGGGAGCT------
---ATGTTCCTTGGGTTCTTGGGAGCA---GCAGGAAGCACTATG---GGCGCAGCGTCA---
ATGGCGCTGACGGTACAG---GCCAGACAA---TTATTG---TCTGGTATAGTGCAACAGCAGAAC---
AATTTG---CTGAGG---GCTATTGAGGCGCAACAACATTTGTTGCAACTCACAGTCTGGGGCATC
>AB588264..1
TTCTACTGCAATACATCACAACTGTTTAATAGTACTTGGAATAGTACTGGGAATAGTAGTACTGAGAATAGTA
TG---

TCAAATGACACTATCACACTCCCATGTAGGATAAAACAAATTATAAATATGTGGCAGGAAGTAGGAAAAGCAA
TGTATGCCCCTCCCATCGCAGGACTAATTAGATGTTCATCAAAT---
ATTACAGGGCTATTATTAGTGAGAGATGGTGGTGGTAATCAGACT----------------------------
----

AACGGGACAGAAATCTTCAGACCTGGGGGAGGAAATATGAAGGACAATTGGAGAAGTGAATTATATAAATATA
AAGTAGTAAAAATTGAACCAGTA---GGAATAGCACCCGACAGGGCAAAGAGAAGAGTGGTG-----------
----------------------------CAGAGAGAAAAAAGGGCA---GTGGGA---ATAGGAGCT------
---GTGTTCCTTGGGTTCTTGGGAGCA---GCAGGAAGCACTATG---GGCGCAGCGTCA---
ATAACGCTGACGGCACAG---GCCAGACAA---TTATTG---TCTGGTATAGTGCAACAGCAGAAC---
AATCTG---CTGAGG---GCCATTGAGGCGCAACAGCATATGTTGCAACTCACAGTCTGGGGCATC
>AB731669..1
TTCTACTGTAATACATCAGCACTGTTTAATTGGAGTATTACTGGGTCAAATATTACAAAAGAC----------
----

```
ATTAATAGGTCAAATACTATCCCATGCAGAATAAAACAAATTATCAACATGTGGCAGAAAGTAGGAAAAGCAA
TATATGCCCCTCCCATCAGCGGGCAAATTAATTGTTCATCAAAT---
ATTACAGGGTTGCTATTAACAAGAGATGGTGGTAAAAGTAATCAGACT-------------------------
-------------------------------------------------------------------------
----------
AATAACACTGAGGTTTTCAGACCTACAGGAGGAAATATGAGAGATAATTGGAGGAGTGAATTATATAAATATA
AAGTAGTAAGGATTGAACCATTA---GGAGTAGCACCCACCAAGGCAAGGAGAAGAGTGGTG-----------
----------------------------CAAAGAGAAAAAAGAGCA---GTGGGAACAATAGGAGCT------
---ATGTTCCTTGGGTTCTTGGGAGCA---GCAGGAAGCACTATG---GGCGCAGCGTCA---
ATAACGCTGACGGTACAA---GCCAGACTA---TTATTG---TCTGGTATAGTGCAACAGCAAAAC---
AATTTG---CTGAGA---GCTATTGAGGCGCAACAACATCTGTTGCAACTCACAGTCTGGGGCATC
>AB565478..1
TTCTACTGTAATACAACAAAGCTGTTTAATAGTACTTGGTATACTAATGGGAATAAGACTGAAGAATCAAAT-
-------------------------------------------------------------------------
----------------------------
AACAATGACACTATTACACTCCCATGCAGAATAAAACAAATTATAAACAGATGGCAGGAAGTAGGAAAAGCAA
TGTATGCCCCTCCCATCCAAGGACGAATTTACTGCTCATCAAAT---
ATTACAGGACTGTTATTAATAAGAGATGGTGGTAACAGTACTGAT----------------------------
-------------------------------------------------------------------------
----------
AACGAGTCTGAGATCTTCAGACCTGGAGGAGGAGATATGAGGGACAATTGGAGAAGTGAATTATATAAATATA
AAGTAGTACAAATTGAGCCATTA---GGAGTAGCACCCACCAAGGCAAAGAGAAGGGTGGTG-----------
----------------------------CAGAGAGAAAAAAGAGCA---GTGGGA---ATAGGAGCT------
---ATGTTCCTTGGGTTCTTGGGAGCA---GCAGGAAGCACTATG---GGCGCAGCGTCA---
CTAACGCTGACGGTACAG---GCCAGACGA---TTATTG---TCTGGTATAGTGCAACAGCAGAAC---
AATCTG---CTGAGA---GCTATTGAGGCGCAACAGCATATGTTGCAACTCACAGTCTGGGGCATC
>JQ316131..2
TTCTACTGTAATACATCAAAGCTGTTTAATAGTACTTGGGCTGTGAATATGACTAACGGGTCAAATATAACTA
AA-----------------------------------------------------------------------
----------------------------
GAAGGAAATACCACAACAATCCCATGCAGAATAAAACATATTATAAACAGGTGGCAAGAAGTGGGAAAAGCAA
TGTATGCCCCTCCCATCGAGGGACAAATTAGATGTACATCAAAT---
ATTACGGGGCTACTATTAACAAGAGATGGGGGGACTCAGAAAAAT----------------------------
--------------------------------------------------------------
GGGAGTAACAATGGGACTCACAACAACACTGAGATCTTCAGACCTGGGGGAGGAAATATGAAGGACAATTGGA
GAAGTGAATTATATAAATATAAAGTAGTAAAAATTGAGCCATTA---
GGAATAGCACCCACCAAGGCAAGGAGAAGAGTGGTG-------------------------------------
--CAGAGAGAAAAAAGAGCA---GTGGGAACAATAGGAGCT---------
ATGTTCCTTGGGTTCTTGGGAACA---GCAGGAAGCACTATG---GGCGCAGCGTCA---
CTGACGCTGACGGTACAG---GCCAGACTA---TTATTG---TCTGGTATAGTGCAACAGCAAAAT---
AATTTG---CTGAGA---GCTATTGAGGCGCAACAGCATCTGTTGCAACTCACAGTCTGGGGCATC
>JQ316133..1
TTCTACTGTAATACAACAAAACTGTTTGATAGTACTTGGAATGGTACTAAAGAGTGGAATGATACTAAAGAG-
-------------------------------------------------------------------------
----------------------------
TCAGATAAAAACATTACACTCCAATGCAGAATAAAACAAATTATAAACAGGTGGCAGGAAGTAGGACAAGCAA
TGTATGCCCCTCCCATCAGCGGACTAATTAGATGTTCGTCAAAT---
ATTACAGGGCTAATATTAACAAGAGACGGGGGGGCTAACGAGACT----------------------------
-------------------------------------------------------------------------
-------
CACACCAACACTGAAACCTTCAGACCTGCAGGAGGAGATATGAAGGACAATTGGAGAAGTGAATTATATAAAT
ATAAAGTAGTAAGAATTGAGCCATTA---GGGATAGCACCCACCAAGGCAAGGAGAAGAGTGGTG--------
-------------------------------CAGAGAGAAAAAAGAGCA---GTGGGAATATTAGGAGCT---
------ATGTTCCTTGGGTTCTTGGGAGCA---GCAGGAAGCACTATG---GGCGCAGCGTCA---
ATGACGCTGACGGTACAG---GCCAGACAA---TTATTG---TCTGGTATAGTGCAACAGCAGAAT---
AATTTG---CTGAGA---GCTATTGATGCGCAACAACATCTGTTGCGACTCACAGTCTGGGGCATT
>DQ295192..1
TTCTACTGTGATACAACACAACTATTTAATAGCACTTGGACATGGAATGGTACTTGGATTAACGGGTCAACTA
AA-----------------------------------------------------------------------
-------------------------
```

GCTGAGAATGACACAATTGTCCTCCCATGCAGAATAAAACAAATTATAAACAGATGGCAGGAAGTAGGAAAAG
CAATGTATGCCCCTCCCATCAGCGGACACATTAATTGCTCATCAAAG---
ATTACAGGACTGCTATTAACAAGAGATGGGGGGATTAAAAACAGCACGAAC----------------------
----

GGGACTCAGACTGAGATCTTCAGACCTGGGGGAGGAGATATGAGGGACAATTGGAGAAGTGAATTATATAAAT
ATAAAGTAGTAAAAATTGAGCCATTA---GGGATAGCACCCACCAAGGCAAGGAGAAGAGTGGTG--------
-------------------------------CAGAGAGAAAAAAGAGCA---GTGGGAACAATAGGAGCT---
------ATGTTCCTTGGGTTCTTGGGAGCA---GCAGGAAGCACTATG---GGCGCAGCGTCA---
CTGACGCTGACGGTACAG---GCCAGACTA---CTATTG---TCTGGTATAGTGCAACAGCAGAAT---
AATTTG---CTGAGA---GCTATTGAGGCGCAACAGCATCTGTTGCAACTCACAGTCTGGGGCATC
>KT878039..2
TTCTACTGTAATACAACAAAACTGTTTAATAGTACTTGGTATAGTAATGGTACTAGGAAAGATCAAAATGAA-
----

GGAGATGACACAATTACACTCCAGTGCAGGATAAAACAAATTATAAACAGGTGGCAAGAAGTAGGAAGGGCAA
TGTACGCCCCTCCCATCAGAGGAATAATTAAATGTTCATCAAAC---
ATTACAGGGCTGCTATTAACAAGAGATGGGGGGAACGGGACTAAT----------------------------
----

GGGACTGAGACCTTCAGACCTGGGGGAGGAAATATGAAAGACAATTGGAGAAGCGAATTATATAAATATAAAG
TAGTAAAAATTGAGCCATTA---GGGATAGCACCCACCAAGGCAAGGAGAAGAGTGGTG--------------
-------------------------CAGAGAGAAAAAAGAGCA---GTGGGGACGCTAGGAGCT---------
GTGTTCCTTGGGTTCTTGGGAGCA---GCAGGAAGCACTATG---GGCGCAGCGTCA---
GTGGCGCTGACGGGACAG---GCCAGACAA---TTATTG---TCTGGTATAGTGCAACAGCAGAAT---
AATTTG---CTGAGA---GCTATTGAGGCGCAACAGCATCTGTTTCAACTCACAGTCTGGGGCATC
>KT878021..2
TTCTACTGTAATACAACACAGTTGTTCAATAGTACTTGGTTTCATAATGGTACTTGGAGTGGTGCTAAT----
----

GGGGATGAGACTATGACACTCCCATGCAGAATAAAACAAATTATAAACAGATGGCAAGAAGTAGGAAAAGCAA
TGTATGCCCCTCCCATCGCAGGATCAATTAATTGCTCATCAAAT---
ATTACAGGACTGTTATTAACAAGAGATGGTGGTATTACCAGCAACAAG-------------------------
----
ACTAAGAACAGCACCGAGACTGAGACCTTTAGACCGGGGGGAGGAGATATGAGGGACAATTGGAGAAGTGAAT
TATACAAATATAAAGTAGTAAAAATTGAACCACTA---
GGAGTAGCACCAACCAAGGCAAAGAGAAGAGTGGTG-------------------------------------
--CGGAGAGAAAAAAGAGCA---GTAACA---CTGGGAGCT---------
GTGTTCCTTGGGTTCTTGGGAGCA---GCAGGAAGCACTATG---GGCGCAGCGTCA---
GTGACGCTGACGGTACAG---GCCAGACTA---TTATTG---TCTGGTATAGTGCAGCAGCAGAAC---
AATCTG---CTGAGG---GCTATTCAGGCGCAACAGCATCTGTTGCAGCTCACAGTCTGGGGCATT
>KT878029..1
TTCTACTGTAATACAACAAGACTGTTTAATAGTACTTGGCATACTAATACTACAGGGTTAAATGAAACTGAAG
GA---

AATAGCACAACTATCACACTCCCATGCAGAATAAAACAAATTATAAACAGATGGCAGGAAGTAGGAACAGCAA
TGTATGCCCCTCCCATCAGCGGACAAATTAGATGTTCATCAAAT---
ATTACAGGGCTGCTATTAACAAGAGATGGGGGGAATAACACTCAT----------------------------
----

GGGAATGGGACTGAGATCTTCAGACCTCTGGGAGGAGATATGAAGGACAATTGGAGAAGTGAATTATATAAAT
ATAAAGTAGTGAGAATTGAGCCATTA---GGGATAGCACCCACCAGGGCAAGGAGAAGAGTGGTG--------
-------------------------------CAGAGAGAAAAAAGAGCA---GTGGGAACGATAGGAGCT---
------ATGTTCCTTGGGTTCTTGGGAGCA---GCAGGAAGCACTATG---GGCGCAGCGTCA---
TTGACGCTGACGGTACAG---GCCAGACTA---TTATTG---TCTGGTATAGTGCAACAGCAGAAT---
AATTTG---CTGAGA---GCTATTGAGGCGCAACAGCATCTGTTGCAACTCACAGTCTGGGGCATC
>KF561441..1
TTCTACTGTAATACAACACCACTGTTTAATAGTATTTGGTATAGTAATAATACTGAAAAGTCATATAATACTG
CA---

GAAAATGACACAATTACCCTCCAATGCAGAATAAGACAAATTATAAAAAGGTGGCAGGAGGTAGGAAAAGCAA
GGTATGCCCCTCTCATCACCTGCCTAATTAGATGCTATTCAAAT---
ATTACAGGGCTGTTATTGGCAGGTGATGGAGGTGAGGCAGGGAAGGATAAC----------------------
----

GGGAATAAGAATGAGACCTTCAGGCCTGGGGGAGGAGATATGAGGGACAATTGGAGAAGTGAATTGTATAAAT
ATAAAGTAGTAGAAATTGAACCATTA---AGGATAGCACCCACCAGGGCAAGGAGAATAGTGGTG--------
-------------------------------CAGAGAGAAAAAAGAGCA---GTGGGAACATTAGGAGCC---
------ATGTTCCTTGGGTTCTTGGGAGCA---GCAGGAAGCACTATG---GGCGCAGCGTCA---
CTGACGCTGACGGTACAG---GCCAAGCAA---GTATTG---TCTGGTATAGTGCGACAGCAGAAT---
AATTTG---CTGAGA---GCTATTGAGGCGCAACAGCGTCTGTTGCAACTCACAGTCTGGGGCATC
>MN043577..1
TTCTACTGTAATACAACACAACTGTTTAATAGTACTTGGTATAGTCCTTGGAATAGTACTAACGGGTCAAATA
TAACT--

GAAAGAAATATCACAACACTCCCATGCAGAATAAAACAAATTATAAACAGATGGCAGGAAGTAGGAAAAGCAA
TGTATGCCCCTCCCATCAGTGGACAAATTAGATGTTCATCAAAT---
ATTACAGGGCTGCTATTAACAAGAGATGGGGGGACTCAGAACAAT----------------------------
----

GGGACTGAGATCTTCAGACCTGGGGGAGGAAATATGAAGGACAATTGGAGAAGTGAATTATATAAATATAAAG
TAGTAAAAATTGAGCCATTA---GGGATAGCACCCACCGGGGCAAAGAGAAGAGTGGTG--------------
-------------------------CAGAGAGAAAAA---ACA---GTGGGAACGATAGGAGCT---------
ATGTTCCTTGGGTTCTTGGGAGCA---GCAGGAAGCACTATG---GGCGTAGCGTCA---
ATGACGCTGACGGTACAG---GCCAGACTA---TTATTG---TCTGGTATAGTGCAACAGCAGAAT---
AATTTG---CTGAGA---GCTATTGAGGCGCAACAGCATCTGTTGCAACTCACAGTCTGGGGCATC
>MN043581..2
TTCTACTGTAATACAACACAACTGTTTAATAGTACTTGGATGATTACTAATAATTGCAATAGTACTGAAGGAT
ACAATAACACT--

GCACGAAATAGCACAATTACCCTCCCATGCAGAATAAAACAAATTATAAACAGGTGGCAGGAAGTAGGAAAAG
CAATGTATGCCCCTCCCATCAGAGGACAAATTAGATGTTCATCAAAT---
ATTACAGGGCTACTATCAACAAGAGATGGGGGGACTGGGAACAAC----------------------------
----

GAGACTGAGATCTTCAGACCTGGGGGAAGAAATATGAGGGACAATTGGAGAAGTGAATTATATATATATAAAG
TAGTAACAATTGAGCCATTA---GGGATAGCACCCACCGGGGCAAAGAGAAGAATGGTG--------------
-------------------------CAAAGAGAAAAAAGAGCA---GTGGGAACGATGGGAGCT---------
ATGTTCCTTGGGTTCTTGGGAGCA---GCAGGAAGCACTATG---GGCGCGACATCA---
ATGACGCTGACGGTACAG---GCCAGACTA---TTATTG---TCTGGTATACTGCATCATCAGAAT---
AATCTG---CTGAAA---GCTATTGAGGCGCAACAGCATCTGTTGCAACTCACAGTCTGGGGCATC
>HQ386180..1
TTCTATTGTAACACAGCACAACTGTTTAATAGTACTTGGAATGCAACAGAAATGCTTAATAGTACAGAATCAA
ATGAC--
-------------------ACA---
AATAGCAACATCAACATCACACTCCAATGCAGAATAAGACAAATTATAAACATGTGGCAGGAAGTAGGAAAAG
CAATGTATGCCCCTCCCATCGAAGGACAAATTAGATGTTCATCAAAT---
ATTACAGGGATACTATTAACAAGAGATGGTGGTCATAGCACGAACGGGACT----------------------
----

GAGAACGGGAACGAGACCTTCAGACCTGGAGGAGGAAACATGAGGGACAATTGGAGAAGTGAATTATATAAAT
ATAAAGTAGTAAAACTTGAACCATTA---GGAATAGCACCCACCAAGGCAAAGAGAAGAGTGGTG--------
-------------------------------CAGAGAGAAAAAAGAGCG---GTGGGAATGCTAGGAGCT---
------ATGTTCCTTGGGTTCTTGGGAGCA---GCAGGAAGCACTATG---GGCGCAGCGTCA---
CTAACGCTGACGGTACAG---ACCAGACAT---TTATTG---GCTGATATAGTGCAACAGCAGAAC---
AATTTG---CTGAGA---GCTATTGAGGCGCAGCAGCATCTGTTGCAACTCACAGTCTGGGGCATC
>EU744081..1
TTCTACTGTAATACAACACAACTGTTTAATAGTACTTGGATGTTTAATGGTACTTGGGAGTCAAATAGCACTG
AGGGA--

AATCCCACAGAAAATATCACACTCCCATGCAGAATAAAACAAATTATAAACCTGTGGCAGGAAGTAGGAAAGG
CAATGTATGCCCCTCCTATCAGGGGACTAATTCAATGTGTATCAAAT---
ATTACAGGGTTGCTATTAACAAGAGATGGTGGGAACAACACGACTGAG-------------------------
----

AATGGACCGAGATTTTCAGACCTGGAGGAGGAGATATGAGGGACAATTGGAGAAGTGAATTATATAAATATA
AAGTAGTAAAAATTGAACCATTA---GGGGTAGCACCCACCAGGGCAAAGAGAAGAGTGGTG-----------
----------------------------CAGAGAGAAAAAAGAGCA---GTGGGAGTGATAGGAGCT------
---ATGTTCCTTGGGTTCTTGGGAGCA---GCAGGAAGCACTATG---GGCGCAGCGTCA---
ATGACGCTGACGGTACAG---GCCAGACAA---TTACTG---TCTGGTATAGTGCAGCAGCAGAAC---
AATCTG---CTGAGG---GCTATTGAGGCGCAACAGCATCTGTTGCAACTCACAGTCTGGGGCATC
>JF320244..3
TTCTACTGTAATACATCACAACTGTTTAATGGAAGTTGGCAACCACCGCTTAATGAAACTATATTTCAATACA
ATGCTACA---
-------------TGGGCA------
AAAAATGACACTAATATCACACTCCCATGCAGAATAAAACAATTTGTAAACATGTGGCAGGAAGTAGGAAAAG
CAATGTATGCCCCTCCCATCAGCGGACCAATCAAATGTGTATCAAAT---
ATTACAGGGTTGCTAATAACAAGAGATGGGGGTTACAACAGTAGTAGC-------------------------
----

AATGAGACCAATGAGACCTTCAGACCTGCAGGAGGAGAAATGAGGGACAATTGGAGAAGTGAATTATATAAAT
ATAAAGTAGTACAAGTTGAACCATTA---GGATTAGCACCCACCAAGGCAAAGAGAAGAGTGGTG--------
-------------------------------CAGAGAGAAAAAAGAGCA---GTGACG---CTGGGAGCT---
------ATGTTCCTTGGGTTCTTGGGAGCA---GCAGGAAGCACTATG---GGCGCAGCGTCA---
ATAACGCTGACGGTACAG---GCCAGACAG---TTATTG---TCTGGTATAGTGCAACAGCAGAAC---
AATCTG---CTGAGG---GCTATTGAGGCGCAACATCATCTGTTGCAACTCACAGTCTGGGGCATA
>JF320226..1
TTCTACTGTAATACCACACCACTGTTTAATAGTACCTGGAATGCCACACAACTGGTTAATATTACTGGGAATA
TCACAGGG---
-------------CTTAGT------
AATAGAACTGAAAATATTACACTCCCATGTAGAATAAAACAAATTATAAACATGTGGCAGGAAGTAGGAAAAG
CAATGTATGCTCCTCCCATCGCAGGACGAATTAAATGTTTATCAAAT---
ATTACAGGTTTACTATTAGTAAGAGATGGTGGCAATAGCAATGAC----------------------------
----

ACGGAGACCTTCAGACCTCAAGGAGGAAATATGAAGGACAATTGGAGAAGTGAATTATATAAATATAAAGTAG
TAAAAATTGAACCATTA---GGAGTAGCACCCACCAAGGCAAAGAGAAGAGTGGTG-----------------
----------------------CAGAGAGAAAAAAGAGCA---GTGGGAGTACTGGGAGCT---------
ATGTTCCTTGGGTTCTTGGGAGCA---GCAGGAAGCACTATG---GGCGCAGCGTCA---
ATAACGCTGACGGCACAG---GCCAGACAA---TTATTG---TCTGGTATAGTGCAACAGCAGAAC---
AATCTG---CTGAGG---GCTATTGAGGCGCAACAGCACCTGTTGCAACTCACAGTCTGGGGCATC
>JF320189..1
TTCTACTGTAATTCATCAGAACTGTTTAATAGTACCTGGACCTGGAATGAGACTGAAGGGTCTATTACTGAAG
GGTCA--

ATTACTAATGGGACAATCACACTCCCATGTAGAATAAGACAAATTATAAACATGTGGCAGAGAGTAGGAAAAG
CAATGTATGCCCCTCCCATCCAAGGACAAATTAGATGTTCATCAAAT---
ATTACGGGGCTGCTATTAACAAGAGATGGTGGTGAAGGTAACACCACC-------------------------
----

AATAACAACGAAACCTTCAGACCTGGAGGAGGTGATATGAGGGACAATTGGAGAAATGAATTATATAAATATA
AAGTAGTACAAATTGAACCATTA---GGAATAGCACCCACCAAGGCAAGGAGAAGAGTGGTG-----------
----------------------------CAGAGAGAAAAACGAGCA---GTGGGA---CTAGGAGCT------
---GTGTTCCTTGGGTTCTTGGGAGCA---GCAGGAAGCACTATG---GGCGCAGCGTCA---
GTGACGCTGACGGTACAG---GCCAGACAA---TTATTG---TCTGGTATAGTGCAACAGCAGAAC---
AATCTG---CTAAAG---GCTATTGAGGCGCAACATCATCTGTTGCAACTCACAGTCTGGGGCATC
>EF210727..1
TTCTACTGTAACTCTACACAGCTGTTTAATAGTACTTGGAACAGCAGCAATTGGAATTGGAATGATACTGAAA
AG---

ACAAATAAAAGTATCACACTCCCATGCAGAATAAAACAAATTATAAACATGTGGCAGGAAGTAGGAAAAGCAA
TGTATGCCCCTCCCATCAGAGGACAAATTAGATGTTCATCAAAT---
ATTACAGGGCTGATGCTAACAAGGGATGGTAGTAATGAGAGTCAC----------------------------
----

AGTGAGATCTTCAGACCAGGAGGAGGAAATATGAGGGATAATTGGAGAAGTGAATTATATAAATATAAAGTAG
TAAAAATTGAACCATTA---GGAGTAGCACCCACCAAGGCAAAGAGAAGAGTGGTG-----------------
----------------------CAGAGAGAAAAAAGAGCA---GTAGTGGGGATAGGAGCT---------
GTGTTCCTTGGGTTCTTGGGAGCA---GCAGGAAGCACTATG---GGCGCAGCGTCA---
ATGATGCTGACGGTACAG---GCCAGACAA---TTATTG---TCTGGTATAGTGCAACAGCAGAAC---
AATTTG---CTGAGG---GCTATTGAGGCGCAACAGCATATGTTGCAACTCACAGTCTGGGGCATC
>EF210729..2
TTTTATTGTAATACAACACGGCTGTTTAATAGTACTTGGAAGAATAATACTTGGACTAAGAATCATATTGAGA
AC---

GAGACTGAAACAATCACACTCCCATGCAAAATAAAACAAATTATAAACAGGTGGCAGGAAGTAGGACAAGCAA
TGTATGCCCCTCCCATCCCAGGATGGATTAACTGTTCATCAAAT---
ATTACAGGGCTACTATTAACAAGAGATGGTGGTGAAAACGGAACC----------------------------
----

ACCGAGACCGAGACCTTCAGACCTGGAGGAGGAGATATGAGGGACAATTGGAGAAGTGAATTATATAAATATA
AAGTAGTTGAAATTCAACCATTA---GGAGTAGCACCCACCACAGCAAAGAGAAGAGTGGTG-----------
----------------------------CAGAGAGAAAAAAGGGCA---GTAACA---CTAGGAGCT------
---ATGTTCCTTGGGTTCTTGGGAACA---GCAGGAAGCACTATG---GGCGCAGCGTCG---
CTGACGCTGACGGTACAG---GCCAGACAA---TTATTG---ACTGGTATAGTGCAACAGCAGAAC---
AATCTG---CTGAGG---GCTATTGAGGCGCAACAGCATATGTTGCAACTCACAGTCTGGGGCATT
>EF210731..2
TTCTACTGTAATCTATCAAAATTGTTTAATAGTACATGGAATGATACTACTTGGAACACGACTGATGAGTCA-
----

AATGACACTTACATCCTGCTCCCATGTAGGATAAAACAAATTATAAACATGTGGCAGACAGTAGGAAAAGCAA
TGTACGCCCCTCCCATCAAAGGAAAAATTAGTTGTTCATCAAAT---
ATTACAGGGCTGCTATTAACAAGAGATGGAGGGAATGACAACAGGACT-------------------------
----

CAGGGGGTCGAGATCTTCAGACCTGGAGGAGGAGATATGAGAGATAATTGGAGAAGTGAATTATATAAATATA
AAGTAGTAAAAATTGAACCATTA---GGAATAGCACCCACCGAGGCAAAAAGAAGAGTGGTG-----------
----------------------------CAGAGAGAAAAAAGAGCA---GTGGGA---ATAGGAGCT------
---GTGTTCCTTGGGTTCCTGGGAGCA---GCAGGAAGCACTATG---GGCGCAGCGGCA---
GTGACGCTGACGGTACAG---GCCAGACAA---TTATTG---TCTGGTATAGTGCAACAGCAGAAC---
AATCTG---CTAAAG---GCCATTGAGGCGCAACAGCATCTGTTGCAACTCACAGTCTGGGGCATC
>EF210735..1
TTCTACTGTAACACATCACACCTATTTAATAGTACTTGGAATGATACTAGTAGTTGGGATAATAGC-------
----

ACTGAAGGAAATATCACACTCCAATGTAGAATAAAACAAATCATAAACAGGTGGCAGGAAGTAGGAAAAGCAA
TGTATGCCCCTCCCATTGCAGGACAAATTAGATGCTCATCAAAT---
ATTACAGGGCTGCTATTAACAAGAGATGGTGGTCAGGGCAGTAAC----------------------------
----

GAGACCGAGGTCTTTAGACCTGGAGGAGGAAATATGAAGGACAATTGGAGAAGTGAACTATATAAATATAAAG
TAGTAAAAGTTGAACCATTA---GGAATAGCACCCACCAAGGCAAGGAGAAGAGTGGTG--------------
-------------------------CAGAAAGAAAAAAGAGCA---ATAACA---CTAGGAGCT---------
ATGTTCCTTGGTTTCTTGGGAGCA---GCAGGAAGCACTATG---GGCGCAGCGTCG---
ATAACGCTGACGGTACAG---GCCAGACAA---TTATTG---TCTGGTATAGTGCAGCAGCAGAAT---
AATTTG---CTGAGG---GCTATTGAGGCGCAACAGCATATGTTGCAACTCACAGTCTGGGGCATC
>MH327746..1
TTCTACTGCAATACAACAAAACTGTTTAACAGTAGTTGGCCAAATAGTACTAGGAATAATACTGAAGAGTCA-
----

AATAGCACTGTTATCACACTCCCATGTAGAATAAAACAAATTATAAACATGTGGCAGGAAGTAGGAAAAGCAA
TGTATGCCCCTCCCATCAGGGGACGAATTAATTGTACATCAAAC---
ATTACAGGGCTACTATTAACAAGAGATGGGGGCAATGGAAATAGC----------------------------
----

ACGAATGAAACCTTCAGACCTCAAGGAGGAAATATAAGGGACAATTGGAGAAGTGAACTATATAAATATAAAG
TAGTAAAAATTGAACCATTA---GGAGTAGCACCTACCAAGGCAAAGAGAAGAGTGGTG--------------
-------------------------CAAAGAGAAAAGAGAGCA---GTGGGA---ATGGGAGCT---------
TGGTTCCTTGGGTTCTTGGCAGCA---GCAGGAAGCACTATG---GGCGCAGCGTCA---
GTGACGTTGACGGTACAG---GCCAGACAA---TTATTG---TCTGGTATAGTGCAGCAGCAAAAT---
AATTTG---CTGCAA---GCTATTGAGGCGCAACAGCATTTGTTGCAACTCACAGTCTGGGGCATC
>KY658690..1
TTCTACTGCAATACAACAAAACTGTTTAACAGTAGTTGGCCAAATAGTACTAGGAATAATACTGAAGAGTCA-
----

AATAGCACTGTTATCACACTCCCATGTAGAATAAAACAAATTATAAACATGTGGCAGGAAGTAGGAAAAGCAA
TGTATGCCCCTCCCATCAGGGGACGAATTAATTGTACATCAAAC---
ATTACAGGGCTACTATTAACAAGAGATGGGGGCAATGGAAATAGC----------------------------
----

ACGAATGAAACCTTCAGACCTCAAGGAGGAAATATAAGGGACAATTGGAGAAGTGAACTATATAAATATAAAG
TAGTAAAAATTGAACCATTA---GGAGTAGCACCTACCAAGGCAAAGAGAAGAGTGGTG--------------
-------------------------CAAAGAGAAAAGAGAGCA---GTGGGA---ATGGGAGCT---------
TGGTTCCTTGGGTTCTTGGCAGCA---GCAGGAAGCACTATG---GGCGCAGCGTCA---
GTGACGTTGACGGTACAG---GCCAGACAA---TTATTG---TCTGGTATAGTGCAGCAGCAAAAT---
AATTTG---CTGCAA---GCTATTGAGGCGCAACAGCATTTGTTGCAACTCACAGTCTGGGGCATC
>MF373127..1
TTCTACTGTAATACAACAAGACTGTTTAATACTACTTGGAATAACACTGAGAAATCAAATCCCACTGAA----
----

GGAAATGGCACAATCACACTCCAATGCAGAATAAAACAAATTATAAACCTGTGGCAGGAAGTAGGAAAAGCAA
TGTATGCCCCTCCCATCAGAGGACAAATTAACTGTTCATCCAAC---
ATTACAGGACTGCTATTAACAACAGATGGTGGTATGAACACCAGT----------------------------
----

AATAACAAGACCTTTATACCTGCAGGAGGAGATATGAGAGACTATTGGAGGAGTGGAGTATTGAAATATGATG
TAGTAATAGTTGAGCCATTA---GGAATAGCACCAACCACGGATATGAGAAGAGTGGTG--------------
-------------------------CAGATACAAAAAAGAGCA---GAGCTA---CTACGAGCT---------
CTGTTGCTTGTGTTCTTGGGAGCA---GCAGGAAGGACGATG---AGCGCAGCGTCG---
ATGACGCTGACGGTACAG---GCCAGACAA---TTATTG---TCTGGTATAGTGCAACAGCAAAAT---
AATTTG---CTGCAG---GCTATTGAGGCGCAACAGCATATGTTGAAACTCACAGTCTGGGGCATC
>MF373129..2
TTCTACTGTAATACAGCAGAACTGTTTAACAGTACTTGGAGGAATGACACTGAAAGGTCAAATACCACTACA-
----

GGAAATGACACAATCACACTTCAATGCACAATAAAACGAATTGTAAACATGTGGCAGGAAGTAGGAAAAGCAA
TGTATGCCCCTCCCATCACAGGACAAATTAATTGTTCATCAAAA---
ATTACAGGGCTACTGTTAACAAGAGATGGTGGTGGGAACCAGAAC----------------------------
----

ACCAGCGAGACTTTCAGACCTGGAGGGGGAAATATGAAAGACAATTGGAGAAGTGAATTATATAAATATAAAG
TAATAAAAATTGAACCATTA---GGAGTAGCACCCACCAAGGCAAAGAGAAGAGTGGTG--------------
-------------------------CAGAGAGAAAAAAGAGCA---GTGGGA---ATAGGAGCT---------
GTGTTCCTTGGGTTCTTGGGAGCA---GCAGGAAGCACTATG---GGCGCAACGTCG---
ATGACGCTGACGGTACAG---GCCAGACAA---TTATTG---TCTGGTATAGTGCAACAGCAGAAT---
AATTTG---CTGAAG---GCTATTGAGGCGCATCAGCATATGTTGCAACTCACAGTCTGGGGCATC
>KP411829..1
TGCTATTGCAAACCATCACTTCTGTTTACTGGCACATACAAGAGTAACATTAATATTTCAAATATCACCCAAT
CCTCACAA---
-------------------TCC---GATGAAAGGTATATTTAATTCTGGCTTGTATGACG----------

ATCAATGTGTGGCAGGTAGTAGGAAAAGCGATCTATTCCCCTCCCATCAGTGGACAAATTAGATGTTCATCAA
AT---ATTACAGGGCTGCTATTAACAAGAGATGGTGGTAGCAACAGCAGTAACGAG-----------------
----

ACCGAGACTGAAATCTTCAGACCTGTGGGAGGAAATATGAGGGACAATTGGAGAAGTGAATTATATAAATATA
AAGTAGTACAAATTGAACCATTA---GGAGTAGCACCCACCAAGGCAAAGAGAAGAGTGGTG-----------
----------------------------CAAAGAGAAAAAAGAGCA---GTGGGA---TTAGGAGCA------
---ATGTTCCTTGGGTTCTTGGGAGCA---GCAGGAAGCACTATG---GGCGCAGCGTCA---
ATAACGCTGACGGTACAG---GCCAGACAA---TTATTG---TCTGGTATAGTGCAACAGCAGAAT---
AATTTG---CTGAGA---GCTATTGAGGCGCAACAGCATCTGTTGCAACTCACAGTCTGGGGCATC
>MF373151..2
CTCTACTGTAATTCCACACAACTATTTAATAGTACTTGGAATGGTACTGAAAGATCAAACTATTCCACTGAG-
----

ACTAATGAAACTATCACACTCCCATGCAGAATAAAACAAATTATAAACATGTGGCAGAGAGTAGGAAGAGCAA
TGTATGCCCCTCCCATCCAAGGACTAATTAGATGTTCATCAAAT---
ATTACAGGGCTGCTGCTAACAAGAGATGGAGGTCAGAATAGTAGT----------------------------
----

AATAGCACTGAGACCTTCAGACCGGGAGGAGGAAACATGAAGGATAATTGGAGAAGTGAATTATATAAATATA
AAGTAATAAAGATTGAACCATTA---GGAGTAGCACCCACCAAGGCAAAGAGAAGAGTGGTG-----------
----------------------------CAAAGAGAAAAAAGAGCA---ATAACA---GTAGGAGCT------
---ATGTTCCTTGGGTTCTTGGGAGCA---GCAGGAAGCACTATG---GGCGCAGCGTCA---
ATGGCGCTGACGGTACAG---GCCAGACAA---TTGTTG---TCTGGTATAGTGCAACAGCAGAAC---
AATCTG---CTGAGG---GCTATTGAGGCGCAACAGCATCTGTTGCAACTCACAGTCTGGGGCATC
>KP411823..1
TTCTACTGTAATACAACACAACTGTTTAATAGTACTTGGAATATCACTGGAGGGTTAAATAACACTGAA----
----

GGAAATGGCACAATCACACTCCAATGCAAAATAAAACAATTTATAAACATGTGGCATGAAGTAGGGAAAGCAA
TGTATGCCCCTCCCATCAGAGGACAGATTACATGTTCATCAAAC---
ATTACAGGGCTGCTATTAACAAGAGATGGTGGTGAGAACCCGGGC----------------------------
----

AACGACACTGATACTTTCACACCTGGAGGGGGAGATATGAAAGACAATTGGAGTAGTGAATTAGATAAATATA
GAGTAATAGGAATAGCACCACTA---CCAGTAGCACCCACCAAGGCAAAGACAAGATTGTTG-----------
----------------------------CAGAGAGATAAAAGAGCA---GTTGGA---ATAGGATCT------
---GTGTTCCTTGTCTTCCTGGTAGCG---GCAGGAAGCACTATG---TCTGCAATGTCA---
ATGACGATGACGCTACAA---GCTCAAGAA---TTACTG---TATGTGACCGAGCGAATGCAAAAA---
AATTTG---CTGAAG---GCTATTGAGGCGCAACAGCATCTGTTGCAACTCACAGTCTGGGGCATC
>MF373162..4
TTCTATTGTAATACAACACAGTTATTTAATAGTACTTGGGAGGCTCATCAGCTTAATCAGACTTGGAATGAGC
CCCTAGGG---

CCAAATGTTACTAATATCACACTCCCATGCAGAATAAAACAAATTATAAACAGGTGGCAGGAAGTGGGAAAAG
CAATGTTTGCCGCTCCCATCAGCGGACGAATTCAATGCAAATCAAAT---
ATTACAGGGTTGTTATTAACAAGAGATGGTGGTACTAACACCAGC----------------------------
----

GACCCCAACAGAACTGAGACCTTCAGACCTGGAGGAGGAGATATGAGAGACAATTGGAGAAGTGAATTATATA
AGTATAAAGTAGTACAAATTGAGCCATTA---GGAATAGCACCCACCAAGGCAAAGAGAGTAGTGGTA-----
----------------------------------AAGAGAGAAAAAAGAGCA---
GTGGGAGTGCTAGGAGCT---------ATGTTCCTTGGGTTCTTGGGAACA---GCAGGAAGCGTTATG---
GGCGCAGCGTCA---ATGACGCTGACGGTACAG---GCCAGACAA---TTATTG---
TCTGGTATAGTGCAACAGCAGAAC---AATCTG---CTGAGA---
GCTATTGAGGCGCAACAACATATGTTGCAACTCACAGTCTGGGGCATC
>KP411824..3
TTCTACTGCAATTCAACACCACTGTTTAATAGCACCTGGGATACTAATGGGACTTGCAAGGGCTCTAATAGT-
----

```
GCTGATTGGAATGTTACTGTCCCATGCATTAGCATCCCAATTACTACAATGGGGCAGAAACTAGAACAAGCAA
TGGACGTGCGTCCCATTGCAGGAAAAGCAATATGTTCATCAAGT---
TTGTCCAAAGGTATCCTTTTAGCCAATTCCCATAATAGCATTATTGTG-------------------------
-------------------------------------------------------------------------
-------------
CCCCGGCTGGTTATGCCTAAAGGTAAAGATAAGAAGTTCAATTGGAACAGGAAAATGTATCAATGTCAAGTAC
AATGTCAAATTGAACCATTA---GGAGTAGCACCCACCATGGCAAAGAGAAGAGTGGTG--------------
-------------------------CAAAGAGAGAAAAGAGAT---TTGGGA---CTAGGAGCT---------
CTGTTCCTTGGGTTCTTGGGAGCA---GCAGGAAGCACTATG---GGCGCAGCGTCA---
ATAACGCTGACGGTACAG---GCCAGACAA---TTATTG---TCTGGTATAGTGCAACAGCAGAAC---
AACTTG---CTGAGG---GCTATTGAGGCGCAACAGCATCTGTTGCAACTCACAGTCTGGGGCATT
>MF373185..1
TTCTACTGTAATTCAACACCACTGTTTAATAGTACTTGGTATGTTAATGGAACTAGGAATAATGATACTGATA
TT-----------------------------------------------------------------------
----------------------------
GGAAAGATAAACATCACACTCTCATGTAGAATAAAACAAATTATAAACATGTGGCAGGAAGTAGGAAAAGCAA
TGTATGCCCCTCCCATCCCAGGAAAAATTCAATGTATATCAAAT---
ATTACAGGGCTGCTATTAACAAGAGATGGAGGTAATACCAGTGAGACCAGG----------------------
-------------------------------------------------------------------------
----------
AACGGGACCGAGATTTTCAGACCTGGAGGAGGAAATATGAGGGACAATTGGAGAAGTGAATTATATAAATACA
AAGTAGTACAAGTTGAACCATTA---GGAATAGCACCCACCAAGGCAAGGAGAAGAGTGGTG-----------
----------------------------CAGAGAGAAAAAAGAGCT---CTGGGA---CTAGGAGCT------
---TTGTTTCTTGGGTTCTTGGGAGCA---GCAGGAAGCACTATG---GGCGCAGCGTCA---
GTGACGCTGACGGTACAG---GCCAGACAA---TTATTG---TCTGGTATAGTGCAGCAGCAGAAC---
AATCTG---CTGAGA---GCTATTGAGGCGCAACAACACATGTTGCAACTCACAGTCTGGGGCATC
>MF373186..1
TTCTACTGCAACACAACACAACTGTTTAATAGTACTTGGAATGATACACAACTGTCTGATAGCACTTGGAATA
ATACAAGA-----------------------------------------------------------------
-------------------------
GGGATAAATGACACTATCACACTCCAATGTAGAATAAGACAAATCATAAACATGTGGCAGAAGGTAGGAAAAG
CAATGTATGCCCCTCCCATCAGTGGACAAATTAGATGTACATCAAAT---
ATTACAGGGCTGATATTAACAAGAGATGGTGGTAATAGTGACAACAAC-------------------------
-------------------------------------------------------------------------
-------
ACCACCCAGCCCGAGATCTTCAGACCTCAAGGAGGAAATATGAAAGACAATTGGAGAAGTGAATTATATAAAT
ATAAGGTAGTAGCAATTGAACCATTA---GGACTAGCACCCACCAAGGCAAAGAGAAGAGTGGTG--------
-------------------------------CAGAGAGACAAAAGAGCA---GTAGGA---CTGGGAGCT---
------CTGTTCCTTGGGTTCTTGGGAGCA---GCAGGAAGCACTATG---GGCGCAGCGTCA---
ATGGCGCTGACGGTACAA---GCCAGACAA---TTATTG---TCTGGTATAGTGCAACAGCAGAAC---
AATTTG---CTGAGG---GCTATTGAGGCGCAACAGCATCTGTTGCAGCTCACAGTCTGGGGCATT
>KY213743..1
TTTTACTGTAATACATCAAAACTGTTTAATAGTACTTGGCTGAATAATAATACAGGGTCAAAT----------
-------------------------------------------------------------------------
----------------------------
GCCACTGAACCTATCATACTCCCATGCAGGATAAAACAAATTGTAAACATGTGGCAGGAAGTAGGAAAAGCAA
TGTATGCCCCTCCCATCAGAGGACAAATTAGATGCATATCAAAT---
ATTACAGGGCTCCTATTAACAAGAGATGGTGGTCAAGAGAACAAAACCGAG----------------------
-------------------------------------------------------------------------
----------
AACCACACCGAGGTCTTTAGACCCACGGGAGGAAATATGAAGGACAATTGGAGAAGTGAATTATATAAATATA
AAGTAGTAAAAATTGAACCATTA---GGAATAGCACCCACCAAGGCAAAAAGAAGAGTGGTG-----------
----------------------------CAGAGAGAAAAAAGAGCA---GTGGGA---ATAGGAGCG------
---ATGTTCCTTGGGTTCTTGAGCGCA---GCAGGAAGCACTATG---GGCGCAGCGTCA---
ATAACGCTGACGGTACAG---GCCAGACAG---TTACTG---TCTGGTATAGTGCAACAGCAAAAC---
AATTTG---CTGAGA---GCTATTGAGGCGCAACAACATATGTTGCAACTCACAGTCTGGGGCATC
>KY213745..1
TTCTACTGCAATACAACACAACTATTTAATAGTTCTTGGAATAATAGTAGTTTGAATAGTACTGAGGGTAATG
GAGGG--------------------------------------------------------------------
----------------------------
```

TCAAATGACACTATCACACTCCCGTGTAGAATAAAACAAATTATAAACATGTGGCAGGAAGTAGGAAAAGCAA
TGTATGCCCCTCCCATTCAAGGACAAATTAGATGCTCCTCAAAT---
ATTACAGGACTATTATTAGTAAGAGATGGTGGTAATAACAGGACG----------------------------
----

GGCGGCAGTGAGACCTTCAGACCTGGAGGAGGAAATATGAAGGACAATTGGAGAAGTGAATTATATAAATATA
AAGTAGTAAAAATTGAACCATTA---GGAATAGCACCCACCAGGGCAAGGAGAAGAGTGGTG-----------
----------------------------CAGAGAGAAAAAAGAGCA---GTGGGA---CTAGGAGCT------
---TTGTTCCTTGGGTTCCTGGGAGCA---GCAGGAAGCACTATG---GGCGCAGCGTCA---
ATGACGCTGACGGTACAG---GCCAGACAA---TTATTG---TCTGGTATAGTGCAACAGCAGAAC---
AATCTG---CTGAAG---GCCATTGAGGCGCAACAGCATATGTTGCAACTCACAGTCTGGGGCATC
>JN248346..2
TTCTACTGTGATTCAACACAACTGTTTAATAGTAGTTGGAATAGTAATTGGTCTAATAATACTGAA-------
----

GGGTCAGACAACATTACACTTCAATGCAGAATAAAACAAATTATAAACATGTGGCAGCAAACAGGAAAGGCAA
TGTATGCCCCTCCCATCAGTGGAATAATTAGCTGTTCATCAAAT---
ATTACAGGGCTGTTATTAACAAGAGATGGTGGTAACAATCAGACT----------------------------
----

AATAATAATGAGACCTTCAGACCTGGAGGAGGAAATATGAAGGACAATTGGAGAAGTGAATTATATAGATATA
AAGTAGTACAAATTGAACCATTA---GGAGTAGCACCCACCATGGCAAAGAGAAGAGTGGTG-----------
----------------------------CAGAGAGAAAAAAGAGCA---GTGGGA---ATAGGAGCT------
---CTATTCCTTGGGTTCTTGGGAGCA---GCAGGAAGCACTATG---GGCGCAGCATCA---
ATAACGCTGACGGTACAG---GCCAGACAA---TTATTG---TCTGGTATAGTGCAACAGCAGAAC---
AATTTG---CTGAGA---GCTATTGAGGCGCAACAGCATATGTTGCAACTCACAGTCTGGGGCATA
>KJ769147..1
TTCTACTGTAACACATCACAACTGTTTAATAGTACTTGGTATAGTAATGGT----------------------
----
-ACTCGAAATGATACT---------
GTAGGAAATGGCAATATCACGCTCCCTTGCAGAATAAAACAAATTGTAAACATGTGGCAGGAAGTAGGAAAAG
CAATGTATGCCCCTCCCATTGGAGGACAAATTAGATGTTCATCAAAT---
ATTACAGGGTTGCTATTAACAAGAGATGGTGGT--

AACAATCAGAGCAACATC---
ACCACCGAGACCTTCAGACCAGGAGGAGGAGATATGAGGGACAATTGGAGAAGTGAACTATATAAATATAAAG
TGGTAAAAATTGAACCATTA---GGAATAGCACCCACCAAGGCAAAGAGAAGAGTGGTG--------------
-------------------------CAGAGAGAAAAAAGAGCA---GTGGGAATGATAGGAGCT---------
ATGTTCCTTGGGTTCTTGGGAGCA---GCAGGAAGCACTATG---GGCGCAGCGTCA---
ATAACGCTGACGGTACAG---GCCAGGCAA---TTGTTG---TCTGGTATAGTGCAACAGCAGAGT---
AATCTG---CTGAGA---GCTATTGAGGCGCAACAGCATATGTTGCAACTCACAGTCTGGGGCATC
>KP109514..1
TTCTACTGTAATACAACACAGCTATTTAATAATACTTGGCTTACTTGGAAGGCCAATGCAAGTGATCCTGAAA
AAAAT--

AACACAAAAAATATCACACTCCCATGCAGAATAAAACAAATTATAAACATGTGGCAGGAAGTAGGAAAAGCAA
TGTATGCCCCTCCCATCGCAGGACAAATTAAATGTGTATCAAAT---
ATTACAGGGTTACTATTAACAAGAGATGGGGGTCAGGACAATAACACT-------------------------
----

AACGGTAACGAGACCTTCAGACCTGGAGGAGGAGATATGAGGGACAATTGGAGGAGTGAATTATATAAATATA
AAGTAGTAAAAATTGAACCATTA---GGAATAGCACCCACTAAGGCAAAGAGAAGAGTGGTG-----------
----------------------------CAGAGAGAAAAAAGAGCA---GCA------CTAGGAGCT------
---ATGTTCCTTGGGTTCTTGGGAATG---GCAGGAAGCACTATG---GGCGCAGCGTCA---
ATAACGCTGACGGCACAG---GCCAGACAA---TTGTTG---TCTGGTATAGTGCAACAGCAGAAC---
AATCTG---CTGAGG---GCTATTGAGGCGCAACAACATTTGTTGCAACTCACAGTCTGGGGCATC
>EU578539..1
TTCTACTGTAATGCAACACAACTGTTTAATAGTACTTGGCCCATTAACGGTACTTGGGAAGGATCA-------
----

```
AATAATACTAATGTCACACTACCATGCAGAATAAAACAAATTATAAACATGTGGCAGAGAGTAGGCAAAGCAA
TGTATGCCCCTCCCATCAGAGGACAAATTAGATGTTCATCAAAT---
ATCACAGGGCTGCTATTAACAAGAGATGGTGGTGGTAGTAACCAA----------------------------
-------------------------------------------------------------------------
-------------
AGCAACGAGACCTTTAGACCTGGAGGAGGAGACATGAAAGACAATTGGAGAAGTGAATTATATAAGTATAAAG
TAGTAAGAATTAAACCATTA---GGAGTAGCCCCCACCAAGGCAAAGAGAAGAGTGGTG--------------
-------------------------CAGAGAGAAAAAAGAGCA---GTGGGAACAATAGGAGCT---------
ATGTTCCTTGGGTTCTTGGGAGCA---GCAGGAAGCACTATG---GGCGCAGCGTCA---
GTGACGCTGACGGTACAG---GCCAGACAA---TTATTG---TCTGGTATAGTGCAACAGCAGAAC---
AATTTG---CTGAGG---GCTATTCAGGCGCAACAGCATCTGTTGCAACTTACGGTCTGGGGCATC
>HQ217760..1
TTCTATTGTAATACAACAGAGCTGTTTAATAGTACTTGGGATGTTAATAGTACTTTGAATGTTAATAATACTT
GGAATAAT-----------------------------------------------------------------
-------------ACTGAA------
GGAGCAAGTGGCAATATCACACTCCCATGCAGAATAAAACAAATTATAAATATGTGGCAGAAAGTAGGCAAGG
CAATGTATGCCCCTCCCATCAGAGGACAAATTAGATGTACATCAAAG---
ATCACAGGGCTGCTATTAACAAGAGATGGT---TATAATAATAAT----------------------------
-------------------------------------------------------------------------
----------
AACAACACCGAGACTTTCAGACCTGGAGGAGGAAATATGAAGGACAATTGGAGAAGTGAGTTATATAAGTATA
AAGTAGTAAAAATTGAACCATTA---GGAGTAGCACCCACCATGGCAAGGAGGAGAGTGGTG-----------
----------------------------CAGAGAGAAAAAAGAGCA---GTGGGAACAATAGGAGCT------
---ATGTTCCTTGGGTTCTTGGGAGCA---GCAGGAAGCACTATG---GGCGCAGCGTCA---
CTGACGCTGACGGTACAG---GCCAGACTA---TTATTG---TCTGGTATAGTGCAACAGCAGAAC---
AATCTG---CTGAGG---GCTATTGAGGCGCAACAGCATCTGTTGCAACTCACGGTCTGGGGCATC
>AF277071..1
TTCTACTGTAATTCAACACAGCTGTTTAATAGTACTTGGAATATTAATGATACTAGGAATGAT----------
-------------------------------------------------------------------------
----------------------------
ACTACAGGGAATATCACACTCCCATGCAGAATAAGACAAATTGTGAACAGGTGGCAGGAAGTAGGCAAAGCAA
TGTATGCCCCTCCCATCAGAGGACAAATTAGCTGTTCATCAAAT---
ATTACAGGGGTGCTATTAACAAGAGATGGTGGTAATCACACGAGC----------------------------
-------------------------------------------------------------------------
-------------
GAAACTGAGATCTTCAGACCTGGAGGAGGAGATATGAGGGACAATTGGAGAAGTGAATTATATAAATATAAAG
TAGTAAAAATTGAACCATTA---GGAAAAGCACCCACCAAGGCAAGGAGAAGAGTGGTG--------------
-------------------------CAAAGAGAAAAAAGAGCA---GTGACA---ATGGGAGCT---------
ATGTTCCTTGGGTTCTTAGGAGCA---GCAGGAAGCACTATG---GGCGCAGCATCC---
ATGACGCTGACGGTACAG---GCCAGGCTA---TTATTG---CCTGGTATAGTGCAACAGCAGAGC---
AATCTG---CTGAGG---GCTATCGAGGCGCAACAACATCTGTTGCAACTCACAGTCTGGGGCATC
>EF593267..1
TTCTACTGTAATACAACAAAGCTGTTTAATAGTACTTGGCATGCTAATGGCACTCGGAATGATACTGGAGGG-
-------------------------------------------------------------------------
----------------------------
TCAAATGACACTATCATACTACCATGCAGAATAAAACAAATTATAAACATGTGGCAGAAAGTAGGCAAAGCAA
TCTATGCCCCTCCCATCAGAGGACAAATTAGATGTGAATCAAAG---
ATCACAGGGCTGCTATTAACAAGAGATGGTGGTGATGATAACAGA----------------------------
-------------------------------------------------------------------------
-------------
ACCACCGAGATCTTCAGACCTGGAGGAGGAGATATGAGGGACAATTGGAGAAGTGAATTATATAAATATAAAG
TAGTAAAAATTGAACCATTA---GGAATAGCACCCACCAAGGCAAGGAGAAGAGTGGTG--------------
-------------------------CAGAGAGAAAAAAGAGCA---GTGGGA---ATAGGAGCT---------
GTGTTCCTTGGGTTCTTGGGAGCA---GCAGGAAGCACTATG---GGCGCAGCGTCA---
ATGACGCTGACGGTACAG---GCCAGACTA---TTATTG---TCTGGTATAGTGCAACAGCAGAAC---
AATTTG---CTGCAG---GCTATTGAGGCGCAACAGCATCTGTTGCAACTCACGGTCTGGGGCATC
>DQ853436..1
TTCTACTGTAATTCAACACGATTGTTTAGTAGTAAGTGGCATGCTAATGATAGTTGGAGTAGTAGTACTTGGA
ACGATACT-----------------------------------------------------------------
-------------------GGA---
```

GAGTCAAATGACACTATCACACTCCCATGTAGAATAAAACAAATTATAAACATGTGGCAGGAAGTAGGAAAAG
CAATGTATGCCCCTCCCATCAAAGGACAAATTCAATGTTCATCAAAT---
ATTACAGGGCTAATATTAACAAGAGATGGTGGTGCAAACACGAAC----------------------------
----

ACGAGCGAGGAGGTCTTCAGACCTGCAGGAGGAAATATGGGGGACAATTGGAGAAGTGAATTATATAAATATA
AAGTAGTAAAAATTGAACCAATA---GGAATAGCACCCACCAAGGCAAAAAGAAGAGTGGTG----------
----------------------------CAGAGAGAAAAAAGAGCA---GTGACA---CTGGGAGCT------
---GTGTTCCTTGGGTTCTTGGGAGCA---GCAGGAAGCACTATG---GGCGCAGCGTCA---
ATAACGCTGACGGTACAG---GCCAGACAA---TTATTG---TCTGGTATAGTGCAGCAGCAGAAC---
AATCTG---CTGAGG---GCTATTGAGGCGCAACAACATCTGTTGCAACTCACAGTCTGGGGCATC
>KT124806..1
TTCTACTGTGATACAACAGAACTGTTTAATAGTACTTGGAGTATTAATGGTACTAGGAATACTACTGAAGGGT
CA---

AATAGCACAGACATCACACTCCAATGTAGAATAAAACAAATTATAAACATGTGGCAGGAAGTAGGGAAAGCAA
TGTATGCCCCTCCCATTAGAGGACAAATTAGATGTTCATCAAAT---
ATTACAGGGCTGCTATTAACAAGAGATGGTGGTAGCGGGAGTGAGACT-------------------------
----

AAAAACGAGACTGAAACCTTCAGACCTGGAGGAGGAAATATGAAGGATAATTGGAGAAGTGAATTATATCAAT
ATAAAGTAGTAAAAATTGAACCATTA---GGAGTAGCACCCACCAGGGCAAAGAGAAGAGTGGTG--------
-------------------------------CAGAGAGAAAAAAGAGCA---GTCACA---CTAGGAGCT---
------GTGTTCCTTGGTTTCTTGGGAGCA---GCAGGAAGCACTATG---GGCGCAGCGTCA---
ATAACGCTGACGGTACAG---GCCAGACAA---TTATTG---TCTGGAATAGTGCAACAGCAAAGC---
AATCTG---CTGAGA---GCTATTGAGGCGCAACAGCATCTGTTGCAACTCACAGTCTGGGGCATC
>EF593290..1
TTTTACTGTGATACAACAGAGCTGTTTAATAGTACTTGGAATAATACTGATAGTACTCGGAATGATACT----
----

GAGTCAGACAAAATCACCCTCCAATGTAGAATAAAACAAATTATAAACATGTGGCAGGAAGTAGGAAAAGCAA
TGTATGCCCCTCCCATCCAAGGACAAATTCAATGTAACTCAAGT---
ATTACAGGGCTACTATTAACAAGAGATGGTGGGAACAACTCCACT----------------------------
----

AATTCCACCGAGATATTCAGACCTGGAGGAGGAGACATGAGGGATAATTGGAGAAGTGAATTATATAAATATA
AAGTAGTAAAAATTGAACCATTA---GGAGTGGCACCCACCAAGGCAAAGCGAAGAGTGGTA-----------
----------------------------CAGAGAGAAAAAAGAGCA---ATAACG---ATGGGAGCT------
---ATGTTCCTTGGGTTCTTGGGAGCA---GCAGGAAGCACTATG---GGCGCAGCGTCA---
ATAGCGCTGACGGCACAG---GCCAGACTA---TTATTG---TCTGGTATAGTGCAACAGCAGAAC---
AATCTG---CTGAGA---GCTATTGAGGCGCAACAGCATATGTTGCAACTCACAGTCTGGGGCATC
>JX863967..2
TTCTACTGTAATACAACACCACTGTTTAATAGCACTTGGAATGAAACTCAACTGTTTAATAGTACTTGGAATG
AAACTGAG---

GCAAATGGAACAATTACACTCCCATGCAGAATAAAACAAATTATAAACATGTGGCAGGAAGTAGGAAAAGCAA
TGTATGCCCCTCCCATCGAAGGACAAATTTACTGTTCTTCAAAC---
ATTACAGGGCTACTGTTATTAAGGGATGGTGGTAATACCACAAAC----------------------------
----

GGGACTGAGATCTTCAGACCTGGAGGAGGAGACATGAGGGACAATTGGAGAAGTGAATTATATAAATATAAAG
TAGTAAAAATTGAACCATTA---GGAGTAGCACCCACTAGGGCAAAGAGAAGAGTGGTG--------------
-------------------------CAGAGAGAAAAAAGAGCA---GTGGGA---ATAGGAGCT---------
ATGTTCCTTGGGTTCTTGGGAATG---GCAGGAAGCACTATG---GGCGCAGCGTCA---
CTAACGCTGACGGTACAG---GCCAGACAA---TTGTTG---TCTGGTATAGTGCAACAGCAGAAT---
AATTTG---CTGAGA---GCTATTGAGGCGCAACAGCACCTGTTGCAACTCACAGTCTGGGGCATC
>KT124764..2
TTCTACTGTAATACCACACAACTGTTTAATAGTACTTGGTATGCAAACAATACAAATAGTACTGAAGGGGGA-
----

AACAGTGGGAATATCACACTCCCATGCAGAATAAAACAATTTATAAACATGTGGCAGGAAGTAGGAAAAGCAA
TGTATGCCCCTCCCATCAGTGGACAGATTAAATGTACATCAAAT---
ATTACAGGGCTGCTATTAACAAGAGATGGTGGTCAAGGCAATCAGACC-------------------------
----

AACAACCAGACTGAGATCTTCAGACCTGTAGGAGGAGATATGAAGGACAATTGGAGAAGTGAATTATATAAAT
ATAAAGTAGTAAGAATTGAACCATTA---GGAATAGCACCCACCAGGGCAAAAAGGAGAGTGGTG--------
-------------------------------CAGAGAGAAAAAAGAGCA---GTGGGA---ATAGGAGCG---
------TTGTTCCTTGGTTTCTTGGGAGCA---GCAGGAAGCACTATG---GGCGCAGCGTCA---
ATGACGCTGACGGTACAG---GCCAGACTA---TTATTG---TCTGGTATAGTGCAACAGCAGAAC---
AATCTG---CTGAGA---GCTATTGAGGCGCAACAGCATCTGTTGCAACTCACAGTCTGGGGCATC
>KT124814..1
TTCTACTGTAATACAACACCATTATTTAATAGTACTTGGAATAATACTAGAAATATCAGTGGTCTTGAAGGGT
CAAAG--

GACAATGGAAATATCACCCTCCAATGCAGAATAAAACAAATTATAAACATGTGGCAGGAAGTAGGAAAAGCAA
TGTATGCCCCTCCCATCAGAGGACAAATTCAATGCTCATCAAAT---
ATTACAGGACTCCTATTAACAAGAGATGGTGGTACTAATGTTACT----------------------------
----

GATAACACCACTGAGATCTTCAGACCTGGAGGAGAAGATATGAGGGACAATTGGAGAAGTGAATTATATAAAT
ATAAAGTAGTACAAATTGAACCATTA---GGAGTAGCACCCACCAAGGCAAGGAGAAGAGTGGTG--------
-------------------------------CAGAGAGAAAAAAGAGCA---GTGGGA---ATAGGAGCT---
------TTGTTCCTTGGGTTCTTGGGAGCA---GCAGGAAGCACTATG---GGCGCAGCGTCA---
GTGACGCTGACGGTACAA---GCCAGACAA---TTATTG---TCTGGTATAGTGCAGCAGCAGAAC---
AATCTG---CTGAGG---GCTATTGAGGCGCAACAGCATATGTTGCAACTCACAGTCTGGGGCATC
>EU578104..1
TTCTACTGTAACACAGCACAACTGTTTAATACTATTTGGAATAATACTTGGAATAGTAATGGGAATGATACTG
AA---

GGGGATAATAAAACAATCATACTCCCGTGCAGAATAAAACAAATTGTAAACAGGTGGCAGGAAGTAGGAAAAG
CAATGTATGCCCCTCCCATCAGCGGAATCATTAACTGTTTATCAAAC---
ATTACGGGGCTGCTATTAACAAGAGATGGTGGAAATAACAGTAGT----------------------------
----

ACCAACGAGACCTTCAGACCTGGAGGAGGAGATATGAGGGACAATTGGAGAAGTGAACTCTATAAATATAAAG
TAGTAAAAATTGAGCCATTA---GGAGTAGCACCCACCAAGGCAAAAAGAAGAGTGGTG--------------
-------------------------CAGAGAGAAAAAAGAGCA---GTGGGAACGTTAGGAGCG---------
ATGTTCCTTGGGTTCTTGGGAGCA---GCAGGAAGCACTATG---GGCGCAGCGTCA---
GTGACGCTGACGGTACAG---GCCAGACAA---TTATTG---TCTGGTATAGTGCAACAGCAGAAT---
AATCTG---TTGAAG---GCTATTGAGGCGCAACAGCATCTGTTGCAGCTCACAGTCTGGGGCATC
>JQ403105..1
TTCTACTGTAATACAACAAAACTGTTTAATAGTACTTGGTATGAAAATGGTACAAATAGTACTTGGTATGCAA
ATGGTACAAAT--
-------AGTACTGGAGAG------
GGAAACAGTGATATTATCACAATCCCATGCAGAATAAAACAATTTATAAACATGTGGCAGGAAGTAGGAAAAG
CAATGTATGCCCCTCCCATCAGTGGACAGATTAGATGTTCATCAAAC---
ATTACAGGGCTGCTATTAACAAGAGATGGTGGTGAGGGCAATCAAACC-------------------------
----

AACAACCAGGAGATCTTCAGACCTGTAGGAGGAGATATGAAAAACAATTGGAGAAGTGAATTATATAAATATA
AAGTAGTAAGAATTGAACCATTA---GGAATAGCACCCACCAAGGCAAAAAGGAGAGTGGTG-----------
----------------------------CAGAGAGAAAAAAGAGCA---GTGGGA---ATAGGAGCG------
---TTGTTCCTTGGGTTCTTGGGAGCA---GCAGGAAGCACTATG---GGCGCAGCGTCA---
ATGACGCTGACGGTACAG---GCCAGACTA---TTATTG---TCTGGTATAGTGCAACAGCAGAAC---
AATCTG---CTGAGA---GCTATTGAGGCGCAACAGCATCTGTTGCAACTCACAGTCTGGGGCATC
>EU578230..2
TTCTACTGTAATACATCAGGACTGTTTAATGGTACTTGGAATGGTACTAGCACTTGGAATGATACTGCAAATT
AT---

```
ACTGCAAATGAGAATATCACACTCCCATGCAGAATAAAACAAATTATAAACATGTGGCAGGAAGTAGGAAAAG
CAATGTATGCCCCTCCCATCAGAGGACAAATTAACTGTTCATCAAAA---
ATTACAGGGCTGCTATTAACAAGAGATGGTGGCCATCACAACGGCAGC-------------------------
-------------------------------------------------------------------------
-------
AACAACGACACAGAAATCTTCAGACCTGGAGGAGGAGATATGAGGGACAATTGGAGAAGTGAATTATATAAAT
ATAAAGTAGTAAAAATTGAACCATTA---GGAGTAGCACCCACCAGGGCAAAAAGAAGAGTGGTG--------
-------------------------------CAGAGAGAAAAAAGAGCA---GTGGGA---ATGGGAGCT---
------TTGTTCCTTGGGTTCTTGGGAGCA---GCAGGAAGCACTATG---GGCGCAGCGTCA---
CTAACGCTGACGGTACAG---GCCAGACAA---TTATTG---TCTGGTATAGTGCAACAGCAGAAC---
AATCTG---CTGAGG---GCTATTGATGCGCAACAACATCTGTTGCAACTCACAGTCTGGGGCATT
>FJ469754..1
TTCTACTGTAATACAACAAGACTGTTTAATAGTAATTGGAATAGCAGTACTTGGAATAACACTGAAGGGTTAA
ATACCACT-----------------------------------------------------------------
-------------AACCTT------
GGAGAAAATGACACAATCATACTCCCATGCAGAATAAAACAGATTATAAACACGTGGCAGAAAGTAGGAAAAG
CAATATATGCCCCTCCCATCGAAGGACAAATTAAATGTATATCAAAT---
ATTACAGGGCTGCTATTATTAAGAGATGGTGGTATAAATGTGAGT----------------------------
-------------------------------------------------------------------------
----------
GAGACCAACGAAACCTTCAGACCTGGAGGAGGAGATATGAAGGATATTTGGAGAAGTGAATTATATAAATATA
AAGTAGTAAAAATTGAACCATTA---GGAATAGCACCCAACAAGGCAAAGAGAAGAGTGGTG----------
----------------------------CAGAGAGAAAAAAGAGCA---GTGGGA---ATAGGAGCT------
---GTGTTCCTTGGGTTCTTGGGAGCA---GCCGGAAGCACTATG---GGCGCAGCGTCA---
ATGACGCTGACGGTACAG---GCCAGACAA---TTATTG---TCTGGTATAGTGCAACAGCAAAGC---
AATTTG---CTGAGG---GCTATTGAGGCTCAACAACATCTGTTGAAACTCACAGTCTGGGGCATC
>FJ469757..2
TTCTACTGTAATTCCTCACAACTGTTCAGTAGTACTTGGAATAGGACTAACGGGACTTGGAATGATGTTGAAG
GGTTAAAT-----------------------------------------------------------------
-------------TACACT------
AATGGAAATGACACAATCACACTCCCATGCAGAATAAAACAATATATAAACATGTGGCAGGAAGTAGGAAAAG
CAATGTATGCCCCTCCCATCAGAGGGCAACTTAATTGTTCATCAAAG---
ATTACAGGGTTGCTATTAACAAGAGATGGTGTGAACGACACCGAC----------------------------
-------------------------------------------------------------------------
-------------
AAAAATGAGACTTTCAGACCTCAAGGAGGGGATATGAAGGACAATTGGAGAAGTGAATTATATAAATATAAAG
TAGTAAAAATTGAACCGTTA---GGGATAGCACCCACCAGGGCAAAGAGAAGAGTGGTG--------------
-------------------------CAAAGAGAAAAAAGAGCA---GTGGGA---TTAGGAGCT---------
ATGTTCCTTGGGTTCTTGGGAGCA---GCAGGAAGCACTATG---GGCGCAGCATCA---
ATAACGCTGACGGTACAG---GCCAGACAA---TTACTG---TCTGGTATAGTGCAACAGCAGAAC---
AATCTG---CTGAGG---GCTATTGAGGCGCAACACCATCTGTTGCAACTCACAGTCTGGGGCATC
>GU330538..2
TTCTACTGTAATACAACAAAACTGTTTTATAGTAATTGGACTATTAATGGGACTATTGTGAGTAATTGGACAT
ATGAAAATGAC--------------------------------------------------------------
-------------------ACT---
GGAACAAATGACACTATCATACTCCCATGTAGAATAAAACAAATTATAAACATGTGGCAGGAAGTAGGAAAAG
CAATGTATGCCCCTCCCATCAAAGGACCAATTACATGTAACTCAAGT---
ATTACAGGGTTGCTATTAACAAGAGATGGTGGTATTAACAGGACCAAT-------------------------
-------------------------------------------------------------------------
----------
AATGAGACCGAGATCTTCAGACCTGTAGGGGGAGATATGAGGGACAATTGGAGAAGTGAATTATATAAATATA
AAGTAGTAGAAATTCAACCATTA---GGAATAGCACCCACCAAGGCAAAGAGAAGAGTGGTG-----------
----------------------------CAGAGAGAAAAGAGAGCG---GTAACA---CTAGGAGCT------
---ATGTTCCTTGGGTTCTTGGGAGCA---GCAGGAAGCACTATG---GGCGCAGCGTCA---
ATGGCGCTGACGGTACAG---GCCAGACAA---TTATTG---TCTGGTATAGTGCAACAGCAGAAC---
AATCTG---CTGAGG---GCTATTGAGGCGCAACAGCACATGTTGCAACTCACAGTCTGGGGCATT
>GU330550..3
TTCTACTGTAATTTAACACAACTGTTTAATAGTACTTGGAATATTACTGACAGATTAAATAATAATGAAACTC
AA-----------------------------------------------------------------------
-------------------------
```

```
GGAAATGACACAACAATTCACCTCCCGTGCAGAATAAAACAAATTATAAACATGTGGCAAGAAGTAGGAAAAG
CAATGTATGCCCCTCCCATCAGGGGGATAATTAGATGTTCATCAAAT---
ATTACAGGGCTGCTATTAACAAGAGATGGTGGTACAAATACCACTGAC-------------------------
-------------------------------------------------------------------------
----------
AATGACACTGAGGTCTTCAGACCTGGAGGAGGAGACATGAGGGACAATTGGAGAAGTGAATTGTATAAATACA
AAGTAATACAAATTGAACCATTA---GGAGTAGCACCCACTAAGGCAAAGAGAAGAGTGGTG-----------
----------------------------CAGAGAGAAAAAAGAGCA---GTGGGA---ATAGGAGCT------
---GTGTTCCTTGGGTTCTTGGGAGCA---GCAGGAAGCACTATG---GGCGCAGCGTCA---
GTGACGCTGACGGTACAG---GCCAGACAA---TTATTG---TCTGGTATAGTGCAGCAGCAGAGC---
AATCTG---CTGAGG---GCTATTGAGGCGCATCAACATCTGTTGCAACTCACAGTCTGGGGCATC
>KT124781..1
TTCTACTGTAATTCAACACCACTGTTTAATAGTGAATGGTCAGATAATAATACTTGGAATAGTGCTGAAGAA-
-------------------------------------------------------------------------
----------------------------
GGAAATGCCACAATCACACTCCAATGCAGAATAAAACAAATTGTAAACATGTGGCAGGAAGTAGGAAGAGCAA
TGTATGCACCTCCCATCAAAGGAGTAATTAGCTGTACATCAAAT---
ATTACAGGACTGATATTAACAAGAGATCATAGTAAGGATAATGTA----------------------------
-------------------------------------------------------------------------
----------------
ACCTTCACCTTCAGACCTGGAGGAGGAAATATGAAGGACAATTGGAGAAGTGAATTATATAAATATAAAGTAG
TAAAAATTGAACCATTA---GGAGTAGCACCCACTACAGCCAAGAGAAGAGTGGTG-----------------
----------------------CAGAGAGAAAGAAGAGCA---GTGGGA---ATAGGAGCT---------
GTGTTCCTTGGGTTCTTGGGAGCA---GCAGGAAGCACTATG---GGCGCAGCGTCA---
ATAACGCTGACGGTACAG---GCCAGACTA---TTATTG---TCTGGTATAGTGCAACAGCAGAAC---
AATCTG---CTGAGA---GCTATTGAGGCGCAACAGCACCTGTTGCAACTCACAGTCTGGGGCATC
>MH060965..1
TTCTACTGTAATTCAACACAACTGTTTAATAGTACTTGGTTTACTGAGGATAATGCGACCGGCACTACC----
-------------------------------------------------------------------------
----------------------------
GGAAATGAAACAATCATACTCCCATGCAGAATAAAACAAATTATAAACATGTGGCAGGAAGTAGGAAGAGCAA
TGTATGCCCCTCCCATCAGAGGACAAATTAACTGTACATCAAAT---
ATTACAGGACTGATATTAACAAGAGATGGTGGTAATAACAATCACACT-------------------------
-------------------------------------------------------------------------
----------
AATGAGAACGAAACCTTCAGACCTGGAGGAGGAGATATGAGGGACAATTGGAGAAGTGAATTATATAAATATA
AAGTAGTAAAAGTTGAACCATTA---GGAATAGCACCCTCTAAGGCAAGGAGAAGAGTGGTG-----------
----------------------------CAGAGAGAAAAAAGAGCA---GCAGGA---CTAGGAGCT------
---TTGCTCCTTGGGTTCTTGAGCGCA---GCAGGAAGCACTATG---GGCGCAGCGTCA---
CTGACGCTGACGGTACAG---GCCAGGCAA---TTATTG---TCTGGTATAGTGCAACAGCAAAAC---
AATTTG---CTGAGG---GCTATTGAGGCGCAACAGCATATGTTGCAACTCACAGTCTGGGGCATT
>FJ469731..1
TTCTACTGCAATTCAACACCACTGTTTAATAAGACTTGGAATAAGAATGATATTAGGAATAATACTACTGGGA
AT-----------------------------------------------------------------------
----------------------------
GATAATGACACTATCACACTCCAATGCAGAATAAAACAAATTATAAACATGTGGCAGGAAGTAGGAAAAGCAA
TGTATGCCCCTCCCATTAGAGGACTAATCAGCTGTTCATCAAAT---
ATTACAGGGCTACTATTAACAAGAGATGGAGGTCATAACACAAGT----------------------------
-------------------------------------------------------------------------
----------
AGGGCAAATGAGACCTTCAGGCCTGGAGGAGGAAATATGAAGGACAATTGGAGAAGTGAATTATATAAATATA
AAGTAGTAAAAAATTGAACCATTA---GGAGTAGCACCCACCAGGGCAAAGAGAAGAGTGGTG----------
----------------------------CAGAGAGAAAAAAGAGCA---GTGGGA---CTAGGAGCT------
---TTATTCCTTGGGTTCTTGGGAGCA---GCAGGAAGCACTATG---GGCGCAGCGTCG---
ATAACGCTGACGGTACAG---GCCAGACAA---TTATTG---TCTGGTATAGTGCAACAGCAGAAC---
AATTTG---CTGAGA---GCTATTGAGGCGCAACAGCATCTGTTGCAGCTCACAGTCTGGGGCATC
>HQ216397..1
TTCTACTGTAATTCAACACTGCTGTTTAATAGTAGTTGGAAGAATGAGACCAATGCTGACGGGACAGAG----
-------------------------------------------------------------------------
----------------------------
```

GGCAATGATACTATTACACTCCAATGCAGAATAAAACAAATCATAAACAGATGGCAGGAAGTAGGAAAAGCAA
TGTATGCCCCTCCCATCGCAGGACAAATTAACTGTTCATCAAAA---
ATTACAGGATTGCTATTAACAAGAGATGGTGGTAATCATACCGCC----------------------------
----

AACACCGAGACCTTCAGACCTGGAGGAGGAGATATGAGAGACAATTGGAGAAGTGAATTATATAAATACAAAG
TAGTAAAAATTGAGCCATTA---GGAATAGCGCCCACCAAGGCAAGGAGAAGAGTGGTG---------------
-------------------------CAAAGAGAAAGAAGAGCA---GTGGGAACATTTGGAGCT---------
TTGTTCCTTGGGTTCTTGGGAGCA---GCAGGAAGCACTATG---GGCGCAGCATCA---
GTGACGCTGACGGTACAA---GCCAGACAA---TTATTG---TCTGGTATAGTGCAACAGCAGAAC---
AATCTG---CTGAAG---GCTATTGAGGCGCAACAACAGCTGTTGCAACTCACAGTCTGGGGCATC
>KT124758..1
TTCTACTGCAACACAACACAACTGTTTAATAGTACTTGGAATGATACACAACTGTCTAATAGCACTTGGAATA
ATACAAGA---

GGGATAAATGACACTATCACACTCCAATGTAAAATAAAACAAATCATAAACATGTGGCAGAAGGTAGGAAAAG
CAATGTATGCCCCTCCCATCAGTGGACAAATTAGATGTTCATCAAAT---
ATTACAGGGCTGATATTAACAAGAGATGGTGGTAATACTGACAACACC-------------------------
----

ACCCAGCCCGAGATCTTCAGACCTCAAGGGGGAAATATGAAGGACAATTGGAGAAGTGAATTATATAAATATA
AGGTAGTAGCAATTGAACCATTA---GGAATAGCACCCACCAAGGCAAAGAGAAGAGTGGTG-----------
----------------------------CAGAGAGACAAAAGAGCA---GTGGGA---ATGGGAGCT------
---CTGTTCCTTGGGTTCTTGGGAGCA---GCAGGAAGCACTATG---GGCGCAGCGTCA---
ATAACGCTGACGGCACAG---GCCAGACAA---TTATTG---TCTGGTATAGTGCAACAGCAGAAC---
AATTTG---CTGAGG---GCTATTGAGGCGCAACAGCATCTGTTGCAGCTCACAGTCTGGGGCATT
>JX863968..1
TTCTATTGTAATTCAACATCACTGTTTAATAGTACTTGGAGGACAAATGAAACTGCAGACTTAAAT-------
----

ACTACAGAAATTATCACACTCCCATGCAGAATAAAACAAATTATAAACATGTGGCAAGAAGTAGGAAAAGCAA
TATATGCCCCTCCCATCGAAGGAAACATTACATGTGTATCAAAT---
ATTACAGGGCTGCTATTAACAAGAGATGGTGGTAACAATAATCAGAGTAAT----------------------
----

GAGACGACTGAGATCTTTAGACCTGGAGGAGGAGATATGAGGGACAATTGGAGAAGTGAATTATATAAATATA
AAGTAGTAAAAATTGAACCACTA---GGAGTAGCACCCACCAAGGCAAAGAGAAGAGTGGTG-----------
----------------------------CAGAGAGAAAAAAGAGCA---GCGGGA---CTAGGAGCT------
---GTGTTTCTTGGGTTCTTGGGAGCA---GCAGGAAGCACTATG---GGCGCAGCGTCA---
ATAACGCTGACGGCACAG---GCCAGGCAA---TTATTG---TCTGGTATAGTGCAACAGCAAAAT---
AACTTG---CTGAGA---GCTATTGAGGCGCAACAACATCTGTTGCAACTCACAGTCTGGGGCATC
>EF593190..1
TTCTACTGTAATACAACACGATTGTTTAATAGTACTTGGGAGGCTAATGGTAATTGGACTGGAACTGAAGGAA
AT---

GATAATGGCACAGTCACACTTCCATGCAGAATAAAACAAATTATAAACATGTGGCAACAAGTAGGAAAAGCAA
TGTATGCCCCTCCCATTGCAGGAAAGATTACATGTAACTCAAGT---
ATTACAGGAATACTATTAACAAGAGATGGAGGTATCACTAACAACACC-------------------------
----

GAGACCCACACCGAGACCTTCAGACCTGGAGGAGGAGATATGAGGGACAATTGGAGAAGTGAATTATATAAAT
ATAAAGTAGTAAAAATTCAACCATTA---GGAGTAGCACCCACCAAGGCAAGAAGAAGAGTGGTG--------
-------------------------------CAGAGAGAAAAAAGAGCA---GTGGGA---TTAGGAGCT---
------ATGTTCCTTGGGTTCTTGGGAGCA---GCAGGAAGCACTATG---GGCGCAGCGTCA---
GTAACGCTGACGGTACAG---GCCAGACAA---TTATTG---TCTGGTATAGTGCAACAGCAGAAC---
AACTTG---CTGAGG---GCTATTGAGGCGCAACAGCATCTGTTGCAACTCACAGTCTGGGGCATC
>MH060681..1
TTCTACTGTAATTCAACACCACTATTTAATAGTACACACAACAGTACTACTACAAGGTCAAAT----------
----

GAAACTGAACCAATCATACTCCAATGTAGAATAAAACAAATTGTAAACATGTGGCAGGAGGTAGGAAAAGCAA
TGTATGCCCCTCCCATTGCAGGACAAATTAACTGTACATCAAAT---
ATTACAGGGCTACTGTTAACAAGAGATGGTGGCAATAATCAGAGTAGAGAA----------------------
----

AATAATACCGAGATCTTCAGACCTGGAGGAGGAAATATGAAGGACAATTGGAGAAGTGAATTATATAAATATA
AAGTAGTAAAAGTTGAACCATTA---GGAGTAGCACCCACCAAGGCAAAAAGAAGAGTGGTG-----------
----------------------------CAAAGAGAAAAAAGAGCA---GTGGGA---TTCGGAGCT------
---TTGTTCCTTGGGTTCTTAGGAGCA---GCAGGAAGCACTATG---GGCGCAGCGTCA---
ATGACGCTGACGGTACAG---GCCAGACAA---TTATTG---TCTGGTATAGTGCAACAGCAGAAC---
AATCTG---CTGAGG---GCTATTGAGGCGCAACAACATCTGTTGCAACTCACAGTCTGGGGCATC
>MH060990..2
TTCTACTGTAATACAACAAAACTGTTTAATAGTACTTGGAATAGTACTGAGAATTTTGAGGGAGGG-------
----

TCAGATAACAATATCACACTCCCATGCAGGATAAAGCAAATTATAAACATGTGGCAGGAAGTAGGAAAAGCAA
TGTATGCCCCTCCCATCGCAGGACAAATTAGATGTTCATCAAAC---
ATTACAGGGCTGATATTAACAAGAGATGGTGGTAGTCATAATGGTAGT-------------------------
----

AATAATAGTGAGACCTTCAGACCTGGAGGAGGAGATATGAGGGACAATTGGAGAAGTGAACTTTATAAATATA
AAGTAGTAAAAATTGAACCATTA---GGAGTAGCACCCACTAAGGCAAAAAGAAGAGTGGTG-----------
----------------------------CAGAGAGAAAAAAGAGCA---GCA------ATAGGAGCT------
---TTGTTCCTTGGGTTCTTGGGAGCA---GCAGGAAGCACTATG---GGCGCAGCGTCA---
GTAACGCTGACGGTACAG---GCCAGACTA---TTGTTG---TCTGGTATAGTGCAACAGCAGAGC---
AATCTG---CTGAAA---GCTATTGAGGCGCAACAGCATCTGTTGCAACTCACAGTCTGGGGCATC
>DQ410526..1
TTCTATTGTAATACAACACCACTGTTTAATAATACTTGGTTTAATAGTACTAATAATTTAAATGAAACAAAGG
AC---

AACAACAACAACACAATCATACTCCAATGCAGAATAAAACAAATTATAAACATGTGGCAGGAAGTAGGAAAAG
CAATGTATGCCCCTCCCATCAGGGGAAACATTAGCTGTTCATCAAAT---
ATTACAGGACTATTATTAACAAGAGACGGTGGTCATAATGTGAGC----------------------------
----

AACACGAACGAGACTTTCAGACCTGGAGGAGGAGATATGAGGGACAATTGGAGAAGTGAATTATATAAATATA
AAGTAGTAAGAATTGAACCATTA---GGAATAGCACCCACCAAAGCAAAGAGAAGAGTGGTG-----------
----------------------------CAAAGAGAAAAAAGAGCA---GTGGGA---TTAGGAGCT------
---GTGTTCATTGGGTTTCTGGGAGCA---GCAGGAAGCACTATG---GGCGCAGCGTCA---
ATGACGCTGACGGTACAG---GCCAGACAA---TTATTG---TCTGGTATAGTGCAGCAGCAGAAC---
AATCTG---CTGAGA---GCTATTGAGGCGCAACAGCATCTGTTGCAACTCACAGTCTGGGGCATT
>EF363127..1
TTCTACTGTAATACAACAGACTTGTTTACTTGGAATGGTACTTGGAATGGTACTGCAGGGGCA----------
----

GATAACAATACAATCACACTCCAATGCAGAATAAGACAAATTATAAACATGTGGCAGGAAGTAGGAAAAGCAA
TGTATGCCCCTCCCATCCCAGGATACCTTAACTGTTCATCAAAT---
ATTACAGGACTGCTATTAACAAGGGATGGTGGTCATAACACGAAT----------------------------
----

ACTACTGAGATCTTCAGACCTGGAGGAGGAGATATGAGGGACAATTGGAGAAGTGAATTATATAAATATAAAG
TAGTAAAAATTGAACCATTA---GGAATAGCACCCACCAAGGCAAGGAGAAGAGTGGTG--------------
-------------------------CAGAGAGAAAAAAGAGCA---GTGGGA---ATAGGAGCT---------
GTGTTCCTTGGGTTCTTGGGAGCA---GCAGGAAGCACTATG---GGCGCAGCGTCA---
ATAACGCTGACGGTACAG---GCCAGGCTA---TTATTG---TCTGGTATAGTGCAACAGCAAAAC---
AACTTG---CTGAGA---GCTATTGAGGCGCAACAGCATCTGTTGCAACTCACAGTCTGGGGCATC
>DQ410205..1
TTCTACTGTAATACATCACAACTGTTTAATAGTAATTGGACTTGGCCTGATACTGAAGGGTCAAATGACACTA
AC---

ACTAACGAAAATATCACACTCCCATGCAGAATAAAACAAATTATAAACATGTGGCAGGAGGTAGGAAAAGCAA
TGTATGCCCCTCCCATCAGAGGACAAATTAGCTGTAAATCAAAT---
ATTACAGGACTGCTATTGACAAGAGATGGTGGTCAGGACGAGAAC----------------------------
----

GGGACCGAGACCTTCAGACCTGGAGGAGGAGATATGAGGGACAATTGGAGAAGTGAATTATATAAATATAAAG
TAGTAAAAATTGAACCATTA---GGAGTAGCACCCACCAAGGCAAAGAGAAGAGTGGTG--------------
-------------------------CAGAGAGAAAAAAGAGCA---TTAGGA---ATAGGAGCC---------
GTGTTCCTTGGGTTCTTGGGAGCA---GCAGGAAGCACTATG---GGCGCAGCGTCA---
ATGACGCTGACGGTACAA---GCCAGACTG---CTATTG---TCTGGTATAGTGCAACAGCAGAAC---
AATTTG---CTGAGG---GCTATTGAGGCGCAACAGCATCTGTTGCAACTCACAGTCTGGGGCATC
>FJ469733..1
TTCTACTGTAATTCAACACAACTGTTTAATAGTACTTGGTATACTAATAATACTATGGAGGGGTCACATGGTA
AT---

GAGACAAATGGCACTATCACTCTCCCATGCAAAATAAGACAAATTGTAAACAAGTGGCAAGAAGTAGGAAAAG
CAATGTATGCCCCTCCCATTAGAGGACTAATTAGATGTTCATCAAAT---
ATTACAGGGCTGCTATTAACAAGAGATGGTGGTCATAATAATAATAAT-------------------------
----

AATACTAATGAGACCTTCAGACCTGGAGGAGGAGATATGAGGGACAATTGGAGAAGTGAATTATATAAATACA
AAGTAGTAAGAATTGAGCCATTA---GGAGTAGCACCCACCAAGGCACAGAGAAGAGTGGTG-----------
----------------------------CAAAGAGAAAAAAGAGCA---GTGGGA---CTAGGAGCT------
---GTGTTCCTTGGGTTCTTGGGAGCA---GCAGGAAGCACTATG---GGCGCAGCGTCA---
ATAACGCTGACGGCACAG---GCCAGACAA---TTATTG---TCTGGTATAGTGCAACAGCAGAAC---
AACCTG---CTGAGA---GCTATTGAGGCGCAACAGCATATGTTGCAACTCACAGTCTGGGGCATA
>JQ403107..1
TTCTACTGTGATACAACACAACTGTTTAATAGCAGCTGGGAGGGGAATGGTACTAAAGATACTATAGAGTCAA
ATGAC--

ACTGGAGGAAATATCACACTCCCATGTAGAATAAAACAGATTATAAACATGTGGCAGGAAGTAGGAAAAGCAA
TGTATGCCCCTCCCATCAGTGGACCAATTAGCTGTTCATCAAAC---
ATTACAGGGCTACTATTAACAAGAGATGGGGGTCATAACAATGACACT-------------------------
----

AACGGGAACGAGACCTTCAGACCTGGAGGAGGAAATATGAAGGACAATTGGAGAAATGAATTATATAAATATA
AAGTAGTAAAAATTGAACCACTA---GGAGTAGCACCCACCAAGGCAAAGAGAAGAGTGGTG-----------
----------------------------CAGAGAGAAAAAAGAGCA---ATGGGA---CTAGGAGCT------
---ATGTTCATTGGGTTCTTGGGAGCA---GCAGGAAGCACTATG---GGCGCAGCGTCA---
GTGACGCTGACGGTACAG---GCCAGACAA---TTACTG---TCTGGTATAGTGCGCCAGCAGAAC---
AATCTG---CTGAGA---GCTATTGAGGCGCAACAGCATATGTTGCAACTCACAGTCTGGGGCATC
>JN024100..1
TTCTACTGTAATACAACAAAACTGTTTAATAGTACTTGGAATAGTACTGAGAATGTGGAGGAAGGG-------
----

CCAGATAACAATATCACACTCCCATGCAGGATAAAGCAAATTATAAACATGTGGCAGGAAGTAGGAAAAGCAA
TGTATGCCCCTCCCATCGCAGGACAAATTAGATGTTCATCAAAT---
ATTACAGGGCTGATATTAACAAGAGATGGTGGTGAGAATAACAAC----------------------------
----

GAGACCGAGACCTTCAGACCTGGAGGAGGAGATATGAGGGACAATTGGAGAAGTGAACTTTATAAATATAAAG
TAGTAAAAATTGAACCATTA---GGAGTAGCACCCACTAAGGCAAAAAGAAGAGTGGTG--------------
-------------------------CAGAGAGAAAAAAGAGCA---GCA------ATAGGAGCT---------
TTGTTCCTTGGTTTCTTGGGAGCA---GCAGGAAGCACTATG---GGCGCAGCGTCA---
ATAACGCTGACGGTACAG---GCCAGACTA---TTGTTG---TCTGGTATAGTGCAACAGCAGAGC---
AATCTG---CTGAAA---GCTATTGAGGCGCAACAGCATCTGTTGCAACTCACAGTCTGGGGCATC
>HQ216601..2
TTCTACTGTGATACAACACAGCTGTTTAATAGTACTTGGAATATTGAGAATGATACGAACAGGTCAAAT----
----

GAAAGTGAAACAATTATACTTCCATGCAAAATAAAGCAAATTATAAACCTGTGGCAGGAAGTAGGAAAAGCAA
TGTATGCCCCTCCCATCAGAGATCAAATTAGATGCTCATCAAAT---
ATTACAGGGCTGCTATTAACAAGAGATGGTGGTAATAATACAACTGAG-------------------------
----

AACAATAACGAGACCTTCAGACCTGGAGGAGGAGATATGAGGGACAATTGGAGAAGTGAACTTTATAAATATA
AAGTAGTAAAAATTGAACCATTA---GGAATAGCACCCACTACTGCAAAAAGAAGAGTGGTG-----------
----------------------------CAGAGAGAAAAAAGAGCG---GCA------CTAGGAGCC------
---TTGTTCCTTGGGTTCTTGGGAGCA---GCAGGAAGCACTATG---GGCGCAGCGTCA---
ATAACGCTGACGGTACAG---GCCAGACTA---TTGTTG---TCTGGTATAGTGCAACAGCAGAAC---
AATCTG---CTGAAG---GCTATTGAGGCGCAACAGCATCTGTTGCAACTCACAGTCTGGGGCATC
>HQ216795..1
TTCTACTGTAACACATCACAGCTGTTTGATAGTACTTGGCTGTTTAATAAGACTGGGGATAATAATACTGAAG
GG---

TCAAATGACACTATCATACTTCCATGCAGAATAAAACAAATTATAAACAGGTGGCAGGAAGTAGGAAAGGCAA
TGTATGCCCCTCCCATCGCAGGACAAATTAGATGTCAATCAAAT---
ATTACAGGGGTGCTACTAACAAGAGATGGGGGTAAGAGTGGGAACGGT-------------------------
----

ACTAATGAGACCGAAACATTCAGACCTGTTGGAGGAAATATGAAGGACAATTGGAGAAGTGAATTGTATAAAT
ATAAGGTAGTAAAGATTGAACCATTA---GGAGTAGCACCCACCAAGGCAAAGAGGCTAGTGGTG--------
-------------------------------CAGAGAGAAAAAAGAGCA---GTGACG---CTGGGAGCT---
------ATGTTCATTGGGTTCTTGGGAGCA---GCAGGAAGCACTATG---GGCGCAGCGTCA---
CTGACGCTGACGGTACAG---GCCAGACAA---TTACTG---TCTGGTATAGTGCAACAGCAGAAC---
AATCTG---CTGAGA---GCTATTGAGGCGCAACAGCATCTGTTGCAACTCACAGTTTGGGGCATT
>JX863965..1
TTCTACTGTAATTCAGCACAACTGTTTAATAGTACTTGGGATAATACTGCTGGGAATAATACAGCTGGA----
----

GAAAATGACACAATCACACTCCCATGCAGAATAAAACAAATTGTAAATAGGTGGCAAGAAGTAGGAAAAGCAA
TGTACGCCCCTCCCATCAGCGGACCAATTAAATGTTCATCAAAT---
ATTACAGGACTGCTATTAACAAGAGATGGTGGTCAAAATACAACT----------------------------
----

AATAACACCGAAATTTTCAGACCAGGAGGAGGAGATATGAGGGACAATTGGAGAAGTGAATTATATAAATATA
AAGTAGTAAAAATTGAACCATTA---GGAGTAGCACCCACTAAGGCAAAGAGAAGAGTGGTG-----------
----------------------------CAGAGAGAAAAAAGAGCA---GTGGGA---ATAGGAGCT------
---ATGTTCCTTGGGTTCTTGGGAGCA---GCAGGAAGCACTATG---GGCGCAGCATCA---
ATAACGCTGACGGTACAG---GCCAGACAA---TTATTG---TCTGGTATAGTGCAACAGCAGAAC---
AATCTG---CTGAGA---GCTATTGAGGCGCAACAGCATCTGTTGCAACTCACAGTCTGGGGCATT
>JF320043..1
TTTTACTGTAATACAACAAAACTATTTAATAGCACTTGGGAGCCTAATAATGTAACTGTCACTGGT-------
----

GATAATGACACAATCACACTCCCATGCAGAATAAAACAAATTGTAAACTTGTGGCAGGAAGTAGGAAGAGCAA
TGTATGCCGCTCCCATCAGAGGACTGATCAGATGTTCATCAAAT---
ATTACAGGGCTACTATTAACAAGAGATGGTGGTGTTGATAACAATCAGACCAAT-------------------
----

GTGACTAATAACACAGAAACCTTCAGACCAGGAGGAGGAGATATGAGGGACAATTGGAGAAGTGAATTATATA
AATATAAAGTAGTAAAAATTGAACCATTA---GGAGTAGCACCCACCAAGGCAAAGAGAAGAGTGGTG-----
----------------------------------CAGAGAGAAAAAAGAGCA---ATGGGA---
TTCGGAGCT---------TTTTTCCTTGGGTTCTTGGGAGCA---GCAGGAAGCACTATG---
GGCGCAGCGTCA---ATAACGCTGACGGCACAG---GCCAGACAA---TTATTG---
TCTGGTATAGTGCAACAGCAAAAC---AATTTG---CTGAGG---
GCTATTGAGGCGCAACAACACCTGTTGCAACTCACAGTCTGGGGCATC
>EU577873..1
TTCTACTGTAATACAACAAAACTGTTTAATAGTACTTGGAATACTACTAGTATTTGGAATAATACTGCAGAGA
ATAATACTACA--

-------------GGGTCA------
AATGAAACTGAACCAATCACACTCCCATGCAGAATAAAACAAATTATAAATATGTGGCAGGAAGTAGGAAAAG
CAATGTATGCCCCTCCCATTAAAGGAAACATTAGCTGTTCATCAAAT---
ATTACAGGATTGATATTAACAAGAGATGGTGGTAACGTTAACGGGACC-------------------------
----

AATGGAACTGAAATCTTTAGACCTGCAGGAGGAAATATGAAGGACAATTGGAGGAGTGAATTATATAAATATA
AAGTAGTAAAAATTGAGCCATTA---GGAGTAGCACCCACCAAGGCAAAGAGAAGAGTGGTG-----------
----------------------------CAGAGAGAAAAAAGAGCA---GTGGGA---TTCGGAGCT------
---GTGTTCCTTGGGTTCTTGGGAGCA---GCAGGAAGCACTATG---GGCGCAGCGTCG---
GTGACGCTGACGGTACAG---GCCAGACAA---CTATTG---TCTGGTATAGTGCAACAGCAAAAC---
AATTTG---CTGAGA---GCTATTGAGGCGCAACATCATCTGTTGCAACTCACCGTTTGGGGCATC
>KT124778..1
TTCTACTGTAATACGTCACAACTGTTTAATAGTACTTGGCTGGGGAATGACACTGAAAGGTTAAACAGAACTG
AAGGGTCA---

GACAGCACTGAAGTAATCACACTCCAGTGTAGAATAAAACAAATTATAAACATGTGGCAGGAAGTAGGAAAAG
CAATGTATGCCCCTCCCATCGCAGGACAAATTAACTGTTCATCAAAT---
ATTACGGGGCTACTATTAACAAGAGATGGTGGTTACAATGATACT----------------------------
----

AGCCAGAACGAGACCTTCAGACCTGGGGGAGGAGATATGAGGGACAATTGGAGAAGTGAATTATATAAATATA
AAGTAGTAAGAATTGAACCATTA---GGAGTAGCCCCCACCAAGGCAAAGAGAAGAGTGGTG-----------
----------------------------CAAAGAGAAAGAAGAGCA---GTGGGA---TTAGGAGCT------
---GTGTTCCTTGGGTTCTTGGGAGCA---GCAGGAAGCACTATG---GGCGCAGCGTCA---
GTGACGCTGACGGTACAG---GCCAGACAA---TTATTG---TCTGGTATAGTGCAGCAGCAAAAC---
AATTTG---CTGAAG---GCTATTGAGGCGCAACACCATCTGTTGCAACTCACAGTCTGGGGCATC
>DQ410562..2
TTCTACTGTAATACAACACTGCTGTTTGATAGTACTTGGGATGATAATAATAGAAGTTTGAATTTGAATAAT-
----

ACTAATAGAAACATCACACTCCCATGCAAGATAAAACAAATTATAAACAGGTGGCAGGAAGTGGGAAAAGCAA
TGTATGCCCCTCCCATTCAAGGAGTAATTCAATGTTCATCAAAT---
ATTACAGGGCTGTTATTAGTAAGAGATGGTGGCAACACTGCTAACAATACT----------------------
----

GCTAACAAAACTGAGACCTTCAGACCTGGAGGAGGAAATATGAAGGACAATTGGAGAAGTGAATTATATAAAT
ATAAAGTAGTAAAAATTGAACCATTA---GGAGTGGCACCCACCAAGGCAAAGAGGAGAGTGGTG--------
-------------------------------CAGAGGGAAAAAAGAGCA---GTGGGA---ATAGGAGCT---
------ATGTTCCTTGGATTCTTAGGAGCA---GCAGGAAGCACTATG---GGCGCAGCGTCA---
ATAACGCTGACGGTACAG---GCCAGGCAA---TTATTG---TCTGGTATAGTGCAACAGCAGAAC---
AATCTG---CTGAGA---GCTATTGAGGCGCAACAGCATCTGTTGCAACTCACAGTCTGGGGCATC
>FJ469717..1
TTCTACTGTAATACAACACAGCTGTTTAATAGTACTTGGTTTACTAATGGCACTTGGATTAATGGTACTGTGT
TAAATACA---
-------------AATGAT------
ACTGACAAGGGAAACATCACACTCCCATGTAAAATAAAACAAATTATAAACATGTGGCAGGAAGTAGGAAAAG
CAATATATGCCCCTCCCATCAAAGGACCAATTAACTGTACATCAAAT---
ATTACAGGGATAATAATAACAAGAGATGGTGGTAATAACACGGGCGAAAATAAA-------------------
----

AATAAAACTTTCACTGAAACTTTCAGACCTGGAGGAGGAAATATGAGGGATAATTGGAAAAGTGAACTATATA
AATATAAAGTAGTACAGATTCAACCAATA---GAAATAGCACCCACCAGGGCAAAGAGAAGAGTGGTG-----
-----------------------------------CAGAGAGAAAAAAGAGCA---
GTGGGAGCGCTGGGAGCT---------GTGTTCCTTGGGTTCTTGGGAGCA---GCAGGAAGCACTATG---
GGCGCAGCGTCA---ATAACGCTGACGGTACAG---GCCAGACAA---TTATTG---
TCTGGTATAGTGCAACAGCAGAAC---AATCTG---CTGAAG---
GCTATTGAGGCGCAACAGCATCTGTTGCAACTCACAGTCTGGGGCATC
>FJ469724..1

TTCTACTGTAATACAACACAACTGTTTAATAGTACTTGGAATAATGATACTTGGAATGATAATAAAACTGAA-
----

GGAAATGGCACAATTACACTCCAATGCAGAATAAAACAATTTATAAACCTGTGGCAGAAAGTAGGAAAAGCAA
TATATGCCCCTCCCATCCAAGGAAATATTAGCTGTTCATCAAAT---
ATTACAGGGCTGCTATTAACAAGGGATGGTGGTGTTAACGGGAGC----------------------------
----

GAGCACAACGAGACACTCAGACCTGGAGGAGGAGATATAAAAGACAATTGGAGAAGTGAATTATATCAATATA
AAGTAGTAAAAATTGAACCATTA---GGAATAGCACCCACTGCAGCAAAGAGAAGAGTGGTG----------
----------------------------CAGAGAGAAAAAAGAGCA---GCAGGA---ATAGGAGCT------
---CTGCTCCTTGGGTTCTTGTCAGCA---GCAGGAAGCACTATG---GGCGCAGTGTCA---
GTGACGCTGACGGTACAG---GCCAGACAA---TTATTG---TCTGGTATAGTGCAACAGCAAAAC---
AATTTG---CTGAGA---GCTATTGAGGCGCAACAGCATCTGTTGCAACTCACGGTCTGGGGCATT
>FJ653159..1
TTCTATTGTAATACAACACAACTGTTTAATAGTAATTGGAATGAGAGTAGTATTAATAGTACTTGGAATAGTA
CTTGGAAG---
-------------TCAAAT------
GACCCAAATGACACCATCACACTCCCATGCAGAATAAAACAAATTATAAACATGTGGCAAGAAGTGGGAAAAG
CAATGTATGCCCCTCCCATCAAAGGTGTAATTCAGTGTTCATCAAAT---
ATTACAGGGCTGCTATTAACAAGAGATGGTGGGAGTAATGAGACT----------------------------
----

AATACCACCGAGACTTTCAGACCTGGGGGAGGAGATATGAGGGACAATTGGAGAAGTGAACTATACAAATATA
AAGTAGTAAAAATTGAACCATTA---GGAGTAGCACCCACCAAGGCAAAAAGAAGAGTGGTG----------
----------------------------CAAAGAGAAAAAAGAGCA---GTGGGAACAATAGGAGCT------
---ATGTTCCTTGGGTTCTTGGGAGCA---GCAGGAAGCACTATG---GGCGCAGCGTCA---
GTGACGCTGACGGTACAA---GCCAGACAA---TTATTG---TCTGGTATAGTGCAGCAGCAGAGC---
AATTTG---CTGCAG---GCTATTAATGCACAACAACATTTGCTGCAACTAACAGTCTGGGGCATC
>FJ653573..1
TTTTACTGTAATACAACACAACTGTTTAATAGTACTTGGAATATGACTGGTACCTGGAATGATACCTGGAATG
GTACTAAA---
-------------AGGTCA------
AATGAAACTGACAATATCACACTCCCATGCAGAATAAAACAAATAGTAAACAGGTGGCAGGAAGTAGGCAAAG
CAATGTATGCCCCTCCCATCAGAGGAAGCATTAGATGTTCATCAAAT---
ATTACAGGGCTGCTATTAACAAGAGATGGGGGGATTCATAATAGCACTGAG----------------------
----

GCAAATAACACTGAGATTTTCAGACCTGGAGGAGGAGACATGAGGGACAATTGGAGAAGTGAATTATATAAGT
ATAAAGTAGTGAAAATTGAACCATTA---GGAGTAGCACCCACCAAGGCAAGGAGAAGAGTGGTG--------
-------------------------------CAGAGAGAAAAAAGAGCA---GTGGGAGCACTAGGAGCT---
------ATGTTCCTTGGGTTCTTGGGAGCA---GCAGGAAGCACTATG---GGCGCAGCGTCA---
CTGACGCTGACGGTACAG---GCCAGACAA---TTGTTG---TCTGGTATAGTGCAACAGCAGAAC---
AACCTG---CTGAGA---GCTATTGAGGCGCAACAGCATCTGTTGCAACTCACAGTCTGGGGCATC
>EU578629..1
TTCTACTGCAATACAACAAAACTGTTCAGTAGTACTTGGAATAGTACTACTTGGGATAGTTGGAATAGTACTG
AAACA--
-------------------AAT---
AGCACTGACAGAAATATCACACTCCCATGCAGAATAAAACAAATTATAAACATGTGGCAGGAAGTAGGAAGAG
CAATGTATGCCCCTCCCATCAGAGGACAAATTAGATGCTCATCAAAT---
ATTACAGGGCTGATATTAACAAGAGATGGTGGTAACAATACAAAT----------------------------
----

ACAAACGAGACCTTCAGACCTCAAGGAGGAAACATGAAGGACAATTGGAGAAGTGAATTATATAAATATAAAG
TAGTAAAAATTGAACCACTA---GGAATAGCACCCACCAAGGCAAAGAGAAGAGTGGTG--------------
-------------------------CAGAGAGAAAAAAGAGCA---GTGGGA---ATGGGAGCT---------
TTGTTCCTTGGGTTCTTGGGAGCA---GCAGGAAGCACTATG---GGCGCAGTGTCG---
ATGACGCTGACGGTACAG---GCCAGACAA---TTATTG---TCTGGTATAGTGCAACAGCAGAAT---
AATCTG---CTGAGA---GCTATTGAGGCGCAACAGCATCTGTTGCAACTCACAGTCTGGGGCATC
>HQ217860..1

TTCTACTGTAATACAACAAAACTATTTAATAGTACTTGGGATATAGAAACAATACTTAAGCATACTAAAAATA
GTAAT--

GACACAAACTCAACTATTACACTCCAATGCAGAATAAAACAAATTGTAAACATGTGGCAGGAAGTAGGAAAAG
CAATGTACGCCCCTCCCATCAGAGGAATAATTAACTGTACATCAAAT---
ATTACAGGGCTGCTATTAACAAGAGATGGGGGTCATAACAACAGC----------------------------
----

ACCAACGAGACCTTCAGACCGGGAGGAGGAGATATGAGAGACAATTGGAGAAGTGAATTATACAAATATAAAG
TAGTAAAAGTTGAACCATTA---GGAATAGCACCCACCAAGGCAAAAAGAAGAGTGGTG--------------
-------------------------CAAAGAGAGAAAAGAGCA---GTGGGA---ATAGGAGCT---------
GTGTTCCTTGGGTTCTTGGGAGCA---GCAGGAAGCACTATG---GGCGCAGCGACA---
GTGACGCTGACGGTACAG---GCCAGACAA---TTATTG---TCTGGTATAGTGCAACAGCAGAGC---
AATTTG---CTGAGA---GCTATTGAGGCGCAACAGCATCTGTTGCAGCTCACAGTCTGGGGCATC
>JF689865..1
TTCTACTGTAATACAACAAAACTGTTTAATAGTACTTGGCCTTGGAATGTTACTGGGTCAAATGTTACTGGG-
----

TTAAATGACACAATCACAATTCCATGCAGAATAAAACAAATTATAAACATGTGGCAGGAAGTAGGAAAAGCAA
TGTATGCCCCTCCCATCCAAGGACAAATTAGATGTTCATCAAAT---
ATTACAGGGCTACTATTAACCAGAGATGGTGGTAATACAAATGAGAGC-------------------------
----

AATACCACCGAGACCTTCAGACCTGGAGGAGGAGATATGAGGGACAATTGGAGAAGTGAATTATATAAATATA
AAGTAGTAAAAATTGAACCATTA---GGAGTAGCACCCACCAAGGCAAAGAGAAGAGTGGTG-----------
----------------------------CAGAGAGAAAAAAGAGCA---GTGGGA---CTAGGAGCT------
---CTGTTCCTTGGGTTCTTGGGAGCA---GCAGGAAGCACTATG---GGCGCAGCGTCA---
ATGACGCTGACGGTACAA---GCCAGGCAA---TTATTG---TCTGGTATAGTGCAACAGCAAAAC---
AATTTG---CTGAGG---GCTATTGAAGCGCAACATCACCTGTTGCAACTCACAGTCTGGGGCATT
>JF689874..1
TTCTACTGTAATACAACAAAGCTGTTTACAAAGCGGTTTGATTGGAATAGGACTGAAGGGTTAAATGACACTG
CAGGAAAT---
-------------------AAC---
ACTGGAGGAAATAACATCATAATCCCATGCAGAATAAAACAAATTATAAACATGTGGCAAGAAGTAGGAAAAG
CAATATATGCCCCTCCCATCAGTGGACGAATTAACTGTACATCAAAC---
ATTACTGGGCTGCTATTAATAAGAGATGGTGGGATAGGCCCGGGGACA-------------------------
----

GACAATACTAGTGAAACCTTCAGACCTCAAGGAGGGAATATAAGAGATAATTGGAGAAGTGAATTATATAAAT
ATAAAGTAGTAAAAATTGAACCATTA---GGACTAGCGCCCACCAAGGCAAGGAGAAGAGTGGTG--------
-------------------------------CAGAGAGAAAAAAGAGCA---ATGGGA---CTAGGAGCT---
------TTGTTCCTTGGGTTCTTGGGAGCA---GCAGGAAGCACTATG---GGCGCAGCGTCA---
CTGACGCTGACGGTACAG---GCCAGACAA---TTATTG---TCTGGTATAGTGCAACAGCAGAAC---
AATTTG---CTGAGA---GCTATTGAGGCGCAACAGCATATGTTGCAACTCACAGTCTGGGGCATC
>JF689875..1
TTCTACTGTAATACAACACAACTGTTTAATAGTACTTGGAATGAGAATAGTACTAAAGAAGGAAATGGA----
----

AATGACACAATAACCACACTCCCATGCAGAATAAAACAAATTATAAACATGTGGCAAGAAGTAGGAAAAGCAA
TGTATGCCCCTCCCATCAGAGGAAACATTAGCTGTACATCAAAT---
ATTACAGGGCTGCTATTAACAAGAGATGGTGGGAATAAT----------------------------------
----

GAGACCGAGACCTTCAGACCTGGAGGAGGAAATATGAAGGATAATTGGAGAAGTGAATTATATAAATATAAAG
TAGTAAAAATTCAACCATTA---GGAGTAGCACCCACCAAGGCAAAGAGAAGAGTGGTG--------------
-------------------------CAGAGAGAAAAAAGAGCA---GTGGGA---CTAGGAGCT---------
GTGTTCCTTGGGTTCTTGGGAGCA---GCAGGAAGCACTATG---GGCGCAGCGTCA---
ATAACGCTGACGGTACAG---GCCAGACAA---TTATTG---TCTGGTATAGTGCAACAGCAGAAC---
AATTTG---CTGAGG---GCTATTGAGGCGCAACAGCATCTGTTGCAACTCACAGTCTGGGGCATT
>JX863990..1

TTCTACTGTAATACAACACAACTGTTTAATAGTACTTGGAATTCTACTACTTTGAATTCTACTAGTACT----
----

GGGAATGGGACCATCACACTCTCATGTAGAATAAAACAAATTATAAACAGGTGGCAGGAAGTAGGAAAAGCAA
TGTATGCTCCTCCCATCAGAGGACAAATTAAGTGTTCATCAAAT---
ATTACAGGGCTACTGTTAACAAGAGATGGTGGTCACACAAACAACACC-------------------------
----

AACAAAACTGAGACCTTTAGACCTGGAGGAGGAGACATGAGGGACAATTGGAGAAGTGAATTATACAAATATA
AAGTAGTAAAAATTGAACCATTA---GGAATAGCACCCACCAAGGCAAGGAGAAGAGTGGTG-----------
----------------------------CAGAGAGAAAAAAGAGCA---ATAGGA---ATAGGAGCT------
---GTGTTCCTTGGGTTCTTGGGAACA---GCAGGAAGCACTATG---GGCGCAGCGGCA---
GTGACGCTGACGGTACAA---GCCAGACAA---TTACTG---TCTGGTATAGTGCAACAGCAAAAC---
AATTTG---CTGAGG---GCTATTGAGGCGCAGCAGCATCTGTTGCAACTCACAGTCTGGGGCATC
>JF320363..1
TTCTACTGTAACACAGCAAAACTGTTTGATACATCACAACTACAACTGTCTAATAGCAGCACTGAA-------
----

GAAAATGACACACTCACAATTCCATGCAGAATAAAACAAGTTATAAACAGGTGGCAGGAAGTAGGAAAAGCAA
TGTATGCCCCTCCCATCAGAGGACAAATTAAATGTGTATCAAAC---
ATTACAGGGCTGCTATTAACAAGAGATGGTGGAAAGACTGAGGATAATACT----------------------
----

ACTAATACTACTGAGGTCTTCAGACCTCAAGGAGGAAATATGAAAGATAATTGGAGAAGTGAATTATATAAAT
ATAAGGTAGTAAGAACTGAACCATTA---GGAGTAGCACCCACCAAGGCAAAGAGAAGAGTGGTG--------
-------------------------------CAGAGAGAAAAAAGAGCA---ATAGGGGGATTAGGAGCG---
------TTGTTCCTTGGGTTCTTGGGAGCA---GCAGGAAGCACTATG---GGCGCAGCGTCA---
ATAACGCTGACGGCACAG---GCCAGACAA---ATATTG---TCTGGTATAGTGCAACAGCAGAAT---
AATTTG---CTGAGA---GCTATTGAGGCGCAACAACATCTGTTGCAACTCACAGTCTGGGGCATC
>JF320036..1
TTCTACTGTAATACAACACAGCTGTTTAATATTAGTAGTACACAACTGTTTAATAGTACTTGGAATAGTACCG
AA---
-------------------GGG---
CTAAACACAAAAAAATATCACACTCCAATGCAGAATAAAACAAATTATAAACATGTGGCAGCAAGTAGGAAAAG
CAATGTATGCCCCTCCCATCAGAGGACCAATTAAATGTATATCAAAT---
ATTACAGGGCTGCTGTTAACAAGAGATGGTGGGAAGATAAATGAGAGTAGG----------------------
----

GCAAATGAGACTGAGACCTTCAGACCTGGAGGAGGAGATATGAGGGACAATTGGAGAAGTGAACTCTATAAAT
ATAAAGTAGTAAAAATTAAACCATTA---GGAGTAGCACCCACTAAGGCAAAAAGAAGAGTGGTG--------
-------------------------------CAGAGAGAAAAAAGAGCA---GTGGGA---CTAGGAGCA---
------TTGTTCCTTGGGTTCTTGGGAGCA---GCAGGAAGCACTATG---GGCGCAGCGTCA---
ATAACGCTGACGGTACAG---GCCAGACAA---TTATTG---TCTGGTATAGTGCAACAGCAGAAC---
AATCTG---CTGAGG---GCTATTGAGGCGCAACAGCACCTGTTGCAACTCACAGTCTGGGGCATC
>JF320502..1
TTCTACTGTAATACAACAAAACTGTTTAATAGCATTTGGAATGAAACACAACTGTTTAATAGTACTTGGAATG
AGACT--

GAAAGAGGAGTCAATATCACACTCCCATGTAGAATAAAACAAATTATAAATATGTGGCAGGAAGTAGGAAAAG
CAATGTATGCCCCTCCCATCAGAGGAATAATTCAATGTACATCAAAC---
ATTACAGGACTGATATTAACAAGAGATGGTGGTAAAAAAAATAATGGGAGTGAA-------------------
----

ACCTCCAATGGGAATGAAACCTTCAGACCTGGAGGAGGAGATATGAGAGACAATTGGAGAAGTGAATTATATA
AATATAAAGTAGTAAAAGTTGAACCATTA---GGAATAGCACCCACTAGGGCAAGGAGAAGAGTGGTG-----
----------------------------------CAGAGAGAGAAAAGAGCA---GTGGGA---
ATAGGAGCT---------GTGTTCCTTGGGTTCTTGGGAGCA---GCAGGAAGCACTATG---
GGCGCAGCGTCA---ATAACGCTGACGGTACAG---GCCAGACAA---TTATTG---
TCTGGTATAGTGCAACAGCAGAAC---AATCTG---CTGAAA---
GCTATTGATGCGCAACAGCATCTGTTGCAACTCACAGTCTGGGGCATC

>JF320151..2
TTCTACTGTAATACAACAGAGCTGTTTAATGATACTTGGCTGAGTAATAATACTTGGCTGTCAAATTATACTG
AGAGTAAT---
-------------------AAT---
AATACTGCTAAAAATATCGTACTCCCATGCAGAATAAAACAAATTATAAACAGATGGCAGGAAGTAGGAAAAG
CAATGTATGCCCCTCCCATCAGTGGACTAATTAGATGCTCATCAAAT---
ATTACAGGGCTGCTCTTAACAAGAGATGGTGGTAATCATAACACTAGT-------------------------
----

AATGACACCGAGACTTTCAGACCAGGAGGAGGAGATATGAGGGATAATTGGAGAAGTGAATTATATAAATATA
AAGTAGTAAAAATTGAGCCATTA---GGAGTAGCACCCACCAAGGCAAGGAGAAGAGTGGTG-----------
----------------------------CAGAGAGAAAAAAGAGCA---GTGGGA---CTAGGAGCT------
---TTGTTCCTTGGGTTCTTGGGAGCA---GCAGGAAGCACTATG---GGCGCAGCATCA---
GTGACGCTGACGGTACAA---GCCAGACAA---TTATTG---TCTGGTATAGTGCAACAGCAAAAC---
AATTTG---CTGAGG---GCTATTGAGGCGCAACAGCATATGTTGCAACTCACAGTCTGGGGCATT
>JF320145..1
TTCTACTGTAATTCAACAAAACTGTTTAGCAGTGCTTGGTATACTAATAATACTAATAGTAGTAATACTGAAG
GG---

TCAAATGACACTGTTATCATACTTCCATGCAAAATAAAACAAATTATAAACATGTGGCAGGAAGTAGGAAGAG
CAATGTATGCCCCTCCCATCGCAGGAAACATTAGCTGTTCATCAAAT---
ATTACAGGACTGCTGTTAACAAGAGATGGTGGTCACACGGAAACTGCC-------------------------
----

AACACCACAGAAATCTTCAGACCTGGAGGAGGAGATATGAGGGACAATTGGAGAAGTGAATTATATAAATATA
AAGTAGTAAAAATTGAACCATTA---GGAGTAGCACCCACCAAGGCAAGGAGAAGAGTGGTG-----------
----------------------------CAGAGAGAAAAAAGAGCA---GTAACA---GTGGGAGCT------
---GTGTTCCTTGGGTTCTTGAGTGCA---GCAGGAAGCACTATG---GGCGCAGCGTCA---
CTGACGCTGACGGTACAG---GCCAGACTA---CTGTTG---TCTGGTATAGTGCAGCAGCAGAGC---
AATCTG---CTGAGG---GCTATTGAGGCGCAACAACAACTGTTGCAACTCACAGTCTGGGGCATC
>HQ908232..2
TTCTACTGTAATACAACAAGGCTGTTTAATAGTACTTGGCAGTCCAATAGTACGGGGAATGATACTGGAGGGT
CA---

AATAACACTGAACCTATCACACTCCTATGCAGAATAAAACAAATTGTAAACAGGTGGCAAGAAGTAGGAAAAG
CAATGTATGCCCCTCCCATCAGAGGACAAATTTACTGTAGATCAAAT---
ATTACAGGGCTGCTATTAACAAGGGATGGTGGTAATGTCCAGAAC----------------------------
----

AACATGACTGAGACCTTCAGACCAGGAGGAGGAGATATGAGGGACAATTGGAGAAGTGAATTATATAAATATA
AAGTAGTAAAAATTCAGCCATTA---GGAATAGCACCCACCGGGGCAAAGAGAAGAGTGGTG-----------
----------------------------CAGAGAGAAAAAAGAGCG---GTGACA---CTGGGAGCT------
---ATGTTCCTTGGGTTCTTGGGAGCA---GCAGGAAGCACTATG---GGCGCAGCGTCA---
ATAACGCTGACGGTACAG---GCCAGACAA---TTACTG---TCTGGTATAGTGCAACAGCAGAAC---
AATCTG---CTGAAG---GCTATTGATGCGCAGCAGCATATGTTGCAACTCACCGTCTGGGGCATC
>KM353665..1
TTCTACTGTAATACAACAAAATTGTTTAATAGTACTTGGTATAATAATAGT----------------------
----
-ACTTGGAATAATACT---------
GGAGGAAATGACACATTCACACTCCCATGTAGAATAAAACAAATTATAAACATGTGGCAGACAGTAGGAAGAG
CAATGTATACCCCTCCCATCAGAGGATTAATTAAGTGTACATCAAAT---
ATTACAGGGCTGATATTAACAAGAGATGGTGGG--

GGTGGTAACGAGAGTAATCAT---------
ACCACAGAGGTCTTCAGACCTGCAGGAGGAGATATGAGGGACAATTGGAGGAGTGAATTATATAAATACAAAG
TAGTAAAAATTGAACCATTA---GGAATAGCACCCACCAGGGCAAAAAGAAGAGTGGTG--------------
-------------------------CAGAGAGAAAAAAGAGCA---GTGGGG---ATGGGAGCT---------
CTGTTCCTTGGGTTCTTAGGAGCA---GCAGGAAGCACTATG---GGCGCAGCGTCA---
ATAACGCTGACGGTACAG---GCCAGACAA---TTATTG---TCTGGTATAGTGCAACAGCAGAGC---
AATTTG---CTGAAG---GCTATTGAGGCGCAACAGCATCTGTTGCAACTCACAGTCTGGGGCATC

```
>JN687750..1
TTCTACTGTGATACAAAAAAGCTGTTTAATAGTACTTGGTGGGTTAATAATACTTGGAAAGGCACTAAAGGGG
ATGAG-------------------------------------------------------------------
-------------------------
ACTAGGAATGGCACAATCGAACTCCCATGCAGAATAAAACAAATTATAAACATGTGGCAGGAAGTAGGAAAAG
CAATGTATGCCCCTCCCATCAGAGGACAAATTCAGTGTTCATCAAAT---
ATTACAGGGCTGCTATTAACAAGAGATGGGGGTAATAATGAAACG---------------------------
------------------------------------------------------------------------
----------
AACGGAACAGAAACCTTCAGACCTGGAGGAGGAGATATGAGGGACAATTGGAGAAGTGAATTATATAAATATA
AAGTAGTAAAAATTGAGCCATTA---GGAATAGCACCCGGCAGGGCAAAGAGAAGAGTGGTG----------
---------------------------CAGAGAGAAAAAAGAGCA---GTGGGA---CTAGGAGCT------
---ATGTTCCTTGGGTTCTTGGGAGCA---GCAGGAAGCACTATG---GGCGCAGCGTCA---
ATAACGCTGACGGTACAG---GCCAGACAA---TTACTG---TCTGGTATAGTGCAACAGCAGAAC---
AACTTG---CTGAGA---GCTATTGAGGCGCAGCAGCATATGTTGCAACTCACAGTCTGGGGCATC
>HM204582..1
TTTTATTGTAATACATCAGAACTGTTTAAAAGTACCTGGAATAGTACTGATACCTTCAACAGTACTGGAGAGA
GA----------------------------------------------------------------------
----------------------------
GGGAATGACACAATCATTCTCCCATGCAAAATAAGGCAAGTTGTAAACATGTGGCAGGAAGTAGGAAAAGCAA
TGTATGCCCCTCCTATCAGAGGTCAAATTAACTGTTCATCAAAT---
ATTACAGGGCTGCTATTAACAAGAGATGGTGGTGATAAAAATAGT---------------------------
------------------------------------------------------------------------
-------------
ACAAACGAGACCTTCAGACCTGGAGGAGGAGATATGAGGGACAATTGGAGAAGTGAATTATATAAATATAAGG
TAGTAAAAATTGAACCATTA---GGAATAGCACCCACCAAGGCAAAGAGAAGAGTGGTG-------------
-------------------------CAAAGAGAAAAAAGAGCA---GTGGGA---ATAGGAGCT--------
TTGTTCCTTGGGTTCTTGGGAGCA---GCAGGAAGCACTATG---GGCGCAGCGTCA---
CTGACGCTGACGGTACAA---GCCAGACTA---TTATTG---TCTGGTATAGTGCAACAGCAGAAC---
AATTTG---CTGAGG---GCTATTGAGGCGCAACAGCATCTGTTGCAACTCACGGTCTGGGGCATC
>HM204589..1
TTCTACTGTAATTCAACACAATTGTTTAATAGTACTTGGGATAAGAATGGTAGTAGAATAAATGGAACTGAAG
GA----------------------------------------------------------------------
----------------------------
GAAAGTGGTACCATCACACTCCCATGCAGAATAAAACAAATTATAAACATGTGGCAGGAAGTAGGAAAAGCAA
TGTATGCCCCTCCCATCAGAGGACAAATTTATTGTTCATCAAAT---
ATTACAGGGATGCTATTAACAAGAGATGGTGGTACTAATGATACTGAC------------------------
------------------------------------------------------------------------
-------
AAAAACGACACCGAGACCTTCAGACCTGGAGGAGGAGATATGAGGGATAATTGGAGAAGTGAATTATATAAAT
ATAAAGTAGTAAAAATTGAGCCATTA---GGAGTAGCACCCACCAAGGCAAAGAGAAGAGTGGTG-------
------------------------------CAGAGAGAAAAAAGAGCA---GTGGGA---TTGGGAGCT---
------CTGTTCCTTGGGTTCTTGGGAGCA---GCAGGAAGCACTATG---GGCGCAGCGTCA---
GTGACGCTGACGGTACAG---GCCAGACAA---TTATTG---TCTGGTATAGTGCAACAGCAGAAC---
AATCTG---CTGAGG---GCTATTGAGGCGCAACAGCATCTGTTGCAACTCACAGTCTGGGGCATC
>KY112061..1
TTCTACTGTAATTCAACACAGCTGTTTAATAGTGCTTGGCCTTGGAATGATACTGAAGGGTCAAATGACGCT-
------------------------------------------------------------------------
----------------------------
AAAAAAGATAATATCACACTCCCATGCAAAATAAAACAAATTATAAACATGTGGCAGGGAGTAGGAAAAGCAA
TGTATGCCCCTCCCATCGAAGGAAAAATTAAATGCTCATCAAAT---
ATTACAGGACTGCTATTAACAAGAGATGGTGGTTATGAGAACAAT---------------------------
------------------------------------------------------------------------
-------
GAGACCAAGCCCGAGATCTTCAGACCTGGAGGAGGAGATATGAGGGACAATTGGAGGAGTGAATTATATAAAT
ATAAAGTAGTAAAAATTGAACCATTG---GGAGTAGCACCTACCAAGGCAAAGAGAAGAGTGGTG-------
------------------------------CAGAGAGAAAAAAGAGCA---TTTGGA---CTAGGAGCT---
------GTGTTCCTTGGGTTCTTGGGAGCA---GCAGGAAGCACTATG---GGCGCAGCATCA---
ATAACGCTGACGGTACAG---GCCAGACAA---TTATTG---TCTGGTATAGTGCAACAGCAGAAC---
AATTTG---CTGAGG---GCTATTGAGGCGCAACAGCATCTGTTGCAACTCACAGTCTGGGGCATC
```

```
>EU576345..1
TTCTACTGTGATACAACAAAACTGTTTAATAGTACTTGGGGGAATAGTACTTGGGGGTATAAAATGACAAATG
AC----------------------------------------------------------------------
------------------------
ACTGCAAAAGACAACATCACACTCCCATGTAGAATAAAACAAATTATAAACATGTGGCAGGAAGTAGGAAAAG
CAATGTATGCCCCTCCCATCAAAGGACAAATTAGATGTACATCAAAT---
ATTACAGGGTTGGTGTTAACAAGAGATGGTGGTGGTGGTAGTGGTACA------------------------
------------------------------------------------------------------------
-------
AATAACACCACAGAGATCTTCAGACCTGGAGGAGGAGATATGAGGGACAATTGGAGAAGTGAATTATATAAAT
ATAAAGTAGTAAGAATTGAACCATTA---GGAATAGCACCCACCAAGGCAAAGAGAAGAGTGGTG-------
------------------------------CAGAGAGAAAAAAGAGCA---GCA------ATAGGAGCT---
------ATGTTCCTTGGGTTCTTGGGAGCA---GCAGGAAGCACTATG---GGCGCAGCGTCA---
ATAACGCTGACGGTACAG---GCCAGACTA---TTATTG---TCTGGTATAGTGCAACAGCAGAAC---
AATCTG---CTGAGG---GCTATTGAGGCGCAACATCAACTGTTGCAACTCACAGTTTGGGGCATC
>EU576383..1
TTCTACTGTAATTCAACACAACTGTTTAACAGTACTTGGCATAGTAACAGTACTTGGAATGATACTGGAAAGT
CAGAA-------------------------------------------------------------------
------------------------
AATAATACAAAAATCATCACACTCCCATGCAGAATAAAACAAATTATAAACATGTGGCAGGAAGTAGGAAAAG
CAATGTATGCCCCTCCTATCAGAGGACAAATTAGATGTTCATCAAAT---
ATTACAGGGCTGATACTAACAAGGGATGGAGGTCATGGAGGTAATGACAAT---------------------
------------------------------------------------------------------------
-------
AACCAGACCACCGAGACCTTCAGACCTGGAGGAGGAAATATGAAGGACAATTGGAGAAGTGAATTATATAAAT
ATAAAGTAGTAAAAATTGAACCATTA---GGAGTAGCACCCACCCCGGCAAAAAGAAGAGTGGTG-------
------------------------------CAGAGAGAAAAAAGAGCA---TTAGTGGGATTCGGAGCT---
------CTGTTCCTTGGGTTCTTGGGAGCA---GCAGGAAGCACTATG---GGCGCAGCGTCA---
ATAACGCTGACGGTACAG---GCCAGACAA---TTACTG---TCTGGTATAGTGCAACAGCAAAAC---
AATTTG---CTGAAG---GCTATTGAGGCGCAACAGCATCTGCTGCAACTCACAGTCTGGGGCATC
>KX129209..1
TTCTACTGTAATTCAACACAACTGTTTAATAGTACGTGGATTGGGAATAGCACTGAAGAGTCAAAT------
------------------------------------------------------------------------
---------------------------
GGCACTGAAAACATCACACTCCCATGTAGAATAAAACAATTTGTAAATCTGTGGCAGGAAGTAGGAAGAGCAA
TGTATGCCCCTCCCATCAGAGGACTAATTAAATGTTCATCAAAT---
ATTACTGGGCTGTTGTTAACGAGAGATGGTGGTCAGTCTGACAATACT------------------------
------------------------------------------------------------------------
-------
ACCAACGGGACCGAGACCTTTAGACCTGGAGGAGGAAATATGAAGGACAATTGGAGAAGTGAATTATATAAAT
ATAAAGTAGTACAAATTGAACCATTA---GGAATAGCACCCACCAAGGCAAAGAGAAGAGTGGTG-------
------------------------------CAGAGAGAAAAAAGAGCA---GTGGGA---CTAGGAGCT---
------TTGTTCCTTGGGTTCTTGGGAGCA---GCAGGAAGCACTATG---GGCGCAGCGTCA---
GTGACGCTGACGGTACAG---GCCAGACAA---TTATTG---TCTGGTATAGTGCAGCAGCAGAAC---
AACTTG---CTGAGA---GCTATTGAGGCGCAACAACATATGTTGCAACTCACAGTCTGGGGCATC
>KM259534..1
TTCTACTGTAATACAACAAAACTGTTTAATAGTACTTGGAATGGGACTAATATT------------------
------------------------------------------------------------------------
-GATATTGGAAACAGT---------
ACGAAAGATGACAATATCACACTCCCATGCAGAATAAAACAAATTATAAATAGGTGGCAAGAGGTAGGAAAAG
CAATGTATGCCCCTCCCATCAGAGGACAAATTAGATGTTCATCAAAC---
ATTACAGGGCTGCTATTAACAAGAGATGGTGGT---------------------------------------
-------------------------------------------------------
AATAGCAATAACACCAACGAG---------
ACCACCGAGACCTTCAGACCTACAGGAGGAAATATGGAGGACAATTGGAGAAGTGAATTATATAAATATAAAG
TAGTGCAAATTCAACCATTA---GGAGTAGCACCCACCGAGGCAAAGAGAAGAGTGGTG-------------
------------------------CAGAGAGAAAAAAGAGCA---GTGGGA---TTAGGAGCT---------
ATGTTCCTTGGGTTCTTGGGAGCA---GCAGGTAGCACTATG---GGCGCAGCGTCA---
ATAACGCTGACGGTACAG---GCCAGACAA---TTACTG---TCTGGTATAGTGCAACAGCAGAAC---
AATTTG---CTGAGG---GCTATTGAGGCGCAACAGCATATGTTGCAACTCACAGTCTGGGGCATC
```

>HQ217957..1
TTCTACTGTAATACATCAGAACTGTTTAATAGTACCTGGAATATAACACACTTTAATAGTACCTGGAATGATA
CT---

GAAGGATCAAATAACATCACACTCCCATGCAGAATAAAACAAATTATAAATATGTGGCAACAAGTAGGAAAAG
CAATGTATGCCCCTCCCATCCAAGGATTAATTCAATGTTCATCAAAT---
ATTACAGGGCTGCTATTAACAAGAGATGGAGGTAATAATAGTACT----------------------------
----

AACGAGATCTTCAGACCTGGAGGAGGGGATATGAGGGACAATTGGAGAAGTGAATTATATAGATATAAAGTAG
TAAGAATTGAACCATTA---GGAGTAGCACCCACCAAGGCAAGGAGAAGAGTGGTG-----------------
----------------------CAAAGAGAAAAAAGAGCA---GTGGGAACAATAGGAGCT---------
ATGTTCCTTGGGTTCTTGGGAGCA---GCAGGAAGCACTATG---GGCGCAGCGTCA---
GTGACGCTGACGGTACAG---GCCAGACAA---TTATTG---TCTGGAATAGTGCAACAGCAAAAC---
AATTTG---CTGAAG---GCTATTGAGGCGCAACAACACCTGTTGCAACTCACAGTCTGGGGCATC
>HQ217984..1
TTCTACTGTAATTCAACACAACTGTTTAATAGTACTTGGACTAGGGAAAATGGTAGTTGGACTAGGAATGATA
CCGCT--
-------------------GCA---
AATGAGACTGAGATTATCACACTCCCATGCAGAATAAAACAAATTATAAACATGTGGCAGGAAGTAGGAAAAG
CAATGTATGCCCCTCCCATCAAAGGACTTATTCAATGTTCATCAAAT---
ATTACAGGATTGTTATTAACAAGAGATGGTGGTAATAATAGCAAC----------------------------
----

ATCAATGAGATCTTTAGACCTGGAGGAGGAGATATGAGGGACAATTGGAGAAGTGAATTGTACAAATATAAGG
TAGTAAAAATTGAACCATTA---GGAGTAGCACCCACCAAGGCAAGGAGGAGAGTGGTG--------------
-------------------------CAAAGAGAGAAAAGAGCA---GTGGGA---CTAGGAGCT---------
CTGTTCCTTGGGTTCTTGGGAGCA---GCAGGAAGCACTATG---GGCGCAGCGTCA---
TTGACGCTGACGGTACAG---GCCAGACAA---TTATTG---TCTGGTATAGTGCAACAGCAGAAC---
AATATG---CTGAGG---GCTATTGAGGCGCAGCAGCACCTGTTGCAACTCACAGTCTGGGGCATC
>JF689886..1
TTCTACTGTAATTCAACACAACTGTTTAATAGTACTTGGACTAAATATGATAATGGAACTTGGGTTAAGAAGA
ATGGCACT---
-------------------GAA---
TGGGTGAATGATACAATCACACTCCCATGCAGAATAAAACAAATTATAAACATGTGGCAGGAAGTAGGAAAAG
CAATGTATGCCCCTCCCATCAGGGGACTAATTAATTGTATATCAAAT---
ATTACAGGGATACTGCTAACAAGAGATGGTGGTCAGGTTAATGGT----------------------------
----

ACCAATGAGACCTTCAGACCAGGAGGAGGAGATATAAAGGATAATTGGAGAAGTGAATTATATAAATATAAAG
TAGTAAAAATTGAACCATTA---GGAGTAGCACCCACCAAGGCAAGGAGAAGAGTGGTG--------------
-------------------------CAGAGGGAAAAAAGAGCA---ATTGGA---ATAGGAGCT---------
TTGTTCCTTGGGTTCTTGGGAGCA---GCAGGAAGCACTATG---GGCGCAGCGTCA---
GTGACGCTGACGGTACAA---GCCAGACAA---TTATTG---TCTGGTATAGTGCAACAGCAGAAC---
AATTTG---CTGAGG---GCTATTGAGGCGCAACAACATCTGTTGCAACTCACAGTCTGGGGCATC
>JF320385..1
TTCTACTGTAATACAACACAACTGTTTAATAGTACTTGGATTCGGAATGATACTAAATGGTCAAGTAACTCTA
CA---

GGAAATGGCACCATTATACTCCCATGCAGAATAAAACAAATTATAAACATGTGGCAGGAAGTAGGAAAAGCAA
TGTATGCCCCTCCTATCAGAGGAAACATTAGCTGTTCATCAAAT---
ATTACAGGGCTGCTATTAACAAGAGATGGGGGCAATAGTAATCAAACGACC----------------------
----

CAGACCAAAGAAACAGAAACCTTCAGACCTGGAGGAGGAGATATGAGGGACAATTGGAGAAGTGAATTATATA
AATATAAAGTAGTAAAAATTGAACCATTA---GGAGTAGCACCCACCAAGGCAAAGAGAAGAGTGGTG-----
----------------------------------CAGAGAGAAAAAAGAGCA---GTGGGA---
ATAGGAGCA---------ATGTTCCTTGGATTCTTGGGAGCA---GCAGGAAGCACTATG---
GGCGCAGCGTCA---CTAACGCTGACGGTACAG---GCCAGACAA---TTATTG---

TCTGGTATAGTGCAGCAGCAGAAC---AATCTG---CTGAGA---
GCTATTGAGGCGCAACAGCATCTGTTGCAACTCACAGTCTGGGGCATC
>JF320539..1
TTCTACTGTAATACAACACAATTGTTCAATAATACTTGGAAAGGTACTGAAGAAGAGAATAGTACTAGGAAT-
----

AGTAATGACACCATCACACTCCCATGCAGAATAAAACAAATTGTAAACATGTGGCAGGAAGTAGGAAAAGCAA
TGTATGCCCCTCCCATCAGAGGACTAATTCAATGTACATCAAAC---
ATTACAGGGATAATATTAACAAGAGATGGTGGTAATAACACGAAT----------------------------
----

ACCACTGAGATCTTCAGACCAGGAGGAGGAGACATGAGGGACAATTGGAGAAGTGAATTATATAAATATAAAG
TAGTAAAAATTGAACCATTA---GGAATAGCACCCACTAGGGCAAAGAGAAGAGTGGTG--------------
-------------------------CAGAGAGAAAAAAGAGCA---GTGGGA---ATAGGAGCT---------
TTGTTCCTTGGGTTCTTGGGAGCA---GCAGGAAGCACTATG---GGCGCAGCGTCA---
ATGACGCTGACGGTACAG---GCCAGACTA---TTATTG---TCTGGTATAGTGCAACAGCAGAAC---
AATCTG---CTGCAG---GCTATTGAGGCGCAACAGCGTCTGTTGCAACTCACAGTCTGGGGCATC
>JF320131..1
TTCTATTGTAATACAACACAACTGTTCAATAGTACTTGGGAGAGCAATATTACTACTGGGAATTGGAGCAAAA
CTGAA--
-------------------GGA---
TCAAATAACACTAACATCACACTCCCATGTAGAATAAGACAAATTGTAAACATGTGGCAGAAAGTAGGAAAAG
CAATGTATGCCCCTCCCATCAGGGGACCAATTCAATGTATATCAGAT---
ATCACAGGGCTGCTATTAACAAGAGATGGAGGTAATAACGGGGACAAT-------------------------
----

AACAACACAGAGACCTTCAGACCTGGAGGAGGAGATATGAGGGACAATTGGAGAAGTGAATTATATAAATATA
AAGTAGTACAAATTGAACCAATA---GGAATAGCACCCACCAAGGCGCAGAGAAGAGTGGTG-----------
----------------------------CAGAGAGAAAAAAGAGCA---GTGGGA---GTAGGAGCT------
---TTTCTCTTTGGGTTCTTGGCAACA---GCAGGAAGCACTATG---GGCGCAGCGTCA---
ATAACGCTGACGGTACAA---GCCAGACAA---TTATTG---TCTGGTATAGTGCAACAGCAGAGC---
AATTTG---CTGAGG---GCTATTGAGGCGCAACAGCATATGTTGCAACTCACAGTCTGGGGCATC
>HM638503..2
TTCTACTGTAATACAACAGGGCTGTTTAGTAGTACTTGGAATGAGAGTACTACTTGGGAAAGGTCAAAT----
----

GACACTGAAATAATCACTCTCCCATGCAGAATAAAACAAATTATAAACATGTGGCAGGCAGTAGGAAAAGCAA
TATATGCCCCTCCCATCAGTGGACAAATTAATTGTTCATCAAAT---
ATTACAGGGCTGCTATTAACAAGAGATGGTGGTTACAATGGTACGAAT-------------------------
----

GACACCCAGACCGAGACCTTCAGACCTCAGGGAGGAAATATGAAAGACAATTGGAGAAGTGAATTATATAAAT
ATAAAGTAGTAAAAATTGAACCATTA---GGACTAGCACCCACCAAGGCAAAGAGAAGAGTGGTG--------
-------------------------------CAGAGAGAAAAAAGAGCA---GTGGGA---ATAGGAGCT---
------ATGTTCTTTGGGCTCTTGGGAGCA---GCAGGAAGCACCATG---GGCGCAGCGTCA---
GTGACGCTGACGGTACAG---GCCAGAAAG---TTGTTG---TCTGGTATAGTGCAACAGCAGAAC---
AATCTG---CTGAGG---GCTATTGAGGCGCAACAGCATATGTTGCAACTCACAGTCTGGGGCATC
>KT124801..1
TTCTACTGTAATACAACCGAACTGTTTAATAGTACTTGGACTAGGAATAATGGTACCGGGTATGATGGGTCTA
ATGGCACT---

GAAGGAAATGACACAATCACACTCCCATGCAGAATAAAACAAATTATAAACATGTGGCAGGAAGTAGGAAAAG
CAATGTATGCCCCTCCTATTAGAGGACAAATTAGATGCTCATCAAAT---
ATTACAGGACTGATATTAACAAGAGATGGGGGTCAAAGAAACGGG----------------------------
----

AGTACTAATGAGACCTTCAGACCAGGAGGAGGAAATATGAGGGACAATTGGAGAAGTGAATTATATAAGTATA
AAGTAATAAAAATTGAACCACTA---GGAGTAGCACCCACCAGGGCAAAGAGAAGAGTGGTG-----------
----------------------------CAGAGAGAAAAAAGAGCA---GTGGGA---ATAGGAGCT------
---GTGTTCCTTGGGTTCTTGGGAGCA---GCAGGAAGCACTATG---GGCGCAGCGTCA---

CTAACGCTGACGGTACAG---GCCAGACAA---TTATTG---TCTGGTATAGTGCAACAGCAAAAC---
AATTTG---CTGAGA---GCTATTGAGGCGCAACAACATATGTTGCAACTCACAGTCTGGGGCATC
>JN687763..1
TTCTACTGCAATACAACACAACTGTTTAATAGTACTTGGAATACTAATGGTACTTGGACAGGAAGTAATACT-
----

AATAGCAGTAACATTACACTCCCATGCAGAATAAAACAAATTGTAAACATGTGGCAGGAAGTAGGAAAAGCAA
TGTATGCCCCTCCCATCAGCGGACTAATTCACTGTTCATCAAAT---
ATTACAGGGCTGATATTAACAAGGGATGGTGGTAGTGGTAATGACACC-------------------------
----

AACAACAACACTGAGACCTTCAGACCTGCAGGAGGAGATATGAGGGACAATTGGAGAAGTGAATTATACAAAT
ATAAAGTAGTAAAAATTGAACCATTA---GGAGTAGCACCCACCAGGGCAAAGAGAAGAGTGGTG--------
-------------------------------CAAAGAGAGAAAAGAGCA---GTGGGA---ATGGGAGCT---
------CTGTTCCTTGGGTTCTTGGGAGCA---GCAGGAAGCACTATG---GGCGCAGCGTCA---
ATGACGCTGACGGTACAG---GCCAGACAA---TTATTG---TCTGGTATAGTGCAACAGCAGAGC---
AATTTG---CTGAGG---GCTATTGAGGCGCAACAGCATCTGTTGCAACTCACAGTCTGGGGCATC
>JQ403073..1
TTCTACTGTAATACAACACAACTGTTTGATGATAATTGGCCTAATGGAACTTGGATGTATAATAGCACT----
----

AGAAATGACACTATCATACTCCCATGCAGGATAAAACAAATTGTAAACATGTGGCAGGAAGTAGGAAAAGCAA
TGTATGCCCCTCCCATCAAAGGACTAATTCACTGTAATTCAAGT---
ATTACAGGGCTGCTATTAACAAGAGATGGTGGCACTAATAACACC----------------------------
----

AACGAGACCTTCAGACCGGGAGGAGGAGACATGAGGGACAATTGGAGAAGTGAATTATATAAATATAAAGTAG
TAAAAATTGAACCATTA---GGAGTAGCACCCACCAAGGCAAAGAGAAGAGTGGTG-----------------
----------------------CAGAGAGAGAAAAGAGCA---GTGGGA---CTAGGAGCT---------
CTGTTCCTTGGGTTCTTGGGAGCA---GCAGGAAGCACTATG---GGCGCAGCGTCA---
ATGGCGCTGACGGTACAG---ACCAGACAA---TTATTG---TCTGGTATAGTGCAACAGCAGAAC---
AATTTG---CTGAGG---GCTATTGAGGCGCAACAGCATCTGTTGCAACTCACAGTCTGGGGCATC
>JQ403078..1
TTCTATTGTAATACAACACAGCTGTTTAATAGTACTTGGAATGGTACTGAGACTGAGAATAATACTACAGGGT
CAAAT--

AACACTGATAATATCAGACTCCCATGCAGAATAAAACAAATTGTAAACAGGTGGCAGGAAGTAGGAAAAGCAA
TGTATGCCCCTCCCATCGAGGGCATAATTAGTTGTTCATCAAAT---
ATTACAGGGCTTTTATTAACCAGAGATGGTGGTAACGGGAGCAGT----------------------------
----

AACGCCGAGATCTTCAGACCTCAAGGAGGAGATATAAGGGACAATTGGAGAAGTGAATTATATAAATATAAAG
TAGTAAAAATTGAGCCACTA---GGAGTAGCACCCACCAAGGCAAAGAGAAGAGTGGTG--------------
-------------------------CAGAGAGAAAAAAGAGCA---GTGGGA---ATAGGAGCT---------
GTGTTCCTTGGGTTCTTGGGAGCA---GCAGGAAGCACTATG---GGCGCAGCGTCA---
ATAACGCTGACGGTACAG---GCCAGACAA---TTATTG---TCTGGTATAGTGCAACAGCAGAAC---
AATCTG---CTGAGG---GCTATTGAGGCGCAACAACATCTGTTGCAACTCACAGTCTGGGGCATC
>JQ610064..1
TTCTATTGCAATACAACAGAACTGTTTACTTGGAATTTAACACAACTGTCTGAGAATTCAACTGGG-------
----

AATGATGCTAATATCACACTCCCATGTAGAATAAAACAAATTATAAACATGTGGCAGGAAGTAGGAAAAGCAA
TGTATGCCCCTCCCATCAGCGGACCAATTAATTGCACATCAAAT---
ATTACAGGACTACTATTAACAAGAGATGGTGGTAATAACAATGACAGGACTAAC------
AATGGCAGTGATAGTAAT---AACAGTACT---

CAGACTGAGATCTTCAGACCTGGAGGAGGAGATATGAAGGACAATTGGAGAAGTGAATTATATAAATATAAAG
TAGTAAGAATTGAACCATTA---GGAGTAGCACCCACCAAGGCAAAGAGAAGAGTGGTG--------------
-------------------------CAAAGAGAAAAAAGAGCG---GTGGGAACGCTAGGAGCT---------
TTGTTCCTTGGGTTCTTGGGAGCA---GCAGGAAGCACTATG---GGCGCAGCGTCA---

ATAACGCTGACGGTACAA---GCCAGACAA---TTATTG---TCTGGTATAGTGCAACAGCAAAGC---
AATTTG---CTGAGA---GCTATTGAGGCGCAACAGCATCTGTTGCAACTCACAGTCTGGGGCATC
>HQ217713..1
TTCTACTGTGATTCAACACCACTGTTTAATAGGACCTGGATGGCTAATGAGACTTTGAATAGTACTACTTTGA
ATAGTACT---
------------------GGA---
GAGTCAAATAAAAATATCACACTCGCATGCAGAATAAAACAAATTATAAACATGTGGCAGCGAGTAGGGAAAG
CAATGTATGCCCCTCCTATCAGAGGACAAATTAGATGTTCATCAAAT---
ATTACAGGGCTGCTGTTAACAAGAGATGGGGGTCACAACAACGAGACTGGA---------------------
----

GATAACAGGACTGAGATCTTCAGACCGGGAGGAGGAGATATGAGGGACAATTGGAGAAGTAAATTATATAAAT
ATAAAGTAGTAAAAATTGAACCATTA---GGAGTAGCACCCACCAAGGCAAAGAGAAGAGTGGTG--------
------------------------------CAGAGAGAAAAAAGAGTA---GCGGGA---TTCGGAGCT---
------TTGTTCCTTGGGTTCTTGGGAGCA---GCAGGAAGCACTATG---GGCGCAGCGTCA---
GTGACGCTGACGGTACAG---GCCAGACAA---TTACTG---TCTGGTATAGTGCAGCAGCAGAAC---
AATTTG---CTGAGG---GCTATTGAGGCGCAACAGCATCTGTTGCAACTCACAGTCTGGGGCATC
>MK383397..2
TTCTACTGTAATTCAACACCACTGTTTAATAGTACTTGGAATGGTACTGACGAGTCAAATCACCATGGAGAA-
----

AATGACACAATCATCACACTCCGCTGCAGAATAAAACAAATTATAAACATGTGGCAGGAAGTAGGAAGAGCGA
TGTATGCCCCTCCCATTAAAGGAAACATTAGCTGTTCATCAAAT---
ATTACAGGGCTGCTATTAACAAGAGATGGTGGTAATGGTAACCGG---------------------------
----

ACTGACAACAACACCGAGATCTTCAGACCTCAGGGAGGAAATATGAAAGATAATTGGAGAAGTGAATTATATA
AATATAAAGTAGTAAGAATTGAACCATTA---GGAATAGCACCCACCAAGGCAAGGAGAAGAGTGGTG-----
---------------------------------CAGAGAGAAAAGAGAGTA---GTGGGA---
ATGGGAGCT---------TTGTTCCTTGGGTTCTTGGGAGCA---GCAGGAAGCACTATG---
GGCGCAGCGTCA---GTGACGCTGACGGTACAA---GCCAGACTA---TTACTG---
TCTGGTATAGTGCAGCAGCAGAAC---AATTTG---CTGAGG---
GCTATTGAGGCGCAACAGCGTCTGTTGCAACTCACAGTCTGGGGCATC
>KT124761..1
TTCTACTGTAATACAACAAAGCTATTTAATAGTACTTGGGATCGGAATGGAACACAGATAAATGACACTATC-
----

ACAAATGACACTATCATACTCCCATGTAGAATAAAACAATTCATAAACAGGTGGCAAGAAGTAGGAAAAGCAA
TGTATGCCCCTCCCATTAGAGGAGACATTAACTGTTCATCACAG---
ATTACAGGGCTGCTATTAACAAGAGATGGTGGTCAGAGCAATGACAGC------------------------
----

AGTATGAATGAGATCTTCAGACCTGGAGGAGGAGATATGAGGGACAATTGGAGAAGTGAATTATATAAATATA
AAGTAGTAAGAATTGAGCCATTA---GGAGTAGCACCCACCAAGGCAAAAAGAAGGGTGGTG----------
---------------------------CAGAGAGAAAAAAGAGCA---GTAACA---CTAGGAGCT------
---GTGTTCCTTGGGTTCTTGGGAGCA---GCAGGAAGCACTATG---GGCGCAGCGTCA---
ATAACGCTGACGGCACAG---GCCAGACAA---TTATTG---TCTGGTATAGTGCAACAGCAGAGC---
AATCTG---CTGAAA---GCTATTGAGGCGCAACAGCGTCTGTTGCAACTCACAGTCTGGGGCATT
>KM353846..1
TTCTACTGTAATACATCAAAGTTGTTTAACAGTACTTGGAATGGCACT------------------------
----
-------AAGGAATACCAG------
GGAGTGAATGGCACACTCACACTCCCATGCAGAATAAAACAAATCATAAACATGTGGCAGAAAGTAGGAAAAG
CAATGTATGCCCCTCCCATCCAGGGAAATATTAATTGCTCATCAAAT---
ATTACAGGGCTGCTATTAATGAGAGATGGTGGTAAC------------------------------------
----
AAGGGGAACGAAACCGATAAT---------
ACTACCGAGATCTTTAGACCTGGAGGAGGAAATATGAGGGACAATTGGAGAAGTGAATTATATAAATATAAAG
TAGTGAGAATTGAACCATTA---GGAGTAGCACCCACCAAGGCAAAAAGAAGAGTGGTG-------------
------------------------CAGAGAGAAAAAAGAGCG---GTAACA---GTGGGAGCT---------

ATGTTCCTTGGGTTCTTGGGAGCA---GCAGGAACCACTATG---GGCGCAGCATCA---
TTGACGCTGACGGTACAG---GCCAGACAA---TTATTG---TCTGGTATAGTGCAACAGCAAAGC---
AATTTG---CTGAGG---GCTATTCAGGCGCAACAGCATCTGTTGCAGCTCACAGTCTGGGGCATC
>KY112135..1
TTCTACTGTAATACAACAGAACTGTTTAATAGTACTTGGGATAAGGATGGGACAAGGCTTAATGACACTGGA-
----

GGAGATGGCAACATCACACTCCCATGTAGAATAAAACAAATTATAAACATGTGGCAGGAAGTAGGAAAAGCAA
TGTATGCCCCTCCCATCAGAGGACAAATTAGCTGTGTATCAAAT---
ATCACAGGGCTACTATTAACAAGAGATGGGGGTGTAGACGATACT----------------------------
----

CAAAACGAAACCTTCAGACCTGGAGGAGGAGATATGAGGGACAATTGGAGAAGTGAATTATATAAATATAAGG
TAGTAAAAATTGAACCATTA---GGAGTAGCACCTAGCAAGGCAAAGAGAAGAGTGGTG--------------
-------------------------CAAAGAGAAAAAAGAGCA---GTGGGAACGTTAGGAGCC---------
ATGTTCCTTGGGTTCTTGGGAGCA---GCAGGAAGCACTATG---GGCGCAGCGTCA---
CTGACGCTGACGGTACAG---GCCAGACAA---TTATTG---TCTGGTATAGTGCAACAGCAGAAC---
AATCTG---CTGAGA---GCTATTGAGGCGCAACAGCATCTGTTGCAACTCACAGTCTGGGGCATC
>JQ403070..2
TTCTACTGTAATACAACACAACTGTTTAATAGTACTTGGCTGTTTAATGATACTTTGAATGATACTCAAGAGG
AGTATGGT---

GAGAATAACGGGACTATCACACTCCCATGCAGAATAAAACAAATTATAAACAGATGGCAGGAAGTAGGAAAAG
CAACATATGCCCCTCCCATCAGAGGACAAATTAAATGTTCATCAAAT---
ATTACAGGGATACTATTAACAAGAGATGGTAGTCAAGGCAACGGG----------------------------
----

AGTGAGACCTTCAGACCAGGAGGAGGAGATATGAGGGACAATTGGAGAAGTGAATTATATAAATATAAAGTAG
TAAAAATTGAGCCATTA---GGAGTAGCACCCACCAAGGCAAGGAGAAGAGTGGTG-----------------
----------------------CAAAGGGAAAAAAGAGCA---GTGGGAACGCTAGGAGCC---------
ATGTTCCTTGGGTTCTTGGGAGCA---GCAGGAAGCACTATG---GGCGCAGCGTCA---
GTGACGCTGACGGTACAG---GCCAGACAA---CTATTG---TCTGGTATAGTGCAACAGCAGAAC---
AACCTG---CTGAGA---GCTATTGAGGCGCAACAGCATCTGTTGCAACTCACAGTCTGGGGCATC
>JQ403031..2
TTCTACTGCAATACAACAAAACTATTTAATAGTACTTGGAATAGTAGTAGTAGTAGTTGGGTTATTGAGGGTC
AGGAAGAG---

TCAAATGACAATATCACACTCCCATGTAGAATAAAACAAATTATAAACATGTGGCAGGGAGTAGGAAAAGCAA
TGTATGCCCCTCCCATCCAAGGAAAAATTAGATGTTCATCAAAC---
ATTACAGGGCTATTATTAGTGAGAGATGGTGGTATTAACAAGACG----------------------------
----

AATGACACTGAAACCTTCAGACCTCAAGGAGGAAATATGATGGACAATTGGAGAAGTGAATTATATAAATATA
AAGTAGTAAAAATTGAACCATTA---GGAATAGCACCCACCAAGGCAAAGAGAAGAGTGGTG-----------
----------------------------CAGAGAGAAAAAAGAGCA---GTGGGA---ATGGGAGCT------
---TTGTTCCTTGGGTTCTTGGGAGCA---GCAGGAAGCACTATG---GGCGCAGCGTCA---
ATGGCGCTGACGGTACAG---GCCAGACAA---TTATTG---TCTGGTATAGTGCAACAGCAGAAC---
AATCTG---CTGAGA---GCCATTGAGGCGCAACAGCATATGTTGCAACTCACAGTCTGGGGCATC
>JQ403085..1
TTCTACTGTAATACATCACAACTGTTTAAGGGTACCTGGTTGACTAATGATACCTGGGCTAATGGTACCTGGA
CAAAT--
-------------GACACT------
AGCAAAAATGACAACATCACACTCCCATGCCGAATAAAACAAATTGTAAACTTGTGGCAGGAAGTAGGAAGAG
CAATGTATGCCCCTCCCATCAGAGGAGAAATTCATTGTATATCAAAT---
ATTACAGGGCTGTTATTAACAAGAGATGGTGGCCAGAATGCAACC----------------------------
----

AACGAGACCTTCAGACCTGTAGGAGGAGATATGAGGGACAATTGGAGAAGTGAATTATATAAATATAAAGTAG
TAAAAATTGAACCATTA---GGAGTAGCACCCACCAAGGCAAAGAGAAGAGTGGTG-----------------
----------------------CAGAGAGAAAAAAGAGCA---GTGGGA---CTAGGAGCT---------

GTGTTCCTTGGGTTCTTGGGAGCA---GCAGGAAGCACTATG---GGCGCAGCGTCA---
CTGACGCTGACGGTACAG---GCCAGGCAA---TTATTG---TCTGGTATAGTGCAACAGCAAAAC---
AACTTG---CTGAGA---GCTATTGAGGCGCAACAGCACCTGTTGCAACTCACAGTCTGGGGCATC
>JQ403094..1
TTCTACTGTAATACATCACGACTATTTAATAGTACTTGGAATACTAGTAGTATTTGGAATACTACTGGAAGGC
CAAATATT---
-------------------ACA---
GGGAGCACTGACATTATCATACTCCCATGCAGAATAAGACAAATTATAAACATGTGGCAGGAAGTAGGAAAAG
CAATGTATGCCCCTCCCATCAGAGGACGAATTCAGTGTAGCTCAAAT---
ATTACAGGGCTGCTATTAACAAGAGATGGTGGTAATACTAATAACTCA-------------------------
----

GACGCACATGAGATCTTCAGACCTGGAGGAGGAGATATGAGGGACAATTGGAGAAGTGAATTATATAAATATA
AAGTAGTAAAAATTGAACCATTA---GGAATAGCGCCCACCAAGGCAAAGAGAAGAGTGGTG-----------
----------------------------CAGAGAGAAAAAAGAGCA---GTGGGA---ATGGGAGCT------
---CTGTTCCTTGGGTTCTTGGGAACA---GCAGGAAGCACTATG---GGCGCAGCGTCA---
GTGACGCTGACGGTACAG---GCCAGACAA---TTATTG---TCTGGTATAGTGCAACAGCAGAGC---
AATCTG---CTGAGG---GCTATTGATGCGCAACAGCATCTGTTGCAGCTCACAGTCTGGGGCATC
>KR423482..3
TTCTACTGTAATACAACGAAACTGTTTAATAGTAGTTGGAATCAGAATGCTACTAATCAGAATGCTACT----
----
-------------AACAAT------
GGAGGAAATGACACAATTACACTCCCATGTAGAATAAAACAGATTATAAACATGTGGCAGGAAGTAGGAAAGG
CAATGTATGCCCCTCCCATAAAAGGATTAATTAGATGTTCATCAAAT---
ATTACAGGGCTGATATTAACAAGAGATGGTGGTAATGACAATAGCACA-------------------------
----

AACAACAACACTGAAATCTTCAGACCGGGAGGAGGAGATATGAGGGACAATTGGAGAAGTGAATTATATAAAT
ACAAAGTAGTAAGAGTTGAACCATTA---GGGATAGCACCCAACAAGGCAAAGAGGAGAGTGGTG--------
-------------------------------CAGAGAGAAAAAAGAGCA---GTGGGG---ATAGGAGCT---
------TTGTTCCTTGGGTTCTTGGGAGCA---GCAGGAAGCACTATG---GGCGCAGCGTCA---
GTGACGCTGACGGTACAA---GCCAGACAA---TTATTG---TCTGGTATAGTGCAGCAGCAGAAC---
AATCTG---CTGAGG---GCTATTCAGGCGCAACAGCAACTGTTGCAGCTCACAGTCTGGGGCATC
>GU733713..2
TTCTACTGTAATACAACTAGACTGTTCAATAGTACTTGGAATGTTAGTACTTGGAATATTAGTCAGACTGAAG
GGTCA--
-------------------TAC---
AACACTGAAGAAAATATCACACTCCCATGCAGAATAAAACAGATTGTAAACATGTGGCAGGAAGTAGGAAAAG
CAATGTATGCCCCTCCCATCAGAGGAATAATTAGATGTTCATCAAAT---
ATTACAGGGATAATGTTAACAAGAGATGGTGGTAAGAATAGGACAGAG-------------------------
----

AACACCACTGAGACTTTCAGACCGGGAGGAGGAAATATGATGGACAATTGGAGAAGTGAACTATATAACTATA
AAGTAGTAAAAATTGAACCATTA---GGGGTAGCACCCACCAAGGCAAAGAGAAGAGTGGTG-----------
----------------------------CAGAGAGAAAAAAGAGCA---GCGGGA---CTAGGAGCT------
---TTGTTCCTTGGGTTCTTGGGAGCA---GCAGGAAGCACTATG---GGCGCAGCGTCA---
CTGACGCTGACGGTACAG---GCCAGACAA---TTATTG---TCTGGTATAGTGCAACAGCAGAAC---
AATTTG---CTGAGA---GCTATTGAGGCGCAACAGCACCTGTTGCAACTCACAGTCTGGGGCATC
>MK164668..1
TTCTACTGTAATACAACAGAACTGTTTAATAGTACTTGGGGTAATAGTAATAATACTAACAATAATACTGGA-
----

GGGGAAGAAACTATCACTCTCCCATGCAGAATCAAACAAATTATAAACATGTGGCAGGAAGTAGGAAAAGCAA
TGTATGCCCCTCCCATCAGAGGACAAATTAGGTGTTCATCAAAG---
ATTACAGGGCTAATATTAACAAGAGATGGTGGCAACGAGAACAACGACAGTGAG-------------------
----

ATCTTCGAGATCTTCAGACCTACAGGAGGAAATATGAGGGACAATTGGAGAAGTGAATTATACAAATATAAAG
TAGTAAAAATTGAACCATTA---GGAATAGCACCCACCAAGGCAAAAAGAAGAGTGGTG--------------
-------------------------CAGAGAGAAAAAAGAGCA---GTGGGAATACTAGGAGCT---------

ATGTTCCTTGGGTTCTTGGGCGCA---GCAGGAAGCACTATG---GGCGCAGCGTCA---
CTGACGCTGACGGTACAG---GCCAGACAA---CTCTTG---TCTGGTATAGTGCAACAGCAGAAC---
AACCTG---CTGAGG---GCTATTGAGGCGCAACATCATCTGTTGCAACTCACAGTCTGGGGCATC
>KC935959..1
TTCTACTGTAATACAACAAAACTGTTTAGTAGTACTTGGAAGTGGGATAATGGTACTTGGAATATTAGTGGGT
TAGATAACAGT--

ACAGAACAAAATATCACACTCCCATGCAGAATAAAACAAATCATAAATATGTGGCAGACAGTAGGAAAAGCAA
TGTATGCCCCTCCCATTAAGGGACAAATTCAATGTATATCAAAT---
ATTACAGGGCTGCTATTAACAAGAGATGGTGGTAATGAAAGTGATACA-------------------------
----

ACCAGGAACAACGAGACCTTCAGACCGGGAGGAGGAGATATGAGGGACAATTGGAGAAGTGAATTATATAAAT
ATAAAGTAGTAGAAGTTGAACCATTA---GGAATAGCACCCACCAAGGCAAAGAGAAGAGTGGTG--------
-------------------------------CAGAGAGAAAAAAGAGCA---GTGACA---CTAGGAGCT---
------CTGTTCCTTGGGTTCTTGGGAGCA---GCAGGAAGCACTATG---GGCGCAGCATCA---
ATGACGCTGACGGTACAG---GCCAGACAA---TTATTG---TCTGGTATAGTGCAACAGCAGAAT---
AATCTG---CTGAGG---GCTATTGAGGCGCAACAGCATCTGTTGCAGCTCACAGTCTGGGGCATC
>KM082006..2
TTCTACTATAACACAACCCAGCTGTTTGATAGTACGTGGTTAACTAATGGTACTAGGATAAGACCAAATACTA
CTGATGGA---

ACTAATGGAAATATCACACTTCCATGCAGAATAAAACAAATTATAAACAGGTGGCAAGAAGTAGGAAAAGCAA
TGTATGCCCCTCCCATCAGAGGACAAATTAGATGTACATCAAAT---
ATTACAGGGTTAATATTAACAAGAGATGGTGGTCATGACGGGAAC----------------------------
----

GAGACAAATGAGAAGGAGACCTTCAGACCTGGAGGAGGAGATATGAGGGACAATTGGAGAAGTGAATTATATA
AATATAAAGTAGTAAAAATTGAGCCAATA---GGAATAGCACCCACCATGGCAAAGAGAAGGGTGGTG-----
----------------------------------CAGAGAGAAAAAAGAGCA---GTAACG---
CTGGGAGCT---------ATGTTCCTTGGGTTCTTGGGAGCA---GCAGGAAGCACTATG---
GGCGCAGCGTCG---ATGACGCTGACGGTACAG---GCCAGACTA---TTATTG---
TCTGGTATAGTGCAACAGCAGAGC---AATCTG---CTGAGA---
GCTATTGAGGCGCAACAGCAACTGTTGCAACTCACAGTCTGGGGCATC
>MG196775..3
TTCTATTGTAATACATCAGAACTGTTTAATAGTGAGTGGCATACTAATTATACATGGAATAGTACTTACAGTA
CAATAAAT---------------------------------GGTACTTGGAATAGTACT---
GACATTGAGGGTGTA---------------------
AACGCTACTGAAAAGATCACACTCCCATGTAGAATAAAACAAATTATAAACAGGTGGCAGGAAGTAGGAAAAG
CAATGTATGCCCCTCCCATCGCAGGAACAATTAAATGTGTATCAAAT---
ATTACGGGGCTGCTGTTAACCAGAGACGGTGGCAACAATGAAACC----------------------------
----

AAAGCCACTGAGGTCTTCAGACCTCAAGGAGGAAATATGAAGGACAATTGGAGAAGCGAATTATATAAATATA
AAGTAGTACAAATTGAACCATTA---GGAGTAGCACCCAACAAGGCAAAGAGAAGAGTGGTG-----------
----------------------------CAGAGAGAAAAAAGAGCA---ATAACA---ATAGGAGCT------
---ATGTTCCTTGGGTTCTTGGGAGCA---GCAGGAAGCACTATG---GGCGCAGCGTCA---
ATGACGCTGACGGTACAG---GCCAGACAA---TTACTG---TCTGGTATAGTGCAACAGCAGAGC---
AATTTG---CTGAGA---GCTATTGAGGCGCAACAGCATCTGTTGCAACTCACAGTCTGGGGCATC
>JQ182826..2
TTCTACTGCAATACATCAGCACTGTTTAATAGTACTTGGAGTACTAAGAATGGTACTCAGGTGTTGAATATAA
CTAATGAC---

ACCAGCACCAACATCACACTCCCATGCAGAATAAAACAAATTATAAACATGTGGCAGGAAGTAGGAAAAGCAA
TGTATGCCCCTCCCATCAGAGGACTAATTAGGTGTACATCAAAC---
ATTACAGGACTACTATTAACAAGAGATGGTGGTAATGGTAATGGCAATACCACG-------------------
----

AATGGGACAGAGACCTTCAGACCTGTAGGAGGAGATATGAAGAATAATTGGAGAAGTGAATTATATAAATATA
AAGTAGTACAAATTCAACCATTA---GGAGTAGCACCCACCAAGGCAAAGAGAAGAGTGGTG-----------

----------------------------CAGAGAGAGAAAAGGGAT---TTGGGA---CTAGGAGCT------
---TTGTTCATTGGGTTCTTGGGAGCA---GCAGGAAGCACTATG---GGCGCAGCGTCA---
ATAACGCTGACGGCACAG---GCCAGACAA---TTGCTG---TCTGGTATAGTGCAACAGCAGAGC---
GATCTG---ATGAAG---GCTATTGAGGCGCAGCAGCATCTGTTGCAACTCACAGTCTGGGGCATC
>KM353922..1
TTCTATTGCGATACAACACAACTGTTTAATAGTAGTTGGCCTTGGAATGGTACA-------------------
----
GAAGAGTCAAATAACACT---------
GAAGGAAATGACACAATCACCCTCCCATGCAAAATAAAACAAATTATAAACCTGTGGCAGGAAGTAGGAAAAG
CAATGTATGCCCCTCCCATCAGAGGACAAATTAATTGCACATCAAAT---
ATTACAGGGTTACTATTAACAAGAGATGGTGGT--

-
AGTACCAGCAATGAGACCGAGATCTTTAGACCTGCAGGAGGAGACATGAGAGATAATTGGAGAAGTGAATTAT
ATAAATACAAAGTAGTAAAAATTGAACCAATA---GGAGTAGCACCCACCAGGGCAAAGAGAAGAGTGGTG--
-------------------------------------CAGAGAGAAAAAAGAGCA---GTGGGA---
TTAGGAGCT---------ATGTTCCTTGGGTTCTTGGGAGCA---GCAGGAAGCACTATG---
GGCGCAGCGTCA---GTGACGCTGACGGTACAG---GCCAGACAA---TTACTG---
TCTGGTATAGTGCAACAGCAGAAC---AATTTG---CTGAGG---
GCTATTGAGGCGCAACATCATCTGTTGCAACTCACAGTCTGGGGCATC
>KM354862..1
TTCTACTGTAATACAACACAACTATTTAATAGTAATTGGACTGCTACTGGA----------------------
----
-AATACAAATAACACT---------
GGAGGAAATGCCACAATCACACTCCCTTGCAGAATAAAACAACTTATAAACATGTGGCAGGGAGTAGGAAAAG
CAATGTATGCCCCTCCAATAAGAGGACTAATTAGATGTTCATCAAAC---
ATTACAGGGCTACTGTTAACAAGAGATGGTGGT--

GGTAGCAATGGAACTAAT---
GGAACTGAGGTCTTCAGACCTCATGGAGGGGATATAAGGGACAATTGGAGAAGTGAATTATATAAATACAAAG
TAGTAAAAATTGAACCATTA---GGAGTAGCACCCAACAAGGCAGAGAGAAGAGTGGTG--------------
-------------------------CAGAGAGAAAAAAGAGCA---GTGACA---ATAGGAGCT---------
GTGTTCCTTGGGTTCTTGGGAGCA---GCAGGAAGCACTATG---GGCGCAGCGTCA---
ATGACGCTGACGGTACAG---GCCAGACAA---CTATTG---TCTGGTATAGTGCAACAGCAAAGC---
AATTTG---CTGAGG---GCTATTGAGGCGCAACAGCACCTGTTGCAACTCACAGTCTGGGGCATC
>KX587241..1
TTCTACTGTAATACGACACAACTGTTTAATAATACTTGGAATGGTACTTGGGGTGAGAATATT----------
----

ACCGGGAATATTATCACACTCCCATGCAGAATAAAACAAATTATAAACATGTGGCAGGAAGTAGGAAAAGCAA
TGTATGCCCCTCCCATCAGAGGAAATATTAGCTGTTCATCAGAT---
ATTACAGGGCTAATATTAACAAGAGATGGTGGTCATGACAATGAGACC-------------------------
----

AACAACACAGCGATCTTCAGACCAGGAGGAGGAAATATGAAGGACAATTGGAGAAGTGAATTATATAAATATA
AAGTAGTAAAAATTGAACCAATA---GGAGTAGCACCCACCAAGGCAAAGAGAAGAGTGGTG-----------
----------------------------CAGAGAGAAAAAAGAGCA---GTGGGA---GTAGGAGCT------
---GTGCTCCTTGGGTTCTTGTCAGCA---GCAGGAAGCACTATG---GGCGCAGCGTCA---
ATAACGCTGACGGCACAG---GCCAGACAA---TTACTG---TCTGGTATAGTGCAACAGCAAAAC---
AATTTG---CTGAGA---GCTATTGAGGCGCAACAACATATGTTGCAACTCACAGTCTGGGGCATC
>KX587299..1
TTCTACTGTAATACAACAAAGCTGTTTAATAGTACTTGGGATAATACAACTGCAGGGTTAAATACC-------
----

ACTGAAAAAATTATCACACTCCCATGCAGAATAAGACAAATTATAAATATGTGGCAGCAGGTAGGAAAAGCAA
TGTATGCCCCTCCTATCAGAGGAAAAATCCAATGCACATCAAAC---
ATTACAGGGTTATTATTAACAAGAGATGGTGGTAACGAGAACGAGACC-------------------------
----

TATAACAATGAGACTTTCAGACCTGGAGGAGGAGATATGAAGGACAATTGGAGAAGTGAATTATATAAATATA

AAGTAGTAAGAATTGAACCATTA---GGAATAGCACCCACCAAGGCAAAAAGAAGAGTGGTG-----------
----------------------------CAGAGAGAAAAAAGAGCA---ATAGGA---ATGGGAGCC------
---TTCTTCCTTGGGTTCTTGGGAGCG---GCCGGAAGCACTATG---GGCGCAGCGTCA---
ATGGCGCTGACGGTACAG---GCCAGACAA---TTATTG---TCTGGTATAGTGCAGCAGCAAAAC---
AACTTG---CTGAGG---GCTATTGAGGCGCAACAGCATCTGTTGCAACTCACAGTCTGGGGCATC
>MG900412..2
TTCTACTGTAATTCAACAAAGCTGTTTAATAGTACTTGGCCTTGGAATGATACTCAAGGGTCACATGAC----
----

ACTAATGGCACACTCACACTCCCATGCAGAATAAAACAAATTATAAACATGTGGCAGGAAGTAGGAAAAGCAA
TGTATGCCCCTCCCATCGAAGGAATAATTAAATGCTCATCAAAC---
ATTACAGGACTGTTATTAACAAGAGATGGTGGTTACGAGAGCAAT----------------------------
----

AAGACTGATGAGATCTTCAGACCTGGAGGAGGAGATATGAGGGACAATTGGAGGAGTGAATTATATAAATATA
AAGTAGTAAAAATTGAACCATTG---GGAGTAGCACCTACCAAGGCAAAGAGGAGAGTGGTG-----------
----------------------------CAGAGGGAAAAAAGAGCA---TTTGGA---CTAGGAGCT------
---GTGTTCCTTGGGTTCCTGGGAGCA---GCAGGAAGCACTATG---GGCGCAGCATCA---
ATAACGCTGACGGTACAG---GCCAGACAA---TTATTG---TCTGGTATAGTGCAACAGCAGAAC---
AATTTG---CTGAGG---GCTATTGAGGCGCAACAGCATCTGTTGCAACTCACAGTCTGGGGCATT
>KC473826..1
TTCTACTGTAATTCAACACAGCTGTTTAGTAGTAATTGGACTAGGGATAATACGTCTAGTAATTGGACTAGGA
AGGATAAC---
----------GATACAGAG------
CCTAATAAGACTGTGATCACACTCCCATGCAGAATAAAACAAATTATAAACATGTGGCAGGAAGTAGGAAAAG
CAATGTATGCCCCTCCTATCAAAGGACTTATTCAATGTTCATCAAAT---
ATTACAGGATTGTTATTAACAAGAGATGGTGGCGACAACAATAGC----------------------------
----

AACACTGAAATCTTTAGACCTGGAGGAGGAGATATGAGGGACAATTGGAGAAGTGAATTATACAAATATAAAG
TAGTAAAAATTGAACCATTA---GGAGTAGCACCCACCAAGGCAAGGAGGAGAGTGGTG--------------
-------------------------CAAAGAGAGAAAAGAGCA---TTGGGA---ATGGGAGCT---------
CTGTTCCTTGGGTTCTTGGGAGCA---GCAGGAAGCACTATG---GGCGCAGCGTCA---
TTGATGCTGACGGTACAG---GCCAGACAA---TTATTG---TCTGGTATAGTGCAACAGCAGAAC---
AATATG---CTGAGG---GCTATTGAGGCGCAGCAGCATCTGCTGCAACTCACAGTCTGGGGCATC
>KC473828..1
TTCTACTGTAATACAACAAAGCTGTTTAATAGCACTCTTAATGACACTTGGAATTTTTGGAAT----------
----

GAAACTGGGGATATCATACTCCCGTGCAGAATAAAACAAATTATAAATCTGTGGCAGAAAGTAGGAAAAGCAA
TGTATGCCCCTCCCATTAAAGGACAGATTAACTGCACATCAAAT---
ATTACAGGGCTACTCTTAATAAGAGATGGCGGTGATAACGGGACT----------------------------
----

GCCAGTGAGACCTTCAGACCTCAAGGAGGAAATATGAAGGACAATTGGAGAAGTGAATTATATAAATATAAAG
TAGCACAAGTTGAGCCAATA---GGAATAGCACCCACCAAGGCAAAGAGAAGAGTGGTG--------------
-------------------------CAGAGAGAAAAAAGAGCA---GTGACT---ATGGGAGCT---------
TTGTTCCTTGGGTTCTTGGGAACA---GCAGGAAGCACTATG---GGCGCAGCGTCG---
ATAACGCTGACGGTACAG---GCCAGACAA---TTACTG---TCTGGTATAGTGCAACAGCAGAGC---
AATCTG---CTGAGA---GCTATTGAGGCGCAACAGCATCTGTTGCAACTCACAGTCTGGGGCATC
>KC473829..1
TTCTACTGTAATACGACAGAACTGTTTAATAGTTCTTGGTATTGGAATGATACGAATGATACTTTAAAGAGC-
----

AATGAAGAAAATATCACACTTCTGTGCAGAATAAAACAAATTGTAAACATGTGGCAGGAAGTAGGAAAAGCAA
TGTATGCCCCTCCCATTAGAGGACCAATTAGCTGTACATCAAAT---
ATTACAGGGATACTCTTAACAAGAGATGGAGGTCAGGGTAATAATACT-------------------------
----

AATGATACTGAAATCTTCAGACCAGGAGGAGGAGATATGAGGGACAATTGGAGAAGTGAATTATATAAATATA

AAGTAGTAAAAATTGAACCATTA---GGAGTAGCACCCACCAGGGCAAAAAGAAGAGTGGTA-----------
----------------------------CAGAGAGAAAAAAGAGCA---ATAGGGGGATTAGGAGCG------
---TTGTTCCTTGGGTTCTTGGGAGCA---GCAGGAAGCACTATG---GGCGCAGCGTCA---
ATGACGCTGACGGTACAA---GCCAGGCTA---TTATTG---ACTGGTATAGTGCAACAGCAAAAT---
AATTTG---CTGAAG---GCTATTGAGGCGCAACAACATCTGTTGCAACTCACAGTCTGGGGCATT
>KC473830..1
TTCTACTGTGATTCAACACAACTGTTTAGTAGTAATTGGACTAGGAATGATAGTAGTAGTAATTGGACTAGGA
GTGATAATGAT--
-------------ACAGAG------
TCTAACAATACTGTGATCACACTCCCATGCAGAATAAGACAAATTATAAACATGTGGCAGGAAGTAGGAAAAG
CAATGTATGCCCCTCCTATCAAAGGACTTATTCAATGTACATCAAAT---
ATTACAGGATTATTATTAACAAGAGATGGTGGTAATGGTAATAAA----------------------------
----

ACGAACGACACTCTGGAGATCTTTAGACCTGGAGGAGGAGATATGAGGGACAATTGGAGAAGTGAATTGTACA
AATATAAAGTAGTAAAAATTGAACCATTA---GGAGTAGCACCCACCAAGGCAAGGAGGAGAGTGGTG-----
----------------------------------CAAAGAGAGAAAAGAGCA---GTGGGA---
CTAGGAGCT---------CTGTTCCTTGGGTTCTTGTCAGCA---GCAGGAAGCACTATG---
GGCGCAGCATCA---TTGACGCTGACGGTACAG---GCCAGACAA---TTACTG---
TCTGGTATAGTGCAACAGCAGAAC---AATATG---CTGAGG---
GCTATTGAGGCGCAACAGCATCTGTTGCAACTCACAGTCTGGGGCATC
>KU677990..1
TTCTACTGTAATACATCAAGACTGTTTAATAGTACTTTGTATAGTAATAATACTTGGAGTAGTAATATTACAG
AA---

TTAAATGGCATTATCACACTCCCATGCAGAATAAAACAAATTGTAAACATGTGGCAGGAAGTAGGAAAAGCAA
TGTATGCCCCTCCCATCAGTGGACAAATTAACTGTTCATCAAAC---
ATTACAGGACTATTATTAACAAGAGATGGTGGTGATGAGGAGAAC----------------------------
----

AAGACTGATGAGATTTTCAGACCTGGAGGAGGAGATATGAGGGACAATTGGAGAAGTGAACTATATAAATATA
AAGTAGTAAAAATTGAACCATTA---GGAGTAGCACCCTCCAGGGCAAAGAGAAGAGTGGTG-----------
----------------------------CAGAGAGAAAAAAGAGCA---GTGGGA---CTAGGAGCT------
---TTGTTCATTGGGTTCTTGGGAGCA---GCAGGAAGCACTATG---GGCGCAGCGTCA---
GTGACGCTGACGGTACAG---GCCAGACAA---TTATTG---TCTGGTATAGTGCAACAGCAGAAC---
AATTTG---CTGAGG---GCTATTGAGGCGCAACATCATCTGTTGCAACTCACAGTCTGGGGCATC
>KF384800..1
TTCTACTGTAATACAACAGGACTGTTTAATAGTACTTGGAATAGTACTAGTACTGAGCTAAATGAC-------
----

ACTACAGGAAATATCAAACTCCCATGCAGAATAAAACAAATTATAAACATGTGGCAGAAAGTAGGAAAAGCAA
TGTATGCCCCTCCCATAGAAGGAGTAATTCAATGTTCATCAAAT---
ATTACAGGGCTAATATTAACAAGAGATGGTGGTGGTGGTAACGATACGATG----------------------
----

GGGAACGAGACCTTCAGACCGGGAGGGGGAGATATGAGGGACAATTGGAGGAGTGAACTATATAGATATAAAG
TAGTAAAAATTGAACCATTA---GGAATAGCACCCACCAAGGCAAAGAGAAGAGTGGTG--------------
-------------------------CAGAGAGAAAAAAGAGCA---GTG------ATAGGAGCG---------
ATGTTCCTTGGGTTCTTGGGAGCA---GCAGGAAGCACTATG---GGCGCAGCGGCA---
GTGACGCTGACGGTACAG---GCCAGACTA---TTATTG---TCTGGTATAGTGCAACAGCAAAAC---
AACTTG---CTGAGA---GCTATTGAGGCGCAACAGCATATGTTGCAACTCACAGTCTGGGGCATT
>KF384801..1
TTTTACTGTAATACATCACAACTGTTTAAGAGTACTTGGTGGACAAATAATACTCGGAATATACCA-------
----

CTACAAGGAAATGTCACACTCCCATGCAAAATAAAGCAAATTATAAACATGTGGCAAGAAGTAGGAAAAGCAA
TGTATGCCCCTCCCATCGGAGGACTAATTAATTGTACATCAAAT---
ATTACAGGGCTGCTATTAACAAGAGATGGTGGTATAACGAATGGG----------------------------
----

ACTGAGACCTTCAGACCTGGAGGGGGAGATATGAGGGACAATTGGAGAAGTGAATTATATAAATATAAAGTAA
TAAAAATTCAACCATTA---GGGATAGCACCCACCAAGGCAAAGAGAAGAGTGGTG-----------------
----------------------CAGAGAGAAAAAAGAGCA---GTGGGA---GTGGGAGCT---------
TTGTTCCTTGGGTTCTTGGGAGCA---GCAGGAAGCACTATG---GGCGCAGCGTCC---
GTAACGCTGACGGTACAG---GCCAGACAA---TTATTG---TCTGGTATAGTGCAACAGCAGAGC---
AATCTG---CTGAGG---GCTATTGAGGCGCAACAACATCTGTTGCAACTCACAGTCTGGGGCATT
>KF384803..1
TTCTACTGTAATTCAACACCACTGTTTAATAGTACCTGGGAAAATGGTACTTGGACGAATGAGAGTGGTATTG
GA---

GGATCAGATAATATCACACTTCCATGCAGAATAAAACAAATTATAAACCTGTGGCAGGAAGTAGGAAAAGCAA
TGTATGCTCCTCCCATCGAAGGACAAATTAGATGTTCATCAAAT---
ATTACAGGGCTGTTATTAACAAGAGATGGTGGTTCTAATCAGACT----------------------------
----

AACGAGACCTTCAGACCTGGAGGAGGAGATATGAGGGACAATTGGAGAAGTGAATTATATAAATATAAAGTAG
TAAAAATTGAACCATTA---GGATTAGCACCCACCAAGGCAAAGAGAAGAGTGGTG-----------------
----------------------CAGAGAGAAAAAAGAGCA---GCAGGA---CTGGGAGCT---------
CTGTTCCTTGGGTTCTTGGGAGCA---GCAGGAAGCACTATG---GGCGCAGCGTCA---
ATGACGCTGACGGTACAG---GCCAGACTA---TTATTG---TCTGGTATAGTGCAACAGCAGAAC---
AATTTG---CTGAGA---GCTATTGAGGCGCAACAGCATCTGTTGCAACTCACAGTCTGGGGCATT
>KF384804..1
TTCTATTGTAATACAACACAACTGTTTAATAGTACTTGGAATGTTACTGATCCTTGGAATTCTCCTGGAGGGT
CAAAT--

AACACTGGAGATATCACACTCCCATGCAGAATAAAACAAATTGTGAACAGGTGGCAGGAAGTAGGAAAAGCAA
TGTATGCCCCTCCAATCTCAGGATATATTAACTGTTCATCAAAA---
ATTACAGGGCTGATATTAACAAGAGATGGTGGTTATAACACCACC----------------------------
----

AATGAGACTGAGATCTTCAGACCTGGAGGAGGAGATATGAGGGACAATTGGAGAAGTGAATTATATAAATATA
AAGTAGTAAGAATTGAACCATTA---GGAATAGCACCTACCACGGCAAAGAGAAGGGTGGTG-----------
----------------------------CAGAGAGAAAAAAGAGCA---GTGGGA---ATGGGAGCG------
---TTGTTCCTTGGGTTCTTGGGAGCA---GCAGGAAGCACTATG---GGCGCAGCGTCA---
ATGATGCTGACGGTACAG---GCCAGACAA---TTATTG---TCTGGTATAGTGCAACAGCAGAAC---
AATCTG---CTGAGG---GCTATTGAGGCGCAACAACATCTGTTGCAACTCACAGTCTGGGGCATC
>KF384807..1
TTCTACTGTAACACAACAACACTGTTCAATAGTACTTGGGATGGGAATGATACTACTGAAAGGTCAAAGAGTA
CTGAAGAA---

AATGCAACAATCATCACAATCCCATGCAGAATAAAACAAATTATAAACATGTGGCAGAAAGTAGGAAAAGCAA
TATATGCCCCTCCCATCAAAGGAAACATTAGTTGTTCATCAAAT---
ATTACAGGGCTGCTATTAACAAGAGATGGTGGGAATAACGAGGAC----------------------------
----

AGCACCAATGAGACCTTCAGACCTCAAGGAGGAGATATGAGGGACAATTGGAGAAGTGAGCTATATAAATACA
AAGTAGTAAGAATTGAACCATTA---GGAATAGCACCCACCAAGGCAAGGAGAAGAGTGGTG-----------
----------------------------CAGAGAGAAAAAAGAGCA---GTGGGA---ATAGGAGCT------
---TTGTTCCTTGGGTTCTTGGGAGCA---GCAGGAAGCACTATG---GGCGCAGCGTCG---
CTGACGCTGACGGTACAG---GCCAGACAA---TTATTG---TCTGGTATAGTGCAACAGCAGAAC---
AATCTG---CTGAGA---GCTATTGAGGCGCAACAGCATATGTTGCAACTCACAGTCTGGGGCATC
>KF384811..1
TTCTACTGTAATTCATCAAAACTGTTTAATAATACTTGGAATGGTACTAACTGGACAGATACATGGTATAATA
GC---

ACAAATGAAACTATCATACTCCCGTGCAGAATAAAACAAGTTATAAACATGTGGCAGGAAGTAGGAAAAGCAA
TGTATGCCCCTCCCATCAGAGGAAAAATTAAATGTAAATCAAAC---
ATTACAGGGCTGTTGTTAACAAGAGATGGCGGTGGTGGGAGCAATGAA-------------------------
----

ACCACCGAGACCTTCAGACCTGGAGGAGGAAATATGAGGGACAATTGGAGAAGTGAATTATATAAATATAAAG
TAGTAAAAATTCAACCAGTA---GGCTTGGCACCCACCAGGGCAAAAAGAAGAGTGGTG--------------
-------------------------CAGAGAGAAAAAAGAGCA---GTAACA---CTGGGAGCT---------
GTGTTCCTTGGGTTCTTGGGAGCA---GCAGGAAGCACTATG---GGCGCAGCGTCA---
ATGACGCTGACGGTACAG---GCCAGACAA---TTATTG---TCTGGTATAGTGCAACAGCAGAGC---
AATTTG---CTGAAA---GCTATTGAGGCGCAACAGCATCTGTTGCAACTCACAGTCTGGGGCATT
>KX156409..2
TTTTACTGTAATACAACAAAACTGTTCAATAGTACTTGGAATAGTAATAGTACTAGCAACAACACTAAAGGA-
----

AATGAGACTATTATCGAAATACCATGCAGGATAAGACAAATTATAAACATGTGGCAGCAAGTAGGAAGAGCAA
TGTATGCCCCTCCCATCAGAGGACTAATTAGATGTTCATCAAAC---
ATTACAGGACTAATATTAACAAGAGATGGTGGTCATAATCAGGCC----------------------------
----

AATGACAGTGAGATCTTCAGACCTGGAGGAGGAGATATGAGGGACAATTGGAGAAGTGAATTATATAAATATA
AAGTAGTAAGAATTGAACCATTA---GGAATAGCACCCACCAAGGCAAAAAGAAGAGTGGTG-----------
----------------------------CAGAGAGAAAAAAGAGCA---GTGGGA---CTAGGAGCT------
---ATGTTCCTGGGGTTCTTAGGAGCA---GCAGGAAGCACTATG---GGCGCAGCGTCA---
GTGACGCTGACGGTACAA---GCCAGACAA---TTATTG---TCTGGTATAGTGCAACAGCAGAAC---
AATTTG---CTGAGA---GCTATTGAGGCGCAACAGCATATGTTGCAACTCACAGTCTGGGGCATT
>KY778615..1
TTCTACTGTAATACATCAAAGTTGTTTAACAGTACTTGGAATGGCACTGAGGAAAACCAGGGA----------
----

GTGAATGGCACACTCACACTCCCATGCAGAATAAAACAAATCATAAACATGTGGCAGAAAGTAGGAAAAGCAA
TGTATGCCCCTCCCATCCAGGGAAATATTAATTGCTCATCAAAT---
ATTACAGGGCTGCTATTAATGAGAGATGGTGGTAACAAGGGGAACGGAACC----------------------
----

GATAATACTACCGAGATCTTTAGACCTGGAGGAGGAAATATGAGGGACAATTGGAGAAGTGAATTATATAAAT
ATAAAGTAGTGAGAATTGAACCATTA---GGAGTAGCACCCACCAAGGCAAAAAGAAGAGTGGTG--------
-------------------------------CAGAGAGAAAAAAGAGCG---GTAACA---GTGGGAGCT---
------ATGTTCCTTGGGTTCTTGGGAGCA---GCAGGAAGCACTATG---GGCGCAGCATCA---
TTGACGCTGACGGTACAG---GCCAGACAA---TTATTG---TCTGGTATAGTGCAACAGCAAAGC---
AATTTG---CTGAAG---GCTATTCAGGCGCAACAGCATCTGTTGCAGCTCACAGTCTGGGGCATC
>KT124768..1
TTCTACTGTAATACATCAGAACTGTTTAATAGTACTTGGTATAACAGTACATACAATGAGACTGCACCA----
----

GGCAATGAAACAATCACACTCCCATGCAAAATAAGGCAGTTTGTAAACAGGTGGCAGGAAGTAGGAAAAGCAA
TGTATGCCCCTCCCATCAGAGGAATAATTAGATGTTTATCAAAT---
ATTACAGGATTGATATTAACAAGAGATGGGGGTATCTATAATAAC----------------------------
----
ACGAATGGGACCATT---
GGGAATGAGACTTTCAGACCTGGAGGGGGAGATATGAGGGACAATTGGAGAAGTGAATTATATAAATATAAAG
TAGTAAAAATTGAACCATTA---GGAGTAGCACCCACCAAAGCAAGGAGAAGAGTGGTG--------------
-------------------------CAGAGAGAAAAAAGAGCA---GTGGGA---TTCGGAGCT---------
ATGTTCCTTGGGTTCTTGGGAGCA---GCAGGAAGCACTATG---GGCGCAGCGTCA---
GTGACGCTGACGGTACAG---GCCAGGCAA---TTATTG---TCTGGTATAGTGCAGCAGCAAAAC---
AACTTG---CTGAGG---GCTATTGAGGCGCAACAGCATATGTTGCAACTCACAGTCTGGGGCATT
>MG196879..1
TTCTACTGTAATACAACGCAACTGTTTAGGGGTAATGGTACTGGTAATGGTACTGGAGAG-------------
----

TCAAGTGACATTGTCACACTCCCATGCAGAATAAAACAAATTGTAAACATGTGGCAGAAAGTAGGAAAAGCTA
TGTATGCCCCTCCCATCAAAGGACTAATTAGATGTTCATCAAAT---
ATTACAGGGCTAATATTAACAAGAGATGGTAGTAATAAAAGTAAA----------------------------
----

```
AGTGATCCAGAGATCTTCAGACCTGCAGGAGGAGATATGAGGGACAATTGGAGAAGTGAATTATATAAATATA
AAGTAGTAAAAATTCAACCATTA---GGAGTAGCACCCACCAAGGCAAAGAGAAGAGTGGTG----------
---------------------------CAGAGAGAAAAAAGAGCA---GTGGGA---ATAGGAGCT------
---GTGTTCCTTGGGTTCTTAGGAGCA---GCAGGAAGCACTATG---GGCGCAGCGTCA---
ATAACGCTGACGGTACAG---GCCAGACAA---TTATTG---TCTGGTATAGTGCAACAGCAGAAC---
AATCTG---CTGAGA---GCTATTGAGGCGCAACAGCATCTGTTGCAACTCACAGTCTGGGGCATC
>MG197013..1
TTCTACTGTAATACAACACAGCTGTTTAATAGCACTTGGAATAGCACGACTAATAATGACACTGAAGGATCA-
------------------------------------------------------------------------
---------------------------
AGTAATGCTACGATCACACTCCCATGTAGAATAAGACAAATTATAAACATGTGGCAGACAGTAGGAAAAGCAA
TGTATGCCCCTCCCATCAGAGGAGAGATTAGCTGTTCATCAAAT---
ATTACAGGGCTGCTGTTAACAAGAGATGGT---AATACCAATCACACT------------------------
------------------------------------------------------------------------
----------
GATGCCACTGAGACCTTTAGACCTGGAGGAGGAGATATGAGGGACAATTGGAGAAGTGAATTATATAAATATA
AAGTGATACAACTTGAACCAATA---GGAGTAGCACCCACCAAGGCAAAGAGAAGAGTGGTG----------
---------------------------CAAAGAGAAAAAAGAGCA---GTGGGGGCAATAGGAGCT------
---ATGTTCCTTGGGTTCTTGGGAGCA---GCAGGAAGCACTATG---GGCGCAGCGTCA---
ATAACGCTGACGGTACAG---GCCAGACTA---TTATTG---TCTGGTATTGTGCAACAGCAAAAC---
AATTTG---CTGAGG---GCTATTGAGGCGCAACAGCATCTGTTGCAACTCACAGTCTGGGGCATC
>MG197046..2
TTCTACTGTAATACCTCAAAACTGTTTAACAGTACATGGCCAAATAATACTAAAAATGCTAATATC-------
------------------------------------------------------------------------
---------------------------
ACTGAAGGAACTATCACACTCCCGTGCAGAATAAAACAAATTATAAACATGTGGCAGGAAGTAGGAAAGGCAA
TGTATGCCCCTCCCATCAGAGGACAACTTAACTGTTCATCAAAT---
ATTACAGGGCTGCTATTAACAAGAGATGGGGGTTATAATCAGGCG---------------------------
------------------------------------------------------------------------
----------
AACAATAGCGAGACCTTCAGACCGGGAGGAGGAGATATGAGGAACAATTGGAGAAGTGAATTATATAAATATA
AAGTAGTAAGAGTTGAACCATTA---GGAGTAGCACCCACCAAGGCAAAGAGAAGAGTGGTG----------
---------------------------CAAAGAGAAAAGAGAGCA---GTGGGA---ATGGGAGCT------
---TTGTTCCTTGGGTTCTTGGGAGCA---GCAGGAAGCACTATG---GGCGCAGCGTCA---
CTAACGCTGACGGTACAA---GCCAGACAA---TTATTG---TCTGGTATAGTGCAACAGCAAAAC---
AATTTG---CTGAGA---GCTATTGAGGCGCAGCAGCATATGTTGCAACTCACAGTCTGGGGCATC
>MG197064..1
TTCTACTGTAATACATCAAAACTGTTTAACAATACCTGGACTAAGGGTACTTGGAATAGTACATGGTCATGGG
CA----------------------------------------------------------------------
---------------------------
AATGAAACTAACATCACACTCCCATGCAGAATAAAACAGATTATAAACATGTGGCAGGAAGTAGGAAAAGCAA
TGTATGCCCCTCCCATCGAAGGAATAATTAGATGTGCCTCAAAC---
ATTACAGGGCTGCTATTAACAAGAGATGGTGGTAAACATGATAATATTACC---------------------
----------------------------------------------------
ATTAATGGTAATGTCATTGACTATAAC------
ACCACCGAGGTCTTCAGACCTGGAGGGGGAGATATGAGGGACAATTGGAGAAGTGAATTATATAAATACAAAG
TAGTACAAATTGAACCATTA---GGAGTAGCACCCACTAAGGCAAAAAGAAGAGTGGTG-------------
------------------------CAGAGAGAAAAAAGAGCA---GTGGTA---CTAGGAGCT---------
TTGTTCCTTGGGTTCTTGGGAGCA---GCAGGAAGCACTATG---GGCGCAGCGTCA---
ATGACGCTGACGGTACAG---GCCAGACTA---TTATTG---TCTGGTATAGTGCAACAGCAGAAC---
AATTTG---CTGAGG---GCTATTGAGGCGCAACAACATCTGTTGCAACTCACAGTCTGGGGCATT
>MG197113..2
TTCTACTGTAACACATCAAAGTTGTTTAATAGCACTTGGAACTATAATAATGCTACAGGAAACACTGAAGGAA
ATAGCACACAA-------------------------------------------------------------
------------------------
GGAAATATCACACTCATCACACTCCCATGCAGAATAAAACAGATTATAAACAGGTGGCAGGAAGTAGGAAAAG
CAATGTATGCCCCTCCCATCAGCGGACAAATTAACTGTTCATCAAAT---
ATTACAGGACTACTATTAACAAGAGATGGTGGTCATAATAACAAT---------------------------
------------------------------------------------------------------------
----------
```

AGCACAATTGAGACCTTTAGACCTACAGGAGGAGATATGAGGAACAATTGGAGAAGTGAATTATATAAATATA
AAGTAGTAAAAATTGAGCCATTA---GGAATAGCACCCACCAAGGCAAAAAGAAGAGTGGTG-----------
----------------------------CAGAGAGAAAAAAGAGCA---GTAGGAACGCTGGGAGCT------
---ATGTTCCTTGGGTTCTTAGGAGCA---GCAGGAAGCACTATG---GGCGCAGCGTCA---
GTGACGTTGACGGTACAG---GCCAGACTA---TTATTG---TCTGGTATAGTGCAGCAGCAGAGC---
AAGCTG---CTGAGG---GCTATTGAGGCGCAACAGCATATGTTGCAACTCACAGTCTGGGGCATC
>KM355159..1
TTCTATTGTAATTTAACAAAACTGTTTAATAGTACTTGGAATAAGACTGAAGGGCCTTGG-------------
----
AATGTTACTGAAGAGTATGGG------------
AATATTACTGACCCTATCATACTCCCATGCAGAATAAAACAAATTATAAACATGTGGCAGAGAGTAGGAAGGG
CAATGTATGCCCCTCCCATCAAAGGCAAACTTCAATGTACATCAAAT---
ATTACAGGGCTCCTATTAACAAGAGATGGTGGT--

-
AAGGTGAATAATGAGACTGAGATCTTTAGGCCTGCAGGAGGAGATATGAGGGACAATTGGAGAAGCGAATTAT
ATAAATATAAAGTAGTAAAAATTGAACCATTA---GGAATAGCACCCACCAGGGCGAAGAGAAGAGTGGTG--
-------------------------------------CAGAGAGAAAAAAGAGCA---GTGGGA---
TTAGGAGCT---------ATGTTCCTTGGGTTCTTGGGAGCA---GCAGGAAGCACTATG---
GGCGCAGCGGCA---GTGACGCTGACGGTACAG---GCCAGACAA---TTATTG---
TCTGGTATAGTGCAGCAGCAGAAC---AATCTG---CTGAAG---
GCTATTGAGGCGCAGCAGCATCTGTTGCAACTCACAGTCTGGGGCATC
>MG196745..1
TTCTACTGTAATTCAACACCACTGTTTAATGGGACTTGGCAGAATATGACGAATATTACT-------------
----

GAAAACTTAAATATCACACTCCCATGCAGAATAAAGCAGATTATAAACATGTGGCAGAAAGTAGGAAAAGCAA
TGTATGCCCCTCCCATCAGTGGAAGTATTCAATGTTCATCAAAC---
ATTACAGGGCTACTGTTAACAAGAGATGGTGGCATCACTAATAACACTAATAGC-------------------
----

AGTAATGATGAGACCTTCAGACCTGGAGGGGGAGATATGAGGGACAATTGGAGAAGTGAATTATATAAATATA
AAGTAGTAAAAATTGAACCTTTA---GGATTAGCACCCACCAGGGCAAGGAGACTAGTGGTG-----------
----------------------------CAAAGAGACAAAAGAGCA---GTGGGA---ATGGGAGCT------
---ATGTTCCTTGGGTTCTTGGGAGCA---GCAGGAAGCACTATG---GGCGCGGCGTCA---
ATGACGCTGACGGTACAG---GCCAGACAA---TTATTG---TCTGGTATAGTGCAACAGCAAAAC---
AACTTG---CTGAAG---GCTATTGAGGCGCAACAGCACCTGTTGCGACTCACAGTCTGGGGCATC
>MG196964..1
TTCTACTGTAATACAACAAAATTGTTTAATAGTACTTGGACTGAGAATAGTACTGAAGGGTCAAAC-------
----

AGCACTGAAATAATCACACTCCCATGCAGGATAAAACAAATTATAAACATGTGGCAGAGAGTAGGGAGAGCAA
TGTATGCCCCTCCCATCAAAGGACCAATTAGATGTTCATCAAAT---
ATTACAGGGCTGCTATTAACAAGAGATGGTGGTCATAATATGACT----------------------------
----

AATGATAGTGAGACCTTCAGACCTGTGGGAGGAGATATGAAGAACAATTGGAGAAGTGAATTATATAAATATA
AAGTAGTAAAAGTCGAACCATTA---GGAGTAGCACCCACCAAGGCAAGGAGAAGAGTGGTG-----------
----------------------------CAGAGAGAGAAAAGAGCA---GTGGGA---ATAGGAGCT------
---GTGTTCCTTGGGTTCTTGGGAGCA---GCAGGAAGCACTATG---GGCGCAGCGTCA---
ATGACGCTGACGGTACAG---GCCAGACAA---CTATTG---TCTGGTATAGTGCAACAGCAGAAC---
AACTTG---CTAAGA---GCTATTGAAGCGCAACAGCATCTGTTGCAGCTCACAGTCTGGGGCATT
>MG197035..1
TTCTACTGTGATACAACACAACTGTTCAATATTACTTGGGATACTAACAACACTAGTAATAGCAATGGTACT-
----

GACGGTGATAAAATCACACTCCCATGCAGAATAAAACAAATTATAAATCTGTGGCAGGGAGTAGGAAGAGCAA
TGTATGCCCCTCCCATCAGCGGACTAATTAGCTGTTCATCAAAT---
ATTACAGGTCTGCTGTTAGTAAGTGATAGTGGTAATCAAACTAAC----------------------------
----

GAGACTGAGACCTTCAGACCTATAGGAGGAGAGATGAGGGACATTTGGAGAAGTGAATTATACAAGTATAAAG
TAGTACAAATTGAACCATTA---GGAGTAGCACCCACCAAGGCAAAGAGAAGAGTGGTG-------------
-------------------------CAGAGAGAAAAAAGAGAG---CTGACA---TTAGGAGCT---------
TTGTTCATTGGGTTCTTGGGGGCA---GCAGGAAGCACTATG---GGCGCAGCGTCG---
ATGACGCTGACGGTACAG---GCCAGACTA---TTATTG---TCTGGTATAGTGCAACAGCAGAAC---
AATCTG---CTGAGA---GCTATTGAGGCGCAACAGCATCTGTTGCAACTCACAGTCTGGGGCATC
>MK214316..1
TTCTACTGTAATACCGCACAACTGTTTAATAGTAGTTGGACTACTACTGAAAAGACAAATAACACTGAAGGAG
GA---

AATGACACAATAATCATGCTCCCTTGCAGAATAAAACAATTTATAAACAGGTGGCAGGAAGTAGGAAAAGCAA
TGTATGCCCCTCCAATCAGCGGACTAATTAGATGTTCATCAAAC---
ATTACAGGGCTACTGTTAACAAGAGATGGTGGTAGCAGCAATAAAACT------------------------
----

AATGAAACTGAGATCTTCAGACCTCATGGAGGGGATATGAGGGACAATTGGAGAAGTGAATTATATAAATATA
AAGTAGTAAAAATTGAACCATTA---GGAATAGCACCTAGCAAGGCAAAGAGAAGAGTGGTG----------
----------------------------CAGAGAGAAAAAAGAGCG---GTGACA---ATAGGAGCT------
---GTGTTCCTTGGGTTCTTGGGAGCA---GCAGGAAGCACTATG---GGCGCAGCGTCA---
CTGACGCTGACGGTACAG---GCCAGACAA---CTATTG---TCTGGTATAGTGCAACAACAAAGC---
AATTTG---TTGAGG---GCTATTGAGGCGCAACAGCACCTGTTGCAACTCACAGTCTGGGGCATC
>KX505616..1
TTCTACTGTAATACAACACAACTGTTTAATAGTACTTGGAAGGAAAATAGTACTGGGAATAATACTGAAGAG-
----

TTAAATGACACTATCACACTCCCATGTAGAATAAAACAAATTATAAACATGTGGCAGGAAGTAGGAAAAGCAA
TGTATGCCCCTCCCATCCAAGGACAAATTAGATGTTCATCAAAT---
ATTACAGGGCTAGTATTAACAAGAGATGGTGGTCATACCAATAAGACTGAG---------------------
----

AACAAGACTGAGGTCTTCAGACCTGTAGGAGGGAATATGAAGGACAATTGGAGAAGTGAATTATATAAATATA
AAGTAGTAAAAATTGAACCATTA---GGAGTAGCACCCACTAAGGCAAAGAGAAGAGTGGTG----------
----------------------------CAGAGAGAAAAAAGAGCA---GCA------ATAGGAGCT------
---GTGTTCCTTGGGTTCTTGGGAGCA---GCAGGAAGCACTATG---GGCGCAGCGTCG---
ATAACGCTGACGGTACAG---GCCAGACTA---TTATTG---TCTGGTATAGTGCAACAGCAGAGC---
AATCTG---CTGAGG---GCTATTGAGGCGCAACAGCAGCTGTTGCAGCTTACAGTCTGGGGCATC
>KR182192..1
TTCTACTGCGATACAACACAGCTGTTTAACAGTACTTGGTGGACTAATGAAACTTGGAAGGATATCGAAGGGT
CA---

AATAACACTAACATCACACTTCCATGCAGGATAAAACAAATTATAAACAGGTGGCAGGGAGTAGGAAAAGCAA
TGTATGCCCCTCCCATCAAAGGACAAATTAGCTGTTCATCAAAT---
ATTACAGGGCTAATATTAACAAGAGATGGTGGTACTCACGAGGAC----------------------------
----

GAGACCCCTGAGATCTTCAGACCTGGAGGAGGAGATATGAGGGACAATTGGAGAAGTGAATTATATAAATATA
AAGTAGTAAAAATTGAGCCATTA---GGAGTAGCACCCACCAAGGCAAAGAGGAGAGTGGTG----------
----------------------------CAGAGAGAAAGAAGAGCA---GTGGGAACGCTGGGAGCT------
---ATGTTCCTTGGGTTCTTGGGAGCA---GCAGGAAGCACTATG---GGCGCAGCATCG---
ATAACGCTGACGGTACAA---GCCAGACAA---TTATTG---TCTGGTATAGTGCAACAGCAGAAC---
AACTTG---CTGAAG---GCTATTGAGGCGCAACAGCACCTGTTGCAACTCACAGTCTGGGGCATC
>KR182242..1
TTCTATTGTAATACAACACCACTGTTTAACAGTACTTGGGTGCTTAACAGTACTTTGAATAGTACTGAAGGGT
CCAATAGT---

ACTGGACAAAATATCACACTCCAATGTAGAATAAAGCAAATTATAAATATGTGGCAGGAGGTAGGAAAAGCAA
TGTTTGCCCCTCCCATCAGAGGATCAATTCAATGTTCATCCAAC---
ATTACAGGGCTGCTACTAACAAGAGATGGTGGTAATGACAATAAC---------------------------
----

AAGACTGAGATCTTTAGGCCTTGGGGAGGAGATATGAAGGACAATTGGAGAAGTGAATTATATAAATATAAAG
TAATAAAAATTGAACCATTA---GGAGTAGCACCCACCAAGGCAAAGAGAAGAGTGGTG--------------
-------------------------CAGAGAGAAAAAAGAGCG---GCAGGA---CTGGGAGCT---------
CTGCTCATTGGGTTCTTGGGAGCA---GCAGGAAGCACAATG---GGCGCAGCGTCA---
GTGACGCTGACGGTACAG---GCCAGACTA---TTATTG---TCTGGTATTGTGCAACAGCAGAGC---
AATCTG---CTGAGA---GCTATTGAGGCGCAACAGCATATGTTGAAGCTCACAGTCTGGGGCATC
>KR182343..1
TTCTACTGTGATACAACAAAACTGTTTAATAGTACTTGGCCTTGGCCTGCTAATAAAACAGATGATGGCACAA
ATGCA--
-------------------ACA---
GCATCAGACAAAAATATCACACTCCCATGCAGAATAAAACAAATTATAAACATGTGGCAGAAAGTGGGAAGAG
CAATATATGCCCCTCCCATCAAAGGACCAATTGAATGTGTATCAAAT---
ATTACAGGGATAATATTAACAAGGGATGGTGATAAGAATAATACC----------------------------
----

TACAATGAGACCTTCAGACCTACAGGAGGAGAAATGAAGGACAATTGGAGAAGTGAATTATATAAATATAAAG
TAATAAAAATTGAACCATTA---GGAATAGCACCTACCAGGGCAAAGAGAAGAGTGGTG--------------
-------------------------CAGAGAGAAAAAAGAGCA---GTGGGA---ATAGGAGCT---------
CTGCTTCTTGGGTTCTTGGGAGCA---GCAGGAAGCACTATG---GGCGCAGCGTCA---
ATGACGCTGACGGTACAA---GCCAGGCAA---TTACTG---TCTGGCATAGTGCAACAGCAGAAC---
AATCTG---CTGAAA---GCTATTGAGGCGCAACAGCAGCTGTTGCAACTCACAGTCTGGGGCATC
>KT986555..1
TTCTACTGTAATACTACACAACTGTTTAATAGTACTTGGAATAAGAATAAAACTAATAATACTTGGGAAAGGA
ATGACACA---
-------------------AAT---
AGTAATGAAACAACAATCACAATCCCATGCAGAATAAAACAAATTATAAACATGTGGCAGGGAGTAGGCAAGG
CAATGTATGCCCCTCCCATCAGAGGATTAATCAGATGTTCATCAAAC---
ATTACAGGACTGATATTAACAAGAGATGGTGGTCAGAGTAATGAAACAGGG----------------------
----

AACAACACCGAGATATTCAGACCTGGAGGAGGAGACATGAGGGACAATTGGAGAAGTGAATTATATAGATATA
AAGTAGTAGAAATTGAACCATTA---GGAATAGCACCCACCAAGGCGAAGAGAAGAGTGGTG-----------
----------------------------CAGAGAGAAAAAAGAGCG---GTCACA---CTAGGAGCT------
---GTGTTCCTTGGGTTCTTGGGAGCA---GCAGGAAGCACTATG---GGCGCAGCGTCA---
ATGACGTTGACGGTACAA---GCCAGACAG---TTATTG---TCTGGTATAGTGCAACAGCAGAAC---
AATCTG---CTAAGA---GCTATTGAGGCGCAACAGCACCTGTTGCAACTCACGGTCTGGGGCATC
>KX505508..1
TTCTATTGTAATTTAACAAAACTGTTTAATAGTACTTGGAATAAGACTGAAGGGCCTTGGAATGTTACTGAAG
AGTATGGG---

AATATTACTGACCCTATCATACTCCCATGCAGAATAAAACAAATTATAAACATGTGGCAGAGAGTAGGAAGGG
CAATGTATGCCCCTCCCATCAAAGGCAAACTTCAATGTACATCAAAT---
ATTACAGGGCTCCTATTAACAAGAGATGGTGGTAAGGTGAATAAT----------------------------
----

GAGACTGAGATCTTTAGGCCTGCAGGAGGAGATATGAGGGACAATTGGAGAAGTGAATTATATAAATATAAAG
TAGTAAAAATTGAACCATTA---GGAATAGCACCCACCAGGGCGAAGAGAAGAGTGGTG--------------
-------------------------CAGAGAGAAAAAAGAGCA---GTGGGA---TTAGGAGCT---------
ATGTTCCTTGGGTTCTTGGGAGCA---GCAGGAAGCACTATG---GGCGCAGCGGCA---
GTGACGCTGACGGTACAG---GCCAGACAA---TTATTG---TCTGGTATAGTGCAGCAGCAGAAC---
AATCTG---CTGAAG---GCTATTGAGGCGCAGCAGCATCTGTTGCAACTCACAGTCTGGGGCATC
>KX027971..1
TTCTACTGTAATACAACACGACTGTTCGAAAGTACTTGGCATGGAAATGAGACTTGGTATGGGAATGAG----
----
-ACTATAGAGCCAGAG---------
GCAAATGACACAATAATCGAACTCCCATGCAGAATAAAACAAATTATAAACATGTGGCAGGAAGTAGGAAAAG
CAATGTACGCCCCTCCCATCAGAGGACCAATTAAATGTTCATCAAAT---
ATTACAGGGCTACTATTAACAAGAGATGGTGGTGATGATCAGTACAGTAAGAAT------
GAGACCTCCTGCAATAAT---

ACTACTGAGACCTTCAGACCTGGAGGAGGGAATATGAAGGACAATTGGAGAAGTGAATTATATAAATATAAAG
TAGTAAAAATAGAACCATTA---GGAGTAGCACCCACCAAGGCAAAAAGAAGAGTGGTG-------------
------------------------CAGAGAGAAAAAAGAGCA---GTAGGA---ATAGGAGCT---------
ATGTTCCTTGGGTTCTTGGGAGCA---GCAGGAAGCACTATG---GGCGCAGCATCA---
ATAACGCTGACGGTACAG---GCCAGACAA---TTATTG---TCTGGTATAGTGCAACAGCAGAAC---
AATCTG---CTAAGG---GCTATTGAGGCGCAACAACATCTGTTGCAACTCACAGTCTGGGGCATC
>KX505446..1
TTCTACTGCAATACAACACAACTATTTAATAGTACTTGGTCAAAAAATAATACTTGGGAGAAGAATGAAGACA
ATTCA---

ATTAATAGTACTATCACACTCCCATGCAGAATAAAACAAATTATAAACATGTGGCAGGAAGTAGGAAAAGCAA
TGTATACCTCCCCAATCAAAGGACAGATTAGCTGTAACTCAAGT---
ATTACAGGGATACTATTAACAAGAGATGGTGGAAGAAATAATAGT---------------------------
----

GACAATGAGACCTTCAGACCTGGAGGAGGAGATATGAGGGACAATTGGAGAAGTGAATTATATAAATATAAAG
TAGTAAAAATTGTACCATTA---GGAATAGCACCCACTGCGGCAAAAAGAAGAGTGGTG-------------
------------------------CAGAGAGAAAAAAGAGCA---GCA------CTAGGAGCT---------
ATGTTCCTTGGGTTCTTGGGAGCA---GCAGGAAGCACTATG---GGCGCAGCATCA---
GTGACGCTGACGGTACAG---GCCAGACTA---TTATTG---TCTGGTATAGTGCAACAGCAGAAC---
AATCTG---CTGAGG---GCTATTGAGGCGCAACATCATCTGTTGCAACTCACAGTCTGGGGCATC
>KT986947..1
TTCTACTGTGATACAACAAAACTATTTAATAGTACTTGGAATAGTACTTGGGACAATAATAGTACTCGGAATA
AT---
AACAGATCAGAGGACAGATCA------------
GAGAGCGATGACACAATCACACTACCATGCAGAATAAGACAAATTATAAACAGATGGCAAGAAGTAGGAAAAG
CAATGTATGCCCCTCCCATCAAAGGACTAATTGAATGTAACTCAACA---
ATTACAGGGCTGCTCTTAGCAAGAGATGGTGGTATCAACAGCACA---------------------------
----

GGGAATGAGACCTTCAGACCTCAAGGAGGAAATATGAGGGACAATTGGAGAAGTGAATTATATAAGTATAAAA
TAGTAAAAATTGAACCATTA---GGAATAGCACCCACTAAGGCAAAAAGAAGAGTGGTG-------------
------------------------CAGAGAGAAAAAAGAGGA---GCGGGA---CTAGGAGCT---------
TTGTTCCTTGGATTCTTGGGAGCA---GCAGGAAGCACTATG---GGCGCAGCGTCA---
GTGACGCTGACGGTACAA---GCCAGACAA---TTATTG---TCTGGTATAGTGCAACAGCAGAAC---
AATCTG---CTGAGG---GCTATTGAGGCGCAGCAGCATATGTTGCAACTCACAGTCTGGGGCATC
>MH264238..1
TTCTACTGTAATTCAACACAGCTGTTTAATAGTACTTGGTATCTTAATGGTACTTGGAATGCAACTGAAGGGT
TAAATAAC--

ACTGAAGGAAATATCACACTCCCATGCAGAATAAAACAAATTATAAACATGTGGCAGCAGGTAGGAAAAGCAA
TGTATGCCCCTCCTATCAGAGGACTAATTAGATGCACATCAAAC---
ATTACAGGGATAATATTAACAAGAGATGGTGGTTACGAGAACAAGACCGATAAC------------------
----

AATACTAATACTGAGATCTTCAGACCTGGAGGAGGAGATATGAGGGACAATTGGAGAAGTGAATTATATAAAT
ATAAAGTAGTAAGAATTGAACCATTA---GGAGTAGCACCCACCAAGGCAAAGAGAAGAGTGGTG--------
------------------------------CAGAGAGAAAAAAGAGCA---GTGGGA---ATAGGAGCC---
------TTGTTCCTTGGGTTCTTGGGAGCG---GCAGGAAGCACTATG---GGCGCAGCGTCA---
ATGACGCTGACGGTACAG---GCCAGACAA---TTATTG---TCTGGTATAGTGCAACAGCAAAAC---
AACTTG---CTGAGG---GCTATTGAGGCGCAACAGCATCTGTTGCAACTCACAGTCTGGGGCATC
>KX587339..1
TTCTATTGTAATACAACAAACCTGTTTAATAGTACATGGCATACTAACGGAACAAATAGCACTGACAAT----
----

GGTACTGACACAATCACAATCCAATGCAGAATAAAACAAATAATAAACACGTGGCAGGAAGTAGGAAAAGCAA
TGTATGCCCCTCCCATCCAAGGACAACTTAACTGTTCATCAAAT---
ATTACAGGGCTGCTATTAACAAGAGATGGTGGTAATGATAGTAATACT------------------------
----

ACCAATAACACTGAGATCTTCAGACCTCAAGGAGGAAATATGAGAGACAATTGGAGAAGTGAATTATATAAAT
ATAAAGTAGTAAAAATTGAACCATTA---GGAGTAGCACCCACCGAGGCAAAGAGAAGAGTGGTG--------
-------------------------------CAGAGAGAAAGAAGAGCA---GTGGGA---CTAGGAGCT---
------ATGTTCCTTGGGTTCTTAGGAGCA---GCAGGAAGCACTATG---GGCGCAGCGTCA---
TTGACGCTGACGGTACAG---GCCAGACAA---TTATTG---TCTGGTATAGTGCAACAGCAGAAC---
AATTTG---CTGAGA---GCTATTGAGGCGCAACAGCATCTGTTGCAACTCACAGTCTGGGGCATT
>KX505536..1
TTCTACTGTAATACAACACAGCTGTTCAATAGTACATGGGATTGTACTGGAACTGCATGTACATTGAATGGA-
----
-ACTGTTCTTAATGGG---------
ATAAATAACACCAATATTACTCTCCCATGCAGGATAAAACAAATTATAAACATGTGGCAAAAAGTAGGAAAAG
CAATGTACAACCCTCCCATCAAAGGAGTAATTCAATGTACATCAAAT---
ATTACAGGGATAATATTAACAAGCGATAATGGTGGGATTGGAAAT----------------------------
----

AATGAAAATGAAACCTTCAGACCTGCAGGAGGAGATATGAGGGACAATTGGAGAAGTGAATTATATAAATATA
AAGTAGTAAGAATTGAGCCATTA---GGAATAGCACCCACCAAGGCAAAAAGGAGAGTGGTG-----------
----------------------------CAGAGAGAAAAAAGAGCA---GTAACA---TTAGGAGCT------
---ATGTTCATTGGGTTCTTGGGAGCA---GCAGGAAGCACTATG---GGCGCAGCGTCA---
GTGACGCTGACGGTACAG---GCCAGACAA---TTGCTG---TCTGGTATAGTGCAACAGCAGAGC---
AATCTG---CTGAAG---GCTATTGAGGCGCAACAGCATCTGTTGCAACTCACAGTCTGGGGCATC
>MG196412..1
TTCTACTGTAATTCATCAAAACTGTTTAATAGTACTTGGGGAAGTACTGACGGGTCAAATAGCACTGAA----
----

ACAAATGGCACAATCATACTCCCATGCAGAATAAGACAAATTATAAACAAGTGGCAGCAAGTAGGAAAAGCAA
TATACGCCCCTCCCATCCAAGGACGAATTAAATGTTTATCAAAT---
ATTACAGGGCTGCTATTAACAAGAGATGGTGGTCATAACAACAGTAGCAAT----------------------
----

GAGAGTAGGAGTGAGATCTTCAGACCTGGAGGAGGAGACATGAGGGACAATTGGAGAAGTGAATTATATAAAT
ATAAAGTAGTAAGAATTGAACCAGTA---GGAATAGCACCCACCAAGGCAAAGAGAAGAGTGGTG--------
-------------------------------CAGAGAGAAAAAAGAGCA---GTGGGAACAATAGGAGCT---
------ATGTTCCTTGGGTTCTTGGGAGCA---GCAGGAAGCACTATG---GGCGCAGCGTCA---
TTGACGCTGACGGTACAG---GCCAGACAA---TTATTG---TCTGGTATAGTGCAACAGCAGAAC---
AATCTG---CTGAAG---GCTATTGAGGCGCAACAGCACCTGTTGCAACTCACAGTCTGGGGCATC
>MK169417..1
TTCTACTGTAATACAACACAATTGTTTAATAGTAGTTGGATTCGGAATGGTACTGATTGGACTTGGAATAGTA
CCACA--

CAAAATGATACTATCATACTCCCATGCAAAATAAAACAAATTATAAACATGTGGCAGGAAGTAGGAAAAGCGA
TGTATGCCCCTCCTATCGAAGGAATAATTAGGTGTGTATCAAAT---
ATTACTGGGTTGCTATTAACAAGAGATGGTGGTCACAACAGCAGC----------------------------
----

AACGGTACTGAGACCTTCAGACCTGCAGGAGGAAATATGAAGGACAATTGGAGAAGTGAATTATACAAATATA
AAGTAGTAAAAGTTGAACCATTA---GGAGTAGCACCCACCAAGGCAAAGAGAAGAGTGGTG-----------
----------------------------CAGAGAGAAAAAAGAGCA---ATAGGAGGACTAGGAGCT------
---GTGTTCCTTGGGTTCTTGGGAGCA---GCAGGAAGCACTATG---GGCGCAGCATCA---
ATGACGCTGACGGTACAG---GCCAGACAA---TTATTG---TCTGGTATAGTGCAGCAGCAGAAC---
AATCTG---CTGAAG---GCTATTGAGGCGCAACAGCATATGTTGCAACTCACAGTCTGGGGCATC
>MK169834..1
TTCTACTGTAATACAACACCACTGTTTAATCCAAATGGAATAAGAAAT-------------------------
----

GACACTGGAAATATCACACTCCAATGCAGAATAAAACAAATTATAAACATGTGGCAGGAAGTGGGAAAAGCAA
TGTATGCCCCTCCCATCCCAGGTCAAATTAGGTGTGTATCAAAT---
ATTACAGGGCTGATATTAACAAGAGATGGTGGTGATAATAACGAG----------------------------
----

ACCGAGGTCTTCAGACCTGGAGGAGGAGATATGAGGGACAATTGGAGAAGTGAATTATATAAATATAAAGTAG
TACAAATTGAACCATTA---GGAGTAGCACCCACCACGGCAAGAAGAAGAGTGGTG-----------------
----------------------CAGAGAGAAAAAAGAGCA---GTAACT---TTTGGAGCT---------
CTGTTCCTTGGGTTCTTGGGAGCA---GCAGGAAGCACTATG---GGCGCAGCGTCA---
ATGACGCTGACGGTACAG---GCCAGGCAA---TTATTG---TCTGGTATAGTGCAGCAGCAGAAC---
AATCTG---CTGAGA---GCTATTGAGGCGCAACAACATATGTTGCAACTCACAGTCTGGGGCATT
>AF025753..1
TTCTACTGTAATACAACAAAACTGTTTAATAGTACTTGGAATAGTACTTGGAATGATACTGAAGGGTCAATTG
CCACTGAA---
-------------GGAAAT------
ACCACTGAAGAAAATATCACACTCCCATGCAGAATAAAACAAATTATAAACATGTGGCAGGAAGTAGGAAAAG
CAATGTATGCCCCTCCCATCGCAGGACAAATTAGATGTTCATCAAAT---
ATTACAGGGCTGCTATTAACAAGAGATGGTGGTAATGGTAATAGCAGC-------------------------
----

AACTACACTTCCGAGATCTTCAGACCGGGAGGAGGAGATATGAGGGACAATTGGAGAAGTGAATTATATAAAT
ATAAAGTAGTAAAAATTGAACCATTA---GGAGTAGCACCCACCAAGGCAAAGAGAAGAGTGGTG--------
-------------------------------CAGAGAGAAAAGAGAGCA---GTGGGA---ATAGGAGCG---
------TTGTTCCTCGGGTTCTTGGGAGCA---GCAGGAAGCACTATG---GGCGCAGCGTCA---
CTGACGCTGACGGCACAG---GCCAGACTA---TTATTG---TCTGGTATAGTGCAACAGCAGAAC---
AATCTG---CTGAGG---GCTATTGAGGCGCAACAGCATCTGTTGCAACTCACAGTCTGGGGCATT
>MH012806..2
TTCTACTGTAATTCAACACAACTGTTTAATAGTACTTGGAGTAGTAATAGTACTGGGAGTAATAGCACTGAA-
----

GAAGAAAGAAATATCACACTCCCATGCAGAATAAAGCAAATTATAAACATGTGGCAGGAAGTAGGAAAAGCAA
TGTATACCCCTCCCATCGGAGGACAAATTCAATGTTCATCAAAT---
ATTACAGGGCTACTATTAACAAGAGATGGTGGTCATAGTGGGAACGAGTCC----------------------
----

GAGAACAGGCCCGAGATCTTCAGACCAGGAGGAGGAGATATGAGGGATAATTGGAGAAGTGAATTATATAAAT
ATAAAGTAGTAAAAGTTGAACCATTA---GGAATAGCACCCACCAAGGCAAAGAGAAGAGTGGTG--------
-------------------------------CAGAGAGAAAAGAGAGCA---GTGGGA---ATAGGAGCT---
------ATGTTCCTTGGGTTCTTGGGAGCA---GCAGGAAGCACTATG---GGCGCAGCGTCG---
GTGACGCTGACGGTACAG---GCCAGACAA---TTATTG---TCTGGTATAGTGCAACAGCAGAAC---
AATCTG---CTGAAG---GCTATTGAGGCGCAACAGCATCTGTTGCAACTCACAGTCTGGGGCATC
>MH012971..1
TTCTACTGTAATTCAACACAACTGTTTAAGAGTATTTGGCATGGGAATGAAACCACGTGGAATACAAAT----
----

AACACTGAAAATATCACACTCCCGTGCAGAATAAAACAATTTATAAACATGTGGCAGGAAGTAGGGAAAGCAA
TGTATGCCCCTCCCATCAGTGGACAAATTAGATGTTCATCAAAT---
ATTACAGGGCTGCTATTAACAAGAGATGGTGGTGCTAAGGAGAAC----------------------------
----

GAGACCACCTACAATGAGACCTTCAGACCTGGAGGAGGAGATATGAGGGACAATTGGAGAAGTGAATTATATA
AATATAAAGTAGTAAGAATTGAACCATTA---GGAGTAGCACCCACCAAGGCAAAGAGAAGAGTGGTG-----
----------------------------------CAGAGAGAAAAAAGAGCA---GTGGGA---
ATAGGAGCT---------CTGTTCCTTGGGTTCTTGGGAACA---GCAGGAAGCACTATG---
GGCGCAGCGTCA---CTGACGCTGACGGTACAG---GCCAGACAA---TTATTG---
ACTGGTATAGTGCAACAGCAGAAC---AACCTG---CTGAGA---
GCTATTGAGGCGCAACAGCATCTGTTGCAACTCACAGTCTGGGGCATC
>MH012542..1
TTCTACTGTAATACAACACAACTGTTTAATAGTACTTGGACTTGGAATGATACTAAAGGGTCAAATGACACTG
GA---

GGAAATGGCACAATCACACTCCCATGCAGAATAAAACAAATTATAAACATGTGGCAGGAAGTAGGAAAAGCAA
TGTATGCCCCTCCCATCCCAGGACAAATTAGATGTTCATCAAAT---
ATTACAGGGCTGCTATTAACAAGAGATGGTGGTAACAATAATCAGAGCAGCGAG-------------------

----

ACCACCGAGGTCTTCAGACCTGGAGGAGGAGATATGAGGGACAATTGGAGAAGTGAATTATATAAATATAAAG
TAGTAAAAATTGAACCATTA---GGAGTAGCACCCACCAAGGCAAAGAGAAGAGTGGTG--------------
-------------------------CAGAGAGAAAAAAGAGCA---GTGGGA---ATAGGAGCT---------
GTGTTCCTTGGGTTCTTGGGAGCA---GCAGGAAGCACTATG---GGCGCAGCGTCA---
ATGACGCTGACGGTACAG---GCCAGACTA---TTATTG---TCTGGTATAGTGCAACAGCAGAAC---
AATTTG---CTGAGG---GCTATTGAGGCGCAACAGCATCTGTTGCAACTCACAGTCTGGGGCATC
>AY835435..1
TTCTACTGTAATACAACACAACTGTTTAATAGTACTTGGTTTAATAATGCTACTTTGAATGTTAATAGTAATG
TTACT--

GAAGGGTCAGAAAATATCACACTCCCATGCAGAATAAGACAAATTGTGAACATGTGGCAAGAAGTAGGAAAAG
CAATGTATGCCCCTCCCATCCAAGGGCAAATTAGATGTTCATCAAAT---
ATTACAGGGCTGCTATTAACAAGAGATGGTGGTGGTAGTAACAGTAGT-------------------------
----

AACACGAGCGAGGAGGTCTTCAGACCTGGAGGAGGAAATATGAGGGACAATTGGAGAAGTGAATTATATAAAT
ATAAAGTAGTAAAAATTGAACCATTA---GGAATAGCACCCACCAAGGCAAAGAGAAGAGTGGTG--------
-------------------------------CAGAGAGAAAAAAGGACA---GTGGGA---ATAGGAGCT---
------TTGTTCCTTGGGTTCTTGGGAGCA---GCAGGAAGCACTATG---GGCGCAGCGTCA---
ATGACGCTGACGGTACAG---GCCAGACAA---TTACTG---TCTGGTATAGTGCAACAGCAGAAC---
AATCTG---CTGAGG---GCTATTCAGGCGCAACAGCATCTGTTGCAGCTCACAGTCTGGGGCATT
>AY331292..1
TTCTACTGTGATACAACAAAACTGTTTAATAGTACTTGGCATGGTAATGAAACATCTTGGAATAATACTGAAA
AG---

TTAAATGACACTATCATACTCCCATGCAGAATAAAACAAATTATAAACATGTGGCAGGAAGTAGGAAAAGCAA
TGTATGCCCCTCCAATCGAAGGACTAATTAACTGTTCATCAAGT---
ATTACGGGGCTGCTATTAACAAGAGATGGTGGTAAGAGCAATAAT----------------------------
----

CAGACTGAGATCTTCAGACCTGGAGGAGGAGATATGAGGGACAATTGGAGAAGTGAACTATATAAATATAAAG
TAGTAAAAATTGAACCAGTA---GGAGTAGCACCCACCAAGGCAAAGAGAAGAGTGGTG--------------
-------------------------CAGAGAGACAAAAGAGCA---GTGGGA---CTAGGAGCG---------
TTGTTCATTGGGTTCTTGGGAGCA---GCAGGAAGCACTATG---GGCGCAGCGTCA---
ATGACACTGACGGTACAG---GCCAGACAA---TTATTG---TCTGGTATAGTGCAACAGCAGAAC---
AACTTG---CTGAGG---GCTATTGAGGCGCAACAACATCTGTTGCAACTCACAGTCTGGGGCATC
>EU289191..1
TTCTACTGTAATACATCACAACTGTTTAATAGTACTTGGAATGGTACTGAAGGGTTTAATAGTACT-------
----

GGGAGTGAAAATATCACACTCCCATGCAGAATAAGACAAGTTGTAAACCTGTGGCAGGAAGTAGGAAAAGCAA
TGTATGCCCCTCCCATCAGGGGAGTAATTAAATGTTCATCAAAC---
ATTACAGGGGTACTATTAACAAGAGATGGTGGTTATGATGATAATAATACC----------------------
----

GCAAACACCAACGAGACCTTCAGACCAGGAGGAGGAGATATGAGGGACAATTGGAGAAGTGAATTATATAAAT
ATAAAGTAGTAAGAATTGAACCATTA---GGAGTAGCACCCACCAGGGCAAAGAGAAGAGTGGTG--------
-------------------------------CAGAGAGAAAAAAGAGCA---GTGACA---CTAGGAGCT---
------TTGTTCCTTGGGTTCTTGGGAGCA---GCAGGAAGCACTATG---GGCGCAGCGTCA---
CTGACGCTGACGGTACAG---GCCAGACTC---TTGTTG---TCTGGTATAGTGCAGCAGCAGAAC---
AATCTG---CTGAGG---GCTATTGAGGCGCAACAACATCTGTTGCAACTCACCGTCTGGGGCATC
>KT124770..1
TTCTACTGTAATACATCAAAACTGTTTAATAGTACCTGGGAGTGGAATAATAGTACCAGAGATTGGAATAATA
CT---
GAAATGTCAAATAACACT---------
GAAGGAAATGGAACTATCACTCTCCAATGCAGAATAAAACAAATTGTAAATATGTGGCAGGAAGTAGGAAAAG
CAATGTATGCCCCTCCCATCAGAGGACAAATTAGATGTTCATCAAAT---
ATTACCGGGTTGCTATTAACAAGAGATGGTGGTTACAACATCAGC----------------------------

----

AATAACAACGAGACCTTCAGACCTGGAGGAGGAGATATGAGGGACAATTGGAGAAGTGAATTATACAGATATA
AAGTAGTAAAAATTGAACCATTA---GGAATAGCACCCACCAAGGCAAGGAGAAGAGTGGTG-----------
----------------------------CAAAGAGAAAAAAGAGCA---GTGGGA---TTAGGAGCT------
---ATGTTCCTTGGGTTCTTGGGAGCA---GCAGGAAGCACTATG---GGCGCAGCATCA---
ATGATGCTGACGGTACAG---GCCAGACAA---TTATTG---TCTGGTATAGTGCAACAGCAGAGC---
AATCTG---CTGCAG---GCTATTGAGGCACAACAGCATCTGTTGAAACTCACAGTCTGGGGCATT
>EU575670..1
TTCTACTGTAATACAACAAAACTGTTTAATAGTACTTGGGTTAATGGTACTAGGAATGATACTGAAGAGTCA-
----

AATAGCACTGAAATCACACTCCCATGCAGAATAAAACAAATCATAAACATGTGGCAGGAAGTAGGAAAAGCAA
TGTATGCCCCTCCTATCAGAGGACAAATTAGATGTTCATCAAAT---
ATCACAGGGCTGCTATTAACAAGAGATGGGGGTGAGGGAGTTGAGAAA-------------------------
----

AACGATACCGAGATCTTTAGACCTGAAGGAGGAGATATGAAGAACAATTGGAGAAGTGAATTATATAAATATA
AAGTAGTACAAATTGAACCATTA---GGAGTAGCACCCACCAGGGCAAAGAGAAGAGTGGTG-----------
----------------------------CAGAGAGAAAAAAGAGCA---GTGGGA---ATAGGAGCT------
---ATGTTCCTTGGGTTCTTGGGAGCA---GCAGGAAGCACTATG---GGCGCAGCGTCA---
ATGGCGCTGACGGTACAA---TCCAGACAA---TTATTG---TCTGGTATAGTGCAACAGCAAAAC---
AACTTG---CTGAGA---GCTATTGAGGCGCAACATCATCTGTTGCAACTCACAGTCTGGGGCATT
>EU575927..1
TTCTACTGTAATACCTCACAACTGTTTAATAGCTCTTGGTATTCAAATGGTACTCGGAATGGTACTGAAGAA-
----

TCAAATGACCTTATCACACTCCCATGCAGAATTAAACAAATTGTAAACATGTGGCAGGAAGTAGGAAAAGCAA
TGTATGCCCCTCCCATTAGAGGACACATTAACTGTACATCAAAT---
ATTACAGGGCTGTTATTAACAAGAGATGGGGGTCAGAGTAATGAAACC-------------------------
----

AAGGACGAGGAGATCTTCAGACCTGGAGGAGGAGATATGAGGGACAATTGGAGAAGTGAATTATATAAATATA
AAGTAGTAAGAATTGAACCATTA---GGAATAGCACCCACCAGGGCAAAGAGAAGAGTGGTG-----------
----------------------------CAGAGAGAAAAAAGAGCA---GTGGGA---ATAGGAGCT------
---CTGTTCCTTGGGTTCTTGGGAGCA---GCAGGAAGCACTATG---GGCGCAGCGTCA---
ATAACGCTGACGGTACAG---GCCAGGCTA---TTAATG---TCTGGTATAGTGCAACAGCAAAAC---
AATCTG---CTGAGA---GCTATTGAGGCGCAACAGCATCTGTTGCAACTCACAGTCTGGGGCATC
>AY835442..1
TTCTACTGTAATACAACACAACTGTTTAATAATACTTGGAATGGTACTGGATGGTCAGATAACTCAGAGAACA
CT---

ACAGGAAACGGAACTATCACACTCCCATGCAGAATAAAACAAATTGTTAACCTGTGGCAGGAAGTAGGAAAAG
CAATGTATGCCCCTCCCATCAGAGGACTAATTAGATGTTCATCAAAT---
ATTACAGGGCTGCTATTAACAAGAGATGGAGGTAATAATACCACCACGAAC----------------------
----

GGGACTCAGACTGAGATATTCAGACCTGGAGGAGGAGATATGAGGGACAATTGGAGAAGTGAATTATATAAAT
ATAAAGTAGTAAAAATTGAACCATTA---GGAGTAGCACCCACTAGGGCAAAGAGAAGAGTGGTG--------
-------------------------------CGGAGAGAAAGAAGAGCA---GCA------CTAGGAGCT---
------TTGTTCCTTGGGTTCTTGGGAGCA---GCAGGAAGCACTATG---GGCGCAGCGTCA---
ATGACGCTGACGGTACAG---GCCAGACTA---TTATTG---TCTGGTATAGTGCAACAGCAGAAT---
AATTTG---CTGAGG---GCTATTGAGGCGCAACAGCATCTGTTGCAACTCACAGTCTGGGGCATC
>DQ853463..1
TTCTACTGTAATACAACACAATTGTTTAATGGTATGTGGCATGCTAATGGTACTTGGAATAGTACTTGGAATG
ATACT--
-------------------GGA---
GGGTCAAATGACACTATCAGACTCCCATGTAGAATAAAACAAATTGTAAACATGTGGCAGGAAGTAGGAAAAG
CAATGTATGCCCCTCCCATCAAAGGACAAATTCAATGTTCATCAAAT---
ATTACAGGGCTGATATTAACAAGAGATGGTGGTAACAGCACGAAC----------------------------

```
-------------------------------------------------------------------------
----------
GAGACCGGGGAGGTCTTCAGACCTGGAGGAGGAAATATGAAGGACAATTGGAGAAGTGAATTATATAAATATA
AAGTAGTAGAAATTGAACCATTA---GGAGTAGCACCCACCAAGGCAAAGAGAAGAGTGGTG-----------
----------------------------CAGAGGGAAAAAAGAGCA---GTGGGATTAATAGGAGCT------
---GTGTTCCTTGGGTTCTTGGGAGCA---GCAGGAAGCACTATG---GGCGCAGCGTCA---
ATAGCGCTGACGGTACAG---ACCAGACAC---TTATTG---TCTGGTATAGTGCAACAGCAGAAC---
AATCTG---CTGAGG---GCTATTGAGGCGCAACAACATCTGTTGCAACTCACAGTCTGGGGCATC
>KT124769..2
TTCTACTGTAATACAGCAGAACTGTTTAATAGTACTTGGTATGCAAATGGTACTTGGTATGCAAATGGTACA-
-------------------------------------------------------------------------
-AATAGTACTGGAGGG---------
GGAAACAAGACTAATATCACACTCCCATGCAGAATAAAACAATTTATAAACATGTGGCAGGAAGTAGGAAAAG
CAATGTATGCCCCTCCCATCAGTGGACAGATTAGATGTGCATCAAAT---
ATTACAGGACTGCTATTAACAAGAGATGGTGGTCAGGGCAATCAGACC-------------------------
-------------------------------------------------------------------------
-------
GACAACCAGACTGAGATCTTCAGACCTGTAGGAGGAGATATGAAGAACAATTGGAGAAGTGAATTATATAAAT
ATAAAGTAGTAAGAATTGAACCATTA---GGAATAGCACCCACCAGGGCAAAAAGGAGAGTGGTG--------
-------------------------------CAGAGAGAAAAAAGAGCA---GTGGGA---ATAGGAGCT---
------TTGTTCCTTGGGTTCTTGGGAGCA---GCAGGAAGCACTATG---GGCGCAGCGTCA---
ATGACGCTGACGGTACAG---GCCAGACTA---TTATTG---TCTGGTATAGTGCAACAGCAGAAC---
AATCTG---CTGAGG---GCTATTGAGGCGCAACAACATCTGTTGCAACTCACAGTCTGGGGCATC
>EU289196..1
TTCTACTGTAATACATCAAAACTGTTTAATAGTACTTGGAATAGTACTGGAGGGTCCATTTCTGAA-------
-------------------------------------------------------------------------
----------------------------
GACTCAGAAAATATCACACTCCCATGCAGAATAAAACAAATTGTAAACATGTGGCAGGAGGTAGGAAAAGCAA
TGTATGCCCCTCCCATCAGAGGACAGATCAGATGTTCATCAAAT---
ATTACAGGGCTGCTATTAACAAGAGATGGTGGTATAAATCAGAGC----------------------------
-------------------------------------------------------------------------
-------------
ATCAGCGAGACCTTCAGACCGGGAGGAGGAGATATGAGGGACAATTGGAGAAGTGAATTATACAAATATAAAG
TAGTAAAAATTGAACCATTA---GGAATAGCACCCACCAAGGCAAGGAGAAGAGTGGTG--------------
-------------------------CAAAGAGAAAAAAGAGCA---GTGGGA---ATAGGAGCT---------
GTGTTCCTTGGGTTCTTGGGAGCA---GCAGGAAGCACTATG---GGCGCAGCGTCA---
CTGACGCTGACGGTACAG---GCCAGACTA---TTATTG---TCTGGTATAGTGCAACAGCAGAAC---
AATCTG---CTGAGG---GCTATTGAGGCGCAACAGCATATGTTGCAACTCACAGTCTGGGGCATC
>EU576141..2
TTCTACTGTAATACAACACCACTGTTTAATAGTACTTGGAATAATACTCGGACTGATGATTATCCTACTGGG-
-------------------------------------------------------------------------
----------------------------
GGGGAACAAAATATCACACTCCAATGCAGAATAAAACAAATTATAAACATGTGGCAGGGAGTAGGAAAAGCAA
TGTATGCTCCTCCCATCAGAGGACAAATTAGATGTTCATCAAAT---
ATTACAGGGCTGCTATTAACAAGAGATGGTGGTAGAGATCAGAAT----------------------------
-------------------------------------------------------------------------
-------------
GGTACTGAGACCTTCAGACCTGGAGGAGGAAATATGAGGGACAATTGGAGAAGTGAATTATATAAATATAAAG
TAGTAAAAATTGAACCATTA---GGAATAGCACCCACCGCGGCAAAGAGAAGAGTGGTG--------------
-------------------------CAGAGAGAAAAAAGAGCA---GTGGGA---CTAGGAGCT---------
TTTATCCTTGGGTTCTTGGGAGCA---GCAGGAAGCACTATG---GGCGCAGCGTCA---
ATGGCGCTGACGGTACAG---GCCAGACTA---TTATTG---TCTGGTATAGTGCAACAGCAAAAC---
AATTTG---CTGAGA---GCTATTGAGGCGCAACAGCATATGTTGCAACTCACAGTCTGGGGCATC
>AY835446..1
TTCTACTGTAATACAGCAGAACTGTTTAATAGTACTTGGTATGCAAATGGCACAATTAGTATTGGAGGGGGA-
-------------------------------------------------------------------------
----------------------------
AACAAGACTAATATCATACTCCCATGCAGAATAAAACAATTTATAAACATGTGGCAGGAAGTAGGAAAAGCAA
TGTATGCCCCTCCCATCAGTGGACAGATTAGATGTTCATCAAAT---
ATTACAGGACTGCTATTAACAAGAGATGGTGGTAGGGGCAATCAGACC-------------------------
```


GACAACCAGACTGAGATCTTCAGACCTGTAGGAGGAGATATGAAAAACAATTGGAGAAGTGAATTATATAAAT
ATAAAGTAGTAAGAATTGAACCATTA---GGAATAGCACCCACCAGGGCAAAAAGGAGAGTGGTG--------
-------------------------------CAGAGAGAAAAAAGAGCA---GTGGGA---ATAGGAGCT---
------TTGTTCCTTGGGTTCTTGGGAGCA---GCAGGAAGCACTATG---GGCGCAGCGTCA---
ATGACGCTGACGGTACAG---GCCAGACTA---TTATTG---TCTGGTATAGTGCAACAGCAGAAC---
AATCTG---CTGAGG---GCTATTGAGGCGCAACAGCATCTGTTGCAACTCACAGTCTGGGGCATC
>EF593282..1
TTCTACTGTAATTCAACACCACTGTTTAGTAGTACTTGGTATACTGGGAATAGTACCAATAGTACTAAAGGGT
TAAAT--

AACGCTACAGAAATTATCACACTCCTATGTAGAATAAAACAAATTATAAACATGTGGCAGGAAGTAGGAAAAG
CAATGTATGCCCCTCCCATCAGAGGACAAATTAGATGCTCATCAAAT---
ATTACAGGGCTGCTATTAACAAGAGATGGTGGTAATAGCAGCAGC----------------------------
----

AATGAAGAGATTTTCAGACCTGCAGGAGGAGATATGAGGGACAATTGGAGAAGTGAATTATATAAATATAAAG
TAGTAAAAATTGAACCATTA---GGAGTAGCACCCACCAAGGCAAAGAGAAGAGTGGTG--------------
-------------------------CAGAGAGAAAAAAGAGCA---GTGGGA---ATAGGAGCT---------
GTGTTCCTTGGGTTCTTGGGAGCA---GCAGGAAGCACTATG---GGCGCAGCGTCA---
GTGACGCTGACGGTACAG---GCCAGACAA---TTATTG---TCTGGAATAGTGCAGCAGCAGAAC---
AATCTG---CTGAGG---GCTATTCAGGCGCAACAGCATCTGTTGCAACTCACAGTCTGGGGCATC
>JQ610043..1
TTCTATTGTAATACAACACCGCTGTTTAGCAGCAGGTGGGGGCCTGACGGCACTTGGAATAGCACTTGGAACA
GTACAAGT---
-------------------ACA---
ACTAACAACGACACTATCACACTCCAATGCAGAATAAAACAAATTATAAACAGGTGGCAGGAAGTAGGAAGAG
CAATGTATGCCCCTCCCATCCAAGGACAAATTAGATGCTCATCAAAT---
ATTACAGGGCTGCTATTAACAAGAGATGGTGGTCATGGTAATGAC----------------------------
----

ACGGATAATACCACCGAGACCTTCAGACCTGGAGGAGGAAATATGAGGGACAATTGGAGAAGTGAACTGTATA
AATATAAAGTAGTAAAAATTGAGCCATTA---GGATTAGCACCCACCAAGGCAAAGAGAAGAGTGGTG-----
----------------------------------CAAAGAGAAAAAAGAGCA---
GTGGGAACGCTAGGAGCT---------ATGTTCCTTGGGTTCTTGGGAGCA---GCAGGAAGCACTATG---
GGCGCAGCGGCA---GTGACGCTGACGGTACAG---GCCAGACAA---TTATTG---
TCTGGTATAGTGCAACAGCAGAAC---AATTTG---CTGAAG---
GCTATTGAGGCGCAACAGCATATGTTGCAACTCACAGTCTGGGGCATC
>EF593212..2
TTCTACTGTAATACAACACCACTGTTTAATAGTACTTGGAATAATACTAATGAAGGGTCAGGTAAC-------
----

TATGAAGGAAATATCACACTCCAATGCAGAATAAAACAAATTATAAATATGTGGCAGCAAGTAGGAAAAGCAA
TGTATGCCCCTCCCATCCAAGGACAAATTTACTGTTCATCAAAT---
ATTACAGGGCTGCTATTAACAAGAGATGGTGGTGGTAATAACGAG----------------------------
----

AGCAGCGAGACCTTCAGACCTGGAGGAGGAGATATGAGGGACAATTGGAGAAGTGAATTATATAAATATAAAG
TAGTAAAACTTGAACCATTA---GGAATAGCACCCACCAGGGCAAAGAGAAGAGTGGTG--------------
-------------------------CAGAGAGAAAGAAGAGCA---GCGGGA---CTAGGAGTT---------
ATGTTCCTTGGGTTCTTGGGAGCA---GCAGGAAGCACTATG---GGCGCAGCGTCA---
ATGGCGCTGACGGTACAA---GCCAGACAA---TTATTG---TCTGGTATAGTGCAACAGCAGAGC---
AATCTG---CTGAGG---GCTATTGAGGCGCAACAACATATGTTGCAACTCACAGTCTGGGGCATC
>EF593300..1
TTCTACTGTAATACAACACAGTTGTTTAATAGTACTTGGAATAGTACTGGGAATAATAATAACAGG-------
----

TCAGGAGAAAATATCACACTCCCATGCAGAATAAAACAATTCATAAACCTGTGGCAAGAAGTAGGAAAAGCAA
TGTATGCTCCTCCCATCAAAGGAGAAATTCAATGCCGATCAAAT---

ATTACAGGGCTAATAATAACAAGAGATGGTGGTAACACTACTGAA----------------------------
----

GAGATCTTCAGACCTGGAGGAGGAGATATGAGGGACAATTGGAGAAGTGAATTATATAAATATAAAGTAGTAA
GAATTGAACCATTA---GGAATAGCACCCACCAAGGCAAAGAGAAGAGTGGTG--------------------
-------------------CAAAGAGAAAAAAGAGCA---GTGGGA---TTCGGAGCT---------
ATGTTCCTTGGGTTCTTGGGAGCA---GCAGGAAGCACTATG---GGCGCAGCGTCA---
ATAGCGCTGACGGCACAG---GCCAGACAA---TTATTG---TCTGGTATAGTGCAACAGCAAAAC---
AATTTG---CTGAGG---GCTATTGAGGCGCAACAGCATATGTTGCAACTCACAGTCTGGGGCATT
>GQ153937..2
TTCTACTGTGATACAACACAACTGTTTAATAGTACTTGGGATAATACTAGTACTTGGAATAATAGTAACACTC
AGAAT--

AAAAATGATAGGAATATCACACTCCAATGCAGGATAAAACAAATTATAAACATGTGGCAGGAAGTAGGAAAAG
CAATGTATGCCCCTCCCATTATGGGACAAATTAGATGTGTATCAAAT---
ATTACAGGGCTGCTATTAACACGGGATGGTGGTAACGGTAGCGAGGCC-------------------------
----

AAAAACGACACGGAGATCTTCAGACCTGGAGGAGGAGATATGAGGGACAATTGGAGAAGCGAATTATATAAAT
ATAAAGTAGTAAAAATTGAACCATTA---GGAGTAGCACCCACCAAGGCAAAGAGAAGAGTGGTG--------
-------------------------------CAGAGAGAAAAAAGAGCA---GTGGGAACGATAGGAGCT---
------ATGTTCCTTGGGTTCTTGGGAGCA---GCAGGAAGCACTATG---GGCGCAGCGTCA---
TTGACGCTGACGGTACAG---GCCAGACAA---TTGTTG---TCTGGTATAGTGCAGCAGCAGAAC---
AATCTG---CTGAGA---GCCATTCAGGCGCAACAGCACCTGTTGCAGCTCACAGTCTGGGGCATT
>MH263520..1
TTCTACTGTAATACAGCAAAGCTATTTAATAGTACATGGTATAGTGCAGGGAATAGCACTGATGGGGATAAAG
GA---

AATAATAACATAATCACACTCCCATGCAGAATAAAACAAATTGTAAACATGTGGCAGGAAGTAGGAAAAGCAA
TGTATGCCCCTCCCATCAGTGGACAAATTAGATGCTCATCAAAT---
ATTACAGGGCTGCTATTAACAAGAGATGGTGGTCATGACAATGACACA-------------------------
----

AACGGGACAGAGATCTTCAGACCTGGAGGAGGGAATATGAAGGACAATTGGAGAAGTGAATTATATAAATATA
AAGTAGTAAGAATTGAACCATTA---GGAGTAGCACCCACCAGGGCAAAGAGAAGAGTGGTG-----------
----------------------------CAGAGAGACAAAAGAGCA---GTGGGA---ATAGGAGCT------
---CTGTTCCTTGGGATCTTGGGAGCA---GCAGGAAGCACTATG---GGCGCAGCGTCA---
ATAACGCTGACGGTACAG---ACCAGACAA---TTATTG---TCTGGTATAGTGCAACAGCAGAAC---
AATTTG---CTGAGG---GCTATTGAGGCGCAACAGCACATGTTGCAACTCACAGTCTGGGGCATT
>KX984437..2
TTCTACTGTAATACATCAAAATTGTTTAGTAGTACTTGGAATGGTACTACCACACTGCCTAGTAGTACTTGGA
ATACTACT---

CAGAATGATACTATCACACTCCCATGCAGAATAAAACAAATTATAAACCTGTGGCAGGAAGTAGGAAAAGCAA
TGTATGCCCCTCCCATCAAAGGACAGATTAAATGTTCATCAAAT---
ATTACAGGGCTGTTATTAACAAGAGATGGTGGTATTACCAACAAC----------------------------
----

AGCATGAATCAGACCTTCAGACCTGGAGGAGGAGATATGAAGGACAATTGGAGAAGTGAATTATATAAATATA
AAGTAGTAAAAATTGAACCATTA---GGAATAGCGCCTACCAAGGCAAAGAGAAGAGTGGTG-----------
----------------------------CAGAGAGAAAAAAGAGCA---GTGGGA---ATAGGAGCT------
---TTGTTCCTTGGGTTCTTGGGAGCA---GCAGGAAGCACTATG---GGCGCAGCGTCA---
ATAACGCTGACGGTACAG---GCCAGACTA---TTATTG---TCTGGTATAGTGCAACAGCAGAAC---
AATTTG---CTGAGG---GCTATTGAGGCGCAACAGCATATGTTGCAACTCACAGTCTGGGGCATC
>KX984699..1
TTCTACTGTAATACAACAAAACTGTTTAGCAGTACTTGGTATGATAATGGAACGTCAAAAAGCACTGAAAGA-
----

TTAAATGACACTATCATACTCCCATGCAGGATAAAACAGATTATAAACATGTGGCAGGAAGTAGGAAAAGCAA
TGTATGCCCCTCCCATCAGAGGACAAATTAACTGTTCATCAAAT---

ATTTCAGGGCTGCTATTAACAAGAGATGGTGGTAATGATAATAACACG-------------------------
----

AACGACATCGAGACATTCAGACCGGGAGGAGGAGATATGAGGGACAATTGGAGAAGTGAATTATATAATTATA
AAGTGGTAAAAATTGAACCATTA---GGAATAGCACCCACTAAGGCAAAGAGAAGAGTGGTG-----------
----------------------------CAAAGAGAAAAAAGAGCG------GGA---ATAGGAGCT------
---TTGTTCCTTGGGTTCTTGGGATTA---GCAGGAAGCACTATG---GGCGCAGCGTCA---
GTGACGCTGACGGTACAA---GCCAGACTA---TTATTG---TCTGGTATAGTGCAACAGCAGAAC---
AATCTG---CTGAGG---GCTATTGAGGCGCAACAGCATCTGTTGCAACTCACGGTCTGGGGCATC
>MH632763..1
TTCTACTGTGATACAACAAAATTGTTTAATAGTACTTGGAATGTTAGTAGTATTTGGAATGTTACT-------
----

GAGGATAAAAATATCACACTCACATGTAGAATAAGACAAATTATAAACAGGTGGCAAGAAGTAGGAAAAGCAA
TGTATGCCCCTCCCATCAGAGGACAAATTAGATGTTCATCAAAT---
ATTACAGGACTGTTATTAACAAGAGATGGTGGTCAGAACACAACA----------------------------
----

AGCGAGACCTTTAGACCTGGAGGAGGAGATATGAGGGACAATTGGAGAAGTGAATTATATAAATATAAAGTAG
TACAAATTAAACCATTA---GGAATAGCACCCACCAGGGCACGGAGAAGAGTGGTG-----------------
----------------------CAGAGAGAGAAAAGAGCA---GTAGGA---CTGGGAGCA---------
ATGTTCCTTGGGTTCTTGGGAGCA---GCAGGAAGCACTATG---GGCGCAGCGTCA---
GTGACGCTGACGGTACAG---GCCAGACAA---TTACTG---TCTGGTATAGTGCAACAGCAGAAC---
AATCTG---CTGAGG---GCTATTGATGCGCAACAGCACCTGTTGCAACTCACAGTCTGGGGCATC
>MH576107..1
TTCTACTGTAATACAACACAACTGTTT------ACTTGGGGGAGTAATAGTACTAACATTACAGGA-------
----

AGTAATAGCACACTCACACTCCCATGCAGAATAAAACAAATTATAAATATGTGGCAGAGAGTAGGAAGAGCAA
TGTATACCCCTCCTATCAGAGGACTAATTCAATGCTCATCAAAT---
ATTACAGGACTACTATTAACAAGAGATGGTGGGTATGGCAATAACAAT-------------------------
----

ACCAATGATACTGAGATCTTCAGACCTGGAGGAGGAGACATGAGGGATAATTGGAGAAGTGAATTATATAAAT
ATAAAGTGGTAAAAATTGAACCATTA---GGAGTAGCACCCACCAAGGCAAGGAGAAGAGTGGTG--------
-------------------------------CAAAGAGAAAAAAGAGCA---GTAGGA---ATAGGAGCT---
------GTGTTCCTTGGGTTCTTGGGAGCA---GCAGGAAGCACTATG---GGCGCAGCGTCA---
ATAACGCTGACGGTACAG---GCCAGACAA---TTACTG---TCTGGTATAGTGCAACAGCAGAAC---
AATTTG---CTGAGG---GCTATTGAGGCGCAACAGCACCTGTTGCAACTCACAGTCTGGGGCATC
>EU616649..1
TTCTACTGCAATACATCAGGACTGTTTAATAGTTCTTGGGATATTAATGGTTCTTGGTATGGTAATAGAGGG-
----

TCAAATGACACAATTACACTCCCATGCAGAATAAAACAATTTATAAACATGTGGCAGGAAGTAGGAAAAGCAA
TGTATGCCCCTCCTATCCAAGGACTAATTAACTGTTTATCAAAT---
ATTACAGGGCTGCTATTAACAAGAGATGGTGGCAATAGCAGTCATAGT-------------------------
----

AATGGGACAGAGACCTTTAGACCTGGAGGAGGAGATATGAGAGATAATTGGAGAAGTGAATTATATAAATATA
AAGTAGTAAAAATTGAACCATTA---GGAGTAGCACCCACCAAGGCAAAGAGAAGAGTGGTG-----------
----------------------------CAGAGAGAAAAAAGAGCA---GTGGGA---ATAGGAGCT------
---TTATTCCTTGGGTTCTTGTCAGCA---GCAGGAAGCACTATG---GGCGCAGCGTCA---
ATGACGCTGACGGTACAG---GCCAGACAA---TTATTG---TCTGGTATAGTGCAACAGCAGAAC---
AATTTG---CTGAGA---GCTATTGAGGCGCAACAGCATATGTTGCAACTCACAGTCTGGGGCATC
>FJ469704..1
TTCTACTGTAACTTAACAAAACTGTTTAATAGTACTTGGGCTTGGAATAGTACTTGGAATAGTACTGAAGGAA
AT---

GAAAATAATACTATCACACTCCCATGTAGAATAAAACAAATTATAAACATGTGGCAGACAGTAGGAAAAGCAA
TGTATGCCCCTCCTATCAGAGGACAAATTACATGCTCATCACAT---

```
ATTACAGGGATACTATTAACAAGAGATGGTGGTAACAGTGATGACACA-------------------------
-------------------------------------------------------------------------
----------
AACTATACTGAGATCTTCAGACCTGCAGGAGGAGATATGAGGGACAATTGGAGAAGTGAATTATATAAATATA
AAGTAGTAAAAATTGAACCATTA---GGAGTAGCACCTACCAAGGCAAAGAGAAGAGTGGTG-----------
----------------------------CAGAGAGAAAAAAGAGCA---GTGGGA---TTAGGAGCT------
---ATGTTCCTTGGGTTCTTGGGAGCA---GCAGGAAGCACTATG---GGCGCAGCGTCG---
CTGACGCTGACGGTACAG---GCCAGGCAG---TTATTG---TCTGGTATAGTGCAACAGCAGAAC---
AATCTG---TTGAGG---GCTATTGAGGCGCAACAGCATCTGTTGCAACTCACAGTCTGGGGCATC
>MN515543..1
TTCTACTGTAATTCAACATCACTGTTTAATAGTACTTGGATGTTTAATAGTACTTGGAATGGCACTGATGGGA
ATGATGCT-----------------------------------------------------------------
-------------------------
GACTCTAGAGAAAATATCACACTCCAATGCAGAATAAAACAAATTATAAACATGTGGCAGGAAGTAGGAAAAG
CAATGTATGCCCCTCCCATCCAAGGGCTAATTAGATGTTCATCAAAT---
ATTACTGGGCTGCTATTAACAAGAGATGGTGGTAATCACAATAACAGT-------------------------
-------------------------------------------------------------------------
----------
AATGGACAGAGACCTTTAGACCTGGAGGAGGAGATATGAGGGACAATTGGAGAAGTGAATTATATAAATATA
AAGTAGTAAAAATTGAACCATTG---GGAGTAGCACCCACCAAGGCAAAGAGAAGAGTGGTG-----------
----------------------------CAGAGAGAAAAAAGAGCA---CTAGGGGGACTAGGAGCT------
---TTGTTCCTTGGGTTCTTGGGAGCA---GCAGGAAGCACTATG---GGCGCAGCGTCC---
CTGACGCTGACGGTACAG---GCCAGACTA---TTATTG---TCTGGTATAGTGCAACAGCAGAAC---
AATCTG---CTGAGA---GCTATTGAGGTGCAACAGCATCTGTTGCAACTCACAGTCTGGGGCATC
>MN515731..1
TTCTATTGTAATACCGCAAAACTGTTTAGTAGTACTTGGCATGCTAATGGTACGTGGAATGGTACTTGGAATA
ATACT--------------------------------------------------------------------
----------------------------
ACAGAGCAAAATATTACAATTCTATGCAGAATAAAACAAATTATAAACATGTGGCAGGAAGTAGGAAAAGCAA
TGTATGCCCCTCCCATCGGAGGAAAAATTAGATGCTCATCAAAT---
ATCACAGGGCTACTATTAGTGAGAGATGGTGGCAACACCACCATC----------------------------
-------------------------------------------------------------------------
-------
GGGAACACCACTGAGATTTTCAGACCTCAAGGAGGAAATATGAAGGACAATTGGAGAAGTGAATTATATAAAT
ATAAAGTAGTACAAATTGAACCAATA---GGAGTAGCACCCACCAAGGCAAAAAGAAGAGTGGTG--------
-------------------------------CAGAGAGAAAAAAGAGCA---GTGGGA---ATAGGAGCT---
------CTGCTCCTTGGGTTCTTGGGAGCA---GCAGGAAGCACTATG---GGCGCAGCGTCA---
ATAACGCTGACGGTACAG---GCCAGACAA---TTATTG---TCTGGTATAGTGCAACAGCAAAAC---
AATTTG---CTGAGA---GCTATTGAGGCGCAACAGCATCTGTTGCAACTCACAGTCTGGGGCATC
>GU728412..1
TTCTACTGTAATACAACACAACTGTTTAATAGTACTTGGGATATAAAGAGTAATATGACAAGGTCAAAT----
-------------------------------------------------------------------------
----------------------------
AACACTGAACCAATCACCCTCCAATGCAGAATAAAGCAAATTATAAACATGTGGCAGGAAGTAGGAAAAGCAA
TGTATGCCCCTCCCATCAGAGGACAAATTAAATGTTCATCAAAT---
ATTACAGGGCTGCTATTAACAAGAGATGGTGGTAATGAGACAGGGAAAGAG----------------------
-------------------------------------------------------------------------
----
AATGATACAGAGAAAGAGACCTTCAGACCTGGAGGAGGAGATATGAGGGACAATTGGAGAAGTGAACTTTATA
AATATAAAGTAGTAAAAATTAAACCATTA---GGAGTAGCACCCACTGCAGCAAAAAGAAGAGTGGTG-----
----------------------------------CAGAGAGAAAAAAGAGCG---GCACTA------
GGAGCC---------TTGTTCCTTGGGTTCTTGGGAGCA---GCAGGAAGCACTATG---GGCGCAGCGTCA-
--GTAACGCTGACGGTACAG---GCCAGACTA---TTATTG---TCTGGTATAGTGCAACAGCAGAAC---
AATCTG---CTGAGA---GCTATTGAGGCGCAACAGCATCTGTTGCAACTCACAGTCTGGGGCATC
>GU727880..1
TTCTACTGTAATACAACACAACTGTTTAATAGTACTTGGAATGGTACACAACTGCCTAATCTTACT-------
-------------------------------------------------------------------------
----------------------------
GGGAATGAAAATATCACACTCCAATGCAGAATAAAGCAAATTATAAATATGTGGCAGGAAGTAGGAAAAGCAA
TATATAACCCTCCCATCCAAGGACTAATTAGATGTTCATCAAAT---
```

ATTACAGGGCTGTTATTAACAAGAGATGGTGGTCATGGGAATAAT----------------------------
----

GACACCGAGATCTTCAGACCAGGAGGAGGAGATATGAGGGACAATTGGAGAAGTGAATTATATAAATATAAAG
TAGTAAAAATTGAACCATTA---GGACTAGCACCCACCACGGCGAAGAGAAGAGTGGTG--------------
-------------------------CAGAGAGAAAAAAGAGCA---GTGGGA---ATAGGAGCA---------
TTGTTCCTTGGGTTCTTGGGAGCA---GCAGGAAGCACTATG---GGCGCAGCGTCA---
ATGACGCTGACGGTACAA---GCCAGACAA---TTATTG---TCTGGTATAGTGCAACAGCAGAAC---
AATCTG---CTGAGG---GCTATTGAGGCGCAACAACATGTATTGCAGCTCACAGTCTGGGGCATC
>DQ886031..1
TTCTACTGTAATACATCACGGCTGTTTAATAGTACTTGGAATATTACTGAAGGGTCAAATAGCACTGCAGGC-
----

TCTAACAAGAATATCACACTCCCATGCAGGATAAAACAAATTATAAACATGTGGCAGGAAGTAGGAAAAGCAA
TGTATGCCCCTCCCATCAGCGGACTAATTAGATGTACATCAAAT---
ATTACAGGGCTGTTACTAACAAGAGATGGTGGTCATGATAATAAAACA-------------------------
----

AATGACACCGAGATCTTCAGACCTGGAGGAGGAAACATGAGGGACAATTGGAGAAGTGAATTATATAAATATA
AAGTAGTAAAAATTGAACCATTA---GGAATAGCACCCACCAAGGCAAGGAGAAGAGTGGTG-----------
----------------------------CAAAGAGACAAAAGAGCA---GTGGGA---ATAGGAGCT------
---ATGTTCCTTGGGATCTTAGGAGCA---GCAGGAAGCACTATG---GGCGCAGCGTCA---
ATAACGCTGACGGTACAG---GCCAGACAA---CTATTG---TCTGGTATAGTGCAACAGCAGAAC---
AATTTG---CTGAGG---GCCATTGAGGCGCAACAGCATCTGTTGCAACTCACAGTCTGGGGCATA
>DQ886032..1
TTCTACTGTAACACAACAAAACTTTTTAATAATAATACTGAAGGGTCAAAAAACACTGAA-------------
----

GACAATACCACACTCACACTCCCATGCAGAATAAAACAGATTATAAACATGTGGCAGGAAGTAGGAAGAGCAA
TGTATGCCCCTCCTATCAAAGGAATAATTAGATGTACATCAAAT---
ATTACAGGGCTGATATTAACAAGAGATGGTGGTCATAACATGAATAACACG----------------------
----

AATGAGACCACCGAGACCTTCAGACCTGGAGGAGGAGATATGAGGGACAATTGGAGAAGTGAATTATATAAAT
ATAAAGTAGTAAGAATTGAACCATTA---GGAATAGCACCTTCCAGGGCAAAGAGAAGAGTGGTG--------
-------------------------------CAGAGAGAAAAAAGAGCA---GTGGGA---ATAGGAGCT---
------GTGTTCCTTGGGTTCTTGGGAGCA---GCAGGAAGCACTATG---GGCGCAGCGTCA---
CTGACGCTGACGGTACAG---GCCAGACAA---TTATTG---TCTGGTATAGTGCAACAGCAGAAC---
AATTTG---CTGAGA---GCTATTGAGGCGCAACAGCATCTGTTGCAGCTCACAGTCTGGGGCATC
>GU367399..1
TTCTACTGCAATACAACAGGACTGTTTAATAGTTCTTGGAATAATGATACTATCTGGAATGATACTAGCACTG
AAGGA--

AGGAGAAATGACACAATCATACTCCCATGCAGAATAAGACAAATTGTAAACATGTGGCAGGAAGTAGGAAAAG
CAATGTATGCCCCTCCTATCCAAGGACTAATTAACTGTTCATCAAAT---
ATTACAGGGCTGCTATTAACAAGAGATGGTGGTAATAGCAACAAT----------------------------
----

AGCTATGAGACCTTTAGACCTGGAGGAGGAGATATGAGGGACAATTGGAGAAGTGAACTATATAAATATAAAG
TAGTAAAAATTGAACCAATA---GGAGTAGCACCCACCAAGGCAAAGAGACGAGTGGTG--------------
-------------------------CAGAGAGAAAAAAGAGGG---GTCGGG---ATCGGAGCT---------
TTGTTCCTTGGGTTCTTGGGAGCA---GCAGGAAGCACTATG---GGCGCAGCGTCA---
ATAACGCTGACGGTACAG---GCCAGACAA---CTATTG---TCTGGTATAGTGCAACAGCAAAAC---
AACTTG---CTGAGG---GCTATTGAGGCGCAACAACATCTGTTGCAACTCACAGTCTGGGGCATC
>GU367403..1
TTCTACTGTAATACATCAAAACTGTTTAATAGTACTTGGGGGTGGAATGTCACTGAAAATGGCACTGAAGTGT
CAAAT--

AACACTGAAGGAAATATCACACTTCCATGCAGAATAAAACAAATTATAAACATGTGGCAAAAAGTAGGAAAAG
CTATATATGCCCCTCCTATCAGAGGACAAATTAGCTGTTTATCAAAT---

```
ATTACAGGGCTGCTATTAACAAGAGATGGTGGTCATAACGAGGGTAAC-------------------------
-------------------------------------------------------------------------
-------
GAGACTAAGACTGAGGTCTTCAGACCTGGAGGAGGAGATATGAGGGACAACTGGAGGAGTGAATTATATAAAT
ATAAAGTAGTAAAAATTGAACCATTA---GGAGTAGCACCCACCAAGGCAAAAAGAAGAGTGGTG--------
-------------------------------CAAAGAGAAAAAAGAGCA---GTAGGA---ATAGGAGCT---
------GTGTTCCTTGGGTTCTTGTCAGCA---GCAGGAAGCACTATG---GGCGCAGCGTCA---
CTGACGCTGACGGTACAG---GCCAGACAA---TTATTG---TCTGGTATAGTGCAACAGCAGAAC---
AATCTG---CTGAGG---GCTATTGAGGCGCAACAGCATCTGTTGCAACTCACAGTCTGGGGCATC
>KP754469..1
TTCTACTGTGATACATCAAAACTGTTTAATAGTACTTGGGATAAGGGTAATTGGAGTACAGGGACAATTGCCA
CTAAC--------------------------------------------------------------------
----------------------------
GGAAATGACACTATCATACTCCAATGCAGAATAAGACAAATTATAAACATGTGGCAGGAAGTAGGAAAAGCAA
TCTATGCCCCTCCTATCAGTGGATACGTTAACTGTACATCAAAT---
ATTACAGGGTTGATATTAACAAGAGATGGTGGTAATGATAATGGTACA-------------------------
--------------------------------------------------------------------
AAAAATGGTACAAAC---
TCCACCGAGACTTTTAGACCTGGAGGAGGAGATATGAGGGACAATTGGAGAAGTGAATTATATAAATATAAAG
TAGTAAAAATTGAACCATTA---GGAATAGCACCCACCAAGGCAAAGAGAAGAGTGGTG--------------
-------------------------CAGAGAGAAAAAAGAGCA---GTGGGG---ATGGGAGCT---------
TTGTTCCTTGGGTTCTTGGGAGCA---GCAGGAAGCACTATG---GGCGCAGCGTCA---
ATAACGCTGACGGTACAA---GCCAGACAA---TTGTTG---TCTGGTATAGTGCAACAGCAGAAC---
AATCTG---CTGAAG---GCTATTGAGGCGCAACAGCATCTGTTGCAACTCACAGTCTGGGGCATC
>KP754471..1
TTCTACTGTAACACATCACAACTGTTTAATAGTACGTGGAATGATACTGAAAGGCCAAATAGCAATGGAGAA-
-------------------------------------------------------------------------
----------------------------
AATGACCAAAATATTACACTCCCTTGCAGAATAAGACAAATTGTAAACATGTGGCAGGAGGTAGGAAAAGCAA
TGTATGCCCCTCCCATCAGAGGACCAATTCAATGCTCATCAAAC---
ATTACAGGGATGATATTAACAAGAGATGGTGGCAATAACAGTGCC----------------------------
-------------------------------------------------------------------------
-------------
CAGAACGAGACCTTCAGACCTGGAGGAGGAGACATGAGGGACAATTGGAGAAGTGAATTATATAAATATAAAG
TAGTAAAAATTGAACCATTA---GGAGTAGCACCCACCAAGGCAAAAAGAAGAGTGGTG--------------
-------------------------CAGAGAGAAAAAAGAGCA---GTGGGA---ATAGGAGCT---------
TTGTTCATCGGGTTCTTGGGAGCA---GCAGGAAGCACTATG---GGCGCAGCGTCA---
ATGACGCTGACGGTACAG---GCCAGACAG---TTACTG---TCAGGTATAGTGCAACAGCAAAAC---
AATTTG---CTGAGA---GCTATTGAGGCGCAACAGCATATGTTGCAACTCACAGTCTGGGGCATC
>FJ469744..3
TTCTACTGTAATTCAACACAACTGTTTGATAGTACTTGGGATAATGGAACAAAAACAAATACCACTGAGGGAA
GAAATAGCGAT--------------------------------------------------------------
-------------GGAGGA------
AATAGCAGCGAACCCATCATACTCCCATGCAGAATAAAACAAATTATAAACATGTGGCAGGAAGTAGGAAAAG
CAATGTATGCCCCTCCCATCAGTGGAAACATTACATGTTCATCCAAT---
ATTACAGGGTTGCTATTAACAAGAGATGGTGGCAAGGGCAATGAGACTCAGGGC-------------------
-------------------------------------------------------------------------
-
TATGGGACTCAGGGCACTGAGATCTTCAGACCTGGAGGAGGAGATATGAGAGACAACTGGAGAAGTGAATTAT
ATAAATATAAAGTAGTAAGAATTGAACCATTA---GGAGTAGCACCCACCAAGCCAAAGAGAAGAGTGGTG--
-------------------------------------CAGAGAGAAAAAAGAGCA---GTGGGA---
TTAGGAGCT---------CTGTTCCTTGGGTTCTTGGGAGCA---GCAGGAAGCACTATG---
GGCGCAGCGTCA---ATGACGCTGACGGTACAG---GCCAGACTA---TTATTG---
TCTGGTATAGTGCAGCAGCAGAAC---AATCTG---CTGAGG---
GCTATTGAGGCGCAACAGCATCTGTTGCAACTCACAGTCTGGGGCATC
>FJ687543..1
TTCTACTGTGACTCAACAAAACTGTTTAATTTTACTTGGAATGGTACTGAAACAAATAACACTGGA-------
-------------------------------------------------------------------------
----------------------------
GGAAGTGATAAAATCATACTCCCATGCAGAATAAAACAAATTATAAAAATGTGGCAGAAAGTAGGACAAGCAA
```

TGTATGCCCCTCCCATCCAAGGAAAAATTTACTGCTCATCAAAT---
ATTACAGGGCTACTATTAACAAGAGATGGTGGCATCAACAATGAACCT-------------------------
----

AACAATACTGAGACCTTCAGACCTGGAGGAGGAAATATGAAGGATAATTGGAGAAGTGAATTATATAAATATA
AAGTAGTACAAATTGAACCATTA---GGAGTAGCACCCACCAAGGCAAAAAGAAGAGTGGTG-----------
----------------------------CAGAGAGAAAAAAGAGCA---GTGGGC---ATAGGAGCT------
---TTGTTCCTTGGGTTCTTGGGAGCA---GCAGGAAGCACTATG---GGCGCAGCGTCA---
ATAACGCTGACGGTACAG---GCCAGACAA---TTATTG---TCTGGTATAGTGCAACAGCAGAAC---
AATCTG---CTGAGG---GCTATTGAGGCGCAACAGCATATGTTGCAACTCACAGTCTGGGGCATC
>KF725982..2
TTCTACTGTAACACAACACCACTGTTTAACAAAACACACCTGGATAGCCTGAATAGCACT-------------
----

ACAGACGGTAATATTACACTTCAATGCAGAATAAAACAAATTATAAACATGTGGCAGGAAGTAGGAAAAGCAA
TGTATGCCCCTCCCATCAGAGGACAAATTAGATGTTCATCAAAT---
ATTACAGGGCTGTTATTAACAAGAGATGGTGGTAATGGTAATGAGAGC-------------------------
----

AGCAACCATGAGACCTTCAGACCTGGGGGAGGAGATATGAGGGACAATTGGAGAAGTGAATTATATAAATATA
AAGTAGTACAAATTGAACCATTA---GGAATAGCACCCACCAAGGCAAAGAGAAGAGTGGTG-----------
----------------------------CAGAGAGAAAAAAGAGCA---GTGGGA---ATAGGAGCT------
---TTGTTCCTTGGGTTCTTGGGAGCA---GCAGGAAGCACTATG---GGCGCAGCGTCA---
GTGACGCTGACGGTACAA---GCCAGACAA---TTACTG---TCTGGTATAGTGCAACAGCAGAAC---
AATTTG---CTGAGG---GCTATTCAGGCGCAACAACATTTGTTGCAACTTACAGTCTGGGGCATC
>KF725986..1
TTCTACTGTAATACAGCACCACTGTTTAATAGTACTTGGAATGATACCAGTGCCTGGAATATAACT-------
----

GATACTAGCCCACTCACAATCCAGTGCAGAATAAGACAAATTATAAATAGGTGGCAGGAAGTAGGAAAATCGA
TGTATGCCCCTCCCATCAGTGGACAAATTAACTGTTCATCAAAT---
ATTACAGGGCTGCTATTAACAAGAGATGGTGGTGGAAACAGGTCC----------------------------
----

CAGGAAAACAGGTCCGAGACCTTCAGACCTGGAGGAGGAGACATGAGGGACAATTGGAGGAGTGAATTATATA
AATATAAAGTAGTAAAAATTGAGCCATTA---GGGATAGCACCCACCAAGGCAAGGAGGAGAGTGGTG-----
----------------------------------CAAAGAGAAAAAAGAGCA---
GTGGGAGCGATAGGAGCT---------ATGTTCCTTGGATTCTTGGGGACA---GCAGGAAGCACTATG---
GGCGCAGCGGCA---TTGACGCTGACGGTACAG---GCCAGACAA---TTATTG---
TCTGGTATAGTGCAACAGCAAAAC---AATTTG---CTGAGG---
GCTATTGAGGCGCAACATCATCTGTTGCAACTCACAGTCTGGGGCATC
>KF725988..2
TTCTACTGTGATACAAAAAAACTGTTTAATAGTACATGGAATTTTAATAATACTTGGAATTCTACTCAAACA-
----

GGCAATGACACAATCACACTCCCATGCAGAATAAAACAAATTATAAACATGTGGCAAGGAGTAGGAAAAGCAA
TGTATGCCCCTCCCATTAGTGGACAAATCAACTGTTTATCAAAT---
ATTACAGGGCTGATATTAACAAGAGATGGTGGTCAGAACGATAGC----------------------------
----

AGTAAGGAGACCTTCAGACCTGCAGGAGGAAATATGAAGGACAATTGGAGAAGTGAATTATATAAATATAAAG
TAGTACAAATTGAACCATTA---GGAGTAGCACCCACCAAGGCAAAGAGAAGAGTGGTG--------------
-------------------------CAAAGAGACAAAAGAGCA---GTGGGA---ATAGGAGCA---------
TTGTTCCTTGGGTTCTTGGGAGCA---GCAGGAAGCACTATG---GGCGCAGCGTCA---
ATGACGCTGACGGTACAG---GCCAGGCAA---TTATTG---TCTGGTATAGTGCAGCAGCAGAAC---
AATCTG---CTGAGG---GCTATTGAGGCGCAGCAGCACCTGCTGCAACTCACAGTCTGGGGCATC
>KU168260..5
TTCTACTGTAATACAACACAACTGTTTAATAGTATTTGGGAAGGTAATAAGACTCAGACAGGTCATAGTACTC
ATAAT--

GCTAGGAATGACACTATCACGCTCCCATGTAGAATAAAACAAATTATAAACATGTGGCAGGAAGTAGGAAAAG
CAATGTATGCCCCTCCCATCCATGGACAAATTAGATGTATATCAAAT---
ATCACAGGTCTGCTATTAACAAGAGACGGTGGTATACACAACAAAACC-------------------------
----

CACATGACTGAGGTCTTCAGACCTGGAGGAGGAAATATGAAGGACAATTGGAGAAGTGAATTATACAAATATA
AAGTAGTACAAATTGAACCATTA---GGGCTGGCACCCACCAAGGCAAAGAGAAGAGTGGTG-----------
----------------------------CAGAGAGAAAAAAGAGCA---GTGGGAGCAATAGGAGCT------
---TTGTTCCTTGGGTTCTTGGGAGCA---GCAGGAAGCACTATG---GGCGCAGCGTCA---
CTGGCGCTGACGGTACAG---ACCAGACAA---TTACTG---TCTGGTATAGTGCAACATCAGAAC---
AATCTG---CTGAGG---GCTATTCAAGCGCAACAGCATCTGTTGCAGCTTACAGTCTGGGGCATC
>KT452481..1
TTCTACTGTAATACAACAAAACTGTTTAACAGTACTTGGATTTGGAATAGTACTGAAGGGTCAAACTCC----
----

AGTGAAGGAAACACAACACTCCAATGCAGAATAAAACAAATTGTAAACATGTGGCAGGAAGTAGGAAAAGCAA
TGTATGCTCCTCCCATCAAAGGAGTAATCAGATGTTCATCAAAT---
ATTACAGGGCTGTTATTAACAAGAGATGGTGGTAGCAAGAACAGTAGCGAGAAC-------------------
----

ACCACCACTAAGGAGACCTTCAGACCTGGAGGAGGAGATATGAGGGATAATTGGAGAAGTGAATTATATAAAT
ATAAAGTAGTAAAAATTGAACCATTA---GGAATAGCACCCACCAAGGCAAGGAGAAGAGTGGTG--------
-------------------------------CAGAGAGAAAAAAGAGCC---GTGGGA---ATAGGAGCA---
------TTGTTCCTTGGGTTCTTGGGAGCA---GCAGGAAGCACTATG---GGCGCAGCGTCA---
CTAACGCTGACGGTACAG---GCCAGACAA---TTATTG---TCTGGTATAGTGCAGCAGCAAAAC---
GATTTG---CTGAGA---GCTATTGAGGCGCAACATCATCTGTTGCAACTCACAGTCTGGGGCATC
>KT452508..1
TTCTACTGTGATACAACAAAACTGTTTGATAGCTCTTGGAATAAAACTGGAAGAATAGATAAAAGGAATAATA
CAGAAGGG---

TCAAATGACACAATCACACTCCCATGCAGAATAAAACAAATTATAAACATGTGGCAGGAAGTAGGAAAAGCAA
TGTATGCTCCTCCCATCAGTGGACGGATTAACTGTACATCAAAT---
ATTACAGGGCTGATATTAACAAGAGATGGTGGTCATAATGTTACA----------------------------
----

ACTAAAGGGGACACCTTCAGACCTGGAGGAGGAAATATGAAGGACAATTGGAGAAGTGAATTATATAAATATA
AAGTAGTAAAAATTGAACCATTA---GGAATAGCACCCACCAAAGCAAGGAGAAGAGTGGTG-----------
----------------------------CAGAGAGAAAAAAGAGCC---GTGGGA---TTAGGAGCT------
---TTGTTCCTTGGGTTCTTGGGAGCA---GCAGGAAGCACTATG---GGCGCAGCGTCA---
ATGGCGCTGACGGTACAG---GCCAGACAA---TTATTG---TCTGGTATAGTGCAACAGCAGAAC---
AATCTG---CTGAGG---GCCATTGAGGCGCAACAGCATCTGTTGCAACTCACAGTCTGGGGCATC
>KT452155..1
TTCTACTGCAATACATCACGACTATTTAATAGTACTTGGTTTAAGAATGATAGTACTTGGTTTAGGAATGATA
CTGGGAAT---
-------------GATACA------
GAAAAGTCAGAAAAGATCATACTTCCATGCAGAATAAAACAAATTATAAATATGTGGCAGGAAGTAGGAAAAG
CAATGTATGCCCCTCCCATCCGAGGACTAATTAGTTGTCCATCAAAT---
ATTACAGGACTGTTATTAACAAGAGATGGTGGTGGAGATAATAACAGA-------------------------
----

ACTAATGAGACTTTCAGACCTCAAGGAGGAGATATGAGGGACAATTGGAGAAGTGAATTATATAAATATAAAG
TAGTAAGAATTGAACCATTA---GGAATAGCACCCACCAAGGCAAGGAGGAGAGTGGTG--------------
-------------------------CAAAGAGAAAAAAGAGCA---GTGGGAACATTAGGAGCT---------
ATGTTCCTTGGGTTCTTGGGAACA---GCCGGAAGCACTATG---GGCGCAGCGTCA---
ATAACGCTGACGGTACAG---GCCAGACAA---TTATTG---TCTGGTATAGTGCAACAGCAAAAT---
AACTTG---CTGAGA---GCTATTGAGGCGCAACAGCATCTGTTGCAACTCACAGTCTGGGGCATC
>U39243..1
TTCTATTGCAATACAACAAAACTGTTTAGTAGTACATATATAGTTAATAATACACATGACAGTAAT-------
----

ACAACAAAAATTTTAAACATCACATGCAGAATAAAACAAATTATAAACATGTGGCAAGGGGTAGGACGAGCAA
TGTATGCCCCTCCCATTGCAGGAAACATAACATGTAAATCAAAT---
ATCACGGGACTACTACTGACACGTGATGGAGGAAATAAGACAGAAAGC-------------------------
----

AATGACACAGAGACATTCAGGCCTCAGGGAGGAGATATGAGGGACAATTGGAGAAATGAATTATATAAATATA
AAGTGGTAGAAGTTAACCCATTA---GGAATAGCACCCACTAAGGCAAAAAGGAGAGTGGTG-----------
----------------------------GACAGAGAAAAAAGAGCA---GTAGTGGACTAGGAGCT-------
---GTGTTCCTTGGGGTCTTGACACCC---GCAGGAAGCACTATG---GGCGCAGCATCA---
ATAACGCTGACGGTACAG---GCCAGACAA---TTATTG---TCGGGCATAGTGCAACAGCAAAAC---
AATTTG---CTGAGG---GCTATAGAGGCGCAACAGCATATGTTGCAACTCACGGTCTGGGGCATT
>U39240..1
TTCTATTGCAATACATCAATACTGTTTAATACAACATACCTGGTTAATAATAATTTGACTGAG----------
----

AAAAATTCAACCATTATGCTCCCATGCAGAATAAAACAAATTATAAACATGTGGCAGGAGGTAGGACGAGCAA
TGTATGCCCCTCCCATTGCAGGACTCATACAATGTAACTCAAGT---
ATCACAGGACTGCTACTGACACGTGATGGAGGAGTGAGAAATGACACAAAT----------------------
----

GAGACAGAGACAGAGACATTCAGACCTGCAGGAGGAGATATGAGGGACAATTGGAGAAGTGAATTATATAAAT
ATAAAGTGGTAGAAATTCAGCCATTA---GGAATAGCACCCACTAAGGCAAAAAGGAGAGTGGTG--------
-------------------------------GAGAGAGAAAAAAGAGCA---GCGGGA---ATAGGAGCT---
------TTGTTCCTTGGGTTCTTGGGAGCA---GCAGGAAGCACTATG---GGCGCAGCGTCG---
ATAACGCTGACGGTACAG---GCCAGACAA---TTATTG---TCGGGTATAGTGCAACAGCAAAAC---
AATTTG---CTGAGA---GCTATAGAGGCGCAACAGCATATGTTACAACTCACAGTCTGGGGCATT
>AF443090..1
TTCTATTGCAATACATCACGCCTGTTTAATAGTACATACAATCCTAATAGTACATACAATAATAGTATAGAAG
GT---

AGGTCAAATGCAACCATTACACTCCAATGCAGAATAAAGCAAATTATAAACCTCTGGCAGGAGGTAGGACGAG
CAATATATGCCCCTCCCATTGCAGGAAACATAACATGTAAATCAAAT---
ATCACAGGATTACTCTTGACACGTGATGGAGGTAATAATTCAACG----------------------------
----

ACAGAGATATTCAGACCTCAAGGAGGGAATATGAAGGACAATTGGAGAAGTGAATTATATAAATATAAAGTGG
TAGAAGTTAAGCCATTG---GGGGTAGCACCCACTAAGGCAAAAAGGAGAGTGGTG-----------------
----------------------GAGAGAGAAAAAAGAGCA---GTAGGA---ATAGGAGCT---------
GTGTTCCTTGGGTTCTTGGGAGCA---GCAGGAAGCACTATG---GGCGCAGCGTCA---
ATGACGCTGACGGTACAA---GCCAGACAA---CTGTTG---TCTGGTATAGTGCAACAGCAAAGC---
AATTTG---CTGAGG---GCTATAGAGGCGCAACAGCATATGTTGCAACTCACAGTCTGGGGCATT
>AF443097..1
TTCTATTGCAATACATCAGAGCTGTTTAATGGTACATACAACAGTACAGGAAATGATTCA-------------
----

AATTCAAACCTCATCACACTCCAATGCAGAATAAAACAAATTATAAACATGTGGCAGAAGGTAGGACGAGCCA
TGTATGCCCCCCCCATTGAAGGAAACATAACATGTATATCAAAT---
ATCACAGGACTATTATTAACACGTGATGGAGGATATGAGGCAAAT----------------------------
----

CATACTGAGATATTCAGACCTATAGGAGGAGATATGAGGGACAATTGGAGAAGTGAATTATATAAATATAAAG
TGGTAGAAATTAAGCCATTA---GGACTAGCACCCACTGAGTCAAAAAGGAGAGTGGTG--------------
-------------------------GAGAGAGAAAAAAGAGCA---GTGGGA---ATAGGAGCT---------
GTGTTCCTTGGGTTCTTGGGAGCA---GCAGGAAGCACTATG---GGCGCGGCGTCA---
ATAACGCTGACGGTACAG---GCCAGACAA---CTGTTG---TCTGGTATAGTGCAACAGCAAAGC---
AATTTG---CTGAGA---GCTATAGAGGCGCAACAACATATGCTGCAACTCACGGTCTGGGGCATT
>AF443109..3
TTCTATTGCAATACATCACTACTGTTTAATAGCAGTTATAATGGTAATAGTAGTTATAATGATACAGGA----
----

AGCAATTCAACCATCACACTCCAATGCAGAATAAAACAAGTTATAAACATGTGGCAGAGGGTAGGACAAGCAA
TATATGCCCCTCCCATTGAAGGAATAATAACATGTAACTCAAGT---
ATCACAGGATTACTCTTAGTGAGGGATGGAGACAATCAAACAAGT----------------------------
----

GATACAGAGACATTCAGACCACAAGGAGGAGAAATGAGGGACAATTGGAGAAGTGAGTTATATAAATATAAAG
TGGTAGAAATTAAGCCATTG---GGAGTAGCACCCACTACGGCAAAAAGGAGAGTGGTG--------------
-------------------------GAGAGAGAAAAAAGAGCT---GTGGGA---ATGGGAGCT---------
ATGATCCTTGGGTTCTTGAGTGCA---GCAGGAAGCACTATG---GGCGCAGCGTCA---
ATAACGCTGACGGTACAG---GCCAGACAA---TTGTTG---TCTGGAATAGTGCAACAGCAAAGC---
AATTTG---CTGAGG---GCTATAGAGGCGCAACAGCATATGTTGCAACTCACAGTCTGGGGCATT
>AF443114..2
TTCTATTGCAACACATCAAGCCTGTTTAATAATACATACCGGCCTACATACTGGCCTAATGGTACAGAA----
----

AGTAATTCAACCATCACACTTCAATGCAGAATAAAACAAATTATAAACATGTGGCAAAAAGTAGGACGAGCAA
TATATGCCCCTCCCATTGCAGGAAAAATAACATGTAAATCAAAC---
ATCACAGGACTACTATTAGTACGTGATGGAGGTAATGGAGGCAACAAT-------------------------
----

ACCGCAACAGAAATATTCAGACCTGGAGGAGGAAATATGAAGGACAATTGGAGAAGTGAATTATATAAATATA
AAGTGGTAGAAATTAAGCCATTG---GGAATAGCACCCACTGGAGCAAAAAGGAGAGTGGTG-----------
----------------------------GGGAGAGAAAAAAGAGCA---GTGGGA---ATAGGAGCT------
---GTGTTCCTCGGGTTCTTGGGAGCA---GCAGGAAGCACTATG---GGCGCGGCGTCA---
ATAACGCTGACGGTACAG---GCCAGACAA---CTGTTG---TCTGGTATAGTGCAACAGCAAAGC---
AATTTG---CTGAAG---GCTATAGAGGCGCAACATCATCTGTTGCAACTCACGGTCTGGGGCATT
>KR861314..2
TTCTATTGTAATACATCAAGACTGTTTGATAGTAATGGCACTTATAATAATACAGAA----------------
----

TCAAACAGCACAATCACACTCCCATGCAGAATAAAACAAATTATAAACATGTGGCAGAAAGTGGGACGAGCAA
TGTATGCCCCTCCCATTGAAGGAATGCTAACATGTAAATCAAAT---
ATCACTGGACTACTATTGACACGAGATGGAGGAGAAGGAAATCAA----------------------------
----

GGGTCAGAGATATTCAGACCTGGAGGAGGAAATATGAGGGACAATTGGAGAAGTGAATTATATAAATATAAAG
TGGTAGAGATTAAACCATTG---GGAGTAGCACCCACTGGGGCAAAAAGGAGAGTGGTG--------------
-------------------------GAGAGAGAAAAAAGAGCA---GTGGGA---ATAGGAGCT---------
GTGCTCCTTGGGTTCTTGGGAGCA---GCAGGAAGCACTATG---GGCGCGGCGTCA---
CTCACGCTGACGGTACAG---GCCAGACAA---TTATTG---TCCGGTATAGTGCAACAGCAAAGC---
AATTTG---CTGAGG---GCCATAGAGGCGCAACAGCATATGTTGCAACTCACAGTCTGGGGCATT
>KR861341..3
TTCTATTGCAATACATCAGGACTGTTTAATGGTACATACCATCCTCAATATAAT-------------------
----
-GATACACAGAATAAT---------
TACACAGACTCAAACATCACACTCCCATGCAGAATAAAACAAATTGTAAATATGTGGCAGGGGGTAGGAAGGG
CAATATATGCCGCTCCCATTGAAGGAAAAATAGTATGTACATCAAAT---
ATCACAGGACTAATATTGACAAGGGATGGAGGAGAAAATAATAGA----------------------------
----

ACAGAAACATTTAGACCTGAAGGAGGAAATATGAGGGACAATTGGAGAAGTGAATTATATAAATATAAAGTGG
TAGAAATTCAGCCATTG---GGAGTGGCACCCACTGAGGCAAAAAGGAGAGTGGTG-----------------
----------------------GAGAGAGAAAAAAGAGCA---GTGGGA---ATAGGAGCT---------
GTGTTCCTTGGGTTCTTGGGAGCA---GCAGGAAGCACTATG---GGCGCGGCGTCA---
ATAACGCTGACGGTACAG---GCCAGACAA---CTGTTG---TCTGGTATAGTGCAACAGCAAAGC---
AATTTG---CTGAGG---GCTATAGAGGCGCAACAGCATATGTTGCAACTCACAGTCTGGGGCATT
>KR861270..1
TTCTATTGCAATACATCAAGACTGTTTACAAACATAGATAATAATAATACAGAAACTGAT-------------
----

```
AATGATACCACCATCACCATCCCATGCAAAATAAAACAAATTATAAACATGTGGCAGAAGGTAGGACGAGCAA
TGTATGCCCCCCCCATTGAAGGAAACATAACATGTAAATCAAAT---
ATCACAGGACTACTATTGACACGTGATGGAGGTAATGAAACAAAC----------------------------
-------------------------------------------------------------------------
----------
TACAACACTGAGATATTCAGACCTGGAGGAGGAGATATGAGGGACAATTGGAGAAGTGAGTTATATAAATATA
AAGTGGTGGAGGTTAGGCCATTA---GGAATAGCACCCACTAAAGCAAAAAGGAGAGTGGTG-----------
----------------------------GAGAGAGAGAAAAGAGCA---GTGGGA---ATAGGAGCT------
---GTGTTCCTTGGGTTCTTGGGAGCA---GCAGGAAGCACTATG---GGCGCGGCATCA---
CTAACGCTGACGGTACAG---GCCAGACAA---TTATTG---TCTGGTATAGTGCAACAGCAAAGC---
AATTTG---CTGAGG---GCGATAGAGGCGCAACAACATATGTTGCAACTCACAGTCTGGGGCATT
>KR861273..1
TTCTATTGCAATACATCTGAGCTGTTTAATAAGCAGTTTAACAATACAAACAGTACAGAAAGTGAGTCAAAT-
-------------------------------------------------------------------------
----------------------------
TCAAATTCAATCATCACACTCCCATGTAGAATAAAACAAATTATAAACATGTGGCAGGAGGTAGGACGAGCGA
TGTATGCCCCTCCCATTGCAGGAAACATAACATGTAAATCAAAT---
ATCACAGGACTACTATTGACACGCGATGGAGGACCTGGAAATAGA----------------------------
-------------------------------------------------------------------------
-------------
AGTAACGAGACATTCAGACCCTTAGGAGGAGATATGAAGGACAATTGGAGAAGTGAATTATATAAGTATAAAG
TGGTAGAGATTCAGCCATTA---GGACTAGCACCCACTAGGGCAAAAAGGAGAGTGGTG--------------
-------------------------GAGAGAGAGAAAAGAGCA---GTGGGA---ATAGGAGCT---------
GTGTTCCTTGGGTTCTTGGGAGCA---GCAGGAAGCACTATG---GGCGCAGCATCA---
ATAACGCTGACGGTACAG---GCCAGACAA---CTGTTG---TCTGGTATAGTGCAACAGCAAAAC---
AATTTG---CTGAGG---GCTATAGAGGCGCAACAACATTTGTTGCAACTCACGGTTTGGGGCATT
>KY658708..1
TTCTATTGCAACACATCACAACTGTTTAATAGTACATACAGTCCCAGTTTTAATGGTACAGAAGGTAAA----
-------------------------------------------------------------------------
----------------------------
TTAAATGGGACCATCACAATCCCATGTAGAATAAAACAAATTATAAACATGTGGCAGAAAGTAGGACAAGCAA
TGTATGCCCCTCCCATTGCAGGAAACCTAACATGTGAATCAAAT---
ATCACAGGATTACTATTGACACGTGATGGAGGAAAAACAGGTTCA----------------------------
-------------------------------------------------------------------------
----------
AATCACCCAGAGATATTCAGACCTGGAGGAGGGGATATGAGGGATAACTGGAGAAGTGAATTATATAAATATA
AAGTGGTAGAAATTAAGCCATTG---GGAGTAGCACCCACTGAGGCAAAAAGGAGAGTGGTG-----------
----------------------------GAGAGAGAAAAAAGAGCA---GTGGGA---ATAGGAGCT------
---GTGTTCCTTGGGTTCTTGGGAGCA---GCTGGAAGCACTATG---GGCGCAGCGTCA---
ATAACGCTGACGGTACAG---GCCAGACAA---TTGTTG---TCTGGTATAGTGCAGCAGCAAAGC---
AATTTG---CTGAGG---GCTATAGAGGCGCAACAGCATCTGTTGCAACTCACAGTCTGGGGCATT
>AF443084..1
TTTTATTGCAATACATCAAAACTGTTTACATACCAGTCTAATAGCACATACGTG-------------------
-------------------------------------------------------------------------
----------------------------
GCTAATAGTACCATCACGCTCCCATGCAAAATAAAACAAATTATAAACATGTGGCAGGAGGTGGGACGAGCAA
TGTATGCACCTCCCATTGCAGGAAACATAACATGTCAATCAAAT---
ATCACAGGACTACTGTTGACACGTGACGGAGGCACAGAGACAGAC----------------------------
-------------------------------------------------------------------------
----------
AATAAGACAGAGACATTCAGACCTGGAGGAGGAGATATGAGGGACAGTTGGAGAAGTGAATTATATAAATATA
AAGTGGTAGAAATTAAGCCATTA---GGAGTAGCACCCACTAAGGCAAAAAGAAGAGTGGTG-----------
----------------------------GAAAGAGAAAAAAGAGCA---GTGGGA---ATAGGAGCT------
---GTACTCCTTGGGTTCTTGGGAGCA---GCTGGAAGCACTATG---GGCGCAGCGTCA---
ATAGCGCTGACGGCACAG---GCCAGACAA---GTGTTG---TCTGGTATAGTGCAACAGCAAAGC---
AATTTG---CTGAGG---GCTATAGAGGCGCAACAGCATATGTTGCAACTCACAGTCTGGGGCATT
>KC899004..1
TTCTATTGTAATACATCAGGCCTGTTTAATGGTACATACAGGCCTAATGGTAATAATAATGGTACTAATGATA
CAATG--------------------------------------------------------------------
----------------------------
```

AGTAATGCAACCATCACAATCCCATGCAGAATAAAGCAAATTATAAACATGTGGCAGGAGGTAGGACGAGCAA
TGTATGCCCCTCCTATTGCAGGGAACATAACATGTAAATCAAAT---
ATCACAGGATACTATTGGTACGTGATGGAGGAGATAATCAGACA-----------------------------
----

GAAGAGATATTCAGACCTGGAGGAGGAGATATGAGGAACAATTGGAGAAGTGAATTATATAAATATAAAGTGG
TAGAAATTAAGCCATTG---GGAATAGCACCCACTGGAGCAAAAAGGAGAGTGGTG-----------------
----------------------GAGAGAGAAAAAAGAGCAGTAGTGGGA---ATAGGAGCT---------
GCGCTCCTTGGGTTCTTGAGTACA---GCCGGAAGCACTATG---GGCGCGGCGTCA---
ATAGCGCTGACGGTACAG---GCCAGACAA---TTGCTG---TCTGGTATAGTGCAACAGCAAAGT---
AATTTG---CTGAGG---GCTATAGAGGCGCAGCAGCATATGTTGCAACTCACGGTCTGGGGCATT
>KF835522..1
TTCTATTGTAATACATCAAGACTGTTTAATAGTACATACAATCCTAAGAGTACAGGAAGTAAT----------
----

TCAAGCTCAATCATCACAATCCCATGCAGAATAAAGCAAATTATAAACATGTGGCAGGAGGTAGGAAGAGCAA
TGTATGCCCCTCCCATTGAAGGAAACATAACATGTAGATCAAAG---
ATCACAGGACTACTATTGGTACGTGATGGAGGAAGCCCAGTGCCAAACAGT----------------------
----

ACAGAGACAGAGACAGAGATATTCAGACCTGGAGGAGGAGATATGAGGGACAATTGGAGAAGTGAATTATATA
AATATAAAGTGGTAGAAATTCAGCCATTG---GGAATAGCACCCACTAAAGCAAAAAGGAGAGTGGTG-----
----------------------------------GAGAGAGAAAAAAGAGCA---GCA------
CTAGGAGCT---------TTGTTCCTTGGGTTCTTGGGAGCA---GCAGGAAGCACTATG---
GGCGCGGCGGCA---GTAACGCTGACGGTACAG---GCCAGACAA---TTATTG---
TCTGGTATAGTGCAACAGCAAAGC---AATTTG---CTGAGG---
GCTATAGAGGCGCAACAGCATATGTTGCAACTCACGGTCTGGGGCATT
>HM215403..1
TTCTATTGTAATACATCAAGCCTGTTTAATGGCACATACAATGGCACATACATGACTAATATGGCTAAT----
----

GATTCATCAGAAATCACAATCCCATGCAGAATAAAGCAAATTGTAAACATGTGGCAGGAGGTAGGACGAGCAA
TGTATGCCCCTCCCATTGCAGGAAACATAACATGTAGATCAAAT---
ATCACAGGACTATTATTGGTACGTGATGGAGGACGATCAGAGGTGTCA-------------------------
----

AATACTACAGAGACATTCAGACCTCAAGGGGGAGATATGAGGGACAATTGGAGAAGTGAATTATATAAATATA
AAGTGGTAGAAATTAAGCCATTA---GGAATAGCACCCACTGGAGCGAAAAGGAGAGTAGTG-----------
----------------------------GAGAGAGAAAAAAGAGCA---GCGGGA---ATAGGAGCT------
---GTGTTCCTTGGGTTCTTGAGTGCA---GCCGGAAGCACTATG---GGCGCGGCGTCA---
TTAACGCTGACGGTACAG---GCCAGACAA---TTGCTG---TCTGGTATAGTGCAACAGCAAAGC---
AATTTG---CTGAGG---GCTATAGAGGCGCAACAGCATATGTTGCAACTCACAGTCTGGGGCATT
>HM215408..1
TTCTATTGTAATACATCAAGACTGTTTAATAGTACATACAGCAATGGTACACTCAGGTTTAATGGTACAGACT
TTAATGGTACA--
-------------ATGGAT------
AATTCAAGCTCAACCATCACAATCCCATGCAGAATAAAGCAATTTATAAACATGTGGCAGGAAGTAGGAAGAG
CAATGTATGCCCCTCCCATTGAAGGAAACATAACATGTAGATCAAAT---
ATCACAGGACTACTATTGGTACGTGATGGAGGAACAAATTTAACAGCAGAA----------------------
----

TTAAATCAGACAGAGATATTTAGACCTGAAGGAGGAAATATGAGGGACAATTGGAAAAATGAATTATATAAAT
ATAAAGTGGTAGAAATTAAGCCATTG---GGACTAGCACCCACTGGAGCAAAAAGGAGAGTGGTG--------
-------------------------------GAGAGACAAAAAAGAGCA---GTGGGA---ATAGGAGCT---
------GTAATCTTTGGGTTCTTGGGAGCA---GCCGGAAGCACTATG---GGCGCGGCGTCA---
ATGGCGCTGACGGTACAG---GCCAGACAA---TTGTTG---TCTGGTATAGTGCAACAGCAAAGC---
AATTTG---CTGAGG---GCTATAGAGGCGCAACAGCATCTGTTGCAACTCACGGTCTGGGGCATT
>HQ699950..2
TTCTATTGTAATACATCAGGCCTGTTTAATAGTACATACATGCCTAATGGTACCTACATGTCTAATGGTACCA
AT---

AGTAATGCAACCATCACAATCCCATGCAGAATAAAGCAAATTATAAACATGTGGCAGGAGGTAGGACGAGCAA
TATATGCCCCTCCCATTGCAGGAAACATAACATGTAAATCAAAT---
ATCACAGGGCTACTATTGGTACGTGATGGAGGACCAGATAATCAG----------------------------
----

ACAGAGATATTCAGACCTGAAGGAGGAGATATGAGGAACAATTGGAGAAGTGAATTATATAAATATAAAGTGG
TAGAAATTAAGCCATTG---GGACTAGCACCCACTGGAGCAAAAAGGAGAGTGGTG-----------------
----------------------GAGAGAGAAAAAAGAGCA---GTGGGA---ATAGGAGCT---------
GTGTTCCTTGGGTTCTTGAGTGCA---GCCGGAAGCACTATG---GGCGCGGCGTCA---
ATAACGCTGACGGTACAG---GCCAGACAA---TTGCTG---TCTGGTATAGTGCAACAGCAAAGT---
AATTTG---CTGAGG---GCTATAGAGGCGCAGCAGCATATGTTGCAACTCACGGTCTGGGGCATT
>HQ699981..1
TTCTATTGTAACACATCAGGCCTGTTTAATAGTACATACATGCCTAGTGGTACATACATAACTAATGATACAA
AT---

AGTAGTTCAGATACAATCACAATCCCATGCAAAATAAAGCAAATTATAAACATGTGGCAGGAGGTAGGACGAG
CAATGTATGCCTCTCCCATTGCAGGAAACATAACATGTACATCAAAT---
ATCACGGGACTACTATTGGTACGTGATGGAGACAAAACAAGTAAT----------------------------
----

ACTACAGAGACATTCAGACCTCAAGGAGGAGACATGAGGGACAATTGGAGGAGTGAATTATATAAATATAAAG
TGGTAGAAATTAAGCCATTG---GGAATAGCACCCACTAAAGCAAAAAGGAGAGTGGTG--------------
-------------------------GAGAGAGAAGAAAGAGCAGCGGGAATAGGAATAGGAGCT---------
TTGTTCCTTGGGTTCTTGAGTGCA---GCCGGAAGCACTATG---GGCGCGGCGTCA---
ATAACGCTGACGGTACAG---GCCAGACAA---TTGTTG---TCCGGTATAGTGCAACAGCAAAGC---
AATTTG---CTGAGG---GCTATAGAGGCGCAACAGCATATGTTGCAACTCACGGTCTGGGGCATT
>KC807928..2
TTCTATTGTAATACAACAAACCTGTTTAATAGTACATACAATAGTACAACAGGCCTGTTTACTTATACATACA
ATGGTACT--ACA---
TACAATGGTAGTGTTACAGAC---------------
AATGATAGCATCCCAATCCAAATCCAATGCAGAATAAAGCAAATTATAAACATGTGGCAGGAGGTAGGACGAG
CAATGTATGCCCCTCCCATTGCAGGGAACATAACATGTAGCTCAAAT---
ATCACAGGACTACTATTGACACGTGATGGAGGAAATACAACTGTGACA-------------------------
----

CAAAAAGAGATATTCAGACCTCAAGGAGGAAATATGAGAGACAATTGGAGAAATGAATTATATAAATATAAAG
TGGTAGAAGTTAAGCCATTG---GGACTAGCACCCACTAAAGCAAAAAGGAGAGTGGTG--------------
-------------------------GAAAGAGAAAAAAGAGCA---GTGGGA---CTAGGAGCT---------
GTGTTCCTTGGGTTCTTGGGAGCA---GCAGGAAGCACTATG---GGCGCGGCGTCA---
ATGACGCTGACGGTACAG---GCCAGACAA---TTGTTG---TCTGGTATAGTGCAACAGCAAAGC---
AATTTG---CTGAGA---GCTATAGAGGCGCAACAGCATATGTTGCAACTCACGGTCTGGGGCATT
>JF683757..3
TTCTATTGCAACACATCAGTACTGTCTAATAGTACCCATAATAGTGGTACTGGATGGCACCTACAATCAAGTG
ATATT--

AATTCTTATGCAACCATCGCGGTTTCATATAGAATAGGACAAATTATAAACATGTGGCGGCAGGTAGGACGAG
CAATCTATGGCCCTCCGGTGGCACGAAAAATGTCAGCCCATCCAAGT---
ACCACAGGCCTATTATTGGTATGTGATGGGGGTGAGAAGAGCTTAAATAGCACA------
AATGGCACAGGTATCACA---AAT---

GCCACAGAGATATTTAGACCTCAAGGAGGAGATATGAAGGACAATTGGAGAAGTGAATTATATAAGTATAAAG
TGGTAGAAATTAAGCCATTG---GGAATAGCACCCACTGAAGCAAGAAGGAGAGTGGTG--------------
-------------------------GAGAGAGAAAAAAGAGCA---GTGGGA---ATAGGAGCT---------
GTGATCCTTGGGTTCTTGGGAGCA---GCAGGAAGCACTATG---GGCGCGGCGTCA---
ATAGCGCTGACGGCACAG---GCCAGAACA---GTAATG---GCTGGTATAGTGCAACAGCAAAGC---
AATTTG---CTGAGG---GCTATAGATGCGCAACAGCATATGTTGCAGCTCACAGTCTGGGGCATT
>L23065..3
TTCTATTGTAATACATCAGCCTTGTTTAATGATTCAATTACAAATGCACCCATTAATAATTCGAGT-------
----

ACAGATGCAAACATCACACTTCAATGCAGAATAAAACAAATTATAAACATGTGGCAGGAGGTAGGACGAGCAA
TGTATGCCCCTCCCATTCAAGGAATAATAGCATGTAAATCAAAT---
ATCACAGGACTGCTACTGACACGTGATGGAGGGACAAATAGCACA----------------------------
----

GACGAGACATTCAGACCTGGAGGAGGAGATATGAGGGACAATTGGAGAAGTGAATTATATAAATACAAAGTGG
TAGAAATTAAGCCATTA---GGACTAGCACCCACTAAGGCAAAAAGGAGAGTGGTG-----------------
----------------------GAGAGAGAAAAAAGAGCA---GCA------CTAGCAGCT---------
GTGTTCCTTGGGTTCTTGGGAGCA---GCCGGAAGCACTATG---GGCGCAGCATCA---
ATAACGCTGACGGTACAG---GCCAGACAA---TTATTG---TCTGGTATAGTGCAACAGCAAAGC---
AATTTG---CTGAAG---GCTATAGAGGTGCATCAGCATATGTTGCAACTCACAGTCTGGGGCATT
>EF531335..1
TTCTATTGCAACACATCAGATCTGTTTAATAGTACATATTTTAATAATACATATTTTAATGGTGTATATAATG
GTACA--

AGAGATAATGGTACCATCACACTCCCTTGCAGAATAAAACAATTTATAAACATGTGGCAGAGGGTAGGAAGAG
CAATGTACGCCCCTCCCATTCAAGGACTTATAACATGTAGGTCAAAT---
ATCACAGGACTACTATTGACACGTGATGGAGGAAACACTATTAACAATACA----------------------
----

AATAACAATACAGAGATATTCAGACCTGGAGGAGGAGATATGAGGGACAATTGGAGAAGTGAATTATATAAAT
ATAAAGTGGTAGAAATTAGGCCATTA---GGAGTAGCACCTACTGATGCAAGAAGGAGAGTGGTG--------
-------------------------------GGGAGAGAAAAAAGAGCA---GTA------ATGGGAGCT---
------TTGTTCCTTGGGTTCTTGGGAGCA---GCAGGAAGCACTATG---GGCGCAGCGTCA---
ATGGCGCTGACGGTACAG---ACCAGACAA---TTATTG---TCTGGTATAGTGCAACAGCAAAGC---
AATTTG---CTGAGG---GCCATAGAGGCGCAACAGCATATGTTGCAACTCACGGTCTGGGGCATC
>KU319529..3
TTCTATTGCGATACATCAAATCTGTTTAATATGACATACTTGTTTAATGATTGGAGTTACGAACATAGTACTG
AC---

TCAGATTTCATCATCATGATCCGCTGCAGAATAACACATATAATAAACATGTGGCAGGTAGTGAGAGCAGTAC
TGTATGCCCCTGCCCTTCGCAGGCAACAGTTAAGCAAGCAATCCATC---
TACGCAGTACTACTATGGACTCGTGATGCAGCAAGTAGTTACTGG----------------------------
----

CGCGAGAAAGAGATATTCAGACCTGCAGGAGGAGATATGAGGGACAATTGGAGAAGTGAATTATATAAATATA
AAGTGGTAGAGATTAAACCATTA---GGAATAGCACCCACTGAGGCAAAAAGGAGAGTGGTG-----------
----------------------------GAGAGAGAAAAAAGAGCA---GTGGGA---ATAGGAGCT------
---GTGTTCCTTGGGTTCTTGGGAGCA---GCAGGAAGCACTATG---GGCGCAGCGTCA---
CTGACGCTGACGGTACAG---GCCAGGCAA---TTGCTG---TCTGGTATAGTGCAACAGCAAAAC---
AATTTG---CTGAGA---GCTATAGAGGCGCAACAGCATATGTTGCAACTCACAGTCTGGGGCATA
>KU319530..1
TTCTATTGCAATACATCAAGACTGTTTAATGGTGCATACATGTATAACAGTACTAATAGTACT----------
----

TCAAATGCAACCATCACACTCCAATGCAGAATAAAGCAAATTATAAACATGTGGCAGGGGGTAGGACGAGCAA
TGTATGCCCCTCCCATTGAAGGAAACATTACATGTAAATCAATA---
TCACAGGATTCTATTGACCCTGATGGAGGATTGGGC-------------------------------------
----
-------------------------CAAAAACCCAAGGTTTCCAACTGGGAGAGGAACTTAGGGACA------
---------------TTGGAAATGGATCCTTTG---GGTTACCCCCTG------GCCAAAAAGGAAGGGGAG-
--------------------------------------AAAAGAAAAAAAAGGACA---GGGGTT---
TTACGAAAA---------AACTGG---------------------GCGCAGAAGCCTATG---
GGCGCAGCATCA---ATAACGCTGACGGTACAG---GCCAGACAA---TTGTTG---
TCTGGTATAGTGCAACAGCAAAGT---AATTTG---CTGAGG---
GCTATAGAGGCGCAACAGCATATGTTGCAACTCACGGTCTGGGGCATT
>KU319539..2
TTCTATTGTAATACATCAAAACTGTTTAATGGTACATACAATAAGAACAATACTAATAGGACTAATACTAAT-
----

AGTACTTCAACCATCACACTTCAATGCAGAATAAAACAAATTATAAACATGTGGCAGGGGGTAGGACAAGCAA
TGTCTGCCCCTCCCATTCAAGGAAACATAACATGTGAATCAAAT---
ATCACAGGACTACTATTGACACGTGATGGAGGAACAACTAATAATAACGAAACAAATAATAACACAGAAGCAA
ATAAT--

AACACAGAAACATTCAGACCTGGAGGAGGAGATATGAGGGACAATTGGAGAAATGGAGGTGAGTTATATAAAT
ATAAAGTGGTAGAAGTTAATCCATTA---GGAATAGCACGCACTGAGGCAAAAAGGAGAGTGGTG--------
-------------------------------CAGAGAGAAAAAAGAGCA---GTGGGA---ATAGGAGCT---
------GTGCTCCTTGGGTTCTTGGGAGCA---GCTGGAAGCACTATG---GGCGCAGCGTCA---
GTAACGCTGACGGTACAG---GCCAGACAA---TTGCTG---TTTGGTATAGTGCAACAGCAAAGC---
AATTTG---CTGAAG---GCTATAGAGGCGCAACAGCATATGTTGCAACTCACGGTCTGGGGCATT
>KU319542..1
TTCTATTGCAATACAACAAAACTGTTTAATAGTACATACAATGCTAATAGTACAAACAGTACT----------
----

TCAAATTCAACCATCACAATCCAATGCAGAATAAAACAAATTATAAACATGTGGCAGGAGGTAGGACGAGCAA
TGTATGCCCCTCCCATTGCAGGAAACATAACATGTAACTCAACT---
ATTACAGGAATACTATTAACACGTGATGGAGGGTATAATATTACC----------------------------
----

AACGACACAAAAACATTCTTACCTGGAGGAGGAGATATGATGAACAATTGGAGAAATGAATTATATAATTATA
AAGTGGTAGAAAATAAACCATTA---GGAATAGCTCCTACTAGTGCAAAGAGGAGAGTGGTG-----------
----------------------------GAGAAAAAAAAAAGAGCA---ATATGGGGAATAGGAGCT------
---GCGCTCCTTGGTTTCTTACGAGCA---GCAGGAAGCACTATG---GGCGCAGCGTCA---
TTGACGCTGACGGTACAG---GCCAGGCAA---TTGTTG---TCTGGTATAGTGCAACAGCAAAGC---
AATTTG---CTGAGG---GCTATAGAGGCGCAACAACATATGTTGCAACTCACGGTCTGGGGCATT
>KU319543..4
TTCTACTGCAATACATCAAATTTGTTTAATAGAACTTACCTGATTAATGGTACCTATAATAATAATAAAACT-
----

TCCATAAGCACCATCACACTCCACTGCAAAATAAAACAAATTATAAATATGTGTCAGGGGGTAGGACAAGCAA
TGTATGCCCCTGCCACTGCAGGAATCATATCATGTATATCAACT---
ATCGCAGTACTACTACTTGCTGGTGATGGAGGCAATGATACAAGT----------------------------
----

GATACAGAGACATTCAGACCTGGAGGAGGAGATATGAGGGACAATTGGAGAAGTGAATTATATAAATATAAAG
TGGTAGAAGTTCACCCATTA---GGAGTAGCACCCACTAGAGCAAAAAGGAGAGTGGTG--------------
-------------------------GAGAGAGAAAAAAGAGCA---GCA------CTAGGAGCT---------
ATGATCATTGGGTTCTTGGGAGCA---GCAGGAAGCACTATG---GGCGCAGCATCA---
ATAACGCTGACGGTACAG---GCCAGGCAA---TTGTTG---TCTGGTATAGTGCAACAGCAAAGC---
AATTTG---CTGAGG---GCTATAGAGGCGCAACAGCATATGTTGCAACTCACAGTCTGGGGCATA
>KU319548..2
TTCTATTGCAATACATCACAACTGTTTAATAGGACATACATGGAAACTACTATTAGCGCTGTGTCA-------
----

ACAAATACAAAAATCACCCTCCTATGCAAAATAAAACAAATTATTCAAATGCTTCAGGGAGTAGGACGAGCAA
TGTATGCCCCTCGCAATGAAAGAAACATAACATGTCTTTCAAAT---
ATCACAGGATTACTATTGACACGTGATGGAGGAATTAACAGTAGC----------------------------
----

ACAACTGAGATATTCAGACCTGGGGGAGGAGATATGAGGGACAATTGGAGAAGTGAATTATATAAATATAAAG
TGGTAGAAATCAAACCATTA---GGAGTAGCACCCACTAAGGCAAAAAGGAGAGTGGTG--------------
-------------------------GAGAGAGAAAAAAGAGCA---GTGGGA---ATAGGAGCT---------
GTGCTCCTTGGGTTCTTGGGAGCA---GCAGGAAGCACTATG---GGCGCAGCATCA---
ATAACGCTGACGGTACAG---GCCAGGCAA---TTGTTG---TCTGGTATAGTGCAACAGCAAAGC---
AATTTG---CTGAGG---GCTATAGAGGCGCAACAGCATATGTTGCAACTCACAGTCTGGGGCATT
>KU319549..1
TTCTATTGCAAAACATCAAAACTGTTTATTAAGACTTGGAAAAGTTGGAACCATCGGAAT-------------
----

GATGGAACCATCATCACGATCCCATGCAGAATAAAACAAATTATACATATGTGGCAGGGAGTAGGACGAGCAA
TGTATGCCCCTCCCATCAGTGGACCAATAACATGTAGATCAAAT---
ATCACAGGACTACTACTGACACGGGATGGAGGCATCAGTGACAATAACAGT----------------------
----

ACCAATACCACAGAGATATTCAGACGTCAAGGAGGAGATATGAAAGACAATTGGAGAAGTGAATTATATAAAT
ATAAAGTGGTAGAAATTAAGCCATTA---GGAATAGCACCCACTAAGGCAAAAAGGAGAATGGTG--------
-------------------------------CAGAGAGAGAGAGGAGAA---AAGAGG---CACGGAGCT---
------ATGTTCCTTGGGTTCTTGGGAGCA---GCCGGAAGCACTATG---GGCGCAGCATCA---
ATAACGCTGACGGTACAG---GCCAGACAA---TTATTG---TCAGGTATAGTGCAACAGCAAAGC---
AATTTG---CTGAGG---GCTATAGAGGCGCAACAGCATATGTTGCAACTCACGGTCTGGGGCATT
>AF219266..3
TTCTATTGCTATACTTCAAGACTGTTTAACAGTACATACCTGTTTAATGGTACTTACCTGGTTAATGATATAA
ATAGT--

ACAGGAAATGCAACCATCATACTCCCATGCAAAATAGAACAAATTATAAACATGTGGCAGGAGGTAGGGCGAG
CAATGTATGCCCCTCCCATTGAAGGAAACATAACATGTAGCTCAAAC---
ATCACAGGACTATTATTGGTACGTGATGGAGGAAAAGATTCAAAT----------------------------
----

GGCACAGAGATATTCAGACCTGGAGGAGGAGATATGAGGGACAATTGGAGAAGTGAATTATATAAATATAAAG
TGGTAGAAATTAAGCCATTG---GGAATAGCACCCACTAAGGCAAAAAGGAGAGTGGTG--------------
-------------------------GAGAGAGAAAAAAGAGCA---GTAGGA---ATAGGAGCT---------
GTGTTCCTTGGGTTCTTGGGAGCA---GCAGGAAGCACTATG---GGCGCGGCGTCA---
ATAACGCTGACGGTACAG---GCCAGACAA---GTGTTG---TCTGGTATAGTGCAACAGCAAAGC---
AATTTG---CTGAGG---GCTATAGAGGCTCAACAGCATATGTTGCAACTCACGGTCTGGGGCATT
>FJ977095..1
TTCTATTGCAATACATCAGAACTGTTTAAGAATGGTACAAATAATGGTACCAATAGTACAGACTCAACA----
----

AATTCAACATCCATCACAATCCAATGCAGAATAAAACAAATTATAAATATGTGGCAGGGGGTAGGTCGAGCAA
TATATGCCCCTCCCATTAAAGGAAACATAACATGTAACTCAAAT---
ATCACAGGACTACTGCTGACACGTGATGGAGGAAAAGGAGAAGAAAGT-------------------------
----

GATAATAAGACAGAGATATTCAGACCTGCAGGAGGAGATATGAGGGACAATTGGAGAAGTGAATTATATAAAT
ACAAAGTGGTAGAAATTCAGCCATTA---GGAGTAGCACCCACTGAGGCGAAAAGGAGAGTGGTG--------
-------------------------------GAGAGAGAAAAAAGAGCA---GTGGGA---ATAGGAGCT---
------GTGTTTCTTGGGTTCTTGGGAGCA---GCAGGAAGCACTATG---GGCGCAGCGTCA---
ATAACGCTGACGGTACAG---GCCAGACAA---TTATTG---TCAGGCATAGTGCAACAGCAAAGT---
AATTTG---CTGAGA---GCTATAGAGGCGCAACAGCATATGTTGCAACTCACGGTCTGGGGCATT
>AY255823..1
TTCTATTGCAATACATCAGCCCTGTTTACAAATTCAACCATC-------------------------------
----

CCAAATACAACCATCACACTCCCATGCAAAATAAAACAAATTATAAATATGTGGCAGGAGGTAGGACGAGCAA
TGTATGCCCCTCCCATTAAAGGAGAGATAACATGTAATTCAACT---
ATCACAGGAATACTACTGACACGTGATGGAGGCAACACCACAAAC----------------------------
----

GAAATCGAAACATTCAGACCTGGAGGAGGAGATATGAGGGACAATTGGAGAAGTGAATTATATAAATACAAAG
TGGTAGAAATTAAGCCATTA---GGAATAGCACCCACGGAGGCAAAAAGGAGAGTGGTG--------------
-------------------------GAGAGAGAAAAAAGAGCA---GCA------CTAGGAGCT---------
GTGTTCCTTGGGTTCTTGGGAGCA---GCCGGAAGCACTATG---GGCGCAGCATCA---
ATGACGCTGACGTTACAG---GCCAGACAA---TTACTG---TCGGGTATAGTGCAACAGCAAAGC---
AATTTG---CTGAGG---GCTATAGAGGCGCATCAGCATATGCTGCAACTCACAGTCTGGGGCATT
>KP109480..2
TTCTATTGCAACACATCAAGCCTGTTTAAGGGTACATATAGGCCTAATGGTACAAGTAATTCCACT-------
----

TCAGGCTCTATCATCACACTCCCATGCTACATAAAACAAGTTATAAACTTGTGGCAGGAGGTAGGACGAGCAA
TATATGCCCCTCCCATTGAAGGAAACATAACATGTATATCAAAT---
ATCACAGGACTACTATTGGTAAGAGATGGAGGAAATCATGAAGAG----------------------------
----

GCAAATACGACAGAGACATTCAGACCTGGAGGAGGAAATATGAGAGACAATTGGAGAAGTGAATTATATAAAT
ATAAAGTAGTAGAAATTAAGCCATTG---GGAGTAGCACCCACTAAAGCAAGAAGGAGAGTGGTG--------
-------------------------------GAGAGAGAAAAAAGAGCA---GTGGGAGTAGGAGCTGTT---
------ATGTTCCTTGGGTTCTTGGGAGCA---GCAGGAAGCACTATG---GGCGCGGCGTCA---
ATAACGCTGACGGTACAG---GCCAGACAA---TTGCTG---TCTGGTATAGTGCAACAGCAAAGC---
AATTTG---CTGAAG---GCTATAGAGGCGCAACAGCATCTGTTGCAACTCACGGTCTGGGGCATT
>KP109483..1
TTCTATTGTAATACATCAAGCCTGTTTAATAGTACATTTAATAGTACATACATGACTAATGATACAGACATGA
AT---

TCAAACTCAACCATCTCAATCCCATGCCGCATAAAACAAATTATAAACATGTGGCAGGAGGTAGGACGAGCAA
TGTATGCCCCTCCCATTGCAGGAAACATAACATGTAAATCAAAT---
ATCACAGGACTACTATTGGTACGAGATGGAGGAAACTCAAATGATACA-------------------------
----

AATGAGCCAGAGATATTCCGACCTCAAGGAGGAGATATGAGGGACAATTGGAGAAGTGAATTATATAAATATA
AAGTGGTAGAAATTAAGCCATTG---GGAGTAGCACCCACTGCAGCAAAAAGGAGAGTGGTG-----------
----------------------------GGAAGAGAAAAAAGAGCA---GTGGGA---CTAGGAGCT------
---ATGATCCTTGGGTTCTTGGGAGCA---GCCGGAAGCACTATG---GGCGCGGCGTCA---
ATAACGCTGACGGTACAG---GCCAGACAA---TTGTTG---TCTGGTATAGTGCAACAGCAAAGC---
AATTTG---CTGAGG---GCTATAGAGGCGCAACAGCATATGTTGCAACTCACGGTCTGGGGCATT
>KY929363..1
TTCTATTGCAATACATCAAAACTGTTCAACAGTACATACTGGCAGAATGGTACATACTCATACTGGAATAATG
GG--
TCATTCCTAGAATGGAATGATACACAAAGCACT------------
CCAAATCGAAGCATCACAATTCCATGCAGAATAAAACAAATTATAAACATGTGGCAGGAGGTAGGACGAGCAA
TGTATGCCCCTCCCATTGCAGGAAAAATAATGTGTAAATCCAAT---
ATCACAGGAATACTATTGGAACGTGATGGAGGAAATACAAATGAGACA-------------------------
----

AATAAGACAGAGACATTCAGACCAATAGGAGGAGATATGAGGAATAATTGGAGAAGTGAATTATATAAATATA
AAGTGGTAGAAATTAAGCCATTG---GGGGTAGCGCCCACTAAGGCAAGAAGGAGAGTGGTG-----------
----------------------------GAGAGAGAAAAAAGAGCA---ATAGGGGGAATAGGAGCT------
---GTATTCCTTGGGTTCTTAGGAGCA---GCAGGAAGCACTATG---GGCGCGGCGTCA---
ATAACGCTGACGGTACAG---GCCAGACAA---TTGTTG---TCTGGTATAGTGCAACAGCAAAGC---
AATTTG---CTGAAG---GCTATAGAGGCGCAACATCATCTGTTGCAACTCACGGTCTGGGGCATT
>KY929365..2
TTCTATTGCAATACATCAGGCCTGTTTAATGCTACATACAATAGTACATCAGGCCTGTTTAATGCTACTAATC
AC---

ACAAATAACACCATCACCATCCCCTGCAGAATAAAACAATTTATAAACATGTGGCAGGGGGTAGGAAAAGCAA
TGTATGCCCCTCCCATTGCAGGGAACATAACATGTACATCAAAT---
ATCACAGGAATACTATTGACATGGGATGGAGGAGAAAATAGAACA----------------------------
----

GATAATGATACAGAGACATTCAGACCTGCAGGAGGAGATATGAGGGACAATTGGAGAAGTGAATTATACAAAT
ATAAAGTGGTAGAAATTCAGCCATTG---GGGGTAGCACCCACTAAAGCAAAAAGGAGAGTGGTG--------
-------------------------------GAGAGAGAAAAAAGAGCA---GTGGGA---CTAGGAGCT---
------GTGTTCCTTGGGTTCTTGGGAGCA---GCAGGAAGCACTATG---GGCGCAGCGTCA---
ATGGCGCTGACGGTACAG---GCCAGACAA---TTGTTG---TCTGGTATAGTGCAACAGCAAAGC---
AATTTG---CTGAGG---GCTATAGAGGCGCAACAACACATGTTGCAACTCACGGTCTGGGGCATT
>KY929369..1
TTCTATTGTAATACATCAGGCCTGTTTAATAGTAATAATGAGACAAACAATAATTCAAGCTTA----------
----

ATCACAATTCCATGCAGAATAAAACAAGTTGTAAACTTGTGGCAGGGGGTAGGACGAGCAATGTATGCTCCTC
CCATAGCAGGAAACATAACATGTAATTCAAAT---
ATCACAGGACTACTATTGGTACGTGATGGAGGAACAGGAAATAAC-------------------------------
----

ACAATAGAGAAATTCAGACCTCAAGGAGGAGATATGAGAGACAATTGGAGGAACGAACTATATAAATATAAAG
TGGTAGAAATTAAACCATTG---GGAGTAGCACCCACTAAAGCAAAAAGGAGAGTGGTG---------------
---------------------------GAGAGAGAAAAAAGAGCAATAGCGGGA---CTAGGAGCT----------
GTGTTCCTTGGGTTCTTGGGAGCA---GCAGGAAGCACTATG---GGCGCGGCGTCA---
ATAACGCTGACGGTACAG---GCCAGACAA---TTGTTG---TCTGGTATAGTGCAACAGCAAAGT---
AATTTG---CTGAGG---GCTATAGAGGCGCAACAGCATCTGTTGCAACTCACGGTCTGGGGCATT
>KY929370..3
TTCTATTGTAATACATCAGGCCTGTTTAATGGTACATACATGTCTAATGGTACTTATAATGAGACAAACAATA
AT--

TCAAGCTCAAACATCACAATTCCATGCAGAATAAAACAAGTTATAAACTTGTGGCAGGGGGTAGGACGAGCAA
TGTATGCTCCTCCCATTGCAGGAAAAATAACATGTAACTCAAAT---
ATCACAGGACTACTATTGGTACGTGATGGAGGAACAGGTAATCAGACA---------------------------
----

AATGATATAGAGACATTCAGACCTCAAGGAGGAGATATGAGAGACAATTGGAGGAGCGAATTATATAAATATA
AGGTGGTAGAAATTAAACCATTG---GGAATAGCACCCACTGAAGCAAAAAGGAGAGTGGTG-----------
------------------------------GGAAGAGAAAAAAAGAGCA---GTGGGA---CTAGGAGCT------
---GTGCTCTTTGGGTTCTTGGGAGCA---GCAGGAAGCACTATG---GGCGCGGCGTCA---
ATAACGCTGACGGTACAG---GCCAGACAA---TTGTTG---TCTGGTATAGTGCAACAGCAAAGC---
AATTTG---CTGATG---GCTATAGATGCGCAACAGCATCTGTTGCAACTCACAGTCTGGGGCATT
>KX756609..1
TTCTATTGCAATACCTCAAAACTGTTTAATATATCAAACCTGTCTTCTAATGATACA-----------------
----

ACAGATAATGGCACAATCATGATCCCATGCAGAATAAAACAAATTATAAACATGTGGCAGGAGGTAGGACGAG
CAATGTATGCTCCTCCCATTGCAGGAAACATAACGTGTAGTTCAAAC---
ATCACCGGACTACTGTTGGAACGTGATGGAGGAAATTCAACTAATTCAACTAAT-------------TCA----
----

AATAGGACAGAGACATTCAGGCCTCAAGGAGGAAATATGAGGAACAATTGGAGAAGTGAATTATATAAATATA
AGGTGGTAGAAATACAACCAGTG---GGAATAGCGCCCACTACAGCAAAAAGGAGAGTGGTG-----------
------------------------------GAGAGAGAAAAAAGAGCA---GTGGGA---ATAGGAGCT------
---ATGTTCCTTGGGTTCTTGGGAGCA---GCAGGAAGCACTATG---GGCGCAGCGTCG---
ATAACGCTGACGGTACAG---GCCAGACAA---CTGTTG---TCTGGTATAGTGCAACAGCAAAGC---
AATTTG---CTGAGA---GCTATAGAGGCGCAACAACATCTGTTGCAACTCACAGTCTGGGGCATT
>MK076687..2
TTCTATGGTAATACAGCAGGCCTGTTTAATAATACATACAATATTACAACAGGCACGCTTACATACAATGGTA
AATCCCTTAAT--

GATTTAAACTCAACCATCACACTCCCATGCAGAATAAAGCAGTTTAAAAATATGTGGCAGGGGGTAGGAAGAG
CAATGTATGCCCCTCCCATTGCAGGAAACATAACATGTACATCAAAT---
ATCACAGGCATAATATTGACACGTGATGGAGGGAATGAGAGTAGTACTGAA------------------------
----

GTTCACAATAATACAGAGACATTCAGACCTCAAGGGGGAAATATGAGAGATAATTGGAGAAGTGAATTATACA
AATATAAAGTGGTAGAAATTAAGCCATTG---GGAGTAGCACCCACTGCAGCAAAAAGGAGAGTGGTG-----
-------------------------------------GAAAAAGAAAAAAGG----------GGC---
CTAGGAGCT---------ATACTCATTGGGTTCTTGGGAACA---GCAGGAAGCACTATG---
GGCGTGGCGTCA---ATAACGCTGACGGTACAG---GCCAGACAA---TTGTTG---
TCTGGTATAGTGCAACAGCAAAGC---AATTTG---CTGAGA---
GCTGTAGAGGCGCAACAACATCTGTTGCAACTCACGGTCTGGGGCATT
>KY713231..1

TTCTATTGCAATACCTCAAGCTTGTTTAGCAGTATAAGCCCTAATAGTACA----------------------
----

AATAATACTGTAATAACAATCCCATGCAGAATAAAACAAATTATAAACATGTGGCAAGGGGTAGGACGAGCAA
TATACGCCCCTCCTATTGAAGGAAACATAACATGTAAATCAAAT---
ATCACAGGAATACTATTGGTACGTGATGGAGGCACAAATACTACA----------------------------
----
-
GAAGAAGGAAAAGAAATAGAAATATTTAGACCTCAAGGAGGAAATATGAAGGACAATTGGAGAAGTGAATTAT
ATAAATATAAAGTGGTAGAAATTAAGCCATTA---GGAGTAGCACCCACTAAAGCAAAAAGGAGAGTGGTG--
-------------------------------------GAGAGAGAAAAAAGAGCAGTAGTGGGG---
CTAGGAGCT---------GCGTTCCTTGGGTTCTTGTCAGCT---GCAGGAAGCACTATG---
GGCGCGGCGTCA---ATAACGCTGACGGTACAA---GCCAGACAA---TTGTTG---
TCTGGTATAGTGCAACAGCAAAGC---AATTTG---CTGAGA---
GCTATAGAGGCGCAACAGCATATGTTGCAACTCACAGTCTGGGGCATT
>KY713235..2
TTCTATAGGAGACAGTCAAAATTGTTTAATCACACATACATGCAGAATGGTACACACAATGGTACAATAAAT-
----

GGTTCAGACCCAATCATAATTCCATGCAGAATAAAGCAAATTATAAACATGTGGCAGGAGGTAGGAAGAGCAA
TGTATGCCTCTCCCATTGCAGGAAACATAACCTGTAAATCAAAT---
ATCACAGGACTACTATTGACACGAGATGGAGGACTCAAAAATGATAGTAGTAAT-------------------
----

GAGACAAAGATAGAGATATTCAGACCTGGAGGAGGAAACATGAAAGACAATTGGAGAAGTGAATTATACAAAT
ATAAAGTGGTAGAAATTAAGCCATTG---GGAATAGCACCCACTGAAGCAAGAAGAAGAGTAGTG--------
-------------------------------CAGAGAGAAAAAAGAGCA---GTGACA---CTAGGAGCT---
------ATGTTCCTTGGGTTCTTGGGAGCA---GCAGGAAGCACTATG---GGCGCGGCGTCA---
ATAACGCTGACGGTACAG---GCCAGACAA---TTGTTG---TCTGGTATAGTGCAACAGCAAAGC---
AATTTG---CTGAGG---GCTATAGAGGCGCAACATCATCTGTTGCAACTCACAGTTTGGGGCATT
>MN703343..1
TTCTATTGTAATACATCAAGACTGTTTAATGGTACATATAATACAACAGAGGAG-------------------
----

TCAAACTCAACCATCACAATCCAATGCAGAATAAAACAAATTATAAACATGTGGCAGGAGGTAGGACGAGCAA
TGTATGCCCCTCCCATTGCAGGAAACATAACATGTGAATCAAAC---
ATCACAGGACTAATATTGGTACGTGATGGAGGAACTCATGAAAATAGT-------------------------
----

AATACTACAGAGATATTCAGACCTGGAGGAGGAGATATGAGGGACAATTGGAGAAGTGAACTATATAAATATA
AAGTGGTAGAAGTTAGGCCTTTG---GGAATAGCACCCACTAAAGCAAAAAGGAGAGTGCTG-----------
----------------------------GAGAGACAAAAAAGAGCA---GTAGGA---ATGGGAGCT------
---GTGTTCCTTGGGATCTTGGGAGCA---GCAGGAAGCACTATG---GGCGCGGCGTCA---
ATAACGCTGACGGTACAG---GCTAGACAA---TTGTTG---TCTGGTATAGTGCAACAGCAAAAC---
AATTTG---CTGAGG---GCTATAGAGGCGCAACAGCACATGTTGCAACTCGCAGTCTGGGGCATT
>MN703361..1
TTCTATTGTAATACATCAGGCCTGTTTAATGAAAGCCGGCTTAACGAAACAGAAGGTGAT-------------
----

TCAAGTAAAAACATCACACTTCCATGTAGAATAAAGCAAATTATAAACATGTGGCAGGCGGTAGGAAAAGCAA
TATATGCCCCTCCCATTGCAGGAAACATAACATGTAAATCAAAT---
ATCACAGGATTACTATTGGAACGTGATGGAGGCAAAGGCAATGCA----------------------------
----

ACAGAGGAAATATTCAGACCTGGAGGAGGAAATATGGGGGACAATTGGAGAAGTGAATTATATAAATATAAAG
TGGTAGAAATTCAACCATTG---GGAATAGCACCTACTGGAGCAAAAAGGAGAGTAGTG--------------
-------------------------GAGAGAGAAAAAAGAGCAGTAGTGGGA---ATAGGAGCT---------
GTGTTCCTTGGGATCTTGAGTGCA---GCAGGAAGCACTATG---GGCGCGGCGGCA---
ATAACGCTGACGGTACAA---GCCAGACAA---TTATTG---TCTGGTATAGTGCAACAGCAAAGC---
AATTTG---CTGAAG---GCTATAGAGGCGCAACAGCATATGTTGCAGCTCACGGTCTGGGGCATT

>AF067159..2
TTCTATTGTAATACATCAGGCCTGTTTAATAGTACATACATGAGTGGTACATACATGAATTCAAGTGCAGACA
TG---

AATTCAAGCTACATCACAATCCCATGCAGAATAAAGCAAATTATAAACATGTGGCAGGAGGTAGGACGAGCAA
TGTATGCCCCTCCCATTGCAGGAAACATAACATGTAAATCAAAT---
ATCACAGGAATACTATTGGAACGTGATGGAGGGTCAGGGTCAAAT----------------------------
----

GGCACAGAGACATTCAGACCTGGAGGAGGAGATATGAGGAACAATTGGAGAAGTGAATTATATAAATATAAAG
TGGTAGAAATTCAGCCATTG---GGAGTAGCACCCACGGAAGCAAAAAGGAGAGTGGTG--------------
-------------------------GAGAGAGGAAAAAGAGCA---GTGGGA---ATAGGAGCT---------
GTGTTCCTTGGGTTCTTGGGAGCA---GCAGGAAGCACTATG---GGCGCGGCGTCA---
ATAACGCTGACGGTACAG---GCCAGACAA---TTGTTG---TCTGGTATAGTGCAACAGCAAAGC---
AATTTG---CTGAGG---GCTATAGAGGCGCAACAGCATCTGTTGCAACTCACGGTCTGGGGCATT
>FJ866133..1
TTCTATTGCAATACATCAGGACTGTTTAATAATAATATTACA-------------------------------
----

AATGCAACCACCACCCTCCCATGCAGAATAAAACAAATTGTAAACATGTGGCAGGGGGTAGGACGAGCAATGT
ATGCCCCTCCCATTGAAGGAAAAATAACATGTAACTCAAAT---
ATCACAGGACTGCTCCTGACACGTGATGGAGGGGTGAATAATGAA----------------------------
----

ACGACAGAGACATTCAGACCTGGAGGAGGAGATATGAAGGACAATTGGAGAAATGAATTATATAAATATAAAG
TGGTAGAAATTCAGCCATTA---GGAATAGCACCCACTGGAGCAAAAAGGAGAGTGGTG--------------
-------------------------GAGAGAGAAAAAAGAGCG---GTGGGA---CTAGGAGCT---------
GTGTTCCTTGGGTTCTTGGGAGCC---GCAGGAAGCACTATG---GGCGCAGCATCA---
ATAACGCTGACGGTACAA---GCCAGACAA---TTATTG---TCGGGTATAGTGCAACAGCAAAAC---
AACTTG---CTGAGA---GCTATAGAGGCGCAACAACATATGTTGCAACTCACAGTCTGGGGCATT
>AB097871..1
TTCTATTGTAATACATCAGGCCTGTTTAATGGGACATACAATGATACATACAGGTCTAATGATACAGAT----
----

AGTAATTCAACCATCACACTCCCATGCAGAATAAAGCAAATTATAAACATGTGGCAGGAGGTAGGACGAGCAA
TGTATGCCCCTCCTATTGCAGGAAACATAACATGTAAATCAAAC---
ATCACAGGACTACTATTGGTACGTGATGGAGGAAATGGTACACAG----------------------------
----

ACATTCAGACCTGGAGGAGGAAATATGAAGGATAATTGGAGAAGTGAATTATATAAATATAAAGTGGTAGAAA
TTCAGCCATTA---GGAATAGCACCCACTGGAGCAAAAAGGAGAGTGGTG-----------------------
----------------GAGAGAGAAAAAAGAGCA---GCA------ATAGGAGCT---------
ATGTTCCTTGGGTTCTTGGGAGCA---GCAGGAAGCACTATG---GGCGCGGCGTCG---
ATGGCGCTGACGGTACAG---GCCAGACAA---TTGTTG---TCTGGTATAGTGCAACAGCAAAGC---
AATTTG---CTGAGG---GCTATAGAGGCGCAACAGCATATGTTGCAACTCACAGTCTGGGGCATT
>KC894073..1
TTCTATTGCAATACATCAAAACTGTTTAATGGTACATACAATGGTACATACACAAATAATGAT----------
----

ACAAACAGCACAATCATACTCCCATGCAGAATAAAACAAATTATAAACATGTGGCAGGAGGTAGGACAAGCAA
TGTATGCCCCTCCCATTGAAGGAATCATAGCATGTAATTCAACT---
ATCACAGGATTATTATTGACACGTGATGGAGGAGACAAGAATGGG----------------------------
----

AGTAAGCCAGAAATATTCAGACCTGGAGGAGGAGATATGAGGGACAATTGGAGGAGTGAATTATATAAATATA
AAGTGGTAGAAATTAAGCCATTG---GGAATAGCACCCACTAAGGCAAAAAGGAGAGTGGTGGAG---AAA--
----------------------GAAAAAACAATTCAAAAAAGAGCA---GTAGGA---ATAGGAGCT------
---GTGTTCCTTGGGTTCTTGGGAGCA---GCAGGAAGCACTATG---GGCGCGGCGTCA---
ATAACGCTGACGGTACAG---GCCAGACAA---TTGTTG---TCTGGTATAGTGCAACAGCAAAGC---
AATTTG---CTGAGG---GCTATAGAGGCGCAACAGCATATGTTGCAACTCACAGTCTGGGGCATT

>FJ444207..1
TTCTATTGCAATACATCAAAACTGTTTACAGAAAGTAATTCAACCTCAAACAGCACC----------------
----

TCAAACACCGTACTCACACTCCCATGCAGAATAAAACAAATTATAAACATGTGGCAGGGGGTAGGACGAGCAA
TGTATGCCCCTCCCATTGAGGGAAATATAACATGTGAATCAAAC---
ATCACAGGATTACTATTGACGCGTGATAGA---ACAGACAATAAA----------------------------
----

ACAGAGACATTTAGACCTGGAGGAGGAGACATGAGGGACAATTGGAGAAGTGAATTATACAAATATAAAGTGG
TAGAAATTCAGCCATTA---GGAGTAGCACCCACTAAGGCAAAAAGGAGAGTGGTG-----------------
----------------------GAGAGAGAAAAAAGAGCA---GTGGGA---ATAGGAGCT---------
GTGTTCCTTGGGTTCTTGGGAGCA---GCAGGAAGCACTATG---GGCGCAGCATCA---
ATAACGCTGACGGTACAG---GCCAGACAA---TTGTTG---TCTGGTATAGTGCAGCAGCAGAGC---
AATTTG---CTGAGG---GCTATAGAGGCGCAACAGCATATGTTGCAACTCACGGTCTGGGGCATT
>KC894078..1
TTCTATTGTAATACATCAAACCTGTTTAATATTAATATTACAGGAAATATTACAGGAAATATGACA-------
----

GGAAATGAAAACATCACACTCCCATGCAGAATAAAACAAATTATAAACATGTGGCAAAGGGTAGGGCAAGCTA
TATATGCCCCTCCCATTGCAGGAAACATAACATGTAACTCAAGT---
ATCACAGGACTACTGTTGACACGTAATGGAGGCAATGATAACAGAACAGGG----------------------
----

AATAATAATACAGAGACATTCAGACCTGGAGGAGGAGATATGAGGGATAATTGGAGAAGTGAATTATATAAAT
ATAAAGTGGTAGAAGTTCAGCCATTG---GGAGTAGCACCCACTGGAGCAAAAAGGAGAGTGGTG--------
-------------------------------GAGAGAGAAAAAAGAGCA---GTA------ATGGGAGCT---
------ATGTTCCTTGGGTTCTTGGGAGCA---GCAGGAAGCACTATG---GGCGCGGCGTCA---
GTGGCGCTGACGGTACAG---GCCAGACAA---CTATTG---TCTGGTATAGTGCAACAGCAAAGC---
AATTTG---CTGAGG---GCTATAGAGGCGCAACAGCATCTGTTGCAACTCACGGTCTGGGGCATT
>KC894079..2
TTCTATTGCAATACATCAGACCTGTTTAATGGTACATACAGAAATGGTACATACAATCATACAGGAAGAAGT-
----

TCAAATGGAACCATCACCCTCCAATGCAAAATAAAACAAATTATAAACATGTGGCAGGAGGTAGGAAGAGCAA
TATATGCCCCTCCCATTGAAGGAGAAATAACATGTAACTCAAAT---
ATCACAGGACTACTATTGCTACGTGATGGAGGTCAATCAAATGAAACA-------------------------
----

AATGACACAGAGACATTCAGACCTGGAGGAGGAGATATGAGGGACAATTGGAGAAGTGAATTATATAAATATA
AAGTAGTAGAAATTAAACCATTG---GGAGTAGCACCCACTGAGGCAAAAAGGAGAGTGGTG-----------
----------------------------GAGAGAGAAAAAAGAGCA---GTGGGA---ATAGGAGCT------
---GTGTTCCTTGGGTTCTTGGGAGCA---GCCGGAAGCACTATG---GGCGCAGCATCA---
ATGACGCTGACGGTACAG---GCCAGGCAA---TTATTG---TCTGGTATAGTGCAACAGCAAAGC---
AATTTG---CTGAGG---GCTATAGAGGCGCAACAGCATATGTTGCAACTCACGGTCTGGGGCATT
>KC894081..1
TTCTATTGTAATACATCAGGACTGTTTAATGGTACATACCACCCTAATGGTACATACAATGAGACAGCAGTTA
AT---

TCAAGCGACACAATCACACTCCAATGCAGAATAAAACAAATTATAAACATGTGGCAAGAGGTAGGAAGAGCAA
TGTATGCTCCTCCCATTGCAGGAAATATAACATGTAATTCAACT---
ATCACAGGACTATTATTGACACGTGATGGAGGAATAAATCAGACA----------------------------
----

GGAGAAGAGATATTCAGACCTGGAGGAGGAGATATGAGGGATAATTGGAGAAATGAATTATATAAATATAAAG
TGGTGGAGATTAAGCCATTA---GGAATAGCACCCACTAAGGCAAAAAGGAGAGTGGTG--------------
-------------------------GAGAGAGAAAAAAGAGCA---GTGGGA---ATAGGAGCT---------
GTGTTCCTTGGGTTCTTGGGAGCA---GCAGGAAGCACTATG---GGCGCGGCGTCA---
ATAACGCTGACGGTACAG---GCCAGACAA---CTGTTG---TCTGGTATAGTGCAACAGCAAAGC---
AATTTG---CTGAGG---GCCATAGAGGCGCAACAGCATATGTTGCAACTCACGGTCTGGGGCATT

>FJ444522..1
TTCTATTGCAATACATCAGGCCTGTTTAATAGTACATATAATCCAAATAGTACATATAATAATTCAAGC----
----

TCAGACACACCACTTAGACTCTCATGTAGAATAAAACAAATTATAAATATGTGGCAGGGGGTAGGAAGAGCAA
TATATGCCCCTCCCATTGAAGGAAGCATAACATGTAACTCAAGT---
ATCACAGGACTACTATTGCTACGTGATGGAGGTCATGACGGTGACTCA-------------------------
----

AATAACACAGAGACATTTAGACCGGGAGGAGGAGATATGAGGGACAACTGGAGAAGTGAATTATATAAATATA
AAGTGGTAGAAATTAAGCCATTG---GGAGTAGCACCCACTGACGCAAAAAGAAGAGTGGTG-----------
----------------------------GAAAGAGAAAAAAGAGCA---GTAGTGGGAATAGGAGCT------
---GTGCTCCTTGGGTTCTTGGGAGCA---GCCGGAAGCACTATG---GGCGCGGCGTCA---
CTAACGCTGACGGTACAG---GCCAGACAA---CTGCTG---TCTGGTATAGTGCAACAGCAAAAC---
AATTTG---CTGAGG---GCTATAGAGGCGCAACAGCATATGTTGCAACTCACGGTCTGGGGCATT
>KC894089..1
TTTTATTGCAATACATCAGCACTGTTTAATGGTACATACAATTCTACATACAATTCTACTAGTAAC-------
----

CCAAACATCACAATCACAATCCAATGCAGAATAAAACAAATTATAAACATGTGGCAGGAGGTGGGACGAGCAA
TGTATGCCGCTCCCATTGCAGGAGACATAACATGTGAATCAAAC---
ATCACAGGACTACTATTGACACGTGATGGAGGAAATACTGCAAAC----------------------------
----

GGGACAGAGATATTCAGACCTGGAGGAGGAGATATGAGGGATAATTGGAGAAGTGAATTATATAAATATAAAG
TGGTAGAAGTTCAGCCATTG---GGAGTAGCACCTACCAAGGCAAAAAGAAGAGTGGTG--------------
-------------------------GGTAGAGAAAAAAGAGCA---GTA------CTGGGAGCT---------
GTGTTCCTTGGGTTCTTGGGAGCA---GCAGGAAGCACTATG---GGCGCGGCGTCA---
ATGGCGCTGACGGTACAG---GCCAGACAA---TTGTTG---TCTGGTATAGTGCAACAGCAAAGT---
AATTTG---CTGAGG---GCTATAGAGGCGCAACAGCATATGTTGCAACTCACAGTCTGGGGCATT
>KC894084..1
TTCTATTGCAATACATCAGAACTGTTTAATAATACATACTGGCCTAATAGTACTGTTACAAATGGAACT----
----

TTAAATGGAACCTTCACACTCCCATGCAAAATAAAACAAATTATAAACATGTGGCAGGAGATAGGAAGAGCAA
TATATGCCCCTCCCATTGCAGGAAACATAACATGTAAATCAAAT---
ATCACAGGACTACTATTAACACGTGATGGAGGTCATAACCCAGAG----------------------------
----

AAGAATGACACAGAGACATTCAGACCTGGAGGAGGAGATATGAGGGATAATTGGAGAAGTGAATTATACAAAT
ATAAAGTAGTAGAAGTCAAGCCATTA---GGAATAGCACCCACGGGGGCAAAAAGGAGAGTGGTG--------
-------------------------------GAGAGAGAAAAAAGAGCA---GTGGGA---ATAGGAGCT---
------GTGTTCCTTGGGTTCTTGGGAGCA---GCAGGAAGCACTATG---GGCGCGGCGTCA---
ATAACGCTGACGGTACAG---GCCAGACAA---TTATTG---TCTGGTATAGTGCAACAGCAAAGC---
AATTTG---CTGAGG---GCTATAGAGGCGCAGCAGCATATGCTGCAACTCACGGTCTGGGGCATT
>KC156114..2
TTCTATTGCAACACATCAAGATTGTTTAATCATACATTTAATGAGACA-------------------------
----

TCAGACGAAGACATCACACTCCAATGTAGGATAAAACAAATTATAAACATGTGGCAGGGGGTAGGACGAGCAA
TATATGCCCCTCCCATTGCAGGAAACATAACATGTAACTCAAAT---
ATCACAGGACTACTATTGACACGTGATGGAGGGAAGAGTAATAGTAGT-------------------------
----

AATCAGACAGAGATATTCAGACCTGCAGGAGGAAATATGAGGGACAATTGGAGAAGTGAATTATATAAATATA
AAGTGGTAGAAATTAAACCATTA---GGAATAGCACCCACTAAGGCGAAAAGGAGAGTGGTG-----------
----------------------------GAGAGAGAAAAAAGAGCG---GCA------ATAGGAGCT------
---ATGTTCCTTGGGTTCTTGGGAGCA---GCAGGAAGCACTATG---GGCGCGGCGTCA---
ATAACGCTGACGGTACAG---GCCAGACAA---TTATTG---TCTGGTATAGTGCAACAGCAAAGC---
AATTTG---CTGAGA---GCTATAGAGGCGCAACAGCATATGTTGCAACTCACGGTCTGGGGCATT

```
>KC156120..1
TTTTATTGCAATACATCAATACTGTGGGATAGTGATAAGAGT-------------------------------
-------------------------------------------------------------------------
----------------------------
AATGACACAAACATCACACTCCCATGCAGAATAAAACAAATTATAAATATGTGGCAGGAGGTAGGACGAGCAA
TGTATGCCCCTCCCATTGCAGGAAGCATAACATGTAAATCAAAA---
ATCACAGGACTACTATTGGTACGTGATGGAGGAATAAATAGCAGC----------------------------
-------------------------------------------------------------------------
----------------
ACAGAGACATTCAGACCTGGAGGAGGAAATATGAAGGACAATTGGAGAAGTGAATTATATAAATATAAAGTGG
TAGAAATTCAGCCATTA---GGAGTTGCACCCACTGGAGCAAAAAGGAGAGTGGTG-----------------
----------------------GAGAGGGAGAAAAGAGCA---GTGGGA---ATAGGAGCT---------
GTGTTCCTTGGGTTCTTGGGAGCA---GCTGGAAGCACTATG---GGCGCAGCGTCA---
ATAACGCTGACGGTACAG---GCCAAACAA---CTGTTG---TCTGGTATAGTGCAACAGCAGAGT---
AATTTG---CTGAGA---GCTATAGAGGCGCAACAGCATATGCTGCAACTCACAGTCTGGGGCATA
>HQ615984..1
TTCTATTGCAATACATCAGGCCTGTTTAATGGTACATTTAATGGTACATACATGCCAAATAGTACAGAAAGTA
CT-----------------------------------------------------------------------
----------------------------
TCAAATACAAACATCACACTCCAATGCAGAATAAAACAAATCATAAACATGTGGCAGCAGGTAGGACGAGCAA
TGTATGCCCCGCCCATTGAAGGAAACATAACATGTAGATCAAAT---
ATCACAGGACTACTATTGACACGTGACGGAGGCGGAAGAGCAAAT----------------------------
-------------------------------------------------------------------------
----------
GATACTGAAGAGATATTCAGGCCTGGAGGAGGAGATATGAGGGACAATTGGAGAAGTGAATTATACAAATATA
AAGTGGTAGAGGTTCAGCCATTG---GGAATAGCACCCACTAAGGCAAAAAGGAGAGTGGTG-----------
----------------------------GAGAGAGAAAAAAGAGCA---GTGGGA---ATGGGAGCT------
---GTGTTCCTTGGGTTCTTGGGAGCA---GCAGGAAGCACTATG---GGCGCGGCGTCA---
ATAACGCTGACGGTACAG---GCCAGACAA---TTGTTG---TCTGGTATAGTGCAACAGCAAAGC---
AATTTG---CTGAGG---GCCATAGAGGCGCAACAGCATATGTTGCAACTCACGGTCTGGGGCATT
>KC156216..1
TTCTATTGCAATACATCAAAACTGTTTAATAGTATACACAATAGTACAGATATGTATAATAGTACAGTT----
-------------------------------------------------------------------------
----------------------------
AGTAATTCAACCATCACACTCCAATGCAAAATAAAACAAATTATAAATATGTGGCAGGGGGTAGGACGAGCAA
TGTATGCCCCTCCCATAGCAGGAAACATAACATGTAAATCAAAT---
ATCACAGGACTACTATTAACACGGGATGGAGGAAACGTGAGTGGA----------------------------
-------------------------------------------------------------------------
-------------
ACAACAGAGACATTCAGACCTGGAGGAGGAAATATGAAGGACAATTGGAGAAGTGAATTATATAAATATAAAG
TGGTGGAAATTCAGCCATTA---GGAATAGCACCCACTAAGGCAAAAAGGAGAGTGGTG--------------
-------------------------GAAAGAGAAAAAAGAGCA---GTGGGA---ATAGGAGCT---------
GTGTTCCTTGGGTTCTTGGGAGTA---GCCGGAAGCACTATG---GGCGCAGCATCA---
ATGACGCTGACGGTACAG---GCCAGACAA---TTGTTG---TCTGGTATAGTGCAACAGCAAAGC---
AATTTG---CTGAGG---GCTATAGAGGCGCAACAGCATATGTTGCAGCTCACGGTCTGGGGCATT
>KC156219..2
TTCTATTGCTATACATCAGAACTGTTTAATGAGACAGACAAACTGCTTAATAAGACAAAAGAGAAA-------
-------------------------------------------------------------------------
----------------------------
GACAATTCAACCATCATACTCCCATGCAGAATAAAACAAATTATAAACATGTGGCAGGGGGTAGGACGAGCAA
TATATGCCCCTCCCATTGAAGGAAATATAACATGTAAGTCAAAT---
ATCACAGGATTACTATTGACACGTGATGGAGGAGAGGACATTAATGGAACT----------------------
-------------------------------------------------------------------------
----------
AATAGAGAAGAGATATTCAGACCTGGAGGAGGAGATATGAAGGATAATTGGAGAAGTGAATTATATAAATATA
AAGTAGTAGAAATTCAGCCATTA---GGAATAGCACCCACTAAGGCAAAAAGGAAAGTGGTG-----------
----------------------------GAGAGAGAAAAAAGAGCA---GCA------ATAGGAGCT------
---GTGTTCCTTGGGTTCTTGGGGGCA---GCAGGAAGCACTATG---GGCGCGGCGTCA---
ATAACGCTGACGGTACAA---GCCAGACAA---TTATTG---TCTGGTATAGTGCAACAGCAAAGC---
AATTTG---CTGAGA---GCTATAGAGGCGCAACAGCATATGTTGCAACTCACGGTCTGGGGCATT
```

>HQ615963..2
TTCTATTGCAATACATCAAGCCTGTTTAATAGGACATATAATAGTACAGATATGGCCAATAGTACAGAA----
----

GCTAACAGTACCATCACAATCCACTGCAGAATAAAACAAATTATAAACATGTGGCAGGAGGTGGGACGAGCAA
TGTATGCCCCTCCCATTAAAGGAAACATAACATGTATATCAAAT---
ATCACAGGACTACTATTAACAAGGGATGGAGGAGATAATAATGAA----------------------------
----

ACGGAGACATTCAGACCTGGAGGAGGAAATCTGAGGGACAATTGGAGAAGTGAATTATATAAATATAAAGTGG
TAGAAGTTCAGCCATTG---GGAGTAGCACCCACTAATTCAAGAAGGAGAGTGGTG-----------------
----------------------GAGAGAGAAAAAAGAGCA---GTGGGA---CTAGGAGCT---------
GTGTTCCTTGGGTTCTTGGGGGCA---GCAGGAAGCACTATG---GGCGCAGCATCA---
ATAACGCTGACGGTACAG---GCCAGACAA---TTATTG---TCTGGTATAGTGCAACAGCAAAGC---
AATTTG---CTGAAA---GCTATAGAGGCTCAACAGCATATGTTGAAACTCACGGTCTGGGGCATT
>HQ615964..2
TTCTACTGCAATACATCAAACCTGTTTAACAGTACATATAACAGTAACAGTACATATAACAGTACAGATACTC
CT---

GAGGATAAAAACATCACACTCCAATGCAGAATAAAACAAATTATAAACATGTGGCAGGGGGTAGGACAAGCAA
TGTATGCCCCTCCCATTGCAGGAAACATAACATGTAAATCAAAT---
ATCACAGGATTATTATTAACACGTGATGGAGGAACCAACACAAAC----------------------------
----

AACACAACAGAAATATTCAGACCTGGAGGAGGAGATATGAGGGACAATTGGAGAAGTGAATTATATAAATATA
AAGTGGTAGAAATTCAACCATTA---GGAGTAGCACCTACTCAGGCAAAAAGGAGAGTGGTG-----------
----------------------------GAGAGAGAAAAGAGG---------GGA---ATAGGAGCT------
---GTGTTCCTTGGGTTCTTGGGAGCA---GCAGGAAGCACTATG---GGCGCAGCATCA---
ATGACGCTGACGGTACAG---GCCAGACAA---TTGTTG---TCTGGTATAGTGCAACAGCAAAGC---
AATTTG---CTGAGG---GCTATAGAGGCGCAACAGCATATGTTGCAACTCACGGTCTGGGGCATT
>HM204613..1
TTCTATTGCGATACATCAAAACTGTTTAATAGTACATACCTGGCTAATGGCACACGCTGGATTAATGATAGTA
CAGAA--
-------------------CAG---
AATACAAGCAAAGTCATCTCACTCCCATGTAGAATAAAACAAATTATAAACATGTGGCAGGAGGTAGGACGAG
CAATGTATGCTCCTCCCATTGAAGGAAACATAAGATGTGAATCAAAT---
ATCACAGGATTGTTATTGACGCGTGATGGAGGAATAGATAACAAC----------------------------
----

AAGAGCGAGATATTCAGACCTGGAGGAGGAGATATGAGGGACAATTGGAGAAGCGAATTATATAAATATAAAG
TGGTAGAAATTAAACCATTG---GGAATAGCACCCACTAAGGCAAAAAGGAGAGTGGTG--------------
-------------------------GAGAGAGAGAAAAGAGCA---GTGGGA---ATAGGAGCT---------
CTGTTCCTTGGGTTCTTGGGAGCA---GCAGGAAGCACTATG---GGCGCGGCGTCA---
ATGACGCTGACGGTTCAG---GCCAGACAA---TTGTTG---TCTGGTATAGTGCAACAGCAAAGC---
AATTTG---CTGAGG---GCTATAGAGGCGCAACAACATATGTTGCAACTCACGGTCTGGGGCATT
>KC187578..1
TTCTATTGCAATACATCAGGACTGTTTAATAGTACATACAATAATGGTACATACACTAGTGCATACAATATT-
----
-------------------ACA---
GAAAATAATTCAAGTATTACACTCCCATGCAGAATAAAGCAGATTATTAACATGTGGCAGGGGGTAGGACGAG
CAATGTATGCCCCTCCCATTGCAGGAAACATAACATGCAGATCAAAT---
ATCACAGGACTACTATTGACACGGGATGGTGGCAATGGAATCAATGTG-------------------------
----

ACCACAGAGATATTCAGACCTGAAGGGGGAAATATGAAGGACAATTGGAGAAGTGAATTATACAAATACAAAG
TGGTAGAAATTCAGCCATTG---GGAATAGCACCCACCAAGGCAAAAAGGAGAGTGGTG--------------
-------------------------GAGAGAGAAAAAAGAGCA---GTGGGA---ATAGGAGCT---------
GTGTTCCTTGGGTTCTTGGGAGCA---GCAGGAAGCACTATG---GGCGCGGCGTCA---
ATAACGCTGACGGTACAG---GCCAGACAA---TTATTG---TCTGGTATAGTGCAACAGCAAAGC---
AATTTG---CTGAGA---GCTATAGAGGCGCAACAGCATATGTTGCAACTCACGGTCTGGGGCATT

```
>KC187323..1
TTCTATTGTAATACATCAAAACTGTTTAATAATACATTTGATGGTACAAACATGATGAATAACAAT------
------------------------------------------------------------------------
----------------------------
ACAGATGAAACCATCACACTCCAATGCAGAATAAAACAAATTATAAACATGTGGCAGGGGGTAGGACGAGCAA
TGTATGCCCCCCCCATTAAGGGAATCATAACATGTAAATCAAAT---
ATCACAGGACTACTATTGACACGTGATGGAGGAAATCACAATGAGACT------------------------
------------------------------------------------------------------------
----------
AATAACACAGAGACATTCAGACCTGCAGGAGGAAATATGAAGGACAATTGGAGAAGTGAATTATATAAATATA
AAGTGGTAGAAATTAAGCCATTA---GGAATAGCACCCACTACGGCAAAAAGGAGAGTGGTG----------
----------------------------GAGAGAGAAAAAAGAGCA---GTGGGA---ATAGGAGCT-----
---GTGTTCCTTGGGTTCTTGGGAGCA---GCAGGAAGCACTATG---GGCGCAGCATCA---
ATAACGCTGACGGTACAG---GCCAGACAA---TTGTTG---TCTGGTATAGTGCAACAGCAAAGC---
AATTTG---TTGAGA---GCTATAGAGGCGCAACAGCATATGTTGCAACTCACAGTCTGGGGCATT
>KC186955..1
TTCTATTGCAATACATCGCAACTGTTTAATAGTACATACCCTAATGAGACATATAATATTAATGTTAATGATA
CA----------------------------------------------------------------------
----------------------------
AATTCAACAGAGATTGTACTCCCATGCAGGATAAAACAAATTATAAACATGTGGCAGGAGGTAGGACGAGCAA
TGTATGCTCCTCCCATTGCAGGAAACATAACATGTAGATCAAAT---
ATCACAGGATTACTATTGACGCGAGATGGAGGACCTAATCAGACC---------------------------
------------------------------------------------------------------------
----------------
ACAGAAACATTCAGACCTGCAGGAGGAGATATGAGGGACAATTGGAGAAGTGAATTATACAAATATAAAGTGG
TAGAAATTAAGCCATTG---GGAATAGCACCCACTGGGGCAAAAAGGAGAGTGGTG----------------
----------------------GAGAGAGGAAAAAGAGCA---GTGGGA---ATAGGAGCT--------
GTGTTCCTTGGGTTCTTGGGAGCA---GCAGGAAGCACTATG---GGCGCGGCGTCA---
CTAACGCTGACGGTACAG---GCCAGACAA---TTGTTG---TCTGGTATAGTGCAACAGCAAAGC---
AATTTG---CTGAGG---GCCATAGAGGCGCAACAGCATCTGTTGCAACTCACAGTCTGGGGCATT
>KC187164..1
TTCTATTGCAATACATCAGGACTGTTTAAAAATAGGATATACAGTAGTACATACAATGATACAGTAAATAATT
CAAGC-------------------------------------------------------------------
-------------------------
TCAGAGAACATCACAATCACACTCCCATGCAGAATAAAACAAATTATAAACATGTGGCAGAGGGTAGGACGAG
CAATGTATGCTCCTCCCATTAAAGGAAGCTTAACATGTAACTCAAGT---
ATCACAGGGTTACTATTGACGAGGGATGGAGGCAGTGGAGACAACACA------------------------
------------------------------------------------------------------------
-------------
AGTACAGAGATCTTCAGACCTGGAGGAGGAGATATGAGGGACAATTGGAGAAGTGAATTATACAAATATAAAG
TGGTAGAAGTTCAACCATTA---GGAATAGCACCCACTAAGGCAAAAAGGAGAGTGGTG-------------
-------------------------GAGAGAGAAAAAAGAGCA---GTGGGA---ATAGGAGCT--------
GTGTTCCTTGGGTTCTTGGGAGCA---GCCGGAAGCACTATG---GGCGCGGTGTCA---
ATAACGCTGACGGTACAG---GCCAGACAA---TTGCTG---TCTGGTATAGTGCAGCAGCAAAGC---
AATTTG---CTGAAG---GCTATAGAGGCGCAACAGCATATGTTGCGACTCACGGTCTGGGGCATT
>KC187620..2
TTCTATTGCAATACATCAGGACTATTTAATGGTACATATACAAACATGAGCAGCACAGGAAAT---------
------------------------------------------------------------------------
----------------------------
GCAAATGCAAACATCACACTTCAATGCAAAATAAAACAAATTATAAACATGTGGCAACAGGTGGGAAAAGCAA
TGTATGCTGCTCCCATTGCAGGAAACATAACATGTAAATCAAAT---
ATCACAGGACTTCTATTGACACGTGATGGAGGAGTAGGAAACAATGAC------------------------
------------------------------------------------------------------------
-------
ACAAATGGCACAGAGACATTCAGACCTGGAGGAGGAGATATGAGGGACAATTGGAGAAGTGAATTATACAAAT
ATAAAGTGGTAGAAATTCAGCCATTA---GGAATAGCACCCACTAAGGCAAAAAGGAGAGTGGTG-------
------------------------------GAGAGAGAAAAAAGAGCA---GTAGGA---ATAGGAGCT---
------TTGTTCCTTGGGTTCTTAGGAGCA---GCCGGAAGCACTATG---GGCGCAGCGTCA---
ATAACGCTGACGGTACAG---GCCAGACAA---TTGTTG---TCTGGTATAGTGCAACAGCAAAGC---
AATTTG---CTGAGA---GCTATAGAGGCGCAACAGCATATGTTGCAACTCACAGTCTGGGGCATT
```

>KY111994..2
TTCTATTGCAATACATCAAATCTGTTTAATGGTACATACAATGGTACATACATAAGTACAAAC----------
----

AGTACTTCAAACATCACGCTTCAATGCAGAATAAAACAAATTATAAACATGTGGCAGGGGGTAGGAAGAGCAA
TGTATGCTCCTCCCATTGCAGGAAACATAACATGTAAATCAAAT---
ATCACAGGGCTACTATTGACACGTGATGGAGGGACCAAAAATAATAGCAACGAA------------------
----

ACAGAGGAGACATTCAGGCCTGCAGGAGGAGATATGAGGGATAATTGGAGAAGTGAATTATATAAATATAAAG
TAGTAGAAATTCAGCCATTA---GGAATAGCACCAACTGGTGCAAAAAGGAGAGTGGTG-------------
------------------------GAGAGAGAAAAAAGAGCA---GCAGGA---CTAGGAGCT---------
TTGTTCCTTGGGTTCTTGGGAGCA---GCAGGAAGCACTATG---GGCGCAGCATCA---
ATAACGCTGACGGTACAG---GCCAGACAA---TTGTTG---TCTGGTATAGTGCAACAGCAAAGC---
AATTTG---CTGAGG---GCTATAGAGGCGCAACAGCATATGTTGCAACTCACGGTCTGGGGCATC
>KC187280..1
TTCTATTGTAATACATCACAATTGTTTAATAGTACACACAAGAGTACAGCCGATCCTACAAGGAGTGAGTCAA
AT--

GAAAATGAAACCATTATACTCCCATGCAAAATAAAACAAATTATAAACATGTGGCAGGAGGTAGGACGAGCAA
TGTATGCCCCTCCCATTGCAGGAAACATAACATGTAAATCAAAT---
ATCACAGGGCTATTATTGACACGTGATGGAGAGTATAATGGGACA---------------------------
----

AACAAAAATGAAACATTCAGACCTGCAGGAGGAGACATGAGGGACAACTGGAGAAGTGAATTATATAAATATA
AAGTGGTAGAAATTCAACCATTG---GGAGTGGCACCCACTAAGGCAAAAAGGAGAGTGGTG----------
---------------------------GAGAGAGAAAAAAGAGCA---ATAACA---ATGGGAGCT------
---GTGTTCCTTGGGTTCTTAGGAGCA---GCAGGAAGCACTATG---GGCGCAGCGGCA---
GTAACGCTGACGGCACAG---GCCAGACAA---CTGTTG---TCTGGTATAGTGCAACAGCAAAGC---
AATTTG---CTGAGG---GCTATAGAGGCACAACAGCATATGTTGCAACTCACAGTCTGGGGCATT
>KC187249..2
TTCTATTGCAATACATCACAACTGTTTAATGGAACATACTATGCAAATGGGACATCCACGTCTAATGATACAG
CAGGTAAT--

TCAAGCGACATCATCATACTCCCATGCAGAATAAAACAAATTATAAATATGTGGCAGAAGGTAGGACGGGCAA
TGTATGCTCCTCCCATTGCAGGAAACATAACATGTCACTCAAAC---
ATCACAGGATTACTATTGACAAGGGATGGTGGAAACAGGAATGGC---------------------------
----

ACAAATGAGACATTCAGACCTGCAGGAGGAGATATGAGGGACAATTGGAGAAGTGAATTATACAAATACAAAG
TGGTAGAAGTTAATCCATTG---GGAGTAGCACCCACTAGGGCAAAAAGGAGAGTGGTG-------------
------------------------GAGAGAGAAAAAAGAGCA---GTGGGA---ATAGGAGCT---------
ATGTTCCTTGGGTTCTTGGGAGCA---GCAGGAAGCACTATG---GGCGCGGCGTCA---
ATAACGCTGACGGTACAG---GCCAGACAG---TTGTTG---TCTGGTATAGTGCAACAGCAAAGC---
AACCTG---CTGAGG---GCCATAGAGGCGCAACAGCATATGTTGCAACTCACGGTCTGGGGCATT
>MF502153..1
TTCTATTGCAATACATCACAACTGTTCAATAGTAGCAGTAATTCAAGCTCAACCAGCAAT------------
----

TCAAACTCAACCATCACCCTCCCATGCAGAATAAAACAAATTATAAACATGTGGCAGGGGGTAGGACGGGCAA
TGTATGCCCCTCCCATTCAAGGAAAAATAACATGTAATTCAAGT---
ATCACAGGATTACTATTGACACGTGATGGGGGACCTCGGAATGGC---------------------------
----

ACAGAGACATTCAGACCTGGAGGAGGAGATATGAGGGACAATTGGAGAAGTGAATTATATAAATATAAAGTGG
TAGAAGTTAAACCATTG---GGAATAGCACCCACTGGAGCAAAAAGGAGAGTGGTG----------------
----------------------GAGAGAGAAAAAAGAGCA---GTGGGA---ATGGGAGCT---------
ATGTTCCTTGGGTTCTTGGCAGCA---GCAGGAAGCACTATG---GGCGTGGCGTCA---
ATAACGCTGACGGTACAG---GCCAGACAA---TTGCTG---TCTGGTATAGTGCAACAGCAAAGT---
AATTTG---CTGAGA---GCCATAGAGGCGCAACAGCATATGTTGCAACTCACAGTCTGGGGCATT

>MF353063..1
TTCTATTGCAATACATCAAGCCTGTTTAATAGGACATATATGGCTAATAGTACAGATATGGCTAATAGTACAG
AAACTAAC---

AGTAACGGAACCATCACAATCCACTGCAGAATAAAACAAATTATAAACATGTGGCAAGGGGTGGGACGAGCAA
TGTATGCCCCTCCCATTGCAGGAAACATAACATGTATATCAAAT---
ATCACAGGACTACTATTGACAAGGGATGGAGGAAACACCAGTATCAGTGAG----------------------
----

AAGACAGAGACATTCAGACCTGAAGGAGGAAATATGAAGGACAATTGGAAAAGTGAATTATATAAATATAAAG
TGGTAGAAGTTAAGCCATTA---GGAGTAGCACCCACTAATGCAAGAAGGAGAGTGGTG--------------
-------------------------GAGAGAGAAAAAAGAGCAGTAGTGGGA---ATGGGAGCT---------
GTGTTCCTTGGGTTCTTGGGAGCG---GCAGGAAGCACTATG---GGCGCAGCATCA---
ATAACGCTGACGGTACAG---GCCAGACAA---TTATTG---TCTGGTATAGTGCAACAGCAAAGC---
AATTTG---CTGAAG---GCTATAGAGGCTCAACAGCATATGTTGAGACTCACGGTCTGGGGCATT
>HM638803..1
TTCTATTGCAATACTGCAAGCCTGTTTAATAGTACATACAATCCTAATCCTAATGGTACAGAAGACAATTCAA
ATTCA--

ACTTCAAATTCAAGCATCACAATCAAATGCAGAATAAAGCAAATTATAAACATGTGGCAGGAGGTAGGAAAAG
CAATGTATGCCCCTCCCGTTGCAGGAAATATAACATGTATATCCAAT---
ATCACAGGACTACTATTGACACGTGATGGAGGAAAGGGAGGTGGCACA-------------------------
----

AGTAATGACACAGAGATATTCAGACCTCAAGGAGGAAATATGAAGGACAATTGGAGAAGTGAATTATATAAAT
ATAAAGTGGTAGAAATTAAACCATTA---GGAGTAGCACCAACTAAGGCAAAAAGGAGAGTGGTG--------
-------------------------------GAGAGAGAAAAAAGAGCA---GTGGGA---CTAGGAGCT---
------GTGCTCTTTGGGTTCTTGGGAGCA---GCAGGAAGCACTATG---GGCGCAGCATCA---
ATAACGCTGACGGTACAG---GCCAGACAA---TTGTTG---TCTGGTATAGTGCAACAGCAAAGC---
AATTTG---CTGAGA---GCTATAGAGGCGCAACAGCATATGTTGCAACTCACGGTCTGGGGCATT
>KC894127..1
TTCTATTGCAATACATCAAGATTGTTTGATCCTAAAGGTACTAATGGTACA----------------------
----

GATAATTCAACCATCCAACTCCCATGCAGAATAAAACAAATTGTAAACATGTGGCAGGGGGTAGGAAGAGCAA
TGTATGCCCCTCCCATTGCAGGAAACATAACATGTAGATCAAAT---
ATCACAGGACTACTATTGGTACGTGATGGAGGCAGAGACGCAAATACC-------------------------
----

ACAGAGCCAGAGATCTTCAGACCTGGAGGAGGAGATATGAGGGACAATTGGAGAAGTGAATTATATAAATATA
AAGTGGTAGAAATTCAGCCATTG---GGAGTAGCACCCACTAGGGCAAAAAGGAGAGTGGTG-----------
----------------------------GAGAGAGAAAAAAGAGCA---GTGGGA---ATAGGAGCT------
---GTGTTCCTTGGGTTCTTGGGAGCA---GCAGGAAGCACTATG---GGCGCGGCGTCA---
ATGACGCTGACGGTACAG---GCCAGACAA---CTGCTG---TCTGGTATAGTGCAACAGCAAAGC---
AATTTG---CTGAGA---GCTATAGAGGCGCAACAGCATATGTTGCAACTCACAGTCTGGGGCATT
>HM639260..1
TTCTATTGCAATACATCAGGACTGTTTAATGAGACATACAATCCTAATGGTACAGAAAGTAAT----------
----

TCAAGCGCAGTCATCACACTCCCATGCAGAATAAAACAAATTATAAACATGTGGCAGGAAGTAGGAAAAGCAA
TATATGCCCCTCCCATTCAAGGAAACATAACATGTAAATCAAAT---
ATCACAGGACTACTATTGGTACGTGATGGAGGAAACTCAAATGGCACA-------------------------
----

AATAACACAGAGATATTCAGACCTGGAGGAGGAGATATGAGGGACAATTGGAGAAGTGAATTATATAAATATA
AAGTGGTAGAAATTAAACCATTG---GGAATAGCACCCACTAGTGCAAAAAGGAGAGTGGTG-----------
----------------------------GAGAGAGAAAAAAGAGCA---GTTGGA---ATAGGAGCT------
---GTGTTCCTTGGGTTCTTGGGAGCA---GCAGGAAGCACTATG---GGCGCGGCGTCA---
ATAACGCTGACGGTACAG---GCCAGACAA---CTGTTG---TCTGGTATAGTGCAACAGCAAAGC---
AATTTG---CTGAGA---GCTATAGAGGCGCAACAGCATATGTTGCAACTCACGGTTTGGGGCATT

>KJ541837..2
TTCTATTGTAATACATCAGGCCTGTTTAATGGTTCATACAATTATAATGCTACAGCA----------------
----
AGTAATTCCAGCTCAGAC---------
AATTCCAGCTCAGTCATCAGAATCCCATGCAGAATAAAGCAAATTATAAACATGTGGCAGGAGGTAGGACGAG
CAATGTATGCCCCTCCCATTGCAGGAAACATAACATGTAAATCAAAT---
ATCACAGGAATACTATTGTATCACAGGACTACTGTTGGAGGA-------------------------------
----
AAGGAAAATGATAAATAC---
TGGAAAGAGACATTCAGACCTACAGGAGGAGATATGAGGGACAATTGGAGAAGTGAATTATATAAATATAAAG
TGGTAGAAATTAAACCATTG---GGGATAGCACCCACTGAAGCAAGAAGGAGAGTGGTG--------------
-------------------------GAAAGAGAAAAAAGAGCA---GTGGGA---GTAGGAGCT---------
ATGTTCCTTGGGTTCTTGGGAGCA---GCAGGAAGCACTATG---GGCGCGGCGTCA---
ATAACGCTGACGGTACAG---GCCAGACAA---TTGTTG---TCTGGTATAGTGCAACAGCAAAGC---
AATTTG---CTGAGG---GCTATAGAGGCGCAACAGCATCTGTTGCAACTCACAGTCTGGGGCATT
>KJ541839..4
TTCTATTGTAATACATCAAAACTGTTTAATGGTACATACATTCCTAATGGTACA-------------------
----
GGAAGTTACTTAAGCAGT---------
AATTCAAGCTCAAACATCACAATTCCATGCAGAATAAAGCAAATTATAAACATGTGGCAGGAGGTAGGACGAG
CAATATATGCCCCTCCCATTGAAGGAAACATAACATGTAGATCAAAT---
ATCACAGGAATAATATTGACACGTGATGGAGGAAAAAATCCAAAATCAA------------------------
----
AATGAGACAGACAAT---ACAACAGAAGACTATTCTAGCGTGGAAGGA---
ATGAAGGACAATTGGAGAAGTGAATTATATAAATATAAAGTGGTAGAAATTCAGCCATTG---
GGAATAGCACCCACTGGAGCAAAAAGGAGAGTGGTG-------------------------------------
--GAGAGAGAAAAAAGGGAT---GTGACA---CTAGGAGCT---------
TTGTTCCTTGGGTTCTTGGGAGCG---GCAGGAAGCACTATG---GGCGCGGCGTCA---
ATAACGCTGACGGTACAG---GCCAGACAA---TTGTTG---TCTGGTATAGTGCGACAGCAAAGC---
AATTTG---CTGAGA---GCTATAGAGGCGCAACAGCATCTGTTGCAACTCACAGTCTGGGGCATT
>KJ541841..3
TTCTATTGTAATACATCAAAACTGTTTAATGGTACATACATGCTTAATGGTACA-------------------
----
GGAAGTTATTTAATCAGT---------
AATTCAAGCTCAAACATCACAATTCCATGCAGAATAAAGCAAATTATAAACATGTGGCAGGAGGTAGGACGAG
CAATATATGCCCCTCCCATTGAAGGAAACATAACATGTAGATCAAAT---
ATCACAGGACTACTATTGACACGTGATGGAGGACAA-------------------------------------
----
TCCAAAATCAAATTAGACAGTCAA---------
TCCACAGAGATATTTAGACCTGGAGGAGGAGATATGAAGGACAATTGGAGAAGTGAATTATATAAATATAAAG
TGGTAGAAATTCAGCCATTG---GGAATAGCACCCACTGGAGCAAAAAGGAGAGTGGTG--------------
-------------------------GAGAGAGAAAAAAGGGAT---GTGACA---CTAGGAGCT---------
TTGTTCCTTGGGTTCTTGGGAGCG---GCAGGAAGCACTATG---GGCGCGGCGTCA---
ATAACGCTGACGGTACAG---GCCAGACAA---TTGTTG---TCTGGTATAGTGCAACAGCAAAGC---
AATTTG---CTGAGA---GCTATAGAGGCGCAACAGCATCTGTTGCAACTCACAGTCTGGGGCATT
>KJ541848..1
TTCTATTGCAACACATCAAAACTGTTTAATAGTACATACATGCCTAAACATGCA-------------------
----
AAGTGCCATACAATCACA---------
AAGGCACACGCAATCATCACAATTCCATGCAGAATAAAACAAATTATAAATATGTGGCAGGAGGTAGGACGAG
CAATGTATGCTCCTCCCATTAAAGGAAAAATAACATGTAATTCAAGT---
ATCACAGGACTGCTATTGACACGTGACGGAAGGGAA-------------------------------------
----
AACAAAAAGAATTCAAAGGGGGGT---------
GATACAGAAACTTTCAGACCTGAAGGAGGAAATATGAAAGACAATTGGAGAAGTGAATTATATAAATATAAGG
TGGTAGAAATTAAGCCATTG---GGAATAGCACCCACTGGAGCAAAAAGGAGAGTGGTG--------------
-------------------------GAGAGAGAAAAAAGAGCA---GTGGGA---CTGGGAGCT---------
CTGTTCCTTGGGTTCTTGGGAGCA---GCAGGAAGCACTATG---GGCGCAGCGTCA---
ATGACGCTGACGGTACAG---GCCAGACAA---TTGTTG---TCTGGTATAGTGCAGCAGCAAAAC---
AATTTG---CTGAGA---GCTATAGAGGCGCAACATCAGTTGTTGCAACTCACGGTCTGGGGCATT

>MF373132..1
TTCTATTGCAATACATCAAACCTGTTTAATAGTAGTAAAACTAATATAACAGCAAAT----------------
----

GAAAATGGAACCATCACACTCCCATGCAGAATAAAACCAATTGAAACCTTGGGGCAGGGGGTAGGAAAAGCAA
TTATTGCCCCCTCCATTAAAGGAAAATTACCTGGTACTTCCAAA---
TTCCCAGGCTTACCACTGGCCCCGAGTAGGGGTGAAAAAATAGGAACCTAT---------------
GGAAATTTGGCA---AAAAGAGAA---

TTTTCCCCGGGGGGGGGAGATATGAGTGACAATTGGAGAAGTGAATTATATAAATATAAAGTGGTAGAAATTA
ATCCATTA---GGAGTAGCACCCACTAAGGCAAAAAGGAGAGTGGTG--------------------------
-------------GAGAGAGAAAAAAGAGCA---GTGGGA---ATAGGAGCT---------
GTGTTCCTTGGGTTCTTGAGTGCA---GCAGGAAGCACTATG---GGCGCAGCGTCA---
ATAACGCTGACGGTACAA---GCCAGACAA---TTGTTG---TCTGGTATAGTGCAACAGCAAAAC---
AATTTG---CTGAGA---GCTATAGAGGCGCAACAACATATGTTGAAACTCACAGTCTGGGGCATT
>MF373137..1
TTCTATTGTAATACATCAGGCTTGTTTAATACATCAGGCCCATTTAATAATAGTGAAACAAATAACACA----
----

GCAAATGTAAACATCACACTCCCATGCAGAATAAAACAAATTATAAACATGTGGCAGGGGGTAGGACAAGCAA
TGTATGCCCCTCCTGTTGCAGGAAACATAACATGTAGATCAAAT---
ATCACAGGAATACTACTGACACGTGATGGAGGAATAAATACCACAACTAAT----------------------
----

AACACATACGAACTATTCAGGCCTACAAGAAGAGACAATAGGGACAATTGGAGAAGTGAATTATATAAATATA
AAGTAGTAGAAATTCAGCCATTA---GGAGTAGCACCCACTGAGGCAAGAAGGAGAGTGGTG-----------
----------------------------GAGAGAGAAAAAAGAGCA---GTGGGA---ATAGGAGCT------
---GTGTTCCTTGGGTTCTTGGGAGCA---GCAGGAAGCACTATG---GGCGCAGCATCC---
ATAACGCTGACGGTACAG---GCCAGACAA---TTATTG---TCTGGTATAGTGCAACAGCAGAAC---
AATCTG---CTGCGG---GCTATAGAGGCGCAACAGCATATGTTGCAACTCACAGTCTGGGGCATT
>MF373139..3
TTCTATTGCAATACATCGGGCCTGTTTAGTATAAATTATACAGAAAATAATACAGATAATATTCAGAGTATGG
AC---

ATCACACTCCCCTGCCCGATCAGACAAATCATAATCATGTGGCAGGGGCTAGAGGTAGCAATATCTACCCCTC
CCCCTAACGGTACAGTATCATGTACTTCTAAT---
TTCACTGGCGTAGTCCTGCTACGTGCTGGAGGGCGTCATCGGGGAAATGAA----------------------
----

AGTGGGACAGAGACATTCAGACCTGCAGGAGGAGATATGAAGGACAATTGGAGAAGTGAATTATATAAATATA
AGGTAGTAGAAATTAAGCCATTA---GGAGTAGCACCCACTAAGGCAAAAAGGAGAGTGGTG-----------
----------------------------GAGAGAGAAAAAAGAGCA---GTGGGA---CTAGGAGCT------
---GTGTTCCTTGGGTTCTTGGGAGCG---GCAGGAAGCACTATG---GGCGCAGCATCA---
ATAACGCTGACGGTACAG---GCCAGACAA---TTATTG---TCTGGTATAGTGCAACAGCAAAAC---
AATTTG---CTGAGG---GCTATAGAGGCGCAACAGCATATGTTGCAACTCACAGTCTGGGGCATT
>KP411839..2
TTCTACTGCAATACATCAAAGCTGTTTAATAGTACATACCCGCCTAACAGTACTGATACGTACATGAACAGTG
CT---

AATACTTCAAACATCATACTTCAATGCAGAATAAAACAAATCATAAACATGTGGCAGGGGGTAGGACAAGCAA
TGTATGCCCCTCCTATTGCAGGGAACATAACATGTGAATCAAAT---
ATCACAGGACTGCTGTTAACACGTGATGGAGGAAACAATCAAACA----------------------------
----

GAGGAGGAGACATTTACACCTGGAGGAGGAGATATGAGAGACAATTGGAGAAGTGAATTATATAAATACAAAG
TGGTAGAAATTAAGCCATTA---GGAATAGCACCCACCAAGGCAAAAAGGAGAGTGGTG--------------
-------------------------GAGAGAGAAAAAAGAGCA---GTGGGA---ATAGGAGCT---------
GTGTTCCTTGGGGTCTTGGGAGCA---GCAGGAAGCACTATG---GGCGCAGCATCA---
ATAACGCTGACGGTACAG---GCCAGACAA---TTGTTG---TCTGGTATAGTGCAGCAGCAAAGC---
AATCTG---CTGAGG---GCTATAGAGGCGCAACAGCATATGTTGCAACTCACAGTCTGGGGCATT

>MF373148..1
TTCTATTGCAATACATCAGAACTGTTTAACGGTACATACAGCAACGGTACATACACGCCTAATAATAATGGGA
GTGAC--

TCAAATTTGACCATCACCATCCCGTGCAGAATAAAACAAGTTGTAAATCTGTGGCAGGGAGTAGGACAAGCAA
TGGATGCCCCTCCCATTGCAGGAGCATATCGTGGGATCAATAT---CCGAGGACTACTATTGCCCGT---
GGAGGAGGACCGAACCCCAGT--

ACCGCGGAGATATTCAGACCTGGAGGAGGAGATATGAGGGACAATTGGAGAAGTGAATTATATAAATATAAAG
TGGTGGAAGTTAACCCATTA---GGAATAGCACCCACTAGAGCAAAAAGGAGAGTGGTG--------------
-------------------------GAGAGAGAAAAAAGAGCA---GTGGGA---ATAGGAGCT---------
GTGTTCCTTGGGTTCTTAGGAGCA---GCAGGAAGCACTATG---GGCGCAGCATCA---
GTAACGCTGACGGTACAA---GCCAGGCAA---TTGTTG---TCTGGTATAGTGCAACAGCAAAGC---
AATTTG---CTGAGG---GCTATAGAGGCGCAACAGCATATGTTGCAACTCACAGTCTGGGGCATT
>KP411836..5
TTCTATTGCAATACATCAAACTTGTTTAATAGTACATACAATAGTACT-------------------------
----

AATGATACCATCACACTCCAATGTAAGATAAAACAAATCATAAACATGTGGCAGGGGGTAGGACGAGCAGCAG
TATTCTCCTCCCCATCGCAGGCAAGACATCACATCTTATACTAC---
TATTACAGCAGTACTGCTGACGACCTGATGGAGGACGATGAAACTAATAAT----------------------
----

ACTGTAGAGACATTCAGACCTGGAGGAGGGGATATGAGAGACAATTGGAGAAGTGAATTGTATAAATATAAAG
TGGTAGAAGTTAAAACATTA---GGAATAGCTCCATCTCATTCAAAAAGGAGAGTGGTG--------------
-------------------------CAGAGAGAAAAAAGAGCA---GTGGGC---CTGGGAGCT---------
ATGTTCCTTGGGTTCTTGGGAGCC---GCAGGAAGCACTATG---GGCGTGGCGTCA---
TTGACGCTGACGGTACAG---GCCAGACAA---TTATTG---TCTGGTATAGTGCAGCAGCAAGGC---
AACTTG---CTGAGG---GCTATAGAGGCGCAACAGCATCTGTTGCAACTCACAGTCTGGGGCATT
>MF373159..1
TTCTATTGCAATACATCAGGGCTGTTTCGAATTAATATTTCTGATGCACATAATGATGCACATAAT-------
----

GACACAAATGTAAACATCACAATTCCATGCAGAATAAAGCAAATTATAAATATGTGGCAGGGGGTAGGACGAG
CAATGTATGCTCCTCCCATTAAAGGAATAATACAATGTAGATCAAAT---
ATCACAGGACTGCTACTGACACGTGATGGTGGTAATGGTGGTAAT----------------------------
----

GAAACAAATTCAGAGACGTTTAGACCTTTAGGAGGAGATATGAAGGACAATTGGAGAAGTGAATTATATAAAT
ACAAAGTAGTAGAAATTCAACCAGTA---GGAATAGCACCCACTAGAGCAAGAAGGAGAGTGGTG--------
-------------------------------GAGAGAGAAAAAAGAGCA---GCA------CTAGGAGCT---
------ATGTTCCTTGGGTTCTTAGGAGCA---GCCGGAAGCACTATG---GGCGCAGCATCA---
ATAACGCTGACGGTACAG---GCCAGACAA---TTATTG---TCTGGTATAGTGCAACAGCAAAAC---
AACTTG---CTGAGA---GCTATAGAGGCGCAACAGCATATGTTGCAACTCACGGTCTGGGGCATT
>MF373175..3
TTTTATTGCAATACATCCAACCTGTTTAATAATAGTATGATA-------------------------------
----

AATCCAAATGTTACCATCACACTCCCATGCAGAATAAAACAGATTATAAACATGTGGCAGCAGGTAGGAAAGG
CAATGTATGCCCCTCCCATTGCAGGAAGAATAACATGTAACTCAAAT---
ATCACAGGACTGCTACTGACACGTGATGGGGGAACAAATCAGACA----------------------------
----

AACAGCACAGAGATATTCAGACCTGGAGGAGGAGACATGAGAGACAATTGGAGAAGTGAATTATATAAATATA
AGGTGGTAGAAATTCAGCCATTA---GGAGTAGCACCCACTAGGGCAAAAAGGAGAGTAGTG-----------
----------------------------GGCAGAGAGAAAAGAGCA---GTGGGA---CTAGGAGCT------
---ATGTTCCTTGGGTTCTTGAGTGCA---GCAGGAAGCACTATG---GGCGCAGCATCA---
CTAACGCTGACGGTACAG---GCCAGACAA---TTGTTG---TCTGGTATAGTGCAACAGCAAAGA---
AATTTG---CTGAGG---GCTATAGAGGTGCATCAGCATATGTTGCAACTCACGGTCTGGGGCATT
>MF373179..4

GTCTATTGCGATACATCAGACCTGCTCACTACATCAGTTAGTGTTCAAAAAGTACTAGTTATCACT-------
----

AGTACAGATGCCATCATACTCCGCATGCCAAATACAACACATTATAAGCATGTGGCAGTGAGTACGACGAGCA
TTGTATGCCCCCCTCCTATGAAAGGCATGCGTAGCAGTACATGCACT---
ATCACAGGTACTACTGACCTCAGGGATGGAAGGACTGACTATCAC----------------------------
----

AGCATGTACATAGTCAGACCTGGAGGAGGAGATATGAGGGACAATTGGAGAAGTGAATTATATAAATATAAAG
TGGTAGAAGTTAAGCCAATA---GGAATAGCACCCACTGGGGCAAAAAGGAGAGTGGTG--------------
-------------------------GAGAGAGAAAAAAGAGCG---GCA------CTAGGAGCT---------
GTGTTCCTTGGGTTCTTGGGAGCA---GCCGGAAGCACTATG---GGCGCAGCATCA---
GTAACGCTGACGGTACAG---GCCAGACAA---TTATTG---TCGGGTATAGTGCAACAGCAGAAC---
AATTTG---CTGAGG---GCTATAGAGGCGCAACAGCATATGTTGCAACTCACGGTCTGGGGCATT
>MF373192..2
TTCTATTGCAATACAACAGCCTTGTTTCATAATAATATT----------------------------------
----

ACAAATTCAACCATCATGCTCCCATGCAGAATAAAACAATTTATAAACCTGTGGCAGGGGGTAGGACGAGCAA
TGTATGCTCCTCCCATTAAAGGACTAATAACATGTAATTCAAGT---
ATCACAGGAATACTACTGACACGAGATGGAGGAGATGTCAATCAAACA-------------------------
----

GCAAATGAAACAGAGACATTCAGACCTGGAGGAGGAGACATGAGGGACAATTGGAGAAGTGAATTATATAAAT
ACAAAGTGGTAGAAATTAAACCATTA---GGAATAGCACCTACCCCGGCAAAAAGGAGAGTGGTG--------
-------------------------------GAGAGAGAAAAAAGAGCA---GCA------CTAGGAGTT---
------ATGTTCCTTGGGTTCTTGGGAGCA---GCTGGAAGCACTATG---GGCGCAGCATCA---
TTAACGCTGACGGTACAG---GCCAGACAA---TTATTG---TCAGGTATAGTGCAACAGCAAAAC---
AATTTG---CTGAGA---GCTATAGAGGCGCAACAGCATATGTTGCAACTCACAGTCTGGGGCATT
>KP411835..1
TTCTATTGCGATACATCAAACCTGTTTAATGAGACAAAATTTAATAGTAGT----------------------
----

ACAAATGAAAACATCACACTCCGATGCAGAATAAAACAAATTATAAACATGTGGCAGAAGGTAGGACGGGCAA
TGTATGCCCCTCCCATTCAAGGAACAATAAAATGTGACTCAAAT---
ATCACAGGATTACTACTAACACGAGATGGAGGCAAAAATGAAAATGAA-------------------------
----

ACACAAGAGATATTCAGACCTGCAGGAGGAGATATGAGGGACAATTGGAGAAGTGAATTATATAAATATAAAG
TGGTAGAAATTAAGCCATTA---GGAATAGCACCCACTGCTGCAAAAAGGAGAGTGGTG--------------
-------------------------GGGAGAGAGAAAAGAGCA---GCA------CTAGGAGCT---------
ATGTTCCTTGGGTTCTTGGGAGCA---GCAGGAAGCACTATG---GGCGCAGCATCA---
ATAACGCTGACGGTACAG---GCCAGACAA---TTATTG---TCTGGTATAGTGCAACAGCAAAGC---
AATTTG---CTGAGA---GCTATAGAGGCGCAACAACATATGTTGCAACTCACAGTCTGGGGCATT
>MF373198..2
TTCTATTGCAATACATCAGGCCTGTTTAATGAGACAAATGGTACATATGCTACA-------------------
----

AATGGTACAACCATCACACTTCCATGCAGAATAAAGCAAATTATAAGGATGTGGCAGGGGGTAGGACAAGCAA
TGTATGCCCCTCCCATTGCAGGAGTAATATCATGTAATTCAAAT---
ATCACAGGACTGCTACTAACCCGTGATGGAGGTAGTGGTCACAAT----------------------------
----

AATAACACAGAGATATTCAGACCTGAAGGAGGAGATATGAGGGATAATTGGAGAAGTGAATTATATAAATACA
AAGTGGTAGAAATTAAGCCATTA---GGGATAGCACCCACTAAGGCAAGAAGGATGGTGGTG-----------
----------------------------CAGAGAGAAAAAAGAGCA---GCA------CTGGGAGCT------
---GTATTCCTTGGGTTCTTGGGAGCA---GCTGGAAGCACTATG---GGCGCAGCATCA---
ATAACGCTGACGGTACAG---GCCAGACAA---TTATTG---TCTGGCATAGTGCAACAGCAAAGC---
AATTTG---CTGAAG---GCTATAGAGGCGCATCAGCATATGCTGCAACTCACAGTTTGGGGCATT
>HM215318..1

TTTTATTGCAATACATCCAACCTGTTTAATAGTACATACATAAACAGCACA----------------------
----

AACATCACATACATCACAATCCCATGCAGAATAAAACAAATAATAAACATGTGGCAGAGGGTAGGACAAGCAA
TGTATGCCCCTCCCATTGCAGGAAACATAACATGTAACTCAAGT---
ATCACAGGACTAATATTGACACGGGATGGAGGAAAAAACAACACA----------------------------
----

AATGAAACATTCAGACCTGGAGGAGGAGATATGAGGGACAATTGGAGAAGTGAATTATATAAATATAAGGTGG
TAGAAATTCAACCATTG---GGAATAGCACCCACCAAGGCAAAAAGGAGAGTGGTG-----------------
----------------------GAGAGAGAAAAAAGAGCA---GTTGGA---CTAGGAGCT---------
TTGTTCCTTGGGTTCTTGGGAGCA---GCAGGAAGCACTATG---GGCGCGGCGGCA---
ATAACGCTGACGGCACAG---GCCAGACAA---GTGTTG---TCTGGCATAGTGCAACAGCAGAGC---
AATTTG---CTGAGA---GCTATAGAGGCGCAACAGCATATGTTGCAACTCACGGTCTGGGGCATT
>KX907415..1
TTCTATTGCAATACATCAGGCTTGTTTAATGAAACAGGCTTGTTTAATGAAACACACAATAGTACC-------
----

ATGAATGATAACATCACACTCCCATGCAGAATAAAACAAATTATAAACATGTGGCAGGAGGTAGGACGAGCAA
TCTATGCCCCTCCCATTGCAGGAAACATAGCCTGTAACTCAAAT---
ATCACAGGACTACTATTGACACGTGATGGAGGCAGCATTAATGGAAGCGAG----------------------
----

GATGAGAATAACACAGAAATATTCAGACCTGGAGGAGGAAACATGAAGGACAATTGGAGAAGTGAATTATATA
AATATAAAGTGGTAGAAATTAAGCCATTG---GGAATAGCACCCACTGCGGCAAAAAGGAGAGTGGTG-----
----------------------------------GAGAGAGAAAAGAGAGCA---GTGGGA---
CTAGGAGCT---------GTGTTCCTTGGGTTCTTGGGAGCA---GCTGGAAGCACTATG---
GGCGCGGCGTCA---ATAACGCTGACGGCACAA---GCCAGACAA---GTGTTG---
TCTGGTATAGTGCAACAGCAAAGC---AATTTG---CTGAGG---
GCTATAGAGGCGCAACAGCATATGTTGCAACTCACGGTTTGGGGCATC
>KX907398..2
TTCTATTGCAATACATCAAAGCTGTTTGATAGTAGTCTGTTTAATAGTACTGAAAGTAATAGTACTGGTAAT-
----

AATTATACTGACATCAAACTCCCATGCAGAATAAAACAAATTATAAACATGTGGCAGGGGGTAGGACGAGCAA
TATATGCCCCTCCCATTGCAGGAAACATAACATGTAAATCAAAT---
ATCACAGGAATATTATTAACACGTGATGGAGGCTATGACAACGCTACA-------------------------
----

AATGGCACGGAGACCTTCAGACCTGGAGGAGGAGATATGAGGGACAATTGGAGAAGTGAATTATATAAATATA
AAGTGGTAGAAATTAAGCCATTG---GGAATAGCACCCACTAAGGCAAAAAGGAGAGTGGTG-----------
----------------------------GAGAGAGAAAAAAGAGCA---GCGGGA---ATAGGAGCT------
---GTGTTCCTTGGGTTCTTGGGAGCT---GCAGGAAGCACTATG---GGCGCGGCGTCA---
ATAACGCTGACGGTACAA---GCCAGACAA---TTGTTG---TCTGGTATAGTGCAACAGCAAAGT---
AATTTG---CTGAAG---GCTATAGAGGCGCAACAACATCTGTTGCAACTCACGGTTTGGGGCATT
>KX907425..1
TTCTATTGCAATACAGTAGGCCTGTTTAATAAAACATACAATAGTACC-------------------------
----

ATGAATGGTACCATCACACTCCCATGCAGAATAAAACAAATTATAAACATGTGGCAGGAGGTAGGACGAGCAA
TCTATGCCCCTCCCATTGAAGGAAACATAACATGTAACTCAAGT---
ATCACAGGACTACTATTGACACGTGATGGAGGCAACACTAATGAA----------------------------
----

AGTGAAGAGATATTCAGACCTGGAGGAGGAAACATGAGGGACAATTGGAGAAGTGAATTATATAAATATAAAG
TGGTAGAAATTAAGCCATTG---GGAGTAGCACCCACTGAGGCAAAAAGGAGAGTGGTG--------------
-------------------------GAGAGAGAAAAGAGAGCA---GTAGGA---CTAGGAGCT---------
GTGTTCCTTGGGTTCTTGGGAGCA---GCTGGAAGCACTATG---GGCGCGGCGTCA---
ATAACGCTGACGGCACAA---GCCAGACAA---TTGTTG---TCTGGTATAGTGCAACAGCAAAGC---
AATTTG---CTGAGG---GCTATAGAGGCGCAACAACATATGTTGCAACTCACGGTTTGGGGCATT

>KX907428..1
TTCTATTGCAACACATCAGGCCTGTTTAATAAAACATACAATGATACC-------------------------
----

ATGAATAGTACCATCACACTCCCATGTAGGATAAAACAAATTGTAAACATGTGGCAGGAGGTAGGACGAGCAA
TCTATGCCCCTCCTATTGCAGGAAACATAACATGTAACTCAAAT---
ATCACAGGACTACTACTGACACGTGATGGAGGCACGAATGTGACA----------------------------
----

AATGGCACACAGATTTTCAGACCTGGGGGAGGAGACATGAGGGACAATTGGAGAAGTGAATTATATAAATATA
AAGTGGTAGAAATTAAACCATTG---GGAATAGCACCCACTACGGCACAAAGGAGAGTGGTG-----------
----------------------------GCGAGAGAAAAAAGAGCA---GTGGGA---CTAGGAGCT------
---GTGTTCCTTGGGTTCTTGGGAGCA---GCTGGAAGCACTATG---GGCGCGGCGTCA---
ATAACGCTGACGGCACAA---GCCAGACAA---TTGCTG---TCTGGTATAGTGCAACAGCAAAGC---
AATTTG---CTGAGA---GCTATAGAGGCGCAACAACATATGTTGCAACTCACGGTTTGGGGCATC
>HQ615976..2
TTCTATTGCAATACATCAAAAATATTTAATAGTATATACCAGGAGAATTACACTAACAGTGCTAATAGTACAG
AA---

AACAACGGCACACTCACACTCCCATGCAGAATAAAACAAATTGTAAACATGTGGCAGGGGGTAGGACGGGCAG
TGTATGCCCCTCCCATTCAAGGGAACATAACATGTAAATCAAAT---
ATCACAGGACTGCTACTGACACGTGATGGAGGAATAAATGAGACA----------------------------
----

GATGAAACATTCAGGCCTATAGGGGGAAATATGAGGGACAATTGGAGAAGTGAATTATATAAATATAAGGTGG
TAGAAATTAAACCATTA---GGAATAGCACCCACTAAGGCAAAAAGGAGAGTGGTG-----------------
----------------------GAGAGAGAAAAAAGAGCG---GTAGGA---ATAGGAGCT---------
GTGCTCTTTGGGTTCTTGGGAGCA---GCAGGAAGCACTATG---GGCGCAGCGTCA---
ATAACGCTGACGGTACAA---GCCAGACAA---TTATTG---TCTGGTATAGTGCAACAGCAAAAC---
AATTTG---CTGAGG---GCTATAGAGGCGCAACAGCATATGTTGCAACTCACGGTCTGGGGCATT
>KF526226..1
TTCTATTGCCATACATCAACCCTGTTTAATAATAATATTACAAATTCA-------------------------
----

ACCAATTCAACCATCACACTCCCATGCAGGATAAAACAAATTATAAACATGTGGCAGGAGGTAGGACGAGCAA
TGTATGCCCCGCCCATTCAAGGAATAATAAGATGTAGATCAAAT---
ATCACAGGACTACTACTAACACGTGATGGAGGCACAGGGACAAATGAC-------------------------
----

ACAAACACAACCGAGACATTTAGACCTGGAGGAGGAGATATGAGGGACAATTGGAGAAGTGAATTATATAAAT
ATAAAGTGGTAGAAATTAAACCATTA---GGAATAGCACCCACTAAGGCAAAAAGGAGAGTGGTG--------
-------------------------------GAGAGAGAAAAAAGAGCA---GCA------CTAGGAGCT---
------TTGTTCCTTGGGTTCTTGGGAGCA---GCAGGAAGCACTATG---GGCGCAGCATCA---
ATAACGCTGACGGTACAG---GCCAGACAA---TTATTG---TCGGGTATAGTGCAACAGCAAAGC---
AATTTG---CTGAGA---GCTATAGAAGCGCAACAGCATATGTTGCAACTCACGGTCTGGGGCATT
>AY463229..2
TTCTATTGCTATACATCAGAATTGTTTAATAACACATACATACCTACATACATGAATAATAAAACAGAT----
----

GATAATTCTACCATCACACTTCCATGCAGAATAAAACAAATTATAAACATGTGGCAGGAGGTAGGACGAGCAA
TGTATGCCCCTCCCATTGCAGGAAACATAACATGTAACTCAAGT---
ATCACAGGACTACTATTGACACGTGATGGAGGCAATAATAATAAT----------------------------
----

GATACTGAAATATTCAGACCTGGAGGAGGAAATATGAAGGATAATTGGAGAAGTGAATTATATAAATATAAAG
TGGTAGAAATTCAGCCACTG---GGAGTAGCACCCACTGGGGCAAAAAGGAGAGTGGTG--------------
-------------------------GAGAGAGAAAAAAGAGCA---GTGGGA---ATAGGAGCT---------
GTGTTCCTTGGGTTCTTGGGAGCA---GCAGGAAGCACTATG---GGCGCGGCGTCA---
ATAACGCTGACGGTACAG---GCCAGACAA---CTGCTG---TCTGGTATAGTGCAACAGCAAAGC---
AATTTG---CTGAGA---GCTATAGAGGCGCAACAACACGTGTTGCAACTCACAGTCTGGGGCATT

```
>JX845587..1
TTCTATTGCAATACAACACAACTGTTTTCAAACAAAAGTGTT-------------------------------
-------------------------------------------------------------------------
-------------------------
CCAACCAACAAAACAATCATACTCCTATGCAGGATAAAACAAATTATAAACATGTGGCAGGGGGTAGGACGAG
CAATGTATGCCCCTCCCATTGCAGGAAACATAACATGTAACTCAAAT---
ATCACAGGACTACTATTGACACGTGATGGAGGAAAAAACAACGCAACAGGG----------------------
-------------------------------------------------------------------------
-------
GGCAACGAAACAGAGATATTCAGACCTGGAGGAGGAAATATGAAGGACAATTGGAGAAGTGAATTATACAAAT
ATAAAGTGGTAGAAATTAAGCCATTA---GGAGTAGCACCCACTAAGGCACAAAGGAGAGTGGTG--------
-------------------------------CAGAGAGAAAAAAGAGCA---GTGGGA---ATAGGAGCT---
------GTGCTCCTTGGGTTCTTGGGAGCA---GCAGGAAGCACTATG---GGCGCGGCGTCA---
ATAACGCTGACGGTACAG---GCCAGACAG---CTGTTG---TCTGGTATAGTGCAGCAGCAAAGC---
AATTTG---CTGAGA---GCTATAGAGGCGCAACATCATATGTTGCAACTCACGGTCTGGGGCATT
>AY463235..3
TTCTATTGCAATACAACGCAACTGTTTAATAGTACATACTATAATAGTACTAATAGTACAGAGGAGACA----
-------------------------------------------------------------------------
----------------------------
GGAAATTCAAACATCACACTTCAATGCAGAATAAAACAAATTATAAACATGTGGCAAGGGGTAGGACAAGCAA
TGTATGCCCCTCCCATTCAAGGAATAATAACATGTAGATCAAAC---
ATCACAGGAATACTACTGGCACGTGATGGAGGAGCCTCAAATAGCACAGAA----------------------
-------------------------------------------------------------------------
----
AACTCAACTAACACAGAGATATTCAGGCCTGGAGGAGGAGATATGAGGGACAATTGGAGAAATGAATTATATA
AATATAAAGTGGTAGAAGTTAAACCATTA---GGAGTAGCACCTACTAGAGCAAAAAGGAGAGTGGTG-----
----------------------------------GAGAGAGAAAAAAGAGCA---GTGGGA---
ATAGGAGCT---------GTGTTCCTTGGGTTCTTGGGAGCC---GCAGGGAGCACTATG---
GGCGCAGCATCA---ATAACGCTGACGGTACAG---GCCAGACAA---TTATTG---
TCGGGTATAGTGCAACAGCAAAGC---AATTTG---CTGAGG---
GCTATAGAGGCGCAACAGCATATGTTGCAACTCACGGTCTGGGGCATT
>DQ369995..2
TTCTATTGCGACACATCACAACTATTTAATAAGACATACAATGTTACAGATGATTCAAACACC----------
-------------------------------------------------------------------------
----------------------------
ACAGACACAAACAACACAATTCAATGCAGAATAAAGCAGATTATAAACATGTGGCAGGAAGTAGGACGAGCAA
TGTATGCCCCTCCCATCGCAGGAAGCATAACATGTACCTCAAAT---
ATTACAGGACTACTATTGACACGTGATGGAGGGGGAGGCACAAATAGCACA----------------------
-------------------------------------------------------------------------
-------
AGTAATACGGAAGAGATATTCAGACCCGGAGGAGGAAATATGAAGGATAATTGGAGAAGTGAATTATATAAAT
ATAAAGTGGTAGAAGTTCAGCCATTA---GGAATAGCTCCCACTAAAGCAAAAAGGAGAGTGGTG--------
-------------------------------GAGAGAGAAAAAAGAGCA---ATAGGA---ATAGGAGCT---
------GTGTTCCTTGGGTTCTTGGGAGCA---GCAGGAAGCACTATG---GGCGCAGCGTCA---
ATAACGCTGACGGTACAG---GCCAGACAA---CTATTG---TCTGGTATAGTGCAACAGCAAAGC---
AACTTG---CTGAGG---GCTATAGAGGCGCAACAGCATATGTTGCAACTCACGGTCTGGGGCATC
>DQ351223..1
TTCTATTGCAATACAACAGGACTGTTTGCTAGTAAAAATGATTCAAACACAACCATCAGT-------------
-------------------------------------------------------------------------
----------------------------
ACAAACGCAACCATCACACTCCCATGTAGAATAAAACAAATTATAAACATGTGGCAGGGGGTAGGACGAGCGA
TGTATGCCCCTCCCATTGCAGGGAACATAACATGTAGATCAAAT---
ATCACAGGATTGTTATTGCTACGTGATGGGGGAGACAATCAGACC----------------------------
-------------------------------------------------------------------------
----------------
ACAGAGACATTCAGGCCTGGAGGAGGAAATATGAAGGACAACTGGAGAAGTGAATTATATAAATATAAAGTAG
TAGAAATTAAGCCATTG---GGAATAGCACCCACGAAGGCAAAAAGGAGAGTGGTG-----------------
----------------------GAGAGAGAAAAAAGAGCA---GCGGGA---ATAGGAGCG---------
ATGATCTTTGGGTTCTTGGGAGCA---GCAGGAAGCACTATG---GGCGCGGCGTCA---
```

ATAACGCTGACGGTACAG---GCCAGACAG---CTGTTG---TCTGGTATAGTGCAGCAGCAAAGC---
AATTTG---CTGAGA---GCTATAGAGGCGCAACAGCATATGTTGCAACTCACGGTCTGGGGCATT
>DQ396388..1
TTCTATTGCAATACATCAGGACTGTTTAATAGTACATACATGCCTAATGGTACAGAAAGCAATTCAACCATC-
----

ACAAAGTCAACCATCACACTCCTATGCAGAATAAAACAAATTATAAACATGTGGCAGCAAGTAGGAAGAGCAA
TGTATGCCCCTCCCATTGCAGGAAACATAACATGTACATCAAAC---
ATCACAGGAATATTATTAGTACGTGATGGAGGCTACAGTAACAAT----------------------------
----

ACTACAGAGATATTCAGACCTGGAGGAGGAAATATGAAGGACAATTGGAGAAGTGAATTATATAAATACAAAG
TGGTAGAAGTTAAGCCATTG---GGAATAGCACCCACTAAGGCAAAAAGGAGAGTGGTG--------------
-------------------------GAGAGAGAAAAAAGAGCA---GCA------CTAGGAGCT---------
ATGTTCCTTGGGTTCTTGGGAGCA---GCAGGAAGCACTATG---GGCGCGGCGTCA---
ATAACGCTGACGGTACAG---GCCAGACAA---TTGTTG---TCTGGTATAGTGCAACAGCAAAGC---
AATTTG---CTGAGG---GCTATAGAGGCGCAACAGCATATGTTGCAACTCACGGTCTGGGGCATT
>DQ351228..1
TTTATTGCAACACATCAAAACTATTTAATGGTACATACGAGAATAGTACAGACAAGGAGTCAAACAGC----
----

ACAAATTCAATCATCACAATTCCATGCAGAATAAAACAAATTATAAACATGTGGCAGGAGGTGGGACGAGCAA
TGTATAACCCTCCCATTGCAGGAAACATAACATGTAAATCAAAT---
ATCACAGGACTACTATTAGTACGTGATGGA---GACAACAATAGT----------------------------
----

ACAGAAACATTCAGACCTCAAGGAGGAAATATGAAGGACAATTGGAGGAGTGAATTATATAAATATAAAGTGG
TGGAAATTAAGCCATTG---GGAATAGCACCCACTGAAGCAAAAAGGAGAGTGGTG-----------------
----------------------GAGAGAGAAAAAAGAGCA---GTAGGA---ATAGGAGCG---------
GTACTCCTTGGGTTCTTGGGAGCA---GCAGGAAGCACTATG---GGCGCGGCGTCA---
ATAACGCTGACGGTACAG---GCCAGACAA---CTGTTG---TCTGGTATAGTGCAACAGCAAAGC---
AATTTG---CTGAGA---GCTATAGAGGCGCAACAGCATATGTTGCAACTCACAGTCTGGGGCATT
>DQ011175..1
TTCTATTGCAATACATCAAAACTATTTAATAGTACATACAATCCTGACCATAATAGTACAGGAAAT-------
----

ATCACAGGGGTCATCACAATTCCATGCAGAATAAAACAAATTATAAACATGTGGCAGGAGGTGGGACGAGCTA
TGTATGCTCCTCCCATTAAAGGAAACATAACATGTAACTCAAGT---
ATCACAGGACTACTATTAACACGTGATGGAGGCAAGTCAAATGACACAATA----------------------
----

GAAAACAAAAATGAAACATTTAGACCTCAAGGAGGAAATATGAAGGACAATTGGAGAAGTGAATTATATAAAT
ATAAGGTGGTACAAATTGAGCCATTG---GGAATAGCACCCACTAAAGCAAAAAGGAGAGTGGTG--------
-------------------------------GAGAGAGAAAAAAGAGCA---GTGGGA---ATAGGAGCT---
------GTGTTCCTTGGGTTCTTGGGAGCA---GCAGGAAGCACTATG---GGCGCGGCGTCA---
ATGGCGCTGACGGTACAG---GCCAGACAA---GTGCTG---TCTGGTATAGTGCAACAGCAAAGC---
AATTTG---CTGAGA---GCTATAGAGGCGCAACAGCATATGTTGCAGCTCACGGTCTGGGGCATT
>AY901971..2
TTCTATTGCAATACATCAAGACTATTTAATAATACATTTAATTATACAGACACAACAGAACTTAACAGCACAG
ACAGC--
-------------------ACA---
GACTCAAACGAGACCATCACAATTCCATGCAGAATAAAACAAATTATAAACATGTGGCAGCAGGTAGGACGAG
CAATGTATGCTCCTCCCATTGCAGGGAACATAACATGTACATCAAAT---
ATCACAGGATTACTATTGGTACGTGATGGAGGCAAGGATAATACA----------------------------
----

GGTACAAATGAGACATTCAGACCTCAAGGAGGAAATATGAAGGACAATTGGAGAAGTGAATTATACAAATATA
AAGTGGTAGAAATTAAACCATTG---GGAATAGCACCTACTGGGGCAAAAAGGAGAGTGGTG-----------
---------------------------GAAAGGGAAAAAAGAGCA---GCG------CTAGGAGCT------
---ATGTTCCTTGGGTTCTTGGGAGCA---GCAGGAAGCACTATG---GGCGCAGCGTCA---

ATAACGCTGACGGTACAG---GCCAGACAA---CTGTTG---TCTGGTATAGTGCAACAGCAAAAC---
AATTTG---CTGAGA---GCCATAGAGGCGCAACAGCATATGTTGCAACTCACAGTCTGGGGCATA
>DQ093597..1
TTCTATTGCAATACATCAAGACTATTTAATAGTACATACTGGGAAGGATCTAATAGTACAGGA----------
----

GGTGACTCAACCATCACAATTCCATGCAGAATAAAACAAATTATAAACATGTGGCAGGAGGTAGGACGAGCAA
TGTATGCCCCTCCCATAGCAGGAAACATAACATGTAACTCAAGT---
ATCACAGGATTACTATTAGTACGTGATGGAGGCCTATCCAAAAATAAC-------------------------
----

ACAAATCAAAATGAAACATTCAGACCTGGAGGAGGAGATATGAAAGACAATTGGAGGAGTGAATTATATAAAT
ACAAAGTGGTAGAAATTAAGCCATTG---GGAATAGCACCCACTAAAGCAAAAAGGAGAGTGGTG--------
-------------------------------GAGAGAGAAAAAAGAGCA---GTGGGA---ATAGGAGCT---
------GTGCTCCTTGGGTTCTTGGGAGCA---GCAGGAAGCACTATG---GGCGCAGCGTCA---
ATAGCGCTGACGGCACAA---GCCAGACAA---GTGTTG---TCTGGTATAGTGCAACAGCAAAGT---
AATTTG---CTGAGG---GCTATAGAGGCGCAACAGCATATGTTGCAACTCACGGTCTGGGGCATT
>AY772692..1
TTCTATTGCAATACAACAAAACTGTTTAATGAAAATGAAACAAGTAAG-------------------------
----

GCAAATGACACAATCATACTCCCATGCAGAATTAAACAAATCATAAACATGTGGCAGACGGTAGGACGAGCAA
TGTATGCTCCTCCCATTGAAGGATACATAAATTGTACATCAAAT---
ATCACAGGAATACTATTGACACGTGATGGAGGAAATAATAAAACAAAT-------------------------
----

AACACAAAAGAGATATTCAGACCTGCAGGAGGAGATATGAGGAACAATTGGAGAAGTGAATTATACAAATATA
AAGTAGTAGAAATTCAGCCATTG---GGAATAGCACCCACTACTGCAAAAAGGAGAGTGGTG-----------
----------------------------GAGAGAGAAAAAAGAGCA---GCA------CTAGGAGCT------
---ATATTCCTTGGGTTCTTGGGAGCA---GCAGGAAGCACTATG---GGCGCGGCGGCA---
ATAACGCTGACGGTACAG---GCCAGACAA---CTGTTG---TCTGGTATAGTGCAACAGCAAAGC---
AATTTG---CTGAGA---GCTATAGAGGCGCAACAGCATCTGTTGCAACTCACGGTCTGGGGCATT
>DQ164107..2
TTCTATTGCAATACATCGGCACTATTTCATAGTCCTACATACAATGAGACAAAAGGT----------------
----

AATTCAACAACCATCACACTTCCATGTAGAATAAAACAAATTATAAACATGTGGCAGGGGGTAGGACGAGCTA
TGTATGCTCCTCCTATTGCAGGAGAAATAACATGTAACTCAAGC---
ATCACAGGACTACTATTGACACGTGATGGAGGAGAAGGAGAGAAT----------------------------
----

CACACAGAGATATTCAGGCCTGGAGGAGGAAATATGAAGGACAATTGGAGAAGTGAATTATATAAATATAAAG
TGGTAGAAATTAAGCCATTG---GGAGTAGCACCCACTAAGGCGGAAAGGAGAGTGGTG--------------
-------------------------AAAAAAGAAAAAAGAGCA---GTGGGA---ATAGGAGCG---------
TTGTTCCTTGGGATCTTGGGAGCA---GCAGGAAGCACTATG---GGCGCGGCGTCA---
ATAACGCTGACGGTACAG---GCCAGACAA---CTGTTG---TCGGGTATAGTGCAACAGCAAAAT---
AATTTG---CTGAGG---GCTATAGATGCGCAACAGCATATGTTGCAACTCACGGTCTGGGGCATT
>DQ093600..1
TTCTATTGCAATACAACAAAACTGTTTAATGAAAGTGAAACACAGTTTGCGGCAAACAACAGT----------
----

GCCAATAGTAACATCACACTCCCATGCAAGATAAAACAAATTATAAACATGTGGCAGCAGGTGGGACGAGCAA
TGTATGCCCCTCCTGTTCAAGGAAACATAATATGTAACTCTAGT---
ATCACAGGACTACTATTGACACGTGATGGAGGAGTTAACAATACT----------------------------
----

AATGAGACATTCAGACCTGGAGGAGGAAATATGAAAGACAATTGGAGAAATGAATTATATAAATATAAAGTGG
TAGAAATTAAGCCATTA---GGAATAGCACCCACTAGGGCAAAAAGGAGAGTGGTG-----------------
----------------------GAGAGAGAAAAAAGAGCA---GTGGGA---CTAGGAGCT---------
GTGATCTTTGGGTTCTTGGGAGCA---GCAGGAAGCACTATG---GGCGCGGCGTCA---

ATAACGCTGACGGTACAG---GCCAAACAA---CTGTTG---TCTGGTATAGTGCAACAGCAAAGC---
AATTTG---CTGAGA---GCTATAGAGGCGCAACAGCATATGTTGCAACTCACGGTCTGGGGCATT
>DQ011166..1
TTCTATTGTAATACAACAGATCTGTTTAATAGTACATTTAATAGTGAATACAATAGTACAGACAGGCATAAT-
----

AATAATACCAATATCACACTCCAATGCAGGATAAAACAAGTGATAAACATGTGGCAGCAGGTAGGACGAGCAA
TGTATGCTCCTCCCATTGCAGGAAACATAACATGTATCTCAAAT---
ATCACAGGATTACTATTGGTGCGTGATGGAGGAGGAAAAAATAACACA-------------------------
----

AATCAAACAGAGGAGATATTCAGACCTGGAGGAGGAGATATGAGGGACAATTGGAGAAGTGAATTATACAAAT
ATAAGGTGGTAGAACTCAAACCATTG---GGAATAGCACCTACTGAGGCAAAGAGGAGAGTGGTG--------
-------------------------------GAGAGAGAAAAAAGAGCA---GTGGGA---ATAGGAGCT---
------GTGTTCCTTGGGTTCTTGAGTGCA---GCAGGAAGCACTATG---GGCGCGGCGTCA---
ATGACGCTGACGGTACAG---GCCAGACAA---TTGTTG---TCTGGTATAGTGCAACAGCAAAAC---
AATTTG---CTGAAG---GCCATAGAGGCGCAACAACATCTGTTGCAACTCACGGTCTGGGGCATT
>DQ011167..1
TTCTATTGCAATACATCAGGACTGTTTAATAAGAGTAATACAGACATGACTAATAAAACAGAA----------
----

GATAATTCAACCATCACACTCCCATGTAGAATAAAACAGATTATAAACATGTGGCAGAACGTAGGACGAGCAA
TATATGCCCCTCCCGTTCAAGGAAACATAACATGTAACTCAAGT---
ATCACAGGACTACTATTGACACGTGATGGAGGTAACGGAAATGACACT-------------------------
----

AATGGCACAGAGATATTCAGACCTGGAGGAGGAGATATGAGGGACAATTGGAGAAGTGAATTATACAAATATA
AAGTGGTAGAAATTAAGCCATTG---GGAATAGCACCCACTAAGGCAAAAAGGAGAGTGGTG-----------
----------------------------GAGAGAGAAAAAAGAGCA---GTGGGA---ATAGGAGCT------
---GTGTTCCTTGGGTTCTTGGGAGCA---GCAGGAAGCACTATG---GGCGCAGCATCA---
ATAACGCTGACGGTACAG---GCCAGACAA---TTGTTG---TCTGGAATAGTGCAACAGCAAAGC---
AATTTG---CTGAGG---GCTATAGAGGCGCAACAGCATATGTTGCAACTCACGGTCTGGGGCATT
>DQ093590..1
TTCTATTGTAATACATCAAAACTGTTTAATAGTACATACAATCCTAATAATACAGAAAGTAAT----------
----

TCAAGCGCAGTAATCACACTCCCATGCAAGATAAAGCAAATTATAAACATGTGGCAGCAGGTAGGGCGAGCAA
TGTATGCTCCTCCCATTGCAGGAAACATAACATGTAACTCAAAT---
ATCACAGGATTACTACTGGTGCGTGATGGGGGAACAATTGGCAATGGC-------------------------
----

ACAAATAACGAGACATTCAGACCTGGAGGAGGAGATATGAGGGACAATTGGAGAAGTGAATTATACAAATATA
AAGTGGTAGAAGTTAAACCATTG---GGAATAGCACCTACTCAGGCAAAAAGGAGAGTGGTG-----------
----------------------------GAGAGAGAAAAAAGAGCA---GTGGGA---CTAGGAGCT------
---GTGTTCCTTGGGTTCTTGGGAGCA---GCAGGAAGTACTATG---GGCGCGGCGTCA---
ATAACGCTGACGGTACAG---GCCAGACAA---CTGTTG---TCTGGCATAGTGCAACAGCAAAGC---
AATTTG---CTGAGG---GCCATAGAGGCGCAACAGCATATGTTGCAACTCACGGTCTGGGGCATT
>DQ011178..1
TTCTATTGCAACACATCAGGCCTGTTTAATACTACAGACCTGTTTAATAATACAGACCTGTCTAATAGTACAG
GA---

AGTAATGAAAACATCACGCTTCCATGCAGAATAAAACAATTTGTGAATATGTGGCAGGAGGTAGGGCGAGCAA
TGTATGCCCCTCCCATTCAAGGAATCATAAATTGTATATCAAAT---
ATCACAGGAATACTATTGCTACGTGATGGAGGAAACACGAGTGCTAAT-------------------------
----

GGAACATTAACAGAGACATTCAGACCTGGAGGAGGAAATATGAAGGACAATTGGAGAAGTGAATTATATAAAT
ATAAGGTGGTAGAAATTAGGCCATTA---GGAGTAGCACCCACCAAGGCAAAAAGGAGAGTGGTG--------
-------------------------------GAGAGAGAAAAAAGAGCA---GTGGGA---ATAGGAGCT---
------GTGCTCTTTGGGTTCTTGGGAGCA---GCAGGAAGCACTATG---GGCGCGGCGTCA---

ATAACGCTGACGGTACAG---GCCAGACAA---CTGCTG---TCTGGTATAGTGCAACAGCAAAGC---
AATTTG---CTGAGG---GCTATAGAGGCGCAACAGCATATGTTGCAACTCACGGTCTGGGGCATT
>AY901980..2
TTTTATTGTAATACATCACAACTGTTTAATAGTACATACAATCCTGCATACAGTCATAATGAGACAGAA----
----

AGTAATTCAACCATCATGCTTCCATGCAGAATAAAACAAATTATAAACATGTGGCAGGAGGTAGGAAAAGCAA
TGTATGCCCCTCCCATTGCAGGAAACATAATATGTAACTCAAGT---
ATCACAGGACTACTATTGACACGTGATGGAGGAAACAATAACACG----------------------------
----

AATACAGAAGAAATATTCAGACCTGGAGGAGGAAATATGAAGGACAATTGGAGAAATGAATTATATAAATATA
AAGTGGTAGAAATTCAGCCATTG---GGAGTAGCACCCACCGAGGCAAAAAGGAGAGTGGTG-----------
----------------------------GAGAGAGAAAAAAGAGCA---GTGGGA---CTGGGAGCT------
---GTGTTCCCTGGGGTCTTGAGTTTG---GCAGGAAGCACTATG---GGCGCAGCGTCA---
ATAACGCTGACGGTACAG---GCCAGGCAA---CTGTTT---GCTGGTATAGTGCAACAGCAAAAT---
AATTTG---CTGAGG---GCTATAGAGGCGCAACAGCATATGTTGCAACTCACAGTCTGGGGCATT
>AY878059..2
TTCTATTGCAATACAACAAAACTGTTTAACATAACCTATAATAACAGTAATTCA-------------------
----

GACAACAATACCATCACGCTCCCCTGCAGAATCAAACAATTTATAAACCTGTGGCAGGAGGTAGGGCGAGCAA
TGTATGCCCCTCCCATTGAAGGAAAAATAACATGTCACTCAAGT---
ATTACAGGACTACTATTGACACGTGATGGAGGAACGAGTGCAAACAATGATACA------------CAA----
----
GCAAGTACAAACAAT---
AATACAGAGACATTCAGACCTGGAGGGGGAGATATGAAAAACAATTGGAGAAATGAATTATATAAATATAAAG
TGGTAGAAATTAAGCCCTTA---GGAGTAGCACCCACTGGAGCAAAAAGGAGAGTGGTG--------------
-------------------------GAGAGAGAAAAAAGAGCA---GTAGGA---ATAGGAGCT---------
GTACTCTTTGGGTTCTTGGGAGCA---GCAGGAAGCACTATG---GGCGCGGCGTCA---
ATAACGCTGACGGTACAG---GCCAGACAA---TTGTTG---TCTGGTATAGTGCAACAGCAAAGC---
AATTTG---CTGAGA---GCTATAGAGGTGCAACAGCATATGTTGCAACTCACGGTTTGGGGCATT
>DQ011174..1
TTCTATTGCAATACGACAGAACTGTTTAATGATAGTAGCTCA-------------------------------
----

GAAAACATCACACTCCCATGCAGAATAAAACAAATTGTAAACATGTGGCAGGGGGTAGGACGAGCAATGTATG
CCCCTCCCATTGAAGGAAGCATAACATGTAATTCAAGT---
ATCACAGGACTACTATTAGAACGTGATGGAGGGGAAAGCTATAAT----------------------------
----

AAAACAGAGATATTCAGGCCTGGAGGAGGAAATATGAAGGACAATTGGAGAAGTGAATTATATAAATATAAAG
TGGTAGAAATTAAGCCATTA---GGAGTAGCACCCACAAGGGCAAAAAGGAGAGTGGTG--------------
-------------------------GAGAGAGAAAAAAGAGCA---GTGGGA---ATAGGAGCT---------
GTGTTCCTTGGGTTCTTGGGGGCA---GCAGGAAGCACTATG---GGCGCGGCGTCA---
ATAACGCTGACGGTACAG---GCCAGACAA---CTGCTG---TCTGGTATAGTGCAACAGCAAAGC---
AATTTG---CTGAGA---GCTATAGAGGCGCAACAGCATATGTTGCAACTCACGGTCTGGGGCATT
>GQ999986..2
TTCTATTGCAATACATCAAAACTTTTTAATAATACATACTATAATGGTACAGGAAATGCTAACAGT-------
----

ACGCATGAAAACATCACACTCCCATGCAGAATAAAACAAATTATAAGGATGTGGCAGAAGGTAGGACAAGCAA
TGTACGCCCCTCCCATTGCAGGAAACATAACATGTACATCAAAT---
ATCACAGGACTACTACTGGTACGTGATGGAGGAGAGGCAAATGGCACA-------------------------
----

AACAACATAGAGACATTCAGACCTGGAGGAGGAGATATGAGGGACAATTGGAGAAGTGAATTATATAAATATA
AAGTGGTAGAAATTCAGCCATTG---GGAATAGCACCCACCAAGGCAAAAAGGAGAGTGGTG-----------
----------------------------GAGAGAGAAAAAAGAGCA---GTGGGA---ATAGGAGCT------
---ATGTTCCTTGGGTTCTTGGGAGCA---GCAGGAAGCACTATG---GGCGCGGCGTCA---

ATGGCGCTGACGGTACAG---TCCAGACAA---CTGTTG---TCTGGTATAGTGCAACAGCAAAGC---
AATTTG---CTGAGG---GCTATAGAGGCGCAACAGCATATGTTGCAACTCACGGTCTGGGGCATT
>GQ999987..2
TTCTACTGCAATACAACAGGACTGTTTAATCATAGTGGGGTAAACACCAAAGAACTGCTTAATAATAATACTG
AG--

TCAAACACAACCATCACACTCCCATGCAAAATAAAACAAATTATAAACATGTGGCAGGAGGTAGGACGGGCAA
TGTATGCCCCTCCCATTGCAGGAAACATAACATGTAACTCAAGT---
ATCACAGGACTACTATTAACACGTGATGGAGGTAATAATAAAAATAAAACAGAA------------------
----

AATGACACAAAGATAGAGATATTCAGACCTGGAGGAGGAAATATGAAGGACAATTGGAGAAGTGAATTATATA
AATATAAAGTGGTAGAAATTCAACCACTG---GGAGTAGCACCCACGGAGGCAAAAAAGGAGAGTGGTG-----
---------------------------------GAGAGAGAAAAAAGAGCA---GTAGGA---
ATAGGAGCT---------GTACTCTTTGGGTTCTTGGGAGCA---GCAGGAAGCACTATG---
GGCGCGGCGTCA---ATAACGCTGACGGTACAG---GCCAGACAA---CTATTG---
TCTGGTATAGTGCAACAGCAAAGC---AATTTG---CTGAGA---
GCTATAGAGGCGCAACAGCATATGTTGCAACTCACAGTCTGGGGCATT
>GQ999977..2
TTCTATTGCAATACATCAGGACTGTTTAATGGTACATACAATGGTACGGGAAATAAT---------------
----

TCAACAGAAACCATCACACTTCAATGCAGAATAAAACAAATTATAAATATGTGGCAGGAGGTAGGACGAGCAA
TGTATGCCCCTCCCATTGCAGGAAACATAACATGTAACTCAAAG---
ATCACAGGACTACTACTAGTACGTGATGGAGGCACATATGGAGAT---------------------------
----

AACAAAACAGAGACATTCAGACCTGGAGGAGGAAATATGAAGAACAATTGGAGAAGTGAATTATATAACTATA
AAGTGGTAGAAATTAAACCATTA---GGAATAGCACCCACTGAGGCAAAAAGGAGAGTGGTG----------
---------------------------GAGAGAGAAAAAAGAGCA---GTAGGA---ATAGGAGCT------
---GTGTTCCTTGGGTTCTTGGGAGTA---GCAGGAAGCACTATG---GGCGCAGCGTCA---
ATAACGCTGACGGCACAG---GCCAGACAA---CTATTG---TCTGGTATAGTGCAACAGCAAAGC---
AATTTG---CTGAGA---GCTATAGAGGCGCAACAGCATATGTTGCAACTCACGGTCTGGGGCATT
>JN687821..1
TTCTATTGCAATACATCACACCTGTTTAGTAATAATGGTACA------------------------------
----

AATTCTATCATCACACTCCCATGCAGGATAAAACAAATAATAAACATGTGGCAGGGGGTGGGACGAGCAATGT
ATGCCCCTCCTATTCAAGGAGACATAACATGTAAATCAAAC---
ATAACAGGAATACTATTGACACGTGATGGAGGAAGAAATGGCAAC---------------------------
----

ACAACAGAGATATTCAGACCTGGAGGAGGAGATATGAGGGACAATTGGAGAAGTGAATTATATAAATATAAAG
TGGTAGAAATTAAGCCATTG---GGAATAGCACCCACTGAAGCTAAAAGGAGAGTGGTG-------------
------------------------GAGAGAGAAAAGAGAGCA---GTGGGA---ATAGGAGCT---------
GTGTTCCTTGGGTTCTTGGGAGCA---GCAGGAAGCACTATG---GGCGCGGCGTCG---
ATAACGCTGACGGTACAG---GCCAGACAA---CTGTTG---TCTGGTATAGTGCAACAGCAAAGC---
AATTTG---CTGAGA---GCCATAGAAGCGCAACAGCATATGTTGCAACTCACAGTCTGGGGCATT
>KC863367..1
TTCTATTGCAATACATCAGGCCTGTTTAATGGTACACTTAATGATACATACACGGCTAATAGTACAAGT----
----

GATAACTCAACCATCACACTCCAATGCAGAATAAAACAAATTATAAACATGTGGCAAGGGGTAGGACAAGCAA
TGTATGCCCCTCCCATTGAGGGAAACATAACATGTAAATCAAAT---
ATCACAGGGCTACTATTGACCCGTGATGGAGGAACAAATACTAGT---------------------------
----

GAGGAGGAGACATTCAGACCTGGAGGAGGAGATATGAGGGACAATTGGAGAAGTGAATTATACAAATATAAAG
TGGTAGAGATTAAGCCATTG---GGAATAGCACCCACTCAGGCAAAAAGGAGAGTGGTG-------------
------------------------GAGAGAGAAAAAAGAGCA---GTGGGA---ATAGGAGCT---------

GTGTTCCTTGGGTTCTTGGGAGCA---GCCGGAAGCACTATG---GGCGCGGCGTCA---
ATAACGCTGACGGTACAG---GCCAGACAA---TTATTG---GCTGGTATAGTGCAACAGCAAAGC---
AATTTG---CTGAGG---GCTATAGAGGCGCAACAGCATATGTTGCAACTCACAGTCTGGGGCATT
>KC894137..1
TTCTATTGCAATACATCAAACCTGTTTAATGATACACATACAGCAAACATCACA-------------------
----

GCAAACATCACACTCACACTCCCATGCAGAATAAAACAAATTATAAACATGTGGCAGGAGGTAGGAAAAGCAA
TGTATGCCCCTCCCATTGCAGGAAACATAACATGTACATCAAAT---
ATCACAGGATTGCTATTGGTACGTGATGGAGGTACAAATAGAACT----------------------------
----

AATGAAACATTCAGACCTGGAGGAGGAGATATGAGGGACAATTGGAGAAGTGAATTATATAAATATAAAGTGG
TAGAAATTCAGCCATTG---GGAATAGCACCCACTAAGGCAAAAAGGAGAGTGGTG-----------------
----------------------GAGAGAGAAAAAAGAGCA---GTGGGA---ATTGGAGCT---------
GTGCTCCTTGGATTCTTGGGAGCA---GCAGGAAGCACTATG---GGCGCGGCGTCA---
ATAACGCTGACGGTACAG---GCCAGACAA---TTGTTG---TCTGGTATAGTGCAACAGCAAAGC---
AATTTG---CTGAAG---GCTATAGAGGCGCAACAGCATCTGTTGCAACTCACAGTCTGGGGCATT
>FJ443911..1
TTCTATTGCAATACAACACAACTGTTTAACAGTACATACAATACCAATGAT----------------------
----

ACAAGGGTAAACATCACACTTCCATGCAGAATAAAACAGATTATCAACATGTGGCAAAGGGTAGGACGAGCAA
TGTATGCTCCTCCCATTGCAGGAAACATAACATGTATATCAAAC---
ATCACAGGACTACTGTTAGTACGTGATGGAGGCAATAGCAGTGAC----------------------------
----

ACAACAGAAACATTCAGACCTGGAGGAGGAAATATGAAGGACAATTGGAGAAGTGAACTATATAAATATAAAG
TGGTAGAAATTCAGCCATTA---GGAGTAGCACCCACTAAAGCAAAAAGGAGAGTGGTG--------------
-------------------------GAGAGAGAAAAAAGAGCA---GTGGGA---ATAGGAGCT---------
GTGTTCCTTGGGTTCTTGGCAACA---GCAGGAGGCACTATG---GGCGCGGCGTCA---
ATAACGCTGACGGTACAG---ACCAGACAA---TTGTTG---TCTGGTATAGTGCAACAGCAAAGC---
AATTTG---CTGAAG---GCTATAGACGCGCAACAGCATCTGTTGCAACTCACAGTCTGGGGCATT
>KT183265..1
TTCTATTGTAATACATCAGGACTGTTTAATGGTACATACAATAATATATACAATACAGACAATGATACAACA-
----

GACAATGGAACCATCACACTCCCATGCAAAATAAAACAAATTATAAACATGTGGCAGGAGGTAGGAAGAGCAA
TATACGCCCCTCCCATTGCAGGAAATCTAACATGTATGTCAAAC---
ATCACAGGACTTCTCTTGGTACGGGATGGAGGTAATCAAAATAAT----------------------------
----

AGCACAGAGGTATTCAGACCTGGAGGAGGAGATATGAGGGACAATTGGAGAAGTGAATTATATAAATATAAAG
TGGTAGAAATTGAGCCATTG---GGAATAGCACCCACTAAGGCAAAAAGGAGAGTGGTG--------------
-------------------------GAGAGAGAAAAAAGAGCA---GTGGGA---ATAGGAGCT---------
GTACTCCTTGGGTTCTTGGGAGCA---GCAGGAAGCACTATG---GGCGCGGCGTCA---
ATAACGCTGACGGTACAG---GCCAGGCAA---CTGTTG---TCTGGTATAGTGCAACAGCAAAAT---
AATTTG---CTGAGA---GCTATAGAGGCGCAACAGCATATGTTGCAGCTCACGGTCTGGGGCATT
>KC894114..1
TTCTATTGTGATACATCAAGACTATTTAATGGTACATACAATGGTACAGATAGTAATCCAAACAATGGTACAG
ATAGT--
-------------GATTCA------
AACTCAAATTCAAACATCACACTTCCATGCAGAATAAAACAGATTATAAACATGTGGCAGGAGGTAGGAAGAG
CAATGTATGCTCCTCCCATTGCAGGAAGCATAACATGTAAATCAAAC---
ATCACAGGATTATTATTGACGCGTGATGGAGGCAAAGAGGAAAATAGC-------------------------
----

ACAACTAACAGAATAGAGATATTCAGACCTGGAGGAGGAAATATGAAGGACAATTGGAGAAGTGAATTATATA
AATATAAAGTAGTAGAAATCAAACCATTG---GGAATAGCACCCACTGGAGCAAAAAGGAGAGTGGTG-----
----------------------------------GAGAGAGAAAAAAGAGCA---GTGGGA---

ATAGGAGCT---------GTGTTCCTTGGGTTCCTGGGAGCG---GCAGGAAGCACTATG---
GGCGCGGCGTCA---ATAACGCTGACGGTACAG---GCCAGACAA---TTGTTG---
TCTGGTATAGTGCAACAGCAAAGC---AATTTG---CTGAAG---
GCTATAGAGGCACAACATCATCTGTTGCAACTCACAGTCTGGGGCATT
>KC894120..1
TTCTATTGCAATACATCAAACCTGTTTAATAATATTAGTACAGGCAAT-------------------------
----

TCTGGTAACATCACACTCCCATGCCAAATAAAACAAATTGTAAGAATGTGGCAGGGAGTGGGACAAGCAATGT
ATGCCCCTCCCATTGCAGGAAGCATAACATGTAGATCAAAT---
ATCACAGGACTATTATTGACACGTGATGGAGGCAAAACAAATTACACA-------------------------
----

GACCTAGAGATATTCAGACCAGGAGGAGGTGATATGAGGGACAATTGGAGAAGTGAATTATATAAATATAAAG
TAGTAGAAATTAAGCCATTA---GGAATAGCACCAACTGGGGCAAAAAGGAGAGTGGTG--------------
-------------------------GAGAGAGAAAAAAGAGCA---GTGGGA---ATAGGAGCT---------
GTACTCCTTGGGTTCTTGGGAGCA---GCAGGAAGCACTATG---GGCGCGGCGTCA---
ATAACGCTGACGGTACAG---GCCAGACAA---TTGTTG---TCTGGTATAGTGCAACAGCAAAGC---
AATTTG---CTGAGA---GCCATAGAGGCGCAACAGCATATGTTGCAACTCACAGTCTGGGGCATT
>KC156115..1
TTCTATTGCAATACATCAAAACTGTTTAATGGTACCATAGTTGATGGTACTACCCTGGTTAATAGTACCATAT
TTAAT--
-------------GATACC------
ATGTCTAATGGAACCATCACACTCCCATGTAGAATAAAACAAATTATAAATATGTGGCAGGAGGTAGGACGAG
CAATCTATGCCCCTCCCATTGCAGGAAACATCACATGTATCTCTAAT---
ATCACAGGACTACTATTGACACGTGATGGTGGAGGTGCCGGGGATGGG-------------------------
----

TCAAATGACACAGAAGAAATATTCAGACCTGGAGGAGGAGATATGAGGGACAATTGGAGAAGTGAATTATATA
AATATAAAGTGGTAGAAATTAAGCCGTTG---GGAATAGCACCCACTAAGGCAAAAAGGAGAGTGGTG-----
----------------------------------GAGAGAGAAAAAAGAGCA---GTGGGA---
ATGGGAGCT---------GTGTTCCTTGGGTTCTTGGGAGCA---GCAGGAAGCACTATG---
GGCGCAGCGGCA---GTAACGCTGACGGTACAG---GCCAGACAA---TTGTTG---
TCTGGAATAGTGCAACAGCAAAGC---AATTTG---CTGAGA---
GCTATAGAGGCGCAACAGCATATGTTGAAACTCACGGTCTGGGGCATT
>KC894124..1
TTTTACTGTAATACAACAAAACTGTTTAATACAACAGAATTTAATGAAAATGAT-------------------
----

TCAAATGATACCATCACACTCCCATGTAGAATAAAACAATTTATAAACATGTGGCAGGGAGTAGGAAGAGCAA
TGTATGCCCCTCCCATTGCAGGAAACATAACCTGTAACTCAAGT---
ATCACAGGAATATTATTGATACATGAAGAAAGGGGTGATAATCAGACAAGG----------------------
----

ATATTCAAACCTATAGGAGGAGATATGAAGAATAATTGGAGAAGTGAACTATACAAATATAAAGTGGTAGAAA
TTAAGCCATTG---GGAATAGCACCCACTGATGCAAAAAGGAGAGTGGTG-----------------------
----------------GAGAGAGAAAAAAGAGCA---GTGGGA---ATAGGAGCT---------
TTGTTCCTTGGGTTCTTGGGAGCA---GCAGGAAGCACTATG---GGCGCGGCGTCA---
GTGACGCTGACGGTACAG---GCCAGACAG---TTGTTG---TCTGGTATAGTGCAACAGCAAAGC---
AATTTG---CTGAGG---GCTATAGAGGCGCAACAGCATATGTTGCAACTCACAGTCTGGGGCATT
>HM204615..1
TTCTATTGCAATACATCAGGCCTGTTTAATGGTACATACAATAGTACAAACAATAGTACAGGA----------
----

GACAATAAGACCATCACACTCCCATGCAGAATAAAACAAATTATAAACATGTGGCAGGGGGTAGGACGAGCAA
TGTATGCCCCTCCCATTGCAGGAAACATAACATGTAAATCAAAT---
ATCACAGGATTACTATTGACACGTGATGGAGGAAATACTCATAAT----------------------------
----

AATACAGAGATATTCAGACCTGGAGGAGGCAATATGAAAGACAATTGGAGAAATGAATTGTATAAATATAAAG

TGGTAGAAATTAAGCCATTA---GGAATAGCACCCACTAGTGCAAAAAGGAGAGTGGTG--------------
-------------------------GCAAGAGAAAAAAGAGCA---GTGGGA---ATAGGAGCT---------
GTGTTCCTTGGGTTCTTGGGAGCA---GCAGGAAGCACTATG---GGCGCGGCGTCA---
ATAACGCTGACGGTACAG---GCCAGACAA---TTATTA---ACTGGTATAGTGCAACAGCAAAGT---
AATTTG---CTGAGG---GCTATAGAGGCGCAACAGCATATGTTGCAACTCACGGTCTGGGGCATT
>FJ443547..1
TTCTATTGCAATACAACAGAACTGTTCAATAATACTAATAAA-------------------------------
----

ACTGGCATACTCCCATGCAAAATAAAACAAATTATAAACATGTGGCAGGGGGTAGGACGAGCAATGTATGCCC
CTCCCATTGAAGGAAACATAACATGTAGGTCAATT---
ATCACAGGACTACTATTGGTACGTGATGGAGGAGAAGATAATAGC----------------------------
----

ACAGAGGAGGTATTCAGACCTGGAGGAGGAAATATAAAGGACAATTGGAGAAGTGAATTATATCAATATAAAG
TGGTAGCAATTAAGCCGTTG---GGAATAGCACCCACGCCGGCAAAAAGGAGAGTGGTGGAC---AGG-----
----------------GAAGTGGTGAAGAGAGGAAAAAGAGCA---GTGGGA---ATAGGAGCT---------
GTGTTCCTTGGGTTCTTGGGAGCA---GCAGGAAGCACTATG---GGCGCGGCGTCA---
ATAACGCTGACGGTACAG---GCCAGACAA---CTGTTG---TCTGGTATAGTGCAACAGCAAAGC---
AATTTG---CTGAGA---GCTATAGAGGCGCAACAGCATATGTTGCAACTCACGGTCTGGGGCATT
>FJ443838..1
TTCTATTGCAATACATCAAAACTGTTTAATGGTATATACAATAATACATACCATAAT----------------
----

ACATCTAGTAATATCATACTCAAATGCAGAATAAAACAAATTATAAACATGTGGCAAAAGGTAGGACGAGCAA
TGTATGCCCCTCCCATTGCAGGAAACATAACATGTACATCAAAT---
ATCACAGGACTACTATTGACACGTGATGGAGATAATAGGACTGAG----------------------------
----

TCAGAGACATTCAGACCTGGAGGAGGAGACATGAGGGACAATTGGAGAAGTGAATTATATAAATACAAGGTGG
TAGAAATTCAACCATTA---GGAGTAGCACCCACTGAGGCAAGAAGGAGAGTGGTG-----------------
----------------------GGGAAAGAAAAAAGAGCAGTAGTAGGA---ATAGGAGCT---------
GTGCTCCTTGGGTTCTTGAGTGCA---GCAGGAAGCACTATG---GGCGCAGCGTCA---
ATAACGCTGACGGTACAG---GCCAGACAA---TTGTTG---TCTGGTATAGTGCAACAGCAAAGC---
AATTTG---CTGAGA---GCTATAGAGGCGCAACAGCATATGTTGCAACTCACGGTCTGGGGCATT
>FJ443865..1
TTCTATTGCAATACATCAAACCTGTTTAATGATAGTACAAAA-------------------------------
----

AGTAATTCAAGCATCACACTCCCATGCAGAATAAAACAAATTATAAACATGTGGCAGAAGGTAGGACGAGCAA
TGTATGCCCCTCCCATTGCAGGAAACATAACATGTAGATCAGAG---
ATCACAGGACTACTATTGGTACATGATGGAGGAACAAATAGCAGT----------------------------
----

AATGAGACATTCAGGCCTGGAGGAGGAGATATGAGGGATAATTGGAGAAGTGAATTATATAAATATAAAGTGG
TAGAAATTAAGCCATTA---GGAGTAGCACCCACTCAGGCAAAAAGGAGAGTGGTG-----------------
----------------------GAGAGAGAAAAAAGAGCA---GTGGGA---ATAGGAGCT---------
GTGTTCCTTGGGTTCTTGGGAGCA---GCAGGAAGCACTATG---GGCGCGGCGTCA---
ATAACGCTGACGGTACAG---GCCAGACAA---TTGTTG---TCTGGTATAGTGCAGCAGCAAAGC---
AATTTG---CTGAGG---GCTATAGAGGCGCAACAGCATATGTTGCAACTCACGGTCTGGGGCATT
>KT183218..1
TTCTATTGCAATACAACAAAGCTATTCACAAACACAACC----------------------------------
----

ACAAATACAACCATCCTAATCCCATGCAGAATAAAACAATTTGTAAACATGTGGCAGGAGGTAGGGCGAGCAA
TGTATGCCCCTCCCATTGCAGGAAACATAACATGTAACTCAAGT---
ATCACAGGACTACTATTGGTACGTGATGGAGGAATTTCAAATGACACAAAT----------------------
----

AACACAACTGAAACATTCAGGCCTGGAGGAGGAAACATGAAGGACAATTGGAGAAGTGAATTATATAGCTATA

```
AAGTGGTAGAACTTAAGCCACTA---GGAGTAGCACCCACTGGAGCGAAAAGGAGAGTGGTGGAG--------
-------------------------ATGGAAAGATCAAAAAGAGCA---GTGGGA---ATAGGAGCT------
---GCACTCCTTGGGTTCTTGGGAGCA---GCAGGAAGCACTATG---GGCGCGGCGTCA---
ATGGCGCTGACGGTACAG---GCCAGACAA---CTGCTG---TCTGGTATAGTGCAACAGCAAAGC---
AATTTG---CTGAGA---GCCATAGAGGCGCAACAGCATATGTTGCAACTCACGGTCTGGGGCATT
>HM623548..1
TTCTATTGCAATACAACAAAACTGTTTAAGGAAGGTGAGTCAAATGCAACAGAT-------------------
-------------------------------------------------------------------------
----------------------------
TTAAATGCAACAATCATACTCCCATGCAGGATAAAACAAATTATAAACATGTGGCAGGGGGTAGGACAAGCAA
TGTATGCCCCTCCCATTGCAGGAAACATAACATGTATCTCAAAT---
ATCACAGGACTACTATTGACACGTGATGGAGGAAGAGACTCCAATAAC-------------------------
-------------------------------------------------------------------------
-------
ACAAATGACACAGAGACATTCAGACCTGGAGGAGGAAATATGAAGGACAATTGGAGAAGTGAATTATATAAAT
ATAAAGTGGTAGAAATTCAGCCATTG---GGAGTAGCTCCCACTGGATCAAAAAGGAGAGTGGTG--------
-------------------------------GAGAGAGAAAAAAGAGCA---GTGGGA---CTGGGAGCT---
------GTACTCCTTGGGTTCTTGGGAGCA---GCAGGAAGCACTATG---GGCGCGGCGTCA---
ATAGCGCTGACGGTACAG---GCCAGACAA---CTGTTG---TCTGGTATAGTGCAACAGCAAAAC---
AATTTG---CTGAGA---GCTATAGAGGCGCAACAGCATATGTTGCAACTCACAGTCTGGGGCATT
>HM623555..3
TTCTATTGCAATACAACAAACCTGTTTTATTATAATGGAAGTGAGAAG-------------------------
-------------------------------------------------------------------------
----------------------------
AATGACACAACCATCACCATCCAATGCAAAATAAAACAAATTATAAACATGTGGCAAGAGGTAGGACGAGCAA
TGTATGCCCCTCCCATTGCAGGAAACATAACATGTAGATCAAAT---
ATCACAGGATTACTATTGACACGTGATGGAGGAAACACGAATAGT----------------------------
-------------------------------------------------------------------------
-------------
ACAGAGGAGATATTCAGACCTGGAGGAGGAAATATGAAGGACAATTGGAGAAGTGAATTATATAAATATAAGG
TGGTAGAAATTCAGCCATTG---GGAATAGCACCCAATAAGGCAAAAAGGAGAGTGGTG--------------
-------------------------GAGAGAGAAAAAAGAGCA---GTAGCA---ATAGGAGCT---------
GTGCTCCTTGGGTTCTTGGGAGTA---GCCGGAAGCACTATG---GGCGCGGCGGCA---
CTAACGCTGACGGTACAG---GCCAGACAA---CTGTTG---TCTGGTATAGTGCAACAGCAAAGC---
AATTTG---CTGAGA---GCTATAGAGGCGCAACAGCATATGTTGCAACTCACAGTCTGGGGCATT
>HM623562..1
TTCTATTGCAATACAACAAAACTGTTTAATGCAAGTGAGACGGCTAGAAATATTAGTAAC-------------
-------------------------------------------------------------------------
----------------------------
CCAAATGAAAATATCACACTCCCATGCAGGATAAAACAAATTATAAACATGTGGCAGGAGGTAGGACGAGCAA
TGTATGCCCCTCCCATTGCAGGAAACATAACATGTAACTCAAAT---
ATCACAGGACTACTACTGACAAGGGATGGAGGAAACTATAGTATCACA-------------------------
-------------------------------------------------------------------------
----------
AATAACACAGAGACATTCAGACCTGGAGGAGGAAATATGAAGGACAATTGGAGAAGTGAATTATATAAATATA
AAGTAGTAGAAGTTAAGCCATTG---GGAGTAGCACCCACTAAAGCAAAAAGGAGAGTGGTG-----------
----------------------------GAGAGAGAAAAAAGAGCA---ATAGGA---ATGGGAGCT------
---GTACTCCTTGGGTTCTTGGGAGCG---GCAGGAAGCACTATG---GGCGCAGCGTCA---
ATAACGCTGACGGTACAG---GCCAGACAA---TTGTTG---TCTGGTATAGTGCAACAGCAAAGC---
AATTTG---CTGAGA---GCCATAGAGGCGCAACAGCGCATGTTGCAACTCACGGTCTGGGGCATT
>HM623568..1
TTCTACTGCAATACATCAAACCTGTTTAATTCAACAAGACTAAAACTGTTTAATTCAAGTGATCCA-------
-------------------------------------------------------------------------
----------------------------
GGCGACAAAACCATCACACTCCAATGCAGAATAAAACAAATTATAAACATGTGGCAGCAGGTAGGACGAGCCA
TGTATGCCCCTCCCATTGAAGGAAAAATATCATGTAACTCAAGT---
ATCACAGGATTACTATTGACACGTGATGGAGGACATAGAAACACAACA-------------------------
-------------------------------------------------------------------------
----------
AACAACACAGAGGTATTCAGACCTGGAGGAGGAGATATGAGGGACAATTGGAGAAGTGAATTATATAAATATA
```

AAGTGGTAGAAATTCAACCATTG---GGAGTAGCTCCCACCAAGGCAAAAAGGAGAGTGGTG-----------
----------------------------GAGAGAGAAAAAAGAGCA---TTGGGA---TTCGGAGCT------
---GTGCTCATTGGGTTCTTGGGAGCA---GCAGGAAGCACTATG---GGCGCGGCGTCA---
ATAACGCTGACGGTACAG---GCCAGACAA---CTGCTG---TCTGGTATAGTGCAACAGCAAAGC---
AATTTG---CTGAGA---GCTATAGAGGCGCAACAGCATATGTTGCAACTCACAGTCTGGGGCATT
>HM623571..2
TTCTATTGCAACACATCAAGACTGTTTAATGGTACATTTAATGAGACAAAGTTTAATAATACTTCAAACAACA
CC---

ACTCCTCAAAACATCACTCTCCAATGCAGAATAAAACAAATCATAAACACGTGGCAGGGGGTAGGACAAGCAA
TATATGCCCCTCCCATTGCAGGAAACATAACATGTAATTCAAGT---
ATCACAGGAATACTATTAACACGTGATGGAGGCCAAGACACAAATGGCACAGAT-------------------
----
-
GAGACCAAAAACGACACAGAGACATTCAGACCTATAGGAGGAGATATGAGGGACAATTGGAGAAGTGAATTAT
ATAAGTATAAAGTGGTAGAAATTCAGCCATTA---GGAATAGCACCCACTAAGGCAAGAAGGAGAGTGGTG--
-------------------------------------GAAAGAGTAAGAAGAGCA---GCA------
CTGGGAGCT---------GTGTTCCTTGGGTTCTTGGGAGCG---GCAGGAAGCACTATG---
GGCGCAGCGTCA---ATAACGCTGACGGTACAG---GCCAGACAA---TTATTG---
TCTGGTATAGTGCAACAGCAGAGT---AATCTG---CTGAGA---
GCTATAGAGGCGCAACAGCATATGTTGCAACTCACGGTCTGGGGCATT
>JN687726..1
TTCTATTGCAATACATCAAATCTGTTTAATACTACAGGACCTACA----------------------------
----

ACAAATGCAACTATCACACTCCCATGCAGAATAAAACAGATTATAAACATGTGGCAGGGGGTAGGACAAGCAA
TGTATGCCTCTCCCATTGCAGGAAATATAACATGTAACTCAAAT---
ATCACAGGATTACTATTGACACGTGATGGAGGTCATAATGGTACA----------------------------
----

TATGGTAATGAGACATTCAGACCTGGAGGAGGAGATATGAGGGACAATTGGAGAAGTGAATTATATAAATATA
AGGTGGTGGAAATTAAACCATTA---GGAATAGCACCCACTGAGGCAAAAAGAAGAGTGGTG-----------
----------------------------CAGAGAGAGAAAAGAGCA---GTGGGA---CTAGGAGCT------
---GTGTTCCTTGGGTTCTTGGGAGCA---GCAGGAAGCACTATG---GGCGCGGCGTCA---
GTGGCGCTGACGGTACAG---GCCAGACAA---CTGTTG---TCTGGTATAGTGCAACAGCAAAGC---
AATTTG---CTGAAA---GCTATAGAGGCGCAACAACATATGTTGCAACTCACGGTCTGGGGCATT
>KC154023..1
TTCTATTGCAATACAACAGGACTGTTTAATGCAACAAACCTCAATGCAACAAAT-------------------
----

CCCAATGAAAACATCACACTCCCATGCAGAATAAAGCAATTTGTAAACATGTGGCAGGGGGTAGGACGAGCAA
TGTATGCCCCTCCTGTTGAAGGAATAATAACATGTAACTCAAGT---
ATCACAGGACTACTGTTGACACGTGATGGGGGAAACACAGACAAT----------------------------
----

AACACAACAGAGACATTCAGACCTGGAGGAGGAAATATGAAGGACAATTGGAGAAGTGAATTATATAAATATA
AAGTAGTAGAAATTCAACCATTG---GGAATAGCACCCACAAACGCAAAAAGGAGAGTGGTG-----------
----------------------------GAGAGAGAAAAAAGAGCA---GTGGGA---ATAGGAGCT------
---GTATTCCTTGGGTTCTTGGGAGCA---GCAGGAAGCACTATG---GGCGCGGCGTCA---
ATAACGCTGACGGTACAG---GCCAGACTA---CTGTTG---TCTGGTATAGTGCAACAGCAAAGC---
AATTTG---CTGAGA---GCTATAGAGGCGCAACAACATATGTTGCAACTCACGGTCTGGGGCATT
>HM204619..1
TTCTATTGCAATACAACACAATTGTTTAATAGTACATATAATGTTACAGATCCGTTTAATAGTACAGAACTG-
----

GGTAATGATACCATCACACTCCCATGCAGAATAAAGCAAATTATAAACATGTGGCAGAGGGTAGGACGAGCAA
TGTATGCTCCTCCCATTGCAGGAAACATAACATGTAGATCAAAT---
ATCACAGGATTGCTATTGGTGCGTGATGGAGGAAATAATAATCAG----------------------------
----

ACAGGGAGAGAGATATTCAGACCTGCAGGAGGGAATATGAAAGATAATTGGAGAAGTGAATTACATAAATATA
AAGTGGTAGAAATTAAGCCATTG---GGAATAGCACCCACTAAGGCAAAAAGGAGAGTGGTG-----------
----------------------------GAGAGAGAAAAAAGAGCA---GTGGGA---CTGGGAGCT------
---GTGTTCTTTGGGTTCTTAGGCGCA---GCAGGAAGCACTATG---GGCGCGGCGTCA---
ATAGCGCTGACGGCACAG---GCCAGACAA---GTGTTG---TCTGGTATAGTGCAACAGCAAAGC---
AATTTG---CTGAGG---GCTATAGAGGCGCAACAACATATGTTGCAACTCACGGTCTGGGGCATT
>JX140666..1
TTTTATTGCAATACAACAAATCTGTCTAATACAAGTCTGTTTAATGACAGTAGT-------------------
----

TCAAACAAAACCATCACGCTCCCATGTAGGATAAAACAAATTATAAACATGTGGCAGGGGGTAGGACGAGCAA
TGTATGCCCCTCCCATTGCAGGGAACATAACATGCAACTCAAGC---
ATCACAGGACTATTATTAACACGTGATGGAGGAAACATGAGTGGCACTGATGAAGGAAAAAACAACACGACTA
AC--
GGGAATAACACGACTAACGGGATTGAAATATTTAGACCTGGAGGAGGAAATATGAAGGACAATTGGAGAAGTG
AATTATATAAATATAAAGTGGTAGAAATTCAGCCATTG---
GGAGTAGCACCCACGAAGGCAAAAAGGAGAGTGGTG-------------------------------------
--GAGAGAGAAAAAAGAGCA---GTGGGA---ATAGGAGCT---------
GTACTCCTTGGGTTCTTGGGAACA---GCAGGAAGCACTATG---GGCGCGGCGTCA---
ATAACGCTGACGGTACAG---GCCAGACAA---CTGTTG---TCTGGTATAGTGCAACAGCAAAGC---
AATTTG---CTGAGA---GCTATAGAGGCGCAACAGCATATGTTGCAACTCACAGTCTGGGGCATT
>JN681255..1
TTCTATTGCAATACATCACAACTGTTTACTGGAAATGGTACAGCTAATAGTACAGGC----------------
----

AATACAACCATCACCACACTCCCATGCAGAATAAAACAAATTATAAACATGTGGCAGGGGGTAGGACGAGCAA
TGTATGCCCCTCCCATTGAAGGAAACATAACATGTAAATCAAAC---
ATCACAGGACTACTATTGACACGTGATGGAGGCAACGACAACAATCAA-------------------------
----

AGTAGAACAGAGACATTTAGACCTGGAGGAGGAAATATGAAGGACAATTGGAGAAGTGAATTATATAAATATA
AAGTGGTAGAAATTAAGCCATTA---GGAATAGCACCCACTGAAGCAAAAAGGAGAGTGGTG-----------
----------------------------GAGAGAGAAAAAAGAGCA---GTGGGA---CTAGGAGCT------
---GTAATCTTTGGGTTCTTGGGAGCA---GCAGGAAGCACTATG---GGCGCGGCGTCA---
ATAACGCTGACGGTACAG---GCCAGACAA---TTGTTG---TCTGGTATAGTGCAACAGCAAAGC---
AATTTG---CTGAAA---GCTATAGAGGCGCAGCAGCATATGTTGCAACTCACAGTCTGGGGCATT
>HQ595752..1
TTCTATTGTAATACATCACAGCTGTTTAATAGGACTGATACAGACATGCAGCAAGACAACACA----------
----

GGAAATGATACAATCACACTCCAATGCAGAATAAAACAAATTATAAACATGTGGCAGGAGGTAGGACGAGCAA
TGTATGCCTCTCCCATTGCAGGAGACATAACATGCAAATCAAAT---
ATCACAGGATTAATATTGACACGTGATGGAGGTAATAGCAGCACAGGGAATGAT-------------------
----

AGTAATAGCCGCACAGAGATATTCAGACCTGCAGGAGGAGATATGAGGGACAATTGGAGAAGTGAATTATATA
AATATAAAGTGGTGGAGATTAAGCCATTA---GGAGTAGCACCCACTCATTCAAAAAGGAGAGTGGTG-----
----------------------------------GAAAGAGAAAAAAGAGGA------------
ATAGGAGCT---------GTGCTCCTTGGGTTCTTGGGAGCA---GCAGGAAGCACTATG---
GGCGCAGCGTCA---ATAGCGCTGACGGTACAG---GCCAGACAA---CTGTTG---
TCTGGTATAGTGCAACAGCAAAGC---AATTTG---CTGAGA---
GCTATAGAGGCGCAACAGCATATGTTGCAACTCACGGTCTGGGGCATT
>HQ615957..1
TTCTATTGCAATACATCAGCACTGTTTAATGGCAATGGCACATCTGTTAATGAT-------------------
----

ACAACTTCAACCATCACACTTCCATGCAGAATAAAACAAATTATAAACATGTGGCAGGAGGTAGGACGAGCAA
TGTATGCCCCTCCCATTGCAGGAAACATAGCATGTGAATCAAAT---
ATCACAGGACTACTATTAGAACGTGATGGAGGTGGAGGCAATGAC----------------------------
----

ACAGAAGAGATATTCAGACCTGGAGGAGGAAATATGAGGGACAATTGGAGAAGTGAACTGTATAAATATAAAG
TGGTAGAAATTCAACCATTA---GGAGTAGCACCCACTAAGACAAAAAGGAGAGTGGTG--------------
-------------------------GAGAGAGAGAAAAGAGCA---GTGGGA---ATAGGAGCT---------
GTGTTCCTTGGGTTCTTGGGAGCA---GCAGGAAGCACTATG---GGCGCAGCATCA---
ATAACGCTGACGGTACAC---GCCAGACAA---TTATTG---TCTGGTATAGTGCAACAGCAAAGC---
AATTTG---CTGAGG---GCTATAGAGGCGCAACAGCATCTGTTGCAACTCACGGTCTGGGGCATT
>HQ595760..2
TTCTATTGTAATACAACAAGACTGTTTAATAATACAGAAAGTAACTCAACCATCACCGGGAAT----------
----

CATTCAGACACTATCACACTCCCATGTAGAATAAAACAATTTATAAACATGTGGCAGGAGGTAGGACGAGCAA
TGTATGCCCCTCCCATTGCAGGAACCATAACATGTATCTCAAAT---
ATCACAGGACTACTATTGGTACGGGATGGAGGCATACCAAATGAG----------------------------
----

AACACAGAAATATTCAGACCTCAAGGAGGAAATATGAAGGACAATTGGAGAAGTGAATTATATAAATATAAAG
TGGTAGAAATTAAGCCATTA---GGAATAGCACCCACTACGGCAAAGAGAAGAGTGGTG--------------
-------------------------CAGAGAGAAAAAAGAGCA---GTGGGA---ATAGGAGCT---------
GTGCTCCTTGGGTTCTTGGGAGCA---GCAGGAAGCACTATG---GGCGCGGCGTCA---
ATAACGCTGACGGTACAG---GCCAGACAT---CTGTTG---TCTGGTATAGTGCAACAGCAAAGC---
AATTTG---CTGAGA---GCTATAGAGGCGCAACAGCATATGTTGCAACTCACAGTTTGGGGCATT
>KT183289..2
TTCTATTGTAATACAACAAAATTGTTTAATAGTACATACAATAGTACATACTATAAGAATAATAATACTAATA
GTACAGAA---

GAAAACTCTACCATCACGCTCACATGCAGAATAAAGCAATTTATAAACATGTGGCAGGAGGTAGGACGGGCAA
TGTATGCCCCTCCCATTGAAGGAAACATAACATGTAGATCAAAC---
ATCACAGGACTACTACTGACGCGTGATGGAGGAAATTCAACAGAT----------------------------
----

ACTCAAACGGAGACATTCAGGCCTGAGGGAGGAAATATGAAGAACAATTGGAGAAGTGAATTATATAAATACA
AAGTGGTAGAAATTAAACCATTA---GGAATAGCACCCACTAGAGCAAAAAGGAGAGTGGTG-----------
----------------------------CAGAGGGAAAAAAGAGCA---GTGGGA---ATAGGAGCT------
---GTGTTCCTTGGGTTCTTGGGAGCA---GCAGGAAGCACTATG---GGCGCAGCATCA---
ATAACGCTGACGGTACAA---GCCAGACAA---CTATTG---TCGGGTATAGTGCAACAGCAAAGC---
AATTTG---CTGAGA---GCTATAGAGGCGCAACAGCATATGTTGCAACTCACGGTCTGGGGCATT
>GU080183..1
TTCTATTGCGATACATCAGCACTGTTTAATAGTACATACAATTCTGCATACAATAAGGAGACTAATAGTACAT
ACAATAAT---
-------------GGGACA------
GTAAGTAATTCAACTATCACACTTCCATGCAGAATAAAGCAAATTATAAACATGTGGCAAGAGGTAGGACGAG
CAATGTATGCCCCCCCCATTGCAGGAAATATAACATGTAAATCAAAT---
ATCACAGGATTACTATTAGTACGTGATGGAGGAGGCACAAATACA----------------------------
----

ACAAATGAGACATTCAGACCTCAAGGAGGAAATATGAAGGACAATTGGAGAAGTGAACTATATAAATATAAAG
TGGTAGAAATTGAGCCATTG---GGAATAGCACCCGATAAGGCAAGAAGGAGAGTGGTG--------------
-------------------------AAGAGAGAAAAAAGAGCA---GTGGGA---ATAGGAGCT---------
TTGTTCCTTGGGTTCTTGGGAGCA---GCAGGAAGCACTATG---GGCGCAGCATCA---
TTAATGCTGACGGTACAG---GCCAGACAA---CTGTTG---TCTGGTATAGTGCAACAGCAAAGC---
AATTTG---CTGAGA---GCTATAGAGGCGCAACAGCATATGTTGCAACTCACGGTCTGGGGCATT
>GU080187..1
TTCTACTGCAATACATCAAGCCTATTCAATATTACATACCATAACAATGGAACAAACACTAGTACATACACAG
AC---

AGCAACAGCATAATTACAATTCCATGCAGAATAAAACAAATTATAAACATGTGGCAGGGGGTAGGACGAGCAA
TGTATGCCTCCCCCATTGCAGGAAACATAACATGTAAATCAAAT---
ATCACAGGAATACTATTAACACGTGATGGAGGCTATCTAGAGAACAATACA----------------------
----

AATAATGACAAAGAAATATTCAGACCTGGAGGAGGAAATATGAAAGACAATTGGAGAAGTGAACTATATAAAT
ATAAAGTGGTAGAAATTAGACCATTA---GGAATAGCACCCACTAAAGCAAAAAGGCTAGTGGTG--------
-------------------------------AAGAGAGAAAAAAGAGCA---GTAACA---CTAGGAGCT---
------CTGTTCCTTGGGTTCTTGGGAACA---GCTGGAAGCGCTATG---GGCGCGGCGTCA---
CTGACGCTGACGGTACAG---GCCAGACAA---TTGTTG---TCTGGTATAGTGCAACAGCAAAAT---
AATTTG---CTGAGG---GCTATAGAGGCGCAACAGCATATGTTGCAACTCACAGTCTGGGGCATT
>HM623598..1
TTCTATTGCAATACCACAAAATTGTTTAATAAGACTATTGCA-------------------------------
----

AACGGAACCATCACACTCCCATGCAGAATAAAACAAATTATAAACATGTGGCAGGAGGTAGGACGAGCAATGT
ATGCCCCTCCCATTGCAGGAAACATAACATGTGAATCAAAT---
ATCACAGGACTATTATTGCTACGTGATGGAGGAAGGATTAATGACACT-------------------------
----

AATAACACAGAAGAGATATTCAGACCTGGAGGAGGAAATATGAAGGACAATTGGAGAAGTGAATTATATAAAT
ATAAAGTGGTAGAAGTTCAGCCATTG---GGAATAGCTCCTACTGAATCAAAAAGGAGAGTGGTG--------
-------------------------------GAGAGAGAAAAAAGAGCA---GTGGGA---ATGGGAGCT---
------GTACTCCTTGGGTTCTTGGGAGCT---GCAGGAAGCACTATG---GGCGCGGCGTCA---
ATAACGCTGACGGCACAG---GCCAGACAA---CTGTTG---TCTGGTATAGTGCAACAGCAAAGC---
AATTTG---CTGAGA---GCTATAGAGGCGCAACAGCATATGTTGCAACTCACGGTCTGGGGCATT
>HM623604..1
TTCTATTGCAATACATCAGGACTGTTTAATAATAGCAAAAGT-------------------------------
----

GATTCAAACATAACCACACTCCCATGCAAAATAAAACAAATTATAAACATGTGGCAGAAGGTAGGACGAGCAA
TGTATGCCCCTCCCATTGCAGGAAACATAACATGTAACTCAAAT---
ATCACAGGACTACTATTGGTACGCGATGGGGGATACAATGTAGACAATCAG----------------------
----

ACAGACAATAAGACAGAGATTTTTAGACCTGGAGGAGGAAATATGAAGGATAATTGGAGAAGTGAATTATATA
AATATAAAGTGGTTGAAATTAAGCCATTA---GGAATAGCACCCACTAAGGCAAAAAGGAGAGTGGTG-----
----------------------------------GAGAGAGAAAAAAGAGCA---GTGGGA---
CTAGGAGCT---------GTGTTCCTTGGGTTCTTGGGAGCA---GCAGGAAGCACTATG---
GGCGCGGCATCA---ATAACGCTGACGGTACAG---GCCAGACAA---CTGTTG---
TCTGGTATAGTGCAACAGCAAAGC---AATTTG---CTGAGA---
GCTATAGAGGCGCAACAGCATATGTTGCAACTCACGGTCTGGGGCATT
>HM623587..1
TTCTATTGCAATACAACAAACCTGTTCAATTTTAATGAAACACAACTTAATGAAATACAACTTAATAATGCAA
CAGAA--
-------------------AAC---
AACACAAGCACCAATATCACACTCACATGCAGAATAAAACAATTTGTAAACATGTGGCAAAGGGTAGGACGAG
CAATGTATGCCCCTCCCATTGCAGGAAATATAACATGTCATTCAAAC---
ATCACAGGACTGCTATTGACACATGATGAAGAAGAAATGAATAACGCAACT----------------------
----

GGGGCAAATAAAACAGAGGTATTCAGACCTATAGGAGGCAATATGAAGGACAATTGGAGAAGTGAATTATACA
AATATAAAGTAGTAGAAATTAAACCATTG---GGAGTAGCACCCACAGAGGCAAAACGGAGAGTGGTG-----
----------------------------------GAGAGAGAAAAAAGAGCA---GCA------
ATAGGAGCT---------ATGTTCCTTGGGTTCCTGGGAGCG---GCAGGAAGCACTATG---
GGCGCGGTGTCA---TTGACGCTGACGGTACAG---GCCAGACAA---CTGCTG---
TCTGGTATAGTGCAACAGCAAAGC---AATTTG---CTGAGA---
GCTATAGAGGCGCAACAACATCTGTTGCAACTCACGGTCTGGGGCATT
>KF114887..1
TTCTACTGCAATACAACATACCTGTTTAATAACAGTGCA----------------------------------
----

TCAGAGGGAAACATCACAATCCCATGCAGAATAAAACAGATTATAAATATGTGGCAGGAGGTAGGACGAGCAA
TGTATGCCCCGCCCGTTGCAGGAAACATAACATGTAAATCAAAT---

ATCACAGGATTACTATTAGTACGTGATGGTGGAAATGAGGACAACAGCACAGAT-------------------
----

GGGAATAAGACAGAGATATTCAGACCTCAAGGAGGAAATATGAAGGACAATTGGAGAAGTGAATTATATAAAT
ATAAAGTGGTAGAAGTTAAGCCATTA---GGAATAGCACCCACTACAGCAAAAAGGAGAGTGGTG--------
-------------------------------GAAAGAGAAAAAAGAGCA---GTCGGA---ATAGGAGCT---
------GTGTTCCTTGGGTTCTTGGGAGCA---GCAGGAAGCACTATG---GGCGCAGCGTCA---
GTGGCGCTGACGGTACAA---GCCAGACAA---TTGTTG---TCTGGTATAGTGCAGCAGCAAAAT---
AATTTG---CTGAGA---GCTATAGAGGCGCAACAGCATATGTTGCAACTCACAGTCTGGGGCATT
>KF725917..1
TTCTATTGCAACACATCACAATTGTTTAATAGTACAGTCAATGGTACAATATCTAATAATACAGGCAAT----
----

AGTACAGACATCATCACACTCCCATGCAGGATAAAACAAATTATAAACATGTGGCAGGGGGTAGGACGAGCAA
TGTATGCCCCTCCCATTGCAGGAAACATAACATGTAAGTCAAAC---
ATCACAGGATTACTATTGACACGTGATGGAGGATATAACACAAGTGGG-------------------------
----

GATAACGAGACATTCAGACCTGCAGGAGGAGATATGAGAGACAATTGGAGAAGTGAATTGTATAAATATAAAG
TAGTAGAAATTCAGCCATTA---GGAATAGCACCCACAGGAGCAAGAAGGAGAGTGGTG--------------
-------------------------GAGAGGCAAAAAAGAGCA---GTAGGA---ATAGGAGCT---------
GTGTTCCTTGGGTTCTTGGGAGTA---GCCGGAAGCACTATG---GGCGCGGCGTCA---
ATGGCGCTGACGGTACAG---GCCAGACAA---TTGTTG---TCTGGTATAGTGCAACAGCAAGGC---
AATTTG---CTGAGA---GCTATAGAGGCGCAACAACACATGTTGCAACTCACAGTCTGGGGCATT
>JN681257..1
TTCTATTGTAATACATCAAAACTGTTTAATGAGACAGAAACGAAGTCA-------------------------
----

AACATCTCAAACATCACACTTCCATGCAGAATAAAACAAATTATAAACATGTGGCAGGGGGTAGGACGAGCAA
TGTATGCCCCTCCTATTGCAGGAAGCATAACATGTAACTCAAAT---
ATCACAGGACTACTATTGACACGTGATGGAGGAGGAGGAAATGAAACT-------------------------
----

AATCACACAGAAACATTCAGACCTACAGGAGGAAATATGAAGGACAATTGGAGAAGTGAATTATATAAATATA
AAGTGGTAGAAATTAAGCCATTG---GGAATAGCACCCACTAGAGCAAAAAGGAGAGTGGTG-----------
----------------------------GAGAAAGAAAAAAGAGCA---GTGGGA---ATAGGAGCT------
---GTGTTCCTTGGGTTCTTGGGGGCA---GCAGGAAGCACTATG---GGCGCGGCGTCA---
ATAACGCTGACGGTACAG---GCCAGACAA---CTGTTG---TCTGGTATAGTGCAACAGCAAAAT---
AATTTG---CTGAGG---GCTATAGAGGCGCAACAGCATATGTTGCAACTCACGGTCTGGGGCATT
>JN681259..1
TTCTATTGCAATACAACAAAACTGTTTAATGAAAGTGAACTAAGTAAG-------------------------
----

TCAAACGAAACAATCACACTCCCATGCAGAATAAAACAAATTATAAACATGTGGCAGGAGGTAGGACGAGCAA
TGTATAACCCTCCCATTGAAGGAAACATAACATGTAACTCAAGT---
ATCACAGGACTATTATTGACATGGGATGGAGGAAATAACACAAAT----------------------------
----

AAAAATCAAACATTCAGACCTGGAGGAGGAGATATGAGGGACAATTGGAGAAGTGAATTATATAAATATAAAG
TGGTAGAAATTAAGCCATTG---GGAGTAGCACCCACTGAGGCAAAAAGGAGAGTGGTG--------------
-------------------------GAGAGAGAAAAAAGAGCA---GTGGGA---ATAGGAGCT---------
GTGCTCTTTGGGTTCTTGGGAGCA---GCAGGAAGCACTATG---GGCGCGGCGTCA---
ATAACGCTGACGGTACAT---GCCAGACAA---CTGTTG---AATGGTATAGTGCAACAGCAAAAC---
AATTTG---CTGAGA---GCTATAGAGGCGCAACAGCATATGTTGCAACTCACGGTCTGGGGCATT
>KC154028..2
TTCTATTGTAACACAACAGAACTGTTTATAATGATTTTAATAATACAGGA-----------------------
----

AAGAATGTAAACATCACACTCCAGTGCAAAATAAAACAAATTATAAACATGTGGCAGCAGGTAGGACGAGCAA
TGTATGCCCCTCCCATTGAAGGAAACATAACATGTACATCAAAT---

ATTACAGGACTACTATTGACATGGGATGGAAACATAACAAATGAAGATAATACA------------TCT----
----

AGTACAAATCATACATTCAGACCTGCAGGAGGAGATATGAGGGACAATTGGAGAAGTGAACTATATAAATATA
AAGTGGTAGAAGTTAAGCCATTG---GGAGTAGCACCCACTAAGGCACAAAGGAGAGTGGTG-----------
----------------------------GAGAGGAAAAAAAGAGCA---TTGGGA---ATAGGGGCT------
---ATGTTCCTTGGGATCTTGGGAACA---GCAGGAAGCACTATG---GGCGCGGCGTCA---
ATAACGCTGACGGTACAG---GCCAGACAA---TTATTG---TCTGGTATAGTGCAACAGCAAAGC---
AATTTG---CTGAGA---GCTATAGAGGCGCAACAGCATCTGTTGCAACTCACGGTCTGGGGCATT
>KF725924..1
TTCTATTGCGATACATCAAATCTGTTCAATGAAAGTAAAAGTTGGTATTCAAACAACAGTACA----------
----

GACGACATAAACATCACACTCCCCTGTAGGATAAAACAAATTGTAAACATGTGGCAGAGAGTAGGACAAGCAA
TATATGCCCCTCCCATTGCAGGAAGCATAACATGTAAATCAAAT---ATCACAGGACTACTATTGGCA-----
----CGCATAACAGATAACACA---

AATGGGACAGAGAAATTTAAACCTGAAGGAGGAAATATGAAGAATAATTGGAGAAGTGAATTATATAAATATA
AAGTGGTAGAAATTCAACCATTA---GGAATAGCCCCCACTGCATCAAAAAGGAGAGTGGTA-----------
----------------------------GAGAGAGAAAAAAGAGCA---GTGGGA---ATAGGAGCT------
---GTGCTCCTTGGGTTCTTGGGAGCA---GCAGGAAGCACTATG---GGCGCGGCGTCA---
ATAACGCTGACGGTACAG---GCCAGACAA---CTGTTG---TCTGGTATAGTGCAACAGCAAAGC---
AATTTG---CTGAGA---GCTATAGAGGCGCAACAGCATATGTTGCAGCTCACAGTCTGGGGCATT
>KF725926..4
TTCTATTGCTATACATCACAACTGTTTAATAGTACATACAAGACATACAATCATAGTTGGACAACA-------
----

GGTAATGCAACCATCACACTTCCATGCAGAATAAAACAAATTATAAACATGTGGCAGGGAGTAGGACAAGCAA
TGTATGCCCCTCCCATTCAAGGAAATATAACATGTAAATCAAAT---
ATCACAGGAATACTATTAGTACGTGATGGAGACAAAAATGATACA----------------------------
----

GAGACATTCAGACCTCAAGGAGGAAATATGAAAGACAATTGGAGAAGTGAATTATATAAATATAAAGTGGTAG
AAATTAAGCCATTA---GGAATTGCACCTACTGGAGCAAAAAGGAGAGTGGTG--------------------
-------------------GAGAGAGAAAAAAGAGCA---GTGGGA---ATAGGAGCT---------
GTACTCCTTGGGTTCTTGGGAGCA---GCAGGAAGCACTATG---GGCGCAGCGTCA---
ATAACGCTGACGGTACAG---GCCAGACAA---TTATTG---TCTGGTATAGTGCAACAGCAGGAC---
AATCTG---CTGAGG---GCTATAGAGGCGCAACAGCATATGTTGCAACTCACGGTCTGGGGCATT
>KF725934..2
TTCTATTGCAATACATCAACCTTGTTTAATAGTACATATGGGTTTAATGGTACATACAATGGGACAACA----
----

AACAATGATACATACACACTCCAATGCAGAATAAAACAAATTATAAACATGTGGCAAAAGGTAGGACAAGCAA
TGTATGCCCCTCCTATTCAAGGAGTAATACAATGTAAATCAAAT---
ATCACAGGACTACTATTGACACGGGATGGAGGTGAAGGAAATAATACAAAT----------------------
----

AATGGTACAGAGACATTCAGACCTGGAGGAGGAGATATGAGGGATAACTGGAGAAGTGAATTATATAAATACA
AGGTGGTAGAAGTTCAGCCATTA---GGAGTAGCACCCACTGCTGCAAGAAGGAGAGTGGTG-----------
----------------------------CAGAGAGAAAAAAGAGCA---GTAACA---CTAGGAGCT------
---ATGATCCTTGGATTCTTGGGAACA---GCAGGAAGCACTATG---GGCGCGGCGTCA---
ATGGCGCTGACGGTACAG---GCCAAACAA---TTGTTG---TCTGGTATAGTGCAACAGCAAAGC---
AATTTG---CTGAAG---GCTATAGAGGCGCAACAGCATATGTTGCAACTCACAGTCTGGGGCATA
>KF725961..1
TTCTATTGTAATACAACAAAGCTGTTTAATAGTACATACTTTCCTAATGGTACATACATGCCTAATGTTACAG
AA---

GATAATGGCACAATCATACTCCCATGCAGGATAAAGCAAATTATAAACATGTGGCAGGCGGTAGGACAAGCAA
TATATGCTCCTCCCATCGCAGGGAACATAACATGTATCTCAAAT---
ATCACAGGATTACTATTGGAGCGTGATGGAGGAAATGAAAGTAACACAGAG----------------------

----

GAGATATTCAGACCTCAAGGAGGAAACATGAGGGACAATTGGAGAAGTGAATTATACAAATATAAAGTGGTAG
AAGTTAAACCATTA---GGAATAGCACCTACTAAGGCAAAAAGGAGAGTGGTG--------------------
-------------------GAGAGAGAAAAAAGAGCA---GTGGGA---CTAGGAGCT---------
GTGTTCCTTGGGTTCTTGGGAGCA---GCAGGAAGCACTATG---GGCGCGGCGTCA---
ATAACGCTGACGGTACAG---GCCAGACAA---TTGCTG---TCTGGTATAGTGCAACAGCAAAAC---
AATTTG---CTGAGA---GCCATAGAGGCGCAACAGCATATGTTGCAACTCACGGTCTGGGGCATT
>KF725984..2
TTCTATTGCAATACAACAAAACTGTTTAATAGGATATATTATCCTAATGGTACATACAATGGTACA-------
----

GAAAATGATACCATCATACTCCCATGTAGAATAAAACAAATTATAAACATGTGGCAAGGGGTAGGACGAGCAA
TGTATGCCCCTCCCATTGCAGGAGAAATAACATGTAACTCAAGT---
ATCACAGGACTACTATTGACACGTGATGGAGGACGTGATGGAGGCAACAATAGC-------------------
----

GCAGAGACAGAGACATTCAGACCTAGCGGAGGAAATATGAGGGACAACTGGAGAAGTGAGTTATATAAATATA
AAGTGGTAGAAATTCAGCCATTA---GGAGTAGCACCCACTAAGGCAAAAAGAAGAGTGGTG-----------
----------------------------GAGAGAGAAAAAAGAGCA---GTGGGG---CTAGGAGCT------
---GTATTCCTTGGGTTCTTGGGAGCA---GCAGGAAGCACTATG---GGCGCGGCGTCA---
ACAGCGCTGACGGTACAG---GCCAGACAA---CTGTTG---TCTGGTATAGTGCAACAGCAGAGC---
AATCTG---CTGAGA---GCCATAGAGGCGCAACAGCATATGTTGCAACTCACGGTCTGGGGCATT
>KF725994..1
TTCTATTGCAATACATCAAAACTGTTTAATAGTAATTTTACAGAGAATTTTACAGAGACTTCTAATAGT----
----

ACAGAGGGTGACATCACACTCCAATGCAGAATAAAACAAATTATTAACATGTGGCAGGGGGTAGGACGAGCAA
TGTATGCCCCTCCCATTGCAGGAACCATAACATGTAACTCAAGC---
ATCACAGGAATACTATTGACACATGATGGT---AACCATTCAGAAGAA-------------------------
----

GAGGTGGTGGTATTCAGACCTGGAGGAGGAGATATGAGGGACAACTGGAGAAGTGAATTATATAAATATAAAG
TGGTAGAAGTTAAACCACTA---GGAATAGCACCCACTCAGGCAAAAAGAAGAGTGGTG--------------
-------------------------GAGAGAGAAAAAAGAGCA---GTGGGA---CTAGGAGCT---------
GTGTTCCTTGGGTTCTTGGGAGCG---GCAGGAAGCACTATG---GGCGCAGCGTCA---
ATAACGCTGACGGTACAG---GCCAGACAA---CTGCTG---TCTGGTATAGTGCAACAGCAAAGC---
AATTTG---CTGAAG---GCTATAGAGGCGCAACAGCATATGTTGCAACTCACGGTCTGGGGCATT
>KF725998..1
TTCTATTGTAATACATCAAACCTGTTTAATGATACAGGGAGTAATTCA-------------------------
----

ACTAATTCAACCATCATACTCCCATGCAGAATAAGACAAATTATAAACATGTGGCAGGGGGTAGGACGAGCAA
TGTATGCCCCTCCCATTAAAGGAAACATAACATGCAACTCAAGC---
ATCACAGGAATACTATTGACACGTGATGGAGGAAAGGACAACAGTACA-------------------------
----

AATGAAACAGAGACATTCAGACCTATAGGAGGAAATATGAGGGACAACTGGAGAAGTGAATTATATAAATATA
AAGTGGTAGAAATCAAGCCATTG---GGAATAGCACCCACTCAGGCAAAAAGAAGAGTGGTG-----------
----------------------------GAGAGAGAAAAAAGAGCA---GCGGGA---GTAGGAGCT------
---GTGCTCTTTGGGTTCTTGGGAGCA---GCCGGAAGCACTATG---GGCGCAGCGTCA---
ATGGCGCTGACGGTACAG---GCCAGACAA---CTGTTG---TCTGGTATAGTGCAACAGCAAAGC---
AATTTG---CTGAAG---GCTATAGAGGCGCAACAACATATGTTGCAACTCACGGTCTGGGGCATT
>MH933706..2
TTCTATTGCAACACATCAAGCTTGTTTAATGGAACATACAGTAATTACACTGATATAAACAGTTCTAGTATT-
----

ATCACACTCCCATGCAGGATAAAACAAATTATAAACATGTGGCAGGCGGTAGGACGAGCAATATATGCCCCTC
CCATCGAAGGGAACATAACATGTAAATCAAAT---
ATCACAGGACTACTATTGACACGTGATGGAGGAAAAGACAATAAT----------------------------

----

AACACAGAGGTATTCAGACCTGGAGGAGGAGAGATGAGGGACAACTGGAGAAATGAATTATATAAATATAAAG
TGGTAGAAATTAAGCCATTA---GGAATAGCACCCACTCATGCAAAAAGGAGAGTGGTG--------------
-------------------------GAGAGAGAAAAAAGAGCA---GTGGGA---ATAGGAGCT---------
TTGTTCCTTGGGTTCTTGGGAGCA---GCAGGAAGCACTATG---GGCGCGGCGTCA---
ATAACGCTGACGGTACAG---GCCAGACAA---CTGTTG---TCTGGTATAGTGCAACAGCAAAGC---
AACTTG---CTGAGG---GCTATAGAGGCGCAACAGCATATGTTGCAACTCACAGTCTGGGGCATT
>MK643550..1
TTCTATTGCAATACATCACAACTGTTTAATGGTACATATACAGGCAATAACACAGAGAACAAT----------
----

TCAAGCGAAACAATCACACTCCCATGCAGGATAAAACAAATTATAAACATGTGGCAGGAGGTAGGACGAGCAA
TGTATGCTCCTCCCATTGAAGGAAAAATAACATGTAACTCAAGC---
ATCACAGGACTACTATTGACAAGTGACAAAGGGGGTATAGGAAAT----------------------------
----

AATCAGACAGAGACATTCAGACCTGGGGGAGGCAATATGAAAGACAATTGGAGCAATGAATTATATAAATATA
AAGTAGTGGAAATTAGACCATTA---GGAGTAGCACCAACTGCTGCAAAGAGGAGAGTGGTG-----------
----------------------------GAGAGAGAAAAAAGAGCA---GTAGGA---ATAGGAGCT------
---GTACTCTTTGGGTTCTTGGGAGCC---GCAGGAAGCACTATG---GGCGCGGCGTCG---
ATAACGCTGACGGTACAA---GCCAGACAA---CTGTTG---TCTGGTATAGTGCAACAGCAAAGC---
AATTTG---CTGAGA---GCTATAGAGGCGCAACAGCATATGTTGCAACTCACAGTTTGGGGCATT
>MN611462..3
TTCTATTGTGATACAACACAACTGTTTAATCAAACACTGTTTAATGGAAGTGATTCAAACAAAACAAACATT-
----

GTCCTCCCCTACATTGTCCTCCCATGCAGAATAAAACAGATTATAAACATGTGGCAGAAGGTAGGACGAGCAA
TATATAATCCTCCCATTGCAGGAAACCTAACATGTAACTCAAGT---
ATCACAGGGCTACTATTGACACGTGATGGAGGAGAGAACATGACA----------------------------
----

CAGGGAGCGGAAACATTCAGACCTACAGGAGGAAATATGAAGGACAATTGGAGAAGTGAATTATATAAATATA
AAGTGGTAGAAATTCAGCCATTG---GGAGTAGCACCCACTGAGGCAAAAAGGAGAGTGATG-----------
----------------------------CAGAGAGAAAGAAGAGCAGTAGTGGGA---ATGGGAGCT------
---GTGCTCCTTGGGTTCTTGGGAGCA---GCAGGAAGCACTATG---GGCGCGGCGTCA---
ATAACGCTGACGGTACAG---GCCAGACAA---CTGTTG---TCTGGTATAGTGCAACAGCAAAGC---
AATTTG---CTGAGA---GCTATAGAGGCGCAACAGCATCTGTTGCAACTCACGGTCTGGGGCATT
>MN097669..1
TTCTATTGTAATACAACACAGTTGTTTAATGATAGCATCTCAAACGCCACTAACCTCAACATCTCAAACGCC-
----

ACTAACATCACACTCACACTCCCATGCAGAATCAAACAAATTATAAACATGTGGCAGGAGGTAGGACGAGCAA
TGTATGCCCCTCCCATCGAAGGAAACATAACATGTGTATCAAAT---
ATCACAGGACTACTACTGCTACGTGATGGCGGAAACACCAATAACACC-------------------------
----

ACTCAGCCAGAGATATTCAGACCTGGAGGAGGAAATATGAAGGATAATTGGAGAAGTGAATTATATAAATATA
AAGTGGTAGAAATTAAGCCATTA---GGAATAGCTCCCACGGAGGCAAAAAGGAGAGTGGTG-----------
----------------------------GAAAGAGAAAAAAGAGCA---GTA------CTAGGAGCT------
---TTGCTCCTTGGGTTCTTGGGAGCA---GCAGGAAGCACTATG---GGCGCGGCGTCA---
ATAGCGCTGACGGTACAG---GCCAGAAAT---CTGTTG---TCTGGTATAGTGCAGCAGCAAAGC---
AATTTG---CTGAGA---GCTATAGAGGCGCAACAGCATATGTTGCAGCTCACGGTCTGGGGCATT
>MN097680..1
TTCTATTGCAATACATCAAACTTGTTTAATAGTACATACAACGGTACAGACATGGCTAATGGTACAGGAAAT-
----

ACTTCAGAGTACATCACACTCCAATGCAGAATAAAACAAATTATAAACATGTGGCAGGAGGTGGGGCGAGCAA
TGTATGCCCCTCCCATTGCAGGAAACATAACCTGTGAATCAAAT---
ATCACAGGACTACTATTGACACGAGATGGAGGAAAAGAAAATAAT----------------------------

```
-------------------------------------------------------------------------
----------------
ACAGAGACATTCAGACCTGAAGGAGGAGATATGAGGAATAATTGGAGAAGTGAATTATATAAATATAAAGTAG
TAGAAATTCAACCATTA---GGAATAGCACCCACTAAGGCAAGAAGGAGAGTGGTG-----------------
----------------------CAGAAAGAGAAAAGAGCA---GTGGGA---ATGGGAGCG---------
TTGTTCCTTGGGTTCTTGGGGGCA---GCAGGAAGCACTATG---GGCGCAGCATCA---
ATAACGCTGACGGTACAG---GCCAGACAA---TTATTG---TCTGGTATAGTGCAACAGCAAAGC---
AATTTG---CTGAGG---GCTATAGAGGCGCAACAGCATATGTTGCAACTCACGGTCTGGGGCATT
>AY162225..1
TTCTATTGCAATACATCTGTACTGTTTAATGGCACATACAGTAATGGCACAAACAGTACA-------------
-------------------------------------------------------------------------
----------------------------
AATACAACAGTCATCACACTCCCATGCAGAATAAAACAAATTATAAACATGTGGCAGGGGGTAGGACGAGCAA
TGTATGCCCCTCCCATTGCAGGAAACATAACATGTAGATCAAAC---
ATCACAGGACTAATATTGACACGTGATGGAGGGCAGGGAGAGAAT----------------------------
-------------------------------------------------------------------------
----------
GACACAAATGAGATATTTAGACCTGCAGGAGGAGATATGAGGGACAATTGGAGAAGTGAATTATACAAATATA
AAGTGGTAGAAATTCAGCCATTA---GGAGTAGCACCCACTAAGGCAAAAAGGAGAGTGGTG-----------
----------------------------GAGAGAGAAAAAAGAGCA---GCT------TTGGGAGCT------
---GTGTTCCTTGGGTTCTTGGGAGCA---GCAGGAAGCACTATG---GGCGCGGCATCA---
ATAATGCTGACGGTACAG---GCCAGACAA---CTGTTG---TCTGGTATAGTGCAACAGCAAAGC---
AATTTG---CTGAGA---GCTGTAGAGGCGCAACAGCATATGTTGCAACTCACGGTCTGGGGCATT
>AF391249..1
TTCTATTGCAATACATCAGAACTGTTTAATAGTACATACATGCCTAATGGTACAGAAAGTAAT----------
-------------------------------------------------------------------------
----------------------------
ACAAGCAAAACCATCATACTCCCATGCAGAATAAAACAAATTATAAATATGTGGCAGGGGGTAGGACGAGCAA
TGTATGCCCCTCCCATTGCAGGAAACATAACATGTCAATCAAAT---
ATCACAGGAATACTATTGACCCGTGATGGAGGAGAAGAGTCAAAGTCA-------------------------
-------------------------------------------------------------------------
----------
AATGGAACAGAGATATTCAGGCCTGCAGGAGGGGATATGAAGGACAATTGGAGAAGTGAATTATATAGATATA
AAGTGGTAGAAATTAAACCATTA---GGAGTAGCACCCACTGAGGCAAAAAGGAGAGTGGTG-----------
----------------------------GAGAGAGAAAAAAGAGCA---GTGGGA---ATAGGAGCT------
---GTGTTCCTTGGGTTCTTGGGAGCA---GCAGGAAGCACTATG---GGCGCGGCGTCA---
ATAACGCTGACGGTACAG---GCCAGACAA---CCGTTT---TCTGGTATAGTGCAACAGCAAAGC---
AATTTG---CTGAGG---GCTATAGAGGCGCAACAGCATATGTTGCAACTCACAGTCTGGGGCATT
>AY522721..1
TTCTATTGCAACACATCAAGGCTGTTTAATGAGACAGTAAAA-------------------------------
-------------------------------------------------------------------------
----------------------------
AATAATGCAACCATCACAATCCCATGCAAAATAAAACAATTTATAAACATGTGGCAAAAGGTAGGACAAGCAA
TGTATGCCCCTCCCATTAAAGGAAACATAACATGTACATCAAAT---
ATCACAGGACTACTATTGACACGTGATGGAGGGAATCACAACAATAGC-------------------------
-------------------------------------------------------------------------
----------
CATAGCACAGAGATATTCAGACCTGGAGGAGGAGACATGAAAGATAACTGGAGAAGTGAATTATATAAATATA
AAGTGGTAGAAATTAAGCCATTG---GGCATAGCACCCACTGGGGCAAAAAGGAGAGTGGTG-----------
----------------------------GAGAGAGAAAAAAGAGCA---GTGGGA---ATAGGAGCT------
---GTGTTCCTTGGGTTCTTGGGAGCA---GCAGGAAGCACTATG---GGCGCAGCGTCA---
GTGACGCTGACGGTACAG---GCCAGACAA---TTGTTG---TCTGGTATAGTGCAACAGCAAAAC---
AATTTG---CTGAGA---GCTATAGAGGCGCAACAACATCTGTTGCAACTCACGGTCTGGGGCATA
>DQ447267..2
TTCTACTGCAATACATCAGGACTGTTTAATAGAAGGTATAATGGTACAGAAAATAATACTTATGATGATACA-
-------------------------------------------------------------------------
----------------------------
GATAATTCAACCATCACAATTCCATGCAGAATAAAACAATTTATAAACATGTGGCAGCAGGTAGGACGGGCAA
TATATGCCCCTCCCATTGCAGGAAACATAACATGTAATTCAAGT---
ATCACAGGATTACTATTGACACGCGATGGAGGAAATCAGACTGAGGAGGAC----------------------
```


AATATCACAAAGACAGAGATATTCAGACCAGGAGGAGGAGATATGAGGGATAATTGGAGAAGTGAACTATATA
AATATAAAGCGGTAGAAATTAAGCCATTA---GGAGTAGCGCCCACTGGAGCAAAAAGGAGAGTGGTG-----
----------------------------------GAGAGAGAAAAAAGAGCA---GTGGGA---
ATAGGAGCT---------GTACTCCTTGGGTTCTTGGGAGCA---GCAGGAAGCACTATG---
GGCGCAGCGTCA---ATAGCGCTGACGGCACAG---GCCAGACAA---GTGTTG---
TCTGGAATAGTGCAACAGCAAAAC---AATTTG---CTGAGA---
GCTATAGAGGCGCAACAGCATATGTTGCAACTCACAGTCTGGGGCATT
>JX905394..1
TTCTACTGCAATACATCAAAACTGTTTAATGGTACATATAATGGTACATATAATGGTACA-------------
----

GAAAATAGCACCATTACCCTCCCATGCAAAATAAAACAAATTATAAACATGTGGCAAGGGGTAGGAAGAGCAA
TGTATGCCCCGCCCATTCAAGGAAATATAACATGTGAATCAAAC---
ATCACAGGACTACTATTGACACGTGATGGAGGCACAAATAATACT----------------------------
----

AATAGTACAGAGACATTCAGACCTGGAGGAGGAAATATGAAGGACAATTGGAGAAGCGAATTATATAAATATA
AAGTGGTAGAAATTAAGCCATTA---GGACTAGCACCCACTAATGCAAAAAGGAGAGTGGTG-----------
----------------------------GAGAGAGAAAAAAGAGCA---GTGGGA---ATAGGAGCT------
---GTGTTCCTTGGGTTCTTGGGAGCA---GCAGGAAGCACTATG---GGCGCAGCATCA---
ATAACGCTGACGGTACAG---GCCAGACAA---TTATTG---TCTGGTATAGTGCAACAGCAAAGC---
AATTTG---CTGAGA---GCTATAGAGGCGCAACAGCATATGTTGCAACTCACGGTCTGGGGCATT
>HQ625577..1
TTCTATTGCAATACATCAGGGCTGTTTAATAGAACATACCTGTCTAATGAAACATACCTGTCTAATGATACAC
TGACTAATGGT--

ACAAATAGGAATATAACACTCCCATGCAAAATAAAACAAATTATAAACATGTGGCAGGGGGTAGGAAAAGCAA
TGTATGCCCCTCCCATTGAAGGAAACATAACATGTAGATCAAAT---
ATCACAGGAATACTATTAACACGTGATGGAGGGAAAAGTGAGGGA----------------------------
----

ACGAAAAATGGTACAGAGATATTCAGACCTGGAGGAGGAGATATGAAGGACAATTGGAGAAATGAATTATATA
AATATAAAGTGGTAGAAATTCAGCCATTA---GGAGTAGCACCCACTAAGGCAAAAAGGAGAGTGGTG-----
----------------------------------GAGAGAGAAAAAAGAGCA---GTGGGA---
CTGGGAGCT---------GTGCTCTTTGGGTTCTTGGGAGCA---GCAGGAAGCACTATG---
GGCGCAGCGTCA---ATAACGCTGACGGTACAG---GCCAGACAA---TTGCTG---
TCTGGTATAGTGCAACAGCAAAGC---AATTTG---CTGAGA---
GCTATAGAGGCGCAACAGCATATGTTGCAACTCACGGTCTGGGGCATT
>HQ625568..1
TTCTATTGCAATACAACAAATCTGTTTAATACAACAGACCTGTTGAGTAACAATAGTACGTTAGAAAAC----
----

AAAACAAAAACCATCACACTCCCATGCAAAATAAAACAAATTATAAACATGTGGCAGGAAGTAGGACGAGCAA
TGTATGCCCCGCCCATTGCAGGAAACATAACATGTAGATCAAAT---
ATCACAGGACTACTATTGACCCGTGATGGAGGGAGCTATAGTGATAGA-------------------------
----
GGGAATAGTAGTGCAAATGAAACA---------
GAAAAAGAAATATTCAGACCAGGAGGAGGAAATATGAAGGACAATTGGAGAAGTGAATTATATAAATATAAGG
TGGTAGAAGTTAAGCCATTG---GGAGTAGCACCCACTGAGGCAAAAAGGAGAGTGGTG--------------
-------------------------GAGAGACAAAAAAGAGCA---GTGGGA---CTAGGAGCT---------
ATGTTCCTTGGGATCTTGGGAATA---GCAGGAAGCACTATG---GGCGCGGCGTCA---
ATCACGCTGACGGTACAG---ACCAGACAA---CTGTTG---TCTGGTATAGTGCAACAGCAAAGC---
AATTTG---CTGAGA---GCTATAGAGGCGCAACAGCATATGTTGCAACTCACGGTCTGGGGCATT
>HQ625588..1
TTCTATTGTAATACATCACAACTGTTTAATGGTGTGTATAATACTTCAACCAGCAGTAATTCAACCGAT----
----

AGTAAGAAAAACATCACACTCCCATGCAGAATAAAACAAATTATAAACATGTGGCAAGAGGTAGGACGAGCAA

TGTATGCCCCTCCCATTGCAGGAAACATAACATGTATCTCAAAC---
ATCACAGGATTACTATTGACACGGGATGGAGGAACAAACAATGAGACT-------------------------
----

AATGGTACAGAGACATTCAGGCCTGGAGGAGGAAATATGAAGGACAATTGGAGAAGTGAATTATATAAGTATA
AGGTAGTGGAAATTCAACCATTA---GGAATAGCACCCACTGATGCAAAAAGGAGAGTGGTG-----------
----------------------------GAGAGACAGAAAAGAGCAGTAATAGGA---CTAGGAGCT------
---ATGTTCCTTGGGATCTTAGGAGTA---GCAGGAAGCACTATG---GGCGCAGCATCA---
ATAACGCTGACGGTACAG---GCCAGACAA---TTGTTG---TCTGGTATAGTGCAACAGCAAAGT---
AATTTG---CTGAAG---GCTATAGAGGCGCAACAGCATATGTTGCAACTCACAGTCTGGGGCATT
>AB254156..1
TTCTATTGCGACACATCAAACCTGTTTAACAGCACATTTAATGTTAACAGCACATTTAATGGTACAGATAATG
CA---

AATAGCACACAGAACATCACACTCCAATGCAGAATAAAACAAATTATAAACATGTGGCAGGAGGTAGGACGAG
CAATGTATGCCCCTCCCATTGCAGGAAACATAACATGTATTTCAAAT---
ATCACAGGAATACTATTAACACGTGATGGA---AATGATAATTAC----------------------------
----

ACAGAGACATTCAGACCTATAGGAGGAAATATGAAGGACAATTGGAGAAGTGAATTATACAAATATAAAGTGG
TAGAAATTAAGCCATTA---GGAGTAGCACCCACCAAGGCAAAAAGGAGAGTGGTG-----------------
----------------------GAGAGACAAAAAAGAGCA---GTGGGA---CTAGGAGCT---------
GTGTTCCTTGGGTTCTTAGGAGCA---GCAGGAAGCACTATG---GGCGCAGCGTCA---
ATAACGCTGACGGTACAG---GCCAGACAA---TTGTTG---TCTGGTATAGTGCAACAGCAAAGC---
AATTTG---CTGAGA---GCTATAGAGGCGCAACAGCACATGTTGCAACTCACAGTCTGGGGCATA
>KY229510..1
TTCTATTGCAAGACATCACGACTATTTAATGGCACATACAGAGGTCCTAATGATACAAAAAGCAAT-------
----

TTAAGTGAGACTATCACACTCCAATGCAAAATAAAACAGATTATAAACACGTGGCAGGGGGTAGGACGAGCAA
TATATGCCCCTCCCATTGCGGGAAACATAACATGTAAATCAAAT---
ATCACAGGACTACTCTTGGTAAGGGATGGAGGGAATAATACAAAT----------------------------
----

GAAGAAATATTTAGACCTGGAGGAGGAGATATGAGGGACAATTGGAGAAGTGAATTATATAAATATAAAGTAG
TAGAAATTAAGCCATTG---GGAATAGCACCCACCAAGGCAAAAAGGAGAGTGGTG-----------------
----------------------GAGAGAGAAAAAAGAGCA---GTGGGA---ATAGGAGCT---------
GTACTCCTTGGGTTCTTGGGAACA---GCAGGAAGCACTATG---GGCGCAGCGTCA---
ATAACGCTGACGGTACAG---GCCAGACAA---GTGTTG---TCTGGTATAGTGCAACAGCAAAGC---
AATTTG---CTGAGG---GCTATAGAGGCGCAGCATCATATGTTGCAACTCACGGTCTGGGGCATT
>HM036864..1
TTCTATTGTAATACATCAGGCCTGTTTAATGGTACATTTAATGGAACATACCCTAATAGTACAGACCCTAAT-
----

GGAAATGATACCATCACACTCCCATGCAGAATAAAACAGTTTATAAACATGTGGCAGAAGGTAGGACGAGCAA
TGTATGCCCCTCCCATTAAAGGAAACATAACATGTAACTCAAAT---
ATCACAGGATTACTGTTGACGAGTGATTATGGAAGAAATGCAAGT----------------------------
----

AAAAACGAGACATTCAGACCTGGAGGAGGAGATATGAGGGACAATTGGAGAAGTGAATTATATAAATATAAAG
TGGTAGAAATTAAACCATTG---GGAGTAGCACCCACTGGAGCAAAAAGGAGAGTGGTG--------------
-------------------------GAGAGAGAAAAAAGAGCA---GTGGGA---ATAGGAGCT---------
GTTCTCCTTGGGTTCTTGGGAGCA---GCAGGAAGCACTATG---GGCGCAGCGTCA---
ATAACGCTGACGGTACAG---GCCAGACAA---TTGTTG---TCTGGTATAGTGCAACAGCAAAGC---
AATTTG---CTGAGG---GCTATAGAGGCGCAACAGCATATGTTGCAACTCACGGTCTGGGGCATT
>DQ422948..2
TTCTATTGCAATACAACAAAACTGTTTCATGAGGGAAGCAGGAAAAAT-------------------------
----

GATAATGATACAATCACACTTCCATGCAGAATAAAACAATTTATAAACATGTGGCAGGAGGTAGGACGAGCAA

TGTATGCACCTCCCATTGCAGGAAACATAACATGTAATTCAAGC---
ATCACAGGAATACTATTGGTACGTGATGGAGGTACCAATAACACA-------------------------
----

AATGAGACATTCAGACCTGGAGGAGGAGATATGAGGGACAATTGGAGAAGTGAATTATATAAATATAAAGTGG
TAGAAATTCAACCATTG---GGAATAGCACCCACTAAGGCAAAAAGGAGAGTGGTG-----------------
---------------------GAGAGAGAAAAAAGAGCA---GTGGGA---CTAGGAGCT---------
GTGTTCCTTGGGTTCTTGGGAGCA---GCGGGAAGCACTATG---GGCGCGGCGTCA---
ATAACGCTGACGGCACAG---GCCAGACAA---TTGCTG---TCTGGTATAGTGCAACAGCAAAGC---
AATTTG---CTGAGA---GCTATAGAGGCGCAACAGCATATGTTGCAACTCACAGTCTGGGGCATT
>EU166653..1
TTCTATTGCAATACATCAAGACTGTTTAATAGTACACACTGGGCTAATGGTACTACAGACATGTCTAAT----
----

GATACTAAGAATATCACCCTCCCATGCAAAATAAAACAAATTATAAACATGTGGCAGGGGGTAGGACGAGCAA
TATATGCCCCTCCCATTGAAGGAAGCATAACATGTAATTCAAGT---
ATCACAGGACTACTATTGACACGTGATGGAGGAAACAGTCATAAT-------------------------
----

ACTACAGAGACATTCAGACCTGGAGGAGGAGATATGAGAGACAATTGGAGAAGTGAATTATATAAATATAAGG
TGGTAGAAATTAAGCCATTA---GGACTAGCACCCACTGAGGCAAAAAGGAGAGTGGTG-------------
-------------------------CAGAGAGAAAAAAGAGCA---GTGGGG---ATAGGAGCT---------
GCATTCCTTGGGTTCTTGGGATTA---GCAGGAAGCACTATG---GGCGCGGCATCA---
ATAACGCTGACGGTACAG---GCCAGACAA---TTATTG---TCTGGTATAGTGCAACAGCAAAGC---
AATTTG---CTGAGA---GCTATAGAGGCGCAACAGCATATGTTGCAACTCACAGTCTGGGGCATT
>KY229617..1
TTCTATTGCGAAACATCAGACCTGTTTAATAGTACATTTAGATTTAATGGTACAGACATACAGACCACATTTA
ATGATACA--

TTGAATGATACACAGATCATCATCAAATGCAGAATAAAGCAAATTATAAACATGTGGCAAGGGGTAGGGCGAG
CAATATATGCCCCTCCCATTGAAGGAAATATAACATGTGAATCAAAT---
ATCACAGGATTAATATTGACACGGGATGGAGGGAATACCACAGAT-------------------------
----

CCAGAGACATTCAGACCTGCAGGAGGAGATATGAGGGACAATTGGAGAAGTGAATTATATAAATATAAAGTGG
TAGAAATTCAGCCATTA---GGAGTAGCACCCACAAAGGCAAAAAGGAGAGTGGTGGAG-------------
---------------------AGAAGAGAAAAAAGAGCA---GTGGGA---ATAGGAGCT---------
GTGTTCCTTGGGTTCTTGGGAGCA---GCAGGAAGTACTATG---GGCGCAGCATCA---
ATAACGCTGACGGTACAG---GCCAGACAA---CTGTTG---TCTGGTATAGTGCAACAGCAGAGC---
AATCTG---CTGAGG---GCTATAGAGGCGCAACAGCATATGTTGCAACTCACGGTCTGGGGCATT
>HM036739..2
TTCTATTGCAATACATCAGGCCTGTTTAATTATACATTTACAGCTAATGATACAAAGATACAGATCACATCTA
ATGAC---

ACACTTAATGATACACTGACCATCAAATGCAGAATAAAGCAAATTATAAACATGTGGCAGGGGGTAGGACGAG
CAATGTATGCCCCTCCCATTGAAGGAATAATAGCATGTAATTCAACT---
ATCACAGGATTAATATTGACACGGGATGGAGGGAAGAATACCAGTAAC----------------------
----

AATGACACAGAGACATTCAGACCTGCAGGAGGAGATATGAGGGACAATTGGAGAAGTGAATTATATAAATATA
AAGTGGTAGAAATTCAGCCATTA---GGAGTAGCACCCACAAAGGCAAAAAGGAGAGTGGTGGAG--------
---------------------------AGAAGAGAAAAAAGAGCA---GTGGGA---ATAGGAGCT------
---GTGTTCCTTGGGTTCTTGGGAGCA---GCAGGAAGCACTATG---GGCGCAGCATCA---
ATAACGCTGACGGTACAG---GCCAGACAA---CTGTTG---TCTGGTATAGTGCAACAGCAGAGC---
AATCTG---CTGAGG---GCTATAGAGGCGCAACAGCATATGTTGCAACTCACGGTCTGGGGCATT
>GU329051..1
TTCTATTGCGATACATCAGGCCTGTTTGATTCAAGCCTGTTGAATAAGACAGAAAATAGTACAAAGACAGAA-
----

AATAATACAAACATCATACTCCAATGCAGAATAAAACAAATTATAAACATGTGGCAAGAGGTAGGAAAAGCAA

TATATGCCCCTCCCATTGAAGGAAACATAACATGTATATCAAAC---
ATCACAGGAATACTATTGACGAGGGATGGAGGAAACAATAACAAT----------------------------
----

GACACAGAGATATTCAGACCTGGAGGAGGGAATATGAAAGACAATTGGAGAAGTGAATTATATAAATATAAAG
TAGTAGAAATTCAGCCATTG---GGAATAGCACCTACCGAGGCAAAGAGAAGAGTGGTG--------------
-------------------------GAGAGAGAAAAAAGAGCA---GTGGGA---ATAGGAGCT---------
GTGTTCCTTGGGTTCTTGGGAGCA---GCAGGAAGCACTATG---GGCGCAGCGTCA---
ATGACGCTGACGGTACAG---GCCAGACAA---TTGTTG---TCTGGTATAGTGCAACAGCAAAGC---
AATTTG---CTGAGA---GCCATAGAGGCGCAACAGCATATGTTGCAACTCACAGTCTGGGGCATT
>GU329105..1
TTCTACTGCAATACATCAGACCTGTTTAAGGGTGAATTTAATGGTACGCGTAATCTAGGAAATGAT-------
----

ACTTCAGGAAACATCACACTCAACTGCAGAATAAAACAAATTATAAACATGTGGCAGGAGGTAGGGCGAGCAA
TGTATGCCCCTCCCATTGCAGGAAATATAACATGTAACTCAAGT---
ATCACAGGACTACTATTGACACGTGATGGAGGAGAAACTAATGAA----------------------------
----

ACTGAGAGTGAGACATTTAGACCTGGAGGAGGAGATATGAGGGACAATTGGAGAAGTGAATTATATAAATATA
AAGTGGTAGAAGTTAATCCATTA---GGAATAGCACCCACTAATGCAAGAAGGAGAGTGGTGACA--------
-------------------AAAGAAGAAGTGAGAAAGAAAAGAGCA---GTGGGA---CTAGGAGCT------
---GTGTTCCTTGGGTTCTTGGGAGCA---GCAGGAAGCACTATG---GGCGCGGCGTCA---
ATAACGCTGACGGTACAG---GCCAGACAA---TTACTG---TCTGGTATAGTGCAACAGCAAAAC---
AATTTG---CTGAGA---GCTATAGAGGCGCAACATCATATGTTGCAACTCACAGTCTGGGGCATC
>GU329116..1
TTCTATTGCAATACATCAGGCCTGTTTAATAAGACATACAATGATACA-------------------------
----

AGTGATAAAAACATCACACTCCCATGCAGGATAAAACAAATTATAAACATGTGGCAGAAGGTAGGACAGGCAA
TGTATGCCCCTCCCATTAGAGGAAACATAACATGTCACTCAAAT---
ATCACAGGGCTATTATTGGTACGTGATGGGGGAAACACAAACAATACTGACACA------------AAT----
----
AATACTGATTCAAAT---
AGAACTGAGATATTTAGACCTGGAGGAGGAGATATGAGAGACAATTGGAGAAGTGAATTGTACAAATATAAAG
TGGTAGAAATTAAGCCATTG---GGAATAGCACCCACTGGAGCAAAAAGGAGAGTGGTG--------------
-------------------------GGGAGAGAGAAAAGAGCA---GCGGGA---ATAGGAGCT---------
TTACTCCTTGGGTTCTTGGGAGCT---GCAGGAAGCACTATG---GGCGCGGCGTCA---
ATAACGCTGACGGTACAG---GCCAGACAA---TTATTG---TCTGGTATAGTGCAACAGCAAAGC---
AATTTG---CTGAGA---GCCATAGATGCGCAACAGCATATGTTGCAACTCACGGTCTGGGGCATT
>GU329220..1
TTCTATTGCAATACATCAAACTTATTCAATAGATCACTGTCTAATGATACAGAAGAAGATACAGAC-------
----

AGTAATAGAACCATCACACTCCCATGTAGAATAAAACAGATTATAAACATGTGGCAGGAAGTAGGAAGAGCAA
TGTATGCCCCTCCCATTGCAGGAAACATAACATGTGTATCAAAT---
ATCACAGGACTACTCCTGGTGCGGGATGGAGGCCATCAAAATAGCACAGAG----------------------
----

AATAATAACACAGAGATATTCAGACCTGGAGGAGGAGATATGAGGGACAATTGGAGAAGTGAATTATATAAAT
ACAAAGTAGTAGAAATTAAACCATTA---GGAATAGCACCTACCAAGGCACGGAGGAGAGTGGTG--------
-------------------------------CAGAGAGAAAAAAGAGCA---GTGGGA---ATAGGAGCT---
------GTACTCCTTGGGTTCTTGGGAACA---GCAGGAAGCACTATG---GGCGCAGCGTCA---
ATAACGCTGACGGTACAG---GCCAGACAA---TTGCTG---TCTGGTATAGTGCAACAGCAAAGC---
AATTTG---CTGAGA---GCTATTGAGGCGCAACAGCATATGTTGCAACTCACAGTCTGGGGCATT
>GU329240..1
TTCTATTGCAATACATCAGATCTGTTTAATTCATCAAAACTGTTTAATACAAACAATACTGAG----------
----

AACTTAACAATAATCACCATTCCATGCAGAATAAAACAAATCATAAACATGTGGCAGGAGGTAGGACGAGCAA

TGTATGCTCCTCCCATTGCAGGAAACATAACATGTACATCAGAC---
ATCACAGGATTACTATTGGTACGTGATGGAGGCACAGAAAGTAGC----------------------------
----

ACAAAAGAGATATTCAGACCTGGAGGAGGAGATATGAGGGATAATTGGAGAAGTGAATTATACAAATATAAAG
TGGTAGAAATTAAGCCATTG---GGAATAGCACCCACCGAGGCAAAAAGGAGAGTGGTG--------------
-------------------------GAGAGAGAAAAAAGAGCA---GTGGGA---CTAGGAGCT---------
TTGTTCCTTGGGTTCTTGGGAGTA---GCAGGAAGCACTATG---GGCGCGGCGTCA---
ATAACGCTGACGGTACAG---GCCAGACAA---CTGTTG---TCTGGTATAGTGCAGCAGCAAAGC---
AATTTG---CTGCAG---GCTATAGAGGCTCAACAGCATATGTTGAAACTCACGGTCTGGGGCATT
>GU329324..1
TTCTATTGCAATACATCAGGGTTGTTTAATGGTACATACAATAGTACAGACAGTAAT----------------
----

TCAAACTCAACCATCACAATTCCATGCAGAATAAAACAAATTATAAACATGTGGCAGGAAGTAGGACGAGCAA
TATATGCCCCTCCCATTGCAGGAAACATAACATGTAACTCAAGT---
ATCACAGGACTACTATTGGTACGTGATGGGGGCAGCAACGCCACT----------------------------
----

AATGAAACATTCAGACCTCAAGGAGGAAATATGAAAGACAATTGGAGAAGTGAATTATATAAATATAAAGTGG
TAGAAGTTAAGCCATTA---GGAATAGCACCAACTGAAGCAAAAAGGAGAGTGGTG-----------------
----------------------GAGAGAGAAAAAAGAGCA---GTAGGA---ATAGGAGCT---------
GTGTTCCTTGGGTTCTTGGGAGCA---GCAGGAAGCACTATG---GGCGCAGCGTCA---
ATAACGCTGACGGTACAG---GCCAGACAA---CTGCTG---TCTGGTATAGTGCAACAGCAAAGC---
AATTTG---CTGAGG---GCTATAGAGGCGCAACAGTATATGTTGCAACTCACAGTCTGGGGCATC
>GU329333..2
TTCTATTGCAATACATCAAATCTGTTTAATAGACACCTGCTCAATAGTAAAGGTATGTCTAATAATACAGAA-
----

AGTAATTCAACAATCATACTCCCATGCAAAATAAAACAAATTGTAAACATGTGGCAGGAGGTAGGACGAGCAA
TGTATGCCTCTCCCATTGCAGGAAACATAACATGTAAATCAAAT---
ATCACAGGACTATTATTGGTACGTGATGGGGGAAGTAGCATAAAT----------------------------
----

CAAACAGAGATATTTAGGCCTCAAGGAGGAGACATGAGGGACAACTGGAGAAGTGAGTTATATAAATATAAAG
TGGTAGAAATTAAACCATTG---GGAGTAGCACCCACTGAGGCAAAAAGGAGAGTGATG--------------
-------------------------CAGAGAGAAAAAAGAGCA---GTGGGA---ATGGCAGCT---------
GTGTTCCTTGGGTTCTTGGGAGCA---GCAGGAAGCACTATG---GGCGCGGCGTCA---
ATAACGCTGACGGTACAG---GCCAGACAA---TTATTG---TCTGGTATAGTGCAACAGCAAAGC---
AATTTG---CTGAGG---GCTATAGAGGCGCAACAGCATCTGTTGCAACTCACGGTCTGGGGCATT
>GU329368..2
TTCTATTGCAATACAACACCACTGTTTAATTACAGTGCAACAGAGCTGTTTAGTAATAGTAGTAAAAACATTA
GT---

GAAAAGGACACCATCACACTCCAGTGCAGAATAAAACAAATTATAAACATGTGGCAGAAAGTAGGACGAGCAA
TGTATGCCCCTCCCATTGCAGGAAACATAACATGTACATCAAAC---
ATCACAGGACTACTATTGGTCCGGGATGGAGGCGTCAACAGAACA----------------------------
----

AATCACAATGAGACATTTAGACCTGGAGGGGGAGACATGAGAGACAATTGGAGAAGTGAATTATATAAATATA
AAGTGGTAGAGATAAAGCCATTG---GGAATAGCACCAACTGAGGCAAAAAGGAGAGTGGTG-----------
----------------------------GAGAGACAAAAAAGAGCA---GTGGGA---CTGGGAGCT------
---ATGTTCCTTGGGTTCTTGGGAGCA---GCAGGAAGCACTATG---GGCGCGGCGTCA---
ATAGCGCTGACGGCACAG---GCCAGACAA---TTGTTG---TCTGGTATAGTGCAGCAGCAAAAC---
AATTTG---CTGCAG---GCCATAGAGGCGCAACAGCATCTGTTGCAACTCACTGTCTGGGGCATT
>MK749242..1
TTCTATTGCAACACATCAGGGCTGTTTAAGGGAATGTACCTGGATAATAGCACAACCACAGAGAATTCA----
----

ACTTCGCCAAACATCACACTCCACTGCAAAATAAAACAATTTATAAACATGTGGCAGGGGGTAGGTCAAGCAA

TGTATGCCCCTCCCATTGCAGGAAACATAAGATGTGAATCAAAT---
ATCACAGGAATACTGTTAACACGTGATGGAGGAGGGGAAAATAAT----------------------------
----

AACACAAAAGAAACCTTCAGACCTGGAGGAGGAGATATGAGGGACATATGGAGAAGTGAGTTATATAAATATA
AAGTGGTAGAAATTAAGCCATTA---GGAGTAGCACCCACTGAGGCAAAAAGGAGAGTTGTG-----------
----------------------------GAGAGAGGAAAAAGAGCA---GTGGGA---GTAGGAGCT------
---GTGATCTTTGGGTTCTTGGGAGCA---GCAGGAAGCACTATG---GGCGCAGCGTCA---
TTAACGCTGACGGTACAG---GCCAGACAA---TTGCTG---TCTGGTATAGTGCAACAGCAAAGC---
AATTTG---CTGAGA---GCCATAGAGGCGCAACAGCATATGTTGCAACTCACGGTCTGGGGCATT
>KU749426..2
TTCTATTGCAACACATCAGGACTGTTTAATGGTATATACAATACTACAAGTAATTCAAGTGAT----------
----

ACAAATAAAAACATCACACTCCAATGCAGGATAAGACAATTTATAAACTTGTGGCAGGAGGTAGGAAAGGCAA
TGTATGCACCTCCCATTGCAGGAAACATCACTTGTAAATCAAAT---
ATCACAGGAATACTATTAACACGTGATGGAGGAGAAGGAAAGAAT----------------------------
----
-
CAAACAAACAGTAGTCAAGAGATATTCAGACCTGGAGGAGGAAATATAAAGGATAATTGGAGAAGTGAACTAT
ACAAATATAAAGTGGTAGAAATTAAACCATTA---GGACTAGCACCCACTCCGGCAAAAAGGAGAGTGGTG--
-------------------------------------GAGAGAGAAAAAAGAGCA---GTGGGA---
ATAGGAGCT---------GTGTTCCTTGGGTTCTTGGGAGCA---GCCGGAAGCACTATG---
GGCGCGGCGTCA---ATAACGCTGACGGTACAG---GCCAGACAA---CTATTG---
TCTGGTATAGTGCAACAGCAAAGC---AATTTG---CTGAGG---
GCTATAGAGGCGCAACAGCATATGTTGCAACTCACGGTCTGGGGCATT
>KR820394..1
TTCTATTGCAATACATCAAGACTATTCAATGATGATGATAATGATACAGCACTGGCTAGTGATACAGCTTTAA
AT---

AAAAATAAAACCATCACACTCCCATGCAAAATAAAACAGATTATAAACATGTGGCAGGAAGTAGGACGAGCAA
TGTATGCCCCTCCCATTGCAGGGAACATAACATGTAACTCAAAC---
ATCACAGGAATACTATTAACACGTGATGGAGGAAATGGAAATCGC----------------------------
----
ACAGGAAATCAGACAGAGGCCAATCGCAAT---------
CGCACAGAGATATTTAGACCTGCAGGAGGAAATATGAAGGACAATTGGAGAAGTGAATTATATAAATATAAAG
TGGTAGAAATTAAGCCATTA---GGAATAGCACCAACTGAAGCAAAAAGGAGAGTGGTG--------------
-------------------------GAGAGAGAAAAAAGAGCA---GTGGGA---ATAGGAGCT---------
GTGTTCCTTGGGTTCTTGGGAGCA---GCAGGAAGCACTATG---GGCGCGGCGTCA---
ATAACGCTGACGGTACAG---GCCAGACAA---CTGCTG---TCTGGTATAGTGCAACAGCAAAGT---
AATTTG---CTGAGG---GCTATAGAGGCGCAACAGCATCTGTTGCAACTCACGGTCTGGGGCATT
>U88822..1
TTCTACTGCAATACATCAGGACTGTTTAATAGTGCATGGAATATATCAGGACATAGTACAGGGTTA-------
----

AATGATACAATCATAACCATCCCATGCAGAATAAAACAAATTATAAACATGTGGCAGGAAGTAGGAAAAGCAA
TGTATGCCCCTCCCATTGAAGGGCAAATTAATTGTTCATCAAAT---
ATTACAGGGCTACTATTGACAAGAGATGGTGGTGCTAATAATACT----------------------------
----

CAGAATGACACCTTTAGACCTGGAGGAGGAGATATGAGAGACAACTGGAGAAGTGAATTATATAAATATAAAG
TAGTAAAAATTGAACCACTA---GGAGTAGCACCCACCAAGGCAAAGAGAAGAGTGGTG--------------
-------------------------GAAAGAGAAAAAAGAGCA---ATAGGA---CTAGGAGCT---------
ATGTTCCTTGGGTTCTTGGGAGCA---GCAGGAAGCACGATG---GGCGCAGCGTCA---
ATGACGCTGACGGTACAG---GCCAGACAA---GTATTG---TCTGGTATAGTGCAACAGCAAAAC---
AATTTG---CTGAGG---GCTATAGAGGCGCAACAGCATCTGTTGCAACTCACGGTCTGGGGCATT
>AJ277820..1
TTCTACTGCAATACATCAAAACTATTTAATAGTACATGGAGTATAGAGAGTAATATTACAGAGTCA-------
----

AATGATAGTACAATCATACTTCCATGCAGAATAAAACAGATTGTAAACATGTGGCAGAGAGTAGGAAAAGCAA
TGTATGCCCCTCCCATTGAAGGAATAATCAGATGTTCATCAAAT---
ATTACAGGGCTACTATTGCTAAGAGATGGTGGGGTAAATAATCAT----------------------------
----

AGTAATGAGACCTTCAGACCTGGAGGAGGAGATATAAGGGACAATTGGAGAAGTGAATTATATAAATATAAAG
TAGTAAGACTTGAACCACTA---GGAGTAGCACCCACCAGGGCAAAGAGAAGAGTGGTG--------------
-------------------------GAAAGAGAAAAAAGAGCA---ATAGGA---TTAGGAGCT---------
ATGTTCCTTGGGTTCTTGGGAGCA---GCAGGAAGCACGATG---GGCGCAGTGTCA---
ATGACGCTGACGGTACAG---GCCAGACTG---TTACTG---TCTGGTATAGTGCAACAGCAAAAC---
AATTTG---CTGAGG---GCTATAGAGGCGCAACAGCATCTGTTGCAACTCACGGTCTGGGGCATT
>AY371156..1
TTCTACTGCAATACATCAGGACTGTTTAATAGAAATATTAGTACAGAGAGTACTGAAAATAGTACAGGA----
----

GTAAATGATACAATAACCATCCCATGTAAAATAAGACAAATTATAAACATGTGGCAGGGAGTAGGAAAAGCAA
TATATGCCCCTCCCATTGAAGGACAAATTAAATGTTCATCAAAT---
ATTACAGGGCTACTATTGACAAGAGATGGTGGTAATACTTCTTAT----------------------------
----

GAGGAGACCTTCAGGCCTGGAGGAGGAAATATGAAGGACAATTGGAGAAGTGAATTATATAAATATAAAGTAG
TAAGAATTGAACCACTA---GGAGTAGCACCCACCAGGGCAAAGAGAAGAGTGGTG-----------------
----------------------GAAAGAGAAAAAAGAGCA---ATAGGA---TTAGGAGCT---------
ATGTTCCTTGGGTTCTTGGGAGCA---GCAGGAAGCACGATG---GGCGCAGCGTCA---
GTGACGCTGACGGTACAG---GCCAGACAA---TTATTG---TCTGGTATAGTGCAACAGCAAAAT---
AATTTG---CTGAGG---GCTATAGAGGCGCAACAGCATCTGTTGCAACTCACGGTCTGGGGCATT
>FJ388945..1
TTCTACTGTAATACATCAAAACTGTTTAATACAACATGGTCTAGTGAAACATGGGAGAATAGTACGGGAAATA
ATACC--

GAATTAAATGGTACGATAACCATCCCATGTACAATAAAACAAATTATAATCATGTGCCAGGGAGTGGGAGTAG
CAATGTATACCCCTCCCCCTGCAGGAATAATTCAGTGTAAATCAAAT---
ATTACAATTACAGGGCTGACAAGAGATGGTGGTAATAATAGTAAT----------------------------
----

TGTAATGAGACCTTCAGGCCTGGAGGAGGAGATATGAGGGACAATTGGAGAAGTGAATTGTATAAATATAAAG
TAGTAAGAATTGAACCATTA---GGAATAGCACCCACCAGAGCAAAGAGAAGAGTGGTG--------------
-------------------------GAAAGAGAAAAAAGAGCA---ATAGGA---TTAGGAGCT---------
ATGTTCCTTGGGTTCTTGGGAGCA---GCAGGAAGCACGATG---GGCGCAGCGTCA---
ATAGCGCTGACGGCACAG---GCCAGACAA---GTGTTG---TCCGGTATAGTGCAACAGCAAAAT---
AATTTG---CTGAGG---GCGATAGAGGCGCAACAACATCTGTTGCAACTCACGGTCTGGGGCATT
>AF219271..1
TTCTACTGTAATACATCAAAACTGTTTAATAGTAATATTAGTACATCAGAAGGGGCTAATAGTACAAAGTCA-
----

AATAATAGAACAATCACACTCCAATGCAGAATAAAACAAATTATAAATATGTGGCAAGGAGTAGGAAAAGCAA
TGTATGCCCCTCCCATTGAAGGACTAATTAGATGTTCATCAAAT---
ATTACAGGGCTACTATTGACAAGAGATGGTGGTTCAAATACTAGT----------------------------
----

TCTCATGAGACCTTCAGACCTGGAGGAGGAAATATGAAGGACAATTGGAGAAGTGAATTATATAAATATAAGG
TAGTAAAAATTGAACCACTA---GAAGTAGCACCCACCAGGGCAAAGAGAAGAGTGGTG--------------
-------------------------GAAAGAGAAAAAAGAGCA---ATAGGA---CTAGGAGCT---------
ATGTTCCTTGGGTTCTTGGGAGCA---GCAGGAAGCACGATG---GGCGCAGCGTCA---
GTGACGCTGACGGTACAG---GCCAGACAG---TTATTG---TCTGGTATAGTGCAACAGCAAAAT---
AATTTG---CTGAGG---GCTATAGAGGCGCAACAACATCTGTTGCAACTCACAGTCTGGGGCATT
>AF219272..1
TTCTACTGCAATACATCAGGACTGTTTAATAGTACATGGGGTTCAGGACTGTCTAAT----------------
----

```
GGTACTGAGAATATCACCATCCCATGCAGAATAAAACAAATTATAAACATGTGGCAGGGAGTAGGAAAAGCAA
TGTATGCCCCTCCCATTGCAGGAATAATCAGCTGTTCATCAAAT---
ATTACAGGGCTACTATTGACAAGAGATGGAGGTACAAATAGTAGT----------------------------
-------------------------------------------------------------------------
-------------CATAACGAGACCTTC---AGACCT---
GGAGATATGAGAGACAATTGGAGAAGTGAATTATATAAATATAAAGTAGTAAGAATTGAACCCCTA---
GGAGTAGCACCCACCAGGGCAAAAAGAAGAGTGGTG-------------------------------------
--GAAAGAGAAAAAAGAGCA---ATTGGA---TTAGGAGCT---------
TTGTTCCTTGGGTTCTTGGGAGCA---GCAGGAAGCACAATG---GGCGCAGCGTCA---
TTGACGCTGACGGTACAG---GCCAGACAA---TTATTG---GCTGGTATAGTGCAACAGCAAAAC---
AATTTG---CTGAGG---GCTATAGAGGCGCAACAGCATCTGTTGCAACTCACGGTCTGGGGCATT
>HQ595791..2
TTCTACTGCGATACATCAAGACTATTTAATAGTACCTGGACGAATAGTACCTGGGGGAACGATACTAGCTCAA
AT-----------------------------------------------------------------------
----------------------------
AGGAATGATACAATCACACTCCCATGCAGGATAAAACAAATTATAAACATGTGGCAGGAAGTAGGAAAAGCAA
TGTATGCCCCTCCCATTCGAGGACTAATCAGATGCTCATCACGC---
ATTACAGGACTATTGTTGACAAGAGATGGTGGTATCTATAATAGTAGT-------------------------
-------------------------------------------------------------------------
----------
CAGGAAGAAGAGACCTTCAGACCGGGAGGAGGAGATATGAGGGACAATTGGAGAAGTGAATTATACAAATATA
AGGTAGTAAGAATTGAACCACTA---GGTCTAGCACCCACCAGGGCAAAGAGAAGAGTGGTG-----------
----------------------------GAAAGAGAAAAAAGAGCA---ATAGGA---CTGGGAGCT------
---GTGTTCCTTGGGTTCTTAGGAGCA---GCAGGAAGCACAATG---GGCGCAGCGTCA---
CTGACGCTGACGGTACAG---GCCAGACAG---TTACTG---TCTGGTATAGTGCAACAGCAAAAC---
AATTTG---CTGAGG---GCTATAGAGGCGCAACAGCATCTGTTGCAACTCACAGTCTGGGGCATT
>KF716476..1
TTCTACTGCAATACATCAAAATTGTTTACAACAAATATAACAAGTAACACT----------------------
-------------------------------------------------------------------------
----------------------------
AGTGAAGGGAACATCACACTCCCATGCAGAATAAAACAAATTATAAACATGTGGCAGGGAGTAGGAGGAGCAA
TGTATGCCCCTCCTAGGGAAGGATTGATCACATGTTCATCAAAT---
ATCACAGGACTACTGTTGACAAGAGATCAAGAGGGCTCAAATAAC----------------------------
-------------------------------------------------------------------------
-------------
ATTAATGAGACCTTCAGACCTGGAGGAGGAAATATGAAAGACAATTGGAGAAGTGAATTATACAAATATAAAG
TAGTAAGAATTGAACCACTA---GGTCTAGCACCCACCAGGGCAAAAAGAAGAGTGGTG--------------
-------------------------GAAAGAGAAAAAAGAGCA---ATAGGC---CTGGGAGCT---------
ATGTTCCTTGGGTTCTTGGGAGCA---GCAGGAAGCACAATG---GGCGCAGCGTCA---
CTGACGCTGACGGTACAG---GCCAGACAG---TTACTG---TCTGGTATAGTGCAACAGCAAAAC---
AATTTG---CTGAGA---GCTATAGAGGCGCAACAGCATCTGTTGCAACTCACAGTCTGGGGCATT
>FJ866137..1
TTCTACTGCAATACATCAGAACTGTTTAATAATACATGGTTAAATAATACAGGGAACTGGTCATCAGAT----
-------------------------------------------------------------------------
----------------------------
AGTACAGGGATAATCATGCTCCCATGCAGAATAAAACAAATTATAAACATGTGGCAGGGAGTAGGAAAAGCAA
TGTATGCCCCTCCCATTGCAGGACTAATCAATTGTTCATCAAAT---
ATTACAGGACTATTGTTGACAAGAGATGGTGGTGCAAATAGTAGT----------------------------
-------------------------------------------------------------------------
-------------
CAGAATGAGACCTTCAGACCGGGAGGAGGAGATATGAGAGACAATTGGAGAAGTGAATTATACAAATATAAAG
TAGTAAAAATTGAACCACTA---GGATTAGCACCCACCAAGGCAAGGAGAAGAGTGGTG--------------
-------------------------GAAAGAGAAAAAAGAGCA---ATAGGA---CTAGGAGCT---------
ATGTTCCTTGGGTTCTTGGGAGCA---GCAGGAAGCACGATG---GGCGCAGCGTCA---
CTGACGCTGACGGTACAG---GCCAGACAG---TTATTG---TCTGGTATAGTGCAACAGCAAAAC---
AATTTG---CTGAGG---GCTATAGAGGCGCAACAGCATCTGTTGCAACTCACAGTCTGGGGCATT
>FJ866135..1
TTCTACTGCAACACATCAAGACTGTTTAATAATAGTGAATGGAAATCAAATAGTAGTACAGGGGGA-------
-------------------------------------------------------------------------
----------------------------
```

```
AATGAAAGTATAATCATACTCCCATGCAGAATAAAACAAATTATAAACATGTGGCAGGGAGTAGGAAGAGCAA
TGTATGCCCCTCCCATTGAAGGACACATCAATTGTTCATCAAAT---
ATTACAGGACTATTGTTGACAAGAGATGGTGGTGTAACTAATCAG----------------------------
-------------------------------------------------------------------------
----------
AGTTCGAATGAGACCTTCAGACCTGGGGGAGGAGACATGAGAGACAATTGGAGAAGTGAATTATACAAATATA
AAGTAGTAAGAATTGAACCACTA---GGTCTAGCACCCACCAGGGCAAAGAGAAGAGTGGTG-----------
----------------------------GAAAGAGAAAAAAGAGCA---ATAGGA---CTGGGAGCT------
---ATGTTCCTTGGGTTCTTGGGAGCA---GCAGGAAGCACGATG---GGCGCAGCGTCG---
CTGACGCTGACGGTACAG---GCCAGACAG---TTATTG---TCTGGTATAGTGCAACAGCAAAAC---
AATTTG---CTGAGG---GCTATAGAGGCGCAACAGCATCTGTTGCAACTCACAGTCTGGGGCATT
>AY322189..1
TTCTACTGCAACACATCAAACCTGTTTAATAGCATTTGGAATGTTACTAATGGTACTAGCCAGCTGGAGTCAA
ATAGC--------------------------------------------------------------------
-------------------ACA---
GAGTTAGCCGGAAATATAACTCTCCCATGCAGAATAAAGCAAATTATAAACATGTGGCAGGGAGTAGGAATAG
CAATGTATGCCCCTCCCATTGCAGGAATAATCAAATGTTCATCAAAT---
ATTACAGGACTATTGTTAACAAGAGATGGTGGTGCAGATAATAGT----------------------------
-------------------------------------------------------------------------
----------
AGTCAGAATGAGACCTTCAGACCAGGAGGAGGAGATATGAGAGACAATTGGAGAAGTGAATTATACAAATATA
AAGTAGTAAGAGTTGAACCACTA---GGTATAGCACCCACCAAGGCAAAGAGAAGAGTGGTG-----------
----------------------------GAAAGAGAAAAAAGAGCA---ATAGGA---CTGGGAGCT------
---ATGTTCCTTGGGTTCTTGGGAGCA---GCAGGAAGCACGATG---GGCGCAGCGTCA---
GTGACGCTGACGGTACAG---GCCAGACAG---TTACTG---TCTGGTATAGTGCAACAGCAAAAC---
AATTTG---CTGAGA---GCTATAGAGGCGCAACAGCATCTGTTGCAACTCACAGTCTGGGGCATT
>FJ866138..1
TTCTACTGCAGTACATCAGAACTGTTTAATAGTACATGGAATACATCAATTAGTAGTACAAGGAATACATCAA
AT-----------------------------------------------------------------------
----------------------------
AGTACAAGGGATATCAGACTCCCATGCAGAATAAAACAAATTATAAACATGTGGCAGGGAGTAGGAAAAGCAA
TGTATGCCCCTCCCATAGAAGGACTAATCAAATGTTCATCAAAT---
ATTACAGGACTATTGTTGGCAAGAGATGGTGATGTAAACAATAAT----------------------------
-------------------------------------------------------------------------
-------------
AGTCAGGAGACCCTTAGACCTGGAGGAGGAGATATGAGAGACAATTGGAGAAGTGAATTATACAAATATAAAG
TAGTAAGACTTGAACCACTA---GGTCTAGCACCCACCAGGGCAAAGAGAAGAGTGGTG--------------
-------------------------GAAAGAGAAAAAAGAGCA---ATAGGA---CTAGGAGCT---------
ATGTTCCTTGGGTTCTTGGGAGCA---GCAGGAAGCACGATG---GGCGCAGCGTCA---
CTGACGCTGACGGTACAG---GCCAGACAG---TTATTG---TCTGGTATAGTGCAACAGCAAAAC---
AATTTG---CTGAGG---GCTATAGAGGCGCAACAGCATCTGTTGCAACTCACAGTCTGGGGCATT
>FJ866141..1
TTCTACTGCAATACATCAAGACTGTTTAATAGTACATGGGGGGATAATAATACATCAAGTGATACA-------
-------------------------------------------------------------------------
----------------------------
GAGGAAGGTAACATCACCATCCCATGTAGAATAAAACAAATTATAAACATGTGGCAAGGAGTAGGAAAAGCAA
TGTATGCCCCTCCCATTGAAGGACTAATCAGATGTTCATCAAAT---
ATTACAGGATTACTGTTAACATATGATGGGGGTGTAAATAATAAT----------------------------
-------------------------------------------------------------------------
----------
AGTCAGAGTGAGATCTTCAGACCTGGAGGAGGAGATATGAGAGACAATTGGAGAAGTGAATTATACAAATATA
AAGTAGTAAGACTTGAACCACTA---GGTCTAGCACCCACCAAGGCAAAAAGAAGAGTGGTG-----------
----------------------------GAAAGAGAAAAAAGAGCA---ATAGGC---CTAGGAGCT------
---ATGTTCCTTGGGTTCTTGGGAGCA---GCAGGAAGCACGATG---GGCGCAGCGTCA---
TTGACGCTGACGGTACAG---GCCAGACAA---TTATTG---TCTGGTATAGTGCAGCAGCAAAAC---
AATCTG---CTGATG---GCTATAGAGGCGCAACAGCATCTGTTGCAACTCACAGTCTGGGGCATT
>GU204938..1
TTCTACTGCAATACAACTGGACTGTTTAATAATAGTGTATGGAAGAATGATAGTGATACAGCAAAT-------
-------------------------------------------------------------------------
----------------------------
```

AGTACAGAGGTAATCAAACTCCCATGCAGAATAAAACAAATTATAAACATGTGGCAGGGAGTAGGCAAAGCAA
TGTATGCCCCTCCCATTGAAGGACAAATCGAATGTACATCAAAT---
ATCACAGGACTATTGCTGACAAGAGATGGTGGTGTAAGTAATAAT----------------------------
----

AGTCAGAATGAAACCTTTAGGCCTGGAGGAGGAGATATGAGAGACAACTGGAGGAGTGAACTATACAAATATA
AGGTAGTAAGAATTGAACCACTA---GGTCTAGCACCCACCAGGGCAAGGAGAAGAGTGGTG-----------
----------------------------GAAAGAGAAAAAAGAGCA---ATAGGA---CTAGGAGCT------
---ATGTTCCTTGGGTTCTTGGGAGCA---GCAGGAAGCACGATG---GGCGCAGCGTCA---
TTGACGCTGACGGTACAG---GCCAGACAG---TTAATG---TCTGGTATAGTGCAACAGCAAAAC---
AATTTG---CTGAGA---GCTATAGAGGCGCAGCAGCATCTGTTGCAACTCACAGTCTGGGGCATT
>HQ659623..1
TTCTACTGCAATACATCAGGACTGTTTAATAATAGTGAGTGGACAGATACGACACCAAATACGACA-------
----

TCAAATGGTACCATCATACTCCCATGCAGAATAAAACAATTTATAAACCTGTGGCAGGGAGTAGGAAGAGCAA
TGTATGCCCCTCCCATTCAAGGACTAATCAGATGTTCATCAAAT---
ATTACAGGACTATTGTTGACAAGAGATGGTGGAAATAATAATAGT----------------------------
----

AGTCTGAATGAGACCTTCAGACCTGGAGGAGGAGATATGAGAGACAATTGGAGAAGTGAATTATACAAATATA
AAGTAGTAAGAATTGAACCACTA---GGTCTAGCACCCACCAGTGCAAAGAGAAGAGTGGTG-----------
----------------------------GAAAGAGAAAAAAGAGCA---ATAGGA---CTGGGAGCT------
---GTGTTCCTTGGGTTCTTGGGAGCA---GCAGGAAGCACGATG---GGCGCAGCATCA---
CTGATGCTGACGGTACAG---GCCAAACAG---CTATTG---TCTGGTATAGTGCAACAGCAAAAC---
AATTTG---CTGAGA---GCTATAGAGGCGCAGCAACATCTGTTGCAACTCACAGTCTGGGGCATT
>KX907406..1
TTCTACTGTAATACATCAGGACTGTTCAATAGTACATGGGATATTACGAATACTAGTAGCCCAAACAAC----
----

ACAGACCCAATTATCACACTACAATGCAGAATAAAACAAATTATAAACATGTGGCAGGGAGTAGGAAGAGCAA
TATATGCCCCTCCCATTGCAGGGCAAATTCTATGTTCATCAAAT---
ATTACAGGGCTACTATTGACAAGAGATGGTGGTGCAGATAATAGC----------------------------
----

AGTCATAATGAGACCTTCAGACCTGGAGGAGGAAATATGAAGGACAATTGGAGAAGTGAATTGTATAAATATA
AAGTAGTAAAAATTGAACCACTA---GGAATAGCACCCACCAAGGCAAGGAGAAGAGTGGTG-----------
----------------------------GAAAGGGAAAAAAGAGCA---ATAGGA---CTAGGAGCT------
---ATGTTCCTTGGGTTCTTGGGAGCA---GCAGGAAGCACGATG---GGCGCAGCGTCA---
GTGACGCTGACGGTACAG---GCCAGACAG---TTACTG---TCTGGTATAGTGCAGCAGCAAAGC---
AATTTG---CTGAGG---GCTATACAGGCGCAACAGCATCTGTTGCAGCTCACTGTCTGGGGCATT
>HQ615975..2
TTCTACTGTAATACAACAAAACTGTTTAATACAAGTGAATCAAATAGGACAGAG-------------------
----

GTAAATGGAACAATCACACTCCCATGCAGAATAAAACAAATTATAAACATGTGGCAGGGAGTAGGGAAAGCAA
TCTATGCCCCTCCCATTGAAGGACACATCAACTGTTTATCAAAT---
ATTACAGGACTATTGTTGACAAGAGATGGTGTTGCAAACAATAAG----------------------------
----

AGTCAGAATGAGACCTTCAGACCTGGAGGAGGAGATATGAGAGACAATTGGAGAAGTGAATTATACAAATATA
AAGTAGTAAGAATTGAACCACTA---GGTCTAGCACCCACCAAGGCAAAGAGAAGAGTGGTG-----------
----------------------------GAAAGAGAAAAAAGAGCA---ATAGGA---CTAGGAGCT------
---TTGTTCCTTGGGTTCTTGGGAGTA---GCAGGAAGCACAATG---GGCGCAGCGTCG---
ATGACGCTGACGGTACAG---GCCAGGCAA---TTATTG---TCTGGTATAGTGCAACAGCAAAAC---
AATTTG---CTGAGG---GCTATAGAGGCGCAACAGCATCTGTTGCAACTCACAGTCTGGGGCATT
>JX512899..1
TTCTACTGCAATACAACAAGACTGTTCAATAGTACATGGAATACAACTACATCAAATAGTACAGAACAA----
----

AATGATACAAGAATACTATTCCCATGCAGAATAAAACAAATTATAAACATGTGGCAGGGAGTAGGAAAAGCAA
TGTATGCTCCTCCCATTGAAGGACTAATCAGATGTACATCAAAT---
ATTACAGGACTATTGTTGGCAAGAGATGGTGGTGCAAGCAATAAT----------------------------
----

AGTCAGACTGAGACCTTCAGACCTGGAGGAGGAGATATGAGAGACAATTGGAGAAGTGAATTGTACAAATATA
AAGTAGTAAGAATTGAGCCACTA---GGTCTAGCACCCACCAGGGCAAAGAGAAGAGTGGTG-----------
----------------------------GAAAGAGAAAAAAGAGCA---ATAGGA---CTGGGAGCT------
---ATGTTCCTTGGGTTCTTGGGAGCA---GCAGGAAGCACGATG---GGCGCAGCGTCA---
CTGACGCTGACGGTACAG---GCCAGACAG---TTATTG---TCTGGTATAGTGCAACAGCAAAAC---
AATTTG---CTGAGG---GCTATAGAGGCGCAACAGCATCTGTTGCAACTCACAGTCTGGGGCATT
>KF986106..1
TTCTACTGCAATACAACAAAACTGTTTAACAGTACATGGCATAACAGTACATGGAAG----------------
----
AATGATACTGCATTAAATACA------------
ACAGGGGATAAGATAATCACACTCCCATGTAGAATAAAACAAATTATAAACATGTGGCAGGGAGTAGGCAAAG
CAATGTATGCCCCTCCCATTGCAGGACTAATCAGATGTTCATCAAAT---
ATTACAGGACTATTGTTAACAAGAGATGGTGGTAAAACTAAT-------------------------------
----
AATCATAGTGAGACC---
CTTAATGAGACCTTCAGACCTGGAGGAGGAGATATGAGAGACAATTGGAGAAGTGAATTATACAAATATAAAG
TAGTAAGACTTGAACCACTA---GGTCTAGCGCCCACCAAGGCAAAGAGAAGAGTGGTG--------------
-------------------------CAGAGAGAAAAAAGAGCA---ATAGGA---CTAGGAGCT---------
ATGTTCCTTGGATTCTTGGGAGCA---GCAGGAAGCACAATG---GGCGCAACGTCA---
CTGACGTTGACGGTACAG---GCCAGACAG---TTATTG---TCTGGTATAGTGCAACAGCAAAAC---
AATCTG---CTGAGG---GCTATAGAGGCGCAACAGCATCTGTTGCAACTCACAGTCTGGGGCATT
>KF986033..1
TTCTACTGTAATACAACAAAACTGTTTAATAACAATAAT----------------------------------
----
-------------------AGT---
ACTAATGAGACAGTAATCACACTCCCATGCAGAATAAAACAAATTATAAACATGTGGCAGGGAGTAGGAAAAG
CAATGTATGCCCCTCCCATTGCAGGAAGCATTCGCTGTTCATCCAAT---
ATTACAGGACTATTGTTGACAAGAGATGGTGGG--

-
AATAAGACTGCTCAGAATGAAACCTTCAGACCTGGAGGAGGAGATATGAGAGACAATTGGAGAAGTGAATTGT
ACAAATATAAAGTAGTAAAAATTGAACCACTA---GGTCTAGCACCCACCAAGGCAAAAAGAAGAGTGGTG--
-------------------------------------GAAAGAGAAAAAAGAGCA---GTAGGA---
CTAGGAGCT---------ATGTTCCTTGGGTTCTTGGGAGCA---GCAGGAAGCACAATG---
GGCGCAGTGTCA---ATGACGCTGACGGTACAG---GCCAGACAG---GTGTTG---
TCTGGTATAGTGCAACAGCAAAAC---AATTTG---CTGAGG---
GCTATAGAGGCGCAACAGCATCTGTTGCAACTCACAGTCTGGGGCATT
>KF716479..2
TTCTACTGCAATACATCAGGACTGTTTAATAGTACATGGCAGGAAAATAGTACTCTATTAAATAAAACAAATT
AT---
-------------------ACA---
GAAGAAAATAAAACAATCACACTCCCATGCAGAATAAAACAAATTATAAATATGTGGCAGAGAATAGGAAAAG
CAATGTATGCCCCTCCCATTGAAGGACTAATCAAATGTTCCTCAAAT---
ATTACAGGACTATTGTTGGAAAGAGATGGTAGTGTTGAAAATAAT----------------------------
----

AGTCAGGAGACTGTCAGACCTGTAGGAGGAGAGATGAGAAACAATTGGAGAAGTGAATTATACAAATATAAAG
TAGTAAGAATTAAACCAATA---GGTCTAGCACCCACCAGGGCAAAGAGAAGAGTGGTG--------------
-------------------------GAAAAACAAAAAAGAGCA---ATAGGA---CTAGGAGCT---------
ATGTTCCTTGGGTTCTTGGGAGCA---GCGGGAAGCACGATG---GGCGCAGCGTCA---
ATGGCGCTGACGGTACAG---GCCAGACAA---GTACTG---TCTGGTATAGTGCAACAGCAAAAC---
AATTTG---CTGAGG---GCTATAGAGGCGCAACAGCATCTGTTGCAACTCACAGTCTGGGGCATT
>AB485651..1
TTCTACTGTAATACATCAAGATTGTTTAATAGTGAATGGAGATACAATACAACAAGTAAGGAATGGAGTTCTA
ATACAACA---

-------------AATAAT------
ACAGGGGTAAATGAAATCAAACTCCCATGCAGAATAAAACAGCTTATAAACATGTGGCAGAGAGTAGGAAAAG
CAATGTATGCCCCTCCCATTGAAGCACTAATTAACTGTTCATCAAAC---
ATTACAGGACTATTGTTGTCAAGAGATGGTGGTGCAAATAATAAT----------------------------
----

AGTCAGAATGAGACCTTCAGACCTGGAGGAGGAGATATGAGAGACAATTGGAGAAGTGAATTGTACAAATATA
AAGTAATAAGAATTGAACCAATA---GGTCTAGCACCCACCAAGGCAAAAAGAAGAGTGGTG-----------
----------------------------GAAAGGGAAAAAAGAGCA---ATAGGA---CTAGGAGCT------
---ATGTTCCTTGGGTTCTTGGGAGCA---GCAGGAAGCACGATG---GGCGCAGCGTCA---
TTGACGCTGACGGTACAG---GCCAGACAG---TTAATG---TCTGGTATAGTGCAACAGCAAAAT---
AATTTG---CTGAGG---GCTATAGAGGCGCAACAACATCTGTTGCAACTCACAGTCTGGGGCATT
>AJ320484..3
TTCTACTGCAATACATCAAAACTGTTTAACAGTGCATGGAATGACAGTACATGGAATATAGGGAATAATAATA
CAGGG--

TCAGATAATGAGACAATCATTATCCCATGCAGAATAAAACAAATTATAAACATGTGGCAGGGAGTAGGAAAAG
CAATGTATGCCCCTCCCATCGAAGGATGGATCAATTGTGCATCAAAT---
ATTACAGGGCTCTTACTGGTAAGGGATGGTGGTGGTGCAAATGAT----------------------------
----

AGTCAGAACGAGACCTTCAGACCTCAAGGAGGAGATATGAGAGACAATTGGAGAAGTGAATTATACAAGTATA
AAGTAGTAAAAATTGAACCACTA---GGAATAGCACCCACCAAGGCAAAGAGAAGAGTGGTG-----------
----------------------------GAAAGAGAAAAAAGAGCA---ATAGGA---CTAGGAGCT------
---ATGTTCCTTGGGTTCTTGGGAGCA---GCAGGAAGCACGATG---GGCGCAGCGTCA---
TTGACGCTGACGGTACAG---GCCAGACAA---TTATTG---TCTGGTATAGTGCAACATCAAAAC---
AATTTG---CTGATG---GCTATAGAGGCGCAACAGCATCTGTTGCAACTCACAGTCTGGGGCATT
>U08805..3
TTCTACTGCAATACATCAAAATTGTTTAATCATAGTGTAGGGGAGAATAATACTCTATCAATTAGCAACGGG-
----

GTAAATACTAACATCACACTCCCATGCAAAATAAAACAAATTGTAAACATGTGCCAGGGAGTAGGAAAAGCAA
TGTATGCCCCTCCCATTCAAGGACTAATCAGATGTTCATCAAAT---
ATTACAGGACTATTGTTGACAAGAGATGGTGGTAATACTAGTCAG----------------------------
----

AATGAGACCTTCAGACCTGGAGGAGGAGATATGAGAGACAATTGGAGAAGTGAATTATACAAATATAAAGTAA
TAAAAATTGAACCACTA---GGTCTAGCACCCACCAGGGCAAAGAGAAGAGTGGTG-----------------
----------------------GCAAGAGAAAAAAGAGCA---ATAGGA---CTAGGAGCT---------
GTGTTCCTTGGGTTCTTGGGAGCA---GCAGGAAGCACAATG---GGCGCAGCGTCA---
CTGACGCTGACGGTACAG---GCCAGACAG---TTAATG---TCTGGTATAGTGCAACAGCAAAAC---
AATTTG---CTGAGG---GCTATAGAGGCGCAACAGCATCTGTTGCAACTCACAGTCTGGGGCATT
>EU852998..1
TTCTACTGCAATACATCAGGACTGTTTAACACTAATGTGACATCAAATAGTACAGCAAAT-------------
----

CCAAATGGGAACATCACACTCCCATGCAGAATAAAACAAATTGTAAACATGTGGCAGGGAGTAGGAAAAGCAA
TATATGCCCCTCCCATTGAAGGACTAATCAACTGCTCATCAAAT---
ATTACAGGACTATTGTTGGCAAGAGATGGTGGTGCAAATAATAAT----------------------------
----

AGTCAGAATGAGACCTTCAGACCTGCAGGAGGAGATATGAGAGACAATTGGAGAAGTGAATTATACAAATATA
AAGTAGTAAGAATTGAGCCAATA---GGTCTAGCACCTGCCAGGGCAAAGAGAAGAGTGGTG-----------
----------------------------GAAAGAGAAAAAAGAGCA---ATAGGA---CTGGGAGCT------
---ATGTTCCTTGGGTTCTTGGGAGCA---GCAGGAAGCACGATG---GGCGCAGCGTCA---
ATAACGCTGACGGTACAG---GCCAGACAA---GTACTG---TCTGGTATAGTGCAACAGCAAAAC---
AATTTG---CTGAGG---GCTATAGAGGCGCAACAGCATCTGTTGCAACTCACAGTCTGGGGCATT
>JX658585..1
TTCTACTGCAATACATCAGGACTGTTTAATAGTAGTGGTATAACAAATAGTACAGGAATGTTGAATAATAGT-
----

GATATATCAAATATCACAATCCACTGCAGAATAAGACAAATTATAAACATGTGGCAGGGAGTAGGAAAAGCAA
TGTATGCCCCTCCCATTAAAGGACTAATCTACTGTTCATCAAAT---
ATTACAGGACTATTGTTAACAAGAGATGGTGCAGATAATAATAGT----------------------------
----

CCGAATGAAGCCTTCAGACCTGAAGGAGGAAATATGAAAGACAATTGGAGGAGTGAATTATACAAATATAAAG
TAGTAAGAATTGAGCCATTA---GGTCTAGCACCCACCAGGGCAAAGAGAAGAGTGGTG--------------
-------------------------GAAAGAGAAAAAAGAGCA---ATAGGA---CTAGGAGCT---------
ATGTTCCTTGGGTTCTTGGGAACA---GCAGGAAGCACGATG---GGCGCAGCGTCA---
CTGACGCTGACGGTACAG---GCCAGACAA---TTATTG---TCTGGTATAGTGCAACAGCAAAAC---
AATTTG---CTGAGG---GCTATAGAGGCGCAACAGCATCTGTTGCAACTCACAGTCTGGGGCATT
>EU852934..1
TTCTACTGCAATACATCACAGCTGTTTAATAGTACATGGGAAGCCAATACGACAGATAAG-------------
----

CTAAATGAGACAATCACACTCCCATGCAGAATAAAACAAATTATAAACATGTGGCAGGGAGTAGGAAAAGCAA
TGTATGCCCCTCCCATTGAAGGACAAATCACATGTTCATCAAAT---
ATTACAGGGCTTTTATTGACAAGAGATGGTGGTACAAATAATAGT----------------------------
----

AGTCAGAATGAGACCTTCAGACCTGGAGGAGGAGATATGAGAGATAATTGGAGAAGTGAATTATACAAATATA
AAGTAGTAAGAATTGAACCACTA---GGTCTAGCACCCACCAGGGCAAAGAGAAGAGTGGTG-----------
----------------------------GAAAGAGAAAAAAGAGCA---ATAGGA---CTGGGAGCT------
---ATGTTCCTTGGGTTCTTGGGAGCA---GCAGGAAGCACGATG---GGCGCAGCGTCA---
CTGACGCTGACGGTACAG---GCCAGACAA---TTATTG---TCTGGTATAGTGCAACAGCAAAAC---
AATTTG---CTGAGA---GCTATAGAGGCGCAACAGCATCTGTTGCAACTCACAGTCTGGGGCATT
>EU852982..1
TTCTACTGCAATACATCAGGACTGTTTAACAATACGTTTAAATGGGAGAATGGCACTTTATTAAATACTACAG
TAAATATT---
-------------ACAGAG------
AATAGTACAGAGAATATCACACTCCCATGCAGAATAAAACAAATTATAAACATGTGGCAGGGAGTAGGAAAAG
CAATGTATGCCCCTCCCATCGCAGGACTAATTAAATGTTCATCAAAT---
ATTACAGGACTATTATTGACAAGGGATGGTGGTACAAATAATAGT----------------------------
----

CAGGATGATGAGACCTTTAGACCTGGAGGAGGAGATATGAGGGACAATTGGAGAAGTGAATTATATAAATATA
AAGTAGTAAAAATTGAACCACTA---GGTCTAGCACCCACCAAGGCAAAGAGAAGAGTGGTG-----------
----------------------------GAAAGAGAAAAAAGAGCA---ATAGGA---CTGGGAGCT------
---TTGTTCCTTGGGTTCTTGGGAGCA---GCAGGAAGCACGATG---GGCGCAGCGTCA---
ATAACGCTGACGGTACAG---GCCAGACAG---TTATTG---TCTGGTATAGTGCAGCAGCAAAAT---
AATTTG---CTGAGA---GCTATAGAGGCGCAACAGCATCTGTTGCAACTCACAGTCTGGGGCATT
>EU853014..2
TTCTACTGCAATACATCAGGACTGTTCAATCATAGTGTATGGAGCAATAATGGTACATGGAATACTACATCAA
TT---

AATAAAACAGAGAATATCATACTCCCATGCAGAATAAAACAAATTATAAACATGTGGCAAAGAGTAGGAAAAG
CAATGTATGCCCCTCCCATTGCAGGAGTAATCAATTGTGTATCAAAT---
ATTACAGGACTATTGTTGATAAGAGATGGTGGTAATACTAATACTAGTCAT----------------------
----

AATAATGGGACTGAGACCTTCAGACCTGGAGGAGGAGATATGAGAGACAATTGGAGAAGTGAATTATACAAAT
ATAAAGTAGTAAGACTTGAGCCACTA---GGTCTAGCACCCACCAGGGCAAAGAGAAGAGTGGTG--------
-------------------------------GAAAGAGAAAAAAGAGCA---GTAGGA---CTAGGAGCT---
------ATGTTCCTTGGGTTCTTGGGAGCA---GCAGGAAGCACGATG---GGCGCAGCGTCA---
CTGACGCTGACGGTACAG---GCCAGACTG---TTATTG---TCTGGTATAGTGCAACAGCAAAAT---
AATTTG---CTGAGG---GCTATAGAGGCGCAACAGCATCTGTTGCAACTCACAGTCTGGGGCATT
>AF484506..1
TTCTACTGCAATACATCAGGACTGTTTAATAGTACATGGGAAAATGGTACATTAAATAGTGCGGAG-------
----

```
----------------------------
GGAAATGATACAATCACACTCCCATGCAGAATAAAACAAATTATAAACATGTGGCAGGAAGTAGGAAAAGCAA
TGTATGCCCCTCCCATTGAAGGACTAATCCAATGTTCATCAAAT---
ATTACAGGACTATTGTTGACAAGAGATGGTGGTGTAAATAATAGT----------------------------
-------------------------------------------------------------------------
-------------
CAGAATGAGACCTTCAGGCCTGGAGGGGGAGATATGAGAGACAATTGGAGAAGTGAATTATACAAATATAAAG
TAGTAAAAATTGAACCACTA---GGTATAGCACCCACCAAGGCAAAGAGAAGAGTGGTG--------------
-------------------------GAAAAAGAAAAAAGAGCA---ATAGGC---CTGGGAGCT---------
ATGTTCCTTGGGTTCTTGGGAGCA---GCAGGAAGCACGATG---GGCGCAGCGTCA---
CTGACGCTGACGGTACAG---GCCAGACAG---TTATTG---TCTGGTATAGTGCAACAGCAAAAC---
AATTTG---CTGAGG---GCTATAGAGGCGCAACAGCATCTGTTGCAACTCACAGTCTGGGGCATT
>AF484513..1
TTCTACTGCAATACATCAGAACTGTTTAATAGTACATGGGGGAATTCTAGTACATCAACTAGTACAGGGGTA-
-------------------------------------------------------------------------
----------------------------
AATAAGAGTACAATCACTATCCCATGCAGAATAAAACAAATTATAAACATGTGGCAGGAAGTAGGAAAAGCAA
TGTATGCCCCTCCCATTCGAGGACTAATCAAATGTTCCTCAAAT---
ATTACAGGACTATTGTTGGTAAGAGATGGTGGTGCAACTAATAAT----------------------------
-------------------------------------------------------------------------
----------
AGTCAGAATGAGACCTTCAGACCTGGAGGAGGAGATATGAGAGACAATTGGAGAAGTGAATTATACAAATATA
AAGTAGTAAGACTTGAACCACTA---GGTCTAGCACCCACCAAGGCAAAGAGAAGAGTGGTG-----------
----------------------------GAAAGAGAAAAAAGAGCA---ATAGGA---CTGGGAGCT------
---ATGTTCCTTGGGTTCTTGGGAACA---GCAGGAAGCACGATG---GGCGCAGCGTCA---
CTGACGCTGACGGTACAG---GCCAGACAG---TTATTG---TCTGGTATAGTGCAACAGCAAAAC---
AATTTG---CTGAGG---GCTATAGAGGCGCAACAGCATCTGTTGCAACTCACAGTCTGGGGCATT
>AF484518..1
TTCTACTGCAATACAACAGTACTGTTTAATAGCACATGGGAAAATAATAGTACATGGGGTAATGAGAGT----
-------------------------------------------------------------------------
----------------------------
GCAATGGGTAATGTCACACTCCCATGCAGAATAAAACAAATTATAAACATGTGGCAGGGAGTAGGAAAAGCAA
TGTATGCCCCTCCCATTGAAGGACTAATCAGCTGCTCATCAAAT---
ATTACAGGATTACTGTTGACAAGAGATGGTGGTGGAAATGAAAGT----------------------------
-------------------------------------------------------------------------
----------
AGTCAGAATGAGACCTTCAGACCTGGAGGGGGAGATATGAGAGACAATTGGAGAAGTGAATTATATAAATATA
AAGTAGTAAGAATTGAACCATTA---GGTCTAGCACCCTCCAAGGCAAAAAGAAGAGTAGTA-----------
----------------------------GAAAGAGAGAAAAGAGCA---ATTGGA---CTAGGAGCT------
---ATGTTCCTTGGGTTCTTGGGAGCA---GCAGGAAGCACGATG---GGCGCAGCGTCA---
CTGACGCTGACGGTACAG---GCCAGACAG---CTACTG---TCTGGTATAGTGCAACAGCAAAAC---
AATTTG---CTGAGG---GCTATAGAGGCGCAACAGCACCTGTTGCAACTCACAGTCTGGGGCATT
>AY304496..1
TTCTACTGCAATACATCAGGACTGTTTAATGGTGTATGGAAGAATAATACATCAGATAATGAAACGGTAAAT-
-------------------------------------------------------------------------
----------------------------
GGAAGTGAGACAATAACAATCCCATGCAGAATAAAACAAATTATAAACACGTGGCAAGGAGTAGGAAGAGCAA
TGTATGCCCCTCCCATTAGAGGACTAATCCGATGTTCATCAAAC---
ATTACAGGACTATTGTTAACAAGAGATGGTGGTACAAATGGTGCT----------------------------
-------------------------------------------------------------------------
----------
AGTCAGAAGGAGACCTTCAGACCCGGAGGAGGAGATATGAGAGACAATTGGAGAAGTGAATTATACAAATATA
AAGTAGTAAAAATTGAACCATTA---GGTCTAGCACCCACCAGGGCTAAAAGAAGAGTGGTG-----------
----------------------------GAAAGAGAAAAAAGAGCA---ATAGGA---CTAGGAGCT------
---ATGTTCCTTGGGTTCTTGGGAACA---GCAGGAAGCACGATG---GGCGCAGTGTCA---
CTGACGCTGACGGTACAG---GCCAGACAG---GTACTG---TCTGGTATAGTGCAGCAGCAAAAC---
AATTTG---CTGAGG---GCCATAGAGGCGCAACAGCATCTGTTGCAACTCACAGTCTGGGGCATT
>AF484480..2
TTCTACTGCAATACATCAAAACTGTTTAATAATACATTTAATACTAGTGATTGGAGGACTTATAATACATCAA
ATAGT--------------------------------------------------------------------
```

ACAGGGAATGAGACAATCACACTCCCATGCAGAATAAAACAAATTATAAACATGTGGCAGGGAGTAGGAAGAG
CAATGTATGCCCCTCCCATTGAGGGACTAATCAGATGTTCATCAAAT---
ATTACAGGACTATTGTTGACAAGAGATGGGGGTAATGCAAATAATACT-------------------------
----

AATCAAAGTGAGACCTTCAGACCTGGAGGAGGAGATATGAGAGACAATTGGAGAAGTGAATTATACAAATATA
AAGTAGTAAGAATTGAGCCAATA---GGTCTAGCACCCACCAAGGCAAGGAGAAGAGTGGTG-----------
----------------------------GAAAGAGAAAAAAGAGCA---ATAGGA---CTAGGAGCT------
---ATGTTCCTTGGGTTCTTGGGAGCA---GCAGGAAGCACGATG---GGCGCAGCGTCA---
CTGACGCTGACGGTACAG---GCCAGACAG---GTGTTG---TCTGGTATAGTGCAACAGCAAAAC---
AACTTG---CTGAAG---GCTATAGAGGCGCAACAGCATCTGTTGCAACTCACAGTCTGGGGCATT
>AF484485..1
TTCTACTGCAATACATCAGAATTGTTTAATAATAGTGTATGGACATCAAATAATACAGGGATGTCAAATAGT-
----

ACAAAGGCAAATATCACACTCCCGTGCAGAATAAAACAAATTATAAACATGTGGCAGGGAGTAGGAAAAGCAA
TGTATGCCCCTCCCATTGCAGGACAAATCAGATGCTCGTCAAAT---
ATTACAGGATTATTGTTGACAAGAGATGGTGGTGCAAATAATAAT----------------------------
----

AGTCAGAATGAGACCTTCAGACCTGGAGGAGGAGATATGAAAGACAATTGGAGAAGTGAATTATACAAATATA
AAGTAGTAAGACTTGAACCACTA---GGTCTAGCACCCACCAAGGCAAAGAGAAGAGTGGTG-----------
----------------------------GAAAGAGAAAAAAGAGCA---ATAGGA---CTGGGAGCT------
---ATGTTCCTTGGGTTCTTGGGAGCA---GCAGGAAGCACGATG---GGCGCAGCGTCG---
CTGACGCTGACGGTACAG---GCCAGACTA---TTATTG---TCTGGTATAGTGCAACAGCAAAAC---
AATTTG---CTGAGG---GCTATAGAGGCGCAACAGCATCTGTTGCAACTCACAGTCTGGGGCATT
>AF484486..1
TTCTATTGCAATACATCAGGACTGTTTAATAGTACATGGAGGAATGATACATCAAGTAGTACATGGAGGAATG
ATACATCAAGT--
-------------AGTACA------
AAGCCAGATGAGACACTCACACTCCCATGCAGGATAAAACAAATTATAAACCTGTGGCAGGAAGTAGGAAGAG
CAATGTATGCCCCTCCCATTGCAGGAAACATCACATGTTCATCAAAT---
GTTACAGGACTATTGTTGACAAGAGATGGTGGTACACATAATAATAAT-------------------------
----

AGTCAGACTGAGACCATCAGGCCTGGAGGAGGAGATATGAGAAACAATTGGAGAAGTGAATTATACAAATATA
AAGTAGTAAGAATTGAGCCAATA---GGCCTAGCACCCACCAAGGCAAAGAGAAGAGTGGTG-----------
----------------------------GAAAGAGAAAAAAGAGCA---ATAGGC---CTGGGAGCT------
---ATGTTCCTTGGGTTCTTGGGAGCA---GCAGGAAGCACGATG---GGCGCAGCGTCA---
CTGACGCTGACGGTACAG---GCCAGACAA---GTATTG---TCTGGTATAGTGCAACAGCAAAAT---
AATTTG---CTGAGG---GCTATAGAGGCGCAACAGCACCTGTTGCAACTCACAGTCTGGGGCATT
>AF484487..1
TTCTACTGTGATACAACAGGACTGTTTAATGGTACAAATAGTACAGGGGGA----------------------
----

GATAATGAGACAATCACACTCCAATGCAGAATAAAACAAATTATAAACATGTGGCAGGGAGTAGGAAGAGCAA
TGTATGCCCCTCCCATTGCAGGGCAAATCTATTGCTCATCAAAT---
ATTACAGGACTATTGCTGACAAGAGATGGTGGTAACAATGATACG----------------------------
----

GGGGAGGAGATTTTCAGACCTGTAGGAGGAGATATGAGAGACAATTGGAGAAGTGAATTATACAAATATAAAG
TAGTAAGAATTGAACCACTA---GGTCTAGCACCCTCCAAGGCAAGGAGAAGAGTGGTG--------------
-------------------------GAAAGAGAAAAAAGAGCA---ATAGGA---CTAGGAGCT---------
GTGTTCCTTGGGTTCTTGGGAGCA---GCAGGAAGCACGATG---GGCGCAGCGTCG---
CTGACGCTGACGGTACAG---GCCAGACAG---TTATTG---TCTGGTATAGTGCAACAGCAAAAC---
AATTTG---CTGCAG---GCTATAGAGGCGCAACAGCATCTGTTGCAACTCACAGTCTGGGGCATT
>AF484519..2
TTCTATTGCAACACATCAGGACTGTTTAATAGTACATGGACATCAAATAGTACATCAAATAGTACAAAGGTAA
AT---

AGTACTGAGCCAATCACACTCCAATGCAGAATAAAACAAATTATAAATATGTGGCAGGGAGTAGGAAGAGCAA
TGTATGCCCCGCCCATTGAAGGACTAATTCATTGTTCATCAAAT---
ATTACAGGACTACTGTTGACAAGAGATGGTGGTACAAATAATACT----------------------------
----

AGTCAGAATGAGACTTTCAGACCTGGAGGAGGAAATATGAGAGACAATTGGAGAAGTGAATTATACAAATATA
AAGTAGTAAGAATTGAACCAATA---GGTCTAGCACCCACCAAGGCAAAGAGGAGAGTGGTG-----------
----------------------------GAAAGAGAAAAAAGAGCA---ATAGGA---CTAGGAGCT------
---ATGTTCCTTGGGTTCTTGGGAACA---GCAGGAAGCACGATG---GGCGCAGCGTCA---
ATAACGCTGACGGTACAG---GCCAGACAG---TTACTG---TCTGGTATAGTGCAGCAGCAAAAC---
AATTTG---CTGAGG---GCTATAGAGGCGCAACAGCATCTGTTGCAACTCACAGTCTGGGGCATT
>AF484494..1
TTCTACTGCAATACATCAGGACTGTTTAATAATAGTGTATGGAATACTAGTGTATGGAGAAATGATAGTACAT
TA---

GATAATGAGACTATCACACTCCCATGCAGAATAAAACAAATTATAAACATGTGGCAAGGAGTAGGAAAAGCAA
TGTATGCCCCTCCCATTGAAGGACGAATCAGTTGTTCATCAAAT---
ATTACAGGACTATTGTTGACAAGAGATGGTGGTGCAAATAATAAT----------------------------
----

AGTCAGAATGAGACCTTCAGACCTGGAGGAGGAGATATGAGAGATAATTGGAGAAGTGAATTATACAAATACA
AAGTAATAAGAATTGAACCACTA---GGTCTAGCACCCACCAAGGCAAAAAGAAGAGTGGTG-----------
----------------------------GAAAGAGAAAAAAGAGCA---ATAGGA---CTAGGAGCT------
---ATGTTCCTTGGGTTCTTGGGAGCA---GCAGGAAGCACGATG---GGCGCAGCGTCA---
CTGACGCTGACGGTACAG---GCCAGACAG---TTATTG---TCTGGTATAGTGCAGCAGCAAAAC---
AATTTG---CTGAGG---GCTATAGAGGCGCAACAACATCTGTTGCAACTCACAGTCTGGGGCATT
>AF484495..2
TTCTACTGCAACACAACAGGATTGTTTAATCATAGTAAATGGAATAATAATAATACATCAGAATGGAATAAT-
----

ACAGATAAGATAATCACACTCCCATGCAGACTAAAACAAATTATAAACATGTGGCAGGGAGTAGGAAGAGCTA
TGTATGCCCCTCCCATTGAAGGAGAAATTAAATGTTCATCAAAT---
ATTACAGGACTATTGTTAACAAGAGATGGAGGTACAAATAGTACT----------------------------
----

CAGAATGAGACCTTCAGACCTGGAGGGGGAGATATGAGAGACAATTGGAGAAGTGAATTATACAAATATAAAG
TAGTAAGAATTGAACCACTA---GGTCTAGCACCCACCAGAGCAAAGAGAAGAGTGGTG--------------
-------------------------CAAAGAGAAAAAAGAGCA---ATAGGA---CTGGGAGCT---------
ATGTTCCTTGGGTTCTTGGGAGCA---GCAGGAAGCACGATG---GGCGCAACGTCG---
ATGGCGTTGACGGTACAG---GCCAGACAA---TTGTTG---TCTGGTATAGTGCAACAGCAAAAC---
AATTTG---CTGAGG---GCTATAGAGGCGCAACAGCATCTGTTGCAACTCACAGTCTGGGGCATT
>AF484497..1
TTCTACTGCAATACATCAGGACTGTTTAATAGTGAATGGACATCGTCTAAAGGTGAATGGACAGGGGTAGAAA
AGAAT--

AGTACAAGCAGCAACAACAACCTCCCATGCAGAATAAAACAAATTATAAACATGTGGCAGGGAGTAGGAAAAG
CTATGTATGCCCCTCCCATTAAAGGACAAATCAGATGTTCATCAAAT---
ATTACAGGACTATTGTTGACAAGAGATGGTGGTGTAAATAATAAT----------------------------
----

AGTCAGGAGACCTTCAGACCTGGAGGAGGAGATATGAGAGACAATTGGAGAAGTGAATTATACAAATATAAAG
TAGTAAGAATTGAACCACTA---GGCCTAGCACCCACCAAGGCAAAAAGAAGAGTGGTG--------------
-------------------------GAAAGAGAAAAAAGAGCA---ATAGGA---CTAGGAGCT---------
TTGTTCCTTGGGTTCTTGGGAGCA---GCAGGAAGCACAATG---GGCGCAGCGTCA---
CTGACGCTGACGGTACAG---GCCAGACAG---GTATTG---GCTGGTATAGTGCAACAGCAAAAC---
AATTTG---CTGAGG---GCTATAGAGGCGCAACAGCATCTGTTGCAACTCACAGTCTGGGGCATT
>AF484498..2
TTCTACTGCAATACAGCAAGATTGTTTAATTATGAATGGAGTAATAATACATTAGCTAGTACAGGGAATGACA
GT---

AAAGGGAATGACAGTATCACACTCCAATGCAGAATAAAACAAATTATAAACATGTGGCAGGGAGTAGGAAAAG
CAATGTATGCCCCTCCCATTGAGGGACTAATCACCTGTTCATCAAAT---
ATTACAGGACTATTGTTGACAAGAGATGGTGGTGCAAATAATAAT----------------------------
----

AGTCAGAATGAGACCTTCAGACCTGGAGGAGGAGATATAAGGGACAATTGGAGAAGTGAATTATACAAATATA
AAGTAGTAAAACTTGAACCACTA---GGTCTAGCACCCACCAGGGCAAAGAGAAGAGTGGTG-----------
----------------------------GAAAGAGAAAAAAGAGCA---ATAGGA---CTAGGAGCT------
---ATGTTCCTTGGGTTCTTGGGAGCA---GCAGGAAGCACGATG---GGCGCAGCGTCC---
CTGACGCTGACGGTACAG---GCCAGACAG---TTATTG---TCTGGTATAGTGCAACAGCAAAAT---
AATTTG---CTAATG---GCTATAGAGGCGCAACAGCATCTGTTGCAACTCACAGTCTGGGGCATT
>AF484499..1
TTCTACTGCAATACATCAAAGCTGTTTAATAGTACATGGACAGGGGGTGGAAATATCACAAATATCACA----
----

AGTATCACAAATATCACACTCCCATGCAGAATAAAACAAATTATAAACATGTGGCAGGGAGTAGGCAAAGCAA
TGTATGCCCCTCCCATTGAAGGACTAATCCAATGTTCATCAAAT---
ATTACAGGACTATTATTGACAAGAGATGGTGGTGCAACTAATGAT----------------------------
----

AGTCAGAGTGAGACCTTTAGACCTGCAGGAGGAGATATGAGAGATAATTGGAGAAGTGAATTATACAAATATA
AAGTAGTAAGAATTGAACCACTA---GGTCTAGCACCCACCAAGGCAAAGAGAAGAACAGTG-----------
----------------------------GAAAGAGAAAAAAGAGCA---ATAGGA---CTAGGAGCT------
---ATGTTCCTTGGGTTCTTGGGAGCA---GCAGGAAGCACGATG---GGCGCAGCGTCA---
CTGACGCTGACGGTACAG---GCCAGACTG---TTATTG---TCTGGTATAGTGCAACAGCAAAAC---
AATTTG---CTGAGG---GCTATAGAGGCGCAACAGCATCTGTTGCAACTCACAGTCTGGGGCATT
>U36871..1
TCCTACTGCAATACATCAAAACTGTTTAATAGTACATGGAGGAATAATAATACATCAAATAGTACAGGGGTAA
ATAGT--
-------------------ACA---
GGGGAAAATGAGATAATCAAACTCCCATGCAGAATAAAACAAATTGTAAATATGTGGCAGGGAGTAGGAAAAG
CAATGTATGCCCCTCCCATTGAAGGGCTAATCAATTGTTCATCAAAT---
ATTACAGGACTATTGTTGACAAGAGATGGTGGTGCCAATAATAGT----------------------------
----

CAGAATGAGACCTTCAGACCTGGAGGAGGAGATATGAGGGACAATTGGAGAAGTGAATTATACAAATATAAAG
TAGTAAAACTTGAACCACTA---GGTCTAGCACCCACCAGGGCAAAGAGAAGAGTGGTG--------------
-------------------------GAAAGAGAAAAAAGAGCA---ATAGGA---CTAGGAGCT---------
ATGTTCCTTGGGTTCTTGGGAGCA---GCAGGAAGCACGATG---GGCGCAGCGTCA---
CTGACGCTGACGGTACAG---GCCAGACAG---TTAATG---TCTGGTATAGTGCAACAGCAAGAC---
AATTTG---CTGAGG---GCTATAGAGGCGCAACAGCATCTGTTGCAACTCACAGTCTGGGGCATT
>EF575364..1
TTCTACTGCAACACATCAGGACTATTTAATGGTACATGGAGAAATGCTACATCAAATAGTACACAG-------
----

CTGAATGGTACAATCACGCTCCCATGCAGAATAAAACAAATTATAAACATGTGGCAAGAAGTAGGAAAAGCAA
TGTATGCCCCTCCCATTGAAGGATTCATCAATTGTTCATCAAAT---
ATTACAGGACTATTGTTGGTAAGAGATGGTGGTAGTAATGATGAT----------------------------
----

AGTCAGAATGAGACCTTCAGACCTGGAGGAGGAGATATGAGAGACAATTGGAGAAGTGAATTATACAAATATA
AGGTAGTAAGAATTGAACCACTA---GGTCTAGCACCCACCAAGGCAAAAAGAAGAGTGGTG-----------
----------------------------GAAAGAGAAAAAAGAGCA---ATAGGA---CTGGGAGCT------
---ATGTTCCTTGGGTTCTTGGGAGCA---GCAGGAAGCACGATG---GGCGCAGCGTCA---
ATAACGCTGACGGTACAG---GCCAGACAG---TTACTG---TCTGGTATAGTGCAACAGCAAAAC---
AATTTG---CTGAGG---GCTATAGAGGCGCAACAGCATCTGTTGCAACTCACAGTCTGGGGCATT
>EF575370..1
TTCTACTGCAATACATCAAAACTGTTTAATAGTACATGGGAGAATGTGAATGTTACTACATCAAATAATACAG
GG---

CCAGATAGTACAATCACACTCCCATGCAGAATAAAACAAATTATAAACATGTGGCAGGGAGTAGGAAAAGCAA
TGTATGCCCCTCCCATTGAAGGGCTAATCAAATGTTCATCAAAT---
ATTACAGGACTATTGTTACTAAGAGATGGTGGTCTAAATACTAGT----------------------------
----

CAGAGTGAAACCTTCAGACCTGGAGGAGGAGATATGAGGGACAATTGGAGAAGTGAATTATACAAATATAAAG
TAGTAAAAATTGAACCAATA---GGTCTAGCACCTACCAAGGCAAGGAGAAGAGTGATG--------------
-------------------------GAAAGAGAAAAGAGAGCA---ATAGGA---CTGGGAGCT---------
GTGTTCCTTGGGTTCTTGGGAGCA---GCGGGAAGCACAATG---GGCGCAGCGTCA---
CTAACGCTGACGGTACAA---GCCAGACAA---GTATTG---TTTGGTATAGTGCAACAGCAAAAC---
AATTTG---CTGAGG---GCTATAGAGGCGCAGCAGCATCTGCTGCAACTCACAGTCTGGGGCATT
>EF575429..1
TTCTACTGCAATACATCAGGACTGTTTAATAATACATTTAATACTAAGAATAGTACAGAGGTAAATAGTAAT-
----

GGGACAGAGGTAATCATACTCCCATGCAGAATAAAACAAATTACAAACATGTGGCAGAAAGTAGGAAAAGCAA
TGTATGCCCCTCCTATTGAAGGACTAATCAGCTGTTCATCAAAT---
ATCACAGGACTATTGTTGACAAGAGATGGTGGTAATATAACTAGT----------------------------
----

CAGAATGAGACCTTCAGGCCTGCAGGAGGAGATATGAGAGATAATTGGAGAAGTGAATTATACAAATATAAAG
TAGTAAGAATCGAACCACTA---GGTCTAGCACCCACCAAGGCAAGGAGAAGAGTGGTG--------------
-------------------------GAAAGGGAAAAAAGAGCA---ATAGGA---CTGGGAGCT---------
TTGTTCCTTGGGTTCTTGGGAACA---GCAGGAAGCACGATG---GGCGCAGCGTCA---
CTGACGCTGACGGTACAG---GCCAGACAG---TTATTG---TCTGGTATAGTGCAACAGCAAAAC---
AATTTG---CTGAGG---GCTATAGAGGCGCAACAGCATCTGTTGCAACTCACAGTCTGGGGCATT
>EF575482..1
TTCTACTGCAATACATCACGCCTGTTTAATAGTACATGGAATAATAATACAGGGGATATAGGGAGTAGTGAT-
----

ACAAATAAGAATATCACAATCCCATGCAGAATAAAACAAATTATAAACATGTGGCAGGGAGTAGGAAAAGCAA
TGTATGCCCCTCCCATCGAAGGAAAAATCCAGTGTAGATCAAAT---
ATTACAGGGCTCTTACTGACAAGAGATGGTGGTGCAGATGCTAAT----------------------------
----

CAGAATGAGACCATCAGACCTGGAGGAGGAGATATGAGAGACAATTGGAGAAGTGAATTATACAAATATAAGG
TAGTAAGAATTGAACCACTA---GGAATAGCACCCACCAAGGCAAAGAGAAGAGTGGTG--------------
-------------------------GAAAGAGAAAAGAGAGCA---ATAGGA---CTAGGAGCT---------
GTGTTCCTTGGGTTCTTGGGAGCA---GCAGGAAGCACGATG---GGCGCAGTGTCA---
TTAACGCTGACGGTACAA---GCCAGACAG---TTATTG---TCTGGTATAGTGCAACAGCAAAGC---
AATTTG---CTGAAG---GCTATAGAGGCGCAACAGCGTCTGTTGCAACTCACAGTCTGGGGCATT
>JQ403079..1
TTCTACTGCAATACATCAGGACTGTTTAATAATATATGGAATGGCACTTGGAATGGCACTTTAAATAGTACA-
----

GGGAGAGAGAATATCACACTCCGATGCAGAATAAAACAAATTATAAACATGTGGCAGGGAGTAGGAAAAGCAA
TGTATGCCCCTCCCATTGAAGGAGTAATTAATTGTACATCAAAT---
ATTACAGGACTAATATTAACAAGAGATGGTGGTGCTAATGAGAGT----------------------------
----

CAGAATAATAATGAGACCTTCAGACCTGGAGGAGGAGATATGAGGGACAATTGGAGAAGTGAGTTATACAAAT
ATAAAGTAGTAAAAAATTGAACCATTA---GGTCTAGCACCCACCAGGGCAAAAAGGAGAGTGGTG-------
-------------------------------GAAAGAGAAAAAAGAGCA---ATAGGA---CTGGGAGCT---
------TTGTTCCTTGGGTTCTTGGGAGCA---GCAGGAAGCACGATG---GGCGCAGCGTCA---
ATGGCGCTGACGGTACAG---GCCAGACAA---GTATTG---TCTGGTATAGTGCAACAGCAAAAC---
AATTTG---CTGAGG---GCTATAGAGGCGCAACAGCATTTGTTGCAACTCACAGTCTGGGGCATT
>AY773338..1
TTCTACTGTAATACATCAAGGCTGTTTAATAATACATGGAATGGTACAACATGGTCAAATAAGACAGAC----
----

ACCAATGGGACAGTCACACTCCCATGCAGAATAAAACAAATTATAAACATGTGGCAGGAAGTAGGAAAAGCAA
TGTATGCCCCCCCCATAGAAGGACTACTTAGATGTTCATCAAAT---
ATTACAGGGTATATATTGACAAGAGATGGTGGTTATACCAGTTCT----------------------------
----

GGCAATGCGACCTTCAGACCTGGCGGAGGAGATATGAGGGACAATTGGAGAAGAGAATTATATACATACAAAG
TAGTACAAATTGGACCAATA---GGAGTAGTGCCCACCAGGGCAAAGAGAAGAGTGGTG--------------
-------------------------GAAAGGGAAAAAAGAGGG---GTT------------------------
------------TTCTTGGGAGCA---GCAGGAAGCACGATG---GGCGCAGCGTCA---
TTGTCGCTGCCGGTACAG---GCCAGACAG---GTATTG---TCTGGTACAGTGCAACAGCAAAGC---
AATTTG---CTCAGG---GCTATATCGGCGCAACAGCATCTGTTGCAACTCACGGTCTGGGGCATT
>EF633445..1
TTCTACTGTAATACATCAGGGCTGTTTAATAGTACATGGAATAGGACAGGGAATAATAGTAGTAGTACAGGG-
----

TCAAATGAGACAATCACACTTCCATGCAGAATAAGACAAATTATAAACATGTGGCAGGGAGTAGGAAAAGCAA
TGTATGCCCCTCCCATAGAAGGACTAATTAGATGTTCATCAAAT---
ATTACAGGGCTAATATTGACAAGGGATGGTGGTGATAATAGTAGT----------------------------
----

TCTCACAATGAGACCTTCAGACCTGGAGGAGGAGATATGAGGGACAATTGGAGAAGTGAATTATATAAATACA
AAGTAGTAAGAATTGAACCAATA---GGATTAGCACCCACCAGGGCAAAGAGAAGAGTGGTG-----------
----------------------------GCAAGGGAAAAAAGAGCA---ATAGGA---CTAGCGAGC------
---TGGTTCCTTGGGTTCTTGGGAGCA---GCAGGAAGCACAATG---GGCGCAGCGTCT---
GTGACGCTGACGGTACAG---GCCAGACAG---TTATTG---TCTGGTATAGTGCAACAGCAAAAC---
AATTTG---CTGAGA---GCTATAGAGGCGCAACAGCATCTGTTGCAACTCACAGTCTGGGGCATT
>FJ900266..1
TTCTACTGCAATACATCAGGATTGTTTAATAACAGTGATACC-------------------------------
----

TCAAACAGAACTATTACTCTCCCATGTAGAATAAAACAAATTGTAAACATGTGGCAGGGAGTAGGACAAGCAA
TGTATGCCGCTCCTGTTGCAGGAAAAATTACCTGTAACTCAAAC---
ATTACAGGCCTACTATTGACAAGAGATGGTGGTAATAATAGTCAT----------------------------
----

ACCGAGACCTTCAGACCTGGGGGAGGAAATATGAAAGACAATTGGAGAAGTGAACTATATAAATATAAAGTAG
TAGAACTTGAACCATTA---GGAGTAGCACCCACCAAGGCAAGAAGAAGAGTGGTGGCG--------------
-------------------AGAGGGAGAGAAAAAAGAGCA---GTGGGA---ATAGGAGCT---------
TTGTTCTTTGGGTTCTTGGGAGCA---GCAGGAAGCACTATG---GGCGCAGCGTCA---
ATAACGCTGACGGTACAG---GCCAGACAA---TTATTG---TCTGGAATAGTGCAACAGCAGAAC---
AATCTG---CTGAGA---GCTATTGAAGCGCAGCAGCATATGTTGCAGCTCACAGTCTGGGGCATT
>FJ900267..1
TTCTACTGCAATACATCAAAATTGTTTAATAATAACACAGAA-------------------------------
----

CCCAATGGCACTATCACTCTTCCATGTAAAATAAAACAAATTGTAAACATGTGGCAGGGAGTAGGACAAGCAA
TGTATGCCGCTCCTATTGCAGGAAACATTACCTGTAACTCAAAT---
ATTACAGGCTTACTATTGACAAGAGATGGTGGTACGAATCAGAGT----------------------------
----

GCTACTGAGACATTCAGACCTGGGGGAGGAAATATGAAAGACAATTGGAGAAGTGAGCTATATAAATATAAAG
TAGTACAAATTGAACCACTA---GGAGTAGCACCCACCAAGGCAAAAAGACGAGTGGTGGAG-----------
----------------------AAAGAGAGACCAAAGAGAGCA---GTGGGA---ATAGGAGCT---------
CTGTTCCTTGGGTTCTTGGGAGCA---GCAGGAAGCACTATG---GGCGCAGCATCA---
ATAACGCTGACGGCACAG---GCCAGACAA---TTATTG---TCTGGAATAGTGCAACAGCAGAAC---
AATCTG---CTGAGG---GCTATTGAAGCGCAGCAGCATCTGTTGCAGCTCACAGTCTGGGGCATT
>DQ189088..1
TTCTACTGCAATACATCAGGACTGTTTAATGACACAGATGAC-------------------------------
----

AATGGCACTATCACTCTCCCATGTCGAATAAAACAAATTGTAAACATGTGGCAGGAAGTAGGACGAGCAAACA
ATCACCCTCCCCATGCAGGAAACATTACCTGTGGTTCAAAT---
ATTACAGGTCTACTATTGACAAGAGATGGTGGTACTGGGAATTGCACTGATGGT-------------------
----
-
GGTACTGGGAATTGCACTGAGACCTTCAGACCTGGGGGAGGAAATATGAAAGATAATTGGAGAAGTGAATTAT
ATAAATACAAAGTAGTAGAAATTGAACCACTA---GGAGTAGCACCCACCAAAGCAAGAAGACAAGTGGTA--
-------------------------------------AAGAGAGAAAAAAGAGCA---GTGGGA---
ATGGGAGCT---------TTGTTCATTGGGTTCTTGGCAGCA---GCAGGAGGCACTATG---
GGCGCGGCGTCA---ATAACGCTGACGGTACAG---GCCAGACAG---TTACTG---
TCTGGAATAGTGCAACAGCAAAAC---AATTTG---CTGAGA---
GCTATTGAAGCGCAACAGCATATGTTGCAGCTCACAGTCTGGGGCATT
>MH746246..1
TTCTACTGCGATACATCAAAATTGTTTAAT---

-AATAATACAAAAGCATCA------
GCATCCAATGGCACTATCACTATCCCATGTAAAATAAAACAAATTATAAACATGTGGCAGGGAGTAGGACAAG
CAATGTATGCCCCTCCCATTGCAGGAAACATTACCTGTCGTTCAAAC---
ATTACAGGCCTACTATTGATAAGAGATGGTGGTAGTAATCAGAAT----------------------------
----

AATTCTGAGGAGATCTTTAGACCTGGGGGAGGAAATATGAAAGACAATTGGAGAAGTGAACTATATAAATATA
AAGTAGTAGAAATTGAGCCGCTA---GGAGTAGCACCCACCAAGGCAAGAAGACCAGTGGTG-----------
----------------------------AGCAGAGAAAAAAGAGCA---GCAGGA---ATAGCAGCG------
---TTGTTCCTTGGGTTCTTGGGAGCA---GCAGGAAGCACTATG---GGCGCAGCGTCA---
ATAACGCTGACGGTACAG---GCCAGACAA---TTATTG---TCTGGAATAGTGCAACAGCAAAAC---
AATTTG---CTGAGG---GCTATTGAAGCGCAACAGCATATGTTGCAGCTCACAGTCTGGGGCATT
>FJ771008..1
TTCTACTGTAATACATCAGGACTGTTTAAATTCAATGCCTCATCCGGTGAG----------------------
----

AATAGTACAAATATCACTCTCCCATGTAGAATAAAACAAGTTATAAACATGTGGCAGGAAGTAGGACGAGCAA
TGTATGCCGCTCCTATTGAAGGAAAAATTTATTGTAGCTCAAAT---
ATTACAGGTCTACTATTGACTAGAGATGGTGGTACTAATGATACT----------------------------
----

AATGGTACTGAGACCTTCAGACCTGGGGGAGGAGATATGAGAGACAATTGGAGAAGTGAATTATATAAATATA
AGGTAGTAAGAATTGAACCACTA---GGAGTAGCACCCACCAAGGCAAAAAGACAAGTGGTG-----------
----------------------------CAGAGAGAAAAAAGAGCA---GTGGGA---CTAGGAGCT------
---GTGTTCCTTGGGTTCTTGGGAGCA---GCAGGAAGCACTATG---GGCGCAGCGTCA---
ATAACGCTGACGGTACAG---GCCAGACAA---TTATTG---TTTGGAATAGTGCAACAGCAGAAC---
AATCTG---CTGAGG---GCTATTGAAGCACAACAGCATATGTTGCAGCTCACAGTCTGGGGCATT
>KJ849782..1
TTCTACTGCAATACATCAGAACTGTTTAATGACACAGGCAAT-------------------------------
----
-------------------GAC---
ACTAGTGCCGCTACTATCACTCTCCCATGTAAAATAAAACAAGTTATAAACATGTGGCAGGAAGTAGGAAAAG
CAGTGTATACTGCTCCCATTGCAGGAAACCTTGCCTGTAACTCAACT---
ATTACAGGTCTACTATTGACAAGAGATGGTGGT--

CAGGACAATAATAGT---
ACTACTGAGATCTTCAGACCTGGGGGAGGAAATATGAAAGACAATTGGAGAAGTGAGTTATATAAATATAAAG
TAGTAGAAATTGAACCACTA---GGAGTAGCACCCACCAGGGCAAAAAGACAAGTGGTG--------------
-------------------------CAGAAGCAAAAAAGAGCA---GTGGGG---CTGGGAGCT---------
ATGTTCCTTGGGTTCTTGGGAGCA---GCAGGAAGCACTATG---GGCGCAGCGTCA---
ATAACGCTGACGGCACAG---GCCAGACAA---GTATTG---TTTGGAATAGTGCAACAGCAGAGC---
AATTTG---CTGAGG---GCTATTGAAGCGCAACAGCACATGTTGCAGCTCACAGTCTGGGGCATT
>KT427774..2

```
TTCTACTGCAATACATCAAAACTGTTTAATGACAATAGCACTCAAAAATCC----------------------
-------------------------------------------------------------------------
----------------------------
AATAATGACACTATCACTCTCCCATGTCGAATAAAACAAATGGTAAACATGTGGCAGGAAGTGGGGCGAGCAA
TGTATGCCGCTCCCATTGCAGGGAACATTACCTGTAATTCAAAT---
ATTACAGGTCTACTATTGGTAAGAGATGGTGGAAATGGTGGAAATAAT-------------------------
-------------------------------------------------------------------------
-------
ACTAATAAGACTGAGATCTTCAGACCTGGGGGAGGAAATATGAAAGACAACTGGAGAAGTGAATTATATAAAT
ATAAAGTAGTAGAAATTCAGCCACTA---GGAGTAGCACCCACCAGGGCAAAAAGACAAGTGGTG--------
-------------------------------AAGAGAGAAAAAAGAGCA---GTGGGA---CTGGGAGCT---
------TTGTTCCTTGGGTTCTTGGGAGCA---GCAGGAAGCACTATG---GGCGCAGCGTCA---
ATAACGCTGACGGTACAG---GCCAGACAA---TTATTG---TCTGGTATAGTGCAACAGCAGAAT---
AATCTG---CTGAGG---GCTATTGAAGCGCAACAGCATCTGTTGCAGCTTACAGTCTGGGGCATT
>MG365764..1
TTCTACTGTAATACATCAGGACTGTTTAATAATAAC-------------------------------------
-------------------------------------------------------------------------
-------------------ACA---
GAATCCAATGACACTATCACTCTCCCATGTCGAATAAGACAAATTGTAAACATGTGGCAGGAAGTAGGAAAAG
CAATGTATGCCGCTCCCATTGCAGGAAACCTTGCCTGCAGCTCAAAC---
ATTACAGGTCTACTATTGACAAGAGATGGTGGTCAGAGTAATGGGACTAGTAAT-------------------
-------------------------------------------------------------------------
----------
GGGACTACTGAGACCTTCAGACCTGGGGGAGGAAATATGAAAGACAATTGGAGAAGTGAATTATATAAATATA
AAGTGGTAGAAATTGAACCACTA---GGAGTAGCACCCACCAAGGCAAGAAGACCAGTGGTG-----------
----------------------------AAGAGAGAAAAAAGAGCA---GTGGGAATAATAGGAGCT------
---TTGTTCCTTGGGTTCTTGGGAGCA---GCAGGAAGCACTATG---GGCGCAGCGTCA---
ATAACGCTGACGGTACAG---GCCAGACAA---TTATTG---TCTGGAATAGTGCAACAGCAGAGC---
AATTTG---CTGAGG---GCTATTGAAGCGCAACAGCATATGTTGCAGCTCACAGTCTGGGGCATT
>KX181923..1
TTCTACTGCAATACATCAAGACTGTTTAATGACACAAAA----------------------------------
-------------------------------------------------------------------------
----------------------------
TTCAATGACACTATCGTTCTCCCATGTCGAATAAGACAACTGGTAAGACTGTGGCAGGAAGTAGGACGAGCAA
TGTATGCCGTTCCCATTGCAGGAAGCATTACCTGTAACTCAACT---
ATTACAGGTCTACTATTGACAAGAGATGGTGGTCAGCATAATGAAACT-------------------------
-------------------------------------------------------------------------
----------
AATAAAACTGAGATCTTCAGACCTGGGGGAGGAAATATGAAAGACAATTGGAGAAGTGAATTATATAAATATA
AAGTAGTAGAAATTGAACCACTA---GGAGTAGCACCCACTGGGGCAAGGAGACAAGTGGTG-----------
----------------------------AAGAGAGAAAAAAGAGCAGTAGTGGGA---ATAGGAGCT------
---GTGCTCATTGGGTTCTTGGGAGCA---GCAGGAAGCACTATG---GGCGCGGCGTCA---
GTGACGCTGACGGTACAG---GCCAGACAA---TTATTG---TCTGGAATAGTGCAACAGCAGAAC---
AATTTG---CTGAAG---GCTATTGACGCGCAACAACATATGTTGCAGCTCACAGTCTGGGGCATT
>KX181926..1
TTTTACTGCAATACATCAGGCCTGTTTAATGACTCAATAATA-------------------------------
-------------------------------------------------------------------------
-------------------------
AATGGCACTGAGAGTATCACTCTCCCATGTCGAATAAAACAAATTGTAAACATGTGGCAGGAAGTAGGACGAG
CAATGTATGCCGTTCCTATTGCAGGAAACATTACCTGTATTTCAAAT---
ATTACAGGTCTGCTATTAACAAGAGATGGTGGTCTGAACAATGATACT-------------------------
-------------------------------------------------------------------------
----------
AATCAGACTGAGACCTTCAGACCTGGGGGGGGAAATATGAAGGACAATTGGAGAAGTGAATTATATAAATATA
AAGTAGTAGAAATTGAACCCCTA---GGGGTAGCCCCCCCCAGGGCAAAAAGACAAGTGGTA-----------
----------------------------AAGAGAGAAAAAAGAGCA---GTGGGA---ATAGGAGCT------
---TTGTTCCTTGGGTTTTTGGGAGCA---GCAGGAAGCAATATG---GGCGCGGGGTCA---
ATGACGCTGACGGTACAG---GCCAGACAA---TTGTTG---TCTGGGATAGTGCAACAGCAAAAC---
AATTTG---CTGAGG---GCTATTGAAGCGCAACAGCATTTGTTGCAGCTCACAGTCTGGGGCATT
>KX181925..3
```

TTCTACTGCAATACATCAGCTCTGTTTAATAATGAAACAGGATCCAATGACACTACAGGA-------------
----

TCCAATAGCACTATCACTCTCCCATGTAGAATAAAACAAGTTGTAAACATGTGGCAGGAAGTAGGGCGAGCAA
TGTATACCGCTCCCATTGCAGGAAGCATTACCTGTAACTCAAAT---
ATTACAGGTCTACTATTGACAAGAGATGGTGGTTATGAAAATAAT----------------------------
----

CAGACTGAGACCTTCAGACCTGGGGGAGGAAATATGAAAGACAATTGGAGAAGTGAATTATATAAATATAAAG
TAGTAGAAATTCAGCCACTA---GGAGTAGCACCCACCAGGGCAAAAAGACCAGTGGTG--------------
-------------------------AGGAGAGAGAAAAGAGCAGCAGTGGGA---ATAGGAGCT---------
ATGTTCCTTGGGTTCTTGGGAGCA---GCAGGAAGCACTATG---GGCGCGGCGTCA---
ATAGCGCTGACGGTACAG---GCCAGACAA---TTATTG---TCTGGCATAGTGCAACAGCAGAAC---
AATCTG---CTGAGG---GCTATTGAAGCGCAACAGCATCTGTTGCAGCTCACAGTCTGGGGCATT
>MK041565..1
TTCTACTGCAATACAACAAATCTATTTGGTGACACAATGGTAAATAAC-------------------------
----

ACAGATGACACAATCACACTCCCATGTAGAATAAAACAAATTGTAAACATGTGGCAGGAAGTGGGACGAGCAA
TGTATGCCAATCCTGTTGCAGGAAACATTACCTGTAACTCAAAT---
ATTACAGGTCTACTATTGACAAGAGATGGTGGTACTAATAAAACA----------------------------
----

GATGAAACTGAGACCTTTAGACCAGGGGGAGGAAATATGAAAGACAATTGGAGAAGTGAATTATATAAATATA
AAGTAGTAAAAATTGAACCACTA---GGAATAGCACCCACCAGGGCAAAAAGACTAGTGGTG-----------
----------------------------CAGAGAGAAAAAAGAGCA---GTAGGA---CTGGGAACT------
---CTGTTCCTTGGGTTCTTGGGAGCA---GCCGGAAGCACTATG---GGCGCAGCGTCA---
ATGACGCTGACGGTACAG---GCCAGACAA---TTATTG---TCTGGAATAGTGCAACAGCAGAGC---
AATCTG---CTGAGG---GCTATTGAAGCGCAACATCACATGTTGCAGCTCACAGTCTGGGGCATT
>AY173958..2
TTCTACTGCAATACATCAGGACTGTTTAATGACACAGGATCC-------------------------------
----

AATGGCACTATCACTCTCCTATGTCGAATAAAACAAATGGTAAACATGTGGCAGGAAGTGGGACGAGCAATGT
ATGCCGCTCCCATCTCAGGAAACATTACCTGTAACTCGAAT---
ATTACAGGTCTACTATTGACAAGAGATGGTGGTCAGAATAATAAT----------------------------
----

CAGACTGAGACCTTCAGACCTGGGGGAGGAAATATGAAAGACAATTGGAGAAGTGAATTATATAAGTATAAAG
TAGTAGAAATTGAACCACTA---GGAGTAGCACCCACCAGGGCAAAAAGACAAGTGGTG--------------
-------------------------AGGAGAGAAAAAAGAGCA---GTGGGA---ATGGGAGCT---------
TTGTTCCTTGGGTTCTTGAGCGCG---GCAGGAAGCACTATG---GGCGCGGCGTCA---
ATAACGCTGACGGTACAG---GCCAGACAA---TTATTG---TCTGGAATAGTGCAACAGCAGAGT---
AATCTG---CTGAGG---GCTATTGAAGCGCAACAGCATCTGTTGCAGCTCACAGTCTGGGGCATT
>JF683771..1
TTCTATTGTAATACATCAGAATTGTTTAATAATAGTAACTCA-------------------------------
----

AACAATGGCACTATCACTCTCCCATGTAAAATAAAACAAATTATAAACATGTGGCAGGGAGTAGGGCAAGCAA
TGTATGCCCCTCCCATTGCAGGAAAAATTACCTGTAACTCAAGT---
ATTACAGGCCTACTATTGACAAGAGATGGTGGTCAAAGTAATAAGAGTAAA----------------------
----

GATAATGGGAGTGAGATCTTCAGACCTGGGAGAGGAAATATAAAAGACAATTGGAGAAGTGAACTATATAAAT
ACAAAGTAGTAGAAATTGAACCACTA---GGAATAGCACCCACCAAGGCAAAAAGACAAGTGGTA--------
------------------------------AAGAGAGAAAAAAGAGCA---GTGGAA---ATAGGAGCT---
------GTGTTCCTTACGTTCTTGGGAGCA---GCAGGAAGCACTATG---GGCGCAGCGTCA---
CTAACGCTGACGGTACAG---GCCAGACAA---TTATTG---TCTGGGATAGTGCAGCAGCAAAAC---
AATTTG---CTGAAG---GCTATTGAAGCGCAACAGCATCTGTTGCAGCTCACAGTCTGGGGCATT
>HM068554..1

TTCTACTGCAATACATCAAAACTGTTTAACAACACAGAATTCAAT----------------------------

----

GTCAGTGACACTATCATTCTCCCATGTCGAATAAAGCAAATTGTAAACATGTGGCAGGAAGTAGGACAAGCAA

TGTATGCTAATCCTATTGCAGGAAACATCACCTGTAACTCAAGT---

ATTACAGGTATACTATTGACAAGAGATGGTGGTAACGGGAATGAAACTGAG----------------------

----

AATAATGATACTGAAATCTTCAGACCTGGGGGAGGAAATATGAAAGACAATTGGAGAAGTGAATTATATAAAT

ATAAAGTAGTAGAAATTGAACCACTA---GGAGTAGCACCCACCAGGGCAAAAAGACAAGTGGTG--------

-------------------------------AAGAGAGAAAAGAGAGCA---GCGGGA---ATGGCAGCT---

------TTGCTCCTTGGGTTCTTGGGCGCA---GCAGGAAGCACTATG---GGCGCAGCGTCA---

ATAACGCTGACGGTACAG---GCCAGACAA---TTATTG---TCTGGAATAGTGCAGCAGCAGAAC---

AATCTG---CTGAGG---GCTATTGAAGCTCAACAGCATATGTTGAAGCTCACAGTCTGGGGCATT

>JX140671..1

TTCTACTGTAATACATCAAAACTGTTTAATGAAACAGAATCCGGTAACACA----------------------

----

CAGAATGACACTATCATACTCCCATGTAGAATAAAACAAATTATAAACCTGTGGCAGCAAGTAGGGCGAGCAA

TGTATGCCGCTCCCATTGCAGGACACATTACATGTAATTCAAGT---

ATTACAGGGCTACTACTAATAAGAGATGGTGGTCAGCATAATAGTAGT-------------------------

----

AATGATGCTGAGATCTTCAGACCTGGGGGAGGAGATATGAGAGACAATTGGAGAAGTGAATTATACAAATATA

AAGTAGTAGAAATTGAACCACTA---GGAGTAGCACCCACCAAGGCAAAAAGACAAGTGGTG-----------

----------------------------AGGAGAGAAAAAAGAGCA---GTGGGA---CTAGGAGCT------

---CTGTTCCTTGGGTTCTTGAGTACA---GCAGGAAGCACTATG---GGCGCAGCGTCA---

ATAACGCTGACGGTACAG---GCCAGACAA---TTATTG---TCTGGAATAGTGCAACAGCAAAAC---

AATTTG---CTGAGG---GCTATTGAAGCGCAACAGCATATGTTGCAGCTCACAGTCTGGGGCATT

>KJ883138..2

TTCTACTGTAATACATCAAAACTGTTTAATGAAAAAGAACTCAATAACACA----------------------

----

CAGAATGACACTATCATACTCCCATGTAGAATAAAACAAGTTATAAACCTGTGGCAGGAAGTAGGGCGAGCAA

TGTATGCCGCTCCCATTGCAGGAAACATTACATGTCATTCAAAT---

ATTACAGGGCTACTATTGACAAGAGATGGTGGTCAAAGGAATGAGACT-------------------------

----

AATACTACTGAGATCTTCAGGCCTGGGGGAGGAGATATGAGAGACAATTGGAGAAGTGAATTATATAAATATA

AAGTAGTAGAAATTGAACCACTA---GGAGTAGCACCCACCAAGGCAAAAAGACAAGTGGTG-----------

----------------------------AGGAGAGAAAAAAGAGCA---GTGGGA---CTAGGAGCT------

---CTGTTCCTTGGGTTCTTGAGCGCA---GCAGGAAGCACTATG---GGCGCGGCGTCA---

ATGACGCTGACGGTACAG---GCCAGACAA---TTATTG---TCTGGAATAGTGCAACAGCAAAAC---

AATTTG---CTGAGG---GCTATTGAAGCGCAACAGCATATGTTGCAGCTCACAGTCTGGGGCATT

>DQ979023..1

TTCTACTGCAATACATCAAAATTGTTTACAAATAACATAAGC-------------------------------

----

TCCAATGGGACTATCACTCTCCCATGTAGAATAAAACAGATTATAAACATGTGGCAGGGAGTAGGACAAGCAA

TGTATACCGCCCCCATTGCAGGAAACATTACATGTAACTCAAAT---

ATTACAGGCTTACTCATAACAAGAGATGGTGGTCAGGAGGGGCGTAAT-------------------------

----

GATACTGAAGAAATATTCAGACCTGGGGGAGGAAATATGAAAGACAATTGGAGAAGTGAACTATATAAATATA

AAGTAGTAGAAATTAAACCACTA---GGAATAGCACCCACCGAGGCAAGGAGACAAGTGGTG-----------

----------------------------AAGAGAGAAAAAAGAGCA---GCAGGA---CTAGGACTT------

---TTGTTCCTTGGGTTCTTGGGAGCA---GCAGGAAGCACTATG---GGCGCAGCGTCA---

ATAACGCTGACGGTACAG---GCCAGACAT---TTATTG---TCTGGAATAGTGCAACAGCAAAAC---

AATTTG---CTGAAG---GCTATTGAAGCACAACAGCATATGTTGCAGCTCACAGTCTGGGGCATT

>DQ979024..1

TTCTATTGCAATACAACAAAATTGTTTAATGGTAGTGGAAACAATGACATTATC-------------------
----

ACTAATGGCACTATCACTATCCCATGTAGAATAAAACAAATTGTAAACATGTGGCAGGGAGTAGGACAAGCAA
TGTATGCCGCTCCCATTGCAGGACAAATTACCTGTAACTCAAAT---
ATTACAGGCATAATATTGACAAGAGATGGTGGTAACAAAAATGAGACTAAT----------------------
----

AAAAATGGGACTGAGATCTTCAGACCTGCAGGAGGAAATATGAAAGACAATTGGAGAAGTGAACTCTATAAAT
ATAAAGTAGTAGAAATTGAACCACTA---GGAGTAGCACCTACCAAGGCAAAAAGACGAGTGGTG--------
-------------------------------ACGAGAGAAAAAAGAGCA---TTGGGA---ATGGGAGCC---
------TTGTTCCTTGGGTTCTTGGGAGCA---GCAGGAAGCACTATG---GGCGCAGCGTCA---
ATAACGCTGACGGTACAG---GCCAGACAA---TTATTG---TCTGGAATAGTGCAACAGCAAAAC---
AATCTG---CTGAAG---GCCATTGAAGCGCAACAGCATCTGTTGCAGCTCACAGTCTGGGGCATT
>AF075703..1
TTCTACTGCAATACATCACTACTGTTTAATAATACAGTACCT-------------------------------
----

AATAATGGCACTATCACTCTCCCATGTCGAATAAAACAATTTGTAAACATGTGGCAGGAAGTAGGACGAGCAA
TGTATGCCGCTCCCATTGCAGGAAACATTACCTGTAACTCAAAT---
ATTACAGGTCTGCTATTGACAAGAGATGGTGGTCAGAGTAATAAT----------------------------
----

AGTGATTCTGAGACCTTCAGACCTGGGGGAGGAGATATGAAAGACAATTGGAGAAGTGAACTATATAAATATA
AAGTAGTAGAAATTGAACCACTA---GGAGTAGCACCCACCAGGCCCAAAAGACCAGTGGTG-----------
----------------------------AGGAGAGAAAGAAGAGCA---GTGGCA---ATAGGAGCT------
---GTATTCCTTGGGTTCTTGAGCGCA---GCAGGAAGCACTATG---GGCGCGGCGTCA---
CTAACGCTGACGGTACAG---GCCAGACAA---TTATTG---TCTGGAATAGTGCAACAGCAGAAC---
AATCTG---CTGCAG---GCTATTGAAGCGCAACAGCATATGTTGCAGCTCACAGTCTGGGGCATT
>KU168276..3
TTCTACTGCAATACATCAGGGCTGTTTAATAATACAGGAACT-------------------------------
----

AATGAAACTATCACTCTCCCATGTCAAATAAAACAAATTGTAAACATGTGGCAGGGAGTAGGACGAGCAATGT
ATGCTGCTCCCATTGCAGGAAACATTACCTGTAGATCAAAC---
ATTACAGGTCTACTATTGACAAGAGATGGTGGTCAGAGTACTAAT----------------------------
----

GGTAGTATTGAGACCTTCAGACCTGGGGGAGGAGATATGAAAGACAATTGGAGAAGTGAACTATATAAATACA
AAGTAGTAGAAATTCAACCACTA---GGAATAGCACCCACCGAGGCAAAAAGACAAGTGGTG-----------
----------------------------AAGAGAGAAAAAAGAGCA---GTGGGA---ATAGGAGCT------
---TTGTTCCTTGGGTTCTTGGGAGCA---GCAGGAAGCACTATG---GGCGCGGCGGCA---
ACAACGCTGACGGTACAG---GCCAGACAA---TTATTG---TCTGGAATAGTGCAACAGCAAAAC---
AATTTG---CTGAGG---GCTATTGAAGCGCAACAGCATATGTTGCAGCTCACAGTCTGGGGCATT
>AJ249238..1
TTCTACTGCGATACATCAGGATTGTTTAATGAAAGTGAAAAG-------------------------------
----

TACAATGGCACTATCATTCTCCCATGTAAAATAAAACAAATTATAAACATGTGGCAGGGAGTAGGACAAGCAA
TGTATTCCGCTCCCATTGCAGGACGCATTAACTGTAACTCAACT---
ATTACAGGCCTACTATTAACAAGAGATGGTGGTCAGAGTAATGATACT-------------------------
----

AATAGGACTGAGACTTTCAGACCTGAGGGAGGAAACATGAAAGACAATTGGAGAAATGAACTATATAAATATA
AAGTAGTAGAAATTGAACCACTA---GGAGTAGCACCCACCAAGGCAAGAAGACGAGTGGTG-----------
----------------------------CAGAGAGAAAGAAGAGCA---GTGGGA---ATAGGAGCT------
---CTGTTCCTTAGATTCTTGGGAGCA---GCAGGAAGCAACATA---GGCGCAGCGTCA---
ATAACGCTGACGGTACAG---GCCAGACAA---TTACTG---TCTGGAATAGTGCAGCAGCAAAAC---
AATTTG---CTGAGG---GCTATTGAAGCGCAACAGCATCTGTTGCAGCTCACAGTCTGGGGCATT
>AY231157..2

TTCTACTGCGATACATCACAGCTGTTTGATAGTACAGTACTT-------------------------------
----

AATGGCACCATCACTCTCCCATGTCGAATAAAACAAATTGTAAACATGTGGCAGGGAGTAGGAAAAGCAATGT
ATGCTGCGCCCATTGCAGGAAGCATTACCCGTCATTCAAAT---
ATTACAGGTCTACTATTGACAAGAGATGGTGGTCAGAATAGTAGG----------------------------
----

AATGAGACCTTCAGACCTGGGGGAGGAGATATGAAAGACAATTGGAGAAGTGAACTATATAAATATAAAGTAG
TAGAAATTGAACCACTA---GGAATAGCACCCACCAGGGCAAAAAGACCAGTGGTG-----------------
----------------------AAGAGAGAAAAAAGAGCG---TTGGGA---ATGGGAGCT---------
TTGTTTCTTGGGTTCTTGAGCGCA---GCAGGAAGCACTATG---GGCGCAGCGTCA---
ATAACGCTGACGGCACAG---GCCAGACAA---TTATTG---TCTGGAATAGTGCAACAGCAGAAC---
AATCTG---CTGAGG---GCTATTGAAGCGCAACAGCATCTGTTGCAGCTCACAGTCTGGGGCATT
>KU168274..1
TTCTATTGCAATACATCAGAGTTGTTTAAAACTAATATA----------------------------------
----

ACCAATAACACTATCACTCTCCCATGTAGAATAAAACAGATTGTAAAAATGTGGCAGGGAGTAGGACAAGCAA
TGTATGCCGCTCCCATTGCAGGAAGCATTACCTGTAACTCAAAT---
ATTACAGGATTACTATTGACAAGAGATGGTGGTCAGGATAATAAAAATGGG----------------------
----

ACTAATAGGACTGAGACCTTCAGACCTGGGGGAGGAAATATGAAAGACAATTGGAGAAGTGAACTATATAAAT
ATAAAGTAGTAGAAATTGAACCACTA---GGAATAGCACCCACCAGGGCAAAAAGACAAGTGGTG--------
-------------------------------CAGAGAGAAAAAAGAGCA---GCGGGA---ATAGGACTT---
------TTGTTCCTTGGGTTCTTGGGAGCA---GCAGGAAGCACTATG---GGCGCAGCGTCG---
ATAACGCTGACGGTACAG---ACCAGACAA---TTATTG---TCTGGAATAGTGCAACAGCAAAAC---
AATTTG---CTGAAG---GCTATTGAAGCGCAACAGCATATGTTGCAGCTCACAGTCTGGGGCATT
>AB485659..1
TTCTACTGCAATACATCAGGACTGTTTAATAGTAGCACGAGCCCCGAAGGAGTGTTTAACACGAGC-------
----

CACAAAGGAAATATCACTCTCCTATGTAGAATAAAACAGATTATAAACATGTGGCAGGGAGTAGGCCAAGCAA
TGTATGCCGCTCCCATTGCAGGAAGCATTACCTGTAACTCAAAT---
ATTACAGGCTTACTATTGACAAGAGATGGTGGTCAGAATAATGAG----------------------------
----

ACTGAGACCTTCAGACCTGGGGGAGGAAATATGAAAGACAATTGGAGAAGTGAACTATATAAATATAAAGTAG
TAGAAATTGAACCACTA---GGAATAGCACCCACCAGGGCACAAAGACAAGTGGTG-----------------
----------------------AAGAGAGAAAAACGAGCA---GCGGGA---TTAGGACTT---------
TTGCTCCTTGGGTTCTTGGGAGCA---GCAGGAAGCACTATG---GGCGCGGCGTCA---
ATAACGCTGACGGTACAG---GCCAGACAA---TTATTG---TCTGGAATAGTGCAACAGCAAAAC---
AATTTG---CTGAAG---GCTATTGAAGCGCAACAGCATCTGTTGCAGCTCACAGTCTGGGGCATT
>HQ236605..2
TTCTACTGCAATACAACACAACTGTTTAACTACACAAGATATTCCAATGGCACTATCATTTTCGGT-------
----

TCCAATGGCACTATCACTCTCCCATGCAGAATAAAACAAATTGTAAACATGTGGCAGGAAGTAGGACGAGCAA
TATATGCCGCTCCAATTGCAGGGAACATCACCTGTCACTCAAAT---
ATTACAGGCCTACTATTGACAAGAGATGGTGGTAGTGGCAATGGTACT-------------------------
----

GACAATAGAAGTGAGATCTTCAGACCTGGAGGGGGAAACATGAAAGACAATTGGAGAAATGAATTATATAAAT
ATAAAGTAGTAAGAATTGAACCACTA---GGAATAGCACCCACCAGGGCACACAGACGAGTGGTG--------
-------------------------------AAGAGACAAAAAAGAGCA---CTCGGA---ATGGGAGCC---
------ATGTTCATTGGGTTCTTGGGAGCA---GCAGGAAGCACTATG---GGCGCAGCGTCA---
GTGACGCTGACGGTACAG---GCCAGACAA---TTGATG---TCTGGAATAGTGCAACAGCAAAAT---
AATTTG---CTGATG---GCCATTAAAGCGCAACAGCATCTGTTGCAGCTCACAGTCTGGGGCATT
>KU168275..1

TTCTACTGCAATACATCAGGGCTGTTTAATAATACAGTACTC-------------------------------
----

AATAACACTACTATCACTCTCCCATGTCGAATAAAACAAATTGTAAACATGTGGCAGGGAGTAGGACAAGCAA
TGTATGCCGCTCCCATTGCAGGAAACATTACCTGTAGATCAAAT---
ATTACAGGTCTATTATTGACAAGAGATGGTGGTGAGAATAATGAT----------------------------
----

ACTGAGACCTTCAGACCTGGGGGAGGAGATATGAAAGACAATTGGAGAAGTGAACTATATAAATACAAAGTAG
TAGAAATTCAACCACTA---GGAATAGCACCCACCAAGGCAAAAAGACAAGTGGTG-----------------
----------------------AAGAGGGAAAAAAGAGCA---GTTGGA---ATGGGAGCC---------
TTGTTCCTTGGGTTCTTGGGAGCA---GCAGGAAGCACTATG---GGCGCGGCGTCA---
GTAACGCTGACGGTACAG---GCCAGACAA---TTATTG---TCTGGAATAGTGCAACAGCAAAGC---
AATTTG---CTGAGG---GCTATTGAAGCGCAACAGCATCTGTTGCAGCTCACAGTCTGGGGCATT
>MH705144..1
TTCTACTGCAACACAAGTAAGCTGTTTAACACAACTTGGCTAAATAATAAAACGGAACACAATGGCATTATCA
ACAGC--

AGCACTGAAATTATGGTGCTTCCGTGTAGAATAAGACAATTTGTAAACATGTGGCAAAGAGTAGGAAGAGCAA
TGTATGCCCCTCCCATTGCAGGGGTAATTCAGTGTAAATCAGAT---
ATCACAGGTCTACTATTGACAAGAGATGGTGGTAATACTGGTAATAAT-------------------------
----
ACAGGAGGTGATGGTAAT------------
AATACCGACATCCTCAGACCTACAGGGGGAGATATGAGAGACAATTGGAGAAGTGAACTATATAAATATAAGG
TAGTAAAACTTGAACCACTA---GGAGTAGCACCCACCAGGGCAAAAAGACAAGTGGTGAAA-----------
-------------------GGAAAAGAAAGAGAAAAAAGAGCA---GTGGGA---CTGGGAGCT---------
ATGTTCCTTGGGTTCTTGGGAGCA---GCAGGAAGCACTATG---GGCGCAGCGTCA---
ATAACGCTGACGGTACAG---GCTAGACTA---TTATTG---TCTGGTATAGTGCAACAGCAAAGC---
AATTTG---CTGAGG---GCTATAGAAGCGCAACAGCATATGTTGCAGCTCACAGTCTGGGGCATT
>AY371158..2
TTCTACTGCGACACAACAGCACTGTTTAACACAACATTACTTAACACAACAATGGAT----------------
----

AATAATGGTACTATCATTATTCCATGTAGAATAAAACAAATTGTAAACGTGTGGCAAAGAGTAGGGCGAGCAA
TGTATGCCCCTCCCATTGCAGGAAAAATTCAGTGCAACTCAAAT---
ATCACAGGTCTACTATTGACAAGAGATGGTGGAAGTAAGGCAAAT----------------------------
----

AACACAGACATCCTCAGACCTATAGGGGGAGAGATGAGAGACAATTGGAGAAGTGAATTATATAAATATAAGG
TAGTACAAATTCAACCACTA---GGAATAGCACCCTCCAGGGCAAAAAGACAAGTGGTG--------------
-------------------------AAGAGAGAAAGAAGAGCA---GTAGGA---ATAGGAGCT---------
GTGTTCCTTGGGTTCTTGGGAGCA---GCAGGAAGCACTATG---GGCGCAGCGTCA---
ATAACGCTGACGGTACAG---GCCAGACAA---TTATTG---TCTGGTATAGTGCAACAGCAAAAC---
AATTTG---CTGAAG---GCTATAGAAGCGCAACAGCATCTGTTGCAGCTCACAGTCTGGGGCATT
>JX140672..3
TTCTACTGCAATACATCAGCTCTGTTTAATACAGAAGCTCTGAAAAATAACACAGGT----------------
----

CAAAATGGCACTACCACCATTTATTGTAGAATAAAACAAATTATAAATATGTGGCAAAGAGTAGGAAAAGCAA
TGTATGCCCCTCCCATTGCAGGAAAAATTCAGTGTAACTCAAGT---
ATCACAGGTCTAATATTAACAATAGATGGT---AATAGAACAAAT----------------------------
----

AATAAGAGTGAGACCCTCAGACCTGGAGGGGGAGATATGAAAGACAATTGGAGAAGTGAACTGTATAAATATA
AGGTAGTGAAAATTGAACCATTA---GGAGTAGCACCCACCAGGGCAAAAAGACGAGTGGTG-----------
----------------------------AGCAGAGAGAAAAGAGCA---GTGGGA---CTAGGAGCT------
---ATGTTCCTTGGGTTCTTGGGAGCA---GCAGGAAGCACTATG---GGCGCGGCGTCA---
ATAACGCTGACGGTACAG---GCCAGACAA---TTGTTG---TCTGGTATAGTGCAACAGCAAAGC---
AATTTG---CTGAAG---GCTATTGAAGCGCAGCAGCACTTGTTGCAGCTCACAGTCTGGGGCATT
>KU749420..1

TTCTACTGTAACACAAGCAGACTGTTTAATAACACACTGTTTAATAACACACAGCTTAATAACACA--------
----

GAAGGAGGGAACATTACTCTTCCATGTAGAATAAAACAATTTGTAAACATGTGGCAAAGAGTAGGAAAAGCAA
TGTATGCCCCTCCCATTGCTGGAAGAATTCAGTGTATCTCAAAT---
ATCACAGGTCTACTATTGACAAGAGATGGTGGTACTAATGATACGTCT------------------------
----

ACAAATGAGACCTTCAGACCTGGAGGGGGAGACATGAGAGATAATTGGAGAAGTGAACTGTATAAATATAAGG
TAGTAAAAATTGAGCCACTA---GGAGTAGCCCCACTAGGGCAAAAAGGCGAGTGGTG--------------
-------------------------CAGAGAGAAAAAAGAGCT---GTGGGA---CTAGGAGCT--------
CTGTTCCTTGGGTTCTTGGGAGCA---GCAGGAAGCACTATG---GGCGCGGCGTCA---
ATAACGCTGACGGTACAG---GCCAGAAAC---TTATTG---TCTGGTATAGTGCAACAGCAGAAT---
AATCTG---CTGAAG---GCTATAGAAGCGCAACAGCATCTGTTGCAGCTCACAGTCTGGGGCATT
>AJ277824..1
TTCTACTGCAACACAGATGAGCTGTTTAATAACAGCTTGTTGAATAACACA---------------------
----

AAAAAAGAAAATATCACTCTTCCATGTAGAATAAAACAATTTGTAAACATGTGGCAAAGGGTAGGACAAGCAA
TGTATGCCCCTCCTATAGCCGGACAAATTCAGTGCAACTCAAGT---
ATCACAGGTCTACTATTGACAAGAGATGGT---AACAAAAATGGG---------------------------
----

TCCGAGACCCTCAGACCTGGAAGGGGAGATATGAGAGACAATTGGAGAAGTGAACTATATAAATATAAGGTAG
TAAAAATTGAACCCCTA---GGAGTAGCACCCACCAGGGCAAAAAGACAAGTGGTG----------------
----------------------AAGAGAGAAAGAAGAGCA---GTGGGA---ATAGGAGCT--------
GTGTTCCTTGGGTTCTTGGGAGCA---GCAGGAAGCACTATG---GGCGCGGCGTCA---
ATAACGCTGACGGTACAG---GCCAGACAA---TTATTG---TCTGGTATAGTGCAACAGCAAAGC---
AATTTG---CTGAAG---GCTATAGAAGCGCAACAGCATCTGTTGCAGCTCACAGTCTGGGGCATT
>AJ249237..3
TTCTACTGCAACACAACTATTCTGTTTAATCACACAAGAGTAAATGACATTCTGTCTAATAATCACACGAGA-
----

GAAAATGACACTATCACTCTTCCATGTAGAATAAAACAAATTGTAAACATGTGGCAAAGAGTAGGACAGGCAA
TGTATGCCCCTCCCATTGCCGGAAAAATTCAGTGTAACTCAAAT---
ATCACAGGTCTACTATTAACAATAGATGGTGGGGAGGGAAATGAG---------------------------
----

TCCGAGACCCTCAGACCTGGAGGGGGAGATATGAGAGACAATTGGAGAAGTGAACTATATAAATATAAGGTAG
TAAAAATTGAACCACTA---GGAGTAGCACCCACCAAGGCAAAAAGACAAGTGGTG----------------
----------------------CAGAGAGAAAAAAGAGCA---GTGGGA---ATGGGAGCT--------
ATGTTCCTTGGGTTCTTGGGAGCA---GCCGGAAGCACTATG---GGCGCGGCGTCA---
ATAACGCTGACGGTACAG---GCCAGAAAC---TTATTG---TCTGGTATAGTGCAGCAGCAAAGC---
AATTTG---CTGAAA---GCTATTGAAGCGCAACAGCATCTGTTGCAGCTCACAGTCTGGGGCATT
>AF377956..1
TTCTACTGCAACACAGTAGCTCTGTTTAATGCAACTAACATGACTAATGCAATGAACAGG------------
----

TCCAATGGCATTATCACTCTTCCATGTAGAATAAGACAAATTGTAAACATGTGGCAAAGAGTAGGACGAGCAA
TGTATGCCGCTCCCATTGCTGGACAAATTCAGTGTAACTCAAGC---
ATCACAGGTCTAATATTGACAAGAGATGGTGGGAAAAATAATACC---------------------------
----

AATAATGACACCCTCAGACCTGGAGGGGGAGATATGAGAGACAATTGGAGAAGTGAACTGTATAAATATAAGG
TAGTAAAAATTGAACCACTA---GGAGTAGCACCCACCAAGGCAAAAAGACAAGTGGTGAAG----------
----------------------AGAGAAAGAGAAAAAAGAGCA---GTGGGA---ATAGGAGCT--------
GTGCTCCTTGGGTTCTTGGGAGCA---GCAGGAAGCACTATG---GGCGCGGCGTCA---
ATGACGCTGACGGTACAG---GCCAGACAA---TTATTG---TCTGGTATAGTGCAACAGCAAAAC---
AATTTG---CTGAAG---GCTATAGAAGCGCAACAGCATCTGTTGCAGCTCACAGTCTGGGGCATT
>JN054264..1

TTCTACTGTAACACAACAAAGCTGTTTAATTCAAGTCTGCTTAACAGTACAAGTACAAATGAAAGTATACCTG
AG--

ATTATTCTTCCATGTAGAATAAAACAAATTATAAACATGTGGCAAAGAGTAGGAAAAGCAGTGTATTCCTCCC
CCATTGCAGGGAAAATTCAGTGTATCTCAAAT---
ATCACAGGTCTATTATTGGTGAGGGATGGTGGTAATACATCCCAC---------------------------
----

AATGACAATGACACCCTCAGACCTATGGGGGGAGATATGAAAGCCAATTGGAGAAGTGAACTGTATAAATATA
AGGTAGTAAAAAATTGAGCCACTA---GGAATAGCACCCTCCAGGGCAAAAAGACGAGTGGTG---------
----------------------------CAGAGAGAGAAAAGAGCT---GTGGGA---ATAGGAGCT-----
---TTGCTCTTTGGGTTCTTGGGAGCA---GCAGGAAGCACTATG---GGCGCAGCGTCA---
CTAACGCTGACGGTACAG---GCCAGACAA---TTATTG---TCTGGTATAGTGCAACAGCAAAGC---
AATTTG---CTGAAG---GCTATAGAAGCGCAACAGCATCTGTTGCAGCTCACAGTCTGGGGCATT
>KF725967..1
TTCTACTGTAATACAACAGATCTGTTTAATATAACAGCTCTGGTTAATAAC---------------------
----

ACAGGGAACAATATCACTATCCCATGTAGAATAAAACAAATTATAAACATGTGGCAAAGAGTAGGACGAGCAA
TGTATGCCCCTCCCATAGCCGGAAGAATTCAGTGTAACTCAACT---
ATCACAGGTCTACTATTAACAAGAGATGGTAGAAATGATACAAAT---------------------------
----

GAGACCCTCAGACCTGGAGGGGGAGATATGAGAGACAATTGGAGAAGTGAGCTATATAAATATAAGGTAGTGA
AAATTGAACCACTA---GGAGTAGCACCCACTGAGGCAAGAAGACAAGTGGTA-------------------
------------------AAGAGAGAAAAAAGAGCA---GTGGGA---ATGGGAGCT---------
GTGCTCTTTGGGTTCTTGGGAACA---GCAGGAAGCACTATG---GGCGCGGCGTCA---
ATAACGCTGACGGTACAG---GCCAGACAA---TTATTG---TCTGGTATAGTGCAGCAGCAAAGC---
AATTTG---CTGAAG---GCTATAGACGCGCAACAGCATCTGTTGCAGCTTACAGTCTGGGGCATT
>AY371121..1
TTCTATTGTAATACATCAGAACTGTTTAATATTAATATATCAAATAGTAGTACAAATAGTACT---------
----

GAGAATGAAACTATCACACTCCCATGTAAAATAAAACAAATTGTAAGAATGTGGCAGAGAGTGGGACAAGCAA
TGTATGCCCCTCCTATCGCCGGAAACATTACATGTAAATCAAAC---
ATTACAGGACTAATATTAACAAGAGATGGTGGAAATACTAGGGAT---------------------------
----

AATACCTCAGAGATCTTCAGACCTGGAGGAGGAGAAATGAGAGACAATTGGAGAAATGAATTATATAATTATA
AAACAGTAAAAATCAAATCATTA---GGAGTAGCACCCACCAGGGCAAGGAGAAGAGTGGTG----------
----------------------------GAGAGAGAAAAAAGAGCA---GTTGGA---ATAGGAGCT-----
---GTCTTCCTTGGGTTCTTAGGAGCA---GCAGGAAGCACTATG---GGCGCGGCGTCA---
ATAACGCTGACGGTACAG---GTCAGACAA---TTATTG---TCTGGCATAGTGCAACAGCAAAAC---
AATTTG---CTGAGG---GCTATAGAGGCGCAGCAGCATCTGTTGCAACTCACAGTCTGGGGCATT
>FJ389367..3
TTCTATTGTAATACATCAGGCCTGTTTAATATATCATATAATAGTACAGAGAATAGTACTTATAATGAA----
----

GGGAATGAGACTTATACACTCCCATGTAAAATAAAACAAATTGTGAGAATGTGGCAAAGAGTGGGACAAGCAA
TGTATGCCCCTCCCATCAAAGGAAACATTACATGTAGATCAAAC---
ATTACAGGACTACTATTAACAAGAGATGGTGGGGAGAATGATAATAAT------------------------
----

AACAGTAGTGAGGTCTTCAGACCTGCAGGAGGAGACATGAGGGACAATTGGAGAAGTGAATTATATAAGTATA
AAATAGTAAAAATAAAACCATTA---GGAGTAGCACCCACCAAGGCAAGGAGAAGAGTGGTG----------
----------------------------GGGAGAGAAAAAAGAGCA---GTAGTTGGACTAGGAGCT-----
---GTTTTCCTTGGGTTCTTAGGAGCA---GCAGGAAGCACTATG---GGCGCGGCGTCA---
ATAACGCTGACGGTACAG---GTCAGACAA---TTATTG---TCTGGCATAGTGCAACAGCAAAGC---
AATTTG---CTGAGA---GCTATAGAGGCACAGCATCATCTGTTGCAACTCACAGTCTGGGGCATT
>FJ389365..1

TTCTATTGTAATACATCAGACTTGTTTAATATCAGTACAACAAATAGT-------------------------
----

ACTAATGATACTATCACACTCCCATGTAAGATAAAACAAATTGTGAGAATGTGGCAGAGAGTGGGACAAGCAA
TGTATGCCCCTCCCATTGCAGGAGAAATTACATGCAGGTCAAAT---
ATTACAGGACTACTATTAACAAGAGATGGTGGGGGTGGAGGTAAT----------------------------
----

CAAACAAATGAAACCTTCAGACTTGCAGGAGGAAATATGAGGGACAATTGGAGAAGTGAATTATATAAGTATA
AAGTAGTAAAGATTAAACCACTA---GGAATAGCACCCACCAAGTCAAGGAGAAGAGTGGTG-----------
----------------------------GAGAGAGAAAAAAGAGCA---GTTGGA---ATAGGAGCT------
---GCCTTCCTTGGGTTCTTAGGAGCA---GCAGGAAGCACTATG---GGCGCGGCGTCA---
ATAACGCTGACGGTACAG---GTCAGACAA---TTATTG---TCTGGCATAGTGCAACAGCAAAGC---
AATTTG---CTGAGA---GCTATAGAGGCGCAGCAACATCTGCTGCAACTCACAGTCTGGGGCATT
>AY231156..1
TTCTATTGTAATACATCAGGACTGTTTAATAGTAGTAATCTGTCTAATAGTAGTAACAAT-------------
----

GAGACCGATATTATAACGCTCCCATGTAGAATAAAACAAATTGTAAGAATGTGGCAGAGAGTGGGACAAGCAA
TGTATGCCCCTCCCATAGAAGGAAACATTACATGTATATCAAAT---
ATTACAGGACTATTATTAACAAGGGATGGTGGGAAGACTAACAGT----------------------------
----

AGTAATGAGACCTTCAGACCTGCAGGAGGAGACATGAGGGACAATTGGAGAAGTGAATTATATAAGTATAAAG
TAGTAAAAATTAAACCACTA---GGAATAGCACCCACCAAGGCAAGGAGAAGAGTGGTG--------------
-------------------------GGGAGAGAAAAAAGAGCA---GTTGGA---ATAGGAGCT---------
GTCCTCCTTGGGTTCTTAGGAACA---GCAGGAAGCACTATG---GGCGCAGCGGCA---
ATAACGCTGACGGTACAG---GTCAGACAA---TTATTG---TCTGGCATAGTGCAACAGCAAAGC---
AATTTG---CTGAGG---GCTATAGAGGCTCAGCAACATATGTTGCAGCTCACAGTCTGGGGCATT
>EF033660..1
TTCTATTGTAATACATCAGGATTGTTTAATAGTAGTAACCTTAAAAGTAGTAACCTGACT-------------
----

GGTAATGAGACCATCACACTCCCATGTAGGATAAAACAAATTGTGAGAATGTGGCAGAGAGTGGGACAAGCAG
TGTATGCCCCTCCCATTGCAGGAAACATTACATGTATATCAAAC---
ATTACAGGACTACTAATAACAAGAGATGGTGGGAATAATAGCAGC----------------------------
----

CAAAATGAGACCTTTAGACCTGCAGGAGGAGACATGAGGGACAATTGGAGAAGTGAATTATATAAGTATAAAG
TAGTAAAAATTAAACCACTA---GGAGTAGCACCCACCAGGGCAAGGAGAAGAGTGGTG--------------
-------------------------GGGAGAGAAAAAAGAGCA---GTTGGA---CTGGGAGCT---------
GTCCTCCTTGGGTTCTTAGGAACA---GCAGGAAGCACTATG---GGCGCAGCGGCA---
GTAACGCTGACGGTACAG---GTCAGACAA---CTATTG---TCTGGCATAGTGCAACAGCAAAGC---
AATTTG---CTGAGG---GCTATAGAGGCTCAGCTACATCTGTTGCAACTCACAGTCTGGGGCATT
>KT022379..1
TTCTATTGTAATACATCTAGATTGTTTAATGATAATATTACACATAATAATAGTGCATCTAATAATAAT----
----

ACTAAGGGAAACATCACACTCCCATGTAAAATAAAACAAATTGTGAGAATGTGGCAAAGAGTGGGACAAGCAA
TGTATGCCCCTCCTATCGCAGGAGAACTTACATGTAAATCAAAC---
ATTACAGGACTATTATTAACAAGAGATGGTGGGACTCATAAAAAT----------------------------
----

AATAAAAGTGAGATCTTCAGACCTGCAGGAGGAGATATGAAGGACAATTGGAGAAGTAAATTATATAAGTATA
AAGTAGTAAAAATTAAACCACTA---GGAGTAGCACCCACCAAAGCAAGGAGAAGAGTGGTG-----------
----------------------------GGAAGAAAAAAAAGAGCA---GTTGGA---CTGGGAGCT------
---GTCTTCCTTGGGTTCTTGGGAGCA---GCAGGAAGCACTATG---GGCGCAGCGTCG---
ATAACGCTGACGGTACAG---GCCAGACAA---TTATTG---TCTGGCATAGTGCAACAGCAAAAC---
AATTTG---CTGAGA---GCTATAGAAGCGCAGCAGCATTTGTTGCAGCTCACAGTCTGGGGCATT
>JN248582..1

TTCTATTGTAACACAACAGGACTGTTTAATAATAGTACATTGGAGCTTAATAACACT----------------
----

AGTAATGGCACTATCACACTCCCATGTAAGATAAAACAAATTGTGAGAATGTGGCAGAGAACTGGACAAGCAA
TGTATGCCCCTCCCATCGCAGGAAATATTACATGTAGATCAAAC---
ATTACAGGACTATTATTAACAAGAGATGGTGGGAATAATGATACT----------------------------
----

CATGAGACCTTCAGACCTGGAGGAGGAGATATGAGGGACAATTGGAGAAGCGAACTATATAAATATAAAATAG
TAAAAATTAAACCACTA---GGAATAGCACCCACCAAGGCAAGGAGAAGAGTGGTG-----------------
----------------------CAGAGGGAAAAAAGAGCAGTAGTTGGA---CTAGGAGCT---------
GTCTTCCTTGGGTTCTTGGGAGCA---GCAGGAAGCACTATG---GGCGCAGCGTCA---
ATAACGCTGACGGTACAG---GTCAGACAA---TTATTG---TCTGGCATAGTGCAACAGCAAAGC---
AATTTG---CTGAGG---GCTATAGAGGCACAGCAACATCTGTTGCAACTCACAGTCTGGGGCATT
>AF069947..1
TTCTATTGTAATACATCAGGATTGTTTAATGATAGTGCATCA-------------------------------
----

AATGACACTATCACACTCCCATGTAAGATAAAACAAATAGTGAGAATGTGGCAAAGAGTGGGACAAGCAATGT
ATGCCCCTCCCATCGCAGGAAACATTACATGTAGATCAAAT---
ATTACAGGGCTACTATTAACAAGAGATGGTGGAAATGATGGGGCTAATAGC----------------------
----

ACAAATCAAAATGAAACCTTCAGACCTGGAGGAGGAGATATGAGGGACAATTGGAGAAGTGAATTATATAAGT
ATAAAATAGTAAGAATTAAACCACTA---GGAATAGCACCCACCAGGGCAAGAAGAAGAGTGGTG--------
-------------------------------GAGAGAGAAAAAAGGGCA---GTTGGA---CTGGGAGCT---
------GTCCTCCTTGGGTTCTTAGGAACA---GCAGGAAGCACTATG---GGCGCGGCGTCA---
ATAACGCTGACGGTACAG---GTCAGACAA---TTATTG---TCTGGCATAGTGCAACAGCAAAGC---
AATTTG---CTGAGG---GCTATAGAGGCGCAGCAACATCTGTTGCAACTCACAGTCTGGGGCATA
>KY229519..2
TTCTATTGTAATACATCAGAACTGTTTAATCCTAATACCCTGAATAGTAATAGTAGT----------------
----

GATCAGTCTAACATCACCCTCCCATGTAAAATAAAACAAATTGTGAGAATGTGGCAGAAAGTGGGACAAGCGA
TGTATGCCCCTCCCATTGCAGGAGAGATTACATGTAGATCAAAC---
ATTACAGGAATACTATTAACAAGAGATGGTGGAAATCACCAGAACATT-------------------------
----

AGTGGAAATGAAACCTTCAGACCTGCAGGAGGAGACATGAGGGACAATTGGAGAAGTGAATTATATAAGTATA
AAGTAGTAAAAATTAAACCACTA---GGAATAGCACCCACCAAGGCAAAAAGAAGAGCGGTG-----------
----------------------------GAGAGAGAAAGAAGAGCA---GTTGGA---ATGGGAGCT------
---TTACTCCTTGGGTTCTTAGGAACA---GCAGGAAGCACTATG---GGCGCAGCGTCA---
ATAACGCTGACGGTACAG---ATCAGACAG---TTGTTG---TCTGGTATAGTGCAACAGCAAAGC---
AATTTG---CTGAGA---GCTATAGAGGCGCAGCAGCATCTGTTGCAGCTTACAGTCTGGGGCATT
>KU168273..1
TTCTATTGCAATACATCAAAACTGTTTAATAGTATCTGGTATGTTAACGGGACAAATGGGACCGTACTAAATG
GAACAATTACT--
-------------GGGACC------
GGACTAAATGGAACAATTACACTGCCATGCAGAATAAAACAAATTGTAAACATGTGGCAGAGAGTAGGACAAG
CAATGTATGCCCCTCCCATTAGGGGAGATATTACATGCGAATCAAAT---
ATTACAGGACTAATATTAACAAGTGACTATGTTAATACAACAGATACA-------------------------
----

AATGGAAGTGTTACCCTCAGACCTACAGGAGGAGACATGAGGGACAATTGGAGAAGTGAATTATATAAATATA
AAGTAGTAAAAATTGAGCCACTA---GGAATAGCACCCTCCAGGGCAAGGAGAAGAGTGGTG-----------
----------------------------GAAAGAGAAAAAAGAGCT---GTGGGA---ATGGGAGCT------
---TTTTTCCTTGGGTTCTTGGGAGCA---GCAGGAAGCACTATG---GGCGCGGCGTCC---
ATGGCGCTGACGGTACAG---GCCAGACAA---TTATTG---TCTGGTATAGTGCAACAGCAAAGT---
AATCTG---CTGAGA---GCTATAGAGGCGCAACAGCATATGTTGCAGCTCACAGTCTGGGGCATT
>AF005496..2

```
TTCTATTGCAATACATCAGGGCTGTTTAATAGTAGTTGGGAAATGCATACTAATTACACATCAAATGACACAA
AG-----------------------------------------------------------------------
----------------------------
GGAAACGAAAACATTACACTGCCATGCAGAATAAAACAAATTGTAAACATGTGGCAGAGAGTAGGACGAGCAA
TGTATGCCCCTCCCATCCAAGGAAACATTATGTGTGTATCAAAT---
ATTACAGGACTAATATTGACAATTGACGAGGGTAACGCGTCTGCA----------------------------
-------------------------------------------------------------------------
-------------
GAAAATTATACCTTCAGACCTGGAGGAGGAGATATGAGGGACAATTGGAGAAGTGAATTGTATAAATATAAAG
TAGTAAAAATTGAACCACTG---GGAATAGCACCCACCAAGACAAGGAGAAGAGTGGTG--------------
-------------------------GAGAGAGAAAAAAGAGCA---GTGGGA---ATGGGAGCT---------
TCTTTCCTTGGGTTCTTGGGAGCA---GCAGGAAGCACTATG---GGCGCGGCGTCA---
ATAACGCTGACGGTACAG---GCCAGGCAA---TTATTG---TCTGGTATAGTGCAGCAGCAAAGC---
AATTTG---CTGAGA---GCTATACAGGCGCGACAGCATATGTTGCAGCTCACGGTCTGGGGCATT
>KY392776..1
TTCTATTGTAATACATCAAAACTGTTCAATAGTAGTGAAAAT-------------------------------
-------------------------------------------------------------------------
----------------------------
TCCAATGCAAACATCACACTCCCATGTAGAATAAGGCAAATTGTAAGGATGTGGCAGAGAGTAGGACAAGCAA
TATATGCCCCTCCCATCGCAGGAAACATTACATGCATGTCAAAT---
ATTACAGGATTAATATTGACACGTGATGGGGGAGTTAACAATAACAATGACAGT-------------------
-------------------------------------------------------------------------
-------
ACTAATAATACAGAAATCTTCAGACCTACAGGAGGAAATATGAAGGACAATTGGAGAAGTGAGCTATATAAAT
ATAAAGTGGTAAAAATTGAGCCACTA---GGAGTAGCACCTACCAGGGCAAAAAGAAGAGTGGTG--------
-------------------------------GAGAGAGAAAAAAGAGCA---GTGGGA---ATGGGAGCT---
------GTGTTCCTTGGGTTCTTGGGAACA---GCAGGAAGCACTATG---GGCGCAGCGTCA---
ATAACGCTGACGGTACAG---GTCAGACAA---TTGTTG---TCTGGCATAGTGCAACAGCAAAGC---
AATTTG---CTAAAG---GCTATAGAGGCTCAACAGCATCTGTTGAGACTCACTGTCTGGGGCATT
>KU168280..1
TTCTATTGCAATACATCACAACTGTTCAATAATAGTTGGAATGGAAGTAGTTGGAATAACAATGCT-------
-------------------------------------------------------------------------
----------------------------
ACAAATGACACTATCACAATTCCATGTAAAATAAGACAAATTGTGAGAATGTGGCAGAGAGTAGGACAAGCCA
TATACGCCCCTCCCATCGCAGGAAACATTACATGTGTATCAAAT---
ATTACAGGATTAATATTAACACGTGATGGGGGACAGGGAGGAAAT----------------------------
--------------------------------------------------------------------
GAGACAAACAAGACT---
GAGGCTGAGACCTTCAGACCTACAGGAGGAGATATGAGGGACAATTGGAGAAGTGAATTATATAAATATAAAG
TAGTGGAGGTTGAGCCACTA---GGAGTAGCACCCACCAGGGCAAAAAGAAGAGTGGTG--------------
-------------------------GGGAGAGAAAAAAGAGCG------GGA---ATAGGAGCT---------
CTGTTCCTTGGGTTCTTGGGAGCA---GCAGGAAGCACTATG---GGCGCAGCGTCA---
ATAACGCTGACGGTACAG---GCCAGACAA---TTGCTG---TCTGGCATAGTGCAACAGCAAAGC---
AATTTG---CTGAAT---GCTATAGAGGCTCAACAGCATCTGTTGAAGCTCACTGTCTGGGGCATT
>GU237072..2
TTCTATTGTAATACATCAAGCCTGTTCAATAGTAGCTGGAGTAGTAAGAATGATACAAATAGC----------
-------------------------------------------------------------------------
----------------------------
ACAGCAAATTACACAATCATCCCATGTAAAATAAGACAAATAGTGAGAATGTGGCAGAAAGTAGGACAAGCAA
TATATGCCCCTCCCATTGAAGGAAACATTACATGCTCATCAAAT---
ATTACAGGATTAATATTGACACGTGATGGAGGAGACAAGAACAGCACTAGC----------------------
-------------------------------------------------------------------------
-------
AACAACAGTACTGAGATCTTTAGACCCACAGGAGGAGATATGAAAGACAATTGGAGAAGTGAATTATATAAAT
ATAAAGTAGTAGAGATTGAGCCACTA---GGAGTAGCACCCACCAGGGCAAAAAGAAGAGTGGTG--------
-------------------------------GAGAGAGAAAAAAGAGCA---CTGGGA---ATAGGAGCT---
------GTGTTCCTTGGGTTCTTGGGAACA---GCAGGAAGCACTATG---GGCGCAGCGTCA---
ATAACGCTGACGGTACAG---GTCAGACAA---TTGTTG---TCTGGCATAGTGCAACAGCAAAGC---
AATTTG---CTGAAG---GCTATAGAGGCTCAACAGCATCTGTTGAAGCTCACTGTCTGGGGCATT
>AJ249239..1
```

TTCTACTGCAATACAACAAAACTGTTTAATGAGACAGGGGAG-------------------------------
----

AATGGTACTATCACACTCCCATGTAGAATAAAGCAGATTATAAACATGTGGCAGAAAGTGGGAAAAGCAATAT
ATGCCCCTCCCATTGCAGGAAGTATTAACTGTAGCTCAAAT---
ATTACAGGAATGATATTGACAAGAGATGGTGGTAATAATACTCAT----------------------------
----

AATGAGACCTTCAGACCTGGAGGAGGAGACATGAGGGACAATTGGAGAAGTGAACTGTATAAATATAAAGTAG
TACAGATTGAACCACTA---GGAATAGCACCCACCAGGGCCAGGAGAAGAGTGGTG-----------------
----------------------CAGAGAGAAAAAAGAGCA---GTAGGA---TTAGGAGCT---------
GTGTTCTTTGGATTCTTGGGAGCA---GCAGGAAGCACTATG---GGCGCGGCGTCA---
ATAACGCTGACGGTACAG---GCCAGACAA---TTATTG---TCTGGTATAGTGCAACAGCAAAGC---
AATTTG---CTGAGA---GCTATAGAAGCGCAACAACATCTGTTACAGCTCACGGTCTGGGGCATT
>MH746262..1
TTCTATTGCAATACAACACAACTGTTTAATGGC--

ACGATTGGCAATATCACACTGCCATGTAGGATAAAACAAATTATAAACATGTGGCAAGGAGTAGGACAAGCAA
TGTATGCTCCTCCCATCAATGGAATACTTAATTGTGTATCAAAT---
ATTACAGGAATACTATTGACAAGAGATGGTGGTAATCATACGAAT----------------------------
----

GAAACTGAGACCTTCAGACCGGGAGGAGGAAATATAAAGGACAATTGGAGAAGTGAATTATATAAATATAAAG
TAGTAGAAATTGAACCACTA---GGAATAGCACCCACCAAGGCAAAGAGAAGAGTGGTG--------------
-------------------------GAGAGAGAAAAAAGAGCA---GTGGGA---ATAGGAGCT---------
ATGATCTTTGGGTTCTTAGGAGCA---GCCGGAAGCACGATG---GGCGCGGCGTCA---
ATAACGCTGACGGTACAG---GCCAGACAA---TTATTG---TCTGGTATAGTGCAGCAGCAAAGC---
AATTTG---CTGAGG---GCTATAGAGGCGCAGCAGCATATGTTGCAACTCACAGTCTGGGGCATT
>U51188..2
TTCTATTGCAATACAACAGCACTGTTTAATAGTACTTGGATAAATGGAACCATGCAGGAGGTTAATGGCACA-
----

AACTCAGGCAATATCACACTTCCATGCAGGATAAAGCAAATTGTAAACATGTGGCAGGAAGTAGGACGAGCAA
TGTATGCTCCTCCCATCAGTGAAGTAATTAATTGTGTATCAAAT---
ATTACAGGAATACTATTGACAAGAGATGGGGGTATTAATCAAAATCAG-------------------------
----

ACTAACAAAAATGAGACCTTCAGACCTGGAGGAGGAAATATAAAGGACAATTGGAGAAGTGAATTATACAAAT
ATAAAGTAGTACAAATTGAACCACTA---GGAATAGCACCCACCAAGGCAAGGAGAAGAGTGGTG--------
-------------------------------GAGAGAGAAAAAAGAGCA---GTGGGA---ATAGGAGCT---
------ATGATCTTTGGGTTCTTAGGAGCA---GCAGGAAGCACTATG---GGCGCGGCGTCA---
ATAACGCTGACGGTACAG---GCCAGACAA---TTATTG---TCTGGTATAGTGCAACAGCAAAGC---
AATTTG---CTGAGG---GCTATAGAGGCGCAGCAGCATCTGTTGCAACTCACAGTCTGGGGCATT
>JX112863..1
TTCTATTGCAATACAACAAAACTGTTTAATAATACTTGCATAAATGGAACCACGAAGGGG-------------
----

TGTAATGGTACTATCATAATTCTATGCAAGATAAAGCAAATTGTAAACATGTGGCAGGGAGTAGGACAAGCAA
TGTATGCTCCTCCCGTCAGAGGAACAATTAATTGTGTATCAAAT---
ATTACAGGAATAATATTGACAAGAGATGGTGGAGTTAATAGGACA----------------------------
----

CATAACGAGACCTTCAGACCTGGAGGAGGAAATATGAAAGATAATTGGAGAAATGAATTATATAAATATAAAA
CAGTACTAATTGAACCACTA---GGAATAGCACCCACCAGGGCAAAGAGAAGAGTGGTG--------------
-------------------------GAGAGAGAAAAAAGAGCA---GTGGGA---ATAGGAGCT---------
ATGATCTTTGGGTTCTTGGGAGCA---GCAGGAAGCACTATG---GGCGCAGCGTCA---
ATAACGCTGACGGTACAG---GCCAGACAA---TTATTG---TCTGGTATAGTGCAACAGCAAAGC---
AATTTG---CTGAGG---GCTATAGAGGCGCAACATCATCTGTTGCAACTCACAGTCTGGGGCATT
>KF835503..1

```
TTCTATTGTAATACATCACAACTGTTTAATACAACACAACTTAAACGGTTTAATAATGGTGGG----------
-------------------------------------------------------------------------
----------------------------
GGTAGTGACACTATCATACTTCCATGTAAGATAAAACAAATTATAAACATGTGGCAGGGAGCAGGACAAGCAA
TGTATGCTCCTCCCATCAGTGGAGCAATTAATTGTTCATCAAAT---
ATTACAGGAATACTATTAACAAGAGATGGTGGTGTTCATAATGGGAGT-------------------------
-------------------------------------------------------------------------
----------
AACGAGACCGAGATCTTCAGACCTGGAGGAGGAAATATAAAGGACAATTGGAGAAGTGAACTATATAAATATA
AAGTAGTAGAAATTGAACCATTA---GGAATAGCACCCACCAAGGCAAGGAGAAGAGTGGTG-----------
----------------------------GAGAGAGAAAAAAGAGCA---GTGGGA---ATAGGAGCT------
---ATGATCTTTGGGTTCTTAGGAGCA---GCAGGAAGCACTATG---GGCGCGGCGTCA---
ATAACGCTGACGGTACAG---GCCAGACAA---TTATTG---TCTGGTATAGTGCAACAGCAAAGC---
AATTTG---CTGAGG---GCTATTGAGGCGCAACAGCATATGTTGCAACTCACAGTCTGGGGCATT
>KF835527..1
TTCTATTGCAACACATCAAACCTGTTTAATACAACATACCTTAATAGTAGCACA-------------------
-------------------------------------------------------------------------
----------------------------
GAAAATAACAATATCACACTTCCATGCAGAATAAAGCAAATTATAAACATGTGGCAGGGAACAGGACAAGCAA
TGTATGCTCCTCCCATCAGTGGAAACATTAGCTGTGTATCAAAT---
ATTACAGGAATACTATTGACAAGAGATGGTGGTGGGAATGGTACAGAGGGG----------------------
-------------------------------------------------------------------------
-------------
ACGAATGAGACCTTCAGACCTGGAGGAGGAAATATGAAGGACAATTGGAGAAATGAATTATATAAATATAAAG
TAGTACAAATTGAACCACTA---GGAATAGCACCCACCAGGGCAAGGAGAAGAGTGGTG--------------
-------------------------GAGAGAGAAAAAAGAGCA---GTGGGA---ATAGGAGCT---------
ATGATCTTTGGATTCTTGGGAGCA---GCAGGAAGCACTATG---GGCGCGGCGTCA---
ATAACGCTGACGGTACAG---GCCAGACAA---TTATTG---TCTGGTATAGTGCAACAGCAAAGC---
AATTTG---CTGAGG---GCTATAGAGGCGCAGCATCATTTGCTGCAACTCACAGTCTGGGGCATT
>JX112809..2
TTCTATTGCAATACAAGCCAACTGTTTAATGCTACTTACGGAACAAATGAAACCACGCGTACG----------
-------------------------------------------------------------------------
----------------------------
CGTAATGACACTATCATACTTCCATGCAGGATAAAGCAAATTATAAACATGTGGCAGGGAGTAGGACAAGCAA
TGTATGCTCCTCCCATCAGTGGAAGAATTAATTGTGTATCAAAT---
ATTACAGGAATACTATTAACAAGAGATGGTGGTGATAATAATAGT----------------------------
-------------------------------------------------------------------------
-------------
ACTAACGAGACCTTCAGACCTGGAGGGGGAGATATAAAGGACAATTGGAGAAGTGAATTATATAAATATAAAG
TAGTACAAATTGAACCACTA---GGAGTAGCACCCACCAGGGCAAAAAGAAGAGTGGTG--------------
-------------------------GAGAGAGAAAAAAGAGCA---GTGGGA---ATAGGAGCT---------
ATGATCTTTGGGTTCTTAGGAGCA---GCAGGAAGCACTATG---GGCGCGGCGTCA---
ATAACGCTGACGGTACAG---GCCAGACAA---TTATTG---TCTGGTATAGTGCAACAGCAAAGC---
AATTTG---CTGAGG---GCTATAGAGGCGCAGCATCATCTGTTGCAACTCACAGTCTGGGGCATT
>JX112813..1
TTCTATTGCAATACAACAAAACTGTTTAACAGTACTTGGACAGGAAATGAAACCATAAATGAGAAT-------
-------------------------------------------------------------------------
----------------------------
GGGACTACTAAAATCATAATTCCATGCAGGATAAAGCAAATTATAAACATGTGGCAAGGAGCAGGACAAGCAA
TATATGCTCCTCCCATCAGTGGAGCAATTAATTGTGTATCAAAT---
ATTACAGGAATACTATTGACAAGAGATGGTGGTGATGGTAATCAT----------------------------
-------------------------------------------------------------------------
-------------
ATAAACGAGACCTTCAGACCTGCAGGAGGAAATATGAGGGACAATTGGAGAAGTGAATTATATAAATATAAAG
TAGTACAAATTGAACCATTA---GGAATAGCACCCACCAGGGCAAAGAGAAGAGTGGTG--------------
-------------------------GAGAGACAAAAAAGAGCA---GTGGGA---ATAGGAGCT---------
ATGATCTTTGGGTTCTTAGGAGCA---GCAGGAAGCACTATG---GGCGCGGCGTCA---
ATAACGCTGACGGTACAG---GCCAGACAA---TTATTG---TCTGGTATAGTGCAACAGCAAAGC---
AATTTG---CTGAGG---GCTATAGAGGTGCAGCAGCATATGTTGCAACTCACAGTCTGGGGCATT
>JX112814..3
```

TTCTATTGCAATACAAGCCAACTGTTTAATAAAACTTACTATGGAAATGAAACTACTTCCATAGGGAATACC-
----

ACGAATAACACTATCGTACTTCCATGCAAGATAAAGCAAATTATAAACATGTGGCAGGGAGTAGGACAAGCAA
TGTATGCTCCTCCCATCAGTGGAAACATTAGTTGTGTATCAAAT---
ATTACAGGAATACTATTAACAAGAGATGGTGGTAATAATAAGTCT----------------------------
----

CAGGTGAACGAGACCTTCAGACCTGGAGGAGGAAATATGAAGGACAACTGGAGAAGTGAATTATATAAATATA
AAGTAGTACAAATTGAACCATTA---GGAATAGCACCCACCAGGGCAAAGAGAAGAGTGGTG-----------
----------------------------GAGAGAGAAAAAAGAGCA---GTGGGA---ATAGGAGCT------
---ATGATCTTTGGGTTCTTAGGAGCA---GCAGGAAGCACTATG---GGCGCGGCGTCA---
ATAACGCTGACGGTACAG---GCCAGACAA---TTATTG---TCTGGTATAGTGCAACAGCAAAGC---
AATTTG---CTGAGG---GCTATAGAGGCGCAGCAGCATCTGCTGCAACTCACAGTCTGGGGCATT
>JX112820..1
TTCTATTGCAATACATCAGGACTGTTTAATAGTACTTGGGAAAGAAATGGAACCATGAGGGGGCATAATAGC-
----

ACTAATGGCACTATCATACTTCCATGCAGGATAAAGCAAATTATAAACATGTGGCAGGGAGTAGGACAAGCAA
TGTATGCTCCTCCCATCGCAGGAAGTATTAATTGTACATCAAAT---
ATTACAGGGATACTATTGACAAGAGATGGTGGTTATGATGCGGAGAAT-------------------------
----

AATGAGACTGAGACTTTCAGACCTGGAGGAGGAAATATAAAAGACAATTGGAGAAGTGAATTATATAAATATA
AAGTAGTACAAATTGAACCACTA---GGAATAGCACCCACCATGGCAAAGAGAAGAGTGGTG-----------
----------------------------GAAAGAGAAAAAAGAGCA---GTGGGA---ATAGGAGTT------
---ATGATCTTTGGGTTCTTAGGAGCA---GCCGGAAGCACTATG---GGCGCAGCGTCA---
CTAACGCTGACGGTACAG---GCCAGACAA---TTATTG---TCTGGTATAGTGCAACAGCAAAGC---
AATTTG---CTGAGG---GCTATAGAGGCGCAGCAGCATATGTTGCAACTCACAGTCTGGGGCATT
>JX112823..1
TTCTATTGCAATACAAGCCAACTGTTTAATGATACAGGAAATGAAACCATAGGGAAT----------------
----

AATAGTAACACTATCATACTTCCATGCAGGATAAAGCAAATTATAAACATGTGGCAGGGAGTAGGACAAGCAA
TGTATGCTCCTCCCATCAGTGGAACAATTAATTGTGTATCAAAT---
ATTACAGGAATACTATTAACAAGAGATGGCGGTACTAATGGTAATAATACTAAT------------AAT----
----

ACCACTAATAATACCGAGACCTTCAGACCTGGGGGAGGAAATATAAAGGACAATTGGAGAAGTGAATTATATA
AATATAAAGTAGTACAAATTGAACCACTA---GGAATAGCACCCACCAGGGCAAAGAGAAGAGTGGTG-----
----------------------------------GACAGAGAAAAAAGAGCA---GTGGGA---
ATAGGAGCT---------ATGATCTTTGGGTTCTTAGGAGCA---GCTGGAAGCACTATG---
GGCGCGGCGTCA---ATAACGCTGACGGTACAG---GCTAGACAA---TTATTG---
TCTGGTATAGTGCAACAGCAAAGC---AATTTG---CTGAGG---
GCTATAGAGGCGCAGCATCATTTGTTGCAACTCACAGTCTGGGGCATT
>JX112826..1
TTCTATTGCAATACAAGCAAACTGTTTAATTTTACTTTCCCAAGAAATGAAACAGGGGAG-------------
----

AATGATACAACTATCATACTTCCATGCAGGATAAAGCAAATTATAAACATGTGGCAGGGAGTAGGACAAGCAA
TGTATGCTCCTCCCATCAGTGGGATAATTAATTGTGTATCAAAT---
ATTACAGGAATACTATTAACAAGAGATGGTGGTCATAATAATAATACT-------------------------
----

ATTACTAATGAGACCTTCAGACCTGGAGGAGGAAATATAAAGGACAATTGGAGAAGTGAATTATACAAATATA
AAGTAGTACAAATTGAACCACTA---GGAATAGCACCCACCAGGGCAAAGAGAAGAGTGGTG-----------
----------------------------GAGAGAGAAAAAAGAGCA---GTGGGA---ATAGGAGCT------
---ATGATCTTTGGGTTCTTAGGAGCA---GCAGGAAGCACTATG---GGCGCGGCGTCA---
ATAACGCTGACGGTACAG---GCCAGACAA---TTATTG---TCTGGTATAGTGCAACAGCAAAGC---
AATTTG---CTGAGG---GCTATAGAGGCGCAGCAGCATATGTTGCAACTCACAGTCTGGGGCATT

>JX112835..2
TTCTATTGCAATACAAACAAACTGTTTAATGTAACTTACAATGAAAAGAATACCACAGGGGGG----------
----

AGTAATGGCACTATCATACTCCTATGCAAGATAAAGCAAATTATAAACATGTGGCAGGAAGTAGGACAAGCAA
TGTATGCTCCTCCCATCAGTGGAAGAATTAATTGTGTATCAAAT---
ATTACAGGAATACTACTAACAAGAGATGGTGGTGGTACTAATAGG----------------------------
----

ACTAATGAGACCTTCAGACCTGGAGGAGGAAATATAAAGGACAATTGGAGAAGTGAATTATATAAATATAAAG
TAGTACAACTTGAACCACTA---GGAATAGCACCCACCAGGGCAAAGAGAAGAGTGGTG--------------
-------------------------GAGAGAGAAAAAAGAGCA---GTGGGA---ATAGGAGCT---------
ATGATCTTTGGGTTCTTGGGAGCA---GCAGGAAGCACTATG---GGCGCGGCGTCA---
ATAACGCTGACGGTACAG---GCCAGACAA---TTATTG---TCTGGTATAGTGCAACAGCAAAGC---
AATTTG---CTGAGG---GCTATAGAGGCGCAGCATCATCTGTTGCAACTCACAGTCTGGGGCATT
>JX112844..1
TTCTATTGCAATACAACAAACCTGTTTAACAGTACTTGGGAAAATGGAACCACGGAGGGTAAT----------
----

GGCACCGAGAAGATCATACTTCCATGCAGGATAAAGCAAATTATAAACATGTGGCAGGGAGCAGGACAAGCAA
TGTATGCTCCTCCCATCAGTGGAACAATTAATTGTGTATCAAAT---
ATTACAGGAATACTATTAACAAGAGATGGTGGTTATAATAATGCG----------------------------
----

GATAACGAGACCTTCAGACCTGGAGGAGGAAATATAAAGGACAATTGGAGAAGTGAATTATATAAATATAAAG
TAGTAGAAATTGAACCACTA---GGAATAGCACCCACCAGGGCAAAGAGAAGAGTGGTG--------------
-------------------------GAGAGAGAAAAAAGAGCA---GTGGGA---ATAGGAGCT---------
ATGATCTTTGGGTTCTTGGGAGCA---GCAGGAAGCACTATG---GGCGCGGCGTCA---
ATAACGCTGACGGTACAG---GCCAGACAA---TTATTG---TCTGGTATAGTGCAACAGCAAAGC---
AATTTG---CTGAGG---GCTATAGAGGCGCAGCAGCATATGCTGCAACTCACAGTCTGGGGCATT
>GU475036..2
TTCTATTGCAATACAACAAAACTGTTTAATAGGAATAATAAC-------------------------------
----

TATAGCACTATCATACTTCCATGCAAGATAAAGCAAATTGTAAGAATGTGGCAGGGAGTAGGACAAGCAATGT
ATGCTCCTCCCATCAGTGGAAGCATAAATTGTGTATCAAAT---
ATTACAGGAATACTATTGACAAGAGATGGTGGTATTCATAATAGT----------------------------
----

AGTGATGAGACCTTCAGACCTGGAGGAGGAAATATAAAGGACAATTGGAGAAGTGAATTATATAAATATAAGG
TAGTAGAAATTGAACCACTA---GGAATAGCACCCACCAAGGCAAAGAGAAGAGTGGTG--------------
-------------------------GAGAGAGAAAAAAGAGCA---GTGGGA---ATAGGAGCT---------
ATGATCTTTGGGTTCTTAGGAGCA---GCAGGAAGCACTATG---GGCGCGGCGTCA---
ATAACGCTGACGGTACAG---GCCAGACAA---TTATTG---TCTGGTATAGTGCAACAGCAAAGC---
AATTTG---CTGAGG---GCTATAGAGGCGCAACAGCATATGTTGCAACTCACAGTCTGGGGCATT
>GU475019..1
TTCTATTGCAATACAACAAATCTGTTTAATAATACTTGCATAAACAATGCAAGCACAAAAGGG----------
----

TGTAATGAAAATATCATACTTCCATGCAGGATAAAACAAATTATAAACATGTGGCAAGGAGTAGGACAAGCAA
TGTATGCTCCTCCCATCAGTGGAAAAATTACTTGTAGATCAAAT---
ATTACAGGAATACTATTGACAAGAGATGGTGGTAATCATAGCACA----------------------------
----

GGTAATGAGACCTTCAGACCTGAAGGAGGAAATATAAAGGACAATTGGAGAAATGAATTATATAAATATAAAG
TAGTAGAAATTGAACCACTA---GGAATAGCACCCACCAGGGCAAAGAGAAGAGTGGTG--------------
-------------------------GAGAGAGAAAAAAGAGCA---GTGGGA---ATAGGAGCT---------
ATGATCTTTGGGTTCTTAGGAGCA---GCAGGAAGCACTATG---GGCGCAGCGTCA---
ATAACGCTGACGGTACAG---GCCAGACAA---TTATTG---TCTGGTATAGTGCAACAGCAAAGC---
AATTTG---CTGAGG---GCTATAGAGGCGCAGCAGCATCTGTTGCAACTCACAGTCTGGGGCATT

>JX960615..1
TTCTATTGCAATACAACAAAGCTGTTCAATAGTACTTGGATAGGAAATGAAACCACGGAGGGAGGTAAT----
----

GATAATGACACTATCATACTTCCATGCAGGATAAAGCAAATTATAAACATGTGGCAAGGAGCGGGACAAGCAA
TGTATGCTCCTCCCATCAGTGGAATAATTAAATGTGTATCAAAT---
ATTACAGGAATACTATTGACAAGAGATGGTGGTGATTATAATAAT----------------------------
----

ACAAATGAGACCTTCAGACCTGGAGGAGGAAATATAAAGGACAATTGGAGAAGTGAATTATATAAATATAAGG
TAGTACAAATAGAACCACTA---GGAGTAGCACCTACCAGGGCAAGGAGAAGAGTGGTG--------------
-------------------------GAGAGAGAAAAAAGAGCA---GTGGGA---ATAGGAGCT---------
ATGATCTTTGGGTTCTTAGGAGCA---GCAGGAAGCACTATG---GGCGCGGCGTCA---
ATAACGCTGACGGTACAG---GCCAGACAA---TTATTG---TCTGGTATAGTGCAACAGCAAAGC---
AATTTG---CTGAGG---GCTATAGAGGCGCAACAGCATATGTTGCAACTCACAGTCTGGGGCATT
>JX960639..1
TTCTATTGCAATACAACAAAATTGTTTAATAGTACAGAAAATGGAACCATGGAGGGG----------------
----

CTTAATGGCACTATTATACTCCCATGCAGGATAAAACAAATTGTAAACCTGTGGCAGGGAGCAGGACAAGCAA
TGTATGCTCCTCCCGTCAGTGGAAAAATTAATTGTGCATCAAAT---
ATTACAGGAATACTATTAACAAGAGATGGTGGTAATAATGCAACA----------------------------
----

ACTAACGAGACCTTCAGACCGGGAGGAGGAAATATAAAGGACAATTGGAGAAGTGAATTATATAAATATAAAG
TAGTACAAATTGAACCACTA---GGAGTAGCACCCACCAGGGCAAAGAGAAGAGTGGTG--------------
-------------------------GATAAAGAAAAAAGAGCA---GTGGGA---ATAGGAGCT---------
ATGATCTTTGGGTTCTTAGGAGCA---GCAGGAAGCACTATG---GGCGCGGCGTCA---
ATAACGCTGACGGTACAG---GCCAGACAA---TTATTG---TCTGGTATAGTGCAACAGCAAAGC---
AATTTG---CTGAGA---GCTATAGAGGCGCAGCATCATCTGTTGCAACTCACAGTCTGGGGCATT
>JX960632..1
TTCTATTGCAATACAACAAGGCTGTTCAATAGTACTTGGATAGGAAATGAGACCATAGGAAATGAGACCATGG
GGGAAGGTAAT--

AATAATAACACTATCATACTTCCATGCAGGATAAAGCAAATTATAAACATGTGGCAAGGAGCAGGACAAGCAA
TATATGCTCCTCCCATCAGTGGAATAGTTAATTGTGTATCAAAT---
ATTACAGGGATACTATTGACAAGAGATGGTGGTCAAGAAGATAAT----------------------------
----

GGAACAAATAGCGAGATTTTCAGACCTGGAGGAGGAAATATAAAGGACAATTGGAGAAGTGAATTATATAAAT
ATAAAGTAGTAGAAATAGAACCACTA---GGAGTAGCACCCACCAGGGCAAAGAGAAGAGTGGTA--------
-------------------------------AATAGAGAAAAAAGAGCA---GTGGGA---ATAGGAGCT---
------ATGATCTTTGGGTTCTTGGGAGCA---GCAGGAAGCACTATG---GGCGCGGCGTCA---
ATAACGCTGACGGTACAG---GCCAGACAA---TTATTG---TCTGGTATAGTGCAACAGCAAAGC---
AATTTG---CTGAGG---GCTATAGAGGCGCAGCAGCATATGTTGCAACTCACAGTCTGGGGCATT
>MH672757..1
TTCTATTGCAATACAACAAAGCTGTTCAATGGTAATGGCACTAAAAATGGTAGTGAGACA-------------
----

AACAGTAGCACTATCATACTTCCATGCAGGATAAAGCAAATTATAAACATGTGGCAAGGAGCAGGACAAGCAA
TGTATGCTCCTCCCATCAGTGGAATAATTAATTGTGTATCAAAT---
ATTACAGGAATACTATTAACAAGAGATGGTGGTAATCATAATAAT----------------------------
----

ACTACCGAGATTTTCAGACCTGGAGGAGGAAATATGGAGGACAATTGGAGAAGTGAATTATATAAATATAAAG
TAGTACAAATAGAACCACTA---GGAGTAGCACCCACCAAGGCAAAGAGAAGAGTGGTG--------------
-------------------------GATAGAGAAAAAAGAGCA---GTGGGA---ATAGGAGCT---------
ATGATCTTTGGGTTCTTGGGAGCA---GCAGGAAGCACTATG---GGCGCGGCGTCA---
ATAACGCTGACGGTACAG---GCCAGACAG---TTATTG---TCTGGTATAGTGCAGCAGCAAAGC---
AATTTG---CTGAGG---GCTATAGAGGCGCAACAGCATATGTTGCAACTCACAGTCTGGGGCATT

```
>JX112798..2
TTCTATTGCAATACAACAAAACTGTTCAATAATGAAACCATG-------------------------------
-------------------------------------------------------------------------
----------------------------
AAAGAGGACACTATCATACTTCCATGCAGGATAAAGCAAATTATAAACTTGTGGCAAGGAGTGGGACAAGCAA
TGTATGCTCCTCCCATCAGTGGAATAATTCAATGTGTATCAAAT---
ATTACAGGAATAATATTAACAAGGGATGGTGGTAATCAAAGTAATAGTAATGTA-------------------
-------------------------------------------------------------------------
-------------
ACTAGAGAGATCTTCAGACCTGGAGGAGGAAATATGAAGGACAATTGGAGAAGTGAATTATATAAATATAAAG
TAGTACAAATTGAACCACTA---GGAGTAGCACCCACCAGGGCAAAGAGAAGAGTGGTG--------------
-------------------------GATAGACAAAAAAGAGCA---GTGGGA---ATAGGAGCT---------
ATGATCTTTGGGTTCTTAGGAGCA---GCAGGAAGCACTATG---GGCGCGGCGTCA---
ATAACGCTGACGGTACAG---GCCAGACAA---TTATTG---TCTGGTATAGTGCAACAGCAAAGC---
AATTTG---CTGAGA---GCTATAGAGGCGCAGCAGCATATGTTGCAACTCACAGTCTGGGGCATT
>JX112799..1
TTCTATTGTAATACAACAAAATTGTTTAATAAGACAGAGAATGGAACCATGGAGGGG----------------
-------------------------------------------------------------------------
----------------------------
TTTAATACCACTATCATACTTCCATGCAGGATAAAGCAAATTGTAAGGATGTGGCAGGGAGTAGAACAAGCAA
TGTATGCTCCTCCCATCAGTGGAAACATTACTTGTAGATCAAAT---
ATTACAGGAATACTATTAACAAGAGATGGTGGTGGTTATGATACA----------------------------
-------------------------------------------------------------------------
-------------
TCTCCCGAAACCTTCAGACCAGGAGGAGGAAATATGAAGGACAATTGGAGAAGTGAATTATATAAATATAAAG
TAGTACAAATTGAACCACTA---GGAATAGCACCCTCCAGGGCAAAGAGAAGAGTGGTG--------------
-------------------------GATAGACAAAAAAGAGCA---GTGGGA---ATAGGAGCT---------
ATGATCTTTGGGTTCTTAGGAGCA---GCAGGAAGCACTATG---GGCGCAGCGTCA---
ATAACGCTGACGGTACAA---GCCAGACAA---TTATTG---TCTGGTATAGTGCAACAGCAAAGC---
AATTTG---CTGAGG---GCTATAGAGGCGCAGCAGCATCTGTTGCAACTCACAGTCTGGGGCATT
>KC870041..1
TTCTATTGCAATACAACAAAACTGTTTAATAAGACTTTCGATAAGAACATAGAA-------------------
-------------------------------------------------------------------------
-AATGACACCATGGAT---------
GGCAGTAATAACACTATCATACTTCCATGCAGGATAAAGCAAATTATAAACATGTGGCAGGGAGTAGGACAAG
CAATGTATGCCCCTCCCATTAGGGGAAAAATTCATTGTGTATCAAAT---
ATTACAGGAATACTATTGACAAGAGATGGTGGT----------------------------------------
-------------------------------------------------------------------------
-------
ACTGATAATAACGAGACCTTCAGACCTGGAGGGGGAAACATAAAAGACAATTGGAGAAGTGAATTATATAAAT
ATAAAGTAGTACAAATTGAACCACTA---GGGGTAGCACCTACCAGGGCAAAGAGAAGAGTGGTG--------
-------------------------------GAAAGAGAAAAAAGAGCA---GTGGGA---ATAGGAGCT---
------ATGATCCTTGGGTTCCTAGGAGCA---GCAGGAAGCACTATG---GGCGCGGCGTCA---
ATAACGCTGACGGTACAG---GCCAGACAA---TTATTG---TCTGGTATAGTGCAACAGCAAAGC---
AATTTG---CTGCGG---GCTATAGAGGCGCAACAGCATATGTTGCAACTCACAGTCTGGGGCATT
>MH672923..1
TTCTATTGCAATACAACAAAATTGTTTAATAGTACAGAAAATGGAACCATGGAGGGG----------------
-------------------------------------------------------------------------
----------------------------
TTTAATACCACTATCATAATTCCATGCAAGATAAAGCAAATTGTAAATATGTGGCAGGGAGTAGGACAAGCAA
TATATGCTCCTCCCATTAGTGGAGTAATTAAGTGTCATTCAAAT---
ATTACAGGAATACTATTAACAAGAGATGGTGGTAATAATAATGCA----------------------------
-------------------------------------------------------------------------
-------------
AATAACGAAACCTTCAGACCGGGAGGAGGAAATATAAAGGACAATTGGAGAAATGAATTATATAAATATAAAG
TAGTACAAATTGAACCACTA---GGAATAGCACCCACCAAGGCAAAAAGAAGAGTGGTG--------------
-------------------------GAGAGAGAAAAAAGAGCA---GTGGGA---ATAGGAGCT---------
ATGATCTTTGGGTTCTTAGGAGCA---GCAGGAAGCACTATG---GGCGCAGCGTCA---
GTAACGCTGACGGTACAG---GCCAGACAA---TTATTG---TCTGGTATAGTGCAACAGCAAAGC---
AATTTG---CTGAGA---GCTATAGAGGCGCAACAGCACCTGTTGCAACTCACAGTCTGGGGCATT
```

```
>MG655209..1
TTCTATTGCGATACAACAAAACTGTTTAATAGTACAGGAAATGGAACCATGGAGGGG----------------
-------------------------------------------------------------------------
----------------------------
CTTAATGGCACCATTATACTTCCATGCAAGATAAAGCAAATTATAAACATGTGGCAGGGAGCAGGACAAGCAA
TATATGCTCCTCCCATCAGTGGAAGAATTGAGTGTAGATCAAAT---
ATTACAGGAATACTATTAACAAGAGATGGTGGTCATAATATAACT----------------------------
-------------------------------------------------------------------------
----------------
AACGAGACCTTCAGACCAGGAGGAGGAAATTTAAAGGACAATTGGAGAAATGAATTATATAAATATAAAGTAG
TACAAATTGAACCACTA---GGAATAGCACCCACCAAGGCAAAGAGAAGAGTGGTG-----------------
----------------------AATAGAGAAAAAAGAGCA---GTGGGA---ATAGGAGCT---------
ATGATCTTTGGGTTCTTAGGAGCA---GCAGGAAGCACTATG---GGCGCGGCGTCA---
ATAACGCTGACGGTACAG---GCCAGACAA---TTATTG---TCTGGTATAGTGCAACAGCAAAGC---
AATTTG---CTGAGA---GCTATAGAGGCGCAGCAGCATCTGTTGCAACTCACAGTCTGGGGCATT
>MG655214..2
TTCTATTGCAATACAACAAAGTTATTTAATAGTACAGAAAATGGAACCAGGGAAGAGGGT-------------
-------------------------------------------------------------------------
----------------------------
CATAATGGCACTATTATACTCCCATGCAGGATAAAGCAAATTGTAAACATGTGGCAGGGAGCAGGCCAAGCAA
TGTATGCTCCTCCCGTCAGAGGAAACATTAATTGTGTATCAAAT---
ATTACAGGACTACTATTAACAAGAGATGGTGGTATTAAGGGAACT----------------------------
-------------------------------------------------------------------------
-------------
CACAACGAGACCTTCAGACCGGGAGGAGGAAATATAAAGGACAATTGGAGAAGTGAATTATATAAATATAAAG
TAGTACAAATTGAACCACTA---GGAGTAGCACCCACCAAGGCAAAAAGAAGAGTGGTG--------------
-------------------------GATAGAGAAAAAAGAGCA---GTAGGA---ATAGGAGCT---------
ATGATCTTTGGGTTCTTAGGAGCA---GCAGGAAGCACTATG---GGCGCGGCGTCA---
ATAACGCTGACGGTACAG---GCCAGACAA---TTATTG---TCTGGTATAGTGCAACAGCAAAGC---
AATTTG---CTGAGA---GCTATAGAGGCGCAGCAGCATATGCTGCAACTCACAGTCTGGGGCATT
>MG655215..1
TTCTATTGCAATACAACACAATTGTTTAATAATAATTCCACG-------------------------------
-------------------------------------------------------------------------
----------------------------
TTTAATACCACTGTCATACTTCCATGCAGGATAAAGCAAATTGTAAATATGTGGCAGGGGGTAGGACGAGCAA
TGTATGCTCCTCCCATCAGTGGAATGATTAATTGTGTATCAAAT---
ATTACAGGAATAATATTAACAAGAGATGGTGGTTATAGTAATACA----------------------------
-------------------------------------------------------------------------
-------------
ACTAACGAAACCTTCAGACCGGGAGGAGGAAATATAAAGGACAATTGGAGAAGTGAACTATATAAATATAAAG
TAGTACAAATTGAACCACTA---GGAATAGCACCCACCAGGGCAAAGAGAAGAGTGGTG--------------
-------------------------ACTAGAGAAAAAAGAGCAGTAGTGGGA---ATAGGAGCT---------
ATGATCTTTGGGTTCTTAGGAGCA---GCAGGAAGCACTATG---GGCGCAGCGTCA---
ATAACGCTGACGGTACAG---GCCAGACAA---TTATTG---TCTGGTATAGTGCAACAGCAAAGC---
AATTTG---CTGAGA---GCTATAGAGGCGCAGCAGCATCTGCTGCAACTCACAGTCTGGGGCATT
>DQ234790..1
TTCTATTGCAATACAACACAACTGTTTGATAGTAGTTGGAATACAACAACAACCAATAGGGAG----------
-------------------------------------------------------------------------
----------------------------
AACCGTAGTAATTTCATACTTCCATGCAGGATAAAACAAATTATAAACATGTGGCAGGAAACAGGAAAAGCAA
TGTATGCTCCTCCCATCAGGGGAAGCATTCAGTGTGTATCAAAT---
ATTACAGGAATACTATTGACAAGAGATGGTGGTAATAATAATGGGTCT-------------------------
-------------------------------------------------------------------------
----------
AACGAGACCGAGACCTTTAGACCTGGAGGAGGAGATATAAGAGACAATTGGAGAAGTGAATTATATAAATATA
AAGTAGTACAAATTGAACCACTA---GGAGTAGCACCCACCAGGGCAAAGAGAAGAGTGGTG-----------
----------------------------GAGAGAGAAAAAAGAGCAGTAGTGGGA---ATAGGAGCT------
---ATGATCTTTGGGTTCTTAGGAGCA---GCAGGAAGCACTATG---GGCGCGGCGTCA---
TTAACGCTGACGGTACAG---GCCAGACAA---TTACTG---TCTGGTATAGTGCAACAGCAAAGC---
AATTTG---CTGAGG---GCTATAGAGGCGCAACAGCATATGTTGCAACTCACAGTCTGGGGCATT
```

>KF859741..2
TTCTATTGCAATACAACAAAATTATTTAATCACACAAACAATGACACCATGCAG-------------------
----

TATAATGACACTATCACACTTCCATGCAGGATAAAGCAAATTATAAACATGTGGCAGAGAGTAGGACAAGCAA
TGTATGCTCCTCCCGTCAGGGGAAGAATTAATTGTGTATCAAAC---
ATTACAGGAATACTATTGACAAGGGATGGTGGTGGGACAAACAGT----------------------------
----

ACGAACGAGATCTTCAGACCTCAAGGAGGAAATATAAAGGACAATTGGAGAAGTAAATTATATAAATATAAAG
TAGTACAAATTGAACCACTA---GGAGTGGCACCCACCAGGGCAAGGAGAAGAGTGGTG--------------
-------------------------GAGAGAGAAAAAAGAGCA---GTGGGA---ATAGGAGCT---------
CTGATCTTTGGGTTCTTAGGAGCA---GCAGGAAGCACTATG---GGCGCGGCGTCA---
ATAACGCTGACGGTACAG---GCCAGACAA---TTATTG---TCTGGTATAGTGCAACAGCAAAGC---
AATTTG---CTGAGG---GCTATAGAGGCGCAGCAGCATCTGTTGCAACTCACAGTCTGGGGCATT
>MH327759..1
TTCTATTGCAATACAACACAATTATTTAATAAC--

ACCAATGGTACTATCATACTTCCATGCAAGATAAAACAAATTATAAACATGTGGCAGGGAGTAGGACAAGCAA
TGTATGCTCCTCCCGTCAGGGGAAAAATTAATTGTGTATCAAAC---
ATTACAGGAATACTATTGACAAGGGATGGTGGGGCAAATAGCACG----------------------------
----

AATGAGACCTTCAGACTTCAAGGAGGAAATATAAAGGACAATTGGAGAAATGAATTATATAAATATAAAGTAG
TACAAATTGAACCACTA---GGAATAGCACCCACCAGGGCACGGCGAAGAGTGGTG-----------------
----------------------GAGAGAGAAAAAAGAGCA---GTGGGA---ATAGGAGCT---------
ATGATCTTTGGGTTCTTAGGAGCA---GCAGGAAGCACTATG---GGCGCGGCGTCA---
ATAACGCTGACGGTACAG---GCCAGACAA---TTATTG---TCTGGTATAGTGCAACAGCAAAGC---
AATTTG---CTGAGG---GCTATAGAGGCGCAGCAGCATCTGTTGCAACTCACAGTCTGGGGCATT
>MH078562..2
TTCTATTGCAATACAACACGATTATTTAATTGCACAAACAATAATAACACG----------------------
----

TGTAATGAAACTATCATACTTCCATGCAAGATAAAGCAAATTATAAACATGTGGCAGGGAGTAGGACAAGCAA
TGTATGCTCCTCCCATCAGGGGAAGAATTAATTGTGTATCAAAC---
ATTACAGGAATACTATTAACAAGGGATGGTGGTCAGACAAACGATACG-------------------------
----

AACTACGAAACCTTCAGACCTGCAGGAGGAAATATAAAGGACAATTGGAGAAATGAATTATATAAATATAAAG
TAATACAAATTGAACCACTA---GGATTGGCACCCACCAGGGCAAGGCGAAGAGTGGTG--------------
-------------------------GAGAGAGAAAAAAGAGCA---GTGGGA---ATAGGAGCT---------
ATGATCTTTGGGTTCTTAGGAGCA---GCAGGAAGCACTATG---GGCGCGGCGTCA---
ATAACGCTGACGGTACAG---GCCAGACAA---TTATTG---TCTGGTATAGTGCAACAGCAAAGC---
AATTTG---CTGAGG---GCTATAGAGGCGCAGCAGCATATGTTGCAACTCACAGTCTGGGGCATT
>MF373160..1
TTCTATTGCAATATAACAAAACTGTTTAATAGTACTTGGGGGAGTAATGGCACTTGGAATGGCACTGAA----
----

TACAATAACGATACCATACTCCCATGCAGGATAAAGCAAATTATAAACATGTGGCAACAACCAGGACAAGCAA
TGTATGCTCCTCCCGTTCAGGGAGTAATTAATTGTATATCAAAT---
ATTACAGGACTAATATTGACAAGAGATGGTGGTATTAATACTACA----------------------------
----

ATTAATGAAACCTTCAGACCTGGAGGAGGAAATATAAAGGACAATTGGAGAAGTGAATTATATAAATATAAAG
TAGTACAAATTGAACCACTA---GGAATAGCACCCACCAAGGCAAAGAGAAGAGTGGTG--------------
-------------------------GAGAGAGAGAAAAGAGCA---GTGGGA---ATAGGAGCT---------
ATGATCTTTGGGTTCTTAGGAGCA---GCCGGAAGCACTATG---GGCGCGGCGTCA---
ATAACGCTGACGGTACAG---GCCAGACAA---TTATTG---ACTGGTATAGTGCAGCAGCAAAGC---
AATTTG---CTGAGA---GCTATAGAGGCGCAACAGCATCTGTTGCAACTCACAGTCTGGGGCATT

>KP411840..2
TTCTATTGCAATACATCAAGGCTGTTTAATAGTACTTACAATGGAACTGAAACCGGGAAAGAG----------
----

GGTAATGGCACTATTATACTTCCATGCAGGATAAAGCAAATTATAAACATGTGGCAGGGAGTGGGACGAGCAA
TGTATGCTCCTCCCATCAGTGGAAGCATTAATTGTACATCAAAT---
ATTACAGGAATACTATTGACAAGCGATTATGGTGGTAATAATACA----------------------------
----

CAGAATGAGACCTTCAGACCTGCAGGAGGAAATATAAAGGACAATTGGAGAAGTGAATTATATAAATATAAAG
TAGTACAAATTGAACCACTA---GGAGTAGCACCCACCAAGGCAAAGAGAAGAGTGGTG--------------
-------------------------GAGAGACAAAAAAGAGCA---GTGGGA---ATAGGAGCT---------
ATGATCTTTGGGTTCTTGGGAGCA---GCAGGAAGCACTATG---GGCGCGGCGTCA---
ATAACGCTGACGGTACAG---GCCAGACAA---TTATTG---ACTGGTATAGTGCAACAGCAAAGC---
AATTTG---CTGAGG---GCTATAGAGGCGCAGCAGCATCTGTTGCAACTCACAGTCTGGGGCATT
>KP411841..3
TGCTACTGCAACCCCCTGCAACTGTTTAATAATAAGACTCATACTAATGGT----------------------
----

ACTAATGGCGCTATTGTACTTCTACGGATAATTAAGATAATGAAAATGATGAACCTGGGAGGAGGAGCAGCA-
--CAGGCTCCGCCTGCTCCTCGAAGTAGTATATGTGTTATTTCA---
GTTTCAAGAATAACACTGATACTAGTGGGTGGTGATGATGATAATAATAAT----------------------
----

AATAACCGCCAGAGCTTCGGACGAGCGGGAGGATGGATGAGGTACGATTATGGATATGAAGTATTACAATATG
AACTAGTACGAATAGTACCA------------CCACCAACAACAACAACCATGGTGGAGATG-----------
-------------------AGAAAAAGAGAAAAAGGAAAAAGAGCA---GTGGTA---ATAAGAGTT------
---TTGATCATCGGAGGAATAGGAACA---GCA------------------GCGGCACCA---
TTATCGCTGACGGCGATG---GCCCAGCAG---ATATTG---TCATGGATATTGAGACAGCGGAGG---
CTGCTG---CTGATG---AGTATTAAGTTGATGGAGCATCAGCAGCAGCTCACCTGCTGGGCCATC
>KP411842..2
TTCTATTGCAATACATCAAGGCTGTTTAATAGTACTTACAATGGAACTGAAAGTACCGAAGGGTCAAATGAG-
----

GGTAATGGCACTATTATACTTCCATGCAGGATAAAGCAAATTATAAACATGTGGCAGGGAGCAGGACGAGCAA
TGTATGCTCCTCCCATCAGTGGAAGCATTAATTGTACATCAAAT---
ATTACAGGAATACTATTGACAAGCGATTATGGTGGTAGTAATACA----------------------------
----

CAGAATGAGACCTTCAGACCTGCAGGAGGAAATATAAAGGACAATTGGAGAAGTGAATTATATAAATATAAAG
TAGTACAAATTGAACCACTA---GGAGTAGCACCCACCAAGGCAAAGAGAAGAGTGGTG--------------
-------------------------GAGAGACAAAAAAGAGCA---GTGGGA---ATAGGAGCT---------
ATGATCTTTGGGTTCTTGGGAGCA---GCAGGAAGCACTATG---GGCGCGGCGTCA---
ATAACGCTGACGGTACAG---GCCAGACAA---TTATTG---ACTGGTATAGTGCAACAGCAAAGC---
AATTTG---CTGAGG---GCTATAGAGGCGCAGCAGCATCTGTTGCAACTCACAGTCTGGGGCATT
>KY213735..1
TTCTATTGCAATACAACAAAACTGTTTAATAGTCCTTGCCTGAAAAATGAAGAAAATGGAACA----------
----

TGTAATAGCACTATCATACTTCCATGTAAGATAAAGCGAATTGTAAGATTGTGGCAGGAAGTAGGTCAAGCAA
TGTATGCTCCTCCCATCAGCGGAATCATTAGGTGTAACTCAACT---
ATTACAGGAATACTATTGACAAGAGATGGTGGTAACCTTAATAAC----------------------------
----

AGTACTAATGAAACCCTCAGACCTGGAGGAGGAAATATAAAGGACAATTGGAGAAGTGAATTATATAAATATA
AAGTAGTACAAATTGAACCACTA---GGAATAGCACCCACCAAGGCAAAAAGAAGAGTGGTG-----------
----------------------------GAGAGAGAAAAAAGAGCA---GTGGGA---ATAGGAGCT------
---ATGATCTTTGGGTTCTTAGGAGCA---GCAGGAAGCACTATG---GGCGCGGCGTCA---
ATAACGCTGACGGTACAG---GCCAGACAA---TTATTG---TCTGGTATAGTGCAACAGCAAAAC---
AATTTG---CTGAGG---GCTATAGAGGCGCAGCAGCATCTGTTGCAACTCACAGTCTGGGGCATT

```
>KY213733..1
TTCTATTGCAATACAACAAAACTGTTTAATAACACTTGCATAAATGGAACAATGGAAAATGAG----------
-------------------------------------------------------------------------
----------------------------
AGTAATAGCACTATCATACTTCCATGCAAGATTAAACAAATTATAAACATGTGGCAGGGAGCAGGACAAGCAA
TGTATGCTCCTCCCATCAGTGGAAGAATTAATTGTATATCAAAT---
ATTACAGGAATACTATTAACAAGAGATGGTCAG----------------------------------------
-------------------------------------------------------------------------
-------
CCGAATGATACGGAGATCTTCAGACCTGGAGGAGGAGATATGAAGGACAATTGGAGAAATGAATTATATAAAT
ATAAAGTAGTACAAATTGAACCACTA---GGAATAGCACCCACCAGGGCAAAGAGAAGAGTGGTG--------
-------------------------------GAGAGAGAAAAAAGAGCA---GTGGGA---ATAGGAGCG---
------ATGATCTTTGGGTTCTTAGGAGCA---GCAGGAAGCACTATG---GGCGCGGCGTCA---
ATAACGCTGACGGTACAG---GCCAGACAG---TTATTG---TCTGGTATAGTGCAACAGCAAAGC---
AATTTG---CTGAGG---GCTATAGAGGCGCAGCAGCATCTGCTGCAACTCACAGTCTGGGGCATT
>KJ952667..1
TTCTATTGCAATACAACAAAACTGTTTAATAATACGTGCATAGGAAATGAAACCATAGAGGGG----------
-------------------------------------------------------------------------
----------------------------
TGTAATGACACTATCATACTTCCATGCAAGATAAAGCAAATTGTGAACATGTGGCAGGGAGCAGGACAAGCAA
TGTATGCTCCTCCCATCAGTGGAATAATTAATTGTGTATCAAAT---
ATTACAGGAATACTATTGACAAGAGATGGTGGTTATAATAATAATACT-------------------------
-------------------------------------------------------------------------
----------
AAGGCTAACGAGACCTTCAGACCTGAAGGAGGAAATATAAAGGACAATTGGAGAAATGAATTATATAAATATA
AAGTAGTAGAAATTGAACCACTA---GGAATAGCACCCACCAGGGCAAAGAGAAGAGTGGTG-----------
----------------------------GAGAGACAAAAAAGAGCA---GTGGGA---ATAGGAGCT------
---ATGATCTTTGGGTTTTTGGGAGCA---GCAGGAAGCACTATG---GGCGCGGCGTCA---
ATAACGCTGACGGTACAG---GCCAGACAA---TTATTG---TCTGGTATAGTGCAACAGCAAAGC---
AATTTG---CTGAGG---GCTATAGAGGCGCAGCAGCATATGTTGCAACTCACAGTCTGGGGCATT
>KJ953089..1
TTCTATTGCAATACATCACAACTGTTTAATAGTACTTGGATAGGAAATGAAACCAAGGAAGAGTGT-------
-------------------------------------------------------------------------
-AATAAATTCAAGAAA---------
GAGTGTAATAGCACTATCATACTTCCATGCAAGATAAAGCAAATTATAAACATGTGGCAGGGAGTAGGACAAG
CAATGTATGCTCCTCCCATTAGAGGAAGAATTAATTGTGTATCAAAT---
ATCACAGGAATACTATTGACAAGAGATGGTGGTAGTATTAATAGGAAT-------------------------
-------------------------------------------------------------------------
----------
TATGAGAATGAGACCTTCAGACCTGGAGGAGGAAATATAAAGGACAATTGGAGAAGTGAATTATATAAATATA
AAGTAGTAGAAATTGAACCACTA---GGAGTAGCACCCACCAAGGCAAAGAGGAGAGTGGTG-----------
----------------------------GAGAGAGAAAAAAGAGCA---GTGGGA---ATAGGAGCT------
---ATGATCTTTGGGTTCTTAGGAGCA---GCAGGAAGCACTATG---GGCGCGGCGTCA---
ATAACGCTGACGGTACAG---GCCAGACAA---TTATTG---TCTGGTATAGTGCAACAGCAAAGC---
AATTTG---CTGAAG---GCTATAGAGGCGCAACAGCATCTGTTGCAGCTCACAGTCTGGGGCATT
>KJ953475..1
TTCTATTGTAATACAACAAAATTGTTTAATAGTACTTGGACAGAAAATGAAACCGAGGAGGGG----------
-------------------------------------------------------------------------
----------------------------
AATAATAACACTATCATACTTCCATGCAAGATAAAGCAAATTATAAACATGTGGCAGGGAGTAGGACAAGCAA
TGTATGCTCCTCCCATCAGTGGAAAAATTAATTGTGTATCAAAT---
ATTACAGGAATACTATTGACAAGAGATGGTGGTAATAATGCGATGAATGAG----------------------
-------------------------------------------------------------------------
-------------
ACCTATGAGACCTTTAGACCTGGAGGAGGAAATATGAAGGACAATTGGAGAAGTGAATTATATAAATATAAAG
TGGTACAAATTGAACCACTA---GGAATAGCACCCACCAGGGCAAGGAGAAGAGTGGTG--------------
-------------------------GAGAGAGAAAAAAGAGCA---GTGGGA---ATAGGAGCT---------
ATGATCTTTGGGTTCTTAGGAGCA---GCAGGAAGCACTATG---GGCGCGGCGTCA---
ATAACGCTGACGGTACAG---GCCAGACAA---TTATTG---TCTGGTATAGTGCAACAGCAAAGC---
AATTTG---CTGAGG---GCTATAGAGGCGCAGCAGCATCTGTTGCAACTCACAGTCTGGGGCATT
```

```
>AY358059..1
TTCTATTGCGATACAACAAAACTGTTTAATAATACTATAAAA-------------------------------
-------------------------------------------------------------------------
-------------------------------
AATGGAACCATAACGCTTCCATGCAAGATAAAGCAAGTTGTAAACATGTGGCAGGGAGCAGGACAGGCAATGT
ATGCTCCTCCCATCAAGGGAAGCATTAATTGTGTATCAAAT---
ATTACAGGAATACTATTGACAAGAGATGGTGGTAATCAAAACGCG----------------------------
-------------------------------------------------------------------------
----------------
ACTGAGATTTTCAGACCTGGAGGAGGAAATATAAGGGACAATTGGAGAAGTGAATTATATAAATATAAAGTGG
TACAAATTGAACCACTA---GGAATAGCACCCACCAGGGCAAAGAGAAGAGTGGTG-----------------
----------------------GAGAGAGAAAAAAGAGCA---GTGGGA---ATAGGAGCT---------
ATGATCTTTGGGTTCTTAGGAGCA---GCAGGAAGCACTATG---GGCGCGGCGTCA---
ATAACGCTGACGGTACAG---GCCAGACAA---TTATTG---TCTGGTATAGTGCAACAGCAAAGC---
AATTTG---CTGAGG---GCTATAGAGGCGCAGCAGCATCTGTTGCAACTCACAGTCTGGGGCATT
>DQ314732..1
TTCTATTGCAATACAACACAACTGTTTAATAGTACTTGGGTAGGAAATGAAACCAAAAAGGGGGATAATGGCA
CTATAAGAAAT--------------------------------------------------------------
-------GAAACCAAAAAG------
GGGGATAATGGCACTATCATACTTCCATGCAGGATAAAGCAAATTATAAACATGTGGCAGGAAACAGGACAAG
CAATGTATGCTCCTCCCATCAGTGGAACAATTAATTGTGTATCAAAT---
ATTACAGGACTACTATTGACAAGAGATGGTGGAGGTAATAATGACTCG-------------------------
-------------------------------------------------------------------------
----------
AGTCAGAACGAGACCTTCAGGCCTGGAGGGGGAAATATAAAGGACAATTGGAGAAGTGAATTATATAAATATA
AAGTAGTAGAAATTGAACCACTA---GGAATAGCACCCACCAGGGCAAAGAGAAGAGTGGTG-----------
----------------------------GAGAGAGAAAAAAGAGCA---GTGGGA---ATAGGAGCT------
---ATGATCTTTGGGTTCTTAGGAGCG---GCAGGAAGCACTATG---GGCGCGGCGTCA---
ATAACGCTGACGGTACAG---GCCAGACAA---TTATTG---TCTGGTATAGTGCAACAGCAAAGC---
AATTTG---CTGAGG---GCTATAGAGGCGCAGCAGCATCTGTTGCAACTCACAGTCTGGGGCATT
>JX447561..1
TTCTATTGCAATACATCAAACCTGTTTGATAGAAATGAAACCACGAGTAAT----------------------
-------------------------------------------------------------------------
----------------------------
GGCACTGGCACTATCATACTTCCTTGCAAGATAAAGCAAATTGTACGCATGTGGCAGAGAACAGGACAAGCAA
TGTATGCTCCTCCCATCAGTGGAAGCATCAATTGTGTATCAAAT---
ATTACAGGAATTCTGTTGACAAGAGATGGTGGTAATGGTAATAATACT-------------------------
-------------------------------------------------------------------------
----------
AATCAGACAGAGATCTTCAGACCTGAGGGAGGAAATATAAAGGACAATTGGAGAAGTGAATTATATAAATATA
AAGTGGTACAAATTGAACCACTA---GGAATAGCACCCACCAGGGCAAGAAGAAGAGTGGTG-----------
----------------------------CAGAGAGAAAAAAGAGCA---GTGGGA---ATAGGAGCT------
---ATGATCTTTGGGTTCTTAGGAGTA---GCAGGAAGCACTATG---GGCGCGGCGTCA---
ATAACGCTGACGGTACAG---GCCAGACAA---TTATTG---TCTGGTATAGTGCAACAGCAAAGC---
AATTTG---CTGAGG---GCTATAGAGGCGCAGCAGCATCTGTTGCAACTCACAGTCTGGGGCATC
>JX447855..2
TTCTATTGCAATACAACACAACTGTTTAATAGTACTTACGGAGAAAATGGAGAAAATGAAACCATGGGGGGG-
-------------------------------------------------------------------------
----------------------------
AATAATGGCACTATCATACTTCCATGTAAGATAAAGCAGATTATAAACATGTGGCAGGGAGTAGGACAAGCAA
TGTACGCTCCTCCCATCAGTGGAAGAATTAATTGTAGATCACAA---
ATTACAGGAATACTATTGATAAGAGATGGTGGTTATGATCGTGTT----------------------------
-------------------------------------------------------------------------
----------------
AACGAGACCTTCAGACCTGAAGGAGGAAATATGAAGGACAATTGGAGAAGTGAATTATATAAATATAAAGTAG
TACAAATTGAACCACTA---GGAGTAGCACCCACCAGGGCAAAGAGAAGAGTGGTG-----------------
----------------------GAGAGAGAAAAAAGAGCA---GTGGGA---ATAGGAGCC---------
ATGATCTTTGGGTTCTTAGGAGCA---GCAGGAAGCACTATG---GGCGCGGCGTCA---
ATAACGCTGACGGTACAG---GCCAGACAA---TTATTG---TCTGGTATAGTGCAACAGCAAAGC---
AATTTG---CTGAGG---GCTATAGAGGCGCAGCAGCATCTGTTGCAACTTACAGTCTGGGGCATT
```

>JX448022..1
TTCTATTGCAATACAACATCACTGTTTAATGATACTTGCATAGGAAATGAAACCAAGGAAGGG----------
----

TGTAATACAACTATCATACTTCCATGCAGGATAAAACAAATTGTAAACATGTGGCAGGGAGTAGGACAGGCAA
TGTATGCTCCTCCCATCAGTGGAAGAATTAATTGTGTATCAAAT---
ATTACAGGAATACTATTGACAAGAGATGGTGGTGTTAATAATGAT----------------------------
----

TCTAGTGAGATCTTCAGACCTGGAGGAGGGGATATAAGAGACAAATGGAGAAGTGAATTATATAAATATAAAG
TAGTACAAATTGAACCATTA---GGAGTAGCACCCACCAGGGCAAAGAGAAGAGTGGTG--------------
-------------------------GAGAGACCAAAAAGAGCA---GTGGGA---ATAGGAGCT---------
ATGATCTTTGGGTTCTTAGGAGCA---GCAGGAAGCACTATG---GGCGCGGCGTCA---
ATAACGCTGACGGTACAG---GCCAGACAA---TTATTG---TCTGGTATAGTGCAACAGCAAAGC---
AATTTG---CTGAGG---GCTATAGAGGCGCAACATCATCTGTTGCAACTCACAGTCTGGGGCATT
>EU743764..1
TTCTATTGCAATACAACACAACTGTTTAATCTTAATAATGCTACTGACAGA----------------------
----

GAAAATAAAAATATCACACTTCCATGCAGGATAAAGCAAATTATAAAAATGTGGCAGAGAGTAGGACAAGCAA
TGTATGCTCCTCCCATCAGGGGAAAAATTAATTGTGTCTCCAAT---
ATTACAGGAATACTATTAACAAGAGATGGTGGTAGTCACAATGATACT-------------------------
----

AATAATAATACTGAGACCTTTAGACCCGCAGGAGGAAATATAAAAGACAATTGGAGAAGTGAATTATATAAAT
ATAAAGTAGTAAGAATTGAACCACTG---GGAATAGCACCCACCAGGGCAAGGAGAAGAGTGGTG--------
-------------------------------GACAGACCGAAAAGAGCA---GTGGGA---ATAGGAGCT---
------ATGATCTTTGGGTTCTTAGGAGCA---GCAGGAAGCACTATG---GGCGCGGCGTCA---
CTAACGCTGACGGTACAG---GCCAGACAA---TTACTG---TCTGGTATAGTGCAACAGCAAAGC---
AATTTG---CTGAGG---GCTATAGAGGCGCAGCAGCATCTGTTGCAACTCACAGTCTGGGGCATT
>EU743781..2
TTCTATTGCGATACAACAAAACTGTTTAATAATAATTGCACACAAAATGCAACCGATCAGGATGAAAAAAAG-
----

TGTAATGGCACTCTCATACTTCCATGCAGGATAAAGCAAATTATAAACATGTGGCAGGGAGTAGGACAAGCAA
TATATGCTCCTCCCGTCCGTGGAAGTATTACTTGTGTATCAAAT---
ATTACAGGAATAATACTGACAAGAGATGGTGGTACTCATAAGAATGGTACT----------------------
----

GCTGATAATACAACCGAGATCTTCAGACCTACAGGAGGAAATATGAAGGACAATTGGAGAAGTGAATTATATA
AATATAAAGTAGTACAAATTGAACCACTA---GGAATAGCACCCACCAGGGCAAAGAGAAGAGTGGTG-----
----------------------------------GAGAGAGAAAAAAGAGCA---GTGGGA---
CTAGGAGCT---------ATGATCTTTGGGTTCTTAGGAGCA---GCTGGAAGCACTATG---
GGCGCGGCGGCA---GTAACGCTGACGGTACAG---GCCAGACAA---TTATTG---
TCTGGTATAGTGCAACAGCAAAGC---AATTTG---CTGAGA---
GCTATAGAGGCGCAACAACATCTGTTGCAACTCACAGTCTGGGGCATT
>JX446699..4
TTCTATTGCAATACATCAAGACTGTTTAATAGCACTTGCTTCAAAAATCATAACACCACTGAGGCCACTCAGG
AGTGTAAT---
----------GACACTATC------
ACAACAGATGGCAAGATCATACTTCACTGCAGGATAAAGCAAATTATAAACATGTGGCAGGGAGCAGGACAAG
CAATGTATGCTCCTCCCATCAGTGGAAGAATTAATTGTGTATCAAAC---
ATTACAGGAATACTATTGACAAGAGATGGTGGTGATACTGGTAAAAATTATACA------------AAT----
----

GGTACCAGGAAAGAGATCTTCAGACCTGGAGGAGGAAATATGAAGGACAATTGGAGAAGTGAATTATATAAAT
ATAAAGTAGTACAAATTGAACCACTA---GGAATAGCACCCACCAGGGCAAAGAGAAGAGTGGTG--------
-------------------------------GAGAGAGAAAAAAGAGCA---GTGGGA---ATAGGAGCT---
------ATGATCTTTGGGTTCTTAGGAGCA---GCCGGAAGCACTATG---GGCGCAGCGTCA---

ATAACGCTGACGGTACAA---GCCAGACAA---TTATTG---TCTGGTATAGTGCAACAGCAAAGC---
AATTTG---CTGAGG---GCTATAGAGGCGCAGCAGCATATGTTGCAACTCACAGTCTGGGGCATT
>JX447312..1
TTCTATTGCAACACAACAAAACTGTTTAATTGGACAGAAGGTGAAACCATAGAGAAT----------------
----

AGCACGGAGAATATCATGCTGCCATGCAAGATAAAGCAAATTATAAACATGTGGCAGGAAACAGGACAAGCAA
TGTATGCTCCTCCCATCAGTGGAATGATTAATTGTGTATCAAAT---
ATTACAGGAATACTATTGACAAGAGATGGTGGTCATGATGGTAATAATACAAAT------------AAT----
----

ACAAATAATAATACCGAGACCTTCAGACCTGGAGGAGGAAATATAAAGGACAATTGGAGAAGTGAATTATATA
AATATAAAGTAGTACAAATTGAACCACTA---GGAATAGCACCCACCAGGGCAAAGAGAAGAGTGGTG-----
----------------------------------GAGAGAGAAAAAAGAGCA---GTGGGA---
ATAGGAGCT---------ATGATCTTTGGGTTCTTGGGAGCA---GCAGGAAGCACTATG---
GGCGCAGCGTCA---ATAACGCTGACGGTACAG---GCCAGACAA---TTATTG---
TCTGGTATAGTGCAACAGCAAAGC---AATTTG---CTGAGA---
GCTATAGAGGCGCAGCAGCATCTGTTGCAACTCACAGTCTGGGGCATT
>JX447646..1
TTCTATTGCAATACAACAAACCTGTTTAATAATACTTGCTTAAATGAAACCAAG-------------------
----

TGTAATGGCACTGTCACACTTCCATGCAGGATAAAGCAAATTATAAACATGTGGCAGGGAGTAGGACAAGCAA
TGTATGCTCCTCCCATCAGGGGAAATATTACTTGTGTATCAAAT---
ATTACAGGAATACTATTGACAAGAGATGGTGGTAATGGGGGGAAC----------------------------
----

GAGACCTTCAGACCTGAAGGAGGAAATATGAAGGACAATTGGAGAAATGAATTATATAAATATAAAGTAGTAC
AAATTGAACCACTA---GGATTAGCACCCACCAAGGCAAAAAGAAGAGTGGTG--------------------
------------------GAGAGAAAAAAAGAGCA---GTGGGA---ATAGGAGCT---------
ATGATCTTTGGGTTCTTAGGAGCA---GCAGGAAGCACTATG---GGCGCGGCGTCA---
ATAACGCTGACGGTACAG---GCCAGACAA---TTATTG---TCTGGTATAGTGCAACAGCAAAGC---
AATTTG---CTGAGG---GCTATAGAAGCGCAGCATCATCTGTTGCAACTCACAGTCTGGGGCATT
>JN944659..1
TTCTATTGTAATACAACAAAACTGTTTAATAGTACTTGGAGAGAAAATGAAACCATAGAGGAGCATAATGGC-
----

ACTAATGGCACTATCATACTTCCATGTAGGATAAAGCAAATTATAAACATGTGGCAGGGAGTAGGACAAGCAA
TGTATGCTCCTCCCATCAGTGGAAAAATTAATTGTATATCAAAT---
ATTACAGGAATACTATTGACAAGAGATGGAGGTCATAATACGAGT----------------------------
----

AACGAGACCTTCAGACCTGGAGGAGGAAATATAAAGGACAATTGGAGAAGTGAATTATATAAATATAAAGTAG
TACAAATTGAACCACTA---GGAATAGCACCCACCAGGGCAAAGAGAAGAGTGGTG-----------------
----------------------GAGAGAGAAAAAAGAGCA---GTGGGA---ATAGGAGCT---------
ATGATCTTTGGGTTCTTAGGAGCA---GCAGGAAGCACTATG---GGCGCGGCGTCA---
ATAACGCTGACGGTACAG---GCCAGACAA---TTACTG---TCTGGTATAGTGCAACAGCAAAGC---
AATTTG---CTGAGG---GCTATAGAGGCGCAACAGCATCTGTTGCAACTCACAGTCTGGGGCATT
>HQ691090..1
TTCTATTGCAATACAACAAAACTGTTTAATAATACCTGCATAAATGAAACCATGAGGGGAAGCAATGAAACCA
GG---

GAGTGTAATAACACTATTACACTTCCATGCAGGATAAAGCAAATTATAAACATGTGGCAGGGAGTAGGACAAG
CAATGTATGCTCCTCCCATCAGTGGAATAATTAGTTGTGTATCAAAT---
ATTACAGGAATACTATTGACAAGAGATGGTGGTAATAATACTGGG----------------------------
----

AATGACGAGATTTTCAGACCTGCAGGAGGAAATATAAGGGACAATTGGAGAAATGAATTATATAAGTATAAAG
TAGTACAAATTGAACCACTA---GGAGTAGCACCCACCAAGGCAAAGAGAAGAGTGGTG--------------
-------------------------GAGAGACAAAAAAGAGCA---GTGGGA---ATAGGAGCT---------

ATGATCTTTGGGTTCTTAGGAGCA---GCAGGAAGCACTATG---GGCGCGGCGTCA---
ATAACGCTGACGGTACAG---GCCAGACAA---CTATTG---TCTGGTATAGTGCAACAGCAGAGC---
AATCTG---CTGAGG---GCTATAGAGGCGCAACATCATCTGTTGCAACTCACAGTCTGGGGCATT
>JX446728..2
TTCTATTGCAATACAACAAAATTGTTTCATAGTAATTGGACAACAACAAATGGCACTATAGAAAGGTACAAT-
----

GACAATGAAACTATCATACTTCCATGCAAGATAAAGCAAATTATAAATATGTGGCAGGGAGTAGGACAAGCAA
TGTATGCTCCTCCCATCAGAGGAATAATTAATTGTACATCAAAT---
ATTACAGGAATATTATTGACAAGAGATGGTGGTCTTCATAATGGGACT-------------------------
----

AACGATACAGAGACATTCAGGCCTGAAGGAGGAAATATAAAGGACAATTGGAGAAGTGAATTATATAAATATA
AAGTAGTACAGATTGAACCATTA---GGAGTAGCACCTACCAGGGCAAGGAGAAGAGTGGTG-----------
----------------------------GAGAGAGAAAAAAGAGCA---GTGGGA---ATAGGAGCT------
---ATGATCTTTGGGTTCCTAGGAGCA---GCAGGAAGCACTATG---GGCGCGGCGTCA---
ATAACGCTGACGGTACAG---GCCAGACAA---TTATTG---TCTGGTATAGTGCAACAGCAAAGC---
AATTTG---CTGAGA---GCTATAGAGGCGCAGCAGCATCTGTTGCAACTCACAGTCTGGGGCATT
>JX447057..3
TTCTATTGCAATACAACACGACTGTTTAATCATTCTCACAATGAAACCTGGAAGGAGAAG-------------
----

TATAATGGCACTATCATACTTCCATGCAAGATAAAGCAAATTATAAACATGTGGCAGGGAGTAGGACAAGCAA
TGTATGCTCCTCCCATCAGTGGAAAGATTGAGTGTGTATCAAAT---
ATTACAGGAATACTATTGACAAGAGATGGTGGTGTTAATCAAACT----------------------------
----

AATGGGACCGAGATCTTCAAACCTGGAGGAGGAAATATAAAGGACAATTGGAGAAGTGAATTATATAAATACA
AAGTAGTACAAATTGAACCACTA---GGATTGGCACCTACCAAGGCAAAGAGAAGAGTGGTG-----------
----------------------------GAGAGAGAAAAAAGAGCA---GTGGGA---ATAGGAGCT------
---ATGATCTTTGGGTTCTTAGGAGCA---GCAGGAAGCACTATG---GGCGCGGCGTCA---
ATAACGCTGACGGTACAG---GCCAGACAA---TTATTG---TCTGGTATAGTGCAACAGCAAAGA---
AATTTG---CTGAGG---GCTATAGAGGCGCAGCAGCATCTGTTGCAACTCACAGTCTGGGGCATT
>JX447480..2
TTCTATTGCAACACAACAAAACTGTTTAATAGTACTTGGAGAGGAAATGAAACCATAGCGGGCTATGAT----
----

AATAACAGCAATATCATACTTCCATGCAGGATAAAGCAAATTATAAACATGTGGCAGGAAGTAGGACAAGCAA
TGTATACTTCTCCCGTCAGTGGAAGAATTAATTGTATCTCAAAT---
ATCACAGGAATACTATTAACAAGAGATGGTGGTGGTAATCATAGC----------------------------
----

AATGAGACCTTTAGACCTGGAGGAGGAAATATGAAGGACAATTGGAGAAGTGAACTATATAAATATAAAGTAG
TACAACTTGAACCACTA---GGAATAGCACCCACCAAGGCAAAGAGAAGAGTGGTG-----------------
----------------------GAGAGAGAAAAAAGAGCA---GTGGGA---ATAGGAGCT---------
ATGATCTTTGGGTTCTTAGGAGCA---GCCGGAAGCACTATG---GGCGCAGCGTCA---
ATAACGCTGACGGTACAG---GCCAGACAA---TTATTG---TCTGGTATAGTGCAACAGCAAAGC---
AATTTG---CTGAGG---GCTATAGAGGCGCAGCATCGTCTGTTGCAACTCACAGTCTGGGGCATT
>HQ691006..1
TTCTATTGCAATACAACACAACTGTTTAATAATAATTGTACAAATGAAACCATGAATGGG-------------
----

TGTGATGGCAATATCACACTTCCATGCAAGATAAAGCAAATTGTAAACATGTGGCAGGGAGTGGGACAAGCAA
TGTATGCTCCTCCCATCAGAGGACAAATTAGTTGTGTGTCAAAT---
ATTACAGGGATACTACTGACAAGAGATGGTGGTCATAATGGTAGT----------------------------
----

ACAAATAATGAGACCTTCAGACCTGGAGGAGGAAATATAAAGGACAATTGGAGAAGTGAATTATATAAATATA
AAGTAGTAGAAATTGAACCACTA---GGGATAGCACCTTCCTGGGCAAGGAGAAGAGTGGTG-----------
----------------------------GAGAGACAAAAAAGAGCA---GTGGGA---ATAGGAGCT------

---ATGATCTTTGGGTTCTTAGGAGCA---GCAGGAAGCACTATG---GGCGCGGCGTCA---
GTAACGCTGACGGTACAG---GCCAGACAA---TTATTG---TCTGGTATAGTGCAACAGCAAAGC---
AATTTG---CTGAGG---GCTATAGAGGCGCAGCAGCATCTGTTGCAACTCACAGTCTGGGGCATT
>JX447303..2
TTCTATTGCAATACATCACAACTGTTTAATTACACAACAGATGCAGACATGAGGAACAAGACAGCCATA----
----

AAAAATAGCACTATCATACTTCCATGCAGGATAAAGCAAATTATAAACATGTGGCAGGGAGTAGGACAAGCAA
TGTATGCTCCTCCCATCAGTGGAATAATTAATTGTGTATCAAAT---
ATTACAGGAATATTATTGACAAGAGATGGTGGTGAGCAGAAGAATGAGAGT----------------------
----

AGGCGAAATGAGACCTTCAGACCTGAAGGAGGAAATATGAAGGACAATTGGAGAAGTGAATTATATAAATATA
AAGTAGTACAAATTGAACCACTA---GGAGTAGCACCCACTAAGGCAAGGAGAAGAGTGGTG-----------
----------------------------GAGAAACAAAAAAGAGCA---GTGGGA---ATAGGAGCT------
---ATGATCTTTGGGTTCTTAGGAGCA---GCAGGAAGCACTATG---GGCGCGGCGTCA---
ATAACGCTGACGGTACAG---GCCAGACAA---TTATTG---TCTGGTATAGTGCAACAGCAAAGC---
AATTTG---CTGAGA---GCTATAGAGGCGCAACATCATATGTTGCAACTCACAGTCTGGGGCATT
>JX447335..2
TTCTATTGCAATACATCACAACTGTTTAATAGTACTGTTAATGGCACTATCACACTTCCTAAT----------
----

GGCACACTTCCATGCAAGATAAAGCAAATTATAAAAATGTGGCAAGGGGTAGGACAAGCAATGTATGCTCCTC
CCATCAATGGAAGCATTAATTGTGTATCAAAC---
ATTACGGGACTACTATTAACAAGGGATGGTGGTAATCAGGGTAATCAT-------------------------
----

ACTAATGATACTGAGACCTTCAGACCTGGAGGAGGAAACATAAAGGACAATTGGAGAAGTGAATTATATAAAT
ATAAGGTGGTACAAATTGAACCACTA---GGAATAGCACCCACCAGGGCAAAGAGAAGAGTGGTG--------
-------------------------------GAAAGAGAAAAAAGAGCA---GTGGGA---ATAGGAGCT---
------ATGATCTTTGGGTTCTTAGGAGCA---GCAGGAAGCACTATG---GGCGCGGCGTCA---
ATAACGCTGACGGTACAG---GCCAGACAA---TTATTG---TCTGGTATAGTGCAACAGCAAAGC---
AATTTG---CTGAGG---GCTATAGAGGCGCAGCAGCATCTGTTGCAACTCACAGTCTGGGGCATT
>JN388161..1
TTCTATTGCAATACAACACAACTGTTTAATAGTACTTGCATGGGAGATATGCCCAAGGATGGGTGTAATGGCA
CTTTC--

AATGGCACCGGCACTATCATACTTCCATGCAGAATAAAGCAATTTATAAACATGTGGCAGGGAGTAGGACAAG
CAATGTATGCTCCTCCCATCAAGGGAAACATTAGTTGTGTATCAAAT---
ATTACAGGAATACTATTAACAAGAGATGGTGGTAATAGGACTCATAAC-------------------------
----

GGGACTAAGGAAGAGATCTTCAGACCTGCAGGAGGAAATATGAAGGACAATTGGAGAAGTGAATTATATAAAT
ATAAAGTAGTACAAATTGAACCACTA---GGAGTAGCACCTACCAAGGCAAAGAGAAGAGTGGTG--------
-------------------------------GATAGACAAAAAAGAGCA---GTGGGA---ATAGGAGCT---
------ATGATCTTTGGGTTCTTGGGAGCA---GCAGGAAGCACTATG---GGCGCAGCGTCA---
ATAACGCTGACGGTACAG---GCCAGACAA---TTATTG---TCTGGTATAGTGCAACAGCAAAGC---
AATTTG---CTGAGA---GCTATAGAGGCGCAACAACATCTGTTGCAACTCACAGTCTGGGGCATT
>GQ916581..5
TTCTATTGCAATACAACAAAACTGTTTAATAGTACCTGGGCCAAGGAGGGGTGGAATGGCACTAATTATAATG
ACACTTATTAT--
----------AATGGCACT------
AATTATACTGGCAATATCACACTTCCATGCAGGATAAAGCAAATTGTAAACATGTGGCAGGGAATAGGACAAG
CAATGTATGCTCCTCCCATCAGTGGAACGATTCAGTGTGTATCAGAA---
ATTACAGGAATACTATTGACAAGAGATGGTGATATTACTAATGCG----------------------------
----

GCTAACGAGACTTTCAGACCTGGAGGAGGAAATATAAAGGACAATTGGAGAAGTGAATTATATAAATATAAAG
TAGTGCAAATTGAACCACTA---GGAATAGCACCCACCAGGGCAAAGAGAAGAGTGGTG--------------
-------------------------GAGAGAGAAAAAAGAGCA---GTGGGA---ATAGGAGCG---------

ATGATCTTTGGGTTCTTAGGAGCA---GCAGGAAGCACTATG---GGCGCGGCGTCA---
ATAACGCTGACGGTACAG---GCCAGGCAA---TTATTG---TCTGGTATAGTGCAACAGCAAAGC---
AATTTG---CTGAGG---GCTATAGAGGCGCAGCAGCATCTGTTGCAACTCACAGTCTGGGGCATT
>JF680932..1
TTCTATTGCAATACAACAAAGATGTTTAATGCGACAGAAAATGGAACCAGGGAAGGG----------------
----

AATAACAATACGATCATAATTCCATGCAGGATAAAGCAAATTGTAAACATGTGGCAGAAAACAGGACAAGCAA
TGTATGCTCCTCCCATCAGGGGAATAATTAATTGTGTATCAAAT---
ATTACAGGAATATTATTAACAAGAGATGGTGGAAATGAGGATAATCAT-------------------------
----

AGTAAGGACAACGAGACCTTCAGACCTGCAGGAGGAAATATAAAGGACAATTGGAGAAGTGAATTATATAAAT
ATAAAGTAGTACAAATTGAACCACTA---GGAATAGCACCCACCAAGGCAAAGAGAAGAGTGGTG--------
-------------------------------GAGAGAGAAAAAAGAGCA---GTGGGA---ATAGGAGCT---
------ATGATCTTTGGGTTCTTAGGAGCA---GCAGGAAGCACTATG---GGCGCGGCGTCA---
ATAACGCTGACGGTACAG---GCCAGACAA---TTATTG---TCTGGTATAGTGCAACAGCAAAGC---
AATTTG---CTGAGG---GCTATAGAGGCGCAACAGCATATGTTGCAACTCACAGTCTGGGGCATT
>KT185951..3
TTCTATTGCAATACAACAAAACTGTTTAATGGTACTTACCCGAAAAATGGAACAGATGGAACA----------
----

AATAATGACACTATCATACTCCCATGTAGGATAAAGCAAATTGTAAGATTGTGGCAGGAAGTAGGTCAAGCAA
TGTATGCTCCTCCCATCAGTGGAAGCATTGAGTGCTACTCAAAT---
ATTACAGGAATACTATTGACAAGAGATGGTGGTAATACTAGCAATAGT-------------------------
----
GATGGTGGTAATATTAGC---
AATAGTGAAACCTTCAGACCTGGAGGAGGAAATATAAAGGACAATTGGAGAAGTGAATTATATAAATATAAAG
TAGTACAAATTGAACCACTA---GGAATAGCACCCACCAAGGCAAAAAGAAGAGTGGTG--------------
-------------------------GAGAGAGAAAAAAGAGCA---GTAGGA---ATAGGAGCT---------
ATGATCTTTGGGTTCTTAGGAGCA---GCAGGAAGCACTATG---GGCGCGGCATCA---
ATAACGCTGACGGTACAG---GCCAGACAA---TTATTG---TCTGGTATAGTGCAACAGCAAAGC---
AATTTG---CTGAGG---GCTATAGAGGCGCAGCAGCATCTGTTGCAACTCACAGTCTGGGGCATT
>KT185973..1
TTCTATTGCAATACATCAAAACTGTTCAACAATACTTGCAGAGAAGATAGCACGAAGGAGGGGTGTAATGAT-
----

ACCATACTGCCATGCAAAATAAAGCAAATTATAAGGATGTGGCAGGGAGTAGGACAAGCAATGTATGCTCCTC
CCATCAGTGGGAGTATCAACTGTATATCAAAT---
ATTACAGGAATACTATTGACAAGAGATGGTGGTTACAACAATAAGAGTAATGGG-------------------
----

AACGAGACTGAGACTTTCAGACCTGGAGGAGGAAATATGAAGGACAATTGGAGAAATGAATTATATAAATATA
AAGTAGTACAAATTGAACCACTA---GGAATAGCACCCACCAAGGCAAAGAGAAGAGTGGTG-----------
----------------------------GAGAGAGAAAAAAGAGCA---GTGGGA---ATAGGAGCT------
---ATGATCTTTGGGTTCTTAGGAGCA---GCAGGAAGCACTATG---GGCGCGGCGTCA---
ATAACGCTGACGGTACAG---GCCAGACAA---TTATTG---TCTGGTATAGTGCAACAGCAAAGC---
AATTTG---CTGAGG---GCTATAGAGGCGCAACAGCATCTGTTGCAACTCACAGTCTGGGGCATT
>KT185837..2
TTCTATTGCAATACAACAAAACTGTTTAATAGTACAGGGAAC-------------------------------
----

TATAATGACACTATCATACTTCCATGCAGGATAAAGCAAATTATAAACATGTGGCAGAGAGTAGGACAAGCAA
TGTATGCTCCTCCCATCAGGGGCAACATTACTTGTGTATCAAAT---
ATTACAGGAATATTATTGACAAGAGATGGTGGTGCGAATCAGACTAATGAG----------------------
----

ACCGATGAGACCTTCAGACCGGGAGGAGGAGATATAAAGGACAATTGGAGAAATGAATTATATAAATATAAAG
TAGTACAAATTGATCCATTA---GGAATAGCACCCACCAAGGCAAAGAGAAGAGTGGTG--------------
-------------------------GAGAGAGAAAAAAGAGCA---GTGGGA---ATAGGAGCT---------

ATGATCTTTGGGTTCTTAGGAGCA---GCAGGAAGCACTATG---GGCGCGGCGTCG---
ATAACGCTGACGGTACAG---GCCAGACAA---TTATTG---TCTGGGATAGTGCAACAGCAAAGT---
AATTTG---CTGAGG---GCTATAGAGGTGCAGCAGCATATGTTGCAACTCACAGTCTGGGGCATT
>MG989500..3
TTCTATTGCAATACAACAAAACTGTTTAATAGGACTTACCCAGGAAATGATACCACTGAGGGG----------
----

CAGAATGGCACTATCATACTCCCATGCAGAATAAAGCAAATTATAAACATGTGGCAGGAAGTAGGACAAGCAA
TGTATGCTCCTCCCATCAGAGGACCGATTAACTGCTCATCAATT---
ATTACAGGAATACTATTGACAAGGGATGGTGGTAGTAGTACTCAAAACGAG----------------------
----

ACTCAAAACGGGACCGAGATCTTCAGACCTGGAGGAGGAAATATAAAGGACAATTGGAGAAGTGAATTATATA
AATATAAAGTAGTACAAATTGAACCACTA---GGAATAGCACCCACCAGGGCAAAGAGAAGAGTGGTG-----
----------------------------------GAGAGGGAAAAAAGAGCA---GTGGGA---
ATAGGAGCT---------ATGATCTTTGGGTTCTTGGGAGCA---GCAGGAAGCACTATG---
GGCGCGGCGTCA---ATAACGCTGACGGTACAG---GCCAGACAG---TTACTG---
TCTGGTATAGTGCAACAGCAAAGC---AATTTG---CTGAGA---
GCTATAGAGGCGCAGCAGCATATGCTGCAACTCACAGTCTGGGGCATA
>MG989502..3
TTCTATTGCAATACAACAAAACTGTTTAATGGTACTTACCCGAAAAATGGAACAGATGGAACA----------
----

AATAATGACACTATCATACTCCCATGTAGGATAAAGCAAATTGTAAGATTGTGGCAGGAAGTAGGTCAAGCAA
TGTATGCTCCTCCCATCAGTGGAAGCATTGAGTGCTACTCAAAT---
ATTACAGGAATACTATTGACAAGAGATGGTGGTAATACTAGCAATAGTGATGGT-------------------
----
-
GGTAATATTAGCAATAGTGAAACCTTCAGACCTGGAGGAGGAAATATAAAGGACAATTGGAGAAGTGAATTAT
ATAAATATAAAGTAGTACAAATTGAACCACTA---GGAATAGCACCCACCAAGGCAAAAAGAAGAGTGGTG--
-------------------------------------GAGAGAGAAAAAAGAGCA---GTAGGA---
ATAGGAGCT---------ATGATCTTTGGGTTCTTAGGAGCA---GCAGGAAGCACTATG---
GGCGCGGCATCA---ATAACGCTGACGGTACAG---GCCAGACAA---TTATTG---
TCTGGTATAGTGCAACAGCAAAGC---AATTTG---CTGAGG---
GCTATAGAGGCGCAGCAGCATCTGTTGCAACTCACAGTCTGGGGCATT
>MG989521..1
TTCTATTGCAATACATCAAAACTGTTCAACAATACTTGCAGAGAAGATAGCACGAAGGAGGGGTGTAATGAT-
----

ACCATACTGCCATGCAAAATAAAGCAAATTATAAGGATGTGGCAGGGAGTAGGACAAGCAATGTATGCTCCTC
CCATCAGTGGGAGTATCAACTGTATATCAAAT---
ATTACAGGAATACTATTGACAAGAGATGGTGGTTACAACAATAAGAGTAATGGG-------------------
----

AACGAGACTGAGACTTTCAGACCTGGAGGAGGAAATATGAAGGACAATTGGAGAAATGAATTATATAAATATA
AAGTAGTACAAATTGAACCACTA---GGAATAGCACCCACCAAGGCAAAGAGAAGAGTGGTG-----------
----------------------------GAGAGAGAAAAAAGAGCA---GTGGGA---ATAGGAGCT------
---ATGATCTTTGGGTTCTTAGGAGCA---GCAGGAAGCACTATG---GGCGCGGCGTCA---
ATAACGCTGACGGTACAG---GCCAGACAA---TTATTG---TCTGGTATAGTGCAACAGCAAAGC---
AATTTG---CTGAGG---GCTATAGAGGCGCAACAGCATCTGTTGCAACTCACAGTCTGGGGCATT
>KU230418..3
TTCTATTGCAATACAACAAGCTTGTTTAATAAAACTTACATAGGAAATGAAACCAGAAATGAAACCCATGATG
GG---

GGGAGTAATAAAACTATTACACTTCCATGCAAGATAAAGCAAATTGTAAGGATGTGGCAGGGAGTAGGACAAG
CAATGTATGCTCCTCCCATCAGTGGAGTAATTAACTGTGTATCAAAT---
ATTACAGGAATACTATTAACAAGAGATGGTGGTCAGGGTGGTCATAATAAT----------------------
----

ACAACTGAGACCGAGACCTTCAGACCTGGAGGAGGAAATATAAAGGATAATTGGAGAAGTGAATTATATAAAT

ATAAAGTAGTACAAATTGAACCACTA---GGAATAGCACCCACCAGGGCAAAGAGAAGAGTGGTG--------
-------------------------------GAGAGAGAAAAAAGAGCA---GTGGGA---ATAGGAGCT---
------ATGATCTTTGGGTTCTTAGGAGCA---GCAGGAAGCACTATG---GGCGCAGCGTCA---
ATAACGCTGACGGTACAG---GCCAGACAA---TTATTG---TCTGGTATAGTGCAGCAGCAAAGC---
AATTTG---CTGAGG---GCTATAGAGGCGCAACAGCATCTGTTGCAACTCACAGTCTGGGGCATT
>MG989579..1
TTCTATTGCAATACAACAAAATTGTTTAATGGAAACCATAGCAAGAGT-------------------------
----

GGTAATGACAGCATCGTACTTCCATGTAAGATAAAGCAGATTATAAACCTGTGGCAGGGAGTAGGACAAGCAA
TGTATGCTCCTCCCATCAGGGGGATAATTAACTGTGTATCAAAT---
ATTACAGGAATACTATTGACAAGAGATGGTGGAGCTCACAATGGGAGT-------------------------
----

AACGAGACTGAAATCTTTAGACCTGGAGGAGGAAATATGAAGGACAATTGGAGAAGTGAACTATATAAATATA
AAGTAGTACAAATTGAACCTCTA---GGAATAGCACCCACCAGGGCAAAGAGAAGAGTGGTG-----------
----------------------------GAGAAAGAAAAAAGAGCA---GTGGGA---ATAGGAGCT------
---ATGATCTTTGGGTTCTTAGGAGCA---GCAGGAAGCACTATG---GGCGCGGCGTCA---
CTAACGCTGACGGTACAG---GCCAGACAA---TTATTA---TCTGGTATAGTGCAACAGCAAAGC---
AATTTG---CTGAGG---GCTATAGAGGCGCAGCAGCATCTGTTGCAACTCACAGTCTGGGGCATT
>MF957493..1
TTCTATTGCAATACAACAAAATTATTTAATGGTACAGAAAATGGAACCATGGAGGGGAATACTGAC-------
----

ACTAATGGCACTATCATCCTTCCATGCAGGATAAAGCAAATTGTAAGAATGTGGCAGGGAGTAGGACAAGCAA
TATATGCTCCTCCCATCAGTGGAAACATTAGTTGTGAATCAAAC---
ATTACAGGAATACTATTGACAAGAGATGGTGGTAGTGGTAGTTATACTAATGAT-------------------
----
-
AGTGATGCTAATAATACAGAAATCTTCAGACCTGAGGGAGGAAATATAAAGGACAATTGGAGAAGTGAATTAT
ACAAATATAAAGTAGTACAAATTGAACCACTA---GGAGTAGCACCCACCAAGGCAAGGAGAAGAGTGGTG--
-------------------------------------GAGAGAGAAAAAAGAGCA---GTGGGA---
ATAGGAGCT---------ATGATCTTTGGGTTCTTAGGAGCA---GCAGGAAGCACTATG---
GGCGCGGCGTCA---ATAACGCTGACGGTACAG---GCCAGACAA---TTATTG---
TCTGGTATAGTGCAACAGCAAAGC---AATTTG---CTGAGG---
GCTATAGAGGCGCAGCAGCATCTGTTGCAACTCACAGTCTGGGGCATT
>MG989524..1
TTCTATTGCAATACAACACAACTTTTTAATAGTACTAGTACTAACAAC-------------------------
----

TCAGAAGGGAATATCATAATTCCATGCAGGATAAAGCAAATTATAAACATGTGGCAGGGAGTAGGGCAAGTAA
TGTATGCTCCTCCTGTCCAAGGGCGAATTAATTGTGCATCAAAT---
ATTACAGGAATACTATTAACAAGAGATGGTGGTCATAACGGGACATCC-------------------------
----

CAGAACGAGACATTTCGACCTGGAGGAGGAAATATAAAGGACAATTGGAGGAGTGAATTATATAAATATAAAG
TAGTACAAATTGAACCACTA---GGAATAGCACCCACCAGGGCAAAGAGAAGAGTGGTA--------------
-------------------------AAGAGAGAAAAAAGAGCA---GTGGGC---ATAGGAGCT---------
ATGATCTTTGGGTTCTTAGGAGCA---GCAGGAAGCACTATG---GGCGCGGCGTCA---
ATAACGCTGACGGTACAG---GCCAGACAA---TTATTG---TCTGGTATAGTGCAACAGCAAAGC---
AATTTG---CTGAGG---GCTATAGAGGCGCAGCAGCATATGTTGCAACTCACAGTCTGGGGCATT
>MG989535..2
TTCTATTGCAATACAACAAAACTGCTTAATAATACTTGTATAAAAAATCACACCCAGAAGGGG----------
----

TGTAATGACAGTATCATACTTCCATGCAGAATAAAGCAGTTCATCAACATGTGGCAGGGAGTAGGACAAGCAA
TGTATGCTCCTCCCATCAGGGGAATCATTAATTGTGTATCAAAT---
ATTACAGGAATACTATTGACAAGAGATGGTGGTAATGTAACTAAT----------------------------
----

ACTAGTGAAATCTTCAGACCAGGGGGAGGAAATATAAAGGACAATTGGAGAAGTGAATTATATAAATATAAAG
TAGTACAAATTGAACCACTA---GGAATAGCACCCACCAGGGCAAAGAGAAGAGTGGTG--------------
-------------------------GAGAGAGAAAAAAGAGCA---GTGGGA---ATAGGAGCT---------
ATGATCTTTGGGTTCTTAGGAGCA---GCAGGAAGCACTATG---GGCGCAGCGTCA---
ATAACGCTGACGGCACAG---GCCAGACAA---TTATTG---TCTGGTATAGTGCAACAGCAAAGC---
AATTTG---CTGAGA---GCTATAGAGGCGCAGCATCATCTGTTGCAACTCACAGTCTGGGGCATT
>MG989548..1
TTCTACTGCAATACAACACAACTATTTAATGGTACTGGT----------------------------------
----

ACTAATAGCACTATCACACTTCCATGCAGGATAAAGCAAATTATAAACATGTGGCAGGGAGTAGGACAAGCAA
TGTATGCTCCTCCCATCAGTGGAACAATCAATTGTATATCAAAT---
ATTACAGGAATATTATTGACAAGAGATGGTGGTCATAATAATGGT----------------------------
----

ACTAATGAGACCTTCAGACCTGGAGGAGGAAATATAAAGGACAATTGGAGAAGTGAATTATATAAATATAAAG
TAGTAGAAATTGAACCACTA---GGAATAGCACCCACCAAGGCAAGGAGAAGAGTGGTG--------------
-------------------------GAGAGAGAAAAAAGAGCA---GTAGGA---ATAGGGGCT---------
ATGATCTTTGGGTTCTTAGGAGCA---GCAGGAAGCACTATG---GGCGCGGCGTCA---
ATAACGCTGACGGTACAG---GCCAGACAA---TTATTG---TCTGGTATAGTGCAACAGCAAAGC---
AATTTG---CTGAGG---GCTATAGAGGCGCAGCAGCATCTGTTGCAACTCACAGTCTGGGGCATT
>MG989534..1
TTCTATTGCAATACATCAAAACTGTTTAATAGAACATATGAGTTTAATAGAACACATGAGGGAAATGATACCA
TG---

AATGATACCACTATCATAATTCCATGCAAAATAAAGCAAATAATAAGAATGTGGCAGGGAGCAGGACAAGCAA
TGTATGCTCCTCCCGTCAGGGGAAAAATTCAGTGTGTATCAAAT---
ATTACAGGAATATTATTGACAAGAGATGGTGGTGGTGGGAATACT----------------------------
----

AGTAATAATGAGACCTTCAGACCTGGAGGAGGAAACATAAAGGACAATTGGAGAAGTGAATTATACAAATATA
AAGTAGTACAAATTGAACCATTA---GGGATAGCACCCACCAGGGCAAAAAGAAGAGTGGTG-----------
----------------------------GAGAAACACAAAAGAGCA---GTGGGA---ATAGGAGCT------
---GTGATCTTTGGGTTCTTAGGAGCA---GCCGGAAGCACTATG---GGCGCGGCGTCA---
ATATCGCTGACGGTACAG---GCCAGACAA---TTATTG---TCTGGTATAGTGCAACAGCAAAGC---
AATTTG---CTGAGA---GCTATAGAGGCGCAACAGCATCTGTTGCAACTCACAGTCTGGGGCATC
>MG989537..1
TTCTATTGCAATACAACAAAACTGTTT--

-GATAATGAAACCACAGGG------
CCGTGTAATGACACCATTATACTTCCATGCAGAATAAAGCAAATTATAAGAATGTGGCAGGGAGTAGGACAAG
CAATGTATGCTCCTCCCATCAGTGGAAGTATTAATTGTGTATCAAAT---
ATTACAGGAATACTATTGACAAGAGATGGTGGTAATCATAATGGG----------------------------
----

AACAATAGTACTGAGACCTTCAGACCTGGAGGAGGAAATATAAAAGACAATTGGAGAAGTGAATTGTTTAAAT
ATAAAGTAGTAGAAATTGAACCACTA---GGAATAGCACCCACCAGGGCAAAGAGAAGAGTGGTG--------
-------------------------------GAAAGAGAAAAAAGAGCA---GTGGGA---CTAGGAGCT---
------ATGATCTTTGGGTTCTTAGGAGCA---GCAGGAAGCACTATG---GGCGCAGCGTCA---
ATAACGCTGACGGTACAT---GCCAGACAA---TTGTTG---TCTGGTATAGTGCAACAGCAAAGC---
AATTTG---CTGAGG---GCTATAGAGGCGCAGCAGCATATGCTGCAACTCACAGTCTGGGGCATT
>JX848346..1
TTCTATTGCAATACAACACAACTGTTTAATAAGACTGACATAGAAAATGAAACCAAGGAGGCTAAT-------
----

GCTAATGGCACTATCATACTTCCATGCAAGATAAAGCAAATTATAAACATGTGGCAGGGAGTAGGACAAGCAA
TGTATGCTCCTCCCATCAGTGGACAAATTAGTTGTGTATCAAAT---
ATTACAGGAATACTATTGACAAGAGATGGTGGTAGTCAGAACGAGAACGGG----------------------
----

ACCACCGAGACCTTCAGACCTGGAGGAGGAAATATAAAGGACAATTGGAGAAGTGAATTATATAAATATAAAG
TAGTACAAATTGAACCACTA---GGAGTAGCACCCACCAGGGCAAAGAGAAGAGTGGTG--------------
-------------------------GAGAGAGAAAAAAGAGCA---GTGGGA---ATAGGAGCT---------
ATGATCTTTGGGTTCTTAGGAGTA---GCAGGAAGCACTATG---GGCGCAGCGTCA---
ATAACGCTGACGGTACAG---GCCAGACAA---TTATTG---TCTGGTATAGTGCAACAGCAAAGC---
AATTTG---CTGAGA---GCTATAGAGGCGCAACAACATCTGTTGCAACTCACAGTCTGGGGCATT
>KJ952820..1
TTCTATTGCAATACAACAGGACTGTTTAATAATTGCACAGGAAATGAAACC----------------------
----

TGTAATGACACTATCATACTTCCATGCAGGATAAAGCAAATTATAAACATGTGGCAGGGAGCAGGACAAGCAA
TGTATGCTCCTCCCATCAGGGGAACAATTAATTGTCTATCAAAT---
ATTACAGGAATAATATTGACAAGAGATGGTGGTAATAATATTAAGAAT-------------------------
----

AACGAGACGGAGATCTTCAGACCTGGAGGAGGAGATATGAAGGACAATTGGAGAAATGAATTATATAAATATA
AAGTAGTACAGATTGAACCACTA---GGAGTAGCACCCACCAGGGCAAAGAGAAGAGTGGTG-----------
----------------------------GAGAGAGAAAAAAGAGCA---GTGGGA---ATAGGAGCT------
---ATGATCTTTGGGTTCTTAGGAGCA---GCAGGAAGCACTATG---GGCGCAGCGTCA---
ATAACGCTGACGGTACAG---GCCAGACAA---TTATTG---TCTGGTATAGTGCAACAGCAAAGC---
AATTTG---CTGAGG---GCTATAGAGGCGCAGCATCATCTGTTGCAACTCACAGTCTGGGGCATT
>HM215432..1
TTCTATTGCAATACATCAAAACTATTTAATAGTACAAAAAATAGTACTTGTGAGGGG----------------
----

TGTAATGGTACTATCATACTTCCATGTAAGATAAAGCAATTTATAAACATGTGGCAGAACCCAGGACAAGCAA
TGTATGCTCCTCCCATCGAGGGAAAAATTAATTGTGTATCAAAT---
ATTACAGGAATACTATTGACAAGAGATGGTGGTGTTAATAGTACA----------------------------
----

AATAACGAGACCTTCAGACCTCAAGGAGGAAATATGAAGGACAATTGGAGAAGTGAATTATATAAATATAAAG
TAGTACAAATTGAACCACTG---GGAATAGCTCCCACCAGGGCAAGGAGAAGAGTGGTG--------------
-------------------------GAGAGAGAAAAAAGAGCA---GTGGGA---ATAGGAGCT---------
ATGATCTTTGGGTTCTTAGGAGCA---GCAGGAAGCACTATG---GGCGCGGCGTCA---
ATAACGCTGACGGTACAG---GCCAGACAA---TTATTG---TCTGGTATAGTGCAACAGCAAAGC---
AATTTG---CTGAGG---GCTATAGAGGCGCAGCAGCATCTGTTGCAACTCACAGTCTGGGGCATT
>AY444805..2
TTTATTGCAATACAACACAGTTGTTTAATAGTACTTGGAGAATAAATGAAACTATGGGGGAGTCTAATGAC-
----

ATAAACTCAAATATCACACTTCCATGCAGGATAAGGCAAATTGTAAAAATGTGGCAGGGAGTAGGACAAGCAA
TGTATGCTCCTCCCATCGATGAAGCAATTCAGTGTGTATCAAAT---
ATTACAGGAATACTATTGACAAGAGATGGTGGTCATAATGTGAGT----------------------------
----

AATGATACTGAGACCTTCAGACCTGGAGGAGGAGATATAAGGGACAATTGGAGAAGTGAATTATATAAATATA
AAGTAGTAGAAATTGAACCACTA---GGAATAGCACCCACCAGGGCAAGGAGAAGAGTGGTG-----------
----------------------------GAGAGAGAAAAAAGAGCA---GTGGGA---ATAGGAGCT------
---ATGATCTTTGGGTTCTTAGGAGCA---GCAGGAAGCACTATG---GGCGCGGCGTCA---
ATAACGCTGACGGTGCAG---GCCAGACAA---TTATTG---TCTGGTATAGTGCAACAGCAAAGC---
AATTTG---CTGAGA---GCTATAGAGGCACAACAGCATCTGTTGCAACTCACAGTCTGGGGCATT
>FJ185251..1
TTCTATTGCAATACAACAAGACTGTTTAATAATACTTGCATAAAAAATGAAACCACGGAGGGG----------
----

TGTGATGGCACTATCACACTGCCATGCAGGATAAAGCAAATTATAAACATGTGGCAGGGAGTAGGACAAGCAA
TATATGCTCCTCCCATCAGAGGAAAAATTAATTGTGTATCAAAT---
ATTACAGGAATACTATTGACAAGAGATGGTGGTGCTCATAATACG----------------------------
----

ACGACTAATGAGACCTTCAGACCTGGGGGAGGAAATATGAGGGACAATTGGGAAAGTGAATTATATAAATATA
AAGTAGTAGAAATTGAACCACTA---GGAATAGCACCCACCAGGGCAAAGAGAAGAGTGGTG-----------
----------------------------GAGATAGAAAAAAGAGCA---GTGGGA---ATAGGAGCT------
---ATGATCTTTGGGTTCTTAGGAGCA---GCAGGAAGCACTATG---GGCGCGGCGTCA---
ATAACGCTGACGGTACAG---GCCAGACAA---TTATTG---TCTGGTATAGTGCAACAGCAAAGC---
AATTTG---CTGAGG---GCTATAGAGGCGCAGCAGCATCTGTTGCAACTCACAGTCTGGGGCATT
>FN432728..1
TTCTATTGCAATACATCAAAACTGTTTAATAGCACTTGGGACAGCAATAGCACTGTTAGCTCAGATGACAGTG
ATACT--

AATGACAATGTCAATATAACTCTCCCATGCAGAATAAAGCAAATTATAAATATGTGGCAGAGAGTAGGACAAG
CAATATATGCCCCTCCCATCGAAGGAGTAATACAGTGTGAATCAAAC---
ATCACAGGACTACTATTAACAAGAGATGGAGGGAATGAGAATAATACAAAC----------------------
----

ACAACTAAAGGAGAAGTTTTCAGGCCTGGAGGAGGAAATATGAGGGACAATTGGAGAAGTGAACTATACAAGT
ATAAAGTAGTAAAAATTGAACCACTA---GGTGTAGCACCCTCACCTGCAAGAAGAAGAGTGGTG--------
-------------------------------CAGAGAGAAAAAAGAGCA---GTCGGC---CTAGGAGCT---
------TTCTTCCTTGGGTTCTTAGGAGCA---GCAGGAAGCACTATG---GGCGCAGCGTCA---
ATAACGCTGACGGTACAG---GCCAGACAG---TTACTG---TCTGGGATAGTGCAACAGCAAAGC---
AATTTG---CTGAGG---GCTATAGAGGCTCAACAACATATGTTGAAACTCACGGTCTGGGGCATT
>HQ912710..1
TTCTATTGCAATACATCAGAACTGTTTAATAGCACTTGGAATAACAATAGCATTGCCAACTCAACAGAT----
----

AGCAAGAGCACCATAACTCTCCAATGCAAAATAAAGCAAATTGTAAATATGTGGCAGAAAGTAGGACAAGCAA
TGTATGCCCCTCCCATCCAAGGAGTAATAAGCTGTAATTCAAGC---
ATTACAGGATTACTATTAACAAGAGATGGAGGGAATAATAATAGT----------------------------
----

ACAAATGAGACCTTCAGGCCTGGAGGAGGAGATATGAGGGACAATTGGAGGAGTGAATTATATAAGTATAAAG
TAGTAAAAATTGAACCAATA---GGTGTAGCACCCACCCATGCAAAAAGAAGGGTGGTG--------------
-------------------------CAGAGAGAAAAAAGAGCA---GTTGGA---CTAGGAGCT---------
GTCCTCTTTGGGTTCTTAGGAGCA---GCAGGAAGCACTATG---GGCGCGGCGTCA---
ATAACGCTGACGGTACAG---GCCAGACAA---TTATTG---TCCGGTATAGTGCAACAGCAGGGC---
AATCTG---CTGAGA---GCTATAGAGGCTCAACAACATCTGTTGAAACTCACGGTCTGGGGCATT
>DQ313242..3
TTCTATTGCAATACACCGATACTGTTTAATAGCACCTGGGAGTACAATAGCACTTGGGGTAACTATAGCTCAA
ATTAC--
-------------------ACA---
GGGTCAAATGACATTATAACTCTCCAATGCAAAATAAAGCAAATTGTAAATATGTGGCAGAAAGTAGGACAAG
CAATGTATGCCCCTCCCATCCCAGGAGAGTTAAGGTGTGAATCAAAC---
ATTACAGGATTATTATTAACAAGGGATGGAGGGACTAATAGTACAAAT-------------------------
----

GAGACTTTCGAGACTTTTAGGCCTGGAGGAGGAGACATGAGGGACAATTGGAGAAGTGAATTATATAAGTATA
AGGTAGTAAAAATTGAACCACTA---GGTGTGGCACCCACCCATGCAAAAAGAAGAGTGGTG-----------
----------------------------CAGAGAGAAAAAAGAGCA---GTTGGA---CTGGGAGCT------
---GTCTTCCTTGGGTTCTTAGGAGCA---GCAGGAAGCACTATG---GGCGCGGCGTCA---
ATAACGCTGACGGTACAG---GCCAGACAA---TTATTG---TCCGGTATAGTGCAACAGCAGAAC---
AATTTG---CTGAGG---GCTATAGAGGCTCAACAACATCTGTTGAAACTCACGGTCTGGGGCATT
>MH746242..1
TTCTATTGCAATACATCAGCACTATTTAATAGTAGTTGGAATAGCACTACTTGGAATAGCACTCGAAATACCA
ATGCTAGCTCA--

AATGACACAGAACCTATAACTCTCCAATGCAGAATAAAACAAATTGTAAATATGTGGCAGAGGATAGGACAAG
CAATGTATGCCCCTCCCATCAAAGGAGAAATAAGGTGTGAATCAAAC---
ATTACAGGACTACTATTAACAAGAGATGGAGGGACAAATAATAGT----------------------------
----

ACAAATGAAACATTCAGACCTGGAGGAGGAGATATGAGGGACAATTGGAGAAGTGAATTATATAAGTATAAGG
TAGTACAAATTGAACCAATA---GGTGTGGCACCCACCCATGCAAAAAGAAGAGTGGTG--------------
-------------------------GAGAGAGAAAAAAGAGCA---GTTGGA---CTGGGAGCT---------
GTCTTCCTTGGGTTCTTAGGAGCA---GCAGGAAGCACTATG---GGCGCGGCGTCA---
ATAACGCTGACGGTACAG---GCCAGACAA---TTATTG---TCTGGTATAGTGCAACAGCAGAGC---
AATTTG---CTGAGG---GCTATAGAGGCTCAACAACATATGTTGAGACTCACGGTCTGGGGCATT
>MH746250..1
TTCTATTGCAATACATCAGGACTATTTAATAGCACTTGGAAATGGAATACTACCACTAGGAATTGGATTGACA
TCTCA--
-AATAACACAGACACTGTC------
TCAAATAACACAAATATAACTCTCCAGTGCAGAATAAAACAATTTGTAAATATGTGGCAGAGGGTAGGACAAG
CAATGTATGCCCCTCCCATCCCAGGAGAGATAAGATGTAACTCAAAC---
ATTACAGGACTACTATTAACAAGAGATGGAGGGAATAACAGTGGT----------------------------
----

GATGCAAATGAGACATTCAGACCTGGAGGAGGAGATATGAGGGACAATTGGAGAAGTGAATTATATAAGTATA
AGGTAGTACAAATTGAACCAATA---GGTCTGGCACCCACCCCTGCAAAACGAAGAGTGGTG-----------
----------------------------GAGAGAGAAAAAAGAGCA---ATTGGA---ATGGGAGCT------
---TTCTTCCTTGGGTTCTTGGCAACA---GCAGGAAGCACTATG---GGCGCAGCGTCA---
ATAACGCTGACGGTACAA---GCCAGACAA---TTATTG---TCTGGTATAGTGCAACAGCAAAGC---
AATTTG---CTGAGG---GCTATAGAGGCTCAACAACATATGTTGAAACTCACGGTCTGGGGCATT
>MH078536..1
CTCTATTGCAATACATCAGGCTTGTTTAATAGCACTTGGGAAAACAATACTAACATA----------------
----

ACCAACTCCAATATAACGCTCAAATGCCAAATAAAGCAAATTATAAGGATGTGGCAGAGAGTAGGACAAGCAA
TGTATGCCCCTCCCATTAAAGGAAATATAACATGTGTATCAAAC---
ATTACTGGACTACTATTAACAAGAGATGGAGGGGGGAATAATACT----------------------------
----

AATGAGACCTTCAGACCTGGAGGAGGAGATATGAGGGACAATTGGAGAAGTGAATTATATAAGTATAAAGTAG
TAAAAATTGAACCACTA---GGTGTAGCACCCACCCATGCGAAGAGAAGAGTGGTG-----------------
----------------------GAGAGAGAAAAAAGAGCA---GTTGGA---CTGGGAGCT---------
GTCTTTCTTGGGTTCTTAGGGGCA---GCAGGAAGCACTATG---GGCGCAGCGTCA---
ATAACGCTGACGGTACAG---GCCAGACAA---TTATTG---TCTGGTATAGTGCAACAGCAGAGC---
AATCTG---CTGAAG---GCTATAGAGGCTCAACAACAGCTGTTGAGACTCACAGTCTGGGGCATT
>MH078539..1
TTCTATTGCGATACATCAGAGCTGTTCAATAGGACTTGGGTGTGGAATGACACTGTTGACAATAACACAGTTA
ACAAT--
-------------AGCACA------
GATGACAATAGCACAATAACTCTCCCATGCAAAATAAAGCAAATTGTAAGGATGTGGCAGAAAGTAGGACAAG
CAATGTATGCCCCTCCCATCCCAGGAGTAATAAGGTGTGACTCAAAC---
ATTACAGGACTAATATTAACAAGAGATGGAGGGAATAGTAATAGTAGTAATAAT-------------------
----

GGCACAAGGGAAGAAACTTTCAGGCCTGGAGGGGGAGACATGAGGGACAATTGGAGAAGTGAATTATATAAGT
ATAAGGTAATACAAATTGAACCATTA---GGAGTAGCACCCACCAAGGCAAGGAGAAGAGTGGTG--------
-------------------------------GAGAGAGAAAAAAGAGCA---GTTGGA---ATGGGAGCT---
------CTTTTCCTTGGGTTCTTAGGAGCA---GCAGGAAGCACTATG---GGCGCGGCGTCA---
ATAACGCTGACGGTACAG---GCCAGACAA---TTATTG---TCTGGTATAGTGCAACAGCAGAGC---
AATCTG---CTGAAA---GCTATAGAGGCTCAACAACAGCTGTTGAGACTCACAGTCTGGGGCATT
>HQ912707..2
TTCTATTGCAATACATCAACACTGTTTAATAGCACTTGGAATGGCAATGACACTGCCAGCTCAAATTACACAG
GA---

TCAAATGATACTATAACTCTCCAATGCAGAATAAAGCAAATTGTAAACATGTGGCAGAGAGTAGGACAAGCCA
TGTATGCCCCTCCCATCAAAGGAAAAATAAAGTGTGAATCAAAC---
ATTACAGGACTACTATTAACAAGAGATGGAGGAAAGAATAGTAGT----------------------------
----

AATACAAGTGAGACCTTCAGACCTGGAGGAGGAGATATGAGGGACAATTGGAGAAGTGAATTATATAAGTATA
AAGTAGTCAAAATTGAACCACTA---GGTGTAGCACCTAACCGTGCAAAAAGAAGAGTGGTG-----------
----------------------------GAGAGAGAAAAAAGAGCAGTTGGAGTTGGACTGGAGCA-------
---GTCTTCCTTGGGTTCTTAGGAGCA---GCAGGAAGCACTATG---GGCGCGGCGTCA---
CTAACGCTGACGGTACAG---GCCAGACAA---TTATTG---TCCGGTATAGTGCAACAGCAGAGC---
AATTTG---CTGAGG---GCTATAAAGGCTCAACAACATCTGTTGAAACTCACGGTCTGGGGCATT
>EU513199..2
TTCTATTGCAATACAACAAAGCTGTTTAATAGCACTTGGAATAATGATAACTACAATGTCAGCGCAAACCTCA
CAGAG--
-------------------GAA---
AACAGAAAGGAAAACATAACTCTCCACTGCAGAATAAAGCAAATTGTAAATATGTGGCAGAGAGTAGGACAAG
CAATATATGCCCCTCCCATCCCAGGAAACATAACTTGTGGATCAAAC---
ATTACTGGGCTACTATTAACAAGAGATGGAGGGAATAATGGTACA----------------------------
----

AATGATACTGAGACCTTCAGGCCTGGAGGAGGAGATATGAGGGACAATTGGAGAAGTGAATTATATAAATATA
AAGTAGTAAAAATTGAACCACTA---GGTGTAGCACCAACCCCTGCAAAAAGAAGAGTGGTG-----------
----------------------------GAAAGAGAAAAAAGAGCA---GTTGGA---ATGGGAGCT------
---TTGATCTTTGGGTTCTTAGGAGCA---GCAGGAAGCACTATG---GGCGCGGCGTCA---
ATGGCGCTGACGGTACAG---GCCAGACAA---TTATTG---TCTGGTATAGTGCAACAGCAGAGC---
AATCTG---CTGAAG---GCTATAGAGGCTCAACAACATCTGTTGAGACTCACGGTCTGGGGCATT
>KU168265..3
TTCTATTGTTATACATCAAAATTATTTAATAGCATTTGGAGGAGCAATGACACTTGGAGGAGCGATGGCAATG
ACCAT--

ACAAAGGAAACCATAACTCTCCAATGCAGAATAAAGCAAATTGTAAATATGTGGCAGAGAGTAGGACAAGCAA
TGTATGCCCCTCCCATCCCAGGAGTAATAAGGTGTGAATCAAAC---
ATTACAGGACTACTATTAACAAGAGATGGAGTAGGGAATAATATG----------------------------
----

ACAAATGAAACCTTCAGGCCTGGGGGAGGAAATATGAAGGACAATTGGAGAAGTGAACTATATAAGTATAAAG
TAGTAAAAATTGAACCACTA---GGTGTAGCACCCTCCTATGCAAGGAGAAGAGTGGTG--------------
-------------------------GAAAGAGAAAAAAGAGCA---GTTGGA---ATAGGAGCT---------
GTCTTCCTTGGGTTCTTAGGAGCA---GCAGGAAGCACTATG---GGCGCGGCGTCA---
CTAACGCTGACGGTACAG---GCCAGAAAA---TTATTG---TCTGGTATAGTGCAACAGCAGAGC---
AATCTG---CTGCAG---GCTATAGAGGCTCAACAACAGCTGTTGAGGCTCACGGTCTGGGGCATT
>MK086123..1
TTCTATTGCAATACAACAGACCTATTT------TCTTGGAATAGTAGTAACCCATGGAAGAACATAACG----
----

TCAAATGACAGTATAACTCTCCAATGCAGAATAAAGCAAATTGTAAGGATGTGGCAGCAAGTAGGACAAGCAA
TGTATGCCCCTCCTATACCAGGAATAATAAGGTGTGAATCAAAC---
ATTACAGGAATACTGTTAACAAGAGATGGAGGAAATAATAATACA----------------------------
----

AATAACACAGAGGTATTCAGGCCTGGAGGAGGAGATATGAGGGACAATTGGAGAAGTGAATTATATAAATATA
AAGTAGTAAAAATTGAACCATTA---GGTGTAGCACCCTCCCGTGCAAGAAGAAGAGTGGTG-----------
----------------------------GAGAGAGAAAAAAGAGCA---GTTGGC---CTGGGAGCT------
---TTCTTCCTTGGGTTCTTAGGAGCA---GCAGGAAGCACTATG---GGCGCGGCGTCA---
ATAACGCTGACGGTACAG---GCCAGACAA---TTATTG---TCTGGTATAGTGCAGCAGCAGAGC---
AATCTG---CTGCGG---GCCATAGAGGCTCAACAACATCTGTTGAAACTCACGGTCTGGGGCATT
>AY371122..1
TTCTATTGCAATACAACAAGCCTGTTTAATAGCACTTGGCATGACAATGACACTGCCAGCTCAAACCAC----
----

ACAAATGGCACTATAACTCTCCAATGCAGAATAAAGCAAATTATAAATATGTGGCAGAGAGTAGGACAAGCAA
TATATGCCCCTCCTATCCCAGGAGAAATAAGGTGTGACTCAAAC---
ATTACAGGACTACTATTAACAAGAGATGGAGGGAATAATAATGTGACG-------------------------
----

AATGGGACTGAGACTTTCAGGCCTGAAGGAGGAGATATGAGAAACAATTGGAGAAGTGAATTATATAAGTATA
AAGTAGTAAAAATTGAACCAATA---GGTGTAGCACCTACCCCTGCAAAAAGAAGAGTGGTG-----------
----------------------------GGGAGAGAAAAAAGAGCA---CTG------GGAGCTGCT------
---GTCTTCCTTGGGTTCTTAGGAGCA---GCAGGAAGCACTATG---GGCGCGGCGTCA---
ATAACGCTGACGGTACAG---GCCAGACAA---CTATTG---TCCGGTATAGTGCAACAGCAGAGC---
AATTTG---CTGAGG---GCTATAGAGGCTCAACAACACCTGTTGAGACTCACGGTCTGGGGCATT
>AY371123..2
TTCTATTGCAATACAACAGACCTATTTAATAGCACTTGGAACGAAACCACAGAGTCAAAT-------------
----

GGCAATATGAATCTAACAATCCCATGCAGAATAAAACAATTTGTGAATTTGTGGCAGAAAGTAGGACAAGCAA
TGTATGCCCCTCCCATCCAAGGAGAAATAAGGTGTGAATCAAAC---
ATTACAGGATTACTATTAACAAGAGATGGAGGGGTAAATAATAAT----------------------------
----

AATAGTACGGAGATATTCAGGCCTGGAGGAGGAGATATGAGGGACAATTGGAGAAGTGAATTATATAAGTATA
AGGTAGTTCAAATTAAACCACTA---GGTGTGGCACCTACCCATGCAAAACGAAGAGTGGTG-----------
----------------------------GAGAGAGAAAAAAGAGCA---GTTGGA---ATAGGAGCT------
---GTTTTCCTTGGGTTCTTAGGAGCA---GCAGGAAGCACTATG---GGCGCGGCGTCA---
ATAACGCTGACGGCACAG---GCCAGACAA---TTATTG---TCTGGTATAGTGCAACAGCAGAGC---
AATCTG---CTGAGG---GCTATAGAGGCTCAACAACATCTGTTGAAACTCACGGTCTGGGGCATT
>AY371124..1
TTCTATTGCAATACATCACAACTGTTTAATAGAACTTGGGGTAATAATAGCACCTGGGGAAACGAGACAGGGG
AG---

GGCAATATAACTATCACTCTCCCATGCAGAATAAAGCAAATTGTAAATATGTGGCAAAGAGTAGGACGAGCAA
TGTATGCCCCTCCCATCCAAGGAGAAATAAGGTGTAACTCAAAC---
ATTACAGGACTACTATTAACAAGAGATGGAGGGAATAATACTAAT----------------------------
----

AGTACAGATGAGATCTTCAGGCCTGGAGGAGGAGATATGAGGGACAATTGGAGAAGTGAATTATATAAGTATA
AGGTAGTAAAAATTGAACCACTA---GGTGTAGCACCCACCCATGCAAAAAGAAGAGTGGTG-----------
----------------------------GAGAGAGAAAAAAGAGCA---GTTGGA---CTGGGAGCT------
---GTTTTCCTTGGGTTCCTAGGAGCA---GCAGGAAGCACTATG---GGCGCGGCGTCA---
ATAACGCTGACGGTACAG---GCCAGACAA---TTATTG---TCTGGTATAGTGCAACAGCAGAGC---
AATTTG---CTGAAG---GCTATAGAGGCTCAACAACATCTGTTGAGACTCACAGTCTGGGGCATT
>AY371131..2
TTCTATTGCGATACATCAGGACTGTTTAATAGCACTTGGGACAGCACTTGGGGCAATAACAATGACAGCTCAG
AG---

GCAAATGGCACTATAACTCTCCAATGCAGAATAAAGCAAATTGTAAATATGTGGCAGAAAGTAGGACAAGCAA
TGTATGCCCCTCCCATCCAAGGAGTAATAAGGTGTGAATCAAAC---
ATTACAGGACTAATATTAACAAGGGATGGAGGGCCTGATAGTCCA----------------------------
----

AATGAGATATTCAGGCCTGGAGGAGGAGATATGAGGGACAATTGGAGAAGTGAATTATATAAGTATAAGGTAG
TACAAATAGAACCAATA---GGTGTGGCACCCACTTATGCAAAGAGAAGAGTGGTG-----------------
----------------------GAGAGAGAAAAAAGAGCA---TTTGGA---GTGGGAGCT---------
GTCTTCCTTGGGTTCTTAGGAGCA---GCAGGAAGCACTATG---GGCGCGGCGTCA---
ATAACGCTGACGGTACAG---GCCAGACAA---TTATTG---TCCGGTATAGTGCAACAGCAAAGC---
AATTTG---CTGAGG---GCTATAGAGGCTCAACAACATCTGTTGAAACTCACGGTCTGGGGCATT
>GU201498..1
TTCTATTGCAATACATCACAACTATTTAATAGCACTTGGGATGGTCCATTTAATAGCACTTGGAATGACAATA
GCACAGAC---
-------------------ACA---
GAGTTAAATGACACTATAACTCTCCAATGCAGAATAAAGCAAATTGTAAATATGTGGCAGAGAGTAGGACAAG
CAATGTATGCCCCTCCCATCCAAGGAGTAATAAGGTGTGAATCAAAC---
ATTACAGGACTGCTATTAACAAGGGATGGAGGGAATAATAGCACA----------------------------
----

AATGAGACATTCAGGCCTGGAGGAGGAGATATGAGGGACAATTGGAGAAGTGAACTATATAAGTATAAGGTAG
TACAAATTGAACCACTA---GGTGTGGCACCCACCCGGGCAAAAAGAAGAGTGGTG-----------------
----------------------GAGAGAGGAAAAAGAGCA---GTAGGA---CTGGGAGCT---------
GTCTTCTTTGGGTTCTTAGGAGCA---GCAGGAAGCACTATG---GGCGCGGCGTCA---
ATAACGCTGACGGTACAG---GCCAGACAA---TTATTG---TCCGGTATAGTGCAACAGCAGAGC---
AATCTG---CTGAGG---GCTATAGAGGCTCAACAACATCTGTTGAAACTCACGGTCTGGGGCATC
>GU201499..2
TTCTATTGCAATACATCAAAACTGTTTAATAGCACTTGGATTAATGGCACTAGCAATTGGATTAATGACACTT
GGACTAAT---
-------------GGCAAT------
GAGGATGATAATAAAATAACTCTCCAATGCAGAATAAAGCAAATTATAAATATGTGGCAGAGAGTAGGACAAG
CAATGTATGCCCCTCCCATCCGAGGAGAAATAAGGTGTCATTCAAAT---
ATTACAGGTCTACTATTAACAAGAGATGGAGGGAATAATAATAGT----------------------------
----

GCTGCAAATGAAACCTTCAGGCCTACAGGAGGAGACATGAGGGACAATTGGAGAAGTGAATTATATAAATATA
AAGTAGTAAAAATTGAACCACTA---GGTGTAGCACCAAACAAGGCAAAAAGAAGAGTGGTG-----------
----------------------------GAGAGAGAAAAAAGAGCA---GTTGGA---ATGGGAGCT------
---TTCTTCCTTGGGTTCTTAGGAGCA---GCAGGAAGCACTATG---GGCGCGGCGTCA---
ATAACGCTGACGGTACAG---GCCAGACAA---TTATTG---TCTGGTATAGTGCAACAGCAGAGA---
AATCTG---CTGCAG---GCTATAGAGGCTCAACAACATCTGTTGAGACTCACGGTCTGGGGCATT
>AY371137..2
TTCTATTGCAATACATCACAACTGTTTAATAGTACTTGGAATAGTACTTATAGCAATAATGCCAACTTA----
----

ACAAATGGCACTATAACTCTCCAATGCAGAATAAAGCAAATTGTACAGATGTGGCAGAGAGTAGGACAAGCAA
TGTATGCCCCTCCCATCCAAGGAGTAATAAGGTGTGAGTCAAAC---
ATAACAGGATTACTATTAACAAGAGATGGAGGGACAAATAATACA----------------------------
----

AATAATACCGAGACATTCAGGCCTGGAGGAGGAGATATGAGGGACAATTGGAGAAGTGAATTATATAAGTATA
AGGTAGTACAAATTGAACCAGTG---GGTGTGGCACCCACCCATGCAAAAAGAAGAGTGGTG-----------
----------------------------GAGAGAGAAAAAAGAGCA---GTTGGA---CTGGGAGTT------
---GCGTTCCTTGGGTTCTTAGGAGCA---GCAGGAAGCACTATG---GGCGCGGCGTCA---
ATAACGCTGACGGTACAG---GCCAGACAA---TTATTG---TCTGGTATAGTGCAACAGCAGAGC---
AATTTG---CTGAAG---GCTATAGAGGCTCAACAACATATGTTGAAACTCACGGTCTGGGGCATT
>AY371132..1
TTCTATTGCAGTACATCAAGACTATTTAATAGCACGTGGGATAATAGCACGTGGAATAACAATACCACT----
----

GACACAGATGACATAACTCTCCCATGCAGAATAAAGCAATTTGTAAATATGTGGCAGAGAGTAGGACAAGCAA
TGTATGCCCCTCCTATCCGAGGAGAAATAAGGTGTGAGTCAAAC---
ATTACAGGACTACTATTAACAAGGGATGGAGGAAATAATAGCACA----------------------------
----

AATGAGACATTCAGGCCTGGAGGAGGAGATATGAGGGACAACTGGAGAAGTGAATTATATAAGTATAAGGTAG
TACAAATTGAACCACTA---GGTGTGGCACCCACCCGTGCAAGGAGAAGAGTGGTG-----------------
----------------------GAGAGAGAAAAAAGAACA---GTAGGA---CTGGGAGCT---------
GTCTTCCTTGGGTTCTTAGGAGCA---GCAGGAAGCACTATG---GGCGCGGCGTCA---
ATAACGCTGACGGTACAG---GCCAGACAA---TTATTG---TCCGGTATAGTGCAACAGCAGAGC---
AATCTG---CTGAGG---GCTATAGAGGCTCAACAACATCTGTTGAAACTCACGGTCTGGGGCATT
>GU201500..1
TTCTATTGCAATACATCAAAACTGTTTAATAGCATTTGGTCTAACAATACATCAAAATCGTTTAATAGCACTT
GGCTTAACAGT--
-------------AGCATT------
GGGAATGACAGTAGCATAACTCTCCAATGCAGAATAAAGCAATTTGTAAGTATGTGGCAGAGAGTAGGACAAG
CAATGTATGCTCCTCCCATCCCAGGAGAACTAAGGTGCGAATCAAAC---
ATTACAGGACTACTATTAACAAGAGATGGAGGAGGTAATAATAGG----------------------------
----

GAAAATGAGACCTTCAGGCCTGGAGGAGGAGATATGAGGGACAATTGGAGAAGTGAATTATATAAGTATAAAG
TAGTAAAAATTGAACCACTA---GGTGTAGCACCCTCCCAGGCAAAAAGAAGAGTGGTG--------------
-------------------------AATAGAGAAAAAAGAGCA---GTTGGA---ATAGGAGCT---------
CTCTTCCTTGGGTTCTTAGGAGCA---GCAGGAAGCACTATG---GGCGCGGCGTCA---
ATGACGCTGACGGTACAG---GCCAGACAA---TTATTG---TCTGGTATAGTGCAACAGCAGAGC---
AATCTG---CTGAAG---GCTATAGAGGCTCAACAACATCTGTTGAGACTCACGGTCTGGGGCATT
>AY371134..1
TTCTATTGCAATACATCACAACTGTTTAATAGCACTTGGAAATCTAATAGCACTTGGAACAGCACTAGGAACA
AT---

AACACTGACATTATAACTCTCCAATGCAGAATAAAGCAAATTGTAAATATGTGGCAGAAAATAGGACAAGCAA
TGTATGCCCCTCCCATCCAAGGAGTAATAGAGTGTAAATCAAAC---
ATTACAGGACTACTATTAACAAGGGATGGAGGGCGAGGGCCTGAT----------------------------
----

AATACAACTGAGATATTCAGGCCTGGAGGAGGAGATATGAGGGACAATTGGAGAAGTGAATTATATAAGTATA
AGGTAGTACAAATAGAGCCAATA---GGTGTGGCACCCACCCATGCAAAAAGAAGAGTTGTG-----------
----------------------------GAGAGAGAAAAAAGAGCA---GTTGGA---CTGGGAGCT------
---GTCTTCCTTGGTTTCTTAGGAGCA---GCAGGAAGCACTATG---GGCGCGGCGTCA---
ATAACGCTGACGGTACAG---GCCAGACAA---TTACTG---TCTGGTATAGTGCAACAGCAGAGC---
AATCTG---CTGAGG---GCTATAGAGGCTCAACAGCATCTGTTGAAACTCACAGTCTGGGGCATT
>AY371136..1
TTCTATTGCAATACATCAAAGCTATTTAATAGCACTTGGACTTGGACCAATGACACTGCCAGCTCAAATGACA
CAAAG--

GGGTTAAATGACACTATAACGCTCCAATGCAGAGTAAGGCAAATTGTAAGAATGTGGCAGAAAGTAGGACAAG
CAATGTATGCCCCTCCCATCCGAGGAGTAATAAGATGTGAGTCAAAC---
ATTACAGGACTACTATTAACAAGGGATGGAGGGACAAATAATAAA----------------------------
----

AATAATGAGACATTTAGGCCTGGAGGAGGAAATATGAGGGACAATTGGAGAAGTGAATTATATAAGTATAAGG
TAGTACAAATTGAACCACTA---GGTGTGGCACCCACCCATGCAAGAAGAAGAGTGGTG--------------
-------------------------CAGAGAGAAAAAAGAGCA---GTTGGA---CTGGGAGCT---------
GTCTTCCTTGGGTTCTTAGGAGCA---GCAGGAAGCACTATG---GGCGCGGCGTCA---
ATAACGCTGACGGTACAG---GCCAGACAA---TTATTG---TCCGGTATAGTGCAACAGCAGAGC---
AATCTG---CTGAGG---GCTATAGAGGCTCAACAGCATCTGTTGAAACTCACGGTCTGGGGCATT
>AY371138..1
TTCTATTGCAATACATCAGAACTATTTAATAGCACTTGGGCACATGACACAAATAACACAGGGTTG-------
----

GATAATGGCAATCTAACTCTCCAATGCAGAATAAAGCAAATTGTAAGAATGTGGCAGAGGACAGGACAAGCAA
TGTTTGCCCCTCCCATCCAAGGAGAAATAAAGTGTGAATCAAAC---
ATTACAGGATTACTATTAACAAGAGATGGAGGGAACAATGGTAGT----------------------------
----

GCAAATGAGACATTCAGGCCTGGAGGAGGAGATATGAGGGACAATTGGAGAAGTGAATTATATAAGTATAAGG
TAGTACAAATTGAACCAATA---GGTGTGGCACCCACCCATGCAAAAAGAAGAGTGGTG--------------
-------------------------GAGAGAGGAAAAAGAGCAGTAGTTGGA---CTGGGAGCT---------
GTCTTCCTTGGGTTCTTAGGAGCA---GCAGGAAGCACTATG---GGCGCAGCGTCA---
ATAACGCTGACGGTACAA---GCCAGACAA---TTATTG---TCTGGTATAGTGCAGCAGCAGAGC---
AATTTG---CTGAGG---GCCATAGAGGCTCAACAACAACTGTTGAAACTCACGGTCTGGGGCATT
>AY371142..2
TTCTATTGCAATACATCAAAACTGTTTAATAGCACTTGGGAAAATGGAACTTTAACCAGCTCAAACCACACAA
GG---

TCAAATGGCACCATAACCCTCCAATGCAAAATAAGGCAAATTGTAAGAATGTGGCAGAGAGTAGGACAAGCAA
TGTATGCCCCTCCCATCGACGGGGACATAAGGTGCGAATCAAAC---
ATTACAGGACTACTATTAACAAGAGATGGAGGGAGTAAAAATAGC----------------------------
----

ACAAATAATGAGACTTTCAGGCCTGGAGGAGGAGATATGAGGGACAATTGGAGAAGTGAATTATATAAGTATA
AAGTAGTAGAAGTTGAACCAATA---GGTGTAGCACCCACCTATGCAAAAAGAAGAGTGGTG-----------
----------------------------GAGAGAGAAAAAAGAGCA---GTTGGA---CTGGGAGCT------
---GTCTTCCTTGGGTTCTTAGGAGCA---GCAGGAAGCACTATG---GGCGCGGCGTCA---
ATAACGCTGACGGTACAG---GCCAGACAA---TTGTTG---TCTGGTATAGTGCAACAGCAGAGC---
AATTTG---CTGAAG---GCTATAGAGGCTCAACAACATCTGTTGAGACTCACGGTCTGGGGCATT
>AY371125..1
TTCTATTGCAATACATCAGGACTATTTAATAGCACTTGGACTTGGAATAGTGATAATAACACTGGATATAGCA
AC---

AACACTAGTGAACTTATAACTCTCCAATGTAGAATAAAGCAGATTATAAATATGTGGCAGAGAGTAGGACAAG
CAATGTATGCCCCTCCCATTAGAGGAATAATAAAATGTGAATCAAAC---
ATTACAGGGCTATTATTAACAAGAGATGGAGGAAATAATAGTACA----------------------------
----

AATGAGACATTCAGGCCTGGAGGAGGAGATATGAGGGACAATTGGAGAAGTGAATTATATAAATATAAGGTAA
TACAAATTGAACCATTA---GGTGTGGCACCCAACAAGGCAAAAAGAAGAGTGGTG-----------------
----------------------GAGAGAGAAAAAAGAGCA---GTGGGA---ATTGGAGCT---------
GTCCTCTTTGGGTTCTTAGGAGCA---GCAGGAAGCACTATG---GGCGCAGCGTCA---
ATAACGCTGACGGTACAG---GCCAGACAA---TTATTG---TCTGGTATAGTGCAACAGCAGAGC---
AATCTG---CTGAGG---GCTATAGAGGCTCAACAACATCTGTTGCAACTCACGGTCTGGGGCATT
>AY371126..1
TTCTATTGCAATACATCAAACCTGTTTAATAGCACTTGGATTAGCAATAGCACTTGGAATAGCAGTAGCAGTA
CA---

GGGTCAGACATTATAACTCTCCAATGCAGAATAAAGCAAATTGTAAATATGTGGCAGAAAGTAGGACAAGCAA
TGTATGCCCCTCCCATCCGAGGAGAAATAAGATGTAACTCGAAC---
ATTACAGGACTACTATTAACAAGGGATGGAGGGGATAGAGGGAATAGTGGT----------------------
----

GGTGCAAATGGGACCGAAACTTTCAGGCCTGGAAGAGAAAATATGAAGGACAATTGGAGAAGTGAATTATATA
AGTATAAGGTAGTAAAAATTGAACCACTA---GGTGTAGCACCCTCCCATGCAAAAAGAAGAGTAGTG-----
----------------------------------GAGAGAGAAAAAAGAGCA---GTTGGA---
ATGGGAGCT---------GTCTTCCTTGGGTTCTTAGGAGCA---GCAGGAAGCACTATG---
GGCGCAGCGTCA---ATAACGCTGACGGTACAG---GCCAGACAA---TTATTG---
TCCGGTATAGTGCAACAGCAGAAC---AATCTG---CTGAGA---
GCTATAGAGGCTCAACAACATCTGTTGAAACTCACGGTCTGGGGCATT
>AY371127..2
TTCTATTGCAACACAACAGACTTATTTAATAGCACTTGGGATAACAGTAGCATTGAGACTAACTATAGCACTG
GG---

AATAACAATGAAACTATAACTCTCCAATGCAGAATAAAACAAATTGTAAATATGTGGCAGAAAGTAGGACAAG
CAATGTATGCCCCTCCCATCCCAGGAGTAATAAGGTGTGAATCAAAC---
ATTACAGGGTTACTATTAACAAGAGATGGAGGGAACAATACTGAGGCA-------------------------
----

AATGAGAATGAGACATTCAGGCCTGGAGGAGGAGATATGAGGGACAATTGGAGAAGTGAATTATATAAGTATA
AGGTAGTACAAATTGAGCCACTA---GGTGTGGCACCCACCCAGGCAAAGAGAAGAGTGGTG-----------
----------------------------CAGAGAGAAAAGAGAGCA---GTTGGA---ATGGGAGCT------
---TTCTTCCTTGGGTTCTTAGGAGCA---GCAGGAAGCACTATG---GGCGCGGCGTCA---
ATAACGCTGACGGTACAG---GCCAGACAA---TTATTG---TCTGGTATAGTGCAACAGCAGAGC---
AATCTG---CTGAGG---GCTATAGAGGCTCAACAACATCTGTTGAAACTCACGGTCTGGGGCATT
>AY371139..1
TTCTATTGCAATACATCAAGCCTGTTTAATAGCATTTGGAGTACCAATAGTACTGGGAATAACAATAGCACT-
----

GGGGATGGCAATATAACTCTCCAATGCAGAATAAAGCAAATTATAAAGATGTGGCAGAGAGTAGGACAAGCAA
TGTATGCCCCTCCCATCCCAGGAGAAATAAGGTGCAACTCAAAC---
ATTACAGGACTACTATTAACAAGAGATGGAGGGGTTAATAATAGT----------------------------
----

ATAAATGAGACCTTCAGGCCTGGAGGAGGAGATATGAGGGACAACTGGAGAAGTGAATTATATAAGTATAAAG
TAGTAAAGATTGAACCACTA---GGTGTAGCACCCACCCGTGCAAAAAGAAGAGTGGTG-------------
-------------------------GGGAGAGAAAAAAGAGCAGTTGTTGGA---CTGGGAGCT--------
GTCTTCCTTGGGTTCTTAGGAGCG---GCAGGAAGCACTATG---GGCGCGGCGTCA---
ATAACGCTGACGGTACAG---GCCAGACAA---TTATTG---TCTGGTATAGTGCAACAGCAGAGC---
AATTTG---CTGAGG---GCTATAGAGGCTCAACAACATCTGTTGAGACTCACGGTCTGGGGCATT
>AY371140..1
TTCTATTGCAATACATCAAAACTGTTTAATAGCACTTGGAGTAACAATGACACTGCCAGCTCAAACCACACAG
GG--

TTAAATAACACTATAACTCTCCAATGCAGAATAAAGCAAATTATAAATATGTGGCAGAAAGTAGGACAAGCAA
TGTATGCCCCTCCCATCCGAGGAGAAATAAGGTGTGAATCAAAC---
ATTACAGGACTACTACTAACAAGAGATGGAGGGAGTAATAATAGT---------------------------
----

ACAAATGAGACTTTCAGGCCTGGAGGAGGAGATATGAGGGACAATTGGAGAAGTGAATTGTATAAGTATAAAG
TAGTAAAAATTGAACCACTA---GGTGTAGCCCCCACCAAGGCAAAAAGAAGAGTGGTG-------------
-------------------------GGGAGAGAAAAAAGAGCA---GTTGTTGGACTGGGAGCT--------
GTCTTCCTTGGGTTCTTAGGAGCA---GCAGGAAGCACTATG---GGCGCGGCGTCA---
ATGACGCTGACGGTACAG---GCCAGACAA---TTGTTG---TCCGGTATAGTGCAACAGCAGAGC---
AATTTG---CTGAGG---GCTATAGAGGCTCAACAACATCTGTTGAAACTCACGGTCTGGGGCATT
>AY371146..1
TTCTATTGCAATACAACAGCACTATTTAATAGCACTTGGTTTGGCAATGAGACTGACAGCTCAAACCATACAA
GG--

TCAAATGACACTATAACTCTCCAATGCAGAATAAAGCAAATTGTAAGGATGTGGCAGAAAGTAGGACAAGCAA
TGTATGCCCCTCCCATCAAAGGAAACATAACGTGTAAATCAAAT---
ATTACAGGAATACTATTAACAAGAGATGGAGGAACTAATAGAACA---------------------------
----

AATGAGACATTCAGGCCTGGAGGAGGAGATATGAGAGACAATTGGAGAAGTGAATTATATAAGTATAAGGTAG
TACAAATTGAACCACTA---GGTGTGGCACCTACCCCTGCAAAAAGAAGAGTGGTG----------------
----------------------CAGAGAGAAAAAAGAGCA---GTTGGA---ATGGGAGCT--------
TTCTTCCTTGGGTTCTTAGGAGCA---GCAGGAAGCACTATG---GGCGCGGCGTCC---
CTGACGCTGACGGTACAG---GCCAGACAA---TTGTTG---TCTGGTATAGTGCAACAGCAGAGC---
AATTTG---CTGAGG---GCTATAGAGGCTCAACAACATCTGTTGAAACTCACAGTCTGGGGCATT
>AY371128..1
TTCTATTGCAATACATCAAAACTGTTCAATAGCATTTGGACTTTGGAGAATGGCAATTGGACTAACAATAGCA
CAGAA---

AATGGAAATGACACTATAACTCTCCAATGCAGAATAAAGCAAATTATAAATATGTGGCAGAGAGTAGGACAAG
CAATGTATGCCCCTCCCATCCCAGGAGTAATAATGTGTAATTCAAAT---
ATCACAGGACTACTATTAACAAGAGATGGAGGGAACAGTGGTGGT---------------------------
----

GCAAATGAGACCTTCAGGCCTGGAGGAGGAGATATGAGAGACAATTGGAGAAGTGAATTATATAAGTATAAAG
TGGTGAAGATTAACCCACTA---GGAGTAGCACCCACCAATGCAAAAAGAAGAGTGGTG-------------
-------------------------GGGAGAGAGAAAAGAGCA---GTTGGA---CTGGGAGCT--------
GTCTTCCTTGGGTTCTTAGGAGCA---GCAGGAAGCACTATG---GGCGCGGCGTCA---
ATAACGCTGACGGTACAG---GCCAGACTA---TTATTG---TCTGGTATAGTGCAACAGCAAAGC---
AATTTG---CTGAAG---GCTATAGAGGCTCAACAACATCTGTTGAGACTCACGGTCTGGGGCATT
>AY371129..2
TTCTATTGCAATACATCAGAACTATTTAATAGCACTTGGGGTAATAGCACTTGGGATAATAGCACTTGGGATA
ACAGC---

TCAAATTATGACAATATAACTCTCCAATGCAGAATAAAGCAAATTATAAATATGTGGCAGAAAGTAGGACAAG
CAATGTATGCCCCTCCCATCCCAGGAGTAATAAGGTGTGAATCAAAC---
ATTACAGGACTACTATTAACAAGGGATGGAGGGAGCAGTAATGGTACA------------------------
----

AATGAGACAAGTGAGACATTCAGGCCTGGAGGAGGAGATATGAGAGACAATTGGAGAAGTGAATTATATAAGT
ACAAGGTAGTACAAATTGAACCATTA---GGTGTGGCACCCTCCCATGCAAAAAGAAGAGTGGTG--------
-------------------------------GAGAGAGGAAAAAGAGCA---GTTGGA---ATGGGAGCT---
------TTCTTCCTTGGGTTCTTAGGAGCA---GCAGGAAGCACTATG---GGCGCAGCGTCA---
CTGACGCTGACGGTACAA---GCCAGACAA---TTATTG---TCTGGTATAGTGCAGCAGCAGAGC---
AATTTG---CTGAAG---GCTATAGAGGCTCAACAACATCTGTTGAAACTCACGGTCTGGGGCATT
>AY371130..1
TTCTATTGTAATACAACAAAACTATTTAACACTGCCAGCTTAAATGACACAGAG-------------------
----

ACAAATGGCACTATAACTCTCCCATGCAGAATAAAACAAATTGTAAATATGTGGCAGAAAGTAGGACAGGCAA
TGTATGCCCCTCCCATCCAAGGAGTAATAAGGTGTGAATCAAAC---
ATTACAGGACTACTATTAACAAGAGATGGAGGGAACAATAGGAGT----------------------------
----

ACAGAAACCTTTAGGCCTGGAGGAGGAGATATGAGGGACAATTGGAGAAGTGAATTATATAAGTATAAAGTAG
TAAAAATTGAACCACTA---GGTGTGGCACCCACCCGTGCAAAAAGAAGAGTGGTG-----------------
----------------------GAGAGAGAAAAAAGAGCA---GTTGGA---ATGGCAGCT---------
GTCTTCTTTGGGTTCTTAGGAGCA---GCAGGAAGCACTATG---GGCGCGGCGTCA---
ATAACGCTGACGGTACAG---GCCAGACAA---TTATTG---TCCGGTATAGTGCAACAGCAGAGC---
AATTTG---CTGAAA---GCTATAGAGGCTCAACAACATATGTTGAGACTCACGGTCTGGGGCATT
>GU201513..1
TTCTATTGCAATACATCAAGACTATTTAATAGTACTTGGACTAGCAACTCTACAACAAATTCAACT-------
----

GCTAATGAAATTATAACTCTCCAATGTAGAATAAAGCAAATTGTAAATATGTGGCAGACGGTAGGACAAGCAA
TGTATGCCCCTCCCATCCCAGGAATAATAAGGTGTGACTCAAAC---
ATTACAGGACTACTATTAACAAGAGATGGAGGGGATACAAATAGA----------------------------
----

ACAAATGAAATATTCAGACCTGGAGGAGGAGATATGAGGGACAATTGGAGAAGTGAGTTATATAAGTATAAGG
TAGTAAAAATTGAACCAATA---GGTGTGGCACCCACCCCTGCAAAAAGAAGAGTGGTG--------------
-------------------------GAAAGAGGAAAAAGAGCA---GTTGGA---CTGGGAGCT---------
GTCTTCCTTGGGTTCTTAGGAGCA---GCAGGAAGCACTATG---GGCGCGGCGTCA---
ATAACGCTGACGGTACAG---GCCAGACAA---TTATTG---TCCGGTATAGTGCAACAGCAGAGC---
AATTTG---CTGAGG---GCTATAGAGGCTCAACAACATCTGTTGAAACTCACGGTCTGGGGCATT
>GU201514..2
TTCTATTGCAATACATCAGAGTTGTTTAATAGCATTTGGAATAACAATGGCAGCATTTGGTATAACAATGACA
CTGCTATCAAT--
----------AACACAGAG------
CCAAAGAATAAGACTCTAACTCTCCAATGCAGAATAAAGCAGGTTATAAAGATGTGGCAGAGAGTAGGACAAG
CAATGTATGCCCCTCCCATCCGAGGAGAAATAAGGTGTGATTCAAAC---
ATTACAGGACTATTATTAACAAGAGATGGAGGGAATAATAGGACA----------------------------
----

GATGAGACCTTCAGGCCTGGAGGAGGAGATATGAGGGACAATTGGAGAAGTGAATTATATAAGTATAAAGTAG
TAAAAATTGAACCACTA---GGTGTAGCACCCACCTATGCAAAAAGAAGAGTGGTG-----------------
----------------------GAGAGAGAAAAAAGAGCA---GTTGGA---ATAGGAGCT---------
GTCCTCCTTGGGTTCTTAGGAGCA---GCAGGAAGCACTATG---GGCGCGGCGTCA---
ATAACGCTGACGGTACAG---GCCAGACAA---TTATTG---TCTGGTATAGTGCAACAGCAGAGC---
AATTTG---CTGAAG---GCTATAGAGGCTCAACAACATCTGTTGAGACTCACGGTCTGGGGCATT
>AY371141..2
TTCTATTGCAATACATCAGGACTGTTTAATAGCAGTTGGGATAGCAATAGCACTGAGGAAACCAATAATTCAG
AA---

AATAATGAAACTATAACTCTCCAATGCAGAATAAAGCAAATTGTAAACATGTGGCAGAGAGTAGGACAAGGAA
TGTATGCCCCTCCCATCCAGGGAATAATAAGGTGTGAATCAAAC---
ATTACAGGACTACTATTAACAAGAGATGGAGGGGAAAATAATAGT----------------------------
----

ACAAATCAGACCTTCAGGCCTGGAGGAGGAGATATGAGGGACAATTGGAGAAGTGAATTATATAAGTATAAAG
TAGTAAAAATTGAACCAATA---GGTGTAGCACCCACCCATGCAAAAAGAAGAGTGGTG--------------
-------------------------GAGAGAGGAAAAAGAGCA---GTTGGA---CTGGGAGCT---------
GTCTTCCTTGGGTTCTTAGGAGCA---GCAGGAAGCACTATG---GGCGCGGCGTCA---
ATAACGCTGACGGTACAG---GCCAGACAA---TTATTG---TCTGGTATAGTGCAACAGCAGAGC---
AATCTG---CTGAAA---GCTATAGAGGCTCAACAACATCTGTTGAAACTCACGGTCTGGGGCATT
>DQ313247..1
TTCTATTGTAATACAACAGATCTGTTTAATAGCACTTGGGCTAATAATAGCAATAAGTGGGACTCAACAGCCC
CA---

GAGAATGGCACTATAACTCTCAAATGCAGAATAAAGCAAATTGTAAATATGTGGCAGAGAGTAGGACAAGCAA
TATATGCCCCTCCCATCGAAGGAGACATAAGGTGTGAATCAAAT---
ATTACAGGACTACTATTAACAAGAGATGGAGGGGAAAATAGTAGT----------------------------
----

GAGCCCAGTGAGACCTTCAGGCCTGGAGGGGGAGATATGAGGGATAATTGGAGAAGTGAATTATATAAGTATA
AAGTAGTAAAAATTGAACCACTA---GGTGTAGCACCTACCCATGCAAAAAGAAGAGTGGTG-----------
----------------------------GAGAGAGGAAAAAGAGCA---GTTGGA---CTGGGAGCT------
---TTCTTCTTTGGGTTCTTAGGAGCA---GCAGGAAGCACTATG---GGCGCGGCGTCA---
ATAACGCTGACGGTACAG---GCTAGACAA---TTATTG---TCTGGTATAGTGCAACAGCAGAGC---
AATTTG---CTGAGA---GCTATAGAGGCTCAACAACATATGTTGAAACTCACGGTCTGGGGCATT
>MK086121..2
TTCTATTGCAATACATCAAATTTATTCAATAGCACTTGGAATAGCACTTGGGGTAATAACACTTGGAATAGCA
GTAGC--

ACTAATATTAATATAACGCTTCAATGCAGAATAAAGCAAATTGTAAATATGTGGCAGACAGTAGGACAAGCAA
TGTATGCCCCTCCCATCCAAGGAGAAATAAGGTGTGAATCAAAC---
ATTACAGGATTACTATTGACAAGAGATGGAGGGGGACAGAGACC----------------------------
----

AATAATACAGAGATCTTCAGGCCTGGAGGAGGAGATATGAGGGACAATTGGAGAAGTGAATTATATAAGTATA
AAGTAGTAAAAATTGAACCACTA---GGTGTAGCACCCACCCATGCAAAAAGAAGAGTGGTG-----------
----------------------------GAGAGAGAAAAAAGAGCA---GTTGGA---CTGGGAGCT------
---GTCTTCCTTGGGTTCTTAGGAGCA---GCAGGAAGCACTATG---GGCGCGGCGTCA---
ATAACGCTGACGGTACAG---GCCAGACAA---TTATTG---TCTGGTATAGTGCAACAGCAGAGC---
AATTTG---CTGAAG---GCTATAGAGGCTCAACAACAGATGTTGAGACTCACGGTCTGGGGCATT
>EU513187..1
TTCTATTGCAATACAACAGCACTATTTAATAGCACTTGGGATAACAATAGTACTAGGGAAGACAATAACTCAA
CA---

GGGAATGACACTATAACTCTCCAATGCAGAATAAAGCAAATTGTGAGGATGTGGCAAAGATCAGGACAAGCAA
TGTATGCCCCTCCCATCCAAGGAGTAATAAAGTGTAAATCAAAT---
ATCACAGGACTACTATTAACAAGGGATGGGGGGAATAATGAAAGT----------------------------
----

ACAAATGAGACCTTCAGACCTGGAGGAGGAGATATGAGGGACAATTGGAGAAGTGAATTATATAAGTATAAAG
TAGTAAAAATTGAACCACTA---GGTGTAGCACCCACCCATGCAAAAAGAAGAGTGGTG--------------
-------------------------CAGAGAGAAAAAAGAGCA---GTAGGA---CTGGGAGCT---------
GTCTTCTTTGGGTTCTTAGGAGCA---GCAGGAAGCACTATG---GGCGCGGCGTCA---
ATAACGCTGACGGTACAG---GCCAGACAA---TTATTG---TCTGGTATAGTGCAACAGCAGAGC---
AATTTG---CTGAAG---GCTATAGAGGCTCAACAGCATCTGTTGAAACTCACGGTCTGGGGCATT
>EU513195..1
TTCTATTGCAATACATCAAGACTGTTTAATAGCACTTGGGGTAGCAATAACACTTGGGATAACAGC-------
----

ACGGAGCAAAATATAACTCTCAGATGCAAAATAAAGCAGATTATAAATATGTGGCAGAGAGTAGGACAAGCAA
TGTATGCCCCTCCCATCCGAGGAGAAATAAGATGTGAATCAAAC---
ATTACAGGACTACTATTAACAAGGATGGAGGGGGTAATAGTAGT----------------------------
----

AGTGCAAATGAGACCTTCAGACCTACAGGAGGAAATATGAAGGACAATTGGAGAAGTGAATTATATAAGTATA
AAGTAGTAAAAATTGAACCACTA---GGTGTAGCACCCACCCGTGCAAAAAGAAGAGTGGTG----------
----------------------------GGGAGAGAAAAAAGAGCA---GCA------CTGGGAGCT------
---GTCTTCCTTGGGTTCTTAGGAGCA---GCAGGAAGCACTATG---GGCGCGGCGTCA---
ATAACGCTGACGGTACAG---GCCAGACAA---TTATTG---TCCGGTATAGTGCAACAGCAGAGC---
AATTTG---CTGAAG---GCTATAGAGGCTCAACAACATCTGTTGAAACTCACGGTCTGGGGCATT
>EU513189..1
TTCTATTGCAATACATCAGGACTATTTAATAGCACTTGGCACAATAGCACTTGGACCAATAGCACCACTGGCT
CAAATGGC---
-------------------ACA---
GAGTCAAATGACACTATAACTCTCCAATGCGAAATAAAGCAATTTATAAATATGTGGCAGAGAGTAGGACGAG
CAATGTATGCCCCTCCCATCCCAGGAGTGATAAGGTGTGAATCAGAC---
ATTACAGGACTACTATTAACAAGAGATGGACCGAATAGTACACAA---------------------------
----

AATGAGACATTCAGGCCTGGAGGAGGAGATATGAGAGACAATTGGAGAAGTGAATTATATAAGTATAAAGTAG
TACAAATTGAACCACTA---GGTGTGGCACCCACCCATGCAAAAAGAAGAGTGGTG----------------
----------------------GAGAGAGAAAAAAGAGCA---GTTGGA---CTGGGAGCT---------
GTCTTCTTTGGGTTCTTGGGAGCG---GCAGGAAGCACTATG---GGCGCGGCGTCA---
ATAACGCTGACGGTACAG---GCCAGACAA---TTATTG---TCTGGTATAGTGCAACAGCAGAGC---
AATTGG---CTGAAA---GCTATAGAGGCTCAACAACATCTGTTGAGACTCACGGTCTGGGGCATT
>EU513196..2
TTCTATTGCAATACATCAGGCCTATTTAATAGCACTTGGTATAGCAATAGCACTAACAGCTCAAATCACACAA
AT--

GGCACAGATGATATAACTCTCCAATGCAGAATAAAGCAAATTGTAAATATGTGGCAGAGAGTAGGACAAGCAA
TGTATGCCCCTCCCATCCGAGGAGAAATAAGGTGTGAATCAAAC---
ATTACAGGAATACTATTAACAAGAGATGGAGGGAATAATGGTAGT---------------------------
----

AAAGCAAATGAGACCTTCAGACCTGGAGGAGGAGACATGAGGGACAATTGGAGAAGTGAATTATATAAATACA
AAGTAGTAAAAATTGAACCAATA---GGTATAGCACCCACCCATGCAAAAAGGAGAGTGGTG----------
----------------------------GAGAGAGAAAAAAGAGCA---GTCGGA---CTGGGAGCT------
---TTCTTCCTTGGGTTCTTAGGAGTA---GCAGGAAGCACTATG---GGCGCGGCGTCA---
ATGACGCTGACGGTACAG---GCCAGACAT---TTATTG---TCTGGTATAGTGCAACAGCAGAGC---
AATCTG---CTGAAG---GCTATAGAGGCTCAACAACAGCTGTTGAGACTCACAGTCTGGGGCATT
>EU513191..1
TTCTATTGCAATACATCAAAACTGTTTAGTAGCACTTGGGATAATAGCACTTGGGGTAATAACACTTGGGGTA
ACAGCACT--
-------------------GGG---
GACAACAATGAGACCATAACCCTCCAATGCAAAATAAAACAAATTATAAATATGTGGCAGCAAGTAGGACGAG
CAATGTATGCCCCTCCCATCCAAGGAGAGATAAGGTGTGAGTCAAAC---
ATTACAGGACTACTATTAACAAGAGATGGAGGGGCTAATAGCAGT---------------------------
----

ACAAATGAGACCTTCAGGCCTGGAGGAGGAGATATGAGGGATAATTGGAGGAGTGAATTATATAAGTATAAAG
TTGTAAAAATTGAACCACTA---GGTGTAGCACCCACCCGTGCAAAAAGAAGAGTGGTG-------------
-------------------------CAGAGAGAAAAAAGAGCG---GTTGGA---CTGGGAGCT---------
GTCTTCATTGGGTTCTTAGGAGCA---GCAGGAAGCACTATG---GGCGCAGCGTCA---
ATAACGCTGACGGTACAG---GCCAGACAA---TTATTG---TCCGGTATAGTGCAACAGCAGAGC---
AATTTG---CTGAAG---GCTATAGAGGCTCAACAACATCTGTTGAGACTCACGGTCTGGGGCATT
>EU513184..2
TTCTATTGCAATACATCAAAACTATTTGATAGCATTTGGAATAGAACTAATGGGTCAAATTACACAAGGTTAG
GTGATGAC--
-------------------ACA---
GAGACAAATGACCCTATAACTCTCCAATGCAGAATAAAGCAAATTGTAAGAATGTGGCAGACAGTAGGACAAG
CAATGTATGCCCCTCCCATCCGGGGAGAAATAAAATGTGAATCAAAC---
ATTACAGGACTGCTATTAACACGAGATGGAGGAAATAATAATAGT---------------------------
----

```
-------------
ATGAATGAGACATTCAGGCCTGGAGGAGGAGATATGAGGGACAATTGGAGAAGTGAATTATATAAGTATAAGG
TAGTACAAATTGAACCACTA---GGTGTGGCACCCACCTTTGCAAAAAGAAGAGTGGTG--------------
-------------------------GAGAGAGGAAAAAGAGCA---GTTGGA---CTGGGAGCT---------
GTTTTCCTTGGGTTCTTAGGAGCT---GCAGGAAGCACTATG---GGCGCGGCGTCA---
ATAACGCTGACGGTACAG---GCCAGACAA---TTACTG---TCTGGTATAGTGCAACAGCAAAGC---
AATTTG---CTGAGG---GCTATAGAGGCTCAACAACATCTGTTGAAACTCACGGTCTGGGGCATA
>EU513193..2
TTCTATTGCAATACATCAGGACTGTTTAATAGCACCTGGTGGGATAGCAACAATAGCACTGAGAATATAACTC
AC-----------------------------------------------------------------------
----------------------------
AGAAAAGAAAATATAACTCTCCAATGCAGAATAAAGCAATTTATAAATATGTGGCAGAGAGTGGGACGAGCAA
TGTATGCCCCTCCCATCCAAGGAGTAATAAGGTGTGAATCAAAC---
ATTACAGGAATACTATTAACAAGAGATGGTGGGGTTAATAATAATAAC-------------------------
-------------------------------------------------------------------------
-------
ACAAACAAAACTGAGACCTTCAGGCCTGGAGGAGGAGATATGAGGGACAATTGGAGAAGTGAATTATATAAGT
ATAAAGTAGTGAAAATTGAACCACTA---GGTGTAGCACCCACCCATGCAAAAAGAAGAGTGGTG--------
-------------------------------GAGAGAGAAAAAAGAGCA---GTTGGA---CTGGGAGCT---
------GTCTTCCTTGGGTTCTTAGGAGCA---GCAGGAAGCACTATG---GGCGCGGCGTCA---
ATAACGCTGACGGTACAG---GCCAGACAG---TTATTG---TCTGGTATAGTGCAACAGCAGAGC---
AATTTG---CTGAAG---GCTATAGAGGCTCAACAACAGCTGTTGAGACTCACAGTCTGGGGCATT
>EU513186..3
TTCTATTGTAATACATCAAGCCTATTTAATTATACATTTAGCAATAGCACTGAGGATAACAACCCA-------
-------------------------------------------------------------------------
----------------------------
AGTAATGCCACAATAACTCTCCAATGCAGAATAAGGCAAGTTGTAAATATGTGGCAGAGAGTAGGACTAGCAA
TGTATGCCCCTCCCATCAAAGGAAAACTAAGATGTGAATCAAAC---
ATTACAGGACTACTATTAACAAGAGATGGAGGGAATAATAATAAT----------------------------
-------------------------------------------------------------------------
-------------
GCAACTGAGACATTCAGGCCTGGAGGAGGAGATATGAGGGACAACTGGAGAAGTGAATTATATAAATATAAGG
TAGTACAAATTGAACCCCTA---GGTGTGGCACCCACCTATGCAAAAAGAAGAGTGGTG--------------
-------------------------GAAAGAGGAAAAAGAGCAGTAGTTGGA---CTAGGAGCT---------
GTCTTCCTTGGGTTCTTAGGAGCA---GCAGGAAGCACTATG---GGCGCGGCGTCA---
ATAACGCTGACGGTACAG---GCCAGACAA---TTATTG---TCTGGTATAGTGCAACAGCAGAGC---
AATCTG---CTGAGG---GCTATAGAGGCTCAACAACAGCTGTTGAAACTCACGGTCTGGGGCATT
>MH705136..2
TTCTATTGCAATACCTCAAATCTATTTGATAGCGCTTGGGAAGCAAACTCAAATCACACAGAGTCAAATGAC-
-------------------------------------------------------------------------
----------------------------
ACAAGGGCAAATATAACTCTCCAATGCAGAATAAGGCAAATTGTAAGGATGTGGCAGAGAGTAGGACAAGCAA
TGTATGCCCCTCCCATCCGAGGAGAAATAAGGTGTGAGTCAAAC---
ATTACAGGACTACTATTAACAAGAGATGGAGGGGATGGGAACAATGCA-------------------------
-------------------------------------------------------------------------
-------------
ACTAATGAGACATTCAGGCCTGGAGGAGGAGATATGAGAGACAATTGGAGAAGTGAACTATATAAATATAAGG
TAGTACAAATTGAACCACTA---GGTGTAGCACCCACCCATGCAAAAAGAAGAGTGGTG--------------
-------------------------GAGAGAGAAAAAAGAGCA---GTGGGA---ATAGGAGCT---------
GTCTTCCTTGGGTTCCTAGGGGCA---GCAGGAAGCACTATG---GGCGCGGCGTCA---
ATAACGCTGACGGTACAG---GCCAGACAA---TTATTG---TCCGGTATAGTGCAACAGCAGAGC---
AATTTG---CTGAGG---GCTATAGAGGCTCAACAACAACTGTTGAAACTCACAGTCTGGGGCATT
>MH460467..3
TTCTATTGTAATACATCAGGACTATTTAATTATAGTTGGAATACATCAGAACCAACCAATAATTGGAATAGTA
GTGCTCAG-----------------------------------------------------------------
----------------------------
GATAACGATACCATAACTCTCCAATGCAGAATAAAGCAAATAGTAAATATGTGGCAGGAAGTAGGACAAGCAA
TGTATGCCCCTCCCATCCAAGGAATAATAAGGTGTGAATCAAAC---
ATTACAGGACTACTATTAACAAGAGATGGAGGGACAAATAATAGT----------------------------
-------------------------------------------------------------------------
```

ACAAATGAGACATTCAGACCTGGGGGAGGAGATATGAGGGACAATTGGAGAAGTGAATTATATAAGTATAAGG
TAGTACAAATTGAACCAATA---GGTTTGGCACCCACCCATGCAAAAAGAAGAGTGGTG-------------
-------------------------CAGAGAGAAAAAAGAGCAGTAATTGGA---ATGGGAGCT---------
GTCTTCCTTGGGTTCTTAGGAGCA---GCAGGAAGCACTATG---GGCGCGGCGTCA---
ATAACGCTGACGGTACAG---GCCAGACAA---TTATTG---TCCGGTATAGTGCAACAGCAGAGC---
AATTTG---CTGAGG---GCTATAGAAGCTCAACAACATCTGTTGAAACTCACGGTCTGGGGCATT
>EU513198..1
TTCTATTGCAATACCTCAAACCTGTTTAATGGCACTTTGTATAACAACACCTGGATGTTTAATGGCACTTTGT
ATAATAATACT--
----------AACGAGAGC------
TCAACATCAGGTACTATAACTATCAAGTGCAGAATAAAGCAAATCATAAATATGTGGCAGACAGTAGGACAAG
CAATGTATGCCCCTCCCATCCCAGGAGAAATAAGGTGTGAATCAAAC---
ATTACAGGATTGCTATTAACAAGAGATGGAGAGGCTGGAAAAAGC----------------------------
----

ACAAATGAGACCTTCAGGCCTATAGGAGGAAATATGAGAGACAATTGGAGAAGTGAATTATACAAATATAAAG
TAGTAAAAATTGAACCACTA---GGTGTAGCACCCACACCTGCAAAAAGAAGAGTGGTG-------------
-------------------------GAGAGAGAAAAAAGAGCA---GTGGGA---CTGGGAGCT---------
GTCTTCCTTGGGTTCTTAGGAGCA---GCAGGAAGCACTATG---GGCGCAGCGTCA---
ATAACGCTGACGGTACAG---GCTAGACAA---TTATTG---TCTGGTATAGTGCAACAGCAAAGC---
AATTTG---CTGAAG---GCTATAGAGGCTCAACAACATCTGTTGAAACTCACGGTCTGGGGCATT
>JF706407..3
TTCTATTGTGATACATCAAGACTGTTTAATAGCACTTGGCCTAATAGCACATGGGACAATAATACTCAAAAGA
ATGAG--

AATGACACAGGGTACTATACTCTCCAATGCAGAATAAAGCAAATTATAAATATGTGGCAGAGAGTAGGACAAG
CAATGTATGCCCCTCCTATCCCAGGAGAAATAAGTTGTGAATCAAAC---
ATTACAGGACTACTATTAACAAGAGATGGAGGGAATAATAATAAT----------------------------
----

AGTATAAATGAGACCTTCAGGCCTGGTGGAGGAGATATGAGGGACAATTGGAGAAGTGAATTATATAAGTACA
AAGTGGTACAAATTGAGCCAATA---GGAATAGCACCCACCCCTGCAAAAAGAAGAGTGGTG----------
----------------------------GAGAGAGAGAAAAGAGCAGTAGTTGGA---CTAGGAGCT------
---GTCTTCCTTGGGTTCTTAGGAGCA---GCAGGAAGCACTATG---GGCGCAGCGTCA---
ATAACGCTGACGGTACAG---GCCAGACAA---TTATTG---TCTGGTATAGTGCAACAGCAGAGC---
AATCTG---CTGCAG---GCTATAGAGGCTCAACAACATCTGTTGAGACTCACGGTCTGGGGCATT
>JF706428..1
TTCTATTGCAATACATCAGGACTATTTAATGGCACTTTGAATGGCACTGAAACTTGGAATAACACTTGGGGTG
ACAGTAGC---
-------------AATGAG------
AATGAAAATGAGAATATAACTATCCAATGCAGAATAAAACAAATTGTAAATATGTGGCAGAGAGTAGGACGAG
CAATGTATGCCCCTCCCATCAAAGGAGTAATAAAGTGTGAATCAAAC---
ATTACAGGACTACTATTAACAAGAGATGGAGAGGGTAATAATAGG----------------------------
----

ACAAATGAGACCTTCAGGCCTGGAGGAGGAGATATGAGGGATAATTGGAGAAGTGAATTATATAAATATAAAG
TAGTAAAAATTGAACCACTA---GGTATAGCACCCACCCATGCAAAAAGAAGAGTGGTG-------------
-------------------------CAGAGAGAAAAAAGAGCA---GTTGGA---CTGGGAGCT---------
GTCTTCCTTGGGTTCTTAGGAGCA---GCAGGAAGCACTATG---GGCGCGGCGTCA---
ATAACGCTGACGGTACAG---GCCAGACAA---TTATTG---TCTGGTATAGTGCAACAGCAGAGT---
AATCTG---CTGAAG---GCTATAGAGGCTCAACAACATCTGTTGAGACTCACAGTCTGGGGCATT
>KU168310..1
TTCTATTGCAATACATCACAACTATTTAATAGCACTTGGAACAACACCAATATCAGCTTAAATGAC-------
----

ACAAATGAAAATATAACTCTCCCATGCAGAATAAAGCAAGTTATAAATATGTGGCAGAGAGTAGGACAAGCAA
TGTATGCCCCTCCCATCGCAGGAACACTAAAGTGTAAATCAAAC---
ATTACAGGATTACTATTAACAAGAGATGGAGGGGAGAGCAATAATACA-------------------------
----

AATACAAATGAGACCTTCAGGCCTGGAGGAGGAGATATGAGGGACAATTGGAGAAGTGAATTATATAAGTATA
AAGTAGTAAAAATTGAACCACTA---GGTGTAGCACCTACCCATGCAAAAAGAAGAGTGGTG-----------
----------------------------GAGAGAGAAAAAAGAGCAGTGTTTGGA---CTGGGAGCT------
---GTCTTCCTTGGGTTCTTAGGAGCA---GCAGGAAGCACTATG---GGCGCGGCGTCA---
ATAACGCTGACGGTACAG---GCCAGACAA---CTATTG---TCTGGTATAGTGCAGCAGCAGAGC---
AATTTG---CTGAGG---GCTATAGAGGCTCAACAACATCTGTTGAAACTCACGGTCTGGGGCATT
>JN687776..2
TTCTATTGTAATACAACAAAACTATTTAATAGCACTTGGACTAATGAGACAAATCACACAGCTAGC-------
----

ATAGATAGAAATATAACTCTCCAATGCAGAATTAAGCAAATTGTAAATATGTGGCAAAGAGTAGGACAAGCAA
TATATGCCCCTCCCATCCCAGGAGTAATAAGGTGTGAATCAAAC---
ATTACAGGACTACTACTAACAAGAGATGGAGGGACAAATAGTTCA----------------------------
----

AATAATGAGACATTCAGGCCTGGAGGAGGAGATATGAGGGACAATTGGAGAAGTGAATTATATAAGTATAAGG
TAGTACAAATTGAACCAATA---GGTGTGGCACCCACCCCTGCAAAAAGAAGAGTGGTG--------------
-------------------------GAGAGAGAAAAAAGAGCA---GTTGGA---GTAGGAGCT---------
TTTATCCTTGGGTTCTTAGGAGCA---GCAGGAAGCACTATG---GGCGCGGCGTCA---
ATAACGCTGACGGTACAA---GCCAGACAA---TTATTG---TCTGGTATAGTGCAACAGCAGAGC---
AATTTG---CTGAAG---GCTATAGAGGCTCAACAACATCTGTTGAAACTCACGGTCTGGGGCATT
>JX140646..2
TTCTATTGCAATACAACAGAATTATTTAATAAGACTTTTAATGATACTGATCTAAAGTTAAATAGCAGTAGCA
TTAAGGAG---

TCAAATGATACTATAACTCTCCAATGTAGAATAAAGCAATTTGTAAGGATGTGGCAGAGAGTAGGACAAGCAA
TGTATGCCTCTCCCATCCCAGGAGTAATAAGGTGTGAATCAAAC---
ATTACAGGTCTACTATTAACAAGAGATGGAGGGGGTAATGAAAGCACA-------------------------
----

AGAGATAATGAGACATTCAGGCCTGGAGGAGGAGATATGAGAGACAATTGGAGAAGTGAATTATATAAGTATA
AGGTAGTACAAATTGAACCAATA---GGAGTGGCACCCACCTATGCAAAAAGAAGAGTGGTG-----------
----------------------------GAGAGAGAAAAAAGAGCA---GTTGGA---CTGGGAGCT------
---GTCTTCCTTGGGTTCTTAGGGGCA---GCAGGAAGCACTATG---GGCGCGGCGTCA---
ATAACGCTGACGGTACAG---GCCAGGCAA---TTATTG---TCTGGTATAGTGCAACAGCAGGGC---
AATCTG---CTGAGG---GCTATAGAGGCTCAACAACACCTGTTGAAACTCACAGTCTGGGGCATT
>JX140647..1
TTCTATTGTAATACATCAGGACTATTTAATAGTAGTTGGACCGCGAACGACATTTTGAATAACAGTACTGCTA
GCTCAAAT---

AACACAGAAGTTATAACTCTCCAATGCAGAATAAAGCAAATTATAAATATGTGGCAGACAGTAGGACAAGCAA
TGTATGCCCCTCCCATCCGAGGAGAAATAAGGTGTGAGTCAAAT---
ATTACTGGACTACTATTAACAAGAGATGGAGGGAATAATATGACA----------------------------
----

AATGAAACCTTCAGGCCTGGAGGAGGGGATATGAGGGACAATTGGAGAAGTGAATTATATAAGTATAAAGTAG
TACAAATTGAACCACTA---GGTGTAGCACCCACCCATGCAAAAAGAAGAGTGGTG-----------------
----------------------GAGAGAGAAAAAAGAGCA---GTTGGA---ATAGGAGCT---------
GTCTTCCTTGGGTTCTTAGGAGCA---GCAGGAAGCACTATG---GGCGCGGCGTCA---
ATAACGCTGACGGTACAA---GCCAGACAA---TTATTG---TCTGGTATAGTGCAACAGCAGAGC---
AATTTG---CTGATG---GCTATAGAGGCTCAACAACATATGTTGAAACTCACTGTCTGGGGCATT
>KF859739..2
TTCTATTGCAGTACATCAGCACTGTTTAATAGCACTTGGCTGTTTAATGAAACTTATAGCACTTGGCATAGCT
CTTGGAACAGT--

AGCGCTGAGAATATAACTCTCCAATGCAGAATAAAGCAATTTGTGAATATGTGGCAGAGAGTAGGGCAAGCAA
TGTATGCCCCTCCCATTCGAGGAATGATAAGGTGTGAATCAAAC---
ATTACAGGACTACTATTAACAAGAGATGGAGGGAATAAAAATACT----------------------------
----

```
----------
AGTGCAAATGAGACCTTCAGGCCTGGAGGAGGAGATATGAAGGACAATTGGAGAAGTGAATTATATAAGTATA
AAGTAATAAAAATAGAACCACTA---GGTGTGGCACCCACCCCTGCAAAAAGAAGAGTGGTG-----------
----------------------------GGAAGAGAAAAAAGAGCA---GTTGGA---CTAGGAGCT------
---GTCTTCTTTGGGTTCTTAGGAGCA---GCAGGAAGCACTATG---GGCGCAGCGTCA---
ATAACGCTGACGGTACAG---GCCAGACAA---TTATTG---TCTGGTATAGTGCAACAGCAGAGC---
AATTTG---CTGAAG---GCTATAGAGGCTCAACAACATCTGTTGAGACTCACGGTCTGGGGCATT
>KU749419..1
TTCTATTGCAGTACATCAAGCCTGTTTAATAGCACTTGGAATAATAGCATTGGGGATAATAGCACTAGGGAT-
-------------------------------------------------------------------------
----------------------------
GACAATAGCACTATTACTCTCCCATGCAGAATAAAGCAAATTGTAAATATGTGGCAGGGAGTAGGACAAGCAA
TGTATGCCCCTCCCATCCCAGGAGAAATAAAGTGTGAATCAAAT---
ATTACAGGACTATTATTGACAAGAGATGGTGGGAGAAATAGCACA----------------------------
-------------------------------------------------------------------------
----------------
AATGAGACCTTCAGGCCTGGAGGAGGAGATATGAGGGACAATTGGAGAAATGAATTATATAAGTATAAAGTAG
TAAAAATTGAACCACTA---GGTGTGGCACCCACCCATGCAAAAAGAAGGGTGGTG-----------------
----------------------GAGAGAGAAAAAAGAGCA---GTTGGA---CTAGGAGCT---------
GTCTTCCTTGGGTTCTTAGGAGCA---GCAGGAAGCACTATG---GGCGCGGCGTCA---
ATAACGCTGACGGTACAG---GCCAGACAA---TTATTG---TCCGGTATAGTGCAACAGCAGAGC---
AATTTG---CTGAGG---GCTATAGAGGCTCAACAACAGGTGTTGAGACTCACGGTCTGGGGCATT
>MK086109..1
TTCTATTGCAATACATCTAACCTATTTAATAGCACTTGGGGGAGCAATACCACTACCAGCTGGAATACCAGCT
CAATTGGC-----------------------------------------------------------------
----------------------------
ACAAATGAAACTATAACTATCCAGTGCAGAATAAAGCAGATTGTAAGAATGTGGCAGAGAGTAGGACAAGCAA
TGTATGCCCCTCCCATCCCAGGAAAAATAAGGTGTAACTCAAGC---
ATTACAGGACTGCTATTAACAAGAGATGGAGGGAATAATAGTAGT----------------------------
-------------------------------------------------------------------------
-------------
GACAATGAGACCTTCAGGCCTGGAGGAGGAGATATGAGGGACAATTGGAGAAGTGAATTATATAAGTATAAAG
TAGTAAAAATTGAACCACTG---GGTGTAGCACCCACCCATGCAAGGAGAAGAGTGGTG--------------
-------------------------GAGAGAGAAAAAAGAGCA---GTTGGA---CTGGGAGCT---------
GTCTTCCTTGGGGTCTTAGGAGCA---GCAGGAAGCACTATG---GGCGCGGCGTCA---
ATAACGCTGACGGTACAG---GCCAGACAA---TTATTG---TCTGGTATAGTGCAACAGCAAAGT---
AATTTG---CTGAGG---GCTATAGAGGCTCAACAGCATATGTTGAGACTCACGGTCTGGGGCATT
>MK086113..1
TTCTATTGCAATACAACAAAACTATTTAATAGCATTTGGAGTAAGAATAGTGGGAATTGGAGTAGTACAAATG
GG-----------------------------------------------------------------------
----------------------------
ACAAATGACACTATAACTCTCCAATGCAGAATAAAACAAATTGTAAGAATGTGGCAGAGAGTAGGACAAGCAA
TGTACGCCCCTCCCATCCGAGGAAACATAACGTGTGGATCAAAC---
ATTACAGGATTACTATTAACAAGAGATGGAGATGGAGGTGCACAT----------------------------
-------------------------------------------------------------------------
----------
AGTCCCAATGAAACATTCAGGCCTGGAGGAGGAAATATGAAGGACAATTGGAGAAGTGAATTATACAAGTATA
AGGTAGTACAAATTAAACCATTA---GGTGTAGCACCCACCCAGGCAAAAAGAAGAGTGGTG-----------
----------------------------GAGAGAGAAAAAAGAGCA---GTTGGA---CTGGGAGCT------
---GTCTTCCTTGGGTTCTTAGGAGCA---GCAGGAAGCACTATG---GGCGCGGCGTCA---
CTGACGCTGACGGTACAG---GCCAGACAA---TTATTG---TCTGGTATAGTGCAGCAGCAGAGC---
AATCTG---CTGCGG---GCCATAGAGGCTCAACAACATCTGTTGAAACTCACGGTCTGGGGCATT
>MK086125..1
TTCTATTGCAATACATCAGGCCTATTCAATAGCACTTGGCACAATAGCACTTGGGATAGTAATGAAACTGCCA
GTGCA--------------------------------------------------------------------
----------------------------
ACAAATGACACTATAACTCTCCGATGCAGAATAAAGCAATTTGTAAATATGTGGCAGAAAGTAGGACGAGCAA
TGTATGCCCCTCCCATTCCAGGAGTAATAAGATGTGAGTCAAAC---
ATTACAGGATTACTATTAACAAGAGATGGAGGAAATAATAATAAT----------------------------
-------------------------------------------------------------------------
```

AGTGTAAATGAGACATTCAGGCCGGGAGGAGGAGATATGAGGGACAATTGGAGAAGTGAATTATATAAGTATA
AAGTAGTACAAATTGAGCCACTA---GGTGTAGCACCCACCCATGCAAGAAGAGTGGTA--------------
----------------------------CAGAGGGAAAAAAGAGCA---GTTGGA---CTGGGAGCT------
---GTCTTCCTTGGGTTCTTAGGAGCA---GCAGGAAGCACTATG---GGCGCGGCGTCA---
ATAACGCTGACGGTACAG---GCCAGACAA---TTATTG---TCTGGTATAGTGCAGCAGCAGAGT---
AATTTG---CTGCGG---GCTATAGAGGCTCAACAACATCTGTTGAAACTCACGGTCTGGGGCATT
>HQ912706..2
TTCTATTGCAATACAACAGAACTATTTAATAGCACTTGGAACGTAACTGCCAGCTCAAACTACACAGAGTCAA
AC---

TCAAATGACAGTATAACTCTCCCATGCAGAATAAAACAAATTGTGAATTTATGGCAGAAAGTAGGACAAGCAA
TGTATGCCCCTCCCATCCAAGGAGAAATAAGGTGTGAATCAAAC---
ATTACAGGATTACTATTAACAAGAGATGGAGGGAATAATAGTACA----------------------------
----

AATGAGACATTCAGGCCTGGAGGAGGAGATATGAGGGACAATTGGAGAAGTGAATTATATAAGTATAAGGTAG
TACAAATTGAACCACTA---GGTGTGGCACCCACCCAGGCAAAAAGAAGAGTGGTG-----------------
----------------------GAGAGAGAAAAAAGAGCA---GTTGGA---ATAGGAGCT---------
GTCTTCCTTGGGTTCTTAGGAGCA---GCAGGAAGCACTATG---GGCGCGGCGTCA---
CTAACGCTGACGGTACAG---GCCAGACAA---TTATTG---TCTGGTATAGTGCAACAGCAGAGC---
AATCTG---CTGAGG---GCTATAGAGGCTCAACAACATCTGTTGAAACTCACGGTCTGGGGCATT
>AM279349..1
TTCTATTGCAATACATCAGGCCTGTTTAATAACACTTTGAATAATAACACTTGGACTTGGGATAATAGCACA-
----

AAGGATGACAATATAACTCTCCGATGCAGGATAAAGCAAATTGTAAATATGTGGCAGAGAGTAGGACAAGCAA
TGTATGCCCCTCCCATCCAAGGAGTGATAAGGTGTGAATCAAAC---
ATTACAGGATTACTATTAACAAGAGATGGAGGGAATAATAATAGC----------------------------
----

ACAACAAATGAGACCTTCAGGCCTGGAGGAGGAGATATGAGGGACAATTGGAGAAGTGAATTATATAAATATA
AGGTAGTAAAAATTGAACCACTA---GGTGTAGCGCCTACCCATGCAAAAAGAAGAGTGGTG-----------
----------------------------GAGAGAGAAAAAAGAGCA---GTTGGA---CTGGGAGCT------
---GTCTTCCTTGGGTTCTTAGGAGCA---GCAGGAAGCACTATG---GGCGCGGCGTCA---
ATAACGCTGACGGTACAG---GCCAGACAA---TTATTG---TCCGGTATAGTGCAACAGCAGAGC---
AATCTG---CTGAAG---GCTATAGAGGCTCAACAACATCTGTTGAGACTCACGGTCTGGGGCATT
>AM279361..3
TTCTATTGCAATACATCAGCACTGTTCAATAGCACTTATGACAATAGCACTTGGAATGACAGTACAAATACCA
GCTCA--

GAGACAAATGACACTATAACTCTCCAATGCAAAATAAAGCAAATTGTAAGAATGTGGCAGAGAGTAGGACAAG
CAATGTATGCCCCTCCCATCCCAGGAATAATAAGGTGTGAATCAAAC---
ATTACAGGACTAATATTAACAAGAGATGGAGGGAATAATAATAGT----------------------------
----

GAAAATCAGACTTTCAGGCCTGGAGGAGGAGATATGAGGGACAATTGGAGAAGTGAATTATATAAGTATAAAG
TAGTAAAAATTGAACCACTA---GGTATAGCACCCACCCATGCAAAAAGAAGAGTGGTG--------------
-------------------------CAGAGAGAAAAAAGAGCA---GTTGGA---ATAGGAGCT---------
GTCTTCCTTGGGTTCTTAGGAGCA---GCGGGAAGCACTATG---GGCGCGGCGTCA---
ATAACGCTGACGGTACAG---GCCAGACAA---TTATTG---TCCGGTATAGTGCAACAGCAGAAC---
AATTTG---CTGAAG---GCTATAGAGGCTCAACAACAGTTGTTGAAACTCACAGTCTGGGGCATT
>AM279360..1
TTCTATTGCAATACAACAAAACTGTTTAATAGCACGTGGAATAGCAGTACTGGGAATAACAATGTCAGC----
----

TCAAATGACACTATAACCCTCCAATGCAAAATAAAGCAAATTGTAAATATGTGGCAAAAAGTAGGACAAGTAA
TGTATGCCCCTCCCATCCCAGGAATAATAAGGTGTGAATCAAAT---
ATTACAGGACTATTATTAACAAGAGATGGAGGAAATAATAATGAAACA-------------------------
----

GAGAATGGGACTGAGACCTTCAGGCCTGGAGGAGGAGATATGAGGGACAATTGGAGAAGTGAATTGTATAAGT
ATAAAGTAGTAAAAATTGAACCACTA---GGTGTAGCACCCACCCATGCAAAAAGAAGAGTGGTG---GAG--
-------------------------------AGGAGAGAAAAAAGAGCA---GTTGGA---CTGGGAGCT---
------GTCTTCTTTGGGTTCTTAGGAGCA---GCAGGAAGCACTATG---GGCGCGGCGTCA---
CTAACGCTGACGGTACAG---GCCAGACAA---TTATTG---TCTGGTATAGTGCAACAGCAGAGC---
AATTTG---CTGAAG---GCTATAGAGGCTCAACAACATCTGTTGAAACTCACGGTCTGGGGCATT
>AM279358..2
TTCTACTGCAATACATCAGCACTGTTTAATAGCACTTGGAATAACACTTTGGATACTAACAATAGCACTCAG-
----

GATAATGACACTATAACTCTCCAATGCAGAATAAAGCAAATTATAAGGATGTGGCAGACAGTAGGACAAGCAA
TGTATGCCCCTCCCATCCAAGGAAAAATAAGGTGTGAATCAAAC---
ATTACAGGACTACTGTTAACAAGAGATGGAGGGAACAGTGATGGT----------------------------
----

GCAAATGAGACCTTCAGGCCTGGAGGAGGAGATATGAGGGACAATTGGAGAAGTGAATTATATAAGTATAAAG
TAGTAAAAATTGAACCACTA---GGTGTAGCACCCACCCGAGCAAAAAGAAGAGTGGTG--------------
-------------------------GAGAGAGAAAAAAGAGCAGTAGTTGGA---CTGGGAGCT---------
GTCTTCCTTGGGTTCTTAGGAGCA---GCAGGAAGCACTATG---GGCGCGGCGTCA---
CTAACGCTGACGGTACAG---GCCAGACAA---TTATTG---TCCGGTATAGTGCAACAGCAGAGC---
AATCTG---CTGAGG---GCTATAGAGGCTCAACAACATATGTTGAAACTCACAGTCTGGGGCATT
>AJ286133..1
TTCTATTGTAATACATCAGAACTATTTAATAGCATTTGGAATATTAATAGCACTTGGAATAACAGTGACACTG
CCAGC--
-------------------TCG---
AAGCCAAATGACACTATAACTCTACAATGCAGAATAAGGCAAATTGTAAGAATGTGGCAGAAAGTAGGACAAG
CAATATATGCCCCTCCTATCCCAGGAGTAATAAGGTGTGAATCAAAC---
ATTACAGGACTACTATTAACAAGAGATGGAGGGGAAGATAATAAT----------------------------
----

GCAAGTGAGACATTCAGGCCTGGAGGAGGAGATATGAGGGACAATTGGAGAAGTGAATTATATAAGTATAAGG
TAGTACAAATTGAACCACTA---GGCGTGGCACCCACCTGGGCAAAAAGAAGAGTGGTG--------------
-------------------------GAGAGAGGAAAAAGAGCA---GTTGGA---CTGGGAGCT---------
GTCTTCCTTGGGTTTTTAGGAGCA---GCAGGAAGCACTATG---GGC---CGGTCA---
ATAACGCTGACGGTACAG---GCCAGACAA---TTATTG---TCCGGTATAGTGCAACAGCAGAGC---
AATTTG---CTGAGG---GCTATAGAGGCTCAACAACATCTGTTGAAACTCACGGTCTGGGGCATT
>AF377955..1
TTCTATTGTAATACATCAGCACTGTTTAATAGTACTTGGTTTAGCAATAGTACTGGGGATAACAATAGTTCAA
CA---

GGGAATGAAACTATAACTCTCCAATGCAGAATAAAGCAAATTGTAAATATGTGGCAGAGAGTAGGACAAGCAA
TGTATGCCCCTCCCATCCAAGGAGTAATAAGGTGTGAATCAAAC---
ATTACAGGATTACTATTGACAAGAGATGGAGGGAATAATAATAGC----------------------------
----

ACAAATGAGACCTTCAGACCTGGAGGAGGAGATATGAGGGACAATTGGAGAAGTGAATTATATAAGTATAAAG
TAGTAAAACTTGAGCCACTG---GGTGTAGCACCCACCCGTGCAAAAAGAAGAGTGGTG--------------
-------------------------GAGAGAGAAAAAAGAGCA---GTTGGA---CTGGGAGCT---------
GTCTTCTTTGGGTTCTTAGGAGCA---GCAGGAAGCACTATG---GGCGCGGCGTCA---
ATAACGCTGACGGTACAG---GCCAGACTA---TTATTG---TCTGGTATAGTGCAACAGCAGAGC---
AATTTG---CTGAAA---GCTATAGAGGCTCAACAGCATCTGTTGAAACTCACGGTCTGGGGCATT
>AM279352..1
TTCTATTGCAATACATCAGGACTCTTTAATAGCACTTGGAATAGCAATGACACTGACAGATCAAACCACACAG
GA---

CCACATAACAATATAACTCTCCAATGCAGAATAAAGCAAATTGTAAATATGTGGCAGAGAGTAGGACAAGCAA
TGTATGCCCCTCCCATCCAAGGCGAAATAAGGTGTGTATCAAAC---
ATTACAGGATTACTACTAACAAGAGATGGAGGGATCAGTAATAAG----------------------------
----

ACAGAGGAGATATTCAGGCCTGGAGGAGGAGATATGAGGGACAATTGGAGAAGTGAATTATATAAGTATAAGG
TAGTACAAATTAAACCACTA---GGTGTGGCACCAGACCGGGCAAAAAGAAGAGTGGTG--------------
-------------------------GGAAGAGAAAAAAGAGCA---GTTGGA---ATGGGAGCT---------
CTTTTCCTTGGGTTCTTAGGAGCA---GCAGGAAGCACTATG---GGCGCGGCGGCA---
ATGACGCTGACGGTACAG---GCCAGACAA---TTATTG---TCTGGTATAGTGCAACAGCAGAGC---
AATTTG---CTGAGG---GCTATAGAGGCTCAACAACATCTGTTGAAACTCACGGTCTGGGGCATT
>AY271690..3
TTCTATTGCAATACATCAGGACTATTTAATAGCACTTGGAGTAGGAATAGCACTGATAACAGCACTTGGAATA
TCAATGACACT--
----------GTCAGCTCA------
AATCACACAGGAAATATAACTCTCCAATGCAGAATAAGGCAAGTTGTAAGAATGTGGCAGAGGGTAGGACAAG
CAATGTATGCCCCTCCCATCCCAGGAGAAATTAAGTGTAAATCAAAC---
ATTACAGGAATACTATTGACAAGAGATGGAGGGACAAAAAATCAGAATGCAAGT-------------------
----
-
GAGGAATTTAATACAACTGAGGTATTCAGACCTGGAGGAGGAGACATGAGGGACAATTGGAGAAGTGAATTAT
ATAAATATAAGGTAGTACAAATTGAACCAATA---GGTGTGGCACCCACCCGTGCAAAAAGAAGAGTGGTG--
------------------------------------CAGAAAGAAAAAAGAGCA---GTTGGA---
CTGGGAGCT---------TTCTTCCTTGGGTTCTTAGGAGTT---GCAGGAAGCACTATG---
GGCGCAGCGTCA---ATAACGCTGACGGTACAG---GCCAGACAA---TTATTG---
TCTGGTATAGTGCAACAGCAGAGC---AATCTG---CTGAAG---
GCTATAGAGGCTCAACAACATATATTGAGACTCACGGTCTGGGGCATT
>HQ700002..2
TTTTATTGCAATACATCAAAGCTGTTTAATAGCACCTGGCCTGGGAATATCACTGGGAATAACTATACCAGCT
CA---

AACTCAAGTGACAATATAGCTCTCCAATGCAGAATAAGACAAATTGTAAATATGTGGCAGAAAGTAGGAAAAG
CAATATATGCTCCTCCCATCCCAGGAGAAATAAAGTGTGAATCAAAC---
ATTACTGGACTACTACTAACAAGAGATGGAGGAAACAATAATGCA----------------------------
----

AGTAATGAGACCTTCAGGCCTGGAGGAGGAGATATGAGGGACAATTGGAGAAGTGAATTATATAAGTATAAAG
TAGTGAGAATTAACCCACTA---GGTATAGCACCCACCCACGCAAAAAGAAGAGTGGTG--------------
-------------------------GAGAGAGAAAAAAGAGCA---GTTGGA---CTGGGAGCT---------
GTCTTCCTTGGGTTCTTAGGAGCA---GCAGGAAGCACTATG---GGCGCGGCGTCA---
ATAACGCTGACGGTACAA---GCCAGACAA---TTATTG---TCTGGTATAGTGCAACAGCAGAGC---
AATCTG---CTGAAG---GCTATAGAGGCTCAACAGCATCTGTTGAAGTTCACAGTCTGGGGCATT
>JF683758..1
TTCTATTGCAATACATCAGGACTGTTTAATAGCATTTGGGATAATAACAGCACTTGGAATGGGAGC-------
----

AACTCAGGCAATATAACTCTCCCATGCAGAATAAGGCAAATTGTAAATATGTGGCAGAGAGTAGGAAAAGCAA
TGTATGCCCCTCCCATCCAAGGAGAAATAAGGTGTGACTCAAAC---
ATTACTGGACTACTATTAGCAAGAGATGGAGGGGATGGTACTGAG----------------------------
----

ACAAATGAGACTTTCAGGCCTATAGGAGGAGATATGAGAGACAATTGGAGAAGTGAATTATATAAGTATAAAG
TAGTAAAAATTGAACCATTA---GGCATAGCACCCACCCGGGCAAAAAGAAGAGTGGTG--------------
-------------------------AGCAGAGAGAAAAGAGCA---GTTGGA---ATGGCAGCT---------
ATCTTCCTTGGGTTCTTAGGAGCA---GCAGGAAGCACTATG---GGCGCGGCGTCA---
ATAACGCTGACGGTACAG---GCCAGACAG---TTATTG---TCTGGTATAGTGCAACAGCAGAGC---
AATCTG---CTGCAG---GCTATAGAGGCTCAACAGCACATGTTGAGACTCACGGTCTGGGGCATT
>JF683786..1
TTCTATTGCAATACATCAAAGTTGTTTAATAGCAGTTGGGGAAACAACACTGCCAGTAACCATACAGAGGCAA
GT---

GGAGAAAATGACACTATAACTATACAATGCAGAATAAAGCAAATTATAAATCTATGGCAGAGAGTAGGACAAG
CAATGTATGCCCCTCCTATCCAAGGAATAATAAGGTGTGAATCAAAC---
ATTACAGGATTACTATTAACAAGAGATGGAGGGAATAATAGTAGT----------------------------

```
-------------------------------------------------------------------------
----------
GGTGCAGATGAGACTTTCAGGCCTGGAGGAGGAGATATGAGAGACAATTGGAGAAGTGAATTATATAAGTATA
AAGTAGTAAAAATTGAACCACTA---GGTGTTGCACCCACCCGTGCAAAAAGAAGAGTGGTG-----------
----------------------------GAAAGACAAAAAAGAGCA---ATTGGA---ATGGGAGCT------
---GTCTTCCTTGGGTTCTTAGGAGCA---GCAGGAAGCACTATG---GGCGCAGCGTCA---
CTATCGCTGACGGTACAG---GCCAGACAA---TTATTG---TCCGGTATAGTGCAACAGCAGAGC---
AATTTG---CTGAAG---GCTATAGAGGCTCAACAACATATGTTGAGACTCACGGTCTGGGGCATT
>JF683792..2
TTCTACTGCAATACAACAAACCTGTTTAATAGCACTTGGAATAGCACTTGGTATAATAATGGGAGTAGCGCCA
AC-----------------------------------------------------------------------
-------------------------
AATATAACTCACACTATAACTCTCCCATGCAGAATAAGGCAAATTGTAAATATGTGGCAGAGAGTAGGACAAG
CAATGTATGCCAATCCCATCCCAGGAGTAATAAGGTGTGAATCAAAC---
ATTACAGGACTACTACTAACAAGAGATGGAGGGAATAATAACTCA----------------------------
-------------------------------------------------------------------------
-------------
AATGAAGAGACCTTCAGGCCTGGAGGAGGAGATATGAGGGACAATTGGAGAAGTGAATTATATAAGTATAAAG
TAGTAAAAATTGAACCACTA---GGTCTAGCACCCACCCATGCAAAAAGAAGAGTGGTG--------------
-------------------------GAAAGAGAAAAAAGAGCAGTTGTTGGA---CTGGGAGCT---------
GTCTTCCTTGGGTTCTTAGGAGCA---GCAGGAAGCACTATG---GGCGCAGCGTCA---
ATAACGCTGACGGTACAG---GCCAGACAA---TTATTG---TCTGGTATAGTGCAACAGCAGAGC---
AATTTG---CTGAAA---GCTATAGAGGCTCAACAACAGCTGTTGAGACTCACAGTCTGGGGCATT
>JF683795..2
TTCTATTGCAATACATCAGGACTATTTAATAGCACTTGGGATAATAGCACTTGGAATACCACTGCAAATTACA
CA-----------------------------------------------------------------------
-------------------------
GAGTCAAGTGACAATATAACTCTCCAATGCAGAATAAAGCAAATTGTAAATATGTGGCAGAGAGCAGGACAAG
CAATATATGCCCCTCCCATCCCAGGAGTAATAAGGTGTGAATCAAAC---
ATTACAGGACTACTATTAACAAGAGATGGAGGAAATGACAGTAGT----------------------------
-------------------------------------------------------------------------
-------------
ACAGAGGAGACCTTCAGGCCTGGAGGAGGAGATATGAGGGACAATTGGAGAAGTGAACTATATAAGTATAAAG
TGGTAAAAATTGAACCACTA---GGTGTAGCACCCACCCGTGCAAAAAGAAGAGTGGTG--------------
-------------------------GAGAGAGGAAAAAGAGCAGTAGTTGGA---CTGGGAGCT---------
GTCTTCCTTGGGTTCTTAGGAGCA---GCAGGAAGCACTATG---GGCGCGGCGTCA---
ATAACGCTGACGGTACAG---GCCAGACAA---TTATTG---TCCGGTATAGTGCAACAGCAGAGC---
AATTTG---CTGAAG---GCTATAGAGGCTCAACAACATATGTTGAAACTCACAGTCTGGGGCATT
>JF683799..1
TTTTATTGCGATACATCAAGACTGTTTAATAGCACTTGGGATACTAGCACTGGGTATGACAATAGCAGCAGA-
-------------------------------------------------------------------------
----------------------------
GACAATGACACTATAACTCTCAAATGCAAAATAAGGCAAGTTGTAAGGATGTGGCAAAGAGTAGCACAAGCAA
TGTATGCCCCTCCCATCCAAGGAGAAATAAGGTGTAACTCAACA---
ATTACAGGACTACTATTAACAAGAGATGGAGGGAATAGTAGTGGT----------------------------
-------------------------------------------------------------------------
-------------
GCAAATGAGACCTTCAGGCCTGGAGGAGGAGATATGAGGGACAATTGGAGAAGTGAATTATATAAGTATAAAG
TAGTAAAGATTGAACCACTA---GGTGTAGCACCCACCCATGCAAAAAGAAGAGTGGTG--------------
-------------------------GAGAGAGAAAAAAGAGCA---GTTGGA---ATAGGAGCT---------
GTCCTCCTTGGGTTCTTAGGAGCA---GCAGGAAGCACTATG---GGCGCGGCGTCA---
ATAACGCTGACGGTACAG---ACCAGACAA---TTATTG---TCTGGTATAGTGCAGCAGCAGAGC---
AATTTG---CTGAAG---GCTATAGAGGCTCAACAACAGCTGTTGAGACTCACGGTCTGGGGCATT
>KT124792..1
TTCTATTGCAATACATCAGGACTGTTTAATAGCACTTGGATCAATGGGAGCTGGGTCAATAATGATACCTGGG
CCAAT--------------------------------------------------------------------
-------------------AAT---
GATACCTCAAAAAATATAACTCTCCAATGCAGAATAAAGCAAATTATAAATATGTGGCAGAGAGTAGGGCAAG
CAATGTATGCCCCTCCCATCAAAGGAGAAATAAGGTGTAGATCAAAC---
ATTACTGGACTACTATTAACAAGAGATGGAGGGAATAATAGTAGT----------------------------
```


GGAAATGAGACATTCAGGCCGGCAGGAGGAGATATGAGAGACAATTGGAGAAGTGAATTATATAAGTATAAGG
TAGTAAAAATTGAACCACTA---GGTGTAGCACCCACACATGCAAGGAGACAAGTAGTG-----------
------------------------GGGAGAGAAAAAAGAGCA---GTTGGA---CTGGGAGCT--------
GTCTTCCTTGGGTTCTTAGGAGCA---GCAGGAAGCACTATG---GGCGCGGCGTCA---
ATAACGCTGACGGTACAG---GCCAGACAA---TTATTG---TCTGGTATAGTGCAGCAGCAGAGC---
AATCTG---CTGAAG---GCTATAGAGGCTCAACAACATATGTTGAGACTCACAGTCTGGGGCATT
>L22939..3
TTCTATTGCAATACATCAAGTCTGTTTAATAGCACTTGGAATAGCAGCTCAATTCACACAAATTACAGCTCAA
AT--

GACACAGACAATATAACTCTCCAATGCAGAATAAAGCAAATTGTAAATATGTGGCAGAAAGTAGGAAAAGCAA
TATATGCCCCTCCCATCTCAGGAGAAATAAGGTGTGAATCAAAC---
ATTACAGGACTAATATTAACAAGAGATGGAGGGAGTAATAATAGT-------------------------
----

ACAAATGAGATCTTCAGGCCTGGAGGAGGAGATATGAGGGATAATTGGAGAAGTGAATTATATAAGTATAAAG
TAGTAAAAATTGAACCACTA---GGTGTAGCACCCACCCGTGCAAAAAGAAGAGTGGTG-----------
------------------------GAGAGAGAAAAAAGAGCA---GTTGGA---CTGGGAGCT--------
TTCTTCCTTGGGTTCTTAGGAGCT---GCAGGAAGCACTATG---GGCGCGGCGTCA---
ATAACGCTGACGGTACAG---GCCAGACAA---TTATTG---TCCGGTATAGTGCAACAGCAGAGC---
AATTTG---CTGAGG---GCTATAGAGGCTCAACAACATCTGTTGAAACTCACGGTCTGGGGCATT
>AY151002..1
TTCTATTGCAATACAGCAGGCCTGTTTAATAGCACTTGGGATGATAACAATAGCACAGGGTCAAACACAAAG-
----

TCAAATGACATTATAACTCTCCAATGTAGAATAAAGCAACTTATAAATATGTGGCAGAGAGTAGGACAGGCAA
TGTATGCCCCTCCCATCCGAGGAATAATAAGGTGTAACTCAAAC---
ATTACTGGATTACTATTAACAAGAGATGGAGGGAATAATAGTACA-------------------------
----

AATGAGACCTTCAGGCCTGGAGGAGGAGATATGAGGGACAATTGGAGGAGTGAATTATATAAATATAAAGTAG
TAAAAATTGAACCACTA---GGTGTAGCACCCACCCCTGCAAAAAGAAGAGTGGTG--------------
----------------------GAGAGAGAAAAAAGAGCA---GCTGGA---CTGGGAGCT---------
GTCTTCCTTGGGTTCTTAGGAGCA---GCAGGAAGCACTATG---GGCGCGGCGTCA---
ATAACGCTGACGGTACAG---GCCAGACAG---TTATTG---TCCGGTATAGTGCAACAGCAAAGC---
AATTTG---CTGAAG---GCTATAGAGGCTCAACAACATATGTTGAGACTCACGGTCTGGGGCATT
>MK177825..1
TTCTATTGCAATACATCACAACTGTTTAATAGCACCTGGAATGAACATAACATTACCAACTCAACCTTTACAG
AG--

GACAATACAACTCTCACTCTCACATGTAGAATAAAGCAATTTATAAATATGTGGCAGAGAGTAGGACAAGCAA
TGTATGCCCCTCCCATCCAAGGAGAAATAAAATGTAATTCAAAC---
ATTACTGGATTACTGTTAACACGGGATGGAGGGAAGAACACTGAG-------------------------
----

AGCAATGAGACCTTCAGGCCTGGAGGAGGGGATATGAGGAACAATTGGAGAAGTGAATTATATAAGTATAAGG
TAGTAGAAATTAAACCACTA---GGTGTAGCACCCTCACCTGCAAAAAGAAGAGTGGTG-----------
------------------------GAGAGAGAAAAAAGAGCA---GCA------CTGGGAGCT--------
GTCTTCCTTGGGTTCTTAGGAGCA---GCAGGAAGCACTATG---GGCGCGGCGTCA---
ATAACGCTGACGGTACAG---GCCAGAAAG---TTATTG---TCTGGTATAGTGCACCAGCAAAGC---
AATTTG---CTGAAG---GCTATAGAGGCTCAACAACATATGTTGAGACTCACGGTCTGGGGCATT
>MK177826..2
TTCTATTGCAATACATCAAAACTGTTTAATAGCACCTGGGAGAATGAAAATAACACTACCAACTCAAACTCTG
CA--

GAGAATATAACTCTCACTCTCCCATGTAGAATAAAGCAAGTCATAAATATGTGGCAGAGAGTAGGACAAGCAA
TGTATGCCCCTCCCATCCAAGGAATAATAAAATGTAATTCAAAC---
ATTACTGGATTACTGCTAACACGGGATGGAGGGAATAACAATACA-------------------------

----

ACGAATGAGACCTTCAGGCCTGGAGGAGGGGATATGAGGGACAATTGGAGAAGTGAATTATACAAGTATAAAG
TAGTAAAAATTGAACCACTA---GGTGTAGCACCCTCCTATGCAAAAAGAAGAGTGGTG-------------
------------------------GAGAGAGAAAAAAGAGCA---GTTGGA---CTAGGAGCT---------
GTCTTCCTTGGGTTCTTAGGAGCA---GCAGGAAGCACTATG---GGCGCGGCGTCA---
ATAACGCTGACGGTACAG---GCCAGACAA---TTATTG---TCTGGTATAGTGCAACAGCAGAGC---
AATCTG---CTGAAG---GCTATAGAGGCTCAACAACAAATGTTGAGACTCACGGTCTGGGGCATT
>KY989950..2
TTCTATTGTAATACATCAAAATTGTTTAATAGCACTTGGAATACCAGCCACACAGAG---------------
----

TCAGGTGACAATATAACTCTCCCATGCCGAATAAAACAAATTGTAAGGATGTGGCAGAATGTAGGACAAGCAA
TGTATGCCCCTCCCATCCCAGGAGAAATAAGGTGTGACTCAAAC---
ATTACTGGACTACTATTAACAAGAGATGGAGGGGAGGAAAATAGGAATGGGACA-----------
AACGGGAATGGG---ACA--

AAGGAGGAGACCTTCAGGCCTGGAGGAGGAGATATGAGGGACAACTGGAGAAGTGAATTATACAAGTATAAAG
TAGTAAAAATTGAACCAGTA---GGTGTAGCACCTACTCATGCAAAAAGAAGAGTGGTG-------------
------------------------GAGAGAGAAAAAAGAGCA---GTA------CTTGGAGCT---------
GTCTTCCTTGGGTTCTTGGGAGCA---GCAGGAAGCACTATG---GGCGCGGCGTCA---
ATAACGCTGACGGTACAG---GCCAGACAA---TTGTTG---TCTGGTATAGTGCAACAGCAAAGC---
AATTTG---CTGAAG---GCTATAGAGGCTCAGCAACAACTGTTGAGGCTCACGGTCTGGGGCATT
>MF157740..1
TTCTATTGCAGTACATCAGAACTGTTTAATAGCACTTGGAAACGGGTTAATAACACTACTACTTGGAATAGCA
CTGCCAAC--

TCAACAAATGACCCTATAACTCTCCGGTGTAGAATAAAGCAAATTGTAAGGATGTGGCAGAGAGTAGAACAAG
CAATGTATGCCCCTCCCATCCGAGGAGAAATAAGGTGTGAGTCAAAC---
ATTACAGGATTACTATTAACAAGAGATGGAGGGCATGATGATGGGACAGAT---------------------
----

GAGACAGAGGAGACCTTCAGGCCTGGAGGAGGAGATATGAGGGACAATTGGAGAAGTGAATTATATAAGTATA
AAGTAGTAAAAATTGAACCACTA---GGTGTAGCACCCACCCATGCAAAAAGAAGAGTGGTG----------
---------------------------GAGAGAGAAAAAAGAGCA---GTTGGA---CTGGGAGCT------
---GTCTTCCTTGGGTTCTTAGGAGCA---GCAGGAAGCACTATG---GGCGCGGCGTCA---
ATAACGCTGACGGTACAG---GCCAGACAA---TTATTG---TCTGGTATAGTGCAACAGCAGAGC---
AATTTG---CTGAGA---GCTATAGAGGCTCAACAACATCTGTTGAGACTCACGGTCTGGGGCATT
>MH742763..1
TTCTATTGCAATACATCAGAATTATTTAACAATAACACAGAATTAAACAATGGCACTGCCGGC---------
----

GTAAATGACACTATAAAACTCCCATGCAGGGTAAAGCAAATTGTAAGAATGTGGCAGAAAGTAGGGCAAGCAA
TGTATGCCCCTCCCATCAAAGGAGTAATAAGGTGTAACTCAAAC---
ATTACAGGACTACTGTTAACAAGGGATGGAGGGAATACTAGCACA---------------------------
----

GAGGAGGAGATATTTAGGCCTGGAGGAGGAGATATGAGGGACAACTGGAGAAGTGAATTATATAAGTATAAGG
TAGTACAAATTGAACCATTA---GGTGTGGCACCCACCCATGCAAAAAGAAGAGTGGTG-------------
------------------------GAGAGAGAAAAAAGAGCA---CTTGGA---ATAGGAGCT---------
GTCTTCATTGGGTTCTTAGGAGCA---GCAGGAAGCACTATG---GGCGCGGCGTCA---
ATAACGCTGACGGTACAG---GCCAGACAA---TTATTG---TCCGGTATAGTGCAACAGCAGAGC---
AATCTG---CTGAGG---GCTATAGAGGCTCAACAACATCTGTTGAAACTCACGGTCTGGGGCATT
>MH742765..2
TTCTATTGCAATACATCAGCACTGTTTAATAGCACTTGGAATAACAGTACTGGCACTTGGACTGACACTAACA
ATAGCAGTTCA---

GAGTCAAATGGCACTATCACTCTCAAATGCAAAATAAAGCAAATTGTAAATATGTGGCAGAGAGTAGGGCAAG
CAATGTATGCCCCTCCCATCGAAGGAATAATAAGGTGTGAATCAAAC---
ATTACAGGACTACTATTAACAAGAGATGGAGGGAATAATAATGCA---------------------------

----

AATGAGACTTTCAGGCCTGGAGGAGGAGATATGAGGGATAATTGGAGAAGTGAATTATATAAATATAAAGTAG
TAAAAATTGAACCACTA---GGTGTAGCACCCACCTATGCAAAAAGAAGAGTGGTG----------------
---------------------GAGAGAGAAAAAAGAGCA---GTTGGA---CTGGGAGCT---------
GTTCTCTTTGGGTTTCTAGGAGCA---GCAGGAAGCACTATG---GGCGCGGCGTCA---
ATAACGCTGACGGTACAG---GCCAGACAA---TTATTG---TCCGGTATAGTGCAACAGCAGAGC---
AATTTG---CTGAGA---GCTATAGAGGCTCAACAACATCTGTTGAAACTCACGGTCTGGGGCATT
>MH742766..1
TTCTACTGCAATACGACAGACCTGTTTAACAGCCAGTGGCCCACTAACAACTCAGAG-------------
----

TCAAATGACACTATAACTCTCCAATGCAGAATAAAGCAATTTATAAATATGTGGCAGAGAGTAGGACAAGCAA
TGTATGCCCCTCCCATCCAAGGAAATATAAGTTGTAGATCAAAC---
ATTACAGGACTACTATTAACAAGAGATGGAGGGAATAATAATAGT--------------------------
----

AAGACAAATGAGACTTTTAGGCCTGGAGGAGGAGATATGAGGGACAATTGGAGAAGTGAATTATATAAGTATA
AAGTAGTAAAAATTGAACCAATA---GGTGTAGCACCCACCCATGCAAAAAGAAGAGTGGTG----------
---------------------------GAGAGAGAAAAAAGAGCA---GCTGGA---CTGGGAGCT------
---GTCTTCCTTGGGTTCTTAGGAGCA---GCAGGAAGCACTATG---GGCGCGGCGTCA---
ATAACGCTGACGGTACAG---GCCAGACAA---TTATTG---TCTGGTATAGTGCAACAGCAGAGC---
AATCTG---CTGAGG---GCTATAGAGGCTCAACAGCATCTGTTGAAACTCACGGTCTGGGGCATT
>MH742768..1
TTCTATTGCAATACAACAAAACTGTTTAATAGCACTTGGTTTAACAATGGCACAAACGCCACAAACAACGAAG
CA--

ATGAATGACACTCTAACTCTCCAATGCAGAATAAAGCAAATTGTGAGAATGTGGCAGACAGTAGGACAAGCAA
TGTATGCCCCTCCCATCTCAGGAGAGATAAGGTGTAATTCAAAC---
ATTACTGGGTTGCTATTAACAAGGGATGGAGGGGAGGATAATAGTACAAAAAAT------------------
----

GAGACAAAGGAGACTTTCAGGCCTGGAGGAGGAGATATGAGGGACAATTGGAGAAGTGAATTATATAAATATA
AAGTAGTAAAAATTGAACCTCTA---GGTGTAGCACCCACCCGTGCAAAGAGAAGAGTGGTG----------
---------------------------GAGAGAGAAAAAAGAGCA---GTTGGA---CTGGGAGCT------
---GTCTTCCTTGGGTTCTTAGGAGCA---GCAGGAAGCACTATG---GGCGCGGCGTCA---
ATAACGCTGACGGTACAG---GCCAGACAA---TTATTG---TCTGGTATAGTGCAACAGCAGAGC---
AATCTG---CTGAAG---GCTATAGAGGCTCAACAACAGCTGTTGAGACTAACAGTCTGGGGCATT
>MH742770..1
TTCTATTGTGATACATCAGGTCTGTTTAATAGCGTTTGGGATAGGAATGGCACTACTTGGAATAGCAACACCA
CAAGT---

GACACTGATGATATAATTCTCTCATGCAGAATAAGACAAATTATAAATATGTGGCAGAGAGTAGGACAAGCAA
TGTATGCCCCTCCCATTAAAGGAGTGATAAACTGTGAATCAAAC---
ATTACAGGGCTACTGCTAACAAGAGATGGAGGGACTAATAGGGGT--------------------------
----

AGTACAAATGAGACTTTCAGGCCTGTAGGAGGAGATATGAGAGACAATTGGAGAAGTGAATTATATAAGTATA
AAGTAGTAAAAATTGAGCCACTA---GGAGTAGCACCCACCAGGGCAAAAAGAAGAGTGGTG----------
---------------------------GAGAGAGAAAAAAGAGCA---GTTGGA---CTGGGAGCT------
---GTCTTCTTTGGGTTCTTAGGAGCA---GCAGGAAGCACTATG---GGCGCGGCGTCA---
ATAACGCTGACGGTACAA---GCCAGACAA---TTATTG---TCTGGTATAGTGCAACAGCAAAGC---
AATTTG---CTGAAG---GCTATAGAGGCTCAACAACATCTGTTGAGACTCACGGTCTGGGGCATT
>MH742762..1
TTCTATTGCAATACATCAGACCTGTTTAATGCATCAAGCACT-----------------------------
----

TGGGGTAACAATATAACTCTCCCATGCAGAATAAAGCAAATTGTAAATATGTGGCAGAAAGTAGGACGAGCAA
TGTATGCCCCTCCCATTCCAGGAGAAATAAGGTGTGAATCAAAC---
ATAACAGGACTACTATTAACAAGAGATGGAGGGAATAATAATAAT--------------------------

----

ACAAATGAGACCTTCAGACCTGGAGGAGGAGATATGAGGGACAATTGGAGAAGTGAATTATATAAGTATAAAG
TAGTAAAAATTGAACCACTA---GGTGTAGCACCCACCCATGCAAAAAGAAGAGTGGTG--------------
-------------------------CAGAGAGAAAAAAGAGCA---GTTGGA---CTGGGAGCT---------
GTCTTCCTTGGGTTCTTAGGAGCA---GCAGGAAGCACTATG---GGCGCGGCGTCA---
ATAACGCTGACGGTACAG---GCCAGACAA---TTGCTA---TCTGGTATAGTGCAACAGCAGAGC---
AATTTG---CTGAAG---GCTATAGAGGCTCAACAACATCTGTTGAGACTCACGGTCTGGGGCATT
>MH000309..1
TTCTATTGCAATACGACAGACCTGTTTAACAGCAAGTGGTTCAGT----------------------------
----

AACAACGGCATTATAACTCTCCAATGCAGAATAAAGCAATTCATAAATATGTGGCAGAGAGTAGGACAAGCAA
TGTATGCCCCTCCCATCTCAGGAATTATAAATTGTACATCAAAC---
ATTACAGGACTATTATTAACCAGAGATGGAGGA--

ACTGAGACTTTTAGGCCTGGAGGAGGAGATATGAGGGACAATTGGAGAAGTGAATTATATAAGTATAAAGTAG
TAAAAATTGAACCAATA---GGAATAGCACCCACCCATGCAAAAAGAAGAGTGGTG-----------------
----------------------GAGAGAGAAAAAAGAGCA---GTTGGA---CTGGGAGCT---------
GTCTTCCTTGGGTTCTTAGGAGCA---GCAGGAAGCACTATG---GGCGCGGCGTCA---
ATAACGCTGACGGTACAG---GCCAGACAA---TTATTG---TCTGGTATAGTGCAACAGCAGAGC---
AATCTG---CTGAGG---GCTATAGAGGCTCAACAACAGCTGTTGAAACTCACAGTCTGGGGCATT
>MH742757..1
TTCTACTGCAATACAACAAACTTGTTTAACATCAGCCTGGGGAACAGTACCACTAACAGCTCAGAG-------
----

TCAAATGACAATATAACTCTCCAATGCAGAATAAAGCAAATTATAAGGATGTGGCAGAGAGTAGGACAAGCAA
TGTATGCCCCTCCCATCCAAGGAGATATAAGTTGTAGATCAAAC---
ATTACAGGACTACTATTAACAAGAGATGGAGGGACAAGAGATGGAGGGAAT----------------------
----

GTGACTAATAATACTGAGATTTTTAGGCCTGAAGGAGGAAATATGAAGGACAATTGGAGAAGTGAATTATATA
AGTATAAAGTAGTAAGAATTGAACCAATA---GGTGTAGCACCCACCCGTGCAAAAAGAAGAACGGTG-----
----------------------------------GGGAGAGAGAAAAGAGCA---GTTGGA---
CTGGGAGCT---------GTCTTCCTTGGGTTCTTAGGAGCA---GCAGGAAGCACTATG---
GGCGCGGCGTCA---ATAACGCTGACGGTACAG---GCCAGACAA---TTATTG---
TCTGGTATAGTGCAACAGCAGAGC---AATCTG---CTGAGG---
GCTATTGAGGCTCAACAGCATCTGTTGAAACTCACAGTCTGGGGCATT
>AY231152..2
TTCTATTGCAATACATCAATACTGTTTAATAGCACTTGGAATACCAATGACACTTGGAATATCAATAACACTG
ACACA--

GAGTCAAATGACACTATAACTATCCAATGCAGAATAAGGCAAGTTGTAAAGATGTGGCAGAAAGTAGGACAAG
CAATGTATGCCCCTCCCATCGAAGGAGTAATAAGTTGTAAATCAAAC---
ATTACAGGACTACTATTAACAAGAGATGGAGGGAATCATAATAAT----------------------------
----

ACAAATGAGACTTTCAGGCCTGGAGGAGGAGATATGAGGGACAATTGGAGAAGTGAATTATATAAGTATAAAG
TAGTAAAAATTGAGCCACTA---GGTGTAGCACCCACCCGTGCAAAAAGAAGAGTGGTG--------------
-------------------------GAGAGAGAAAAAAGAGCAGTATTTGGA---CTGGGAGCT---------
GTTTTCCTTGGGTTCTTAGGAGCA---GCAGGAAGCACTATG---GGCGCGGCGTCA---
ATAACGCTGACGGTACAG---GCCAGACAA---TTATTG---TCCGGTATAGTGCAACAGCGGAGC---
AATTTG---CTGAGG---GCTATAGAGGCTCAACAACATCTGTTGAAACTCACAGTCTGGGGCATT
>AY231153..1
TTCTATTGCAATACATCAAAACTGTTTGATAGCACTTGGTATAGCAATGATAGCACTTGGTATAGCAATGACA
CCAGCTTAAAC--
-------------CACACA------
GAGTCAAATGGCACTATAACTCTCCAATGCAGAATAAAGCAAATTATAAATATGTGGCAAAAGGTAGGGCGAG
CAATGTATGCCCCTCCCATCCAAGGAGTAATAAGGTGTGATTCAAAC---

ATTACAGGACTACTATTAACAAGAGATGGAGGGGATAATAATACT----------------------------
----

AAAAATGAGACCTTCAGGCCTGGAGGAGGAGATATGAGGGACAATTGGAGAAGTGAATTATATAAGTATAAAG
TAGTAAAAATTGAACCACTA---GGTGTAGCACCCACCCATGCAAAAAGAAGAGTGGTG--------------
-------------------------GAGAGAGAAAAAAGAGCA---GTTGGA---CTGGGAGCT---------
GCCTTCCTTGGGTTCTTAGGAGCA---GCAGGAAGCACTATG---GGCGCGGCGTCA---
ATAACGCTGACGGTACAA---GCCAGACAA---TTATTG---TCCGGTATAGTGCAACAGCAGAGC---
AATCTG---CTGAAA---GCTATAGAGGCTCAACAACAGATGTTGAGACTCACGGTCTGGGGCATT
>MH742764..1
TTCTATTGCAATACATCAATACTGTTTAATAGCACTTGGAATAATAGCACTTGGGATAATAACACTAGAGATA
ACAAT--
-------------AACAAT------
GCCACAAATGATACTATGACTCTCCAATGCAGAATAAAGCAAATTGTAAGAATGTGGCAGACAGTAGGACAAG
CAATGTATGCCCCTCCCATCCAAGGAACAATAAAGTGTGAGTCAAAC---
ATTACAGGACTACTATTAACAAGAGATGGAGGAAATAATAATAAT----------------------------
----

ACAAATGAGACTTTCAGGCCTGGAGGAGGAGATATGAGGGACAATTGGAGAAGTGAATTATATAAGTATAAAG
TAGTAAAACTTAAACCACTA---GGTGTAGCACCCACCCCTGCAAAAAGAAGAGTGGTG--------------
-------------------------ATGAGAGAAAAAAGAGCA---GCA------CTGGGAGCT---------
ATTTTCCTTGGGTTCTTAGGAGCA---GCAGGAAGCACTATG---GGCGCGGCGTCA---
ATAACGCTGACGGTACAG---GCCAGACAA---TTATTG---TCCGGTATAGTGCAACAGCAGAGC---
AATTTG---CTGAGA---GCTATAGAGGCTCAACAACATATGTTGAAACTCACGGTCTGGGGCATT
>AF321079..2
TTCTATTGCAATACAGCATCACTGTTTAATAGCACTTGGTATAGCAATGGCAGCTCAAACTACGCAGGG----
----

TCAAATGGGAATATAACTCTCCAATGCAGAATAAAGCAAATTGTAAATATGTGGCAGAGAGTAGGACAAGCAA
TGTATGCCCCTCCCATCCAAGGAGTAATAAGGTGTGTATCAAAC---
ATTACTGGACTACTATTAACAAGAGATGGAGGGAATAATAATAGT----------------------------
----

AGTACAAATGAGACCTTTAGGCCTGGAGGAGGAGACATGAGAGACAATTGGAGAAGTGAATTATATAAGTATA
AAGTAGTAAAAATTGAACCACTA---GGTGTAGCACCCTCCCGTGCAAAAAGAAGAGTGGTG-----------
----------------------------GAGAGAGAAAAAAGAGCA---ATCGGA---CTGGGAGCT------
---GTTTTCCTTGGGTTCTTAGGAGCA---GCAGGAAGCACTATG---GGCGCGGCGTCA---
ATAACGCTGACGGTACAG---GCCAGACAA---TTATTG---TCCGGTATAGTGCAACAGCAGAGC---
AATCTG---CTGAAG---GCTATAGAGGCTCAACAACATATGTTGAAACTCACGGTCTGGGGCATT
>AB286856..2
TTCTATTGCTATACATCAGACCTGTTTAATAGCACTTGGCCCAATAACACTGCAGAGTCAAATAGCACAGGGT
CAATA--

AGGTCAAATGAGACTATAACTCTCAAATGCAGAATAAAGCAAATTATAAATATGTGGCAGAGAGTAGGACAAG
CAATATATGCCCCTCCCATCAGAGGAGAAATAAGGTGTGATTCAAAC---
ATTACAGGACTAATATTAACAAGAGATGGAGGGAATAATAGTGAGAGT-------------------------
----

GCAAATGGAACTGAAATTTTCAGGCCTGGAGGAGGAGATATGAGGGACAATTGGAGAAGTGAATTATATAAGT
ATAAAGTAGTAAAAATTGAACCACTA---GGATTAGCACCCACTAGAGCAAAAAGAAGAGTGGTG--------
-------------------------------CAGAGAGAAAGAAGAGCA---GTTGGC---CTGGGAGCT---
------TTATTCATTGGGTTCCTAGGAGCA---GCAGGAAGCACTATG---GGCGCGGCGTCA---
TTAACGCTGACGGTACAG---GCCAGACAA---TTACTG---TCTGGTATAGTGCAACAGCAAAGC---
AATTTG---CTGAGG---GCTATAGAGGCTCAACAACATCTGTTGAAACTCACAGTCTGGGGCATT
>AB286857..1
TTCTATTGCAATACATCAGGACTGTTTAATAGCACTTGGGAGAATAATACTAGCACAGCCAGCTCAACAAATG
TTAGCTCA---
-------------------ATA---
AATGACACTGACATTATAGAACTCAAATGCAGAATAAAGCAAATTGTAAATATGTGGCAGAAAGTAGGACAAG
CAATGTATGCTCCTCCCATTCCAGGAGTAATAAGGTGTGATTCGAAC---

ATTACAGGACTACTATTAACAAGAGATGGAGGGAAGAATAGTAGT----------------------------
----

GCTACAAATGAGACCTTCAGGCCTGGAGGGGGAGATATGAGAGACAATTGGAGAAGTGAATTATATAAATATA
AAGTAGTAAAAATTGAACCACTG---GGTGTAGCACCCACCCATGCAAAAAGAAGAGTGGTG-----------
----------------------------GAGAGAGAAAAAAGAGCA---GTTGGA---CTAGGAGCT------
---GTACTCCTTGGGTTCTTAGGAGCA---GCAGGAAGCACTATG---GGCGCGGCGTCA---
ATAACGCTGACGGTACAG---GCCAGACAA---TTATTG---TCTGGTATAGTGCAACAGCAGAGC---
AATCTG---CTGAGA---GCTATAGAGGCTCAACAACAGCTGTTGAGACTCACGGTCTGGGGCATT
>AB286859..2
TTCTATTGTAATACAACAACACTGTTTAATAGCACTTGGGATGACAATAACACTGCCAGCTCAAACTACACAG
GG---

TCAAATGGCACTATAACTCTCCAATGCAGAATAAAGCAAATTATAAATATGTGGCAGAAAGTAGGACAAGCAA
TGTATGCCCCTCCCATCCAAGGAGTAATAAGGTGTAACTCAAGC---
ATTACTGGACTACTATTAACAAGAGATGGAGGAAGTAATAGTAGT----------------------------
----

ACAAATGAAACTTTCAGGCCTGGAGGAGGAGACATGAGGGACAATTGGAGAAGTGAATTATATAAGTATAAAG
TAGTAAAAATTGAACCACTG---GGTGTAGCACCCACCCATGCAAAAAGAAGAGTGGTG--------------
-------------------------GAGAGAGAAAAAAGAGCA---GTTGGA---CTGGGAGCT---------
GTCTTCCTTGGGTTCTTAGGAGCA---GCAGGAAGCACTATG---GGCGCAGCGTCA---
CTAACGCTGACGGTACAG---GCCAGACAA---TTATTG---TCTGGTATAGTGCAACAGCAAAGC---
AATTTG---CTGAAG---GCTATAGAGGCTCAACAACATCTGTTGAAACTCACAGTCTGGGGCATT
>AB286862..2
TTCTATTGCAATACAACAAATTTGTTTAATAGCACTTGGGAAAAGAAAAAGGGCACTTGGAATGATACCACTG
CCAAC--

CCACCAAATGGCACTATAACTCTCCAATGCAGAATAAAGCAAATTGTAAATATGTGGCAGAGAGTAGGACAAG
CAATATATGCCCCTCCCATCCCAGGAATAATAAGTTGTAATTCAAGC---
ATCACAGGATTACTATTAACAAGAGATGGGGGTGAGGGGAATGAAACT-------------------------
----

AGGAAAAATGAGACCTTCAGACCTGGAGGAGGAGATATGAGGGACAATTGGAGAAGTGAATTATATAAGTATA
AAGTGGTAAAAATTGAACCACTA---GGTGTGGCACCTACCTATGCAAAAAGAAGAGTGGTG-----------
----------------------------GAGAGAGAAAAAAGAGCA---GTTGGA---ATGGGAGCT------
---GTCTTACTTGGGTTCTTAGGAGCA---GCAGGAAGCACTATG---GGCGCAGCGTCA---
ATAACGCTGACGGTACAG---GCCAGACAA---TTATTG---TCCGGTATAGTGCAACAGCAGAGC---
AATCTG---CTGAAA---GCTATAGAGGCTCAACAACAACTGTTGAGACTCACGGTCTGGGGCATT
>AB286863..3
TTCTATTGCTATACATCAGAACTGTTTAATAGCACTTGGAATAATAATACTGGCAGCCCAAACAGC-------
----

ACAAATGACACTATAACTCTTCAATGCAAAATAAAGCAAATTGTAAATATGTGGCAGAGGGTAGGGCAAGCGA
TGTATGCCCCTCCCATCCCAGGAGAGCTAAGGTGTGACTCAAAC---
ATTACTGGACTACTATTAACAAGAGATGGAGGGAATACTAATAATACA-------------------------
----

AATACAACTGAAACCTTCAGGCCGGGAGGGGGAGATATGAGGGACAATTGGAGAAGCGAACTATACAAGTATA
AAGTAGTACAAATTGAACCAATA---GGAGTAGCACCCACCCCGGCAAAAAGAAGAGTGGTG-----------
----------------------------GAGAGAGAAAAAAGAGCA---GTTGGA---CTGGGAGCT------
---GTCTTCCTTGGGTTCTTAGGAGCA---GCAGGAAGCACTATG---GGCGCAGCGTCA---
ATAACGCTGACGGTACAG---GCCAGACAA---TTATTG---TCCGGTATAGTGCAACAGCAGAGC---
AATTTG---CTGAGA---GCTATAGAGGCTCAACAACATCTGTTGAAACTCACGGTCTGGGGCATT
>AB231895..2
TTCTATTGCAATACATCAGGACTGTTTAATAGTACTTGGTATAGCGATACCACTGCAAACCAAACAGAG----
----

TCAAATGACACCATAACTCTACAATGCAGAATAAAGCAAATTATAAATATGTGGCAGACAGTAGGACAGGCAA
TGTATGCCCCTCCCATCCAAGGAGTAATAAGGTGTGAATCAAAC---

ATCACTGGATTACTATTAACAAGAGATGGAGGGAATAAGAATCAT----------------------------
----

ACAAATGAGACCTTCAGGCCTGGAGGAGGAGATATGAGAGACAATTGGAGAAGTGAATTATATAAGTATAAAG
TAGTAAAGATTGAACCAATA---GGTGTAGCACCCTCTCCTGCAAAAAGAAGAGTGGTG--------------
-------------------------GGAAGAGAAAAAAGAGCA---GTCGGA---CTGGGAGCT---------
GTCTTCCTTGGGTTCTTAGGAGCA---GCAGGAAGCACTATG---GGCGCGGCGTCA---
ATGACGCTGACGGTACAA---GCCAGACAA---TTATTG---TCTGGTATAGTGCAACAGCAGAGC---
AATTTG---CTGAAG---GCTATAGAGGCTCAACAACATCTGTTGAAACTCACGGTTTGGGGCATT
>AB231896..1
TTCTATTGCGATACATCAGGGCTGTTTAATAGCACTTGGGATAACATTACCAGTACAAATGGCACAAAGGTAA
AT---

GAAACTGAAACTATAACTCTCCCATGCAGAATAAAACAAATTGTAAGAATGTGGCAGAGAGTAGGACAAGCAA
TGTATGCCCCTCCTATCCAAGGAGAACTACGGTGTGAATCAAAT---
ATTACAGGACTACTATTAACAAGAGATGGAGGGAATAATAATCGT----------------------------
----

ACAAATGAGACCTTCAGGCCTATAGGAGGAGATATGAAGGACAATTGGAGAAGTGAATTATATAAATATAAAG
TGGTAAAAATTGAACCACTA---GGTATAGCACCCTCCAGGGCAAAAAGGAGAGTGGTG--------------
-------------------------GAGAGAGAAAAAAGAGCA---GTTGGA---CTGGGAGCT---------
GTCTTCCTTGGGTTCTTAGGAGCA---GCAGGAAGCACTATG---GGCGCGGCGTCA---
TTAACGCTGACGGTACAG---GCCAGACAA---TTATTG---TCCGGTATAGTGCAACAGCAAAGC---
AATTTG---CTGAGG---GCTATAGAGGCTCAACAACATCTGTTGAAACTCACAGTCTGGGGCATT
>AB231898..1
TTCTATTGCAATACATCAAGACTGTTTAATAGCAATTGGAAAAACGGTACTGCCAGCTCAAATGGCACT----
----

GCAAATGACATTATAACTCTCCAATGCAGAATAAGGCAAATTATAAATATGTGGCAGAAAGTAGGAAAAGCAA
TGTATGCCCCTCCCATCCCAGGAGTAATAAGGTGTGAGTCAAAC---
ATTACAGGACTACTATTAACAAGAGATGGAGGGAAAAATACTAGT----------------------------
----

GGTGTAAATGAGACTTTCAGACCTGAAGGAGGAAATATGAAAGACAATTGGAGAAGTGAATTATATAAGTATA
AAGTAATAAAAATTGAACCACTA---GGTGTAGCACCCACCCGTGCAAGAAGAAGAGTGGTG-----------
----------------------------GGAAGAGAAAAAAGAGCA---ATAGGTGGACTGGGAGCT------
---GCCCTCCTTGGGTTCCTAGGAGCA---GCAGGAAGCACTATG---GGCGCGGCGTCA---
ATAACGCTGACGGTACAG---GCCAGACAA---TTATTG---TCTGGTATAGTGCAACAGCAGAGC---
AATCTG---CTGAGG---GCTATAAAGGCTCAACAAGAACTGTTGAGACTCACGGTCTGGGGCATT
>AB049811..3
TTCTATTGCAATACATCAGAACTGTTTAATAGGAATTACAATAGGTCTAGCAATAGCACTTGGAATTACAATA
GTTCTAGCAAG--
-------------GGCACA------
GAGTCAAATGGCACTATAATTCTCCAATGCAGAATAAAGCAAATTGTAAATATGTGGCAGAGAGTAGGACAAG
CAATATATGCCCCTCCCATCCCAGGAGTAGTAAGGTGTGTCTCAAAC---
ATTACAGGACTACTATTAACAAGAGATGGAGGGAATGGTAATAGT----------------------------
----

ACAAATGAGACTTTCAGACCGGGAGGAGGAGATATGAGGGACAATTGGAGAAGTGAATTATATAAGTATAAAG
TAGTAAAAATTGAACCACTA---GGTGTAGCACCCACCCATGCAAAAAGAAGAGTGGTG--------------
-------------------------GGGAGAGAAAAAAGAGCA---GTTGGA---CTGGGAGCT---------
GTCTTCCTTGGGTTCTTAGGAGCT---GCAGGAAGCACTATG---GGCGCAGCGTCA---
ATAACGCTGACGGTACAG---GCCAGACAA---TTATTG---TCTGGTATAGTGCAACAGCAGAGC---
AATCTG---CTGAGG---GCTATAGAAGCTCAACAACATATGTTGAAGCTCACGGTCTGGGGCATT
>AB485633..1
TTCTATTGTAAGACATCGAGACTGTTTAATAGCACTTGGCAGTTTAATAGCACTTGGAAGAGCATTCGGGATG
GC---

AATGACAATGACACTATAACTCTCCCATGCAGAATAAGACAAATTGTACATATGTGGCAGAGAGTAGGACAAG
CAATGTATGCCCCTCCCATTCGAGGAGTAATAAACTGTCTATCAAGC---

ATTACAGGACTACTGCTAACAAGAGATGGTGGTGGGAATGGGAAT----------------------------
----

AGTACAAAGGAGATCTTCAGGCCTGGAGGAGGAGATATGAGGGACAATTGGAGAAGTGAATTATATAAGTATA
AGGTAGTGAAAATTGAACCACTA---GGTGTAGCACCCACCCATGCAAAAAAAAGAGTGGTG-----------
----------------------------GAGAGAGAAAAGAGAGCA---GTTGGA---CTGGGAGCT------
---GTCTTCCTTGGGTTCTTAGGAGCA---GCAGGAAGCACTATG---GGCGCGGCGTCA---
ATAACGCTGACGGTACAG---GCCAGACAA---TTATTG---TCCGGTATAGTGCAACAGCAGAGC---
AATCTG---CTGAAG---GCTATAGAGGCTCAACAACATCTGTTGAGACTCACGGTCTGGGGCATT
>HQ385450..1
TTCTATTGCAATACATCAGAACTGTTTACTAGCACTTGGCCCAATGAGAATAATAGCACCGAGACCAATAATA
CTGCCAAC---
-------------------TTA---
AATAATACAGACACTATAACTCTCCAATGCAGAATAAAGCAAATTGTAAGAATGTGGCAGACAGTGGGACAAG
CAATGTATGCCCCTCCCATCAAAGGGGTAATAAATTGTAAATCAAAC---
ATTACAGGACTATTATTAATAAGAGATGGAGGGAAAAATAATAGT----------------------------
----

GAAAATGAGACTTTCAGGCCTGGAGGAGGAGATATGAGGGACAATTGGAGAAGTGAATTATATAAGTATAAAG
TAGTAAAGATTGAACCAATA---GGTGTAGCACCCACCCATGCAAAAAGAAGAGTGGTG--------------
-------------------------GAGAGAGAAAAAAGAGCA---GTTGGA---ATGGGAGTG---------
TTTATCCTTGGGTTCTTAGGAGCA---GCAGGAAGCACTATG---GGCGCGGCGTCA---
ATAACGCTGACGGTACAG---GCCAGACAA---TTATTG---TCTGGTATAGTGCAACAGCAGAGC---
AATTTG---CTGAGG---GCTATAGAGGCTCAACAACATCTGTTGAAACTCACAGTCTGGGGCATC
>HQ385451..1
TTCTATTGCAATACATCAGGACTGTTTAATAGCACCTGGGTGAATAACAGTAGCACTGAGGGTGCCAGTAACA
ATGCCAGC---
-------------------TCA---
GATGCAAATGACACTATAACTCTCCAATGCAGGATAAAGCAAATTATAAATCTGTGGCAGAGAGTAGGAAAAG
CAATGTATGCCCCTCCCATCCCAGGAATAATAAGGTGTGACTCAAAC---
ATTACAGGACTATTATTAACAAGAGATGGAGGGTATAATAATAAT----------------------------
----

AGTAGAAATGAGACTTTCAGACCTGGAGGAGGAGATATGAGGGACAATTGGAGAAGTGAATTATATAAGTATA
AAGTAGTAAAAATTGAACCACTA---GGTATAGCACCCACCAAGGCAAGGAGAAGAGTGGTG-----------
----------------------------GAGAGAGAAAAAAGAGCA---GTTGGC---ATGGGAGCT------
---CTCTTCCTTGGGTTCTTAGGAGCA---GCAGGAAGCACTATG---GGCGCGGCGTCA---
ATAACGCTGACGGTACAG---GCCAGACAA---TTATTG---TCTGGTATAGTGCAACAGCAGAGC---
AATTTG---CTGAGA---GCTATAGAGGCTCAACAACATCTGTTGAAACTCACGGTCTGGGGCATT
>HQ385460..1
TTCTACTGCAATTCATCAGCACTGTTTGATACAACATGGAACGCCACTACATGGAATAATACCATAGAGTCAA
ATAATACC---
-------------------ACA---
GGGCCAAACGATACAATCACACTCCACTGCAGGATAAAACAGATTATAAACATGTGGCAGACAGTAGGACAAG
CAATGTACGCCCCTCCCATCCGAGGAGAAATAAGGTGTGAATCAAAC---
ATTACAGGACTACTATTAACAAGAGATGGAGGGAATGGTAATAGTAGT-------------------------
----

GGTGGTGCAAATGAGACTTTTAGGCCTGGAGGAGGAGATATGAGGGACAATTGGAGGAGTGAATTATATAAGT
ATAAAGTAGTAAAAATTGAACCACTA---GGTGTAGCACCCACCCATGCAAAGAGAAGAGTGGTG--------
-------------------------------GAGAGAGAAAAAAGAGCA---ATTGGA---CTGGGAGCT---
------TTCTTCCTTGGGTTCTTAGGAACA---GCAGGAAGCACTATG---GGCGCAGCGTCA---
ATAACGCTGACGGTACAG---GCCAGACAA---TTATTG---TCTGGTATAGTGCAACAGCAAAGC---
AATTTG---CTGATG---GCTATAGAGGCTCAACAACATATGTTGAAACTCACTGTCTGGGGCATT
>HQ385446..2
TTCTATTGCAATACATCAGGACTGTTTAACATTTGGGAGAAC-------------------------------
----

AATGACAATATAACTCTCCCATGCAGAATAAAGCAAATTATAAATATGTGGCAGAAAGTAGGACAAGCAATGT
ATGCCCCTCCCATTGCAGGAAAAATAGAGTGTGAGTCAAAC---

ATTACAGGACTACTGCTAACAAGAGATGGAGGAAATAATAATAGTACA-------------------------
----

AATAAAAGTGAGACTTTCAGGCCTGGAGGAGGAGACATGAGGGACAATTGGAGAAGTGAATTATATAAGTATA
AGATAGTAAAAATTGAACCACTA---GGTGTGGCACCCACTCATGCAAAAAGAAGAGTGGTG-----------
----------------------------GGGAGAGAAAAAAGAGCA---GTTGGA---CTGGGAGCT------
---GTTCTCTTTGGGTTCTTAGGAGCA---GCAGGAAGCACTATG---GGCGCGGCGTCA---
ATAACGCTGACGGTACAG---GCCAGACAA---CTATTG---TCTGGTATAGTGCAACAGCAGAGC---
AATCTG---CTGAAA---GCTATAGAGGCTCAACAACATCTGTTGAAACTCACAGTCTGGGGCATT
>HQ385447..2
TTCTATTGCAATACAACAGCACTGTTTTATGGTACATTGTATAACAATGACACTTGGGATAGAAATAGCACTT
GGGAT--
-------------------AAC---
AACAACTCAACAAATATAACTCTCCAATGCAAAATAAAGCAAATTATAAGAATGTGGCAGAGAGTAGGGCAAG
CAATGTATGCCCCTCCCATCCGAGGAAAAATATGGTGTGAATCAAAC---
ATTACAGGAATACTATTAACAAGAGATGGGGGAATGATAATGCA-----------------------------
----

AATGAGACCTTCAGGCCTGGAGGAGGAGATATGAGGGACAATTGGAGAAGTGAATTATATAAATATAAAGTAG
TAAAAATTGAGCCACTA---GGTGTAGCACCCACCCATGCAAAAAGAAGAGCGGTG-----------------
----------------------GATAGAGAAAAAAGAGCA---GTTGGA---CTGGGAGCT---------
TTTTTCCTTGGGTTCTTAGGAGCA---GCAGGAAGCACTATG---GGCGCGGCGTCA---
ATAACGCTGACGGTACAG---GCCAGACAA---TTATTG---TCTGGTATAGTGCAACAGCAGAGC---
AATCTG---CTGAAG---GCTATAGAGGCTCAACAACATCTGTTGAGACTCACGGTCTGGGGCATT
>HQ385462..1
TTCTATTGCAATACATCAGGACTGTTTAATAGCACTTGGCCAGAGGCAAATGCCACACAGTCAAATACCACAC
AG---

TCAAATAGCACTATAACTCTCCCATGCAGAATAAAGCAAATTGTAAATATGTGGCAGAAAGTAGGACAAGCAA
TGTATGCCCCTCCCATCCAAGGAATAATAAAGTGTGAATCAAAC---
ATTACAGGGTTATTGTTAACAAGAGATGGGGGAAATAATAGTGGAAAT-------------------------
----

AATGGTGAACCTGATATTTTCAGGCCCGAAGGAGGAGATATGAGAGATAATTGGAGAAGTGAATTATATAAGT
ATAAAGTAGTAAAAATTGAACCACTG---GGTATAGCACCCAGCCATGCAAAAAGAAGAGTGGTG--------
-------------------------------GAGAGAGAAAAAAGAGCA---GTTGGA---CTGGGAGCT---
------GTCTTCCTTGGGTTCTTAGGAGCA---GCAGGAAGCACTATG---GGCGCGGCGTCA---
ATAACGCTGACGGCACAC---GCCAAACAA---TTACTG---TCTGGTATAGTGCAACAGCAGAGT---
AATCTG---CTGAGG---GCTATAGAGGCTCAACAACATCTGTTGAAACTCACGGTCTGGGGCATT
>HQ385455..2
TTCTATTGCAATACAACAGCCCTGTTTAACACAACTTGGATCAATGGCACTGACAGCTCAAACAAC-------
----

AGCACAGAGTCTATAACGATCCAATGCAGAATAAAGCAAATTATAAACATGTGGCAGAAAGTAGGACAAGCAA
TATATGCCCCTCCTATCCCAGGAACAATAAGGTGTGAATCAAAC---
ATTACAGGACTATTATTAACAAGAGATGGAGGAAAGAATAGAACA----------------------------
----

AATAATGAGACTTTCAGGCCTGAAGGAGGAGATATGAGGGACAATTGGAGAAGTGAATTATATAAGTATAAAG
TAGTAAAAATTGAACCACTA---GGTATAGCACCTACCTATGCAAAAAGAAGAGTGGTG--------------
-------------------------GAGAGAGAAAAAAGAGCA---GTTGGA---CTGGGAGCT---------
GTCTTCCTTGGGTTCCTAGGAGCA---GCAGGAAGCACTATG---GGCGCGGCGTCA---
GTGACGCTGACGGTACAG---GCCAGACAA---TTATTG---TCTGGTATAGTGCAGCAGCAAAGC---
AATTTG---CTGAAG---GCTATAGAGGCTCAACAACATCTGTTGAGACTCACAGTCTGGGGCATT
>HQ385457..1
TTCTATTGCAATACATCAGAACTGTTTAATAGTACTTGGAATAACAATAGCACTGGGGATAACAAT-------
----

AGCTCAACACCAATAATTCTCCAATGCAAAATAAAGCAAATTATAAGAATGTGGCAGAAAGTAGGACAAGCAA
TGTATGCCCCTCCCATCCCAGGAGAAATAAGGTGTGAATCAAAC---

ATTACTGGACTACTATTAACAAGAGATGGAGGGAATAATAATAAT----------------------------
----

ACAAATGAAACATTCAGGCCTGGGGGAGGAGATATGAGAGACAATTGGAGAAGTGAATTATATAAGTATAAAG
TAGTAAAAATTGAACCACTA---GGTGTAGCACCTACCCGTGCAAAAAGAAGAGTGGTG--------------
-------------------------GAGAGAGAAAAAAGAGCA---GTTGGA---ATGGGAGCT---------
TTATTCCTTGGGTTCTTAGGAGCA---GCAGGAAGCACTATG---GGCGCGGCGTCA---
ATAACGCTGACGGTACAG---GCCAGACAA---TTATTG---TCCGGTATAGTGCAACAGCAGAGC---
AATTTG---CTGAAG---GCTATAGAGGCTCAACAGCATCTGTTGAGACTCACGGTCTGGGGCATT
>HQ385449..1
TTCTACTGCAATACATCAAGACTGTTTAATAGCACTTGGAATAACATTAATAGCACTGGGTATAACCTCACAG
GG---

TCACAGGAAAATATAACTCTCCAATGCAGAATAAAGCAAATTGTAAGAATGTGGCAGAGAGTAGGACAAGCAA
TGTATGCCCCTCCCATCCGAGGAGAAATAAGGTGTAACTCAAAC---
ATTACTGGACTACTATTAACAAGAGATGGAGGGAATGAAAATAAT----------------------------
----

AATACAAACGAGACCTTCAGGCCTGGAGGAGGAGATATGAGGGACAATTGGAGAAGTGAATTATATAAGTATA
AAGTAGTAAAAATTGAACCACTA---GGTGTAGCACCCACCCGTGCAAGAAGAAGAGTGGTG-----------
----------------------------GAGAGAGAAAAAAGAGCA---GTTGGA---CTGGGAGCT------
---GTCTTCCTTGGGTTCTTAGGAGCA---GCAGGAAGCACTATG---GGCGCGGCGTCA---
ATAACGCTGACGGTACAG---GCCAGACAA---TTATTG---TCCGGTATAGTGCAACAGCAAAGC---
AATTTG---CTGAAG---GCTATAGAGGCTCAACAACATCTGTTGAGACTCACGGTCTGGGGCATT
>HQ385454..2
TTCTATTGCTACACATCAAACCTGTTTAACAGTACTTGGGATGACAACAGTACTTGGGATAACAATACTACTT
GGGATAAC---
-------------AGCACG------
ACAGAGGACACCAACATAACTCTCCAATGCAGGATAAAGCAAATTGTAAATATGTGGCAGAGAGTAGGACAAG
CAATATATGCCCCTCCCATCCAAGGAGACATAAAGTGTGAATCAAAC---
ATTACTGGATTATTGTTAACAAGAGATGGAGAGAATAATACTATG----------------------------
----

AATGAGACTTTCAGGCCTGGAGGAGGAGACATGAGGGACAATTGGAGAAGTGAATTATATAAATATAAAGTAG
TAAAAATTGAACCACTA---GGTGTAGCACCCACCCATGCAAAAAGAAGAGTGGTG-----------------
----------------------GAGAGAGAAAAAAGAGCA---GTTGGA---CTAGGAGCT---------
GTCCTCTTTGGGTTCTTAGGAGCA---GCAGGAAGCACTATG---GGCGCGGCGTCA---
ATAACGCTGACGGTACAG---GCCAGACAA---TTATTG---TCTGGTATAGTGCAACAGCAGAGC---
AATTTG---CTGAAG---GCTATAGAGGCTCAACAACATCTGTTGAAACTCACGGTCTGGGGCATT
>MH078541..1
TTCTACTGTAATACATCAGCACTGTTTAATAGCACTTGGAATTGGAATAATAACACTTGGGGGACCAATGAGA
GCAGCTCACAG--
-------------GCAAAT------
GACACTGGTGAACCTATAACTCTCCAATGCAGAATAAAGCAAATTATAAATATGTGGCAGAAAGTAGGACGAG
CAATGTATGCCCCTCCCATCCCAGGAGTCATAAGGTGTGAGTCAAAC---
ATTACAGGCCTACTGCTAACAAGAGATGGAGGAAGTACTAATAGT----------------------------
----

ACAAATGAGACTTTCAGGCCTGGAGGAGGAGACATGAGGGATAATTGGAGGAGTGAATTATATAAGTATAAAG
TAGTAAAAATTGAACCACTA---GGTGTAGCACCTACTCGTGCAAGAAGAAGAGTGGTG--------------
-------------------------GGAAGAGAAAAAAGAGCA---GTTGGA---CTGGGAGCT---------
GTTTTCCTTGGGTTCTTAGGAGCA---GCAGGAAGCACTATG---GGCGCAGCGTCA---
CTAACGCTGACGGTACAG---GCCAGACAA---TTATTG---TCTGGTATAGTGCAACAGCAGAGT---
AATCTG---CTGAGG---GCTATAGAGGCTCAACAACAGATGTTGAGGCTCACGGTCTGGGGCATT
>MH078542..3
TTCTATTGCAATACATCAAAATTGTTTAATAGTACTTGGAATGAGAATAGTACTTGGAATAGTAGTCAG----
----

AGTAATGAGAATATAACCATCCCATGCAGAATAAGACAAATTGTACATCTGTGGCAGAGAGTAGGACAAGCAA
TGTATGCCCCTCCCATTCCGGGAGTAATAACCTGTACATCAAAT---

ATTACAGGGCTACTGCTAACAAGAGATGGTGGGAATAATAATAGT----------------------------
----

ACAAATGAGACCATCAGGCCTCAAGGAGGAAATATGAAGGACAATTGGAGGAGTGAATTATACAAATATAAAG
TAGTAAAAATTGAACCAATA---GGAGTAGCACCCACCAAGGCAAAGAGAAGAGTGGTG--------------
-------------------------GAGAGAGAAAAAAGAGCA---GTTGGA---CTGGGAGCT---------
GTCTTCCTTGGGTTCTTAGGAGCA---GCAGGAAGCACTATG---GGCGCAGCGTCA---
ATAACGCTGACGGTACAG---GCCAGACAA---TTATTG---TCTGGTATAGTGCAACAGCAAAGC---
AATTTG---CTGAAG---GCTATAGAGGCTCAACAGCAGCTGTTGAGACTCACAGTCTGGGGCATT
>MH078544..2
TTCTATTGTAATACAACAAAATTGTTTAGTAGCACTTGGGCTAATAATAGCACTTTGGGTAATAGCACTGAGG
ATAAC--

AACAATACTACTATAACTCTCCAATGTAGAATAAAGCAAATTGTGAATATGTGGCAGAGAGTAGGACAAGCAA
TGTATGCCCCTCCCATTCCAGGAGAAATAAGTTGTGTATCGAAC---
ATTACTGGACTTTTATTAACAAGAGATGGAGAGGGTAGTAATAGG----------------------------
----

ACAAATGAGACCTTCAGGCCTGGAGGAGGGGATATGAGGGACAATTGGAGAAGTGAATTATATAAGTATAAAA
TAGTAAAAATTGAACCACTA---GGTATAGCACCTACTCATGCAAGAAGGAGAGTGGTG--------------
-------------------------GAGAGAGAAAAAAGAGCA---GTTGGA---CTGGGAGCT---------
TTCTTCCTTGGGTTCTTAGGAGCA---GCAGGAAGCACTATG---GGCGCGGCGTCA---
ATAACGCTGACGGTACAA---GCCAGACAA---TTATTG---TCTGGTATAGTGCAACAGCAGAGC---
AATCTG---CTGAAG---GCTATAGAGGCTCAACAACATCTGTTGAAACTCACGGTCTGGGGCATT
>MK397970..2
TTCTATTGCAATACATCACAGTTGTTTAATAGCACTTGGGAGAACGGGGAATTAAATGGCAGTATA-------
----

TCAGATGGCAATATAACGATCCCCTGCAGAATAAAGCAAATTATAAATATGTGGCAGAGAGTAGGACAAGCAA
TGTATGCCCCTCCCATCCAAGGAGAGATAAGGTGTCAATCAAAC---
ATTACTGGACTAATATTAACAAGAGATGGAGGTAAAAATGAGACA----------------------------
----

CATACTGAGACCTTCAGGCCTGGAGGAGGAGATATAAGGGACAATTGGAGAAGTGAATTATATAAATATAAAG
TAGTAAAAATTGAACCACTA---GGAGTAGCACCCACCAGGGCAAAGAGAAGAGTGGTG--------------
-------------------------GAAAGAGAAAAAAGAGCA---GTTGGA---CTGGGAGCT---------
GTCTTCCTTGGGTTCTTAGGAGCA---GCAGGAAGCACTATG---GGCGCAGCGTCA---
ATAACGCTGACGGTACAA---GCCAGACAA---CTATTG---TCCGGTATAGTGCAACAGCAGAAC---
AATTTG---CTGAAG---GCCATAGAGGCTCAACAACAGATGTTGAGACTCACGGTATGGGGCATT
>KF561435..1
TTCTATTGCAATACATCAAAATTGTTTAATAGCACTTGGGACAACAATAGCACTTGGGACAACAATAGCACTT
GGGAAAACAAT--
----------AGCAATACC------
AACTCAAGTGGCACTATAACTTTCCAATGCAAAATAAAGCAAATTGTAAATATGTGGCAGAGAGTAGGACAAG
CAATGTATGCCCTTCCCATCCAAGGAGTAATAAGGTGTGAGTCAAAT---
ATTACAGGATTATTGTTAACAAGAGATGGAGGGAATAAGAATAAT----------------------------
----

AGTAATAATGAGACTTTCAGACCTGGAGGAGGAGATATGAGGGATAATTGGAGAAGTGTATTATATAAGTATA
AAGTAGTAAAAATTGAACCACTG---GGTGTAGCACCCACCCATGCAAGAAGAAGAGTGGTG-----------
----------------------------GGGAGAGAAAAAAGAGCA---GTCGGA---CTGGGAGCT------
---GTCCTCTTGGGATTCTTAGGAGCA---GCAGGAAGCACTATG---GGCGCGGCGTCA---
CTAACGCTGACGGTACAG---GCCAGACAA---TTATTG---TCCGGTATAGTGCAACAGCAGAGC---
AATTTG---CTAAGG---GCTATAGAGGCTCAACAACATCTGTTGAAACTCACGGTCTGGGGCATT
>DQ168577..1
TTCTATTGCAATACATCAAAACTGTTTAATAGCACTGGGGATACCAATACCACTGCCAAC-------------
----

TCAACAGGAACTATAGAGCTCCAATGCAGAATAAAGCAAATTGTAAATATGTGGCAGAAAGTAGGACAAGCAA
TATATGCCCCTCCCATCCAAGGAATAATAAGGTGTGATTCAAAC---

ATTACAGGACTACTATTAACAAGAGATGGAGGGATTGAGAATGCA----------------------------
----

GATAATGAGACCTTCAGGCCGGGAGGAGGAGATATGAGGGACAATTGGAGAAGCGAATTATATAAGTATAAAG
TAGTAAAAATTGAACCACTA---GGTGTAGCACCCACAAAAGCAAAAAGAAGAGTGGTG--------------
-------------------------GAGAGAGAAAAAAGAGCA---GTC------GTTGGAGCT---------
CTCTTCCTTGGGTTCTTAGGAGCA---GCAGGAAGCACTATG---GGCGCGGCGTCA---
ATAACGCTGACGGTACAG---GCCAGACAA---TTATTG---TCCGGTATAGTGCAACAGCAGAGC---
AATTTG---CTGAGA---GCTATAGAGGCTCAACAACATCTGTTGAAACTCACGGTCTGGGGCATT
>DQ168578..1
TTCTATTGTAATACATCAAACCTGTTTAATAGCACTTGGAATACCAGCTCAATCCACACAGAGCAAAATGCA-
----

ACTAATGAAACTATAACTCTCCAATGCAAAATAAAGCAAATTGTAAATATGTGGCAGAGAGCAGGACAAGCAA
TATATGCCCCTCCCATCCAAGGAGAAATAAGGTGTGACTCAAAC---
ATTACAGGAATATTATTAACAAGAGATGGAGGAAATAATAATAGT----------------------------
----

ACAAATGAGACCTTCAGGCCTGGAGGAGGAGATATGAGAGACAATTGGAGAAGTGAATTATATAAGTACAAAG
TAGTAAAACTTGAACCACTA---GGTATAGCACCCACCCATGCAAAAAGAAGAGTGGTG--------------
-------------------------GAGAGAGAAAAAAGAGCA---GTA------CTGGGAGCT---------
GTCTTCATTGGGTTCTTAGGAGCA---GCAGGAAGCACTATG---GGCGCGGCGTCA---
ATAACGCTGACGGTACAG---GCCAGACAA---TTATTG---TCCGGTATAGTGCAACAGCAAAGC---
AATTTG---CTGCAG---GCTATAGAGGCTCAACAACATCTGTTGAGACTCACGGTCTGGGGCATT
>JN248580..1
TTCTACTGCAATACATCAGCACTGTTTAACAACAGCTTAACA-------------------------------
----

AATGGAACTATAACTCTCCCATGCAGAATAAAGCAAATTGTAAATATGTGGCAGAGAGTAGGACTAGCAATGT
ATGCCCCTCCCATCCAAGGAAACATAACATGTAATTCAAAC---
ATTACAGGACTACTATTAACAAGAGATGGAGGGAATAATAGGAATAATGAG----------------------
----

AATAGTAGTGAACCTGAGACCTTCAGGCCGGGAGGAGGAGATATGAGGGATAATTGGAGAAGTGAATTATATA
AGTATAAAGTAGTAAAAATCGAACCACTA---GGTGTAGCACCTACTAAAGCAAAAAGAAGAGTGGTG-----
----------------------------------GAGAGAGAAAAAAGAGCA---GCCGGA---
CTGGGAGCT---------TTTTTCCTTGGGTTCTTAGGAGCA---GCAGGAAGCACTATG---
GGCGCGGCGTCA---ATAACGCTGACGGTACAG---GCCAGACAA---TTATTG---
TCTGGTATAGTGCAACAGCAGAGC---AATCTG---CTGAGA---
GCTATAGAGGCTCAACAACATCTGTTGAAACTCACGGTCTGGGGCATT
>KX389630..1
TTCTATTGCAATACATCATCACTGTTTAACGGCACCTGGAGGGCCAATGGCACTGACAATAACACTTTCAAC-
----

TTAAATGACACTATAATTATCCCATGCAGAATAAAACAAATTGTAAACATGTGGCAGAAAGTAGGACAAGCAA
TGTATGCCCCTCCCATCAAAGGAGTAATAAGTTGTATTTCAAAC---
ATTACGGGATTACTATTAACAAGAGATGGAGGGGAGCGTAACGAT----------------------------
----

AGTACAAATGAGACCTTCAGGCCTGGCGGAGGAGATATGAGGGACAATTGGAGAAGTGAATTATATAAGTATA
AAGTAGTAAAAATTGAACCATTA---GGTGTAGCACCCACCTCTGCAAAAAGAAGAGTGGTG-----------
----------------------------GAGAGAGAAAAAAGAGCA---GCTGGA---CTAGGAGCT------
---TTCTTCCTTGGGTTCTTAGGAGCA---GCAGGAAGCACTATG---GGCGCGGCGTCG---
ATGACGCTGACGGTACAA---GCCAGACAA---TTATTG---TCTGGTATAGTGCAACAGCAAAGT---
AATTTG---CTGAAA---GCTATAGAGGCTCAACAACAGATGTTGAGACTCACAGTCTGGGGCATT
>KX389629..1
TTCTATTGCAACACATCAGGACTATTTAATAGCAGTTGGCCTAACAATAGCACTTGGAATAATATA-------
----

ACAAATGACAATATAACTCTCCAATGCAGAATAAAGCAATTTGTCAATATGTGGCAGAGAGTAGGACAAGCAA

TGTATGCCCCTCCCATCCAAGGAGTAATAAGGTGTGAATCAAAC---
ATTACAGGACTACTATTAACAAGAGATGGAGGGTATGATAATAGTACC------------------------
----

AATAACACAGAAACATTCAGGCCTGCAGGAGGAGATATGAGAGATAATTGGAGAAGTGAATTGTATAAGTATA
AAGTAGTAAAAATTGAACCGCTG---GGTGTAGCACCCACCCATGCAAAAAGAAGAGTGGTG----------
----------------------------GAGAGAGAAAAAAGAGCA---GTAGGA---CTGGGAGCT------
---GTCTTCCTTGGGTTCTTAGGAGCA---GCAGGAAGCACTATG---GGCGCGGCGTCA---
ATAACGCTGACGGTACAG---GCCAGACAA---TTATTG---TCTGGTATAGTGCAACAGCAAGGC---
AATTTG---CTGAAG---GCCATAGAGGCTCAACAACAGCTGTTGAGACTCACGGTCTGGGGCATT
>JN248585..2
TTCTATTGCAATACATCAGGACTGTTTAATAGTAATTGGCATAGAAATACTACTAATGATACTTGGTATACTA
ACGAC---
-------------------ACA---
GCAAACAGCAGCACTATAACTATCCAATGCAGAATAAAACAAATTGTAAACATGTGGCAGAGAGTAGGACAAG
CAATATATGCTCCTCCCATTGCAGGAAAAATAGAGTGTAAATCAAAC---
ATTACTGGACTATTATTAATAAGAGATGGGGGAATACGAATACTAGC-------------------------
----

AGTACAAATGAGACTTTCAGACCTGTAGGAGGAGAGATGAGAGACAATTGGAGAAGTGAATTATATAAGTATA
AAGTAGTAAAAATTGAACCACTA---GGTGTAGCACCCACCTATGCAAAAAGAAGAGTGGTG----------
----------------------------GAGAGAGAAAAAAGAGCA---GTTGGA---ATGGGAGCT------
---TTCTTCTTTGGGTTCTTAGCAGCA---GCAGGAAGCACCATG---GGCGCGGCGTCA---
ATAACGCTGACGGTACAG---GCCAGACAA---TTATTG---ACTGGTATAGTGCAACAGCAGAGC---
AATCTG---CTGAAG---GCTATAGAGGCTCAACAACAGCTGTTGAGACTCACAGTCTGGGGCATT
>JN248589..1
TTCTATTGCAATACATCAGACCTGTTTAATAGCAGTTGGGATAATAACAATAACACTGATAGCTCAAACCACA
CAGGG---

TCAGGTAACGATACCATAACTCTCCAATGCAGAATAAAGCAAATTATAAATATGTGGCAGAGAGTAGGACAAG
CAATATATGCCCCTCCCATCCAAGGAGTAATAAACTGTGAATCAAAC---
ATTACAGGGCTAATATTAACAAGAGATGGAGAGAATAATACTAAG---------------------------
----

GCAAATGAAACCTTCAGGCCTGGAGGAGGAGATATGAGGGACAATTGGAGAAGTGAATTGTATAAGTACAAGG
TAGTAAAAATTGAACCACTA---GGTGTAGCACCCACCCGTGCAAGAAGAAGAGTGGTG-------------
-------------------------GAGAGAGAAAAAAGAGCG---GTTGGA---CTGGGAGCT---------
GTCTTCCTTGGGTTCTTAGGAGCA---GCAGGAAGCACTATG---GGCGCGGCGTCA---
CTGACGCTGACGGTACAG---ACCAGACAA---TTATTG---TCTGGTATAGTGCAACAGCAGAGC---
AATTTG---CTGAGA---GCTATAGAGGCTCAACAGCATCTGTTGAAACTCACGGTCTGGGGCATT
>JN248590..1
TTCTATTGCAACACATCAAAACTGTTTAATAGCACTTGGTTGTCTAATAGCACTTGGAACACTACTGGCAAC-
----

CAAAATGAAAATATAACACTCCAATGTAGGATAAAGCAAATTATAAATATGTGGCAGAGAGTAGGACAAGCAA
TATATGCCCCTCCTATCCAAGGAGAAATAAAATGTGACTCAAAC---
ATTACTGGACTACTATTAACAAGAGACGGAGGGGAGAGTAACAGT---------------------------
----

ACAGAGGAGACCTTCAGGCCTGGAGGAGGAGATATGAGGGACAATTGGAGAAGTGAATTATATAAGTATAAAG
TAGTAAAAATTGAACCACTA---GGTATAGCACCCACCCATGCAAAAAGAAGAGTGGTG-------------
-------------------------GAGAGAGAAAAAAGAGCA---GTTGGA---CTGGGAGCT---------
GTCTTCCTTGGGTTCCTAGGAGCA---GCAGGAAGCACTATG---GGCGCGGCGTCA---
ATAACGCTGACGGTACAA---GCCAGACAA---TTATTG---TCCGGCATAGTGCAACAGCAAAGC---
AATTTG---CTGAAG---GCTATAGAGGCTCAACAACATCTGTTGAAACTCACGGTCTGGGGCATT
>JN248592..1
TTCTATTGCAATACAACACCACTGTTTAAGAGCGTTTGGAATAAAAATGGGAGCACTTGGAACAGCAATACCA
CTAACAGT--
-------------------ACA---
GAGTCAAATAACACTATAACCCTCCAGTGCAAAATAAAGCAAATTATAAATCTGTGGCAGAGAGTAGGACAAG

```
CAATATATGCCCCCCCCATCCCAGGAGTAATAAGGTGTGAATCAAAT---
ATTACAGGACTACTATTAACAAGAGATGGGGGAAATATTGATGAT----------------------------
-------------------------------------------------------------------------
----------
GATGCAAATGAGACTTTCAGGCCGGGAGGAGGAGATATGAGGGACAATTGGAGAAGTGAATTATATAAGTATA
AAGTAGTAAAAATTGAACCACTA---GGTGTAGCACCCACCCGTGCAAGAAGAAGAGTGGTG-----------
----------------------------GGGAGAGAAAAAAGAGCA---GTTGGA---CTAGGAGCT------
---GTCTTCCTTGGGTTCTTAGGAGCA---GCAGGAAGCACTATG---GGCGCAGCGTCA---
GTAACGCTGACGGTACAA---GCCAGACAA---TTATTG---TCTGGTATAGTGCAACAGCAAAGC---
AATTTG---CTGAGG---GCGATAGAGGCTCAACAACAGTTGTTGAAACTCACGGTCTGGGGCATT
>KX389617..2
TTCTATTGCAATACAACAGGACTGTTTAATAGCACGTGGACTAGCAGTGGCCATGGCACTTATAACATCACTG
ACAGCTCA-----------------------------------------------------------------
-------------------AAA---
AACCTCACAGAGGACATTACACTCCAATGCAGAATAAAGCAAATTATAAATCTGTGGCAGAAAGTAGGGCAAG
CAATATATGCCCCTCCCATCCCAGGAGTAATAAGTTGCAACTCAAAC---
ATTACTGGACTACTATTGACAAGAGATGGAGGGAATAATAATGAT----------------------------
-------------------------------------------------------------------------
-------------
TCAACAGAGACCTTCAGACCTGGAGGAGGAGACATGAGGGACAATTGGAGAAGTGAATTATATAAGTATAAAG
TAGTAAAAATTGAACCACTA---GGTATAGCACCTACCCATGCAAAAAGAAGAGTGGTG--------------
-------------------------GAAAGAGAAAAAAGAGGA---TTTGGA---ATGGGAGCT---------
GTCTTCCTTGGGTTCTTAGGAGCA---GCAGGAAGCACTATG---GGCGCAGCGTCA---
ATAACGCTGACGGTACAG---GCCAGACAA---TTATTG---TCCGGTATAGTGCAACAGCAGAGC---
AATCTG---CTGAAG---GCTATTGAGGCTCAACAGCATCTGTTGAAACTCACAGTCTGGGGCATT
>KX389614..2
TTCTATTGCAATACATCAAAACTGTTTGATAGCACTTGGCCTACAAATAGCACT-------------------
-------------------------------------------------------------------------
----------------------------
TCAAATGGCACTATAACTCTTCAATGCAGAATAAAGCAAATAATCAATATGTGGCAGAGAGTAGGACAGGCAA
TGTATGCCCCTCCCATCAAAGGAGCACTAAATTGTTCTTCAAAC---
ATTACAGGACTACTATTAACAAGAGATGGAGGGATAAGGAATGATACA-------------------------
-------------------------------------------------------------------------
----------
GATAAGAATGAGACTTTCAGGCCGGGAGGAGGAGATATGAGGGACAATTGGAGAAGTGAATTATATAAATATA
AAGTAGTAAAAATTGAACCACTA---GGTGTAGCACCCACCCCTGCAAGAAGAAGAGTGGTG-----------
----------------------------GAGAGAGGAAAAAGAGCA---GTCGGA---ATGGGAGCT------
---GTCTTCCTTGGGTTCTTGGGAGCA---GCAGGAAGCACTATG---GGCGCAGCGTCA---
ATAACGCTGACGGTACAG---GCCAGACAA---TTATTG---TCTGGTATAGTGCAACAGCAGAGC---
AATCTG---CTGAGA---GCTATAGAGGCTCAACAACATCTGTTGAAACTCACGGTCTGGGGCATT
>KX389610..1
TTCTATTGCAATACATCAAAACTGTTTAATAGCAGTTGGAGTAACGACAGTAACACTGACAACAATGACAATA
ACACTGAC-----------------------------------------------------------------
----------------------------
AACTCTAGCATTATAACTCTCCAATGCAGAATAAGGCAAATTGTAAATATGTGGCAGAGAGTAGGACAAGCAA
TGTATGCCCCTCCCATCCCAGGAGTAATTAGTTGTAATTCAAGC---
ATTACAGGACTATTATTAACAAGAGATGGGGGAAATAAGAGTGAA----------------------------
-------------------------------------------------------------------------
----------------
AACGAGACCTTCAGGCCTGGAGGAGGAAATATGAGGGACAATTGGAGAAGTGAATTATATAAGTATAAGGTAG
TAAGAATTGAACCATTA---GGTGTAGCACCCACCCATGCAAAAAGAAGAGTGGTG-----------------
----------------------GAGAGACAAAAAAGAGCA---GTTGGA---CTGGGAGCT---------
GTCCTCTTTGGGTTCTTAGGAGCG---GCAGGAAGCACTATG---GGCGCGGCGTCA---
ATAACGCTGACGGTACAG---GCCAGACAA---TTATTG---TCCGGTATAGTGCAACAGCAGAGT---
AATCTG---CTGAAA---GCTATAGAGGCTCAACAACATCTGTTGAGACTCACGGTCTGGGGCATT
>KX389643..1
TTCTATTGCAACACATCGAACCTGTTTAATAGCAGTTGGGAGAACGGTACCAGATTAAACAACACAAAG----
-------------------------------------------------------------------------
----------------------------
TCAAATGGCACTATAATTCTCCAATGTAGAATAAAGCAAATTGTAAATATGTGGCAGAAAGTGGGACAAGCAA
```

TGTATGCCCCTCCTATCCCAGGAATAATAAGGTGTGAGTCAAAC---
ATCACTGGACTATTATTAACAAGAGATGGAGGGGAAAATAGTTCA---------------------------
----

ACTGAGACTTTCAGACCTGAAGGAGGAAATATGAAGGACAATTGGAGAAGTGAATTATATAAGTATAAAGTTG
TAAAAATTGAACCACTA---GGTGTAGCACCCTCCCGTGCAAAAAGAAGAGTGGTG-----------------
----------------------GAGAGAGAAAAAAGAGCA---GTTGGA---ATAGGAGCT---------
GTCTTCCTTGGGTTCTTAGGAGCA---GCAGGAAGCACTATG---GGCGCGGCGTCA---
ATAACGCTGACGGTACAG---GCCAGACAA---TTATTG---TCTGGTATAGTGCAACAGCAGAAT---
AATTTG---CTGAAA---GCTATAGAGGCTCAACAACAGATGTTGAGACTCACGGTCTGGGGCATT
>KX389644..3
TTCTATTGCAATACATCAGGACTGTTTAATAACACGTTTAATCATGAAACGTTTGATAGCAATATGACGACTG
ATAAC---

AGCACAGGAAATATAACTCTCCCATGTAGAATAAGACAAATTGTAAATATGTGGCAGAGAGTAGGACAAGCAA
TGTATGCCCCTCCCATCAGCGGAGTAATAAGGTGTAATTCAAAT---
ATTACTGGACTAATATTAACAAGAGATGGAGGGAATAACAGTACA---------------------------
----

AATGAGACCTTTAGGCCTGGAGGAGGGGATATGAGGGACAATTGGAGAAGTGAATTATATAAATATAAGGTAG
TAAAAATTGAACCAATA---GGTGTGGCACCCACCTATGCAAGAAGAAGAGTGGTG-----------------
----------------------CAGAGAGAAAAAAGAACA---GTTGGA---CTGGGAGCT---------
GTCCTCTTTGGGTTCTTAGGAGCA---GCAGGAAGCACTATG---GGCGCGGCGTCA---
ATAACGCTGACGGTACAG---GCCAGACAA---TTATTG---TCTGGTATAGTGCAACAGCAGAGC---
AATCTG---CTGAGG---GCTATAGAGGCTCAACAACATATGTTGAAACTCACAGTCTGGGGCATT
>KY953197..5
TTCTATTGCAACACAACACAACTGTTTAATAGTACTTGGGGACTGATTAATAATACTATTTGGGGAAGCTACA
ATAGTAGCTAC---AAT---
-------AGTAGCCAGATA------
AAGGAAAATGATACCATAATTCTCCCATGCAGACTAAAGCAAATTGTAAGAATGTGGCAGAGAGTAGGACAAG
CAATGTATGCCCCTCCTATCCAGGGAGTAATAAAGTGTTATTCAAAC---
ATTACAGGATTACTGTTAACGAGCGATGGAGGGTTTAGGAATACA---------------------------
----

AGTGAGATCGTCAGGCCTGGAGGAGGAGACATGAGGGACAATTGGAGAAGTGAATTATATAAATACAAAGTAG
TAAAAATTATGCCACTA---GGTGTAGCACCCTCCTATGCAAAAAGAAGAGTGGTG-----------------
----------------------CAGAGAGAAAAAAGAGCA---GTTGGA---ATGGGAGCT---------
ATGTTCGTTGGGTTCTTAGGAGCT---GCAGGAAGCACTATG---GGCGCGGCGTCA---
ATAACGCTGACGGTACAG---GTCAGGCAA---TTATTG---TCTGGCATAGTGCAACAGCAGAGA---
AATTTG---CTGAAT---GCTATAGAGGCTCAACAACAGCTGTTGAGACTCACGGTCTGGGGCATT
>KY953198..2
TTCTATTGTGATACATCAAACCTGTTTAATAGCACTTGGGATAATATCACTAGGAACAATAGCACTTGGGACA
AGAGC---
-------------AATACA------
GAGTCAAATGACACTATAACACTCCAATGCAAAATAAAACAGATTGTAAATATGTGGCAGAGAGTAGGACAAG
CAATATATGCCCCTCCCATCGCAGGAGAAATAAGGTGTGAATCAAAT---
ATTACTGGATTATTATTAACAAGAGATGGAGGGGATATAAATGAG---------------------------
----

ACTGAGATCTTCAGGCCTGGAGGGGGAGATATGAGGGACAATTGGAGAAGTGAATTATATAAGTATAAAGTAG
TAAAAATTGAACCCCTA---GGTATAGCACCTACTCATGCAAAAAGAAGAGTGGTG-----------------
----------------------GGGAGAGAAAAAAGAGCA---GTTGGA---CTGGGAGCT---------
GTCTTCTTTGGGTTCTTAGGAGCA---GCAGGAAGCACTATG---GGCGCGGCGTCA---
ATAACGCTGACGGTACAG---GCCAGACAA---TTATTG---TCTGGTATAGTGCAACAGCAGAGC---
AATCTG---CTGAAG---GCTATAGAGGCTCAACAGCATCTGTTGAAACTCACGGTCTGGGGCATT
>KX389647..1
TTCTATTGCAATACATCAGGACTGTTTAATAGCGCTTGGAGGATTAATGGCACTGTTAACCATACAGAATCAA
ATACCAACCAT---

ACAAATAACACCATAATTCTCCCATGCAGAATAAAGCAAATTGTAAATATGTGGCAAAGGGTAGGACAAGCAA

TATATGCCCCTCCCATCCGAGGAATCATAAGGTGTGTATCAAAC---
ATTACAGGACTATTATTAACAAGAGATGGAGGGAATGAGGCAAAT----------------------------
----

AATAGTGAGACCTTCAGACCTGGAGGAGGGGATATGAGGGACAATTGGAGAAGTGAATTATATAAGTATAAAG
TAGTAAAAATTGAACCACTG---GGTGTAGCACCCACCCATGCAAAAAGAAGAGTGGTG--------------
-------------------------GAGAGAGAAAAAAGAGCA---GTTGGA---ATGGGAGCT---------
CTTCTCTTTGGGTTCTTAGGAGCA---GCAGGAAGCACTATG---GGCGCGGCGTCA---
ATAACGCTGACGGTACAA---GCCAGACAA---TTATTG---TCCGGTATAGTGCAACAGCAGAGC---
AATTTG---CTGAGA---GCTATAGAGGCTCAACAACATCTGTTGAAACTCACAGTCTGGGGCATT
>KX389649..1
TTCTATTGCAATACATCAGGACTGTTTAGTAGCGTTTGGAAGAACGAT-------------------------
----

ACCAATGCAACGATAACTCTCCAATGCAGAATAAAGCAAATTATCAATATGTGGCAGAGAGTAGGACAAGCAA
TGTATGCCCCTCCCATCCCAGGAGCAATAAGGTGTGACTCAAAC---
ATTACAGGACTACTATTACTAAGAGATGGAGGAGATAATAATAGT----------------------------
----

ACAAATGAGACTTTCAGGCCTGGAGGAGGAAATATGAAGGACAATTGGAGAAGTGAGTTATATAAGTATAAAG
TAGTAAAAGTTGAACCACTA---GGTATAGCACCTACCCGTGCAAAAAGAAGAGTGGTG--------------
-------------------------ATGAGAGAAAAAAGAGCA---GTTGGA---CTGGGAGCT---------
GCCTTCCTTGGGTTCTTAGGAGCA---GCAGGAAGCACTATG---GGCGCGGCGTCA---
ATAACGCTGACGGCACAG---GCCAGACAA---TTACTG---TCCGGTATAGTGCAACAGCAGAGC---
AATCTG---CTGCAA---GCTATAGAGGCTCAACAGCATCTGTTGAAACTCACTGTCTGGGGCATT
>AF069933..1
TTCTATTGCAGTACATCAAAACTTTTTAATAGCACTTGGAATAACAGTAACATTAGCAGCTCAAATAACACAG
AT---

TCAAATGACACTATAACTATCCAATGCAGAATAAAGCAAATTATAAATATGTGGCAGAGAGTAGGACAGGCAA
TGTATGCCCCTCCCATCCGAGGAGAAATAAGGTGTGAATCAAAC---
ATTACAGGACTACTATTAACAGGAGATGGAGGGGAAAATAATGGGACA-------------------------
----

AACGGGACAGAGACATTCAGGCCTGGAGGAGGAGATATGAGGGACAATTGGAGAAGTGAATTATATAAATATA
AGGTAGCACAAATTGAACCAATA---GGTGTGGCACCCACCCATGCAAAAAGAAGAGTGGTG-----------
----------------------------GAGAGAGAAAAAAGAGCA---GTTGGA---CTGGGAGTT------
---GCCTTCCTTGGGTTCTTAGGAGCA---GCAGGAAGCACTATG---GGCGCAGCGTCA---
ATAACGCTGACGGTACAG---GCCAGACAA---TTATTG---TCTGGTATAGTGCAACAGCAGAGC---
AATTTG---CTGAAG---GCTATAGAGGCTCAACAACATCTGTTGAAACTCACGGTCTGGGGCATT
>AF069941..2
TTCTATTGCAATACATCAGGACTGTTTAATAGCACTTGGAATAATAACAATTGGAATACCACTAGCAATAACA
GCTCA--

AAGTCAAATGAAATTATAAAACTCCAATGCAGAATAAGGCAAATTGTAAATATGTGGCAGAGAGTAGGACAAG
CAATGTATGCCCCTCCCACCCTAGGAGTAATAAGGTGTGAATCAAAC---
ATTACAGGACTACTATTAACAAGAGATGGAGGGAAGCATAATAGT----------------------------
----

ACAAATGAGACTTTCAGGCCTGGAGGAGGAGATATGAGGGACAATTGGAGAAGTGAATTATATAAGTATAAAG
TAGTAAAAATTGAAGCACTA---GGTGTAGCACCTACCTATGCAAAAAGAAGAGTGGTG--------------
-------------------------GAGAGAGAAAAAAGAGCA---GTTGGA---CTGGGAGCT---------
GTCTTCCTTGGGTTCCTAGGAGCA---GCAGGAAGCACTATG---GGCGCGGCGTCA---
ATAACGCTGACGGTACAG---GCCAGACAA---TTATTG---TCCGGTATAGTGCAACAGCAGAGC---
AATCTG---CTGAGG---GCTATAGAGGCTCAACAACATCTGTTGAAACTCACAGTCTGGGGCATT
>L39106..1
TTCTATTGCAATACATCAAAACTGTTTAATAGCACTTGGGATAATAGCAATAGCACTGCCAACCACACAGGG-
----

TCAAATGACACTATAACTCTCCAATGCAGAATAAAGCAAATTGTAAATATGTGGCAGAAAGTAGGACAAGCAA

TGTATGCCCCTCCCATCCAAGGAATAATAAGATGTGATTCAAAC---
ATTACAGGACTACTATTAACAAGAGATGGAGGGAATAATAGTACA----------------------------
----

AATGAAACCTTCAGGCCTGGAGGAGGAGATATGAGGGACAATTGGAGAAGTGAATTATATAAGTATAAAGTAG
TAAAAATTGAACCACTA---GGTGTAGCACCCACCCGTGCAAAAAGAAGAGTGGTG-----------------
----------------------GAGAGAGAAAAAAGAGCG---GTTGGA---CTGGGAGCT---------
GTCTTCCTTGGGTTCTTAGGAGCA---GCAGGAAGCACTATG---GGCGCGCGGTCA---
ATAACGCTGACGGTACAG---GCCAGACAA---TTATTG---TCCGGCATAGTGCAACAGCAGAGC---
AATTTG---CTGAAA---GCTATAGAGGCTCAACAACATCTGTTGAAACTCACGGTCTGGGGCATT
>KX232598..2
TTCTATTGTAATACAACAGGACTATTTAATAGCGCTTGGCAATTTAATAGCGCTTGGCAGGAGAATGACACTG
AA---

ACAGAGGGAAATATAACTCTTCCATGCAAAATAAAGCAAATTATAAATATGTGGCAGAGGGTAGGACAAGCAA
TTTATGCCCCTCCCATCCAAGGAGTAGTAAAGTGTGAGTCAAAC---
ATTACTGGACTACTATTGACAAGAGATGGGGGAATTAAGAATAGT----------------------------
----

ACGAATGAAACTTTCAGGCCTGGAGGAGGAGATATGAGGGACAATTGGAGAAGTGAATTATATAAGTATAAAG
TAGTAAAAATTGAACCATTA---GGTGTAGCACCCACCCGTGCAAAAAGAAGAGTGGTG--------------
-------------------------GAGAGAGAAAAAAGAGCA---ATTGGTGGACTGGGAGCT---------
GCATTCCTTGGGTTCTTAGGAGCA---GCAGGAAGCACTATG---GGCGCGGCGTCA---
ATAACGCTGACGGTACAG---GCCAGACAA---TTATTG---TCTGGTATAGTGCAACAGCAGAGG---
AATTTG---CTGAAG---GCTATTGAGGCTCAACAACAGATGTTGAGACTCACGGTCTGGGGCATT
>KY658712..3
TTCTATTGTAATACAACAAAACTATTTAATAGCACTTGGAATGCAACAAATCCATCTAATAGCAGCTAT----
----

GGCAACGAAACTATAACTCTTCCATGCAGAATAAAGCAAATTATAAATATGTGGCAGAGGGTAGGACAAGCAA
TTTATGCCCCTCCCATCCAAGGAATAATAAAGTGTGAGTCAAAC---
ATTACTGGACTACTATTGACAAGAGATGGGGGAACAACTAATAGCTCG-------------------------
----

AAGAATGAAACTTTCAGGCCTGGAGGAGGAGACATGAGGGATAATTGGAGAAGTGAATTATATAAGTATAAAG
TAGTAAAAATTGAACCATTA---GGTATAGCACCCACCCCTGCAAGAAGGAGAGTGGTG--------------
-------------------------GAGAGAGAAAAAAGAGCA---ATTGGT---GTGGGAGCT---------
GTATTCCTTGGGTTCTTATCAGCA---GCAGGAAGCACTATG---GGCGCGGCGTCA---
ATAACGCTGACGGTACAG---GCCAGACAA---TTATTG---TCTGGTATAGTGCAACAGCAAAGT---
AATTTG---CTGAAG---GCTATTGAGGCTCAACAACAGATGTTGAGACTCACGGTCTGGGGCATT
>KX232616..2
TTCTATTGTGATACAACACAACTATTTAATAGCACTTGGAATACAACACAACTCAACAATGACAACGAGACAG
AG---

GACAATACAACTCTTACTCTTCCATGCAGAATAAAGCAAATTGTAAATATGTGGCAGAGGGTAGGACAAGCAA
TTTATGCCCCTCCCATCCCAGGAATAATAGAGTGTGAATCAAAC---
ATTACTGGACTACTGTTGACAAGAGATGGGGGAAATAGTGATAGC----------------------------
----

TCGAATGAAACTTTTAGGCCTGGAGGAGGAGACATGAGAGACAATTGGAGAAGTGAATTATATAAATATAAAG
TAGTAAAAATTGAACCACTA---GGAGTAGCACCCACCAGGGCAAAGAGAAGAGTGGTG--------------
-------------------------GAGAGAGAAAAAAGAGCA---GTTGGA---CTAGGAGCT---------
TTGTTCATTGGGTTCTTAGGAGCA---GCAGGAAGCACTATG---GGCGCGGCGTCA---
GTGACGCTGACGGTACAG---GCCAGACAA---TTATTG---TCTGGTATAGTGCAACAGCAAAGC---
AATTTG---CTGAGG---GCTATTGAGGCTCAACAACAGATGTTGAGACTCACGGTCTGGGGCATT
>KX232622..2
TTCTATTGTAATACAACAAAACTATTTAATAGCACTTGGAATAGCAATGGCACTTGGAATAGCACTGGGAAGG
ACAATAGCACT--
-------------------GGA---
ACAGGGAACGATACAATAACTCTTCCATGCAAAATAAAGCAAATTATAAATATGTGGCAGAGGGTAGGACAAG

CAATTTATGCCCCTCCTATCCCAGGAGTAATACAGTGTGAGTCAAAC---
ATTACTGGACTACTATTGACAAGAGATGGGGGAGTTAATAGTACT----------------------------
----

ACAAATGAAACTTTCAGGCCTGGAGGAGGAGACATGAGGGACAATTGGAGAAGTGAATTATATAAGTATAAAG
TAGTAAAAATTGAACCAGTA---GGTGTAGCACCCAACCGTGCAAGAAGGAGAGTGGTG--------------
-------------------------GAGAGAGAAAAAAGAGCA---ATTGGA---ATGGGAGCT---------
TTTTTCCTTGGGTTCTTAGGAGCA---GCAGGAAGCACTATG---GGCGCGGCGTCA---
ATAACGCTGACGGTACAC---GCCAGACAA---TTATTG---TCTGGTATAGTGCAACAGCAAAGT---
AATTTG---CTGAAG---GCTATTGAGGCTCAACAACAGATGTTGAGACTCACGGTCTGGGGCATT
>KP411845..2
TTCTATTGTAATACTTCAGGCCTGTTTAACAGCACTTGGGATGGCACAGCACAAAGCAACACAGAT-------
----

GATGATGACACTATCCTTCTCCCGCGAAAAATAAATATAATTATAAATATGAGGAGAAGAGTAGGACTATCAA
TATCTGCTCCTCCTAGACGAGGAACAAGGAGGTGTACTTCTATT---
ATTACAGTACTACTATTAACAGGAGGTGGGAGTACTACTAATACT----------------------------
----
-------------AATGATGACATCTTG---
CCTGCAGGAGGAGAAAAGATGAACTACTGTAGAAGAGAACAATATATATATAAAGTAGTAATAATAGCAGCAT
TA---GTAGCAGCACCCCCCCCCTCCTCAAGAAGAGTAGTG--------------------------------
-------CTGAGAAAAAAAAGAGCA---GCAGTA---GTAGTAGCA---------
GTCGTCTTTGTCGTCTTAAGAGCA---GCAGGAAGCAGTATG---
ATCGCGTCGTCGATAATGATGATGACAGTAGAC---GCATACCAG---TGTTTG---
TCTGGTATAGTGCAACAGCAGTAC---AATTTG---CTGTAT---
GCTAATGAGGTTCAACAACATCTGTTGAAACTCTCATTTTGGGGCATT
>MF373200..1
TTCTATTGCAATACATCAGCATTGTTTAATAGCACTTGGGTTAATAACACTTGGAATAACGAGATCAACGAG-
----

ACCAACAAGACCATAACTCTCCAATGCAGGATAAAGCAAATTATAAATATGTGGCAGAGAGTAGGACAAGCAA
TGTATGCCCCTCCCATCCCAGGAGCAATAAAATGTGAATCAAAC---
ATTACAGGACTACTACTGACAAGAGATGGAGGAAATAATAATACA----------------------------
----

ACTGAGACTTTTAGGCCTGGAGGAGGAGACATGAGGGACAATTGGAGAAGTGAATTATATAAGTATAAAGTAG
TAAAAATTGAACCACTA---GGTGTAGCACCCAGTCGTGCAAAAAGAAGAGTGGTG-----------------
----------------------GAGAGAGAAAAAAGAGCAGTTGTTGGA---ATGGCAGCT---------
GTTTTCCTTGGTTTCTTAGGAGCA---GCAGGAAGCACTATG---GGCGCGGCGTCA---
ATAACGCTGACGGTACAG---GCCAGACAA---TTATTG---TCTGGTATAGTGCAACAGCAGAGC---
AATCTG---CTGAAG---GCTATAGAGGCTCAACAACATATGTTGAGACTCACGGTCTGGGGCATT
>AF107770..2
TTCTATTGCAATACATCAAATCTGTTTAATAGAACTTGGAATCATAATGGCACTTGGAATGCACCAGGACCGT
TTAATGACACT--
GAGGATAAAACAATAAATGGC------------
ACTGAGGATAAAACAATAACTCTCCAATGCAGAATAAAGCAAATTGTGCGTATGTGGCAGAAAGTAGGACAAG
CAATGTATGCCCCTCCCATCCCAGGAGAAATAAGGTGTGAATCAAAC---
ATTACAGGACTACTATTAACAAGAGATGGAGGGAATGATAATAAT----------------------------
----

AATACAGAGACCTTCAGGCCTGGAGGAGGAGATATGAGGGACAATTGGAGAAGTGAATTATATAAATATAAAG
TAGTAAAAATTGAACCACTA---GGTGTAGCACCCTCCCATGCAAAAAGAAGAGTGGTG--------------
-------------------------GAGAGAGAAAAAAGAGCACTTGTTGGA---CTGGGAGCT---------
TTCTTCTTTGGGTTCTTAGGAGCA---GCAGGAAGCACTATG---GGCGCGGCGTCA---
ATAACGCTGACGGTACAG---GCCAGACAA---TTATTG---TCTGGTATAGTGCAACAGCAGAGC---
AATCTG---CTGAAG---GCTATAGAGGCTCAACAACATCTGTTGAGACTCACGGTCTGGGGCATT
>HM215281..1
TTCTATTGCAATACAACACCACTGTTTAATAGCACTTGGTACAGCAATAACACTGCCAATAGCTCAAACAGCA
CA---

GAGTTAAATGGAACTATAACTCTCCAATGCAGAATAAAGCAAATTGTAACTATGTGGCAAAGGGTAGGACAGG
CAATGTATGCCCCTCCCATCCCAGGAGAAATAAGGTGTGAATCAAAC---
ATTACTGGACTACTATTAACAAGAGATGGAGGGGGGAATAATACA----------------------------
----

AATACAAATGAGACCTTTAGGCCTGGAGGAGGAGATATGAGGGACAATTGGAGAAGTGAATTATATAAGTATA
AAGTAGTAAAAATTGAACCACTA---GGTGTAGCACCCACCCCTGCAAAAAGAAGAGTGGTG-----------
----------------------------GAGAGAGAAAAAAGAGCA---GTTGGA---CTGGGAGCT------
---GTCTTCTTTGGGTTCTTAGGAGCA---GCAGGAAGCACTATG---GGCGCGGCGTCA---
TTAACGCTGACGGTACAG---GCCAGACAA---TTATTG---TCCGGTATAGTGCAACAGCAAAGT---
AATTTG---CTGAAG---GCTATAGAGGCTCAACAACATCTGTTGAAACTCACAGTCTGGGGCATT
>HM215292..1
TTCTATTGCGATACATCAAAACTGTTTAATAGCACTTGGACCAAAAATAGCACTTGGAACACTACAGAA----
----

TTAGGTGGCAATATAACGCTCCAGTGCAAAATAAAGCAAATTATAAATACGTGGCAAAAAGTAGGACTAGCAA
TGTATGCCCCTCCCATCCAAGGAATAATAAGGTGTGATTCAAAC---
ATTACAGGACTACTATTAACAAGAGATGGAGGGAATAATAATGCA----------------------------
----

AGTGAGACTGAGACCTTCAGGCCTGGAGGAGGAGATATGAGGGACAATTGGAGAAGTGAATTATATAAATACA
AAGTAGTAAAAATTAAACCACTA---GGTGTCGCACCCACCCATGCAAAAAGAAGAGTGGTG-----------
----------------------------GAGAGAGAAAAAAGAGCA---GTTGGA---CTGGGAGCT------
---GTTTTCCTTGGGTTCTTAGGAGCA---GCAGGAAGCACTATG---GGCGCGGCGTCA---
ATAACGCTGACGGTACAG---GCCAGACAA---TTATTG---TCCGGTATAGTGCAACAGCAGAGC---
AATTTG---CTGCAA---GCTATAGAGGCTCAACAACATCTGTTGAAACTCACGGTCTGGGGCATT
>KT223759..2
TTCTATTGCAATACATCAAAGCTGTTTAATAGCACTTGGAATGCCACTGACATCAATAGCAATGTCAGCACAG
ACTCAGGG---

AAAAATGACACTATAACTCTCCCATGCAGAATAAAGCAAATTATAAGGATGTGGCAGAGCGTAGGGCAAGCAA
TGTATGCCCCTCCTATCCAAGGAATAATAAAGTGTGAATCAAAC---
ATTACAGGACTACTATTAACAAGAGATGGAGGGAAGGAAAACAAT----------------------------
----

AGTACAAATGAGACTTACAGGCCAGGAGGAGGAGACATGAGGGACAATTGGAGAAGTGAATTATATAAGTATA
AAGTAGTGAAAATTGAACCATTT---GGTATAGCACCCACCCTTGCAAGGAGAAGAGTGGTG-----------
----------------------------GAGAGAGAAAAAAGAGCA---GTTGGA---CTGGGAGCT------
---GTCTTCCTTGGGTTCTTAGGAGCA---GCAGGAAGCACTATG---GGCGCGGCGTCA---
GTGACGCTGACGGTACAA---GCCAGACAA---TTGTTG---TCTGGTATAGTGCAACAGCAGAGC---
AATCTG---CTGAAG---GCTATAGAGGCTCAACAACACTTGTTGAGACTCACAGTCTGGGGCATT
>KT223760..2
TTCTATTGCAACACATTAGGACTGTTTAATAGCACTTGGAACTGGGAATGGCACTTGGATAACAATAGCTCAA
TT---

AGCTATGCTCGTGTAACTCTCCAATGCAAAATAAAGCAAATTGTAAATATGTGGCAGAGAGTAGGACAAGCAA
TGTATGCCCCTCCCATCCCAGGGGAACTAAGGTGTGATTCAAAT---
ATTACAGGACTATTATTAACAAGAGATGGGGGA--

-
AAAATAATACTAATGAATGAGACCTTCAGGCCTGGAGGAGGAGATATGAGGGACAATTGGAGAAGTGAATTAT
ATAAGTATAAAGTAGTAAAATTTGAACCACTA---GGTGTAGCACCTACCCATGCAAAAAGAAGAGTGGTG--
-------------------------------------CAGAGAGAAAAAAGAGCA---GTTGGA---
ATGGGAGCT---------CTCTTCCTTGGGTTCTTAGGAGCA---GCAGGAAGCACTATG---
GGCGCGGCGTCA---ATAACGCTGACGGTACAG---GCCAGACAA---TTATTG---
TCTGGTATAGTGCAACAGCAGAGT---AATCTG---CTGAAG---
GCTATAGAGGCTCAACAACAGCTGTTGAGACTCACAGTCTGGGGCATT
>AJ251056..1
TTCTATTGCAATACAGCAGAACTGTTTAATAGCACTTGGGCTAGCAATACTAATGGCATTTGGGCTAGCAATA
TAAATGCTAGC--

----------AATAACAAG------
GACGCAAATGACACTATAACTCTCAAATGCAAAATAAAACAAATTATAAATATGTGGCAGAAAGTAGGACAAG
CAATATATGCCCCTCCCATCGAAGGAGTAATAAGGTGTGATTCAAAC---
ATTACAGGAATACTATTAACAAGAGATGGAGGGGATAATACTAAT----------------------------
----

GGTGATGAGACTTTCAGGCCTGGAGGAGGAGATATGAGGGACAACTGGAGAAGTGAACTATATAAGTATAAAG
TAGTAAAAATTGAACCACTA---GGTGTAGCACCCTCCCATGCAAAAAGAAGAGTGGTG--------------
-------------------------GAGAGAGAAAAAAGAGCA---GTTGGA---CTGGGAGCT---------
GTCTTCCTTGGGTTCTTAGGAGCA---GCAGGAAGCACTATG---GGCGCAGCGTCA---
ATAACGCTGACGGTACAG---GCCAGACAA---TTATTG---TCTGGTATAGTGCAACAGCAGAAC---
AATCTG---CTGAAG---GCTATAGAGGCTCAACAACATTTGTTGAAACTCACGGTCTGGGGCATT
>AJ251057..2
TTCTATTGCAATACATCAGGACTGTTTAATGGCACTTGGAATGGCACTAGCAGCTCAAACCACACATGG----
----

GTAAATGACACTATAACTCTCCAATGCAGAATAAGGCAAATTGTAAATATGTGGCAGAGAGTAGGACGAGCAA
TGTATGCCCCTCCCATCCAGGGAGAAATAAAGTGTGTATCAAAC---
ATTACTGGACTACTATTAACAAGAGATGGAGGGGATAATAATAGT----------------------------
----

ACAAATGAGACTTTCAGGCCTGGAGGAGGAGATATGAGGGACAATTGGAGAAGTGAATTATATAAATATAAAG
TAGTAAAAATTGAACCACTG---GGTGTAGCACCCTACCGTGCAAAAAGGAGAGTGGTG--------------
-------------------------GAGAGAGAAAAAAGAGCA---GTTGGA---ATAGGAGCT---------
GTCCTCCTTGGGTTCTTAGGAGCA---GCAGGAAGCACTATG---GGCGCGGCGTCA---
ATGACGCTGACGGTACAG---GCCAGACAA---TTATTG---TCTGGTATAGTGCAACAGCAGAGC---
AATCTG---CTGAGG---GCTATAGAGGCTCAACAACATCTGTTGAAACTCACAGTCTGGGGCATT
>HM215264..2
TTCTATTGCAATACATCAGAACTGTTTAATAGCACTTGGACTAGCAATAACACTTGGGGTAGCAGTAGCTTA-
----

AATCACACAAATATAACTCTCCAATGCAGAATAAAGCAAATTATAAGAATGTGGCAGAGAGTAGGACAAGCAA
TGTATGCCCCTCCTATCAAAGGGGAAATAAGGTGTGAATCAAAC---
ATTACTGGACTACTATTAATAAGAGATGGA---ATTGTTAATAGT----------------------------
----

ACAAATGAGACCTTCAGGCCTGGAGGAGGAGATATGAGGGACAATTGGAGAAGTGAATTATATAAATATAAAG
TAGTAAAAATTGAACCACTA---GGGGTAGCACCTAACCGTGCAAAAAGAAGAGTGGTG--------------
-------------------------GAGAGAGAAAAAAGAGCA---GTTGGA---CTGGGAGCT---------
TTCTTTCTTGGGTTCTTAGGAGCA---GCAGGAAGCACTATG---GGCGCGGCGTCA---
ATGACGCTGACGGTACAG---GCCAGACAA---TTATTG---TCCGGTATAGTGCAACAGCAGAGC---
AATTTG---CTGAAG---GCTATAGAGGCTCAACAACATCTGTTGAGACTCACGGTCTGGGGCATT
>HM215290..1
TTCTATTGTAATACATCAGCACTGTTTAATAGCACTTGGAATGGCACTGACAGTGCAAACAAGACAAAG----
----

TCAAATGAAACTATAACTCTCCAATGCAAAATAAAGCAAATTATAAATATGTGGCAGAGAGTAGGACAAGCAA
TGTATGCCCCTCCCATCCAAGGAGAAATAAAGTGTACATCAAAC---
ATTACAGGACTACTACTAACAAGAGATGGAGGGAATACTAGTGGT----------------------------
----

GCAAATGAGACTTTCAGGCCTGGAGGAGGAGACATGAGGGACAATTGGAGAAGTGAGTTATATAAATATAAAG
TAGTAAAAATTGAGCCACTA---GGTGTAGCACCCACCCCTGCAAAAAGAAGAGTGGTG--------------
-------------------------GGGAGAGAAAAAAGAGCA---GTTGGA---CTGGGAGCT---------
GTCTTCCTTGGGTTCTTAGGAGCA---GCAGGAAGCACTATG---GGCGCGGCGTCA---
ATAACGCTGACGGTACAG---GCCAGACAA---TTATTG---TCTGGCATAGTGCGACAGCAGAGC---
AATCTG---CTGAGG---GCTATAGAGGCTCAACAACATCTGTTGAAACTCACGGTCTGGGGCATT
>HM215291..1
TTCTATTGCAATACATCAAAACTATTTAATAGCACTTGGAATAGCACTGAAAATAACAATACCAGTGAC----
----

ACAAATGGTATTATAACTCTCCAATGCAGAATAAAGCAAATTGTAAATATATGGCAGAGAGTAGGACAAGCAA
TGTATGCCCCTCCTATCCAAGGAGTAATAAGGTGTGAATCAAAC---
ATTACAGGACTACTATTAACAAGAGATGGAGGGGATAATGATAGT----------------------------
----

ACAAATGAGACCTTCAGGCCTGGAGGAGGAGATATGAGGGACAATTGGAGAAGTGAATTATATAAGTATAAGG
TAGTACAAGTTGAACCACTA---GGTGTGGCACCCACCCATGCAAGAAGAGAGTGGTG--------------
-------------------------GAGAGAGGAAAAAGAGCA---GTTGGA---GTGGGAGCT---------
GTTTTCCTTGGGTTCTTAGGAGCA---GCAGGAAGCACTATG---GGCGCGGCGTCA---
ATAACGCTGACGGTACAG---GCCAGACAA---TTATTG---TCTGGTATAGTGCAACAGCAGAGC---
AATTTG---CTGAAG---GCTATAGAGGCTCAACAACATCTGTTGAAACTCACGGTCTGGGGCATT
>HM215293..1
TTCTATTGCAATACATCAAAGCTGTTTAATAGCACTTGGGGTAACAATACCACTAACAGCTCATCAGAATCAA
AT---

AACAATGACACTCTAATTCTCCAATGCAGAATAAGACAAATTGTAAGGATGTGGCAGAGAGTAGGACAAGCAA
TGTATGCCCCTCCCATCAGAGGAGAAATAAGGTGTGAGTCAAAC---
ATTACAGGGCTACTATTAACAAGAGATGGAGGGATTAATAATACA----------------------------
----

AATAATGAGACCTTCAGGCCTGGAGGAGGAGATATGAGGGACAATTGGAGAAGTGAATTATATAAGTATAAAG
TAGTAAAAATTGAACCACTA---GGTGTAGCACCTACCCATGCAAAAAGAAGAGTGGTG--------------
-------------------------GAGAGAGAAAAAAGAGCA---GTTGGA---CTGGGAGCT---------
TTCTTCCTTGGGTTCTTAGGAGCA---GCAGGAAGCACTATG---GGCGCGGCGTCA---
ATAACGCTGACGGTACAA---GCCAGACAA---TTACTG---TCTGGTATAGTGCAACAGCAGAGC---
AATCTG---CTGAGA---GCAATTGAGGCTCAACAACATCTGTTGAGACTCACGGTCTGGGGCATT
>JX447154..3
TTCTATTGCAATACATCAGGACTGTTTAATAGCACTTGGAATCACAGGAGCACTTGGAATAGCACTGATCACA
ATATCTCT---
-------------------GAC---
AGCTCAAACCCCAATATAACTCTCCAATGCAGAATAAAGCAAGTTATAAATATGTGGCAGAGAGTAGGACAAG
CAATGTATGCCCCTCCCATCCCAGGAGTAATAAGGTGTGAATCAAAC---
ATTACTGGACTGTTATTAACAAGAGATGGAGGAAATGAGAGTAGT----------------------------
----

ACAAATGAGACTTTCAGACCTGGAGGAGGAGACATGAGGGACAATTGGAGAAGTGAATTATATAAGTATAAAG
TAGTAAAAATTGAACCAGTG---GGTGTAGCACCCTCCCGTGCAAAAAGAAGAGTGGTG--------------
-------------------------GAGAGAGAAAAAAGAGCA---GTTGGA---CTGGGAGCT---------
GTCTTCCTTGGGTTCTTAGGAGCA---GCAGGAAGCACTATG---GGCGCGGCGTCG---
GTGACGCTGACGGTACAG---GCCAGACAG---TTATTG---TCTGGTATAGTGCAACAGCAGAGC---
AATTTG---CTGAGG---GCTATAGAGGCTCAACAACATTTGTTGAAACTCACGGTCTGGGGCATT
>KF716488..1
TTCTATTGCAACACCTCAGGACTGTTTAATAGCAGCTTCAATAGTAGCAGCATCAATGACACAGACACAGAG-
----

TTAACTGGCAATATAACTCTTCCATGCAGAATAAAACAGATTGTAAATATGTGGCAGAAGGTAGGAAAAGCAA
TGTATGCCCCCCCTATTGAAGGAATAATAAGGTGTGAGTCAAAC---
ATTACAGGACTGATATTAACAAGAGATGGAGGGGGCAATACAACA----------------------------
----

GAGGAGACGGAGACCTTCAGGCCTGGAGGAGGAGATATGAGGGACAATTGGAGAAATGAATTATATAAATATA
AGGTAGTGAGAATTGACCCAATA---GGTGTAGCACCCACTAAGGCAAAAAGAAGAGTGGTG-----------
----------------------------GAGAGAGGAAAAAGAGCA---GTTGGA---CTGGGAGTT------
---GCCTTCCTTGGGTTCTTAGGAGCA---GCAGGAAGCACTATG---GGCGCGGCGTCA---
ATAACGCTGACGGTACAG---GCCAGACAA---TTATTG---TCTGGTATAGTGCAACAGCAGAGC---
AATCTG---TTGAGA---GCTATAGAGGCTCAACAACATCTGTTGAAACTCACAGTCTGGGGCATT
>AY444811..3
TTCTATTGCAATACATCGGGACTGTTTAATAGTACTTTTAATAACACTCAGGCCAACTCAAGTTACACCTCAG
GG---

ACAAATGATACTATACTTCTCCAATGCAGAATAAGGCAAATTATAAGAATGTGGCAGAGAGTAGGACAAGCAA
TGTATGCCCCTCCCATCCAAGGAGAAATAAGGTGTGAATCAAAC---
ATTACTGGACTACTATTAACAAGAGATGGAGGGGATACTAGTGAT----------------------------
----

AATAGTACCGAGACCTTCAGGCCTGGAGGAGGAGATATGAGGGACAATTGGAGAAGTGAATTATATAAGTATA
AAGTAGTAAAAATTGAACCAATA---GGTATAGCACCCACCAAGGCAAAAAGAAGAGTGGTG-----------
----------------------------CAGAGAGAAAAAAGAGCA---GTTGGA---ATAGGAGCT------
---GTCCTCTTTGGGTTCTTGGGAGCA---GCAGGAAGCACTATG---GGCGCAGCGTCA---
ATAACGCTGACGGTACAG---GCCAGACAA---TTATTG---TCCGGTATAGTGCAACAGCAAAGC---
AATTTG---CTGAAG---GCCATAGAGGCTCAACAACATCTGTTGAGACTCACAGTCTGGGGCATT
>DQ869018..1
TTCTACTGCAATACAACAGCACTGTTTAATAGCACTTGGGATAGCAATACCACTGCCAGCGAAGAGTCAAATA
GC---

ACCATAGATGACATTATAACTCTCCAATGCAGAATAAAGCAAGTTGTAAACATGTGGCAGAAAGTAGGACAAG
CAATGTATGCCCCTCCCATTGCAGGAGAAATAAGGTGTGAATCAAAC---
ATTACAGGACTACTATTAACAAGAGATGGAGGGGATAATAATAAC----------------------------
----

AGTGAGACCTTCAGACCTGGAGGAGGAGATATGAGGGACAATTGGAGAAGTGAATTATATAAATATAAAGTAG
TAAAAATTGAACCGCTA---GGAGTAGCACCCTCCAGGGCAAAAAGAAGAGTGGTG-----------------
----------------------GAGAGAGAAAAAAGAGCA---GTTGGA---CTGGGAGCT---------
GTCTTCCTTGGGTTCTTAGGAACA---GCAGGAAGCACTATG---GGCGCGGCGTCA---
ATAACGCTGACGGTACAG---GCCAGACAA---TTATTG---TCCGGTATAGTGCAACAGCAGAGC---
AATCTG---CTGAAG---GCCATAGAGGCTCAACAGCAACTGTTGAGACTCACGGTCTGGGGCATT
>JF320297..1
TTCTATTGCAATACATCAGAACTATTTAATAGCAGTTGGAATAATGACACTGCCAGCTCAAATAACACAGGG-
----

TCAAATGACACTATAACTCTCCAATGCAGAATAAAGCAAATTGTAAATATGTGGCAGAAGGTAGGACAAGCAA
TGTATGCCCCCCCCATCAAAGGAGTAATAAGGTGTGAATCAAAC---
ATTACAGGACTAATATTAACAAGAGATGGAGGGGATAATAATAATAAT-------------------------
----

AGTGTAAATGAGACCTTCAGGCCTGGAGGAGGAGATATGAGGGACAATTGGAGAAGTGAATTATATAAGTATA
AAGTAGTAAAAATTGAACCACTA---GGTATAGCGCCCACCCATGCAAGAAGAAGAGTGGTG-----------
----------------------------CAGAGAGAAAAAAGAGCA---GTTGGA---CTAGGAGCT------
---CTCTTCCTTGGGTTCTTAGGAGCA---GCAGGAAGCACTATG---GGCGCGGCGTCA---
ATAACGCTGACGGTACAG---GCCAGACAA---TTATTG---TCCGGTATAGTGCAACAGCAGAGC---
AATCTG---CTGAAA---GCTATAGAGGCTCAACAACATCTGTTGAAACTCACGGTCTGGGGCATT
>AY736840..1
TTCTATTGCAATACATCAATACTGTTTAATAGCACTTGGGACAATAGCACTGTCAACACAAATAATATCACAG
AG---

CCAAATGATACTATAACCCTCCAATGCAGAATAAAGCAAATTATAAATATGTGGCAGAAAGTAGGGCAAGCAA
TGTATGCCCCTCCCATCAAAGGAATCATAAGGTGTGATTCAAAC---
ATTACTGGATTACTATTAACAAGAGATGGAGGGAATAATAATAGT----------------------------
----

ACAAATGAGACCTTCAGACCTGGAGGAGGAGATATGAGGGACAATTGGAGAAGTGAATTATATAAGTATAAAG
TAGTAAAAATTGATCCAATA---GGGGTGGCTCCCACCCATGCAAAAAGAAGAGTGGTG--------------
-------------------------GAGAAAGAAAAAAGAGCA---GTTGGA---CTGGGAGCT---------
GTCTTCATTGGGTTCTTAGGAGCT---GCAGGAAGCACTATG---GGCGCGGCGTCA---
ATAACGCTGACGGTACAA---GCCAGACAA---TTATTG---TCTGGTATAGTGCAACAGCAAAGC---
AATTTG---CTGAAG---GCTATAGAGGCTCAACAACATCTGTTGAGACTCACAGTCTGGGGCATT
>KU168266..2
TTCTATTGCAATACATCAGGCCTGTTTAATAGTACTTGGAATGTGACCACCAGCCTCAACCAGAGTGAA----
----

GCGGACGTAAATATAACTCTCCCATGCAAAATAAAACAAATTGTACGTATGTGGCAGGGAGTAGGACAAGCAA
TGTATGCCCCTCCCATTCAAGGAGTAATTAACTGTACATCAAAC---
ATCACAGGGCTACTGCTAACAAGAGACGGGGGGAGGGATAATGGT---------------------------
----

GCAAATGAGACGTTCAGACCCGGAGGAGGAAATATAAAGGACAATTGGAGAAGTGAATTATATAAATATAAGG
TAGTAAAAATTGAACCACTA---GGTGTAGCACCCACCCGTGCAAAAAGAAGAGTGGTG-------------
------------------------GAGAGAGAAAAAAGAGCA---GTTGGA---CTGGGAGCT---------
GTCCTCTTTGGGTTCTTAGGAGCA---GCAGGAAGCACTATG---GGCGCGGCGTCA---
ATAACGCTGACGGTACAG---GCCAGACAA---TTATTG---TCTGGTATAGTGCAACAGCAGAAC---
AATCTG---CTGAGG---GCTATAGAGGCTCAACAGCAAATGTTGAAACTTACGGTCTGGGGCATT
>KU168267..1
TTCTATTGCAATACATCAGATCTGTTCGATAGCACTTGGACTTCAAACCACACCTCAAACCACACAGGGTCAG
ATGAGACC--
------------------ATA---
ACGTCAAATGACACTATAATTCTCCAATGTAGAATAAAGCAAATTGTAAGGATGTGGCAGACAGTAGGACAAG
CAATGTATGCCCCTCCCATCCCAGGAGAAATAAGGTGTGAATCAAAC---
ATTACTGGACTACTATTAACAAGAGATGGAGGGCAAACAGAAAAT---------------------------
----

AATAATGAGACCTTCAGGCCTGGAGGAGGAAATATGAAGGACAATTGGAGAAGTGAATTATATAAGTATAAAG
TAGTAAAAATTGAACCACTA---GGTGTAGCACCCACCCCTGCAAGAAGAAGAGTGGTG-------------
------------------------CAGAGAGAAAAAAGAGCA---GTTGGACTACTGGGAGCT---------
GTCTTCCTTGGGTTCTTAGGAGCA---GCAGGAAGCACTATG---GGCGCGGCGTCA---
ATAACGCTGACGGTACAG---GCCAGACAA---TTACTG---TCTGGTATAGTGCAACAGCAAAGC---
AATTTG---CTGAAA---GCTATAGAGGCTCAACAACATCTGTTGAGACTCACGGTCTGGGGCATT
>MF510462..1
TTCTATTGCAATACATCAGACCTGTTTAATAGCACTTGGGATAACAATACCAACAGTTCAAACTTCACA----
----

GGTAATGACACTATAACTCTCCAATGCAGAATAAAGCAATTTGTAAATATGTGGCAGAGGGTAGGACAAGCAA
TGTATGCCCCTCCCATCGAAGGAAGAATAAGATGTGAATCAAAT---
ATTACTGGACTACTATTAACAAGAGATGGAGGAGAAGGTAATAAT---------------------------
----

AGGACAAATGAAACCTTCAGGCCTGGAGGAGGAGATATGAGGGACAATTGGAGAAGTGAATTATATAAGTATA
AAGTAGTAAAAATTGAACCACTA---GGTGTAGCACCCACCCATGCAAAAAGAAGAGTGGTG----------
----------------------------CAGAGAGAAAAAAGAGCA---GTGGGA---CTGGGAGCT------
---GTCTTCCTTGGGTTCTTAGGAGCG---GCAGGAAGCACTATG---GGCGCGGCGTCA---
ATAACGCTGACGGTACAG---GCCAGACAA---TTATTG---TCTGGTATAGTGCAACAGCAGAGC---
AATTTG---CTGAAG---GCTATAGAGGCTCAACAACATCTGTTGAAACTCACAGTCTGGGGCATT
>AF082485..1
TTCTACTGTAATACAACAAAACTGTTTGATAGCACTTGGAATGGTACTGAAGAGTTAGATAACACT-------
----

GAAGGAGATATAGTCACACTCCCATGCAGAATAAAACAAATTATAAACATGTGGCAGGAAGTAGGAAAAGCAA
TGTATGCCCCTCCCATCGCGGGACAAATTAGATGTTCCTCAAAT---
ATTACAGGACTGCTATTAACAAGAGATGGTGGTTACCAGAGCAAT---------------------------
----

GTCACCGAGATCTTCAGACCTGGAGGAGGAGATATGAGGGACAATTGGAGAAGTGAATTATATAAATATAAAG
TAGTAAAAATTGAACCATTA---GGAGTAGCGCCCACCAGGGCAAAGAGAAGAGTGGTG-------------
------------------------CAGAGAGAGAAAAGAGCA---GTGGGA---ATAGGAGCT---------
GTGTTCCTTGGGTTCTTGGGAGCA---GCAGGAAGCACTATG---GGCGCGGCGTCA---
ATAACGCTGACGGTACAG---GCCAGACAA---TTATTG---TCTGGTATAGTGCAACAGCAGAAT---
AATCTG---CTGAGG---GCTATTGAGGCGCAACAACATCTGTTGCAACTCACAGTCTGGGGCATC
>FJ388917..2
TTCTATTGCAATACAACAAAACTGTTTAATAGTACACTGAAAAATGGTACATTCATTCCGGGTACAAAT----
----

TCTAATTCAAACATCACACTCCAATGCAGACTAAAACAATTTGTAAGGATGTGGCAGGAAGTGGACAAGCAA
TGTACTCCTCCCCAATGGAAGGGAAAATTTACTGCAGCTCAAAT---
ATTACAGGACTAATATTAACAAGAGATGGTGGTAATAATGGTGATGAC------------------------
----

AATGGTACTGAGACCTTCAGACCTGGAGGAGGAGACATGAGGGACAATTGGAGAAGTGAACTATATAAATATA
AAGTAGTAAGGATTGAACCAATA---GGAGTAGCACCCACTGAAGCAAGGAGACGGGTGGTG----------
---------------------------CAGAGAGAAAAACGAGCA---GTGGGA---ATAGGAGCC------
---GTGTTCCTTGGGTTCTCGGGAGCA---GCAGGAAGCACTATG---GGCGCAGCGTCA---
TTGACGCTGACGGTACAG---GCCAGACAA---TTATTG---TCTGGTATAGTGCAGCAGCAGAGC---
AATCTG---CTGAGG---GCTATAGAGGCTCAACAACATCTGTTGAGACTCACAGTCTGGGGCATT
>JN687742..1
TTCTATTGTAGCACAACAGCACTGTTTAATAGTACCTGGACGTATAATGGTACCTGGACTACCAATGGCAGCA
CTGAG---

ACAGATGACACAATCATTACACTGCAATGCAGAATAAGACAAATTGTAAACCTGTGGCAGGCAGTGGGACAAG
CAATGTACGCCCCCCCCATTGCAGGAAACATTACCTGCAGCTCAAAT---
ATTACAGGAATGATATTGACAAGAGATGGTACTAATAGTACTAAT---------------------------
----

AATGATACCTTCAGACCTGGAGGAGGAGACATGAGGGACAATTGGAGAAGTGAACTATATAAATATAAAGTAG
TAAAGATTGAACCAATA---GGAGTAGCACCCACTAGGGCAAGGAGACGAGTGGTG----------------
---------------------CAGAGAGAAAAACGAGCA---GTAGGA---CTGGGAGCT---------
GTGTTCCTTGGGTTCTTGGGAGCA---GCAGGAAGCACTATG---GGCGCAGCGTCA---
ATAACGCTGACGGTACAG---GCCAGACAA---TTATTG---TCTGGCATAGTGCAACAGCAGAGC---
AATCTG---CTAAGG---GCTATAGAGGCTCAACAACATCTGTTGAAACTCACAGTCTGGGGCATT
>JX140648..1
TTCTATTGCAACACAACAGAACTGTTTAATAGTACCTGGACACTGAATAATAGTACCAATAGCAGCATTGAGA
CA--

AATGGCACAACTATTATACTGCCATGCAGAATAAGACAATTTATAAACATGTGGCAGGAAGTGGGACAAGCAA
TGTACGCCCCCCCCATTGAAGGAAGCATTAAATGCAGCTCAAAT---
ATTACAGGAATGATACTGACAAGAGATGGTGGTAATAGTACTACT---------------------------
----

AATGAGACCTTCAGACCTGGAGGAGGAAACATGAGGGACAATTGGAGAAGTGAACTATATAAATATAAAGTAG
TAAAGATTGAACCAGTA---GGAGTAGCACCCACTAAGGCAAGGAGACGAGTGGTG----------------
---------------------CAGAGAGAAAAACGAGCA---GTAGGA---CTGGGAGTG---------
ATGTTCCTTGGGTTCTTGGGAGCA---GCAGGAAGCACTATG---GGCGCAGCGTCA---
ATGACGCTGACGGTACAG---GCCAGACAA---TTATTG---TCTGGCATAGTGCAACAGCAGAGC---
AATTTG---CTAAGG---GCTATAGAGGCTCAACAACATCTGTTGAAACTCACAGTCTGGGGCATT
>JN687748..1
TTCTATTGCAACACATCACAGCTGTTTTTAGGTAATGGTACAAATAGTACAAACACAAATAAT---------
----

ACAAATTCAACTATTGAACTCAAATGCAGAATAAGACAAATTGTAAACATGTGGCAGGGAGTGGGACAAGCAA
TGTACGCCCCACCCATTGCAGGGAGCATTAGCTGCAGCTCAAAT---
ATTACAGGATTAATATTGACAAGAGATGGTGGTAATGATACTGGG---------------------------
----

ACGAATGAGACCTTCAGACCTGGAGGAGGAGACATGAGGGACAATTGGAGAAGTGAACTATATAAATATAAAA
TAGTAAAGATTGAACCAGTA---GGAGTAGCACCCACTAGGGCCAGGAGACGAGTGGTG-------------
------------------------CAGAGAGAAAAACGAGCA---GTGGGA---ATAGGAGCT---------
GTGCTCCTTGGGTTCTTGGGAGCA---GCAGGAAGCACTATG---GGCGCAGCGTCA---
ATAACGCTGACGGTACAG---GCCAGACAA---TTATTG---TCTGGCATAGTGCAACAGCAAGGC---
AATTTG---CTGAGG---GCTATAGAAGCTCAACAACATCTGTTGAGGCTTACGGTCTGGGGCATT
>AF119820..1
TTCTACTGCAATACATCAGGACTGTTTAATAGTACATACATGTTTAATAGTACAAATAGGACAAACACAACCA
AT--

GGAACAAACTCAACCATCACGCTCCCATGCAGAATAAAACAAATTGTAAGAATGTGGCAGGGAGTAGGACAAG
CAATGTACGCCCCTCCCATTGCAGGGAGCATTAACTGCAGCTCAGAT---
ATTACAGGAATAATATTGACAAGAGATGGTGGTATAAGTAATAATAATGAG----------------------
----

ACTAATGATAATGAGACCCTCAGACCTGGAGGAGGAGACATGAGGGACAATTGGAGAAGTGTACTATATAAAT
ATAAAGTAGTAAAGATTGAACCAGTG---GGAATAGCACCCACGGGGGCAAGGAGACGAGTGGTG--------
-------------------------------CAGAAAGAAAAACGAGCA---GTGGGA---CTAGGAGCT---
------TTGTTCCTTGGGTTCTTGGGAGCA---GCAGGAAGCACTATG---GGCGCAGCGTCA---
CTAACGCTGACGGTACAG---GCCAGACAA---TTATTG---TCTGGTATAGTGCAACAGCAGAGC---
AATCTG---CTGAGG---GCTATAGAGGCTCAGCAACATCTGCTGAAACTCACGGTCTGGGGCATT
>KU168269..1
TTCTATTGTAATACATCCAATCTGTTTAATAGTAATATAACAGAGAATGATACAACAAAT-------------
----

AGTAATGACAATATCACACTCCCATGTAGGATAAAACAAATTGTAAGAATGTGGCAGAGAGTGGGACAAGCAA
TGTATGCCCCTCCCATTGCAGGAAACATTACATGTAGATCAAAC---
ATTACAGGAATACTATTAACAAGAGATGGTAATGGTGGGAATGATACC-------------------------
----

AATAGCAATGAGACTTTCAGACCTGGAGGAGGAGATATGAGGGACAATTGGAGAAGTGAATTATACAACTATA
AAGTAGTAAAAATTAAACCACTA---GGAGTAGCACCCACCCATGCAAGAAGAAGAGTGGTG-----------
----------------------------GGGAGAGAGAAAAGAGCA---GTTGGA---CTAGGAGCT------
---CTTCTCCTTGGGTTCTTGGGAACA---GCAGGAAGCACTATG---GGCGCAGCGTCA---
ATAACGCTGACGGTACAG---GCCAGACAA---TTATTG---TCTGGTATAGTGCAACAGCAAAGT---
AATTTG---CTGCAA---GCTATAGAGGCGCAACAGCATCTGTTGCAGCTCACAGTCTGGGGCATT
>AB286852..1
TTCTATTGTAATACATCAAAACTATTTAATAGTACTTTTTCTAATGATAATTCCACAGATAATGATACCTCTT
TA---

GAAAATAATGGTACCATCACACTCCCATGTAAGATAAAACAAATTGTAAGAATGTGGCAGAGAGTGGGACAGG
CAATGTATGCCCCTCCCATTGAAGGAAACATTACATGTATATCAAAC---
ATTACAGGACTAATATTAACAAGAGATGGGCACAATGGGCACAATCAG-------------------------
----

AGTAATAATGAGACCTTCAGACCTACAGGAGGAGATATGAGGGACAATTGGAGAAGTGAATTATATAAGTATA
AAGTAGTAAGAATTAAACCACTA---GGAATAGCACCCACCAAGGCAAGAAGAAGAGTAGTG-----------
----------------------------GGCAGAGAGAAAAGAGCA---GTTGGA---CTGGGAGCT------
---GTTTTCCTTGGGTTCTTAGGAGCG---GCAGGAAGCACTATG---GGCGCAGCGTCA---
ATAACGCTGACGGTACAG---GTCAGACAA---TTATTG---TCTGGCATAGTGCAGCAGCAAAGC---
AATTTG---CTGAGA---GCTATAGAAGCGCAGCAGCATCTGTTGCAGCTCACAGTCTGGGGCATT
>AF069934..1
TTTTATTGTAATACATCAGAGCTGTTTACTAATACATCAACTAAGAATGGG----------------------
----

ACCAATGAAACACCCACACTCCCATGTAAGATAAAACAAATTGTAAGAATGTGGCAGAGAGTGAGACAAGCAA
TGTATGCCCTTCCCATTGCAGGAAACATTACGTGTACATCAAAC---
ATTACAGGACTACTATTAACAAGAGATGGG---AATAATGACAGT----------------------------
----

CACACTGAGACCTTCAGACCTGGAGGGGGAGATATGAGGGACAATTGGAGAAGTGAATTATATAAGTATAAAG
TAGTAAAAATTAAACCACTA---GGAATAGCACCCACCTGGGCGAGGAGAAGAGTAGTG--------------
-------------------------GGGAGAGAGAAAAGAGCA---GTTGGA---CTGGGAGCT---------
GTTTTCCTTGGGTTCTTAGGGACA---GCAGGAAGCACTATG---GGCGCAGCGTCA---
ATAACGCTGACGGTACAG---GTCAGACAA---TTATTG---TCTGGCATAGTGCACCAGCAAAGC---
AATTTG---CTGAGA---GCTATAGAGGCGCAGCAGCATCTGTTGCAACTCACAGTCTGGGGCATT
>AJ288981..1
TTTTATTGTAATACATCACAACTGTTTAACAGTAGTAACCTTAATAATAATAGCTCAGAT-------------
----

AATAATGGGACCATCACACTCCCATGTAGGATAAATCAAATTATAAGAATGTGGCAAAGAGTGGGACAAGCAA
TGTATGCCCCTCCCATTGCAGGAAACATTACATGTACATCAAAC---
ATTACAGGACTACTATTAACAAGAGATGGGCATAATGACACTGAA----------------------------
----

ATCTTCAGACCTGGAGGAGGAAATATGAAGGACAATTGGAGAAGTGAATTATATAAGTATAAAGTAGTAAAAA
TTAAACCACTA---GGGATAGCACCCACCAAGGCAAGGAGAAGAGTAGTG-----------------------
----------------GGGAGAGAGAAAAGAGCAGTTGTTGGA---CTGGGAGCT---------
GTTTTCCTTGGGTTCTTAGGAACG---GCAGGAAGCACTATG---GGCGCAGCGTCA---
ATAACGCTGACGGTACAG---GTCAGACAA---TTATTG---TCTGGCATAGTGCACCAGCAAAGC---
AATTTG---CTGAGA---GCTATAGAGGCGCAGCAGCATCTGTTGCAACTCACAGTCTGGGGCATT
>EU363837..1
TTCTATTGTAATACATCAGGCCTGTTTAATGGTACATACATGTCTAATGGTACACAAAGTAAT----------
----

TCAAGCTCAATCATCACGATTCCATGCAGAATAAAGCAAATTATAAACATGTGGCAGGAGGTAGGACGGGCAA
TGTATGCCCCTCCCATTGAAGGAAACATAACATGTAAATCAAAT---
ATCACAGGACTACTATTGGTACGTGATGGAGGAACGACAAATACTATA-------------------------
----

AATGATACAGAGATATTCAGACCTGGAGGAGGAGACATGAGGGACAATTGGAGAAGTGAATTATATAAATATA
AAGTGGTAGAAATTAAGCCATTA---GGAGTAGCACCCACTGCAGCAAAAAGGAGAGTGGTG-----------
----------------------------GAGAGAGAAAAAAGAGCA---GTGGGA---CTAGGAGCT------
---GTGTTCCTTGGGTTCTTGGGTGCA---GCAGGAAGCACTATG---GGCGCGGCGTCA---
ATAACGCTGACGGTACAA---GCCAGACAA---TTATTG---TCTGGTATAGTGCAACAGCAAAGC---
AATTTG---CTGAGA---GCTATAGAGGCGCAACATCATCTGTTGCAACTCACAGTCTGGGGCATT
>KC492737..1
TTCTATTGTAATACATCAAGACTGTTTAATGGTACATACATGTTTAATGGTACAAGGGGTAATTCAAGC----
----

TCAAACTCAACCATCACAATCCCATGCAGAATAAAGCAAATTATAAATATGTGGCAGCAGGTAGGACGAGCAA
TGTATGCCCCTCCCATTGAAGGAAACCTAACATGTAGATCAAAT---
ATCACAGGATTACTATTGGTACGTGATGGAGGAGACAACACAAAT----------------------------
----

AAAACAGAGATATTCAGACCTCAAGGGGGAGATATGAGGGACAATTGGAGAAGTGAATTATATAAATATAAAG
TAGTAGAAATTAAGCCATTG---GGAGTAGCACCCACTGCAGCAAAAAGGAGAGTGGTG--------------
-------------------------GAGAGAGAAAAAAGAGCA---GTGGGA---ATAGGAGCT---------
GTGTTCCTTGGGTTCTTGGGAGCA---GCAGGAAGCACTATG---GGCGCGGCGTCA---
ATAACGCTGACGGTACAG---GCCAGACAA---TTGCTG---TCTGGTATAGTGCAACAGCAAAGC---
AATTTG---CTGAGG---GCTATAGAGGCGCAACAACATCTGTTGCAACTCACGGTCTGGGGCATT
>KF835494..1
TTCTATTGTAATACATCAGGCCTGTTTAATAGTACATACATGTTCAATAATACAGACATATTTAATGGTACAG
GAAAT--

AGTTCAAAAGTCATCACGATTCCATGCAGAATAAAGCAAATTATAAATATGTGGCAGGAGGTAGGACGAGCAA
TGTATGCCCCTCCCATTGAAGGAAACATAACATGTAACTCAACT---
ATCACAGGACTACTATTGGTACGTGATGGAGGAAAGCATAATAATAGT-------------------------
----

AATACTACAGAAACATTCAGACCTGGAGGAGGAGATATGAAGGACAATTGGAGAAGTGAATTATACAAATATA
AAGTGGTAGAAATTCAGCCATTG---GGAGTAGCACCCACTGGAGCAAAAAGGAGAGTGGTG-----------
----------------------------GAGAGAGAGAAAAGAGCA---GCAGGA---CTAGGAGCT------
---GTGTTCCTTGGGTTCTTGGGAGCA---GCAGGAAGCACTATG---GGCGCGGCGTCA---
TTAACGCTGACGGTACAG---GCCAGACAA---TTGCTG---TCTGGTATAGTGCAACAGCAAAGC---
AATTTG---CTGAAG---GCTATAGAGGCGCAACAGCATCTGTTGCAACTCACAGTCTGGGGCATT
>KF835511..1
TTCTATTGTAATACATCAGACCTGTTTAATGGTACATATAATGGTACATACATACATAATGATACACAAGGT-
----

AATTCAACTGTCTTTACAATCCAATGCAGAATAAAGCAAATTATAAATATGTGGCAGGAGGTAGGACGAGCAA
TGTATGCTCCTCCCATTGCAGGAAATATAACATGTAAATCAAT---
ATCACAGGACTACTATTGGTACGTGATGGAGGAAATACTAGTACA----------------------------
----

GAAGAGATATTCAGACCTGGAGGAGGAGATATGAAAAACAATTGGAGAAGTGAATTATATAAATATAAAGTGG
TAGAAATTAAGCCATTG---GGAATAGCACCCACTGCAGCAAAAAGGAGAGTGGTG-----------------
----------------------GAGAGAGAAAAAAGAGCAGTAATGGGA---ATAGGAGCT---------
GTGTTCCTTGGGTTCTTGGGAGCA---GCAGGAAGCACTATG---GGCGCGGCGTCA---
ATAACGCTGACGGTACAG---GCCAGACAA---TTGCTG---TCTGGTATAGTGCAACAGCAAAGC---
AATTTG---CTGAAG---GCTATAGAGGCGCAACATCATCTGTTGCAACTCACGGTCTGGGGCATT
>JF346907..1
TTCTATTGTAATACATCAGGCCTGTTCAATAGTACATACCTGTTCAATAGTACATACATGTCTAATGGTAGTT
CAAACTCA---
-------------------AAC---
AACACAAACTCAACCATCACAATCCCATGCAGAATAAAGCAAATTATAAATATGTGGCAGGAGGTAGGACGAG
CAATGTATGCCCCTCCCATTGCAGGAAACATAACATGTAAATCAAAT---
ATCACAGGACTACTATTGGTCCGTGATGGAGGAGATCAAAAATCAAATGAGACA------------GAG----
----
-
AATGGGACAGAGACTGAGGAGACATTTAGACCTGGAGGAGGAGATATGAGGGACAATTGGAGAAGTGAATTAT
ATAAATATAAAGTGGTAGAAATTAAGCCATTG---GGAATAGCACCCACTAAAGCAAAAAGGAGAGTGGTG--
-------------------------------------GAGAGAGAAAAAAGGGGA------------
CTAGGAGCT---------GTGTTCCTTGGGTTCTTAGGAGCA---GCAGGAAGCACTATG---
GGCGCGGCGTCA---ATAACGCTGACGGTACAG---GCCAGACAA---TTGCTG---
TCTGGTATAGTGCAACAGCAAAGC---AATTTG---CTGAAG---
GCTATAGAGGCGCAACAGCATCTGTTGCAACTCACGGTCTGGGGCATC
>JF346913..2
TTCTATTGTAATACAACAGGCCTGTTTAAGGGTACATACAACCCTAATTACACAAACCCTAATGGTACAGGGA
GT---

AATTCAAGCTCAACCATCACAATACCATGCAAAATAAAGCAAATTATAAATATGTGGCAGGAGGTAGGACGAG
CAATGTATGCCCCTCCCATTGCAGGAAACATAACATGTAAATCAAAT---
ATCACAGGACTGCTATTGGTACGTGATGGAGGATACACAAATAAGACA-------------------------
----

AATCATACAGAGATATTCAGGCCGGGAGGAGGAGATATGAAGGATAATTGGAGAAGTGAATTATATAAATATA
AAGTGGTAGAAATCAAGCCATTG---GGAGTAGCACCCACTAAAGCAAAAAGGAGAGTGGTG-----------
----------------------------GAGAGAGAAAAAAGAGCA---GCA------TTAGGAGCT------
---GTGTTCCTTGGGTTCTTGGGAGCA---GCAGGAAGCACTATG---GGCGCGGCGTCA---
ATGGCGCTGACGGTACAG---GCCAGACAA---TTGCTG---TCTGGTATAGTGCAACAGCAAAGC---
AATTTG---CTGAGG---GCTATAGAGGCGCAACAACATCTGTTGCAACTCACGGTATGGGGCATT
>EU363847..2
TTCTATTGTAATACCTCAAAACTGTTTAATGGTACATACAACAATGGTACATACAATGGTACATACGGT----
----

ACGGAAAGTAATATCACAATCCCATGCAGAATAAAGCAAATTATAAACATGTGGCAGGAGGTAGGACGAGCAA
TGTATGCTCCTCCCATTGCAGGAAACATAACATGTAACTCAACT---ATCACAGGCTACTAT---
GTACGTGATGGAGGAACAGGAAATGGGACA---

GATAATGGTACAGAGATATTCAGACCTGGAGGAGGAGATATGAGAAACAATTGGAGAAGTGAATTATATAAAT
ATAAAGTGGTAGAAATTAAGCCATTG---GGAGTAGCACCCACTGAAGCAAAAAGGAGAGTGGTG--------
-------------------------------GAGAGAGAAAAAAGAGCA---GTGGGA---CTAGGAGCT---
------GTGTTCCTTGGGTTCTTGGGAGCA---GCAGGAAGCACTATG---GGCGCGGCGTCA---
ATAACGCTGACGGTACAA---GCCAGACAA---TTGTTG---TCTGGTATAGTGCAACAGCAAAGC---
AATTTG---CTGAGA---GCTATAGAGGCGCAACAGCATCTGTTGCAACTCACAGTCTGGGGCATT
>KF835500..1
TTCTATTGTAATACATCAGGCATATTTAATAGTACATACAATGAGACATACCAAGGTAACTCA----------
----

ACTAACTTAAACATCACAATTCCATGCAAAATAAAGCAAATTATAAACATGTGGCAGGGGGTAGGACGAGCAA
TGTATGCCCCTCCCATTAAAGGAAACATAACATGTAGATCAAAT---
ATCACAGGACTACTACTGACACGTGATGGAGGAATAAATGTAACAAAT-------------------------
----

AGAACAGATCAAGAGATATTCAGACCTGCAGGAGGAGATATGAGGGACAATTGGAGAAGTGAATTATATAAAT
ATAAAGTGGTAGAAATTAAGCCATTG---GGGGTAGCACCCACTACAGCAAAAAGGAGAGTGGTG--------
-------------------------------GAGAGAGAAAAAAGAGCA---GTGGGA---CTGGGAGCC---
------GTGTTCCTTGGGTTCTTGGGAGCA---GCAGGAAGCACTATG---GGCGCAGCGTCA---
ATAACGCTGACGGTACAA---GCCAGACAA---TTGTTG---TCTGGTATAGTGCAACAGCAAAGC---
AATTTG---CTGAAA---GCTATAGAGGCGCAACAACATCTGTTGCAACTCACGGTCTGGGGCATT
>KF835507..1
TTCTATTGTAATACATCAGGCCTGTTTAATGGTACATACAGACAGCCTAATGGTACATACGTGTTTAAAGGAG
ATTCA--
-------------------AAC---
TCAAACATTACAACCATTACACTCCCATGCAGAATAAAGCAAATTATAAACATGTGGCAGGAGGTAGGACGAG
CAATGTATGCCCCTCCCATTGCAGGAAAAATAACATGTAAATCAAAT---
ATCACAGGACTACTGTTGGTACGTGATGGAGGGTCAAATGATACA----------------------------
----

GAGACAAATAAAACAGAGACATTTAGACCTCAAGGAGGAGATATGAGGGACAATTGGAGAAGTGAATTATATA
AATATAAAGTGGTAGAAATTAAGCCATTG---GGAATAGCACCCACTGGGGCAAAAAGGAGAGTGGTG-----
----------------------------------GAGAGAGAAAAAAGAGCA---GTGGGA---
CTAGGAGCT---------GTGTTCCTTGGGTTCTTGGGAGCA---GCAGGAAGCACTATG---
GGCGCGGCATCA---ATAACGCTGACGGTACAG---GCCAGACAA---CTGTTG---
TCTGGTATAGTGCAACAGCAAAGC---AATTTG---CTGAAA---
GCTATAGATGCGCAACAGCATCTGTTGCAACTCACGGTCTGGGGCATT
>KF835509..2
TTCTATTGTAATACATCAGGCCTGTTTAACAGTACATACAATCCTAATGGTACAAATTATAAT----------
----

TCAAACTCAACCATCACAATCCCATGCAGAATAAAGCAAATTATAAACATGTGGCAGGGGGTAGGACGAGCAA
TGTATGCCCCTCCCATTGCAGGAAACATAACATGTAGATCAAAT---
ATCACAGGACTACTATTGTTACGTGATGGAGGAACTTCAGATTCAAGT-------------------------
----

AATACAACAGAGATATTCAGACCTGGAGGAGGGGATATGAGGGACAATTGGAGAAATGAATTATATAAATATA
AAGTGGTAGAAATTCAGCCATTG---GGAATAGCACCCACTGGAGCAAAAAGGAGAGTGGTG-----------
----------------------------GAGAGAGAAAAAAGAGCA---GTGGGA---CTAGGAGCT------
---ATGTTCATTGGGTTCTTGGGAGCA---GCAGGAAGCACTATG---GGCGCGGCGTCA---
ATAACGCTGACGGTACAA---GCCAGACAA---TTGTTG---TCTGGTATAGTGCAACAGCAAAGC---
AATTTG---CTGAGA---GCTATAGAGGCGCAACAGCATCTGTTGCAACTCACTGTCTGGGGCATT
>KF835534..1
TTCTATTGTAATACATCAGGCCTGTTTAATAGTACATACATGTCTAATGGTACATACATGCCTAATGGT----
----

ACAGGAACAAACATCACAATTCCATGCAGAATAAAACAAATTATAAACATGTGGCAGGAGGTAGGACGAGCAA
TGTATGCCCCTCCCATTGCAGGAAACATAACATGTAGGTCAAAT---
ATCACAGGATTACTCTTGGTACGTGATGGAGGAAAGAGAAATGGTACA-------------------------
----

AATAATACAGAGATATTCAGACCTGGAGGAGGAGATATGAGAGACAATTGGAGAAATGAATTATATAAATATA
AAGTGGTAGAAATTCAGCCATTG---GGAGTAGCACCCACTAGAGCAAAAAGGAGAGTGGTG-----------
----------------------------GAGAGAGAAAAAAGAGCAGTAGTAGGA---CTAGGAGCT------
---GTGTTCCTTGGGTTCTTGGGAGCA---GCAGGAAGCACTATG---GGCGCGGCGTCA---
ATAACGCTGACGGTACAG---GCCAGACAA---CTGTTG---TCTGGTATAGTGCAACAGCAAAGC---
AATTTG---CTGAAA---GCTATAGAGGCGCAACAGCATCTGTTGCAACTCACGGTCTGGGGCATT
>KF835545..1

TTCTATTGTGATACGTCAGACCTGTTTAATGATACATACAATGACAAGTTTAATGGTACAACAGGTGAT----
----

TCAAACAAAACCATCACAATCCAATGCAGAATAAAGCAAATTATAAACATGTGGCAGGAGGTAGGACGAGCAA
TGTATGCCCCTCCCATTGCAGGAAACATAACATGTAGATCAAAT---
ATCACAGGACTACTATTGGTACGTGATGGAGGTAATGGGACAGAGACA-------------------------
----

AATGGCACAGAAACATTCAGACCTGGAGGAGGAGATATGAGGGACAATTGGAGAAATGAACTATATAAATATA
AAGTGGTAGAAATTCAGCCATTG---GGAGTAGCACCCACTGCAGCAAAAAGGAGAGTTGTG-----------
----------------------------GAGAGAGAAAAAAGAGCA---ATAGGA---TTTGGAGCT------
---ATGTTCCTTGGGTTCTTGGGAGCA---GCAGGAAGCACTATG---GGCGCGGCGTCA---
ATAACGCTGACGGTACAA---GCCAGACAA---TTGCTG---TCTGGTATAGTGCAACAGCAAAGT---
AATTTG---CTAAGA---GCTATAGAGGCGCAACAGCATCTGTTGCAACTCACTGTCTGGGGCATT
>HQ699961..1
TTCTATTGCAATACATCAGGCCTGTTTAATGGTAATATTACAAAAGATAACTCAAGCTCAGATAAT-------
----

TCAAGCTCAGTCATCATACTCCCATGCAAAATAAAGCAAATTATAAATATGTGGCAGGAGGTAGGACGAGCAA
TGTATGCCCCTCCCATTGAAGGAAACATAACATGTAAATCAAAT---
ATCACAGGACTACTCTTAGTACGTGATGGGGGATACACAAATGGCACA-------------------------
----

GAGAGTAATAACGAGACATTCAGACCAGGAGGAGGAGATATGAGAGACAATTGGAGAAGTGAATTATATAAAT
ATAAGGTGGTAGAAATTAAGCCATTG---GGAATAGCACCCACTAAATCAAAAAGGAGAGTGGTG--------
-------------------------------GAGAAAGAAAAAAGAGCA---GTGGGA---CTGGGAGCT---
------GTGTTCCTTGGGTTCTTGGGAGCT---GCAGGAAGCACTATG---GGCGCGGCGTCA---
ATAACGCTGACGGTACAA---GCCAGACAA---TTGCTG---TCTGGTATAGTGCAACAGCAAAGC---
AATTTG---CTGAGA---GCTATGGAGGCGCAGCATCATCTGTTGCAACTCACAGTCTGGGGCATT
>HM215416..1
TTCTATTGTAATACATCAGGCCTGTTTAATAGTACGTACAATGGTACATACAGGGGTACAGGAGATAAT----
----

TCAAGCTCAATCATCACAATCCCATGCAGAATAAGGCAAATTATAAACATGTGGCAGAAGGTAGGACGAGCAA
TGTATGCCCCTCCCATTGAAGGAAACATAGCATGTAACTCAAGT---
ATCACAGGACTACTGTTGGTACGTGATGGAGGAAGCCCAAATAATCCAGAG----------------------
----

ATTCCAAATAATACAGAGATATTCAGACCTGGAGGAGGAGACATGAAGGACAATTGGAGAAATGAATTATATA
AATATAAAGTGGTAGAAATTCAACCATTG---GGAGTAGCACCCACTGAAGCAAAAAGGAGAGTGGTG-----
----------------------------------CAGAGAGAAAAAAGAGCA---GTGGGA---
ATAGGAGCT---------GTGTTCCTTGGGTTCTTGGGAGCA---GCAGGAAGCATTATG---
GGCGCGGCGTCA---ATAACGCTGACGGTACAA---GCCAGACAA---CTGTTG---
TCTGGTATAGTGCAACAGCAAAGC---AATTTG---CTGAGA---
GCTATAGAGGCGCAACAGCACATGTTGCAACTCACGGTCTGGGGCATT
>KF250369..1
TTCTATTGTAATACATCAAGACTGTTTAATAGTACATACAATAGTACATTAGACCTGTCTAGT----------
--AGT---
ACATACAATAGTACAAAAGAT---------------
AATTCAAGCTCCCCCATCACAATCTCATGCAGAATAAAGCAAATTATAAACATGTGGCAGGAGGTAGGACGAG
CAATGTATGCCCCTCCCATTGCAGGAAGCATAACATGTAAATCAAAT---
ATCACAGGACTACTATTGGTGCGTGATGGAGGA--

-
ATAGGACCAAAAGATACAGAGACATTCAGACCTGGAGGAGGAGATATGAGAGACAATTGGAGAAATGAATTAT
ATAAATATAAAGTGGTAGAAATTCAGCCATTG---GGAGTAGCACCCACTGAAGCAAAAAGGAGAGTGGTG--
-------------------------------------GAGAGAGAAAAAAGAGCA---GTGGGA---
CTAGGAGCT---------GTGTTCCTTGGGTTCTTGGGAGCA---GCAGGAAGCACTATG---
GGCGCGGCGTCA---ATAACGCTGACGGTACAG---GCCAGACAA---TTGTTG---

TCTGGTATAGTGCAACAGCAAAGC---AATTTG---CTGAGA---
GCTATAGAGGCGCAACAACATATGTTGCAACTCACGGTCTGGGGCATT
>KU820846..2
TTCTATTGTAATACATCAGGCCTGTTTAATGGTACATACAATGATACATACGAGCTAAATGGTACAGAAAGT-
----

AATTCAAGCTTAATCACAATCCCATGCAGAATAAAGCAAATTATAAACATGTGGCAAAAGGTGGGACGAGCAA
TGTATGCCCCTCCCATTCAAGGAAACATTACATGTAAATCAAAT---
ATCACAGGACTACTATTAGTACGTGATGGAGGAGCAAATCAGACA----------------------------
----

AATGATACAGAGATATTCAGGCCTGGAGGAGGAAATATGAAGGACAATTGGAGAAGTGAATTATATAAATATA
AAGTGGTAGAAGTTAAGCCAGTA---GGAATAGCCCCCACTAAAGCAAAAAGGAGAGTGGTG-----------
----------------------------GAGAGAGAAAAAAGAGCA---GCG------CTAGGAGCT------
---GTGTTCCTTGGGTTCTTGGGAGCA---GCAGGAAGCACTATG---GGCGCGGCGTCA---
ATAACGCTGACGGTACAA---GCCAGACAA---TTGTTG---TCTGGTATAGTGCAACAGCAAAGC---
AATTTG---CTGAGA---GCTATAGAGGCGCAACAGCATATGTTGCAACTCACGGTCTGGGGCATT
>AJ866553..3
TTCTATTGCAATACATCAGACCTGTTTAATAGCATTTGGAATAGCACTAGGGAAAATACAAATAACACAAATG
CA---

AATTACACAGATTACATTATCCCATGCAGAATAAAGCAAATTGTGAGAATGTGGCAGAGAGTAGGACAAGCAA
TGTATGCTCCTCCCATCCCAGGAGTAATAAGCTGTACATCCAAT---
ATTACAGGACTAATAATAACAAGAGATGGTGGGAGTAATAACACT----------------------------
----

GGGAGTGAAACCTTCAGACCTACAGGAGGAAATATGAAAAATAATTGGAGAAGTGAATTATATAAGTATAAAG
TAGTAGAAATTGAACCACTA---GGAGTAGCACCCACCAGGGCAAGGAGAAGAGTGGTG--------------
-------------------------CAGAGAGAAAAAAGAGCA---GTTGGA---ATGGGAGCT---------
TTGTTCCTTGGGTTCCTAGGAGCA---GCAGGAAGCACTATG---GGCGCAGCGTCA---
ATAACGCTGACGGTACAG---GCCAGACAA---TTACTG---TCTGGTATAGTGCAGCAGCAGAGC---
AATCTG---CTGAAG---GCTATAGAGGCTCAACAGCATATGTTGAGACTCACAGTCTGGGGCATA
>AY093603..2
TTCTATTGCAATACAACAAACCTATTTAATTGGAATAACACTAAGGATATCATGCCAAAT-------------
----

GGCACAAAGAATTACTTTATCCAATGCAAAATAAAGCAAATTGTGAGAATGTGGCAGAGAGTAGGACAAGCAA
TGTATGCCCCTCCTATCCCAGGAATAATAGAGTGTGTATCAAAC---
ATTACAGGACTAGTATTAACAAGAGATGGTGGGACTGGCAATACA----------------------------
----

AGTGAGACCTTCAGACCTACAGGAGGAAATATGAAGGACAATTGGAGAAGTGAATTATATAAGTATAAAGTAG
TAAAAATTGAACCACTA---GGAGTGGCACCCACCAGGGCAAAGAGAAGAGTGGTG-----------------
----------------------GAGAGAGAAAAAAGAGCA---GTTGGA---ATGGGAGCT---------
CTTTTCCTTGGGTTCTTAGGAGCA---GCAGGAAGCACTATG---GGCGCGGCGTCA---
ATGACGCTGACGGTACAG---GCCAGACAA---TTATTG---TCTGGTATAGTGCAACAGCAGAGC---
AATCTG---CTGAAG---GCTATAGAGGCTCAACAGCATCTGTTAAAACTCACAGTCTGGGGCATT
>AY093604..2
TTCTATTGCGATACATCAGGCCTGTTTAATAGCACGTGGAAAAATGGCACTAATATACAGGGGTCAAATGGCA
CT---

GGATCAAATTACACACAGATCCAGTGCAAAATAAAGCAAATTGTGAGAATGTGGCAGAGAGTAGGACAAGCAA
TGTATGCCCCTCCCATCCCAGGAGTAATAGGGTGTATATCAAAC---
ATTACAGGACTAATATTAACAAGAGATGGTGGGGACAATACAGGT----------------------------
----

GATAATGAAACCTTCAGACCTATAGGAGGAAATATGAAGGACAATTGGAGAAGTGAATTATATAAGTATAAAG
TAGTAAAACTTGAACCACTA---GGAGTAGCACCCACCCCGGCAAAGAGAAGAGTGGTG--------------
-------------------------GAGAGAGAAAAAAGAGCA---GTTGGA---ATGGGAGCT---------
CTTTTCCTTGGGTTCTTAGGAGCA---GCAGGAAGCACTATG---GGCGCAGCGTCA---

ATAACGCTGACGGTACAG---GCCAGACAA---TTATTG---TCTGGCATAGTGCAACAGCAGAGC---
AATCTG---CTGAAG---GCTATAGAGGCTCAACAGCATTTGTTGAAACTCACGGTCTGGGGCATT
>AY093607..1
TTCTATTGCAATACATCAAGCCTATTTAATAGCACTTGGGATGAAAATAACACTAAAAGTGCACAGGGGCCAA
AT---

GGCACGGCCAATATCACAATCCCATGTAAAATAAGACAAATTGTGAGAATGTGGCAGAGAGTAGGACAAGCAA
TGTATGCCCCTCCCATCCCAGGAGTAATAAATTGTATATCAAAC---
ATTACAGGACTAATATTAATAAGAGATGGGGGGAATGAGAGCGCT----------------------------
----

ACAAATGAGACCTTCAGACCTATAGGAGGAGATATGAAGAATAATTGGAGAAGTGAATTATATAAGTATAAAG
TAGTAAAAATTGAACCACTA---GGAGTGGCACCCACCAGGGCAAGAAGAAGAGTGGTG--------------
-------------------------CAGAGAGAAAAAAGAGCA---GTTGGA---ATGGGAGCT---------
CTGTTCCTTGGGTTCTTAGGAGCA---GCAGGAAGCACTATG---GGCGCAGCGTCA---
ATAACGCTGACGGTACAG---GCTAGACAA---TTATTG---TCTGGTATAGTGCAACAGCAGAGC---
AATCTG---TTGAAG---GCTATAGAGGCTCAACAGCATCTGTTGAAACTCACGGTCTGGGGCATT
>AF289548..1
TTCTACTGCAATACATCAGAACTGTTTAATAGTACATGGACATCAAATAATACAAGTACAGGGGTA-------
----

AATGGGAGTACAATCACACTCCCATGCAGAATAAGACAAATTATAAATATGTGGCAGGGAGTAGGAAAAGCAA
TATATGCCCCTCCCATTGCAGGACTGATCAATTGTTCATCAAAT---
ATTACAGGACTAGTGTTGACAAGAGATGGTGGTGCGAATAATAAT----------------------------
----

AGTCAGAATGAGACCTTCAGACCTGGAGGAGGAGATATGAGGGACAATTGGAGAAGTGAATTATACAAATATA
AAGTAGTAAAAATTGAACCAATA---GGTCTAGCACCCACCACGGCAAAGAGAAGAGTGGTG-----------
----------------------------GAAAGAGAAAAAAGAGCA---ATAGGA---CTGGGAGCT------
---GTGTTCCTTGGGTTCTTGGGAGCA---GCAGGAAGCACAATG---GGCGCGGCGTCG---
CTGACGCTGACGGTACAG---GCCAGACAA---GTATTG---TCTGGTATAGTGCAGCAACAAAAC---
AATTTG---CTGAGG---GCTATAGAGGCGCAACAGCATCTGTTGCAACTCACAGTCTGGGGCATT
>AF289549..1
TTCTACTGCAATACAACAATACTGTTTAATAATAGTGAATGGACATCAAATAATACAGGTAATACAAGTTCAG
GG---

GTAAATGATAGTACAATCATAATCCCATGCAGAATAAAACAAATTGTAAACATGTGGCAGAAAGTAGGAAAAG
CAATATATGCCCCTCCCATTGCAGGACTAATCAGCTGTTCATCAAAC---
ATAACAGGACTATTGTTGACAAGAGATAGAGATGGTGGTGCAAATAAT-------------------------
----

ACTAGTCAGAATGAGACCTTCAGACCTGGAGGAGGAGATATGAGAGACAATTGGAGAAGTGAATTATACAAAT
ACAAAGTAGTAAAAATTGAACCACTA---GGTCTAGCACCCACCAAGGCAAAGAGAAGAGTGGTG--------
-------------------------------GAAAGAGAAAAAAGAGCA---ATAGGA---CTGGGAGCT---
------ATGTTCCTTGGGTTCTTGGGAGCA---GCAGGAAGCACGATG---GGCGCAGCGTCG---
CTGACGCTGACGGTACAG---GCCAGACAG---TTATTG---TCTGGTATAGTGCAACAGCAAAAC---
AATTTG---CTGAAG---GCTATAGAGGCGCAACAGCATCTGTTGCAACTCACAGTCTGGGGCATT
>AF289550..1
TTCTACTGCAATACATCAAAACTGTTTAATAGCAGTATAGAGATATCAAATACAAGTATAGGT----------
----

AATACAAGTATAATCAAACTCCCATGCAAAATAAAACAAATTATAAACATGTGGCAGGGAGTAGGAAAAGCAA
TATATGCCCCTCCCATTGCAGGATTAATCAACTGTTCATCAAAT---
ATTACAGGACTATTGTTAACAAGAGATGGTGGTGCAAATAATAGTAAT-------------------------
----

CAGAGTGGGGAGATCTTCAGACCTGGAGGAGGAGATATGAGAGACAATTGGAGAAGTGAATTATATAAATATA
AAGTAGTAAGGATTGAACCACTA---GGTCTAGCACCCACCAAGGCAAAGAGAAGAGTGGTG-----------
----------------------------GAAAGAGAAAAAAGAGCA---ATAGGA---CTGGGAGCT------
---GTGTTCCTTGGGTTCTTGGGAGCA---GCAGGAAGCACAATG---GGCGCAGCGTCG---

TTGACGCTGACGGTACAG---GCCAGGCAG---TTATTG---TCTGGTATAGTGCAACAGCAAAAC---
AATTTG---CTGAGG---GCTATAGAGGCGCAACAGCATATGTTGCAACTCACAGTCTGGGGCATT
>AY371149..2
TTCTATTGTAATACATCAGGCCTGTTTAATAGCAGTTGGAGTAGGGAGAATGGCAATGGCACAGACAATTGGA
ATATCACAACA--
----------AACCACACG------
AGCTCCAATGACACTATAACTCTCCCATGCAAAATAAAGCAAATTGTACATATGTGGCAGAGAGTAGGACAAG
CAATGTATGCCCCTCCCATCCGAGGAGAAATAAGGTGTGTGTCAAAC---
ATTACAGGAATACTATTAACAAGAGATGGTGGGAGAGATACAAAT----------------------------
----

CATACTACCGATACCTTCAGACCTACAGGAGGAGATATGAGGGACAATTGGAGAAGTGAATTATATAGGTATA
AAGTAGTGGCAATTGAACCACTA---GGAGTAGCACCCACCAGGGCAAGAAGAAGAGTGGTG-----------
----------------------------GAGAGAGAAAAAAGAGCA---GTAGGG---ATAGGAGCT------
---GTGCTCCTTGGGTTCTTGGGAACA---GCAGGAAGCACTATG---GGCGCAGCGTCA---
ATAACGCTGACGGTACAA---GCCAGACAA---TTATTG---TCTGGCATAGTGCAACAGCAAGGC---
AATTTG---CTGAAG---GCTATCGAGGCTCAACAGCAACTGTTGAGACTCACTGTCTGGGGCATT
>KP718935..1
TTCTATTGCAATACATCAAACCTGTTTAATAGCAGTTGGAAAATTGGAAATAGCACTGCAAATAACACTGGC-
----

ACAAATGACACTATAACTCTCCAATGTAGAATAAAGCAAATTGTACATATGTGGCAGAGAGTAGGACAAGCAA
TGTATGCCCCTCCCATTCCAGGAAAAATAAGGTGTGAATCAAAC---
ATTACAGGACTACTATTAACAAGAGATGGTGGGAATAATAGTGCA----------------------------
----

AATGAAACCTTCAGACCTACAGGAGGAGATATGAAGGACATTTGGAGAAGTGAATTATATAAGTATAAAGTAG
TGGAAATTAAACCACTA---GGAGTAGCACCCACCAGGGCAAAAAGAAGAGTGGTG-----------------
----------------------GAGAGAGAAAAAAGAGCA---GTTGGA---CTAGGAGCT---------
GTACTCCTTGGGTTCTTGGGAACA---GCAGGAAGCACTATG---GGCGCGGCGTCA---
ATAACGCTGACGGTACAG---GCCAGACAA---TTATTG---TCTGGTATAGTGCAACAGCAAAGC---
AATTTG---CTGAAA---GCTATTGAGGCTCAACAGCAACTGTTGAAACTCACTGTCTGGGGCATT
>KP718938..1
TTCTATTGCAATACATCACAACTGTTTAATAGCACTTGGGATAACAATACAACTTGGGATAACAATACAACAC
AGAATAAT---

AGCAGTAAGAATATAACTCTTCCATGCAAAATAAAGCAAATTGTAAGGATGTGGCAGAGAGTAGGACAAGCAA
TGTATGCCCCTCCCATCCAAGGAGAAATAAAGTGTGAATCAAAC---
ATTACAGGACTAATATTAACAACAGATGGT---ACGGGAAATAGC----------------------------
----

ACAAATGAGACCGTCAGACCTGCAGGAGGAGATATGAGGGACAATTGGAGAAGTGAGCTATATAAGTATAAAG
TAGTGGAGATTAAACCACTA---GGAGTAGCACCCACCAGGGCAAAAAGAAGAGTGGTG--------------
-------------------------GAGAGAGAAAAAAGAGCA---GTGGGA---CTAGGAACT---------
TTGCTCCTTGGGTTCTTGGGAACA---GCAGGAAGCACTATG---GGCGCAGCGTCA---
ATAACGCTGACGGTACAG---GCCAGACAA---TTATTG---TCTGGCATAGTGCAACAGCAAAGC---
AATTTG---CTGAAG---GCTATTGAAGCTCAACAGCAACTGTTGAAGCTCACTGTCTGGGGCATT
>KY658700..1
TTCTATTGCAACACAACAAAGCTGTTTAATAGCACTTGGCAGTTTAATGATACTAATGGTACTTGGCGGGAAA
ATGGCACT---
-------------------GAA---
GACAAAAATGACACTATATATCTTCCATGCAGAATAAAGCAGATTGTAAGACTATGGCAGACAGTAGGACAAG
CAATGTATGCCCCTCCCATCCGAGGAGAAATAAGGTGTAATTCAAAT---
ATTACAGGGTTGCTATTAACAAGAGATGGTGGACAGTCAAATGAGTCA-------------------------
----

AAGGTTAATGAGACCTTCAGACCTACAGGAGGAAATATGAAAGACAATTGGAGAAGTGAATTATATAGGTATA
AGGTGGTGGAAATTAAACCACTA---GGAGTAGCACCCACCAAGGCAAGAAGAAGAGTGGTG-----------
----------------------------CAGAGAGAAAAAAGAGCA---GTGGGA---ATGGGAGCT------
---GTACTCCTTGGGTTCTTGGGAGCA---GCAGGAAGCACTATG---GGCGTAGCGTCA---

ATAACGCTGACGGTACAA---GCCAGACAA---TTATTG---TCTGGTATAGTGCAACAGCAAAGC---
AATTTG---CTGAGG---GCTATTGAGGCTCAACAGCAATTGTTGAGACTCACTGTCTGGGGCATT
>AF069945..1
TTCTATTGTAATACATCAAGTCTGTTTAATAGCACTTGGGAAAATGAAAATACAACTAGCAATGGGACTGAG-
----

GAAAATGGCACTATAACTCTCCCATGCAGAATAAAGCAAATTGTAAATCTGTGGCAGAGAGTAGGACAAGCAA
TGTATGCCCCTCCCATCCAAGGAAACTTAACATGTAACTCAAAC---
ATTACAGGGCTACTATTAACAAGAGATGGTGGGAAAAATAACACT----------------------------
----

CATAATGAGACCTTCAGACCTACAGGAGGAGATATGAAGGACAATTGGAGAAGTGAATTATATAAATATAAAG
TGGTAGAAATTAAACCACTA---GGAGTAGCACCCACCAGGGCAAAAAGAAGAGTGGTG--------------
-------------------------GAGAGAGAAAAAAGAGCA---CTGGGA---ATAGGAGCT---------
GTGCTCCTTGGGTTCTTGGGGACA---GCAGGAAGCACTATG---GGCGCGGCGTCA---
ATAACGCTGACGGTACAG---GCCAGACAA---TTATTG---TCTGGCATAGTGCAACAGCAAAGC---
GATTTG---CTGAAG---GCTATAGATGCACAACAGCAACTGCTAAAACTCACTGTCTGGGGCATA
>AF408631..3
TTCTACTGCAATACATCAAAACTGTTTAATGACACAACAACC-------------------------------
----

AATGACACTATTATTCTCCCATGCAAAATAAAACAAATTGTAAATATGTGGCAGGAAGTGGGACGAGCAATGT
ATGCCGCTCCCATCGCAGGAAGCATTACCTGTAGATCAAAT---
ATTACAGGTCTACTATTGACAAGAGATGGTGGTCAGACTAATATTACTAATCAG------------ACT----
----
-
GAGACTAATCAGACTACTGAGATCTTCAGACCTGGGGGAGGAGATATGAGAGACAATTGGAGAAGTGAATTAT
ATAAATATAAAGTAGTAGAAATTGAGCCACTA---GGAGTAGCACCCACCAGGGCAAGAAGACAAGTGGTG--
-------------------------------------AGAAGAGAAAAAAGAGCA---GTGGGA---
ATGACAGCT---------GTGTTCTTTGGGTTCTTGGCAGCA---GCAGGAAGCACTATG---
GGCGCAGCGTCA---ATAACGCTGACGGCACAG---GCCAGACAA---TTATTG---
TCTGGAATAGTGCAACAGCAGAAC---AATCTG---CTGAGG---
GCTATTGAAGCGCAACAGCATCTGTTGCAGCTCACAGTCTGGGGCATT
>AF408629..1
TTCTACTGCAATACATCAAAACTGTTTGATGAAGGACTGTTTAATACAACAGAT-------------------
----

GACAATGACACTATTATTCTCCCATGTCGAATAAAACAAATTGTAAACATGTGGCAGGAAGTGGGACGAGCAA
TGTATGCCGCTCCCATTGCAGGAAACATTAACTGTAACTCAACT---
ATTACAGGTCTAATATTGACAAGAGATGGTGGGAAAAATACTACT----------------------------
----

GGGAATGAGACCTTCAGACCTGGGGGAGGAAATATGAAAGACAATTGGAGAAGTGAATTATATAAATATAAGG
TGGTAGAAATTCAACCACTA---GGAGTAGCACCCACCACGGCAAAGAGACAAGTGGTG--------------
-------------------------CAGAGAGAAAGAAGAGCA---GTGGGA---ATGGGAGCT---------
TTGTTCCTTGGGTTCTTGGGAGCA---GCAGGAAGCACTATG---GGCGCGGCGTCA---
ATAACGCTGACGGTACAG---GCCAGACAA---TTATTG---TCTGGAATAGTGCAACAGCAAAAC---
AATTTG---CTGAGG---GCTATTGAAGCGCAACAGCATCTGTTGCAGCTCACAGTCTGGGGCATT
>AY037266..2
TTCTACTGTGATACATCAAAACTGTTTCATGACACGCTGATTAATACCAATACATCCGATGACACT-------
----

GCTAATGGCACTATAACTCTCCCATGTAGAATAAAACTAATTGTAAACCTGTGGCAGGAAGTGGCACGAGCAA
TGTATGCCGCTCCCATTGCAGGAAGCGTTACCTGTAACTCAAGT---
ATTACAGGTCTACTATTGACAAGAGATGGTGGTCAGAAGAATAGT----------------------------
----

ACTAATGACACCTTCAGACCTGGGGGAGGAAATATGAGGGACAATTGGAGAAGTGAATTATATAAATATAAAG
TAGTAGAAATTAAACCATTA---GGAATAGCTCCCACCAAGGCAAAAAGACAAGTGGTG--------------
-------------------------AAGAGAGAAAGAAGAGCA---GTGGGA---ATAGGAGCG---------

TTGTTCCTTGGGTTCTTGGGAACA---GCAGGAAGCACTATG---GGCGCGGCGTCA---
ATAACGCTGACGGTACAG---GCCAGACAA---TTATTG---TCTGGAATAGTGCAACAGCAGAAC---
AATTTG---CTGAGG---GCTATTGAAGCGCAACAGCATCTGTTGCAGCTCACAGTCTGGGGCATT
>AF385936..1
TTCTACTGCAATACATCAGGACTGTTTAATGATACAATATCC------------------------------
----

AATGATACTATTATTCTCCCATGTAAAATAAAACAAATTGTAAACCTGTGGCAGGAAGTGGGACGAGCAATGT
ATGCCGCTCCCATTGAAGGAAACATTACCTGTAACTCAAAT---
ATTACAGGTCTACTATTGACAAGAGATGGTGGTCTTAATGAGACT---------------------------
----

AATCAGACTGAAACCTTCAGACCTGGGGGAGGAAATATGAAAGACAATTGGAGAAGTGAATTATATAAATATA
AAGTGGTAGAAATTGAACCACTA---GGAGTAGCACCCACCAGGGCAAAAAGACAAGTGGTG----------
---------------------------ACGAGAGAAAAAAGAGCA---GTGGGA---ATAGGAGCT------
---TTGTTCCTTGGGTTCTTGGGAGCA---GCAGGAAGCACTATG---GGCGCGGCGTCA---
GTGACGCTGACGGTACAG---GCCAGACAA---TTATTG---TCTGGAATAGTGCAACAGCAAAAC---
AACTTG---CTGCAG---GCTATTGAAGCGCAACAGCATCTGTTGCAGCTCACAGTCTGGGGCATT
>JN054280..1
TTCTACTGCAATACATCAAAACTGTTTAATAACACAAAAATT------------------------------
----

AATGGCACTATTACTCTCCCATGTCGAATAAAACAAATTGTAAACCTGTGGCAGGAAGTGGGACGAGCAATGT
ATGCCGCTCCCATTGCAGGAAACATTACCTGTAACTCAAAT---
ATTACAGGTCTGCTATTAACAAGAGATGGGGGACAAAGTTATAATAGTACTAAT-----------AAC----
----
-
AGTACTATTAATAGGACTGAGATCTTCAGACCTGGGGGAGGAAATATGAAAGACAATTGGAGAAATGAATTAT
ATAAATATAAAGTGGTAGAAATTGAACCACTA---GGAATAGCACCCACCAAGGCAAAAAGACAAGTGGTG--
------------------------------------AAGAGAGAAAAAAGAGCA---
GTAGGAGTAATAGGAGCT--------ATGTTCCTTGGGTTCTTGGGAGCA---GCAGGAAGCACTATG---
GGCGCAGCGTCA---ATAACGCTGACGGTACAG---GCCAGACAA---TTATTG---
TCTGGAATAGTGCAACAGCAAAAC---AATTTG---CTGAGG---
GCTATTGAAGCGCAACAGCATCTGTTGCAGCTCACAGTCTGGGGCATT
>JN235963..2
TTCTACTGCAATACATCAGGACTGTTTAATAATGACACAGTA------------------------------
----

TCCAATGGCACTATTACTCTCCCATGTCGAATAAAACAAATTATAAACATGTGGCAGGAAGTGGGACGAGCAA
TGTATGCCAATCCCATTGCAGGAAACATTACCTGTAACTCAAAT---
ATTACAGGTCTACTATTGACAAGAGATGGTGGTGGTCATAGTAATGAA------------------------
----

ACTAATCAGACTGAGATCTTCAGACCTGGGGGAGGAAATATGAAAGACAATTGGAGAAGTGAATTATATAAAT
ATAAAGTAGTAGAAATTGAACCACTA---GGAGTAGCACCCACCAGGGCAAAAAGACAAGTGGTG-------
------------------------------AAGAGAGAAAAAAGAGCA---GTGGGA---CTAGGAGCT---
------GTGTTCATTGGGTTCTTGGGAGCA---GCAGGAAGCACTATG---GGCGCAGCGTCA---
GTAACGCTGACGGTACAG---GCCAGACAA---TTATTG---TCTGGAATAGTGCAACAGCAGAGC---
AATTTG---CTGAAG---GCTATTGAAGCGCAACAGCATCTGTTGCGGCTCACAGTCTGGGGCATT
>AY037272..1
TTCTACTGCAATACATCAGGACTGTTTAATGAAAGCCTGATTAATGTCACA---------------------
----

CAACCCAATGACATTACTCTCCCATGTCGAATAAAACAAATTGTAAACCTGTGGCAGAAAGTGGGACGAGCAA
TGTATGCCGCTCCCATTGCAGGAAGCATTATCTGTATCTCAAAT---
ATTACAGGTCTACTATTGACAAGAGATGGTGGTCAGAATAATAGT---------------------------
----

ACTCCTGAGATCTTCAGACCTGGGGGAGGAAATATGAAAGACAATTGGAGAAGTGAATTATATAAATATAAAG
TAGTAGAAATTGAACCACTA---GGAATAGCACCCACCGGGGCAAAAAGACAAGTGGTA-------------

-------------------------AAGAGAGAAAAAAGAGCA---ATAGGA---ATGGGAGCT---------
TTGTTCCTTGGGTTCTTGGGAGCA---GCAGGAAGCACTATG---GGCGCGGCGTCA---
ACAGCGCTGACGGTACAG---GCCAGACAG---TTATTG---TCTGGAATAGTGCAACAGCAGAAC---
AATTTG---CTGAGA---GCTATTGAAGCGCAACAGCATCTGTTGCAGCTCACAGTCTGGGGCATT
>AY371154..1
TTCTATTGCAATACATCAGCCCTGTTTAATAGCGCTTGGAGAAATGATACTAACAGCACGCAGGTTTCGGGG-
----

TCAAATGACAATATAACTCTCCCATGCAGAATAAAGCAATTTGTAAAGCTATGGCAGAGGGTGGACAAGCAA
TGTATGCCCCTCCCATCCAAGGAGTAATAAGGTGCGAATCAAAC---
ATTACAGGGCTACTATTAACAAGAGATGCTGAGGGTAATAATAGA---------------------------
----

ACAAATGAGACCTTCAGACCTGGAGGAGGAGATATGAGGGATAATTGGAGAAGTGAATTATATAAATATAAAG
TAGTAAAAATTGAACCACTA---GGAGTAGCACCCAGCAGAGCAAGGAGAAGAGTGGTA-------------
-------------------------GCAAGAGAAAAAAGAACA---GTTGGA---CTAGGAGCT--------
GTCTTCCTTGGGTTCTTAGGAGCA---GCAGGAAGCACTATG---GGCGCAGCGTCA---
CTGACGCTGACGGTACAG---GCCAGACAA---TTATTG---TCTGGCATAGTGCAACAACAGAAC---
AATCTG---CTGATG---GCTATAGAGGCTCAACAGCACCTGTTGAAACTCACGGTCTGGGGCATT
>DQ845388..1
TTCTATTGCAATACATCAGCCCTGTTTAATAGCACTTGGGAAGGGAACAATACCAGCACGCAAGGGTCAAATG
ACACA---

GGGTCAAATGACACTATAACTCTCCCATGCAGAATAAAACAATTTGTAAATATATGGCAGAGAGTAGGACAAG
CAATATATGCCCCCCCCATCCAAGGAGTAATAAGGTGTGACTCAAAC---
ATTACAGGACTACTATTAACAAGAGATGGTGGGGATAATGATACTACT------------------------
----

AGGGCAAATGAGACCTTCAGACCTGGAGGAGGAGATATGAAGGACAATTGGAGAAGTGAATTATATAAATACA
AAGTAGTAAAAATTGAACCACTA---GGAGTAGCACCCACCAAGGCAAAGAGAAGAGTGGTG----------
---------------------------GCAAGAGAAAAAAGAGCA---GCA------CTGGGAGCT------
---GTCTTCCTTGGGTTCTTAGGAGCA---GCAGGAAGCACTATG---GGCGCGGCGTCA---
ATAACGCTGACGGTACAG---GCCAGACAA---TTATTG---TCTGGCATAGTGCAACAGCAGAGC---
AATCTG---CTGATG---GCTATACAGGCTCAACAGCATATGTTGAAACTTACGGTCTGGGGCATT
>DQ845386..1
TTCTATTGCAATACATCAGAACTGTTTAATAGTGCATGGAGAAATGGGACCAGAGTGCGGGGGTCAACTAAC-
----

ACAAGTGAAAATATAACTCTCCCATGCAGAATAAAGCAAATTGTAAAATTATGGCAGAGAGTAGGGCAGGCAA
TATATGCCCCTCCCATCCAAGGAGTAATAAGGTGTGAATCAAAC---
ATTACAGGGCTACTATTAACAAGAGATGGTGGGAATAATAATGAGACA------------------------
----

AATAAGACTGAGACCTTCAGACCTGGAGGAGGAGATATGAAGGACAATTGGAGAAGTGAATTATATAAATATA
AAGTAGTAAAAATTGAACCACTA---GGAGTAGCACCCAGCAGAGCAAGGAGAAGAGTGGTG----------
---------------------------GCAAGAGAAAAAAGAGCA---GTTGGA---CTAGGAGCT------
---GTCTTCCTTGGGTTCTTAGGAGCA---GCAGGAAGCACTATG---GGCGCGGCGTCA---
TTAACGCTGACGGTACAG---GCCAGACAA---TTGTTG---TCTGGTATAGTGCAACAGCAGAAT---
AACCTG---CTGATG---GCTATAGAGGCTCAACAGCATATGTTGAAACTCACAGTCTGGGGCATT
>DQ845387..1
TTCTATTGTAATACATCAGAACTGTTTAATAGCACTTGGACAAATGACACTAACAGCACGCAGGGGTCAAAT-
----

GACACAACGCCCATTATTCTCCCATGCAGAATAAAGCAAATTATAAATATGTGGCAGAGAGTAGGACAAGCAA
TATATGCCCCTCCCATTGAAGGAGTAATAAGGTGTGAATCAAAC---
ATTACAGGACTACTATTAACAAGAGATGGGGGTAATAACAGAACA---------------------------
----

AGTGAGATCTTCAGACCTGGAGGAGGGGATATGAGGGACAACTGGAGGAGTGAATTATATAAATATAAAGTAG
TAAAAATTGAGCCACTA---GGAGTAGCACCCAGCAAGGCAAGGAGAAGAGTGGTG----------------

---------------------ACGAGAGAAAAAAGAGCA---GTTGGA---ATAGGAGCT---------
GTCTTCCTTGGGTTCTTAGGAGCA---GCAGGAAGCACTATG---GGCGCGGCGTCA---
TTAACGCTGACGGTACAG---GCCAGAAAC---TTATTG---TCTGGTATAGTGCAACAGCAGAAC---
AACCTG---CTGAGG---GCTATAGAGGCTCAACAGCAACTGTTGAAACTCACGGTCTGGGGCATT
>MH705141..1
TTCTACTGCAATACATCAGGCCTGTTTAATAGCAGTTGGGAAAATGACAATGCCAGCACAGGGTCAAATAACA
CAGAG---

TCAAATGCCAATATAACTCTCCCATGCAAAATAAAGCAATTTGTAAATGTATGGCAGAGAGTAGGACAAGCAA
TGTATGCCCCTCCCATTGCAGGATTAATAAGATGTGAATCAAAT---
ATTACAGGACTATTATTAACAAGAGATAATGAGGGTAATAACAGG---------------------------
----

ACAAATGAGACCTTCAGACCCGGAGGAGGAGACATGAAGGACAATTGGAGAAGTGAATTATATAAGTATAAAG
TAGTAAAAATTGAACCACTA---GGAGTAGCACCCAGCAAGGCAAGGAGAAGAGTGGTG-------------
------------------------GCAAGAGAAAAAAGAGCAGTTGTTGGA---CTAGGAGCT---------
GTCTTCATTGGGTTCTTAGGAGCA---GCAGGAAGCACTATG---GGCGCGGCGTCA---
TTAACGCTGACGGTACAG---GCCAGACAA---TTATTG---TCTGGCATAGTGCAACAACAGAAC---
AATCTG---TTGATG---GCTATAGAGGCTCAACAGCATTTGTTGAAGCTCACGGTCTGGGGCATT
>KP718924..1
TTCTATTGCAATACATCAGGCCTGTTTAATAGTACTTGGGAATCTAACAATACCAGCACGTCGGGGTCAAATG
ACACAGGG--

TCAAATGACACTATAACTCTCCCATGCAGAATAAAGCAATTTGTAAAGATGTGGCAGAGAGTAGGACAAGCAA
TATATGCCCCTCCCATCCCAGGAGTAATACAGTGTAATTCAAAC---
ATTACAGGACTACTATTAGCAAGAGATGGTGGGGATGATAATACA---------------------------
----

ACAAATGAGACCTTCAGACCTATAGGAGGAGATATGAAGGACAATTGGAGAAGTGAATTATATAAATATAAAG
TAGTAAAAATTGAACCATTA---GGAGTAGCACCCAGCAAGGCAAGGAGAAGAGTGGTA-------------
------------------------GCAAGAGAAAAAAGAGCA---ATCGGAGGACTAGGGGCT---------
GTCTTCCTTGGGTTCTTAGGAGCA---GCAGGAAGCACTATG---GGCGCGGCGTCA---
CTGACGCTGACGGTACAG---GCCAGACTA---TTATTG---TCTGGCATAGTGCAACAGCAGAAC---
AATCTG---CTGAGG---GCTATAGAGGCTCAACAACATCTGTTGAAACTCACGGTCTGGGGCATT
>KP718926..2
TTCTATTGCAATACAACAAAACTGTTTAATAGCACTTGGGACAACTACACGCAAGGGTCAAATGAC------
----

ACAGAGGACCCTATAGTTCTCCCATGCAGAATAAAGCAATTTGTAAATATATGGCAGAGAGTAGGACAAGCAA
TATATGCCCCTCCCATCCAAGGAGTAATAAATTGTAGCTCAAAC---
ATTACAGGACTACTATTAACAAGAGATAATGATGAGGGTAGTAAT---------------------------
----

AGGGCAAATGAGACCTTCAGACCTGGAGGAGGAGATATGAGGGACAATTGGAGAAGTGAGTTATATAAATATA
AAGTAGTAAAAATTGAACCACTA---GGAGTAGCACCCAGCAGGGCAAGGAGAAGAGTGGTG----------
---------------------------GCAAGAGAAAAAAGAGCAATAGTTGGA---ATAGGAGCT------
---CTCTTCCTTGGGTTCTTAGGAGCA---GCAGGAAGCACTATG---GGCGCGGCGTCA---
CTAACGCTGACGGTACAG---GCCAGACAA---TTATTG---TCTGGCATAGTGCAACAGCAGAGC---
AATCTG---CTGAGG---GCTATAGAGGCTCAACAGCATCTGTTGAAACTCACGGTCTGGGGCATT
>AF460972..1
TTCTATTGCAATACATCAGCCCTGTTTAATAGCACTTGGGAAAATGACACTGTCAGCACGCAAGGGTCAAATG
ACACA---

GGGTCAAATGACACTATAACTCTCCCATGCAGAATAAAGCAATTTGTAAACATGTGGCAGAGAGTAGGACAAG
CAATGTATGCCCCTCCCATCCAAGGAATAATAAGGTGTGAATCAAAC---
ATTACAGGACTACTATTATCAAGAGATGGTGGTGTGGGTAATAAT---------------------------
----

AGGACAAATGAGACATTCAGACCTGGAGGAGGAAATATGAAAGACAATTGGAGAAGTGAATTATATAAGTATA
AAGTAGTAAAAATTGAACCATTA---GGAGTAGCACCCAGCAGAGCGAGGAGAAGAGTGGTG----------

----------------------------GCAAGAGAAAAAAGAGCA---GTAGTTGGACTAGGGGCT------
---GTCTTCCTTGGGTTCTTAGGAGCA---GCAGGAAGCACTATG---GGCGTGGCGTCA---
CTAACGCTGACGGTACAG---GCCAGACAA---TTATTG---TCTGGCATAGTGCAACAACAGAAC---
AATCTG---CTGATG---GCTATAGAGGCTCAACAGCATCTGTTGAAACTCACGGTCTGGGGCATT
>AF460974..1
TTCTATTGCAATACATCAACCCTGTTTAATAGCACTTGGGCAAATGACACAAAGGCAAATGACACAGGG----
----

TCAAATGACACTATAACTCTCCCATGCAGAATAAAGCAATTTGTAAATAAGTGGCAAAGAGTAGGACAATCAA
TGTATGCCCCTCCCATCCAAGGAACAATAAGGTGTGACTCAAAC---
ATTACAGGACTACTATTAACAAGAGATGGGGGTGATAATAGCCTA----------------------------
----

AATGAGACCTTCAGACCTGGAGGAGGAGATATGAGGGACAATTGGAGAAGTGAATTATATAAATATAAAGTAG
TAAAAATTGAACCACTA---GGAGTAGCACCCAGCAAGGCAAGGAGAAGAGTGGTG-----------------
----------------------ACAAGAGAAAAAAGAGCA---GTGGGA---CTAGGGGCT---------
GTCTTCCTTGGGTTCTTAGGAGCA---GCAGGAAGCACTATG---GGCGCGGCGTCA---
CTAACGCTGACGGTACAG---GCCAGACAA---TTATTG---TCTGGCATAGTGCAACAACAGAAC---
AATCTG---CTGAGG---GCTATAGAGGCTCAACAGCATCTGTTGAAACTCACGGTCTGGGGCATT
>AF450097..1
TTCTATTGTAATACAACAAAATTGTTTAACAGTACTTGGACTGTGAATGGAACTACAAATACTACAAAT----
----

AACAATGACACAATCACACTCCCATGCAAAATAAAACAAATTATAAACATGTGGCAGGAAGTAGGAAAAGCAA
TGTATGCCCCTCCCATCAGCGGTCAAATTAGATGCTCATCAAAT---
ATTACAGGGCTGCTATTAGTAAGAGATGGTGGTAATAGTAACGAG----------------------------
----

ACCGAGATCTTCAGACCTGGAGGAGGAAATATGAAGGACAATTGGAGAAGTGAATTATATAAATATAAAGTAG
TAAAAATTGAACCATTA---GGAGTAGCACCCACCAGGGCAAGGAGAAGAGTGGTG-----------------
----------------------CAAAGAGAAAAAAGAGCA---GTGGGA---ATAGGAGCT---------
TTGCTCATTGGGTTCTTGGGAGCA---GCAGGAAGCACTATG---GGCGCAGCGTCA---
ATGACGTTGACGGTACAG---GCCAGACAA---TTACTG---TCTGGTATAGTGCAACAGCAGAAC---
AATCTG---CTGAGG---GCTATTGAGGCGCAACAGCATATGTTGCAACTCACAGTCTGGGGCATA
>JN248351..1
TTCTACTGTAATACATCACAACTGTTTAATAGTACATGGAATAATACTGCAGGAAATTCTAATAATGAT----
----

ACCAATACCACTATCACGCTCCCTTGCAGAATAAGACAAATTGTAAATATGTGGCAGGAAGTAGGAAGAGCAA
TGTATGCCCCTCCCATTGAAGGACAAATTAGCTGTTCATCAAAT---
ATTACAGGGTTGCTATTAACAAGAGATGGTGGTCATAACAATGTTAGC-------------------------
----

AATACCACCGAGACCTTCAGACCTGGAGGAGGAGATATGAGGGACAATTGGAGAAGTGAATTATATAAATATA
AAGTAGTAAAAATTGAACCATTA---GGAGTAGCACCCACCAAGGCAAAGAGGAGAGTGGTG-----------
----------------------------CAAAGAGAAAAAAGAGCA---GTGGGAACGATAGGAGCT------
---GTGTTCCTTGGGTTCTTGGGAGCA---GCAGGAAGCACTATG---GGCACAGCGTCA---
ATAACGCTGACGGTACAG---GCCAGACAA---TTGTTG---TCTGGTATAGTGCAACAGCAGAGA---
AATTTG---CTGAGG---GCCATTGAGGCGCAACAGCATCTGCTGCAACTCACAGTCTGGGGCATC
>KT022406..2
TTCTATTGCAATACATCAAGCCTGTTTAATGGCACATGGAACAGGGCTAACTACACAATGGAG----------
----

AATGACACTATTATGAATATCACATGCAGAATAAAACAAATTATAAACATGTGGCAGAGAGTAGGACAAGCAA
TGTATGCCCCTCCCATTGCAGGAGTAATAAAGTGTACATCAAAC---
ATTACAGGAATAATATTGACAAGAGATGGTGGGAACGGGAACAACAGT-------------------------
----

ACCAACAGTACAGAGACCTTCAGGCCTGGAGGAGGAGATATGAGAGACAATTGGAGAAGTGAATTATATAAGT
ATAAAGTAGTAAAAATTGAACCATTA---GGAGTAGCACCCACCAAGGCAAAGAGAAGAGTGGTG--------

------------------------------GAGAGAGAAAAAAGAGCA---GTTGGA---CTGGGAGCT---
------GTCTTCCTTGGGTTCTTGGGAGCA---GCAGGAAGCACTATG---GGCGCAGCGTCA---
ATAACGCTGACGGTACAG---GCCAGACAA---TTATTG---TCTGGCATAGTGCAACAGCAAAGC---
AATTTG---CTGAAG---GCTATAGAGGCTCAACAACATCTGTTGAGACTCACGGTCTGGGGCATT
>AY945736..1
TTCTATTGCAATACATCAGACCTGTTTAATAGCACGTGGACCAACGACACGTGGGGGAAAATGGCACTGCCA
ACGAC---
------------------TCA---
AAGTCAAATGACACTATAATGCTCCCATGCAGGATAAAACAAATTATAAACATGTGGCAGAGAGTAGGACAAG
CAATGTATGCCCCTCCCATTGCAGGAATAATAAAGTGTACATCAAAC---
ATTACAGGAATAATATTGACAAGAGATGGTGGAGAGAACGATAGT---------------------------
----

ACAGAGACCTTCAGACCTGGAGGAGGAGATATGAGAGACAATTGGAGAAGTGAATTATATAAGTATAAAGTAG
TAAAACTTGAACCACTA---GGAGTAGCACCCACCAAGGCCAGGAGAAGAGTGGTG----------------
---------------------GAGAGAGACAAAAGAGCA---GTTGGA---CTGGGAGCT---------
TTCTTCCTTGGGTTCTTGGGAGCA---GCAGGAAGCACTATG---GGCGCAGCGTCA---
CTAACGCTGACGGTACAG---GCCAGACAA---TTATTG---TCTGGCATAGTGCAACAGCAAAGC---
AATTTG---CTGAAG---GCTATAGAGGCTCAACAACATCTGTTGAGACTCACAGTCTGGGGCATT
>AF286239..1
TTCTATTGCGATACATCAGGCCTGTTTAATAGCACGTGGCCTGCCAATGCATCAAGGGAGAATGAGGAG----
----

AAAGATCGCAATGTGACTCTCCCATGCAGAATAAAACAAATTGTAAACATGTGGCAGAGAGTAGGACGAGCAA
TGTATGCCCCTCCCATTAACGGAACAATAAAGTGTACATCCAAC---
ATTACAGGAATGATATTAACAAGAGATGGG---AACAGTGGGGGCAAT------------------------
----

GCAACCAATGAGACCTTCAGACCTGGAGGAGGAGATATGAGGGACAATTGGAGAAGTGAATTATATAAGTATA
AAGTAGTAAAACTAGAACCACTA---GGAGTAGCACCTACCAGGGCAAGAAGAAGAGTGGTG----------
---------------------------GAGAGGGAAAAAAGAGCA---GCA------GTTGGACTG------
---TTCTTCCTTGGGTTCTTGGGAGCA---GCAGGAAGCACTATG---GGCGCGGCGTCA---
GTGACGCTGACGGTACAG---GCCAGACAA---TTATTG---TCTGGCATAGTGCAACAGCAAAGC---
AATTTG---CTGAAG---GCTATAGAGGCTCAACAGCATATGTTGAGACTCACGGTCTGGGGCATT
>EU581825..1
TTCTACTGCAATACATCAGGATTGTTTAATAACTCAGTATTC------------------------------
----

AATAATAACACTATCACTCTCCCATGTCGAATAAAACAAATTGTAAACATGTGGCAGGAAGTGGGACGAGCAA
TGTATGCCGCTCCCATTGCAGGAAACATTGCCTGTAGCTCAAAT---
ATTACAGGTCTACTATTGACAAGAGACGGTGGTTGTCAGAACCAGAGCAAT---------------------
----

ACTAACTGTACAGAGACCTTCAGACCTTGGGGAGGAAATATGAAAGACAATTGGAGAAGTGAATTATATAAAT
ATAAAGTAGTAGAAATTGAACCATTA---GGAATAGCACCCACCAAGGCAAAGAGAAGAGTGGTG-------
------------------------------CAGAGAGACAAAAGAGCA---GTGGGA---TTGGGAGCT---
------ATGTTCCTTGGGTTCTTGGGAGCA---GCAGGAAGCACTATG---GGCGCAGCGTCA---
ATAACGCTGACGGTACAA---GCTAGACAA---TTATTG---TCTGGAATAGTGCAACAGCAAAAC---
AATTTG---CTGAGG---GCTATTGAAGCGCAACAGCATATGTTGCAGCTCACAGTCTGGGGCATT
>AY037281..1
TTCTACTGCAATACATCAAAACTGTTTAATGACTCCCGGGATAATGACACAGAA------------------
----

TCCAATGACACTATCATTCTCCCATGTCGAATAAAACAAATTGTAAACAGGTGGCAGGAAGTAGGACAAGCAA
TGTATGCCGCTCCCATTGCAGGAAACATTGTTTGTAACTCAAAT---
ATTACAGGTCTACTATTGACAAGAGATGGTGGTCAGGGAAATGGT---------------------------
----

AGTGGACCAGAAATCTTCAGACCTGAGGGAGGAAATATGAAAGACAATTGGAGAAGTGAATTATATAAATACA
AAGTAGTAGAAATTGAACCATTA---GGAGTAGCACCCACCAAGGCAAAGAGAAGAGTGGTG----------

----------------------------CAGAGAGAAAAAAGAGCA---GTGGGA---ATGGCAGCT------
---GTGTTCATTGGGTTCTTGGGAGCA---GCAGGAAGCACTATG---GGCGCAGCGTCA---
ATAACGCTGACGGTACAG---GCCAGACAA---TTATTG---TCTGGAATAGTGCAACAGCAGAGC---
AATCTG---CTGAGG---GCTATTGAAGCGCAACAGCATATGTTGCAGCTCACAGTCTGGGGCATT
>EU581827..1
TTCTACTGCAATACATCAGGATTGTTTAATGACACAGTATCT-------------------------------
----

AATGGCACTATCACTCTCCCATGTAAAATAAAACAAATTGTAAACATGTGGCAGGAAGTGGGAAGAGCAATGT
ATGCCGCTCCCATTGCAGGAAAAATTACCTGTAACTCAAGT---
ATTACAGGTCTTCTATTGACAAGAGATGGTGGTAGTAATACAACA----------------------------
----

GGCAACACAGAGACCTTCAGACCTGGGGGAGGAGATATGAGAGACAATTGGAGAAGTGAATTATATAAATATA
AAGTAGTAGAAATTCAACCAATA---GGAGTAGCACCCACCCCGGCAAAAAGAAGAGTGGTG-----------
----------------------------CAAAGAGAAAAAAGAGCA---GCA------TTAACAACT------
---ATGTTCCTTGGGTTCTTGGGAGCA---GCAGGAAGCACTATG---GGCGCAGCGTCA---
ATAGCGCTGACGGTACAG---GCCAGACAA---TTATTG---TCTGGAATAGTGCAACAGCAAAGC---
AATTTG---CTGAGA---GCTATTGAAGCGCAACAACATATGTTGCAGCTCACAGTCTGGGGCATT
>KP718931..2
TTCTATTGTGATACAACACAACTGTTTAATAGTACCTGGGATAAGAGTACCTGGAATAACACCAATAGCAGCT
ATGAGGGA---

AATGGAGATGATACTATTACATTGCAGTGCAAAATAAAACAAATTGTAAACATGTGGCAGAGAGTAGGACAAG
CAATGTATGCCCCTCCCATCGCGGGAAATATTACATGTGTATCAAAT---
ATTACAGGACTAATATTGACAAGTGACTTTGTTAATGCAACTACA----------------------------
----

GATGTGACCTTCAGACCTGGAGGAGGAGATATGAGGGACAATTGGAGAAGTGAATTATATAAATATAAAGTGG
TGAAAATTGAACCACTA---GGAGTAGCACCCTCTAAGGCAAAGAGAAGAGTGGTG-----------------
----------------------GAGAGAGAAAAAAGAGCA---ATTGGA---ATAGGAGCT---------
GTCTTCCTTGGGTTCTTAGGAGCA---GCAGGAAGCACTATG---GGCGCGGCGTCA---
ATAACGCTGACGGTACAG---GCTAGACAA---TTATTG---TCTGGTATAGTGCAACAGCAAAGC---
AATTTG---CTGAGG---GCTATAGAGGCTCAACAGCATCTGTTGAAACTCACGGTCTGGGGCATT
>AF377959..2
TTTTATTGTAATACAACAAACCTGTTTACATGGGATAGCACCACCAATGGCACCAATGGCAACTACACAGTAA
GT---

AATAATGATACTATTACACTGCAATGCAGAATAAAACAAATTATAAACATGTGGCAGAGAGTAGGACAAGCAA
TGTACGCTCCTCCCATCGAAGGAAGCATTACATGTGTATCAAAT---
GTTACAGGACTAATATTGACAAGTGACTTTGTTAATAAGTCAACA----------------------------
----

GAGGAGGTGACCTTCAGACCTGGAGGGGGAGATATGAGAGACAATTGGAGAAGTGAATTATATAAATATAAAG
TAGTGAAAATTGAACCACTA---GGAGTAGCACCCACTAAGGCAAAGAGAAGAGTGGTG--------------
-------------------------GGGAGAGAAAAAAGAGCA---GTTGGA---CTAGGAGCT---------
GTCTTCCTTGGGTTCTTAGGAGCA---GCAGGAAGCACTATG---GGCGCGGCGTCA---
ATAACGCTGACGGTACAG---GCTAGACAA---TTATTG---TCTGGTATAGTGCAACAGCAAAGC---
AATTTG---CTGAGG---GCTATAGAGGCTCAACAGCATCTGTTGAAACTCACGGTCTGGGGCATT
>EU743963..2
TTCTATTGCAATACATCAGGACTGTTCAATAGCACTTGGACTGACAACACGACAGAATTAAATAGCACGGATG
GC---

AATATAACTCATATAATTCTTCCGTGCAGAATAAAGCAAATTATAAATATGTGGCAGAGAGTAGGACAAGCAA
TGTATGCCCCTCCCATCCCAGGAATAATAAGGTGTGAGTCAAAC---
ATTACAGGATTACTATTGACAAGAGATGGTGGGGTTACTGATAACAGT-------------------------
----

ACAGAAGATGAGACTTTCAGACCTGGAGGAGGAGATATGAGAGACAATTGGAGAAGTGAATTATATAAATATA
AAGTAGTACAAATTGAACCACTA---GGAGTAGCACCCACCAAGGCAAGGAGAAGGGTGGTG-----------

----------------------------GAGAGAGAAAAAAGAGCA---GTTGCTGGACTTGGAGCT------
---GTCCTCTTTGGGTTCTTAGGAGCA---GCAGGAAGCACTATG---GGCGCGGCGTCA---
ATAACGCTGACGGTACAG---GCCAGACAA---TTATTG---TCTGGCATAGTGCAACAGCAAAGC---
AATTTG---CTGAAT---GCTATAGAGGCTCAACAGAATCTGTTGAGACTCACGGTCTGGGGCATT
>AY371165..1
TTCTATTGTAATACAGCAAGCCTGTTTAACAGTACTTGGGGACCAGGGTCAAATAACACGAATGACCCTAGTA
AC---

ACGAGTGACACTATAATTCTCCCGTGCAGAATAAAGCAAATTATAAATATGTGGCAGAGAGCAGGACAAGCAA
TGTATGCCCCTCCCATCCCAGGAATAATAAAGTGTGAATCAAAC---
ATTACAGGACTACTATTGACAAGAGATGGTGGGATAATAATAGT-----------------------------
----

ACAAATGAGACTTTCAGACCTGGAGGAGGAGATATGAGAGACAATTGGAGAAGTGAATTATTTAAATATAAAG
TAGTAAAAATTGAACCACTA---GGAGTAGCACCCACCAAGGCAAAGAGAAGAGTGGTG--------------
-------------------------GAAAGAGAAAAAAGAGCA---GTTGGA---TTTGGAGCT---------
GTCTTCCTTGGGTTCTTAGGAGCA---GCAGGAAGCACTATG---GGCGCGGCGTCA---
ATAACGCTGACGGTACAG---GCCAGACAA---TTATTG---TCTGGCATAGTGCAACAGCAAAGC---
AATTTG---CTGAGG---GCTATAGAGGCTCAACAGCATATGTTGAGACTCACAGTCTGGGGCATT
>JN864049..1
TTCTATTGCAATACATCAGGCCTGTTTAATAGCACTTGGCATAATAATGACACTGGCAGTGGGGCAGAGTACC
AGAGC--

ACGAATGATAGTATAACTCTCCCGTGCAAAATAAAGCAAATTATAAATATGTGGCAGAGAGTAGGACAAGCAA
TGTATGCTCCTCCCATCCCAGGAATAATAGAGTGTAAGTCAAAC---
ATTACAGGACTACTGTTGACACGAGATGGTGGAAATGGTAGTGAC----------------------------
----

AGTGCAAATGAGACTTTCAGACCTGGAGGAGGAGATATGAGAGACAATTGGAGAAGTGAATTATATAAATATA
AAGTAGTAAAAATTGAACCACTA---GGAGTAGCACCCACTAAGGCAAAGAGAAGGGTGGTGGAA---AGA--
-------------------AAGGTGGTGGAGAGAAAGAAAAGAGCA---ATTGGA---TTTGGAGCT------
---GTCTTCCTTGGGTTCTTAGGAGCA---GCCGGAAGCACTATG---GGCGCGGCGTCA---
ATAACGCTGACGGTACAG---GCCAGACAA---TTATTT---TCTGGCATAGTGCAGCAGCAAAGC---
AATTTG---CTGAGG---GCTATAGAGGCTCAACAACAAATGTTGAAACTCACGGTCTGGGGCATT
>JN864050..1
TTCTATTGCAATACATCAGGCCTGTTTAATAGCACTTGGTATCCAAATGGCAGCATGACAGGGTATACAAATG
ACACAAAT---

GACAATGGCACCATAATTCTCCCTTGCAGAATAAAGCAGGTTATAAATATGTGGCAGAGAGCAGGAAAAGCAA
TGTATGCCCCTCCCATCCCAGGAGTAATAAGGTGTGAGTCAAAC---
ATTACAGGACTACTATTAACAAGAGATGGTGGGATAAACAGTACA----------------------------
----

GAGGAGACTTTTAGACCTGGAGGAGGAGATATGAGAGACAATTGGAGAAGTGAATTATATAAATATAAGGTAG
TAAAAATTGAACCACTA---GGAGTAGCACCCACCCACTCAATGAGAAGAGTGGTG-----------------
----------------------CAGAGAGAAAAAAGAGCA---GTTGGA---TTGGGAGCT---------
GTCTTCCTTGGGTTCTTAGGAGCA---GCAGGAAGCACTATG---GGCGCGGCGTCA---
ATAACGCTGACGGCACAG---GCCAGACAA---TTATTG---TCTGGCATAGTGCAACAGCAAAGC---
AATTTG---CTGAGG---GCTATAGAGGCTCAACAGAATATGTTGAGACTCACGGTCTGGGGCATT
>JN864051..1
TTCTATTGCAATACATCAGGCCTGTTTAATAGCACTTGGAATTGGAATGACACTGTCAACACGACAGAGAACA
ATGAT--

ACAAACGGCACTATAACTCTCCAGTGCAGAATAAAGCAAATTATAAATATGTGGCAGAGAGTAGGACAAGCAA
TGTATGCCCCTCCCATCCCAGGAGTAATACGATGTGAGTCAAAC---
ATTACAGGAATACTATTGACAAGGGATGGTGGGACTAATGACAGT----------------------------
----

AGTCTAAATGAGACTTTCAGACCTGGGGGAGGAGATATGAAAGACAATTGGAGAAGTGAATTATATAAATATA
AAGTAGTAAAAATTGAACCACTA---GGAGTAGCGCCCACCAAGGCAAGGAGAAGGGTGGTG-----------

----------------------------CAGAGAGAAAAAAGAGCA---ATTGGA---TTTGGAGCT------
---TTCTTCCTTGGGTTCTTAGGAGCA---GCAGGTAGCACTATG---GGCGCGGCGTCA---
CTGACGCTGACGGTACAG---GCCAGACAA---TTATTA---TCTGGCATAGTGCAACAGCAAAAC---
AATTTG---CTGAGG---GCTATAGAGGCTCAACAACAGATGTTGAGACTCACAGTCTGGGGCATT
>JN864058..1
TTCTATTGCAATACATCAGGCCTGTTCAATAGCACTTGGAATAATAGCACTTGGAATACAACAGACATGTTTA
ATAGCACT---
-------------------GGG---
AATGAGAATGAGAACATAACTCTCCCATGCAGAATAAAGCAAATTATAAATATGTGGCAGAGAACAGGACAAG
CAATGTATGCCCCTCCCATCCCAGGAATAATAAAGTGTGAGTCAAAC---
ATTACAGGACTACTATTGACAAGAGATGGTGGGATTAATAACAAT----------------------------
----

TCAAATGAGACTTTTAGACCTGGAGGAGGAGATATGAAAGACAATTGGAGAAGTGAATTATACAAATATAAAG
TAGTAAAAATTGAACCACTA---GGAGTAGCACCCACCAGGGCAAGGAGAAGGGTGGTG--------------
-------------------------CAGAGAGAAAAAAGAGCA---GTTGGA---TTGGGAGCT---------
GTCTTCCTTGGGTTCTTAGGAGCA---GCAGGAAGCACTATG---GGCGCGGCGTCA---
CTAACGCTGACGGTACAG---GCCAGACAA---TTGTTG---TCTGGCATAGTGCAACAGCAAAAC---
AATTTG---CTGCAG---GCTATAGAGGCTCAACAGCAGATGTTGAGACTCACGGTCTGGGGCATT
>FM877780..1
TTCTATTGTAATACATCAGGCCTGTTTAATAGCACTTGGAAAAATGGAGTTTGGGAAAGGGGCAGGATGAACC
AGACA--
-------------------AAT---
GACACGAATGAAAACATAACACTCCCATGCAGGATAAAACAAATTGTAAACATATGGCAGGAAGTAGGACAAG
CAATGTATGCCCCTCCAGTTGCAGGAGAGATACAGTGTGTAACAAAC---
ATTACAGGAATAATATTGACAAGAGATGGGGGAATTAATAATGAG----------------------------
----

ACTACTGAGACCTTCAGACCTGGAGGAGGAAATATGAAGGACAATTGGAGAAGTGAGTTATACAAATATAAAG
TAGTAAAACTTGAACCACTA---GGAGTAGCACCCGACAAGGCAAAGAGAAGAGTGGTG--------------
-------------------------GAGAGAGAAAAAAGAGCA---GTTGGA---CTGGGAGCT---------
CTTTTCCTTGGGTTCTTAGGAACA---GCAGGAAGCACTATG---GGCGCGGCGTCA---
ATAACGCTGACGGTACAG---GCCAGACAA---TTATTG---TCTGGCATAGTGCAACAGCAAAGC---
AATTTG---CTGAGG---GCTATAGAGGCTCAACAGCATGTGTTGAAGCTCACCGTCTGGGGCATT
>FM877781..2
TTCTATTGTGATACATCAGGACTGTTTAATAGCAGTTGGCCAATTAATAGCAGCTGGCCAACTAATAACAGTG
AG---

ACAAATGAAACAATAACACTCCCATGCAGGATAAAACAAATTGTAAACATGTGGCAAGGAGTAGGACAAGCAA
TGTATGCCCCTCCTATCGAAGGAGAGATACAGTGTATATCAAAT---
ATTACAGGAATAATATTGACAAGAGATGGGGGAAATAACAGTAAC----------------------------
----

ACTGAAATCTTCAGACCTGGAGGAGGAAATATGAAGGACAATTGGAGAAGTGAATTATATAAGTATAAGGTAG
TTCAAATTGAACCGCTG---GGAGTAGCACCCAGCGAGGCAAGGAGAAGAGTGGTG-----------------
----------------------CAGAGAGAAAAAAGAGCA---GTAGGA---ATGGCAACT---------
CTTTTCCTTGGGTTCTTGGGAACA---GCAGGAAGCACTATG---GGCGCAGCGTCA---
ATAACGCTGACGGTACAG---GCCAGAAAA---TTATTG---TCTGGCATAGTGCAACAGCAAAGC---
AATTTG---CTGAAG---GCTATAGAAGCTCAACAGCATATGTTGAAGCTCACAGTCTGGGGCATT
>FM877782..1
TTCTATTGTAACACATCAGGCCTGTTTAATAGAACTTGGAAAGATGGCAATTGGACCACCCAGGAAAATGGG-
----

ACAAATGAAAACATAACACTCCCATGCAGGATAAAACAAATTGTACGCATGTGGCAAGAAGTAGGACAAGCAA
TGTATGCCCCTCCTGTCGCAGGAATGATACAGTGTATATCAAAT---
ATTACAGGATTAATATTGACAAGAGATGGGGGGAAAGATAAGAAT----------------------------
----

GATACTGAGATCTTCAGACCTGGAGGAGGAAATATGAAAGACAATTGGAGAAGTGAATTATATAAGTATAAAG
TAGTACAAATTGAGCCACTA---GGATTAGCACCTACCAAGGCAAGGAGAAGAGTGGTG--------------

-------------------------GAGAGAGAAAAAAGAGCA---GTTGGA---ATGGGAGCT---------
CTTTTCCTTGGGTTCTTGGGAGCA---GCCGGAAGCACTATG---GGCGCGGCGTCA---
ATAACGCTGACGGTACAG---GCCAGACAA---TTGCTG---TCTGGCATAGTGCAACAGCAAAGC---
AATTTG---CTGAGG---GCTATAGAAGCTCAACAGCATATGTTGAAGCTCACAGTCTGGGGCATT
>AM851091..2
TTCTATTGCGATACAAAAGAACTGTTTAATAGTACCTGGAATAAGAGCACCAATGGCACCTGGACTAAGAACA
GCACCACTGGC--
-------------GAGACA------
GGGAACACAGCAAATATTACACTGCAATGCAGAATAAAACAAATTGTAAACATGTGGCAGAGAACAGGACAAG
CAATATATGCCCCTCCTATTAGAGGAATAATTCAGTGTGAATCAAAT---
ATTACAGGGATACTATTGACAAGAGATAGG---CATAATCATACT----------------------------
----

AATGAGACCTTCAGACCTGGAGGAGGAAATATGAAGGACAATTGGAGAAATGAATTATATAAATATAAAGTAG
TAGAAATTGAACCACTA---GGAATAGCACCCACCAGGGCAAAAAGGAGAGTGGTG-----------------
----------------------GAGAGAGAAAAAAGAGCA---GTGGGA---ATAGGAGCT---------
TTGATCTTTGGGTTCTTAGGAGCA---GCAGGAAGCACTATG---GGCGCGGCGTCA---
ATAACGCTGACGGTACAT---GCCAGACAA---TTATTG---TCTGGTATAGTGCAACAGCAAAGC---
AATTTG---CTGAGG---GCTATAGAGGCGCAACAGCATATGTTGCAACTCACAGTCTGGGGCATT
>JF804811..2
TTCTACTGTAATACATCACTGCTGTTTAATACTACTTGGGAGAAGCATAATACCACTCAACCTTGGATAATGA
AAAATGAACCT--
-------------GGGTAT------
GATGTGAATAGGAATATCACACTCCACTGCAGAATAAAACAAATTATAAACAGGTGGCAGGAAGTAGGAAAAG
CAATGTATGCCCCTCCCATCAGTGGAATAATTAACTGTTTATCAAAT---
ATTACAGGGCTGTTACTAACAAGAGATGGAGGTCCAAACGGGACC----------------------------
----

CAAAATGAGACCTTCAGACCTCAAGGAGGAGATATGAGAGACAATTGGAGAAGTGTATTATATAAATATAAAG
TAGTAAAAATTGAGCCATTA---GGGGTAGCACCCACCAGGGCAAAGAGAAGAGTGGTG--------------
-------------------------CAGAGAGAAAAAAGAGCA---GTGGGA---CTGGGAGTC---------
TTGTTCCTTGGGTTCTTGGGAGCA---GCAGGAAGCACTATG---GGCGCAGCATCA---
ATAACGCTGACGGTACAG---GCCAGACAG---TTATTG---TCTGGTATAGTGCAACAGCAAAAC---
AATTTG---CTGAGG---GCTATTGAGGCGCAACAGCATCTGTTGCAGCTCACAGTCTGGGGCATC
>EF158043..2
TTCTATTGTAATACATCAGGCCTGTTTAATAGCACTTGGAATATCAATGGCAGCGGAGGGTTTAATAGC----
----

ACAAATGACAACATAACTCTCCCATGTAGAATAAAGCAAATTATAAATATGTGGCAGAGAACAGGACAAGCAA
TGTATGCCCCTCCCATCAAAGGAGAAATCAGGTGTGTATCAAAC---
ATTACAGGACTATTGTTGACAAGAGATGGTGGTGGTAATGCGAGT----------------------------
----

CAGAATGAGACCTTCAGACCTGGAGGAGGAGATATGAGAGACAATTGGAGAAGTGAATTATACAAATATAAAG
TAGTAAAAATTGAACCACTA---GGAGTAGCACCCACCGGGGCAAAGAGAAGAGTGGTG--------------
-------------------------GAGAGAGAAAAAAGAGCA---GTTGGA---CTAGGAGCT---------
GTGTTCATTGGGTTTTTAGGAGCA---GCCGGAAGCACTATG---GGCGCGGCGTCA---
GTGACGCTGACGGTACAG---GCCAGGCAA---TTATTG---TCTGGCATAGTGCAACAGCAAAGC---
AATTTG---CTGAGG---GCTATAGAGGCTCAACAGCATGTGTTGAGGCTCACGGTCTGGGGCATT
>EF158040..2
TTCTATTGTGATACATCAGGTCTGTTTAATAGCGCTTGGAATGGCACCAATGGCAACGAAATGTCAAATATC-
----

ACAAATGGCACTATAACTCTCCAATGCAGAATAAAGCAAATTGTAAATATGTGGCAGAGAGTAGGACAGGCAA
TGTATGCCCCTCCCATCCCAGGAGTAATCAGGTGTGTATCAAAC---
ATTACCGGACTATTGTTAACAAGAGATGGTGGTCAGGATAATGGG----------------------------
----

AGTAGTGAGATCTTCAGACCTGGAGGAGGAGATATGAGAGACAATTGGAGAAGTGAATTATATAAATATAAAG
TAGTACAACTTGAACCACTA---GGAGTAGCACCCACTAAGGCAAGGAGAAGAGTGGTG--------------

```
-------------------------GAGAGAGAAAAAAGAGCA---GTTGGA---ATAGGAGCT---------
GTGTTCATTGGGTTCTTAGGAGCA---GCAGGAAGCACTATG---GGCGCAGCGTCA---
ATGACGCTGACGGTACAG---GCCAGACAA---TTATTG---TCTGGCATAGTGCAACAGCAAAGT---
AATTTG---CTGAGG---GCTATAGAGGCTCAACAGCATATGTTGAAACTCACGGTCTGGGGCATT
>EF158041..1
TTCTATTGTGATACATCAGGCCTGTTTAACAGTACCTGGAATATCACTGACATAGGAGAGCCAAATAGCACAA
AT-----------------------------------------------------------------------
-------------------------
AGCACAAATGACACTATAACTCTCCCATGCAGAATAAAGCAAATTATAAATATGTGGCAGAGAACAGGACAAG
CAATATATGCCCCTCCCATCCGAGGAGAAATCAGGTGTGTATCAAAC---
ATTACAGGACTATTGTTGACAAGAGATGGTGGTACTAATAACAGT----------------------------
-------------------------------------------------------------------------
----------
AGTCTGAATGAGACCTTCAGACCTGGAGGAGGAGATATGAGAGACAACTGGAGAAGCGAATTATACAAATATA
AAGTAGTAAAACTTGAACCACTA---GGAGTAGCACCCACCAGGGCAAAGAGAAGAGTGGTG-----------
----------------------------GAGAGAGAAAAAAGAGCA---GTCGGA---CTGGGAGCT------
---ATGTTCATTGGGTTCTTAGGAGCA---GCAGGAAGCACTATG---GGCGCGGCGTCA---
ATGACGCTGACGGTACAG---GCCAGACAA---TTATTG---TCTGGCATAGTGCAACAGCAAAGC---
AATTTG---CTGAGG---GCTATAGAGGCTCAACAGCATATGTTGAAACTCACGGTCTGGGGCATT
>EF158042..1
TTCTATTGTAATACATCAGGACTGTTTAACAGCACTTGGAAAATCAATGCCAGCGAAGAGTTAAATAGCACAA
AT-----------------------------------------------------------------------
----------------------------
GACAATGTAACTATAACTCTCCCATGCAGAATAAAGCAAATTATAAATATGTGGCAGAGAACAGGACAGGCAA
TGTATGCCCCTCCCATCCGAGGAGAAATTAGGTGTGTATCAAAC---
ATTACAGGAATATTGTTGACCAGAGATGGTGGTAGAGTTAATGAA----------------------------
-------------------------------------------------------------------------
----------
AGTCTAAATGAGACCTTCAGACCTGGAGGAGGAGATATGAGAGACAATTGGAGAAGTGAATTATACAAATATA
AAGTAGTAAAAATTGAACCACTA---GGAGTAGCACCCACCAGGGCAAAGAGAAGAGTGGTG-----------
----------------------------GAGAGAGAAAAAAGAGCA---GTTGGA---CTGGGAGCT------
---GTGTTCATTGGGTTCTTAGGAGCA---GCAGGAAGCACTATG---GGCGCGGCGTCA---
GTGACGCTGACGGTACAG---GCCAGACAA---TTATTG---TCGGGCATAGTGCAACAGCAAAGC---
AATTTG---CTGAGG---GCTATAGAGGCTCAACAGCATATGTTGAAACTCACGGTCTGGGGCATT
>GQ477443..2
TTCTATTGTGATACATCAGGCCTGTTTAATAGCACTTGGAATATCAGTGGCAGCGAAGAGTTAAATAGC----
-------------------------------------------------------------------------
----------------------------
ACAAATGACATAATAACTCTCCCATGCAGAATAAAGCAAATTATAAATCTATGGCAGAGAATAGGGCAAGCAA
TGTATGCCCCTCCCATCCGAGGAGAAATTAGGTGTGAATCAAGC---
ATTACAGGACTATTGTTGACAAGAGATGGTGGTGTTAATGGGAGT----------------------------
-------------------------------------------------------------------------
-------------
CAGAATGAAACCTTCAGACCTGGAGGAGGAGATATGAGAGACAATTGGCGAAGTGAATTATACAAATATAAAG
TAGTAGAACTTGAACCACTA---GGAGTAGCTCCCACCAGGGCAAAGAGAAGAGTGGTG--------------
-------------------------GAGAGAGAAAAAAGAGCA---GTCGGA---CTAGGAGCT---------
ATGTTCATTGGGTTCTTAGGAGCA---GCAGGAAGCACTATG---GGCGCGGCGTCA---
ATGACGCTGACGGTACAG---GCCAGACAA---TTATTG---TCTGGCATAGTGCAACAGCAAAGT---
AATTTG---CTGAGG---GCTATAGAGGCTCAACAGCATATGTTGAAACTCACGGTCTGGGGCATT
>GQ477442..3
TTCTATTGTAATACATCAGGCCTGTTTAATAGCACTTGGAATATCAATGGCAGTGAAGGGGTAAATAGC----
-------------------------------------------------------------------------
----------------------------
ACAAATGTCAATATAACTCTCCCATGCAGAATAAAGCAATTTATAAATATGTGGCAGAGAACAGGACAAGCAA
TGTATGCCCCTCCCATTCAAGGAGAAATCAGGTGTGTATCAAAC---
ATTACAGGACTATTGTTGACAAGAGATGGTGGTAATGGTAATGACAGT-------------------------
-------------------------------------------------------------------------
----------
CAGGCTAATGAGACCTTCAGACCTGGAGGAGGAGATATGAGAGACAATTGGAGAAGTGAATTATACAAATATA
AAGTAGTAAAACTTGAACCACTA---GGAGTAGCACCCACCAGGGCAAGGAGAAGAGTGGTG-----------
```

----------------------------GAGAGAGAAAAAAGAGCA---GTTGGA---ATAGGAGCT------
---GTGTTCATTGGGTTCTTAGGAGCA---GCAGGAAGCACTATG---GGCGCGGCGTCA---
ATGACGCTGACGGTACAG---GCCAGACAA---TTATTG---TCTGGCATAGTGCAACAGCAAAGC---
AATTTG---CTGAAG---GCTATAGAGGCTCAACAGCATATGTTGAGACTCACGGTCTGGGGCATT
>GQ477446..1
TTCTATTGTAATACATCAGGCCTGTTTAACAATAGCCTGTTTAACAGCAGCGGAGAGTCA-------------
----

GATAGCACAAATATAACTCTCCCATGCAGAATAAAGCAAATTATAAATATGTGGCAGAGAACAGGACAAGCAA
TGTATGCCCCTCCCATCCGAGGAGAAATCAGGTGTGTATCAAAC---
ATTACAGGACTATTGTTGACAAGAGATGGTGGTGCTAATGAGAAT----------------------------
----

GAGAGTGAGACCTTCAGACCTGGAGGAGGAGATATGAGAGATAATTGGAGAAGTGAATTATACAAATATAAAG
TAGTAAAAATTGAACCACTA---GGAGTAGCACCCACCAAGGCAAAGAGAAGAGTGGTG--------------
-------------------------GAGAGAGAAAAAAGAGCA---GTTGGA---CTGGGAGCT---------
TTGTTCATTGGGTTCTTAGGAGCA---GCAGGAAGCACTATG---GGCGCGGCGTCG---
GTGACGCTGACGGTACAG---GCCAGACAA---TTATTG---TCTGGCATAGTGCAACAGCAAAGC---
AATTTG---CTGAAG---GCTATAGAGGCTCAACAGCATATGTTGAGACTCACGGTCTGGGGCATT
>GQ477448..4
TTCTATTGTAATACATCAGGCCTGTTTAACAGCACTTGGGATAATAGCACTTGGTATAACAATGGCAGCCAAA
GGTCA--
-------------------AAT---
AGCACAAGTGGCATTATAACTCTCCCATGCAGAATAAAGCAAATTATAAATATGTGGCAGAGAACAGGACAAG
CAATGTATGCCCCTCCCATTCAAGGAGAAATCAAGTGTATATCAAAC---
ATTACAGGACTATTGTTGACAAGAGATGGTGGTAATCATGATGGTAAT-------------------------
----

GAGAGTCAGGAGACCTTCAGACCTGGAGGAGGAGATATGAGAGACAATTGGAGAAGTGAATTATACAAATATA
AAGTAGTAAAAATTGAACCACTA---GGAGTAGCACCTACCAGGGCAAGGAGAAGAGTGGTG-----------
----------------------------GAGAGAGAAAAAAGAGCA---GTTGGA---CTAGGAGCT------
---GTGTTCATTGGGTTCTTAGGAGCA---GCAGGAAGCACTATG---GGCGCGGCGTCA---
GTGACGCTGACGGTACAG---GCCAGACAA---TTGCTG---TCTGGCATAGTGCAACAGCAAAGC---
AATTTG---CTAAAG---GCTATAGAGGCTCAACAGCAGATGTTGAGACTCACAGTCTGGGGCATT
>AB703608..3
TTCTATTGTAATACATCAGGCCTGTTTAACAGCACTTGGAATACCAATGGCAGCGAAGGGTCAACAGAC----
----

ACAAGTGGCAATATAACTCTCCAATGCAGAATAAAGCAAATTATAAATATGTGGCAGAGAACAGGACAAGCAA
TATATGCCCCTCCCATCCGAGGAGAAATCATGTGTAAATCAAAC---
ATTACAGGACTATTGTTAACAAGAGATGGTGGTGGTGGTAATCAG----------------------------
----

AGTCAGAATGAGACCTTCAGACCTGGAGGAGGAGATATGAGAGACAATTGGAGAAGTGAATTATACAAATATA
AAGTAGTAAAAATTGAACCACTA---GGAGTAGCACCCACCAAGGCAAAGAGAAGAGTGGTG-----------
----------------------------CAGAGAGAAAAAAGAGCA---GTTGGA---CTGGGAGCT------
---TTGTTCATTGGGTTCTTAGGAGCA---GCAGGAAGCACTATG---GGCGCGGCGTCA---
GTGACGCTGACGGTACAG---GCCAGACAA---TTATTG---TCTGGCATAGTGCAACAGCAAAAC---
AACTTG---CTGAGG---GCTATAGAGGCTCAACAGCATATGTTGAAACTCACGGTCTGGGGCATT
>AB703615..1
TTCTATTGTAATACATCAAACCTGTTTAGTAGCACTTGGTTTATCAATGGCAGCGGAGAGTCACATAATAAAA
ATGGC--

ACTAATGACACTATAACTCTCCAATGCAGAATAAAGCAAATTATAAATCTGTGGCAGAGAACAGGACAAGCAA
TGTATGCCCCTCCCATCCAAGGAGAAATTAGGTGTGTATCAAAC---
ATTACAGGACTGTTGTTGACAAGAGATGGTGGTGTTAATGTGAGC----------------------------
----

AATGAGACTGAAACCTTCAGACCTGGAGGAGGAGATATGAGAGACAATTGGAGAAATGAATTATACAAATATA
AAGTAGTAAAAATTGAACCACTA---GGAGTAGCACCCACCAGGGCAAAGAGAAGAGTGGTG-----------

---------------------------GAGAGAGAAAAAAGAGCA---GTTGGA---CTAGGAGCT------
---GTGTTCATTGGGTTCTTAGGAGCA---GCAGGAAGCACTATG---GGCGCGGCGTCA---
GTGACGCTGACGGTACAG---GCCAGACAA---TTATTG---TCTGGCATAGTGCAACAGCAAAGC---
AATTTG---CTGAGG---GCTATAGAGGCTCAACAGCATATGTTGAAACTCACGGTCTGGGGCATT
>AB703609..2
TTCTATTGTAATACATCAGGCCTGTTTAACAGTACTTGGAATATCAATGGCAGCGGAGGGTTAGATAAC----
----

ACAAATGATAATATAACTCTCCAATGCAGAATAAAGCAAATTATAAATATGTGGCAGAGAACAGGGCAAGCAA
TGTATGCCCCTCCCATCCGAGGAGAAATCAGGTGTGTATCAAAC---
ATTACCGGACTATTGTTGACAAGAGATGGTGGTAATAATAGTGAG---------------------------
----

AGTCAGGAGACCTTCAGACCTGGAGGAGGAGATATGAGAGACAATTGGAGAAGTGAATTATACAAATATAAAG
TAGTAAAAATTGAACCAATA---GGAGTAGCACCCACCAAGGCAAAAAGAAGAGTAGTG-------------
------------------------GAGAGAGAAAAAAGAGCA---GTTGGA---CTGGGAGCT---------
ATGTTCATTGGGTTCTTAGGAGCA---GCAGGAAGCACTATG---GGCGCGGCGTCA---
GTGACGCTGACGGTACAG---GCCAGACAA---TTATTG---TCTGGCATAGTGCAACAGCAAGGC---
AACTTG---CTGAGG---GCCATCGAGGCTCAACAGCATATGTTGAGACTCACAGTCTGGGGCATT
>AB703611..1
TTCTATTGTAATACGTCAGGTCTGTTTAACAGTACTTGGAATGTCACTGGCAGCAAAGAGTCACATAGC----
----

CCAAATGATACTATAACTCTCCCATGCAGAATAAAGCAAATTATAAATATGTGGCAGAGAACAGGACAAGCAA
TGTATGCCCCTCCCATCAAAGGAGAAATCAGGTGTGTATCAAAC---
ATTACAGGACTATTGTTGACAAGAGATGGTGGGGTTAATAACAGC---------------------------
----

CAGAATGAGACCTTCAGACCTGGAGGAGGGAATATGAGAGACAATTGGAGAAGTGAATTATACAAATATAAAG
TAGTGAAAATTGAACCACTA---GGAGTAGCACCCACCAGGGCAAAGAGAAGAGTGGTG-------------
------------------------GAGAGAGAAAAAAGAGCA---GTAGGA---CTGGGAGCT---------
GTGTTCATTGGGTTCTTAGGAGCA---GCAGGAAGCACTATG---GGCGCAGCGTCA---
ATGACGCTGACGGTACAG---GCCAGACAA---TTATTG---TCTGGCATAGTGCAACAGCAAAGT---
AATTTG---CTGAAG---GCTATAGAGGCTCAACAGCATATGTTGAAACTCACGGTCTGGGGCATT
>AB703612..3
TTCTATTGTAATACATCAGGCCTGTTTAATAGCACTTGGCCTATCAATGGTAGCAGCGAAGGGAAAAATAGC-
----

ACAGATGACACTATAACTCTCCCACGCAGAATAAAGCAGATTATAAATATGTGGCAGAGAACAGGACAAGCAA
TGTATGCCCCTCCCATTAAAGGAGAAATCAGATGTGTATCAAAC---
ATTACAGGAATATTGTTGACAAGAGATGGTGGTGAGGGGAATCAGACTGAGGGG------
AATCAAACTGAGGGGAAC--

CGGACCGAGACCTTCAGACCTGGAGGAGGAGATATGAGAGACAATTGGAGAAGTGAATTATACAAATATAAAG
TAGTAGAACTTGAACCACTA---GGAGTAGCACCCACCAAGGCAAAGAGAAGAGTGGTG-------------
------------------------GAGAGAGAAAAAAGAGCA---GTTGGA---CTAGGAGCT---------
GTGTTCATTGGGTTCTTGGGAGCA---GCAGGAAGCACTATG---GGCGCGGCGTCA---
ATGACGCTGACGGTACAG---GCCAGACAA---TTATTG---TCTGGCATAGTGCAACAGCAAAGC---
AATTTG---CTGAGG---GCTATAGAGGCTCAACAGCAGACGTTGAGACTCACGGTCTGGGGCATT
>AB703613..2
TTCTATTGTAATACATCGAGCCTGTTTAACAGCACTTGGTATAACAATGGCAGCAAAGAGTTAAATAGC----
----

GCAAATGGCATTATAAAACTCCCATGTAGAATAAAGCAAATTATAAATACGTGGCAGAGAACAGGACAAGCAA
TGTATGCCCCTCCCATCCGAGGAGAAATTAGGTGTGAATCAAAC---
ATTACAGGACTATTGTTGACAAGAGATGGTGGAAATGATACTAAT---------------------------
----

CAGACTGAGACCTTCAGACCTGGAGGAGGAGATATGAGAGACAATTGGAGAAGTGAATTATACAAATATAAAG
TAGTAAAAATTGAACCACTA---GGAGTAGCACCCACCAAGGCAAAGAGAAGAGTGGTG-------------

-------------------------GAGAGAGAAAAAAGAGCA---TTTGGA---CTGGGAGCT---------
TTGTTCGTTGGGTTCTTAGGAGCA---GCAGGAAGCACTATG---GGCGCGGCGTCA---
GTGACGCTGACGGTACAG---GCCAGACAA---TTATTG---TCTGGCATAGTGCAACAGCAAAGC---
AATTTG---CTGAGG---GCTATAGAGGCTCAACAGCATATGTTGAAACTCACGGTCTGGGGCATT
>AB703614..1
TTCTATTGTAATACATCAGGCCTGTTTAACAGCGCTTGGAATATCACTGGCAGCAGCGAAGGGTTAAATAGT-
----

ACAAATGACACTATAACTCTCCCATGCAGAATAAAGCAAATTATAAATATGTGGCAGAGAATAGGACAAGCAA
TGTATGCCCCTCCCATCCAAGGAGTAATCAGGTGTGAATCAAAC---
ATTACAGGACTATTGTTGACACGAGATGGTGGTAATAATAGGAATGAGAGT----------------------
----

GAAAATGAGACTGAGACCTTCAGACCTGGAGGGGGAGATATGAAAGATAATTGGAGAAGTGAATTATACAAAT
ATAAAGTAGTAAAACTTGAACCACTA---GGAGTAGCACCCACCAGGGCAAAGAGAAGAGTGGTG--------
-------------------------------GAGAGAGAAAAAAGAGCA---GCTGGA---CTGGGAGCT---
------GTGTTCATTGGGTTCTTAGGAGCA---GCAGGAAGCACTATG---GGCGCGGCGTCA---
ATGACGCTGACGGTACAG---GCCAGACAA---TTATTG---TCTGGCATAGTGCAACAGCAAAGC---
AATTTG---CTGAGG---GCTATAGAGGCTCAACAGCATATGTTGAAACTCACGGTCTGGGGCATT
>EF087995..1
TTCTATAGCGATACATCAGGCATGTTTAATAGCACTTGGGATAACAATAGCACTTGGAATAACAATAGCAGCA
TGACAGGG---
-------------TCAAAT------
AACACAAATGACAATATAACACTCCCGTGTAGAATAAAGCAAGTTATAAATATGTGGCAGAGAGTAGGACAAG
CAATGTATGCCCCTCCCATCGCAGGAATAATAAGGTGTGAGTCAAAT---
ATTACAGGACTACTATTGACAAGAGATGGTGGGAATACTAGCAGT----------------------------
----

ACAAATGAGACTTTCAGACCAGGAGGAGGAGATATGAGGGACAATTGGAGAAGTGAATTATATAAGTATAAGG
TAGTACAAATTGAACCACTA---GGTGTGGCACCCACCCGTGCAAAAAGAAGGGTGGTG--------------
-------------------------GAGAGAGAAAAAAGAGCAGTTGTTGGA---CTGGGAGCT---------
GTCTTCCTTGGGTTCTTAGGAGCA---GCAGGAAGCACTATG---GGCGCGGCGTCA---
ATAACGCTGACGGTACAG---GCCAGACAA---TTACTG---TCCGGTATAGTGCAACAGCAAAGC---
AATTTG---CTGAGG---GCTATAGAGGCTCAACAACATCTGTTGAAACTCACGGTCTGGGGCATT
>EF087994..1
TTCTATTGCAATACATCAGGCCTGTTTAATAGCACTTGGGGGTATAACAATAGCAACGTGACAAGGTTAAATA
AC---

ACGAATGACACTATAATTCTCCCGTGTAAAATAAAGCAAATTATAAAGATGTGGCAGAGAGTAGGACAAGCAA
TGTATGCCCCTCCCATCGCAGGAAGGATAAGGTGTGAGTCAAAT---
ATTACAGGACTACTATTGACAAGAGATGGTGGGAAAAATAACAGT----------------------------
----

ACAAATGAGACTTTCAGACCAGGAGGAGGAGATATGAGGGACAATTGGAGGAGTGAATTATATAAGTATAAGG
TAGTACAAATTGAACCACTA---GGTGTGGCACCCACCCGAGCAAAGAGAAGGGTGGTG--------------
-------------------------GAGAGAGAAAAAAGAGCA---GTTGGA---CTGGGAGCT---------
GTCTTCCTTGGGTTCTTAGGAGCA---GCAGGAAGCACTATG---GGCGCGGCGTCA---
ATAACGCTGACGGTACAA---GCCAGACAA---TTACTG---TCCGGTATAGTGCAACAGCAAAGC---
AATTTG---CTGAGG---GCTATAGAGGCTCAACAACATCTGTTGAAACTCACGGTCTGGGGCATT
>EF116594..1
TTCTACTGCAATACATCAGGCCTGTTCAATAGTACATGGAACACTACTACCAGCACGCAGATTCCAAATAGGA
CT---

ACAGATGGGAATATAACTCTCCCTTGCAGAATAAAGCAAATTGTCAATATGTGGCAGAGAGTAGGACAAGCAA
TGTATGCCCCTCCCATCCCAGGAGTAATAAGGTGTGAGTCCAAC---
ATTACAGGACTAATGTTAACAAGAGATGGTGGGATTAACAAAACA----------------------------
----

GATAATGAGACCTTCAGACCTGGGGGAGGAGATATGAGGGATAATTGGAGAAGTGAATTATATAAGTATAAAG
TAGTAAAAATTGAACCAATA---GGAATAGCACCCACCAAGGCAAGGAGAAGAGTGGTG--------------

-------------------------GAGAGAGAAAAAAGAGCA---GTTGGA---CTGGGAGCT---------
GTCTTCCTTGGGTTCTTAGGAGCA---GCAGGAAGCACTATG---GGCGCGGCGTCT---
ATAACGCTGACGGTACAG---GCTAGACAA---TTATTG---TCTGGCATAGTGCAACAGCAGAGC---
AACCTG---CTAAGG---GCTATAGAGGCTCAACAACATCTGTTGAAACTCACGGTCTGGGGCATT
>KP718917..2
TTCTATTGCAATACAACAAAACTGTTCAATCACCTGTTCAATAATAGTGAGTGGGACAATGCAGCTAACAGC-
----

ACGAATGAGAGTATAACTCTCCCTTGCAGAATAAAGCAAATTATAAATATGTGGCAGAGAGTAGGACAAGCAA
TGTATGCCCCTCCCATCCCAGGAGAAATAAGGTGTAGATCTAAT---
ATTACAGGACTATTGTTAACAAGAGATGGTGGAAGCAACAATGCA----------------------------
----

ACTAATGAAACCTTCAGACCTGGAGGAGGAGACATGAGGGACAATTGGAGAAGTGAATTATATAAGTATAAGG
TAGTGAAAATTGAACCAATA---GGAGTAGCACCCACCGGGGCAAGGAGAAGAGTGGTG--------------
-------------------------CAGAGAGAAAAAAGAGCA---GTTGGC---CTAGGAATT---------
GCCTTCCTTGGGTTCTTAGGAGCA---GCAGGAAGCACTATG---GGCGCGGCGGCC---
ATAACGCTGACGGTACAG---GCCAGACAA---TTGTTG---TCTGGCATAGTGCAACAGCAGAGC---
AATCTG---CTAAGG---GCTATAGAGGCTCAACAACACATGTTGAGACTCACGGTCTGGGGCATT
>AF377957..2
TTCTATTGCGATACAACAGGCCTGTTCAATAGTAGTTGGAGCTACAATGGCACGAGTTGGAATAAGACCACTA
CAAT---
-------------------GGC---
ACTACAAATGGCAATATAACTCTCCATTGCAGAATAAAACAAATTATAAATATGTGGCAGAGAGTAGGACAAG
CAATGTATGCCCCTCCCATCCGAGGAGTAATAAGGTGTGAATCTAAC---
ATTACAGGATTGCTATTAACAAGAGATGGTGGGACAAACAATAGT----------------------------
----

AACACTACTGAAACCTTCAGACCTGGAGGAGGAGATATGAGGGACAATTGGAGAAGTGAATTATATAAGTATA
AAGTAGTAAAAATTGAACCAATA---GGAGTAGCACCTACCGGGGCAAGGAGAAGAACGGTG-----------
----------------------------GAGAGAGAGAAAAGAGCA---GTTGGA---CTAGGAGCT------
---GTTTTTCTTGGATTCTTAGGAGCA---GCAGGAAGCACTATG---GGCGCGGCGTCT---
GTGACGCTGACGGTACAG---GCCAGACAA---TTATTG---TCTGGCATAGTGCAACAGCAGAGC---
AATCTG---CTGAGG---GCTATAGAGGCTCAACAACATCTGTTGAAACTCACGGTCTGGGGCATT
>JF683745..2
TTCTATTGCAATACAACAAACCTGTTCAATAGTGTTTGGACAGACGGGAAATGGAATAATACCATGAACAGCA
GTCAA--

TCAGATAGTAATAATATAACTCTCCATTGCAAAATAAAACAAATTGTAAGGATGTGGCAAAGAGTAGGACAAG
CAATGTATGCCCCTCCCATCCGAGGAGTAATAAACTGTATATCCAAC---
ATTACAGGACTGCTATTAACAAGAGATGGTGGGCTAAGTAATAGG----------------------------
----

ACAAATGAGACCTTCAGACCTGGAGGAGGAGATATGAGGGACAATTGGAGAAGTGAATTATATAAGTATAAAG
TAGTAAAAATTGATCCACTA---GGAGTAGCACCCTCCAGGGCAAGGAGAAGAGTGGTG--------------
-------------------------GAGAGAGAAAAAAGAGCA---GTTGGA---CTGGGAGCT---------
ATGTTCTTTGGATTCTTAGGAGCA---GCAGGAAGCACTATG---GGCGCGGCGTCC---
TTAACGCTGACGGTACAG---GCCAGACAA---TTATTG---TCTGGCATAGTGCAACAGCAGAGC---
AATCTG---CTGAGG---GCTATAGAGGCTCAACAGCATCTGTTGAAACTCACGGTCTGGGGCATT
>FJ213783..2
TTCTACTGCAATACATCAAAACTGTTTAATGACACAGCTTCC-------------------------------
----

AATAATGGTACCATCACTCTCCCATGTCGAATAAAACAAATTGTAAACATGTGGCAGGAAGTGGGACGAGCAA
TGTATACCGCTCCCATTGCAGGAGACATTACCTGTAGATCAAAT---
ATTACAGGTCTACTATTGACAAGAGATGGTGGTCAGAAAAATAATGGAAGTTGT-------------------
----
-
ACAGAGGCAACTTGTACACAGATCTTCAGACCTCAGGGAGGAAATATGAAAGACAATTGGAGAAGTGAATTAT
ATAAATATAAAGTAGTAGAAATTGAACCATTA---GGAATAGCACCCACCAGGGCAAAGAGAAGAGTGGTG--

-------------------------------------CAGAGAGAAAAAAGAGCA---GTGGGA---
ATGGCAGCT---------GTGTTCATTGGGTTCTTGGGAGCA---GCAGGAAGCACTATG---
GGCGCGGCGTCA---ATAACGCTGACGGTACAG---GCCAGACAA---TTATTG---
TCTGGAATAGTGCAACAGCAGAAC---AATCTG---CTGAGG---
GCTATTGAAGCGCAACAGCATATGTTGCAGCTCACAGTCTGGGGCATT
>FJ213780..1
TTCTACTGCAATACATCAAAACTGTTTGATAACATAGAAAAC-------------------------------
----

AATGGAACTATCATTCTCCCATGTAAAATAAAACAAATAGTAAACATGTGGCAAGAAGTGGGACGAGCAATGT
ATGCCGCTCCCATTGCAGGAAACATTGTCTGTAACTCAACT---
ATTACAGGTCTACTCTTAACAAGAGATGGTGGTCATGGTAATGGCACA-------------------------
----

ATCAATAAGACAGAGATCTTCAGACCTGGGGGAGGAAATATGAAAGACAATTGGAGAAGTGAATTATATAAAT
ATAAAGTAGTAGAAATTGAACCATTA---GGAATAGCACCCACCAGGGCAAGGAGAAGAGTGGTG--------
-------------------------------CAGAGAGATAAAAGAGCA---GTGGGA---ATGGCAGCT---
------ATGTTCCTTGGGTTCTTGGGAGCA---GCAGGAAGCACTATG---GGCGCAGCGTCA---
ATAACGCTGACGGTACAG---GCCAGACAA---TTATTG---TCTGGAATAGTGCAACAGCAAAGC---
AATTTG---CTGAGG---GCTATTGAAGCGCAACAGCATATGTTGCAGCTCACAGTCTGGGGCATT
>EU735535..1
TTCTACTGCAATACATCAAATCTGTTTAATGACACGATAGGC-------------------------------
----

AATGGCACTATAATTCTCCCATGTAAAATAAGACAAATTGTAAACATGTGGCAGGAAGTGGGACGAGCAATGT
ATGCTGCTCCCATTGCAGGAAACATTGCCTGTAACTCAACT---
ATTACAGGTCTACTATTGACAAGAGATGGTGGTAGTGGAAGTAATGGT-------------------------
----

ACTAATCAAAATGAGACCTTCAGACCTGGGGGAGGAAATATGAAAGACAATTGGAGAAGTGAATTATACAAAT
ATAAAGTAGTAGAAATTGAACCACTA---GGAATAGCACCCACCAGGGCACAAAGACAAGTGGTG--------
-------------------------------AAGAGAGAAAAAAGAGCA---GCA------CTAGGAGTT---
------GTGTTCCTTGGGTTCTTGGCAGCA---GCAGGAAGCACTATG---GGCGCGGCGTCA---
ATAACGCTGACGGTACAG---GCCAGACAA---TTACTG---TCTGGAATAGTGCAACAGCAGAAT---
AATCTG---CTGAAG---GCTATTGATGCGCAACAGCATCTGTTGCAGCTCACAGTCTGGGGCATT
>EU735538..2
TTCTACTGCAATACATCAGGACTGTTTAATGACACAGAAATTGAAGCTAATCACACAAATGCTAAT-------
----

GATAATGACACTATCATTCTCCCATGTAAAATAAAACAGATGATAAACATGTGGCAGGAAGTAGGACGAGCAA
TGTATGCTGCCCCCGTGGCAGGAAACATTATCTGTAACTCAAAT---
ATTACAGGACTACTATTAACAAGAGATGGTGGTAATAAGACTCATAAT-------------------------
----

GATACTAGGAATGAGATCTTTAGACCTGGAGGAGGAAATATGAAGGACAATTGGAGAAGTGAATTATATAAAT
ATAAAGTAGTAAGAATTGAGCCATTA---GGAGTAGCACCCACCAGGGCAAGGAGAAGAGTGGTG--------
-------------------------------CAGAGAGAAAAAAGAGCA---GTAACA---ATGGGAGCT---
------ATGTTCCTTGGGTTCTTGGGAGCA---GCAGGAAGCACTATG---GGCGCAGCGTCA---
CTAACGCTGACGGTACAG---GCCAGACAA---TTATTG---TCTGGTATAGTGCAACAGCAGAAC---
AATCTG---CTGAAG---GCTATTGAAGCGCAACAACATCTGTTGCAGCTCACAGTCTGGGGCATT
>EU735537..1
TTCTACTGCAATACATCAGGATTGTTTGATAATAACACAGGGACTGAC-------------------------
----

ACTAATGACACTATCACTCTCCCATGTAAAATAAAACAAATAGTAAATATGTGGCAAGAAGTAGGACGAGCAA
TGTATGCCGCCCCCATTGCAGGAAACATTACCTGTAACTCAAGT---
ATTACAGGACTACTATTGACAAGAGATGGTGGTCAGAATAATGAGACT-------------------------
----

AATAATACTGAGACCTTCAGACCTGGGGGAGGAAATATGAAGGACAATTGGAGAAGTGAATTATATAAATATA

AAGTAGTAAAAATTGAGCCATTA---GGAGTAGCACCCACCAGGGCAAGGAGAAGAGTGGTG-----------
----------------------------CAGAGAGAGAAAAGAGCA---GTGACG---CTGGGAGCT------
---ATGTTCCTTGGGTTCTTGGGAGCA---GCAGGAAGCACTATG---GGCGCAGCGGCA---
ACAACGCTGACGGTACAG---GCCAGACAA---TTATTG---TCTGGTATAGTGCAGCAGCAAAGC---
AATTTG---CTGAGG---GCTATTGAAGCGCAACAGCATATGTTGCAGCTCACAGTCTGGGGCATT
>EU170155..2
TTCTACTGCAATACAACAGAACTGTTTAATGACACAGAATCC-------------------------------
----

AATAACACTATCATTACTCTCCCATGTCGAATAAAACAAATTATAAACATGTGGCAGGAAGTGGGACGAGCAA
TGTATGCTGCTCCCATTGCAGGAAACATTACCTGTTCATCAAAT---
ATTACAGGTCTACTATTGACAAGAGATGGTGGTCTGGCTAATCAGACT-------------------------
----

GGAAATCAGAATGACACCTTCAGACCTGGGGGAGGAGATATGAGAGACAATTGGAGAAGTGAATTATATAAAT
ATAAAGTAGTAGAAATTGAGCCATTA---GGAATAGCACCCACCACAGCAAAGAGAAGAGTGGTG--------
-------------------------------CAGAGAGAAAAAAGAGCA---GTG------------------
------------------------------------GGAAGCACTATG---GGCGCAGCGTCA---
GTAACGCTGACGGTACAG---GCCAGACAA---TTATTG---TCTGGTATAGTGCAACAGCAGAAC---
AATTTG---CTGAAG---GCTATTGAGGCGCAACAGCACCTGTTGCAACTCACAGTCTGGGGCATC
>EU170139..1
TTCTACTGTAATACAACAATACTGTTTAATGAAACAGGATTTAATGACACAAGA-------------------
----

TCCAATGACACCATTACTCTCCCATGTCGAATAAAACAGATTATAAACATGTGGCAGGAAGTGGGACGAGCAA
TGTATACTGCTCCCATCGCAGGAAACATTGCTTGTTCATCAAAT---
ATTACAGGTCTACTATTGACAAGAGATGGTGGTAAGACTACTGAC----------------------------
----

AATCAGACTGACACCTTCAGACCTGGGGGAGGAGATATGAGAGACAATTGGAGAAGTGAATTATATAAATATA
AAGTAGTAGAAATTGAGCCATTA---GGAATAGCACCCAATAAAGCAAGGAGAAGAGTGGTG-----------
----------------------------CAGAGAGAAAAAAGAGCA---GTGGGG------------------
--GGCGCAGCGTCA---
GTAACGCTGACGGTACAG---GCCAGACAA---TTATTG---TCTGGTATAGTGCAACAGCAGAAT---
AATTTG---CTGAGG---GCTATTGAGGCGCAACAGCACATGTTGCAACTCACAGTCTGGGGCATC
>FN392874..1
TTCTATTGCAATACATCAGGCCTATTTAATAGCACTTGGTCTGACAAAAATAATACAGACAGCAATAGCACGG
AG---

TCAAATAGCACTATAACTCTTCCATGCAGAATAAAGCAAATTATAAATATGTGGCAGAGAGTAGGACGAGCAA
TGTATGCTCCTCCCATCCGAGGAATAATAAAGTGTACTTCAAAC---
ATTACAGGACTACTATTAACAAGAGATGGTGGTGTTAATAATAGTACA-------------------------
----

AATGAGACCTTCAGACCAGGAGGAGGAGATATGAGGGACAATTGGAGAAGTGAATTATATAAGTATAAAGTAG
TAAAAATTGAACCACTA---GGAGTAGCACCCACAAAGGCAAGGAGAAGAGTGGTG-----------------
----------------------GAGAGAGAAAAAAGAGCA---GTTGGA---CTGGGAGCT---------
TTCTTCCTTGGGTTCTTAGGAGCA---GCAGGAAGCACTATG---GGCGCAGCGGCA---
GTAACGCTGACGGTACAG---GCTAGACAA---TTATTG---TCTGGCATAGTGCAACAGCAGAGC---
AATCTG---CTGAGG---GCTATAGAGGCTCAGCAGCATCTGTTGAAACTCACGGTCTGGGGCATT
>FN392876..2
TTCTATTGCAATACATCAGGCCTGTTTAATACCACTTGGTACAACAATGGTTCAGACACTTGGTACAATGGTA
CAAAT--

AGCACGGAGCCAATAACTCTTCAATGCAGAATAAAGCAAATTATAAATATGTGGCAGAGAGTAGGACAAGCAA
TGTATGCCCCTCCCATCCCAGGAATAATACAATGTAACTCAAGC---
ATTACAGGAATACTATTAACAAGAGATGGTGGTAACAATAGTACA----------------------------
----

AATGAGACCTTCAGACCTGGAGGAGGAGATATGAGGGACAATTGGAGAAGTGAATTATATAGGTATAAAGTAG

TACAAGTTGAACCACTA---GGAGTAGCACCCACCAGGGCAAAGAGAAGAGTGGTG-----------------
----------------------GAGAGAGAAAAAAGAGCA---GTTGGA---ATAGGGGCT---------
GTCTTCCTTGGGTTCTTAGGAGCA---GCAGGAAGCACTATG---GGCGCAGCGTCA---
ATAACGCTGACGGTACAG---GCCAGACAA---TTATTG---TCTGGCATAGTGCGACAGCAGAAC---
AATCTG---CTGAAG---GCTATAGAGGCTCAACAGCATCTGTTGAAACTCACGGTCTGGGGCATT
>EU448295..1
TTCTATTGTAATACATCACAATTGTTTAATAGCACTTGGAGCCACAATGATACAGGCAGCACGAAGGAG----
----

TACAATGGCACTATACAACTTCCATGCAGAATAAAGCAAATTATAAATATGTGGCAGAGAGCAGGACGAGCAA
TGTTTGCCCCTCCCATCCAAGGAGCAATAAGGTGTGAATCAAAC---
ATTACAGGACTATTATTAACAAGAGATGGTGGTAATGCAAATGAGGCT-------------------------
----

AGAAATGAGACTGAGATCTTCAGACCTGGAGGAGGAGATATGAGGGACAATTGGAGAAGTGAATTATATAAGT
ATAAAGTAGTACAAATTGAGCCACTA---GGAGTAGCACCCACCAGGGCAAGGAGAAGAGTGGTG--------
-------------------------------GAGAGAGAAAAAAGAGCAGTTGTTGGA---CTGGGAGCT---
------GTCTTCATTGGGTTCTTAGGAGCA---GCAGGAAGCACTATG---GGCGCAGCGGCA---
GTGACGCTGACGGTACAG---GCCAGGCAA---TTATTG---TCTGGCATAGTGCAACAGCAGAGC---
AATCTG---TTGAGG---GCTATAGAGGCTCAACAGCATCTGTTGAAGCTCACGGTCTGGGGCATT
>DQ358801..2
TTCTACTGCAATACATCAGGACTGTTTCAGTTTAATGACACAGAT----------------------------
----

TCCAATGACACTATCATTCTCCCATGTCGAATAAAACAAATTGTAAACATGTGGCAGGAAGTGGGGCGAGCAA
TGTATGCCGCTCCCATTGCAGGAAACATTACCTGTAACTCAAAT---
ATTACAGGTCTACTATTGACAAGAGATGGTGGTCAGGATAATGGGACT-------------------------
----

AATGATATTGAGACCTTCAGACCTGGGGGAGGAAATATGAAAGACAACTGGAGAAGTGAATTATATAAATATA
AAGTAGTAGAAATTAATCCATTA---GGAATAGCACCCTCCAGGGCAAAAAGACAAGTGGTG-----------
----------------------------CAGAGAGAAAAAAGAGCA---GCGGGA---ATAGCAGTT------
---ATGCTCTTTGGGTTCTTGGGAGCA---GCAGGAAGCACTATG---GGCGCGGCGTCA---
ATGGCGCTGACGGTACAG---GCCAGACAA---TTATTG---TCTGGCATAGTGCAACAGCAGAAC---
AATCTG---CTGAGA---GCTATTGAAGCGCAACAGCATCTGTTGCAGCTCACAGTCTGGGGCATT
>DQ358802..2
TTCTACTGCGATACATCAACACTGTTTAATGACAATGACACAGTA----------------------------
----

TCCAATGACACTATCACTCTCCCATGTAAAATAAAACAAATTGTAAACATGTGGCAGGAAGTGGGGCGAGCAA
TGTATGCCGCTCCCATTGCAGGAGAAATTACCTGTAGATCAAAT---
ATTACAGGTCTACTATTGACAAGAGATGGTGGCCGAAATATTAGTACT-------------------------
----

GATCAGCCTGAGATCTTCAGACCTGGGGGAGGAAATATGAAAGACAACTGGAGAAGTGAATTATATAAATATA
AAGTAGTAGAAATTGAGCCACTA---GGAGTAGCACCCACCGAGGCAAAAAGACAAGTGGTG-----------
----------------------------AAGAGAAAAAAAAGAGCA---GCGGGA---ATAACAGCT------
---TTGTTCCTTGGGTTCTTGGGAGCA---
GCAGGAAGCACTATGGGCGCAGCCTCAATGACGCTGACGGTACAGGACCAG---ACGAATTAT---
TGGACTTGGCCATATTGCAGTCCGCGGGAACAA---TCTGCT---GAGAGG---
ACTATTGAAGCGCAACAGCATCTGTTGCAGCTCACAGTCTGGGGCATT
>MG365771..3
TTCTACTGCAATACATCAAAACTGTTTAATAATGACACAGGATCCAATGACACTACAGGA-------------
----

TCCAATGACACTATCACTCTCCCATGTAGAATAAAACAAGTTGTAAACATGTGGCAGGAAGTGGGGCGAGCAA
TGTATGCCGCTCCCATTGCAGGAAGAATTACCTGTAACTCAAAT---
ATTACAGGTCTACTATTGACAAGAGATGGTGGTCACACTAGTACTAATGAGACT-------------------
----

AATCAGACTGAGACCTTCAGACCTGAGGGAGGAAATATGAAAGACAATTGGAGAAGTGAATTATATAAATATA
AAGTAGTAGAAATTCAGCCACTA---GGAGTAGCACCCTCCAGGGCAAAAAGACCAGTGGTG-----------
----------------------------CAGAGAGAAAAAAGAGCA---GTGGGA---CTAGGAGCT------
---ATGTTCCTTGGGTTCTTGGGAGCA---GCAGGAAGCACTATG---GGCGCGGCGTCA---
ATAACGCTGACGGTACAG---GCCAGACAA---TTATTG---TCTGGCATAGTGCAACAGCAGAAC---
AATCTG---CTGAGG---GCTATTGAAGCGCAACAGCATCTGTTGCAGCTCACAGTCTGGGGCATT
>HM026456..1
TTCTACTGCAATACATCAGGACTGTTTAATGACACAGGATCC-------------------------------
----

AACAATGAGACTATCATTCTCCCATGTAGAATAAAACAAATGATAAACATGTGGCAGGAAGTGGGGCGAGCAA
TGTATGCCGCCCCCGTTGCAGGAAACATTACCTGTAACTCGAAT---
ATTACAGGTCTACTATTGACAAGAGATGGTGGTCCTGGTAATAAG----------------------------
----

ACTCAAATCTTCAGACCTGGGGGAGGAGATATGAAAGACAACTGGAGAAGTGAATTATACAAATATAAAGTAG
TAGAAATTGAGCCACTA---GGAATAGCACCCACCAAGGCAAAAAGACAAGTGGTG-----------------
----------------------ACGAGAGAAAAAAGAGCA---GTGGGA---CTGGGAGCG---------
TTGTTCCTTGGGTTCTTGGGAGCA---GCAGGAAGCACTATG---GGCGCGGCGTCA---
ATAACGCTGACGGTACAG---GCCAGACAA---TTATTG---GCTGGCATAGTGCAACAGCAAAAC---
AATTTG---CTGAGG---GCTATTGAAGCGCAACAGCATCTGTTGCAGCTCACAGTCTGGGGCATT
>HM026459..1
TTTTACTGCAATACATCAGGACTGTTTAATAATAATATCACACAA----------------------------
----

TCTAAGGACATTATCACCCTCCCATGTCGAATAAAACAAGTGGTAAACCTGTGGCAGGAAGTGGGGCGAGCAA
TGTATGCCGCCCCCATTGAAGGAATAATTACCTGTAACTCAAGT---
ATTACAGGTCTACTATTGACAAGAGATGGTGGTCAGGGTAATAGTACT-------------------------
----

GATAATAACACTGAGATCTTTAGACCTGGGGGGGGAAATATGAAAGACAACTGGAGAAGTGAATTATATAAAT
ATAAAGTAGTAGAAATTGAGCCACTA---GGAGTAGCACCCACCGGGGCAAAAAGACAAGTGGTG--------
-------------------------------AAGAGAGAGAAAAGAGCA---GTAGGGCTACTGGGAGCT---
------TTGTTCCTTGGGTTCTTGGGAACA---GCAGGAAGCACTATG---GGCGCAGCGTCA---
ATGGCGCTGACGGTACAG---GCCAGACAA---TTATTG---TCTGGCATAGTGCAACAGCAAAAC---
AATTTG---CTGAGG---GCTATTGAAGCGCAACAACATCTGTTGCAGCTCACAGTCTGGGGCATT
>HM026460..1
TTCTACTGCAACACATCAAGACTGTTTAATGACACAGGATCCAGA----------------------------
----

TCCAATGACACTATCATTCTCCCATGTCGAATAAAACAAATTGTAAACATGTGGCAGGAAGTGGGGCGAGCAA
TGTATGCCGCTCCCATTGCAGGAAATATTACCTGTAATTCAAAT---
ATTACAGGTCTACTATTGACAAGAGATGGTGGTAATCAGACTGAGAGT-------------------------
----

AATAATAATGAGATCTTCAGACCTGGGGGAGGAAATATGAAAGACAACTGGAGAAGTGAGTTATATAAATATA
AGGTAGTAGAAATTGAGCCACTA---GGAATAGCACCCACCAAGGCAAGAAGACGAGTGGTGGCGAGAGAA--
-------------AAAAGAGCAGTGGTGGCGAGAGAAAAAAGAGCA---GTAGGA---CTAGGAGCT------
---ATGTTCATTGGTATCTTGGGAGCA---GCAGGAAGCACTATG---GGCGCGGCGTCA---
ATAACGCTGACGGTACAG---GCCAGACAA---TTATTG---TCTGGCATAGTGCAACAGCAGAGT---
AATCTG---CTGAGG---GCTATTGAAGCGCAACAGCATCTGTTGCAGCTCACAGTCTGGGGCATT
>AF192135..1
TTCTATTGTAATACATCAGGATTGTTTAATAAATCATTATTGAATGAGACTAGTAATGAGACTACAGATGGT-
----

GCAAATAATACTATCACACTCACATGTAGAATAAAGCAAATTGTAAGAATGTGGCAGAGAGTAGGACAAGCAA
TATATGCCCCTCCCATTGCAGGAAACATTACATGCACATCAAAT---
ATTACAGGATTGCTATTGACACGTGATGGGGGATATACAAATAATACT-------------------------
----

AATGGAACAGAGATTTTCACACCTACAGGAAGAAATATGAGGGACAATTGGAGAAGTGAGTTATATAAATATA
AAGTGGTGAAGATTGAGCCAATA---GGAGTAGCACCCACCAGGGCAAAAAGAAGAGTGGTG-----------
----------------------------GGGAGAGAAAAAAGAGCA---GTAGGAATAATGGGAGCT------
---ATGTTCCTTGGGTTCTTGGGAACA---GCAGGAAGCACTATG---GGCGCAGCGTCA---
ATAACGCTGACGGTACAG---GCCAGACAA---TTGTTG---TCTGGTATAGTGCAACAGCAAAGC---
AATTTG---CTGAAG---GCTATAGAGGCTCAACAGCACCTGTTGAGACTCACTGTCTGGGGCATT
>JN417239..1
TTCTATTGCAATACATCACACCTGTTTAATAGCACTTGGAATAACAGCACACAGGTGTCAAATAACACACAG-
----

TTAAATGACACTATAACTCTCCAATGCAGAATAAAACAAATTATAAACATGTGGCAGGGAGTAGGAAAAGCAA
TGTATGCCCCTCCCATTGAAGGAAAGATTACATGTTTATCAAAT---
ATTACAGGACTAATATTAACAAGAGATGGTGGTGCAAATAATAAT----------------------------
----

AGTCAGAATGAGACTTTCAGACCTGCAGGAGGAGATATGAGAGACAATTGGAGAAGTGAATTATACAAATATA
AAGTAGTAAGACTTGAACCACTA---GGTCTAGCACCCACCGGGGCAAGGAGAAGAGTGGTG-----------
----------------------------GCAAGAGAAAAAAGAGCA---ATAGGA---CTAGGAGCT------
---ATGTTCCTTGGGTTCTTGGGAGCA---GCAGGAAGCACAATG---GGCGCAGCGTCA---
CTGACGCTGACGGTACAG---GCCAGAAAA---TTATTG---TCTGGTATAGTGCAGCAGCAAAAC---
AATTTG---CTGAGG---GCTATAGAGGCGCAACAACATTTGTTGCAACTCACAGTCTGGGGCATT
>JN882655..1
TTCTATTGCAATACATCAGGACTGTTTAATAGTACTTGGAATGACACTAGTACTTGGAATGAC----------
----

ACTGATAGGAATGTAACTCTCCCATGCAGAATAAAGCAATTTATAAATATGTGGCAGAGAGTAGAGCGAGCAA
TGTATGCCCCTCCCATCCGAGGAGACATAAAATGTGCATCAAAC---
ATCACTGGACTACTACTAACAAGAGATGGAGGGAATAATAATAGT----------------------------
----

ACAAATGAGACCTTCAGGCCTGCAGGAGGAGATATGAGAGACAATTGGAGAAGTGAATTATATAAGTATAAGG
TAGTAAAAATTGAACCATTA---GGAATAGCACCCACCAGGGCAAAAAGGAGAGTAGTG--------------
-------------------------GGGAGAGAGAAAAGAGCA---GTTGGA---ATGGGAGCT---------
GTCTTCCTTGGGTTCTTGGGAGCA---GCAGGAAGCACTATG---GGCGCGGCGTCA---
ATAACGCTGACGGTACAG---GCCAGACAA---TTATTG---TCTGGTATAGTGCAACAGCAAAAC---
AATTTG---CTGAAG---GCTATAGAGGCTCAACAACATATGTTGAGACTCACAGTTTGGGGCATT
>KC898985..1
TTCTATTGTAACACATCAGGCCTGTTTAATAGTACATACATACTTAATGGTACATACATGTACAGGGCTAATG
GTACAGACAAG---AAT---
ACGACTGATGGTACAATTAGT---------------
AATCCAAACTCAACCATCACAATCCCATGTAGAATAAAGCAAATTGTAAACATGTGGCAGGAGGTGGGACAAG
CAATGTATGCCTCTCCCATTGCAGGAAACATAACATGTAAATCAAAT---
ATCACAGGACTACTATTGGTACGTGATGGAGGTGGAGAGAAAAAT----------------------------
----

GATACAGAAATCTTCAGACCTGGAGGAGGAGATATGAGGGACAATTGGAGAAGTGAATTATATAAATATAAAG
TGGTAGAAATTCAGCCATTG---GGGGTAGCACCCACTAAAGCAAAAAGGAGAGTAGTG--------------
-------------------------GAGAGAGAAAAGAGAGCA---GTGGGA---ATAGGAGCT---------
GTGTTCCTTGGGATCTTGAGTGTA---GCTGGAAGCACTATG---GGCGCGGCGTCA---
ATGGCGCTGACGGTACAG---GCCAGACAA---TTGCTG---TCTGGTATAGTGCAACAGCAGAAC---
AATTTG---CTGAGG---GCTATAGAGGCGCAACAGCATATGTTGCAACTCACGGTCTGGGGCATT
>KC899008..1
TTCTATTGTGATACATCAGGCCTGTTTAATGGTACATATGGTACATACGATCCTAATGGCACAGAAGGTAATT
CAAGC--
-------------TCAGAA------
GGTAATTCAAAATTCATCATACTTCCATGCAGAATAAAGCAAATTATAAACATGTGGCAGGAGGTAGGACGAG
CAATGTATGCCCCTCCCATTGAGGGAAACATAACATGTAAATCAAAT---
ATCACAGGACTACTATTGGTACGTGATGGAGGAACTCAAAATGAAACA-------------------------
----

AAAAATGAAGAGATATTCAGACCTGAAGGAGGAGATATGAGGGACAATTGGAGAAGTGAATTATATAAATATA
AAGTGGTAGAAATTAAGCCATTG---GGAGTAGCACCCACTGAAGCAAAAAGGAGAGTAGTG-----------
----------------------------GAGAGAGAAAAAAGAGCA---GTGGGA---CTAGGAGCT------
---GTGTTCCTTGGGTTCTTGGGTGCA---GCCGGAAGCACTATG---GGCGCAGCGTCA---
GTAGCGCTGACGGTACAG---GCCAGACAA---TTGCTG---TCTGGTATAGTGCAACAGCAAAAC---
AATTTG---CTGAGG---GCTATAGAGGCGCAACAGCATATGTTGCAACTCACGGTCTGGGGCATT
>JX679207..1
TTCTATTGTAATACATCAAGACTATTTAATAGTACATACATGTCTAATGGTACATACACATACACAATTAATA
GT---

AACATCTCAAACATCACAATTCCATGCAGAATAAAGCAAATTGTAAACATGTGGCAGGAGGTAGGACGAGCAA
TGTATGCCCCTCCCATTGCAGGAAATATAACATGTAAATCAAAT---
ATCACAGGACTACTATTGGTACGTGATGGAGGATCAAATGAGACT----------------------------
----

CAGCCAGAGATATTCAGACCTGGAGGAGGAGATATGAGGGACAATTGGAGAAGTGAATTATATAAATATAAAG
TGGTAGAAATTAAGCCATTG---GGACTAGCACCCACTACTGCAAAAAGGAGAGTAGTG--------------
-------------------------GAGAGAGAAAAAAGAGCA---GTGGGA---ATAGGAGCT---------
GTGTTCCTTGGGTTCTTGGGTGCA---GCCGGAAGCACTATG---GGCGCGGCGTCA---
ATAACGCTGACGGTACAG---GCCAGACAA---TTGTTG---TCTGGGATAGTGCAACAGCAAAGC---
AATTTG---CTGAAG---GCTATAGAGGCGCAACAGCATCTGTTGCAACTCACGGTCTGGGGCATT
>JN230353..1
TTCTATTGCAATACATCAGCACTATTTAATAGCAGTTGGAATAGCACTGGGAATAGCACTGGGGTGAAT----
----

ATCACTGACCCTATAAGACTCCCATGCAAAATAAAGCAAATTATAAATATGTGGCAGAGAGTAGGACAAGCAA
TATATGCCCCTCCTATCCCAGGAGTAATAAGGTGTAATTCAAAC---
ATTACAGGAATACTATTAACAAGAGATGGAGGGACTGGGACTAACAGT-------------------------
----

ACAAATGAGACCGAGACCTTCAGACCTGGAGGAGGAGACATGAGGGATAATTGGAGAAGTGAATTATATAAGT
ATAAAGTAGTAAAAATTGAACCACTA---GGTGTAGCACCCACCGGGGCAAAACGAAGAGTGGTG--------
-------------------------------GAGAGAGGAAAAAGAGCAGTCGTCGGA---CTGGGAGCT---
------GTCTTCCTTGGGTTCTTAGGAACA---GCAGGAAGCACTATG---GGCGCAGCAGCA---
TTAACGCTGACGGTACAG---GCCAGACAA---TTATTG---TCTGGTATAGTGCAACAGCAGAAC---
AATCTG---CTGAGG---GCTATAGAGGCTCAACAGCACATGTTGAAACTCACGGTCTGGGGCATT
>KC899009..1
TTCTATTGTAATACATCAGGTCTGTTTAATGGAACATACATGCCTAATGGTACAATGGATAGTCCA-------
----

AGCACAATCCCATGCAGAATAAAGCAAATTATAAACATGTGGCAGGAGGTAGGGCGAGCAATGTATGCCCCTC
CTATTGAAGGAAACATAACATGTCAATCAAAT---
ATCACAGGAATACTATTGGTACGGGATGGAGAGTTAAATAGGACA----------------------------
----

GAAGAGATATTCAGACCTGGAGGAGGAGATATGAGGAACAATTGGAGAAGTGAATTATATAAATATAAAGTGG
TAGAAATTAAGCCATTG---GGAATAGCACCCACTGAAGCAAAAAGGAGAGTGGTG-----------------
----------------------GAGAGAGAAAAAAGAGCA---GTGGGA---ATAGGAGCT---------
GTGTTCCTTGGGTTCTTGGGAGCA---GCAGGAAGCACTATG---GGCGCGGCGTCA---
ATAACGCTGACGGTACAG---GCCAGACAA---TTGTTG---TCTGGGATAGTGCAACAGCAAAGC---
AATTTG---CTGAGG---GCTATAGAGGCGCAACAGCATATGTTGCAACTCACGGTCTGGGGCATT
>KC870043..1
TTCTATTGTAATACATCAGGCCTGTTTAAGGGTACATACACTAATGGT-------------------------
----
----------ACATTAAGT------
AATTCAAGCTCAGACATCACAATCCCATGCAGAATAAAGCAAATTATAAACATGTGGCAGGAGGTAGGAAGAG
CAATGTATGCCCCTCCCATTGAAGGAAACATAACATGTAAATCAAAT---
ATCACAGGACTACTATTGGTACGTGATGGAGGT--

-

ACTTCTAATAATAATACAGAGGTATTTAGACCTGGAGGAGGAGATATGAGGGACAATTGGAGAAGTGAATTAT
ATAAATATAAAGTGGTAGAAATTAAGCCATTG---GGAGTAGCACCCACTGGAGCAAAAAGGAGAGTGGTG--
-----------------------------------GAGAGAGAAAAAAGAGCA---GTGGGA---
ATAGGAGCT---------GTGTTCCTTGGGTTCTTGGGAGCA---GCAGGAAGCACTATG---
GGCGCGGCGTCA---ATAACGCTGACGGTACAG---GCCAGACAA---TTGTTG---
TCTGGCATAGTGCAACAGCAAAAC---AATTTG---CTGAAG---
GCTATAGAGGCGCAACATCATCTGTTGCAACTCACAGTCTGGGGCATT
>JN251903..1
TTCTACTGCAATACATCAGGACTGTTTAATAACACACTGTTAAATGTAAATGAAACAGGA------------
----

TCTAATAACACTATCACTCTCCCATGTCGAATAAAACAAATTGTAAACATGTGGCAGGAAGTAGGGCGAGCAA
TGTATGCCGCTCCCATTGCAGGAAACATTACCTGTAACTCAAAT---
ATTACAGGTCTACTATTGACAAGAGATGGTGGTCAGACTAATGAGACT------------------------
----

AATGGGAATGAGACTGTCAGGCCTGGGGGAGGAAATATGAAAGACAATTGGAGAAATGAATTATATAAATATA
AAGTAGTAGAAATTGAACCTCTA---GGAGTAGCACCCACCAAGGCAAAAAGACAAGTGGTG----------
--------------------------CAGAGAGAAAAAAGAGCA---ATAGGA---ATAGGAGCT------
---ATGTTCCTTGGGTTCTTGGGAGCA---GCAGGAAGCACTATG---GGCGCGGCGTCA---
ATAACGCTGACGGTACAG---GCCAGACAA---TTATTG---TCTGGAATAGTGCAACAGCAGAAC---
AATTTG---CTGCAG---GCTATTGAAGCGCAACAGCATCTGTTGCAGCTTACAGTCTGGGGCATC
>AB845347..1
TTCTATTGCAATACAACAAAACTGTTTAATAATACTTGCATAAGAAATAAAACCATGGAGGGG---------
----

TGTAGTGGCAATATCACTATCCCATGCAAGATAAAACAAATTATAAACATGTGGCAGGGCATAGGACAAGCAA
TGTATGCTCCTCCCATCAGTGGAAAAATTAATTGTGTATCAAAT---
ATTACAGGAATACTATTGACAAGAGATGGTGGTGCTAATAATACA---------------------------
----

AATAATGAGACCTTCAGACCTGGAGGAGGAGATATGAAGGACAATTGGAGAAGTGAATTATATAAATATAAAG
TAGTAGAAATTGAACCACTA---GGAATAGCACCCACCAGGGCAAAGAGAAGAGTGGTG-------------
-----------------------GAGAGAGAAAAAAGAGCA---GTGGGA---ATAGGAGCT---------
ATGATCTTTGGGTTCTTAGGAGCA---GCAGGAAGCACTATG---GGCGCGGCGTCA---
ATAACGCTGACGGTACAG---GCCAGACAA---TTATTG---TCTGGTATAGTGCAACAGCAAAGC---
AATTTG---CTGAGG---GCTATAGAGGCGCAGCATCATCTGTTGCAACTCACAGTCTGGGGCATC
>KU749388..1
TTCTACTGTGACTCCACTCCACTGTTTAATAGTACTTGGAATGGTACTGAAGAAATAAATGGCACTGAA----
----

GGAAATGACACGATCACACTCAAATGCAGAATAAAACAAATTATAAACATGTGGCAGAGAGTAGGAAGAGCAA
TGTATGCCCCTCCCATTCAAGGACAAATTAGCTGTTCATCAAAT---
ATTACAGGGATTCTATTAACAAGAGATGGTGGTAGTGGCAACGAG---------------------------
----

ACGAACAACAAGACAGAGACCTTCAGACCTGGAGGAGGAGATATGAGGGACAATTGGAGAAGTGAATTATATA
AATATAAAGTAGTAAAAATTGAACCATTA---GGAGTGGCACCAACCCATGCAAAGAGAAGAGTGGTG-----
---------------------------------CAGAGAGAAAAAAGAGCA---ATAGGA---
CTAGGAGCT---------ATGTTCCTTGGGTTCTTGGGAGCA---GCAGGAAGCACTATG---
GGCGCAGCGTCA---ATAACGCTGACGGTACAG---GCCAGACAA---TTATTG---
TCTGGTATAGTGCAGCAGCAGAAT---AATCTG---CTGAGG---
GCTATTGAAGCGCAACAGCATATGTTGCAACTCACAGTCTGGGGCATC
>DQ358811..1
TTCTACTGTAACTCAACACAACTGTTTAATAGTACTTGGTATGGTAATGAAACTTGGAATGGTACTGAAAAAT
TCAAT---

GAGGAAAATGACACAATCAGCCTCCCATGCAGAATAAAACAAATTATAAACATGTGGCAAGGAGTAGGAAAAG
CAATGTATGCCCCTCCCATCCAAGGACAAATTAGATGTACATCAAAT---
ATTACAGGGCTGATGTTAACAAGAGATGGTGGTCATAACAATGACACG------------------------

----

CACCAGATGACAGAGACCTTCAGACCTGGAGGAGGAGACATGAGGGACAATTGGAGAAGTGAATTATATAAAT
ATAAAGTAGTAAGAATTGAACCATTA---GGAATAGCACCCACCAAGGCAAAGAGAAGAGTGGTG--------
-------------------------------CAGAGAGAAAAAAGAGCA---ATAGGA---ATAGGAGCT---
------ATGCTCCTTGGGTTCTTGGGAGCA---GCAGGAAGCACTATG---GGCGCAGCATCA---
ATAACGCTGACGGTACAG---GCCAGACTA---TTATTG---TCTGGTATAGTGCAACAGCAAAAC---
AATTTG---CTGAGG---GCTATTGAGGCGCAACAGCATCTGTTGCAACTCACAGTCTGGGGCATC
>KJ671535..1
TTTTATTGCAATACATCAAGATTGTTTACTGGCACAGTGGTCAATGGCACT----------------------
----
-CTCACTAATGCCACT---------
ATCACTAATGGCACTATCACTCTCCCCTGTCGAATAAAACAAATTGTAAACATGTGGCAAGAAGTAGGACGAG
CAATGTATGCCGCTCCTGTTGCAGGAAGCATTAACTGTACATCAAAT---
ATTACAGGGCTACTATTGACAAGAGATGGGGGT--

AAGGATAATCAGACTAAT---
AAGAATGAGATCTTTAGACCTGGAGGAGGAAATATGAAAGATAATTGGAGAAGTGAATTATATAAATATAAAG
TGGTAGAAATTGAACCACTA---GGAGTAGCACCCACCAGGGCAAAGAGGCAAGTGGTG--------------
-------------------------AAGAGAGAAAGAAGAGCA---GTGGGA---ATAGGAGCT---------
TTGTTCCTTGGGTTCTTGGGAGCA---GCAGGAAGCACTATG---GGCGCAGCGTCA---
ATGACGCTGACGGTACAA---GCTAGACTA---TTATTG---TCTGGTATAGTGCAACAGCAGAAC---
AATCTG---CTGAGA---GCTATTGAGGCACAACAGCATCTGTTGCAACTCACAGTCTGGGGCATT
>KJ671537..1
TTCTATTGCAATACATCAGGACTGTTTAATGACTCAAAATTCAATGACTCAAAATTC----------------
----
AATGGCAATTTCTCTTCC---------
AATGACACTAGCACTATCACTATCCCATGTAGAATAAAACAAGTCATAAACATGTGGCAGGAAGTAGGACGAG
CAATGTATGCTGCTCCTATTGCAGGAAACATTATCTGTAACTCAAAT---
ATTACAGGTCTACTATTGACAAGAGATGGTGGT--

-
CAAACAAATAATACAAACGAGACCTTCAGACCTGGGGGAGGAAATATGAAAGACAATTGGAGAAGTAAGTTAT
ATAAATATAAAGTAGTAGAAGTTGAACCACTA---GGAGTAGCACCCACCAAGGCACAAAGACGAGTGGTA--
-------------------------------------AAGAGAGACAAAAGAGCA---GTGGGA---
ATGGGAGCT---------TTGTTCATTGGGTTCTTGAGCGCA---GCAGGAAGCACTATG---
GGCGCAGCGTCA---GTGACGCTGACGGTACAG---GCCAGACAA---TTATTA---
TCTGGTATAGTGCAACAGCAGAAC---AACTTG---CTGAGA---
GCTATTGAGGCGCAACAGCATCTGTTGCAACTCACAGTCTGGGGCATT
>MK341078..1
TTTTATTGTAATACTTCACAACTGTTTAATAGTACTTGGATCAATAGTATTTGG-------------------
----

AGCAATGACACAATCATACTTCCATGCAGAATAAGACAAATTATAAACTTGTGGCAGGAAGTAGGAAGAGCAA
TGTATGCCCCTCCCATTAGTGGACCTATTAAATGTTCATCAAAT---
ATTACAGGTCTACTACTAACAAGGGATGGGGGTCAGAGTAATAAC----------------------------
----

GAGACTGAGACCTTCAGACCTGGGGGAGGGAATATGAAAGACAATTGGAGAAGTGAATTATATAAATATAAAG
TAGTAGAACTTAAACCACTA---GGAGTAGCACCCACCAAGGCAAAAAGACAAGTGATA--------------
-------------------------GGGAGACAAAAAAGAGGG------------CTAGGCGCT---------
GTGTTCCTTGGGTTCTTGGGAGCA---GCAGGAAGCACTATG---GGCGCAGCGTCG---
CTGACGCTGACGGTACAA---GCCAGACTA---TTATTG---TCTGGTATAGTGCAACAGCAGAAC---
AATCTG---CTGAGG---GCCATTGAGATGCAACAACATCTGTTGAAACTCACAGTCTGGGGCATT
>GU595149..1
TTCTATTGTAATACAGCACAACTGTTTAATAGTACTTGGACGAATGGTACTGAACAGACAAATAGCACTATA-
----

GGAAATGACACAATCACACTCCCATGCAAAATAAAACAAATTATAAACTTGTGGCAGGAAGTAGGAAAAGCAA
TGTATGCCCCTCCCATCAGTGGACTAATTAGATGTTCATCAAAT---

ATTACAGGTCTACTACTGACAAGGGATGGGGGTCAGAATAATGAT----------------------------
----

ACTAAGAATGAGACCTTCAGACCTGGGGGAGGGGATATGAAAGACAATTGGAGAAGTGAATTATATAAATATA
AAGTAGTAGAAATTAAACCACTA---GGAGTAGCACCCACCAAGGCAAAAAGACAAGTGGTG-----------
----------------------------GGGAGACAAAAAAGAGGA------------CTAGGAGCT------
---GTGTTCCTTGGGTTCTTGGGAGCA---GCAGGAAGCACTATG---GGCGCAGCGTCA---
ATAACGCTGACGGTACAG---GCCAGACTA---TTATTG---TCTGGTATAGTGCAACAGCAGAAC---
AATCTG---CTGAGG---GCTATTGAGGCGCAACAGCATCTGTTGCAACTCACAGTCTGGGGCATC
>AY037271..1
TTCTACTGCAATACATCAAAACTGTTTAATAACAACACAGAA-------------------------------
----

TTCAATAACACTATTATTCTCCCATGTCGAATAAAACAGATGGTAAACATGTGGCAGGAAGTGGGACGAGCAA
TGTATGCCGCTCCCATTGCAGGAAACATTAACTGTAGCTCAAAT---
ATTACAGGTTTACTATTGACAAGAGATGGTGGTATAAATATAACA----------------------------
----

AGTGGTCAGGAGACCTTCAGACCTGGGGGAGGAAATATGAAAGACAATTGGAGAAGTGAATTATATAAATATA
AAGTAGTAGAAATTGAACCATTA---GGAGTAGCACCCACCAAGGCAAAGAGAAGAGTGGTG-----------
----------------------------CAGAGAGAAAAAAGAGCA---GTGGGA---ATGGGAGCT------
---TTGTTCCTTGGGTTCTTGGGAGCA---GCAGGAAGCACTATG---GGCGCGGCGTCA---
ATAACGCTGACGGTACAG---GCCAGACAA---TTATTG---TCTGGAATAGTGCAACAGCAGAAC---
AATTTG---CTAAGG---GCTATTGAAGCGCAACAGCATCTGTTGCAGCTCACAGTCTGGGGCATT
>MF372649..1
TTCTATTGCAATACATCAGCACTGTTTAATAGCACTTGGAATTCCACTTCAATTGCAAATTTCACAGAG----
----

GAAAATGGCACTATTACTATCCAATGCAGAATAAAGCAATTTATAAATATGTGGCAGAGAGTAGGAAGAGCAA
TGTATGCCCCTCCCATCAAAGGAAAGATACAATGTAACTCAACC---
ATAACAGGACTCATATTAACAAGAGATGGAGGTAATGGTAATAGC----------------------------
----

AGTGCAAATGAGACATTCAGACCTGGAGGAGGAGACATGAGGGACAATTGGAGAAGTGAATTATACAAGTACA
AAGTAGTAAAAATTGAACCATTA---GGTGTAGCACCCACCAAGGCAAAAAGAAGAGTGGTG-----------
----------------------------GAGAGAGAAAAAAGAGCG---GTTGGA---CTGGGAGCT------
---GTTTTCCTTGGGTTCTTAGGAGCA---GCAGGAAGCACTATG---GGCGCGGCGTCA---
ATAACGCTGACGGTACAA---GCCAGACAA---TTATTG---TCTGGTATAGTGCAACAGCAGAGC---
AATCTG---CTGAAG---GCTATAGAGGCTCAACAACAGCTGTTGAGACTCACAGTCTGGGGCATT
>MH666154..3
TTCTATTGCAATACATCAGGACTGTTTGATAGTACTTGGAATACATCAAGACTGTCTAATGGTACTTGGAATG
AT---
-ACATCAAGAGAATCTAAT------
AACTCAGTCAATAACATAACTCTTCAATGCAGAATAAGGCAAATTGTAAGAATGTGGCAGAGAGTAGGGCAAG
CAATGCATGCCCCTCCCATCCGAGGAGAAATAAGATGTGTATCAAAC---
ATTACTGGACTACTATTAACAAGGGATGGAGGGACGACTAATGAGACAGAG----------------------
----

ACAGAGGAGATCTTCAGGCCTGCAGGAGGAGATATGAGAGACAATTGGAGAAGTGAATTATATAAGTATAAAG
TAGTAAAAATTGAACCACTA---GGTGTAGCACCCAACTATGGAAGAAGAAGAGTGGTG--------------
-------------------------GAGAGAGAAAAAAGAGCA---GTTGGA---CTGGGAGCT---------
GTCTTCCTTGGGTTCCTAGGAGCA---GCAGGAAGCACTATG---GGCGCGGCGTCA---
ATAACGCTGACGGTACAG---GCCAGGCAG---TTATTG---TCTGGTATAGTGCAACAGCAAAGC---
AATTTG---CTGAAG---GCTATAGAGGCTCAACAACAGCTGTTGAGACTCACAGTCTGGGGCATT
>MH666157..2
TTCTACTGCAATACATCAGAACTGTTTAATAGTACTTGGAATGCCAATGGTAGCAATGATAGCCTAAAC----
----

CACACTGGCAACATAACTCTCAATTGCAGAATAAAGCAAATTATAAATATGTGGCAGAGAGTAGGGCAAGCAA
TTTATGCCCCTCCCATCCGAGGAGTAATAAGGTGTGAATCAAAC---

ATTACTGGAATATTATTAACAAGAGATGGAGGGAAAAGTAATGCT----------------------------
----

ACAAATGAGACCTTCAGGCCTGCAGGAGGAGATATGAGAGACAATTGGAGAAGTGAATTATATAAGTATAAAG
TAGTAAAAATTGAACCACTA---GGTGTAGCACCCACCCCTGCAAAAAGAAGAGTAGTG--------------
-------------------------GGGAGAGAAAAAAGAGCA---GTTGGA---CTGGGAGCT---------
GTCTTCCTTGGGTTCCTGGGAGCA---GCAGGAAGCACTATG---GGCGCGGCGTCA---
ATAACGCTGACGGTACAG---GCCAGACAA---TTATTG---TCTGGTATAGTGCAACAGCAGAGC---
AATTTG---CTGAAG---GCTATAGAGGCTCAACAGCATATGTTGAGACTCACAGTTTGGGGCATT
>MH666161..2
TTCTATTGCAATACATCAGGACTGTTTAATAGTACTTGGAACAATACTTGGAAAGATACATCAGAATAT----
----

AATGGATCTACCATAACTCTCCAATGCAGAATAAGGCAAATTGTAAGGATGTGGCAGAGAGTAGGGCAAGCAA
TGTATGCCCCTCCCATCCAAGGAGAAATAAGATGTGCATCAAAC---
ATTACTGGACTACTATTAACAAGAGATGGAGGGGATAATAGTGGG----------------------------
----

ACAAATGAGACCTTCAGGCCTGAGGGAGGAGATATGAAAGACAATTGGAGAAGTAAATTATATAAATATAAAG
TAGTGAAAATTGAACCACTA---GGTGTAGCACCCACCTATGGAAGAAGGAGAGTGGTG--------------
-------------------------GGGAGAGAAAAAAGAGCA---GTTGGA---CTGGGAGCT---------
GTCTTCTTTGGGTTCCTAGGAGCA---GCAGGAAGCACTATG---GGCGCGGCGTCA---
ATAACGCTGACGGTACAG---GCCAGACAA---TTATTG---TCTGGTATAGTGCAACAGCAAAGC---
AATTTG---CTGAAG---GCTATAGAGGCTCAACAACAACTGTTGAGACTCACAGTCTGGGGCATT
>MH666156..1
TTCTATTGTAATACATCAGGACTGTTTAATAGTACTTGGAGCAATACGAGCAATACATCAGGACCTAATACC-
----

TCAGACGCTGACATAACTCTCCAATGCAGAATAAGGCAAATTATAAGGATGTGGCAGAGAGTAGGGCAGGCAA
TATATGCCCCTCCCATCCAAGGAGAAATAAGGTGTGTATCAAAC---
ATTACTGGACTACTATTAACAAGGGATGGAGGGACTGATAATAAT----------------------------
----

ACAGATGAGACCTTCAGGCCTGCAGGAGGAGATATGAGAGATAATTGGAGAAGTGAATTATATAAATATAAAG
TAGTAAAAATTGAACCACTA---GGTGTAGCACCCACCCCTGCAAGAAGAAGAGTGGTG--------------
-------------------------GGGAGAGAAAAAAGAGCA---GTT------CTGGGAGCT---------
GTCTTCCTTGGGTTCCTAGGAGCA---GCAGGAAGCACTATG---GGCGCGGCGTCA---
ATAACGCTGACGGTACAG---GCCAGACAA---TTATTG---TCTGGTATAGTGCAACAGCAAAGT---
AATTTG---CTGAAG---GCTATAGAGGCTCAACAACAGCTGTTGAGACTCACAGTCTGGGGCATT
>MH666155..1
TTCTATTGCAATACATCAGGACTGTTTAATAGTACTTGGAATAGTACTTGGACCAATACATCAGGCATTAAT-
----

AGCTCAGAACCTATAATTCTCCAATGCAGGATAAGGCAAATTATAAGAATGTGGCAGAGAGTAGGGCAAGCAA
TGTATGCCCCTCCCATCCGAGGAGAAATAAGGTGTGAATCAAAC---
ATTACTGGACTACTATTAACAAGGGATGGAGGGAATGATAATACA----------------------------
----

AATGAGACCTTCAGGCCTGCAGGAGGAGATATGAGAGACAATTGGAGAAGTGAATTGTATAAGTATAAAGTAG
TAAAAATTGAACCACTA---GGTGTAGCACCCGGCCAGGCAAGGAGAAGAGTAGTG-----------------
----------------------GGGAGAGAAAAAAGAGCA---GTTGGA---ATAGGAGCT---------
GTCTTCCTTGGGTTCCTAGGAGCA---GCAGGAAGCACTATG---GGCGCGGCGTCA---
ATAACGCTGACGGTACAG---GCCAGGCAG---TTATTG---TCTGGGATAGTGCAACAGCAAAGC---
AATTTG---CTGAAG---GCTATAGAGGCTCAACAACATATGTTGAGACTCACAGTCTGGGGCATT
>KF850149..1
TTCTATTGTAATACAACAAAATTGTTTAATAGTACTTGGAAGGGAAATGAA----------------------
----
-ACCATGGAGAGGGAT---------
AATAAGACTGAGATCATCATACTTCCATGCAGGATAAAGCAAATTATAAACATGTGGCAGGGAGCAGGACAAG
CAATGTATGCTCCTCCCATCAAGGGATTAATTAATTGTACATCAAAT---

ATTACAGGAATACTATTAACAAGAGATGGTGGT--

CATAATGGTACAAATGAGACCTTCAGACCTGGAGGAGGAAATATAAAGGACAATTGGAGAAGTGAATTATATA
AATATAAAGTAGTAGAACTTGAACCACTA---GGGGTAGCACCCACCAGGGCAAAGAGAAGAGTGGTG-----
----------------------------------GAAAGAGAAAAAAGAGCA---GTGGGA---
ATAGGAGCT---------ATGATCTTTGGGTTCTTAGGAGCA---GCAGGAAGCACTATG---
GGCGCGGCGTCA---ATAACGCTGACGGTACAG---GCCAGACAA---TTATTG---
TCTGGTATAGTGCAACAGCAAAGC---AATTTG---CTGAGG---
GCTATAGAGGCGCAACAGCGTATGTTGCAACTCACAGTCTGGGGCATT
>MG518476..1
TTCTATTGTAATACAACAAAATTGTTTAATAGTACTTGGAAGGGAAATGAAACCATGGAGAGGAATAAT----
----

AACACTGACACTATCATACTTCCATGCAGGATAAAGCAAATTATAAACCTGTGGCAGGGAGTAGGACAAGCAA
TGTATGCTCCTCCCATCAGGGGAGCAATTAATTGTGTATCAAAT---
ATTACAGGAATACTATTAACAAGAGATGGTGGTCATAATAGTACA----------------------------
----

AATGAGACCTTCAGACCTGGAGGAGGAAATATAAAGGACAATTGGAGAAGTGAATTATATAAATATAAAGTAG
TAGAGATTGAACCACTA---GGAATAGCACCCACCAGGGCAAAGAGAAGAGTGGTG-----------------
----------------------GAGAGAGAAAAAAGAGCA---GTGGGA---ATAGGAGCT---------
ATGATCTTTGGGTTCTTAGGAGCA---GCCGGAAGCACTATG---GGCGCGGCGTCA---
CTAACGCTGACGGTACAG---GCCAGACAA---TTATTG---TCTGGTATAGTGCAACAGCAAAGC---
AATTTG---CTGAGG---GCTATAGAGGCGCAACAGCATATGTTGCAACTCACAGTCTGGGGCATT
>AY371145..2
TTCTATTGCAATACATCAGGACTATTTAATAGCACTTGGGAAATGAACACAACCAGCTTAGGG----------
----

TTAAATGATACTATAATTCTCCAGTGCAGAATAAAGCAAATTGTAAATATGTGGCAGAGAGTAGGACAAGCAA
TGTACGCCCCTCCCATCCCAGGAAAAATAAGGTGTCAGTCAAAC---
ATTACAGGACTACTATTGACAAGAGATGGTGGGACTAGTAACAATACAGAG----------------------
----

ACTAGTAACAGTACAGAGACTTTCAGACCTGGAGGAGGAGATATGAGAGATAATTGGAGAAGTGAATTATATA
AATATAAAGTAGTAAAAATTGAACCACTA---GGAGTAGCACCTACCAGGGCAAAGAGGAGGGTGGTG-----
----------------------------------GAGAGAGAAAAAAGAGCA---GTTGGA---
CTGGGAGCT---------GTCTTCCTTGGGTTCTTAGGAGCA---GCAGGAAGCACTATG---
GGCGCGGCGTCA---ATAACGCTGACGGTACAG---GCCAGACAA---TTATTG---
TCTGGCATAGTGCAACAGCAAAGC---AATTTG---CTGAGG---
GCTATAGAGGCTCAACAACATATGTTGAGACTCACGGTCTGGGGCATT
>KP718921..1
TTCTATTGTAATACAACAGAACTATTTAATAGCACTTGGGAGAGTAGCAATAGCACTAACAGCTCAAGTGCA-
----

ACAAATGGAACTATAACTCTCAAATGCAGGATAAAGCAAATTATAAATATGTGGCAGAGAGTAGGACAAGCAA
TGTATGCCCCTCCCATCCAAGGAATAATAAGGTGTGAATCAAAC---
ATTACAGGACTACTATTAACAAGAGATGGAGGAGATACTAATAGT----------------------------
----

AATAATAGTGAGATCTTCAGGCCTGGAGGAGGAGATATGAGGGACAATTGGAGAAGTGAATTATATAAGTATA
AAGTAGTAAAAATTGAACCAATA---GGTATAGCACCCACCCATGCAAAAAGAAGAGTGGTG-----------
----------------------------GAGAGAGAAAAAAGAGCA---GTTGGACTACTGGGAGCT------
---GCCTTCCTTGGGTTCTTAGGAGCA---GCAGGAAGCACTATG---GGCGCAGCGTCA---
ATAACGCTGACGGTACAG---GCCAGACAA---TTATTG---TCTGGCATAGTGCAACAGCAAAGC---
AATTTG---CTGAGG---GCTATAGAGGCTCAACAACAGCTGTTGAAACTCACAGTCTGGGGCATT
>KP718922..1
TTCTATTGCCATACATCAAACCTATTTAATAGCACTTGGAAAGAGAACACAACAGAGTCAAGTGGCAATATA-
----

ACAAGTGGCAATATAACTCTCCAGTGCAGAATAAAGCAAATTGTAAATATGTGGCAGAGAGCAGGAAAAGCAA
TGTATGCCCCTCCCATTCCAGGAGTGATAGAGTGTAGGTCAAAC---
ATTACAGGGCTCCTATTACAAAGAGATGGAGGGAAAAATAATACAAATAAT----------------------
----

ACCTTATGGCCTGGAGGAGGAGATATGAGGGATAATTGGAGAAGTGAGTTATACAAATATAAAGTAGTGAAAA
TTGAACCACTA---GGAGTAGCACCCACCAGGGCAAGGAGAAGGGTGGTG-----------------------
----------------CAGAGAGAAAAAAGAGCA---GTTGGA---ATAGGAGCT---------
GTCTTCCTTGGGTTCTTAGGAACA---GCAGGAAGCACTATG---GGCGCAGCGGCA---
GTAACGCTGACGGTACAG---GCCAGACAA---TTATTG---TCTGGTATAGTGCAACAGCAAAGC---
AATTTG---CTGAAG---GCTATAGAGGCTCAACAACAACTGTTGAGACTCACGGTCTGGGGCATT
>JF683748..1
TTCTATTGTAACACATCAGGCCTGTTTAATAGCACTTGGACTGCCAATGGTGGCAGGCTGCGGGAAGATAGTG
CA---

AAGTCAAACAGCACTATAACTCTCCAATGTAGAATAAAGCAAATTATAAATATGTGGCAGAGAGCAGGACAAG
CAATATATGCCCCTCCCATCCAAGGAGTAATAAGGTGTGAATCAAAC---
ATTACAGGGCTAATATTAACAAGAGATGGTGGGGGGAGTAATGAT----------------------------
----

ACAGCGGAAACCTTCAGACCTGGAGGAGGAGATATGAGGGACAATTGGAGAAGTGAATTATATAAGTATAAAG
TAGTAAAAATTGAACCATTA---GGAGTAGCACCCACCAAGGCAAGGAGAAGAGTGGTG--------------
-------------------------GAGAGAGAAAAAAGAGCA---GTTGGA---CTGGGAGCT---------
GTCTTCATTGGGTTCTTGGGAGCA---GCAGGAAGCACTATG---GGCGCGGCGTCA---
GTGACGCTGACGGTACAG---GCCAGGCAA---TTATTG---TCTGGCATAGTGCAACAGCAAAGC---
AATTTG---CTGAAG---GCTATAGAGGCTCAACAGCAGCTGTTGAGACTCACAGTTTGGGGCATT
>MK086114..3
TTCTATTGCAATACAACAAACCTGTTTAACAGCATTTGGAATTACAATAGCACTGGGAATCACAGTGACAGCA
TGACAAATAAC--

ACGGATGGCAATATAATTCTCCAGTGCAGAATAAAGCAAATTATAAATATGAGGCAGAGAGCAGGAAAAGCAA
TATATGCCCCTCCCATTCGAGGAAGAATACAGTGTGAGTCAAAC---
ATTACAGGAATACTATTGACAAGAGATGGTGGGAATAATGCTAGT----------------------------
----

AATTCAAATGAGACTTTTAGACCTGGAGGAGGAGATATGAGAGACAATTGGAGAAGTGAATTATATAAATATA
AAGTAGTAAAAATTGAACCACTA---GGAGTAGCACCCACCAAGGCAAGAAGGAGAGTGGTG-----------
----------------------------GAGAGAGAAAAAAGAGCA---TTTGGA---TTGGGAGCT------
---ATGTTCCTTGGGTTCTTGGGAACA---GCAGGAAGCACTATG---GGCGCAGCGTCG---
CTAACGCTGACGGTACAG---GCCAGACAA---TTATTG---TCTGGCATAGTGCAACAGCAAAGC---
AATTTG---CTGAGA---GCTATAGAGGCTCAACAGAATATGTTGAGACTCACAGTCTGGGGCATT
>KX159285..2
TTCTACTGCGATACAACAAAATTGTTTAATGAGAGACAGAAAATGGATCACGGAAGA----------------
----

GCTATAATGGCTACTATCTTACATCCATGTAGGATAAAACAAATTGTAAACATCTGGCAGCGAGTGGGACAAG
CAACGTGCTCCTCCCATGCTGCAGCAAACATATCATGTCAGTCAAAT---
ATCACAGGACTACTATTAGTACGTGATGGAGGAAATGATACAGAG----------------------------
----

GAGACAGAGACATTCAGGCCTGGAGGAGGAGATATAAAGGACAATTGGAGAAGTGAATTATATAAATATAAAG
TAGTACAAATTGAACCATTA---GGAATAGCACCCACCAAAGCAAAGAGAAGAGTGGTA--------------
-------------------------GACAGAGAAAAAAGAGCA---GTGGGA---ATAGGAGCT---------
ATGATCTTTGGGTTCTTAGGAGCA---GCAGGAAGCACTATG---GGCGCGGCGTCA---
ATAACGCTGACGGTACAG---GCCAGACAA---TTATTG---TCTGGTATAGTGCAACAGCAAAAC---
AATTTG---CTGAGA---GCTATAGAGGCGCAACAACATCTGTTGCAACTCACAGTCTGGGGCATT
>MN452901..1
TTCTATTGTAATACATCAAGCCTGTTTAATGGTACATACAATAGCAGTAGGCCAAACAGTACT----------
----

TCAAACTCAATCATCATAATCCCATGCAGAATAAAGCAAATTATAAATATGTGGCAAGGAGTAGGACAAGCAA
TGTACGCTCCTCCCATCAGTGGAACAATTAATTGTGTATCAAAT---
ATTACAGGAATCTTATTGACAAGGGATGGTGGTAATGATACCGTGAGTAGG----------------------
----

GAAAATGAGACCTTCAGACCTGGAGGAGGCAATATAAAGGACAATTGGAGAAATGAATTATATAAATATAAAG
TAGTAGAAATTGAACCACTA---GGAATAGCACCCACCAGGGCAAAGAGAAGAGTGGTG--------------
-------------------------GAGAGAGAAAAAAGAGCA---GTGGGA---ATAGGAGCT---------
ATGATCTTTGGGTTCTTAGGAGCA---GCAGGAAGCACTATG---GGCGCAGCGTCA---
ATAACGCTGACGGTACAG---GCCAGACAA---TTATTG---ACTGGTATAGTGCAACAGCAAAGC---
AATTTG---CTGAGG---GCTATAGAGGCGCAACATCATCTGTTGCAACTCACAGTCTGGGGCATT
>AY371167..1
TTCTATTGCAATACATCAGGCCTGTTTAATAGCACTTGGAATGACACTGGCAGCATGCCAGAGTCAAATAAC-
----

ACGAATGGCAATATAACTCTCCAGTGCAGAATAAAGCAGATTATAAATATGTGGCAGAGAACAGGACAAGCAA
TGTATGCCCCTCCCATCCCAGGAACAATAAGGTGTGAGTCAAAC---
ATTACAGGACTACTACTGACAAGAGATGGTGGGACTCCTAACAGT----------------------------
----

ACAAATGAAACTTTCAGACCTGGAGGAGGAGATATGAGAGACAATTGGAGAAGTGAATTATATAAATATAAAG
TAGTAAAACTTGAACCACTA---GGAGTAGCACCCACCAGGGCAAAGAGAAGAGTGGTG--------------
-------------------------CAGAGAGAAAAAAGAGCG---GTTGGA---TTGGGAGCT---------
GTCTTCCTTGGGTTCTTAGGAGCA---GCAGGAAGCACTATG---GGCGCGGCGTCA---
ATAACGCTGACGGTACAG---GCCAGACAA---TTATTG---TCTGGCATAGTGCAACAGCAAAGC---
AATTTG---CTGAAG---GCTATAGAGGCTCAACAGCATCTGTTGAGACTCACAGTCTGGGGCATT
>FJ388953..1
TTCTATTGTAACACATCAGGCCTGTTTAATAGCACTTGGCATGCCAATGACAGTTTGCAAGAGTCAAATGACA
CA---

ACAATAAAGAAGAATATAACTCTCCAATGCAGAATAAAGCAGATTATAAATCTGTGGCAGAGAGCAGGACAAG
CAATGTATGCCCCTCCCATCCCAGGAGTAATAAAGTGTGTATCAAAC---
ATAACAGGACTAATATTAACAAGAGATGGTGGAGGTAATAACACAGCT-------------------------
----

CGTGATAATGAAACCTTCAGACCTGGAGGAGGAGATATGAGGGACAATTGGAGAAGTGAATTATATAAGTATA
AAGTAGTAAAAATTGAGCCACTA---GGAGTAGCACCCACCAGGGCAAGGAGGAGAGTGGTG-----------
----------------------------GAGAGAGAAAAAAGAGCA---GTTGGA---ATAGGAGCT------
---GTCTTCATTGGGTTCTTGGGAGCA---GCAGGAAGCACTATG---GGCGCGGCGTCA---
GTGACGCTGACGGTACAG---GCCAGGCAA---TTATTG---TCTGGCATAGTGCAACAGCAAAGC---
AATTTG---CTGAGG---GCTATAGAGGCTCAACAACATCTGTTGAAACTCACAGTCTGGGGCATT
>KR017779..1
TTCTATTGCAATACATCAGGACTGTTTAATAGCACTTGGAAGATTAATAGCACTTGGAATAGC----------
----

ACTAATGGCACTATAACTCTCCTGTGCAGAATAAAGCAAATTGTAAACATGTGGCAGAGAGCAGGACAAGCAA
TGTATGCCCCTCCCATCCCAGGAGTAATAAGATGTGAATCAAAC---
ATTACAGGGATACTATTGACAAGAGATGGCGGGGAGGATTCTAAT----------------------------
----

AAGACTAATGAAACAGAAATTTTCAGACCTGGAGGAGGAGATATGAGAGACAATTGGAGAAGTGAATTATATA
AATATAAAGTAGTAAAAATTGAACCACTA---GGAGTAGCACCCACCGAGGCAAAGAGAAGGGTGGTG-----
----------------------------------GAGAGAGAAAAAAGAGCA---GTTGGA---
CTGGGAGCT---------GTTTTCCTTGGGTTCTTAGGAGCA---GCAGGAAGCACTATG---
GGCGCGGCGTCA---ATAACGCTGACGGTACAG---GCCAGACAA---TTATTG---
TCTGGTATAGTGCAACAGCAAAGC---AATTTG---CTGAGG---
GCCATAGAAGCTCAACAGCATGTGTTGAGACTCACAGTCTGGGGCATT
>U76035..1
TTCTATTGCAATACATCAGGCCTGTTTAATAGCACTTGGAAAAATAGCACCAGCATAAATGACACGGTG----
----

TCAAATGGCACTATAACTCTCCCATGCAGAATAAAGCAAATTGTAAATATGTGGCAAAGAGTAGGACAAGCAA
TGTATGCCCCTCCCATCAAAGGAGTAATAAAGTGTGAATCAAAC---
ATTACAGGAATACTATTAACAAGAGATGGTGTGGGTAATAACACA----------------------------
----

GCAAATGAGACCTTCAGACCTGGAGGAGGAGATATGAGGGACAATTGGAGAAGTGAATTATATAAGTATAAAG
TAGTAAAGATTGAACCACTA---GGAGTAGCACCCACCAAGGCAAAGAGAAGAGTGGTG--------------
-------------------------GCGAGAGAAAAAAGAGCA---ATTGGA---ATGGGAGCT---------
TTCTTCCTTGGGTTCTTAGGAGCA---GCAGGAAGCACTATG---GGCGCGGCGTCA---
ATAACGCTGACGGTACAT---GTCAGACAA---TTATTG---TCTGGTATAGTGCAGCAGCAGAGC---
AATCTG---CTGAGG---GCTATAGAGGCTCAACAGCATCTGTTGAAACTCACAGTCTGGGGCATT
>MH327751..1
TTCTATTGCAATACAACAAAATTGTTTAATTGCACAGACAATAATAACACATACACA----------------
----

TGTAATGACACTATCGTACTTCCATGCAAGATAAAGCAAATTATAAACATGTGGCAAGGAGTAGGACAAGCAA
TGTATGCTCCTCCCGTCAGGGGAATAATTAATTGTGTATCAAAC---
ATTACAGGAATACTATTGACAAGGGATGGTGGGACAAATAGCACT----------------------------
----

AATAGAAATGAGACCTTCAGACCTCAAGGAGGAAATATAAAGGACAATTGGAGAAGTGAATTATATAAATATA
AAGTAGTACAAATTGAACCACTA---GGAGTGGCACCCACTAGGGCAAGGCGAAGAGTGGTG-----------
----------------------------GAGAGAGAAAAAAAGAGCA---GTGGGA---ATAGGAGCT-----
---ATGATCTTTGGGTTCTTAGGAGCA---GCAGGAAGCACTATG---GGCGCGGCGTCA---
ATAACGCTGACGGTACAG---GCCAGACAA---TTATTG---TCTGGTATAGTGCAACAGCAAAGC---
AATTTG---CTGAGG---GCTATAGAGGCGCAGCAGCATCTGTTGCAACTCACAGTCTGGGGCATT
>KP411826..1
TTCTACTGTAACACAACAGAACTGTTTAACAGTACTTGGAATAGCACTGAAGAGTCAAGTACCCCTAAA----
----

GGAAATGACACAATTACACTCCAATGTAGAATAAAACAAATTATAAACATGTGGCAGGAAGTAGGAAAAGCAA
TGTATGCCCCTCCCATCAGAGGACAAATTAATTGTTCATCAAAC---
ATCACAGGGCTACTGTTAACAAGAGATGGCGATCAGAACGACAGT----------------------------
----

GAGACTTTCAGACCTGGAGGAGGAAATATGAAAGACAATTGGAGAAGTGAATTATATAAATATAAAGTAATAA
AAATTGAACCACTA---GGAATAGCACCCACCAGGGCAAAGAGAAGAGTGGTG--------------------
-------------------GAGAGAGAAAAAAGAGCA---GTGGGA---ATAGGAGCT---------
ATGATCTTTGGGTTCTTGGGAGCA---GCAAGAAGCACTATG---GGCGCAGCGTCA---
CTGACGCTGACGGTACAG---GCCAGACAA---TTATTG---TCTGGTATAGTGCAACAGCAGAAT---
AATTTG---CTGAAG---GCTATTGAGGCGCAACAGCATCTGTTGCAACTCACAGTCTGGGGCATT
>AY358069..2
TTCTATTGCAACACAACAGATCTGTTCAACACAACAAAACTAAATGAAACCACGTTAAATAATAGC-------
----

ACTAATAGCACTATCATACTTCCATGCAGAATAAAGCAAATTATAAACATGTGGCAGGGAGTAGGACAAGCAA
TGTATGCTCCTCCCATCAGAGGAAAGATTAATTGTGTATCAAAT---
ATTACAGGAATACTATTGACAAGAGATGGTGGTAATAATAATCAGACTAATAAT-------------------
----

CAGACTAACAAGACCGAGACCTTCAGACCTGGAGGAGGAAATATGAAGGACAATTGGAGAAATGAATTATATA
AATATAAAGTAGTACAAATTGAACCACTA---GGAATAGCACCCACCAAGGCAAAAAGAAGAGTGGTG-----
----------------------------------GAGAGAGAAAAAAGAGCA---GTGGGA---
ATAGGAGCT---------ATGATCTTTGGGTTCTTAGGAGCA---GCAGGAAGCACTATG---
GGCGCGGCGTCA---ATAACGCTGACGGTACAG---GCCAGACAA---TTATTG---
TCTGGTATAGTGCAACAGCAAAGC---AATTTG---CTGAGG---
GCTATAGAGGCGCAGCAGCATCTGTTGCAACTCACAGTCTGGGGCATT
>MG989522..1

TTCTATTGCAATACATCACGACTGTTTAATAATACTTGTGCAAAAAATGTAACCCAGGAAGGG----------
----

TGTAATGAAACTATCATACTCCCATGCAGGATAAAGCAAATAGTAAACATGTGGCAGAGAGCAGGACAAGCAA
TGTATGCTCCTCCCATCAGTGGACAAATTAATTGCATATCAAAT---
ATTACAGGAATACTGTTGACAAGAGACGGTGGTAAGGACGGTGATAATGGT----------------------
----

ACTCATAATGGGAGCGAGATCTTCAGACCTACAGGAGGAAATCTAAGGGACAATTGGAGAAATGAATTATATA
AATATAAGGTAGTGCAAATTGAACCACTA---GGAATAGCACCCACCAAGGCAAAGAGAAGAGTAGTG-----
----------------------------------GAGAGAGAAAAAAGAGCAGTAGTGGGA---
ATAGGAGCT---------ATGATCTTTGGGTTCTTAGGAGCA---GCAGGAAGCACTATG---
GGCGCGGCGTCA---ATAACGCTGACGGTACAG---GCCAGACAA---TTATTG---
TCTGGTATAGTGCAACAGCAAAGC---AATTTG---CTGAGA---
GCTATAGAGGCGCAACAGCATATGTTGCAACTCACAGTCTGGGGCATC
>MG989595..1
TTCTACTGTAATACAACACAACTGTTTAATAGTACTTGGTATGGGAATGATTCTGACAAGAATAATAACAATA
ATAAT--

GACAATGACACAATCATACTCCCATGTAAAATAAGACAAATTGTAAACATGTGGCAGGAAGTAGGAAAAGCAA
TATATGCTCCTCCTATCAGTGGACAAATTAACTGTTCATCAAAG---
ATTACAGGGCTGTTATTAACAAGAGATGGTGGTAATGGTAACGGTACC-------------------------
----

AGGAATGAAACCTTCAGACCTGGAGGAGGAAATATGAGGGACAATTGGAGAAGTGAATTATATAAATATAAAG
TAGTAAAAATTGAACCATTA---GGAGTAGCACCCACTAAGGCAAAGAGAAGAGTGGTG--------------
-------------------------CAGAGAGAAAAAAGAGCT---GTAACA---CTGGGAGCT---------
GTGTTCCTTGGGTTCTTGGGAGCA---GCAGGAAGCACTATG---GGCGCAGCGTCA---
TTGACACTGACGGTACAG---GCCAGACAA---TTATTG---TCTGGTATAGTGCAACAGCAGAAC---
AATCTG---CTGAGG---GCTATTGAGGCGCAACATCATCTGTTGCAACTCACAGTCTGGGGCATT
>MG989556..1
TTCTATTGCAATACATCAAAACTGTTTAATAGTACTTGGAATGGAACAAACAATACAGGAGGG----------
----

GATACTGGCACTATCATACTTCCATGTAGGATAAAGCAAATTATAAACATGTGGCAGCAAACAGGACAAGCAA
TGTATGCTCCTCCCATCAGTGGACCAATTAATTGTATGTCAAAT---
ATTACAGGAATACTATTGACAAGAGATGGTGGAGTTAATAATAGT----------------------------
----

CATACTAATGAAACCTTCAGACCTGGAGGAGGAGATATGAAAAATAATTGGAGAAATGAATTATATAAATATA
AAGTAGTACAAATTGACCCACTA---GGAATAGCACCCACCAAGGCAAAGAGGAGAGTGGTG-----------
----------------------------GAGAGAGAAAAAAGAGCA---GTGGGA---ATAGGAGCT------
---ATGATCTTTGGGTTCTTAGGAGCA---GCAGGAAGCACTATG---GGCGCGGCGTCA---
ATAACGCTGACGGTACAG---GCCAGACAA---TTATTG---TCTGGTATAGTGCAACAGCAAAGC---
AATTTG---CTGAGG---GCTATAGAGGCGCAGCAGCATCTGTTGCAACTCACAGTCTGGGGCATT
>MG989612..1
TTCTACTGTAATACAACAAAACTGTTTAATAGTACTTGGAATAATACTGGAGAGTCAAATGAGATCTCTACA-
----

GGAAATGACACCATCACACTCCCATGCAGAATAAAACAAATTATAAACATGTGGCAGCAAGTAGGGAAAGCAA
TGTATGCTCCTCCCATCAGTGGTCAAATTAACTGCTCATCAAAT---
ATTACAGGGCTACTATTATTAAGAGACGGTGGTAACCAGAACAATAGCGCC----------------------
----

AACACAGAGACATTCAGACCGGGAGGAGGAGACATGAGGGACAATTGGAGAAGTGAATTATATAAATATAAAG
TAGTAAAAATTGAACCATTA---GGAGTAGCACCAACCAAGGCAAGAAGAAGAGTGGTG--------------
-------------------------CAGAGAGACAAAAGAGCA---GCA------CTAGGAGCG---------
GTGTTCCTTGCGTTCTTGGGAGCA---GCAGGAAGCACTATG---GGCGCAGCGTCA---
ATAACGCTGACGGTACAG---GCCAGACAA---TTACTG---TCTGGTATAGTGCAACAGCAGAAC---
AATTTG---CTGAGA---GCTATTGAGGCGCAACAGCATATGTTGCAACTCACAGTCTGGGGCATA

```
>KF250395..2
TTCTATTGTAATACATCACAACTGTTTAATAGTACATGGAAGAATCACACTGGTACATATGAGACGAATGATA
CAAGTAACAAT--------------------------------------------------------------
----------------------------
CCAAACATCACAATCACAATTCCATGCAGAATAAAGCAAGTTATAAACATGTGGCAGGAGGTCGGGCGAGCAA
TGTATGCCCCTCCCATTCAAGGAACCATAACATGTAAATCAAAT---
ATCACAGGGCTACTATTGACACGTGATGGAGGAAATGGAAATATGACA-------------------------
-------------------------------------------------------------------------
----------
AATGAGACAGAGACATTCAGACCTGGAGGAGGAGATATGAGGGACAATTGGAGAAGTGAATTATATAAATATA
AAGTAGTAGAAATTAAGCCATTG---GGAGTAGCACCCACTGGAGCAAAAAGGAGAGTGGTG-----------
----------------------------GAGAGAGAAAAAAGAGCA---GTGGGA---ATAGGAGCT------
---GTGTTCCTTGGGTTCTTGGGAGCA---GCAGGAAGCACTATG---GGCGCGGCGTCA---
ATAACGCTGACGGTACAG---GCCAGACAA---TTGTTG---TCTGGTATAGTGCAACAGCAAAGT---
AATTTG---CTGAGG---GCTATAGAGGCGCAACAACATATGTTGCAACTCACGGTCTGGGGCATT
>KF250398..1
TTCTATTGTAATACATCAGGCCTGTTTAATGAGACAGACATGCTGCGTATTACA-------------------
-------------------------------------------------------------------------
----------------------------
GAAGGTAATATCACCACAATCCCATGCAGAATAAAGCAAATTATAAACATGTGGCAGAGGGTAGGAAAAGCAA
TGTATGCCTCTCCCATCAAAGGAAACATAACATGTAAATCAAAT---
ATCACAGGACTACTATTGGTACATGATCAAGTA------AATTCA----------------------------
-------------------------------------------------------------------------
-------------
ACCAATATGACATTCAGACCTGAAGGAGGAGATATGAAGAACAATTGGAGAAGTGAATTATATAAATATAAAG
TAGTAGAAATTAATCCATTG---GGAATAGCACCCACTGCAGCAAAAAGGAGAGTGGTGGAG---AGA-----
-------------------GAAAATAAGAGAGAAAAAAGAGCG---GTGGGA---CTAGGAGCT---------
TTGTTCCTTGGGTTCTTGGGAGCA---GCAGGAAGCACTATG---GGCGCGGCGTCA---
ATAACGCTGACGGTACAA---GCCAGACAA---TTGCTG---TCTGGTATAGTGCAACAGCAAAAC---
AATTTG---CTGATG---GCTATAGAGGCGCAACAGCATATGTTGCAACTCACAGTCTGGGGCATT
>MG519330..1
TTCTATTGCAATACAACAAACCTGTTTAATAATGATTGCATAAACAATACCACAGCGGGGGGG----------
-------------------------------------------------------------------------
----------------------------
TGTAATAAGACTATCATACTTCCATGCAAGATAAAACAAATTATAAACATGTGGCAAGGAGTAGGACAAGCAA
TGTATGCTCCTCCCATCCAGGGAATAATTAATTGTGTATCAAAT---
ATTACAGGAATACTATTGACAAGAGATGGTGGTATTCATAACACAAGCGCA----------------------
-------------------------------------------------------------------------
-------------
GGTAACGAGACCTTTAGACCAGGAGGAGGAAATATGAAGGACAATTGGAGAAATGAATTATATAAATATAAAG
TAGTAGAAATTGAACCACTA---GGAATAGCACCCACCAGGGCAAAGAGAAGAGTGGTG--------------
-------------------------GAAAGACAAAAAAGGGCA---GTGGGA---ATAGGAGCT---------
ATGATCTTTGGGTTCTTAGGAGCA---GCAGGAAGCACTATG---GGCGCGGCGTCA---
ATAACGCTGACGGTACAG---GCCAGACAA---TTATTG---TCTGGTATAGTGCAACAGCAAAGC---
AATCTG---CTAAGG---GCTATAGAGGCGCAGCAGCATCTGTTGCAACTCACAGTCTGGGGCATT
>MN336531..1
TTCTATTGCAATACATCAAAATTGTTT---AGCACCAGGGAAGGG----------------------------
-------------------------------------------------------------------------
----------------------------
CTTAATAGCACTATCATACTTCCATGCAGGATAAAACAAATTGTAAACATGTGGCAGGGAGCAGGACAAGCAA
TGTATGCTCCTCCCATCAAAGGAAGCATTAATTGTTATTCAAAT---
ATTACAGGAATACTATTAACAAGAGATGGTGGTAATAATAGTACG----------------------------
-------------------------------------------------------------------------
-------------------
GAGACCTTCAGACCAGGAGGAGGAAATATGAAGGACAATTGGAGAAATGAATTATATAAATATAAAGTAGTAG
AAATTGAACCACTA---GGAGTAGCACCCACCAGGGCAAAGAGGAGAGTGGTG--------------------
-------------------GATAGAGAAAAACGAGCA---GTGGGA---ATAGGAGCT---------
ATGATCTTTGGGTTCTTAGGAGCA---GCAGGAAGCACTATG---GGCGCAGCGTCA---
ATAACGCTGACGGTACAG---GCCAGACAA---TTATTG---TCTGGTATAGTGCAGCAGCAAAGC---
AATTTG---CTGAGA---GCTATAGAGGCGCAGCAGCATCTGTTGCAACTCACAGTCTGGGGCATT
```

>KU820848..1
TTCTATTGTAATACATCGAAACTGTTTAATGGTACATACAATACAACAAGTAATTCAAGTAATGGTACATACA
ATACAACA---
-------------------AGT---
AATTCAAGTTCAGTCATCACAATCCCATGCAAAATAAAACAAATTATAAACATGTGGCAGGAGGTAGGACGAG
CAATGTATGCTCCACCCATTGAAGGAAATATAACATGTAGATCAAAT---
ATTACAGGGTTGCTATTAACAAGAGATGGTGGTAGT-------------------------------------
----

AATACCACCGAGACATTCAGACCTCAAGGAGGAGATATGAGAGACAATTGGAGAAGTGAATTATATAAATATA
AAGTAGTAAAAATTGAACCATTA---GGAGTAGCACCCACCAGGGCAAAGAGAAGAGTAGTG-----------
----------------------------CAGAGAGAAAAAAGAGCA---GTGGGA---ATAGGAGCT------
---GTGTTCCTTGGGTTCTTGGGAGCA---GCAGGAAGCACTATG---GGCGCAGCGTCA---
ATAACGCTGACGGTACAG---GCCAGACAA---TTGTTG---TCTGGTATAGTGCAACAGCAAAAC---
AATTTG---CTGAGA---GCTATAGAGGCGCAACAACATATGTTGCAACTCACGGTCTGGGGCATT
>MH327758..1
TTTTATTGCAATACATCAGGCCTGTTTAATAAGACATACCCGTCTAATAGTACAGTTAATAATACAGAA----
----

ACTGATTCGATCATCACGATCCCATGCAAAATAAAGCAAATTATAAATATGTGGCAGGAGGTGGGACGAGCAA
TGTATGCTCCTCCCATTGCAGGAAACATAACATGTAGGTCAAAT---
ATCACAGGACTACTATTAGTACGTGATGGAGGATTAAATAATACA----------------------------
----

ACTGAGGTATTCAGGCCTCAAGGAGGAGATATGAGGGACAATTGGAGAAGTGAATTATATAAATATAAAGTGG
TAGAGATTAAGCCATTG---GGAATAGCACCCACTGGAGCAAAAAGGAGAGTGGTG-----------------
----------------------GAGAGAGAAAAAAGAGCA---GTGGGA---ATAGGAGCT---------
GCGTTCCTTGGGTTCTTGGGAGTA---GCAGGAAGCACTATG---GGCGCGGCGTCA---
GTGACGCTGACGGTACAG---GCCAGACAA---TTGCTG---TCTGGTATAGTGCAACAGCAAAGC---
AATTTG---CTGAGG---GCTATAGAGGCGCAACAGCATATGTTGCAACTCACGGTCTGGGGCATT
>KF250401..2
TTCTATTGTAATACATCAGGCCTGTTTAATAGTACATACTCGTCTAATAGTACATACAATGATACAGGA----
----

AGTAATGAAACCATCACAATCCCATGCAAACTAAAGCAAATTATAAACATGTGGCAGAAGGTAGGACGAGCAA
TGTATGCCCCTCCCATTGCAGGAAACATAACATGTAACTCAAAT---
ATCACAGGACTACTGTTGGTACGTGATGGAGGAGGAACAAATAAT----------------------------
----

TCAGAGGAGATATTCAGACCTCAAGGAGGAAATATGAAGGACAATTGGAGAAGTGAATTATATAAATATAAAG
TAGTAAAAATTGAACCATTA---GGATTGGCACCCACCCCTGCAAAGAGAAGCGTGGTG--------------
-------------------------CAGAGAGAAAAAAGAGCA---GTGACA---CTAGGAGCT---------
TTGTTCCTTGGGTTCTTGGGAACA---GCAGGAAGCACTATG---GGCGCGGCGTCA---
ATAACGCTGACGGTACAG---GCCAGACAA---TTGTTG---TCTGGTATAGTGCAACAGCAAAGC---
AATTTG---CTGATG---GCTATAGAGGCGCAACATCATATGTTGCAACTCACGGTCTGGGGCATT
>AJ508597..1
TTCTATTGTAATACATCAGGACTGTTTAATGGTACAGAACTGCTTAATAGTACATCT----------------
----

AATAATACTGCAATCACACTCCGATGTAGAATAATACGAATTATAAGGATGTGGCAGAGAGTGGGACAAGCCA
TTTACGCCCCTCCCATTGAAGGATACATTACATGTACATCAAAT---
ATAACAGGATTACTATTTACAAGAGATGGA---AATAACAATGAC----------------------------
----

AGTGAGACCTTCAGACCTGGAGGAGGAGATATGAGGGACAATTGGAGAAGTGAATTATATAAGTATAAAGTAG
TAAAAGTTAAACCACTA---GGAATAGCACCCAACAAGGCAAGGAGAAGAGTAGTG-----------------
----------------------GGGAGAGAGAAAAGAGCA---GTTGGA---CTGGGAGCT---------
GTTTTCCTTGGGTTCTTAGGAACA---GCCGGAAGCACTATG---GGCGCGGCGTCA---
ATAACGCTGACGGTACAG---GTCAGACAA---TTATTG---TCTGGCATAGTGCAACAGCAAAGC---
AATTTG---CTGAGA---GCTATAGAGGCGCAGCAGCATCTGTTGCAACTCACAGTCTGGGGCATT

>AJ508595..3
TTCTATTGTAATACATCAGGCCTGTTTAATATGTCAGGGAGTAGTAATATATCAGGGAAT------------
----

AGTAATGAGACCATCACACTCCCATGTAGGATAAAACAAATTGTAAGGATGTGGCAGAGAGTGGACAAGCCA
TTTATGCCCCTCCCATCGCAGGATACATTACATGCACATCAGAC---
ATTACAGGACTACTATTTACAAGAGACGGGGGTCATCACAGCAGT---------------------------
----

AATGAGACCTTCAGACCGGAAGGAGGAGATATGAGGAACAATTGGAGAAGTGAATTATATAAGTATAAAGTAG
TAAAAATTAAACCACTA---GGAATAGCACCCACCAGAGCAAGGAGAAGAGTAGTG----------------
----------------------GGGAGAGAGAAAAGAGCA---GTTGGA---CTGGGAGCT---------
GTGTTCCTTGGGTTCTTAGGAACA---GCAGGAAGCACTATG---GGCGCAGCGTCA---
ATAACGCTGACGGTACAG---GCCAGACAA---TTATTG---TCTGGCATAGTGCAACAGCAAAGC---
AATTTG---CTGAGA---GCTATAGAGGCGCAGCAGCATCTGTTGCAACTCACAGTCTGGGGCATT
>KU168299..1
TTCTATTGCAATACAACAGCATTGTTTAATAGCTCTTGGGATGCCAACACACTAAGTAATAGCACT-------
----

GTAAATGACACTATAACTCTCCAATGCAGAATAAAGCAAATTGTAAATATGTGGCAGAAAGTAGGACAAGCAA
TGTATGCCCCTCCCATCCCAGGAATAATAAAGTGTGAATCAAAC---
ATTACAGGGTTACTATTAACAAGAGATGGAGGGAACACAGATAAT---------------------------
----

ATAACTGAGACTTTCAGGCCTGGAGGAGGAAATATGAGGGACAATTGGAGGAGTGAATTGTATAAATATAAAG
TAGTAAAAATTGAACCATTA---GGTGTAGCACCCACCCATGCAAAAAGAAGAGTGGTG-------------
------------------------GGAAGAGAAAAAAGAGCA---GTTGGA---CTGGGAGCT---------
GTCTTCCTTGGGTTCTTAGGAGCA---GCAGGAAGCACTATG---GGCGCGGCGTCA---
ATAACGCTGACGGTACAA---GCCAGACAA---TTATTG---TCCGGTATAGTGCAACAGCAGAGC---
AATCTG---CTAAGG---GCTATAGAGGCTCAACAACATATGTTGAAGCTCACGGTCTGGGGCATT
>AJ866554..1
TTCTATTGCAATACAACAAAACTGTTTAATAGCACTTGGAATAGTTGGGATAATAGCACTTGGAATAGTACCA
GCTCAAACGAC---
-------------ACATGG------
CCAAAGGGGTCAAATATAACTCTCCAATGCAGAATAAAGCAAATTATAAATATGTGGCAGAGAGTAGGACAAG
CAATGTATGCCCCTCCCATCCCAGGAGAAATAAAGTGTGTATCAAAC---
ATTACTGGACTAATATTAACAAGAGATGGAGGGAATAATAATAGT---------------------------
----

GCAAGTGAGACCTTCAGGCCTGGAGGAGGAGATATGAGGGACAATTGGAGAAGTGAATTATATAAATATAAAG
TAGTAAAAATTGAACCACTA---GGAGTAGCACCCACTAGAGCGAAGAGAAGAGTGGTG-------------
------------------------GAGAGAGAAAAAAGAGCA---GTTGGA---CTGGGAGCT---------
TTCTTCTTTGGGTTCCTAGGAGCA---GCAGGAAGCACTATG---GGCGCGGCGTCA---
ATAACGCTGACGGCACAG---GCCAGACAA---TTATTG---TCTGGCATAGTGCAACAGCAGAGC---
AATCTG---CTGAAG---GCTATAGAGGCTCAACAGCATCTATTGAAACTCACGGTCTGGGGCATT
>AJ866556..1
TTCTATTGCAATACATCAGCACTGTTTAATAGCACTTGGGTAGGGGTTAATGGCAATTGGAGCAATACCAGCT
CAAACAAC--
-------------------ACA---
GGGTTAAATGACATTATAACTCTTCGATGCAGAATAAAACAAATTATAAATATGTGGCAGAGAGTAGGACAAG
CAATGTATGCCCCTCCCATCCAAGGAGAACTAAGGTGTGACTCAAAC---
ATTACTGGATTACTATTAACAAGAGATGGAGAGGGTGATACTAAG---------------------------
----

GAAAATGAGACCTTCAGGCCTGGAGGAGGAGATATGAGGGACAATTGGAGAAGTGAATTATATAAGTATAAAG
TAGTAAAAATTAAACCACTA---GGTGTAGCACCCACCCGTGCAAAAAGGAGAGTGGTG-------------
------------------------GAGAGAGAAAAAAGAGCA---GTTGGA---CTGGGAGCT---------
GTCTTCCTTGGGTTCTTAGGAGCA---GCAGGAAGCACTATG---GGCGCGGCGTCA---
ATAACGCTGACGGTACAG---GCCAGACAA---TTATTG---TCCGGTATAGTGCAACAGCAGAAC---
AATTTG---CTGAAG---GCTATAGAGGCTCAACAACATTTGTTGAGACTCACGGTCTGGGGCATT

>AJ866558..2
TTCTATTGCAGTACATCAGCATTGTTTAATAGCACCTGGATGATGGTTAATGGCACCTGGAGCGACACTGCAA
ATGACACAGAG--
-------------GCAAAT------
AACAATATAACTCATATAACTCTCCAATGCAAAATAAAGCAAATTGTAAATATGTGGCAGAGAGTAGGACAAG
CAATATATGCCCCTCCTATCCAAGGAAAAATAAGTTGTAATTCAAGC---
ATTACAGGACTGCTATTAACAAGAGATGGAGGAGGGAATAATACT----------------------------
----

GCAAATGAAACTTTCAGGCCTGGAGGAGGAGATATGAGGGACAATTGGAGAAGTGAATTATATAAGTATAAAG
TAGTAAAAAATTGAACCACTA---GGTGTGGCACCCACCCATGCAAAAAGAAGAGTGGTG-------------
-------------------------GGAAGAGAGAAAAGAGCG---GTTGGA---CTGGGAGCT---------
GTCTTCCTTGGGTTCTTAGGAGCA---GCAGGAAGCACTATG---GGCGCGGCGTCA---
ATAACGCTGACGGTACAG---GCCAGACAA---TTATTG---TCCGGTATAGTGCAACAGCAGAGT---
AATTTG---CTGAGG---GCTATAGAGGCTCAACAACAACTGTTGAAACTCACGGTCTGGGGCATT
>AJ866555..1
TTCTACTGCAATACAACACAACTGTTTAATAGCGCTTGGTTTAATGGCACTTGGGTATACAACAATAACACCA
ATGTC--

AGCTCAGGGTCAAATATAACTCTCCCATGCAGCATAAAGCAAATTGTGAGAATGTGGCAGAAAGTAGGACAAG
CAATGTATGCCCCTCCCATCCCAGGAATAATAAGGTGTGAGTCAAAT---
ATTACAGGACTAATCTTAACAAGAGATGGGGGGAACGATAGCAGT----------------------------
----

GTAAATGAGACCTTCAGGCCTGGAGGAGGAGATATGAGGGACAATTGGAGAAGTGAATTATATAAGTATAAAG
TAGTAAAAAATTGAACCACTA---GGTGTAGCACCCACCCCTGCAAAAAGAAGAGTGGTG-------------
-------------------------CAGAGAGAAAAAAGAGCAGTAGTTGGA---ATGGGAGCT---------
CTTTTCCTTGGGTTCTTAGGAGCA---GCAGGAAGCACTATG---GGCGCAGCGTCA---
GTAACGCTGACGGTACAG---GCCAGACAA---TTATTG---TCCGGTATAGTGCAACAGCAGAGC---
AATTTG---CTGAGG---GCTATAGAGGTTCAACAACATCTGTTGAAACTCACGGTCTGGGGCATT
>AF184155..1
TTCTATTGCAATACATCAGGACTGTTTAATAGCACTTGGTATAAAAATAGCACTTGGTATAGCAATAGCACTG
CCAGCTCAAAC--
-------------CACACA------
GAGTTAAATAGCACTATAACTCTCCAATGCAAAATAAAGCAAATTATAAATATGTGGCAGAAAGTAGGACAAG
CAATGTATGCCCCTCCCATCCAAGGAGTAATAAGATGTGATTCAAAC---
ATTACAGGACTACTATTAACAAGAGATGGAGGGAGTAATAATAAT----------------------------
----

ACACCTGAGATCTTCAGGCCTGGAGGAGGAAATATGAGGGACAATTGGAGAAGTGAATTATATAAGTATAAAG
TAGTAAAAAATTGAACCACTA---GGTGTAGCACCCACCCGTGCAAAAAGAAGAGTGGTG-------------
-------------------------GAGAGAGAAAAAAGAGCA---GTTGGA---CTGGGAGCT---------
GTCTTCCTTGGGTTCTTAGGAGCA---GCAGGAAGCACTATG---GGCGCAGCGTCA---
ATAACGCTGACGGTACAG---GCCAGACAA---TTATTG---TCCGGTATAGTGCAACAGCAGAGC---
AATTTG---CTGAGA---GCTATAGAGGCTCAACAACATCTGTTGAAACTCACGGTCTGGGGCATT
>HQ385445..3
TTCTATTGCAATACATCAAGCCTATTTAATAGCACTTGGAATACATCAAGCATTAGCATTGAGAATAGTACCG
AT---

TACTCAAATGAAACTATAACTCTCCAATGCAGAATAAAGCAAATTATAAATATGTGGCAGACAGTAGGACAAG
CAATATATGCCCCTCCCATCCCAGGAATAATAAGCTGTAATTCAACT---
ATTACTGGACTACTATTAACAAGAGATGGAGGAAATACTAATAGT----------------------------
----

ACGAATGAAACCTTCAGGCCTGGGGGAGGAGATATGAGGGACAATTGGAGAAGTGAATTATATAAGTATAAAG
TAGTGAAAATTGAACCACTA---GGTGTAGCACCCACTCATGCAAAAAGAAGAGTGGTG-------------
-------------------------GAGAGAGAAAAAAGAGCA---GTTGGA---CTGGGAGCT---------
GTCTTCCTTGGGTTCTTAGGAGCA---GCAGGAAGCACTATG---GGCGCGGCGTCA---
ATGGCGCTGACGGTACAG---GCCAGACAA---TTATTG---TCCGGTATAGTGCAACAGCAGAGC---
AATTTG---CTGAAG---GCTATAGAGGCTCAACAGCATCTGTTGAGACTCACGGTCTGGGGCATT

>GU201497..2
TTCTATTGCAATACATCAAGACTGTTTAATAGCACTTGGTATAATGACACTAGAAGGCCCAACTACACAGAG-
----

TTAAATGGCACTATAACCCTCCAATGCAGAATAAAGCAAATTATAAATATGTGGCAGAGAACAGGACAAGCAA
TATATGCCCCTCCCATCCCAGGAGAAATAAGGTGTAATTCAAAC---
ATTACAGGAATACTATTAGTAAGAGATGGAGGGGTTAATACTAGT----------------------------
----

ACAAATGAGACCTTCAGGCCTGCAGGAGGAGATATGAGGGATAATTGGAGAAGTGAATTATATAAGTATAAAG
TAGTAAAAATTGAACCACTA---GGTGTAGCACCCACCCATGCAAGAAGAAGAGTGGTG--------------
-------------------------CAGAGAGAAAAAAGAGCA---GTTGGA---CTGGGAGCT---------
GTCTTCCTTGGGTTCTTAGGAGCA---GCAGGAAGCACTATG---GGCGCGGCGTCA---
ATAACGCTGACGGTACAA---GCCAGACAA---TTATTG---TCCGGTATAGTGCAACAGCAGAGC---
AATCTG---CTGAGA---GCTATAGAGGCTCAACAACATATGTTGAGACTCACGGTCTGGGGCATT
>JN864047..2
TTCTATTGCAATACATCAGGCCTGTTTAATGGCACTTGGGATAAATCTTACAACATGACAGAG----------
----

TTGACTGGCACTATAATTCTCCCGTGCAGAATAAAGCAAATTATAAATATGTGGCAGAGAGTGGGACAAGCAA
TGTATGCCCCTCCCATCCCAGGAAGAATAAAGTGTGAGTCAAAT---
ATTACAGGGCTACTATTGACAAGAGATGGTGGAAGTAATAATGAT----------------------------
----

ACTAGTGAGATTTTTAGACCTGGAGGAGGAGACATGAGAGATAATTGGAGAAGTGAACTATATAAATATAAAG
TAGTAAAAATTGAACCAATA---GGAGTAGCACCCACCAAGGCAAGAAGAAGGGTGGTG--------------
-------------------------GAGAGAGAAAAAAGAGCA---ATTGGA---TTTGGAGCT---------
GCCTTCATTGGGTTCTTAGGAGCA---GCCGGAAGCACTATG---GGCGCGGCGTCA---
ATAACGCTGACGGTACAG---GCCAGACAA---TTATTG---TCTGGCATAGTGCAACAGCAAAGC---
AATTTG---CTGAAG---GCTATAGAGGCTCAACAGCATATGTTGAGACTCACGGTCTGGGGCATT
>JN864052..2
TTCTATTGCAATACATCACAACTATTTAATGGCACTTGGTCTAATAACACTGGGAATGGGAATGCTACCAGCT
CA---

AATGAAACTATAATAACTCTTCCATGTAGAATAAAGCAAATTATCAATATGTGGCAGAGAGTAGGACAAGCAA
TGTATGCCCCTCCCATCCGAGGAGGAATAAGGTGCGACTCAAAC---
ATTACAGGATTACTATTAACAAGAGATGGAGGAACGAATAGTACA----------------------------
----

CATCAGACATTCAGGCCTGGAGGAGGAGATATGAGAAACAATTGGAGAAGTGAATTATATAAGTATAAGGTAG
TACAAATTGAACCAATA---GGTGTGGCACCCACCCCTGCAAAAAGAAGAGTGGTG-----------------
----------------------GAAAGAGAAAAAAGAGCA---GTAGGA---CTGGGAGCT---------
GTCTTCCTTGGGTTCTTAGGAGCA---GCAGGAAGCACTATG---GGCGCAGCGTCA---
ATAACGCTGACGGTACAG---ACCAGACAA---TTATTG---TCCGGTATAGTGCAGCAGCAGAGC---
AATCTG---CTGAGG---GCTATAGAGGCTCAACAGCATCTGTTGAAACTCACAGTCTGGGGCATT
>JN864053..2
TTCTATTGCAATACAACAAACCTGTTTAATAGCACTTGGTATAACAATGACACTAGTGCTTGGTATAACAATG
GCACAGGACCA--
TACAATGGCACAGGACTA---------
TGGATAAATGGCACTATAATTCTCTCGTGTAGAATAAAGCAAATTGTAAACATGTGGCAAAGAGTAGGACAAG
CAATGTATGCTCCCCCCATCCGAGGAAGAATAGAGTGTCAATCAAAC---
ATTACAGGACTACTGTTGACAAGAGATGGTGGGGAGCATAATAATACTAATAAT------
AATAATACTAATAAT--

AATACTGAGATTTTTAGGCCTGGAGGAGGAGATATGAGAGACAATTGGAGAAGTGAATTATATAAATATAAAG
TAGTAAAAATTGAACCACTA---GGAATAGCACCCACCAAGGCAAGGAGAAGAGTGGTG--------------
-------------------------CAGAGAGAAAAAAGAGCA---GTTGGA---TTTGGAGCT---------
GTCTTCCTTGGGTTCTTAGGAGTA---GCCGGAAGCACTATG---GGCGCGGCGTCA---
ATGGCGCTGACGGTACAG---GCCAAACAA---TTATTG---TCTGGCATAGTGCAACAGCAAAGC---
AATTTG---CTGAGG---GCTATAGAGGCTCAACAGCAGATGTTGAGACTCACGGTCTGGGGCATT

```
>DQ826727..2
TTCTATTGCAATACATCAGGCTTATTTAATAGTAGTTGGACAGGAAATAACACAGAAAATAACACTGCA----
-------------------------------------------------------------------------
----------------------------
CTGAATGGCACTATAACTCTCCCGTGCAGAATAAAACAAATTGTAAACATGTGGCAGAGAGTAGGACGAGCAA
TGTATGCCCCTCCCATCCCAGGAGTAATAAGGTGCACATCAAAC---
ATTACAGGATTAATACTGACAAGGGATGGTGGAAACCATGGAAATAGT-------------------------
-------------------------------------------------------------------------
-------
AGTTATAAAAATGAAACCTTCAGACCTGGAGGAGGAGACATGAGGGACAATTGGAGAAGTGAATTATATAAGT
ATAAGGTAGTACAAATTGAACCAGTA---GGTGTGGCACCCACCCATGCAAGAAGAAGAGTGGTG--------
-------------------------------GAGAGAGGAAAAAGAGCA---GTTGGA---CTGGGAGCT---
------GTCTTCCTTGGGTTCTTAGGAGCA---GCAGGAAGCACTATG---GGCGCGGCGTCA---
ATAACGCTGACGGTACAG---GCCAGACAA---TTACTG---TCCGGTATAGTGCAACAGCAGAGC---
AATTTG---CTGAAG---GCTATAGAGGCTCAACAACAGCTGTTGAAACTCACGGTCTGGGGCATT
>MH078538..1
TTCTATTGCAATACATCAGGACTGTTCAATAAGAATTGGACATATGATAACAGCAATATCACCTCAAATAAC-
-------------------------------------------------------------------------
----------------------------
ACAGAATCAACTATAACTCTCCCATGCAACATAAGGCAAATTGTAAAGATGTGGCAGAGAGTAGGACGAGCAA
TGTATGCCCCTCCTATCGCAGGAAACATAAGTTGCAACTCAAAC---
ATTACTGGATTACTCTTAACAAGAGATGGAGGGAATAATACACAT----------------------------
-------------------------------------------------------------------------
-------
AAGAATAAGACCGAGACCTTCAGGCCTGCAGGAGGAGATATGAGGGACAATTGGAGAAGTGAATTATATAAAT
ATAAAGTAGTAAAAATTGAACCATTA---GGTATAGCACCCTCCAAGGCAAAAAGAAGAGTGGTG--------
-------------------------------GAGAGAGAAAAAAGAGCA---GTTGGA---ATGGGAGCT---
------GTCTTCTTTGGGTTCTTAGGAGCA---GCAGGAAGTACTATG---GGCGCGGCGTCA---
ATAACGCTGACGGCACAG---GCCAGGCAA---CTATTG---TCTGGTATAGTACAACAGCAGAGT---
AATCTG---CTAAGG---GCTATAGAGGCTCAACAAAACATGTTGAGACTCACGGTCTGGGGCATT
>AY371143..1
TTCTATTGCAGTACATCACCACTGTTTAATAGCACTTGGGACAGTAATAACACTGCCAGTTCAAACCACACAG
AG-----------------------------------------------------------------------
----------------------------
TCAAATGACATTATAACTCTCCAATGCAGAATAAAGCAAATTATAAATATGTGGCAGAGGGTGGGACAAGCAA
TGTATGCCCCTCCCATCAGTGGAAACATAACTTGTGAATCAAAC---
ATAACAGGACTACTATTAACAAGAGATGGAGGGGCTACTAATAGT----------------------------
-------------------------------------------------------------------------
----------
ACAACAAATGAGACCTTCAGGCCTGGAGGAGGAGATATGAGGGACAATTGGAGAAGTGAATTATATAAGTATA
AAGTAGTAAAAATTGAACCACTA---GGTGTAGCACCCACCCCTGCAAGAAGAAGAGTGGTG-----------
----------------------------CAGAGAGAAAAAAGAGCA---GTTGGA---CTGGGAGCT------
---GTTTTCCTTGGGTTCTTAGGAGCA---GCAGGAAGCACTATG---GGCGCGGCGTCA---
GTGACGCTGACGGTACAG---GCCAGACAA---TTATTG---TCTGGTATAGTGCAGCAGCAGCGC---
AATCTG---CTGCAG---GCTATAGAGGCTCAACAACATCTGTTGAAACTCACGGTCTGGGGCATT
>KF716465..1
TTCTATTGCAATACATCACAACTGTTTAATGGCACTTGGTTTAATGACACTTGGAACAACAATACCAATGACA
AC-----------------------------------------------------------------------
----------------------------
AGCTCAGAATTTATAACTCTCCAATGCAGAATAAAGCAAATTGTAAATATGTGGCAGAGAGTAGGACAAGCAA
TGTATGCCCCTCCCATCCCAGGAGTAATAAGTTGTAATTCAAAC---
ATTACTGGACTACTATTAATAAGAGATGGAGGGAATAATAATATT----------------------------
-------------------------------------------------------------------------
-------------
ATGAAGGAGACCTTCAGGCCTGGAGGAGGAGATATGAGGGACAATTGGAGAAGTGAATTATATAAATATAAAG
TAGTAAAAATTGAACCACTA---GGTGTAGCACCCACCCATGCAAAAAGAAGAGTGGTG--------------
-------------------------GAGAGAGAAAAAAGAGCA---GTTGGA---CTGGGAGCT---------
GTCTTCTTTGGGTTATTAGGAGCA---GCAGGAAGCACTATG---GGCGCGGCGTCA---
ATCACGCTGACGGTACAG---GCCAGACAA---TTATTG---TCCGGTATAGTGCAACAGCAGAGC---
AATTTG---CTGAAG---GCTATAGAGGCTCAACAACATATGTTGAGACTCACGGTCTGGGGCATT
```

>FJ670515..2
TTTTATTGCAATACATCAAGCCTGTTTAATAGTACTTGGTACGGTAATGGCACTAACAATACAACTGACACGG
GG---

TCCAATGATACTATAAGTCTCCCATGCAGGATAAAACAAATTGTACATATGTGGCAGAGAGTAGGACAAGCAA
TGTATGCCCCTCCAATTCAAGGAATAATAAACTGTAAATCAAAC---
ATTACAGGGCTACTGCTAGTAAGAGATGGTGGGAATGGGAATGGTACA-------------------------
----

AATGAGACCGAGACGTTCAGACCTGGAGGAGGAAACATGAAGGACAATTGGAGAAGTGAATTATATAAATATA
AAGTAGTAAAAATTGAACCACTA---GGAGTAGCACCCACCAGGGCAAAGAGAAGAGTGGTG-----------
----------------------------GGGAGAGAAAAAAGAGCA---GTTGGA---CTGGGAGCT------
---GTCTTCCTTGGGTTCTTAGGAGCA---GCCGGAAGTACTATG---GGCGCGGCGTCA---
ATAACGCTGACGGCACAG---GCCAGACAA---TTATTG---TCTGGCATAGTACAACAGCAGAGC---
AATCTG---CTGAGG---GCTATAGAGGCTCAACAGCACATGTTGAGACTCACGGTCTGGGGCATT
>KU685587..1
TTCTATTGCAGTACATCAGGATTGTTTAATATGACTTGGTATAAGAACAGCTCAGAGTCAAATAGCACTGAC-
----

AGGCCTGGAAATATAACTCTCCAATGCAGAATAAAGCAATTTATAAATATGTGGCAGAGAGTAGGGCAAGCAA
TGTATGCCCCTCCCATCAGAGGAGAAATAAGTTGTGTGTCAAAC---
ATTACTGGAATACTATTAACAAGAGATGGAGGGAAAAATAGTACT----------------------------
----

GAAAAGGAGATCTTCAGGCCTGGAGGAGGAGACATGAGGGACAATTGGAGAAGTGAATTATACAAGTATAAAG
TAGTAGAAATTGAACCACTA---GGTGTAGCACCCACCCGTGCAAAAAGAAGAGTGGTG--------------
-------------------------GAGAGAGAAAAAAGAGCA---GCA------CTAGGAGCT---------
GTCTTCATTGGGTTCTTGGGAGCA---GCAGGAAGCACTATG---GGCGCGGCGTCA---
GTGGCGCTGACGGTACAG---GCCAGACAA---TTATTG---TCTGGTATAGTGCAACAGCAAAGC---
AATTTG---CTGAAA---GCTATAGAGGCTCAACAGCAACTGTTGAAACTCACAGTCTGGGGCATT
>KR067669..1
TTCTATTGTAATACATCAAAACTGTTCAATAGCACTTGGCCGGTTAATAGCACTTGGCATAATAGAACTAACA
TTGACAGT---

TCAAATGGCACAATAACTCTCCAATGCAGAATAAAGCAAGTTATAAATATGTGGCAGAAAGTAGGGCAAGCCA
TGTATGCCCCTCCCATCCAAGGAGAAATAAGGTGTGACTCAAAC---
ATTACTGGACTACTATTAACAAGAGATGGAGGGAGTAGTAATGAT----------------------------
----

ATGAGTGAGACCTTCAGGCCTGGAGGAGGAGATATGAAGGACAATTGGAGAAGTGAATTATATAAGTATAAAG
TAGTAAAAATTGAACCAATA---GGGGTGGCACCCACCCCTGCAAAAAGAAGAGTGGTG--------------
-------------------------GAGAGAGAAAAAAGAGCA---GTAGGA---CTGGGAGCT---------
GTCTTCTTTGGGTTCTTAGGAGCA---GCAGGAAGCACTATG---GGCGCGGCGTCA---
ATAACGCTGACGGTACAG---GCCAGACAG---TTATTG---TCTGGTATAGTGCAACAGCAAAGC---
AATTTG---CTGAGG---GCTATAGAGGCTCAACAACAGCTGTTGAGACTCACGGTCTGGGGCATT
>KR067670..2
TTCTATTGCAATACATCAAAACTGTTTAGTGGCTCTTGGAATGATAGCTCTTTGAATGATAGTAATACAAATG
GAAAC--

TCAAGTGAAACTACTATAATTCTCCCATGCAAAATAAGACAAATTGTACGTATGTGGCAGACAGTAGGACAAG
CAATGTATGCCCCTCCCATTCAAGGAGCAATAAATTGTACATCAAAC---
ATTACAGGGCTACTGCTAACGAGAGATGGTGGGGATGAACCAGAG----------------------------
----

AATGAGACGTTCAGACCTGGAGGAGGAGATATGAGGGATAATTGGAGAAGTGAATTATATAAATATAAAGTAG
TAAAAATTGAACCACTA---GGAGTGGCACCCACCAGGGCAAAGAGAAGAGTGGTG-----------------
----------------------GGGAGAGAGAAAAGAGCA---ATTGGA---ATAGGAGCT---------
GTCTTCCTTGGGTTCTTAGGAGCA---GCAGGAAGCACTATG---GGCGCGGCGTCA---
GTAACGCTGACGGTACAG---GCCAGACAA---TTATTG---TCCGGTATAGTGCAACAGCAAAAC---
AATTTG---CTGAGG---GCTATAGAGGCTCAACAGCAGTTGTTGAAACTCACGGTCTGGGGCATT

```
>KR067667..1
TTCTATTGCAATACATCAAACTTGTTTAACAGTACTTGGTATAGTAACGGTACCAACAGTACTGAGAAAAAT-
-------------------------------------------------------------------------
----------------------------
AATGACACCAACATAACTCTCCAATGTAGAATAAGACAAATTGTAAATATGTGGCAGAGGGTAGGACAAGCAA
TGTATGCCCCTCCCATCAGAGGAAACATAACTTGTATATCAAAC---
ATTACAGGACTGTTGTTAACAAGAGATGGAGGGGATAATAGTACA----------------------------
-------------------------------------------------------------------------
----------
AATGCAACTGAGACTTTCAGGCCTGGAGGAGGAGATATGAGGGACAATTGGAGAAGTGAATTATATAAATATA
AAGTAGTACAAGTTGAACCGCTA---GGAGTAGCACCCAACAGGGCAAAAAGAAGAGTGGTG-----------
----------------------------CAGAGAGAAAAAAGAGCA---GTTGGA---CCGGGAGCT------
---GTCTTCATTGGGTTCTTAGGAGCT---GCAGGAAGCACTATG---GGCGCGGCGTCA---
ATGACGCTGACGGTACAG---GCCAGACAA---TTATTG---TCTGGTATAGTGCAGCAGCAAAGT---
AATTTG---CTGAGG---GCTATAGAGGCTCAACAGCAGGTGTTGAGACTCACGGTCTGGGGCATT
>KR067671..1
TTCTATTGTAATACAGCAGAGTTGTTTAATAGCACTTGGAATAATGATACTATGAGCTCAAATGACACTAGC-
-------------------------------------------------------------------------
----------------------------
TCAAATGACACTATAACTCTCCAATGCAGAATAAGGCAAATTATAAAGATGTGGCAGAAAGTAGGACAAGCAA
TGTATGCCCCTCCCATCCCAGGAGAAATAAGGTGTGAATCAAAC---
ATTACTGGACTACTATTAACAAGGGATGGAGGGAATGGGAATAAT----------------------------
-------------------------------------------------------------------------
----------
GATCCAAATGAGACCTTCAGGCCTGGAGGAGGAGATATGAGGGACAATTGGAGAAGTGAATTATATAAGTATA
AAGTAGTAAAAATTGAACCACTA---GGTGTAGCGCCCACCCATGCAAGAAGAAGAGTGGTG-----------
----------------------------GAAAGAGAAAAAAGAGCA---GTTGGA---CTGGGAGCT------
---GTCCTCTTTGGATTCTTAGGAGCA---GCAGGAAGCACTATG---GGCGCGGCGTCA---
ATAACGCTGACGGTACAA---GCCAGACAA---TTATTG---TCCGGTATAGTGCAGCAGCAGAGC---
AATCTG---CTGAAG---GCTATAGAGGCTCAACAACATTTGTTGAGACTCACGGTCTGGGGCATT
>MH078547..1
TTCTATTGCAATACATCAGCATTATTTAATAGCACTTGGAATAAGCCTTGGAATAATTCAATGAATAACACTG
AGGGAGGG-----------------------------------------------------------------
----------------------------
TCAAATGACATTCTTACTCTCAAATGCAAGATAAGGCAAGTTGTAAGAATGTGGCAAAGAGTGGGACAAGCAA
TGTATGCCAATCCCATCCCAGGAGTAATAAGGTGTGAATCAAAC---
ATAACAGGACTACTATTAACAAGAGATGGAGGGAATAACAATAGT----------------------------
-------------------------------------------------------------------------
-------------
ACAAATGAAACTTTTAGGCCTGGAGGAGGAGATATGAGGGACAATTGGAGAAGTGAATTATATAAGTATAAAG
TAGTAAAAATTGAACCATTA---GGTGTAGCACCTACCCGTGCAAGAAGAAGAGTGGTG--------------
-------------------------CAGAGAGAAAAAAGAGCA---GTTGGA---ATGGCAGCT---------
GTCTTCTTTGGGTTCTTGGGAGCA---GCAGGAAGCACTATG---GGCGCGGCGTCA---
ATAACGCTGACGGTACAA---GCCAGACAA---TTATTG---TCTGGTATAGTGCAACAGCAGAGC---
AATCTG---CTGCGG---GCTATAGAGGCTCAACAACATCTGTTGAAACTCACGGTCTGGGGCATT
>MH078549..1
TTTTATTGCAATACATCAAGCCTGTTTAATAGCAGATGGTATAATGGAACTAACACTGCAAACAACACAGATG
AC-----------------------------------------------------------------------
----------------------------
AATGCTGATAATATAACACTCCCATGCAGACTAAAACAAGTGATAAGAATGTGGCAGAAAGTAGGGCAAGCAA
TGTATGCCCCTCCTATCAAAGGAGAAATAAGGTGTGTCTCAAAT---
ATTACTGGACTACTATTAACAAGAGATGGAGGGTATGGAGGGAAT----------------------------
-------------------------------------------------------------------------
----------
AGTACAGATGAGACCTTCAGGCCTGGAGGAGGAGATATGAGGGACAATTGGAGGAGTGAATTATATAAGTATA
AAGTAGTAAAAATCGAACCACTA---GGGGTGGCACCCACCCGAGCAAAAAGAAGAGTGGTG-----------
----------------------------GAGAGAGAAAAAAGAGCA---GTTGGA---ATGGCAGCT------
---GTCTTCCTTGGGTTCTTAGGAGCA---GCAGGAAGCACTATG---GGCGCGGCGTCA---
CTGACGTTGACGGTACAA---GCCAGACAA---TTATTG---TCCGGTATAGTGCAACAGCAAAGC---
AATTTG---CTGAAG---GCTATAGAGGCTCAACAACAGATGTTGAGACTCACGGTCTGGGGCATT
```

>MH078550..2
TTCTATTGCAACACAGCAGGCCTGTTTAATGGTACTTGGGCTAATGATACTTGGGATCAAGGAAATGGCACGA
AA---

GAAAATGACACTATAACTCTCCAATGCAAAATAAAACAAGTTGTACATATGTGGCAGAGAGTAGGACAAGCAA
TGTATGCCCCTCCCATTCCAGGAGAAATAAGATGTGGATCAAAC---
ATTACAGGGCTACTGCTAACAAGAGATGGTGGGAATACTAATAAT------------------------------
----

ACAGAGGAGACGTTCAGACCTGGAGGAGGAAATATGAAAGACAATTGGAGAAGTGAATTATATAAATATAAAG
TAGTACAAATTGAACCAATA---GGAATAGCACCCACCAAGGCAAAGAGAAGAGTGGTG----------------
-------------------------GAGAGAGAAAAAAAGAGCA---ATTGGA---CTGGGAGCT----------
GTCTTCCTTGGGTTCTTAGGAGCA---GCAGGAAGTACTATG---GGCGCGGCGTCA---
ATAACGCTGACGGTACAG---GCCAGACAA---TTATTG---ACTGGTATAGTACAACAGCAGAGC---
AATCTG---CTGAGG---GCTATAGAGGCTCAACAGCACATGTTGAAGCTCACAGTCTGGGGCATT
>KX389616..1
TTCTATTGCAACACATCAAAACTGTTTAATAGTATTTGGGATAACAATGCCACAAAAAATAATATTACTGACA
AAAATGGT---

ATTAATGATACTATAACTCTCCCATGCAGAATAAGACAAATTGTACATATGTGGCAGAGAGTAGGACAAGCAA
TGTATGCTCCTCCCATTCCAGGAGTAATAAACTGTACATCAAAC---
ATTACAGGGATATTGCTAACAAGAGATGGTGGCAATAATAGTGAT------------------------------
----

ACAAATGAGACATTCAGACCTGGAGGAGGAGATATGAGAGATAATTGGAGAAGTGAATTGTATAAATATAAAG
TAGTAAAAATTGAACCACTA---GGAGTAGCACCCACCAATGCAAAGAGAAGAGTGGTG----------------
-------------------------GAGAGAGAAAAAAAGAGCA---GTTGGA---CTGGGAGCT----------
GTCTTCCTTGGGTTCTTAGGAGCA---GCAGGAAGTACTATG---GGCGCGGCGTCA---
ATGGCGCTGACGGCACAG---GCCAGACAA---CTATTG---ACTGGTATAGTACAACAGCAGAAC---
AATCTG---CTGAGG---GCTATAGAGGCTCAGCAGCAAATGTTGAGACTCACAGTCTGGGGCATT
>KU168304..2
TTCTATTGCAATACATCAGGACTATTTAATAGCACTTGGAGTAATCTAAATGACACAAATGGCACAAATCCAG
AGCCAAAT---

GACACTCACACTCTAACTATCCAATGCAGAATAAAGCAAATTGTAAGAATGTGGCAGAGGGTAGGACAAGCAA
TGTATGCCCCTCCCATCGAAGGAGAAATAAGGTGTGAATCAAAC---
ATTACAGGATTACTATTAACAAGAGATGGAGGAGCAAATAATAAT------------------------------
----

AATACAAAGGAGACATTCAGGCCTGGAGGAGGAGATATGAGGGACAATTGGAGAAGTGAATTATATAAGTATA
AGGTAGTACAAATTGAACCAATA---GGTGTGGCACCCACCCATGCAAAAAGAAGAGTGGTG-------------
----------------------------GAGAGAGGAAAAAAGAGCA---GTTGGA---ATAGGAGCT-------
---GTCTTCCTTGGGTTCTTAGGAGCA---GCAGGAAGCACTATG---GGCGCAGCATCA---
ATGACGCTGACGGTACAG---GCCAGACAA---TTATTG---TCCGGTATAGTGCAGCAGCAAAGC---
AATTTG---CTGAGG---GCTATAGAGGCTCAACAACAGCTGTTGAAGCTCACAGTCTGGGGCATT
>EU513188..3
TTCTATTGCAATACATCAGAACTGTTTAATAGCAATTGGACTAATACTAATAACAATACCAATAATGCCAGCT
CA---

GAGGCAAATGGCAATATAACTCTCACATGCAAAATAAAGCAAATTATAAATATGTGGCAAAGAGTGGGACAAG
CAATGTATGCCCCTCCCATCCAGGGAATAATAAGGTGTGAATCAAAC---
ATTACAGGATTATTATTAACAAGAGATGGAGGGTTTAGGAATGATACA---------------------------
----

AATGAGACCTATGAGGCCTTCAGGCCTGGAGGAGGAGATATGAGGGACAATTGGAGAAGTGAACTATATAAGT
ATAAAGTAGTAAAAATTGAACCACTA---GGTGTAGCACCCACTCATGCAAAAAGAAGAGCGGTG----------
-------------------------------CAGAGAGAAAAAAAGAGCA---GTTGGG---CTGGGAGCT----
------GTCTTCCTTGGGTTCTTAGGAGCA---GCAGGAAGCACTATG---GGCGCGGCGTCA---
ATGACGCTGACGGTACAG---GCCAGACAA---TTATTG---TCCGGTATAGTGCAACAGCAGAGT---
AATCTG---CTGAGG---GCTATAGAGGCTCAACAACATCTGTTGAGACTCACGGTCTGGGGCATT

>EU513185..1
TTCTATTGCAGTACATCAAATCTATTTAATAGTACCTGTACAAATAATAACGGCACTGCAAATTGTACAAATA
CAAATGGG---
-------------------ACA---
GCATTAAATGAAACTATAACTCTCCAATGCAGAATAAAACAAATTGTAAGGATGTGGCAGAGAGTAGGACAAG
CAATGTATGCCCCTCCCATCCAAGGAGTAATAAGGTGTGAATCAAAC---
ATTACAGGAATAATATTAACAAGGGATGGAGGGGATAGTAATGATACA-------------------------
----

AATGGAAATGAGACATTCAGGCCTGGAGGAGGAGATATGAGGGACAATTGGAGAAGTGAATTATATAAGTATA
AAGTAGTGCAACTTGAACCATTA---GGTGTGGCACCTACCCAGTCAAGAAGAAGAGTGGTG-----------
----------------------------GAGAGAGAAAAAAGAGCA---GTTGGA---ATGGGAGCT------
---TTGTTCCTTGGGTTCTTAGGAGCA---GCAGGAAGCACTATG---GGCGCGGCGTCA---
GTGACGCTGACGGTACAG---GCCAGACAA---TTATTG---TCTGGTATAGTGCAACAGCAAAGC---
AATTTG---CTGAGG---GCTATAGAGGCTCAACAGCATCTGTTGAAACTCACAGTCTGGGGCATT
>EU513194..1
TTCTATTGCAATACATCAGGACTGTTTAACAGCACTTGGTGGAATAACAATAACAGT----------------
----

TCAAATAACATTACCACTCTCCAATGCAGAATAAAGCAGATTGTGAATCTGTGGCAGAGAGTAGGAAAAGCAA
TGTATAACCCTCCCATCCAAGGAGTAATAAGGTGCGAATCAAAC---
ATTACAGGGCTACTATTAACAAGAGATGGAGGGCTTAATGATAGT----------------------------
----

AGTACAAATGAAACCTTCAGGCCTGGAGGAGGAGATATGAGGGACAATTGGAGAAGTGAATTGTATAGGTACA
AAGTAGTAAAAATTGAACCATTA---GGAGTAGCACCCTCCAGGGCAAGGAGAAGAGTGGTG-----------
----------------------------CAGAGAGAAAAAAGAGCA---ATTGGA---ATGGGAGCT------
---GTCTTCCTTGGGTTCTTAGGAGCA---GCAGGAAGCACTATG---GGCGCGGCGTCA---
ATAACGCTGACGGTACAG---GCCAGACAA---TTATTG---TCTGGTATAGTGCGACAGCAAAGC---
AATTTG---CTGAAG---GCTATAGAGGCTCAACAACATCTGTTGAAACTCACGGTCTGGGGCATT
>EU513183..1
TTCTATTGCAATACATCAAGCCTGTTTAATAGCACTTGGAATTGGAATGGCACTAGCATACAGGGGCCAAATG
ACACG--

CAGTCAAATGGCACTATAACACTCCCATGCAGAATAAAACAAATTGTAAATATGTGGCAGAGAGCAGGACAAG
CAATGTATGCCCCTCCCATCCCAGGAATAATAAAGTGTGAGTCAAAC---
ATTACAGGACTACTATTGGTAAGAGATGGTGGGAATAATAGTGGT----------------------------
----

TCAAATGAAACCTTCAGACCTACAGGAGGAGATATGAGAGACAATTGGAGAAGTGAACTATATAAATATAAAG
TAGTAGAAATTGAACCACTA---GGAGTAGCACCCACCAGGGCAAAAAGAAGAGTGGTG--------------
-------------------------GAGAGAGAAAAAAGAGCA---GTTGGA---TTGGGAGCT---------
GTCTTCCTTGGGTTCTTAGGAGCA---GCAGGAAGCACTATG---GGCGCGGCGTCA---
ATAACGCTGACGGTACAG---GCCAGACAA---TTATTG---TCTGGCATAGTGCAACAGCAAAGC---
AATTTG---CTGAGG---GCTATAGAGGCTCAACAACATCTGTTGAAACTCACGGTCTGGGGCATT
>KU168307..2
TTCTATTGCAATACATCAGACCTGTTTGATAGCACATGGCCTAACAATAGTAGTAACAATAGCACTAAC----
----

AATAGCACTACCATAACTCTCAAATGCAGAATAAAGCAAATTGTAAATATGTGGCAGAGAACAGGGCAAGCAA
TGTATGCCCCTCCCATCCCAGGACTAATAAGGTGTGAGTCAAAC---
ATTACAGGACTGCTATTGACAAGAGATGGAGGGGATAATAACAGT----------------------------
----

ACAAATGAGACTTTCAGACCTGGAGGAGGAGATATGAGAGATAATTGGAGAAGTGAATTATATAACTATAAAG
TAGTAAAAATTGAACCAGTA---GGCATAGCACCCACCAAGGCAAAAAGAAGGGTGGTG--------------
-------------------------GAGAGAGAAAAAAGAGCA---GTTGGA---ATAGGAGCT---------
GTCTTCCTTGGGTTCTTAGGAGCA---GCAGGAAGCACTATG---GGCGCGGCGTCA---
ATAACGCTGACGGTACAG---GCCAGACAA---TTATTG---TCTGGCATAGTGCAACAGCAAAGT---
AATTTG---CTGAGA---GCTATAGAGGCTCAACAACAGTTGTTGAGACTCACAGTCTGGGGCATT

>AM279362..2
TTCTATTGCAATACATCAGGCCTATTTAATAGCATTTGGTCTTGGAATACTAGCACTTGGAATTATACCAACT
CAAATTCC---
-------------ACAGGG------
TTAGATAATGACACTATAACTCTCCAATGCAGAATAAAGCAAATTGTAAATATGTGGCAGAGAGTAGGACAAG
CAATGTATGCCCCTCCCATCAAAGGAATGATAAGGTGTGAATCAAAC---
ATTACAGGATTACTATTAACAAGAGATGGAGGGAAGACTAATGATACT-------------------------
----

AATACAACTGAGACATTCAGGCCTGGAGGAGGAGATATGAGGGATAATTGGAGAAGTGAATTATATAAGTATA
AGGTAGTACAAATTAAACCATTG---GGTGTGGCACCCACCCATGCAAAAAGAAGAGTGGTG-----------
----------------------------GAGAGAGAAAAAAGAGCA---GTTGGA---CTGGGAGTT------
---GCCTTCCTTGGGTTCTTAGGAGCA---GCAGGAAGCACTATG---AGCGCAGCGTCC---
ATAACGCTGACGGTACAG---GCCAGACAA---TTATTG---TCTGGTATAGTGCAGCAGCAAAGC---
AATTTG---CTGAGG---GCTATAGAGGCTCAACAACATCTGTTGAAACTCACGGTCTGGGGCATT
>AM279356..2
TTCTATTGCAATACATCAAAACTATTTAATAGCACTTGGTATAGCAATGGTAGTGCCAGCTTAGATCACACAA
AT---

AAAGATGAAAATATAACTCTCCTATGTAGAATAAGGCAAATTGTAAATATGTGGCAGAGAGTAGGACAAGCAA
TGTATGCCCCTCCCATCGAAGGAGTAATAAGGTGTGAATCAAAC---
ATTACAGGATTGCTATTAACAAGAGATGGAGGGAAAGATAATAGTACA-------------------------
----

AATAGTACACAGGAGATATTCAGGCCTGGAGGAGGAGATATGAGGGACAATTGGAGAAGTGAATTATATAAGT
ATAAGGTAGTACAAATTGAACCAATA---GGTGTAGCACCCACACATGCAAAAAGAAGAGTGGTG--------
-------------------------------GAGAGAGAAAAAAGAGCA---GTTGGA---CTGGGAGTT---
------TTCTTCCTTGGGTTCTTAGGAGCT---GCAGGAAGCACTATG---GGCGCGGCGTCA---
ATAACGCTGACGGCACAG---GCCAGACAA---TTATTG---TCCGGTATAGTGCAACAGCAAAGC---
AATTTG---CTGAGG---GCTATAGAGGCTCAACAACATATGTTGAAACTCACGGTCTGGGGCATT
>AM279363..1
TTCTATTGCAATACATCAGGATTATTTAATAGTACTTGGAAATCTAATAGCACTTGGGACAATAACACTGACA
CAGGG--

TCAAACAATATAACTCTCACCCTCCAATGCAGAATAAAGCAAATTGTAAATATGTGGCAGAGGGTAGGACAAG
CAATGTATGCCCCTCCCATCCCAGGAGTAATAAGGTGTGACTCAAAC---
ATTACAGGACTCCTATTAACAAGGGATGGAGGGAATACTAGCACA----------------------------
----

GATGAAACATTCAGGCCTGGAGGAGGAGATATGAGGGACAACTGGAGAAGTGAATTATATAAGTATAAGGTAG
TACAAATTGAACCAATA---GGTGTGGCACCCACCCATGCAAAAAGAAGAGTGGTG-----------------
----------------------GAGAGAGAAAAAAGAGCA---GTAGGA---CTGGGAGCT---------
GTCCTCATTGGGTTCTTAGGAGCA---GCAGGAAGCACTATG---GGCGCGGCGTCA---
ATAACGCTGACGGTACAG---GCCAGACAA---TTATTG---TCTGGTATAGTGCAACAGCAGAGT---
AATCTG---CTGAAG---GCTATAGAGGCTCAACAACATCTGTTGAAACTCACAGTCTGGGGCATT
>KM606636..1
TTCTATTGTAATACATCAAATCTGTTTAATAAAACCTTTAACCACAACTCAACAGGGTCAAATTCAACAGAG-
----

TCAGATAGGACTATAACTCTCCCATGCAGAATAAAGCAAATTATAAATATGTGGCAGAAAGTAGGACAAGCAA
TGTATGCCCCTCCCATCCGAGGAGTAATAAAGTGTGAATCAAAC---
ATTACAGGACTACTATTAACAAGAGATGGAGGGGTGAATAATGGT----------------------------
----

AGTACAAACGAGACTTTCAGGCCTGGAGGAGGAGACATGAGGGACAATTGGAGAAGTGAATTATATAAGTATA
AAGTAGTAAAAATTGAACCACTA---GGTGTGGCACCCACCCCTGCAAAAAGAAGAGTGGTG-----------
----------------------------GAGAGAGAAAAAAGAGCA---ATTGGA---ATAGGAGCT------
---GTCTTCCTTGGGTTCTTAGGAGCA---GCAGGAAGCACTATG---GGCGCAGCGTCA---
ATAACGCTGACGGTACAG---GCCAGACAA---TTACTG---TCTGGTATAGTGCAACAGCAGAGC---
AATCTG---CTGAGA---GCTATAGAGGCTCAACAACATATGTTGAAACTCACGGTCTGGGGCATT

>AB052867..2
TTCTATTGCAATACATCAAGACTGTTTAATAGTACTTGGAATGACAATAGCACTTGGAATGACAGTAGCTCAA
ATTAC--
-------------------ACA---
GAGTCAAATGACACTATAACTCTCCAATGCAGAATAAAGCAAATTATAAATATGTGGCAGGGAGTAGGACAAG
CAATGTATGCCCCTCCCATTAGAGGAGTGATAAGGTGTGAATCAAAT---
ATTACAGGACTACTATTAACAAGAGATGGAGGGATTAATAATAGT----------------------------
----

ACAAATGAGACCTTCAGACCTGGAGGAGGAGATATGAGAGACAATTGGAGAAGTGAATTATACAAGTATAAAG
TAGTAAAACTTGAACCACTA---GGAGTGGCACCCACCAGGGCAAGGAGAAGAGTGGTA--------------
-------------------------ACAAGAGAAAAAAGAGCA---GTTGGA---ATGGGAGCT---------
TTGTTCCTTGGGTTCTTAGGAGCA---GCAGGAAGCACTATG---GGCGCGGTGTCA---
ATGACGCTGACGGTACAG---GCCAGACAA---TTATTG---TCCGGTATAGTGCAACAGCAGAGC---
AATCTG---CTAAAA---GCTATAGAGGCTCAGCAGCATCTGTTGAAACTCACGGTCTGGGGCATT
>HQ385444..2
TTCTATTGCAATACATCAGACCTGTTTAATAGTACTTGGCTAAACTCAACTGTGGAA----------------
----

TCAAATGAGACTATAATTCTCCCATGCAAAATAAAACAAATTATAAATATGTGGCAGAGAGTAGGACAAGCAA
TGTATGCACCTCCCATTCAAGGAGTAATAAACTGTGTATCAAAC---
ATTACAGGGATACTGCTAGTAAGAGATGGTGGGAATAATAATAGT----------------------------
----

ACAGCTGAGACATTCAGACCTGGAGGAGGAGATATGAAGGACAATTGGAGAAGTGAATTGTATAAATATAAAG
TAGTAAAAATTGAACCACTA---GGAGTAGCACCTACCAGGGCAAAGAGACGAGTGGTG--------------
-------------------------GAGAGAGAAAAAAGAGCA---GTTGGA---CTGGGAGCT---------
GTCTTCTTTGGGTTCTTAGGAGCA---GCAGGAAGTACTATG---GGCGCAGCGTCA---
ATAACGCTGACGGTACAG---GCCAGACAA---TTATTG---TCTGGTATAGTACAACAGCAGAGT---
AATTTA---CTGAAG---GCTATAGAGGCTCAACAACATCTGTTGAAACTCACGGTCTGGGGCATT
>KR067668..1
TTCTATTGCAATACATCAGGACTGTTTAATAGCGCTTGGAACAGTAGCACTTGGAACAATACCCTGACCAAC-
----

AATACTGGCAATATAACGCTCCACTGCAGAATAAAGCAAATTATAAATAAGTGGCAGAGAGTAGGACAAGCAA
TGTATGCCCCCCCCATCCCAGGAGTAATAAGGTGTGAGTCAAAC---
ATCACAGGATTACTACTAACAAGAGATGGAGGAAACAATAATAGG----------------------------
----

ACAAATGAGACTTTCAGGCCTGAAGGAGGAGATATAAGGGACAATTGGAGGAGTGAATTATATAAGTATAAAG
TAGTAAAAATTGAACCAGTA---GGAGTAGCACCCACCAGGGCAAGGAGAAGAGTGGTG--------------
-------------------------GAAAGAGAGAAAAGAGCA---GTCGGA---CTGGGAGCT---------
GTCTTCCTTAGGTTCTTAGGAGCA---GCAGGAAGCACCATG---GGCGCGGCGTCA---
ATAACGCTGACGGTACAG---GCCAGACAA---TTATTG---TCTGGTATAGTGCAACAGCAAAGT---
AATTTG---CTGAAG---GCTATAGAGGCTCAACAACACCTGTTGAGACTCACGGTCTGGGGCATT
>KU749413..1
TTCTATTGTAATACAACAAGCCTGTTTAATAGTACTTGGTATAATAATGAGACTGAGAACACAAATAGCACGG
GA---

TCAAATGAAACCATAACACTCCCATGCAGAATAAAGCAAATTGTAAATGTATGGCAGAGAGTAGGACAAGCAA
TATATGCCCCTCCCATCCAAGGACTAATACAATGTAACTCAAAT---
ATTACGGGGATACTATTAACAAGAGATGGTGGGAATAACGGATCA----------------------------
----

TCTAATGAAACTTTCAGGCCTGGAGGAGGAGATATGAGGGACAATTGGAGAAGTGAATTATATAAGTATAAAG
TAGTGAAAATTGAACCATTA---GGTGTAGCACCCACCCATGCAAAAAGAAGAGTGGTG--------------
-------------------------CAGAGAGAAAAAAGAGCA---ATTGGA---ATAGGAGCT---------
GTGCTCATTGGGTTCTTGGGAGCA---GCCGGAAGCACTATG---GGCGCGGCGTCC---
ATAACGCTGACGGTACAG---GCCAGACAA---TTATTG---TCTGGTATAGTGCAACAGCAAAGC---
AATTTG---CTGAGG---GCTATAGAGGCTCAGCAACATATGCTGAAACTCACAGTCTGGGGCATT

```
>KX232596..3
TTCTATTGTAATACAACACAGCTATTTAATAGCACTTGGAATATAACAGGACTATCTAATAGCAATTGGACAG
GTAAT--------------------------------------------------------------------
----------------------------
GACACTAGCAATATAACTCTTCCATGCAGAATAAAGCAAGTTGTAAATATGTGGCAGAGGGTAGGACAAGCAA
TTTATGCCCCTCCCATCCCAGGAATAATACAGTGTCAGTCAAAC---
ATTACTGGACTACTATTGACAAGAGATGGGGAAATAATAATAAT-----------------------------
-------------------------------------------------------------------------
----------
AGTTCGAATGAAACTTTCAGGCCTGGAGGAGGAGACATGAGGGACAATTGGAGAAGTGAGTTATATAAGTATA
AAGTAGTAAAAATTGAGCCATTA---GGTGTAGCACCCACTCCTGCAAAAAGGAGAGTGGTG-----------
----------------------------GAGAGAGAAAAAAGAGCA---ATTGGTGGACTGGGAGCT------
---GTATTCCTTGGGTTCTTAGGAGCA---GCAGGAAGCACTATG---GGCGCGGCGTCA---
ATAACGCTGACGGTACAG---GCCAGACAA---TTATTG---TCTGGTATAGTGCAACAGCAGAGT---
AATTTG---CTAAAG---GCTATTGAGGCTCAACAACAGATGTTGAGACTCACGGTCTGGGGCATT
>KY658718..2
TTCTATTGTAATACAACAGGACTATTTAATAGCACTTGGATGGAAAATAGCAGCATTTGGGAAAGCAATGGCA
ATAAA--------------------------------------------------------------------
----------------------------
ACAAATGAAACTATAACTCTTCCATGCAGAATAAAGCAAATTGTAAATATGTGGCAGAGAGTGGGACAAGCAA
TTTATGCCCCTCCCATCCCAGGAATAATACAGTGTGAGTCAAAC---
ATTACTGGACTACTATTGACAAGAGATGGGGAAATTAATAGTTCA----------------------------
-------------------------------------------------------------------------
----------------
AATGAAACTTTCAGGCCTGGAGGAGGAGACATGAGGGACAATTGGAGAAGTGAATTATATAAGTATAAAGTAG
TAAAAATTGAACCAGTA---GGTGTAGCACCCACCCGTGCAAGAAGGAGAGTGGTG-----------------
----------------------GAGAGAGAAAAAAGAGCA---ATTGGTGTACTGGGAGCT---------
GCATTCCTTGGGTTCTTAGGAGCA---GCAGGAAGCACTATG---GGCGCGGCGTCA---
ATAACGCTGACGGTACGG---GCCAGACAA---TTATTG---TCTGGTATAGTGCAACAGCAGAGT---
AATTTG---CTGAAG---GCTATTGAGGCTCAACAACAGATGTTGAGACTCACGGTCTGGGGCATT
>KX232617..3
TTCTATTGTAATACAACAAAACTATTTAATAGCACTTGGAAAGGAAATATCACTTGGGAGAGCAATGGCAATA
AA-----------------------------------------------------------------------
----------------------------
ACAAATGACAGTATAACTCTTCCATGCAGAATAAAGCAAATTGTAAATATGTGGCAGAGGGTAGGACAAGCAA
TTTATGCCCCTCCCATCCCAGGAGTAATAACGTGTGAGTCAAAC---
ATTACTGGACTACTATTGACAAGAGATGGGGGAAATAATAATAATAGA-------------------------
-------------------------------------------------------------------------
-------------
AATAATGAAACTTTCAGGCCTGGAGGAGGAGACATGAGGGACAATTGGAGAAGTGAATTATATAAGTATAAAG
TAGTAAAAATTGAACCATTA---GGTGTAGCACCCACCCCTGCAAGAAGGAGAGTGGTG--------------
-------------------------GAGAGAGAAAAAAGAGCA---ATTGGTGGACTGGGAGCT---------
GTATTCTTTGGGTTCTTAGGAGCA---GCAGGAAGCACTATG---GGCGCGGCGTCA---
ATAACGCTGACGGTACAG---GCCAGACAA---TTATTG---TCTGGTATAGTGCAACAGCAGAGT---
AATTTG---CTGAAG---GCTATAGAGGCTCAACATCAAATGTTGAGACTCACGGTCTGGGGCATT
>KX232623..1
TTCTATTGTAATACAACAGGCCTGTTTAATAGTACTTGGCCTAGGAATGAGAATGAGACTAGGGAAGCAAATA
GCACAGGG-----------------------------------------------------------------
----------------------------
TCAGATGACATCATAACACTCCCATGCAGAATAAAGCAAATTATAAATGTATGGCAGAGAGTAGGGCAAGCAA
TATATGCCCCTCCTATCCCAGGACTAATAAGATGTAACTCAAGT---
ATTACGGGGCTACTATTAGTAAGAGATGGTGGAAATAATAACACA----------------------------
-------------------------------------------------------------------------
-------------
GATAATGAAACCTTCAGACCTGGAGGAGGAAATATGAAGGACAATTGGAGAAGTGAATTGTATAAGTATAAAG
TAGTAAAACTTGAACCACTA---GGAGTAGCACCCACCAGGGCAAAGAGAAGAGTGGTG--------------
-------------------------GAGAGAGAAAAAAGAGCA---TTTGGA---TTAGGAGCT---------
GTATTCATTGGGTTCTTGGGAGCA---GCCGGAAGCACTATG---GGCGCGGCGTCT---
GTAACGCTGACGGTACAG---GCCAGGCAA---TTATTG---TCTGGCATAGTGCAACAGCAAAGC---
AATTTG---CTGAGG---GCTATAGAGGCTCAGCAGCAGATGCTGAAACTCACGGTCTGGGGCATT
```

>KX232627..2
TTCTATTGTAATACAACAAAACTATTTAATAGTACTTGGGGCAATGGCAGTGAACCAGAG-------------
----

GTAAACAATACCATAACCCTGCCATGCAGAATAAAGCAATTTGTAAATATGTGGCAGAGGGTAGGACAAGCAA
TTTATGCCCCTCCCATCCCAGGAAGAATAAAGTGTGAATCAAAC---
ATTACTGGACTACTATTGACAAGGGATGGGGGACAGAATAATAGG----------------------------
----

TCAAATGAAACTTTCAGGCCTGGAGGAGGAGATATGAGGGACAATTGGAGAAGTGAATTATATAAGTATAAAA
TAGTAAAAATTGAACCATTA---GGTGTAGCACCCACCCATGCAAGAAGGAGAGTGGTG--------------
-------------------------GGGAGAGAAAAAAGAGCA---ATTGGTGGACTGGGAGCT---------
GTATTCCTTGGGTTCTTAGGAGCA---GCAGGAAGCACTATG---GGCGCAGCGTCA---
ATAACGCTGACGGTACAG---GCCAGACAA---TTATTG---TCTGGTATAGTGCAACAGCAAAGT---
AATTTG---CTGAAG---GCTATTGAGGCTCAACAACAGATGTTGAGACTCACAGTCTGGGGCATT
>HM215282..1
TTCTATTGTAATACATCAAGCCTGTTTAATAGCACTTGGTATAACAATAGCAATAACAACAATGCCAGCTCAA
ACCCC--
-------------------ACA---
GAGTTAAATGGCACTATAACTCTCCCATGCAGAATAAAGCAAATTGTAAATATGTGGCAGAAAGTAGGACAAG
CAATGTATGCCCCTCCCATCCCAGGAATAATAGAGTGTAAATCAAAC---
ATTACAGGATTGTTATTAACAAGAGATGGAGGGAATAGTAATGGT----------------------------
----

GCAAATGAAACTTTCAGGCCTGGAGGAGGAGATATAAGGGACAATTGGAGAAGTGAATTATATAAGTATAAAG
TAGTAAAAATTGAACCACTA---GGTGTAGCACCCACCCATGCAAGAAGAAGAGTGGTG--------------
-------------------------GGGAGAGAAAAAAGAGCA---GTAGGA---CTGGGAGCT---------
GTCATCTTTGGGTTCTTAGGAGCA---GCAGGAAGCACTATG---GGCGCGGCGTCA---
ATAACGCTGACGGTACAG---GCCAGACAA---TTATTG---TCTGGTATAGTGCAACAGCAAAGC---
AATTTG---CTGCAG---GCTATAAAGGCTCAACAACATCTGTTGAGGCTCACGGTCTGGGGCATT
>KR017777..2
TTCTATTGCAATACATCAAAGCTGTTTGAAGGTTGGATTTGGAATCATAGCAATGGGAGTTGGGAAAGGAATA
ATAAA--
ACAAATGATGAAAATAAA---------
ACAGAGGATGGAAATATAATTCTCCCATGCAAAATAAAGCAAATAGTAAGAATGTGGCAGAGAGCAGGACAGG
CAATGTATGCCCCTCCCATCAAAGGATTAATAAATTGTAAATCAAAC---
ATTACAGGACTACTATTAACAAGGGATGGA---ACACATAATGGG----------------------------
----

ACCAACGAGACCTTCAGGCCTATAGGAGGAGAGATGAGGGACAATTGGAGAAGTGAATTATATAAATATAAAG
TAGTGGAAATTAAACCACTA---GGAGTAGCACCCACCGAGGCAAGGAGAAGAGTGGTG--------------
-------------------------CAGAGAGAAAAAAGAGCA---GTGGGA---ATAGGAGCT---------
GTGCTCCTTGGGTTCTTAGGAGCA---GCAGGAAGCACTATG---GGCGCGGCGTCA---
ATAACGCTGACGGTACAG---GCCAGACAA---TTATTG---TCTGGTATAGTGCAACAGCAGAGC---
AATTTG---CTGAAG---GCTATAGAGGCTCAACAACATCTGTTGAGACTCACGGTCTGGGGCATT
>KU749421..3
TTCTATTGTAATACATCAAAGCTATTTAATAGCACCTGGAATAGCACTGGGGAGAATCAGAAAATT-------
----

AGGAATGGGACTCTCATCCTCCCATGCAGAATAAAGCAAATTGTACGGATGTGGCAGAGAGTAGAACAAGCAA
TGTATGCCCCTCCCATCCAAGGAGAAATAAGATGTCATTCAAAT---
ATTACAGGAATACTGTTAACGAGAGATGGCGGGGGTAACGAGACA----------------------------
----

AAGGAGACCTTCAGACCTGTAGGAGGAAATATGAAAGACAATTGGAGAAGTGAATTATATAAGTATAAAGTAG
TAAAAATTGAGCCAATA---GGAATAGCACCCTCCAGGGCACGAAGAATAGTGGTG-----------------
----------------------GGGAGAGAAAAAAGAGCA---GTTGGA---CTGGGAGCT---------
GTCTTCCTTGGGTTCTTAGGAGCA---GCAGGAAGCACTATG---GGCGCGGCGTCA---
GTGACGCTGACGGTACAG---GCCAGACAA---TTATTG---TCGGGCATAGTGCAACAGCAAAGC---
AATTTG---CTGATG---GCTATAGAGGCTCAACAACATATGTTGAAACTCACGGTCTGGGGCATT

>GU201504..1
TTCTATTGTGATACAGCAGAACTATTTAATAGTACTTGGGAAAGGGAAAATGGCACTCGGAGAAAT-------
----

AGCACTGGGAATATAAATCTCCAGTGCAGAATAAAGCAAATTGTAAATATGTGGCAGAGAACAGGACAAGCAA
TGTATGCCCCTCCCATTCCAGGAATAATAGAATGTAAGTCAAAC---
ATTACAGGACTACTATTGACAAGAGATGGTGGAGCTAATAACAGT----------------------------
----

GCAGAGGAGACTTTCAGACCTGGAGGAGGAGATATGAGAGACAATTGGAGAAGTGAATTATATAAGTATAAAG
TAGTAAAAATTGAACCACTA---GGGGTAGCACCCACCCATGCAAAAAGAAGAGTGGTG--------------
-------------------------GAGAGAGAAAAAAGAGCA---GTTGGA---CTGGGAGCT---------
GTCTTCCTTGGGTTCTTAGGAGCA---GCAGGAAGCACTATG---GGCGCGGCGTCA---
ATAACGCTGACGGTACAG---GCTAGACAA---TTATTG---TCTGGCATAGTGCAACAGCAAAGC---
AATTTG---CTGAGG---GCTATAGAGGCTCAACAGCATATGTTGAGACTCACGGTCTGGGGCATT
>KR017773..1
TTCTATTGCAATACATCAGGCCTATTTAATAGCACTTGGAATAGCACTGGGAATAATAATAATATC-------
----

AGCTCAGACAATATCACTCTCCCATGCAGAATAAAGCAAATTGTGAGGCTATGGCAGAGAGTAGGACAAGCAA
TGTATGCCCCTCCCGTCTCAGGAGTAATAAGATGTGAATCAAAC---
ATTACAGGACTACTATTAACAAGAGATGGAGGGAAGAAAAATAAGAGT-------------------------
----

AGTGACCCTGAAACCTTCAGACCTGGAGGAGGAGATATGAAGGACAATTGGAGAAGTGAATTATATAAGTATA
AAGTAGTAAAAATTGAACCATTA---GGAGTAGCACCCACCAAGGCAAAAAGAAGAGTGGTG-----------
----------------------------GAGAGAGAAAAAAGAGCA---GCTGGA---CTGGGAGCT------
---GTCCTCCTTGGGTTCTTAGGAGCA---GCAGGAAGCACTATG---GGCGCGGCGTCA---
ATAACGCTGACGGTACAG---GCCAGACAA---TTATTG---TCTGGCATAGTGCAACAGCAAAGC---
AATTTG---CTGAGA---GCTATAGAGGCTCAACAGCAGATGTTGAGACTCACGGTCTGGGGCATT
>KR017772..1
TTCTATTGCGATACATCAGATCTGTTTAATAGCACTTGGGACAATAATACTGTCAGCCCAAACGACACAGAG-
----

GCAAAAGACAATATAACCCTCCAGTGCAGAATAAAGCAGATTGTAAATATGTGGCAGAGAGTAGGACAAGCAA
TATATGCCCCTCCCATCCGAGGAGTAATAAGGTGTGAATCAAAC---
ATTACTGGACTACTATTAGTAAGAGATGGAGGAATTGATAATAGG----------------------------
----

ACAAATGAGACCTTCAGGCCTGGAGGAGGAGATATGAGGGACAATTGGAGAAGTGAATTATATAAGTATAAAG
TAGTAAAAATTGAACCACTA---GGTGTAGCACCCACTAGGGCAAAAAGAAGAGTGGTG--------------
-------------------------GAGAGAGAAAAAAGAGCA---GTTGGA---ATAGGAGCT---------
GTCCTCCTCGGGTTCTTAGGAGCA---GCAGGAAGCACTATG---GGCGCGGCGTCA---
CTAACGCTGACGGTACAG---GCCAGACAA---TTATTG---TCTGGTATAGTGCAACAGCAAAGC---
AATTTG---CTGAAG---GCTATAGAGGCTCAACAACATCTGTTGAAACTCACGGTCTGGGGCATT
>GQ372986..1
TTCTATTGCAATACAACAGATCTGTTTAATAGCGCCTGGAATGCCAGCTCAACCTGGCCCAAC----------
----

TCAACAGGTAATATAACTCTCCAATGCAGAATAAAGCAAATTATAAATATGTGGCAGAGGGTAGGACAAGCAA
TGTATGCCCCTCCCATCCCAGGAGAAATAAGGTGTGAATCAAAC---
ATTACAGGACTACTCTTAACAAGAGATGGAGGGACTAACACTAACAGT-------------------------
----

AGTGGTGCAAACGAGACTTTCAGGCCTGGAGGAGGAGATATGAGAGACAATTGGAGAAGTGAATTATATAAAT
ATAAAGTAGTAAAAATTGAACCACTA---GGAGTAGCACCCTCCAAGGCAAAGAGAAGAGTGGTG--------
-------------------------------GAGAGAGAAAAAAGAGCA---GTTGGA---TTTGGAGCT---
------GTCTTCCTTGGGTTCTTGGGAGCA---GCAGGAAGTACTATG---GGCGCGGCGTCA---
ATAACGCTGACGGTACAG---GCCAGACAA---TTATTG---TCTGGGATAGTACAACAGCAGAGC---
AATCTG---CTGAGG---GCTATAGAGGCTCAACAGCAACTGTTGAAACTCACGGTCTGGGGCATT

```
>AB231894..1
TTCTACTGCAATACAGATGAACTGTTTAATAGCACTTGGAATAGTAGCACTTGGAATAGTAGCAGTACTACC-
-------------------------------------------------------------------------
----------------------------
GATAGCTCAAATATAACTCTCCACTGCAGAATAAAACAAATTATAAATATGTGGCAGACAGTAGGACGAGCAA
TGTATGCCCCTCCCATCCCAGGAATAATACAGTGTAACTCAAAC---
ATTACAGGATTGCTATTAACAAGAGATGGAGGAAAGGATAATAGTACTGAG----------------------
-------------------------------------------------------------------------
-------
ACTAATAATACTGAGACTTTCAGGCCTGGAGGAGGAGACATGAGGGACAATTGGAGAAGTGAATTATATAAAT
ATAAAGTAGTAAAAGTTGAACCAATA---GGTGTAGCACCCACCAAGGCAAAAAGAAGAGTGGTG--------
-------------------------------GAGAGAGAAAAAAGAGCA---GTTGGA---ATAGGAGCT---
------GTCTTCCTTGGGTTCTTAGGAGCA---GCAGGAAGCACTATG---GGCGCGGCGTCA---
ATAACGCTGACGGTACAA---GCCAGACAA---TTATTG---TCTGGTATAGTGCAACAGCAGAGC---
AATTTG---CTGAGA---GCTATAGAGGCTCAACAACAGATGTTGAGACTCACGGTCTGGGGCATT
>AY521632..1
TTCTATTGCAATACATCAGGCCTGTTTAATAGTAGTTGGAGTTGGAATGACACTACAAATTTAACGGAA----
-------------------------------------------------------------------------
----------------------------
TCAAATGGTACTATAAATCTCCCATGTAAAATAAAACAAATTATACGTATGTGGCAGAGAGTAGGGCAAGCAG
TGTATGCCCCTCCCATCCAAGGAGTAATACGCTGTAAATCAAAC---
ATTACAGGGCTACTGCTAACAAGAGATGGTGGGAATGATAATGAT----------------------------
-------------------------------------------------------------------------
----------
AATACAACTGAGAAGTTTAGACCTGGAGGAGGAAATATGAAGGACAATTGGAGAAGTGAATTATATAAATATA
AAGTAGTAAAAATTGAACCACTA---GGAGTAGCACCCACCAAGGCAAAAAGAAGAGTGGTG-----------
----------------------------GAGAGAGAAAAAAGAGCA---GTTGGA---CTGGGAGCT------
---GTCTTCCTTGGGTTCTTAGGCGCA---GCTGGAAGTACTATG---GGCGCGGCGTCA---
ATAACGCTGACGGCACAG---GCCAGACAA---TTACTG---TCTGGCATAGTACAACAGCAGAGT---
AATCTG---CTGAGA---GCTATAGAGGCTCAACAGCACCTGTTGAAACTCACGGTCTGGGGCATT
>AY521633..1
TTCTATTGCAATACATCAGGCCTGTTTAATAGTACTTGGATTCGGTATGCCAATGGCACCGTGCAGAACAATG
GCACG--------------------------------------------------------------------
-------------------------
GAGCCAAATGGTATTATTCCTCTCCAATGCAGAATAAGACAAGTTGTACGTATGTGGCAGAAAGTACGACAAG
CAATATATGCCCCTCCCATTAGAGGAGTAATATGCTGTGTATCCATC---
ATTGCAGGGCTACTACTAACAAGAGATGGTGGGGATAATAATACT----------------------------
-------------------------------------------------------------------------
----------------
ACCGAGAAGTTCAGACCTGGAGGAGGAGATATGAGGGACAATTGGAGAAGTGAATTATATAAATATAAAGTAG
TAAAAATTGAACCACTA---GGAGTAGCACCCACCAAGGCAAAAAGAAGAGTGGTG-----------------
----------------------GGGAGAGAAAAGAGAGCA---GTTGGA---CTGGGAGCT---------
GTCCTCTTTGGGTTCTTAGGAGCA---GCAGGAAGTACTATG---GGCGCGGCGTCA---
ATAACGCTGACGGCACAG---GCCAGACAA---TTATTG---TCTGGCATAGTACAACAGCAGAGC---
AATCTG---CTGAGG---GCTATAGAGGCTCAACAGCACCTGCTGAAACTCACAGTCTGGGGCATT
>JN864055..1
TTCTATTGCGATACAACAGAATTATTTAATAGTACTTGGGAGACTAATAACACTGCCAGCTCCAATACC----
-------------------------------------------------------------------------
----------------------------
ACAAATGGAACTATAACTCTCCAATGCAGAATAAAGCAAATTATAAATATGTGGCAGAGAGTAGGACAAGCAA
TGTATGCCCCTCCCATCCGAGGAGTAATAAGGTGTGAATCAAAC---
ATTACAGGACTACTATTAACAAGAGATGGAGGGACAACTGAGACA----------------------------
-------------------------------------------------------------------------
----------
AATAATAAAGAGACATTCAGGCCTGCAGGAGGAGATATGAGGGACAATTGGAGAAGTGAATTATATAAGTATA
AGGTAGTACAAATTGAACCACTA---GGTGTGGCACCCACCCATGCAAAAAGAAGAGTGGTG-----------
----------------------------GAGAGAGGAAAAAGAGCA---GTTGGA---ATAGGAGCT------
---GCTTTCCTTGGGTTCTTATCAGCA---GCAGGAAGCACTATG---GGCGCGGCGTCA---
ATAACGCTGACGGTACAG---GCCAGACAA---TTATTG---TCTGGTATAGTGCAACAGCAAAGC---
AATTTG---CTGAAG---GCTATAGAGGCTCAACAACATCTGTTGAAACTCACAGTCTGGGGCATT
```

>FJ388896..1
TTCTATTGCAATACATCAGGCCTGTTTAATAGCTCTTGGTATAACAATGGCACTGCCATCTCAAACCACACAG
GGTCAAAT---
-------------GGCACT------
GGGTCAAATGGCACTATCACTCTCCAATGCAGAATAAAGCAAGTTATAAATATGTGGCAGAAAGTAGGACAGG
CAATGTATGCCCCTCCCATCCCAGGAATAATAAGATGTGACTCAAAC---
ATTACTGGACTACTATTAACAAGAGATGGAGGGAGTAATAATAATACAAAT----------------------
----

GAGACTAAGACTAAGATCTTCAGGCCTGGAGGAGGAGATATGAGGGACAATTGGAGAAGTGAATTATACAAGT
ATAAAGTAGTAGAAATTGAACCACTA---GGTGTAGCACCCACCCCTGCAAGAAGAAGAGTGGTG--------
-------------------------------CAGAGAGAAAAAAGAGCA---GTTGGA---ATGGGAGCT---
------TTGCTCTTTGGGTTCTTAGGAGCA---GCAGGAAGCACTATG---GGCGCGGCGTCA---
ATAACGCTGACGGTACAG---GCCAGACAA---TTACTG---TCTGGTATAGTGCAACAGCAGAGC---
AATCTG---CTGAAG---GCTATAGAGGCTCAACAACAGATGTTGAGACTCACGGTCTGGGGCATT
>DQ926899..2
TTCTATTGCAATACATCAGAACTGTTTAATAGCACTTGGGGCAATAACACCTGGGACAATAACACTGCC----
----

AGCTCAGAAAATATAACTCTCCAATGCAGAATAAAACAATTTATAAATATGTGGCAGAGAGTAGGAAGAGCAA
TGTATGCCCCTCCCATCCGAGGAATAATAAGTTGTAAATCAAAC---
ATTACTGGACTACTATTAACAAGAGATGGAGGGAAATATAATGATAGC-------------------------
----

AATGCAACTCAGATCTTCAGGCCTGGAGGAGGAGATATGAGGGACAATTGGAGAAGTGAATTATATAAGTATA
AAGTAGTAAAAATTGAACCACTA---GGTGTAGCACCCACACATGCAAGAAGAAGAGTGGTG-----------
----------------------------GGGAGAGACAAAAGAGCA---GTTGGA---CTGGGAGCT------
---TTCTTCCTTGGGTTCTTAGGAGCA---GCAGGAAGCACTATG---GGCGCAGCGTCA---
ATAACGCTGACGGTACAA---GCCAGACAA---TTATTG---TCTGGTATAGTGCAACAGCAAAGC---
AATTTG---CTGAAA---GCTATAGAGGCTCAACAACAGCTGTTGAGACTCACGGTCTGGGGCATT
>JN882651..1
TTCTATTGCAATACGACAAGCCTGTTTAACAACAGCCAGTGGCCATTTAATGGGAGTAGTAACCCTGTC----
----

CCTAATGGCACCATAACTCTCCAATGCAGAATAAAGCAATTCATAAATATGTGGCAGAGAGTAGGACAAGCAA
TGTATGCCCCTCCCATCCCAGGAAATATAAGTTGTATGTCAAAC---
ATTACAGGACTACTATTAATAAGAGATGGAGGGAATGGTAATAGT----------------------------
----

ACAAATGAGACTTTTAGGCCTGGAGGAGGAGATATGAGGGACAATTGGAGAAGTGAATTATATAAGTATAAAG
TAGTAAAAATTGAACCAATA---GGTATAGCACCCACCCATGCAAGAAGAAGGGTGGTG--------------
-------------------------GGGAGAGACAAAAGAGCA---GCTGGA---CTGGGAGCT---------
GTCTTCCTTGGGTTCTTAGGAGCA---GCAGGAAGCACTATG---GGCGCGGCGTCA---
ATAACGCTGACGGTACAG---GCCAGACAA---TTATTG---TCTGGTATAGTGCAACAGCAGAGC---
AATCTG---CTGAAG---GCTATTGAGGCTCAACAGCATCTGTTGAAACTCACGGTCTGGGGCATT
>MH666158..1
TTCTATTGCGATACATCAGGACTGTTTAATAGTTCTTGGAATGGCACTAGTCCTAGGAATGATACT-------
----

ACCAATAGCACTATAACTCTCCAATGCAGAATAAAGCAAATTATCAATATGTGGCAGAGAGTAGGGCAAGCAA
TGTATGCCCCTCCCATCCGAGGGGTAATAAGGTGTGAATCAAAC---
ATTACTGGACTATTATTAACAAGAGATGGAGGGAATAAAAATAAT----------------------------
----

AGTGATCCTGAGATCTTCAGGCCTGCAGGAGGAGATATGAGAGACAATTGGAGAAGTGAATTATATAAGTATA
AAGTAGTGAAAATTGAACCACTA---GGTATAGCACCCAACCGTGCAAAAAGAAGAGTAGTG-----------
----------------------------GGGAGAGAAAAAAGAGCA---GTTGGA---CTGGGAGCT------
---GTCTTCCTTGGGTTCCTAGGAGCA---GCAGGAAGCACTATG---GGCGCGGCGTCA---
ATAACGCTGACGGTACAG---GCCAGACAA---TTACTG---TCCGGTATAGTGCAACAGCAGAGC---
AATCTG---CTGAAG---GCTATAGAGGCTCAACAACAGCTGTTGAGACTCACAGTTTGGGGCATC

>JN882653..1
TTCTATTGCAATACATCAATACTGTTTAATGGTACATTTAATGGTACAGAATTTAATGATACAGAAATTAAC-
----

AATACAACCACACTCCGACTCCCATGCAGAATAAAACAGATTGTAAACATGTGGCAGGGAGTAGGAAAAGCAA
TGTATGCCCCTCCCATTGAAGGAAAAATCAACTGTTTCTCAAAT---
ATTACAGGGCTACTATTAGTAAGAGATGGAGGGACTAATAATAGT----------------------------
----

ACAAATGAGACCTTTAGACCTGGAGGAGGAGATATGAGGGACAATTGGAGAAGTGAATTATATAAGTATAAAG
TAGTAAAAATTGAACCATTA---GGAGTAGCACCCACCAGGGCAAGGAGAAGAGTGGTG--------------
-------------------------GAGAGAGAAAAAAGAGCA---ATTGGA---ATGGGAGCT---------
CTTTTCCTTGGGTTCTTAGGAGCA---GCAGGAAGCACTATG---GGCGCAGCGTCA---
ATAACGCTGACGGTACAG---GCCAGACAA---TTATTG---TCTGGCATAGTGCAACAGCAGAGC---
AATCTG---CTGAGA---GCTATAGAGGCTCAGCAGCAAATGTTGAGGCTCACGGTCTGGGGCATT
>JF683752..2
TTCTATTGCAATACATCAGGACTGTTTAATAGTACTTGGAATACCACTGATACTTGGAATAACACTTATAGC-
----

CACAATGACACTATAACTCTCCAATGCAGAATAAAGCAATTTGTAAATATGTGGCAGAGAGTAGGGCGGGCAA
TGTATAACCCTCCCATCCCAGGAGAAATAAAGTGTGCATCAAAC---
ATTACTGGACTACTATTAACAAGAGATGGAGGGAGTAATAATATT----------------------------
----

ACAAATGAGATCTTCAGGCCTGCAGGAGGAGATATGAGAGACAATTGGAGAAGTGAACTATATAAGTATAAAG
TAGTAAAAATTGAACCACTG---GGTGTAGCACCCACCCCTGCAAAAAAAAGAGTAGTG--------------
-------------------------GGGAGAGAGAAAAGAGCA---GTTGGA---CTGGGAGCT---------
GTCTTCCTTGGGTTCCTAGGACCA---GCAGGAAGCACTATG---GGCGCGGCGTCA---
ATAACGCTGACGGTACAG---GCCAGACAA---TTATTG---TCTGGTATAGTGCAACAGCAGAGC---
AATTTG---CTGAAG---GCTATAGAGGCTCAACAACATATGTTGAGACTCACAGTCTGGGGCATT
>MH666162..1
TTCTACTGCAATACATCAGGACTGTTTAATAGTACTTGGAATAGTACTTGGATCAACTTAGATAAC-------
----

TTAAATGGCAGCATAACTCTCCAATGCAAAATAAAGCAATTTATAAATATGTGGCAGAGAGTAGGGCAAGCAA
TGTATGCCCCTCCCATCAAAGGAATGATACAGTGTAGATCAAAC---
ATTACTGGACTAATATTAACAAGAGATGGAGGGAATACTGAGAATGCT-------------------------
----

ACTGATAATGATACAGAGATCTTCAGGCCTACAGGAGGAGATATGAGAGACAATTGGAGAAGTGAATTATATA
AGTATAAAGTAGTAAAAATTGAACCACTA---GGTGTAGCACCCACCCATGCAAGAAGAAGAGTGGTG-----
----------------------------------GAGAGACAAAAAAGAGCA---GTTGGA---
CTGGGAGCT---------GTCTTCCTTGGGTTTCTAGGAGCA---GCAGGAAGCACTATG---
GGCGCAGCGTCA---ATAACGCTGACGGTACAG---GCCAGACAA---TTATTG---
TCTGGTATAGTGCAACAGCAGAGA---AATTTG---CTGCAA---
GCTATAGAGGCTCAACAACATATGTTGAGACTCACAGTCTGGGGCATT
>JF683766..1
TTCTACTGCAATACATCAACACTATTTAATAGAACATGGAATGGTACCACATGGAAT----------------
----

AACACAGCAAATAGTACAATCCCATGTAGAATAAAACAAATTATAAACATGTGGCAGGGAGTAGGAAAAGCAA
TGTATGCCCCTCCCATTGAAGGACAAATAAATTGTACATCAAAT---
ATTACAGGGCTACTACTGACAGTAGATGGTGGTAATAATACTAAT----------------------------
----

AATTCAGAGACATTCAGACCTGGAGGAGGAGATATGAAAGACAATTGGAGAAGTGAATTATATAAGTATAAGG
TAGTACAAATTGAACCAATA---GGTGTGGCACCCACCCATGCAAAAAGAAGAGTGGTG--------------
-------------------------GAGAGAGAAAAAAGAGCA---GTTGGA---CTGGGAGCT---------
GTCTTCCTTGGGTTCTTAGGAGCA---GCAGGAAGCACTATG---GGCGCGGCGTCA---

ATAACGCTGACGGTACAG---GCCAGACAA---TTATTG---TCTGGTATAGTGCAACAGCAGAGC---
AATCTG---CTGAGG---GCTATAGAGGCTCAACAACATCTGTTGAAACTCACAGTCTGGGGCATT
>AB231897..2
TTCTATTGCAATACATCAACACTGTTTAATAGCACTTGGATTAGGAATAGCTCAAATAGCACTTGGAATAGCT
CTGCCTCGAAC--
-------------AGCACA------
GAGTTAAATAGCAATATAACTCTTCAGTGCAGAATCAAGCAAATTGTAAGAATGTGGCAGAGAGTAGGACAAG
CAATGTATGCCCCTCCAATCGCAGGAGACATAAGGTGTGAATCAAAC---
ATTACTGGACTACTATTAACAAGAGATGGAGGGCATGAGACTGAG----------------------------
----

AATAAGACTGAGATCTTCAGGCCTGGAGGAGGAGATATGAGGGACAATTGGAGAAGTGAATTATATAAGTATA
AAGTAGTAAAAATTAAACCATTA---GGTATAGCACCCACCCATGCAAAAAGAAGAGTGGTG-----------
----------------------------ACGAGAGAAAAAAGAGCA---GTTGGA---CTGGGAGCT------
---GTCTTCCTTGGGTTCTTAGGAACA---GCAGGAAGCACTATG---GGCGCAGCGTCA---
CTAACGCTGACGGTACAG---GCCAGACAA---TTATTG---TCCGGTATAGTGCGACAGCAAAAC---
AATTTG---CTGAAG---GCTATAGAGGCTCAACAACAGCTGTTGAGACTCACGGTCTGGGGCATT
>AB480046..2
TTCTATTGCAATACATCAGGCCTGTTTAATAGTACCTGGTATGGAAATGGCAATGACACCACAAATGGCACGA
TTCTA--
-------------------CAT---
GGTAATAGAACTCATATAACTCTCCCATGCAGAATAAGACAAATTGTACATATGTGGCAGAGAGTAGGACAAG
CAATGTATGCCCCTCCCATTAAAGGAATAATAAAATGTACATCAAAC---
ATTACAGGGCTACTGCTAACAAGAGATGGTGGGAATAATAATAGT----------------------------
----

ACAAATGAGACATTCAGACCTGGGGGAGGAAATATGAAGGACAATTGGAGAAGTGAATTATATAGATATAAAG
TAGTAAAAATTGAACCACTA---GGAGTAGCACCCACCAGGGCAAAAAGAAGAGTGGTG--------------
-------------------------GAGAGAGAAAAAAGAGCAATTGTTGGA---CTGGGAGCT---------
GTCTTCTTTGGGTTCTTAGGAGCA---GCAGGAAGTACTATG---GGCGCGGCGTCA---
ATAACGCTGACGGTACAG---GCCAGACAA---TTATTG---TCTGGTATAGTACAACAGCAGAAC---
AATCTG---CTAAGG---GCCATAGAGGCTCAACAGCAACTGTTGAAACTCACGGTCTGGGGCATT
>MK086119..1
TTCTATTGCAATACAACAGACCTATTT------TCTTGGAATAGTAGTAGCCCATGGAAGAACATAACG----
----

TCAAATGACAGTATAACTCTCCAATGCAGAATAAAGCAAATTGTAAGGATGTGGCAGCAAGTAGGACAAGCAA
TGTATGCCCCTCCTATACCAGGAATAATAAGGTGTGAATCAAAC---
ATTACAGGAATACTGTTAACAAGAGATGGAGGAAATAATAATACA----------------------------
----

AATAACACAGAGGTATTCAGGCCTGGAGGAGGAGATATGAGGGACAATTGGAGAAGTGAATTATATAAATATA
AAGTAGTAAAAATTGAACCATTA---GGTGTAGCACCCTCCCGTGCAAGAAGAAGAGTGGTG-----------
----------------------------GAGAGAGAAAAAAGAGCA---GTTGGC---CTGGGAGCT------
---TTCTTCCTTGGGTTCTTAGGAGCA---GCAGGAAGCACTATG---GGCGCGGCGTCA---
ATAACGCTGACGATACAG---GCCAGACAA---TTATTG---TCTGGTATAGTGCAGCAGCAGAGC---
AATTCTGCTGACGGGCTC---ATAGAGGCTCAACAACATCTGTTGAAACTCACGGTCTGGGGCATT
>MK086120..1
TTCTATTGCAATACAACAGACCTATTT------TCTTGGAATAGTAGTAGCCCATGGAAGAACATAACG----
----

TCAAATGACAGTATAACTCTCCAATGCAGAATAAAGCAAATTGTAAGGATGTGGCAGCAAGTAGGACAAGCAA
TGTATGCCCCTCCTATACCAGGAATAATAAGGTGTGAATCAAAC---
ATTACAGGAATACTGTTAACAAGAGATGGAGGAAATAATAATACA----------------------------
----

AATAACACAGAGGTATTCAGGCCTGGAGGAGGAGATATGAGGGACAATTGGAGAAGTGAATTATATAAATATA
AAGTAGTAAAAATTGAACCATTA---GGTGTAGCACCCTCCCGTGCAAGAAGAAGAGTGGTG-----------
----------------------------GAGAGAGAAAAAAGAGCA---GTTGGC---CTGGGAGCT------
---TTCTTCCTTGGGTTCTTAGGAGCA---GCAGGAAGCACTATG---GGCGCGGCGTCA---

ATAACGCTGACGGTACAG---GCCAGACAA---TTATTG---TCTGGTATAGTGCAGCAGCAGAGC---
AATCTG---CTGCGG---GCCATAGAGGCTCAGCAACATCTGTTGAAACTCGCGGTCTGGGGCATT
>KR017771..1
TTCTACTGTAATACAACAAATCTGTTTAATAATCTGTTTAATAATACAGAA---------------------
----

GACAATGGTACAATCACTATTCCATGTAGAATAAAGCAAATTATAAACATGTGGCAAAGAGTAGGACGAGCAA
TGTATGCCCCTCCCATAGCCGGAAAAATTCAGTGTAACTCAACT---
ATCACAGGTCTACTATTAACAAGAGATGGTGGTAATACTAATGTG---------------------------
----

ACTGTGACTGAGATCCTCAGACCTGGAGGGGGAGATATGAGAGACAATTGGAGAAGTGAACTGTATAAATATA
AGGTAGTGAAAATTGAACCACTA---GGAGTAGCACCCACCAGGGCAAAAAGACAAGTGGTA----------
---------------------------ACCAGAGCAAAAAGAGAC---ATGGGA---ATAGGAGCT------
---ATGCTCCTTGGGTTCTTGGGAGCA---GCAGGAAGCACTATG---GGCGCGGCGTCA---
ATAACGCTGACGGTACAG---GCCAGACAA---CTATTG---TCTGGTATAGTGCAACAGCAAAAT---
AATTTG---CTGAAG---GCTATAGAAGCGCAACAGCATCTGTTGCAGCTTACAGTCTGGGGCATT
>MK086117..1
TTCTATTGCAATACAACAGACCTATTT------TCTTGGAATAGTAGTAGCCCATGGAAGAACATAACG---
----

TCAAATGACAGTATAACTCTCCAATGCAGAATAAAGCAAATTGTAAGGATGTGGCAGCAAGTAGGACAAGCAA
TGTATGCCCCTCCTATACCAGGAATAATAAGGTGTGAATCAAAC---
ATTACAGGAATACTGTTAACAAGAGATGGAGGAAATAATAATACA---------------------------
----

AATAACACAGAGGTATTCAGGCCTGGAGGAGGAGATATGAGGGACAATTGGAGAAGTGAATTATATAAATATA
AAGTAGTAAAAATTGAACCATTA---GGTGTAGCACCCTCCCGTGCAAGAAGAAGAGTGGTG----------
---------------------------GAGAGAGAAAAAAGAGCA---GTTGGC---CTGGGAGCT------
---TTCTTCCTTGGGTTCTTAGGAGCA---GCAGGAAGCACTATG---GGCGCGGCGTCA---
ATAACGCTGACGATACAG---GCCAGACAA---TTATTG---TCTGGTATAGTGCAGCAGCAGAGC---
AATCTG---CTGCGG---GCCATAGAGGCTCAACAACATCTGTTGAAACTCACGGTCTGGGGCATT
>MK086118..1
TTCTATTGTAATACATCAGAACTATTTAATAGCACATGGAATAGTAGCACTCCTAGCACT------------
----

GGAAATAGAACTATAACTCTCCAATGCAAAATAAAGCAAATTGTAAGAATGTGGCAAAAGGTAGGACAAGCAA
TGTATGCTCCTCCCATTAGTGGAAATATAAGTTGTAACTCAAAC---
ATTACAGGATTGCTATTAACAAGAGATGGAGTGGACAATAATAGT---------------------------
----

ACAAATGAGACATTCAGGCCTGGAGGAGGAGATATGAGGGACAATTGGAGAAGTGAATTATATAAGTATAAGG
TAGTACAAATTAAACCATTA---GGTGTGGCACCCACCCAGGCAAAAAGAAGAGTGGTG-------------
------------------------CAGAGAGAAAAAAGAGCA---ATTGGA---CTGGGAGCT---------
GTGTTCATTGGGTTCTTAGGAGCA---GCAGGAAGCACTATG---GGCGCGGCGTCA---
ATAACGCTGACGGTACAG---GCCAGAGAA---TTATTG---TCTGGTATAGTGCGACAGCAGAGC---
AATCTG---CTGCAG---GCTATAGAGGCTCAACAGCATCTGTTGAAACTCACAGTCTGGGGCATT
>MK086115..1
TTCTATTGCAATACAACAAGACTATTTAATAGCATTTGGAGTAGGAATGAGACTAAGAATGACAATGACACTT
GGAGTAAT--
-ATCACTAATAGCACTGCC------
TGGACAAATGACACTATAACTCTCCAATGCAGAATAAAGCAAATTGTAAGAATGTGGCAGAGAGTAGGACAAG
CAATGTACGCCCCTCCCATCCAAGGAGAAATAAAGTGTGAGTCAAAC---
ATTACAGGATTACTATTAACAAGAGATGGAGGTGCACATGGTGAC---------------------------
----

AATGAGACATTCAGGCCTGGAGGAGGAAATATGAAGGACAATTGGAGAAGTGAATTATATAAGTATAAGGTAG
TACAAATTAAACCATTA---GGTGTAGCACCTACACATGCAAAAAGAAGAGTGGTG----------------
---------------------GAGAGAGAAAAAAGAGCA---GTTGGA---CTGGGAGCT---------
GTCTTCCTTGGGTTCTTAGGAGCA---GCAGGAAGCACTATG---GGCGCGGCGTCA---

ATAACGCTGACGGTACAG---GCCAGACAA---TTATTG---TCTGGTATAGTGCAACAGCAGAGC---
AATTTG---CTGAGG---GCTATAGAGGCTCAACAACATCTGTTGAAACTCACAGTCTGGGGCATT
>MK086126..2
TTCTATTGTAATACAACACCACTATTTAATAGCACTTGGAAGAGCAATGACACTGCCAGTTCAAAC-------
----

CCAAATGACACTATAACTCTCCAATGCAGAATAAAACAAATTGTAAATATGTGGCAGAAAGTAGGACAAGCAA
TGTATGCCCCTCCCATCTCAGGAAACATAATGTGTAACTCAACC---
ATTACAGGAATACTATTAACAAGAGATGGAGGGATTAATAATACA----------------------------
----

GAGGAGGAGGAGACGTTCAGGCCTGGAGGAGGAGATATGAGGGACAATTGGAGAAGTGAATTATATAAGTATA
AGGTAGTAAAAATTGAGCCACTA---GGTGTGGCACCCACCTATGCAAAAAGAAGAGTGGTG-----------
----------------------------GAGAGAGAAAAAAGAGCA---GTTGGA---CTGGGAGCT------
---GTCTTCCTTGGGTTCTTAGGAGCA---GCAGGAAGCACTATG---GGCGCGGCGTCG---
ATAACGCTGACGGTACAG---GCCAGACAA---TTATTG---TCTGGTATAGTGCAACAGCAGAGC---
AATCTG---CTGAAG---GCTATAGAGGCTCAACAACATCTGTTGAAACTCACGGTCTGGGGCATT
>JN864057..1
TTCTATTGCAATACAACAAGACTGTTTAATAGCATGTGGATCAAAAATGTCAGCAGTGACACTTGGAATAACA
ACATCACTAGT--

GATAACAATGGCACTATAACTCTCCAATGCAGAATAAAGCAAATTGTAAATATGTGGCAAAGAGTAGGACAAG
CAATGTATGCCCCTCCCATCCAAGGAGAATTAAGGTGTGAATCAAAC---
ATTACAGGACTACTGTTAACAAGAGATGGAGGGGATACTAATAAT----------------------------
----

ACAGAGACCTTCAGGCCTGGAGGAGGAGATATGAAGGACAATTGGAGAAGTGAATTATACAAGTATAAAGTAG
TGAAAATTGAACCACTA---GGTATAGCACCCACCCCTGCAAAAAGAAGAGTGGTG-----------------
----------------------GAGAGAGAAAAAAGAGCA---GTTGGA---CTAGGAGCT---------
GTTTTCCTTGGGTTCTTAGGAGCA---GCAGGAAGCACTATG---GGCGCGGCGTCA---
ATAACGCTGACGGTACAG---GCCAGACTA---TTAATG---TCTGGTATAGTGCAACAGCAGAGC---
AATTTG---CTGAAG---GCTATAGAGGCTCAACAACAGCTGTTGAGACTCACGGTCTGGGGCATT
>MK086116..1
TTCTACTGTAATACAACAGTTCTGTTTAATACCACAGGG----------------------------------
----

TTAAAAGACAGTATCACTATTCCATGTAAAATAAAACAAATCATAAACATGTGGCAAAGAGTAGGACGAGCAA
TGTATGCCCCTCCCATTGCAGGAAAAATTCAATGTAACTCAAGT---
ATTACAGGTCTACTCTTAACAAGAGATGGTGTTAATGATACAAAA----------------------------
----

AATGAAACCCTCAGACCTGGAGGGGGAGATATGAGAGACAATTGGAGAAGTGAACTGTATAAATATAAGGTAG
TGCAAATTGAACCACTA---GGAGTAGCACCCACCAGGGCACAAAGACGAGTGGTA-----------------
----------------------CAGAGAGAAAAAAGAGCAGTAGTGGGA---CTAGGAGCT---------
ATGTTCATTGGGTTCTTGGGAGCA---GCAGGAAGCACTATG---GGCGCGGCGTCA---
ATAACGCTGACGGTACAG---GCCAGACAA---TTATTG---TATGGTATAGTGCAACAGCAGAGC---
AATCTG---CTGAGG---GCTATAGAAGCGCAACAGCATCTGTTGCAGCTCACAGTCTGGGGCATT
>AJ866557..1
TTCTATTGCAATACATCACAACTGTTTAACAGTACTTGGAGTAACAATGGCACTGCCATCCACACAGGG----
----

TCAAATGATACTATAACTCTCCAATGCAGAATAAAGCAAATTGTAAGGATGTGGCAGAGAGTAGGACAAGCAA
TGTATGCCCCTCCTATCCCAGGAATAATAAGGTGTGAATCAAAC---
ATTACAGGCCTACTATTAACAAGAGATGGAGGGAATAGTAATAATAGT-------------------------
----

AATACAAATGAGACTTTCAGGCCTGGAGGAGGAGATATGAGGGACAATTGGAGAAGTGAATTATATAAGTATA
AAGTAGTAAGAATTGAACCACTA---GGTGTAGCACCCTCCCGTGCAAGAAGAAGAGTGGTG-----------
----------------------------GAGAGAGAAAAAAGAGCA---GTTGGA---ATAGGAGCT------
---GTTTTCCTTGGGTTCTTAGGAGCA---GCAGGAAGCACTATA---GGCGCAGCATCA---

GTAACGCTGACGGTACAG---GCCAGACAA---CTATTG---TCTGGTATAGTGCACCAGCAGAGC---
AATCTG---CTGAAG---GCTATAGAGGCTCAACAACAGCTGTTGAGACTCACAGTCTGGGGCATT
>MH667256..1
TTCTATTGTAATACATCAAACCTGTTTAATAGCACTTGGAACAATAGCACTTGGGATAACAATAACACT----
----

GGGAATGAGAATATAACTCTCCAATGCAAAATAAAGCAATTTGTAAATATGTGGCAGAGGGTAGGAAGAGCAA
TGTATGCCCCTCCCATCCGAGGAGTAATAAAGTGTGTATCACAC---
ATTACAGGACTAATATTAACAAGAGATGGAGGAAATAATAATACA---------------------------
----

CAGGAGGAGATTTTTAGGCCTGGAGGAGGAGATATGAGGGACAATTGGAGAAGTGAATTATATAAATATAAAG
TAGTAAAAATTAAACCACTA---GGAGTAGCACCCACTATGGCTAAAAGAAGAGTGGTG-------------
------------------------GAGAGAGAAAAAAGAGCA---GTTGGA---ATAGGAGCT---------
GTCTTCCTTGGGTTCTTAGGAGCA---GCAGGAAGCACTATG---GGCGCGGCGTCA---
GTGACGCTGACGGTACAG---GCCAGACAA---TTATTG---TCTGGCATAGTGCAACAGCAAAGC---
AATTTG---CTGAAG---GCTATAGAGGCTCAACAACAGCTGTTGAGACTCACGGTCTGGGGCATT
>FJ388944..1
TTCTATTGCAATACAACATTACTATTTGATAGCACTTGGTATGGAAATGGCTCTGACATCGACAAAATAAATA
AAACA---

ATGTCAAATGAAACTATAATTCTCCCATGCAGAATAAAGCAAATTGTACGCATGTGGCAGAGAGTAGGACAAG
CAATGTACGCCCCTCCCATCCCAGGAGAAATAAGGTGTGAGTCAAAC---
ATTACAGGACTACTATTGACAAGAGATGGTGGGGGTAACGGTAGC---------------------------
----

AGTACAAATGAGACCTTCAGGCCTGGAGGAGGAGATATGAGAGACAATTGGAGAAGTGAATTATATAAGTATA
AGGTAGTAAAAATTGAACCACTA---GGTGTAGCACCCAACAAGGCAAGAAGGAGAGTGGTG----------
----------------------------GAGAGAGAAAAAAGAGCA---GTTGGA---ATAGGAGCT------
---GTCTTCCTTGGGTTCTTAGGAGCG---GCAGGAAGCACTATG---GGCGCGGCGTCA---
ATAACGCTGACGGTACAG---GCCAGGCAA---TTATTG---TCTGGTATAGTGCAACAGCAGAGT---
AATTTG---CTGAAG---GCTATAGAGGCTCAACAACACTTGTTGAGACTCACGGTCTGGGGCATT
>DQ313254..2
TTCTATTGCAATACATCAGGTCTGTTTAATAGTACTTGGTATCGGAATGGCACTGCCATCAGGCAGAACGGCA
CG--

GGGTTAAATGATACTATAACTCTCCCATGCAGAATAAGGCAAATTGTACGTACATGGCAGAGAGTAGGACAAG
CAATGTATGCCCCTCCCATTCAAGGAGTAATAAAATGTGAATCAAAC---
ATTACGGGGCTACTGTTAACAAGAGATGGTGGGAATAATAGTAGTAAT------------------------
----

AATGACACTGAGACCTTCAGACCTGGAGGAGGAGATATGGAAGACAATTGGAGAAGTGAATTATACAACTATA
AGGTAGTAAAAATTAAACCATTA---GGAATAGCACCCACCAAGGCAAGGAGAAGAGTAGTG----------
----------------------------GGGAGAGAGAAAAGAGCA---GTTGGA---CTGGGAGCT------
---GTTTTCCTTGGGTTCTTAGGGACA---GCAGGAAGCACTATG---GGCGCAGCGTCA---
ATAACGCTGACGGTACAG---GTCAGACAA---TTATTG---TCTGGCATAGTGCACCAGCAAAGC---
AATTTG---CTGAGA---GCTATAGAGGCGCAGCAGCATCTGTTGCAGCTCACAGTCTGGGGCATT
>AM279364..2
TTCTATTGCAATACATCAGGCCTGTTTAATAGTACTTGGAATGACACTAGCATCAATGAGAACTACACGGAT-
----

CCAAATGCAACTCTTATTCTCCTATGCAGAATAAAACAAATTGTGCGTATGTGGCAGAGAGTAGGGCAAGCAA
TGTATGCCCCTCCCATTCCAGGAGTAATACAGTGTGTATCAAAC---
ATTACAGGGCTACTGCTAACAAGAGATGGTGGGAATAATACTAAT---------------------------
----

AGGACAGATGAGACGTTCAGACCTGGAGGAGGAGATATGAAGGACAATTGGAGAAATGAGTTATATAAGTATA
AAGTAGTAAAAATTGAACCACTA---GGAGTAGCACCCACCAAGGCAAGGAGAAGAGTGGTG----------
---------------------------CAGAGAGAAAAAAGAGCA---ATTGGA---ATGGGAGCT------
---CTTTTCCTTGGGTTCTTAGGAACA---GCAGGAAGCACTATG---GGCGCGGCGTCA---

ATAACGCTGACGGTACAG---GCCAGGCAA---TTATTG---TCTGGCATAGTGCAACAGCAGAGC---
AATCTG---CTGATG---GCCATAGAGGCTCACCAGCATTTGTTGAAACTCACGGTCTGGGGCATT
>KR017778..1
TTCTATTGCAATACATCACGATTGTTTAATAGCACTTGGACTGGGAATAACACTACTTGGAGTATCAGCAGCG
AGAACGATACT---

GGGAAAGATGATCCCATAATTCTCCCATGTAGAATAAAGCAAATTATAAATATGTGGCAGAGAGTAGGACAAG
CAATATACACTCCTCCTATTCAAGGAGAACTAAGGTGTGACTCAAAC---
ATTACAGGACTATTGTTAACAAGAGATGGTGGGAATAACAATGAC-------------------------------
----

ACAGAGATCTTCAGACCTATAGGAGGAAATATGAAGGACAATTGGAGAAGTGAATTATATAAGTATAAAGTGG
TGGAGATTAAGCCACTA---GGAATAGCACCCACCAAGGCAAAAAGAAGAGTGGTG--------------------
-----------------------GAGAGAGAAAAAAGAGCA---GTAGGA---ATAGGAGCT---------
GTACTCCTTGGGTTCTTGGGAGCA---GCAGGAAGCACTATG---GGCGCGGCGTCA---
ATAACGCTGACAGTGCAG---GCCAGACAA---TTATTG---TCTGGCATAGTGCAACAGCAAAAC---
AACTTG---CTGAAG---GCTATAGAAGCACAACAGCAGCTGTTAAAACTCACTGTCTGGGGCATA
>EU513192..1
TTCTATTGTAATACATCAGACCTGTTTAATTGGGAGAATGACACCTTGCAGGGGCCAAATAAGGCTGTG----
----

TCAAATGATAATATAACTCTCCCATGCAGAATAAAGCAAATTATAAATATGTGGCAGAGAGTAGGACAAGCAA
TATATGCCCCTCCTATCCAAGGAGTAATAAGGTGTAACTCAAAC---
ATTACAGGACTACTATTAACAAGAGATGGTGGGAACAATAGTAAT-------------------------------
----

CCGACTGAGACCTTCAGACCTGAAGGAGGGGATATGAGGGACAATTGGAGGAGTGAATTATATAAGTATAAAG
TAGTAAAAATTGAACCACTA---GGAGTAGCACCCACCAAGGCAAGGAGAAGAGTGGTG-----------------
--------------------------ACGAGAGAAAAAAGAGCA---ATTGGA---ATGGGAGCT---------
GTCCTCTTTGGGTTCTTAGGAGCA---GCAGGAAGCACTATG---GGCGCGGCGTCA---
ATAACGCTGACGGTACAG---GCCAGACAA---TTATTG---TCTGGCATAGTGCAACAACAGAAC---
AACCTG---CTGAGG---GCTATAGAGGCTCAACAGCAACTGTTGAAACTCACAGTCTGGGGCATT
>AY894995..2
TTCTATTGTAATACAACAAAATTATTTAATGGTATCTGGAATAAAACAGGTAGTACCACATATAACTATACAA
ATAGC---

ACAGAGAATGATACTATTACACTGCCATGCAGAATAAAACAAGTTGTAAGAATGTGGCAGAGGACAGGACAAG
CAATGTATGCCCCTCCTATCCAGGGAATAATAAGGTGTGCATCTAAC---
ATTACAGGAATAATATTAACAAGAGATGGTGGGAATAACAATAAT-------------------------------
----

GCAACTGAGACCTTCAGACCTGGAGGAGGAAATATGAAAGACAATTGGAGAAGTGAATTATATAAATATAAGG
TAGTGAAAATTGAACCACTA---GGAGTAGCACCCACTAGGGCAAGGAGGAGAGTAGTG-----------------
--------------------------GGGAGAGAAAAAAGAGCA---GTTGGA---CTAGGAGCT---------
GTCTTCCTTGGGTTCTTGGGA------------------------GGCGCAAAATCA---
ATGACGCTGACGGTACAG---GCCAGACAA---TTATTG---TCTGGTATAGTGCAACAGCAAAGC---
AATTTG---CTGAGG---GCTATAGAGGCTCAACAACAGATGTTGAGACTCACGGTCTGGGGCATT
>AY371163..1
TTCTATTGCAATACATCAGGCCTGTTTAATAGCACTTGGAGAAACATTAATGGCACTTGGAATAGCACTGGCA
ACACA---

GAGTTAAATAGCACTATAATTCTCCTGTGTAGGATAAAACAAATTGTAAATATGTGGCAGAGAACAGGACAAG
CAATGTATGCCCCTCCCATCCCAGGAGTGATAAGGTGTGAGTCAAAC---
ATTACAGGACTACTATTGACAAGAGATGGTGGGAATGAGAATAATATTAAT-------------------------
----

ACAAATACAACTGAAACCTTCAGACCTGGAGGAGGAGATATGAGAGACAATTGGAGAAGTGAATTATATAAAT
ATAAAGTAGTAAAAATCGAACCACTA---GGAGTAGCACCCACCAAGGCAAAGAGAAGAGTGGTG--------
--------------------------------GAGAGAGAAAAAAGAGCA---GTTGGA---TTGGGAGCT---
------GTTTTCCTTGGGTTCTTAGGAGCA---GCAGGAAGCACTATG---GGCGCGGCGTCA---

ATAACGCTGACGGTACAG---GCCAGACAA---TTATTG---TCTGGCATAGTGCAACAGCAAAGC---
AATTTG---CTGAGG---GCTATAGAGGCTCAACAGCATCTGTTGAGACTCACGGTCTGGGGCATT
>FM877779..1
TTCTATTGTAATACATCAAGCCTGTTTAATAGCACGTACTTGAATATAACA----------------------
----

AAGGGAAACATAACAATGCTCCCATGCAGGATAAAACAAATTGTAAACATGTGGCAGGGAGTAGGACGAGCAA
TATATGCCCCTCCTATTGCAGGAAAGATACAGTGTGTATCTAAT---
ATAACAGGACTAATATTGACAAGAGATGGGGGAAATGAAGATAAT----------------------------
----

AATACTGAAACCTTCAGACCTGGAGGAGGAAATATGAAAGACAATTGGAGAAGTGAATTATATAAGTATAAAG
TAGTAAAAATTGAACCACTA---GGAATAGCACCCACCAAGGCAAAGAGAAGAGTGGTG--------------
-------------------------GAGAGAGAAAAAAGAGCA---GTTGGA---CTGGGAGCT---------
CTTTTCTTTGGGTTCTTAGGAGCA---GCAGGAAGCACTATG---GGCGCAGCGTCA---
ATAACGCTGACGGTACAG---GCCAGACAA---TTATTG---TCTGGCATAGTGCAACAGCAAAGC---
AATTTG---CTGAGG---GCTATAGAGGCTCAACAGCACATGTTGAAGCTCACAGTCTGGGGCATT
>FM877778..1
TTCTATTGTAATACATCAGGACTGTTTAATAGCACTTGGCTGGTTAATAGCACTAGGGAGAATGGCACGAGC-
----

ACCATGGGAAACATAACACTCCCATGCAGGATAAAACAAATTGTAAACATGTGGCAGGGAGTAGGACAAGCAA
TGTATGCCCCTCCTGTCGCAGGAATGATACAGTGTGTATCAAAT---
ATTACAGGACTAATATTAACAAGAGATGGGGGAAGTAATGATAATAGTACT----------------------
----

GATAATAATACTGAGACCTTCAGACCTACAGGAGGAAATATGAAAGACAATTGGAGAAGTGAATTATACAAAT
ATAAAGTAGTACAACTTGAACCACTA---GGATTAGCACCCACCGAGGCAAAAAGAAGAGTGGTG--------
-------------------------------GAGAGAGAAAAAAGAGCA---GTTGGA---ATGGGAGCT---
------CTTTTCCTTGGGTTTTTAGGAACA---GCAGGAAGCACTATG---GGCGCGGTGTCA---
ATGACGCTAACGGTACAG---GCCAGACAA---TTATTG---TCTGGCATAGTGCAACAGCAAAGC---
AATTTG---CTGAGG---GCTATAGAGGCTCAACAGCATATGTTGAAACTCACAGTCTGGGGCATT
>FM877783..1
TTCTATTGTAATACATCAGGCCTGTTTAATAACGAGTGGAATAGCACTTGGAAGAATAGCACTTGG-------
----

GAAAATGGCACCATAACACTCCCATGCAGGATAAAACAAATAGTAAATATGTGGCAGGGAGTAGGACAAGCAA
TGTATGCCCCTCCTGTCGCAGGAATGATACAGTGTGCATCAAAT---
ATTACAGGACTAATATTGACAAGAGATGGGGGAGAGAATAATAGT----------------------------
----

AGTAAGGAGACCTTCAGACCTGGAGGAGGAGATATGAAGGACAATTGGAGAAGTGAATTATATAAGTACAAAG
TAGTACAACTTGAACCATTA---GGAGTAGCACCCACCAAGGCAAAAAGAAGAGTGGTG--------------
-------------------------GAGAGAGAAAAAAGAGCA---ATGGGA---ATGGCAGCT---------
ATGTTCCTTGGGTTCTTAGGAGCA---GCAGGAAGCACTATG---GGCGCGGCGTCA---
ATAACGCTGACTGTACAG---GTCAGACAA---TTATTG---TCTGGAATAGTGCAACAGCAAAGC---
AATTTG---CTGAGA---GCTATAGAGGCTCAACAGCATATGTTGCAGCTCACAGTCTGGGGCATT
>FM877784..1
TTCTATTGCAATACCTCAGGTCTGTTTAATAGCACTTGGAATGGAACTACAAGTGGGTCAAATGTCAAT----
----

ACAACTGGCAATATAACACTACCATGCAAAATAAAACAAATTATAAACATGTGGCAGAGAGTAGGACAAGCAA
TGTATGCTCCTCCCATCCCAGGAATAATACAGTGTATATCAAAT---
ATTACAGGGTTATTAATAACAAGAGATGGTGGAAATAATGCAACT----------------------------
----

AATGAAACGGAGATCTTCAGACCTGGAGGAGGAGATATGAGGGACAATTGGAGAAGTGAATTATATAAATATA
AAGTAGTAGAAATTGAACCACTA---GGAGTAGCACCCACCAAAGCAAAGAGAAGGGTGGTG-----------
----------------------------CAGAGAGAAAAAAGAGCA---GTAGGA---ATAGGAGCT------
---GTGTTCCTTGGGTTCCTAGGAGCA---GCAGGAAGCACTATG---GGCGCGGCGTCA---

ATGACGCTGACGGTACAG---GCCAGACAA---CTATTG---TCGGGCATAGTGCAACAGAAAAAC---
AACTTT---CTGAAG---GCTATAGAGGCTCAACAGCACCTGTTGAAACTCACGGTCGGGGGCATT
>KT022402..1
TTCTATTGTAACACATCAGGCCTTTTTAATAGCAAGTGGCATAGGGATAACAATAGCAGCATTGGGTATAGCA
ATAATAGCACA--

GAGTCAAATGACACTATAACTCTCCAATGCAGAATAAAGCAATTTATAAAACTGTGGCAGAGAGTAGGACAAG
CAATGTATGCCCCTCCCATCCCAGGAATAATAAGGTGTGACTCAAAT---
ATTACAGGTCTCATATTAACAAGAGATGGGGGAAATAACAATGCA----------------------------
----

AGCAATGAAACCTTCAGACCTGGGGGAGGAGATATGAGGGACAATTGGAGAAGTGAACTATATAAGTATAAAG
TAGTAAAAATTGAACCACTA---GGGTTAGCACCCTCCAGGGCAAGGAGAAGAGTGGTG--------------
-------------------------GAGAGAGAAAAAAGAGCA---GTTGGA---CTGGGAGCT---------
GTATTCCTTGGGTTCTTGGGAGCA---GCTGGAAGCACTATG---GGCGCAGCGGCA---
ATAACGCTGACGGCACAG---GCCAGACAA---TTATTG---TCTGGTATAGTGCAGCAGCAAAGC---
AATTTG---CTGCAA---GCTATAGAGGCTCAACAGCAACTGTTGAGACTCACGGTCTGGGGCATT
>KT022407..1
TTCTATTGCAATACATCAAGCTTGTTTAATAGCACATGGAACGGCACTGCACGGGATAACAAAAAC-------
----

TCAAATGATACTATGACTCTCCCATGCAAAATAAAACAAATTATAAACATGTGGCAGAGAGTAGGACAAGCAA
TATATGCCCCTCCCATTGCAGGAATGATACAGTGTACATCAAAC---
ATTACAGGAATAATATTGATAAGAGATGGGGGTAATCTCAGTGCG----------------------------
----

AGCAATGAGACCTTCAGACCTGGAGGAGGAGATATGAGGGACAATTGGAGAAGCGAATTATATAAGTATAAAG
TAGTAAAAATTGAACCACTA---GGAGTGGCACCCACCAGGGCAAGGAGAAGAGTGGTG--------------
-------------------------GAGAGAGAAAAAAGAGCA---GTTGGA---ATAGGAGCT---------
GTCTTCCTTGGGTTCTTAGGAGCA---GCAGGTAGCACTATG---AGCGCGGCGTCA---
ATAACGCTGACGGTACAG---GCCAGACAA---TTATTG---TCTGGCATAGTGCAACAGCAAAGC---
AATTTG---CTGAGG---GCTATAGAGGCTCAACAGCATCTGTTGAAACTCACGGTCTGGGGCATT
>KT022386..1
TTTTATTGTAACACATCAGGTCTGTTCAATAGCTCTTTCAAGAGCTCTTGGAAGGTTAATGTGTCAAAT----
----

GACAATAGCACTATAACTCTCCCATGCAGAATAAAACAAATTGTAAACATGTGGCAGAGAGTAGGACGTGCAA
TGTATGCCCCTCCCATTGCAGGAGTAATAAAATGTGAGTCAAAC---
ATTACAGGACTAATATTAACAAGAGATGGAGGGAAAGTGGACAAT----------------------------
----

GACAATGACACTGAGACCTTCAGACCTGGAGGAGGAGATATGAGGGACAATTGGAGAAGTGAATTATATAAGT
ATAAAGTAGTAAAACTTGAACCACTA---GGAGTAGCACCCACCGAGGCAAGGAGAAGAGCGGTG--------
-------------------------------CAGAGAGAAAAAAGAGCA---ATTGGA---CTGGGAGCT---
------TTCTTCCTTGGGTTCTTGGGAACA---GCAGGAAGCACTATG---GGCGCGGCGTCA---
CTAACGCTGACGGTACAG---GCCAGGCAA---TTATTA---TCTGGCATAGTGCAACAGCAAAGC---
AACTTG---CTGAAG---GCTATAGAGGCTCAACAACATATGTTGAGACTCACGGTCTGGGGCATT
>KU749429..2
TTCTATTGTGATACATCAATCCTGTTTAATAGCACTTGGTATCACAATGGCAGTATCTTGCAGAAGTCAAAT-
----

AGCACGGATAATATAATTCTCCCATGCAGAATAAAGCAAATTATAAATATGTGGCAGAGAGCAGGACAAGCAA
TATATGCCCCTCCCATCAAAGGACCAATAGTGTGTGAATCAAAT---
ATTACAGGACTAATATTAACAAGAGACGGTGGCACGGATGACAAT----------------------------
----

ACAACTGAAACTTTCAGACCTGGAGGAGGAGATATGAGGGACAATTGGAGAAGTGAATTATATAAGTATAAAG
TAGTAAAAATTGAACCATTA---GGATTAGCACCCTCCAGGGCAAGGAGAAGAGTGGTG--------------
-------------------------GAGAGAGAAAAAAGAGCA---GCGGGG---CTAGGAGCT---------
GTCTTCATTGGGTTCTTAGGAATG---GCAGGAAGCACTATG---GGCGCGGCGTCG---

ATGACGCTGACGGTACAG---GCCAGACAA---TTATTG---TCTGGTATAGTGCAACAGCAAAGC---
AATTTG---CTGAGG---GCTATAGAGGCTCAACAGCAGATGTTGAGACTCACGGTCTGGGGCATT
>KU749427..1
TTTTATTGCAATACAACAGAGTTGTTTAATGGCACATGGAGTAATGGCACTGAATGGAGCAAGGGCAATGTCA
GC---

ACAAATGGCAATATAACTATCCCATGCAGAATAAAACAAATTATAAACATGTGGCAGAGAGTAGGAAAAGCAA
TGTATGCTCCTCCCATCCCAGGAGTAATAAGGTGTGTATCAAAC---
ATTACAGGACTAATATTAACAAGAGATGGTGATACTAGCAGTGCA----------------------------
----

GATGAAACCTTCAGACCTGGAGGAGGAGATATGAGAGACAATTGGAGAAGTGAATTGTATAAGTATAAAGTAG
TAAAGATTGAACCACTA---GGAGTAGCACCTACCAGGGCAAAGAGAAGAGTGGTG-----------------
----------------------CAGAGAGACAAAAGAGCA---GTGGGA---ATGGGAGCT---------
TTATTCCTTGGGTTCTTGGGAGCA---GCAGGAAGCACTATG---GGCGCAGCGTCA---
ATAACGCTGACGATACAG---GCCAGACAA---TTATTG---TCTGGCATAGTGCAACAGCAAAGC---
AATTTG---CTGAAA---GCTATAGAGGCTCAGCAGCAACTGTTGAGGCTCACGGTCTGGGGCATT
>AF457088..1
TTCTATTGTAACACATCAGGCCTGTTTAATAATAGTGTTTGGAATAGCACTACCGTCACGCAGGAGTCAAATA
GCACG--

GAGTCAAATGGCAATATAACTCTCAATTGCAGAATAAAGCAAATTATAAATATGTGGCAGAGAGTAGGACAAG
CAATGTATGCCCCTCCTATTCCAGGAGTAATACAGTGTGTATCAAAC---
ATTACAGGACTAATATTAACAAGAGATGGTGGGAAGAATAACAGT----------------------------
----

ACAAATGAAACCTTCAGACCTGGAGGAGGAGATATGAGGGACAATTGGAGAAGTGAACTATATAAATATAAAG
TAGTAAAAATTGAACCACTA---GGAGTAGCACCCACCAGGGCAAAGAGAAGAGTGGTG--------------
-------------------------GAGAGAGAAAAAAGAGCC---GTTGGA---ATAGGAGCT---------
GTATTCCTTGGGTTCTTGGGAGCA---GCAGGAAGCACTATG---GGCGCGGCGTCA---
ATAACGCTGACGGTACAG---GCCAGACAA---TTATTG---TCTGGCATAGTGCAACAGCAAAGC---
AATTTG---CTGAGA---GCTATAGAGGCTCAACAACATCTGTTGAAACTCACGGTCTGGGGCATT
>FJ623492..1
TTCTATTGCAATACATCAGGCCTGTTTAATAGCACATGGAATAGCACATGGGAGAATAACATTAATGAC----
----

ACGAAGCCAAATATAACTCTCCCATGCAGAATAAAGCAAATTATAAATATGTGGCAGAGAACAGGACAAGCAG
TATATGCTCCTCCCATCCAAGGAGTAATAAGGTGTATATCAAAC---
ATTACAGGACTAATATTAACAAGAGATGGTGGGAATAATAGTAGG----------------------------
----

ACAAATGAAATCTTCAGACCTGGAGGAGGAGATATGAGAGACAATTGGAGAAGTGAATTATATAAGTATAAAG
TAGTAAAGATTGAACCACTA---GGAGTAGCACCCACCAAGGCAAGGAGAAGAGTGGTG--------------
-------------------------CAGAGAGAAAAAAGAGCA---GTTGGA---ATAGGAACT---------
GTCCTCCTTGGGTTCTTAGGAGCA---GCCGGAAGCACTATG---GGCGCGGCGTCA---
ATAACGCTGACGGTACAG---GCCAGACAA---TTATTG---TCTGGCATAGTGCAACAGCAAAGC---
AATTTG---CTGAAG---GCTATAGAGGCTCAACAGCAGATGTTGAGACTCACTGTCTGGGGCATT
>AY945739..1
TTCTATTGTAATACATCAGGTCTGTTCAATAGCACTTGGTATGCCAATAGCAGCATGCAAGGGTCAAATGACA
CG---

GAGTTAAATGGCACTATAACTCTCCCATGCAGAATAAAACAAATTATAAATATGTGGCAAAGAGTAGGACAAG
CAATGTATGCCCCTCCCATCCAAGGAGTAATAAGATGTAAATCAAAT---
ATCACAGGACTACTGTTAACAAGAGATGGAGGGAAGGGAAATGAC----------------------------
----

ACTGAAACTTTCAGACCCGGAGGAGGAGATATGAGGGACAATTGGAGAAGTGAATTATATAAGTACAAAGTAG
TAAAAATTGAACCATTA---GGAGTAGCACCCAACAAGGCAAAGAGAAGAGTGGTG-----------------
----------------------GAGAGAGAAAAAAGAGCA---GTTGGA---ATAGGAGCT---------
GTCTTCCTTGGGTTCTTAGGAGCA---GCAGGAAGCACTATG---GGCGCAGCGTCA---

CTAACGCTGACGGTACAG---GCCAGACAA---TTATTG---TCTGGCATAGTGCAACAGCAAAGC---
AATTTG---CTGAGG---GCTATAGAGGCTCAACAACATATGTTGAAACTCACGGTCTGGGGCATT
>KC473836..1
TTCTACTGTAACACATCAGACCTGTTTAATAGCACTTGGAATAGCACTGAGTCAAATAGCACAGAGGCAGAGT
CA---

AACAATGACACTATAACTCTTCCATGCAGAATAAAGCAAATTATAAATATGTGGCAGAGAGTAGGACAAGCAA
TATACGCCCCTCCCATCCCAGGAGTAATAAAGTGTAAATCAAAC---
ATTACAGGACTAATATTAACAAGAGATGGGGGTATTAACAATAGT----------------------------
----

AGCAGCTCCGAGACCTTCAGACCTGGAGGGGGAGATATGAGGGACAATTGGAGAAGTGAATTATATAAGTATA
AAGTAGTACAAATTGAACCATTA---GGAGTAGCACCCTCCAGGGCAAGGAGAAGAGTGGTG-----------
----------------------------CAGAGAGAAAAAAGGGCA---GTAACC---CTAGGAGCT------
---CTATTCCTTGGGTTCTTGGGAACA---GCAGGAAGCACTATG---GGCGCAGCGTCA---
GTAACGCTGACGGTACAG---GCCAGGCAA---TTATTG---TCTGGTATAGTGCAACAGCAGAGC---
AATCTG---CTGAGG---GCTATTGAGGCGCAACATCATCTGTTGCAACTCACAGTCTGGGGCATT
>EU220698..1
TTCTATTGTAATACATCACAACTGTTTAATAGCACTTGGGACAAAAATGGCACTTGGATCAATGGCAGCGCAG
AC---

ACAACAAATGGAACTATCACTCTCCCGTGCAGAATAAAGCAAATTATAAATATGTGGCAGCGAGTAGGAAAAG
CAATGTATGCCCCTCCCATCCAAGGAGTAATAGAATGTAGATCAGAG---
ATTACAGGACTACTATTAACAAGAGATGGTGGGAATGAGAGCAGT----------------------------
----

TCAGCAGAGACCTTCAGACCTGGAGGAGGAAATATGAAGGACAATTGGAGAAGTGAATTATATAAGTATAAAG
TAGTAAAACTAGAACCACTA---GGAGTAGCACCCACCAAGGCAAGGAGAAGAGTGGTG--------------
-------------------------GAGAGAGAAAAAAGAGCA---GCT------CTAGGAGCA---------
GTCCTTTTTGGGTTCTTAGGAGCA---GCAGGAAGCACTATG---GGCGCAGCGTCA---
ATAACGCTGACGGTACAG---GCCAGAAAA---TTATTG---TCTGGCATAGTGCAACAGCAAAGC---
AATTTG---CTGATG---GCTATAGAGGCTCAACAGCATATGTTGAAACTCACGGTCTGGGGCATT
>DQ083238..1
TTCTATTGTAATACATCAGAACTGTTTAATAGCACTTGGAATTCCAGTACGCAGGGGACAAATAACACAGAG-
----

CTAAATGACACGATAACTCTCCCATGCAGAATAAAGCAAGTTATAAATATGTGGCAGAGAGTGGGACAAGCAA
TGTATGCCCCTCCCATCAAAGGAATAATAAAGTGTGTATCAAAC---
ATTACAGGACTAATATTAACAAGAGATGGTGGGATTAATAGTACA----------------------------
----

AATGGAACTGAAACCTTCAGACCTGGAGGAGGAGATATGAGGGACAATTGGAGAAGTGAATTATATAAATATA
AGGTAGTACAAATTGAACCACTA---GGAGTAGCACCCACCAAGGCAAGGAGAAGAACGGTG-----------
----------------------------GGGAGAGAAAAAAGAGCA---ATTGGA---CTAGGAGCT------
---GTGTTCCTTGGGTTCTTAGGAGCA---GCAGGAAGCACTATG---GGCGCAGCATCA---
ATAACGCTGACGGTACAG---GCCAGACAA---TTATTG---TCTGGTATAGTGCAACAGCAAAGC---
AATTTG---CTGAGG---GCTATAGAGGCTCAACAACAAATGTTGAGACTCACGGTCTGGGGCATT
>AF067156..2
TTCTATTGTAATACATCAGGCCTGTTTAATGGCACTTGGAATGCCAGCATGCAGGAGTCAAATAGCACGGAG-
----

TCAAATGAAACTATAATTCTCCCATGCAGAATAAAACAAATTATAAATATGTGGCAGAGAGTAGGACAAGCAA
TGTATGCCCCTCCCATTCAAGGAATAATAAAGTGTGTATCAAAC---
ATTACAGGACTAATATTAACAAGAGATGGT---AAAAGTAGTAAC----------------------------
----

AGTACAGATGAAATCTTCAGACCTGGAGGAGGAGATATGAGGGACAATTGGAGAAGTGAATTATATAAGTATA
AAGTAGTAAAAATTGAACCACTA---GGAGTAGCACCCACCAGGGCAAGGAGAAGAGCGGTG-----------
----------------------------GGGAGAGAAAAAAGAGCA---GTTGGA---ATAGGAGCT------
---GTCTTCCTTGGGTTCTTAGGAGCA---GCAGGAAGCACTATG---GGCGCAGCGTCA---

ATAACGCTGACGGTACAG---GCCAGACAA---TTATTG---TCTGGCATAGTGCAACAGCAAAGC---
AATTTG---CTGAGG---GCTATAGAGGCTCAACAACATCTGTTGAAACTCACGGTCTGGGGCATT
>AF457087..1
TTCTATTGTAACACATCAGGCCTGTTTAATAGCACTTGGAATGACACTGCCATCGAGGCTAAGTCAAATAACA
CG---

GAGACAGACGGTATTATAACTCTCCAATGCAGAATAAAGCAACTTATAAATATGTGGCAGAGGGTAGGACAAG
CAATATATGCCCCTCCCATCCCAGGATTAATAAGGTGTGAATCAAAC---
ATTACAGGGCTAATATTAACAAGAGATGGTGGGAATAATGGTGGGAATAAT----------------------
----

AGCAGTGAAAAAAATGAAACCTTCAGACCTGGAGGAGGAGATATTAGGGACAATTGGAGAAGTGAATTATATA
AGTATAAAGTAGTAAAAATTGAACCACTA---GGAGTAGCACCCACCAAGGCAAGGAGAAGAGTGGTG-----
----------------------------------CAGAGAGAAAAAAGAGCA---ATTGGA---
CTGGGAGCT---------GTCTTCATTGGGTTCTTAGGAGCA---GCAGGAAGCACTATG---
GGCGCGGCGTCA---ATAACGCTGACGGTACAG---GCCAGACAA---TTATTG---
TCTGGCATAGTGCAACAGCAAAGC---AATTTG---CTGAGG---
GCTATAGAGGCTCAACAGCATCTGTTGAAACTCACAGTCTGGGGCATT
>KT022391..2
TTCTATTGTGATACATCAGGCCTGTTTAATAGCGCTTGGGCAGCCAATAGCACTTGGACAGCCAATGACACTG
AC---
-AGAAATCAGGTAGAT---------
AACAATAATGACACCATAACTCTCCCATGTAAAATAAAGCAAATTATAAATATGTGGCACAGAGTAGAACAAG
CAATATATGCCCCTCCCATTCCAGGAGTAATAAGGTGTGAATCAAAC---
ATTACAGGACTACTATTAACAAGAGATGGTGGGGTTAGGGTTGAC----------------------------
----
-
AATACAAGTAATACAAGTGAAACCTTCAGACCTGGAGGAGGAGATATGAAGGATAATTGGAGAAGTGAATTAT
ATAAGTATAAAGTAGTAGAAATTGAACCACTA---GGAGTAGCACCCACCAGGGCAAAGAGAAGAGTGGTG--
-------------------------------------GAGAGAGAAAAAAGAGCA---GTTGGA---
ATAGGAGCT---------GTTTTCATTGGGTTCTTAGGAGCA---GCAGGAAGCACTATG---
GGCGCGGCGTCA---GTGACGCTGACGGTACAG---GCCAGACAA---GTATTG---
TCTGGCATAGTGCAACAGCAAAGC---AATTTG---CTAAGG---
GCTATAGAGGCTCAACAACATCTGTTGAAACTCACGGTCTGGGGCATT
>KT022393..1
TTTTATTGTAACACATCAGGCCTGTTTAATAGCAGTTGGGCAAATAACACAGAGTCAAACAACACAGAG----
----

TTAAACAGAACTATAACGCTCCCATGCAGAATAAAGCAAATTATAAATATGTGGCAGAGAACAGGACAAGCAA
TGTATGCCCCTCCCATCCAAGGAGTAATAAGGTGTGAATCAAAC---
ATTACAGGATTAATATTGACAAGAGATGGTGGGGATAATAGTATA----------------------------
----

AATGAAACCTTCAGACCGGGAGGAGGAGATATGAGGGATAATTGGAGAAGTGAATTATATAAGTATAAAGTAG
TAAAAATTGAACCACTA---GGAGTAGCACCCACCAGGGCAAAGAGAAGAGTGGTG-----------------
----------------------GAGAGAGAAAAAAGAGCA---GTTGGA---ATAGGAGCT---------
GTCTTCCTTGGGTTCTTAGGAGCA---GCAGGAAGTACTATG---GGCGCGGCGTCA---
ATGACGCTGACGGTACAG---GCCAGGCAA---TTATTG---TCTGGAATAGTACAACAGCAAAGT---
AATTTG---CTGAGG---GCTATAGAGGCTCAACAGCATCTGTTGAAGCTCACGGTCTGGGGCATT
>KT022395..1
TTTTATTGTAATACATCAGGACTGTTTAATGAGAGTTGGGTAAATGGCACTGAACCAAGTAGCAATGATACT-
----

GATATGACTCCCATAACTCTCCAATGCAGAATAAAGCAAATTATAAATATGTGGCAGAGAATAGGACAGGCAC
TGTATGCCCCTCCCATCCCAGGAGAAATAAGGTGTAACTCAAAC---
ATTACCGGACTACTATTAGTAAGAGATGGAGGGTCTCTTAACAGT----------------------------
----

ACAAATGAAACCTTCAGACCTGGAGGAGGAGACATGAGGGACAATTGGAGAAGTGAATTATATAAGTATAAAG
TAGTAAAAATTGAACCACTA---GGAGTAGCACCCAACAAGGCAAAGAGAAGAGTGGTG--------------

-------------------------GAAAGAGAAAAAAGAGCA---GCTGGA---ATAGGAGCT---------
TTGCTCATTGGGTTCTTAGGAGCA---GCAGGAAGCACTATG---GGCGCGGCGTCA---
ATAACGCTGACGGTACAG---GCCAGACAA---TTGTTG---TCTGGCATAGTGCAACAGCAAAGC---
AATTTG---CTGAGG---GCTATAGAGGCTCAACAGCATCTGTTGAAACTCACGGTCTGGGGCATT
>KT022408..2
TTCTATTGTAATACATCAGGCCTGTTTAATAGTACTTGGAATAGCACAGAGCGGTCTAGAAATAGCACTAACA
GCACAGGG---

GAAGGTAGAAATATAACTCTCCAATGCAGAATAAAGCAAATTATAAATATGTGGCAGAGAGTAGGACAAGCAA
TGTATGCCCCTCCCATCCAAGGAGAAATAAAGTGTGTATCAAAC---
ATTACAGGAATACTATTAACAAGAGATGGTGGGAACAATACAAGT----------------------------
----

CAAAATGAAACCTTTAGACCTGGAGGAGGAGATATGAGGGACAATTGGAGAAGTGAATTATATAAGTATAAAG
TAGTACAAATTGAACCACTA---GGAGTAGCACCCACCAGGGCAAGGAGAAGAGTGGTG--------------
-------------------------CAGAGAGAAAAAAGAGCA---GTTGGA---CTGGGAGCT---------
GTCTTCATTGGGTTCTTAGGAGCA---GCAGGAAGCACTATG---GGCGCAGCGTCA---
ATAACGCTGACGGTACAA---GCCAGACAA---TTATTG---TCTGGCATAGTGCAACAGCAAAGC---
AATTTG---CTGAGG---GCTATAGAGGCTCAACAGCATCTGTTGAAACTCACAGTCTGGGGCATT
>KT022413..3
TTCTATTGTAATACATCAAGCCTGTTTAATAGCGCTTGGAATCACACTCACAATGACAGCATACAGGGG----
----

TCAAATGACACTCTAACTCTCCCATGTAGAATAAAGCAAATTATAAATATGTGGCAGAGAGTAGGACAAGCAA
TGTATGCCCCTCCCATCCAAGGAATAATAAAGTGTGAATCAAAC---
ATTACAGGACTAATATTAACAAGAGATGGTGGTGGGAGTAATAAC----------------------------
----

AGTACAGATGAAATCTTCAGACCGGGAGGAGGAAATATGAGGGACAATTGGAGAAGTGAATTATATAAGTATA
AAGTAGTAAAAATTGAACCAATA---GGAGTAGCACCCACCAAGGCAAGGAGAAGGGTGGTG-----------
----------------------------GAGAGAGAAAAAAGAGCA---GTTGGA---ATAGGAGCT------
---GTTTTCCTTGGGTTCTTGGGAGCA---GCAGGAAGCACTATG---GGCGCGGCGTCA---
ATAACGCTGACGGTACAG---GCCAGACAA---TTATTG---TCTGGCATAGTGCAACAGCAAAGC---
AATTTG---CTGAGG---GCTATAGAGGCTCAACAACAGATGTTGAGACTCACGGTCTGGGGCATT
>FJ623489..1
TTCTATTGTGACACATCAGGCCTATTTAATAGCACTTGGTATGGGAATGGGACCAACACAACGGAACCAGATA
GC---

ACAAATGACAATATAACTCTCCCATGCAGAATAAAGCAAATTATAAATATGTGGCAGAGAGTAGGACAAGCAA
TGTACGCCCCTCCCATCCCAGGAGCAATAAGCTGTACCTCAAAC---
ATTACAGGATTATTATTAACAAGAGATGGTGGGTATAATAGCACT----------------------------
----

AATGAAACCTTCAGACCTGGTGGAGGAGATATGAGGGACAATTGGAGAAGTGAATTATTTAAGTACAAGGTAG
TACAAATTAAACCACTA---GGAGTAGCACCCACCGAGGCAAGGAGAAGAGTGGTG-----------------
----------------------CAGAGAGAAAAAAGAGCA---GTTGGA---CTAGGAGCT---------
TTCTTCATTGGGTTCTTAGGAGCA---GCTGGAAGCACTATG---GGCGCGGCGTCA---
ATAACGCTGACGGTACAG---GCCAGACAA---CTGTTG---TCTGGCATAGTGCAACAGCAAAGC---
AATTTG---CTGAGG---GCTATAGAGGCTCAACAGCATCTGTTGAAACTCACAGTCTGGGGCATT
>HM215276..2
TTCTATTGTGATACATCAATCCTGTTTAATAGCACTTGGTATCACAATGGCAGTATCTTGCAGAAGTCAAAT-
----

AGCACGGATAATATAATTCTCCCATGCAGAATAAAGCAAATTATAAATATGTGGCAGAGAGCAGGACAAGCAA
TATATGCCCCTCCCATCAAAGGACCAATAGTGTGTGAATCAAAT---
ATTACAGGACTAATATTAACAAGAGACGGTGGCACGGATGACAAT----------------------------
----

ACAACTGAAACTTTCAGACCTGGAGGAGGAGATATGAGGGACAATTGGAGAAGTGAATTATATAAGTATAAAG
TAGTAAAAATTGAACCATTA---GGATTAGCACCCTCCAGGGCAAGGAGAAGAGTGGTG--------------

-------------------------GAGAGAGAAAAAAGAGCA---GCGGGG---CTAGGAGCT---------
GTCTTCATTGGGTTCTTAGGAATG---GCAGGAAGCACTATG---GGCGCGGCGTCG---
ATGACGCTGACGGTACAG---GCCAGACAA---TTATTG---TCTGGTATAGTGCAACAGCAAAGC---
AATTTG---CTGAGG---GCTATAGAGGCTCAACAGCAGATGTTGAGACTCACGGTCTGGGGCATT
>KF716469..3
TTCTATTGTAATACATCAGGCCTGTTTAATCATACATACAATCACACGCAGGAGGCAAATGGCACGGGG----
----

TCAAATGGCAATATAACTCTCCCATGCAGAATAAAGCAAATTATAAATATGTGGCAGAGAACAGGACAAGCAA
TGTATGCCCCTCCCATCCAAGGAGTAATAAAATGTGTATCAAAT---
ATTACAGGACTACTATTAACAAGAGATGGTGGGGGAAATAACAGGACAGAG----------------------
----

GAAATCTTTAGACCTGGAGGAGGAGATATGAGGGACAATTGGAGAAGTGAATTATATAAGTATAAAGTAGTAA
AAATTGAACCACTG---GGAGTAGCACCTACCACGGCAAGGAGAAGAGTGGTG--------------------
-------------------GGAAGAGAAAAAAGAGCA---GTTGGA---CTAGGAGCT---------
GTCTTCTTTGGGTTCTTAGGAGCA---GCCGGAAGCACTATG---GGCGCGGCGTCA---
ATAACGCTGACGGCACAG---GCCAGACAA---TTATTG---TCTGGCATAGTGCAACAGCAGAGC---
AATCTG---CTGAGG---GCTATAGAGGCTCAACAACATCTGTTGAAACTCACGGTCTGGGGCATT
>KF716471..2
TTCTATTGTAATACATCAGGCCTGTTTAATCATACATACAATGACACGCAGGAGGCAAACAGCACGGGG----
----

TTAAATGGCAATATAACTCTCCCATGCAGAATAAAGCAAATTGTAAATATGTGGCAGAGAGCAGGACAAGCAA
TGTATGCCCCTCCCATCCAAGGAGTAATAAAATGTGTATCAAAT---
ATTACAGGACTACTATTAACAAGAGATGGTGGGGGAAATAGCAGT----------------------------
----

ACAAAGGAAATCTTTAGACCTGGAGGAGGAGATATGAGGGACAACTGGAGAAGTGAATTATATAAGTATAAAG
TAGTAAAAATTGAGCCACTG---GGGGTAGCACCTACCGAGGCACGGAGAAGAGTGGTG--------------
-------------------------GGAAGAGAAAAAAGAGCA---GTTGGA---ATAGGAGCT---------
GTCTTCCTTGGGTTCTTAGGAGCA---GCAGGAAGCACTATG---GGCGCGGCGTCA---
ATAACGCTGACGGTACAG---GCCAGACAA---TTATTG---TCTGGCATAGTGCAACAGCAGAGC---
AATCTG---CTGAGG---GCTATAGAGGCTCAACAACATCTGTTGAAACTCACGGTCTGGGGCATT
>KU921793..1
TTCTATTGTAACACATCAAGCCTGTTTAATAGCACTTGGGGTAATACCAGTGTGCCAGAGTTAAATAGCACGG
AGGCAAATAGC---ACG------GAG---

CAAAACAATATAACAATAACACTCCAATGCAGAATAAAGCAAATTATAAACATGTGGCAGAGAGCAGGACAAG
CAATGTATGCCCCTCCCATCCAAGGAGAGATAAGGTGTATATCAAAC---
ATTACAGGATTAATATTAACAAGAGATGGTGGGAATAATAATAAC----------------------------
----

AGCACAAATGAAACCTTCAGACCTGGAGGAGGAGATATGAGGGACAATTGGAGGAGTGAATTATATAAGTATA
AAGTGGTAAAAATTGAACCAATA---GGAATAGCACCCACCAGGGCAAAAAGAAGAGTGGTG-----------
----------------------------CAGAGAGAGAAAAGAACA---GTAGGA---CTAGGAGCT------
---GTCTTCATTGGGTTCTTAGGAGCA---GCAGGAAGCACTATG---GGCGCGGCGTCA---
GTGACGCTGACGGTACAG---GCTAGACAA---CTATTG---TCTGGCATAGTGCAACAGCAAAGC---
AATTTG---CTGAGG---GCTATAGAGGCTCAACAGCATCTGTTGAAACTCACGGTCTGGGGCATT
>KU921796..2
TTCTATTGTGACACATCAGGCCTGTTTAATAGCACTTGGGATAGCAATAACAGCGAGCAAGAGTCAAATAGCA
CTGAGTTA---

AACAATATAACTCTAACTCTCCACTGCAGAATAAAGCAAATTATAAATATGTGGCAGAGAGCAGGACAAGCAA
TGTATGCCCCTCCCATCCAAGGAGTGATAAGATGTGTATCAAAC---
ATTACAGGATTAATATTAACAAGAGATGGTGGGGATCATAATAAC----------------------------
----

AGCGCAAATGAAACCTTCAGACCTGGAGGAGGAGATATGAGGGACAATTGGAGGAGTGAATTATATAAGTATA
AAGTAGTAAAAATTGAACCAATA---GGAATAGCACCCACCAAGGCAAGAAGAAGAGTGGTG-----------

----------------------------CAGAGAGAGAAAAGAGCA---ATAGGA---ATGGGAGCC------
---GTCTTCCTTGGGTTCTTAGGAGCA---GCAGGAAGCACTATG---GGCGCAGCGTCA---
GTAGCGCTGACGGTACAG---GCCAAACAA---CTATTG---TCTGGCATAGTGCAACAGCAAAAC---
AATTTG---CTGAGG---GCTATAGAGGCTCAACAGCATATGTTGAAACTCACGGTCTGGGGCATT
>KU921839..1
TTCTATTGTAATACATCAGGACTGTTTAATAGCACTTGGAATTCCACTAACATGCAGGAGCTAGATAGCACAG
AG---

GCAAATGGTACTATAATACTCCCATGCAAAATAAAGCAAATCATAAATATGTGGCAGAGAGTAGGACAAGCAA
TGTATACCCCTCCCATACCGGGAGTAATAAATTGTGAATCAAAC---
ATCACAGGATTAATATTAACAAGGGATGGTGGCCCTAATAACGAT----------------------------
----

ACAGAAATCTTCAGACCAGGAGGAGGAGATATGAGGGACAATTGGAGAAGTGAATTATACAAGTATAAAGTAG
TAAAAATTGAACCATTA---GGAGTAGCACCCACCAAAGCAAGGAGAAGAGTGGTG-----------------
----------------------GAGAGAGAAAAAAGAGCA---GTTGGA---ATAGGAGCT---------
GTTCTCGTTGGGTTCTTGGGAGCA---GCAGGAAGCACTATG---GGCGCGGCGTCA---
ATAACGCTGACGGCACAG---GCCAGGCAA---TTATTG---TCTGGCATAGTGCAACAGCAAAGC---
AATTTG---CTGAGG---GCTATCGAGGCTCAACAACAGCTGTTGAAACTCACGGTCTGGGGCATT
>KU749431..2
TTCTATTGTGATACAACAAGCCTGTTTAATAGTACTCACTCCCCTAACGACACGCAGGGTGACCAGGAGTCAA
AT---

TCAAATGACACTATAACTCTCCCATGCAGAATAAAGCAAATTATAAATATGTGGCAGAGAACAGGACAAGCAA
TATATGCCCCTCCCATCGAAGGAGATATAAGGTGTAGCTCAAAC---
ATTACAGGATTATTATTAACAAGAGATGGTGGGATTAATGTGAGC----------------------------
----

ACTACTAATGAAACCTTCAGACCTAGTGGAGGAGATATGAGGGACAATTGGAGAAGTGAATTATATAAGTACA
AGGTAGTAAGAATTAAACCACTA---GGAGTAGCACCCACCAAGGCAAGGAGAAGAGTGGTG-----------
----------------------------GAGAGAGAAAAAAGAGCA---GTTGGA---ATGGGAGCT------
---TTCTTCCTTGGGTTCTTAGGAGCA---GCAGGAAGCACTATG---GGCGCAGCGTCA---
ATAACGCTGACGGTACAG---GCCAGAAAC---TTATTG---TCTGGCATAGTGCAACAGCAAAAC---
AATTTG---CTGAGG---GCTATAGAGGCTCAACAACATCTGTTGAAACTCACAGTCTGGGGCATT
>FJ396022..1
TTCTATTGTAACACATCAGGCCTGTTTAATAGCACTTGGTATAATGGCAGCATGTCAAATAGCACGGAG---
----

TCAAATGACAATATAACACTCCAATGCAGAATAAAGCAAATTATAAATATGTGGCAGAGAGTAGGACAAGCAA
TGTATGCCCCTCCCATCCAAGGAGAAATAAAGTGTGTATCAAAC---
ATTACAGGGCTAATATTAACAAGAGATGGTGGTGACAGCACAAAT----------------------------
----

GAAACCTTCAGACCTGGAGGAGGAGATATGAGGGACAATTGGAGAAGTGAATTATATAAATATAAAGTGGTAG
AAATTAAACCATTA---GGAGTAGCACCCACTGAGGCAAAAAGGAGAGTGGTG--------------------
-------------------GAGAGAGAAAAAAGAGCA---GTGGGA---ATAGGAGCT---------
GTGTTCCTTGGGTTCTTGGGAGCA---GCAGGAAGCACTATG---GGCGCAGCGTCA---
ATAACGCTGACGGTACAG---GCCAGACAA---TTATTG---TCTGGCATAGTGCAACAGCAAAGC---
AATTTG---CTGAGG---GCTATAGATGCTCAACAGCATATGTTGAAACTCACGGTCTGGGGCATT
>AF457064..1
TTCTATTGTAACACATCAGGCCTGTTTAATAGCACTTGGAATGACAGTGACAGCATGCAGGGGCCAAATAGCA
CG---

AAGTCAAATGGCACTATAACTCTCCCATGCAGAATAAAGCAAATTATAAATATGTGGCAGAGAGCAGGACAAG
CAATATATGCCCCTCCCATCCCAGGAATAATAAGGTGTGAATCAAAC---
ATTACAGGCCTAATATTAACAAGAGATGGTGGGGATAATAGCAGT----------------------------
----

ACAAATGAAACCTTCAGACCTGGAGGAGGAGATATGAGGGACAATTGGAGAAGTGAATTATATAAGTATAAAG
TAGTAAAAATTGAACCACTA---GGAGTAGCACCCACCGAGGCAAAGAGAAGAGTGGTG--------------

```
------------------------GAGAGAGAAAAAAGAGCA---GTTGGA---ATAGGAGCT---------
GTCTTCATTGGGTTCTTAGGAGCA---GCAGGAAGCACTATG---GGCGCGGCGTCA---
ATGACGCTGACGGTACAG---GCCAGACAA---TTATTG---TCTGGCATAGTGCAACAGCAAAGC---
AATTTG---CTGAGG---GCTATAGAGGCTCAACAGCAACTGTTGAAACTCACGGTCTGGGGCATT
>KU749428..1
TTCTATTGCAACACATCACAGCTGTTTAATTCATCACTGCTCAATAGTACAGAAAGTATTAATGAAACAAGT-
------------------------------------------------------------------------
---------------------------
GGTAATGAAACAATCACACTCCCATGCAGAATAAAACAGATTATAAACATGTGGCAGGAGGTAGGGCGGCAA
TGTATGCCCCTCCCATTAGAGGAATAATAACATGTAAATCAAAT---
ATCACAGGACTACTACTGACACGTGATGGAGGAAATGAGAAAAAT---------------------------
-------------------------------------------------------------------
AACTCAGAGGCAAAT---
AACACAGAGATATTCAGGCCTGGAGGAGGAAATATGAAGGACAATTGGAGAAGTGAATTATATAAGTATAAAG
TAGTAAAAGTTGAACCACTA---GGAGTAGCACCCACCAAGGCAAAGAGAAGAGTGGTG-------------
------------------------GAGAGAGAAAAAAGAGCA---GTAGGA---ATAGGAGCT---------
GTCTTCATTGGGTTCTTAGGAGCA---GCCGGAAGCACTATG---GGCGCAGCGTCG---
GTGACGCTGACGGTACAG---GCCAGACAA---TTATTG---TCTGGCATAGTGCAACAGCAAAGT---
AATTTG---CTGAGG---GCTATAGAGGCTCAACAACATTTGTTGAAACTCACGGTCTGGGGCATT
>KX983685..2
TTCTATTGTAATACATCAGGCCTGTTTAATAACACATGGGCTAATAACAATTGGACTGACAGCACAAATTAC-
------------------------------------------------------------------------
---------------------------
ACAAATGACAATATAACTCTCCAATGCAGAATAAAGCAAATTATAAATATGTGGCAGAGGACAGGACAAGCAA
TGTATGCCCCTCCCATCCAAGGAATAATAATGTGTAGGTCAAAC---
ATTACAGGCCTACTATTAACAAGAGATGGAGGGAATACAAATGATACA------------------------
------------------------------------------------------------------------
----------
AATGCAACTGAAACCTTCAGACCTGGGGGAGGAAATATGAGGGATAATTGGAGAAGTGAGCTATATAAATATA
AAGTAGTAAAAGTTGAACCACTA---GGAGTAGCACCCACCGAGGCAAGAAGAAGAGTGGTG----------
---------------------------CAGAGAGAAAAAAGAGCA---GTTGGA---CTGGGAGCT------
---GCCCTCCTTGGGTTCTTAGGAGCA---GCAGGAAGCACTATG---GGCGCGGCGTCA---
ATAACGCTGACGGTACAG---GCCAGACTA---TTATTG---TCTGGTATAGTGCAACAGCAAAGC---
AATTTG---CTGAGG---GCTATAGAGGCTCAACAGCATATGTTGAAGCTCACAGTCTGGGGCATT
>U88823..1
TTCTATTGTAATACATCAGGCTTGTTTAATAGCACCTGGAGTAAAAGAAATGGCACCTGGCAGTCAAATGGC-
------------------------------------------------------------------------
---------------------------
ACAGAATTAAATATAACACTCCCATGCAGAATAAAGCAAATTATAAATATGTGGCAGAGGACAGGACAAGCAA
TGTATGCCCCTCCCATCCAAGGAGTAATAAGCTGTGTATCAAAC---
ATTACAGGACTACTATTAACAAGAGATGGTGGAAATAATAATACT---------------------------
------------------------------------------------------------------------
------------
ACAACTGAAACCTTCAGACCTGGAGGAGGAGATATGAGGGATAATTGGAGAAGTGAACTATATAAATATAAAG
TAGTAAAAATTGAACCACTA---GGAGTAGCACCCACCAGGGCAAAGAGGAGAGTGGTG-------------
------------------------GAGAGAGAAAAAAGAGCA---GTTGGA---CTGGGAGCT---------
GTCTTCATTGGGTTCTTAGGAGCA---GCAGGAAGCACTATG---GGCGCGGCGTCA---
ATAACGCTGACGGTACAG---GCCAGACAA---TTATTG---TCTGGCATAGTGCAACAGCAAAGC---
AATTTG---CTGAGG---GCTATAGAGGCTCAACAGCATCTATTAAAACTCACAGTCTGGGGCATT
>MF373141..1
TTTTATTGTAATACATCAGTACTTTTTAACAGAAGTATAACAGGCAATATCACAAACCTAACAGAAGTA----
------------------------------------------------------------------------
---------------------------
AACAGTACAAACATCACACTCCCATGCAGAATAAAACAAATTATAAACATGTGGCAGGGGGTAGGACGAGCAA
TGTATGCCCCTCCCATTCAAGGATTCATAAAATGTGAATCAAAT---
TTCACAGGACTACTCCTGACACGTGATGGAGGGAATATTAGTACA---------------------------
------------------------------------------------------------------------
------------
GAGAACGAGACATTCAGGCCGGAAGGAGGAAATATGAAGGACAATTGGAGAAGTGAATTATATAAATATAAAG
TGGTAGAAATTAAACCATTA---GGAATAGCACCCCATAAGGCAAAAAGAAGAGTGGTG-------------
```

-------------------------GAGAGAGAAAAAAGAGCA---GTGGGA---ATAGGAGCT---------
GTGTTCCTTGGGTTCTTGGGAGCA---GCAGGAAGCACTATG---GGCGCAGCATCA---
CTAACGCTGACGGTACAG---GCCAGACAA---TTATTG---TCGGGTATAGTGCAACAGCAGAGC---
AATCTG---CTGAGG---GCTATTGAGGCGCAACAGCATATGTTGCAACTCACAGTCTGGGGCATT
>MF373155..1
TTCTATTGTAATACAACAGGCCTGTTTAATAGCACTTGGAATGATACCACTTTGAACAGCACCACTGAGTTAA
ACAAC--

ACATATGAAAATATAACTCTTCCATGTAGAATAAAGCAAATTATAAACATGTGGCAGAGAGCAGGAAAAGCAA
TGTATGCCCCTCCCATCCCAGGAATAATAAGGTGTGAGTCAAAC---
ATTACAGGACTACTGTTAACAAGCGATGGTGGGAATAAAAGTACT----------------------------
----

ACAAATGAAACCTTCAGACCTGGAGGAGGAGATATGAGGGACAATTGGAGAAGTGAATTATATAAGTATAAAG
TAGTAAAACTTGAACCACTA---GGAGTAGCACCTGAAAGGGCAAAAAGAAGAGTGGTG--------------
-------------------------GCGAGAGAAAAAAGAGCA---GTTGGA---CTGGGAGCT---------
GTCTTCATTGGGTTCTTAGGAGCA---GCAGGAAGCACTATG---GGCGCGGCGTCA---
CTGACGCTGACGGTACAG---GCCAGACAA---TTATTG---TCTGGTATAGTGCAACAGCAAAGC---
AATTTG---CTGAAG---GCTATAGAGGCTCAACAGCACCTGTTGAGACTCACGGTCTGGGGCATT
>AF071474..1
TTCTATTGTGATACATCAGGGCTGTTTAATAGCACCTGGCCGTTTAATAGCACAAACAGCACAGGG-------
----

CCAAATGGCACTATAACTCTCCAATGCAGAATCAAGCAAATTATAAGGATGTGGCAGAGAACAGGACAAGCAA
TATATGCCCCTCCCATCCCAGGAGAAATAAACTGTGTATCAAAC---
ATTACAGGACTACTATTAACAAGAGATGGTGGGAATAATATTACA----------------------------
----

AATGAAACCTTCAGACCTGGAGGAGGAGATATGCAGGACAATTGGAGAAGTGAATTATATAAGTATAAGGTAG
TACAAATTGAACCACTA---GGAGTAGCACCCACCAAGGCAAGGAGAAGAGTGGTG-----------------
----------------------GAGAGAGAAAAAAGAGCA---GTTGGC---ATGGGAGCT---------
GTCTTCATTGGGTTCTTAGGAGCA---GCAGGAAGCACTATG---GGCGCAGCGTCA---
GTAACGCTGACGGTACAG---GCCAGACAA---TTATTG---TCTGGCATAGTGCGACAGCAAAGC---
AATTTG---CTGAGA---GCTATAGAGGCTCAACAACATTTGTTGAAACTCACAGTCTGGGGCATT
>AY253318..2
TTCTATTGTAACACATCAGGCCTGTTTAATAGCACTTGGAATAGCAATGCCAGCATGCAGACGTCAAATGGTA
CG---

GGGTTAAATGACACTATAATTCTTCTATGTTGTATAATACAACTTATTATTATGTGGCGGAGAGCAGGACGAG
CAGTATTTGCCCCTCCTATCCTACGAGGAGTAATGTGGGGATCATAC---
ATCACAGGGCTACTATTATCAACAGATGGTGGGGATGGTACACAT----------------------------
----

GAAGAACTCTTCGTACCTGGACGAGGAGATATGAAGGTCAAGGGCAGAAGCAAATTCTATTTGTATAAAGTTG
TAAAAATTGAACCTGTA---CGTGTAGCACCCACCCCTGCCAACACAAAAGTGGTG-----------------
----------------------GAGAGAGAAAAAAGAGCA---GTTGCA---ATAGGAACT---------
GGATTTGTTGGGTTTTTAGGATCA---GGAGCAAGCACTATG---GGTGCGGCGTCA---
ATAACGCTGACGGTACAG---GCCAGACAA---TTATTG---TCTGGCATAGTGCAACAGCAAAGC---
AATTTG---CTGAGG---GCTATAGAGGCTCAACAACATCTGTTGAAGCTTACGGTCTGGGGCATT
>AY253315..1
TTCTATTGCAACACAACAGACCTGTTTAATTGGAATAATGACACCAGGCAGAAGCGAAATAGAACAGAG----
----

TCAGATAACACTATAACTCTCCAATGCAGAATAAAGCAAATTATAAATATGTGGCAGAGAACAGGACAAGCAA
TATATGCCCCTCCCATCCAAGGAGCAATAAGGTGTGACTCAAAC---
ATTACAGGACTAATATTAGCAAGAGATGGGGGTAACACAAATGAA----------------------------
----

AGTGAAGAGGAAATCTTCAGACCTATAGGAGGAGATATGAGGGACAATTGGAGAAGTGAATTATATAAGTACA
AAGTAGTGAAAATTGAGCCACTA---GGAGTAGCACCCACCACGGCAAAGAGAAGAGTGGTG-----------

----------------------------GAGAGAGAAAAAAGAGCA---GTTGGA---CTGGGAGCT------
---GTCTTCCTTGGGTTCTTAGGAGCA---GCAGGAAGCACTATG---GGCGCAGCATCA---
CTGACGCTGACGGTACAG---GCCAGACAA---TTATTG---TCTGGCATAGTGCAACAGCAAAGC---
AATTTG---CTGAGG---GCTATAGAGGCTCAACAACATCTGTTGAGACTCACAGTCTGGGGCATT
>AY253319..1
TTCTATTGCAACACATCAGGCCTGTTTGATAGCATTTGGAATGACACTGACAGCATGCAGGGGTTAGATAGCA
TG---

GAGTCAGATGGCACTATAACTCTCCAATGCAGAATAAAGCAAATTATAAATATGTGGCAGAGAGCAGGACAAG
CAATATATGCCCCTCCCATCCCAGGAGTAATAGAGTGTACATCAAAC---
ATTACAGGACTAATATTAACAAGAGATGGTGGGAATAGTAGCAGT----------------------------
----

ACAAATGAAACCTTCAGACCTGGAGGAGGAGATATGAGGGACAATTGGAGAAATGAATTATATAAGTATAAGG
TAGTAAAAATTGAGCCACTA---GGAGTAGCACCCACCAAGGCAAAAAGAAAAGTGGTG--------------
-------------------------GAGAGAGAAAAAAGAGCA---GTTGGA---ATAGGAGCT---------
GTTTTCCTTGGGTTCTTGGGAGCA---GCAGGAAGCACTATG---GGCGCGGCGTCA---
ATAACGCTGACGGTACAG---GCCAGACAA---TTATTG---TCTGGCATAGTGCAACAGCAAAGC---
AATTTG---CTGAGG---GCTATAGAGGCTCAACAGCATCTGTTGAAACTCACGGTCTGGGGCATT
>AY734552..1
TTCTATTGTAATACATCAGGCCTGTTTAATGGCATTTGGAACAATACCAGCACGCAGGAGTCAAATAGCACGG
AA---

TCAAATGACACTATAACTATTCCATGCAGAATAAAGCAAATTATAAATATGTGGCAGAGAGTAGGACAAGCAA
TGTATGCCCCTCCCATCCAAGGAATGATAAGGTGTGAATCAAAC---
ATTACAGGACTACTGTTAACAAGAGATGGTGGGAATGAGAGCAGT----------------------------
----

ACAAATGAAACCTTCAGACCTGGAGGAGGAGATATGAGGGACAATTGGAGAAGTGAATTATATAAGTATAAAG
TAGTAAAAATTGAACCGTTA---GGAGTAGCACCCACCAGGGCAAAGAGAAGAGTGGTG--------------
-------------------------GGAAGAGAAAAAAGAGCA---GTTGGA---ATAGGAGCT---------
GTCTTCCTTGGGTTCTTAGGAGCA---GCCGGAAGCACTATG---GGCGCGGCGTCA---
ATAACGCTGACGGTACAG---GCCAGACAA---TTATTG---TCTGGTATAGTGCAACAGCAAAGC---
AATTTG---CTGAAG---GCTATAGAGGCTCAACAACATATGTTGAGACTCACAGTCTGGGGCATA
>AY734553..1
TTCTATTGCAACACATCAAGCCTGTTTAATAGCACTTGGAATGGCACTACTGATAATGGCACTGGCAGCACAA
AC---

AGCGCAAATAGCAATATAACTCTCCAATGCAAAATAAAGCAAATTATAAATATGTGGCAGAGAGCAGGACAAG
CAATATATGCCCCTCCCATCCCAGGAGTGATAAAGTGTGTATCAAAC---
ATTACAGGACTAATATTAACAAGAGATGGTGGGAATAATAACAGT----------------------------
----

ACAAATGAAACCTTCAGACCAGGAGGAGGAGATATGAGGGACAATTGGAGAAGTGAATTATATAAGTATAAGG
TAGTAAAAATTGAACCACTA---GGAGTAGCACCCACCAGGGCAAAGAGAAGAGTGGTG--------------
-------------------------GAGAGAGAAAAAAGAGCA---GTCGGA---ATAGGAGCT---------
GTTCTCCTTGGGTTCTTAGGAACA---GCAGGAAGCACTATG---GGCGCGGCGTCA---
ATAACGCTGACGGTACAG---GCCAGACAA---TTATTG---TCTGGCATAGTGCAACAGCAAAGC---
AATTTG---CTGAGG---GCTATAGAGGCTCAACAACACATGTTGAAACTCACAGTCTGGGGCATT
>AY734562..1
TTCTATTGTGATACATTTGAACTGTTTAATAGCACTTGGAATCTCACTGCCAGCATGCAGGGGTCAAATAGA-
----

ACGGAGGGCAATATAACACTCCCGTGCAGAATAAAACAAATTGTAAACATGTGGCAGGGGGTAGGACGAGCAA
TGTATGCCCCTCCCATTGCAGGAAATATAACATGTAGATCAAAC---
ATCACAGGACTATTATTGACAAGTGATCATGGTAATGGTAGTCCA----------------------------
----

ACAAATGAGACATTCAGACCTGGAGGAGGAAATATGAGGGACAATTGGAGAAATGAATTATATAAATACAAAG
TGGTAGAAATTAAGCCATTA---GGAATAGCACCCACTAAGGCAAAAAGGAGAGTGGTG--------------

-------------------------GAGAGAGAAAAAAGAGCA---GTGGGA---ATAGGAGCT---------
GTGCTCCTTGGGTTCTTGGGAGCA---GCAGGAAGCACTATG---GGCGCGGCGTCA---
ATAACGCTGACGGTACAG---GCCAGACAA---TTATTG---TCTGGTATAGTGCAACAGCAAAGC---
AATTTG---CTGAAG---GCTATAGAGGCGCAACAACATATGTTGCAACTCACAGTCTGGGGCATT
>AY734554..1
TTCTATTGTAACACATCAGGACTGTTTAATAGCACTTGGAATAGCAATGACAACGTGCAGGAGTCAAATAGCA
CA---

AAGTCAAACGACACTATAACTCTCCAATGCAGAATAAAACAAATTATAAATATGTGGCAGAGGGTAGGACGAG
CAATGTATGCCCCTCCCATCCCAGGAGTAATAAGGTGTAATTCAAAC---
ATTACAGGACTAATATTAACAAGAGATGGTGGGAATGAGAGCAGT----------------------------
----

ATAAATGAAACCTTCAGACCTGGAGGAGGAGATATGAGAGACAATTGGAGAAGTGAATTATATAAGTATAAAG
TAGTAAAAATTGAACCACTA---GGAGTAGCACCCACCAGGGCAAGGAGAAGAGTGGTG--------------
-------------------------GAGAGAGAAAAAAGAGCA---GTTGGA---ATAGGAGCT---------
GTCTTCATTGGGTTCTTAGGAGCA---GCAGGAAGCACTATG---GGCGCGGCGTCA---
ATAACGCTGACGGTACAG---GCCAGACAA---TTACTG---TCTGGCATAGTGCAACAGCAAAGC---
AATTTG---CTGAGG---GCTATAGAGGCTCAACAGCATCTGTTGAAACTCACGGTCTGGGGCATT
>AY734555..1
TTCTATTGTAACACATCAGGCCTGTTTAATAGCACTTGGAATACTAACACTTTGAATAGCAGTAACAGCATGG
AG---

GAGTCAAATGACAATATAACTCTCCAATGTAGAATAAAGCAAATTATAAATATGTGGCAGAGAGCAGGACAAG
CAATGTATGCCCCTCCCATCCCAGGAATAATAAGGTGTGAATCAAAC---
ATTACAGGACTAATATTAATGAGAGATGGTGGGAATAATAATACA----------------------------
----

AATGAGACATTCAGACCTGGAGGGGGAGATATGAGGGACAATTGGAGAAGTGAATTATACAAATATAAAGTAG
TAGAAATTAAGCCATTA---GGAATAGCACCCAACAGGGCAAAAAGGAGAGTGGTG-----------------
----------------------GAGAGAGAAAAAAGAGCA---GTTGGA---CTGGGAGCT---------
TTGTTCCTTGGGTTCTTAGGAGCA---GCAGGAAGCACTATG---GGCGCGGCGTCA---
ATAACGCTGACGGTACAG---GCCAGAAAT---TTACTG---TCTGGCATAGTGCAGCAGCAAAGC---
AATTTG---CTGAGG---GCTATAGAGGCTCAACAGCAGATGTTGAGACTCACGGTCTGGGGCATT
>KX907342..2
TTCTACTGCAATACATCAGGTCTGTTTAATTATACAGAAGGTACTTTAAATAGTACAATCAAT----------
----

AGTACAGAACCAATCACACTCCCATGCAGGATAAAACAAATTATAAACATGTGGCAGGGGGTAGGACGAGCAA
TGTATGCCCCGCCCATTGCAGGAACCATAACATGTAGATCAAAC---
ATTACAGGACTACTATTGACACGTGATGGAGGATGTCATAATAAT----------------------------
----

TGTACAGAGAAATTCAGACCTGGAGGAGGAGATATGAGGGACAATTGGAGAAGTGAATTATATAAATATAAAG
TGGTAGAAATCAAACCATTA---GGAATAGCACCCACTAGGGCAAAAAGGAGAGTGGTG--------------
-------------------------GAGAGAGAAAAAAGAGCA---GTGGGA---ATAGGAGCT---------
GTGTTCCTTGGGTTCTTGGGAGCA---GCAGGAAGCACTATG---GGCGCAGCATCA---
ATAACGCTGACGGTACAG---GCCAGACAA---TTACTG---TCTGGTATAGTGCAACAGCAAAGC---
AATTTG---CTGAGG---GCTATAGAGGCGCAACAGCATATGTTGCAACTCACAGTCTGGGGCATT
>KX907377..1
TTCTATTGCAACACCACAGAACTGTTTAATAGAACATACTGGTCAAATGAGACAGGAAGCAATTCAAACAATT
CAAGCAGT---

TCAAATGACACCATCACACTCCCATGCAGAATAAAACAAATTATAAACATGTGGCAGAGAGTAGGACAAGCAA
TGTACGCCCCTCCCATTGCAGGAAACATAACATGTGAATCAAAC---
ATTACAGGGCTACTGTTAACAAGAGATGGTGAGAAT-------------------------------------
----

AACGGTACAGAAACTTTTAGACCTGGAGGAGGAGATATGAGGGACAATTGGAGAAGTGAATTATATAAGTATA
AAGTAGTAAAAATTGAACCACTA---GGAGTGGCACCCACCAGGGCAAAAAGGAGAGTGGTG-----------

----------------------------GAGAGAGAAAAAAGAGCA---GTTGGA---ATAGGAGCT------
---GTCCTCTTTGGGTTCTTAGGAACA---GCAGGAAGCACTATG---GGCGCGGCGTCA---
ATGACGCTGACGGTACAG---GCCAGACAA---TTATTG---TCTGGCATAGTGCAACAGCAAAGC---
AATTTG---CTGAGG---GCTATAGAGGCTCAACAACATATGTTGAAACTCACGGTCTGGGGCATT
>KX907379..2
TTCTATTGTAATACATCAAGCCTGTTTAATAGCACTTGGGACAACAGTAGCATCACACAGAATTACACGGAG-
----

TCAAATGAGACTATAACTCTCCAGTGCAGAATAAAACAAATTGTAAACGCGTGGCAGAGAGTAGAACAAGCAA
TATATGCCCCTCCCATCCAAGGAGTAATAAGGTGTGACTCAAAC---
ATTACAGGACTACTATTAACAAGAGATGGTGGTGGTAATAACAGT----------------------------
----

AGTGAAAACGAAACCTTTAGACCTGGAGGAGGAGATATGAGGGACAATTGGAGAAGTGAATTATATAAGTATA
AAGTAGTAAAACTTGAACCACTA---GGAGTAGCACCTACCAAGGCAAAAAGAAGAGTGGTG-----------
----------------------------GAAAGAGAAAAAAGAGCA---GTCGGA---ATAGGAGCT------
---GTTTTCCTTGGGTTCTTAGGAGCA---GCAGGAAGCACTATG---GGCGCGGCGTCA---
ATGACGCTGACGGTACAG---GCCAGACAA---TTATTG---ACTGGCATAGTGCAACAGCAAAGC---
AATTTG---CTGAGG---GCTATAGAGGCTCAACAACACCTGTTGAAACTCACGGTCTGGGGCATT
>KX907409..2
TTCTATTGTAACACATCATCCCTGTTTAATAGTACTTGGCAGGTCAGCGGGCAGGGGTTAAATAGCACGGAG-
----

TTAGGTGACACTATAACTCTCCAATGCAGAATAAAGCAAATTATAAATATGTGGCAGAGAGCAGGACAAGCAA
TATATGCCCCTCCCATCCCAGGAGTAATAAGGTGCGAATCAAAC---
ATAACAGGACTAATATTAACAAGTGATTATGGGAATAGTAGCAGT----------------------------
----

GACAATGAAACCTTCAGACCTACAGGAGGAGATATGAGGGACAATTGGAGAAGTGAATTATATAAGTATAAAG
TAGTGAAAATTGAACCAATA---GGAGTAGCACCCACCAAAGCAAGAAGAAGAGTGGTG--------------
-------------------------CAGAGAGAAAAAAGAGCA---GTTGGA---CTGGGAGCT---------
GTCTTCATTGGGTTCTTAGGAGCA---GCAGGAAGCACTATG---GGCGCGGCGTCA---
GTGACGCTGACGGTACAG---GCCAGACAA---TTATTA---TCTGGCATAGTGCAACAGCAAAGC---
AATTTG---CTGAGG---GCTATAGAGGCTCAACAGCATATGTTGAAACTCACAGTCTGGGGCATT
>KX907362..1
TTCTATTGTGACACATCAGACCTGTTTAATAGCACTTGGCCTAGCAATACCAATAACAGCATACAGGAGTCAA
ATAGCACA---

GAGCCAAACGCCACTATAACACTCCCATGCAGAATAAAACAAATTATAAATATGTGGCAGAGAGCAGGACAAG
CAATGTATGCCCCTCCCATCCCAGGAATGATAAAGTGTAACTCAAAC---
ATTACAGGACTCATATTAACAAGAGATGGTGGAAATGATAGCAGT----------------------------
----

ACAGATGAAATCTTCAGACCTGGAGGAGGAGACATGAGGGACAATTGGAGAAGTGAATTATATAAGTATAAAG
TAGTACAAATTGAACCACTA---GGAGTAGCACCTACCAGGGCAAGGAGAAGAGTGGTG--------------
-------------------------CAGAGAGAAAAAAGAGCA---GTTGGA---ATGGGAGCT---------
GTGCTCATTGGGTTCTTAGGAGCA---GCAGGAAGCACTATG---GGCGCAGCGTCA---
GTAACGCTGACGGTACAG---GCCAGACAA---TTATTG---TCTGGCATAGTGCAACAGCAAAGC---
AATTTG---CTGAGG---GCTATAGAGGCTCAACAGCATCTGTTGAAACTCACAGTCTGGGGCATT
>KX907381..1
TTCTATTGTAATACATCAGGTCTGTTTAATAGCACTTGGTCTAACAGCACGCAGGCGTCAAATGACACAGAG-
----

TCAAATGACACTATAACTCTCCCATGCAGAATAAAGCAAATTATAAATATGTGGCAGAGAGTAGGACAAGCAA
TGTATGCCCCTCCCATCCAAGGAATAATAAACTGTATATCAAAT---
ATTACAGGACTAATATTAACAAGAGATGGTGGGAATAACAATGGA----------------------------
----

ACTGAAACCTTCAGACCTGGAGGAGGAGACATGAGGGACAATTGGAGAAGTGAATTATATAAGTATAAAGTAG
TAAAAATTGAACCACTA---GGAGTAGCACCCACCAGGGCAAAGAGAAGAGTGGTG-----------------

----------------------GAGAGAGAAAAAAGAGCA---GTAGGA---ATAGGAGCT---------
GTCCTCTTTGGGTTCTTAGGAGCA---GCAGGAAGCACTATG---GGCGCGGCGTCA---
ATAACGCTGACGGTACAG---GCCAGACAA---TTATTG---TCTGGTATAGTGCAACAGCAAAGC---
AATTTG---CTGAGG---GCCATAGAGGCTCAACAACAACTGTTGAAACTCACAGTCTGGGGCATC
>KX907386..1
TTCTATTGTAATACATCAAACCTGTTTAATGACACTTGGCCTATGTCAAATAACACGGAG-------------
----

TCAGATGACAACATAACCCTCCAATGCAGGATAAAGCAAATTATAAATATGTGGCAGAGAGCAGGACAAGCAA
TGTATGCCCCTCCCATTGCAGGAAACATAACATGTATATCAAAT---
ATTACAGGACTACTGTTAACAAGAGATGGTGGGGGGAATAGCAGT----------------------------
----

ACTGGTAATGAAACCTTCAGACCTGGAGGAGGAAATATGAAGGACAATTGGAGAAGTGAATTATATAAGTATA
AAGTAGTACAACTTGAACCCCTA---GGAGTAGCACCTACCAGGGCAAGAAGAAGAGTGGTG-----------
----------------------------CAGAGAGAAAAAAGAGCAGTAGTTGGA---CTGGGAGCT------
---GTTTTCATTGGGTTCTTAGGAGCA---GCAGGAAGCACTATG---GGCGCGGCGTCA---
ATAACGCTGACGGTACAG---GCCAGACAA---TTATTG---TCTGGTATAGTGCAACAGCAAAGC---
AATTTG---CTGAGG---GCTATAGAGGCTCAACAGCATATGTTGAAACTCACAGTCTGGGGCATT
>KX907429..1
TTCTATTGCAACACATCAGGTTTGTTTAATAGCACTTGGGATATCAATGACACTGCCAGCGTGCAGGTGTCAA
ATGGCACG---

AAGTCAAACAGCACTATAACTCTCCAATGCAGAATAAAGCAAATTATAAATATGTGGCAAAGAGTAGGGCAAG
CAATATATGCCCCTCCCATCCAAGGAATGATAAGGTGTGTATCAAAT---
ATTACAGGACTAATATTAACAAGAGATGGTGGGAATGATAGCAGG----------------------------
----

ACAAATGAAACCTTCAGACCTGGAGGAGGAGATATGAGGGACAATTGGAGAAGTGAATTATATAAGTATAAGG
TAGTAAAACTTGAGCCATTA---GGAATAGCACCCAACAGGGCAAAGAGAAGAGTGGTG--------------
-------------------------GAGAGAGAAAAAAGAGCA---GTTGGA---ATAGGAGCT---------
GTCCTCTTTGGGTTCTTGGGAGCA---GCAGGAAGCACTATG---GGCGTGGCGTCA---
ATGACGCTGACGGTACAG---GCCAGACAA---TTATTG---TCTGGCATAGTGCAACAGCAAGGC---
AATTTG---CTGAGG---GCTATAGAGGCTCAACAGCATCTGTTGAAACTCACAGTCTGGGGCATT
>HQ615972..2
TTTTATTGTAACACATCAGGCCTGTTTAATAGCACTTGGAGTTTTAATAGCACTGGAAATAATTACAGCATGA
ATAGCACCAAT--
-------------GACACT------
ATAACTAACGACACTATAATTCTCCAATGTAGAATAAAGCAAATTATAAATATGTGGCAGAGAGTAGGACAGG
CAATGTATGCCCCTCCCATTCGAGGAGTAATAAGGTGTGAATCAAAC---
ATTACAGGACTAATATTAACAAGAGATGGAGGTAATAATGGGTCA----------------------------
----

AATGAAATCTTCAGACCTGGAGGAGGAGATATGAGGGACAATTGGAGAAGTGAATTATATAAATATAAAGTAG
TAAAAATTGAGCCACTA---GGAGTAGCACCCACCAAGGCAAAAAGAAGAGTGGTG-----------------
----------------------GAGAGAGCAAAAAGAACA---GTAGGA---CTGGGAGCT---------
GTCTTCCTTGGGTTCTTAGGAGCA---GCAGGAAGCACTATG---GGCGCAGCGTCA---
ATAACGCTGACGGCACAG---GCCAGACAA---TTATTG---TCTGGCATAGTGCAACAGCAAAGC---
AATTTG---CTGAGG---GCTATAGAGGCTCAACAGTATCTGTTGAAGCTCACAGTCTGGGGCATT
>HM204625..1
TTCTATTGTGACACATCAGGCCTGTTTGATAGCCAGGCAAATAGA----------------------------
----

ACAAATGACACTATAACTCTCCAATGCAGAATAAAGCAAATTATAAATATGTGGCAGAGAGTAGGACAAGCAA
TGTATGCCCCTCCCATTAAAGGAGTAATAAATTGTACATCACAT---
ATTACAGGATTAATATTAACAAGAGATGGTGGTAATGATACAACT----------------------------
----

GAAACCTTCAGACCTGGAGGAGGAGATATGAGGGACAATTGGAGAAGTGAACTATATAAGTATAAAGTAGTAA
AAATTGAGCCACTA---GGAGTAGCACCCACCAGGGCAAGGAGAAGAGTGGTG--------------------

-------------------GGGAGAGAAAAAAGAACA---GTTGGA---CTAGGAGCT---------
GTCCTCTTTGGGTTCTTAGGAGCA---GCAGGAAGCACTATG---GGCGCGGCGTCA---
ATAACGCTGACGGTACAG---GCCAGACAA---TTATTG---TCTGGCATAGTGCAACAGCAAAGC---
AATTTG---CTGAAG---GCTATAGAGGCTCAACAGCATATGTTGAGACTCACGGTCTGGGGCATT
>KY658699..1
TTCTATTGTAACACATCAGGCCTGTTTGATAACAACACGCTGACAAATACA----------------------
----

ACGCAGGAAAATATAACTCTCCCATGCAGAATAAAGCAAATTATAAATATGTGGCAGAGAGCAGGACAAGCAA
TATATGCCCCTCCCATTAAAGGAGTAATAAAGTGTGAATCAAAT---
ATTACAGGATTAATATTAACAAGAGATGGTGGTAATACTAATGATAGCGAT----------------------
----

GAAATTGAAACCTTCAGACCTGGAGGAGGAGATATGAGGGACAATTGGAGAAGTGAACTATATAAGTATAAAG
TAGTAAAAATTGAGCCACTA---GGAGTAGCACCCACCAAGGCAAGGAGAAGAGTGGTG--------------
-------------------------GGGAGAGAAAAAAGAGCA---GTTGGA---ATAGGAGCT---------
GTCCTCTTTGGGTTCTTAGGAGCA---GCAGGAAGCACTATG---GGCGCGGCGTCA---
ATAACGCTGACGGTACAG---GCCAGACAA---TTATTG---TCTGGCATAGTGCAGCAGCAAAGC---
AATTTG---CTGCAG---GCTATAGAGGCTCAACAGCATATGTTGAGACTCACGGTCTGGGGCATT
>AF361876..1
TTCTATTGTAATACAACAGACCTGTTTAATAGCACCAGGTACCGGGTTAATAGCACTTGGAATATGCAGGGGT
CAAATAGT---
-------------------ACG---
GAGTCCAATGACACTATAACTCTCCAATGCAGAATAAAGCAAATCATAAATATGTGGCAGAGAGCAGGACAAG
CAATGTATGCCCCTCCCATTGAAGGAAACATAACATGTATGTCAAAC---
ATTACAGGACTACTGTTAACAAGAGATGGTGGGAATAATAACAGT----------------------------
----

ACAAATGAAACCTTCAGACCTGAAGGAGGAGATATGAGGGACAATTGGAGAAGTGAATTATATAAGTATAAAG
TAGTAAAACTTGAACCACTA---GGAGTAGCACCTACCAGGGCAAAAAGAAGAGTGGTG--------------
-------------------------GAAAGAGAAAAAAGAGCA---GTTGGA---CTGGGAGCT---------
GTCTTCCTTGGGTTCTTAGGAGCA---GCAGGAAGCACTATG---GGCGCGGCGTCA---
ATAACGCTGACGGTACAG---GCCAGAAAT---TTATTG---TCTGGTATAGTGCAACAGCAAAAC---
AATTTG---CTGAGG---GCTATAGAGGCTCAACAGCATATGTTGAAACTCACGGTCTGGGGCATT
>AF361879..1
TTCTATTGTAATACTTCAGGCCTGTTTAATAGCACTTGGAACATTACCAGCACACAGAAGTCAAATAGCACGG
AG---

TCAAATAATGACACTATAACTCTCCAATGCAGAATAAAGCAAATTATAAATATGTGGCAGAGAGTAGGACAAG
CAATGTATGCCCCTCCCATCCCAGGAGCAATAGGGTGTAAATCAAAC---
ATTACAGGACTATTGTTAACAAGAGATGGTGGGGAGAATAACAGT----------------------------
----

GAAAATGAAACCTTCAGACCTGGAGGAGGAAATATGAGGGACAATTGGAGAAGTGAATTATATAAGTATAAAG
TAGTAAAACTTGAACCACTA---GGAGTAGCACCTACCAAGGCAAAAAGAAGAGTGGTG--------------
-------------------------GAGAGAGAAAAAAGAGCA---GTTGGA---CTAGGAGCT---------
GTCTTCATTGGGTTCTTAGGAGCA---GCAGGAAGCACTATG---GGCGCGGCGTCA---
ATAACGCTGACGGTACAG---GCCAGACAA---TTATTG---TCTGGCATAGTGCAACAGCAAAGC---
AATTTG---CTGAAG---GCTATAGAGGCTCAACAACAATTGTTGAGACTCACGGTCTGGGGCATT
>HM215279..1
TTCTATTGCAATACATCAGACCTATTTAATGGCACGTTTAATGAGACATCAGGCCAGTTTAAT----------
----

AGCACATTTAATAGTACACTCCAGTGCAGAATAAAACAAATTATAAACATGTGGCAGGAAGTCGGACAAGCAA
TGTATGCCCCTCCCATTGCAGGAAGCATAACATGTATATCGAAT---
ATCACAGGACTAATATTAACAAGAGATGGTGGGAATACTAACAGT----------------------------
----

ACAAAGGAAACCTTCAGACCTGGAGGAGGAAATATGAGGGACAATTGGAGAAGTGAATTATATAAGTATAAAG
TAGTAAAAATTGAACCACTA---GGAGTAGCACCCACCAAGGCAAGGAGAAGAGTGGTG--------------

-------------------------GAGAGAGAAAAAAGAGCA---GTAGGA---ATAGGAGCT---------
GTCTTCATTGGGTTCTTAGGCGCA---GCAGGAAGCACTATG---GGCGCGGCGTCA---
ATAACGCTGACGGTACAG---GCCAGACAA---TTATTG---TCTGGTATAGTGCAACAGCAAAGC---
AATTTG---CTGAGG---GCTATAGAGGCTCAACAGCATCTGTTGAAACTCACGGTCTGGGGCATT
>HM215315..1
TTCTATTGTGATACATCAGCCCTGTTTAATAGCACTTGGAATAACAGCACGAGGAACAGCATGGAGTGG----
----

TTAAATGACACTATAACTCTCCAATGCAAAATAAAGCAAATTATAAATATGTGGCAGAGAGCAGGACAAGCTA
TGTACGCCCCTCCCATCCAAGGAACAATAAGTTGTAACTCAAAC---
ATTACAGGACTACTGTTAACAAGAGATGGGGGAAGGACACCACA-----------------------------
----

AATGAAACCTTTAGACCTGGAGGAGGAGACATGAGGAACAATTGGAGAAGTGAATTATATAAGTATAAAGTAG
TAAAACTTGAACCACTA---GGAGTAGCACCTACCAAGGCAAAAAGAAGAGTGGTG-----------------
----------------------GAGAGAGAAAAAAGAGCA---GTTGGA---ATAGGAGCT---------
GTCCTCATTGGGTTCTTAGGAGCA---GCAGGAAGCACTATG---GGCGCGGCGTCA---
ATAACGCTGACGGTACAG---GCCAGACAA---TTATTG---TCTGGTATAGTGCAACAGCAAAGC---
AATTTG---CTGAGG---GCTATAGAGGCTCAACAGCAACTGTTGAAACTCACGGTCTGGGGCATT
>HM215322..2
TTCTATTGTGATACATCAGACCTGTTTAATAGCACTTGGAATTATACTAGTATCACGTCAAATAGCACGGAG-
----

TCAAATGACACTATAACTCTCCAATGCAGAATAAAGCAAATTATAAATATGTGGCAGAGAGAAGGACAAGCAA
TGTATGCCCCTCCCATTGAAGGAAACATAACATGTATGTCAAAC---
ATTACAGGACTACTGTTAGTAAGAGATGGTGGGATTAATAACAGG----------------------------
----

ACAGATGAAATCTTCAGACCTGGAGGAGGAAATATGAAAGACAATTGGAGAAGTGAATTATATAAGTATAAAG
TAGTAAAACTTGAACCACTA---GGAGTAGCACCTACCAGGGCAAAAAGAAGAGTGGTG--------------
-------------------------GAGAGAGAAAAAAGAGCA---GTTGGA---CTGGGAGCT---------
GTCTTCATTGGGTTCTTAGGAGCA---GCAGGAAGCACTATG---GGCGCGGCGTCA---
ATAACGCTGACGGTACAG---GCCAGAAAT---TTATTG---TCTGGTATAGTGCAACAGCAAAAC---
AATTTG---CTGAGG---GCTATAGAGGCTCAACAGCATATGTTGAAACTCACAGTCTGGGGCATT
>AF484491..1
TTCTATTGTAATACATCAGGTTTGTTTAATAGCTCCTGGGCAAATAACACTGACAATAGCAATAACACTGTCA
AT---

AGCACGGACATTATAACTCTCCCATGCAGAATAAAGCAAATTGTAAATATGTGGCAAAGAACAGGACAAGCAA
TGTATGCCCCTCCCATCAAAGGAGTGATAAGGTGTGAATCAAAT---
ATTACAGGACTACTATTAACAAGAGATGGTGGGGCTACTAGCATT----------------------------
----

ATAAATGAAACCTTCAGACCTGGAGGAGGAAATATGAGGGACAATTGGAGAAGTGAATTATATAAGTATAAAG
TAGTACAAATTGAACCACTA---GGAGTAGCACCCACCAGGGCAAGGAGAAGAGTGGTG--------------
-------------------------GAGAGGGGAAAAAGAGCA---ATTGGA---ATAGGAGCT---------
GTATTCCTTGGGTTCTTAGGAACA---GCAGGAAGCACTATG---GGCGCGGCGTCA---
ATAACGCTGACGGTACAG---GCCAGACAA---TTACTG---TCTGGTATAGTGCAACAGCAAAGC---
AATTTG---CTGAGG---GCTATAGAGGCTCAACAGCATATGTTGAAACTCACGGTCTGGGGCATT
>AF484501..3
TTTTATTGTAACACATCAGGCCTGTTTAATCACACTTACACTTGGTATAATGGCAGCTTGCAGGAGTCAAATA
GC---

ACGGGGTCAGACAACATAACTCTCCAATGCAGAATAAAGCAAATTATAAATATGTGGCAGAGAGTGGGACAAG
CTATGTATGCCCCTCCCATTCGAGGAGTAATACAGTGTGAATCAAAC---
ATTACAGGACTAATATTAACAAGAGATGGTGGAAATAATAATGAT----------------------------
----

AATACAACTGAAATCTTCAGACCTGGAGGAGGAGATATGAGGGACAATTGGAGAAGTGAATTATATAAGTATA
AAGTAGTAACAATTGAACCACTA---GGAGTAGCACCCACCCAGGCAAGGAGAAGAGTGGTG-----------

----------------------------GAGAGAGAAAAAAGAGCA---GTGGGA---ATAGGAGCT-------
---GTCCTCCTTGGGTTCTTAGGAACA---GCAGGAAGCACTATG---GGCGCGGCGTCC---
ATGACGCTGACGGTACAG---GCCAGACAA---TTATTG---TCTGGCATAGTGCAACAGCAAAGC---
AATTTG---CTGAAG---GCTATAGAGGCTCAACAGCAACTGTTGAGACTCACGGTCTGGGGCATT
>FJ647147..1
TTCTATTGCAATACATCAGGCCTGTTTAACAGCACTTGGGAGTTTAACAACCCCTTTTAATGAAACTGAGTGGC
CA--

CAAAATAAAACTATAATTCTCCAATGCAGAATAAAGCAAATTGTAAATATGTGGCAGAGAGTAGGACAGGCGA
TGTATGCCCCTCCCATCAAAGGAGTAATAAGGTGTAATTCAACC---
ATTACAGGACTATTCTTAACAAGAGATGGTGGAAATACTAGCAGT-----------------------------
----

ACAAATGAGACCTTCAGGCCTGGAGGAGGAGATATGAGGGACAATTGGAGAAGTGAATTGTATAAATATAAAG
TAATAAAAATTGAACCAATA---GGAGTAGCACCCACCAGGGCAAAAAGAAGAGTGGTG---------------
-------------------------GAAAGAGAAAAAAGAGCA---GTTGGA---CTGGGAGCT----------
GTTTTCCTTGGGTTCTTAGGAGCA---GCAGGAAGCACTATG---GGCGCAGCGTCA---
ATAACGCTGACGGTACAG---GCCAGACAA---TTATTG---TCTGGCATAGTGCAACAGCAAAGC---
AATTTG---CTGAGG---GCTATAGAGGCTCAACAGCATCTGTTGAAACTCACAGTCTGGGGCATT
>KT183118..2
TTCTACTGCAATACATCAGGCCTGTTTAATGACACATTTATGCCCAATAATACGGCTAATAGTACTAAGAATG
AAACA---

CAGGAAAAGAATATAACTATCTCATGCAAAATAAAACAAATTATAAGAATGTGGCAAAGAGTAGGACAAGCAA
TGTATGCCCCTCCCATTGAAGGATTAATAAAATGTGAATCAAAC---
ATTACAGGAATACTATTGACAAGAGATGGTGGGAACAATACTAAC-----------------------------
----

AGTACAGAGGTCTTCAGACCTGGAGGGGGAAATATGAAGGACAAGTGGAGAAGTGAATTATATAATTATAAGG
TAGTAAAAATTGAACCACTA---GGAGTAGCACCCACCGGGGCAAGGAGAAGAGTGGTG---------------
-------------------------GAGAGAGAGAAAAGAGCAGTAGTTGGA---CTGGGAGCT----------
GTCTTCCTTGGGTTCTTAGGAGCA---GCAGGAAGCACTATG---GGCGCGGCGTCA---
TTAACGCTGACGGTACAG---GCCAGGCAA---TTATTG---GCTGGCATAGTGCAACAGCAAAGC---
AATTTG---CTGCAG---GCTATAGAGGCTCAACAACATATGTTGAAACTCACTGTCTGGGGCATT
>GU201611..1
TTCTATTGTAACACAACAGACTTGTTTAATAGCAGTTGGGCTAACAATGGCAGCAGGCTGGGGCCAAATGGTA
CA--

GAGTCAGACATTATAACTATCCAATGCAGAATAAAGCAAATTATAAATATGTGGCAAAGAGTAGGACAAGCAA
TATATGCCCCTCCCAGAAAAGGAGTAATAAGGTGTGACTCAAAC---
ATTACAGGACTGATATTAACAAGAGATGGTGGTAGCAATAACACA-----------------------------
----

AATAATGAAACCTTCAGACCTGGAGGAGGAGACATGAGGGACAATTGGAGAAGTGAATTATATAAGTATAAAG
TAGTAAAACTTGAACCACTA---GGGGTAGCACCTACCCCGGCAAAAAGAAGAGTGGTG---------------
-------------------------GAGAGAGAGAAAAGAGCA---GTTGGA---CTAGGAGCT----------
GTTTTCATTGGGTTCTTAGGAGCA---GCAGGAAGCACTATG---GGCGCGGCGTCA---
GTAACGCTGACGGTACAG---GCTAGACAA---TTATTG---TCTGGCATAGTGCAGCAGCAAAGT---
AATTTG---CTGAGG---GCTATAGAGGCTCAACAACATATGTTGAAACTCACGGTCTGGGGCATT
>GU329121..2
TTCTATTGCAACACATCAGGCCTGTTTAATAGCAGTTGGAAAATTGAGAATGGCAATGCCACGCTAAATAGCA
CG--

TGGCCAAATGGCACCATAACTCTTCAATGCAGAATAAAGCAAATTATAAATATGTGGCAAAGGACAGGACAAG
CAATATATGCCCCTCCCATCCAAGGAGTAATAAGGTGTGACTCAAAC---
ATTACAGGACTACTATTAACAAGAGATGGTGGGGATAATAATGGT-----------------------------
----

ACAGAAACCTTCAGACCTGGAGGAGGAGATATGAGGGACAATTGGAGAAGTGAGTTATATAAATATAAAGTAG
TAAAAATTGAGCCACTA---GGAGTAGCACCCACCAGGGCAAAGAGAAGAGTGGTG------------------

---------------------GAGAGAGAAAAAAGAGCA---GTAGGA---ATAGGAGCT---------
GTGTTCCTTGGGTTCTTAGGAGCA---GCAGGAAGCACTATG---GGCGCGGCGTCA---
ATAACGCTGACGGTACAG---GCCAGACAA---TTATTG---TCTGGCATAGTGCAACAGCAAAGC---
AATTTG---CTGAGG---GCTATAGAGGCTCAACAGCATATGTTGAAACTCACGGTCTGGGGCATT
>JF680922..3
TTCTATTGCAACACATCAGGACTGTTTAATAGCACTTGGTTTCAGAATGGCACTAACATTACACAGGGG----
----

TCAGATGGCAATATAACTCTTCATTGCAGAATAAAGCAAATCATAAATATGTGGCAGAGAGCAGGACAAGCAA
TATATGCCCCTCCCATCAAGGGAGTAATAAAGTGTGTATCAAAC---
ATTACAGGACTAATATTAACAAGAGATGGTGGTACCAATGTGACG---------------------------
----

AATGGAACTGAAATCTTCAGGCCTGGAGGGGGAGATATGAAGGATAATTGGAGAAGTGAATTATATAAATATA
AAGTGGTAGAAATTAAACCATTA---GGAGTAGCACCCACCAAGGCAAAAAGGAGAGTGGTG-----------
---------------------------GGAAGAGAAAAAAGAGCA---GTAGGA---ATAGGAGCT------
---GTGTTCCTTGGGTTCTTGGGAGCA---GCAGGAAGCACTATG---GGCGCAGCGTCA---
ATAACGCTGACGGTACAG---GTCAGACAA---TTATTG---TCTGGCATAGTGCAACAGCAAAAC---
AATTTG---CTGAGG---GCTATAGAGGCTCAACAGCATCTGTTGAGACTCACAGTCTGGGGCATT
>AF457056..3
TTCTATTGTGATACATCAGGCCTGTTTAATTACACGTTTTATCCCAATGGCAGCTTGCAGGGGGAATCAAATG
AC--

ACGCAGGAGAAGATAACTCTCCCATGCAGAATAAAGCAAATTATAAATATGTGGCAGAGAACAGGGCAAGCAA
TATATGCCCCTCCCATCCAAGGAGTAATAAGATGTGTATCAAAC---
ATTACAGGAATACTATTAACAAGAGATGGTGGAGATACTGGGGAC---------------------------
----

AACAATACTGAAACCTTCAGACCTGGAGGAGGAGATATGAGGGACAATTGGAGAAGTGAATTATATAAGTATA
AAGTAGTACAAATTGAACCACTA---GGAGTAGCACCCACCAGGGCAAGGAGAAGAGTGGTG-----------
---------------------------GGAAGAGAAAAAAGAGCA---GTTGGA---ATAGGAGCT------
---GTTTTCCTTGGGTTCTTAGGAGCA---GCAGGAAGCACTATG---GGCGCGGCGTCA---
ATAACGCTGACGGTACAG---GCCAGACAA---TTGTTG---TCTGGTATAGTGCAACAGCAAAGC---
AATTTG---CTGAGG---GCTATAGAGGCTCAACAACATCTGTTGAAACTCACGGTCTGGGGCATT
>KT022400..2
TTCTATTGTAACACAGCAGGCCTGTTTAATAGCACTTGGAATAGTAGCACTTATAGTAACAATGCCAGCGGGC
AGGAG---

TCAAATGACACTATAACTCTCCAATGCAGAATAAAGCAAATTATAAATATGTGGCAGAGAGCAGGACAAGCAA
TATATGCCCCTCCCATCGAAGGAGTAATAAAGTGTGTATCAAAC---
ATTACAGGACTAATATTAACAAGAGATGGTGGGAATGCTAACAGT---------------------------
----

ACAAATGAAACCTTCAGACCTGGAGGAGGAGATATGAGGGACAATTGGAGAAGTGAATTATATAAGTATAAAG
TAGTAGAACTTGAACCACTA---GGAGTAGCACCCACCAAGGCAAAGAGAAGAGTGGTG-------------
------------------------GAGAGAGAAAAAAGAGCA---GTTGGA---CTGGGAGCT---------
GTTCTCATTGGGTTCTTAGGAGCA---GCAGGAAGCACTATG---GGCGCAGCGTCA---
GTGACGCTGACGGTACAG---GCCAGACAA---CTATTG---TCTGGCATAGTGCAACAGCAAAGC---
AATTTG---CTGATG---GCTATAGAGGCTCAACAGCAACTGTTGAAACTCACAGTCTGGGGCATT
>KT022401..1
TTCTATTGCAACACAAATGAATTGTTTAATAGCACTTGGTTTTTGAACACTACTAATACCAGCACGTGGGAGT
CAAATGGG--

ACGAGGTCAGAAAATATAACTCTCAAATGCAGAATAAAGCAAATTATAAATATGTGGCAGAGAGCAGGACAAG
CAATGTATGCCCCTCCCATCCGAGGAGTAATAAACTGTACATCAAAC---
ATTACAGGACTAATATTAACGAGAGATGGTGGGGTTAATAACAAT---------------------------
----

ACAAATGAAATCTTCAGACCTGGAGGAGGAGATATGAGGGACAATTGGAAAAGTGAATTATATAAATATAAGG
TAGTAAAAATTGAACCACTA---GGAGTAGCACCCACCAAGGCAAAGAGAAGAGTGGTG-------------

-------------------------GAGAGAGAAAAAAGAGCA---GTTGGA---ATAGGAGCT---------
GTATTCATTGGGTTCTTAGGAGCA---GCAGGAAGCACTATG---GGCGCGGCGTCA---
ATAACGCTGACGGTACAG---GCCAGACAA---TTATTG---TCTGGCATAGTGCAACAGCAAAGC---
AATTTG---CTGAAG---GCTATAGAGGCTCAACAGCATCTGTTGAGACTCACGGTCTGGGGCATT
>KT022414..2
TTCTATTGCAATACATCAAGACTGTTTAATAGCATTTGGGAGCATAATAGCACTTGGACCAATACACGGGGA-
----

TCAAATAGCACAATAACTCTCCCATGCAGAATAAAGCAAATTATAAATATGTGGCAGAGAGTAGGACAAGCAA
TGTATGCTCCTCCCATCGCAGGAGCAATACAGTGTGCATCAAAT---
ATTACAGGACTAATATTAACAAGAGATGGTGGAAATCATAACAGG----------------------------
----

ACAAATGAAACCTTCAGACCTGGAGGAGGAGATATGAGGGACAATTGGAGAAGTGAATTATATAAGTATAAAG
TAGTAAAAATTGAACCACTA---GGAGTAGCACCCACCAGGGCAAAGAGAAGAGTGGTG--------------
-------------------------GAGAGAGAAAAAAGAGCA---GTTGGA---CTAGGAGCT---------
GTTTTCATTGGGTTCTTAGGAGCA---GCAGGAAGCACTATG---GGCGCAGCGTCA---
GTGACGCTGACGGTACAA---GCCAGGCAA---TTACTG---TCTGGCATAGTGCAACAGCAAAGC---
AATTTG---CTGAGG---GCTATAGAGGCTCAACAGCACCTGTTGAGACTCACGGTCTGGGGCATT
>KU749430..1
TTCTATTGCGATACAGCAAAACTGTTTAATAGCACGTGGGAGTACAATGGCACTGAATACAACATGACCAAAG
GG---

TCAAATGGAAATCTGACTCTCCCATGCAGAATAAAACAAATTGTACACATGTGGCAGAGAGTAGGACAAGCAA
TGTATGCCCCTCCCATCCCAGGAATAATAAAGTGTGAATCAAAC---
ATCACAGGACTAATATTAACAAGAGATGGTGGGAAGAATACCAGT----------------------------
----

AATGAGACCTTCAGACCTGGAGGAGGAGATATGAGGGATAATTGGAGAAGTGAATTATATAAGTATAAAGTAG
TAAAACTTGAACCACTA---GGAGTAGCACCCACCGCAGCAAGGAGAAGAGTGGTG-----------------
----------------------CAGAGAGAAAAAAGAGCA---GTTGGA---ATGACAGCT---------
GTCTTCCTTGGGTTCTTAGGAGCA---GCAGGAAGCACTATG---GGCGCGGCGTCA---
ATAACGCTGACGGTACAG---GCCAGACAA---CTGTTG---TCTGGCATAGTGCAACAGCAAAGC---
AATTTG---CTGAAG---GCTATAGAGGCTCAACAGCATATGTTGAGACTCACGGTCTGGGGCATT
>KF716470..1
TTCTATTGTAATACATCAGGCCTATTTAATAGTACTTGGCATTGGAATAATACTGATACTAATGGCACCAATG
GT---

ACTAATGATACTATAACTCTCCCATGCAGAATAAAGCAAATTATAAATATGTGGCAGAGGGTAGGACAAGCAA
TATATGCCCCTCCCATCCCAGGGGTAATAAAATGTGTATCAGAC---
ATTACAGGACTCCTATTAACAAGAGATGGTGGGGTCAATAATAGC----------------------------
----

AGCAATAATGAAACCTTCAGACCTGGAGGAGGGGATATGAGGGACAATTGGAGAAGTGAATTATATAAGTATA
AGGTAGTAAAAATTGAACCACTA---GGAGTAGCACCCACCAAGGCAAAGAGAAGAGTGGTG-----------
----------------------------GAGAGAGAAAAAAGAGCA---GTTGGA---CTGGGAGCT------
---GTCTTCATTGGGTTCTTGGGAGCA---GCAGGAAGCACTATG---GGCGCGGCGTCA---
ATAACGCTGACGGTACAG---GCCAGGCAA---TTATTG---TCTGGCATAGTGCAACAGCAAAGC---
AATTTG---CTGAGG---GCTATAGAGGCTCAACAACATCTGTTGAAACTCACAGTCTGGGGCATT
>KU921717..2
TTCTATTGTAACACATCAAGCCTGTTTAATAGCACTTGGAATAGCACTTGGAATGGCAATGACAGCAGCACA-
----

GAAAATGGCACTATAACTTTACAATGCAAAATAAAGCAAATCATAAATATGTGGCAGAGAGTAGGACAAGCAA
TGTATGCCCCCCCCATTCAAGGAGTCATAAATTGTAAATCAAAC---
ATTACAGGATTGATATTAACAAGGGATGGTGGAAACACTACCAGC----------------------------
----

AGTGAGGAAATTTTCAGACCTGGAGGGGGAGATATAAGGGATAATTGGAGAAGTGAATTATATAAGTATAAAG
TAGTAAAAATTGAGCCACTA---GGAGTAGCACCCACCAGGGCAAGGAGAAGAGTGGTG--------------

```
-------------------------AAGAGAGAAAAAAGAGCA---GTTGGA---CTGGGAGCT---------
TTTTTCCTTGGGTTCTTAGGAGCA---GCGGGAAGCACTATG---GGCGCGGCGTCA---
ATAACGCTGACGGTACAG---GCCAGGCAA---TTATTG---TCTGGCATAGTGCAACAGCAAAGC---
AATTTG---CTGAGG---GCTATAGAGGCTCAACAACATCTGTTGAGACTCACGGTCTGGGGCATT
>KU921730..1
TTCTATTGTAATACATCAAAACTGTTTAATAGCACTTGGTATAATTCCACTAATACCACTTGGGAGGATGAC-
-------------------------------------------------------------------------
----------------------------
GGCACACAGAATATAACTCTCCCATGCAGAATAAAGCAAATTATAAATCTGTGGCAGAGAACAGGACGAGCAA
TGTATGCCCCTCCCATCCGAGGAGTAATACAGTGTAGGTCAAAT---
ATTACAGGGCTAATATTAACAAGAGATGGTGGGAATGATAATGATACC-------------------------
-------------------------------------------------------------------------
----------
AGTAACAATGAAACCTTCAGACCTGGAGGTGGAAATATGAGGGACAATTGGAGAAGTGAATTATATAAGTATA
AAGTAGTACAAATTGAACCAATA---GGAATAGCACCCACCAAGGCAAGGAGGATAGTGGTA-----------
----------------------------GGGAGAGAAAAAAGAGCA---GTTGGA---CTAGGAGCT------
---GTCTTCCTTGGGTTCTTAGGAGCA---GCCGGAAGCACTATG---GGCGCAGCGTCA---
ATGACGCTGACGGTACAG---GCCAGACAG---TTATTG---TCTGGCATAGTGCAACAGCAAAGC---
AATTTG---CTGAGG---GCTATAGAGGCTCAACAGCATCTGTTGAAACTCACAGTCTGGGGCATT
>KU921766..3
TTCTACTGCAATACATCAAATCTGTTTAATGGTACATGGATAGTAAATAATACAAATAGTACAAATAATGCAG
ATACA--------------------------------------------------------------------
----------------------------
TCAAATAGTAATATTACACTCCAATGCAGAATAAAACAAATTATAAACAGGTGGCAGGAAGTAGGAAAAGCAA
TCTATGCCCCTCCTATTGCAGGACTAATAAAATGTTCATCAAAT---
ATTACAGGAATATTGTTAACAAGAGATGGTGGTGCAAGTCATAAT----------------------------
-------------------------------------------------------------------------
----------
AATCAGAGTGAGACCCTCAGACCTGGAGGAGGAAATATGAAAGATAATTGGAGAAGTGAATTATACAAATATA
AAGTAGTAAGGCTTGAACCAATA---GGTCTAGCACCCACCAGGGCAAAAAGAAGAGTGGTG-----------
----------------------------GAAAGAGAAAAAAGAGCA---ATAGGA---CTGGGAGCT------
---ATGTTCCTTGGGTTCTTGGGAGCA---GCAGGAAGCACAATG---GGCGCAGCGTCG---
GTGACGCTGACGGTACAG---GCCAGACAG---TTATTG---TCTGGCATAGTGCAACAGCAAAAT---
AATTTG---CTGAGG---GCTATAGAGGCGCAACAGCATATGTTGCAACTCACAGTCTGGGGCATT
>AF075702..1
TTCTATTGTAATACATCAGGCCTGTTTAATAGCACTTGGGTAAATGGCAGCAGGGAATCAAATAGCACA----
-------------------------------------------------------------------------
----------------------------
GATAATGATACTATAACTCTCCCGTGTAGAATAAAGCAAATTATAAATATGTGGCAGAGAGTAGGACAAGCAA
TGTATGCCCTTCCCATCCGAGGAGTAATAAGGTGTGAATCAAAC---
ATTACAGGATTAATATTAACAAGAGATGGTGGGAATAATACCAGT----------------------------
-------------------------------------------------------------------------
-------------
ACAAATGAAACCATCAGACCTGCAGGAGGAGATATGAGGGACAATTGGAGAAGTGAATTATATAAATATAAAG
TAGTAAAAATTGAACCACTA---GGAGTAGCACCCACCAAGGCAAGGAGAAGAGTGGTG--------------
-------------------------GAGAGAGAAAAGAGAGCA---GTTGGA---ATAGGAGCT---------
GTGTTCCTTGGGTTCTTAGGAGCA---GCAGGAAGCGCTATG---GGCGCAGCGGCA---
GCAACGCTGACGGTACAG---GCCAGGCAA---TTATTG---TCTGGCATAGTGCAACAGCAAAGC---
AATTTG---CTGAAG---GCTATAGAGGCTCAACAGCATCTGTTGAAACTCACGGTCTGGGGCATT
>HQ659612..1
TTTTATTGTAATACATCAGACATGTTTAATAGCACTTGGAATTGGAATAGCAGTGCCAACATGATGGAGTCAA
ATAGCACCAGC--------------------------------------------------------------
-GTGCAGGGGCCAAAT---------
ATAACTGAGCAAAATATAACTCTCCAATGCAGAATAAAGCAAATTATAAATATGTGGCAGAGAGCAGGACAAG
CAATATATGCCCCTCCTATCAAAGGAGTAATAAGGTGTGAATCAAAC---
ATTACAGGGCTAATATTAACAAGAGATGGGGGAAAAAGTAACAACACA-------------------------
-------------------------------------------------------------------------
----------
AATAATACTGAAACCTTCAGACCTACAGGAGGAGATATGAGGGACAATTGGAGAAGTGAATTATACAAATATA
AAGTGGTGGAAATTAAACCATTA---GGAGTAGCACCCACTGAGGCAAAAAGGAGAGTGGTG-----------
```

----------------------------AAGAGAGGAAAAAGAGCA---GTAGGA---ATAGGAGCT------
---GTATTCCTTGGGTTCTTGGGAGCA---GCAGGAAGCACTATG---GGCGCAGCGTCA---
ATAACGCTGACGGTACAG---GCCAGACAA---TTATTG---TCTGGCATAGTGCAACAGCAAAGC---
AATTTG---CTGAGG---GCTATAGAGGCTCAACAGCATCTGTTGAAGCTCACAGTCTGGGGCATT
>KX907397..2
TTCTATTGTAATACATCAGGCCTGTTTAATAGCACGTGGAACAATAGCATGTGGAACAACATGCTGACTTGGA
ATGCC--

ACGGGGAAGTACATTATGACTCTCCCATGTAAAATAAAACAAATTATAAATATGTGGCAGGGAGTAGGAAAAG
CAATGTATGCCCCTCCCATTGAAGGACTAATTCAATGCTCATCAAAT---
ATTACTGGACTATTATTAACAAGAGATGGGGGTGTAAATAATGAT----------------------------
----

AGTCAGAGTGAGACCTTCAGACCTGGAGGGGGAGATATGAGAGACAATTGGAGAAGTGAATTATACAAATATA
AAGTAGTAAGAATTGAACCAATA---GGTCTGGCACCTACCAGGGCAAAAAGAAGAGTGGTG-----------
----------------------------GAAAGAGAAAAAAGAGCA---ATAGGA---CTGGGAGCT------
---ATGTTCCTTGGGTTCTTGGGAGCA---GCAGGAAGCACGATG---GGCGCAGCGTCA---
CTGACGCTGACGGTACAA---GCCAGAGAG---TTATTG---TCTGGTATAGTGCAACAGCAAAAT---
AATTTG---CTGAGA---GCTATAGAGGCGCAACAGCATCTGTTGCAACTCACTGTCTGGGGCATT
>KX907350..1
TTCTATTGCAATACATCAAACCTGTTTAACGGCACTTGGAATGGTAGCACTTGGGATAGCCCTTGGAATAGGA
ATAAC--

AGCAATGAAAATATAACACTCCAATGCAGAATAAAACAAATTATAAATATGTGGCAGAGAGCAGGACAAGCAA
TGTATGCCCCTCCCATCCAAGGAGTAATAAGGTGTGAATCAAAC---
ATCACAGGACTACTATTAACACGTGATGGAGGAGAAAATAATAAT----------------------------
----

ACTACAGAGACATTCAGGCCGGTAGGAGGAAATATGAAAGACAACTGGAGAAGTGAATTATATAAGTATAAAG
TAGTAAAAATTGAACCACTA---GGAGTAGCACCCACTAGGGCAAGGAGAAGAGTGGTG--------------
-------------------------GAGAGAGAGAAAAGAGCA---GTTGGA---ATAGGAGCT---------
GTGTTCCTTGGGTTCTTGGGAGCA---GCAGGAAGCACTATG---GGCGCAGCGTCA---
ATAACGCTGACGGTACAG---GCCAGACAA---TTATTG---ACTGGCATAGTGCAACAGCAAAGC---
AATTTG---CTGAGG---GCTATAGAGGCTCAACAGCAAATGTTGAGACTCACAGTCTGGGGCATT
>KX907351..1
TTTTATTGTAACACATCAGGCTTGTTTAATAGCTCTTGGTCTTGGAATGGTACCAATAACACGGAGTCAGGTA
AC---

ATAAATGTCAATATAACTCTCCCATGCAGAATAAAGCAAATTATAAATATGTGGCAGAGAGTAGGACAAGCAA
TGTATGCCCCTCCCATTCAAGGAGTGATACAGTGTGTATCAAAC---
ATTACAGGACTAATATTAACAAGAGATGGTGGGAATAATAGCAGT----------------------------
----

ACAAATGAAATCTTCAGACCTGGAGGAGGAGATATGAGGGACAATTGGAGAAGTGAATTATATAAGTATAAAG
TAGTAAGAATTGAACCATTA---GGAGTAGCACCCACTAAGGCAAAAAGGAGAGTGGTG--------------
-------------------------GAGAGAGAAAAAAGAGCA---GTGGGA---ATAGGAGCT---------
GTGTTCCTTGGGTTCTTGGGAGCA---GCAGGAAGCACTATG---GGCGCAGCGTCA---
ATAACGCTGACGGTACAG---GCCAGACAA---TTATTG---TCTGGTATAGTGCAACAGCAAAGC---
AATTTG---CTGAGG---GCTATAGAAGCGCAACAGTACCTGTTGAAGCTCACGGTCTGGGGCATT
>KX907353..1
TTCTATTGTAACACATCAAAGTTGTTTGACAGCAGTTGGCCAAAAACAAATAGCACGGTGCAGGAGTCAAAT-
----

AGCACAGACAATATAACGCTCCAATGTAGAATAAAGCAAATTATAAATATGTGGCAGAGAGTAGGACAAGCAA
TGTATGCCCCTCCCATTGCAGGAGTAATAAGGTGTGAATCAAAC---
ATTACAGGACTAATACTAACAAGAGATGGTGGAAGTAATACCAAT----------------------------
----

GCAAGTGAAACCTTCAGACCTGGAGGAGGAGATATGAGGGACAATTGGAGAAGTGAATTATATAAGTACAAAG
TAGTAAAAATTGAACCACTA---GGAGTAGCACCCACCAGGGCAAAGAGAAGAGTGGTG--------------

-------------------------GAGAGAGAAAAAAGAGCA---GTTGGA---ATAGGAGCT---------
GTATTCATTGGGTTCTTAGGAGCA---GCAGGAAGCACTATG---GGCGCAGCGTCA---
ATAACGCTGACGGTACAG---GCCAGACAA---TTATTG---GCTGGCATAGTGCAACAGCAAAGT---
AATTTG---CTGAGG---GCTATAGAGGCTCAACAGCATCTGTTGAAACTCACGGTCTGGGGCATT
>KX907419..1
TTTTATTGTGACACATCAAACCTGTTTAATAGCAATTGGACTGTGACTGACAGCGAACCGAAGCTAAATAGCA
CGGAG--

CCAAACGACAATATAACTCTCCAATGCAGAATAAAGCAAATTATAAGGATGTGGCAGAGAGTAGGACAAGCAA
TGTATGCCCCTCCCATCCCAGGAATAATAGAGTGTAAATCAAAC---
ATTACAGGACTAATACTAACAAGAGATGGTGGGCCTCCTAATACA----------------------------
----

ACTGAGACCTTCAGACCTGGAGGAGGAGATATGAGGGACAATTGGAGAAGTGAATTATATAAATATAAGGTAG
TAAAAATTGAACCAATA---GGAGTAGCACCCACCGGGGCACGGAGAAGAGTGGTG-----------------
----------------------GGGAGAGAAAAAAGAGCA---GTAGGA---ATAGGAGCT---------
GTGTTCCTTGGGTTCTTGGGAGCA---GCAGGAAGCACGATG---GGCGCAGCGTCA---
GTGACGCTGACGGTACAG---GCCAGACAG---TTACTG---TCTGGTATAGTGCAACAGCAAAGC---
AATTTG---CTGAGG---GCTATAGAGGCTCAACAGCATCTGTTGAGACTCACAGTCTGGGGCATT
>KX907370..1
TTCTACTGCAATACAACAAATTTGTTTAATGCAACAAAATGGTTTAATAGGAGTATAGAGGAAAGTGCAAATG
CAAATGAC---
-------------------ACA---
AGTGAGAATTCCACACTCACACTCCCATGCAGAATAAAACAAATTATAAACATGTGGCAGGGAGTAGGAAAAG
CAATGTATGCCCCTCCCATTGCAGGGCAAATCAAATGTTCATCAAAT---
ATTACAGGACTATTGTTGACAAGAGATGGT---AATAATAGTCAT----------------------------
----

GAGACCTTCAGACCTGGGGGAGGAGATATGAGAGACAATTGGAGAAGTGAATTATACAAATATAAAGTAGTAA
AAATTGAACCAATA---GGTCTAGCACCCACCAGGGCAAAGAGAAGAGTGGTG--------------------
-------------------GAAAGAGAAAAAAGAGCA---ATAGGA---CTGGGAGCT---------
GTGTTCCTTGGGTTCTTGGGGGCA---GCAGGAAGCACAATG---GGCGCAGCGTCG---
CTAACGCTGACGGTACAG---GCCAGGCAA---GTATTG---TCTGGTATAGTGCAACAGCAAAAC---
AATTTG---CTGAGG---GCTATAGAGGCGCAACAGCATCTGTTGCAACTCACAGTCTGGGGCATT
>HM215280..1
TTCTACTGCAATACATCACAACTGTTTAATACTACATGGAGTAAAAATGGTACATCAAATAGTACAGGGTCA-
----

AATAATAGTACAATCATACTCCCATGCAGAATAAAACAAATTATAAATATGTGGCAGAGAACAGGACAAGCAA
TGTATGCCCCTCCCATCCCAGGAGTAATAAGGTGTGTATCAAAC---
ATTACAGGACTAATTTTAACAAGAGATGGTGGAAATAATAACAAA----------------------------
----

ACAGATGAAACTCTCAGACCTGGAGGTGGAGATATGAGGGACAATTGGAGAAGTGAATTATATAAGTATAAAG
TAGTAAAACTTGAACCACTA---GGAGTAGCACCCACCAGGGCAAAGAGAAGAGTGGTG--------------
-------------------------GGGAGAGAAAAAAGAGCA---GTTGGA---ATAGGAGCT---------
GTTTTCATTGGGTTCTTAGGAGCA---GCAGGAAGCACTATG---GGCGCAGCGTCA---
GTAACGCTGACGGTACAG---GCCAGACAA---TTATTG---TCTGGCATAGTGCAACAGCAAAGC---
AATTTG---CTGAGG---GCTATAGAGGCTCAACAGCATATGTTGAAACTCACGGTCTGGGGCATT
>KF716489..1
TTCTATTGTAATACATCAGGCCTGTTTAATAGTACTTGGTATCCTAATAGTACTTTGAATGATAGTACTGGAA
AG---

TATAATAAGACAATAACACTCCCATGTAGAATAAAGCAAATTGTAAACATGTGGCAAAGAGTAGGACAAGCAA
TATATACCCCTCCCATTGCAGGAATAATAAAGTGTGTATCAAAC---
ATTACAGGACTATTATTAACAAGAGATGGTGGTATGAGTAACACAAAT-------------------------
----

AACACAACTGAAATCTTCAGACCTGGAGGAGGAGATATGAGGGACAATTGGAGAAGTGAATTGTATAAGTATA
AAGTAGTAGAAATTAAACCACTA---GGAATAGCCCCTAGCAAGGCAAAGAGAAGAGTGGTG-----------

----------------------------GAGAGAGAAAAAAGAGCA---GTTGGA---ATAGGAGCT------
---GTACTCTTTGGGTTCTTAGGAGCA---GCAGGAAGCACTATG---GGCGCAGCGTCA---
ATAACGCTGACGGTACAG---GCCAGACAA---TTATTG---TCTGGCATAGTGCAACAGCAAAGC---
AATTTG---CTGAGA---GCTATAGAGGCTCAACAGCATATGTTGAAACTCACAGTCTGGGGCATT
>EU110096..1
TTCTATTGTAACACATCAGGCTTGTTTAATAGCACTTGGAATAGCAATAGCACTTGGAATAGCACTGCCACGC
AGGAGTCAAAT--
-------------AACACG------
GAGTTAAATGGCAATATAACTCTCCCATGCAGAATAAAGCAAATTGTAAGAATGTGGCAGAGAGTAGGACAAG
CAATGTATGCCCCTCCCATTGCAGGAGTAATACAGTGTAAATCAAAC---
ATTACAGGAATGATATTGACAAGAGATGGTGGAAATAGTACAGAGACT-------------------------
----

TCCAATAATAATGAGACTTTCAGACCTGGAGGAGGAGATATGAGAGATAATTGGAGAAGTGAATTATATAAGT
ATAAAGTAGTAAAACTTGAACCACTA---GGAGTAGCACCCACAAAAGCAAGGAGAAGAGTGGTG--------
-------------------------------GAGAGAGAGAAAAGAGCA---GTGGGA---CTGGGAGCT---
------CTCTTCCTTGGGTTCTTAGGAGCA---GCCGGAAGCACTATG---GGCGCGGCGTCA---
CTAACGCTGACGGGACAG---GCCAGACAA---TTATTG---TCTGGCATAGTGCAACAGCAAAGC---
AATTTG---CTGCAG---GCTATAGAGGCTCAACAGCATCTGTTGAAACTCACAGTCTGGGGCATT
>DQ912822..1
TTCTATTGTAACACATCAGGTCTGTTTAATAGCACTTGGAACAAAACTGACAGTGGCAGCATGCAGGGATCAA
ATAGC--

ACAACAAGTGACGTTATAACTCTCCCATGCAGAATAAAACAAATTGTAAATATGTGGCAGAGAGCAGGACAAG
CAATGTATGCCCCTCCCATTCCAGGAGTAATAAGGTGTGTATCAAAC---
ATTACAGGACTACTATTAACAAGAGATGGTGGGAATGATAATACT----------------------------
----

ACCGAAATTTTCAGACCTGGAGGAGGAAATATGAGGGACAATTGGAGAAGTGAATTATATAAGTATAAAGTAG
TAAAAATTGAACCACTA---GGAGTAGCACCCACCAGGGCAAAGAGAAGAGTGGTG-----------------
----------------------GAGAGAGAAAAAAGAGCA---GTTGGA---ATAGGAGCT---------
GTCCTCATTGGGTTCTTAGGAGCA---GCAGGAAGCACTATG---GGCGCGGCGTCA---
ATAACGCTGACGGTACAG---GCCAGACAA---TTATTG---TCTGGTATAGTGCAACAGCAAAGC---
AATTTG---CTGAGA---GCTATAGAGGCTCAACAGCATATGTTGAGACTCACGGTCTGGGGCATT
>AF457058..2
TTCTATTGTAATACATCAGGACTGTTCAATAGTACTGTGAATACTTTGAATATTAGCACTTGGAATTACAATG
AG---

TCAGATAACACTACTATACAACTCCCATGCAGAATAAAACAAATTATAAACATGTGGCAGGGAGTGGGACAAG
CAATGTATGCCCCTCCCATTGCAGGAGTCATCAAATGTTCATCAAAT---
ATTACAGGACTATTGTTGACAAGAGATGGTGGTGCAAATAGTACC----------------------------
----

AGTCAGAAAGAAACCTTCAGACCTGGAGGGGGAGACATGAGAGACAATTGGAGAAGTGAATTATATAAATATA
AAGTAGTAAGAATTGAACCACTA---GGTCTAGCACCCACCAGGGCAAGAAGAAGAGTGGTG-----------
----------------------------GAAAGAGAAAAAAGAGCA---ATAGGA---CTGGGAGCT------
---ATGTTCCTTGGGTTCTTGGGAGCA---GCAGGAAGCACGATG---GGCGCAGCGTCA---
CTGACGCTGACGGTACAT---GCCAGACAG---TTATTA---TCTGGTATAGTGCAACAGCAAAAC---
AATTTG---CTGAGG---GCTATAGAGGCGCAACAACATCTGTTGCAACTCACAGTCTGGGGCATT
>AF457082..1
TTCTATTGCAACACATCAGACCTGTTTAATAGCAATTGGACTTGGGATAGTAATAATGGCACTATGAAGGAGG
TAAATAGC---
-------------------ACG---
GGGTCAAATGGCACTATAACTCTCCAATGCAGAATAAAGCAAATTATAAATATGTGGCAGAGAGCAGGACAAG
CAATATATGCCCCTCCCATCAAAGGAGTAATAAACTGTGTATCAAAT---
ATTACAGGACTAATATTAACAAGAGATGGTGGGGATAATAGTAGT----------------------------
----

ACAGAAACCTTCAGACCTGGAGGAGGAGATATGAGGGACAATTGGAGAAGTGAATTATATAAGTATAAAGTAG
TAAAAATTGAACCACTA---GGAGTAGCACCCACCAGGGCAAAGAGAAGAGTGGTG-----------------

----------------------GAGAGAGAAAAAAGAGCA---GTTGGA---ATAGGAGCT---------
GTCTTCCTTGGGTTCTTGGGAGCA---GCAGGAAGCACTATG---GGCGCGGCGTCA---
ATAACGCTGACGGTACAG---GCCAGACAA---TTATTA---TCTGGCATAGTGCAACAGCAAAGC---
AATTTG---CTGAGG---GCTATAGATGCTCAACAACATCTGTTGAAGCTCACGGTCTGGGGCATT
>KT022385..1
TTCTATTGTAATACAACAGCCCTGTTTAATAGCATTTGGAATGATACTGGCCTTAATAATAAAACTTGGGATA
CAAAT--

ACAAATGATACTATAATCCCATGCAGAATAAAGCAAATTATACGAATGTGGCAGAGAGTAGGACAAGCAATGT
ACGCCCCTCCCATCCCGGGAGTGATAAAGTGTATATCTAAC---
ATTACAGGGCTACTATTAACAAGAGATGGTGGACAGGGCAATAAT----------------------------
----

GAAACTGAAACCTTCAGACCTGGAGGAGGAAATATGAAGGACAATTGGAGAAGTGAATTATATAAGTATAAAG
TAGTAAAAATTGAACCACTA---GGAGTAGCACCCACCAGGGCAAGGAGAAGAGTGGTG--------------
-------------------------GAGAGAGAAAAGAGAGCA---GTTGGA---CTGGGAGCT---------
GTATTCATTGGGTTCTTAGGAGCA---GCAGGAAGCACTATG---GGCGCAGCGTCA---
ATAACGCTGACGGCACAG---GCCAGACAA---TTGTTG---TCTGGTATAGTGCAACAGCAAAGC---
AATTTG---CTGAGG---GCTATAGAGGCTCAACAGCATCTGTTGAAACTCACAGTCTGGGGCATT
>KT022387..1
TTCTATTGTAATACAACAGAACTGTTTAATAGCACCTGGTTTAATAGCACTTGGGACAGCACGCAGGCGCCAA
ATAGCACG---

GATTCAAATGACACTATAACCCTCCAATGCAGAATAAAACAAATTATAAATATGTGGCAGAGAGTAGGACAAG
CAATGTATGCCCCTCCCATCCCAGGAGTAATACAGTGTGTATCAAAC---
ATTACAGGATTACTATTAACAAGAGATGGTGGGGATAATAACAGT----------------------------
----

ACAAATGAAACCTTCAGACCTGGAGGAGGAGATATGAGGGACAATTGGAGAAGTGAATTATATAAGTATAAAG
TAGTAAAAATTGAACCACTA---GGAGTAGCACCCACCGGGGCACAGAGAAGAGTGGTG--------------
-------------------------GAAAGACAAAAAAGGGCA---GTTGGA---ATAGGAGCT---------
GTGTTCCTTGGGTTCTTAGGAGCA---GCAGGAAGCACTATG---GGCGCGGCGTCA---
ATAACGCTGACGGTACAG---GCCAGACTA---TTATTG---TCTGGCATAGTGCAACAGCAAAGC---
AATTTG---CTGAGG---GCTATAGAGGCTCAGCAACATCTGTTGAAACTCACAGTCTGGGGCATT
>KT022388..1
TTCTATTGTGACACATCCAGCCTGTTTAATAGCACTTGGGATACCGATGACACCAGGCGAGAGTTAAATAGCA
CAGAG--

TCAAATGACACTATAACACTCCAATGCAGAATAAAGCAAATTATAAATATGTGGCAGAGAGTAGGACAAGCAA
TGTATGCCCCTCCCATCCAAGGAATAATAAGGTGTAAATCAAAA---
ATTACAGGACTAATATTAACAAGAGATGGTGGTAAGAATAGCAGT----------------------------
----

ACCAATGAAATCTTCAGACCTGGAGGAGGAGATATGAGGGACAATTGGAGAAGTGAATTATATAAGTATAAAG
TAGTAAAACTTGAACCATTA---GGAGTAGCACCCACAAGGGCAAAAAGAAGAGTGGTG--------------
-------------------------GAGAGAGAAAAAAGAGCA---GTTGGA---ATAGGAGCT---------
GTCTTCCTTGGGTTCTTAGGAGCA---GCAGGAAGCACTATG---GGCGCAGCGTCA---
ATAACGCTGACGGCACAG---GCCAAACAA---TTATTG---TCTGGCATAGTGCAACAGCAAAGC---
AATTTG---CTGAGG---GCTATAGAGGCTCAACAACATCTGTTGAAACTCACAGTCTGGGGCATT
>KT022389..2
TTCTACTGCAATACATCAGCACTGTTTAATAGGACTTATAATATATCAGAACTGGGTAATACTAATAATATAT
CAGATACTAGT--

AATACATCAAAGGTTATCCCACTCCCATGCAGAATAAGACAAATTATAAACATGTGGCAGGAAGTAGGCAAAG
CAATATATGCCCCTCCCATTGCAGGAATAATCAGATGTTCATCAAAT---
ATTACAGGACTATTGATGACAAGGGATGGTGGTGAATATAATAAT----------------------------
----

AGTCAGAATGAAACCTTCAGACCTGGAGGGGGAGATATGAGAGACAACTGGAGAAGTGAATTATATAAATATA
AAGTAGTAAAACTCGAACCATTA---GGTCTAGCACCCACCAGGGCAAAGAGAAGAGTGGTG-----------

----------------------------GACAGAGAAAAAAGAGCA---ATAGGA---CTAGGAGCT------
---ATGTTCCTTGGGTTCTTGGGAGCA---GCAGGAAGCACGATG---GGCGCAGCGTCA---
CTGACGCTGACGGTACAG---GCCAGACAG---TTATTG---TCTGGTATAGTGCAACAGCAAAAT---
AATTTG---CTGAGA---GCTATAGAGGCGCAACAGCATATGTTGCAACTCACAGTCTGGGGCATT
>KT022390..1
TTCTATTGCAATACATCAGGACTGTTTAATACATTAAATAGTACAGGGATA----------------------
----

AATGAGACTACAATCACACTCCCATGCAGGATAAAACAAATTATAAACATGTGGCAGGGAGTAGGAAAAGCAA
TGTATGCCCCTCCCATTGAAGGACTAATCCACTGTTCATCAAAC---
ATTACAGGACTATTATTGACAAGAGATGGTGGTAATGATGGTGCA----------------------------
----
-
AATATTACTAATCAGAATGAGACTTTTAAACCTGAAGGAGGAGATATGAGAGACAATTGGAGAAGTGAATTAT
ACAAATATAAAGTAGTAAAAATTGAACCACTA---GGTCTAGCACCCACCAAGGCAAAAAGAAGAGTGGTG--
-------------------------------------GAAAGACAAAAAAGAGCA---ATAGGA---
CTAGGAGCT---------GTGTTCCTTGGGTTCTTGGGAGCA---GCAGGAAGCACAATG---
GGCGCAGCGTCA---CTGACGCTGACGGTACAG---GCCAGACAG---TTATTG---
TCTGGTATAGTGCAACAGCAAAAC---AATTTG---CTGAGG---
GCTATAGAGGCGCAACAGCATCTGTTGCAACTCACAGTCTGGGGCATT
>KT022392..1
TTCTATTGTAACACATCAGGCCTGTTTAATGGCACTGTCAAACAGGAGTCAAATGGCACAGAG----------
----

TCAAATGATACTATAATTCTCCAATGCAAAATAAAGCAAATTATAAATATGTGGCAGAGAGTAGGACAAGCAA
TATATGCCCCTCCCATCCCAGGAGTAATAAGGTGTGTCTCAAAC---
ATTACAGGACTAATATTAACAAGAGATGGTGTTAATAGCAGTGCA----------------------------
----

AATGAAACCTTCAGACCTGGAGGAGGGGATATGAGGGACAACTGGAGAAGTGAATTATATAAGTATAAAGTAG
TAAAAATTGAACCACTA---GGAGTAGCACCCACCAGGGCAAAAAGAAGAGTGGTG-----------------
----------------------GAGAGAGAAAAAAGAGCA---GTTGGA---ATAGGAGCT---------
GTTTTCATTGGGTTCTTGGGAGCA---GCAGGAAGCACTATG---GGCGCGGCGTCA---
ATGACGCTGACGGTACAG---GCCAGACAA---TTATTG---TCTGGCATAGTGCAACAGCAAAGC---
AATTTG---CTGAGG---GCTATAGAGGCTCAACAACATCTGTTGAAACTCACGGTCTGGGGCATT
>KT022394..1
TTCTACTGCAATACATCAAGACTGTTCAATAGTACATGGGTATTAAATAGTACATCAAATAGTACATGGACAT
TAAATAGTACA--
----------ACGGGAGAT------
AGCAGTACATCAAATATCACAATCCCATGCAGAATAAAACAAATTATAAACAGGTGGCAGGAAACAGGAACAG
CAATATACGCCCCTCCCATTGAAGGACTAATTCACTGTTCATCAAAT---
ATTACAGGACTACTATTAACAAGAGATGGTGGGAACCCTAATGAGACAAGG----------------------
----

GAGAATGAAACCTTTAGACCAGAAGGAGGAAATATGAAAGACAATTGGAGAAGTGAATTATACAAATATAAAG
TAGTAAGACTTGAACCACTA---GGTCTAGCACCCACCAAGGCAAAGAGAAGAGTGGTG--------------
-------------------------GAAAGAGAAAAAAGAGCA---ATAGGA---CTAGGAGCT---------
ATGTTCCTTGGGTTCTTGGGAGCA---GCAGGAAGCACGATG---GGCGCAGCGTCA---
CTGACGCTGACGGTACAG---GCCAGACAG---TTATTG---TCTGGTATAGTGCAACAGCAAAAC---
AATTTG---CTGAGG---GCTATAGAGGCGCAACAGCATATGTTGCAGCTCACAGTCTGGGGCATT
>KT022396..1
TTCTATTGCAGCACATCAGGCCTGTTTAATGGCACTTGGACTTGGACTAATGACACTGGGAGTAATAACAATG
TCAGCCAGCAA--

GAGTTAAATGACACTATAACTCTCCCATGCAAAATAAAGCAAGTTATAAATATGTGGCAAAGGGTGGGACAAG
CAATATATGCCCCTCCCATCCCAGGAGTAATAAGGTGTGAATCAAAC---
ATTACAGGACTAATATTATCAAGAGATGGTGGAGATAATAACAGT----------------------------
----

ACAAATGAAACCTTCAGACCTGGAGGAGGAGATATGAGGGACAATTGGAGAAGTGAATTATATAAGTATAAAG

TAGTAAAAATTGAACCACTA---GGAGTAGCACCCACCAGGGCAAAGAGAAGAGTGGTG--------------
-------------------------GAGAGAGAAAAAAGAGCA---GTTGGA---CTGGGAGCT---------
GTTTTCATTGGGTTCTTAGGAGCA---GCAGGAAGCACTATG---GGCGCGGCGTCA---
GTGACGCTGACGGTACAG---GCCAGACAA---TTATTG---TCTGGCATAGTGCAACAGCAAAGC---
AATTTG---CTGAGG---GCTATAGAGGCTCAACAACATCTGTTGAAACTCACGGTCTGGGGCATT
>KT022409..1
TTCTACTGTAATACAACAGGCCTGTTTAATAGCACTTGGAAGTTTAATAGCACTTGGGAGCAAGGGTCAAATA
CCACGGAG---

ACAAATGAAACTATAACTCTCCAATGCAGAATAAAGCAAATCATAAACATGTGGCAGACAGTAGGACAAGCAA
TGTATGCTCCCCCCATCCCAGGAGTAATAAAGTGTAGATCAAAC---
ATTACAGGATTAATATTAACAAGAGATGGTGGGAATAATAACAAT----------------------------
----
GGAAGTGGAAATAATAACAAT---------
GGAAGTGAAATCTTCAGACCTGGAGGAGGAGATATGAGAGACAATTGGAGAAGTGAATTATATAAGTATAAAG
TAGTAAAAATTGAACCCCTA---GGAGTAGCACCCACCAGGGCAAAGAGAAGAGTGGTG--------------
-------------------------GAGAGAGAAAAAAGAGCA---GTTGGA---ATAGGAGCT---------
GTCTTCCTTGGGTTCTTAGGAGCA---GCAGGAAGCACTATG---GGCGCGGCGTCA---
ATAACGCTGACGGTACAG---GCCAGACAA---TTATTG---TCTGGCATAGTGCAACAGCAAAGC---
AATTTG---CTGAAG---GCTATAGAGGCTCAACAACATCTGTTGAAACTCACGGTCTGGGGCATT
>KT022410..1
TTCTATTGTAACACATCAAGCCTGTTTAATAGCACTTGGAATTTAACTGCCAACAGGCAGGGACCAAATAGCA
CGGAG--

TCAACTGACACCATAACTCTACAGTGCAGAATAAAGCAAATTATAAATATGTGGCAGAGAACAGGACAAGCAA
TATATGCCCCTCCAATCCAGGGAGTAATAAGGTGTGCATCAAAC---
ATTACAGGACTACTATTAACAAGAGATGGTGCT--

GGCAGTGACAGTGAAACCTTCAGACCTACAGGAGGAGATATGAGAGACAATTGGAGAAGTGAATTATATAAAT
ATAAAGTAGTAAAAATTGAACCATTA---GGAGTAGCACCCACCAGGGCAAGGAGAAGAGTGGTG--------
-------------------------------GGGAGAGAAAAAAGAGCA---GTTGGA---ATAGGAGCT---
------GTATTCCTTGGGTTCCTAGGAGCA---GCAGGAAGCACTATG---GGCGCGGCGTCA---
ATAACGCTGACGGTACAG---GCCAGACAA---TTGTTG---TCTGGCATAGTGCAACAGCAAAGC---
AATTTG---CTGAAG---GCTATAGAGGCTCAACAACAGCTGTTGAGACTCACTGTCTGGGGCATT
>KT022411..1
TTCTACTGTAACACATCAAATCTGTTTAATAGCACATGGACTAATAGTACATGGAATATAACAGAAGAGGCTA
AT---

AGTACAGAGCCAATTATACTCCAATGCAGAATAAAGCAAATTATAAATCTGTGGCAGAGAGTAGGACAAGCAA
TGTATGCTCCTCCCATCCAAGGAGTAATAAGGTGTGAATCAAAC---
ATTACAGGACTAATATTAACAAGAGATGGTGGGCATAATAAGACA----------------------------
----

AATGAAACCTTCAGACCTGGAGGAGGAGACATGAGGGATAATTGGAGAAGTGAATTATACAAATATAAGGTGG
TAAAAATCGAGCCACTA---GGAGTAGCACCCACAAGGGCAAGGAGAAGAGTGGTG-----------------
----------------------CAGAGAGAAAAAAGAGCA---GTTGGA---ATAGGAGCT---------
GTCTTCCTTGGGTTCTTGGGAGCA---GCAGGAAGCACTATG---GGCGCAGCGTCA---
ATAACGCTGACGGTACAG---GCTAGACAA---TTATTG---TCTGGCATAGTGCAACAGCAAAGC---
AATTTG---CTGAAG---GCTATAGAGGCTCAACAACATCTGTTGAAACTCACGGTCTGGGGCATT
>FJ623491..1
TTCTATTGTAACACCTCAGGCCTGTTTAATAGCGCTTGGTCGGGCAATACCAGTAATACCAGAACCAATAGT-
----

GCAGAAGCCAATATAACTCTCCAATGCAGGATAAAGCAAGTTATAAATATGTGGCAGAGAGTAGGACAAGCAA
TATATGCCCCTCCCATCTCAGGAGTAGTAAGGTGTAAATCAAAT---
ATTACAGGACTACTATTAACAAGAGATGGTGGGGGTAATGGTACA----------------------------
----

AATGAAACCTTTAGACCTGGAGGAGGAGATATGAGGGACAATTGGAGAAGTGAATTATATAAGTATAAGGTAG

TAAAAATTGAACCACTA---GGAGTAGCACCCACCAGGGCAAAGAGAAGAGTGGTG-----------------
----------------------GAGAGAGAAAAAAGAGCA---GTTGGA---ATAGGAGCT---------
GTTTTCATTGGGTTCTTGGGAGCA---GCAGGAAGCACTATG---GGCGCGGCGTCA---
ATAACGCTGACGGTACAG---GCTAGACAA---TTATTG---TCTGGCATAGTGCAACAGCAAAGC---
AATTTG---CTGAGG---GCTATAGAGGCTCAACAACATCTGTTGAAACTCACGGTCTGGGGCATT
>KP174771..1
TTCTATTGTAACACATCAGGCCTATTTAATAGCACGTGGGATGCGAATGCCACCATGAATGCCAACAGTACGG
AGTCA--

AATGACACAACTATCACTCTCCAATGCAGAATAAAGCAAATTATAAATATGTGGCAGAGAACAGGACAAGCAA
TGTATGCCCCTCCCATCCCAGGAGAAATAAGGTGTGAATCAAAC---
ATTACAGGACTAATACTAACAAGAGATGGGGGAAGTAATAATACA----------------------------
----

AATGAGACCTTCAGACCTGGAGGAGGAGATATGAGGGACAATTGGAGAAGTGAATTATATAAGTATAAAGTAG
TAAAAATTGAACCACTA---GGAGTAGCACCCACCAGGGCAAAAAGGAGAGTGGTG-----------------
----------------------GAGAGACAAAAAAGAGCA---GTTGGC---ATAGGAGCT---------
GTCTTCCTTGGGTTCTTAGGAGCA---GCAGGAAGCACTATG---GGCGCGGCGTCA---
ATAACGCTGACGGTACAG---GCCAGACAA---TTATTG---TCTGGTATAGTGCAGCAGCAAAGC---
AATTTG---CTGAGG---GCTATAGAGGCTCAACAACATCTGTTGAAACTCACAGTCTGGGGCATT
>KU921707..1
TTCTACTGTAATACATCGAACCTGTTTAATAGCACTTGGTACAGTAACGGTTCGATGCCAAATATC-------
----

ACTACTGGCAATATAACTCTCCCGTGCAGAATAAAACAAATTATAAAGATGTGGCAGAGAGCAGGACAAGCAA
TATATGCCCCTCCCATCAAAGGAGTAATAAGGTGTGACTCAAAC---
ATTACAGGATTACTACTAACAAGAGATGGTGGGAGTACTAACAGT----------------------------
----

ACAAATGAAACCTTCAGACCTGCAGGAGGAGACATGAGGGACAATTGGAGAAGTGAATTATATAAATATAAAG
TAGTAAAAATTGAACCACTA---GGAGTAGCACCCACCAAGGCAAAGAGAAGAGTGGTG--------------
-------------------------GAAAGAGAAAAAAGAGCAATTATTGGA---CTGGGAGCT---------
GTATTCGTTGGGTTCTTAGGAGCA---GCAGGAAGCACTATG---GGCGCGGCGTCA---
GTGACGCTGACGGTACAG---GCCAGACAA---TTATTG---TCTGGCATAGTGCAACAGCAAAGC---
AATTTG---CTGATG---GCTATAGAGGCTCAACAACATTTGTTGAAACTCACAGTCTGGGGCATT
>KU921719..1
TTCTACTGTAATACATCAGGCTTGTTTAATAGCACTTGGTTTAGCAATGTCAGCTTGCAAGAAAATAGCACAG
AG---

TCAAGTGGCAATATAACTCTCCACTGCAGAATAAAGCAAGTTATAAATATGTGGCAGAGAGTAGGACAAGCAA
TGTATGCCCCTCCCATCTCGGGAAAAATAAGGTGTGAATCAAAC---
ATTACAGGACTAATATTAACAAGGGATGGTGGGAATAGTGACAATACA-------------------------
----

AGTACAGATGAGACCTTCAGACCTGCAGGAGGAGATATGAGGGACAATTGGAGAAGTGAATTATACAAGTATA
AAGTAGTACAAATTGAACCACTA---GGAGTAGCACCCACCAGGGCAAAAAGAAGAGTGGTG-----------
----------------------------GAGAGAGGAAAAAGAGCA---GTTGGG---CTGGGAGCT------
---GTGTTCATTGGGTTCTTGGGAGCA---GCAGGAAGCACGATG---GGCGCAGCGTCC---
CTGACGCTGACGGTACAG---GCCAGACAA---TTATTG---TCTGGTATAGTGCAACAGCAAAGC---
AATTTG---CTGAGG---GCTATAGAGGCTCAACAAAGTCTGTTGAAACTCACAGTCTGGGGCATT
>KU921749..2
TTCTACTGCAATACATCAAGCCTGTTTAGGAGTACATGGGAAAATAGTACTACATTAAATAGTACAGGG----
----

GGAAATGAGACAATTACACTCCAATGCAGAATAAAACAGATTATAAACATGTGGCAGGGAGTAGGGAAGGCAA
TGTATGCCCCTCCTATTGAAGGACTAATCTCCTGTTCATCCAAT---
ATTACAGGGCTATTATTAACGAGAGATGGT---AATAAAAATAGT----------------------------
----

CAGAAGAATGAGACCTTCAGACCTGGAGGAGGAGATATGAGGGACAATTGGAGAAGTGAATTATATAAATATA

AAGTAGTAAAAATTGAACCACTA---GGAGTAGCACCCACCGGGGCACGGAGAAGAGTGGTG-----------
----------------------------GGAAGAGAAAAAAGAGCA---GTTGGA---ATAGGAGCG------
---GTCCTCCTTGGGTTCTTAGGAGCA---GCAGGAAGCACTATG---GGCGCGGCGTCA---
ATAACGCTGACGGTACAG---GCCAGAGAA---TTACTG---TCTGGCATAGTGCAACAGCAAAGT---
AATTTG---CTGAGG---GCTATAGAGGCTCAACAACAACTGTTGAAACTCACGGTCTGGGGCATT
>KU921798..3
TTCTATTGTAACACATCAGGCCTGTTTCATGGCAAATGGTCTAATGGCACTTGGACTAGGAATGACACT----
----

GCAAACGACACTATAAGGCTCCCATGCAGAATAAAGCAAATTATAAATATGTGGCAGAGAGCAGGACAAGCAA
TATATGCCCCTCCCATAAAAGGAGTAATAACATGTAGATCAAAT---
ATTACAGGACTACTATTAACAAGAGATGGTGGAACGAATAGTACA----------------------------
----

AATGAAACCTTCAGACCTGGAGGAGGAGATATGAGGGACAATTGGAGAAGTGAATTATATAATTATAAAGTAG
TAAAAATTGAACCACTA---GGAGTAGCACCCTCCAAGGCAAAGAGAAGAGTGGTG-----------------
----------------------GAGAGAGAAAAAAGAGCA---GTC---------GGAATG---------
GTCCTCATTGGGTTCTTAGGAGCA---GCAGGAAGCACTATG---GGCGCAGCGGCA---
GTAACGCTGACGGAACAG---GCCAGACAA---TTATTG---TCTGGCATAGTGCAACAGCAAAGC---
AATTTG---CTGAAG---GCTATAGAGGCTCAACAGCATCTGTTGAAACTCACGGTCTGGGGCATT
>KU921799..1
TTCTATTGTGACACATTAGGCCTGTTTAAAGGCACTTGGTCTAATGGCACTTGGTCTAATGGCAATTGGACTA
GGAATGACACT--

GGCAGCAACAACACTATAATTCTCCCATGCAGAATAAAGCAAATTATAAAGATGTGGCAGAGAACAGGACAAG
CAATATATGCCCCTCCCATAGAAGGAGTAATAAGATGTGAATCAAAT---
ATTACAGGACTAATATTAACAAGAGATGGTGGAAAGAATGAGCTA----------------------------
----

AATGAAACCTTCAGACCTGGAGGAGGAAATATGAAGGACAATTGGAGAAGTGAATTATATAAATATAAAGTAG
TAAAAATTGAACCACTA---GGAGTAGCACCCACCAGGGCAAAGAGAAGAGTGGTG-----------------
----------------------GAGAGAGAAAAAAGGGGA---GTTGGA---ATAGGAGTT---------
GTCTTCTTTGGGTTCTTAGGAGCA---GCAGGAAGCACGATG---GGCGCAGCGGCA---
CTAACGCTGACGGTACAG---GCCAGACAA---TTATTG---TCTGGCATAGTGCAACAGCAAAGC---
AATTTG---CTGAAG---GCTATAGAGGCTCAACACCATCTGTTGAAACTCACAGTCTGGGGCATT
>KU921805..1
TTCTATTGTGACACATCAAGACTGTTTAATGGCGCTTGGTCTGGGAATGACACTAGCATTGAACAGGAGCAAA
AT---

GGAACCGACCCTATAACTCTCCCATGCAGAATAAAGCAAATTATAAATATGTGGCAGAGAACAGGACAAGCAA
TATATGCCCCTCCCATTGCAGGAGTAATAAGATGTGAATCAAAT---
ATTACAGGATTAATATTAACAAGAGATGGGGGACTTAATAATACAAAT-------------------------
----

AAAGATAATGAAACCTTCAGACCTACAGGAGGAAATATGAGGGACAATTGGAGAAGTGAATTATATAAATATA
AGGTAGTAAAAATTGAACCACTA---GGAGTCGCACCCACCAGAGCAAGGAGAAGAGTGGTG-----------
----------------------------GAGAGAGAAAAAAGAGCA---GTTGGA---ATAGGAGCT------
---GTCTTCCTTGGGTTCTTAGGAGCA---GCAGGAAGCACTATG---GGCGCAGCGGCA---
GTGACGCTGACGGTACAG---GCCAGACAA---TTATTG---TCTGGTATAGTGCAACAGCAAAGC---
AATTTG---CTGCAG---GCTATAGAGGCTCAACAGCATCTGTTGAAACTCACAGTCTGGGGCATT
>KU921813..1
TTCTATTGTAATACATCAAGCTTGTTTGGTAGCACTTGGGTCCCTAATAGCACTGTGGACACCAACAGTACCA
ACAGC--

ACGCAGGGGACAACTATAACTCTCCAATGCAGAATAAAACAAATTATAAACATGTGGCAGAGAGTAGGAAGAG
CCATGTATGCCCCTCCCATTGCAGGAACAATCAGCTGTTCATCAAAT---
ATTACAGGACTATTGTTGACAAGAGATGGTGGTGCAAACAGTAGT----------------------------
----

CATAATGAGACTTTCAGACCTGGAGGGGGAGATATAAGAGACAATTGGAGAAGTGAATTATACAAATACAAAG

TAGTAAGAATTGAACCATTA---GGACTAGCACCCACCAGGGCAAAGAGAAGAGTGGTG--------------
-------------------------GAAAGAGAAAAAAGAGCA---ATAGGA---CTAGGAGCT---------
GTGTTCCTTGGGTTCTTGGGAGCA---GCAGGAAGCACGATG---GGCGCAGCGTCA---
TTGACGCTGACGGTACAG---GCCAGACAA---TTATTG---ACTGGTATAGTGCAACAGCAAAAC---
AATTTG---CTGAGG---GCTATAGAGGCGCAACAGCATTTGTTGCAACTCACAGTCTGGGGCATT
>AF407149..1
TTCTATTGTAATACATCAGGCCTGTTTGATAGCACTTGGAATGACACTGACAGCAGACAG-------------
----

GAAAATGGCACTATAACTCTCCCATGCAGAATAAAGCAAATTATAAATATGTGGCAGAGAACAGGACAAGCAA
TATATGCCCCTCCCATCCAAGGAGCAATAAGGTGTGTATCAAAC---
ATTACAGGACTAATATTAACAAGAGATGGTGGGAACAATAACAGT----------------------------
----

ACAAATGAAACCTTCAGACCTGGAGGAGGAGATATGAGGGACAATTGGAGAAGTGAATTATATAAGTATAAAG
TAGTAAAAATTGAACCACTA---GGAGTAGCACCCACCAAGGCAAGGAGAAGAGTGGTG--------------
-------------------------GGGAGAGAAAAAAGAGCA---GTTGGA---ATAGGAGCT---------
GTCTTCCTTGGGTTCTTAGGAGCA---GCAGGAAGCACTATG---GGCGCGGCGTCA---
ATAACGCTGACGGTACAG---GCCAGACAA---TTACTG---TCTGGCATAGTGCAACAGCAAAGC---
AATTTG---CTGAAA---GCTATAGAGGCTCAACAGCATCTGTTGAGACTCACGGTCTGGGGCATT
>AF457059..1
TTCTACTGTAACACATCAAGCCTGTTTAATAGCACTTGGAATAGTACTAGCACTGGGCACAATGCCAGCGGGC
AG---

GAGATAAATGACACTATAATTCTCCAATGCAGAATAAAGCAAATTATAAATATGTGGCAGAGAATAGGACAAG
CAATGTATGCCCCTCCCATCAAAGGAGCAATATGGTGTAGATCAAAC---
ATTACAGGACTAATATTAACAAGAGATGGTGGGGAGGACAATAGC----------------------------
----

AGTACTGAAACCTTCAGACCTGGAGGAGGAAACATGAAGGATAATTGGAGAAGTGAATTATATAAGTATAAAG
TAGTAAAACTTGAACCATTA---GGAGTGGCACCCACCAGGGCAAAGAGAAGAGTGGTG--------------
-------------------------GAGAGAGAAAAAAGAGCA---GCTGGA---CTGGGAGCT---------
GTTTTCATTGGGTTCTTAGGAGCA---GCCGGAAGCACTATG---GGCGCAGCGTCA---
GTAACGCTGACGGTACAG---GCCAGACAG---TTATTG---TCTGGCATAGTGCAACAGCAAAAC---
AATTTG---CTGAAG---GCTATAGAGGCTCAACAACATCTGTTGAAACTCACGGTCTGGGGCATT
>AF457073..1
TTCTATTGTAACACATCAGACCTGTTTAATAGCACCTGGAACAGCACCACACTGGAGTCAAACACCACAGGG-
----

TCAAATGGCACTTTAATTCTCCAATGCAGAATAAAGCAAATTATAAATATGTGGCAGAGAGTAGGACAAGCAA
TGTATGCCCCTCCCATCAAAGGAGTAATAAGGTGTAATTCAACA---
ATTACAGGGCTACTATTAACAAGGGATGGTGGAAAGGATAACAGTAGT-------------------------
----

GAGAGTAGTGAAGAAATCTTCAGACCTGAAGGAGGAAATATGAGGGACAATTGGAGAAATGAATTATATAAGT
ATAAAGTAGTAAAAATTGAACCACTA---GGAGTAGCACCCACCAGGGCAAAGAGAAGAGTGGTG--------
-------------------------------GGGAGAGAGAAAAGAGCA---GTTGGA---CTGGGAGCT---
------GTTTTCCTTGGGTTCTTAGGAGCA---GCAGGAAGCACTATG---GGCGCGGCGTCA---
ATAACGCTGACGGTACAG---GCCAGACAA---TTATTG---TCTGGCATAGTGCAACAGCAAAGC---
AATTTG---CTGAGA---GCTATAGAGGCTCAACAGCATCTGTTGAAACTCACGGTCTGGGGCATT
>DQ208501..2
TTCTATTGTAATACATCAGGCCTGTTTAATAGCACTTGGAGTTGGAATCACACTGTCAGCAGCATGCAGGAAG
CAAATGGC---
-------------------ACG---
GAGTCAAATGGCACTATAACCCTCCAATGCAGAATAAAGCAAATTATAAATATGTGGCAGAGAGTAGGACGAG
CAATGTATGCCCCTCCCATCGAAGGAATAATAAGGTGTGAATCAAAC---
ATTACAGGACTAATATTAACAAGAGATGGTGGGGTTAATAACAGT----------------------------
----

ACAAATGAAACCTTCAGACCTGGAGGAGGAGATATGAGGGACAATTGGAGAAGTGAATTATATAAGTATAAGG

TAGTAAAAATTGAACCACTA---GGAGTAGCACCCACCCCGGCGAAGAGAAGAGTGGTG--------------
-------------------------GAGAGAGAAAAAAGAGCA---GTCGGA---ATAGGAGCT---------
GTCTTCCTTGGTTTCTTAGGAGCA---GCAGGAAGCACTATG---GGCGCGGCGTCA---
ATAACGCTGACGGTACAG---GCCAGACAA---TTATTG---TCTGGCATAGTGCAACAACAAAGC---
AATTTG---CTGAGG---GCTATAGAGGCTCAACAGCAGATGTTGAGACTCACGGTCTGGGGCATT
>DQ187009..1
TTCTATTGTAATACATCAGGCCTGTTTTCCAGCACGCAGGAG-------------------------------
----

TCAAGTGATCCTATAACTCTCCCATGCAGAATAAAGCAAATTATAAATATGTGGCAGAGAGTAGGACAAGCAA
TGTATGCTCCTCCCATCCAAGGAGAAATAAGGTGTAAATCAAAC---
ATTACAGGAATAATATTAACAAGAGATGGTGGGCTTGGTACTACA----------------------------
----

AATGAAACCTTCAGACCTGGAGGAGGAGACATGAGGGACAATTGGAGAAGTGAGTTATATAAGTATAAAGTAG
TAAAAATTGAACCACTA---GGAGTAGCACCCACCAGGGCAAGGAGAAGAGTGGTG-----------------
----------------------GAGAGAGAGAAAAGAGCA---GTTACA---ATAGGAGCT---------
GTTCTCCTTGGGTTCTTGGGAACA---GCAGGAAGCACTATG---GGCGCAGCGTCA---
CTAACGCTGACGGTACAG---GCCAGACTA---TTATTG---TCTGGCATAGTGCAACAGCAAAGC---
AATTTG---CTGAGG---GCTATAGAGGCTCAACAACATCTGTTGAAACTCACAGTCTGGGGCATT
>AF075701..1
TTCTATTGTAACACAACAGGCCTGTTTAATAGCACGTGGAATGACACTGCCACAGAGCAGAAG----------
----

CCAAACGACACTATAAGACTCCAATGCAGAATAAAGCAAATTATAAATATGTGGCAAAGAGCAGGACGAGCAA
TATATGCCCCTCCCATCCAAGGAGTAATAAATTGTGTATCAGAC---
ATTACAGGACTAATATTAACAAGAGATGGTGGGGTTAATAATACA----------------------------
----

AATGAAACCTTCAGACCTGGAGGGGGAGATATGAGGGACAATTGGAGAAGTGAATTATATAAGTATAAAGTAG
TAAAAATTGAACCACTA---GGAGTAGCACCCACCAGGGCAAAGAGAAGAGTGGTG-----------------
----------------------GAGAGAGAAAAAAGAGTA---GCTGGA---CTAGGAGCT---------
GTCTTCATTGGGTTCTTAGGAGCA---GCAGGAAGCACTATG---GGCGCGGCGTCA---
ATAACGCTGACGGTACAG---GCCAGACAA---TTGTTG---TCTGGCATAGTGCAACAGCAAAAC---
AATTTG---CTGAAG---GCTATAGAGGCTCAACAACATCTGTTGAAACTCACGGTCTGGGGCATT
>AF071473..1
TTCTATTGTAATACATCAGGCCTGTTTAATAGCACTTGGAATAACACTGACAGCATGCAGGAGTCACATAGCA
CG---

GAGACAAATGACACTATAACTCTCCCATGCAGAATAAAGCAAATTATAAATATGTGGCAGAGAGTAGGACAAG
CAATGTATGCCCCTCCCATCCAAGGAGTAATAAAGTGTACATCAAAC---
ATTACAGGACTGATATTAACAAGAGATGGTGGGGGTAATAACAGC----------------------------
----

ATAAATGCAACCTTCCGACCTGGAGGAGGAGATATGAGGGACAATTGGAGAAGTGAATTATATAAGTATAAAG
TAGTAAAAATTGAACCAGTA---GGAATAGCACCCAACAGGGCAAAGAGAAGAGTGGTG--------------
-------------------------GAGAGAGAAAAAAGAGCA---GTTGGA---ATAGGAGTT---------
GTCTTCTTTGGGTTCCTGGGAGCA---GCAGGAAGCACTATG---GGCGCAGCGTCA---
ATAACGCTGACGGTACAG---GCCAGACAA---TTATTG---TCTGGCATAGTGCAACAGCAAAGC---
AATTTG---CTGAGG---GCTATAGAGGCTCAACAACATCTGTTGAAACTCACGGTCTGGGGCATT
>AY253316..1
TTCTACTGCAATACATCAGAACTGTTTAATAGTACATGGAGCATGAACAATAATACATCAAATAGTACAGGG-
----

GTAAATGAGAATATCACACTCCAATGCAGGATAAAACAAATTATAAACATGTGGCAGGGAGTAGGAAAAGCAA
TATATGCTCCTCCCATTGAAGGAATAATCAGCTGTTCATCAAAT---
ATTACAGGACTAATATTAACAAGAGATGGTGGGAATAATAACAGTACA-------------------------
----

AATGAAAATGAAACCTTTAGACCTGGAGGAGGAGATATGAGGGACAATTGGAGAAGTGAATTATATAAGTATA

AAGTAGTAAAAATTGAACCACTA---GGAGTAGCACCCACCAGGGCAAAGAGAAGAGTGGTG-----------
----------------------------GAGAGAGAAAAAAGAGCA---GTTGGA---ATAGGAGCT------
---GTCCTCTTTGGGTTCTTAGGAGCA---GCAGGAAGCACTATG---GGCGCAGCGTCA---
ATAACGCTGACGGCACAG---GCCAGACAA---TTATTG---TCTGGTATAGTGCAACAGCAAAGC---
AATTTG---CTGAGG---GCTATAGAGGCTCAACAGCATGTGTTGAGACTCACGGTCTGGGGCATT
>KX907376..3
TTCTATTGTAATACATCAGGCCTGTTTAGTAGCACTTGGAATGTCACTTACAATAATAACACTTGGAATGCCA
CTCAGGTG---
-------TCAAATAGCACA------
GAGCCAAATGGCACTATAGTGATCCCATGCAGAATAAAGCAAATTATAAATATGTGGCAGAGAACAGGACAAG
CAATGTATGCCCCTCCCATTCCAGGAAAAATACAGTGCATATCAAAC---
ATTACAGGACTACTATTAACAAGAGATGGTGGGGACACTAGCAGT----------------------------
----

ATGAATGAAACCTTCAGACCTGGAGGAGGAGATATGAGGGACAATTGGAGAAGTGAATTATATAAATATAAAG
TAGTAAAAATTGAACCATTA---GGAGTAGCACCCACCAGGGCGCAGAGACGAGTGGTG--------------
-------------------------GGAAGAGAAAAAAGAGCA---GTTGGA---CTGGGAGCT---------
GTCTTCCTTGGGTTCTTAGGAGCA---GCAGGAAGCACTATG---GGCGCAGCGTCA---
CTGACGCTGACGGTACAG---GCCAGACAA---TTATTG---TCTGGCATAGTGCAGCAGCAAAAT---
AATTTG---CTGAGG---GCTATAGAGGCTCAACAACAACTGTTGAAACTCACAGTCTGGGGCATT
>KX907355..2
TTCTATTGTAACACATCAGGCCTGTTTAGCAGCATTAGCCGATTTAATAACAATAGCGAAATAAATAGCACAG
GG---

TTAAACGGCACTATAACTCTCCAATGCAAAATAAAGCAAATTATAAGAATGTGGCAGAGAACAGGACAAGCAA
TGTATGCCCCTCCCATCCCAGGAGTAATAAGGTGTCAATCAAAC---
ATTACAGGAATAATATTAACAAGAGATGGTGGGATTAATAACACA----------------------------
----

AATGAAACCTTCAGACCTGGAGGAGGAGATATGAGGGACAATTGGAGAAGTGAATTATACAAATATAAAGTAG
TAAAAATTGAACCAATA---GGAGTAGCACCCACCAGGGCAAAGAGAAGAGTGGTG-----------------
----------------------GAGAGAGAAAAAAGAGCA---GTTGGA---ATAGGAGCT---------
GTCTTCATTGGGTTCTTAGGAGCA---GCAGGAAGCACTATG---GGCGCGGCGTCA---
ATAACGCTGACGGTACAA---GCCAGACAA---TTATTG---TCTGGTATAGTGCAACAGCAAAGT---
AATTTG---CTGAGG---GCTATAGAAGCTCAACAGCATCTGTTGAAACTCACAGTCTGGGGCATT
>KX907361..1
TTCTATTGTAACACATCAAAGTTATTTAATAATGGCACTGCCAAC----------------------------
----

ATAGATGACTCTATAACTCTCCCATGCAAAATAAAGCAAATTATAAGGATGTGGCAGAGAACAGGACAAGCAA
TGTATGCCCCTCCTATCCAAGGAACAATAGAGTGTATATCAAAC---
ATTACAGGACTGCTATTAACAAGAGATGGTGGGAGTGATAGCAAT----------------------------
----

ACAAATGAAACCTTCAGACCTGGAGGAGGAGATATGAGGGACAATTGGAGAAGTGAATTATATAAGTATAAGG
TAGTAAAAATTGAACCACTA---GGAGTAGCACCCACCAAGGCAAAGAGAAGAGTGGTG--------------
-------------------------GAGAGAGAAAAAAGAGCA---GTTGGA---ATAGGAGCT---------
GTCCTCTTTGGGTTCTTAGGAGCA---GCAGGAAGCACTATG---GGCGCGGCGTCA---
TTAGCGCTGACGGAACAG---GCCAGGCAA---TTATTG---TCTGGCATAGTGCAACAGCAAAGT---
AATTTG---CTGAGG---GCTATAGAGGCTCAACAGAATATGTTGAGGCTCACAGTCTGGGGCATT
>HQ615969..1
TTCTACTGCAATACATCAGGACTATTTAATAGTACATGGTTAGCAAATGGGACACCAAATAGTACAGGGATA-
----

AATGAGAGTACAATTACACTCCCATGCAGAATAAAACAAATTATAAATATGTGGCAGAGAACAGGACAAGCAA
TATATGCCCCTCCCATCCAAGGAGTAATAAGGTGTGAATCAAAT---
ATTACAGGACTAATATTAACAAGAGATGGTGGGGATACTAATGGA----------------------------
----

ACAGATGAAACCTTCAGGCCTGGAGGAGGAGATATGAGAGACAATTGGAGAAGTGAATTATACAAATATAAGG

TAGTAAAAATTGAACCACTA---GGAGTAGCACCCACCAGGGCAAGGAGAAGAGTGGTG--------------
-------------------------GACAGAGAAAAAAGAGCA---GTTGGA---TTAGGAGCT---------
GTCTTCCTTGGGTTCTTAGGAGCA---GCAGGAAGCACTATG---GGCGCAGCGTCA---
ATAACGCTGACGGTACAG---GCTAGACAA---TTATTG---TCTGGCATAGTGCGGCAGCAAAGC---
AATTTG---CTGCAG---GCTATAGAGGCTCAACAGCAGCTGTTGAAACTTACGGTCTGGGGCATT
>KY658698..1
TTCTATTGTAATACATCAGGCCTGTTTAACAGTGCTTGGAATAACACTAACCTCTTGACAAATAGCACAGGG-
----

ACAAATGGCACTATAACTCTCCCATGCAGGATAAAGCAAATTATAAACATGTGGCAGAGAGTAGGGCAAGCAA
TGTATGCCCCTCCCATCCAAGGAGTAATAAAGTGTGTATCAAAC---
ATTACAGGACTAATATTAACAAGAGATGGAGGGGATAATAACAGT----------------------------
----

ACAAGGGAAATCTTCAGACCTGGAGGAGGAGATATGAGGGACAATTGGAGAAGTGAATTATATAAGTATAAAG
TAGTACAACTTGAACCATTA---GGAGTAGCACCCACCAGGGCAAAGAGAAGAGTGGTG--------------
-------------------------GAGAGAGAAAAAAGAGCA---GTCGGA---ATGGCAGCT---------
GTTTTCATTGGGTTCTTAGGAGCA---GCAGGAAGCACTATG---GGCGCGGCGTCA---
ATAACGCTGACGGTACAG---GCCAGACAA---TTATTG---TCTGGCATAGTGCAACAGCAAAGC---
AATTTG---CTGAGG---GCTATAGAGGCTCAACAACATCTGTTGAAACTCACAGTCTGGGGCATT
>AF442565..1
TTCTATTGTAACACATCAGGCCTGTTTAATAGCACTTGGGAGGGCAACAATACCAGCATATTAAATGGCACGG
GG---

TCAAACGACAATACAACTCTCCCATGCAGAATAAAGCAAATTATAAATATGTGGCAGAGAGTAGGACAAGCAA
TATATGCCCCTCCCATCCCAGGAGTAATAAAGTGTGTGTCAAAT---
ATTACAGGACTAATATTAACAAGAGATGGTGGTAATGATGGTAGT----------------------------
----

AATGAGACCTTCAGACCTGGAGGAGGAGATATGAGGGACAATTGGAGAAGTGAATTATATAAGTATAAAGTAG
TAAAAATTGAACCAATA---GGAGTAGCACCCACCAGGGCAAAGAGAAGAGTGGTG-----------------
----------------------GAGAGAGAAAAAAGAGCA---GTTGGA---CTGGGAGCT---------
GTCTTCATTGGGTTCTTAGGAGCA---GCAGGAAGCACTATG---GGCGCGGCGTCA---
ATAACGCTGACGGTACAG---GCCAGGCAA---TTATTG---TCTGGCATAGTGCAACAGCAAAGC---
AATTTG---CTGAGG---GCTATAGAGGCTCAACAGCATCTGTTGAAACTCACTGTCTGGGGCATT
>AF442570..3
TTCTACTGTAACACATCAAACCTGTTTAATAGCACTTGGAATAATGACAATGCCACACAGTTAAATAGCACGG
AG---

TCAAACGAAACTATAACTCTTCAATGCAAAATAAAGCAAATTATAAATATGTGGCAGAGAGTAGGACAAGCAG
TATATGCCCCTCCCATTCAAGGAGTAATAAAATGTGAATCAAAC---
ATTACAGGAATAATATTAACAAGAGATGGTGGAAATAATAGCAGT----------------------------
----

GCAGAGGAGACCTTCAGACCTGGAGGAGGAGATATGAGGGACAATTGGAGAAGTGAATTATATAAATATAAAG
TAGTAAAAATTGAACCACTA---GGAGTAGCACCCACCAAGGCAAGGAGAAGAGTGGTG--------------
-------------------------GAGAGAGAAAAAAGAGCA---GTTGGA---ATAGGAGCT---------
GTTTTCATTGGGTTCTTAGGAGCA---GCTGGAAGCACTATG---GGCGCGGCGTCA---
GTGACGCTGACGGTACAG---GCCAGACAA---TTATTG---TCTGGCATAGTGCAACAGCAAAGC---
AATTTG---CTGAGG---GCTATAGAGGCTCAACAGCAAATGTTGAAACTCACAGTCTGGGGCATT
>AF442566..1
TTCTATTGTAACACATCAGGCCTGTTTAATAGCACTTGGAGTAATGGCACTAGGAAGGACAATGACAGCAGGC
AGAAA--

ACAAATGACACTATAATAACTCTCCAATGCAGAATAAAGCAAATTATAAATTTGTGGCAGAGAGCAGGACAAG
CAATATATGCCCCTCCCATCGAAGGAATAATAAGATGTAACTCAAAC---
ATTACAGGACTAATATTAACAAGAGATGGGGGCGAATCTAATAACAAA-------------------------
----

ACTGAAGAAACTGAAACCTTCAGACCTGGAGGAGGAGATATGAGGGACAATTGGAGAAGTGAATTATATAAGT

ATAAAGTAGTAAAAATTGAACCACTA---GGAGTAGCACCCACCAGGGCAAGGAGAAGAGTGGTG--------
-------------------------------CAGAGAGAAAAAAGAGTA---GCT------TTTGGAGCT---
------GTCTTCATTGGGTTCTTAGGAGCA---GCAGGAAGCACTATG---GGCGCAGCGTCA---
ATAGCGCTGACGGCACAG---GCCAGACAG---TTATTG---TCTGGTATAGTGCAACAGCAAAGC---
AATTTG---CTGAGG---GCTATAGAGGCTCAACAGCATATGTTGAAACTCACGGTCTGGGGCATT
>HM027851..1
TTCTATTGTAACACAACAAACCTGTTCAATAGCACCTGGGAGAACGGCAAGCAGACATCAAATAGCACA----
----

AACAATGACACTATAACTCTCCCATGTAGAATAAAACAAATTGTAAATATGTGGCAGAGAACAGGACAGGCAA
TATATGCCCCTCCCATCCAAGGAATAATAAGGTGTAACTCAACC---
ATTACAGGACTACTATTAACAAGAGATGGTGGGAATAATAACAAT----------------------------
----

ACAAATGACACCTTCAGACCTGGAGGAGGAGATATGAGGGACAATTGGAGAAGTGAATTATATAAATATAAAG
TAGTACGAATTGAACCATTA---GGAGTAGCACCCACCGAGGCAAGGAGAAGAGTGGTG--------------
-------------------------GGGAGAGAAAGAAGAGCA---GTTGGA---CTGGGAGTT---------
TTCTTCCTTGGGTTCTTAGGAGCA---GCAGGAAGCACTATG---GGCGCAGCGTCA---
ATAACGCTGACGGTACAG---GCCAGACAG---TTACTG---TCTGGCATAGTGCAACAGCAAAAT---
AATTTG---CTGAGA---GCTATAGAGGCTCAACAGCATCTGTTGAGACTCACAGTCTGGGGCATT
>HM027850..1
TTCTATTGTAACACATCAGATCTGTTTAATGGCAGTTGGAATAACACAATGCAGGGGTCAAATAACACGGGG-
----
----------------------------TCAAATGACACTATAACTCTCAAATGTAGAATA------------
----
--ATAACAGTACAA---------------------AATGAAACTAAA--------------------------
----

ACCTTTAGACCTACAGGAGGAGATATGAGGGACAATTGGAGAAGTGAATTATATAAATATAAGGTAGTAAAAA
TTGAGCCACTA---GGAGTAGCACCCACCAAGGCAAAGAGAAGAGTGGTG-----------------------
----------------GAGAGAGAAAAAAGAGCA---GTTGGA---CTAGGAGCT---------
GTCTTCCTTGGGTTCTTAGGAGCA---GCAGGAAGCACTATG---GGCGCGGCGTCA---
ATGACGCTGACGGTACAG---GCCAGACAA---TTATTG---TCTGGTATAGTGCAACAGCAAAAC---
AATTTG---CTGAAG---GCTATAGAGGCTCAACAACATCTGTTGAAACTCACAGTCTGGGGCATT
>HM027864..2
TTCTACTGTAATACATCAGGCCTATTTAATGGCAGTTGGAGTAGAAATGATACTACAAATGACACAGAG----
----

TCAAATGACACTATAACTCTACAATGTAGAATAAAACAATTTATAAATATGTGGCAAAGAGTAGGACAAGCAA
TGTATGCCCCTCCCATTAAAGGACCAATCACGTGTAACTCAAAT---
ATAACAGGACTATTGTTGACAAGGGATGGTAGTGGTAATCAAAAT----------------------------
----

AATGATACCGAGACCTTTAGACCTGGAGGAGGAAATATGAGGGACAATTGGAGAAGTGAATTATACAAATATA
AAGTAGTAAAAATTGAACCACTA---GGAGTAGCACCCACCAGGGCAAAGAGAAGAGTGGTG-----------
----------------------------GAGAGAGAAAAAAGAGCA---GTTGGA---ATAGGAGCT------
---GTCCTCTTTGGGTTCCTAGGAGCA---GCAGGAAGCACTATG---GGCGCAGCGTCA---
ATAACGCTGACGGTACAG---GCCAGACAA---TTATTG---TCTGGCATAGTGCAACAGCAAAGC---
AATTTG---CTGAGG---GCTATAGAGGCTCAACAACATATGTTGAAACTCACAGTCTGGGGCATT
>HM027870..1
TTCTATTGTAATACATCAGGCCTGTTTAATAGCACTTGGGATAGCAATGAGCTTAATAGCACTTTGAATGGCA
CT---

GAGTCAAATGACACTATAACTCTCCCATGCAGAATAAAACAAATTATAAATATGTGGCAGAGAGTAGGACAGG
CAATGTATGCCCCTCCCATCCCAGGAATAATAAGGTGTGAATCAAAC---
ATTACAGGACTAATATTAACAAGAGATGGTGGAAACATTACCAAC----------------------------
----

AATACAGAAATCTTCAGACCGGGGGGAGGAGATATGAGGGACAATTGGAGAAGTGAATTATATAAGTATAAAG
TAGTAAAAATTGAACCACTA---GGAGTAGCACCTACCAAAGCAAAGAGAAGAGTGGTG--------------

-------------------------GAGAGAGAAAAAAGAGCA---GTTGGA---CTAGGAGCT---------
GTCTTCATTGGATTCTTAGGAGCA---GCAGGAAGCACTATG---GGCGCGGCGTCA---
ATAACGCTGACGGTACAG---GCCAGACAA---TTATTG---TCTGGCATAGTGCAACAGCAAAGC---
AATTTG---CTGAGA---GCTATAGAGGCTCAACAACATCTGTTGAAGCTCACGGTCTGGGGCATT
>JX658625..1
TTCTACTGTAATACATCAAAACTGTTTAATAATAGTATAGGGAGGAATGCTACTACATCAAAT----------
----

AGTACAGAATCTATAACACTCCCATGCAAAATAAAACAAATTATAAACATGTGGCAGGGAGTAGGCAAAGCAA
TGTATGCCCCTCCCATTGAAGGACTAATCAAATGTGAATCAAAT---
ATTACAGGGCTATTGTTGACAAGAGATGGTGGTAATACTAATAAT----------------------------
----

CAGAATGAGACCTTCAGACCTGGAGGAGGAGATATGAGAGACAATTGGAGAAGTGAATTATACAAATATAAAG
TAGTAAGAATTGAACCACTA---GGTCTAGCACCCACCAGGGCAAAGAGAAGAGTGGTG--------------
-------------------------GAAAGAGAAAAAAGAGCA---ATAGGA---CTGGGAGCT---------
TTGTTCCTTGGGTTCTTGGGAGCA---GCAGGAAGCACAATG---GGCGCAGCGTCG---
CTGACGCTGACGGTACAG---GCCAGACAA---GTACTG---TCTGGTATAGTGCAGCAACAAAAT---
AATTTG---CTGAGA---GCTATTGAGGCGCAACAGCATCTGTTGCAACTCACAGTCTGGGGCATT
>HM027827..1
TTCTACTGTAATACATCAGGCCTGTTTAATGGCACCTGGAATAGTAATGATACTATAAATGAAACAGAG----
----

ACAAATGACATTATAATTCTCCAATGTAGAATAAAACAAATTATAAATATGTGGCAAAGAGTAGGACAAGCAA
TGTATGCCCCTCCCATTAAAGGACCAATCACGTGTAACTCAAAT---
ATAACAGGACTATTGTTGACAAGAGATGGTGGTGAAGAGAAAGAGAAA-------------------------
----

CAGAATAATGAGACCTTTAGACCTGAAGGAGGAAATATGAAGGACAATTGGAGAAGTGAATTATACAAATATA
AAGTAGTAAAAATTGAACCACTA---GGAGTAGCACCCACCAGGGCAAAGAGAAGAGTGGTG-----------
----------------------------GAGAGAGAAAAAAGAGCA---GTTGGA---ATAGGAGCT------
---GTCCTCTTTGGGTTCCTAGGAGCA---GCAGGAAGCACTATG---GGCGCAGCGTCA---
ATAACGCTGACGGTACAG---GCCAGGCAA---TTATTG---TCTGGCATAGTGCAACAGCAAAGC---
AATTTG---CTGAGG---GCTATAGAGGCTCAACAACATATGTTGAAACTCACAGTCTGGGGCATT
>HM215273..2
TTCTATTGTAATACATCAGGATTGTTTAATAGCACTCATCCTATCAACAGGACGACAAATAGCACAGAG----
----

AACAATGGAACTATAACTCTCCCATGCAGAATAAAACAAATTATAAATATGTGGCAGAGGGCAGGACAAGCAA
TATATGCCCCTCCCATCCCAGGAATAATAAGGTGTACATCAAAC---
ATTACAGGACTATTATTAACAAGAGATGGTGGGAATCATAATGATACT-------------------------
----

AACAATACTGAAACCTTCAGACCTGGAGGAGGAGATATGAAAGATAATTGGAGAAGTGAACTGTATAAATATA
AAGTAGTAAAAATTGAACCACTA---GGTCTAGCACCCACCAAGGCAAAAAGAAGGGTGGTG-----------
----------------------------AAAAGAGAAAAAAGAGCA---ATAGGA---CTAGGAGCT------
---TTGTTCCTTGGGTTCTTGGGAGCA---GCAGGAAGCACGATG---GGCGCAGCGTCA---
CTGACGCTGACGGTACAG---GCCAGACAA---GTACTG---TCTGGTATAGTGCAACAGCAAAAC---
AATTTG---CTGATG---GCTATAGAGGCGCAACAGCATCTGTTGCAACTCACAGTCTGGGGCATT
>HM215274..2
TTCTATTGTAATACATCAAGCTTGTTTAATAGCACCTGGAACAAGAACAGTGACACCTGGAACACCACCAGGA
ACGTG--

CCTAAGTTAGAACCTATAACACTCCCATGCAGAATAAAGCAAATTATAAATATGTGGCAGCGAACAGGACAAG
CAATGTATGCCCCTCCCATCCAGGGAGTAATAAGGTGTAACTCAACC---
ATTACAGGACTCCTATTAACAAGAGATGGTGGGCTTAATAATGAA----------------------------
----

ACCTATGAAACCTTCAGACCTGGAGGAGGAGATATGAGGGACAATTGGAGAAGTGAGTTATACAAATATAAAG
TAGTAAGAATTGAACCAATA---GGAATAGCACCCACCAAGGCAAAGAGAAGAGTGGTG--------------

-------------------------GAGAGAGAAAAAAGAGCA---GTTGGA---ATGGCAGCT---------
GTATTCATTGGGTTCTTGGGAGCA---GCAGGAAGCACTATG---GGCGCGGCGTCA---
ATAACGCTGACGGTACAG---GCCAGACAA---TTATTG---TCTGGCATAGTGCGACAGCAAAAC---
AAGTTG---CTGAGA---GCTATAGAGGCTCAGCAGCATCTGCTGAGACTCACGGTCTGGGGCATT
>JX236674..1
TTCTATTGCAACACATCAGACCTGTTTAATAGCAGTTGGAAGAACAGTACCAGCATACATAACAGTACC----
----

AGCAATGACACTATAACTATCCCATGCAGGATAAAGCAAATTATAAATATGTGGCAGAGAGCGGGAAAAGCAA
TATATGCCCCTCCCATCCAAGGAGTAATAAAGTGTGAATCCAAC---
ATTACAGGACTAATATTAACAAGAGATGGTGGAATTAGTAACAGTACA-------------------------
----

AATAAAAATGAAACCTTCAGACCTGGAGGGGGAGATATGAGGGACAATTGGAGAAGTGAATTATATAAGTATA
AAGTAGTAAAAATCGAGCCAATA---GGAATAGCACCCACCAGGGCAAGGAGAAGAGTGGTG-----------
----------------------------GAGAGAGAGAAAAGAGCA---GTTGGA---CTAGGAGCT------
---GTTTTCATTGGGTTCTTAGGAGCA---GCAGGAAGCACTATG---GGCGCGGCGTCA---
ATAACGCTGACGGTACAG---GCCAGACAA---TTATTG---TCTGGCATAGTGCAACAGCAGAGT---
AATCTG---CTGAAG---GCTATAGAGGCTCAACAGCAGCTGTTGAGACTCACGGTCTGGGGCATT
>KF859746..1
TTCTATTGTAATACATCAAGCCTGTTCAATAGCACGTGGCCTGCTAATGACAGCATGCAGGCAAATAGCACGG
AAAAT--

AGCACGGAGATGATAAAACTCTCATGCAACATAAAGCAAATTATAAATATGTGGCAGAGAGTAGGACAAGCAA
CATATGCCCCTCCCATCAAAGGAATAATAAAGTGTGAATCAAAC---
ATTACAGGATTAATATTAACAAGAGATGGTGGGAATAATAACAATGGA-------------------------
----

AACAATAAAAGTGAAACCTTCAGACCTGGAGGAGGAGATATGAGGGATAATTGGAGAAGTGAATTATATAAGT
ATAAGGTAGTAAGGATTGAACCATTA---GGAGTAGCACCCACCAGGGCAAAAAGACGAATGGTG--------
-------------------------------GAGAGAGAAAAAAGAGCA---GTTGGC---CTGGGAGCT---
------GTCTTCTTTGGGTTCTTAGGAGCA---GCAGGAAGCACTATG---GGCGCGGCGTCA---
ATAACGCTGACGGTACAG---GCCAGACAA---TTATTG---TCTGGCATAGTGCAACAGCAAAGC---
AATTTG---CTGAGA---GCTATAGAGGCTCAACAGCAGCTGTTGAGACTCACAGTCTGGGGCATT
>KF859747..1
TTCTACTGTGATACATCAGGCCTGTTTAATAGTACTTGGAATACCACTGACACTGGCAGCATAAACTCAAAT-
----

AGTACAGATAATATAACTCTCCCATGCAGAATAAAGCAAATTGTAAATATGTGGCAGAGAGTAGGACGAGCAA
TGTACGCCCCTCCCATCCAGGGAATAATAAGGTGTGAATCAAAC---
ATTACAGGATTACTATTAACAAGAGATGGTGGTAACAGTACAAAT----------------------------
----

GAAACCTTCAGACCTGGGGGAGGAGATATGAAAGACAATTGGAGAAGTGAATTATACAAATATAAAGTAGTAA
GAATTGAACCACTA---GGAATAGCACCCAACAGGGCCAGGAAAAGAGTGGTG--------------------
-------------------GGAAGAGAAAAAAGAGCA---GTTGGA---ATTGGAGCT---------
GTATTCCTTGGGTTCTTAGGAGCA---GCAGGAAGCACTATG---GGCGCGGCGTCA---
ATAACGCTGACGGTACAG---GCCAGACAA---TTATTG---TCTGGCATAGTGCAACAGCAAAGC---
AATTTG---CTGAGG---GCTATAGAGGCTCAACAGCAGATGTTGAAACTCACAGTCTGGGGCATT
>KP109491..1
TTCTATTGTAATACATCAGACCTGTTTAATAGCACCTGGCCCAGGGATGCCTGGAATAGCAGCAACCAGACTG
GGACAAGG---

GACAATATAAATATAACTCTCCAATGTAGAATAAAGCAAATTATAAATATGTGGCAGAGAACAGGACAAGCAA
TATATGCCCCTCCCATCCCAGGAAACATAACTTGTGTATCAAAC---
ATTACAGGACTAATATTAACAAGAGATGGTGGGGCTAATAATGAA----------------------------
----

GGCAATGAAACCTTCAGACCTGGAGGAGGAGATATGAGGGACAATTGGAGAAGTGAATTATATAAGTATAAAG
TAGTAAAAATTGAACCACTA---GGAGTGGCACCCACCAAGGCAAGGAGAAGAGTGGTG--------------

-------------------------CAGAGAGAAAAAAGAGCA---GTTGGA---ATGGGAGCT---------
TTGTTCATTGGGTTCTTAGGAGCA---GCAGGGAGCACTATG---GGCGCGGCGTCA---
ATAACGCTGACGGTACAG---GCCAGACAA---TTATTG---TCTGGTATAGTGCAACAGCAAAGC---
AATTTG---CTGAGG---GCTATAGAGGCTCAACAACATCTGTTGAAGCTCACAGTCTGGGGCATT
>KF716487..2
TTCTATTGTAATACAACAAAATTGTTTGATAGCACTTGGAACAAAACCACTTGGAACACAACGAGCACAGGGG
AGACAAAT---
----------AACACAGGG------
GAGACAAATGACACTATAACTATCCCATGTAGAATAAAGCAAATTATAAATATGTGGCAGAGGACAGGACAAG
CAATATATGCCCCTCCCATTCAAGGAATAATAAGGTGTGACTCAAAC---
ATTACAGGACTACTATTAACAAGAGATGGTGGGCCTGGTAATACA----------------------------
----

ACTGAAATCTTCAGACCTGGAGGAGGAGATATGAGGGATAATTGGAGAAGTGAACTATATAAATATAAAGTAG
TAAAAGTTGAACCATTA---GGAGTAGCACCCACCAGGGCAAAGAGAAGAGTGGTG-----------------
----------------------GAGAGAGAAAAAAGAACA---GTTGGA---CTGGGAGCT---------
GTTTTCCTTGGGTTCTTGGGAGCA---GCAGGAAGCACTATG---GGCGCGGCGTCA---
ATAACGCTGACGGTACAG---GCCAGACAG---TTATTG---TCTGGCATAGTGCAACAGCAAAGT---
AATTTG---CTGAGG---GCTATAGAGGCTCAACAGCATCTGTTGAAACTCACGGTCTGGGGCATT
>KF716490..1
TTCTATTGTAATACAACAAACCTGTTTAATACTACTTGGAGTAATGGCAGCCTGGTTAATATTACGGAGTCAA
ATAAT--
-------------------GCT---
ACAAATAATGCTACGATTCTTCTCCCATGCAGAATAAAGCAAATTATAAATATGTGGCAGAGAGTAGGACAAG
CAATGTATGCCCCTCCCATCCCAGGAGTAATACAGTGTACATCAAAC---
ATTACAGGACTATTATTAACAAGAGATGGTGGGAATATTACTAAC----------------------------
----

AATACAGAGATCTTTAGACCTGGAGGGGGAGATATGAGGGACAATTGGAGAAGTGAACTATATAAATATAAAG
TAGTAAGAATTGAACCATTA---GGAGTAGCACCCACCGGGGCAAAGAGAAGAGTGGTG--------------
-------------------------GAGAGAGAAAAAAGAGCAATTGCTGGA---ATAGGAGCT---------
GTCCTCTTTGGGTTCTTAGGAGCA---GCAGGAAGCACTATG---GGCGCGGCGTCA---
CTAACGCTGACGGTACAG---GCCAGACAA---TTATTG---TCTGGCATAGTGCAACAGCAAAGC---
AATTTG---CTGCAG---GCTATAGAGGCTCAACAGCATCTGTTGAAACTCACGGTCTGGGGCATT
>KF716482..3
TTCTATTGTGACACATCAGGCCTGTTTAATAGCAGCTGGGATGGCAACAGCGAGGATAACAATACTCAG----
----

GGAACTGACACTATAACTCTCCAATGCAAAATAAAGCAAATTATAAGGATGTGGCAAAGAACAGGACAAGCAA
TGTATGCCCCTCCCATCCAAGGAACAATAAAGTGTCAATCAAAC---
ATTACAGGGCTATTATTGACAAGAGATGGGGGGAGTGAAAGTACA----------------------------
----

AATGAAAGTGAAACCTTCAGACCTGGAGGAGGAAATATGAGGGACAATTGGAGAAGTGAATTATATAAATATA
AAGTAGTAAAGATTGAACCACTA---GGAGTAGCACCCACCAGGGCAAAGAGAAGAGTGGTG-----------
----------------------------GAGAGAGAAAAAAGAGCA---GTTGGA---CTGGGAGCT------
---GTCTTCATTGGGTTCTTAGGAGCA---GCAGGAAGCACTATG---GGCGCAGCGTCA---
GTGACGCTGACGGTACAG---GCCAGACAA---TTATTG---TCTGGTATAGTGCAACAGCAAAAC---
AATTTG---CTGAGG---GCTATAGAGGCTCAACAGCATCTGTTGAAACTCACGGTCTGGGGCATT
>KP109493..2
TTCTATTGCAACACAACAAGGCTGTTTAATAGCACTTGGAGTAATGACACTTGGGATAATAGCACTTGGGATA
AA---

TCAAACAACATTATAAATCTCCAATGCAGAATAAAGCAAATCATAAATATGTGGCAGAGAACAGGACGAGCAA
TGTATGCCCCTCCCATTCAAGGAGTAATAAGGTGTGAATCAAAC---
ATTACAGGACTAATATTAACAAGAGATGGTGGGAATAGTAACGAG----------------------------
----

ACAGAAATCTTCAGACCTGGGGGAGGAGATATGAGGGACAATTGGAGAAGTGAATTATATAAGTATAAAGTAG
TAAAAATTGAACCACTA---GGAGTAGCACCCACCGAGGCAAAGAGAAGAGTGGTG-----------------

----------------------GAGAGAGAAAAAAGAGCAGCTGTTGGA---CTGGGAGCG---------
TTCTTCATTGGGTTCTTAGGAGCA---GCAGGAAGCACTATG---GGCGCGGCGTCA---
ATAACGCTGACGGTACAG---GCCAGACAA---TTATTG---TCTGGCATAGTGCAACAGCAAAGC---
AATTTG---CTGAGG---GCTATAGAGGCTCAACAGCATATGTTGAAACTCACGGTCTGGGGCATT
>KY580490..1
TTCTATTGTGATACAGCAGAACTGTTTGATAGCGCTTGGAATAGTAGTAGCAATTGGACTATAACTGACAAGT
CA---

AATAATACCAATATAACCCTCCAGTGCAGAATAAAGCAAATTATAAATATGTGGCAGAGAGTAGGACAAGCAA
TGTATGCCCCTCCCATCCCAGGAGTAATAAGGTGTACATCAAAC---
ATTACAGGACTAATATTAACAAGAGATGGTGGGGAGACTAACAATGCA-------------------------
----

ACTGAAGAAACCTTCAGACCGGGAGGAGGAGACATGAGGGACAATTGGAGAAGTGAATTATATAAATATAAAG
TAGTAAAAATTGAACCACTA---GGAGTAGCACCCACCGGGGCAAAAAGAAGAGTGGTG--------------
-------------------------GAGAGAGAAAAAAGAGCA---ATTGGA---ATAGGAGCT---------
GTCTTCATTGGGTTCTTAGGAGCA---GCAGGAAGCACTATG---GGCGCAGCGTCA---
ATAACGCTGACGGTACAG---GCCAGACAA---TTATTG---TCTGGCATAGTGCAACAGCAAAGC---
AATTTG---CTGAAG---GCTATAGAGGCTCAACAACATCTGTTGAGACTCACAGTCTGGGGCATT
>KP109497..1
TTCTACTGTAATACATCAGGGTTGTTTAATAGTACATGGAGAAGGAACAGTACAAATGGTACATGGGAAAGTG
ATATATCAGGG--

GTAAATGAGAGTACAATCACACTCCCATGCAGAATAAAACAAATTATAAACATGTGGCAGGGGGTAGGAAGAG
CAATGTATGCCCCTCCCATTCCAGGATCAATTAATTGTTCATCAAAT---
ATTACAGGATTATTGTTGACAAGAGATGGTGGTACAAACAGTAGT----------------------------
----

CAGAATGACACCTTCAGACCTGGAGGAGGAGATATGAGGGACAATTGGAGAAGTGAATTATACAAATATAAAG
TAGTAAGAATTGAACCAATA---GGTCTGGCACCCACCAAGGCACAAAGAAGAGTGGTG--------------
-------------------------GCAAGAGAAAAAAGAGCA---ATAGGA---CTGGGAGCT---------
ATGTTCCTTGGGTTCTTGGGAGCA---GCAGGAAGCACGATG---GGCGCAGCGTCA---
GTGACGCTGACGGTACAG---GCCAGACAG---TTATTG---TCTGGTATAGTGCAACAGCAAAAC---
AATTTG---CTGAGG---GCTATAGAGGCGCAACAGCATCTGTTGCAACTCACAGTCTGGGGCATT
>KF716484..1
TTCTATTGTAACACATCAGGTCTGTTTAATAGCACATGGGAAAGGAATATGACATCAAATAGCACAGAG----
----

TCAAATGACACCATAACTCTCCAATGCAGAATAAAGCAGATTGTAAACATGTGGCAGAGAGTAGGACAAGCAA
TATATGCCCCTCCCATTCCAGGAGAAATAAGGTGTAGATCAAAC---
ATTACAGGACTAATATTAACAAGAGATGGTGGGGGGCAGAACGATACAGGA----------------------
----

GGAGGAGGAAACGAAACCTTCAGACCTGGAGGAGGAGATATGAGGGACAATTGGAGAAGTGAATTATACAAGT
ATAAAGTAGTAAAGATTGAACCACTA---GGAGTAGCACCCACCAAGGCAAAGAGAAGAGTGGTG--------
-------------------------------GCGAGAGAAAAAAGAGCA---ATTGGA---CTGGGAGCT---
------GCGTTCCTTGGGTTCTTAGGAGCA---GCAGGAAGCACTATG---GGCGCAGCGTCA---
ATAACGCTGACGGTACAG---GCCAGAGCA---TTATTG---TCTGGCATAGTGCAACAGCAAAGC---
AATTTA---CTGAAG---GCTATAGAGGCTCAACAGCAACTGTTGAAACTCACGGTCTGGGGCATT
>AY352657..2
TTCTACTGCAATACATCAGGACTGTTTAATAATAGTGTATGGAGGAATGATTATCCATCAAATAGTACAGGAG
TA---

AAGGAGAGCGTAATCACACTCCCATCCAAAATAAAACAAATTATAAACCTGTGGCAGGGAGTAGGAACAGCAA
TGTATCCCCCTCCCATTGAAGGACTAATCAAATGTTCATCAAAT---
ATTACAGGACTATTATTGACAAGAGATGGTGGTGTCAATAATAGT----------------------------
----

CAGAATGAGACCTTCAGACCTGGAGGAGGAGATATGAGGGACAATTGGAGAAGTGAATTATACAAATATAAAG
TAGTAAGACTTGAACCACTA---GGTCTGGCACCTACCAGGGCAAAGAGAAGAGTGGTG--------------

-------------------------GAAAGAGAAAAAAGAGCA---ATAGGA---CTAGGAGCT---------
ATGTTCCTTGGGTTCTTGGGAGCA---GCAGGAAGCACGATG---GGCGCAGCGTCA---
CTGACGCTGACGGTACAG---GCCAGACTG---TTATTG---TCTGGTATAGTGCAACAGCAAAAC---
AATTTG---CTGATG---GCTATAGAGGCGCAACAGCATCTATTGCAACTCACAGTCTGGGGCATT
>U36883..1
TTCTACTGCAATACATCAGGACTGTTTAATGGTACATGGAGTAATGGTACATGGAGTAATGATACATCAAATA
GTGCA--

AATGGAAATGGTACAATCACTCTCCCATGTAGAATAAAACAAATTATAAACATGTGGCAGGGAGTAGGAAAAG
CAATGTATGCCCCTCCCATTAAAGGACGAATCCAATGTGAATCAAAT---
ATTACAGGACTATTGTTGACAAGAGATGGCGGTCAGACCAATGAT----------------------------
----

AGTACTGAGACCTTCAGACCTGGAGGAGGAGATATGAGGGACAATTGGAGAAGTGAATTATACAAATATAAAG
TAGTAAGAGTTGAACCACTA---GGTCTAGCACCCACCAGGGCAAAGAGAAGAGTGGTG--------------
-------------------------GAAAGAGAAAAAAGAGCA---ATAGGA---CTAGGAGCT---------
GTGTTCCTTGGGTTCTTGGGAGCA---GCAGGAAGCACGATG---GGCGCAGCGTCA---
CTAACGCTGACGGTACAG---GCCAGACAG---TTAATG---GCTGGTATAGTGCAACAGCAGAAC---
AATTTG---CTGAGG---GCTATAGAGGCGCAACAGCACCTGTTGCAACTCACAGTCTGGGGCATT
>KJ579955..1
TTCTATTGCAACACATCAGGCCTATTTAATAGCATTTGGGAGAATGGCACT----------------------
----
-TTGAAAAGCAATGAC---------
AGCATGTCAAACAACATAACTCTCCAATGCAGAATAAAGCAAATTATAAATATGTGGCAGAGAACAGGACAAG
CAATGTATGCCCCTCCCATCCCAGGAGTAATAAGGTGTGAGTCAAAC---
ATTACAGGATTAATATTAACAAGAGATGGTGGG--

-
AATAATAACAATGAAAGTGAAACCTTCAGACCTGGAGGAGGAGATATGAGGGACAATTGGAGAAGTGAATTAT
ATAAGTATAAAGTAGTAAAAATTGAACCACTA---GGAGTAGCACCCACCAAAGCAAGGAGAAGAGTGGTG--
-------------------------------------GAGAGAGAAAAAAGAGCAGTTGTTGGA---
ATAGGAGCT---------GTCTTCTTTGGGTTCTTAGGAGCA---GCAGGAAGCACTATG---
GGCGCGGCGTCA---CTAACGCTGACGGTACAG---GCCAAGCAA---TTATTG---
TTTGGCATAGTGCAACAGCAAAGC---AATTTG---CTGAGG---
GCTATAGAGGCTCAACAGCATCTGTTGAAACTCACGGTCTGGGGCATT
>AF484503..1
TTCTATTGTAACACATCAGGCCTGTTTAATAACAGTTGGAATGGCACGCAGATGTCAAATAGCATGAAG----
----

CCAAATGACACCATAACTCTCCAATGCAGGATAAAGCAAATTGTAAATATGTGGCAGAGAGCAGGACAAGCAA
TATATGCCCCTCCTATCCGAGGAGTAATAAGGTGTGTATCAAAC---
ATTACAGGACTGATATTAACAAGAGATGGTGCTGAGGATAAGGAC----------------------------
----

AGTACAAAGGAAACCTTCAGACCTGCAGGAGGAGATATGAGGGACAATTGGAGAAGCGAATTATATAAATATA
AAGTAGTAAAAATTGAACCACTA---GGAGTAGCACCCACCAAGGCAAAGAGAAGAGTGGTG-----------
----------------------------GAGAGAGAAAAAAGAGCA---GTTGGA---ATAGGAGCT------
---GTCTTCCTTGGGTTCTTAGGAGCA---GCCGGAAGCACTATG---GGCGCAGCGTCA---
ATAACGCTGACGGTACAG---GCCAGACAA---TTGTTG---TCTGGCATAGTGCAACAGCAAAGC---
AATTTG---CTGAAG---GCTATAGAGGCTCAACAGCATCTGTTGAAACTCACGGTCTGGGGCATT
>AF484521..1
TTCTACTGCAATACATCACAACTATTTAATAGAACATGGAATGATAGTATATCAAATAATGCATGGAAGAATA
ATAGT--
-------------------ACA---
GGGGAAAATGACACAATCACTCTCCCATGCAGAATAAAACAAATTATAAACATGTGGCAGGAAGTAGGAAAAG
CAATGTATGCCCCTCCCATTAAAGGTCTAATCAGATGTTCCTCAAAT---
ATTACAGGACTATTGTTAGTAAGAGATGGTGGAAATAATAGTAGT----------------------------
----

CAGAATGAAACCTTCAGACCTGGAGGAGGAGATATGAGAGACAATTGGAGAAGTGAATTATACAAATATAAAG

TAGTAAGAATTGAACCACTA---GGTCTAGCACCCACCAAGGCAAAGAGGAGAGTGGTA-------------
------------------------GAAAGAGAAAAAAGAGCA---ATAGGA---CTGGGAGCT---------
GTATTCCTTGGGTTCTTGGGAGCA---GCGGGAAGCACGATG---GGCGCAGCGTCA---
CTGACGCTGACGGTACAG---GCCAGAAAT---CTATTG---TCTGGTATAGTGCAACAGCAAAAT---
AATTTG---CTGAGG---GCTATAGAGGCGCAACAGCATCTGTTGCAACTCACAGTCTGGGGCATT
>AF484522..1
TTCTACTGCAATACATCAGGACTGTTTAACAATAGTGTATGGACATCAAATAGTACGATAGGG---------
----

GCAAATGGGACAATCACACTCCCATGCAGAATAAAACAATTATAAATATGTGGCAGGGAGTAGGAAAAGCAA
TGTATGCCCCTCCCATTGAAGGACAAATCAACTGTTCATCAACT---
ATTACAGGACTATTGTTGACAAGAGATGGTGGTGTAAAAAATAAT---------------------------
----

AGTCAGAACGAGACCTTCAGACCTGGAGGGGGAGATATGAGAGACAACTGGAGAAATGAATTATACAAATATA
AAGTAGTAAGAATTGAACCACTA---GGTCTAGCACCCACCAAGGCAAGGAGAAGAGTGGTG----------
---------------------------GAAAGAGAAAAAAGAGCA---ATAGGA---CTGGGAGCT------
---GTGTTCCTTGGGTTCTTGGGAGCA---GCAGGAAGCACGATG---GGCGCAGCGTCG---
CTGACGCTGACGGTACAG---GCCAGACAG---TTACTG---TCTGGTATAGTGCAACAGCAAAAC---
AATTTG---CTGAGG---GCTATAGAGGCGCAACAGCATTTGTTGCAACTCACAGTTTGGGGCATT
>AF484482..1
TTCTACTGCAATACATCAGAACTGTTTAATAGTGAATGGAAAAATGGGACTACATCAAACAGTACA------
----

AGAAATAGGACAATCACACTCCCATGCAGAATAAAACAAATCATCAACATGTGGCAGGGAGTAGGAAGAGCAA
TGTATGCCCCTCCCATTAAAGGACTAATCAATTGTTCATCAAAT---
ATTACAGGACTATTGTTAACAAGAGATGGTGGTACAAATCAAAGT---------------------------
----

GAGGAGACTTTCAGACCTGGGGGAGGAGATATGAGAGACAATTGGAGAAATGAATTATACAAATATAAAGTAG
TAAGAATTGAACCACTA---GGTCTAGCACCCACCAGGGCAAAAAGAAGAGTGGTG----------------
---------------------GAAAGAGAAAAAAGAGCA---ATAGGA---CTAGGAGCT---------
TTGTTCCTTGGGTTCTTGGGAACA---GCAGGAAGCACGATG---GGCGCAGCGTCA---
CTGACGCTGACGGTACAG---ACCAGACAA---GTATTG---TCTGGTATAGTGCAACAGCAAAAC---
AATTTG---CTGAGG---GCTATAGAGGCGCAACAGCATCTGTTGCAACTCACAGTCTGGGGCATT
>AF484479..1
TTCTATTGTAACACATCAGGGTTGTTTAATAGCACTTGGACGGGTAACAACGCTGCCAACAGC---------
----

ACGGAGGGCAATATAACTCTCCCGTGCAGAATAAAGCAAATTATAAATATGTGGCAGAGAGTAGGACAAGCAA
TGTATGCCCCTCCCATCCAAGGAGTAATAAAATGTGTATCAAAT---
ATTACAGGAATATTATTAACAAGAGATGGTGGATCAAATGGAACT---------------------------
----

GGAAATGAAACCTTCAGACCTGGAGGAGGAGATATGAGGGACAATTGGAGAAGTGAACTATATAAGTATAAAG
TAGTAAAAGTTGAGCCACTA---GGGGTAGCACCCACCAGGGCAAGGAGAAGAGTGGTG-------------
------------------------GAGAGAGAAAAAAGAGCA---GTTGGA---ATAGGAGCT---------
GTATTCCTTGGGTTCTTAGGAGCA---GCAGGAAGCACTATG---GGCGCGGCGTCA---
ATAACGCTGACGGTACAG---GCCAGACAA---TTATTG---TCTGGCATAGTGCAGCAGCAAAGC---
AATTTG---CTGAGG---GCTATAGAGGCTCAACAGCATTTGTTGAGACTCACAGTCTGGGGCATT
>AF484517..1
TTCTATTGTAACACATCAGGCCTGTTTAACAGCACTTGGACAAATGGCACTATGCAGGGGTTAAAT------
----

GGCACAGGAAACATAACACTCTCATGCAGAATAAAACAAATTATAAACATGTGGCAGGGAGTAGGAAAAGCAA
TGTATGCCCCTCCCATTGAAGGACTAATCCAATGTTCATCAAAT---
ATTACAGGACTATTGTTGACAAGAGATGGTGGTAATAATAATAGT---------------------------
----

CAAAATGAGACCTTCAGACCTGGAGGAGGAGATATGAGAGACAATTGGAGAAGTGAATTATATAAATATAAAG

```
TAGTAAGAGTTGAACCAATA---GGTCTAGCACCCACCAAGGCAAAGAGAAGAGTGGTG--------------
-------------------------GAAAGAGAAAAAAGAGCA---ATTGGA---CTGGGAGCT---------
GTGTTCCTTGGGTTCTTGGGAGCA---GCAGGAAGCACGATG---GGCGCAGCGTCA---
ATGACGCTGACGGTACAG---GCCAGACAG---GTATTG---TCTGGTATAGTGCAACAGCAAAAT---
AATTTG---CTGAGG---GCTATAGAGGCGCAACAGCATCTGTTGCAACTCACAGTCTGGGGCATT
>AF484488..1
TTCTATTGTAATACATCAGGTCTGTTTAATAGTACTTGGAATAGTACTAAGGAAAATGATACTGACAGC----
-------------------------------------------------------------------------
----------------------------
GGGAGTGGCACCATAACTCTCCCATGCAAAATAAAGCAAATTATAAATATGTGGCAGAGAGTAGGACAAGCAA
TATATGCCCCTCCCATCAGAGGAGTAATAAGGTGTGATTCAAAC---
ATTACAGGACTACTGTTAACAAGAGATGGAGGGGATAGTAACAAT----------------------------
-------------------------------------------------------------------------
----------------
AATGAAACCTTCAGACCTGGAGGGGGAGATATGAGGGACAATTGGAGAAGTGAATTATATAAGTATAAAGTAG
TAAAAATTGAGCCAATA---GGAGTAGCACCCACCAGGGCAAAGAGAAGAGTGGTG-----------------
----------------------CAGAGAGAAAAAAGAGCA---GTTGGA---ATAGGAGCT---------
GTATTCCTTGGGTTCTTGGGAGCA---GCAGGAAGCACTATG---GGCGCAGCGTCA---
ATAACGCTGACGGTACAG---GCCAGACAA---TTATTG---TCTGGCATAGTGCAACAGCAAAGC---
AATTTG---CTGATG---GCTATAGAGGCTCAACAGCATCTGTTGAAACTCACGGTCTGGGGCATT
>AF484492..2
TTCTATTGTAACACATCAAAGTTGTTTAATAGCACTTGGGGGGAAAATAGCACTTGGGGGGAAAATAGCACTA
GCATA--------------------------------------------------------------------
-------------------------
GAGTTAAATGACACTATAACTCTCCCGTGCAAAATAAAGCAAATTATAAATATGTGGCAGAGAACAGGACAAG
CAATATATGCCCCTCCCATCCAAGGAGTGATAAGGTGCGAGTCAAAT---
ATTACAGGACTATTGTTAACAAGAGATGGTGGTAAAAATAATAGT----------------------------
-------------------------------------------------------------------------
----------
AATCAGAATGAAACCTTCAGACCTGGAGGAGGAGATATGAGGGACAACTGGAGAAGTGAGTTATATAAGTATA
AAGTAGTAAAAATTGAACCTTTG---GGAGTAGCACCCACCAAGGCAAGGAGAAGAGTGGTG-----------
----------------------------GGAAGAGAAAAAAGAGCA---GTAGGA---CTAGGAGCT------
---TTGTTCCTTGGGTTCTTGGGAGCA---GCAGGAAGCACTATG---GGCGCAGCGTCA---
ATAACGCTGACGGTACAA---GCCAGACAA---TTATTG---TCTGGCATAGTGCAACAGCAAAAC---
AATTTG---CTGAGG---GCTATAGAGGCTCAACAGCATCTGTTGAAACTCACGGTCTGGGGCATT
>AF484496..2
TTCTATTGTAATACATCAGGCCTGTTTAATAGCACCTGGGACAGCAAGACAAATTACACGGAGTCAAAT----
-------------------------------------------------------------------------
----------------------------
AGCACTACTAATATAACTCTTCCATGCAGAATAAAGCAAATTATAAATATGTGGCAGAGAGTAGGACAAGCAA
TGTATGCCCCTCCCATCCAAGGAGTAATAAATTGTGTGTCAGAC---
ATTACAGGACTACTATTAGTAAGAGATGGTGGTCCTGGAAATACA----------------------------
-------------------------------------------------------------------------
----------------
TCTGAAACCTTCAGACCTGGAGGAGGAAATATGAGGGACAATTGGAGAGGTGAATTATATAAGTATAAAGTAG
TACAAATTGAACCACTA---GGAGTAGCACCCACCAGGGCAAAGAGAAGAGTGGTG-----------------
----------------------GAGAGAGAAAAAAGAGCA---GTTGGA---ATAGGAGCT---------
GTATTCCTTGGGTTCTTGGGAGCA---GCAGGAAGCACTATG---GGCGCGGCGTCA---
ATAACGCTGACGGTACAG---GCCAGACTA---TTATTG---TCTGGTATAGTGCAACAGCAAAAT---
AATTTG---CTGAGG---GCTATAGAGGCTCAACAGCATCTGTTGAAACTCACAGTCTGGGGCATC
>KJ948659..1
TTCTATTGTAACACATCAGAACTGTTTAATAGCACTTGGAATGACACTAAC----------------------
-------------------------------------------------------------------------
-------TGGCAGGGATCA------
AATGACACGGAGAACATAACGCTCCCATGCAGAATAAAGCAAATTATAAATATGTGGCAGAGAACAGGACAAG
CGATATATGCCCCTCCCATCCAAGGAGTAATACAGTGTAAATCAAAC---
ATTACAGGACTAATATTAACAAGAGATGGGGGG----------------------------------------
-------------------------------------------------------------------------
-
AAGAATAACAGTAATAATGAAACCTTTAGACCTATAGGAGGAGATATGAGGGACAATTGGAGAAGTGAATTGT
```

ATAAGTATAAAGTAGTAAAAATTGAACCCTTA---GGAGTAGCACCCACTAAGGCAAAGAGAAGAGTGGTG--
-------------------------------------GAGAGAGAAAAAAGAGCA---GTGGGA---
CTAGGAGCT---------GTTTTCCTCGGGTTCTTAGGAGCA---GCAGGAAGCACTATG---
GGCGCGGCGTCA---ATAACGCTGACGGCACAG---GCCAGACAA---CTATTG---
TCTGGCATAGTGCAACAGCAAAGC---AATTTG---CTGAGG---
GCTATAGAGGCTCAACAGCATCTGTTGAAGCTCACGGTCTGGGGCATT
>JF683744..3
TTCTACTGCAATACATCACGGCTATTTAATAATAGCGCAGCATCCAAT-------------------------
----

GGTACTATCCCTATCACAATCCCATGTATCATAAACATGTGGCAGGGAGTGGGGCAGGCAATGTATCAGGCAA
TCTATGCTGGAAACATTGCAGGAAAAATAAATAGTACATCCATT---ATT------------
TCAGCAAGAGATGGGGCCAGAGATGGAGGCAATGAT-------------------------------------
----
AGTAACTATACTGAAACCTTCAGGCCTGGGGGAGGAAATATGAAAGACAATTGGAGAAGTGAGCTATATAAAT
ACAAAGTAGTAGAAATTAAACCACTA---GGAGTAGCGCCCACCAGGGCAAAAAGACAAGTGGTG--------
-------------------------------AGAAGAGAAAAAAGAGCG---GCA------CTAGGAGCT---
------TTGTTCCTTGGGTTCTTGGGAGCA---GCAGGAAGCACTATG---GGCGCGGCGTCA---
ATAACGCTGACGGTACAG---GCCAGACAA---TTATTG---TCTGGAATAGTGCAACAGCAAAAT---
AATTTG---CTGAGA---GCTATTGAAGCGCAACAACATCTGTTGCAGCTCACAGTCTGGGGCATT
>AY371164..2
TTCTATTGTAATACATCAGGCCTGTTTAATAGCACTTGGCCCAATGGCACTGGGCCTAATAATGGCACTTGGA
GC---

AGTGAAAGTGACATTATACAACTCCCATGCAAAATAAAGCAAATTATAAATGTGTGGCAGAGAACAGGACAAG
CAATATATGCCCCTCCCATCCCAGGAGTAATAAGGTGTGAATCAAAC---
ATTACAGGACTACTTTTAACAAGAGATGGTGGGAATGGTGGGAATGAT-------------------------
----

ACCAATCACACTGAGATCTTCAGACCTGGAGGAGGAAATATGAAGGACAATTGGAGAAGTGAATTATATAAAT
ATAAAGTAGTAAAAATTGAACCATTA---GGAATAGCACCCACCAGGGCAAAGAGAAGAGTGGTG--------
-------------------------------GAGAGAGAAAAGAGAGCA---GTCGGA---ATAGGAGCT---
------GTGTTCATTGGGTTCTTGGGAGCA---GCAGGAAGCACTATG---GGCGCAGCGTCA---
ATAACGCTGACGGCACAG---GCCAGACAG---TTATTG---TCTGGTATAGTGCAACAGCAAAGC---
AATTTG---CTAAGG---GCTATAGAGGCTCAACAGCAACTGTTGAAACTCACGGTCTGGGGCATT
>KP718932..1
TTCTATTGCAATACATCAGGCCTGTTTAATAGCACGTGGAATAACACTGGCAGCATGCAAGACAATAATAGTG
AC---

ACTACTGGCACTATAATTCTCCCGTGCAACATAAAGCAAATTGTAAACATGTGGCAGAGAGTAGGACAAGCAA
TGTATGCCCCTCCCATCCCAGGAATAATAAAATGTGACTCAAAC---
ATTACAGGGATGTTATTGACAAGGGATGGCGGTCTTAATGATAGT----------------------------
----

GCAGAGACTTTCAGACCTGGAGGAGGAGATATGAGAGACAATTGGAGAAGTGAATTATATAAATATAAAGTAG
TAAAAATTGAACCACTA---GGAGTAGCACCCACCAGGGCAAAGAGAAGGGTGGTG-----------------
----------------------GAGAGAGAAAAAAGAGCA---GTTGGA---CTGGGAGCT---------
GTTTTCCTTGGGTTCTTAGGAGCA---GCAGGAAGCACTATG---GGCGCGGCGTCA---
ATAACGCTGACGGTACAG---GCCAGACAA---TTATTG---TCTGGTATAGTGCAACAGCAAAGC---
AATTTG---CTGAGG---GCCATAGAAGCTCAACAGCATATGTTGAGACTCACAGTCTGGGGCATT
>GU332510..2
TTCTACTGTAACACAACCCAGCTGTTTAATAACATAAGAGGA-------------------------------
----

AATGACACTATCACCCTACCATGTAGAATAAAACAAATTGTAAATATGTGGCAGGGGGTAGGACAAGCAATGT
ATGCTTCTCCCATTGCCGGAAAAATTCAGTGTAACTCAACT---
ATCACAGGTCTCATATTGACAAGAGATGGTGGTAATACCACCAAT----------------------------
----

GGTACCGACACCCTCAGACCTGGAGGGGGAAATATGAAAGACAATTGGAGAAGTGAATTGTATAAATATAAGG

TAGTGAAAATTGAGCCAATA---GGAGTAGCACCCACCAAGGCAAAAAGACAAGTGGTG-------------
------------------------AGGAGAGAAAAAAGGGCA---GTGGGA---ATGGGAGCT---------
GTGTTCCTTGGGTTCTTAGGAGCA---GCAGGAAGCACTATG---GGCGCAGCGTCA---
ATAACGCTGACGGTACAG---GCCAGACAA---TTATTG---TCCGGTATAGTGCAACAGCAGAGC---
AATCTG---CTGAGG---GCTATAGAGGCTCAACAACATCTGTTGAAGCTCACGGTCTGGGGCATT
>EU191618..3
TTCTATTGCAGTACATCAGGCCTGTTTAATAGCACTTGGGAAAATGGCACTCAGGTGGCAAATGACACGGGG-
----

TCAAATGACACTATAACTCTCCCATGCAGAATAAAGCAAATTGTAAGAATGTGGCAGAGAGTAGGACAAGCAA
TGTATGCCCCTCCCATCCAAGGAATGATAAGATGTGAATCAAAC---
ATTACAGGATTAATATTAACAAGAGATGGTGTTGGGAATAACAGT---------------------------
----

ACAAATGAAACCTTCAGACCTGGAGGAGGAGATATGAGGGACAATTGGAGAAGTGAATTATATAAGTATAAAG
TAGTAAAAATTGAACCATTA---GGAGTAGCACCCAGCAGGGCAAAGAGAAGAGTGGTG-------------
------------------------GCGAGAGAAAAAAGAGCA---GTTGGA---CTGGGAGCT---------
GTTTTCCTTGGGTTCTTAGGAGCA---GCAGGAAGCACTATG---GGCGCAGCGTCA---
CTAACGCTGACGGTACAG---GCCAGACAA---TTATTG---TCTGGCATAGTGCAGCAGCAGAGC---
AATCTG---CTAAGG---GCTATAGAGGCTCAACATCATCTGTTGCAGCTCACTGTCTGGGGCATT
>EF025170..1
TTCTATTGTAATACATCAGGCCTGTTTAATAGTACTTGGCCTAATGGCACTCGGAGCGAAGAGGACAAT----
----

GAAACTGACATTATAACTCTCCCATGCAGAATAAAGCAAATTATAAATATGTGGCAGAGAACAGGACAAGCAA
TATATGCCCCTCCCATCCAAGGAGTAATAAGGTGTGAATCAAAC---
ATTACAGGACTACTTTTAACAAGAGATGGTGGGAATGATAGCAAT---------------------------
----

CACACTGAGATCTTCAGACCTGGAGGGGGAAATATGAAGGACAACTGGAGAAGTGAATTATATAAGTATAAAG
TAGTAAAAATTAACCCATTA---GGGGTAGCACCCACCAGGGCAAGAAGAAGAGTGGTG-------------
------------------------GGAAGAGAAAAAAGAGCA---GTTGGA---CTGGGCGTC---------
GTCCTCCTTGGGTTCTTAGGAACA---GCAGGAAGCACTATG---GGCGCGGCGTCA---
ATAACGCTGACGGTACAG---GTCAGACAA---TTATTG---TCTGGCATAGTGCAACAGCAAAGC---
AATTTG---CTGAGG---GCTATAGAGGCGCAGCAACATCTGCTGCAACTCACAGTCTGGGGCATT
>JX422197..1
TTCTATTGCAATACATCAGGACTGTTTAATAGCACTTGGAAGAACAATGGCAATGGCACTTGGAGGGAAAATA
GCACTGGGAT--

GATAATACAGGCAACATAACTCTCCAATGTAGAATAAAGCAAATTGTAAATATGTGGCAGAAAGTAGGACAAG
CAATGTATGCCCCTCCCATCCAAGGAGTAATAAGGTGTGAATCAAAC---
ATTACTGGGCTATTATTAACAAGAGATGGAGGAAATACTAATAATACA------------------------
----

ATTAATGAAACCTTCAGACCTGGAGGGGGAGATATGAGGGACAATTGGAGAAGTGAATTATATAAGTATAAAG
TAGTAAAAATTGAACCACTA---GGTGTAGCACCCACCCGTGCAAAAAGAAGAGTGGTG-------------
------------------------GAGAGAGAAAAAAGAGCA---GTAGGA---CTGGGAGCT---------
GTCTTCCTTGGGTTTCTAGGAGCA---GCAGGAAGCACTATG---GGCGCGGCGTCA---
ATAACGCTGACGGTACAG---GCTAGACAA---TTATTG---TCTGGTATAGTGCAACAGCAGAGC---
AATTTG---CTGAAG---GCTATAGAGGCTCAACAACAGCTGTTGAGACTCACAGTCTGGGGCATT
>AF457062..1
TTCTATTGTAATACATCAAGCCTGTTTAATAGCACCTGGCCCTGGGAGAAGTTAAACAGCACAGATAATAGTA
GC--

ACAGATGACAATATAACTCTCACATGCAGAATAAAGCAAATTATAAATATGTGGCAGAGAGTAGGACAAGCAA
TATATGCCCCTCCTATCCCAGGAGAAATAAGATGTGTATCAAAC---
ATTACAGGACTAATATTAACAAGAGATGGTGGGGATAATAGCAGT---------------------------
----

ACAAATGAAACCTTCAGACCTGGAGGAGGAAATATGAGGGACAATTGGAGAAGTGAATTATATAAGTATAAAG

TAGTAAAAATTGAACCACTA---GGAGTAGCACCCACCACGGCAAGGAGAAGAGTGGTG-------------
------------------------CAGAGAGAAAAAAGAGCA---GTTGGA---ATAGGAGCT---------
GTTTTCATTGGGTTCTTAGGAGCA---GCAGGAAGCACTATG---GGCGCAGCGTCA---
ATAACGCTGACGGTACAG---GCCAGACAA---TTATTG---TCGGGCATAGTGCAACAGCAAAGC---
AATTTG---CTGAGG---GCTATAGAGGCTCAACAGCATCTGTTGAAACTCACAGTCTGGGGCATT
>KC186563..1
TTCTATTGCAACACATCAGGCCTGTTTAATAGCACTTGGAGTAATACTACTGTGGCAAATAATACGGGG----
----

GTAAATGGAACTATAATACTCCCATGCAGAATAAAACAAATTATAAATATGTGGCAGAGAGCAGGACAAGCAA
TATATGCCCCTCCCATCCAAGGATTAATAAATTGTGTATCAAAC---
ATTACAGGACTACTATTAACAAGAGATGGTCAGAAGAATGGTAGT---------------------------
----

GACAATGATACCTTTAGACCTATAGGAGGAAATATGAAGGACAATTGGAGAAGTGAATTATATAAATATAAAG
TGGTGGAGATTAGACCATTA---GGAGTAGCACCCACCAGGGCAAAAAGAAGAGTGGTG-------------
------------------------GAGAGAGAAAAAAGAGCA---GTGGGA---ATAGGAGCT---------
GTGCTCTTTGGGTTCTTGGGAGCA---GCAGGAAGCACTATG---GGCGCGGCGTCA---
ATAACGCTGACGGTACAG---GCCAGACAA---TTGTTG---ACTGGCATAGTGCAACAGCAAAGC---
AATTTG---CTGCAG---GCTATAGAGGCACAACAGCATCTATTAAAGCTCACTGTCTGGGGCATT
>GU201508..1
TTCTATTGCAATACATCACTCCTGTTTAACAGCACCTGGATGGTGAATGGCACTGGGGTCACCAATGGCAATA
AT--

GTCACCAATGACATTATAACACTCCCATGCAGAATAAAGCAAATTATAAATATGTGGCAGAGGGTAGGACAAG
CAATGTACGCCCCTCCCATCCGAGGAATCATAAATTGTGTATCAAAC---
ATTACAGGACTACTCTTAACAAGAGATGGTGGTAGCAGCCAAAAC---------------------------
----

GGATCTGAGACCTTCAGACCTGGAGGAGGAGATATGAGAGACAATTGGAGAAGTGAATTATATAAGTATAAAG
TAGTAAAAATTGACCCAATA---GGAGTAGCACCCACCAGGGCAAAAAGAAGAGTGGTG-------------
------------------------GAGAGAGAAAAAAGAGCA---CTTGTTGGACTGGGAGCT---------
GTCTTCCTTGGGTTCTTAGGAACA---GCAGGAAGCACTATG---GGCGCGGCATCA---
CTAACGCTGACGGTACAG---GCCAGACAA---TTATTG---TCTGGCATAGTGCAACAGCAGAGC---
AATCTG---CTGAGG---GCTATAGAGGCTCAACAGCATTTGTTGAGACTCACGGTCTGGGGCATT
>KP718927..2
TTCTATTGCAATACAACACAACTGTTTAATAGTACCTGGCAAGAGGGCACCAACAACAGCAATGCAGGT----
----

AATGATACTACTATTACACTGCCATGCAGAATAAAACAAATTGTAAACATGTGGCAGAGAGTAGGACAAGCAA
TGTATGCCCCTCCCATCCAAGGAGCGATAAAGTGTGTATCTAAC---
ATTACAGGACTAATATTGACAAGAGATGGTGGGAATAATAGGACA---------------------------
----

AATGAGACCATCAGACCTGGAGGAGGGGATATGAGGGACAATTGGAGAAGTGAATTATATAAATATAAAACAG
TGAGAATTGAACCACTA---GGAATAGCACCCACTCATGCAAGGAGAAGAATGGTG----------------
---------------------AACAGAGAAAAAAGAGTA---GCTGGA---ATGGGAGCT---------
GTCTTCTTTGGGTTCTTAGCAGCA---GCAGGAAGCACTATG---GGCGCAGCGTCA---
ATAACGCTGACGGTACAG---GCCAGACAA---CTATTG---TCTGGTATAGTGCAACAGCAAAGC---
AATTTG---CTGAAT---GCTATAGAGGCTCAACAGCAGCTGTTGAAACTCACGGTCTGGGGCATT
>KP718919..2
TTCTATTGCAATACATCGGGCCTGTTTAATAGCACTTGGGGAACTGGGGACTACACTGCAGGAAATGACACTG
TA--

GGAAATGACACTATAACTCTCCGGTGCAGAATAAAGCAAATTATAAATATGTGGCAGAAAGTAGGACAAGCAA
TGTATGCCCCTCCCATCCCAGGAGTAATAAGGTGTGAGTCAAAT---
ATTACAGGACTACTCTTGACAAGAGATGGTGGGAATAATAATAGT---------------------------
----

ATAAATGAGACTTTCAGACCTGGAGGAGGAGATATGAGAGACAATTGGAGAAGTGAATTATATAAATATAAAG

TAGTAAAAATTGAACCACTA---GGAATAGCACCTACCCAGGCAAAGAGAAGGGTGGTG--------------
-------------------------GAGAGAGAAAAAAGAGCA---GTTGGA---TTAGGAGCT---------
TTCTTCCTTGGGTTCTTAGGAGCA---GCTGGAAGCACTATG---GGCGCAGCATCA---
CTAACGCTGACGGTACAG---GCCAGACAA---TTATTG---TCTGGCATAGTGCAACAGCAAAGC---
AATTTG---CTAAGG---GCTATAGAGGCTCAACAACAGATGTTGAGACTCACGGTCTGGGGCATT
>KY359380..1
TTTTATTGCAATACATCAGGCCTGTTTACTAATGGCACAAATATAACT-------------------------
----

ACAAATATAAATATAACTCTCCCGTGCAGAATAAAACAAATTATAAATATGTGGCAGAGAGTAGGACAAGCAA
TGTATGCCCCTCCCATCGCCGGAGTAATAAGATGTGACTCAAAC---
ATTACAGGACTACTGTTAACAAGAGATGGTGGAATTAACAATGAC----------------------------
----

AGTAATACTGAGGTCTTCAGGCCTACAGGAGGAGATATGAGGGACAATGGGAGAAGTGAATTATACAAATATA
AAGTAGTAAAACTTGAACCCCTA---GGAGTAGCACCCACCAGGGCAAGGAGAAGAGTGGTG-----------
----------------------------GGAAGAGAAAAAAGAGCA---GTTGGA---CTGGGAGCT------
---GCCTTCCTTGGGTTCTTAGGAGCA---GCAGGAAGCACTATG---GGCGCAGCGTCA---
CTAACGCTGACGGTACAG---GCCAGACAA---TTATTG---TCTGGCATAGTGCAACAGCAAAGC---
AATTTG---CTGAGG---GCTATAGAGGCTCAACAACATATGTTGAAACTCACAGTCTGGGGCATT
>KJ849789..2
TTCTATTGCAATACATCAGGCCTGTTTAATAGCACTTGGAATAACACACAGCCA-------------------
----
-GATAACACAACAGAT---------
AACACAACAGAGTTCATAACTCTGCCATGCAGAATAAAGCAAATCATAAATATGTGGCAGAGAGTAGGACAAG
CAATGTATGCCCCTCCCATTCAAGGAATAATAAATTGTACATCAAAT---
ATTACAGGGCTAATATTAACAAGAGACGGT---

-
AATCCTAATGCGAGTAGTGAAACCTTCAGACCTGGAGGAGGGGATATGAGGGACAATTGGAGAAGTGAACTAT
ATAAGTATAAAGTAGTACAAATTGAACCACTA---GGAGTAGCACCCACCTGGGCAAGGAGAAGAGTGGTG--
-------------------------------------CAGAGAGAAAAAAGAGCA---GTTGGA---
CTGGGAGCT---------GTCTTCCTTGGGTTCTTGGGAGCA---GCAGGAAGCACTATG---
GGCGCAGCGTCA---ATGACGCTGACGGTACAG---GCCAGACAA---TTATTG---
TCTGGTATAGTGCAACAGCAGAGC---AATCTG---CTGAAG---
GCTATAGAGGCTCAACAACATCTGTTGAAACTCACAGTCTGGGGCATT
>FJ388929..3
TTCTATTGCAATACATCAGGCCTGTTTAATAGTACTTACAATGGGACAGAGGAGGCAAATGAAAATGACACTA
AG---

GCAAATGACACTATAATACTTCCATGCAGAATAAAGCAATTTATAAATATGTGGCAGAGAGTAGGAAAAGCAA
TGTATGCCCCTCCCATTCAAGGAGTAGTACAGTGTAATTCAAGC---
ATTACAGGATTATTATTAACAAGAGATGGTGGTAGTAAAAATGAG----------------------------
----

ACTGGGAAACATTCAGACCTGGAGGAGGAAATATGAGAGACAATTGGAGAAGTGAATTATATAAGTATAAAG
TAGTAAGGATTGAACCACTA---GGAGTAGCACCCACCAAGGCAAAGAGAAGAGTGGTG--------------
-------------------------GAGAGAGAAAAAAGAGCA---GTCGGA---CTGGGAGCG---------
GTTTTCCTTGGGTTCTTAGGAACA---GCAGGAAGCACTATG---GGCGCAGCGTCA---
ATAACGCTGACGGTACAG---GCCAGACAG---CTATTA---TCTGGCATAGTGCAACAGCAGAGA---
AATCTG---CTGCAG---GCTATAGAGGCTCAACAGCATCTGTTGAAACTCACAGTTTGGGGCATT
>EU191612..1
TTCTATTGCGATACAACAAAATTGTTTACAAACTTGTCTACAAATAGCACGGGGTTGGATGATAATAGCACGG
GG---

TCGAATGATACCATAATTCTCCCGTGCAGAATAAAACAAGTTATAAATATGTGGCAGAGAGTAGGACAAGCAA
TGTATGCCCCTCCTATCCGAGGAGTAATAAGGTGTAACTCTAAC---
ATTACAGGACTACTATTAACAAGAGATGGTGGGAATGGGAATAAC----------------------------
----

AGTACAGAGGAGATTTTCAGACCTGGAGGAGGAGATATGAGGGATAATTGGAGAAGTGAATTATATAAGTATA
AAGTAGTAAAATTGAACCACTG---GGAGTAGCACCCACCAGGGCAAGGAGAAGAGTGGTG------------
----------------------------CAGAGAGAAAAAAGAGCA---GTTGGA---CTGGGAGCT------
---GTCTTCCTTGGATTCTTAGGAGCA---GCAGGAAGCACTATG---GGCGCGGCGTCC---
ATAACGCTGACGGTACAG---GCCAGACAA---TTATTG---TCTGGCATAGTGCAACAGCAGAGC---
AATTTG---CTGAGG---GCTATAAAGGCTCAACAACATCTGTTGAAACTCACGGTCTGGGGCATT
>AF069672..2
TTCTATTGTAATACATCAAGCCTGTTTAACAGCACGTGGAGTAACGATAACAACACTCAGGGGTCAAATAGCA
CA---

GAGACAAAAGGCACTATAACTCTCCCATGCAGAATAAAGCAAATTATAAATATGTGGCAGAGAGCAGGAAAAG
CAATGTATGCCCCTCCCATCCAAGGAGTAATAAGGTGTGAATCAAAC---
ATCACAGGACTAATATTAACAAGAGATGGTGGAGATGCCGGGGAA----------------------------
----

AATGAAACCTTCAGACCTGGAGGAGGAGATATGAGGGACAATTGGAGAAGTGAGTTATATAAGTATAAAGTAG
TAAAAATTGAACCACTA---GGAGTAGCACCCACCCAGGCAAGGAGAAGAGTGGTG-----------------
----------------------AAGAGAGAAAAAAGAGCA---GTTGGA---CTAGGAGCT---------
GTCTTCATTGGGTTCTTAGGAGCA---GCAGGAAGCACTATG---GGCGCGGCGTCA---
ATAACGCTGACGGTACAG---GCCAGACAA---TTATTG---TCTGGCATAGTGCGACAGCAAAGT---
AATTTG---CTGAAG---GCTATAGAGGCTCAACAACATCTGTTGAAACTCACGGTCTGGGGCATT
>AF411965..2
TTCTACTGCAATACATCAAGACTGTTTAATAGCACATTTGATAACACATCTCTGGTTAATGGCACGGGTAATA
GTACCAAC---
-------------ACACAG------
GGGGCAAATGACACTATATATATTCCATGCAGAATAAAACAGATTATAAACATGTGGCAAAGAGTGGGACAAG
CAATGTATGCCCCTCCCATTGCAGGAGTAATAAAATGTGAATCAAAC---
ATTACAGGAATGCTATTAACAAGAGACGGTGGGTCTGAAAGTAAC----------------------------
----

GGTACAGAGGTCTTCAGACCTGGAGGAGGAGATATGAAGGACAATTGGAGAAGTGAATTATATAATTATAAAG
TAGTAAAAATTGAACCACTA---GGAGTAGCACCCACCAGGGCAAAGAGAAGAGTGGTG--------------
-------------------------GAGAGAGAAAAAAGAGCA---GCTGGA---ATAGGAGCT---------
GTCCTCCTTGGCTTCTTAGGAGCA---GCAGGAAGCACTATG---GGCGCGGCGTCA---
ATAACGCTGACGGTACAG---GCCAGGAAA---TTATTG---ACTGGCATAGTGCGGCAGCAAAGC---
AATTTG---CTGCAG---GCTATAGAGGCTCAACAACATCTGTTGAAACTCACGGTCTGGGGCATT
>U86778..1
TTCTATTGCAATACATCAGGCCTGTTTAATAGTACATACAATAGGGCATACACGACTAATGGTACAGGA----
----

AATGACTCAACCATCACACTCCCATGCAGAATAAAACAAATTATAAACATGTGGCAGGAGGTAGGGCGAGCAA
TATATGCCCCTCCCATTGCAGGAAACATAACATGTATATCAAAT---
ATCACAGGACTATTATTGCTACGTGATGGAGGAAAAGAAGACAAT----------------------------
----

AAAACAGAGATGTTCAGACCTGGAGGAGGAGATATGAGGGACAATTGGAGAAGTGAATTATATAAATATAAAG
TGGTAGAAATTAAACCATTG---GGAATAGCACCCACTAAGCCACCCAGGAGAGTGGTG--------------
-------------------------GAGAGAGGTGGAAGAACA---GTGGGA---ATAGGAGCT---------
GTGTTCCTTGGGTTCTCGGGAGCA---GCAGGAAGCACTATG---GGCGCGGCGTCA---
ATAACGCTGACGGTACAG---GCCAGACAA---CTGTTG---TCTGGTATAGTGCAACAGCAAAGC---
AATTTG---CTGAAG---GCTATAGAGGCTCAACAGCATCTGTTGAAACTCACGGTCTGGGGCATT
>L22955..1
TTCTATTGCAATACATCAAACCTGTTTAATAGTACATACAATCCTAATAGTACAGAAAGTAAT----------
----

TCAGACTTAAACATCACACTCCCATGCAGAATAAAACAAATTATAAACATGTGGCAGAGAGTAGGACGAGCAA
TGTATGCCCCTCCCATTGCAGGAGTAATAAAGTGTACATCAAAC---
ATTACAGGAATAATATTGTCAAGAGATGGTGGGAACAGTGGGAACAGT-------------------------
----

ACCAATGGGACTGAGACCTTCAGACCTGGAGGAGGAGATATGAGGGACAATTGGAGAAGTGAATTATATAAGT
ATAAAGTAGTAAAACTAGAACCACTA---GGAGTAGCACCCACCAAGCCAAGGAGAAGAGTGGTG--------
-------------------------------GAGAGAGAAAAAAGAGCA---GTTGGA---CTGGGAGCT---
------GTCTTCCTTGGGTTCTTGGGAGCA---GCAGGAAGCACTATG---GGCGCGGCATCA---
ATAACGCTGACGGTACAG---GCCAGACAA---CTGTTG---TCTGGTATAGTGCAACAGCAAAGC---
AATTTG---CTGAAG---GCTATAGAGGCTCAACAGCATCTGTTGAAACTCACGGTCTGGGGCATT
>MK086111..1
TTCTATTGTAATACATCAAGCCTGTTCGATAGTGCTTGGCTTAATAATGAAACTGGCATCGGGCAAAACATCA
CAGAG--

TCAAATAATACTATAACTCTCCAATGCAGAATAAAACAATTTATAAATATGTGGCAGAGAGTAGGGCAAGCAC
TGTATGCCCCTCCTATTCCAGGAAACATAACCTGTAGATCAAAC---
ATTACAGGGCTGCTACTAACAAGAGATGGTGGGAATAATACGAGTGATAATGGT-------------------
----

ACAGTTATTGAAACATTCAGACCTGGAGGAGGAGATATGAGGGACAATTGGAGAAGTGAATTATATAAATATA
AAGTAGTAAAACTTGAACCACTA---GGAATAGCACCCACCGAGGCAAGGAGAAGAGTGGTG-----------
----------------------------GAGAGAGAAAAAAGAGCA---ATTGGA---CTAGGAGCT------
---TTTTTCCTTGGGTTCTTGGGAGCA---GCAGGAAGCACTATG---GGCGCAGCGTCA---
CTAACGCTGACGGTACAG---GCCAGACAA---TTATTG---TCTGGTATAGTGCAACAGCAAAAC---
AATTTG---CTGAAG---GCTATAGAGGCTCAGCAGCTCATGTTGAAACTCACGGTCTGGGGCATT
>HQ385818..1
TTCTACTGTAATACATCACAATTGTTTAATAGTACTTGGAAGAATGGTACTTTTGCCAATGGCACTGAA----
----

GGAAATGGCACAATCACACTCCCATGCAGAATAAAGCAATTTATAAACATGTGGCAACAAGTAGGAAAAGCAA
TGTATGCCCCTCCCATCAGTGGACAAATTAACTGCTCATCAAAT---
ATTACAGGGCTACTATTAACAAGAGATGGTGGTAGTGGTAACAGCACA-------------------------
----

GAGAACGAGACTGAGATCTTCAGACCTGCAGGGGGAGATATGAGGGACAATTGGAGAAGTGAATTATATAAAT
ATAAAGTAGTACAAGTTGAGCCATTA---GGATTAGCACCCACCAGGGCAAAGAGAAGAGTGGTG--------
-------------------------------CAGAGAGAAAAAAGAGCA---ATAGGC---CGGGGAGCT---
------GCCTTCCTTGGGTTCTTAGGAGCA---GCAGGAAGCACTATG---GGCGCGGCGTCA---
ATGACGCTGACGGTACAG---GCCAGACAA---TTATTG---TCTGGCATAGTGCAACAGCAAAGC---
AATTTG---CTGAGG---GCCATAGAGGCTCAGCAACATATGCTGAAACTCACGGTCTGGGGCATT
>MN485971..1
TTCTATTGCAATACATCAGGCCTGTTTAATAGTATTTGGAATAGTAGCACTTGGAATAATAATAGCACTGGGA
ATAACAGCACA---CAG------GAG---

TCAAATGACACTATAACTCTCCAATGCAGTATAAAACAAATTATAAACATGTGGCAGGGAGTAGGAAAAGCAA
TGTATGCCCCTCCCATTGAAGGAAAGATTGCATGTTTATCACAA---
ATTACAGGACTAATATTAACAAAAGATGGTGGTGCAAAAAATAAT----------------------------
----

AGTCAGAATGAGACTTTCAGACCTGCAGGAGGAGATATGAGAGACAATTGGAGAAGTGAATTATACAAATATA
AAGTAGTAAGACTTGAACCACTA---GGTCTAGCACCCACCAGGGCAAGGAGAAGAGTGGTG-----------
----------------------------GTAAGAGAAAAAAGAGCA---ATAGGA---CTAGGAGCT------
---ATGTTCCTTGGGTTCTTGGGAGCA---GCAGGAAGCACAATG---GGCGCAGCGTCA---
CTGACGCTGACGGTACAG---GCCAGACAA---TTACTG---TCTGGTATAGTGCAACAGCAGAAC---
AATTTG---CTGAGG---GCTATTGAGGCGCAACAGCATATGTTGCAACTCACAGTCTGGGGCATC
>AJ277825..1
TTCTATTGTAATACATCAGGCCTGTTTAATAGCACTTGGAGCCTTAACAGCAGCGCGCCAGATGACACAGAA-
----

TCAAATGACACTATAACTCTCCCATGCAGAATAAAGCAAATTATAAACATGTGGCAAAGGGCAGGACGAGCAA
TATATGCCCCTCCCATCCAAGGAATAATAAGGTGTAATTCAAGC---
ATTACAGGACTACTATTAACAAGAGATGGTGGGGATAACAATACG----------------------------
----

CATAATGAAACCTTCAGACCTGGAGGTGGAAATATGAGGGATAATTGGAGAAGTGAACTATATAAATATAAAG
TAGTAAAAATTGAACCACTA---GGAGTAGCACCCACCAAGGCAAAAAGAAGAGTGGTG--------------
-------------------------GAGAGAGAAAAAAGAGCA---GTTGGA---CTGGGAGCT---------
GTCTTCATTGGGTTCTTAGGAGCA---GCAGGAAGCACTATG---GGCGCGGCGTCA---
ATAACGCTGACGGTACAG---GCCAGACAA---TTATTG---TCTGGCATAGTGCGACAGCAAAGC---
AATTTG---CTGAGG---GCTATAGAGGCTCAACAGCATCTGTTGAAACTCACGGTCTGGGGCATT
>HQ595799..3
TTCTATTGTAACACATCAAGACTGTTTAATAGCACTTGGCATGATAACAGCACGCTAGAGTCAAATCAAACAG
AG---

TCAAACGATACTATAATACTCCCATGCAGAATAAAACAAATTATAAACATGTGGCAGAGAGCAGGACAAGCAA
TGTATGCCCCCCCCATTCAAGGAGTGATAAGGTGTGTATCAAAT---
ATTACAGGACTAATATTAACAAGAGATGGTGGGGATAATAACAGC----------------------------
----

GCAAACGAAACCTTCAGACCTGGAGGAGGAGATATGAGGGACAATTGGAGGAGTGAATTATATAAATATAAAG
TAGTAAAAATTGAACCAATA---GGAATAGCACCCACCGAGGCAAGGAGAAGAGTGGTG--------------
-------------------------CAGAGAGAGAAAAGAGCA---GTAGGA---CTAGGAGCT---------
GTTTTCATTGGGTTCTTAGGAGCA---GCAGGAAGCACTATG---GGCGCGGCGTCA---
ATGACGCTGACGGTACAG---GCCAGACAA---TTATTG---TCTGGCATAGTGCAACAGCAAAGC---
AATTTG---CTGAAG---GCTATAGAAGCTCAACAGCATCTGTTGAAACTCACGGTCTGGGGCATT
>AY322191..1
TTCTATTGTACTACCTCAGGCCTGTTTAATGGCACTTGGGACAGCAAGCAGAAGTCAAATAGCACGGAG----
----

TCAGATGACACTATAACTCTCCCATGCAAAATAAAGCAAATTATAAATATGTGGCAGAGGGTGGGACAAGCAA
TGTATGCCCCTCCTATCCAAGGAATAATAAGTTGTGTATCAAAC---
ATTACAGGACTAATATTAACAAGAGATGGTGGGGGTAATAACAGT----------------------------
----

AGCAATGAAACCTTCAGACCTGGAGGAGGAGATATGAGAGACAATTGGAGAAGTGAATTATATAAGTATAAAG
TAGTAAAAATTGAACCACTA---GGAGTAGCACCCACCAGGGCAAGGAGAAGAGTGGTG--------------
-------------------------GGGAGAGAAAAAAGAGCA---GTTGGA---ATGGGAGCT---------
GTCTTCCTTGGGTTCTTAGGAGCA---GCAGGAAGCACTATG---GGCGCGGCGTCA---
ATAACGCTGACGGTACAG---GCCAGACAA---TTATTG---TCTGGCATAGTGCAACAGCAAAGC---
AATTTG---CTGAGA---GCTATAGAGGCTCAACAACATCTGTTGAAACTCACGGTCTGGGGCATT
>JN977603..1
TTCTATTGCAACACATCACAGCTGTTTAATTCATCACTGCTCAATAGTACAGAAAGTATTAATGAAACAAGT-
----

GGTAATGAAACAATCACACTCCCATGCAGAATAAAACAGATTATAAACATGTGGCAGGAGGTAGGGCGGGCAA
TGTATGCCCCTCCCATTAGAGGAATAATAACATGTAAATCAAAT---
ATCACAGGACTACTACTGACACGTGATGGAGGAAATGAGAAAAATAACTCA----------------------
----

GAGGCAAATAACACAGAGATATTCAGGCCTGGAGGAGGAAATATGAAGGACAATTGGAGAAGTGAATTATATA
AGTATAAAGTAGTAAAAGTTGAACCACTA---GGAGTAGCACCCACCAAGGCAAAGAGAAGAGTGGTG-----
----------------------------------GAGAGAGAAAAAAGAGCA---GTAGGA---
ATAGGAGCT---------GTCTTCATTGGGTTCTTAGGAGCA---GCCGGAAGCACTATG---
GGCGCAGCGTCG---GTGACGCTGACGGTACAG---GCCAGACAA---TTATTG---
TCTGGCATAGTGCAACAGCAAAGT---AATTTG---CTGAGG---
GCTATAGAGGCTCAACAACATTTGTTGAAACTCACGGTCTGGGGCATT
>AY887516..2
TTCTATTGTGATACAAAAAACCTGTTTAATAGCACTTGGAACGACAGTAACATCAACATCACACAGGAGTCAA
ATGAC--
-------------------ACA---
GATTCAAAAGGCAATATAACTCTCCAGTGCAGAATAAAGCAAATTATAAATATGTGGCAGAGAGCAGGACAAG
CAATATATGCCCCTCCCATCCCGGGAGTAATAAGATGTGAGTCGAAC---
ATTACAGGAATAATATTAACAAGAGATGGTGGGAATGTTAGCAGT----------------------------
----

CAAAATGAAACCTTCAGACCTGGAGGAGGAGATATGAGGGACAATTGGAGAAGTGAATTATATAAGTATAAAG
TAGTAAAAATTGAACCACTA---GGAGTAGCACCCTCCAAGGCAAAGAGAAGAGTGGTG-----------
------------------------GAGAGAGAAAAAAGAGCA---GTCGGA---ATAGGAGCT-------
GTTTTCCTTGGGTTCTTAGGAGCA---GCAGGAAGCACTATG---GGCGCGGCGTCA---
CTGACGCTGACGGTACAG---GCCAGACAA---TTATTG---ACTGGCATAGTGCAACAGCAAAGC---
AATTTG---CTGAGG---GCTATAGAGGCTCAACAACATCTGTTGAAACTCACAGTCTGGGGCATT
>HQ540690..2
TTCTATTGTAATACATCAGGCCTGTTTAATAGCACTTGGAACTCTACTAATGGAAATTGGACTCAAAACGAAA
CTGAC---

AATAAGACTGAAACTATAAGTCTCCCATGCAGAATAAAGCAAATTATAAACATGTGGCAGAGAGTAGGACAAG
CAATGTATGCCCCTCCCATACAAGGAGTAATAAGCTGTAACTCAAGC---
ATTACAGGACTGCTACTAACAAGAGATGGTGGTTACAATAATAGC-------------------------
----

AATAATGAAACCTTCAGACCTGTAGGAGGAAACATGAAGGACAATTGGAGAAGTGAATTATATAAGTATAAAG
TAGTAAAAATTGAGCCACTA---GGAATAGCACCCACCAGGGCAAAAAGGAGAGTGGTG-----------
------------------------GAGAGAGAAAAAAGAGCA---GTGGGA---TTCGGAGCT-------
TTGTTCCTTGGGTTCCTGGGAGCA---GCAGGAAGCACTATG---GGCGCAGCATCA---
ATAACGCTGACGGTACAG---GCCAGACAA---TTATTG---TCGGGTATAGTGCAACAGCAAAAC---
AATTTG---CTGAGG---GCTATAGAGGCGCAACAGCATATGTTGCAACTCACAGTCTGGGGCATT
>DQ093606..2
TTCTACTGCAATACATCAAGATTGTTTAATAGCACATTTAAGGTCAAGGACACAGATAATAATGCTGGGGACA
AC--

ACAGAAGAGGATATAACTATCCCATGCAGAATAAAACAAATTATAAACATGTGGCAGAGAGTAGGACAAGCAA
TGTATGCCCCTCCCATTGCAGGAGTAATAAAATGTGAATCAAGC---
ATCACAGGAATACTATTGACAAGAGATGGGGGGGAGTCTGATAAC-------------------------
----

AAAACAGAAGAGGTCTTCAGACCTGAAGGAGGAAATATGAAGGACAATTGGAGAAGTGAATTATATAATTATA
AGGTAGTAAAAATTGAACCACTA---GGAGTAGCACCCACCAAGGCAAAGAGAAGAGTGGTG--------
---------------------------GAGAGAGAAAAAAGAGCAGTAGTTGGA---CTGGGAGCT----
---GTTTTCCTTGGGTTCTTAGGAGCA---GCAGGAAGCACTATG---GGCGCAGCATCG---
ATAACGCTGACGGTACAG---GCCAGGCAA---TTATTG---ACTGGCATAGTGCGGCAGCAAGGC---
AATTTG---CTGCAG---GCTATAGAGGCTCAACAACATCTGTTGAAGCTCACTGTCTGGGGCATT
>HQ540691..3
TTCTACTGCAATACATCAGGCCTGTTTAAGGGCACATTTCAGGTTAATGACACGGGTAATAGTAACAGCACAC
AG--

GGGGCAAATGACACCATAACTATCCCATGCAGAATAAAACAGATTATAAATATGTGGCAAAGAGTGGGACAAG
CAATGTATGCCCCTCCCATTGAAGGAGTAATAAAATGTAAGTCAAAC---
ATTACAGGAATGCTATTGACAAGAGACGGCGGGGCTGAAAGCAAT-------------------------
----

ACAGAGGTCTTCAGACCTGGGGGAGGAGATATGAAGGACAATTGGAGAAGTGAATTATATAATTATAAAGTAG
TAAAAATTGAACCAGTA---GGAGTAGCACCCGCCAAGGCAAAGAGAAGAGTGGTG--------------
---------------------GAGAGAGAAAAAAGAGCA---GTTGGA---ATAGGAGCT---------
GTCCTCCTTGGGTTCTTAGGAGCA---GCAGGAAGCACTATG---GGCGCAGCGTCA---
ATAACGCTGACGGTACAG---GCCAGACAA---TTATTG---ACTGGCATAGTGCGGCAGCAAAGC---
AATTTG---CTGCAG---GCTATAGAGGCTCAACAACATCTGTTGAAACTCACGGTCTGGGGCATT
>HQ595773..1
TTCTATTGCAATACATCAAAACTGTTTAATAGCACTTGGAAGAATGACACTTGGGAGTCAAATAGCACAGAG-
----

TCAAATGGCACTATATATCTTAATTGCAGAATAAAACAAATTATAAACATGTGGCAGGGAGTAGGAAAAGCAA
TGTATGCCCCTCCCATTGAAGGAATGATTACATGTATATCAAAT---
ATTACAGGGATATTATTAACAAGAGATGGTGGTGCAAATAATAAT-------------------------
----

AGTCAGAATGAGACTTTCAGACCTGGAGGAGGAGATATGAGAGACAATTGGAGAAGTGAATTATATAAATATA
AAGTAGTAAGGCTTGAACCACTA---GGTCTAGCACCCACCAAGGCAAGGAGAAGAGTGGTG-----------
----------------------------ACAAGAGAAAAAAGAGCA---ATAGGA---CTAGGAGCT------
---ATGTTCCTTGGGTTCTTGGGAGCA---GCAGGAAGCACAATG---GGCGCAGCGTCA---
CTGACGCTGACGGTACAG---GCCAGGCAG---TTATTG---TCTGGTATAGTGCAGCAGCAAAAC---
AATTTG---CTGATG---GCTATAGAGGCGCAACAACATTTGTTGCAACTCACAGTCTGGGGCATT
>HQ595793..1
TTCTATTGCAATACATCAGGCCTGTTTAATAGCACTTGGAATAACAGCACACAGGTGTCAAATGACACACAG-
----

TTAAATGACACTATAACTCTCCAATGCAGGATAAAACAAATTATAAACTTGTGGCAGGGAGTAGGAAAAGCAA
TGTATGCTCCTCCCATTGAAGGAAATATTACATGTTTATCAAAT---
ATTACAGGACTAATATTAACAAGAGATGGTGGTGCAAATACTACT----------------------------
----

AGTCAGAATGAGACTCTCAGGCCTGCAGGAGGAGATATGAGAGACAATTGGAGAAGTGAATTATACAAATATA
AAGTAGTAAGACTTGAACCACTG---GGTCTAGCACCCACCAGGGCAAGGAGAAGAGTGGTG-----------
----------------------------GCAAGAGAAAAAAGAGCA---ATAGGA---CTAGGAGCT------
---GTGTTCCTTGGGTTCTTGGGAGCA---GCAGGAAGCACAATG---GGCGCAGCGTCA---
CTGACGCTGACGGTACAG---GCCAGACAA---TTATTG---TCTGGTATAGTGCAGCAGCAAAAC---
AATTTG---CTGAGG---GCTATAGAGGCGCAACAACACTTGTTGCAACTCACAGTCTGGGGCATT
>L22942..1
TTCTACTGCAATACATCAAAACTGTTTAATAGTGTATGGGGAAATTCAAGTAATGTGACAAAGGTAAATGGGA
CA---

AAGGTAAAAGAGACAATCACACTCCCATGTAAAATAAAACAAATTATAAACATGTGGCAGGAAGTAGGACGAG
CAATGTATGCCCCTCCCATTGAAGGACTCATCAACTGTTCATCAAAT---
ATTACAGGACTATTGTTGACAAGAGATGGTGGTGAAAATAATAGT----------------------------
----

AGTCAGAATGAGACCTTCAGACCTGGAGGAGGAGATATGAGAGACAATTGGAGAAGTGAATTATACAAATATA
AAGTAGTAAAAATTGAACCACTA---GGTCTAGCACCCACCAGCGCAAAGAGAAGAGTGGTG-----------
----------------------------GAAAGAGAAAAAAGAGCA---ATAGGA---CTGGGAGCT------
---ATGTTCCTTGGGTTCTTGGGAGCA---GCAGGAAGCACGATG---GGCGCAGCGTCA---
ATGACGCTGACGGTACAG---GCCAGACAG---TTAATG---TCTGGTATAGTGCAACAGCAAAAC---
AATTTG---CTGAGG---GCTATAGATGCGCAACAGCATCTGTTGCAACTCACAGTCTGGGGCATT
>DQ208482..1
TTCTACTGTAACACATCAGAACTGTTTAATAGTGTATGGGCAAATGATAGTACAGAAAATAATAGTACATGGA
AC---

AATGAGAGTATAATCACACTCCCATGCAGAATAAAACAATTTATAAACATGTGGCAGGGAGTAGGACGAGCAA
TGTATGCCCCTCCCATTGAAGGATACATCAACTGTTCATCAAAT---
ATTACAGGACTATTGCTGACAAGAGATGGTGGTATAAATAATAATAGT-------------------------
----

CATGATAATGAGACCTTCAGACCTGGAGGAGGAGATATGAGAGACAATTGGAGAAGTGAATTATACAAATATA
AAGTAGTAAAAATTGAACCACTA---GGTCTAGCACCCACCAAGGCAAGGAGAAGAGTGGTG-----------
----------------------------GAAAAAGAAAAAAGAGCA---ATAGGA---CTAGGAGCT------
---ATGTTCCTTGGGTTCTTGGGAGCA---GCAGGAAGCACGATG---GGCGCAGCGTCA---
CTGGCGCTGACGGGACAG---GCCAGACAG---TTAATG---TCTGGTATAGTGCAACAGCAAAAC---
AATTTG---CTGAGG---GCTATAGATGCGCAACAGCATCTGTTGCAACTCACAGTCTGGGGCATT
>U36866..2
TTCTACTGCAATACATCAGACCTGTTTAATAGTACATGGGAGAGTAATAATACATCAAATAGTACAGGGTCA-
----

AATAATGGTACAATCATACTTCCATGCAGAATAAAACAAATTATAAACCTGTGGCAGCAAGTAGGAAAAGCAA
TGTATGCCCCTCCCATCGAAGGAATAATCACGTGTAGATCAAAT---
ATTACAGGGCTATTACTGACAAGAGATGGGGGTAGTAATAATAGT----------------------------
----

CATAACGAGACCTTCAGACCTGGAGGAGGAGATATGAGAGACAATTGGAGAAGTGAATTATACAAATATAAAG
TAGTAAAAATTGAACCACTA---GAAGTAGCACCCACCAGGGCAAAGAGAAGAGTGGTG--------------
-------------------------GAGAGAGAAAAAAGAGCA---GTTGGA---ATAGGAGCT---------
GTCTTCATTGGGTTCTTAGGAGCA---GCAGGAAGCACTATG---GGCGCGGCGTCA---
ATAACGCTGACGGTACAG---GCCAGACAA---TTATTG---TCTGGCATAGTGCAACAGCAAAGT---
AATTTG---CTGAGG---GCTGTAGAGGCTCAACAGCATATGTTGAAACTCGCGGTCTGGGGCATT
>AY444799..1
TTCTATTGTGACACATCAGGCCTGTTTAATAGCACGTGGAATGTCAATACCAGCGTGCAGCAGTCAAATATCA
CGGGC--

AATATCACGGACAATATAACTCTCCCATGCAGAATAAAGCAAATTATAAATATGTGGCAGAGAGCAGGACAAG
CAATGTATGCCCCTCCCATCCAAGGAAAAATAAAGTGTGTATCAAAC---
ATTACAGGACTAATATTAACAAGAGATGGTGGAGGTAATAGCAGT----------------------------
----

GCAAATGAAACCTTTAGACCTGGAGGAGGAGATATGAGGGACAATTGGAGAAGTGAATTATATAAGTATAAAG
TAGTAAGAATTGAACCACTA---GGAGTAGCACCCACCAAGGCAAAAAGAAGAGTGGTG--------------
-------------------------CAGAGAGAAAAAAGAGCA---ATTGGA---ATAGGAGCT---------
GTCCTCATTGGGTTCTTAGGAGCA---GCCGGAAGCACTATG---GGCGCAGCGTCA---
ATAACGCTGACGGTACAG---GCCAAACAA---TTATTG---TCTGGTATAGTGCAACAGCAAAGC---
AATTTG---CTGAGG---GCTATAGAGGCTCAACAGCAGCTGTTGAAACTCACAGTCTGGGGCATT
>AY371160..1
TTCTATTGTAATACATCAGGCTTGTTTAACAGCACTTGGTATAAGGAGACTGCCCCTGTCAGCAGAACAGGGT
CAAAT--

GACACAAGTGACAATATAACTCTCCCGTGTAGAATAAAGCAAATTATAAATATGTGGCAGAGAACAGGACAAG
CAATGTATGCCCCTCCCATCCCAGGAATAATAAGGTGTGAGTCAAAC---
ATTACAGGACTATTATTGACAAGAGATGGTGGGATTAGCAATAAC----------------------------
----

AGTAATGAGACTTTCAGACCTACAGGAGGAGAGATGAAAGATAATTGGAGAAGTGAATTATATAAATATAAAG
TAGTAAAAATTGAACCACTG---GGAGTAGCACCCACCAAGGCAAGGAGAAGGGTGGTG--------------
-------------------------GAGAGAGAAAAAAGAGCA---GTTGGA---CTTGGAGCT---------
GTCTTCCTTGGGTTCTTAGGAGCA---GCCGGAAGCACTATG---GGCGCGGCGTCA---
ATAACGCTGACGGTACAG---GCCAGACAA---TTATTG---TCTGGCATAGTGCAACAGCAAAGT---
AATTTG---CTGAAG---GCTATAGAGGCTCAACAGAATCTGTTGAGACTCACAGTCTGGGGCATT
>MK086110..1
TTCTATTGCAACACATCAAACTTGTTC------AATTGGAATGGTACTTGGAATGGTAACACAACA-------
----

GGCAATGACACGATAACTCTCCAGTGCAGAATAAAGCAAATTATAAATATGTGGCAGAGGGTAGGACAAGCAA
TGTATGCCCCTCCCATTCCAGGAGAAATAAAGTGTGAGTCAAAC---
ATTACAGGACTACTATTGACAAGAGATGGTGGGAACAGTACAGAT----------------------------
----

AATGAGACTTTCAGACCTGGAGGAGGAGATATGAGAGACAATTGGAGAAGTGAATTATATAAATATAAAGTAG
TAAAAATTGAACCACTA---GGAGTAGCACCCAACAAGGCACGGAGGAGAGTGGTG-----------------
----------------------GGGAGAGAAAAAAGAGCA---GTTGGA---CTAGGAGCT---------
GTCTTCCTTGGGTTCTTGAGTGCA---GCAGGAAGCACTATG---GGCGCGGCGTCA---
CTGACGCTGACGGTACAG---GCCAGGCAA---TTATTG---TCTGGCATAGTGCAACAGCAAAAC---
AATTTG---CTGAGG---GCAATAGAGGCTCAACAGCATATGTTGAGACTCACGGTCTGGGGCATT
>AF377958..1
TTCTATTGCGATACATCAAAACTGTTTAATAGTACTTGGGACAAGAATGGCACGTGGGTTAATGGCCACACAG
GG---

TCAGATAATGACAATATAACTCTCCAATGCAGAATAAAACAAATTATAAATATGTGGCAGAGAGTAGGACAAG
CAATGTATGCCCCTCCCATCCCAGGAGTAATAAGGTGTGAATCTAAC---
ATTACAGGACTACTATTAACAAGAGATGGAGGGAATAATAATAAT----------------------------
----

AGTGCAAATGAGACCTTCAGGCCTGGAGGAGGAGATATGAGGGACAATTGGAGAAGTGAATTATATAAGTATA
AGGTAGTAGAAATTGAACCACTA---GGTGTAGCACCCAACCATGCAAGAAGAAGAGTGGTG-----------
----------------------------GAGAGAGAAAAAAGAGCA---GTTGGA---CTGGGAGCT------
---GTCTTCCTTGGGTTCTTAGGAGCA---GCAGGAAGCACTATG---GGCGCAGCGTCA---
ATAACGCTGACGGTACAG---GCCAGACAA---TTATTG---TCTGGTATAGTGCAACAGCAGAGC---
AATTTG---CTGAAA---GCTATAGAGGCTCAACAACATATGTTGAGACTCACGGTCTGGGGCATT
>MH705142..3
TTCTATTGCAATACATCAGCCCTGTTTAATAGCAAGTGGGAAAGTGAATACAATGCCAGCGAGAATGAACAAA
AG---

AGTGACACTAATATAACTATTCCATGTAGAATAAAGCAATTTGTAAATGTATGGCAGAGAGTAGGACAAGCAA
TGTATGCCCCTCCAGTCCAAGGAGAAATAAGATGTGACTCAAAC---
ATTACAGGACTACTATTAGAAAGAGAATATGGGGATCATCATAATGGG-------------------------
----

ACAAATGAGACTTTCAGACCTACAGGAGGAGACATGATGAACAATTGGAGAAGTGAATTATATAAGTATAAAG
TAGTAAAAGTTGAACCACTA---GGAGTAGCACCCAGCGGAGCAAGGAGAAGAGTGGTG--------------
-------------------------GCAAGAGAAAAAAGAGCA---CTTGGAGGACTAGGGGCT---------
GTCTTCCTTGGGTTCTTAGGAGCA---GCAGGAAGCACTATG---GGCGCGGCGTCA---
CTGACGCTGACGGTACAG---GCCAGACAA---TTACTG---TCTGGTATAGTGCAACAGCAGAGC---
AATCTG---CTAAGG---GCTATAGAGGCTCAACAACATATGTTGAAACTCACAGTCTGGGGCATT
>AY371162..1
TTCTATTGCAATACAACACAACTGTTTAATAGTACCTGGGAAAATGACACCCTGAAGGGAGCGGCA-------
----

GGTAATGATACTATTACACTGCCATGCAGAATAAAACAAATTGTAAACATGTGGCAGAGAGTAGGACAAGCAA
TGTATGCCCCTCCCATCCAAGGAGTAATACAGTGTGTATCAAAC---
ATTACAGGACTAATATTGACAAGAGATGGTGGGAGTAATAACAAT----------------------------
----

ACAAATGAGACCATCAGACCTGGAGGAGGGGAGATGAGGGACAATTGGAGAAGTGAATTATATAAATATAAAG
TAGTACAAATTGAACCACTA---GGAGTAGCACCCACCAGGGCAAGGAGAAGAGTGGTG--------------
-------------------------CAGAGAGAAAAAAGAGCA---GTTGGA---TTGGGAGCT---------
CTTTTCCTTGGGTTCTTAGGAGCA---GCAGGAAGCACTATG---GGCGCAGCGTCA---
ATAACGCTGACGGTACAG---GCCAGACAA---TTATTG---TCTGGCATAGTGCAACAGCAGGGC---
AATCTG---CTGAGG---GCTATAGAGGCTCAACAACATCTGTTGAAGCTCACGGTCTGGGGCATT
>MH705135..1
TTCTATTGTGATACATCAAGCCTGTTTAATAGCACTTGGTCCTGGGAAAATGGCAATTGGTCTTGGAATAATA
ATGACACGACC--
----------ACCATGAAC------
GAGATAAATACAAATGTGACTATCCCATGCAGAATAAAGCAAATAGTAAATATGTGGCAGAGAGTAGGACAAG
CAATGTATGCCCCTCCCATCCCAGGAATAATAAATTGTGTATCAAAC---
ATTACAGGACTATTATTGACAAGAGATGGTGGGAGTAATAACAGT----------------------------
----

ACAAATGAGACTTTTAGACCTGGAGGAGGAGATATGAGGGACAATTGGAGAAGTGAATTATATAAGTATAAAG
TAGTAAAAATTGAACCACTA---GGAGTAGCACCCACTAGGGCCAGGAGACGAGTGGTG--------------
-------------------------CAGAGAGAAAAACGAGCA---ATGGGA---ATAGGAGCT---------
GTGCTCCTTGGGTTCTTGGGAGCA---GCAGGAAGCACTATG---GGCGCGGCGTCA---
ATAACGCTGACGGTACAG---GCCAGACAA---TTATTG---TCTGGTATAGTGCAACAGCAAAGC---
AATTTG---CTGAGG---GCTATAGAGGCTCAACAGCATCTGTTGAAACTCACGGTCTGGGGCATT
>AF069932..1
TTCTATTGCAATACATCAGGCTTGTTTAATAGCACTTGGACAAATGGGACAAACAGCACGGGG----------
----

ACTAATGACAATATAACTCTTCCATGCAGAATAAAGCAAATTATAAATATGTGGCAGAGAGTAGGACAAGCAA
TGTATGCCCCTCCCATCAAAGGAGTAATAGAGTGTAAATCAAAT---
ATTACAGGAATACTATTAATGAGAGATGGTGGTGCTAATGATACA----------------------------
----

GCTAACAGTGAGACATTCAGACCTGGAGGAGGAGATATGAAGGACAATTGGAGAAGTGAATTATATAAGTATA
AAGTAGTAAAAATTGAACCACTA---GGAGTAGCACCCACCAGGGCAAAGAGAAGAGTGGTG-----------
----------------------------GAGAGAGAAAAAAGAGCA---GTTGGA---CTGGGGGCT------
---GTCTTCCTTGGGTTCTTAGGAGCA---GCAGGAAGCACTATG---GGCGCGGCGTCA---
TTAACGCTGACGGTACAG---GCCAGACAA---TTATTG---TCTGGCATAGTGCAACAGCAGAGC---
AATCTG---CTGAAG---GCTATAGAGGCTCAACAGCATCTGTTGAAACTCACAGTCTGGGGCATT
>AF069939..1
TTCTATTGCAACACATCAGGACTGTTTAATAGCACTTGGGATAACAACAACATTGCCAGCTCAAACCACACAG
GG---

TCAAATGATACTATAACTCTCCAATGCAGAATAAAGCAAATTATAAATCTGTGGCAGAAAGTAGGACAAGCAA
TGTATGCCCCTCCCATCCCAGGAATAATAAGGTGTGAATCAAAT---
ATTACTGGACTCCTATTAACAAGAGATGGAGGGAATAATAATAGT----------------------------
----

ACAAATGAGACCTTCAGACCTGGAGGAGGAGATATGAGGGACAACTGGAGAAGTGAATTATATAAGTATAAAG
TAGTAAAAATTGAACCACTA---GGTGTAGCACCCACCAAGGCAAAAAGAAGAGTGGTG--------------
-------------------------GCGAGAAAAAAAAGAGCA---GTTGGA---CTGGGAGCT---------
GTCTTCCTTGGTTTCTTAGGAGCT---GCAGGAAGCACTATG---GGCGCAGCGTCA---
ATAGCGCTGACGGTACAG---GCCAGACAA---TTATTG---TCCGGCATAGTGCAACAGCAGAGC---
AATCTG---CTGAGG---GCTATAGAGGCTCAACAGCATATGTTGAAACTCACGGTCTGGGGCATT
>KT427849..1
TTCTACTGCAACACAATACAGCTGTTTAATAGTACTTGGTTCAATGAGAATAGTACTTGGTTCAATGAGAGTG
GTACAGGG---

TCAAACACTGAAAATATCACACTCCCATGCAGAATAAAGCAAATTATAAATAGGTGGCAGGAAGTAGGAAAAG
CAATGTATGCCCCTCCCATCAGTGGACGAATTAACTGTTCATCAAAT---
ATTACAGGGCTGCTATTGGTAAGAGATGGTGGTGATGGGAACGCG----------------------------
----

ACAGCAGAAATCTTCAGACCTGGAGGAGGAGATATGAGGGACAATTGGAGAAGTGAATTATATAAATATAAAG
TAGTACAAATAGAACCATTA---GGAATAGCACCCACCAAGGCGAGGAGAAGAGTGGTG--------------
-------------------------CAGAGAGAAAAAAGAGCA---GTGGGAACGATAGGAGCT---------
ATGTTCCTTGGGTTCTTGGGAACA---GCAGGAAGCACTATG---GGCGCAGCGTCA---
GTAGCGCTGACGGTACAG---GCCAGACAA---TTATTG---TCTGGTATAGTGCAACAGCAAAAT---
AATTTG---CTGAAG---GCTATTGAGGCGCAACATCATCTGTTGCAACTCACAGTCTGGGGCATT
>KT427790..1
TTCTATTGCAATACATCAGGACTGTTTAATGGTACATACAGACCTAATATTACAGGAAACCATAGTGAAATA-
----

GAAAATTCGACCATTACAATCCCATGCAGAATAAAACAAATTATAAATATGTGGCAGGAGGTAGGAAAAGCAA
TGTATGCCCCTCCCATTCAAGGAATAATAACATGTAGATCAAAT---
ATCACAGGACTACTACTAACACGGGATGGAGGAGGCAATGTCAAT----------------------------
----

GACAGCACAGAGACATTCAGGCCTGGAGGAGGAGATATGAGGGACAATTGGAGAAGTGAATTATATAAATATA
AAGTGGTAGAAATTAAACCATTA---GGAATAGCACCCACTGCGGCAAAAAGGAGAGTGGTG-----------
----------------------------GAGAGAGAAAAAAGAGCA---GTGGGA---ATAGGAGCT------
---ATGATCTTTGGGTTCTTGGGAGCA---GCAGGAAGCACTATG---GGCGCAGCATCA---
ATAACGCTGACGGTACAA---GCCAGACAA---TTATTG---TCTGGGATAGTGCAACAGCAAAGC---
AATTTG---CTGAGA---GCTATAGAGGCGCAACAGCATATGTTGCAACTCACGGTCTGGGGCATT
>KF835539..1
TTCTATTGTAATACTTCAGGCCTGTTTAATGGTACATACCTGCCTAATGGTACATACATGCCTAATGATATAC
AAGAT--

ACAAACTCAACCATCACGATTCCATGCAGAATAAAGCAAATTATAAATATGTGGCAGGAGGTAGGACGAGCAA
TGTATGCCCCTCCCATTGAAGGAAACATAACATGTAAATCAAAT---
ATCACAGGACTACTATTGGTACGTGATGGAGGAAACCAAGAAAAAACAGGAAT-------------AAT----
----

-
ACAACAAATGATACAACAGAGACATTCAGACCTCAAGGAGGAGATATGAGGGATAATTGGAGAAGTGAATTAT
ATAAATATAAAGTGGTAGAAATTAAGCCATTG---GGAATAGCACCCACTAAAGCAAAAGGAGAGTAGTG--
-----------------------------------GAGAGAGAAAAAAGAGCA---GTGGGA---
CTAGGAGCT---------GTGTTCCTTGGGTTCTTGGGAGCA---GCAGGAAGCACTATG---
GGCGCGGCGTCA---ATAACGCTGACGGTACAG---GCCAGACAA---TTGCTG---
TCTGGTATAGTGCAACAGCAAAGC---AATTTG---CTGCAA---
GCTATAGAGGCGCAACAGCATCTGTTGCAACTCACGGTCTGGGGCATT
>KC898983..1
TTCTATTGTAATACATCAAAGCTGTTTAATGGCACATACAATGGCACAGACATGACTAATATGATTAAT----
----

AGTTCAGCAGTAATCATAATCACATGCAGAATAAAGCAAATTGTAAACATGTGGCAGGAGGTAGGACGAGCAA
TGTATGCCCCTCCCATTGCAGGAAACATAACATGTAGATCAAAT---
ATCACAGGACTATTATTGGTACGTGATGGAGGAAAATCAAATACT---------------------------
----

ACAGAGACATTCAGACCTCAAGGGGGAGATATGAGGGACAATTGGAGAAGTGAATTATATAAATATAAAGTGG
TAGAAATTAAGCCATTA---GGAATAGCACCCACTGAAGCAAAAGGAGAGTAGTG-----------------
---------------------GAGAGAGAAAAAAGAGCA---GCGGGA---ATAGGAGCT---------
GTGTTCCTTGGGTTCTTGAGTGCA---GCCGGAAGCACTATG---GGCGCGGCGTCA---
GTAACGCTGACGGTACAG---GCCAGACAA---TTGCTG---TCTGGTATAGTGCAACAGCAAAGC---
AATTTG---CTGAGG---GCTATAGAGGCGCAACAGCATATGTTGCAACTCACGGTCTGGGGCATT
>HM776938..2
TTCTATTGTAATACATCAGGCCTGTTTAATAGTACATACTGGCCTAATGGTACATACATGTATTATAAAACAA
AC--

AATGATTCAATCATCACAATCCCATGCAGAATAAAGCAAATTATAAACATGTGGCAGCAGGTAGGAAGAGCAA
TGTATGCCCCTCCCATTGCAGGAAGAATAACATGTAACTCAAAT---
ATCACAGGACTACTATTGGTACGTGATGGAGGCCTAGGTAATACTACA------------------------
----

GATAATACTACAGAGACGTTCAGACCCCAAGGAGGAAATATGAGGGACAATTGGAGAAGTGAACTATATAAAT
ATAAAGTGGTAGAAATTCAGCCGTTG---GGAATAGCACCCACTAAAGCAAAGAGGAGAGTGGTG--------
------------------------------GAGAGAGAAAAAAGAGCA---GTGGGA---ATAGGAGCT---
------GTGTTCCTTGGGTTCTTGGGAGCA---GCAGGAAGCACTATG---GGCGCGGCGTCA---
ATAACGCTGACGGTACAG---GCCAGACAA---TTGCTG---TCTGGTATAGTGCAACAGCAAAGC---
AATTTG---CTGAAG---GCTATAGAGGCGCAACAGCATATGTTGCAACTCACGGTCTGGGGCATT
>KF250381..1
TTCTATTGTAATACATCAGGTCTGTTTAATGGTACATACAATAGTACAAGA---------------------
----
-------TACAATAATACA------
AGGGAGAATGGAACCATCACAATCCCATGTAGAATAAAGCAAATTATAAACATGTGGCAAGAGGTAGGACGAG
CAATGTATGCCCCTCCCATTAAAGGAAACATAACATGTAAATCAAAT---
ATCACAGGACTACTATTGGTACGTGATGGAGGA-------------------------------------
----
ACAAATCAAAGTAAT---
ACAACAGAGATATTCAGACCTGGAGGAGGGGATATGAGGGACAATTGGAGAAGTGAATTATATAAATATAAAG
TGGTAGAAATTAAGCCATTG---GGAGTAGCACCCACTAAAGCAAAAAGGAGAGTGGTG-------------
------------------------GAGAGAGAAAAAAGAGCA---GTGGGA---TTGGGAGCT---------
ATGTTCCTTGGGTTCTTGGGAGCA---GCAGGAAGCACTATG---GGCGCAGCGTCA---
ATAACGCTGACGGTACAA---GCCAGACAA---TTGTTG---TCTGGTATAGTGCAACAGCAAAGC---
AATTTG---CTGAGA---GCTATAGAGGCGCAACAGCATCTGTTGCAACTCACTGTCTGGGGCATT
>KF250382..1
TTCTATTGTAATACATCAGGCTTGTTTAATGGTACATACCATAATGGTACATAC------------------
----
ACATTTAATGGTACAGAA---------
GGTAATGCAAGCTCAATCATCATTCCATGCAGAATAAAGCAAATTATAAATATGTGGCAAGAGGTAGGACGAG
CAATGTATGCCCCTCCCATTGAAGGAAAATTAGAATGTAAATCAAAT---
ATCACAGGACTACTATTGGTACGTGATGGAGGG--------------------------------------

----

ATAAATACTACAGAGGAGACATTCAGACCTGGAGGAGGAGATATGAGGGACAATTGGAGAAATGAATTATATA
AATATAAAGTGGTAGAAGTTCAGCCATTG---GGAATAGCACCCACTAAAGCAAAAAGGAGAGTGGTG-----
----------------------------------GAGAGAGAAAAAAGAGCA---GCGGGA---
CTGGGAGCT---------GTGTTCCTTGGGTTCTTGGGAGCA---GCAGGAAGCACTATG---
GGCGCGGCGTCA---ATAACGCTGACGGTACAG---GCCAGACAA---TTGTTG---
TCTGGTATAGTGCAACAGCAAAGC---AATCTG---CTGAGA---
GCTATAGAGGCGCAACAACATCTGTTGCAACTCACTGTCTGGGGCATT
>KC898988..2
TTCTATTGTAATACATCAAGCCTGTTTAATAGTACATACACGAATGGTACATACATGTTTAATGGTACGATAG
GTAAT--

TCAAGTTCAGACATCACAATTCAATGCAAAATAAAGCAAATTATAAACATGTGGCAGGAGGTAGGACGAGCAA
TGTATGCCCCTCCCATTAAAGGAAACATAACATGTAAATCAAAT---
ATCACAGGACTACTATTGGTACGTGATGGAGGAAATGGGACAAAT----------------------------
----

CAGACAGAGACATTCAGACCTGGAGGAGGAGATATGAGAGACAACTGGAGAAGTGAATTATATAAATATAAAG
TGGTAGAAATTAAGCCATTG---GGAGTAGCACCCACTAAGGCAAAAAGGAGAGTGGTG--------------
-------------------------GAGAGAGAAAAAAGAGCA---GTGGGA---ATAGGAGCT---------
GTGTTCCTTGGGTTCTTAGGTGCA---GCCGGGAGCACTATG---GGCGCGGCGTCA---
ATAACGCTGACGGTACAG---GCCAGACAA---TTGTTG---TCTGGTATAGTGCAACAGCAAAGC---
AATTTG---CTGAGG---GCTATAGAGGCGCAACAGCATATGTTGCAACTCACGGTCTGGGGCATT
>KC899010..1
TTCTACTGTAATACAACAAAACTGTTTAATAATACTTGGAATAGTACTAAACGAGACTTTGAGAAACCTTGGA
GTGATGATAAC--

AGAACAGGCATTATCACAATCCCATGCAGAATAAAACAAATTATAAACATGTGGCAGGGAGTAGGAAAAGCAA
TGTATGCCCCTCCCATTGCAGGAAACATAACATGTAACTCCACT---
ATCACAGGACTACTGTTGGTACGTGATGGAGGAAATACTACAGGGAAAAATGAT-------------------
----

ACAAATAATATAGAGATATTCAGGCCTCAAGGAGGAAATATGAAGGACAATTGGAGAAGTGAATTATATAAAT
ATAAAGTAGTAAAAATTGAACCATTG---GGAGTAGCACCCACCAAGGCAAAGAGAAGAGTGGTG--------
-------------------------------CAGAGGGAAAAAAGAGCA---GTGGGGGCGTTTGGAGCT---
------ATGTTCCTTGGGTTCTTGGGAGCA---GCAGGAAGCACTATG---GGCGCAGCGTCA---
ATAACGCTGACGGTACAG---GCCAGACAA---TTGTTG---TCTGGTATAGTGCAACAGCAGAGA---
AATTTG---CTGAGG---GCTATTGAGGCGCAACAACATCTGTTGCAACTCACAGTCTGGGGCATC
>KC807955..1
TTCTATTGTAATACATCAAGCCTGTTTAATAGTACATACAATGGTACATACATGTTTAATGGTACAATGGTTG
ATAATATA---
-------------AATAGG------
ACTTCAGACTTAAACTTCACAATCCCATGCAGGATAAAGCAAATTATAAATCTGTGGCAGGAGGTAGGACGAG
CAATGTATGCCCCTCCCATTGAAGGAAGAATAACATGTAAATCAAAT---
ATCACAGGACTACTATTGACACGTGATGGAGGGAATGGGACAGAGACA-------------------------
----

AATCAGACAGAGATATTCAGACCTGGAGGAGGAGATATGAGGGACAATTGGAGAAGTGAATTATATAAATATA
AAGTAGTAGAAATTAAGCCATTG---GGAGTAGCACCCACTACAGCAAAAAGGAGAGTGGTG-----------
----------------------------GAGAGAGAAAAAAGAGCA---GTGGGA---ATAGGAGCT------
---GTGTTCCTTGGGGTCTTGGGAGTA---GCCGGAAGCACTATG---GGCGCGGCGTCA---
ATAACGCTGACGGTACAG---GCCAGACAA---TTGCTG---TCTGGTATAGTGCAACAGCAAAAT---
AATTTG---CTGAGA---GCTATAGAGGCGCAACAGCATCTGTTGCAACTCACGGTCTGGGGCATT
>KC807982..1
TTCTATTGTAATACATCAAAACTGTTTAATAGTACATACATGCCTACATACAATGATACAGAGAATAGT----
----

TCAAATGAAAGCATCACAATCCCATGCAGAATAAAGCAAATTATAAATATGTGGCAGAAGGTAGGACGAGCAA
TGTATGCCCCTCCCATTGCAGGAATCATAACATGTAAATCAAAT---

ATCACAGGACTACTATTGGCACGTGATGGAGGAAAAGGTAATGAGACAGAT----------------------
----

AATAAGACAGAAATATTTAGACCTCAAGGAGGAAATATGAGGGACAATTGGAGAAATGAATTATATAAATATA
AAGTGGTAGAAATCAAGCCATTG---GGAATAGCACCAACTGGAGCAAAAAGGAGAGTGGTG-----------
----------------------------GACAGACAAAAAAGAGCA---GTGGGA---ATAGGAGCT------
---GTGTTCCTTGGGTTCTTGGGAGCA---GCAGGAAGCACTATG---GGCGCGGCGTCA---
ATAACGCTGACGGTACAG---GCCAGAAAA---TTGCTG---TCTGGTATAGTGCAACAGCAAAGC---
AATTTG---CTGAGA---GCTATAGAGGCGCAACAGCACATGTTGCAACTCACGGTCTGGGGCATT
>KF250399..1
TTCTATTGTAATACATCAAAACTGTTTAATGGTACATACAATGATACAGACATGTCTAATGGTACAGAA----
----

AGTAATTCAACCATCACAATTCCATGCAGAATAAAGCAAATTATAAACATGTGGCAGGGGGTAGGACGAGCAA
TGTATGCCCCTCCCATTGAAGGAAACATAACATGTAAATCAAAT---
ATCACAGGACTACTATTGGTACGTGATGGAGGAGAAGAGACAAAT----------------------------
----

GTTACAAATAACACAGAGACATTCAGACCTCAAGGAGGAGATATGAAGGACAATTGGAGAAGTGAATTATATA
AATATAAAGTGGTAGAAATTAAGCCATTG---GGAATAGCACCCACTGGAGCAAAAAGGAGAGTGGTG-----
----------------------------------GAGAGAGAAAAAAGAGCA---GTAGGA---
ATAGGAGCT---------GTGTTCCTTGGGTTCTTGGGAGCA---GCAGGAAGCACTATG---
GGCGCGGCGTCA---ATAACGCTGACGGTACAG---GCCAGACAA---TTGTTG---
TCTGGTATAGTGCAACAGCAAAGC---AATTTG---CTGAGA---
GCTATAGAGGCGCAACAACATCTGTTGCAACTCACGGTCTGGGGCATT
>KF250405..1
TTCTATTGTAATACATCAGGCCTGTTTAATGGTACATACAACAATACAGCCAATAATTCAAGC----------
----

TCAAACAGCACAATCATAATCCCATGTAGAATAAAGCAAATTATAAATATGTGGCAGGAGGTAGGACGAGCAA
TGTATGCCCCTCCCATTAAAGGAAACATAACATGTAAATCAAAT---
ATCACAGGAATATTATTGATCCGTGATGGAGGAAATGGAAATGAGACA-------------------------
----

AATGAAACAGAGACATTCAGACCTCAAGGAGGAGATATGAGGGACAATTGGAGAAGTGAATTATATAAATATA
AAGTAGTAAAAATAGAACCATTA---GGAGTAGCACCCACCAAGGCAAAAAGAAGAGTGGTG-----------
----------------------------CAGAGAGAAAAAAGAGCA---GTAGGA---ATAGGAGCT------
---GTGTTCCTTGGGTTCTTGGGAGCA---GCAGGAAGCACTATG---GGCGCGGCGTCA---
ATAACGCTGACGGTACAG---GCCAGACAA---TTGTTG---TCTGGTATAGTGCAACAGCAAAGC---
AATTTG---CTGAGG---GCTATAGATGCGCAACAACATATGTTGCAACTCACAGTCTGGGGCATC
>MK792287..2
TTCTATTGCAATACATCAGGCCTGTTTAATAGTACATACTGGTCTAATGGTACATACAAGATCAATGATACAT
ACAAAGTT---
-AATGATACAGAAAGT------------
AAGTCAATCATCTTCCAAATCCCATGCAGAATAAAGCAAATTGTAAACATGTGGCAGGAGGTAGGAAGAGCAT
TTTATGCCCCTCCCATTGAAGGAAACATAACATGTAAATCAAAT---
ATCACAGGACTACTATTGGTACGTGATGGAGGGCAGCCAAAAAATGGT-------------------------
----

ACAAATAGTACAGAAGAGATATTCAGACCTATAGGAGGAAATATGAAGGACAATTGGAGAAGTGAATTATACA
AGTATAAAGTGGTAGAAATTCAACCATTG---GGAGTAGCACCCACTACAGCAAAAAGGAGAGTGGTG-----
----------------------------------GGAAGAGAAAAAAGAGCAGTATTTGGA---
ATAGGAGCT---------GTGTTCCTTGGGTTCTTGGGAGCA---GCAGGAAGCACTATG---
GGCGCGGCGTCA---ATAGCGCTGACGGTACAG---GCCAGACAA---TTGCTG---
TCTGGTATAGTGCAACAGCAAAAC---AATTTG---CTGAGA---
GCTATAGAGGCGCAACAGCATATGTTGCAACTCACGGTCTGGGGCATT
>EU000514..1
TTCTACTGTAATACATCAAAACTGTTTAATAGTACTTGGAACAGTAGTAGTACTTGGACTACT----------
----

ACAGGAAATATCACCACAATCCCATGCAGAATAAAGCAAATTGTAAATATGTGGCAGGAAGTAGGACGAGCAA
TGTATGCCCCTCCCATTGAAGGACTAATTAGATGTACATCAAAT---
ATTACAGGGTTACTATTAACAAGAGATGGTGGTTATGAGAACAAGACA-------------------------
----

GGGAACAGGACAGAGACCTTCAGACCTGCAGGAGGAAATATGAGGGACAATTGGAGAAGTGAATTATATAAAT
ATAAAGTAGTAAAAATTGAACCATTA---GGAGTAGCACCCACCAAGGCAAAGAGAAGAGTGGTG--------
-------------------------------CAAAGAGAAAAAAGAGCA---GTGGGAGCGATAGGAGCT---
------GTGTTCCTTGGGTTCTTGGGAGCA---GCAGGAAGCACTATG---GGCGCAGCGGCA---
ATAACGCTGACGGTACAG---GCCAGACAA---TTGATG---TCTGGTATAGTGCAACAGCAGAGA---
AATTTG---CTGAGA---GCTATTGAGGCGCAACAGCATCTGTTGCAACTCACAGTCTGGGGCATT
>AB097873..1
TTCTATTGTAATACAACACAACTGTTTAATAGTAGTTGGAATGAGACTGATATTGGGAATAATACTAGTACTG
GG---

AATGATACTACTATCATGCTCCCTTGCAGAATAAAACAAGTTGTAAACATGTGGCAGGAAGTAGGAAAAGCAA
TGTATGCCCCTCCCATCAGAGGACTAATTAAATGTTCATCAAAT---
ATTACAGGGTTACTATTGACAAGAGATGGTGGTCATAATGAGACT----------------------------
----

GAGACTGAGACCTTCAGACCTGGAGGAGGAGATATGAGGGACAATTGGAGAAGTGAATTATATAAATATAAAG
TAGTAAAAATTGAACCATTA---GGAGTAGCACCCACCAGGGCAAAGAGAAGAGTGGTG--------------
-------------------------CAGAGAGAAAAAAGAGCA---GTGGGAATGATGGGAGCT---------
ATGTTCCTTGGGTTCTTGGGAACA---GCAGGAAGCACTATG---GGCGCAGCGTCA---
ATAACGCTGACGGTACAG---GCCAGACAA---TTGTTG---TCTGGTATAGTGCAACAGCAGAGA---
AATTTG---CTGAGG---GCTATTGAGGCGCAACAGCATATATTGCAACTCACAGTCTGGGGCATC
>MF373189..1
TTCTACTGTAATACATCAAACCTGTTTAATACATCAAACCTGTTTAATACTGATGATAATGCAACTGGA----
----

AAAAGTGACACTATCACTCTCACATGCCCAAGAAAACTAATTATAAACATGTGGCAGGAATTAGGGAAAGGGA
AGGATGCGCCTCCCATTCCAAGCCAAAGACAATGTACATCTTCC---
ATTACCGGACTGCTATTGCTAATAGATGGAGGTTTAGACAACACC----------------------------
----

AACAACGAGACCTTCAGACCTGGAGGAGGAGATATAAGAGATAATTGGAAAAGTGGAATATATAAATATTATG
CATTAAAAATAGAACCATTA---GGAATATCACCCAACACTGCAACGAAAACAGTGATG--------------
-------------------------CTAAGGGAACAAAGAGTA---ATGAGA---ACAGGAGCT---------
GTGTTCCTTGGGTTCTTGGGAGCA---GCAGGAAGCACTATG---GGCGCAGCGTCA---
ATAACGCTGACGGTACAG---GCCAGGCTA---TTATTG---TCTGGTATAGTGCAGCAGCAGAGC---
AATTTG---CTGAGG---GCTATTGAGGCGCAACAACATCTGTTGCAACTCACAGTCTGGGGCATC
>KT183077..1
TTCTACTGTAATACATCAAAACTGTTTAATACTACACTGTTTAATAAGAATAGTACTACACTGACTTGGAATG
ATACTGAC---

ACTGCAGAAAATATTACAATCCCATGCAGAATAAAACAAATTATAAATATGTGGCAGGAAGTAGGAAAAGCAA
TATATGCTCCTCCCATCAGTGGACTAATTAGATGCTCATCAAAT---
ATTACAGGACTGCTATTAATAAGAGATGGGGGTAATGATAATCAG----------------------------
----

ACTGAGATCTTTAGACCTGGAGGAGGAGATATGAGGGATAACTGGAGAAGTGAATTATACAAATATAAAGTAG
TAGAAATTAAGCCATTA---GGAGTAGCACCCACCAAGGCAAAGAGAAGAGTGGTG-----------------
----------------------CAGAGAGAAAAAAGAGCA---ATAATAGGGATGGGAGCT---------
GTGCTCTTTGGGTTCTTGGGAGCA---GCAGGAAGCACTATG---GGCGCAGCGTCA---
ATAACGCTGACGGTACAG---GCCAGACAA---TTACTA---TCTGGTATAGTGCAACAGCAAAAC---
AATTTG---CTGAGA---GCTATTGAGGCGCAACAACATCTGTTGCAACTCACAGTCTGGGGCATT
>KJ849787..3
TTCTACTGCAATACATCAGGACTGTTTAATTACTTCAATAATAACACCAAT----------------------
----
-AATAACAAATCCTCT---------

TCTGATGACACTCATATCACTCTCCCATGTCGAATAAAACAAATTATAAACATGTGGCAGGAAGTAGGAAGAG
CAATGTATGCTAATCCCATTGCAGGAAACATTACCTGTAGATCAAAT---
ATTACAGGCCTACTATTGACTAGAGATGGTGGT--

CATAATGATAATACTACTAAT---------
CGGACTGAGATCTTTAGACCTGGGGGAGGAAATATGAAAGACAATTGGAGAAGTGAATTATATAAATACAAAG
TAGTAGAAATTGAACCACTA---GGAGTAGCACCCACAAAGGCAAAAAGACAAGTGGTG--------------
-------------------------AGCAGACACAGAAGAGCA---GTGGGA---TTTGGAGCT---------
GTGTTCCTTGGGTTCTTGTCGGCA---GCAGGAAGCACTATG---GGCGCAGCGTCA---
GTGACGCTGACGGTACAG---GCCAGGCAA---TTATTG---TCTGGTATAGTGCAACAGCAGAAC---
AATTTG---CTGAAA---GCTATTGAGGCGCAACAGCATTTGTTGCAACTCACAGTCTGGGGCATT
>KJ849800..2
TTCTACTGCAATACATCACAACTGTTTAATGAGACA-------------------------------------
----

ATAAGCAATGACACTATCAAACTCCCATGTCGAATAAAACAAATTGTAAACATGTGGCAAGAAGTAGGAAAAG
CAATGTATGCCACTCCCATTGAAGGAAACATTACCTGTAACTCAAAC---
ATTACAGGTCTACTATTGACAAGGGATGGTGGT--

CAGAATAATGATACCAAT---
AAGACTGAGACCTTCAGACCTGGGGGAGGAAATATGAAGGACAATTGGAGAAGTGAATTATATAAATATAAAG
TAGTAGAAATTCAACCACTA---GGAATAGCACCCACCAAGGCAAAAAGACAAGTGGTG--------------
-------------------------AAAAAAGAAAAAAGAGCA---ATAGGA---CTAGGAGCT---------
ATGTTCCTTGGGTTCTTGGGAGCA---GCAGGAAGCACTATG---GGCGCAGCGTCA---
ATAACGCTGACGGTACAG---ACCAGACAA---TTAATG---TCTGGTATAGTGCAACAGCAAAGC---
AATTTG---CTGAGG---GCTATTGAGGCGCAACAGCATCTGTTGCAACTCACGGTTTGGGGCATC
>KY968400..1
TTCTACTGTAATACAACACAACTATTTAATACTACTTGGAAAAATAATAATACTGAAGAGACAAATAGCACTA
AA---

GGAAATGACACTATCACACTCCCATGCAGAATAAAACAGATCATAAATATGTGGCAGGAAGTAGGAAAAGCAA
TGTATGCCCCTCCTATCAGCGGAAAAATTAGATGCTCATCAAAT---
ATTACAGGACTGCTACTAACAAGAGATGGTGGTATTCATAACAATAGC-------------------------
----

AATGGGACTGAGACCTTCAGACCTGGGGGAGGAAATATGAAGGATAATTGGAGAAGTGAATTATATAAATATA
AAGTAGTACAAATTGAACCATTA---GGAGTAGCACCCACCAGGGCAAAGCGAAGAGTGGTG-----------
----------------------------CAGAGAGAAAAAAGAGCA---GCAGGA---ATGGGAGCT------
---TTGTTCCTTGGGTTCTTGGGAGCA---GCAGGAAGCACTATG---GGCGCAGCGGCA---
GTGACGCTGACGGTACAG---GCCAGACAA---TTATTG---TCTGGTATAGTGCAACAGCAAAAC---
AATTTG---CTGATG---GCTATTGAGGCGCAACAGCATCTGTTGCAACTCACAGTCTGGGGCATC
>KY968405..1
TTCTACTGTAATACAACACCACTGTTTAATAGCACTTGGAATAGGACTAATAGCACTTGGAATAGGAATGGGA
ATAGT--

ACTAATGGTACAATCACACTCCAATGCAGAATAAAACAAATTATAAACATGTGGCAGGAAGTAGGAAAAGCAA
TGTATGCCCCTCCCATCCAAGGACTAGTTAACTGTTCATCAAAT---
ATTACAGGTCTACTATTGACAAGAGATGGTAGTCAG-------------------------------------
----

AATGATACTGAGACCTTCAGACCTGGGGGAGGAGATATGAAAGACAATTGGAGAAGTGAATTATATAAATATA
AAGTAGTAAGAATTGAACCATTA---GGAGTAGCACCAACAAAGGCAAAGAGAAGAGTGGTG-----------
----------------------------CAGAGAGAAAAAAGAGCA---GTGGGA---CTAGGAGCT------
---ATGTTCCTTGGGTTCTTGGGAGCA---GCAGGAAGCACTATG---GGCGCAGCGTCA---
GTGACGCTGACGGTACAG---GCCAGACAA---TTATTG---TCTGGTATAGTGCAACAGCAGAAC---
AATCTG---CTGAGA---GCTATTGAGGCGCAACAACATCTGTTGCAACTCACAGTCTGGGGCATC
>MH078535..2
TTTTACTGCAATACAGCAGGATTGTTTAATAACATAAAA----------------------------------
----

AATAATGGAACAATCATACTCCCTTGCAGAATAAAACAAATAATAAACATGTGGCAGGAAGTAGGAAAAGCAA
TGTATGCTCCTCCCATCAGCGGACGAATCAACTGTTCATCAAAT---
ATTACAGGTCTAATATTGACAAGAGATGGTGGCTATCAAAATGATAGT-------------------------
----

AATGCTAGTGAAATCTTCAGACCTGGAGGAGGAGATATGAGGGACAATTGGAGAAGTGAATTATATAAATATA
AAGTAGTAAAAGTTGAACCATTA---GGGATAGCACCCACCAAGGCAAAGAGAAGAGTGGTG-----------
----------------------------CAGAGAGAAAGAAGAGCA---GTGGGA---CTAGGAGCT------
---GTGTTCCTTGGGTTCTTGGGAGCA---GCAGGAAGCACTATG---GGCGCAGCGTCA---
ATAACGCTGACGGTACAG---GCCAGACAA---TTATTG---TCTGGTATAGTGCAGCAGCAGAAC---
AATCTG---CTGAGG---GCTATTGAGGCGCAACAGCACCTGTTGCAACTCACAGTCTGGGGCATC
>AF408628..3
TTCTACTGTAACTCAACACCGCTGTTTAATAGTACTTGGCATACAAATGGTACTAGTACTTTAGAGTCAAATG
CCACT--

GAAGGAAACGACACAATCATACTCCCATGCAGAATAAAACAAATTATAAACAGGTGGCAGGAAGTAGGAAAAG
CAATGTATGCCCCTCCCATCAGAGGAATAATTAACTGTTTATCAAAT---
ATTACAGGGCTGCTGTTAATAAGAGATGGTGGTAATCAGAATCAGACA-------------------------
----

GGGGATCAGACAGAGACCTTCAGACCTGGAGGAGGAAATATAAAAGACAATTGGAGAAGTGAATTATATAAAT
ATAAAGTAGTAGAAATTGAACCATTA---GGAGTAGCACCCACCAAGGCAAAGAGAAGAGTGGTG--------
-------------------------------CAGAGAGAAAAAAGAGCA---GCG------ATAGGAGCT---
------ATATTCCTTGGGTTCTTGGGAGCA---GCAGGAAGCACTATG---GGCGCAGCGTCA---
ATAACGCTGACGGTACAG---GCCAGACTA---TTATTG---TCTGGTATAGTGCAACAGCAGAAC---
AATTTG---CTGAGG---GCTATTGAGGCACAACAACATCTGTTGCAACTCACAGTCTGGGGCATC
>AY037278..1
TTCTACTGCAATACATCAGGACTGTTTAATAACACAAAAGAA-------------------------------
----

AATGGCACTATTATTCTCCCATGTAGAATAAAACAAATTGTAAACATGTGGCAGGAAGTGGGACAAGCAATGT
ATGCCGCTCCCATTGCAGGAAACATTTCCTGTAACTCAAAT---
ATTACAGGTCTACTATTGACAAGAGATGGTGGTCAGAATAATGATACT-------------------------
----

AATGGAACTGAGATCTTCAGACCTGGGGGAGGAAATATGAAAGACAATTGGAGAAATGAATTATATAAATATA
AGGTAGTAGAAATTGAACCACTA---GGAATAGCACCCACCAGGGCAAAGAGACGAGTGGTG-----------
----------------------------GAGAGAGAAAAAAGAGCA---GTGGGA---ATAGGAGCT------
---TTGTTCCTTGGGTTCTTGGGAGCA---GCAGGAAGCACTATG---GGCGCAGCGTCA---
ATAACGCTGACGGTACAG---GCCAGGCAA---TTATTG---TCTGGAATAGTGCAACAGCAGAAC---
AATCTG---CTGAGA---GCTATTGAAGCGCAACAACATATGTTGCAGCTCACAGTCTGGGGCATT
>AY037283..1
TTCTACTGCAATACATCAAGACTGTTTAATGACACACTGTTTAATGATACAGAA-------------------
----

TCCAATGAAACTATTATTCTCCCATGTAAAATAAAACAAGTTGTAAACCTGTGGCAGGAAGTGGGACGAGCAA
TGTATGCCGCTCCCATTGCAGGAGCCATTACCTGTAACTCAACT---
ATTACAGGTCTATTATTGACAAGAGATGGTGGTCAGAGTAATGATACTAAT----------------------
----

GGTACTAAGATTGAGACCTTCAGACCTGGGGGAGGAAATATGAAAGACAATTGGAGAAGTGAATTATATAAAT
ATAAAGTAGTAGAAATTGAACCAATA---GGAGTAGCACCCACCAAGGCAAAAAGACAAGTGGTG--------
-------------------------------AGGAGAGAAAAAAGAGCA---GCA------TTGGGAGCG---
------TTGCTCCTTGGGTTCTTGGGAGCA---GCAGGAAGCACTATG---GGCGCGGCGTCA---
ATAACGCTGACGGTACAG---GCCAGACAA---TTATTG---TCTGGAATAGTGCAACAGCAAAAC---
AATTTG---CTGAGG---GCTATTGAAGCGCAACAGCATCTGTTGCAGCTCACAGTCTGGGGCATT
>DQ358803..2
TTCTACTGTAATACAACACAACTGTTTAATAGTACTTGGGATAGTACTGCAGGGAGCAACACTGAT-------
----
----------------------------GGAAGTGACGCCATCATACTCCTATGCCGAATA---

AACAATTATACATGTGGCAGAAGTAGGAAAAGC---
ATGTTTGCCCCTCCCATCAGCGGACAAATCAGATGCTCATCAAAT---
ATTACAGGTATACTATTGACAAGAGATGGTGGTCAGAATAATGATACT------------------------
----

AATGAGACTGAGATCTTCAGACCTGGAGGAGGAGATATGAGAGACAATTGGAGGAGTGAATTATATAGATATA
AAGTGGTAAAAATTGAGCCATTA---GGAGTAGCACCCACCTTGGCAAAGAGAAGAGTGGTG----------
---------------------------CAGAGAGAAAAAAGAGCA---GTGGGG---ATAGGAGCT------
---TTGTTCCTTGGGTTCTTGGGAGCA---GCAGGAAGCACTATG---GGCGCAGCGTCA---
ATAACGCTGACGGTACAG---GCCAGACAA---TTATTG---TCTGGTATAGTGCAACAGCAGAAC---
AATTTG---CTGAGG---GCTATTGAGGCGCAACAACATCTGTTGCAACTCACAGTCTGGGGCATC
>DQ358804..1
TTCTACTGCAATACATCAGGACTGTTTGATGAAACAAAATTTAATGACACAGAATCC---------------
----

AATAACGCCACTATCACTCTCCTATGTCGAATAAAACAAATTGTAAACATGTGGCAGGAAGTAGGACGAGCAA
TGTATGCCAATCCCATTGCAGGAAGCATTACCTGTAACTCAACT---
ATCACAGGTCTATTATTGACAAGAGATGGTGGTCAGAATACTACG---------------------------
----

AATAGTACTGAGATCTTCAGACCTGGGGGAGGAAATATGAAAGACAATTGGAGAAGTGAATTATATAAATATA
AAGTAGTAGAAATTGAACCACTA---GGAATAGCACCCTCCAGGGCAAAAAGACAAGTGGTG----------
---------------------------AAGAGAGAAAAAAGAGCA---GTTGGA---CTAGGAGCT------
---ATGTTCATTGGGTTCTTGGGAGCA---GCAGGAAGCACTATG---GGCGCGGCGTCA---
ATAACGCTGACGGTACAG---GCCAGACAA---TTACTG---TCTGGAATAGTGCAGCAGCAAAAT---
AATTTG---CTGAGG---GCTATTGAAGCGCAACAGCATCTGTTGCAGCTCACAGTCTGGGGCATT
>DQ358806..1
TTCTACTGCAATACATCAGGACTGTTTAATGACACAGTACCC------------------------------
----

AATGGCACTATCACTCTCCCATGTCGAATAAAACAAATTGTAAATATGTGGCAAGGAGTGGGGCAAGCAATGT
ATGCCGCTCCCATTGCAGGAAATATTACCTGCAGATCAAAT---
ATTACAGGTATACTATTGACAAGAGATGGTGGTGATGCTAATCAGAATGAT---------------------
----

CCCAATGGAAATGAGACCTTTAGACCTGGGGGAGGAAATATGAAAGACACCCGGAGAAGTGAATTATATAAAT
ATAAAGTAGTAAAAATTGAGCCATTA---GGAATAGCACCCACCAAGGCAAAGAGAAGAGTGGTG-------
------------------------------CAGAGAGAAAAAAGAGCA---GTGGGCACGTTAGGAGCT---
------ATGTTCCTTGGGTTCTTGGGAGCA---GCAGGAAGCACTATG---GGCGCAGCGTCA---
ATAACGCTGACGGTACAG---GCCAGGCAA---TTATTG---TCTGGTATAGTGCAACAGCAAAAC---
AATTTG---CTGAGG---GCTGTTGAAGCGCAACAACATCTGTTGCAACTCACAGTCTGGGGCATC
>DQ358812..2
TTTTACTGCAATACATCAGAACTGTTTAATAACACAAAAAAC------------------------------
----

AATGGCACCATCATTCTCCCATGTAAAATAAAACAAATTGTAAACATGTGGCAGAGAGTAGGACGAGCAATGT
ATGCCAATCCCATTGCAGGAAAAATTAACTGTAACTCAAAT---
ATTACAGGTCTATTATTGACAAGAGATGGTGGTAATCAGGCTAATGAGACTAGT-----------GGT----
----
-
ATTCAGACTAATGGGACTGAGATCTTCAGACCTGGAGGAGGAAATATGAGAGACAATTGGAGAAGTGAATTAT
ATAAATATAAAGTAGTACAAATTAAACCACTA---GGAATAGCACCCACCAGGGCAAAAAGACGAGTGGTG--
------------------------------------CAGAGAGCAAGAAGAGCA---GTGGGA---
ATAGGAGCT---------CTTTTCATTGGATTCTTAGGAGCA---GCAGGAAGCACTATG---
GGCGCGGCGTCA---ATAACGCTGACGGTACAG---GCCAGACAA---TTATTG---
TCTGGAATAGTGCAACAGCAAAAC---AATTTA---CTGCAG---
GCTATTGAAGCGCAACAGCATCTGTTGCAGCTCACAGTCTGGGGCATT
>MG365770..2
TTCTACTGCAATACATCAGGGCTGTTTAATGAAACAAAC---------------------------------
----

TTTAATGACACTATCACTCTCCCATGTCGAATAAAACAAATTGTAAACATGTGGCAGGAAGTAGGACGAGCAA
TGTATGCCAATCCCATTAAAGGAAACATTACCTGCAGCTCAAAT---
ATTACAGGTCTAATATTGACAAGAGATGGTGGTCAGAAGAATGATACT-------------------------
----

AATCAGACTGAGACCTTCAGACCTGGGGGAGGAAATATGAAAGACAATTGGAGAAGTGAATTATACAAATATA
AAGTAGTAGAAATTGAACCACTA---GGAGTAGCACCCACCAAGGCAAAAAGACAAGTGGTG-----------
----------------------------ACGAGACAAAAAAGAGCA---GCA------ATAGGAGCT------
---TTGTTCCTTGGGTTCTTGAGTGCA---GCAGGAAGCACTATG---GGCGCGGCGTCA---
ATAACGCTGACGGTACAG---GCCCGACAA---TTATTG---TCTGGGATAGTGCAACAGCAGAGC---
AATTTG---CTGAGG---GCTATTGAAGCGCAACAACATCTGTTGCAGCTCACAGTCTGGGGCATA
>JN692449..2
TTCTACTGTAATACAACACAACTGTTTAATAGTACTTGGCATTTGAATGCCACTGGAAAGTCTAATTACACAG
AA---

GGAAATGACACAATCACACTCCCATGCAGAATAAAACAAATTGTAAACATGTGGCAAGAAGTAGGAAAAGCAA
TGTATGCCCCTCCCATCAGAGGACAAATTAGCTGTTCATCAAAT---
ATTACAGGACTGCTATTAACAAGAGATGGTAGTAACAACACCAAC----------------------------
----

GAAAGCGAGATCTTCAGACCTGGAAGAGGAAATATAAAGAACAATGGAAGGAGTGAACTATACAAATATAAAG
TAGTAAAAATTCAGCCATTA---AGAGTAGCACCTACCAAGGCAAAGAGAAGAGTGGTG--------------
-------------------------CAGAGAGAAAAAAGAGCA---GTGACC---CTAGGAGCT---------
GTGTTCCTTGGGTTCTTGGGAGCA---GCAGGAAGCACTATG---GGCGCAGCGTCG---
CTGACGCTGACGGTACAG---GCCAGACAA---TTATTG---TCTGGTATAGTGCAGCAGCAAAAC---
AATTTG---CTGAGA---GCTATTGAAGCGCAACAACATATGTTGCAGCTCACAGTCTGGGGCATC
>EF637052..2
TTCTACTGCAATACATCAGGACTATTTAATAACGCACTGTTTAATGACACAGGAGTCAATAATGGC-------
----

AGTGATGACAATATCATTCTCCCATGTCGAATAAAACAAATTGTAAACATGTGGCAGGAAGTAGGACGAGCAA
TGTATACTGCTCCTATTGCAGGAAACATTACCTGTAGATCAAAT---
ATTACAGGTCTTCTATTGACAAGGGATGGTGGTAATAATGGTGAT----------------------------
----

AATCAGACTCAGATTTTCAGACCTGGGGGAGGAAACATGAAAGACAATTGGAGAAGTGAATTATATAAATATA
AAGTAGTAGAGATTGAACCACTA---GGAGTAGCACCCACCAGGGCAAAAAGACAAGTGGTG-----------
----------------------------ACAAGAGAAAGAAGAGCA---GCA------GTGGGACTG------
---TTTTTCCTTGGGTTCTTGGGAGCA---GCAGGAAGCACTATG---GGCGCAGCGTCA---
CTGACGCTGACGGTACAG---GCCAGACTA---TTACTG---TCTGGTATAGTGCAGCAGCAGAGC---
AATCTG---CTGAGA---GCTATTGAGGCGCAACAGCATCTGTTGCAACTCACAGTCTGGGGCATT
>MH938678..2
TTCTACTGTAATACAACAAAGCTGTTTAATAGTACTTGGAATGGCACTAATAGTATTAAT-------------
----

AAGACTGGTACTATCACAATCCCATGCAGAATAAAACAAATTATAAACAGGTGGCAGGAAGTAGGAAAAGCAA
TATATGCCCCTCCCATTGGAGGACAAATTAGATGTTCATCAAAT---
ATTACAGGGCTGCTAATAACAAGAGATGGTGGTCATAATCAGACT----------------------------
----

GCTGAAACCTTCAGACCTGGAGGAGGAGATATGAAAGACAACTGGAGAAATGAATTATATAAATATAAAGTAG
TAGAAATTGAGCCACTA---GGAATAGCACCCTCCAAGGCAAAGAGAAGAGTAGTG-----------------
----------------------CAGAGAGAAAAGAGAGCA---GTGACA---TTAGGAGCT---------
ATGTTCCTTGGGTTCTTGGGAGCA---GCAGGAAGCACTATG---GGCGCAGCGTCA---
ATGACGCTGACGGTACAG---GCCAGACAA---TTATTG---TCTGGTATAGTGCAGCAGCAGAAC---
AATTTG---CTGAAG---GCTATTGAAGCGCAACAGCATATGTTGCAGCTCACAGTCTGGGGCATT
>JN692456..1
TTCTACTGTAATACATCAGCACTGTTTAATAGTACTTGGCCGTCTAATAGTACTTGGAATGATACTGACAATA
AA---

GGAAATGATACTATCATCATACTCCCATGCAGAATAAAACAAATTATAAACAGGTGGCAGGAAGTAGGAAAAG
CAATGTATGCCCCTCCCATCGCAGGACAAATTAGATGTTCATCAAAC---
ATTACAGGGCTGCTATTAACAAGAGATGGTGGTAACAACAGCACC----------------------------
----

AATGAAACCTTCAGACCAGGAGGAGGAGATATGAGGGACAATTGGAGAAGTGAATTATTTAAATATAAAGTAA
TAAGAATTGAACCATTA---GGAATAGCCCCCACTCAGGCAAAGAGAAGAGTGGTG-----------------
----------------------CAGAGAGAAAAAAGAGCA---GCA------CTAGGAGCT---------
ATGTTCCTTGGGTTCTTGGGAGCA---GCAGGAAGCACTATG---GGCGCAGCATCA---
ATAACGCTGACGGTACAG---GCCAGACAA---TTATTG---TCTGGTATAGTGCAACAGCAGAAC---
AATCTG---CTGAGA---GCTATTGAGGCGCAACAACATCTGTTGCAACTCACAGTCTGGGGCATC
>JF804808..1
TTCTACTGTAATACAACACAACTGTTTAATAGTACTTGGACTTGGAATAATAGCAATAGTACTTGGAATAGGA
ATGATAGCACT--
-------AATACTTGGGAT------
AGTGCTAATGAAAATATCACACTCCCATGCAGAATAAAACAAATTATAAACAGGTGGCAGGAAGTAGGAAAAG
CAATGTATGCCCCTCCCATCAAAGGACAAATTAGATGTTCATCAAAT---
ATTACGGGGCTGATATTAACAAGAGATGGTGGGTCCAATCACAGC----------------------------
----

TCCACCGAGACCTTCAGACCTGGAGGGGGAGATATGAGGGATAATTGGAGAAGTGAGTTATATAAATATAAAG
TAGTAAAAATTGAGCCATTA---GGAATAGCCCCCAACAAGGCAAAAAGAAGAGTGGTG--------------
-------------------------CAGAGAGAAAAAAGAGCA---GTGGGAGTGATAGGAGCT---------
ATGTTCCTTGGGTTCTTGGGAGCA---GCAGGAAGCACTATG---GGCGCAGCGTCA---
ATGACGCTGACGGTACAG---GCCAGACAA---CTGTTG---TCTGGTATAGTGCAACAGCAAAAT---
AATTTG---CTGAGA---GCTATTGAGGCGCAACAGCATCTGTTGCAGCTCACAGTCTGGGGCATC
>HM026455..1
TTCTACTGCAATACATCAGAATTGTTTACTGACAAAAAATTCAATGCC-------------------------
----

ACCCATGGCACTATCACTCTCCCATGTCGAATAAAACAAATTGTAAACCTATGGCAGGAAGTAGGAAGAGCAA
TATATGCCGCTCCCATTGAAGGAAACATTACCTGTAATTCAAAT---
ATTACAGGTCTACTACTAACAAGAGATGGTGGTCAGAACAGTAGT----------------------------
----

GCTAATGAGACCTTCAGACCTGGGGGAGGAGATATGAGAGACAATTGGAGAAGTGAATTATATAAATATAAAG
TAGTAGAAATTGAACCACTA---GGAATAGCACCCACCAAGGCAAAAAGAAGAGTGGTG--------------
-------------------------CAGAGAGAAAAAAGAGCA---GTGGGA---ATAGGAGCG---------
TTGTTCATTGGGTTCTTGGGAGCA---GCAGGAAGCACTATG---GGCGCAGCGTCA---
ATAACGCTGACGGTACAG---GCCAGACAA---TTATTG---TCTGGAATAGTGCAGCAGCAGAAC---
AACCTG---CTGAGG---GCTATTGAAGCGCAACAGCATCTGTTGCAGCTCACAGTCTGGGGCATT
>HQ236610..1
TTCTACTGTAATACATCAGGACTGTTTAATACATCAAGACTGATTATTAATGACACTTCCCCT----------
----

CTCAATGACACTATCATTCTCCCATGTAGAATAAAACAAATTGTAAACATGTGGCAGGGAGTAGGAAGAGCAA
TGTATGCTAATCCAATTGCAGGAAACATTACCTGTAACTCAAAT---
ATTACAGGGCTACTATTGACAAGAGATGGTGGAAAGAAAGATAAT----------------------------
----

CAGACTGACGAGATCTTCAGACCTGGGGGAGGAAATATGAAAGACAATTGGAGAAGTGAATTATATAAATATA
AAGTAGTAGAAATTGAACCACTA---GGAATAGCTCCCACAAAGGCAAAAAGACAAGTGGTG-----------
----------------------------CGGAGAGACAAAAGAGCA---GTGGGA---TTTGGAGCT------
---TTGTTCCTTGGGTTCTTGTCAGCA---GCAGGAAGCACTATG---GGCGCAGCGTCA---
GTGACGCTGACGGTACAG---GCCAGGCAA---TTGTTG---TCTGGTATAGTGCAACAGCAGAAT---
AATTTG---CTGAAA---GCTATTGAGGCGCAACAGCATCTGTTGCAACTCACAGTCTGGGGCATT
>KT427871..1
TTCTACTGTAACACATCAAAACTGTTTAGTAGTACTTGGAATAGTAGTACTTTGAATAGTACTGGAGAGTCAA
ATAAC--

ACTGAGGACACAATTATACTCCCATGCAGAATAAAACAAATTATAAACATGTGGCAGAGAGTAGGAAAAGCAA
TGTATGCCCCTCCCATCCAAGGACAAATTAACTGTTCATCAAAT---
ATTACAGGGATGATATTAACAAGAGATGGGGGTCATAACACGAGC----------------------------
----

GAGACGACCGAAATCTTCAGACCTGGAGGAGGAAATATGAAGGACAATTGGAGAAGTGAATTATATAAATATA
AAGTAGTACAAATTGAACCATTA---GGAATAGCACCCACCAGGGCTAAGAGAAGAGTGGTG-----------
----------------------------CAGAGAGAAAAAAGAGCA---GTAGGA---CTAGGAGCT------
---TTGTTCCTTGGGTTCTTAGGAGCA---GCGGGAAGCACTATG---GGCGCAGCGTCA---
ATAACGCTGACGGTACAG---GCCAGACAA---TTATTG---TCTGGTATAGTGCAACAGCAAAAT---
AATTTG---CTGAGG---GCTATTGAGGCGCAACAGCATATGTTGCAACTCACAGTCTGGGGCATT
>KT427779..1
TTCTACTGCAATACATCACAGCTGTTTAATGACACAGAATCC-------------------------------
----

AATAATAGCATTATTACTCTCCCATGTAGAATAAAACAAATAGTAAACATGTGGCAGGAAGTAGGAAAGGCAA
TGTATGCCGCTCCCATTGCAGGAAACATTACCTGTAACTCAAAT---
ATTACAGGTCTGCTACTAACAAGAGATGGTGGCCAGGATAATAATACT-------------------------
----

AATCAGAATGAGACCTTCAGACCTGGGGGAGGAGATATGAGAGATAATTGGAGAAGTGAGTTATATAAATATA
AAGTAGTAGAAATTGAACCATTA---GGAATAGCACCCACCAGGGCAAGAAGACGAGTGGTG-----------
----------------------------CAGAGAGAGAAACGAGCA---GTGGGA---CTAGGAGCT------
---GTGTTCCTTGGATTCTTAGGAGCA---GCAGGAAGCACTATG---GGCGCAGCATCA---
ATAACGCTGACGGTACAG---GCCAGACAA---TTATTG---TCTGGAATAGTGCAACAGCAGAAC---
AATTTG---CTGAGG---GCTATTGAAGCGCAACAGCATCTGTTGCAGCTCACAGTCTGGGGCATT
>KT427651..1
TTCTACTGTAACACATCCCGACTATTTGATGATAGTTGGAATGGTACTTGGTCAAAGAACACT----------
----

ACTATAGAAAATATCACACTCCCATGCAGAATAAAACAAGTTGTAAACCTGTGGCAGCAAGTAGGAAAAGCAA
TGTATGCCGCTCCCATCAAAGGACTACTTAACTGTACATCAAAT---
ATTACAGGGCTGATATTAGCAAGAGATGGTGGTTATAATGTTGAT----------------------------
----

AACAATACAGAGATCTTCAGACCTGCAGGAGGAGATATGAGGGACAATTGGAGAAGTGAATTATATAAATATA
AAGTAGTAAAAATTGAGCCATTA---GGAGTAGCACCCACCAAGGCAAAGAGAAGAGTGGTG-----------
----------------------------CAGAGAGAAAAAAGAGCA---GTAACA---TTAGGAGCT------
---ATGTTCCTTGGGTTCTTGGGAGCA---GCAGGAAGCACTATG---GGCGCAGCGTCA---
ATGACGCTGACGGTACAG---GCCAGACTA---TTATTG---TCTGGTATAGTGCAGCAGCAGAAC---
AATTTG---CTGAGA---GCTATTGAGGCGCAACAGCATCTGTTGCAACTCACAGTCTGGGGCATT
>KJ849794..1
TTCTACTGCAATACAACACTATTGTTTAACGAAGAATTAAATAACACTGGC----------------------
----
-------ACTGATGACACT------
GGCACTAACGACACTATCATTCTCCCATGTAAAATAAAACAAATAATAAACATGTGGCAGGAAGTGGGACGAG
CTATGTACGCTGCTCCCATTGCAGGAAACATTACCTGTTACTCAAAT---
ATTACAGGTCTACTATTGGTAAGAGATGGTGGTAATACT----------------------------------
----
AATGATGGTAATACTACTAGTGGTAAT---------
AAGACCGAGATCTTCAGACCTGAGGGAGGAAATATGAAAGACAATTGGAGAAGTGAACTATATAAATATAAAG
TAATAGAAATAGAACCACTA---GGGGTAGCACCCACCAAGGCAAAAAGACAAGTGGTG--------------
-------------------------AAGAGAGAAAAAAAGAGCA---ATAGGA---ATGGGAGCT--------
TTGTTCCTTGGGTTCTTGGGAGCA---GCAGGAAGCACTATG---GGCGCAGCGTCA---
GTAACGCTGACGGTACAG---GCCAGACAA---TTATTG---TCTGGTATAGTGCAACAGCAGAAC---
AATCTG---CTGAGG---GCTATTGAAGCGCAACAGCATCTGTTGCAGCTCACAGTCTGGGGCATC
>KT427660..2
TTCTACTGTAATACAACACAGCTGTTTAATAGTACAATAGTTAATAATACAATGCAGAATAAGACT-------
----

GGGAATGGTAATATCACACTCCCATGCACAATAAAACAAATTATAAATATGTGGCAGAAAGTAGGGAAAGCAA
TGTATGCCCCTCCCATCAGAGGACAAATTAGCTGTACATCAAAT---
ATTACAGGTCTACTATTGACTAGAGATGGGGATCAGAATGACAGT---------------------------
----

GAGACCTTCAGACCTCAGGGAGGAAATATGAAAGATAATTGGAGAAGTGAATTATATAAATACAAAGTAGTAA
GAATTGAGCCATTA---GGAGTAGCACCCACCAAGGCAAAGAGAAGAGTGGTG-------------------
-------------------CAGAGAGAAAAAAGAGCA---GTGGGAACATTAGGAGCT---------
ATGTTCCTTGGGTTCTTGGGAGCA---GCAGGAAGCACTATG---GGCGCAGCGGCA---
CTGACGCTGACGGTACAG---GCCAGACTA---TTATTG---TCTGGTATAGTGCAACAGCAAAAC---
AATTTG---CTGAGA---GCTATTGAGGCGCAACAGCATCTGTTGCAACTCACAGTCTGGGGCATC
>KX181927..1
TTCTATTGCAATACATCAGGCCTGTTCAATATGTCCCATAACAATGGCAATGAC------------------
----

ACAACCACTGTCACTATCACCCTCCCGTGTCGAATAAAACAAATTGTAAACATGTGGCAGGAGGTAGGACGAG
CAATGTATGCCGCTCCCATTGCAGGAAGCATTACCTGCAAATCAAAT---
ATTACAGGTCTGCTATTGACAAGAGATGGTAGTAGCAATAATAGTACT------------------------
----

CATAATAACACTGAGACCTTCAGACCTGGGGGAAAGAATATAAAAGACAATTGGAGAAGTGAACTGTATAAAT
ATAAAGTAATAGAAATTGAACCACTA---GGAATAGCACCCACCAAGGCAAAAAGACGAGTGGTG--------
------------------------------ACGAGAGAAAAAAGAGCA---GTAGGA---ATAGGAGCT---
------TTGTTCCTTAAGTTCTTAGGAGCA---GCAGGAAGCACTATA---GGCGCAGCGTCA---
GTGACGCTGACGGTACAG---GCCAGACAA---TTATTG---TCTGGTATAGTGCAACAGCAGAAC---
AATTTG---CTGAGA---GCTATTGAGGCGCAACAGCATATGTTGCAACTCACAGTCTGGGGCATT
>MK041580..1
TTCTACTGCAATACATCAGGACTGTTTAATGAGACA------------------------------------
----

GAATTCAATGGTACTATCACTCTCCCATGTCGAATAAAACAAATTGTAAACATGTGGCAGGAAGTAGGAAAAG
CAATGTATGCCGCTCCCATCGCAGGAAACATTACCTGCAACTCAAAT---
ATTACAGGTCTACTATTGACAAGAGATGGTGGTCAGAATAATAAT---------------------------
----

GCTACTGAGATCTTCAGACCTGGGGGAGGAAATATGAAAGACAATTGGAGAAGTGAATTATATAAATATAAAG
TAGTAGAAATTGAACCACTA---GGAGTAGCACCCACCAGGGCAAAAAGACAAGTGGTG-------------
-------------------------AGGAGAGAAAAAAGGGCA---GTGGGA---ATAGGAGCT---------
TTGTTCCTTGGGTTCTTGGGAGCA---GCAGGAAGCACTATG---GGCGCAGCGTCA---
CTAACGCTGACGGTACAG---GCCAGACAA---TTATTG---TCTGGAATAGTGCAACAGCAGAGC---
AATTTG---CTGAGG---GCTATTGAAGCGCAACAGCATATGTTGCAGCTCACAGTCTGGGGCATT
>AF005495..2
TTCTACTGCAATACATCAGGACTGTTTAATGACACAGTAGAC------------------------------
----

AATGGCACTATCACTCTCCCATGTCGAATAAAGCAAATTGTAAATATGTGGCAGGAAGTGGGGCGAGCAATGT
ATGCCGCTCCCATTGCAGGAAACATTACCTGTAGCTCAAAT---
ATTACAGGTCTACTATTGACAAGAGATGGTGGTCAGAATAATCAG---------------------------
----

ACGGAGGAGACCTTCAGACCTGGGGGAGGAAATATGAAAGACAACTGGAGAAGTGAATTATATAAATATAAAG
TAGTAGAAATTGAGCCATTA---GGAGTAGCACCCACCAAGGCAAAAAGACAAGTGGTG-------------
-------------------------AAGAGAGAAAAAAGAGCA---GTGGGA---ATGGGAGCT---------
TTGTTCCTTGGGTTCTTGGGAGCA---GCAGGAAGCACTATG---GGCGCAGCGTCA---
ATAACGCTGACGGCACAG---GCCAGACAA---TTATTG---TCTGGCATAGTGCAACAGCAGAAT---
AATTTG---CTGAGG---GCTATTGAAGCGCAACAGCATCTGTTGCAGCTCACAGTCTGGGGCATT
>AY455784..1
TTCTACTGCAATACATCAGGACTGTTTAATAACACAAGATCC------------------------------
----

```
-------------------------------
AATGGCACTATCACTCTCCCATGTCGAATAAAACAAATTATAAATATGTGGCAGGAAGTAGGACGAGCAATGT
ATGCCCCTCCCGTTGCAGGAAACATTACCTGTAACTCAAAT---
ATTACAGGTCTACTATTGACAAGAGATGGTGGTAAACAGAATGGTACT-------------------------
-------------------------------------------------------------------------
----------
AATCAGACTGAGATCTTCAGACCTGGGGGAGGAGATATGAGAGACAATTGGAGAAGTGAATTATATAAATATA
AAGTAGTAGAAATTGAACCACTA---GGAGTAGCACCCACCAAGGCAAAAAGACAAGTGGTG-----------
----------------------------AAGAGAGAAAAAAGAGCA---ATAGGA---ATAGGAGCT------
---GTACTCCTTGGGTTCTTGGGAAGA---GCAGGAAGCACTATG---GGCGCGGCGTCA---
ATAACGCTGACGGCACAG---GCCAGACAA---GTATTG---TCTGGAATAGTGCAACAGCAGAAC---
AATCTG---CTGAGG---GCTATTGAAGCGCAACAGCATCTGTTGCAGCTCACAGTCTGGGGCATT
>AY455785..1
TTCTACTGCAATACATCAAAACTGTTTAATAACATAGGAGAC-------------------------------
-------------------------------------------------------------------------
-------------------------------
AATGGCACTATCATTCTCCCATGTCGAATAAAACAAATTATAAACATGTGGCAGGAAGTAGGACGAGCAATGT
ATGCCGCTCCCATTGCAGGAAACATTACCTGTAACTCAAAT---
ATTACAGGTTTACTATTGACAAGAGATGGTGGTCAGACTAATGAA----------------------------
-------------------------------------------------------------------------
----------
ACTAATACTGAGACCTTCAGACCTGGGGGAGGAAATATGAAAGACAATTGGAGAAGTGAATTATATAAATATA
AAGTAGTAGAAATTGAACCAATA---GGAATAGCACCCACCAGGGCAAAAAGACGAGTGGTG-----------
----------------------------CAGAAAGAAAAAAGAGCA---GTGGGA---ATGGGAGCT------
---TTGTTCCTTGGGTTCTTGGGAGCA---CAAGGAAGCACTATG---GGCGCGGCGTCA---
ATAGCGCTGACGGTACAG---GCCAGACAA---TTATTG---TCTGGAATAGTGCAACAGCAGAAC---
AATTTG---CTGAGG---GCTATTGAAGCGCAACAGCACCTGTTGCAGCTCACAGTCTGGGGCATT
>AY455780..1
TTCTATTGCGACACATCACAACTGTTTAATAACACAAAAGTC-------------------------------
-------------------------------------------------------------------------
-------------------------------
AATGACACTATCACCCTCCCGTGTAGAATAAAACAAATTGTAAACATGTGGCAGGAAGTAGGACGAGCAATGT
ATGCCGCTCCTATTGCAGGAAACATTACCTGTAATTCAAGT---
ATTACAGGTCTACTATTGACAAGAGATGGTGGTCAGGCTAATAGG----------------------------
-------------------------------------------------------------------------
----------------
ACTGAGACCTTCAGACCTGGGGGAGGAAATATGAAAGACAATTGGAGAAGTGAATTATATAAATATAAAGTAG
TAGAAATTGAACCACTA---GGAGTAGCACCCACCGGGGCAAGAAGACAAGTGGTG-----------------
----------------------AGGAGAGAAAGAAGAGCA---GTGGGAATGCTAGGAGCT---------
ATGTTCCTTGGGTTCTTGGGAGCA---GCAGGAAGCACTATG---GGCGCAGCGTCA---
ATATCGCTGACGGTACAG---GCCAGACAA---TTATTG---TCTGGTATAGTGCAACAGCAGAAC---
AATCTG---CTGAGA---GCTATTGAGGCGCAACAGCATATGTTGCAACTCACAGTCTGGGGCATT
>AY771592..3
TTCTACTGTGATACAACACAACTGTTTAATAGTACTTGGAATGATACTTGGTATAATAATGTGAATGAT----
-------------------------------------------------------------------------
----------------------------
ACTAGTGAAAATATCACACTCCCATGCAGAATAAAACAAATTATAAACAAGTGGCAGGAAGTAGGAAAAGCAT
TTTATGCCCCTCCTATCAGCGGACAAATTAGATGTTCATCAAAT---
ATTACAGGTATACTATTGACAAGAGATGGTGGTCAGAATAGTCAG----------------------------
-------------------------------------------------------------------------
----------------
AATGAGACCTTCAGACCAGGAGGAGGAAATATGAAGGACAATTGGAGAAGTGAATTATATAAATATAAAGTAG
TAAGAGTTGAGCCATTA---GGAATAGCACCCACCAGGGCAAAGAGAAGAGTGGTG-----------------
----------------------CAGAGAGAAAGAAGAGCA---GTGGGAACACTAGGAGCT---------
ATGTTCCTTGGGTTCTTGGGAGCA---GCAGGAAGCACTATG---GGCGCAGCGTCA---
ATGACGCTGACGGTACAG---GCCAGACTA---CTATTG---TCTGGTATAGTGCAACAGCAGAAC---
AATCTG---CTGAGA---GCTATTGAGGCGCAACAGCATATGTTGCAGCTCACAGTCTGGGGCATC
>AY771593..5
TTCTACTGTAATTCAACTCAACTGTTCAATAGTACTTGGCTCAATAATACTTGGTATAATACTACTTGGGAAG
GAAT---------------------------------------------------------------------
```

AGCACTGAAGACAATATCACACTCCCATGCAGAATAAAACAAATTATAAACCTGTGGCAGAAAGTAGGAAAAG
CAATGTATGCCCCTCCTATCAGAGGACAAATTAGATGCTCATCAAAT---
ATTACAGGTCTAATATTGACAAGAGATGGTGGTCGTCAGAATGATACT-------------------------
----

AATGCTACTGACACCTTAACAGCTGGAGGAGGAAATATGAACGACTGTTGGAGAGGTGAATTTTATTATTATA
AAGTAGTAATAGTTGCGCCAATA---GGAGCACCCCCCCCCCAGGCAAAGAGAATAGTGGTG-----------
----------------------------CAAAAAGAAAAAAGAGCG---GTGGGAGTAATAGGAGCT------
---ATGTTCCTTGGGTTCTTGGGAGCA---GCAGGAAGCACTATG---GGCGCAGCGTCA---
ATGACGCTGACGGTACAG---GCCAGGTCA---TTATTG---TCTGGTATAGTGCAACAGCAGAAC---
AATCTG---CTGAGG---GCTATTGAGGCGCAACAGCATCTGTTGCAACTCACAGTCTGGGGCATC
>AY536233..1
TTCTACTGCAATACATCAGGACTGTTTAATGAAACAAGGTTTAATAACACAACA-------------------
----

TCCAATGACACCATTATTCTCCAATGTAAAATAAGACAAATAGTAAACCTGTGGCAGGAAGTGGGACGAGCAA
TGTATGCCGCTCCCATTGAAGGAAATATTACCTGTAACTCAAAT---
ATTACAGGTCTACTATTGACAAGAGATGGTGGTAATACTAGTCAGGCT-------------------------
----

AATAAGACTGAGACCTTCAGACCTGGGGGAGGAAATATGAGAGACAATTGGAGAAGTGAATTATATAAATATA
AAGTAGTAGAAATTGAACCACTA---GGAATAGCACCCACCAAGGCAAAAAGACAAGTGGTG-----------
----------------------------CAGAGAGAAAAAAGAGCAGTAGTGGGA---ATAGGAGCT------
---GTTCTCCTTGGGTTCTTGGGAGCA---GCAGGAAGCACTATG---GGCGCAGCGTCA---
ATAACGCTGACGGTACAG---GCCAGACAA---TTATTG---TCTGGAATAGTGCAACAGCAGAGC---
AATTTG---CTGAGG---GCTATTGATGCGCAACAGCATATGTTGCAGCTCACAGTCTGGGGCATT
>AY536238..1
TTCTACTGCAATACATCAGAACTGTTTAATAACACAGAAACCAATAGCACAAACACCAATGGCACAGAA----
----

ACCAATGGCACTATTATTCTCCCATGTAGAATAAAACAAATTGTGAACATGTGGCAGGAAGTGGGACGAGCAA
TGTATGCCAATCCCATTGCAGGAAACATTACCTGCAACTCAAAT---
ATTACAGGTCTACTATTGACAAGAGATGGTGGTAATACTAGTAAT----------------------------
----

AATACTGAGACCTTCAGACCTGGGGGAGGAGATATGAGAGACAATTGGAGAAGTGAATTATATAAATATAAGG
TAGTAGAAATTCAACCATTA---GGAATAGCACCCACCAAGGCAAGGAGACCAGTGGTG--------------
-------------------------AAGAGAGAAAAAAGAGCA---GTGGGA---CTAGGAGCT---------
TTGATCATTGGGTTCTTGGGAGCA---GCAGGAAGCACTATG---GGCGCAGCGTCA---
ATGGCGCTGACGGTACAG---GCCAGACAA---TTATTG---TCTGGAATAGTGCAACAGCAAAAC---
AATTTG---CTGAGA---GCTATTGAAGCGCAACAACATATATTGCAGCTCACAGTCTGGGGCATT
>KU749408..1
TTCTACTGCAATACATCAGGACTGTTTAATAACTCACTGCTCAATGACACAAAATCCAATAATGACACAGGA-
----

TCCAATAATACTATTACTCTCCCATGTAGAATAAGACAAATTGTAAACCTGTGGCAGGAAGTAGGACGAGCAA
TGTATGCCGCTCCCATTGCAGGAAACATTACCTGTAACTCAAAT---
ATTACAGGTATACTATTGACAAGAGATGGTGGTCAGAATAATAAGAATGAGACC------------TTC----
----

AAGAATGAGACCTTCAGACCTGGGGGAGGAAATATGAGAGACAATTGGAGAAGTGAATTATATAAATATAAAG
TAGTAGAAATTGAACCACTA---GGAGTAGCACCCACCAAGGCAAAAAGACAAGTGGTG--------------
-------------------------AAGAGAGAAAAAAGAGCA---GTAGGA---ATGGGAGCT---------
TTGTTCCTTGGGTTCTTGGGAGCA---GCAGGAAGCACTATG---GGCGCAGCGTCA---
GTAGCGCTGACGGTACAG---GCCAGACAA---TTATTG---TCTGGAATAGTGCAACAGCAGAAC---
AATTTG---CTGAGG---GCTATTGAAGCGCAACAGCATATGTTGCAGCTCACAGTCTGGGGCATT
>FJ853621..1
TTCTACTGTAATACATCAGAACTGTTTAATAACACAATATCC-------------------------------
----

AAAAATGGCACTATCATTCTCCCATGTCGAATAAAACAAATTATAAACATGTGGCAGGAAGTGGGGCGAGCAA
TGTATGCCGCTCCCATTGCAGGAAACATTACCTGTAACTCAAAT---
ATTACAGGTCTACTATTGGTAAGAGATGGTGGTAATCGTAATCAGACA-------------------------
----

AATAAGACTGAGACCTTCAGACCTGGGGGAGGAAATATGAAGGACAATTGGAGAAGTGAATTATATAAATATA
AAGTAGTAGAAATTGAACCACTA---GGAATAGCACCCACCAAGGCAAAAAGACAAGTGGTA-----------
----------------------------ACGAGAGAAAAAAGAGCA---GTGGGA---CTGGGAGCT------
---CTGTTCCTTGGGTTCTTGGGAGCA---GCAGGAAGCACTATG---GGCGCGGCGTCA---
ATAACGCTGACGGTACAG---GCCAGACAA---TTATTG---TCTGGAATAGTGCAACAGCAGAGC---
AATCTG---CTGAGA---GCTATTGAAGCGCAACAGCATATGTTGCAGCTCACAGTCTGGGGCATT
>JX140661..2
TTCTATTGCAATACATCAGGACTGTTTAATAACATAAAATT--------------------------------
----

AATGGTACTATTACTCTCCCATGTCGAATAAAACAAATTGTAAACATGTGGCAGGAAGTGGGACGAGCAATGT
ATGCCAATCCCATTGCAGGAAACATTACCTGTAGCTCAATT---
ATTACCGGTCTGTTATTGACAAGAGATGGTGGTAATCAGACTGGTAATAAT----------------------
----

AGTTATATTGAGACCTTCAGACCTGGGGGAGGAAATATGAAAGACAATTGGAGAAGTGAATTATATAAATATA
AAGTAGTAGAAATTGAACCACTA---GGAGTAGCACCCACCAGGGCAAAAAGACAAGTGGTG-----------
----------------------------AAGAGAGAAAAGAGAGCA---CTAGGA---CTAGGAGCT------
---TTGTTCCTTGGGTTCTTGGGAGCA---GCAGGAAGCACTATG---GGCGCAGCGTCA---
GTGACGCTGACGGTACAG---GCCAGACAA---TTATTG---TCTGGAATAGTGCAGCAGCAGAAC---
AATTTG---CTGAAG---GCCATTGAAGCGCAGCAGCATCTGTTGCAGCTCACAGTCTGGGGCATT
>FJ904244..3
TTCTATTGCGATACATCTAAATTGTTTAAAGTGTTTAATGACACCAATGGCACAAAAGCCAATAAT-------
----

GACACAGACCCTATCATTCTCCCATGTCGAATAAAACAAATTGTAAACATGTGGCAAGAAGTAGGACGAGCAA
TGTATGCTAATCCCATTGAGGGAAACATCACCTGTAGATCAAAT---
ATTACAGGTCTAATATTGACAAGAGATGGTGGTAATCATAATAATGATAGT----------------------
----

CAGGATAATCAGACTGAAATCTTTAGACCTGGAGGAGGAAATATGAAAGACAATTGGAGAAGTGAATTATATA
AATATAAAGTAGTAGAAATTGAACCACTA---GGAGTAGCACCCACCAGGGCAAAAAGGCAAGTGGTG-----
----------------------------------AAGAGAGAAAAAAGAGCA---ATGGGA---
CTAGGAGCT---------TTGTTCCTTGGGTTCTTGGGAGCA---GCAGGAAGCACTATG---
GGCGCAGCGTCA---GTGACGCTGACGGTACAA---GCCAGACAA---TTACTG---
TCTGGTATAGTGCAACAGCAGAAC---AATCTG---CTGAGA---
GCTATTGAGGCACAACAGCATCTGTTGCAACTCACAGTCTGGGGCATT
>AB480301..2
TTCTACTGCAATACATCAGGATTGTTTAATGACACAGAAACC-------------------------------
----

AATGATACTATCATTCTCCCATGTAAAATAAAACAAATTATAAACATGTGGCAGGAAGTGGGACGAGCAATGT
ATGCCGCTCCCATTGCAGGAAACATTACCTGTAACTCAAGT---
ATTACAGGTCTACTATTGACAAGAGATGGTGGTCAGAAAAATAAT----------------------------
----

ACTAATGAGACCTTCAGACCTGGGGGAGGAAATATGAAAGACAATTGGAGAAGTGAATTATATAAATATAAAG
TAGTAGAAATTCAACCACTA---GGAGTAGCACCCACCAGGGCAAGAAGATTGGTGGTG--------------
-------------------------AAGAGAGAAAAAAGAGCA---GTGGGA---ATGGGAGCT---------
ATGTTCCTTGGGTTCTTGGGAGCA---GCAGGAAGCACTATG---GGCGCAGCATCA---
ATAACGCTGACGGTACAG---GCCAGACAA---TTATTG---TCTGGAATAGTGCAACAGCAAAAC---
AATTTG---CTGAAG---GCTATTGAAGCGCAACAGCATCTGTTGCAGCTCACAGTCTGGGGCATT
>JN251899..2

TTCTACTGCAATACATCAGGACTGTTTAATGACACAAGACTGTTAAATGACACA-------------------
----

TCCAATAACACTATTGTTCTACCATGTCAAATAAAACAAATTGTAAACTTATGGCAGGAAGTAGGACGAGCAA
TGTATGCCGCTCCCATTGCAGGAAATATTACCTGTAGCTCAAAT---
ATTACAGGTCTACTATTGACAAGAGATGGTGGTCACAGTAATAAT----------------------------
----

ACTAATGAGACCTTCAGACCTGGGGGAGGAAACATGAAAGACAATTGGAGAAGTGAATTATATAAATATAAAG
TAGTAGAAATTGAACCACTA---GGAGTAGCACCCACCAAGGCAAAAAGACAAGTGGTG--------------
-------------------------AAGAGAGAAAAAAGAGCA---GTGGGA---ATAGGAGCT---------
GTTCTCCTTGGGTTCTTGGGAGCA---GCAGGAAGCACTATG---GGCGCAGCGTCA---
GTAGCGCTGACGGTACAG---GCCAGACAA---TTATTG---TCTGGAATAGTGCAACAGCAGAAC---
AATCTG---CTGAGG---GCTATTGAAGCGCAACAGCATATGTTGCAGCTCACAGTCTGGGGCATT
>JN251905..1
TTCTACTGTGATACATCAAAACTGTTTAATGAGACAAAATTCAATGACACTAGTGAA----------------
----

GGTAGTCCTAATATTACTCTCCCATGTCGAATAAAACAAATTGTAAACATGTGGCAGGAAGTGGGGCGAGCAA
TGTATGCCGCTCCCATTGCAGGAGCCATTACCTGTAACTCAAAT---
ATTACAGGTCTCCTATTGACAAGAGATGGTGGTAATACTACTAAT----------------------------
----

CAGACTATTGAGATCTTCAGACCTGGGGGAGGAGATATGAAAGACAATTGGAGAAGTGAATTATATAAATATA
AAGTAGTAGAAATTGAACCATTA---GGAATAGCACCCACCAAGGCAAAGAGACGAGTGGTG-----------
----------------------------CAGAGAGAAAGAAGAGCA---GTGGGA---ATGGCAGCT------
---GTACTCCTTGGGTTCTTGGGAGCA---GCAGGAAGCACTATG---GGCGCAGCGTCA---
ATAACGCTGACGGTACAG---GCCAGACAA---TTATTG---TCTGGGATAGTGCAACAGCAGAGC---
AATTTG---CTGAGG---GCTATTGAAGCGCAACAGCATCTGTTGCAGCTCACAGTCTGGGGCATT
>AY781128..1
TTCTACTGCAATACAACACAACTGTTTAATGACGCAGTATCT-------------------------------
----

AGTGACACTATTATTCTCCCATGTAAAATAAAGCAAATTATAAACATGTGGCAGGAAGTGGGACGAGCAATGT
ATGCCGCTCCCATTGCAGGAAATATTACCTGTAACTCAAAC---
ATTACAGGTCTATTATTGGTAAGAGATGGTGGTAATAATAATCAGACT-------------------------
----

AATAATACTGAGACCTTTAGACCTGGGGGAGGAAATATGAAAGACAATTGGAGAAATGAATTATATAAATATA
AAGTAGTAGAAATTGAACCACTA---GGAGTAGCACCCACCAGGGCAAAAAGACAAGTGGTG-----------
----------------------------AAGAGAGAAAAAAGAGCA---GCAGGA---ATAGGAGCT------
---GTGCTCATTGGGTTCTTGGGAGCA---GCTGGAAGCACTATG---GGCGCGGCGTCA---
ATGACGCTGACGGTACAG---GCCAGACAA---TTATTG---TCTGGAATAGTGCAACAGCAGAGC---
AATCTG---CTGAGG---GCTATTGAAGCGCAACAGCATCTGTTGCAGCTCACAGTCTGGGGCATT
>JN235953..1
TTCTACTGCAATACATCAGGACTGTTTAATGACACAATATCC-------------------------------
----

AATGACACTATCATCCTCCCGTGTCGAATAAAACAAATTGTAAACATGTGGCAGGAAGTGGGACGAGCAATGT
ATGCCGCTCCCATTGCAGGAAACATTACCTGTAGCTCAAAT---
ATTACAGGTCTACTATTGACAAGAGATGGTGGTCAGAATAATGGA----------------------------
----

ACAGAGACCTTCAGACCTGGAGGAGGAGATATGAGGGACAATTGGAGAAGTGAATTATATAAATATAAAGTAG
TAGAAATTGAACCATTA---GGAATAGCACCCACCAAGGCAAAGAGAAGAGTGGTG-----------------
----------------------CAGAGAGAAAAAAGAGCAGTAATGGGA---TTAGGAGCT---------
GTGTTCCTTGGGTTCTTGGGAGCA---GTAGGAAGCACTATG---GGCGCAGCATCA---
CTAACGCTGACGGTACAG---GCCAGACAA---TTATTG---TCTGGTATAGTGCAACAGCAAAAC---
AATTTG---CTGAGG---GCTATTGAGGCGCAACAGCATATGTTGCAACTCACAGTCTGGGGCATC
>KF726017..1

TTCTACTGTAATACAACAAAACTGTTTAATAGTACTTGGAGACTGCTTAATAGTACTTGGAATAGTACTGAAG
GGTTAAAT---
-------------GACACT------
GTAGGAAATGACACAATCACACTCCCATGCAGAATAAAACAAATTGTAAATTTGTGGCAGGAAGTAGGAAGAG
CAATGTATGCCCCCCCCATCAGTGGAAACATTAGATGTTCATCAAAT---
ATTACAGGTCTATTACTGACAAGGGATGGGGGTCAGGGTAATTCAAGTAAT----------------------
----

AGTACTACGGAGACCTTCAGACCTGGGGGAGGAAATATGAAAGACAATTGGAGAAGTGAACTATATAAATATA
AAGTAGTAGAAATCAAACCACTA---GGAGTAGCACCCACCAAGGCAAGAAGACAAGTGGTG-----------
----------------------------AGGAGAGAAAAAAGAGGC------------CTAGGAGCT------
---GTGTTCCTTGGGTTCTTAGGAGCA---GCAGGAAGCACTATG---GGCGCAGCGTCA---
ATAACGCTGACGGTACAG---GCCAGACTA---TTATTG---TCTGGTATAGTGCAACAGCAGAAC---
AATCTG---CTGAGG---GCTATTGAGGCGCAACAGCATCTGTTGCAACTCACAGTCTGGGGCATC
>MK086130..1
TTCTACTGTAACACAACAAGGCTGTTT------GAGGAGAATAATAATGCAACACAGAAAAAT----------
----

AATGAAACTATTATCACTCTTCCCTGTAGAATAAGACAACATGTAAACATGTGGCAAAGAGTGGGACGAGCAA
TGTATGCCCCCCCCATTGCTGGAATAATTCAGTGTAACTCAAAT---
ATCACAGGTCTACTATTAACAAGAGATGGT---AATAACACTGGA----------------------------
----

AATAATGACACCCTCAGACCTGGAGGGGGAGATATGAGAGACAATTGGAGAAGTGAACTGTATAAATATAAGG
TAGTAAAAATTGAACCACTA---GGAATAGCACCCTCCAAGGCAAAAAGACGAGTGGTG--------------
-------------------------CAGAGAGAAAAAAGAGCA---GTGGGA---ATGGGAGCT---------
ATGTTCCTTGGGTTCTTGGGAACA---GCAGGAAGCACTATG---GGCGCGGCGTCA---
ATGGCGCTGACGGTACAG---GTCAGACAA---TTATTG---TCTGGTATAGTGCAGCAGCAAAGC---
AATTTG---CTGAAG---GCTATAGAAGCGCAACAGCATCTGTTGCAGCTCACAGTCTGGGGCATT
>U39253..1
TTCTACTGCAATACATCAAAACTGTTTAGTACATCAAATAGTACATGGGTA----------------------
----

AACAAGACTACAATCACACTCCCATGCAGAATAAGACAAATTATAAACATGTGGCAGAAAGTAGGAAAAGCAA
TGTACGCCCCTCCCATTGAAGCACTAATCAACTGTTCATCAAAT---
ATTACAGGACTATTGTTGACAAGAGATGGTGGTGTAAATAATAGT----------------------------
----

CAGGAGGAGATCTTCAGGCCTGGAGGTGGAGATATGAGAGACAATTGGAGGAGTGAATTATACAAATATAAAG
TAATAAGAATTGAACCAATA---GGTCTAGCACCCACCAGGGCAAAGAGAAGAGTGGTG--------------
-------------------------GAAAGAGAAAAAAGAGCA---ATAGGA---CTGGGAGCT---------
GTGTTCCTTGGGTTCTTGGGAGCA---GCAGGAAGCACTATG---GGCGCAGCATCA---
ATAACGCTGACGGTACAA---GCCAGACAA---TTATTG---TCAGGTATAGTGCAACAGCAAGAT---
AATTTG---CTGAGG---GCTATAGAGGCGCAACAGCATATGTTGCAACTCACAGTCTGGGGCATT
>AY074891..1
TTCTACTGTAACACATCAGGACTGTTTAATAGTATAAGTATAAGTGAATCAAATAGTACAGGG----------
----

GTAAATGGTACAATCACACTCCGATGCAGAATAAAACAAATTATAAACATGTGGCAGGGAGTAGGAAAAGCAA
TATATGCCCCTCCCATTGAGGGACTAATCCAATGTTCATCAAAT---
ATTACAGGACTATTGTTGACAAGAGATGGTGGTAATAATAGTCAG----------------------------
----

ACTGAGACTGAGACCTTCAGACCTGGAGGAGGAGATATGAGAGATAATTGGAGAAGTGAATTATACAGATATA
AAGTAGTAAGAATTGAACCAATA---GGTCTAGCACCCACCAGGGCAAAAAGAAGAGTGGTG-----------
----------------------------ACAAGAGAAAAAAGAGCA---ATAGGA---CTGGGAGCT------
---ATGTTCCTTGGGTTCTTGGGAGCA---GCAGGAAGCACGATG---GGCGCAGCGTCG---
CTGACGCTGACGGTACAG---GCCAGACAG---TTATTG---TCTGGTATAGTGCAACAGCAAAAC---
AATTTG---CTGAGA---GCTATAGAGGCGCAACAGCATCTGTTGCAACTCACAGTCTGGGGCATT
>KU921741..1

TTCTACTGCAAGACATCAGAACTGTTTAATAGTACATGGACATTAAATAGTACAAGTGAAGGGACATCAAATA
GTACAAAG---
-------------------ATA---
ACAAATAGTACAAATATAACACTCCCGTGCAGAATAAAACAAATTATTAACATGTGGCAGGGAGTAGGAAAAG
CAATGTATGCCCCTCCCATTGAAGGACTTATCAAATGCTCATCAAAT---
ATTACAGGAATATTGTTGACAAGAGATGGTGGTACAGATAATCAGACAAATGAG-------------------
----

ACCTTCAGTGAGACCTTCAGACCTGGAGGAGGAGATATGAGGGACAATTGGAGAAGTGAATTATACAAATATA
AAGTAGTAAAAATTGAACCAATA---GGTCTAGCACCCACCAGGGCAAAAAGGAGAGTGGTG-----------
---------------------------GAAAGAGAAAAAAGAGCA---ATAGGA---CTGGGAGCT-------
---ATGTTCCTTGGGTTCTTGGGAGCT---GCAGGAAGCACGATG---GGCGCAGCGTCG---
GTGACGCTGACGGTACAG---GCCAGACAG---CTAATG---TCTGGTATAGTGCAACAGCAAAGC---
AATTTG---CTGAGG---GCTATAGAGGCGCAACAACATATGTTGCAACTCACAGTCTGGGGCATT
>MF373183..3
TTCTATTGCAATACATCAAGCCTGTTTCATGAGAACATCACTGCAAACAGCACAAACAGCACAGAT-------
----

GTAAATAAAAACATCACACTTCAATGCAAAATCAGACAAATTGTAAGAATGTGGCAAGGGATAGGACAAGCAA
TGTATGCCCCTCCCATTGAAGGACTAATAAATTGTAAATCAAAT---
ATCACAGGGCTGTTGCTGACACGTGATGGAGGCCCCAACAACCGG----------------------------
----

ACTAATGAAACATTTAGACCTCAAGGGGGAAATATGAGGGACAATTGGAGAAGTGAATTATATAAATACAAAG
TGGTAGAAATTAAGCCATTA---GGACTAGCACCCACTGGGGCACAGAGGAGAGTGGTG--------------
-------------------------GGAAGAGAAAAAAGAGCA---GCAACA---CTAGGAGCT---------
TTGTTCCTTGGGTTCTTGGGAGCA---GCCGGAAGCACTATG---GGCGCAGCGTCA---
ATAACGCTGACGGTACAG---GCCAGACAA---TTATTG---TCTGGTATAGTGCAACAGCAAAGC---
AATTTG---CTGAGA---GCCATAGAGGCGCAACAGCATATGTTGCAACTCACAGTCTGGGGCATT
>AY253309..2
TTCTACTGCAATACATCAGGGCTATTTAATAATAGTGAGTGGACATCAAATAGTACAAATAGTACATCAAATA
GTACA--

GGGGGAGATGATGTAATCACACTCCAATGCAAAATAAAACAGATCATAAACATGTGGCAGGGAGTAGGAAAAG
CAATGTATGCCCCTCCCATTGAAGGCCAAATCAGATGTTCATCAAAT---
ATTACAGGACTATTGTTAACAAGAGATGGTGGTCAGAATAAGAGT----------------------------
----

CAGGAGGAGACCTTCAGACCTGGAGGAGGAAATATGAGAGACAATTGGAGAAGTGAATTATACAAATATAAAG
TAGTAAGGATTGAACCACTA---GGTCTAGCACCCACCAGGGCACAAAGAAGAGTGGTG--------------
-------------------------CAAAGAGAAAAAAGAGCA---ATAGGC---CTGGGAGCT---------
ATGTTCCTTGGGTTCTTGGGAGCA---GCAGGAAGCACGATG---GGCGCGGCGTCG---
CTGACGCTGACGGTACAG---GCCAGACAG---TTATTG---TCTGGTATAGTGCAACAGCAAAAC---
AATTTG---CTGAGG---GCTATAGAGGCGCAACAACATTTGTTGCAACTCACAGTCTGGGGCATT
>HQ615974..4
TTCTACTGCAATACAACACCACTGTTTAATAATAGTGTATGGTCAAGTAATACAAGTATAGATACAAGTACAA
ATAATACA---
-------------------GAG---
GGAAATAATGGGACAATCATACTCCAATGTAGAATAAAACAAATTGTAAACATGTGGCAAAAAGTAGGAAAAG
CAATATATGCCCCTCCCATTGCAGGACTAATCAATTGTCACTCAAAT---
ATTACAGGAATATTGTTGACAAGAGATGGTGGTAACAATATTAATCAGACTAAG------
AACAATAGTAGTCAGACTGAGAGC---

AATAGGAGTCAGACTGAGACCTTCAGACCTGGAGGAGGAGATATGAGAGACAATTGGAGAAGTGAATTATATA
AATATAAAGTAGTAAGAATTGAACCAATA---GGTATAGCACCCACCAAGGCAAAGAGAAGAGTGGTG-----
----------------------------------GAAAGAGAAAAAAGAGCA---ATAGGA---
CTGGGAGCT---------ATGTTCCTTGGGTTCTTGGGAGCA---GCAGGAAGCACAATG---
GGCGCAGCGTCG---GTGACGCTGACGGTACAG---GCCAGACAG---TTATTG---
TCTGGTATAGTGCAACAGCAGAGC---AATTTG---CTGAGG---
GCTATAGAGGCGCAACAGCATCTGTTGCAACTCACAGTCTGGGGCATT

>AF484500..1
TTCTACTGCAATACATCAGGGATATTTAATAGTACATGGGAGGCTAATAATACAGCAAATAGTACAGGG----
----

GTAGATAAGAATATCACACTCTCATGCAGAATAAAACAAATTATAAACATGTGGCAGGGAGTAGGAACAGCAA
TGTATGCCCCTCCCATTGAAGGACTAATTAAATGTACATCAAAT---
ATTACAGGACTATTGTTAACAAGAGATGGTGGTGCAAATAATAAT----------------------------
----

AGTCAGAATGAGACCTTCAGACCTGGAGGGGGAGATATGAGAGACAATTGGAGAAGTGAATTATACAAATATA
AAGTAGTAAGAATTGAACCACTA---GGTCTAGCACCCACCAGGGCAAAGAGAAGAGTGGTG-----------
----------------------------GAAAGAGAGAAAAGAGCA---ATAGGA---CTGGGAGCT------
---ATGTTCCTTGGGTTCTTGGGAGCA---GCAGGAAGCACGATG---GGCGCAGCGTCA---
CTGACGCTGACGGTACAT---GCCAGACAG---TTATTG---TCTGGTATAGTGCAACAGCAAAAT---
AATTTG---CTGAGG---GCTATAGAGGCGCAGCAGCATCTGTTGCAACTCACAGTCTGGGGCATT
>MF373188..1
TTCTACTGCAATACATCAGAGCTGTTTAATGAAACAGAAAAC-------------------------------
----

TCCAATGGCACTATTATTCTCCCATGTAGAATAAAACAAATGATAAACATGTGGCAGGAAGTGGGACGAGCAA
TGTATGCCGCTCCTATTGCAGGAAACATTGTCTGTAACTCATCT---
ATTACAGGTCTACTATTGACAAGAGATGGGGGTAATAATGATAAT----------------------------
----

AATAACACTGAGATCTTCAGGCCTGGAGGAGGAGATATGAGGGACAATTGGAGAAGTGAATTATACAAATATA
AAGTGGTAGAAATTCAACCATTA---GGAATAGCACCCACCAAGGCAAAAAGGAGAGTGGTG-----------
----------------------------GAGAGAGAAAAAAGAGCA---GCA------TTGGGAGCT------
---TTGTTCCTTGGGTTCTTGGGAGCA---GCCGGAAGCACTATG---GGCGCAGCATCA---
ATAACGCTGACGGTACAG---GCCAGACAA---TTATTG---TCTGGGATAGTGCAACAGCAGAGC---
AATCTG---CTGAGG---GCTATTGAAGCGCAACAGCATCTGTTGCAGCTCACAGTCTGGGGCATT
>EU166607..2
TTCTATTGCAACACATCAGAACTGTTTAATGATACATACAATAATACTACT----------------------
----

CCAAATGGGACCATCACACTCCAATGCAGAATAAAACAAATTATAAACATGTGGCAGGGAGTAGGACAAGCAA
TGTATGCCGCCCCCATTGCAGGAAACATTATCTGTAACTCAAGT---
ATTACGGGGCTACTATTGACAAGAGATGGTGGTAAAAATAAAACT----------------------------
----

GGAGAAGAGATATTCAGACCTGGAGGAGGAGATATGAGGGACAATTGGAGAAGTGAACTCTATAAATATAAAG
TAGTAGAAATTCAACCACTA---GGAGTAGCACCCACCAGGGCAAGAAGACAAGTGGTG--------------
-------------------------AGAAGAGAAAAAAGAGCA---GTGGGA---ATAGGAGCC---------
TTGTTCCTTGGGTTCTTGGGAGCA---GCAGGAAGCACTATG---GGCGCAGCATCA---
ATAACGCTGACGGTACAG---GCCAGACAA---GTGCTG---TCTGGTATAGTGCAACAGCAAAAC---
AATTTG---CTGAGA---GCTATAGAGGCGCAACAGCATATGTTGCAACTCACAGTCTGGGGCATT
>KY392768..1
TTCTACTGCGACACATCAGGATTATTTAATGTTACATATGAGGTCAATAGTTCAAACCAAATTAATAATGCT-
----

ACTAATAATAACATCACACTTCCATGTAAAATAAAACAATTTATAAACATGTGGCAGAAGGTAGGACGAGCAA
TGTATGCCCCTCCTATTGAAGGAATAATAACATGTGTATCAAAT---
ATTACAGGTCTACTATTGACTCGTGATGGAGGTCAAAATAACTCA----------------------------
----

GACACAGAGACCTTCAGACCAACAGGGGGAAATATGAAGGACAATTGGAGAAGTGAATTATATAAATATAAAG
TAGTAGAAATTAAACCACTA---GGAGTAGCACCCACCAAGGCAAGAAGGAGAGTGGTG--------------
-------------------------GAGAGAGAAAAAAGAGCA---GCGGGA---CTGGGAGCT---------
GTGATCATTGGGTTCTTGGGAGCA---GCAGGAAGCACTATG---GGCGCAGCGTCA---
CTAACGCTGACGGTACAG---GCCAGACAA---TTATTG---TCTGGTATAGTGCAACAGCAAAAT---
AATTTG---CTGAAA---GCTATAGAGGCGCAACAGCATCTGTTGCAGCTCACAGTCTGGGGCATT

```
>AB286849..4
TTCTATTGCAATACATCAGGCCTGTTTAATAGTACATATTACAGCCTGTTTAATAGTACATATTACAATGGTA
CATAC--------------------------------------------------------------------
-------------------------
AATAATAATACCAACATCACCCTCCAATGCAGGATAAGACAAATTGTAAACATGTGGCAAGAGGTAGGAAAAG
CAATGTATGCTGCTCCCATTCAAGGAAGCATAACATGTGCATCAAAC---
ATCACAGGGTTACTCTTGACACGCGACGGAGGCAATGGAGAGACA----------------------------
-------------------------------------------------------------------------
----------
AATGGGACAGAGATTTTCAGACCTCAAGGAGGAAATATAAAGGACAATTGGAGAAGTGAATTATATAAATATA
AAGTAGTAGAAGTTGAACCACTA---GGAGTAGCACCCACTAAGGCAAAAAGGAGAGTGGTG-----------
----------------------------GAGAGAGAAAAAAGAGCA---GTGGGA---CTGGGAGCT------
---GCGTTCTTGGGGTTCTTAGGAGCA---GCAGGAAGCACTATG---GGCGCAGCGTCA---
CTAGCGCTGACGGTACAG---GCCAGACAA---TTACTG---TCTGGTATAGTGCAACAGCAAAAT---
AATTTG---CTGAGA---GCTATAGAGGCGCAACAACATATGTTGCAACTCACAGTCTGGGGCATT
>AJ277819..2
TTCTACTACAACACAACAGATCTGTTTAATAACATGGAAAAAACT----------------------------
-------------------------------------------------------------------------
----------------------------
AACAATAACACTATCATTCTCCCATGTAAAATAAGACAATTTGTAAACATGTGGCAAAGAGTGGGACGAGCCA
TGTATGCCCCTCCCATTGCCGGACAAATTCAGTGTAATTCAAAG---
ATCACAGGTCTACTATTGACAAGAGATGGTGGAGAGAATACAAAT----------------------------
-------------------------------------------------------------------------
-------------
GGGTCCGAGACCCTCAGGCCTGGAGGAGGAGATATGAGAGACAATTGGAGAAGTGAACTATATAAATATAAGG
TAGTAAGAATAGAACCACTA---GGAGTAGCACCTACCAAGGCAAAAAGACAAGTGGTG--------------
-------------------------CAGAGAGGAAAAAGAGCA---GTGGGA---ATAGGAGCT---------
GTACTCTTTGGGTTCTTAGGAGCA---GCAGGAAGCACTATG---GGCGCGGCGTCA---
ATAACGCTGACGGTACAG---GCCAGACAA---TTATTG---TCTGGTATAGTGCAACAGCAGAAT---
AATTTG---CTGAAG---GCTATAGAAGCGCAACAGCATCTGTTGCAGCTCACAGTCTGGGGCATT
>MN485986..1
TTCTACTGCAACACCTCACAACTGTTT------ACATGGTCAGATAATAGCACAGGGATA-------------
-------------------------------------------------------------------------
----------------------------
AATGATAGCACAATCGTACTCTCTTGTAGAATAAAACAAATTATAAATATGTGGCAGGAAGTAGGAAAAGCTA
TGTATGCCCCTCCCATTGCAGGAAAAATCAGTTGTTCATCAAAA---
ATTACAGGGCTGTTATTGACAAGGGATGGTGGTAAAGAGACTAACGAGACCTCC-------------------
-------------------------------------------------------------------------
-------
GCTCACGAGATAGAGATCTTCAGACCTGCAGGAGGAGATATGAGGGACAATTGGAGAAGTGAATTATATAAAT
ATAAAGTAGTAAGAATTGAACCCCTT---GGAATAGCACCCACTAGGGCAAAAAGAAGAGTGGTG--------
-------------------------------GAAAGAGAAAAAAGAGCA---ATAGGA---CTAGGAGCT---
------ATGTTCCTTGGGTTCTTGGGAGCA---GCAGGAAGCACTATG---GGCGCGGCGTCA---
ATAACGCTGACGGTACAG---GCCAGACAA---TTATTG---TCTGGTATAGTGCAACAGCAAAAT---
AATTTG---CTGAGA---GCTATAGAGGCGCAACAGCATCTGTTGCAACTCACGGTCTGGGGCATT
>FJ670527..2
TTCTACTGCAACACATCATCCCTGTTTAATGGGACATGGACTAATAATACATGGCACAATATTACA-------
-------------------------------------------------------------------------
----------------------------
TGGAACACAAATATGACAATCCCATGCAAAATAAGGCAAATTGTAAACATGTGGCAAGGAGTAGGAAAAGCAA
TGTATGCCCCTCCCATCGAAGGACACATCAACTGTTCATCAAAT---
ATTACAGGACTACTATTGATAAGAGATGGGGGTAAAGGACAGAATCAG-------------------------
-------------------------------------------------------------------------
-------
AGTAATCAGAGTGAGACTTTCAGACCTGGAGGAGGAGATATGAGGGACAATTGGAGAAGTGAATTATATAAAT
ATAAAGTAGTAAAAATTGAACCTCTA---GGAATAGCACCCACCAAGGCAAGGAGAAGAGTGGTG--------
-------------------------------CAAAGAGAAAAAAGAGCA---ATAGGA---CTAGGAGCT---
------ATGTTCCTTGGGTTCCTGGGAGCA---GCAGGAAGCACGATG---GGCGCAGCGTCA---
GTGACGCTGACGGTACAG---GCCAGACAA---TTGATG---TCTGGTATAGTGCAACAGCAAAAC---
AATTTG---CTGAGA---GCTATAGAGGCGCAACAGCATCTGTTGCAACTCACAGTCTGGGGCATT
```

>FJ388908..1
TTCTACTGCAATACATCAAAACTGTTTAATAGGACATGGGATACAGGGAATACTACAGAACAGGGTATTAGTA
TA---

GGGTCAAATGATATAATCATACTCCCATGCAAACTAAAACAAATTATAAACATGTGGCAGGAAGTAGGAAAAG
CAATGTATGCCCCTCCCATTGAAGGAGAAATCAAATGCTCATCAAAT---
ATTACAGGGCTACTATTAACAAGAGATGGGGGAAATATTAATGGAAGT-------------------------
----

AGTAATCAGACAGAGATCTTCAGACCTGGAGGAGGAGATATGAGAGACAATTGGAGAAGTGAATTATATAAAT
ATAAAGTAGTAACAATTGAACCAATA---GGACTAGCACCCACCAAGGCAAAAAGAAGAGTGGTG--------
-------------------------------GAAAGAGAAAAAAGAGCA---ATAGGG---TTAGGAGCT---
------ATGTTCCTTGGGTTCTTGGGAGCA---GCCGGAAGCACGATG---GGCGCAGCGTCA---
GTGACGCTGACGGTACAG---GCCAGACAA---TTGTTG---TCTGGTATAGTGCAGCAGCAAAAC---
AATTTG---CTGAGG---GCTATAGAGGCGCAACAGCACTTGTTGCAACTCACGGTCTGGGGCATT
>KX579838..1
TTCTACTGCAATACATCAGAACTGTTTAATTGGGATAATAGTACAAAGAGTACTGTAAATGGA----------
----

ACAAAATTAAATATAACTATCCACTGTAAAATAAAACAAATTATAAACATGTGGCAGGGAGTAGGAAGAGCAA
TGTATGCCCCTCCCATCGAAGGACAAATTCAATGTATATCAAAT---
ATTACAGGGCTACTATTGACAAGAGATGGTGGTAGTGATTCTAAT----------------------------
----

GAGACCTTCAGGCCTGGAGGAGGAGATATGAGAGACAATTGGAGAAGTGAATTATATAAATATAAAGTAGTAA
GAGTTGAGCCATTA---GGAGTAGCACCCACCAGGGCAAAGAGAAGAGTGGTG--------------------
-------------------GACAGAGAAAAAAGAGCA---ATAGGA---TTAGGAGCT---------
ATGTTCCTTGGGTTCTTGGGAGCA---GCAGGAAGCACTATG---GGCGCAGCGTCA---
GTGACGCTGACGGTACAG---GCCAGACAA---TTATTG---TCTGGTATAGTGCAACAGCAAAAT---
AATTTG---CTGAGG---GCTATAGAGGCGCAACAGCATCTGTTGCAACTCACGGTCTGGGGCATT
>MN337382..1
TTCTACTGCAATACATCAGGACTGTTTAATAGAGCATTTAATAATACAACATGGGATACTAGTACAGGGAGTG
CTAGA--

TTAAATAATACAATAACCATCCTATGTAGAATAAAACAAATTATAAACATGTGGCAGGGAGTAGGAAGAGCAA
TGTATGCCCCTCCCATTGAAGGACAAATTAAATGTTCATCAAAT---
ATTACAGGGTTACATTTGACAGCAGATTGGGTTGGAAATGAGTCTGTG-------------------------
----

TCTAGAGTAATTTACAGGCCTGGAGGAGGAAATATGAAGGACAATTGGAGAAGTGAATTATATAAATATAAAG
TAGTAAAAATTGAACCACTA---GGAGTAGCACCCACCAGGGCAAGGAGAAGAGTGGTG--------------
-------------------------GAAAGAGAAAAAAGAGCA---ATAGGA---CTAGGAGCT---------
ATGTTCCTTGGGTTCTTGGGAGCA---GCAGGAAGCACGATG---GGCGCAGCGTCA---
GTGACGCTGACGGTACAA---GCCAGACAA---CTGTTG---TCTGGTATAGTGCAACAGCAAAAT---
AACTTG---CTGAGG---GCTATAGAGGCGCAACAGCATCTGTTGCAACTCACGGTCTGGGGCATT
>AF286236..1
TTCTATTGCAACACATCAGAGCTGTTTACTGGTATTTGGAATGGTACTTGGGATAAGAATTGCACTAGCACTG
AGAGT--

AATTGCACTGGAAATATTACACTCCCATGCAGGATAAAACAAGTGGTAAGAACATGGCAGGGAGTAGGACAAG
CAATGTATGCCCCTCCTATCGAAGGGACAATTAGGTGCTCATCAAAT---
ATTACAGGTCTACTATTGACAAGAGATGGTGGTAATGGCAATGCA----------------------------
----

ACTCAAAATGAGACCTTTAGACCTGGAGGAGGAGACATGAAAGATAATTGGAGAAGTGAATTGTATAAGTATA
AAGTAGTAAAAATTGAACCACTA---GGAGTAGCACCCACCAGGGCAAAAAGAAGAGTGGTG-----------
----------------------------GAGAGAGAAAAAAGAGCA---GTGGGG---ATGGGAGCT------
---TTGTTTCTCGGGTTCTTGGGAGCA---GCCGGAAGCACTATG---GGCGCGGCGTCA---
ATGGCGCTGACGGCACAG---GCCAGACAA---TTATTG---TCTGGTATAGTGCAGCAGCAAAAC---
AATTTG---CTGAGG---GCTATAGAGGCGCAACAGCATCTGTTGCAACTCACAGTCTGGGGCATT

>MN617377..1
TTCTACTGTAATACATCACAACTGTTTAGTAGTACTTGGGATAATAATGGTACTGAGATTAATACTACAGGAA
CTACA--

GGAAATGACACTATCATTCTCCCTTGCAGAATAAAACAACTTGTAAACATGTGGCAGGAAGTAGGGAAAGCAA
TGTATGCCCCTCCCATTAGTGGAATAATTAGATGTTCATCGAAT---
ATTACAGGATTGCTATTAACAAGAGATGGTGGTAACAATCAGAGCACT-------------------------
----

ATCAATGAGACTTTCAGACCTGGAGGAGGAGACATGAGAGACAATTGGAGAAGTGAATTATATAAATATAAAG
TAGTAAAAATTGAACCATTA---GGAGTAGCACCCACCAAGGCAAAGAGAAGAGTGGTG--------------
-------------------------CAAAGAGAAAAAAGAGCA---GTGGGAACTATAGGAGCT---------
ATGTTCCTTGGGTTCTTGGGAGCA---GCAGGAAGCACAATG---GGCGCAGCGTCA---
ATAACGCTGACGGTACAG---GCCAGACAA---TTGTTG---TCTGGTATAGTGCAACAGCAGAGA---
AATCTG---CTGAGG---GCTATTGAGGCGCAACAGCATCTGTTGCAACTCACAGTCTGGGGCATC
>MH705158..1
TTCTATTGCAATACATCAGGCCTGTTTAATAGTAGTTGGAACGAAAGTGACACTGCAAATGACACTATAACGC
TCCCGTGCAAAATAAAACAAATTGTAAGGATGTGGCAGAGAGTAGGACAAGCAATGTATGCCCCTCCCATCCA
AGGGGTAATGAGGTGTGAATCTAACATTACAGGACTACTATTAACAAGAGATGGTGGGAATGAGGGCAATGAA
AGTAGTGAGACGTGCAGACCTGGAGGAGGAGATATGAGGGATAATTGGAGAAGTGAACTATATAAATATAAAG
TAGTAAAAATTGAACCACTAGGAGTAGCACCCACCAGGGCAAAGAGAAGAGTGGTGGAGAGAGAAAAAAGAGC
AGTTGGACTGGGAGCTGTCTTCATTGGGTTCTTAGGAGCAGCAGGAAGCACTATGGGCGCGGCGTCAGTGACG
CTGACGGTACAGGCCAGACAATTATTGTCTGGCATAGTGCAACAGCAAAACAATTTGCTGAGGGCTATAGAGG
CTCAACAGCATCTGTTGAAACTCACGGTCTGGGGCATT
>MH705151..1
TTCTATTGCAATACATCAGACCTGTTTAATAGCACTTGGCGGAAACTGGCAAATGGCACTTGGAGCAACATAA
ATAATGCAACGTCAAATGACACTATAACTCTCCAATGCAAAATAAAGCAAATTGTAAACATGTGGCAGAGAGT
AGGACAAGCAATGTATGCCCCTCCCATCCAAGGAGTAATACAGTGTGTATCAAACATTACAGGACTACTCTTA
ACAAGAGATGGTGGGAGTAGTAACAGGGCAAATGAGACCTTCAGACCTGGAGGAGGAGATATGAGGGACAATT
GGAGAAGTGAATTATATAAGTATAAAGTAGTAAAAATTGAACCACTAGGAGTAGCACCCACCAGGGCAAGGAG
AAGAGTGGTGGAGAGAGAAAAAAGAGCAATTGGAATGGGAGCTCTTTTCCTTGGGTTCTTAGGAACAGCAGGA
AGTACTATGGGCGCGGCGTCAATAACGCTGACGGTACAGGCCAGACAATTATTGTCTGGTATAGTACAACAGC
AGAGCAATCTGCTGAGGGCTATAGAGGCTCAACAGCATCTGTTGAAACTCACAGTCTGGGGCATT
>MH705157..1
TTCTATTGTAATACATCAAGCCTGTTTAATAGCACTTGGAAAGAGGAGAATGGCACTGTCAGCATGCAGAATG
ACACGGAGTCAACTGACCCTATAACTCTCCAATGTAGAATAAAGCAAGTTATAAACATGTGGCAGAGAGTAGG
ACAAGCAATATATGCCCCTCCCATCCAAGGAGTAATAAGATGTGTATCAAACATTACAGGACTACTATTAACA
AGAGATGGTGGGAGTAATAATAATACTTCAAATGAAACCTTCAGACCTGGAGGAGGAGATATGAGGGACAATT
GGAGAAGTGAATTATATAAGTATAAAGTAGTAAAAATTGAACCACTAGGAGTAGCACCCACCAAGGCAAAGAG
AAGAGTGGTGGAGAGAGAAAAAAGAGCAGTTGGACTGGGAGCTGTCTTCCTTGGGTTCTTAGGAGCAGCAGGA
AGTACGATGGGCGCGGCGTCAATAACGCTGACGGTACAGGCCAGACAATTATTGTCTGGCATAGTGCAACAGC
AGAGCAATCTGCTGAGGGCTATAGAGGCTCAACAGCAACTGTTGAGACTCACGGTCTGGGGCATT
>MH705153..2
TTCTATTGCGATACATCAGGCCTGTTTAATGGCACTTGGAGCAATACATCAGGCCTGATTAATAGCACTTGGA
GCGAGAATGACACTATAACTCTCCCATGTAGAATAAAGCAAATTGTAAATATGTGGCAGAGAGTAGGACAAGC
AATGTATGCCCCTCCCATCCAAGGAATAATAAATTGTACATCAAACATTACAGGACTACTGTTAACAAGAGAT
GGTGGGAATAATAACAGTATAAATGAGCCTGAGACCTTCAGACCTGGAGGAGGAGATATGAGGGACAATTGGA
GAAGTGAACTATATAAGTATAAAGTAGTAAAAATTCAACCACTAGGAGTAGCACCCACCAGGGCAAAGAGAAG
AGTGGTGGAGAGAGAAAAAAGAGCAGTTGGCATGGGAGCTCTTCTCCTTGGGTTCTTAGGAGCAGCAGGAAGC
ACGATGGGCGCGGCGTCAATAACGCTGACGGTACAGGCCAGACAATTATTGTCTGGTATAGTGCAACAGCAGA
GCAATCTGCTGAGGGCTATAGAGGCTCAACAGCATCTGTTGAAACTCACGGTCTGGGGCATT
>MH705133..1
TTCTATTGCAATACGTCAGGCCTGTTTAATAGCACTTGGAAGAATGGCACTAAAGAGGGAAATGACACTGCAA
AGTTAAGTGACACTATAACTCTCCCATGCAGAATAAAGCAAATTGTAAGGATGTGGCAGAGAACAGGACAAGC
AATGTATGCCCCTCCCATCCCAGGAGTAATAAATTGTGTCTCTAACATTACAGGGATACTATTAACAAGGGAT
GGTGGGAATAGTAGTAATGCCAATGAAACCTTCAGACCTGGAGGAGGAAATATGAAGGACAATTGGAGAAGTG
AACTATATAAGTATAAAGTAGTAAAAATTGAACCAATAGGAGTAGCACCCACCAGGGCAAAGAAAAGAGTGGT
GGAGAGAGAAAAAAGAGCAGTTGGACTGGGAGCTGTCTTCCTTGGGTTCTTAGGAGCAGCAGGAAGTACTATG
GGCGCGGCGTCAATAACGCTGACGGTACAGGCCAGACAATTATTGTCTGGCATAGTACAACAGCAAAGCAATT
TGCTGAGGGCTATAGAGGCTCAACAGCATATGTTGAGACTCACAGTCTGGGGCATT

>JQ403028..1
TTCTACTGCAATACAACAGAACTGTTTAATAGCACGTGGAATGACACGAGCAACACATCGCAGGGGACAAATA
ACAATAGTACTATTACTCTCCCATGCAGAATAAAGCAATTTGTAAATATGTGGCAGAGAGTAGGACAAGCAAT
GTATGCCCCTCCCATCAAAGGAAAAATACAGTGCATATCAAATATTACAGGTCTACTATTAACAAGAGATGGG
GGTAAGGGTAAAAGCAATATTACAGAGATCTTCAGACCTGAAGGAGGAAATATGAAGGACAACTGGAGAAGTG
AATTATATAAATATAAAGTAGTAAAAATTGAACCACTAGGAGTAGCACCCACCAAGGCAAAGAGAAGAGTGGT
GGAGAGAGAAAAAAGAGCAGTTGGACTGGGAGCTGTGTTCCTTGGGTTCTTAGGAGCAGCCGGAAGTACTATG
GGCGCGGCGTCAATAACGCTGACGGCACAGGCCAGGCAATTATTGTCTGGTATAGTACAACAGCAGAGCAATC
TGCTGAGGGCTATAGAGGCTCAACAGCATCTGTTGAAACTCACGGTCTGGGGCATT
>KX389622..1
TTCTATTGCAATACATCAGACTTGTTTAATAGCAATTGGACAAATGGGAATTGGACAAATGGGATAGGCAGCA
CGGAGACTAATGACACTATAACTCTTCCATGCAGAATAAAGCAGATTATAAGAATGTGGCAGAGAGTAGGACA
AGCAATGTATGCCCCTCCCATCAAAGGGGTAATAGAGTGTAAATCAAATATTACAGGAATAATATTAGTAAGA
GATGGTGGTAAGAATAACACAGACAAGGAGACATTTAGACCTGGAGGAGGAGATATGAGGGACAATTGGAGAA
GTGAACTATACAAATATAAAGTAGTAAAAATTGAACCATTAGGAGTAGCACCCACCAGGGCAAAAAGAAGAGT
GGTGGAGAGAGAAAAAAGAGCAGTTGGAATAGGAGCTGTCTTCCTTGGGTTCTTAGGAGCGGCAGGAAGCACT
ATGGGCGCAGCGTCAATAACGCTGACGGTACAGGCCAGACAATTATTGTCTGGCATAGTGCAACAGCAGAGCA
ATCTGCTGAGGGCTATAGAGGCTCAACAACATCTGTTGAAACTCACAGTCTGGGGCATT
>DQ396400..1
TTCTATTGCAATACATCAGGCCTGTTTAACAGAACTTGGAATGACACCGCTTGGAATGAAACAACTTGGAATA
ACATAAATTCAAATGGAAGTATAACTCTCCAATGTAGAATAAAGCAAATTTTAAATATGTGGCAGAGAGTAGG
ACAGGCGATGTATGCCCCTCCCATCCCAGGAGAAATAAGGTGTGAATCAAACATTACAGGACTAATCTTAACA
AGAGACGGGGGGCTTGAGCTTAACAATACGAATAATGAGACCTTCAGGCCTGGAGGAGGAGATATGAGGGACA
ATTGGAGAAGTGAATTGTATAAATACAAAGTAATAAAAATTGAACCACTAGGAGTAGCACCCACCAGGGCAAA
AAGAAGAGTGGTGGAGAGAGAAAAAAGAGCAGTTGGACTGGGAGCTGTTTTCCTTGGGTTCTTAGGGGCAGCA
GGAAGCACTATGGGCGCAGCGTCAATAACGCTGACGGTACAGGCTAGGCAATTATTGTCTGGCATAGTGCAAC
AGCAAAGCAATTTGCTGAGGGCTATAGAGGCTCAACAACATTTGTTGAAACTCACAGTCTGGGGCATT
>FM165645..2
TTCTACTGTAACACATCAGGCCTGTTTAATAGCAGTTGGGAAAATGACACTGGCAGCACGCAGGAGTCAAATA
GAACAAAGTCAAATGACACTATAACTCTCCCATGCAGAATAAAGCAAATTATAAATATGTGGCAGAGGGCAGG
ACAAGCAATATATGCCCCTCCCATCCCAGGGGTAATACAGTGTCAGTCAAACATTACAGGACTACTATTAACA
AGAGATGGTGGGAATAACAATACAACTAATGAAACCTTCAGACCTGGAGGAGGAAATATGAGGGATAATTGGA
GAAGTGAACTATATAAATATAAAGTAGTAAAAATTGAACCACTAGGAGTAGCACCCACCACGGCAAGGAGAAG
AGTGGTGGAGAGAGAAAAAAGAGCAGTGGGACTGGGAGCTCTGTTCATTGGGTTCTTAGGAGCCGCAGGAAGC
ACTATGGGCGCGGCGTCAGTGACGCTGACGGTACAGGCCAGACAATTATTGTCTGGCATAGTGCAACAGCAAA
ACAATTTGCTGAGGGCTATTGAGGCTCAACAGCATCTGTTGAAACTCACGGTCTGGGGCATT
>FM165628..2
TTCTACTGCAACACATCAAGACTGTTTAATAGCAGTTGGGAACCATTAGGCCAGTATGATGACAGTCAGAGCA
ATGACACAAATATAACTCTCCCATGCAGAATAAAGCAAATTATAAGGATGTGGCAGAGGACAGGACAAGCAAT
ATATGCCCCTCCCATCCAAGGGGAAATAACGTGTAGATCAAATATTACAGGACTACTATTAACAAGAGATGGT
GGAAATAAAACTAATGAAACCTCCGAAACCTTCAGACCTGGAGGAGGAGATATGAGAGATAATTGGAGAAGTG
AACTATATAAATATAAAGTAGTAAAAATTGAACCACTAGGAGTAGCACCCACCAAGGCAAAAAGAAGAGTGGT
GGAGAGAGAAAAAAGAGCAGTGGGACTGGGAGCTGTTCTCATTGGGTTCTTAGGAACAGCAGGAAGCACTATG
GGCGCGGCGTCAGTGACGCTGACGGTACAGGCCAGACAATTATTGTCTGGCATAGTGCAACAGCAAAACAATT
TGCTGAGGGCTATAGAGGCTCAACAGCATCTGTTGAAACTCACGGTCTGGGGCATT
>EU191615..1
TTCTACTGCAACACATCAACACTGTTTAATAGCAGTTGGCCATTAAACCAGTCTAACAGTTGGAGCAATGGCG
GAAATGACACAAATATAACTCTCCCATGCAGAATAAAGCAAATTATAAGGATGTGGCAGAGGACAGGACAAGC
AATATATGCCCCTCCCATCGAAGGGGTAATAATGTGTAGGTCAAATATTACAGGACTCCTATTAACAAGAGAT
GGTGGAAATGAAGCTAATAAAACTAATGAAACTGCTGAAATCTTCAGACCTGGAGGAGGGGATATGAGAGATA
ATTGGAGAAGTGAACTATATAAATATAAAGTAGTAAAAATTGAACCACTAGGAGTAGCACCCACCAGGGCAAA
AAGAAGAGTGGTGGAGAGAGAAAAAAGAGCAGTGGGACTGGGAGCTGTTCTCATTGGGTTCTTAGGAACAGCA
GGAAGCACTATGGGCGCGGCGTCAGTGACGCTGACGGTACAGGCCAGACAATTATTGTCTGGCATAGTGCAAC
AGCAAAACAATTTGCTGAGGGCTATAGAGGCTCAACAGCATCTGTTGAAACTCACGGTCTGGGGCATT
>MH746258..1
TTCTATTGTAACACATCAGGCCTGTTTAATAGCACATGGTCTAAAAATGGCACACAGCAGGTAGACAACATAA
CCAATCCTATAATTCTCCAATGCAGAATAAAGCAGATTATAAATATGTGGCAGAGAGCAGGACAAGCAATATA
TGCCCCTCCCATCCAAGGACTAATAAGGTGTGAATCAAACATTACAGGACTAATATTAACAAGAGATGGTGGA
AATGGGAATACAACAAACAATGAAACCGAAACCTTCAGACCTGGAGGAGGAGATATGAGGGACAATTGGAGAA
GTGAATTATATAAGTATAAGGTAGTACAAGTTGAACCACTAGGAGTAGCACCCACCAAGGCAAAAAGAAGAGT

GGTGGAGAGAGAAAAAAGAGCAGTTGGACTAGGAGCTGTATTCATTGGATTCTTAGGAGCAGCAGGAAGCACT
ATGGGCGCGGCGTCTGTGACGCTGACGGTACAGGCCAGGCAATTATTGTCTGGTATAGTGCAACAGCAAAGCA
ATTTGCTGAGGGCTATAGAGGCTCAACAGCATTTGTTGAAACTCACAGTCTGGGGCATT
>MH746253..1
TTCTATTGTAATACATCAGCCTTATTTAATAGCACTTGGAATGATACCAATAATACGGGATCAAATACTGACA
ATAACACCACGGGGTCAAATGAAGTTATAACTCTCCCTTGCAGAATAAAACAAATTATACGTATGTGGCAGAG
AACAGGACAAGCAATGTATGCCCCTCCCATTTCAGGAATAATAAATTGTACATCAAACATTACAGGACTAATA
TTGACAAGAGATGGTAGTAATGGTGATAATAACAGTGAAACCTTCAGACCTGGAGGAGGAGATATGAGGGACA
ATTGGAGAAGTGAATTATATAAGTATAAAGTAGTAAAAATTGAACCATTAGGGGTAGCACCCACCAGGGCAAG
GAGAAGAGTGGTGCAGAGAGAAAAAAGAGCAGTTGGAATAGGAGCTGTCTTCCTTGGGTTCTTAGGAGCAGCA
GGAAGCACTATGGGCGCAGCGTCAATAACGCTGACGGTACAGGCCAGACAATTATTGTCTGGCATAGTGCAAC
AGCAAAACAATTTGCTGAGGGCTATAGAGGCTCAACAACAGCTGTTGAAACTCACAGTCTGGGGCATT
>MH746251..1
TTCTATTGCAATACATCAGCCCTGTTCAATAGTACTTGGAATGGCACTGTCACAAATGGCACACAAGCAAATG
GAACTATAATTCTACCATGCAGAATAAAGCAAATTATAAGAATGTGGCAGAGAGTAGGACAAGCAATGTATGC
CCCTCCTATCAGAGGAGTGATAAGGTGTGAATCAAACATTACAGGACTAATACTAACAAGAGATGGTGGAGGT
GGAACTAATAGCAGCAATGAGACCTTCAGGCCTACAGGAGGAGATATGAGGGACAATTGGAGAAGTGAACTAT
ATAAGTATAAAGTAGTAAAAATTGAACCAATAGGATTAGCACCTACCGAGGCAAGGAGGAGAGTGGTGGAGAG
AGAAAAAAGAGCAATTGGACTGGGAGTTGCCTTCCTTGGGTTCTTAGGAGCAGCAGGAAGTACTATGGGCGCG
GCGTCATTGACGCTGACGGCACAAGCCAGACAATTATTGTCTGGTATAGTGCAACAGCAAAGCAATTTGCTGA
GGGCTATAGAGGCTCAGCAGCATCTGCTGAAACTCACAGTCTGGGGCATT
>KU168256..1
TTCTATTGCAATACATCAGGCCTGTTTAATAGCACTTGGCATGTCAATAATACATCAAGCCTGGTTAATAGCA
CTGGGAACATAACTCTCCCATGCAGAATAAAGCAAATTATAAATATGTGGCAGAGAGTAGGACAAGCAATGTA
TGCCCCTCCTATTCCAGGAATAATAAAGTGTGAGTCAAACATTACAGGACTACTATTGATAAGAGATGGTGGA
ATTAATAATAACAGTATGAATGAGACCTTCAGACCTGGAGGAGGAGATATGAGAGACAATTGGAGAAGTGAAT
TATATAAATATAAAGTAGTACAAATTGAACCACTAGGAGTAGCACCTACCAAGGCAAAGAGAAGAGTGGTGGA
GAGAGAAAAAAGAGCAGTTGGATTTGGAGCTGTCTTCCTTGGGTTCTTAGGAGCAGCAGGAAGCACTATGGGC
GCGGCGTCAATAACGCTGACGGCACAGGCCAGACAATTATTGTTTGGCATAGTGCAACAGCAAAGCAATTTGC
TGAGGGCTATAGAGGCTCAACAGCATCTGTTGAAACTCACAGTCTGGGGCATT
>KU168305..1
TTCTATTGTAATACATCAGGCCTGTTTAATAGCACTTGGAAGACTAATGGCACTAGCACTTGGAAGGAGACTA
ATGGCACTGGGAGCCAGGAAAATAGCAATGACAATATAACTCTCCCATGCAGAATAAAGCAAATTATAAATAT
GTGGCAGAGAGCAGGACAAGCAATGTATGCCCCTCCCATCGCAGGAGTAATAAAGTGTAACTCAAACATTACA
GGACTACTATTAACAAGAGATGGGGGAAGTAATAACAATAGCCAAGAGGAGACTTTCAGACCTGGAGGAGGAA
ATATGAAGGATAACTGGAGAAGTGAATTATATAGGTACAAAGTAGTACAAATTGAACCCCTAGGAATAGCACC
CACCAGGGCAAGGAGAAGAGTGGTGGGGAGAGAAAAAAGAGCAGTTGGACTGGGAGCTGTCTTCATTGGGTTC
TTAGGAGCAGCTGGAAGCACTATGGGCGCAGCGTCAATAACGCTGACGGCACAGGCCAGACAATTATTGTCTG
GCATAGTGCAACAGCAAAGCAATTTGCTGAGGGCTATAGAGGCTCAACAGCATATGTTGAAACTCACGGTCTG
GGGCATT
>KP718918..2
TTCTATTGTAATACATCAGGCCTGTTTAATAGCACTTGGTTTAGTAATGGGAGCAGGGGCAATGAGAGCATGG
AAGATGACACCAATATAACTCATATAACTCTCCCATGCAGAATAAGGCAAATTGTGAATATGTGGCAGAGAGC
AGGACAAGCAATGTATGCCCCTCCCATCCAAGGAGTAATAAAGTGTACATCAAATATTACAGGAATACTATTA
ACAAGAGATGGTGGATATAATATTAGCAATAACGACACCGAGATTTTCAGACCTGGAGGAGGAAATATGAAGG
ACAATTGGAGAAGTGAATTATATAAATATAAAGTAGTAAAAGTTGAACCACTAGGAATAGCACCCACTAGGGC
AAAGAGAAGAGTGGTGGAGAGAGAAAAAAGAGCAGTTGGAATAGGAGCTGTGTTCATTGGGTTCCTAGGAGCA
GCAGGAAGCACTATGGGCGCAGCGTCAATAACGCTGACGGCACAGGCCAGACAGTTATTGTCTGGAATAGTGC
AACAGCAAAGCAATTTGCTGAGGGCTATAGAGGCTCAACAGCATTTGTTGAAACTCACGGTCTGGGGCATT
>KP718928..1
TTCTATTGTAATACATCAGGCCTGTTTAATAGCACTTGGCATAGTAATGGCACTGCAAATGGCACTGAAAATG
CCACTATAACTCTCCCATGCAGGATAAAGCAGATTATAAATCTATGGCAGAGAGTAGGGCAAGCAATGTATGC
CCCTCCTATCCCAGGAATAATAAATTGTACATCAAACATTACAGGACTACTATTAACAAGAGATGGTGGGAAT
GCTAGTGAGGAAACTGAGACCTTCAGACCTGGAGGAGGAAATATGAAGGACAATTGGAGGAGTGAATTATATA
AATATAAAGTAGTAAAGATTGAACCACTAGGAGTAGCACCCACCAGGGCAAAGAGAAGAGTGGTGGAGAGAGA
AAAAAGAGCAGTCGGAATAGGAGCTGTGTTCATTGGGTTCTTAGGAGCAGCAGGAAGCACTATGGGCGCAGCG
TCAATAACGCTGACGGCACAGGCCAGACAGTTACTGTCTGGTATAGTGCAACAGCAAAGCAATTTGCTGAGGG
CTATAGAGGCTCAACAGCACATGTTGAAACTCACAGTCTGGGGCATT
>AM279343..1

TTCTATTGTAATACATCAGGCCTGTTTAATAGCACTTGGTTTAATAGCACTTGGAATGACACTATCAGCAATA
ATAACACAGGGTCAAATGACACCATATATCTCCCATGCAGAATAAAGCAAATTATAAATATGTGGCAAAGGAC
AGGACAAGCAATGTATGCCCCTCCCATCCGAGGAATAATACAGTGTAATTCAACCATTACAGGACTACTATTA
ACAAGAGATGGTGGGATTAACAACAACAGTACAAATGAAACCTTCAGACCTGGAGGAGGAAATATGAGGGATA
ATTGGAGAAGTGAACTATATAAATATAAAGTAGTAAAAATTGAACCACTAGGAGTAGCACCCACCAAGGCAAG
GAGAAGAGTGGTGGAGAGAGAAAAAAGAGCAGTTGGACTGGGAGCTGTATTCATTGGGTTCTTAGGAGCAGCA
GGAAGCACTATGGGCGCGGCGTCAATAACGCTGACGGTACAGGCCAGACAATTATTGTCTGGCATAGTGCAAC
AGCAAAACAATTTGCTGAGGGCTATAGAGGCTCAACAGCATCTGTTGAAACTCACGGTCTGGGGCATT
>AM279348..2
TTCTATTGCAATACATCAGGCCTGTTTGCTCAGGAGTTAAAAAATGACACAAATGACAATAACACAATAATTC
TCCTATGCAAAATAAAGCAAATTATAAATATGTGGCAGAGAGTAGGACAAGCAATGTATGCCCCTCCCAGAGC
AGGAGTAATAAGGTGTGTATCAAACATTACAGGACTACTGTTAACAAGAGATGGTGGAAAAAATGACAATGGA
ACTAATGGAACTGAGATATTCAGGCCTGGAGGAGGAGATATGAGGGACAATTGGAGAAGTGAATTATATAAGT
ATAAAGTAGTAAGAATTGAACCAGTAGGGGTAGCACCCACCAGGGCAAAGAGAAGAGTGGTGGAGAGAGAGAA
AAGAGCAGTTGGACTGGGAGCTGTCTTCCTTGGGTTCTTAGGAGCAGCAGGAAGCACTATGGGCGCGGCGTCA
CTGACGCTGACGGTACAGGCCAGGCAATTATTGTCTGGCATAGTGCAACAGCAGAGCAATTTGCTGAGGGCTA
TAGAGGCTCAACAACATATGTTGAGACTCACAGTCTGGGGCATC
>FJ388894..1
TTCTATTGTAACACATCAAGCCTGTTTAATAGCACCTGGAATATTAATACTATTTGGAATAACACTATGTCAA
ACAGCACTGAGTCAAACGGCACTATAGTGCTCCCATGCAGAATAAAGCAAATTGTAAATGTGTGGCAGAGAGC
AGGACAAGCAATATACGCCCCTCCCATCCAAGGAATAATACAGTGTGTCTCAAACATTACAGGACTAATATTA
ACAAGAGATGGTGGGAATAATAATAACACAAATAATACTGAAATCTTCAGACCTGGAGGAGGAGATATGAGGG
ACAATTGGAGAAGTGAATTATATAAATATAAGGTAGTAAAAATTGAACCATTAGGAGTAGCACCCACCAAGGC
AAGGAGAAGAGTGGTGGGGAGAGAAAAAAGAGCAGTTGGACTGGGAGCTGTCTTCCTTGGGTTCTTAGGAACA
GCAGGAAGCACTATGGGCGCGGCGTCAATAACGCTGACGGTACAGGCCAGGCAATTATTGTCTGGCATAGTGC
AACAGCAAAGCAATTTGCTGAGGGCTATAGAGGCTCAACAACATCTGTTGAAACTCACTGTCTGGGGCATT
>FJ388903..3
TTCTATTGTAACACATCGGGCCTGTTTAATAGCCAATATAACACATCAGGCCTGGTTAATAACACGCAGGAGT
CAGGAAGCACAGAGTCAAATGACACTGAGTCAATTGTCACAGACAATATAACTCTCCAAATCAGAATAAAGCA
AATTATAAATCAGTGGCAAAGAGTAGCTCAAGCAACATATGCCCCTCCCATCCCAGGAGTAATAAAATGTGTA
TCAAACATTACAGGACTAATATTAACTAGAGATGGTGGGAGTAGTAGCGGAAATGAAACCTTCAGACCTGGAG
GAGGAGATATGAGGGACAATTGGAGAAGTGAATTATATAAGTATAAAGTAGTAAAAATTGAACCACTAGGAGT
AGCACCCACCAGGGCAAAGAGAAGAGTGGTGGAGAGAGAAAAAAGAGCAGTTGGAATAGGAGCTGTCTTCATT
GGGTTCTTAGGAGCAGCAGGAAGCACTATGGGCGCGGCGTCAATGACGCTGACGGTACAGGCCAGGCAATTAT
TGTCTGGCATAGTGCAACAGCAAAGTAATTTGCTGAGGGCTATAGAGGCTCAACAGCATCTGTTGAAACTCAC
GGTTTGGGGCATT
>FJ388909..1
TTCTATTGTAATACATCAGGCCTGTTTAATAGCACTTGGAATAATGACACCTGGAATGGCAATGACACACAAG
AGTCAAATGAAACCTCGTCAAATGGCACCATAATTCCACCATGCAAAATAAAACAAATTGTAAATATGTGGCA
GAGAGTACAACAAGCAATGTATCCCCCTCTCGTCGAAGGAGTAATAAGGTGTGAATCCATCATTACAGGACTA
CTATCAACAAGAGATGGTGGGATTAACAGCAGGACAAATGAAACCTTCAGACCTGGGGGAGGAGATATGAGGG
ACAATTGGAGAAGTGAACTATATAAATATAAGGTAGTAAAAATTGAACCACTAGGAGTAGCACCCACCACGGC
AAAGAGAAGAGTGGTGGAGAGAGAAAAAAGAGCAGCTGGACTGGGAGTTATGTTCCTTGGGTTCTTAGGAGCA
GCCGGAAGCACTATGGGCGCGGCGTCAATAACGCTGACGGTACAGGCCAGACAGTTATTATCTGGTATAGTGC
AACAGCAAAGCAATTTGCTGAAGGCTATAGAGGCTCAACAGCACCTGTTGAGACTCACGGTCTGGGGCATT
>FJ388925..1
TTCTATTGTAACACATCAGGCTTGTTTAATAGCACTTGGAATGCCACTGCCAGCATGCAGGAGTCAAATAGCA
CAGAGTCAAACGACACTACTATAACTCTCCCATGCAGAATAAAGCAAATTATAAATATGTGGCAGAGAGCAGG
ACAAGCAATATATGCCCCTCCCATCCGAGGAGTAATAAGGTGTGAATCAAACATTACAGGACTAATATTAACA
AGAGATGGAGGGAATAGTAACAGCACAAATGAAACCTTCAGACCTGGAGGAGGAGATATGAGGGATAATTGGA
GAAGTGAATTATATAAGTATAAAGTAGTAAAAATTGAACCACTAGGAGTAGCACCCACCAGGGCAAGGAGAAG
AGTGGTGGAGAGAGAAAAAAGAGCAGTTGGAATAGGAGCTGTCTTCATTGGGTTCTTAGGAGCAGCAGGAAGC
ACTATGGGCGCGGCGTCAATAGCGCTGACGGTACAGGCCAGGCAATTATTGTCTGGCATAGTGCAACAGCAAA
GCAATTTGCTGAGGGCTATAGAGGCTCAACAGCATATGTTGAAACTCACGGTCTGGGGCATT
>FJ388932..1
CTCTATTGTAACACATCAGGCCTGTTTAATAGCACTTGGCCTGCCAATAACAGTACGCAGGGGTCAAATAGCA
CAGAGTCAAACGACACCGTAATTCTCCAATGCAGAATAAAGCAAATTATAAATATGTGGCAAAGAGTAGGACA
AGCAATATATGCCCCTCCCATACCAGGAGAAATAAAGTGTGTATCAAACATTACAGGACTAATATTAACAAGA
GATGGTGGGAGTAGCAATAATACAGAAAATGAAACCTTCAGACCAGGAGGAGGAGATATGAGGGACAATTGGA
GAAGTGAATTATATAAGTATAAAGTAGTAAAAATTGAACCACTAGGAGTAGCACCCACCAGGGCAAAGAGAAG

AGTGGTGCAGAGAGAAAAAAGAGCAGTATTAGGTGGACTGGGAGCTGTCTTCGTTGGGTTCTTGGGAGCAGCA
GGAAGCACTATGGGCGCGGCGTCAGTGACGCTGACGGTACAGGCCAGGAAATTATTGACTGGCATAGTGCAAC
AGCAAAGTAATTTGCTGAGGGCTATCGAGGCTCAACAGCATCTGTTGAAACTCACGGTCTGGGGCATT
>FJ388938..1
TTCTATTGTAACACATCAGGCCTGTTTAATAGCACTTGGAATGGCACTACCATGCAGGAGTCAAATAACACAG
AGTCAAACGACACTGAGTCAAACGACATTATAACTCTCCGATGCAGAATAAGGCAAATTATAAGAATGTGGCA
GAGAGCAGGACAAGCAATGTATGCCCCACCCATCCAAGGAATAATAAAGTGTGAATCAAACATTACAGGACTA
ATATTAGCAAGAGATGGTGGGAATAGAAATGAAACTGAAACCTTCAGACCTGGAGGAGGAGACATGAGGGACA
ATTGGAGAAGTGAATTATATAAGTATAAAGTAGTAAAAATTGAACCATTAGGAGTAGCACCCACCAAGGCAAG
GAGAAGAGTGGTGCAGAGAGAAAAAAGAGCAGTTGGAATAGGAGCTGTCTTCATTGGGTTCTTAGGAGCAGCA
GGAAGCACTATGGGCGCGGCGTCAATAACGCTGACGGTACAGGCCAGGCAACTATTGTCTGGCATAGTGCAAC
AGCAAAGTAATTTGCTGAAGGCTATAGAGGCTCAACAACATCTGTTGAAACTCACGGTTTGGGGCATT
>FJ388942..1
TTCTATTGTAACACATCAGGCCTGTTTAATAGCTCTTGGCTGAAGTCAAATAGAACAGGGCCTAGCACAGAGC
CAAACAACACTGAGCCAAACAACACTATTATAACTCTCCCATGCAAAATAAAGCAAATTATAAATATGTGGCA
GAGAACAGGACAAGCAATGTATGCCCCTCCCATCCCAGGAGTAATAAGGTGTGAATCAAACATTACAGGACTA
ATATTAACAAGAGATGGTGGGAATAGTAGCAATAGCAATGAAACCTTCAGACCTGGAGGAGGAAATATGAGGG
ACAATTGGAGAAGTGAATTATATAAGTATAAAGTAGTAAAAATTGAACCACTAGGAGTAGCACCCACCAGGGC
AAGGAGAAGAGTGGTGGAGAGAGAAAAAAGAGCAGTTGGAATAGGAGCTGTCTTCATTGGGTTCTTAGGAGCA
GCAGGAAGCACTATGGGCGCGGCGTCAATGACGCTGACGGTACAGGCCAGGCAATTATTGTCTGGCATAGTGC
AACAGCAAAGCAATTTGCTGAGGGCTATAGAGGCTCAACAGCATATGTTGAAACTCACGGTCTGGGGCATT
>FJ388946..2
TTTTATTGTAATACATCAGGCCTGTTTAATAGAACTTGGTCTACCAATACCAGCATGCAGGTGTCAAATAGCA
CAGAGTTAAATGGCCCTATAATTCTCCAATGCAGAATAAAGCAAATTATAAATATGTGGCAAAGAGTAGGACA
AGCAATATATGCCCCTCCCATCCCAGGAGTAATAAGGTGTGAATCAAACATTACAGGACTAATATTAACAAGA
GATGGTGGAAATAATAATACCGGAAATCAAACCTTCAGACCTGTAGGAGGAGATATGAAGGACAATTGGAGAA
GTGAATTATATAAGTATAAAGTAGTAAAAATTGAACCACTAGGAGTAGCACCCTCCAAGGCAAAGAGAAGAGT
GGTGGAGAGAGAAAAAAGAGCAATTGGAATAGGAGCTGTCTTCATTGGGTTCTTAGGAGCAGCAGGAAGCACT
ATGGGCGCGGCGTCAGTGACGCTGACGGTACAGGCCAGGCAATTATTGTCTGGCATAGTGCAACAGCAAAGTA
ATTTGCTGAGGGCTATAGAGGCTCAACAGCATCTGTTGAACCTCACGGTCTGGGGCATT
>JF683737..1
TTCTATTGTAACACATCAGGCCTGTTTAATAGCACGTGGAATGCTAGTGACATCACGCAGGGGTTAAATGACA
CAATAACTCTCCAATGCAGAATAAAGCAAATTATAAATATGTGGCAGAGAGTAGGGCAGGCAATGTATGCCCC
TCCCATCCAGGGAGAAATAAGGTGTGAATCAAACATTACAGGACTAATATTAACAAGAGATGGTGGGAATGGG
AATGGAACAAATGAAACCTTCAGACCTGGAGGAGGAGATATGAGGGACAATTGGAGAAGTGAATTATATAAGT
ATAAAGTAGTAAAAATTGAACCACTAGGAGTAGCACCCACCAGGGCAAAGAGAAGAGTGGTGGAGAGAGAAAA
AAGAGCAGTTGGACTGGGAGCTGTATTCATTGGGTTCTTAGGAGCAGCAGGAAGCACTATGGGCGCGGCGTCA
GTGACGCTGACGGTACAGGCCAGGCAATTATTGTCTGGCATAGTGCAACAGCAAAGCAATTTGCTGAGGGCTA
TAGAGGCTCAACAGCATTTGTTGAAACTCACAGTCTGGGGCATT
>JF683759..1
TTCTATTGTGACACATCAGGCCTGTTTAATAGCACTTGGAATACCACTGACACCATGCACGAGGAGACTAATA
CAGAGGCAAATAGTACTATTACTCTCCCATGCAGAATAAAGCAAATTATAAATATGTGGCAGAGAGCAGGACA
AGCAATATATGCCCCTCCCATCCCAGGAGTAATACAGTGTAAATCAAACATTACAGGACTAATATTAACAAGA
GATGGTGGGAGTAATAGCAGCACAAATGAAACCTTCAGACCTGGAGGAGGAGATATGAGGGACAATTGGAGAA
GTGAATTATATAAGTATAAAGTAGTAAAAATTGAACCACTAGGAGTAGCACCCACCAAGGCAAGGAGAAGAGT
GGTGCAGAGAGAAAAAAGAGCAGTAGGACTGGGAGCGGTATTCATTGGGTTCTTAGGAGCAGCTGGAAGCACC
ATGGGCGCGGCGTCAATAACGCTGACGGTACAGGCCAGGCAATTATTGTCTGGTATAGTGCAACAGCAAAGCA
ATTTGCTGAGGGCTATAGAGGCTCAACAGCATCTGTTGAAACTCACAGTCTGGGGCATT
>JF683760..1
TTCTATTGTAACACCTCAAAGTTGTTTAATAGCACTTGGGATCCTAATAATACCAGCACGCAGGAGTCAAATA
GCACAAATGATAATATAATTCTCCCATGCAGAATAAGGCAAATTATAAATATGTGGCAGAGAGCAGGACAAGC
AATATATGCCCCTCCCATCCAAGGAATAATAAACTGTGTATCAAACATTACAGGATTAATATTAACAAGAGAT
GGGGGTGTAGGTAACAACGCAAAGGAAGTCTTCAGACCTGGAGGAGGAGATATGAGGGACAATTGGAGAAGTG
AATTATATAAGTATAAAGTAGTAAAAATTGAGCCACTAGGAGTAGCACCCACTGGGGCAAGGAGAAGAGTGGT
GCAGAGAGAAAAAAGAGCAGTTGGACTGGGAGCTGTTTTCATTGGGTTCTTGGGAGCAGCAGGAAGCACTATG
GGCGCGGCGTCAATGACGCTGACGGTACAGGCCAGACAATTATTGTCTGGCATAGTGCAACAGCAAAGCAATT
TGCTGAGGGCTATAGAGGCTCAACAGCATATGTTGAGACTCACGGTCTGGGGCATT
>JF683761..1
TTCTATTGTAACACCTCAAAGCTGTTTAATAGCACTTGGAATAATACCAGCACGCAGGAGTCAAATAGCACTA
ATCCAAATGATAATATAACTCTCCCATGCAGAATAAAGCAAATTATAAATATGTGGCAAAGAGCAGGACAAGC

AATATATGCCCCTCCCATCCAAGGAAAAATAAACTGTGTATCAAACATTACAGGATTAATATTAACAAGAGAT
GGGGGGGGTAACAGCACAGAGGAAGTCTTCAGACCTGGAGGAGGAGATATGAGGGACAATTGGAGAAGTGAAT
TATATAAGTATAAAGTAGTAAAAATTGAGCCAATAGGAGTAGCACCCACTGAGGCAAGGAGAAGAGTGGTGCA
GAGAGAAAAAAGAGCGGTTGGACTGGGAGCTGTTTTCATTGGGTTCTTGGGAGCAGCAGGAAGCACTATGGGC
GGGGCGTCAATGACGCGGACGGTACAGGCCAGACAATTATTGTCTGGCATAGTGCAACAGCAAAGCAATTTGC
TGAGGGCTATAGAGGCTCAACAGCATATGTTGAGACTCACGGTCTGGGGCATT
>JF683767..1
TTCTATTGTAATACATCAGGTCTGTTCAATAGCTCTTGGAATATCAATGGCAGCAGGTCAAATAGCACAAGGT
CAAATGGCAATATAACTCTCCCATGCAGAATAAAGCAAATTATAAATATGTGGCAAAGAACAGGACAGGCAAT
GTATGCCCCTCCCATCCAAGGAGTAATAAGGTGTGTATCAAACATTACAGGACTACTGTTAACAAGAGATGGA
GGGGGTAACAATACAAATGAAACTGAAATTTTCAGACCTGGAGGAGGAGATATGAGGGGCAATTGGAGAAGTG
AATTATATAAGTATAAAGTAGTAAAAATTGACCCACCAGGAGTAGCACCCAACACAGCAAGGAGAAGAGTGGT
GGGGAGAGAAAAAAGAGCAGCTGGAATAATAGGAGCTGTCTTCCTTGGGTTCTTAGGAGCAGCAGGAAGCACT
ATGGGCGCGGCGTCAGTGACGCTGACGGTACAGGCCAGACAATTATTGTCTGGTATAGTGCAACAGCAAAGCA
ATTTGCTGAGGGCTATAGAGGCTCAACAACACCTGTTGAAACTCACGGTCTGGGGCATT
>JF683779..2
TTCTACTGTAACACATCAGGCCTGTTTAATAGCACTTGGTATACCAATAACAGCATGCAGCCAAATTACACAA
AAACTATAACTTTGCCATGCAGAATAAAGCAAGTTATAAATATGTGGCAAAGAGTAGGACAAGCAATGTATGC
CCCTCCCATCCCAGGAATAATACAATGTGTATCAAACATTACAGGACTAATATTAACAAGAGATGGTGGGCAT
AATAATAGTGCAAATGAGACCTTCAGACCTGGAGGAGGAGATATGAGGGACAATTGGAGAAGTGAATTATATA
AGTATAAAGTAGTGAAAGTTGAACCATTAGGAGTAGCACCCACCAGGGCAAGGAGAAGAGTGGTGGAGAGAGA
AAAAAGAGCAGTTGGAATAGGAGCTGTCTTCCTTGGGTTCTTAGGAGCAGCAGGAAGCACTATGGGCGCGGCG
TCAGTGACGCTGACGGTACAGGCCAGGCAATTATTGTCTGGCATAGTGCAACAGCAAAGCAATTTGCTGAGGG
CTATAGAGGCTCAACAACATATGTTGAAACTCACGGTCTGGGGCATT
>JF683782..1
TTCTATTGTAATACATCAAAATTGTTTAATACAACTTGGAATGCCAATGGCAGCGTACAGGAGTCAGTCAACA
CTGCAGGCAACACTACTATATTTCTCCCATGCAGAATAAAGCAAATTATAAGGATGTGGCAGAGAGCAGGACA
AGCAATATATGCCCCTCCCATCCAGGGAAGAATAAGGTGTGAATCAAACATTACAGGACTACTGTTAACAAGA
GATGGTGGAGGTAATGCAAACAACACAGAAATCTTTAGACCTGGAGGAGGAGATATGAGGGACAATTGGAGAA
GTGAATTATATAAGTACAAGGTAGTAAAAATTGAACCACTAGGAGTAGCACCCACCAAGGCAAGGAGAAGAGT
GGTGCAGAGAGAAAAAAGAGCAGTTGGACTAGGAGCTGTCTTCCTTGGGTTCTTAGGAGCAGCAGGAAGCACT
ATGGGCGCGGCGTCAATAACGCTGACGGTACAGGCCAGGCAATTATTGTCTGGCATAGTGCAACAGCAAAGCA
ATTTGCTGAGGGCTATTGAGGCTCAACAGCAGATGTTGAGACTCACGGTCTGGGGCATT
>JF683783..1
TTCTATTGTAATACATCAAAATTGTTTAATACAACTTGGAATGTCACTGACAGCATAGAAGAGTCAGGCAACA
CTATTATAGTCCTCCCATGCAAAATAAAGCAAATTATAAATATGTGGCAGAGAGCAGGACAAGCAATATATCC
CCCTCCCATCCGGGGAATAATAAGGTGTGAATCCAACATTACAGGACTATTATTAACAAGAGATGGTGGAGAT
ACTGAAAACACCACAGAAATCTTTAGACCTGGAGGAGGAGATATGAGGGACAATTGGAGAAGTGAATTATATA
AGTATAAAGTAGTAAAAATTGAACCACTAGGAGTAGCACCCACCGAGGCAAGGAGAAGAGTGGTGCAGAGAGA
AAAAAGAGCAGTTGGAATAGGAGCTGTCTTCCTTGGGTTCTTAGGAGCAGCAGGAAGCACTATGGGCGCGGCG
TCAATAACGCTGACGGTACAGGCCAGGCAATTATTGTCTGGCATAGTGCAACAGCAAAGCAATTTGCTGAGGG
CTATAGAGGCTCAACAGCAGATGTTGAGACTCACGGTCTGGGGCATT
>JF683789..1
TTCTATTGTAACACCTCAAAGCTGTTTAATAGCATTTGGCATCCTAATGATACCATGCAGGAGGAGACTAATA
CAAATCCAAATGCTAATATAACTCTCCCATGCAGAATAAAGCAAATTATAAATCTGTGGCAAAGAGTAGGGAA
AGCAATATATGCCCCTCCCATCCGAGGAAGAATAGAGTGTGTATCAAACATTACAGGATTAATAATAACAAGA
GATGGTGGAAATAACACAAATAACACTGAAATCTTCAGACCTGCAGGAGGAGACATGAGGGACAATTGGAGAA
GTGAATTATATAAATATAAAGTAGTACAAATTGAGCCACTAGGAATAGCACCCACTGAGGCAAGGAGAAGAGT
GGTGCAGAGAGAAAAAAGAGCAATTGGACTAGGAGCTATGTTCATTGGGTTCTTAGGAGCAGCAGGAAGCACT
ATGGGCGCGGCGTCAGTGACGCTGACGGTACAGGCCAGGCAATTATTGTCTGGCATAGTGCAACAGCAAAGCA
ATTTGCTGATGGCTATAGAGGCTCAACAGCATCTGTTGAAACTCACAGTCTGGGGCATT
>FJ670523..1
TTCTATTGTAACACATCAGGCCTGTTTAATAGCACTTGGCCTAATAGCACTTGGAATGCCAATACCAGCATAC
AGGAGTCAAATAGCACAGACAATATAACTCTCCAATGCAGAATAAAGCAAATTATAAATCTGTGGCAGAGAGC
AGGAAAAGCAATATATGCCCCTCCCATCCAAGGAGCAATACAGTGTATATCAAACATTACAGGACTAATATTA
ACAAGAGATGGAGGTAATAATGGAACTAATAACACTGAGATCTTCAGACCTGGAGGAGGAGATATGAGAGATA
ATTGGAGAAGTGAATTATATAAGTATAAGGTAGTAAAAATTGAACCATTAGGAGTAGCACCCTCCAGGGCAAA
GAGAAGAGTAGTGGGGAGAGAAAAAAGAGCAGTTACAATAGGAGCTGTCTTCCTTGGGTTCTTAGGAGCAGCA
GGAAGCACTATGGGCGCGGCGTCACTGACGCTGACGGTACAGGCCAGGCAATTATTGTCTGGCATAGTGCAAC
AGCAAAGCAATTTGCTGCAGGCTATAGAGGCTCAACAGCATCTGTTGAAACTCACGGTCTGGGGCATT

>AF219261..2
TTCTATTGTAATACATCAGGCCTGTTTAATAGCACTTGGGTGTCAAACAACACTGGGAAATATTATAGTAATA
GCACTAGGGAAAATCCAAATATAACTCTCCCATGCAGAATAAAACAAATTATAAATATGTGGCAGAGAGCAGG
ACAAGCAATATATGCCCCTCCCATCGAAGGAGTAATAAGGTGTGAATCAAACATTACAGGACTACTATTAACA
AGAGATGGTGGGGATAAGAACAACACAAGTGAAATCTTCAGACCTGGAGGAGGAAATATGAAGGACAACTGGA
GAAGTGAATTATATAAGTATAAAGTAGTAAAAATTGAACCATTAGGAGTAGCACCCACCCCGGCAAGGAGAAG
AGTGGTGCAGAGAGAAAAAAGAGCAGTATTTGGGCTGGGAGCTGTCTTTCTTGGGTTCTTAGGAGCAGCAGGA
AGCACTATGGGCGCGGCGTCGATAACGCTGACGGTACAGGCCAGACAATTATTGTCTGGCATAGTGCAACAGC
AAAGTAATTTGCTGAGGGCTATAGAGGCTCAACAGCATTTGTTGAAACTCACGGTCTGGGGCATT
>AF219265..2
TTCTATTGTAATACATCAGGCCTGTTTAATAGCACTTGGAGTAATAGCACTTGGAGTTCCAATGCCAGCACGC
AGGGGTCAGAAAATGACACTATAACTCTCCCATGCAGAATAAAACAAATTATAAATATGTGGCAGAGAGCAGG
ACAAGCAATGTATGCCCCTCCCATTCAAGGAGTAATAAGGTGTGATTCAAATATTACAGGACTAATATTAACA
AGAGATGGTGGGGTTACAAATGAAACCGAAACCTCCAGACCTGGAGGAGGAGATATGGGGGACAATTGGAGAA
GTGAATTATATAAATATAAAGTAGTGAAAATTGAACCACTAGGAGTAGCACCCACCAGGGCAAAAAGAGTAGT
GGTGGGGAGAGAAAAAAGAGCAATTGGAATAGGAGCTGTTTTCATTGGGTTCCTAGGAGCAGCAGGAAGCACT
ATGGGCGCGGCGTCAATGACGCTGACGGTACAGGCCAGACAATTATTGTCTGGCATAGTGCAACAGCAAAGCA
ATTTGCTGAGGGCTATAGAGGCTCAACAACATCTGTTGAAACTCACGGTCTGGGGCATT
>HM204635..1
TTCTATTGTAATACATCACACCTGTTTAATAGCGCTTGGTCTGTCAATGACACCGTGCAGGAGTCAAATAGCA
CGGAGTCAAATGACACTATAACTCTCCAATGCAGAATAAAGCAAATTATAAATATGTGGCAGAGAACAGGACA
AGCAATGTATGCCCCTCCCATCCCAGGAGTAATAAGATGTAACTCAAACATTACAGGACTAATATTAACAAGA
GATGGTGGGAATAATAATAGTTCAAATGAAAATGAAACCTTCAGACCTGGAGGAGGAGATATGAGAGACAATT
GGAGAAGTGAATTATATAAGTATAAAGTAGTAAAAATTGAACCACTAGGAGTAGCACCCACCAAGGCAAGGAG
AAGAGTGGTGCAGAGAGAAAAAAGAGCAGTTGGACTGGGAGCTGTCTTCATTGGGTTCTTAGGAGCAGCAGGA
AGCACTATGGGCGCGGCGTCAGTGACGCTGACGGTACAGGCCAGACAATTATTGTCTGGCATAGTGCAACAGC
AAAGCAATTTGCTGAGGGCTATAGAGGCTCAACAACATCTGTTGAAACTCACAGTCTGGGGCATT
>HM204648..2
TTTTATTGTAACACATCAGGCCTGTTTACTGGCACTTGGAATCAGACTAATAGTACTTGGAATAGTGGCACTT
GGACTAACACTACTGGCAATATAACTCTCCAATGCAGAATAAAGCAGGTCATAAGAATGTGGCAGAGAGTAGG
ACAAGCAATGTATGCCCCTCCCATCCCAGGAGTAATAAGGTGTGAATCAAACATTACAGGACTAATATTAACA
AGAGATGGGGAAGGTAACAGTAGCATAAAAGATGAAACCTTCAGACCTGGAGGAGGGAATATGAAGGACAATT
GGAGAAGTGAATTATATAAATATAAAGTAGTAAAAATTGAACCAATAGGAGTAGCACCCACCAGGGCAAAGAG
AAGAGTGGTGGAGAGAGAAAAAAGAGCAGTTGGACTAGGAGCTGTCTTCATTGGGTTCTTAGGAGCAGCAGGA
AGCACTATGGGCGCAGCGTCAATAACGCTGACGGTACAGGCCAGGCAATTGTTGTCTGGCATAGTGCAGCAGC
AAAGCAATTTGCTGAAGGCTATTGAGGCTCAACAACAGTTGTTGAGACTCACGGTCTGGGGCATT
>HQ595782..1
TTCTATTGCAATACGTCTAAACTGTTTAATAGCACGTGGAAAAATGAAAGCAGGCAGGGGTCAAATGACATTA
TAACTCTCCCATGCAGAATAAAGCAAATTATAAGGATGTGGCAAAGAGTAGGGCAAGCAATGTATGCCCCTCC
CATCCCAGGAGTAATAGAGTGTAATTCAAACATTACAGGGCTACTATTGACAAGAGATGGTGGTAATAGAACC
GAAAATGAGACCTTCAGACCTGAGGGAGGAGATATGAGAAACAATTGGAGAAGTGAATTATATAAGTATAAAG
TAGTAAGAATTGAACCATTGGGAGTAGCACCCACCAGGGCAAAAAGAAGAGTGGTGGAGAGAGAAAAAAGAGC
AGCTGGATTGGGAGCTGTCTTCCTTGGGTTCTTAGCAGCAGCAGGAAGTACTATGGGCGCAGCGTCAATAACG
CTGACGGCACAGGCCAGACAATTATTGTCTGGCATAGTACAACAGCAAAGCAATTTGCTGAGGGCTATAGAGG
CTCAACAGCAACTGTTGAGGCTCACGGTCTGGGGCATT
>HQ595784..2
TTCTATTGCAACACAGCAGGTCTGTTTAATAGCAGCTGGGGGAACAATGACACTGACAGCAACCAGACAGCAA
AGATAAATATAACTCTCCAATGCAGAATAAAGCAAATTGTAAGGATGTGGCAGAGAGTAGGACAAGCAATGTA
TGCCCCTCCCATCGCAGGAACGATAAACTGTTTATCAAATATTACAGGACTACTATTAACAAGAGATGGGGGG
ACAAATAAGACGTGGTCTCACAATGAGACCTTCAGACCTGAAGGAGGAAATATGAAGGACAATTGGAGAAGTG
AATTATATAGGTATAAAGTAGTAAAAATTGAACCACTAGGAGTAGCACCCACCGAGGCAAGGAGAAGAGTGGT
GGAGAGAGGAAAAAGAGCAGTTGGAATAGGAGCTGTTCTCTTTGGGTTCTTAGGAGCAGCAGGAAGCACTATG
GGCGCGGCGTCACTAACGCTGACGGTACAGGCCAGACAATTACTGTCTGGCATAGTGCAACAGCAGAGCAATC
TGCTGAGGGCTATAGAGGCTCAACAGCATCTGTTGAAGCTCACAGTCTGGGGCATT
>Y13718..1
TTCTATTGTAACACATCAAGACTGTTTAATAGCACTTGGAATGACACTACCAGTATGCTAAATGACACGAAGC
CAAACGACACTATAACTCTCCCATGCAGAATAAAGCAAATTATAAATATGTGGCAAAGAGCAGGACAAGCAAT
ATATGCCCCTCCCATCCAAGGAGTAATAAGGTGTGAATCAAACATTACAGGACTAATATTAACAAGAGATGGT
GGGGGTAATAGCAGTGTAAGTAGTGAAACTTTCAGACCTGGAGGAGGAGATATGAGAGACAATTGGAGAAGTG
AATTATATAAGTATAAAGTAGTAAAAATTGAACCACTAGGAGTAGCACCCACCAAGGCAAAGAGAAGAGTGGT

GGAGAGAGAAAAAAGAGCAGTTGGACTAGGAGCTGTTTTCATTGGGTTCTTAGGAGCAGCAGGAAGCACTATG
GGCGCGGCGTCAATAACGCTGACGGTACAGGCCAGACAATTATTGTCTGGCATAGTGCAACAGCAAAGCAATT
TGCTGAGGGCTATAGAGGCTCAACAGCATCTGTTGAAACTCACGGTCTGGGGCATT
>Y13717..1
TTCTATTGTAACACATCAGGCCTGTTTAATAGCACTTGGGATAGCAGTGCCAGCATGCAAGAGTCAAATAGCA
CGGCCAATATAACTCTCCCATGCAGAATAAGGCAAATTATAAATCTGTGGCAGAGAACAGGACAAGCAATATA
TGCCCCTCCCATCCAAGGAGTAATAAGGTGTGTATCAGACATCACAGGACTAATATTAACAAGAGATGGTGGG
GGTAATGGTACTGAAAGTAAAAAAGAAACTTTCAGACCTGGAGGAGGAGATATGAGGGACAATTGGAGAAGTG
AATTATATAAGTATAAAGTAGTAAAAATTGAACCACTAGGAGTAGCACCCACCAAGGCAAAGAGAAGAGTGGT
GGAGAGAGAAAAAAGAGCAGTTGGAATGGGAGCTGTCCTCCTGGGGTTCTTAGGAGCAGCAGGAAGCACTATG
GGCGCGGCGTCAATAACGCTGACGGTACAAGCCAGACAATTATTGTCTGGCATAGTGCAACAGCAAAGCAATT
TGCTGAGGGCTATAGAGGCTCAACAGCAGGTGTTGAGACTCACGGTCTGGGGCATT
>HQ385459..2
TTCTATTGCGATACATCAGGCCTGTTTAATAGTATTTGGAATAGCACTGCCATCAGCCAGAATGTCGAGGAGT
CAAATGATACTATAACTCTCCCATGCAAAATAAGACAAATTGTAAATATGTGGCAGAGAGTAGGACAAGCAAT
ATATGCCCGTCCCATTCAAGGAATAATAAACTGTACATCAAACATTACAGGGCTACTGCTAACAAGAGATGGT
GGGGATAATGATAATAAAACTGAGACTTTCAGACCTGGAGGAGGAAATATGAAGGACAATTGGAGAAGTGAGT
TATATAGATATAAAGTAGTAAAAATTGAACCACTAGGAGTAGCACCCACCAAGGCAAAGAGAAGAGTGGTGGA
GAGAGAAAAAAGAGCAGTTGGACTAGGAGCTGTCATCTTTGGGTTCCTGGGAGCAGCCGGAAGTACTATGGGC
GCGGCGTCAATAACGCTGACGGCACAGGCCACACAATTATTGTCTGGTATAGTACAACAGCAAAGCAATCTGC
TGAGGGCTATAGAGGCTCAACAGCACCTGTTGAAACTCACGGTCTGGGGCATT
>HQ385452..1
TTCTATTGCAATACATCAGGCCTGTTTTCTGGCTCTTGGTATAATGGCACTTGGGCTAATGACTCTTGGAGTG
CTGGTCCTGGGACCAATGATAATATAACTCTCCCATGCAAAATAAAGCAAATCATAAGAATGTGGCAGAGAGT
GGGACAAGCAATGTATGCCCCTCCCATCCAAGGAGAAATAAATTGTGTATCAAACATCACAGGACTATTACTA
ACAAGAGATGGTGGTAATAGTAGTGAAGAGATCTTCAGACCTGGAGGAGGAGATATAAGAGACAATTGGAGGA
GTGAATTATATAAATATAAAGTGGTAAAAATTGAACCATTAGGAGTAGCGCCCACCAGGGCAAGGAGAAGAGT
GGTGGGAAGAGAAAAAAGAGCAGTTGGACTGGGAGCTGTCTTCCTTGGGTTCTTAGGAGCAGCAGGAAGCACT
ATGGGCGCGGCGTCAATCACGCTGACGGTACAGGCCAGGCAATTATTGTCTGGCATAGTGCAACAGCAGAGCA
ATCTGCTGAGGGCTATAGAGGCTCAACACCATCTGTTGAAACTCACGGTCTGGGGCATT
>HQ385453..1
TTCTACTGCGACACAACAGGCCTGTTTAATAACACAGAGAAAAATGACACTATAATGCTCCCATGCAAAATAA
GACAAATTGTACGTATGTGGCAGAGAGTAGGACAAGCAATGTATGCCCCTCCCATTCCAGGAGTAATAAACTG
TCTATCAAACATTACAGGGCTACTGCTGACAAGAGATGGTGGGGATAGTACTAATAATACTGAGAAATTCAGA
CCAGGAGGAGGAGATATAAGGGACAATTGGAGAAGTGAATTATATAAATATAAAGTAGTAAAAATTGAACCAC
TAGGAGTAGCACCTACCAAGGCAAAGAGAAGAGTGGTGGAGAGAAGAAAAAGAGCAGGACTAGCAGCTGTCTT
CCTTGGGTTCTTAGGAGCAGCAGGAAGCACTATGGGCGCAGCGTCAATAACGCTGACGGTACAGGCCAGACAA
TTATTGTCTGGTATAGTGCAACAGCAAAACAATTTGCTGAAGGCTATAGAGGCTCAACAACAGATGTTGAGAC
TCACGGTCTGGGGCATT
>KT152846..2
TTCTATTGTAATACATCAAACCTGTTTAATAGCACTTGGACGAAAAATGACAGCATGCAGGGGTTAAATCACA
CGGAGTCAAATGACAATATAACTCTCCCATGCAGACTAAGGCAAATTATAAAAATGTGGCAGAGAGTAGGACA
AGCAATGTATGCCCCTCCCATCCAAGGAATAATAAAGTGTGAATCAAACATTACAGGGCTAATATTAACAAGA
GATGGTGGGAATAACAACAGTGCAGAGGAAACCTTCAGACCTGGAGGAGGAGATATGAGGGACAATTGGAGAA
GTGAATTATATAAGTATAAAGTAGTACAAATTGAACCACTAGGGGTAGCACCCACCAAGGCAAGGAGAAGAAC
GGTGGGGAGAGAAAAAAGAGCAGTTGGAATGGGAGCTGTTTTCCTTGGGTTCTTAGGAGCAGCAGGAAGCACT
ATGGGCGCAGCGTCAATAACGCTGACGGTACAGGCCTGGCAATTATTGTCTGGCATAGTGCAACAGCAAAGCA
ATTTGCTGAGGGCTATAGAGGCTCAACAACATCTGTTGAAACTCACGGTCTGGGGCATT
>KT152841..1
TTCTATTGTGATACATCAGCCCTGTTTAATGGCACTTGGAATAATACCAGCATGAAGGAGTCAAATAGCACGG
AATCAAATGACACTATAACTCTCCCATGCAGAATAAAGCAAATTATAAATATGTGGCAGAGAGTAGGACAAGC
AATGTATGCCCCTCCCATCCAAGGAATAATAAAGTGTGAATCAAACATTACAGGACTACTATTAACAAGAGAT
GGTGGTGGGGATAACAGTACAAATGAGGAAACCTTCAGACCTGGAGGAGGAGATATGAGGGACAATTGGAGAA
GTGAATTATATAGGTATAAAGTAGTAAAAATTGAACCACTAGGAGTAGCACCCACCAGGGCAAGGAGAAGAAC
GGTGGGGAGAGAAAAAAGAGCAGTGGAAATAGGAGCTGTCTTCCTTGGGTTCTTAGGAGCAGCAGGAAGCACT
ATGGGCGCAGCGTCAATAACGCTGACGGTACAGGCCAGACAATTATTGTCTGGCATAGTGCAACAGCAAAACA
ATTTGCTGAGGGAAGAGGCTCAACAACATTTGTTGAAACTCACGGTCTGGGGCATA
>KT152842..1
TTCTATTGTAATACATCAGCTCTGTTTAATGGCACTTGGGGGCAAAATGCCAGCATGGAGTCAAATGGCACTA
TAAATGACACTATAATTCTCCCATGCAGACTAAAGCAAATTGTAAGGATGTGGCAGAGAGTAGGACAAGCAAT

GTATGCCCCTCCCATCCAAGGAGAAATAAATTGTGTATCAAACATTACAGGACTAATATTAACAAGAGATGGT
GGGAATAATACAGAGGAAATCTTCAGACCTGGAGGAGGAGATATGAGGGACAATTGGAGAAGTGAATTATATA
AGTATAAAGTAGTAAAAATTGAACCACTAGGAGTAGCACCCACCAGTGCAAGGAGAAGAACGGTGGGGAGAGA
AAAAAGAGCAGTTGGAATAGGAGCTGTCTTCCTTGGGTTCTTAGGAGCAGCAGGAAGCACTATGGGCGCGGCG
TCAATAACGCTGACGGTACAGGCCAGACAATTATTGTCTGGCATAGTGCAACAGCAAAGCAATTTGCTGAGGG
CTATAGAGGCTCAACAACATCTGTTGAAACTCACAGTCTGGGGCATT
>KT152844..1
TTCTACTGTAATACATCAGCCCTGCTTAATGGCACTTGGCAAAATGCCAGCATGCAGGAGTCAAATAGCACAG
AGTCAAATGACACCATAACTCTCCCATGCAGAATAAAGCAAGTTATAAATATGTGGCAGAGAGTAGGACAAGC
AATGTATGCCCCTCCCATCCAAGGAATAATAAAGTGCGTATCAAACATCACAGGACTAATATTAACAAGAGAT
GGTGGGAATAGTAACAGTAGTACAGTTGAAACCTTCAGGCCTGGAGGAGGAGATATGAGGGACAATTGGAGAA
GTGAATTATATAAGTATAAAGTAGTAAAAATTGAACCACTAGGAGTAGCACCCACCAGGGCAAGGAGAAGAAC
GGTGGAGAGAGAAAAAAGAGCAGTTGGAATAGGAGCTGTCTTCCTTGGGTTCTTAGGAGCAGCAGGAAGCACT
ATGGGCGCAGCGTCAATAACGCTGACGGTACAGGCCAGACAATTATTGTCTGGCATAGTGCAACAGCAAAGTA
ATTTGCTGAGGGAAATAGAGGCTCAACAACATCTGTTGAAACTCACGGTCTGGGGCATT
>AY736809..2
TTCTATTGTAATACATCAGGCCTGTTCAATAGCACTTGGCCTGTCAATGCCAGTGAGCAGGAGTCAAATAGCA
CAGAGTCAGATGGCATTATAACTCTCCGATGCAGAATAAAGCAAATCATAAATATGTGGCAGAGAACAGGACA
AGCAATGTATGCCCCTCCCATTCAAGGAATAATAAAGTGTGTATCAAACATTACAGGACTACTGTTAACAAGA
GATGGTGGGAATAGTACTGTCAATGAAACTTTCAGACCTGGAGGAGGAGATATGAGGGACAATTGGAGAAGTG
AATTATATAAGTATAAAGTAGTAAAAATTGAACCACTAGGAGTAGCACCCACCAAGGCAAGGAGAAGAGTGGT
GGGGAGAGAAAAAAGAGCAGTTGGAATAGGAGCTGTTTTCCTTGGGTTCTTAGGAGCAGCTGGAAGCACTATG
GGCGCGGCGTCAATAACGCTGACGGTACAGGCCAGACAATTACTGTCTGGCATAGTGCAACAGCAAAACAATT
TGCTGAGGGCTATAGAGGCTCAACAACAACTGTTGAAACTCACGGTCTGGGGCATT
>AY736810..2
TTTTATTGTAATACATCAGACCTGTTTAATAGTACTTGGAACATTGAAGGCACTGCCAGCATAAATGGCACGG
AGTCAAATGACAATATAACTCTCCCATGCAGAATAAAGCAAATTATAAATATGTGGCAGAGAGTAGGACAAGC
AATGTATGCCCCTCCCATCCAAGGAGTAATAAGGTGTCAATCAAACATTACAGGAATACTATTAACAAGAGAT
GGTGGGAATACTGGGAATAATAGCAGGACAAATGAAACCTTTAGACCTGGAGGAGGAGATATGAGGGACAATT
GGAGAAGTGAATTATATAAGTATAAAGTAGTAAAAATTGAACCACTGGGAGTAGCACCCACCAAGGCAAAGAG
AAGAGTGGTGGAGAGAGAAAAAAGAGCAGTTGGACTGGGAGCTGTATTCATTGGGTTCTTAGGAGCAGCCGGA
AGCACTATGGGCGCGGCGTCAATAACGCTGACGGTACAGGCCAGACAATTATTGTCTGGCATAGTGCAACAGC
AAAGCAATTTGCTGAGGGCTATAGAGGCTCAACAGCATTTGTTGAAACTCACGGTCTGGGGCATT
>AY736812..1
TTCTATTGTAATACATCAGGTCTGTTCAATAGCACCTGGAAGGCCAACAATGGCACCTGGAAGGCCAACATAT
CAGAGTCAAATAACACGGAGATAACTCTCCAATGCAGAATAAAGCAAATTATAAATATGTGGCAGAGAACAGG
ACAAGCAATATATGCCCCTCCCATCCAGGGAGTGATAAGGTGTGAATCAAACATCACAGGACTACTGTTAACA
AGAGATGGTGGGGAGGGGAACAATGAAAGTGAGATCTTCAGACCTGGAGGAGGAGATATGAGGGACAACTGGA
GAAGTGAATTATATAAGTATAAAGTAGTAAAAATTGAACCACTAGGAGTAGCACCCACCAGGGCAAGGAGAAG
AGTGGTGGGAAGAGAAAAAAGAGCAGTTGGAATAGGAGCTGTTTTCCTTGGGTTCTTAGGAGCAGCAGGAAGC
ACTATGGGCGCGGCGTCAATAACGCTGACGGTACAGGCCAGGCAATTATTGTCTGGCATAGTGCAACAGCAAA
GCAATTTGCTGAGGGCTATAGAGGCTCAACAACATATGTTGAAACTCACGGTCTGGGGCATT
>AY736815..1
TTCTATTGTAATACATCAGGTCTGTTTAATAGCACCTGGGACAACAACACCGACCCAACCAACACAACGTCAA
ATGACACTATCACTCTCCAATGCAGAATAAAGCAATTTATAAATATGTGGCAGAGAACAGGGCAAGCAATGTA
TGCCCCTCCCATCCAAGGGGTAATAAGGTGTGATTCCAACATTACAGGACTACTATTAACAAGAGATGGTGGA
GTAGGTAACAATACAACTGAAACCTTCAGACCTGGAGGAGGAGATATGAGGGACAATTGGAGAAGTGAATTAT
ATAAGTATAAAGTAGTAAAAATTGAACCACTAGGAGTAGCACCCACCGGGGCAAAGAGAAGAGTGGTGGAGAG
AGAAAAAAGAGCAGTTGGAATAGGAGCTGTTTTCATTGGGTTCTTAGGAGCAGCAGGAAGCACTATGGGCGCG
GCGTCAGTAACGCTGACGGTACAGGCCAGACAATTATTGTCTGGCATAGTGCAACAGCAAAGCAATTTGCTGA
GGGCTATAGAGGCTCAACAGCATCTGTTGAAACTCACGGTCTGGGGCATT
>AY736813..1
TTCTATTGTGATACATCAGGCCTGTTTAATAGCAGTTGGAATAATAACACTTGGAAGGAGTCAAATAGCACGG
GGTCAAATGACACTATAACTCTCCTATGCAGAATAAAACAAATTATAAATATGTGGCAGAGAACAGGACAAGC
AATGTATGCCCCTCCCATCCCAGGGTTAATAAGCTGTAAATCAAACATTACAGGTATAATATTGACAAGAGAT
GGTGGGAATGGTAACAGTACAAATGAAACCTTCAGACCTGGAGGAGGAGATATGAGGGACAATTGGAGAAGTG
AACTATATAGGTATAAAGTAGTACAAATTGAACCACTAGGAGTAGCACCCACCAGGGCAAGGAGAAGAGTGGT
GCAGAGAGAAAAAAGAGCAGTAGGAATAGGAGCTGTCTTCCTTGGGTTCTTAGGAGCAGCAGGAAGCACTATG
GGCGCAGCGTCAATAACGCTGACGGTACAGGCCAGACAATTATTGTCTGGCATAGTGCAACAGCAAAGCAATT
TGCTGAGGGCTATAGAGGCTCAACAACATCTGTTGAAACTCACGGTCTGGGGCATT

>AY736816..1
TTCTATTGTGACACATCAGGCCTGTTTACTTGGAATAGCACTTGGAATGGCACTGAGTCAAACAACACAAAGT
CAAACGACACTATAACTCTCCAATGCAGAATAAAGCAAATTATAAATATGTGGCAGAGAGTAGGACAAGCAAT
ATATGCCCCTCCCATCAAAGGAGTAATAAGGTGTGAATCAGACATTACAGGACTTATACTAACAAGAGATGGT
GGGAATACTAACAGTACAGAGGAAACCTTCAGACCTGGAGGAGGAGATATGAGGGACAATTGGAGAAGTGAAT
TATATAAGTATAAAGTAGTAAAAATTGAACCACTAGGAGTAGCACCCACCAAGGCAAAGAGAAGAGTGGTGGG
GAGAGGAAAAAGAGCAGTTGGAATAGGAGCTGTCTTCCTTGGGTTCTTAGGAGCAGCCGGAAGCACTATGGGC
GCGGCGTCAATAACGCTGACGGTACAGGCCAGACAATTATTGTCTGGCATAGTGCAACAGCAAAGCAATTTGC
TAAGGGCTATAGAGGCTCAACAGCATCTGTTGAAACTCACGGTCTGGGGCATT
>AF457053..2
TTCTATTGTAATACATCAGGCCTGTTTAACAGCACCTGGCACTATAACAGCAATGGTACTTGGCACAATAACA
GCACAAATAGCATGGACTCAAATGAAACTATAACTCTCACATGCAGAATAAAGCAAATTATAAATATGTGGCA
GAGAGTAGGAAAAGCAATGTATGCTAATCCCATCCAAGGAATAATAAAGTGTGAATCAAACATTACAGGACTA
CTCTTAACAAGAGATGGTGGGAATACTAGCAGTACAAATGAAACCTTCAGACCTGGAGGAGGAGATATGAGGG
ACAATTGGAGAAGTGAATTATACAAATATAAAGTAGTAAAGATTGAGCCACTAGGAGTAGCACCCACCAGGGC
ACAGAGAAGAGTGGTGGGGAGAGAAAAAAGAGCAATTGGAATAGGAGCTGTCTTCCTTGGGTTCTTAGGAGCA
GCAGGAAGCACTATGGGCGCGGCGTCAATGACGCTGACGGTACAGGCCAGACAATTATTGTCTGGCATAGTGC
AACAGCAAAGCAATTTGCTGAGGGCTATAGAGGCTCAACAACAACTGTTGAAACTCACGGTCTGGGGCATT
>AF457055..1
TTCTATTGTAATACAACAAACCTGTTTAATAGCACCTGGAGAGGAAATAAAACTGTCAGCAATAGCACAGAGT
TAGATGAAAATGGCACTATAACTCTCCCATGCAGAATAAAGCAAATTATAAATATGTGGCAGAGAACAGGACA
AGCAATGTATGCCCCTCCCATCCAAGGAGTAATAAAGTGTGTATCAAACATTACAGGACTACTATTAACAAGA
GATGGTGGAAGTGATAACAATATAAGTAGTGAAACCTTCAGACCAGGAGGAGGAGATATGAGGGACAATTGGA
GAAGTGAATTATATAAGTATAAAGTAGTGAAACTTGAACCACTAGGAGTAGCACCCAACAAGGCAAGGAGAAG
AGTGGTGGAGAGAGAAAAAAGAGCAGTTGGACTGGGAGCTGTATTCATTGGGTTCTTAGGAGCAGCAGGAAGC
ACTATGGGCGCGGCGTCAATAACGCTGACGGCACAGGCCAGACAATTATTGTCTGGCATAGTGCAACAGCAAA
GCAATTTGCTGAAGGCTATAGAGGCTCAACAGCATCTGTTGAAACTCACGGTCTGGGGCATT
>AF457070..1
TTCTATTGTAATACATCAGGCCTGTTTAATAGCACTTGGAATAGCACTGGGAATGACACTTGGAGTGGCAATG
CCAGCACGCAGGAGTCAAATGACACTATAACTCTCCCATGCAGAATAAAGCAAATTGTAAATATGTGGCAGAG
AATAGGACAAGCAATGTATGCCCCTCCCATCCAAGGAGTAATAAGGTGTGAATCAAACATTACAGGACTACTA
TTAACAAGAGATGGTGGGAACAATGAAAGTGAAACCTTCAGACCTGGAGGAGGAAATATGAGGGACAATTGGA
GAAGTGAATTATATAGGTACAAAGTAGTGAAAATTGAACCACTAGGAGTAGCACCCACCAGGGCAAAAAGAAG
AGTGGTGGGGAGAGAAAAAAGAGCAGTTGGAATAGGAGCTGTGTTCCTTGGGTTCTTAGGAGCAGCAGGAAGC
ACTATGGGCGCGGCGTCAGTAACGCTGACGGTACAGGCCAGACAATTATTGTCTGGCATAGTGCAACAGCAAA
GCAACTTGCTGAGGGCTATAGAGGCTCAACAACATCTGTTGAAACTCACGGTCTGGGGCATT
>AF457077..1
TTCTATTGTAATACATCAGGCCTGTTTAATAGCACTTGGTCTTGGAATGACACTGCAAATGGCACTGCAATGG
AGCCAAATAGCACTATAACTCTCCCATGCAGAATAAAGCAAATTATAAATATGTGGCAGAGAGTAGGACAAGC
AATGTATGCCCCTCCCATCCCAGGAGTAATAAGTTGTGTATCAAACATTACAGGACTAATATTAGTAAGAGAT
GGAGGGAATAATGTTACAAATGAAACCTTCAGACCTGGAGGAGGAGATATGAGGGACAATTGGAGAAGTGAAT
TATATAAGTATAAGGTAGTAAAAATTGAACCACTAGGAGTAGCACCCACCAAGGCAAAGAGAAGAGTGGTGGA
GAGAGAAAAAAGAGCAGTTGGAATAGGAGCTGTCTTCCTTGGGTTCTTAGGAGCAGCAGGAAGCACTATGGGC
GCGGCGTCAATAACGCTGACGGTACAGGCCAGACAATTATTGTCTGGTATAGTGCAACAGCAAAGCAATTTGC
TGAGGGCTATAGAGGCTCAACAACATCTGTTGAAACTCACAGTCTGGGGCATT
>AF457080..2
TTCTATTGTAATACATCGGGCCTGTTTAATAGCACCTACAATGGCACTGACATCGCTGAAAATAGCACGGAGT
CAAATGACACTATAGTTCTCCCATGCAGAATAAAGCAAATTATAAGGATGTGGCAGCGAGTAGGACAAGCAAT
GTATGCCCCTCCCATCCCAGGAATAATAAGATGTGACTCAAACATTACAGGATTACTATTAACAAGAGATGGT
GGGGATGATAATGAAACTGAAACCTTTAGACCTGGAGGAGGAGATATGAGGGATAATTGGAGAAGTGAATTAT
ATAAGTATAAAGTAGTAAAAATTGAACCACTAGGAGTAGCACCCACCAGGGCAAAAAGAAGAGTGGTGGAGAG
AGAAAAAAGAGCAGTTGGAATAGGGGCTTTTGTCCTTGGGTTCTTAGGAGCAGCAGGAAGCACTATGGGCGCA
GCATCAATAACGCTGACGGTACAGGCCAGACAATTATTGTCTGGCATAGTGCAACAGCAAAGCAATTTGCTGA
GGGCTATAGAGGCTCAACAACAAATATTGAGACTCACGGTCTGGGGCATT
>AF457081..1
TTCTATTGTAATACATCAGGCCTGTTTAATAGCACTTGGACAAATGACAGTATCAGCACAAATAGCACAAAGT
CAAATGACACTATAACTCTCCCATGCAGAATAAAGCAAATTATAAATATATGGCAGAGAGTGGGACAAGCAAT
GTATGCCCCTCCCATCCAAGGAGTAATACAGTGTGTATCAAACATTACAGGACTACTATTAACAAGAGATGGT
GGGGATAACAGTAGTACGAATGAAACCTTCAGACCTGGAGGAGGAGATATGAGGGACAATTGGAGAAGTGAAT
TATATAAGTATAAAGTAGTAAAAATTGAACCACTAGGAGTAGCACCCACCAGGGCAAGGAGAAGAGTGGTGGA

GAGAGAGAAAAGAGCAGTTGGACTGGGAGCTGTTTTCATTGGGTTCTTGGGAGCAGCAGGGAGCACTATGGGC
GCAGCGTCAATAACGCTGACGGTACAGGCCAGACAATTATTGTCTGGCATAGTGCAACAGCAAAGCAATTTGC
TGAGGGCTATAGAGGCTCAACAACATCTGTTGAAACTCACGGTCTGGGGCATT
>AF457083..1
TTCTATTGTAATACATCAAGTCTGTTTAATAGTACTTGGAATAACAGTACTTGGAATACCAGCAGGCAGGAGG
CAAATAATAGCCCGGGGTCAAACGACACTATAACTCTCAAATGCAGAATAAAGCAAATTATAAATATGTGGCA
GAGAGCGGGACAAGCAATATATGCCCCTCCCATCCAAGGAGTAATAAGGTGTAACTCAAACATTACAGGACTA
ATATTAACAAGAGATGGTGGGGGTAATAACAATACAAATGAAACCTTCAGACCTGGAGGAGGAGATATGAGGG
ACAATTGGAGAAGTGAATTATATAAGTACAAAGTAGTAAAAATTGAACCACTAGGAGTAGCACCCACCAGGGC
AAAGAGAAGAGTGGTGGAGAGAGGAAAAAGAGCAGTTGGAATAGGAGCTGTCCTCATTGGGTTCTTAGGAGCA
GCAGGAAGCACTATGGGCGCGGCGTCAATAACGCTGACGGCACAGGCCAGACAATTATTGTCTGGCATAGTGC
AACAGCAAAACAATTTGCTGAGGGCTATAGAGGCTCAACAACATCTGTTGAGACTCACGGTCTGGGGCATT
>AF457084..1
TTCTATTGTGACACATCAGGCCTGTTTAATAGCAGTTGGACTGCTAGCAATGATAGCATGCAGGAGGCACATA
GCACAGAGTCAAATATAACTCTCCAATGCAGAATAAAGCAAATTATAAATATGTGGCAGAGAGCAGGACAAGC
AATGTATGCCCCTCCCATTCCAGGAATAATAAGGTGTGAATCAAACATTACAGGACTAATATTAACAAGAGAT
GGTGGTGAGGGTAATAACAGTACAAATGAAACCTTCAGACCAGTAGGAGGAAATATGAGGGACAATTGGAGAA
GTGAATTATATAAGTATAAAGTAGTAAAAGTTGAACCACTAGGAGTAGCACCCACCAAGTCAAGGAGGAGAGT
GGTGGAGAGAGAAAAAAGAGCAGTTGGACTAGGAGCTGTGTTCATTGGGTTCTTAGGAGCAGCAGGAAGCACT
ATGGGCGCGGCGTCAATGACGCTGACGGTACAGGCCAGACAATTATTGTCTGGCATAGTGCAACAGCAAAGCA
ATTTGCTGAGGGCTATAGAGGCTCAACAGCATCTGTTGAAACTCACGGTTTGGGGCATT
>AF457086..3
TTCTATTGTAATACAACAAAACTGTTTAATAGCACTTGGAGTCATAATGACAGCACGCAAGAGTCAAATAGCA
CAAAGTCAAATGACACTATAACTCTCCCATGCAGAATAAAGCAAATTATAAATATGTGGCAGAGAGTAGGACA
AGCAATATATGCCCCTCCCATACAAGGAATAATAAGGTGTGAATCAAATATTACAGGACTAATATTAACAAGA
GATGGAGGTACCAATAGTACAAATGAAACCTTCAGACCTGGAGGAGGAGATATGAGGGACAATTGGAGAAGTG
AATTATATCAGTATAAAGTAGTAAAAATTGAACCACTAGGAGTAGCACCTACCAGGGCAAAGAGAAGAGTGGT
GGAGAGAGCAAAAAGAGCAGTTGGAATAGGAGCTGTCTTCCTTGGGTTCTTAGGAGCAGCAGGAAGCACTATG
GGCGCGGCGTCAATAACGCTGACGGTACAGGCCAGACAATTATTGTCTGGCATAGTGCAACAGCAAAGTAATT
TGCTGAGGGCTATAGAGGCTCAACAGCATCTGTTGAAACTCACGGTCTGGGGCATT
>AF457089..1
TTTTATTGCAATACATCAGATCTGTTTAATAGTACTTGGCCTGTCAATGCCAGCATGGAGTCAAATAGCACGG
AGTCAAATGACAATATAACACTCCAATGCAGAATAAAGCAAATTATAAATATGTGGCAGAAAGTAGGACAAGC
AATGTATGCCCCTCCCATACAAGGAGTAATAAGGTGTGAATCAAACATTACAGGACTACTATTAACAAGAGAT
GGTGGATCTAACAGTACAGAAGAAACCTTCAGACCTGGAGGAGGAGATATGAGGGACAATTGGAGAAGTGAAT
TATATAAGTATAAAGTAGTGAAAATTGAGCCATTAGGGGTAGCACCCACCAGGGCAAAGAGAAGAGTGGTGGA
GAGAGAAAAAAGAGCGGTTACACTAGGAGCTGTATTCATTGGGTTCTTAGGAGCAGCAGGAAGCACTATGGGC
GCAGCGTCAGTAACGCTGACGGTACAGGCCAGACAATTACTGTCTGGCATAGTGCAACAGCAAAGCAATTTGC
TGAGGGCTATAGAGGCTCAACAGCATATGTTGAAACTCACGGTCTGGGGCATT
>EU110088..2
TTCTATTGTAACACATCACGCCTGTTTAATAGCACCTGGTGGGCCAATGGCACCAGGACGGGCTACAACAGCA
CTGATGGCAATATAACACTCCCATGCAGAATAAAGCAAATTATAAATATGTGGCAGAGAGTAGGACAAGCAAT
GTATGCCCCTCCCATCCCAGGAAGAATAAGTTGTGAATCAAACATTACAGGACTAATATTAACAAGAGATGGT
GGGAGTGATAATACAACTGAAACCTTCAGACCTGGAGGAGGAGATATGAGGGACAATTGGAGAAGTGAATTAT
ATAAGTATAAAGTAGTAGAAGTTGAACCACTAGGAGTAGCACCCACAGAGGCAAGGAGAAGAGTGGTGCAGAG
AGAAAAAAGAGCAGTCGGACTGGGAGCTGTCTTCATTGGGTTCTTGGGAGCAGCAGGAAGCACTATGGGCGCG
GCGTCAGTGACGCTGACGGTACAGGCCAGACAATTATTGTCTGGTATAGTGCAACAGCAAAGCAATTTGCTGA
GGGCTATAGAGGCTCAACAACATCTGTTGAAACTCACGGTCTGGGGCATT
>EU110092..1
TTCTATTGTGATACATCAGCTCTGTTCAATAGCACTTGGAATGCCACTGAGTCAAATGACACAAACTCCACAG
AATCAAACTCAAATGACACTATAACTCTCCCATGCAGAATAAAACAAATTATAAATATGTGGCAGAGAATAGG
ACAAGCAATGTATAACCCTCCCATCCAAGGAAAAATAAAGTGTGAATCAAACATTACAGGACTATTGTTAACA
AGAGATGGTGGAGAAAATAGAGAAAATAATGACAGTAGTGAAATCTTCAGACCAGGAGGAGGAGATATGAAGG
ACAATTGGAGAAGTGAATTGTATAAGTATAAAGTAGTAAAAATTGAACCACTAGGAGTAGCACCCACCAGGGC
AAGGAGAAGAGTGGTGGGGAGAGAAAAAAGAGCAGTTGGAATAGGAGCTGTCTTCCTTGGGTTCTTAGGAGCA
GCAGGAAGCACTATGGGCGCGGCGTCAATAACGCTGACGGTACAGGCCAGACAATTATTGTCTGGCATAGTGC
AACAGCAAAGCAATTTGCTGAGGGCTATAGAGGCTCAACAACATCTGTTGAAACTCACAGTCTGGGGCATT
>EU110094..1
TTCTATTGTAATACATCAGGCCTGTTTAGTAGCACTTGGAGTTGGAATGACACTACCAAAACAATGGAATCAA
ATAGCACGGTGTCAGATGGCACTATAAATCTCCCATGCAGAATAAAGCAAATTATAAATATGTGGCAGAGAGT

AGGGCAAGCAATGTATGCCCCTCCCATCCAAGGAGCAATAAGGTGTACATCAAACATTACAGGACTAATATTA
ACAAGAGATGGTGGGAAAGATGACAGGAATAATGAAACCTTCAGACCTGGAGGAGGAGATATGAGGGACAATT
GGAGAAGTGAATTATATAAATATAAAGTAGTAAAAATTGAACCACTAGGAGTAGCACCCACCAGGGCAAAGAG
AAGAGTGGTGGAGAGAGAAAAAAGAGCAGTTGGAATAGGAGCTGTCTTCCTTGGGTTCTTAGGAGCAGCAGGA
AGCACTATGGGCGCGGCGTCAATAACGCTGACGGTACAGGCCAGACAATTATTGGCTGGCATAGTGCAACAGC
AAAGCAATTTGCTGAGGGCTATAGAGGCTCAACAACATCTGTTGAAACTCACGGTCTGGGGCATT
>KT022360..2
TTCTATTGTAACACATCAGGCTTGTTTAATAACACTTGGGTGTTTAATAGCATTTGGGATAATAGCACTCAGG
AGTCCAATAGCACAGAGTCAAATAAAACTATAATTCTCCAATGCAGAATAAAGCAAATTATAAATATGTGGCA
GAGAGCAGGACAAGCAATATATGCTCCTCCCATTCCAGGAGTAATAAGGTGTGAATCAAACATTACAGGACTA
ATATTAACAAGAGATGGTGGAAATAATAACACGATAAATGAAACATTTAGACCTGGAGGAGGAGACATGAGGG
ACAATTGGAGAAGTGAATTATATAAGTACAAAGTAGTAAAAATTGAACCACTAGGAGTAGCACCTACCAAGGC
ACGGAGAAGAGTGGTGGAGAGAGAAAAAAGAGCAGTTACAATAGGAGCTATGTTCATTGGGTTCTTAGGAGCA
GCAGGAAGCACTATGGGCGCGGCGTCAATGACGCTGACGGTACAGGCCAGACAATTATTGTCTGGCATAGTGC
AACAGCAAAGCAATTTGCTGAGGGCTATAGAGGCTCAACAGCATCTGTTGAAACTCACGGTCTGGGGCATC
>KT022361..3
TTCTATTGTAATACATCAGGCCTGTTTAATTACAGCTACAGCATGCAGGAGTCAAATGATACGGAGGCAAATG
GCACTATAACTCTCCAATGCAAAATAAAACAAATTATAAATATGTGGCAGAGAGTAGGACAAGCAATGTACGC
CCCTCCCATCCAAGGAGTAATACAGTGTAAATCAAACATTACAGGACTAATATTAACAAGAGATGGTGGAAAT
AGTACTAATGGTACAGAAATCTTCAGACCTGGAGGAGGAGATATGAGAGACAATTGGAGAAGTGAGTTATATA
AGTATAAAGTAGTACAACTTGAACCACTAGGAGTAGCACCCACCAAGGCAAAGAGAAGAGTGGTGGAAAGAGA
AAAAAGAGCAGTTGGAATAGGAGCTGTCTTCCTTGGGTTCTTAGGAACAGCAGGAAGCACTATGGGCGCGGCG
TCACTGACGCTGACGGTACAAGCCAGACTATTATTGTCTGGCATAGTGCAACAGCAAAGCAATTTGCTGAGGG
CTATAGAGGCTCAACAATATCTGTTGAAACTCACGGTCTGGGGCATT
>KT022363..1
TTCTATTGTGACACCTCGAAGCTGTTTAATAGCTCTTGGAATGAGAGCACTTGGGATGAAAATAGCACGAAGC
CTAACAACAATGAAACTATAATTCTCCCATGCAGAATAAAGCAAATCATAAATATGTGGCAGAGAACAGGACA
AGCAATATATGCCCCTCCCATTGAAGGAATAATAAAGTGTGACTCAAACATTACAGGACTAATATTAACAAGA
GATGGTGGGAGGATTAACACAACTAATGAAACTTTCAGACCTGGAGGAGGAGATATAAGGGACAATTGGAGAA
GTGAATTATATAAATATAAAGTAGTAAAAATTGAGCCACTAGGAGTAGCACCCTCCAGGGCAAGGAGAAGAGT
GGTGGGGAGAGAAAAAAGAGCAGTTGGAATAGGAGCTGTCTTCCTTGGGTTCTTAGGAGCAGCAGGAAGCACT
ATGGGCGCGGCGTCACTAACGCTGACGGTACAGGCCAGGCAATTATTGTCTGGCATAGTGCAACAGCAAAGCA
ATTTGCTGAGGGCTATAGAGGCTCAACAGCATATGTTGAAACTCACGGTCTGGGGCATT
>KT022364..1
TTTTATTGTGATACCTCAGGCTTGTTTAATAGTACTTGGGAAATTGAAAACAATACTGCCAGAATAAAGCCAA
ATAACACGGGGTTAAATATAACGCTCCCGTGCAGAATAAAGCAAATTATAAATATGTGGCAAAGGGTAGGACA
AGCAATGTATGCCCCTCCCATCAAAGGAGTAATAAGGTGTGTATCAAACATCACAGGACTACTGCTAACAAGA
GATGGTGGGAGTAAAAATAGAAGTAATGAAACCTTCAGACCTGGAGGAGGAGATATGAGGGACAATTGGAGAA
GTGAATTATATAAATATAAAGTAGTAAAAATTGAACCACTAGGAGTAGCACCCTCCAGGGCAAAGAGGAGAGT
GGTGGAGAGAGAGAAAAGAGCAGTTGGAATAGGAGCTGTATTCCTTGGGTTCTTAGGAGCAGCAGGAAGCACT
ATGGGCGCGGCGTCACTGACGCTGACGGTACAGGCCAGACAATTATTGTCTGGCATAGTGCAACAGCAAAGCA
ATTTGCTGAGGGCTATAGAAGCTCAACAGCATCTGTTGAAGCTTACAGTCTGGGGCATT
>KT022365..1
TTCTATTGTGATACGTCAGGCTTATTCAGTAGCACCTGGCAATTCAATGGGAGTACCTGGAACAACACAGATT
CAAACAGCACAGGGTCAGATGACACTATAGAACTCCCATGCAGAATAAAGCAAATTATAAATATGTGGCAGAG
AGTAGGACGAGCAATGTATGCCCCTCCCATCCAAGGAGTAATAAGTTGTGTATCAAACATCACAGGACTAATA
TTAACAAGAGATAGTAATAACAGAACTAATGAAACCTTCAGACCTGGAGGAGGAGACATGAGAGACAATTGGA
GAAGTGAATTATATAAGTATAAAGTAGTAAAAATTGAACCACTAGGAGTAGCACCCACCAAGGCAAAAAGAAG
AGTGGTGGAGAGAGAAAAAAGAGCAGTTGGAATAGGAGCTGTCTTCCTTGGGTTCTTAGGAGCAGCAGGAAGC
ACTATGGGCGCGGCGTCAATAACGCTGACGGTACAGGCCAGACAATTATTGTCTGGCATAGTGCAACAGCAAA
GCAATTTGCTGAGGGCTATAGAGGCTCAACAACATCTGTTGAAACTCACAGTCTGGGGCATT
>KT022367..1
TTCTATTGTAATACATCAGGTCTATTTAATAGCAGTTGGAATGATACTGAAATCGGGCGGGAGATAAATAGGA
CGGAGTCAAATGACACTATAATTCTCCCATGCAGAATAAAGCAAATTATAAATATGTGGCAGAGAGTAGGACA
AGCAATATATGCCCCTCCCATCCAAGGAGTAATAAGGTGTGAATCAAACATTACAGGACTAATATTAACAAGG
GATGGTGGGGGTAATAACAGAAAAAATGAAACCTTCAGACCTGGAGGAGGAGATATGAGGGACAATTGGAGAA
GTGAATTATATAAGTATAAAGTAGTAAAAATTGAACCAATAGGAATAGCACCCACCAGGGCAAAGAGAAGAGT
GGTGGAGAGAGAAAAAAGAGCAATTGGAATGGGAGCTGTATTCATTGGGTTCTTAGGAGCAGCAGGAAGCACT
ATGGGCGCGGCGTCAATAACGCTGACGGTACAGGCCAGACAATTGTTGTCTGGCATAGTGCAACAGCAAAACA
ATTTGCTGAGGGCTATAGAGGCTCAACAACATCTGTTGAAGCTCACGGTCTGGGGCATT

>KT022368..2
TTCTATTGTAATACAACAGACCTGTTTGATAGCATTTGGCTTTACAATGCCAGCATGCATAATGCAAATAGCA
CGATAACTCTCCAATGCAGAATAAAGCAAATTATCAATATGTGGCAGAGAGTAGGACAAGCAATGTATGCCCC
TCCCGTCGAAGGAAAAATACAGTGCAAATCAAACATTACAGGACTAATATTAACAAGAGATGGTGGGGTTAAC
GATACAAATGGAACCGAAATCTTCAGACCTGGAGGAGGGGATATGAGGGACAATTGGAGAAGTGAATTATATA
AGTATAAAGTAGTAAAAATTGAACCACTAGGAGTAGCACCCACCAGGGCAAGGAGAAGAGTGGTGCAGAGAGA
AAAAAGAGCAGTTGGAATAGGAGCTGTATTCCTTGGGTTCTTAGGAGCAGCAGGAAGCACTATGGGCGCAGCG
TCAGTGACGCTGACGGTACAGGCCAGAAGATTATTGTCTGGCATAGTGCAACAGCAAAGCAATTTGCTGAAGG
CTATAGAAGCTCAACAGCATCTGTTGAAGCTCACGGTCTGGGGCATT
>KT022369..2
TTCTATTGTAATACATCAGGCCTGTTTAATAGCACTTGGAATTATACTAATAGCATGCAGCAGTCAAATAGCA
CAGAGTCAAACGAAACTATAACTCTCCAATGCAGAATAAAGCAAATTATAAATATGTGGCAGAGAGTGGGGCA
AGCAATATATGCCCCTCCCATCCCAGGGGTAATAAGGTGTGAATCAAACATTACAGGACTAATATTGACAAGA
GATGGTGGGAGTAATAACAGTACAAATGAAACCTTCAGACCTGGAGGAGGGAATGTGAGGGACAATTGGAGAA
GTGAATTATATAAGTATAAAGTAGTAAAAATTGAACCACTAGGAGTAGCACCCACCAGGGCAAAGAGAAGAGT
GGTGGAGAGAGAAAAAAGAGCAGTTGGACTAGGAGCTGTCTTCATTGGGTTCTTGGGAACAGCAGGAAGCACT
ATGGGCGCGGCGTCAATAACGCTGACGGTACAGGCCAGACAATTATTGTCTGGTATAGTGCAACAGCAAGGCA
ATTTGCTGAGAGCTATAGAAGCTCAACAACAACTGTTGAGACTCACGGTCTGGGGCATT
>FJ866117..1
TTTTATTGTAATACATCAGGCCTGTTTAATAGTACTTGGTCACCTGACAATAGTACTGGGACTAGTACTGGGA
ATAATGACACTGCCAGCAATGGCACTATAACTCTCCCATGTAGAATAAAGCAGATTATAAATATGTGGCAGAG
AGTAGGACAAGCAATGTATGCCCCTCCCATCCAAGGAGTAATAAGGTGTGACTCAAACATTACAGGACTACTA
TTAACAAGAGATGGTGGGAGTGGGAATAATACCACAGAGATCTTCAGACCTGGAGGAGGAGATATGAGGGACA
ATTGGAGAAGTGAATTATATAAGTATAAAGTAGTAAGAATCGAACCTCTAGGAGTAGCACCCACCAGGGCAAA
GAGAAGAGTGGTGGAGAGAGAAAAAAGAGCAGTTGGAATAGGAGCTGTATTCATTGGGTTCTTAGGAGCAGCA
GGAAGCACTATGGGCGCAGCGTCAATAACGCTGACGGTACAGGCCAGACAATTATTGTCTGGCATAGTGCAAC
AGCAAAGCAATTTGCTGAGGGCTATAGAGGCTCAACAGCATTTGTTGAAGCTCACAGTCTGGGGCATT
>KT022370..3
TTCTATTGTAATACATCAGGCCTGTTTAATAGAGCTTACACTAACAGCACTTACAATAACAGCACGCAGGACA
ATGACAATAACAGCACGCAGGACAATGCAACTATAACTCTCCCATGCAGAATAAGGCAAATTATAAATATGTG
GCAGAGAGCAGGGAAAGCAATGTATGCCCCTCCCATCCAAGGAGTAATAAACTGTGTATCAAACATTACAGGA
CTAATTTTAACAAGAGATGGTGGAAAGAGTAACAGTACGGAGGGGGAAATCTTCAGACCTGGGGGAGGAGATA
TGAGGGATAATTGGAGAAGTGAATTATATAAGTATAAAGTAGTAAAAATTGAACCACTAGGAGTAGCACCTAC
CAAGGCAAGGAGAAGAGTGGTGGGGAGAGAGAAAAGAGCAGTTGGACTAGGAGCTGTCTTCATTGGGTTCTTA
GGAGCAGCCGGAAGCACTATGGGCGCGGCGTCAGTGACGCTGACGGTACAGGCCAGGCAATTATTGTCTGGCA
TAGTGCAACAGCAAAGCAATTTGCTGAGGGCTATAGAGGCTCAACAACATCTGTTGAAACTCACGGTCTGGGG
CATT
>KT022372..2
TTCTATTGTGATACAGCAGGCCTGTTTAATAGCACTTGGAGTCACAATGACAGCATACAGGGGACAAATAGCG
CAGAGTCAAATGAAACTATAACTCTCCCATGCAGAATAAAGCAAATTATAAATATGTGGCAGAGAGCAGGACA
AGCAATGTATGCCCCTCCCATCCCAGGAGTAATAAAGTGTGTATCAAACATTACAGGACTAATATTAACAAGA
GATGGTGGGAAGAACAATACAGAGGAAGTCTTCAGACCTGGAGGAGGAGATATGAGGGATAATTGGAGAAGTG
AATTATATAAGTATAAAGTAGTAAAAATTGAACCACTAGGAGTAGCACCCACCAAGGCAAGGAGAAGAGTGGT
GGGGAGAGAGAAAAGAGCAGTTGGAGTAGGAGCTGTCTTCATTGGGTTCTTAGGAGCAGCCGGAAGCACTATG
GGCGCGGCGTCAGTGACGCTGACGGTACAGGCCAGACGATTATTGTCTGGCATAGTGCAACAGCAAAGCAATT
TGCTGAAGGCTATAGAGGCTCAACAACACCTGTTGAAACTCACGGTCTGGGGCATT
>KT022373..1
TTCTATTGTAACACATCAAGCTTGTTTAGGGGCGCTTGGAATAGTACAACTGCAACACTTAATAGCACTGGGA
ATAGCAATGACACTATAACTCTTCCATGCAGAATAAAGCAAATTATAAATATGTGGCAGAGAGCAGGACAAGC
AATATATGCCCCTCCCATAAAAGGAGTAATAAGGTGTAACTCAAATATTACAGGACTAATATTAACAAGAGAT
GGTGGGGGTAGTGATAATAGAACTGAAACTTTTAGACCTGGAGGAGGAGATATGAGAGACAATTGGAGAAGTG
AATTATATAAGTATAAAGTAGTTAAAATTGAACCACTAGGAGTAGCACCCACCAGGGCAAAGAGAAGAGTGGT
GGAGAGAGAAAAAAGAGCAGTTGGAATAGGAGCTGTCTTCATTGGGTTCTTAGGAGCAGCAGGAAGCACTATG
GGCGCGGCGTCGATAACGCTGACGGTACAGGCCAGACAATTATTGTCTGGCATAGTGCAACAGCAAAGAAATT
TGCTGAGGGCTATAGAGGCTCAACAACATCTGTTGAAACTCACGGTCTGGGGCATT
>KT022374..1
TTCTATTGTAATACAACAAAGCTGTTCAATAGCTCTTGGTCTGCCAATGCCAGTGAGTCAAATAGTACAGATG
GCAATAGTACAGATGGCACTATAACTCTCCCATGCAAAATAAAGCAAATTATAAATATGTGGCAGAGAGTAGG
ACGAGCAATGTATGCCAATCCCATCCAAGGAAAAATAGAGTGTGCATCAAACATTACAGGACTAATATTAACA
AGAGATGGTGGGAATGTGAGCAGGGAAAATGAAACCTTCAGACCTGGAGGAGGAGATATGAGGGACAATTGGA

GAAGTGAATTATATAAGTATAAAGTAGTAAAGATTGAACCACTAGGAGTAGCACCCCATACGGCAAGGAGAAG
AGTGGTGGGGAGAGAAAAAAGAGCAGTTGGAATGGGAGCTGTCTTCCTTGGGTTCTTAGGAGCAGCAGGGAGC
ACTATGGGCGCAGCGTCACTAACGCTGACGGTACAGGCCAGACAATTATTGTCTGGCATAGTGCAACAGCAAA
GCAATTTGCTGAGGGCTATAGAGGCTCAACAACATCTGTTGAAACTCACAGTCTGGGGCATT
>KT022375..1
TTCTATTGTAATACATCAAAACTGTTTAATAGCACTTGGCCACAGTCAAACCACACGGAAGACAATGGAAATC
TAACTCTCCCATGCAGAATAAAACAACTTATAAACCTGTGGCAAAGAGCAGGACAAGCAATGTATGCCCCTCC
CATACCAGGAAATATAAGTTGTGTATCAAACATTACAGGACTACTATTGACAAGAGATGGTGGGAACACTGGG
AACAATACAGAGGGAAATAAAACTGAAATCTTCAGACCTGGAGGAGGAAATATGAGGGACAATTGGAGAAGTG
AATTATATAAGTATAAAGTAGTGAAAATTGAACCAATAGGAATAGCACCCACCAAGGCAAAGAGAAGAGTGGT
GGAGAGAGAAAAAAGAGCAGCACTTGCTGGAGCTTTCTTTCTTGGGTTCTTAGGAACAGCAGGAAGCACTATG
GGCGCGGCGTCAATAGCGCTGACGGTACAGGCCAGGCAATTATTGTCTGGCATAGTGCAACAGCAAAGCAATT
TGCTGAGGGCTATAGAGGCTCAACAGCAATTGTTGAGACTCACAGTCTGGGGCATT
>KT022376..1
TTCTATTGTAATACATCAGGCCTGTTTGATAGCACTTGGTCGTTTAATAGCACACAGGAGTCAAATAACACGG
GGGAAAATGATACTATAATTCTCCCATGCAGAATAAAGCAAATTATAAACATGTGGCAGAGAGTAGGACAAGC
AATGTATGCCCCTCCCATCCCAGGAGTAATACAGTGTAACTCAACCATTACAGGGCTAATATTAACAAGAGAT
GGTGGGGAGGATAATAATAACACAGAAACCTTCAGACCTGCAGGAGGAGATATGAGGGACAATTGGAGAAGTG
AATTATATAAATATAAAGTAGTAAAACTTGAACCACTAGGAGTAGCGCCCACCAAGGCAAAGAGAAGAGTGGT
GGAGAGAGAAAAAAGAGCAGTTGGACTAGGAGCTGTCTTCCTTGGGTTCTTAGGAGCAGCAGGAAGCACTATG
GGCGCGGCGTCAATAACGCTGACGGTACAGGCCAGGCAATTATTGTCTGGCATAGTGCAACAGCAAAGCAATT
TGCTGAGGGCTATAGAGGCTCAACAACATATGTTGAAACTCACAGTCTGGGGCATT
>KT022377..1
TTCTATTGTAATACATCAGGGCTGTTCAATAGTACTTGGAATACCACGAAGGTAAATAGCACAGAGTCAAATG
GCACTATAACTCTCCCATGCAGAATAAAGCAAATTATAAATATGTGGCAAAGAACAGGACAAGCAATGTATGC
CCCTCCCATCAAAGGAATAATAAATTGTGCATCAAATATTACAGGACTACTATTAACAAGAGATGGTGGGGAT
AATAACAGTACAAATGAAACCTTCAGACCTGGAGGAGGAGATATGAGGGACAATTGGAGAAGTGAATTATATA
AGTATAAAGTAGTAAAAATTGAACCACTAGGGGTAGCACCCACCGAGGCAAGGAGAAGAGTGGTGGAGAGAGA
AAAAAGAGCATTTGTTGGAATAGGAGCTGTCTTCCTTGGGTTCTTAGGAGCAGCCGGCAGCACTATGGGCGCG
GCGTCATTAACGCTGACGGTACAGGCCAGACAATTATTGTCTGGTATAGTGCAACAGCAAAACAATTTGCTGA
GGGCTATAGAGGCTCAACAACATCTGTTGAAACTCACGGTCTGGGGCATT
>FJ866116..2
TTCTACTGTGATACATCAGGACTGTTTAATAGCTCTTGGAATAATACATCGGGCCAGTTTAATAGCACTTGGA
CTGATGGCTACAATAGAAAGAGCGAGAATATAACTCTCCAATGCAGAATAAAGCAATTTATAAATATGTGGCA
GAGAACAGGGCAAGCAATGTATGCCCCTCCCATCCCAGGAATAATAAAATGTGAATCAAACATTACAGGACTA
ATACTAACAAGAGATGGTGGGCAGGCTAACAAAACAGAGGAAACCTTCAGACCTGGAGGAGGAGATATGAGGG
ACAATTGGAGAAGTGAATTATTTAGGTATAAAGTAGTAAAAATTGAACCACTAGGAGTAGCACCCACCAAGGC
AAAGAGAAGAGTGGTGGAGAGAGAGAAAAGAGCAGTAATTGGCATGGGAGCTGTTTTCCTTGGGTTCTTAGGA
GCAGCAGGAAGCACTATGGGCGCGGCGTCACTGACGCTGACGGTACAGGCCAGACAATTATTGTCTGGCATAG
TGCAACAGCAAGGCAATTTGCTGAGGGCTATAGAGGCTCAACAACATCTGTTGAGACTCACGGTCTGGGGCAT
T
>FJ866118..1
TTCTATTGTAATACAACAGGACTGTTCAATCTCACTTGGAATGGCACTGACAGCACAAATAGAAATGAGTCAG
GCAATATAACTCTCCCATGCAGAATAAAGCAAATTATAAATATGTGGCAAAGAGTAGGACGAGCAATATATGC
CCCTCCCATCCCAGGAGTAGTAAAGTGTAAATCAAATATTACAGGATTAATATTAACAAGGGATGGTGGAGAC
GACGAGAATAACACTGAAACCTTCAGACCTGGAGGAGGAAATATGAGGGACAATTGGAGAAGTGAATTATATA
AATATAAAGTAGTAAAAATTGAACCACTAGGGGTAGCACCCACCAAGGCAAGGAGGAGAACGGTGGAGAGAGA
AAAAAGAGCAGTTGGAATAGGAGCTGTCTTCCTTGGGTTCTTAGGAGCAGCAGGAAGCACTATGGGCGCGGCG
TCAATAACGCTGACGGTACAGGCCAGACAATTATTGGCTGGCATAGTGCAACAGCAAAGCAATTTGCTGAGGG
CTATAGAGGCTCAACAACAGATGTTGAGACTCACAGTCTGGGGCATT
>FJ866120..2
TTCTATTGTAATACATCACTCCTGTTTGAAAGCATTTGGTATGGCAATGAAAGCATAAATGCCACAGAAGAGG
CAAATGACACTATAATAACTCTCCGATGCAGAATAAAGCAATTTATAAAAATGTGGCAGAGAGTAGGACAAGC
AATGTATGCCCCTCCCATCCCAGGAGCAATCAGGTGTAATTCAAACATTACAGGACTAATATTAACAAGAGAT
GGAGGGCTTAATGACAATGGAGCTGATGGAAGAAATGAAACCTTCAGACCTGGAGGAGGAGATATGAGGGACA
ATTGGAGAAGTGAATTATATAAGTATAAAGTGGTAGAAATTGAACCACTAGGAGTAGCCCCCACTCAGGCAAG
AAGAAGAGTGGTGCAGAGAGAAAAAAGAGCAGTTGGAATAGGAGCTGTCTTCATTGGGTTCTTAGGAGCAGCA
GGAAGCACTATGGGCGCAGCGTCAATAGCGCTGACGGCACAGGCCAGACAATTATTGTCTGGCATAGTGCAAC
AGCAAAGCAATTTGCTGAGGGCTATAGAGGTTCAACAACAGTTGTTGAAACTCACGGTCTGGGGCATT
>FJ866121..2

TTCTACTGTAACACATCAAAACTGTTTAATAGTACTTGGCATTACAATAACAGCACTGGGTATAATAATGGCA
GCCTACAGGAGGAAAATAACACAGAGATAGTTCTCCTATGCAGAATAAAGCAAATTATAAATCTGTGGCAGAG
GGTAGGAAAGGCAATGTATGCCCCTCCCATCCAAGGAAGCATAAAGTGTAACTCAAACATTACAGGACTACTA
TTAATCAGGGATGGTGGGGTTAATACTACTAATGAAACCTTCAGACCTGGAGGAGGAGATATGAGGGACAATT
GGAGAAGTGAATTATATAAATATAAAGTAATAAAAATTGAACCACTAGGAATAGCACCCACCAGGGCAAAGAG
AAGAGTGGTGGAGAGAGAAAAAAGAGCAGTTGGACTAGGAGCTGTTTTCGTTGGGTTCTTAGGAGCAGCAGGA
AGCACTATGGGCGCAGCGTCAGTGACGCTGACGGTACAGGCCAGACAGTTATTGTCTGGTATAGTGCAACAGC
AAAGCAATTTGCTGAGGGCTATAGAGGCTCAACAACATATGTTGAAACTCACGGTCTGGGGCATT
>KT022380..1
TTCTATTGTAATACATCAGGCTTGTTTAATAGGACTTGGAAAAAGGGGATAATCAAGGGCACTGCCAATAACA
CGGAGTCAGACCCTATAGTGCTCCAATGCAGAATAAAGCAAATTATAAATATGTGGCAGAGAGTAGGACGAGC
AATGTATGCCCCTCCCATCCCAGGAATAATAAAATGTGTATCAAACATTACAGGACTAATATTAACAAGAGAT
GGTGGGAATAGTAATGGAGATAATGAAACCTTCAGACCTGGAGGAGGAGATATGAGGGACAATTGGAGAAGTG
AATTATATAAGTATAAAGTAGTAAAAGTTGAGCCATTAGGAGTAGCACCCACCAGGGCAAGGAGGAGAGTGGT
GGCGAGAGAAAAAAGAGCAGTAGTTGGACTGGGAGCTGTTTTCATTGGGTTCTTAGGAGCAGCAGGAAGCACT
ATGGGCGCGGCGTCAATAACGCTGACGGTACAGGCCAGACAATTATTGTCTGGCATAGTGCAACAGCAAAGCA
ATTTGCTGAGGGCTATAGAGGCTCAACAACATCTGTTGAAACTCACGGTCTGGGGCATT
>KT022381..4
TTCTATTGTAATACATCAGGCCTGTTTAATTACACCTATCCATCAAATCACAGTGATAAGTCAAATAACACTA
TAAATGAGACTATAACTCTCCCATGCAGAATAAAACAAATTATAAGAATGTGGCAGAGAACAGGACAAGCAAT
GTATGCCCCTCCCATCCCAGGAATAATTAAGTGTGAATCAAACATTACAGGACTAATATTAACAAGAGATGGT
TTTAATGGCACGAATGAAACCTTCAGGCCTGTAGGAGGAGATATGAGGGACAATTGGAGAAGTGAATTATATA
AGTATAAAGTAGTAAGAATTGAACCACTAGGAGTAGCACCCACCAGGGCAAAGAGAAGAGTGGTGGGAAGAGA
GAAAAGAGCAGTTGGAATGGGAGCTGTATTCCTTGGGTTCTTAGGAGCAGCAGGAAGCACTATGGGCGCGGCG
TCAATAACGCTGACGGTACAGGCCAGACAATTATTGTCTGGCATAGTGCAACAGCAAAGCAATTTGCTGAAGG
CAATAGAGGCTCAACAACATCTGCTGAGACTCACGGTCTGGGGCATC
>KT022382..2
TTCTATTGTAATACATCAGGCCTGTTCAATAGTAGCTGGGGTCCAAATGACACGAAATCAGATAACTCAACTA
CAAATTACACTCTCCCATGCAGAATAAAGCAAATTGTAAGGATGTGGCAGAAAATAGGACAAGCAATGTATGC
CCCTCCAATCGAGGGAGAAATAAATTGTGTATCAAACATTACAGGACTATTATTAACAAGAGATGGTGGGCAT
AACAGTATTAACAATGAAACTGAAACATTCAGACCTGGAGGAGGAGATATGAAGGACAATTGGAGAAGTGAAT
TATATAAGTATAAAGTAGTAAAAATTGAACCACTAGGAGTAGCACCCACCAGGGCAAAGAGAAGAGTGGTGGG
GAGAGAAAAAAGAGCAGTTGGAATAGGAGCTGTTTTCCTTGGGTTCTTGGGAGCAGCAGGAAGCACTATGGGC
GCGGCGTCAATAACGCTGACGGTACAGGCCAGACAATTATTGTCTGGCATAGTGCAGCAGCAAAACAATTTGC
TGAGGGCTATAGAGGCTCAACAACATCTGTTGAAACTCACGGTCTGGGGCATT
>KT022383..1
TTCTATTGTAATACATCAGGCCTGTTTAATAGCACTTGGAACAGCCCTTGGAATAACACTGGGGAACAAGAAG
GCAATATAACTCTCCCATGCAGAATAAAGCAAATTATAAATATGTGGCAGAGAGTAGGACAAGCAATATATGC
CCCTCCCATCCCAGGAGTAATAAAGTGTGAATCGAACATTACAGGGCTACTATTAACAAGAGATGGTGGGAAC
ACTAACAGTAGTACTGAAACCTTCAGACCTGGAGGAGGAGATATGAGGGACAATTGGAGAAGTGAATTATATA
AATATAAAGTAGTAAAAGTTGAACCACTAGGAGTAGCACCCACCAGGGCAAGGAGAAGAGTGGTGGGGAGAGA
AAAAAGAGCAGTTGGAATAGGAGCTGTCTTCCTTGGGTTCTTAGGAGCAGCAGGAAGCACTATGGGCGCGGCG
TCAATAACGCTGACGGTACAGGCCAGACAATTATTGTCTGGCATAGTGCAACAGCAAAGCAATTTGCTGAGGG
CTATAGAGGCTCAACAACATATGTTGAAACTCACGGTCTGGGGCATT
>FJ623487..1
TTCTATTGTAATACATCAAGACTGTTTAATAGCACTTGGAACAATGGCACTTGGAACAATACCAACATGCAGG
AGTCAAATAGCACGAAGTCAGAAAATGGCACTATAACTCTCCCATGCAGAATAAAGCAAATTATAAATATGTG
GCAGAGAACAGGACAAGCAATGTATGCCCCTCCCATCCGAGGAGTAATAAGATGTGAATCAAACATTACAGGA
CTAATATTAACAAGAGATAGTGTGAATAATAGCAAAGACAATGAAACCTTCAGACCAGGAGGAGGAGATATGA
GGGACAATTGGAGAAGTGAATTATATAAGTATAAAGTAGTAAAAGTTGAACCACTAGGAGTAGCACCCACCAC
GGCAAGGAGAAGAGTGGTGGGAAGAGAAAAAAGAGCAGTGGGAATAGGAGCTGTATTCCTTGGGTTCTTGGGA
GCAGCAGGAAGCACGATGGGCGCGGCGTCAATGACGCTGACGGTACAGGCCAGACAATTATTGTCTGGTATAG
TGCAACAGCAAAGCAATTTGCTGAGGGCTATAGAGGCTCAACAACACCTGTTGAAACTCACGGTCTGGGGCAT
T
>FJ623481..2
TTTTATTGTAATACATCAGGCCTGTTTAATAGCACTTGGAATCACACTGACATAAAGGGGTCAAATAGCACGG
AGTCAAGTGAAACTATAACTCTCACATGCAGAATAAAGCAAATTATAAATATGTGGCAGAGAGTAGGGCAAGC
AATGTATGCCCCTCCCATACCAGGAGCAATAAGGTGTGAATCAAACATTACAGGAATACTCTTAACAAGAGAT
GGTGGGAATAGCAAGAACAGCACAACAGAAATCTTCAGACCTGGAGGAGGAAACATGCGGGACAATTGGAGAA
GTGAATTATATAAGTATAAAGTAGTACAAATTGAACCACTAGGAGTAGCACCTACCAGGGCAAAGAGAAGAGT

GGTGGAGAGAGAGAAAAAAGAGCAATTGGAATAGGAGCTGTATTCATTGGGTTCTTAGGAGCAGCAGGAAGCACT
ATGGGCGCGGCGTCAATAACGCTGACGGTACAGGCCAGACAATTATTGACTGGCATAGTGCGACAGCAAAGCA
ATTTGCTGAGGGCTATAGAGGCTCAACAACATCTGTTGAAACTCACGGTCTGGGGCATT
>FJ623475..1
TTTTACTGTGATACATCAGGCCTGTTTAATAGCACTTGGGGAGCCACTGACAGCATGCATGGGGCAAATGACA
CGGAGTCAAATGACACTATAACTCTCCCATGTAGGATAAAACAAATTATAAATAGGTGGCAGAGAGTAGGACA
AGCAATGTATGCCCCTCCCATCCAAGGAGTAATAAAGTGCAAATCAGACATTACAGGAATAATATTAACAAGA
GATGGTGGGAATAATAATGACAGTAGAAATGAAACCTTCAGACCTGGAGGAGGAGATATGAGGGACAATTGGA
GAAGTGAATTGTATAAGTATAAGGTGGTAAAGATTGAACCACTAGGAGTAGCACCCACCAGGGCAAAGAGAAG
AGTGGTGGAGAGAGGAAAAAGAGCAGTTGGGATAGGAGCTGTTTTCCTTGGATTCTTAGGAGCAGCCGGAAGC
ACTATGGGCGCGGCGTCAATAACGCTGACGGTACAGGCCAGGCAATTATTGTCTGGCATAGTGCAACAGCAAA
GCAATTTGCTGAGGGCTATAGAGGCTCAACAACATATGTTGAAACTCACGGTCTGGGGCATA
>FJ623476..2
TTCTATTGTAATACATCAGGCTTGTTTAATAGCACTTGGAATATCACTGACAATCTCAACATAACTCAGTCAA
ATGAGAACATAACTCTCCCATGCAAAATAAAGCAAATTATAAATATGTGGCAGAGAGTAGGACAAGCAATATA
TGCCCCTCCCATCCCAGGAAGGGTAAGTTGTGTCTCAAACATTACAGGACTAATATTAACAAGAGATGGTGGG
AACAATAACAGTACAAATGAAACCTTCAGGCCTGGAGGAGGAGATATGAGGGACAATTGGAGAAGTGAATTAT
ATAAGTATAAAGTAGTAAGAATTGAACCACTAGGAGTAGCACCTACCCAGGCAAAGAGAAGAGTGGTGGGAAG
AGAAAAAAGAGCAATTGGAATAGGAGCTGTCTTCCTTGGGTTCTTAGGAGCAGCAGGAAGCACTATGGGCGCG
GCGTCAATAACGCTGACGGTACAGGCCAGACAGTTATTGTCTGGCATAGTGCAACAGCAAAACAATTTGCTGA
GGGCTATAGAGGCTCAACAACAACTGTTGAAACTCACGGTCTGGGGCATT
>FJ623485..3
TTTTATTGTAATACATCAGGCCTGTTTAATAGCACTTGGTATCACAATAGCACTTGGAATGATCAAACTGACG
AGAGGTCAAATGACACTATAACTCTCCAATGCAGAATAAAGCAAATTATAAATATGTGGCAGAGAGTAGGACA
AGCAATATATGCCCCTCCTATCCAAGGAACAATAAGGTGTGAATCAAACATTACAGGACTAATATTAACAAGA
GATGGTGGGGAGGATAGCAATACAACAACTGAAACCTTCAGACCTAGCGGAGGAGATATGAAGGACAATTGGA
GAAGTGAATTATATAAATATAAAGTAGTAAAAATTGAACCACTAGGAGTAGCACCCTCCAGGGCAAGGAGAAG
AGTGGTGGGAAGAGAAAAAAGAGCAGTTGGAATAGGAGCTGTCTTCCTTGGGTTCTTAGGAGCAGCAGGAAGC
ACTATGGGCGCAGCGTCAATAACGCTGACGGTACAGGCCAGACAATTACTGTCTGGCATAGTGCAACAGCAAA
GCAATTTGCTGAGGGCTATAGAGGCTCAACAACATATGTTGAGACTCACGGTCTGGGGCATT
>FJ623488..1
TTCTATTGTAACACATCAGGTCTGTTTAATAGCACTTGGGATAACAATACTGGCAATTGGTCTAGCAATAGCA
GCAGCATGCAGGGGTCAAATAGCACAGATGATACTATAACTCTCCCATGCAGAATAAAGCAAATTATAAATAT
GTGGCAGAGAACAGGACAAGCAATGTATGCCCCCCCCATCCAAGGGATAATAAGGTGTGAATCAAACATCACA
GGGATAATATTAACAAGAGATGGTGGGGGTACTGAGGATAATACTACAGAAGTCTTCAGACCTGGAGGAGGAG
ATATGAGGGACAATTGGAGAAGTGAATTATATAAATATAAAGTAGTACAAGTTGAACCAGTAGGAATAGCACC
CTCCAAGGCAAGGAGAAGAGTGGTGCAGAGAGAAAAAAGAGCAATTGGAATGGGAGCTGTCTTCCTTGGGTTC
TTAGGAGCAGCAGGAAGCACTATGGGCGCGGCGTCAATGACGCTGACGGTACAGGCCAGAAATTTATTGTCTG
GCATAGTGCAACAGCAGAGTAATTTGCTGAGGGCTGTAGAGGCTCAACAACAACTGTTGAGACTCACGGTCTG
GGGCATT
>FJ623478..4
TTCTATTGTAATACATCAGGCCTGTTTAATCACACTTACCTGCCTAAGAGCACTTACCTGCCTAATAGCACTT
GGAATAACAGTACGGGATCAAATGACAGTATAACTCTTCCATGTAGAATAAAGCAAATTATAAATATGTGGCA
GAGAGTAGGACAAGCAATGTATGCCCCTCCTATCCAAGGAGTAATAAAGTGTAAATCAGATATTACAGGACTA
ATATTGACAAGAGATGGTGGGAATAGTAACAGTGATAATGAAACCTTCAGACCTGGAGGAGGAGATATGAGGG
ACAATTGGAGAAGTGAATTATATAAGTATAAAGTAGTAAAAATTGAACCACTAGGAGTAGCACCCACCAGGGC
AAAGAGAAGAGTGGTGGAGAGAGAAAAAAGAGCAGTTGGAATAGGAGCTGTTTTCATTGGGTTCTTAGGAGCA
GCAGGAAGCACTATGGGCGCGGCGTCAATAACGCTGACGGTACAGGCTAGACAATTATTGTCTGGCATAGTGC
AACAGCAAAGCAATTTGCTGAGGGCTATAGAGGCTCAACAACATATGTTGAAACTCACGGTCTGGGGCATT
>FJ623482..1
TTCTATTGTAATACATCAGACCTGTTTAATGGCAATAGCACTAGGAATGGCACTGGGAATAGCAACAGCACGC
CGGTAACTGGCAATATAACTCTCCCATGCAGAATAAAGCAAATTATAAATATGTGGCAGAGAGTAGGACAAGC
AATGTATGCCCCGCCCATCCAAGGGGTAATAAGGTGTGTATCAAACATTACAGGATTAATATTGACAAGAGAT
GGTGGGAAACAGAATAATAATAGTAATAGTAATAGTAGTGATATTGAAGTCTTCAGACCTGGAGGAGGAGATA
TGAGGGACAATTGGAGAAGTGAATTATATAAGTATAAAGTAGTAAAAATTGAACCACTAGGAATAGCACCTAC
CAAGGCAAAAAGAAGAGTGGTGGAGAGAGAAAAAAGAGCAATTGGAATAGGAGCTGTCCTCCTTGGGTTCTTA
GGAACAGCAGGAAGCACTATGGGCGCGGCGTCAATAGCGCTGACGGTACAGGCCAGACAATTAATGTCTGGCA
TAGTGCAACAGCAAAGCAATTTGCTGAGGGCTATAGAGGCTCAACAGCATATGTTGAAACTCACAGTCTGGGG
CATT
>FJ623477..1

TTCTATTGTGATACATCAGGCCTGTTTAATAGCACCTGGAATAATAGCACCTGGAATAATAATGACACTGCCC
AGTCAAATGACACAATAACTCTCCCATGCAGAATAAAACAAATTGTAAATATGTGGCAGAGAACAGAACAAGC
AATGTATGCCCCTCCCATCCAAGGAGTAATAAGGTGTGAATCAAACATCACAGGACTACTATTAACAAGAGAT
GGTGGGATTAATAACACAAATGAAACCTTCAGACCTGGAGGAGGAGATATGAGGGACAATTGGAGAAGTGAAC
TATATAAGTATAAAGTAGTAAAAATTGAACCACTGGGAGTAGCACCCTCCAGGGCAAGGAGAAGAGTGGTGGG
GAGAGAAAAAAGAGCAGTTGGAATAGGAGCTTTATTCCTTGGATTCTTGGGCGCAGCAGGAAGCACTATGGGC
GCGGCGTCAATGACGCTGACGGTACAGGCCAGACAATTATTGTCTGGCATAGTGCAACAGCAAAGCAATTTGC
TGAAGGCTATAGAGGCTCAACAACATCTGTTGAGACTCACGGTCTGGGGCATT
>FJ623486..3
TTCTATTGTAATACATCAGAACTATTTAATAGCACTTGGAATCTTAATAGCACTTGGAATCACACTGGCAGCA
TACCGGAGTCAAATAGCACGGAGTCAAATGGCACTATAACCCTCAAATGCAGAATAAAGCAAATTATAAATAT
GTGGCAGAGAGTAGGGCAAGCAATGTATGCCCCTCCCATCAAAGGGACAATAAGGTGTAAATCAAACATCACA
GGACTAATATTAACAAGAGATGGTGGGATAATAACAGTACAAAGGAAACCTTCAGGCCTGGAGGAGGAGATA
TGAGGGACAATTGGAGAAGTGAATTATATCAGTATAAAGTAGTAAAACTTGAACCACTAGGAGTAGCACCCAC
CAGGGCAAGGAGAAGAGTGGTGGGGAGAAAAAAAAGAGCAGTTGGACTAGGAGCTGTTTTCATTGGGTTCTTG
GGAGCAGCAGGAAGCACTATGGGCGCGGCGTCAATGACGCTGACGGTACAAGCTAGACAATTATTGTCTGGCA
TAGTGCAACAGCAAAGCAATTTGCTGAGGGCTATAGAGGCTCAACAACATCTGTTGAAACTCACGGTCTGGGG
CATT
>FJ623479..1
TTCTATTGTAATACATCAGACCTGTTTAATGGTACTTGGAATAATAGCACTTTGAATGGCACTGCCAAGCAGG
AGGTAAATGACAGCACTATAACGATCTCATGCAGGATAAAGCAATTTATAAATATGTGGCAGAGAACAGGACG
AGCAATGTATGCCCCTCCCATTCCAGGAGTAATAAGTTGTAACTCAAGCATTACAGGATTAATATTAACAAGA
GATGGTGGGGATGATAACAATAATAATGAAACATTCAGACCTGGAGGAGGAGATATGAGGGACAATTGGAGAA
GTGAATTATATAAGTATAAAGTAGTAAAAATTGAACCACTAGGAGTAGCACCCACCAAGGCAAGGAGAAGAGT
GGTGGAGAGAGAAAAAAGAGCAGTTGGAATAGGAGCTGTACTCCTTGGGTTCTTAGGAGCAGCAGGAAGCACT
ATGGGCGCGGCGTCAATAACGCTGACGGTACAGGCCAGACAATTATTGTCTGGCATAGTGCAACAGCAAAGCA
ATTTGCTGAAGGCTATAGAGGCTCAACAACAACTGTTGAAACTCACAGTCTGGGGCATT
>HM215275..1
TTCTATTGTAATACATCAGGTCTGTTCAATAGCACTTGGGAGGCCAATAGTACTGCCAATACCAGCACGACAA
ATATAACTCTCCCATGCAAAATAAAGCAACTTGTAAGAATGTGGCAGAGGACAGGACAAGCATTGTATGCCCC
TCCCATCGAAGGAGTAATAAAATGTGTATCAAACATTACAGGACTAATATTAACAAGAGATGGTGGGGGTAAT
AACAGTGCAACAAATGAAACCTTCAGACCTGGAGGAGGAGATATGAGGGACAATTGGAGAAGTGAATTATATA
AGTATAAAGTAGTACAAATTGAACCACTAGGAGTTGCACCCACCGAGGCAAGGAGAAGAGTGGTGAAGAGAGA
AAAAAGAGCAGTTCCTGGACTGTTTTTCCTTGGGTTCTTAGCAGCAGCAGGAAGCACTATGGGCGCGGCGTCA
ATAACGCTGACGGTACAGGCCAGACAATTATTGTCTGGCATAGTGCAACAGCAAAGCAATTTGCTGAGGGCTA
TAGAGGCTCAACAGCATCTGTTGAAACTCACGGTCTGGGGCATT
>KT252544..1
TTCTATTGTAACACATCAGGCCTGTTTAATAGTACTTGGGATGGGAATAGCACTGAGGCAAACATTACGCAGT
CAAATGAGACTATAACTCTCCCATGCAAAATAAAGCAAATTGTAAATATGTGGCAGAGAGTGGGACGAGCAAT
GTATGCCCCTCCTATCCCAGGAGTAATAAAGTGTGAATCAAACATTACAGGGCTAATACTGACAAGAGATGGT
GGGAATGAAACAAATGGAACAGAAATCTTCAGACCTGTAGGAGGAGACATGAGGGACAATTGGAGAAGTGAAT
TATATAAGTATAAAGTAGTACAAATTAAACCACTAGGAGTAGCACCCACCAAGGCAAAGAGAAGAGTGGTGCA
GAGAGAAAAAAGAGCAGTTGGAATGGGAGCTGTTTTCTTTGGGTTCTTAGGAGCAGCTGGAAGCACTATGGGC
GCGGCGTCAATAACGCTGACGGTACAGGCCAGACAATTATTGACTGGCATAGTGCAACAGCAAAGCAATTTGC
TGAGGGCTATAGAGGCTCAACAGCATCTGTTGAAACTCACTGTCTGGGGCATT
>HQ540689..1
TTCTACTGTAATACAACAAACCTGTTTAATAGTAGCATTCCCTTCAACGCCAGCGAGAGGGCAAATAACACCA
ATAGCACAGATGACATTATAACTCTCCAATGCAGAATAAAGCAAATTGTAAGGATGTGGCAAAGAGTAGGACA
AGCAATGTATGCCCCTCCCATCCCAGGAGTAATAAGGTGTGAATCAAACATTACAGGACTTATGTTAACATGG
GATGGTGGGAGTAAAAATATTACTGAGGGCAATAGAACTGAAACCTTTAGACCTGGAGGAGGAGACATGAGGG
ACAATTGGAGAAGTGAATTATATAAATATAAAGTAGTAAAAATTGAACCACTAGGAGTTGCACCCACCAAGGC
AAGGAGAAGAGTGGTGGGAAGAGAAAAAAGAGCAGTTGGAATAGGAGCTGTTTTCCTTGGGTTCTTAGGAGCA
GCAGGAAGCACTATGGGCGCGGCGTCAGTGACGCTGACGGTACAGGCCAGACAATTATTGTCTGGTATAGTGC
AACAGCAAAGCAATTTGCTGAGAGCTATAGAGGCTCAACAACAGTTGTTGAAACTCACGGTCTGGGGCATT
>KU921708..2
TTCTATTGTAATACAGTAAATCTGTTCAATAGCTCTTGGGGTGCCAACGCCACTGAGTCAAATAGCACAGGGT
TAAATAATCAAACTATACATCTCCCATGCAGAATAAAGCAAATTATAAATATGTGGCAGAGAACAGGACAAGC
AATGTATGCCCCTCCCGTCGAAGGAATAATAAGTTGTGTATCAGACATTACAGGACTACTGTTAACAAGAGAC
GGAGGAGAAAATACAACACATGAAATTTTCAGACCTGGAGGAGGAGATATGAGGGACAATTGGAGAAGTGAAT
TATATAAGTATAAAGTAGTAAAAATTGAACCCCTAGGAGTAGCACCCACCAAGGCAAGGAGAAGAGTGGTGCA

GAGAGAAAAAAGAGCAGTTGGAATGGGAGCTGTCTTCCTTGGGTTCTTAGGAGCAGCAGGAAGCACTATGGGC
GCAGCGTCAATAACGCTGACGGTACAGGCCAGACAATTATTGTCTGGTATAGTGCAACAGCAAAGCAATTTGC
TGAGGGCTATAGAGGCTCAACAGCATCTGTTGAAACTCACTGTCTGGGGCATT
>KU921712..2
TTCTATTGTAATACATCAAAGCTGTCTAACAGCACATTTCCACTTAATAACACTCAGAATAACAGCACGCAGA
CAAACAGTACTATAACTCTCCCATGCAGAATAAAGCAAATTATAAATATGTGGCAGAGAACAGGAAAAGCAAT
ATATGCCCCTCCCATCTCAGGACCAATAAAGTGTACATCAAACATTACAGGACTAATATTAACAAGAGATGGT
GGAAATGATAATAATAATGATACCAATGGAAATGAAACCTTCAGACCTGGAGGAGGAGATATGAGGGACAATT
GGAGAAGTGAATTATATAAGTATAAAGTAGTAAAACTTGAACCACTAGGAGTAGCACCCACCAGGGCAAGAAG
AAGAGTGGTGCAGAGAGAGAAAAGAGCAATTGGACTGGGAGCTGTATTCATTGGGTTCTTAGGAGCAGCAGGG
AGCACTATGGGCGCGGCGTCAGTGACGCTGACGGTACAGGCCAGAGCCTTATTGTCTGGCATAGTGCAACAGC
AAAGCAATTTGCTGAGGGCTATAGAAGCTCAACAACATCTGTTGAAGCTCACGGTCTGGGGCATT
>KU921727..2
TTCTATTGTGACACATCAAGCCTGTTTAATAGCACTTGGAATGAGAATGACAATGTCAGCAGAACAGAGTCAA
ATAGTACAGAAGATATAACTCTCCAATGCAGAATAAAACAAATTGTAAATATGTGGCAGAGAGTAGGACAAGC
GATGTATGCCCCTCCCATCCAAGGAATAATAAAGTGTGAATCAAACATAACAGGACTAATATTAACAAGAGAT
GGTGGGAATCATAATAGTACAAATGAAACCTCCAGAAATGAAACCTTCAGACCTGGAGGAGGAGATATGAGGG
ACAATTGGAGAAGTGAATTATATAAGTATAAGGTAGTAAAAATTGAACCTCTAGGAGTAGCACCCACCAAGGC
AAAGAGAAGAGTGGTGGAGAGAGAAAAAAGAGCAGTTGGAATAGGAGCTGTCTTCCTTGGATTCTTGGGAGCA
GCAGGAAGCACTATGGGCGCGGCGTCAATAACGCTGACGGCACAGGCCAGACAATTATTGTCTGGTATAGTGC
AACAGCAAAGCAATTTGCTGAGGGCTATAGAGGCTCAACAGCATCTGTTGAGACTCACAGTCTGGGGCATT
>KU921732..1
TTCTATTGTAATACATCAGGCCTTTTTGATGACACTTGGAATGGCACGAAGTCAAATGACACTATAACACTCC
CATGCAGAATAAAGCAAGTTATAAATATGTGGCAGAGGACAGGACAAGCAATGTATGCCCCTCCCATCCAAGG
AGTAATAAGGTGCGAATCAAACATCACAGGACTAATATTAACAAGAGATGGTGGGCATAACATTACAAATGAA
ACCTTCAGACCTACAGGAGGAAATATGAGGGACAATTGGAGAAGTGAATTATATAAGTATAAAGTAGTAAGAA
TTGAACCACTAGGAGTAGCACCCACCTCGGCAAAGAGAAGAGTGGTGGAGAGAGAAAAAAGAGCAGTAGTTGG
AATGGGAGCTGTCTTCATTGGGTTCTTGGGAGCAGCAGGAAGCACTATGGGCGCGGCGTCAATGACGCTGACG
GTACAGGCCAGACAATTATTGTCTGGCATAGTGCAACAGCAAAGCAATTTGCTGAGGGCTATAGAGGCTCAAC
AGCATTTGTTGAAGCTCACGGTCTGGGGCATT
>KU921734..1
TTCTATTGTAACACATCAGGTCTGTTTAATAGCACGTGGAATAAGGATAATAACACCTTGAATGGCGCTGTAA
ATAACATAACAGACAATATAACCCTCCAATGCAGAATAAAACAAATTATAAATATGTGGCAGAGAGCAGGACA
AGCAATATATGCTCCTCCCATCCCAGGAGTAATAAGGTGCACCTCAAATATTACAGGATTAATATTAACAAGA
GATGGTGGGAAAGATAGCAATGAAAGTGCTGAAACCTTCAGACCTGGAGGAGGAGATATGGGGGACAACTGGA
GAAGTGAATTATATAAGTATAAGGTAGTAAAAATTGAACCACTAGGAATAGCACCCAACAGGGCAAAGAGAAG
AGTGGTGGAGAGAGAAAAAAGAGCAATCTTGGGACTGGGAGCTGTTTTCGTTGGGTTCTTAGGAGCAGCAGGA
AGCACTATGGGCGCGGCGTCACTGACGCTGACGGTACAGGCCAGAAACTTATTGTCTGGCATAGTGCAACAGC
AAAACAATTTGCTGAAGGCTATAGAGGCTCAACAGCATCTGTTGAAACTCACGGTCTGGGGCATT
>KU921738..2
TTCTATTGTAACACATCAGAGCTGTTTAATAGCACTTGGAATAGGACTGACCCTGGCACTTGGAATAACACCA
GCACGCAGGAGTCAAATAGCACAGATCAAAAAATAACTCTCCAATGCAGCATAAAGCAAATTATAAATATGTG
GCAAAGGGTAGGACGAGCAATGTATGCCCCTCCCATCCCAGGACCAATACAGTGTAGATCAAACATTACAGGA
CTAATATTAGTGAGAGATGGTGGGAATACGAATACTAGCAAGGAAACCTTTAGACCTGCAGGAGGGGATATGA
GGGACAATTGGAGAAGTGAATTATATAAATATAAAGTAGTAAAAATTGAACCACTAGGAGTAGCACCCACCAA
GGCAAAGAGAAGAGTGGTGGAGAGAGGAAAAAGAGCAGTTGGACTAGGAGCTGTCTTCATTGGGTTCTTAGGA
GCAGCAGGAAGCACTATGGGCGCAGCGTCAATAACGCTGACGGTACAGGTCAGACAATTATTGTCTGGCATAG
TGCGACAGCAAAGCAATTTGCTGAGGGCTATAGAAGCTCAACAACATCTGTTGAAACTCACGGTCTGGGGCAT
T
>KU921746..1
TTCTATTGTAACACATCAGGTCTGTTCAACAGCACTTGGGATGATACCGCCATACAGGAAAATATCACGGAGA
TCACGGAGTCAAATGACACAATAACTCTCCAATGCAGAATAAGGCAATTTATAAGGATGTGGCAGAGGACAGG
ACAGGCAATGTATGCCCCTCCCATACCAGGAGTAATAAGGTGTGAATCAAACATTACAGGACTAATATTAACA
AGAGATGGTGGGCATAGTAATAGTACTAGTAATGAAACCTTCAGACCTGGAGGAGGAGATATGAGGGACAATT
GGAGAAGTGAATTATATAAGTATAAAGTAGTAAAAATTGAACCACTAGGAGTAGCACCCTCCAGGGCAAGGAG
AAGAGTGGTGGGAAGAGAAAAAAGAGCAGTTACAATAGGAGCCGTCTTCCTTGGGTTCTTAGGAGCAGCCGGA
AGCACTATGGGCGCGGCGTCAATAACGCTGACGGTACAGGCCAGGCAATTATTGTCTGGTATAGTGCAACAGC
AAAGCAATTTGCTGAAGGCTATAGAGGCTCAACAACATCTGTTGAGACTCACGGTCTGGGGCATT
>KU921752..1

TTCTATTGTAACACATCAGGCCTGTTTAATAGCACTTGGAATGACACCAGCAATGGGAATGACACCAACATAT
CAAATAGCACGGAGAAGAATGAGAATATAACTCTCCAATGCAGAATAAAGCAAATTATAAATATGTGGCAAAG
AGTAGGAAAAGCAATGTATGCTCCTCCCATCCCAGGAATAATAAAATGTGAATCAAACATTACAGGACTAATA
TTAACAAGAGATGGGGGAAAGGAGGAAAACGAAAGTGAAACCTTCAGACCTGGAGGAGGAGATATGAGGGACA
ATTGGAGAAGTGAATTATATAAGTATAAAGTAGTAAAAATTGAACCACTAGGAGTAGCACCCACCAGGGCAAG
GAGAAGAGTGGTGGAGAGAGAAAAAAGAGCAGTTGGAATGGGAGCTTTGTTCATTGGGTTCTTAGGAGCAGCT
GGAAGCACTATGGGCGCGGCGTCGATGACGCTGACGGTACAGGCTAGACAACTATTGTCTGGCATAGTGCAAC
AGCAAAGCAATTTGCTGAGGGCTATAGAGGCTCAACAGCATCTGTTGAAACTCACAGTCTGGGGCATT
>KU921756..1
TTCTATTGTAACACATCAAACCTGTTTAATAGTACTTGGCTTAACAATGGCACATTAGGTAGTGACAATGGCA
TATCAGATAGTAAAAACATAACTCTCCCATGCAAAATAAGGCAAATTATCAGAATGTGGCAGAGAGTAGGACA
AGCAATATATGCCCCTCCCATCCAAGGAGTAATAAGGTGTAAATCAGACATTACAGGACTAATATTAACAAGA
GATGGTGGAGTTAATAACAATATAACTGAAACCTTCAGACCTGGAGGAGGAGATATGAGGGACAATTGGAGAA
GTGAGTTATATAAATATAAAGTAGTAAAAATTGAACCACTAGGAGTAGCACCCACCAGGGCAAGGAGAAGAGT
GGTGGGAGAGAAAAAAGAGCAGTTGGAGTAGGAGCTGTATTCCTTGGGTTCTTAGGAGCAGCAGGAAGCACT
ATGGGCGCGGCGTCAATAACGCTGACGGTACAGGCCAGACAATTATTGTCTGGCATAGTGCAACAGCAAAGCA
ATTTGCTAAGGGCTATAGAGGCTCAACAGCATCTGTTGAAACTCACAGTCTGGGGCATT
>KU921759..1
TTCTATTGCAGTACAAAAGGCCTGTTTAATAGCACTTGGAATGCTAATGGCACCAGGAATGACTTAGGGAGCT
CGGAATCAGATGGCACTATAACTCTCCAGTGCAGAATAAAACAAATTATAAATATGTGGCAGAGAACAGGACA
AGCAATGTATGCCCCTCCCATCGCAGGAGTAATAAGTTGTAATTCAACCATTACAGGACTACTATTAACAAGA
GATGGTGGGAACAATAGTACAAGTGAAACTGAAACCTTTAGACCTGGAGGAGGAGATATGAGGGACAATTGGA
GAAGTGAATTATATAAGTATAAAGTAGTAAAAATTGAACCATTAGGAGTAGCACCCACCAGGGCGCGGAGAAG
AGCGGTGGGGAGAGAAAAAAGAGCAGTTGGACTGGGAGCTGTTTTTCTTGGGTTCTTAGGAGCAGCAGGAAGC
ACTATGGGCGCGGCGGCAGTGACGCTGACGGTACAGGCCAGACAATTATTGTCTGGCATAGTGCAACAGCAAA
GCAATTTGCTGAGAGCTATAGAGGCTCAACAACATCTGTTGAAACTCACTGTCTGGGGCATT
>KU921772..1
TTCTATTGTAACACATCAGGCTTGTTTAATAGCAGTTGGAATAGCACCAGCAAGGAGCTAAATAGCACCAACA
GGGAGCTAAATAACATGACTGTAACTCTCCAATGTAGAATAAAACAAATTATAAATATGTGGCAGAGAGTGGG
ACAAGCAATGTATGCCCCTCCCATCCCAGGAGTAATAAAGTGTGTATCAAACATTACAGGATTAATATTAACA
AGGGATGGTGGGCTTAATCCTACAGAGAACGAAACCGAAACCTTCAGACCTGGAGGAGGAGATATGAGAGACA
ATTGGAGAAGTGAATTATATATGTATAAAGTAGTGAAAATTGAACCACTAGGAGTAGCACCCACCAGGGCAAA
GAGAAGAGTGGTGGAGAGAGAAAAAAGAGCAGTTGGAATAGGAGCTGTTTTCCTTGGGTTCTTGGGAGCAGCA
GGAAGCACTATGGGCGCGGCGTCAATAACGCTGACGGTACAAGCCAGACAATTATTGTCTGGCATAGTGCAAC
AGCAAAGCAATTTGCTGAGGGCTATAGAGGCTCAACAGCATCTGTTGAAACTCACAGTCTGGGGCATT
>KU921780..1
TTCTATTGTAATACATCAGGCTTATTTAATGGCACTTGGACCAATGCTAGCATACAAGAAAATAGCACGGAGT
CAAATATAACTCTCCAATGCAGAATAAAACAAATTATAAATATGTGGCAGACAGTAGGACGAGCAATGTATGC
TCCTCCCATTGAAGGAGCAATAAGGTGTGTATCAAACATTACAGGACTAATATTAACCAGAGATGGTGGGGAT
AACACAACTACAACTGAAATCTTCAGACCTGGAGGAGGAAATATGAAGGACAATTGGAGAAGTGAATTATATA
AGTATAAAGTAGTAAAAATTGAACCATTAGGAGTAGCACCCACCAAGGCAAAGAGAAGAGTGGTGGAGAGAGA
AAAAAGAGCAGTTGGACTGGGAGCTGTCTTCCTTGGGTTCTTAGGAGCAGCAGGAAGCACTATGGGCGCGGCG
TCAGTAACGCTGACGGTACAGGCCAGACAATTATTGTCTGGCATAGTGCAACAGCAAAACAATTTGCTGAGGG
CTATCGAGGCTCAACAACATCTGTTGAAACTCACGGTCTGGGGCATT
>KU921783..1
TTCTATTGTAATACAACAGGCCTGTTTAATAGCACTTGGGGGAATGGCACTTGGAATGCCAGCAGCCAGGGGG
AGTCAAATGACACTATACTCCTCCAATGCAAAATAAAGCAAATTATAAATATGTGGCAGAGAGTAGGACAAGC
AATGTATGCTCCTCCCATCCCAGGAGTAATAAGGTGTGAATCAAACATTACAGGACTAATATTAACAAGAGAT
GGTGGGGGGGATGACAATGGAACCGAAACCTTCAGACCTGGAGGAGGAGATATGAGGGATAATTGGAGAAGTG
AATTATATAAGTATAAAGTAGTAAAAATTGAACCACTAGGAGTAGCACCCACCAGGGCAAGGAGAAGAGTGGT
GGAGAGAGAGAAAAGAGCAGTTACAATAGGAGCTGTTTTTCTTGGGTTCTTAGGAACAGCAGGAAGCACTATG
GGCGCGGCGTCAATAGCGCTGACGGCACAGGCTAGACAATTATTGTCTGGCATAGTGCAACAGCAAAGCAATT
TGCTGAGGGCTATAGAGGCTCAACAACATATGTTGAGACTCACGGTCTGGGGCATT
>KU921785..1
TTCTATTGTAACACATCAGACCTGTTTAATAGCACTTGGTATGGCAATAACACCATGCAGGGATTAAATGGCA
CAAATGACACTATAATTCTCAAATGCAGAATAAAGCAAATTATAAATATGTGGCAGAGAACAGGACAAGCAAT
ATATGCCCCTCCCATCCCAGGAGTAATAGAGTGTGTATCAAACATAACAGGACTAATATTGACAAGAGATGGT
GGGGGTAGTGAGAGCAGGACAGAGGAAACATTTAGACCTGGAGGAGGAGATACGAGGGACAATTGGAGAAGTG
AATTATATAAGTATAAAGTAGTAAAAATTGAACCACTAGGAGTAGCACCCACCAGGGCAAAAAGAAGAGTGGT
GGAGAGAGAAAAAAGAGCAATTGGAATAGGAGCTGTCTTCATTGGGTTCTTAGGAGCAGCAGGAAGCACTATG

GGCGCGGCGTCAATAACGCTGACGGTACAGGCCAGACAATTATTGTCTGGTATAGTGCAACAGCAAAGCAATT
TGCTGAGGGCTATAGAGGCTCAACAGCATCTGTTGAAACTCACAGTCTGGGGCATT
>KU921788..1
TTCTATTGTGATACATCAAGCCTGTTTAACAGCACTTGGGAACCAAACAATGTTAGTAGCAATTTAAATGGCA
CTATAACTCTCCAATGCAGAATAAAGCAAGTCATAAATATGTGGCAGAGAGTAGGACAAGCAATGTATGCCCC
TCCCATCCCAGGAAACATAACATGTAGATCAAACATTACAGGAATAATATTAACAAAAGATAAGGATGGTATA
AATAATTCAGATGAAACCTTGAGGCCAGGAGGAGGAGATATGAGGGACAATTGGAGAAGTGAATTATATAAGT
ATAAAGTGGTAAAAATTGAACCACTAGGAGTAGCACCCACCAAGGCAAGGAGAAGAGTGGTGGAGAGAGAAAA
AAGAGCAGTTGGACTAGGAGCTGTTTTCCTTGGGTTCTTAGGAGCAGCAGGAAGCACTATGGGCGCAGCGCCA
ATAACGCTGACGGTACAGGCCAGACAATTGTTGTCTGGCATAGTGCAACAGCAAAGCAATTTGCTGAGGGCTA
TAGAGGCTCAACAACAGCTGTTGAAGCTCACGGTCTGGGGCATT
>KU921804..1
TTCTATTGTGATACATCAAGCCTGTTTACTGGTACTTGGAATGGTACTACTTGGATGAATAGCACTGACACTG
AAAATATAACTCTTCCATGCAGAATAAAGCAAATTATAAATATGTGGCAGAGGGTAGGGAGAGCAATGTATGC
CCCTCCCATCCAAGGAAAAATAAATTGTACATCAAACATTACAGGACTACTATTAACAAGAGATGGTGGGAAA
ACTAATAATTCATCTGAAACCTTCAGACCAGGAGGAGGAGATATGAGGGACAATTGGAGAAGTGAATTATATA
AGTATAAAGTAGTAAAGATTGAACCACTAGGAGTAGCACCCACCAGGGCAAGAAGGAGAGTGGTGGAAAGAGA
AAAAAGAGCAGTTGGACTGGGAGCTGTATTCATTGGGTTCTTGGGAGCAGCAGGGAGCACTATGGGCGCGGCG
TCAGTGACGCTGACGGTACAGGCCAGACGATTACTATCTGGCATAGTGCAGCAGCAAAGCAATTTGCTGAGGG
CTATAGAGGCTCAACAGCATCTGTTGAAACTCACGGTCTGGGGCATC
>KU921831..3
TTCTATTGTAATACATCACGCCTGTTTAGTTACAGGTGCACGTTCGAAAATGAGACTAATACTTACACCTGCA
CTAACACCATGCATGAGTTCAATAGCACGGCAAATATAACTCTCCAATGCAGACTAAAGCAGATCATAAGGAT
GTGGCAGAGGACAGGACAAGCAATGTATGCCCCTCCCATCCCAGGGCAAATAATGTGTAGATCAAACATTACA
GGGTTAATATTAACAAGAGATGGTGGGGTTGATAACAATACAGATGCTACCGAAATCTTCAGACCTGGAGGAG
GAGATATGAAGGACAATTGGAGAAGTGAATTATATAAGTATAAAGTAGTAAAAATTGAACCACTAGGAGTAGC
ACCCACCAGGGCAAGGAGAAGAGTGGTGGGGAGAGAAAAAAGAGCAGTTGGACTGGGAGCTGTTTTCATTGGG
TTCTTAGGAGCAGCAGGAAGCACTATGGGCGCGGCGTCAGTGACGCTGACGGTACAGGCCAGACAATTATTGT
CTGGTATAGTGCAACAGCAAAGCAATTTGCTGAAGGCTATTGAGGCTCAACAACATCTGTTGAGGCTCACGGT
CTGGGGCATT
>KU921835..2
TTCTATTGTAACACATCAGAACTGTTTAATAGCACTTGGAGTTGGAATAACACTTGGAATAACACTCATGAGC
CAAATGGCACTATAACTCTCCAATGCAGAATAAAGCAAATTATAAATATGTGGCAGAGAGTAGGACAAGCAAT
GTATGCCCCTCCCATACAAGGAGAAATAAAGTGTGAATCAAACATCACAGGACTAATATTAACAAGAGATGGT
ATGGGTAACAATGTATCAAATGAAACCTTCAGACCTGGAGGAGGAGATATGAGGGACAACTGGAGAAGTGAGT
TATATAAATATAAAGTAGTAAAAATTGAACCATTAGGAGTAGCACCCACTGGGGCAAGGAGAAGGGTGGTGCA
GAGAGAAAAGAGAGCGGTTGGAATAGGAGCTGTCTTCCTTGGGTTCTTAGGAGCAGCAGGAAGCACTATGGGC
GCAGCGTCATTAACGCTGACGGTACAGGCCAGACAATTACTGTCTGGCATAGTGCAGCAGCAAAGCAATTTGC
TGAGGGCTATAGAGGCTCAACAACATATGTTGAAACTCACGGTCTGGGGCATT
>KU921841..2
TTCTATTGCAACACATCAGGCCTGTTTAATAGCACTGACATGAGTTCAAATAACACAGAGTTAAATGACACTA
TAAGACTCCCATGCAGAATAAAACAAATTATACGTATGTGGCAGAAAGTAGGACAAGCAATGTATGCCCCTCC
CATTCAAGGAGTGATAAGATGTAGCTCAAACATTACAGGAATACTATTAACAAGAGATGGTGGGATAGAAAAT
GAAACAGAAATCTTCAGACCTGGAGGAGGAAACATGAAGGACAATTGGAGAAGTGAATTATATAAGTATAAAG
TAGTAAAAATTGAACCACTAGGAGTAGCACCCACCTCGGCAAGGAGAAGAGTGGTGGAGAGAGAAAAAAGAGC
AGTTGGACTGGGAGCTGTATTCATTGGGTTCTTAGGAGCAGCAGGAAGCACTATGGGCGCGGCGTCAATGACG
CTGACGGTACAAGCCAGACAATTATTGTCTGGCATAGTGCAACAGCAAAGCAATTTGCTGAGGGCTATAGAGG
CTCAACAGCATCTGTTGAAACTCACGGTCTGGGGCATC
>KU921852..1
TTCTATTGTAATACATCAGGCTTGTTTAATAGCACTTGGAATGACACTGTCATGTTTAATAGCACTGGGAATG
ACACTGTCGGCAGGGAGTCAAATGAAACTATAACACTCCAATGCAGGATAAAACAAATTATAAATATGTGGCA
GAGAACAGGACAAGCAATATATGCCCCTCCTATCCCAGGAGTAATAAGGTGTGTATCAAACATTACAGGACTA
ATATTAACAAGAGATGGTGGGAATACGGCAGGGACAGGTTCTGAAACCTTCAGACCTGGAGGAGGAGATATGA
GGGACAATTGGAGAAGTGAATTATATAAGTATAAAGTAGTAAAAGTTGAACCACTAGGAGTAGCACCCACCAG
GGCACAGAGAAGAGTGGTGCAGAGGGAAAAAAGAGCAGTTGGAATAGGAGCTGTCTTCATTGGGTTCTTAGGA
GCAGCAGGAAGCACTATGGGCGCGGCGTCAATAACGCTGACGGCACAGGCCAGACAATTATTGTCTGGTATAG
TGCAACAGCAAAGCAATTTGCTGAGGGCTATAGAGGCTCAACAGCATCTGTTGAAACTCACAGTCTGGGGCAT
C
>KF716474..1

TTCTATTGTGACACATCAGGCCTGTTTAATAGCACTTGGAATGGTACGGCCAGCAATGTCAGCATACCTGACA
CGGTGACAAATGGCACTATAACACTCCCATGCAAAATAAAGCAAATTATAAATATGTGGCAGAGAGTAGGACA
AGCAATGTATGCCCCTCCCATTGCAGGAGTAATAAGGTGTGATTCAAACATTACAGGGATGATATTAACAAGA
GATGGTGGTGATAACAGGACAGAAACCTTCAGACCTGGAGGAGGAGATATGAGGGACAATTGGAGAAGTGAAT
TATATAAATATAAAGTAGTAAAAGTTGAACCACTAGGAGTAGCACCTACCAGGGCAAGAAGAAGAGTGGTGGA
GAGAGAAAAAAGAGCAGTAGGAATAGGAGCTGTTTTCCTTGGGTTCTTAGGAGCAGCAGGAAGCACTATGGGC
GCAGCGTCAATAACGCTGACGGTACAGGCCAGACAATTATTGTCTGGTATAGTGCAACAGCAAAGTAATTTGC
TGAGGGCTATAGAGGCTCAACAACAGCTGTTGAAACTCACAGTCTGGGGCATT
>KF716475..1
TTCTACTGTAATACATCAGGCCTGTTTAATAGCACTTGGTCCCTTAACAACACTTGGTCCCTTAACAGCACGC
AGTCAAATGACACAGGGATAAATGATACCATAACTCTCCCATGCAGAATAAAGCAAATTATAAATATGTGGCA
GAGAGTAGGACAAGCAATGTATGCCCCTCCCATCCAAGGAGTAATACAGTGTGTATCAAACATTACAGGACTA
CTGTTAACAAGAGATGGGGGGAATGTGAATAGCAGTAATGGCAATGAAATCTTCAGACCTGGAGGAGGAGATA
TGAGGGACAATTGGAGAAGTGAATTATATAAGTATAAAGTAGTAAAAATTGAACCACTAGGAGTAGCACCCAC
CAGGGCAAAGAGAAGAGTGGTGGGAGAGAGAAAAAAGAGCAATTGGTGGGCTAGGAGCTGTTTTCCTTGGGTTC
TTAGGAATGGCAGGAAGCACTATGGGCGCGGCGTCAATGACGCTGACGGTACAGGCCAGAAACTTATTGTCTG
GCATAGTGCAACAGCAAAGCAATTTGCTGAGGGCTATAGAGGCTCAACAACATCTGTTGAAACTCACAGTCTG
GGGCATT
>AY322184..1
TTCTATTGTAACACATCAGATCTGTTTAATGAGTCAAATAGTACGGAGTTAAACGACACTATAACTCTCCAAT
GCAGAATAAAGCAAATTATAAATATGTGGCAGAGAGCAGGACAAGCAATATATGCCCCTCCCATCCCAGGAGT
AATAAGGTGTGTATCAAACATTACAGGACTATTGTTAACAAGGGATGGTGGGATTAATACCAGTACAAGTGAA
ACCTTCAGACCTGGAGGAGGAGATATGAGGGACAATTGGAGAAGTGAATTATATAGGTATAAAGTAGTAAAAA
TTGAACCACTAGGAGTAGCACCCACCAGGGCAAAGAGAAGAGTGGTGGAGAGAGAAAAAAGAGCAGTTGGAAT
AGGAGCTGTTTTCATTGGGTTCTTAGGAGCAGCAGGAAGCACTATGGGCGCGGCGTCAATAACGCTGACGGTA
CAGGCCAGACAATTATTGTCTGGCATAGTGCAACAGCAAAGCAATTTGCTGAGGGCTATAGAGGCTCAACAAC
ATCTGTTGAAACTCACGGTCTGGGGCATT
>AF539405..1
TTCTATTGTAACACATCAGGCCTGTTTAATAGCACTTGGAATGGCAGTGAGGCAAATAGCACGCCACACGAGA
ATATAACTCTCCAATGCAGAATAAAGCAAATTATAAATATGTGGCAGAGAGCAGGACAAGCAATATATGCCCC
TCCCATCCCAGGAGTAATAAAATGTAATTCAAACATTACAGGACTAATATTAACAAGAGATGGTGGGGTTAAT
AGCAGTGCAAATGAAACCTTCAGACCTGGAGGAGGAGATATGAGGGACAATTGGAGAAGTGAATTATATAAGT
ATAAAGTAGTAAAAATTGAACCACTAGGAGTAGCACCCACCAGGGCAAAGAGAAGAGTGGTGGAGAGAGAAAA
AAGAGCAGTTGGAATAGGAGCTGTCTTCATTGGGTTCTTAGGAGCAGCAGGAAGCACTATGGGCGCGGCGTCA
ATAACGCTGACGGTACAGGCCAGACAATTATTGTCTGGCATAGTGCAACAGCAAAGCAATTTGCTGAGGGCTA
TAGAGGCTCAACAACATCTGTTGAAACTCACGGTCTGGGGCATT
>L22943..1
TTCTATTGTAATTCATCAGGCCTGTTTAATAGCACTTGGACCAACATGCAGGAGTCAAATAGCACGGAGTCAA
ATGACACTATAACTCTCCCATGCAGAATAAAAATTATAAACATGTGGCAGAGAGCAGGACAAGCAATGTATGC
CCCTCCCATCCAAGGAATAATAAAGTGTGTATCAAACATTACAGGACTAATATTAACAAGAGATGGTGGGGAT
AATAACAGTGAAAGTGAAACCTTCAGACCTGGAGGAGGAGATATGAGGGACAATTGGAGAAGTGAATTATATA
AGTATAAAGTAGTAAAAATTGAACCACTAGGAGTAGCACCCACCAAGGCAAAGAGAAGAGTGGTGGAGAGAGA
AAAAAGAGCAGTTGGAATAGGAGCTGTCTTCCTTGGGTTCTTAGGAGCAGCAGGAAGCACTATGGGCGCGGCG
TCAATAACGCTGACGGTACAGGCCAGACAATTATTGTCTGGCATAGTGCAACAGCAAAGCAATTTGCTGAGGG
CTATAGAGGCTCAACAACATCTGTTGAAACTCACGGTCTGGGGCATT
>KX168075..1
TTCTATTGTAACACATCAGGCCTGTTTAATAGCACTTGGGAGTTTAATAGCACTTGGGAGTTTAATAGCACTT
GGGAGTTTAATAGCACTTGGAATAATTCAAATAGCACAGAGAATATAACTCTCCAATGCAGAATAAAGCAAAT
TGTAAATATGTGGCAAAGAGCAGGACAAGCAATATATGCCCCTCCCATACCAGGAGTAATAAGGTGTGAATCA
AATATTACAGGACTAATATTAACAAGAGATGGTGGGGATAATAATAGTACAAATAATACAAGTGAAACCTTCA
GACCTGGAGGAGGAGATATGAGGGACAATTGGAGAAGTGAATTATATAAGTATAAAGTAGTAAAAATTGAACC
AATAGGAGTAGCACCCACCAGGGCAAAAAGAAGAGTGGTGGAGAGAGAAAAAAGAGCAGTTGGAATAGGAGCT
GTCTTCATTGGGTTCTTAGGAGCAGCAGGAAGCACTATGGGCGCAGCGTCAGTAACGCTGACGGTACAGGCCA
GACAGTTATTGTCTGGTATAGTGCAACAGCAAAGCAATTTGCTGAGGGCTATAGAGGCTCAACAGCATCTGTT
GAAACTCACGGTCTGGGGCATT
>AF004885..2
TTCTATTGTAATACATCAGGCTTGTTTAATAGCACTTGGTATGTTAATAGCACTTGGAATGACACTGACAGCA
CTCAGGAGTCAAATGACACTATAACTCTCCCATGCAGAATAAAGCAAATTATAAATATGTGGCAGAGAGCAGG
ACAAGCAATGTATGCCCCTCCCATCCCAGGAGTAATAAAGTGTGAATCAAACATTACAGGACTACTATTAACA
AGGGATGGTGGAAAGGATAACAATGTAAATGAAACCTTCAGACCTGGAGGAGGAGATATGAGGGACAATTGGA

GAAGTGAATTATATAAGTATAAAGTAGTAGAAATTGAACCACTAGGAGTAGCACCTACCAGGGCAAAGAGAAG
AGTGGTGGAGAGAGAAAAAAGAGCAGTTGGAATAGGAGCTGTCTTCCTTGGGTTCTTAGGAGCAGCAGGAAGC
ACTATGGGCGCAACGTCAATAACGCTGACGGTACAGGCCAGACAATTATTGTCTGGCATAGTGCAACAGCAAA
ACAATTTGCTGAGGGCTATAGAGGCTCAACAACATCTGTTGAAACTCACGGTCTGGGGCATT
>AF407151..2
TTCTATTGTAACACATCAGGCCTGTTTAACAGAGCTTATGGCAATGTCAGCAAGCAAGAGCCAAATAGCTCCG
GCAATATAACACTCCAATGCAGAATAAAGCAAGTTATAAATATGTGGCAGAGAGCAGGACAAGCAATATATGC
CCCTCCCATACAAGGAGTAATAAGATGTGACTCAAATATTACAGGACTAATATTAACAAGAGATGGTGGGAAA
GATAGTAATATAACTGAAACATTCAGACCTGGAGGAGGAGATATGAGGGACAATTGGAGAAGTGAATTATATA
AGTATAAAGTAGTAAAAATTGAACCACTAGGAGTAGCACCCACCAGGGCAAAGAGAAGAGTGGTGGAGAGAGA
AAAAAGAGCAGTTGGAATAGGAGCTGTCTTCATTGGGTTCTTAGGAGCAGCAGGAAGCACTATGGGCGCGGCG
TCAATAACGCTGACGGCACAGGCCAGAAAATTATTGTCTGGCATAGTGCAACAGCAAAGCAATTTGCTGAGGG
CTATAGAGGCTCAACAACATCTGTTGAAACTCACGGTCTGGGGCATT
>AF407162..2
TTCTATTGCAATACAACAAACCTGTTTAATAGCACTTGGAATCACACTGCCAGCATGAATAGCACAGAGTCAA
ATGACACTATAATTCTCCCATGCAGAATAAAACAAATTATAAATATGTGGCAGAGAGTAGGACAAGCAATGTA
TGCCCCTCCCATTCGAGGAGTAATAAGGTGTGAATCAAACATTACAGGACTAATATTAACAAGAGATGGTGGG
AATACTACCGGTACAAGTGAAATCTTCAGACCTGGAGGTGGAGATATGAGGGACAATTGGAGAAGTGAATTAT
ATAAGTATAAAGTAGTAAAAATTGAACCACTAGGAGTAGCACCCACCAGGGCAAAGAGAAGAGTGGTGGAGAG
AGAAAAAAGAGCAGTTGGAATAGGAGCTGTCTTCATTGGGTTCTTAGGAGCAGCAGGAAGCACTATGGGCGCG
GCGTCAATAACGCTGACGGTACAGGCCAGACAATTATTGTCTGGCATAGTGCAACAGCAAAGCAATTTGCTGA
GGGCTATAGAGGCTCAACAGCATCTGTTGAAACTCACGGTCTGGGGCATT
>AF407156..1
TTCTATTGCAATACATCAAAACTGTTTAATAGCAGGTGGGAGAACAATGGCACCGCCAACATGTTGAAGAATG
ACACAGGGTCAAATGAGACTACTCTGATTCTCCGATGCAGAATAAAACAAATTATAAATATGTGGCAGAGAGT
AGGACAAGCGATGTATGCCCCTCCCATCCAAGGAGTAATAAATTGTACATCAAACATCACAGGACTAATATTA
ACAAGAGATGGTGGTGGTGAAAACGATACAGAGACCTTCAGACCTGGAGGAGGAGATATGAGGGACAATTGGA
GAAGTGAATTATATAAGTATAAAGTAGTAAAACTTGAACCACTAGGAGTAGCACCCACCATGGCAAAGAGAAG
AGTGGTGGAGAGGGAAAAAAGAGCAGTTGGAATGGCAGCTGTCTTCCTTGGGTTCTTGGGAACAGCAGGGAGC
ACTATGGGCGCGGCGTCACTAACGCTGACGGTACAGGCCAGACAATTATTGTCTGGCATAGTGCAACAGCAAA
GCAATTTGCTGAAGGCTATAGAGGCTCAACAGCATCTGTTGAGACTCACGGTCTGGGGCATT
>KT008652..1
TTCTATTGTAACACATCAGGCCTGTTTAATAGCACTTGGGGGCACAATGCCAGCAGGCAGGAGAATAGCACGG
AGTCAAGTGACACTATAACTCTCCCATGCAAAATAAAGCAAATTATAAATATGTGGCAGAGAGCAGGGCAAGC
AATATATGCCCCTCCCATCCCAGGAGTAATAAGGTGTGAATCAAACATTACAGGACTAATATTAACAAGAGAT
GGTGGGAACAACAACAGTACAAGTGAAACCTTCAGACCTGGAGGAGGAAATATGAGGGACAATTGGAGAAGCG
AATTATATAAGTATAAAGTAGTAAAAATTGAACCACTAGGAGTAGCACCCACCAGGGCAAAGAGAAGAGTGGT
GGGGAGAGAAAAAAGAGCAGTTGGAATAGGAGCTGTTTTCCTTGGGTTCTTAGGCGCAGCAGGAAGCACTATG
GGCGCAGCGTCAGTAACGCTGACGGTACAGGCCAGACAATTATTGTCTGGCATAGTGCAACAGCAAAGCAATT
TGCTGAGGGCTATAGAGGCTCAACAACATCTGTTGAAACTCACGGTCTGGGGCATT
>AF407157..2
TTCTATTGTAACACATCAGGCCTGTTTAATGGCATTTGGAATGGCACGCAGGAGCCAAATAGAACGGAGTCAA
ACGATACTATAACACTCCAATGCAGAATAAAGCAAATTATAAATATGTGGCAAAGAGTAGGACAAGCAATATA
TGCCCCTCCCATTCAAGGAGAAATAAGGTGTGAGTCAAACATTACAGGACTAATATTAACAAGAGATGGTGGG
ATAATTAACGGTACAGAGGAGACCTTCAGACCTGGAGGAGGAGATATGAGGGACAATTGGAGAAGTGAATTAT
ATAAGTATAAAGTAGTAAAGATTGAACCATTAGGAGTAGCACCCACCAAGGCAAAGAGAAGAGTGGTGGAGAG
GGAAAAAAGAGCAGTTGGATTCGGAGCTTTCTTCCTTGGGTTCTTAGGAGCAGCAGGAAGCACTATGGGCGCG
GCGTCAATAACGCTGACGGTACAGGCTAGACAATTATTGTCTGGCATAGTGCAACAGCAAAACAATTTGCTGA
GGGCTATAGAGGCTCAACAGCATCTGTTGAAACTCACGGTCTGGGGCATT
>FJ866112..1
TTCTATTGTAATACATCAGAACTATTTAATAGCACTTGGACTTGGAAAGGCAATAATGACAGCATGCAGGGGT
TAAATAGCACGGGGTTAAACAACACTATAACTCTCCACTGCAGCATAAAGCAAATTATAAATATGTGGCAGAG
AGTAGGACAAGCAATGTATGCCCCTCCCATCCAAGGAGTAATAAAGTGTGAATCAAACATTACTGGACTACTA
TTAACAAGAGATGGTGGGAATAATAACATAAACGAGACCCTCAGACCTGGAGGAGGAGATATGAGGGACAATT
GGAGAAGTGAATTATATAAGTATAAAGTAGTAAAAATTGAACCACTAGGAGTAGCACCCACCAAGGCAAAGAG
AAGAGTGGTGGAGAGAGAAAAAAGAGCAGTTGGAATAGGAGCTGTCTTCCTTGGGTTCTTAGGAGCAGCAGGA
AGCACTATGGGCGCAGCGTCAATAACGCTGACGGCACAGGCCAGAATATTATTGTCTGGCATAGTGCAGCAGC
AAAGCAATTTGCTGAAGGCTATAGAGGCTCAACAGCATCTGTTGAGGCTCACGGTCTGGGGCATT
>AY322190..2

TTCTATTGTAATACATCAGGCCTGTTTAATAGCATTTGGAATCACACTAATGGCACTTGGAATAGCACGGAGT
TAAATGGCAATATAACTCTCAATATAACTCTCCCATGCAGAATAAACCAAATTATAAATATGTGGCAGAGAGT
AGGACAAGCAATATTTGCCCCTCCCATCCAAGGAGTAATACAGTGTGTATCAAACATTACAGGACTAATATTA
ACAAGAGATGGAGGGAATAATAACACTACAAATGAAACCTTCAGACCTGGAGGAGGAGATATGAGGGACAATT
GGAGAAGTGAATTATATAAGTATAAAGTAGTAAAAATTGAACCACTAGGAGTAGCACCCACCAGGGCACAGAG
AAGAGTGGTGCAGAGAGAAAAAAGAGCAATTGGAATGGGAGCTGTCTTCATTGGGTTCTTAGGAGCAGCAGGA
AGCACTATGGGCGCGGCGTCAATAACGCTGACGGTACAGGCCAGACAATTATTGTCTGGTATAGTGCAACAGC
AAAGCAATTTGCTGAGGGCTATAGAGGCTCAACAGCATCTGTTGAAACTCACGGTCTGGGGCATT
>AY322193..1
TTCTATTGTAACACATCAAGCCTATTTAATGGCACTTGGACGGGCAATGACAGCAGGCAGGAGTCAAATAGCA
GCGACACTATAACTCTTCCATGCAGAATAAAACAAATTATAAATATGTGGCAGAGGGTAGGACAAGCAATATA
TGCCCCTCCCCTACAAGGAGTAATCAGATGTGATTCAAATATTACAGGACTAATCTTAACAAGAGATGGTGGG
AGTCCGGATAATGAAACCTTCAGACCTGTTGGAGGAGATATGAGGGACAATTGGAGAAGTGAATTATATAAGT
ATAAAGTAGTAAAAATTGAACCATTAGGAGTAGCACCCACCAGGGCAAAGAGAAGAGTGGTGGAGAGAGAAAA
AAGAGCAGTTGGAATAGGAGCTGTCCTCATTGGGTTCTTAGGAGCAGCAGGAAGCACTATGGGCGCGGCGTCA
ATAACGCTGACGGTACAGGCCAGACAATTATTGTCTGGCATAGTGCAACAGCAAAGCAATTTGCTGAGGGCTA
TAGAGGCTCAACAGCATCTGTTGAAACTCACGGTCTGGGGCATT
>FJ396018..1
TTTTATTGTAATACATCAGGCCTGTTTAATAGTACTTGGGATGACAATAATACTAGGAATAATGAGACTGCCA
GAAATAATACTATAACTCTCCCATGTAGAATAAAGCAAATTATAAATATGTGGCAGAGGGTAGGACAAGCAAT
GTATGCCCCTCCCATCAAAGGAGTAATAAGGTGTGAATCAAACATTACAGGACTAATATTAACAAGAGATGGT
GGACTTAATAACAATACAGAGACCTTCAGACCTGGAGGAGGAGATATGAGGGACAATTGGAGAAGTGAATTAT
ATAAGTATAAAGTAGTAAAAATTGAACCACTAGGAGTAGCACCCACCAGGGCAAGGAGAAGAGTGGTGGGAAG
AGAAAAAAGAGCAGTTGGAATAGGAGCTGTCTTCCTTGGGTTCTTAGGAGCAGCAGGAAGCACTATGGGCGCG
GCGTCAATAACGCTGACGGTACAGGCCAGACAATTATTGTCTGGCATAGTGCAACAGCAAAGCAATTTGCTGA
GGGCTATAGAGGCTCAACGACATCTGTTGAAACTCACGGTCTGGGGCATT
>AY736811..1
TTCTATTGTAATACATCAGGCCTGTTTAATAGCACATATAGCGGGAACGAAACTGTCAGCAATAGCACAGAGT
ACAACATAACTCTCCCATGCAAAATAAAGCAAATTATAAATATGTGGCAGAGAACAGGACAAGCAATGTATGC
CCCTCCCATCCAAGGAGTAATAAGCTGTGTGTCAAACATTACAGGACTACTATTAACAAGAGATGGTGGTAAT
GGTGGTAATAACAATACAACTGAGACCTTCAGACCTGGAGGAGGAAATATGAAGGACAATTGGAGAAGTGAAT
TATATAAGTATAAAGTAGTAAAAATTGAACCACTAGGAGTAGCACCCACCAGGGCAAAGAGAAGAGTGGTGGA
GAGAGAAAAAAGAGCAGTTGGACTGGGAGCTGTATTCCTTGGGTTCTTAGGAGCAGCAGGAAGCACTATGGGC
GCGGCGTCCATAACGCTGACGGTACAGGCCAGACAATTACTGTCTGGCATAGTGCAACAGCAAAGCAATTTGC
TGAGGGCTATAGAGGCTCAACAGCATCTGTTGAAACTCACGGTCTGGGGCATT
>AY736814..1
TTCTATTGCAACACATCAGGCCTGTTTAATAGCACTTGGGATGATAACATTAAGAATGATACTAACAGCACAC
AGGGGTCAAATAGCACGGAGATAATTCTCCCATGCAAAATAAAGCAAATTGTAAGGATGTGGCAGAGAACAGG
ACAAGCAATGTATGCTCCTCCAATCCCAGGAGTAATAAGGTGTGTATCAAACATTACAGGACTAATACTAACA
AGAGATGGTGGGAATACTAGCAGCATAAATGAAACCTTTAGACCTGGAGGAGGAGATATGAGGGACAACTGGA
GAAGTGAATTATATAAGTATAAAGTAGTAAAAATTGAACCACTAGGAGTGGCACCCACCAAGGCAAGGAGAAG
AGTGGTGGAGAGAGAAAAAAGAGCAGTTGGAATAGGAGCTGTCCTCTTTGGTTTCTTAGGAGCAGCCGGAAGC
ACTATGGGCGCGGCGTCAATAACGCTGACGGTACAGGCCAGACAATTATTGGCTGGCATAGTGCAACAGCAAA
GCAATTTGCTGAGGGCTATAGAGGCTCAACAGCAAATGTTGAGACTCACGGTCTGGGGCATT
>AF457075..1
TTCTATTGTGATACATCAAGCCTGTTTAATGGCACTTTAGAGGCCAATGCCACCGTGCAAAACTTCAATAGCA
CAGAGCATATAATTCTCCCATGCAAAATAAAGCAAATTATAAATATGTGGCAGAGAGCAGGACAAGCAATGTA
TGCCCCTCCCATCCAAGGAGTAATAAATTGTGTGTCAAACATTACAGGACTAATATTAACAAGAGATGGTGGG
GATAATAATAATAGTACAAATGAAACATTCAGACCTGGAGGAGGAGATATGAGGGACAATTGGAGAAGTGAAT
TATATAAGTATAAAGTAGTAAAAATTGAACCACTAGGAGTAGCACCAACCAAGGCAAAAAGAAGAGTGGTGGA
GAGAGAAAAAAGAGCAGTAATTGGACTGGGAGCTGTTTTCATTGGGTTCTTAGGAGCAGCAGGAAGCACTATG
GGCGCGGCGTCAATAACGCTGACGGTACAGGCCAGACAATTATTGTCTGGTATAGTGCAACAGCAAAGCAATT
TGCTGAGGGCTATAGAGGCTCAACAACATCTGTTGAAACTCACGGTCTGGGGCATT
>DQ208458..1
TTCTATTGTAACACATCAGGCCTGTTCAATAGCACTTGGATTAGCAATACCAGCGTGCAGGGGTCAAATAGCA
CGGGGTCAAATGACAGTATAACTCTCCCATGCAGAATAAAGCAAATTATAAATATGTGGCAGAGAATAGGACA
AGCAATGTATGCCCCTCCCATCCAAGGAGTAATAAGATGTGTATCAAACATTACAGGGCTAATATTAACAAGA
GATGGTGGGAGTACTAATAGTACAACTGAAACCTTCAGACCTGGAGGAGGAGATATGAGGGACAATTGGAGAA
GTGAATTATATAAGTATAAAGTAGTAAAAATTGAACCACTAGGAGTAGCACCCACCAGGGCAAAGAGAAGAGT
GGTGGGGAGAGAAAAAAGAGCAGTTGGAATAGGAGCTGTCTTCCTTGGGTTCTTAGGAGCAGCAGGAAGCACT

ATGGGCGCGGCGTCAATGACGCTGACGGTACAGGCCAGAAATTTATTATCTGGCATAGTGCAACAGCAAAGCA
ATTTGCTGAGGGCTATAGAGGCTCAACAACATCTGTTGAAACTCACGGTCTGGGGCATT
>DQ208467..2
TTCTATTGTAATACATCAGGCCTGTTTAATAGCAATTGGAATTACACTAACAGCACGCAGGAGTCAAATAGCA
CGGAGTCAAATGGCACTATAACTCTCCAATGCAGAATAAAGCAAATTATAAATATGTGGCAGAGAGTAGGACA
AGCAATGTATGCCCCTCCTATCCCAGGAGTAATAAGGTGTGTATCAAACATTACAGGACTAATATTAACAAGA
GATGGCGGGGACAACAACAGTATAACTGAAACCTTCAGACCTGGAGGAGGAGATATGAGGGACAATTGGAGAA
GTGAATTATATAAGTATAAAGTAGTAAAAATTGAACCACTAGGAGTAGCACCCACCAGGGCAAGGAGAAGAGT
GGTGGGAAGAGAAAAAAGAGCAGTTGGAATAGGAGCTGTCTTCATTGGGTTCTTAGGAGCAGCCGGAAGCACG
ATGGGCGCGGCGTCAATAACGCTGACGGTACAGGCCAGACAATTATTGTCTGGCATAGTGCAACAGCAAAGCA
ATTTGCTGAGGGCTATAGAGGCTCAACAACATCTGTTGAAACTCACGGTCTGGGGCATT
>DQ208448..1
TTCTATTGTAACACATCAGACTTGTTTAATAGCACTTGGTATGGGAATGGGACCAGCATGCGGGAGTCAAATA
GCACGGAGTCAAACGACACTATAATTCTCCGATGCAGAATAAAGCAAATTATAAATATGTGGCAGAGAGTAGG
ACAAGCAATATATGCCCCTCCCATCCCAGGAGTAATAAACTGTGTATCAAACATCACAGGACTAATATTAACA
AGAGATGGTGGGAATAATAATAATGAAACTGAAACCTTCAGACCTGGGGGAGGAGATATGAGGGACAATTGGA
GAAGTGAATTATATAAGTATAAAGTAGTAAAAATTGAACCACTAGGAGTAGCACCCACCAGGGCAAGGAGAAG
AGTGGTGGAGAGAGAAAAAAGAGCAGTAGTTGGAATAGGAGCTGTCTTCCTTGGGTTCTTAGGAGCAGCAGGA
AGCACTATGGGCGCGGCGTCAATAACGCTGACGGTACAGGCCAGACAATTATTGTCTGGCATAGTGCAACAGC
AAAGCAATTTGCTGAGGGCTATAGAGGCTCAACAACATCTGTTGAAACTCACAGTCTGGGGCATT
>KU749411..1
TTCTATTGTAATACAACAGGCCTATTTAATAGTACTTGGAATACAACAGGCCCATTTAATAATACTTGGCCTG
ATGATGTCAATATAGTACTCCCATGCAAAATAAAGCAAATTATAAATGTATGGCAGAGAGTAGGACAAGCAAT
ATATGCCCCTCCCATCCAAGGACTAATACAATGTAACTCAAATATTACGGGGCTACTATTAACAAGAGATGGT
GGGAATAATGGCACACTTAATGAAACCTTCAGACCTGGAGGAGGAAATATGAAGGACAATTGGAGAAGTGAAC
TGTATAAATATAAAGTAGTAAAAATTGAACCACTAGGAGTAGCACCCACCAGGGCAAAGAGAAGAGTGGTGGA
GAGAGGAAAAAGAGCAATTGGAATAGGAGCTGTGCTCATTGGGTTCTTGGGGGCAGCCGGAAGCACTATGGGC
GCGGCGTCTGTAACGCTGACGGTACAGGCCAGACAATTATTGTCTGGCATAGTGCAACAGCAAAGCAATTTGC
TGAGGGCTATAGAGGCTCAGCAACAGATGCTGAAACTCACAGTCTGGGGCATT
>KX232594..1
TTCTATTGTAATACAACAGGACTGTTTAATAGTACTTGGTATAAAAATGGAACGACTGAGGGAACAAATAGCA
CGGGGTCAAATGACACTATAACACTCCCATGCAGAATAAAGCAAGTTATAAATGTATGGCAGAGAGTAGGACA
AGCAATATATGCCCCTCCCATCCCAGGGATAATAAGGTGTGAATCAAATATTACGGGGCTACTATTAACAAGA
GATGGTGGAAATAATACAAGTAATGAAACCTTCAGACCTGGAGGAGGAAATATGAAGGACAATTGGAGAAGTG
AACTGTATAAGTATAAAGTAGTAAAACTTGAACCACTAGGAGTAGCACCCACCAAAGCAAAGAGAAGAGTGGT
GGAGAGAGAAAAAAGAGCAATTGGATTTGGAGCTTTCTTCCTTGGGTTCTTGGGAGCAGCTGGAAGCACTATG
GGCGCGGCGTCCGTAACGCTGACGGTACAGGCCAGACAATTATTGTCTGGCATAGTGCAACAGCAAAGCAATT
TGCTGAGGGCTATAGAGGCTCAGCAGCATATGCTGAAACTCACGGTCTGGGGCATT
>KX232597..1
TTCTATTGTAATACAACAGGTCTGTTTAATAGTACTTGGCCTAGTAATGAGACTAAGGAAGTAAATAACACGG
AGTCAAATGACATTATCACACTCCCATGCAGAATAAAGCAAATTGTAAATGTATGGCAAAGAGTAGGACAAGC
AATATACGCCCCTCCCATCAAAGGACTAGTACAGTGTAATTCAAGTATTACGGGGCTACTATTAAACAGAGAT
GGTGGGAATAATAACACAGATGAAACCTTCAGACCTGGAGGAGGAAATATGAAGGACAATTGGAGAAGTGAAC
TATATAAGTATAAAGTAGTAAAAATTGAACCACTAGGAGTAGCACCCACCAGGGCAAGGAGAAGAGTGGTGGA
GAGAGAAAAAAGAGCAGCTGGACTAGGAGCTTTATTCATTGGGTTCCTGGGAGCAGCCGGAAGCACTATGGGC
GCAGCGTCTGTAACGCTGACGGTACAGGCCAGACAATTATTGTCTGGCATAGTGCAACAGCAAAGCAATTTGC
TGAGGGCTATAGAGGCTCAGCAGCAGATGCTGAGACTCACAGTCTGGGGCATT
>KX232609..1
TTCTATTGTGATACAACAAACCTGTTTAATAGTACTTGGTTTGATAATGGGACTACTGAAGCAAATAGCACGG
GGTCAAGTGACATTATAACACTCCCATGCAGAATAAAACAAATTGTAAATGTATGGCAGAGAGTAGGACAAGC
AATATATGCCCCTCCCATCAGAGGACTAATAAAGTGTGAATCAAATATTACGGGGCTACTATTAACCAGAGAT
GGTGGGCATAATAGCACAAATGCAAGTAGTGAAACCTTCAGACCTGGAGGAGGAAATATGAAGGACAATTGGA
GAAGTGAACTATATAAGTATAAAGTAGTAAAAATTGAACCACTAGGAGTAGCACCCACCAGAGCAAGGAGAAG
AGTGGTGGAGAGAGAAAAAAGAGCAATTGGAATAGGAGCTGTACTCTTTGGGTTCTTGGGAGCAGCCGGAAGC
ACTATGGGCGCGGCGTCCATAACGCTGACGGTACAGGCCAGACAATTATTGTCTGGCATAGTGCAACAGCAAA
GCAATTTGCTGAGGGCTATAGAGGCTCAGCAGCATCTGCTGAAACTCACGGTCTGGGGCATT
>KX232613..1
TTCTATTGTAATACATCAGCCCTGTTTAATAGCACTTGGAGAGACAACCAGACAGATAACCAAACACATACAA
ATGACACTGACATTATAACTCTCCCATGCAGAATAAAGCAAGTTATAAACATGTGGCAGAGGACAGGACAAGC
AATGTATGCCCCTCCTATCCAAGGAGTAATAAAGTGTACATCAAATATTACAGGACTATTATTAACAAGAGAT

GGGGGTGCTAACACAACAAATGAAACTTTCAGACCTACAGGAGGAGATATGAAAGACAATTGGAGAAGTGAAT
TATACAAGTATAAAGTAGTAAAAATTGAACCACTAGGAGTAGCACCCACCAGGGCAAAGAGAAGAGTGGTGGA
GAGAGAAAAAAGAGCAGTTGGACTGGGAGCTGTATTCATTGGGTTCTTGGGAGCAGCAGGAAGCACTATGGGC
GCGGCGTCAATCACGCTGACGGTACAGGCTAGACAATTATTGTCTGGCATAGTGCAACAGCAAAGCAATTTGC
TGAGGGCTATAGAGGCTCAACAACATATGTTGAAGCTCACAGTCTGGGGCATT
>KX232614..1
TTCTATTGTAATACAACAAACCTGTTTAATAGTACTTGGCCTGAGAATAAAAATAGCACAGGGTCAAATGACA
CAGGGTCAAATGGCACTATAACTCTCCAGTGCAAAATAAAGCAAATTATAAATGTATGGCAGAGAGTAGGACA
AGCAATATATGCCCCTCCCATCCCAGGAGCAATAAGATGTAACTCAAATATTACGGGGCTACTATTAACAAGA
GATGGTGGGAATAATAACACAAATGAAACCTTCAGACCTGGAGGGGGAAATATGAAGGACAATTGGAGAAGTG
AACTGTATAAATATAAAGTAGTAAAACTTGAACCACTAGGAGTAGCACCCACCAAGGCAAAGAGAAGAGTGGT
GGAGAGAGAAAAAAGAGCATTCGGACTAGGAGCTGTATTCATTGGGTTCTTGGGAGCAGCCGGAAGCACTATG
GGCGCGGCGTCTGTAACGCTGACGGTACAGGCCAGACAATTATTGTTTGGCATAGTGCAACAGCAAAGCAATT
TGCTGAGGGCTATAGAGGCTCAGCAACACCTGCTGAAACTCACGGTCTGGGGCATT
>KX232618..2
TTCTATTGTAATACATCAGGCCTGTTTAATAGTACTTGGTCTAATAATGAGACTGAGGGAGCGGGGTCAAATG
ACACTATAACACTCCCATGCAGAATAAAGCAAATTGTAAATGTATGGCAGAGAGTAGGACAAGCAATATATGC
CCCTCCCATCGAAGGACTAATAAAATGTAACTCAAATATTACGGGGCTACTATTGACAAGAGATGGTGGGGAT
TATAACACATCTACAAATAATGAAACCTTCAGACCTGGAGGAGGAAATATGAAGGACAATTGGAGAAGTGAAC
TGTATAAGTATAAAGTAGTAAGACTTGAACCACTAGGAGTAGCACCCACCAAAGCGAAGAGAAGAGTGGTGGA
GAGACAAAAAAGAGCAATTGGAATAGGAGCTGTGTTCATTGGGTTCTTGGGAGCAGCTGGAAGCACTATGGGC
GCGGCGTCCGTAACGCTGACGGTACAGGCCAGACAATTATTGTCTGGCATAGTGCAACAGCAAAGCAATTTGC
TGCAGGCTATAGAGGCTCAGCAACATCTGCTGAAACTCACAGTCTGGGGCATT
>KX232620..2
TTCTATTGTAATACAACAAGCCTGTTTAATAGTACTTGGCCTCATAATGAGACGAAAATAAATAGCACGGAAT
CAAATGACAATGGCACTATAACACTCCCATGCAGAATAAAGCAAATTGTAAATATATGGCAGAGAGTAGGACA
AGCAATGTATGCCCCTCCCATCAAAGGACTAATAAGGTGTAATTCAACTATTACGGGGCTACTATTAACAAGA
GATGGGGGTGAGACTAATGGCACAAATGAAACCTTTAGACCTGGAGGAGGAGATATGAGGGATAATTGGAGAA
GTGAACTGTATAAGTATAAAGTAGTAAAAATTGAACCACTAGGAATAGCACCCACCAAAGCAAGGAGAAGAGT
GGTGGAGAGAGAGAAAAAAGAGCAATTGGACTAGGAGCTGTATTCATTGGGTTCTTGGGAGCAGCCGGAAGCACT
ATGGGCGCGGCGTCTGTAACGCTGACGGTACAGGCCAGACAATTATTGTCTGGCATAGTGCAACAGCAAAGCA
ATTTGCTGAAGGCTATAGAGGCTCAGCAGCAACTGCTGAAACTCACGGTCTGGGGCATT
>KX232624..1
TTCTATTGTAATACAACAGGCCTGTTTAATAGTACTTGGTATAATAATGGAACTGAGGAAGCAAATAACACGG
GGTCAAATGACACTATAACACTCCCATGCAGAATAAAACAAATTATAAATGTATGGCAAAGAGTAGGACAAGC
AATATATGCCCCTCCCATCGAAGGACTAATAAGGTGTAATTCAACTATTACGGGGCTACTATTAACAAGAGAT
GGGGGTAATAACACAAATGACACAAGTGAAACCTTCAGACCTGGAGGAGGAGATATGAGGGATAATTGGAGAA
GTGAACTGTATAAGTATAAAGTAGTAAAAATTGAACCACTAGGAATAGCACCCACCAAAGCAAGGAGAAGAGT
GGTGGAGAGGGAAAAAAGAGCAGTTGGACTAGGAGCTGTATTCATTGGGTTCTTGGGAGCAGCCGGAAGCACT
ATGGGCGCGGCGTCCGTGACGCTGACGGTACAGGCCAGGCAATTATTGTCTGGCATAGTGCAACAGCAAAGCA
ATTTGCTGAGGGCTATAGAGGCTCAGCAACATCTGCTGAAACTCACAGTCTGGGGCATT
>KX232626..1
TTCTATTGTGATACAACAGGCCTGTTTAATAGTACTTGGCCTAACAATGAAACTGGGGAAGTAAATAGCACGG
GATCAAACGATATTATAACACTCCCATGCAGAATAAAGCAAGTTGTAAATGTATGGCAGAGAGTGGGACAAGC
AATATATGCCCCTCCCATCCAAGGACTAATAAAATGTAACTCAAATATTACGGGGATAATATTAACAAGAGAT
GGTGGGAATCTTAATTCTACAAATGAAACTTTCAGACCTGGAGGAGGAAATATGAAGGACAATTGGAGAAGTG
AACTGTATAAGTATAAAGTAGTAAAACTTGAACCACTAGGAGTAGCACCCACCAGAGCAAAGAGAAGAGTGGT
GGAGAGAGAAAAAAGAGCAATTACCATAGGAGCTGTGCTCATTGGGTTCTTGGGAGCAGCCGGAAGCACTATG
GGCGCGGCGTCTGTAACGCTGACGGTACAGGCCAGACAATTATTGTCTGGCATAGTGCAACAGCAAAGCAATT
TGCTAAGGGCTATAGAGGCTCAGCAACATCTGCTGAAACTCACAGTCTGGGGCATT
>KU749423..1
TTCTATTGTAATACATCAGGCCTGTTCAATAGCACCTGGTATCCCAATGGCACCAACATAACTAACCCAGGAC
CAGAGAATATAACCCTCCCATGCAGAATAAAGCAAATTGTAAATATGTGGCAGAGAGTAGGGAGAGCAATGTA
TGCTCCTCCCATCAAAGGAGTAATACAATGTGTATCAAACATTACAGGACTACTATTAACAAGAGATGGTGGG
AATAATAATAATACAACTGAAACCTTCAGACCAGGAGGAGGAGATATGAGGGACAATTGGAGAAGTGAATTAT
ATAAGTATAAAGTAGTACAAGTTGAACCACTAGGAATAGCACCCACTAGGGCAAGGAGAAGAGTGGTGGAGAG
AGAAAAAAGAGCAGTTGGAATGGGAGCTCTTTTCATTGGGTTCCTAGGAGCAGCCGGAAGCACTATGGGCGCG
GCATCAATGATGCTGACGGTACAGGCCAGACAATTATTGTCTGGCATAGTGCAACAGCAAAGCAATTTGCTGA
GGGCTATTGAAGCTCAACAGCATATGTTGAAGCTCACGGTCTGGGGCATT
>KX983643..1

TTCTATTGTAATACATCAGAACTGTTTAATAGCACTTGGGGAGAGGAAGGGCGAAAGGACAATATCACGAAGT
CAAATGCCACTATAACTCTCCCATGTAGAATAAAGCAAGTCATCAATATGTGGCAGAGAGCAGGACGAGCAAT
GTATGCCCCTCCCATCCAAGGAGTAATAAATTGTGCATCAAACATTACAGGACTACTACTAACAAGAGACGGT
GGGAATACTAATACTAGCGATCTACCTGAAATCTTCAGACCAGGAGGAGGAGATATGAGGGACAATTGGAGAA
GTGAATTATATAAGTATAAGGTAGTAAAAATTGAACCACTAGGAGTAGCACCCACCAAGGCAAGGAGAAGAGT
GGTGGAGAGAGAAAAAAGAGCAGTTGGACTGGGAGTTGTCTTCCTTGGGTTCTTAGGAGCAGCAGGAAGCACT
ATGGGCGCGGCGTCAATAACGCTGACGGTACAGGCCAGACAATTATTGTCTGGCATAGTGCAGCAGCAAAGCA
ATTTGCTGAAGGCTATAGAGGCTCAACAGCATCTGTTGAAGCTCACAGTCTGGGGCATT
>KX983542..2
TTCTATTGTGATACAACAGGCCTGTTTAATAGCACTTGGAAGAATGGCACTCTCAACATGCAGGAATTAACTA
TCACAGTCACAAACTACACAAATCTCACAGACAGTATAAATATAACTCTTCCATGTAGAATAAAGCAAATTAT
AAATATGTGGCAGAGAGTAGGACAGGCAATGTATGCTCCTCCCATCCAAGGAGTAATAAAATGTGTGTCAAAC
ATTACGGGACTACTATTAACGAGAGATGGGGGGAATAATGAGACAGAAACCTTCAGACCTGGAGGAGGAAATA
TGAAGGACAATTGGAGAAGTGAATTATATAAGTATAAAGTAGTAAAAGTTGAGCCTATAGGAGTAGCACCCAC
CAGGGCAAGGAGAAGAGTGGTGGAGAGAGAAAAAAGAGCAGTTGGAATAGGAGCTGTTTTCATTGGGTTCTTA
GGAGCAGCAGGAAGCACTATGGGCGCGGCGTCAATAACGCTGACGGTACAGGCCAAACAATTATTGTCTGGCA
TAGTGCAACAGCAAAGCAATTTGCTGAGGGCTATAGAGGCTCAACAGCATCTGTTGAAACTCACGGTCTGGGG
CATT
>KX983566..2
TTCTATTGTAATACATCAAGCCTGTTTAATAGCATTTGGGTTAATGGCACTAGGAATGACACTGTCAGCAATG
GAACTTATATAACTCTCCCATGCAGAATAAAGCAAATTATAAATATGTGGCAGAGAGTAGGGCAAGCAATGTA
TGCCCCTCCCATTAGAGGAGTAATAAAGTGTGAATCAAACATTACAGGACTACTGTTAACAAGAGATGGTGGG
GAGAATAATAGTACCAATGAAACCTTCAGACCTGGAGGAGGAGATATGAGGGACAATTGGAGAAGTGAATTAT
ATAAGTACAAAGTAGTAAAAATTAAACCACTAGGAGTAGCACCCACCAAGGCAAAGAGAAGAGTGGTGGAGAG
AGAAAAAAGAGCAGTAGTTGGACTGGGAGCTGTCTTCCTTGGGTTCTTAGGAGCAGCAGGAAGCACTATGGGC
GCAGCGTCAATAACGCTGACGGTACAGGCCAGACAATTATTGTCTGGCATAGTGCAACAGCAAAGCAATTTGC
TGAGGGCTATAGAGGCTCAACAACATCTGTTGAAACTCACGGTCTGGGGCATT
>JX236677..1
TTCTATTGTAATACATCAGCCCTGTTTGATAGCACTTGGCGGTTTAATGGCATTGGCAGTGGAAATGACACTG
TCAATGCCACTAGAAGCGAGAATGATACTATAAATCTCCCATGCAGGATAAAGCAAATTATAAATATGTGGCA
GAGAGCAGGACAGGCAATGTATGCCCCTCCCATCCAAGGAGTAATAAGGTGTGACTCAAACATTACAGGTCTA
TTATTAACAAGAGATGGTGGGAGGGGGAATGATAATAACACTGAGGTCTTCAGACCTGGAGGAGGAAATATGA
AGGACAATTGGAGGAGTGAATTATATAAGTACAAAGTAGTAAAAATTGAACCATTAGGAGTAGCACCCACCAG
GGCAAGGAGAAGAGTGGTGGAGAGAGGAAAAAGAGCAGTTGGAATAGGAGCTGTTTTCATTGGGTTCTTAGGA
GCAGCAGGAAGCACTATGGGCGCAGCGTCAATAACGCTGACGGTACAGGCCAGGCAATTATTGTCTGGCATAG
TGCAACAGCAAAGCAATTTGCTGAGGGCTATAGAGGCTCAACAGCATCTGTTGAGACTCACAGTCTGGGGCAT
T
>JX236678..2
TTCTATTGTAACACATCAACCCTGTTTAGTAGCACTTGGGAGAACGGTACGGAACTAAACAGCACGAAGTCAA
ATGACACTATAACTCTCCCATGCAGAATAAAGCAAATTATAAATATGTGGCAGAGAGTAGGACAAGCAATATA
TGCTCCTCCAATCGAAGGAGAAATAAGGTGTCAATCAAATATTACAGGACTAATATTAACAAGAGATGGTGGT
GAGGATATTAACAGTACAAGTGAAACCTTCAGACCTGGAGGAGGAGATATGAGGGACAATTGGAGAAGTGAAT
TATATAAGTATAAGGTAGTAAAAATTGAACCACTAGGAGTAGCACCCACCAGGGCAAGAAGAAGAGTGGTGGA
GAGAGAAAAAAGAGCAGTTGGAATAGGAGCTGTCTTCATTGGGTTCTTAGGAGCAGCAGGAAGCACTATGGGC
GCGGCGTCAATAACGCTGACGGTACAGGCCAGACAATTATTGTCTGGCATAGTGCAACAGCAAAGCAATTTGC
TGAGGGCTATAGAGGCTCAACAACATCTGTTGAAGCTCACGGTCTGGGGCATT
>KU749424..1
TTTTATTGTAATACATCAGAACTGTTTAATAGCACTTGGGTATACAATGCATCAGAACTTAATGGCACTTGGG
TAAACAATGCCAGCATGCCAGGATCAAATAGCACAAGTGAAAATATAACTCTCCACTGCAGAATAAAGCAAAT
TATAAATATGTGGCAGAGAGCAGGAAAAGCAATGTACGCCCCTCCCATCTCAGGAAACATAACGTGTGTTTCA
AACATTACAGGACTCATATTAACAAGAGATGGTGGGAATACCAATACAACTGAAATCTTCAGACCTGAAGGAG
GAGATATGAGAGACAATTGGAGAAGTGAATTATATAAGTATAAAGTAGTAAAAATTGAACCACTAGGAGTAGC
ACCCACCAAGGCAAGGAGGAGAGTGGTGGAGAGAGAAAAAAGAGCAATTGGAATAGGAGCTGTCCTCTTTGGG
TTCTTAGGAGCAGCAGGAAGCACTATGGGCGCGGCGTCAATAACGCTGACGGTACAGGCCAGACAATTATTGT
CTGGCATAGTGCAACAGCAAAGCAATTTGCTGAGGGCTATAGAGGCTCAACAACATCTGTTGAAACTCACAGT
CTGGGGCATT
>MF565934..1
TTCTATTGCAATACATCAAGCCTGTTTAATAGTACTTGGGATGGGAATAGCACTACTAACAGCACGCAGAAGC
CAAATGGAACTATAACTCTCCCATGCAGGATAAAGCAAATTATAAATATGTGGCAGAGAACAGGGCAAGCAAT
GTATGCCCCTCCCATCCCAGGAAAAATAAGGTGTGACTCAAACATCACAGGACTAATATTAACAAGAGATGGT

GAAAATAATAACACTGAAAGTGAAACCTTTAGACCTGAAGGAGGAGACATGAGGAACAATTGGAGAAGTGAGT
TATATAAGTATAAAGTAGTAAAGATTGACCCACTAGGAGTAGCACCCACCGGGGCAAAGAGAAGAGTGGTGGA
GAGAGAAAAAAGAGCAGTTGGAATAGGAGCTGTCCTCTTTGGGTTCTTAGGAGCAGCAGGAAGCACTATGGGC
GCAGCGTCTCTAACGCTGACGGTACAGGCCAGACAATTATTGTCTGGCATAGTGCAACAGCAAAGCAATTTGC
TGAGGGCTATAGAGGCTCAACAACATCTGTTGAGACTCACGGTCTGGGGCATT
>KX983624..1
TTCTACTGTAATACATCAGGCCTGTTTAATAGCCTCTGGATAAATGGCACCATGCTGAATGGTACCCTGAAGA
ATTCACCCAGCACAAATGACACCATAACTCTCCCATGCAGAATAAAGCAAATCATAAACATGTGGCAGAAAGT
AGGACAAGCAATGTATGCCCCTCCCATCCCAGGAAGCATAACGTGTGTATCAAACATTACAGGACTAATATTA
ACAAGAGATGGTGGGAATACTAACAGGACAGATGAAATCTTCAGACCTGAAGGGGGAAATATGAGGGACAATT
GGAGAAGTGAATTATATAAGTATAAAGTAGTAAAAATTGAACCACTAGGAGTAGCACCCACTAGGGCAAAGAG
AAGAGTGGTGGAGAGAGAAAAAAGAGCAGTTGGACTGGGAGCTGTCTTCATTGGGTTCTTAGGAGCAGCAGGA
AGCACTATGGGCGCGGCGTCAATAACGCTGACGGTACAGGCCAGAAACTTATTGTCTGGCATAGTGCAACAGC
AAAGCAATTTGCTGAGGGCTATTGAGGCTCAACAGCATCTGTTGAAACTCACAGTCTGGGGCATT
>KF716472..1
TTCTATTGTAACACAACAGGTCTGTTTAATAGCACTTGGAGTCGGAACACAACAGATAATAACACTTGGTTTA
GGAACAATGCTAGCAGTAGCACAGGGTTAAATGGCACAATAACTCTCCAATGCAAAATAAAGCAAATTATAAA
TATGTGGCAGAGAACAGGACGAGCAATGTATGCCCCTCCCATACCAGGAACAATAATGTGTAACTCAAGCATC
ACAGGACTGCTATTAACAAGAGATGGTGGGAATAGTGGGAACAACAGTAATGAAACCTTCAGGCCTGGAGGAG
GAGATATGAAAGACAATTGGAGAAGTGAATTATATAAGTATAAGGTAGTAAAAATTGAACCACTAGGAGTAGC
ACCCACCAGGGCAAAGAGAAGAGTGGTGGAGAGAGAAAAAAGAGCAGTTGGAATGGGAGCTCTTTTCATTGGG
TTCTTGGGAGCAGCAGGAAGCACTATGGGCGCGGCGTCAGTGACGCTGACGGTACAGGCCAGACAATTATTGT
CTGGTATAGTGCAACAGCAAAGCAATTTGCTGAAGGCTATAGAGGCTCAACAACATCTGTTGAGACTCACAGT
CTGGGGCATT
>AB253421..1
TTTTATTGTGATTCATCAGGCCTGTTTAATAGCACTTGGGATAACACTAACATCACGCAGCCAAATAGCACAG
GGTCAAATGACACCATAACTCTCCAATGCAGAATAAAGCAAATTATAAATATGTGGCAGAGAGCAGGACAGGC
AATGTATGCCCCTCCCATCCCAGGAGTAATAAGTTGTGTATCAAACATTACAGGATTACTATTAACAAGAGAT
GGTGGGATTACCAGTGCAAATGAAACCTTCAGACCTGGAGGAGGAGATATGAGGGACAATTGGAGAAGTGAAT
TATATAAGTATAAGGTAGTAAAACTTGAACCACTAGGAGTAGCACCCACCAGGGCAAGGAGAAGAGTGGTGGA
GAGAGAAAAAAGAGCAGTTGGAATAGGAGCTGTATTCATTGGTTTCTTAGGAGCAGCAGGAAGCACTATGGGC
GCGGCGTCAATGACGCTGACGGTACAGGCCAGACAATTATTGTCTGGCATAGTGCAACAGCAAAGCAATTTGC
TGAGGGCTATAGAGGCTCAACAACATCTGTTGAAACTCACGGTCTGGGGCATT
>AB287377..1
TTCTATTGTAATACATCAGGCCTGTTTAATAGCACCTGGAATAATAATAATACCAGGGAGAAGATAAATGGTA
CAGAGTCAAATAGCACTATAACTCTCCATTGCAGAATAAAGCAAATTATAAATAGGTGGCAGGAAGTAGGACA
AGCAATGTATGCCCCTCCCATCCCAGGAGTAATAAATTGTAGATCAAACATTACAGGACTAATATTAACAAGA
GATGGTGGGGATGGGGATAACAATACGGAAATCTTCAGACCTGGAGGAGGAAATATGAAGGACAATTGGAGAA
GTGAATTATATAAGTATAAAGTAGTAAAAATTGAACCACTGGGAGTAGCACCCACCAGGGCTAAGAGAAGAGT
GGTGGAGAGAGCAAAAAGAGCAGTTGGAATAGGAGCTGTTTTCCTTGGGTTCTTAGGAGCAGCAGGAAGCACT
ATGGGCGCGGCGTCAATAACGCTGACGGTACAGGCCAGACAATTATTGTCTGGCATAGTGCAACAGCAAAGCA
ATTTGCTGAGGGCTATAGAGGCTCAACAACATCTGTTGAAACTCACGGTCTGGGGCATT
>AB287379..1
TTCTATTGTAATACATCAGGCCTGTTTAATAGCACTTGGACCAATGAAAGCATGCAAGTAAATAGCACAGAAT
CAAATGACACTATAACTCTCCCATGCAGAATAAAGCAAATTATAAATATGTGGCAGAGGGCAGGACAAGCAAT
ATATGCCCCTCCCATCGAAGGAGTAATAAGGTGTAGCTCAAACATTACAGGACTACTATTAACAAGAGATGGT
GGGGTTAACAATACAGTCAATGAAACCTTCAGACCTGGAGGAGGAAATATGAGGGATAATTGGAGAAGTGAAC
TATATAAATATAAAGTAGTAAAAATTGAACCACTAGGAGTAGCACCCACCAGGGCAAAAAGAAGAGTGGTGGA
GAGAGAAAAAAGAGCAGTTGGACTGGGAGCTGTCTTCATTGGGTTCTTAGGAGCAGCAGGAAGCACTATGGGC
GCGGCGTCAATAACGCTGACGGTACAGGCCAGACAATTATTGTCTGGCATAGTGCAACAGCAAAACAATTTGC
TGAGGGCTATAGAGGCTCAACAACATCTGTTGAAACTCACGGTCTGGGGCATT
>AY713406..1
TTCTATTGTAATACATCAAGCCTGTTTAATAGCACTTGGAATAACACTGCCAGCATGCAGAAGTCAAATAGCA
CGGGGTCAAATGACACTATAACTCTCCAATGCAGAATAAAGCAAATTATAAGAATGTGGCAAAGAACAGGACA
GGCAATGTATGCCCCTCCCATCAAAGGAGATATAAGGTGTGAATCAAACATTACAGGACTAATATTAACAAGA
GATGGTGGGAATGATAGCAGTCCAGATGAAATCTTCAGACCTGGAGGAGGAGATATGAGGGACAATTGGAGAA
GTGAATTATATAAGTATAAAGTAGTACAAATTGAACCTCTAGGAGTAGCACCCACCAAGGCAAGAAGAAGAGT
GGTGCAGAGAGAGAGAAAAGAGCAATTGGAATAGGAGCTGTCTTCCTTGGGTTCTTAGGAGCAGCAGGAAGCACT
ATGGGCGCGGCGTCAATAACGCTGACGGTACAGGCCAGACAATTATTGTCTGGCATAGTGCAACAGCAAAGCA
ATTTGCTGAGGGCTATAGAGGCTCAACAACATCTGTTGAAACTCACGGTCTGGGGCATT

>L07082..1
TTCTATTGTAATACAACAGGCCTGTTTAATAGCACCTGGAAAAATGTAAATGGCACCATGCAGGAGTCAAGCA
GCACAAAGTTAAATGACACTATAACACTCACATGCAGAATAAAGCAAATTATAAATATGTGGCAGAGAGCAGG
ACAGGCAATGTATGCCCCTCCCATCGAAGGAATAATAAGGTGTGACTCAAACATTACAGGACTACTATTAACA
AGAGATGGTGGGGTTGGGGATAACAGTACAAATGGAACTGAAACATTCAGACCTGGAGGAGGAAATATGAGGG
ACAATTGGAGAAGTGAATTATATAAGTATAAAGTAGTAAAAATTGAACCACTAGGAGTAGCACCCACCACGGC
AAAAAGAAAAGTGGTGGAGAGAGAAAAAAGAGCAATTGGAATAGGAGCTGTCTTCCTTGGGTTCTTAGGAGCA
GCAGGAAGCACTATGGGCGCGGCGTCAATAACGCTGACGGTACAGGCCAGACAATTATTGTCTGGCATAGTGC
AACAGCAAAGCAATTTGCTGAGGGCTATAGAGGCTCAACAGCATCTGTTGAAACTCACAGTCTGGGGCATT
>MF373167..1
TTCTATTGTAATACATCAGGCCTGTTTAATAGCACTTGGAATGAAACTACCATGCAGGAGTCAAATGGAACAG
AGTCAAACGACAGCATAACTCTCCCATGCAGAATAAAGCAGATTGTAAATATGTGGCAGAGAACAGGACAAGC
AATGTATGCCCCTCCCATCCAAGGAGTAATACAGTGTGTATCAAATATTACAGGACTAATATTAATGAGAGAT
GGTGGGAATAGTAGTAGGGAAAATGAAACCTTCAGACCTGGAGGAGGAGATATGAGGGACAATTGGAGAAGTG
AATTATATAAGTATAAAGTAGTAAAAATTGAACCACTAGGAGTAGCACCCACCAGGGCAAGGAGAAGAGTGGT
GCAGAGAGAAAAAAGAGCGGTTGGACTGGGAGCTGTTTTCATTGGGTTCTTAGGAGCAGCAGGAAGCACTATG
GGCGCAGCGTCAGTGACGCTGACGGTACAGGCCAGGCAATTATTGTCTGGCATAGTGCAACAGCAAAGCAATT
TGCTGAGGGCTATAGAGGCTCAACAGCATCTGTTGAGACTCACGGTCTGGGGCATT
>MF373168..1
TTCTATTGTGATACATCAGACCTGTTTAATAGCATTTGGCCGTTTAATAGCACTCGGGGACACAATGAGACTG
AAAGATCAAATAGCACAGAGACAAATAACACTATTATAAAACTTCCATGTAGAATAAAACAAATTATAAATAT
GTGGCAGAGAGTAGGACAAGCAATATTTGCCCCTCCCATCCAAGGAGTAATACAGTGTGTATCAAACATTACA
GGACTAATATTAACAAGAGATGGAGGGAATAATAACACTACAAATGAAACCTTCAGACCTGGAGGAGGAGATA
TGAGGGACAATTGGAGAAGTGAATTATATAAGTATAAAGTAGTAAAAATTGAACCACTAGGAGTAGCACCCAC
CAGGGCACAGAGAAGAGTGGTGCAGAGAGAAAAAAGAGCAATTGGAATGGGAGCTGTCTTCATTGGGTTCTTA
GGAGCAGCAGGAAGCACTATGGGCGCGGCGTCAATAACGCTGACGGTACAGGCCAGACAATTATTGTCTGGTA
TAGTGCAACAGCAAAGCAATTTGCTGAGGGCTATAGAGGCTCAACAGCATCTGTTGAAACTCACGGTCTGGGG
CATT
>MF373181..2
TTCTACTGTAATACATCAGGCCTGTTTAACAGTAGTTGGGGAAATGGCACTAACAACAGCAGCACTGGGGGGC
AAACTATAACTCTCCCATGCAAAATAAAGCAAATTGTAAATATGTGGCAGAGGACAGGACAAGCAATGTATGC
CCCTCCTATCGAAGGAGTAATAAGGTGTGTATCAAACATTACAGGAATACTATTAACTAGAGATGGTGGGCCT
AATAATAGTACAAATGAAACCTTCAGACCGGGAGGAGGAAATATGAAGGACAATTGGAGAAGTGAATTATATA
ACTATAAAGTAGTACAAATTAACCCATTAGGAATAGCACCCACCAAGGCAAAGAGAAGAGTGGTGGAGAGAGA
AAAAAGAGCAGTAGTTGGACTGGGAGCTTTGTTCATTGGGTTCCTAGGAGCTGCAGGAAGCACTATGGGCGCG
GCGTCAATGACGCTGACGGTACAGGCCAGACAATTATTGTCTGGCATAGTGCAACAGCAAAGCAATTTGCTGA
GAGCTATAGAGGCTCAACAGCATGTGTTGAGACTCACGGTCTGGGGCATT
>MF373182..1
TTCTATTGTAATACATCAGGCCTGTTTAATAGCAGTTGGGGAGTAAACAACACTGATGGCAATATAACTAATG
GCACGATAACTCTCCCCTGCAAAATAAAACAATTTATAAATATGTGGCAGAGAGTAGGACAAGCAATGTATGC
CCCTCCCATCGAAGGGGCAATAAGTTGTAATTCAACCATCACAGGACTACTATTAACAAGAGATGGTGGGTAC
AACAACAGCACTAACCAAATCGACAAAATCTTCAGACCTGCAGGAGGAGATATGCAGGACAACTGGAGAAGTG
AATTATATAAGTATAAAGTAGTAAAAATTGAACCATTAGGAGTAGCACCCACCTGGGCAAGGAGAAGAGTGGT
GCAGAGAGAAAAAAGAGCAGTTGGACTGGGAGCTGTCTTTCTTGGGTTCTTAGGAGCAGCAGGAAGCACTATG
GGCGCGGCGTCGATAACGCTGACGGTACAGGCCAGACAATTATTGTCTGGCATAGTGCAACAGCAAAGCAATT
TGCTGATGGCTATAGAGGCTCAACAGCATTTGTTGAAACTCACAGTCTGGGGCATT
>AF069670..1
TTCTATTGTAATACATCAGGCCTGTTTAATAGCACTTGGAGCCAAAATGACACTGGGGTATCAAATAGCACGG
AGTCAAATGACACTATAATTCTTCCATGCAGAATAAAACAAATTATAAATATGTGGCAGAGAGCAGGACAAGC
AATATATGCCCCTCCCATCCCAGGAATAATAAGGTGTGAATCAAACATTACAGGGCTACTGTTAACAAGAGAT
GGTGGGGTTGTTAACAGTACAAATGAAACCTTCAGACCTGGAGGAGGAAATATGAAGGACAATTGGAGAAGTG
AATTATATAAGTATAAAGTAGTAAAAATTGAACCACTAGGAGTAGCACCCACCAGGGCAAGGAGAAGAGTGGT
GCAGAGAGAAAAAAGAGCAGTTGGACTGGGAGCTTTATTCATTGGGTTCTTAGGAGCAGCAGGAAGCACTATG
GGCGCGGCGTCAATAACGCTGACGGTACAGGCCAGACAATTATTATCTGGCATAGTGCAACAGCAAAGTAATT
TGCTGAGGGCTATAGAGGCTCAACAGCATCTGTTGAAACTCACTGTCTGGGGCATT
>AF069671..1
TTCTATTGTAATACATCAGGCCTGTTTAATAGCACTATTTTGAATAGCACTAAGATGAATGACAATGCCAGCA
GGGAGTCGTATGATGACACTATAACCCTCCAGTGCAGAATAAAGCAAATTATAAATATGTGGCAGAGAGTAGG
ACAAGCAATGTATGCCCCTCCCATCCGAGGAGCAATAAGATGTAAATCAAACATTACAGGACTATTATTAACA
AGAGATGGTGGAAATAGTAACAGTAGTACAAATGAAACCTTCAGACCTGGAGGAGGAGATATGAGAGATAATT

GGAGAAGTGAATTATATAAATATAAAGTAGTAAAAATTGAACCACTAGGAGTAGCACCCACCAAGGCAAAGAG
AAGAGTGGTGGAGAGAGAAAAAAGAGCAGTTGGAATAGGAGCTGTCTTCCTTGGGTTCTTAGGAGCAGCAGGA
AGCACTATGGGCGCGGCGTCAATAACGCTGACGGTACAGGCCAGACAATTATTGTCTGGCATAGTGCAACAGC
AAAGCAATTTGCTGAGGGCTATAGAGGTTCAACAACATCTGTTGAAACTCACGGTCTGGGGCATT
>AF069673..1
TTCTATTGTAATACATCAGGCCTGTTTAATAGTACTTGGAATGGCACTGACAGCATGCAGAAGTTAAATAGCA
CGGGCAATATAACTCTCCCATGCAGAATAAAACAAATTATAAATATGTGGCAGAGAGCAGGACAAGCAATATA
TGCCCCTCCCATCCAAGGAGTAATAAGGTGTGAATCAAACATTACAGGACTAATATTAACAAGAGATGGTGGG
AATGATAACAATGAAAGTGAAATCTTCAGGCCTGGAGGAGGAGACATGAGGGACAATTGGAGAAGTGAATTAT
ATAAGTATAAGGTAGTAAAAATTGAACCACTAGGAGTAGCACCCACCAAGGCAAAAAGAAGAGTGGTGGAGAG
AGAAAAAAGAGCAATTGGAATAGGAGCTGTCTTCATTGGGTTCTTGGGAGCAGCAGGAAGCACTATGGGCGCA
GCGTCAATAACGCTGACGGTACAGGCCAGGCAATTATTGTCTGGCATAGTGCAACAGCAAAGCAATTTGCTGA
TGGCTATAGAGGCTCAACAGCATCTGTTGAAACTCACGGTCTGGGGCATT
>AF107771..2
TTCTATTGTAATACATCAGGTCTGTTTAATAGTAGTTGGAATGAAAATGACACCAAAGTAAATTACAACACTG
AGTCAAATGACACTATAACTCTCCAATGCAGAATAAAGCAAATTATAAATATGTGGCAGAGAACAGGACAAGC
AACGTATGCCCCTCCCATCCCAGGGGTAATACAGTGTAGATCAAACATTACAGGACTACTATTAACAAGGGAT
GGTGGGGTAACTAACAATACAAATAATGAAACCTTCAGACCTGGAGGAGGAGATATGAGGGACAATTGGAGAA
GTGAATTATACAAGTATAAAGTAGTAAAACTTGAACCACTAGGAGTAGCACCCACCAAGGCACAGAGAAGAGT
GGTGAAGAGAGAAAAAAGAGCAGTTGGACTGGCAGCTGTATTCTTTGGGTTCTTAGGAGCAGCAGGAAGCACT
ATGGGCGCGGCGTCAATAACGCTGACGGTACAGGCCAGACAATTATTGTCTGGTATAGTGCAACAGCAAAACA
ATTTGCTGAGGGCTATAGAGGCTCAACAGCATATGTTGAGACTCACGGTCTGGGGCATT
>HQ659604..1
TTTTATTGTGATACATCAGGCCTGTTTAATAGCACATGGGGGGGAAATAGCACTGACAGCATACAGGAGTCAA
ATAGCACAGAGTCAAATGACACTATAATTCTCCCATGCAGAATAAAGCAAATTATAAATATGTGGCAGAGAGT
AGGACAAGCAATGTATGCCCCTCCCATCCAAGGAGTAATAAAGTGTATATCAAATATTACAGGACTTCTATTA
ACAAGAGATGGTGGGGATAATAACAGTGCAAATGAAACCTTCAGACCTACAGGAGGAAATATGAGGGACAATT
GGAGAAGTGAATTATATAAGTATAAAGTAGTAAAAATTGAACCACTAGGAGTAGCACCCACCCCGGCAAAGAG
AAGAGTGGTGCAGAGAGAAAAAAGAGCAGTTGGAATAGGAGCTGTATTCATTGGGTTCTTAGGAGCAGCCGGA
AGCACTATGGGCGCGGCGTCAATAACGCTGACGGTACAGGCTAGACAATTATTGTCTGGCATAGTGCAACAGC
AAAGCAATTTGCTGAGGGCTATAGAGGCTCAACAACATCTGTTGAAACTCACGGTCTGGGGCATT
>HQ659597..1
TTCTATTGCAACACATCAGAATTGTTTAATAGCACTTGGGATAATAGCACTTGGGATAATAGCACTTGGGATA
ATAACATGCATGAGTCGAATAGCACGGAGAATATAACTCTCCAATGTAGGATAAAGCAAATTATAAATATGTG
GCAGAGAGCAGGACAAGCAATATATGCCCCTCCCATCAAAGGAGTAATAGGGTGTAAATCAAACATTACAGGA
CTAATATTAACAAGAGATGGTGGGGAGAAGGGTAATAACAGTATCAATGAAACCTTCAGACCTGGAGGAGGAG
ATATGAGGGACAATTGGAGAAGTGAATTATACAAGTATAAAGTAGTAAAAATTGAACCACTAGGAGTAGCACC
CACCAAGGCAAAGAGAAGAGTGGTGGAGAGAGAAAAAAGAGCAGTTGGAATAGGAGCTGTCTTCCTTGGATTC
TTAGGAACAGCAGGAAGCACTATGGGCGCGGCGTCAGTAACGCTGACGGTACAGGCCAGACAATTATTGTCTG
GCATAGTGCAACAGCAAAGCAATTTGCTGAGGGCTATAGAGGCTCAACAGCATCTGTTGAAACTCACAGTCTG
GGGCATT
>HQ697994..3
TTCTATTGCTACACATCAGGCCTGTTTAATTATACCTTTAATGATACCAGCACAAATAGCACGGAGTCGAAGT
CAAATGACACTATAACTCTCCAATGCAGAATAAAGCAAATTATAAATATGTGGCAGAGAGCAGGACAAGCAGT
ATATGCCCCTCCCATACCAGGGATAATAAGGTGTGAATCAAACATTACAGGACTAATATTAACAAGAGATGGT
GGGAATAATAATAGTAATACAAATGAAACCTTCAGACCTGGAGGAGGAGATATGAGGGACAATTGGAGAAGTG
AATTATATCGGTATAAAGTAGTAAAAATTGAACCAATAGGAGTAGCACCTACCACGGCAAAGAGAAGAGTGGT
GGAGAGAGAAAAAAGAGCAGTTGGAATAGGAGCTGTCTTCCTTGGGTTCTTAGGAGCAGCAGGAAGCACTATG
GGCGCGGCGTCAATAACGCTGACGGTACAGGCCAGACAATTATTGTCTGGCATAGTGCAACAGCAAAGCAATT
TGCTGAGGGCTATAGAGGCTCAACAGCATCTGTTGAAACTCACGGTCTGGGGCATT
>HQ698029..1
TTCTATTGCAACACGGCAGGCCTGTTTGATAGCATTTGGAATGGTAGCATTGGGAATAACACTGACAATAGTA
GCATGGAGTTAAATGACGATAATGACACTATAACTCTCCCATGCAGAATAAAGCAAATTATAAATATGTGGCA
GAGAGTAGGACAAGCAATATATGCCCCTCCCATCCAAGGAGTAATAAGGTGTGACTCAAATATTACAGGGTTA
ATATTAACAAGAGATGGTGGGGATAATAATAGTGCAAATGAAACCTTCAGACCTGTAGGAGGAGATATGAGGG
ACAATTGGAGAAGTGAATTATATAAGTATAAAGTAGTAAAAATTGAGCCACTAGGAGTAGCACCCACCAGGGC
AAAGAGAAGAGTGGTGGGGAGAGAAAAAAGAGCAGTTGGACTAGGAGCTGTCTTCATTGGGTTCTTAGGAGCA
GCAGGAAGCACTATGGGCGCAGCGTCAGTAACGCTGACGGTACAGGCCAGACAATTATTGTCTGGCATAGTGC
AACAGCAAAGCAATTTGCTGAAGGCTATAGAGGCTCAACAGCATCTGTTGAGACTCACAGTCTGGGGCATT
>AY253305..2

TTCTATTGTAACACATCAGGCCTGTTTAATGGCCTGTTTAATGGCACCTGGAATGGCAATCACACAAACAGCA
CGGAGTTAAATAGCACTATAATTCTCCAATGCAAAATAAAGCAAATTATAAATATGTGGCAGAGAGCAGGACA
AGCAATATATGCCCCTCCCATCCAAGGAGTAATAAAGTGTGTATCAAATATTACAGGACTAATATTAACAAGA
GATGGTGAGAGGAACAATAGTCTAAATGAAACCTTCAGACCTGGAGGAGGAGATATGAGGGACAATTGGAGAA
GTGAATTATATAAGTATAAAGTAGTAAAAATTGAACCACTAGGAGTAGCACCTACCAGGGCAAAGAGAAGAGT
GGTGGAGAGAGAAAAAAGAGCAGTCGGACTGGGAGCTGTCTTCATTGGGTTCTTAGGAGCAGCAGGAAGCACT
ATGGGCGCGGCGTCAGTAACGCTGACGGTACAGGCCAGACAATTATTGTCTGGCATAGTGCAACAGCAAAGCA
ATTTGCTGAGGGCTATAGAGGCTCAACAGCATCTGTTGAAACTCACGGTCTGGGGCATT
>AY253314..1
TTCTATTGTAATACATCAGGCCTGTTTAATAGCACTTGGACTACCAACAACATGCAGGAGTCAAATGACACTA
GCAATGGCACTATAACTCTCCCATGCAGAATAAAGCAAATTATAAGGATGTGGCAGAGAGTAGGACAAGCAAT
GTATGCCCCTCCCATTGAAGGAGTAATAAGATGTGAATCAAACATTACAGGACTAATACTAACAAGAGATGGC
GGGAATAATAACAGTGCAAATGAAACCTTTAGACCTGGAGGAGGAGATATAAGGGACAATTGGAGAAGTGAAT
TATATAAGTATAAAGTAGTAAAAATTGAACCACTAGGAGTAGCACCCACCAGGGCAAAGAGAAGAGTGGTGGA
GAGAGAGAAAAGAGCAGTTGGAATAGGAGCTGTCTTCCTTGGGTTCTTAGGAGCAGCAGGAAGCACTATGGGC
GCGGCGTCAATAACGCTGACGGTACAAGCCAGACAATTATTGTCTGGCATAGTGCAGCAGCAAAGCAATTTGC
TGAGGGCTATAGAGGCTCAACAACAGCTGTTGAAACTCACGGTCTGGGGCATT
>KX907352..1
TTCTATTGTAATACATCAGGCCTGTTTAATAGCACTTGGCTATTTAACAAGTCAAATGAGAATAAGTCAAATG
AGTCAGATGTCAATATAACACTCCCATGCAGAATAAAGCAAATTATAAATATGTGGCAGAGAGCAGGACAAGC
AATGTATGCCCCTCCCATCCCAGGAGTAATAAAGTGTGAATCAAACATTACAGGACTACTATTAACAAGAGAT
GGTGGGAATAACAACAATTCAAATGAGACCTTCAGACCTGGAGGAGGAGATATGAGGGACAATTGGAGAAGTG
AATTATATAAGTATAAAGTAGTCAAAATTGAACCACTAGGAGTAGCACCCACCAGGGCAAGGAGAAGAGTGGT
GGGAAGAGAAAAAAGAGCAGTTGGAATAGGAGCTGTTTTCCTTGGGTTCTTAGGAGCAGCAGGAAGCACTATG
GGCGCGGCGTCAATAACGCTGACGGTACAGGCCAGACAATTATTGTCTGGCATAGTGCAACAGCAAAGCAATT
TGCTGAGGGCTATAGAGGCTCAACAACATCTGTTGAAACTCACAGTCTGGGGCATT
>KX907348..1
TTCTATTGTAACACATCAGGTCTGTTTAATAGCACTTGGTATGGCAATGGCAGCAAGCTGGAGTCAAATAGCA
CGGGGTCAGACGACAATATAACTCTCCACTGTAGAATAAAGCAAATTATAAATATGTGGCAAAGAGCAGGACA
AGCAATATATGCCCCTCCCATCCCAGGAGTAATAAACTGTCTATCAAACATTACAGGACTAATATTAACAAGA
GATGGTGGGGACAATAACAGTACAAATGAAACCTTCAGACCTGGAGGAGGAGATATGAGGGACAATTGGAGAA
GTGAATTATATAAGTATAAAGTAGTAAAAATTGAACCATTAGGAGTAGCACCCACCAGGGCAAAGAGAAGAGT
GGTGGAAAGAGAAAAAAGAGCAGTTGGACTGGGAACTGTTCTCTTTGGGTTCTTAGGAGCAGCAGGAAGCACT
ATGGGCGCGGCGTCAGTAACGCTGACGGTACAGGCCAGAAAGTTACTATCTGGCATAGTGCAACAGCAAAGCA
ATTTGCTGAGGGCTATAGAGGCTCAACAGCATCTGTTGAAACTCACAGTCTGGGGCATT
>KX907336..1
TTCTATTGTAATACATCAGGCCTGTTTAATAGCACTTGGAACAGCACTGGGTCAAGAAATGACAGTGGCAATA
TAACTCTCCCATGCAGAATAAAGCAAATTGTAAATATGTGGCAGAGGACAGGACAAGCAATATACGCCCCTCC
CATCCAAGGAAGAATACAGTGTGTGTCAAATATTACAGGACTACTATTGACAAGAGATGGAGGGATTAACAAC
AGTGAGGAAACCTTCAGACCTGGAGGAGGAAATATGAGAGATAATTGGAGAAGTGAACTATATAAATATAAAG
TAGTAAAAATTGAACCACTAGGAGTAGCACCCACCAGGGCAAAGAGAAGAGTGGTGGAGAGAGAAAAAAGAGC
AGTAGTTGGACTGGGAGCTGTCTTCCTTGGGTTCTTAGGAGCAGCAGGAAGCACTATGGGCGCGGCGTCACTG
ACGCTGACGGTACAGGCCAGACATTTATTGTCTGGCATAGTGCAACAGCAAAACAATTTGCTGAGGGCTATAG
AGGCTCAACAGCATCTGTTGAAACTCACGGTCTGGGGCATT
>KX907372..1
TTCTATTGTAATACATCAGGCCTGTTTAATAGCAGTTGGAACATTAATGGCAGTCGGGAGTCAAATAAAACAC
AGTCAAATGGCACTATAACTCTCCCATGCAGAATAAAGCAAATTATAAATATGTGGCAGAGAGCAGGACAAGC
AATATATGCTCCTCCCATCCAAGGAGTAATAAAGTGTGTATCAAACATTACAGGACTAATATTAACAAGAGAT
GGTGGGAATAATAATAACAGTTCAAATGAAACTTTCAGACCTGGAGGAGGAGATATGAGGGACAATTGGAGAA
GTGAATTGTATAAGTATAAGGTAGTAAAAATTGAACCACTAGGGGTAGCACCCACCAGGGCAAAGAGAAGAGT
GGTGGAGAGAGAAAAAAGAGCAGTTGGAATAGGAGCTGTTTTCCTTGGGTTCTTAGGAGCAGCAGGAAGCACT
ATGGGCGCGGCGTCAATAACGCTGACGGTACAGGCCAGACAATTACTGTCTGGCATAGTGCAACAGCAGAGCA
ATTTGCTGAGGGCTATAGAGGCTCAACAACAGTTGTTGAAACTCACGGTCTGGGGCATT
>KX907383..1
TTCTATTGTGACACATCAAGCCTGTTTAATATCACTTGGGATAGAAGTAACGTCACGCAGGAGTTAAATAACA
CAAAGTTAAATGACACTATAATTCTCCAATGCAGGATAAAGCAAATTATAAATATGTGGCAGAGAGCAGGACA
AGCAATGTATGCCCCTCCCATCCCAGGAATAATAAAGTGTGAATCAAACATTACAGGACTAATATTAACAAGA
GATGGGAGTGAGAATGAAACTGAAACCTTCAGACCTGGAGGAGGAGACATGAGGGACAATTGGAGAAGTGAGT
TATATAAGTATAAAGTGGTAAAAATTGAACCACTAGGAGTAGCACCCACCAAGGCAAGGAGAAGAGTGGTGGA
GAGAGAGAAAAGAGCAGTCGGACTAGGAGCTGTCTTCCTTGGGTTCTTAGGAGCAGCAGGAAGCACTATGGGC

GCAGCGTCAATAACGCTGACGGTACAGGTCAGACAATTGTTGTCTGGCATAGTGCAACAGCAAAACAATTTGC
TGAGGGCTATAGAAGCTCAACAGCATCTGTTGAAGCTCACGGTCTGGGGCATT
>KX907389..2
TTCTATTGTAACACATCAATCCTGTTTAATAGCACTTGGAATAGCACTAGCACTTGGAATATCTCTGAGAGTG
TAAATAGCAGCAGTCAGGAGTCAAATGACACTATAACTCTCCAATGCAGAATAAAGCAAATTATAAATATGTG
GCAGAGAACAGGACAAGCAATGTATGCCCCTCCCATCCAAGGAGTAATAAAGTGCATATCAAATATTACAGGA
CTAATATTAACAAGAGATGGTGGGGGTGATGACAATACAACTGAAACCTTCAGACCTGGAGGAGGAGATATGA
GGGACAATTGGAGAAGTGAATTATATAAGTATAAAGTAGTAAAAGTTGAACCACTAGGAATAGCACCCACCAG
GGCAAGGAGAAGAGTGGTGGAGAGAGAAAAAAGAGCAGTTGGAATAGGAGCTGTTTTCATTGGGTTCTTAGGA
GCAGCAGGAAGCACTATGGGCGCGGCGTCAATAACGCTGACGGCACAGGCCAGACAACTATTGGCTGGCATAG
TGCAACAGCAAAGCAATTTGCTGAGGGCTATAGAGGCTCAACAGCATCTGTTGAAACTCACAGTCTGGGGCAT
T
>KX907341..2
TTCTATTGTAATACATCAGGCCTGTTTAATAGTACTTGGGATGACACCTCGAATCATAATACAAGTGGCACGG
AGTCAAATGCCAATATAACTCTCCAGTGTAGAATAAAGCAAATTATAAATATGTGGCAGAGAGCAGGACAAGC
AATATATGCCCCTCCCATCCAAGGAATAATAAAGTGTGAATCAAACATTACAGGACTGATATTAACAAGAGAT
GGTGGAGATAACAATAACACTACCACCGAAACCTTCAGACCTGGAGGAGGAGATATGAGGGACAATTGGAGAA
GTGAATTATATAAGTATAAAGTAGTAAAAATTGAACCACTAGGAGTAGCACCCACCAAGGCAAGGAGACGAGT
GGTGCAGAGAGAAAAAAGAGCAGTTGGAATGGCAGCTTTATTCTTTGGGTTCTTAGGAGCAGCAGGAAGCACT
ATGGGCGCGGCGTCCATAACGCTGACGGTACAGGCCAGACAATTATTGTCTGGCATAGTGCAACAGCAAAGCA
ATTTGCTGAGGGCTATAGAGGCTCAACAGCATATGTTGAGACTCACGGTCTGGGGCATT
>KX907347..1
TTCTATTGTAATACATCAGGCCTGTTTAATAGCACTTGGCTGTCTAATAGTACTAACAGCACGGATAACACGG
GGACAAATGACACTATAACTCTCCAATGCAGAATAAAGCAAATTATAAATATGTGGCAAAGAGTAGGACAAGC
AATGTATGCCCCTCCCATCCCAGGAGTAATAAGGTGTGAATCAAACATTACAGGACTAATGTTAACAAGAGAT
GGTGGGAATAATGCTACCAATGAAACCTTTAGACCTGGAGGAGGAGATATGAGGGACAATTGGAGAAGTGAAT
TATATAAGTATAAAGTAGTAAAAATTGAACCACTAGGAGTAGCACCCACCAAGGCAAAGAGAAGAGTGGTGGA
GAGAGAAAAAAGAGCAGTTGGAATAGGAGCTGTCTTCCTTGGGTTCTTAGGAGCAGCAGGAAGCACTATGGGC
GCGGCGTCAATAACGCTGACGGTACAGGCCAGACAATTATTGTCTGGCATAGTGCAACAGCAAGGCAACTTGC
TGAGGGCTATAGAGGCTCAACAACATCTGTTGAAACTCACAGTCTGGGGCATT
>KX907364..2
TTCTATTGTAACACATCAGCCCTGTTCAATAGCAGTTGGAGTAGTGTAAATGACAGCGGGAACATTAGCCCAG
AGCCAAACAAAAATATAACCCTCCGATGCAGAATAAAGCAAATTATAAATATGTGGCAGAGAGTAGGACAAGC
AATATATGCCCCTCCCATCCAAGGAGTAATAAGGTGTGACTCAAACATTACGGGATTAATATTAACAAGCGAT
TATGGAGGGACTAACAGTACACATGAAATCTTCAGACCTGCAGGAGGAAATATGAGGGACAATTGGAGAAGTG
AATTATATAAGTATAAAGTAGTACAAATTGAACCAATAGGAGTAGCACCCATCAGGGCAAAGAGAAGAGTGGT
GGAGAGAGAAAAAAGAGCAGTCGGACTGGGAGCTGTCTTCCTTGGTTTCTTAGGAGCAGCAGGAAGCACTATG
GGCGCGGCGTCAATGACGCTGACGGTACAGGCCAGACAATTATTGTCTGGTATAGTGCAACAGCAAAGCAATT
TGCTGAGGGCTATAGAGGCTCAACAGCATCTGTTGAAACTCACGGTCTGGGGCATT
>KX907412..3
TTCTATTGTAACACATCAGGCCTGTTTCATAGCACTTGGTTTAATAGCACTTGGTTTAATAGCACTTGGGAAG
ACAATCAGGCAAATAGCACAAATGACACTATAGTACTCCCATGCAGAATAAAGCAAATTATTAATATGTGGCA
GAGAGCAGGACAAGCAATATATGCCCCTCCCATCCCAGGGGAAATAAGGTGTGAATCAAACATTACAGGGCTA
ATGTTAACAAGAGATGGTGGGAATAATAACAATAACAGGACAAATGAAACCAAAAATGAAACCTTCAGACCTG
GAGGAGGAGATATGAGGGACAATTGGAGAAGTGAATTATATAAGTATAAAGTAGTAAAAATTGAACCACTAGG
ACTAGCACCCACCGGGGCAAAGAGAAGAGTGGTGGAGAGAGAAAAAAGAGCAGTTGGAATAGGAGCTGTCTTC
CTTGGGTTCTTAGGAGCAGCAGGAAGCACTATGGGCGCGGCGTCAATAACGCTGACGGTACAGGCCAGACAAT
TATTGTCTGGCATAGTGCAACAGCAAAGCAATTTGCTGAAGGCTATAGAGGCTCAACAACATCTGTTGAAACT
CACGGTCTGGGGCATT
>KX907414..1
TTCTATTGTAACACATCAGGCCTGTTTAATAGCACTTGGAGTAACACTACCACTTGGGAAAACATTACCGAAA
GGCAGGAACCAAGCAGCAACAAGACAAACGCAACTATAACTCTCCAATGCAGAATAAAGCAAATTATAAATAT
GTGGCAGAGAACAGGACGAGCAATGTATGCCCCTCCCATCCCAGGAATAATAAGTTGTAACTCAAATATTACA
GGACTAATATTAACAAGAGATGGTGGAAATAACAGTACAAATGAAACCTTCAGACCTGGAGGAGGAGACATGA
GGGACAATTGGAGAAGTGAATTATATAGGTATAAAGTAGTAAAAATTGAACCACTAGGAGTAGCACCCACTGA
GGCAAAGAGAAGAGTGGTGGAGAGAGGAAAAAGAGCAATTGGAATAGGAGCTGTTTTCATTGGGTTCTTAGGA
GCAGCAGGAAGCACTATGGGCGCGGCGTCAATAACGCTGACGGTACAGGCCAGACAATTATTGTCTGGCATAG
TGCAACAGCAAAACAATTTGCTGAGGGCTATAGAGGCTCAACAGCATCTGTTGAAACTCACGGTCTGGGGCAT
T
>KX907423..2

TTCTATTGTAACACATCAGGCCTGTTTAATAGCATTTGGGTTAAGGGCCAGGAAAGCAATCAGTCAAATAGCG
CAAATGACACTATAGTACTCCCATGCAGAATAAAGCAAATTATAAATATGTGGCAGAGAGCAGGACAAGCAAT
ATATGCCCCTCCCATCCCAGGGGAAATAAAGTGTGTATCAAACATTACAGGGCTAATATTAACAAGAGATGGT
GGGAATAACGGGATAAATGAAACCTTCAGACCTGGAGGAGGAGATATGAGGGACAATTGGAGAAGTGAATTAT
ATAAGTATAAAGTAGTAAAAATTGAACCACTAGGAGTAGCACCCACCAGGGCAAAGAGAAGAGTGGTGGAGAG
AGAAAAAAGAGCAGTTGGAATAGGAGCTGTCTTCCTTGGGTTCTTAGGAGCAGCAGGAAGCACTATGGGCGCG
GCGTCAATAACGCTGACGGTACAGGCCAGACAATTATTGTCTGGCATAGTGCAACAGCAAAGCAATTTGCTGA
AGGCTATAGAGGCTCAACAACATCTGTTGAGACTCACGGTCTGGGGCATT
>KX907431..1
TTCTATTGTAACACAACAGACCTGTTTAATAGCACCTGGAATGAGACCAGCATACAGGAAAATAGCACAGAGA
CAAACAACGAGACTATAACTCTCCAATGCAGAATAAAGCAAATTATAAATATGTGGCAGAGAGCAGGACAAGC
AATATATGCCCCTCCCGTACCAGGAGTAATAAGGTGTGACTCAAATATTACAGGGCTAATATTAACAAGAGAT
GGTGGGACTAATAATACAAATGAAACATTCAGGCCTGTAGGAGGAGATATGAAGGACAATTGGAGAAGTGAAT
TATATAAGTATAAAGTAGTAAAAATTGAACCACTAGGGGTAGCACCCACCAGGGCAAAGAGAAGAGTGGTGGA
GAGAGAAAAAAGAGCAGTTGGAATAGGAGCTGTCTTCCTTGGGTTCTTAGGAGCAGCAGGAAGCACTATGGGC
GCAGCATCAATAACGCTGACGGTACAGGCCAGACAATTATTGTCTGGCATAGTGCAACAGCAAAGCAATTTGC
TGAGGGCTATAGAGGCTCAACAACAACTGTTGAAACTCACAGTCTGGGGCATT
>HQ615968..1
TTCTATTGCAACACATCAGACCTATTTAATAGCACTTGGGAAAATAACCCTGTCAGCCAGGAGTCAAATAGCA
CAAGGTCAAACGACACTATAACTCTCCAATGCAAAATAAGGCAAATTATAAGAATGTGGCAGAGAGTAGGACA
AGCAATGTATGCCCCTCCCATCCAAGGAGTAATAAACTGTACATCAAATATTACAGGACTAATATTAACAAGA
GATGGTGGGAATAATAATAACAGTGGAAATGAAACCTTCAGACCTGCAGGAGGAGATATGAGGGACAATTGGA
GAAGTGAATTATATAAGTATAAAGTAGTAAAAATTGAACCACTAGGAGTAGCACCCACCGGGGCAAAGAGAAG
AGTGGTGGAGAGAGAAAAAAAGAGCAGTTGGAATAGGAGCTGTCTTCATTGGGTTCTTAGGAGCAGCAGGAAGC
ACTATGGGCGCAGCGTCAATAACGCTGACGGTACAGGCCAGACAATTATTGTCTGGCATAGTGCGGCAGCAAA
GCAATTTGCTGCAGGCTATAGAGGCTCAACAGCATCTGTTGAAACTCACGGTCTGGGGCATT
>HQ615970..2
TTCTATTGTAATACATCAAGCCTGTTTAATAGTACTTGGAATTGGAATCACACAAACAGCTTGCAGGATTCAA
ATAGCACAGAGACAAATGGCACTATAATTCTCAAATGCAGAATAAAACAAATTATAAGGATGTGGCAGAGAGT
AGGACAAGCAATGTATGCCCCTCCTATCCCAGGAGTAATAAGGTGTGTATCAAACATTACAGGATTAATATTA
ACAAGCGATGGCGGGAATAAGACCAGTACAACAGAGACCTTCAGACCTGGGGGAGGAGATATGAGGGACAATT
GGAGAAGTGAATTATATAAGTATAAAGTAGTCAAAATTGAACCACTAGGAGTAGCACCCACCAGGGCAAAGAG
AAGAGTGGTGGAGAGAGAAAAAAGAGCAGTTGGAATAGGAGCTGTCTTCCTTGGGTTCTTAGGAGCAGCAGGA
AGCACTATGGGCGTGGCGTCAATGACGCTGACGGTACAGGCCAGGCAATTATTGTCTGGCATAGTGCAACAGC
AAAGCAATTTGCTGAGGGCTATAGAGGCTCAACAGCATCTGTTGAAACTCACAGTCTGGGGCATT
>HQ615971..2
TTTTATTGTAATACATCAAAACTGTTTAATAGCAGTATATCCTACAATGGCAGTACAAAGTCAGTAGAGTCAA
TAAACAGTAACGAGACAATAAGTCTCCCATGCAGAATAAAGCAAATTATAAATATGTGGCAGAGAGCAGGACA
AGCAATATATGCCCCTCCCATCCGAGGAGTAATAAAGTGTAAGTCAGACATTACAGGACTACTATTAACAAGA
GATGGTGGGGTTACCAACACAAATGAGACCTTTAGACCTGGAGGAGGAAATATGAGGGATAATTGGAAAAGTG
AACTATATAAATATAAAGTAGTAAAAATTGAACCATTAGGAGTGGCACCCTCTCCTGCAAAGAGAAGAGTGGT
GCAGAGAGAAAAAAGAGCAGTAGGACTGGGAGCTGTTTTCATTGGGTTCTTAGGAGCAGCAGGAACCACTATG
GGCGCGGCGTCAATGACGCTGACGGTACAGGCCAGACAATTATTGTCTGGCATAGTGCAACAGCAAGGCAATT
TGCTGAGGGCTATAGAGGCTCAACAGCATCTGTTGAAACTCACGGTCTGGGGCATT
>HM204620..2
TTCTATTGCAACACATCAGGATTGTTTAATAGCAGATGGGAAAATGACACGCAACCAAATAACACGCAGCCAA
ACGGCACTATAACTCTCCCATGCAGAATAAAGCAAATTATAAATATGTGGCAGAGAACAGGACAAGCAATATA
TGCCCCTCCCATCAGAGGAGTAATAAGTTGTCAATCAAACATTACAGGACTACTACTAACAAGAGATGGGGGG
GGGAAGAATACTAGTGATAATGAAACCTTCAGACCTGGAGGAGGAGATATGAGGGACAATTGGAGAAGTGAAT
TATATAAGTATAAAGTAGTAAAAATTGAACCATTAGGAGTAGCACCCAACAGGGCAAGGAGAAGAGTGGTGGA
GAGAGAAAAAAGAGCAGTTGGAATAGGAGCTGTGTTCCTTGGGTTCTTGGGAGCAGCAGGAAGCACTATGGGC
GCAGCGTCAATGACGCTGACGGTACAGGCTAGACAATTATTGTCTGGTATAGTGCAACAGCAAAGCAATTTGC
TGAGGGCTATAGAGGCTCAACAGCATCTGTTGAAACTCACAGTCTGGGGCATT
>HM204621..2
TTCTATTGTAATACATCAAACCTGTTTAATAGCACTTGGGGTAATGGCAGTTACAGCACCAATGACACAGGGG
ACGCAAATTCAAATATAACTATCCAATGCAGAATAAAGCAAATTGTAAGGATGTGGCAGAGGACAGGACAAGC
AATGTATGCCCCTCCCATCAAAGGAATAATAAGATGTATGTCAAATATTACAGGCCTACTATTAACAAGAGAT
GGTGGGATTAATAGGACGAATGAAACCTTCAGACCTATAGGAGGAGATATGATGGATAATTGGAGAAGTGAGC
TATATAAATATAAAGTAGTAAGAATTGAACCAATAGGAGTAGCACCCAACAGGGCAAAGAGAAGAGTGGTGGA
GAGAGAAAAAAGAGCAGTCTTTGGAATGGGAGCTGTCTTCCTTGGGTTCTTAGGAGCAGCCGGAAGCACTATG

GGCGCAGCGTCGATAACGCTGACGTTACAGGCCAGACAATTGTTGTCTGGTATAGTGCAACAGCAAAGCAATT
TGCTGAGGGCTATAGAGGCTCAACAGCATCTGTTGAAACTCACGGTCTGGGGCATT
>KY658695..2
TTCTATTGTAACACATCAAGTTTGTTTAAAGGCACTTGGAATAATGGCACTTGGAATGATAGCATGTCAGAGG
TAAATAGCACAGAGTCAAATGACAACATAACTCTCCCATGCAGAATAAAGCAAATTATAAATATGTGGCAGAA
AGTAGGGCAAGCAATGTATGCCCCACCCATCCAGGGAGTAATAAGGTGTGAATCAAACATTACTGGACTAATA
TTAACAAGAGATGGTGGGAATAGTAGCGGTCAGAATGAAACCTTTAGACCTGGAGGAGGAGATATGAGGGACA
ATTGGAGAAGTGAATTATATAAGTATAAAGTAGTAAAAATTGAACCACTAGGAGTAGCACCCACCAAGGCAAG
AAGAAGAGTGGTGCAGAGAGAAAAAAGAGCAGTTGGAATGGGAGCACTTTTCATTGGGTTCTTAGGAGCAGCA
GGAAGCACTATGGGCGCGGCGTCAATAACGCTGACGGTACAGGCCAGACAATTATTGTCTGGCATAGTGCAAC
AGCAAAGCAATTTGCTGAAGGCTATAGAGGCTCAACAGCATCTGTTGAGACTCACTGTCTGGGGCATT
>KY658696..1
TTCTACTGTAATACATCAGGCCTGTTTAATAGCAATAGCACAGCAAATAGCACAGCAAAGTTTAATAGCAATA
GCACAGCAAATAGCACAGCAAATATAACTCTCCCATGCAGAATAAAACAAATTGTAAATATGTGGCAGAGGCC
AGGACAAGCAATGTATGCCCCTCCAATCCAAGGGCCAATAAGTTGTAGGTCAAACATTACAGGATTACTAATA
ACAAGAGATGGTGGAGAAAACAATGGAACGAATGAAACCTTCAGACCTGGAGGAGGAGAGATGAGGGATATTT
GGAGAAGTGAACTATATAAATATAAAGTAGTGAAAATTGAACCATTAGGAGTAGCACCCACCAGGGCAAGGAG
AAGAGTGGTGGAGAGAGAAAAAAGAGCAGTTGGACTGGGAGCTGTCTTCATTGGGTTCTTAGGAGCAGCCGGA
AGCACTATGGGCGTGGCGTCAATAACGCTGACGGTACAGGCCAGACAATTATTGTCTGGTATAGTGCAGCAGC
AAAACAATTTGCTGAGGGCTATAGAGGCTCAACAGCATATGTTGAAACTCACAGTCTGGGGCATT
>AF361872..2
TTCTATTGTAATACAACAAGCCTGTTTAATAGCACCTGGTATCACAAGAACAGCACGCAGGGGTCAAATGACA
CGGGGTCAGATGACACTATAACTCTCCCATGCAAAATAAAACAAATTGTAAGGATGTGGCAGAGAGCAGGACA
AGCAATGTATGCCCCTCCCATTCCAGGAGTAATAAGGTGTGAATCAAACATTACAGGACTAATATTAACAAGA
GATGGGGGGAGTGAGGACAATACCACTGAAATCTTCAGACCTGGAGGAGGAAATATGAGGGACAATTGGAGAA
GTGAATTATATAAGTATAAAGTAGTAAAAATTGAACCACTAGGAGTAGCACCCACCAAGGCAAAGAGAAGAGT
GGTGGGAAGAGAAAAAAGAGCAGTTGGACTGGGAGCTGTCTTCCTTGGGTTCTTAGGAGCAGCCGGAAGCACT
ATGGGCGCGGCGTCAGTAACGCTGACGGTACAGGCCAGACAATTATTGTCTGGCATAGTGCAACAGCAGAACA
ATTTGCTGAGGGCTATAGAGGCTCAACAACATCTGTTGAAACTCACAGTCTGGGGCATC
>AF361873..1
TTCTATTGTAACACATCAGGCCTGTTTAACAGCACTTGGCATAGCAATGCCAGCATACAAGAGCCAAACAGCA
CAGAGTCAAATGACACTATTATAACTCTCCAATGTAGAATAAAACAAATTATAAATATGTGGCAGAGAACAGG
AAAAGCAATATATGCTCCTCCCATCCAAGGAATAATAAGGTGTGAATCAAACATTACAGGACTAATATTAACA
AGAGATGGTGGGAGTAATAATAGCACCAATGAAACCTTCAGACCTGGAGGAGGAGATATGAGGGACAATTGGA
GAAGTGAATTATATAAGTATAAAGTAGTAAAAATTGAACCACTAGGAGTAGCACCCTCCTGGGCAAGGAGAAG
AGTGGTGCAGAGAAAAAAAAGAGCAGTTGGAATAGGAGCTGTCTTCCTTGGGTTCTTAGGAGCAGCTGGAAGC
ACTATGGGCGCGGCGTCAATAACGCTGACGGTACAGGCCAGACAATTATTGTCTGGCATAGTGCAACAGCAAA
GCAATTTGCTGAGGGCTATAGAGGCTCAACAGCATCTGTTGAAACTCACGGTCTGGGGCATT
>HM027846..2
TTCTATTGTAACACATCAGGTCTGTTTAATAGCACGTGGAATACTACCAACATGTGGGAGCAAAATAGCACGG
AGTCAAACAATAATACTATATATCTTCAATGCAGAATAAAGCAAATTATCAATATGTGGCAGAGAGTAGGACA
AGCAATATATGCCCCTCCCATCCCAGGAATGATAAGGTGTGTCTCAAATATTACAGGACTAATATTAACAAGG
GATGGTGGGAATATTAGTGAGAATAATGCCAGTGAAATCTTCAGACCAGGAGGAGGAGATATGAGGGACAATT
GGAGAAGTGAATTATACAAGTATAAAGTAGTAAAAATTGAACCACTAGGAGTAGCACCAACCCCGGCAAGGAG
AAGAGTGGTGGAGAGAGAAAAAAGAGCAGTCGGAATAGGAGCTGTCTTCCTTGGGTTCTTAGGAGCAGCAGGA
AGCACTATGGGCGCGGCGTCAATAACGCTGACGGCACAGGCCAGACAATTATTGTCTGGTATAGTGCAACAGC
AAAGCAATTTGCTGAGGGCTATAGAGGCTCAACAGCATCTGTTGAAACTCACGGTCTGGGGCATT
>JX877522..1
TTCTATTGTGACACATCAGAACTATTTAATAGCACTTGGAATGACGCTGCCATTATGAGGGAATTAAATAGAA
CGGAGTCAAACGACACCATAACTCTCCAATGCAAAATAAAGCAAATTATAAATATGTGGCAGAGAGCAGGACG
AGCAATGTATGCCCCTCCCATCCGAGGAGTAATAAGATGTGAGTCAAATATTACAGGACTAATATTAACAAGA
GATGGTGGGAGGGATGATAACAATACAACTGAAACCTTCAGACCTGGAGGAGGAGATATGAGGGACAATTGGA
GAAGTGAATTATATAAGTATAAAGTAGTAAAACTTGAGCCACTAGGAATAGCACCCACCAAGGCAAAGAGAAG
AGTGGTGGAGAGAGAAAAAAGAGCAGTTGGACTGGGAGCTGTTTTCATTGGGTTCTTAGGAGCAGCAGGAAGC
ACTATGGGCGCGGCGTCAATAACGCTGACGGTACAGGCCAGACAATTATTGTTTGGCATAGTGCAACAGCAAA
GCAATTTGCTGAGGGCTATAGAGGCTCAACAGCATATGTTGAAACTCACAGTCTGGGGCATT
>HM215272..1
TTCTATTGTAATACATCAAGCCTATTTAATAGTACTTGGTGGAGCAACAACAGCAACAACACTGACAGTGAGG
AGTCAAATAGCATGGGGTTAAATGACACTATAACTCTCCAATGCAGAATAAAGCAAATTATAAATATGTGGCA
GAGAGTAGGACAAGCAATGTATGCCCCTCCCATCCAAGGAGTAATAAGGTGTGAGTCAAACATCACAGGAATA

CTATTAACAAGAGATGGTGGGGATAATAACAATGAAAGTGAAACCTTCAGACCTGTAGGAGGAGATATGAGGG
ACAATTGGAGAAGTGAATTATATAAATATAAAGTAGTAAAAATTGAACCATTAGGAGTAGCACCCACAGAGGC
AAGGAGAAGAGTGGTGGGAAGAGGAAAAAGAGCAGTTGTTGGAATGGGAGCTATGTTCCTTGGGTTTTTGGGA
GCAGCTGGGAGCACTATGGGCGCGGCGTCAATAACGCTGACGGTACAGGCCAGACAATTATTGTCTGGCATAG
TGCAACAGCAAAGCAATTTGCTGAGGGCTATAGAGGCTCAACAGCATCTGTTGAAACTCACTGTCTGGGGCAT
T
>HM027824..1
TTCTATTGTAACACATCAGGTCTGTTTAATAGCACGTGGAATGGTACCCAAATGATGGAGCCAAATAGCACAG
AAAACATTAATGATACTATAACTCTTAAATGCAGAATAAAGCAAATTATACATATGTGGCAGAGAGTAGGACA
AGCAATATATGCCCCTCCCATCAAAGGAGTGATAAGATGTAACTCAAACATTACAGGACTAATATTAACAAGA
GATGGTGGGACTGAAGGGAATAGCAGTGAAACCTTCAGACCGGGAGGAGGTGATATGAGGGACAATTGGAGAA
GTGAATTATATAAGTATAAAGTAGTAAAAATTGAACCACTAGGAGTAGCACCTACCAAGGCAAGGAGAAGAGT
GGTGGAGAGAGAAAAAAGAGCAATCGGACTGGGAGCTGTCTTCCTTGGGTTCTTAGGAGCAGCAGGAAGCACT
ATGGGCGCGGCGTCAATAACGCTGACGGCACAGGCCAGACAATTATTGTCTGGCATAGTGCAACAGCAAAGCA
ATTTGCTGCAGGCTATAGAGGCTCAACAGCATCTGTTGAAACTCACAGTCTGGGGCATT
>JX236669..1
TTCTACTGTAATACATCAGGCCTGTTTAATAGCACTTGGAACATTGCTGGGAACAGGACAAATGACACGAAGT
CAAATGAAACTATAACTCTCCCATGCAGAATAAAGCAAATTGTAAATGTGTGGCAGAGAGTAGGACAAGCAAT
ATATGCCCCTCCCATCGCAGGAGTAATAAGATGTAACTCAAACATTACAGGATTACTATTAGTAAGAGATGGT
GGAGCTACTAACAATACTGATGAAACCTTCAGGCCTGGAGGAGGAAATATGAGGGACAATTGGAGAAGTGAAT
TATATAAGTATAAAGTAGTAAAAATTGAACCACTAGGAGTAGCACCCACCAGGGCAAGGAGAAGAGTGGTGGA
GAGAGAAAAAAGAGCAGTTGGACTGGGAGCTGTATTCATTGGGTTCTTAGGAGCAGCAGGAAGCACTATGGGC
GCGGCGTCAGTGACGCTGACGGTACAGGCCAGACAATTATTGTCTGGCATAGTGCAACAGCAAAGCAATTTGC
TGAGGGCTATAGAGGCTCAACAGCATCTGTTGAAGCTCACGGTCTGGGGCATT
>JX236671..2
TTCTATTGTAATACAGCAGGTCTGTTCAATAGAAATTGGACTTGGAGTAAGGAGAATGGGACCAGAATGCAGA
ATAAAACGGATTCAAATGGCACTATAACTCTCCAATGCAGAATAAAGCAAATTATAAATATGTGGCAGAGAAC
AGGACAGGCAATGTATGCCCCTCCCATCCAAGGAGTAATAAAATGTGCATCAAACATTACAGGACTACTGTTA
ACAAGGGATGGTGGAGATAACAAAAACAATAGTACCAATAGTACCAATCAAAATAACACTGAAATCTTTAGAC
CTATAGGAGGAGATATGAGGAATAATTGGAGAAGTGAATTATATAAGTATAAAGTAGTAAAAATTGAACCACT
AGGAGTAGCACCTACCACGGCAAGGAGAAGAGTGGTGGGAAGAGAAAAAAGAGCAGTACTAGGTGGACTAGGA
GCTGTATTCCTTGGGTTCTTAGGAGCAGCCGGAAGCACTATGGGCGCAGCGTCAATAACGCTGACGGTACAGG
CCAGGCAATTATTGTCTGGCATAGTGCAACAGCAAAGCAATTTGCTGAGGGCTATAGAGGCTCAACAACATCT
GTTGAAACTCACGGTCTGGGGCATT
>JX236676..1
TTCTATTGTAACACATCAAGCCTATTTAATAGCATTTGGAATGGCAGTAAATCAAATAACACGGAGCCAGATA
GCACCATAACTCTCCAATGCAGAATAAAGCAAATTATAAATATGTGGCAGAGAGTAGGACAAGCAATGTATGC
CCCTCCCATCCAAGGAGTAATAAGGTGTGAGTCAAACATTACAGGGCTAATATTAACAAGAGATGGTGGGAGT
AATAGCAGTAGTACAAATGAAACCTTCAGACCTGGAGGAGGAGATATGAGAGACAATTGGAGAAGTGAATTAT
ATAAGTACAAAGTAGTAAAAATTGAACCACTAGGAGTAGCACCCACCAAGGCAAAGAGAAGAGTGGTGGAAAG
AGAAAAAAGAGCAGTTGGAATAGGAGCTGTTTTCCTTGGGTTCTTAGGAGCAGCAGGAAGCACTATGGGCGCA
GCGTCAATAACGCTGACGGTACAGGCCAGACAATTATTGTTTGGCATAGTGCAACAGCAAAGTAATTTGCTGA
GGGCTATAGAGGCTCAACAGCATCTGTTGAAACTCACGGTCTGGGGCATT
>KF716478..2
TTCTATTGTAATACATCAAGCCTGTTTAATAGTACTTGGGTATATGGTAATGGAAGCTGGGGGTCAAATAGCA
CGGGGTTAAATAGTACAATGATAAATAATACTATTTTCAGTGATACCATAATTCTTCAATGCAGAATAAAGCA
AATTATAAATATGTGGCAGAGAGCAGGACAAGCAATATATGCCCCTCCCATCAAAGGAGTAATAAGCTGTAGA
TCAAACATCACAGGACTAATATTAACAAGAGATGGTGGGAGTAACAGCAATGCAAGTACAAATGGAAGTGAAA
CCTTCAGACCTGGAGGAGGAGATATGAGGGACAATTGGAGAAGTGAATTATATAAGTATAAAGTAGTAAAAGT
TGAACCACTAGGAATAGCACCCTCCAAGGCAAGGAGAAGAGTGGTGGCGAGAGAAAAAAGAGCAGTTGGAATA
GGAGCTGTACTCCTTGGGTTCTTAGGAACAGCAGGAAGCACTATGGGCGCAGCGTCAATAACGCTGACGGTAC
AGGCCAGAAAATTATTGTCTGGCATAGTGCAACAGCAAAGCAATTTGCTGAGGGCTATAGAGGCTCAACAGCA
TCTGTTGAAACTCACTGTCTGGGGCATT
>KP109490..1
TTCTACTGTAATACATCAGGCCTGTTCAATAGCACTTGGAATAACACCTCGCAGGCAAATAGCTCGGAGTTAA
AGGGCAATATAACTCTCCCATGCAAAATAAAACAAATTATCAGGATGTGGCAAAGAACAGGACAAGCAATGTA
TGCCCCTCCCATCCAAGGAGTAATAAATTGTGTATCAAACATTACAGGACTACTATTAACAAGAGATGGTGGG
GAGGAGGACAATAGTACTGAAACCTTCAGACCTGGAGGAGGAGATATGAGGGATAATTGGAGAAGTGAATTAT
ATAAGTATAAAGTAGTAAAAATTGAACCAGTAGGAGTAGCACCCACCAAGGCAAGGAGAAGGGTGGTGGAGAG
AGAAAAAAGAGCAGTTGGAATAGGAGCTGTCCTCCTTGGATTCTTAGGAGCAGCAGGAAGCACTATGGGCGCG

GCGTCAATAACGCTGACGGTACAGGCCAGACAGTTATTGTCTGGTATAGTGCAGCAGCAAAGCAATTTGCTGA
GGGCTATAGAGGCTCAACAACAACTGTTGAAACTCACGGTCTGGGGCATT
>JF680921..1
TTCTATTGTAACACATCAGGCCTGTTTAATAGCACTTGGACTAAGAATAGCAGCACTAACACTTGGAATAGTA
CTGACATCATGTGGGGGGTAAATAACAGTGACCATAATATAACTCTCCAATGCAAAATAAAGCAAATTATAAC
CATGTGGCAGAGGACAGGGCAGGCAATGTACGCCCCTCCCATCCCAGGAATAATAAACTGTGTGTCAAACATT
ACAGGACTACTATTAACAAGAGATGGTGGTGAGGAGAGTAACAGTAGAGATAATGAAACCTTCAGACCTATAG
GAGGAAATATGAAGGATAATTGGAGAAGTGAACTATATAAATATAAAGTAGTAAAAATTGAACCACTAGGAGT
AGCACCCACCAAGGCAAAGAGAAGAGTGGTGCAGAGAGAAAAAAGAGCAGTTGGACTGGGAGCTGTCTTCATT
GGGTTCTTAGGAGCAGCCGGAAGCACTATGGGCGCGGCGTCAATAACGCTGACGGTACAGGCCAGACAATTAT
TGTCTGGCATAGTGCAACAGCAAAACAATTTGCTGAGGGCTATAGACGCTCAACAGCATCTGTTGAAGCTCAC
GGTCTGGGGCATT
>KF859745..2
TTCTATTGTAACACATCAGGCTTGTTTAATAGCACTTGGAACAATAGCACTCAAGAGTCTAATAGCACGGAGA
CTATAACTCTCCCATGCCGAATAAAGCAAATTGTAAATATGTGGCAGAGAGCAGGACAAGCCATATATGCCCC
TCCCATCAAAGGAATAATAAATTGTAGATCAAACATTACAGGACTAATATTAACAAGAGATGGTGGACATAAT
AATACAAATGAAACCTTCAGGCCTGGAGGAGGAGATATGAGGGACAATTGGAGAAGTGAATTATATAAGTATA
AAGTAGTAAAAGTTGAACCACTAGGAGTAGCACCCACCACGGCAAGGAGAAGAGTGGTGCAGAGAGAAAAAAG
AGCAGTATTTGGACTGGGAGCTGTCTTCATTGGGTTCTTAGGAGCAGCAGGAAGCACTATGGGCGCGGCGTCA
ATAACGCTGACGGTACAGGCCAGACAATTATTGTCTGGCATAGTGCAACAGCAAAGCAATTTGCTGAGGGCTA
TAGAGGCTCAACAGCATCTGTTGAAACTCACGGTCTGGGGCATT
>KF716486..2
TTCTATTGTAATACATCAAGTCTGTTTAATAGTACTTGGGACCTTAATGCTACTAGGGAAGATAGCACGGAGA
ACATAACTCTCCCATGCAGAATAAAGCAAATCATAAATATGTGGCAAAGAGTAGGACAAGCAATGTATGCCCC
TCCCATCGAAGGAATAATAAGATGTGACTCAAACATTACAGGACTACTCTTAACAAGAGATGGTGGGAATAGT
AGTACAAATGAAACTCAGGAAATCTTCAGACCTATAGGAGGGGATATGAAAGATAATTGGAGAAATGAATTAT
ACAAGTATAAAGTAGTAAAAATTGAACCTCTGGGAGTAGCACCCTCCTGGGCAAGGAGAAGAGTGGTGGAGAG
AGGAAAAAGAGCAGTTGGAATAGGAGCTGTATTCCTTGGGTTCTTAGGAGCAGCAGGGAGCACTATGGGCGCA
GCGTCAATAACGCTGACGGTACAGGCCAGGCAATTATTGTCTGGCATAGTGCAACAGCAAAGCAATTTGCTGA
AGGCTATAGAAGCTCAACAACAACTGTTGAGACTCACGGTCTGGGGCATT
>M62320..1
TTCTATTGCAACACATCAGGCCTGTTTAATAGCATTTGGAATGGCAGCATGTCAAATGACATGGGGCCAAATG
GCACTATAACTCTCCAATGTAGAATAAAGCAAATTATAAATATGTGGCAGAGAGTAGGACAAGCAATGTATGC
CCCTCCCATCCAAGGAGTAATAAGGTGTGAATCAAACATTACAGGACTACTATTAACAAGAGATGGGGGTACT
AACAATACAAAAAATGAGACCTTCAGACCTGGAGGAGGAGATATGAGGGACAATTGGAAAAGTGAATTATATA
AGTATAAAGTAGTAAAAATTGAACCACTAGGAGTAGCACCCACCAGGGCAAAGAGAAGAGTGGTGGAGAGAGA
AAAAAGAGCAGTTGGACTAGGAGCTATCTTCCTTGGGTTCTTAGGAGCAGCTGGAAGCACAATGGGCGCGGCG
TCAATAACGCTGACGGTACAGGCCAGACAATTATTGTCTGGTATAGTGCAACAGCAGAGCAATCTGCTGAGGG
CTATAGAGGCTCAACAGCATCTGTTGAAACTCACTGTCTGGGGCATT
>L22957..2
TTCTATTGTAATACAGCAGGCCTGTTTAATAGTACTTGGAATAAAAATGACAGCATAAAGTCAAATGACACAA
AGTCAAATGACATTATAACTCTTCAATGCAGAATAAAGCAAATTATAAATATGTGGCAGAGAGTAGGACGAGC
AATGTATGCCCCTCCCATCAAAGGAGTAATAAGGTGTGAATCAAACATTACAGGACTACTATTAACAAGAGAT
GGTGGAAATGGGAGCAATACAAATGAAACCTTCAGACCAGGAGGAGGAGATATGAGGGACAATTGGAGAAATG
AATTATATAAGTATAAAGTAGTACAAATTGAACCATTAGGAGTAGCACCTACCCCAGCAAAGAGAAGAGTGGT
GCAGAGAGAAAAAAGAGCAGTTGGACTAGGAGCTGTATTCATTGGGTTCTTAGGAGCAGCAGGAAGCACTATG
GGCGCGGCGTCAATAACGCTGACGGTACAGGCCAGACAATTATTGTCTGGCATAGTGCAACAGCAAAGCAATT
TGCTGAGAGCTATAGAGGCTCAACAGCATCTGTTGAAACTCACAGTCTGGGGCATT
>L22951..1
TTCTATTGTGACACATCAGGCCTGTTTAATAGCACTTGGAATGGCAATACCAGCAAGCAGGAGTCAAGTAGCA
CAGAGTCAAATGGCAATATAACTCTCCAATGCAGAATAAAGCAAATTATAAATATGTGGCAGAGAGCAGGAAA
AGCAATATATGCCCCTCCTATCAAAGGAGTAATAAGGTGTGTATCAAACATTACAGGACTAATATTAACAAGA
GATGGTGGGGGTAATAACAGTGAAAATGAAACCTTCAGACCTGGAGGAAGAGATATGAGGGACAATTGGAGAA
GTGAATTATATAAGTATAAAGTAGTAAAAATTGAACCACTAGGAGTAGCACCCACCAGGGCAAGGAGGAGAGT
GGTGGAGAGAGAAAAAAGAGCAGTTGGAGTAGGAGCTGTCTTCCTTGGGTTCTTAGGAGCAGCAGGAAGCACT
ATGGGCGCGGCGTCAATAACGCTGACGGTACAGGCCAGACAATTATTGTCTGGCATAGTGCAACAGCAAAGCA
ATTTGCTGAAGGCTATAGAGGCTCAACAGCATCTGTTGAAACTCACGGTCTGGGGCATT
>AB253429..1
TTCTATTGTAATACATCAGGCCTGTTTAATAGTACTTGGGTAAATGGCACTACCAGCAGCATGTCAAATGGCA
CTATAACTCTCCCATGCAGAATAAAGCAAATTATAAATATGTGGCAGAGAGTAGGACAAGCAATGTATGCCCC

TCCCATCCAAGGAGTAATAAAGTGTGAATCAAACATTACAGGACTAATATTAACAAGAGATGGTGGGGTTAAT
AGCAGTGACAGTGAAACCTTCAGACCTGGAGGAGGAGATATGAGGGATAATTGGAGAAGTGAATTATATAAGT
ATAAGGTAGTAAAAATTGAACCACTAGGAGTAGCACCCACCAAGGCAAGGAGAAGAGTGGTGGAGAGAGAAAA
AAGAGCAGTTACACTGGGAGCTGTATTCATTGGGTTCTTAGGAACAGCTGGAAGCACAATGGGCGCGGCGTCA
ATAACGCTGACGGTACAGGCCAGAAAATTATTGTCTGGCATAGTGCAACAGCAAAGCAATTTGCTGAGGGCTA
TAGAGGCTCAACAGCATCTGTTGAAACTCACTGTCTGGGGCATT
>AY494973..1
TTCTATTGTAATACATCAGGCCTGTTTAATAGCACTTGGCCTTGGAATGAGACAAATAGCGAGACAAATAACA
CGAACGCTACTATAGTGCTCCCATGCAGAATAAAACAAATTGTAAGAATGTGGCAGAGAGTAGGACAAGCAAT
GTATGCCCCTCCCATCCAAGGAGTAATAAAGTGTACATCAAACATTACAGGACTACTATTAATAAGAGATGGT
GGGGTTGGTAACAATACAAATGGAACTGAAACCTTCAGACCTGGAGGAGGAAATATGAGGGACAATTGGAGAA
GTGAATTATATAAGTATAAAGTAGTAAAAATTGAACCACTAGGAGTAGCACCCACCGGGGCAAAAAGAAGAGT
GGTGGAGAGAGAAAAAAGAGCAGTTGGAATAGGAGCTGTGTTCATTGGGTTCTTAGGAGCAGCTGGAAGCACT
ATGGGCGCAGCATCAGTAACGCTGACGGTACAGGCCAGACAATTGTTGTCTGGCATAGTGCAACAGCAAAGCA
ATTTGCTGAGGGCTATAGAGGCTCAACAGCATATGTTGAAACTCACGGTCTGGGGCATT
>EU853062..2
TTCTATTGTAATACATCAGACCTGTTTAATAGCACTTGGTACTTAAATGGCACTAGCACTTGGTACAATGGCA
CTGCAAATAACGCACCGTTAAATGGTAATATAACTCTCCCATGCAGAATAAAGCAAATCATAAGGATGTGGCA
GAGAACAGGACAAGCAATGTATGCCCCTCCCATTCAAGGAAAAATAGAATGTGTATCAAACATTACAGGACTC
CTATTAGTAAGAGATGGTGGGAATGATGAGAATAACAACACAAGAAATAACACCCTCAAAGATGAAAAATTCA
GACCTGGAGGAGGAGATATGAAGGACAATTGGAGAAATGAATTATATAGGTATAAAGTAGTAAGAGTTGAACC
ACTAGGAGTAGCACCCACCAAGGCAAGGAGAAGAGTGGTGGGGAGAGAAAAAAGAGCAGTTGGAATAGGAGCT
GTGTTCCTTGGGTTCTTAGGAGCAGCAGGAAGCACTATGGGCGCGGCGTCAATAACGCTGACGGAACAGGCCA
GACAATTATTGTCTGGTATAGTGCAACAGCAAAGCAATTTGCTGAGGGCTATAGAGGCTCAACAGCAACTGTT
GAAACTCACAGTCTGGGGCATT
>EU852958..1
TTCTATTGTAATACATCAGGCCTGTTTAATAGCACCTGGGATAAAAATACCACCGTGCAGGAGTCAAATAGCA
CGGGGTCAAATGACACTATAACTCTCCAATGTAGAATAAAGCAAATTATAAATATGTGGCAGGAAGTAGGACG
AGCAATGTATGCTCCTCCCATCAAAGGAGTAATAAGGTGTGAATCAAACATTACAGGAATGATATTAACAAGA
GATGGGGGTAATACTACCGGGAAAAATGAGATCTTTAGGCCTGGAGGAGGAGATATGAGGGATAATTGGAGAA
GTGAATTGTATAAGTATAAAGTAGTAAAAATTGAACCACTAGGAGTAGCACCCACCGAGGCAAAGAGAAGAGT
GGTGGAGAGAGAAAAAAGAGCAATTGGTGGAATAGGAGCTGTCTTCCTTGGGTTCTTAGGAGCAGCAGGAAGC
ACTATGGGCGCAGCGTCAATAACGCTGACGGCACAGGCCAGACAATTATTGTCTGGTATAGTGCAACAGCAAA
GCAATTTGCTGAGGGCTATAGAGGCTCAACAGCATCTGTTGAAACTCACGGTCTGGGGCATT
>EU852954..1
TTCTATTGTAACACATCAGGCCTGTTTAATAGCACCTGGTATACAAATAATACCAGCGTGCAGGAGACAAATA
GCACGGGGACAAATGACACTATAATTCTCCAATGTAGAATAAAGCAAATTATAAATATGTGGCAGGAAGTAGG
ACGAGCCATGTATGCTCCTCCCATCAGGGGAGTAATAAGGTGTGAATCAAACATTACAGGAATGATATTAACA
AGAGATGGGGGTGTGAGTGTTAACAAGACAACTGAGACTGAGGTCTTCAGACCTGGAGGAGGAGATATGAGGG
ATAATTGGAGAAGTGAATTGTATAAGTATAAAGTAGTAAAAATTGAACCACTAGGAGTAGCACCTACCAAGGC
AAAGAGAAGAGTGGTGGAGAGAGAAAAAAGAGCAATTACTGGAATAGGAGCTGTCTTCCTTGGGCTCTTAGGA
GCAGCAGGAAGCACTATGGGCGCAGCGTCAATAACGCTGACGGCACAGGCCAGACAATTATTGTCTGGTATAG
TGCAACAGCAAAGCAATTTGCTGAGGGCTATAGAGGCTCAACAACATCTGTTGAAACTCACGGTCTGGGGCAT
T
>AF484507..1
TTCTATTGTAATACATCACAATTGTTTAATAGCATTTGGAATGTCACTAACAGCACAGAGTCAGAGAATAGCA
CGGGGTCAGATGACACTATAACTCTTCCATGCAGAATAAAGCAAATTATAAAGATGTGGCAGAGAACAGGACA
AGCAATATATGCCCCCCCCATCCAAGGAGTAATAAGGTGTGAATCAAACATTACAGGACTATTATTAACAAGA
GATGGTGGGAATAACAATACAGGTAATGAAACCTTCAGACCTGCAGGAGGAGATATGAAGGACAATTGGAGAA
GTGAATTATATAAGTACAAAGTAGTAAAAATTGAACCACTAGGAGTAGCACCCACCAAGGCAAGAAGGAGAGT
GGTGGAGAGAGAAAAAAGAGCAGTTGGAATAGGAGCTGTATTCCTTGGGTTCTTAGGAACAGCAGGAAGCACT
ATGGGCGCGGCGTCAATAACGCTGACGGTACAGGTCAGACAATTATTGTCTGGCATAGTGCAACAGCAAGGCA
ATTTGCTGAGGGCTATAGAGGCTCAACAACAGCTGTTGGAACTCACGGTCTGGGGCATT
>AF484508..2
TTCTACTGTAACACATCAAGCCTGTTTAATGGCACTTGGAATGGCACTGAGAACATAACGGAGTCAAATAGCA
TGAACGACTCTATAATACTCCAATGCAGAATAAAGCAAATTATAAATATGTGGCAGAGAGCAGGACAAGCAAT
ATATGCCCCTCCCATCCCAGGAGTAATAAGGTGTCAATCAAACATTACAGGACTAATATTAACAAGAGATGGT
GGAAATAACAGTACAAATGAAACCTCCAGACCTGGAGGGGGAGATATGAGGGACAACTGGAGAAGTGAATTAT
ATAAGTATAAAGTAGTAAAACTTGAACCACTAGGAGTAGCACCCACCAGGGCAAAGAGAAGAGTGGTGGAGAG
AGAGAAAAGAGCAGTTGGACTAGGAGCTGTTTTCTTTGGGTTCTTAGGAGCAGCAGGAAGCACTATGGGCGCG

GCGTCAATAACGCTGACGGTACAGGCCAGACAATTATTGTCTGGTATAGTGCAACAGCAAAGCAATTTGCTGA
GGGCTATAGAGGCTCAACAGCATCTGTTGAAACTCACGGTCTGGGGCATT
>AF484509..1
TTCTATTGTGACACATCAGGCCTGTTTAATAGCACTTGGTATAATACTTGGAATAGCACTGAATCAAAAAGCA
CGGAGTCAAACGACATTACTCTCCAATGCAGAATAAAGCAAATTATAAATATGTGGCAGAGAGCAGGACAAGC
AATATATGCCCCTCCCATCCAAGGAGTAATAATGTGTAAATCAAATATTACAGGACTAATATTAACAAGAGAT
GGTGGAGGTAATAGCAGTGAACCTGAAACCTTCAGACCTGGAGGAGGAGACATGAGGGACAATTGGAGAAGTG
AATTGTATAAGTATAAAGTAGTAAAAATTGAACCACTAGGAGTAGCACCCACCAAGGCAAGGAGAAGAGTGGT
GCAGAGAGAAAAAAGAACAGTTGGAATAGGAGCTGTGTTCCTTGGGTTCTTAGGAGCAGCAGGAAGCACTATG
GGCGCGGCGTCAATAACGCTGACGGCACAGGCCAGAGCATTACTGTCTGGCATAGTGCAACAGCAAAGCAATT
TGCTGAGGGCTATAGAGGCTCAACAACATCTGTTGAGACTCACGGTCTGGGGCATT
>AF484512..2
TTCTATTGTGATACAACAGGACTGTTTAATAGCAATTGGACTTGGAATGACACTGCCAGCATAATGGAGTCAA
ATAGCATGGAGTCAACTCAAAATATAACACTCCAATGCAGAATAAAGCAGATTATAAATATGTGGCAGAGAGC
AGGTAAAGCAATGTATGCCCCTCCCATCCAAGGAACAATAAGGTGTGAATCAAACATTACAGGACTACTGTTA
ACAAGAGATGGTGGGGATAATAGAGATAATAGAACTGAAACCTTCAGACCTGGAGGAGGAGATATGAGGGATA
ATTGGAGAAGTGAATTATATAAGTATAAAGTAGTCAAAATTGAACCACTAGGAGTAGCACGCACCAGGGCAAA
GAGAAGAGTGGTGGAGAGAGAAAAAAGAGCAGTTGGAATAGGAGCTGTCTTCCTTGGGTTCTTAGGAGCAGCA
GGAAGCACTATGGGCGCGGCGTCAATAACGCTGACGGTACAGGCCAGACTATTATTGTCTGGTATAGTGCAAC
AGCAAAGCAATTTGCTGAGGGCTATAGAGGCTCAACAACATTTGTTGAAGCTCACGGTCTGGGGCATT
>EU853030..1
CTCTATTGTAATACATCAGGCCTGTTTAATAGCACTTGGAATAACACTGTCAGCACGGAGTTAAATAGCAAGG
AGGCAAATAACAATATAACTCTCCCATGCAAAATAAAGCAAATTATAAACATGTGGCAAAGAACAGGACAAGC
AATGTATGCCCCTCCCATCCGAGGAGTAATAAATTGTGTGTCAAACATTACAGGACTAATATTAACAAGAGAT
GGTGGGCCTAACGATACACATGAAAATGAAACCTTCAGACCTGGAGGAGGAAATATGAGGGACAATTGGAGAA
GTGAATTATATAAATATAAAGTAGTAAAAATTGAACCATTAGGAGTAGCACCCACCAGGGCAAAGAGAAGAGT
GGTGGAAAGAGAAAAAGGAGCAGTTGGAATAGGAGCTGTCTTCCTTGGGTTCTTAGGAGCAGCAGGAAGCACT
ATGGGCGCGGCGTCAATAACGCTGACGGTACAGGCCAGACAATTGTTGTCTGGCATAGTGCAACAGCAAAGCA
ATTTGCTGAGGGCTATAGAGGCTCAACAACATCTGTTGAAACTCACGGTCTGGGGCATT
>AF484478..1
TTCTATTGTAACACAACAAACCTGTTTAATAACATTTGGAGTAATGCCAGCATGCAGAAGGCAAATAGCATAA
ACGACACTATAACTCTCCAATGCAGAATAAAGCAAATTATAAATATGTGGCAGAGAGTAGGACAAGCAATATA
TGCCCCTCCCATCCAAGGAGTGATAAAGTGTGAATCAAACATTACAGGACTAATATTAACAAGAGATGGTGGG
GGTAATAACAATACAAATGAAACCTTCAGACCTGGAGGAGGAGATATGAGGGACAATTGGAGAAGTGAATTAT
ATAAGTATAAAGTAGTAAAAATTGAACCACTAGGAGTAGCACCTACCAGGGCAAAGAGAAGAGTGGTGGAGAG
AGAAAAAAGAGCAGTTGGAATGGGAGCTGTCCTCATTGGGTTCTTAGGAGCAGCAGGAAGCACTATGGGCGCG
GCGTCAATAACGCTGACGGTACAGGCCAGACAATTATTGTCTGGCATAGTGCAACAGCAAAGCAATTTGCTGA
GGGCTATAGAGGCTCAACAGCATCTGTTGAAACTCACGGTCTGGGGCATT
>AF484493..2
TTCTATTGTAATACATCAGGCCTGTTTAATAATACTTACACTACTGACAGCATAGAGTCAAATGACACGGAGC
CAGATGCAAATATAACTCTTCCATGCAGAATAAAGCAAATTATAAATATGTGGCAGAGAGTAGGACAAGCAAT
ATATGCCCCTCCCATCCAAGGAGTAATAAAGTGTGAGTCAAACATCACAGGACTACTATTAACAAGAGATGGT
GGTACTAACAGTACTAAAAGTACTGAAACCTTCAGACCTGGAGGAGGAGATATGAGGGACAATTGGAGAAGTG
AATTATATAAGTATAAAGTAGTACAAGTTGAACCAATAGGAGTAGCACCCACCAGGGCAAAGAGAAGAGTGGT
GGAGAGAGAAAAAAGAGCAGTTGGAATAGGAGCTGTACTCCTTGGGTTCTTAGGAGCAGCTGGAAGCACAATG
GGCGCGGCGTCAATAACGCTGACGGTACAGGCCAGACAGTTATTGTCTGGCATAGTGCAGCAGCAAAGCAATT
TGCTGAGGGCTATAGAGGCTCAACAGCAACTGTTGAGACTCACTGTCTGGGGCATT
>AB098330..2
TTCTATTGTAATACATCAGGCCTGTTCAATGGCACCTGGAAGCCAAACAGCACAGAGTCAAACAACACAACGC
CAAATGACACTATAACTCTTACATGCAGAATAAAGCAAATTATAAATATGTGGCAGAAAGTAGGACAAGCAAT
GTATGCCCCTCCCATTCAAGGAGTAATAAGGTGTGAATCAAACATTACAGGACTACTATTAACAAGAGATGGA
GGGATTAACAGTATAAATGAAACCTTCAGACCTGGAGGAGGAAATATGAGGGACAATTGGAGAAGTGAATTAT
ATAAGTATAAAGTAGTAAAAATTGAACCACTAGGAGTAGCACCCTCCAGGGCAAAGAGAAGAGTGGTGGAGAG
AGAAAAAAGAGCAGTTGGAATAGGAGCTGTCTTCCTTGGGTTCTTAGGAGCAGCTGGAAGCACTATGGGCGCG
GCGTCAATAACGCTGACGGCACAGGCCAGACAATTATTGTCTGGTATAGTGCAACAGCAAAGCAATTTGCTGA
GGGCTATAAAGGCTCAACAGCATATGTTGAAGCTCACGGTCTGGGGCATT
>GU367412..5
TTCTATTGTGATACATCAGGCCTGTTTAATAGTACTTGGAATGACACTGCCACCAATCAGACTTTTGATGCAA
ATAGCAATATAACTCTTCAATGTAAAATAAGACAAATTGTACGTATGTGGCAGAGAATAGGACAAGCATTGTA
TGCCCCTCCCATTCAAGGAGTAATAAGCTGTAAATCAAACATTACAGGGCTGCTACTAACAAGAGATGGTATG

AATGAGAGTAGTGAGACGTTCAGACCTCAAGGAGGAAATATGAAGGACAATTGGAGAAGTGAATTATATAAAT
ATAAAGTAGTAAAAATTGAACCACTAGGAATAGCACCCACCAGGGCAAAGAGAAGAGTGGTGGAGAGAGAAAA
AAGAGCAGTTGGACTGGGAGCTGTCTTCTTTGGGTTCTTGGGAGCAGCAGGAAGTACTATGGGCGCGGCGTCA
ATAACGCTGACGGCACAGGCCAGACAATTATTGTCTGGTATAGTACAACAGCAGAGCAATCTGCTGAGGGCTA
TAGAGGCTCAACAGCAACTGTTGAAACTCACAGTCTGGGGCATT
>FJ647148..1
TTCTATTGTAATACATCAGGTCTGTTTAGTAGCAGGTGGTTCACTAATGGCACTAACAGCACGGAGTCAAATG
GCACAGGCAATATAACTCTCCAATGCAGGATAAAGCAAATTATAAATATGTGGCAGAGAGTAGGACAAGCAAC
GTACACCCCTCCCATCCAAGGAGAAATAAGGTGTAGATCAAACATTACAGGACTACTATTAACAAGAGATGGT
GGGATTAACACAACAGAGGAAATCTTCAGACCTGGAGGGGGAAATATGAAGGACAATTGGAGAAGTGAATTAT
ATAAGTATAAAGTAGTAAAAATTGAACCACTAGGAGTAGCACCATCCAAGGCAAAGAGAAGAGTGGTGGGAAG
AGAAAAAAGAGCAGTTGGACTGGGAGCTGTATTCATTGGGTTCTTGGGAGCAGCAGGAAGCACTATGGGCGCG
GCGTCAGTGACGCTGACGGTACAGGCCAGACAATTATTGTCTGGCATAGTGCAGCAGCAAAGCAATTTGCTGA
GGGCTATAGAGGCTCAACAGCATCTGTTGAAACTCACAGTCTGGGGCATT
>KT183312..1
TTCTATTGTGACACATCAGGTCTGTTTAATGGCAGTTGGAGTGAAAATAGCACTTGGAATAGCACTATGGAGG
CAAATAGCACAATTATACTCCCATGTAGAATAAAGCAAATTATAAATATGTGGCAGAGAACAGGACGAGCAAT
GTATGCCCCTCCCATCCAAGGAACAATACAGTGTACATCAAACATCACAGGATTAATATTAACAAGAGATGGT
GGTGAGAAAAACAATAGCACTGAAGAAATCTTCAGACCTGGAGGAGGAGATATGAGGGACAATTGGAGGAGTG
AATTATATAAATATAAAGTAGTAAAAATTGAACCACTAGGAGTAGCACCCACCAGGGCAAAGAGAAGAGTGGT
GGAGAGAGAAAAAAGAGCAGTTACACTAGGAGCTGTATTCATTGGGTTCTTAGGAGCAGCAGGAAGCACTATG
GGCGCGGCGTCAGTGACGCTGACGGTACAGGCCAGACAATTATTGTCTGGCATAGTGCAACAGCAAAGCAATT
TGCTGAGGGCTATAGAGGCTCAACAGCATATGTTGAAACTCACGGTCTGGGGCATT
>KC863311..1
TTCTATTGTAATACAACAGACCTGTTTAAGGGTACTTGGCATCCTGATAATAAAACTTGGAGTGATCCTTGGA
ATGCCAGCATGAAGTCAAATAACACATCAAATGTCAATATAACTATCCTATGCAAGATAAAGCAAATTGTAAG
GATGTGGCAGAGAGTAGAACAAGCAATGTATGCCCCTCCCATCCAAGGAGTAATAAGCTGTTCATCAAACATT
ACAGGACTACTATTAACAAGCGATGGTGGTAGGGATAATAGCAATAATACTGGTAAGGATAATAGCAATAAAA
ATAATACTGAAACCTTCAGACCTGGAGGAGGAAATATGAGGGACAATTGGAGAAGTGAATTATATAAGTATAA
AGTAGTAAAAATTGAACCACTAGGAGTAGCACCTACCCCGGCAAGGAGAAGAGTGGTGCAGAGAGAAAAAAGA
GCAGTTGGCCTAGGAGCTGTCTTCATTGGGTTCTTAGGAGCAGCAGGAAGCACTATGGGCGCGGCGTCAGTGA
CGCTGACGGTACAGGCCAGACAATTATTGACTGGTATAGTGCGACAGCAAAGCAATTTGCTGAAGGCTATAGA
GGCTCAACAACATCTGTTGAGACTCACAGTCTGGGGCATT
>HM623589..3
TTCTACTGCAACACATCAAGCTTGTTTAATAGGACATTTCCGGCCAACTACACGTATAATAGGACTGCTGACA
GCACACAGGGGAATACAACTATAACTATCCCATGCAGAATAAAACAAATTATAAACATGTGGCAGAGAGTAGG
ACAAGCAATGTATGCCCCTCCCATTGAAGGAGTAATAAGATGTGAATCAAACATTACAGGAATACTATTGACA
AGAGATGGTGGGAATGAGACAAATGTAGAAGTCTTCAGACCAGAGGGAGGAAATATGAGGGACAATTGGAGAA
GTGAATTATATAATTACAAGGTAGTAAGAATTGAACCACTAGGAGTAGCACCCACCAGGGCAAGGAGGAGAGT
GGTGGAGAGAGAAAAAAGAGCAGTTGGACTGGGAGCTGTCTTCCTTGGGTTCTTAGGAGCAGCAGGAAGCACT
ATGGGCGCGGCGTCAATAACGCTGACGGTACAGGCCAGGCAATTATTGGCTGGCATAGTGCAACAGCAAAGCA
ATTTGCTGCAAGCTATAGAGGCTCAACAGCATCTGTTGAAACTCACTGTCTGGGGCATT
>MH705163..1
TTCTATTGCAATACAACAGCCCTGTTTAATAGCACGTGGGAGAATGAGAATGACACGGCCAACATACAGAAGA
ATGGCACGGAGTCAAATGACACTATAACACTCCCATGCAGAATAAAACAAATTATAAACATGTGGCAGAGAGT
AGGACGAGCAATGTATGCCCCTCCCATTGCAGGAGTAATAAAGTGTACATCAACCATTACAGGAATAATATTG
ACAAGAGATGGTGGGGATAGTGGGAACAATACCAGGGAGGAGATCTTCAGACCTGGAGGAGGAGATATGAGGG
ACAATTGGAGAAGTGAATTATATAAGTATAAAGTAGTAAAACTTGAACCACTAGGAGTAGCACCCACCAAGGC
AAGGAGAAGAGTGGTGGAGAGAGAAAAAAGAGCAGTTGGACTGGGAGCTGTCTTCCTTGGGTTCTTGGGAGCA
GCAGGAAGCACTATGGGCGCAGCGTCAATAACGCTGACGGTACAAGCCAGACAATTATTGTCTGGCATAGTGC
AACAGCAAGGCAATTTGCTGAAGGCTATAGAGGCTCAACAGCATCTGTTGAAACTCACGGTCTGGGGCATT
>AF286241..1
TTCTATTGTAATACAACAAGCCTGTTTAATAGCACATGGAAGAATGGCGCCACCATACAGGAGAATAGCACGG
AGACAAATGGAATTATGACTCTCCCATGCAGAATAAAACAAATTGTAGACATGTGGCAGGAAGTAGGACAAGC
AATGTATGCCCCTCCCATTGCAGGAGTAATATATTGTACATCAAACATTACAGGAATAATATTGACAAGAGAT
GGTGGGAGCAGTAACACCAATAGTGAGATCTTTAGGCCTGGAGGAGGAGATATGAGGGACAATTGGAGAAGTG
AATTATATAAGTATAAAGTAGTAAAAATTGAACCACTAGGAGTAGCACCCTCCAGGGCAAAGAGAAGAGTGGT
GGAGAGAGAAAAAAGAGCAGTGGGAATAGGAGCTGTTTTCCTTGGGTTCTTGGGAGCTGCAGGAAGCACTATG
GGCGCGGCGTCAATAACGCTGACGGTACAGGCCAGACAGTTATTATCTGGCATAGTGCAACAGCAAAGCAATT
TGCTGAAGGCTATAGAGGCTCAACAGCATCTGTTGAAACTCACAGTCTGGGGCATT

>AF286238..2
TTCTATTGCAATACAACAGGCCTGTTTAATAGCACATGGGAAAATGGCACTAACAAACAGAATTACACGGAGT
CAAATGACACTATAACGCTCCAATGCAGAATAAAACAAATTATAAACATGTGGCAGAGAGTAGGCCGAGCAAT
GTATGCCCCTCCCATTGCAGGAGTAATAAAGTGTACATCAAACATTACAGGAATGATATTGACAAGAGACGGT
GGGAAGAACAGTATTAATGAGACCTTCAGACCTGGAGGAGGAGATATGAGGGACAATTGGAGAAGTGAATTAT
ATAAGTATAAAGTAGTAAAAATTGAACCACTAGGAATAGCACCCACAGAGGCAAGGAGAAGAGTGGTGCAGAG
AGAAAAAAGAGCAGTTGGATTGGGAGCTGTTTTCCTTGGGTTCTTGGGAGCAGCAGGAAGCACTATGGGCGCG
GCGTCAATAACGCTGACGGTACAGGCCAGACAATTATTGACTGGCATAGTGCAACAGCAAAGCAATTTGCTGA
AGGCTATAGAGGCTCAACAACAGATGTTGAGGCTCACAGTCTGGGGCATT
>AF286237..2
TTCTATTGCAATACAACAGGCCTGTTTAATGGTACGTGGTGGAACAATGGTACGTGGAACGGGCCCTACACAC
CTAATAACACCAATGGAAGTATAATCCTCCCATGCAGAATAAAACAAATTATAAACATGTGGCAGAGAGTAGG
AAGAGCAATGTATGCCCCTCCCATTGCAGGAATAATAAAGTGTACATCAAACATTACAGGAATAATATTGACA
AGAGATGGTGGTAACAATGGGACTAATGAGACCTTCAGACCTGGAGGAGGAGATATGAGGGACAATTGGAGAA
GTGAATTATATAAATATAAAGTAGTAAAACTTGAACCACTAGGAGTAGCACCTACCAGGGCAAAAAGAAGAGT
GGTGGAGAGAGAAAAAAGAGCAGTTGGACTGGGAGCTGTCTTCCTTGGGTTCTTGGGAGCAGCAGGAAGCACT
ATGGGCGCGGCGTCACTAACGCTGACGGTACAGGCCAGACAATTATTGTCTGGTATAGTGCAACAGCAAAGCA
ATTTGCTGCAGGCTATAGAAGCTCAACAGCATCTGTTGAAACTCACAGTCTGGGGCATT
>AY521629..3
TTCTATTGCAATACATCAGGTCTGTTTAATAGTACTTGGAATGACACGGCCATCAATGACACTATCAACGTGC
AGAATTCAACTTACAATATAACTCTCCCATGCAGAATAAGACAAATTGTACATATGTGGCAGAGAGTAGGACA
AGCAATGTATGCCCCTCCCATTCAAGGGGTAATAAACTGTACATCAAACATTACAGGGCTACTGTTAACAAGG
GATGGTGGGGATAATAGTAGTACAAATGAGACATTCAGACCTGGAGGAGGAAATATGAAGGACAATTGGAGAA
GTGAATTATATAAATATAAAGTAGTAAAAATTGAACCACTAGGAGTAGCACCCACCAAGGCAAAGAGGAGAGT
GGTGGAGAGAGAAAAAAGAGCAGTTGGACTAGGAGCTGTCTTCCTTGGGTTCTTAGGAGCAGCAGGAAGTACT
ATGGGCGCGGCGTCAATGACGCTGACGGTACAGGCCAGACAATTATTGTCTGGCATAGTACAACAGCAGAGCA
ATCTGCTGAGGGCTATAGAGGCTCAACAGCACATGTTGAAACTCACAGTCTGGGGCATT
>AY521631..2
TTCTATTGCAATACATCAGGCCTGTTTAATAGTACTTGGTATAATAGTACTTGGAATAACACTGACAGCACAC
AGAAATCAAATGGCACGGAGAAAATAACTCTCCCATGCAGAATAAAACAAGTTATAAAAATGTGGCAAAGAGT
AGGACAAGCAATGTATGCCCCTCCCATTCAAGGAGTAATAAAATGTGTATCAAACATTACAGGGATACTGCTA
ACAAGAGATGGGGGGAACCTTAATAGCTCATTTGAGACGTTCAGACCTGGAGGAGGAAATATGAAGGACAATT
GGAGAAGTGAATTATATAAATATAAAGTAGTAAAAATTGAACCACTAGGAGTAGCACCCACCAAGGCAAAGAG
AAGAGTGGTGGAGAGAGAAAAAAGAGCGGTCGGACTGGGAGCTGTCTTCCTTGGGTTCTTAGGAGCAGCAGGA
AGTACTATGGGCGCGGCGTCAATAACGCTGACGGCACAGGCCAGACAATTATTGTCTGGTATAGTACAACAGC
AGAGCAATCTGCTGAGGGCTATAGAGGCTCAACAGCAAATGTTGAAACTCACGGTCTGGGGCATT
>AY521630..1
TTCTATTGCAATACATCAGGTCTGTTTAATAGTACTTGGAATAGCACTACCATCAAGCAGAATTTAACTAACA
ATGACACGGGGGTAATAAATCTCCCATGCAGAATAAGACAAATTGTACATATGTGGCAGAGAGTAGGACAAGC
AATGTATGCCCCTCCCATTAAAGGGGTAATAAACTGTACATCAAACATTACAGGGCTACTGCTAACAAGGGAT
GGTGGGGATAATAATAGTACAGATGAGAAGTTCAGACCTGGAGGAGGAGATATGAGGGACAATTGGAGAAGTG
AATTATATAAATATAAAGTAGTAAAAATTGAACCACTAGGAGTAGCACCCACCAGGGCAAAGAGAAGAGTGGT
GCAGAGAGAAAAAAGAGCAGTTGGACTAGGAGCTGTCTTCTTTGGGTTCTTAGGAGCAGCAGGAAGTACTATG
GGCGCGGCGTCAATAACGCTGACGGCACAGGCCAGACAATTATTGTCTGGCATAGTACAACAGCAGAGCAATC
TGCTGAGGGCTATAGAGGCTCAACAGCACTTGTTGAAACTCACAGTCTGGGGCATT
>AM000053..1
TTCTATTGCAATACATCAGGCCTATTTAATAGCAGTTGGGCGCCCAACGGCAGTTGGATCAACAACATTGGCA
GTAGGGAAAATGACACTATGACTCTGCCATGCAGAATAAAACAAATTATAAATATGTGGCAGAGAGTAGAACA
AGCAATGTATGCCCCTCCTATCAAAGGAAACATAACTTGTGTATCAAACATTACAGGACTACTGTTAACAAGA
GATGGTGGAACTAATGAAACTAGGGAGAATGAGACCTTCAGACCTATAGGAGGAGAGATGAGAGACAATTGGA
GAAGTGAATTATATAAGTATAAAGTAGTAAAAATTGAACCACTAGGAGTAGCACCCACCAAGGCAAAGAGAAG
AGTGGTGGAGGGGAGAGAAAAAAGAGCAGTTGGAATGGGAGCTTTCTTCCTTGGGTTCTTAGGAGCAGCAGGA
AGCACTATGGGCGCGGCGTCAATAACGCTGACGGTACAGGCCAGACAATTATTGGCTGGTATAGTGCAACAGC
AAAGCAATTTGCTGAAGGCTATAGAGGCTCAACAACATCTGTTGAGACTCACAGTCTGGGGCATT
>AM000054..2
TTCTATTGTAATACATCAAGCCTGTTTAATAGCTCTTGGCCCATTAACAGCACTACCAGCAATAACAGCACTG
CCAGTAATGAGACCATAATTCTGCCATGTAGAATAATACAAATTATAAATATGTGGCAGAGACTAGGACAGGC
AATGTATGCCCCTCCCATCAAAGGATTAATAAATTGTGTATCAAACATTACAGGACTACTGTTGACAAGGGAT
GGTGGAGATAGGAACAGTAGTTATGAGACCTTCAGACCTATAGGAGGAAATATGAAGGACAATTGGAGAAGTG
AACTGTATAAGTATAAAGTAGTAAAAATTGAACCACTAGGAGTAGCACCTACCAAGGCAAGGAGGAGAGTGGT

GGAGAGAGAAAAAAGAGCAGTTGGAATAGGAGCTTTCTTCCTTGGGATCTTAGGAGCAGCAGGAAGCACTATG
GGCGCGGCGTCAATAACGCTGACGGTACAAGCCAGGCAAGTATTGTCTGGTATAGTGCAACAGCAGAGCAATT
TGCTGAAGGCTATAGAGGCTCAACAACATTTGTTGAAACTCACAGTCTGGGGCATC
>FJ388892..2
TTCTATTGCAATACAACAGACCTGTTTAATAGCACTTGGTACACTAATGACACTGGCACAAATAGCACAGGGG
CCAATGAAAATGGGGCCAATGAAAATATAACTTTACCATGCAGAATAAAGCAAATTATAAATATGTGGCAGAG
AGTAGGGCAAGCAATGTATGCCCCTCCTATCAAAGGAAGTATAAGGTGTGAATCAAACATTACAGGACTACTA
CTAACAAGAGATGGTGGAAGTGGAAATGATAGCTACAATGAGACCTTCAGACCTATAGGAGGAAATATGAGGG
ATAATTGGAGAAGTGAACTATATAAGTATAAAGTAGTAGAAATTGAACCAATAGGAGTAGCACCTACCAAGGC
AAGGAGAAGAGTGGTGGGGAGAGAAAAAAGAGCAATTGGACTGGGAGCTGCCTTCCTTGGGTTCTTAGGAGCA
GCAGGAAGCACTATGGGCGCGGCGTCAATAACGCTGACGGTACAGGCCAGACAATTATTGTCTGGCATAGTGC
AACAGCAAAGCAATTTGCTGAGGGCTATAGAGGCTCAGCAGCATCTGCTGAAACTCACAGTCTGGGGCATT
>JF683763..1
TTCTATTGCAATACAACAGACCTGTTCAGTAGCACTTGGGACGAAGATGACACTGACATCACTCTCGCCCAGG
ACAATGGAACTATAACTCTGCCATGCAGAATAAAGCAAATTATAAATATGTGGCAGAGAGTAGGACAAGCAAT
GTATGCCCCTCCTATCAAAGGAAGTATAAGGTGTGAATCAAACATTACAGGACTACTACTAACAAGAGATGGT
GGAGCTAATAGCAGTAATGAGACCTTCAGACCTATAGGAGGAGATATGAGAGACAATTGGAGAAGTGAACTAT
ATAAATATAAAGTGGTACAAATTGAACCAATAGGAATAGCACCTACCAGGGCAAGGAGAAGAGTGGTGGAGAG
AGAAAAAAGAGCAATTGGACTGGGAGCTGCCTTCCTTGGGTTCTTAGGAGCAGCAGGAAGTACTATGGGCGCG
GCGTCACTGACGCTGACGGTACAGGCCAGAAAATTATTGTCTGGCATAGTGCAACAGCAAAACAATTTGCTGA
GGGCTATAGAGGCTCAGCAGCATCTGCTGAAACTCACGGTCTGGGGCATT
>JF683798..1
TTCTATTGCGATACAACAAACCTGTTCAATAGCACTTGGGACGGAAATAGCACTGTCACAAATATCACACAGA
CCAATGAAAATATAACTTTACCATGCAAAATAAGGCAAATTATAAGGATGTGGCAGAGAGTAGGACAAGCAAT
GTATGCCCCTCCTATCAAAGGAATTATAAGGTGTGAATCAAACATTACAGGACTACTGCTAACAAGAGATGGT
GGAAATAAAACCAATAGCAACAATGAGACCTTCAGACCTATAGGAGGAGATATGAGGGACAATTGGAGAAGTG
AACTATATAAGTATAAAGTAGTAAAAGTGGAACCAATAGGAGTAGCACCTACCAGAGCAAGGAGAAGAGTGGT
GGAGAGAGAAAAAAGAGCAATAATTGGACTGGGAGCTGCCTTCATTGGGTTCTTAGGAGCAGCAGGAAGCACT
ATGGGCGCGGCGTCAATAACGCTGACGGTACAGGCCAGACAATTATTGTCTGGCATAGTGCAACAGCAAAGCA
ATTTGCTGAGGGCTATAGAGGCTCAGCAGCATCTGCTGAAACTCACGGTCTGGGGCATT
>EF545108..1
TTCTATTGCAATACAACAGGCCTGTTCAATAATGCCAGCAACAATGGTACAAACCGCACAGACCCTATAATTC
TCCCATGCAGAATAAAGCAGATTATAAATATGTGGCAAAGAGTAGGGCAGGCAATGTATGCTCCTCCTATCAA
AGGAATCATAAGGTGTGAATCAAACATTACAGGACTACTATTAACAAGAGATGGTGGGAATAAAAATGGAAGC
AACGGGACCGAGACCTTCAGACCTATAGGAGGAGATATGAGGGACAATTGGAGAAGTGAATTATATAAGTATA
AAGTAGTAAAAATTGAACCAATAGGAGTAGCACCTACCAGGGCAAAGAGAAGAGTGGTGGAGAGAGAAAAGAG
AGCAATTGGACTGGGAGCTGCCTTCCTTGGGTTCTTAGGAGCAGCAGGAAGTACTATGGGCGCGGCGTCAATA
ACGCTGACGGTACAGGCCAGACAATTACTGTCTGGCATAGTACAACAGCAAAGCAATTTGCTGAGGGCTATAG
AGGCTCAGCAGCATCTGCTGAAGCTCACGGTCTGGGGCATT
>GU481566..1
TTCTATTGCAATACAACAGCCCTGTTCAATAGCACTTGGGATGAAAATAGCACTGTCACAAATAGCACAACGG
CCAATGAAAATATAACTCTGCCATGCAGAATAAAGCAAATTATAAATATGTGGCAGAGAGTAGGACAAGCAAT
GTATGCCCCTCCTATCGCAGGAATTATAAGGTGTGAATCAAACATTACAGGACTACTAATAACAAGAGATGGT
GGAACTAATAGCAGCAATGAGACTGAGACCTTCAGACCTACAGGAGGAAATATGAGGGACAATTGGAGAAGTG
AACTATATAAGTATAAAGTAGTACAAATTGAACCAATAGGAGTAGCACCTACCAAGGCAAAGAGAAGAGTGGT
GGAGAGAGAAAAAAGAGCAATTGGACTGGGAGCCGCCTTCCTTGGGTTCTTAGGAGCAGCAGGAAGTACTATG
GGCGCGGCGTCACTGACGCTGACGGTACAGGCCAGACAATTATTGTCTGGCATAGTACAACAGCAAAGCAATT
TGCTGAGGGCTATAGAGGCTCAGCAGCATCTGCTGAAACTCACGGTCTGGGGCATT
>GU481615..1
TTCTATTGCAATACAACAGAACTGTTCAATAGCACTTGGAACACAAGCAGCACTGTCACAAATAGCACACAGG
CCAATGAAACTATAAGTCTACCATGCAAAATAAAGCAAATTATAAGGATGTGGCAGAGAGTAGGACAAGCAAT
GTATGCCCCTCCTATCAACGGAACTATAAGGTGTGAATCAAACATTACAGGACTACTACTAACAAGAGATGGT
GGAGGTGAAAATAATAGCAGCAATGAGACCTTCAGACCTGCAGGAGGAGATATGAAGGACAATTGGAGAAGTG
AACTATATAAGTATAAAGTAGTAGAAATTGAACCAATAGGATTAGCACCTACCAAGGCAAGGAGAAGAGTGGT
GGAGAGAGAAAAAAGAGCAATTGGACTGGGAGCTGCCTTCCTTGGGTTCTTAGGAGCAGCAGGAAGTACTATG
GGCGCGGCGTCATTGACGCTGACGGTACAGGCCAGACAATTATTGTCTGGCATAGTGCAACAGCAAAGCAATT
TGCTGAGGGCTATAGAGGCTCAGCAGCATCTGCTGAAACTCACAGTCTGGGGCATT
>HQ896489..1
TTCTATTGCAATACAACAGACCTGTTCACAAATAGCACTCTCACAGATAACACAGAGACCAATGAAACTATAA
CTCTGCCATGCAGAATAAAGCAAATCATAAATATGTGGCAGAGAGTAGGACAAGCAATGTATGCCCCTCCTAT

CAGAGGAAGTATAAGGTGTAATTCAAACATTACAGGACTACTGCTAATAAGAGATGGTGGAGCTAATAGCAGC
AATGAGACCTTCAGACCTATAGGAGGAAATATGAGGGACAATTGGAGAAGTGAACTATATAAGTATAAAGTAG
TAAAAATTGAACCAATAGGAGTAGCACCTACCAAGGCAAAGAGAAGAGTGGTGGAGAGAGAAAAAAGAGCAAT
TGGACTGGGAGCCGCCTTCCTTGGGTTCTTAGGAACAGCAGGAAGCACTATGGGCGCGGCGTCACTGACGCTG
ACGGTACAAGCCAGACAATTATTGTCTGGCATAGTGCAACAGCAAAGCAATTTGCTGAGGGCTATAGAGGCTC
AGCAACATCTGCTGAAACTCACGGTCTGGGGCATT
>JQ292897..1
TTCTATTGCGATACAACAGCCCTGTTCAATAGCACTTGGGAAAATGGCACTGTTATAAATAGCACACAGGCCA
ATGAGACTATAACTCTACCATGCAGAATAAAGCAAATTATAAATATGTGGCAGAGAGTAGGACGAGCAATGTA
TGCCCCTCCTATCAGAGGAATTATAAGGTGTGAATCAAACATTACAGGACTACTACTAACAAGAGATGGTGGA
GGTGGAAATAATAGCAGCAATGAGACCTTCAGACCTATAGGAGGAAATATGGAGGACAATTGGAGAAGTGAAC
TATATAAGTATAAAGTAGTAGAAATTGAACCAATAGGAGTAGCACCTACCAAGGCAAAGAGAAGAGTGGTGGA
GAGGGAAAAAAGAGCAATTGGACTGGGAGCTGCCTTCCTTGGGTTCTTGGGAGCAGCAGGAAGCACTATGGGC
GCGGCGTCACTAACGCTGACGGTACAGGCCAGACAATTATTGTCTGGCATAGTGCAACAGCAAAGCAATTTGC
TGAGGGCTATAGAGGCTCAGCAGCATCTGCTGAAACTCACGGTCTGGGGCATT
>JQ292899..1
TTCTATTGCAATACAACAGACCTGTTCAATTGGGACGGAAATGGCAATGTTACAAACAGCACACAGGCCGATG
GAAATATAACTCTACCATGCAGAATAAAGCAAATTATAAATATGTGGCAGAGAGTAGGACAAGCAATGTATGC
CCCTCCTATCAAAGGAAATATAACGTGTGTATCAAACATTACAGGACTACTATTAACAAGAGATGGTGGAGGT
GGAATTAATAGTAGCAATGAGACCTTCAGACCTATAGGAGGAGATATGAGGGACAATTGGAGAAGTGAACTAT
ATAAGTATAAAGTAGTAAAAATTGAACCAATAGGAGTAGCACCTACCAAGGCAAGGAGAAGAGTGGTGGAGAG
AGAAAAAAGAGCAATTGGACTGGGAGCTGCCTTCCTTGGGTTCTTGGGAGCAGCAGGAAGTACTATGGGCGCG
GCGTCAATAACGCTGACGGTACAGGCCAGACAATTATTGTCTGGCATAGTGCAACAGCAAAGCAATTTGCTGA
GGGCTATAGAGGCTCAGCAGCATATGCTGAAACTCACGGTCTGGGGCATT
>HQ385841..2
TTCTATTGCTATACAACAAAACTGTTCAATAGCACTTGGAACGGAAATGACACTGTTACAAATAGCACACAGG
CCAATGAAAATATAACTATACCATGCAGAATAAAGCAAATTATAAATATGTGGCAGAGAGTAGGACAAGCAAT
GTATGCCCCTCCTATCAAAGGAAATATAAGCTGTACATCAAACATTACAGGACTATTACTAACAAGAGATGGT
GGAACTAATAGCAGTAATGAGACCTTCAGACCTATAGGAGGAGATATGAGGGACAATTGGAGAAGTGAACTAT
ATAAGTATAAAGTAGTACAAATTGAACCAATAGGAGTAGCACCTACCAGGGCAAAGAGAAGAGTGGTGGAGAG
AGAAAAAAGAGCAATTGGACTGGGAGCTGCCTTCCTTGGGTTCTTGGGAGCAGCAGGAAGTACTATGGGCGCG
GCGTCAATAACGCTGACGGTACAGGCCAGACAATTATTGTCTGGCATAGTACAACAGCAAAGCAATTTGCTGA
GGGCTATAGAGGCTCAGCAGCATCTGCTGAAACTCACGGTCTGGGGCATT
>HQ385843..1
TTCTATTGCAATACAACAGCCCTGTTCAATAGTACTTGGCACGAAAATAGCACTGATACAAATAGCACACAGG
ACAATGAAACTATAACTCTACCATGCAGAATAAAGCAAATTATAAATATGTGGCAGAGAGTAGGACAAGCAAT
GTATGCCCCTCCTATCGAAGGAAATATAAGCTGTACATCAAACATTACAGGACTACTACTAACAAGGGATGGT
GGAGCTAATAGCAGCAATGAGACCTTCAGACCTATAGGAGGAGATATGAGGGACAATTGGAGAAGTGAACTAT
ATAAGTATAAAGTAGTACAAATTGAACCAATAGGAGTAGCACCTACCAAGGCAAAGAGAAGAGTGGTGGAGAG
AGAAAAAAGAGCAATTGGACTGGGAGCTGCCTTCCTTGGGTTCTTGGGAGCAGCAGGAAGCACTATGGGCGCG
GCGTCAATAACGCTGACGGTACAGGCCAGACAATTATTGTCTGGCATAGTGCAACAGCAAAGCAATTTGCTGA
GGGCTATAGAGGCTCAGCAGCATCTGCTGAAACTCACGGTCTGGGGCATT
>HQ385846..1
TTCTATTGCAATACAACAGAACTGTTCAATAGCACTTGGGGCAATGGCACTGTCACAAATAGCACAGGGGACA
ATGAAAATATAATTCTACCATGCAGAATAAAGCAAATCATAAGGATGTGGCAGAGAGTAGGACAAGCAATGTA
TGCCCCTCCTATCAAAGGAATTATAAAGTGTAAATCAAACATTACAGGACTACTACTAACAAGAGATGGTGGA
GGTGGAAATAGCAGCAATGAGACCAGTGAAACCTTCAGGCCTATAGGAGGAGATATGAGGGACAATTGGAGAA
GTGAACTATACAAGTATAAAGTAGTACAAATTGAACCAATAGGAGTAGCACCTACCAGGGCAAAGAGAAGAGT
GGTGGAGAGAGAAAAAAGAGCAATTGGACTGGGAGCTGCCTTCCTTGGGTTCTTAGGAGCAGCAGGAAGCACT
ATGGGCGCGGCGTCAATAACGCTGACGGTACAGGCCAGACAATTATTGTCTGGCATAGTGCAACAGCAAAGCA
ATTTGCTGAGAGCTATAGAGGCTCAGCAGCATCTGCTGAAACTCACGGTCTGGGGCATT
>GU481434..2
TTCTATTGCTATACAACAAACCTGTTCAATAGCACTTGGGGCGAAAATAGCACTGATATAAATAGCACACAGG
CCAATGAAAATATAACTCTACCATGCAGAATAAAGCAAATTATAAATATGTGGCAGAGAGTAGGACAAGCAAT
GTATGCCCCTCCTATCAAAGGAAGTATAATGTGTACATCAAACATTACAGGGCTATTACTAACAAGAGATGGT
GGAGGTGGAGCTAATAGCAGCAATGAGACCTTCAGACCTATAGGAGGAAATATGAGGGACAATTGGAGAAGTG
AACTATATAAGTATAAAGTAGTAAAAATTGAACCAATAGGAGTAGCACCTACCACGGCAAGGAGAAGAGTGGT
GGAGAGAGAAAAAAGGGCAATTGGACTGGGAGCTGCCTTCCTTGGGTTCTTGGGAGCAGCAGGAAGCACTATG
GGCGCGGCGTCACTAACGCTGACGGTACAGGCCAGACAATTATTGTCTGGCATAGTGCAGCAGCAAAGCAATT
TGCTGAAGGCTATAGAGGCTCAGCAGCATCTGCTGAAACTCACGGTCTGGGGCATT

>GU481486..1
TTCTATTGCAATACAACAGACCTGTTCAATAGCACTTGGAACGAAACTGACATTGATGCACAGGCCAATGAAG
TCAATGAAACTATAACTCTACCATGCAGAATAAAGCAAATTATAAATATGTGGCAGAGAGTAGGGCAAGCAAT
GTATGCCCCTCCTATCAAAGGAAGTATAAGGTGTGAATCAAACATTACAGGACTACTACTAACAAGAGATGGT
GGAAGACTTAATGGCAGCAATGAGACCTTTAGACCTATAGGAGGAGATATGAGGGACAATTGGAGAAGTGAAC
TATATAAGTATAAAGTAGTACAAATTGAACCAATAGGAGTAGCACCTACCAAGGCAAAGAGAAGAGTGGTGGA
GAGAGAAAAAAGAGCAATTGGACTGGGAGCTGCCTTCCTTGGGTTCTTGGGAGCAGCAGGAAGTACTATGGGC
GCGGCGTCAATAACGCTGACGGTACAGGCCAGACAATTATTGTCTGGCATAGTGCAACAGCAAAGCAATTTGC
TGAGGGCTATAGAGGCTCAGCAGCATCTGCTGAAACTCACGGTCTGGGGCATT
>GU481644..1
TTCTATTGCAATACAACAAACCTGTTCAATAGCACTTGGAATGAAACAGACATTGTTACAAATATCACACAGG
GCAATGGAACTATAACTCTACCATGCAGAATAAAGCAAATTATAAATATGTGGCAGAGAGTAGGACAAGCAAT
GTATGCCCCTCCTATCAAAGGAAATATAACGTGTACATCAAACATTACAGGACTACTACTAACAAGAGATGGT
GGAACTAATAGCAGCAATGAGACCTTCAGACCTATAGGAGGAGATATGAGGGACAATTGGAGAAGTGAACTAT
ATAAATATAAAGTAGTACAAATTGAACCAATAGGAGTAGCACCTACCAGGGCAAGGAGAAGAGTGGTGGAGAG
AGAAAAAAGAGCAATTGGACTGGGAGCTGCCTTCCTTGGGTTCTTGGGAGCAGCAGGAAGCACTATGGGCGCG
GCGTCACTAACGCTGACGGTACAGGCCAGACAATTACTGTCTGGCATAGTGCAACAGCAAAGCAATTTGCTGA
GGGCTATAGAGGCTCAGCAGCATCTGCTGAAACTCACGGTCTGGGGCATT
>JQ292891..1
TTCTATTGCAATACAACAGACCTGTTCAATAGCACTTGGGACAATAGCACTGTCACAAATAGCACAGGGACCA
ATGAAACTATAACTCTGCCATGTAGAATAAAGCAAATTATAAATATGCGGCAGAGAGTAGGACAAGCAATGTA
TGCCCCTCCTATTAGAGGAAGTATAAGGTGTGAATCAAACATTACAGGACTACTACTAATAAGAGATGGTGGA
GCTAATAGCAGCAATGAGACCTTCAGACCTATAGGAGGAAATATGAGGGACAATTGGAGAAGTGAACTATATA
AGTATAAAGTAGTAAAAATTGAACCAATAGGAGTAGCACCTACCAAGGCAAGGAGAAGAGTGGTGGAAAGAGA
AAAAAGAGCAATTGGACTGGGAGCCGCCTTCCTTGGGTTCTTAGGAGCAGCAGGAAGCACTATGGGCGCGGCG
TCAATGACGCTGACGGTACAGGCCAGACAATTATTGTCTGGCATAGTGCAACAGCAAAGCAATTTGCTGAGGG
CTATAGAGGCTCAGCAGCATCTGCTGAAACTCACGGTCTGGGGCATT
>HQ385820..1
TTCTATTGCAATACAACAAACCTGTTCAATAGCACTTGGGACACAAATATCACAGAGGCCAATATCACAGAGG
CCAATGAAACTATAATTCTACCATGCAAAATAAAGCAAATTATAAATATGTGGCAGAGAGTAGGAAAAGCAAT
GTATGCCCCTCCTATCAAAGGAAGTATAAGGTGTGAATCAAACATTACAGGACTACTACTAACAAGAGATGGT
GGAACTAATAGCAGCAATAGCAATGAGACCTTCAGACCTATAGGAGGAAATATGAGGGACAATTGGAGAAGTG
AACTATATAAATATAAAGTAGTAAAAATTGAACCAATAGGAGTAGCACCTACCAAGGCAAAGAGAAGAGTGGT
GGAGAGAGAAAAAAGAGCAATTGGGTTGGGAGCTGCCTTCCTTGGGTTCTTAGGAGCAGCAGGAAGCACTATG
GCCGGCGGGTCAATAACGCTGACGGTACAGGCCAGACAATTATTGTCTGGCATAGTGCAACAGCAAAGCAATT
TGCTGAGGGCCATAGAGGCTCAGCAACATATGCTGAAACTCACGGTCTGGGGCATT
>JF952018..1
TTTTATTGCAATACAACAGCCCTGTTCAATAGCACTTGGGACAATAGTAGCATTGCCGAAAATAGCACAGAGG
ACAATGCAACTATAACTCTACCATGCAGAATAAAGCAAATTATAAATATGTGGCAGAGAGTAGGGCAAGCAAT
GTATGCCCCTCCTATCAAAGGAAGTATAAGGTGTGAATCAAATATTACAGGACTATTACTAACAAGAGATGGT
GGAGATGCCGATGGAAATGGAACTAATAGCAACAATGAAACCTTCAGACCTACAGGAGGAGATATGAGGAACA
ATTGGAGAAGTGAACTATATAAATATAAAGTAGTACAAATTGAACCAATAGGGGTAGCACCTACCACGGCAAA
GAGAAGAGTGGTGGAGAGAGAAAAAAGAGCAATTGGACTGGGAGCTGCCTTCCTTGGGTTCTTAGGAGCAGCA
GGAAGCACTATGGGCGCGGCGTCAATAACGCTGACGGTACAGGCCAGACAATTATTGTCTGGCATAGTGCAAC
AGCAAAGCAATTTGCTGAGGGCTATAGAGGCTCAGCAGCATCTGCTGAAACTCACGGTCTGGGGCATT
>HQ338112..1
TTCTATTGCAATACAACAGAACTGTTCAATAGCACTTGGGACAATAGCACTTGGGACAAAAATGACACTAGCA
CAAATATCACACAGGTCATAACTCTGCCATGCAGAATAAAACAAATTATAAATATGTGGCAGAGAGTAGGACA
AGCAATGTATGCCCCTCCTATTAAAGGAGTTATGAGGTGTGAATCAAACATTACAGGACTACTACTAACAAGA
GATGGTGGAGCTAATAGCAGCAATGAGACCTTCAGACCTATAGGAGGAGATATGAGGGACAATTGGAGAAGTG
AACTATATAAGTATAAAGTAGTAGAAATTGAACCAATAGGAGTAGCACCTACCAGGGCAAAGAGAAGAGTGGT
GGAGAGAGAAAAAAGAGCAATTACACTGGGAGCTGCCTTCCTTGGGTTCTTAGGAGCAGCAGGAAGCACTATG
GGCGCGGCGTCATTGACGCTGACGGTACAGGCCAGACAATTATTGTCTGGCATAGTGCAACAGCAAAGCAATT
TGCTGAGGGCTATAGAGGCTCAGCAGCATCTGCTGAAACTCACGGTCTGGGGCATT
>GU481277..1
TTCTATTGCAATACAGCAGACCTGTTCAATAGCACTTGGGACACAAATAGCACACAGACCAATAGAAATATAA
CTGCCAGTGAAAATATAACTCTACCATGCAGAATAAAGCAAATTATAAATATGTGGCAGAGAGTAGGACAAGC
AATGTATGCCCCTCCTATCAAAGGAATTATAATGTGTACATCAAACATTACAGGACTACTCCTAACAAGAGAT
GGTGGAGGTGAAACTAATAGCAGCAATGAGACCTTCAGACCTATAGGAGGAGACATGAGGGACAATTGGAGAA
GTGAACTATATAAATATAAAGTAGTAAAAATTGAACCAATAGGGGTAGCACCTACCAGGGCAAAGAGAAGAGT

GGTGGAGAGAGAAAAAAGAGCAATTGGACTGGGAGCTGCCTTCCTTGGGTTCTTGGGAGCGGCAGGAAGTACT
ATGGGCGCGGCGTCAATAACGCTGACGGTACAGGCCAGACAATTATTGTCTGGCATAGTACAACAGCAAAGCA
ATTTGCTGAGGGCTATAGAGGCTCAGCAGCATCTGTTGAAGCTCACGGTCTGGGGCATT
>GU481544..1
TTCTATTGCAATACAACAAAACTGTTCAATAGCTCTTGGGGCGAAAATGGCACAAATAGCATCGAGACCAATG
AAACTATAATTCTACCATGCAGAATAAAGCAAATTATAAATATGTGGCAGAGAGTAGGACAAGCAATGTATGC
CCCTCCTATCAAAGGAATTATAAAGTGTGAATCAAACATTACAGGACTACTACTAATCAGAGATGGTGGAACT
AACAGCAGTAATGAGACCTTCAGACCTATAGGAGGAGATATGAGGGACAATTGGAGAAGTGAACTATATAAGT
ATAAAGTAGTAAAAATTGAACCAATAGGAGTAGCACCTACCAAGGCAAGGAGAAGAGTGGTGGAGAGAGAAAA
AAGAGCAATTGGACTGGGAGCTGCCTTCCTTGGGTTCTTAGGAGCAGCAGGAAGCACTATGGGCGCGGCATCA
ATAACGCTGACGGTACAGGCCAGACAATTATTGTCTGGCATAGTGCAACAGCAAAGCAATTTGCTGAGGGCTA
TAGAGGCTCAGCAACATCTGCTGAAACTCACGGTCTGGGGCATT
>GU481661..1
TTCTATTGCGATACAACAAACCTGTTCAATAGCACTTGGGAAGAAAAAAGCACTGTTACAAATAGCACCAATG
ACACTATAACTCTACCATGCAGAATAAAGCAAATTATAAATATGTGGCAGAGAGTAGGACAAGCAATGTATGC
CCCTCCTATCCAAGGAAATATAAGTTGTGAATCAAACATTACAGGACTACTACTAACAAGAGATGGTGGAAGT
GAAAATAATGAGACCTTCAGACCTATAGGAGGAGATATGAGGGACAATTGGAGAAGTGAATTATACAAGTATA
AAGTAGTAAAAATTGAACCAATAGGAGTAGCACCTACCAGGGCAAGGAGAATAGTGGTGAAGAGAGAAAAAAG
AGCAATTGGACTGGGAGTTGCCTTCCTTGGGTTCTTAGGAGCAGCAGGAAGCACTATGGGCGCAGCGTCACTA
ACGCTGACGGTACAGGCCAGACAATTATTGTCTGGCATAGTGCAACAGCAAAGCAATTTGCTGAGGGCTATAG
AGGCTCAGCAACATCTGCTGAAACTCACGGTCTGGGGCATT
>KF716491..1
TTCTATTGCAATACAACAAACCTGTTCGATAGCACTTGGAATGACACTGACACAAATAGCACTGACACAAATA
GCACAGGGGCCAATGAAATTATAACTCTACCATGCAGAATAAGGCAAATTATAAATATGTGGCAGAGAGTAGG
ACAAGCAATGTATGCCCCTCCTATCAAAGGAGTTATAAGGTGTGAATCAAACATTACAGGACTACTACTAACA
AGAGATGGTGGAACTAATAGCAGCAATGAGAATGAGATCTTCAGACCTATAGGAGGAGATATGAGGGACAATT
GGAGAAGTGAACTATATAAGTATAAAGTAGTAGAAGTTGAACCAATAGGAGTAGCGCCTACCAAGGCAAGGAG
AAGAGTGGTGGAGAGAGAAAAAAGAGCAATTGGACTGGGAGCTGCCTTCCTTGGGTTCTTAGGAGCAGCAGGA
AGTACTATGGGCGCGGCGTCAATAACGCTGACGGTACAGGCCAGACAATTATTGTCTGGCATAGTGCAACAGC
AAAGCAATTTGCTGAAAGCTATAGAGGCTCAGCAGCATCTGCTGAAACTCACGGTCTGGGGCATT
>KF716492..1
TTCTATTGCAATACAACAGAACTGTTCAATAGCACTTGGAAAGAAAATAGCACTATCACAAATAACACAACAG
CCAATGACACTATAACTCTACCATGCAGAATAAAGCAAATTATAAATATGTGGCAGAGAGTAGGACAAGCAAT
GTATGCCCCTCCTATCAAAGGAAGTATAAGGTGTAAATCAAACATTACAGGACTACTACTAACAAGAGATGGT
GGAAGTGAACCTAATAGCAGTGAGGAAATCTTCAGACCTATAGGAGGAAATATGAGGGACAATTGGAGAAGTG
AACTATATAAGTATAAAGTAGTAAAAATTGAACCAATAGGAGTAGCACCTACCAAGGCAAAGAGAAGAGTGGT
GGAGAGAGAAAAAAGAGCAATAATTGGACTGGGAGCTGCCTTCCTTGGGTTCTTAGGAGCAGCAGGAAGCACT
ATGGGCGCGGCGTCACTGACGCTGACGGTACAGGCCAGACAATTATTGTCTGGCATAGTGCAACAGCAAAGCA
ATTTGCTGAGGGCTATAGAGGCTCAGCAGCATCTGCTGAAACTCACGGTCTGGGGCATT
>FJ864679..1
TTCTATTGTAATACAACAGAACTGTTCAATAGCACTTGGTACAAAAATGGCACTAACACAACTAGCACACAGA
CCAGTGAAAACATAACTCTGCCATGCAGAATAAAGCAAATTGTAAGGATGTGGCAGAGAGTAGGACAAGCAAT
GTATGCCCCTCCTATCAGTGGAATTATAAAGTGTGAATCAAACATTACAGGATTAATACTAACAAGAGATGGT
GGAGATAATAGTAGCAGCAATGAGACCTTCAGACCTGAAGGAGGAGACATAAGGGACAATTGGAGAAATGAAC
TATATAAGTATAAAGTAGTAGAAATTGAACCAATAGGAGTAGCACCTACTGAGGCAAGGAGAAGAGTGGTGCA
GAGAGAAAAAAGAGCAATTGGACTGGGAGCTGCCTTCATTGGGTTCTTAGGAGCAGCAGGAAGCACTATGGGC
GCAGCGTCACTGACGCTGACGGTACAGGCCAGAAAATTATTGTCTGGCATAGTGCAACAGCAAAGCAATTTGC
TGAGGGCTATAGAGGCTCAGCAGCATCTGCTGAAACTCACGGTCTGGGGCATT
>JQ292893..1
TTCTATTGCAATACCACAGACCTGTTCAATAGCACTTGGGACAAAAATGACACTATCACAAATACCCCAGAGG
TCAATGGAACTACTATAACTCTGCCATGCAGAATAAAGCAAATTGTAAATATGTGGCAGAGAGTAGGACAAAC
AATGTATGCCCCTCCTATCAAAGGAAGTATAAGGTGTGAATCAAACATTACAGGACTACTGCTAATAAGAGAT
GGTGGAGAAACTAATAGCAGCAGTGAGACCTTCAGACCTGTAGGAGGAGATATGAGGGATAATTGGAGAAGTG
AACTATATAAGTATAAAGTAGTAAAAATTGAACCAATAGGAGTAGCACCTACCAGGGCAAAGAGAAGAGTGGT
GGAGAGAGAAAAAAGAGCAATTGGACTGGGAGCCGCCTTCCTTGGGTTCTTAGGAGCAGCAGGAAGTACTATG
GGCGCGGCGTCACTGACGCTGACGGTACAGGCCAGACAATTATTGTCTGGCATAGTGCAACAGCAAAGCAATT
TGCTGAGGGCTATAGAGGCTCAGCAGCATCTGCTGAAACTCACGGTCTGGGGCATT
>JF430898..1
TTCTATTGCAATACAACAGACCTGTTCAATAGCACTTGGAGGAATGACACTGTCGCAAATATCACGGCCAATG
GAACTATAACTCTGCCATGCAGAATAAAGCAAATTATAAATATGTGGCAGAGAGTAGGACAAGCAATGTATGC

CCCTCCTATCGAAGGAAATATAAGTTGTGAATCAAACATTACAGGGCTACTACTAATAAGAGATGGTGGAACT
GATGACAGCAATGAGACTGAGTCCTTCAGACCCATAGGAGGAGATATGAGGGACAATTGGAGAAGTGAACTAT
ATAAATATAAAGTGGTACAAATTGAACCAATAGGAGTAGCACCTACCAAGGCAAGGAGAAGAGTGGTGGAGAG
AGAAAAAAGAGCAATTGGACTGGGAGCTGCCTTCCTTGGGTTCTTAGGAGCAGCAGGAAGCACTATGGGCGCG
GCGTCATTGACGCTGACGGTACAGGCCAGACAATTATTGTCTGGCATAGTGCAACAGCAAAACAATTTGCTGA
GGGCTATAGAGGCTCAGCAGCATCTGCTGAAACTCACGGTCTGGGGCATT
>HQ834967..1
TTTTATTGCAATACAACAGGCCTGTTCAATAGCACTTGGGGCAATAGCACTGACGCAAATAGCAATGAAAATA
TAACTCTACCATGCAGAATAAAGCAAATTATAAATATGTGGCAGAGAGTAGGACAAGCAATGTATGCCCCTCC
TATCAGCGGAAATATAAGTTGTGTATCAAACGTTACAGGACTACTACTAGTAAGAGATGGTGGAGGTGGAAAT
AGTAGCAACAATGAGACCTTCAGACCTATAGGAGGAGATATGAGGGACAATTGGAAAAGTGAACTATATAAGT
ATAAAGTAGTAGAAATTGAACCAATAGGAGTAGCACCTACCAAGGCAAAGAGAAGGGTGGTGCAGAGAGAAAA
AAGAGCAATTGGAGGACTGGGAGCTGCCCTCCTTGGATTCTTAGGAGCAGCAGGAAGCACTATGGGCGCGGCG
TCACTGACGCTGACGGTACAGGCCAGACAATTATTGTCTGGCATAGTGCAACAGCAAAGCAATTTGCTGAGGG
CTATAGAGGCTCAGCAGCATCTGCTGAAACTCACCGTCTGGGGCATT
>JX500696..1
TTCTATTGTAATACAACAAAACTGTTCAATAGCACTTGGGACAAAAATAGTACTTGGAATGAAAATAGCACTG
CCAATGAAAATATAACTCTGCCATGCAGGATAAAGCAAATTATAAATATGTGGCAGAGAGTAGGACAAGCAAT
GTATGCCCCTCCTATCAAAGGAGTTATAATGTGTAAATCAAACATTACAGGTCTACTCCTAACAAGAGATGGT
GGAGGTGGAGCTAATAGCAGCAATGAGACTTTCAGACCTATAGGAGGAAATATGAGGGACAATTGGAGAAGTG
AACTATATAAGTATAAAGTAGTACAAATTGAACCAATAGGAGTAGCACCTACCAGGGCAAAGAGAAGAGTGGT
GGAGAGAGAAAAAAGAGCAATTGGACTGGGAGCCGCTTTCCTTGGGTTCTTAGGAGCAGCAGGAAGCACTATG
GGCGCGGCGTCACTGACGCTGACGGTACAGGCCAGACAATTATTGTCTGGCATAGTGCAACAGCAAAGCAATT
TGCTGAGGGCTATAGAGGCTCAGCAGCAGCTGCTGAAACTCACGGTCTGGGGCATT
>JX500695..1
TTCTATTGCAATACAACAGGCCTGTTCAATAGCACTTGGGACACAAATGGCACTGTCACAAGTAGCACAAATG
GAACAAATAAAACTATAACTCTGCCATGCAGAATAAAACAAATTATAAATATGTGGCAGAGAGTAGGACAAGC
AATGTACGCCCCTCCTATCAAAGGAAGTATAAGGTGTGAATCAAACATTACAGGTCTACTACTAATAAGAGAT
GGTGAAAGGAATAGCAGCAATGAGACCTTTAGACCTATAGGAGGAAATATGAGGGACAATTGGAGGAGTGAAC
TATATAAGTATAAAGTAGTACAAATTGAACCAATAGGACTAGCACCTACCAGGGCAAAGAGAAGAGTGGTGGA
GAGAGAAAAAAGAGCAATTGGACTGGGAGCCGCCCTCCTTGGGTTCTTAGGAGCAGCAGGAAGCACTATGGGC
GCGGCGTCAATGACGCTGACGGTACAGGCCAGACAATTATTGTCTGGCATAGTGCAACAGCAAAGCAATTTGC
TGAGGGCTATAGAGGCTCAGCAGCATCTGCTGAAACTCACGGTCTGGGGCATT
>JX500694..1
TTCTATTGCAATACAACAGAACTGTTCAATAGCACTTGGGACGGAAATGGCACTGCCACAAATAACACAGAGG
ACAATGTAACTCTGCCATGCAGAATAAAGCAAATTATAAATATGTGGCAGAGAGTAGGACAAGCAATGTATGC
CCCTCCTATCAGAGGAAGTATAAAGTGTGTATCAAACATTACAGGACTACTACTAATAAGAGATGGTGGAGGT
GGAAATAATAGCAACAATGAGACCTTCAGACCTATAGGAGGAAATATGAGGGACAATTGGAGAAGTGAACTAT
ATAAGTATAAGGTAGTACAAATTGAACCAATAGGAATAGCACCTACCACAGCAAAAAGAAGAGTGGTGGAGAG
AGAAAAAAGAGCAATTGGACTGGGAGCTGCCTTCCTTGGGTTCTTAGGAGCAGCAGGAAGTACTATGGGCGCG
GCGTCAATAACGCTGACGGTACAGGCCAGACAATTATTGTCTGGTATAGTACAACAGCAAAGCAATTTGCTGA
GGGCTATAGAGGCTCAGCAGCATCTGCTGAAACTCACGGTCTGGGGCATT
>MH603967..1
TTCTATTGCAATACAACACAACTGTTCGATAGCACGTGGGAAGAGAATGCCAATATCACAAATACAAATAATA
CACAGAATAATGAAAATATAACTCTACCATGCAGAATAAAGCAAATTATAAGGATGTGGCAGGGGGTAGGACA
AGCAATGTATGCCCCTCCTATAAGAGGAGAAATAAGGTGTGAATCAAACATTACAGGATTACTACTAACAAGA
GATGGTGGAGGTACAAATAGCAGCAACAATGAGACCTTCAGACCTATAGGAGGAGACATGAGGGACAATTGGA
GAAGTGAACTATATAAGTATAAAGTAGTACAAATTGAACCCATAGGAGTAGCACCTACCAAGGCAAAGAGAAG
AGTGGTGGAGAGAGAAAAAAGAGCAATTGGACTGGGAGCTGCCTTCCTTGGGTTCTTAGGAGCAGCAGGAAGC
ACTATGGGCGCGGCGTCAATAACGCTGACGGTACAGGCCAGACAATTATTGTCTGGCATAGTGCAACAGCAAA
GCAATTTGCTGAGGGCTATAGAGGCTCAGCAGCATCTGCTGAAACTCACGGTCTGGGGCATT
>MH604120..1
TTTTATTGTAATACAACAGACCTGTTCAATAGCGCTTGGGAAAATGGCAATGCTACAAATATCACACAGGCCA
ATGACAATGAAACTATAATTCTACCATGCAGAATAAGGCAAATTATAAATATGTGGCAAAGAGTAGGACAGGC
AATGTATGCCCCTCCTATCAGAGGAAATATAACGTGTATATCAAATATTACAGGACTACTACTAACAAGAGAT
GGTGGAGGTGGAAATAATAGCAACAATGAGACCTTCAGACCTATAGGAGGAGATATGAGGGACAATTGGAGAA
GTGAACTATATAAATATAAAGTAGTACAAATTGAACCAATAGGAATAGCACCTAACAGGGCAAAGAGAAGAGT
GGTGGAGAGAGAAAAAAGAGCAATTGGACTGGGAGCTGCCTTCCTTGGGTTCTTGGGAGCAGCAGGAAGTACT
ATGGGCGCGGCGTCGCTAACGCTGACGGTACAGGCCAGAAAATTATTGTCTGGCATAGTGCAACAGCAAAACA
ATTTGCTGAAGGCTATAGAGGCTCAGCAGCATCTGCTGAGACTCACGGTCTGGGGCATT

>MH604308..1
TTCTATTGCAATACAACAGACCTGTTCAATAGCACTTGGGACGGAAATAGCACTAGAGATGAAAATGGCAATG
ATACAGATAGCACACAGAACAATAGACCTATAACTCTGCCATGCAGAATAAAGCAAATTATAAATATGTGGCA
GAGAGTAGGACAAGCAATGTATGCCCCTCCTATAAAAGGAATTATAAGGTGTACATCAAACATTACGGGACTA
CTACTAATAAGAGATGGTGGAGGCGGAGCTAATAGCAGCAATGAGACCTTCAGACCTATAGGAGGAGATATAA
GGGACAATTGGAGAAGTGAACTATTTAAGTATAAAGTAGTACAAATTGAACCAATAGGAGTAGCACCTACCAG
GGCAAAGAGAAGAGTGGTGGAGAGAGAAAAAAGAGCAATTGGACTGGGAGCTGCCTTCCTTGGGTTCTTGGGA
GCAGCAGGAAGTACTATGGGCGCGGCGTCAATAACGCTGACGGTACAGGCCAGACAATTATTGTCTGGCATAG
TACAACAGCAAAGCAATTTGCTGAGGGCTATAGAGGCTCAGCAGCATCTGCTGAAACTCACGGTCTGGGGCAT
T
>MH604056..1
TTCTATTGCAATACAACAGCCCTGTTCAATTGGAACGGCAATGAAACTGTTACAAATAGCACACAGGCCAATG
AAACTATAACTTTACCATGCAGAATAAAGCAAATTATAAATATGTGGCAGAGAGTAGGACAAGCAATGTATGC
CCCTCCTATCAAAGGAAGTATAAGGTGTACATCAAACATTACAGGACTGCTGCTAACAAGAGATGGTGGAACT
AACAGCAGCAACAGCACTGAGATCTTCAGACCTATAGGAGGAAATATGAAGGACAATTGGAGAAGTGAACTAT
ATAAATATAAAGTAGTAAAAATTGAACCAATAGGAGTAGCACCTACCAAGGCAAGGAGAAGAGTGGTGGAGAG
AGAAAAAAGAGCAATTGGACTGGGAGCTGCCTTCCTTGGGTTCTTGGGAGCAGCAGGAAGCACTATGGGCGCG
GCGTCAATAACGCTGACGGTACAGGCCAGACAATTATTGTCCGGCATAGTGCAACAGCAAAGCAATTTGCTGA
GGGCTATAGAGGCTCAGCAGCATCTGCTGAAACTCACGGTCTGGGGCATT
>MH604408..1
TTCTATTGCAATACAACAAACCTGTTCAATAGCACGTGGAACGAAACTGACAGTGTTACAAATAGCACACAGG
CCAATAATGAAACTATAATTTTACCATGCAGGATAAAGCAAATTATAAGGACGTGGCAGAGAGTAGGACAAGC
AATGTATGCCCCTCCTATCAAAGGAAGGATAAGGTGTGAATCAAACATTACAGGACTACTACTAACAAGAGAT
GGTGGAGGTAAAAGTAATAATGAGACAAGTAATAATGAGACCTTCAGACCTATAGGAGGAGATATGAGGGACA
ATTGGAGAAGTGAACTATATAAATATAAAGTAGTAGAAATTGAACCAATAGGAGTAGCACCTACCAAGGCAAA
GAGAAGAGTGGTGGAGAGACAAAAAAGAGCAATTGGACTGGGAGCTGCCTTCCTTGGGTTCTTAGGAGCAGCA
GGAAGCACTATGGGCGCGGCGTCACTAACGCTGACGGTACAGGCCAGACAATTATTGTCTGGCATAGTGCAAC
AGCAAAGCAATTTGCTGAGGGCTATAGAGGCTCAGCAGCATCTGCTGAAACTCACGGTCTGGGGCATT
>MH604109..1
TTCTACTGCAGTACAGCAGCCCTGTTCAATAGCACTTGGGACGGAAATACCACTATTGCAAATAACACACAGA
ACAATGAAAATATAACTATACCATGCAGAATAAAGCAAATTATCAATATGTGGCAGAGAGTAGGACAAGCAAT
GTATGCCCCTCCTATACAAGGAGTTATAACTTGTGAATCAAACATTACAGGACTACTACTAACAAGAGATGGT
GGAGTTAATAGCAGCAATGAAACCTTCAGGCCTATAGGAGGAGATATGAGGGACAATTGGAGAAGTGAATTAT
ATAAGTATAAAGTAGTAAAAGTTGAACCAATAGGAGTAGCACCTACCACGGCAAGGAGAAGAGTGGTGGGGAG
AGAAAAAAGAGCAATTGGACTGGGAGCTGCCCTCCTTGGGTTCTTGGGAGCAGCAGGAAGCACTATGGGCGCG
GCGTCAATAACGCTGACGGTACAGGCCAGACAATTATTGTCTGGCATAGTGCAACAGCAAAGCAATTTGCTAA
GGGCCATAGAGGCTCAGCAGCATCTGCTGAAACTCACGGTCTGGGGCATT
>MH604508..2
TTCTATTGCTATACAACAGGGCTGTTCAATAGCACTTGGGACGAAAATAGCACTAGCACAAATATCACACAGA
AAAATGAAAATATAACTATACCATGCAGAATAAAGCAAATTATAAATATGTGGCAGAGAGTAGGACAAGCAAT
GTATGCCCCTCCTATCAGAGGAAATATAAGATGTGTATCAAACATTACAGGACTACTACTAACAAGAGATGGT
GGAGATGGAGATAATAGCAGTGAGACCTTCAGACCTATAGGAGGAGATATGAGGGACAATTGGAGAAGTGAAC
TATATAAATATAAAGTAGTACAAATTGAACCCATAGGAGTAGCACCTACCAAGGCAAGGAGAAGAGTGGTGGA
GAGAGAAAAAAGAGCAATTGGACTGGGAGCTGCCTTCCTTGGGTTCTTAGGAGCGGCAGGAAGCACTATGGGC
GCGGCGTCAATAACGCTGACGGTACAGGCCAGACAATTATTGTCTGGCATAGTGCAACAGCAAAGCAATTTGC
TGAGGGCTATAGAGGCTCAGCAGCATCTGCTGAAACTCACGGTCTGGGGCATT
>MH604524..1
TTCTATTGCAATACAACAAACCTGTTCAATAGTGCTTGGAAAAATGGCAGTGCTACAAATAGCACACAAGCCA
ATGGAACAACTATAACTCTACCATGCAGAATAAAGCAAATTATAAATATGTGGCAGAGAGTAGGACAAGCAAT
GTATGCCCCTCCTATCAAAGGAAGTATAAGGTGTGAATCAAACATTACAGGACTACTACTAACAAGAGATGGT
GGAGGTGAAACTAATAGCAGCGATGAAACCGAAACCTTCAGACCTATAGGAGGAGATATGAGGAATAATTGGA
GAAGTGAATTATATAAGTATAAAGTAGTAAAAATTGAACCAATAGGAGTAGCACCTACCAGGGCAAGGAGAAG
AGTGGTGGAGAGAGAAAAAAGAGCAGTTGGACTGGGAGCTGCCTTCCTTGGGTTCTTGGGAGCAGCAGGAAGC
ACTATGGGCGCGGCGTCAATGACGCTGACGGTACAGGCCAGAAAATTATTGTCTGGCATAGTGCAACAGCAAA
GCAATTTGCTGAGGGCTATAGAGGCTCAGCAGCATCTGCTGAAACTCACGGTCTGGGGCATT
>MH604541..1
TTCTATTGCAATACAACAAAGCTGTTCAATAGCACTTGGGAAAATAGCACTTGGGAAAATGGCACTGTTGCAA
ATAGCACACAGGGGACAATGACACTATAACTATACCATGCAGAATAAAGCAAATTATAAATATGTGGCAAAGAGT
AGGACAAGCAATGTATGCCCCTCCTATCAAAGGAATTATAAGGTGTGAATCAAACATTACAGGACTACTACTA
ACAAGAGATGGTGGAGGTGGAACTAATAGCAGCAATGAGACCTTCAGACCTATAGGAGGAGACATGAGGGACA

ATTGGAGAAGTGAACTATATAAGTATAAAGTAGTAAAAATTGAACCAATAGGAGTAGCACCTACCAGGGCAAA
GAGAAGAGTGGTGGAGAGAGAAAAAAGGGCATTTGGACTGGGAGCTGCCTTCCTTACGTTCTTGGGAGCAGCA
GGAAGCACTATGGGCGCGGCGTCAATAACGCTGACGGTACAGGCCAGACAATTATTGTCTGGCATAGTGCAAC
AGCAAAGCAATTTGCTGAGGGCTATAGAGGCTCAGCAGCATCTGCTGAAACTCACGGTCTGGGGCATT
>MH330347..1
TTCTATTGCAATACCACATACCTGTTCAATAGCACTTGGGATGGAAATAGCACTGTAAATAGCACAGGGGGCG
ATAGAAATATAACTTTACCATGCAGAATAAAGCAAATTATAAATATGTGGCAGAGAGTAGGACAAGCAATGTA
TGCCCCTCCTATCCGAGGAAACATAAGTTGTGAATCAAACATCACAGGACTACTCCTAATAAGAGATGGTGGA
GGTGGAAATAATAAGACTAATGAGACCTTCAGACCTATAGGAGGAGATATGAGGGACAATTGGAGAAGTGAAC
TATATAAGTATAAAGTAGTAAAAATTGAACCAATAGGGGTAGCACCTACCCTGGCCAGGAGAAGAGTGGTGGA
GAGAGAAAAAAGAGCAATAATTGGACTGGGAGCTGCCTTCATTGGGTTCTTAGGAGCAGCAGGAAGCACTATG
GGCGCGGCGTCACTGACGCTGACGGTACAGGCCAGACAATTATTGTCTGGCATAGTGCAACAGCAAAGCAATT
TGCTGAGGGCTATAGAGGCTCAGCAGCATCTGCTGAAACTCACGGTCTGGGGCATT
>MH330348..1
TTCTATTGCAATACAACAAACCTGTTCAACATAAATAGCACTGACACAAATATCACACAGGACAATGAAACTA
TAACTCTACCATGCAGAATAAAGCAAATTATAAATATGTGGCAGAGAGTAGGACAAGCAATGTATGCCCCTCC
TATCAAAGGAAGTATAATGTGTGAATCAAACATTACAGGACTACTACTAACAAGAGATGGTGGAGGTGACAGA
AATGATACTAATAGTACTGAGACCTTCAGACCTATAGGAGGAGATATGAGGGACAATTGGAGAAGTGAACTAT
ATAAGTATAAAGTAGTAAAAATTGAACCAATAGGAGTAGCACCTACCAGGGCAAAGAGAAGAGTGGTGGGGAG
AGAAAAAAGAGCAATTGGACTGGGAGCCGCCTTCCTTGGGTTCTTAGGAGCAGCAGGAAGCACTATGGGCGCG
GCGTCACTGACGCTGACGGTACAGGCCAGACAATTATTGTCTGGCATAGTGCAACAGCAAAGCAATTTGCTGA
GGGCTATAGAGGCTCAGCAGTATATGCTGAAACTCACGGTCTGGGGCATT
>MH330349..1
TTCTATTGCAATACAACAGCCCTGTTCAATAGCACTTGGAGTGAAAATGGCACTGCCATAAATAGCACAGAGG
CCAATGGAACTATAACTCTGCCATGCAGAATAAGGCAAATTATAAATATGTGGCAGAGAGTAGGACAAGCAAT
GTATGCCCCTCCTATCAGAGGAATTATAAGGTGTGAATCAAACATTACAGGACTACTGCTAACACGAGATGGT
GTAGCTAATAGCAGCAATGAGACCTTCAGACCTATAGGAGGAGATATGAGGAACAATTGGAGAAGTGAACTAT
ATAAGTATAAAGTAGTAAAAATTGAACCAATAGGAGTAGCACCTACCAGGGCAAAGAGAAGAGTGGTGGAGAG
AGAAAAAAGAGCAATTGGACTGGGAGCCGCCTTCCTTGGGTTCTTAGGAGCAGCAGGAAGCACTATGGGCGCG
GCGTCACTGACGCTGACGGTACAGGCCAGACAACTCTTGTCTGGCATAGTGCAACAGCAAAGCAATTTGCTGA
GGGCTATAGAGGCTCAGCAGCATCTGCTGAAACTCACGGTCTGGGGCATT
>MH330350..1
TTTTATTGTAATACAACAGAACTGTTCAATAGTACTTGGGACGGAAATAACACTGTTACAAATAGCACACAGG
ACAATGGCACTATAACTCTACCATGCAGAATAAGGCAAATTATAAATATGTGGCAGAGAGTAGGACAAGCAAT
GTATGCCCCTCCTATCAGAGGAAATATAAGTTGTGAATCAAACATTACAGGACTACTACTAACAAGAGATGGT
GGAGTTAATACCAGCAATGAGACCTTCAGACCTACAGGAGGAGATATGAGGAACAATTGGAGAAGTGAACTAT
ATAAGTATAAAGTAGTACAAATTGAACCAATAGGAGTAGCACCTACCAAGGCAAAGAGAAGAGTGGTGGAGAG
AGGAAAAAGGGCAATTGGACTGGGAGCTGCCTTCCTTGGGTTCTTGGGAGCAGCAGGAAGTACTATGGGCGCG
GCGTCAATAACGCTGACGGTACAGGCCAGACAATTATTGTCTGGCATAGTACAACAGCAAAGCAATTTGCTGA
TGGCTATAGAGGCTCAGCAGCATCTGCTGAAACTCACGGTCTGGGGCATT
>MH330351..1
TTCTATTGCAATACAACAAACCTGTTCAATAGCACTTGGGACAAAAATAGTACTGTCACAAATAGCACAAACA
GCACAGAAACTATAACTCTACCATGCAGAATAAAGCAAATTATAAATATGTGGCAGAGAGTAGGACAAGCAAT
GTATGCCCCTCCTATCAAAGGAAGTATAAGGTGTGAATCAAACATTACAGGACTAATATTAATAAGAGATGGT
GGAAATAATAGCAGCAATGAGACCTTCAGACCTATAGGAGGAAATATGAGGGACAATTGGAGAAGTGAACTAT
ATAAATATAAAGTAGTAGAGATTGAACCAATAGGAATAGCACCTACCAAGGCAAGAAGGAGAGTGGTGGAGAG
AGAAAAAAGAGCAATTGGACTGGGAGCTGCCTTCCTTGGGTTCTTAGGAGCAGCAGGAAGCACTATGGGCGCG
GCGTCAATAACGCTGACGGTACAGGCCAGACAATTATTGTCTGGCATAGTGCAACAGCAAAGCAATTTGCTGA
GGGCTATAGAAGCTCAGCAGCATCTGCTGAAGCTCACGGTCTGGGGCATT
>MH330352..1
TTCTATTGCAATACAACACAACTGTTCAATAGCACTTGGCCTTGGAATGACACTGTCACAAATAGCACAGAGA
CCAGTGAAAATATAACTCTACCATGCAGAATAAAGCAAATTATAAATATGTGGCAGAGAGTAGGACAAGCAAT
GTATGCCCCTCCTATCAGAGGAAGTATAAGGTGTGAATCAAACATTACAGGACTACTACTAATAAGAGATGGT
GGAACTAATAGCACCAATGAGACCTTCAGACCTATAGGAGGAGATATGAGGGACAATTGGAGAAGTGAACTAT
ATAAGTATAAAGTAGTACAAATTGAACCAATAGGAGTAGCACCTACCAAGGCAAAGAGAAGAGTGGTGGAGAG
AGAAAAAAGAGCAATTGGACTGGGAGCTGCCTTCCTTGGGTTCTTAGGAGCAGCAGGAAGCACTATGGGCGCG
GCGTCAATAACGCTGACGGTACAGGCCAGACAATTATTGTCTGGCATAGTGCAACAGCAAAGCAATTTGCTGA
GGGCTATAGAGGCTCAGCAGCATCTGCTGAAACTCACGGTCTGGGGCATT
>MH330353..1

TTCTATTGCAATACAACAGACCTGTTCGATAGCACTTGGGATGGAAATAACACAGATAACACTGTCACAAATA
TCACGGGCAATGGAACTATGACTCTGCCATGCAGAATAAGGCAAATTGTAAGGATGTGGCAGAGAGTAGGACA
AGCAATGTATGCCCCTCCTATTGAAGGGAAAATAAGGTGTGAATCAAACATTACAGGACTACTACTACTAAGA
GATGGTGGAATTAATAGCAGCAATGAGACCTTCAGACCTATAGGAGGAGATATGAGGGACAATTGGAGAAGTG
AGCTATATAAGTATAAAGTAGTAAAAATTGAACCAATAGGAGTAGCACCTACCCAGGCAAGGAGAAGAGTGGT
GGCGAGAGAAAAAAGAGCAATTGGATTAGGAGCTGCCTTCCTTGGGTTCTTAGGAGCAGCAGGAAGCACTATG
GGCGCGGCGTCAATAACGCTGACGGTACAGGCCAGACAATTATTGTCTGGCATAGTGCAACAGCAAAGCAATT
TGCTGAAGGCTATAGAGGCTCAGCAGCATCTGCTGAAACTCACAGTCTGGGGCATT
>MH330354..1
TTCTATTGCAATACAACAAGCCTGTTCAATAGCACTTGGGATGAAAATAGCACTGTCACAAACACAGAGGCCA
ATGGAACTATAATTCTGCCATGCAGAATAAAGCAAATTATAAATATGTGGCAAAGAGTAGGACAAGCAATGTA
TGCCCCTCCTATCAGAGGAAGGATAAGGTGTGAATCAAACATTACAGGACTACTAATAACAAGAGATGGTGGA
GGTGGAAATAATAGCACCAATGAAACCTTCAGACCTATAGGAGGAGATATGAGGGACAATTGGAGAAGTGAAC
TATATAAGTATAAAGTAGTAAAAATTGAACCAATAGGAGTAGCACCTACCAGGGCAAAGAGAAGAGTGGTGGA
GAGAGAAAAAAGAGCAATTGGACTGGGAGCCGCCTTCCTTGGGTTCTTAGGAGCAGCAGGAAGCACTATGGGC
GCAGCGTCAATGACGCTGACGGTACAGGCCAGACAATTATTGTCTGGCATAGTGCAACAGCAAAGCAATTTGC
TGAGGGCTATAGAGGCTCAGCAGCATCTGCTGAAACTCACGGTCTGGGGCATT
>MH330355..1
TTCTATTGCAATACAACAAGGCTGTTCAATAGCTCTTGGGGCGAAAATAGCACTGTCACAAATAGCACAGATG
TCAATGGAACTATAATTCTGCCATGCAGAATAAAACAAATTATAAATATGTGGCAGAGAGTAGGACAAGCAAT
GTATGCCCCTCCTATCCCAGGAAATATAACATGTATATCAAACATTACAGGACTACTACTAGTAAGAGATGGT
GGACAGAACAGTAGTAACACCAGTGAAACCTTCAGACCCATAGGAGGAGATATGAAGCACAATTGGAGAAGTG
AACTATATAAGTATAAAGTAGTAGAAATTGAACCAATAGGAGTAGCACCTACCAAGGCAAAGAGAAGAGTGGT
GGAGAGAGAAAAAAGAGCAATTATTGGACTGGGAGCTGCCTTCATTGGGTTCTTAGGAGCAGCAGGAAGCACT
ATGGGCGCGGCGTCAATAACGCTGACGGTACAGGCCAGACAATTACTGTCTGGCATAGTGCAACAGCAAAGCA
ATTTGCTGAGGGCTATAGAGGCTCAGCAGCATCTGCTGAAACTCACGGTCTGGGGCATT
>MH330356..1
TTCTATTGCAATACAACAGACCTGTTTAATAGCACTTGGGAGGAAAATAGCACTGTTACAAATAACACAAATG
CCAATGAAAATATAACTCTGCCATGCAGGATAAAGCAAATTATAAATTTGTGGCAGAGAGTAGGGCGAGCAAT
GTATGCCCCTCCTATCAGAGGAATTATAAGGTGTAACTCAACTATTACAGGACTACTACTAACAAGAGATGGT
GGAAATGGAAATAGTAGCAACAATGAGACTGAGACCTTCAGACCTATAGGAGGAAATATGAGGGACAATTGGA
GAAGTGAACTATATAAGTATAAAGTAGTAGAAATTGAACCAATAGGAGTAGCACCTACCAGGGCAAGGAGAAG
AGTGGTGCAGAGAGAAAAAAGAGCAGTTGGACTGGGAGCCGCCTTCCTTGGGTTCTTAGGAGCAGCAGGAAGC
ACTATGGGCGCGGCGTCACTGACGCTGACGGTACAGGCCAGACAATTATTGTCTGGCATAGTGCAACAGCAAA
GTAATTTGCTGAGGGCTATAGAGGCTCAGCAGCATCTGCTGAAACTCACGGTCTGGGGCATT
>MG902950..1
TTCTATTGCAATACAACAAACCTGTTCAGTAGCACTTGGAACGACACTATCACAAATTTCACAGAGACCAATG
AAACTATAATTCTGCCATGCAGAATAAGGCAATTTATAAAGATGTGGCAGAGAGTAGGACAAGCAATGTATGC
CCCTCCTATCAAAGGGAGCATAAGGTGTGAATCAAACATTACAGGACTACTACTAACAAGAGATGGTGGAAAT
GGAACTACTGACAGCAATGAGACCTTCAGACCTATAGGAGGAGATATGAGGGACAATTGGAGAAGTGAACTAT
ATAAGTATAAAGTAGTACAAATTGAACCAATAGGAATAGCACCTACCAAGGCAAAGAGAAGAGTGGTGGAGAG
AGAAAAAAGAGCAATTGGACTGGGAGCCGCTTTCCTTGGGTTCTTAGGAGCAGCAGGAAGTACTATGGGCGCG
GCGTCAATAACGCTGACGGCACAGGCCAGACAATTATTGTCTGGTATAGTACAACAGCAAAGCAATTTGCTGA
GGGCTATAGAGGCTCAGCAGCATCTGCTGAAACTCACGGTCTGGGGCATT
>MG902951..1
TTCTATTGCAATACAACAGACCTGTTCAATAGCACTTGGGGAAATAACACAAATAGCACAGGGACCAATGAAA
CTATAACTCTGCCATGCAGAATAAAGCAAATTATAAATATGTGGCAGAGAGTAGGACAAGCAATGTATGCCCC
TCCTATCCGAGGAATTATAAAGTGTGAATCAAACATTACAGGACTACTACTAACAAGAGATGGTGGAGGTGAA
GCTAATAATAGCGAGAGCGAGACCTTCAGACCTATAGGAGGAGATATGAGGGACAATTGGAGAAGTGAACTAT
ATAAGTATAAAGTAGTAGAAATTGAACCAATAGGAGTAGCACCTACCAGGGCAAAGAGAAGAGTGGTGCAGAG
AGAAAAAAGAGCAATTGGACTGGGAGCCGCCCTCCTTGGGTTCTTAGGAGCAGCAGGAAGCACTATGGGCGCG
GCGTCAATAACGCTGACGGTACAGGCCAGACAATTATTGTCTGGTATAGTGCAACAGCAAAGCAATTTGCTGG
ATGCTATAGAGGCTCAGCAGCATCTGCTGAGACTCACGGTCTGGGGCATT
>MH330337..1
TTCTATTGCAATACAACAGACCTGTTCAATAGCACTTGGGACGAAAATGACACTGTTACAAATAGCACACAGG
CCAATGAAACTATAATTCTACCATGCAGGATAAAGCAAATTATAAATATGTGGCAGAGAGTAGGACAAGCAAT
GTATGCCCCTCCTATCAAAGGAAGTATAAGGTGTGAATCAAACATTACAGGACTAATACTAACAAGAGATGGT
GGAACTAATAGCAGCAATGAGACCTTCAGACCTATAGGAGGAAATATGAGGGACAATTGGAGAAGTGAACTAT
ATAAGTATAAAGTAGTAAAAATTGAACCAATAGGAGTAGCACCTACCAAGGCAAAGAGAAGAGTGGTGGAAAG
AGAAAAAAGAGCAATTGGACTGGGAGCTGCCTTCCTTGGGTTCTTGGGAGCAGCAGGAAGTACTATGGGCGCG

GCGTCAATAACGCTGACGGTACAGGTCAGACAATTATTGTCTGGCATAGTGCAACAGCAAAGCAATTTGCTGA
GGGCTATAGAGGCTCAGCAGCATCTGCTGAAACTCACGGTCTGGGGCATT
>MH330338..1
TTCTATTGCGATACAACAAAACTGTTCAATAGCACTTGGAACGAAAATGGCACTAGGACAGATACAGATACCA
CACAGGATGAAAATATAACTATACCATGCAGAATAAAGCAAATTATAAATATGTGGCAGAGAGTAGGACAAGC
AATGTATGCCCCTCCCATCAGAGGAACTATAATGTGTATATCAAACATTACAGGGCTACTCCTAACAAGAGAT
GGTGGAGCTAATAACAGCAATGAAACCTTCAGACCTATAGGAGGAGATATGAGAGACAATTGGAGAAGTGAAC
TATATAAGTATAAAGTAGTAGAAATTGAACCAATAGGGGTAGCACCTACCAAGGCAAAGAGAAGAGTGGTGGA
GAGAGAAAAAAGAGCAGTTGGACTGGGAGTTGCCTTCCTCGGGTTCTTAGGAGCAGCAGGAAGTACTATGGGC
GCGGCGTCACTGACGCTGACGGTACAGGCCAGACAATTATTGTCTGGCATAGTGGAACAGCAAAGCAATTTGC
TGAGGGCTATAGAGGCTCAGCAGCATCTGCTGAAACTCACGGTCTGGGGCATT
>MH330339..1
TTCTATTGCAATACAACAGCACTGTTCAATAGCACTTGGGGAGGAAATAACACACAGGGCAATGAAAGTATAA
CTCTACCATGCAGAATAAAGCAAATTATAAGGATGTGGCAGAGAGTAGGACAAGCAATGTATGCCCCTCCTAT
CCAAGGAAGGATAAGGTGTGAATCAAATATTACAGGACTACTATTAACAAGAGATGGTGGAGTAACTAATAGC
AGCAATGGGACTGAGACCTTCAGACCTACAGGAGGAGATATGAGGGACAATTGGAGAAGTGAACTATATAAGT
ATAAAGTGGTAGAAATTGAACCAATAGGAATAGCACCTACCAGGGCAAGGAGAAGAGTGGTGCAGAGAGAAAA
AAGAGCAATTACACTGGGAGCTGCCTTCCTTGGGTTCTTGGGAGCAGCAGGAAGTACTATGGGCGCGGCGTCA
ATAACGCTGACGGTACAGGCCAGGCAATTATTGTCTGGTATAGTACAACAGCAAAGCAATTTGCTAAAGGCTA
TAGAGGCTCAGCAGCATCTGCTGAAACTCACGGTCTGGGGCATT
>MH330340..1
TTCTATTGCAATACAACAGACCTGTTCAATAGCATTTGGGAAAACGGCAATGTCACAAATAGCACAGAGACCA
ATGAAACTATAACTCTACAATGCAGAATAAAGCAAATTATAAATATGTGGCAAAGAGTAGGACAAGCAATGTA
TGCCCCTCCTATCAGTGGAAGCATAAGGTGTGAATCAAACATTACAGGACTAATACTAATAAGAGATGGTGGA
TATAATAGCAGCAATGAGACCTTCAGACCTATAGGAGGAAATATGAAGGACAATTGGAGAAGTGAACTATATA
AGTATAAAGTAGTAAAAATTGAACCAATAGGAGTAGCACCTACCAGGGCAAAGAGAAGAGTGGTGGAGAGAGA
AAAAAGAGCAATCGGACTGGGAGCTGCCTTCCTTGGGTTCTTAGGAGCAGCAGGAAGTACTATGGGCGCGGCG
TCACTGACGCTGACGGTACAAGCCAGACAATTATTGTCTGGCATAGTGCAACAGCAAAGCAATTTGCTGAGGG
CTATAGAGGCTCAGCAACATCTGCTGAAACTCACGGTCTGGGGCATT
>MH330341..1
TTCTATTGCAATACAACAGCCCTGTTCAATAGCACTTGGCACGAAAATGACACTGTTACAAATAGCACACAGA
ACAATGAAACTATAATTCTACCATGCAGAATAAAGCAAATTATAAATATGTGGCAGAGAGTAGGACAAGCAAT
GTATGCCCCTCCGATCAGAGGAAGTATAAGGTGTGAATCAAACATTACAGGACTACTACTAACAAGAGATGGT
GGGGCTAATAGCAGCAATGAGACCTTCAGACCTATAGGAGGAGATATGAGGGACAATTGGAGAAGTGAACTAT
ATAAGTATAAAGTAGTAAAAATTGAACCAATAGGAGTAGCACCTACCAGGGCAAAGAGAAGAGTGGTGGAAAG
AGAAAAAAGAGCAATTGGACTGGGAGTTGCCTTCCTTGGGTTCTTGGGAGCAGCAGGAAGCACTATGGGCGCG
GCGTCAATAACGCTGACGGTACAGGCCAGACAATTATTGTCTGGCATAGTGCAACAGCAAAGCAATTTGCTGA
GGGCTATAGAGGCTCAGCAGCATCTGCTGAAACTCACGGTCTGGGGCATT
>MH330342..1
TTCTATTGCAATACAACAGCCCTGTTCAATAACACTGACACAAATAGCACAGAGACCAGTGGAAATATAACTA
TACCATGTAGAATAAAGCAAATTATAAGAATGTGGCAGAGAGTAGGACAAGCAATGTATGCCCCTCCTATCGA
AGGAAATATAACGTGTGTATCAAACATTACAGGTCTACTACTAACAAGAGATGGTGGGGTAAATAACACCAAT
GAGACCTTCAGACCCATAGGAGGAGATATGAGGGACAATTGGAGAAGTGAACTATATAAGTATAAAGTAGTAG
AAATTGAACCAATAGGAGTAGCACCTACCAGGGCAAAGAGAAGAGTGGTGGAGAGAGAAAAAAGAGCAATTGG
ACTGGGAGCTGCCTTCCTTGGGTTCTTAGGAGCAGCAGGAAGCACTATGGGCGCGGCGTCAATGACGCTGACG
GTACAGGCCAGACAATTATTGTCTGGCATAGTGCAGCAGCAAAGCAATTTGCTGCAGGCTATAGAGGCTCAGC
AGCATCTGCTGAGACTCACGGTCTGGGGCATT
>MH330344..1
TTCTATTGTAATACAACAAAACTGTTCAATAGCACTTGGGACAAAAATAGCACAAGTACAAATATCACACAGA
CAAATGGAAATATAACTCTACCATGCAGAATAAAGCAAATTATAAATATGTGGCAGAGAGTAGGACAAGCAAT
ATATGCCCCTCCTATCAAAGGAAATATAATGTGTGTATCAAACATTACAGGACTACTACTAATAAGAGATGGG
GGAGGTGGAAATAATAGCATCAATGAAACTTTCAGACCTATAGGAGGAGATATGAGGGACAATTGGAGAAGTG
AACTATATAAGTATAAAGTAGTACAAATTGAACCAATAGGGGTAGCACCTACCAGGGCAAGGAGAAGAGTGGT
GCAGAGAGAAAAAAGAGCAATTGGACTGGGAGCCGCCTTCCTCGGGTTCTTAGGAGCAGCAGGAAGCACTATG
GGCGCAGCGTCACTGACGCTGACGGTACAGGCCAGACAATTATTGTCTGGCATAGTGCAACAGCAAAGCAATT
TGCTGAGGGCTATAGAGGCTCAGCAGCATCTGCTGAAACTCACGGTCTGGGGCATT
>MH330345..1
TTCTATTGCAATACAACAGCCCTGTTCAATAGCATTTGGAACGGAAATGACAATGGCACAAATAGTACAGAGG
ACACTGACCCTATAATTCTACCATGCAGAATAAAGCAAATTGTAAATATGTGGCAGAGAGTAGGACAAGCAAT
GTATGCCCCTCCTATCCCAGGAAATATAAGTTGTACATCAAACATTACAGGACTACTACTAACAAGAGATGGA

ATTAATGGCAGCAATGAGACCTTCCGACCTATAGGAGGAGACATGAGGGACAATTGGAGAAGTGAACTATATA
AGTATAAAGTAGTAAAAATTGAACCAATAGGAGTAGCACCTACCAAGGCAAAGAGAAGAGTGGTGGAGAGAGA
AAAAAGAGCAATTGGACTGGGAGCTGCCTTCCTTGGGTTCTTAGGAGCAGCAGGAAGCACTATGGGCGCGGCG
TCAATAACGCTGACGGTACAGGCCAGACAATTATTGTCTGGCATAGTGCAACAGCAAAGCAATTTGCTGAGGG
CTATAGAGGCTCAGCAGCATCTGCTGAAACTCACGGTCTGGGGCATT
>MH330346..1
TTCTATTGCAATACATCAGACCTGTTCACACATAATATCACAGGGGCCAATGAAACAGGGGCCAATGGAACTA
TAACTATACCATGCAAAATAAAACAAATTATAAATATGTGGCAGAGAGTAGGACAAGCAATGTATGCCCCTCC
TATCAGAGGAATTATAAGGTGTGAATCAAACATTACAGGACTATTACTAACAAGAGATGGTGGAAAGAGCGAG
AATGAGACCTTCAGACCTATAGGGGGAGATATGAGGGACAATTGGAGAAGTGAACTATATAAGTATAAAGTAG
TAGAAATTGGACCAATAGGAGTAGCACCTACCAGGGCACAGAGGAAAGTGGTGAAGAGAGAAAAAAGAGCAAT
TATTGGACTGGGAGCTGCCTTCCTTGGGTTCTTAGGAGCAGCAGGAAGCACTATGGGCGCGGCGTCATTGACG
CTGACGGTACAGGCCAGACAATTATTGTCTGGCATAGTGCAACAGCAAAGCAATTTGCTGAGGGCTATAGAGG
CTCAGCAGCATCTGCTGAAACTCACGGTCTGGGGCATT
>HQ616082..1
TTCTATTGCAATACAACAAACCTGTTCAATGGCACTTGGTCAAAAAGCACAACGGCCAATGCAACTGCCAATG
AAACTATAACTCTACCGTGCAGAATAAAGCAAATTATAAATATGTGGCAGAGAGTAGGACAAGCAATGTATGC
CCCTCCTATCATAGGCAATATAAGCTGTGAATCAAACATTACAGGACTGCTACTGACAAGAGATGGTGGAACA
ACTAATAGCAGCGATGAGACCTTCAGACCTATAGGAGGAAATATGAGGGACAATTGGAGAAGTGAACTATATA
AGTATAAAGTAGTAATAATTGATCCAATAGGAGTAGCACCTACCAGGGCAAAGAGAAGAGTGGTGGAGAGGGA
AAAAAGAGCAATTGGACTGGGAGTTGCCTTCCTTGGGTTCTTAGGAGCAGCAGGAAGCACTATGGGCGCGGCG
TCACTGACGCTGACGGTACAGGCCAGACAATTATTGTCTGGTATAGTGCAACAGCAAAGCAATTTGCTGAGGG
CTATAGAGGCTCAGCAGCATCTGCTGAAACTCACGGTCTGGGGCATT
>DQ823357..1
TTCTATTGCAATACAACAGAACTGTTCAATAGCACTTGGGGCGAAAATGGCACTATCACAAATAGCACAGAGA
CCAGTGAAAATATAACTCTACCATGCAGAATAAAGCAAATTATAAATATGTGGCAGAGAGTAGGACAAGCAAT
GTATGCCCCTCCTATCAGAGGAAATATAAGTTGTGTATCAAACATTACAGGACTACTACTAACAAGAGATGGT
GGAACTAATAGCAGCACTGAGACCTTCAGACCTACAGGAGGAGATATGAGGGACAATTGGAGAAGTGAACTAT
ATAAGTATAAAGTAGTAGAAATTGAACCAATAGGAGTAGCACCTACCAAGGCAAAGAGAAGAGTGGTGGAGAG
AGAAAAAAGAGCAATAATTGGACTGGGAGCTGCCTTCCTTGGGTTCTTAAGTGCAGCAGGAAGCACTATGGGC
GCAGCGTCACTGACGCTGACGGTACAGGCCAGACAATTATTGTCTGGCATAGTGCAACAGCAAAGCAATTTGC
TGAGGGCTATAGAGGCTCAGCAGCATTTGCTGAGACTCACGGTCTGGGGCATT
>KU749399..1
TTCTATTGCAATACAACAGGCCTGTTCAATAGTACTTGGGACAATAGTACTTGGGACAAAAATAGCACTGTCA
CAAATGGCACCGAGGACAATATAACTCTACAATGCAAAATAAAGCAAATTATAAGGATGTGGCAGAGAGTAGG
ACAAGCAATGTATGCCCCTCCTATCAGAGGAAATATAAAGTGTACATCAAACATTACAGGACTACTACTAATA
AGAGATGGTGGAAATGGAAATAATAGCAACAATGAGACCTTCAGACCGACAGGAGGAGATATGAGAAATAATT
GGAGAAGTGAACTATATAAGTATAAAGTAGTAGAAGTTGAACCAATAGGAATAGCACCTACCAAGGCAAAGAG
AAGAGTGGTGGAGAGAGAAAAAAGAGCAATAGTTGGACTGGGAGCTGCCTTCCTTGGGTTCTTAGGAGCAGCA
GGAAGTACTATGGGCGCGGCGTCAATAACGCTGACGGTACAGGCCAGACAATTACTATCTGGCATAGTACAAC
AGCAGAGCAATCTGCTGAGGGCTATAGAGGCTCAGCAGCATCTGCTGAAACTCACGGTCTGGGGCATT
>KU749401..1
TTCTATTGCAATACAACAGACCTGTTCAATAGCACTTGGAACGGCAGCAATAGCACTGCCATGAATAGCTCAG
GCAATGAAAATATAACCATACACTGCAGAATAAAGCAAATTATAAATATGTGGCAGAGAGTAGGACAAGCAAT
GTATGCCCCTCCTATCAGAGGAAATATAACATGTGTATCAAACATTACAGGGCTACTACTAATAAGAGATGGG
GGAAATGGAAATAATAGCAACAATGAGACCTTCAGACCTATAGGAGGAGATATGAGGGATAATTGGAGAAGTG
AACTATATAAATATAAAGTAGTAAAAATTGAACCAATAGGAGTAGCACCTACCAAGGCAAGGAGAAGAGTGGT
GGGGAGAGAAAAAAGAGCAATTGGACTGGGAGCTGCCTTCCTTGGGTTCTTAGGAGCAGCAGGAAGCACTATG
GGCGCGGCGTCACTGACGCTGACGGTACAGGCCAGAAAATTGCTATCTGGCATAGTGCAACAGCAAAGCAATT
TGCTGAGGGCTATAGAGGCTCAGCAGCAACTGCTGAGACTCACAGTCTGGGGCATT
>KY658681..1
TTCTATTGCAATACAACAGCCCTGTTCAATAGCACTTGGAACACCACTTGGAACGAAAATAGCACTGTTGAAA
GTAGTACAGGGGACAATGAAAATATAACTATACCATGCAGAATAAAGCAAATTATAAATATGTGGCAGAGAGT
AGGACAAGCAATGTATGCCCCTCCTATCAAAGGAATTATAAAGTGTGAATCAAACATTACAGGACTACTACTG
GTAAGAGATGGTGGAAACACTAATAGCAGCAATGAGACCTTCAGACCTATAGGAGGAGATATGAGGGACAATT
GGAGAAGTGAACTATATAAGTATAAGGTAGTAAAAATTGAACCAATAGGAGTAGCACCAACCAAGGCACAGAG
AAGAGTGGTGGGGAGAGAAAAAAGAGCAATTGGACTGGGAGTTGCCTTCCTTGGGTTCTTAGGAGCAGCAGGA
AGCACTATGGGCGCAGCGTCAATAACGCTGACGGTACAGGCCAGACAATTACTGTCTGGCATAGTGCAACAGC
AAAGCAATTTGCTGAAGGCTATAGAGGCTCAGCAGCATCTGCTGAAACTCACAGTCTGGGGCATT
>KU749402..1

TTCTATTGCAATACATCAAAACTGTTCAATAGCACTTGGGACGAAAATAGCACTGCCATAAATAGTACAGAGG
ACAGTGGAATTATAACTATACCATGCAGAATAAGGCAAGTTATAAATATGTGGCAGAGAGTAGGACAAGCAAT
GTATGCCCCTCCTATCAGAGGAAGTATAAGGTGTGAATCAAACATTACAGGATTAATACTAACAAGAGATGGT
GGAAATATTAGTAGCACCAATGAGACCTTCAGACCTACAGGAGGAGATATGAGGGACAATTGGAGAAGTGAAC
TATACAAGTATAAAGTAGTAAGCATTGAACCAATAGGAGTAGCACCTACCAAGGCAAGGAGAAGAGTGGTGCA
GAGAGAAAAAAGAGCAGTTGGACTGGGAGCTGCCTTCCTTGGGTTCTTAGGAGCAGCAGGAAGCACTATGGGC
GCGGCGTCATTGACGCTGACGGTACAGGCCAGAAAATTATTGTCTGGCATAGTGCAACAGCAAAACAATTTGC
TGAAGGCTATAGAGGCTCAGCAGCATCTGCTGAAACTCACAGTCTGGGGCATT
>KU749403..1
TTCTATTGTAATACAACAGACCTGTTCAATAGCACTTGGGACAGAAATGACACTGATGCAAATAGTACACAGG
TTGATAGTACACAGGTTGATGTAACTATAACTCTACCATGCAGAATAAAGCAAATTGTAAGGATGTGGCAGAG
AGTAGGACAAGCAATGTATGCCCCTCCTATCGGAGGAGTTATAAGGTGTGAATCAAACATTACAGGACTACTA
CTAACAAGGGATGGTGGAGCTAATAATAGCACTAATGAGACCTTTAGACCTCTAGGAGGAGATATGAGGGACA
ATTGGAGAAGTGAACTATATAAGTATAAAGTAATACAAGTTGAACCAATAGGAGTAGCACCTACCCCAGCAAA
GAGAAGAGTGGTGGAGAGACAAAAAAGAGCAATTGGACTGGGAGCTGCCTTCCTTGGGTTCTTAGGAGCAGCA
GGAAGTACTATGGGCGCGGCGTCAATAACGCTGACGGTACAGGCCAGACAATTATTGTCTGGCATAGTACAAC
AGCAAAGTAATTTGCTGAGAGCTATAGAGGCTCAGCAGCATCTGCTGAAACTCACAGTCTGGGGCATT
>KU749404..1
TTCTATTGCAATACAACAGAGCTGTTCAATAGCACTGAGGACAAAAATGGCACTGTCACAAATAGCAATGAAA
ATATAACTCTACCATGCAGGATAAAGCAAATTATAAATATGTGGCAGAGAGTAGGACAAGCAATGTATGCCCC
TCCTATCAGAGGAAATATAACTTGTGAATCAAACATTACAGGACTACTACTAACAAGAGATGGAGATGATAAT
AGCAGTGAGACCTTCAGACCTATAGGAGGAAATATGAGGGACAATTGGAGAAGTGAACTATATAAATATAAAG
TAGTCCAAATTGAACCAATAGGAGTAGCACCTACCAAGGCAAGGAGAAGAGTGGTGGGGAGAGAAAAAAGAGC
AATTGGACTGGGAGCTGCCTTCCTTGGGTTCTTAGGAGCAGCAGGAAGCACTATGGGCGCGGCGTCACTGACG
CTGACGGTACAGGCCAGACAATTATTGTCTGGTATAGTGCAACAGCAAAGCAATTTGTTGAGGGCTATAGAGG
CTCAGCAGCATCTGCTGAAACTCACAGTCTGGGGCATT
>KU749405..1
TTCTATTGCAATACAACAGACTTGTTCAATTGGGATAGAAATAGCACTGTCCCAAATAACACAAGTAGCAATA
TAACTCTGCCATGCAGAATAAAGCAAATTATAAATATGTGGCAGAGAGTAGGACAATCAATGTATGCCCCTCC
TATCAGCGGAAAGATAAGGTGTGAATCAAACATTACAGGACTACTACTAGTAAGAGATGGTGGAGATAATAAT
AGCACCAATGAGACCTTCAGACCTATAGGAGGAGAAATGAGGCACAATTGGAGAAGTGAACTATATAAGTATA
AAGTAGTAGAAATTGAACCAATAGGAGTAGCACCTACCAAGGCAAGGAGAAGAGTGGTGGGAAGAGAAAAAAG
AGCAGCCTTTGGACTGGGAGCTGTCTTCCTTGGGTTCTTAGGAGCAGCAGGAAGCACTATGGGCGCGGCGTCA
CTGACGCTGACGGTACAGGCCAGGCAATTATTGGCTGGCATAGTGCAACAGCAAAGCAATTTGCTGAGGGCTA
TAGAGGCTCAGCAGCATCTGCTGAAACTCACAGTCTGGGGCATT
>KY658682..1
TTCTATTGCAATACAACAGAACTGTTCAATAGCACTTGGAATGGCACTGACACAAACAGCACAGAGGACAATA
ATGTAAATATAACTATACCATGCAGAATAAAGCAAATTATAAATATGTGGCAGAGAGTAGGACGAGCAATGTA
TGCCCCTCCTATCAGAGGAAATATAACTTGTGAATCAAACATTACAGGACTACTACTAACAAGAGATGGTGGT
AAAGAGAAAAATGACAACGAAACCTTTAGACCTATAGGAGGAAATATGAAGGACAATTGGAGAAGTGAACTAT
ATAAGTATAAAGTAGTAGAAATTAACCCAATAGGAGTAGCACCTACCCCGGCAAAGAGAAGAGTGGTGGAGAG
AGAAAAAAGAGCAGTTAGCCTAGGAGTTGCCTTCCTTGGGTTCTTAGGAGCAGCAGGAAGTACTATGGGCGCG
GCGTCACTGACGCTGACGGTACAGGCCAGACAACTATTGTCTGGCATAGTACAACAGCAAAGCAATTTGCTGA
GGGCTATAGAGGCTCAGCAGCAATTGCTGAAACTCACGGTCTGGGGCATT
>KU749407..1
TTCTATTGCAATACAACAGAACTGTTCAATAGCACTTGGGACGACAATAGCACTGTTGCAAATATCACAAAGA
CCAATGAAAATATAACTCTACCATGCAGACTAAGGCAAATCATAAAGATGTGGCAGAGAGTAGGACAAGCAAT
GTATGCCCCTCCTATCCAAGGAGTTATAAAGTGTGAATCAAACATTACAGGACTACTACTAACAAGAGATGGT
GGAGATGGAACTAATAACAGCAATAATGAGATTTTCAGACCTATAGGAGGAAATATGAGGGACAATTGGAGAA
GTGAACTATATAAGTATAAAGTAGTAGAAATTGAACCAATAGGAGTAGCACCTACCAAGGCAAAGAGAAGAGT
GGTGCAGAGAGAAAAAAGAGCAGTTATTGGACTGGGAGCTGCCTTCCTTGGGTTCTTAGGAGCAGCAGGAAGC
ACTATGGGCGCGGCGTCAATAACGCTGACGGTACAGGCCAGACAATTACTGTCTGGCATAGTGCAACAGCAAA
GCAATTTGCTGAGGGCTATAGAGGCTCAGCAGCATCTGCTGAAACTCACGGTCTGGGGCATT
>AF082486..1
TTCTATTGCAATACAACAGACCTGTTCAATAGCACTTGGGACGAACATGGCACTGTCACAAATAGCACAATGG
CCAATGGAACTATAACTCTACCATGCAGAATAAAGCAAATTATAAATATGTGGCAGAGAGTAGGACAAGCAAT
GTATGCCCCTCCTATTAAAGGAAGTATAAGGTGTGAATCAAACATTACAGGACTACTATTAACAAGAGATGGT
GGAAGTGGAACTAATAGCAGCAATGAGACCTTCAGACCTATAGGAGGAAATATGAGGGATAATTGGAGAAGTG
AACTATATAAGTATAAAGTAGTAAAAATTGAACCCATAGGATTAGCACGTACCAGGGCAAAGAGAAGAGTGGT
GGAGAGAGAAAAAAGAGCCATTGGACTGGGAGCTGCCTTCCTTGGGTTCTTAGGAGCAGCAGGAAGTACTATG

GGCGCGGCGTCAATGACGCTGACGGTACAGGCCAGACAATTATTGTCTGGCATAGTGCAACAGCAAAGCAATT
TGCTGAGGGCTATAAAGGCTCAGCAGCATCTGCTGAAACTCACGGTCTGGGGCATT
>KY968402..1
TTCTACTGTAACACAACAAAACTGTTTAATAGTACTTGGTCTAATAATGATACTGGGGAGGGTACTGCAGATT
CAAATAACGAAACTATCACAATCCCATGCAGAATAAAACAAATTATAAACATGTGGCAACAAGTAGGAAAAGC
AATGTATGCCCCTCCCATCAGTGGAAACATTAGTTGCTCATCAAATATTACAGGGTTACTGTTAACAAGAGAT
GGTGGAGATAATGATGACCTAAACGGGACCGAGACCTTCAGACCTCAAGGAGGAAACATGAAGGACAATTGGA
GAAGTGAATTATATAAATATAAAGTAGTAAAAATTGAACCATTAGGAGTAGCACCCACCAAGGCAAAGAGAAG
AGTGTTGCAGAGAGACAAAAGAGCAGTGGGACTAGGAGCTATGTTCCTTGGGTTCTTGGGAGCAGCAGGAAGC
ACTATGGGCGCAGCGTCAATAGCGCTGACGGTACAGACCAGACAATTATTGTCTGGTATAGTGCAACAGCAGA
ACAATATGCTGAGGGCTATTGAGGCGCAACAGCATATGTTGCAACTCACTGTCTGGGGCATC
>KY968404..2
TTCTACTGTAATACAACACAACTGTTTTATAGTACTTGGTTGTCTAATGGTAGTAGTTTGACAAATAACACTG
AAGGAAATGACACTATCACACTCCCATGCAGAATAAAACAGATTATAAATATGTGGCAGCAAGTAGGAAAAGC
AATGTATGCCCCTCCCATCAAAGGACTAATTAGATGCTCATCGAATATTACAGGACTGCTACTAACAAGAGAT
GGTGGTAGTCATAACGAGAGCAATGAGACTGAGACATTCAGACCTGGGGGAGGAGATATGAGGGACAATTGGA
GAAGTGAATTATACAAATATAAAGTAGTACAAATTGAACCATTAGGAATAGCACCCACCAAGGCAAAGAGAAG
AGTGGTGCAGAGAGAAAAAAGAGCAGTGGGACTGGGAGCTTTGTTCCTTGGGTTCTTGGGAACAGCAGGAAGC
ACTATGGGCGCAGCGTCATTGACGCTGACGGTACAGGCCAGACAATTATTGTCTGGTATAGTGCAACAGCAGA
ATAATCTGCTGAGAGCTATTGAGGCGCAACAACATCTGTTGCAGCTCACAGTCTGGGGCATC
>KY968408..1
TTCTACTGTAATACAACAAAACTGTTTAATAAGACTTGGAATGGTACTTGGCTTAATAATGAGAATACTACAC
AGAATATCACACTCCCATGCAGAATAAAACAAATTATAAACAGGTGGCAGGAAGTAGGAAAAGCAATGTATGC
CCCTCCCATCAAAGGACAAATTAGCTGCTCATCAAATATTACAGGGCTGCTATTAACAAGAGATGGTGATAAC
GAGACTGGGTCGGAGACCTTCAGACCTGGAGGAGGAGATATGAGGGACAATTGGAGAAGTGAATTATATAAAT
ATAAAGTAGTAAAAATTCAGCCATTAGGAGTAGCACCCACCAAGGCAAAGAGAAGAGTGGTGCAAAGAGAAAA
AAGAGCAGTGGGAATAGGAGCTGTGTTCCTTGGGTTCTTGGGAGCAGCAGGAAGCACTATGGGCGCAGCGTCA
GTAACGCTGACGGTACAGGCCAGACAATTATTGTCTGGTATAGTGCAGCAGCAGAACAATTTGCTGAGGGCTA
TTGAGGCGCAACAGCATCTGTTGCAACTCACAGTCTGGGGCATC
>DQ383749..1
TTCTACTGTAATACATCACAACTGTTTAATAGTACTTGGCCTTGGAATGGGAATGCGACTACAGATAATACTA
CAGGAGTAAATATCACACTCCCATGCAGAATAAAACAAATTATAAACATGTGGCAGGAAGTAGGAAAAGCAAT
GTATGCCCCTCCTATTAAAGGACAAATTAGATGTTCATCAAATATTACAGGGCTGCTATTAACAAGAGATGGT
GGTCATAACGAGACCAACGACACTGAGACCTTCAGACCTGGAGGAGGAAATATGAAGGATAATTGGAGAAGTG
AATTATATAAATATAAAGTAGTAAAAATTGAACCATTAGGAATAGCACCCACCACGGCAAAGAGAAGAGTGGT
GCAGAGAGAGAAAAGAGCAATAACAATAGGAGCTATGTTACTTGGTTTCTTGGGAACAGCAGGAAGCACTATG
GGCGCAGCGTCAATCACGCTGACGGTACAGGCCAGACAATTATTGTCTGGTATAGTGCAACAGCAGAACAATC
TGCTGAGGGCTATTGAGGCGCAACAGCATCTGTTGCAACTCACAGTCTGGGGCATC
>MH078532..1
TTCTACTGTAACACATCAAAGCTGTTTAACAGTACTTGGCATAGGAATGGTACTTGGAATGGTACTAGTACTG
AGAATAATACAATCACAATCCCATGCAGAATAAAACAAATTATAAACAGGTGGCAGGAAGTAGGAAAAGCAAT
GTATGCCCCTCCCATCAGAGGAAATATTACATGTACATCTAATATTACAGGACTGTTATTAATAAGAGATGGT
GGTAACATTAACACCACGAATGAGACCGAGATCTTCAGACCTGGAGGAGGAGACATGAGGGATAATTGGAGAA
GTGAATTATATAAATATAAAGTAGTAAGAATTCAGCCATTAGGAATAGCACCCACCGAGGCAAAGAGAAGAGT
GGTGCAAAGAGAAAAGAGAGCGGTGGGAACACTAGGAGCTGTGTTCCTTGGGTTCTTGGGAGCAGCAGGAAGC
ACTATGGGCGCAGCGGCAACAACGCTGACGGTACAGGCCAGACAGTTACTGTCTGGCATAGTGCAACAGCAAA
ACAATTTGCTGAGAGCTATTGAGGCGCAACAGCATACGTTGCAACTCACAGTCTGGGGCATT
>AF538307..1
TTCTACTGTGATTCAACACAACTGTTTAATAGTACTTGGCATAATAGTACTTGGAATAATAATAAAGAGTCAA
ATAGCACTGATCCAATCACACTTCCATGCAGAATAAAACAAAATATAAACATGTGGCAGGAAGTAGGAAAAGC
AATGTATGCCCCTCCCATCAGAGGACAAATTAAATGTTCATCAAACATTACAGGGCTAATATTAACAAGAGAT
GGTGGTAATAACACAGACAATAACAACACCGAAACCTTCAGACCTGGAGGAGGAAATATGAAGGACAATTGGA
GAAGTGAATTATATAAATATAAAGTAGTAAAAATTGAACCATTAGGAGTAGCACCCACCAAGGCAAGGAGAAG
AGTGGTGCAGAGAGAGAAAAGAGAAGTAGGACTAGGAGTTATGTTCCTTGGGTTCTTGGGAGCAGCAGGAAGC
ACTATGGGCGCAGCGTCACTGAGGCTGACGGTACAGGCCAGACAATTATTGTATGATATAGTGCAACAGCAAA
ACAATTTGCTGAGAGCTATTGAGGCGCAACATCATCTGTTGCAACTCACAGTCTGGGGCATC
>GU367404..1
TTCTATTGTAATACAACACAACTGTTTAACAGTACTTGGAGAAAGGAGTCAAATAACACTGGAGGAAATATCA
CACGAAATATCACGCTCCCATGTAGAATAAGACAGATTGTAAACATGTGGCAGGAAGTAGGAAAAGCAATATA
TGCCCCTCCCATTGAAGGACTAATTAGATGTTTATCAAATATTACAGGGCTGCTATTAACAAGAGACGGTGGT

AATCAGAATGGGACTAATGACACTGAAATCTTCAGACCTGGAGGGGGAGATATGAGGGACAATTGGAGAAGTG
AATTATATAAATATAAAGTAGTAAAAATTGAACCCTTAGGAATAGCACCCACCAAGGCAAAGAGAAGAGTGGT
GCAAAGAGAAAAAAGAGCAGTGGGAGCATTAGGAGCTATGTTCCTTGGGTTCTTGGGAGCAGCAGGAAGCACT
ATGGGCGCAGCGTCAGTAACGCTGACGGTACAGGCCAGACAATTATTGTCTGGTATAGTGCAACAGCAGAATA
ATCTGCTGAAGGCTATTGAGGCGCAACAGCATCTGTTGCAACTCACAGTCTGGGGCATC
>AF146728..1
TTCTACTGTAATTCAACACAACTGTTTAATAGTACTTGGTTTAATAGTACTGGGAATGATACTGAAAGGGCAA
CTAACAATACAGAAAATATCACACTCCCATGTAGAATAAAACAAATTATAAACATGTGGCAGAAAGTAGGAAA
AGCAATGTATGCCCCTCCCATCAACGGACAGATTAGATGTTCATCAAATATTACAGGGCTGATACTAACAAGA
GATGGTGGTAATCAAGAGAACAAGACCGAGATCTTCAGACCTGGAGGAGGAGATATGAGGGACAATTGGAGAA
GTGAATTATATAAATATAAAGTAGTAAGAATTGAACCATTAGGAGTAGCACCCACCAAGGCAAAGAGAAGAGT
GGTGCAGAGAGAAAAAAGAGCAGTGGGAATGATAGGGGCAATGATCCTTGGGTTCTTGGGAGCAGCAGGAAGC
ACTATGGGCGCAGCGTCACTGGCGCTGACGGTACAGACCAGGCAATTGTTGTCTGGTATAGTGCAACAGCAGA
ACAATCTGCTGAGGGCTATTGAGGCGCAACAGCATCTGTTGCAACTCACAGTCTGGGGCATC
>MN485974..1
TTCTATTGTAATACAACAAAACTGTTTAATGGTACTTGGAATGGTACTTGGCTATCGGGGAACTGGCCAAATG
AAAATATCACACTCCCATGTAGAATAAAACAAATTGTAAACATGTGGCAAGAAGTAGGAAAAGCAATGTATGC
CCCTCCCATCCAAGGACAGATTAGATGTGACTCAAATATTACAGGGCTGCTATTAACAAGAGATGGTGGTTCC
GGGAATGGAACTGAGAATAACACTGAGATCTTCAGACCTGGAGGAGGAAATATGAGAGACAATTGGAGAAGTG
AATTATATAAATATAAAGTAGTAAAAATTGCACCATTAGGAATAGCACCCACCAAGGCAAAGAGAAGAGTGGT
GCAGAGAGAAAAAAGAATAGGAACGCTAGGAGCTGTGTTCCTTGGGTTCTTGGGAACAGCAGGAAGCACTATG
GGCGCAGCGTCACTGACGCTGACGGTACAGGCCAGACAATTATTGTCTGGTATAGTGCAACAGCAGAACAATT
TGCTGAGGGCTATTGAGGCGCAACATCATCTGTTGCAACTCACAGTCTGGGGCATC
>MN485990..1
TTCTACTGTGATTCAACAGAACTATTTAATAGTACTTGGAATTTTAATAATAATACTTGGTATGAGAACTCAA
ATAACACAAGTGACAAAGAAAACATCACACTCCCATGTAGAATAAAACAAATTAGAAACTTGTGGCAGGAAGT
AGGAAAAGCAATGTATGCCCCTCCCATCCAAGGGCTAATTACATGTCAATCAAATATTACTGGGGTGCTACTA
ACAAGAGATGGTGGTATTAATAACACCAGGAACGAGACCTTTAGACCTGGAGGAGGAAATATGAAAGACAATT
GGAGAAGTGAACTATACAAATATAAAGTAATAAAAATTGAACCATTAGGAGTGGCACCCACCAAGGCAAAGAG
AAGAGTGGTGCAGAGAGAAAAAAGAGCAGTGGGAACAATAGGAGCTGTGTTCCTTGGTTTCTTGGGAACAGCA
GGAAGCACTATGGGCGCAGCGTCAATAGCGCTGACGGCACAGGCCAGACAATTATTGTCTGGTATAGTGCAAC
AGCAAAACAATTTGCTGAGAGCTATTGAGGCGCAACAACATCTGTTGCAACTCACAGTCTGGGGCATC
>MN485995..1
TTCTACTGTAATACAACACAACTGTTTAATAGTACTTGGTGGTCTAATAGTACTGGGAATATTACTGGACAGC
CAGAAAATATCACTCTCCCATGCAGAATAAAACAAATCATAAACTTGTGGCAAGAGGTAGGAAAAGCAATGTA
TGCCCCTCCCATCCAAGGACTAATTAGATGTTCATCAAATATTACAGGGTTACTATTAACAAGAGATGGTGAT
AATCAAACTAAAGAGACTGAGATCTTTAGACCGGGAGGAGGAGATATGAGGGACAATTGGAGAAGTGAATTAT
ATAAATATAAAATAGTAAAAATTGAGCCATTGGGAATAGCACCCACCAGGGCAAAGAGAAGAGTGGTGCAAAG
AGAAAAAAGAGCAGTGGGAATAGGAGCTCTGTTCCTTGGGTTCTTGGGAGCTGCAGGAAGCACTATGGGCGCA
GCGTCGGTGACGCTGACGGTACAGGCCAGACAATTATTGTCTGGTATAGTGCAACAGCAAAACAACTTGCTGA
GAGCTGTTGAGGCGCAACAACATATGTTGCAACTCACAGTCTGGGGCATC
>MN486034..1
TTCTACTGTAATACAACAGAGCTATTTAATAGGAATATTACTGAAGAGTCAAACACTAATGGAAATAGCAGTA
GCGCAGGAAATATCACACTCCAATGCAGAATAAAACAGATTATAAACATGTGGCAGGAAGTAGGAAAAGCAAT
GTATGCCCCTCCCATCAGAGGACAAATTCAATGTATATCAAATATTACAGGACTAATATTAACAAGAGATGGT
GGTAAGACAAATAATGAGACAAATGGGACTGAGATTTTCAGACCTGGAGGAGGAAATATGAGAGACAATTGGA
GAAGTGAATTATATAAATATAAGGTAGTAAAAATTGAACCATTAGGAGTAGCACCCACCAGGGCAAAGAGAAG
AGTGGTGCAGAGAGAAAAAAGAGCAGTGGGAATAGGAGCTGTGTTCCTTGGGTTCTTGGGAGCAGCAGGAAGC
ACTATGGGCGCAGCGTCAATAACGCTGACGGTACAGGCCAGACAATTATTGTCTGGTATAGTGCAACAGCAAA
ACAATTTGCTGAGAGCTATTGAGGCGCAACAGCATCTGTTGCAACTCACAGTCTGGGGCATC
>DQ358805..1
TTCTACTGTAATACAACGAAGCTGTTTACTGGTACTTTGAATAGCACTGGGACTTGGCATACAGAAAACCAGA
CTGATACTATCACACTCCCATGCAAAATAAAACAAATTATAAACAGGTGGCAGGAAGTAGGAAAAGCAATGTA
TGCCCCTCCCATTAAAGGACAAATTAGCTGTTCATCAAATATTACAGGGATACTATTAACACGAGATGGTGGT
GGGAATGGGTCCGACACCACCGCCTCCTTAACACCAGGAGGAGGAGATATGAGGAACAGTTGGAGAAATGAAT
TATATAATTATGAGGTGGTAAAAATTCCACCATTAGGAGTACCACCCACCGAGGCGAGGAGAAGAGTGGTGCA
GAGAGAAAAAAGAGCAGTGGGGGCACTAGGAGCTATGTTCCTTGGGTTCTTGGGAACAGCAGGAAGCACTATG
GGCGCAGCGTCACTGACGCTGACGGTACAGGCCAGACAATTATTGTCTGGTATAGTGCAACAGCAGAATAATT
TGCTGAGAGCTATTAAGGCGCAACAGCATATGTTGCAACTCACAGTCTGGGGCATC
>JN692439..3

TTCTACTGTGATACCTCACAAATATTTAATAGTAATTGGAATAGTTCTTCAAATTTTAGTCATCATGAAAATT
CAAATGAAAATATCACACTCCAATGCAGAATAAAACAAATTATAAACAGGTGGCAGGAAGTAGGAAGAGCAAT
GTATGCCCCTCCCATCCGAGGACTAATTAGATGTTCATCAAATATTACAGGGCTGCTATTAACAAGAGATGGT
GGTAAGAATGATAACAATGGGACTGAGAATGGGACTGAGACCTTCAGACCTGGAGGGGGAGATATGAGGGACA
ATTGGAGAAGTGAATTATATAAATATAAAGTAGTTCAAATTGAGCCAGTAGGAATAGCACCCACCAAGGCAAA
GAGAAGAGTGGTGCAAAGAGAAAAAAGAGCAGTAGGAGTAGGAGCTGTGTTCCTTGGGTTCTTGGGAACAGCA
GGAAGCACTATGGGCGCAGCGTCACTCACGCTGACGGTACAGGCCAGACAATTGTTGTCTGGTATAGTGCAAC
AGCAGAACAATCTGCTGAAGGCTATTGATGCGCAACAGCATCTGTTGCAGCTCACAGTCTGGGGCATC
>JN692462..1
TTCTACTGTAATACATCACCACTGTTTAATAGTACTTGGAAAATTGGAACTAATAAGTCTATAGAAAATGGAA
CTATCACACTCCCATGCAGAATAAAACAAATTATAAACAGGTGGCAGGAAGTAGGAAAAGCAATGTATGCCCC
TCCCATCGAAGGACCAATTAGATGTTCATCAAATATTACAGGGCTGCTATTAACAAGAGATGGTGGTGAAGAC
AACAATGGGTCCGAGACCTCCAATGAGACCTACACCTCTGGACCAGGAGATATGAATATGAATTGCAATTGTG
AATTATATTTATATAAAGATATGGAAATGGAGCCTGTACCATTAGCACCCACCCCGACCAAGGCAAAGAGAAG
AGTGGTGCAGAGAGAAAAAAGAGCAGTGGGAGTAGGAGCTGTGTTCCTTGGGTTCTTGGGAGCAGCAGGAAGC
ACTATGGGCGCAGCGTCAGTGGCGCTGACGGCACAAGCCAGACAATTATTGTCTGGTATAGTGCAACAGCAGG
ACAATCTGCTGAGGGCTATTGAGGCGCAACAGCATCTGTTGCAACTCACAGTCTGGGGCATC
>JN692465..1
TTCTATTGTAATACAACAGAGCTGTTTAAGGGTACTTGGAATGGTACTTGGAATGGTACTACTTGGGCTAATA
GTACTTGGGCTAATGGTACTGGGAATATGAACATCACACTCCCATGCAGAATAAGACAAATTATAAACATGTG
GCAGGAGGTAGGAAAAGCAATGTATGCCCCTCCCATCAGCGGACCCATTAACTGTACATCAAATATTACAGGG
ATGATATTAACAAGAGATGGTGGTGATGGTAACGGGAGTCAGGAGATCTTCAGACCTGGAGGAGGAGATATGA
GGGACAATTGGAGAAGTGAATTATATAAATATAAAGTAATAAAAATTGAACCATTAGGAGTAGCACCCACCAG
GGCAAAGAGAAGAGTGGTGCAGAGAGAAAAAAGAGCAGCTATAGGAGCTGTGCTCATTGGGTTCTTGGGAGCA
GCAGGAAGCACTATGGGCGCAGCGTCAATAACGCTGACGGTACAAGCCAGGCAATTATTGTCTGGTATAGTGC
AACAGCAGAGCAATCTGCTGAGAGCTATTGAGGCGCAACAACACCTGTTGCAACTCACTGTCTGGGGCATC
>JN692473..1
TTCTACTGTAATACATCAAAGCTGTTTAATAGGAATTGGACTGATAATAGTAATAATACTGGAAATGATACTG
GGAATAATACTATCACACTCCCATGCAGAATAAAACAAATTATAAACAGATGGCAGGAAGTAGGAAAGGCAAT
GTATGCCCCTCCCATTCGAGGACTAATTAGATGTTCATCAAATATTACAGGGCTGATATTAACAAGAGATGGT
GGTAATCATAACAATAGTAACGGTAGTGAAATCTTCAGACCTGGGGGAGGAGATATGAGGGACAATTGGAGAA
GTGAATTATATAAATATAAAGTAATAAAAATTGAGCCATTAGGAATAGCACCCACCAAGGCAAAGAGAAGAGT
GGTGCAGAGAGAAAAAAGAGCAGTGGGAACGATAGGAGCTATGTTCCTTGGGTTCTTGGGAGCAGCAGGAAGC
ACTATGGGCGCAGCGGCAGTGACGCTGACGGTACAGGCCAGACAATTATTGTCTGGTATAGTGCAACAGCAGA
ACAATCTGCTGAGAGCTATTGAGGCGCAACAGCATATGTTGCAACTCACAGTCTGGGGCATC
>JN692474..2
TTTTACTGTAATACAACACAGCTGTTTAATAGCACTTGGCTGTTTAACAGCACTTGGAATCAAAGTGCTAATG
ATGCAGACAATATCACACTCCCATGCAGAATAAAACAAATAGTAAACAGATGGCAGGAAGTAGGAAAAGCAAT
GTATGCCCCTCCCATCAAAGGACAAATTAGATGTTCATCAAATATTACAGGGCTGCTCTTAACAAGAGATGGT
GATAATAACACCAACAACAACGAGACCTTTAGACCTGGAGGAGGAGATATGAGGGACAATTGGAGAAGTGAAT
TATATAAATATAAAGTAGTAAAAATTGAGCCATTAGGAATAGCACCCACCAAGGCAAAAAGAAGAGTGGTGCA
GAGAGAAAAAAGAGCAGTGGGAACAATAGGAGCTGTGTTCCTTGGGTTCTTGGGAGCAGCAGGAAGCACTATG
GGCGCAGCGTCAGTGACGCTGACGGTACAGGCCAGACTATTATTGTCTGGTATAGTGCAACAGCAGAACAATC
TGCTGAAGGCTATTGAAGCGCAACATCATCTGTTGCAGCTCACAGTCTGGGGCATC
>JN692480..1
TTCTACTGTAACACAGCACAGCTGTTTAATAATACTTGGCTGTCCAATAATACCTGGCTACAGAATAATACTT
GGATTAATGGCACTATCACACTCCCATGCAGAATAAGACAAATTGTAAACAGGTGGCAGGAAGTAGGGAGAGC
GATGTATGCTCCTCCCATCGCAGGAATAATCAACTGTGTATCAAACATTACAGGGCTGATATTAACAAGAGAT
GGAAACATAACAACAAATAGTACCTACTCTGAAAACAATACTGAAATCTTCAGACCTGGAGGAGGAGATATGA
GGGACAATTGGAGAAGTGAATTATATAAATATAAAGTGGTAACAATTGAGCCAGTGGGAATAGCACCCACCGA
GGCAAGGAGAAGAGTGGTGCAGAGAGAAAAAAGAGCAGTGGGAACGATAGGAGCTATGTTCCTTGGGTTCTTG
GGAGCAGCAGGAAGCACTATGGGCGCAGCGTCACTAACGCTGACGGTACAGGCCAGAATATTATTGTCTGGTA
TAGTGCAACAGCAAAACAATTTGCTGAGAGCTATTGAGGCGCAACAACATATGTTGCAACTCACAGTCTGGGG
CATC
>HQ377423..2
TTCTACTGTGATACAAAACCACTGTTCAATAGTACTTGGAATGATACTACAGGGTCAATTCACATTAATGGAA
CTATCACACTCCGATGCAGAATAAAACAAATTATAAACATGTGGCAGGGAGTAGGAAAAGCAATGTATGCCCC
TCCCATCAGAGGACTAATTAACTGTACATCAAATATTACAGGGCTGCTATTAACAAGAGATGGTGGTAAAGAG
AACAGGACCACCGAGACCTTTAGACCTCAAGGAGGAAATATGAAAGACAATTGGAGAAGTGAACTATATAAAT
ATAAAGTAGTAAAAATTGAACCATTAGGAATAGCACCCACCAAGGCAAAGCGAAGAGTGGTACAAAGAGAAAA

AAGAGCAATAGGGGTAATAGGAGCTATGTTCCTTGGGTTCTTGGGAACAGCAGGAAGCACTATGGGCGCAGCG
TCACTGACGCTGACGGTACAGGCCAGACAATTATTGTCTGGTATAGTGCAACAGCAGAACAATTTGCTGAGAG
CTATTGAGGCGCAACAACATCTGTTGCAACTCACAGTCTGGGGCATC
>KT427681..1
TTCTACTGTAATACATCAACGCTGTTTAATTGGACTGAAAATTCAAATAACAATGCAAGTGACAATATGACAA
TCCCATGCAGAATAAGACAAATTGTAAACAGGTGGCAGGAAGTAGGAAAAGCAATGTATGCCCCTCCCATCAG
CGGACAGATTAGCTGTTTATCAAATATCACAGGGCTGATATTAACAAGAGATGGTGGTATCAACAGCAGCAGC
AGCAATGAGACCTATGAGACCTTCAGACCTGCAGGAGGAAATATGAAAGATAATTGGAGAAGTGAATTATATA
AATATAAAGTAGTAAAAGTTGAGCCATTAGGAGTAGCACCCACCAAGGCAAAGAGAAGAGTGGTGCAGAGAGA
AAAAAGAGCAATAACACTAGGAGCCATGTTCCTTGGGTTCTTGGGAGCAGCAGGAAGCACTATGGGCGCAGCG
TCGATGACGCTGACGGTACAGGCCAGACTATTATTGTCTGGTATAGTGCAACAGCAGAACAACCTGCTGAGAG
CTATTGAGGCGCAACAACATCTGTTGCAACTCACAGTCTGGGGCATT
>KT427732..1
TTCTACTGTAAGACAACACAGCTGTTTAATAGCACTTGGTTAAATACTAGTATTGAGTATAATAATACTGAAG
GGGCTGGAGTACCAAATATCACACTCCAATGCAGAATAAAACAAATTATAAACAGATGGCAGGGAGTAGAAAG
AGCAATATATGCCCCTCCCATCCCGGGACAAATTAGATGTACATCAAATATTACAGGGCTGATATTGACACGG
GATGGTAATGGTAATCAAACAGATAGCACTAACGAAACCTTCAGACCAATAGGAGGAAATATGCAGGACAATT
GGAGAAGTGAATTATATAAATATAAAGTGGTAAAAATTGAGCCAGTAGGAATAGCACCCACCAAGGCAAAGAG
AAGAGTGGTGCAGAGAGAAAAAAGAGCAGTGGGAACACTAGGAGCTATGTTCCTTGGGTTCTTGGGAGCAGCA
GGAAGCACTATGGGCGCAGCGTCAGTGACGCTGACGGTACAGGCCAGACTATTATTGTCTGGTATAGTGCAAC
AGCAGAACAACCTGCTGAAGGCTATTGAGGCGCAACAGCATTTGTTGCAACTCACAGTCTGGGGCATC
>KT427713..1
TTCTATTGTAATACAACAAACCTGTTCAACAGTACTTGGAATGACACTACTGAAGGAAATAACACAGGAAATA
TAGGAAATATCACACTCCCGTGCAGAATAAAACAAATTATAAACATGTGGCAGCAAGTAGGAAAAGCAATGTA
TGCCCCTCCCATCGCAGGACAAATTAGCTGTTCATCAAATATTACAGGGTTGTTACTAACAAGAGATGGTGGT
AATAATGATTCTAATAATTATGAAATCTTCAGGCCTGGAGGAGGAGATATGAGGGACAATTGGAGAAGTGAAT
TATACAAATATAAAGTAGTAAAAATTGAACCATTAGGAGTAGCACCCACTAAGGCAAAGAGAAGAGTGGTGCA
GAGAGAAAAAAGAGCGGCACTAGGAGCTTTGTTCCTTGGGTTCTTGGGAGCAGCAGGGAGCACTATGGGCGCA
GCGGCAGTAACGCTGACGGTACAGGCCAGAACATTATTGTCTGGTATAGTGCAGCAGCAAAGGAATTTGCTGA
GAGCTATTGAGGCGCAACAACATCTGTTGCAACTCACAGTCTGGGGCATC
>KT427870..2
TTCTACTGTAATACATCAGGACTGTTTAATAGTACTTGGAATAGGGATGGTAATTGGTCTCAGGATGATACTA
ATGGGTCAAATGAAACAATCACACTCCCATGCAGAATAAAACAAGTTATAAACAGGTGGCAGGAAGTAGGAAA
AGCAATGTATGCCCCTCCCATCAGCGGAAGGATTTTCTGTTCATCAAACATTACAGGGCTACTCTTAGTAAGA
GATGGTGGTAACCAGAGCCACGAGAGTGAGACCTTCCGACCTGGAGGAGGAGATATGAGGGACAATTGGAGAA
GTGAATTATATAAATATAAAGTAGTAAAAATTGAGCCATTAGGAATAGCACCCACCAAGGCAAAGAGAAGAGT
GGTGCAGAGAGAAAAAAGAGCAGTGGGAACGCTAGGAGCTATGTTCCTTGGGTTCTTGGGAACAGCAGGAAGC
ACTATGGGCGCAGCGTCACTGACGCTGACGGTACAGGCCAGACAATTATTGTCTGGTATAGTGCAACAGCAGA
ACAATCTGCTGAAGGCTATTGAGGCGCAACATCATCTGTTGCGACTCACAGTCTGGGGCATA
>KT427707..1
TTCTACTGTAATACAACAGGGCTGTTTAATAGTAATAATACTTGGAATAATACTAATAATAATAGTAGGAATA
ATGAAACATATACCATACCATGCAGAATAAAGCAAATTATAAACAGGTGGCAAGAAGTAGGAAAAGCAATGTA
CGCCCCTCCCATCAAAGGAATAATTCACTGTACATCAAATATTACAGGGCTGCTATTAACAAGAGATGGTGGG
AATAGTTCAGAAAATGAAACTTTCAGACCTGCAGGAGGAGATATGAGGGACAATTGGAGAAGCGAATTATATA
AATATAAAGTAGTAAAAATTGAGCCAATAGGACTAGCACCTACCAAGGCACAGAGAAGAGTGGTGCAGAGAGA
AAAAAGAGCAGTGACACTGGGAGCTATGTTCCTTGGGTTCTTAGGAGCAGCAGGAAGCACTATGGGCGCAGCG
TCAATAACGCTGACGGTACAGGCCAGACAATTGCTGTCTGGTATAGTGCAACAGCAGAGTAATCTGCTGAGGG
CTATTGAGGCGCAACAGCATATGTTGCAACTCACAGTCTGGGGCATC
>KT427873..1
TTCTACTGTAATTCAACACAACTGTTTAATAGTACCTGGGATACTAATGCAACCTTGAATGCTACAGACAGCC
AAGATGAACTTAACAAAACTATCACACTCCCATGTAGAATAAAACAAATTATAAACATGTGGCAGGAAGTAGG
AAAAGCAATGTATGCCCCTCCCATTAGAGGGCTAATTAGATGTACAACAAATATTACAGGGCTACTATTAACA
AGAGATGGTGGCACAGGCAATAAGACTCAAAATAACACTGAGGTCTTCAGACCTGGAGGAGGAGATATGAAGG
ATAATTGGAGGAGTGAGTTATATAAATATAAAGTAGTAAAAATTGAACCATTAGGAGTAGCACCTACCAAGGC
CAAGAGAAGAGTGGTGCAGAGAGAAAAAAGAGCAGTGGGAACAATAGGAGCTATGTTCCTTGGGTTCTTGGGA
GCAGCAGGAAGCACTATGGGCGCAGCGTCAATAACGCTGACGGCACAGGCCAGACAATTATTGTCTGGTATAG
TGCAGCAGCAGAATAATCTGCTGAGGGCTATTGAGGCGCAACAACATCTGTTGCAACTCACAGTCTGGGGCAT
C
>KT427695..1

TTCTACTGCAATACATCGGTGCTGTTTAATGATACTTGGCTGAATAATAATAAGACTGAACACCAAAATATCA
CGCTCCCATGCAAAATAAAACAGATTATAAACATGTGGCAGGAAGTAGGAAAAGCAATGTATGCCCCTCCCAT
CAGTGGACCAATTAGGTGTTCATCAAATATCACAGGGCTGCTATTAACAAGAGATGGTGGTAATAGCACAAAT
AATAACGAGACCTTCAGACCTGGAGGAGGAGATATGAGGGACAATTGGAGAAGTGAATTATATAAATATAAAG
TAGTAAAACTTGAGCCATTAGGATTAGCACCCACCAAGGCAAAAAGGAGAGTGGTGCAGAGAGAAAAAAGAGC
AGTGGGAACGCTAGGAGCTATGTTCCTTGGGTTCTTGGGAACAGCAGGAAGCACTATGGGCGCAGCATCACTA
ACGCTGACGGTACAGGCCAGACTATTATTGTCTGGCATAGTGCAACAGCAGAGCAATTTGCTGAGGGCTATTG
AGGCGCATCAGCATATGTTGCAACTCACAGTCTGGGGCATC
>KT427785..1
TTTTACTGTAATACAACACAACTATTTAATAGTACTTGGGTTAATGATACTTTTAGTACTTGGGTTAATAATA
CTTGGGTTAATGGTACTGTAAAGTCAATTAGTAATAAAACAAATATTAATATTACACTTCCATGCAGAATAAA
GCAAATTATAAACATGTGGCAGGAAGTAGGAAAAGCAATGTATGCCCCTCCCATCAAGGGACCAATTAGCTGT
TCATCAAATATTACAGGACTGCTATTAACAAGAGATGGGGGAAATAGTGATAATAGTAGCAAAAATGAGACAA
ATGAGACCGAGACTTTTAGACCTGGAGGAGGAAATATGAAAGACAATTGGAGAAGTGAATTATATAAATATAA
AGTAGTGCAAATTGAACCATTAGGAATAGCACCCACCAGGGCAAAAAGAAGAGTGGTGCAAAGAGAGAAAAGA
GCAGTGGGAATAGGAGCATTGTTCATTGGGTTCTTGGGAGCAGCAGGAAGCACTATGGGCGCAGCGTCAATGA
CGCTGACGGTACAGGCCAGACAATTATTGTCTGGTATAGTGCAACAGCAGAACAACCTGCTGAGGGCTATTCA
GGCGCAACAGCATTTGTTGCAGCTCACAGTCTGGGGCATC
>KJ849814..2
TTCTACTGCAATACAACACAGCTATTTAATTGGACAATTGGCAAGTCAAATATAACCCAAAATGATAGTGAAA
TTATTACAATCCAATGCAGAATAAAACAGATTATAAATAAGTGGCAGGAAGTAGGAAAATCAATATATGCCCC
TCCTATCAGAGGAAATATTAGCTGTTCATCAAATATTACAGGACTGATATTAACAAGAGATGGTGGTAATCAC
AATGAAACTAAAAACAAGACTAAGACTGAGACTTTCAGACCTGGAGGAGGAGATATGAGGGACAATTGGAGAA
GTGAATTATATAAATATAAGGTAGTGAAAATTCAGCCATTAGGAATAGCACCCACCAGGGCAAGGCGAAGAGT
GGTGCAGAGAGAAAAAAGAGCAGTGGGAACACTAGGAGCTATGTTCCTTGGGTTCTTGGGAGCAGCAGGAAGC
ACTATGGGCGCAGCGTCAATAACGCTGACGGTACAGGCCAGACAATTGTTGTCTGGTATAGTGCAGCAGCAGA
ACAATCTGCTGAAAGCTATTGAGGCGCAACAGCATCTGTTGCAACTCACAGTCTGGGGCATC
>KT427771..1
TTCTACTGTAATACAACACAACTGTTTAATAGTACTTGGCAAAGTAATAGTACTGGAAATAGTACTGAGAATA
ATAGCACAATAATCACACTCCCCTGTAGGATAAAACAAGTTATAAACATGTGGCAGGAGGTAGGAAAAGCAAT
GTATGCCCCTCCCATCAGTGGACTAATTCAATGTACATCAAATATTACAGGGCTGCTGTTAACAAGAGATGGT
GGGTCTGATGGTGAATCTAATAAAACAGAGAGTGAGATCTTCAGACCTGGAGGAGGAGATATGAGGGACAATT
GGAGAAGTGAATTATATAAATATAAAATAGTAAAAATTGAACCATTAGGAGTAGCGCCCACCAAGGCAAAGAG
AAGAGTGGTGCAGAGAGAAAAGAGAGCACTAGGGGGACTAGGAGCTGTATTCCTTGGGTTCTTGGGAGCAGCA
GGAAGCACTATGGGCGCAGCGTCAATAACGCTGACGGTACAGGCCAGACAATTATTGTCTGGTATAGTGCAGC
AGCAGAACAATCTGCTGAGGGCTATTGATGCGCAACGGCACATGTTGCAACTCACAGTCTGGGGCATC
>KT427753..1
TTCTACTGTAATACAACAAAACTGTTCAATAGTACTTGGAAGTGGAATAATGGTACTTGGAATAGGAATGATA
CTGAAGGACAAGATAACACTGGAGAAACTATTACACTTCCATGCAGAATAAAACAAATCATAAACATGTGGCA
GGAAGTAGGAAAAGCAATGTATGCCCCTCCCATCAGAGGACAAATTAATTGCACATCAAATATTACAGGACTG
CTATTAACAAGGGATGGTGGTAATAATAATAATAGCAAGAATAACGAGACTTTCAGACCTGGAGGAGGAAATA
TGAAGGACAATTGGAGAAGTGAATTATATAAATATAAAGTAGTACAAATTGAACCATTAGGAATAGCACCCAC
CAAGGCAAAAAGGAGAACGGTGCAGAGAGAAAAAAGAGCAGTGGGAATAGGAGCTGTGCTCTTTGGGTTCTTG
GGAGCAGCAGGAAGCACTATGGGCGCAGCGTCAGTAACGCTGACGGTACAGGCCAGACAATTGTTGTCTGGTA
TAGTGCAACAGCAGAGCAATCTGCTGAAGGCTATTCAGGCGCAACAGCATCTGTTGCAGCTCACAGTCTGGGG
CATC
>KT427749..3
TTCTACTGTAATACAACAGAACTGTTCTATAGTAATTGGACGTGGAATGGTACTTGGAATAGGAATGATACTG
AAGGATTACAAAATAAAACTATTATACTTCCATGCAGAATAAAACAAATCATAAACATGTGGCAGGAAGTAGG
AAAAGCAATGTATGCCCCTCCCATCAGAGGACAAATTAATTGCACATCAAATATTACAGGACTGCTATTAATA
AGAGATGGTGGTAATGATACTGGAACTAGTGGGACTGAAATTTTCAGACCTCAAGGAGGAAACATGAAAGACA
ATTGGAGAAGTGAATTATATAAATATAAAGTAGTACAAATTGAACCATTAGGAATAGCACCCACCAAGGCAAA
GAGAAGAACGGTGCAGAGAGAAAAAAGAGCAGTGGGATTTGGAGCTGTGATCTTTGGGTTCTTGGGAGCAGCA
GGAAGCACTATGGGCGCAGCGTCAATAACGCTGACGGTACAGGCCAGACAATTGTTGTCTGGTATAGTGCAAC
AGCAGAGCAATTTGCTGAAGGCTATTCAAGCGCAACAGCATCTGTTGCAGCTCACAGTCTGGGGCATC
>KT427826..1
TTTTACTGTAATACAACAAAACTGTTTAATACTACGTGGAATATTACTGGAGAAGGAAACAACAATCTCACTA
ATGAAACTATCACACTCCCATGCAGAATAAAACAGTTCATAAACATGTGGCAGGAAGTAGGAAAAGCAATGTA
TGCCCCTCCTATCAGGGGACAAATTCACTGCTCATCAAATATTACAGGGCTACTATTAACAAGAGATGGTGGT
AATAGTAATAATGAGTCGGAGACCTTCAGACCGGGAGGAGGAGATATGAGGGACAATTGGAGGAGTGAATTAT

ATAAATATAAAGTAGTAAAAATTGAACCATTAGGAATAGCACCCACCAAGGCAAGAAGGAGAGTGGTGCAAAG
AGAAAAAAGAGCAATAGGAGGAATAGGAGTTGTGATCCTTGGGTTCTTGGGAGCAGCAGGAAGCACTATGGGC
GCAGCGTCGCTAACGCTGACGGTACAGGCCAGACAATTATTGTCTGGTATAGTGCAACAGCAAAGCAATTTGC
TGAGAGCTATTGAGGCGCAACAGCGTTTGTTGCAACTCACTGTCTGGGGCATC
>KJ849821..1
TTCTACTGTGATACAGCAAAGCTGTTTAACAGTACTTGGGAGAGGCGTAATGACACGAACACTTGGGAGTCGA
ACAATACAATACCAAATGAGAATATCACACTCCCATGCAGAATAAAACAAATTATAAACAGGTGGCAGGAAGT
AGGAAAAGCAATGTATGCCCCTCCCATTGCAGGACGAATTGAATGTACATCAAATATTACAGGGCTACTATTA
ACAAGAGATGGTGGTTCTAACAATAGTAGTAACGATACCTTCAGACCAGGAGGAGGAGATATGAAGGACAATT
GGAGAAGTGAATTATATAAATATAAAGTAGTACAAATTCAGCCATTAGGAGTAGCACCCACCAGGTCAAGGAG
AAGAGTGGTGCAGAGAGAAAAAAGAGCAGTAACACTGGGAGCTATGTTCCTTGGGTTCTTGGGAGCAGCAGGA
AGCACTATGGGCGCAGCGTCAGTGACGCTGACGGTACAGGCCAGACAATTATTGTCTGGTATAGTGCAGCAGC
AAAGCAATTTGCTGAGGGCTATTGATGCGCAACAGCACATGTTGCAACTCACAGTCTGGGGCATC
>KT427811..1
TTTTACTGTAATACATCAAGACTGTTTAACAGTATTTGGAACAGTACCTGGCAGGATAATACAGCTGAAGAGT
CAAATAATACTGACATCATACTCCATTGCAGAATAAAACAAATTATAAACAGATGGCAGGAAGTAGGAAAAGC
AATGTATGCCCCTCCCATCAAGGGAAAAATTCAATGTTCATCAAATATTACAGGGCTGCTATTAGTAAGAGAT
GGTGGTAACGAGAACACTAGTAACACCGAGACCTTCAGACCTGGAGGAGGAGACATGAGGGACAATTGGAGAA
GTGAATTATATAAATATAAAGTAGTGCAAATTGAGCCAGTAGGAATAGCACCCACCAAGGCAAGAAGAAGAGT
GGTGCAGAGAGAAAAAAGAGCAGTAACAATAGGAGCTATGTTCCTTGGGTTCTTGGGAGCAGCAGGAAGCACT
ATGGGCGCAGCGTCAATCGCGCTGACGGGACAGGCCAGACAATTATTGTCTGGTATAGTGCAACAGCAGAATA
ATCTGCTGAAGGCTATTGAGGCGCAACAACATATGTTGCAACTCACAGTCTGGGGCATC
>KT427796..1
TTCTATTGTAATACATCACAGCTGTTCAATAGTACTTGGAATTTCACTAGTGCTAGGAATGAGACTGAAGAGT
CAAATGACACTATCACACTCCCATGTAGGATAAAACAAATTATAAATAAGTGGCAAGAAGTAGGAAAAGCAAT
ATATGCTCCTCCTATCCAAGGACGAATTTACTGTTCATCAAACATTACAGGGTTGCTATTAACAAGAGATGGG
GGTGAAAACAACCTGACCAATGAGACTTTTAGACCTGGAGGAGGAGATATGAGGGACAATTGGAGAAGTGAAC
TGTATAAATATAAAGTAGTAAAAATTGAGCCATTAGGACTAGCACCCACCAAGGCAAAGAGAAGAGTGGTGCA
GAGAGAAAAAAGAGCAGTGGGAACGCTGGGAGCAATGTTCCTTGGGTTCTTGGGAACAGCAGGAAGCACTATG
GGCGCAGCGTCGCTGACGCTGACGGTACAGGCCAGACAATTATTGTCTGGTATAGTGCAACAGCAAAACAATT
TGCTGAAGGCTATTGAGGCGCAACAGCATCTGTTGCAACTCACAGTCTGGGGCATT
>KX181909..1
TTCTACTGTAATACAGCAGAGCTGTTTAGTAATACTTGGTTAAATAAGAGTGGCAATAACGATGGAACTATCA
CACTTCCATGCAGAATAAAACAAATTATAAACAGATGGCAGGAAGTAGGAAAAGCAATGTATGCCCCTCCCAT
CAGGGGATTAATTGAATGTTCATCAAATATTACAGGGCTGATACTAACAAGAGATGGTGGTAATAATAGTGCA
AACAATGCTAGTGAAACCTTCAGACCCGGAGGGGGAAATATGAAGGACAATTGGGGAAGTGAATTATATAAAT
ATAAAGTAGTAAAAATTCAGCCCTTAGGAATAGCACCCACCAGGGCAAAAAGAAGAGTGGTGCAAAGAGAAAA
AAAAGCAGTAACGCTAGGAGCTATGTTCCTTGGGTTCTTGGGAACAGCAGGAAGCACTATGGGCGCAGCGTCA
GTGACGCTGACGGTACAGGCCAGACAGTTACTGTCTGGTATAGTGCAACAGCAGAGCAATCTGCTGAAGGCTA
TTGAGGCGCAACAGCATCTGTTGCAACTCACAGTCTGGGGCATC
>KX181908..2
TTTTACTGTGATACAACAAAGCTGTTTAATAGTACTTGGAATAATACTGGAGAGTCAAATTACACGGAGGGAA
ATGCCACTATCATACTTCCATGCAGAATAAAACAAATTATAAACAGATGGCAGGAAGTAGGAAAAGCAATGTA
TGCCCCTCCCCTCAGTGGAAATATTAGCTGTTTATCAAATATTACAGGGGTACTATTAACAAGAGATGGGGGT
AATGAAACAAATGCCCCTGAGACCTTCAGACCTCAAGGGGGAAATATAAAGGATAATTGGGGAAGTGAATTAT
ATAAATATAAAGTAGTAAAAATTGAACCCTTAGGAGTAGCCCCCCCCAAGGCAAAGAGAAGAGTGGGGCAAAG
AGAAAAAAGAGCAGTGGGAATAGGAGCTATGTTCCTTGGGTTCTTGGGAGCAGCAGGAAGCAATATGGGCGCA
GCATCACTAACGCTGACGGTACAGGCCAGACTATTATTGTCTGGTATAGTGCAACAGCAAAACAATTTGCTGA
GAGCTATTGAGGCGCAACAGCATCTGTTGCAGCTCACAGTCTGGGGCATC
>KX181898..2
TTCTACTGTAATACATCACAGCTGTTTAATTGGACTGAAAGAAATAGAAATAGCTCAGATGGGACAGGGTCAA
ATGAGACTGAGACCATTACACTCCCATGCAGAATAAAACAAATTATAAACAGATGGCAGGAAGTAGGAAAAGC
AATGTATGCCCCTCCCATCAAAGGAAATATTAGCTGTTCATCAAATATTACTGGGCTGCTATTAACAAGAGAT
GGTGGTACAAACCTGACTGGGTCTAACGGGACCGAGACCTTCAGACCTCAAGGAGGAAATATGAAGGATAATT
GGAGAAGTGAATTATATAAATATAAAGTAGTAAGAATTGAGCCAGTAGGAATAGCACCCACCGAGGCAAAGAG
AAGAGTGGTGCAAAGAGAAAAAAGAGCAGTGGGAACACTAGGAGCTATGTTCCTTGGGTTCTTGGGAGCAGCA
GGAAGCACTATGGGCGCAGCGTCAATGACGCTGACGGTACAGGCCAGACAATTATTGTCTGGTATAGTGCAAC
AGCAAAACAATCTGCTGAAAGCTATTGAGGCGCAACATCATCTGTTGCAACTCACAGTCTGGGGCATC
>MK041573..1

TTCTACTGTAATACAACAAAACTATTTAATAGTACTTGGGATGGGTCAAACATCATAGGAAATGATACAAATG
ACACAGTGATCACACTCCCATGCAGAATAAAACAAATTATAAACATGTGGCAGCGAGTAGGAAAAGCAATGTA
TGCCCCTCCCATCAGAGGACAAATTAGATGTTCATCAAATATTACAGGGCTGCTGTTAACAAGAGATGGTGGA
AATCATACAAGTGGGAATGAGACCTTCAGACCAGGAGGAGGAGATATGAGGGACAATTGGAGAAGTGAATTAT
ATAAATATAAAGTAGTAAAAATTGAACCATTGGGAGTAGCACCCACCAGGGCAAAGAGAAGGGTGGTGCAGAG
AGAAAAAAGAGCAGTAGGACTAGGAGCTTTGTTCCTTGGGTTCTTGGGAGCAGCAGGAAGCACTATGGGCGCA
GCGTCACTAACGCTGACGGTACAGGCCAGACAGTTATTGTCTGGTATAGTGCAACAGCAGAACAACTTGCTGA
GGGCTATTGAGGCGCAACAGCACATGTTGCAACTCACAGTCTGGGGCATC
>MK041575..1
TTCTACTGTAATACAACACAGCTGTTTAATAATACTTGGAATGATACTGCTTGGAATGATACTAGCTGGTCAA
ATGGCACTATCACACTCCCATGCAGAATAAAACAAATTATAAACAGGTGGCAGGAAGTAGGAAAAGCAATGTA
TGCCCCTCCCATCAGCGGACAAATTAATTGTTCATCAAATATTACAGGACTGTTATTAACAAGAGATGGTGGT
AATGACAATGACACGAATACGAATGAGACCGAGATCTTCAGACCTGCAGGAGGAGATATGAGGGACAATTGGA
GAAGTGAATTATATAAATATAAAGTAGTAAAAATTGAACCATTAGGAATAGCACCCACCAGGGCAAAGAGAAG
AGTGGTGCAGAGAGAAAAAAGAGCAGTGACATTAGGAGCTGTGTTCCTTGGGTTCTTGGGAGCAGCAGGAAGC
ACTATGGGCGCAGCGTCAGTGACGCTGACGGTACAGGCCAGACTATTATTGTCTGGTATAGTGCAACAGCAGA
GCAATTTGCTGAGAGCCATTCAGGCGCAACAGCATCTGCTGCAACTCACAGTCTGGGGCATC
>MG571983..1
TTCTACGGTAATACATCAAACCTGTTTAATAATAATATTACAGGGATTATAAAGAATGGGACTATCATACTCC
CATGCAGAATAAAACAAATTATAAACAGGTGGCAGGAAGTAGGAAGAGCAATGTATGCCCCTCCCATCAGAGG
ACTAATCAACTGTACATCAAATATTACAGGGCTGCTATTAACAAGAGATGGTGGTGGTGGTAATGAGAGTCAT
AACGAGACCTTCAGACCTGGAGGAGGAGATATGAGGGACAATTGGAGAAGTGAATTATATAAATATAAAGTAG
TAAGAATTGAGCCATTAGGAATAGCACCCACCAAGGCACAGAGAAGAGTGGTGCAAAGAGAAAAAAGAGCAGT
GGGAGCACTAGGAGCTATGTTCCTTGGGTTCTTGGGAGCAGCAGGAAGCACTATGGGCGCAGCGTCAATAACG
CTGACGGCACAGGCCAGACAAGTATTGTCTGGTATAGTGCAACAGCAAAACAATTTGCTGAGGGCTATTGAGG
CGCAACAGCATTTGTTGCAACTCACAGTCTGGGGCATC
>MG571987..3
TTCTACTGTAATACAACATCACTGTTTAATAGTACTTGGCTGCTTAATGATACTGGAAATGTTACTGTGCCAA
ACAAAACGGACGAAATTATCATACTCCCATGCAGAATAAAACAAATTATAAATATGTGGCAAAAAGTAGGAAG
AGCAATGTATGCCCCTCCCATCAGAGGTTATATTAACTGTTCATCAAATATTACAGGGCTGCTATTAGTAAGG
GATGGTGGTAATAATAATCAGTCTAATCAGGCTGAAACCTTCAGACCTGGAGGAGGAGATATGAGGGACAATT
GGAGAAGTGAATTATATAAATATAAAGTAGTAAAAATTGAGCCATTAGGAATAGCACCCACCAAGGCAAAGAG
AAGAGTGGTGCAGAGAGAAAAAAGAGCAGTGGGGACACTAGGAGCTGTGTTCCTTGGGTTCTTGGGAGCAGCA
GGGAGCACTATGGGCGCAGCGTCACTAACGCTGACGGTACAGGCTAGACTATTATTGTCTGGTATAGTGCAAC
AGCAGAACAATTTGCTGAGAGCTATTGAGGCGCAACAGCATATATTGCAACTCACAGTCTGGGGCATC
>MG571990..1
TTCTACTGTAATACAACAAAACTATTTAATAGTACTTGGGATGGGTCAAACATCATAGGAAATGATACAAATG
ACACAGTGATCACACTCCCATGCAGAATAAAACAAATTATAAACATGTGGCAGCGAGTAGGAAAAGCAATGTA
TGCCCCTCCCATCAGAGGACAAATTAGATGTTCATCAAATATTACAGGGCTGCTGTTAACAAGAGATGGTGGA
AATCATACAAGTGGGAATGAGACCTTCAGACCAGGAGGAGGAGATATGAGGGACAATTGGAGAAGTGAATTAT
ATAAATATAAAGTAGTAAAAATTGAACCATTGGGAGTAGCACCCACCAGGGCAAAGAGAAGGGTGGTGCAGAG
AGAAAAAAGAGCAGTAGGACTAGGAGCTTTGTTCCTTGGGTTCTTGGGAGCAGCAGGAAGCACTATGGGCGCA
GCGTCACTAACGCTGACGGTACAGGCCAGACAGTTATTGTCTGGTATAGTGCAACAGCAGAACAACTTGCTGA
GGGCTATTGAGGCGCAACAGCACATGTTGCAACTCACAGTCTGGGGCATC
>MG572002..1
TTCTATTGTAATTCATCACAGCTGTTTAATACAACTTGGATAAATGGCACTACAGGAAATAGAACTAATGAAA
ATGACACACTTATCACACTCCCATGCAGAATAAAACAAATTATAAACAGATGGCAGGAAGTAGGAACAGCAAT
ATATGCCCCTCCCATTGAAGGCATGATTAAATGTTCATCAAATATTACGGGGGTGCTATTAACAAGAGATGGT
GGTTATGATAACAATAGCACGCGCCAGAACGAGACCTTCAGACCTGGAGGGGGAAATATGAAGGACAATTGGA
GAAGTGAATTATATAAATATAAAGTAGTAAAAGTTGAGCCATTAGGACTAGCACCCACCAAGGCAAAGAGAAG
AGTGGTGCAAAGAGAAAAAAGAGCGGTAACATTAGGAGCTATGTTCCTTGGGTTCTTGGGAGCAGCAGGAAGC
ACTATGGGCGCAGCGTCAATGACGCTGACGGTACAGGCCAGACAATTGTTGTCTGGTATAGTGCAACAGCAGA
ACAATCTGCTGAGAGCTATTGAGGCGCAACAGCATATGTTGCAACTCACAGTCTGGGGCATA
>JF320427..1
TTCTACTGCAATACAACAGCACTGTTTAATAGTACATGGAACGGTACAGAGGGGATAAGTAATGCTACAGGAA
ATATCACACTCCCATGCAGAATAAAACAAGTTATAAACAGATGGCAGGAAGTAGGAAAAGCAATGTATGCCCC
TCCCATTAAAGGAGTAATTAGATGTGTATCAAATATCACAGGGATACTATTAACGAGAGATGGGGGTAATAAT
AATAATACTAATCACACCGAGATATTCAGACCTGGAGGAGGAGATATGAGAGACAATTGGAGAGGTGAATTAT
ATAAATATAAGGTAGTAAAAATTGAACCATTAGGAGTAGCACCCACCAAGGCAAAGAGAAGAGTGGTGCAGAG
AGAAAAAAGAGCAGTGGGAATAGGAGCTTTGTTCCTTGGGTTCTTGGGAGCAGCAGGAAGCACGATGGGCGCA

GCGTCAGTGACGCTGACGGTACAGGCCAGACAATTATTGTCTGGTATAGTGCAACAGCAGAGCAATTTGCTGA
GGGCTATTGAGGCGCAACAACATATGTTGCAACTCACAGTCTGGGGCATC
>GU562236..3
TTCTACTGCAATACAACGCAGCTGTTTAATAGTACTTGGTATAGTAATGGTACTTGGTCTTGGAATGGTACTA
CTTGGAATGGTACTAGAGAGACAAATAGTACGGAAGGAAACGACACAATGATCACACTCCCATGCAGAATAAA
ACAATTTATAAACATGTGGCAGGCAGTAGGAAAAGCAATATATGCCCCTCCCATCAGTGGACTAATCAAATGT
ACATCAAATATTACAGGGCTGTTAATAACAAGAGATGGGGGTCATGAAAGTAATAAAAGTAACTATGGTTATG
AAAGTAGTAAAAGTGAGAATGAGACCTTCAGACCTGGAGGAGGAAATATGAGGGATAATTGGAGAAGTGAATT
GTATAAATATAAAATAGTAAGAATTGAACCATTAGGACTAGCACCCACCAAGGCAAAGAGAAGAGTGGTGCAG
AGAGAAAAGAGAGCCGCACTAGGAGCCATGTTCCTTGGGTTCTTGGGAGCAGCAGGAAGCACTATGGGCGCAG
CGTCACTGACGCTGACGGTACAGGCCAGACAATTATTGTCTGGTATAGTGCAACAGCAGAGCAATTTGCTGAG
GGCTATTGAGGCGCAACAGCATATGTTGCAACTCACGGTCTGGGGCATC
>JQ403024..1
TTCTACTGTAATACAACACAACTGTTTAATAGAGATTGGAATGATACTAGTACTATTAACAGTACTGAAAATA
GAAATATCACACTCCCATGTAGAATAAAACAAATTATAAACATGTGGCAGGAAGTAGGAAAAGCAATGTATGC
CCCTCCCATCCAAGGACAAATTAGATGTTCATCAAATATTACAGGGTTGCTATTAACAAGAGATGGTGGTAAT
GACAATACCACTAACACCACTGAGATCTTCAGACCTGTAGGAGGAAATATGAGGGACAATTGGAGAAGTGAAT
TATACAAATATAAAGTAGTACAAATTGAACCATTAGGATTAGCACCCACCAAGGCAAAGAGAAGAGTGGTGCA
GAGAGAAAAAAGAGCAGTAACGTTAGGAGCTATGTTCCTTGGGTTCTTGGGAGCAGCAGGAAGCACTATGGGC
GCAGCGTCAATGACGCTGACGGTACAGGCCAGACAATTATTGTCTGGTATAGTGCAGCAGCAAAATAATTTGC
TGAAGGCTATTGAGGCGCAACATCATCTGTTGCAACTCACAGTCTGGGGCATC
>JQ403041..2
TTCTACTGTAATACAACACGACTGTTTAATAGTACTTGGAATAGTACCGAATCAAATAATACTATAGGAAATG
GCACAATCACACTCCCATGCAGAATAAAACAAATTGTAAACATGTGGCAGGAAGTAGGAAAAGCAATGTATGC
CCCTCCCATCAGAGGACAAATTAGATGTTCATCAAATATTACAGGGCTGATATTAACAAGAGATGGTGGTCAT
GGTGGTCATAATAAGAATACCACCAACACCACAGAGATCTTCAGACCTGGAGGAGGAGATATGAGGGACAATT
GGAGAAGTGAATTATATAAATATAAAGTAGTAAAACTTGAACCATTAGGAATAGCCCCCACCTCGGCAAGGAG
AAGAGTGGTGCAGAGAGAAAAAAGAGCAGTGGGAATAGGAGCTATGTTCCTTGGGTTCTTGGGAGCAGCAGGA
AGCACTATGGGCGCAGCGTCAGTGACGCTGACGGTACAAGCCAGACTATTATTGTCTGGTATAGTGCAACAGC
AGAACAATTTGCTGAAGGCTATTGAGGCGCAACAGCATATGTTGCAACTCACAGTCTGGGGCATC
>KC797225..3
TTCTACTGTAATACATCACAATTGTTTAATAGTACTTGGAATGCTACTGGTACTCAGAATACTACTGGTACTC
AGAATGAGACTAAAATAGGCAATATCACACTCCCATGTAAAATAAAACAAATTATAAACAGGTGGCAAGAAGT
AGGAAAAGCAATGTATGCCCCTCCCATCAAAGGACTAATTAACTGTGTATCAAATATTACAGGGATACTATTA
ACAAGAGATGGTGGTCATTCTAAGAATACAAATAGTACAGATAATGATACCGAGGTCTTCAGACCTGGAGGAG
GAGATATGAGGGACAATTGGAGAAGTGAATTATATAAATATAAAGTGGTAAAAATTGAGCCATTAGGAGTAGC
ACCCACCAAGGCAAAGAGAAGAGTGGTGCAGAGAGAAAAAAGAGCAGTGACGCTAGGAGCCATGTTCCTTGGG
TTCTTGGGAGCAGCAGGAAGCACTATGGGCGCAGCGTCGCTGACGCTGACGGTACAGGCCAGACAATTATTGT
CTGGTATAGTGCAACAGCAGAACAACCTGCTGAGGGCTATTGAGGCGCAACAGCATATGTTGCAACTCACAGT
CTGGGGCATC
>HQ699978..2
TTCTACTGTAATACATCACAACTGTTTAATAGTACTTATAATGGTACTTGGAGTGGTACTACAGGAAATGATA
CTATCACGCTCCCTTGCAGAATAAAACAAATTGTAAACAGGTGGCAGGAAGTAGGGAAAGCAATGTATGCCCC
TCCCATTCAAGGAATAATTAGATGTTCATCAAATATTACAGGGTTGCTATTAACAAGAGATGGTGGTAAGAAT
GAGAGCGATAGCAATACCACCGAGACCTTCAGACCTGGAGGAGGAAATATGAGAGACAATTGGAGGAGTGAAT
TATATAAATATAAAGTAGTAAAAATTGAACCATTAGGAGTAGCACCCACCAAGGCAAAAAGAAGAGTGGTGCA
GAGAGAAAAAAGAGCAGTGGGAACGATAGGAGCTATGTTCCTTGGGTTCTTGGGAGCAGCAGGAAGCACTATG
GGCGCAGCGTCAATAACGCTGACGGTACAGGCCAGACAATTGTTGTCTGGTATAGTGCAACAGCAGAGAAATT
TGCTGAGGGCTATTGAGGCGCAACAGCATCTGTTGCAACTCACAGTCTGGGGCATC
>EU131792..1
TTCTACTGTAATACATCAGAGCTGTTTAAAAGTACTTGGAATAATACTAGTACTGGGAATGATCCTATCACGC
TCCCTTGCAGAATAAAGCAAATTGTAAACATGTGGCAAGAAGTAGGGAAAGCAATGTATGCTCCTCCCATTGA
AGGACAAATTAGATGTTTATCAAATATTACAGGATTGCTATTAACAAGGGATGGTGGTCATAATGGGAGCGAG
ACCAACGAGACCTTCAGACCTGCAGGAGGAAATATGAGAGACAATTGGAGAAGTGAATTATATAAATATAAAG
TAGTAAAAATTGAACCATTAGGAGTAGCACCCACCAAGGCAAAGAGAAGAGTGGTGCAGAGAGAAAAAAGAGC
AGTGGGAACAATAGGAGCTATGTTCCTTGGGTTCTTGGGAGCAGCAGGAAGCACTATGGGCGCAGCGTCAATG
ACGCTGACGGTACAGGCCAGACAAATGTTGTCTGGTACAGTGCAACAGCAAAGAAATTTGCTGAGGGCTATTG
AGGCGCAACAGCATCTGTTGCAACTCACAGTCTGGGGCATC
>EU131803..1

TTCTACTGTAATACATCAAAATTATTTAATAGTACTTGGTATAATAATGGTACTTGGAATAATGCTAGTACTG
GGAATGATACTATCACGCTCCCTTGCAGAATAAAACAAATTGTAAACATGTGGCAGGAAGTAGGGAAAGCAAT
GTATGCCCCTCCCATTGCAGGACAAATTAGATGTTCATCAAAGATTACAGGGTTGCTATTAACAAGAGATGGT
GGTAATGAGAACAAGACTAAGGACAACAACACCGAGACTGAGACCTTTAGACCTGGGGGAGGAGATATGAGAG
ACTATTGGAGAAGTGAATTATATAAATATAAAGTAGTAAAAATTGAACCATTAGGAGTAGCACCCACCAAGGC
AAAGAGAAGAGTGGTGCAGAGAGAAAAAAGAGCAGTGGGAACAATAGGGGCTATGTTCCTTGGGTTCTTGGGA
GCAGCAGGAAGCACTATGGGCGCAGCGTCAATAACGCTGACGGTACAGGCCAGACAATTGTTGTCTGGTATAG
TGCAACAGCAGAGAAATTTGCTGATGGCTATTGAGGCGCAACAACATTTGTTGCAACTCACAGTCTGGGGCAT
C
>EU363829..1
TTCTACTGTAATACAACAAAACTGTTTAATAGTATTTGGTATAATAATGGTACTTCGAGTGGTACTACAGGAA
ATGAAACTATCATACTCCCTTGCAGAATAAAACAAATTGTAAACATGTGGCAGAAAGTAGGGAGAGCAATGTA
TGCCCCTCCCATTGAAGGAAAAATTAGCTGTGTATCAAATATCACAGGGTTGCTATTAACAAGAGATGGTGGT
CAGAATGAGAACAATACCGAAACCTTCAGACCTGAAGGAGGAAATATGAAAGACAATTGGAGAAGTGAATTAT
ATAAATATAAAGTAGTAAAAATTGAACCATTAGGAGTAGCACCCACCAAGGCAAAGAGAAGAGTGGTGCAAAG
AGAAAAAAGAGCAGTGGGAGCAATAGGAGCTATGTTCCTTGGGTTCTTAGGAGCAGCAGGAAGCACTATGGGC
GCAGCGTCAATAACGCTGACGGTACAGGCCAGACAATTGTTGTCTGGCATTGTGCAACAGCAGAGAAATTTGC
TAAGGGCTATTGAGGCGCAACAGCATCTGTTGCAACTCACAGTCTGGGGCATT
>HM215413..1
TTCTACTGTAATACAGCACAACTGTTTAATAGTACTTGGAATACTACTAAGATTTGGGGGAATACTACAGAAG
GTAACAATAGCACTATCATGCTCCCTTGCAAAATAAAGCAAATTATAAACCTGTGGCAGGAAGTAGGAAAAGC
AATATATGCCCCTCCCATCGCAGGGCGAATTAGATGTGTATCAAATATTACAGGGTTGCTATTAACAAGAGAT
GGTGGTCATAATGGGAACGGGTCCAACGAGACCGAGACCTTCAGACCTGCAGGAGGAGATATGAGGGACAATT
GGAGAAGTGAATTATATAAATATAAAGTAGTAAAAATTGAACCAATAGGATTAGCACCCACCAAGGCAAAGAG
AAGAGTGGTGCAGAGAGAAAAAAGAGCAATGGGAATAGGAGCTATGTTCCTTGGGTTCTTGGGAGCAGCAGGA
AGCACTATGGGCGCAGCGTCAATAACGCTGACGGTGCAGGCCAGACGATTATTGTCTGGTATAGTGCAACAGC
AAAGAAATTTGCTGAGGGCTATTGAGGCGCAACAACATCTGTTGCAACTCACCGTCTGGGGCATC
>EU131802..1
TTCTACTGTAATACATCACAACTGTTTAATAGTACTTGGAATGATACTAGTACTTGGAATGGTAATACAGGAA
CTACTGTCACGCTCCCTTGCAGAATAAAACAAATTGTAAACATGTGGCAGAAAGTAGGGAAAGCCATGTACGC
CCCTCCCATTCAAGGACAAATTAGATGTTCATCAAATATTACAGGGTTGCTATTAACAAGAGATGGTGGTAAT
AATGAGAGCGAGAATACCGAGACCTTCAGACCTGCAGGAGGAAATATGAGAGATAATTGGAGAAGTGAATTAT
ATAAATATAAAGTAGTAAAAATTGAACCATTAGGAGTAGCCCCCACCAAGGCAAAGAGAAGAGTGGTGCAGAG
AGAAAAAAGAGCAGTGGGATTGATAGGAGCTATGTTCCTTGGGTTCTTGGGAGCAGCAGGAAGCACTATGGGC
GCAGCGTCAATAACGCTGACGGTACAGGCCAGGCAATTATTGTCTGGTATAGTGCAACAGCAGAGAAATTTGC
TGATGGCTATTGAGGCGCAACAGAATATGTTGCGACTCACAGTCTGGGGCATC
>EU131804..1
TTCTACTGTAACACATCACAACTGTTTAATAGTACTTGGTATAGTAATGGTACTAGGAAGGATCCTACAGGAA
ATGGCACTATCACGCTCCCTTGCAGAATAAAACAAATTATAAACATGTGGCAGCAAGTAGGGAAAGCAATGTA
TGCACCTCCCATTAAAGGACAAATTAGATGTTCATCAAATATTACAGGGTCGCTATTAACAAGAGATGGTGGT
TATAATGAGAGCAACACCACCGAGACCTTCAGACCTGGAGGAGGAGATATGAGAGACAATTGGAGAAGTGAAT
TATATAAATATAAAGTAGTAAAAATTGAACCATTAGGAGTAGCACCCACCAAGGCAAAGAGAAGAGTGGTGCA
GAGAGAAAAAAGAGCAGTAGGACTAGGAGCTATGTTCCTTGGGTTCTTGGGAGCAGCAGGAGGCACCATGGGC
GCAGCGTCAATAACGCTGACGGTACAGGCCAGACAATTGTTGTCTGGTATAGTGCAACAGCAGAGAAATTTGC
TGAGTGCTATTGAGGCGCAACAACGTTTGTTGCAACTCACAGTCTGGGGCATC
>JF932472..1
TTCTACTGTAATACAGCACAACTGTTTAATAGTACTTGGACAGGAAATGATACTAGCATTTGGAATGATACTA
CAGGAAATGACACTATCACACTCCCTTGCAGAATAAAACAAATTATAAACATGTGGCAGGAAGTAGGGAAAGC
AATGTATGCCCCTCCCATTGCAGGACAAATTAGATGTTCATCAAATATTACAGGGTTGCTATTAACAAGAGAT
GGTGGTAATCATGAGAACAACACTGAGACCTTCAGACCTGGAGGAGGAGATATGAGAGACAATTGGAGAAGTG
AATTATATAGATATAAAGTAGTAAAAATTGAACCATTAGGAGTAGCACCCACCAAGGCAAAGAGAAGAGTGGT
GCAGAGAGAAAAAAGAGCAGTGGGAACGCTAGGAGCTATGTTCCTTGGGTTCTTGGGAGCAGCAGGAAGCACT
ATGGGCGCAGCGTCAATAACGCTGACGGTACAGGCCAGACAATTGTTGTCTGGTATAGTGCAACAGCAGAGAA
ATTTGCTGAGGGCTATTGAGGCGCAACAGCATCTGTTGCAACTCACAGTCTGGGGCATC
>JF932474..3
TTCTACTGTAATACATCACAACTGTTTAATAGTACTTGGCCTTGGAATAATGATACTAGTGTTTGGGATGATA
CTACAGAAAATGACATTATCACGCTCCCTTGCAGAATAAAACAAATTATAAACATGTGGCAGGAAGTAGGGAA
AGCAATGTATGCCCCTCCCATTCAAGGACAACTTAACTGTTCATCAAATATTACAGGGTTGCTATTAACAAGA
GATGGTGGTTATCATAATGAGAGCAATAACACCGAGACCTTCAGACCTGGAGGAGGAGATATGAGAGACAATT
GGAGAAGTGAATTATATAAATATAAAGTAGTAAAAATTGAACCATTAGGAGTAGCACCCACCAAGGCAAAGAG

AAGAGTGGTGCAAAGAGAAAAAAGAGCAGTGGGAGCGATAGGAGCTATGTTCCTTGGGTTCTTGGGAGCAGCA
GGAAGCACTATGGGCGCAGCGTCAATAACGCTGACGGTACAGGCCAGACAACTGTTGTCTGGTATAGTGCAAC
AGCAGAGAAATTTGCTGAGGGCTATTGAGGCGCAACAGCATCTGTTGCAACTCACAGTCTGGGGCATT
>HQ215554..1
TTCTACTGTAATACAACAGGGCTGTTTAATAGTACTTTGACTAATGATACCTGGAATGATAATCGGTCATATA
ACAATGGAACAGGTAACATCACACTCCACTGCAGAATAAAACAAATTATAAACAGGTGGCAGGAAGTAGGAAA
AGCAATGTATGCCCCTCCCATCAGTGGACCAATTAGCTGTTTATCAAATATTACAGGACTGATATTAACAAGA
GATGGAGGTAACAATACCGGGAATCAATCTGATACTGAAACCTTCAGACCTGGGGGAGGGGATATGAAGGACA
ATTGGAGAAGTGAATTATATAAATATAAAGTAGTACAAATTGAGCCAATAGGATTAGCACCCACCAGGGCAAG
GAGAAGAGTGGTGCAGAGAGAAAAAAGAGCAGTGGGAACATTAGGAGCTATGTTCCTTGGGTTCTTGGGAGCA
GCAGGAAGCACTATGGGCGCAGCGTCAGTCACGCTGACGGTACAGGCCAGACAATTACTGTCTGGTATAGTGC
AACAGCAGAACAATTTGCTGAAAGCTATTGAGGCGCAACAACATATGTTGCAACTCACAGTCTGGGGCATC
>JF346917..1
TTCTACTGTAATACATCACAACTGTTTAATAGTACTTGGGATATTAATAGTAATGGGACTAATAATACAAATA
GCAATATCAATATCACACTCCCTTGCAAAATAAAACAAATTGTAAACATGTGGCAGGAAGTAGGGAAAGCAAT
GTACGCCCCTCCCATTAGAGGACAAATTAGATGTTCATCACAAATTACAGGGTTGTTATTAACAAGAGATGGT
GGTACTAATAATAATCATACCAACGAAACCTTCAGACCTGCAGGGGGAGACATGAGAGACAATTGGAGAAGTG
AATTATATAAATATAAAGTAGTAAAAATTGAACCATTAGGAATAGCACCCACCAAGGCAAAGAGAAGAGTGGT
GCAGAGAGAAAAAAGAGCAGTGGGAACAATAGGAGCTATGTTCCTTGGGTTCTTAGGAGCAGCAGGAAGCACT
ATGGGCGCAGCGTCAGTAACGCTGACGGTACAGGCCAGACAATTATTGTCTGGTATAGTGCAACAGCAGAGAA
ATCTGCTGAGGGCTATTGAGGCGCAACAGCGTGTGTTGCAACTCACAGTCTGGGGCATC
>JX960599..1
TTCTACTGCAATACAACACAACTGTTTAATAGTACTTGGAATACTTGGGATAATATTACTGGGAATAGTAATG
AGAATAATACACAGAACAATATCACACTTCCATGTAGAATAAAACAAATTATAAACATGTGGCAGGGAGTAGG
AAAAGCAATGTACGCCCCTCCCATCGCAGGACGAATTAAATGTTCATCAGATATTACAGGACTATTATTAGTG
AGAGATGGTAATAACAATAACAATACAGATAACAAGACAGAAACTTTCAGACCTCAGGGAGGAAACATGAAGG
ACAATTGGAGAAGTGAATTATATAAATATAAAGTAGTAAAAATTGAACCAATAGGAATAGCACCCACTACGGC
AAGGAGAAGAGTGGTGCAGAGAGAAAAAAGAGCAGTGACACTAGGAGCTATGTTCCTTGGGTTCTTAGGAGCA
GCAGGAAGCACAATGGGCGCAGCGTCAATAACGCTGACGGTACAGGCCAGACAATTACTGTCTGGTATAGTGC
AACAGCAGAACAATTTGCTGAGGGCCATTGAGGCGCAACAGCATATGTTGCAACTCACAGTCTGGGGCATC
>JF932497..2
TTCTACTGTAATACATCACGACTGTTTAGTAGTACATGGTATACTAATGATACTCAGAATGGTACTACAGGAA
ATGACACTATCACACTCCCTTGCAGAATAAAACAAATTGTAAACATGTGGCAGGAAGTAGGAAAAGCAATGTA
TGCCCCTCCCGTTGAAGGACTAATTAGATGTTCATCAAATATTACAGGGTTGCTATTAACAAGAGATGGTGGT
TATAATGTAAGCAACAACACCGAGATCTTCAGACCTGGAGGAGGAGATATGAGGGACAATTGGAGAAGTGAAT
TATATAAATATAAAGTAATAAAAATTGAACCATTAGGAGTAGCACCCACCAAGGCAAAGAGAAGAGTGGTGCA
AAGAGAAAAAAGAGCAGTGGGAATAGGAGCTATGTTCCTTGGGTTCTTGGGAGCAGCAGGAACCACTATGGGC
GCAGCGTCAATGACGCTGACGGTACAGGCCAGACAATTGTTGTCTGGTATAGTGCAACAGCAGGGAAATCTGC
TGAGGGCTATTGAGGCGCAACAGCATATGTTGCAACTCACAGTCTGGGGCATT
>KX693642..1
TTCTACTGTAATACATCACAACTGTTTAATAGTACTTGGGATGTTAATGACACTAGTAATGATACTACAGGAA
ATGAAACTATCACGCTCCCTTGCAGAATAAAACAAATTATAAACATGTGGCAGGGAGTAGGGAAAGCAATGTA
TGCCCCTCCCATTAAAGGAAAAATTAGATGTTCATCAAATATTACAGGGTTGATATTAACAAGAGATGGTGGT
GTAAATGAGAGCGAGTTCAATGAGACCTTCAGACCTCAAGGAGGAAATATGAAAGACAATTGGAGAAGTGAAT
TGTATAAATATAAAGTAGTAAAAATTGAACCATTAGGAGTAGCACCCACCAAGGCAAAGAGAAGAGTGGTGCA
GAGAGAAAAAAGAGCAGGAATAGGAGCTCTGTTCCTTGGGTTCTTGGGAGCAGCAGGAAGCACTATGGGCGCA
GCGTCAATAACGCTGACGGTACAGGCCAGACAATTGATGTCTGGTATAGTGCAACAGCAGAGAAATTTGCTGA
GAGCTATTGAGGCGCAACAGCATCTGTTGCAACTCACAGTCTGGGGCATC
>KX693171..1
TTTTACTGTAATACATCACAACTGTTTGACAGTATTTGGAATGCTACTACTGGTACTTGGAATGGTACTACAG
GAGGTGACAATATCACGCTCCCTTGCAGGATAAAACAAATTGTAAACATGTGGCAGGAAGTAGGGAAAGCAAT
GTATGCCCCTCCCATTAGAGGACAAATTTATTGTTCATCAAGTATTACAGGGTTGCTATTAACAAGAGATGGT
GGTACGAGTAAGAATGAGAGCAATACCACCGAGACCTTCAGACCTGGAGGAGGAGATATGAGGGACAATTGGA
GAAGTGAATTATATAAATACAAAGTAGTAAAACTTGAACCACTAGGAGTAGCACCCACCGAGGCAAAGAGAAG
AGTGGTGCAGAGAGAGAAAAAAGAGCAGTGGGAGCGATAGGAGCTATGTTCCTTGGGTTCTTGGGAGCAGCAGGA
AGCACTATGGGCGCAGCGTCAATAACGCTGACGGTACAGGCCAGACAATTATTGTCTGGTATAGTGCAACAGC
AAAGAAATTTGCTGAGGGCTATTGAGGCGCAACAGCATATGTTGCAACTCACAGTCTGGGGCATC
>KX693309..1
TTCTACTGTAATACATCAAAACTGTTTAATAGTACTTGGAGTAAGACTAGTACTTGGAATGATACTACAGGAA
ATGACACTATCACGCTCCCTTGCAGAATAAAACAAATTGTAAACATGTGGCAGGAAGTAGGGAAAGCAATGTA

TGCCCCTCCCATTCAAGGACTAATTAACTGTTCATCAAATATTACCGGGTTGCTATTAACAAGAGATGGTGGT
AATAATACTGAGAGCAACACCACCGAGACCTTCAGACCTACAGGAGGAAATATGAGAGACAATTGGAGAAGTG
AATTATATAAATATAAAGTAGTACAAATTGAACCATTAGGAGTAGCACCCACCAAGGCAAAGAGAAGAGTGGT
GCAGAGAGAAAAAAGAGCAGTGGGAACAATAGGAGCTATGTTCCTTGGGTTCTTGGGAGCAGCAGGAAGCACT
ATGGGCGCAGCGTCGGTAACGCTGACGGTACAGGCCAGACAATTGTTGTCTGGTATAGTGCAACAGCAGAGAA
ATTTGCTGAGGGCTATTGAGGCGCAACAGCATCTGTTGCAACTCACAGTCTGGGGCATT
>KX693362..1
TTCTACTGTAATACATCAAAACTGTTTAATAGTACTTGGAATGATAATAGTACTTGGAATGATAATGATAAGA
CAGGAGATGACAATCTCACGCTCCCTTGTAGAATAAAACAAATTGTAAACATGTGGCAGGAAGTAGGGAAAGC
AATGTATGCCCCTCCCATTAAAGGACAAATTAGATGTTCATCAAATATTACAGGGTTGCTATTAACAAGAGAT
GGTGGTCAGAATGTGAGCAATACCACCGAGACCTTCAGACCTGCAGGAGGAGATATGAGAGACAATTGGAGAA
GTGAATTATATAAATATAAAGTAGTAAAAATTGAACCATTAGGAGTAGCACCTACCAAGGCAAAGAGAAGAGT
GGTGCAGAGAGAAAAAAGAGCAGTGGGAACGATAGGAGCTATGTTCCTTGGGTTCTTGGGAGCAGCAGGAAGC
ACGATGGGCGCAGCGTCAATAACGCTGACGGTACAGGCCAGACAATTGTTGTCTGGTATAGTGCAACAGCAGA
GAAATTTGCTGAGGGCTATTGAGGCGCAACAGCATCTGTTGCAACTCACAGTCTGGGGCATC
>KX693411..2
TTTTACTGTAATACATCAAAACTGTTTAATAGTACTTGGAGTAATAATAATCCTTGGAATACTACTAGTATTC
AAAATGATACTATCACGCTCCCTTGCAGAATAAAACAAATTGTAAACATGTGGCAGGAAGTAGGGAAAGCAAT
GTATGCCCCTCCCATTGCAGGACCAATTAATTGTTCATCAAATATTACGGGGTTGCTATTAACAAGAGATGGT
GGTAAAGGTGGGGATGTGAGCAACTACACCGAGACCTTCAGACCTGCAGGGGGAAATATGAGAGACAATTGGA
GAAGTGAATTATATAGATATAAAGTAGTAAAAATTGAACCATTAGGAGTAGCACCCACCAAGGCAAAGAGAAG
AGTGGTGCAGAGAGAAAAAAGAGCAGTGGGAGCAATAGGAGCTATGTTCCTTGGGTTCTTGGGAGCAGCAGGA
AGCACTATGGGCGCAGCGTCAATAACGCTGACGGTACAGGCCAGACAATTGTTGTCTGGTATAGTGCAACAGC
AGAGAAATTTGCTGAGGGCTATTGAGGCGCAACAGCATCTGTTGCAACTCACAGTCTGGGGCATC
>KR914676..3
TTCTACTGTAACACAACACCACTGTTTAATAGTAGTTGGTACAAGTCAAATTGGAAAAGTCAAAGGACAAAGG
AGATTGGAGGAAGGGAGACAATCACACTCCAATGTAGAATAAAACAAATTGTAAACATGTGGCAGGAAGTAGG
ACGAGCAATGTATGCCCCTCCCATCAAAGGACAAATTAGATGTTCATCAAATATTACAGGGGTGCTATTAACA
AGAGATGGTGGTAATACCACCAATCACTCCGAGATCTTCAGACCTGGAGGAGGAAATATGAAGGACAATTGGA
GAAGTGAATTATATAAATATAAAGTAGTACAAATTGAACCATTAGGAATAGCACCCACCAAGGCAAAGAGAAG
AGTGGTGCAGAGAGAAAAAAGAGCAATAGGATTCGGAGCTGTGTTCCTTGGGTTCTTGGGAGCAGCAGGAAGC
ACTATGGGCGCAGCGTCAGTAACGCTGACGGTACAAGCCAGACAATTATTGTCTGGTATAGTGCAACAGCAGA
GCAATTTGCTGAATGCTATTCAGGCGCAACAGCATCTGTTGCAACTCACAGTCTGGGGCATT
>FJ388899..1
TTCTACTGCAATACAACAGAACTGTTTAATAGTACTTGGAATAGTACTACTTGGAATAGTAATAGTACTACAA
ATGCAAATGCAACAAATACAGCTAACCTAGTACTCCCATGCAGAATAAAACAGATTGTAAACATGTGGCAGAA
AGTAGGAAAAGCAATGTATGCCCCTCCCATCAAAGGAATAATTAGATGTTCATCAAATATTACAGGGATAATA
TTAACAAGAGATGGTGGTAATAGTAATCAGACCAACACCACAGAGATTTTCAGACCTGTAGGAGGAAATATGA
AGGACAATTGGAGAAGTGAATTATATAAATATAAAGTAGTACAAATTGAACCATTAGGAGTAGCACCCACCAA
GGCAAAAAGAAGAGTGGTGCAGAGAGAAAAAAGAGCAGTGACATTAGGAGCTATGTTCCTTGGGTTCTTGGGA
GCAGCAGGAAGCACTATGGGCGCAGCGTCAATAACGCTGACGGTACAGGCCAGACAATTACTGTCTGGTATAG
TGCAACAGCAGAGCAATCTGCTGAGGGCTATTGAGGCGCAACAGCACCTGTTGCAACTCACAGTCTGGGGCAT
C
>FJ388916..3
TTCTACTGTAATACAACAGGGCTGTATAATAGTACTTGGCAGTTTAATAGTACTTGGAATACTACTTGGAATC
ATACTGACCCTAATCATACTGACCCTATCACACTACCATGCAGAATAAGACAAATTGTAAACAGGTGGCAGGA
AGTAGGAAAAGCAATGTATGCCCCTCCCATCAGCGGACAAATTAGCTGTTCATCAAACATTACAGGGCTACTA
TTAACAAGAGATGGTGGTAGTAATGAGAGCAGAACCAACGAGACCTTTAGACCTGGAGGAGGAGATATGAGGG
ACAATTGGAGAAGTGAATTATATAAATATAAAGTAGTAAGAATTCAACCATTAGGAATAGCACCCACCAAGGC
AAAAAGAAGAGTGGTGCAAAGAGAAAAAAGAGCAGTGGGAACGCTGGGAGCTATGTTCCTTGGGTTCTTGGGA
GCAGCAGGAAGCACTATGGGCGCAGCGTCACTGACGCTGACGGTACAGGCCAGACAATTATTGACTGGTATAG
TGCAACAGCAGAACAATCTGCTGAGAGCTATTGAGGCGCAACAGCATATGTTGCAACTCACAGTCTGGGGCAT
C
>JF683738..2
TTCTACTGTGATACTACAAAGCTGTTTAATAGTACTTGGACTGGGAATGAAACTTGGACTGGGAATGAAACTG
AAGAGTCAAATAATAGTACTATCACACTCCCATGCAGAATAAAACAAATTATAAACAGATGGCAGGAAGTAGG
AAAAGCAATGTATGCCCCTCCCATCAGAGGAGAAATTAAATGTACATCAAATATTACAGGGCTACTATTAACA
AGAGATGGTGGTAATGGTAATGGGACTCAGAATCAGACCGAGATCTTCAGACCTGGAGGAGGAGACATGAAGG
ACAATTGGAGAAGTGAATTATATAAATATAAAGTAGTAAAAATTGAGCCATTAGGAATAGCACCTACCAAGGC
AAAAAGAAGAGTGGTGCAGAGAGAAAAAAGAGCAATAGGACTGGGAGCTGTGTTCATTGGGTTCTTGGGAGCA

GCAGGAAGCACTATGGGCGCAGCGTCGGTGACGCTGACGGTACAGGCCAGACAGTTATTGTCTGGTATAGTGC
AACAGCAAAACAATTTGCTAAAAGCTATTGAGGCGCAACAGCATCTGTTGCAACTCACAGTCTGGGGCATC
>JF683756..1
TTCTACTGTAATACATCAAAGCTGTTTAATAGTACTTGGACTAATAGTAGTAGTACTTGGAATAATACTGAAG
AGTCAAATGACACTATCACACTCCCATGTAAAATAAAACAAATTATAAACATGTGGCAGGAAGTAGGAAGAGC
AATGTATGCCCCTCCCATCAGTGGGCAAATTAGGTGCTCATCAAATATTACAGGGCTACTATTAACAAGAGAT
GGTGGTCATAACGGAACCAATAACGAGACCGAGATCTTCAGACCTGCAGGAGGAAATATGAAGGACAATTGGA
GAAGTGAATTATATAAATATAAAGTAGTGAAAATTGAGCCATTAGGAGTAGCACCCACCAGGGCAAAAAGAAG
AGTGGTGCAGAGAGAAAAAAGAGCAGTGGGAATGGGAGCTTTGCTCCTTGGGTTCTTGGGAGCAGCAGGAAGC
ACTATGGGCGCAGCATCGGTGATGCTGACGGTACAGGCCAGACAATTATTGTCTGGTATAGTGCAACAGCAGA
ACAATCTGCTGAGAGCTATTGAGGCGCAACAACATATGTTGCAACTCACAGTCTGGGGCATT
>JF683764..1
TTCTACTGTGATACAACACAACTGTTTAATAGTACTTGGTTTATGAATGGGACTAATACAACTACCCTTACCG
CAAATGCCACAATCCCAAGCCGAATAAAACAAATTATACACAGGAGGCAGGAAGTAGGAAGAGCAATGTATGT
CCCTGCCCTCAGAGGACCAATTACATGTATATCAAATATGACAGGGACGTTATTAACAAGAGATGGTGGTCAT
AACAATGGCATTAATGACATTGAGACCTTCAGACCAGGAGGAGGAGATATGAGGGACAATTGGAGAAGTGAAT
TATATAAATATAAAGTAGTAAGAGTTGAACCATTAGGAATAGCACCCACCAGGGCAAAGAGAAGAGTGGTGCA
GAGAGAAAAAAGAGCAGCACTAGGAGCTATGTTCCTTGGGTTCTTGGGAGCAGCAGGAAGCACTATGGGCGCA
GCGTCAATAACGCTGACGGTACAGGCCAGACAATTGTTGTCTGGTATAGTGCAACAGCAAAACAATTTGCTGA
GAGCTATTGAGGCGCAACAACACATGTTGCAACTCACAGTCTGGGGCATC
>JF683773..1
TTCTACTGTAATACATCACAACTGTTTAATAGTACTTGGACACCTAATGGTACTTGGACATTTAATGCTGCTA
GTGGGGCTTCAGGATTCAAGGGCACTATCACACTCCCATGTAGAATAAAACAAATTATAAACATGTGGCAGGA
AGTAGGAAAAGCAATGTATGCCCCTCCCATCAGTGGACTAATTAGATGCTCATCAAATATTACAGGACTGCTA
TTAACAAGAGATGGTGGTATAAACAATACCAATACCACCGAGATCTTCAGACCTGTAGGAGGAAATATGAAGG
ACAATTGGAGAAGTGAATTATATAAATATAAAGTAGTACAAGTTGAACCAATAGGAATTGCACCCACCAAGGC
AAAGAGAAGAGTGGTGCAGAGAGAAAAAAGAGCAGTTACACTAGGAGCTGTGTTCCTTGGGTTCTTGGGAGCA
GCAGGAAGCACTATGGGCGCAGCGTCAATGACGCTGACGGTACAGGCCAGGCAATTATTGTCTGGTATAGTGC
AACAGCAGAACAATCTGCTGAGAGCTATTCAGGCGCAACAGCATCTGTTGCAACTCACAGTCTGGGGCATC
>JQ416158..1
TTCTACTGTAATACATCACAACTGTTTAATAGTATTTGGCCTTGGAATAGTACTGCAAACAACACTGGAGGAA
ATGACACAATCACACTCCCATGCAGGATAAGACAAATTGTAAATAGGTGGCAGGAAGTAGGAAAAGCAATGTA
TGCCCCTCCCATCAGTGGACAAATTAGATGTTCATCAAATATTACAGGGCTGCTATTAACAAGAGATGGAGGT
CAGACTAACAACCAGACTGGGAACCAGACCGAGATTTTCAGACCTGGAGGAGGAGATATAAAAGATAATTGGA
GAAGTGAATTATACAAATATAAAGTAGTACAAATTGAACCATTAGGAGTAGCTCCCACTAAGGCAAGGAGAAG
AGTGGTGCAGAGAGAAAAAAGAGCACTAACACTGGGAGCTGTGTTCCTTGGGTTCTTGGGAACAGCAGGAAGC
ACTATGGGCGCAGCGTCACTAACGCTGACGGTACAGGCCAGACAATTATTGTCTGGTATAGTGCAGCAGCAGA
ACAATCTGCTGAGAGCTATTGAGGCGCAACAACATTTGTTGCAACTCACAGTCTGGGGCATC
>KT124803..1
TTCTACTGTAATACAACAAGCCTGTTTAATAGTACTTGGTATGCAAATGGTACAAATAGCACTTGGTCTGATG
AGTCAGATGTTATCACACTTCCATGCAGAATAAGACAAATTGTAAACATGTGGCAGGGGGTAGGAAAAGCAAT
GTATGCCCCTCCCATCAGAGGACAGATTAATTGTACATCAAATATTACAGGGCTAATATTAACAAGAGATGGT
GGGAACAAGAATGGCACTAATGAGACCTATGAGACCTTCAGACCTGCAGGAGGAAATATGAAGGACAATTGGA
GAAGTGAATTATATAAATATAAAGTAGTAAAGATTGAACCATTAGGAATAGCACCCACCAAGGCAAAGAGAAG
AGTGGTGCAAAGAGAAAAAAGAGCAATAGGAATGGGAGCCTTGTTCCTTGGGTTCTTGGGAGCAGCAGGAAGC
ACTATGGGCGCAGCGTCAATGACGCTGACGGTACAGGCCAGACAGTTATTGTCTGGTATAGTGCAACAGCAAA
ACAATTTGCTGAGAGCTATTGAGGCGCAACAACACCTGTTGCAACTCACAGTCTGGGGCATC
>KT124789..1
TTCTACTGTAATACAACAGAGCTGTTTAACGGTACTTGGAATGGTACTTTCTTGAATGTTACTACAGGGCGAG
AGAATATCACACTCCCATGCAGAATAAAACAGATTATAAACAGGTGGCAGGAAGTAGGAAAAGCAATGTACGC
CCCTCCCATCAGAGGACCAATTAGATGCTCATCAAATATTACAGGGCTGATATTAACAAGAGATGGTGGTCAG
AATGCGAGTGCGACCAACGAAACCTTCAGACCGGGAGGAGGAGATATGAGGGACAATTGGAGAAGTGAATTAT
ATAAATATAAAGTAGTAAAAATTGAGCCATTAGGAATAGCACCCACCAAGGCAAGGAGGAGAGTGGTGCAGAG
AGAAAAAAGAGCAGTGACACTAGGAGCTATGTTCCTTGGGTTCTTGGGAGCAGCAGGAAGCACTATGGGCGCA
GCTTCATTGACGCTGACGGTACAGGCCAGACTATTATTGTCTGGTATAGTGCAACAGCAAAACAACTTGCTGA
GGGCTATTGAGGCGCAGCAGCATCTGCTGCAACTCACAGTCTGGGGCATC
>KT124805..1
TTTTACTGTGATACAACACAACTGTTCAACAGTACGTGGGATACAAATAATACTGGGGGAGTGAATGATACTG
ACGAGCCAATTACACTCCCATGCAGGATAAAACAATTTATAAACATGTGGCAGGAAGTAGGGAAAGCAATGTA
TGCCCCTCCCATCCAAGGAATAATTCACTGTTCATCAAATATTACAGGACTACTATTAACAAGAGATGGTGGT

AAGACAGAGAACGGGACAGAGAATGTTACTGAGATCTTTAGACCTGGAGGAGGAGACATGAGGGACAATTGGA
GAAGTGAATTATATAAATATAAAGTAGTAAAAATTGAACCATTAGGAGTAGCACCCACCAGGGCAAAGAGAAG
AGTGGTGCAGAGAGAAAAAAGAGCAGTGACACTGGGAGCTGTGTTCCTTGGGTTCTTGGGAGCAGCCGGAAGC
ACTATGGGCGCAGCGTCACTGACGCTGACGGTACAGGCCAGACAATTATTGTCTGGTATAGTGCAACAGCAGA
ACAATTTGCTGAGAGCTATTGAGGCGCAACACCATCTGTTGCAACTCACAGTCTGGGGCATT
>KT124763..1
TTCTACTGTAATTCATCGACACTGTTTAATAGTACTTGGAATGAAACTATTGCAGGGATTACCAATGCAAATG
AAACTATCACACTCCCATGCAGAATAAGGCAAATTATAAACATGTGGCAGGAAGTAGGAAAAGCAATGTATGC
CCCTCCCATTAGTGGAATAATTAGATGTTCATCAAATATTACAGGGCTGCTATTAACAAGAGATGGTGGTAAT
AATGGTAATCATAGCGAGAATAATACCATTGAGATCTTCAGACCTGGAGGAGGAGATATGAGGGATAATTGGA
GAAGTGAATTATATAAATATAAAGTAGTACAAATTGAACCAGTAGGAGTAGCACCTACCAAGGCAAAGAGAAG
AGTGGTGCAGAGAGAAAAAAGAGCAGTGGGACTAGGAGCTTTGTTCCTTGGGTTCTTGGGAGCAGCAGGAAGC
ACTATGGGCGCAGCGTCAATAACGCTGACGGTACAGGCCAGACAATTATTGTTTGGTATAGTGCAACAGCAGA
ACAATCTGCTGAGAGCTATTGAGGCGCAACAGCACCTGTTGCAACTCACAGTCTGGGGCATC
>KT124779..1
TTCTACTGTGATACAACACAACTGTTTAATAGTACTTGGAGTAATGCTACTTGGAATGAGACTACAGGTTCAA
ATGACACGATTACACTCCAATGTAAAATAAAACAAATTATAAACAGGTGGCAGGAAGTAGGAAAAGCAATGTA
TGCCCCTCCCATCCGAGGACAAATAAAGTGTTCATCAAATATTACAGGGTTGCTATTAACAAGAGATGGTGGT
AACGAGACTAACGGGACCGAGGTCTTCAGACCTATAGGAGGAGATATGAAAGACAATTGGAGAAGTGAATTAT
ATAAATATAAAGTAGTTCAAATTGAACCATTAGGAATAGCACCCACCAAGGCAAAGAGAAGAGTGGTGCAGAG
AGAAAAAAGAGCGGTGACACTGGGAGCTATGTTCCTTGGGTTCTTGGGAGCAGCGGGAAGCACTATGGGCGCA
GCGTCAGTGGCGCTGACGGTACAGGCCAGACAATTATTGTCTGGTATAGTGCAACAGCAGAACAATATGCTGA
GGGCTATTGAGGCACAACAGCATATGTTGCAACTCACAGTCTGGGGCATC
>KT124787..2
TTCTACTGTGATACAACACAGCTGTTTAATAGCAATAGCACTTGGCTGTTTAATAACACTTGGAATAGTACTG
AGAATAATAATACTGAGAATAATAATATCACACTCCAGTGCAGAATAAGACAAATTGTAAACATGTGGCAGAA
AGTAGGAAAAGCAATGTATGCCCCTCCCATCAGAGGACAAATTTACTGTGCATCACAAATAACAGGGCTGCTA
TTAACAAGAGATGGTGGTAGTAGTGATCAGAACAACACCGAGATCTTCAGACCTGCAGGAGGAAATATGAGGG
ACAATTGGAGAAGTGAATTATATAAATATAAAGTAGTAAAAATTAAACCATTAGGAGTAGCACCCACCAAGGC
AAAGAGAAGAGTGGTGCAGAGAGAAAAAAGAGCAGTAACGCTAGGAGCTATGTTCCTTGGGTTCTTGGGAGCA
GCGGGAAGCACTATGGGCGCAGCGGCAATGACGCTGACGGTACAGGCCAGACAATTATTGTCTGGTATAGTGC
AACAGCAGAATAATCTGCTGAGAGCTATTGAGGCGCAACAGCATCTGTTGCAACTCACAGTCTGGGGCATC
>KC596067..1
TTCTACTGTAATACAACACAATTGTTTAACAGTACTTGGAATACTACTGAAGATTTAAATAAAACTAGAACTG
AAGTAAATGACACAATCACACTCCCATGTAGAATAAGACAAATTATAAACAGATGGCAGGAAGTGGGAAAAGC
AATGTATGCCCCTCCCATCAGCGGAAACATTAGTTGTTCATCAAACATCACAGGGCTGCTATTAACAAGAGAT
GGTGGTCAGAATAATGTGAGCAACACCACAGAGATCTTCAGACCTGGAGGAGGAGATATGAGGGACAATTGGA
GAAGTGAATTATATAAATATAAAATAATAAAAATCGAACCATTAGGAATAGCACCCACCAAGGCAAGGAGAAG
AGTGGTGCAGAGAGAGAAAAGAGCAGCACTAGGAGCTATGTTCCTTGGGTTCTTAGGAGCAGCAGGAAGCACT
ATGGGCGCAGCGTCAGTGACGCTGACGGTACAGGCCAGACAATTATTGTCTGGTATAGTGCAACAGCAAAGTA
ACTTGCTGAAAGCTATTGAGGCGCAACAGCATCTGTTGCAACTCACAGTCTGGGGCATC
>EF514700..2
TTTTACTGTAATACGTCAAAACTGTTTGATAGTACCTGGAATGAAACTAGTATTTGGAATTATACCACTACTA
ATGATACCACAAAAAATGACACACTCACACTCCCATGTAAAATAAAACAAATTATAAACATGTGGCAGGAAGT
AGGAAAAGCAATGTATGCCCCTCCCATCAGAGGACAAATCAGATGCTCATCAAATATTACAGGGCTGTTATTA
ACAAGAGATGGTGGTCAGGGTAACGGGACCGATAACACCACCGAGATCTTCAGACCTGCAGGAGGAGATATGA
GGGACAATTGGAGAAGTGAATTATATAAATATAAAGTAGTAAGAATTGAACCATTAGGAGTAGCACCCACCAA
GGCAAAGAGAAGAGTGGTGCAGAGAGAAAAAAGAGCAGTGGGACTGGGAGCTTTGTTCCTTGGGTTCTTGGGA
ACAGCAGGAAGCACTATGGGCGCAGCATCACTAACGCTGACGGTACAGGCCAGACAATTGTTGTCTGGTATAG
TGCAACAGCAGAACAATCTGCTGAGAGCTATTGAGGCGCAACAACATCTGTTGCAACTCACAGTCTGGGGCAT
T
>EU839598..1
TTCTACTGTAATACAACAAAACTGTTTAACAATACTGGACAATTAGATAAAGAAGAAGATGAAGGAGAAGAAG
GCAATATCACACTTCCATGCAGAATAAGGCAATTCGTAAACAGGTGGCAAGAAGTAGGAAAAGCAATGTATGC
CCCTCCCATCAGTGGACAAATTAGATGTTCATCAAATATTACAGGGCTACTATTAACAAGAGATGGTGGTGGT
AGTGGTAATAGTGAGAATGAGACCTTCAGACCTGGGGGAGGAGATATGAAAGACAATTGGAGAAGTGAGTTAT
ATAAATATAAAGTAGTAAAAATTCAGCCATTAGGAATAGCACCCAGCAAGGCAAAGAGAAGAGTGGTGCAAAG
AGAAAAAAGAGCAGTGACACTAGGAGCTATGTTCCTTGGGTTCTTAGGAGCAGCAGGAAGCACTATGGGCGCA
GCGTCAGTAACGCTGACGGTACAGGCCAGACTATTATTGTCTGGTATAGTGCAACAGCAGAACAATCTGCTGA
GGGCTATTGAGGCGCAACAACATCTGTTGCAACTCACGGTCTGGGGCATC

>KC595194..1
TTTTACTGTAATTCAACACAACTGTTTAACAGTGTTTGGAAGGCTAACAGTACTTGGAATAGTACTGAAGGGT
CAAATAACAATGCAGGAAATATCACACTCCCATGCAGAATAAAACAAATTATAAACATGTGGCAGGAAGTAGG
AAAAGCAATGTATGCCCCTCCCATCAGAGGCCAAATTAGCTGTTCATCAAATATTACAGGGCTACTATTAACA
AGAGATGGTGGTAACATCACTAACGGGACCGAGATCTTCAGACCTGGAGGAGGAGATATGAGGGACAATTGGA
GAAGTGAATTATATAAATATAAAGTAGTAAAAATTGAACCATTAGGAATAGCACCCACCAAGGCAAAGAGAAG
AGTGGTGCAGAGAGAGAAAAGAGCAGTGGGAATAGGAGCTATGTTCCTTGGGTTCTTGGGAGCAGCAGGAAGC
ACTATGGGCGCGGCGTCAATGACGTTGACGGTACAGGCCAGACTATTATTGTCTGGTATAGTGCAACAGCAGA
ACAATTTGCTAAGGGCTATTGAGGCGCAACATCATCTGTTGCAACTCACAGTCTGGGGCATC
>KC595151..1
TTCTACTGTGATACAACAAAATTGTTTAATAGTACTTGGGATATTAATGATACTTGGAATGGTACTAACGGGC
CAGATAACACTATCATACTCCCATGCAGAATAAAACAAATTATAAACATGTGGCAGGAAGTAGGAAAAGCAAT
GTATGCCCCTCCCATCAGAGGACGAATTAGGTGTGAATCAAATATTACAGGGCTGCTATTAACAAGAGATGGT
GGTGATGATAGCAGTAACCAGACCGAGATCTTCAGACCTGGAGGAGGAGATATGAGGGACAATTGGAGAAGTG
AATTATATAAATATAAAGTAGTAAAAATTCAACCATTAGGAATAGCACCCAACAAGGCAAAGAGAAGAGTGGT
GCAGAGAGAAAAAAGAGCAGTGGGAATAGGAGCTTTGTTCCTTGGGTTCTTGGGAGCAGCAGGAAGCACTATG
GGCGCAGCGTCAATGACGTTGACGGTACAGGCCAGACAATTATTGTCTGGTATAGTGCAACAGCAGAACAATC
TGCTGAGGGCTATTGAGGCGCAACAGCATCTGTTGCAACTCACAGTCTGGGGCATC
>EU786679..1
TTCTACTGTAATACAACACAGCTGTTTAATAGTACTTGGGGTATGAATACGACTAAAGGGACAAATACCACTG
AAGGAGATATCATACTCCCATGCAGAATAAAACAAATTATAAACATGTGGCAGGAAGTAGGAAAAGCAATGTA
TGCCCCTCCCATCAGCGGGCCAATTACATGTACATCAAATATTACAGGGCTGCTACTAACAAGAGATGGGGGT
CAGAGCAAGAACGAAAGTAACGAGACAGAGATCTTCAGACCAGGAGGAGGAGACATGAAGGACAATTGGAGAA
GTGAATTATATAAATATAAAGTAGTAAAAATTGAACCATTAGGAATAGCACCCACCAAGGCAAAAAGAAGAGT
GGTGCAAAGAGAAAAAAGAGCAGTGGGACTAGGAGCTGTGTTCCTTGGGTTCTTGGGAGCAGCAGGAAGCACT
ATGGGCGCAGCGTCAATAACGCTGACGGTACAGGCCAGACAATTATTGTCTGGTATAGTGCAACAGCAGAACA
ACTTGCTGAGGGCTATTGATGCGCAACAGCATATGTTGCAGCTCACAGTCTGGGGCATC
>KC473841..1
TTCTACTGTAATACATCACAACTGTTTAGTAGTACTTGGAATAATACTGTAGGGTTAAATACTACAGGAAATG
ATACTATCACACTCCACTGCAGAATAAGACAAATTATAAACCTGTGGCAGAGAGTAGGAAAAGCAATGTATGC
CCCTCCCATTAGAGGACAAATTAGTTGTTCATCAAACATTACAGGGTTGCTATTAACAAGAGATGGTGGTAAC
AACAATCAGAGCAATAACACCGAAATCTTCAGACCTGGAGGAGGAGATATGAGGGACAATTGGAGAAGTGAAT
TATATAAATATAAAGTAGTAAGAATTGAACCACTAGGAGTAGCACCCACCAAGGCAAAGAGAAGAGTGGTGCA
GAGAGAAAAAAGAGCAGTGGGACTAGGAGCTGTGCTCTTTGGGTTCTTGGGAGCAGCAGGAAGCACTATGGGC
GCAGCGTCAGTGACGCTGACGGTACAGGCCAGACAATTATTGTCTGGTATAGTGCAACAGCAAAATAATTTGC
TGAAAGCTATTGAGGCGCAACAGCATCTGTTGCAACTCACAGTCTGGGGCATC
>KT200357..1
TTCTACTGTAATACAACACAGCTGTTCAATAGTACTTGGGAGCCAGGGAAGAATACTACTACAGAGGGAAATG
ACACTATCACGCTCCCATGCAGAATAAAACAGATTATAAACAGGTGGCAGGAAGTAGGAAAAGCAATGTATGC
CCCTCCCATCAGAGGACGAATTAATTGTACATCAAATATTACAGGGCTGTTATTAACAAGAGATGGTGGTAAC
AATAATAACCAGACAAATAGGACAGAGACCTTTAGACCTCAAGGAGGGAATATGAAGGACAATTGGAGAAGTG
AATTATATAAATATAAAGTAGTACAAGTTGAGCCATTAGGAATAGCACCCACCAAGGCAAGGAGAAGAGTGGT
GCAGAGAGAAAAAAGAGCAGTGGGACTAGGAGCTGTGTTCCTTGGGTTCTTAGGAGCAGCAGGAAGCACTATG
GGCGCAGCGTCAATAACGCTGACGGTACAGGCCAGACAATTGTTGTCTGGTATAGTGCAACAGCAGAACAACC
TGCTGAGGGCTATTGAAGCGCAACAACATATGTTGCAGCTCACAGTCTGGGGCATC
>KP109518..1
TTCTACTGTAATACAACACAACTGTTTAATAGTACCTGGAATGATACTAGTATTAGTGAAAGGTCAAATACTA
CTAAAAACATCACGCTTCCATGCAGAATAAAACAAATTATAAACATGTGGCAAGAAGTAGGGAAAGCAATGTA
TGCCCCTCCCATCCCAGGACCAATTCAATGTAGATCAAATATTACAGGGATATTATTAACAAGAGATGGTGGT
AATACTAGTGATAGCCAGAATGAAACCTTCAGACCTGGAGGAGGAAATATGAAGGACAATTGGAGAAGTGAAT
TATATAAATATAAAGTAGTAGAAATTGAACCATTAGGAGTGGCACCCACCAGGGCAAGGAGAAGAGTGGTGCA
GAGAGAAAAAAGAGCAGTGGGAGTAGTAGGAGCTATGTTCCTTGGGTTCTTGGGAGCAGCAGGAAGCACTATG
GGCGCAGCGTCAATGACGTTGACGGTACAGGCCAGACTATTACTGTCTGGTATAGTGCAGCAGCAGAACAATC
TGCTGAGAGCTATTGAAGCGCAACAGCATCTGTTGCAGCTCACAGTCTGGGGCATT
>KY465969..2
TTCTACTGTAATACAACACAGCTGTTTAATAGCACTTGGAATAAAACTGTAGTTGAGAATAATAATTATGGTT
CTGAGAATGGTACTATCACACTCCAGTGCAGAATAAGACAAATTGTAAACAGGTGGCAGGAAGTAGGAAAAGC
AATGTATGCCCCTCCCATCAGAGGAGTAATTAACTGTTCATCAAGTATTACAGGGCTGTTATTAACAAGGGAT
GGTGGTAATAGTAAGAACACCAACAGGTCATCTGAGATCTTCAGACCTGGAGGAGGAGATATGAGGGACAATT
GGAGAAGTGAATTATACAAATATAAAGTAGTAAAGATTGAACCATTAGGAGTAGCACCCACCAAGGCAAAGAG

GAGAGTGGTGCAGAGAGAAAAAAGAGCAGTGGGAGCATTAGGAGCTATGTTCCTTGGGTTCTTGGGAACAGCA
GGAAGCACTATGGGCGCAGCGTCACTGACGCTGACGGTACAAGCCAGACAACTATTGTCTGGTATAGTGCAAC
AGCAGAACAATCTGCTGCAGGCTATCGAGGCGCAACATCATCTGTTGCAACTCACCGTCTGGGGCATC
>KC595204..2
TTCTACTGTAACTCAACACAGCTGTTTAATAGTACTTGGAATAGTACTGGGAATCAGAATAATATTGAAGGGT
TAAATGACACAATCACACTCCCATGCAGAATAAAACAAATTATAAACATGTGGCAGGAAGTAGGAAGAGCAAT
GTATGCCCCTCCCATTAGAGGACAAATTAGATGTTCATCAAATATTACAGGGCTGTTATTAACAAGAGATGGT
GGTCATAACACGACAGAGACCGAGATCTTCAGACCTGGAGGAGGAGATATGAGGGACAATTGGAGAAGTGAAT
TATATAAATATAAAGTAGTAAAAATTGAACCATTAGGAGTAGCACCCACCAGAGCAAAGAGAAGAGTGGTGCA
GAGAGAAAAAAGAGCAGTGGGAATAGGAGCTGTGTTCCTTGGGTTCTTGGGAGCAGCAGGAAGCACTATGGGC
GCAGCGTCAGTGACGCTGACGGTACAGGCCAGACTATTATTGTCTGGTATAGTGCAACAGCAGAATAATTTGC
TGAAGGCTATTGAGGCGCAACAGCATCTGTTGCAACTCACAGTCTGGGGCATC
>DQ141342..1
TTCTACTGTAATACAACACAACTGTTTAATAGTACTTGGCCTAAGAATGGTACCAGCGAGTTAAATACCACTG
ACCCATTCATACTCCCATGTAAAATAAAACAAATTATAAACATGTGGCAGAAAGTAGGAAAAGCAATGTATGC
CCCTCCCATCCGTGGACAGATTAGATGCTCATCAAATATTACAGGGCTGCTATTACCAAGAGATGGTGGTAAC
AACAACAATGGTAGTAACAACCGCAGTCCTGAGATCTTCAGACCTGGAGGAGGAGATATGAGGGATAATTGGA
GAAGTGAATTATACAAATATAAAGTAGTAAAAATTCAACCATTAGGAGTAGCACCCACCAAGGCAAAGAGAAG
AGCGGTGCATAGAGACAAAAGAGCAGTGGGACTCGGAGCTGTGTTCCTTGGATTCTTGGGAGCAGCAGGAAGC
ACTATGGGCGCAGCGTCACTGACGTTGACGGTACAGGCCAGACAATTATTGTCTGGTATAGTGCAACAGCAAA
ACAACTTGCTGAGGGCTATTGAGGCGCAACAGCATCTGTTGCAACTCACAGTCTGGGGCATC
>DQ141343..2
TTCTACTGTGATACATCACAACTATTTAATAGTACTTGGAATAGTAGTACTTTGAATAATTTTACTAACGGGT
CAAATATAACTAATGAAACTTTCACACTCCCATGCAGAATAAAACAATTTATAAACATGTGGCAGGAAGTAGG
AAAAGCAATGTATGCCCCTCCTATCAGAGGAGTAATTAACTGTTTATCAAATATTACAGGGCTGCTATTAACA
AGAGATGGTGGTAATAATCATAGTGACACCAATCAGGAGATCTTCAGGCCTGCAGGAGGAGATATGAGGGACA
ATTGGAGAGGTGAATTATATAAGTATAAAGTAGTAAGAGTAGAACCATTAGGGATAGCACCCACCAAGGCAAA
GAGAAGAGTGGTGCAGAGAGAAAAAAGAGCAGTGGGACTAGGAGCTTTGTTCCTTGGGTTCTTGGGAGCAGCA
GGAAGCACTATGGGCGCAGCATCAATGACGCTGACGGTACAGGCCAGACAATTATTGTCTGGTATAGTGCAAC
AGCAGAACAACCTGCTGAGGGCTATTGAGGCGCAACAGCATATGTTGCAACTCACAGTCTGGGGCATC
>DQ448819..1
TTCTACTGTAATACAACACAACTGTTTAATAGTACTTGGTATGCTAGTAATAATACTTGGAAGGGTACTAACA
TTACAGGCAATATCACACTCCCATGCAAAATAAAACAAATTATAAACATGTGGCAGGAAGTAGGAAAAGCAAT
GTATGCCCCTCCCATCAGAGGACAAATTAGATGCTCATCAAATATTACAGGGTTACTATTAACAAGAGATGGG
GGTCAGGGAAACAACACCGACAACGACACTGAGATCTTCAGACCTGGAGGAGGAAATATGAGGGACAATTGGA
GAAGTGAATTATATAAATATAAAGTAGTAAGAATTGAACCATTAGGACTAGCACCCACCAGGGCAAAGAGAAG
AGTGGTGCAGAGAGAGAAAAGAGCAGTGGGACTAGGAGCGTTGTTCATTGGGTTCTTGGGAGCAGCAGGAAGC
ACTATGGGCGCAGCGTCAGTGACGTTGACGGTACAGGCCAGACAATTACTGTCTGGTATAGTGCAACAGCAGA
ATAATTTGCTGATGGCTATTGAGGCGCAACAGCATATGTTGAAACTCACGGTCTGGGGCATT
>EF531331..1
TTTTACTGTAATACAACACAACTATTTAATAGTACTTGGAATATAACAAATCTGTTTAATAGTACTTGGAATA
GTACTGAAGGGTCAAATAACACTAAAAGAAATGGCACAATCATACACCCATGCAGAATAAAACAGATTATAAA
CATGTGGCAGAAAGTAGGAAAAGCAATGTATGCCCCTCCCATCGAAGGAATAATTAGATGTGAATCAAAAATT
ACAGGGATGATACTAACAAGAGATGGTGGTCTTGGTCGTAACATTAGCAACGAGACCGAGATCTTCAGACCTG
GAGGAGGAGATATGAGGGACAATTGGAGAAGTGAATTGTATAAATATAAAGTAGTAAAAATAGAACCCTTAGG
AATAGCACCCTCCAAGGCAAAGAGAAGAGTGGTGCAGAGAGAAAAAAGAGCAATAGGATTCGGAGCTATGTTC
CTTGGGTTCTTGGGAGCAGCAGGAAGCACTATGGGCGCAGCGTCACTAACGCTGACGGTACAGGCCAGACAAT
TACTGTCTGGTATAGTGCAACAGCAGAACAATTTGCTGAGGGCTATTGAGGCGCAACATCATCTGTTGCAACT
CACAGTCTGGGGCATC
>MH000288..1
TTCTATTGTAATACAACACAACTGTTTGATAGTACTTGGAATGATACTACAGACACAATCACACTCCCATGCA
AAATAAGACAAATTGTAAACTTGTGGCAGGAAGTAGGAAAAGCAATGTATGCCCCTCCCATCAGTGGAATAAT
TCAGTGTACATCAAATATTATAGGGTTGCTATTAACAAGAGACGGTGGTACCGAAATCTTCAGACCAGGAGGA
GGAGATATGAGGGACAATTGGAGAAGTGAATTATATAAATATAAAGTAGTACAAGTTGAACCATTAGGAATAG
CACCCACCAAGGCAAAGAGAAGAGTGGTGCAGAGAGAAAAAAGAGCAGTGGGAACGCTAGGAGCTGTATTCCT
CGGGTTCTTGGGAGCAGCAGGAAGCACTATGGGCGCAGCGTCAACAGCGCTGACGGTACAGGCCAGACAATTA
TTGTCTGGTATAGTGCAACAGCAGAACAATCTGCTGAAGGCTATTGAGGCGCAACAGCATTTGTTGCAGCTCA
CAGTCTGGGGCATC
>KC699040..1

TTCTATTGTGATACAGCACAACTGTTTAATAGTACTTGGCCTAAGAATAGCACCAGTAGCACCAATAGCACCG
GTAAAGGGGGGCCAACAACTATCACACTCCCATGCAGAATAAGACAAATTGTAAACATGTGGCAGGAAGTAGG
AAAAGCAATGTATGCTCCTCCCATCAGCGGAAACATCACATGTTCATCAAATATTACAGGGCTGCTATTAACA
AGAGATGGGGGTAATAAGAATGAGACGACCAACAGGACTCAGACCTTCAGACCTATAGGAGGAGATATGAGGG
ACAATTGGAGAAGTGAATTATATAAATATAAAGTAGTAAAAATTGAACCATTAGGAATAGCACCCACCAAGGC
AAAGAGAAGAGTGGTGCAGAGAGAAAAAAGAGCAGTGGGAGTAGGAGCTTTGTTCCTTGGGTTCTTGGGAGCA
GCAGGAAGCACTATGGGCGCAGCGTCAGTGACGTTGACGGTACAAGCCAGACAATTATTGTCTGGTATAGTGC
AACAGCAGAACAATCTGCTGAGAGCTATTGAGGCGCAACAGCATATGTTGCAACTCACAGTCTGGGGCATC
>KC699032..1
TTTTACTGTAATACAACACAGCTGTTTAATAGTACTTGGAATGCTAATGGTACTAGTCGGGATGGTACTACAG
GGAATGATACTATCACAATCCAATGCAGAATAAAGCAAATTATAAACAGGTGGCAGGAGGTAGGAAAAGCAAT
GTATGCCCCTCCCATCCAAGGACAAATTTACTGTTCATCAAATATTACAGGGCTTCTATTAACAAGAGATGGG
GGTAATGACAATAACACTAGCACTAATGAGACCTTCAGACCTGCAGGAGGAAATATGAAGGACAATTGGAGAA
GTGAATTATATAAATATAAAGTAGTAAAAATTGAGCCATTAGGACTGGCACCCACCAGGGCAAGGAGAAGAGT
GGTGCAGAGAGAAAAAAGAGCAGTGGGACTAGGAGCTATGTTCCTTGGGTTCTTGGGAGCAGCAGGAAGCACT
ATGGGCGCAGCGTCATTGACGCTGACGGTACAGGCCAGGCAATTATTGTCTGGTATAGTGCAACAGCAAAACA
ATTTGCTAAGAGCTATTGAGGCGCAACAGCATATGTTGCAACTCACAGTCTGGGGCATC
>KC699037..2
TTTTATTGTAATACATCACAACTGTTTAATAGTAGTTGGACCAATTACACTAATGGTACTTGGGACAATGACA
CCATAATAGATAACACTACCACTGGAGTAAATATCACACTCCCATGCAGAATAAAACAAATTATAAACATGTG
GCAGAAAGTAGGAAAAGCCATGTATGCCCCTCCCATCAGAGGACTAATTCAATGTACATCAAATATTACAGGA
ATACTATTAACAAGAGATGGAGGTAATAGGAATGAGAGCAATGGCAAAAGCAATGAGACCTTCAGACCTGTAG
GGGGAGATATGAGGGACAATTGGAGAAGTGAATTATATAAATATAAAGTAGTAAAAATTAAACCATTAGGAAT
AGCACCCACCAGGGCAAAGAGAAGAGTGGTACAGAGAGAAAAAAGAGCAGTGGGAACGTTGGGAGCCATGTTC
CTTGGGTTCTTGGGAGCAGCAGGAAGCACTATGGGCGCAGCGTCACTAACGCTGACGGTACAAGCCAGACAAT
TATTGTCTGGTATAGTGCAACAGCAAAGCAATTTGCTGAGGGCTATTGAGGCGCAGCAGCACATGTTGCAACT
CACAGTCTGGGGCATT
>MH000292..1
TTCTATTGTAACACAACACAGCTATTTAATAGTACTTGGATGCCTAATGGTGGCACTATCACACTCCCATGCA
GAATAAAACAAATTATAAACAGGTGGCAGGAAGTAGGAAAAGCAATGTATGCCCCTCCCATTAGTGGACAAAT
TAACTGCTCATCAAACATTACAGGGTTACTATTAATAAGAGATGGTGGTAACGAGACCTTCAGACCTGGAGGA
GGAAATATGAAAGACAATTGGAGAAGTGAATTATATAAATATAAAGTAGTACAAATTCAGCCATTAGGAGTAG
CACCCACCAAGGCAAAGAGAAGAGTGGTGCAGAGAGAAAAAAGAGCAGTGGGAACGCTAGGAGCTATGTTCCT
TGGGTTCTTGGGAGCAGCAGGAAGCACTATGGGCGCAGCGTCAGTGACGCTGACGGTACAGGCCAGACAATTA
TTGTCTGGTATAGTGCAACAGCAAAACAACTTGCTGAAAGCTATTGAGGCGCAACACCATCTGTTGCAACTCA
CAGTCTGGGGCATT
>KC699006..1
TTCTACTGTAATACAACACAACTGTTTAATAGTACTTGGAATAGTACTTGGGATATTAATAGTACTATAGAGT
CAAATAACACTGGAGAATATATCACACTCCCATGTAGAATAAAACAAATTATAAACATGTGGCAGGAAGTAGG
AAAAGCAATGTATGCCCCTCCCATCAAAGGACAAATTAGATGTTCATCAAACATTACAGGGCTGCTATTAACA
AGAGATGGTGGTAATAACAATAGTAGCAACACCACCGAGACCTTCAGACCTGGAGGAGGAGATATGAGGGACA
ATTGGAGAAGTGAATTATATAAATATAAAGTAGTAAAAATTGAACCATTAGGAGTAGCACCCACCAAGGCAAA
GAGAAGAGTGGTGCAGAGAGAAAAAAGAGCAGTGGGAATAGGAGCCGTGTTCCTTGGGTTCTTGGGAGCAGCA
GGAAGCACTATGGGCGCAGCGTCAATAACGCTGACGGTACAAGCCAGACTATTATTGTCTGGTATAGTGCAAC
AGCAGAACAATCTGCTGAGGGCCATTGAGGCGCAACATCATCTGTTGCAACTCACAGTCTGGGGCATC
>KC699007..1
TTCTACTGTAATTCAACACAACTGTTTAATAGTACTTGGAGTGAAGGGTCAAATAACACTGAAGGAAATAACA
CTGAAGGAAATGACACAATCACACTCCCATGCAGAATAAAACAAATTATAAACATGTGGCAGAACGTAGGAAA
AGCAATGTATGCCCCTCCCATCAGCGGACAAATTAGATGTTCATCAAATATTACAGGGCTGATATTAACAAGA
GATGGTGGTAATAACCCAAACGGGACCGAGATCTTCAGACCTGGAGGAGGAGATATGAGGGACAATTGGAGAA
GTGAATTATATAAATATAAAGTAGTAAAAATTGAACCATTAGGAGTAGCACCTACCAAGGCAAAGAGAAGAGT
GGTGCAGAGAGAAAAAAGAGCAGCGGGACTAGGAGCTGTGTTCCTTGGGTTCTTGGGAGCAGCAGGAAGCACT
ATGGGCGCAGCGTCAATAACGCTGACGGTACAGGCCAGACGATTATTGTCTGGTATAGTGCAACAGCAGAACA
ATTTGCTGAGGGCTATTGAGGCGCAACATCATCTATTGCAACTCACAGTCTGGGGCATC
>KC699025..1
TTCTACTGTAATACAACAAAACTGTTTAATAGTACTTGGAAGGAAAATGGCACCATTGAAGACGATGGTACAA
AGGATACTAACATCACACTCCCATGCAAAATAAAACAAATTATAAACAGGTGGCAGGCAGTAGGAAAAGCAAT
GTATGCCCCTCCCATCAGAGGACAAATTAAATGTACATCAAATATTACAGGGTTGCTGTTAGTAAGAGATGGT
GGTCATAACACTGACAACGAGACCTTCAGACCTGGAGGGGGAGACATGAAGGACAATTGGAGAAGTGAACTAT
ATAAATATAAAGTAGTAAAAGTTGAACCATTAGGAATAGCACCCACCAAGGCAAAGAGAAGAGTGGTGCAGAG

AGACAAAAGAGCAGTGGGAACACTAGGAGCAATGTTCCTTGGGTTCTTGGGAACAGCAGGAAGCACTATGGGC
GCAGCGTCAGTGGCGCTGACGGTACAGGCCAGACAATTACTGTCTGGTATAGTGCAACAGCAAAACAATTTGC
TGAAGGCTATTGAGGCGCAACAGCATCTGTTGCAACTCACAGTCTGGGGCATC
>JF706474..1
TTCTACTGTAATTCAGCACCACTGTTTAATAGTACTTGGAATAGTATTGAGGGTAGTACTGAGGGTAAGAATG
AAAGCAGTGGAGGAAATATCACACTCCTATGTAGAATAAAACAAATTATAAACATGTGGCAGGAAGTAGGAAA
AGCAATGTATGCCCCTCCTATCAGAGGAGTAATTCAATGCTCATCAAATATTACAGGGCTGCTATTAACAAGA
GATGGTGGTAGTGGTAATAGTAGCAATGAGAACGAGACCGAGACCTTCAGACCTGGGGGGGAAATATGAAGG
ACAATTGGAGAAGTGAATTATATAAATATAAAGTAGTACAAATTGAACCATTAGGAATAGCACCCACCAAGGC
AAAGAGAAGAGTGGTGCAGAGAGAAAAAAGAGCGGTGGGAACACTAGGAGCTGTGTTCCTTGGGTTCTTGGGA
GCAGCAGGAAGCACTATGGGCGCAGCGTCGGTAACGCTGACGGTACAGGCCAGACAATTATTGTCTGGTATAG
TGCAACAGCAGAACAATTTGCTGAAGGCTATTGAGGCGCAACAGCATCTGTTGCAACTCACAGTCTGGGGCAT
C
>HM204630..1
TTCTACTGTAATACAACAAAACTGTTTAATAGTACTTGGGGGAATAATACTGCAGGACAAGGTAATAACACTA
TCACACTCCCATGCAGAATAAAACAAATTATAAACTTGTGGCAGGAAGTAGGAAAAGCAATGTATGCCCCTCC
CATTAGTGGACACATTTACTGTTCATCAAATATTACAGGGCTACTATTAACAAGAGATGGTGGTACTACCAAC
ATAACCAATGAAACTGAGATCTTCAGACCTGGAGGGGGAGATATGAGGGACAATTGGAGAAGTGAATTATATA
AATATAAAGTAGTAAAAGTTGAACCATTAGGAATAGCACCCACCAAGGCGAAGAGAAGAGTGGTGCAGAGAGA
AAAAAGAGCAGTGGGAATAGGAGCTGTGTTCCTTGGGTTCTTGGGAGCAGCAGGAAGCACTATGGGCGCAGCG
TCACTGACGCTGACGGTACAGGCCAGACAATTATTGTCTGGTATAGTGCAACAGCAGAACAATTTGCTGAGAG
CTATTGAGGCGCAACAGCATCTGTTGCAACTCACAGTCTGGGGCATC
>HM204631..2
TTTTACTGTAATTCATCACAACTGTTTAATAGTACTTGGAATGCCACTAGTACTGGGAATAACAGTATAGGGT
TAAATGGCACTATCACACTCCCATGTAAAATAAAACAGATTATAAACAGGTGGCAGGAAGTAGGAAGAGCAAT
GTATGCCCCTCCCATTCAAGGAATAATTAGATGTGAATCAAATATTACAGGGCTACTGTTAACAAGAGATGGT
GGTAATATAACTAGTAATGGGAACGAGACCTTCAGACCCATAGGAGGAAATATGAAGGACAATTGGAGAAGTG
AACTCTATAAATATAAAGTAGTACAAATTGAACCCTTAGGAATAGCACCCACCAAGGCAAAGAGAAGAGTGGT
GCAGAGAGAAAAAAGAGCAGTGGGAACAATAGGAGCTATGTTCCTTGGGTTCTTGGGAGCAGCAGGAAGCACT
ATGGGCGCAGCGTCGGTGGCGCTGACGGTACAGGCCAGACAATTATTGTCTGGTATAGTGCAGCAGCAGAACA
ACCTGCTGAGGGCTATTGAGGCGCAACAGCACATGTTGCAACTCACAGTCTGGGGCATT
>HM204636..1
TTCTATTGTAAAACAACAGAGCTGTTTAATAGTACTTGGCCAGGGAATAGTACTGTGAATAATGCTACAGAAT
CAAATGACAATGACACTATCACACTCCCATGCAGAATAAAACAAATTATAAACAGGTGGCAGGAAGTAGGAAA
AGCAATGTATGCCCCTCCCATCCAAGGAAACATTAGCTGTTCATCAAACATTACAGGACTGCTATTAACAAGA
GATGGTGGTAAAGATCATAACTCCACCGAGACCTTTAGACCTGGAGGAGGAGATATGAGAGACAATTGGAGAA
GTGAATTATACAAATATAAAGTAGTAAGAATTGAGCCATTAGGAGTAGCACCCACCAAGGCAAGGAGAAGAGT
GGTGCAGAGAGAAAAAAGAGCAGCACTGGGAGCTGTGTTCCTTGGATTCTTGGGAGCAGCAGGAAGCACTATG
GGCGCAGCGTCAGTGGCGCTGACGGTACAGGCCAGACAATTATTGTCTGGTATAGTGCAACAGCAAAACAATT
TGCTGAGGGCTATTGAGGCGCAACAACATCTGTTGCAACTCACAGTCTGGGGCATC
>HM204637..1
TTCTACTGTAATACAACACAGTTGTTTAATAATACACAGTGGGTTAATAGTAATAGTACAAATAGCAATGACA
CCATCACACTCCCATGCAGAATAAAACAAATTATAAACAGATGGCAGGAAGTAGGAAAAGCAATGTATGCCCC
TCCCATCGCGGGACAAATTCACTGTTCATCAAATATTACAGGGCTAATATTAACAAGAGATGGTGGTAATAAA
AATGACACCAACGGGACTGAGACTTTCAGGCCTGGAGGAGGAGATATGAGGGACAATTGGAGAAGTGAATTAT
ATAAATATAAAGTAGTAAAAATTGAACCATTAGGAATAGCACCCACCAAGGCAAGGAGAAGAGTGGTGCAGAG
AGAAAAAAGAGCAGTAACTTTAGGAGCTATGTTCCTTGGGTTCTTGGGAGCAGCAGGAAGCACTATGGGCGCA
GCGTCAATGACGCTGACGGTACAAGCCAGACAATTATTGTCTGGTATAGTGCAACAGCAAAACAATTTGCTGA
GAGCTATTGAGGCGCAACAGCACCTGTTGCAACTCACAGTCTGGGGCATC
>HM204644..2
TTCTACTGCAACACATCAAAACTGTTTAATAGTAGTTGGAGTACTAATGGAACATTGTTAAATGGAACGTGGG
TAAATGAGACTAGAATAAATGACACAATCATACTCCCATGCAAAATAAAACAAATTATAAACATGTGGCAGAA
AGTAGGAAAAGCAATGTATGCTCCCCCCATCCAAGGAATAATTAACTGCTCATCAAATATTACAGGACTGCTA
TTAACAAGAGATGGTGGTCATAATCAAAATAGCACCAATGACACAGAGACCTTCAGACCAGGAGGAGGAGATA
TGAGGGACAATTGGAGAAGTGAATTATATAAATATAAAGTAGTAAGAATTGAACCATTAGGAATAGCACCCAC
CAAGGCAAAGAGAAGAGTGGTGCAGAGAGAAAAAAGAGCAGTGGGATTCGGAGCTATGTTCCTTGGGTTCTTG
GGAGCAGCAGGAAGCACTATGGGCGCAGCGTCAATAACGCTGACGGTACAGGCCAGACAATTGCTGTCTGGTA
TAGTGCAACAGCAAAACAATTTGCTGAGGGCTATTGAGGCGCAACAGCATATGTTGCAACTCACAGTCTGGGG
CATC
>JF680923..1

TTCTACTGTAACACAACAAAACTGTTTAATAGTACCTGGGAGAATAATAGTACTTGGGGGAATAATACTACAG
GTACTATCACACTCCCATGCAGAATAAAACAAATTGTAAACATGTGGCAAGAAGTAGGAAAAGCAATGTATGC
CCCTCCCATCCAAGGACAAATTAGATGCTCATCAAATATTACAGGACTGTTATTAACAAGAGATGGTGGTAAT
AATAATAGCACAAATCAGAGCGAGACCTTCAGACCTGGAGGAGGAAATATGAAGGACAATTGGAGAAGTGAAT
TATATAAATATAAAGTAGTAAAAATTGAGCCATTAGGAATAGCACCCACCAGGGCAAAAAGAAGAGTGGTGCA
GAGAGAAAAAAGAGCAGCCATAGGAGCTGTGTTCCTTGGGTTCTTGGGAGCAGCAGGAAGCACTATGGGCGCA
GCGTCAGTGACGCTGACGGTACAGGCCAGACAATTATTGTCTGGTATAGTGCAACAGCAGAACAATTTGCTGA
AGGCTATTGATGCGCAACAGCATCTGTTGCAGCTCACAGTCTGGGGCATC
>JF680924..1
TTCTACTGCAATACAACATTACTGTTCAATAGTGATTGGTATCCTAATGATACTAGCACAGGTACTCCAGGGT
CAGATGGGAATGACTCACTCATACTCCCATGCAGAATAAGACAAATTGTAAACATGTGGCAGGAAGTAGGAAA
AGCAATGTATGCTCCTCCCATCAAAGGACAAATTAGGTGTCATTCAAATATTACAGGATACTACTAACAAGA
GATGGTGGGATTGACAACAACAGGACTAATGAGACCTTTAGACCTGGAGGAGGAGATATGAGGGACAATTGGA
GAAGTGAATTATATAAATATAAAGTAGTAAAACTTGAACCATTAGGATTAGCACCCACCAAGGCAAAGAGAAG
AGTGGTGCAGAGAGAAAAAAGAGCAGTGGGACTAGGAGCTATGTTCCTTGGGTTCTTGGGAGCAGCAGGAAGC
ACTATGGGCGCAGCGTCAATGACGCTGACGGTACAGGCCAGACAATTATTGTCTGGTATAGTGCAACAGCAGA
ACAATCTGCTGAAGGCTATTGAGGCGCAACAGCATATGTTGCAACTCACAGTCTGGGGCATC
>JF680933..1
TTCTATTGCAATACAACACAACTGTTTAATACTACTTGGAATATTACTAATAATATTAATGTTACTAGGAATG
ATAGTGACAACAGCATTATCACACTCCCATGCAGAATAAAACAAATTATAAACATGTGGCAGGAAGTAGGAAA
AGCAATGTACGCCCCTCCCATCAGAGGACAGATTAGATGTTCATCAAAAATTACAGGGCTACTATTAACAAGA
GATGGTGGTAATACAAGCATTGCGAACGGGTCTGAGATCTTCAGACCTGGAGGAGGAAATATGAAGGACAATT
GGAGAAGTGAATTATACAAATATAAAGTAGTAAAAGTTCAACCATTAGGAATAGCACCCACCAAGGCAAAAAG
AAGAGTGGTCCAGAGAGAAAAAAGAGCAATAACACTAGGAGCTGTTTTCCTTGGGTTCTTGGGAGCAGCAGGA
AGCACTATGGGCGCAGCGTCAATAACGCTGACGGTACAGGCCAGACAATTATTGTCTGGTATAGTGCAGCAGC
AGAGCAACTTGCTGAGGGCTATTGAGGCGCAACAACATATGTTGCAACTCACAGTCTGGGGCATC
>HQ595797..1
TTCTACTGTAATTCAACACAACTGTTTAGTAGTACTTGGATGGCTAATGGTACTTCAAATGGTACCGTAGGGA
ATGACACTATCATACTCCCATGTAAAATAAAACAATTTGTAAACATGTGGCAGCGAATAGGACAAGCAGTGTA
TGCCCCTCCCATTAGAGGATACATTAATTGTTCATCAAATATTACAGGACTGCTATTAACAATAGATGGTGAC
AGGAATGACACCCAGAGTGAGACCTTCAGACCTGGAGGAGGAGATATGAGGGACAATTGGAGAAGTGAATTAT
ATAAATATAAAGTAGTAAAAATTGAACCATTAGGAATAGCACCCACTAAGGCAAAGAGAAGAGTGGTGCAGAG
AGAAAAAAGAGCAGTAACGCTGGGAGCTATGTTCCTTGGATTCTTGGGAGCAGCAGGAAGCACTATGGGCGCA
GCGTCAATGACGCTGACGGTACAGGCCAGACTATTACTGTCTGGTATAGTGCAACAGCAGAGCAATCTGCTGA
GGGCTATTCAGGCGCAACAGCATCTGTTGCAACTTACAGTCTGGGGCATC
>AJ535611..1
TTCTACTGTAATACAACACAGCTGTTTAATAGTACTTGGAATATTACACAGCTGTTGAATAGTACTGAGTCAA
ATAACACTGGCAATATCACACTCCCATGCAGAATAAGACAAATTGTAAACAGGTGGCAGGAAGTAGGAAAAGC
AATGTATGCCCCTCCCATCAGCGGACAAATTAGTTGTTCATCAAATATTACAGGGTTGCTATTAACAAGAGAT
GGAGGTAACAGTAGCTACAATGAGACCGAGACCTTCAGACCAGGAGGAGGAAATATGAGGGACAATTGGAGAA
GTGAATTATATAAATATAAAGTAGTAAGAATTGAACCAATAGGAGTAGCACCCACCAGGGCAAAGAGAAGAGT
GGTACAGAGAGAAAAAAGAGCAATAACGCTGGGAGCTGTGCTCCTTGGGTTCTTGGGAGCAGCAGGAAGCACT
ATGGGCGCAGCGTCAATAACGCTGACGGTACAGGCCAGACAGTTATTGTCTGGTATAGTGCAGCAGCAGAACA
ATCTGCTGAGGGCTATTGAGGCGCAACAGCACCTGTTGCAACTCACAGTCTGGGGCATC
>AJ286339..1
TTCTACTGTAGTACAACAGAACTGTTTAATGACACTTGGGATAAGACTAGGAATAATACTGGAGAGGCAGATA
ACAGCACAATCACACTCCCCTGCAGAATAAAACAAATTATAAACAAGTGGCAAGAAGTAGGAAAAGCAATGTA
TGCCCCTCCCATCAGAGGACAAATTAAGTGTACATCAAATATTACAGGGCTAGTCTTAACAAGAGATGGTGGT
AATGAGACTCATAATGAGACTGAGATCTTCAGACCTGGAGGAGGAGATATGAGGGACAATTGGAGAAGTGAAT
TATACAAATATAAAGTAGTAAAAATTGAGCCATTAGGAGTAGCACCCACCAAGGCAAGGAGAAGAGTGGTGCA
GAGAGAAAAAAGAGCAGTGGGAACAATAGGAGCTATGTTCCTTGGGTTCTTGGGAGCAGCAGGAAGCACTATG
GGCGCAGCGCCAATGACGCTGACGGTACAGGCCAGACAATTATTGTCTGGTATAGTGCAGCAGCAGAACAATC
TGCTGAAGGCTATTGAGGCGCAACAGCATCTGTTGCAACTCACAGTCTGGGGCATC
>DQ207942..1
TTCTATTGTAATACAACACAGCTGTTCAATAATACTTGGCTGAATAATGATACTGGGAATAGCACTAAGAATA
ATGCTACAATCACACTCCCGTGCAGAATAAAACAAATTATAAACAGATGGCAGGAAGTAGGAAAAGCAATGTA
TGCCCCTCCCATCAGAGGACTAATTAGATGTTCCTCAAATATTACAGGGCTACTATTAGTAAGAGATGGTGGT
AATAGTAGTGCTGATCAGGAGATCTTCAGACCTGGGGGAGGAAATATAAAGGACAATTGGAGAAGTGAACTAT
ATAAATATAAAGTAGTAAAACTTGAGCCATTAGGAGTAGCACCCACCAAGGCAAAGAGAAGAGTGGTGCAGAG
AGAAAAAAGAGCAGTGGGAGTGTTTGGAGCCATGTTCCTTGGGTTCTTGGGAGCAGCAGGAAGCACTATGGGC

GCAGCGTCAATAACGCTGACGGTACAGGCCAGACAATTATTGTCTGGTATAGTGCAACAGCAGAGCAATTTGC
TGAGAGCTATTGAGGCGCAACAGCATCTGTTGCAACTCACAGTCTGGGGCATT
>MH078546..1
TTCTACTGTGATTCAACACAACTGTTCAATAGTACTTGGTGGACTAATGGTACTGAGAACGGTACTAGAGGGA
CAATTGGCACCATCACACTCCAATGCAGAATAAAACAAATTATAAACATGTGGCAGAAAATAGGAAAAGCAAT
GTATGCCCCTCCCATCAAAGGACAAATTTATTGTTCATCAAATATTACAGGGATAATATTAACAAGAGATGGT
GGTAGGGACAATAATACTAATGGCACTGAGACCTTCAGACCTGGAGGAGGAAATATGAAAGACAATTGGAGAA
ATGAATTATATAAATATAAAGTAGTAAAACTTAAGCCATTAGGAATAGCACCCACCAAGGCACGGAGAAGAGT
GGTGCAAAGAGAAAAAAGAGCAGTGGGAATAGGAGCTCTGTTCCTTGGGTTCTTGGGAGCAGCAGGAAGCACT
ATGGGCGCAGCGTCAGTGACGTTGACGGTACAGGCCAGACAATTGTTGTCTGGTATAGTGCAACAGCAACACA
ACTTGCTGAATGCTATTGAGGCGCAACAACACCTGTTGCAACTCACAGTCTGGGGCATC
>EU839600..1
TTCTACTGTAATACATCAAAGCTATTTAACAATACTTGGAATACTACTAATACTAAGAATTGGACTGGTTTTA
TCATACTCCCATGCAGAATAAAACAAATTATAAATATGTGGCAAAAAGTAGGCAAAGCAATGTATGCCCCTCC
CATCAGGGGACAAATCAAATGTATATCAGCTATTACAGGGATGCTATTAACAAGAGATGGTGGGAACAACAAC
GAGAATAAGACTGTAGGGAATAGGACCGAGATCTTCAGACCTACAGGAGGAAATATGAAAGATAATTGGAGAA
GTGAATTATACAGCTATAAAGTAGTAAAAATTGAACCATTAGGATTAGCACCCACCAGGGCGAAAAGAAGAGT
GGTGCAGAGAGAAAAAAGAGCAGTGGGAGCACTGGGAGCTATGTTCCTTGGGTTCTTGGGAGCAGCAGGAAGC
ACTATGGGCGCAGCGTCATTGACGCTGACGGTACAGGCCAGACAATTACTGTCTGGTATAGTGCAACAGCAAA
ATAATTTGCTAAGGGCTATTGAGGCGCAACAGCATCTGTTGCAACTCACAGTCTGGGGCATC
>MK303349..1
TTCTACTGTGATACAACAGAACTGTTTAATAGTACTTGGAATAATAGTGTTGGAGGGTTAAATAACAGTAACA
GTAATACTATCACACTCCCATGTAGAATAAAACAAATGGTAAACATGTGGCAGAGAGTAGGAAGAGCAATGTA
TGCCCCTCCCATCAAAGGACTAATTAGCTGTTCATCAGATATTACAGGGCTGCTATTAACAAGAGATGGTGGT
AACTATAGTGATAACAGTAATAACACTGAGACCTTCAGACCAGGAGGAGGAGATATGAAAGACAATTGGAGAA
GTGAATTATATAAATATAAAGTAGTAGAAATTAAACCACTAGGAGTAGCGCCCACCAAGGCAAAAAGAAGAGT
GGTGCAAAGAGAAAAAAGAGCAGTGGGAATAGGAGCCTTGTTCCTTGGGTTCTTGGGAGCAGCAGGAAGCACT
ATGGGCGCAGCGTCAATAACGCTGACGGCACAGGCCAGACAATTATTGTCTGGTATAGTGCAACAGCAGAACA
ATCTGCTGAGGGCTATTGAGGCGCAACAGCAGCTATTGCAACTCACAGTCTGGGGCATC
>EF694037..1
TTCTACTGTATTACAACACAACTGTTTAATAGTACTTGGTGGAATGATACTGAAAGGATAAATAACATTGAAG
AAAACACAGAAAATATCACACTCCCATGCAGAATAAGGCAGATTGTAAACCTGTGGCAGACAGTAGGAAAAGC
AATGTATGCCCCTCCCATCAGAGGACAAATTAGATGTTCATCAAATATTACGGGGCTGCTATTAACAAGAGAT
GGTGGTAATCCCAATACCACGGAAGAGAGCTTCAGACCTGGAGGAGGAGATATGAGGGACTATTGGAGAAGTG
AATTATATAAATATAAAGTAGTAAAAATTGAACCATTAGGAGTAGCACCCACCAAGGCAAAGAGAAGAGTGGT
GCAAAGAGAAAAAAGAGCAGTAGGAATAGGAGCTGTGTTCCTTGGGTTCTTGGGAGTAGCAGGAAGCACTATG
GGCGCAGCGTCAATGACGCTGACGGTACAGGCCAGACTATTATTGTCTGGTATAGTGCAACAGCAGAATAACC
TGCTGAGGGCTATTGAGGCGCAACAGCATCTGTTGCAACTCACAGCCTGGGGCATC
>JF680930..2
TTCTATTGTAATACAACGAAACTGTTTAGCAGTAGTTGGAATGAAACTACAAATAGCACTGAAAATGGCACTG
AAAATGACACAATCACACTTCAATGCAGAATAAAACAAATTATAAACATGTGGCAGGAAGTAGGAAAAGCAAT
GTATGCCCCTCCCATCAAAGGTCCAATTAGATGCTCATCAAATATTACAGGGCTAATATTAACAAGAGATGGT
GGTCATAACAATAACACGAACGAGACTGAGATATTCAGACCTGGAGGAGGAGATATGAGGGACAATTGGAGAA
GTGAATTATACAAATATAAAGTAGTAAGAATTGAACCCTTGGGAGTAGCACCCACCAAGGCAAAGAGAAGAGT
GGTGCAGAGAGATAAAAGAGCAGTGGGAGTATTCGGAGCTATGTTCCTTGGGTTCTTGGGAGCAGCAGGAAGC
ACTATGGGCGCAGCGTCAGTGGCGTTGACGGTACAGGCCAGACAATTATTGTCTGGTATAGTGCAACAGCAGA
ATAATCTGCTGAGAGCTATTGAGGCGCAACAACATCTGTTGCAACTCACAGTCTGGGGCATC
>AB287372..1
TTCTACTGTAATTCAACACCACTGTTTAGTAGTAATTGGACTAAGAATACTACTACCGGGTCTTGGATTGGCA
GATATGGATCAAATGAGACTGATGCAAATATTACACTCATATGCAGAATAAAACAAATTATAAACATGTGGCA
GGGGGTAGGAAAAGCAATGTATGCCCCTCCCATCAGTGGACCAATCAGATGTTCATCAAATATTACAGGGCTG
CTATTAACAAGAGATGGTGGTGAGGATAAGAACAGCACTAAAGAGACCTTCAGACCCCTAGGAGGAAATATGA
AAGACAATTGGAGAAGTGAATTATATCAATATAAAGTAGTAGGAATTGAACCATTAGGAGTAGCACCCACCAA
GGCAAGGAGAAGAGTGGTGCAGAGAGAAAAAAGAGCAGTGGGAACGCTGGGAGCTATGTTCCTTGGGTTCTTG
GGAGCAGCAGGAAGCACTATGGGCGCAGCGTCAATGGCGCTGACGGTACAGGCCAGACAATTATTGTCTGGTA
TAGTGCAACAGCAGAACAATTTGCTGAGGGCTATTGAGGCGCAACAACATTTGTTGCAACTCACAGTCTGGGG
CATC
>AB588264..1
TTCTACTGCAATACATCACAACTGTTTAATAGTACTTGGAATAGTACTGGGAATAGTAGTACTGAGAATAGTA
TGTCAAATGACACTATCACACTCCCATGTAGGATAAAACAAATTATAAATATGTGGCAGGAAGTAGGAAAAGC

AATGTATGCCCCTCCCATCGCAGGACTAATTAGATGTTCATCAAATATTACAGGGCTATTATTAGTGAGAGAT
GGTGGTGGTAATCAGACTAACGGGACAGAAATCTTCAGACCTGGGGGAGGAAATATGAAGGACAATTGGAGAA
GTGAATTATATAAATATAAAGTAGTAAAAATTGAACCAGTAGGAATAGCACCCGACAGGGCAAAGAGAAGAGT
GGTGCAGAGAGAAAAAAAGGGCAGTGGGAATAGGAGCTGTGTTCCTTGGGTTCTTGGGAGCAGCAGGAAGCACT
ATGGGCGCAGCGTCAATAACGCTGACGGCACAGGCCAGACAATTATTGTCTGGTATAGTGCAACAGCAGAACA
ATCTGCTGAGGGCCATTGAGGCGCAACAGCATATGTTGCAACTCACAGTCTGGGGCATC
>AB731669..1
TTCTACTGTAATACATCAGCACTGTTTAATTGGAGTATTACTGGGTCAAATATTACAAAAGACATTAATAGGT
CAAATACTATCCCATGCAGAATAAAACAAATTATCAACATGTGGCAGAAAGTAGGAAAAGCAATATATGCCCC
TCCCATCAGCGGGCAAATTAATTGTTCATCAAATATTACAGGGTTGCTATTAACAAGAGATGGTGGTAAAAGT
AATCAGACTAATAACACTGAGGTTTTCAGACCTACAGGAGGAAATATGAGAGATAATTGGAGGAGTGAATTAT
ATAAATATAAAGTAGTAAGGATTGAACCATTAGGAGTAGCACCCACCAAGGCAAGGAGAAGAGTGGTGCAAAG
AGAAAAAAGAGCAGTGGGAACAATAGGAGCTATGTTCCTTGGGTTCTTGGGAGCAGCAGGAAGCACTATGGGC
GCAGCGTCAATAACGCTGACGGTACAAGCCAGACTATTATTGTCTGGTATAGTGCAACAGCAAAACAATTTGC
TGAGAGCTATTGAGGCGCAACAACATCTGTTGCAACTCACAGTCTGGGGCATC
>AB565478..1
TTCTACTGTAATACAACAAAGCTGTTTAATAGTACTTGGTATACTAATGGGAATAAGACTGAAGAATCAAATA
ACAATGACACTATTACACTCCCATGCAGAATAAAACAAATTATAAACAGATGGCAGGAAGTAGGAAAAGCAAT
GTATGCCCCTCCCATCCAAGGACGAATTTACTGCTCATCAAATATTACAGGACTGTTATTAATAAGAGATGGT
GGTAACAGTACTGATAACGAGTCTGAGATCTTCAGACCTGGAGGAGGAGATATGAGGGACAATTGGAGAAGTG
AATTATATAAATATAAAGTAGTACAAATTGAGCCATTAGGAGTAGCACCCACCAAGGCAAAGAGAAGGGTGGT
GCAGAGAGAAAAAAGAGCAGTGGGAATAGGAGCTATGTTCCTTGGGTTCTTGGGAGCAGCAGGAAGCACTATG
GGCGCAGCGTCACTAACGCTGACGGTACAGGCCAGACGATTATTGTCTGGTATAGTGCAACAGCAGAACAATC
TGCTGAGAGCTATTGAGGCGCAACAGCATATGTTGCAACTCACAGTCTGGGGCATC
>JQ316131..2
TTCTACTGTAATACATCAAAGCTGTTTAATAGTACTTGGGCTGTGAATATGACTAACGGGTCAAATATAACTA
AAGAAGGAAATACCACAACAATCCCATGCAGAATAAAACATATTATAAACAGGTGGCAAGAAGTGGGAAAAGC
AATGTATGCCCCTCCCATCGAGGGACAAATTAGATGTACATCAAATATTACGGGGCTACTATTAACAAGAGAT
GGGGGGACTCAGAAAAATGGGAGTAACAATGGGACTCACAACAACACTGAGATCTTCAGACCTGGGGGAGGAA
ATATGAAGGACAATTGGAGAAGTGAATTATATAAATATAAAGTAGTAAAAATTGAGCCATTAGGAATAGCACC
CACCAAGGCAAGGAGAAGAGTGGTGCAGAGAGAAAAAAGAGCAGTGGGAACAATAGGAGCTATGTTCCTTGGG
TTCTTGGGAACAGCAGGAAGCACTATGGGCGCAGCGTCACTGACGCTGACGGTACAGGCCAGACTATTATTGT
CTGGTATAGTGCAACAGCAAAATAATTTGCTGAGAGCTATTGAGGCGCAACAGCATCTGTTGCAACTCACAGT
CTGGGGCATC
>JQ316133..1
TTCTACTGTAATACAACAAAACTGTTTGATAGTACTTGGAATGGTACTAAAGAGTGGAATGATACTAAAGAGT
CAGATAAAAACATTACACTCCAATGCAGAATAAAACAAATTATAAACAGGTGGCAGGAAGTAGGACAAGCAAT
GTATGCCCCTCCCATCAGCGGACTAATTAGATGTTCGTCAAATATTACAGGGCTAATATTAACAAGAGACGGG
GGGGCTAACGAGACTCACACCAACACTGAAACCTTCAGACCTGCAGGAGGAGATATGAAGGACAATTGGAGAA
GTGAATTATATAAATATAAAGTAGTAAGAATTGAGCCATTAGGGATAGCACCCACCAAGGCAAGGAGAAGAGT
GGTGCAGAGAGAAAAAAGAGCAGTGGGAATATTAGGAGCTATGTTCCTTGGGTTCTTGGGAGCAGCAGGAAGC
ACTATGGGCGCAGCGTCAATGACGCTGACGGTACAGGCCAGACAATTATTGTCTGGTATAGTGCAACAGCAGA
ATAATTTGCTGAGAGCTATTGATGCGCAACAACATCTGTTGCGACTCACAGTCTGGGGCATT
>DQ295192..1
TTCTACTGTGATACAACACAACTATTTAATAGCACTTGGACATGGAATGGTACTTGGATTAACGGGTCAACTA
AAGCTGAGAATGACACAATTGTCCTCCCATGCAGAATAAAACAAATTATAAACAGATGGCAGGAAGTAGGAAA
AGCAATGTATGCCCCTCCCATCAGCGGACACATTAATTGCTCATCAAAGATTACAGGACTGCTATTAACAAGA
GATGGGGGGATTAAAAACAGCACGAACGGGACTCAGACTGAGATCTTCAGACCTGGGGGAGGAGATATGAGGG
ACAATTGGAGAAGTGAATTATATAAATATAAAGTAGTAAAAATTGAGCCATTAGGGATAGCACCCACCAAGGC
AAGGAGAAGAGTGGTGCAGAGAGAAAAAAGAGCAGTGGGAACAATAGGAGCTATGTTCCTTGGGTTCTTGGGA
GCAGCAGGAAGCACTATGGGCGCAGCGTCACTGACGCTGACGGTACAGGCCAGACTACTATTGTCTGGTATAG
TGCAACAGCAGAATAATTTGCTGAGAGCTATTGAGGCGCAACAGCATCTGTTGCAACTCACAGTCTGGGGCAT
C
>KT878039..2
TTCTACTGTAATACAACAAAACTGTTTAATAGTACTTGGTATAGTAATGGTACTAGGAAAGATCAAAATGAAG
GAGATGACACAATTACACTCCAGTGCAGGATAAAACAAATTATAAACAGGTGGCAAGAAGTAGGAAGGGCAAT
GTACGCCCCTCCCATCAGAGGAATAATTAAATGTTCATCAAACATTACAGGGCTGCTATTAACAAGAGATGGG
GGGAACGGGACTAATGGGACTGAGACCTTCAGACCTGGGGGAGGAAATATGAAAGACAATTGGAGAAGCGAAT
TATATAAATATAAAGTAGTAAAAATTGAGCCATTAGGGATAGCACCCACCAAGGCAAGGAGAAGAGTGGTGCA
GAGAGAAAAAAGAGCAGTGGGGACGCTAGGAGCTGTGTTCCTTGGGTTCTTGGGAGCAGCAGGAAGCACTATG

GGCGCAGCGTCAGTGGCGCTGACGGGACAGGCCAGACAATTATTGTCTGGTATAGTGCAACAGCAGAATAATT
TGCTGAGAGCTATTGAGGCGCAACAGCATCTGTTTCAACTCACAGTCTGGGGCATC
>KT878021..2
TTCTACTGTAATACAACACAGTTGTTCAATAGTACTTGGTTTCATAATGGTACTTGGAGTGGTGCTAATGGGG
ATGAGACTATGACACTCCCATGCAGAATAAAACAAATTATAAACAGATGGCAAGAAGTAGGAAAAGCAATGTA
TGCCCCTCCCATCGCAGGATCAATTAATTGCTCATCAAATATTACAGGACTGTTATTAACAAGAGATGGTGGT
ATTACCAGCAACAAGACTAAGAACAGCACCGAGACTGAGACCTTTAGACCGGGGGGAGGAGATATGAGGGACA
ATTGGAGAAGTGAATTATACAAATATAAAGTAGTAAAAATTGAACCACTAGGAGTAGCACCAACCAAGGCAAA
GAGAAGAGTGGTGCGGAGAGAAAAAAGAGCAGTAACACTGGGAGCTGTGTTCCTTGGGTTCTTGGGAGCAGCA
GGAAGCACTATGGGCGCAGCGTCAGTGACGCTGACGGTACAGGCCAGACTATTATTGTCTGGTATAGTGCAGC
AGCAGAACAATCTGCTGAGGGCTATTCAGGCGCAACAGCATCTGTTGCAGCTCACAGTCTGGGGCATT
>KT878029..1
TTCTACTGTAATACAACAAGACTGTTTAATAGTACTTGGCATACTAATACTACAGGGTTAAATGAAACTGAAG
GAAATAGCACAACTATCACACTCCCATGCAGAATAAAACAAATTATAAACAGATGGCAGGAAGTAGGAACAGC
AATGTATGCCCCTCCCATCAGCGGACAAATTAGATGTTCATCAAATATTACAGGGCTGCTATTAACAAGAGAT
GGGGGGAATAACACTCATGGGAATGGGACTGAGATCTTCAGACCTCTGGGAGGAGATATGAAGGACAATTGGA
GAAGTGAATTATATAAATATAAAGTAGTGAGAATTGAGCCATTAGGGATAGCACCCACCAGGGCAAGGAGAAG
AGTGGTGCAGAGAGAAAAAAGAGCAGTGGGAACGATAGGAGCTATGTTCCTTGGGTTCTTGGGAGCAGCAGGA
AGCACTATGGGCGCAGCGTCATTGACGCTGACGGTACAGGCCAGACTATTATTGTCTGGTATAGTGCAACAGC
AGAATAATTTGCTGAGAGCTATTGAGGCGCAACAGCATCTGTTGCAACTCACAGTCTGGGGCATC
>KF561441..1
TTCTACTGTAATACAACACCACTGTTTAATAGTATTTGGTATAGTAATAATACTGAAAAGTCATATAATACTG
CAGAAAATGACACAATTACCCTCCAATGCAGAATAAGACAAATTATAAAAAGGTGGCAGGAGGTAGGAAAAGC
AAGGTATGCCCCTCTCATCACCTGCCTAATTAGATGCTATTCAAATATTACAGGGCTGTTATTGGCAGGTGAT
GGAGGTGAGGCAGGGAAGGATAACGGGAATAAGAATGAGACCTTCAGGCCTGGGGGAGGAGATATGAGGGACA
ATTGGAGAAGTGAATTGTATAAATATAAAGTAGTAGAAATTGAACCATTAAGGATAGCACCCACCAGGGCAAG
GAGAATAGTGGTGCAGAGAGAAAAAAGAGCAGTGGGAACATTAGGAGCCATGTTCCTTGGGTTCTTGGGAGCA
GCAGGAAGCACTATGGGCGCAGCGTCACTGACGCTGACGGTACAGGCCAAGCAAGTATTGTCTGGTATAGTGC
GACAGCAGAATAATTTGCTGAGAGCTATTGAGGCGCAACAGCGTCTGTTGCAACTCACAGTCTGGGGCATC
>MN043577..1
TTCTACTGTAATACAACACAACTGTTTAATAGTACTTGGTATAGTCCTTGGAATAGTACTAACGGGTCAAATA
TAACTGAAAGAAATATCACAACACTCCCATGCAGAATAAAACAAATTATAAACAGATGGCAGGAAGTAGGAAA
AGCAATGTATGCCCCTCCCATCAGTGGACAAATTAGATGTTCATCAAATATTACAGGGCTGCTATTAACAAGA
GATGGGGGGACTCAGAACAATGGGACTGAGATCTTCAGACCTGGGGGAGGAAATATGAAGGACAATTGGAGAA
GTGAATTATATAAATATAAAGTAGTAAAAATTGAGCCATTAGGGATAGCACCCACCGGGGCAAAGAGAAGAGT
GGTGCAGAGAGAAAAAACAGTGGGAACGATAGGAGCTATGTTCCTTGGGTTCTTGGGAGCAGCAGGAAGCACT
ATGGGCGTAGCGTCAATGACGCTGACGGTACAGGCCAGACTATTATTGTCTGGTATAGTGCAACAGCAGAATA
ATTTGCTGAGAGCTATTGAGGCGCAACAGCATCTGTTGCAACTCACAGTCTGGGGCATC
>MN043581..2
TTCTACTGTAATACAACACAACTGTTTAATAGTACTTGGATGATTACTAATAATTGCAATAGTACTGAAGGAT
ACAATAACACTGCACGAAATAGCACAATTACCCTCCCATGCAGAATAAAACAAATTATAAACAGGTGGCAGGA
AGTAGGAAAAGCAATGTATGCCCCTCCCATCAGAGGACAAATTAGATGTTCATCAAATATTACAGGGCTACTA
TCAACAAGAGATGGGGGGACTGGGAACAACGAGACTGAGATCTTCAGACCTGGGGGAAGAAATATGAGGGACA
ATTGGAGAAGTGAATTATATATATATAAAGTAGTAACAATTGAGCCATTAGGGATAGCACCCACCGGGGCAAA
GAGAAGAATGGTGCAAAGAGAAAAAAGAGCAGTGGGAACGATGGGAGCTATGTTCCTTGGGTTCTTGGGAGCA
GCAGGAAGCACTATGGGCGCGACATCAATGACGCTGACGGTACAGGCCAGACTATTATTGTCTGGTATACTGC
ATCATCAGAATAATCTGCTGAAAGCTATTGAGGCGCAACAGCATCTGTTGCAACTCACAGTCTGGGGCATC
>HQ386180..1
TTCTATTGTAACACAGCACAACTGTTTAATAGTACTTGGAATGCAACAGAAATGCTTAATAGTACAGAATCAA
ATGACACAAATAGCAACATCAACATCACACTCCAATGCAGAATAAGACAAATTATAAACATGTGGCAGGAAGT
AGGAAAAGCAATGTATGCCCCTCCCATCGAAGGACAAATTAGATGTTCATCAAATATTACAGGGATACTATTA
ACAAGAGATGGTGGTCATAGCACGAACGGGACTGAGAACGGGAACGAGACCTTCAGACCTGGAGGAGGAAACA
TGAGGGACAATTGGAGAAGTGAATTATATAAATATAAAGTAGTAAAACTTGAACCATTAGGAATAGCACCCAC
CAAGGCAAAGAGAAGAGTGGTGCAGAGAGAAAAAAGAGCGGTGGGAATGCTAGGAGCTATGTTCCTTGGGTTC
TTGGGAGCAGCAGGAAGCACTATGGGCGCAGCGTCACTAACGCTGACGGTACAGACCAGACATTTATTGGCTG
ATATAGTGCAACAGCAGAACAATTTGCTGAGAGCTATTGAGGCGCAGCAGCATCTGTTGCAACTCACAGTCTG
GGGCATC
>EU744081..1
TTCTACTGTAATACAACACAACTGTTTAATAGTACTTGGATGTTTAATGGTACTTGGGAGTCAAATAGCACTG
AGGGAAATCCCACAGAAAATATCACACTCCCATGCAGAATAAAACAAATTATAAACCTGTGGCAGGAAGTAGG

AAAGGCAATGTATGCCCCTCCTATCAGGGGACTAATTCAATGTGTATCAAATATTACAGGGTTGCTATTAACA
AGAGATGGTGGGAACAACACGACTGAGAATGGGACCGAGATTTTCAGACCTGGAGGAGGAGATATGAGGGACA
ATTGGAGAAGTGAATTATATAAATATAAAGTAGTAAAAATTGAACCATTAGGGGTAGCACCCACCAGGGCAAA
GAGAAGAGTGGTGCAGAGAGAAAAAAGAGCAGTGGGAGTGATAGGAGCTATGTTCCTTGGGTTCTTGGGAGCA
GCAGGAAGCACTATGGGCGCAGCGTCAATGACGCTGACGGTACAGGCCAGACAATTACTGTCTGGTATAGTGC
AGCAGCAGAACAATCTGCTGAGGGCTATTGAGGCGCAACAGCATCTGTTGCAACTCACAGTCTGGGGCATC
>JF320244..3
TTCTACTGTAATACATCACAACTGTTTAATGGAAGTTGGCAACCACCGCTTAATGAAACTATATTTCAATACA
ATGCTACATGGGCAAAAAATGACACTAATATCACACTCCCATGCAGAATAAAACAATTTGTAAACATGTGGCA
GGAAGTAGGAAAAGCAATGTATGCCCCTCCCATCAGCGGACCAATCAAATGTGTATCAAATATTACAGGGTTG
CTAATAACAAGAGATGGGGGTTACAACAGTAGTAGCAATGAGACCAATGAGACCTTCAGACCTGCAGGAGGAG
AAATGAGGGACAATTGGAGAAGTGAATTATATAAATATAAAGTAGTACAAGTTGAACCATTAGGATTAGCACC
CACCAAGGCAAAGAGAAGAGTGGTGCAGAGAGAAAAAAGAGCAGTGACGCTGGGAGCTATGTTCCTTGGGTTC
TTGGGAGCAGCAGGAAGCACTATGGGCGCAGCGTCAATAACGCTGACGGTACAGGCCAGACAGTTATTGTCTG
GTATAGTGCAACAGCAGAACAATCTGCTGAGGGCTATTGAGGCGCAACATCATCTGTTGCAACTCACAGTCTG
GGGCATA
>JF320226..1
TTCTACTGTAATACCACACCACTGTTTAATAGTACCTGGAATGCCACACAACTGGTTAATATTACTGGAATA
TCACAGGGCTTAGTAATAGAACTGAAAATATTACACTCCCATGTAGAATAAAACAAATTATAAACATGTGGCA
GGAAGTAGGAAAAGCAATGTATGCTCCTCCCATCGCAGGACGAATTAAATGTTTATCAAATATTACAGGTTTA
CTATTAGTAAGAGATGGTGGCAATAGCAATGACACGGAGACCTTCAGACCTCAAGGAGGAAATATGAAGGACA
ATTGGAGAAGTGAATTATATAAATATAAAGTAGTAAAAATTGAACCATTAGGAGTAGCACCCACCAAGGCAAA
GAGAAGAGTGGTGCAGAGAGAAAAAAGAGCAGTGGGAGTACTGGGAGCTATGTTCCTTGGGTTCTTGGGAGCA
GCAGGAAGCACTATGGGCGCAGCGTCAATAACGCTGACGGCACAGGCCAGACAATTATTGTCTGGTATAGTGC
AACAGCAGAACAATCTGCTGAGGGCTATTGAGGCGCAACAGCACCTGTTGCAACTCACAGTCTGGGGCATC
>JF320189..1
TTCTACTGTAATTCATCAGAACTGTTTAATAGTACCTGGACCTGGAATGAGACTGAAGGGTCTATTACTGAAG
GGTCAATTACTAATGGGACAATCACACTCCCATGTAGAATAAGACAAATTATAAACATGTGGCAGAGAGTAGG
AAAAGCAATGTATGCCCCTCCCATCCAAGGACAAATTAGATGTTCATCAAATATTACGGGGCTGCTATTAACA
AGAGATGGTGGTGAAGGTAACACCACCAATAACAACGAAACCTTCAGACCTGGAGGAGGTGATATGAGGGACA
ATTGGAGAAATGAATTATATAAATATAAAGTAGTACAAATTGAACCATTAGGAATAGCACCCACCAAGGCAAG
GAGAAGAGTGGTGCAGAGAGAAAAACGAGCAGTGGGACTAGGAGCTGTGTTCCTTGGGTTCTTGGGAGCAGCA
GGAAGCACTATGGGCGCAGCGTCAGTGACGCTGACGGTACAGGCCAGACAATTATTGTCTGGTATAGTGCAAC
AGCAGAACAATCTGCTAAAGGCTATTGAGGCGCAACATCATCTGTTGCAACTCACAGTCTGGGGCATC
>EF210727..1
TTCTACTGTAACTCTACACAGCTGTTTAATAGTACTTGGAACAGCAGCAATTGGAATTGGAATGATACTGAAA
AGACAAATAAAAGTATCACACTCCCATGCAGAATAAAACAAATTATAAACATGTGGCAGGAAGTAGGAAAAGC
AATGTATGCCCCTCCCATCAGAGGACAAATTAGATGTTCATCAAATATTACAGGGCTGATGCTAACAAGGGAT
GGTAGTAATGAGAGTCACAGTGAGATCTTCAGACCAGGAGGAGGAAATATGAGGGATAATTGGAGAAGTGAAT
TATATAAATATAAAGTAGTAAAAATTGAACCATTAGGAGTAGCACCCACCAAGGCAAAGAGAAGAGTGGTGCA
GAGAGAAAAAAGAGCAGTAGTGGGGATAGGAGCTGTGTTCCTTGGGTTCTTGGGAGCAGCAGGAAGCACTATG
GGCGCAGCGTCAATGATGCTGACGGTACAGGCCAGACAATTATTGTCTGGTATAGTGCAACAGCAGAACAATT
TGCTGAGGGCTATTGAGGCGCAACAGCATATGTTGCAACTCACAGTCTGGGGCATC
>EF210729..2
TTTTATTGTAATACAACACGGCTGTTTAATAGTACTTGGAAGAATAATACTTGGACTAAGAATCATATTGAGA
ACGAGACTGAAACAATCACACTCCCATGCAAAATAAAACAAATTATAAACAGGTGGCAGGAAGTAGGACAAGC
AATGTATGCCCCTCCCATCCCAGGATGGATTAACTGTTCATCAAATATTACAGGGCTACTATTAACAAGAGAT
GGTGGTGAAAACGGAACCACCGAGACCGAGACCTTCAGACCTGGAGGAGGAGATATGAGGGACAATTGGAGAA
GTGAATTATATAAATATAAAGTAGTTGAAATTCAACCATTAGGAGTAGCACCCACCACAGCAAAGAGAAGAGT
GGTGCAGAGAGAAAAAAGGGCAGTAACACTAGGAGCTATGTTCCTTGGGTTCTTGGGAACAGCAGGAAGCACT
ATGGGCGCAGCGTCGCTGACGCTGACGGTACAGGCCAGACAATTATTGACTGGTATAGTGCAACAGCAGAACA
ATCTGCTGAGGGCTATTGAGGCGCAACAGCATATGTTGCAACTCACAGTCTGGGGCATT
>EF210731..2
TTCTACTGTAATCTATCAAAATTGTTTAATAGTACATGGAATGATACTACTTGGAACACGACTGATGAGTCAA
ATGACACTTACATCCTGCTCCCATGTAGGATAAAACAAATTATAAACATGTGGCAGACAGTAGGAAAAGCAAT
GTACGCCCCTCCCATCAAAGGAAAAATTAGTTGTTCATCAAATATTACAGGGCTGCTATTAACAAGAGATGGA
GGGAATGACAACAGGACTCAGGGGGTCGAGATCTTCAGACCTGGAGGAGGAGATATGAGAGATAATTGGAGAA
GTGAATTATATAAATATAAAGTAGTAAAAATTGAACCATTAGGAATAGCACCCACCGAGGCAAAAAGAAGAGT
GGTGCAGAGAGAAAAAAGAGCAGTGGGAATAGGAGCTGTGTTCCTTGGGTTCCTGGGAGCAGCAGGAAGCACT

ATGGGCGCAGCGGCAGTGACGCTGACGGTACAGGCCAGACAATTATTGTCTGGTATAGTGCAACAGCAGAACA
ATCTGCTAAAGGCCATTGAGGCGCAACAGCATCTGTTGCAACTCACAGTCTGGGGCATC
>EF210735..1
TTCTACTGTAACACATCACACCTATTTAATAGTACTTGGAATGATACTAGTAGTTGGGATAATAGCACTGAAG
GAAATATCACACTCCAATGTAGAATAAAACAAATCATAAACAGGTGGCAGGAAGTAGGAAAAGCAATGTATGC
CCCTCCCATTGCAGGACAAATTAGATGCTCATCAAATATTACAGGGCTGCTATTAACAAGAGATGGTGGTCAG
GGCAGTAACGAGACCGAGGTCTTTAGACCTGGAGGAGGAAATATGAAGGACAATTGGAGAAGTGAACTATATA
AATATAAAGTAGTAAAAGTTGAACCATTAGGAATAGCACCCACCAAGGCAAGGAGAAGAGTGGTGCAGAAAGA
AAAAAGAGCAATAACACTAGGAGCTATGTTCCTTGGTTTCTTGGGAGCAGCAGGAAGCACTATGGGCGCAGCG
TCGATAACGCTGACGGTACAGGCCAGACAATTATTGTCTGGTATAGTGCAGCAGCAGAATAATTTGCTGAGGG
CTATTGAGGCGCAACAGCATATGTTGCAACTCACAGTCTGGGGCATC
>MH327746..1
TTCTACTGCAATACAACAAAACTGTTTAACAGTAGTTGGCCAAATAGTACTAGGAATAATACTGAAGAGTCAA
ATAGCACTGTTATCACACTCCCATGTAGAATAAAACAAATTATAAACATGTGGCAGGAAGTAGGAAAAGCAAT
GTATGCCCCTCCCATCAGGGGACGAATTAATTGTACATCAAACATTACAGGGCTACTATTAACAAGAGATGGG
GGCAATGGAAATAGCACGAATGAAACCTTCAGACCTCAAGGAGGAAATATAAGGGACAATTGGAGAAGTGAAC
TATATAAATATAAAGTAGTAAAAATTGAACCATTAGGAGTAGCACCTACCAAGGCAAAGAGAAGAGTGGTGCA
AAGAGAAAAGAGAGCAGTGGGAATGGGAGCTTGGTTCCTTGGGTTCTTGGCAGCAGCAGGAAGCACTATGGGC
GCAGCGTCAGTGACGTTGACGGTACAGGCCAGACAATTATTGTCTGGTATAGTGCAGCAGCAAAATAATTTGC
TGCAAGCTATTGAGGCGCAACAGCATTTGTTGCAACTCACAGTCTGGGGCATC
>KY658690..1
TTCTACTGCAATACAACAAAACTGTTTAACAGTAGTTGGCCAAATAGTACTAGGAATAATACTGAAGAGTCAA
ATAGCACTGTTATCACACTCCCATGTAGAATAAAACAAATTATAAACATGTGGCAGGAAGTAGGAAAAGCAAT
GTATGCCCCTCCCATCAGGGGACGAATTAATTGTACATCAAACATTACAGGGCTACTATTAACAAGAGATGGG
GGCAATGGAAATAGCACGAATGAAACCTTCAGACCTCAAGGAGGAAATATAAGGGACAATTGGAGAAGTGAAC
TATATAAATATAAAGTAGTAAAAATTGAACCATTAGGAGTAGCACCTACCAAGGCAAAGAGAAGAGTGGTGCA
AAGAGAAAAGAGAGCAGTGGGAATGGGAGCTTGGTTCCTTGGGTTCTTGGCAGCAGCAGGAAGCACTATGGGC
GCAGCGTCAGTGACGTTGACGGTACAGGCCAGACAATTATTGTCTGGTATAGTGCAGCAGCAAAATAATTTGC
TGCAAGCTATTGAGGCGCAACAGCATTTGTTGCAACTCACAGTCTGGGGCATC
>MF373127..1
TTCTACTGTAATACAACAAGACTGTTTAATACTACTTGGAATAACACTGAGAAATCAAATCCCACTGAAGGAA
ATGGCACAATCACACTCCAATGCAGAATAAAACAAATTATAAACCTGTGGCAGGAAGTAGGAAAAGCAATGTA
TGCCCCTCCCATCAGAGGACAAATTAACTGTTCATCCAACATTACAGGACTGCTATTAACAACAGATGGTGGT
ATGAACACCAGTAATAACAAGACCTTTATACCTGCAGGAGGAGATATGAGAGACTATTGGAGGAGTGGAGTAT
TGAAATATGATGTAGTAATAGTTGAGCCATTAGGAATAGCACCAACCACGGATATGAGAAGAGTGGTGCAGAT
ACAAAAAAGAGCAGAGCTACTACGAGCTCTGTTGCTTGTGTTCTTGGGAGCAGCAGGAAGGACGATGAGCGCA
GCGTCGATGACGCTGACGGTACAGGCCAGACAATTATTGTCTGGTATAGTGCAACAGCAAAATAATTTGCTGC
AGGCTATTGAGGCGCAACAGCATATGTTGAAACTCACAGTCTGGGGCATC
>MF373129..2
TTCTACTGTAATACAGCAGAACTGTTTAACAGTACTTGGAGGAATGACACTGAAAGGTCAAATACCACTACAG
GAAATGACACAATCACACTTCAATGCACAATAAAACGAATTGTAAACATGTGGCAGGAAGTAGGAAAAGCAAT
GTATGCCCCTCCCATCACAGGACAAATTAATTGTTCATCAAAAATTACAGGGCTACTGTTAACAAGAGATGGT
GGTGGGAACCAGAACACCAGCGAGACTTTCAGACCTGGAGGGGGAAATATGAAAGACAATTGGAGAAGTGAAT
TATATAAATATAAAGTAATAAAAATTGAACCATTAGGAGTAGCACCCACCAAGGCAAAGAGAAGAGTGGTGCA
GAGAGAAAAAAGAGCAGTGGGAATAGGAGCTGTGTTCCTTGGGTTCTTGGGAGCAGCAGGAAGCACTATGGGC
GCAACGTCGATGACGCTGACGGTACAGGCCAGACAATTATTGTCTGGTATAGTGCAACAGCAGAATAATTTGC
TGAAGGCTATTGAGGCGCATCAGCATATGTTGCAACTCACAGTCTGGGGCATC
>KP411829..1
TGCTATTGCAAACCATCACTTCTGTTTACTGGCACATACAAGAGTAACATTAATATTTCAAATATCACCCAAT
CCTCACAATCCGATGAAAGGTATATTTTAATTCTGGCTTGTATGACGATCAATGTGTGGCAGGTAGTAGGAAA
AGCGATCTATTCCCCTCCCATCAGTGGACAAATTAGATGTTCATCAAATATTACAGGGCTGCTATTAACAAGA
GATGGTGGTAGCAACAGCAGTAACGAGACCGAGACTGAAATCTTCAGACCTGTGGGAGGAAATATGAGGGACA
ATTGGAGAAGTGAATTATATAAATATAAAGTAGTACAAATTGAACCATTAGGAGTAGCACCCACCAAGGCAAA
GAGAAGAGTGGTGCAAAGAGAAAAAAGAGCAGTGGGATTAGGAGCAATGTTCCTTGGGTTCTTGGGAGCAGCA
GGAAGCACTATGGGCGCAGCGTCAATAACGCTGACGGTACAGGCCAGACAATTATTGTCTGGTATAGTGCAAC
AGCAGAATAATTTGCTGAGAGCTATTGAGGCGCAACAGCATCTGTTGCAACTCACAGTCTGGGGCATC
>MF373151..2
CTCTACTGTAATTCCACACAACTATTTAATAGTACTTGGAATGGTACTGAAAGATCAAACTATTCCACTGAGA
CTAATGAAACTATCACACTCCCATGCAGAATAAAACAAATTATAAACATGTGGCAGAGAGTAGGAAGAGCAAT
GTATGCCCCTCCCATCCAAGGACTAATTAGATGTTCATCAAATATTACAGGGCTGCTGCTAACAAGAGATGGA

GGTCAGAATAGTAGTAATAGCACTGAGACCTTCAGACCGGGAGGAGGAAACATGAAGGATAATTGGAGAAGTG
AATTATATAAATATAAAGTAATAAAGATTGAACCATTAGGAGTAGCACCCACCAAGGCAAAGAGAAGAGTGGT
GCAAAGAGAAAAAAGAGCAATAACAGTAGGAGCTATGTTCCTTGGGTTCTTGGGAGCAGCAGGAAGCACTATG
GGCGCAGCGTCAATGGCGCTGACGGTACAGGCCAGACAATTGTTGTCTGGTATAGTGCAACAGCAGAACAATC
TGCTGAGGGCTATTGAGGCGCAACAGCATCTGTTGCAACTCACAGTCTGGGGCATC
>KP411823..1
TTCTACTGTAATACAACACAACTGTTTAATAGTACTTGGAATATCACTGGAGGGTTAAATAACACTGAAGGAA
ATGGCACAATCACACTCCAATGCAAAATAAAACAATTTATAAACATGTGGCATGAAGTAGGGAAAGCAATGTA
TGCCCCTCCCATCAGAGGACAGATTACATGTTCATCAAACATTACAGGGCTGCTATTAACAAGAGATGGTGGT
GAGAACCCGGGCAACGACACTGATACTTTCACACCTGGAGGGGGAGATATGAAAGACAATTGGAGTAGTGAAT
TAGATAAATATAGAGTAATAGGAATAGCACCACTACCAGTAGCACCCACCAAGGCAAAGACAAGATTGTTGCA
GAGAGATAAAAGAGCAGTTGGAATAGGATCTGTGTTCCTTGTCTTCCTGGTAGCGGCAGGAAGCACTATGTCT
GCAATGTCAATGACGATGACGCTACAAGCTCAAGAATTACTGTATGTGACCGAGCGAATGCAAAAAAATTTGC
TGAAGGCTATTGAGGCGCAACAGCATCTGTTGCAACTCACAGTCTGGGGCATC
>MF373162..4
TTCTATTGTAATACAACACAGTTATTTAATAGTACTTGGGAGGCTCATCAGCTTAATCAGACTTGGAATGAGC
CCCTAGGGCCAAATGTTACTAATATCACACTCCCATGCAGAATAAAACAAATTATAAACAGGTGGCAGGAAGT
GGGAAAAGCAATGTTTGCCGCTCCCATCAGCGGACGAATTCAATGCAAATCAAATATTACAGGGTTGTTATTA
ACAAGAGATGGTGGTACTAACACCAGCGACCCCAACAGAACTGAGACCTTCAGACCTGGAGGAGGAGATATGA
GAGACAATTGGAGAAGTGAATTATATAAGTATAAAGTAGTACAAATTGAGCCATTAGGAATAGCACCCACCAA
GGCAAAGAGAGTAGTGGTAAAGAGAGAAAAAAGAGCAGTGGGAGTGCTAGGAGCTATGTTCCTTGGGTTCTTG
GGAACAGCAGGAAGCGTTATGGGCGCAGCGTCAATGACGCTGACGGTACAGGCCAGACAATTATTGTCTGGTA
TAGTGCAACAGCAGAACAATCTGCTGAGAGCTATTGAGGCGCAACAACATATGTTGCAACTCACAGTCTGGGG
CATC
>KP411824..3
TTCTACTGCAATTCAACACCACTGTTTAATAGCACCTGGGATACTAATGGGACTTGCAAGGGCTCTAATAGTG
CTGATTGGAATGTTACTGTCCCATGCATTAGCATCCCAATTACTACAATGGGGCAGAAACTAGAACAAGCAAT
GGACGTGCGTCCCATTGCAGGAAAAGCAATATGTTCATCAAGTTTGTCCAAAGGTATCCTTTTAGCCAATTCC
CATAATAGCATTATTGTGCCCCGGCTGGTTATGCCTAAAGGTAAAGATAAGAAGTTCAATTGGAACAGGAAAA
TGTATCAATGTCAAGTACAATGTCAAATTGAACCATTAGGAGTAGCACCCACCATGGCAAAGAGAAGAGTGGT
GCAAAGAGAGAAAAGAGATTTGGGACTAGGAGCTCTGTTCCTTGGGTTCTTGGGAGCAGCAGGAAGCACTATG
GGCGCAGCGTCAATAACGCTGACGGTACAGGCCAGACAATTATTGTCTGGTATAGTGCAACAGCAGAACAACT
TGCTGAGGGCTATTGAGGCGCAACAGCATCTGTTGCAACTCACAGTCTGGGGCATT
>MF373185..1
TTCTACTGTAATTCAACACCACTGTTTAATAGTACTTGGTATGTTAATGGAACTAGGAATAATGATACTGATA
TTGGAAAGATAAACATCACACTCTCATGTAGAATAAAACAAATTATAAACATGTGGCAGGAAGTAGGAAAAGC
AATGTATGCCCCTCCCATCCCAGGAAAAATTCAATGTATATCAAATATTACAGGGCTGCTATTAACAAGAGAT
GGAGGTAATACCAGTGAGACCAGGAACGGGACCGAGATTTTCAGACCTGGAGGAGGAAATATGAGGGACAATT
GGAGAAGTGAATTATATAAATACAAAGTAGTACAAGTTGAACCATTAGGAATAGCACCCACCAAGGCAAGGAG
AAGAGTGGTGCAGAGAGAAAAAAGAGCTCTGGGACTAGGAGCTTTGTTTCTTGGGTTCTTGGGAGCAGCAGGA
AGCACTATGGGCGCAGCGTCAGTGACGCTGACGGTACAGGCCAGACAATTATTGTCTGGTATAGTGCAGCAGC
AGAACAATCTGCTGAGAGCTATTGAGGCGCAACAACACATGTTGCAACTCACAGTCTGGGGCATC
>MF373186..1
TTCTACTGCAACACAACACAACTGTTTAATAGTACTTGGAATGATACACAACTGTCTGATAGCACTTGGAATA
ATACAAGAGGGATAAATGACACTATCACACTCCAATGTAGAATAAGACAAATCATAAACATGTGGCAGAAGGT
AGGAAAAGCAATGTATGCCCCTCCCATCAGTGGACAAATTAGATGTACATCAAATATTACAGGGCTGATATTA
ACAAGAGATGGTGGTAATAGTGACAACAACACCACCCAGCCCGAGATCTTCAGACCTCAAGGAGGAAATATGA
AAGACAATTGGAGAAGTGAATTATATAAATATAAGGTAGTAGCAATTGAACCATTAGGACTAGCACCCACCAA
GGCAAAGAGAAGAGTGGTGCAGAGAGACAAAAGAGCAGTAGGACTGGGAGCTCTGTTCCTTGGGTTCTTGGGA
GCAGCAGGAAGCACTATGGGCGCAGCGTCAATGGCGCTGACGGTACAAGCCAGACAATTATTGTCTGGTATAG
TGCAACAGCAGAACAATTTGCTGAGGGCTATTGAGGCGCAACAGCATCTGTTGCAGCTCACAGTCTGGGGCAT
T
>KY213743..1
TTTTACTGTAATACATCAAAACTGTTTAATAGTACTTGGCTGAATAATAATACAGGGTCAAATGCCACTGAAC
CTATCATACTCCCATGCAGGATAAAACAAATTGTAAACATGTGGCAGGAAGTAGGAAAAGCAATGTATGCCCC
TCCCATCAGAGGACAAATTAGATGCATATCAAATATTACAGGGCTCCTATTAACAAGAGATGGTGGTCAAGAG
AACAAAACCGAGAACCACACCGAGGTCTTTAGACCCACGGGAGGAAATATGAAGGACAATTGGAGAAGTGAAT
TATATAAATATAAAGTAGTAAAAATTGAACCATTAGGAATAGCACCCACCAAGGCAAAAAGAAGAGTGGTGCA
GAGAGAAAAAAGAGCAGTGGGAATAGGAGCGATGTTCCTTGGGTTCTTGAGCGCAGCAGGAAGCACTATGGGC

GCAGCGTCAATAACGCTGACGGTACAGGCCAGACAGTTACTGTCTGGTATAGTGCAACAGCAAAACAATTTGC
TGAGAGCTATTGAGGCGCAACAACATATGTTGCAACTCACAGTCTGGGGCATC
>KY213745..1
TTCTACTGCAATACAACACAACTATTTAATAGTTCTTGGAATAATAGTAGTTTGAATAGTACTGAGGGTAATG
GAGGGTCAAATGACACTATCACACTCCCGTGTAGAATAAAACAAATTATAAACATGTGGCAGGAAGTAGGAAA
AGCAATGTATGCCCCTCCCATTCAAGGACAAATTAGATGCTCCTCAAATATTACAGGACTATTATTAGTAAGA
GATGGTGGTAATAACAGGACGGGCGGCAGTGAGACCTTCAGACCTGGAGGAGGAAATATGAAGGACAATTGGA
GAAGTGAATTATATAAATATAAAGTAGTAAAAATTGAACCATTAGGAATAGCACCCACCAGGGCAAGGAGAAG
AGTGGTGCAGAGAGAAAAAAGAGCAGTGGGACTAGGAGCTTTGTTCCTTGGGTTCCTGGGAGCAGCAGGAAGC
ACTATGGGCGCAGCGTCAATGACGCTGACGGTACAGGCCAGACAATTATTGTCTGGTATAGTGCAACAGCAGA
ACAATCTGCTGAAGGCCATTGAGGCGCAACAGCATATGTTGCAACTCACAGTCTGGGGCATC
>JN248346..2
TTCTACTGTGATTCAACACAACTGTTTAATAGTAGTTGGAATAGTAATTGGTCTAATAATACTGAAGGGTCAG
ACAACATTACACTTCAATGCAGAATAAAACAAATTATAAACATGTGGCAGCAAACAGGAAAGGCAATGTATGC
CCCTCCCATCAGTGGAATAATTAGCTGTTCATCAAATATTACAGGGCTGTTATTAACAAGAGATGGTGGTAAC
AATCAGACTAATAATAATGAGACCTTCAGACCTGGAGGAGGAAATATGAAGGACAATTGGAGAAGTGAATTAT
ATAGATATAAAGTAGTACAAATTGAACCATTAGGAGTAGCACCCACCATGGCAAAGAGAAGAGTGGTGCAGAG
AGAAAAAAGAGCAGTGGGAATAGGAGCTCTATTCCTTGGGTTCTTGGGAGCAGCAGGAAGCACTATGGGCGCA
GCATCAATAACGCTGACGGTACAGGCCAGACAATTATTGTCTGGTATAGTGCAACAGCAGAACAATTTGCTGA
GAGCTATTGAGGCGCAACAGCATATGTTGCAACTCACAGTCTGGGGCATA
>KJ769147..1
TTCTACTGTAACACATCACAACTGTTTAATAGTACTTGGTATAGTAATGGTACTCGAAATGATACTGTAGGAA
ATGGCAATATCACGCTCCCTTGCAGAATAAAACAAATTGTAAACATGTGGCAGGAAGTAGGAAAAGCAATGTA
TGCCCCTCCCATTGGAGGACAAATTAGATGTTCATCAAATATTACAGGGTTGCTATTAACAAGAGATGGTGGT
AACAATCAGAGCAACATCACCACCGAGACCTTCAGACCAGGAGGAGGAGATATGAGGGACAATTGGAGAAGTG
AACTATATAAATATAAAGTGGTAAAAATTGAACCATTAGGAATAGCACCCACCAAGGCAAAGAGAAGAGTGGT
GCAGAGAGAAAAAAGAGCAGTGGGAATGATAGGAGCTATGTTCCTTGGGTTCTTGGGAGCAGCAGGAAGCACT
ATGGGCGCAGCGTCAATAACGCTGACGGTACAGGCCAGGCAATTGTTGTCTGGTATAGTGCAACAGCAGAGTA
ATCTGCTGAGAGCTATTGAGGCGCAACAGCATATGTTGCAACTCACAGTCTGGGGCATC
>KP109514..1
TTCTACTGTAATACAACACAGCTATTTAATAATACTTGGCTTACTTGGAAGGCCAATGCAAGTGATCCTGAAA
AAAATAACACAAAAAATATCACACTCCCATGCAGAATAAAACAAATTATAAACATGTGGCAGGAAGTAGGAAA
AGCAATGTATGCCCCTCCCATCGCAGGACAAATTAAATGTGTATCAAATATTACAGGGTTACTATTAACAAGA
GATGGGGGTCAGGACAATAACACTAACGGTAACGAGACCTTCAGACCTGGAGGAGGAGATATGAGGGACAATT
GGAGGAGTGAATTATATAAATATAAAGTAGTAAAAATTGAACCATTAGGAATAGCACCCACTAAGGCAAAGAG
AAGAGTGGTGCAGAGAGAAAAAAGAGCAGCACTAGGAGCTATGTTCCTTGGGTTCTTGGGAATGGCAGGAAGC
ACTATGGGCGCAGCGTCAATAACGCTGACGGCACAGGCCAGACAATTGTTGTCTGGTATAGTGCAACAGCAGA
ACAATCTGCTGAGGGCTATTGAGGCGCAACAACATTTGTTGCAACTCACAGTCTGGGGCATC
>EU578539..1
TTCTACTGTAATGCAACACAACTGTTTAATAGTACTTGGCCCATTAACGGTACTTGGGAAGGATCAAATAATA
CTAATGTCACACTACCATGCAGAATAAAACAAATTATAAACATGTGGCAGAGAGTAGGCAAAGCAATGTATGC
CCCTCCCATCAGAGGACAAATTAGATGTTCATCAAATATCACAGGGCTGCTATTAACAAGAGATGGTGGTGGT
AGTAACCAAAGCAACGAGACCTTTAGACCTGGAGGAGGAGACATGAAAGACAATTGGAGAAGTGAATTATATA
AGTATAAAGTAGTAAGAATTAAACCATTAGGAGTAGCCCCCACCAAGGCAAAGAGAAGAGTGGTGCAGAGAGA
AAAAAGAGCAGTGGGAACAATAGGAGCTATGTTCCTTGGGTTCTTGGGAGCAGCAGGAAGCACTATGGGCGCA
GCGTCAGTGACGCTGACGGTACAGGCCAGACAATTATTGTCTGGTATAGTGCAACAGCAGAACAATTTGCTGA
GGGCTATTCAGGCGCAACAGCATCTGTTGCAACTTACGGTCTGGGGCATC
>HQ217760..1
TTCTATTGTAATACAACAGAGCTGTTTAATAGTACTTGGGATGTTAATAGTACTTTGAATGTTAATAATACTT
GGAATAATACTGAAGGAGCAAGTGGCAATATCACACTCCCATGCAGAATAAAACAAATTATAAATATGTGGCA
GAAAGTAGGCAAGGCAATGTATGCCCCTCCCATCAGAGGACAAATTAGATGTACATCAAAGATCACAGGGCTG
CTATTAACAAGAGATGGTTATAATAATAATAACAACACCGAGACTTTCAGACCTGGAGGAGGAAATATGAAGG
ACAATTGGAGAAGTGAGTTATATAAGTATAAAGTAGTAAAAATTGAACCATTAGGAGTAGCACCCACCATGGC
AAGGAGGAGAGTGGTGCAGAGAGAAAAAAGAGCAGTGGGAACAATAGGAGCTATGTTCCTTGGGTTCTTGGGA
GCAGCAGGAAGCACTATGGGCGCAGCGTCACTGACGCTGACGGTACAGGCCAGACTATTATTGTCTGGTATAG
TGCAACAGCAGAACAATCTGCTGAGGGCTATTGAGGCGCAACAGCATCTGTTGCAACTCACGGTCTGGGGCAT
C
>AF277071..1
TTCTACTGTAATTCAACACAGCTGTTTAATAGTACTTGGAATATTAATGATACTAGGAATGATACTACAGGGA
ATATCACACTCCCATGCAGAATAAGACAAATTGTGAACAGGTGGCAGGAAGTAGGCAAAGCAATGTATGCCCC

TCCCATCAGAGGACAAATTAGCTGTTCATCAAATATTACAGGGGTGCTATTAACAAGAGATGGTGGTAATCAC
ACGAGCGAAACTGAGATCTTCAGACCTGGAGGAGGAGATATGAGGGACAATTGGAGAAGTGAATTATATAAAT
ATAAAGTAGTAAAAATTGAACCATTAGGAAAAGCACCCACCAAGGCAAGGAGAAGAGTGGTGCAAAGAGAAAA
AAGAGCAGTGACAATGGGAGCTATGTTCCTTGGGTTCTTAGGAGCAGCAGGAAGCACTATGGGCGCAGCATCC
ATGACGCTGACGGTACAGGCCAGGCTATTATTGCCTGGTATAGTGCAACAGCAGAGCAATCTGCTGAGGGCTA
TCGAGGCGCAACAACATCTGTTGCAACTCACAGTCTGGGGCATC
>EF593267..1
TTCTACTGTAATACAACAAAGCTGTTTAATAGTACTTGGCATGCTAATGGCACTCGGAATGATACTGGAGGGT
CAAATGACACTATCATACTACCATGCAGAATAAAACAAATTATAAACATGTGGCAGAAAGTAGGCAAAGCAAT
CTATGCCCCTCCCATCAGAGGACAAATTAGATGTGAATCAAAGATCACAGGGCTGCTATTAACAAGAGATGGT
GGTGATGATAACAGAACCACCGAGATCTTCAGACCTGGAGGAGGAGATATGAGGGACAATTGGAGAAGTGAAT
TATATAAATATAAAGTAGTAAAAATTGAACCATTAGGAATAGCACCCACCAAGGCAAGGAGAAGAGTGGTGCA
GAGAGAAAAAAGAGCAGTGGGAATAGGAGCTGTGTTCCTTGGGTTCTTGGGAGCAGCAGGAAGCACTATGGGC
GCAGCGTCAATGACGCTGACGGTACAGGCCAGACTATTATTGTCTGGTATAGTGCAACAGCAGAACAATTTGC
TGCAGGCTATTGAGGCGCAACAGCATCTGTTGCAACTCACGGTCTGGGGCATC
>DQ853436..1
TTCTACTGTAATTCAACACGATTGTTTAGTAGTAAGTGGCATGCTAATGATAGTTGGAGTAGTAGTACTTGGA
ACGATACTGGAGAGTCAAATGACACTATCACACTCCCATGTAGAATAAAACAAATTATAAACATGTGGCAGGA
AGTAGGAAAAGCAATGTATGCCCCTCCCATCAAAGGACAAATTCAATGTTCATCAAATATTACAGGGCTAATA
TTAACAAGAGATGGTGGTGCAAACACGAACACGAGCGAGGAGGTCTTCAGACCTGCAGGAGGAAATATGGGGG
ACAATTGGAGAAGTGAATTATATAAATATAAAGTAGTAAAAATTGAACCAATAGGAATAGCACCCACCAAGGC
AAAAAGAAGAGTGGTGCAGAGAGAAAAAAGAGCAGTGACACTGGGAGCTGTGTTCCTTGGGTTCTTGGGAGCA
GCAGGAAGCACTATGGGCGCAGCGTCAATAACGCTGACGGTACAGGCCAGACAATTATTGTCTGGTATAGTGC
AGCAGCAGAACAATCTGCTGAGGGCTATTGAGGCGCAACAACATCTGTTGCAACTCACAGTCTGGGGCATC
>KT124806..1
TTCTACTGTGATACAACAGAACTGTTTAATAGTACTTGGAGTATTAATGGTACTAGGAATACTACTGAAGGGT
CAAATAGCACAGACATCACACTCCAATGTAGAATAAAACAAATTATAAACATGTGGCAGGAAGTAGGGAAAGC
AATGTATGCCCCTCCCATTAGAGGACAAATTAGATGTTCATCAAATATTACAGGGCTGCTATTAACAAGAGAT
GGTGGTAGCGGGAGTGAGACTAAAAACGAGACTGAAACCTTCAGACCTGGAGGAGGAAATATGAAGGATAATT
GGAGAAGTGAATTATATCAATATAAAGTAGTAAAAATTGAACCATTAGGAGTAGCACCCACCAGGGCAAAGAG
AAGAGTGGTGCAGAGAGAAAAAAGAGCAGTCACACTAGGAGCTGTGTTCCTTGGTTTCTTGGGAGCAGCAGGA
AGCACTATGGGCGCAGCGTCAATAACGCTGACGGTACAGGCCAGACAATTATTGTCTGGAATAGTGCAACAGC
AAAGCAATCTGCTGAGAGCTATTGAGGCGCAACAGCATCTGTTGCAACTCACAGTCTGGGGCATC
>EF593290..1
TTTTACTGTGATACAACAGAGCTGTTTAATAGTACTTGGAATAATACTGATAGTACTCGGAATGATACTGAGT
CAGACAAAATCACCCTCCAATGTAGAATAAAACAAATTATAAACATGTGGCAGGAAGTAGGAAAAGCAATGTA
TGCCCCTCCCATCCAAGGACAAATTCAATGTAACTCAAGTATTACAGGGCTACTATTAACAAGAGATGGTGGG
AACAACTCCACTAATTCCACCGAGATATTCAGACCTGGAGGAGGAGACATGAGGGATAATTGGAGAAGTGAAT
TATATAAATATAAAGTAGTAAAAATTGAACCATTAGGAGTGGCACCCACCAAGGCAAAGCGAAGAGTGGTACA
GAGAGAAAAAAGAGCAATAACGATGGGAGCTATGTTCCTTGGGTTCTTGGGAGCAGCAGGAAGCACTATGGGC
GCAGCGTCAATAGCGCTGACGGCACAGGCCAGACTATTATTGTCTGGTATAGTGCAACAGCAGAACAATCTGC
TGAGAGCTATTGAGGCGCAACAGCATATGTTGCAACTCACAGTCTGGGGCATC
>JX863967..2
TTCTACTGTAATACAACACCACTGTTTAATAGCACTTGGAATGAAACTCAACTGTTTAATAGTACTTGGAATG
AAACTGAGGCAAATGGAACAATTACACTCCCATGCAGAATAAAACAAATTATAAACATGTGGCAGGAAGTAGG
AAAAGCAATGTATGCCCCTCCCATCGAAGGACAAATTTACTGTTCTTCAAACATTACAGGGCTACTGTTATTA
AGGGATGGTGGTAATACCACAAACGGGACTGAGATCTTCAGACCTGGAGGAGGAGACATGAGGGACAATTGGA
GAAGTGAATTATATAAATATAAAGTAGTAAAAATTGAACCATTAGGAGTAGCACCCACTAGGGCAAAGAGAAG
AGTGGTGCAGAGAGAAAAAAGAGCAGTGGGAATAGGAGCTATGTTCCTTGGGTTCTTGGGAATGGCAGGAAGC
ACTATGGGCGCAGCGTCACTAACGCTGACGGTACAGGCCAGACAATTGTTGTCTGGTATAGTGCAACAGCAGA
ATAATTTGCTGAGAGCTATTGAGGCGCAACAGCACCTGTTGCAACTCACAGTCTGGGGCATC
>KT124764..2
TTCTACTGTAATACCACACAACTGTTTAATAGTACTTGGTATGCAAACAATACAAATAGTACTGAAGGGGGAA
ACAGTGGGAATATCACACTCCCATGCAGAATAAAACAATTTATAAACATGTGGCAGGAAGTAGGAAAAGCAAT
GTATGCCCCTCCCATCAGTGGACAGATTAAATGTACATCAAATATTACAGGGCTGCTATTAACAAGAGATGGT
GGTCAAGGCAATCAGACCAACAACCAGACTGAGATCTTCAGACCTGTAGGAGGAGATATGAAGGACAATTGGA
GAAGTGAATTATATAAATATAAAGTAGTAAGAATTGAACCATTAGGAATAGCACCCACCAGGGCAAAAAGGAG
AGTGGTGCAGAGAGAAAAAAGAGCAGTGGGAATAGGAGCGTTGTTCCTTGGTTTCTTGGGAGCAGCAGGAAGC
ACTATGGGCGCAGCGTCAATGACGCTGACGGTACAGGCCAGACTATTATTGTCTGGTATAGTGCAACAGCAGA
ACAATCTGCTGAGAGCTATTGAGGCGCAACAGCATCTGTTGCAACTCACAGTCTGGGGCATC

>KT124814..1
TTCTACTGTAATACAACACCATTATTTAATAGTACTTGGAATAATACTAGAAATATCAGTGGTCTTGAAGGGT
CAAAGGACAATGGAAATATCACCCTCCAATGCAGAATAAAACAAATTATAAACATGTGGCAGGAAGTAGGAAA
AGCAATGTATGCCCCTCCCATCAGAGGACAAATTCAATGCTCATCAAATATTACAGGACTCCTATTAACAAGA
GATGGTGGTACTAATGTTACTGATAACACCACTGAGATCTTCAGACCTGGAGGAGAAGATATGAGGGACAATT
GGAGAAGTGAATTATATAAATATAAAGTAGTACAAATTGAACCATTAGGAGTAGCACCCACCAAGGCAAGGAG
AAGAGTGGTGCAGAGAGAAAAAAGAGCAGTGGGAATAGGAGCTTTGTTCCTTGGGTTCTTGGGAGCAGCAGGA
AGCACTATGGGCGCAGCGTCAGTGACGCTGACGGTACAAGCCAGACAATTATTGTCTGGTATAGTGCAGCAGC
AGAACAATCTGCTGAGGGCTATTGAGGCGCAACAGCATATGTTGCAACTCACAGTCTGGGGCATC
>EU578104..1
TTCTACTGTAACACAGCACAACTGTTTAATACTATTTGGAATAATACTTGGAATAGTAATGGGAATGATACTG
AAGGGGATAATAAAACAATCATACTCCCGTGCAGAATAAAACAAATTGTAAACAGGTGGCAGGAAGTAGGAAA
AGCAATGTATGCCCCTCCCATCAGCGGAATCATTAACTGTTTATCAAACATTACGGGGCTGCTATTAACAAGA
GATGGTGGAAATAACAGTAGTACCAACGAGACCTTCAGACCTGGAGGAGGAGATATGAGGGACAATTGGAGAA
GTGAACTCTATAAATATAAAGTAGTAAAAATTGAGCCATTAGGAGTAGCACCCACCAAGGCAAAAAGAAGAGT
GGTGCAGAGAGAAAAAAGAGCAGTGGGAACGTTAGGAGCGATGTTCCTTGGGTTCTTGGGAGCAGCAGGAAGC
ACTATGGGCGCAGCGTCAGTGACGCTGACGGTACAGGCCAGACAATTATTGTCTGGTATAGTGCAACAGCAGA
ATAATCTGTTGAAGGCTATTGAGGCGCAACAGCATCTGTTGCAGCTCACAGTCTGGGGCATC
>JQ403105..1
TTCTACTGTAATACAACAAAACTGTTTAATAGTACTTGGTATGAAAATGGTACAAATAGTACTTGGTATGCAA
ATGGTACAAATAGTACTGGAGAGGGAAACAGTGATATTATCACAATCCCATGCAGAATAAAACAATTTATAAA
CATGTGGCAGGAAGTAGGAAAAGCAATGTATGCCCCTCCCATCAGTGGACAGATTAGATGTTCATCAAACATT
ACAGGGCTGCTATTAACAAGAGATGGTGGTGAGGGCAATCAAACCAACAACCAGGAGATCTTCAGACCTGTAG
GAGGAGATATGAAAAACAATTGGAGAAGTGAATTATATAAATATAAAGTAGTAAGAATTGAACCATTAGGAAT
AGCACCCACCAAGGCAAAAAGGAGAGTGGTGCAGAGAGAAAAAAGAGCAGTGGGAATAGGAGCGTTGTTCCTT
GGGTTCTTGGGAGCAGCAGGAAGCACTATGGGCGCAGCGTCAATGACGCTGACGGTACAGGCCAGACTATTAT
TGTCTGGTATAGTGCAACAGCAGAACAATCTGCTGAGAGCTATTGAGGCGCAACAGCATCTGTTGCAACTCAC
AGTCTGGGGCATC
>EU578230..2
TTCTACTGTAATACATCAGGACTGTTTAATGGTACTTGGAATGGTACTAGCACTTGGAATGATACTGCAAATT
ATACTGCAAATGAGAATATCACACTCCCATGCAGAATAAAACAAATTATAAACATGTGGCAGGAAGTAGGAAA
AGCAATGTATGCCCCTCCCATCAGAGGACAAATTAACTGTTCATCAAAAATTACAGGGCTGCTATTAACAAGA
GATGGTGGCCATCACAACGGCAGCAACAACGACACAGAAATCTTCAGACCTGGAGGAGGAGATATGAGGGACA
ATTGGAGAAGTGAATTATATAAATATAAAGTAGTAAAAATTGAACCATTAGGAGTAGCACCCACCAGGGCAAA
AAGAAGAGTGGTGCAGAGAGAAAAAAGAGCAGTGGGAATGGGAGCTTTGTTCCTTGGGTTCTTGGGAGCAGCA
GGAAGCACTATGGGCGCAGCGTCACTAACGCTGACGGTACAGGCCAGACAATTATTGTCTGGTATAGTGCAAC
AGCAGAACAATCTGCTGAGGGCTATTGATGCGCAACAACATCTGTTGCAACTCACAGTCTGGGGCATT
>FJ469754..1
TTCTACTGTAATACAACAAGACTGTTTAATAGTAATTGGAATAGCAGTACTTGGAATAACACTGAAGGGTTAA
ATACCACTAACCTTGGAGAAAATGACACAATCATACTCCCATGCAGAATAAAACAGATTATAAACACGTGGCA
GAAAGTAGGAAAAGCAATATATGCCCCTCCCATCGAAGGACAAATTAAATGTATATCAAATATTACAGGGCTG
CTATTATTAAGAGATGGTGGTATAAATGTGAGTGAGACCAACGAAACCTTCAGACCTGGAGGAGGAGATATGA
AGGATATTTGGAGAAGTGAATTATATAAATATAAAGTAGTAAAAATTGAACCATTAGGAATAGCACCCAACAA
GGCAAAGAGAAGAGTGGTGCAGAGAGAAAAAAGAGCAGTGGGAATAGGAGCTGTGTTCCTTGGGTTCTTGGGA
GCAGCCGGAAGCACTATGGGCGCAGCGTCAATGACGCTGACGGTACAGGCCAGACAATTATTGTCTGGTATAG
TGCAACAGCAAAGCAATTTGCTGAGGGCTATTGAGGCTCAACAACATCTGTTGAAACTCACAGTCTGGGGCAT
C
>FJ469757..2
TTCTACTGTAATTCCTCACAACTGTTCAGTAGTACTTGGAATAGGACTAACGGGACTTGGAATGATGTTGAAG
GGTTAAATTACACTAATGGAAATGACACAATCACACTCCCATGCAGAATAAAACAATATATAAACATGTGGCA
GGAAGTAGGAAAAGCAATGTATGCCCCTCCCATCAGAGGGCAACTTAATTGTTCATCAAAGATTACAGGGTTG
CTATTAACAAGAGATGGTGTGAACGACACCGACAAAAATGAGACTTTCAGACCTCAAGGAGGGGATATGAAGG
ACAATTGGAGAAGTGAATTATATAAATATAAAGTAGTAAAAATTGAACCGTTAGGGATAGCACCCACCAGGGC
AAAGAGAAGAGTGGTGCAAAGAGAAAAAAGAGCAGTGGGATTAGGAGCTATGTTCCTTGGGTTCTTGGGAGCA
GCAGGAAGCACTATGGGCGCAGCATCAATAACGCTGACGGTACAGGCCAGACAATTACTGTCTGGTATAGTGC
AACAGCAGAACAATCTGCTGAGGGCTATTGAGGCGCAACACCATCTGTTGCAACTCACAGTCTGGGGCATC
>GU330538..2
TTCTACTGTAATACAACAAAACTGTTTTATAGTAATTGGACTATTAATGGGACTATTGTGAGTAATTGGACAT
ATGAAAATGACACTGGAACAAATGACACTATCATACTCCCATGTAGAATAAAACAAATTATAAACATGTGGCA
GGAAGTAGGAAAAGCAATGTATGCCCCTCCCATCAAAGGACCAATTACATGTAACTCAAGTATTACAGGGTTG

CTATTAACAAGAGATGGTGGTATTAACAGGACCAATAATGAGACCGAGATCTTCAGACCTGTAGGGGGAGATA
TGAGGGACAATTGGAGAAGTGAATTATATAAATATAAAGTAGTAGAAATTCAACCATTAGGAATAGCACCCAC
CAAGGCAAAGAGAAGAGTGGTGCAGAGAGAAAAGAGAGCGGTAACACTAGGAGCTATGTTCCTTGGGTTCTTG
GGAGCAGCAGGAAGCACTATGGGCGCAGCGTCAATGGCGCTGACGGTACAGGCCAGACAATTATTGTCTGGTA
TAGTGCAACAGCAGAACAATCTGCTGAGGGCTATTGAGGCGCAACAGCACATGTTGCAACTCACAGTCTGGGG
CATT
>GU330550..3
TTCTACTGTAATTTAACACAACTGTTTAATAGTACTTGGAATATTACTGACAGATTAAATAATAATGAAACTC
AAGGAAATGACACAACAATTCACCTCCCGTGCAGAATAAAACAAATTATAAACATGTGGCAAGAAGTAGGAAA
AGCAATGTATGCCCCTCCCATCAGGGGGATAATTAGATGTTCATCAAATATTACAGGGCTGCTATTAACAAGA
GATGGTGGTACAAATACCACTGACAATGACACTGAGGTCTTCAGACCTGGAGGAGGAGACATGAGGGACAATT
GGAGAAGTGAATTGTATAAATACAAAGTAATACAAATTGAACCATTAGGAGTAGCACCCACTAAGGCAAAGAG
AAGAGTGGTGCAGAGAGAAAAAAGAGCAGTGGGAATAGGAGCTGTGTTCCTTGGGTTCTTGGGAGCAGCAGGA
AGCACTATGGGCGCAGCGTCAGTGACGCTGACGGTACAGGCCAGACAATTATTGTCTGGTATAGTGCAGCAGC
AGAGCAATCTGCTGAGGGCTATTGAGGCGCATCAACATCTGTTGCAACTCACAGTCTGGGGCATC
>KT124781..1
TTCTACTGTAATTCAACACCACTGTTTAATAGTGAATGGTCAGATAATAATACTTGGAATAGTGCTGAAGAAG
GAAATGCCACAATCACACTCCAATGCAGAATAAAACAAATTGTAAACATGTGGCAGGAAGTAGGAAGAGCAAT
GTATGCACCTCCCATCAAAGGAGTAATTAGCTGTACATCAAATATTACAGGACTGATATTAACAAGAGATCAT
AGTAAGGATAATGTAACCTTCACCTTCAGACCTGGAGGAGGAAATATGAAGGACAATTGGAGAAGTGAATTAT
ATAAATATAAAGTAGTAAAAATTGAACCATTAGGAGTAGCACCCACTACAGCCAAGAGAAGAGTGGTGCAGAG
AGAAAGAAGAGCAGTGGGAATAGGAGCTGTGTTCCTTGGGTTCTTGGGAGCAGCAGGAAGCACTATGGGCGCA
GCGTCAATAACGCTGACGGTACAGGCCAGACTATTATTGTCTGGTATAGTGCAACAGCAGAACAATCTGCTGA
GAGCTATTGAGGCGCAACAGCACCTGTTGCAACTCACAGTCTGGGGCATC
>MH060965..1
TTCTACTGTAATTCAACACAACTGTTTAATAGTACTTGGTTTACTGAGGATAATGCGACCGGCACTACCGGAA
ATGAAACAATCATACTCCCATGCAGAATAAAACAAATTATAAACATGTGGCAGGAAGTAGGAAGAGCAATGTA
TGCCCCTCCCATCAGAGGACAAATTAACTGTACATCAAATATTACAGGACTGATATTAACAAGAGATGGTGGT
AATAACAATCACACTAATGAGAACGAAACCTTCAGACCTGGAGGAGGAGATATGAGGGACAATTGGAGAAGTG
AATTATATAAATATAAAGTAGTAAAAGTTGAACCATTAGGAATAGCACCCTCTAAGGCAAGGAGAAGAGTGGT
GCAGAGAGAAAAAAGAGCAGCAGGACTAGGAGCTTTGCTCCTTGGGTTCTTGAGCGCAGCAGGAAGCACTATG
GGCGCAGCGTCACTGACGCTGACGGTACAGGCCAGGCAATTATTGTCTGGTATAGTGCAACAGCAAAACAATT
TGCTGAGGGCTATTGAGGCGCAACAGCATATGTTGCAACTCACAGTCTGGGGCATT
>FJ469731..1
TTCTACTGCAATTCAACACCACTGTTTAATAAGACTTGGAATAAGAATGATATTAGGAATAATACTACTGGGA
ATGATAATGACACTATCACACTCCAATGCAGAATAAAACAAATTATAAACATGTGGCAGGAAGTAGGAAAAGC
AATGTATGCCCCTCCCATTAGAGGACTAATCAGCTGTTCATCAAATATTACAGGGCTACTATTAACAAGAGAT
GGAGGTCATAACACAAGTAGGGCAAATGAGACCTTCAGGCCTGGAGGAGGAAATATGAAGGACAATTGGAGAA
GTGAATTATATAAATATAAAGTAGTAAAAATTGAACCATTAGGAGTAGCACCCACCAGGGCAAAGAGAAGAGT
GGTGCAGAGAGAAAAAAGAGCAGTGGGACTAGGAGCTTTATTCCTTGGGTTCTTGGGAGCAGCAGGAAGCACT
ATGGGCGCAGCGTCGATAACGCTGACGGTACAGGCCAGACAATTATTGTCTGGTATAGTGCAACAGCAGAACA
ATTTGCTGAGAGCTATTGAGGCGCAACAGCATCTGTTGCAGCTCACAGTCTGGGGCATC
>HQ216397..1
TTCTACTGTAATTCAACACTGCTGTTTAATAGTAGTTGGAAGAATGAGACCAATGCTGACGGGACAGAGGGCA
ATGATACTATTACACTCCAATGCAGAATAAAACAAATCATAAACAGATGGCAGGAAGTAGGAAAAGCAATGTA
TGCCCCTCCCATCGCAGGACAAATTAACTGTTCATCAAAAATTACAGGATTGCTATTAACAAGAGATGGTGGT
AATCATACCGCCAACACCGAGACCTTCAGACCTGGAGGAGGAGATATGAGAGACAATTGGAGAAGTGAATTAT
ATAAATACAAAGTAGTAAAAATTGAGCCATTAGGAATAGCGCCCACCAAGGCAAGGAGAAGAGTGGTGCAAAG
AGAAAGAAGAGCAGTGGGAACATTTGGAGCTTTGTTCCTTGGGTTCTTGGGAGCAGCAGGAAGCACTATGGGC
GCAGCATCAGTGACGCTGACGGTACAAGCCAGACAATTATTGTCTGGTATAGTGCAACAGCAGAACAATCTGC
TGAAGGCTATTGAGGCGCAACAACAGCTGTTGCAACTCACAGTCTGGGGCATC
>KT124758..1
TTCTACTGCAACACAACACAACTGTTTAATAGTACTTGGAATGATACACAACTGTCTAATAGCACTTGGAATA
ATACAAGAGGGATAAATGACACTATCACACTCCAATGTAAAATAAAACAAATCATAAACATGTGGCAGAAGGT
AGGAAAAGCAATGTATGCCCCTCCCATCAGTGGACAAATTAGATGTTCATCAAATATTACAGGGCTGATATTA
ACAAGAGATGGTGGTAATACTGACAACACCACCCAGCCCGAGATCTTCAGACCTCAAGGGGGAAATATGAAGG
ACAATTGGAGAAGTGAATTATATAAATATAAGGTAGTAGCAATTGAACCATTAGGAATAGCACCCACCAAGGC
AAAGAGAAGAGTGGTGCAGAGAGACAAAAGAGCAGTGGGAATGGGAGCTCTGTTCCTTGGGTTCTTGGGAGCA
GCAGGAAGCACTATGGGCGCAGCGTCAATAACGCTGACGGCACAGGCCAGACAATTATTGTCTGGTATAGTGC
AACAGCAGAACAATTTGCTGAGGGCTATTGAGGCGCAACAGCATCTGTTGCAGCTCACAGTCTGGGGCATT

>JX863968..1
TTCTATTGTAATTCAACATCACTGTTTAATAGTACTTGGAGGACAAATGAAACTGCAGACTTAAATACTACAG
AAATTATCACACTCCCATGCAGAATAAAACAAATTATAAACATGTGGCAAGAAGTAGGAAAAGCAATATATGC
CCCTCCCATCGAAGGAAACATTACATGTGTATCAAATATTACAGGGCTGCTATTAACAAGAGATGGTGGTAAC
AATAATCAGAGTAATGAGACGACTGAGATCTTTAGACCTGGAGGAGGAGATATGAGGGACAATTGGAGAAGTG
AATTATATAAATATAAAGTAGTAAAAATTGAACCACTAGGAGTAGCACCCACCAAGGCAAAGAGAAGAGTGGT
GCAGAGAGAAAAAAGAGCAGCGGGACTAGGAGCTGTGTTTCTTGGGTTCTTGGGAGCAGCAGGAAGCACTATG
GGCGCAGCGTCAATAACGCTGACGGCACAGGCCAGGCAATTATTGTCTGGTATAGTGCAACAGCAAAATAACT
TGCTGAGAGCTATTGAGGCGCAACAACATCTGTTGCAACTCACAGTCTGGGGCATC
>EF593190..1
TTCTACTGTAATACAACACGATTGTTTAATAGTACTTGGGAGGCTAATGGTAATTGGACTGGAACTGAAGGAA
ATGATAATGGCACAGTCACACTTCCATGCAGAATAAAACAAATTATAAACATGTGGCAACAAGTAGGAAAAGC
AATGTATGCCCCTCCCATTGCAGGAAAGATTACATGTAACTCAAGTATTACAGGAATACTATTAACAAGAGAT
GGAGGTATCACTAACAACACCGAGACCCACACCGAGACCTTCAGACCTGGAGGAGGAGATATGAGGGACAATT
GGAGAAGTGAATTATATAAATATAAAGTAGTAAAAATTCAACCATTAGGAGTAGCACCCACCAAGGCAAGAAG
AAGAGTGGTGCAGAGAGAAAAAAGAGCAGTGGGATTAGGAGCTATGTTCCTTGGGTTCTTGGGAGCAGCAGGA
AGCACTATGGGCGCAGCGTCAGTAACGCTGACGGTACAGGCCAGACAATTATTGTCTGGTATAGTGCAACAGC
AGAACAACTTGCTGAGGGCTATTGAGGCGCAACAGCATCTGTTGCAACTCACAGTCTGGGGCATC
>MH060681..1
TTCTACTGTAATTCAACACCACTATTTAATAGTACACACAACAGTACTACTACAAGGTCAAATGAAACTGAAC
CAATCATACTCCAATGTAGAATAAAACAAATTGTAAACATGTGGCAGGAGGTAGGAAAAGCAATGTATGCCCC
TCCCATTGCAGGACAAATTAACTGTACATCAAATATTACAGGGCTACTGTTAACAAGAGATGGTGGCAATAAT
CAGAGTAGAGAAAATAATACCGAGATCTTCAGACCTGGAGGAGGAAATATGAAGGACAATTGGAGAAGTGAAT
TATATAAATATAAAGTAGTAAAAGTTGAACCATTAGGAGTAGCACCCACCAAGGCAAAAAGAAGAGTGGTGCA
AAGAGAAAAAAGAGCAGTGGGATTCGGAGCTTTGTTCCTTGGGTTCTTAGGAGCAGCAGGAAGCACTATGGGC
GCAGCGTCAATGACGCTGACGGTACAGGCCAGACAATTATTGTCTGGTATAGTGCAACAGCAGAACAATCTGC
TGAGGGCTATTGAGGCGCAACAACATCTGTTGCAACTCACAGTCTGGGGCATC
>MH060990..2
TTCTACTGTAATACAACAAAACTGTTTAATAGTACTTGGAATAGTACTGAGAATTTTGAGGGAGGGTCAGATA
ACAATATCACACTCCCATGCAGGATAAAGCAAATTATAAACATGTGGCAGGAAGTAGGAAAAGCAATGTATGC
CCCTCCCATCGCAGGACAAATTAGATGTTCATCAAACATTACAGGGCTGATATTAACAAGAGATGGTGGTAGT
CATAATGGTAGTAATAATAGTGAGACCTTCAGACCTGGAGGAGGAGATATGAGGGACAATTGGAGAAGTGAAC
TTTATAAATATAAAGTAGTAAAAATTGAACCATTAGGAGTAGCACCCACTAAGGCAAAAAGAAGAGTGGTGCA
GAGAGAAAAAAGAGCAGCAATAGGAGCTTTGTTCCTTGGGTTCTTGGGAGCAGCAGGAAGCACTATGGGCGCA
GCGTCAGTAACGCTGACGGTACAGGCCAGACTATTGTTGTCTGGTATAGTGCAACAGCAGAGCAATCTGCTGA
AAGCTATTGAGGCGCAACAGCATCTGTTGCAACTCACAGTCTGGGGCATC
>DQ410526..1
TTCTATTGTAATACAACACCACTGTTTAATAATACTTGGTTTAATAGTACTAATAATTTAAATGAAACAAAGG
ACAACAACAACAACACAATCATACTCCAATGCAGAATAAAACAAATTATAAACATGTGGCAGGAAGTAGGAAA
AGCAATGTATGCCCCTCCCATCAGGGGAAACATTAGCTGTTCATCAAATATTACAGGACTATTATTAACAAGA
GACGGTGGTCATAATGTGAGCAACACGAACGAGACTTTCAGACCTGGAGGAGGAGATATGAGGGACAATTGGA
GAAGTGAATTATATAAATATAAAGTAGTAAGAATTGAACCATTAGGAATAGCACCCACCAAAGCAAAGAGAAG
AGTGGTGCAAAGAGAAAAAAGAGCAGTGGGATTAGGAGCTGTGTTCATTGGGTTTCTGGGAGCAGCAGGAAGC
ACTATGGGCGCAGCGTCAATGACGCTGACGGTACAGGCCAGACAATTATTGTCTGGTATAGTGCAGCAGCAGA
ACAATCTGCTGAGAGCTATTGAGGCGCAACAGCATCTGTTGCAACTCACAGTCTGGGGCATT
>EF363127..1
TTCTACTGTAATACAACAGACTTGTTTACTTGGAATGGTACTTGGAATGGTACTGCAGGGGCAGATAACAATA
CAATCACACTCCAATGCAGAATAAGACAAATTATAAACATGTGGCAGGAAGTAGGAAAAGCAATGTATGCCCC
TCCCATCCCAGGATACCTTAACTGTTCATCAAATATTACAGGACTGCTATTAACAAGGGATGGTGGTCATAAC
ACGAATACTACTGAGATCTTCAGACCTGGAGGAGGAGATATGAGGGACAATTGGAGAAGTGAATTATATAAAT
ATAAAGTAGTAAAAATTGAACCATTAGGAATAGCACCCACCAAGGCAAGGAGAAGAGTGGTGCAGAGAGAAAA
AAGAGCAGTGGGAATAGGAGCTGTGTTCCTTGGGTTCTTGGGAGCAGCAGGAAGCACTATGGGCGCAGCGTCA
ATAACGCTGACGGTACAGGCCAGGCTATTATTGTCTGGTATAGTGCAACAGCAAAACAACTTGCTGAGAGCTA
TTGAGGCGCAACAGCATCTGTTGCAACTCACAGTCTGGGGCATC
>DQ410205..1
TTCTACTGTAATACATCACAACTGTTTAATAGTAATTGGACTTGGCCTGATACTGAAGGGTCAAATGACACTA
ACACTAACGAAAATATCACACTCCCATGCAGAATAAAACAAATTATAAACATGTGGCAGGAGGTAGGAAAAGC
AATGTATGCCCCTCCCATCAGAGGACAAATTAGCTGTAAATCAAATATTACAGGACTGCTATTGACAAGAGAT
GGTGGTCAGGACGAGAACGGGACCGAGACCTTCAGACCTGGAGGAGGAGATATGAGGGACAATTGGAGAAGTG
AATTATATAAATATAAAGTAGTAAAAATTGAACCATTAGGAGTAGCACCCACCAAGGCAAAGAGAAGAGTGGT

GCAGAGAGAAAAAAGAGCATTAGGAATAGGAGCCGTGTTCCTTGGGTTCTTGGGAGCAGCAGGAAGCACTATG
GGCGCAGCGTCAATGACGCTGACGGTACAAGCCAGACTGCTATTGTCTGGTATAGTGCAACAGCAGAACAATT
TGCTGAGGGCTATTGAGGCGCAACAGCATCTGTTGCAACTCACAGTCTGGGGCATC
>FJ469733..1
TTCTACTGTAATTCAACACAACTGTTTAATAGTACTTGGTATACTAATAATACTATGGAGGGGTCACATGGTA
ATGAGACAAATGGCACTATCACTCTCCCATGCAAAATAAGACAAATTGTAAACAAGTGGCAAGAAGTAGGAAA
AGCAATGTATGCCCCTCCCATTAGAGGACTAATTAGATGTTCATCAAATATTACAGGGCTGCTATTAACAAGA
GATGGTGGTCATAATAATAATAATAATACTAATGAGACCTTCAGACCTGGAGGAGGAGATATGAGGGACAATT
GGAGAAGTGAATTATATAAATACAAAGTAGTAAGAATTGAGCCATTAGGAGTAGCACCCACCAAGGCACAGAG
AAGAGTGGTGCAAAGAGAAAAAAGAGCAGTGGGACTAGGAGCTGTGTTCCTTGGGTTCTTGGGAGCAGCAGGA
AGCACTATGGGCGCAGCGTCAATAACGCTGACGGCACAGGCCAGACAATTATTGTCTGGTATAGTGCAACAGC
AGAACAACCTGCTGAGAGCTATTGAGGCGCAACAGCATATGTTGCAACTCACAGTCTGGGGCATA
>JQ403107..1
TTCTACTGTGATACAACACAACTGTTTAATAGCAGCTGGGAGGGGAATGGTACTAAAGATACTATAGAGTCAA
ATGACACTGGAGGAAATATCACACTCCCATGTAGAATAAAACAGATTATAAACATGTGGCAGGAAGTAGGAAA
AGCAATGTATGCCCCTCCCATCAGTGGACCAATTAGCTGTTCATCAAACATTACAGGGCTACTATTAACAAGA
GATGGGGGTCATAACAATGACACTAACGGGAACGAGACCTTCAGACCTGGAGGAGGAAATATGAAGGACAATT
GGAGAAATGAATTATATAAATATAAAGTAGTAAAAATTGAACCACTAGGAGTAGCACCCACCAAGGCAAAGAG
AAGAGTGGTGCAGAGAGAAAAAAGAGCAATGGGACTAGGAGCTATGTTCATTGGGTTCTTGGGAGCAGCAGGA
AGCACTATGGGCGCAGCGTCAGTGACGCTGACGGTACAGGCCAGACAATTACTGTCTGGTATAGTGCGCCAGC
AGAACAATCTGCTGAGAGCTATTGAGGCGCAACAGCATATGTTGCAACTCACAGTCTGGGGCATC
>JN024100..1
TTCTACTGTAATACAACAAAACTGTTTAATAGTACTTGGAATAGTACTGAGAATGTGGAGGAAGGGCCAGATA
ACAATATCACACTCCCATGCAGGATAAAGCAAATTATAAACATGTGGCAGGAAGTAGGAAAAGCAATGTATGC
CCCTCCCATCGCAGGACAAATTAGATGTTCATCAAATATTACAGGGCTGATATTAACAAGAGATGGTGGTGAG
AATAACAACGAGACCGAGACCTTCAGACCTGGAGGAGGAGATATGAGGGACAATTGGAGAAGTGAACTTTATA
AATATAAAGTAGTAAAAATTGAACCATTAGGAGTAGCACCCACTAAGGCAAAAAGAAGAGTGGTGCAGAGAGA
AAAAAGAGCAGCAATAGGAGCTTTGTTCCTTGGTTTCTTGGGAGCAGCAGGAAGCACTATGGGCGCAGCGTCA
ATAACGCTGACGGTACAGGCCAGACTATTGTTGTCTGGTATAGTGCAACAGCAGAGCAATCTGCTGAAAGCTA
TTGAGGCGCAACAGCATCTGTTGCAACTCACAGTCTGGGGCATC
>HQ216601..2
TTCTACTGTGATACAACACAGCTGTTTAATAGTACTTGGAATATTGAGAATGATACGAACAGGTCAAATGAAA
GTGAAACAATTATACTTCCATGCAAAATAAAGCAAATTATAAACCTGTGGCAGGAAGTAGGAAAAGCAATGTA
TGCCCCTCCCATCAGAGATCAAATTAGATGCTCATCAAATATTACAGGGCTGCTATTAACAAGAGATGGTGGT
AATAATACAACTGAGAACAATAACGAGACCTTCAGACCTGGAGGAGGAGATATGAGGGACAATTGGAGAAGTG
AACTTTATAAATATAAAGTAGTAAAAATTGAACCATTAGGAATAGCACCCACTACTGCAAAAAGAAGAGTGGT
GCAGAGAGAAAAAAGAGCGGCACTAGGAGCCTTGTTCCTTGGGTTCTTGGGAGCAGCAGGAAGCACTATGGGC
GCAGCGTCAATAACGCTGACGGTACAGGCCAGACTATTGTTGTCTGGTATAGTGCAACAGCAGAACAATCTGC
TGAAGGCTATTGAGGCGCAACAGCATCTGTTGCAACTCACAGTCTGGGGCATC
>HQ216795..1
TTCTACTGTAACACATCACAGCTGTTTGATAGTACTTGGCTGTTTAATAAGACTGGGGATAATAATACTGAAG
GGTCAAATGACACTATCATACTTCCATGCAGAATAAAACAAATTATAAACAGGTGGCAGGAAGTAGGAAAGGC
AATGTATGCCCCTCCCATCGCAGGACAAATTAGATGTCAATCAAATATTACAGGGGTGCTACTAACAAGAGAT
GGGGGTAAGAGTGGGAACGGTACTAATGAGACCGAAACATTCAGACCTGTTGGAGGAAATATGAAGGACAATT
GGAGAAGTGAATTGTATAAATATAAGGTAGTAAAGATTGAACCATTAGGAGTAGCACCCACCAAGGCAAAGAG
GCTAGTGGTGCAGAGAGAAAAAAGAGCAGTGACGCTGGGAGCTATGTTCATTGGGTTCTTGGGAGCAGCAGGA
AGCACTATGGGCGCAGCGTCACTGACGCTGACGGTACAGGCCAGACAATTACTGTCTGGTATAGTGCAACAGC
AGAACAATCTGCTGAGAGCTATTGAGGCGCAACAGCATCTGTTGCAACTCACAGTTTGGGGCATT
>JX863965..1
TTCTACTGTAATTCAGCACAACTGTTTAATAGTACTTGGGATAATACTGCTGGGAATAATACAGCTGGAGAAA
ATGACACAATCACACTCCCATGCAGAATAAAACAAATTGTAAATAGGTGGCAAGAAGTAGGAAAAGCAATGTA
CGCCCCTCCCATCAGCGGACCAATTAAATGTTCATCAAATATTACAGGACTGCTATTAACAAGAGATGGTGGT
CAAAATACAACTAATAACACCGAAATTTTCAGACCAGGAGGAGGAGATATGAGGGACAATTGGAGAAGTGAAT
TATATAAATATAAAGTAGTAAAAATTGAACCATTAGGAGTAGCACCCACTAAGGCAAAGAGAAGAGTGGTGCA
GAGAGAAAAAAGAGCAGTGGGAATAGGAGCTATGTTCCTTGGGTTCTTGGGAGCAGCAGGAAGCACTATGGGC
GCAGCATCAATAACGCTGACGGTACAGGCCAGACAATTATTGTCTGGTATAGTGCAACAGCAGAACAATCTGC
TGAGAGCTATTGAGGCGCAACAGCATCTGTTGCAACTCACAGTCTGGGGCATT
>JF320043..1
TTTTACTGTAATACAACAAAACTATTTAATAGCACTTGGGAGCCTAATAATGTAACTGTCACTGGTGATAATG
ACACAATCACACTCCCATGCAGAATAAAACAAATTGTAAACTTGTGGCAGGAAGTAGGAAGAGCAATGTATGC

CGCTCCCATCAGAGGACTGATCAGATGTTCATCAAATATTACAGGGCTACTATTAACAAGAGATGGTGGTGTT
GATAACAATCAGACCAATGTGACTAATAACACAGAAACCTTCAGACCAGGAGGAGGAGATATGAGGGACAATT
GGAGAAGTGAATTATATAAATATAAAGTAGTAAAAATTGAACCATTAGGAGTAGCACCCACCAAGGCAAAGAG
AAGAGTGGTGCAGAGAGAAAAAAGAGCAATGGGATTCGGAGCTTTTTTCCTTGGGTTCTTGGGAGCAGCAGGA
AGCACTATGGGCGCAGCGTCAATAACGCTGACGGCACAGGCCAGACAATTATTGTCTGGTATAGTGCAACAGC
AAAACAATTTGCTGAGGGCTATTGAGGCGCAACAACACCTGTTGCAACTCACAGTCTGGGGCATC
>EU577873..1
TTCTACTGTAATACAACAAAACTGTTTAATAGTACTTGGAATACTACTAGTATTTGGAATAATACTGCAGAGA
ATAATACTACAGGGTCAAATGAAACTGAACCAATCACACTCCCATGCAGAATAAAACAAATTATAAATATGTG
GCAGGAAGTAGGAAAAGCAATGTATGCCCCTCCCATTAAAGGAAACATTAGCTGTTCATCAAATATTACAGGA
TTGATATTAACAAGAGATGGTGGTAACGTTAACGGGACCAATGGAACTGAAATCTTTAGACCTGCAGGAGGAA
ATATGAAGGACAATTGGAGGAGTGAATTATATAAATATAAAGTAGTAAAAATTGAGCCATTAGGAGTAGCACC
CACCAAGGCAAAGAGAAGAGTGGTGCAGAGAGAAAAAAGAGCAGTGGGATTCGGAGCTGTGTTCCTTGGGTTC
TTGGGAGCAGCAGGAAGCACTATGGGCGCAGCGTCGGTGACGCTGACGGTACAGGCCAGACAACTATTGTCTG
GTATAGTGCAACAGCAAAACAATTTGCTGAGAGCTATTGAGGCGCAACATCATCTGTTGCAACTCACCGTTTG
GGGCATC
>KT124778..1
TTCTACTGTAATACGTCACAACTGTTTAATAGTACTTGGCTGGGGAATGACACTGAAAGGTTAAACAGAACTG
AAGGGTCAGACAGCACTGAAGTAATCACACTCCAGTGTAGAATAAAACAAATTATAAACATGTGGCAGGAAGT
AGGAAAAGCAATGTATGCCCCTCCCATCGCAGGACAAATTAACTGTTCATCAAATATTACGGGGCTACTATTA
ACAAGAGATGGTGGTTACAATGATACTAGCCAGAACGAGACCTTCAGACCTGGGGGAGGAGATATGAGGGACA
ATTGGAGAAGTGAATTATATAAATATAAAGTAGTAAGAATTGAACCATTAGGAGTAGCCCCCACCAAGGCAAA
GAGAAGAGTGGTGCAAAGAGAAAGAAGAGCAGTGGGATTAGGAGCTGTGTTCCTTGGGTTCTTGGGAGCAGCA
GGAAGCACTATGGGCGCAGCGTCAGTGACGCTGACGGTACAGGCCAGACAATTATTGTCTGGTATAGTGCAGC
AGCAAAACAATTTGCTGAAGGCTATTGAGGCGCAACACCATCTGTTGCAACTCACAGTCTGGGGCATC
>DQ410562..2
TTCTACTGTAATACAACACTGCTGTTTGATAGTACTTGGGATGATAATAATAGAAGTTTGAATTTGAATAATA
CTAATAGAAACATCACACTCCCATGCAAGATAAAACAAATTATAAACAGGTGGCAGGAAGTGGGAAAAGCAAT
GTATGCCCCTCCCATTCAAGGAGTAATTCAATGTTCATCAAATATTACAGGGCTGTTATTAGTAAGAGATGGT
GGCAACACTGCTAACAATACTGCTAACAAAACTGAGACCTTCAGACCTGGAGGAGGAAATATGAAGGACAATT
GGAGAAGTGAATTATATAAATATAAAGTAGTAAAAATTGAACCATTAGGAGTGGCACCCACCAAGGCAAAGAG
GAGAGTGGTGCAGAGGGAAAAAAGAGCAGTGGGAATAGGAGCTATGTTCCTTGGATTCTTAGGAGCAGCAGGA
AGCACTATGGGCGCAGCGTCAATAACGCTGACGGTACAGGCCAGGCAATTATTGTCTGGTATAGTGCAACAGC
AGAACAATCTGCTGAGAGCTATTGAGGCGCAACAGCATCTGTTGCAACTCACAGTCTGGGGCATC
>FJ469717..1
TTCTACTGTAATACAACACAGCTGTTTAATAGTACTTGGTTTACTAATGGCACTTGGATTAATGGTACTGTGT
TAAATACAAATGATACTGACAAGGGAAACATCACACTCCCATGTAAAATAAAACAAATTATAAACATGTGGCA
GGAAGTAGGAAAAGCAATATATGCCCCTCCCATCAAAGGACCAATTAACTGTACATCAAATATTACAGGGATA
ATAATAACAAGAGATGGTGGTAATAACACGGGCGAAAATAAAAATAAAACTTTCACTGAAACTTTCAGACCTG
GAGGAGGAAATATGAGGGATAATTGGAAAAGTGAACTATATAAATATAAAGTAGTACAGATTCAACCAATAGA
AATAGCACCCACCAGGGCAAAGAGAAGAGTGGTGCAGAGAGAAAAAAGAGCAGTGGGAGCGCTGGGAGCTGTG
TTCCTTGGGTTCTTGGGAGCAGCAGGAAGCACTATGGGCGCAGCGTCAATAACGCTGACGGTACAGGCCAGAC
AATTATTGTCTGGTATAGTGCAACAGCAGAACAATCTGCTGAAGGCTATTGAGGCGCAACAGCATCTGTTGCA
ACTCACAGTCTGGGGCATC
>FJ469724..1
TTCTACTGTAATACAACACAACTGTTTAATAGTACTTGGAATAATGATACTTGGAATGATAATAAAACTGAAG
GAAATGGCACAATTACACTCCAATGCAGAATAAAACAATTTATAAACCTGTGGCAGAAAGTAGGAAAAGCAAT
ATATGCCCCTCCCATCCAAGGAAATATTAGCTGTTCATCAAATATTACAGGGCTGCTATTAACAAGGGATGGT
GGTGTTAACGGGAGCGAGCACAACGAGACACTCAGACCTGGAGGAGGAGATATAAAAGACAATTGGAGAAGTG
AATTATATCAATATAAAGTAGTAAAAATTGAACCATTAGGAATAGCACCCACTGCAGCAAAGAGAAGAGTGGT
GCAGAGAGAAAAAAGAGCAGCAGGAATAGGAGCTCTGCTCCTTGGGTTCTTGTCAGCAGCAGGAAGCACTATG
GGCGCAGTGTCAGTGACGCTGACGGTACAGGCCAGACAATTATTGTCTGGTATAGTGCAACAGCAAAACAATT
TGCTGAGAGCTATTGAGGCGCAACAGCATCTGTTGCAACTCACGGTCTGGGGCATT
>FJ653159..1
TTCTATTGTAATACAACACAACTGTTTAATAGTAATTGGAATGAGAGTAGTATTAATAGTACTTGGAATAGTA
CTTGGAAGTCAAATGACCCAAATGACACCATCACACTCCCATGCAGAATAAAACAAATTATAAACATGTGGCA
AGAAGTGGGAAAAGCAATGTATGCCCCTCCCATCAAAGGTGTAATTCAGTGTTCATCAAATATTACAGGGCTG
CTATTAACAAGAGATGGTGGGAGTAATGAGACTAATACCACCGAGACTTTCAGACCTGGGGGAGGAGATATGA
GGGACAATTGGAGAAGTGAACTATACAAATATAAAGTAGTAAAAATTGAACCATTAGGAGTAGCACCCACCAA
GGCAAAAAGAAGAGTGGTGCAAAGAGAAAAAAGAGCAGTGGGAACAATAGGAGCTATGTTCCTTGGGTTCTTG

GGAGCAGCAGGAAGCACTATGGGCGCAGCGTCAGTGACGCTGACGGTACAAGCCAGACAATTATTGTCTGGTA
TAGTGCAGCAGCAGAGCAATTTGCTGCAGGCTATTAATGCACAACAACATTTGCTGCAACTAACAGTCTGGGG
CATC
>FJ653573..1
TTTTACTGTAATACAACACAACTGTTTAATAGTACTTGGAATATGACTGGTACCTGGAATGATACCTGGAATG
GTACTAAAAGGTCAAATGAAACTGACAATATCACACTCCCATGCAGAATAAAACAAATAGTAAACAGGTGGCA
GGAAGTAGGCAAAGCAATGTATGCCCCTCCCATCAGAGGAAGCATTAGATGTTCATCAAATATTACAGGGCTG
CTATTAACAAGAGATGGGGGGATTCATAATAGCACTGAGGCAAATAACACTGAGATTTTCAGACCTGGAGGAG
GAGACATGAGGGACAATTGGAGAAGTGAATTATATAAGTATAAAGTAGTGAAAATTGAACCATTAGGAGTAGC
ACCCACCAAGGCAAGGAGAAGAGTGGTGCAGAGAGAAAAAAGAGCAGTGGGAGCACTAGGAGCTATGTTCCTT
GGGTTCTTGGGAGCAGCAGGAAGCACTATGGGCGCAGCGTCACTGACGCTGACGGTACAGGCCAGACAATTGT
TGTCTGGTATAGTGCAACAGCAGAACAACCTGCTGAGAGCTATTGAGGCGCAACAGCATCTGTTGCAACTCAC
AGTCTGGGGCATC
>EU578629..1
TTCTACTGCAATACAACAAAACTGTTCAGTAGTACTTGGAATAGTACTACTTGGGATAGTTGGAATAGTACTG
AAACAAATAGCACTGACAGAAATATCACACTCCCATGCAGAATAAAACAAATTATAAACATGTGGCAGGAAGT
AGGAAGAGCAATGTATGCCCCTCCCATCAGAGGACAAATTAGATGCTCATCAAATATTACAGGGCTGATATTA
ACAAGAGATGGTGGTAACAATACAAATACAAACGAGACCTTCAGACCTCAAGGAGGAAACATGAAGGACAATT
GGAGAAGTGAATTATATAAATATAAAGTAGTAAAAATTGAACCACTAGGAATAGCACCCACCAAGGCAAAGAG
AAGAGTGGTGCAGAGAGAAAAAAGAGCAGTGGGAATGGGAGCTTTGTTCCTTGGGTTCTTGGGAGCAGCAGGA
AGCACTATGGGCGCAGTGTCGATGACGCTGACGGTACAGGCCAGACAATTATTGTCTGGTATAGTGCAACAGC
AGAATAATCTGCTGAGAGCTATTGAGGCGCAACAGCATCTGTTGCAACTCACAGTCTGGGGCATC
>HQ217860..1
TTCTACTGTAATACAACAAAACTATTTAATAGTACTTGGGATATAGAAACAATACTTAAGCATACTAAAAATA
GTAATGACACAAACTCAACTATTACACTCCAATGCAGAATAAAACAAATTGTAAACATGTGGCAGGAAGTAGG
AAAAGCAATGTACGCCCCTCCCATCAGAGGAATAATTAACTGTACATCAAATATTACAGGGCTGCTATTAACA
AGAGATGGGGGTCATAACAACAGCACCAACGAGACCTTCAGACCGGGAGGAGGAGATATGAGAGACAATTGGA
GAAGTGAATTATACAAATATAAAGTAGTAAAAGTTGAACCATTAGGAATAGCACCCACCAAGGCAAAAAGAAG
AGTGGTGCAAAGAGAGAAAAGAGCAGTGGGAATAGGAGCTGTGTTCCTTGGGTTCTTGGGAGCAGCAGGAAGC
ACTATGGGCGCAGCGACAGTGACGCTGACGGTACAGGCCAGACAATTATTGTCTGGTATAGTGCAACAGCAGA
GCAATTTGCTGAGAGCTATTGAGGCGCAACAGCATCTGTTGCAGCTCACAGTCTGGGGCATC
>JF689865..1
TTCTACTGTAATACAACAAAACTGTTTAATAGTACTTGGCCTTGGAATGTTACTGGGTCAAATGTTACTGGGT
TAAATGACACAATCACAATTCCATGCAGAATAAAACAAATTATAAACATGTGGCAGGAAGTAGGAAAAGCAAT
GTATGCCCCTCCCATCCAAGGACAAATTAGATGTTCATCAAATATTACAGGGCTACTATTAACCAGAGATGGT
GGTAATACAAATGAGAGCAATACCACCGAGACCTTCAGACCTGGAGGAGGAGATATGAGGGACAATTGGAGAA
GTGAATTATATAAATATAAAGTAGTAAAAATTGAACCATTAGGAGTAGCACCCACCAAGGCAAAGAGAAGAGT
GGTGCAGAGAGAAAAAAGAGCAGTGGGACTAGGAGCTCTGTTCCTTGGGTTCTTGGGAGCAGCAGGAAGCACT
ATGGGCGCAGCGTCAATGACGCTGACGGTACAAGCCAGGCAATTATTGTCTGGTATAGTGCAACAGCAAAACA
ATTTGCTGAGGGCTATTGAAGCGCAACATCACCTGTTGCAACTCACAGTCTGGGGCATT
>JF689874..1
TTCTACTGTAATACAACAAAGCTGTTTACAAAGCGGTTTGATTGGAATAGGACTGAAGGGTTAAATGACACTG
CAGGAAATAACACTGGAGGAAATAACATCATAATCCCATGCAGAATAAAACAAATTATAAACATGTGGCAAGA
AGTAGGAAAAGCAATATATGCCCCTCCCATCAGTGGACGAATTAACTGTACATCAAACATTACTGGGCTGCTA
TTAATAAGAGATGGTGGGATAGGCCCGGGGACAGACAATACTAGTGAAACCTTCAGACCTCAAGGAGGGAATA
TAAGAGATAATTGGAGAAGTGAATTATATAAATATAAAGTAGTAAAAATTGAACCATTAGGACTAGCGCCCAC
CAAGGCAAGGAGAAGAGTGGTGCAGAGAGAAAAAAGAGCAATGGGACTAGGAGCTTTGTTCCTTGGGTTCTTG
GGAGCAGCAGGAAGCACTATGGGCGCAGCGTCACTGACGCTGACGGTACAGGCCAGACAATTATTGTCTGGTA
TAGTGCAACAGCAGAACAATTTGCTGAGAGCTATTGAGGCGCAACAGCATATGTTGCAACTCACAGTCTGGGG
CATC
>JF689875..1
TTCTACTGTAATACAACACAACTGTTTAATAGTACTTGGAATGAGAATAGTACTAAAGAAGGAAATGGAAATG
ACACAATAACCACACTCCCATGCAGAATAAAACAAATTATAAACATGTGGCAAGAAGTAGGAAAAGCAATGTA
TGCCCCTCCCATCAGAGGAAACATTAGCTGTACATCAAATATTACAGGGCTGCTATTAACAAGAGATGGTGGG
AATAATGAGACCGAGACCTTCAGACCTGGAGGAGGAAATATGAAGGATAATTGGAGAAGTGAATTATATAAAT
ATAAAGTAGTAAAAATTCAACCATTAGGAGTAGCACCCACCAAGGCAAAGAGAAGAGTGGTGCAGAGAGAAAA
AAGAGCAGTGGGACTAGGAGCTGTGTTCCTTGGGTTCTTGGGAGCAGCAGGAAGCACTATGGGCGCAGCGTCA
ATAACGCTGACGGTACAGGCCAGACAATTATTGTCTGGTATAGTGCAACAGCAGAACAATTTGCTGAGGGCTA
TTGAGGCGCAACAGCATCTGTTGCAACTCACAGTCTGGGGCATT
>JX863990..1

TTCTACTGTAATACAACACAACTGTTTAATAGTACTTGGAATTCTACTACTTTGAATTCTACTAGTACTGGGA
ATGGGACCATCACACTCTCATGTAGAATAAAACAAATTATAAACAGGTGGCAGGAAGTAGGAAAAGCAATGTA
TGCTCCTCCCATCAGAGGACAAATTAAGTGTTCATCAAATATTACAGGGCTACTGTTAACAAGAGATGGTGGT
CACACAAACAACACCAACAAAACTGAGACCTTTAGACCTGGAGGAGGAGACATGAGGGACAATTGGAGAAGTG
AATTATACAAATATAAAGTAGTAAAAATTGAACCATTAGGAATAGCACCCACCAAGGCAAGGAGAAGAGTGGT
GCAGAGAGAAAAAAGAGCAATAGGAATAGGAGCTGTGTTCCTTGGGTTCTTGGGAACAGCAGGAAGCACTATG
GGCGCAGCGGCAGTGACGCTGACGGTACAAGCCAGACAATTACTGTCTGGTATAGTGCAACAGCAAAACAATT
TGCTGAGGGCTATTGAGGCGCAGCAGCATCTGTTGCAACTCACAGTCTGGGGCATC
>JF320363..1
TTCTACTGTAACACAGCAAAACTGTTTGATACATCACAACTACAACTGTCTAATAGCAGCACTGAAGAAAATG
ACACACTCACAATTCCATGCAGAATAAAACAAGTTATAAACAGGTGGCAGGAAGTAGGAAAAGCAATGTATGC
CCCTCCCATCAGAGGACAAATTAAATGTGTATCAAACATTACAGGGCTGCTATTAACAAGAGATGGTGGAAAG
ACTGAGGATAATACTACTAATACTACTGAGGTCTTCAGACCTCAAGGAGGAAATATGAAAGATAATTGGAGAA
GTGAATTATATAAATATAAGGTAGTAAGAACTGAACCATTAGGAGTAGCACCCACCAAGGCAAAGAGAAGAGT
GGTGCAGAGAGAAAAAAGAGCAATAGGGGGATTAGGAGCGTTGTTCCTTGGGTTCTTGGGAGCAGCAGGAAGC
ACTATGGGCGCAGCGTCAATAACGCTGACGGCACAGGCCAGACAAATATTGTCTGGTATAGTGCAACAGCAGA
ATAATTTGCTGAGAGCTATTGAGGCGCAACAACATCTGTTGCAACTCACAGTCTGGGGCATC
>JF320036..1
TTCTACTGTAATACAACACAGCTGTTTAATATTAGTAGTACACAACTGTTTAATAGTACTTGGAATAGTACCG
AAGGGCTAAACACAAAAAATATCACACTCCAATGCAGAATAAAACAAATTATAAACATGTGGCAGCAAGTAGG
AAAAGCAATGTATGCCCCTCCCATCAGAGGACCAATTAAATGTATATCAAATATTACAGGGCTGCTGTTAACA
AGAGATGGTGGGAAGATAAATGAGAGTAGGGCAAATGAGACTGAGACCTTCAGACCTGGAGGAGGAGATATGA
GGGACAATTGGAGAAGTGAACTCTATAAATATAAAGTAGTAAAAATTAAACCATTAGGAGTAGCACCCACTAA
GGCAAAAAGAAGAGTGGTGCAGAGAGAAAAAAGAGCAGTGGGACTAGGAGCATTGTTCCTTGGGTTCTTGGGA
GCAGCAGGAAGCACTATGGGCGCAGCGTCAATAACGCTGACGGTACAGGCCAGACAATTATTGTCTGGTATAG
TGCAACAGCAGAACAATCTGCTGAGGGCTATTGAGGCGCAACAGCACCTGTTGCAACTCACAGTCTGGGGCAT
C
>JF320502..1
TTCTACTGTAATACAACAAAACTGTTTAATAGCATTTGGAATGAAACACAACTGTTTAATAGTACTTGGAATG
AGACTGAAAGAGGAGTCAATATCACACTCCCATGTAGAATAAAACAAATTATAAATATGTGGCAGGAAGTAGG
AAAAGCAATGTATGCCCCTCCCATCAGAGGAATAATTCAATGTACATCAAACATTACAGGACTGATATTAACA
AGAGATGGTGGTAAAAAAAATAATGGGAGTGAAACCTCCAATGGGAATGAAACCTTCAGACCTGGAGGAGGAG
ATATGAGAGACAATTGGAGAAGTGAATTATATAAATATAAAGTAGTAAAAGTTGAACCATTAGGAATAGCACC
CACTAGGGCAAGGAGAAGAGTGGTGCAGAGAGAGAAAAGAGCAGTGGGAATAGGAGCTGTGTTCCTTGGGTTC
TTGGGAGCAGCAGGAAGCACTATGGGCGCAGCGTCAATAACGCTGACGGTACAGGCCAGACAATTATTGTCTG
GTATAGTGCAACAGCAGAACAATCTGCTGAAAGCTATTGATGCGCAACAGCATCTGTTGCAACTCACAGTCTG
GGGCATC
>JF320151..2
TTCTACTGTAATACAACAGAGCTGTTTAATGATACTTGGCTGAGTAATAATACTTGGCTGTCAAATTATACTG
AGAGTAATAATAATACTGCTAAAAATATCGTACTCCCATGCAGAATAAAACAAATTATAAACAGATGGCAGGA
AGTAGGAAAAGCAATGTATGCCCCTCCCATCAGTGGACTAATTAGATGCTCATCAAATATTACAGGGCTGCTC
TTAACAAGAGATGGTGGTAATCATAACACTAGTAATGACACCGAGACTTTCAGACCAGGAGGAGGAGATATGA
GGGATAATTGGAGAAGTGAATTATATAAATATAAAGTAGTAAAAATTGAGCCATTAGGAGTAGCACCCACCAA
GGCAAGGAGAAGAGTGGTGCAGAGAGAAAAAAGAGCAGTGGGACTAGGAGCTTTGTTCCTTGGGTTCTTGGGA
GCAGCAGGAAGCACTATGGGCGCAGCATCAGTGACGCTGACGGTACAAGCCAGACAATTATTGTCTGGTATAG
TGCAACAGCAAAACAATTTGCTGAGGGCTATTGAGGCGCAACAGCATATGTTGCAACTCACAGTCTGGGGCAT
T
>JF320145..1
TTCTACTGTAATTCAACAAAACTGTTTAGCAGTGCTTGGTATACTAATAATACTAATAGTAGTAATACTGAAG
GGTCAAATGACACTGTTATCATACTTCCATGCAAAATAAAACAAATTATAAACATGTGGCAGGAAGTAGGAAG
AGCAATGTATGCCCCTCCCATCGCAGGAAACATTAGCTGTTCATCAAATATTACAGGACTGCTGTTAACAAGA
GATGGTGGTCACACGGAAACTGCCAACACCACAGAAATCTTCAGACCTGGAGGAGGAGATATGAGGGACAATT
GGAGAAGTGAATTATATAAATATAAAGTAGTAAAAATTGAACCATTAGGAGTAGCACCCACCAAGGCAAGGAG
AAGAGTGGTGCAGAGAGAAAAAAGAGCAGTAACAGTGGGAGCTGTGTTCCTTGGGTTCTTGAGTGCAGCAGGA
AGCACTATGGGCGCAGCGTCACTGACGCTGACGGTACAGGCCAGACTACTGTTGTCTGGTATAGTGCAGCAGC
AGAGCAATCTGCTGAGGGCTATTGAGGCGCAACAACAACTGTTGCAACTCACAGTCTGGGGCATC
>HQ908232..2
TTCTACTGTAATACAACAAGGCTGTTTAATAGTACTTGGCAGTCCAATAGTACGGGGAATGATACTGGAGGGT
CAAATAACACTGAACCTATCACACTCCTATGCAGAATAAAACAAATTGTAAACAGGTGGCAAGAAGTAGGAAA
AGCAATGTATGCCCCTCCCATCAGAGGACAAATTTACTGTAGATCAAATATTACAGGGCTGCTATTAACAAGG

GATGGTGGTAATGTCCAGAACAACATGACTGAGACCTTCAGACCAGGAGGAGGAGATATGAGGGACAATTGGA
GAAGTGAATTATATAAATATAAAGTAGTAAAAATTCAGCCATTAGGAATAGCACCCACCGGGGCAAAGAGAAG
AGTGGTGCAGAGAGAAAAAAGAGCGGTGACACTGGGAGCTATGTTCCTTGGGTTCTTGGGAGCAGCAGGAAGC
ACTATGGGCGCAGCGTCAATAACGCTGACGGTACAGGCCAGACAATTACTGTCTGGTATAGTGCAACAGCAGA
ACAATCTGCTGAAGGCTATTGATGCGCAGCAGCATATGTTGCAACTCACCGTCTGGGGCATC
>KM353665..1
TTCTACTGTAATACAACAAAATTGTTTAATAGTACTTGGTATAATAATAGTACTTGGAATAATACTGGAGGAA
ATGACACATTCACACTCCCATGTAGAATAAAACAAATTATAAACATGTGGCAGACAGTAGGAAGAGCAATGTA
TACCCCTCCCATCAGAGGATTAATTAAGTGTACATCAAATATTACAGGGCTGATATTAACAAGAGATGGTGGG
GGTGGTAACGAGAGTAATCATACCACAGAGGTCTTCAGACCTGCAGGAGGAGATATGAGGGACAATTGGAGGA
GTGAATTATATAAATACAAAGTAGTAAAAATTGAACCATTAGGAATAGCACCCACCAGGGCAAAAAGAAGAGT
GGTGCAGAGAGAAAAAAGAGCAGTGGGGATGGGAGCTCTGTTCCTTGGGTTCTTAGGAGCAGCAGGAAGCACT
ATGGGCGCAGCGTCAATAACGCTGACGGTACAGGCCAGACAATTATTGTCTGGTATAGTGCAACAGCAGAGCA
ATTTGCTGAAGGCTATTGAGGCGCAACAGCATCTGTTGCAACTCACAGTCTGGGGCATC
>JN687750..1
TTCTACTGTGATACAAAAAAGCTGTTTAATAGTACTTGGTGGGTTAATAATACTTGGAAAGGCACTAAAGGGG
ATGAGACTAGGAATGGCACAATCGAACTCCCATGCAGAATAAAACAAATTATAAACATGTGGCAGGAAGTAGG
AAAAGCAATGTATGCCCCTCCCATCAGAGGACAAATTCAGTGTTCATCAAATATTACAGGGCTGCTATTAACA
AGAGATGGGGGTAATAATGAAACGAACGGAACAGAAACCTTCAGACCTGGAGGAGGAGATATGAGGGACAATT
GGAGAAGTGAATTATATAAATATAAAGTAGTAAAAATTGAGCCATTAGGAATAGCACCCGGCAGGGCAAAGAG
AAGAGTGGTGCAGAGAGAAAAAAGAGCAGTGGGACTAGGAGCTATGTTCCTTGGGTTCTTGGGAGCAGCAGGA
AGCACTATGGGCGCAGCGTCAATAACGCTGACGGTACAGGCCAGACAATTACTGTCTGGTATAGTGCAACAGC
AGAACAACTTGCTGAGAGCTATTGAGGCGCAGCAGCATATGTTGCAACTCACAGTCTGGGGCATC
>HM204582..1
TTTTATTGTAATACATCAGAACTGTTTAAAAGTACCTGGAATAGTACTGATACCTTCAACAGTACTGGAGAGA
GAGGGAATGACACAATCATTCTCCCATGCAAAATAAGGCAAGTTGTAAACATGTGGCAGGAAGTAGGAAAAGC
AATGTATGCCCCTCCTATCAGAGGTCAAATTAACTGTTCATCAAATATTACAGGGCTGCTATTAACAAGAGAT
GGTGGTGATAAAAATAGTACAAACGAGACCTTCAGACCTGGAGGAGGAGATATGAGGGACAATTGGAGAAGTG
AATTATATAAATATAAGGTAGTAAAAATTGAACCATTAGGAATAGCACCCACCAAGGCAAAGAGAAGAGTGGT
GCAAAGAGAAAAAAGAGCAGTGGGAATAGGAGCTTTGTTCCTTGGGTTCTTGGGAGCAGCAGGAAGCACTATG
GGCGCAGCGTCACTGACGCTGACGGTACAAGCCAGACTATTATTGTCTGGTATAGTGCAACAGCAGAACAATT
TGCTGAGGGCTATTGAGGCGCAACAGCATCTGTTGCAACTCACGGTCTGGGGCATC
>HM204589..1
TTCTACTGTAATTCAACACAATTGTTTAATAGTACTTGGGATAAGAATGGTAGTAGAATAAATGGAACTGAAG
GAGAAAGTGGTACCATCACACTCCCATGCAGAATAAAACAAATTATAAACATGTGGCAGGAAGTAGGAAAAGC
AATGTATGCCCCTCCCATCAGAGGACAAATTTATTGTTCATCAAATATTACAGGGATGCTATTAACAAGAGAT
GGTGGTACTAATGATACTGACAAAAACGACACCGAGACCTTCAGACCTGGAGGAGGAGATATGAGGGATAATT
GGAGAAGTGAATTATATAAATATAAAGTAGTAAAAATTGAGCCATTAGGAGTAGCACCCACCAAGGCAAAGAG
AAGAGTGGTGCAGAGAGAAAAAAGAGCAGTGGGATTGGGAGCTCTGTTCCTTGGGTTCTTGGGAGCAGCAGGA
AGCACTATGGGCGCAGCGTCAGTGACGCTGACGGTACAGGCCAGACAATTATTGTCTGGTATAGTGCAACAGC
AGAACAATCTGCTGAGGGCTATTGAGGCGCAACAGCATCTGTTGCAACTCACAGTCTGGGGCATC
>KY112061..1
TTCTACTGTAATTCAACACAGCTGTTTAATAGTGCTTGGCCTTGGAATGATACTGAAGGGTCAAATGACGCTA
AAAAAGATAATATCACACTCCCATGCAAAATAAAACAAATTATAAACATGTGGCAGGGAGTAGGAAAAGCAAT
GTATGCCCCTCCCATCGAAGGAAAAATTAAATGCTCATCAAATATTACAGGACTGCTATTAACAAGAGATGGT
GGTTATGAGAACAATGAGACCAAGCCCGAGATCTTCAGACCTGGAGGAGGAGATATGAGGGACAATTGGAGGA
GTGAATTATATAAATATAAAGTAGTAAAAATTGAACCATTGGGAGTAGCACCTACCAAGGCAAAGAGAAGAGT
GGTGCAGAGAGAAAAAAGAGCATTTGGACTAGGAGCTGTGTTCCTTGGGTTCTTGGGAGCAGCAGGAAGCACT
ATGGGCGCAGCATCAATAACGCTGACGGTACAGGCCAGACAATTATTGTCTGGTATAGTGCAACAGCAGAACA
ATTTGCTGAGGGCTATTGAGGCGCAACAGCATCTGTTGCAACTCACAGTCTGGGGCATC
>EU576345..1
TTCTACTGTGATACAACAAAACTGTTTAATAGTACTTGGGGGAATAGTACTTGGGGGTATAAAATGACAAATG
ACACTGCAAAAGACAACATCACACTCCCATGTAGAATAAAACAAATTATAAACATGTGGCAGGAAGTAGGAAA
AGCAATGTATGCCCCTCCCATCAAAGGACAAATTAGATGTACATCAAATATTACAGGGTTGGTGTTAACAAGA
GATGGTGGTGGTGGTAGTGGTACAAATAACACCACAGAGATCTTCAGACCTGGAGGAGGAGATATGAGGGACA
ATTGGAGAAGTGAATTATATAAATATAAAGTAGTAAGAATTGAACCATTAGGAATAGCACCCACCAAGGCAAA
GAGAAGAGTGGTGCAGAGAGAAAAAAGAGCAGCAATAGGAGCTATGTTCCTTGGGTTCTTGGGAGCAGCAGGA
AGCACTATGGGCGCAGCGTCAATAACGCTGACGGTACAGGCCAGACTATTATTGTCTGGTATAGTGCAACAGC
AGAACAATCTGCTGAGGGCTATTGAGGCGCAACATCAACTGTTGCAACTCACAGTTTGGGGCATC
>EU576383..1

TTCTACTGTAATTCAACACAACTGTTTAACAGTACTTGGCATAGTAACAGTACTTGGAATGATACTGGAAAGT
CAGAAAATAATACAAAAATCATCACACTCCCATGCAGAATAAAACAAATTATAAACATGTGGCAGGAAGTAGG
AAAAGCAATGTATGCCCCTCCTATCAGAGGACAAATTAGATGTTCATCAAATATTACAGGGCTGATACTAACA
AGGGATGGAGGTCATGGAGGTAATGACAATAACCAGACCACCGAGACCTTCAGACCTGGAGGAGGAAATATGA
AGGACAATTGGAGAAGTGAATTATATAAATATAAAGTAGTAAAAATTGAACCATTAGGAGTAGCACCCACCCC
GGCAAAAAGAAGAGTGGTGCAGAGAGAAAAAAGAGCATTAGTGGGATTCGGAGCTCTGTTCCTTGGGTTCTTG
GGAGCAGCAGGAAGCACTATGGGCGCAGCGTCAATAACGCTGACGGTACAGGCCAGACAATTACTGTCTGGTA
TAGTGCAACAGCAAAACAATTTGCTGAAGGCTATTGAGGCGCAACAGCATCTGCTGCAACTCACAGTCTGGGG
CATC
>KX129209..1
TTCTACTGTAATTCAACACAACTGTTTAATAGTACGTGGATTGGGAATAGCACTGAAGAGTCAAATGGCACTG
AAAACATCACACTCCCATGTAGAATAAAACAATTTGTAAATCTGTGGCAGGAAGTAGGAAGAGCAATGTATGC
CCCTCCCATCAGAGGACTAATTAAATGTTCATCAAATATTACTGGGCTGTTGTTAACGAGAGATGGTGGTCAG
TCTGACAATACTACCAACGGGACCGAGACCTTTAGACCTGGAGGAGGAAATATGAAGGACAATTGGAGAAGTG
AATTATATAAATATAAAGTAGTACAAATTGAACCATTAGGAATAGCACCCACCAAGGCAAAGAGAAGAGTGGT
GCAGAGAGAAAAAAGAGCAGTGGGACTAGGAGCTTTGTTCCTTGGGTTCTTGGGAGCAGCAGGAAGCACTATG
GGCGCAGCGTCAGTGACGCTGACGGTACAGGCCAGACAATTATTGTCTGGTATAGTGCAGCAGCAGAACAACT
TGCTGAGAGCTATTGAGGCGCAACAACATATGTTGCAACTCACAGTCTGGGGCATC
>KM259534..1
TTCTACTGTAATACAACAAAACTGTTTAATAGTACTTGGAATGGGACTAATATTGATATTGGAAACAGTACGA
AAGATGACAATATCACACTCCCATGCAGAATAAAACAAATTATAAATAGGTGGCAAGAGGTAGGAAAAGCAAT
GTATGCCCCTCCCATCAGAGGACAAATTAGATGTTCATCAAACATTACAGGGCTGCTATTAACAAGAGATGGT
GGTAATAGCAATAACACCAACGAGACCACCGAGACCTTCAGACCTACAGGAGGAAATATGGAGGACAATTGGA
GAAGTGAATTATATAAATATAAAGTAGTGCAAATTCAACCATTAGGAGTAGCACCCACCGAGGCAAAGAGAAG
AGTGGTGCAGAGAGAAAAAAGAGCAGTGGGATTAGGAGCTATGTTCCTTGGGTTCTTGGGAGCAGCAGGTAGC
ACTATGGGCGCAGCGTCAATAACGCTGACGGTACAGGCCAGACAATTACTGTCTGGTATAGTGCAACAGCAGA
ACAATTTGCTGAGGGCTATTGAGGCGCAACAGCATATGTTGCAACTCACAGTCTGGGGCATC
>HQ217957..1
TTCTACTGTAATACATCAGAACTGTTTAATAGTACCTGGAATATAACACACTTTAATAGTACCTGGAATGATA
CTGAAGGATCAAATAACATCACACTCCCATGCAGAATAAAACAAATTATAAATATGTGGCAACAAGTAGGAAA
AGCAATGTATGCCCCTCCCATCCAAGGATTAATTCAATGTTCATCAAATATTACAGGGCTGCTATTAACAAGA
GATGGAGGTAATAATAGTACTAACGAGATCTTCAGACCTGGAGGAGGGGATATGAGGGACAATTGGAGAAGTG
AATTATATAGATATAAAGTAGTAAGAATTGAACCATTAGGAGTAGCACCCACCAAGGCAAGGAGAAGAGTGGT
GCAAAGAGAAAAAAGAGCAGTGGGAACAATAGGAGCTATGTTCCTTGGGTTCTTGGGAGCAGCAGGAAGCACT
ATGGGCGCAGCGTCAGTGACGCTGACGGTACAGGCCAGACAATTATTGTCTGGAATAGTGCAACAGCAAAACA
ATTTGCTGAAGGCTATTGAGGCGCAACAACACCTGTTGCAACTCACAGTCTGGGGCATC
>HQ217984..1
TTCTACTGTAATTCAACACAACTGTTTAATAGTACTTGGACTAGGGAAAATGGTAGTTGGACTAGGAATGATA
CCGCTGCAAATGAGACTGAGATTATCACACTCCCATGCAGAATAAAACAAATTATAAACATGTGGCAGGAAGT
AGGAAAAGCAATGTATGCCCCTCCCATCAAAGGACTTATTCAATGTTCATCAAATATTACAGGATTGTTATTA
ACAAGAGATGGTGGTAATAATAGCAACATCAATGAGATCTTTAGACCTGGAGGAGGAGATATGAGGGACAATT
GGAGAAGTGAATTGTACAAATATAAGGTAGTAAAAATTGAACCATTAGGAGTAGCACCCACCAAGGCAAGGAG
GAGAGTGGTGCAAAGAGAGAAAAGAGCAGTGGGACTAGGAGCTCTGTTCCTTGGGTTCTTGGGAGCAGCAGGA
AGCACTATGGGCGCAGCGTCATTGACGCTGACGGTACAGGCCAGACAATTATTGTCTGGTATAGTGCAACAGC
AGAACAATATGCTGAGGGCTATTGAGGCGCAGCAGCACCTGTTGCAACTCACAGTCTGGGGCATC
>JF689886..1
TTCTACTGTAATTCAACACAACTGTTTAATAGTACTTGGACTAAATATGATAATGGAACTTGGGTTAAGAAGA
ATGGCACTGAATGGGTGAATGATACAATCACACTCCCATGCAGAATAAAACAAATTATAAACATGTGGCAGGA
AGTAGGAAAAGCAATGTATGCCCCTCCCATCAGGGGACTAATTAATTGTATATCAAATATTACAGGGATACTG
CTAACAAGAGATGGTGGTCAGGTTAATGGTACCAATGAGACCTTCAGACCAGGAGGAGGAGATATAAAGGATA
ATTGGAGAAGTGAATTATATAAATATAAAGTAGTAAAAATTGAACCATTAGGAGTAGCACCCACCAAGGCAAG
GAGAAGAGTGGTGCAGAGGGAAAAAAGAGCAATTGGAATAGGAGCTTTGTTCCTTGGGTTCTTGGGAGCAGCA
GGAAGCACTATGGGCGCAGCGTCAGTGACGCTGACGGTACAAGCCAGACAATTATTGTCTGGTATAGTGCAAC
AGCAGAACAATTTGCTGAGGGCTATTGAGGCGCAACAACATCTGTTGCAACTCACAGTCTGGGGCATC
>JF320385..1
TTCTACTGTAATACAACACAACTGTTTAATAGTACTTGGATTCGGAATGATACTAAATGGTCAAGTAACTCTA
CAGGAAATGGCACCATTATACTCCCATGCAGAATAAAACAAATTATAAACATGTGGCAGGAAGTAGGAAAAGC
AATGTATGCCCCTCCTATCAGAGGAAACATTAGCTGTTCATCAAATATTACAGGGCTGCTATTAACAAGAGAT
GGGGGCAATAGTAATCAAACGACCCAGACCAAAGAAACAGAAACCTTCAGACCTGGAGGAGGAGATATGAGGG
ACAATTGGAGAAGTGAATTATATAAATATAAAGTAGTAAAAATTGAACCATTAGGAGTAGCACCCACCAAGGC

AAAGAGAAGAGTGGTGCAGAGAGAAAAAAGAGCAGTGGGAATAGGAGCAATGTTCCTTGGATTCTTGGGAGCA
GCAGGAAGCACTATGGGCGCAGCGTCACTAACGCTGACGGTACAGGCCAGACAATTATTGTCTGGTATAGTGC
AGCAGCAGAACAATCTGCTGAGAGCTATTGAGGCGCAACAGCATCTGTTGCAACTCACAGTCTGGGGCATC
>JF320539..1
TTCTACTGTAATACAACACAATTGTTCAATAATACTTGGAAAGGTACTGAAGAAGAGAATAGTACTAGGAATA
GTAATGACACCATCACACTCCCATGCAGAATAAAACAAATTGTAAACATGTGGCAGGAAGTAGGAAAAGCAAT
GTATGCCCCTCCCATCAGAGGACTAATTCAATGTACATCAAACATTACAGGGATAATATTAACAAGAGATGGT
GGTAATAACACGAATACCACTGAGATCTTCAGACCAGGAGGAGGAGACATGAGGGACAATTGGAGAAGTGAAT
TATATAAATATAAAGTAGTAAAAATTGAACCATTAGGAATAGCACCCACTAGGGCAAAGAGAAGAGTGGTGCA
GAGAGAAAAAAGAGCAGTGGGAATAGGAGCTTTGTTCCTTGGGTTCTTGGGAGCAGCAGGAAGCACTATGGGC
GCAGCGTCAATGACGCTGACGGTACAGGCCAGACTATTATTGTCTGGTATAGTGCAACAGCAGAACAATCTGC
TGCAGGCTATTGAGGCGCAACAGCGTCTGTTGCAACTCACAGTCTGGGGCATC
>JF320131..1
TTCTATTGTAATACAACACAACTGTTCAATAGTACTTGGGAGAGCAATATTACTACTGGGAATTGGAGCAAAA
CTGAAGGATCAAATAACACTAACATCACACTCCCATGTAGAATAAGACAAATTGTAAACATGTGGCAGAAAGT
AGGAAAAGCAATGTATGCCCCTCCCATCAGGGGACCAATTCAATGTATATCAGATATCACAGGGCTGCTATTA
ACAAGAGATGGAGGTAATAACGGGGACAATAACAACACAGAGACCTTCAGACCTGGAGGAGGAGATATGAGGG
ACAATTGGAGAAGTGAATTATATAAATATAAAGTAGTACAAATTGAACCAATAGGAATAGCACCCACCAAGGC
GCAGAGAAGAGTGGTGCAGAGAGAAAAAAGAGCAGTGGGAGTAGGAGCTTTTCTCTTTGGGTTCTTGGCAACA
GCAGGAAGCACTATGGGCGCAGCGTCAATAACGCTGACGGTACAAGCCAGACAATTATTGTCTGGTATAGTGC
AACAGCAGAGCAATTTGCTGAGGGCTATTGAGGCGCAACAGCATATGTTGCAACTCACAGTCTGGGGCATC
>HM638503..2
TTCTACTGTAATACAACAGGGCTGTTTAGTAGTACTTGGAATGAGAGTACTACTTGGGAAAGGTCAAATGACA
CTGAAATAATCACTCTCCCATGCAGAATAAAACAAATTATAAACATGTGGCAGGCAGTAGGAAAAGCAATATA
TGCCCCTCCCATCAGTGGACAAATTAATTGTTCATCAAATATTACAGGGCTGCTATTAACAAGAGATGGTGGT
TACAATGGTACGAATGACACCCAGACCGAGACCTTCAGACCTCAGGGAGGAAATATGAAAGACAATTGGAGAA
GTGAATTATATAAATATAAAGTAGTAAAAATTGAACCATTAGGACTAGCACCCACCAAGGCAAAGAGAAGAGT
GGTGCAGAGAGAAAAAAGAGCAGTGGGAATAGGAGCTATGTTCTTTGGGCTCTTGGGAGCAGCAGGAAGCACC
ATGGGCGCAGCGTCAGTGACGCTGACGGTACAGGCCAGAAAGTTGTTGTCTGGTATAGTGCAACAGCAGAACA
ATCTGCTGAGGGCTATTGAGGCGCAACAGCATATGTTGCAACTCACAGTCTGGGGCATC
>KT124801..1
TTCTACTGTAATACAACCGAACTGTTTAATAGTACTTGGACTAGGAATAATGGTACCGGGTATGATGGGTCTA
ATGGCACTGAAGGAAATGACACAATCACACTCCCATGCAGAATAAAACAAATTATAAACATGTGGCAGGAAGT
AGGAAAAGCAATGTATGCCCCTCCTATTAGAGGACAAATTAGATGCTCATCAAATATTACAGGACTGATATTA
ACAAGAGATGGGGGTCAAAGAAACGGGAGTACTAATGAGACCTTCAGACCAGGAGGAGGAAATATGAGGGACA
ATTGGAGAAGTGAATTATATAAGTATAAAGTAATAAAAATTGAACCACTAGGAGTAGCACCCACCAGGGCAAA
GAGAAGAGTGGTGCAGAGAGAAAAAAGAGCAGTGGGAATAGGAGCTGTGTTCCTTGGGTTCTTGGGAGCAGCA
GGAAGCACTATGGGCGCAGCGTCACTAACGCTGACGGTACAGGCCAGACAATTATTGTCTGGTATAGTGCAAC
AGCAAAACAATTTGCTGAGAGCTATTGAGGCGCAACAACATATGTTGCAACTCACAGTCTGGGGCATC
>JN687763..1
TTCTACTGCAATACAACACAACTGTTTAATAGTACTTGGAATACTAATGGTACTTGGACAGGAAGTAATACTA
ATAGCAGTAACATTACACTCCCATGCAGAATAAAACAAATTGTAAACATGTGGCAGGAAGTAGGAAAAGCAAT
GTATGCCCCTCCCATCAGCGGACTAATTCACTGTTCATCAAATATTACAGGGCTGATATTAACAAGGGATGGT
GGTAGTGGTAATGACACCAACAACAACACTGAGACCTTCAGACCTGCAGGAGGAGATATGAGGGACAATTGGA
GAAGTGAATTATACAAATATAAAGTAGTAAAAATTGAACCATTAGGAGTAGCACCCACCAGGGCAAAGAGAAG
AGTGGTGCAAAGAGAGAAAAGAGCAGTGGGAATGGGAGCTCTGTTCCTTGGGTTCTTGGGAGCAGCAGGAAGC
ACTATGGGCGCAGCGTCAATGACGCTGACGGTACAGGCCAGACAATTATTGTCTGGTATAGTGCAACAGCAGA
GCAATTTGCTGAGGGCTATTGAGGCGCAACAGCATCTGTTGCAACTCACAGTCTGGGGCATC
>JQ403073..1
TTCTACTGTAATACAACACAACTGTTTGATGATAATTGGCCTAATGGAACTTGGATGTATAATAGCACTAGAA
ATGACACTATCATACTCCCATGCAGGATAAAACAAATTGTAAACATGTGGCAGGAAGTAGGAAAAGCAATGTA
TGCCCCTCCCATCAAAGGACTAATTCACTGTAATTCAAGTATTACAGGGCTGCTATTAACAAGAGATGGTGGC
ACTAATAACACCAACGAGACCTTCAGACCGGGAGGAGGAGACATGAGGGACAATTGGAGAAGTGAATTATATA
AATATAAAGTAGTAAAAATTGAACCATTAGGAGTAGCACCCACCAAGGCAAAGAGAAGAGTGGTGCAGAGAGA
GAAAAGAGCAGTGGGACTAGGAGCTCTGTTCCTTGGGTTCTTGGGAGCAGCAGGAAGCACTATGGGCGCAGCG
TCAATGGCGCTGACGGTACAGACCAGACAATTATTGTCTGGTATAGTGCAACAGCAGAACAATTTGCTGAGGG
CTATTGAGGCGCAACAGCATCTGTTGCAACTCACAGTCTGGGGCATC
>JQ403078..1
TTCTATTGTAATACAACACAGCTGTTTAATAGTACTTGGAATGGTACTGAGACTGAGAATAATACTACAGGGT
CAAATAACACTGATAATATCAGACTCCCATGCAGAATAAAACAAATTGTAAACAGGTGGCAGGAAGTAGGAAA

AGCAATGTATGCCCCTCCCATCGAGGGCATAATTAGTTGTTCATCAAATATTACAGGGCTTTTATTAACCAGA
GATGGTGGTAACGGGAGCAGTAACGCCGAGATCTTCAGACCTCAAGGAGGAGATATAAGGGACAATTGGAGAA
GTGAATTATATAAATATAAAGTAGTAAAAATTGAGCCACTAGGAGTAGCACCCACCAAGGCAAAGAGAAGAGT
GGTGCAGAGAGAAAAAAGAGCAGTGGGAATAGGAGCTGTGTTCCTTGGGTTCTTGGGAGCAGCAGGAAGCACT
ATGGGCGCAGCGTCAATAACGCTGACGGTACAGGCCAGACAATTATTGTCTGGTATAGTGCAACAGCAGAACA
ATCTGCTGAGGGCTATTGAGGCGCAACAACATCTGTTGCAACTCACAGTCTGGGGCATC
>JQ610064..1
TTCTATTGCAATACAACAGAACTGTTTACTTGGAATTTAACACAACTGTCTGAGAATTCAACTGGGAATGATG
CTAATATCACACTCCCATGTAGAATAAAACAAATTATAAACATGTGGCAGGAAGTAGGAAAAGCAATGTATGC
CCCTCCCATCAGCGGACCAATTAATTGCACATCAAATATTACAGGACTACTATTAACAAGAGATGGTGGTAAT
AACAATGACAGGACTAACAATGGCAGTGATAGTAATAACAGTACTCAGACTGAGATCTTCAGACCTGGAGGAG
GAGATATGAAGGACAATTGGAGAAGTGAATTATATAAATATAAAGTAGTAAGAATTGAACCATTAGGAGTAGC
ACCCACCAAGGCAAAGAGAAGAGTGGTGCAAAGAGAAAAAAGAGCGGTGGGAACGCTAGGAGCTTTGTTCCTT
GGGTTCTTGGGAGCAGCAGGAAGCACTATGGGCGCAGCGTCAATAACGCTGACGGTACAAGCCAGACAATTAT
TGTCTGGTATAGTGCAACAGCAAAGCAATTTGCTGAGAGCTATTGAGGCGCAACAGCATCTGTTGCAACTCAC
AGTCTGGGGCATC
>HQ217713..1
TTCTACTGTGATTCAACACCACTGTTTAATAGGACCTGGATGGCTAATGAGACTTTGAATAGTACTACTTTGA
ATAGTACTGGAGAGTCAAATAAAAATATCACACTCGCATGCAGAATAAAACAAATTATAAACATGTGGCAGCG
AGTAGGGAAAGCAATGTATGCCCCTCCTATCAGAGGACAAATTAGATGTTCATCAAATATTACAGGGCTGCTG
TTAACAAGAGATGGGGGTCACAACAACGAGACTGGAGATAACAGGACTGAGATCTTCAGACCGGGAGGAGGAG
ATATGAGGGACAATTGGAGAAGTAAATTATATAAATATAAAGTAGTAAAAATTGAACCATTAGGAGTAGCACC
CACCAAGGCAAAGAGAAGAGTGGTGCAGAGAGAAAAAAGAGTAGCGGGATTCGGAGCTTTGTTCCTTGGGTTC
TTGGGAGCAGCAGGAAGCACTATGGGCGCAGCGTCAGTGACGCTGACGGTACAGGCCAGACAATTACTGTCTG
GTATAGTGCAGCAGCAGAACAATTTGCTGAGGGCTATTGAGGCGCAACAGCATCTGTTGCAACTCACAGTCTG
GGGCATC
>MK383397..2
TTCTACTGTAATTCAACACCACTGTTTAATAGTACTTGGAATGGTACTGACGAGTCAAATCACCATGGAGAAA
ATGACACAATCATCACACTCCGCTGCAGAATAAAACAAATTATAAACATGTGGCAGGAAGTAGGAAGAGCGAT
GTATGCCCCTCCCATTAAAGGAAACATTAGCTGTTCATCAAATATTACAGGGCTGCTATTAACAAGAGATGGT
GGTAATGGTAACCGGACTGACAACAACACCGAGATCTTCAGACCTCAGGGAGGAAATATGAAAGATAATTGGA
GAAGTGAATTATATAAATATAAAGTAGTAAGAATTGAACCATTAGGAATAGCACCCACCAAGGCAAGGAGAAG
AGTGGTGCAGAGAGAAAAGAGAGTAGTGGGAATGGGAGCTTTGTTCCTTGGGTTCTTGGGAGCAGCAGGAAGC
ACTATGGGCGCAGCGTCAGTGACGCTGACGGTACAAGCCAGACTATTACTGTCTGGTATAGTGCAGCAGCAGA
ACAATTTGCTGAGGGCTATTGAGGCGCAACAGCGTCTGTTGCAACTCACAGTCTGGGGCATC
>KT124761..1
TTCTACTGTAATACAACAAAGCTATTTAATAGTACTTGGGATCGGAATGGAACACAGATAAATGACACTATCA
CAAATGACACTATCATACTCCCATGTAGAATAAAACAATTCATAAACAGGTGGCAAGAAGTAGGAAAAGCAAT
GTATGCCCCTCCCATTAGAGGAGACATTAACTGTTCATCACAGATTACAGGGCTGCTATTAACAAGAGATGGT
GGTCAGAGCAATGACAGCAGTATGAATGAGATCTTCAGACCTGGAGGAGGAGATATGAGGGACAATTGGAGAA
GTGAATTATATAAATATAAAGTAGTAAGAATTGAGCCATTAGGAGTAGCACCCACCAAGGCAAAAAGAAGGGT
GGTGCAGAGAGAAAAAAGAGCAGTAACACTAGGAGCTGTGTTCCTTGGGTTCTTGGGAGCAGCAGGAAGCACT
ATGGGCGCAGCGTCAATAACGCTGACGGCACAGGCCAGACAATTATTGTCTGGTATAGTGCAACAGCAGAGCA
ATCTGCTGAAAGCTATTGAGGCGCAACAGCGTCTGTTGCAACTCACAGTCTGGGGCATT
>KM353846..1
TTCTACTGTAATACATCAAAGTTGTTTAACAGTACTTGGAATGGCACTAAGGAATACCAGGGAGTGAATGGCA
CACTCACACTCCCATGCAGAATAAAACAAATCATAAACATGTGGCAGAAAGTAGGAAAAGCAATGTATGCCCC
TCCCATCCAGGGAAATATTAATTGCTCATCAAATATTACAGGGCTGCTATTAATGAGAGATGGTGGTAACAAG
GGGAACGAAACCGATAATACTACCGAGATCTTTAGACCTGGAGGAGGAAATATGAGGGACAATTGGAGAAGTG
AATTATATAAATATAAAGTAGTGAGAATTGAACCATTAGGAGTAGCACCCACCAAGGCAAAAAGAAGAGTGGT
GCAGAGAGAAAAAAGAGCGGTAACAGTGGGAGCTATGTTCCTTGGGTTCTTGGGAGCAGCAGGAACCACTATG
GGCGCAGCATCATTGACGCTGACGGTACAGGCCAGACAATTATTGTCTGGTATAGTGCAACAGCAAAGCAATT
TGCTGAGGGCTATTCAGGCGCAACAGCATCTGTTGCAGCTCACAGTCTGGGGCATC
>KY112135..1
TTCTACTGTAATACAACAGAACTGTTTAATAGTACTTGGGATAAGGATGGGACAAGGCTTAATGACACTGGAG
GAGATGGCAACATCACACTCCCATGTAGAATAAAACAAATTATAAACATGTGGCAGGAAGTAGGAAAAGCAAT
GTATGCCCCTCCCATCAGAGGACAAATTAGCTGTGTATCAAATATCACAGGGCTACTATTAACAAGAGATGGG
GGTGTAGACGATACTCAAAACGAAACCTTCAGACCTGGAGGAGGAGATATGAGGGACAATTGGAGAAGTGAAT
TATATAAATATAAGGTAGTAAAAATTGAACCATTAGGAGTAGCACCTAGCAAGGCAAAGAGAAGAGTGGTGCA
AAGAGAAAAAAGAGCAGTGGGAACGTTAGGAGCCATGTTCCTTGGGTTCTTGGGAGCAGCAGGAAGCACTATG

GGCGCAGCGTCACTGACGCTGACGGTACAGGCCAGACAATTATTGTCTGGTATAGTGCAACAGCAGAACAATC
TGCTGAGAGCTATTGAGGCGCAACAGCATCTGTTGCAACTCACAGTCTGGGGCATC
>JQ403070..2
TTCTACTGTAATACAACACAACTGTTTAATAGTACTTGGCTGTTTAATGATACTTTGAATGATACTCAAGAGG
AGTATGGTGAGAATAACGGGACTATCACACTCCCATGCAGAATAAAACAAATTATAAACAGATGGCAGGAAGT
AGGAAAAGCAACATATGCCCCTCCCATCAGAGGACAAATTAAATGTTCATCAAATATTACAGGGATACTATTA
ACAAGAGATGGTAGTCAAGGCAACGGGAGTGAGACCTTCAGACCAGGAGGAGGAGATATGAGGGACAATTGGA
GAAGTGAATTATATAAATATAAAGTAGTAAAAATTGAGCCATTAGGAGTAGCACCCACCAAGGCAAGGAGAAG
AGTGGTGCAAAGGGAAAAAAGAGCAGTGGGAACGCTAGGAGCCATGTTCCTTGGGTTCTTGGGAGCAGCAGGA
AGCACTATGGGCGCAGCGTCAGTGACGCTGACGGTACAGGCCAGACAACTATTGTCTGGTATAGTGCAACAGC
AGAACAACCTGCTGAGAGCTATTGAGGCGCAACAGCATCTGTTGCAACTCACAGTCTGGGGCATC
>JQ403031..2
TTCTACTGCAATACAACAAAACTATTTAATAGTACTTGGAATAGTAGTAGTAGTAGTAGTTGGGTTATTGAGGGTC
AGGAAGAGTCAAATGACAATATCACACTCCCATGTAGAATAAAACAAATTATAAACATGTGGCAGGGAGTAGG
AAAAGCAATGTATGCCCCTCCCATCCAAGGAAAAATTAGATGTTCATCAAACATTACAGGGCTATTATTAGTG
AGAGATGGTGGTATTAACAAGACGAATGACACTGAAACCTTCAGACCTCAAGGAGGAAATATGATGGACAATT
GGAGAAGTGAATTATATAAATATAAAGTAGTAAAAATTGAACCATTAGGAATAGCACCCACCAAGGCAAAGAG
AAGAGTGGTGCAGAGAGAAAAAAGAGCAGTGGGAATGGGAGCTTTGTTCCTTGGGTTCTTGGGAGCAGCAGGA
AGCACTATGGGCGCAGCGTCAATGGCGCTGACGGTACAGGCCAGACAATTATTGTCTGGTATAGTGCAACAGC
AGAACAATCTGCTGAGAGCCATTGAGGCGCAACAGCATATGTTGCAACTCACAGTCTGGGGCATC
>JQ403085..1
TTCTACTGTAATACATCACAACTGTTTAAGGGTACCTGGTTGACTAATGATACCTGGGCTAATGGTACCTGGA
CAAATGACACTAGCAAAAATGACAACATCACACTCCCATGCCGAATAAAACAAATTGTAAACTTGTGGCAGGA
AGTAGGAAGAGCAATGTATGCCCCTCCCATCAGAGGAGAAATTCATTGTATATCAAATATTACAGGGCTGTTA
TTAACAAGAGATGGTGGCCAGAATGCAACCAACGAGACCTTCAGACCTGTAGGAGGAGATATGAGGGACAATT
GGAGAAGTGAATTATATAAATATAAAGTAGTAAAAATTGAACCATTAGGAGTAGCACCCACCAAGGCAAAGAG
AAGAGTGGTGCAGAGAGAAAAAAGAGCAGTGGGACTAGGAGCTGTGTTCCTTGGGTTCTTGGGAGCAGCAGGA
AGCACTATGGGCGCAGCGTCACTGACGCTGACGGTACAGGCCAGGCAATTATTGTCTGGTATAGTGCAACAGC
AAAACAACTTGCTGAGAGCTATTGAGGCGCAACAGCACCTGTTGCAACTCACAGTCTGGGGCATC
>JQ403094..1
TTCTACTGTAATACATCACGACTATTTAATAGTACTTGGAATACTAGTAGTATTTGGAATACTACTGGAAGGC
CAAATATTACAGGGAGCACTGACATTATCATACTCCCATGCAGAATAAGACAAATTATAAACATGTGGCAGGA
AGTAGGAAAAGCAATGTATGCCCCTCCCATCAGAGGACGAATTCAGTGTAGCTCAAATATTACAGGGCTGCTA
TTAACAAGAGATGGTGGTAATACTAATAACTCAGACGCACATGAGATCTTCAGACCTGGAGGAGGAGATATGA
GGGACAATTGGAGAAGTGAATTATATAAATATAAAGTAGTAAAAATTGAACCATTAGGAATAGCGCCCACCAA
GGCAAAGAGAAGAGTGGTGCAGAGAGAAAAAAGAGCAGTGGGAATGGGAGCTCTGTTCCTTGGGTTCTTGGGA
ACAGCAGGAAGCACTATGGGCGCAGCGTCAGTGACGCTGACGGTACAGGCCAGACAATTATTGTCTGGTATAG
TGCAACAGCAGAGCAATCTGCTGAGGGCTATTGATGCGCAACAGCATCTGTTGCAGCTCACAGTCTGGGGCAT
C
>KR423482..3
TTCTACTGTAATACAACGAAACTGTTTAATAGTAGTTGGAATCAGAATGCTACTAATCAGAATGCTACTAACA
ATGGAGGAAATGACACAATTACACTCCCATGTAGAATAAAACAGATTATAAACATGTGGCAGGAAGTAGGAAA
GGCAATGTATGCCCCTCCCATAAAAGGATTAATTAGATGTTCATCAAATATTACAGGGCTGATATTAACAAGA
GATGGTGGTAATGACAATAGCACAAACAACAACACTGAAATCTTCAGACCGGGAGGAGGAGATATGAGGGACA
ATTGGAGAAGTGAATTATATAAATACAAAGTAGTAAGAGTTGAACCATTAGGGATAGCACCCAACAAGGCAAA
GAGGAGAGTGGTGCAGAGAGAAAAAAGAGCAGTGGGGATAGGAGCTTTGTTCCTTGGGTTCTTGGGAGCAGCA
GGAAGCACTATGGGCGCAGCGTCAGTGACGCTGACGGTACAAGCCAGACAATTATTGTCTGGTATAGTGCAGC
AGCAGAACAATCTGCTGAGGGCTATTCAGGCGCAACAGCAACTGTTGCAGCTCACAGTCTGGGGCATC
>GU733713..2
TTCTACTGTAATACAACTAGACTGTTCAATAGTACTTGGAATGTTAGTACTTGGAATATTAGTCAGACTGAAG
GGTCATACAACACTGAAGAAAATATCACACTCCCATGCAGAATAAAACAGATTGTAAACATGTGGCAGGAAGT
AGGAAAAGCAATGTATGCCCCTCCCATCAGAGGAATAATTAGATGTTCATCAAATATTACAGGGATAATGTTA
ACAAGAGATGGTGGTAAGAATAGGACAGAGAACACCACTGAGACTTTCAGACCGGGAGGAGGAAATATGATGG
ACAATTGGAGAAGTGAACTATATAACTATAAAGTAGTAAAAATTGAACCATTAGGGGTAGCACCCACCAAGGC
AAAGAGAAGAGTGGTGCAGAGAGAAAAAAGAGCAGCGGGACTAGGAGCTTTGTTCCTTGGGTTCTTGGGAGCA
GCAGGAAGCACTATGGGCGCAGCGTCACTGACGCTGACGGTACAGGCCAGACAATTATTGTCTGGTATAGTGC
AACAGCAGAACAATTTGCTGAGAGCTATTGAGGCGCAACAGCACCTGTTGCAACTCACAGTCTGGGGCATC
>MK164668..1
TTCTACTGTAATACAACAGAACTGTTTAATAGTACTTGGGGTAATAGTAATAATACTAACAATAATACTGGAG
GGGAAGAAACTATCACTCTCCCATGCAGAATCAAACAAATTATAAACATGTGGCAGGAAGTAGGAAAAGCAAT

GTATGCCCCTCCCATCAGAGGACAAATTAGGTGTTCATCAAAGATTACAGGGCTAATATTAACAAGAGATGGT
GGCAACGAGAACAACGACAGTGAGATCTTCGAGATCTTCAGACCTACAGGAGGAAATATGAGGGACAATTGGA
GAAGTGAATTATACAAATATAAAGTAGTAAAAATTGAACCATTAGGAATAGCACCCACCAAGGCAAAAAGAAG
AGTGGTGCAGAGAGAAAAAAGAGCAGTGGGAATACTAGGAGCTATGTTCCTTGGGTTCTTGGGCGCAGCAGGA
AGCACTATGGGCGCAGCGTCACTGACGCTGACGGTACAGGCCAGACAACTCTTGTCTGGTATAGTGCAACAGC
AGAACAACCTGCTGAGGGCTATTGAGGCGCAACATCATCTGTTGCAACTCACAGTCTGGGGCATC
>KC935959..1
TTCTACTGTAATACAACAAAACTGTTTAGTAGTACTTGGAAGTGGGATAATGGTACTTGGAATATTAGTGGGT
TAGATAACAGTACAGAACAAAATATCACACTCCCATGCAGAATAAAACAAATCATAAATATGTGGCAGACAGT
AGGAAAAGCAATGTATGCCCCTCCCATTAAGGGACAAATTCAATGTATATCAAATATTACAGGGCTGCTATTA
ACAAGAGATGGTGGTAATGAAAGTGATACAACCAGGAACAACGAGACCTTCAGACCGGGAGGAGGAGATATGA
GGGACAATTGGAGAAGTGAATTATATAAATATAAAGTAGTAGAAGTTGAACCATTAGGAATAGCACCCACCAA
GGCAAAGAGAAGAGTGGTGCAGAGAGAAAAAAGAGCAGTGACACTAGGAGCTCTGTTCCTTGGGTTCTTGGGA
GCAGCAGGAAGCACTATGGGCGCAGCATCAATGACGCTGACGGTACAGGCCAGACAATTATTGTCTGGTATAG
TGCAACAGCAGAATAATCTGCTGAGGGCTATTGAGGCGCAACAGCATCTGTTGCAGCTCACAGTCTGGGGCAT
C
>KM082006..2
TTCTACTATAACACAACCCAGCTGTTTGATAGTACGTGGTTAACTAATGGTACTAGGATAAGACCAAATACTA
CTGATGGAACTAATGGAAATATCACACTTCCATGCAGAATAAAACAAATTATAAACAGGTGGCAAGAAGTAGG
AAAAGCAATGTATGCCCCTCCCATCAGAGGACAAATTAGATGTACATCAAATATTACAGGGTTAATATTAACA
AGAGATGGTGGTCATGACGGGAACGAGACAAATGAGAAGGAGACCTTCAGACCTGGAGGAGGAGATATGAGGG
ACAATTGGAGAAGTGAATTATATAAATATAAAGTAGTAAAAATTGAGCCAATAGGAATAGCACCCACCATGGC
AAAGAGAAGGGTGGTGCAGAGAGAAAAAAGAGCAGTAACGCTGGGAGCTATGTTCCTTGGGTTCTTGGGAGCA
GCAGGAAGCACTATGGGCGCAGCGTCGATGACGCTGACGGTACAGGCCAGACTATTATTGTCTGGTATAGTGC
AACAGCAGAGCAATCTGCTGAGAGCTATTGAGGCGCAACAGCAACTGTTGCAACTCACAGTCTGGGGCATC
>MG196775..3
TTCTATTGTAATACATCAGAACTGTTTAATAGTGAGTGGCATACTAATTATACATGGAATAGTACTTACAGTA
CAATAAATGGTACTTGGAATAGTACTGACATTGAGGGTGTAAACGCTACTGAAAAGATCACACTCCCATGTAG
AATAAAACAAATTATAAACAGGTGGCAGGAAGTAGGAAAAGCAATGTATGCCCCTCCCATCGCAGGAACAATT
AAATGTGTATCAAATATTACGGGGCTGCTGTTAACCAGAGACGGTGGCAACAATGAAACCAAAGCCACTGAGG
TCTTCAGACCTCAAGGAGGAAATATGAAGGACAATTGGAGAAGCGAATTATATAAATATAAAGTAGTACAAAT
TGAACCATTAGGAGTAGCACCCAACAAGGCAAAGAGAAGAGTGGTGCAGAGAGAAAAAAGAGCAATAACAATA
GGAGCTATGTTCCTTGGGTTCTTGGGAGCAGCAGGAAGCACTATGGGCGCAGCGTCAATGACGCTGACGGTAC
AGGCCAGACAATTACTGTCTGGTATAGTGCAACAGCAGAGCAATTTGCTGAGAGCTATTGAGGCGCAACAGCA
TCTGTTGCAACTCACAGTCTGGGGCATC
>JQ182826..2
TTCTACTGCAATACATCAGCACTGTTTAATAGTACTTGGAGTACTAAGAATGGTACTCAGGTGTTGAATATAA
CTAATGACACCAGCACCAACATCACACTCCCATGCAGAATAAAACAAATTATAAACATGTGGCAGGAAGTAGG
AAAAGCAATGTATGCCCCTCCCATCAGAGGACTAATTAGGTGTACATCAAACATTACAGGACTACTATTAACA
AGAGATGGTGGTAATGGTAATGGCAATACCACGAATGGGACAGAGACCTTCAGACCTGTAGGAGGAGATATGA
AGAATAATTGGAGAAGTGAATTATATAAATATAAAGTAGTACAAATTCAACCATTAGGAGTAGCACCCACCAA
GGCAAAGAGAAGAGTGGTGCAGAGAGAGAAAAGGGATTTGGGACTAGGAGCTTTGTTCATTGGGTTCTTGGGA
GCAGCAGGAAGCACTATGGGCGCAGCGTCAATAACGCTGACGGCACAGGCCAGACAATTGCTGTCTGGTATAG
TGCAACAGCAGAGCGATCTGATGAAGGCTATTGAGGCGCAGCAGCATCTGTTGCAACTCACAGTCTGGGGCAT
C
>KM353922..1
TTCTATTGCGATACAACACAACTGTTTAATAGTAGTTGGCCTTGGAATGGTACAGAAGAGTCAAATAACACTG
AAGGAAATGACACAATCACCCTCCCATGCAAAATAAAACAAATTATAAACCTGTGGCAGGAAGTAGGAAAAGC
AATGTATGCCCCTCCCATCAGAGGACAAATTAATTGCACATCAAATATTACAGGGTTACTATTAACAAGAGAT
GGTGGTAGTACCAGCAATGAGACCGAGATCTTTAGACCTGCAGGAGGAGACATGAGAGATAATTGGAGAAGTG
AATTATATAAATACAAAGTAGTAAAAATTGAACCAATAGGAGTAGCACCCACCAGGGCAAAGAGAAGAGTGGT
GCAGAGAGAAAAAAGAGCAGTGGGATTAGGAGCTATGTTCCTTGGGTTCTTGGGAGCAGCAGGAAGCACTATG
GGCGCAGCGTCAGTGACGCTGACGGTACAGGCCAGACAATTACTGTCTGGTATAGTGCAACAGCAGAACAATT
TGCTGAGGGCTATTGAGGCGCAACATCATCTGTTGCAACTCACAGTCTGGGGCATC
>KM354862..1
TTCTACTGTAATACAACACAACTATTTAATAGTAATTGGACTGCTACTGGAAATACAAATAACACTGGAGGAA
ATGCCACAATCACACTCCCTTGCAGAATAAAACAACTTATAAACATGTGGCAGGGAGTAGGAAAAGCAATGTA
TGCCCCTCCAATAAGAGGACTAATTAGATGTTCATCAAACATTACAGGGCTACTGTTAACAAGAGATGGTGGT
GGTAGCAATGGAACTAATGGAACTGAGGTCTTCAGACCTCATGGAGGGGATATAAGGGACAATTGGAGAAGTG
AATTATATAAATACAAAGTAGTAAAAATTGAACCATTAGGAGTAGCACCCAACAAGGCAGAGAGAAGAGTGGT

GCAGAGAGAAAAAAGAGCAGTGACAATAGGAGCTGTGTTCCTTGGGTTCTTGGGAGCAGCAGGAAGCACTATG
GGCGCAGCGTCAATGACGCTGACGGTACAGGCCAGACAACTATTGTCTGGTATAGTGCAACAGCAAAGCAATT
TGCTGAGGGCTATTGAGGCGCAACAGCACCTGTTGCAACTCACAGTCTGGGGCATC
>KX587241..1
TTCTACTGTAATACGACACAACTGTTTAATAATACTTGGAATGGTACTTGGGGTGAGAATATTACCGGGAATA
TTATCACACTCCCATGCAGAATAAAACAAATTATAAACATGTGGCAGGAAGTAGGAAAAGCAATGTATGCCCC
TCCCATCAGAGGAAATATTAGCTGTTCATCAGATATTACAGGGCTAATATTAACAAGAGATGGTGGTCATGAC
AATGAGACCAACAACACAGCGATCTTCAGACCAGGAGGAGGAAATATGAAGGACAATTGGAGAAGTGAATTAT
ATAAATATAAAGTAGTAAAAATTGAACCAATAGGAGTAGCACCCACCAAGGCAAAGAGAAGAGTGGTGCAGAG
AGAAAAAAGAGCAGTGGGAGTAGGAGCTGTGCTCCTTGGGTTCTTGTCAGCAGCAGGAAGCACTATGGGCGCA
GCGTCAATAACGCTGACGGCACAGGCCAGACAATTACTGTCTGGTATAGTGCAACAGCAAAACAATTTGCTGA
GAGCTATTGAGGCGCAACAACATATGTTGCAACTCACAGTCTGGGGCATC
>KX587299..1
TTCTACTGTAATACAACAAAGCTGTTTAATAGTACTTGGGATAATACAACTGCAGGGTTAAATACCACTGAAA
AAATTATCACACTCCCATGCAGAATAAGACAAATTATAAATATGTGGCAGCAGGTAGGAAAAGCAATGTATGC
CCCTCCTATCAGAGGAAAAATCCAATGCACATCAAACATTACAGGGTTATTATTAACAAGAGATGGTGGTAAC
GAGAACGAGACCTATAACAATGAGACTTTCAGACCTGGAGGAGGAGATATGAAGGACAATTGGAGAAGTGAAT
TATATAAATATAAAGTAGTAAGAATTGAACCATTAGGAATAGCACCCACCAAGGCAAAAAGAAGAGTGGTGCA
GAGAGAAAAAAGAGCAATAGGAATGGGAGCCTTCTTCCTTGGGTTCTTGGGAGCGGCCGGAAGCACTATGGGC
GCAGCGTCAATGGCGCTGACGGTACAGGCCAGACAATTATTGTCTGGTATAGTGCAGCAGCAAAACAACTTGC
TGAGGGCTATTGAGGCGCAACAGCATCTGTTGCAACTCACAGTCTGGGGCATC
>MG900412..2
TTCTACTGTAATTCAACAAAGCTGTTTAATAGTACTTGGCCTTGGAATGATACTCAAGGGTCACATGACACTA
ATGGCACACTCACACTCCCATGCAGAATAAAACAAATTATAAACATGTGGCAGGAAGTAGGAAAAGCAATGTA
TGCCCCTCCCATCGAAGGAATAATTAAATGCTCATCAAACATTACAGGACTGTTATTAACAAGAGATGGTGGT
TACGAGAGCAATAAGACTGATGAGATCTTCAGACCTGGAGGAGGAGATATGAGGGACAATTGGAGGAGTGAAT
TATATAAATATAAAGTAGTAAAAATTGAACCATTGGGAGTAGCACCTACCAAGGCAAAGAGGAGAGTGGTGCA
GAGGGAAAAAAGAGCATTTGGACTAGGAGCTGTGTTCCTTGGGTTCCTGGGAGCAGCAGGAAGCACTATGGGC
GCAGCATCAATAACGCTGACGGTACAGGCCAGACAATTATTGTCTGGTATAGTGCAACAGCAGAACAATTTGC
TGAGGGCTATTGAGGCGCAACAGCATCTGTTGCAACTCACAGTCTGGGGCATT
>KC473826..1
TTCTACTGTAATTCAACACAGCTGTTTAGTAGTAATTGGACTAGGGATAATACGTCTAGTAATTGGACTAGGA
AGGATAACGATACAGAGCCTAATAAGACTGTGATCACACTCCCATGCAGAATAAAACAAATTATAAACATGTG
GCAGGAAGTAGGAAAAGCAATGTATGCCCCTCCTATCAAAGGACTTATTCAATGTTCATCAAATATTACAGGA
TTGTTATTAACAAGAGATGGTGGCGACAACAATAGCAACACTGAAATCTTTAGACCTGGAGGAGGAGATATGA
GGGACAATTGGAGAAGTGAATTATACAAATATAAAGTAGTAAAAATTGAACCATTAGGAGTAGCACCCACCAA
GGCAAGGAGGAGAGTGGTGCAAAGAGAGAAAAGAGCATTGGGAATGGGAGCTCTGTTCCTTGGGTTCTTGGGA
GCAGCAGGAAGCACTATGGGCGCAGCGTCATTGATGCTGACGGTACAGGCCAGACAATTATTGTCTGGTATAG
TGCAACAGCAGAACAATATGCTGAGGGCTATTGAGGCGCAGCAGCATCTGCTGCAACTCACAGTCTGGGGCAT
C
>KC473828..1
TTCTACTGTAATACAACAAAGCTGTTTAATAGCACTCTTAATGACACTTGGAATTTTTGGAATGAAACTGGGG
ATATCATACTCCCGTGCAGAATAAAACAAATTATAAATCTGTGGCAGAAAGTAGGAAAAGCAATGTATGCCCC
TCCCATTAAAGGACAGATTAACTGCACATCAAATATTACAGGGCTACTCTTAATAAGAGATGGCGGTGATAAC
GGGACTGCCAGTGAGACCTTCAGACCTCAAGGAGGAAATATGAAGGACAATTGGAGAAGTGAATTATATAAAT
ATAAAGTAGCACAAGTTGAGCCAATAGGAATAGCACCCACCAAGGCAAAGAGAAGAGTGGTGCAGAGAGAAAA
AAGAGCAGTGACTATGGGAGCTTTGTTCCTTGGGTTCTTGGGAACAGCAGGAAGCACTATGGGCGCAGCGTCG
ATAACGCTGACGGTACAGGCCAGACAATTACTGTCTGGTATAGTGCAACAGCAGAGCAATCTGCTGAGAGCTA
TTGAGGCGCAACAGCATCTGTTGCAACTCACAGTCTGGGGCATC
>KC473829..1
TTCTACTGTAATACGACAGAACTGTTTAATAGTTCTTGGTATTGGAATGATACGAATGATACTTTAAAGAGCA
ATGAAGAAAATATCACACTTCTGTGCAGAATAAAACAAATTGTAAACATGTGGCAGGAAGTAGGAAAAGCAAT
GTATGCCCCTCCCATTAGAGGACCAATTAGCTGTACATCAAATATTACAGGGATACTCTTAACAAGAGATGGA
GGTCAGGGTAATAATACTAATGATACTGAAATCTTCAGACCAGGAGGAGGAGATATGAGGGACAATTGGAGAA
GTGAATTATATAAATATAAAGTAGTAAAAATTGAACCATTAGGAGTAGCACCCACCAGGGCAAAAAGAAGAGT
GGTACAGAGAGAAAAAAGAGCAATAGGGGGATTAGGAGCGTTGTTCCTTGGGTTCTTGGGAGCAGCAGGAAGC
ACTATGGGCGCAGCGTCAATGACGCTGACGGTACAAGCCAGGCTATTATTGACTGGTATAGTGCAACAGCAAA
ATAATTTGCTGAAGGCTATTGAGGCGCAACAACATCTGTTGCAACTCACAGTCTGGGGCATT
>KC473830..1

TTCTACTGTGATTCAACACAACTGTTTAGTAGTAATTGGACTAGGAATGATAGTAGTAGTAATTGGACTAGGA
GTGATAATGATACAGAGTCTAACAATACTGTGATCACACTCCCATGCAGAATAAGACAAATTATAAACATGTG
GCAGGAAGTAGGAAAAGCAATGTATGCCCCTCCTATCAAAGGACTTATTCAATGTACATCAAATATTACAGGA
TTATTATTAACAAGAGATGGTGGTAATGGTAATAAAACGAACGACACTCTGGAGATCTTTAGACCTGGAGGAG
GAGATATGAGGGACAATTGGAGAAGTGAATTGTACAAATATAAAGTAGTAAAAATTGAACCATTAGGAGTAGC
ACCCACCAAGGCAAGGAGGAGAGTGGTGCAAAGAGAGAAAAGAGCAGTGGGACTAGGAGCTCTGTTCCTTGGG
TTCTTGTCAGCAGCAGGAAGCACTATGGGCGCAGCATCATTGACGCTGACGGTACAGGCCAGACAATTACTGT
CTGGTATAGTGCAACAGCAGAACAATATGCTGAGGGCTATTGAGGCGCAACAGCATCTGTTGCAACTCACAGT
CTGGGGCATC
>KU677990..1
TTCTACTGTAATACATCAAGACTGTTTAATAGTACTTTGTATAGTAATAATACTTGGAGTAGTAATATTACAG
AATTAAATGGCATTATCACACTCCCATGCAGAATAAAACAAATTGTAAACATGTGGCAGGAAGTAGGAAAAGC
AATGTATGCCCCTCCCATCAGTGGACAAATTAACTGTTCATCAAACATTACAGGACTATTATTAACAAGAGAT
GGTGGTGATGAGGAGAACAAGACTGATGAGATTTTCAGACCTGGAGGAGGAGATATGAGGGACAATTGGAGAA
GTGAACTATATAAATATAAAGTAGTAAAAATTGAACCATTAGGAGTAGCACCCTCCAGGGCAAAGAGAAGAGT
GGTGCAGAGAGAAAAAAGAGCAGTGGGACTAGGAGCTTTGTTCATTGGGTTCTTGGGAGCAGCAGGAAGCACT
ATGGGCGCAGCGTCAGTGACGCTGACGGTACAGGCCAGACAATTATTGTCTGGTATAGTGCAACAGCAGAACA
ATTTGCTGAGGGCTATTGAGGCGCAACATCATCTGTTGCAACTCACAGTCTGGGGCATC
>KF384800..1
TTCTACTGTAATACAACAGGACTGTTTAATAGTACTTGGAATAGTACTAGTACTGAGCTAAATGACACTACAG
GAAATATCAAACTCCCATGCAGAATAAAACAAATTATAAACATGTGGCAGAAAGTAGGAAAAGCAATGTATGC
CCCTCCCATAGAAGGAGTAATTCAATGTTCATCAAATATTACAGGGCTAATATTAACAAGAGATGGTGGTGGT
GGTAACGATACGATGGGGAACGAGACCTTCAGACCGGGAGGGGGAGATATGAGGGACAATTGGAGGAGTGAAC
TATATAGATATAAAGTAGTAAAAATTGAACCATTAGGAATAGCACCCACCAAGGCAAAGAGAAGAGTGGTGCA
GAGAGAAAAAAGAGCAGTGATAGGAGCGATGTTCCTTGGGTTCTTGGGAGCAGCAGGAAGCACTATGGGCGCA
GCGGCAGTGACGCTGACGGTACAGGCCAGACTATTATTGTCTGGTATAGTGCAACAGCAAAACAACTTGCTGA
GAGCTATTGAGGCGCAACAGCATATGTTGCAACTCACAGTCTGGGGCATT
>KF384801..1
TTTTACTGTAATACATCACAACTGTTTAAGAGTACTTGGTGGACAAATAATACTCGGAATATACCACTACAAG
GAAATGTCACACTCCCATGCAAAATAAAGCAAATTATAAACATGTGGCAAGAAGTAGGAAAAGCAATGTATGC
CCCTCCCATCGGAGGACTAATTAATTGTACATCAAATATTACAGGGCTGCTATTAACAAGAGATGGTGGTATA
ACGAATGGGACTGAGACCTTCAGACCTGGAGGGGGAGATATGAGGGACAATTGGAGAAGTGAATTATATAAAT
ATAAAGTAATAAAAATTCAACCATTAGGGATAGCACCCACCAAGGCAAAGAGAAGAGTGGTGCAGAGAGAAAA
AAGAGCAGTGGGAGTGGGAGCTTTGTTCCTTGGGTTCTTGGGAGCAGCAGGAAGCACTATGGGCGCAGCGTCC
GTAACGCTGACGGTACAGGCCAGACAATTATTGTCTGGTATAGTGCAACAGCAGAGCAATCTGCTGAGGGCTA
TTGAGGCGCAACAACATCTGTTGCAACTCACAGTCTGGGGCATT
>KF384803..1
TTCTACTGTAATTCAACACCACTGTTTAATAGTACCTGGGAAAATGGTACTTGGACGAATGAGAGTGGTATTG
GAGGATCAGATAATATCACACTTCCATGCAGAATAAAACAAATTATAAACCTGTGGCAGGAAGTAGGAAAAGC
AATGTATGCTCCTCCCATCGAAGGACAAATTAGATGTTCATCAAATATTACAGGGCTGTTATTAACAAGAGAT
GGTGGTTCTAATCAGACTAACGAGACCTTCAGACCTGGAGGAGGAGATATGAGGGACAATTGGAGAAGTGAAT
TATATAAATATAAAGTAGTAAAAATTGAACCATTAGGATTAGCACCCACCAAGGCAAAGAGAAGAGTGGTGCA
GAGAGAAAAAAGAGCAGCAGGACTGGGAGCTCTGTTCCTTGGGTTCTTGGGAGCAGCAGGAAGCACTATGGGC
GCAGCGTCAATGACGCTGACGGTACAGGCCAGACTATTATTGTCTGGTATAGTGCAACAGCAGAACAATTTGC
TGAGAGCTATTGAGGCGCAACAGCATCTGTTGCAACTCACAGTCTGGGGCATT
>KF384804..1
TTCTATTGTAATACAACACAACTGTTTAATAGTACTTGGAATGTTACTGATCCTTGGAATTCTCCTGGAGGGT
CAAATAACACTGGAGATATCACACTCCCATGCAGAATAAAACAAATTGTGAACAGGTGGCAGGAAGTAGGAAA
AGCAATGTATGCCCCTCCAATCTCAGGATATATTAACTGTTCATCAAAAATTACAGGGCTGATATTAACAAGA
GATGGTGGTTATAACACCACCAATGAGACTGAGATCTTCAGACCTGGAGGAGGAGATATGAGGGACAATTGGA
GAAGTGAATTATATAAATATAAAGTAGTAAGAATTGAACCATTAGGAATAGCACCTACCACGGCAAAGAGAAG
GGTGGTGCAGAGAGAAAAAAGAGCAGTGGGAATGGGAGCGTTGTTCCTTGGGTTCTTGGGAGCAGCAGGAAGC
ACTATGGGCGCAGCGTCAATGATGCTGACGGTACAGGCCAGACAATTATTGTCTGGTATAGTGCAACAGCAGA
ACAATCTGCTGAGGGCTATTGAGGCGCAACAACATCTGTTGCAACTCACAGTCTGGGGCATC
>KF384807..1
TTCTACTGTAACACAACAACACTGTTCAATAGTACTTGGGATGGGAATGATACTACTGAAAGGTCAAAGAGTA
CTGAAGAAAATGCAACAATCATCACAATCCCATGCAGAATAAAACAAATTATAAACATGTGGCAGAAAGTAGG
AAAAGCAATATATGCCCCTCCCATCAAAGGAAACATTAGTTGTTCATCAAATATTACAGGGCTGCTATTAACA
AGAGATGGTGGGAATAACGAGGACAGCACCAATGAGACCTTCAGACCTCAAGGAGGAGATATGAGGGACAATT
GGAGAAGTGAGCTATATAAATACAAAGTAGTAAGAATTGAACCATTAGGAATAGCACCCACCAAGGCAAGGAG

AAGAGTGGTGCAGAGAGAAAAAAGAGCAGTGGGAATAGGAGCTTTGTTCCTTGGGTTCTTGGGAGCAGCAGGA
AGCACTATGGGCGCAGCGTCGCTGACGCTGACGGTACAGGCCAGACAATTATTGTCTGGTATAGTGCAACAGC
AGAACAATCTGCTGAGAGCTATTGAGGCGCAACAGCATATGTTGCAACTCACAGTCTGGGGCATC
>KF384811..1
TTCTACTGTAATTCATCAAAACTGTTTAATAATACTTGGAATGGTACTAACTGGACAGATACATGGTATAATA
GCACAAATGAAACTATCATACTCCCGTGCAGAATAAAACAAGTTATAAACATGTGGCAGGAAGTAGGAAAAGC
AATGTATGCCCCTCCCATCAGAGGAAAAATTAAATGTAAATCAAACATTACAGGGCTGTTGTTAACAAGAGAT
GGCGGTGGTGGGAGCAATGAAACCACCGAGACCTTCAGACCTGGAGGAGGAAATATGAGGGACAATTGGAGAA
GTGAATTATATAAATATAAAGTAGTAAAAATTCAACCAGTAGGCTTGGCACCCACCAGGGCAAAAAGAAGAGT
GGTGCAGAGAGAAAAAAGAGCAGTAACACTGGGAGCTGTGTTCCTTGGGTTCTTGGGAGCAGCAGGAAGCACT
ATGGGCGCAGCGTCAATGACGCTGACGGTACAGGCCAGACAATTATTGTCTGGTATAGTGCAACAGCAGAGCA
ATTTGCTGAAAGCTATTGAGGCGCAACAGCATCTGTTGCAACTCACAGTCTGGGGCATT
>KX156409..2
TTTTACTGTAATACAACAAAACTGTTCAATAGTACTTGGAATAGTAATAGTACTAGCAACAACACTAAAGGAA
ATGAGACTATTATCGAAATACCATGCAGGATAAGACAAATTATAAACATGTGGCAGCAAGTAGGAAGAGCAAT
GTATGCCCCTCCCATCAGAGGACTAATTAGATGTTCATCAAACATTACAGGACTAATATTAACAAGAGATGGT
GGTCATAATCAGGCCAATGACAGTGAGATCTTCAGACCTGGAGGAGGAGATATGAGGGACAATTGGAGAAGTG
AATTATATAAATATAAAGTAGTAAGAATTGAACCATTAGGAATAGCACCCACCAAGGCAAAAAGAAGAGTGGT
GCAGAGAGAAAAAAGAGCAGTGGGACTAGGAGCTATGTTCCTGGGGTTCTTAGGAGCAGCAGGAAGCACTATG
GGCGCAGCGTCAGTGACGCTGACGGTACAAGCCAGACAATTATTGTCTGGTATAGTGCAACAGCAGAACAATT
TGCTGAGAGCTATTGAGGCGCAACAGCATATGTTGCAACTCACAGTCTGGGGCATT
>KY778615..1
TTCTACTGTAATACATCAAAGTTGTTTAACAGTACTTGGAATGGCACTGAGGAAAACCAGGGAGTGAATGGCA
CACTCACACTCCCATGCAGAATAAAACAAATCATAAACATGTGGCAGAAAGTAGGAAAAGCAATGTATGCCCC
TCCCATCCAGGGAAATATTAATTGCTCATCAAATATTACAGGGCTGCTATTAATGAGAGATGGTGGTAACAAG
GGGAACGGAACCGATAATACTACCGAGATCTTTAGACCTGGAGGAGGAAATATGAGGGACAATTGGAGAAGTG
AATTATATAAATATAAAGTAGTGAGAATTGAACCATTAGGAGTAGCACCCACCAAGGCAAAAAGAAGAGTGGT
GCAGAGAGAAAAAAGAGCGGTAACAGTGGGAGCTATGTTCCTTGGGTTCTTGGGAGCAGCAGGAAGCACTATG
GGCGCAGCATCATTGACGCTGACGGTACAGGCCAGACAATTATTGTCTGGTATAGTGCAACAGCAAAGCAATT
TGCTGAAGGCTATTCAGGCGCAACAGCATCTGTTGCAGCTCACAGTCTGGGGCATC
>KT124768..1
TTCTACTGTAATACATCAGAACTGTTTAATAGTACTTGGTATAACAGTACATACAATGAGACTGCACCAGGCA
ATGAAACAATCACACTCCCATGCAAAATAAGGCAGTTTGTAAACAGGTGGCAGGAAGTAGGAAAAGCAATGTA
TGCCCCTCCCATCAGAGGAATAATTAGATGTTTATCAAATATTACAGGATTGATATTAACAAGAGATGGGGGT
ATCTATAATAACACGAATGGGACCATTGGGAATGAGACTTTCAGACCTGGAGGGGGAGATATGAGGGACAATT
GGAGAAGTGAATTATATAAATATAAAGTAGTAAAAATTGAACCATTAGGAGTAGCACCCACCAAAGCAAGGAG
AAGAGTGGTGCAGAGAGAAAAAAGAGCAGTGGGATTCGGAGCTATGTTCCTTGGGTTCTTGGGAGCAGCAGGA
AGCACTATGGGCGCAGCGTCAGTGACGCTGACGGTACAGGCCAGGCAATTATTGTCTGGTATAGTGCAGCAGC
AAAACAACTTGCTGAGGGCTATTGAGGCGCAACAGCATATGTTGCAACTCACAGTCTGGGGCATT
>MG196879..1
TTCTACTGTAATACAACGCAACTGTTTAGGGGTAATGGTACTGGTAATGGTACTGGAGAGTCAAGTGACATTG
TCACACTCCCATGCAGAATAAAACAAATTGTAAACATGTGGCAGAAAGTAGGAAAAGCTATGTATGCCCCTCC
CATCAAAGGACTAATTAGATGTTCATCAAATATTACAGGGCTAATATTAACAAGAGATGGTAGTAATAAAAGT
AAAAGTGATCCAGAGATCTTCAGACCTGCAGGAGGAGATATGAGGGACAATTGGAGAAGTGAATTATATAAAT
ATAAAGTAGTAAAAATTCAACCATTAGGAGTAGCACCCACCAAGGCAAAGAGAAGAGTGGTGCAGAGAGAAAA
AAGAGCAGTGGGAATAGGAGCTGTGTTCCTTGGGTTCTTAGGAGCAGCAGGAAGCACTATGGGCGCAGCGTCA
ATAACGCTGACGGTACAGGCCAGACAATTATTGTCTGGTATAGTGCAACAGCAGAACAATCTGCTGAGAGCTA
TTGAGGCGCAACAGCATCTGTTGCAACTCACAGTCTGGGGCATC
>MG197013..1
TTCTACTGTAATACAACACAGCTGTTTAATAGCACTTGGAATAGCACGACTAATAATGACACTGAAGGATCAA
GTAATGCTACGATCACACTCCCATGTAGAATAAGACAAATTATAAACATGTGGCAGACAGTAGGAAAAGCAAT
GTATGCCCCTCCCATCAGAGGAGAGATTAGCTGTTCATCAAATATTACAGGGCTGCTGTTAACAAGAGATGGT
AATACCAATCACACTGATGCCACTGAGACCTTTAGACCTGGAGGAGGAGATATGAGGGACAATTGGAGAAGTG
AATTATATAAATATAAAGTGATACAACTTGAACCAATAGGAGTAGCACCCACCAAGGCAAAGAGAAGAGTGGT
GCAAAGAGAAAAAAGAGCAGTGGGGGCAATAGGAGCTATGTTCCTTGGGTTCTTGGGAGCAGCAGGAAGCACT
ATGGGCGCAGCGTCAATAACGCTGACGGTACAGGCCAGACTATTATTGTCTGGTATTGTGCAACAGCAAAACA
ATTTGCTGAGGGCTATTGAGGCGCAACAGCATCTGTTGCAACTCACAGTCTGGGGCATC
>MG197046..2
TTCTACTGTAATACCTCAAAACTGTTTAACAGTACATGGCCAAATAATACTAAAAATGCTAATATCACTGAAG
GAACTATCACACTCCCGTGCAGAATAAAACAAATTATAAACATGTGGCAGGAAGTAGGAAAGGCAATGTATGC

CCCTCCCATCAGAGGACAACTTAACTGTTCATCAAATATTACAGGGCTGCTATTAACAAGAGATGGGGGTTAT
AATCAGGCGAACAATAGCGAGACCTTCAGACCGGGAGGAGGAGATATGAGGAACAATTGGAGAAGTGAATTAT
ATAAATATAAAGTAGTAAGAGTTGAACCATTAGGAGTAGCACCCACCAAGGCAAAGAGAAGAGTGGTGCAAAG
AGAAAAGAGAGCAGTGGGAATGGGAGCTTTGTTCCTTGGGTTCTTGGGAGCAGCAGGAAGCACTATGGGCGCA
GCGTCACTAACGCTGACGGTACAAGCCAGACAATTATTGTCTGGTATAGTGCAACAGCAAAACAATTTGCTGA
GAGCTATTGAGGCGCAGCAGCATATGTTGCAACTCACAGTCTGGGGCATC
>MG197064..1
TTCTACTGTAATACATCAAAACTGTTTAACAATACCTGGACTAAGGGTACTTGGAATAGTACATGGTCATGGG
CAAATGAAACTAACATCACACTCCCATGCAGAATAAAACAGATTATAAACATGTGGCAGGAAGTAGGAAAAGC
AATGTATGCCCCTCCCATCGAAGGAATAATTAGATGTGCCTCAAACATTACAGGGCTGCTATTAACAAGAGAT
GGTGGTAAACATGATAATATTACCATTAATGGTAATGTCATTGACTATAACACCACCGAGGTCTTCAGACCTG
GAGGGGGAGATATGAGGGACAATTGGAGAAGTGAATTATATAAATACAAAGTAGTACAAATTGAACCATTAGG
AGTAGCACCCACTAAGGCAAAAAGAAGAGTGGTGCAGAGAGAAAAAAGAGCAGTGGTACTAGGAGCTTTGTTC
CTTGGGTTCTTGGGAGCAGCAGGAAGCACTATGGGCGCAGCGTCAATGACGCTGACGGTACAGGCCAGACTAT
TATTGTCTGGTATAGTGCAACAGCAGAACAATTTGCTGAGGGCTATTGAGGCGCAACAACATCTGTTGCAACT
CACAGTCTGGGGCATT
>MG197113..2
TTCTACTGTAACACATCAAAGTTGTTTAATAGCACTTGGAACTATAATAATGCTACAGGAAACACTGAAGGAA
ATAGCACACAAGGAAATATCACACTCATCACACTCCCATGCAGAATAAAACAGATTATAAACAGGTGGCAGGA
AGTAGGAAAAGCAATGTATGCCCCTCCCATCAGCGGACAAATTAACTGTTCATCAAATATTACAGGACTACTA
TTAACAAGAGATGGTGGTCATAATAACAATAGCACAATTGAGACCTTTAGACCTACAGGAGGAGATATGAGGA
ACAATTGGAGAAGTGAATTATATAAATATAAAGTAGTAAAAATTGAGCCATTAGGAATAGCACCCACCAAGGC
AAAAAGAAGAGTGGTGCAGAGAGAAAAAAGAGCAGTAGGAACGCTGGGAGCTATGTTCCTTGGGTTCTTAGGA
GCAGCAGGAAGCACTATGGGCGCAGCGTCAGTGACGTTGACGGTACAGGCCAGACTATTATTGTCTGGTATAG
TGCAGCAGCAGAGCAAGCTGCTGAGGGCTATTGAGGCGCAACAGCATATGTTGCAACTCACAGTCTGGGGCAT
C
>KM355159..1
TTCTATTGTAATTTAACAAAACTGTTTAATAGTACTTGGAATAAGACTGAAGGGCCTTGGAATGTTACTGAAG
AGTATGGGAATATTACTGACCCTATCATACTCCCATGCAGAATAAAACAAATTATAAACATGTGGCAGAGAGT
AGGAAGGGCAATGTATGCCCCTCCCATCAAAGGCAAACTTCAATGTACATCAAATATTACAGGGCTCCTATTA
ACAAGAGATGGTGGTAAGGTGAATAATGAGACTGAGATCTTTAGGCCTGCAGGAGGAGATATGAGGGACAATT
GGAGAAGCGAATTATATAAATATAAAGTAGTAAAAATTGAACCATTAGGAATAGCACCCACCAGGGCGAAGAG
AAGAGTGGTGCAGAGAGAAAAAAGAGCAGTGGGATTAGGAGCTATGTTCCTTGGGTTCTTGGGAGCAGCAGGA
AGCACTATGGGCGCAGCGGCAGTGACGCTGACGGTACAGGCCAGACAATTATTGTCTGGTATAGTGCAGCAGC
AGAACAATCTGCTGAAGGCTATTGAGGCGCAGCAGCATCTGTTGCAACTCACAGTCTGGGGCATC
>MG196745..1
TTCTACTGTAATTCAACACCACTGTTTAATGGGACTTGGCAGAATATGACGAATATTACTGAAAACTTAAATA
TCACACTCCCATGCAGAATAAAGCAGATTATAAACATGTGGCAGAAAGTAGGAAAAGCAATGTATGCCCCTCC
CATCAGTGGAAGTATTCAATGTTCATCAAACATTACAGGGCTACTGTTAACAAGAGATGGTGGCATCACTAAT
AACACTAATAGCAGTAATGATGAGACCTTCAGACCTGGAGGGGGAGATATGAGGGACAATTGGAGAAGTGAAT
TATATAAATATAAAGTAGTAAAAATTGAACCTTTAGGATTAGCACCCACCAGGGCAAGGAGACTAGTGGTGCA
AAGAGACAAAAGAGCAGTGGGAATGGGAGCTATGTTCCTTGGGTTCTTGGGAGCAGCAGGAAGCACTATGGGC
GCGGCGTCAATGACGCTGACGGTACAGGCCAGACAATTATTGTCTGGTATAGTGCAACAGCAAAACAACTTGC
TGAAGGCTATTGAGGCGCAACAGCACCTGTTGCGACTCACAGTCTGGGGCATC
>MG196964..1
TTCTACTGTAATACAACAAAATTGTTTAATAGTACTTGGACTGAGAATAGTACTGAAGGGTCAAACAGCACTG
AAATAATCACACTCCCATGCAGGATAAAACAAATTATAAACATGTGGCAGAGAGTAGGGAGAGCAATGTATGC
CCCTCCCATCAAAGGACCAATTAGATGTTCATCAAATATTACAGGGCTGCTATTAACAAGAGATGGTGGTCAT
AATATGACTAATGATAGTGAGACCTTCAGACCTGTGGGAGGAGATATGAAGAACAATTGGAGAAGTGAATTAT
ATAAATATAAAGTAGTAAAAGTCGAACCATTAGGAGTAGCACCCACCAAGGCAAGGAGAAGAGTGGTGCAGAG
AGAGAAAAGAGCAGTGGGAATAGGAGCTGTGTTCCTTGGGTTCTTGGGAGCAGCAGGAAGCACTATGGGCGCA
GCGTCAATGACGCTGACGGTACAGGCCAGACAACTATTGTCTGGTATAGTGCAACAGCAGAACAACTTGCTAA
GAGCTATTGAAGCGCAACAGCATCTGTTGCAGCTCACAGTCTGGGGCATT
>MG197035..1
TTCTACTGTGATACAACACAACTGTTCAATATTACTTGGGATACTAACAACACTAGTAATAGCAATGGTACTG
ACGGTGATAAAATCACACTCCCATGCAGAATAAAACAAATTATAAATCTGTGGCAGGGAGTAGGAAGAGCAAT
GTATGCCCCTCCCATCAGCGGACTAATTAGCTGTTCATCAAATATTACAGGTCTGCTGTTAGTAAGTGATAGT
GGTAATCAAACTAACGAGACTGAGACCTTCAGACCTATAGGAGGAGAGATGAGGGACATTTGGAGAAGTGAAT
TATACAAGTATAAAGTAGTACAAATTGAACCATTAGGAGTAGCACCCACCAAGGCAAAGAGAAGAGTGGTGCA
GAGAGAAAAAAGAGAGCTGACATTAGGAGCTTTGTTCATTGGGTTCTTGGGGGCAGCAGGAAGCACTATGGGC

GCAGCGTCGATGACGCTGACGGTACAGGCCAGACTATTATTGTCTGGTATAGTGCAACAGCAGAACAATCTGC
TGAGAGCTATTGAGGCGCAACAGCATCTGTTGCAACTCACAGTCTGGGGCATC
>MK214316..1
TTCTACTGTAATACCGCACAACTGTTTAATAGTAGTTGGACTACTACTGAAAAGACAAATAACACTGAAGGAG
GAAATGACACAATAATCATGCTCCCTTGCAGAATAAAACAATTTATAAACAGGTGGCAGGAAGTAGGAAAAGC
AATGTATGCCCCTCCAATCAGCGGACTAATTAGATGTTCATCAAACATTACAGGGCTACTGTTAACAAGAGAT
GGTGGTAGCAGCAATAAAACTAATGAAACTGAGATCTTCAGACCTCATGGAGGGGATATGAGGGACAATTGGA
GAAGTGAATTATATAAATATAAAGTAGTAAAAATTGAACCATTAGGAATAGCACCTAGCAAGGCAAAGAGAAG
AGTGGTGCAGAGAGAAAAAAGAGCGGTGACAATAGGAGCTGTGTTCCTTGGGTTCTTGGGAGCAGCAGGAAGC
ACTATGGGCGCAGCGTCACTGACGCTGACGGTACAGGCCAGACAACTATTGTCTGGTATAGTGCAACAACAAA
GCAATTTGTTGAGGGCTATTGAGGCGCAACAGCACCTGTTGCAACTCACAGTCTGGGGCATC
>KX505616..1
TTCTACTGTAATACAACACAACTGTTTAATAGTACTTGGAAGGAAAATAGTACTGGGAATAATACTGAAGAGT
TAAATGACACTATCACACTCCCATGTAGAATAAAACAAATTATAAACATGTGGCAGGAAGTAGGAAAAGCAAT
GTATGCCCCTCCCATCCAAGGACAAATTAGATGTTCATCAAATATTACAGGGCTAGTATTAACAAGAGATGGT
GGTCATACCAATAAGACTGAGAACAAGACTGAGGTCTTCAGACCTGTAGGAGGGAATATGAAGGACAATTGGA
GAAGTGAATTATATAAATATAAAGTAGTAAAAATTGAACCATTAGGAGTAGCACCCACTAAGGCAAAGAGAAG
AGTGGTGCAGAGAGAAAAAAGAGCAGCAATAGGAGCTGTGTTCCTTGGGTTCTTGGGAGCAGCAGGAAGCACT
ATGGGCGCAGCGTCGATAACGCTGACGGTACAGGCCAGACTATTATTGTCTGGTATAGTGCAACAGCAGAGCA
ATCTGCTGAGGGCTATTGAGGCGCAACAGCAGCTGTTGCAGCTTACAGTCTGGGGCATC
>KR182192..1
TTCTACTGCGATACAACACAGCTGTTTAACAGTACTTGGTGGACTAATGAAACTTGGAAGGATATCGAAGGGT
CAAATAACACTAACATCACACTTCCATGCAGGATAAAACAAATTATAAACAGGTGGCAGGGAGTAGGAAAAGC
AATGTATGCCCCTCCCATCAAAGGACAAATTAGCTGTTCATCAAATATTACAGGGCTAATATTAACAAGAGAT
GGTGGTACTCACGAGGACGAGACCCCTGAGATCTTCAGACCTGGAGGAGGAGATATGAGGGACAATTGGAGAA
GTGAATTATATAAATATAAAGTAGTAAAAATTGAGCCATTAGGAGTAGCACCCACCAAGGCAAAGAGGAGAGT
GGTGCAGAGAGAAAGAAGAGCAGTGGGAACGCTGGGAGCTATGTTCCTTGGGTTCTTGGGAGCAGCAGGAAGC
ACTATGGGCGCAGCATCGATAACGCTGACGGTACAAGCCAGACAATTATTGTCTGGTATAGTGCAACAGCAGA
ACAACTTGCTGAAGGCTATTGAGGCGCAACAGCACCTGTTGCAACTCACAGTCTGGGGCATC
>KR182242..1
TTCTATTGTAATACAACACCACTGTTTAACAGTACTTGGGTGCTTAACAGTACTTTGAATAGTACTGAAGGGT
CCAATAGTACTGGACAAAATATCACACTCCAATGTAGAATAAAGCAAATTATAAATATGTGGCAGGAGGTAGG
AAAAGCAATGTTTGCCCCTCCCATCAGAGGATCAATTCAATGTTCATCCAACATTACAGGGCTGCTACTAACA
AGAGATGGTGGTAATGACAATAACAAGACTGAGATCTTTAGGCCTTGGGGAGGAGATATGAAGGACAATTGGA
GAAGTGAATTATATAAATATAAAGTAATAAAAATTGAACCATTAGGAGTAGCACCCACCAAGGCAAAGAGAAG
AGTGGTGCAGAGAGAAAAAAGAGCGGCAGGACTGGGAGCTCTGCTCATTGGGTTCTTGGGAGCAGCAGGAAGC
ACAATGGGCGCAGCGTCAGTGACGCTGACGGTACAGGCCAGACTATTATTGTCTGGTATTGTGCAACAGCAGA
GCAATCTGCTGAGAGCTATTGAGGCGCAACAGCATATGTTGAAGCTCACAGTCTGGGGCATC
>KR182343..1
TTCTACTGTGATACAACAAAACTGTTTAATAGTACTTGGCCTTGGCCTGCTAATAAAACAGATGATGGCACAA
ATGCAACAGCATCAGACAAAAATATCACACTCCCATGCAGAATAAAACAAATTATAAACATGTGGCAGAAAGT
GGGAAGAGCAATATATGCCCCTCCCATCAAAGGACCAATTGAATGTGTATCAAATATTACAGGGATAATATTA
ACAAGGGATGGTGATAAGAATAATACCTACAATGAGACCTTCAGACCTACAGGAGGAGAAATGAAGGACAATT
GGAGAAGTGAATTATATAAATATAAAGTAATAAAAATTGAACCATTAGGAATAGCACCTACCAGGGCAAAGAG
AAGAGTGGTGCAGAGAGAAAAAAGAGCAGTGGGAATAGGAGCTCTGCTTCTTGGGTTCTTGGGAGCAGCAGGA
AGCACTATGGGCGCAGCGTCAATGACGCTGACGGTACAAGCCAGGCAATTACTGTCTGGCATAGTGCAACAGC
AGAACAATCTGCTGAAAGCTATTGAGGCGCAACAGCAGCTGTTGCAACTCACAGTCTGGGGCATC
>KT986555..1
TTCTACTGTAATACTACACAACTGTTTAATAGTACTTGGAATAAGAATAAAACTAATAATACTTGGGAAAGGA
ATGACACAAATAGTAATGAAACAACAATCACAATCCCATGCAGAATAAAACAAATTATAAACATGTGGCAGGG
AGTAGGCAAGGCAATGTATGCCCCTCCCATCAGAGGATTAATCAGATGTTCATCAAACATTACAGGACTGATA
TTAACAAGAGATGGTGGTCAGAGTAATGAAACAGGGAACAACACCGAGATATTCAGACCTGGAGGAGGAGACA
TGAGGGACAATTGGAGAAGTGAATTATATAGATATAAAGTAGTAGAAATTGAACCATTAGGAATAGCACCCAC
CAAGGCGAAGAGAAGAGTGGTGCAGAGAGAAAAAAGAGCGGTCACACTAGGAGCTGTGTTCCTTGGGTTCTTG
GGAGCAGCAGGAAGCACTATGGGCGCAGCGTCAATGACGTTGACGGTACAAGCCAGACAGTTATTGTCTGGTA
TAGTGCAACAGCAGAACAATCTGCTAAGAGCTATTGAGGCGCAACAGCACCTGTTGCAACTCACGGTCTGGGG
CATC
>KX505508..1
TTCTATTGTAATTTAACAAAACTGTTTAATAGTACTTGGAATAAGACTGAAGGGCCTTGGAATGTTACTGAAG
AGTATGGGAATATTACTGACCCTATCATACTCCCATGCAGAATAAAACAAATTATAAACATGTGGCAGAGAGT

AGGAAGGGCAATGTATGCCCCTCCCATCAAAGGCAAACTTCAATGTACATCAAATATTACAGGGCTCCTATTA
ACAAGAGATGGTGGTAAGGTGAATAATGAGACTGAGATCTTTAGGCCTGCAGGAGGAGATATGAGGGACAATT
GGAGAAGTGAATTATATAAATATAAAGTAGTAAAAATTGAACCATTAGGAATAGCACCCACCAGGGCGAAGAG
AAGAGTGGTGCAGAGAGAAAAAAGAGCAGTGGGATTAGGAGCTATGTTCCTTGGGTTCTTGGGAGCAGCAGGA
AGCACTATGGGCGCAGCGGCAGTGACGCTGACGGTACAGGCCAGACAATTATTGTCTGGTATAGTGCAGCAGC
AGAACAATCTGCTGAAGGCTATTGAGGCGCAGCAGCATCTGTTGCAACTCACAGTCTGGGGCATC
>KX027971..1
TTCTACTGTAATACAACACGACTGTTCGAAAGTACTTGGCATGGAAATGAGACTTGGTATGGGAATGAGACTA
TAGAGCCAGAGGCAAATGACACAATAATCGAACTCCCATGCAGAATAAAACAAATTATAAACATGTGGCAGGA
AGTAGGAAAAGCAATGTACGCCCCTCCCATCAGAGGACCAATTAAATGTTCATCAAATATTACAGGGCTACTA
TTAACAAGAGATGGTGGTGATGATCAGTACAGTAAGAATGAGACCTCCTGCAATAATACTACTGAGACCTTCA
GACCTGGAGGAGGGAATATGAAGGACAATTGGAGAAGTGAATTATATAAATATAAAGTAGTAAAAATAGAACC
ATTAGGAGTAGCACCCACCAAGGCAAAAAGAAGAGTGGTGCAGAGAGAAAAAAGAGCAGTAGGAATAGGAGCT
ATGTTCCTTGGGTTCTTGGGAGCAGCAGGAAGCACTATGGGCGCAGCATCAATAACGCTGACGGTACAGGCCA
GACAATTATTGTCTGGTATAGTGCAACAGCAGAACAATCTGCTAAGGGCTATTGAGGCGCAACAACATCTGTT
GCAACTCACAGTCTGGGGCATC
>KX505446..1
TTCTACTGCAATACAACACAACTATTTAATAGTACTTGGTCAAAAAATAATACTTGGGAGAAGAATGAAGACA
ATTCAATTAATAGTACTATCACACTCCCATGCAGAATAAAACAAATTATAAACATGTGGCAGGAAGTAGGAAA
AGCAATGTATACCTCCCCAATCAAAGGACAGATTAGCTGTAACTCAAGTATTACAGGGATACTATTAACAAGA
GATGGTGGAAGAAATAATAGTGACAATGAGACCTTCAGACCTGGAGGAGGAGATATGAGGGACAATTGGAGAA
GTGAATTATATAAATATAAAGTAGTAAAAATTGTACCATTAGGAATAGCACCCACTGCGGCAAAAAGAAGAGT
GGTGCAGAGAGAAAAAAGAGCAGCACTAGGAGCTATGTTCCTTGGGTTCTTGGGAGCAGCAGGAAGCACTATG
GGCGCAGCATCAGTGACGCTGACGGTACAGGCCAGACTATTATTGTCTGGTATAGTGCAACAGCAGAACAATC
TGCTGAGGGCTATTGAGGCGCAACATCATCTGTTGCAACTCACAGTCTGGGGCATC
>KT986947..1
TTCTACTGTGATACAACAAAACTATTTAATAGTACTTGGAATAGTACTTGGGACAATAATAGTACTCGGAATA
ATAACAGATCAGAGGACAGATCAGAGAGCGATGACACAATCACACTACCATGCAGAATAAGACAAATTATAAA
CAGATGGCAAGAAGTAGGAAAAGCAATGTATGCCCCTCCCATCAAAGGACTAATTGAATGTAACTCAACAATT
ACAGGGCTGCTCTTAGCAAGAGATGGTGGTATCAACAGCACAGGGAATGAGACCTTCAGACCTCAAGGAGGAA
ATATGAGGGACAATTGGAGAAGTGAATTATATAAGTATAAAATAGTAAAAATTGAACCATTAGGAATAGCACC
CACTAAGGCAAAAAGAAGAGTGGTGCAGAGAGAAAAAAGAGGAGCGGGACTAGGAGCTTTGTTCCTTGGATTC
TTGGGAGCAGCAGGAAGCACTATGGGCGCAGCGTCAGTGACGCTGACGGTACAAGCCAGACAATTATTGTCTG
GTATAGTGCAACAGCAGAACAATCTGCTGAGGGCTATTGAGGCGCAGCAGCATATGTTGCAACTCACAGTCTG
GGGCATC
>MH264238..1
TTCTACTGTAATTCAACACAGCTGTTTAATAGTACTTGGTATCTTAATGGTACTTGGAATGCAACTGAAGGGT
TAAATAACACTGAAGGAAATATCACACTCCCATGCAGAATAAAACAAATTATAAACATGTGGCAGCAGGTAGG
AAAAGCAATGTATGCCCCTCCTATCAGAGGACTAATTAGATGCACATCAAACATTACAGGGATAATATTAACA
AGAGATGGTGGTTACGAGAACAAGACCGATAACAATACTAATACTGAGATCTTCAGACCTGGAGGAGGAGATA
TGAGGGACAATTGGAGAAGTGAATTATATAAATATAAAGTAGTAAGAATTGAACCATTAGGAGTAGCACCCAC
CAAGGCAAAGAGAAGAGTGGTGCAGAGAGAAAAAAGAGCAGTGGGAATAGGAGCCTTGTTCCTTGGGTTCTTG
GGAGCGGCAGGAAGCACTATGGGCGCAGCGTCAATGACGCTGACGGTACAGGCCAGACAATTATTGTCTGGTA
TAGTGCAACAGCAAAACAACTTGCTGAGGGCTATTGAGGCGCAACAGCATCTGTTGCAACTCACAGTCTGGGG
CATC
>KX587339..1
TTCTATTGTAATACAACAAACCTGTTTAATAGTACATGGCATACTAACGGAACAAATAGCACTGACAATGGTA
CTGACACAATCACAATCCAATGCAGAATAAAACAAATAATAAACACGTGGCAGGAAGTAGGAAAAGCAATGTA
TGCCCCTCCCATCCAAGGACAACTTAACTGTTCATCAAATATTACAGGGCTGCTATTAACAAGAGATGGTGGT
AATGATAGTAATACTACCAATAACACTGAGATCTTCAGACCTCAAGGAGGAAATATGAGAGACAATTGGAGAA
GTGAATTATATAAATATAAAGTAGTAAAAATTGAACCATTAGGAGTAGCACCCACCGAGGCAAAGAGAAGAGT
GGTGCAGAGAGAAAGAAGAGCAGTGGGACTAGGAGCTATGTTCCTTGGGTTCTTAGGAGCAGCAGGAAGCACT
ATGGGCGCAGCGTCATTGACGCTGACGGTACAGGCCAGACAATTATTGTCTGGTATAGTGCAACAGCAGAACA
ATTTGCTGAGAGCTATTGAGGCGCAACAGCATCTGTTGCAACTCACAGTCTGGGGCATT
>KX505536..1
TTCTACTGTAATACAACACAGCTGTTCAATAGTACATGGGATTGTACTGGAACTGCATGTACATTGAATGGAA
CTGTTCTTAATGGGATAAATAACACCAATATTACTCTCCCATGCAGGATAAAACAAATTATAAACATGTGGCA
AAAAGTAGGAAAAGCAATGTACAACCCTCCCATCAAAGGAGTAATTCAATGTACATCAAATATTACAGGGATA
ATATTAACAAGCGATAATGGTGGGATTGGAAATAATGAAAATGAAACCTTCAGACCTGCAGGAGGAGATATGA
GGGACAATTGGAGAAGTGAATTATATAAATATAAAGTAGTAAGAATTGAGCCATTAGGAATAGCACCCACCAA

GGCAAAAAGGAGAGTGGTGCAGAGAGAAAAAAGAGCAGTAACATTAGGAGCTATGTTCATTGGGTTCTTGGGA
GCAGCAGGAAGCACTATGGGCGCAGCGTCAGTGACGCTGACGGTACAGGCCAGACAATTGCTGTCTGGTATAG
TGCAACAGCAGAGCAATCTGCTGAAGGCTATTGAGGCGCAACAGCATCTGTTGCAACTCACAGTCTGGGGCAT
C
>MG196412..1
TTCTACTGTAATTCATCAAAACTGTTTAATAGTACTTGGGGAAGTACTGACGGGTCAAATAGCACTGAAACAA
ATGGCACAATCATACTCCCATGCAGAATAAGACAAATTATAAACAAGTGGCAGCAAGTAGGAAAAGCAATATA
CGCCCCTCCCATCCAAGGACGAATTAAATGTTTATCAAATATTACAGGGCTGCTATTAACAAGAGATGGTGGT
CATAACAACAGTAGCAATGAGAGTAGGAGTGAGATCTTCAGACCTGGAGGAGGAGACATGAGGGACAATTGGA
GAAGTGAATTATATAAATATAAAGTAGTAAGAATTGAACCAGTAGGAATAGCACCCACCAAGGCAAAGAGAAG
AGTGGTGCAGAGAGAAAAAAGAGCAGTGGGAACAATAGGAGCTATGTTCCTTGGGTTCTTGGGAGCAGCAGGA
AGCACTATGGGCGCAGCGTCATTGACGCTGACGGTACAGGCCAGACAATTATTGTCTGGTATAGTGCAACAGC
AGAACAATCTGCTGAAGGCTATTGAGGCGCAACAGCACCTGTTGCAACTCACAGTCTGGGGCATC
>MK169417..1
TTCTACTGTAATACAACACAATTGTTTAATAGTAGTTGGATTCGGAATGGTACTGATTGGACTTGGAATAGTA
CCACACAAAATGATACTATCATACTCCCATGCAAAATAAAACAAATTATAAACATGTGGCAGGAAGTAGGAAA
AGCGATGTATGCCCCTCCTATCGAAGGAATAATTAGGTGTGTATCAAATATTACTGGGTTGCTATTAACAAGA
GATGGTGGTCACAACAGCAGCAACGGTACTGAGACCTTCAGACCTGCAGGAGGAAATATGAAGGACAATTGGA
GAAGTGAATTATACAAATATAAAGTAGTAAAAGTTGAACCATTAGGAGTAGCACCCACCAAGGCAAAGAGAAG
AGTGGTGCAGAGAGAAAAAAGAGCAATAGGAGGACTAGGAGCTGTGTTCCTTGGGTTCTTGGGAGCAGCAGGA
AGCACTATGGGCGCAGCATCAATGACGCTGACGGTACAGGCCAGACAATTATTGTCTGGTATAGTGCAGCAGC
AGAACAATCTGCTGAAGGCTATTGAGGCGCAACAGCATATGTTGCAACTCACAGTCTGGGGCATC
>MK169834..1
TTCTACTGTAATACAACACCACTGTTTAATCCAAATGGAATAAGAAATGACACTGGAAATATCACACTCCAAT
GCAGAATAAAACAAATTATAAACATGTGGCAGGAAGTGGGAAAAGCAATGTATGCCCCTCCCATCCCAGGTCA
AATTAGGTGTGTATCAAATATTACAGGGCTGATATTAACAAGAGATGGTGGTGATAATAACGAGACCGAGGTC
TTCAGACCTGGAGGAGGAGATATGAGGGACAATTGGAGAAGTGAATTATATAAATATAAAGTAGTACAAATTG
AACCATTAGGAGTAGCACCCACCACGGCAAGAAGAAGAGTGGTGCAGAGAGAAAAAAGAGCAGTAACTTTTGG
AGCTCTGTTCCTTGGGTTCTTGGGAGCAGCAGGAAGCACTATGGGCGCAGCGTCAATGACGCTGACGGTACAG
GCCAGGCAATTATTGTCTGGTATAGTGCAGCAGCAGAACAATCTGCTGAGAGCTATTGAGGCGCAACAACATA
TGTTGCAACTCACAGTCTGGGGCATT
>AF025753..1
TTCTACTGTAATACAACAAAACTGTTTAATAGTACTTGGAATAGTACTTGGAATGATACTGAAGGGTCAATTG
CCACTGAAGGAAATACCACTGAAGAAAATATCACACTCCCATGCAGAATAAAACAAATTATAAACATGTGGCA
GGAAGTAGGAAAAGCAATGTATGCCCCTCCCATCGCAGGACAAATTAGATGTTCATCAAATATTACAGGGCTG
CTATTAACAAGAGATGGTGGTAATGGTAATAGCAGCAACTACACTTCCGAGATCTTCAGACCGGGAGGAGGAG
ATATGAGGGACAATTGGAGAAGTGAATTATATAAATATAAAGTAGTAAAAATTGAACCATTAGGAGTAGCACC
CACCAAGGCAAAGAGAAGAGTGGTGCAGAGAGAAAAGAGAGCAGTGGGAATAGGAGCGTTGTTCCTCGGGTTC
TTGGGAGCAGCAGGAAGCACTATGGGCGCAGCGTCACTGACGCTGACGGCACAGGCCAGACTATTATTGTCTG
GTATAGTGCAACAGCAGAACAATCTGCTGAGGGCTATTGAGGCGCAACAGCATCTGTTGCAACTCACAGTCTG
GGGCATT
>MH012806..2
TTCTACTGTAATTCAACACAACTGTTTAATAGTACTTGGAGTAGTAATAGTACTGGGAGTAATAGCACTGAAG
AAGAAAGAAATATCACACTCCCATGCAGAATAAAGCAAATTATAAACATGTGGCAGGAAGTAGGAAAAGCAAT
GTATACCCCTCCCATCGGAGGACAAATTCAATGTTCATCAAATATTACAGGGCTACTATTAACAAGAGATGGT
GGTCATAGTGGGAACGAGTCCGAGAACAGGCCCGAGATCTTCAGACCAGGAGGAGGAGATATGAGGGATAATT
GGAGAAGTGAATTATATAAATATAAAGTAGTAAAAGTTGAACCATTAGGAATAGCACCCACCAAGGCAAAGAG
AAGAGTGGTGCAGAGAGAAAAGAGAGCAGTGGGAATAGGAGCTATGTTCCTTGGGTTCTTGGGAGCAGCAGGA
AGCACTATGGGCGCAGCGTCGGTGACGCTGACGGTACAGGCCAGACAATTATTGTCTGGTATAGTGCAACAGC
AGAACAATCTGCTGAAGGCTATTGAGGCGCAACAGCATCTGTTGCAACTCACAGTCTGGGGCATC
>MH012971..1
TTCTACTGTAATTCAACACAACTGTTTAAGAGTATTTGGCATGGGAATGAAACCACGTGGAATACAAATAACA
CTGAAAATATCACACTCCCGTGCAGAATAAAACAATTTATAAACATGTGGCAGGAAGTAGGGAAAGCAATGTA
TGCCCCTCCCATCAGTGGACAAATTAGATGTTCATCAAATATTACAGGGCTGCTATTAACAAGAGATGGTGGT
GCTAAGGAGAACGAGACCACCTACAATGAGACCTTCAGACCTGGAGGAGGAGATATGAGGGACAATTGGAGAA
GTGAATTATATAAATATAAAGTAGTAAGAATTGAACCATTAGGAGTAGCACCCACCAAGGCAAAGAGAAGAGT
GGTGCAGAGAGAAAAAAGAGCAGTGGGAATAGGAGCTCTGTTCCTTGGGTTCTTGGGAACAGCAGGAAGCACT
ATGGGCGCAGCGTCACTGACGCTGACGGTACAGGCCAGACAATTATTGACTGGTATAGTGCAACAGCAGAACA
ACCTGCTGAGAGCTATTGAGGCGCAACAGCATCTGTTGCAACTCACAGTCTGGGGCATC
>MH012542..1

TTCTACTGTAATACAACACAACTGTTTAATAGTACTTGGACTTGGAATGATACTAAAGGGTCAAATGACACTG
GAGGAAATGGCACAATCACACTCCCATGCAGAATAAAACAAATTATAAACATGTGGCAGGAAGTAGGAAAAGC
AATGTATGCCCCTCCCATCCCAGGACAAATTAGATGTTCATCAAATATTACAGGGCTGCTATTAACAAGAGAT
GGTGGTAACAATAATCAGAGCAGCGAGACCACCGAGGTCTTCAGACCTGGAGGAGGAGATATGAGGGACAATT
GGAGAAGTGAATTATATAAATATAAAGTAGTAAAAATTGAACCATTAGGAGTAGCACCCACCAAGGCAAAGAG
AAGAGTGGTGCAGAGAGAAAAAAGAGCAGTGGGAATAGGAGCTGTGTTCCTTGGGTTCTTGGGAGCAGCAGGA
AGCACTATGGGCGCAGCGTCAATGACGCTGACGGTACAGGCCAGACTATTATTGTCTGGTATAGTGCAACAGC
AGAACAATTTGCTGAGGGCTATTGAGGCGCAACAGCATCTGTTGCAACTCACAGTCTGGGGCATC
>AY835435..1
TTCTACTGTAATACAACACAACTGTTTAATAGTACTTGGTTTAATAATGCTACTTTGAATGTTAATAGTAATG
TTACTGAAGGGTCAGAAAATATCACACTCCCATGCAGAATAAGACAAATTGTGAACATGTGGCAAGAAGTAGG
AAAAGCAATGTATGCCCCTCCCATCCAAGGGCAAATTAGATGTTCATCAAATATTACAGGGCTGCTATTAACA
AGAGATGGTGGTGGTAGTAACAGTAGTAACACGAGCGAGGAGGTCTTCAGACCTGGAGGAGGAAATATGAGGG
ACAATTGGAGAAGTGAATTATATAAATATAAAGTAGTAAAAATTGAACCATTAGGAATAGCACCCACCAAGGC
AAAGAGAAGAGTGGTGCAGAGAGAAAAAAGGACAGTGGGAATAGGAGCTTTGTTCCTTGGGTTCTTGGGAGCA
GCAGGAAGCACTATGGGCGCAGCGTCAATGACGCTGACGGTACAGGCCAGACAATTACTGTCTGGTATAGTGC
AACAGCAGAACAATCTGCTGAGGGCTATTCAGGCGCAACAGCATCTGTTGCAGCTCACAGTCTGGGGCATT
>AY331292..1
TTCTACTGTGATACAACAAAACTGTTTAATAGTACTTGGCATGGTAATGAAACATCTTGGAATAATACTGAAA
AGTTAAATGACACTATCATACTCCCATGCAGAATAAAACAAATTATAAACATGTGGCAGGAAGTAGGAAAAGC
AATGTATGCCCCTCCAATCGAAGGACTAATTAACTGTTCATCAAGTATTACGGGGCTGCTATTAACAAGAGAT
GGTGGTAAGAGCAATAATCAGACTGAGATCTTCAGACCTGGAGGAGGAGATATGAGGGACAATTGGAGAAGTG
AACTATATAAATATAAAGTAGTAAAAATTGAACCAGTAGGAGTAGCACCCACCAAGGCAAAGAGAAGAGTGGT
GCAGAGAGACAAAAGAGCAGTGGGACTAGGAGCGTTGTTCATTGGGTTCTTGGGAGCAGCAGGAAGCACTATG
GGCGCAGCGTCAATGACACTGACGGTACAGGCCAGACAATTATTGTCTGGTATAGTGCAACAGCAGAACAACT
TGCTGAGGGCTATTGAGGCGCAACAACATCTGTTGCAACTCACAGTCTGGGGCATC
>EU289191..1
TTCTACTGTAATACATCACAACTGTTTAATAGTACTTGGAATGGTACTGAAGGGTTTAATAGTACTGGGAGTG
AAAATATCACACTCCCATGCAGAATAAGACAAGTTGTAAACCTGTGGCAGGAAGTAGGAAAAGCAATGTATGC
CCCTCCCATCAGGGGAGTAATTAAATGTTCATCAAACATTACAGGGGTACTATTAACAAGAGATGGTGGTTAT
GATGATAATAATACCGCAAACACCAACGAGACCTTCAGACCAGGAGGAGGAGATATGAGGGACAATTGGAGAA
GTGAATTATATAAATATAAAGTAGTAAGAATTGAACCATTAGGAGTAGCACCCACCAGGGCAAAGAGAAGAGT
GGTGCAGAGAGAAAAAAGAGCAGTGACACTAGGAGCTTTGTTCCTTGGGTTCTTGGGAGCAGCAGGAAGCACT
ATGGGCGCAGCGTCACTGACGCTGACGGTACAGGCCAGACTCTTGTTGTCTGGTATAGTGCAGCAGCAGAACA
ATCTGCTGAGGGCTATTGAGGCGCAACAACATCTGTTGCAACTCACCGTCTGGGGCATC
>KT124770..1
TTCTACTGTAATACATCAAAACTGTTTAATAGTACCTGGGAGTGGAATAATAGTACCAGAGATTGGAATAATA
CTGAAATGTCAAATAACACTGAAGGAAATGGAACTATCACTCTCCAATGCAGAATAAAACAAATTGTAAATAT
GTGGCAGGAAGTAGGAAAAGCAATGTATGCCCCTCCCATCAGAGGACAAATTAGATGTTCATCAAATATTACC
GGGTTGCTATTAACAAGAGATGGTGGTTACAACATCAGCAATAACAACGAGACCTTCAGACCTGGAGGAGGAG
ATATGAGGGACAATTGGAGAAGTGAATTATACAGATATAAAGTAGTAAAAATTGAACCATTAGGAATAGCACC
CACCAAGGCAAGGAGAAGAGTGGTGCAAAGAGAAAAAAGAGCAGTGGGATTAGGAGCTATGTTCCTTGGGTTC
TTGGGAGCAGCAGGAAGCACTATGGGCGCAGCATCAATGATGCTGACGGTACAGGCCAGACAATTATTGTCTG
GTATAGTGCAACAGCAGAGCAATCTGCTGCAGGCTATTGAGGCACAACAGCATCTGTTGAAACTCACAGTCTG
GGGCATT
>EU575670..1
TTCTACTGTAATACAACAAAACTGTTTAATAGTACTTGGGTTAATGGTACTAGGAATGATACTGAAGAGTCAA
ATAGCACTGAAATCACACTCCCATGCAGAATAAAACAAATCATAAACATGTGGCAGGAAGTAGGAAAAGCAAT
GTATGCCCCTCCTATCAGAGGACAAATTAGATGTTCATCAAATATCACAGGGCTGCTATTAACAAGAGATGGG
GGTGAGGGAGTTGAGAAAAACGATACCGAGATCTTTAGACCTGAAGGAGGAGATATGAAGAACAATTGGAGAA
GTGAATTATATAAATATAAAGTAGTACAAATTGAACCATTAGGAGTAGCACCCACCAGGGCAAAGAGAAGAGT
GGTGCAGAGAGAAAAAAGAGCAGTGGGAATAGGAGCTATGTTCCTTGGGTTCTTGGGAGCAGCAGGAAGCACT
ATGGGCGCAGCGTCAATGGCGCTGACGGTACAATCCAGACAATTATTGTCTGGTATAGTGCAACAGCAAAACA
ACTTGCTGAGAGCTATTGAGGCGCAACATCATCTGTTGCAACTCACAGTCTGGGGCATT
>EU575927..1
TTCTACTGTAATACCTCACAACTGTTTAATAGCTCTTGGTATTCAAATGGTACTCGGAATGGTACTGAAGAAT
CAAATGACCTTATCACACTCCCATGCAGAATTAAACAAATTGTAAACATGTGGCAGGAAGTAGGAAAAGCAAT
GTATGCCCCTCCCATTAGAGGACACATTAACTGTACATCAAATATTACAGGGCTGTTATTAACAAGAGATGGG
GGTCAGAGTAATGAAACCAAGGACGAGGAGATCTTCAGACCTGGAGGAGGAGATATGAGGGACAATTGGAGAA
GTGAATTATATAAATATAAAGTAGTAAGAATTGAACCATTAGGAATAGCACCCACCAGGGCAAAGAGAAGAGT

GGTGCAGAGAGAAAAAAGAGCAGTGGGAATAGGAGCTCTGTTCCTTGGGTTCTTGGGAGCAGCAGGAAGCACT
ATGGGCGCAGCGTCAATAACGCTGACGGTACAGGCCAGGCTATTAATGTCTGGTATAGTGCAACAGCAAAACA
ATCTGCTGAGAGCTATTGAGGCGCAACAGCATCTGTTGCAACTCACAGTCTGGGGCATC
>AY835442..1
TTCTACTGTAATACAACACAACTGTTTAATAATACTTGGAATGGTACTGGATGGTCAGATAACTCAGAGAACA
CTACAGGAAACGGAACTATCACACTCCCATGCAGAATAAAACAAATTGTTAACCTGTGGCAGGAAGTAGGAAA
AGCAATGTATGCCCCTCCCATCAGAGGACTAATTAGATGTTCATCAAATATTACAGGGCTGCTATTAACAAGA
GATGGAGGTAATAATACCACCACGAACGGGACTCAGACTGAGATATTCAGACCTGGAGGAGGAGATATGAGGG
ACAATTGGAGAAGTGAATTATATAAATATAAAGTAGTAAAAATTGAACCATTAGGAGTAGCACCCACTAGGGC
AAAGAGAAGAGTGGTGCGGAGAGAAAGAAGAGCAGCACTAGGAGCTTTGTTCCTTGGGTTCTTGGGAGCAGCA
GGAAGCACTATGGGCGCAGCGTCAATGACGCTGACGGTACAGGCCAGACTATTATTGTCTGGTATAGTGCAAC
AGCAGAATAATTTGCTGAGGGCTATTGAGGCGCAACAGCATCTGTTGCAACTCACAGTCTGGGGCATC
>DQ853463..1
TTCTACTGTAATACAACACAATTGTTTAATGGTATGTGGCATGCTAATGGTACTTGGAATAGTACTTGGAATG
ATACTGGAGGGTCAAATGACACTATCAGACTCCCATGTAGAATAAAACAAATTGTAAACATGTGGCAGGAAGT
AGGAAAAGCAATGTATGCCCCTCCCATCAAAGGACAAATTCAATGTTCATCAAATATTACAGGGCTGATATTA
ACAAGAGATGGTGGTAACAGCACGAACGAGACCGGGGAGGTCTTCAGACCTGGAGGAGGAAATATGAAGGACA
ATTGGAGAAGTGAATTATATAAATATAAAGTAGTAGAAATTGAACCATTAGGAGTAGCACCCACCAAGGCAAA
GAGAAGAGTGGTGCAGAGGGAAAAAAGAGCAGTGGGATTAATAGGAGCTGTGTTCCTTGGGTTCTTGGGAGCA
GCAGGAAGCACTATGGGCGCAGCGTCAATAGCGCTGACGGTACAGACCAGACACTTATTGTCTGGTATAGTGC
AACAGCAGAACAATCTGCTGAGGGCTATTGAGGCGCAACAACATCTGTTGCAACTCACAGTCTGGGGCATC
>KT124769..2
TTCTACTGTAATACAGCAGAACTGTTTAATAGTACTTGGTATGCAAATGGTACTTGGTATGCAAATGGTACAA
ATAGTACTGGAGGGGGAAACAAGACTAATATCACACTCCCATGCAGAATAAAACAATTTATAAACATGTGGCA
GGAAGTAGGAAAAGCAATGTATGCCCCTCCCATCAGTGGACAGATTAGATGTGCATCAAATATTACAGGACTG
CTATTAACAAGAGATGGTGGTCAGGGCAATCAGACCGACAACCAGACTGAGATCTTCAGACCTGTAGGAGGAG
ATATGAAGAACAATTGGAGAAGTGAATTATATAAATATAAAGTAGTAAGAATTGAACCATTAGGAATAGCACC
CACCAGGGCAAAAAGGAGAGTGGTGCAGAGAGAAAAAAGAGCAGTGGGAATAGGAGCTTTGTTCCTTGGGTTC
TTGGGAGCAGCAGGAAGCACTATGGGCGCAGCGTCAATGACGCTGACGGTACAGGCCAGACTATTATTGTCTG
GTATAGTGCAACAGCAGAACAATCTGCTGAGGGCTATTGAGGCGCAACAACATCTGTTGCAACTCACAGTCTG
GGGCATC
>EU289196..1
TTCTACTGTAATACATCAAAACTGTTTAATAGTACTTGGAATAGTACTGGAGGGTCCATTTCTGAAGACTCAG
AAAATATCACACTCCCATGCAGAATAAAACAAATTGTAAACATGTGGCAGGAGGTAGGAAAAGCAATGTATGC
CCCTCCCATCAGAGGACAGATCAGATGTTCATCAAATATTACAGGGCTGCTATTAACAAGAGATGGTGGTATA
AATCAGAGCATCAGCGAGACCTTCAGACCGGGAGGAGGAGATATGAGGGACAATTGGAGAAGTGAATTATACA
AATATAAAGTAGTAAAAATTGAACCATTAGGAATAGCACCCACCAAGGCAAGGAGAAGAGTGGTGCAAAGAGA
AAAAAGAGCAGTGGGAATAGGAGCTGTGTTCCTTGGGTTCTTGGGAGCAGCAGGAAGCACTATGGGCGCAGCG
TCACTGACGCTGACGGTACAGGCCAGACTATTATTGTCTGGTATAGTGCAACAGCAGAACAATCTGCTGAGGG
CTATTGAGGCGCAACAGCATATGTTGCAACTCACAGTCTGGGGCATC
>EU576141..2
TTCTACTGTAATACAACACCACTGTTTAATAGTACTTGGAATAATACTCGGACTGATGATTATCCTACTGGGG
GGGAACAAAATATCACACTCCAATGCAGAATAAAACAAATTATAAACATGTGGCAGGGAGTAGGAAAAGCAAT
GTATGCTCCTCCCATCAGAGGACAAATTAGATGTTCATCAAATATTACAGGGCTGCTATTAACAAGAGATGGT
GGTAGAGATCAGAATGGTACTGAGACCTTCAGACCTGGAGGAGGAAATATGAGGGACAATTGGAGAAGTGAAT
TATATAAATATAAAGTAGTAAAAATTGAACCATTAGGAATAGCACCCACCGCGGCAAAGAGAAGAGTGGTGCA
GAGAGAAAAAAGAGCAGTGGGACTAGGAGCTTTTATCCTTGGGTTCTTGGGAGCAGCAGGAAGCACTATGGGC
GCAGCGTCAATGGCGCTGACGGTACAGGCCAGACTATTATTGTCTGGTATAGTGCAACAGCAAAACAATTTGC
TGAGAGCTATTGAGGCGCAACAGCATATGTTGCAACTCACAGTCTGGGGCATC
>AY835446..1
TTCTACTGTAATACAGCAGAACTGTTTAATAGTACTTGGTATGCAAATGGCACAATTAGTATTGGAGGGGGAA
ACAAGACTAATATCATACTCCCATGCAGAATAAAACAATTTATAAACATGTGGCAGGAAGTAGGAAAAGCAAT
GTATGCCCCTCCCATCAGTGGACAGATTAGATGTTCATCAAATATTACAGGACTGCTATTAACAAGAGATGGT
GGTAGGGGCAATCAGACCGACAACCAGACTGAGATCTTCAGACCTGTAGGAGGAGATATGAAAAACAATTGGA
GAAGTGAATTATATAAATATAAAGTAGTAAGAATTGAACCATTAGGAATAGCACCCACCAGGGCAAAAAGGAG
AGTGGTGCAGAGAGAAAAAAGAGCAGTGGGAATAGGAGCTTTGTTCCTTGGGTTCTTGGGAGCAGCAGGAAGC
ACTATGGGCGCAGCGTCAATGACGCTGACGGTACAGGCCAGACTATTATTGTCTGGTATAGTGCAACAGCAGA
ACAATCTGCTGAGGGCTATTGAGGCGCAACAGCATCTGTTGCAACTCACAGTCTGGGGCATC
>EF593282..1

TTCTACTGTAATTCAACACCACTGTTTAGTAGTACTTGGTATACTGGGAATAGTACCAATAGTACTAAAGGGT
TAAATAACGCTACAGAAATTATCACACTCCTATGTAGAATAAAACAAATTATAAACATGTGGCAGGAAGTAGG
AAAAGCAATGTATGCCCCTCCCATCAGAGGACAAATTAGATGCTCATCAAATATTACAGGGCTGCTATTAACA
AGAGATGGTGGTAATAGCAGCAGCAATGAAGAGATTTTCAGACCTGCAGGAGGAGATATGAGGGACAATTGGA
GAAGTGAATTATATAAATATAAAGTAGTAAAAATTGAACCATTAGGAGTAGCACCCACCAAGGCAAAGAGAAG
AGTGGTGCAGAGAGAAAAAAGAGCAGTGGGAATAGGAGCTGTGTTCCTTGGGTTCTTGGGAGCAGCAGGAAGC
ACTATGGGCGCAGCGTCAGTGACGCTGACGGTACAGGCCAGACAATTATTGTCTGGAATAGTGCAGCAGCAGA
ACAATCTGCTGAGGGCTATTCAGGCGCAACAGCATCTGTTGCAACTCACAGTCTGGGGCATC
>JQ610043..1
TTCTATTGTAATACAACACCGCTGTTTAGCAGCAGGTGGGGGCCTGACGGCACTTGGAATAGCACTTGGAACA
GTACAAGTACAACTAACAACGACACTATCACACTCCAATGCAGAATAAAACAAATTATAAACAGGTGGCAGGA
AGTAGGAAGAGCAATGTATGCCCCTCCCATCCAAGGACAAATTAGATGCTCATCAAATATTACAGGGCTGCTA
TTAACAAGAGATGGTGGTCATGGTAATGACACGGATAATACCACCGAGACCTTCAGACCTGGAGGAGGAAATA
TGAGGGACAATTGGAGAAGTGAACTGTATAAATATAAAGTAGTAAAAATTGAGCCATTAGGATTAGCACCCAC
CAAGGCAAAGAGAAGAGTGGTGCAAAGAGAAAAAAGAGCAGTGGGAACGCTAGGAGCTATGTTCCTTGGGTTC
TTGGGAGCAGCAGGAAGCACTATGGGCGCAGCGGCAGTGACGCTGACGGTACAGGCCAGACAATTATTGTCTG
GTATAGTGCAACAGCAGAACAATTTGCTGAAGGCTATTGAGGCGCAACAGCATATGTTGCAACTCACAGTCTG
GGGCATC
>EF593212..2
TTCTACTGTAATACAACACCACTGTTTAATAGTACTTGGAATAATACTAATGAAGGGTCAGGTAACTATGAAG
GAAATATCACACTCCAATGCAGAATAAAACAAATTATAAATATGTGGCAGCAAGTAGGAAAAGCAATGTATGC
CCCTCCCATCCAAGGACAAATTTACTGTTCATCAAATATTACAGGGCTGCTATTAACAAGAGATGGTGGTGGT
AATAACGAGAGCAGCGAGACCTTCAGACCTGGAGGAGGAGATATGAGGGACAATTGGAGAAGTGAATTATATA
AATATAAAGTAGTAAAACTTGAACCATTAGGAATAGCACCCACCAGGGCAAAGAGAAGAGTGGTGCAGAGAGA
AAGAAGAGCAGCGGGACTAGGAGTTATGTTCCTTGGGTTCTTGGGAGCAGCAGGAAGCACTATGGGCGCAGCG
TCAATGGCGCTGACGGTACAAGCCAGACAATTATTGTCTGGTATAGTGCAACAGCAGAGCAATCTGCTGAGGG
CTATTGAGGCGCAACAACATATGTTGCAACTCACAGTCTGGGGCATC
>EF593300..1
TTCTACTGTAATACAACACAGTTGTTTAATAGTACTTGGAATAGTACTGGGAATAATAATAACAGGTCAGGAG
AAAATATCACACTCCCATGCAGAATAAAACAATTCATAAACCTGTGGCAAGAAGTAGGAAAAGCAATGTATGC
TCCTCCCATCAAAGGAGAAATTCAATGCCGATCAAATATTACAGGGCTAATAATAACAAGAGATGGTGGTAAC
ACTACTGAAGAGATCTTCAGACCTGGAGGAGGAGATATGAGGGACAATTGGAGAAGTGAATTATATAAATATA
AAGTAGTAAGAATTGAACCATTAGGAATAGCACCCACCAAGGCAAAGAGAAGAGTGGTGCAAAGAGAAAAAAG
AGCAGTGGGATTCGGAGCTATGTTCCTTGGGTTCTTGGGAGCAGCAGGAAGCACTATGGGCGCAGCGTCAATA
GCGCTGACGGCACAGGCCAGACAATTATTGTCTGGTATAGTGCAACAGCAAAACAATTTGCTGAGGGCTATTG
AGGCGCAACAGCATATGTTGCAACTCACAGTCTGGGGCATT
>GQ153937..2
TTCTACTGTGATACAACACAACTGTTTAATAGTACTTGGGATAATACTAGTACTTGGAATAATAGTAACACTC
AGAATAAAAATGATAGGAATATCACACTCCAATGCAGGATAAAACAAATTATAAACATGTGGCAGGAAGTAGG
AAAAGCAATGTATGCCCCTCCCATTATGGGACAAATTAGATGTGTATCAAATATTACAGGGCTGCTATTAACA
CGGGATGGTGGTAACGGTAGCGAGGCCAAAAACGACACGGAGATCTTCAGACCTGGAGGAGGAGATATGAGGG
ACAATTGGAGAAGCGAATTATATAAATATAAAGTAGTAAAAATTGAACCATTAGGAGTAGCACCCACCAAGGC
AAAGAGAAGAGTGGTGCAGAGAGAAAAAAGAGCAGTGGGAACGATAGGAGCTATGTTCCTTGGGTTCTTGGGA
GCAGCAGGAAGCACTATGGGCGCAGCGTCATTGACGCTGACGGTACAGGCCAGACAATTGTTGTCTGGTATAG
TGCAGCAGCAGAACAATCTGCTGAGAGCCATTCAGGCGCAACAGCACCTGTTGCAGCTCACAGTCTGGGGCAT
T
>MH263520..1
TTCTACTGTAATACAGCAAAGCTATTTAATAGTACATGGTATAGTGCAGGGAATAGCACTGATGGGGATAAAG
GAAATAATAACATAATCACACTCCCATGCAGAATAAAACAAATTGTAAACATGTGGCAGGAAGTAGGAAAAGC
AATGTATGCCCCTCCCATCAGTGGACAAATTAGATGCTCATCAAATATTACAGGGCTGCTATTAACAAGAGAT
GGTGGTCATGACAATGACACAAACGGGACAGAGATCTTCAGACCTGGAGGAGGGAATATGAAGGACAATTGGA
GAAGTGAATTATATAAATATAAAGTAGTAAGAATTGAACCATTAGGAGTAGCACCCACCAGGGCAAAGAGAAG
AGTGGTGCAGAGAGACAAAAGAGCAGTGGGAATAGGAGCTCTGTTCCTTGGGATCTTGGGAGCAGCAGGAAGC
ACTATGGGCGCAGCGTCAATAACGCTGACGGTACAGACCAGACAATTATTGTCTGGTATAGTGCAACAGCAGA
ACAATTTGCTGAGGGCTATTGAGGCGCAACAGCACATGTTGCAACTCACAGTCTGGGGCATT
>KX984437..2
TTCTACTGTAATACATCAAAATTGTTTAGTAGTACTTGGAATGGTACTACCACACTGCCTAGTAGTACTTGGA
ATACTACTCAGAATGATACTATCACACTCCCATGCAGAATAAAACAAATTATAAACCTGTGGCAGGAAGTAGG
AAAAGCAATGTATGCCCCTCCCATCAAAGGACAGATTAAATGTTCATCAAATATTACAGGGCTGTTATTAACA
AGAGATGGTGGTATTACCAACAACAGCATGAATCAGACCTTCAGACCTGGAGGAGGAGATATGAAGGACAATT

GGAGAAGTGAATTATATAAATATAAAGTAGTAAAAATTGAACCATTAGGAATAGCGCCTACCAAGGCAAAGAG
AAGAGTGGTGCAGAGAGAAAAAAGAGCAGTGGGAATAGGAGCTTTGTTCCTTGGGTTCTTGGGAGCAGCAGGA
AGCACTATGGGCGCAGCGTCAATAACGCTGACGGTACAGGCCAGACTATTATTGTCTGGTATAGTGCAACAGC
AGAACAATTTGCTGAGGGCTATTGAGGCGCAACAGCATATGTTGCAACTCACAGTCTGGGGCATC
>KX984699..1
TTCTACTGTAATACAACAAAACTGTTTAGCAGTACTTGGTATGATAATGGAACGTCAAAAAGCACTGAAAGAT
TAAATGACACTATCATACTCCCATGCAGGATAAAACAGATTATAAACATGTGGCAGGAAGTAGGAAAAGCAAT
GTATGCCCCTCCCATCAGAGGACAAATTAACTGTTCATCAAATATTTCAGGGCTGCTATTAACAAGAGATGGT
GGTAATGATAATAACACGAACGACATCGAGACATTCAGACCGGGAGGAGGAGATATGAGGGACAATTGGAGAA
GTGAATTATATAATTATAAAGTGGTAAAAATTGAACCATTAGGAATAGCACCCACTAAGGCAAAGAGAAGAGT
GGTGCAAAGAGAAAAAAGAGCGGGAATAGGAGCTTTGTTCCTTGGGTTCTTGGGATTAGCAGGAAGCACTATG
GGCGCAGCGTCAGTGACGCTGACGGTACAAGCCAGACTATTATTGTCTGGTATAGTGCAACAGCAGAACAATC
TGCTGAGGGCTATTGAGGCGCAACAGCATCTGTTGCAACTCACGGTCTGGGGCATC
>MH632763..1
TTCTACTGTGATACAACAAAATTGTTTAATAGTACTTGGAATGTTAGTAGTATTTGGAATGTTACTGAGGATA
AAAATATCACACTCACATGTAGAATAAGACAAATTATAAACAGGTGGCAAGAAGTAGGAAAAGCAATGTATGC
CCCTCCCATCAGAGGACAAATTAGATGTTCATCAAATATTACAGGACTGTTATTAACAAGAGATGGTGGTCAG
AACACAACAAGCGAGACCTTTAGACCTGGAGGAGGAGATATGAGGGACAATTGGAGAAGTGAATTATATAAAT
ATAAAGTAGTACAAATTAAACCATTAGGAATAGCACCCACCAGGGCACGGAGAAGAGTGGTGCAGAGAGAGAA
AAGAGCAGTAGGACTGGGAGCAATGTTCCTTGGGTTCTTGGGAGCAGCAGGAAGCACTATGGGCGCAGCGTCA
GTGACGCTGACGGTACAGGCCAGACAATTACTGTCTGGTATAGTGCAACAGCAGAACAATCTGCTGAGGGCTA
TTGATGCGCAACAGCACCTGTTGCAACTCACAGTCTGGGGCATC
>MH576107..1
TTCTACTGTAATACAACACAACTGTTTACTTGGGGGAGTAATAGTACTAACATTACAGGAAGTAATAGCACAC
TCACACTCCCATGCAGAATAAAACAAATTATAAATATGTGGCAGAGAGTAGGAAGAGCAATGTATACCCCTCC
TATCAGAGGACTAATTCAATGCTCATCAAATATTACAGGACTACTATTAACAAGAGATGGTGGGTATGGCAAT
AACAATACCAATGATACTGAGATCTTCAGACCTGGAGGAGGAGACATGAGGGATAATTGGAGAAGTGAATTAT
ATAAATATAAAGTGGTAAAAATTGAACCATTAGGAGTAGCACCCACCAAGGCAAGGAGAAGAGTGGTGCAAAG
AGAAAAAAGAGCAGTAGGAATAGGAGCTGTGTTCCTTGGGTTCTTGGGAGCAGCAGGAAGCACTATGGGCGCA
GCGTCAATAACGCTGACGGTACAGGCCAGACAATTACTGTCTGGTATAGTGCAACAGCAGAACAATTTGCTGA
GGGCTATTGAGGCGCAACAGCACCTGTTGCAACTCACAGTCTGGGGCATC
>EU616649..1
TTCTACTGCAATACATCAGGACTGTTTAATAGTTCTTGGGATATTAATGGTTCTTGGTATGGTAATAGAGGGT
CAAATGACACAATTACACTCCCATGCAGAATAAAACAATTTATAAACATGTGGCAGGAAGTAGGAAAAGCAAT
GTATGCCCCTCCTATCCAAGGACTAATTAACTGTTTATCAAATATTACAGGGCTGCTATTAACAAGAGATGGT
GGCAATAGCAGTCATAGTAATGGGACAGAGACCTTTAGACCTGGAGGAGGAGATATGAGAGATAATTGGAGAA
GTGAATTATATAAATATAAAGTAGTAAAAATTGAACCATTAGGAGTAGCACCCACCAAGGCAAAGAGAAGAGT
GGTGCAGAGAGAAAAAAGAGCAGTGGGAATAGGAGCTTTATTCCTTGGGTTCTTGTCAGCAGCAGGAAGCACT
ATGGGCGCAGCGTCAATGACGCTGACGGTACAGGCCAGACAATTATTGTCTGGTATAGTGCAACAGCAGAACA
ATTTGCTGAGAGCTATTGAGGCGCAACAGCATATGTTGCAACTCACAGTCTGGGGCATC
>FJ469704..1
TTCTACTGTAACTTAACAAAACTGTTTAATAGTACTTGGGCTTGGAATAGTACTTGGAATAGTACTGAAGGAA
ATGAAAATAATACTATCACACTCCCATGTAGAATAAAACAAATTATAAACATGTGGCAGACAGTAGGAAAAGC
AATGTATGCCCCTCCTATCAGAGGACAAATTACATGCTCATCACATATTACAGGGATACTATTAACAAGAGAT
GGTGGTAACAGTGATGACACAAACTATACTGAGATCTTCAGACCTGCAGGAGGAGATATGAGGGACAATTGGA
GAAGTGAATTATATAAATATAAAGTAGTAAAAATTGAACCATTAGGAGTAGCACCTACCAAGGCAAAGAGAAG
AGTGGTGCAGAGAGAAAAAAGAGCAGTGGGATTAGGAGCTATGTTCCTTGGGTTCTTGGGAGCAGCAGGAAGC
ACTATGGGCGCAGCGTCGCTGACGCTGACGGTACAGGCCAGGCAGTTATTGTCTGGTATAGTGCAACAGCAGA
ACAATCTGTTGAGGGCTATTGAGGCGCAACAGCATCTGTTGCAACTCACAGTCTGGGGCATC
>MN515543..1
TTCTACTGTAATTCAACATCACTGTTTAATAGTACTTGGATGTTTAATAGTACTTGGAATGGCACTGATGGGA
ATGATGCTGACTCTAGAGAAAATATCACACTCCAATGCAGAATAAAACAAATTATAAACATGTGGCAGGAAGT
AGGAAAAGCAATGTATGCCCCTCCCATCCAAGGGCTAATTAGATGTTCATCAAATATTACTGGGCTGCTATTA
ACAAGAGATGGTGGTAATCACAATAACAGTAATGGGACAGAGACCTTTAGACCTGGAGGAGGAGATATGAGGG
ACAATTGGAGAAGTGAATTATATAAATATAAAGTAGTAAAAATTGAACCATTGGGAGTAGCACCCACCAAGGC
AAAGAGAAGAGTGGTGCAGAGAGAAAAAAGAGCACTAGGGGGACTAGGAGCTTTGTTCCTTGGGTTCTTGGGA
GCAGCAGGAAGCACTATGGGCGCAGCGTCCCTGACGCTGACGGTACAGGCCAGACTATTATTGTCTGGTATAG
TGCAACAGCAGAACAATCTGCTGAGAGCTATTGAGGTGCAACAGCATCTGTTGCAACTCACAGTCTGGGGCAT
C
>MN515731..1

TTCTATTGTAATACCGCAAAACTGTTTAGTAGTACTTGGCATGCTAATGGTACGTGGAATGGTACTTGGAATA
ATACTACAGAGCAAAATATTACAATTCTATGCAGAATAAAACAAATTATAAACATGTGGCAGGAAGTAGGAAA
AGCAATGTATGCCCCTCCCATCGGAGGAAAAATTAGATGCTCATCAAATATCACAGGGCTACTATTAGTGAGA
GATGGTGGCAACACCACCATCGGGAACACCACTGAGATTTTCAGACCTCAAGGAGGAAATATGAAGGACAATT
GGAGAAGTGAATTATATAAATATAAAGTAGTACAAATTGAACCAATAGGAGTAGCACCCACCAAGGCAAAAAG
AAGAGTGGTGCAGAGAGAAAAAAGAGCAGTGGGAATAGGAGCTCTGCTCCTTGGGTTCTTGGGAGCAGCAGGA
AGCACTATGGGCGCAGCGTCAATAACGCTGACGGTACAGGCCAGACAATTATTGTCTGGTATAGTGCAACAGC
AAAACAATTTGCTGAGAGCTATTGAGGCGCAACAGCATCTGTTGCAACTCACAGTCTGGGGCATC
>GU728412..1
TTCTACTGTAATACAACACAACTGTTTAATAGTACTTGGGATATAAAGAGTAATATGACAAGGTCAAATAACA
CTGAACCAATCACCCTCCAATGCAGAATAAAGCAAATTATAAACATGTGGCAGGAAGTAGGAAAAGCAATGTA
TGCCCCTCCCATCAGAGGACAAATTAAATGTTCATCAAATATTACAGGGCTGCTATTAACAAGAGATGGTGGT
AATGAGACAGGGAAAGAGAATGATACAGAGAAAGAGACCTTCAGACCTGGAGGAGGAGATATGAGGGACAATT
GGAGAAGTGAACTTTATAAATATAAAGTAGTAAAAATTAAACCATTAGGAGTAGCACCCACTGCAGCAAAAAG
AAGAGTGGTGCAGAGAGAAAAAAGAGCGGCACTAGGAGCCTTGTTCCTTGGGTTCTTGGGAGCAGCAGGAAGC
ACTATGGGCGCAGCGTCAGTAACGCTGACGGTACAGGCCAGACTATTATTGTCTGGTATAGTGCAACAGCAGA
ACAATCTGCTGAGAGCTATTGAGGCGCAACAGCATCTGTTGCAACTCACAGTCTGGGGCATC
>GU727880..1
TTCTACTGTAATACAACACAACTGTTTAATAGTACTTGGAATGGTACACAACTGCCTAATCTTACTGGGAATG
AAAATATCACACTCCAATGCAGAATAAAGCAAATTATAAATATGTGGCAGGAAGTAGGAAAAGCAATATATAA
CCCTCCCATCCAAGGACTAATTAGATGTTCATCAAATATTACAGGGCTGTTATTAACAAGAGATGGTGGTCAT
GGGAATAATGACACCGAGATCTTCAGACCAGGAGGAGGAGATATGAGGGACAATTGGAGAAGTGAATTATATA
AATATAAAGTAGTAAAAATTGAACCATTAGGACTAGCACCCACCACGGCGAAGAGAAGAGTGGTGCAGAGAGA
AAAAAGAGCAGTGGGAATAGGAGCATTGTTCCTTGGGTTCTTGGGAGCAGCAGGAAGCACTATGGGCGCAGCG
TCAATGACGCTGACGGTACAAGCCAGACAATTATTGTCTGGTATAGTGCAACAGCAGAACAATCTGCTGAGGG
CTATTGAGGCGCAACAACATGTATTGCAGCTCACAGTCTGGGGCATC
>DQ886031..1
TTCTACTGTAATACATCACGGCTGTTTAATAGTACTTGGAATATTACTGAAGGGTCAAATAGCACTGCAGGCT
CTAACAAGAATATCACACTCCCATGCAGGATAAAACAAATTATAAACATGTGGCAGGAAGTAGGAAAAGCAAT
GTATGCCCCTCCCATCAGCGGACTAATTAGATGTACATCAAATATTACAGGGCTGTTACTAACAAGAGATGGT
GGTCATGATAATAAAACAAATGACACCGAGATCTTCAGACCTGGAGGAGGAAACATGAGGGACAATTGGAGAA
GTGAATTATATAAATATAAAGTAGTAAAAATTGAACCATTAGGAATAGCACCCACCAAGGCAAGGAGAAGAGT
GGTGCAAAGAGACAAAAGAGCAGTGGGAATAGGAGCTATGTTCCTTGGGATCTTAGGAGCAGCAGGAAGCACT
ATGGGCGCAGCGTCAATAACGCTGACGGTACAGGCCAGACAACTATTGTCTGGTATAGTGCAACAGCAGAACA
ATTTGCTGAGGGCCATTGAGGCGCAACAGCATCTGTTGCAACTCACAGTCTGGGGCATA
>DQ886032..1
TTCTACTGTAACACAACAAAACTTTTTAATAATAATACTGAAGGGTCAAAAAACACTGAAGACAATACCACAC
TCACACTCCCATGCAGAATAAAACAGATTATAAACATGTGGCAGGAAGTAGGAAGAGCAATGTATGCCCCTCC
TATCAAAGGAATAATTAGATGTACATCAAATATTACAGGGCTGATATTAACAAGAGATGGTGGTCATAACATG
AATAACACGAATGAGACCACCGAGACCTTCAGACCTGGAGGAGGAGATATGAGGGACAATTGGAGAAGTGAAT
TATATAAATATAAAGTAGTAAGAATTGAACCATTAGGAATAGCACCTTCCAGGGCAAAGAGAAGAGTGGTGCA
GAGAGAAAAAAGAGCAGTGGGAATAGGAGCTGTGTTCCTTGGGTTCTTGGGAGCAGCAGGAAGCACTATGGGC
GCAGCGTCACTGACGCTGACGGTACAGGCCAGACAATTATTGTCTGGTATAGTGCAACAGCAGAACAATTTGC
TGAGAGCTATTGAGGCGCAACAGCATCTGTTGCAGCTCACAGTCTGGGGCATC
>GU367399..1
TTCTACTGCAATACAACAGGACTGTTTAATAGTTCTTGGAATAATGATACTATCTGGAATGATACTAGCACTG
AAGGAAGGAGAAATGACACAATCATACTCCCATGCAGAATAAGACAAATTGTAAACATGTGGCAGGAAGTAGG
AAAAGCAATGTATGCCCCTCCTATCCAAGGACTAATTAACTGTTCATCAAATATTACAGGGCTGCTATTAACA
AGAGATGGTGGTAATAGCAACAATAGCTATGAGACCTTTAGACCTGGAGGAGGAGATATGAGGGACAATTGGA
GAAGTGAACTATATAAATATAAAGTAGTAAAAATTGAACCAATAGGAGTAGCACCCACCAAGGCAAAGAGACG
AGTGGTGCAGAGAGAAAAAAGAGGGGTCGGGATCGGAGCTTTGTTCCTTGGGTTCTTGGGAGCAGCAGGAAGC
ACTATGGGCGCAGCGTCAATAACGCTGACGGTACAGGCCAGACAACTATTGTCTGGTATAGTGCAACAGCAAA
ACAACTTGCTGAGGGCTATTGAGGCGCAACAACATCTGTTGCAACTCACAGTCTGGGGCATC
>GU367403..1
TTCTACTGTAATACATCAAAACTGTTTAATAGTACTTGGGGGTGGAATGTCACTGAAAATGGCACTGAAGTGT
CAAATAACACTGAAGGAAATATCACACTTCCATGCAGAATAAAACAAATTATAAACATGTGGCAAAAAGTAGG
AAAAGCTATATATGCCCCTCCTATCAGAGGACAAATTAGCTGTTTATCAAATATTACAGGGCTGCTATTAACA
AGAGATGGTGGTCATAACGAGGGTAACGAGACTAAGACTGAGGTCTTCAGACCTGGAGGAGGAGATATGAGGG
ACAACTGGAGGAGTGAATTATATAAATATAAAGTAGTAAAAATTGAACCATTAGGAGTAGCACCCACCAAGGC
AAAAAGAAGAGTGGTGCAAAGAGAAAAAAGAGCAGTAGGAATAGGAGCTGTGTTCCTTGGGTTCTTGTCAGCA

GCAGGAAGCACTATGGGCGCAGCGTCACTGACGCTGACGGTACAGGCCAGACAATTATTGTCTGGTATAGTGC
AACAGCAGAACAATCTGCTGAGGGCTATTGAGGCGCAACAGCATCTGTTGCAACTCACAGTCTGGGGCATC
>KP754469..1
TTCTACTGTGATACATCAAAACTGTTTAATAGTACTTGGGATAAGGGTAATTGGAGTACAGGGACAATTGCCA
CTAACGGAAATGACACTATCATACTCCAATGCAGAATAAGACAAATTATAAACATGTGGCAGGAAGTAGGAAA
AGCAATCTATGCCCCTCCTATCAGTGGATACGTTAACTGTACATCAAATATTACAGGGTTGATATTAACAAGA
GATGGTGGTAATGATAATGGTACAAAAAATGGTACAAACTCCACCGAGACTTTTAGACCTGGAGGAGGAGATA
TGAGGGACAATTGGAGAAGTGAATTATATAAATATAAAGTAGTAAAAATTGAACCATTAGGAATAGCACCCAC
CAAGGCAAAGAGAAGAGTGGTGCAGAGAGAAAAAAGAGCAGTGGGATGGGAGCTTTGTTCCTTGGGTTCTTG
GGAGCAGCAGGAAGCACTATGGGCGCAGCGTCAATAACGCTGACGGTACAAGCCAGACAATTGTTGTCTGGTA
TAGTGCAACAGCAGAACAATCTGCTGAAGGCTATTGAGGCGCAACAGCATCTGTTGCAACTCACAGTCTGGGG
CATC
>KP754471..1
TTCTACTGTAACACATCACAACTGTTTAATAGTACGTGGAATGATACTGAAAGGCCAAATAGCAATGGAGAAA
ATGACCAAAATATTACACTCCCTTGCAGAATAAGACAAATTGTAAACATGTGGCAGGAGGTAGGAAAAGCAAT
GTATGCCCCTCCCATCAGAGGACCAATTCAATGCTCATCAAACATTACAGGGATGATATTAACAAGAGATGGT
GGCAATAACAGTGCCCAGAACGAGACCTTCAGACCTGGAGGAGGAGACATGAGGGACAATTGGAGAAGTGAAT
TATATAAATATAAAGTAGTAAAAATTGAACCATTAGGAGTAGCACCCACCAAGGCAAAAAGAAGAGTGGTGCA
GAGAGAAAAAAGAGCAGTGGGAATAGGAGCTTTGTTCATCGGGTTCTTGGGAGCAGCAGGAAGCACTATGGGC
GCAGCGTCAATGACGCTGACGGTACAGGCCAGACAGTTACTGTCAGGTATAGTGCAACAGCAAAACAATTTGC
TGAGAGCTATTGAGGCGCAACAGCATATGTTGCAACTCACAGTCTGGGGCATC
>FJ469744..3
TTCTACTGTAATTCAACACAACTGTTTGATAGTACTTGGGATAATGGAACAAAAACAAATACCACTGAGGGAA
GAAATAGCGATGGAGGAAATAGCAGCGAACCCATCATACTCCCATGCAGAATAAAACAAATTATAAACATGTG
GCAGGAAGTAGGAAAAGCAATGTATGCCCCTCCCATCAGTGGAAACATTACATGTTCATCCAATATTACAGGG
TTGCTATTAACAAGAGATGGTGGCAAGGGCAATGAGACTCAGGGCTATGGGACTCAGGGCACTGAGATCTTCA
GACCTGGAGGAGGAGATATGAGAGACAACTGGAGAAGTGAATTATATAAATATAAAGTAGTAAGAATTGAACC
ATTAGGAGTAGCACCCACCAAGCCAAAGAGAAGAGTGGTGCAGAGAGAAAAAAGAGCAGTGGGATTAGGAGCT
CTGTTCCTTGGGTTCTTGGGAGCAGCAGGAAGCACTATGGGCGCAGCGTCAATGACGCTGACGGTACAGGCCA
GACTATTATTGTCTGGTATAGTGCAGCAGCAGAACAATCTGCTGAGGGCTATTGAGGCGCAACAGCATCTGTT
GCAACTCACAGTCTGGGGCATC
>FJ687543..1
TTCTACTGTGACTCAACAAAACTGTTTAATTTTACTTGGAATGGTACTGAAACAAATAACACTGGAGGAAGTG
ATAAAATCATACTCCCATGCAGAATAAAACAAATTATAAAAATGTGGCAGAAAGTAGGACAAGCAATGTATGC
CCCTCCCATCCAAGGAAAAATTTACTGCTCATCAAATATTACAGGGCTACTATTAACAAGAGATGGTGGCATC
AACAATGAACCTAACAATACTGAGACCTTCAGACCTGGAGGAGGAAATATGAAGGATAATTGGAGAAGTGAAT
TATATAAATATAAAGTAGTACAAATTGAACCATTAGGAGTAGCACCCACCAAGGCAAAAAGAAGAGTGGTGCA
GAGAGAAAAAAGAGCAGTGGGCATAGGAGCTTTGTTCCTTGGGTTCTTGGGAGCAGCAGGAAGCACTATGGGC
GCAGCGTCAATAACGCTGACGGTACAGGCCAGACAATTATTGTCTGGTATAGTGCAACAGCAGAACAATCTGC
TGAGGGCTATTGAGGCGCAACAGCATATGTTGCAACTCACAGTCTGGGGCATC
>KF725982..2
TTCTACTGTAACACAACACCACTGTTTAACAAAACACACCTGGATAGCCTGAATAGCACTACAGACGGTAATA
TTACACTTCAATGCAGAATAAAACAAATTATAAACATGTGGCAGGAAGTAGGAAAAGCAATGTATGCCCCTCC
CATCAGAGGACAAATTAGATGTTCATCAAATATTACAGGGCTGTTATTAACAAGAGATGGTGGTAATGGTAAT
GAGAGCAGCAACCATGAGACCTTCAGACCTGGGGGAGGAGATATGAGGGACAATTGGAGAAGTGAATTATATA
AATATAAAGTAGTACAAATTGAACCATTAGGAATAGCACCCACCAAGGCAAAGAGAAGAGTGGTGCAGAGAGA
AAAAAGAGCAGTGGGAATAGGAGCTTTGTTCCTTGGGTTCTTGGGAGCAGCAGGAAGCACTATGGGCGCAGCG
TCAGTGACGCTGACGGTACAAGCCAGACAATTACTGTCTGGTATAGTGCAACAGCAGAACAATTTGCTGAGGG
CTATTCAGGCGCAACAACATTTGTTGCAACTTACAGTCTGGGGCATC
>KF725986..1
TTCTACTGTAATACAGCACCACTGTTTAATAGTACTTGGAATGATACCAGTGCCTGGAATATAACTGATACTA
GCCCACTCACAATCCAGTGCAGAATAAGACAAATTATAAATAGGTGGCAGGAAGTAGGAAAATCGATGTATGC
CCCTCCCATCAGTGGACAAATTAACTGTTCATCAAATATTACAGGGCTGCTATTAACAAGAGATGGTGGTGGA
AACAGGTCCCAGGAAAACAGGTCCGAGACCTTCAGACCTGGAGGAGGAGACATGAGGGACAATTGGAGGAGTG
AATTATATAAATATAAAGTAGTAAAAATTGAGCCATTAGGGATAGCACCCACCAAGGCAAGGAGGAGAGTGGT
GCAAAGAGAAAAAAGAGCAGTGGGAGCGATAGGAGCTATGTTCCTTGGATTCTTGGGGACAGCAGGAAGCACT
ATGGGCGCAGCGGCATTGACGCTGACGGTACAGGCCAGACAATTATTGTCTGGTATAGTGCAACAGCAAAACA
ATTTGCTGAGGGCTATTGAGGCGCAACATCATCTGTTGCAACTCACAGTCTGGGGCATC
>KF725988..2

TTCTACTGTGATACAAAAAAACTGTTTAATAGTACATGGAATTTTAATAATACTTGGAATTCTACTCAAACAG
GCAATGACACAATCACACTCCCATGCAGAATAAAACAAATTATAAACATGTGGCAAGGAGTAGGAAAAGCAAT
GTATGCCCCTCCCATTAGTGGACAAATCAACTGTTTATCAAATATTACAGGGCTGATATTAACAAGAGATGGT
GGTCAGAACGATAGCAGTAAGGAGACCTTCAGACCTGCAGGAGGAAATATGAAGGACAATTGGAGAAGTGAAT
TATATAAATATAAAGTAGTACAAATTGAACCATTAGGAGTAGCACCCACCAAGGCAAAGAGAAGAGTGGTGCA
AAGAGACAAAAGAGCAGTGGGAATAGGAGCATTGTTCCTTGGGTTCTTGGGAGCAGCAGGAAGCACTATGGGC
GCAGCGTCAATGACGCTGACGGTACAGGCCAGGCAATTATTGTCTGGTATAGTGCAGCAGCAGAACAATCTGC
TGAGGGCTATTGAGGCGCAGCAGCACCTGCTGCAACTCACAGTCTGGGGCATC
>KU168260..5
TTCTACTGTAATACAACACAACTGTTTAATAGTATTTGGGAAGGTAATAAGACTCAGACAGGTCATAGTACTC
ATAATGCTAGGAATGACACTATCACGCTCCCATGTAGAATAAAACAAATTATAAACATGTGGCAGGAAGTAGG
AAAAGCAATGTATGCCCCTCCCATCCATGGACAAATTAGATGTATATCAAATATCACAGGTCTGCTATTAACA
AGAGACGGTGGTATACACAACAAAACCCACATGACTGAGGTCTTCAGACCTGGAGGAGGAAATATGAAGGACA
ATTGGAGAAGTGAATTATACAAATATAAAGTAGTACAAATTGAACCATTAGGGCTGGCACCCACCAAGGCAAA
GAGAAGAGTGGTGCAGAGAGAAAAAAGAGCAGTGGGAGCAATAGGAGCTTTGTTCCTTGGGTTCTTGGGAGCA
GCAGGAAGCACTATGGGCGCAGCGTCACTGGCGCTGACGGTACAGACCAGACAATTACTGTCTGGTATAGTGC
AACATCAGAACAATCTGCTGAGGGCTATTCAAGCGCAACAGCATCTGTTGCAGCTTACAGTCTGGGGCATC
>KT452481..1
TTCTACTGTAATACAACAAAACTGTTTAACAGTACTTGGATTTGGAATAGTACTGAAGGGTCAAACTCCAGTG
AAGGAAACACAACACTCCAATGCAGAATAAAACAAATTGTAAACATGTGGCAGGAAGTAGGAAAAGCAATGTA
TGCTCCTCCCATCAAAGGAGTAATCAGATGTTCATCAAATATTACAGGGCTGTTATTAACAAGAGATGGTGGT
AGCAAGAACAGTAGCGAGAACACCACCACTAAGGAGACCTTCAGACCTGGAGGAGGAGATATGAGGGATAATT
GGAGAAGTGAATTATATAAATATAAAGTAGTAAAAATTGAACCATTAGGAATAGCACCCACCAAGGCAAGGAG
AAGAGTGGTGCAGAGAGAAAAAAGAGCCGTGGGAATAGGAGCATTGTTCCTTGGGTTCTTGGGAGCAGCAGGA
AGCACTATGGGCGCAGCGTCACTAACGCTGACGGTACAGGCCAGACAATTATTGTCTGGTATAGTGCAGCAGC
AAAACGATTTGCTGAGAGCTATTGAGGCGCAACATCATCTGTTGCAACTCACAGTCTGGGGCATC
>KT452508..1
TTCTACTGTGATACAACAAAACTGTTTGATAGCTCTTGGAATAAAACTGGAAGAATAGATAAAAGGAATAATA
CAGAAGGGTCAAATGACACAATCACACTCCCATGCAGAATAAAACAAATTATAAACATGTGGCAGGAAGTAGG
AAAAGCAATGTATGCTCCTCCCATCAGTGGACGGATTAACTGTACATCAAATATTACAGGGCTGATATTAACA
AGAGATGGTGGTCATAATGTTACAACTAAAGGGGACACCTTCAGACCTGGAGGAGGAAATATGAAGGACAATT
GGAGAAGTGAATTATATAAATATAAAGTAGTAAAAATTGAACCATTAGGAATAGCACCCACCAAAGCAAGGAG
AAGAGTGGTGCAGAGAGAAAAAAGAGCCGTGGGATTAGGAGCTTTGTTCCTTGGGTTCTTGGGAGCAGCAGGA
AGCACTATGGGCGCAGCGTCAATGGCGCTGACGGTACAGGCCAGACAATTATTGTCTGGTATAGTGCAACAGC
AGAACAATCTGCTGAGGGCCATTGAGGCGCAACAGCATCTGTTGCAACTCACAGTCTGGGGCATC
>KT452155..1
TTCTACTGCAATACATCACGACTATTTAATAGTACTTGGTTTAAGAATGATAGTACTTGGTTTAGGAATGATA
CTGGGAATGATACAGAAAAGTCAGAAAAGATCATACTTCCATGCAGAATAAAACAAATTATAAATATGTGGCA
GGAAGTAGGAAAAGCAATGTATGCCCCTCCCATCCGAGGACTAATTAGTTGTCCATCAAATATTACAGGACTG
TTATTAACAAGAGATGGTGGTGGAGATAATAACAGAACTAATGAGACTTTCAGACCTCAAGGAGGAGATATGA
GGGACAATTGGAGAAGTGAATTATATAAATATAAAGTAGTAAGAATTGAACCATTAGGAATAGCACCCACCAA
GGCAAGGAGGAGAGTGGTGCAAAGAGAAAAAAGAGCAGTGGGAACATTAGGAGCTATGTTCCTTGGGTTCTTG
GGAACAGCCGGAAGCACTATGGGCGCAGCGTCAATAACGCTGACGGTACAGGCCAGACAATTATTGTCTGGTA
TAGTGCAACAGCAAAATAACTTGCTGAGAGCTATTGAGGCGCAACAGCATCTGTTGCAACTCACAGTCTGGGG
CATC
>U39243..1
TTCTATTGCAATACAACAAAACTGTTTAGTAGTACATATATAGTTAATAATACACATGACAGTAATACAACAA
AAATTTTAAACATCACATGCAGAATAAAACAAATTATAAACATGTGGCAAGGGGTAGGACGAGCAATGTATGC
CCCTCCCATTGCAGGAAACATAACATGTAAATCAAATATCACGGGACTACTACTGACACGTGATGGAGGAAAT
AAGACAGAAAGCAATGACACAGAGACATTCAGGCCTCAGGGAGGAGATATGAGGGACAATTGGAGAAATGAAT
TATATAAATATAAAGTGGTAGAAGTTAACCCATTAGGAATAGCACCCACTAAGGCAAAAAGGAGAGTGGTGGA
CAGAGAAAAAAGAGCAGTAGTGGGACTAGGAGCTGTGTTCCTTGGGGTCTTGACACCCGCAGGAAGCACTATG
GGCGCAGCATCAATAACGCTGACGGTACAGGCCAGACAATTATTGTCGGGCATAGTGCAACAGCAAAACAATT
TGCTGAGGGCTATAGAGGCGCAACAGCATATGTTGCAACTCACGGTCTGGGGCATT
>U39240..1
TTCTATTGCAATACATCAATACTGTTTAATACAACATACCTGGTTAATAATAATTTGACTGAGAAAAATTCAA
CCATTATGCTCCCATGCAGAATAAAACAAATTATAAACATGTGGCAGGAGGTAGGACGAGCAATGTATGCCCC
TCCCATTGCAGGACTCATACAATGTAACTCAAGTATCACAGGACTGCTACTGACACGTGATGGAGGAGTGAGA
AATGACACAAATGAGACAGAGACAGAGACATTCAGACCTGCAGGAGGAGATATGAGGGACAATTGGAGAAGTG
AATTATATAAATATAAAGTGGTAGAAATTCAGCCATTAGGAATAGCACCCACTAAGGCAAAAAGGAGAGTGGT

GGAGAGAGAAAAAAGAGCAGCGGGAATAGGAGCTTTGTTCCTTGGGTTCTTGGGAGCAGCAGGAAGCACTATG
GGCGCAGCGTCGATAACGCTGACGGTACAGGCCAGACAATTATTGTCGGGTATAGTGCAACAGCAAAACAATT
TGCTGAGAGCTATAGAGGCGCAACAGCATATGTTACAACTCACAGTCTGGGGCATT
>AF443090..1
TTCTATTGCAATACATCACGCCTGTTTAATAGTACATACAATCCTAATAGTACATACAATAATAGTATAGAAG
GTAGGTCAAATGCAACCATTACACTCCAATGCAGAATAAAGCAAATTATAAACCTCTGGCAGGAGGTAGGACG
AGCAATATATGCCCCTCCCATTGCAGGAAACATAACATGTAAATCAAATATCACAGGATTACTCTTGACACGT
GATGGAGGTAATAATTCAACGACAGAGATATTCAGACCTCAAGGAGGGAATATGAAGGACAATTGGAGAAGTG
AATTATATAAATATAAAGTGGTAGAAGTTAAGCCATTGGGGGTAGCACCCACTAAGGCAAAAAGGAGAGTGGT
GGAGAGAGAAAAAAGAGCAGTAGGAATAGGAGCTGTGTTCCTTGGGTTCTTGGGAGCAGCAGGAAGCACTATG
GGCGCAGCGTCAATGACGCTGACGGTACAAGCCAGACAACTGTTGTCTGGTATAGTGCAACAGCAAAGCAATT
TGCTGAGGGCTATAGAGGCGCAACAGCATATGTTGCAACTCACAGTCTGGGGCATT
>AF443097..1
TTCTATTGCAATACATCAGAGCTGTTTAATGGTACATACAACAGTACAGGAAATGATTCAAATTCAAACCTCA
TCACACTCCAATGCAGAATAAAACAAATTATAAACATGTGGCAGAAGGTAGGACGAGCCATGTATGCCCCCCC
CATTGAAGGAAACATAACATGTATATCAAATATCACAGGACTATTATTAACACGTGATGGAGGATATGAGGCA
AATCATACTGAGATATTCAGACCTATAGGAGGAGATATGAGGGACAATTGGAGAAGTGAATTATATAAATATA
AAGTGGTAGAAATTAAGCCATTAGGACTAGCACCCACTGAGTCAAAAAGGAGAGTGGTGGAGAGAGAAAAAAG
AGCAGTGGGAATAGGAGCTGTGTTCCTTGGGTTCTTGGGAGCAGCAGGAAGCACTATGGGCGCGGCGTCAATA
ACGCTGACGGTACAGGCCAGACAACTGTTGTCTGGTATAGTGCAACAGCAAAGCAATTTGCTGAGAGCTATAG
AGGCGCAACAACATATGCTGCAACTCACGGTCTGGGGCATT
>AF443109..3
TTCTATTGCAATACATCACTACTGTTTAATAGCAGTTATAATGGTAATAGTAGTTATAATGATACAGGAAGCA
ATTCAACCATCACACTCCAATGCAGAATAAAACAAGTTATAAACATGTGGCAGAGGGTAGGACAAGCAATATA
TGCCCCTCCCATTGAAGGAATAATAACATGTAACTCAAGTATCACAGGATTACTCTTAGTGAGGGATGGAGAC
AATCAAACAAGTGATACAGAGACATTCAGACCACAAGGAGGAGAAATGAGGGACAATTGGAGAAGTGAGTTAT
ATAAATATAAAGTGGTAGAAATTAAGCCATTGGGAGTAGCACCCACTACGGCAAAAAGGAGAGTGGTGGAGAG
AGAAAAAAGAGCTGTGGGAATGGGAGCTATGATCCTTGGGTTCTTGAGTGCAGCAGGAAGCACTATGGGCGCA
GCGTCAATAACGCTGACGGTACAGGCCAGACAATTGTTGTCTGGAATAGTGCAACAGCAAAGCAATTTGCTGA
GGGCTATAGAGGCGCAACAGCATATGTTGCAACTCACAGTCTGGGGCATT
>AF443114..2
TTCTATTGCAACACATCAAGCCTGTTTAATAATACATACCGGCCTACATACTGGCCTAATGGTACAGAAAGTA
ATTCAACCATCACACTTCAATGCAGAATAAAACAAATTATAAACATGTGGCAAAAAGTAGGACGAGCAATATA
TGCCCCTCCCATTGCAGGAAAAATAACATGTAAATCAAACATCACAGGACTACTATTAGTACGTGATGGAGGT
AATGGAGGCAACAATACCGCAACAGAAATATTCAGACCTGGAGGAGGAAATATGAAGGACAATTGGAGAAGTG
AATTATATAAATATAAAGTGGTAGAAATTAAGCCATTGGGAATAGCACCCACTGGAGCAAAAAGGAGAGTGGT
GGGGAGAGAAAAAAGAGCAGTGGGAATAGGAGCTGTGTTCCTCGGGTTCTTGGGAGCAGCAGGAAGCACTATG
GGCGCGGCGTCAATAACGCTGACGGTACAGGCCAGACAACTGTTGTCTGGTATAGTGCAACAGCAAAGCAATT
TGCTGAAGGCTATAGAGGCGCAACATCATCTGTTGCAACTCACGGTCTGGGGCATT
>KR861314..2
TTCTATTGTAATACATCAAGACTGTTTGATAGTAATGGCACTTATAATAATACAGAATCAAACAGCACAATCA
CACTCCCATGCAGAATAAAACAAATTATAAACATGTGGCAGAAAGTGGGACGAGCAATGTATGCCCCTCCCAT
TGAAGGAATGCTAACATGTAAATCAAATATCACTGGACTACTATTGACACGAGATGGAGGAGAAGGAAATCAA
GGGTCAGAGATATTCAGACCTGGAGGAGGAAATATGAGGGACAATTGGAGAAGTGAATTATATAAATATAAAG
TGGTAGAGATTAAACCATTGGGAGTAGCACCCACTGGGGCAAAAAGGAGAGTGGTGGAGAGAGAAAAAAGAGC
AGTGGGAATAGGAGCTGTGCTCCTTGGGTTCTTGGGAGCAGCAGGAAGCACTATGGGCGCGGCGTCACTCACG
CTGACGGTACAGGCCAGACAATTATTGTCCGGTATAGTGCAACAGCAAAGCAATTTGCTGAGGGCCATAGAGG
CGCAACAGCATATGTTGCAACTCACAGTCTGGGGCATT
>KR861341..3
TTCTATTGCAATACATCAGGACTGTTTAATGGTACATACCATCCTCAATATAATGATACACAGAATAATTACA
CAGACTCAAACATCACACTCCCATGCAGAATAAAACAAATTGTAAATATGTGGCAGGGGGTAGGAAGGGCAAT
ATATGCCGCTCCCATTGAAGGAAAAATAGTATGTACATCAAATATCACAGGACTAATATTGACAAGGGATGGA
GGAGAAAATAATAGAACAGAAACATTTAGACCTGAAGGAGGAAATATGAGGGACAATTGGAGAAGTGAATTAT
ATAAATATAAAGTGGTAGAAATTCAGCCATTGGGAGTGGCACCCACTGAGGCAAAAAGGAGAGTGGTGGAGAG
AGAAAAAAGAGCAGTGGGAATAGGAGCTGTGTTCCTTGGGTTCTTGGGAGCAGCAGGAAGCACTATGGGCGCG
GCGTCAATAACGCTGACGGTACAGGCCAGACAACTGTTGTCTGGTATAGTGCAACAGCAAAGCAATTTGCTGA
GGGCTATAGAGGCGCAACAGCATATGTTGCAACTCACAGTCTGGGGCATT
>KR861270..1
TTCTATTGCAATACATCAAGACTGTTTACAAACATAGATAATAATAATACAGAAACTGATAATGATACCACCA
TCACCATCCCATGCAAAATAAAACAAATTATAAACATGTGGCAGAAGGTAGGACGAGCAATGTATGCCCCCCC

CATTGAAGGAAACATAACATGTAAATCAAATATCACAGGACTACTATTGACACGTGATGGAGGTAATGAAACA
AACTACAACACTGAGATATTCAGACCTGGAGGAGGAGATATGAGGGACAATTGGAGAAGTGAGTTATATAAAT
ATAAAGTGGTGGAGGTTAGGCCATTAGGAATAGCACCCACTAAAGCAAAAAGGAGAGTGGTGGAGAGAGAGAA
AAGAGCAGTGGGAATAGGAGCTGTGTTCCTTGGGTTCTTGGGAGCAGCAGGAAGCACTATGGGCGCGGCATCA
CTAACGCTGACGGTACAGGCCAGACAATTATTGTCTGGTATAGTGCAACAGCAAAGCAATTTGCTGAGGGCGA
TAGAGGCGCAACAACATATGTTGCAACTCACAGTCTGGGGCATT
>KR861273..1
TTCTATTGCAATACATCTGAGCTGTTTAATAAGCAGTTTAACAATACAAACAGTACAGAAAGTGAGTCAAATT
CAAATTCAATCATCACACTCCCATGTAGAATAAAACAAATTATAAACATGTGGCAGGAGGTAGGACGAGCGAT
GTATGCCCCTCCCATTGCAGGAAACATAACATGTAAATCAAATATCACAGGACTACTATTGACACGCGATGGA
GGACCTGGAAATAGAAGTAACGAGACATTCAGACCCTTAGGAGGAGATATGAAGGACAATTGGAGAAGTGAAT
TATATAAGTATAAAGTGGTAGAGATTCAGCCATTAGGACTAGCACCCACTAGGGCAAAAAGGAGAGTGGTGGA
GAGAGAGAAAAGAGCAGTGGGAATAGGAGCTGTGTTCCTTGGGTTCTTGGGAGCAGCAGGAAGCACTATGGGC
GCAGCATCAATAACGCTGACGGTACAGGCCAGACAACTGTTGTCTGGTATAGTGCAACAGCAAAACAATTTGC
TGAGGGCTATAGAGGCGCAACAACATTTGTTGCAACTCACGGTTTGGGGCATT
>KY658708..1
TTCTATTGCAACACATCACAACTGTTTAATAGTACATACAGTCCCAGTTTTAATGGTACAGAAGGTAAATTAA
ATGGGACCATCACAATCCCATGTAGAATAAAACAAATTATAAACATGTGGCAGAAAGTAGGACAAGCAATGTA
TGCCCCTCCCATTGCAGGAAACCTAACATGTGAATCAAATATCACAGGATTACTATTGACACGTGATGGAGGA
AAAACAGGTTCAAATCACCCAGAGATATTCAGACCTGGAGGAGGGGATATGAGGGATAACTGGAGAAGTGAAT
TATATAAATATAAAGTGGTAGAAATTAAGCCATTGGGAGTAGCACCCACTGAGGCAAAAAGGAGAGTGGTGGA
GAGAGAAAAAAGAGCAGTGGGAATAGGAGCTGTGTTCCTTGGGTTCTTGGGAGCAGCTGGAAGCACTATGGGC
GCAGCGTCAATAACGCTGACGGTACAGGCCAGACAATTGTTGTCTGGTATAGTGCAGCAGCAAAGCAATTTGC
TGAGGGCTATAGAGGCGCAACAGCATCTGTTGCAACTCACAGTCTGGGGCATT
>AF443084..1
TTTTATTGCAATACATCAAAACTGTTTACATACCAGTCTAATAGCACATACGTGGCTAATAGTACCATCACGC
TCCCATGCAAAATAAAACAAATTATAAACATGTGGCAGGAGGTGGGACGAGCAATGTATGCACCTCCCATTGC
AGGAAACATAACATGTCAATCAAATATCACAGGACTACTGTTGACACGTGACGGAGGCACAGAGACAGACAAT
AAGACAGAGACATTCAGACCTGGAGGAGGAGATATGAGGGACAGTTGGAGAAGTGAATTATATAAATATAAAG
TGGTAGAAATTAAGCCATTAGGAGTAGCACCCACTAAGGCAAAAAGAAGAGTGGTGGAAAGAGAAAAAAGAGC
AGTGGGAATAGGAGCTGTACTCCTTGGGTTCTTGGGAGCAGCTGGAAGCACTATGGGCGCAGCGTCAATAGCG
CTGACGGCACAGGCCAGACAAGTGTTGTCTGGTATAGTGCAACAGCAAAGCAATTTGCTGAGGGCTATAGAGG
CGCAACAGCATATGTTGCAACTCACAGTCTGGGGCATT
>KC899004..1
TTCTATTGTAATACATCAGGCCTGTTTAATGGTACATACAGGCCTAATGGTAATAATAATGGTACTAATGATA
CAATGAGTAATGCAACCATCACAATCCCATGCAGAATAAAGCAAATTATAAACATGTGGCAGGAGGTAGGACG
AGCAATGTATGCCCCTCCTATTGCAGGGAACATAACATGTAAATCAAATATCACAGGGATACTATTGGTACGT
GATGGAGGAGATAATCAGACAGAAGAGATATTCAGACCTGGAGGAGGAGATATGAGGAACAATTGGAGAAGTG
AATTATATAAATATAAAGTGGTAGAAATTAAGCCATTGGGAATAGCACCCACTGGAGCAAAAAGGAGAGTGGT
GGAGAGAGAAAAAAGAGCAGTAGTGGGAATAGGAGCTGCGCTCCTTGGGTTCTTGAGTACAGCCGGAAGCACT
ATGGGCGCGGCGTCAATAGCGCTGACGGTACAGGCCAGACAATTGCTGTCTGGTATAGTGCAACAGCAAAGTA
ATTTGCTGAGGGCTATAGAGGCGCAGCAGCATATGTTGCAACTCACGGTCTGGGGCATT
>KF835522..1
TTCTATTGTAATACATCAAGACTGTTTAATAGTACATACAATCCTAAGAGTACAGGAAGTAATTCAAGCTCAA
TCATCACAATCCCATGCAGAATAAAGCAAATTATAAACATGTGGCAGGAGGTAGGAAGAGCAATGTATGCCCC
TCCCATTGAAGGAAACATAACATGTAGATCAAAGATCACAGGACTACTATTGGTACGTGATGGAGGAAGCCCA
GTGCCAAACAGTACAGAGACAGAGACAGAGATATTCAGACCTGGAGGAGGAGATATGAGGGACAATTGGAGAA
GTGAATTATATAAATATAAAGTGGTAGAAATTCAGCCATTGGGAATAGCACCCACTAAAGCAAAAAGGAGAGT
GGTGGAGAGAGAAAAAAGAGCAGCACTAGGAGCTTTGTTCCTTGGGTTCTTGGGAGCAGCAGGAAGCACTATG
GGCGCGGCGGCAGTAACGCTGACGGTACAGGCCAGACAATTATTGTCTGGTATAGTGCAACAGCAAAGCAATT
TGCTGAGGGCTATAGAGGCGCAACAGCATATGTTGCAACTCACGGTCTGGGGCATT
>HM215403..1
TTCTATTGTAATACATCAAGCCTGTTTAATGGCACATACAATGGCACATACATGACTAATATGGCTAATGATT
CATCAGAAATCACAATCCCATGCAGAATAAAGCAAATTGTAAACATGTGGCAGGAGGTAGGACGAGCAATGTA
TGCCCCTCCCATTGCAGGAAACATAACATGTAGATCAAATATCACAGGACTATTATTGGTACGTGATGGAGGA
CGATCAGAGGTGTCAAATACTACAGAGACATTCAGACCTCAAGGGGGAGATATGAGGGACAATTGGAGAAGTG
AATTATATAAATATAAAGTGGTAGAAATTAAGCCATTAGGAATAGCACCCACTGGAGCGAAAAGGAGAGTAGT
GGAGAGAGAAAAAAGAGCAGCGGGAATAGGAGCTGTGTTCCTTGGGTTCTTGAGTGCAGCCGGAAGCACTATG
GGCGCGGCGTCATTAACGCTGACGGTACAGGCCAGACAATTGCTGTCTGGTATAGTGCAACAGCAAAGCAATT
TGCTGAGGGCTATAGAGGCGCAACAGCATATGTTGCAACTCACAGTCTGGGGCATT

>HM215408..1
TTCTATTGTAATACATCAAGACTGTTTAATAGTACATACAGCAATGGTACACTCAGGTTTAATGGTACAGACT
TTAATGGTACAATGGATAATTCAAGCTCAACCATCACAATCCCATGCAGAATAAAGCAATTTATAAACATGTG
GCAGGAAGTAGGAAGAGCAATGTATGCCCCTCCCATTGAAGGAAACATAACATGTAGATCAAATATCACAGGA
CTACTATTGGTACGTGATGGAGGAACAAATTTAACAGCAGAATTAAATCAGACAGAGATATTTAGACCTGAAG
GAGGAAATATGAGGGACAATTGGAAAAATGAATTATATAAATATAAAGTGGTAGAAATTAAGCCATTGGGACT
AGCACCCACTGGAGCAAAAAGGAGAGTGGTGGAGAGACAAAAAAGAGCAGTGGGAATAGGAGCTGTAATCTTT
GGGTTCTTGGGAGCAGCCGGAAGCACTATGGGCGCGGCGTCAATGGCGCTGACGGTACAGGCCAGACAATTGT
TGTCTGGTATAGTGCAACAGCAAAGCAATTTGCTGAGGGCTATAGAGGCGCAACAGCATCTGTTGCAACTCAC
GGTCTGGGGCATT
>HQ699950..2
TTCTATTGTAATACATCAGGCCTGTTTAATAGTACATACATGCCTAATGGTACCTACATGTCTAATGGTACCA
ATAGTAATGCAACCATCACAATCCCATGCAGAATAAAGCAAATTATAAACATGTGGCAGGAGGTAGGACGAGC
AATATATGCCCCTCCCATTGCAGGAAACATAACATGTAAATCAAATATCACAGGGCTACTATTGGTACGTGAT
GGAGGACCAGATAATCAGACAGAGATATTCAGACCTGAAGGAGGAGATATGAGGAACAATTGGAGAAGTGAAT
TATATAAATATAAAGTGGTAGAAATTAAGCCATTGGGACTAGCACCCACTGGAGCAAAAAGGAGAGTGGTGGA
GAGAGAAAAAAGAGCAGTGGGAATAGGAGCTGTGTTCCTTGGGTTCTTGAGTGCAGCCGGAAGCACTATGGGC
GCGGCGTCAATAACGCTGACGGTACAGGCCAGACAATTGCTGTCTGGTATAGTGCAACAGCAAAGTAATTTGC
TGAGGGCTATAGAGGCGCAGCAGCATATGTTGCAACTCACGGTCTGGGGCATT
>HQ699981..1
TTCTATTGTAACACATCAGGCCTGTTTAATAGTACATACATGCCTAGTGGTACATACATAACTAATGATACAA
ATAGTAGTTCAGATACAATCACAATCCCATGCAAAATAAAGCAAATTATAAACATGTGGCAGGAGGTAGGACG
AGCAATGTATGCCTCTCCCATTGCAGGAAACATAACATGTACATCAAATATCACGGGACTACTATTGGTACGT
GATGGAGACAAAACAAGTAATACTACAGAGACATTCAGACCTCAAGGAGGAGACATGAGGGACAATTGGAGGA
GTGAATTATATAAATATAAAGTGGTAGAAATTAAGCCATTGGGAATAGCACCCACTAAAGCAAAAAGGAGAGT
GGTGGAGAGAGAAGAAAGAGCAGCGGGAATAGGAATAGGAGCTTTGTTCCTTGGGTTCTTGAGTGCAGCCGGA
AGCACTATGGGCGCGGCGTCAATAACGCTGACGGTACAGGCCAGACAATTGTTGTCCGGTATAGTGCAACAGC
AAAGCAATTTGCTGAGGGCTATAGAGGCGCAACAGCATATGTTGCAACTCACGGTCTGGGGCATT
>KC807928..2
TTCTATTGTAATACAACAAACCTGTTTAATAGTACATACAATAGTACAACAGGCCTGTTTACTTATACATACA
ATGGTACTACATACAATGGTAGTGTTACAGACAATGATAGCATCCCAATCCAAATCCAATGCAGAATAAAGCA
AATTATAAACATGTGGCAGGAGGTAGGACGAGCAATGTATGCCCCTCCCATTGCAGGGAACATAACATGTAGC
TCAAATATCACAGGACTACTATTGACACGTGATGGAGGAAATACAACTGTGACACAAAAAGAGATATTCAGAC
CTCAAGGAGGAAATATGAGAGACAATTGGAGAAATGAATTATATAAATATAAAGTGGTAGAAGTTAAGCCATT
GGGACTAGCACCCACTAAAGCAAAAAGGAGAGTGGTGGAAAGAGAAAAAAGAGCAGTGGGACTAGGAGCTGTG
TTCCTTGGGTTCTTGGGAGCAGCAGGAAGCACTATGGGCGCGGCGTCAATGACGCTGACGGTACAGGCCAGAC
AATTGTTGTCTGGTATAGTGCAACAGCAAAGCAATTTGCTGAGAGCTATAGAGGCGCAACAGCATATGTTGCA
ACTCACGGTCTGGGGCATT
>JF683757..3
TTCTATTGCAACACATCAGTACTGTCTAATAGTACCCATAATAGTGGTACTGGATGGCACCTACAATCAAGTG
ATATTAATTCTTATGCAACCATCGCGGTTTCATATAGAATAGGACAAATTATAAACATGTGGCGGCAGGTAGG
ACGAGCAATCTATGGCCCTCCGGTGGCACGAAAAATGTCAGCCCATCCAAGTACCACAGGCCTATTATTGGTA
TGTGATGGGGGTGAGAAGAGCTTAAATAGCACAAATGGCACAGGTATCACAAATGCCACAGAGATATTTAGAC
CTCAAGGAGGAGATATGAAGGACAATTGGAGAAGTGAATTATATAAGTATAAAGTGGTAGAAATTAAGCCATT
GGGAATAGCACCCACTGAAGCAAGAAGGAGAGTGGTGGAGAGAGAAAAAAGAGCAGTGGGAATAGGAGCTGTG
ATCCTTGGGTTCTTGGGAGCAGCAGGAAGCACTATGGGCGCGGCGTCAATAGCGCTGACGGCACAGGCCAGAA
CAGTAATGGCTGGTATAGTGCAACAGCAAAGCAATTTGCTGAGGGCTATAGATGCGCAACAGCATATGTTGCA
GCTCACAGTCTGGGGCATT
>L23065..3
TTCTATTGTAATACATCAGCCTTGTTTAATGATTCAATTACAAATGCACCCATTAATAATTCGAGTACAGATG
CAAACATCACACTTCAATGCAGAATAAAACAAATTATAAACATGTGGCAGGAGGTAGGACGAGCAATGTATGC
CCCTCCCATTCAAGGAATAATAGCATGTAAATCAAATATCACAGGACTGCTACTGACACGTGATGGAGGGACA
AATAGCACAGACGAGACATTCAGACCTGGAGGAGGAGATATGAGGGACAATTGGAGAAGTGAATTATATAAAT
ACAAAGTGGTAGAAATTAAGCCATTAGGACTAGCACCCACTAAGGCAAAAAGGAGAGTGGTGGAGAGAGAAAA
AAGAGCAGCACTAGCAGCTGTGTTCCTTGGGTTCTTGGGAGCAGCCGGAAGCACTATGGGCGCAGCATCAATA
ACGCTGACGGTACAGGCCAGACAATTATTGTCTGGTATAGTGCAACAGCAAAGCAATTTGCTGAAGGCTATAG
AGGTGCATCAGCATATGTTGCAACTCACAGTCTGGGGCATT
>EF531335..1
TTCTATTGCAACACATCAGATCTGTTTAATAGTACATATTTTAATAATACATATTTTAATGGTGTATATAATG
GTACAAGAGATAATGGTACCATCACACTCCCTTGCAGAATAAAACAATTTATAAACATGTGGCAGAGGGTAGG

AAGAGCAATGTACGCCCCTCCCATTCAAGGACTTATAACATGTAGGTCAAATATCACAGGACTACTATTGACA
CGTGATGGAGGAAACACTATTAACAATACAAATAACAATACAGAGATATTCAGACCTGGAGGAGGAGATATGA
GGGACAATTGGAGAAGTGAATTATATAAATATAAAGTGGTAGAAATTAGGCCATTAGGAGTAGCACCTACTGA
TGCAAGAAGGAGAGTGGTGGGGAGAGAAAAAAGAGCAGTAATGGGAGCTTTGTTCCTTGGGTTCTTGGGAGCA
GCAGGAAGCACTATGGGCGCAGCGTCAATGGCGCTGACGGTACAGACCAGACAATTATTGTCTGGTATAGTGC
AACAGCAAAGCAATTTGCTGAGGGCCATAGAGGCGCAACAGCATATGTTGCAACTCACGGTCTGGGGCATC
>KU319529..3
TTCTATTGCGATACATCAAATCTGTTTAATATGACATACTTGTTTAATGATTGGAGTTACGAACATAGTACTG
ACTCAGATTTCATCATCATGATCCGCTGCAGAATAACACATATAATAAACATGTGGCAGGTAGTGAGAGCAGT
ACTGTATGCCCCTGCCCTTCGCAGGCAACAGTTAAGCAAGCAATCCATCTACGCAGTACTACTATGGACTCGT
GATGCAGCAAGTAGTTACTGGCGCGAGAAAGAGATATTCAGACCTGCAGGAGGAGATATGAGGGACAATTGGA
GAAGTGAATTATATAAATATAAAGTGGTAGAGATTAAACCATTAGGAATAGCACCCACTGAGGCAAAAAGGAG
AGTGGTGGAGAGAGAAAAAAGAGCAGTGGGAATAGGAGCTGTGTTCCTTGGGTTCTTGGGAGCAGCAGGAAGC
ACTATGGGCGCAGCGTCACTGACGCTGACGGTACAGGCCAGGCAATTGCTGTCTGGTATAGTGCAACAGCAAA
ACAATTTGCTGAGAGCTATAGAGGCGCAACAGCATATGTTGCAACTCACAGTCTGGGGCATA
>KU319530..1
TTCTATTGCAATACATCAAGACTGTTTAATGGTGCATACATGTATAACAGTACTAATAGTACTTCAAATGCAA
CCATCACACTCCAATGCAGAATAAAGCAAATTATAAACATGTGGCAGGGGGTAGGACGAGCAATGTATGCCCC
TCCCATTGAAGGAAACATTACATGTAAATCAATATCACAGGATTCTATTGACCCTGATGGAGGATTGGGCCAA
AAACCCAAGGTTTCCAACTGGGAGAGGAACTTAGGGACATTGGAAATGGATCCTTTGGGTTACCCCCTGGCCA
AAAAGGAAGGGGAGAAAAGAAAAAAAAGGACAGGGGTTTTACGAAAAAACTGGGCGCAGAAGCCTATGGGCGC
AGCATCAATAACGCTGACGGTACAGGCCAGACAATTGTTGTCTGGTATAGTGCAACAGCAAAGTAATTTGCTG
AGGGCTATAGAGGCGCAACAGCATATGTTGCAACTCACGGTCTGGGGCATT
>KU319539..2
TTCTATTGTAATACATCAAAACTGTTTAATGGTACATACAATAAGAACAATACTAATAGGACTAATACTAATA
GTACTTCAACCATCACACTTCAATGCAGAATAAAACAAATTATAAACATGTGGCAGGGGGTAGGACAAGCAAT
GTCTGCCCCTCCCATTCAAGGAAACATAACATGTGAATCAAATATCACAGGACTACTATTGACACGTGATGGA
GGAACAACTAATAATAACGAAACAAATAATAACACAGAAGCAAATAATAACACAGAAACATTCAGACCTGGAG
GAGGAGATATGAGGGACAATTGGAGAAATGGAGGTGAGTTATATAAATATAAAGTGGTAGAAGTTAATCCATT
AGGAATAGCACGCACTGAGGCAAAAAGGAGAGTGGTGCAGAGAGAAAAAAGAGCAGTGGGAATAGGAGCTGTG
CTCCTTGGGTTCTTGGGAGCAGCTGGAAGCACTATGGGCGCAGCGTCAGTAACGCTGACGGTACAGGCCAGAC
AATTGCTGTTTGGTATAGTGCAACAGCAAAGCAATTTGCTGAAGGCTATAGAGGCGCAACAGCATATGTTGCA
ACTCACGGTCTGGGGCATT
>KU319542..1
TTCTATTGCAATACAACAAAACTGTTTAATAGTACATACAATGCTAATAGTACAAACAGTACTTCAAATTCAA
CCATCACAATCCAATGCAGAATAAAACAAATTATAAACATGTGGCAGGAGGTAGGACGAGCAATGTATGCCCC
TCCCATTGCAGGAAACATAACATGTAACTCAACTATTACAGGAATACTATTAACACGTGATGGAGGGTATAAT
ATTACCAACGACACAAAAACATTCTTACCTGGAGGAGGAGATATGATGAACAATTGGAGAAATGAATTATATA
ATTATAAAGTGGTAGAAAATAAACCATTAGGAATAGCTCCTACTAGTGCAAAGAGGAGAGTGGTGGAGAAAAA
AAAAAGAGCAATATGGGGAATAGGAGCTGCGCTCCTTGGTTTCTTACGAGCAGCAGGAAGCACTATGGGCGCA
GCGTCATTGACGCTGACGGTACAGGCCAGGCAATTGTTGTCTGGTATAGTGCAACAGCAAAGCAATTTGCTGA
GGGCTATAGAGGCGCAACAACATATGTTGCAACTCACGGTCTGGGGCATT
>KU319543..4
TTCTACTGCAATACATCAAATTTGTTTAATAGAACTTACCTGATTAATGGTACCTATAATAATAATAAAACTT
CCATAAGCACCATCACACTCCACTGCAAAATAAAACAAATTATAAATATGTGTCAGGGGGTAGGACAAGCAAT
GTATGCCCCTGCCACTGCAGGAATCATATCATGTATATCAACTATCGCAGTACTACTACTTGCTGGTGATGGA
GGCAATGATACAAGTGATACAGAGACATTCAGACCTGGAGGAGGAGATATGAGGGACAATTGGAGAAGTGAAT
TATATAAATATAAAGTGGTAGAAGTTCACCCATTAGGAGTAGCACCCACTAGAGCAAAAAGGAGAGTGGTGGA
GAGAGAAAAAAGAGCAGCACTAGGAGCTATGATCATTGGGTTCTTGGGAGCAGCAGGAAGCACTATGGGCGCA
GCATCAATAACGCTGACGGTACAGGCCAGGCAATTGTTGTCTGGTATAGTGCAACAGCAAAGCAATTTGCTGA
GGGCTATAGAGGCGCAACAGCATATGTTGCAACTCACAGTCTGGGGCATA
>KU319548..2
TTCTATTGCAATACATCACAACTGTTTAATAGGACATACATGGAAACTACTATTAGCGCTGTGTCAACAAATA
CAAAAATCACCCTCCTATGCAAAATAAAACAAATTATTCAAATGCTTCAGGGAGTAGGACGAGCAATGTATGC
CCCTCGCAATGAAAGAAACATAACATGTCTTTCAAATATCACAGGATTACTATTGACACGTGATGGAGGAATT
AACAGTAGCACAACTGAGATATTCAGACCTGGGGGAGGAGATATGAGGGACAATTGGAGAAGTGAATTATATA
AATATAAAGTGGTAGAAATCAAACCATTAGGAGTAGCACCCACTAAGGCAAAAAGGAGAGTGGTGGAGAGAGA
AAAAAGAGCAGTGGGAATAGGAGCTGTGCTCCTTGGGTTCTTGGGAGCAGCAGGAAGCACTATGGGCGCAGCA
TCAATAACGCTGACGGTACAGGCCAGGCAATTGTTGTCTGGTATAGTGCAACAGCAAAGCAATTTGCTGAGGG
CTATAGAGGCGCAACAGCATATGTTGCAACTCACAGTCTGGGGCATT

>KU319549..1
TTCTATTGCAAAACATCAAAACTGTTTATTAAGACTTGGAAAAGTTGGAACCATCGGAATGATGGAACCATCA
TCACGATCCCATGCAGAATAAAACAAATTATACATATGTGGCAGGGAGTAGGACGAGCAATGTATGCCCCTCC
CATCAGTGGACCAATAACATGTAGATCAAATATCACAGGACTACTACTGACACGGGATGGAGGCATCAGTGAC
AATAACAGTACCAATACCACAGAGATATTCAGACGTCAAGGAGGAGATATGAAAGACAATTGGAGAAGTGAAT
TATATAAATATAAAGTGGTAGAAATTAAGCCATTAGGAATAGCACCCACTAAGGCAAAAAGGAGAATGGTGCA
GAGAGAGAGAGGAGAAAAGAGGCACGGAGCTATGTTCCTTGGGTTCTTGGGAGCAGCCGGAAGCACTATGGGC
GCAGCATCAATAACGCTGACGGTACAGGCCAGACAATTATTGTCAGGTATAGTGCAACAGCAAAGCAATTTGC
TGAGGGCTATAGAGGCGCAACAGCATATGTTGCAACTCACGGTCTGGGGCATT
>AF219266..3
TTCTATTGCTATACTTCAAGACTGTTTAACAGTACATACCTGTTTAATGGTACTTACCTGGTTAATGATATAA
ATAGTACAGGAAATGCAACCATCATACTCCCATGCAAAATAGAACAAATTATAAACATGTGGCAGGAGGTAGG
GCGAGCAATGTATGCCCCTCCCATTGAAGGAAACATAACATGTAGCTCAAACATCACAGGACTATTATTGGTA
CGTGATGGAGGAAAAGATTCAAATGGCACAGAGATATTCAGACCTGGAGGAGGAGATATGAGGGACAATTGGA
GAAGTGAATTATATAAATATAAAGTGGTAGAAATTAAGCCATTGGGAATAGCACCCACTAAGGCAAAAAGGAG
AGTGGTGGAGAGAGAAAAAAGAGCAGTAGGAATAGGAGCTGTGTTCCTTGGGTTCTTGGGAGCAGCAGGAAGC
ACTATGGGCGCGGCGTCAATAACGCTGACGGTACAGGCCAGACAAGTGTTGTCTGGTATAGTGCAACAGCAAA
GCAATTTGCTGAGGGCTATAGAGGCTCAACAGCATATGTTGCAACTCACGGTCTGGGGCATT
>FJ977095..1
TTCTATTGCAATACATCAGAACTGTTTAAGAATGGTACAAATAATGGTACCAATAGTACAGACTCAACAAATT
CAACATCCATCACAATCCAATGCAGAATAAAACAAATTATAAATATGTGGCAGGGGGTAGGTCGAGCAATATA
TGCCCCTCCCATTAAAGGAAACATAACATGTAACTCAAATATCACAGGACTACTGCTGACACGTGATGGAGGA
AAAGGAGAAGAAAGTGATAATAAGACAGAGATATTCAGACCTGCAGGAGGAGATATGAGGGACAATTGGAGAA
GTGAATTATATAAATACAAAGTGGTAGAAATTCAGCCATTAGGAGTAGCACCCACTGAGGCGAAAAGGAGAGT
GGTGGAGAGAGAAAAAAGAGCAGTGGGAATAGGAGCTGTGTTTCTTGGGTTCTTGGGAGCAGCAGGAAGCACT
ATGGGCGCAGCGTCAATAACGCTGACGGTACAGGCCAGACAATTATTGTCAGGCATAGTGCAACAGCAAAGTA
ATTTGCTGAGAGCTATAGAGGCGCAACAGCATATGTTGCAACTCACGGTCTGGGGCATT
>AY255823..1
TTCTATTGCAATACATCAGCCCTGTTTACAAATTCAACCATCCCAAATACAACCATCACACTCCCATGCAAAA
TAAAACAAATTATAAATATGTGGCAGGAGGTAGGACGAGCAATGTATGCCCCTCCCATTAAAGGAGAGATAAC
ATGTAATTCAACTATCACAGGAATACTACTGACACGTGATGGAGGCAACACCACAAACGAAATCGAAACATTC
AGACCTGGAGGAGGAGATATGAGGGACAATTGGAGAAGTGAATTATATAAATACAAAGTGGTAGAAATTAAGC
CATTAGGAATAGCACCCACGGAGGCAAAAAGGAGAGTGGTGGAGAGAGAAAAAAGAGCAGCACTAGGAGCTGT
GTTCCTTGGGTTCTTGGGAGCAGCCGGAAGCACTATGGGCGCAGCATCAATGACGCTGACGTTACAGGCCAGA
CAATTACTGTCGGGTATAGTGCAACAGCAAAGCAATTTGCTGAGGGCTATAGAGGCGCATCAGCATATGCTGC
AACTCACAGTCTGGGGCATT
>KP109480..2
TTCTATTGCAACACATCAAGCCTGTTTAAGGGTACATATAGGCCTAATGGTACAAGTAATTCCACTTCAGGCT
CTATCATCACACTCCCATGCTACATAAAACAAGTTATAAACTTGTGGCAGGAGGTAGGACGAGCAATATATGC
CCCTCCCATTGAAGGAAACATAACATGTATATCAAATATCACAGGACTACTATTGGTAAGAGATGGAGGAAAT
CATGAAGAGGCAAATACGACAGAGACATTCAGACCTGGAGGAGGAAATATGAGAGACAATTGGAGAAGTGAAT
TATATAAATATAAAGTAGTAGAAATTAAGCCATTGGGAGTAGCACCCACTAAAGCAAGAAGGAGAGTGGTGGA
GAGAGAAAAAAGAGCAGTGGGAGTAGGAGCTGTTATGTTCCTTGGGTTCTTGGGAGCAGCAGGAAGCACTATG
GGCGCGGCGTCAATAACGCTGACGGTACAGGCCAGACAATTGCTGTCTGGTATAGTGCAACAGCAAAGCAATT
TGCTGAAGGCTATAGAGGCGCAACAGCATCTGTTGCAACTCACGGTCTGGGGCATT
>KP109483..1
TTCTATTGTAATACATCAAGCCTGTTTAATAGTACATTTAATAGTACATACATGACTAATGATACAGACATGA
ATTCAAACTCAACCATCTCAATCCCATGCCGCATAAAACAAATTATAAACATGTGGCAGGAGGTAGGACGAGC
AATGTATGCCCCTCCCATTGCAGGAAACATAACATGTAAATCAAATATCACAGGACTACTATTGGTACGAGAT
GGAGGAAACTCAAATGATACAAATGAGCCAGAGATATTCCGACCTCAAGGAGGAGATATGAGGGACAATTGGA
GAAGTGAATTATATAAATATAAAGTGGTAGAAATTAAGCCATTGGGAGTAGCACCCACTGCAGCAAAAAGGAG
AGTGGTGGGAAGAGAAAAAAGAGCAGTGGGACTAGGAGCTATGATCCTTGGGTTCTTGGGAGCAGCCGGAAGC
ACTATGGGCGCGGCGTCAATAACGCTGACGGTACAGGCCAGACAATTGTTGTCTGGTATAGTGCAACAGCAAA
GCAATTTGCTGAGGGCTATAGAGGCGCAACAGCATATGTTGCAACTCACGGTCTGGGGCATT
>KY929363..1
TTCTATTGCAATACATCAAAACTGTTCAACAGTACATACTGGCAGAATGGTACATACTCATACTGGAATAATG
GGTCATTCCTAGAATGGAATGATACACAAAGCACTCCAAATCGAAGCATCACAATTCCATGCAGAATAAAACA
AATTATAAACATGTGGCAGGAGGTAGGACGAGCAATGTATGCCCCTCCCATTGCAGGAAAAATAATGTGTAAA
TCCAATATCACAGGAATACTATTGGAACGTGATGGAGGAAATACAAATGAGACAAATAAGACAGAGACATTCA
GACCAATAGGAGGAGATATGAGGAATAATTGGAGAAGTGAATTATATAAATATAAAGTGGTAGAAATTAAGCC

ATTGGGGGTAGCGCCCACTAAGGCAAGAAGGAGAGTGGTGGAGAGAGAAAAAAGAGCAATAGGGGGAATAGGA
GCTGTATTCCTTGGGTTCTTAGGAGCAGCAGGAAGCACTATGGGCGCGGCGTCAATAACGCTGACGGTACAGG
CCAGACAATTGTTGTCTGGTATAGTGCAACAGCAAAGCAATTTGCTGAAGGCTATAGAGGCGCAACATCATCT
GTTGCAACTCACGGTCTGGGGCATT
>KY929365..2
TTCTATTGCAATACATCAGGCCTGTTTAATGCTACATACAATAGTACATCAGGCCTGTTTAATGCTACTAATC
ACACAAATAACACCATCACCATCCCCTGCAGAATAAAACAATTTATAAACATGTGGCAGGGGGTAGGAAAAGC
AATGTATGCCCCTCCCATTGCAGGGAACATAACATGTACATCAAATATCACAGGAATACTATTGACATGGGAT
GGAGGAGAAAATAGAACAGATAATGATACAGAGACATTCAGACCTGCAGGAGGAGATATGAGGGACAATTGGA
GAAGTGAATTATACAAATATAAAGTGGTAGAAATTCAGCCATTGGGGGTAGCACCCACTAAAGCAAAAAGGAG
AGTGGTGGAGAGAGAAAAAAGAGCAGTGGGACTAGGAGCTGTGTTCCTTGGGTTCTTGGGAGCAGCAGGAAGC
ACTATGGGCGCAGCGTCAATGGCGCTGACGGTACAGGCCAGACAATTGTTGTCTGGTATAGTGCAACAGCAAA
GCAATTTGCTGAGGGCTATAGAGGCGCAACAACACATGTTGCAACTCACGGTCTGGGGCATT
>KY929369..1
TTCTATTGTAATACATCAGGCCTGTTTAATAGTAATAATGAGACAAACAATAATTCAAGCTTAATCACAATTC
CATGCAGAATAAAACAAGTTGTAAACTTGTGGCAGGGGGTAGGACGAGCAATGTATGCTCCTCCCATAGCAGG
AAACATAACATGTAATTCAAATATCACAGGACTACTATTGGTACGTGATGGAGGAACAGGAAATAACACAATA
GAGAAATTCAGACCTCAAGGAGGAGATATGAGAGACAATTGGAGGAACGAACTATATAAATATAAAGTGGTAG
AAATTAAACCATTGGGAGTAGCACCCACTAAAGCAAAAAGGAGAGTGGTGGAGAGAGAAAAAAGAGCAATAGC
GGGACTAGGAGCTGTGTTCCTTGGGTTCTTGGGAGCAGCAGGAAGCACTATGGGCGCGGCGTCAATAACGCTG
ACGGTACAGGCCAGACAATTGTTGTCTGGTATAGTGCAACAGCAAAGTAATTTGCTGAGGGCTATAGAGGCGC
AACAGCATCTGTTGCAACTCACGGTCTGGGGCATT
>KY929370..3
TTCTATTGTAATACATCAGGCCTGTTTAATGGTACATACATGTCTAATGGTACTTATAATGAGACAAACAATA
ATTCAAGCTCAAACATCACAATTCCATGCAGAATAAAACAAGTTATAAACTTGTGGCAGGGGGTAGGACGAGC
AATGTATGCTCCTCCCATTGCAGGAAAAATAACATGTAACTCAAATATCACAGGACTACTATTGGTACGTGAT
GGAGGAACAGGTAATCAGACAAATGATATAGAGACATTCAGACCTCAAGGAGGAGATATGAGAGACAATTGGA
GGAGCGAATTATATAAATATAAGGTGGTAGAAATTAAACCATTGGGAATAGCACCCACTGAAGCAAAAAGGAG
AGTGGTGGGAAGAGAAAAAAGAGCAGTGGGACTAGGAGCTGTGCTCTTTGGGTTCTTGGGAGCAGCAGGAAGC
ACTATGGGCGCGGCGTCAATAACGCTGACGGTACAGGCCAGACAATTGTTGTCTGGTATAGTGCAACAGCAAA
GCAATTTGCTGATGGCTATAGATGCGCAACAGCATCTGTTGCAACTCACAGTCTGGGGCATT
>KX756609..1
TTCTATTGCAATACCTCAAAACTGTTTAATATATCAAACCTGTCTTCTAATGATACAACAGATAATGGCACAA
TCATGATCCCATGCAGAATAAAACAAATTATAAACATGTGGCAGGAGGTAGGACGAGCAATGTATGCTCCTCC
CATTGCAGGAAACATAACGTGTAGTTCAAACATCACCGGACTACTGTTGGAACGTGATGGAGGAAATTCAACT
AATTCAACTAATTCAAATAGGACAGAGACATTCAGGCCTCAAGGAGGAAATATGAGGAACAATTGGAGAAGTG
AATTATATAAATATAAGGTGGTAGAAATACAACCAGTGGGAATAGCGCCCACTACAGCAAAAAGGAGAGTGGT
GGAGAGAGAAAAAAGAGCAGTGGGAATAGGAGCTATGTTCCTTGGGTTCTTGGGAGCAGCAGGAAGCACTATG
GGCGCAGCGTCGATAACGCTGACGGTACAGGCCAGACAACTGTTGTCTGGTATAGTGCAACAGCAAAGCAATT
TGCTGAGAGCTATAGAGGCGCAACAACATCTGTTGCAACTCACAGTCTGGGGCATT
>MK076687..2
TTCTATGGTAATACAGCAGGCCTGTTTAATAATACATACAATATTACAACAGGCACGCTTACATACAATGGTA
AATCCCTTAATGATTTAAACTCAACCATCACACTCCCATGCAGAATAAAGCAGTTTAAAAATATGTGGCAGGG
GGTAGGAAGAGCAATGTATGCCCCTCCCATTGCAGGAAACATAACATGTACATCAAATATCACAGGCATAATA
TTGACACGTGATGGAGGGAATGAGAGTAGTACTGAAGTTCACAATAATACAGAGACATTCAGACCTCAAGGGG
GAAATATGAGAGATAATTGGAGAAGTGAATTATACAAATATAAAGTGGTAGAAATTAAGCCATTGGGAGTAGC
ACCCACTGCAGCAAAAAGGAGAGTGGTGGAAAAAGAAAAAAGGGGCCTAGGAGCTATACTCATTGGGTTCTTG
GGAACAGCAGGAAGCACTATGGGCGTGGCGTCAATAACGCTGACGGTACAGGCCAGACAATTGTTGTCTGGTA
TAGTGCAACAGCAAAGCAATTTGCTGAGAGCTGTAGAGGCGCAACAACATCTGTTGCAACTCACGGTCTGGGG
CATT
>KY713231..1
TTCTATTGCAATACCTCAAGCTTGTTTAGCAGTATAAGCCCTAATAGTACAAATAATACTGTAATAACAATCC
CATGCAGAATAAAACAAATTATAAACATGTGGCAAGGGGTAGGACGAGCAATATACGCCCCTCCTATTGAAGG
AAACATAACATGTAAATCAAATATCACAGGAATACTATTGGTACGTGATGGAGGCACAAATACTACAGAAGAA
GGAAAAGAAATAGAAATATTTAGACCTCAAGGAGGAAATATGAAGGACAATTGGAGAAGTGAATTATATAAAT
ATAAAGTGGTAGAAATTAAGCCATTAGGAGTAGCACCCACTAAAGCAAAAAGGAGAGTGGTGGAGAGAGAAAA
AAGAGCAGTAGTGGGGCTAGGAGCTGCGTTCCTTGGGTTCTTGTCAGCTGCAGGAAGCACTATGGGCGCGGCG
TCAATAACGCTGACGGTACAAGCCAGACAATTGTTGTCTGGTATAGTGCAACAGCAAAGCAATTTGCTGAGAG
CTATAGAGGCGCAACAGCATATGTTGCAACTCACAGTCTGGGGCATT
>KY713235..2

TTCTATAGGAGACAGTCAAAATTGTTTAATCACACATACATGCAGAATGGTACACACAATGGTACAATAAATG
GTTCAGACCCAATCATAATTCCATGCAGAATAAAGCAAATTATAAACATGTGGCAGGAGGTAGGAAGAGCAAT
GTATGCCTCTCCCATTGCAGGAAACATAACCTGTAAATCAAATATCACAGGACTACTATTGACACGAGATGGA
GGACTCAAAAATGATAGTAGTAATGAGACAAAGATAGAGATATTCAGACCTGGAGGAGGAAACATGAAAGACA
ATTGGAGAAGTGAATTATACAAATATAAAGTGGTAGAAATTAAGCCATTGGGAATAGCACCCACTGAAGCAAG
AAGAAGAGTAGTGCAGAGAGAAAAAAGAGCAGTGACACTAGGAGCTATGTTCCTTGGGTTCTTGGGAGCAGCA
GGAAGCACTATGGGCGCGGCGTCAATAACGCTGACGGTACAGGCCAGACAATTGTTGTCTGGTATAGTGCAAC
AGCAAAGCAATTTGCTGAGGGCTATAGAGGCGCAACATCATCTGTTGCAACTCACAGTTTGGGGCATT
>MN703343..1
TTCTATTGTAATACATCAAGACTGTTTAATGGTACATATAATACAACAGAGGAGTCAAACTCAACCATCACAA
TCCAATGCAGAATAAAACAAATTATAAACATGTGGCAGGAGGTAGGACGAGCAATGTATGCCCCTCCCATTGC
AGGAAACATAACATGTGAATCAAACATCACAGGACTAATATTGGTACGTGATGGAGGAACTCATGAAAATAGT
AATACTACAGAGATATTCAGACCTGGAGGAGGAGATATGAGGGACAATTGGAGAAGTGAACTATATAAATATA
AAGTGGTAGAAGTTAGGCCTTTGGGAATAGCACCCACTAAAGCAAAAAGGAGAGTGCTGGAGAGACAAAAAAG
AGCAGTAGGAATGGGAGCTGTGTTCCTTGGGATCTTGGGAGCAGCAGGAAGCACTATGGGCGCGGCGTCAATA
ACGCTGACGGTACAGGCTAGACAATTGTTGTCTGGTATAGTGCAACAGCAAAACAATTTGCTGAGGGCTATAG
AGGCGCAACAGCACATGTTGCAACTCGCAGTCTGGGGCATT
>MN703361..1
TTCTATTGTAATACATCAGGCCTGTTTAATGAAAGCCGGCTTAACGAAACAGAAGGTGATTCAAGTAAAAACA
TCACACTTCCATGTAGAATAAAGCAAATTATAAACATGTGGCAGGCGGTAGGAAAAGCAATATATGCCCCTCC
CATTGCAGGAAACATAACATGTAAATCAAATATCACAGGATTACTATTGGAACGTGATGGAGGCAAAGGCAAT
GCAACAGAGGAAATATTCAGACCTGGAGGAGGAAATATGGGGGACAATTGGAGAAGTGAATTATATAAATATA
AAGTGGTAGAAATTCAACCATTGGGAATAGCACCTACTGGAGCAAAAAGGAGAGTAGTGGAGAGAGAAAAAAG
AGCAGTAGTGGGAATAGGAGCTGTGTTCCTTGGGATCTTGAGTGCAGCAGGAAGCACTATGGGCGCGGCGGCA
ATAACGCTGACGGTACAAGCCAGACAATTATTGTCTGGTATAGTGCAACAGCAAAGCAATTTGCTGAAGGCTA
TAGAGGCGCAACAGCATATGTTGCAGCTCACGGTCTGGGGCATT
>AF067159..2
TTCTATTGTAATACATCAGGCCTGTTTAATAGTACATACATGAGTGGTACATACATGAATTCAAGTGCAGACA
TGAATTCAAGCTACATCACAATCCCATGCAGAATAAAGCAAATTATAAACATGTGGCAGGAGGTAGGACGAGC
AATGTATGCCCCTCCCATTGCAGGAAACATAACATGTAAATCAAATATCACAGGAATACTATTGGAACGTGAT
GGAGGGTCAGGGTCAAATGGCACAGAGACATTCAGACCTGGAGGAGGAGATATGAGGAACAATTGGAGAAGTG
AATTATATAAATATAAAGTGGTAGAAATTCAGCCATTGGGAGTAGCACCCACGGAAGCAAAAAGGAGAGTGGT
GGAGAGAGGAAAAAGAGCAGTGGGAATAGGAGCTGTGTTCCTTGGGTTCTTGGGAGCAGCAGGAAGCACTATG
GGCGCGGCGTCAATAACGCTGACGGTACAGGCCAGACAATTGTTGTCTGGTATAGTGCAACAGCAAAGCAATT
TGCTGAGGGCTATAGAGGCGCAACAGCATCTGTTGCAACTCACGGTCTGGGGCATT
>FJ866133..1
TTCTATTGCAATACATCAGGACTGTTTAATAATAATATTACAAATGCAACCACCACCCTCCCATGCAGAATAA
AACAAATTGTAAACATGTGGCAGGGGGTAGGACGAGCAATGTATGCCCCTCCCATTGAAGGAAAAATAACATG
TAACTCAAATATCACAGGACTGCTCCTGACACGTGATGGAGGGGTGAATAATGAAACGACAGAGACATTCAGA
CCTGGAGGAGGAGATATGAAGGACAATTGGAGAAATGAATTATATAAATATAAAGTGGTAGAAATTCAGCCAT
TAGGAATAGCACCCACTGGAGCAAAAAGGAGAGTGGTGGAGAGAGAAAAAAGAGCGGTGGGACTAGGAGCTGT
GTTCCTTGGGTTCTTGGGAGCCGCAGGAAGCACTATGGGCGCAGCATCAATAACGCTGACGGTACAAGCCAGA
CAATTATTGTCGGGTATAGTGCAACAGCAAAACAACTTGCTGAGAGCTATAGAGGCGCAACAACATATGTTGC
AACTCACAGTCTGGGGCATT
>AB097871..1
TTCTATTGTAATACATCAGGCCTGTTTAATGGGACATACAATGATACATACAGGTCTAATGATACAGATAGTA
ATTCAACCATCACACTCCCATGCAGAATAAAGCAAATTATAAACATGTGGCAGGAGGTAGGACGAGCAATGTA
TGCCCCTCCTATTGCAGGAAACATAACATGTAAATCAAACATCACAGGACTACTATTGGTACGTGATGGAGGA
AATGGTACACAGACATTCAGACCTGGAGGAGGAAATATGAAGGATAATTGGAGAAGTGAATTATATAAATATA
AAGTGGTAGAAATTCAGCCATTAGGAATAGCACCCACTGGAGCAAAAAGGAGAGTGGTGGAGAGAGAAAAAAG
AGCAGCAATAGGAGCTATGTTCCTTGGGTTCTTGGGAGCAGCAGGAAGCACTATGGGCGCGGCGTCGATGGCG
CTGACGGTACAGGCCAGACAATTGTTGTCTGGTATAGTGCAACAGCAAAGCAATTTGCTGAGGGCTATAGAGG
CGCAACAGCATATGTTGCAACTCACAGTCTGGGGCATT
>KC894073..1
TTCTATTGCAATACATCAAAACTGTTTAATGGTACATACAATGGTACATACACAAATAATGATACAAACAGCA
CAATCATACTCCCATGCAGAATAAAACAAATTATAAACATGTGGCAGGAGGTAGGACAAGCAATGTATGCCCC
TCCCATTGAAGGAATCATAGCATGTAATTCAACTATCACAGGATTATTATTGACACGTGATGGAGGAGACAAG
AATGGGAGTAAGCCAGAAATATTCAGACCTGGAGGAGGAGATATGAGGGACAATTGGAGGAGTGAATTATATA
AATATAAAGTGGTAGAAATTAAGCCATTGGGAATAGCACCCACTAAGGCAAAAAGGAGAGTGGTGGAGAAAGA
AAAAACAATTCAAAAAAGAGCAGTAGGAATAGGAGCTGTGTTCCTTGGGTTCTTGGGAGCAGCAGGAAGCACT

ATGGGCGCGGCGTCAATAACGCTGACGGTACAGGCCAGACAATTGTTGTCTGGTATAGTGCAACAGCAAAGCA
ATTTGCTGAGGGCTATAGAGGCGCAACAGCATATGTTGCAACTCACAGTCTGGGGCATT
>FJ444207..1
TTCTATTGCAATACATCAAAACTGTTTACAGAAAGTAATTCAACCTCAAACAGCACCTCAAACACCGTACTCA
CACTCCCATGCAGAATAAAACAAATTATAAACATGTGGCAGGGGGTAGGACGAGCAATGTATGCCCCTCCCAT
TGAGGGAAATATAACATGTGAATCAAACATCACAGGATTACTATTGACGCGTGATAGAACAGACAATAAAACA
GAGACATTTAGACCTGGAGGAGGAGACATGAGGGACAATTGGAGAAGTGAATTATACAAATATAAAGTGGTAG
AAATTCAGCCATTAGGAGTAGCACCCACTAAGGCAAAAAGGAGAGTGGTGGAGAGAGAAAAAAGAGCAGTGGG
AATAGGAGCTGTGTTCCTTGGGTTCTTGGGAGCAGCAGGAAGCACTATGGGCGCAGCATCAATAACGCTGACG
GTACAGGCCAGACAATTGTTGTCTGGTATAGTGCAGCAGCAGAGCAATTTGCTGAGGGCTATAGAGGCGCAAC
AGCATATGTTGCAACTCACGGTCTGGGGCATT
>KC894078..1
TTCTATTGTAATACATCAAACCTGTTTAATATTAATATTACAGGAAATATTACAGGAAATATGACAGGAAATG
AAAACATCACACTCCCATGCAGAATAAAACAAATTATAAACATGTGGCAAAGGGTAGGGCAAGCTATATATGC
CCCTCCCATTGCAGGAAACATAACATGTAACTCAAGTATCACAGGACTACTGTTGACACGTAATGGAGGCAAT
GATAACAGAACAGGGAATAATAATACAGAGACATTCAGACCTGGAGGAGGAGATATGAGGGATAATTGGAGAA
GTGAATTATATAAATATAAAGTGGTAGAAGTTCAGCCATTGGGAGTAGCACCCACTGGAGCAAAAAGGAGAGT
GGTGGAGAGAGAAAAAAGAGCAGTAATGGGAGCTATGTTCCTTGGGTTCTTGGGAGCAGCAGGAAGCACTATG
GGCGCGGCGTCAGTGGCGCTGACGGTACAGGCCAGACAACTATTGTCTGGTATAGTGCAACAGCAAAGCAATT
TGCTGAGGGCTATAGAGGCGCAACAGCATCTGTTGCAACTCACGGTCTGGGGCATT
>KC894079..2
TTCTATTGCAATACATCAGACCTGTTTAATGGTACATACAGAAATGGTACATACAATCATACAGGAAGAAGTT
CAAATGGAACCATCACCCTCCAATGCAAAATAAAACAAATTATAAACATGTGGCAGGAGGTAGGAAGAGCAAT
ATATGCCCCTCCCATTGAAGGAGAAATAACATGTAACTCAAATATCACAGGACTACTATTGCTACGTGATGGA
GGTCAATCAAATGAAACAAATGACACAGAGACATTCAGACCTGGAGGAGGAGATATGAGGGACAATTGGAGAA
GTGAATTATATAAATATAAAGTAGTAGAAATTAAACCATTGGGAGTAGCACCCACTGAGGCAAAAAGGAGAGT
GGTGGAGAGAGAAAAAAGAGCAGTGGGAATAGGAGCTGTGTTCCTTGGGTTCTTGGGAGCAGCCGGAAGCACT
ATGGGCGCAGCATCAATGACGCTGACGGTACAGGCCAGGCAATTATTGTCTGGTATAGTGCAACAGCAAAGCA
ATTTGCTGAGGGCTATAGAGGCGCAACAGCATATGTTGCAACTCACGGTCTGGGGCATT
>KC894081..1
TTCTATTGTAATACATCAGGACTGTTTAATGGTACATACCACCCTAATGGTACATACAATGAGACAGCAGTTA
ATTCAAGCGACACAATCACACTCCAATGCAGAATAAAACAAATTATAAACATGTGGCAAGAGGTAGGAAGAGC
AATGTATGCTCCTCCCATTGCAGGAAATATAACATGTAATTCAACTATCACAGGACTATTATTGACACGTGAT
GGAGGAATAAATCAGACAGGAGAAGAGATATTCAGACCTGGAGGAGGAGATATGAGGGATAATTGGAGAAATG
AATTATATAAATATAAAGTGGTGGAGATTAAGCCATTAGGAATAGCACCCACTAAGGCAAAAAGGAGAGTGGT
GGAGAGAGAAAAAAGAGCAGTGGGAATAGGAGCTGTGTTCCTTGGGTTCTTGGGAGCAGCAGGAAGCACTATG
GGCGCGGCGTCAATAACGCTGACGGTACAGGCCAGACAACTGTTGTCTGGTATAGTGCAACAGCAAAGCAATT
TGCTGAGGGCCATAGAGGCGCAACAGCATATGTTGCAACTCACGGTCTGGGGCATT
>FJ444522..1
TTCTATTGCAATACATCAGGCCTGTTTAATAGTACATATAATCCAAATAGTACATATAATAATTCAAGCTCAG
ACACACCACTTAGACTCTCATGTAGAATAAAACAAATTATAAATATGTGGCAGGGGGTAGGAAGAGCAATATA
TGCCCCTCCCATTGAAGGAAGCATAACATGTAACTCAAGTATCACAGGACTACTATTGCTACGTGATGGAGGT
CATGACGGTGACTCAAATAACACAGAGACATTTAGACCGGGAGGAGGAGATATGAGGGACAACTGGAGAAGTG
AATTATATAAATATAAAGTGGTAGAAATTAAGCCATTGGGAGTAGCACCCACTGACGCAAAAAGAAGAGTGGT
GGAAAGAGAAAAAAGAGCAGTAGTGGGAATAGGAGCTGTGCTCCTTGGGTTCTTGGGAGCAGCCGGAAGCACT
ATGGGCGCGGCGTCACTAACGCTGACGGTACAGGCCAGACAACTGCTGTCTGGTATAGTGCAACAGCAAAACA
ATTTGCTGAGGGCTATAGAGGCGCAACAGCATATGTTGCAACTCACGGTCTGGGGCATT
>KC894089..1
TTTTATTGCAATACATCAGCACTGTTTAATGGTACATACAATTCTACATACAATTCTACTAGTAACCCAAACA
TCACAATCACAATCCAATGCAGAATAAAACAAATTATAAACATGTGGCAGGAGGTGGGACGAGCAATGTATGC
CGCTCCCATTGCAGGAGACATAACATGTGAATCAAACATCACAGGACTACTATTGACACGTGATGGAGGAAAT
ACTGCAAACGGGACAGAGATATTCAGACCTGGAGGAGGAGATATGAGGGATAATTGGAGAAGTGAATTATATA
AATATAAAGTGGTAGAAGTTCAGCCATTGGGAGTAGCACCTACCAAGGCAAAAAGAAGAGTGGTGGGTAGAGA
AAAAAGAGCAGTACTGGGAGCTGTGTTCCTTGGGTTCTTGGGAGCAGCAGGAAGCACTATGGGCGCGGCGTCA
ATGGCGCTGACGGTACAGGCCAGACAATTGTTGTCTGGTATAGTGCAACAGCAAAGTAATTTGCTGAGGGCTA
TAGAGGCGCAACAGCATATGTTGCAACTCACAGTCTGGGGCATT
>KC894084..1
TTCTATTGCAATACATCAGAACTGTTTAATAATACATACTGGCCTAATAGTACTGTTACAAATGGAACTTTAA
ATGGAACCTTCACACTCCCATGCAAAATAAAACAAATTATAAACATGTGGCAGGAGATAGGAAGAGCAATATA
TGCCCCTCCCATTGCAGGAAACATAACATGTAAATCAAATATCACAGGACTACTATTAACACGTGATGGAGGT

CATAACCCAGAGAAGAATGACACAGAGACATTCAGACCTGGAGGAGGAGATATGAGGGATAATTGGAGAAGTG
AATTATACAAATATAAAGTAGTAGAAGTCAAGCCATTAGGAATAGCACCCACGGGGGCAAAAAGGAGAGTGGT
GGAGAGAGAAAAAAGAGCAGTGGGAATAGGAGCTGTGTTCCTTGGGTTCTTGGGAGCAGCAGGAAGCACTATG
GGCGCGGCGTCAATAACGCTGACGGTACAGGCCAGACAATTATTGTCTGGTATAGTGCAACAGCAAAGCAATT
TGCTGAGGGCTATAGAGGCGCAGCAGCATATGCTGCAACTCACGGTCTGGGGCATT
>KC156114..2
TTCTATTGCAACACATCAAGATTGTTTAATCATACATTTAATGAGACATCAGACGAAGACATCACACTCCAAT
GTAGGATAAAACAAATTATAAACATGTGGCAGGGGGTAGGACGAGCAATATATGCCCCTCCCATTGCAGGAAA
CATAACATGTAACTCAAATATCACAGGACTACTATTGACACGTGATGGAGGGAAGAGTAATAGTAGTAATCAG
ACAGAGATATTCAGACCTGCAGGAGGAAATATGAGGGACAATTGGAGAAGTGAATTATATAAATATAAAGTGG
TAGAAATTAAACCATTAGGAATAGCACCCACTAAGGCGAAAAGGAGAGTGGTGGAGAGAGAAAAAAGAGCGGC
AATAGGAGCTATGTTCCTTGGGTTCTTGGGAGCAGCAGGAAGCACTATGGGCGCGGCGTCAATAACGCTGACG
GTACAGGCCAGACAATTATTGTCTGGTATAGTGCAACAGCAAAGCAATTTGCTGAGAGCTATAGAGGCGCAAC
AGCATATGTTGCAACTCACGGTCTGGGGCATT
>KC156120..1
TTTTATTGCAATACATCAATACTGTGGGATAGTGATAAGAGTAATGACACAAACATCACACTCCCATGCAGAA
TAAAACAAATTATAAATATGTGGCAGGAGGTAGGACGAGCAATGTATGCCCCTCCCATTGCAGGAAGCATAAC
ATGTAAATCAAAAATCACAGGACTACTATTGGTACGTGATGGAGGAATAAATAGCAGCACAGAGACATTCAGA
CCTGGAGGAGGAAATATGAAGGACAATTGGAGAAGTGAATTATATAAATATAAAGTGGTAGAAATTCAGCCAT
TAGGAGTTGCACCCACTGGAGCAAAAAGGAGAGTGGTGGAGAGGGAGAAAAGAGCAGTGGGAATAGGAGCTGT
GTTCCTTGGGTTCTTGGGAGCAGCTGGAAGCACTATGGGCGCAGCGTCAATAACGCTGACGGTACAGGCCAAA
CAACTGTTGTCTGGTATAGTGCAACAGCAGAGTAATTTGCTGAGAGCTATAGAGGCGCAACAGCATATGCTGC
AACTCACAGTCTGGGGCATA
>HQ615984..1
TTCTATTGCAATACATCAGGCCTGTTTAATGGTACATTTAATGGTACATACATGCCAAATAGTACAGAAAGTA
CTTCAAATACAAACATCACACTCCAATGCAGAATAAAACAAATCATAAACATGTGGCAGCAGGTAGGACGAGC
AATGTATGCCCCGCCCATTGAAGGAAACATAACATGTAGATCAAATATCACAGGACTACTATTGACACGTGAC
[truncated: 599,239 more chars]
