# Supplementary material for: Extending the Coding Potential of Viral Genomes with Overlapping Antisense ORFs: A Case for the De Novo Creation of the Gene Encoding the Antisense Protein ASP of HIV-1
Source: Viruses. 2022 Jan 14;14(1):146. doi: 10.3390/v14010146 (PMC8781085; doi:10.3390/v14010146)
Supplement: Supplementary file 1 [file viruses-14-00146-s001.zip › Supplementary File S2B.pdf]

Supplementary File S2B.

Supplementary File S2B is a complement of Supplementary File S2A. The latter contains a control set of 1753 *env/asp* overlaps in which the *asp* ORF is delimited by a start and stop codons at canonical positions but that have lost the ability to encode a full-length ASP protein because of the presence of one or more internal *stop* codons. Supplementary File S2B contains the non-overlapping coding region of the 1735 *env* sequences. It includes the region of *env* outside the antisense overlap with *asp*, as well as the region outside the sense overlap with genes *vpu* and *tat/rev*. The non-overlapping coding region of *env* is reported in the alignment format with gaps and it consists of 2436 nucleotide positions. Considering the 3822 nucleotide positions of the multiple alignment of *env* (see paragraph 2.1 in Materials and Methods), the non-overlapping coding region is comprised between nucleotide positions 151-1815, 2683-3264 and 3634-3822. All sequences belong to the group M of HIV-1. The non-overlapping coding region of *env* is also reported in the format without gaps.

>MH705158
GCT---GCA--
GAAAACTTGTGGGTTACTGTCTACTATGGGGTACCTGTGTGG---AAAGACGCAGAG---
ACCACCTTATTTTGTGCATCAGATGCTAAAGCATATGATACAGAA---GTGCATAAT----------------
--------------GTCTGGGCTACA---CATGCCTGTGTACCCACAGAC---
CCCAATCCACAAGAAATACATTTGGAA---------AATGTGACAGAAGAGTTTAAC----------------
--------------------------ATGTGGAAAAATAACATGGTAGAGCAGATGCATACAGATATA---
ATCAGTCTATGGGACCAAAGCCTA---AAACCATGTGTAAAGTTA---
ACCCCTCTCTGTGTTACTTTAGATTGCACCAATAACATCACCAATAGCAACGATAACAACACT----------
----
---CAAAGTGAAATGAAA---
AACTGCTCTTTCAATATGACCACAGAATTAAGG---------------------------
GATAAGAAACGGAAAGTGTATTCACTT---TTTTATAGACTTGATATAGTACAAATTAATGAC----------
----

AATAATAATAGCAGTGAGTATAGATTAATAAATTGTAATACCTCAGCCATTACACAA---
GCTTGTCCAAAGGTGACCTTTGAGCCAATTCCCATACATTATTGTGCCCCAGCTGGTTTTGCAATTCTAAAGT
GTAAA---GATATAGAGTTCAAT------GGAACAGGACCATGCAAGAAT-----------------------
-GTCAGC---ACAGTACAATGCACACATGGAATCAAGCCAGTAATATCAACT---
CAACTGCTGTTAAATGGCAGTCTAGCAGAACAAAAG------------
GTAAGGATTAGATCTGAAAATATCACAAACAAT---------GCCAAAACCATAATAGTACAATTTGAC----
-----------CAGCCTGTAGAA---------ATTAATTGTACCAGACCTAACAACAATACAAGAAAA-----
-AGTGTACGTATA------------GGACCAGGACAAACA---TTC------TATGCAACAGGTGAC------
---------ATAATAGGGGATATA------------AGACAAGCAGTGTGT---AATGTCAGTAAAACA---
GAATGGAATAACACTTTACAACAGGTAGCTACACAATTAAGA---------------AAATACTTTGGG----
-----------------------------------AACGCAACAATAATCTTTGCT------
AACTCCTCAGGAGGAGATTTAGAAATTACA---ACACATAGTTTTAATTGTGCAGGAGAA---
TTTAAACAGCTCCAGGCAAGAGTCCTGGCTGTGGAAAGATACCTAAAAGATCAACAGCTCCTAGGAATTTGGG
GCTGCTCTGGAAAACTCATCTGCACCACTACTGTGCCCTGGAACTCCAGTTGGAGT-----------------
----------------------------AATAAATCTCAGAGTGAGATATGG------GAT------
AACATGACCTGGCTGCAATGG---GATAAAGAAATTAGCAATTACACAGACATAATATAT---
ACTCTAATTGAAGAAGCGCAAAACCAGCAGGAA--

AAGAATGAACAAGACTTATTGGCATTGGATAAGTGGGCAAATCTGTGGAATTGGTTTGAAATATCAAATTGGC
TGTGGTATATAAAAATATTTATAATGATAGTAGGAGACTTAATAGAATTAAAAATAGTTTTTGCT---
GTGCTTTCTATAATA---------AATAGAGTTAGGCAGGGATACTCACCT------
TTGTCGTTTGTTATTAGTTTGCTTGATACCATAGCAATAGCAGTAGCTGGC---
TGGACAGATAGGGTTATAGAAATAGGACAAGGAATTTGTAGAGCT----------------------------
--------ATTCGCAATATA------CCTAGAAGAATCAGACAAGGCTTAGAACGGGCTTTGCTA---TAA
>MH705151
GCT---ACA--
GGAAACTTGTGGGTCACGGTATATTATGGGGTACCTGTGTGG---AAAGACGCAGAG---

ACCACCCTATTTTGTGCGTCAGATGCTAAAGCATATGATACAGAA---GTGCATAAT----------------
--------------GTCTGGGCTACA---CATGCCTGTGTACCCACAGAC---
CCCAGCCCACAAGAAATAGACCTGGAA---------AATGTAACAGAAAAGTTTAAC----------------
--------------------------ATGTGGAAAAAATAACATGGTAGAGCAGATGCATATGGATATA---
ATTAGTCTATGGGATCAAAGCCTA---AAGCCATGTGTAAAGTTA---
ACCCCTCTCTGTGTTACTTTAGATTGTAGCCATAGCATCACCACCATCAATAGCACCACCATCAAT-------
----
----------------------------AGCAACAGCACAAGTGATATATCAGGAGAAATAAAA---
AACTGCTCTTTCAATATGACCACAGAACTAAGA---------------------------
GATAAGACAAAGAAAGTATATTCACTT---TTTTATAGACTTGATGTGGTACAAATTGATGAA----------
----

AATAATGGTAGTAATAACAGTGAGTATAGACTAATAAATTGTAATACCTCAGCCATTACACAG---
GCTTGTCCAAAGGTAACCTTTGAGCCAATTCCCATACATTATTGTGCCCCAGCTGGTTTTGCGATTCTAAAGT
GTAAG---GAGGAGAATTTCAAT------GGAACAGGGATATGCAGGAAT-----------------------
-GTCAGC---ACAGTACAATGCACACATGGAATTAAGCCAGTAGTATCAACT---
CAACTGCTGTTAAATGGCAGCCTAGCAGAAGGAGAG------------
GTAGTGATTAGATCTGAAAACTTCACAAACAAT---------GCCAAAATCATAATAGTACAGTTTAAT----
-----------GAGTCTGTACAA---------ATTAATTGTACCAGACCTAACAACAATACAAGAAGA-----
-AGTTTCCGTATA------------GGACCAGGACAGACA---TTC------TATGCAACAGGTGAA------
---------ATAATAGGAAATATA------------AGACAAGCACATTGT---AATGTCAGTAAAACA---
GACTGGGAGAGCACTTTACAAAAGGTAGCTGCACAATTAGGC---------------AAAGAATTTAAA----
-----------------------------------ACCAACACAACAATCTTTGCT------
AACTCCTCAGGAGGGGATATAGAAATTACA---ACACATAGTTTTAATTGTAGAGGGGAA---
TTTAAACAGCTCCAGGCAAGAGTCCTGGCTGTGGAAAGATACCTAAAGGATCAACAGCTCCTAGGAATTTGGG
GTTGCTCTGGAAAACTCATCTGCACCACTACTGTGCCCTGGAACTCTAGTTGGAGT-----------------
----------------------------AATAGATCTCAGGAGGAAATATGG------AAC------
AACATGACCTGGCTGCAATGG---GAAAAAGAAATTAGCAATTACACAGACATAATATAT---
AGTCTACTTGAAGAATCACAGAACCAGCAGGAA--

AAGAATGAACAAGACTTATTGGCATTGGACAAGTGGGCAAGTCTGTGGAATTGGTTTGACATAACAAATTGGC
TATGGTATATAAAAATATTTATAATGATAGTAGGAGGCTTAATAGGATTAAGAATAGTATTTGCT---
GTGCTTTCTATAATA---------AATAGAGTTAGGCAGGATACTCACCT------
CTCTCGTTTGCTATTAATTTGCTTGATACCACAGCAGTAGCAGTAGCTGAG---
TGGACAGATAGGATTATAGAAATAGGACAAAGACTTGCTAGAGCT----------------------------
--------ATTCTCAACATA------CCTAGAAGAATCAGACAGGGCTTAGAAAGGGCTTTGCTA---TAA
>MH705157
GCT---GCA--
GAAAACTTGTGGGTCACGGTCTACTATGGGGTACCTGTGTGG---AGAGACGCAGAT---
ACCACCCTATTTTGTGCATCAGATGCTAAAGCACATGATACAGAG---GTGCATAAT----------------
--------------ATCTGGGCTACA---CATGCCTGTGTACCCACAGAC---
CCCAACCCACAAGAAATAAAGTTGGAC---------AATGTGACAGAAGAGTTTAAC----------------
--------------------------ATGTGGAAAAAATAACATGGTAGAGCAGATGCATGAAGATATA---
ATCAGTCTATGGGACCAAAGCCTA---ACGCCATGTGTAAAGTTA---
ACCCCTCTCTGTGTTACTTTAGATTGTAGCCATAACATCACCAGAAACAACACC-------------------
----
----------------------------AATAGCACATTAGAGGAGATGAAACAAGAAATAAAA---
AACTGCTCTTTCAATATGACCACAGAACTAAGA---------------------------
GATAAGAAACAGAAAGTATATGCACTT---TTTTATAGACTTGATGTAGTGCAAATTAATGAAAATAATGAT-
----

AATGGTAGTGATCATAACCAATATAGACTAATAAATTGTAATACCTCAGCCATTACACAG---
GCTTGTCCCAAGGTGACCTTTGAGCCAATTCCCATACATTATTGTGCCCCAGCTGGTTTTGCGATTCTAAAGT
GTAAG---GATAAGAAGTTCAAT------GGAACAGGGCCATGCAAGAAT-----------------------
-GTCAGC---ACAGTGCAATGTACACATGGAATCAAGCCAGTAGTATCAACT---
CAACTGCTGTTAAATGGCAGTCTAGCAGAAGAACAG------------
GTAATGATTAGATCTGAAAATATCACAGACAAT---------GCCAAAACCATAATAGTACAGCTTGCC----
-----------ACGCCTGTAGAA---------ATTAATTGTACCAGGCCAAACAACAATACAAGAAAA-----
-AGTGTACGGATA------------GGACCAGGACAAACA---TTC------TATGCAACAGGTGAC------
---------ATAATAGGGAATATA------------AGACAAGCACATTGT---GAAGTTAGTAAAGAG---

AAATGGCAGGATGCTTTACAACAGGTAGCTAAACAATTGATA---------------AAACACTTTAAG----
-----------------------------------AACAAAACAATAAACTTTACT------
GACTCCTCAGGAGGGGATTTAGAAATTACA---ACACATAGCTTTAATTGTGGAGGAGAA---
TTTAAACAGCTCCAGGCAAGAGTCCTGGCTGTGGAAAGATACCTAAAGGATCAACAGCTCCTAGGAATTTGGG
GCTGCTCTGGAAAACTCATCTGCACCACTTCTGTGCCCTGGAACAATAGTTGGAGT-----------------
----------------------------AATAAATCTCAGAATGAAATATGG------GAC------
AACATGACTTGGCTGCAATGG---GATAAAGAAATTAGCAATTACACACAAATAATATAT---
AATCTACTTGAAGAATCGCAGAACCAGCAGGAA--

AAGAATGAACAAGACTTATTGGCATTGGACAAATGGGCAAATCTGTGGACTTGGTTTGACATCTCAAATTGGC
TATGGTATATAAAAATATTTATAATGGTAGTAGGAGGCTTAATAGGATTAAGAATAGTTTTTGAT---
GGGCTTTCTATAATA---------CGTAGAGTTAGGCAGGATACTCACCT------
TTGTCGTTTGCTATTACTTTGCTTGACACTGCAGCAGTAGTGGTAGCTGGG---
TGGACAGATAGGGTTATAGAAGTAGGACAAAGAGTGGGGAGAGCT----------------------------
--------GTTCTCCATATA------CCTAGAAGAATCAGACAAGGCTTCGAAAGGGCTTTGCTA---TAA
>MH705153
GCT---GCA--
GAAAACTTGTGGGTCACGGTCTACTATGGGGTACCTGTGTGG---AAAGACGCAGAT---
ACCACCCTATTTTGTGCATCAGATGCTAAAGCATATGATGCAGAA---GTACATAAT----------------
--------------GTCTGGCTACA---CATGCCTGTGTACCCACAGAC---
CCCAATCCACAAGAAATAAATTTGGTC---------AATGTGATAGAAGAGTTTAAC----------------
--------------------------ATGTGGAAAAATAACATGGTAGAGCAGATGCATGTAGACATA---
ACCAGTCTATGGACCAAAGTCTA---AAGCCATGTGTAAAGTTA---
ACCCCTCTCTGCGTTACATTAGACTGTAGCCATAACATCACCACTAATAACACCAATAAA-------------
----
-------------------------ATCACAAACAGCACAATTCACATGATAGAAGAAATAAAA---
AACTGCACATACAATACAACCACAATACTAAGA---------------------------
GATAAAAAACAGCAAGTATATTCACTT---TTTTATAGACTTGATGTAGTACAAATTAATAAAAAGAAT----
----

GGTAATAACAGTGAACAGTATAGGCTAATAAATTGTAATACCTCAGCCATTACACAG---
GCTTGTCCCAAGGTGACCTTTGAGCCAATTCCCATACATTATTGTGCCCCAGCTGGTTTTGCGATTCTAAAGT
GTAAG---GATGAGGAGTTCAAT------GGAACAGGGCCATGCAAGAAT-----------------------
-GTCAGC---ACCGTCCAATGTACACATGGAATCAAGCCAGTAGTATCAACT---
CAACTGCTGTTAAATGGCAGTCTAGCAGAGAAGGAG------------
GTAAGGATTAGATCTGAAAATCTCAAAGACAAT---------ACCAAAACCATAATAGTACAGCTTGAC----
-----------AAGCCTGTAATA---------ATTAATTGTACCAGACCTAACAATAATACAAGACAA-----
-AGTATACGTATA------------GGACCAGGACAAGCA---TTC------TATGCAACAGGTGCC------
---------ATAATAGGAAATATA------------AGACAAGCATATTGT---AATGTCAGTAGAACA---
GAATGGAATCAAACTTTACAAAAGGTAGGTACACAATTAGGA---------------AAATACTTTAAG----
-----------------------------------AACAAAACAATAATCTTTACT------
AACTCCTCAGGAGGGGATATAGAAATTACA---ACACATAGCTTTAATTGTGGGGGAGAA---
TTTAAACAGCTCCAGGCAAGAGTCCTGGCTGTGGAAAGATACCTACAGGATCAACAGCTCCTAGGAATTTGGG
GCTGCTCTGGAAAACTCATCTGCACCACTACTGTGCCCTGGAATTCTAGTTGGAGT-----------------
----------------------------AATAAATCTCTGAGTGAAATATGG------GAT------
AACATGACCTGGTTGCAATGG---GATAAAGAAATTAGTAATTACACACAAAAAATATAT---
ACTCTAATTGAAGAATCGCAGAACCAGCAGGAA--

AAGAATGAACAAGAGTTATTGGAATTGGACAAGTGGGCAAATCTGTGGAATTGGTTTGACATCTCAAATTGGC
TATGGTATATAAAAATATTTATAATGATAGTAGGAGGCTTAGTAGGATTAAGAATAGTTTTTGCT---
GTGCTTTCTGTAATA---------AATAGAGTTAGGCAGGATACTCACCT------
TTGTCGTTTGCTATTACTTTGCTTGACGCTACAGCAATAGCAGTAGCTGGG---
TGGACAGATAGGGCTATTGAATTAGGACAAAGAATTTGTAGAGCT----------------------------
--------ATTCTTCACATA------CCTGTAAGAATCAGACAAGGCTTAGAAAGGGCTTTGCTA---TAA
>MH705133
GCT---GCA--
GAAAACTTGTGGGTTACGGTTTACTATGGGGTACCTGTGTGG---AAAAATGCGGAG---
ACCACCCTCTTCTGTGCATCAGAAGCTAAAGCATATGAGAAAGAA---GTGCATAAT----------------
--------------GTCTGGCTACA---CATGCCTGTGTGCCCACAGAC---
CCCAACCCACGAGAAATAAATTTGGAC---------AATGTGACAGAAGAGTTTAAC----------------

--------------------------ATGTGGAAGAATAACATGGTCGAGCAGATGCATACAGATATA---
ATCAGTCTATGGGACCAAAGCCTA---AAGCCATGTGTAAAGTTA---
ACCCCTCTCTGTGTTACTTTAAATTGTAGCCATAACATCACCAGAAACATTACCGATATC-------------
----
----------------------------GGCAACAGCACAATTAATATGAGAGAAGAAATAAAA---
AACTGCTCTTTCAATGTGACCACAGCAATAAAA---------------------------
GATAGGAGAGAGAAAGTATATTCACTT---TTTTATAGACTTGATGTAGTACAAATGAATGAA----------
----

AATAATGGTAGTAAACAGTATAGATTAATAAATTGTAATACCTCAGCCATTACACAA---
GCTTGTCCAAAGGTATCCTTTGAGCCAATTCCCATACATTATTGTGCCCCAGCTGGTTTTGCGATTCTAAAGT
GTAAT---GATAAGCAGTTCAAT------GGAACAGGGCTATGCAAAAAT-----------------------
-GTTAGC---ACAGTACAATGCACACATGGAATTAAGCCAGTAGTATCAACT---
CAACTGCTGCTAAATGGCAGTCTAGCAGAAAAAAAG-----------
GTAATGATTAGATCTGAGAATTTCTCAGACAAT---------GCCAAAACCATAATAGTACAGCTTGTC----
-----------AAGCCTGTAAAA---------ATTAATTGTACAAGACCTAACAACAATACAAGAAGG-----
-AGTGTACATATA------------GGACCAGGACAAGCA---TTC------TATGCAACAGGTGAC------
---------ATAATAGGGGACATA------------AGACAGGCATATTGT---ACAGTCAATAGAACA---
GCATGGAATGAAACTTTGAAAGAGGTAGCTATGCAATTAGGT---------------AACAATCTC-------
-----------------------------------AACAAAACAATAATCTTTACT------
AATCACTCCGGAGGGGATTTAGAAATTACA---ACACATAGTTTTAATTGTGGAGGAGAA---
TTTAAACAGCTCCAGGCAAGAGTCCTGGCTGTGGAAAGATACCTAAAGGATCAACAGCTCCTAGGAATTTGGG
GTTGCTCTGGAAAACTCATCTGCACCACTGCTGTGCCCTGGAACTCTAGTTGGAGT-----------------
----------------------------AATAAATCTCAGAATGAGATATGG------GAG------
AACATGACCTGGCTGCAATGG---GATAAAGAAATTAGCAATTACACAAACATAATATAT---
AGTTTAATTGAAGACTCGCAGAACCAGCAGGAA--

AAGAATGAACAAGACTTATTAGCACTGGACAAGTGGGAAATCTGTGGAATTGGTTTGACATATCAAATTGGC
TGTGGTATATAAGAATATTTATAATGATAGTAGGAGGCTTAATAGGATTAAGAATAGTTTTTGCT---
GTGCTTGCTGTAATA---------AATAGAGTTAGGCAGGGATACTCACCT------
TTGTCGTTTGCTATTAATTTGCTTGATACCACAGCAGTAGCAGTAGCTGGG---
TGGACAGATAGGGTTATAGAAATAGGACAAGGAGTTGGTAGAGCT----------------------------
--------ATTCTCCACATA------CCTAGAAGAATCAGACAGGGCTTGGAAAGGGCTTTGCTA---TAA
>JQ403028
GCT---ACA--
GAAAAATTGTGGGTCACGGTCTACTATGGGGTGCCTGTGTGG---AAAGACGCAGTG---
ACCACCCTATTTTGTGCATCAGATGCTAAAGCATATGATACAGAA---GTGCACAAT----------------
--------------GTCTGGGCTACA---CATGCCTGTGTACCCACAGAC---
CCCAACCCACACGAAATACCTTTAGCT---------AATGTGACAGAAGAGTTTAAC----------------
--------------------------ATGTGGAAAAATAACATGGTAGAGCAAATGCATACAGACATA---
ATCAGTCTATGGGACCAAAGCCTA---AAGCCATGTGTAAAGTTA---
GCCCCTCTCTGTGTAACTTTAGAGTGTAATGATGCCAACAGAACAAACATCAAT-------------------
----
-------------------------------------ATAACTGAAGAAGAATTTAAAGTAAAA---
AACTGCTCTTTCAATATGACCACAATTCTAAGA---------------------------
GATAAGAAACAGAAAATGCATGCACTT---TTTTATAGAACTGATGTAGTACAAATTGGAAGT----------
----

AATAGTAGTGAAAGATATAAGTATAGACTAATAAATTGTAATACCTCAGCTATCACACAG---
GCTTGTCCAAAGGTATCCTTTGAGCCAATTCCCATACATTATTGTGCCCCAGCTGGTTTTGCGATTCTAAAAT
GTAAT---GATAAGGAATTCAAT------GGAACAGGGCCATGCATGAAT-----------------------
-GTCAGT---ACAGTACAATGCACACATGGAATCAAACCAGTAGCGTCAACT---
CAGCTGCTGTTAAATGGCAGTCTAGCAGAAGGAAAA-----------
ATAATGATTAGATCTGAAAATCTCACAGACAAT---------GCCAAAATCATAATAGTGCAGCTTAAT----
-----------GAGTCTGTATCA---------ATTAATTGTACCAGACCTAACAACAATACAAGAAAA-----
-AGTTGGCATATA------------GGACCAGGACAGGCT---TTC------TATGCAATGGACGAC------
---------ATACTAGGGGACATA------------AGAAAAGCACAGTGT---AATGTCAACAGAACA---
GCATGGGAAAACACTTTACAAAAGATAGCTACACAATTAAGA---------------ATTATGTAC-------
-----------------------------------AACAAAACAATAGAATTTAAG------
AATTCCTCAGGAGGGGATTTAGAAATTACA---ACACATAGTTTTAATTGTGGAGGAGAA---

TTTAAACAGCTCCAGGCACGAGTCCTGGCTGTAGAAAGATACCTACAGGATCAACAGCTCCTAGGAATTTGGG
GTTGCTCTGGAAAACTCATCTGCACCACTAATGTGCCCTGGAACTCCAGTTGGAGT-----------------
----------------------------AATAAATCTCAGGAAGACATATGG------GGT------
AACATGACCTGGTTACAATGG---GAGAAAGAAATTAGCAATTACACATCCATAATATAT---
GAGCTACTTGAAGAATCACAGAACCAGCAGGAA--

AAGAATGAACAAGACTTATTGGCATTGGACAAGTGGGCAAGTCTGTGGAATTGGTTTAACATATCAAATTGGC
TATGGTATATAAAAATATTCATAATGATAGTAGGAGGCTTAATAGGATTAAGAATAGTTTTTACT---
GTGCTTTCTATAATA---------AATAGAGTTAGGCAGGGCTACTCACCT------
TTGTCATTTGCTATTACTTTGCTTGATGCTACAGCAATAACAGTAGCTGGG---
TGGACAGATAGGGTTATAGAAATAGGACAAAGATTTTGCAGAGCT----------------------------
--------ATACTCCATATA------CCTAGAAGAATCAGACAGGGCTTAGAAAGGGCTTTAATA---TAA
>KX389622
GCT---ACA--
GACAACTTGTGGGTCACAGTCTATTATGGGGTACCGGTATGG---AAAGATGCAGAG---
ACCACCTTATTTTGTGCATCAGATGCTAAAGCACATCATACAGAA---GCACATAAT----------------
--------------GTCTGGGCTACA---CATGCTTGTGTACCCACAGAC---
CCCGACCCACAAGAAATAAATCTGGAA---------AATGTGACAGAAAAGTTTAAC----------------
--------------------------ATGTGGAAAAATAAGATGGTAGATCAGATGCATGAGGATATA---
ATCAGTATATGGGATCAAAGCCTA---AAGCCATGTGTAAAGTTA---
ACCCCTCTCTGCGTCACTTTAGAATGCAGTAAGGTCACAATCAATAACAGTACCACCCAC-------------
----
-------------------AACACCGCCCCAAACAACAACAACACGATAGAAAAAGAAATGAGA---
AACTGTACCTACAATATGACCACAGAAGTAATA---------------------------
GATAAGAAAAAGAAAGTACATTCACTT---TTTTATAGGCTTGATATAGTACCAATAACAAATGAAACCACG-
----

AATGGTAGCCAGTATAGACTAATAAACTGTAATACCTCAGCCATTACACAG---
GCTTGCCCAAAGGTAAATTTTGAGCCAATTCCAATACATTATTGTGCCCCAGCTGGTTTTGCGATTCTAAAAT
GTAAG---GATAAGAATTTCAAT------GGAACAGGGACATGCAGGAAT-----------------------
-GTCAGC---ACAGTACAATGCACACATGGAATTAAGCCAGTAGTCTCAACT---
CAACTGCTGTTAAATGGCAGTCTAGCGGAGGAAAAC------------
ATAAGGATTAGCTCTGAAAATCTCACAGATAAT---------GCTAAAATCATAATAGTACAGCTAAAT----
-----------GAGTCTGTACCA---------ATTAATTGTATTAGACCTAGCAACAATACAAGAGAA-----
-AGTATACGTATA------------GGACCAGGACAAGCA---TTC------TATGCAGCAGGTGAG------
---------ATAATAGGGGACATA------------AGACAAGCACATTGT---AATGTCAGTAGAACT---
GAATGGAATAACACTTTACAAAAGGTAGCTGGCGAATTAAAA---------------AGACTTTTTAAC----
-----------------------------------ACAACRACAATAGTCTTTCAA------
AATTCCTCAGGAGGGGATTTGGAAATTACA---ACACATAGTTTTAATTGTGGAGGAGAA---
TTTAAACAGCTCCAGGCAAGAGTCCTGGCGATAGAAAGATACCTAAAGGATCAACAGCTCCTAGGAATTTGGG
GCTGCTCTGGAAAACTCATCTGCACCACTACTGTGCCCTGGAATTCTAGTTGGAGT-----------------
----------------------------AATAAATCTTATGATGACATATGG------AAT------
AATATGACCTGGATGGAATGG---GATAGAGAAGTTAGTAATTACACAAACATAATATAT---
TCTCTGATTGAAGAATCGCAGAACCAGCAGGAA--

AAGAATGAACAAGACTTATTGYCATTGGACAAGTGGGCAAGTCTGTGGAACTGGTTTGACATATCACACTGGC
TATGGTATATAAGAATATTTATAATGATAGTAGGGGGCTTGATAGGTTTAAGAATAATTTTTGCT---
GTGCTTTCTGTAGTG---------AATAGAGTCAGGCAGGGATACTCACCT------
CTGTCATTGGCTATTAGCTTGCTCAATACCACAGCAATAGCAGTAGCTGAG---
TGGACAGATAGGGTTATAGAAATAGCACAAAGCATTGGTAGAGCC----------------------------
--------TTTATCAACATA------CCTAGAAGAATCAGACAGGGCTTAGAAAGGGCATTGCTA---TAA
>DQ396400
------TCA--
GACAACCTGTGGGTCACGGTCTATTATGGAGTACCTGTGTGG---AAAGATGCAGAT---
ACCACTCTATTTTGTGCATCAGATGCTAAAGCATATAGTAAAGAA---GTACATAAT----------------
--------------GTCTGGGCTACA---CATGCCTGTGTACCCACAGAC---
CCAAACCCACAAGAAATGAATTTGGAA---------AATGTAACAGAAAAGTTTAAC----------------
--------------------------ATGTGGAAAAATGGCATGGTAGAACAAATGCATACAGATATA---
ATCAGTTTATGGACCAAAGCCTA---ACACCATGTGTAAAGTTA---
ACCCCTCTCTGTGTTACTTTAAATTGTACTAACAGCAACTTCACTAGGAATAGCAACCTCACTGATGAT----

----
----------------ACTGATAACAACCCCAATAATACTATTAATATGGAAAATGAAATAAAA---
AATTGCTCTTTCAATGTGACTACAGAAATAAGA--------------------------
GATAAGAGGCAAAAAGCATATGCACTT---TTTTATAAACTAGATGTAGTACAAATTGATAAT----------
----

AATAGTAGTGAGTATATACTAATAAATTGTAATACCTCAGCCATTACACAG---
GCTTGTCCAAAGGTAACCTTTGACCCAATTCCCATACATTATTGTGCCCCAGCTGGTTTTGCGATTCTAAAAT
GTAAT---GATAAGCAATTCAAT------GGATCAGGGATATGCAAGAAT----------------------
-GTCAGC---ACAGTACAATGCACACATGGAATCAAGCCAGTAGTATCAACT---
CAATTGTCGTTAAATGGCAGTCTAGCAGAGGAAGAC-----------
ATAATGATTAGATCTGAAAATATCACAGACAAT---------ACCAAAAACATAATAGTACAGTTTAAT----
-----------GAGTCTGTAAGC---------ATTAATTGTACCAGACCTGGCAACAATACAAGAAAA-----
-AGTATACGTATA------------GGGCCAGGACAAACA---TTC------TTTGCAACAGGCGAC------
---------ATCATTGGGGATATA------------AGACAAGCACATTGT---AATGTCAGCAAAAG----
GAATGGATGAAAGTTCTGCAACAGGTAGTTAGAAAATTAAGA---------------AAGGAATTTGAA----
-----------------------------------AACAAAACAATAGTCTTTGAT------
AATCCCTCAGGAGGGGATTTAGAAATTACA---ATGCATAGTTTTAATTGTAAAGGAGAG---
TTTAAACAGCTCCAGGCAAGAGTCCTGGCTGTGGAAAGATACCTAAAGGATCAACAGCTTCTAGGAATTTGGG
GCTGCTCTGGAAAGATCATCTGCACCACTACTGTGCCCTGGAACTCTAGTTGGAGT----------------
----------------------------AATAAATCCTATGATGACATATGG------GAT------
AACATGACCTGGATGCAATGG---GAAAAGGAAATTGGCAATTACACAGAATTAATATAT---
AGTCTACTTGAAGAATCGCAGGTCCAGCAGGAA---------------------------------------
----
AAGAATGAACAAGAATTATTGGCATTGGACAAGTGGGCAAGTCTATGGAATTGGTTTGACATATCAAGATGGC
TGTGGTATATAAAAATATTCATAATGATAGTAGGAGGCTTAATAGGATTGAGAATAATTTTTGCT---
GTACTTTCTATGCTA---------AATAGAGTTAGGCAGGGTTACTCACCT------
CTGTCATTTGCTATCTCTTTGCTTGATACTATAGCAATAACAGTAGCTGGC---
TGGACAGGTAGGGTTATAGAAATAGGACAAAGAATTGTTAGAGCT---------------------------
--------ATTCTCCACATA------CCTAGAAGAATTAGACAGGGCTTAGAAAGAGCTTTGCAA---TAA
>FM165645
GCT---ACA---
ACAAACTTGTGGGTTACTGTCTACTATGGGGTACCTGTGTGG---AAAGATGCAGAG---
ACCACCTTATTTTGTGCATCAGATGCTAAAGCATATGATACAGAA---GTGCATAAT---------------
--------------ACCTGGGCTACA---CATGCCTGTGTACCCACGGAC---
CCCAACCCACAAGAAATACAGTTGGAA---------AATGTGACAGAAAAGTTTAAC---------------
-------------------------ATGTGGAAAAATAACATGGTAGAACAGATGCATGAGGATATA---
ATCAGTCTATGGGACCAAAGCCTA---AAACCATGTGTAAAGTTA---
ACCCCTCTCTGTGTCACTTTAGAATGCCATAACTACAAAGGCAATGGCACCAATAATACA------------
----
------------------------TATATCAACGGAACGGATGCCATGCAAGGAGAAATAAAA---
AACTGCTCTTACAATATGACCACAGAATTAAGG--------------------------
GATAAGAAACAGAAAGTGTATTCACTT---
TTTTATAGACTTGATATAGTACCAATTAGTGAGAAAAATAGTAAC---------------------------
----
AGTAATAGTAACAATAGTAGCAGTAAGTATAGATTAATAAATTGTAATACCTCAGCTATTACACAA---
GCTTGTCCAAAGGTATCCTTTGAGCCAATTCCCATACATTATTGTGCCCCAGCTGGTTTTGCGATTCTAAAGT
GTAAT---GATATAGATTTCAAT------GGAACAGGACTATGCAAGAAT----------------------
-GTCAGC---ACAGTACAATGCACACATGGAATCAGGCCAGTAGTATCAACT---
CAACTGCTGTTAAATGGCAGTCTAGCAGAAAAAGAG-----------
GTAAGGATTAGATCTGAAAATATCACAAACAAT---------GCCAAAACCATAATAGTACAACTTGAT----
-----------CAGCCTGTAAAT---------ATTACTTGTATGAGACCTAACAACAATACAAGAAAA-----
-AGTGTACATATA------------GGACCAGGACAAGCA---TTT------TATGCAACAGGTGAC------
---------ATAATAGGGAACATA------------AGAGAAGCACATTGT---AATGTCAGTAGAGAA---
GATTGGAACAATACTTTACAAAAGGTAGCCACACAATTAAGA---------------AAACACTTTGGG----
-----------------------------------AACAAAACAATAATCTTTGCT------
AAGTCCTCAGGGGGGGATTTAGAAATTACA---ACACATAGTTTTAATTGTGAAGGAGAA---
TTTAAACAGCTCCAGGCAAGAGTCCTGGCTGTGGAAAGATACCTACATGATCAGCAGCTCCTAGGAATTTGGG
GCTGCTCTGGAAAACTCATCTGCACCACTAATGTGCCTTGGAACGATAGTAGGAGT----------------
----------------------------AATAAATCACTAAATGACATATGG------AAT------

AATATGACCTGGATGCAATGG---GATGAAGAAATTAGCAATTACACACAAATAATATAT---
AATCTACTTGAAAAATCGCAGAATCAGCAGGAA--

AAAAATGAACAAGAGTTATTAGCATTGGACAAGTGGGCAAGTTTGTGGAATTGGTTTAACATCTCAAACTGGC
TATGGTATATAAGAATATTTATAATAATAGTAGGCGGCTTAATAGGGTTAAGAATAGTTTTTGCT---
GTGCTTTCTATAATA---------AATAGAGTTAGGCAGGGATACTCACCT------
CTGTCGTTTGCTACTAGTCTGCTTGATACCATAGCAATAGTAGTAGCTGGC---
TGGACAGATAGGGCTATAGAAATCACACAGAACATTTGTAGAGGT----------------------------
--------ATTCTCCACATA------CCTAGAAGAATCAGACAAGGCTTTGAAAGGGCTTTGCTA---TAA
>FM165628
GCT---ACA--
ACAAACTTGTGGGTTACTGTCTACTATGGGGTACCTGTGTGG---AGAGATGCAGAG---
ACCACCTTATTTTGTGCATCAGATGCTAAAGCATATGATACAGAG---GTGCATAAT----------------
--------------ATCTGGGCTACA---CATGCTTGTGTACCCACAGAC---
CCCGACCCACAAGAAATAATTTTGAAA---------AATGTGACAGAAGAGTTTAAC----------------
-------------------------ATGTGGAAAAATAACATGGTAGAACAGATGCATGCAGATATA---
ATCAGTCTATGGGATCAAAGCCTA---AAACCATGTGTACAGTTA---
ACCCCTCCCTGTGTTACTTTAGAGTGCAATGATTATAATAGCACCAAATCCAACAAT----------------
----
-------------------------------ACGGCTCCCACTGAAACGCCAGAAGAAGTAAAA---
AACTGCACTTTCAATACAACCACAGAATTAAGG---------------------------
GGCAAGAAAAAGAAAGAGTATTCACTT---TTTTATAGACTTGATATAGTACAGATTAATGAAGAC-------
----

AATAGTAGTGATAGCAGGAAGTATAGATTAATAAATTGCAATACCTCAGCCATTACACAG---
GCTTGTCCAAAGGTATCCTTTGAGCCAATCCCCATACATTATTGTGCCCCAGCTGGTTTTGCGATTCTAAAGT
GTAAT---GATATAGAGTTCAAT------GGAACAGGACCATGCAAGAAT-----------------------
-GTCAGC---ACAGTACAATGCACACATGGAATCAAGCCAGTAGTATCAACT---
CAACTGCTGTTGAATGGCAGTCTAGCAGAACAAGAC------------
ATAAGGATTAGATCTGAAAATATCTCACAAAAT---------CACAAAACCATAATAGTACAATTTAAT----
-----------CAGCCGGTAGCA---------ATTAATTGTACCAGACTTAGAAACAATACAAGAAGA-----
-AGTATACATATA------------GGACCAGGACAAGCA---TTC------TATGCAACAGGAGCA------
---------ATAATAGGGGACATA------------AGAAGTGCACATTGT---AATACCAGTAGCAAA---
GCTTGGAATAAGACTTTACACAGGGTAGCCACACAGTTAAGA---------------AAACACTTT-------
-----------------------------------AACGGAACAATAATCTTTACT------
AATGCCTCAGGGGGGGATTTAGAAATTACA---ACACATAGTTTTAATTGTGGAGGAGAA---
TTTAAACAGCTCCAGGCAAGAGTCCTGGCTATGGAAAGATACCTACATGATCAACAGCTCCTAGGAATTTGGG
GCTGCTCTGGAAGACTCATCTGCACCACTAATGTGCCCTGGAACGCTAGTTGGAGT-----------------
----------------------------AATAGATCTCAAAATGAGATATGG------AAT------
AATATGACCTGGTTGCAATGG---GATAAAGAAGTTGGCAATTACACAGACATAATATAT---
GATCTACTTGAAAAATCGCAGAACCAGCAGGAA--

AAGAATGAACAAGACTTATTGGCATTAGACAAGTGGGCAAGTCTGTGGAATTGGTTTAATATCTCAAAATGGC
TGTGGTATATAAAAATATTTATAATGATAGTAGGCGGCTTAATAGGGTTAAGAATAGTTTTTGCT---
ATGCTTTCTATAATA---------AATAGAGTTAGGCAGGGATACTCACCT------
CTGTCGTTTGCTACTAGTCTGCTTGATACCATAGCCATAGTAGTAGCTGGC---
CGGACAGATAGGGTTATAGAAATAACACAAAGCCTTTGTAGAGCT----------------------------
--------ATTCTCAACATA------CCTAGAAGAATCAGACAGGGCCTTGAAAGGGCTTTGCTA---TAA
>EU191615
GCT---ACA--
ACAAACTTGTGGGTTACTGTCTACTATGGGGTACCTGTGTGG---AGAGATGCAGAG---
ACCACCCTATTTTGTGCATCAGATGCTAAAGCATATGCTACAGAG---GTGCATAAT----------------
--------------GTCTGGGCTACA---CATGCTTGTGTACCCACAGAC---
CCTGACCCACAAGAAGTAATTATGGAA---------AATGTGACAGAAGAGTTTAAC----------------
-------------------------ATGTGGAAAAATAACATGGTAGAACAGATGCATGCAGATATA---
ATCAGTCTATGGGATCAAAGCTTA---AAACCATGTGTACAGTTA---
ACCCCTCTCTGTGTTACTTTAGAGTGCGCTAACTATACTGGCACCGCCTACAATTGCACCGCCTGCAAAAAT-
----
-------------GGCACCAGCACCATGGTTCCCCCTATAGTTGAAACGCCAGGAGAAATACAA---
AACTGCTCTTTCAATATAACCACAGAATTAAGG---------------------------

GATAAGAAAAAGAAAGTACATTCACTT---TTTTATAGAGTTGATATAGTACAGATTGATAATAGTAGT----
----

AGTAACAGTAATACCAGTAAGTATAGATTAATAAATTGCAATACCTCAGCCATTACACAG---
GCTTGTCCAAAGGTATCCTTTGAGCCAATCCCCATACATTATTGTGCCCCAGCTGGTTTTGCGATTCTAAAGT
GTAAT---GATATAGAGTTCAAT------GGAACAGGACCATGCAAGAAT-----------------------
-GTCAGC---ACAGTACAATGCACACATGGAATCAAGCCAGTAGTATCAACT---
CAACTGCTGTTAAATGGCAGTCTAGCAGAAAAAGAG------------
ATAAGGATTAGATTTGAAAATATCACAGACAAT---------GCCAAAGCTATAATAGTACAATTTAAT----
-----------CAGTCGGTAGAA---------ATTAATTGTACCAGGCCTAACAACAATACAAGAACA-----
-AGTGTACATATA------------GGACCAGGACAAGCA---TTC------TATGCAACAGGTGAC------
---------ATAATAGGGGACATA------------AGAAATGCACATTGT---AATGTCAGTAGAAAG---
AATTGGACTGAGGCTTTATACAGGGTAGCCACACAATTAAGA---------------AAACACTTT-------
-----------------------------------AACAGAACAATAAACTTTACT------
AGTGCCTCAGGGGGGGATTTAGAACTTACA---ACACATACTTTTAATTGTGGAGGAGAA---
TTTAAACAGCTCCAGGCAAGAGTCCTGGCTATGGAAAGATACCTACATGATCAACAGCTCCTAGGAATTTGGG
GCTGCTCTGGAAGACTCATCTGCACCACTAATGTGCCCTGGAACACTAGTTGGAGT-----------------
----------------------------AATAAATCTCAAGATGAGATATGG------AAT------
AATATGACCTGGTTGCAATGG---GATAAAGAAGTTGGCAATTACACAGACATAATATAT---
GATCTACTTGAAAAATCGCAGAACCAGCAGGAA--

AAGAATGAACAAGAATTATTGGCATTAGACAAGTGGGCAAGTCTGTGGAATTGGTTTAATATCTCAAATTGGC
TGTGGTATATAAAAATATTTATAATGATAGTAGGTGGCTTAATAGGGTTAAGAATAGTTTTTGCT---
GTGCTTGCTATAATA---------AATAGAGTTAGGCAGGGATACTCACCT------
CTGTCGTTTGCTACTAGTCTGCTTGATACCATAGCAATAGCAGTAGCTGGC---
TGGACAGATAGGGTTATAGAAATAACACAAAACCTTTGTAGAGCT----------------------------
--------ATTCTCAACATA------CCTAGAAGAATCAGACAGGGCCTTGAAAGAGCTTTGCTA---TAA
>MH746258
ACT---GCA--
GACCACTTGTGGGTTACTGTATACTATGGGGTACCTGTGTGG---AGAGATGCAGAA---
ACCACCCTATTTTGTGCATCAGATGCGAAAGCATATAAAACAGAA---ATGCATAAT----------------
--------------GTATGGGCTACA---CATGCCTGTGTACCTACAGAC---
CCCAACCCACAAGAAATAAAAATGGCA---------AATGTGACAGAGGAGTTTAAC----------------
--------------------------ATGTGGAAAAATAACATGGTAGAACAGATGCATAAAGATATA---
ATCAGTCTATGGGACCAAAGCCTA---AAGCCATGTGTACATTTA---
ACCCCTCTCTGTGTAACTTTGAAATGTATAGATGTCCAAAATAGCACCACCAATAGCACC-------------
----
-------------------------AAGCATACTGTCTCCCCTGAAATGAACGGAGAAATAAAA---
AACTGCTCTTTCAACGTGACCACAGTATTAAGG---------------------------
GACAAGCAACAGAAAGTACATTCACTT---
TTTTATAGACTTGATGTAGTACCAATTGATAGCAATAGTAATAGTAGTAGC----------------------
----
AATAGTAGTAATAGTGATAATAGTAAGGAGTATAGATTAATAAATTGTGATACCTCAGCCATTACACAG---
GCTTGTCCAAAGATATCTTTTGATCCAATTCCCATACATTATTGTGCTCCAGCTGGTTTTGCAATTCTAAAGT
GTAAT---GATAAGGAGTTCAAT------GGAACAGGACCATGCAGGAAT-----------------------
-GTCAGC---ACAGTACAATGCACACATGGAATCAGGCCAGTAGTATCAACT---
CAGCTGCTGCTAAATGGCAGTCTAGCAGAAAATAAG------------
ACAATGATTAGATCTGAAAATATCACAAACAAT---------GCCAAAAACATAATAGTACAACTTACT----
-----------AAGCCTGTAAAC---------ATTACTTGTATCAGGCCTAACAACAACACAAGAACA-----
-AGTGTACATATA------------GGACCAGGACAAGCA---TGG------TATTCAACAGGTGAC------
---------ATAATAGGGGATATA------------AGGAAAGCATATTGT---GAAGTCAATAAGACA---
GAATGGAATAGAACCTTGCACGAGGTAGCCAACCAATTAAGA---------------ACATACTTTGGG----
--------------------------------GAGAACAAAACAATAATCTTTAAT------
AGCTCCTCAGGAGGAGATCTAGAAATTACA---ACACATAGTTTTAATTGTGGAGGAGAA---
TTCAAACAGCTCCAGGCAAGAGTCCTTGCTGTGGAAAGGTATCTAAGGGATCAACAGCTCCTAGGAATTTGGG
GCTGCTCTGGAAAACTCATCTGCACCACTACTGTGCCCTGGAACTCCACTTGGAGT-----------------
----------------------------AATAAATCCTATGAGTACATCTGG------AAC------
AACATGACCTGGCTACAATGG---GATAGAGAAGTTAGCAATTATACACAAATAATATAT---
GATCTAATTGAAGAATCACAGACCCAGCAGGAA--

ATAAATGAAAAAGACTTATTGGCATTGGACAAGTGGGCAAGTCTGTGGAATTGGTTTGAGATCTCAAATTGGC
TGTGGTATATAAAAATATTTATAATGATAGTAGGAGGCTTAATAGGATTAAGAATAGTTTTTGCT---
GTGCTTTCTGTAATA---------AAAAGAGTTAGGCAGGGATACTCACCC------
CTGTCGTTGGCTATTAGTTTGTTTGACACCACAGCAATAGCAATAGCTGGC---
TGGACAGATAGAGTTATAGAAGTAGGACAAGGAATTTGTAGAGCT----------------------------
--------ATTCGCAACGTA------CCTAGAAGAATCAGACAGGGCTTAGAAAGAGCTTTGCTA---TAA
>MH746253
GCT---ACA--
GAACAATTGTGGGTTACTGTCTACTATGGGGTGCCAGTGTGG---AGAGACGCAGAT---
ACCACCTTATTTTGTGCATCAGATGCTAAAGCAACCAGTGCAGAA---AAACATAAT----------------
--------------GTCTGGGCTACA---CATGCCTGTGTACCAACAGAC---
CCCAACCCACAAGAAGTACATCTGAAA---------AATGTGACAGAAGAGTTTAAC----------------
--------------------------ATGTGGAAAAATAACATGGTAGAGCAGATGCATACAGATATA---
ATTAGTCTATGGGACCAAAGCCTA---AAGCCATGTGTACAACTA---
ACCCCTCTCTGCGTCACTTTAAATTGTAGCAATGTCAATATCACTAACAGCGATGCCAAAAGCAATAGC----
----
-------------------------AACAATGTCACTAGCTTAGAGGACAAGGGAGAAATAAAA---
AACTGCTCTTTCAATATGACCACAGAGCTAAGG---------------------------
GATAAGGAAAAGAAGGTATATTCACTT---TTTTATAAACTTGACATAGTACCTCTTAATAATAATATTACT-
----

GATAGTAATGATAGTAGTGGGGAATATAGATTAATAAATTGCAATACCTCAGCCATTACACAG---
GCTTGTCCAAAGGTATCCTTTGAGCCAATTCCCATACATTATTGTGCCCCAGCTGGTTTTGCGATCCTAAAAT
GTAAT---GAGGAGAAGTTCAAT------GGAACAGGGCCATGCAAAAAT-----------------------
-GTTAGC---ACAGTACAGTGCACACATGGAATCAAGCCAGTAGTATCAACT---
CAACTGCTGCTAAATGGTAGTCTGGCAGAAAAAGAT------------
GTAATTATTAGATCTGAAAATATCACAAACAAT---------GTCAAAAATATAATAGTACAATTTGCC----
-----------AAACCTGTACAA---------ATTAATTGTACCAGACCTAACAACAATACAAGAACA-----
-AGTATACGTATA------------GGACCAGGACAAGCA---TTC------TATGGAACAGGTGAC------
---------ATAATAGGGGACATA------------AGAAAAGCACATTGT---AATGTCAGGAAGTCA---
GACTGGAATGACACTTTACAACAGGTAGTTAAGCAACTAAGG---------------ACACACTTTGGG----
-----------------------------------AACAAAGCAATAAGATTTAAT------
AGCTCCTCAGGAGGAGATCTAGAAATCACA---ACACATAGTTTTAATTGTGCAGGAGAA---
TTTAAACAGCTCCAGGCAAGAGTCCTGGCTGTGGAAAGATACCTAAAGGATCAACAGCTCCTAGGAATTTGGG
GCTGCTCTGGAAAACTCATCTGCACCACTAATGTGCCTTGGAATCAAACTTGGAGT-----------------
----------------------------AATAAATCTCAGACTGACATATGG------GAC------
AACATGACCTGGCTGCAATGG---GATAAAGAAGTTAGTAATTACACAGAGGTAATATAT---
AAACTACTTGAAGAATCACAAAACCAGCAGGAA--

AAGAATGAACAAGACTTATTGGCATTGGACAAGTGGGCAAATCTGTGGAATTGGTTTAACATATCACAATGGC
TGTGGTATATAAAAATATTTATAATGATAGTAGGAGGCTTAATAGGCTTAAGAATAGTTTTTGCA---
GTGCTTGCTATAATA---------AAGAAAGTTAGGCAGGGCTACTCACCT------
CTGTCGTTTGCTATTAGTTTGTTTGATTTCCTAGCAGTAACAATAGCTGGC---
TGGACAGATAGGATTATAGAAATAGGACAAGCAATTGGTAGAGCT----------------------------
--------ATCCTCCACATA------CCTAGAAGAATTAGACAGGGTTTTGAAAGAGCTTTGCTA---TAA
>MH746251
GCT---GCA--
GGAAAATTGTGGGTCACAGTATACTATGGGGTACCTGTGTGG---AGAGATGCAGAC---
ACCACTCTATTTTGTGCATCAGATGCTAAAGCTTATGATAAAGAA---GTACACAAT----------------
--------------GTCTGGGCTACA---CATGCCTGTGTACCCACAGAC---
CCTGACCCACAAGAGATAATTTTGGAA---------AATGTGACAGAAAGATTTGAC----------------
--------------------------ATGTGGAAAAATAACATGGTGGAGCAAATGCAAACAGATATA---
ATTAGTCTCTGGGACCAAAGCCTA---AAGCCATGTGTAAAGTTA---
ACCCCTCTATGCGTTACTTTAAATTGTACCACCCCAACATTCAAAAACCTTAGCCCTAACATCACTAGCTTTA
ACAGCAGTAGC--
----------------------------CTTAACCACAATGAAAGTGTGGTTGAAGAAATGAAA---
AACTGCTCTTTCAACATGACCACAGAACTTAGA---------------------------
GATAAACAGAAAAAGGTATATTCACTT---
TTCTATAGACTTGATATAGTACCACTTGATAGTAATAGTAGTTGGCAA-------------------------
----

TCTAATGCTAGTGTGCAGTATAGACTAATAAATTGTAATACATCAACCATAACACAG---
GCCTGTCCTAAGGTAACCTTTGAGCCAATTCCTATACATTATTGTGCCCCAGCTGGTTTTGCGATTCTAAAGT
GCAGG---GATCCAAATTTTAAT------GGAATAGGGCCATGCAAGAAT----------------------
-GTCAGC---ACAGTACAATGCACACATGGAATCAAGCCAGTAGTGTCAACT---
CAACTGCTGTTAAATGGCAGTCTAGCAGAGAAAGAG-----------
GTAATAATTAGATCTAAAAATATCACAGACAAT---------AGCCAAAACATAATAGTACAGCTTACT----
-----------GAACCTGTAAAC---------ATCACTTGTATCAGACCTGGCAACAATACAAGAACA-----
-TTTATACGTATAGGA---CCAGGAGGACCAGGAGCAGCC---TTC------TATACAACAAAGGAT------
---------GTAATAGGGGACCCA------------AGGAAAGCACATTGT---AATGTCAACAGCACA---
GCATGGAATAACACTTTACATAAGATAAGTACACAATTAAGA---------------AAATACTTTAAT----
----------------------------------AATAGAACAATAATCTTTAAT------
AGCTCCTCAGGAGGGGATGTAGAAGTTACA---ACTCATAGTTTCAATTGTGGAGGAGAA---
TTTAAACAGCTCCAGGCAAGACTCCTGGCTGTGGAAAGTTACCTAAAGGATCAGCAGCTCCTAGGAATTTGGG
GTTGCTCTGGAAAACTCATCTGCACCACTAATGTGCCTTGGAACTCTAGTTGGAGT-----------------
----------------------------AGTAAAAATCTGAGTCAAATATGG------GAT------
AACATGACCTGGATGCAATGG---GACAAGGAAGTTATCAATTACACAAACACAATATAT---
AATCTACTTGAACAATCGCAAAACCAGCAGGAA---------------------------------------
----
AAGAATGAACAAGATTTATTGGCATTAGATAAGTGGGCAAATCTGTGGAGTTGGTTTGACATATCAAATTGGT
TATGGTATATAAAAATATTTATAATGATAGTAGGAGGCTTAATAGGATTAAGAATAGTTTTTGCT---
GTGCTTTCTCTAATA---------AATAGAGTCAGGCAGGGATACTCACCC------
TTGTCATTGGCTATTAATCTGATAGATACCACAGCAATAGTAGTAGCTGGG---
TGGACAGATAGGGTTATAGAAGCAATACAAGGATTGTGTAGAGCT----------------------------
--------ATTAGTAACATA------CCTAGGAGAATCAGACAGGGCGCAGAAAAAGCTTTGCAA---TAA
>KU168256
GCT--
GGCCAATTGTGGGTCACGGTCTACTATGGGGTACCTGCGTGG---AAAGATGCAGTG---
ACCACCCTATTTTGTGCATCAGATGCCAAAGCATATGATAAAGAA---GTGCATAAT----------------
--------------GTTTGGGCCACA---CATGCCTGTGTACCCACAGAT---
CCCAACCCACAAGAAATAAAATTGGTT---------AATGTAACAGAAGAGTTTAAT---------------
-------------------------ATGTGGAAAAATCCCATGGTAGAGCAGATGCATACAGATATA---
ATCGGTCTATGGGATCAAAGCTTA---AAGCCATGTGTACAGTTA---
ACCCCTCTCTGTGTTACCTTAGATTGCCATGACAATATCACCAGTGACAATATCACCAATAATGGC-------
----
-------------AACACCACCAATAGTCCCAACATCACAAATGAGATGGGAGGAGAAATAAAA---
AACTGCTCTTTCAATATGACCACAGAACTAAGA--------------------------
GATAAGAGGAAGAAAGTACATTCACTT---TTTTATAGACTTGATATAGTAGAAATCGGTAAT----------
----

AGTAGTAGTAGTAATAATCATGGCCAATATAGATTAATAAATTGTAATACCTCAGCCATTACACAG---
GCTTGTCCAAAGGTAACCTTTGAGCCAATTCCCATACATTATTGTGCCCCAGCTGGTTTTGCGATTCTAAAGT
GTACA---GAGAAAAGGTTCAAT------GGAACAGGGCCATGCAGGAAT----------------------
-GTCAGC---ACAGTACAATGCACACATGGAATCAAACCAGTAGTATCAACT---
CAGCTACTGTTAAATGGCAGTTTAGCAGAAGAAAAT-----------
GTAACTATCAGATCTGAAAATATCACAGACAAT---------GCAAAAACATTAATAGTGCAGCTTGTC----
-----------AAGCCTGTAAAT---------ATTACTTGTATCAGACCTAACAACAATACAAGAAAA-----
-GGTATAGGTATA------------GGTCCAGGACAAACC---TTC------TATGCAGCAGATGCT------
---------ATAATAGGGAATATC------------AGACACGCATATTGT---GTGGTCAATAAAACA---
GAATGGAATAAAACTTTACAACAGGTAGTGCACAAATTAAGA---------------GAACATTGG-------
----------------------------------AACAAGACAATAATCTTTGCT------
CCCTCCTCAGGGGGGGATATAGAAATTACA---ACACATAGTTTTAATTGTGGAGGAGAA---
TTCAAACAGCTCCAGGCACGAGTCCTGGCTGTGGAAAGATACCTAAAGGATCAACAGCTCCTAGGACTTTGGG
GCTGCTCTGGAAAACTCATCTGCACCACTGCTGTGCCTTGGAACTCTAGTTGGAGT-----------------
----------------------------AACAAATCTCAAGAGCAGATATGG------GAA------
AACATGACCTGGCAGCAATGG---GAAAAAGAAATTAGTAATTACACAAGCATAATATAT---
GATCTACTTGAAGACTCGCAGAACCAGCAGGAA---------------------------------------
----
AAGAATGAACAAGAGTTATTGGCATTGGACAAGTGGACAAATCTGTGGAATTGGTTTGACATATCAAAATGGC
TGTGGTATATTAAAATATTTATAATGATAGTAGGAGGCTTAATAGGATTAAGAATAGTTTTTGCT---
GTGCTTGCTATCTTA---------AATAGAGTTAGGCAGGGTTACTCACCT------

TTGTCATTTGCTATTAATTTGCTTGATACTATAGCAGTAGCAGTAGCAGGG---
TGGACAGATAGGGTTATAGAAATAGGACAAAGAGCTTGTAGGGCT----------------------------
--------CTTCTCAACATA------CCTAGAAGAATAAGACAGGGCTTCGAAAGGGCTTTGCTG---TAA
>KU168305
GTT---GCA--
GAAGAGCGGTGGGTTACTGTCTACTATGGAGTGCCTGTGTGG---AGAGACGCAGAG---
ACTACCTTATTTTGCGCCTCAGATGCTAAAGCATATGATACAGAA---ATGCATAAT----------------
--------------GTCTGGGCTACA---CATGCATGTGTGCCCACAGAC---
CCCAACCCACAAGAAATACTTTTGGGA---------AATGTGACAGAGGATTTTAAC----------------
--------------------------ATGTGGAAAAATAACATGGTGGAACAGATGCATACAGATATA---
ATTAGTCTATGGGATCAAAGTCTA---AAGCCATGTGTAAAGTTA---
ACCCCTCTCTGTGTCACTCTACATTGTAGAAACATCCCCAATGTGACTAGT----------------------
----
--ATGACA---
AACTGCTCTTACAATACGACCACAGAATTAAGG---------------------------
GATAAGAGACAGAAAGTGTACTCACTT---TTTTATAAGCTTGACRTAGTACCAATTAATGAA----------
----

AATAATAGCCAGTATRTATTAATAAATTGTAATACCTCAGCCATKACACAA---
GCTTGTCCAAAGGTAACCTTTGAGCCAATTCCCATACATTACTGTGCCCCAGCTGGTTTTGCGATTCTAAAGT
GTAAG---GATCCAGAATTCAAT------GGAACAGGGCCATGCAAGAAT-----------------------
-GTCAGC---TCAGTACAATGCACACATGGGATCAAGCCAGTAGTATCAACA---
CAACTGCTGCTCAATGGCAGTCTAGCAGAAGACAAG------------
GTAATGATTAGATCTGAAAATATCACAGACAAT---------GCCAAAACCATAATAGTACAATTTAAC----
-----------CAGTCTGTAGAA---------ATTAATTGTACCAGACCTAATAACAATACAAGGAAA-----
-AGTATACGTATA------------GGACCAGGACAATCA---TTC------TATGCAACA---GAC------
---------ATAATAGGGGATATA------------AGACAAGCATCTTGT---ACTGTCAAGAGAGCA---
GATTGGGAGAAAACTTTACAAGAAGTAGTCAAACAACTAGGA---------------GAACATTTTAAA----
--------------------------------AACAGAACAACAATAAAATTTAAT------
AGCTCCTCAGGAGGGGATTTAGAAGTTACA---ACACATAGTTTTAATTGTGGAGGAGAA---
TTTAAACAGCTCCAGGCAAGAGTCCTGGCTGTGGAAAGATACCTAAAGGATCAACAGCTCTTAGGAATTTGGG
GCTGCTCTGGAAAACTCATCTGCACCACTAATGTGCCCTGGAACTCTAGTTGGAGT-----------------
----------------------------AATAAGACTCAGAATGAGATATGG------AAT------
AACATGACCTGGCTGCAATGG---GATAAAGAAATTAACAATTACACACAAGAAATATAT---
AGACTAATTGAAGAATCGCAGAACCAACAGGAA--

AAGAATGAACAGGACTTATTGGCATTGGACAAGTGGTCAAATTTGTGGAATTGGTTCGACATATCAAAGTGGC
TGTGGTATATAAGAATATTTATAATGATAGTAGGAGGCTTAATAGGATTAAGAATAGTTTTTGCT---
GTGCTTTCTATAATA---------AATAGAGTTAGGCAGGGATACTCACCT------
CTATCGTTTGCTGTTAATTTGCTTGATACCATAGCAATAGCAGTAGCTGGC---
TGGACAGATTGGGTTATAGAAATAGGACAGAGAATTTGCAGAGCT----------------------------
--------GTGATCAACATA------CCTACAAGAATTAGACAGGGCTTCGAAAGGGCTTTACTA---TAA
>KP718918
GTT---GCA--
GAAAACTTGTGGGTTACTGTCTACTATGGGGTGCCTGTATGG---AGAGATGCAGAG---
ACCACTTTATTTTGCGCCTCAGATGCTAAAGCATATGATACAGAA---GTGCATAAT----------------
--------------GTCTGGGCTACA---CATGCCTGTGTGCCCACAGAC---
CCCAACCCACAAGAACTACCTTTGAAA---------AATGTGACAGAGGATTTTAAC----------------
--------------------------ATGTGGAAAAATAGCATGGTAGAGCAAATGCATACAGATATA---
ATCAGTTTATGGGATCAAAGTCTA---AAGCCATGTGTAAAGTTA---
ACCCCTCTCTGTGTTACCTTAAATTGTAGCAACATCAACAAAAATGTCACCAAT-------------------
----
--GGGACTGACATAGATATGATGAAA---
AACTGTTCTTTCAATATGACCACAGAAATAAGG---------------------------
GATAAGAAACAGAAAGTGTATTCACTT---TTTTATAAGCTTGATATAGTACCAATTAGTGAA----------
----

GGTAATAGTACAAGTAATAACAGCCAGTATATATTAATAAATTGTAATACTTCAGCCATTACACAA---
GCCTGTCCAAAGGTAAGCTTTGAGCCAATTCCTATACATTACTGTGCTCCAGCTGGTTTTGCGATTCTAAAGT
GTAGA---GATCCAAAGTTCAAT------GGAAAAGGGCCATGCAAAAAT-----------------------

-GTCAGC---ACAGTACAATGCACACATGGAATCAAGCCAGTAGTATCAACA---
CAACTGTTGCTGAATGGCAGTCTAGCAGAACAAGAG------------
GCAATGATTAGGTCTGAAAATATCACAGACAAT---------ACCAAAACCATAATAGTACAACTTAAC----
-----------CAGTCTGTAATA---------ATTAATTGTACCAGACCTAACAACAATACAAGAAAA-----
-AGTGTACGTATA------------GGACCAGGACAATCA---TTC------TATGCAACAGGTGAC------
---------ATAATAGGGGATATA------------AGACAAGCACATTGT---AATGTCAGTAAAGGA---
GACTGGAATAAAGCTCTACAAGAAGTAGTCACACAACTAGGG---------------GAACATTTGGG----
-----------------------------------AACAAAACAATAAAATTCAAT------
AGCTCCGCAGGAGGGGATTTAGAAATCACA---ACACATAGTTTTAATTGTGGAGGAGAA---
TTTAAACAGCTCCAGGCAAGAGTCCTGGCTATGGAAGGATACCTAAGGGATCAACAGCTCCTAGGAATTTGGG
GCTGCTCTGGAAAACTCATCTGCACCACTACTGTGCCCTGGAACTCTAGTTGGAGT-----------------
----------------------------AATAAATCTTACACAGAGATATGG------AAT------
AACATGACCTGGCTGCAATGG---GATAAAGAAGTTAGCAATTACACACAAGAAATATAT---
AAACTAATTGAAGAATCGCAGAACCAACAGGAA--

AAGAATGAACAGGACTTATTGGCATTGGACAAGTGGGCAAATTTGTGGAATTGGTTTGACATATCAAATTGGC
TGTGGTATATAAAAATATTTATAATGATAGTAGGAGGCTTAATAGGATTAAGAATAGTTTTTGCT---
GTGCTTGCTATAATA---------AATAGAGTTAGGCAGGATACTCACCT------
CTGTCGTTGGCTGTTAATTTGCTTGATACCATAGCAATAGCGGTAGCTGGC---
TGGACAGATAGGATTATAGAAGTAGGACAGAGAATTGGTAGAGCT----------------------------
--------ATAATCCATATA------CCTGTAAGAATAAGACAGGGCTTAGAAAGGGCTTTGCTA---TAG
>KP718928
GTT---GCA--
GGAAACTTGTGGGTTACTGTTTACTATGGGGTGCCTGTGTGG---AGAGACGCAGAG---
ACCACCTTATTTTGCGCCTCAGATGCTAAAGCATATGATACAGAA---ATGCATAAT----------------
--------------GTCTGGGCTACA---CATGCCTGTGTGCCCACAGAC---
CCCGACCCACAAGAAATAAATTTGGAA---------AATGTGACAGAGGATTTTAAC----------------
--------------------------ATGTGGAAAAATAGCATGGTAGAACAGATGCATACAGATATA---
ATCAGTTTATGGATCAAAGCCTA---AAGCCATGTGTAAAATTA---
ACCCCTCTCTGCGTTACCTTAAATTGTAGCAGCATCATCACCAATAGCACTGTCAAT----------------
----
---GAGACTAGACAAGGAATGAGA---
AACTGCTCTTTCAATACAACCACAGCATTAAGG---------------------------
GATAAGAGACAGAGAGTGTATTCACTT---TTTTATAAGCTTGATATAGTACCCATTAATGGA----------
----

GATAATAGTACAAATACTTATATGTTAATAAATTGTAATACCTCAGCCATTACACAA---
GCTTGTCCAAAGGTAACCTTTGAGCCAATTCCCATACATTACTGTACCCCAGCTGGTTTTGCGATTCTAAAGT
GTAAG---GATCCAACATTCAAT------GGAACAGGGCCATGCAGAAAT-----------------------
-GTCAGC---TCAGTACAATGCACACATGGAATCAAGCCAGTAGTATCAACA---
CAACTGCTGCTAAATGGCAGTCTAGCTGAACAAAAG------------
GTAATGATTAGGTCTGAAAATATCACAGATAAT---------ACAAAAACCATAATAGTACAACTTAAC----
-----------CAGTCTGTAGTA---------ATTAATTGCTCCAGGCCTAACAACAATACAAGAAGA-----
-AGTATACGTATA------------GGACCAGGGCAATCA---TTC------TATGCAACAGGTGAA------
---------ATAATAGGGGATATA------------AGAGAAGCACGTTGC---AATGTCAATGGAACA---
GAATGGAATAAAGCTTTACAAAAAGTAGTCATACAACTAAGA---------------GAACATTTGGG----
-----------------------------------AACAAAACAATAAAATTTAAT------
AGCTCCTCAGGAGGGGATTTGGAAATAACA---ACACATAGTTTTAATTGTGCAGGAGAA---
TTTAAACAGCTCCAGGCAAGAGTCCTGGCTGTGGAAAGATACCTAAAGGATCAACAGCTCCTAGGAATTTGGG
GCTGCTCTGGAAAACTCATCTGCACCACTAATGTGCCCTGGAACTCTAGTTGGAGT-----------------
----------------------------AATAAGTCTCAAGATGATATATGG------AAA------
AACATGACCTGGCTGCAATGG---GATAGAGAAATTAGTAATTACACACAAGAAATATAT---
AAACTAATTGAAGAATCGCAGAACCAACAGGAA--

AAGAATGAACAGGACTTATTGGCATTGGACAAGTGGGCAACTTTGTGGAGTTGGTTTGACATATCAAATTGGC
TGTGGTATATAAGAATATTTGTAATAATAGTAGGGGGCTTAATAGGATTAAGAATAGTTTTTGCT---
GTGCTTTCTATAATA---------AAGAGAGTTAGGCAGGATACTCACCT------
CTGTCGTTTGCTGTTAATTTGCTTGATACCATAGCAATAGCAGTAGCTGGC---
TGGACAGATTGGGTTATAGAACTAGGACAGAGAATTTGTAGAGCT----------------------------
--------ATTAGAAATATA------CCTAGAAGAATTAGACAGGGCCTCGAAAGGGCTTTGCTA---TAA

>AM279343
ACT---GCA--
GAAAAATTGTGGGTCACTGTCTACTATGGGGTACCTGTGTGG---AAAGATGCAGAC---
ACCACCTTATTTTGTGCGTCAGATGCTAAAGCATATGATACAGAA---GTGCATAAT----------------
--------------GTCTGGGCTACA---CATGCCTGTGTACCCACAGAC---
CCTGACCCACGAGAAATGCCTTTGAAA---------AATGTAACAGAAGAGTTTAAC----------------
--------------------------ATGTGGAAAAATAACATGGTAGAGCAGATGCATACAGATATA---
ATCAGTCTATGGGACCAGAGCCTA---AAACCATGTGTAAAGTTA---
ACCCCTCTCTGTGTTACTTTAGATTGCCATGACTCTATATTTAACACCACTAGCAATAGTACCACTAACAGTA
CT---
----------AATAGCCCCAGGAATGACACCGCAGAAAGAATAGAAATGCAAAGAGAAATACAA---
AACTGCTCTTACAATATGACCACAGAATTAAGG---------------------------
GATAAGAAAAAGAAAATGTATTCACTT---TTTTATAGACTTGATTTAGTACAAATTAATGATGATAGCAGT-
----

AATAACAGTACATATAGTAACAGTACGTATAGATTAATAAATTGTAATACCTCAGCCATTACACAG---
GCTTGTCCAAAGGTATCCTTTGAGCCAATTCCCATACATTATTGTGCCCCAGCTGGTTTTGCGATTCTAAAGT
GTAAT---AATATAGAGTTCAAT------GGAACAGGACCATGCACGAAT-----------------------
-GTCAGC---ACAGTACAATGCACACATGGAATCAGGCCAGTAATATCAACT---
CAACTGCTGTTAAATGGCAGTCTAGCAGAAGGAGAG------------
GTACGAATTAGATCTGAAAATATCACAAACAAT---------GCCAAAACCATAATAGTACAACTTGAT----
-----------GAGGGTGTACAT---------ATTAATTGTACCAGACCTAACAACAATACAAGAAAA-----
-AGTGTACATATA------------GCACCAGGACAGGCA---TTC------TATGCAACAGGGGCC------
---------ATAATAGGGGACATA------------AGAAGAGCATATTGT---CAGATCAATGGAACA---
AGATGGAATGAAACTTTGCACAAGATAGTCAAACAATTAAGA---------------AAACACTTTAAC----
-----------------------------------AACAGAACAATAATCTTTAAT------
AGTTCCTCAGGAGGGGATTTAGAAATTACA---ACACATAGTTTCAATTGTGGAGGAGAA---
TTTAAACAGCTCCAGGCAAGACTCCTGGCTGTGGAAAGATACCTAAAGGATCAACAGCTCCTAGGAATTTGGG
GCTGCTCTGGAAAACTCATCTGCACCACTAATGTGCCTTGGAACTCTAGTTGGAGT-----------------
----------------------------AATAAATCTCAGAGTGAGATATGG------GAC------
AACATGACCTGGCTGCAGTGG---GATAAAGAAATTAGCAATTACACAGACATAATATAT---
AATCTAATTGAAGAATCGCAGATCCAGCAGGAA--

AAGAATGAACAAGACTTATTGGCATTGGACAAGTGGGCAAATCTGTGGAATTGGTTTGACATAACAAACTGGC
TGTGGTATATAAAAATATTTATAATGATAGTAGGAGGCTTAATAGGATTAAGAATAGTTTTTGCT---
GTGCTTGCTATAATA---------AATAGAGTTAGGCAGGGATACTCACCT------
CTGTCGTTTGCTATCAATTTGCTTGACACCATAGCAGTAGCAGTAGCTGGC---
TGGACAGATAGGGTGATAGAAATAGGACAAAGAATTTGTAGAGCT----------------------------
--------ATTCGCAACATA------CCTAGAAGAATCAGACAAGGCGCTGAAAGAGCTTTAGTA---TAA
>AM279348
AAT---GCT---GCA------------------------------------
GACAACTTGTGGGCCACGGTCTACTATGGGGTACCTGTGTGG---AAAGATGCGGAA---
ACCACCCTATTTTGTGCATCAGATGCTAAAGCATATAAAACAGAA---ATGCATAAT----------------
--------------GTCTGGGCTACA---CATGCTTGTGTACCCACAGAC---
CCCAACCCACAAGAAATAAAGTTGGAT---------AATGTGACAGAAGAATTTAAC----------------
--------------------------ATGTGGAAAAATAACATGGTAGAGCAGATGCATGCAGATATA---
ATCAGCCTATGGGACCAAAGCCTA---AAACCATGTGTACAGTTA---
ACCCCTCTCTGTGTTACTTTAAATTGTAGTGATGTCAAAGTCAATACCACCAAGAATAATGGCACTAATAGT-
----
-------------AGTAATGTCCTTGTCATCAATGGTACTGAAAACGGACAGGAAGAACTAAAA---
AACTGCTCTTACAATGTGACTACAGAATTAAGA---------------------------
GATAAGAAACAGAGAGTATATTCACTT---TTCTATAGACTTGATATAGTACCAATTAATGAAGATAATAAA-
----

AGTAGTGATAATAGTAGTGGGGAATATAGACTAATAAATTGTAATACCTCAGCCATTACACAG---
GCTTGTCCCAAGGTATCCTTTGAGCCAATTCCCATACATTTCTGTGCCCCAGCTGGTTTTGCGATTCTAAAAT
GTAGA---GATAAAAATTACAAT------GGAACAGGGCTATGCAAGAAT-----------------------
-GTCAGC---ACAGTACAATGCACACATGGAATCAAGCCAGTGGTATCAACT---
CAATTGCTGTTAAATGGTAGTCTAGCAGAAGGAGAA------------
ATAAAGGTTAGATCTGAGAATTTCACAAACAAT---------GCTAAAACTATAATAGTACAGCTTGAC----

-----------CAGCCTGTAATA---------ATTAATTGTACCAGACCTAACAACAATACAAGAAGA-----
-AGTGTACGTATA------------GGACCAGGACAAGCA---TAC------TATGCAACAGGAGAA------
---------ATAATAGGGGATATA------------AGAAAAGCATATTGT---ACTGTCAATAAAACA---
GCATGGAATGAAACAAAACACAAGGTAATGGAAAAATTAAGA---------------GGAATATAC-------
-----------------------------------CACAGACCAATAAAGTTTAAT------
AGCTCCTCAGGAGGGGATTTAGAAATTACA---ACACATATGTTTAATTGTGGGGGAGAA---
TTTAAACAGCTCCAAGCAAGAGTCCTGGCTGTGGAAAGATACCTAAGGGATCAACAGCTCCTAGGAATTTGGG
GCTGCTCTGGAAAACTCATCTGCACCACTAATGTGCCCTGGAACTCCAGTTGGAGT-----------------
----------------------------AATAAATCTTATGAGGATATATGG------GAC------
AACATGACTTGGCTGCAATGG---GATAAAGAGATTAGCAATTACACAGCACTAATATAT---
GATCTAATTGAGGAATCACAGAACCAGCAGGAA--

AAGAATGAAAAGGACTTATTAGAGTTGGACAAGTGGGCAAGTCTGTGGAATTGGTTTAGCATATCACAATGGC
TATGGTATATAAAAATATTCATAATGGTAGTGGGGGGTTTAATAGGATTAAGAATAATTTTTGCT---
GTGCTTTCCTTAGTA---------AATAGAGTTAGGCAGGGATACTCACCT------
TTGTCGTTTGCTATTAACTTGCTTGATACTATAGCAATAGCAGTAGGTGGG---
TGGACAGATAGTCTTATAGAATTAACACAAAGAATTGGTAGAGCT----------------------------
--------ATTCTCCACATA------CCTAGAAGAATCAGACAGGGCTTCGAAAGGGCTTTGCTA---TAA
>FJ388894
GCT---GTA--
GAAAACACGTGGGTTACTGTCTACTATGGGGTACCTGTGTGG---AGAGATGCAGAG---
ACCACCTTATTTTGTGCATCAGATGCGAAAGCATATAAATCAGAA---GTGCATAAT----------------
--------------GTCTGGGCTACG---CATGCCTGTGTACCTACAGAC---
CCTAACCCACAAGAAATAAATTTGGAA---------AATGTGACAGAAAAATTTAAC----------------
--------------------------ATGTGGAAAAATAACATGGTAGAGCAAATGCATATAGATATA---
ATCAGTCTATGGGACGAGAGCCTA---AAGCCATGTGTAAAGTTA---
ACCCCTCTCTGTGTTACTTTAGAGTGTAGCAACCATGTCATGAGTACCAATGCCAGTACCAAT----------
----
----------------------GCCACTGCCAATATCAACTCTGACATGAGAGAAGAAATAAAA---
AACTGCTCTCACAACATAACCACAGAACTAAGG---------------------------
GATAAGAAACAGAAAGTACATTCACTT---TTTTATAGGCTTGATATAGTACCAATTAATAGTAATAAT----
----

GATAATAATAATACTAGCAAGGAATATAGATTAATAAATTGTAATACCTCAGCCATTACACAA---
GCTTGTCCAAAGGTATCTTTTGAACCAATTCCCATACATTATTGTGCTCCAGCTGGTTTCGCGATTCTAAAGT
GTAAT---GATAAGATGTTCAAT------GGAACAGGAATATGCAGGAAT-----------------------
-GTCAGC---ACAGTACAATGCACACATGGAATCAGGCCAGTAGTATCAACT---
CAGCTGCTGTTAAATGGCAGTTTAGCAGAAAAGGAG------------
ATAATGATTAGATCTGAAAATATCACAAACAAT---------GCCAAAAACATAATAGTACAACTTACC----
-----------AAGCCTGTAAAC---------ATTACTTGTATCAGACCTAACAACAATACAAGAAAA-----
-AGTATACATATA------------GGACCAGGACAAGCG---TTC------TATGCAACAGGTGAC------
---------ATAATAGGGGATATC------------AGACAAGCACATTGT---AATGTCAGTGGAACA---
GTGTGGTATGAAACTTTGCAAAATGTAACCGAACAATTGAGA---------------AAACACTTTATG----
-----------------------------------AACAAAACAATAAACTTTACT------
AAATCCTCAGGAGGGGATCCAGAAATTACA---ACACATAGTTTTAATTGTGGAGGAGAA---
TTTAAACAGCTCCAGGCAAGAGTCCTGGCTGTGGAAAGGTACCTAAGGGATCAACAGCTCCTAGGAATTTGGG
GCTGCTCTGGAAAACTCATCTGCACCACTAATGTACCTTGGAACTCTACTTGGAGT-----------------
----------------------------AATAAATCCCTAGATGACATATGG------AAT------
AACATGACCTGGCTGCAATGG---GATGAAGAGATTAACAATTATACAGAACAAATATAT---
AGACTAATTGAAGAATCGCAGAACCAGCAGGAA--

AAGAATGAAAAAGAATTATTAGAATTGGACAAGTGGGCAAGTCTGTGGACTTGGTTTGACATCACAAAATGGC
TGTGGTATATAAAAATATTTATAATGATAGTAGGAGGCTTAATAGGATTAAGAATAGTTTTTGCT---
GTGCTTTCTATAATA---------AATAGAGTTAGGCAGGGATACTCACCT------
CTGTCGTTGGCTATTAATTTGTTTAATACCATAGCAATAACAGTAGCTGGC---
TGGACAGATAGGGTTATAGAAATAGGACAAAGACTTTGTAGAGCT----------------------------
--------ATCTTCAACATA------CCTAGAAGAATTAGACAGGGCTTAGAAAGGGCTTTGCTA---TAA
>FJ388903
ACT---GCA--
GAAAACTTGTGGGTTACTGTCTACTATGGGGTACCTGTATGG---AAAGAAGCAGAC---

ACCACTTTATTTTGTGCATCAGATGCGAAAGCATATAAGACAGAA---GTGCATAAT----------------
--------------GTCTGGGCTACA---CATGCCTGTGTACCTACAGAC---
CCCAACCCACAAGAAATAAATATGGAA---------AATGTGACAGAAGAGTTTAAC----------------
--------------------------ATGTGGAAAAATAACATGGTAGAGCAGATGCATACAGATATA---
ATCAGTCTATGGGACCAAAGCCTA---AAACCATGTGTAGAGTTA---
ACCCCTCTCTGTGTTACTTTAGAATGTAGAAATGTCTATACTACCAATATCACC-------------------
----
-------------------------------AATATCACCAAGGAAATGCAAGGAGAAATAAAA---
AACTGCTCTTTCAACATGACCACAGAATTAAGG---------------------------
GATAAGAGACAGAAAGTATATTCACTT---TTTTATAAACTTGATATAGTACAAATTAATAGTAACAGTAGT-
----

AATAGTAGTAGTGATAGTAGTGAATATAGATTAATAAATTGTAATACCTCAGCCATTACACAG---
GCTTGTCCAAAGGTATCTTTTGAGCCAATTCCCATACATTATTGTGCTCCAGCTGGTTTTGCGATTCTAAAGT
GTAAT---GACAAGGAGTTCAAT------GGAACAGGACTGTGCAGGAAT-----------------------
-GTCAGC---ACAGTACAATGCACACATGGAATCAGGCCAGTAGTATCAACT---
CAATTGCTGTTAAATGGCAGTCTAGCAGAAAACAAG------------
ACAATGATTAGATCTGAAAATATCACAAACAAT---------GCTAAAAACATAATAGTACAATTTACC----
-----------GAGCCTGTAAAC---------ATTACTTGTATCAGACCTAACAACAATACAAGAAAA-----
-AGTGTACGTATA------------GGACCAGGACAAGCA---TTC------TATGCAGCAGGTGAC------
---------ATAATAGGGGATATA------------AGACAAGCACATTGT---AATGTCAGTAAAGAA---
AAATGGAATAGAACTTTGCAAGAGGTAGCCCAACAATTAGGA---------------ATACACTTTGAG----
-----------------------------------AACAAAACAATAATCTTTGCT------
AACTCCTCAGGAGGGGATCTAGAAATTACA---ACACATAGTTTTAATTGTGGAGGGGAA---
TTTAAACAGCTCCAGGCAAGAGTCCTGGCTCTGGAAAGATACCTAAGGGATCAACAGCTCCTAGGAATTTGGG
GCTGCTCTGGAAAACTCATCTGCACCACTACTGTGCCCTGGAACTCTACTTGGAGT-----------------
----------------------------AATAAATCCCTAGATGAGATATGG------GAT------
AATATGACCTGGCTGCAATGG---GATAAAGAAATTAACAATTATACACAATTAATATAT---
ACTCTAATTGAAGAGTCGCAGAACCAGCAGGAA--

ATAAATGAAAAAGAATTATTGGAATTGGACAAGTGGGCAAATCTGTGGAATTGGTTTGACATCACAAGCTGGC
TGTGGTATATAAGAATATTTATAATGATAGTAGGAGGCTTAATAGGATTAAGAATAGTTTTTGCT---
GTGCTTGCTATAATA---------AATAGAGTTAGGCAGGGATACTCACCT------
TTGTCGTTGGCTATTAATTTGCTTGATACCATAGCAATAGCAGTAGCTGGC---
TGGACAGATAGGGTTATAGAAAGAGGACAAAGCATTTGTAGA-------------------------------
--------------------------------ACAATCAGATAGGGCTTCGAAAGGGCTTTGCTG---TAA
>FJ388909
GTT---ACA--
GGAAACTTGTGGGTTACTGTCTACTATGGGGTACCTGTGTGG---AAAGATGCAGAC---
ACCACCTTATTTTGTGCATCAGATGCTAAAGCATATGACACAGAA---GTGCATAAT----------------
--------------GTCTGGGCTACA---CATGCCTGTGTGCCCACAGAC---
CCCAACCCACAAGAGATAAATTTGGAG---------AATGTGACAGAAGAGTTTAAC----------------
--------------------------ATGTGGAAAAATAACATGGTAGAGCAGATGCATACAGATATA---
ATCAGTCTATGGGACCAAAGTCTA---AAGCCATGTGTACAGTTA---
ACCCCTCTCTGCGTTACGTTAAATTGTAGAGATGATGACGACATCAATGTCGCCAATAGCACAGACACC----
----
-------------------ACTAACGAAGTCAAAAACGGATCTGCCAATATCACAGATGAAAAA---
AACTGCTCTTTCACTATGACCACTGACCTAATA---------------------------
GATAAGCTAATTGAAGTATTTTCTGTT---TTTTATAGACTTGATGTTGTACATATTGATAAA----------
----

AATAGTAATAACAGTGGGTATAGATTAATAAATTGAAATACCACAACCGTTACACAG---
GCTGGTCCAAAGGTATCCTTTGATCCAATTCCCATACATTATTGGGCCCCTGCTGGTTTTGCGATTCTAAAGT
GTATT---GAGAAGGACTTCAAT------GGAACAGGAACATGCAAGAAT-----------------------
-GTCAGC---TCAATACACTGTACACATGGAATCAAGCCAGTAGTATCAACT---
CAACTGCTGTTAAATGGCAGTCTGGCAGAAGGAGAG------------
GTAAAGATTAGATCTGAAAATCTCACAAACAAT---------GCCAAAACCATAATAGTACAGTTTGTT----
-----------GAGCCTGTACAC---------ATTACTTGTATCAGACCTTACAACAATACAAGAAAA-----
-GGTATACATATA------------GGACCAGGGCAAGCA---TTC------TATGCAATGAGTAAC------
---------ATAATAGGAGATATA------------AGACAAGCACATTGT---AACGTCAGTAAAACA---

AAATGGAATGCAACGTTATCCCAGGTAGCCAAACAATTAGGG---------------AATAAGTTTAAC----
-----------------------------------AACAGAACAATAATCTTTAAT------
AGCTCCTCAGGAGGGGATATAGAACTTACA---AGACATAGTTTTAACTGTGGAGGAGAG---
TTTAAACAGCTCCAGGCAAGAGTCCTGGCTGTGGAAAGATACCTAAAGGATCAACAGCTCCTAGGAATTTGGG
GCTGCTCTGGAAAACTCATCTGCACCACTACTGTGCCCTGGAACTCTAGTTGGAGT-----------------
----------------------------AATAGATCTCAGGAGGAAATATGG------AAT------
AACATGACCTGGCTGCAATGG---GAGAAAGAAATTGGCAATTACACAGGTATAATATAT---
AGACTACTTGAAGAATCGCAGAACCAGCAGGAA--

AAGAATGAACAAGACTTATTGGCATTGGACAAGTGGGCAAGTCTGTGGAATTGGTTTAGCATATCAAACTGGC
TATGGTATATTAAAATATTTATAATGATAGTAGGAGGCTTAATAGGATTAAGAATAGTTTTTACT---
GTGCTTTCTATAATA---------AATAGAGTTAGGCAGGGATACTCACCC------
TTGTCGTTTGCTGTTAATTTGGTTGATACTATAGCAATAGCAGTAGCTGGC---
TGGACAGATAGGGTTATAGAAATAGGACAAAGAGTTGGTAGAGCT----------------------------
--------ATTCTCAACATA------CCTAGAAGAATTAGACAGGGCTTAGAAAGGGCTTTGCTA---TAA
>FJ388925
GCT---GCA--
GAAAACTTGTGGGTTACTGTCTACTATGGGGTACCTGTGTGG---AAAGAAGCAAAG---
ACCACCTTATTTTGTGCATCAGATGCGAAAGCATATAAAACAGAA---GTGCATAAT----------------
--------------GTCTGGGCTACA---CATGCCTGTGTCCCTACAGAC---
CCCAACCCACAAGAAATAAATCTGGAA---------AATGTGACAGAAGAGTTTAAC----------------
--------------------------ATGTGGAAAAATAACATGGTAGAGCAAATGCATACAGATATA---
ATCAGTCTATGGGACCAAAGCCTA---AAGCCATGTGTAAAGTTA---
ACCCCTCTCTGTGTTACTTTGAATTGTAGCCATAATGTCACTAAAGTCACCAATAATGGCACT----------
----
----------------------GCCACTGCTAACATAACAGGAGAAATGAAAGGAGAAATAAAA---
AACTGCTCTTTCAATATAACCACAGAATTAAGG---------------------------
GATAAGACACAGAAAATATATTCACTT---TTTTATAGACTTGATGTAGTAGAAATTGATAAG----------
----

AATAGTAGTAATTATAGATTAATAAATTGTAATACCTCAGCCATTACACAA---
GCTTGTCCAAAGGTAACTTTTGAGCCCATTCCCATACATTATTGTGCTCCAGCTGGTTTTGCAATTTTAAAGT
GTAAT---GATAAGGAGTTCAAT------GGAACAGGACCATGCAGGGAT-----------------------
-GTCAGC---ACAGTACAATGCACACATGGAATCAAGCCAGTAGTATCAACT---
CAACTGCTGTTAATGGCAGTCTAGCAGAAAATAAG-------------
ACAATGATTAGATCTGAAAATATCACAAACAAT---------GCCAAAAACATAATAGTACAACTTACT----
-----------GAGCCTGTAAAC---------ATTACTTGTATCAGACCTAACAACAATACAAGAAAA-----
-AGTGTACATATA------------GGACCAGGACAGGCA---TTC------TATGCAACAGGTGAA------
---------ATAATAGGGGATATA------------AGACAAGCACATTGT---AATGTCAGTGAAACA---
GAATGGAATAACACTTTGCAAAAGGTAACCAACCAATTAAGA---------------ACATACTTTGGG----
-----------------------------------AACAAAACAATAATCTTTGCT------
AACTCCTCAGGAGGGGATCCAGAAATTATA---ACACATAGTTTTAATTGTGGAGGAGAA---
TTTAAACAGCTCCAGGCAAGAGTCCTGGCTGTGGAAAGATACTTAAGGGATCAACAGCTCCTAGGAATTTGGG
GCTGTTCTGGAGAACTCATCTGCACCACTAATGTGCCCTGGAACTCTACCTGGAGT-----------------
----------------------------AATAAATCCCTGAATGAGATATGG------GAC------
AACATGACCTGGCTGCAATGG---GATAAAGAAATTAGCAATTATACACAATTAATATAT---
GATCTAATTGAAGAATCGCAGAACCAGCAGGAA--

AAAAATGAACAAGACTTATTGGCATTGGACAAGTGGGCAAGTCTGTGGAGTTGGTTTAACATCTCAAAATGGC
TGTGGTATATAAAAATATTTATAATGATAGTAGGAGGCTTAATAGGATTAAGAATAGTTTTTGCT---
GTGCTTTCTATAATA---------CGTAGAGTTAGGCAGGGATACTCACCT------
GTGTCGTTTGCTATTAATTTGCTTGATACCATAGCAATAGCAGTAGCTGGC---
TGGACAGATAGGGTTATAGAAATAGGACAAGGAATTGGTAGAGCT----------------------------
--------ATTATCAACATA------CCTAGAAGAATCAGACAGGGCCTTGAAAGGGCTTTGCTA---TAA
>FJ388932
GCT---ACA---GAA------------------AAA---GTT---GCA---
GAAAATTTGTGGGTTACTGTCTATTATGGGGTACCTGTATGG---AAAGAAGCAAAC---
ACCACTTTATTTTGTGCATCAGATGCAAAAGCATATAAAACAGAA---GTGCATAAT----------------
--------------GTATGGGCTACA---CATGCCTGTGTACCTACAGAC---
CCCAACCCACAAGAAATAGATATAAAA---------AATGTGACAGAAAAGTTTAAC----------------

--------------------------ATGTGGAAAAATAACATGGTAGAGCAGATGAATACAGATATA---
ATCAGTCTATGGGACCAAAGCCTA---AAGCCATGTGTAAAGTTA---
ACCCCTCTCTGTGTTACTTTAGAATGTAGAGATGCCTCTAATACCACTAATGCC-------------------
----
-------------------------------ACTTTCACCGAGGGAATGGATAGAGAAATAAAA---
AACTGCTCTTACAACATGACCACAGAATTAAGG---------------------------
GATAAGAGACAGAAAGTATCTTCACTT---TTTTATAGACTTGATATAGTACCAATTGATAGTAATAGTAGG-
----

AATAGTAGTAGTAGTAATAGTAGTGAATATAAATTAATAAATTGTAGTACCTCATCCATTACACAA---
GCTTGTCCAAAGGTATCTTTTGAGCCAATTCTCATACATTATTGTGCTCCAGCTGGTGTTGCGATTCTAAAGA
GTAAT---GATAAGGAGGTCAAT------GGAGCAGGACCATGCAGGAAT-----------------------
-GTCGGC---ACAGTACAATGCAGACCTGGAATCAGGCCAGTAGTATCAACA---
CAACTGTTGTTAAATGGCAGTCTAGCAGAAAGCAAG------------
ATAATGATTAGATCTGAAAATATCTCAAACAAT---------GCTAAAAACATACTAGTACAATTTACT----
-----------GAGCCTGTAAAC---------ATTACTTGTATCAGACCTGGCAACAATACAAGAAGA-----
-GGTGTACATATA------------GGACCAGGACAGGCA---TTC------TATGCAGTAGGTGAC------
---------ATAATAGGAAATATA------------AGACAAGCACATTGT---AATGTCAGTAGAAGA---
AAATGGAGGAAACGTTGCAAAAGGTAGCCAGCCAATTAAGA----------------ACATACTTTGTG----
-----------------------------------AACAAAACAATAATCTTTACT------
AAATCCTCAGGAGGGGATCTAGAAATTACA---ACACATAGTTTTAATTGTGGAGGAGAA---
TTTAAACAGCTCCAGGCAAGAGTCCTGGCTCTGGAAAGATACCTAAGGGATCAACAGCTCCTAGGAATTTGGG
GCTGCTCTGGAAAACTCATCTGCACCACTAATGTGCCCTGGAACTCTACTTGGAGT-----------------
-------------------------AAGAATAAATCCCACGATGAGATATGG------AAT------
AATATGACCTGGCTGCAATGG---GAGAAAGAAATTGACATATATACACAAGACATATAT---
ACTCTAATTGAAGAGTCGCAGAACCAGCAGGAA--

ATAAATGAAAAGGAATTATTGGAATTGGACAAGTGGGCAAATCTGTGGAATTGGTTTAACATCACAAACTGGC
TGTGGTACATAAAAATATTTATAATAATAGTAGGAGGTTTAATAGGATTAAGAATAGTTTTTGCT---
GTGCTTGCTGTAATA---------AATAGAGTTAGGCAGGGGTACTCACCT------
TTGTCGTTTGCTATTAATTTGCTTGATACCATAGCAATAGCAGTAGCTGGC---
TGGACAGATAGGGTTATAGAAGTAGGACAAAGCATTTGTAGAGCT----------------------------
--------ATTTTCAACATA------CCTAGA---------------------------------------
>FJ388938
GCT---GCA--
GACAACTTGTGGGTTACTGTCTACTATGGGGTACCTGTGTGG---AAAGACGCAAAG---
ACCACTTTATTTTGTGCATCAGATGCGAAAGCATATAAAACAGAG---ATGCATAAT----------------
--------------GTCTGGGCTACA---CATGCCTGTGTACCTACAGAC---
CCCAACCCACAAGAAATAAATTTGGAA---------AATGTGACAGAAGAATTTAAC----------------
--------------------------ATGTGGAAAAATAACATGGTAGAGCAGATGCATACAGATATA---
ATCAGTCTATGGGACCAAAGCCTA---AAGCCATGTGTAAAGTTA---
ACCCCTCTCTGTGTTACTTTAAATTGCACCAATATCAAGAATATCACT-------------------------
----
-------------------------------------GACGATGAAATGAGAAGAGAAATAAAA---
AACTGCTCTTTCAACATGACCACAGAACTAAGG---------------------------
GATAAGCAAAAGAAAGTATATTCACTT---
TTTTATAGACTTGATGTAGTACCAATTAGGAATAATAAGAATAATAGT-------------------------
----
AGTGAGACTAATAGTAGTGAGGCTGGTGAATATAGATTAATAAATTGTAATACTTCAGCCATTACACAG---
GCTTGTCCAAAGGTATCTTTTGAGCCAATTCCCATACATTATTGTACTCCAGCTGGTTTTGCGATTCTAAAGT
GTAAT---GATAAGGAGTTCAAT------GGAACAGGATTATGCAGGAAT-----------------------
-GTCAGC---ACAGTACAATGCACACATGGAATCAGGCCAGTAGTATCAACT---
CACCTGCTGCTAAATGGCAGTCTAGCAGAAAATAGG------------
ACAATGATTAGATCTGAAAATATCACAAACAAT---------GCCAAAAACATAATAGTACAACTTACT----
-----------GAGCCTGTAAAC---------ATTACTTGTATCAGACCTAACAATAATACAAGAAAA-----
-AGTGTACATATA------------GGACCAGGACAAGCA---TTC------TATGCAACAGATGAC------
---------ATAATAGGGGATATA------------AGAAAAGCACATTGT---AATGTCAGTAGAACA---
AAATGGAATAATACTTTGAAAGAGGTAGTCAACCAATTAAGA---------------AAATACTTTGGG----
--------------------------------AACAACAAAACAATAATCTTTAAT------
AGCTCCTCAGGAGGGGATCTAGAAATTACA---ACACATAGTTTTAACTGTGGAGGAGAA---

TTTAAACAGCTCCAGGCACGAGTCCTGGCTGTGGAAAGATACCTAAAGGATCAACAGCTCCTAGGAATTTGGG
GCTGCTCTGGAAAACTCATCTGCACCACTAATGTGCCCTGGAACTCTACTTGGAGT-----------------
----------------------------AATAAAACCCAGGAGGAGATATGG------GAC------
AACATGACCTGGCTGCAATGG---GATAAAGAAATTAGCAATTATACAAAAACCATATAT---
GAGTTAATTGAAAAATCGCAAAACCAGCAGGAA--

ATAAATGAACAAGACTTATTAGCATTGGACAAGTGGGCAAACCTGTGGACTTGGTTTGACATCTCAAACTGGC
TGTGGTATATAAGAATATTTGTAATGATAGTAGGAGGCTTAATAGGATTAAGAATAGTTTTTGCT---
GTGCTCAATATAATA---------AATAGAGTTAGGAAGGGATACTCACCT------
CTGTCGTTTGCTATTAGTTTGCTTGATACCATAGCAATAGCAGTAGCTGGC---
TGGACAGATAGGGTTATAGAAATAGGACAAAGAATTTGTAGAGCT----------------------------
--------ATTTGCAACATA------CCTAGAAGAATCAGACAGGGCCTCGAAAGGGCTTTGCTA---TAA
>FJ388942
GCT---GCA--
GAAAACTTGTGGGTTACTGTCTACTATGGGGTACCTGTGTGG---AAAGACGCAGAG---
ACCACCTTATTTTGTGCATCAGATGCGAAAGCATATGACACAGAA---GTGCATAAT----------------
--------------GTCTGGGCTACA---CATGCCTGTGTACCCACGGAC---
CCCAACCCACAAGAAATAAATTTGGAA---------AATGTGACAGAAGAGTTTAAC----------------
--------------------------ATGTGGAAAAATAACATGGTAGAACAAATGCATACAGATATA---
GTCAGTCTATGGGACCAAAGCCTA---AAGCCATGTGTAGAGTTA---
ACCCCTCTCTGTGTTACTTTGAATTGTAGCCATAATGTCACTGCGAATGACACCGTT----------------
----
-------------------------------AGCATAAAAGAAGGAATGGAAGGAGAAATAAAA---
AACTGCTCTTTCAATGTAACCACAGAACTAAGG---------------------------
GATAAGACACAGAAAGTATATTCACTT---TTTTATAGACCTGATGTAGAACAAATTGATAAT----------
----

AGTAGTAGTTATAGATTAATAAATTGTAATACCTCAGCCATTGCAAAA---
GCTTGTCCAAAGGTAACTTTTGAGCCAATTCCCATACATTATTGTGCTCCAGCTGGTTTTGCAATTTTAAAGT
GTAAT---GATAAGGAGTTTAAT------GGAACAGGACCATGCAGGAAT-----------------------
-GTCAGC---ACAGTACAATGCACACATGGAATCAGGCCAGTAGTATCAACT---
CAACTGCTGTTAAATGGCAGTCTAGCAGAAAATAAG------------
ACAATGATTAGATCTGAAAATATCACAAGCAAT---------ACCAAAAACATAATAGTACAACTTACC----
-----------GAGCCTGTAAAT---------ATTACTTGTATCAGACCTAACAACAATACAAGAAAA-----
-AGTATACATATG------------GGACCAGGACGAGCA---TTC------TATGCAACAGGTGAA------
---------ATAATAGGGGACATA-----------AGGCAAGCACATTGT---AATGTCAGTAGAGCA---
AAATGGAATAACACTTTGCAAAAGGTAGTTGACCAATTAAGA---------------ACATACTTTGGG----
-----------------------------------AACAAAACAATAACCTTTACT---
AGCAACGCCCCAGGTGGGGATCTAGAAATTACA---ACACATAGTTTTAACTGTGGAGGAGAA---
TTTAAACAGCTCCAGGCAAGAGTCCTAGCTGTGGAAAGATACCTAAGGGATCAACAGCTCCTAGGAATTTGGG
GCTGCTCGGGAAAACTCATCTGCACCACTAATGTGCCCTGGAACTCTACTTGGAGT-----------------
----------------------------AATAAATCCCTGGATGACATATGG------GAA------
AACATGACCTGGATGCAGTGG---GATAAAGAAATTAGCAATTATACACAATTAATTTAT---
AATCTAATTGAAGAATCGCAGAACCAGCAGGAA--

AAAAATGAAAAAGATTTATTGGCATTGGACAAGTGGGCAAGTCTGTGGAATTGGTTTAACATCTCAAACTGGC
TGTGGTATATAAAAATATTTATAATGATAGTAGGAGGCTTAATAGGGTTAAGAATAGTTTTTGCT---
GTGCTTTCTATAATA---------CGTAGAGTTAGGCAGGGATACTCACCT------
CTGTCGTTTGCTATTAATTTGTTTGACACCATAGCAATAGCAGTAGCTGGC---
TGGACAGATAGAGTTATAGAAATAGGACAAAGAATTTGTAGAGCT----------------------------
--------ATTATCAACATA------CCTAGAAGAATCAGACAGGGCCTTGAAAGGGCTTTGTTA---TA-
>FJ388946
GCT---ACA--
GAAAATTTGTGGGTTACTGTCTACTATGGGGTACCTGTATGG---AGAGAAGCAGAC---
ACCACTTTATTTTGTGCATCAGATGCGAAAGCATATGAACCAGAA---GTGCATAAT----------------
--------------GTCTGGGCTACA---CATGCCTGTGTACCTACAGAC---
CCCAACCCACAAGAAATAAATATGAAA---------AATGTGACAGAAGAGTTTAAC----------------
--------------------------ATGTGGAAAAATAACATGGTAGAGCAAATGCATACAGATATA---
ATCAGTTTATGGGACCAAAGCCTA---AAGCCATGTGTAAAGTTA---
ACCCCTCTCTGTGTTACTTTAGAATGTAGAGATGTAAATGTCAATAGTACCAATACTACTACCAATGCC----

----
----------------ACCATTGAAAGGAGAGGAGAAATAGCTGAAGAGCCAGGAGAAATAAAA---
AACTGCTCTTTCAACATGACCACAGAATTAAGG---------------------------
GATAAGAGACAGAAAGTATACTCACTT---TTTTATAGACTTGATATAGTACCAATTAATAGTAATAGTATT-
----

AATAGTAGTAGTAGTGATAGTAGTGAATATAGATTAATAAATTGTAATACCTCAGCCATTACACAG---
GCTTGTCCAAAGGTATCTTTTGAGCCAATTCCCATACATTATTGTGCTCCAGCCGGTTTTGCGATTCTAAAGT
GTAAT---GATAAGGAGTTCAAT------GGAACAGGACTATGCAGGAAT-----------------------
-GTCAGC---ACAGTACAATGCACACATGGAATCAGGCCAGTAGTATCAACT---
CAACTGCTGTTAAATGGCAGCCTAGCAGAAAACAGG------------
ACAATGATTAGATCTGAAAATATCTCAGACAAT---------GCTAAAAACATAATAGTACAATTTACT----
-----------GAGCCTGTAAAC---------ATTACTTGTATCAGACCTAACAACAATACAAGAAAA-----
-AGTGTACGTATA------------GGACCAGGACAAGCA---TTC------TATGCAACAGGTGAC------
---------ATAATAGGGAATATA------------AGACAAGCATATTGT---GAGGTCAATGGAAGT---
CAATGGAATAAAGCTTTGCAAAAGGTAGTCAACCAATTAAAA---------------ACACACTTTGAG----
--------------------------------AACGCAACAACAATAACCTTTAAT------
AAATCCTCAGGAGGGGATCTAGAAATTACA---ACACATAGTTTCAATTGTGGAGGAGAA---
TTTAAACAGCTCCAGGCAAGAGTCCTGGCTCTGGAAAGATACCTAAGGGATCAACAGCTCCTAGGAATTTGGG
GCTGCTCTGGAAAACTCATCTGCACCACTAATGTGCCCTGGAACTCTACTTGGAGT-----------------
----------------------------AATAAATCCCTAGATGAGATATGG------AAG------
AATATGACCTGGCTGCAATGG---GATAAAGAAATTAACAATTATACACAATTAATATAT---
AATTTAATTGAAGAATCGCAGAACCAGCAGGCA--

ATAAATGAAAAGGAATTGTTGGAATTGGACAAGTGGGCAAGTCTGTGGAGTTGGTTTGACATCACAAATTGGC
TGTGGTATATAAAAATATTTATAATGATAGTAGGAGGCTTAATAGGATTAAGAATAGTTTTTGCT---
GTGCTTGCTGTAATA---------AATAGAGTTAGGCAGGGATACTCACCC------
TTGTCGTTCACTATTAATTTTCTTGATACCATAACAATAATAGTAGCTGCC---
TGGACAGATAAGATTATAAAAATAATACAAAACATCTGTAGA-------------------------------
----
>JF683737
GCT---ACA---
AACATGTGGGTTACTGTATACTATGGGGTACCTGTGTGG---AAAGATGCAGAG---
ACCACCTTATTTTGTGCATCAGATGCGAAAGCATATAAAACAGAA---GTGCATAAT----------------
--------------GTCTGGCTACA---CATGCCTGTGTACCTACAGAC---
CCCAACCCACAAGAAATAAATATGGAA---------AATGTGACAGAAGAGTTTAAC----------------
--------------------------ATGTGGAAAAATAACATGGTAGAACAGATGCATACAGATATA---
ATTAGTCTATGGGACCAAAGTCTA---AAGCCATGTGTAAAGTTA---
ACCCCTCTCTGTGTAACTTTAGAATGTAGCAATGTCACCACCAATATCACC----------------------
----
--GGAGAAATGAAAGAGGAAATAAAC---
AGCTGCTCTTTCGACGTGACCACAGTACTAAGG---------------------------
GATAAAAGAAAGAAATCTTATTCCCTC---TTTTATATACTTGATATACCACCTGTTAATAGTAATAGTAAT-
----

AGTGATAATAAAAATAGTATGGAGTATAGATTAATAAATTGTAATACCTCAGTCATTACACAG---
GGTTGTCCAAAGGTATCTTTTGATCCAATTCCCATACATTATTGTGCTCCTGCTGGTTTTGCGATTCTAAAGT
GTGAT---GATAAGGAGTTCAAT------GGAACAGGACCATGCAATAAT-----------------------
-GTCAGC---ACACAACAATGCACACATGGAATCAGGCCAGTAGTGTCAACT---
CAGTTGCTGTTAAATGGTAGTTTAGCAGAAAATAAG------------
ACAATGATTAGATCTGAAAATATTACAAACAAT---------GCCAAAAACATAATAGTACAACTTACC----
-----------AAGCCTGTAAAC---------ATTACTTGTATCAGACCTGGCAATAATACAAGACAA-----
-AGTATACATATA------------GGACCAGGACAAGCC---TTC------TATTCAAGAGATGAT------
---------ATAACAGGGGACATA------------AGAAAAGCACATTGT---GAAGTCAATGGAACA---
GAATGGGATAAAACTTTGCAAGAGGTAGCAAAACAATTAAGA---------------ACATACTTTGAG----
-----------------------------------AACAAAACAATAATCTTTAAT------
GGCTCTTCAGGAGGAGATCTAGAAATTACA---ACACATAGTTTTAATTGTAGAGGAGAA---
TTTAAACAGCTCCAGGCAAGAGTCCTGGCTGTGGAAAGATACCTAAAGGATCAACAGCTCCTAGGAATTTGGG
GCTGCTCTGGAAAACTCATCTGCACCACTAATGTGCCCTGGAACTCCACTTGGAGT-----------------
----------------------------AATAAATCCCTGGATGAGATATGG------GAC------

AACATGACCTGGCTGCAATGG---GATAGAGAAATTAGCAATTATACACAAATAATATAT---
AATCTAATTGAAGAATCGCAGACCCAGCAGGAA--

ATGAATGAAAAAGACTTATTGGCATTGGACAAGTGGGCAAATCTGTGGAGTTGGTTTGACATCTCAAATTGGC
TGTGGTATATAAAAATATTTATAATGATAGTAGGAGGCTTAATAGGATTAAGAATAGTTTTTGCT---
GTGCTTTCTGTAATA---------AAGAAAGTTAGAAAGGATACTCACCT------
CTGTCATTTGCTATTAGTCTGCTTAATACCACGGCAATAGTAATAGCTGGC---
TGGACAGATAGAGTTATAGAAATAATACAAAGAATTTATACAGCT----------------------------
--------ATTTTCAACATA------CCTAGAAGAATCAGACAGGGCTTAGAAAGGGCTTTGCTA---TAA
>JF683759
GCT---GCA--
GGAAACTTGTGGGTTACTGTCTACTATGGGGTACCTGTGTGG---AAAGATGCAGAC---
ACCACCTTATTTTGTGCATCAGATGCGAAAGCAATTAAAACAGAA---GTGCATAAT----------------
--------------GTCTGGGCTACA---CATGCCTGTGTACCTACAGAC---
CCCAACCCACAAGAAATAAATTTGGAA---------AATGTGACAGAAGATTTTAAC----------------
--------------------------ATGTGGAAGAATAACATGGTAGAGCAGATGCAGACAGATATA---
ATCAGTCTATGGACCAAAGCCTA---AAGCCATGTGTAAAGTTA---
ACCCCTCTTTGTGTTACTCTAGATTGTAGCCACAATATCACTTCTAATAATACTGATAAT-------------
----
----------------------------AATCCTAATAATACCAAAGTGGAAGTGGAAATGAGA---
AACTGCTCTTTCAACACGACCACAGAAATAAGG---------------------------
GATAGGACGCAGAAAGTATATTCACTT---TTTTATAAAGTTGATGTAGTAGAAATTGATAGTAAT-------
----

AGTAATAATAGTAATACTAATAATTATAGATTAATAAATTGTAATACCTCAGTAATTACACAG---
GCTTGTCCAAAGGTAACTTTTGAGCCAATCCCCATACATTATTGTGCTCCAGCTGGTTTTGCAATCCTAAAGT
GTAAT---GATAAGGAGTTCAAT------GGAACAGGACCATGCAAGAAT-----------------------
-GTCAGC---TCAGTACAATGCACACATGGAATCAAGCCAGTAGTATCAACT---
CAACTGTTGTTAAATGGTAGTCTAGCAGAAAATAAA------------
ACAATGATTAGATCTGAAAATATAACAAACAAT---------GCCAAAAACATAATAGTACAACTTACT----
-----------AAGCCTGTACAA---------ATTAATTGTACCAGACCTAACAACAATACAAGAAAA-----
-AGTGTACGTATA------------GGACCAGGACAAGCA---TTC------TATGCAACAGGTGAA------
---------ATAATAGGGGATATA------------AGACAAGCACATTGT---AATGTCAGTAAGGCA---
GAATGGCATGGAGCTTTGCAAGAGGTAGGCAAACAATTAGGA---------------ACATATTTTAAG----
-----------------------------------AACAAAACAATAATCTTTACT------
AACTCATCAGGAGGGGATCTAGAAATTACT---ACACATAGTTTTAATTGTGGAGGAGAA---
TTTAAACAGCTCCAGGCAAGAGTCCTGGCTGTGGAAAGATACCTAAAGGATCAACAGCTCCTAGGAATTTGGG
GCTGCTCTGGAAAACTCATCTGCACCACTAATGTGCCCTGGAACTCTACTTGGAGT-----------------
----------------------------AATAAATCCCAGGATGAGATATGG------GAC------
AACATGACCTGGTTGCAATGG---GATAAAGAAATTAGCAATTATACACAAGCAATATAT---
ACTCTAATTGAAGAATCTCAAAATCAGCAGGAA--

ATGAATGAAAAAGAATTATTGGAATTGGACAAGTGGGCAAGTCTGTGGAATTGGTTTGACATCTCAAACTGGC
TGTGGTATATAAAAATATTCATAATGATAGTAGGAGGTTTAATAGGGTTAAGAATAGTTTTTGCT---
GTGCTTTCTATAGTA---------AATAGAGTTAGGCAGGATACTCACCT------
TTGTCATTCGCTATTAATTTGGTTGATACCATAGCAATAACAGTAGCTGGC---
TGGACAGATAGGGTTATAGAAGTAGGACAAAGAATTTGCAGAGCT----------------------------
--------ATTATCAACATA------CCTAGAAGAATCAGACAGGGCCTCGAAAGGGCTTTGCTA---TAA
>JF683760
GCT---GCA--
GAAAAAACGTGGGTTACTGTCTACTATGGGGTACCTGTTTGG---AAAGATGCAGAG---
ACCACCTTATTTTGTGCATCAGATGCAAAAGCCTATAAAACAGAA---GTGCATAAT----------------
--------------GTTTGGGCTACA---CATGCCTGTGTACCTACAGAC---
CCTAACCCACAAGAAATGCATTTGGAA---------AATGTGACAGAAGAGTTTAAC----------------
--------------------------ATGTGGAAAAATAACATGGTAGAGCAGATGCATACAGATATA---
ATCCGTCTATGGACCCAAGCCCA---CAGCCCTGTGTAAAGTTA---
ACCCCTCTCTGTGTTACTTTAAATTGTAGCTACAATATCACTAGTAATGTCACTGTTGAC-------------
----
----------------------------ATGAATGATACCATTAACATGAGAGAAGAAATAAAA---
AACTGTTCTTTCTATACAACCACCGAAATAAGG---------------------------

GATAATAGACAGAAAGTATATTCACTT---TTTTATAGACTTGATATAGTACCAATTGGTGAT----------
----

AGGAATAATAATCATAGTTATAGATTAATAAATTGTAATACCTCAGCCATTACACAG---
GCTTGTCCAAAGGTAACTTTTGAGCCAATTCCCATACATTATTGTGCTCCAGCTGGTTTTGCAATTCTAAAGT
GTAAT---GATAAGAATTTCAAT------GGAGCAGGATCATGCAAGAAT-----------------------
-GTCAGC---ACAGTACAATGCACACATGGAATCAAGCCAGTAGTATCAACT---
CAACTGCTGTTAAATGGCAGTCTAGCAGAAAATGAG------------
ACAATGATTAGATCTGAAAATATCACAAACAAT---------GTCAAAAACATCATAGTACAATTTAAT----
-----------GAGTCTGTAAAA---------ATTAATTGTACCAGACCGAACAACAATACAAGAAAA-----
-AGTGTACATATA------------GGACCAGGACAAGCA---TTC------TATGCAACAGGTGAA------
---------ATAGTAGGGGATATA------------AGACAAGCACGTTGT---GAGGTCAATAAAACA---
GAATGGAATAAAGCTTTAAAAAGGGTAGCCCACCAATTAGAA---------------AAATACTTTAAG----
------------------------------------AACAAAACAATAGCCTTTAAT------
AAATCCCCAGGAGGAGATCTAGAAATTACA---ACACATAGTTTTAATTGTGGAGGGGAA---
TTTAAACAGCTCCAGGCAAGAGTCCTGGCTGTGGAAAGATACCTAGGGGATCAACAGCTCCTAGGAATTTGGG
GCTGCTCTGGAAAACTCATCTGCACCACTAATGTACCCTGGAACTCTACTTGGAGT-----------------
----------------------------AATAAGACCCGCAGAGAGATATGG------GAC------
AACATGACTTGGATGCAATGG---GATAAGGAAATTAGCAATTATACACAAGTAATATAT---
ACTCTAATTGAAGAATCGCAGAACCAGCAAGAA---

AAAAATGAGCAAGACTTATTGGCATTGGGCAAGTGGGACAGTCTGTGGAGTTGGTTTAGCATCACAAACTGGC
TGTGGTATATAAAAATATTTATAATGATAGTAGGAGGCTTAATAGGATTAAGAATAGTTTTTGCT---
GTAATTTCTATAATA---------CATAGAGTTAGGCAGGGATACTCACCT------
TTGTCATTTGATATTAGTTTGCTTGATTCCATAGCAATAGCAGTAGCTGCC---
GGGACAGATAGGGTTATAGAAATAGGACAGAGAATTTGTAGAGCT-----------------------------
--------ATTGTTATCATA------CATAGTAGAATCAGATATGGCCTTGAAAGAGCTTTGCTA---TAA
>JF683761
GCT---GCA--
GATAAAATGTGGGTTACTGTCTACTATGGGGTACCTGTTTGG---AAAGATGCAGAG---
ACCACCTTATTTTGTGCATCAGATGCAAAAGCATATAAAACAGAA---GTGCATAAT----------------
--------------GTTTGGGCTACA---CATGCCTGTGTACCTACAGAC---
CCTAACCCACAAGAAATGCATTTGGAA---------AATGTGACAGAAGAGTTTAAC----------------
--------------------------ATGTGGAAAAATAACATGGTAGAGCAGATGCATACAGATATA---
ATCAGTCTATGGGACCAAAGCCTA---AAGCCATGTGTAAAGTTA---
ACCCCTCTCTGTGTTACTTTAAATTGTAGCTACATCACTAGTAATGTCACTGCTGACATCACTAGTAAT----
----
----------------GTCACTGCTGACATGAATGATGCCATTAACATGACAGCAGAAATAAGA---
AACTGTTCTTTCAATATAACCACAGAGATAAGG---------------------------
GATAAGATACAGAAAGTATATTCACTT---TTTTATAGACTTGATATAGTACCAATTGATAGG----------
----

AATGATAGTAATGCTAGTTATAGATTAATAAATTGTAATACCTCAGCCATTACACAG---
GCTTGTCCAAAGGTAACTTTTGAGCCAATTCCCATACATTATTGTGCTCCAGCTGGTTTTGCAATTCTAAAGT
GTAAT---GATAAGGATTTCAAT------GGAACAGGACCATGCAGGAAT-----------------------
-GTCAGC---ACAGTACAATGCACACATGGAATCAAGCCAGTAGTATCAACT---
CAACTGCTGTTAAATGGCAGTCTAGCAGAAAATGAG------------
ACAATGATTAGATCTGAAAATATCACAAACAAT---------GCCAAAAACATAATAGTACAACTTAAT----
-----------GAGCCTGTAAAA---------ATTAATTGTACCAGACCTAACAACAATACAAGAAAA-----
-AGTATACGTATA------------GGACCAGGACAAGCA---TTC------TATGCAACAGGTGAC------
---------ATAGTAGGGAATATA------------AGACAAGCACATTGT---GAGGTCAATAAAACA---
GAATGGAATAAAGCTTTAAAAAGGGTAGCCGACCAATTAAGA---------------AAATACTTT-------
------------------------------------AACAAAACAATAGCCTTTAAT------
AAATCCTCAGGAGGAGATCTAGAAATTACA---ACACATAGTTTTAATTGTGGAGGGGAA---
TTTAAACAGCTCCAGGCAAGAGTCCTGGCTGTGGAAAGATACCTAGGGGATCAACAGCTCCTAGGACTTTGGG
GCTGCTCTGGAAAACTCATCTGCACCACTAATGTACCCTGGAACTCTACTTGGAGT-----------------
----------------------------AATAAGTCCCACAAAGCCATATGG------GAC------
AACATGACTTGGATGCAATGG---GATAAAGAAATTAGCGATTATACACAAGTAATATAT---
ACTCTAATTGAAGAATCGCAGAACCAGCAAGAA---

```
AAAAATGAGCAAGACTTATTGGCATTGGACAAGTGGGACAGTCTGTGGAGTTGGTTTAGCATCACAAAATGGC
TGTGGTATATAAAAATATTTATAATGATAGTAGGAGGCTTAATAGGATTAAGAATAGTTTTTGCT---
GTAATTTCTATAATA---------CGTAGAGTTAGGCAGGGATACTCACCT------
TTGTCATTTGCTATTAGTTTGCTTGATACCACAGCAATAGCAGTAGCTGGC---
TGGACAGATAGGGTTATAGAAACAGGACAGACAATTTGTAGAGCT----------------------------
--------ATTGTTAACATA------CCTAGAAGAATCAGACAGGGTCTTGAAAGAGCTTTGCTA---TAA
>JF683767
GCT---GCA------------------------------------------
CAAAACTTGTGGGTTACTGTCTATTATGGGGTACCTGTGTGG---AAAGATGCAGAG---
ACCACCTTATTTTGTGCATCAGATGCTAAAGCATATGAGACAGAA---AAGCATAAT----------------
--------------GTCTGGGCTACA---CATGCCTGTGTGCCCACAGAC---
CCCAACCCACAAGAAATACCTTTGGAA---------AATGTGACAGAAAATTTTAAC----------------
--------------------------ATGTGGAAAAATAATATGGTAGAGCAGATGCATACAGATATA---
ATCAGTCTATGGGATCAAAGTCTA---CAGCCATGTGTAAAGTTA---
ACCCCTCTCTGCGTTACTTTAAATTGTACAAATGTCAGAAATGTCACAGGAAATAGCACGGGTAGCAGCTTC-
-------------------------------------------------------------------------
----------------ACGGGGGGGCCTGAAGAACTAAAAACGGGGGGGCCTGAAGAACTAAAA---
AACTGCTCTTTCAATATGACCACAGTACTAAGG---------------------------
GATAAGAAACAGAAAGTATATTCACTT---
TTTTATAGACTTGATATAGTACAAATAGATAAGGATAATAATAGT----------------------------
-----------------------------------------------------
TCTGGTAATAATAGTTATAATGAATACAGATTAATAAATTGTAATACCTCAGCCATTACACAA---
GCCTGCCCAAAAGTAAGCTTTGAACCAATTCCCATACATTATTGTGCTCCAGCTGGTTTTGCGATCCTAAAGT
GTAGG---AATAATTCATTCGAT------GGAACAGGGCCATGCAATAAT-----------------------
-GTCAGC---ACAGACCAATGTACTCATGGAATCAAGCCAGTAGTATCAACT---
CAACTGCTGTTAAATGGCAGTCTAGCAGAAGAAAAG------------
ATAATGATTAGATCTGAAAACATCACAAACAAT---------GCCAAAACTATAATAGTACAACTTGTC----
-----------AAACCTGTGACA---------ATTAATTGTACCAGACCTAATAACAATACAAGAAAA-----
-ATTGTACGTATAGGA------CCAGGAGGACCAGGACAA---GCA------TTCTATACAAATGAC------
---------ATAATAGGGGATATA------------AGACGAGCATATTGT---ACTGTCAATGCATCA---
GAGTGGAATGAAACTTTAGAACAAGTAGTTGAACAGTTGAGA---------------GTACACTTTGGG----
-----------------------------------AACAAAACAATAAACTTTACT------
AAACCCTCAGGAGGGGATCTAGAAATCACA---ACACATAGTTTTAATTGTGGAGGAGAA---
TTTAAACAGCTCCAGGCAAGAGTCCTGGCTGTGGAAAGATACCTAAAGGATCAACAGCTCCTAGGAATTTGGG
GCTGCTCTGGAAAGCTCATCTGCCCCACTAATGTGCCTTGGAACTCTAGTTGGAGT-----------------
----------------------------AATAAATCTATGGAGGACATATGG------GAC------
AATATGACCTGGCTGCAATGG---GAGAAAGAAATTAGCAACTACACAGACATAATATAT---
AACCTAATTGAAGAATCACAGAACCAGCAGGAA----------------------------------------
-----------------------------
AAGAATGAACAAGACTTATTGGCATTGGACAAGTGGGCAAATTTGTGGAATTGGTTTGAAATATCAAAATGGC
TGTGGTATATAAAAATATTTATAATGATAGTAGGAGGCTTAATAGGATTAAGAATAGTTTTTGCA---
GTGCTTTCTATAATA---------AATAGAGTTAGGCAGGGATACTCACCT------
TTGTCATTTGCTACTAATTTGTTTGATACCATAGCAATAGCAATAGCTGGC---
TGGACAGATAGAATTATAGAAATAGGGCAAAGAATTGTTAGAGCT----------------------------
--------TTTCTCAACATA------CCCAGAAGAATCAGACAGGGCTTTGAAAGGGCTTTATTA---TAA
>JF683779
GCT---GAA------------------------------------------
AATAACTTGTGGGTTACTGTCTACTATGGGGTACCTGTATGG---AAAGAAGCAGAC---
ACCACCTTATTTTGTGCATCAGATGCGAAAGCATATAAAACAGAA---GTGCATAAT----------------
--------------GTCTGGGCTACA---CATGCCTGTGTACCTACAGAC---
CCCAACCCACAAGAAATAAATATGGCA---------AATGTGACAGAAAAGTTTAAC----------------
--------------------------ATGTGGAAAAATAACATGGTAGAGCAGATGCATACAGATATA---
ATCAGTCTCTGGGACCAAAGCCTG---AAGCCATGTGTAAAGTTA---
ACCCCTCTCTGTGTTACTTTAAATTGTAACAATGTCAACAATACCAGTAATACCAATAAT-------------
-------------------------------------------------------------------------
-------------------------ACCACCAATGTCACCGCTGAAATGCGAGAAGAGATAAAA---
AACTGCTCTTTCAATGCAACCACAATACTAAGG---------------------------
GATAAGACACAGAGAATACATTCACTT---TTTTATAGACTTGATATAGTACCAATTAATAGT----------
-------------------------------------------------------------------------
-------------
```

AATAATAGTAATAATAGTGAAAATAGATTAAAAAATTGTAATACCTCATCCATTACTCGG---
GATTGCTCAAACGTATCTTTTGATCGACTTCCCGTACCTTATGGTGCTCCTGCTGGTTTTGCGAGTCTTCAAT
GTGAT---GTTAAGGATTTAAAT------GGAACAGGAATAGGTAACAAT----------------------
-GTCACC---AGAATACATTGCACTGATGGAATCAGGCCAGTGGTATCAACT---
CAACTGCTGTTGAATGGCAGTCTAGCAGAAAATAGG-----------
ACAATGATTAGATCTGAAAATATCACAAACAAT---------GCCAAAAACATAATAGTACAATTTAAC----
-----------ACGACTGTAAAC---------ATTACCTGTATCAGACCTAACAACAATACAAGAACA-----
-AGTGTACGTATA------------GGACCAGGACAAGCA---TTC------TATGCAACAGGTGAC------
---------ATAATAGGGGATATA------------AGACAAGCACATTGT---AATGTCAGTGGAGAA---
CAATGGAATAAAGCTTTGCAAAAGGTAGTCAAAGAATTAAGA---------------ATACATTTT-------
-----------------------------------AACAAAACAATAATCTTTACG------
AAATCCTCAGGAGGGGATCCAGAAATTACA---ACACATAGTTTTAATTGTGGAGGAGAA---
TTTAAACAGCTCCAGGCAAGAGTCCTGGCTCTGGAAAGATACCTACAGGATCAACAGCTCCTAGGAATTTGGG
GCTGCTCTGGAAAACTCATCTGCACCACTAATGTGCCCTGGAACTCTACTTGGAGT-----------------
----------------------------AATAAAAACTTGACTCAAATATGG------GAT------
AACATGACCTGGCAGCAATGG---GATAAAGAAATTGACAATTATACACAAGTAATATAT---
AATCTACTTGAAGAATCGCAGAACCAGCAGGAA---------------------------------------
----
ATGAATGAAAAAGACTTATTGGAATTGGACAAATGGGCAAATCTATGGAATTGGTTTAGCATCACAAACTGGC
TGTGGTATATAAGATTATTTATAATGATAGTAGGAGGCTTAATAGGATTAAGAATAGTTTTTGCT---
GTGCTTTCTGTAATA---------AATAGAGTTAGGCAGGGATACTCACCT------
TTGTCTTTTGCTATTAGTTTGGTTGATACCATAGCAGTAGTAGTAGCTGGC---
TGGACAGATAGGGTTATAGAAATAGGACAAAGAATTGGTAGAGCT----------------------------
--------ATCTTTAACATA------CCTAGAAGAATCAGACAGGGCTTAGAAAGGGCTTTGCTA---TAA
>JF683782
GCT---GTA---
GAAAAAACGTGGGTTACTGTCTACTATGGGGTACCTGTGTGG---AAAGATGCAGAA---
ACCACCTTATTTTGTGCATCAGATGCAAAAGCATATACAACAGAA---ATGCATAAT----------------
--------------GTCTGGGCTACA---CATGCCTGTGTACCTACGGAC---
CCCAACCCACAGGAAATAAATTTGGAA---------AATGTGACAGAAGAATTTAAC----------------
-------------------------ATGTGGAAACTTAACATGGTAGAGCAAATGCATACAGATATA---
ATCAGTCTATGGGATCAAAGCCTA---AAGCCATGTGTAAAGTTA---
ACCCCTCTCTGTGTCACTTTAGATTGTGGATATAATGTCAGTATTAATAACACCTAT----------------
----
--------------------------------AGCATAAATGATACCATGGAAACAGATTTAAGA---
AACTGCTCTTTCGATGTAACCACGAAATTAAGG--------------------------
GATAAGAGACAGAATGTATATTCACTT---TTTTATAGACTTGATATAGTACACATTGATAGT----------
----

AATAGTAATAATAGCCATTATAGATTAATAAATTGTAATACCTCAGCCATTACACAA---
GCTTGTCCAAAGGTATCTTTTGAACCAATTCCTATACATTATTGTGCTCCAGCTGGTTTTGCGATTCTAAAGT
GTAAT---GATAAGGAGTTCAGT------GGAAAAGGACCATGCAAGAAT----------------------
-GTTAGC---ACGGTACATTGCACACATGGAATCAGGCCAGTAGTATCAACT---
CAACTGCTGTTAAATGGCAGTCTAGCAGAAAATAGG-----------
ACAATGATTAGATCTGAAAATATCACAAACAAT---------GCCAAAAACATAATAGTACAACTTACT----
-----------GAGCCTGTAAAC---------ATTACTTGTATCAGACCTAGCAACAATACAAGAAAA-----
-AGTGTACATATA------------GGACCAGGACAAGCA---TTC------TTTGCAACAGGTGAT------
---------ATCATAGGGGACATA------------AGAAAAGCCCATTGT---GAGGTCAATGAAACA---
GAATGGAATAAAGCTTTAACAAAGGTAGTTGAACAATTAAGA---------------ACATACTTTAGG----
-----------------------------------AACAAAACAATAACCTTTAAA------
GGCCCCTCAGGAGGGGATCTAGAAATTACA---ACACATAGTTTTAATTGTGGAGGAGAA---
TTTAAACAGCTCCAGGCAAGAGTCCTGGCTGTGGAAAGATACCTAGGGGATCAACAGCTCCTAGGAATTTGGG
GCTGCTCTGGAAAACTCATCTGCACCACTAATGTGCCCTGGAACTCTACTTGGAGT-----------------
----------------------------AATAAATCCCTGGATAAGATATGG------AAC------
AACATGACCTGGATGCAATGG---GACAAAGAAATTAGCAATTATACACAATTAATATAT---
ACTCTAATTGAACAATCGCAGAACCAGCAGGAA---------------------------------------
----
ATAAATGAACAAGAATTATTGGAATTGGACAAGTGGGCAAATCTGTGGAATTGGTTTGACATCTCACACTGGT
TGTGGTATATAAAAATATTTATAATGATAGTAGGAGGCTTAATAGGATTAAGAATAGTTTTTGCT---
GTGCTTTCTATAATA---------CGTAGGGTTAGGCAGGGATACTCACCT------

TTGTCATTTGCTATTAGTTTGCTTGATACCATAGCAATAGCAGTAGCTAAC---
TGGACAGATAGAATTATAGAAATAGGACAAGGAATTTGTAGAGCT----------------------------
--------ATTATCAACATA------CCTAGAAGAATCAGACAGGGCCTTGAAAGGGCTTTGCTA---TAA
>JF683783
GCT---GTA--
GAAAAAACGTGGGTTACTGTCTACTATGGGGTACCTGTGTGG---AAAGATGCAGAG---
ACCACCTTATTCTGTGCATCGGATGCAAAAGCATATACAACAGAA---GCGCATAAT----------------
--------------ATCTGGGCTACA---CATGCCTGTGTACCTACGGAC---
CCCAACCCACAGGAAATAAATTTGGAA---------AATGTGACAGAAGAGTTTAAC----------------
--------------------------ATGTGGAAACTTAACATGGTAGAGCAGATGCATACAGATATA---
ATCAGTCTATGGGATCAAAGCCTA---AAGCCATGTGTAAAATTA---
ACCCCTCTCTGTGTTACTTTAGATTGTAGACATAATGTCACTATACATAATAATGTCACTAATAATAAT----
----
-------------------AATATCACCAGTGGCATAAATGATACCATTGCCACAGAAGTAAAA---
AACTGCTCTTTCAATACAACCACAGAATTAAAA---------------------------
GATAAGAGACAGAACGTATATTCACTT---TTTTATAGACTTGATATAGTACAAATTGATAGT----------
----

AATAGTAATAATAGTCATTATAGATTAATAAATTGTAATACCTCAACCATTACACAA---
GCTTGTCCAAAAGTAACTTTTAAGCCAATTCCTATACATTATTGTGCTCCAGCTGGTTTTGCAATTCTAAAGT
GTAAT---GATAAAAAGTTCAGT------GGAAAAAGACCATGCCGAAAT-----------------------
-GTTAGC---ACAGTACAATGGACTCATGGAATCAGGCCAGTAGTATCAACT---
CAACTGCTGTTAAATGGCAGTCTAGCAGAAAATAGG------------
ACAATGATTAGATCTGAAAATATCACAAACAAT---------GCCAAAAACATAATAGTACAACTTACT----
-----------GAACCTGTAAAC---------ATTACTTGTATCAGACCTAGCAACAATACAAGAAAA-----
-AGTATACGTATA------------GGACCAGGACAAGCA---TTC------TATGCAACAGGTGAT------
---------ATTATAGGGAATATA------------AGAAAAGCCCATTGT---GAGGTCAATGGAACA---
GAATGGAATAAAGCTTTAAAAGGGGTAGTTGAACAATTAAGA---------------ACATACTTTAGT----
-----------------------------------AACAAAACAATAGCCTTTAAC------
GACTCCTCAGGAGGGGATCTAGAAATTACA---ACACATAGTTTTAATTGTGGAGGAGAA---
TTTAAACAGCTCCAGGCAAGAGTCCTGGCTGTGGAAAGATACCTAGGGGATCAACAGCTCCTAGGAATTTGGG
GCTGCTCTGGAAAACTCATCTGCACCACTAATGTGCCCTGGAACTCTACTTGGAGT-----------------
----------------------------AATAAATCCCTGGATAAGATATGG------AAC------
AACATGACCTGGATGCAATGG---GACAAAGAAATTAGCAATTATACACAATTAATATAT---
ACTCTAATTGAGGAATCGCAGAACCAGCAGGAA--

ATAAATGAACAAGACTTATTGGCATTGGACAAGTGGGCAAATCTGTGGAATTGGTTTGACATCTCACACTGGT
TGTGGTATATAAAAATATTTATAATGATAGTAGGAGGCTTAATAGGATTAAGAATAGTTTTTGCT---
GTGCTTTCTATAATA---------CGTAGAGTTAGGCAGGGATACTCACCT------
TTGTCATTTGCTATTAATTTGTTTGACACCATAGCAATAGCAGTAGCTGGC---
TGGACAGATAGAGTTATAGAAATAGTACAAGGAATTGGTAGAGCT----------------------------
--------ATTATCAATATA------CCTAGAAGAATCAGACAGGGCCTTGAAAGGGCTTTGCTA---TAA
>JF683789
GCT---GCA--
AGAAAGTTGTGGGTTACTGTCTACTATGGGGTACCTGTGTGG---AAAGATGCAAAA---
ACCACCTTATTTTGTGCATCAGATGCAAAAGCATATTCAACAGAA---AAGCATAAT----------------
--------------GTCTGGGCTACA---CATGCCTGTGTACCCACAGAC---
CCTAACCCACAAGAAATGCATTTGGAA---------AATGTAACAGAAGAGTTTAAC----------------
--------------------------ATGTGGAAAAATAACATGGTAGAGCAGATGCATACAGATATA---
ATCAGTCTATGGGACCAAAGTCTA---AAGCCATGTGTAAAGTTA---
ACCCCTCTCTGTGTTACTTTAAATTGTAGCTACAATATCACTAGTAAGGTCAATAAGACTTTCATC-------
----
----------------------GATGACATTAATAGTACCATTGACATGAAAACAGAAATAAAA---
AACTGCTCTTTCAATACAACCACAAAAATAAAG---------------------------
AATAAGGTTCAGAAAGTACATTCACTT---TTTTATAAACTTGATATAGTACCAATGGGTAGT----------
----

GATAATAGTAGTTATAGATTAATAAATTGTAATACCTCAACCATTACACAG---
GCTTGTCCAAAGATAACTTTTGAGCCTATTCCCATACATTATTGTGCTCCAGCTGGTTTTGCGATTCTAAAGT
GTAAT---GATAATAATTTCAAT------GGAACAGGACCATGCACGAGT-----------------------

-GTCAGC---GCAGTACAATGCCCACGTGGAATCAAGCCAGTAGTATCAACT---
CAATTGCTGATAAATGGCAGTCTAGCAGAAAATGAG------------
ACAATGATTAGATCTGAAAATATCACAAACAAT---------GCCAAAAACATAATAATACAACTTAAG----
-----------GAGCCTGTAAGA---------ATTAATTGTACCAGACCTAACAACAATACAAGGAAA-----
-AGTGTACGTATA------------GGACCAGGACAAGCA---TTC------TATGCAACAGATGAA------
---------ATAATAGGGGATATA------------AGACAGGCACGTTGT---GAGGTCAATGAAACA---
GAATGGAATAGAACTTTGCATGAAGTAGTCAAACAATTAAGA---------------ACATACTTTGTG----
-----------------------------------AACAAAACAATAGCCTTTAAT------
AAATCCTCAGGAGGAGATCTAGAAATTACA---ACACATAGTTTTAATTGTGGAGGAGAA---
TTTAAACAGCTCCAGGCAAGAGTCCTAGCTGTGGAAAGATACCTAAAGGATCAACAGCTCCTAGGAATTTGGG
GTTGCTCTGGAAAACTCATCTGCACCACTAATGTGCCCTGGAACAAAACTTGGAGT-----------------
----------------------------AATAGAACCAAGGAAGAGATATGG------AAC------
AACATGACTTGGATGCAATGG---GAAAAAGAAATTGACAATTATACAGGATTAATATAT---
AACCTAATTGAAAAATCACAGAACCAGCAGGAA---

ATAAATGAAAAAGAATTATTGGAATTGGACAAGTGGGACAGTCTGTGGAGTTGGTTTAGCATCACAAACTGGC
TGTGGTATATAAAAATATTTATAATGATAGTAGGAGGCTTAATAGGATTAAGAATAGTTTTTGCT---
GTGCTTTCAATAATA---------CGTAGAGTTAGGCAGGGATACTCACCT------
TTGCCGTTTGCTATTAATTTGCTTGATACCATAGCAATAGCAGTAGCTGGC---
TGGACAGATACGGTTATAGAAATAGGACAAAGAATTGGTAGAGCT----------------------------
--------ATTCTCAACATA------CCTAGAAGAATCAGACAGGGCTTAGAAAGGGCTTTGCTA---TAA
>FJ670523
GCT---GCA--
GATAACTTGTGGGTTACTGTCTATTATGGGGTACCTGTGTGG---AAAGATGCAGAC---
ACCACCTTATTTTGTGCATCAGATGCGAAAGCATATGACACAGAA---GTGCATAAT----------------
--------------GTTTGGGCTACA---CATGCCTGTGTACCCACAGAC---
CCCAACCCACAAGAAATACCATTGGTA---------AATGTGACAGAAGAGTTTAAC---------------
--------------------------ATGTGGAAAAATAACATGGTAGAGCAGATGCATACAGATATA---
ATCAGTCTATGGGACCAAAGTCTA---AAGCCATGTGTAAAGTTA---
ACCCCTCTTTGTGTTACTTTAAACTGTGGCCATAATGACACCCTCACTAATRACACT----------------
----
----------------------------GCCCCCGTCAATAACACCATGAGAGAAGAAGTAAGA---
AACTGCTCTTACAATATGACCACAGAACTAAGG--------------------------
GATAAGCAAAAGAAAGTGTTTTCACTC---TTTTATAAACTTGATGTAGTATCAATTAATAAT----------
----

AGTAGTAGTCAGTATAGATTAATAAATTGTAATACCTCAGCCATTACACAG---
GCTTGTCCAAAAGTATCTTTTGAGCCAATTCCCATACATTATTGTGCTCCAGCTGGTTTTGCAATTCTAAAGT
GTAGA---GATAACAAGTTCAAT------GGAACAGGACCATGTAGGAAT-----------------------
-GTCAGC---ACAGTACAATGCACACATGGAATCAGGCCAGTAGCATCAACT---
CAACTRCTGTTAAATGGTAGTCTAGCAGAAGAAGAG------------
GTAATGATTAGATCTGAAAATATCAGCAACAAT---------GCCAAAAACATAATAGTACAACTTAAG----
-----------GAGCCTGTAAAC---------ATTACTTGTGCCAGACCTGGCAACAATACAAGAACA-----
-AGTGTACGTATA------------GGACCAGGACAGGCA---TTC------TATGCAACAGGTGAC------
---------ATAATAGGGGATATA------------AGACAAGCACATTGT---AATGTCAGTAGAGCA---
GCATGGAATAAAACTTTGCAAGAGGTAGTCAAGCAATTAAGA---------------AAATACTTTGTG----
-----------------------------------AACGAAACAATAACGTTTACT------
AACTCCTCAGGAGGGGATCCAGAAATTGTA---ACACATAGTTTTAATTGTGGAGGAGAA---
TTTAAACAGCTCCAGGCAAGAGTCCTGGCTCTGGAAAGATACCTAAAGGATCAACAGCTCCTAGGAATTTGGG
GATGCTCTGGGAAAATCATCTGCCCCACTGCTGTTCCCTGGAACTATACTTGGAGT-----------------
-------------------------AATAATAAATCCTATGACGAGATATGG------AAA------
AACATGACCTGGCTGCAATGG---GATAAAGAAGTYAGCAGTTATACAGACCAAATATAT---
AATCTAATTGAAAAATCGCAGAACCAGCAGGAA---

ATAAATGAACAAGACTTATTGGCATTGGACAAGTGGGCAGACCTGTGGAATTGGTTTGACATTTCAAAATGGC
TGTGGTATATAAGAATATTCATAATGATAGTAGGAGGCTTAATAGGATTAAGGATAGTTTTTGCT---
GTGCTTTCTATAATA---------AAAAGAGTTAGGCAGGGATACTCACCT------
TTGTCGTTTGCCATTAGTTTGCTTGACACCACAGCAATAGTAATAGCTGGC---
TGGACAGATAGGATTATAGAAATAGGACAAGGAATTGGTAGAGCT---------------------------
--------ATCCTCAACGTA------CCTAGAAGAATCAGACAGGGCCTAGAAAGGTCGTTGTTA---TAA

>AF219261
ACT---ACA--
GAAAACTTGTGGGTTACTGTCTATTATGGGGTACCTGTGTGG---AAAGACGCAGAG---
ACCACCCTATTTTGTGCATCAGATGCAAAAGCATATGATACAGAA---ATGCATAAT----------------
--------------GTCTGGGCTACA---CATGCCTGTGTACCCACAGAC---
CCCAACCCACAAGAAATATATTTGGAA---------AATGTAACAGAAGAGTTTAAT----------------
--------------------------ATGTGGAAAAATAACATGGTAGAGCAGATGCATACAGATATA---
ATCAGTCTATGGGATGAAAGCCTA---AAGCCATGTGTACAGTTA---
ACCCCTCTCTGCGTTACTTTAAATTGTAGCAATGCCAATGCCAACTCTATCAATGCCAATGCCACC-------
----
----------------------TCTACCAATGCCACTGCCGAGAACGAGAAGGGAGAAATAAAA---
AACTGCTCTTTCAATATGACCACAGAACTAAGG---------------------------
GATAAGAAAAAGAAAGTATATTCACTT---TTTTATAGACTTGATGTAATACCACTTAATGGAACG-------
----

GAGAATGAGACTTACACTGAGTATAGGTTAATAAATTGTAATACCTCAGCCATTACACAA---
GCTTGTCCAAAGGTATCCTTTGAGCCAATCCCCATACATTATTGTGCTCCAGCTGGTTTTGCGATCCTAAAGT
GTAGG---GATGAGAAGTTCAAT------GGAACAGGGCCATGCACGAAT-----------------------
-GTTAGC---ACAGTACAGTGCACACATGGAATCAGGCCAGTAGTATCAACT---
CAACTGCTGTTAAATGGCAGTCTGGCAAAAGAAGAG------------
GTAAGAATTAGATCTGAAAATATCACAAATAAT---------GTCAAAACTATAATAGTACAACTTGTC----
-----------AAGCCTGTAAAC---------ATTACCTGTATCAGACCTAACAATAATACAAGAAAG-----
-AGTATACATTTA------------GGACCAGGACGAGCA---TTC------TATGCAACAGGTGAC------
---------ATAATAGGGAATATA------------AGAAAAGCACATTGT---ATTGTCAATGAATCA---
GAATGGAATGAAGCTTTACAACAGGTAGCTACACAATTAGGA---------------AAATACTTTGAG----
-----------------------------------AACAAAACGATAAATTTTACT------
AGCCCCTCAGGAGGGGATCTAGAAGTCACA---ACACATAGTTTTAATTGTGGAGGAGAA---
TTTAAACAGCTCCAGGCAAGAGTCCTGGCTGTGGAAAGATACCTAAAGGATCAACAGCTCCTAGGAATTTGGG
GCTGCTCTGGAAAACTCATCTGCACCACTAATGTGCCCTGGAACTCTACTTGGAGT-----------------
----------------------------AATAAATCTTATAGTGAGATATGG------GAT------
AACATGACCTGGATGCAGTGG---GATAAAGAAATTAGCAATTACACACAAACAATATAT---
AATCTAATTGAAAAATCACAGATCCAGCAGGAA--

AAGAATGAACAGGATTTATTGGCATTGGACAAGTGGACAAATCTGTGGACTTGGTTTGACATATCAAATTGGC
TGTGGTATATAAAAATATTTATAATGATAATAGGAGGCTTAATAGGATTAAGAATAGTTTTTGCA---
GTGCTTTCTGCAATA---------AATAGAGTTAGGCAGGGATACTCACCT------
TTGTCGTTTGCTAGTGATTTGCTTGATACCATAGCAATAGCAGTAGCTGGC---
TGGACAGATAGGGTTATAGAAATAGGACAAAGAATTGGTAGAGCT----------------------------
--------ATTCTCAACATA------CCTAGAAGAACCAGACAGGGCTTAGAAAGGGCTTTGGTA---TAA
>AF219265
GCT---GCA--
GAAAACTTGTGGGTTACTGTCTACTATGGGGTACCAGTGTGG---AAAGATGCAGAG---
ACTACCTTATTTTGTGCATCAGATGCTAAAGCATATGAGACAGAA---AAGCATAAT----------------
--------------GTTTGGGCTACA---CATGCCTGTGTACCCACGGAC---
CCCAACCCACAAGAAATACATTTGGAA---------AATGTGACAGAAGAGTTTAAC----------------
--------------------------ATGTGGAAAAATAACATGGTAGAGCAGATGCATACAGATATA---
ATCAGTCTATGGGACCAAAGCCTA---AAGCCATGTGTAAAGCTA---
ACCCCTCTCTGCGTTACTTTACATTGTAACAATGCCAATTTCACAAGTGCCAACAACATG-------------
----
----------------------------AGTACCGACATTACGAGGGATAGGGGAGAAATAAAA---A-
CTGCTCTTTCAATATGACCACAGAGCTAAGG---------------------------
GATAAGCAACAGAAGGTATATTCACTT---
TTTTATAGGCTTGATGTAGTACAAATTAATGAGAATCAAGGTAATAGT-------------------------
----
AGTAACAATGAGTATAGATTAATAAATTGTAATACCTCAGCCATTACACAA---
GCTTGTCCAAAGGTAACCTTTGAGCCAATTCCCATACATTATTGTGCCCCAGCTGGTTTTGCGATCCTAAAGT
GTAAG---GATGAGAAGTTCAAT------GGAACAGGGCCACGCAAGAAT-----------------------
-GTCAGC---ACAGTACAATACACACATGGAATCAAGCCAGTAGTATCAACT---
CAACTGCTGTTAAATGGCAGTCTAGCAAAAGAAAAG------------
GTAAAAATTAGATGTGAAAATATCACAAACAAT---------GCCAAAACTATAATAGTACAACTGGTC----

-----------GAGCCTGTGCAA---------ATTAATTGTACCAGACCTAACAACAATACAAGAAGA-----
-AGTGTACGTATA------------GGACCAGGACAGGCA---TTT------TAT---ACAACAGAC------
---------ATAATAGGGGATATA------------AGACAAGCCCATTGT---AATGTCAGTAGATCA---
GCATAGAATAAAACTTTACAAAAGGTAGCTGAGCAATTAGGA---------------GGATACTTTGGG----
------------------------------------AACAAAACAATAATATTTGCT------
AACGCCTCAGGAGGGGATCTAGAAATCACA---ACACATAGTTTTAATTGTGGAGGAGAA---
TTTAAACAGCTCCAGGCAAGAGTCCTGGCTGTGGAAAGATACCTAAAGGATCAACAGCTCCTAGGAATTTGGG
GCTGCTCTGGAAAACTCATCTGCACCACTAATGTGCCCTGGAACTCTAGTTGGAGT----------------
-----------------------------AATAAATCTGAGAGTGAGATATGG------GAG------
AACATGACCTGGCTGCAATGG---GATAAAGAAATTAGCAATTACACACAGATAATATAT---
ACCCTAATTGAAGAATCGCAGAACCAGCAGGAA--

AAGAATGAACTAGACTTATTGGCACTGGACAAGTGGGCAAATCTGTGGAATTGGTTTGACATATCAAAGTGGC
TGTGGTATATAAAAATATTTATAATGATAGTAGGAGGCTTAATAGGATTAAGAATAGTTTTTGCA---
GTACTTGCTGTAATA---------AATAGAGTTAGGCAGGGGTACTCACCT------
TTGTCGTTTGCTATTAATTTGGTTGATACCATAGCAATAGTAGTAGCTGGC---
TGGACAGATAGGGTTATAGACATAGGACAAGGAATTGGTAGAGCT---------------------------
--------ATTCTCCACATA------CCTAGAAGAATCAGACAGGGCTTAGAAAGAGCTTTGCTA---TAA
>HM204635
GCT---GTA--
GAAAACTTGTGGGTTACGGTCTATTATGGGGTACCTGTGTGG---AAAGACGCAACT---
ACCACCTTATTTTGTGCATCAGATGCGAGAGCATATGATACAGAA---GTACATAAT----------------
--------------GTCTGGGCTACA---CATGCCTGTGTACCTACAGAC---
CCCAATCCACAAGAAATAAAGTTGGAA---------AATGTGACAGAAGAATTTAAC---------------
--------------------------ATGTGGAAAAATAACATGGTAGAGCAGATGCATACAGATATA---
ATCAGTCTCTGGGACCAAAGCTTA---CAGCCATGTGTAAAATTA---
ACTCCTCTCTGCGTTACTTTAAATTGTACAAATGTCACCAATAATTCCACCAATAATTCC------------
----
-----------------------------ACTAACAATGTCACCATAGAAGACATAGGACTAACA---
AACTGCTCTTACAATATGACCACAGAATTAAGG--------------------------
GATAAGAAACAGAAAGTATATTCACTT---TTTTATAAACTGGATGTAGTACAAATTAATGACAACAAT----
----

AACAGTAGTAATAATTATAGTTTGTATAGATTAATAAATTGTAATACCTCAGCCATTACACAA---
GCTTGTCCAAAGGTAACCTTTGATCCAATTCCCATACATTATTGTGCTCCAGCTGGTTTTGCGATTTTAAAGT
GTAAT---AATAAGCAGTTCAAT------GGAACAGGGCCATGCAAGAAT----------------------
-GTCAGC---ACAGTACAATGCACACATGGAATCAAGCCAGTAGTATCAACT---
CAACTGCTGCTAAATGGTAGTCTAACAGAAGGGGAG-----------
ATAATGATTAGATCTGAAAATATTACAAACAAT---------GCTAAAAACATAATAGTACAATTTAACACG-
--------TCTACGGCTGTAGCA---------ATCAATTGTACCAGACCTAACAATAATACAAGAAAA-----
-AGTGTACGTATA------------GGACCAGGACAAGCA---TTC------TATGCAACAGGTGAT------
---------ATAATAGGGGATATA------------AGACAGGCACATTGT---AACGTCAGTAGAACA---
CAATGGAATAAAACTTTGCAAGAGGTAGCTAACAAACTAAGA---------------GAACATTTTAAG----
------------------------------------AACAAAACAATAATATTTAAT------
ACCTCCTCAGGAGGGGATCTAGAAATAACA---ACACATAGTTTTAATTGTGGAGGAGAA---
TTTAAACAGCTCCAGGCGAGAATCCTGGCTGTGGAAAGATACCTAAAGGATCAACAGCTCCTAGGAATTTGGG
GCTGCTCTGGAAAACTCATCTGCACCACTAATGTGCCCTGGAACTCTAGTTGGAGT----------------
-----------------------------AATAAAACCCAGGCTGAAATATGG------GAA------
AACATGACCTGGCTGCAATGG---GATAAAGAAATTAGCAATTACACACAGATAATATAT---
GAACTAATTGAAGAATCGCAGAACCAGCAGGAA--

AAGAATGAACAAGACTTGTTGGCATTGGACAAGTGGGCAAGTCTATGGACTTGGTTTGATATAACAAAATGGC
TGTGGTATATAAAAATATTTATAATGATAGTAGGAGGCTTAATAGGATTAAGAATAGTTTTTGCT---
GTGCTTTCTATAATA---------AATAGAGTTAGGCAGGGGTACTCACCT------
TTGTCATTCGCTGTTAGTTTGTTTGATACCATAGCAATAGCAGTAGCTGAG---
TGGACAGATAGGGTTATAGAAACAGGTCACAGACTTTGTAGAGCT---------------------------
--------ATCTTCAACATA------CCTAGAAGAATCAGACAGGGCCTCGAAAGGGCTTTGCTA---TAA
>HM204648
GCT---ACA--
GAGAACTTGTGGGTTACTGTCTACTATGGGGTACCTGTGTGG---AAAGATGCAGAG---

ACCACCTTATTTTGTGCATCAGATGCAAAAGGATATGATACAGAA---GTGCATAAT----------------
--------------GTCTGGCTACA---CATGCCTGTGTACCTACAGAC---
CCCAACCCACAAGAAATAAATTTGGAA---------AATGTGACAGAGAATTTTAAC----------------
--------------------------ATGTGGAAAAATAACATGGTAGAGCAGATGCATACAGATATA---
ATCAGTCTTTGGGACCAAAGCCTA---AAGCCATGTGTAAAGTTA---
ACCCCTATCTGTGTTACTTTAAATTGTACGGATGACGTCTTTTTCGTCAAAACTACCAGCAATGACACGTACA
AC---
-------------AACACGCAACAAATGCAACAAATCAGCAATAAAATGAAAGGAGAAATAAAA---
AACTGCTCTTTCAATACGACCACAGTCTTAAGA---------------------------
GATAAGACACAGAAAATACATTCACTT---
TTTTATAGACTGGATGTAATACCATTTGGAAACAACAGTGTATCATCTAAAAACAGCAGTGTAGTAAAAAATA
CAACATTAACT---------
ACAAGTGCACCTACAGGAAACAACAATCAATCTACTAACAGCAGCCAGTATAGCCTGTATACATTAATAAATT
GTAATACCTCAACCATTACACAG---
GCTTGTCCAAAGGTATCCTTTGAGCCAATTCCCATACATTATTGTGCCCCAGCTGGTTTTGCGATTCTAAAGT
GTAAT---GATAAAGAGTTCAAT------GGAACAGGGCTATGCAAGAAT-----------------------
-GTCAGC---TCAGTACAATGCACACATGGAATCAAGCCAGTAGTGTCAACT---
CAGCTGCTGTTAAATGGCAGTCTAGCAGAAGGAGAA------------
ATAATAATTAGTTCTGAAAATCTCACAGACAAT---------ACCAAAAACATAATAGTACATCTTAAT----
-----------AAGACTGTAAAC---------ATTACTTGTATCAGGCCTAACAACAATACAAGAAAA-----
-AGTGTACACATA------------GGACCAGGACAAGCA---TAC------TATGCAACAGGTGAC------
---------ATAATAGGAAATATA------------AGACACGCATATTGT---CTTGTCAATAAATCA---
GAATGGATTACTGCTTTGCGAGGGGTAGTCGAACGATTAGGA---------------AGACTAGCGGAC----
-----------------------------------ATAACATCAATAACCCTTAAT------
AACTCTGCAGGAGGAGATCTAGAAATTACA---TCACATAGCTTTATTTGTGGAGGAGAA---
TTTAAACAGCTCCAGGCAAGAGTCCTGGCTCTGGAAAGATACCTAAGGGATCAACAGCTCCTAGGAATTTGGG
GCTGCTCTGGAAAACTCATCTGCACCACTACTGTGCCCTGGAACTCTAGTTGGAGT-----------------
----------------------------AATAAATCCCAGAGTGAGATATGG------GGA------
AACATGACCTGGCTACAATGG---GACAAAGAAATTAGCAATTACACAAATATAATATAT---
GATCTAATTGAAAAATCACAGAACCAGCAGGAA--

AAGAATGAACAAGACTTATTGGCATTGGACAAGTGGGACAGTCTGTGGAATTGGTTTGACATAACAAACTGGC
TGTGGTATATAAAAATATTCATAATAATAATAGGAAGCTTAATAGGATTAAGAATAGTCTTTGCT---
GTGCTTGCTGTAATA---------AATAGAGTTAGGCAGGATACTCACCC------
TTGTCGTTCGCTATTAATTTGCTTGATACCATAGCAATAGTAGTAGCTGGG---
TGGACAGATAGGATTATAGAAATAGGACAAAGACTTTGTAGAGCT----------------------------
--------ATCCTTAATATA------CCTAGAAGAATCAGACAGGGCTTTGAAAGGGCTTTGCTA---TAA
>HQ595782
AAT---GCA--
GACCGTTTGTGGGTAACAGTCTACTATGGGGTACCTGTGTGG---AAAGATGCAGAG---
ACCACTTTATTCTGTGCATCAGATGCCAAAGTATATGATAAAGAA---GTGCATAAT----------------
--------------GTCTGGCCACA---CATGCCTGCGTACCCACAGAC---
CCCAACCCACAAGAAATAGGTTTAGCC---------AATGTGACAGAAGAGTTTAAC----------------
--------------------------ATGTGGAAAAACCACATGGTAGAGCAGATGCATACAGATATA---
ATCAGTCTATGGGACCAAAGCCTA---AAGCCATGTGTAAAGTTA---
ACCCCTCTCTGCGTTACCTTAAATTGTAGCAATTCCATAACCCCTCTCGGCGGTACCTCAAAT----------
----
-------------------------AATAGCAGTTCCAATAATAGTTTCAAGGAAGAAATGAAA---
AACTGCTCTTTCAATATGACTACAGAACTAAGG---------------------------
GATAGGAAACAGACAGTATATTCACTT---TTTCATAAGCTTGATTTAGTAGAAATTAAGGGAAAT-------
----

AATAATAGCAGTAATGAGACTTATATACTAACAAATTGTAATACATCAGTTATTACACAA---
GCTTGTCCAAAAGTGACTTTTGACCCAATACCCATACATTATTGTGCCCCAGCTGGTTTTGCGATTTTAAAAT
GTAGA---GATGAAGAGTTTAAG------GGAACAGGGTCATGCAAGAAT-----------------------
-GTTAGC---TCAGTACAATGCACACATGGAATTAAACCAGTAGTCTCAACT---
CAACTGCTGTTAAATGGTAGTCTAGCAAAGGGAAAT------------
GTAAGCATCAGATCGGAAAATATCACAGACAAC---------GCCAAGACCATAATAGTACAGCTTGCC----
-----------ACGCCTGTAAAA---------ATTAATTGTACTAGACCTAACAACAATACCAGAAGA-----
-AGTATACATATA------------GGACCAGGACAAGCA---TTC------TATGCAACT---GAC------

---------ATAATAGGGGATATA------------AGACAAGCACACTGT---AATCTCAGTGGAGAA---
GAATGGCATGACGCTTTACAAAAGGTAGTAAAAAAATTAAGGGAA---------GAGAAATCCTTTAAG----
-----------------------------------AACAAAGCAATAATCTTCACT------
ACCCACTCAGGTGGGGATTTAGAAGTTACA---ACACATACTTTTAATTGTAGAGGAGAA---
TTTAAACAGCTCCAGGCAAGAGTCCTGGCTGTGGAAAGATACCTAAAGGATCAACAGCTCCTAGGAATTTGGG
GCTGCTCTGGAAAGCTCATCTGCACCACTGATGTGCCCTGGGAATCTGAGTGGAGT-----------------
----------------------------AATAAATCTCAGGAAGAGATATGG------GAT------
AACATGACCTGGCTGCAATGG---GATAGAGAAATTAGCAATTACACACAAACAATATAT---
AGTCTAATTGAGAAATCGCAGAACCAGCAGGAA--

AAGAATGAACAAGACTTATTGGCAATGGACAAGTGGTCAACCCTGTGGAATTGGTTTGACATATCAAACTGGT
TATGGTATATTAGAATATTTATAATGATAGTAGGAGGCTTAATAGGATTAAGGATAGTTTTTGCT---
GTGCTTGCTGTAATA---------AATAGAGTTAGGCAGGGATACTCACCT------
CTGTCGTTTGCTATTACTTTGCTTGATGCCACAGCAGTAACAGTAGCAGGG---
TGGACAGATAGGATTATAGAACTAGGACACAGAGCTGGTAGAGCT----------------------------
--------TTTCTCCATATA------CCTAGAAGAATCAGACAGGGCTTTGAAAGGGCTTTACTA---TAA
>HQ595784
GTT---AAA--
GCAAACTGGTGGGTCACAGTCTACTATGGAGTACCTGTGTGG---AGAGATGCGGAT---
ACCATCCTATTTTGTGCATCAGATGCTAAAGCATATGATAAAGAA---GTGCATAAT----------------
--------------GTCTGGGCTACA---CATGCTTGTGTACCCACAGAC---
CCCAACCCACGAGAAATAAATTTGGAA---------AATGTGACAGAAAATTTTAAT----------------
--------------------------ATGTGGAAAAATAACATGGTAGAGCAAATGCACACAGATATA---
ATCAGCCTATGGGACCAAAGCCTA---AAGCCATGTGTACAGTTA---
ACCCCTCTCTGTGTTACTCTAAACTGTAGTGGTGTAAACAAACCCAACCAGACT-------------------
----
-------------------------------GAAATGGTAAGGGAAATGGTAAGGGAAATGAAA---
AACTGCTCTTACAATATGACCACAGAACTAAAA---------------------------
GATAAGAAACAACAAGTATATTCACTT---TTCTATAAACTTGATGTAGTATCAGTAGATGGTCAGAATGAT-
----

TCGGACCTGGTCAATGAGAGTAGCCCATATAGACTAGTAAATTGTAATACCTCAACCATTACACAG---
GCTTGTCCAAAGGTATCCTTTGAGCCAATTCCCATACATTATTGTGCTCCAGCTGGTTTTGCGATTCTAAAAT
GTAAT---GATAAGGAGTTTAAG------GGAACAGGGCTATGCAAGAAT-----------------------
-GTCAGT---ACAGTACAATGCACACATGGAATTAGACCAGTAGTATCAACC---
CAACTACTGTTAAATGGCAGCCTAGCAGAAGGAGAG------------
GTAATGATTAGGTCTGAAAATTTCACAAACAAT---------GCCAAAACCATAATAGTACAGTTTAAT----
-----------GAGTCTGTACCA---------ATTAATTGTAGCAGACCCTCTAATAATACAAGGAAA-----
-AGTATACCTATA------------GGACCAGGAAGAGCA---TTC------TTTGCAACAGGTGAC------
---------ATAACAGGAGATATA------------CGACGGGCACATTGT---ACTGTCAATGGAACA---
AAATGGAAGCAAACTTTACAACTGGTAGTTGAACAATTAAGA---------------AAACAGTATAAC----
-----------------------------------ACGAGCAAAATAATCTTTAAT------
AAATCCTCTGGAGGGGATATAGAAATTACA---TCACATAGTTTTAACTGTGGAGGAGAA---
TTTAAACAGCTCCAGGCAAGAGTCCTGGCTGTGGAAAGATACCTACAGGATCAACAGCTCCTAGGACTTTGGG
GTTGCTCTGGAAAACTCATCTGCACCACTACTGTGCCTTGGAACTCTAGTTGGAGT-----------------
-------------------------AGTAAAACAGATCTAAAGGATATATGG------GAA------
AATATGACCTGGCTGCAATGG---GATAAAGAAATTAGCAATTACTCAGCAGTAATATAT---
AAACTACTTGAACAATCGCAGAACCAGCAGGAA--

AAGAATGAACAAGACTTATTGGCATTGGACGAATGGGCAAATCTATGGAATTGGTTTGACATATCACATTGGC
TGTGGTATATAAAAATATTTATAATGATAGTAGGAGGCTTAATAGGATTAAGAATTGTTTTTGCT---
GTACTTTCAGTAATA---------AATAGAGTTAGGCAGGGATACTCACCT------
GTGTCATTTGCTACTTCTTTGCTTGATGCTACAGCAATAGCAACAGCTGAG---
TGGACAGATAGGGCTATAGAAATAGGACAAGGGATTTGTAGAGCT----------------------------
--------ATTATCCACATA------CCTAGAAGAATCAGACAAGGCCTCGAAAGAGCTTTACT-------
>Y13718
GCT---GCA--
GGAAACTTGTGGGTTACTGTCTACTATGGGGTACCTGTGTGG---AAAGACGCAGAG---
ACCACCTTATTCTGTGCATCAGATGCGAAAGCATATACCACAGAG---GTGCATAAT----------------
--------------GTCTGGGCTACA---CATGCCTGTGTACCTACAGAC---

CCCAACCCACAAGAAATAAATTTGGAA---------AATGTCACAGAAAAGTTTAAC----------------
--------------------------ATGTGGAAAAATAACATGGTAGAGCAGATGCATACAGATATA---
ATCAGTCTATGGACCAAAGCCTA---CAGCCATGTGTAAAGTTA---
ACCCCTCTCTGCGTTACTTTAAATTGTAGCGATGTCAATGTCAATGCCACCAATTACAAT-------------
----
----------------------------GTCAATGATACCATAAACATGAAAGAAGAAATAAGA---
AACTGCTCTTTCAATATGACCACAGAATTAAGG---------------------------
GATAGGAAACAGAAAGTATATTCACTT---TTTTATAGACTGGATGTAGTACAAATGAATAAT----------
----

AGCAATAATAGCAATCAGTATAGATTAATAAATTGTAATACCTCAGCCATTACACAA---
GCTTGTCCAAAGGTATCCTTTGAGCCAATTCCCATACATTATTGTGCCCCAGCCGGTTATGCGATTCTAAAGT
GTAAG---GATAAGGAGTTCAAT------GGAACAGGGCTATGCAAGAAT-----------------------
-GTCAGC---ACAGTACAATGCACACATGGAATCAAGCCAGTAGTATCAACC---
CAACTGCTGTTAAATGGCAGTCTAGCAGAAAGCAAG------------
GTAATGATTAGATCTGAAAATATCACAAACAAT---------GCCAAAAACATACTAGTACAACTTACC----
-----------AGTCCTGTAAAC---------ATTAGTTGTATCAGACCTAACAACAATACAAGAAAA-----
-AGTGTACGTATA------------GGACCAGGACAAGCA---TTC------TATGCAACAGGTGAA------
---------ATCATAGGGAATATA------------AGACAAGCATATTGT---AATGTCAATAGATCA---
GAATGGAATGAAGCTCTGCGGGAGGTAGTCAAACAATTAAGA---------------ACATACTTT-------
-----------------------------------AACAAAACAATAATCTTTGAT------
AACTCCTCAGGAGGGGATCTAGAAATAACA---ACACATAGTTTTAATTGTGGAGGAGAA---
TTTAAACAGCTCCAGGCAAGAGTCCTGGCTCTGGAAAGATACCTAAGGGATCAACAGATCCTAGGAATTTGGG
GCTGCTCTGGAAAACTCATCTGCACCACTAATGTGCCCTGGAACTCTAGTTGGAGT-----------------
----------------------------AATAAATCCCAGAGTGAGATATGG------GAA------
AACATGACCTGGCTGCAATGG---GATAAAGAAATTAGCAATTACACACACATAATATAT---
ACTTTACTTGAGGAATCGCAGATCCAGCAGGAA--

AAGAATGAACAAGACTTATTGGCATTGGACAAGTGGGCAAATCTGTGGAATTGGTTTGACATAACAAACTGGC
TGTGGTATATAAAAATATTTATAATGATAGTAGGAGGCTTAATAGGATTAAGAATAGTTTTTACT---
GTGCTTTCTATAATA---------AATAGAGTTAGGCAGGATACTCACCT------
TTGTCGTTCGCTATTAATTTGCTTGATACCATAGCCATAATAATAGCTGGG---
TGGACAGATAGGGTTATAGAAATAGGACAAAGATTTTGTAGAGCT----------------------------
--------ATTCTCAACATA------CCTAGAAGAATCAGACAGGGCTTTGAAAGGGCTTTGCTA---TAA
>Y13717
GCT---GCA--
GACAACTTGTGGGTTACTGTTTACTATGGGGTACCTGTGTGG---AAAGATGCAGAG---
ACCACCTTATTTTGTGCATCAGATGCAAAAGCATATGAGACAGAA---ATGCATAAT----------------
--------------GTCTGGGCTACA---CATGCCTGCGTACCTACAGAC---
CCCAGCCCACAAGAAATAAATTTGGAA---------AATGTGACAGAAGAGTTTAAC----------------
--------------------------ATGTGGAAAAATAACATGGTAGAGCAGATGCATACAGATATA---
ATCAGTCTATGGACCAAAGCCTA---AAGCCATGTGTAAAGTTA---
ACCCCTCTCTGCGTTACTTTAGAATGCTTTAATGTCCACAAGACCAATAAGACC-------------------
----
-------------------------------AATATCACCGAGGACATGATAGGAGAAATAAAA---
AATTGCTCTTTCAATATGACCACAGAATTAAGG---------------------------
GATAAGAAACAGAAAGTACATTCACTT---TTTTATAAACTGGATGTAGTAGAAATGAGTAATGGCAGC----
----

GTGAGTAATGGCAGCGGTCAGTACAGACTAATAAATTGTAACACCTCAGCCATTACACAA---
GCTTGTCCAAAGGTATCTTTTGAGCCAATTCCCATACATTATTGTGCCCCAGCTGGTTTTGCGATTCTAGAGT
GTAAG---AATAAGAAATTCAAT------GGAACAGGGCCATGCACGAAT-----------------------
-GTCAGC---ACAGTACAATGCACACATGGAATCAAGCCAGTAGTATCAACT---
CAACTGCTGTTAAATGGCAGTCTAGCAGAAGAAGAG------------
GTAATGATTAGATCTGAAAATATCACAAACAAT---------GCCAAAAACATAATAGTACAATTTACC----
-----------ACGTCTGTAAAA---------ATTAATTGTACCAGATTTAACAACAATACAAGAAGA-----
-AGTATACGTATA------------GGACCAGGACAAGCA---TTC------TATGCAACAAATGAC------
---------ATAATAGGGAATATA------------AGACAAGCACAATGT---GATGTCAATAGAACA---
GAATGGAATAAAGCTTTGCAAGGGGTAGTCAAACAATTACAA---------------AAATACTTTGGG----
-----------------------------------AACAAAACAATAATCTTTACT------

AACTCCTCAGGAGGGGATCTAGAAATAACA---ACACATAGTTTTAATTGTGGAGGAGAA---
TTTAAACAGCTCCAGGCAAGAGTCCTGGCTGTGGAAAGATACCTAAAGGATCAACAGCTCCTAGGAATTTGGG
GCTGCTCTGGAAAACTCATCTGCACCACTAATGTGCCTTGGAACTCTAGTTGGAGT-----------------
----------------------------AATAAATCCCAGAGTGAGATATGG------GAC------
AACATGACCTGGCTGCAATGG---GATAGAGAAATTAGCAATTACACACAGATAATATAT---
AATCTACTTGAAGACTCGCAGAACCAGCAGGAA--

AAGAATGAACAAGACTTATTGGCATTGGACAAGTGGGCAAATCTGTGGACTTGGTTTGACATATCAAACTGGC
TGTGGTATATAAAAATATTCATAATGATAGTAGGAGGCTTAATAGGATTAAGAATAGTTTTTGCT---
GTGCTTTCTTTAATA---------AATAGAGTTAGGCAGGGATACTCACCT------
TTGTCGTTCGCTATTAATTTGCTTGATACCATAGCGATAGCAGTAGCTGGG---
TGGACAGATAGGGTTATAGAATTAGGACAAAGACTTTATAGAGCT----------------------------
--------ATTCTCAACATA------CCTAGAAGAATCAGACAGGGCTTCGAAAGGCTTTGCTA---TAA
>HQ385459
GCT---ACA--
GAAAATTTGTGGGTCACGGTCTACTATGGGGTACCGGTGTGG---AGAGACGCAGAG---
ACCATCCTATTCTGCGCATCAGATGCTAAAGCATATGAAAAAGAA---GTGCATAAT----------------
--------------GTTCGGGATACA---CACGCCTGTGTACCCACAGAC---
CCCAACCCACAAGAAATAGAATTAGAA---------AATGTGACAGAAGAGTTTAAC----------------
--------------------------ATGGGGAAGAATAACATGGTGGAGCAGATGCATACAGATATA---
ATCAGTCTATGGGACCAAAGCTTA---AAGCCATGTGTAAAGTTA---
ACCCCTATCTGCGTTACTTTAAACTGCCACAACATCACCACCAATGACACGGGTACGGGTAATGTC-------
----
-------------------ACGGGTACGGGTAATGTCACGGATAACAACATAGGGGAAATGACA---
AACTGCTCATTCAATATTACCACAGAAATAAGA---------------------------
GATAAGAGACAGAATGTATATTCACTT---TTTTATAAACTTGATGTAGTACCAATTAATGGAAAT-------
----

GGTAGTGGAAATGATAGTGAGTATAGGCTAATAAATTGTAATACCTCAACCATTACACAG---
GCTTGTCCAAAGGTATCCTTTGAGCCAATTCCCATACATTATTGTGCCCCAGCTGGTTTTGCGATTCTAAAGT
GTAGG---GATAAGAACTTCAAT------GGAACAGGGCCATGCAGAAAT-----------------------
-GTCAGC---TCAGTACAATGCACACATGGAATCAAGCCAGTAGTATCAACT---
CAACTGCTGTTGAATGGCAGCCTAGCAGAAGGAGAT------------
GTAGTGATTAGATCTGAAAATCTCACAAACAAT---------GCCAAAACCATAATAGTACAGCTTAAT----
-----------GAGTCTGTACCA---------ATTAATTGTACCAGACCCAACAACAATACAAGAAAA-----
-AGTGTACGTATA------------GGACCAGGACAAGCA---TTA------TATGCAACA---GAC------
---------ATCATAGGGAATATA------------AGACAAGCACATTGT---AATATCAGTAAACAA---
AATTGGAATACAGCTTTACAAAAAATAGCTGCACAGTTAAGA---------------CAACACTTT-------
-----------------------------------TACAGAACAATAAACTTTACT------
AAATCCTCAGGAGGGGATTTAGAAATTACA---ACACATAGTTTTAATTGTAGAGGAGAA---
TTTAAACAGCTCCAGGCAAGAGTCCTGGCGCTGGAAAGGTACCTAAGGGATCAACAGCTCCTAGGAATTTGGG
GTTGCTCTGGAAAACTCATCTGCACCACTACTGTGCCCTGGAACTCTAGTTGGAGT-----------------
----------------------------AATAAATCTCAGAATGAAATATGG------GAG------
AATATGACCTGGCTCAATGGG---ATAAAGAAATTAAGCAATTACACAGACATAATATAT---
GATCTAATTGCGGAATCGCAGAACCAGCAAGAA--

AAGAATGAAAAAGACTTATTGGCATTGGACAAGTGGGCAAGTCTGTGGAATTGGTTTGACATATCAAAGTGGT
TATGGTATATAAAATTATTTATAATGATAGTAGGGGGCTTAATAGGATTAAGAATAGTTTTTACT---
GTGCTTTCTATAATA---------AATAGAGTTAGGCAGGGATACTCACCT------
CTGTCATTTGCTATTACTTTGCTTAATGCTACAGCAATAGCAGTAGCTGAG---
GGGACAGATAGGGTTATAGAAGTAGGACAAAGGGCTGGTAGAGCT----------------------------
--------CTTCTCCACATA------CCTAGAAGAATCAGACAAGGATGGAAAAGGGCTTTGCTA---TAA
>HQ385452
GCT---ACA--
GGAAAATTGTGGGTCACAGTCTACTATGGGGTACCAGTGTGG---AGAGACGCAGAG---
ACCACCCTATTTTGTGCATCAGATGCTAAAGCATATGATACAGAA---GTGCATAAT----------------
--------------GTCTGGGCTACA---CATGCTTGTGTACCCACAGAC---
CCCAGCCCACAAGAAGTAGATTTAACA---------AATGTGACAGAAAATTTTAAC----------------
--------------------------ATGTGGAAAAATAACATGGTAGAGCAGATGCATACAGATATA---
ATCAGTCTATGGGACCAAAGTCTG---AAGCCATGTGTAAAGTTA---

ACCCCTCTCTGCGTAACTCTAAATTGTTCTAACATCACCAGCACCAGCAGCAATAACAACAGCAGTAACATCA
CTAATGCCAGC---
----AGCAGCAGCAGCAACAACAGCAGTAACAGTACTGTAGATGAAAGAAACAAAGTAATGACA---
AACTGCTCTTTCAATATGACCACAGAACTAAGA--------------------------
GATAAGATACAGAAAGTACATTCACTT---TTTTATAGGTCTGATGTAGTAGGAATTAATGGT----------
----

AATAAGGAGTATAGACTAATAAATTGTAATACCTCAACCATTACACAA---
GCTTGTCCAAAAGTATCCTTTGAGCCCATTCCCATACATTATTGTGCCCCAGCTGGTTTTGCGATTCTAAAGT
GTAAG---GATAAGGAGTTCAAT------GGAACAGGGCCATGCAAGAAT----------------------
-GTCAGC---TCAGTACAATGCACACATGGAATCAAGCCAGTAGTATCAACT---
CAACTACTGTTAAATGGCAGTCTGGCAGAAGAAGGG-----------
ATAAGGATTAGATCTGAAAATCTCACAAATAAT---------GCCAAAATCATAATAGTACAGCTTACT----
-----------AAGCCTGTAAGA---------ATTAATTGTACCAGACCTTACAACAATACAAGAAAA-----
-AGTATACGTATA------------GGACCAGGACAATCA---TGG------TATGCAACAGGGGAC------
---------ATAATAGGGAATATA-----------AGACAAGCATTTTGT---AATGTCAGTAAAACA---
GACTGGAACACAGCTTTACAACAGGTAGCTGCACAATTAGGA---------------AGGCACTTTAAT----
-----------------------------------AATAAAAAGATAACCTTTAAA------
AACTCCTCAGGAGGGGATTTAGAAATTACC---ACACATAGCTTTAATTGTAGAGGAGAA---
TTTAAACAGCTCCAGGCACGAGTCCTGGCTGTGGAAAGATTCCTAAAGGATCAACAGCTCCTAGGAATTTGGG
GATGCTCTGGAAAACTCATCTGCACCACTAATGTGCCCTGGAACTCTAGTTGGAGT----------------
----------------------------AATAAATCTCAGGAGCAAATATGG------AAT------
AATATGACCTGGCTGCAATGG---GATAGAGAAATTGACAATTACACACAAATAATATAT---
AATCTAATTGAAGAATCGCAAAACCAGCAAGAA---

AAGAATGAGCAAGACTTATTGGCATTGGACAAGTGGGCAAGTTTGTGGACTTGGTTTGACATATCAAATTGGC
TATGGTATATAAAGATATTCATAATGATAGTAGGAGGCTTAATAGGATTAAGAATAGTTTTTGCT---
GTGCTTTCTATAATG---------AATAGGGTTAGGCAGGGATACTCACCT------
CTATCATTTGCTATTTCTTTGCTTGATACTGTAGCAGTAGCAGTAGCTGGG---
TGGACAGATAGGGTTATAGAAATAGGACAAAGAGTTGGTAAAGCT----------------------------
--------ATTATCCACATA------CCTAGAAGAATCAGACAGGGCTTAGAAAGGGCTTTGCTA---TAA
>HQ385453
GCT---GAA---
GAGTTGTGGGTTACGGTCTTCTATGGAGTACCTGTGTGG---AGAGACGCAGAG---
ACCACCCTATTTTGTGCATCAGATGCTAAAGCATATGATACAGAG---GTACATAAT----------------
--------------ATTTGGGCTACA---CATGCCTGTGTGCCCACAGAT---
CCTAACCCACAAGAAATAGGTTTGGAA---------AATGTGACAGAAGAATTTAAC----------------
--------------------------ATGTGGAAAAATAACATGGTGGAGCAGATGCATACAGATATA---
ATCAGTCTATGGGACCAAAGCTTA---AAGCCATGTGTACAGTTA---
ACCCCTCTCTGCGTTACTCTAAACTGTAGCCACAATATCAGTACCACTAATAGCACCAGTACG----------
----
----------------------GGCAATATCAGTACCATTAATAGCACCAGTGCGGACATGAGA---
AACTGCTCGTTCAATATGACCACAGAACTAAGA--------------------------
GATAAGAAACAGAATGTATATTCACTT---TTTTATAAACTTGATGTAGTACAAATAGGGAAT----------
----

AGTAGTAGTAGTGAGTATAGACTAATAAATTGTAATATCTCAACCATTACACAG---
GCTTGTCCAAAAGTATCCTTTGAGCCGATTCCCATACATTATTGTGCCCCAGCTGGTTATGCGATCCTCAAGT
GTAGG---GAGAAGAACTTCAAT------GGAACAGGGCCATGCACAAAT----------------------
-GTTAGC---ACAGTACAATGCACACATGGAATCAAGCCAGTAGTGTCAACT---
CAACTGCTGTTGAATGGCAGTCTAGCAGAAGGAGAT-----------
GTAATGATTAGATCTGAAAATATCACAGACAAT---------GCCAAAACCATAATAGTACAGTTTAAT----
-----------GAGTCTGTAATG---------ATTAATTGTACCAGACCTAACAACAATACAAGAAGA-----
-AGTGTACGTATA------------GGACCAGGGCAAACA---TTC------TATGCAACAGGTGAA------
---------ATAATAGGGGATATA-----------AGACGAGCACATTGT---AATGTCAGTGGAGAA---
CAATGGAGGAAAGCTTTACAAAATGTAAAGGTACAGTTACGC---------------AAATACTTT-------
-----------------------------------AACACAACAATAGAATTTGCT------
AATTCTTCAGGAGGGGATTTAGAAATTACA---ACACATAGCTTTAATTGTAGAGGAGAA---
TTCAAACAGCTTCAGGCAAGAGTCCTAGCTCTGGAAAGATACCTAAGGGATCAACAGCTCCTAGGAATTTGGG
GCTGCTCTGGAAAACTTATCTGCACCACTAATGTACCTTGGAACTCTAGTTGGAGT----------------

----------------------------AATAAAACTTATAATAGCATATGG------GAC------
AACATGACCTGGTTGCAATGG---GATAAAGAAATTAACAATTACACAAACACAATATAT---
ACTCTAATTGAGAAAGCGCAGAACCAGCAGGAA--

AAGAATGAACAAGACTTATTGGCATTGGACAAATGGGCAAGTCTGTGGAATTGGTTTGACATAACAAACTGGC
TGTGGTATATAAGAATATTTATAATGATAGCAGGAGGTTTAATAGGATTAAGGATAGTTTTTGCT---
GTACTTTCTATAATA---------AATAGAGTTAGGAAGGGATACTCACCT------
CTATCGTTTGCTATTAATTTGCTTGATACAATAGCAATAGCAGTAGCTAAC---
TGGACAGATAGAGTTATAGAAATAGGACAAAGAGTTGGTAGAGCT----------------------------
--------ATTTGCAACATA------CCTAGAAGAATTAGACAGGGTTTAGAAAGGCTTTTGCTA---TAA
>KT152846
GCT---GCA--
GAAAACTTGTGGGTTACTGTCTACTATGGGGTACCTGTGTGG---AAAGATGCAGAG---
ACCACCTTATTTTGTGCATCAGATGCGAAAGCATATAGTACAGAA---AAGCATAAT----------------
--------------GTCTGGGCTACA---CATGCCTGTGTACCCACAGAC---
CCCAACCCACAAGAAATACATTTGGAA---------AATGTGACAGAAGACTTTAAC----------------
--------------------------ATGTGGAAAAATAACATGGTAGAGCAGATGCATACAGACATA---
ATCAGTCTATGGGACCAAAGCCTA---AAGCCATGTGTTAAGTTA---
ACCCCTCTCTGTGTTACTTTAGATTGTGAAACTGTCAGCTGCTCTAATTGT----------------------
----
---AGTGTCAGGGATGAAATAAAA---
AACTGCTCTTTCAATGCAACCACAGAATTAAGG---------------------------
GATAAAACACAGAAGGTACATTCACTT---TTTTATAAACTTGATATAGTACCAATTGATCAA----------
----

GGTAATAGTAGTAATAGTAGTTATAGATTAATAAATTGTAATACCTCAGCCATTACACAG---
GCCTGCCCAAAGGTAACCTTTGAGCCAATTCCCATACATTATTGTGCCCCAGCTGGTTTTGCGATCCTAAAGT
GTAAG---GATGAGAAATTCAAT------GGAACAGGGCCATGCAAGAAT-----------------------
-GTCAGT---ACAGTACAATGCACACATGGGATCAAGCCAGTGGTATCAACT---
CAACTGCTGTTAAATGGCAGTCTAGCAAAAGGAGAA------------
ATAAGAATTAGATCTGAAAATATCACAAACAAT---------GACAAAACTATAATAGTACAGCTTGTC----
-----------AATCCTGTGAGA---------ATTAATTGTACCAGACCTAACAACAATACAAGAAAA-----
-AGTATACATATA------------GGACCAGGACAAGCA---TTC------TAT---ACAAGTGAC------
---------ATAATAGGGGATATA------------AGAGAAGCACATTGT---AATGTCAGTAGATCA---
GAATGGAATAAAACTTTAAAAGAAGTAAGTGAACAATTAGGA---------------CAATACTTTGGG----
-----------------------------------AACAAAACAATAAGATTTGCT------
AACTCCTCAGGAGGGGATATAGAAATCACA---ACACATAGTTTTAATTGTGGAGGAGAA---
TTTAAACAGCTCCAGGCAAGAGTCCTGGCTGTGGAAAGATACCTAAGGGATCAACAGCTCCTAGGAATTTGGG
GCTGCTCTGGAAAACTCATCTGCACCACTAATGTGCCCTGGAACTCTAGTTGGAGT-----------------
----------------------------AATAAATCTCTAGGGGAGATATGG------GAG------
AACATGACCTGGCTGCAATGG---GATAAAGAAATTAGCAATTACACAAGCCTAATATAT---
AACCTAATTGATGAATCGCAGAACCAGCAGGAA--

AAGAATGAACAAGACTTATTGGCATTGGACCAGTGGGCAAATCTGTGGAGTTGGTTTGACATATCAAAATGGC
TGTGGTATATAAAAATATTTATAATGATAATAGGAGGATTAATAGGATTAAGAATAGTTTTTGCA---
GTGCTTTCTTTAATA---------AATAGAGTTAGGCAGGGATACTCACCT------
TTGTCGTTTGCTATTAGTTTGCTTGATACCATAGCAATAGCAGTAGCTGGC---
TGGACAGATAGGATTATAGAAATAGGACAAAGAATTGGTAGAGCT----------------------------
--------ATCCTCAACATA------CCTAGAAGAATCAGACAGGGCTTTGAAAGAGCTTTGCTT---TAA
>KT152841
GCT---GCA--
GAAAAATTGTGGGTTACTGTTTACTATGGGGTACCTGTGTGG---RAAGACGCAGAG---
ACCACCTTATTTTGTGCATCAGATGCTAAAGCATATGATACAGAA---AAGCATAAT----------------
--------------GTCTGGGCTACA---CATGCCTGTGTRCCCACAGAC---
CCCAACCCACAAGAAATACATTTGGAA---------AATGTGACAGAAAACTTTAAC----------------
--------------------------ATGTGGAAAAATAAAATGGTAGAGCAGATGCATACAGACATA---
ATCAGTCTATGGGACCAAAGCCTA---AAGCCATGTGTAAAGTTA---
ACCCCTCTCTGTGTCACTTTAAATTGTAGCAATGTC-------------------------------------
----
----------------------GGTAGCACGAACATCACGGGGCGAGATGGGGAAGAAATAAAA---

AACTGTTCTTTCAATATGACCACAGAACTAAGG--------------------------
GATAAGAGACAGAAGGTATATTCACTT---TTCTATAGACTTGATGTAGTACCAATTGAAGAAGGTAAT----
----

AGTAGTAATAGTAGTTTCAAGGAGTATAGATTAATAAATTGTAATACCTCAGCCATTACACAG---
GCTTGTCCAAAGGTAACCTTTGAGCCAATTCCCATACATTATTGTACCCCAGCTGGTTTTGCGATCATAAAGT
GTAAG---GATGAGAAGTTCAAT------GGAACAGGGCCATGCAAGAAT----------------------
-GTCAGT---ACAGTACAATGCACACATGGAATCAAGCCAGTAGTATCAACT---
CAACTGCTGTTAAATGGCAGTCTAGCAGAAAGAGGA-----------
GTAAAAATTAGATCTGAAAATATCACAGACAAT---------GTCAAAACTATAATAGTACAACTTGAC----
-----------AAGCCTGTGAGA---------ATTATTTGTACCAGACCTAACAACAATACAAGAAAA-----
-AGTATACGTATA------------GGACCAGGACAAGCA---TTC------TAT---ACAAGTGAC------
---------ATAATAGGGGATATA------------AGACAAGCACATTGT---AATGTCAGTAGAYCA---
AAATGGAATTACACTTTACAAAAGGTAGGTGAACAATTAGAA---------------AAATACTTTGTG----
-----------------------------------AACAGAACAATAAGATTTAAT------
AAATCCTCAGGAGGGGATCTAGAAATCACA---ACACATAGTTTTAATTGTGGAGGAGAA---
TTTAACAGACTCCAGGCAAGAGTCCTGGCTGTGGAAAGATACCTAAAGGATCAACAGCTCCTAGGAATTTGGG
GCTGCTCTGGAAAACTCATCTGCACCACTAATGTGCCCTGGAACTCTAGTTGGAGT-----------------
----------------------------AATAAAACTCAGCAGGAGATATGG------GAT------
AACATGACCTGGTTGCAATGG---GATAAAGAAATTAGCAATTACACAGAAATGATATAT---
CAACTAATTGAAGAATCGCAGAACCAGCAGGAA---------------------------------------
----
AAGAATGAACAAGACTTATTGGCATTGGACAAGTGGGCAAATCTGTGGAATTGGTTTGACATATCAAATTGGC
TGTGGTATATAAAAATATTTATAATGATAGTAGGAGGATTAGTAGGATTAAGAATAGTTTTTGCT---
GTACTTTCTGTAATA---------AATAGAGTTAGGCAGGGATACTCACCT------
CTGTCATTTGCCATTAATTTGCTTGATACCATAGCAATAGTAGTAGCTGGC---
TGGACAGATAGGATTATAGAAATAGGACAAGGAATTGTTAGAATT---------------------------
--------ATTCTCCACATA------CCTAGAAGA---------GGCTTAGAAGGAGCTTTGCTA---TAA
>KT152842
GCT---GCA---
GGAAACTTGTGGGTCACTGTTTACTATGGGGTACCTGTGTGG---AAAGAAGCAGAG---
ACCACCTTATTTTGTGCATCAGATGCTAAAGCATATAAGAAAGAA---GTGCATAAT----------------
--------------GTVTGGGCTACA---CATGCCTGTGTACCCACAGAC---
CCCAACCCACAAGAAATACATTTGGAA---------AATGTGACAGAAGACTTTAAC---------------
--------------------------ATGTGGACAAATAACATGGTAGAGCAGATGCATACAGACATA---
ATCAGTCTATGGGATCAAAGCCTA---AAGCCATGTGTAAAGTTA---
ACCCCTCTCTGTGTTACTCTGAATTGTAGTAATGTCAATGTCAATAAC------------------------
----
-------------------------ACGCAGAACTTCACGGGGTGGGACAGGGAACAAATAAAA---
AACTGCTCTTTCAATATGACCACAGAACTAAGG--------------------------
GACAAGAGTCAGAAGGTATATTCACTT---TTTTATAGACTAGATGTAGTACAAAGTCAAGGT----------
----

AATAGTAGTAATAGTAGTTATAGGGAGTATAGATTAATAAATTGTAATACCTCAGCCATTACGCAG---
GCTTGCCCAAAGGTATCCTTTGAGCCAATTCCCATACATTATTGTGCCCCAGCTGGTTTTGCGATCCTAAAGT
GTAAT---GATGAGAAGTTCAAT------GGAACAGGGCCATGCAGGAAT----------------------
-GTCAGT---ACAGTACAATGCACACATGGAATCAAGCCGGTAGTATCAACT---
CAACTACTGTTAAATGGCAGTCTAGCAAAA---AAT-----------
ATAACAATTAGATCTAAAAATATCACAGACAAT---------GTCAAAAATATAATAGTACAACTTGCC----
-----------ACTCCTGTGGAA---------ATTAATTGTACCAGACCTAACAACAATACAAGAAGA-----
-AGTGTACGTATA------------GGACCAGGACAAGCA---TTC------TATACAAATAGC---------
---------ATAATAGGGGATATA------------AGACAAGCATATTGC---AGTGTCAATAGATCA---
GAATGGAATAAAACTTTAAGCCAGGTAGGTGAACAATTAGGA---------------AAATACTTTAAG----
-----------------------------------AACAAAACAATAAAATTTGAT------
AAGTCCTCAGGAGGAGATCTAGAAATCACA---ACACATAGTTTTAATTGTAGAGGAGAA---
TTTAAACAGCTCCAGGCAAGAGTCCTGGCTGTGGAAAGATACCTAAGGGATCAACAGCTCCTAGGAATTTGGG
GTTGCTCTGGAAAACTCATCTGCACCACTAATGTGCCCTGGAACTCTAGTTGGAGT-----------------
----------------------------AATAAGACTCAAGATGATATATGG------AAT------
AAAATGACCTGGATGCAATGG---GAGAAAGAAATTAGCAATTACACAGACATAATATAT---
AGCCTAATTGAAGAATCGCAGAACCAGCAGGAA---------------------------------------

----
AAGAATGAACAAGACTTATTGGCATTGGACAAGTGGGCAAATCTGTGGAATTGGTTTGACATATCAAATTGGC
TGTGGTATATAAAAATATTTATAATGATAGTAGGAGGATTAATAGGATTAAGAATAGTTTTTACT---
GTGCTTTCTGTAATA---------AATAGAGTTAGGCAGGGATACTCACCT------
TTGTCATTTGCTATTAGTTTAATTGATACCATAGCAATAGTAGTAGCTGGC---
TGGACTGATAGGATTATAGAAATAGGACAAACATTTGGTAGAGCT----------------------------
--------ATTCTCCACATA------CCTAGAAGAATCAGACAGGGCTTAGAAAGAGCTTTGCTA---TAA
>KT152844
GCT---GCA--
AGCAACTTGTGGGTTACTGTCTACTATGGGGTACCTGTGTGG---AAAGAAGCAGAT---
ACCACTTTATTTTGTGCATCAGATGCTAAAGCATATGAGACAGAA---AAGCATAAT----------------
--------------GTCTGGGCTACA---CATGCCTGTGTACCCACAGAC---
CCCAGCCCACAAGAAATACATTTGGAA---------AATGTGACAGAAAACTTTAGC----------------
--------------------------ATGTGGAAAAATAAAATGGTAGATCAGATGCATACAGATATA---
ATCAGTCTATGGACCAAAGCCTA---AAGCCATGTGTAGAGTTA---
ACCCCTCTCTGTGTTACTTTAAGTTGTAGCAATGTCAATAACAACACG-------------------------
----
-------------------------------AACATCACAGGGTGGGGCAAGGAAGAAATAAAA---
AACTGCTCTTTCAATATGACCACAGAACTAAGG---------------------------
GATAAGAGACAGAAGGCATATTCACTT---TTTTATAGACTTGATGTAGTACCAATTAATGAAGGTGAT----
----

AGTAGTAATAGTAGTTATAAGGAGTATAGATTAATAAATTGTAATACCTCAGCCATTACACAG---
GCTTGTCCAAAAGTAACCTTTGAGCCAATTCCCATACATTATTGTGCCCCAGCTGGTTTTGCGATCCTAAAGT
GTAAT---GATAAGGAGTTCAAT------GGAACAGGACCATGCAAGGAT-----------------------
-GTCAGT---ACAGTACAATGCACACATGGAATCAAGCCAGTAGTATCAACC---
CAACTACTGTTAAATGGCAGCCTAGCAGAAGAAAAA------------
GTAATAATTAGATCTGAAAATATCACAAACAAT---------GTCAAAACTATAATAGTACAACTTGCC----
-----------ACGCCTGTAAAA---------ATTAGTTATGCCAGACCTAACAACAATACAAGAAAG-----
-AGTGTACGTATA------------GGACCAGGACAGGCA---TTC------TAT---ACAAGTGAC------
---------ATAATAGGGGATATA------------AGACAAGCACATTGT---AATGTCAGTAGATCA---
GAATGGAATAAAACTTTAAACAAGGTAGGTGAACAACTAGGA---------------AGACACTTTGTG----
-----------------------------------AACAAAACAATAAAATTTACT------
AATTCCTCAGGAGGGGATCTAGAAATCACA---ACACATAGTTTTAATTGTGGAGGAGAA---
TTTAAACAGCTCCAGGCAAGAGTCCTGGCTGTGGAAAGGTACCTAAGGGATCAACAGCTCCTAGGAATTTGGG
GCTGCTCTGGAAAGCTCATCTGCACCACTAATGTGCCTTGGAACTCTAGTTGGAGT-----------------
----------------------------AATAAATCTCTAGAAGAGATATGG------GAC------
AACATGACCTGGCTACAATGG---GAAACAGAAATTAGCAATTACACAGAGCTAATATAT---
AACCTAATTGAGGAATCGCAGAACCAGCAGGAA--

AAGAATGAACAAGACTTATTAGCATTGGACAAGTGGGCAAATCTGTGGAATTGGTTTGACATATCAAAATGGC
TGTGGTATATAAAAATATTTATAATGATAGTAGGAGGATTAATAGGATTAAGAATAGTTTTTGCT---
GTGCTTTCTGTAATA---------AATAAAGTTAGGCAGGGATACTCACCT------
CTCTCATTTGCTATTAGTTTGGTTGATACCATAGCAATAGTAGTAGCTGGC---
TGGACAGATAGGATTATAGAAATAGGACAAGGAATTGGTAGAGCT----------------------------
--------ATTCTCCACATA------CCTAGAAGAATCAGACAGGGCTTAGAAAGAGCTTTGCTA---TAA
>AY736809
GCT---GCA--
GACAACTTGTGGGTTACTGTCTATTATGGGGTACCTGTGTGG---AAAGATGCAGAG---
ACCACCTTATTTTGTGCATCAGACGCTAAAGCATATGAGACAGAA---AAACATAAT----------------
--------------GTCTGGGCTACA---CATGCCTGTGTACCCACAGAC---
CCCAGCCCACAAGAAATACCTTTGAAA---------AATGTGACAGAAAAGTTTAAC----------------
--------------------------ATGTGGAAAAATAACATGGTAGAGCAGATGCATACAGATATA---
ATCAGTCTATGGACCAAAGCCTA---AAGCCATGTGTACAGTTA---
ACCCCTCTCTGCGTCACTCTAAATTGTAGTGACGTCACCACCAATAACACCAAGGTCAATACCACCAGT----
----
-------------------GCCCCTACCACCACTGCCACTGTCAACAATGACAATGACATGAAA---
AACTGCTCTTACAATATAACCACAGAACTAAGG---------------------------
GATAAGAAACAGAAAAGGTATTCACTC---TTTTATAGACTTGATGTAGAACTAATAAATAAGAAT-------
----

AATAGTAATAGCACTGAGTATGTATTAATAAATTGTAAAACCTCAACCCTCACACAG---
GCTTGTCCAAAGGTATCCTTTGAGCCAATTCCCAAACATTATTGTGCCCCAGCTGGTTTTGCGATTCTAAAAT
GTAAG---GATAAGGAGTTCAAT------GGAACAGGGCTATGCAAGAAT-----------------------
-GTCAGC---ACAGTACAATGCACACATGGAATCAAGCCAGTAGTGTCTACT---
CAACTACTATTAAATGGCAGTTTAGTGGAAAAAAGG------------
GTAATGATTAGATCTGCAAATATCACAGACAAT---------ACCAAAAACATAATAGTACAACTTAAG----
-----------GAGCCTGTAGAA---------ATTAATTGTACCAGACCTAACAACAATACAAGAAAA-----
-GGTGTACATATA------------GGGCTAGGAAGAAGA---TTC------TATACAACACAA---------
---------GTAGTAGGGGATATA------------AGACAAGCATATTGT---AATGTCAGTAAATCA---
AAATGGAATGATACTTTGAAAAAAGTAGTTTACCAATTAAGA---------------AAATACTTT-------
-----------------------------------AACAAAACAATAATCTTTAAC------
TCCTCCTCAGGAGGGGATGTAGAAATTACA---ACACATATGTTTACTTGTGGAGGAGAG---
TTTAAACAGCTCCAGGCACGAGTCCTGGCTGTGGAAAGATACCTAAGGGATCAACAGCTCCTAGGAATTTGGG
GCTGCTCTGAAAAACTCATCTGCACCACTAATGCGCCTTGGAACTCCAGTTGGAGT-----------------
----------------------------AATAAGTCTCAGAGTGAGATATGG------GAC------
AACATGACCTGGTTGCAATGG---GATAGAGAAATTAACAATTATACAGACATAATATAT---
AGACTACTTGAAGATTCGCAAAACCAGCAGGAA--

AAGAATGAACAAGACTTATTGGCATTGGACAAGTGGTCAAGCCTGTGGACTTGGTTTGACCTATCAAACTGGC
TGTGGTATATAAAAATATTCATAATGATAGTAGGAGGTTTAATAGGATTAAGAATAGTGTTTGCT---
GTGCTTGCTGTAATA---------AATAGAGTTAGGCAGGGATACTCACCT------
TTGTCGTTCGCTGTTAGCTTGCTTGATACCATAGCAATAGTAGTAGCTGGC---
TGGACAGATAGGGTTTTAGAAATAGGACAAAGAATTGGTAGAGCT----------------------------
--------TTTATTAACATA------CCTAGAAGAATTAGACAGGGCTTTGAAAGGGCTTTGCTG---TAA
>AY736810
ACT---GCA--
GAAGACTTGTGGGTTACTGTCTACTATGGGGTACCTGTGTGG---AAAAACGCAGAG---
ACCACCCTATTTTGTGCATCAGATGCTAAGGCATATAAGACAGAA---GTGCATAAT----------------
--------------GTCTGGGCTACA---CATGCCTGTGTACCCACAGAC---
CCCAACCCACAAGAAATACATTTGGAA---------AATGTGACAGAAGAGTTTAAT----------------
--------------------------GTGTGGAAAAATAACATGGTAGAGCAGATGCATACAGACATA---
ATCAGTTTATGGGACCAAAGCCTA---AAGCCATGTGTAAAGTTA---
ACCCCTCTCTGCGTTACTTTAAACTGTATCAATGCCAACGTTACCAACAGCAGCATG----------------
----
-------------------------------ACCAACAGCAGCATGATGGAAGGAGAAATAAAA---
AACTGTTCTTACAATATGACCACAGAACTAAGG---------------------------
GATAAGAAAAGGAAAGTATTTTCGCTT---TTTTATAAACTTGATGTAGTACCAATGAATGAA----------
----

AATAATAGTGAGTATAGATTAATAAATTGTAATACCTCAGCCATTACACAG---
GCTTGTCCAAAGGTAACCTTTGAGCCAATTCCCATACATTATTGTGCCCCAGCTGGTTTTGCAATTCTAAAGT
GTAAG---GATGAGGAGTTCAAT------GGAACAGGGTTATGCAAGAAT-----------------------
-GTCAGC---ACAGTACAATGCACACATGGAATCAAGCCGGTAGTGTCAACT---
CAACTACTGTTAAATGGCAGTCTAGCAGAAAAAGAG------------
ATAAGAATTAAATCTGAAAACATCTCAGACAAT---------GCTAAAACCATAATTGTACAACTTACC----
-----------AAGCCTGTACTA---------ATCAATTGTGCCAGACCTAGCAACAATACAAGAAAA-----
-AGTGTACATATA------------GGACCAGGACAAGCA---TTC------TATGCAACAGGTGCT------
---------ATAACAGGAGATATA------------AGACAAGCATATTGT---GTAGTCAATAGAACA---
CAATGGAATGACACTTTAGGACAGGTAGCGATACAATTAAGA---------------AAACACTGG-------
-----------------------------------AACACAACAATAATCTTTAAT------
GAACCCTCAGGAGGGGATTTGGAAATTACA---ACACATAGTTTTAATTGTGGAGGGGAA---
TTTAAACAGCTCCAGGCAAGAGTCCTGGCTGTGGAAAGATACCTAAAGGATCAACAGCTACTAGGAATTTGGG
GCTGCTCTGGAAAACTCATCTGCACCACTAATGTGCCTTGGAACTCTAGTTGGAGT-----------------
----------------------------AATAAATCTTACAATGAGATATGG------GAA------
AACATGACCTGGCTGCAATGG---GATAAAGAAATTAACAATTACACAGAACTAATATAT---
AGCCTAATTGAAGAATCGCAGAACCAGCAGGAA--

AAGAATGAACAAGACTTATTGGCATTGGACAAGTGGGCAAGTCTGTGGAATTGGTTTGACATATCCAACTGGC
TGTGGTATATAAAAATATTTATAATGATAGTAGGAGGCTTAATAGGATTAAGAATAGTTGTTGCT---

GTGCTTTCTGTAATA---------AATAGAGTTAGGCAGGATACTCACCA------
CTATCATTTGCTATTAACTTGCTCAATACCATAGCAATAGCAGTAGCTGGC---
TGGACAGATAGAGTTATAGAAATAGGACAAGGAATTGGTAGAGCT----------------------------
--------ATTCTCCACATA------CCTAGAAGAATCAGGCAGGGCTTCGAAAGGGCTTTATTA---TAA
>AY736812
GCT---GCA--
GACAACTTGTGGGTTACTGTCTATTATGGGGTACCTGTGTGG---AAAGATGCAGAG---
ACCACCTTATTTTGTGCATCAGATGCTAAAGCATATGAGACAGAA---AAGCATAAT----------------
--------------GTTTGGGCTACA---CATGCCTGTGTGCCCACAGAC---
CCCAACCCACAAGAAATACCTTTGGAA---------AATGTGACAGAAGAGTTTAAC----------------
--------------------------ATGTGGAAAAATAAAATGGTAGAACAAATGCATACAGATATA---
ATCAGTCTATGGGACCAAAGCCTA---CAGCCATGTGTAAAGTTA---
ACCCCTCTCTGTGTTACTTTAAATTGTACGGATGCTACTAATGGTACGATTGGC-------------------
----
-------------------------------AACATCACCGATGAAATGAAGGGAGAAATAAAA---
AACTGCTCTTTCAATATAACCACAGAAATAAGG---------------------------
GATAAGAAACAGAAAGTATATTCACTT---TTTTATAGACTTGATGTAGTACCAATAGAGCCAGATAGTAGT-
----

AATAGTAGTAGAAACAGTAGTGAGTATAGATTAATAAATTGTAATACCTCAGCCATTACACAA---
GCCTGCCCAAAGGTAAGCTTTGAGCCAATTCCCATACATTATTGTGCCCCAGCTGGTTTTGCGATCCTGAAGT
GTAGG---GATAAAGAGTTCAAT------GGAACAGGGAAATGCAAGAAT-----------------------
-GTCAGC---ACAGTCCAATGCACACATGGAATCAAGCCAGTAGTATCAACT---
CAACTGCTGTTAAATGGCAGTCTAGCAGAAGGAGAG------------
GTAAGAATTAGATCTGAAAATATCACAAACAAT---------GCCAAAACTATAATAGTACAACTTGTC----
-----------GAGCCTGTGAGA---------ATTAATTGTACTAGACCTAATAACAATACAAGAGAG-----
-AGTGTGCGTATA------------GGGCCAGGACAAGCA---TTC------TTTGCAACAGGTGAC------
---------ATAATAGGGGATATA------------AGACAAGCACATTGT---AATGTCAGTAGATCA---
CAATGGAATAAGACTTTACAACAGGTAGCTGCACAATTAGGA---------------GAACACTTTAAA----
-----------------------------------AACAAAGCAATAACATTTAAC------
AGTTCCTCAGGAGGAGATCTAGAAATCACA---ACACATAGTTTTAATTGTGGAGGAGAA---
TTTAAACAGCTCCAGGCAAGAGTCCTTGCTGTGGAGAGATACCTAAGGGATCAACAGCTCCTAGGAATTTGGG
GCTGCTCTGGAAAACTCATCTGCACCACTAATGTGCCCTGGAACTCTAGTTGGAGT-----------------
----------------------------AATAAATCTCATGATGAAATATGG------AAC------
AACATGACCTGGCTGCAATGG---GATAAAGAAATTAGCAATTACACAAACCTAATATAT---
AGTCTAATTGAAGAATCGCAAAACCAGCAGGAA--

AAGAATGAACAAGATTTATTGGCATTGGACAAGTGGGCAAGTCTGTGGAATTGGTTTGACATATCAAAGTGGC
TGTGGTATATAAAAATATTTATAATGATAGTAGGAGGTTTAATAGGATTAAGAATAGTTTTTGCT---
GTGCTTGCTGTAATA---------AAGAGAGTTAGGCAGGATACTCACCT------
GTGTCATTTGCTATTAATTTGGTTGATACTATAGCAATAGCAGTAGCTGGC---
TGGACAGATAGGGTTATAGAAATAGGACAAGGAATTGGTAGAGCT----------------------------
--------TTTCTCCACATA------CCTAGAAGAATCAGACAGGGCTTAGAAAGGGCATTGCTG---TAA
>AY736815
GTT---GTA--
GGAAACTTGTGGGTTACTGTCTACTATGGGGTACCTGTGTGG---AAAGAGGCAGAC---
ACCACCTTATTTTGTGCATCAAATGCTAGAGCATATGATACAGAA---GTGCATAAT----------------
--------------GTTTGGGCTACG---CATGCCTGTGTACCTACAGAC---
CCCAACCCACAAGAAATAGATTTGGAA---------AATGTGACAGAAGAGTTTAAC----------------
--------------------------ATGTGGAAAAATAACATGGTAGAGCAAATGCATACAGATATA---
ATTAGTCTATGGGACCAATTCCTA---AAACCATGTGTAAAGTTA---
ACCCCTCTCTGCGTTACTTTAGATTGTGGCTATAATGTAACCAACTTGACT----------------------
----
----------------------------------TCCACCAGTAACATGAAAGAAGACATAACA---
AACTGCTCTTACAATATGACCACAGAAATAAGG---------------------------
GATAGGAAACAGAAAGTGTATTCACTT---TTTTATAGGCTTGATATAGTACCAATTGATGAAGAAAAG----
----

AATAATAACAGTGTTACTAGTCCGTATAGATTAATAAATTGTAATACCTCAGCCATTACACAG---
GCCTGTCCTAAGGTATCCTTTGAACCAATTCCCATATATTATTGTGCCCCAGCTGGTTTTGCGATTCTAAAAT

GTAAG---GATGCAGAGTTCAAT------GGAACAGGGCCATGCAAGAAT-----------------------
-GTCAGC---ACAGTACAATGTACACATGGAATCAGGCCAGTAATATCAACT---
CAACTACTGTTAAATGGCAGTCTAGCAGAGAATGGG------------
ACAAAGATTAGATCTGAAAACATCACAAACAAT---------GCCAAAACCATAATAGTACAGCTTAAT----
-----------GAGACTGTACAA---------ATTAATTGTACCAGACCTAACAACAATACAAGAAGA-----
-AGTGTACGTATA------------GGACCAGGACAAGCA---TTC------TATGCAACAGGTGGC------
---------ATAACAGGGGATATA------------AGACGAGCATATTGT---AATGTCAGTAGACAA---
AAATGGGAACAAGCATTAAAAGGGGTAGTCATACAATTAAGA---------------AAACACTTTAAT----
-----------------------------------AATAAAACAATAACCTTTACC------
AATTCCTCAGGAGGGGATGTAGAAATTACA---ACACATAGTTTTAATTGTGGAGGAGAA---
TTTAAACAGCTCCAGGCAAGAGTCCTGGCTGTGGAAAGATACCTAAGGGATCAACAGCTCCTAGGAATTTGGG
GCTGCTCTGGAAAACTCATCTGCACCACTAATGTGCCCTGGAACTCTAGTTGGAGT-----------------
----------------------------AATAAATCTCAGGAAGAGATATGG------GGT------
AACATGACCTGGCTGCAATGG---GATAAAGAAATTAGCAAGTACACACAAACAATATAT---
GACTTATTTGAAGAATCGCAGAACCAGCAGGAA--

AAGAATGAACAAGACTTATTGGCATTGGACAAGTGGGCAAATTTGTGGACTTGGTTTGACATAACAAATTGGC
TGTGGTATATAAAAATATTTATAATGATAGTAGGAGGCTTAATAGGATTAAGAATAGTTTTTTCT---
GTGCTTTCTGTAATA---------AATAGAGTTAGGCAGGATACTCACCT------
CTGTCGTTTGCTATCAATTTGATTGATACCATAGCAATAGCAGTAGCTGGC---
TGGACAGATAGAATTATAGAAGCAGGACAAAGACTTTGTAGAGCT----------------------------
--------ATACTTAACATA------CCTAGAAGAATCAGACAGGGTGCCGAAAGGCTTGGCTA---TAA
>AY736813
GCT---ACA--
GACAACTTGTGGGTTACTGTCTATTATGGGGTACCAGTGTGG---AAAGACGCAGAG---
ACCACCTTATTTTGTGCATCAGATGCTAAAGCATATGCGACAGAA---AAGCATAAT----------------
--------------GTCTGGCTACA---CATGCCTGTGTACCCACAGAC---
CCCAACCCACAAGAAATACATTTGGAA---------AATGTGACAGAAGAATTTAAC----------------
--------------------------ATGTGGAAAAATAACATGGTAGAACAGATGCATACAGATATA---
ATCAGTCTATGGGACCAAAGCCTA---AAACCATGTGTAAAGTTA---
ACCCCTCTCTGCGTTACTTTAAATTGTAGCAATGCCAATGTAAGGTATAGCAATGCC----------------
----
-------------------------------ACTGTTAACAACACCATCAAGGATGAAATAAAA---
AACTGCTCTTTCAATACAACCACAGCACTAAGG---------------------------
GATAAGAGACAGAAAGTATATTCACTT---TTTTATAGACTTGATATAGTACAAATTGATAATAGTAGT----
----

AGTGATAGTAGTAGTAGTGAGTATAGATTAATAAATTGTAATACCTCAGCCATTACACAA---
GCTTGTCCAAAGGTAACCTTTGAGCCAATTCCCATACATTATTGTGCCCCAGCTGGTTTTGCAATCCTAAAGT
GTAAA---GATGAGGAGTTCAAT------GGAACAGGGCCATGCAAGAAT-----------------------
-GTCAGC---ACAGTACAATGCACACATGGAATCAAGCCAGTAGTATCAACT---
CAACTGCTGCTAAATGGCAGTCTAGCAAAAAGAGAG------------
GTAAAAATTAGAACTGAAAATATCACAAACAAT---------GCCAAAAATATAATAGTACAATTTGTT----
-----------AATCCTGTGGAA---------ATTAATTGTACCAGACCTAACAACAATACAAGAAAA-----
-AGTATACATATA------------GGACCAGGACAAGCA---TTC------TATGCAACAGGTGAC------
---------ATAATAGGGGATATA------------AGACAAGCACATTGT---AATGTCAGTAGATCA---
TCCTGGAATAAGACTTTACAACAGGTAGCTAAACAATTAGGA---------------ACATACTTTAAG----
-----------------------------------AACAAAACAATAGTATTTAAT------
ACATCCTCAGGAGGGGATCCTGAAATCACA---ACACATAGTTTTAACTGTGCAGGAGAA---
TTTAAACAGCTCCAGGCAAGAGTCCTGGCTGTGGAGAGATACCTAAGGGATCAACAGCTCCTAGGAATTTGGG
GCTGCTCTGGAAAACTCATCTGCACCACTAATGTGCCCTGGAACTCTAGTTGGAGT-----------------
----------------------------AATAAATCTTATAATGACATATGG------GAT------
AACATGACCTGGCTGCAATGG---GATAAAGAAATTCACAATTACACACAATTAATATAT---
AACCTAATTGAAGAATCGCAGAACCAGCAGGAA--

AAGAATGAACAAGACTTATTGGCATTGGACAAGTGGGCAAATCTGTGGAATTGGTTTAACATAACACATTGGC
TGTGGTATATAAAAATATTTATAATGGTAGTAGGAGGCTTAATAGGATTAAGAATAGTTTTTGCT---
GTGCTTTCGATAATA---------AATAGAGTTAGGCAGGATACTCACCT------
TTGTCGTTTGCTGTTAATTTAGTTGATACCATAGCAATAGCAGTAGCTGGC---

TGGACAGATAGGATTATARAAATAGGACAAGGAATTGGTAGAGCT----------------------------
--------ATTCGCCACATA------CCTAGAAGAATCAGACAGGGTTTAGAAAGAGCTTTGCTA---TAA
>AY736816
GCT---ACA---
GAACACTTGTGGGTTACTGTCTACTATGGGGTACCAGTGTGG---AAAGACGCAGAG---
ACCACCTTATTTTGTGCCTCAGACGCTAAAACATATGAACCAGAA---AAGCATAAT---------------
--------------GTCTGGGCTACA---CATGCCTGTGTACCCACAGAC---
CCCAACCCACAAGAAATACATTTGGAA---------AATGTGACAGAAAAGTTTAAC---------------
-------------------------ATGTGGAAAAACAAAATGGTAGAACAAATGCATACAGATATA---
ATCAGTCTATGGGATCAAAGCCTA---AAACCATGTGTAAAGTTA---
ACCCCTCTCTGCGTTACTTTAAATTGTAACAACACATTCCCCAAGAATAATACC------------------
----
-----------------------------------ATCGATGATAGCATGAAAGAAGCAATAACA---
AACTGCTCTTTCAATATGACCACAGAATTAAGG--------------------------
GATAAGAGCCAGGAAGTACATTCACTT---TTTTATAAACTGGATGTAGTACCTATGAATGGC----------
----

AATCAGTATAGATTAATAAATTGTAATACCTCAGTCATTAAACAA---
GCTTGTCCAAAAGTATCCTTTGAGCCAATTCCCATACATTATTGTGCCCCAGCTGGTTTTGCGATTCTAAAGT
GTAAT---GATAAGGAGTTCAAT------GGAACAGGGCCATGCAAGAAT----------------------
-GTCAGC---ACAGTACAATGCACACATGGAATCAAGCCAGTGGTGTCAACT---
CAACTGCTGTTAAATGGCAGTCTAGCAGAAGAAAAG------------
ATAATAATTAGATCTGAAAATATCACAAATAAT---------GCCAAAAACATAATAGTACAACTTAAA----
-----------AAGCCTGTAAGA---------ATTAATTGTACTAGACCTAGCAACAATACAAGAAAA-----
-AGTGTACGTATA------------GGACCAGGCCAAGTA---TTC------TATGCAACA---GAC------
---------ATAATAGGGAACATA-----------AGACAAGCACATTGT---AATGTCAGTAAATCA---
GAATGGAATGACACTTTACAACAGGTAGTTGAACAATTAAGA---------------GGATACTTTAAG----
-----------------------------------AACAAAACAATAATATTTACT------
AACTCCTCAGGAGGGGATCTAGAAATCACA---ACACATAGTTTTAATTGTAGAGGAGAA---
TTTAAACAGCTCCAGGCAAGAGTCCTGGCTCTGGAAAGATACCTAAAGGATCAACAGCTCCTAGGAATTTGGG
GCTGCTCTGGAAAACTCATCTGCACCACTAATGTGCCCTGGAACTCTAGTTGGAGT----------------
----------------------------AATAAAACCCAGGAAGAGATATGG------GGG------
AACATGACCTGGCTGCAATGG---GACAAAGAAATTAGCAATTACACACATATAATATAT---
AATCTAATTGAAGAATCGCAGAACCAGCAGGAA--

AAGAATGAACAAGACTTATTGGCATTGGACAAGTGGGCAAATTTGTGGAATTGGTTTGACATAACAAACTGGC
TGTGGTATATAAAAATATTTATAATGATAGTAGGAGGCTTAATAGGATTAAGAATAGTTTTTGCT---
GTGCTTTCTACAATA---------AATAGAGTTAGGCAGGGATACTCACCT------
TTGTCGTTCGCTATTAATTTGGTCAATACCATAGCAATAGCAGTAGCTGGC---
TGGACAGATAGGGTTATAGAAATAGGACAAGGAATTGGTAGAGCT----------------------------
--------ATTCTCCACATA------CCTAGAAGAATTAGACAGGGCTTAGAAAGGGTTTTGCTA---TAA
>AF457053
GCT---GCA---
GAACACTTGTGGGTTACTGTCTACTATGGGGTACCTGTGTGG---AAAGAAGCAGAA---
ACCACCTTATTTTGTGCATCAGAAGCTAAAGCATATGATCCAGAA---CAGCATAAC---------------
--------------GTCTGGGCTACA---CATGCCTGTGTACCCACAGAC---
CCCAACCCACAAGAAATAAATTTGATA---------AATGTGACAGAAAAGTTTAAC---------------
-------------------------ATGTGGAAAAATACCATGGTAGAGCAAATGCATACAGATATA---
ATCAGTCTATGGGACGAAAGTCTA---AAGCCATGTGTAAAGTTA---
ACCCCTCTCTGTGTTACTTTACGTTGTAACAATACCAATGTCAATGTCAATAACACG---------------
----
------------------------------ATTGACAACAGCACGGACAGGGGGGAAATGAAA---
AACTGCTCTTTCAAAGTGACCACAGAGCTAAGG--------------------------
GATAAGGAACAGAAGGTACATTCACTT---TTTTATAGACTTGACTTAGTACAATTGGATAATACAACG----
----

CCAGAAAATAGTAGTAACAAGGAGTATAGATTAATAAATTGTAATACCTCAGCCATCACACAG---
GCTTGCCCAAAGGTATCTTTTGAGCCAATTCCCATACATTATTGTGCCCCAGCTGGTTATGCGATCCTAAAGT
GTAAT---GATAAGAAGTTCAAT------GGAACAGGGCCATGCACGAAT----------------------
-GTCAGC---ACAGTACAATGCACACATGGAATCAGGCCAGTAGTATCAACT---

CAACTGCTGTTAAATGGAAGTCTAGCAGAAGAAGAG------------
GTAATAATTAGATCTGAAAACATCACAGACAAT---------GCCAAAACTATAATAGTACAACTTACT----
-----------GAGCCTGTAAAA---------ATTAATTGTACCAGACCTAACAACAATACAAGAAGG-----
-AGTATAACTATG------------GGACCAGGAAAAGCA---TTC------TATACAAATGAC---------
---------ATCATAGGAAATATA------------AGACAAGCATATTGT---ACTGTCAATAGATCA---
GAATGGAATAACACTTTACAAAAGGTAGCTACACAATTAAGA---------------GAACACTTTGAA----
-----------------------------------AACAAAACAATAATATTTACT------
CACTCCTCAGGAGGGGATCTAGAAGTCACA---ACACATATGTTTAATTGTGGAGGAGAA---
TTCAAACAGCTCCAGGCACGAGTCCTGGCTGTGGAAAGATACCTACAGGATCAACAGCTCCTAGGAATTTGGG
GATGCTCTGGAAAACTCATCTGCCCCACTAATGTGCCCTGGAACTCTACTTGGAGT-----------------
----------------------------AATAAATCTTATAGTGAGATATGG------GAT------
AACATGACCTGGCTGCAATGG---GATGAAGAAGTTAGCAATTACACACAAATAATATAT---
GACCTAATTGAAAAATCGCAGAACCAGCAGGAA--

AAGAATGAACAAGACTTATTGGCATTGGACAAGTGGGCAAGTCTGTGGAGTTGGTTTAGCATATCAAACTGGC
TGTGGTATATAAGAATATTTATAATGGTAGTAGGAGGTTTAATAGGATTAAGAATAGTTTTTGCT---
GTGTTTTCTGTAATA---------AATAGAGTTAGGCAGGGATACTCACCT------
CTGTCATTTGCTACTAATTTGATAGATACTATAGCAATAGTAGTAGCTGGC---
TGGACAGATAGGGTGATAGAAATAGGACAGAGCATTGGTAGAGCT----------------------------
--------ATCCTCCACATA------CCTAGAAGAATCAGACAGGGCTTTGAAAGAGCTTTGCTA---TAA
>AF457055
ATG---GCA--
CAAAACTTGTGGGTTACTGTCTATTATGGGGTACCTGTGTGG---AGAGATGCAGAC---
ACCACCCTATTTTGTGCATCAGATGCTAAAGCATATAAGACAGAA---GTGCATAAT----------------
--------------GTCTGGGCTACA---CATGCCTGTGTGCCCACAGAC---
CCCAACCCACAAGAAATATATTTGACA---------AATGTGACAGAAAATTTTAAC----------------
--------------------------ATGTGGAAAAATAAAATGGTAGAGCAGATGCATGAAGACATA---
ATTAGTCTATGGGACCAAAGCCTA---AAGCCATGTGTACAGTTA---
ACCCCTCTCTGCGTTACGTTAAACTGTAGCGATGTCACGAATAACACCCTCAGGAATGCCACTGTGAAT----
----
----------------GCCAATGCCAATGCCACTGTCACTGGGGACATGGAAGGAGAAATGAAA---
AACTGCTCTTACAATATGACCACAGCAGTAAGG---------------------------
GATAAGCAAAAGAAAGTATACTCACTT---TTTTATAGGCTAGATGTAGTGCAAATTAGCAATAAT-------
----

AGTAGCAGTAGTAGTGAGTATAGATTAATAAATTGTAATACCTCAGCCATTACACAA---
GCTTGTCCAAAAGTAACCTTTGAGCCAATTCCCATACATTATTGTGCCCCAGCTGGTTTTGCGATCCTAAAGT
GTAAT---GAAGAAGGGTTCAAT------GGAACAGGACCATGCAAAAAT-----------------------
-GTCAGC---ACAGTACAATGCACACATGGAATCAAGCCAGTAGTATCAACT---
CAACTGCTGTTAAATGGCAGTCTAGCAGAAAAAGGG------------
GTAATAATCAGATCTGAAAACATCTCAAACAAT---------GCTAAAACCATAATAGTACAACTTGCC----
-----------GAGCCTGTAACA---------ATTAATTGTACCAGACCTAACAACAATACAAGAAAA-----
-GGTATACATATA------------GGACCAGGACGAGCA---TTC------TATGCAACAGGTGAC------
---------ATAATAGGGGATATA------------AGAAAAGCATATTGT---AATGTCAGTAGAACA---
CAATGGAATAAAACTTTGGCACAGGTAGCTGCACAATTAACA---------------AAATATTGG-------
-----------------------------------AACAAAACAATAAACTTTACT------
AGCTCCTCAGGAGGGGATGTAGAAATTACA---ACACATAGTTTTAATTGTGGGGGAGAA---
TTTAAACAGCTCCAGGCAAGAGTCCTGGCTCTGGAAAGATACCTAAAGGATCAACAGCTCCTAGGAATTTGGG
GCTGCTCTGGAAAACTCATCTGCACCACTAGTGTGCCCTGGAACTCTAGTTGGAGT-----------------
----------------------------AATAAATCTTTTGAGCAAATATGG------AAT------
AACATGACCTGGTTGGAATGG---GATAAAGAAGTTAGCAATTACACACAAATAATATAT---
GAGCTACTTGAAGTATCGCAGAACCAGCAAGAA--

AAGAATGAACAAGACTTATTGTCATTGGACAAATGGGCAAGTCTGTGGAATTGGTTTGACATATCAAAATGGC
TGTGGTATATAAAAATATTTATAATGATAGTAGGAGGCTTAATAGGCTTAAGAATAGTTTTTGCT---
GTGCTTTCTATAATA---------AATAGAGTTAGGCAGGGATACTCACCT------
TTGTCATTTGCTATTAATTTGTTAGATACTATAGCAATAGCAGTAGCTGGC---
TGGACAGATAGGGTTATAGAAATAGGACAAAGAATTGGTAGAGCT----------------------------
--------ATTCTCAACATA------CCTAGAAGAATCAGACAGGGCGCTGAAAGAATCTTAGTA---TAA
>AF457070

AGT---GTT---ACA------------------------------------
GAAGACTTGTGGGTTACTGTCTACTATGGGGTACCTGTGTGG---AAAGATGCAGAG---
ACCACCCTATTTTGTGCATCAGATGCTAAAGCATATGAGACAGAA---AAGCATAAT----------------
--------------GTATGGGCTACA---CATGCCTGTGTACCCACAGAC---
CCCAACCCACAAGAAATACATTTGGAA---------AATGTAACAGAAGAGTTTAAC----------------
--------------------------ATGTGGAAAAATAACATGGTAGAGCAGATGCATACAGATATA---
ATCAGTTTATGGGACCAAAGCTTA---AAGCCATGTGTAAAGTTA---
ACCCCTCTCTGTGTTACTCTAAATTGTACCAATGCCAATGCCAAGAATGGCACC-------------------
----
----------------------------------AACGATTTGAAGGACCAGGAAGAAATAAAA---
AACTGCTCTTTCAACATAACCACAGAGCTAAGG---------------------------
GATAAGAGGAGGAAGGTATATTCACTT---TTTTATAAACTTGATATAGTACAGATTAATGAAAATCAA----
----

GGTAATAGAAGTAACAATAGTGAGTATAGATTAATAAATTGTAATACCTCAGCAATTACACAG---
GCTTGCCCAAAGGTAACTTTTGAGCCAATTCCCATACATTATTGTGCCCCAGCTGGTTTTGCGATCCTAAAGT
GTAAG---GATGAGGAGTTCAAT------GGAACAGGGCCATGCAAGAAT-----------------------
-GTCAGC---TCAGTCCAATGCACACATGGAATCAGGCCAGTAGTATCAACT---
CAACTGTTGTTAAATGGTAGTCTAGCAAAAGGAAAG------------
GTAAAAATTAGATCTGAAAATATCGCAGACAAT---------GTCAAAACTATAATAGTACAACTTACT----
-----------GAGCCTGTAAAC---------ATTACTTGTATCAGGCCTAATAACAATACAAGAACA-----
-AGTGTACGTATA------------GGACCAGGACAAACA---TTC------TATGCAACAGGTGAC------
---------ATAATAGGGAATATA------------AGACAAGCACAGTGT---ACTGTCAATAGAACA---
AAATGGAATAGCGCTTTACACCAGGTAGTTACACAATTAGGA---------------CACTTTGTC-------
-----------------------------------ACCAAAACTATAAAATTTAAT------
GAATCCTCAGGAGGGGATTTAGAAATCATA---ACACATAGTTTTAATTGTGGAGGAGAA---
TTTAAACAGCTCCAGGCAAGAGTCCTTGCTGTGGAAAGATACCTAAGGGATCAACAGCTCCTAGGAATTTGGG
GCTGCTCTGGAAAACTCATCTGCACCACTACTGTGCCCTGGAACTCTAGTTGGAGT-----------------
----------------------------AATAAATCTCTGGGGGAGATATGG------GAA------
AACATGACCTGGCTGCAATGG---GAAAAAGAAATTAGCAATTATACAGACATAATATAT---
AGCCTAATTGAAGAATCGCAGAACCAGCAGGAA--

AAGAATGAACAAGACTTATTGGCATTGGATAAGTGGGCAAGTCTGTGGAATTGGTTTGACATATCAAAGTGGC
TGTGGTATATAAGAATATTTATAATAATAGTAGGAGGCTTAATAGGATTAAGAATAGTTTTTGCT---
GTGCTTTCTATAATA---------AATAGAGTTAGGCAGGGGTACTCACCT------
TTGTCGTTTGCTATTAGTTTGGTTGATACCATAGCAATAGTAGTAGCTGGC---
TGGACAGACAGGGTACTAGAA------------ATTGGTAGAGCT----------------------------
--------ATCCTTCACATA------CCTAGAAGAATTAGACAGGGTCTTGAAAGAGCTTTGCTA---TAA
>AF457077
GCT---AAA--
GACAACTTGTGGGTTACTGTTTATTATGGGGTACCTGTGTGG---AAAGAAGCAGAG---
ACCACTTTATTTTGTGCATCAGATGCTAAAGCATATAAGACAGAA---ATGCATAAT----------------
--------------GTCTGGGCTACA---CATGCCTGTGTACCCACAGAC---
CCCAACCCACAAGAAATACATTTAGAA---------AATGTGACAGAAGAATTCAAC----------------
--------------------------ATGTGGAAAAACAACATGGTAGAACAAATGCATACAGATATA---
ATCAGTCTGTGGGACCAAAGCCTA---AAGCCATGTGTAAAGCTA---
ACCCCTCTCTGCGTTACTTTAAATTGTAGCAATATAGACAATAGCACGAATGTCAAC----------------
----
----------------------------ATCACAAGAGAAGAGGAAGAAATAAAAAACTTAAAA---
AATTGCTCTTTCAATATGACCACAGAACTAAGG---------------------------
GATAAAAGACAGAAAGTAAATTCACTT---TTTTATAGACTTGATATAGTAAAAATTAATGAA----------
----

AGTAGTAAAAGTAGTGAGTATAGATTAATAAATTGTAATACCTCAGCCATTACACAG---
GCGTGCCCAAAGGTATCCTTTGAGCCAATTCCTATACATTATTGTGCCCCAGCTGGTTTTGCGATCTTAAAGT
GTAAG---GATGAGAAATTCAAT------GGAACAGGGCCATGCAAAAAT-----------------------
-GTCAGC---ACAGTCCAATGCACACATGGAATCAAGCCAGTAGTATCAACT---
CAACTGTTGTTAAATGGCAGTCTAGCAGGA---GAG------------
GTAAAAATTAGATCTGAAAATATCACAAACAAT---------GCCAAAAATATAATAGTACAACTTGAC----
-----------AAACCTGTGAAA---------ATTAATTGTACCAGACCTAACAACAATACAAGAAAA-----

-AGTGTC---TATGCA---GGTGAC------
---------ATAATAGGGGATATA------------AGACAGGCACATTGT---AATGTCAATGCATCA---
GATTGGAGTGAAGCTTTACGAAAGGTAGTTCTAAC--------------------------------------
----
TCTTCAGGAGGAGATCTAGAAGTCACA---ACACATAGTTTTAATTGTGGAGGAGAA---
TTTAAACAGCTCCAGGCAAGAGTCCTGGCTGTGGAAAGATACCTAAGGGATCAACAGCTCCTAGGAATTTGGG
GCTGCTCTGGAAAACTCATCTGCACCACTAATGTGCCCTGGAACTCTAGTTGGAGT-----------------
----------------------------AATAAATCTCTAAATGAGATATGG------GAT------
AACATGACCTGGCTGCAATGG---GATAAGGAAATTAGCAATTACACACAAATAATATAT---
AATCTAATTGAAAAATCGCAGAACCAGCAGGAA--

AAGAATGAACAGGAATTATTGGCATTGGACAAGTGGGCAGGCCTGTGGAATTGGTTTGACATAACAAAGTGGC
TGTGGTATATAAAAATATTTATAATGATAGTAGGAGGCTTAATAGGATTAAGAATAGTTTTTGCT---
GTGCTTGCTATAATA---------AATAGAGTTAGGCAGGGATACTCACCT------
TTGTCGTTTGCTATCAATTTGGTTAATACCATAGCAATAGTAGTAGCTGGC---
TGGACAGATAGGGTTATAGAAATAGCACAAGGAGTTGGTAGAGCT----------------------------
--------ATTCTCCACATA------CCTAGAAGAATCAGACAGGGCTTAGAAAGGGCTTTGCTA---TAA
>AF457080
GCT---GCA--
GAAAACTTGTGGGTTACTGTCTATTATGGGGTACCTGTGTGG---AAAGATGCAGAG---
ACCACCTTATTTTGTGCATCAGAGGCTAAAGGATATGAAACAGAA---AAGCATAAT----------------
--------------GTCTGGGCTACA---CATGCCTGTGTACCCACAGAC---
CCCAACCCACAAGAAATACACCTGAAA---------AATGTGACAGAAGGGTTTAAC----------------
--------------------------ATATGGAAAAATAAAATGGTAGAGCAGATGCATGCAGATATA---
ATCAGTCTATGGGACCAAAGCCTA---AAGCCATGTGTAAAGTTA---
ACCCCTCTCTGTGTTACTTTGGATTGTGTCACTTACAATGGCACAAATGCCACAGGG----------------
----
----------------------------AGAGTGGACAACAGCACAATGAGGGAAGAAGTAAAA---
AACTGCACTTTCAATGTGACCACAGAGATTAGG---------------------------
GATAAGAGAAAGAAGGAACATTCACTT---TTTTATAGAGTTGATTTAGTACAAATTGATGAAATGCAA----
----

GGTAATAATAGTGACAGTAAATATAGACTAATAAATTGTAATACCTCAGCCATCACACAG---
GCCTGCCCAAAGGTATCCTTTGAGCCAATTCCCATACATTATTGTGCCCCAGCTGGTTTTGCGATCCTAAAAT
GTAAT---GATATGAATTACAAG------GGACAAGGGCCATGCCAGAAT-----------------------
-GTCAGT---ACAGTACAATGCACACATGGAATCAAGCCAGTAGTATCAACT---
CAACTGTTGTTAAATGGCAGTTTAGCAGAACAAGAG------------
ATAGCAATTAGATCTGAAAATATCACAGACAAT---------GCCAAAACTATAATAGTACAACTTGTC----
-----------AATCCTGTGAGA---------ATTAATTGCACCAGGCCTAACAACAATACAAGAAGA-----
-GATATAGGTATA------------GGACCAGGACAAACA---TTC------TATGCAGCAGGTGCC------
---------ATAATAGGAGATATA------------AGACAAGCATATTGT---AATGTCAGTAGAGCA---
GCATGGAATAAAACTTTACAAGAGGTAGCTAAACAATTAAGA---------------CAAGAATTTAAT----
-----------------------------------AACAAAACAATAAGATTTGCT------
AACCACTCAGGAGGAGATCTAGAAATCACA---ACACATAGTTTTAATTGTGGAGGAGAA---
TTTAAACAGCTCCAGGCACGAGTCCTGGCTGTGGAAGCATACCTAAGGGATCAACAGCTCCTAGGAATTTGGG
GCTGCTCTGGAAAACTCATCTGCACCACTAATGTGCCCTGGAACTCTAGTTGGAGT-----------------
----------------------------AATAAATCTTATAATGAAATATGG------GAT------
AACATGACCTGGCTGGAATGG---GAGAGAGAAATTAGCAATTACACAGATGTAATATAT---
AACCTACTTGAAGCATCGCAGAACCAGCAGGAA--

AAGAATGAACAAGACTTATTGGCATTGGACAAGTGGGCAAGTCTGTGGAGTTGGTTTAGCATAACAAATTGGC
TGTGGTATATAAAAATATTTATAATGATAGTAGGAGGCTTAATAGGATTAAGAATAGTTTTTGCT---
GTGCTTTCTATAATA---------AAAAGAGTTAGGCAGGGATACTCACCT------
TTGTCATTCGCTATTAATTTGGTTGATACTATAGCAATAGTAGTAGCTGGC---
TGGACAGATAGAGTTATAGAAATAGGACAAAGAATTGGTAGAGCT----------------------------
--------ATTCTCCACATA------CCTAGAAGAATCAGACAGGGCTTTGAAAGAGCTTTGCTA---TAA
>AF457081
ACT---GCA--
GAAAATTTGTGGGTTACTGTTTACTATGGGGTACCTGTGTGG---AAAGATGCAGAG---
ACCACCTTATTTTGTGCAACAGATGCTAAAGCAGTAAGTACAGAA---GCGCATAAT----------------

--------------GTCTGGCTACA---CATGCCTGTGTACCCACAGAC---
CCCGACCCACAAGAAATATATTTGGAA---------AATGTGACAGAAAATTTTAAC----------------
--------------------------ATGTGGAAAAATAACATGGTAGAACAGATGCATACAGATATA---
ATCAGTTTATGGACCAAAGCCTA---AAGCCATGTGTAAAGTTA---
ACCCCTCTCTGTGTTACTTTAAACTGTACAAATGCCAACACCACCACCACGAAT-------------------
----
----------------------------------GGCACCATCACAAACGGGGAAGAAATAAAA---
AACTGCTCTTATAATATAACCACAGAACTAAGG---------------------------
GATAAGAGACAGAAAGTATATTCACTT---
TTTTATAAACTTGATGTAGTACAACTTAATGAGGGTAATAGTAGT----------------------------
----
AATAATAGTAGCAATAATGAGTATAAGGAGTATAGATTAATAAATTGTAATACCTCAGCCATTACACAA---
GCTTGTCCAAAGGTATCCTTTGAGCCAATTCCCATACATTATTGTGCACCAGCTGGTTTTGCGATCCTAAAGT
GCAAG---GATAAGAAGTTCAAT------GGAACAGGGCCATGCAGGAAT-----------------------
-GTCAGC---ACAGTACAGTGCACACATGGAATCAAACCAGTAGTATCAACT---
CAACTGCTGTTAAATGGCAGCCTAGCAGAAGAAGAG------------
GTAATAATTAGATCTGAAAATATCACAAATAAC---------GCCAAAACCATAATAGTACAATTTACC----
-----------AAGCCTGTGATA---------ATTAATTGTACCAGACCTAACAACAATACAAGAAAA-----
-AGTATACGAATA------------GGACCAGGACAAGCA---TTC------TATGCAACAGGTGAC------
---------ATAATAGGGGATATA------------AGACAGGCACATTGT---AATGTCAGTAGATCA---
GACTGGAATGGAACTTTAAGCAATGTAACTGCAGAATTAAGA---------------AAACACTTTGGG----
-----------------------------------AACAAAACAATAATCTTTAAT------
AGCTCCTCAGGGGGGGATTTAGAAATTACA---ACACATAGTTTTAATTGTGGAGGAGAA---
TTTAAACAGCTCCAGGCAAGAGTCCTGGCTGTGGAAAGATACCTAAAGGATCAACAGCTCCTAGGAATTTGGG
GCTGCTCTGGAAAACTCATCTGCACCACTACTGTGCCCTGGAATTCTAGTTGGAGT-----------------
----------------------------AATAAATCTGAGAGTGAGATATGG------GAA------
AACATGACCTGGCTGCAATGG---GATAAAGAGATTAGCAATTACACAGACATAATATAT---
AGTCTAATTGAAGAAGCGCAAAACCAGCAGGAA--

AAAAATGAACAAGACTTATTGTCATTGGACAGTTGGGCAACTCTATGGAATTGGTTTGACATAACAAGGTGGC
TGTGGTATATAAAAATATTTATAATAATAGTAGGAGGCTTAATAGGACTAAGAATAGTTTCTACT---
ATACTTTCTGTAGTA---------AATAGAGTTAGGCAGGGATACTCACCT------
TTGTCATTTGCTATTAATTTGGTTGATACCATAGCAATAGCAGTAGCTGGC---
TGGACAGATAGGATTATAGAAATAGCACAAGGAATTGGTAGAGCT----------------------------
--------ATTTTCTACATA------CCTAGAAGAATCAGACAGGGCTTAGAAAGGGCATTGCTA---TAA
>AF457083
GCT---GCA--
GAAAACTTGTGGGTTACTGTCTACTATGGGGTACCTGTGTGG---AAAGACGCAGAG---
ACCACCTTATTTTGTGCATCAGATGCGAAAGCACATGATACAGAA---GCGCATAAT----------------
--------------ATCTGGCTACA---CATGCCTGTGTACCTACGGAC---
CCCAACCCACAAGAAATAAATTTGGAA---------AATGTGACAGAAGAGTTTAAC----------------
--------------------------ATGTGGAAAAATAGCATGGTAGAGCAGATGCATATGGATATA---
ATCAGTCTATGGACCAAAGCCTA---AAGCCATGTGTAAAGTTA---
ACCCCTCTCTGCGTTACTTTAGACTGTAGCGATGTTAGCAATGGTACCATCAAAGCCAAT-------------
----
----------------------------ACCACTATAGACAGTGAAATGCAAGGAGAAATAAAA---
AACTGCTCTTTCAATATGACCACAGAATTAAGA---------------------------
GATAAGAGACAGAAAATGTATTCACTT---TTTTATAGATTGGATGTAGTACAGCTTAATGACAGC-------
----

TACAGCAATGACAGTCAGTATAGATTAATAAATTGTAATACCTCAGCCATTACACAG---
GCTTGTCCAAAGGTATCCTTTCAGCCAATTCCCATACATTATTGTGCCCCAGCTGGTTTTGCGATTCTAGAGT
GTAAG---GATAAGGAGTTCAAT------GGAACAGGGAAATGCAAGAAT-----------------------
-GTCAGC---ACAGTACAATGCACACATGGAATCAAGCCAGTAGTATCAACT---
CAACTGCTGTTAAATGGCAGTTTAGCAGAAGGAAAG------------
GTAAGGATTAGATCTGAAAATATCACAAACAAT---------GCCAAAAACATAATAGTACAATTTACC----
-----------AAGCCTGTACAA---------ATTAATTGTACCAGACCTAACAACAATACAAGAAAA-----
-AGTGTACATATA------------TGACCAGGACAAGCA---TTC------TATGCAACAGGTGAC------
---------ATAATAGGAACATA------------AGACAAGCACATTGT---AATGTCAGTAGAGGA---
GAATGGAATAGAACTTTGCACGAAGTAGGCCAACAATTAAGA---------------ACATACTTTGGG----

```
--------------------------------AATAACACAACAATAATTTTTGCC------
AGCTCCTCAGGAGGGGATCTAGAAATAACA---ACACATAGTTTTAATTGTGGAGGAGAA---
TTTAAACAGCTCCAGGCAAGAGTCCTGGCTGTGGAAAGATACCTAAAGGATCAACAGCTCCTAGGAATTTGGG
GCTGCTCTGGAAAACTCATCTGCACCACTAATGTGCCCTGGAACTCTAGTTGGAGT-----------------
----------------------------AATAAATCCCAGAAGGAGATATGG------GAC------
AACATGACCTGGCTGCAATGG---GATAAAGAAATTAACAATTACACACAGATAATATAT---
AATCTACTTGAAGAATCGCAGAACCAGCAGGAA----------------------------------------
-----------------------------
AAGAATGAACAAGACTTATTGGCATTGGACAAGTGGGCAAATCTATGGAATTGGTTTGACATATCAAACTGGC
TGTGGTATATAAAAATATTTATAATGATAGTAGGAGGCTTAATAGGATTAAGAATAGTTTATGCT---
GTGCTTTCTGTAATA---------AATAGAGTTAGGCAGGGATACTCACCT------
TTGTCGTTCGCTATTAATTTGCTTGATACCATAGCAATAGCAGTAGCTGGG---
TGGACAGATAGGGTTATAGAAATAGGACAAAGACTTTGTAGA-------------------------------
----------------------------------------GCAGGGCAGTGAAAGGGCTTTGCTA---TAA
>AF457084
GTT---GCA------------------------------------------
GAAAAATCGTGGGTTACTGTCTACTATGGGGTACCTGTGTGG---AGAGATGCAGAG---
ACCACCTTGTTTTGTGCATCAGATGCGAAAGCACATGATAAGGAA---GTGCATAAT----------------
--------------GTTTGGGCTACA---CATGCCTGTGTACCTACAGAC---
CCCAACCCACAAGAAATGATTTTGGAA---------AATGTGACAGAAGATTTTAAC----------------
--------------------------ATGTGGAAAAATAGCATGGTAGAGCAGATGCATACAGATATA---
ATCAGTCTATGGGACCAAAGCCTA---AAGCCATGTGTAAAGTTA---
ACCCCTCTCTGTGTTACTTTAAATTGTAGTGATAGCAATATCACCTCTAATAGCACCTCTAATAGC-------
-------------------------------------------------------------------------
----------------------ACCAAGGACAGTGCCACCCTTGATATGAAAAGCGAAATACAA---
AACTGCTCTTTCAATATGACCACAGAACTAAGG---------------------------
GATAAGAAACAGAAAGTATATTCACTT---TTTTATAGACTTGATGTAGTACAAATTAATGAA----------
-------------------------------------------------------------------------
----------------------
AATAGTAGTGACTATAGATTAATAAATTGTAATACCTCAGCCATCACACAA---
GCATGCCCAAAGGTAACCTTTGAGCCAATTCCCATACATTATTGTGCCCCAGCTGGTTTTGCGATTCTAAAGT
GTAAT---GATAAGAAATTCAAT------GGAACAGGACCATGCACTAAT-----------------------
-GTCAGC---ACAGTCCAATGCACACATGGAATCAAGCCAGTAGTAACAACT---
CAACTGCTGTTAAATGGCAGTCTAGCAGAAGAAGAA------------
GTAATGATTAGATCTGAAAATATTACAGAAAAT---------GCCAAAAACATAATAGTACAATTTAAA----
-----------GAGCCTGTACAA---------ATTATTTGTATCAGACCTGGCAACAATACAAGAAAA-----
-AGTGTGCATATA------------GGACCAGGACAAGCA---TTC------TATGCAACAGGTGAC------
---------ATAATAGGGGATATA------------AGACAAGCACATTGT---AATGTCAGTAGAGAG---
TTATGGAATAAAACTTTGCAAGAAGTAGCCACCCAATTAAGA---------------AAACACTTTAGG----
--------------------------------AACAACACAAAGATAATCTTTACT------
AACTCCTCAGGAGGAGATGTAGAAATAACA---ACACATAGTTTTAATTGTGGAGGAGAA---
TTTAAACAGCTCCAAGCACGAGTCCTGGCTGTGGAAAGATACCTAAGGGATCAACAGCTCCTAGGAATTTGGG
GCTGCTCTGGAAAACTCATCTGCACCACTAATGTGCCCTGGAATTCTAGTTGGAGC-----------------
----------------------------AATAAATCCCTAGATGAGATATGG------GAA------
AACATGACCTGGATGCAATGG---GATAAAGAAGTTAGCAATTACACACAGATGATATAT---
AATCTACTTGAAGAATCGCAGAACCAGCAGGAA----------------------------------------
-----------------------------
AAGAATGAACAAGAATTATTGGCATTAGACAAGTGGGCAAATCTGTGGAATTGGTTTAACATATCAAACTGGC
TGTGGTATATAAAAATATTTATAATGATAGTAGGAGGCTTAATAGGATTAAGAATAGTTTTTGCT---
GTGCTTTCTGTAATA---------AATAGAGTTAGGCAGGGATACTCACCT------
TTGTCATTCGCCATTAGTTTGGTTGATTCCATAGCAATAGCAGTAGCTGGC---
TGGACAGATAGGATTATAGAAATAGTACAAGCAATTGGTAGAGCT----------------------------
--------ATTCTTCACATA------CCTAGAAGAATCAGACAGGGCTTAGAAAGGGCTTTGATA---TAA
>AF457086
ACT---GCA------------------------------------------
GACAACTTGTGGGTTACTGTCTACTATGGGGTACCTGTGTGG---AAAGATGCAGAG---
ACCACCTTATTCTGTGCATCAGATGCTAAAGCATATGAGACAGAA---AAGCATAAT----------------
--------------GTCTGGGCTACA---CATGCCTGTGTACCCACAGAC---
CCCAACCCACAAGAGATACATATGGAA---------AATGTAACAGAACAGTTTAAC----------------
--------------------------ATGTGGAAAAATAACATGGTAGAGCAGATGCATACAGATATA---
```

ATCAGTCTATGGGATCAAAGCTTA---AAGCCATGTGTAAAATTA---
ACCCCTCTCTGTGTCACTTTAGAGTGCGGTAATGTCACCCTTGATGGCACCAAGACTAATATC---------
----
-------------------------ACTAATGCCACTACTAAATATCTGAAGGAAGAAATAACA---
AACTGCTCTTACAATGTGACCACAGAAATAAGG--------------------------
GATAGGACACAGAAAGTATATTCACTT---TTTTATAGACTTGATGTAGTACCAATTGATAGCGGTAAT----
----

AGTAGTGGTAATAGTAGTCAGTATAGATTAATAAATTGTAATACCTCAGCCGTTACACAA---
GCTTGTCCAAAGGTAACCTTTGAGCCAATTCCCATACATTACTGTGCCCCAGCTGGTTTTGCGATTCTAAAGT
GTAAT---GATAAGGAGTTCAAT------GGAACAGGGATATGCAAGAAT----------------------
-GTCAGC---ACAGTACAATGCACACATGGAATCAAGCCAGTGGTATCAACT---
CAATTGCTGTTAAATGGCAGTCTAGCAGAAGGAAAG-----------
ATAAAAATTAGATCTGAAAACATCACAAACAAT---------GCTAAAAACATAATAGTACAATTCAAT----
-----------GAGTCTGTAATA---------ATTAATTGTACCAGACCTAACAACAATACAAGAAAA-----
-AGTGTACGTATA------------GGACCAGGACAAGCA---TTC------TATGCAACAGGTGCC------
---------ATAACAGGGGATATA------------AGACAAGCACATTGT---AATGTTAGTAGAAAA---
GAATGGAATACCACTTTGCAAAGGGTAGTCAACCAATTAAGG---------------ACATACTTTGGA----
-----------------------------------AACAGAACAATAAGATTTGCT------
GCCTCCTCAGGAGGGGATATAGAAGTCACA---ACACATAGTTTTAATTGTGGAGGAGAG---
TTTAAACAGCTCCAGGCAAGAATCCTGGCTGTGGAAAGATACCTAAGGGATCAACAGCTCCTAGGAATTTGGG
GCTGCTCTGGAAAACTCATCTGCACCACTAATGTGCCTTGGAACTCCAGTTGGAGT----------------
----------------------------AATAAAACCCTGAGTGAGATATGG------GAT------
AACATGACCTGGCTAGAATGG---GATAAAGAAATTAGCAATTACACACAGATAATATAT---
AATCTAATTGAACAATCGCAGAACCAGCAGGAA---------------------------------------
----
AAGAATGAACAAGACTTACTGGCATTGGACAAGTGGGCAAATCTGTGGAATTGGTTTGACATATCAAACTGGC
TGTGGTATATAAGAATATTTATAATGATAGTAGGAGGCTTAATAGGATTAAGAATAGTTTTTGCA---
GTGCTTTCTATAATA---------AATAGAGTGAGGCAGGGATACTCACCT------
CTGTCATTTGCTGTTAGTTTGATTGATACCATAGCAATAAGAGTAGCTAAC---
TGGACAGATAGGATTATAGAGTTAGGACTAAGATTTTGTAGAGCT---------------------------
--------ATTCTCCACATA------CCTAGAAGAATTAGACAGGGCTTCGAAAGGGCTTTGCTA---TAA
>AF457089
ACT---GCA---------------------------------------
GAAAAGTTGTGGGTTACTATCTACTATGGGGTACCTGTGTGG---AAAGATGCAGAT---
ACCACCCTATTTTGTGCATCAGATGCTAAAGCATATGAGAAAGAA---GTGCATAAT---------------
--------------GTCTGGGCTACA---CATGCCTGTGTGCCCACAGAC---
CCCAACCCACAAGAAATACTTTTGAAA---------AATGTGACAGAAGAGTTTAAC---------------
--------------------------ATGTGGAAAAATAACATGGTAGAGCAGATGCATACAGATATA---
ATCAGTTTATGGGACCAAAGTCTA---CAGCCATGTGTAAAGTTA---
ACCCCTCTCTGTGTTACTTTAAACTGTACCAATGYCAACAACTGTACCAATGCCAACAACTGTACC-------
----
-------------------CATGCCAACAGCAGCATCACGAATGACATGGCAGGAGAAATAAAA---
AACTGCTCTTTCAATATGACCACAGAACTAAGG--------------------------
GATAAGAAACAGAAAGTATATTCACTT---TTCTATAAACTTGATATAGTACAAATTAATGGAAATAAT----
----

GGTAGTGAGTATAATGGTAGTGAGTATAGATTAATAAATTGTAATACCTCAGCCATTACACAG---
GCTTGTCCAAAGGTATCCTTTGAGCCAATTCCCATACATTATTGTGCCCCAGCTGGTTTTGCGATCCTAAAGT
GTAAT---GATGAGGAGTTCAAT------GGAACAGGGCCATGCAAAAAT----------------------
-GTCAGC---ACAGTACAATGCACACATGGAATCAAGCCAGTAGTATCAACT---
CAACTGCTGTTAAATGGCAGTCTAGCAAAAAATAAT-----------
GTAACTATTAGATCTGCAAATATCTCAGACAAT---------GCTAAAATCATAATAATACAACTTAAT----
-----------ACGCCTGTAACA---------ATTAATTGTACCAGACCTAACAACAATACAAGAAAA-----
-AGTGTACGTATA------------GGACCAGGACAAGCA---TTC------TATACAACA---GAT------
---------ATAATAGGGGATATA------------AGACAAGCACATTGT---AATGTCAGTAAATCA---
GAATGGAATAAAACTTTACAACAGATAGTTATACAATTAAGA---------------AAGCACTGG-------
-----------------------------------AACAAAACAATAAACTTTACT------
GAACCCTCAGGAGGGGATTTAGAAATTACA---ACACATAGTTTTAATTGTGGAGGAGAA---
TTTAAACAGCTCCAGGCAAGAGTCCTGGCTGTGGAGAGATACCTAAGGGATCAACAGCTCCTGGGAATTTGGG

GCTGCTCTGGAAAGCTCATCTGCACCACTAGTGTGCCCTGGAACTCTAGTTGGAGT-----------------
----------------------------AATAAATCTTACAATGAGATATGG------GAT------
AACATGACCTGGCAGCAATGG---GAAAGAGAAATTGAAAATTACACAGACTACATATAT---
AACCTGATTGAAGAATCGCAAAACCAGCAGGAA--

AAGAATGAACAAGACTTATTAGCATTGGACAAGTGGGCAAGTCTGTGGAGTTGGTTTAGCATATCAAAATGGC
TGTGGTATATAAAAATATTTATAATGATAGTAGGAGGCTTAATAGGATTAAGGATAGTTTTTGCT---
GTGCTTTCTGTAATA---------AATAGAGTTAGGCAGGGATACTCACCT------
CTATCATTTGCTATCAATTTGCTTGATACCATAGCAATAGCAGTAGCTGGC---
TGGACAGATAGGGTTATAGAAATAGGACAAAGAATTGGTAGAGCC----------------------------
--------ATTCTCAACATA------CCTAGAAGAGTCAGGCAGGGCTTCGAAAGGGCTTTGCTA---TAA
>EU110088
GCT---GAA---
AGAAACTTGTGGGTTACTGTCTACTATGGGGTACCTGTGTGG---AAAGACGCAGAG---
ACCACCTTATTTTGTGCCTCGGATGCTAAAGCATATAAGACAGAA---AAGCATAAT----------------
--------------GTCTGGGCTACA---CATGCTTGTGTACCCACAGAC---
CCCAACCCACAAGAAATACATTTGGAA---------AATGTGACAGAAAATTTTAAC----------------
-------------------------ATGTGGAAAAATAACATGGTAGAACAAATGCATACAGATATA---
ATCAGTCTATGGGACCAAAGYCTA---AAGCCATGTGTAAAGTTA---
ACCCCTCTCTGCGTTACTTTAAGGTGTAGTGACTATAATGGCACCAGTTATGTCAACAGTACTAGTAGTAAAC
ATAATGCC---
-------ACCAGTAATGGGACACACTCCTACAATATCTCCGCAGAAATGCTAGGAGAAATAAAA---
AACTGCTCTTACAAKATGACCACAGAACTAAGG--------------------------
GATAAGACAAAGAAAGTATATTCACTT---TTTTATAGACCTGATATAGTACCACTTAATGGT----------
----

AATAGTAGTAGTAATAAGTATACATTAATAAATTGTAATACCTCAGCCATTAAACAA---
GCTTGTCCAAAGGTATCCTTTGAGCCAATTCCTATRCATTATTGTGCCCCAGCTGGTTTTGCGATTCTAAAGT
GTAAT---AATAAAACATTCAAT------GGAACAGGGCCATGCAATAAT----------------------
-GTCAGT---ACAGTACAATGCACACATGGAATCAAGCCAGTAGTATCAACT---
CAGCTGCTGTTAAATGGCAGTCTAGCAGAAGAACAG------------
ATAATGATTAGGTCTGAAAATATCACAAATAAT---------GCTAAAAACATACTAGTACAATTTACT----
-----------AGCCCTGTGAAA---------ATTAATTGTACCAGACCTAACAACAATACAAGAAAA-----
-AGTGTACGCATA------------GGACCAGGCCAAGCA---TTC------TATGCAACAGGTGAC------
---------ATAATAGGGGATATA------------AGACAAGCACATTGT---AATATCAGTCAAGCA---
GAGTGGAATGAAACTTTGGGAAADCTAACCACCCAATTAAGA---------------ACACACTTTGGG----
-----------------------------------AACAAAACAATAAATTTTACT------
AGTGCCTCAGGAGGGGATCTAGAAATAACA---ACACATAGTTTTAACTGTAGAGGAGAA---
TTTAAACAGCTCCAGGCAAGAGTCCTGGCTGTGGAAAGATACCTAAAGGATCAACARCTCCTAGGAATTTGGG
GCTGCTCTGGAAAACTCATCTGCACCACTAATGTGCCTTGGAACTCTAGTTGGAGT----------------
----------------------------AATAGATCCCTAAATGAGATATGG------AAT------
AACATGACCTGGCTACAATGG---GATAGAGAAGTTAGCAATTACACACAGCTAATATAT---
GATCTACTTGAAGAATCGCAGATCCAGCAGGAA--

AAGAATGAACAAGACTTATTGGCATTGGACAAGTGGGCAAGTCTGTGGAATTGGTTTGACATATCAAAATGGC
TGTGGTATATAAGAATATTTATAATGATAGTAGGAGGTTTAATAGGATTAAGAATAGTTTTTGCT---
GTGATTTCTATAATA---------AATAGAGTTAGGCAGGGATACTCACCT------
TTGTCATTCGCTATTAGTTTGGTTGATACCATAGCAGTAGTAGTAGCTGGC---
TGGACAGATAGGGTTATAGAAATAGGACAAACAATTGGTAGAGCT----------------------------
--------ATCCTCCAYATA------CCTAGAAGAATTAGACAGGGCTTCGAAAGGGCTTTGCTA---TAA
>EU110092
ACT---AAA---
GAAAACTTGTGGGTTACTGTCTATTATGGGGTACCTGTGTGG---AAAGATGCAGAA---
ACCACCTTATTTTGTGCATCAGATGCTAAAGCATATGAGAAAGAA---CAGCATAAT----------------
--------------GTCTGGGCTACA---CATGCCTGTGTACCCACAGAC---
CCCAACCCACAAGAAATACATTTAGAC---------AATGTGACAGAAGACTTTAAC----------------
-------------------------ATGTGGGAAAATAACATGGTAGAGCAGATGCATACAGATATA---
ATCAGCCTATGGGACCAAAGCCTA---AAGCCATGTGTAAAATTA---
ACCCCTCTTTGTGTTACTTTACATTGTACAAATGTAACTGTCACTAATGAT---------------------
----

----------------------------------AAAACGGTAAAGAACCAGCAAGAATTAAAA---
AACTGTTCTTTCAATGTAACTACAGAATTAAGG---------------------------
GATAAGAAAAAGAAAGTATATTCACTT---TTTTATAGACTTGATGTAGTACAAATTAATGAGAAT-------
----

AGTAGTAGTAATAGTAGTGAGTATAGATTAATAAATTGTAATACCTCAGCCATTACCCAG---
GCTTGCCCAAAGGTAAGCTTTAATCCAATTCCCATACATTATTGTGCCCCAGCTGGTTTTGCGATCCTAAAGT
GTAGG---GATGAGGAGTTCAAT------GGAACAGGGCCATGCAAGAAT-----------------------
-GTCAGC---TCCGTCCAATGCACACATGGAATCAGGCCAGTAGTATCAACT---
CAATTGCTGTTAAATGGTAGTCTAGCAAATGAAAAG------------
ATAATAATTAGATCTAAAAATATCACAGACAAT---------GGTAAAAATATAATAGTACAACTTGGC----
-----------AAACCTGTGAAA---------ATTAATTGTACCAGACCCAGTAACAATACAAGAAAA-----
-AGTGTGCATATA------------GGGCCAGGACAGGCA---TTC------TATGCAACAGGTGCC------
---------ATAATAGGGGATATA------------AGACAAGCACATTGT---AATATCAGTAGAGCA---
GACTGGACTGAGACTTTACGAAATGTGAGTAAACAATTAAGA---------------AAACACTTTGAG----
-----------------------------------AATAAAACAATAAAGTTTGAA------
AAATCCTCAGGAGGGGATCCAGAAGTCACA---ACACATAGTTTTAATTGTGGAGGAGAA---
TTTAAACAGCTCCAGGCAAGAGTCCTGGCTGTGGAAAGATACCTAAAGGATCAACAGCTCCTAGGAATTTGGG
GATGCTCTGGAAGACTCATCTGCCCCACTAATGTGCCCTGGAATTCTAGTTGGAGT-----------------
----------------------------AATAAATCTTTTGATGAGATATGG------GAC------
AACATGACTTGGCTGCAATGG---GAGAAGGAAATTAGCAATTACACACAAAAAATATAT---
ACACTAATTGAAGAATCGCAGAACCAGCAGGAA--

AAGAATGAACAAGACTTATTGGCATTGGACAAGTGGGCAAGTCTGTGGARTTGGTTTGACATATCAAGATGGC
TGTGGTATATAAAAATATTTATAATGATAGTAGGAGGCTTAATAGGATTAAGAATAGTTTTTGCT---
GTGCTTTCTATAATA---------AGGAGAGTTAGGCAGGGATACTCACCT------
TTGTCATTTGCTATTAATTTGTATGATACCATAGCAATAGCAGTAGCTGGC---
TGGACAGATAGGGTTATAGAAGTAGGACAAAGAGCTGGTAGAGCT----------------------------
--------TTTCTTAACATA------CCTAGAAGGATCAGACAGGGYTTAGAAAGGCTTTGCTA---TAA
>EU110094
GCT---ACA--
GACAACTTGTGGGTTACTGTCTACTATGGGGTACCTGTGTGG---AAAGACGCAAAG---
ACCAGCTTATTTTGTGCATCAGATGCTAAAGCCTATCAGACAGAA---AAGCATAAT----------------
--------------GTCTGGGCTACA---CATGCCTGTGTACCCACAGAC---
CCCAACCCACAAGAAATATATTTGGCA---------AATGTGACAGAAGAGTTTAAC----------------
--------------------------ATGTGGAAAAATAACATGGTAGAGCAGATGCATACAGATATA---
ATCAGTCTATGGACCAAAGCCTA---AAGCCATGTGTAAAATTA---
ACCCCTCTCTGTGTTACTTTACATTGTGAAAATGTTAATAAGAGTGTCAAC----------------------
----
-------------------------------------ATCACGGGGAGTGGGGAAGAAATAAAA---
AACTGCTCTTTCAATATGACCACAGAACTAAAG---------------------------
GATAAGAGACAGCAAGTATATTCACTT---TTTTATAGACCAGATATAGTAGAAATTAATGAAAAT-------
----

CAGAGCAAGGATAATAACAAGTATAGATTAATAAATTGTAATACCTCAGCCATTACACAA---
GCGTGCCCAAAGGTATCCTTTGAGCCAATTCCAATACATTATTGTGCTCCAGCTGGTTTTGCAATCCTAAAGT
GTAGA---GATGAGGAGTTCAAT------GGAGCAGGGCCATGCAAGAAT-----------------------
-GTCAGC---ACAGTCCAATGCACACATGGAATCAAGCCAGTAGTATCAACT---
CAACTGCTGTTAAATGGCAGTCTAGCAGAAAAAGAG------------
GTAAAAATTAGATCTAGAAATATCACAGATAAT---------GCCAAAAACATAATAGTACAATTTRYT----
-----------GAGCCTGTRRAT---------ATTRCTTGTATCAGACCTAACAACAATACAAGAAAA-----
-AGTATACGTATA------------GGACCAGGACAAGCA---TTC------TATGCAACAGGTGAC------
---------ATAACAGGGGATATT------------AGACAGGCACATTGT---AATGTCACTGGATCA---
AATTGGACTAAGGCTTTACAACAGGTAGCTACACAATTAAGA---------------AACTATGTG-------
-----------------------------------AACAAAACAATAATATTTGCT------
AGCCCCGCAGGAGGGGATCTAGAAATCACA---ACACATAGTTTTAATTGTGGAGGAGAA---
TTTAAACAGCTCCAGGCACGAGTCCTKGCTGTGGAAAGATACCTAARRGATCAACAGCTCCTAGGAATTTGGG
GCTGCTCTGGAAGGCTCATCTGCACCACTAATGTGCCCTGGAACCAGAGTTGGAGT-----------------
----------------------------AATAAATCTCTAGATGAGATATGG------AAT------
AACATGACCTGGCTGCAGTGG---GATAGAGAAATTAACAATTACACACAACTAATATAT---

CAACTAATTGAGGAATCACAAAACCAGCAGGAA---------------------------------------
----
AAGAATGAAAAAGACTTATTGGAGTTGGACAAGTGGGCAAATCTGTGGACTTGGTTTGACATATCAAACTGGC
TGTGGTATATAAAAATATTTATAATGATAGTAGGAGGCTTGATAGGGTTAAGAATAGTTTTTGCT---
GTGTTTTCTATAATA---------AATAGAGTTAGGCAGGGATACTCACCT------
TTGTCGTTTGCTATCAATTTACTTGATACTATAGCAGTAGCAGTAGCTGGG---
TGGACAGATAGGGTTATAGAACTAGGACAAAGACTTTGTAGAGCT---------------------------
--------ATTCTCCACGTA------CCTAGAAGAATCAGACAGGGCTTTGAAAGGGCTTTGCTA---TAA
>KT022360
GCT---GCA--
GAAAAATTGTGGGTTACTGTCTATTATGGGGTACCTGTGTGG---AAAGATGCAGAG---
ACCACCTTATTTTGTGCATCAGATGCGAAAGCATATAAGACAGAA---GTGCATAAT----------------
--------------GTATGGGCTACA---CATGCCTGTGTGCCCACAGAC---
CCCAACCCACAAGAAATAAATTTGGGA---------AATGTGACAGAAGAGTTTAAC----------------
-------------------------ATGTGGAAAAATAACATGGTAGAGCAAATGCACACAGATATA---
ATCAGTCTATGGGACCAAAGCTTA---AAGCCATGTGTAAAGTTG---
ACCCCTCTCTGCGTCACTTTAGATTGT---AACTACAATGTCACCAATATCACCAAT----------------
----
---GAAATGAATGGAGAAATGAGA---
AACTGCTCTTACAATATGACCACAGAATTAAGG--------------------------
GATAAGACACAGAAAGTATATTCACTT---TTTTATAAACTAGATATAGTACAAGCTAATAATAAT-------
----

GATAGTAGGCAGTATAGATTAATAAACTGTAATACCTCAGCCATTACACAA---
GCTTGTCCAAAGGTATCTTTTGAACCAATTCCCATACATTATTGTGCCCCAGCTGGTTTTGCGATTCTAAAGT
GTAAT---GATAAGAAGTTCAAT------GGAACAGGGCTATGCAAAAAT----------------------
-GTCAGC---ACAGTACAATGCACACATGGAATCAAGCCAGTAGTATCAACT---
CAACTGTTGTTAAATGGCAGTCTGGCAGAAACAGAG-----------
ATAATGATCAGATCTGAGAATATCACAAACAAT---------GTCAAAAACATAATAGTACAATTGACT----
-----------CAGCCTGTACCA---------ATTAATTGTACCAGACCTAACAACAATACAAGAAAA-----
-AGTATCCGAATA------------GGACCAGGACAAGCA---TTC------TATGCAACAGATGGC------
---------ATAATAGGGGATATA------------AGACAAGCACATTGT---AATGTCAGTGAAGCA---
GCTTGGAACAGAACGTTGCATCAGATAGCCACCCAATTAAGA---------------ACACACTTT-------
-----------------------------------AACAGAACAATAATCTTTGAT------
AAACCCATAGGAGGGGATCTAGAAATAACA---ACACATAGTTTTAATTGTGGAGGAGAA---
TTTAAACAGCTCCAGGCAAGAGTCCTGGCTGTGGAAAAATACCTAAAGGATCAACAGCTCCTAGGAATTTGGG
GCTGCTCTGGAAAACTCATCTGCACCACTAATGTGCCTTGGAATTCTAGTTGGAGT-----------------
-----------------------------AATAAATCCCAGAGTGAGATATGG------GAC------
AACATGACCTGGCTGCAATGG---GATAAAGAAATTAGCAATTACACACAGACAATATAT---
GAACTAATTGAACAATCGCAGAACCAGCAGGAA---------------------------------------
----
AAAAATGAACAAGACTTATTGGCATTGGACAAGTGGGCAAATTTGTGGAATTGGTTTGACATATCAAGATGGC
TGTGGTATATAAGAATATTTATAATGATAGTAGGAGGCTTGATAGGATTAAGAATAGTTTTTGCT---
GTGCTTTCTGTAATA---------AATAGAGTTAGGCAGGGATACTCACCT------
CTGTCGTTCGCTACTAGTTTGATTGACACCATAGCAATAGTAATAGCTGGG---
TGGACAGATAGGCTTCTAGAATTAGGACAAAGACTTTGTAGAGCT---------------------------
--------TTTCTCAACATA------CCTAGAAGGATCAGACAGGGCTTGGAAAGGGCGTTACTA---TAA
>KT022361
ACT---GCA--
GAAAAATTGTGGGTCACTGTCTATTATGGGGTACCTGTGTGG---AGAGATGCAGAG---
ACTACCTTATTTTGTGCATCAGATGCTAAAGCATATGAGGCAGAA---AAGCATAAT----------------
--------------GTCTGGGCTACG---CATGCCTGTGTACCCACAGAC---
CCCAACCCACAAGAAATACATTTGGAA---------AATGTGACAGAAAATTTTAAC----------------
-------------------------ATGTGGACAAATTACATGGTAGAGCAGATGCATACAGATATA---
ATCAGTCTATGGGATCAAAGCCTA---AAACCATGTGTAAAGTTA---
ACCCCTCTCTGCGTTACTTTAAATTGTAGCGATATCACTGCCAATATCACTGCC-------------------
----
-------------------------------------TCATTTGCCAACGTCACGGACATAAAA---
AACTGCTCTTTCAATATAACCACAGAACTAAGG--------------------------
GATAAGACAAAGAAAGAATATTCACTT---TTTTATAGACTTGATATAGTAGAAATTAATGAA----------

----

AATAGTAGTGAATATAGATTAATAAATTGTAATACCTCAGCCGTTACACAG---
GCATGCCCAAAGGTATCCTTTGAACCAATTCCCATACATTATTGTGCTCCAGCTGGTTTTGCGATCCTAAAGT
GTAAG---GATGAGGATTTCAAT------GGAACAGGGCCATGCAAGAAT-----------------------
-GTCAGC---ACAGTCCAATGCACACATGGAATCAAGCCAGTAGTATCAACT---
CAACTGCTGTTAAATGGCAGTCTAGCAAGAACA---------------
ATAAAAATTAGATCTGAAAATATAACAGACAAT---------GCAAAAAATATACTAGTACAACTTGCC----
-----------CAGGCCGTTAGA---------ATTAATTGTACCAGACCTAACAATAACACAAGAAAA-----
-AGTATACGTATA------------GGACCAGGACAAGCA---TTC------TTTGCAACAGGTGGT------
---------ATAATAGGGAATATA------------AGACAAGCACATTGT---AATGTTAGTAGATCA---
GAATGGAGGAGAACTTTACAAGAGGTAGCTAAAGAATTAAAG---------------AAACACTTTGGG----
--------------------------------AACGAAACAACAATAAAGTTTAAT------
AAATCCTCAGGAGGGGATCTAGAAATCACA---ACACATAGTTTTAATTGTGGAGGAGAA---
TTTAAACAGCTCCAGGCAAGAGTCCTGGCTGTGGAAAGATACCTAAAGGATCAACAGCTCCTAGGAATTTGGG
GCTGCTCTGGAAAACTCATCTGCACCACTAATGTGCCCTGGAACTCTAGTTGGAGT-----------------
----------------------------AATAAATCTTACAGTGAAATATGG------GAT------
AATATGACCTGGCTGCAATGG---GATAAAGAAGTTAGCAACTACACACAAATAATATAT---
GCCCTAATTGAAGAATCGCAGAACCAGCAGGAA--

AAGAATGAACAAGACTTATTAGCATTGGACAAGTGGGCAAATCTGTGGAATTGGTTTGGTATATCAAACTGGC
TGTGGTATATAAAAATATTTATAATGATAGTAGGAGGCTTAATAGGATTAAGAATAGTTTTTGCT---
ATACTTTCTGTAATA---------AATAGAGTTAGGCAGGGATACTCACCC------
TTGTCGTTTGCTCTTAATTTGGTTGATACTATAGCAATAGTAGTAGCTGGC---
TGGACAGATAGGATTATAGAAATAGGACAAGCAATTGGTAGAGCT----------------------------
--------ATTCTCCACATA------CCTAGAAGAATCAGACAGGGCATTGAAAGGGTATTGCTA---TAA
>KT022363
GCT---GCA--
GAAAACTTGTGGGTAACTGTCTATTATGGGGTACCTGTGTGG---AAAGATGCAGAG---
ACCACCTTATTTTGTGCATCAGATGCGAAAGCATATGAGACAGAA---AAGCATAAT----------------
--------------GTCTGGGCTACA---CATGCTTGTGTACCCACAGAC---
CCCAACCCACAGGAAATAAATTTGAAA---------AATGTGACAGAAGGGTTTAAC----------------
--------------------------ATGTGGAAAAATAACATGGTAGAGCAGATGCATACAGATATA---
ATCAGTCTATGGGACCAAAGCCTA---CGGCCATGTGTAAAGTTA---
ACCCCTCTCTGTGTTACTTTAGATTGTAATAATGCCACTGCCACCAAT-------------------------
----
-------------------------ATTACCAGGATTGACAAGGACATGGAAGGAGAAATAAAG---
AACTGCTCTTTCAATATGACCACAGAAGTGAGG---------------------------
GATAAAAAGCATAAAGTATATTCACTT---TTTTATAGGACAGATGTAGTAGAAATGAATAGT----------
----

AGTAGTCAGTATAGATTAATAAATTGTAATACCTCAGCCATTACACAA---
GCTTGTCCAAAGGTATCCTTTGAACCAATTCCCATACATTATTGTGCCCCAGCTGGTTTTGCAATTCTAAAAT
GTAAG---GATAAGGAGTTCAAT------GGAAAAGGGATATGCAAAAAT-----------------------
-GTCAGC---ACAGTACAATGCACACATGGAATCAAGCCAGTAGTGTCAACT---
CAACTGCTGTTAAATGGCAGCCTAGCAGAAGAAAAG------------
GTAATGATTAGATCTGAAAATATCACAAACAAT---------GTTAAAAACATAATAGTACAATTTGCC----
-----------GAGCCTGTATGG---------ATTAATTGTACCAGACCTGGCAACAATACAAGAAAA-----
-AGTGTACATATA------------GGACCAGGACAAGCT---TTC------TATGCAACAGGTGAC------
---------ATAATAGGAGATATA------------AGAAAGGCACATTGT---AATGTCAGTGAAGCA---
GAATGGAAAAGAGCTTTACAGAAGGTAGCCAAACAATTAGGA---------------GCACACTTTGTG----
-----------------------------------AGTAAAACAATAATCTTTACT------
AAATCCTCAGGAGGAGATCTAGAAATAACA---ACACATAGTTTTAATTGTGGAGGAGAA---
TTTAAACAGCTCCAGGCAAGAGTCCTGGCTGTGGAAAGATACCTAAAGGATCAACAGCTCCTAGGAATTTGGG
GCTGCTCTGGAAGGCTCATCTGCACCACTAATGTGCCCTGGAACTCTAGTTGGAGT-----------------
----------------------------AATAAATCCATGAATGAGATATGG------AAT------
AACATGACCTGGCTGCAATGG---GATAAAGAAATTGACAATTATACACAGATAATATAT---
AACCTCATTGAAGAATCGCAGAACCAGCAGGAA--

AAGAATGAACAAGACTTATTGGCACTGGACAAGTGGGCATCTCTGTGGAATTGGTTTGACATATCAAACTGGC

TGTGGTATATAAAAATATTTATAATGATAGTAGGAGGCTTAATAGGATTAAGAATAGTTTTTGCT---
ATACTTTCTGTAATA---------AATAGAGTTAGGCAGGATACTCACCT------
TTGTCGTTCGCTATTAATTTGCTTGATACCATAGCAATAGCAGTAGCTGGG---
TGGACAGATAGGATTATAGAAATAGGACAAAGATTTTGCAGAGCT----------------------------
--------ATTCTCCACATA------CCTAGAAGAATCAGACAGGGCTTTGAAAGGACTTTGCTA---TAA
>KT022364
ACT---GCA--
GAAAACTTGTGGGCTACTGTCTACTATGGGGTACCTGTGTGG---AAAGAAGCAGAG---
ACCACCCTATTTTGTGCATCAGATGCTAAAGCATATCAGACAGAA---GTGCATAAT----------------
--------------GTCTGGGCTACA---CATGCCTGTGTACCCACAGAC---
CCCAGCCCACAAGAAATAGAATTGAAA---------AATGTAACAGAAGAGTTTAAC----------------
--------------------------ATGTGGAAAAATAGCATGGTAGAGCAGATGCATACAGATATA---
ATCAGTTTATGGGACCAAAGCCTA---AAGCCATGTGTAAAGTTA---
ACCCCTCTCTGTGTTACTTTAAACTGTACTAATGCCAAGGCCAACAACACGAATGACACT-------------
----
----------------------------GTCAGCAGCATGGGGGACATGGAAGGAGAAATAAAA---
AACTGCTCTTTCAATATGACCACAGAACTAAGG---------------------------
GATAAGAAGCAGAAAGTATATTCACTT---
TTTTATAAACTTGATGTAGTACAAATTAATGGAAATAATGGTACT----------------------------
----
AATAATGATAGCAGTGAGTATAGATTAATAAATTGTAATACCTCAGCCATTACGCAG---
GCTTGTCCAAAGGTATCCTTTGAGCCAATTCCAATACATTATTGTGCCCCGGCTGGTTTTGCGATCCTAAAGT
GTAAG---GATGAGGAGTTTAAT------GGAACAGGGCCATGCAAGAAT-----------------------
-GTCAGC---ACAGTACAATGCACACATGGAATCAAGCCAGTAGTATCAACT---
CAACTGCTGTTAAATGGCAGTCTAGCAAAAGAAAAG------------
ATAATAATTAGATCTGAAAATATCTCAGACAAT---------GCTAAAACCATAATAATACAACTTACC----
-----------ACGCCTGTAACA---------ATTGAGTGTATCAGACCCAACAACAACACAAGACAA-----
-GGTGTACATATA------------GGGCCAGGACAGGCA---TTC------TATACAACAGGT---------
---------ATCATAGGGGATATA------------AGAAAAGCATATTGT---GAGGTCAATAAATCA---
GAATGGGATCAAACTTTACAACAGGTAGTTACACAATTAAGA---------------GAACATTGG-------
-----------------------------------AACGCAACAATAATCTTTAAT------
AAACCCTCAGGAGGGGACTTAGAAATTACA---ACACACAGTTTTAATTGTGGAGGAGAA---
TTTAAACAGCTCCAGGCAAGAGTCCTGGCTATAGAAAGATACCTAAAGGATCAACAGCTCCTAGGGATTTGGG
GCTGCTCTGGAAAACTCATCTGCACCACTACCGTGCCCTGGAACTCTAGTTGGAGT-----------------
----------------------------AATAAATCTTATGAGGATATATGG------GAT------
AACATGACCTGGCTGCAATGG---GATAAAGAAATTAGCAATTACACAGAAATAATATAT---
AGGCTAATTGAAGAATCACAGAACCAGCAGGAA--

AAGAATGAACAAGACTTATTGGCATTGGATAAGTGGGCAAGTCTGTGGAGTTGGTTTGACATAACAAGATGGC
TGTGGTATATAAGGATATTTATAATGATAGTAGGAGGCATAATAGGCTTAAGAATAATTTTTGCA---
GTGCTTGCTATAATA---------AATAGAGTTAGGCAGGATACTCACCT------
CTATCGTTTGCTATTAATTTGCTTGATACCATAGCAATAGCAGTAGCTGGC---
TGGACAGATAGGGTTATAGAAATAGTACAAAGATTTGGTAGAGCT----------------------------
--------ATTCTCAACGTA------CCTACAAGAATCAGGCAGGGCTTCGAAAGGGCTTTACTA---TAA
>KT022365
GTT---ACA--
GGAAACTTGTGGGTTACTGTCTACTATGGGGTACCTGTGTGG---AAAGATGCAGAG---
ACCACCTTATTTTGTGCATCAGATGCTAAAGCATATGAGACAGAA---AAGCATAAT----------------
--------------GTCTGGGCTACA---CATGCCTGTGTACCCACAGAC---
CCCAACCCACAAGAAATACATTTGAAA---------AATGTAACAGAAGAGTTTAAC----------------
--------------------------ATGTGGAAAAATAACATGGTAGAACAGATGCATACAGATATA---
ATCAGTCTATGGGATGAAAGCCTA---AAGCCATGTGTAAAGTTA---
ACCCCTCTCTGTGTTACGTTAGAGTGTAATGACAACATCACCACCAATAGCACTAACACCATTAGCAAG----
----
-------------------ACCAGCGCCAGCACTACTAGTAGCCCCATGGAAGGAGAAATGAAA---
AACTGCTCTTTCAATATAACCACAGAATTAAGG---------------------------
GATAAGAAAAAGAAAGTACATTCACTT---
TTTTATAGACTTGATATAGTAGAAATTAAGGAAAATCAAGATAAAAGT-------------------------
----
AATAGTAGTAGTAATAGTAGTAGTCAGTATAGTCAGTATAGATTAATAAATTGTAATACCTCAGCCATTACAC

AA---
GCTTGTCCAAAGGTAAGCTTTGAGCCAATTCCCATACATTATTGTGCCCCAGCTGGTTTTGCAATTCTAAAGT
GTAAG---GATGTAGAGTTCAAT------GGAACAGGACCATGTAAGAAA-----------------------
-GTCAGC---ACAGTACAATGCACACATGGAATCAGGCCAGTAGTATCAACT---
CAACTGCTGTTAAATGGCAGTCTAGCAGAAGGCAAG------------
GTAATGATTAGATCTGCAAATATCTCAAGCAAT---------GCCAAAAACATAATAGTACAATTCAAC----
-----------GAGTCTGTAAGA---------ATTGAGTGTATTAGACCTAGTAACAATACAAGAAAA-----
-AGTGTACGGATA------------GGACCAGGACAAACA---TTC------TATGCAACAGGTGAC------
---------ATAATAGGGGATATA------------AGAAAAGCATATTGT---AATGTCAGTAGAGAA---
AATTGGAATGAAGCATTACAAAAGGTAGTAGAACAATTAGGA---------------ACACACGTT-------
-----------------------------------AACGGAACAATAAAATTTGCT------
AACTCCTCAGGAGGGGATCTAGAAATCACA---ACACATAGTTTTAATTGTGGAGGAGAA---
TTTAAACAGCTCCAGGCAAGAGTCCTAGCTGTGGAAAGTTACCTAAGGGATCAACAGCTCCTAGGAATTTGGG
GCTGCTCTGGAAAACTCATCTGCACCACTACTGTGCCTTGGAACTCTAGTTGGAGC-----------------
----------------------------AATAAATCTTATAATGAGATATGG------GAG------
AACATGACCTGGCTGCAATGG---GATAAAGAGATTAGCAATTACACACAAATAATATAT---
AACCTAATTGAAGAATCACAGAACCAGCAGGAG--

AAAAATGAACAAGAATTATTGGCATTGGATAAGTGGGCAAATCTGTGGAATTGGTTTAACATATCAAATTGGC
TGTGGTATATAAGATTATTTATAATGATAGTAGGAGGCTTAATAGGATTAAGAATAGTTTTTGCT---
GTGCTTTCTGTAATA---------AATAGAGTTAGGCAGGGATACTCACCT------
CTATCGTTTGCTATTAAGTTAATTGATACTATAGCAATAGCAGTAGCTGGC---
TGGACAGATAGGGTTATAGAATTAGGACAAAGACTTTGTAGAGCT----------------------------
--------ATTCTCCATATA------CCTAGAAGAATCAGACAGGGCTTAGAAAGAGCTTTGCTA---TAA
>KT022367
ACT---GCA--
GAAAACTTGTGGGTTACAGTCTACTATGGGGTACCTGTGTGG---AGAGATGCAGAG---
ACCACCTTATTTTGTGCATCAGATGCTAAAGCATATAAAACAGAA---AGGCATAAT----------------
--------------GTATGGCTACA---CATGCCTGTGTACCCACAGAC---
CCCAACCCACAAGAAATACATTTGGAA---------AATGTGACAGAAGATTTTAAT----------------
--------------------------ATGTGGAAAAATGGCATGGTAGAGCAGATGCATACAGATATA---
ATCAATCTATGGGACCAAAGCCTA---AAGCCATGTATTAAATTA---
ACCCCTCTCTGTGTTACTTTAAATTGTAGCGACTTCAAGACCAATAACACCATAGGCAATGTTACC-------
----
--ACAGAAATAAAA---
AATTGCTCTTTCAATATGACCACAGAATTAAGG---------------------------
GATAGGAGGCAGCAAGTATATTCACTT---TTTTATAAACTGGATGTAGTACAAATTGATGGC----------
----

AATAATAGTCAGTATAGATTAATAAATTGTAATACCTCAGCCATTACACAA---
GCTTGTCCAAAGGTATCCTTTCAACCAATTCCCATACATTATTGTGCTCCAGCTGGTTTTGCGATTCTAAAGT
GTAAG---GAAAAAGAGTTCAAT------GGAACAGGACCATGCAGGAAT-----------------------
-GTCAGC---ACAATACAATGCACACATGGAATTAAGCCAGTAGTGTCAACT---
CAACTGCTGTTAAATGGCAGCCTAGCAGAAGGAAAG------------
GTAATGATTAGGTCTGAAAATATCACAAACAAT---------GCCAAAAATATAATAATACAATTTAAT----
-----------CAGACTGTACAA---------ATTAATTGCTCCAGACCTAACAACAATACAAGAAAA-----
-AGTGTACGTATA------------GGACCAGGACAAGCA---TTC------TATGCAACAGGTGAC------
---------ATAATAGGGAACATA------------AGACAGGCACATTGC---AATGTCAGTAAAGCA---
GCATGGGAAAAAACTTTACAACAGATAGCTAAACAATTAAGA---------------GGGCACTTTGAG----
-----------------------------------AATAAAACAATAATATTTGCT------
AACTCCTCAGGAGGGGATCTGGAAGTCACA---ACACACAGTTTTAATTGTGGAGGAGAA---
TTTAAACAGCTCCAGGCACGAGTCCTGGCTGTGGAAAGATACCTAAGGGATCAACAGCTCCTAGGAATTTGGG
GTTGCTCTGGAAAACTCATCTGCACCACTAATGTGCCCTGGAACTCTAGTTGGAGT-----------------
----------------------------AATAAAAATCAGAGTGAAATATGG------GAT------
AACATGACCTGGCTGCAATGG---GATAAAGAAGTTAGCAATTACACATATATAATATAT---
GATCTACTTGAAAAATCACAGAACCAGCAGGAA--

AAGAATGAACAAGACTTATTGGCATTGGACAAGTGGGCAAATCTGTGGAATTGGTTTGACATATCAAACTGGC
TGTGGTATATAAAAATATTTATAATGATAGTAGGAGGCTTAATAGGATTAAGAATAGTTTTTGCT---
GTGCTTTCTATAATA---------AATAGAGTTAGGCAGGGCTACTCACCT------

TTGTCATTCGCTGTTAATTTGGTAGATAACATAGCAATAGCAGTAGCTGGC---
TGGACAGATAGGGTTATAGAAATAGGACAAAGAATTGGTAGAGCT----------------------------
--------ATCCTCAACATA------CCTAGAAGAATCAGACAGGGCTTGGAAAGTGCTTTGCTA---TAA
>KT022368
GCT---ACA--
GAAAACTTGTGGGTTACTGTCTACTATGGGGTACCAGTGTGG---AAAGATGCAGAT---
ACCACCTTATTTTGTGCATCAGATGCTAAAGCATATGAGTCAGAA---AAGCATAAT----------------
--------------GTCTGGGCTACA---CATGCCTGTGTACCCACAGAC---
CCCAACCCACAAGAAATAAATTTGGAA---------AATGTGACAGAAAATTTTAAT----------------
--------------------------ATGTGGAAAAATAACATGGTAGAGCAGATGCATACAGATATA---
ATCAGTCTATGGGATCAAAGCCTA---AAGCCGTGTGTAAAGATA---
ACCCCTCTCTGCGTTACTTTAAGTTGTAGCTATAATGTTACCTTCAACAGTACCAATATCACTGCT-------
----
---GAAGAAAGAGAAGAGATAAAA---
AACTGCTCTTACAATGTGACCACAGAAATAAGG---------------------------
GATAAGAAACAGAAAGTATATTCACTC---TTTTATAAACTTGATATAGTACAAATTGAAAATAGTAATAAT-
----

AATAGTAGTAATAGTAGTATAATGTATAGATTAATAAATTGTAATACCTCAGCCATTACACAG---
GCTTGCCCAAAGGTATCCTTTGAGCCAATTCCCATACATTATTGTGCTCCAGCTGGTTTTGCGATCCTAAAGT
GTAAG---GATGAAAAGTTCAAT------GGAACAGGACCATGCAAGAAT-----------------------
-GTCAGT---ACAGTTCAGTGCACACATGGAATCAAGCCAGTGGTATCAACT---
CAACTGCTGTTAAATGGCAGTCTAGCTGAAAGAGAG------------
ATAAAAATTAGATCTGAAAATATCACAAACAAT---------GGTAAAACTATAATAGTGCAACTTACC----
-----------GAGCCTGTGGAA---------ATTGATTGTATCAGACCTTACAACAATACAAGGAAA-----
-AGTGTGCGTATA------------GGACCAGGACAAGCA---TTC------TATGCAACAGGTGAC------
---------ATAATAGGGGATATA------------AGAAAAGCATCTTGT---ACTGTCAATAGAACA---
AAATGGAATGGTACTTTACATCAAGTAGCTGACAAATTAAAG---------------AACTACTTTAGG----
-----------------------------------AACAGAACAATAAGATTTGCT------
AACTCCTCAGGAGGGGATTTGGAAATCACA---ACACATAGTTTTAACTGTAGGGGAGAA---
TTTAAACAGCTCCAGGCAAGAGTCCTGGCTGTGGAAAGATACCTAAAGGATCAACAGCTCCTAGGAATTTGGG
GCTGCTCTGGAAAACTCATCTGCACCACTAATGTGCCCTGGAACTCTAGTTGGAGT-----------------
----------------------------AATAAATCCCAGAATGAGATATGG------GAA------
AACATGACCTGGCTACAATGG---GATAAAGAAATTGGCAACTACACACAGCTAATATAT---
AGTCTACTTGAAGAATCACAAAACCAGCAGGAA--

AAGAATGAACAAGACTTATTGTCATTGGACAAGTGGGCAAATCTATGGAATTGGTTTGACATATCAAACTGGC
TGTGGTACATAAAAATATTTATAATGATAGTAGGAGGCTTAATAGGATTAAGAATAGTTTTTGCT---
GTGCTTTCTATAATA---------AATAGAGTTAGGCAGGGATACTCACCT------
TTGTCGTTCGCTATTAATTTGTTTGATACCATAGCAATAGTAGTAGCTGGC---
TGGACAGATAGAGCTATAGAAATAGGACAAGTAGTTGGTAGAGCT----------------------------
--------ATTCTCCACATA------CCTAGAAGAATCAGACAGGGCCTTGAAAGAGCTTTGCTA---TAA
>KT022369
ACT---GCA--
GAAAACTTGTGGGTTACTGTCTACTATGGGGTACCTGTGTGG---AAAGATGCAGAT---
ACCACCTTATTTTGTGCATCAGATGCGAAAGCATATAAGACAGAA---AAGCATAAT----------------
--------------GTCTGGGCTACA---CATGCCTGTGTGCCCACAGAC---
CCCAACCCACAAGAAATAAATTTGAAA---------AATGTGACAGAAGAGTTTAAC----------------
--------------------------ATGTGGAAAAATAACATGGTAGAGCAGATGCATACAGATATA---
ATCAGTTTATGGGACCAAAGCCTA---CAGCCATGTGTAAAGTTA---
ACCCCTCTCTGCGTTACATTAAAGTGCTCTGATGCCAATTTCACCTCTAATAATAATGCC-------------
----
----------------------------ACCAATATCACCGCTGAGATGAAGGAAGAAATAAAA---
AACTGCTCTTTCAATATGACCACAGAATTAAGG---------------------------
GATAAGAAACAGAAAGTATATTCACTT---TTTTATAGACTGGATGTAGTACCAGTTGATGAA----------
----

GTTGATGAGAATGATACGTATAGATTAATAAATTGTAATACCTCAGCCATTACACAA---
GCTTGTCCAAAGGTATCCTTTGAACCAATTCCCATACATTATTGTGCCCCAGCTGGTTTTGCAATTCTAAAGT
GTAAT---GATAAGAAGTTCAAT------GGAACAGGGCCATGTAGAAAT-----------------------

-GTCAGC---ACAGTACAATGCACACATGGAATCAAGCCAGTAGTATCAACT---
CAACTGCTGTTAAATGGCAGTCTAGCAGAAGAAGAG-----------
GTAATGATCAGATCTGAGAATATTACAAACAAT---------GCCAAGAACATAATAGTACAATTCACC----
-----------CAGCCTGTAACA---------ATTAATTGTACCAGACCTCACAACAATACAAGAAAA-----
-GGTGTACATATA------------GGACCAGGACAAGCA---TTC------TATGCAAGAGGGGAC------
---------ATAATAGGGGATATA------------AGACAAGCACATTGT---AACCTCAGTAAAGCA---
GCATGGAATAACACTTTGCGCCAGGTAGCCATATCATTAGGC---------------AAACACTTTAGG----
---------------------------------AATAAAACAATAATCTTTGCT------
AACTCCACAGGGGGGGATCTAGAAATAACA---ACACATAGTTTTAATTGTGGAGGAGAA---
TTTAAGCAGCTCCAGGCCAGAATCCTGGCTGTGGAAAGATACCTAAGGGATCAACAGCTCCTAGGAATTTGGG
GCTGCTCTGGAAAACTCATCTGCACCACTAATGTGCCCTGGAATTCTAGTTGGAGT-----------------
---------------------------AATAAATCTTACGATGAGATATGG------GAC------
AACATGACCTGGCTGCAATGG---GATAAAGAAGTTAGCAATTACACACACATAATATAT---
GAACTAATTGAAAAAATCGCAGAACCAGCAGGAA--

AAGAATGAACAAGACTTATTGGCATTGGGCAAGTGGGCAAATCTGTGGAATTGGTTTAACATATCAAACTGGC
TGTGGTATATAAGAATATTTATAATAATAGTAGGAGGCTTAATAGGATTAAGAATAGTTTTTACT---
GTGCTTTCTGTAATA---------AATAGAGTTAGGCAGGGATACTCACCT------
TTGTCTTTCGCTATTAATTTGCTTGATACCATAGCGATAAGAGTAGCTGGG---
TGGACAGATAGGGTTATAGAAATAGGACAAAGGCTTTGTAGAGCT----------------------------
--------ATCCTCAACGTA------CCTGTCAGAATCAGACAGGGCCTTGAAAGAGCTTTGCTA---TAA
>FJ866117
ACT---GCA---
GAAGACTTGTGGGTTACTGTCTACTATGGGGTACCTGTGTGG---AAAGATGCAGAG---
ACCACCCTATTTTGTGCATCAGATGCTAAAGCCTATGAAACAGAA---AGGCATAAT----------------
--------------GTCTGGGCTACA---CATGCCTGTGTACCCACAGAC---
CCCAACCCACAAGAAATACCTTTGGAA---------AATGTAACAGAAGAATTTAAC---------------
-------------------------ATGTGGAAAAAATAACATGGTAGAGCAGATGCATACAGATATA---
ATTAGTTTATGGGACCAAAGCCTA---AAGCCATGTGTAAAGTTA---
ACTCCTCTCTGTGTTACTTTAAGCTGTACCAATGCCAAGGGTACTACTTTAAAAGACACC------------
----
---------------------------AATAATGTTACCATCAACACGACAGAAGAAATAAAA---
AACTGCTCTTTCAATATGACCACAGAATTAAGG--------------------------
GATAAGAAACAGAAAGTATATTCACTT---TTTTATAAACTTGATGTAGTACCAATTAATGAAAGT-------
----

AATAATAGTAATACCAGTGAGTATAGATTAATAAATTGTAATACCTCAGCCATTACACAG---
GCTTGTCCAAAGGTAACCTTTGAGCCAATTCCAATACATTATTGTGCCCCAGCTGGGTTTGCGATTCTAAAGT
GTAGA---GATAAGGAGTTCAAT------GGAACAGGGCTATGCAAGAAT-----------------------
-GTTAGC---ACAGTACAATGCACACATGGAATCAAACCAGTAGTATCAACT---
CAACTGCTGTTAAATGGCAGTCTAGCAGAAAAGGAG-----------
GTAAGAATTAGCTCTGAAGATATTTCAAACAAT---------GCTAAAACCATTATAGTACAACTTGCC----
-----------ACGCCTGTAGCA---------ATTAATTGTACTAGACCTAACAATAATACAAGAAAA-----
-AGTATACGTATA------------GGACCAGGACAAGCA---TTC------TATGCAACA---GAT------
---------ATAATAGGGAACATA------------AGACAAGCATATTGC---GAGGTCAATAGATCA---
GAATGGAATGAAGCTTTACGCAAGGTAGCTGTGCAATTAAGA---------------AAGCACTACGGG----
---------------------------------AACACAACAATAAAATTTACT------
GCACCCTCAGGAGGGGATTTAGAAATTACA---ACACATAGTTTTAATTGTGGAGGAGAA---
TTTAAACAGCTCCAGGCAAGGGTCCTGGCTGTGGAAAGATACCTAAAGGATCAACAGCTTCTAGGAATTTGGG
GCTGCTCTGGAAAACTCATCTGCACTACTAATGTGCCCTGGAACTCTAGTTGGAGT-----------------
---------------------------GATAAATCTTACAGTGAAATATGG------GAT------
AACATGACCTGGCTGCAATGG---GATAAAGAAATTAACAATTATACAGAAGAGATATAT---
AGTCTAATTGAAAAAGCGCAGACCCAGCAAGAA---

AAGAATGAACAAGACTTATTGGCATTGGACAAGTGGGCAAGTCTGTGGAATTGGTTTGACATATCAAAATGGC
TGTGGTATATAAAAATATTTATAATGATAGTAGGAGGCTTAATAGGATTAAGAATAGTTTTTGCT---
GTGCTTGCTGTAATA---------AATAGAGTTAGGCAGGGATACTCACCA------
CTATCATTTGCTATTAACTTGGTTGATACCATAGCAATAGGAGTAGCTGGC---
TGGACAGATAGGGTGATAGAAGTAGGACAAAGAATTGGTAGAGCT---------------------------
--------ATTCTCCATATA------CCTAGAAGAATAAGACAAGGCTTTGAAAGGGCTTTACTA---TAA

>KT022370
GTT---GCA---------------------------------------
GGAAACTTGTGGGTTACGGTCTACTATGGCGTACCAGTGTGG---AAAGATGCAGAT---
ACCACCTTATTTTGTGCATCAGATGCTAAAGCATATGAGACAGAA---AAGCATAAT---------------
--------------GTCTGGGCTACA---CATGCCTGTGTACCCACAGAC---
CCCAACCCACAAGAAATACCTTTGGAA---------AATGTGACAGAAGAGTTTAAC---------------
--------------------------ATGTGGAAAAATAACATGGTAGAGCAGATGCATGCAGATATA---
ATCAGTCTATGGGACCAAAGCCTA---AAACCATGTGTACAGTTA---
ACCCCGCTCTGCGTTACTTTACATTGTGTCAATGTCAATGCCACAAATAGTACCACAGCC------------
----
------------------------------AAAAACATAACAGGGGACAGTGATGAACTAAGA---
AACTGCTCTTTCAATATGACCACAGAGCTAAGG---------------------------
GATAAGACAAAGCAGGTATATTCACTT---TTTTATAGACTTGATGTAGTACCAATTCAAGATAAT-------
----

AGTAGTGATGAATATAGATTAATAAATTGTAATACCTCAACCATTACACAA---
GCTTGTCCTAAGGTATCTTTTGAGCCAATTCCCATACATTATTGTGCCCCAGCTGGTTTTGCGATCCTAAAGT
GTAGG---GATGAGGAGTTCAAT------GGAACAGGGCCATGCAAGAAT----------------------
-GTCAGC---TCAGTACAATGCACACATGGAATCAAGCCAGTAGTATCAACT---
CAACTGCTGTTAAATGGCAGCCTAGCAAAAAAAGAG-----------
GTAAAAATTAGATCTGAAAATATCACAAACAAT---------GTCAAAACTATAATAGTACAACTTGTT----
-----------GATCCTGTGGAA---------ATTAATTGTACCAGGCCTAATAACAATACAAGAAGA-----
-AGTGTACGTATA------------GGACCAGGACAAACA---TTC------TATGCAACAGGTGCA------
---------ATAATAGGGCCGATA------------AGAAATGCATATTGT---ACTGTCAATGAATCA---
AAATGGAATGCAACTTTAAAAAAGGTAGCTGGACAGTTAAGA---------------AAACACTTTGGG----
-----------------------------------AACAAAACAATAAGGTTTAAT------
AGCTCTTCAGGAGGAGATCTGGAAATCACA---ACACATAGTTTTAATTGTGGAGGAGAA---
TTTAAACAGCTCCAGGCAAGAGTCCTGGCTGTGGAAAGATACCTAAAAGATCAACAGCTCCTAGGAATTTGGG
GCTGCTCTGGAAAACTCATCTGCACCACTAATGTGCCCTGGAATTCTAGTTGGAGT----------------
---------------------------AATAAATCTCAGAATGAGATATGG------GAG------
AACATGACCTGGCTACAATGG---GATAAAGAAATTAGCAATTACACTGACATAATATAT---
AACCTAATTGAAGAATCGCAGAACCAGCAAGAA---------------------------------------
----
AAGAATGAACAAGAATTATTGGCATTGGACAAGTGGGCAAGTCTGTGGGATTGGTTTAAAATATCAAACTGGC
TGTGGTATATAAAAATATTTATAATGATAGTAGGAGGTTTAATAGGATTAAGAATAGTTTTTGCT---
GTGCTTTCTATAATA---------AATAGAGTTAGGCAGGGATACTCACCT------
TTGTCATTTGCTATTAGTTTGCTTAATACCATAGCAATAGTAGTAGCTGGC---
TGGACAGATAGGCTTATAGAAATAGGACAAGGAATTGGTAGAGCT---------------------------
--------ATTCTTCATATA------CCTAGAAGAATCAGACAGGGTTTAGAAAGAGCTTTGCTA---TAA
>KT022372
GTT---GCA---------------------------------------
GGAAACTTGTGGGTTACGGTCTACTATGGGGTACCAGTGTGG---AGAGATGCAGAT---
ACCACCTTATTTTGTGCATCAGATGCTAAAGCATATGAGAGAGAA---GTGCATAAT---------------
--------------GTCTGGGCTACA---CATGCCTGTGTACCCACAGAC---
CCTAGCCCACAAGAAATAAATATGAAA---------AATGTGACAGAAGAGTTTAAC---------------
--------------------------ATGTGGAAAAATAACATGGTAGAGCAGATGCATACAGATATA---
ATCAGTCTATGGGACCAAAGCCTA---AAACCATGTGTAAAGTTA---
ACTCCTCTCTGCGTTACTTTACATTGTGTCAATATCACCATTACCAACACCACAAAC---------------
----
---------------------------AGCACCATAGACAGCGTAATGAAAGATGAAATAAAA---
AACTGCTCTTACAATATGACCACAGAGATAAGG---------------------------
GATAAGAAACAGAAGATGTATTCACTT---TTTTATAAACTTGATGTAGTACCAATTAATGAA---------
----

AGTCAAGGTAATAATAGTGATGAGTATAGATTAATAAATTGTAATACCTCAGCCATTACACAG---
GCTTGTCCAAAGGTATCCTTTGAGCCAATTCCCATACATTATTGTGCCCCAGCTGGTTTTGCGATCCTAAAGT
GTGCA---GATGAGAATTTCAAT------GGAACAGGGCCATGCAATAAT----------------------
-GTCAGC---ACAGTACAATGCACACATGGAATCAGACCAGTAGTATCAACT---
CAACTACTGTTAAATGGCAGTCTAGCAAAAACGGGG-----------
ATAAAGATTAGATCCGAAAATATCACAAATAAT---------GCCAAAAGTATAATAGTACAATTGAAC----

-----------AAGTCGGTGGAA---------ATTACTTGTATCCGGCCTAACAACAATACAAGAAAA-----
-AGTGTACATATA------------GGACCAGGACAAGCA---TTC------TTTGCAACAGGTGGG------
---------ATAATAGGGGATATA------------AGACAAGCGCATTGT---AATGTCAGTAAATCA---
CGTTGGAATACAACCCTACAAGGAGTAGTTGAACAATTAAGA---------------AAACACTTTAGG----
-----------------------------------AACACAACAATAAAGTTTAAT------
ACCTCCTCAGGAGGAGATCTAGAAGTCACA---ACACATAGTTTTAATTGTGGAGGAGAA---
TTTAAACAGCTCCAGGCAAGAGTCCTGGCTGTGGAAAGATACCTAAGAGATCAACAGCTCCTAGGAATTTGGG
GCTGCTCTGGAAAACTCATCTGCCCCACTAATGTGCCCTGGAATTCTAGTTGGAGT----------------
----------------------------AACAAAACTCAGAGTGAAATATGG------GAG------
AACATGACCTGGCTAGAATGG---GATAAAGAAATTAGCAATTACACTGAAATAATATAT---
AAACTAATTGAAGAATCGCAGAACCAGCAGGAA--

AAGAATGAACAAGAATTATTAGCATTGGACAAGTGGGCAAGTCTGTGGACTTGGTTTGACATATCAAAATGGC
TGTGGTATATAAAAATATTTATAATGATAGTAGGAGGTTTAATAGGATTAAGAATAGTTTTTGCT---
GTGCTTTCTATAATA---------AATAGAGTTAGGCAGGGATACTCACCT------
TTGTCATTTGCTATTAGTTTGTTTGATACCATAGCAGTAGCAGTAGCTGGC---
TGGACAGATAGGATTATAGAAATAGGACAAAGAATTGGTAGAGCT---------------------------
--------ATCCTCCACATA------CCTAGAAGAATCAGACAGGGCTTCGAAAGAGCTTTGCTA---TAA
>KT022373
GCT---GCA---
GAAAACTTGTGGGTTACTGTCTACTATGGGGTGCCTGTGTGG---AAAGACGCAGAG---
ACCACCCTATTTTGTGCATCAGATGCTAAAGCATATGATACAGAA---GTGCATAAT---------------
--------------ATCTGGGCTACA---CATGCCTGTGTACCCACAGAC---
CCCGACCCACAAGAAATAAACTTAGAA---------AATGTGACAGAAGAGTTTAAT---------------
--------------------------ATGTGGAAAAATAACATGGTAGAGCAGATGCACACAGATATA---
ATCAGTCTATGGGACCAAGGACTA---AAGCCATGTGTAAAGTTA---
ACCCCTCTCTGCGTTACTCTAGATTGTAGCAATAATGTCACCAATACCACTAAT------------------
----
---GACATGAAAGAAGAAATAAAA---
AACTGCTCTTTCAATGTGACCACAGTAATAAGG-------------------------
GATAAGAGAGACAGAAAGTATATTCACTT---TTTTATAGACTTGATGTAGTACCAATTGATGGT---------
----

AATAGTGATAGTAATCAGTATAGATTAATAAATTGTAACACCTCAGCCATTACACAG---
GCTTGTCCAAAGGTAACCTTTGAGCCAATTCCCATACACTATTGTGCCCCAGCTGGTTTTGCGATTCTAAAGT
GTAAG---GATAAGGAGTTTAAT------GGAACAGGGCCATGCAAGAAT----------------------
-GTCAGC---ACAGTACAATGCACACATGGAATCAGGCCAGTGGTATCAACT---
CAGCTGCTATTAAATGGCAGCCTAGCAGAAGACAAG-----------
ATAATAATTAGATCTGAAAATATCACAAACAAT---------GCTAAAAACATTATAGTACAATTTAAC----
-----------AGACCTGTAAAA---------ATTAATTGTACCAGACCTAACAACAATACAAGAAAA-----
-GGTGTACATATA------------GGACCAGGACAAACA---TTC------TATGCAACAGGTGAA------
---------ATAATAGGGGAGATA------------AGACAAGCACATTGT---AATATTAGTGGAACA---
GAATGGAATACAACTTTGCAAAAGGTAGCCAAACAATTAAGT---------------GCATACTTTAAG----
-----------------------------------AACAAAACAATAATTTTTGCA------
AACTCCTCAGGAGGGGATTTAGAAATTACA---ACACATAGTTTTAATTGTGGAGGAGAA---
TTTAAACAGCTCCAGGCAAGAGTCCTGGCTCTGGAAAGATACCTAAAGGATCAACAGCTCCTAGGAATTTGGG
GCTGCTCTGGAAAACTCATCTGCACCACTAATGTGCCCTGGAACTCTAGTTGGAGT----------------
----------------------------AATAAATCCCATGATGAGATATGG------GGA------
AACATGACCTGGCTACAATGG---GAGAGAGAAATTAGCAATTACACACAGACAATATAT---
GATCTAATTGAAGACTCGCAGAACCAGCAGGAA--

AAAAATGAACAAGACTTATTAGCATTGGACAAGTGGGCAAGTCTGTGGAATTGGTTTGACATATCAAACTGGC
TGTGGTATATAAAGATATTTATAATAATAATAGGAGGCTTAATAGGATTAAGAATAGTTTTTGCA---
GTGCTTTCTGTAATA---------AATAGAGTTAGGCAGGGATACTCACCT------
TTGTCATTTGCTATTAATTTGTTTGATACCATAGCAATAGCAGTAGCTGGC---
TGGACAGATAGGGTTATAGAACTAGGGCAAAGGCTTGGTAGAGCT---------------------------
--------ATCTTGCACGTA------CCTAGAAGAATTAGGCAGGGCGCAGAAAGAGCTTTGCTA---TAA
>KT022374
GCT---GGA---
GGAAGCTTGTGGGTTACTGTCTACTATGGGGTACCTGTGTGG---AGAGACGCAGAA---

ACCACCTTATTTTGTGCATCAGATGCTAAAGCATATGATACAGAA---AAGCATAAT----------------
--------------ATCTGGGCTACA---CATGCCTGTGTACCCACAGAC---
CCCGACCCACAAGAAATACCTTTGAAA---------AATGTGACAGAAGATTTTAAC----------------
--------------------------ATGTGGAAAAATAATATGGTAGAGCAAATGCATACAGATATA---
ATCAGTCTATGGGATCAAAGTCTA---AAGCCTTGTGTACAGTTA---
ACCCCTCTCTGTGTCACTTTAAATTGTACAAATGCAGGAAATGATACTAATACCAATACT-------------
----
-------------------------------ACCATAAATGATGACATGCGTGGAGAACTAAAA---
AACTGCTCTTTTAATGTGACCACAGAAATAAGG---------------------------
GATAAAAAACAGAAAGTACATGCACTT---TTTTATAAACTTGATGTAGTACAAATAGATAAGGCTAAT----
----

AGTAGTGATGGTAATGGTAGTGGTGACTACAGATTAATAAATTGTAACACCTCAGTCATTACACAG---
GCTTGCCCAAAGATAAAATTTGAGCCAATTCCCATACATTATTGTGCTCCAGCTGGTTTTGCGATCCTAAAGT
GTAGG---AATACGACATTCAAT------GGAACAGGGCCATGCAATAAT-----------------------
-GTCAGC---ACAGTCCAATGCACACATGGAATCAAGCCAGTAGTAACAACT---
CAACTGCTATTAAATGGCAGTCTAGCAGAGGGAGAG------------
GTAATAATTAGATCTGAAAATATCTCAAACAAT---------GCCAAAACTATAATAGTACAACTTGCC----
-----------GAATCTGTGACA---------ATTAATTGTACCAGACCTGGTAATAATACAAAAAGA-----
-AGTGTACATATA------------GGACCAGGACAAGCA---CTC------TAT---ACAAATAAC------
---------ATAATAGGAAATATA------------AGAGAAGCATATTGT---AAGGTCAATGGATCA---
CAATGGAATAACACTCTACGACAGGTAGCTGACAAATTAAGA---------------GAATACTTTAAC----
-----------------------------------GACAAAACAATAATATTTAAG------
CATTCCTCAGGAGGGGACCTAGAAATCACA---ACACATAGTTTTACTTGTGGAGGAGAA---
TTTAAACAGCTTCAGGCAAGAATCCTTGCTGTGGAAAGATACCTAAGGGATCAACAGCTCCTAGGAATTTGGG
GCTGCTCTGGAAAACTCATCTGCACCACTAATGTGCCGTGGAACTCTAGTTGGAGT-----------------
----------------------------AATAAATCTCTGCAGGACATATGG------GAT------
AACATGACCTGGCAGCAATGG---GATAAAGAAATTAGCAATTACACAGAAATAATATAT---
AGGCTAATTGAGGAATCGCAGAACCAGCAGGAA--

AAGAATGAACAAGACTTATTGGCATTGGACAAGTGGGCAAGTTTGTGGAATTGGTTTGACATAACAAATTGGC
TGTGGTATATAAAAATATTTATAATGATAGTAGGAGGCTTAATAGGATTAAGAATAGTTTTTACT---
GTGCTTTCTATAATA---------AATAAAGTTAGGCAGGGATACTCACCT------
TTGTCATTTGCTATTAGTTTGATTGATACCATAGCAATAGTAGTAGCTGGC---
TGGACAGATAGGGTTATAGAAGTAGGACAAGGACTTGGTAGAGCT----------------------------
--------TTTCTCAACATA------CCTAGAAGAGTCAGACAGGGATTGGAAAGGGCTTTGCTT---TAA
>KT022375
GCT---GTA--
GATAAGTTGTGGGTTACTGTCTATTATGGGGTACCTGTGTGG---AAAGATGCAGAG---
ACCACCCTATTTTGTGCATCAGATGCTAAAGCATATAATAGAGAA---GCACATAAT----------------
--------------GTCTGGGCTACA---CATGCCTGTGTACCCACAGAC---
CCCGACCCACAAGAAATACCTTTGGAA---------AATGTGACAGAAGAGTTTAAC----------------
--------------------------ATGTGGAAAAATAACATGGTAGAACAAATGCATACAGATATA---
ATCAGCCTATGGGACCAAAGCCTA---AAGCCATGTGTAAAGTTA---
ACCCCTCTCTGTGTTACCTTAAAATGCACCAATGCTAAAATTATCAATAATGAGACC----------------
----
---AAGTTTAACGAAGAACTAAAA---
AACTGCTCTTTCAATATGACCACAGAACTAAGG---------------------------
GATAAGACACAGAAAGTATATTCACTT---TTTTATAGACTTGATGTAGTACAAATAAATGCT----------
----

AATGGTAGTAATAATGATAGTTATAGATTAATAAATTGTAATACCTCAGCTATTAAACAA---
GCTTGTCCAAAGGTATCCTTTGAGCCAATTCCCATACATTATTGTGCTCCAGCTGGTTTTGCGATCCTAAGGT
GTAAT---GATAATAAGTTCAAC------GGAACAGGAACATGCACTAAT-----------------------
-GTCAGC---ACAGTACAATGCACACATGGAATCAAGCCAGTAGTATCAACT---
CAACTACTGTTAAATGGCAGCCTGGCAGAAGAAGAG------------
GTAATAATTAGATCTGAAAATATCTCAGACAAC---------GCTAAAACCATAATAGTACAACTTGTC----
-----------AAGCCTGTAAGC---------ATCAGATGTATCAGAGTTAGCAACAAGACAAGAACA-----
-AGTATACGTATA------------GGACCAGGACAAACA---TTC------TATGCAACAGGTGCC------
---------ATAATAGGAGACATA------------AGAGAAGCACATTGT---ACGGTCAATGGATCA---

GAATGGAAAGGAACACTTCAACAGATAGTTACACAATTAAGA--------------AAATATTGGAAC----
-----------------------------------ACAACAACAATAAGCTTCAAT------
AGCTCCTCAGGAGGGGATTTAGAAGTTACA---ACACATAGTTTCAATTGTGGAGGAGAG---
TTTAAACAGCTCCAGGCAAGAGTCCTGGCTGTGGAGAGATACCTAAGAGATCAACAGCTCCTAGGAATTTGGG
GTTGCTCTGGAAAACTCATCTGCACCACTAATGTGCCCTGGAACGCTAGTTGGAGT----------------
----------------------------AATAAAACTCAGGATGAGATATGG------GAT------
AGCATGACCTGGCTGCAATGG---GATAAAGAAATTGACAATTATACACAAACAATATAT---
GAGCTACTTGAAGCATCGCAGAACCAGCAGGAA--

AAGAATGAACAAGAGTTATTGGCATTGGACAAGTGGGCAAATCTGTGGAGTTGGTTTAACATAACAAACTGGC
TGTGGTATATAAGAATATTTATAATGATAGTAGGAGGATTAATAGGCTTAAGAATAGTTTTTGCT---
GTGCTTGCTTTAATA---------AATAGAGTTAGGCAGGGATACTCACCT------
CTGTCGTTTGCTATTAATTTACTTGATACCATAGCAATAGCAGTAGCTGGC---
TGGACAGATAGGATTATAGAAGTAGCACAAGGACTTGGTAGAGCT---------------------------
--------ATCCTTAACATA------CCTAGAAGAATCAGGCAGGGCGCCGAAAGGGCTTTATTA---TAA
>KT022376
------ACA--
GAAAACTTGTGGGTCACTGTTTACTATGGGGTACCTGTGTGG---AAAGATGCAGAA---
ACCACCTTGTTTTGTGCATCGGATGCTAAAGCATATGAGACAGAA---AAGCATAAT---------------
--------------GTCTGGGCTACA---CATGCCTGTGTACCCACAGAC---
CCCAACCCACAGGAAATACATTTGGAA---------AATGTGACAGAAGAATTTAAC---------------
--------------------------ATGTGGAAAAATAACATGGTAGAGCAGATGCATACAGATATA---
ATCAGTCTATGGGATCAAAGCCTC---AAGCCATGTGTAAAGTTA---
ACCCCTCTCTGTGTTACTTTAAATTGTGGCGATCCGTCTGCCAACATC------------------------
----
-------------------------------------ACGGGGGATGGCAAGGAAGAAATAAAA---
AACTGCTCTTTCAATATGACCACAGAATTAAGA-------------------------
GATAAGAAGCAAAAAGTATATTCACTG---
TTTTATAGACTTGATATAGTAGAAATTAATGAAAGACAGGGTAATGAA------------------------
----
AGTCAGAGTAATAGTAGTAGTAAGTATAGATTAATAAACTGTAATACCTCAGCCATTACACAA---
ACGTGCCCAAAGGTAACTTTTGACCCAATTCCTATACATTATTGTGCTCCAGCTGGTTTTGCGATCCTAAAGT
GTAAG---GATGAGGAGTTCAGT------GGAGCAGGGCCATGCAAGAAT----------------------
-GTCAGC---ACAGTCCAATGCACACATGGAATCAAGCCAGTAGTATCAACT---
CAACTGCTGTTAAATGGCAGTCTAGCAGGAAAAGAG-----------
GTAAAAATTAGATCTGAAAATATAACAAACAAT---------GCCAAAAATATAATAGTACAACTTGTC----
-----------AAACCCGTGCCA---------ATTAATTGTACTAGACCTAGCAACAACACAAGGAAA-----
-AGTGTATGGATA------------GGACCAGGACAAGCA---TTC------TATGCAACAGGTGAC------
---------ATAATAGGAGATATA------------AGAAAAGCACATTGT---AATGTCAGTAGATCA---
CAATGGAATAACACTTTACAAGAGGTAGTTATACAATTAAGG--------------AAACATTTTGGG----
-----------------------------------AACAAAACAATAAAGTTTGCT------
AACTCCTCAGGAGGGGATCTAGAAATCACA---ACACACAGTTTTAATTGTAGAGGAGAA---
TTTAAACAGCTCCAGGCAAGAGTCCTGGCTGTGGAAAGATACCTAAAGGATCAACAGCTCCTAGGAATTTGGG
GCTGCTCTGGAAGACTCATCTGCACCACTAATGTGCCCTGGAACTCTAGTTGGAGT----------------
----------------------------AACAAATCTTACAGTGAGATATGG------GAT------
AACATGACCTGGCTGCAATGG---GATAAAGAAGTTAGCAACTATACACAGATAATATAT---
AAGCTAATTGAAGAATCACAGAACCAGCAGGAA--

AAGAATGAACAAGACCTATTGGCATTGGACAAGTGGGCAAGTCTGTGGACTTGGTTTGATATATCAAAATGGC
TGTGGTATATAAGAATATTTATAATGATAGTAGGAGGCTTAATAGGATTAAGAATAGTTTTTGCT---
GTGCTTTCTATAATA---------AATAGAGTTAGGCAGGGATACTCACCT------
TTGTCGTTTGCTATTAATTTGGTTGATACCATAGCAATAGTAGTAGCTGGC---
TGGACAGATAGGATTATAGAAATAGGACAAATACTTGGTAGAGCT---------------------------
--------ATTCTCCACATA------CCTAGAAGAATCAGACAGGGCCTCGAAAGGGCTTTGCTA---TAA
>KT022377
GCT---GCA--
AATAACTCGTGGGTTACTGTCTACTATGGGGTACCTGTGTGG---AAAGACGCAGAG---
ACCACCTTATTTTGTGCCTCAGATGCTAAAGCATATGAGAGGGAG---AAGCATAAC---------------
--------------GTCTGGGCTACA---CATGCCTGTGTGCCCACAGAC---
CCCAACCCACAAGAAATACCTTTGGAA---------AATGTGACAGAAGATTTTAAC---------------

--------------------------ATGTGGAAAAATAATATGGTAGAACAGATGCATACAGATATA---
ATCAGTCTATGGGACCAAAGCTTA---AAGCCATGTGTAACGTTA---
ACTCCTCTCTGTGTTACATTAAATTGTAGCACTGCAACCCCTCTCAATGCAACTAGCAATGCC----------
----
---ACGGGAGGGAACGAAATGAAA---
AACTGCTCTTACAATATGACCACAGAACTATGG---------------------------
GATAAGAAACAGAGAGTATATTCACTC---TTTTATAGAACTGATGTAGTACAAATAGGTCAGGGT-------
----

AATAGTACTGAGTATAGATTAATAAATTGTAATACCTCAGCCACTATGCAA---
GCTTGTCCAAAAGTAAGCTTTGAGCCAATTCCCATACATTATTGTGCCCCAGCTGGTTTTGCAATCCTAAAGT
GTAGG---GATGAGGAATTCAAT------GGAACAGGGCCATGCAAGAAT-----------------------
-GTCAGC---ACAGTCCAATGCACACATGGAATCAAGCCAGTAGTATCAACT---
CAACTGCTGTTAAATGGCAGTCTAGCAGAAGGAAAG-----------
GTAAAAATTAGATCTGAGAATATCACAAACAAT---------GCCAAAACTATAATAGTACAACTTATC----
-----------AAGCCTGTGCTA---------ATTAATTGTACCAGACCTAGTAACAATACAAGAAGA-----
-AGTGTACATATA------------GGACCAGGACGAGCA---TTC------TATACGACAGGTGAA------
---------ATAATAGGGGATATA------------AGACAAGCACATTGT---AATGTCACTAGATCA---
GCATGGAATGAAGCGCTACAACGGGTAGTTGCACAATTAAGA---------------GAACACTTTAAG----
-----------------------------------AATAAAACAATAATATTTACT------
AACTCATCAGGAGGGGATCTGGAAATCACA---ACACATAGTTTTAATTGTGGAGGAGAA---
TTTAAACAGCTCCAGGCAAGAGTCCTGGCTGTGGAAAAGTACCTAAAGGATCAACAGCTCCTAGGAATTTGGG
GCTGCTCTGGAAAACTCATCTGCACCACTAATGTGCCCTGGAACTCTAGTTGGAGT-----------------
----------------------------AATAGAACTCTAGATGACATATGG------GGG------
AACATGACCTGGCTGCAATGG---GATAAAGAAATTAGCAATTACACAAACACAATATAT---
GACTTAATTGAAAAAGCGCAGAACCAGCAGGAA--

CAGAATGAACAGGATTTATTGGCATTGGACAAGTGGGCAAATCTATGGAATTGGTTTGACATATCAAAGTGGC
TGTGGTATATAAAAATATTTATAATGATAATAGGAGGCTTAATAGGATTAAGAATAGTTTTTGCT---
GTGCTTTCTATAATA---------AATAGGGTTAGGCAGGGATACTCACCT------
TTGTCGTTCGCTATCAGTTTGATTGATACCATAGCAATAGTAGTAGCTGGC---
TGGACAGATAGGGTTATAGAAGTAGGACAAAGAATTGGTAGAGCT----------------------------
--------TTTCTCAACATA------CCTAGAAGAATCAGACAAGGCTTCGAAAGGGCTTTGCTA---TAA
>FJ866116
GTT---GCA--
GGAAACTTGTGGGTTACTGTCTACTATGGGGTACCTGTGTGG---AGAGACGCAGAG---
ACCACCTTATTTTGTGCATCAGATGCTAAAGCATATGACACAGAA---GTGCATAAT----------------
--------------GTCTGGGCTACA---CATGCCTGTGTACCCACAGAC---
CCCAACCCACAAGAAATAAAGATGGAG---------AATGTGACAGAAAATTTTAAC----------------
--------------------------ATGTGGAAAAATAACATGGTAGAACAAATGCATGCTGATATA---
ATCAGTCTATGGGACCAAAGCCTA---CAGCCATGTGTAAAGTTA---
ACCCCTCTCTGCGTTACTCTAAATTGTGCCTATAATGTCACGATTTTCAATACCACGGGTGCCAATGTCACGA
ATAAA--
-------GAGACACCAACAAACATAGGGACACCAACAAACATAGGGACAAGGGGAGAAATGAGA---
AACTGCTCTTTCAATGTGACCACAGAAATAAGG---------------------------
GATAAGAAAAAGAAAGTATACTCACTT---
TTTTATAAACAGGATATAGTACAAATTAATGAAAATCAAGGTAATAGTAGTGAAAAGAATAATAGTAGTATTA
TTAGTGCTGAAAATAATAATACTAATAGTAATAATAGTGTAAGTAATAATAGTAGTAAGAATAGTAATAAAGA
TGAGTATATATTAATAAATTGTAATACCTCAGCCATTACACAG---
GCATGCCCAAAGGTATCTTTTGAGCCAATTCCCATACATTATTGTGCCCCAACTGGTTTTGCGATCCTAAAGT
GCAAT---GAGGAGGAGTTCAAT------GGAACAGGGCCATGCAACAAT-----------------------
-GTCAGC---ACAGTCCAGTGCACACATGGTATCAGGCCAGTAGTGTCAACT---
CAACTACTGTTAAATGGCAGTCTAGCAAAAGATGAG-----------
ATAAAATTTAGATCTGAAAATCTCACAAATAAT---------GCCAAAAATATAATAGTACAACTTAAC----
-----------GAAACTGTGAGC---------ATTACTTGTATGAGACCTAACAACAATACAAGAAAA-----
-AGTGTGCGTATA------------GGTCCAGGACAAGTA---TTC------TATGCAACAGGTGAC------
---------ATAATAGGGGATATA------------AGACAAGCACATTGT---AATGTTAGTAGATTA---
AACTGGACTAGTACTTTACAAAGAATAGCTACACAATTAGGG---------------AACATCTTTAAC----
-----------------------------------AAAACAACAATAACATTTGCT------
AACTCCTCAGGAGGGGATCTAGAAATCACA---ATGCATAGTTTTAATTGTGGAGGAGAA---

TTTAAACAGCTCCAGGCAAGAGTCCTGGCTGTGGAAAGATACCTAAGGGATCAACAGCTCCTAGGAATTTGGG
GCTGCTCTGGAAAACTCATCTGCACCACTAGTGTGCCTTGGAACACTAGTTGGAGT-----------------
-------------------------AATAATAGATCTCTGGAACAGATATGG------GAT------
AACATGACCTGGATGCAATGG---GATAAAGAAATTAGCAATTACACACAAGAAATATAT---
AGGCTGATTGAAATATCGCAGAACCAGCAAGAA--

GAAAATGAAAAAGACTTATTGGAATTGGACAAGTGGGCAAGTCTGTGGAATTGGTTTGACATATCAAATTGGC
TATGGTATATAAGAATATTTATAATGATAGTAGGAGGCTTAATAGGATTAAGAATAGTTTTTGCT---
GTGCTTTCTATAATA---------AATAGCGTTAGGCAGGGGTACTCACCT------
TTGTCATTTGCTATTAATTTGTTTAATACAATAGCAATAGCAGTAGCTGGC---
TGGACAGATAGGGTTATAGAAGTAGGACAAGTAGTTGGTAGAGCT----------------------------
--------ATTCTCCACATA------CCTAGAAGGATCAGACAGGGCTTTGAAAGGGCTTTGCTA---TAA
>FJ866118
GCT---ACA---
AACTTGTGGGTTACTGTCTACTATGGGGTACCTGTGTGG---AAAGATGCAGAG---
ACCACCTTATTTTGTGCATCAGATGCTAAAGCATATGATAAAGAA---GTACATAAT----------------
--------------GTCTGGGCTACA---CATGCCTGTGTACCCACAGAC---
CCCAACCCACAAGAAGTACATTTGGAA---------AATGTGACAGAAGAGTTTAAC----------------
--------------------------ATGTGGAAAAATTATATGGTAGAGCAGATGCATACAGATATA---
ATTAGTCTATGGGACCAAAGCCTA---CAGCCCTGTGTAAAGTTA---
ACCCCTCTCTGTGTTACTTTAAATTGTAGCCATAATATAAACCATAATGAGACCACTTTCTCT----------
----
-------------------------AATGACACCAATCTCCCCAATATCACCGAAGAGATGAGA---
AACTGCTCTTTCAATATGACCACAGAAGTAAGG---------------------------
GATAGGCAAAAGCAAGTATATTCACTT---TTTTATAGACTTGATATAGTACAAATTAATGAAGATCAA----
----

AAAGAGGGTAGTAAGGGTAGGTATAGATTGATAAATTGTAATACCTCAGCCATTACACAG---
GCTTGTCCAAAGGTATCCTTTGAGCCAATTCCCATACATTTTTGTGCCCCAGCTGGTTTTGCGATTCTAAAGT
GTAAG---GATAAAGAGTTCAAT------GGAACAGGGCCATGTAAGAAT-----------------------
-GTCAGC---ACAGTACAATGCACACATGGAATCAGGCCAGTAGTATCAACT---
CAGTTGCTGTTAAATGGCAGTCTAGCAGAAGGGAAG------------
GTAATGATTAGATCTGAAAATTTCACAAACAAT---------GCCAAAAATATAATAGTACAATTTAAC----
-----------GAGTCTGTAGAA---------ATTGAGTGTCTCAGACCTAACAACAATACAAGAGAA-----
-AGTATACATCTA------------GGACCAGGACGAGCA---TTC------TATGCAAGAGGTGGA------
---------ATAATAGGAGATATA-----------AGACAAGCACATTGT---ATTGTCAATAAAACA---
AACTGGAACAAAGCTCTGAAAGAGGTAGCCAAGCAATTAAGA---------------ACAATATTT-------
-----------------------------------AACAAAACAATAAAATTTACT------
AACTCCTCAGGAGGGGATATAGAAATCACA---ACACATAGTTTTAATTGTAGAGGAGAA---
TTTAAACAGCTCCAGGCAAGAGTCCTGGCTGTGGAAAGATACCTAAAGGATCAACAGCTCCTAGGACTTTGGG
GCTGCTCTGGAAGAATCATCTGCACCACTAATGTGCCCTGGAACTCTAGTTGGAGT-----------------
----------------------------AATAAATCTTATGATGAGATATGG------GAT------
AGCATGACCTGGCTGCAATGG---GATAGAGAAATTAGCAACTACTCAGACATAATATAC---
AACCTAATTGAAGAATCGCAGATCCAGCAGGAA--

AAGAATGAACAAGACTTATTGGCATTGGACAATTGGGCAAGTCTGTGGAATTGGTTTAACATAACAAAATGGC
TGTGGTATATAAAAATATTTATAATGATAGTAGGAGGCTTAATAGGATTAAGAATAGTCTTTACT---
GTGATATCTATGATA---------AATAGAGTTAGGCAGGGATACTCACCT------
TTGTCGTTCGCTATTAGTTTGTTTGATACCTTAGCAATAGTAGTAGCTGGC---
TGGACAGATAGGATTATAGAACTAGGACAAAGATTTTGTAGAGCT----------------------------
--------ATTCTCAACGTA------CCTACAAGAATCAGACAGGGCGCAGAAAGGGCTTTGCTA---TAA
>FJ866120
GCT---GCA--
GAAAACTTGTGGGTTACTGTATACTATGGGGTACCTGTGTGG---AAAGAAGCAGAT---
ACCACCCTATTTTGTGCATCAGATGCTAAAGCATATGCGACAGAA---AAGCACAAT----------------
--------------GTCTGGGCTACA---CATGCCTGTGTACCCACAGAC---
CCCAACCCACAAGAAATATATTTGGAA---------AATGTGACAGAAGAGTTTAAC----------------
--------------------------ATATGGAAAAATAACATGGTAGAGCAGATGCATGCAGATATA---
ATCAGTCTATGGGATCAAAGCCTA---AAGCCATGTGTACAGTTA---
ACCCCTCTCTGTGTTACTTTAAATTGTACAAATGTCAATGGCACGGAGGACAACGTCACTAGGAGCGATAAT-

----
-------------ACCACTAATAGCAATGCCACTGCTAGCCCAACAGTAAGGGAAGACATGAAA---
AACTGCTCTTTCAATATAACTACAGAAGTTAGG--------------------------
GATAAGATACGGCAGGTATATTCCCTT---TTTTATAAAGTTGATGTAACACCAATTGATGAAAGG-------
----

AAAAATGATACCACAGAGTACAGACTAATAAACTGTAATACCTCAACAATTACACAG---
GCCTGCCCAAAGGTAACCTTTGAGCCAATTCCCATACATTATTGTGCCCCTGCTGGTTTTGCGATCCTAAAGT
GTAGA---GATGAGGAGTTCAAT------GGAACAGGGCCATGCAAGAAT----------------------
-ATCAGC---ACAGTACAATGCACACATGGAATCAAGCCAGTAGTCTCAACT---
CAACTGCTGTTAAATGGCAGTCTATCAGAAAAGGGG-----------
ATAAAAATTAGATCTGAAAATATCACAAACAAT---------GCCAAATCAATAATAGTACAATTTAAT----
-----------GAGACTGTGGAA---------ATTAATTGTACTAGACCTAACAACAATACAAGAAAA-----
-AGTATAAGTATA------------GGACCAGGACGAGCA---TTC------TATGCAACAGGGGAC------
---------ATAACAGGAGACATA------------AGACAAGCACATTGT---AATGTCAGTGGAGCA---
AAATGGAATAGAACTTTGAGAATGGTAGCCAACCAACTAAGA---------------AAACACTTT-------
----------------------------------AACAAAACAATAATCTTTACT------
AAACACTCAGGAGGAGATTTAGAAATCACA---ACACACAGTTTTAATTGTGGAGGAGAA---
TTTAAGCAGCTCCAGGCACGAGTCCTGGCTGTGGAAAGATACCTAAAGGATCAACAGCTCCTAGGAATTTGGG
GCTGCTCTGGAAAACTCATCTGCACCACTAATGTGCCCTGGAACTCTAGTTGGAGT-----------------
---------------------------AATAGATCTCTGGCGGATATATGG------AAT------
AACATGACCTGGTTGCAATGG---GACAAAGAAGTTAGCAATTACACAAACATAATATAT---
ACCTTGCTTGAAGAATCACAGAACCAGCAGGAA---------------------------------------
----
AAGAATGAACAAGATTTATTGGCATTGGACAAATGGGCAAATCCGTGGAGTTGGTTTAGCATAACAAATTGGC
TGTGGTATATAAAAATATTTATAATGATAGTAGGAGGCTTAATAGGATTAAGAATAGTTTTTGCT---
GTGCTTTCTATAATA---------AATAGAGTTAGGCAGGGATACTCACCT------
CTGTCATTCGCTATTAACTTGCTTGATACCATAGCAATAGCAGTAGCTGGC---
TGGACAGATAGGGTTATAGAGATAGCACAAAGAATTGGTAGGGCT---------------------------
--------ATCCGCCACATA------CCTAGAAGAATCAGACAGGGCTTAGAAAGAATTCTGCTA---TAA
>FJ866121
GCT---GAC---
AACTGGTGGGTTACTGTCTACTATGGGGTACCTGTGTGG---AAAGATGCAAAG---
ACCACCTTATTTTGTGCATCAGATGCTAAAGCATATGAGACAGAA---AAGCATAAT---------------
--------------GTCTGGGCTACA---CATGCCTGTGTACCCACAGAC---
CCCAACCCACAAGAAATACATTTGGTA---------AATGTAACAGAAGAGTTTAAC---------------
-------------------------ATGTGGAAAAATGGCATGGTAGAGCAGATGCATAAAGACATA---
ATCAGTCTATGGGACCAAAGCCTA---CAGCCATGTGTAAAGTTA---
ACCCCTCTCTGTGTTACTTTAAATTGTGGCCATAATGTCACAATCCATAATAATGTCACAATCCATAATAATG
TCACAATCCAT---
----AATAACACAGCCACTAATAGCACGAGTGCCACATCCCCCTCGATCAGGGAAGAAGTAAGA---
AACTGCTCTTTCAATGTAACCACAGAAATAAGG--------------------------
GATAAGACAAAGAAGGTATATTCATTG---TTTTATAGACTTGATTTAGTACAAATTAATGAAAAA-------
----

CAAAGTAATAGTAGTGAGTATATATTAATAAATTGTAATACCTCAGCCATTACACAG---
GCTTGCCCAAAGGTATCCTTTGAACCAATTCCCATACACTATTGTGCTCCAGCTGGTTTTGCGATCCTAAAGT
GTAAT---GATAAGAATTTCAAT------GGAACAGGGCAGTGCAAGAAT----------------------
-GTCAGT---ACAGTACAATGCACACATGGAATCAAGCCAGTAGTATCAACT---
CAACTGCTGTTAAATGGCAGTCTAGCAGAAGAAAAG-----------
GTAAAAATTAGATCTGAGAATTTCACAAACAAT---------GCCAAAACTATAATAGTACAACTTGCC----
-----------AAACCTGTGAAC---------ATTACTTGCACCAGACCTAGCAACAATACAAGAAGA-----
-AGTGTACGTATA------------GGACCAGGACAAGCA---TTC------TATGCAACAGGGGAT------
---------ATAATAGGGGATATA------------AGAAAAGCATATTGT---ACTATCAATAGAACA---
GAGTGGAATCAAACTTTACAACAGGTAGCTACACAATTAGGA---------------AAACAATGGGGG----
----------------------------------AACAAAACAATAATCTTTACT------
AACTCCTCAGGGGGAGATCCAGAAATAACA---ACACATAGTTTTAATTGTGGAGGAGAA---
TTTAAACAGCTCCAGGCAAGACTCCTGGCTGTGGAAAGATACCTAAAGGAACAACAGCTCCTAGGAATTTGGG
GCTGCTCTGGGAAAGTCATCTGCACCACTAATGTGCCCTGGAACGCTAGTTGGAGT----------------
---------------------------AATAAATCTTATAATGAAATATGG------GAT------

```
AACATGACCTGGATGCAATGG---GATAGAGAAATTAGTAATTACACAGACATAATATAT---
GACCTACTTGAAGTATCGCAGGTCCAGCAGGAA----------------------------------------
-----------------------------
AAGAATGAACAAGACTTATTGGCATTGGACAAATGGGCAAGTCTGTGGAGTTGGTTCAGTATAACAAACTGGC
TGTGGTATATAAGAATATTTATAATGATAGTAGGAGGATTAATAGGATCAAGAATAGTTTTTGCT---
GTGCTTTCTATAATA---------AATAGAGTTAGGCAGGGATACTCACCT------
TTGTCATTGGCTGTTAATTTGCTTGATACTGTAACAATAGTAGTAGCTGGC---
TGGACAGATAGGGTTATAGAAATAGGACAAAGAATTGGTAGAGCT----------------------------
--------ATCCTCAACATA------CCTAGGAGAATTAGACAGGGTGTAGAAAGAGCTTTGCTA---TAA
>KT022380
ACT---GCA------------------------------------------
GACAACTTGTGGGTTACTGTCTACTATGGGGTACCTGTGTGG---AAAGACGCTGAC---
ACCACCTTATTTTGTGCATCAGATGCTAAAGCATATGAGACAGAA---AAGCATAAT----------------
--------------GTCTGGGCTACA---CATGCCTGTGTACCGACAGAC---
CCCAACCCACAAGAAATAAAGTTGGAA---------AATGTGACAGAAGAGTTTAAC----------------
--------------------------ATGTGGAAGAATGGCATGGTAGAGCAGATGCATACAGATATA---
ATCAGTTTATGGACCAAAGCCTA---AAGCCATGTGTACAGTTA---
ACCCCTCTCTGTGTTACTTTAAATTGTAGCCATAATGTCACTGTCATAAATGGCACAAGTGGTGCCAATACCA
CAAGTGCCCCA--------------------------------------------------------------
-------------------------------------------ATTGTCAAAGAAGAAATGAAA---
AACTGCTCTTTCAATACTACCACAGAAATATGG---------------------------
GATAAGAGAAGGAAGGAATATTCACTT---TTTTATAAACTTGATATAGTACAAATTAATGGTAAT-------
-------------------------------------------------------------------------
----------------------
GACAGTAGTGAGTATAGATTAATAAATTGCAATACCTCAACCATTAAACAG---
GCTTGTCCAAAGGTAACCTTTGAGCCAATTCCCATACATTATTGTGCTCCAGCTGGTTTTGCGATTCTAAAGT
GTACT---GATAAAGAATTCAAG------GGAACAGGGAAATGTGAAAAT-----------------------
-GTCAGC---ACAGTACAATGTACACATGGAATCAAGCCAGTAGTATCAACT---
CAACTGCTGTTAAATGGCAGCCTAGCAGAAGGAAAG------------
GTGATGATTAGATCTGAAAATATTACAAACAAT---------GCTAAAAACATAATAGTACAATTTAAT----
-----------AGTTCTGTAGGG---------ATTAATTGTACCAGACCTGGCAACAATATAAGAAAA-----
-AGTGTACGTATA------------GGACCAGGACAAGTA---TTT------TATGCAATGGGTGAC------
---------ATAATAGGGGATATA------------AGAAAAGCATATTGC---AATGTCAGTAGAGCA---
GAATGGAACAAAACTTTGCAAGAAGTAGCCGAACAATTACAA---------------ACATACTTTGAG----
-----------------------------------AACAAAACAATAATTTTTGAT------
AACTCCTCAGGAGGGGATGTAGAAATTACA---ACACATACTTTTAATTGTGGAGGAGAA---
TTTAAACAGCTCCAGGCAAGAGTCCTGGCTCTGGAAAGATACCTAAAGGATCAACAGCTCCTAGGAATTTGGG
GCTGCTCTGGAAAACTCATCTGCCCCACTAGTGTGCCCTGGAATTCTAGTTGGAGT-----------------
----------------------------AATAAATCTCTTAGTGAGATATGG------GAT------
AACATGACCTGGCTGCAATGG---GATAAAGAAATCAGCAATTACACACAAATAATATTT---
GACCTAATTGAAGAATCGCAGAACCAGCAGGAA----------------------------------------
-----------------------------
AAGAATGAACAAGACTTATTGGCATTAGACAAGTGGGCAAGTCTGTGGAATTGGTTTGACATATCAAGGTGGC
TGTGGTATATAAGAATATTTATAATGATAGTAGGAGGCTTAATAGGGTTAAGAATAGTTTTTGCT---
GTGCTTTCTATAATA---------AATAGAGTTAGGCAGGGATACTCACCT------
CTGTCGTCTGCTATTAATTTAGTTGATACCATAGCAATAGCAGTAGCTGAC---
TGGACAGATAGGGTTATAGAAATAGGACAACAAATTGGTAGAGGT----------------------------
--------ATCCTCCACATA------CCTAGAAGAATCAGGCAGGGCTTTGAAAGAGCTTTACTA---TAA
>KT022381
GCT---GCA------------------------------------------
GGAAACTTGTGGGTTACTGTTTACTATGGGGTACCTGTGTGG---AAAGATGCAGAG---
ACCACCCTATTTTGTGCATCAGATGCTAAAGCATATGTGCAAGAA---AAGCATAAT----------------
--------------GTATGGGCTACA---CATGCCTGTGTACCCACAGAC---
CCCAACCCACAAGAAATACATTTGGAA---------AATGTGACAGAAGAGTTTGAC----------------
--------------------------ATGTGGAAAAATAAGATGGTAGAGCAGATGCATACCGATATA---
ATCAGTCTATGGACCAAAGCCTA---AAGCCATGTGTACAGTTA---
ACCCCTCTCTGTGTTACTCTAAATTGTAGCAACGCCAACATCACAAGTAGCAATAATACCACTTCA-------
-------------------------------------------------------------------------
----------------------AGTAACAACACTGCCGGGGTGGAGAACAAGGAAGAAGTAAAA---
AACTGCTCTTTCAATATGACCACAGAGATAAGG---------------------------
```

GATAAGAAGAGGAAGGTCTATTCACTT---TTTTATAGACTTGATATAGTACCAATTAATCAAGATAAT----
----

AGTAGTAATAATGATAGTAATAGTGAGTATAGGTTAATAAATTGTAATACCTCAGTCATTACACAG---
GCTTGCCCAAAGGTGACTTTTGAGCCAATTCCCATACATTATTGTGCCCCAGCTGGTTTTGCGATCCTAAAGT
GTAAG---GATAAGGAGTTCAAT------GGAACAGGGCCATGCAAAAAT-----------------------
-GTCAGC---ACGGTGCAGTGCACACATGGAATCAAGCCAGTAGTATCAACT---
CAACTGCTGTTAAATGGCAGTCTAGCAGAAGGAGGG------------
ATAAGAATTAGATCTAAAAATATTACAGACAGT---------GGCAAAAATATAATAGTACAACTTGTT----
-----------AAGCCTGTAAAA---------ATTGATTGTACCAGACTTGGCAACAATACAAGAACA-----
-AGTATACATATA------------GGACCAGGAAAAGCA---TTC------TATGCAAGAGGTGAA------
---------GTAATAGGAGATATA------------AGACAGGCATTTTGT---AAGGTCAATAAATCA---
GCTTGGAATGAAACTCTTCACCAGGTAGCTGAACATTTAAGA---------------ACATACTTTAAG----
-----------------------------------AACAAAACGATAATATTTAAT------
AGCTCCACAGGAGGGGATCTAGAAATCACA---ACACATAGCTTTAATTGTGCAGGAGAA---
TTTAAACAGCTCCAGGCAAGAGTCCTTGCTGTGGAAAGATACCTAAGGGATCAACAGCTCCTAGGAATTTGGG
GCTGCTCTGGAAAACTCATCTGCACCACTAATGTGCCCTGGAACTCTAGTTGGAGT-----------------
----------------------------AATAAATCACAGGAGGAGATATGG------GAC------
AACATGACCTGGCTGCAATGG---GATAGAGAAATTAGTAATTACACAGACATAATATAC---
AGACTAATTGAAGAATCGCAGAACCAGCAGGAA--

AAGAATGAACAAGACTTATTGGCATTGGACAAGTGGGCAAGTCTGTGGAATTGGTTTGACATATCACGGTGGC
TGTGGTATATAAAAATATTTATAATGATAGTAGGAGGCTTAATAGGATTAAGAATAGTTTTTGCT---
GTGTTTTCAATAATA---------AATAGAGTTAGGCAGGGATACTCACCT------
CTGTCGTTTGCTATTAGTTTGATTGACACCATAGCAATAGTAGTAGCTGGC---
TGGACAGACAGAATCATAGAAATAGGACAGGGAATCGGTAGAGCT----------------------------
--------ATTCTCCACATA------CCTAGAAGAATCAGACAGGGCTTAGAAAGAGCTTTATTG---TAA
>KT022382
ACT---GCA--
GGAAACTTGTGGGTCACTGTCTATTATGGGGTACCTGTGTGG---AGAGACGCAGAG---
ACCACCTTATTTTGTGCATCAGATGCTAAAGCATATGAGGAAGAA---AAGCATAAT----------------
--------------GTCTGGCTACA---CATGCCTGTGTACCCACAGAC---
CCCAACCCACAAGAAATACATTTGGAA---------AATGTGACAGAAAATTTTAAC----------------
--------------------------ATGTGGAAAAACAACATGGTAGAACAGATGCATACAGATATA---
ATCAGTCTATGGGACCAAGGCCTA---AAGCCATGTGTAAAATTA---
ACCCCTCTCTGCGTTACCTTAAATTGTACCGAGGCC-------------------------------------
----
----------------------------AGTGTCAGTTTCGATGGGGACAAGGAAGAAATAAAA---
AACTGCTCTTTCAATATGACTACAGAGTTAAGG---------------------------
GATAAGAAACAGCAGGTGTATTCACTT---TTTTATAGACTTGATATAGTACCAATTAATGAAGAGAAA----
----

AATATCACAGAGTATAGATTAATAAATTGTAATACCTCAGCCATTACGCAA---
GCTTGCCCAAAGGTATCCTTTACGCCAATTCCCATACATTATTGTGCCCCGGCTGGTTTTGCGATCCTAAAGT
GTAAG---GATGAGGAGTTCAAT------GGAACAGGGCCATGCAAGAAT-----------------------
-GTCAGC---ACAGTACAATGCACACATGGAATCAAGCCAACAGTATCAACT---
CAATTGCTGTTGAATGGTAGTCTAGCAGAAAAAGGG------------
GTAAGAATTAGATCTGAAAACATCACAGACAAT---------GGCAAAAATATAATCGTACGACTTGAC----
-----------AAGCCTGTAATA---------ATTAATTGTACCAGACCTTACAACAATACAAGAAAA-----
-AGTGTACGTATA------------GGACCAGGACAAGCA---TTC------TATGCAACAGGTGAG------
---------GTAACAGGAGACATA------------AGAAAAGCATTTTGT---GAGATCAATAAGACA---
AAATGGGATGAAACTTTAAACAAGGTAGCTCAAGAATTAAGA---------------AAATACTTTAAG----
--------------------------------AACACAACAACAATAAAATTTACT------
AACTCCTCAGGAGGGGATGTAGAAATCACA---ACACATAGTTTTAATTGTAGAGGAGAA---
TTTAAACAGCTCCAGGCAAGAGTCCTGGCTGTGGAAAGATACCTAAAGGATCAACAGCTCCTAGGAATTTGGG
GCTGCTCTGGAAAACTCATTTGCACCACTAATGTGCCCTGGAATTCTAGTTGGAGT-----------------
----------------------------AATAAAACTCAGAGTGAGATATGG------GAA------
AACATGACCTGGCTGCAATGG---GATAAAGAAATTAGCAATTACACAGAAATAATATAT---
AGCCTAATTGAAGAATCGCAGAACCAGCAGGAA--

AAGAATGAACAAGATTTATTGGCATTGGACAAGTGGGCAAATCTGTGGAATTGGTTTGACATATCAAAATGGC
TGTGGTATATAAAAATATTTATAATGATAGTAGGAGGCTTAATAGGATTAAGAATAATTTTTGCT---
GTGCTTTCTGTAATA---------AATAGAGTTAGGCAGGGGTACTCACCT------
TTGTCGTTCGCTATTAATTTAGTTGATACTATAGCAATAGTGGTAGCTGGC---
TGGACAGATAGGGTTATAGAAATAGGACAAAGGTTTGGTAGAGCT---------------------------
--------ATTATCCACATA------CCTAGAAGAATCAGACAGGGCCTAGAAAGAGCTTTGCAA---TAA
>KT022383
GCT---GCA---
GAAAACTTGTGGGTTACTGTCCACTATGGGGTACCTGTGTGG---AAAGATGCAGAC---
ACCACCTTATTTTGTGCATCAGACGCTAAAGCATATGAGAGGGAA---AAGCATAAT---------------
--------------GTCTGGGCTACA---CATGCCTGTGTACCCACAGAC---
CCCAACCCACAAGAAATACATATGGGA---------AATGTGACAGAAGAATTTAAC----------------
--------------------------ATGTGGAAAAATAACATGGTAGAGCAGATGCATACAGACATA---
ATCAGTCTATGGGACCAAAGCCTA---CAGCCCTGTGTAAAGTTA---
ACCCCTCTCTGTGTTACTTTAAATTGTAGCAATGCCACGTTCACAAATGCCAATGTCACAAATGCCACC----
----
---GAAGAAATAAAA---
AACTGCTCTTTTAGTATGACCACAGAGCTAAAG--------------------------
GATAAGAAACAGAAAGTATATTCACTT---TTCTATAGACTTGATGTAGTAAAAATTAATGAT----------
----

AAGGATGCTAGTATTGGGTATAGATTAATAAATTGTAATACCTCAGCCATTACACAA---
GCTTGCCCAAAAGTAACCTTTGAGCCAATTCCCATACATTATTGTGCTCCAGCTGGTTTTGCGATCCTAAAGT
GTAAG---GATGAAGAGTTCAAT------GGAACAGGGCCATGCAAGAAT-----------------------
-GTTAGT---ACAGTACAATGCACACATGGAATTAAACCAGTAGTATCAACT---
CAACTGCTGTTAAATGGCAGTCTAGCAGAAAAGGAG-----------
GTAAAAATTAGATCTGAAAATATCACAAACAGT---------GCTAAAGTTATAATAGTACAACTGGAC----
-----------AAGCCTGTGAAT---------ATTACTTGTATCAGACCTAACAACAATACAAGAACA-----
-AGTATACCGATG------------GGACCAGGAAAAGCA---TTC------TACGCAATAGGCGAC------
---------ATAATAGGGGATATA------------AGACAAGCACATTGT---AATGTCAGTAGATCA---
GTCTGGAATAACACTTTAGGAAAGGTAGCCCAACAATTAAGC---------------AAATACTTTGGA----
------------------------------------AACAATACAATAAAATTTGCT------
AGGTCCTCAGGAGGGGATCTAGAAATCACA---ACACATAGTTTTAATTGTGGAGGAGAA---
TTTAAACAGCTCCAGGCAAGAGTCCTGGCTCTAGAAAGATACCTAAAGGATCAACAGCTCCTAGGAATTTGGG
GCTGCTCTGGAAAACTCATCTGCACCACTAATGTGCCCTGGAACTCTAGTTGGAGT-----------------
----------------------------AATAAATCTTACAATGAAATATGG------GAC------
AGCATGACCTGGCTACAATGG---GATAAAGAAATTAGCAATTACACACACATGATATAT---
ACCCTAATTGAAGAATCACAGAACCAGCAGGAG---

AAGAATGAACAAGACTTATTGGCATTGGATAAGTGGGCAAATCTATGGAATTGGTTTGACATAACAAACTGGC
TGTGGTATATAAGAATATTTATAATGATAGTAGGAGGATTAGTAGGATTAAGGATAGTTTTTGCT---
GTGCTTGCTGTAATA---------AATAGAGTTAGGCAGGGATACTCACCT------
TTGTCATTTGCTATTAATTTGTTTGATACCATAGCAATAGCAGTAGCTGGC---
TGGACAGATAGGGTTATAAAAATAGGACAGGCAATTGGTAGAGCT---------------------------
--------ATTCTCCACATA------CCTAGAAGAATCAGACAGGGCTTAGAAAGGGCTTTGCAA---TAA
>FJ623487
AGT---GTT---GCA-----------------------------------
GAAAACTTGTGGGTTACTGTCTACTATGGGGTACCTGTGTGG---AGAGACGCAGAA---
ACCACCTTATTTTGTGCATCAGATGCTAAAGCATATGAGACAGAA---AAGCATAAT---------------
--------------GTCTGGGCCACA---CATGCCTGTGTACCCACAGAC---
CCCAACCCACAAGAAATACGTTTGGCA---------AATGTGACAGAAGAGTTTAAC----------------
--------------------------ATGTGGAAAAATAACATGGTAGAGCAGATGCATACAGATATA---
ATCAGTCTATGGGACCAAAGTCTA---AAGCCATGTGTACAGTTA---
ACCCCTCTCTGTGTTACTTTAAATTGTACAAATGCCCATAAGACTACAAATGATACTACACAGGAT-------
----
-------------------AATACTACAAATGCCACTCCAACACTCGAGCCAGGCGAAATAAAA---
AACTGCTCTTTCAATATGACCACAGAGCTAAGG--------------------------
GATAAGAAACAGAAGGTATATGCACTT---
TTTTATAGACTTGATGTAGTACAAATTAATAATGATACACAAAGT---------------------------
----

ACTAATGGTACAAGTGATGGTACAGGTGAGTATAGATTAATAAATTGTAATACCTCAGCCATTACACAG---
GCTTGTCCAAAGGTAACTTTTGAGCCAATTCCCATACATTATTGTGCCCCAGCTGGTTTTGCGATCCTAAAGT
GTAAT---GATAAGAAGTTCAAT------GGAACAGGGCCATGCAAGAAT-------------------------
-GTCAGC---TCAGTACAATGCACACATGGAATCAGGCCAGTAATATCAACT---
CAACTGCTGTTAAATGGCAGTCTAGCAGAAGAAGAG------------
ATAAAAATTAGATCTGAAAATATCACAAGTAAT---------AGCAAAAATATCATAGTCCAATTTGTC----
-----------AAGCCTGTGAAC---------ATTACTTGTATCAGGCCTAACAATAATACAAGAAAA-----
-GGTATACGGATA------------GGACCAGGACAAGCA---TTT------TATACAACA---GAG------
---------ATAATAGGGGACATA------------AGACAAGCATATTGT---ACTATCAATGGATCA---
GAATGGAATAACACTTTACGAGAGGTAGCTGAACAATTAAGC---------------AATAGGACAAATATT-
---------------------------------------ACAAAAATAATATTTGCT------
AACCCCTCAGGAGGGGATCTAGAAATCATG---ACACATACTTTTAATTGTGGAGGAGAA---
TTCAAACAGCTCCAGGCAAGAGTCCTGGCTGTGGAAAGATACCTAAGGGATCAACAGCTCCTAGGAATTTGGG
GCTGCTCTGGAAAACTCATCTGCACCACTAATGTGCCTTGGAACTCTAGTTGGAGC-----------------
-----------------------------AATAAATCTCAGGAAGAGATATGG------GAT------
AACATGACCTGGTTGCAATGG---GATAAAGAAATTGACAACTATACACAATTCATATAT---
GACCTACTTGAGGAATCGCAGAATCAGCAGGAA--

AAGAATGAACAAGACCTATTGGCATTAGACAAGTGGGCAGGTCTGTGGAATTGGTTTAGCATATCAAATTGGC
TGTGGTATATAAGAATATTTATAATGATAGTAGGAGGCTTAATAGGATTAAGAATAGTCTTTACT---
GTGCTTGCTATAATA---------AATAGAGTTAGGCAGGGATACTCACCT------
TTGTCGTTCGCTATTAATTTGGTTGATACCATAGCAATAGTAGTAGCTGGT---
TGGACAGATAGGGCTATAGAAATAGTACAAAGAATTGGTAGAGCA----------------------------
--------ATTCTCCACATA------CCTAGAAGAATTAGACAGGGCTTTGAAAGAGCTTTGCTA---TAA
>FJ623481
GCT---GTA---
GGAAACTTGTGGGTTACTGTCTACTATGGGGTACCTGTGTGG---AAAGATGCAAAA---
ACCACCCTATTTTGTGCATCAGATGCTAAAGCATATGATACAGAA---GTGCATAAT----------------
--------------GTCTGGGCTACA---CATGCCTGTGTACCCACAGAC---
CCCAACCCACAAGAAATACATTTGGGA---------AATGTGACAGAAGAATTTAAC----------------
--------------------------ATGTGGAAAAATAACATGGTAGAGCAGATGCATACAGATATA---
ATCAGTTTATGGGACCAAAGTCTA---AAGCCATGTGTAAAGTTA---
ACCCCTCTCTGCGTTACTTTAAATTGTACTAATATCATGGGAAACAGTAACAGCACGGGA-------------
----
-------------------------AACAGTAGCAACACGAAAATTGAAGAAATAGAAATGAAA---
AACTGCTCTTTCAATGTGACCACAGAATTAAGG---------------------------
GACAAAAGAAGAAAAGAATATTCACTT---
TTTTATAAAATTGATATAGTACCACTTGATAAAAAAAGTAATAATAAT--------------------------
----
AATAGTAGTAGTAGTAATAGTAGTAGTGAGTATAGATTAATAAACTGTAATACCTCAGCCATTACACAA---
GCTTGCCCAAAGGTATCCTTTGAGCCAATTCCCATACATTATTGTGCCCCAGCTGGTTTTGCGATCCTAAAGT
GTAAT---GATGAACAGTTCAAT------GGAACAGGGGAATGCAAGAAT-------------------------
-GTCAGT---ACAGTACAATGCGCACATGGAATCAAACCAGTAGTATCAACT---
CAACTGCTGTTAAATGGCAGCCTAGCAGAAAAAGAA------------
GTAAAAATTAGATCTGAAAATATCACAGATAAT---------GCCAAAACCATAATAGTACAACTTAGC----
-----------AAGCCTGTGCAA---------ATTAAATGTACCAGACCTAACAACAATACAAGAAAA-----
-GGTATACATCTA------------GGACCAGGACAAGCA---TTC------TTTGGAACAGAAAAA------
---------ATAACAGGGAATATA------------AGAAGCGCACATTGT---AATGTCAGTAGATCA---
AAATGGAATGACACTTTGAAACAGGTAGCTGAACAATTAGAA---------------AAACACTTTAGG----
------------------------------------AACAAAACAATAAGCTTTAAT------
AGCTCCTCAGGAGGGGATCTAGAAATCACA---ACACATAGTTTTAATTGTGGAGGAGAA---
TTTAAACAGCTCCAGGCAAGAGTCCTGGCTGTGGAAAGATACCTAAAGGATCAACAGCTCCTAGGAATTTGGG
GATGCTCTGGAAAACTCATCTGCACCACTACTGTGCCCTGGAACTCTAGTTGGAGT-----------------
-----------------------------AATAAATCTCATGATGAGATATGG------GGA------
AACATGACCTGGCTGCAATGG---GAGAAAGAAATTAGCAACTACACAGACACAATATAT---
GACCTAATTGAAAAATCACAGAACCAGCAGGAA--

ATAAATGAAAAAGATTTATTGGCATTGGACAAGTGGGCAAATCTGTGGAACTGGTTTGACATATCAAGATGGC
TGTGGTATATAAAAATTTTTATAATGATAGTAGGAGGCTTAATAGGATTAAGAATAATTTTTGCT---
GTGCTTTCTATAATA---------AATAGAGTTAGGCAGGGATACTCACCT------

CTGTCGTTTGCTATTAATTTGGTTGACCACATAGCAATAGCAGTAGCTGGC---
TGGACAGATAGAGTTATAGAAGTAGGACGAGGGATTGGTAGAGCT----------------------------
--------CTTCTTCACATA------CCTAGAAGAATCAGACAGGGCTTAGAAAGGTTTTTGCTA---TAA
>FJ623475
GCT---GTA--
GAAAACTTGTGGGTTACTGTATACTATGGGGTACCTGTGTGG---AAAGATGCAGAG---
ACCACCTTATTTTGTGCATCAGATGCTAAAGCATATGAGGAAGAA---CAGCATAAT----------------
--------------GTCTGGGCTACA---CATGCCTGTGTACCCACAGAC---
CCCAACCCACAAGAAATACATTTGGAA---------AATGTGACAGAAAATTTTAAC----------------
--------------------------ATGTGGAAAAATAACATGGTAGAGCAGATGCATACAGATATA---
ATCAGCCTATGGGACCAAAGTCTA---AAGCCATGTGTAAAGCTA---
ACTCCTCTCTGCGTTACTTTAAATTGCAAGGAGGTTAATGTCACAGCC-------------------------
----
-------------------------------------GCAAACAGCACGGAAGAAATGATGAAA---
AACTGCTCTTTCAATATAACCACAGAGATAAGG---------------------------
GATAAGAGAAAGAAGGTCTATTCACTT---TTTTATAGACTTGATATAGTACCAATTAGTGCAAAT-------
----

AGGAATAATAGTACCACTGAGTATAGATTAATAAATTGTAATACCTCAGCCGTTACACAG---
GCTTGCCCAAAGGTATCCTTTCATCCAATTCCTATACATTATTGTGCCCCTGCTGGTTTTGCGATCCTAAAGT
GTCAG---GATGAGGAGTTCAAT------GGAACAGGGCCATGCAAGAAT-----------------------
-GTCAGT---ACAGTACAATGCACACATGGAATCAAGCCAGTAGTATCAACT---
CAACTGCTGTTAAATGGCAGTCTAGCAGGAAAAGAG------------
GTAAAAATTAGATCTGAAAATATCACAAACAAT---------GTCAAAACTATAATAGTACAACTTAAC----
-----------GAGTCTGTGGAA---------ATTGATTGTATCAGACCTAACAACAATACAAGAACA-----
-AGTATACCTATA------------GGACCAGGACAGGCA---TTC------TATGCAATAGGTGAC------
---------ATAATAGGGAAAATA------------AGACAAGCACATTGC---AATGTCAGTAGGTCA---
AAATGGAATAAAACTTTACAAAGGGTTGTAACCCAATTAAGA---------------AAAAACTTTAGA----
--------------------------------AACACAACAACAATAATATTTGAT------
AAACCCTCAGGAGGGGACCTAGAAATCACA---ACACATAGTTTTAATTGTGGAGGAGAA---
TTTAAACAGCTCCAGGCAAGAGTCCTGGCTGTGGAAAGATATCTAAGGGATCAACAGCTCCTAGGAATTTGGG
GCTGCTCTGGAAAACTCATCTGCACCACTAATGTGCCCTGGAACTCTAGTTGGAGT-----------------
----------------------------AATAAATCTCAGGAGGAGATATGG------GGA------
AACATGACCTGGCTGCAATGG---GATAAAGAAATCAGCAATTACACACAAATAATATAT---
AACTTAATTGAAGAATCGCAGAACCAGCAGGAA--

AAGAATGAACAAGACTTATTGGCATTTGACAAGTGGGCAAGTCTGTGGACTTGGTTTGACATATCAAATTGGC
TGTGGTATATAAAAATATTTATAATGATAATAGGAGGTTTGATAGGATTAAGAATAGTTTTTGCA---
GTGCTTGCTTTAATA---------AATAGAGTTAGGCAGGGGTACTCACCT------
TTGTCGTTTGCTATTAATTTGGTTGATACTATAGCAATAGCAGTAGCTAAC---
TGGACAGATAGAGTTATAGAAATAGGACAAAGAATTGGTAGAGCT----------------------------
--------ATTCTCCACATA------CCTAGAAGAATCAGACAGGGCTTAGAAAGAGCTTTGCTA---TAA
>FJ623476
GCT---GTA--
GAAAATTTGTGGGTTACAGTCTACTATGGGGTACCTGTATGG---AAAGATGCGAAG---
ACCATCTTATTTTGTGCATCAGATACTAAAGCATATGAGGTAGAA---ATGCATAAT----------------
--------------GTCTGGGCTACA---CGTGCCTGTGTACCCACAGAC---
CCTAACCCACAAGAAATACATCTGGAA---------AATGTGACAGAAGAGTTTAAC----------------
--------------------------ATATGGGAAAATGACATGGTAGAGCAGATGCATACAGATATA---
ATCAGTCTATGGGACGAAAGCCTA---AAGCCATGTGTAAAGTTA---
ACCCCTCTATGTGTCACTTTACATTGTACTAATGTCACTGACAAGAGTGGCAAT-------------------
----
----------------------------------GTCACTGTCAATGGCACGGATGAAATGAAA---
AACTGCTCTTTCAACATGACCACAGAGCTAAGG---------------------------
GATAAGAGAAAGAAGGAATATTCACTT---TTTTATAGACTTGATATATTACAAATTAATGAAAATCAA----
----

GGTAATAGTAGTAAGAATGATGAGTATAGATTAATAAATTGTAATACCTCAGCCATTACACAG---
GCTTGTCCAAAGGTATCTTTTGAGCCAATTCCCATACATTTTTGCGCCCCAGCTGGTTTTGCGATCCTAAAGT
GTAAG---GATGAGAATTTCAAT------GGAACAGGGCCATGTAGAAAT-----------------------

-GTCAGC---ACAGTACAATGCACACATGGGATCAAGCCAGTAGTATCAACT---
CAGCTGCTGTTAAATGGCAGCCTAGCAGAAAGAGAA------------
GTAAAAATTAGATCTCAAAATATCACAAACAAT---------GCCAAAAATATAATAGTACAACTTGTC----
-----------ACGCCTGTGCAA---------ATTAATTGTACAAGACCTAATAATAATACAAGAAAA-----
-AGTATACATATA------------GGACCAGGAAAAGTA---TTC------TATGCAAGT---GAC------
---------ATAATAGGGGATATA------------AGAAAAGCACATTGT---ATTGTCAATAGGTCA---
GAATGGAATAAGACGTTACAACAGGTAGCTACACAATTAGGA---------------ACATACTTTAAG----
-----------------------------------AATAAAACCATAATATTTGAT------
AGATCCTCAGGAGGGGATATAGAAATCACA---ACACATAGTTTTAATTGTAGAGGAGAA---
TTTAAACAGCTCCAGGCAAGAGTCCTGGCTGTGGAAAGATACCTAAGGGATCAACAGCTCCTAGGAATTTGGG
GCTGCTCTGGAAAACTCATCTGCACCACTAATGTGCCCTGGAACTCTAGTTGGAGT-----------------
----------------------------AATAAATCTCAGAGTGAGATATGG------GGG------
AACATGACCTGGCTGCAATGG---GATAGAGAAATTAGTAAGTACACAGACATAATATAT---
GGCCTAATTGAAGAATCGCAGAACCAGCAGGAA--

AAGAATGAACAAGACTTATTGGCATTGGATAAGTGGGCAAATCTGTGGAATTGGTTTGACATATCAAATTGGC
TGTGGTATATAAAAATATTTATAATGATAGTAGGGGGCTTAATAGGATTAAGAATAGTTTTTGCT---
GTGCTTTCTATAATA---------CGTAGAGTTAGGCAGGGCTACTCACCT------
TTGTCATTTGCTATTAATTTGATAGATACTATAGCAATAGCAGTAGCTGGA---
TGGACAGATAGGGTTATAGAAATAGGACAAGGAATTGGTAGAGCT----------------------------
--------ATCCTCCACATA------CCTAGAAGAATCAGACAGGGCTTCGAAAGAGCTTTGCTA---TAA
>FJ623485
GTT---GCA--
GAAAACTTGTGGGTTACTGTCTACTATGGGGTACCTGTGTGG---AAAGATGCAGAC---
ACCACCTTATTTTGTGCATCAGATGCTAAAGCTTATGAGACAGAA---AAGCATAAT----------------
--------------GTCTGGGCTACA---CATGCCTGTGTACCCACAGAC---
CCCAACCCACAAGAAATACATTTGGAT---------AATGTGACAGAAGAGTTTAAC----------------
--------------------------ATGTGGAAAAATAACATGGTAGAGCAGATGCATACAGATATA---
ATCAGTCTATGGGACCAAAGCCTA---AAGCCATGTGTAAAGTTA---
ACCCCTCTCTGCGTTACTTTAAATTGTACAACTGCCAACATCAATGCCACT----------------------
----
----------------------------------GCTAACAGCACGATCAAGGAAGAAATAAAA---
AACTGCTCTTTCAGTGTAACCACAGAGCTAAAA---------------------------
GATAAGAAACGGAAGGTATATTCACTT---TTTTATAAACTTGATATAGTAGAACTTAATGAAAATCAAGGT-
----

AAGAGTAGTGATAATAGTAGCAAGGAGTATAGATTAATAAATTGTAATACCTCAGCCATTACACAG---
GCTTGCCCAAAGGTGACCTTTGAGCCAATTCCCATACATTATTGTGCCCCAGCTGGTTTTGCGATCCTAAAAT
GTACA---GATAAAGAGTTCAAT------GGAGCAGGGCCATGCAAGAAT-----------------------
-GTCAGC---ACAGTACAATGCACACATGGAATCAAGCCAGTAGTATCAACT---
CAACTGCTGTTAAATGGCAGTCTAGCAGAAGAACAA------------
ATAAGAATTAGATCTGAAAATATCACAAACAAT---------GCCAAAAATATAATAGTACAACTTGTC----
-----------AAGCCTGTAGAA---------ATTAATTGTACCAGACCTAGCAACAATACAAGAACC-----
-AGTATACGTATA------------GGACCAGGACAAGCA---TTC------TATGCAACAGGTGAC------
---------ATAACAGGGGATATA------------AGACAAGCACATTGT---AATGTCAATAAATCA---
CAATGGAATACAGCTTTACAAAAGGTAGTTGAACAATTAAGA---------------AACCACTTT-------
-----------------------------------AACAAAACAATAATATTTAAT------
GAATCTTCAGGAGGGGATATGGAAATCACA---ACACATAGTTTTAATTGTGGAGGAGAA---
TTCAAACAGCTCCAGGCAAGAGTCCTGGCTGTGGAAAGCTACCTAAAGGATCAACAGCTCCTAGGAATTTGGG
GCTGCTCTGGAAGACTCATCTGCACCACCAATGTGCCCTGGAACACTAGTTGGAGT-----------------
----------------------------AATAAATCTTATAATGAGATATGG------GAA------
AACATGACCTRGCTGCAATGG---GATAAAGAGATTAGCAATTACACACAAATAATATAT---
RACCTAATTGAAGACTCGCAGAACCAGCAGGAA--

AAGAATGAACTAGATTTATTGGCATTGGACAAGTGGGCAAGTCTGTGGAATTGGTTTGACATAACAAATTGGC
TATGGTATATAAAAATATTTATAATAATAGTAGGAGGTTTAATAGGATTAAGAATAGTTTTTGCT---
GTGCTTGCTATAATA---------AATAGAGTTAGGCAGGGATACTCACCT------
TTGTCATTTGCTATTAAGTTGGTTGATGCCATAGCAATAGCAGTAGCTGGC---
TGGACAGACAGGGTTATAGAAATAGGACAAAGAATTGGTAGAGCT----------------------------
--------ATTATCAACATA------CCAAGAAGAATCAGACAGGGCGCTGAAAGAGCTTTGCTA---TAA

```
>FJ623488
ACA---GCA------------------------------------------
GAAAAATTGTGGGTCACTGTTTACTATGGGGTACCTGTGTGG---AAAGACGCAGAG---
ACCACTTTATTTTGTGCATCAGATGCTAAAGGATATAGTACAGAA---AAGCATAAT----------------
--------------GTCTGGGCTACA---CATGCCTGCGTGCCCACAGAC---
CCCAACCCACAAGAAATATCTTTGGAA---------AATGTGACTGAAAAGTTTAAC----------------
--------------------------ATGTGGAAAAATAATATGGTAGAACAGATGCATGAAGATATC---
ATTAGTCTATGGGATCAAAGCCTA---AAGCCATGTGTAAAGTTA---
ACCCCTCTCTGTGTTACTCTAAATTGTACCAATGTCAATGTCACAACTAATGCCACT----------------
-------------------------------------------------------------------------
-------------------------------GATACCACTATCAATGGTGATGGTGGCATGAGG---
AACTGCTCTTACAATATGACCACAGAGCTAAGG---------------------------
GATAAGAAACGGAAGGTTTATTCACTT---
TTCTATAGGCCTGATATAGTACCAATTAATCAAGATAATAGTAGTAAT-------------------------
-----------------------------------------------
AATAATAATAGTAGTAATAGTACTAGTGAGTATAGATTAATAAATTGTAATACTTCAACCATTACACAG---
GCTTGTCCAAAGGTATCCTTTGAGCCAATTCCCATACACTATTGTGCTCCAGCTGGTTTTGCGATCCTGAAGT
GTAAA---GAAATTAATTTCAAT------GGAACAGGGCCATGCAAGAAT-----------------------
-GTCAGC---ACAGTACAATGCACACATGGAATTAAGCCAGTAGTATCAACT---
CAGCTGCTGTTAAATGGAAGTCTAGCAGAAGGAAAG------------
ATAGTGATTAGATCTGAAAATATCACAAACAAT---------GCCAAAAACATACTAGTACAATTTGCC----
-----------AAGTCTGTGCAA---------ATTAACTGTACCAGACCTAACAACAACACAAGAACA-----
-AGTATACGTATA------------GGACCAGGACAATCA---TTC------TATGCAACAGGTGGC------
---------ATAATAGGGGATATA------------AGACAAGCACATTGC---AATGTCAGTAGAACA---
GCATGGAAAGAAACTTTGAAAGGGGTAGTTGAACAATTAAGA---------------ACATACTTTAAT----
-----------------------------------AATAAGACAATAATATTTAAT------
GAATCCTCAGGAGGGGATCTAGAAGTCACA---ACACATAGTTTCAATTGTGGAGGAGAA---
TTTAAACAGCTCCAGGCAAGAGTCCTGGCTGTGGAAAGATACCTAAGGGATCAACAGCTCCTAGGAATTTGGG
GCTGCTCTGGAAAACTCATCTGCACCACTAATGTGCCCTGGAACTCTAGTTGGAGT-----------------
----------------------------AATAAATCCCTGAATGAGATATGG------GAC------
AACATGACCTGGCTGCAATGG---GATAAAGAAGTTAGCAATTACACACAGATAATATAT---
AATTTACTTGAGGAATCACAGAACCAGCAAGAA----------------------------------------
-----------------------------
AAGAATGAGCAAGACTTATTGGAATTGGATAAGTGGGCAAGTCTGTGGAATTGGTTTAGCATATCAAACTGGC
TGTGGTATATAAAAATATTTATAATGATAGTAGGAGGCTTAATAGGATTAAGAATAGTTTTTGCT---
GTACTTTCTGTAATA---------AATAGAGTTAGGCAGGGATACTCACCT------
CTCTCGTTTGCTATTAGTTTGCTTGATACCATAGCTATAGCAGTAGCTGGG---
TGGACAGATAGGGTTATAGAAGCAGGACAAATATTATTAGGA-------------------------------
------------------------------------------AGGGCTTAGAAAGGGCTTTGCTA---TAA
>FJ623478
GCT---GCA------------------------------------------
GACAACTTGTGGGTTACTGTCTACTATGGGGTACCTGTGTGG---AAAGATGCAAAG---
ACCACCTTATTTTGTGCATCAGATGCTAAAGCATATGCTACAGAA---AGGCATAAT----------------
--------------GTTTGGGCTACA---CATGCCTGTGTACCCACAGAC---
CCCAACCCACAAGAAATGCCTTTAAAA---------AATGTGACAGAAGACTTTAAC----------------
--------------------------ATGTGGAAAAACAACATGGTAGAGCAGATGCATACAGATATA---
ATCAGTCTATGGGACCAAAGCCTA---AAGCCATGTGTAAAGTTA---
ACCCCTCTCTGTGTTACTTTACATTGTACCAATGTCAATGTCACTAGCAATGTCACTAGC-------------
-------------------------------------------------------------------------
-------------------------AGCAATGCCACTACCGAAGGAAATGGGGAAGAAATGAAA---
AACTGCTCTTTCAATGTGACCACAGAACTAAGG---------------------------
GATAAGAGACAGAAAGTATATTCACTC---TTTTATAAGCTTGATATAGTACAAATTAATGCAAT-------
-------------------------------------------------------------------------
----------------
CAGGGTAATAGTAGTGACTATAGATTAATAAATTGTAATACCTCAGCCATTACACAG---
GCATGCCCAAAAGTATCCTTTGAGCCAATTCCCATACATTATTGTACTCCAGCTGGTTTTGCGATCCTAAAGT
GTAAT---GATAAGGAGTTTAAT------GGAACAGGGCCATGCAAGAAT-----------------------
-GTCAGT---ACAGTTCAATGCACACATGGAATCAAGCCAGTAGTATCAACT---
CAACTGCTGTTAAATGGCAGTCTAGCAGAAGAACAG------------
ATAAGGATTAGATCTGCAAATATCACAGACAAT---------ACCAAAAACATAATAGTACAATTTACT----
```

-----------GAGCCTGTAAAA---------ATTGATTGTACCAGACCTAACAACAACACAAGAAGA-----
-AGTGTGTCTATA------------GGACCAGGACAAGCA---TTC------TATGCAATGGGTGAC------
---------GTAATAGGGGATATA------------AGAAAAGCATATTGT---CAGGTCAATAAAACA---
CAATGGAATATAACTTTACAACAGGTAGCTAACCAATTAAGA---------------GAACACTTTAAG----
------------------------------------AACAAAACAATAATATTTACT------
AGCCCCTCAGGAGGGGATATAGAAATCACA---ACACATAGTTTTAATTGTGGAGGAGAA---
TTCAAACAGCTCCAGGCACGAGTCCTGGCTGTGGAAAGATACCTAAGGGATCAACAGCTCCTAGGAATTTGGG
GCTGCTCTGGAAAACTCATCTGCACCACTAATGTGCCCTGGAACTCTAGTTGGAGT----------------
-----------------------------AATAAATCCTATAATGACATATGG------GAC------
AATATGACCTGGCTGCAATGG---GATAAAGAAATTAACAATTACACACAGATAATATAT---
GATTTAATTGAGGAATCGCAGAAACAGCAGGAA--

AAGAATGAACAAGAGTTACTGGCATTGGACAAGTGGGCAAGTCTGTGGAATTGGTTTGACATAACAAGATGGC
TGTGGTATATAAAAATATTTATAATGATAGTAGGAGGTTTAATAGGATTAAGAATAGTTTTTGCA---
GTGCTTTCTATAATC---------AATAGAGTTAGGCAGGGATACTCACCT------
TTATCGTTTGCTGTTAGTTTGGTTGATACTATAGCAATAGCAGTAGCTGGC---
TGGACAGATAGGGTTATAGAAATAGGACAAGCAGTTGGTAGAGCT----------------------------
--------ATTATCCACATA------CCTAGAAGAATCAGACAGGGCTTAGAAAGGGCTTTGCTA---TAA
>FJ623482
GCT---GAG--
GAAAATTTGTGGGTTACTGTCTACTATGGGGTGCCTGTGTGG---AGGGACGCAGAC---
ACCACCCTATTTTGTGCATCAGATGCTAAAGGATATAATACAGAA---GCGCATAAT----------------
--------------ATATGGGCTACA---CATGCCTGTGTACCTACAGAC---
CCCAACCCACAAGAAATGAAGTTGAAA---------AATATAACAGAAAATTTTAAC----------------
---------------------------ATGTGGAAGAATAATATGGTAGAGCAGATGCATACAGACATA---
ATCAGTCTATGGGACCAAGGCCTA---AAGCCATGTGTAAAGTTA---
ACCCCTCTCTGTGTTACTCTAGATTGTAGCTATAATGTCACCAGTAATTCCACCGAA---------------
----
-----------------------------AATAATATCACCGAGAGCATGAGAGGAGAAATAAAA---
AACTGCTCTTTCAATGTGACCACAGTATTAAGG--------------------------
GATAAGAGTCAGAAAGTACATTCGCTT---TTTTATAGAGTTGATATAGTACCAATTGATGAAAAG-------
----

AAGAATAATAGCAGTGAGTATAGATTAATAAATTGTAACACCTCAGCCATTACACAG---
GCTTGTCCAAAAGTATCCTTTGAGCCAATTCCCATACATTATTGTGCCCCAGCTGGTTTTGCGATTCTAAAGT
GTAAT---GATACGAATTTCAAT------GGAACAGGGGAGTGCAAGAAT-----------------------
-GTCAGC---ACAGTACAATGCACACATGGAATCAAGCCAGTAGTATCAACA---
CAACTGCTGTTAAATGGCAGTCTAGCAGAAAAAGAG------------
ATAACAATTAGATCCGAAAATATCACAGACAAT---------GCTAAAAACATACTAGTACAATTTGAC----
-----------ACACCTGTAAAA---------ATCAATTGTACCAGACCTTACAAAAATATAAGGGAA-----
-GGTGTGCGTATA------------GGACCAGGACAAGCA---TTC------TTTAAAATGGGTGGC------
---------ATAACAGGGGATATA------------AGAAAAGCATATTGT---GAGATTAATAAAACA---
GAGTGGAATGCAACTCTGGAAAGGGTAGCTGACAAATTAAGA---------------GCACACTTTAAC----
------------------------------------AGCAGTATAATAAGCTTTAAA------
AACGCATCAGGAGGGGATCTAGAAATTACA---ACACATAGTTTTAATTGTGGAGGAGAA---
TTTAAACAGCTCCAGGCAAGAGTCCTGGCTGTGGAAAGATACCTAAAGGATCAACAGCTCCTAGGAATATGGG
GCTGCTCTGGAAAACTCATCTGCACCACCAATGTGCCCTGGAACTCTAGTTGGAGT----------------
-----------------------------AATAAATCCTTGGATAAGATATGG------GAC------
AAAATGACCTGGCTAGACTGG---GATAAAGAAATTAGCAATTACTCAGAGTTAATATAT---
AATCTAATTGAAGAAGCACAGAACCAGCAGGAC--

AAAAATGAACAAGACTTATTGGCATTAGACAAATGGGCAAGTCTGTGGAATTGGTTTGACATCACAAAATGGC
TGTGGTATATAAGGATATTTATAATGATAGTAGGAGGCTTAATAGGATTAAGAATAGTTTTTGCA---
GTGCTTTCTGTAATA---------AATAGAGTTAGGCAGGGATACTCACCT------
TTGTCATTTGCTATTAGTTTGCTTGATACCATAGCAATAGCCGTAGCGGGT---
TGGACAGATAGGATTATAGAGCTAGGACTAAGACTTTGCAGAGCT----------------------------
--------ATTCTCCACATA------CCTAGAAGAATCAGACAGGGCATCGAAAGGGCTTTGATA---TAA
>FJ623477
ACT---GCA--
GAAAACTTGTGGGTTACTGTCTATTATGGGGTACCTGTGTGG---AAAGACGCAGAG---

ACCACCTTATTTTGTGCATCAGATGCTAAAGGATATAAGACAGAA---ATGCATAAT----------------
--------------GTTTGGGCTACA---CATGCCTGTGTACCCACAGAC---
CCCAACCCACAAGAAATATATTTGGAA---------AATGTGACAGAAGAGTTTAAC----------------
--------------------------ATATGGAAAAATCACATGGTAGAGCAGATGCATACAGATATA---
ATCAGTCTATGGGACCAAAGCCTA---AAGCCATGTGTAAAGTTA---
ACCCCTCTCTGCGTTACTTTAAATTGTAGCAAGGTCAATGGCAAGGTCAATGTC-------------------
----
-------------------------------ACGAACCATGACAATGCCACTGAAGAAATGAGA---
AACTGCTCTTACAGTGTAACCACAGAAATAAGG---------------------------
GATAAGAAAAAGAAGGTATATTCACTT---
TTTTATAGACTTGATATAGTACAAATTAATGAGAATCAAGGTAAC----------------------------
----
AGTAGTCAAGGTAATAGTAGTAACAATGCCTATAGATTAATAAATTGTAACACCTCAGTCATTACACAG---
GCTTGCCCAAAGGTTTCTTTTGAGCCAATTCCCATACATTATTGTGCCCCAGCTGGTTTTGCGATCCTAAAAT
GTAGG---GATGAGGAGTTCAAT------GGAACAGGGCCATGCAAAAT------------------------
-GTTAGC---TCAGTACAATGCACACATGGAATTAAGCCAGTAGTATCAACT---
CAACTGCTGTTAAATGGCAGTCTAGCAGAAAAAGAG------------
GTAAAGATTAGATCTGAGAATATCACAAACAAT---------ATCAAGACTATAATAGTACAGTTTACC----
-----------AAGCCTGTAAGG---------ATTAATTGTACCAGACCTAACAACAATACAAGAGAA-----
-AGTATACGTATA------------GGACCAGGACAAGCA---TTC------TATGCAACAGGTGAC------
---------ATAATAGGGAAGATA------------AGAAAAGCATATTGT---AATGTCAGTAGATCA---
GATTGGAATAGCAGTTTACAACAGGTAGCTAAACAGTTAGGC---------------AAATACTTTAGG----
-----------------------------------AACAAAACAATAATATTTACT------
AACTCATCAGGAGGGGATATAGAAATCACA---ACACATAGTTTTAATTGTGGAGGAGAA---
TTTAAGCAGCTCCAGGCACGAGTCCTGGCTGTGGAAAGATACCTAAGGGATCAACAGCTCCTAGGCATTTGGG
GCTGCTCTGGAAGACTCATCTGCACCACTAATGTGCCCTGGAACTCCAGTTGGAGT-----------------
----------------------------AATAAATCTTATAATGAGATATGG------GAT------
AACATGACCTGGCTACAATGG---GATAAAGAAGTTAGCAATTACACAGACATAATATAT---
CAACTAATTGAAGAATCGCAGAATCAGCAGGAA--

AAAAATGAGCAAGACTTATTGGCATTGGACAAATGGGCAAGTCTGTGGAGTTGGTTTGACATAACAAAGTGGC
TGTGGTATATAAAAATATTTATAATGATAGTAGGAGGCTTAATAGGATTAAGAATAATTTTTGCT---
GTGCTTGCTGTAATA---------AATAGAGTTAGGCAGGGATACTCACCT------
TTGTCATTTGCTATAAGTTTGGTTGATACCATAGCAATAGCAGTAGCTGGC---
TGGACAGATAGGATTATAGAAATAGTGCAAGGAATTGGTAGAGCT----------------------------
--------ATTCTGCATATA------CCCACAAGAATTAGACAGGGCCTAGAAAGAGCTTTGCTA---TAA
>FJ623486
ACT---GCA--
GAAAACTTATGGGTTACTGTCTACTATGGGGTACCTGTATGG---AAAGACGCAGAG---
ACCACCTTATTTTGTGCATCAGATGCTAAAGCATATAAGACAGAA---ATGCATAAT----------------
--------------GTCTGGGCTACA---CATGCCTGTGTACCCACAGAC---
CCCAACCCACAAGAAATACATTTGGAA---------AATGTGACAGAAGAATTTAAC----------------
--------------------------ATGTGGAAAAATAACATGGTAGAGCAGATGCATACAGATATA---
ATCAGCTTATGGGACCAAAGCCTA---AAGCCATGTGTAAAGTTA---
ACCCCTCTCTGCGTTACTTTACATTGTGGAACTGTCACCAAC-------------------------------
----
---GACACGGAGGGAGAAAGAAAA---
AACTGCTCTTTCAATATGACCACAGAGCTAAGG---------------------------
GATAAAAAACAGAAGGTATATGCTCTT---TTTTATAGACTTGATATAGTAAAAATTAATGAAAGT-------
----

CAAGGTAATAGTAGTGAATATAGATTAATAAATTGTAATACCTCAGCCATTACACAA---
GCTTGCCCAAGGGTATCCTTTGAGCCAATCCCCATACATTATTGTGCCCCAGCTGGTTTTGCGATCTTAAAGT
GTAAG---GATGAGGAGTTCAAT------GGAACAGGGCCATGCAAGAAT------------------------
-GTCAGC---ACAGTACAATGCACACATGGAATCAAGCCAGTAGTATCAACT---
CAACTGTTGTTAAATGGTAGTCTAGCAAAAACAAAG------------
GACAGAATTAGCTCTGAAAATATCACAAACAAT---------GCCAAAAATATAATAGTACAACTTATT----
-----------GAGCCTGTGACA---------ATTAATTGTACCAGACTTGCCAATAATACAAGAAAA-----
-AGTATACGTATA------------GGACCAGGACAATCA---TTC------TATGCAATGGGTGAC------
---------ATAATAGGGGAGATA------------AGAAACGCACAATGT---GAGGTCAGTAAATCA---

CAGTGGAACAAAACCTTACAAAGGGTAGCTAGAAGATTAGGA--------------ACATACTTTCAG----
----------------------------------AACAAAACAATAATATTTGAT------
AAACCCTCAGGAGGGGATCTAGAAATCACA---ACACATAGTTTTAATTGTGGAGGAGAA---
TTTAAACAGCTCCAGGCAAGAGTCCTGGCTGTGGAAAGCTACCTAAGGGATCAACAGCTCCTAGGAATTTGGG
GCTGCTCTGGAAAACTCATCTGCACCACTAATGTGCCCTGGAACTCTAGCTGGAGT----------------
---------------------------AATAAATCTTACAATGAGATATGG------GAT------
AACATGACCTGGCTGCAATGG---GATAAAGAAGTTAGCAATTATACACAGATAATATAT---
ACCCTAATTGAAGAATCGCAGAACCAGCAAGAA---------------------------------------
----
AAAAATGAACAAGACTTATTGGCATTAGACAAGTGGGCAAGTCTGTGGAATTGGTTTAGCATATCAAAATGGC
TGTGGTATATAAAAATATTTATAATGATAGTAGGAGGCTTAATAGGATTAAGAATAGTTTTTGCT---
GTGCTTTCTGTAATA---------AATAGAGTTAGGCAGGGGTACTCACCT------
TTATCGTTTGCTATTAATTTGGTTGATACTATAGCAATAGTAGTAGCTGGT---
TGGACAGATAGGGTTATAGAAATAGGACAAGTAATTGGTAGAGCT---------------------------
--------TTTCTCCACATA------CCTAGAAGAATCAGACAAGGCCTTGAAAGAGCTTTGCTT---TAA
>FJ623479
GCT---GCA--
GAAAATTTGTGGGTTACTGTCTACTATGGGGTACCTGTGTGG---AAAGACGCAGAT---
ACCACCTTATTCTGTGCATCAGATGCTAAAGCATATGTCACAGAA---AAGCATAAT---------------
--------------GTCTGGGCTACA---CATGCTTGTGTACCCACAGAC---
CCCAACCCACAAGAAATACATTTGGGA---------AATGTGACAGAAGACTTTAAT---------------
-------------------------ATGTGGAAAAATAACATGGTAGAGCAAATGCATACAGATATA---
ATCAGCCTATGGGACCAAAGCCTA---AAGCCCTGTGTACAGTTA---
ACTCCTCTCTGTGTCACCTTAAATTGTACAAATGCAAATGCCACCGGTAATGCC------------------
----
-------------------------------ACTGTCGACAAAAATATGGAAGGAGAAATAAAA---
AACTGCTCTTACAATATGACCACAGAAGTAAGG--------------------------
GATAAGCAAAAGAAAGTATATTCACTT---TTTTATAGACTTGATATAGTACAAATTAGCAAGCAA-------
----

AGTAGTAATGATAGTAGTAAATATAGATTAATAAATTGTAATACCTCAGCCATTACACAA---
GCTTGTCCAAAGGTAACCTTTGAGCCAATTCCCATACACTATTGTGCCCCAGCTGGTTTTGCGATTCTAAAAC
GTAAT---GAGAACAACTTCAAG------GGGACAGGGCCATGCAATAAT----------------------
-GTCAGC---ACAGTACAATGCACACATGGAATAAAGCCAGTAGTGTCAACT---
CAACTGCTGTTGAATGGCAGTCTAGCAGAAGAAAAG-----------
GTAATGATTAGATCTGAAAATATCACAAACAAT---------GCCAAGAACATAATAGTACAATTTACC----
-----------AAACCTGTAAGG---------ATTAATTGTACCAGACCTAACAACAATACAAGAAGG-----
-AGTATACATATA------------GGACCAGGACGAGCA---TTC------TATGCAGCAGGTGAC------
---------ATAATAGGGGATATA-----------CGACAAGCACATTGT---AATGTCAATTACACT---
GAATGGAATGACACTTTGAAAGAAGTAGCCCAACAATTAGGA--------------ACATACTTTAAT----
----------------------------------AACAAAACAATAATATTTAAG------
AGCTCCGCAGGAGGGGATCCAGAGATCACA---ACACATAGCTTTAATTGTGGAGGAGAA---
TTTAAACAGCTCCAGGCAAGAGTCCTGGCTGTGGAAAGATACCTAAAGGATCAACAGCTCCTAGGACTTTGGG
GCTGCTCTGGAAAACTCATCTGCACCACTAATGTGCCCTGGAACTCTAGTTGGAGT----------------
---------------------------AATAAAACTTATAAGGACATATGG------GAC------
AACATGACCTGGCTGCAATGG---GATAAAGAAATTAGCAATTACACACAGATAATATAT---
GACCTAATTGAAGAATCACAGAACCAGCAGGAA---------------------------------------
----
AAGAATGAACAAGACTTATTGTCATTGGACAAATGGGCAAGTTTGTGGAATTGGTTTGATATAACAAGATGGC
TGTGGTACATAAAAATATTTATCATAATAGTAGGAGGCTTAATAGGATTAAGAATAGTTTTTGCT---
GTAATTGCTGTAATA---------AATAGAGTTAGGCAGGGGTACTCACCT------
TTGTCTTTCGCTAGTAGTTTGCTTGATACCATAGCAATAGTAGTAGCTGGC---
TGGACAGATAGGGTTATAGAACTAGGACAAAGAATTGGTAGAGGT---------------------------
--------CTTCTCCACATA------CCTAGAAGAATCAGACAGGGTTTAGAAAGGGCCCTGTTA---TAA
>HM215275
GCT---GTA--
GACAACCTGTGGGTTACTGTCTATTATGGGGTACCTGTGTGG---AAAGATGCAGAA---
ACCACCTTATTTTGTGCATCAGATGCTAAAGCATATGAGACAGAG---CAGCATAAT---------------
--------------GTATGGGCTACA---CATGCCTGTGTACCCACAGAC---
CCCAACCCACAGGAAATGCCTTTGAAA---------AATGTGACAGAACAGTTTAAC---------------

--------------------------ATGTGGAAAAATAACATGGTAGAGCAGATGCATACAGATATA---
ATTAGTCTATGGGACCAAAGCCTA---AAGCCATGTGTGGAGTTA---
ACCCCTCTCTGCGTCACGTTAAATTGTAGTACCATCACCCTCAATAACACTGACACC----------------
----
----------------------------CACAGCACCAATAACACAGACATAGACGCAATGAGA---
AACTGCTCTTTCAATGTGACCACAGAATTAAGG---------------------------
GATAAGACAAAGAGAGTGTATTCACTT---TTTTATAGACTTGATATAGTACAAATAGATGGAAAT-------
----

AATAGCAATGGTAGTGGTGAATATAGATTAATAAACTGTAACACCTCAGCCATTACACAG---
GCTTGTCCAAAGGTATCCTTTGAGCCAATTCCCATACATTATTGTGCCCCAGCTGGCTTTGCGATTCTAAAGT
GTAAT---GAAAAAGAGTTTAAT------GGAACAGGGCCATGCAAGAAT-----------------------
-GTCGCC---ACAGTACAATGCACACATGGAATCAAGCCAGTAGTATCAACT---
CAACTACTGTTAAATGGCAGTCTAGCAGAAGGAAAA-----------
GTAATGATCAGGTCTGAAAATATCACAGACAAT---------AGCAAAAAATATCATAGTACAATTTAAC----
-----------AAGACTGTGACA---------ATTAATTGTACCAGACCTAATAACAAGACAAAAATA---
AGAAGTATACATATA------------GGACCAGGACGAGTA---TTC------CAGGCAACACAA-------
--------------ACAGGGGATATG------------AGAAAAGCATTTTGT---ACAGTCAATAAAACA--
-GAATGGGATAAAACTTTGGAAGAAGTAGCCAAGCAATTAAGA---------------GCACACTTTAAC---
------------------------------------AACAAGACAATAATCTTTAAG------
AGCTCCTCGGGAGGGGATATAGAAATCACA---ACACACAGTTTTAATTGTGGAGGAGAA---
TTTAAACAGCTCCAGGCAAGAGTCCTGACTGTGGAAAGATACCTAAGGGATCAACAGCTCCTAGGAATTTGGG
GCTGCTCTGGAAAACTCATCTGCACCACTAATGTGCCCTGGAATTCTAGTTGGAGT-----------------
----------------------------AATAAATCCCAGACGGAGATATGG------GAT------
AACATGACCTGGCTACAATGG---GATAAAGAAATTAGCAATTACACACAGCAAATATAT---
AAACTACTTGAGGAATCGCAGAACCAGCAGGAA--

CAGAATGAACAAGACTTATTGGCATTAGACAAGTGGGCAAGTCTGTGGAATTGGTTTGAAATATCAAACTGGC
TGTGGTATATAAAAATATTTATAATGATAGTAGGAGGCTTGATAGGATTAAGAATAGTTTTTGCT---
GTGCTTTCTATAATA---------AATAGAGTTAGGCAGGGATACTCACCT------
CTGTCGTTCGCCATTAATTTGTTTGATACCATAGCAATAGTAGTAGCTGGC---
TGGACAGATAGGGTTATAGAATTAGGACAAAGAATTGTTAGAGGT----------------------------
--------TTTCTACACATA------CCTAGAAGAATTAGACAGGGCCTTGAAAGAGCTTTGCTG---TAA
>KT252544
GCT---ACA--
GAAAAATTGTGGGTCACAGTCTATTATGGGGTACCTGTGTGG---AGAGATGCAGAT---
ACCACCTTATTTTGTGCATCAGATGCTAAAGCATATGATCCAGAA---AAGCATAAT----------------
--------------GTCTGGGCCACA---CATGCCTGTGTACCCACAGAC---
CCCAACCCACAAGAGATACAGTTGAAC---------AATGTGACAGAAGAATTTAAC----------------
--------------------------ATGTGGGAAAATAACATGGTAGAGCAGATGCATAAAGATATA---
ATCAGTCTATGGGACCAAAGCCTA---AAGCCATGTGTTCAGCTA---
ACCCCTCTCTGCGTTACCTTAAACTGTAGCCATAAGATTCTCATCAATTTCACCGAGAAAAATGTCACT----
----
---GACATGAGA---
AACTGCTCTTATAATATAACCACAGAATTAAGG---------------------------
GATAAGACACAGGAAGTATATTCACTC---
TTCTATAGACTTGATATAGTGCCAGTTAATGAAAAACAAAGTAAT----------------------------
----
AGTGGTAGTAATAGTAGTAATAGTGAGTATAGATTAATAAATTGTAATACCTCAGCCATTAAACAA---
GCTTGTCCAAAGGTATCCTTTGAGCCAATTCCCATACATTATTGTGCCCCAGCAGGTTTTGCGATTCTGAAGT
GTAAT---GATAAGGATTTCAAT------GGAACAGGGCCATGCAAGAAG-----------------------
-GTCAGC---ACAGTACAATGCACACATGGAATCAAACCAGTAGTATCAACT---
CAACTGCTGTTAAATGGCAGCCTAGCAGAAGGAAAC-----------
GTAACAATTAGATCTAAAAACATCACAGACAAT---------ACTAAAAATATAATAGTACAATTTAAA----
-----------AAACATGTAGTA---------ATTAATTGTACCAGACCTAACAACAATACAAGGAGA-----
-AGTGTACGTATA------------GGACCAGGACAAACA---TTC------TATGCAACAGGTGGC------
---------ATAATAGGGGACATA------------AGACAAGCACATTGT---AATGTCAGCGGATTA---
GCATGGAATCAGACTTTACAACAGGTAGCTAAACAGTTAAGA---------------AGATACTTTGTG----
-----------------------------------AACAAAACAATAAACTTTACT------
AGCCCCTCAGGAGGGGATCTAGAAATCACA---ACACATACTTTTAATTGTGGAGGAGAA---

TTTAAACAGCTCCAGGCAAGAGTCCTGGCTGTGGAAAGATACCTAAGAGATCAACAGCTCCTAGGAATTTGGG
GATGCTCTGGAAAACTCATCTGCACCACTAATGTGCCCTGGAACACTAGTTGGAGT-----------------
----------------------------AATAAATCCCAAGAACAGATATGG------GGA------
AACATGACCTGGCTGCAATGG---GATAAAGAAGTTAGCAATTACACAGACCTAATATAT---
ACCCTACTTGAAGAATCGCAGAACCAGCAGGAC--

AAGAATGAACAGGAATTATTGGCATTGGACAAGTGGGCAAATCTATGGAGCTGGTTTGACATATCAAATTGGC
TGTGGTATATAAAGATATTTATAATGATAGTAGGAGGCTTAATAGGATTAAGAATAGTTATGGCT---
ATAATTTCTATAATG---------AATAGAGTTAGGCAGGGATACTCACCC------
TTGTCATTTGCTATTAGTTTAGTTGATACCATAGCAATAGCAGTAGCTGGC---
TGGACAGATAGAGTTATAGAAATAGGACAAGCAATTGGTAGAGGT----------------------------
--------ATTCTCCATATA------CCTAGAAGAATCAGACAAGGCTTTGAAAGGGCTTTGCTA---TAA
>HQ540689
CCT---GCA--
GAAAACTTGTGGGTTACTGTCTACTATGGGGTACCTGTGTGG---AGAGAAGCAGAT---
ACCACCTTATTTTGTGCATCAGATGCTAAAGCATATGAGACAGAA---AAGCATAAT----------------
--------------GTCTGGGCTACA---CATGCCTGTGTACCCACAGAC---
CCCAACCCACAAGAAATACATTTGGCT---------AATGTGACAGAAAATTTTGAC----------------
--------------------------ATGTGGAAAAATAACATGGTAGAGCAAATGCATACAGATATA---
ATCAGTCTATGGACCAAAGTTTA---CAGCCATGTGTAAAGTTA---
ACCCCTCTCTGCGTTACTATGAATTGTAGCGAGCCTAAATTTAACAACACAAACTTTAACAGT----------
----
-------------------------ACAGGAAGTAACAGCACAGGAATCCAGGAAGAAATGAGA---
AATTGCTCTTTTAATATGACCACAGAGCTAAAG---------------------------
GATAAGAAAAAGGAAGTATATTCACTT---TTCTATAGACTTGATATAGTACAAATAGATAGTAAA-------
----

AAAGGAAATAGTAGTGATTATAGATTAATAAATTGTAATACCTCAGCCGTTAAACAG---
GCCTGCCCAAAGGTATCCTTTAATCCAATTCCCATACATTATTGTGCTCCAGCTGGTTTTGCGATTCTAAAGT
GTAGG---GATGAAGATTTCAAT------GGAACAGGGCCATGCAAGAAT-----------------------
-GTTAGC---ACAGTACAATGCACACATGGAATTATGCCAGTAGTATCAACT---
CAACTGCTGTTAAATGGCAGTCTAGCAAAAGAAAAT------------
GTACAAATTAGATCTGAAAATATTTCAAACAAT---------GCCAAGATAATACTAGTACAACTTGCC----
-----------CATCCTGTGAGA---------ATTAATTGTACCAGACCTGGCAACAATACAAGAAAA-----
-AGTATACATATG------------GGACCAGGACAAGCA---TTT------TATGCAAGAGGGGAT------
---------GTAATAGGAGATATA------------AGACAAGCATATTGT---AATGTCAGTAGCTCA---
GAGTGGAGTAACACTTTATACAAGGTAGCTGAACAATTAAGG---------------AAACATTATGGG----
--------------------------------AATGAAACAACAATAAAATTTACT------
AACCACTCAGGAGGGGATCTAGAAGTCACA---ACACATAGTTTTAATTGTGGAGGAGAA---
TTTAAACAGCTCCAGGCAAGAGTCCTGGCTCTGGAAAGATACCTAAAGGATCAACAGCTCCTAGGAATTTGGG
GCTGCTCTGGAAGACTCATCTGCACCACTAATGTGCCCTGGAACTCTAGTTGGAGT-----------------
----------------------------AATAAATCTTATAATGAGATATGG------GAC------
AACATGACCTGGCTACAATGG---GATAGAGAAATTGAAAATTACACACAAATAATATAT---
GGCCTAATTGAAGAATCGCAGAACCAGCAGGAA--

AAGAATGAACAAGACTTATTGTCCTTAAACAAATGGGCAGATCTGTGGAGTTGGTTTAACATAACAAATTGGC
TGTGGTATATAAAAATATTTATAATGATAGTAGGAGGCTTAATAGGATTAAGAATAGTTTTTGCT---
GTGCTTTCAGTAATA---------AATAGAGTTAGGCAGGGATACTCACCT------
TTGTCGTTTGCTATTAGTTTGGTTGATACTATAGCAATAGTAGTAGCTGGC---
TGGACAGATAGGGCTATAGAAATAGGACAAAGAATTGGTAGAGCT----------------------------
--------ATCCTTCACATA------CCTATAAGAATCAGACAGGGCTTAGAAAGAGCTTTGCTA---TAA
>KU921708
GCT---AAA--
AAATCTTTGTGGGTGACTGTCTATTATGGGGTACCTGTGCGG---AAAGATGCAGAG---
ACCACCTTGTTTTGTGCCTCAGATGCTAAAGCATATGAGGCAGAG---AAGCATAAT----------------
--------------ATCTGGGCTACA---CATGCCTGTGTACCCACAGAC---
CCCGACCCACAAGAAATACCTTTGGAA---------AATGTGACAGAAGAGTTTAAC----------------
--------------------------ATGTGGAAAAATAACATGGTAGAGCAGATGCATACAGATATA---
ATTAGTCTATGGGATCAAAGCCTA---AAACCATGTGTACAGTTA---
ACCCCTCTCTGTGTTACTTTGAATTGTTCAAGTGTCATGACAAATGTCACGAGCAATAACACGAAT-------

----
----------------------GGCCACAGTAGAATCATGGAAGACGGCAGGGAAGAAATAACA---
AGCTGCTCTTTCAATATGACCACAGAACTAAGG---------------------------
GATAAGAGAAAGAAAGTATATTCACTT---TTTTATAAACTTGATGTAGTACAAATAAATAAT----------
----

AGTAGTAGTGAGTACAGATTAATAAATTGTAATACCTCAGCCATCACACAG---
GCTTGTCCAAAGGTAAACTTTGAGCCAATTCCCATACATTATTGTGCTCCAGCTGGTTATGCGATCCTAAAAT
GTAGG---GATGAGGAATTCAAT------GGAACAGGGCTATGCAGGAAT-----------------------
-GTCAGC---ACAGTCCAATGCACACATGGAATCAAGCCAGTGGTATCAACT---
CAACTGTTGCTAAATGGCAGTCTATCAGAGAGCGGG------------
GTAAGAATTAGATCTGAGACTATCACAGACAAT---------GGCAAAAATATCATAGTACAACTTGCC----
-----------AAGCCTGTAAAA---------ATTAATTGCACCAGACCTAATAACAATAAAAGAAGA-----
-AGTGTGCATATA------------GGACCAGGACGAGCA---TTC------TATGCAAAT---GAT------
---------ATAATAGGGGATATA------------AGAAAAGCATCTTGC---AGTGTCAATGAATCA---
GAATGGAATGAAGCTTTAAGACAAGTAGCTAAACAATTAAGA---------------ATCCACTTT-------
-----------------------------------AACAAAATAATACAATTTGTG------
AATCACTCAGGAGGAGATCTAGAAGTCACA---ACACATAGTTTTAATTGTAGAGGAGAA---
TTTAAACAGCTTCAGGCAAGAGTCCTGGCTTTGGAAAGATACCTAAGGGATCAACAGCTCCTAGGAATTTGGG
GCTGCTCTGGAAAACTCATCTGCACCACTAATGTGCCTTGGAACTCTAGTTGGAGT-----------------
----------------------------AATAAATCTCAGGAAGAAATATGG------CAA------
AGCATGACCTGGCTGCAATGG---GATAAAGAAATTAGTAATTACACAGACACAATATAT---
AGACTAATTGAAGAATCACAGAACCAGCAGGAA--

AAGAATGAACAAGACTTATTGGCATTTGACCAGTGGGCAAATCTGTGGAATTGGTTTGACATATCAAATTGGC
TGTGGTATATAAAAATATTTATAATGATAGTAGGAGGCTTAATAGGATTAAGAATAGTTTTTGCT---
GTGCTTTCTATAATA---------AAAAGAGTTAGGCAGGGATACTCACCT------
TTGTCGTTGGCTATTAGTTTGCTTGATACCATAGCAATAGTAGTAGCAGGC---
TGGACAGATAGGGTTATAGAAGTAGGACAAAGACTTGTTAGAGCT----------------------------
--------ATTCTCAATATA------CCTAGAAGAATCAGACAGGGCGCTGAAAGGGCTTTGCTA---TAA
>KU921712
GCT---ATA---AAA---------------------------------
AAGGAGGACAAGTGGGTTACTGTCTACTATGGGGTACCTGTATGG---AAAGACGCAGAG---
ACTACCTTATTTTGTGCATCAGATGCTAAAGCATATAATACAGAA---GCACATAAT----------------
--------------GTCTGGCTACA---CATGCCTGTGTACCCACAGAC---
CCCAACCCACAAGAAATACAATTGGAA---------AATGTGACAGAAAAGTTTAAC----------------
--------------------------ATGTGGAAAAATAACATGGTAGAGCAGATGCATACAGATATA---
ATCAGTCTGTGGGACCAAAGCCTA---AAGCCATGTGTACAGTTA---
ACCCCTCTCTGCGTCACTTTAAATTGTAGCAATATCAATAGCACGAGTAACAATAGCACG-------------
----
-------------------------AGTGGAAACAGCACGGTCAGCAACAAGGAAGAACTAAAA---
AACTGCTCTTTCAATGTGACTACAGAGATAAGG---------------------------
GATAAGAAAAAGAAGGAATATTCACTT---TTTTATAAACTTGATGTAGTACCAATTAGTAGT----------
----

ACTAATAGTAATAGTAGTAGTGGTGAGTATAGATTAATAAATTGTAATACCTCAACCATAACACAG---
GCCTGCCCAAAAGTAACCTTTGAGCCAATTCCCATACATTATTGTGCCCCAGCTGGTTTTGCGATCCTAAAGT
GTAAG---GATGAGAATTTCACT------GGAATAGGGCTATGCAACGAT-----------------------
-GTCAGC---ACAGTACAATGCACACATGGAATCAGGCCAGTAGTATCAACC---
CAGCTGCTGTTAAATGGCAGTCTAGCAAGAGAAAGG------------
GTAAAAATTAGATCTGAAAATATCACAGACAAT---------GGCAAAAGTATAATAGTACAACTTGTC----
-----------GACCCTGTGAAC---------ATTACTTGTACCAGACCTAGTAACAATACAAGAAAA-----
-AGTATACATATA------------GGACCAGGACAAGCA---TTC------TATGCAACAGGAGGC------
---------ATAATAGGGGACATA------------AGACAAGCACACTGT---AATATCAGTGAATTA---
GCATGGAATAAAACTCTACAAGGGGTAGTTGAACAATTAAGA---------------GGACACTTTAAG----
-----------------------------------AATAAAACAATAATATTTGCT------
AGCTCCTCAGGGGGGGATCTAGAAATCACA---ACACACAGCTTTAATTGTGCAGGAGAG---
TTTAAACAGCTCCAGGCAAGAGTCCTTGCTGTGGAAAGATACCTAAGGGATCAACAGCTCCTAGGAATTTGGG
GATGCTCTGGAAAACTCATCTGCACCACTAATGTGCCCTGGAATAATAGTTGGAGT-----------------
----------------------------AATAAAACCCTGGAGGACATATGG------GAC------

AACATGACCTGGTTACAGTGG---GATAAAGAAATTAACAATTACACAGAAATAATATAT---
AGACTAATTGAGGAATCGCAGAACCAGCAGGAG--

AAGAATGAACAAGATTTATTAGCACTGGATAAGTGGGCAAGTCTGTGGAGTTGGTTTGACATAACAAATTGGC
TGTGGTATATAAAAATATTTATAATGATAGTGGGAGGCTTAATAGGATTAAGAATAATTTTTGCT---
GTGCTTTCTATAATA---------AATAGAGTTAGGCAGGGATACTCACCT------
CTGTCATTTGCTGTTAGTTTGTTTGATACCCTAGCAATAGTAATAGCTGGC---
TGGACAGATAGGGTTATAGAAATAGGACAAAGGCTTGGTAGAGCT----------------------------
--------ATTCTCCACATA------CCTAGAAGAATTAGACAGGGCATTGAAAGGGCATTGCTA---TAG
>KU921727
GCT---AAA--
GAAGACTTGTGGGTTACTGTCTACTATGGGGTACCTGTGTGG---AAAGACGCAGAG---
ACCACCTTATTCTGTGCATCAGATGCAAAAGCATATGAAAAGGAA---GTGCATAAT----------------
--------------GTCTGGGCTACA---CATGCCTGTGTACCTACAGAC---
CCCAACCCACAAGAAATAAAGTTAGAA---------AATGTGACAGAAAAATTTAAC----------------
--------------------------ATGTGGAAAAATAACATGGTAGAGCAGATGCATGAAGATATA---
ATCAGTCTATGGGACCAAAGCCTA---AAGCCATGTGTAAAATTA---
ACCCCTCTCTGCGTCACTTTAGATTGTAGCAATGTCAACAACAGTACCAGTAACACCACTATCTCCCCTGCTA
CCTCCCCT---
---GACATGAGGGACGAAATAAAA---
AACTGTTCTTTCAATATAACCACAGAATTAAGG---------------------------
GATAAGAAACAGAAAGTGTATTCACTT---TTTTATAAACTGGATGTAGTGCAAATTGACAACAGTCAGAAC-
----

AACAGTCAGAACAGTCAGTATAGATTAATAAATTGTAATACCTCAGCCATTACACAG---
GCTTGTCCAAAAGTAACTTTTGAGCCAATTCCCATACATTATTGTGCCCCAGCTGGTTATGCGATTCTAAAGT
GTAAT---GATAAGGAGTTCAAT------GGAACAGGGTTATGCAAAAAT-----------------------
-GTCAGC---ACAGTACAATGCACACATGGAATCAAGCCAGTAGCATCAACT---
CAACTGCTGTTAAATGGCAGTCTAGCAGAATCAAAG------------
GTAATGATTAGATCTGAAAATCTCACAGACAGT---------AACAAAAACATAATAGTACAATTTAAC----
-----------GAGTCTGTACCA---------ATTACTTGTATCAGACCTAACAACAATACAAGAAAA-----
-AGTGTACGTATA------------GGACCAGGACAAGCA---TTC------TATGCAACAGGTGAT------
---------ATAATAGGGGATATA------------AGACAAGCACATTGT---AATATCACTAAAATA---
AGATGGGATAAAGCTTTGCACCAGGTAGGCAAACAATTAAGA------------GCGGCCGCCTTTGGA----
-----------------------------------AACAAAAAAATAATCTTTGAT------
AGCTCCACAGGGGGGGATCCAGAAATAACA---ACACATAGTTTTAATTGTGGAGGAGAA---
TTTAAACAGCTCCAGGCAAGAGTCCTGGCTGTGGAGAGCTATCTAAAGGATCAACAGCTCCTAGGAATTTGGG
GCTGCTCTGGAAAACTCATCTGCACCACTGCTGTGCCCTGGAACTCTAGTTGGAGT-----------------
----------------------------AATAAATCCCAGAGTGAGATATGG------AAC------
AACATGACCTGGATGCAATGG---GATCGAGAAATTAGTAATTACACAGAGCTAATATAT---
GATCTAATTGAAAAATCGCAAAACCAGCAAGAA--

ATAAATGAACGAGAATTATTGGCATTGGACAAGTGGGCAACTCTGTGGAATTGGTTTGACATATCAAAATGGC
TGTGGTATATAAAAATATTTATCATGATAGTAGGAGGCTTAATAGGATTAAGAATAGTTTTTGCT---
GTGCTTTCTATCATA---------AATAGAGTTAGGCAGGGATACTCACCT------
TTGTCGTTCGCTGTTAGTTTGCTGAATACCATAGCAATAGTAGTAGCTGGG---
TGGACAGATAGAGTTATAGAAGTAGGACAAGGATTGTTTAGAGCT----------------------------
--------ATTATCCACATA------CCTAGAAGAATCAGACAAGGCTTCGAAAGAGCTTTGCTA---TAA
>KU921732
GCT---GCA--
GAAAACTTGTGGGTTACTGTCTACTATGGGGTACCTGTGTGG---AAAGACGCAAAC---
ACCACTTTATTTTGTGCATCAGATGCTAAAGCATATAGTACAGAG---AAGCATAAT----------------
--------------GTGTGGGCTACA---CATGCCTGTGTACCCACAGAC---
CCCAACCCACAAGAAATAAATTTGAAA---------AATGTGACAGAAGAGTTTAAC----------------
--------------------------ATGTGGAAAAATAACATGGTAGAACAAATGCATACAGATATA---
ATTAGCCTATGGGATCGAAGCCTA---AAGCCATGTGTAAAGTTA---
ACTCCTCTCTGCGTTACTTTAGAGTGTAATAATGTTAATATTACTAATAATGTTACTAATAATGTTACTTAT-
----
----------------------AATGTTGTTGACAACAGCACCGATATGAAAGAAGAAATAAAA---
AACTGCTCTTTCAATATGACCACAGAATTAAGA---------------------------

GATAAGCAACAGAAAGTATATTCACTT---TTTTATAGGCTAGATATAGTACAACTTAATAGC----------
----

AGCCAGGAGAATAGCAGTCAGTATATATTAATAAATTGTAAAACCTCAACCATTACACAG---
GCTTGTCCAAAGGTATCCTTTGAGCCAATTCCCATACATTATTGTGCCCCAGCTGGTTTTGCGATTCTAAAGT
GTAAT---GATAAGGAGTTCAAT------GGAACAGGGCCATGCAAGAAT----------------------
-GTCAGC---ACAGTACAATGCACACATGGGATCAAACCAGTAGTATCAACT---
CAACTGCTGTTAAATGGCAGTCTAGCAGAGGGAAAG-----------
ATAATGATTAGATCTGAGAATTTCACAGACAAT---------GCCAAAAACATACTAGTACAACTTAAC----
-----------CAGACTGTAAAA---------ATTAATTGTACCAGACCTAACAATAATACAAGAAAA-----
-AGTGTACATATA------------GGACCAGGACAGGCA---TTG------TATACAACAAATAAC------
---------ATAATAGGGAATATA------------AGACAAGCACATTGT---AATGTCAGTAGACAA---
CAATGGAATAAAACTTTGCAAGAGGTAAGAGAACAATTAAGA---------------CAAATCTTT-------
-----------------------------------AACAAAACAATAACATTTAAT------
ACCTCCTCACAAGGGGATATAGAAATCATG---ACACATAGTTTTAATTGTGGAGGAGAA---
TTTAAACAGCTCCAGGCAAGAGTCCTGGCTGTGGAAAGATACCTAAAGGATCAACAGCTCCTAGGTATTTGGG
GCTGCTCTGGAAAACTCATCTGCACCACCAATGTGCCCTGGAACTCTAGTTGGAGT-----------------
----------------------------AATAAATCCAAGAAAGAGATATGG------GAA------
AACATGACCTGGCTGCAATGG---GATAAAGAAATTAGCAATTACACACAGATCATATAT---
GATCTACTTGCAGACTCGCAGATCCAGCAGGAA---------------------------------------
----
AAGAATGAACAAGACTTATTGGCATGGGACAAGTGGGCAAATCTGTGGACTTGGTTTGACATAACAAAATGGC
TGTGGTATATAAAAATAGTTATAATGATAGTAGGAGGCTTAATAGGATTAAGAATAGTTTTTGCT---
GTACTTTCTATAATA---------AATAGAGTTAGGCAGGGATACTCACCT------
TTGTCATTTGCTATTAATTTGTATGATACTATAGCAGTAGCAGTAGCTGGC---
TGGACAGATAGGATTATAGAAATAGTACAAATAATTGGTAGAGCT---------------------------
--------CTTCTCCACGTA------CCTAGAAGAATCAGGCAGGGCTTCGAAAGGGCATTGTTA---TAA
>KU921734
GCT---GGA---
GGAAACTTGTGGGTCACTGTCTACTATGGAGTACCTGTATGG---AAAGACGCAGAG---
ACCACCTTATCTTGTGCATCAGATGCAAAAGCATATGATAAGGAA---GTGCATAAT----------------
--------------GTCTGGGCTACA---CATGCCTGTGTACCCACAGAC---
CCCAACCCACAAGAAATACATTTGGAA---------AATGTGACAGAAGAGTTTAAC---------------
--------------------------ATGTGGAAAAATAACATGGTAGAGCAGATGCAGACAGATATA---
ATCAGCCTTTGGGACCAAAGCCTA---CAGCCATGTGTAAAATTA---
ACCCCTCTCTGTGTTACTTTACATTGTACCAATTATTATGGTAATAGCACCGAGGTCAAT------------
----
------------------------AATAGCACTGTCAAAAGCACCATGGAAGGGGAAATGAAA---
AACTGCTCTTTCAATGCGACCACAGAATTAAGG--------------------------
GATAAGAAACGGAAAGTACATGCACTT---TTTTATAGACTTGATGTAACACAATTGGATGAT---------
----

GATAATAGTACTTATAGATTAATAAATTGTAATACCTCAGCCATTACACAG---
GCTTGCCCAAAAGTAACCTTTGAACCAATTCCCATACACTATTGTACTCCAGCTGGTTTTGCAATTCTAAAGT
GTAAT---GATAAAGAATTTAAT------GGAACAGGAGTATGTAAGAAT----------------------
-GTCAGC---ACAGTACAATGCACACATGGAATCAAGCCAGTAGTATCAACT---
CAGTTGCTGTTAAATGGCAGTCTAGCAGAAGGAAAG-----------
GTAATAATTAGATCTGAAAACATCACAAACAAT---------GCCAAGAACATAATAGTACAATTTAGA----
-----------GAACCTGTGAAC---------ATTACGTGTATCAGACCTAACAACAATACAAGGACA-----
-AGTATACGTATA------------GGACCAGGACAAGCA---TTC------TATGCAACAGGTGAC------
---------ATAATAGGGGATATA------------AGACAAGCACATTGT---AATGTTAGTAGACAA---
GCCTGGAATGAAACTTTGGGACAGGTAGTCGAGCAATTAAGA---------------ACACACTTTGGG----
-----------------------------------AACAAAACAATAAAATTTACT------
AGCTCCTCAGGAGGGGATTTAGAAATTACA---ACACACAGTTTTAATTGTGGAGGAGAA---
TTTAAACAGCTCCAGGCACGAGTCCTGGCTGTGGAAAGATACCTAAAGGATCAACAGCTCCTAGGAATTTGGG
GCTGCTCTGGAAAACTCATCTGCACCACTACCGTGCCCTGGAACTCTAGTTGGAGT-----------------
----------------------------AATAAAACCATGGGTGAGATATGG------GAT------
AACATGACCTGGATGCAATGG---GATAAAGAAATTGACAACTATACACAGTTGATATAT---
AGTCTACTTGAGGAATCGCAGAACCAGCAGGAA---------------------------------------
----

AAGAATGAACAAGATTTATTGGCATTGGACAAGTGGGCAAATCTGTGGAATTGGTTTAACATATCAAATTGGC
TGTGGTATATAAAGATATTTATAATGATAGTAGGGGGCTTAATAGGATTAAGAATAGTTTTTACA---
GTGCTTTCTATGGTA---------AATAGAGTTAGGCAGGGATACTCACCT------
TTGTCTTTTGCTACTAATTTGCTTGATACCACAGCAATAGCAACAGCTAAC---
TGGACAGATAGGGTTATAGAAGCAATATCAAGGCTGTGTAGAGCT---------------------------
--------ATTTGCAACATA------CCTGA-AGAATTAGACAGGGCTTAGAAAGGGTTTTGCTA---TAA
>KU921738
ACT---GCA---------------------------------------
GAAAACTTGTGGGTTACTGTCTACTATGGGGTGCCTGTATGG---AAAGATGCAGAG---
ACCACCTTATTCTGTGCATCAGATGCGAGAGCATATGATACAGAA---GTGCATAAT---------------
--------------GTCTGGGCTACA---CATGCCTGTGTACCTACAGAC---
CCCGACCCACAAGAAATAGATTTGGAA---------AATGTGACAGAAGAGTTTAAC---------------
-------------------------ATGTGGAAAAATAACATGGTAGAACAGATGCATGAAGATATA---
ATCAGTCTATGGGACCAAAGCCTA---AAGCCCTGTGTAAAATTA---
ACCCCTCTCTGCGTTACTTTGAATTGCAGCAATATCAATATCACTAGAACCAACAGTACCAATAGT-------
----
---------------------AACAGTAGTTCCATAAATAACCAAGGAGAAAGAGAAATGACA---
AACTGCTCGTTCAATGCAACCACAGAATTAAGG-------------------------
GATAAGCAACAGAGAATATATTCACTT---TTTTATAAACTAGATGTAGTACAAATTGGGAAC---------
----

GACAGCCACCAGTATAGATTAATAAATTGTAATACCTCAGCCATTAAACAA---
GCTTGTCCAAAGGTATCCTTTAAGCCAATTCCCATACATTATTGTGCCCCTGCTGGTTTTGCGATTCTAAAGT
GTAAG---GATCAGAAGTTCAAT------GGAACAGGACCATGCAGGAAT----------------------
-GTCAGC---TCTGTACAATGCACACATGGAATCAGGCCAGTAGCATCAACT---
CAACTGCTGTTAAATGGCAGTCTAGCAGAAGAAGAG-----------
GTAATGATTAGATCTGAAAATATCACAAACAAT---------GCCAAAAACATAATAGTACAATTTACA----
-----------AAGCCTGTAAAT---------ATTACTTGTAGCAGGCCTAACAACAATACAAGAAAA-----
-AGTGTACGTATA-----------GGACCAGGACAAGTA---TTC------TATACAACA---GAC------
---------ATAATAGGAAAAATA-----------AGACAAGCACATTGT---AATATCAGTAAAAAA---
GACTGGGATGATACTTTGGGAAAGGTAGTCCAAAAATTAGGA--------------ACATACTTT-------
-----------------------------------AACACAACAATAATTTTTAAT------
AAATCCTCAGGAGGGGATCTAGAAATAACA---ACACATAGTTTTAATTGTGGAGGAGAA---
TTTAAGCAGCTCCAGGCACGAGTCCTGGCTGTGGAAAGATACCTAAAGGATCAACAGCTCCTAGGAATTTGGG
GCTGCTCTGGAAAACTCATCTGCACCACTAATGTGCCTTGGAACACGAGTTGGAGC----------------
---------------------------AATAAAAGCTATAATGAGATATGG------GGC------
AATATGACCTGGCTGCAATGG---GATAAAGAAATTAACAATTACACACAGTTAATATAT---
AGTCTAATTGAAGAATCGCAGAACCAGCAAGAA---------------------------------------
----
AAGAATGAACAAGACTTATTGTCATTGGACAAGTGGGCAGATCTGTGGAATTGGTTTGACATAACAAAGTGGC
TGTGGTATATAAAAATATTTATAATGATAGTAGGAGGCTTAATAGGATTAAGAATAGTTTTTGCT---
GTGCTTTCTATGATA---------AATAGAGTTAGGCAGGGATACTCACCT------
TTGTCGTTCGCTAGTAATTTGCTTGATACCATAGCAATAGTAGTAGCTGGC---
TGGACAGATAGAGTTATAGAAATAGGACAAAGAATTGGTAGAGCT---------------------------
--------ATTCTCCATATA------CCTAGAAGAATCAGACAGGGCTTAGAAAGCGCTTTGCTA---TAA
>KU921746
GCT---GCA---------------------------------------
GAAAACTTGTGGGTTACTGTTTACTATGGGGTACCTGTGTGG---AAAGATGCAATG---
ACCACCTTGTTTTGTGCCTCAGATGCTAAAGCATATGACCTAGAA---AAACATAAT---------------
--------------GTCTGGGCCACG---CATGCCTGTGTACCCACAGAC---
CCCAACCCACAAGAAATACCTTTGGGA---------AATGTGACAGAAAAATTTAAC---------------
-------------------------ATGTGGAAAAATGAAATGGTAGAACAAATGCATACAGATATA---
ATCAGTCTATGGGACCAAAGCCTA---AAGCCATGTGTAACGTTA---
ACCCCTCTCTGCGTTACATTACATTGTACAAATGCCACTGTCATGAAGGCCAATACCATGAATAGC-------
----
---ACCAGCTGGGAGGAAATGAAA---
AACTGCACTTACAATATGACCACAGAACTAAGG-------------------------
GATAAGAAAAAGCAAGCATATTCACTT---TTTTATAAACTTGATGTAGAACCAATAGATCAGGGCAATAGT-
----

AGTAACAATATTAATAGTAGCAAAGAGTATAGACTAATAAATTGTAATACCTCAGCCACTGCACAA---
GCTTGCCCAAAGGTAAGCTTTGAGCCAATTCCAATACATTATTGTGCCCCAGCTGGTTTTGCGATCCTAAAGT
GCAGG---GAGAAGGATTTCAAT------GGAACAGGGATATGCAAGAAT----------------------
-GTCAGC---ACAGTCCAATGCACACATGGAATCAAGCCAGTAGTATCAACA---
CAACTACTGTTAAATGGCAGTCTAGCAGAAGGAAAT-----------
GTAACAATTAGATCTGAAAATATCACAGACAAT---------GCCAAAAATATACTAGTACAATTTACC----
-----------AAGTCTGTAGTA---------ATTAATTGTACCAGACCTAGTAACAATACAAGAAGA-----
-AGTGTAAGGATA------------GGACCAGGACAAGCA---TTC------TATACAACAGGTGAC------
---------ATAATAGGGGATATA------------AGACAAGCACATTGT---AATGTCAGTAGGTCA---
GCATGGAATAAGACTCTACAACAGGTAGCTACACAATTAAGG---------------GAATACTTT-------
-----------------------------------AACAAAACAATAATATTTAAT------
AGTTCCTCAGGAGGGGATCTAGAAATCACA---ACACATAGTTTTAATTGTGAAGGAGAA---
TTTAAACAGCTCCAGGCACGAGTCCTGGCTGTGGAAAGATACCTAAGGGATCAACAGCTCCTAGGAATTTGGG
GCTGCTCTGGAAAACTCATCTGCCCCACTAATGTGCCCTGGAACTCTAGTTGGAGT-----------------
----------------------------AATAAGTCTTATAGTGACATATGG------GAC------
AACATGACCTGGCTGCAATGG---GATAAAGAAATTAACAATTACACAGAACAAATATAT---
AGATTAATTGAAGAATCGCAAAATCAGCAGGAA---------------------------------------
----
AAGAATGAACAAGACTTATTGGCATTGGACAAGTGGGCAAATCTATGGAATTGGTTTGATATATCAAATTGGC
TGTGGTATATAAGAATATTTATAATGATAGTAGGAGGCTTAATAGGATTAAGAATAGTTTTTGCT---
GTGTTTTCTATAATA---------AATAGAGTTAGGAAGGGATACTCACCT------
CTGTCATTTGCTACTAATTTGTTTGATACCTTAGCAATAGCAGTAGCTGGC---
TGGACAGATAGGGTTATAGAATTAGGACTAGCACTCATTAGAGCT-----------------------------
--------ATTTTCCACATA------CCTAGAAGAATCAGACAAGGCTTAGAAAGATTTTTGCTA---TAA
>KU921752
GCT---GCA---
GGAAATTTGTGGGTTACTGTCTACTATGGGGTACCAGTGTGG---AAAGATGCAGAA---
ACCACCTTATTTTGTGCATCAGATGCTAAAGCATATTCAACAGAA---AAACATAAT----------------
--------------ATCTGGGCTACA---CATGCCTGTGTACCCACAGAC---
CCCGACCCACAAGAACTATATTTGGAA---------AATGTGACAGAAAAGTTTAAC----------------
--------------------------ATGTGGAACAATAGCATGGTAGAGCAGATGCATACAGATATA---
ATCAGTTTATGGGATGAAAGCTTA---AAGCCATGTGTAAAGTTA---
ACCCCTCTCTGTGTTACTTTAAATTGTAGCAATGTCACTGTCACCAATAGCACTGGCAGTGGCACCAATGGCA
CTTTC--
-------------------------GATAGAAATTACACCAATGAAATGGGGGAAGAAATAAAA---
AACTGCTCTTTCAATATAACCACAGAGATAAGG---------------------------
GATAGACAGCGGAAAATACATTCACTC---TTTTATAGAATTGATATAGTACCAATTAATGAAAAAGGAAAA-
----

GACAATAATAGTAGTGAGTATAGATTAATAAATTGTAATACCTCAGCCATTACACAG---
GCTTGTCCAAAGGTATCCTTTGAGCCAATTCCCATACATTTTTGTGCCCCAGCTGGTTTTGCGATTCTAAAGT
GTAAT---GATAAGAACTTCAAT------GGAACAGACACATGCAAGAAT----------------------
-GTCAGC---ACTGTACAATGCACACATGGAATCAAGCCAGTAGTATCAACT---
CAATTACTGTTAAATGGCAGTCTAGCAGAAGAAAAG-----------
GTAATGATTAGGTCTAAAAATTTCTCAGACAAT---------GCTAAAAACATAATAGTACAACTTGTC----
-----------CAGCCTATACTA---------ATTAATTGCACCAGACCTAACAACAATACAAGAAAG-----
-AGTATACATATA------------GGACCAGGACAAGCA---TTC------TATGCAAGTGATAAA------
---------ATAATAGGAGATATA------------AGACATGCATATTGT---ACTGTCAATAAAACA---
GAATGGAATGAAGCCTTAAGACAGGTAGCTATACAATTAAGA---------------CCACACTTTAAT----
-----------------------------------AACGGAACAATAATATTTAAT------
AGCTCCTCAGGAGGGGACCTAGAAATCACA---ACACATAGCTTTAATTGTGGAGGAGAA---
TTTAAACAGCTCCAGGCAAGAGTCCTGGCTGTGGAAAGATACCTAAGGGACCAACAGCTCCTAGGAATTTGGG
GCTGCTCTGGAAAACTCATCTGCACCACTAATGTGCCCTGGAACTCTAGTTGGAAT-----------------
----------------------------AATAGATCCGAGGCTGACATATGG------GAA------
AACCTGACCTGGCTGGATTGG---GATAAAGAAATTAGCAATTACACAGGCATAATATAT---
GATTTACTTGAAAAATCGCAGAACCAGCAGGAA---------------------------------------
----
AAAAATGAACAAGAATTGTTGGCATTGGACAAGTGGGCAAATCTGTGGAATTGGTTTAACATATCAAACTGGC
TGTGGTATATAAAAATATTTATAATGATAGTAGGAGGTTTAATAGGATTAAGAATAGTTTTTGCT---
GTGCTTTCTATAATA---------AATAGAGTTAGGCAGGGATACTCACCT------

TTGTCATTCGCTATTAGTTTGGTTGATACCATAGCAATAGTAGTAGCTGGC---
TGGACAGATAGGATTATAGAAATAGGACAAAGAATTGGTAGAGCT----------------------------
--------ATCCTCCACATA------CCTAGAAGAATCAGACAGGGCTTTGAAAGGCTTTGCTA---TAA
>KU921756
ACT---GCA--
GAAAACTTGTGGGTTACTGTCTACTATGGGGTACCGGTGTGG---AAAGACGCAGAG---
ACCACCCTATTTTGTGCATCAGATGCTAAAGCATATAAGGCAGAA---GTGCATAAT----------------
--------------GTCTGGCTACA---CATGCCTGTGTACCCACAGAC---
CCCAACCCACAAGAAATACCTTTGGAT---------AATGTGACAGAAAACTTTAAC----------------
--------------------------ATGTGGAAAAATAATATGGTAGAGCAGATGCATATGGATATA---
ATAAGTCTATGGGACCAAAGCCTA---AAGCCATGTGTAAAGTTA---
ACCCCTCTCTGTGTTACTTTAAACTGTTTCAATGCCACTGTTACAAATTCCACG-------------------
----
--GATGAAATGAAA---
AACTGCTCTTTCAACGTAACAACAGAAATAAGG---------------------------
GATAAGAAACAGAACACATATTCACTT---TTTTATAAACTTGATATAGTACCAATTAATAAG----------
----

AGTAGCAGTAGGTATATATTAATAAATTGTAATACCTCAGCCATTACACAA---
GCCTGTCCAAAGGTAACCTTTGAGCCAATTCCCATACATTATTGTGCCCCAGCTGGTTTTGCGATCCTGAAGT
GTAGG---GATGAGGAGTTCAAT------GGAACAGGGCCATGCAAGAAT-----------------------
-GTTAGT---ACAGTACAATGTACACATGGAATCAAGCCTGTAGTATCAACT---
CAATTGCTGTTAAATGGCAGTCTAGCAGAAAAAGAG------------
GTAAAAATTAGATCTGAAAACATCTCAAACAAT---------GCTAAGGTTATAATAGTACAGTTTAAC----
-----------GAGTCTGTACCA---------ATTACTTGTGTCAGACCTAACAACAATACAAGAAGA-----
-AGTATACATATA------------GGACCAGGACAAGCA---TTC------TATGCAACA---GAC------
---------ATAATAGGAAATATA------------AGACAGGCATATTGT---AAAGTCAATAAAACA---
GAATGGAATAAAGCCTTACAAAAGGTAGTTAGTCAATTACAA---------------AAACATTGG-------
-----------------------------------AACAGGACAATAATCTTTGAA------
GAAAACTCGGGAGGAGATTTAGAAACTGCA---ACACATAGTTTTAATTGTGCAGGAGAA---
TTTAAACAGCTCCAGGCAAGAGTCCTGGCTGTGGAAAGATACCTAAGGGATCAACAGCTCCTAGGAATTTGGG
GCTGCTCTGGAAGACTCATCTGCACCACTAATGTGCCCTGGAACTCTAGTTGGAGT-----------------
----------------------------AATAAATCTTACAATGAAATATGG------GAT------
AACATGACCTGGCTGCAATGG---GACAAAGAAGTTAGCAATTACACTGACACAATATAT---
AGGCTAATTGAAGAATCGCAGAACCAGCAGGAA--

AAAAATGAACAAGACTTATTAGCATTAGACAAATGGGCAAATCTGTGGAATTGGTTTGACATATCAAAATGGC
TGTGGTATATAAAAATATTCATAATGATAGTAGGAGGCTTAATAGGATTAAGAATAGTTATGGCT---
GTGCTTTCTATAATA---------AATAGAGTTAGGCAGGATACTCACCT------
TTGTCATTCGCTACTAATTTACTTGATACCATAGCAATAGTAGTAGCTGGC---
TGGACAGATAGGGGTATAGAAATATTACAAAGAATTGGTAGGGCT----------------------------
--------TTTCTCAACGTA------CCTAGAAGAATCAGGCAGGGCTTAGAAAGGGCTTTACTA---TAA
>KU921759
GCT---GCA--
GATAATTTGTGGGTTACTGTCTACTATGGGGTACCTGTGTGG---AAAGAAGCAGAG---
ACCACTTTATTTTGTGCATCAGATGCTAAAGCATATGTGGAAGAA---AAGCATAAT----------------
--------------GTCTGGCTACA---CATGCCTGTGTACCTACAGAC---
CCTAGCCCACAAGAAATACTTTTGGAA---------AATGTGACAGAAAAGTTTAAT----------------
--------------------------ATGTGGAAAAATCCCATGGTAGAGCAGATGCATACAGATATA---
ATCAGTCTATGGGACCAAAGTCTA---CAGCCATGTGTAAAGTTA---
ACCCCTCTCTGTGTCACTTTATATTGTACTAACACAAACATCACTAACAACAGTACCAGTAACATC-------
----
---ACGGACAGTGGAGAAATGAAA---
AATTGCTCTTTCAATATGACCACAGAACTAAGA---------------------------
GATAGGCAGCGGAAAGTAACTTCACTT---TTTTATAAACTAGATATAGTAGAAATTGGTAAT----------
----

AGTAGTATTAGTAATAATGGTAGCGGTGAATATAGATTAATAAATTGTAATACCTCAGCCATTACACAA---
GCTTGCCCTAAAGTAAGCTTTGAGCCAATTCCCATACATTATTGTGCCCCAGCTGGTTTTGCGATCTTAAAGT
GTAAT---AATGAGAGTTTCAAT------GGAACAGGGCCATGCAATAAT-----------------------

-GTTAGC---ACAGTTCAGTGCACACATGGAATCAAGCCAGTAGTATCAACT---
CAACTACTGTTAAATGGTAGTCTAGCAAAAGGAGAG-----------
GTAATAATTAGATCTGAAAATATTACAGACAAT---------GCTAAAACTATAATAGCACAACTTGAC----
-----------ACGCCTGTGAAC---------ATTACTTGTATCAGACCTAATAACAATACAAGAGAA-----
-GGTGTACATATA------------GGACCTGGAAAAGCA---TTC------TATACAACAAAC---------
---------ATAATAGGAAATATA------------AGACAAGCACATTGT---AATGTCAGTAAGTCA---
GACTGGAATAGAACTTTACACAAGGTAGCTACACAATTAGGA---------------AAATACTTTCAA----
-----------------------------------AACAAAACAATAATATTTAAT------
AGCTCCTCAGGAGGAGATCTAGAAATCACA---ACACATATGTTTAATTGTAGAGGAGAA---
TTTAAACAGCTCCAGGCAAGAGTCCTGGCTGTGGAAAGATACCTAAGGGATCAACAGCTCCTAGGAATTTGGG
GCTGCTCTGGAAAACTCATCTGCACCACTAATGTGCCCTGGAATTCTAGTTGGAGT-----------------
----------------------------AATAAATCTCAGAGTGATATATGG------GAC------
AACATGACCTGGCAGCAATGG---GAGAAAGAAATTGAAAATTACACAGAGGAGATATAT---
AGACTAATTGAAGACTCACAAAACCAGCAGGAA--

AAGAATGAACAAGACTTATTGGCATTAGACAAGTGGGCAAATCTGTGGAATTGGTTTAGCATATCAAATTGGC
TGTGGTATATAAGAATATTTATAATGATAGTAGGAGGCTTAATAGGATTAAGAATAGTTTTTACT---
GTAATTTCTATAATA---------AATAGAGTTAGGCAGGGCTACTCACCT------
TTGTCATTTGCTATTAATTTACTTGATACCATAGCAATAACAGTAGCTGGC---
TGGACAGATAGGATTATAGAAATAGCACAAATAATAGGTAGAGGT----------------------------
--------ATCCTCCACATA------CCTAGAAGAATCAGACAGGGTATTGAAAGGGCTTTGCTA---TAA
>KU921772
GCT---ACA--
GAAAACTTGTGGGTTACTGTTTACTATGGGGTACCTGTGTGG---AAAGATGCAGAG---
ACCACCTTGTTTTGTGCATCAGATGCTAAAACATATGAGACAGAA---AAGCATAAT----------------
--------------GTCTGGGCTACA---CATGCCTGTGTACCTACAGAC---
CCCAACCCACAAGAAATAAATTTAGAA---------AATGTGACAGAGGAGTTTAAC---------------
--------------------------ATGTGGAAAAATAACATGGTAGAGCAGATGCATACAGATATA---
ATCAGCCTATGGGACCAAGGCCTA---AAGCCATGTGTAAAATTA---
ACTCCTCTCTGTGTTACTCTAAAATGCAGCAATGTCACCAATAATAGCACCCCA-------------------
----
---GACATGCAAGAAGAAATAAAA---
AACTGCTCTTTTAATATGACCACAGAACTAAGG--------------------------
GATAAGAGAAAGAAAGTATATTCACTT---TTTTATAAAATTGATATAGTGCAAATTAATGAA----------
----

AATAATAGTAGTAATAATAGTGAGTATAGGTTAATAAATTGTAATACCTCAGCCATTACACAG---
GCTTGTCCAAAGGTATCCTTTGAACCAATTCCCATACATTATTGTGCCCCAGCTGGTTTTGCGATTCTAAAGT
GTAAG---GATAAGGAGTTCAAT------GGAACAGGGCTATGCAAGAAT-----------------------
-GTCAGC---ACAGTACAGTGCACACATGGAATCAAGCCAGTAGTATCAACT---
CAATTGTTGTTAAATGGCAGTTTAGCAGAAGGAAAG-----------
GTAGTGATTAGATCTGAAAATTTCACAAACAAT---------GCCAAAAACATACTAGTGCAATTTCGT----
-----------CAACCTGTAGAA---------ATTAATTGTACCAGACCTAACAACAATACAAGAAAA-----
-AGCATACATATA------------GGACCAGGACAAGCC---TTC------TATGCAACAGGGGAC------
---------ATAATAGGGCCAATC------------AGACAAGCTCATTGT---ACGGTCAATGGAACA---
GAATGGAATAAAGCCTTGCAAGAGGTAGTCACCCAATTAAGA---------------GAAAAATTTAAT----
-----------------------------------AATAAAACAATAATCTTTAAT------
AGCTCCACAGGGGGGGATCCAGAAATAACA---ACACATAGTTTTAATTGTGGAGGAGAA---
TTTAAACAGCTCCAGGCAAGAGTCCTAGCTGTGGAAAGATACCTAAGGGATCAGCAGCTCCTAGGAATTTGGG
GCTGCTCTGGGAAACTCATCTGCACCACTAATGTGCCTTGGAACTCTAGTTGGAGT-----------------
----------------------------AATAAATCTCAGGAGGAAATATGG------GGT------
AACATGACCTGGCTGCAATGG---GACAAAGAAATTGAAAATTATACACAAATAATATAC---
ACTCTCCTTGAAGAATCGCAGAATCAGCAGGAA--

AAGAATGAACAAGATTTATTAGCATTAGACAAGTGGGCAAGTCTGTGGAATTGGTTTGACATATCACACTGGC
TGTGGTATATAAGAATATTTATCATAATAGTAGGAGGCTTAATAGGATTAAGAATAGTTTTTGCA---
GTGCTTTCTGTAATA---------AATAGAGTTAGGCAGGGATACTCACCT------
TTGTCATTTGCTATTAATTTGCTTGATACTATAGCAATAGCAGTAGCTGGT---
TGGACAGACAGGGCTATAAAAATAGGACAAAGATTGTGTAGAGCT--------------------------
--------ATTTTACACATA------CCTAGAAGAGTCAGACAGGACTTGGAAAGGGCTTTGCAA---TAA

>KU921780
GCT---GCA--
GGAAAATTGTGGGTTACTGTCTACTATGGGGTGCCTGTGTGG---AAAGATGCAGAG---
ACCATCTTGTTTTGTGCATCAGATGCTAAATCATACTCCACAGAG---AAACATAAT----------------
--------------GTCTGGCCACT---CATGCCTGTGTACCCACAGAC---
CCCAACCCACAAGAAATACATTTGAAA---------AATGTGACAGAAGAGTTTAAC----------------
--------------------------ATGTGGAAAAATAAAATGGTAGATCAAATGCATACAGATATA---
ATCAGTCTATGGGAAGAAAGTCTA---AAACCATGTGTAAAGTTA---
ACCCCCCTCTGTGTTACTTTAAATTGTAGAAATATCAATATCAACAGCACCAAC-------------------
----
-------------------------ATCACACAGGGAGGAATAGATGTTAGGGAAGAAATAAAA---
AACTGCTCTTTCAATATGACCACAGAGCTAAGG---------------------------
GATAAGAAAAAGAAAGAATATTCACTG---
TTTTATAAACTTGATATAGTACAAATTAATGAAAATCAAGGTAAT----------------------------
----
AGTAGTAGTGATAACAGTAGTGAGTATAGATTAATAAATTGTAATACCTCAGCCATTACACAG---
GCCTGCCCAAAGGTAACTTTTGAGCCAATTCCCATACACTATTGTGCCCCAGCTGGTTTTGCGATCCTAAAGT
GTAAT---GATGAGGAGTTCAAT------GGAACAGGGCCATGTAAGAAT-----------------------
-GTCAGC---ACAGTACAATGCACACATGGAATCAAGCCAGTAGTATCAACC---
CAACTGCTGTTAAATGGCAGTCTGGCAAAAACAAAT---------
GAGGTAATAATTAGATCTGAAAATATCTCAGACAAT---------GTCAAAAACATAATAGTACAGTTTGTC-
--------------AAGCCTGTGAAA---------ATTAATTGTACCCGACCTAACAACAATACAAGAAGA--
----AGTATACATATA------------GGACCAGGACAAGCA---TTC------TTTGCAAGGACAAAT---
------------GTAATAGGGGATATA------------AGACAAGCATATTGT---ACTGTCAATAGATCA-
--GAATGGAATACAACTTTGCAAGGGGTAGCTAAACAATTAATC---------------AAATACTTTAGG--
----------------------------------AACGCAACAACAATAAAATTTAAT------
AGCTCCTCAGGGGGGGATCCAGAAGTCACA---ACACATGCTTTTAATTGTGGAGGAGAA---
TTTAAACAGCTCCAGGCAAGAGTCCTGGCTGTGGAAAGATACCTAAAGGATCAACAGCTCCTAGGAATTTGGG
GTTGCTCTGGAAAACTCATCTGCACCACTAATGTGCCCTGGAACTCTAGTTGGAGT-----------------
----------------------------AATAAATCTCAGCAGGACATATGG------GAG------
AACATGACCTGGCTGCAATGG---GACAAAGAAATTAGCAATTACACAAGCATAATATAT---
GACCTAATTGAAGAATCGCAAAACCAGCAGGAA--

AAGAATGAACATGACTTATTGGCATTGGACAAGTGGGCAAATCTGTGGAATTGGTTTGATATATCAAATTGGC
TATGGTATATAAAAATATTTATAATGATAGTAGGAGGTTTAATAGGATTAAGAATAGTTTTTACT---
GTGCTTTCTATAATA---------AATAGAGTTAGGCAGGGGTACTCACCT------
TTGTCATTTGCTATTAATTTGGTTGATACCATAGCAATAGCAGTAGCTGGC---
TGGACAGATAGGATTATAGAAATAGGACAAAGAATTGGTAGAGCT----------------------------
--------ATCCTCCACATA------CCTGTAAGAATTAGACAGGGCTTAGAAAGAGCTTTGCTA---TAA
>KU921783
GGA---GCA--
GAAAACTTGTGGGTTACTGTCTACTATGGGGTACCTGTGTGG---AAAGACGCAGAG---
ACCACCTTATTTTGTGCATCAGATGCTAAGGCATATGAGACAGAG---AAGCATAAT----------------
--------------GTCTGGCTACA---CATGCCTGTGTACCCACAGAC---
CCCAACCCACAAGAAATACCTTTGGAA---------AATGTGACAGAAGAGTTTAAC----------------
--------------------------ATGTGGAAGAATAACATGGCAGAGCAAATGCATACAGATGTA---
ATCAGTCTATGGGATCAAAGTCTA---CAGCCATGTGTAAAGTTA---
ACCCCTCTCTGTGTTACTTTAAACTGTACCAACATCACAAGAAGCAACATCACGGGGGACAGTGTAAGCAACA
GCACGGGGGACAGTGTAAGCAACAGC---
-------------------ACGGGGGACAGTGTAAGCAACATCACGGGGGACGGTGGATTGACA---
AACTGCTCTTACAATGTAACCACAGACCTAAGA---------------------------
GATAAGAGACAGAAAGTATATTCACTT---TTTTATAAACTTGATGTAGTACAAATGAATAAA----------
----

AGTAGTAACACAGAGTATAGATTAATAAATTGTAATACTTCAGCCATGACACAA---
GCCTGTCCAAAGGTAACCTTTGAGCCAATTCCCATACATTATTGTGCCCCAGCTGGTTTTGCGATCCTAAGGT
GTAAT---AATAAGACGTTCAAT------GGAACAGGGCCATGCAATAAT-----------------------
-GTCAGC---TCAGTACAATGCACACATGGAATCAAGCCAGTAATATCAACT---
CAACTGCTGTTAAATGGCAGTCTGGCAGAAGGAGAG------------
GTGATAATTAGATCTGAAAATATCACAGACAAT---------GCCAAAAACATAATAGTACAATTTAAC----

```
-----------GATTCAGTAGCA---------ATTAATTGTACCAGACCTAACAACAATACAAGAAAA-----
-AGCATAAGGATA------------GGACCAGGACAAACA---TTC------TATGCAACAGGTGGC------
---------ATAATAGGGAATATA------------AGAGCAGCATATTGT---AATGTCAGTAAAACA---
GCATGGAATGGCGCTTTAAAACAGGTAGCTGCCCAATTAGGA---------------AAGTACTTTAAG----
-----------------------------------AACAAAACAATAAGATTTGAT------
AACTCCTCAGGAGGGGATCTAGAAATCACA---ACACATAGTTTTAATTGTGGAGGAGAA---
TTTAAACAGCTCCAGGCAAGAGTCCTGGCTGTGGAAAGATACCTAAGGGATCAACAGCTCCTAGGAATTTGGG
GCTGCTCTGGAAAACTCATCTGCACCACTAACGTGCCCTGGAATTCTAGTTGGAGT-----------------
----------------------------AATAAATCTCAAAGTGAGATATGG------GAC------
AACATGACCTGGCTGCAATGG---GATAAAGAGATTAGCGCTTACACAGACACAATATAT---
AACCTACTTGAAAAATCGCAGAATCAGCAGGAA----------------------------------------
-----------------------------
AAGAATGAACAAGACTTATTGGCATTGGATAAGTGGGCAAATCTATGGAATTGGTTTGACATATCAAGATGGC
TGTGGTATATAAAAATATTTATAATGATAGTAGGAGGCTTAATAGGATTAAGAATAGTTTTTGCT---
GTGCTTTCTATAATA---------AATAGAGTTAGGCAGGATACTCACCC------
TTATCGTTCACTGTTAGGTTGTTTGATACCATAGCAATAGCAGTAGCTGGC---
TGGACAGATAGGGTTATAGTCCTAGGACAAGGAGTTGGTAGAGCT----------------------------
--------ATTTTCCACATA------CCTAGAAGGATCAGACAGGGCCTTGAAAGAGCTTTGCTA---TAA
>KU921785
GCT---GCA------------------------------------------
GACAACTTGTGGGTTACTGTCTATTATGGGGTACCTGTATGG---AGAGACGCAGAT---
ACCACCTTATTTTGTGCATCAGATGCGAAAGCATATGATCCAGAA---GTGCATAAT----------------
--------------ATCTGGCTACA---CATGCCTGTGTGCCCACAGAC---
CCTGACCCACAAGAAATAAATTTGGAA---------AATGTAACAGAAAAGTTCAAC----------------
--------------------------ATGTGGAAAAATAACATGGTAGAGCAGATGCATGTAGATATA---
ATCAGTCTATGGGACCAAAGCCTA---AAGCCATGTGTAAAGTTA---
ACCCCTCTCTGCGTTACTTTAAATTGTAGCAATATCAATAACCCCAGTAGCTCTACTGGT-------------
-------------------------------------------------------------------------
----------------------------GCCAAAATCCCCAGTGACATGAGAGAAGAAATAAAA---
AACTGCTCTTTCAATATGACTACAGAAGTAAGG---------------------------
GATAAGAGACAGAAGATATATTCACTT---TTTTATAAATTGGATATAGTACAATTTAATAAC----------
-------------------------------------------------------------------------
-------------------------
AGCAATCAGTATAGACTAATAAATTGTAATACCTCAGTCATTACACAA---
GCTTGTCCAAAGGTATCCTTTCAGCCAATTCCTATACATTATTGTGCCCCAGCTGGTTTTGCGATTCTAAAGT
GTAAG---GATAAACAGTTCAAT------GGAACAGGGTTTTGCAAGAAT-----------------------
-GTCAGT---ACAGTACAATGCACACATGGAATCAAGCCAGTAGTATCAACT---
CAACTGCTGTTAAATGGCAGTCTAGCAGAAGGACAA------------
GTAATGATTAGATCTGAAAATATCACAAACAAT---------GTCAAAAACATAATAGTACAGTTAGAT----
-----------AAGCCTGTAAGA---------ATTACATGTGTCAGACCTAACAACAATACAAGAAGA-----
-AGTGTACATATA------------GGACCAGGACAAGCA---TTC------TATGCAACAGGTGAC------
---------ATCATAGGGGATATA------------AGACAAGCACATTGT---ATTGTCAATAGAACA---
GACTGGAATAGAACTTTGCACAAGGTGAGCGAACAATTAAGA------------ACAACATACTTTAAG----
-----------------------------------AACAAAACAATAATCTTTGCT------
AACTCCACAGGAGGGGATCTAGAAATAACA---ACACATAGTTTTAATTGTAGAGGAGAG---
TTTAAACAGCTCCAGGCAAGAATCCTGGCTGTGGAAAGATACCTAAAGGATCAACAGCTCCTAGGAATTTGGG
GATGCTCTGGAAAACTTATCTGCACCACTAATGTGCCCTGGAACTCTAGTTGGAGT-----------------
----------------------------AATAGATCCCAGAGTGAGATATGG------GAA------
AACATGACCTGGCTGCAATGG---GATAAAGAAATTAGTAATTACACAGAGATAATATAT---
AAATTAATTGAAGAATCGCAGAACCAGCAGGAA----------------------------------------
-----------------------------
AAGAATGAACAAGACTTATTGGCACTGGACAAGTGGGCAAATCTGTGGAATTGGTTTGACATATCAAATTGGC
TGTGGTATATAAAAATATTTATAATGATAGTAGGAGGCTTAATAGGATTAAGAATAGTTTTTGCT---
GTGCTTTCTGTAATA---------AATAGAGTTAGGCAGGATACTCACCT------
TTGTCGTTCGCTATCAACTTGCTTGATACCATAGCAGTAGTAGTAGCTGAG---
TGGACAGATAGGGTTATAGAAGTAGGACAAAGACTTGGCAGAGCT----------------------------
--------ATCCGCAACATA------CCTAGAAGAATCAGACAGGGCGCAGAAAGGGCTTTGCTA---TAA
>KU921788
ACT---GCA------------------------------------------
GAAAACTCGTGGGTTACTGTCTATTATGGGGTACCTGTGTGG---AAAGATGCAAAA---
```

ACCACTTTGTTTTGTGCATCAGATGCTAAAGCATATAAGACAGAA---AAGCATAAT----------------
--------------GTCTGGGCTACA---CATGCCTGTGTACCCACAGAC---
CCCAACCCACAAGAAGTACATTTGGAA---------AATGTGACAGAAAAGTTTAAT----------------
--------------------------ATGTGGAAAAATAACATGGTAGAGCAGATGCATACAGATATA---
ATCAGTCTATGGGATCAAAGCCTA---AAGCCATGTGTAAAGTTA---
ACTCCTCTCTGCGTTACTTTACATTGTACCAACATCACCATTTGTAACAACAGCATCAACATCACAGAAACCA
CACATTGTACTAACATCACCAGTACC---
----------TCAAATAATACCACAAATGCCACGGTAGACACAGAAGCAATACAAAACATGAAA---
AACTGCTCTTTCAATGTGACCACACTATTAAGG---------------------------
GATAAGCAACAGAAGGTAGCTTCACTT---TTTTATAGACTTGATATAGTACCAATTGATAAT----------
----

AGTGGTAGTGATGAGTATAGATTAATAAATTGTAATACCTCAGCCATTACACAA---
GCCTGCCCAAAGGTAACCTTTGAACCAATTCCCATACATTATTGTGCTCCAGCTGGTTTTGCGATCCTAAAAT
GTAAT---GATAAGGAGTTCAAT------GGAACAGGGCCATGCAAGAAT-----------------------
-GTCAGC---TCAGTACAATGCACACATGGAATCAAGCCAGTAGTATCAACT---
CAACTGCTGCTAAATGGCAGTCTAGCAGAAGGAGAG------------
ATGATGATTAGATCTGAAAATATCTCAAGTAAT---------ACCAAAAATATTATAGTACAACTTAAC----
-----------CATGCTGTGGAA---------ATTAATTGTACCAGACCTGGTAACAATACAAGAACA-----
-GGTATACATATA------------GGACCAGGACGAGCA---TTT------TATGCAACAAATACC------
---------CCAATAGGGGATATA------------AGACAAGCACATTGT---AATATCAGTGGATCA---
GAATGGAATAACACTTTACAAAAGGTAGTTGACAAATTAAGA---------------AAATACTTGAGA----
--------------------------------AATGACACAAAAATAATATTTGAG------
CCCCCCTCAGGAGGGGATCTAGAAATCACA---ACACATAGTTTTAATTGTGGAGGGGAA---
TTTAAACAGCTCCAGGCAAGAGTCCTGGCTCTGGAAAGATACCTACAGGATCAACAACTCCTAGGAATTTGGG
GCTGCTCTGGAAAACTCATCTGCACCACTAATGTGCCTTGGAACAACAGTTGGAGT-----------------
----------------------------AATAAATCTCTAGATACGATATGG------AAC------
AACATGACCTGGCTAGAATGG---GATAGAGAAATTAGCAATTACACAGACATAATATAT---
CAACTAATTGAAGAATCACAAAACCAGCAGGAA--

AAGAATGAACAAGACTTATTGGCATTGGACAAATGGGCAAATCTATGGAATTGGTTTAACATAACACAGTGGC
TGTGGTATATAAAAATATTTATAATGATAGTAGGAGGCTTAATAGGATTAAGAATAGCTTTTGCT---
GTGCTTTCTATAGTA---------AATAGAGTTAGGCAGGGATACTCACCT------
TTGTCGTTTGCTATTAGCTTGATTGATACTATAGCAATAGCAGTAGCTGGC---
TGGACAGATAGGGCTATAGAAATAATACAAGGAATTGGTAGAGCT----------------------------
--------TTTCTTCACGTA------CCTAGAAGGATCAGACAAGGCTTCGAAAGAGCTCTGCTA---TAA
>KU921804
AAT---GCA--
GAACAGTTGTGGGTTACTGTACACTATGGGGTACCTGTGTGG---AAAGACGCAGAT---
GCCACTCTATTTTGTGCATCAGATGCTAAAGCATATGAGACAGAA---AAGCATAAT----------------
--------------GTCTGGGCTACC---CATGCCTGTGTACCCACAGAC---
CCCAGCCCACGAGAATTACCTTTGGGA---------GGTGTGAAGGAAGAATTTAAC----------------
--------------------------ATGTGGAAAAATAATATGGTAGAGCAGATGCATACAGATATA---
ATCAGTCTATGGGACCAAAGCCTA---AAGCCATGTGTAAAGTTA---
ACCCCTCTCTGCGTCACGTTAAACTGTACCAACATCAGCACGGTGGGC-------------------------
----
-------------------------AACCGCACGGTGGAAGGAAACATGATAGGAAAAATAAAA---
AACTGCTCTTTTGATATGACCACAGAACTAATG---------------------------
GATAGGAAAAAGAAAGTATATTCACTC---TTTTATAAACTTGATATAGTGCCAATTGATGAA----------
----

AGGAATAGCAGCAATAGCAGCAATGAGTATAGATTAATAAATTGTAATACCTCAACCATTACACAG---
GCTTGTCCAAAGGTATCCTTTGAGCCAATTCCCATACATTACTGTGCCCCAGCTGGTTTTGCGATCCTAAAGT
GTAAT---GAGGATGAGTTCAAT------GGAACAGGGCTTTGCACAAAT-----------------------
-GTCAGT---ACAGTACAATGCACACATGGAATCAAGCCAGTAGTATCAACT---
CAACTGTTGTTAAATGGCAGTCTAGCAGAAACGGAG------------
GTACAAATTAGATCTGAAAATTTCTCAGACAAT---------GCTAAAACCCTAATAGTACAACTTAGA----
-----------GAACCTGTAAAC---------ATTACTTGCATCAGACCTAACAACAATAGAAGGACA-----
-AGCATACATATG------------GGACCAGGACGAGCA---TTC------TATGCAACT---GAT------
---------ATAATAGGAGATATA------------AGAGAAGCGCGTTGT---AATGTTAATGGATCA---

GATTGGAAGGAAACTTTACAAAAGGTAGCTGAACAATTAAGA---------------AAACATTACAGG----
-----------------------------------AACAAAACAATAACCTTTAGC------
AACTCCTCAGGAGGGGATTTAGAAATTACA---ATGCATACTTTTAATTGTGGAGGAGAA---
TTTAAACAGCTCCAGGCAAGAGTCCTTGCTGTGGAAAGATACCTAAGGGATCAACAGCTCCTAGGAATTTGGG
GCTGCTCTGGAAAACTCATATGCACCACTAATGTGCCCTGGAACTCTAGTTGGAGT----------------
----------------------------AATAAATCTTACGATGAAATATGG------GGA------
AACATGACCTGGCTACAATGG---GAGAGAGAAATTAGCAATTACACAGAAATAATATAT---
GGGCTGATTGAAGAATCGCAAAAACAGCAGGAA--

AAGAATGAACAAGACTTATAGGCATTGGACAAGTGGGCAAGTCTGTGGAATTGGTTTGACATAACACAATGGC
TGTGGTATATATAAAAATATTTATAATGATAGTAGGAGGCTTAATAGGGTTAAGAATAGTTGTTGCT---
ATAAGCTCTGTAGTG---------AATAGAGTTAGGCAGGGATACTCACCT------
TTGTCATTCGCTATCAATTTGTTTGATACCATAGCAATAGCAGTAGCTGAG---
GGAACAGATAGAGTTATAGAAATAGTACAAAGAGCTTGCAGAGCT---------------------------
--------TTTTTCAACATA------CCTAGAAGAATCAGACAGGGCGCAGAGAAGATTTTCCTA---TAA
>KU921831
GCT---GTA---
CAAAACTTGTGGGTTACTGTCTACTATGGGGTGCCTGTGTGG---CAAGATGCAGAA---
ACCACCTTATTTTGTGCATCAGATGCTAAAGCATATAAGACAGAA---GTGCATAAT----------------
--------------GTTTGGGCAACA---CATGCCTGTGTACCCACAGAC---
CCCAACCCACAAGAGGTGCATTTGAAA---------AATGTGACAGAAGAGTTTAAC---------------
--------------------------ATGTGGAAAAATAAAATGGTAGATCAAATGCATGAAGATATA---
ATCAGTCTATGGGACCAAAGCCTA---AAGCCATGTGTAAAGTTA---
ACCCCTCTCTGTGTCACTTTAAGTTGTGGCGAGAGTAATTTCAACAAC------------------------
----
-------------------------ACCATGAACGTCAGTAGGGGGGAAATGGTAGAAACAAAA---
AACTGCTCTTACAATATGACCACAGAACTAAGG--------------------------
GATAAAACACAGAAAATACATTCGATC---TTTTATAGACTTGATTTAGTACAAATTAATAAACCTGAGAAT-
----

AATAGTAGTAGTAATTATAGTGAGTATAGATTAATAAATTGTAATACCTCAACCATTACACAG---
GCTTGTCCAAAGGTAACCTTTGAGCCAATTCCTATACATTATTGTGCCCCAGCTGGTTTTGCGATCCTAAAAT
GTAAT---GAGAAGACGTTCAAT------GGAACAGGGCCATGTAATAAT----------------------
-GTCAGC---ACAGTACAATGCACACATGGAATCAGACCAGTAGTATCAACT---
CAACTGCTGTTAAATGGTAGTCTAGCAGAAGAAGAG------------
ATAACAATTAGATCTGAAAATATCACAGACAAT---------GGCAAAAACATATTAGTTCAACTTAAA----
-----------GAGCCTGTGGAA---------ATTAATTGTACCAGACCTGGCAACAATACAAGAAAA-----
-AGTATACATATA------------GGACCAGGACGATCA---TAC------TATGCAACAGATGAC------
---------ATAATAGGGGATATA------------AGGAAAGCATTTTGT---ACTGTTAAGGCAACA---
GTATGGAATGAAACTTTACAACAGGTAGCTAGACAATTAGGC---------------GAACATTTTGAG----
-----------------------------------AACAGAACAATAACCTTTAAT------
AACTCTGCAGGAGGGGATTTAGAAATCACA---ACACATAGTTTTAATTGTCAAGGAGAA---
TTTAAACAGCTCCAGGCAAGAGTCCTGGCTCTGGAAAGATACCTAAAGGATCAACAGCTCCTAGGAATTTGGG
GCTGCTCTGGAAAACTCATCTGCACCACTAATGTGCCCTGGAACTCTAGTTGGAGT----------------
----------------------------AATAAATCTATGGATGAGATATGG------GAC------
AACATGACCTGGCTGCAATGG---GATAAAGAAATTAGCAATTACACACAGAAAATATAT---
AGCCTAATTGAGGAAGCGCAGAACCAGCAGGAA--

GAGAATGAAAAAGACTTATTGGCATTGGACAAGTGGGCAAGTCTGTGGAATTGGTTTGAAATATCAAAGTGGC
TATGGTATATAAAAATATTTATAATGATAGTAGGAGGCTTAATAGGATTAAGAATAGTTTTTACT---
GTGCTTTCAGTAATA---------AATAGAGTTAGGCAGGGATACTCACCT------
CTGTCGTTCGCTGTTAATTTGTTTGATACCATAGCAATAGCAGTAGCTAAC---
GGGACAGATAGGGTTATAGAAATAGGACAAAGATTTGGTAGAGGA---------------------------
--------ATTCTCCATATA------CCTAGAAGAATCAGACAGGGCTTAGAAGTAGCTTTGCTA---TAA
>KU921835
GTC---ACA---
GGAAGGTATTGGGTCACAGTTTACTATGGGGTACCTGTGTGG---AAAGATGCAGAG---
ACCACCTTATTTTGTGCATCAGATGCTAAAGCATATGAAAGAGAA---AAACATAAT----------------
--------------GTCTGGGCTACA---CATGCCTGTGTACCCACAGAC---
CCCAACCCACAAGAAGTACCTCTGGGA---------GATGTGACAGAAAACTTTAAC---------------

--------------------------ATGTGGAAGAATGGCATGGTGGAGCAGATGCATACAGATATA---
ATCAGTCTATGGGACCAAAGCCTA---ACACCATGTGTAAAATTA---
ACCCCTCTCTGTGTGACTTTAAATTGTTCTAATGCCACGGTCACCAATGCTACTAACACT------------
----
----------------------------TACAATTATACCCTGGTGACAACAGACGAAGTAAAA---
AACTGCTCTTTCAATATGACCACAGAGATAAGG--------------------------
GATAAGAAAAGGAAAGTATATTCACTT---
TTTTATAAGCTTGATATAGTACAAATTAATGAAAATCAAAATGAT---------------------------
----
AGTAGTAAAAATAATAGTAATAGCCAGGAGTATAGATTAATAAATTGTAATACCTCAGCCATTACACAG---
GCTTGTCCAAAAATATCATTTGAGCCAATTCCCATACATTATTGTGCCCCAGCTGGTTTTGCGATCCTAAAGT
GCACT---AATAAGACATTCAAT------GGCACAGGGCCATGTAGTAAT----------------------
-GTCAGC---ACAGTACAATGTACACATGGAATTAAGCCAGTAGTATCAACT---
CAACTGCTGTTAAATGGCAGTCTAGCAGAAGGAGAG-----------
GTAAGGATTAGATCTGAAAATATCTCAGACAAT---------AGCAAAAACATAATAGTACAATTTACC----
-----------AAGCCTGTCAAC---------ATTACTTGTATCAGACCTAGCAACAAGACAAGAACA-----
-AGTGTACATATA------------GGACCAGGACGAGCC---TTC------TATGCAACAGGTAGC------
---------ATAATAGGGGATATA------------AGAAAAGCACATTGT---AATGTTAGTAGAACA---
GCATGGAATAAGACTTTAGGAGAGGTAGCTACACAATTAAGC---------------AAATACTTTGGA----
-------------------------------AACGAAACAACAATAAGATTTGAT------
AAACACGCAGGAGGGGATCCAGAAATAACA---ACACATAGCTTTAATTGTGGAGGAGAA---
TTTAAACAGCTCCAGGCAAGAGTCCTGGCTGTGGAAAGTTACCTAAAGGATCAACAGCTCCTAGGAATTTGGG
GCTGCTCTGGAAAACTCATCTGCACCACTAATGTGCCCTGGAACTCTAGTTGGAGT-----------------
---------------------------AATAAATCCTATGATGAGATATGG------GGT------
AATATGACCTGGCTACAATGG---GAGAAAGAGATTAGCAATTACACAGATATAATATAT---
GATCTACTTGAAGAATCGCAGAACCAGCAGGAA---------------------------------------
----
AAGAATGAACAAGACTTATTAGCGTTGGACAAGTGGGCAAGCCTGTGGAATTGGTTTAGCATATCAAACTGGC
TGTGGTATATAAAAATATTTATAATGATAGTAGGAGGCTTGATAGGATTAAGAATAGTTTTTACT---
GTGTTTAATATGATA---------AATAGAGTTAGGCAGGGATACTCACCT------
TTGTCATTTGCCATTAGTTTGTTTGATACCATAGCAATAGCAGTAGCTGGC---
TGGACGGATAGGGTTATAGAAGTAGGACAAAGAATTGGTAGGGCT---------------------------
--------ATCCTCCACATA------CCTAGAAGAATTAGACAAGGCTTAGAAAGAGCATTGCTA---TAA
>KU921841
ACT---ACA---------------------------------------
GGAAACTTGTGGGTTACAGTTTACTATGGGGTACCTGTGTGG---AAAGATGCAGAG---
ACTACCTTATTTTGTGCATCAGATGCCAAAGCACATGAAAAAGAA---GTGCATAAT----------------
-------------GTCTGGGCTACA---CATGCCTGTGTACCCACAGAC---
CCCAACCCACAAGAGATACCCCTGAAA---------AATGTGACAGAAAAGTTTAAC----------------
------------------------ATGTGGAAAAATAACATGGTAGAGCAGATGCATACAGATATA---
ATCAGTCTATGGGACCAAAGCCTA---AAGCCATGTGTAAAGTTA---
ACCCCTCTCTGTGTTACTTTAAATTGTACCAATGCCACTATTACCAATACCAATGTTACCAATGCC-------
----
-----------------------AATGGCACGGTGGACAGGATACCAGTGGAAGAAATAAAA---
AACTGCTCTTTCAATATGACCACAGAACTAAGG-------------------------
GATAAGAGACAGAAAGTATTTTCACTT---
TTTTATAGACTTGATGTAGTAAAGATTAGTGGCCAGAATGAAACT---------------------------
----
ATTAATGGCAGTGGGCAGTATAGACTAATAAATTGTAATACCTCAGTCATTACACAG---
GCTTGTCCAAAGGTAACCTTTGAACCAATTCCCATACATTATTGTGCTCCAGCTGGTTTTGCGATCCTAAAGT
GTAGG---GAGAAGGATTTCAAT------GGAACAGGGCCATGCAAGAAT----------------------
-GTCAGC---TCAGTACAATGCACACATGGAATCAGGCCAGTAGTATCAACT---
CAACTGCTGTTAAATGGCAGCCTAGCAGAAGGAAAT-----------
GTAACAATTAGATCTGAAAATATCTCAAACAAT---------GCTAAGATCATAATAGTACAACTTAAC----
-----------ACATCTGTAGAA---------ATTAATTGTACCAGACCTGGCAACAACACGAGGAAA-----
-AGTGTACATATA------------GGACCAGGACAAGCA---TTT------TATGCAACAGGTGAA------
---------GTAATAGGGGATATA------------AGAAACGCACATTGT---AATGTCAGTAGATCA---
CAATGGAATGCCACTTTAGAAAAGGTAAAGGAACAGTTAAAA---------------AAGCACTTTCCA----
---------------------------------CACAAAACAATAAAATTTGAT------
AGCCCCGCAGGAGGGGATTTAGAAATTACA---ACACATACTTTTAATTGTGGAGGAGAG---

TTTAAACAGCTCCAGGCAAGAGTCCTAGCTGTGGAAAGATACCTAAAGGATCAACAGCTCCTAGGCATTTGGG
GCTGCTCTGGAAAACTCATCTGCACCACTAATGTGCCTTGGAACTCCAGTTGGAGT----------------
----------------------------AATAAAACTGAGAAGGAGATATGG------AAT------
AACATGACCTGGCTGCAATGG---GATAAAGAAATTGACAATTACACAGATTTAATATAT---
CACTTACTTGAGGAATCGCAGAACCAGCAGGAA---------------------------------------
----
AAGAATGAACAAGATCTATTGGCATTGGACAAATGGGCAAATCTGTGGAATTGGTTCGATATATCACGATGGC
TGTGGTACATAAGAATATTTATAATGATAGTAGGAGGCTTAATAGGATTAAGACTAGTTTTTGCT---
ATGTTTTCTGTAATA---------AATAGAGTTAGGCAGGGATACTCACCT------
TTGTCGTTTGCTATTAGTTTACTTAATGCCACAGCAATAGCAGTAGCTGAG---
GGGACAGATAGGATTATAGAAATAGGACAAAGAGTTGGTAGAGTT---------------------------
--------ATTCGCAACGTA------CCTAGAAGAATCAGGCAGGGCGCTGAAAGGCTTTTGTTA---TAA
>KU921852
GCT---TCA---
AATAAATTGTGGGTTACCGTCTACTATGGGGTACCTGTGTGG---AGAGACGCAGAG---
ACCATCTTATTTTGTGCCTCAGATGCTAAAGCATATGAGACAGAA---AAGCATAAT---------------
--------------GTCTGGGCTACA---CATGCCTGTGTACCCACAGAC---
CCCAACCCACAAGAAATAGATTTGGAA---------AATGTGACAGAAGATTTTAAC---------------
--------------------------ATGTGGAAAAATAACATGGTAGAGCAGATGCAGACAGATATA---
ATCAGTCTATGGGACCAAAGCCTA---AAGCCATGTGTAAGGTTA---
ACCCCTCTCTGTGTTACTTTAGATTGTAGCCATAATGTCAGTTATAGTAGTCTCAATAAC------------
----
-------------------------AGTAGCTCCAATGACACAGGGATACTAGAAGAAATAAGA---
AACTGCTCTTACAATACAACCACAGAATTAAGG--------------------------
GATAAGAAACAGAAAGTATATTCACTT---TTTTATAAACTAGATGTAGTAAAACTTGACAAAGAT------
----

AGTAGCAGCAGCAAGGAGTATAGATTAATAAATTGTAATACTTCAGCCATTACACAA---
TCTTGTCCAAAGGTAACCTTTGAGCCAATTCCCATACATTATTGTGCCCCAGCTGGTTTTGCGATCCTAAAGT
GTAAA---GAAAAGAATTTCACT------GGAATAGGGCCATGTAAGAAT----------------------
-GTCAGC---ACAGTACAATGCACACATGGAATCAAGCCAGTAGTATCAACT---
CAACTACTGCTAAATGGCAGTCTAGCAGAAGAAGAG-----------
GTAATGATTAGATCTGAAAATATCACAAATAAT--------GCCAAAAACATAATAGTACAATTTATT----
-----------AAGCCTGTGAAG--------ATTAATTGCACCAGACCTGGCAACAATACAAGAACA-----
-AGTATAAGAATA-----------GGACCAGGACAAGCA---TTC------TATGCAACAGGTGCC------
---------ATAACAGGGGATATA------------AGGAAAGCATATTGT---ACTGTGAATAGATCA---
GATTGGAATAACGCTTTACAAAAGGTAGTTGGTCAACTAAAA--------------AGGCATTTT-------
----------------------------------AACAAAACAATAATATTTGCT------
AACTCATCAGGAGGGGATCTAGAAATTACA---ACACATAGTTTTAATTGTGGAGGAGAA---
TTTAAACAGCTCCAGGCGAGAGTCCTGGCTGTGGAGAGATACCTAAGGGATCAACAGCTCCTAGGAATTTGGG
GCTGCTCTGGAAAACTCATCTGCACCACTAATGTTCCCTGGAACTCCAGTTGGAGT----------------
----------------------------AATAAATCCCTAGATGAAATATGG------GAC------
AACATGACCTGGATGCAGTGG---GATAGAGAAATTAACAATTACACACAGATAATATAT---
ACTCTACTTGAAGAATCACAGAACCAGCAGGAA---------------------------------------
----
AAGAATGAACAAGACTTATTGGCATTAGACAAGTGGGCAAGTTTGTGGAATTGGTTTGACATATCAAACTGGC
TGTGGTATATAAGGATATTTATAATAATAGTAGGAGGCTTAATAGGATTAAGAATAGTTTTTGCT---
GTGCTTTCTGTAATA---------AATAGAGTTAGGCAGGGATACTCACCT------
TTGTCGTTCGCTATTAATTTGATTGATACCACAGCAATAGTAGTAGCTGGG---
GGGACAGATAGGATTATAGAAATAGTACAAAGAATTGTTAGAGCT---------------------------
--------ATTCTTAACATA------CCCACACGAATAAGACAGGGCTTTGAAAGGGCTTTGCTA---TAA
>KF716474
GCT---GCA---
GAAAACTTGTGGGTTACTGTCTACTATGGGGTACCTGTGTGG---AAAGATGCAGAG---
ACCACCTTATTTTGTGCATCAGATGCTAAAGCCTATGAGACAGAA---AAGCATAAT---------------
--------------GTTTGGGCCACA---CATGCTTGTGTACCCACAGAC---
CCCAACCCACAAGAAATAGATTTGGAA---------AATGTGACAGAAGACTTTAAC---------------
--------------------------ATGTGGAAAAATAACATGGTAGAGCAGATGCATACAGATATA---
ATCAGCCTATGGGATCAAGGCCTA---AAACCATGTGTAAAATTA---
ACTCCTCTCTGTGTTACCTTACATTGTGAGAAGTATATAAAGAACATTACTAACCAGAATGAA---------

----
------------------ACCAGTAATAACCAGAGTGAAATCAGTGGAGAAGGAGAACTAAAA---
AATTGTTCTTACAATATAACCACAGAGCTAAGG--------------------------
GATAAGAAACAGAAGGTATATTCACTT---TTTTATAGACTTGATATAGTGAAAATTAATGAAGGTAAT----
----

AGTAGTGACAATGGAAGTTATAGATTAATAAATTGTAATACCTCAGCCATTACAAAG---
GCTTGTCCAAAAGTATCCTTTGAGCCAATTCCCATACATTATTGTGCCCCAGCTGGTTTTGCGATCCTAAAGT
GTAAG---GATAAGGACTTCAAT------GGAACAGGGCCATGCAAGAAT----------------------
-GTCAGT---ACAGTACAATGCACTCATGGGATCAGGCCAGTAGTATCAACT---
CAACTGCTGTTAAATGGCAGTCTAGCAGAAGGAGAG-----------
GTAAAGATTAGATCTGAAAATATCACAAACAAT---------GCCAAAAACATAATAGTACAACTTAGA----
-----------GAGCCTGTAAGA---------ATTAATTGTACCAGACCTAACAACAATACAAGAAAG-----
-AGTGTACGTATA------------GGGCCAGGACAAGCA---TTC------TTTGCAACAGGTGAG------
---------GTAATAGGAAAAATA------------AGAAAAGCATATTGT---AATGTTAGTGGAGTA---
GAATGGAATAAAACTTTGCAAAAGGTAGTTAGACAATTAAAA---------------GAGTATTTT-------
----------------------------------AATAAAACAATATCATTTAAT------
AACTCCGCAGGAGGGGATATGGAAATAACA---ACACATAGTTTTAATTGTGGAGGAGAA---
TTTAAGCAGCTCCAGGCAAGAGTCCTGGCGATAGAAAGATACCTAAAGGATCAACAGCTCCTAGGAATTTGGG
GCTGCTCTGGAAAACTCATCTGCACCACTAATGTGCCTTGGAACTCCAGTTGGAGT-----------------
---------------------------AATAAATCTCAGGAAGAGATATGG------GAT------
AACATGACCTGGCTGCAATGG---GAGAGAGAAATTAGTAATTACACAGACTACATATAT---
ACCCTACTTGAAGAATCACAGAACCAGCAGGAA---------------------------------------
----
AAGAATGAACAAGACTTATTGGCATTGGACAAGTGGGCAAATCTGTGGAATTGGTTTAACATATCAAATTGGC
TGTGGTATATAAAAATATTTATAATGATAGTAGGAGGCTTAATAGGATTAAGAATAGTTTTTGCT---
GTGCTTTCTATAATA---------AATAGAGTTAGGCAGGGATACTCACCT------
TTGTCGTTCGCTATTAATTTGCTTGATAATATAGCAATAGCAGTAGCTGGC---
TGGACAGATAGGGTTATAGAACTAGGACAAAGATTTTGTAGAGCT----------------------------
--------ATTTTACACATA------CCTAGAAGAATCAGACAGGGCTTAGAGAGAGCTTTGCTA---TAG
>KF716475
GCT---GCA---
CAAAACTTGTGGGTTACTGTCCACTATGGGGTACCTGTGTGG---AAAGATGCAGAC---
ACCATCTTATTTTGTGCATCAGATGCTAAAGCATATGAAACAGAA---AAGCATAAT---------------
-------------GTCTGGGCTACA---CATGCCTGCGTACCCACAGAC---
CCCAACCCACGTGAAGTATATTTGGAA---------AATGTGACAGAAGATTTTAAT---------------
-------------------------ATGTGGAAAAATAGCATGGTAGAGCAGATGCATACAGATATA---
ATCAGTCTATGGGACCAAAGCCTA---AAGCCATGTGTAAAGTTA---
ACCCCACTCTGTGTTACTTTAAATTGTAGCAATGCCAATGTCAGTGTCAGTAATGTCAGTGTCACTAAT----
----
----------GTCACTGTCAATAACACAGAGGTCAACTTACGGGAGGAAGGAGGAGAAATAAAA---
AACTGTTCTTTCAATATAACCACAGAACTAAGG--------------------------
GATAAGAAAAAGAAGGTATATTCACTT---TTTTATAGATCTGATGTAGTATCAATGAATAGCAAT-------
----

GAGAGTAGTGAGTATAGATTGATAAATTGTAATACCTCAACCATTACACAA---
GCCTGCCCAAAGGTATCCTTTCAGCCAATTCCCATACATTATTGTGCCCCAGCTGGTTTTGCAATCCTAAAGT
GTAGG---AACGAGACGTTCGAT------GGAACAGGATCATGCAAAAAT----------------------
-GTCAGC---ACAGTACAATGCACACATGGAATCAAGCCAGTAGTATCAACT---
CAACTGCTGTTAAATGGCAGTCTAGCAGAAGGAAAA---------
CAGGTAATAATTAGATCTGAAAATATCACAGACAAT---------GCCAAAATTATAATAGTACAACTTGCC-
--------------AGTCCCGTGCAA---------ATTAATTGTACCAGGCCTAATAACAATACAAGCACA--
----GGTATACATATA------------GGGCCAGGACAAGCA---TTC------TATGCAAGAAATGCC---
------------ATAATAGGGGATATA------------AGAAAAGCACATTGT---AATGTCAGCAAATCA-
--AAATGGAATGACACTTTACGACAGGTAGCTGAACAATTAAGA---------------AAATACTTTAAA--
---------------------------------AATGATACAACAATAGTGTTTAAG------
AACTCCTCAGGAGGGGATCTAGAAATCACA---ACACATAGTTTTAATTGTGGAGGGGAA---
TTTAAACAGCTCCAGGCAAGAGTCCTGGCCATGGAAAGATACCTAAGGGATCAACAGCTCCTAGGAATTTGGG
GCTGCTCTGGAAAGGTCATCTGCAACACTAATGTGCCCTGGAACTCTAGTTGGAGT----------------
---------------------------AATAAATCTTACAATGAGATATGG------GAC------

GAAATGACCTGGCAGCAATGG---GAGAAAGAAATTAGCAATTACACAGACATAATATAT---
AACCTACTTGAAGAATCGCAAAACCAGCAGGAA--

AAGAATGAACAAGATTTATTGGCATTCGACAAATGGGCAAATCTATGGAGTTGGTTTAAAATAACAAATTGGC
TGTGGTATATAAAAATATTTATAATGATAGTAGGAGGCTTAATAGGATTAAGAATAGTTTTTGCT---
GTGCTCTCTGTAATA---------AATAAAGTTAGGCAGGGATACTCACCC------
TTGTCATTTGCTACTAGCTTGCTTGATACTGTAGCAATAGCAGTAGCTGGC---
TGGACAGATAGGGTTATAGAAATAAGACAAAGAATTGGTAGAGCT----------------------------
--------ATCCTCCACATA------CCTAGAAGAATCAGACAGGGCTTTGAAAGAGCTTTGCTA---TAA
>AY322184
GCT---GCA--
GAAAACTTGTGGGTTACTGTCTACTATGGGGTACCTGTGTGG---AAAGACGCAGAG---
ACCACCTTATTTTGTGCATCAGATGCGAAAGCATATGATACAGAA---GCACATAAT----------------
--------------GTCTGGGCTACA---CATGCCTGTGTACCTACAGAC---
CCCAACCCACAAGAAATAGATTTGGAA---------AATGTGACAGAAGAGTTTAAC----------------
--------------------------ATGTGGGAAAATAACATGGTAGAGCAGATGCATACAGATATA---
ATCAGTCTATGGGACCAAAGCCTA---AAACCATGTGTAAAGTTA---
ACCCCTCTCTGCGTTACTTTAGATTGTAGCTATGATGTCACCATTGCCACT----------------------
----
----------------------------------ATCACCAATGATGTGAGAGAAGAAATAAAA---
AACTGCTCTTTCAATATGACTACAGAATTAAGG---------------------------
GATAAGACACAGAAAGTATATTCACTT---TTTTATAGACTTGATGTAGTACCAATTGATGAAGAG-------
----

AATAACAGTAGTAACAAGGAGTATAGATTAATAAATTGTAATACCTCAGCCATTACACAA---
GCGTGCCCAAAGGTATCCTTTCAGCCAATTCCCATACATTATTGTGCTCCAGCTGGTTTTGCGATCCTAAAGT
GTAAG---GATAAGGAGTTCAAT------GGAACAGGGCCATGCCAGAAT-----------------------
-GTCAGC---ACAGTCCAATGCACACATGGAATCAAGCCAGTAGTATCAACT---
CAACTGCTGTTAAATGGCAGTCTAGCAGAAGGAAAG------------
GTAATGATTAGATTTGAAAATATCACAAACAAT---------GCCAAAAACATAATAGTACAACTTACC----
-----------GAGCCTGTGAAA---------ATTAATTGTACCAGACCTAACAACAATACAATTACA-----
-AGTATACGTATA------------GGACCAGGACAAGCA---TTC------TATGCAACAGGAAGC------
---------ATAATAGGGAATATA------------AGACAAGCACATTGT---ACTGTCAATAGATCA---
GAATGGAATGACACTTTACAACAGGTGAAGTCACAATTAGGA---------------GAATACTTTGTG----
-----------------------------------AACAAAACAATAAAATTTGCT------
AACTCCTCAGGAGGGGATCCAGAAATCACA---ACACATAGTTTTAATTGTGGAGGAGAA---
TTTAAACAGCTCCAGGCAAGAGTCCTGGCTGTGGAAAGATACCTAAAGGATCAACAGCTCCTAGGAATTTGGG
GCTGCTCTGGAAAACTCATCTGCACCACTAATGTGCCCTGGAACTCTAGTTGGAGT-----------------
----------------------------AATAAATCCCAGGAGGAGATATGG------GAA------
AACATGACCTGGCTGCAATGG---GATAAAGAAATTAGCAATTACACAGAGATAATATAT---
AATCTACTTGAAGAATCGCAGAACCAGCAGGAA--

AAGAATGAACAAGACTTATTGGCATTGGACAAGTGGGCAAATCTGTGGACTTGGTTTAACATATCAAACTGGC
TGTGGTATATAAAAATATTTATAATGATAGTAGGAGGCTTAATAGGATTAAGAATAGTTTTTGCT---
GTGCTTTCTGTAATA---------AATAGAGTTAGGCAGGGATACTCACCT------
TTGTCGTTCACAATTAATTGGATTGATACCATAGCCATAGTAGTAGCTGGC---
TGGACAGATAGGGTTATAGAAATAGGACAAGAAATTGGTAGAGCT----------------------------
--------ATTTTCCACATA------CCTAGAAGAATCAGACAGGGTCTCGAAAGGGCTTTGCTA---TAA
>AF539405
ACT---GCA--
GAAAACTTGTGGGTTACTGTCTACTATGGGGTACCTGTGTGG---AGAGACGCAGAG---
ACCACCTTATTTTGTGCATCGGATGCTAAAGCATATAAGACAGAA---ATGCATAAT----------------
--------------GTCTGGGCTACA---CATGCCTGTGTACCCACAGAC---
CCCAACCCACAAGAAATACATTTGGAA---------AATGTGACAGAAGAGTTTAAC----------------
--------------------------ATGTGGAAAAATAACATGGTAGAGCAGATGCATACAGATATA---
ATCAGTCTGTGGGACCAAAGCCTA---AAGCCATGTGTAAAGTTA---
ACCCCTCTCTGTGTTACTTTAGATTGTAGTGACTATAATGGCACCAATATC----------------------
----
-------------------------------------ACCGGTGACATGACAAGGAAATAAAA---
AACTGCTCTTTCAATATAACCACAGAATTAAGG---------------------------

GATAAGAAACAGAAAGTACATTCACTT---TTTTATAGACTAGATGTAGTACAAATTAATAATAGC-------
----

AGTAACAGCAATAACAGTCAGTATAGATTAATAAATTGTAATACCTCAGCCATTACACAA---
GCTTGTCCAAAGGTATCCTTTGAGCCAATTCCCATACATTATTGTGCCCCAGCTGGTTTTGCGATTCTAAAAT
GTAAT---GATAAGGAGTTCAGT------GGAACAGGGCTATGCAGGAAT----------------------
-GTCAGC---ACAGTACAATGCACACATGGAATCAAGCCAGTAGTATCAACT---
CAACTGCTGTTAAATGGCAGTCTAGCAGAAGGAAAG-----------
GCAATGATTAGATCTGAAAATATCACAAACAAT---------GCCAAAAACATATTAGTACAATTTACC----
-----------ACGCCTGTAGAA---------ATTAATTGTACCAGACCTAACAACAATACAAGAAAA-----
-AGTGTACATATA------------GGACCAGGACAAGCA---TTC------TATGCAACAGGGGAC------
---------ATAATAGGGGATATA------------AGACAAGCATATTGT---AATGTCAATGGAACA---
GCATGGAAGGAAAATTTGAAAGAGGTAGCCGACCAATTAAGA---------------ACATATTTTAGG----
-----------------------------------AACACAACAATAAAGTTTGCT------
AACTCCACAGGAGGGGATCTAGAAATAACA---ACACATAGTTTTAATTGTGGAGGAGAA---
TTTAAACAGCTCCAGGCAAGAGTCCTGGCTGTGGAAAGATACCTAAGGGACCAACAGCTCCTAGGAATTTGGG
GCTGCTCTGGAAAACTCATCTGCACCACTAATGTGCCCTGGAACTCTAGTTGGAGT-----------------
----------------------------AATAAATCCCAGAGTGAGATATGG------GAA------
AACATGACCTGGCTGCAATGG---GATAAAGAGATTAGCAATTACACACAGATAATATAT---
ACTCTACTTGAAGAATCGCAGAACCAGCAGGAA---

AAGAATGAACAAGACTTATTGGCATTGGACAAGTGGGCAAATCTGTGGAATTGGTTTGACATAACAAACTGGC
TGTGGTATATAAAAATATTTATAATGATAGTAGGAGGCTTAATAGGATTAAGAATAGTTTTTGCT---
GTGCTTGCTTTAGTA---------AATAGAGTTAGGCAGGGGTACTCACCT------
TTGTCGTTTGCTATTAATTTGATTGATACCATAGCAATAACAGTAGCTGGC---
TGGACAGATAGGGTTATAGAAATAGGACAAGAAATTGGTAGAGCT----------------------------
--------ATTCTCCACATA------CCTAGAAGAATCAGACAGGGCTTTGAAAGGGCTTTGCTA---TAA
>L22943
GCT---GCA---
GAAAACTTGTGGGTTACTGTCTACTATGGGGTACCTGTGTGG---AAAAACGCAGAG---
ACCACCTTATTTTGTGCATCAGATGCTAAAGCATATGAAACAGAA---AAACATAAT----------------
--------------GTCTGGGCTACA---CATGCCTGTGTACCCACAGAC---
CCCAACCCACAAGAAATACATTTGGAA---------AATGTGACAGAAGAGTTTAAC---------------
--------------------------ATGTGGAAAAATAACATGGTAGAGCAGATGCATACAGATATA---
ATCAGTCTATGGGATCAAAGCCTA---AAGCCATGTGTTAAGTTA---
ACCCCTCTCTGCGTTACCTTAAATTGTAGCAATGTCAATGTCACTGCCACGAGT------------------
----
-------------------------------GGCACAACACACGGGGGACGGGGGAGAATGAAA---
AACTGCTCTTACAATATAACCACAGAGCTAAGG--------------------------
GATAGGAGACAGAAGGTATATTCACTT---TTTTATAGACTTGATGTAGTACCAATTAATGAAAAT-------
----

AATAGCAGTAACAATGAGTATAGATTAATAAATTGTAATACCTCAGCCATTACACAA---
GCTTGCCCAAAGGTAACCTTTGAGCCAATTCCCATACATTATTGTGCCCCAGCTGGTTTTGCGATCCTAAAGT
GTAAA---GATAAGAAGTTCAAT------GGAACAGGGCCATGCACGAAT----------------------
-GTCAGC---ACAGTACAATGCACACATGGAATCAAGCCAGTAGTATCAACT---
CAACTGCTGTTAAATGGCAGTCTAGCAGAAGGAGAG-----------
GTAATGATTAGATCTGAAAATATCACAAACAAT---------GCCAAAAATATAATAGTACAATTTGCC----
-----------GAGCCTGTAAAA---------ATTAATTGTACCAGACCTAACAACAATACAAGAATG-----
-AGTATACGTATA------------GGACCAGGACAAGCA---TTC------TATGCAACAGGTGAC------
---------ATAATAGGGAACATA------------AGACAAGCACATTGT---AATGTCAGTAGAGCA---
GAATGGAATACAACTTTGCAAAAGGTAGTCACAAAATTAAGG--------------GAATACTTTGGG----
--------------------------------AACAACAAAACAATAAAATTTGCT------
AACTCCTCAGGAGGGGATCTAGAAATCACA---ACACATAGTTTTAATTGTGGAGGAGAA---
TTTAAACAGCTCCAGGCAAGAGTCCTGGCTGTGGAAAGATACCTAAGGGATCAACAGCTCCTAGGAATTTGGG
GCTGCTCTGGAAAAATCATCTGCACCACTAATGTGCCCTGGAACTCTAGTTGGAGC----------------
----------------------------AATAAATCTCAGAGTGAGATATGG------GAG------
AACATGACCTGGCTGCAATGG---GATAAAGAAATTAGCAATTACACAGACATAATATAT---
AATCTACTTGAAGAATCGCAGAACCAGCAGGAA---

AAGAATGAACAAGACTTATTGGCATTGGACAAGTGGGCAAATCTATGGAATTGGTTTGACATATCAAACTGGC
TGTGGTATATAAAAATATTTATAATGATAGTAGGAGGCTTAATAGGATTAAGAATAGTTTTTACT---
GTGCTTTCTATAATA---------AATAGAGTTAGGCAGGGATACTCACCC------
TTGTCGTTCGCTAGTAATTTGGTTGATACCATAGCAATAGTAGTAGCTGGC---
TGGACAGATAGGGTTATAGAAATAGCACAAGGAATAGGTAGAGCT---------------------------
--------ATTCTCCACATA------CCTAGAAGAATCAGACAGGGCTTTGAAAGAGCTTTGCTA---TAA
>KX168075
GCT---GCA---------------------------------------
GGAAACTTGTGGGTTACTGTCTACTATGGGGTGCCTGTGTGG---AAGGACGCAGAG---
ACCACTCTATTCTGTGCATCAGATGCTAAAGCATATGATGCAGAA---GTGCATAAT---------------
--------------ATCTGGGCTACA---CATGCCTGTGTACCTACAGAC---
CCCAACCCACAAGAAATAAATTTGGAA---------AATGTGACAGAAGAGTTTAAC---------------
------------------------ATGTGGAAAAATAACATGGTAGAACAGATGCATACAGATATA---
ATCAGTCTATGGGACCAAGGCCTA---AAGCCATGTGTAAAGTTA---
ACCCCTCTCTGTGTTACTTTAGATTGCCATAATGTCACCCATATCAATATCACCAGT---------------
----
--GACATGAAGGAAGAAATAACA---
AACTGCTCTTACAATGTGACCACAGTAATAAGG-------------------------
GATAAGAAACAGAAAGTATTCTCACTT---TTTTATAGACTTGATGTAGTACAAATTGGAGGT---------
----

AATAATAGGACTAATAGTCAGTATAGATTAATAAACTGTAACACCTCAGCCATTACACAG---
GCTTGTCCAAAGGTAACCTTTGAGCCAATTCCCATACATTATTGTGCCCCAGCTGGTTTTGCGATTCTAAAGT
GTAAG---GATGAGAAGTTCAAT------GGAACAGGGCTATGTAAAAAT----------------------
-GTCAGC---ACAGTACAATGCACACATGGAATCAAGCCAGTAGTATCAACT---
CAACTGCTGTTAAATGGCAGTCTAGCAGAAGGAAAG-----------
GTAAGAATTAGATCTGAAAATATCACAAACAAT---------GCCAAAAACATAATAGTACAACTTGCC----
-----------AGCCCTGTAACA---------ATTAATTGTATCAGACCTAACAACAATACAAGAAAA-----
-AGTGTACACCTA------------GGACCAGGACAAGCA---TTC------TATGCAACAGGTGGC------
---------ATAATAGGGAAGATA------------AGACAAGCACATTGT---AATGTTAGTAGAAAA---
GAATGGAATAACACTTTGCAAAGGGTAGCCAACCAATTAAGA---------------CCATACTTTAAG----
-----------------------------------AACAACACAATAACCTTTGCT------
AGCTCATCAGGAGGGGATCTAGAAATTACA---ACTCATAGTTTTAATTGTGGAGGAGAA---
TTTAAACAGCTCCAGGCAAGAGTCCTAGCTGTGGAAAGATACCTAAAGGATCAACAGCTCCTAGGAATTTGGG
GCTGCTCTGGAAAACTCATCTGCACCACTAATGTGCCCTGGAACTCTAGTTGGAGT----------------
---------------------------AATAAATCCCAGGATGAGATATGG------GGA------
AACATGACCTGGCTGCAATGG---GATAAAGAAGTTAGCAATTACACACAGATAATATAT---
ACTCTAATTGAAGAATCGCAAAACCAGCAAGAA---------------------------------------
----
AAAAATGAACAAGACTTATTAGCATTGGACAAGTGGGCAAGTCTGTGGAATTGGTTTAACATCTCACAATGGC
TGTGGTATATAAAGATATTTATAATAATAGTAGGAGGCTTAATAGGATTAAGAATAGTTTTTGCA---
GTGCTTTCTGTGATA---------AATAGAGTTAGGCAGGGATACTCACCT------
TTGTCATTTGCTATTAATTTGTTTGATACCATAGCAATAGCAGTAGCTGGC---
TGGACAGATAGGGTTATAGAACTAGGACAAAGACTTTGTAGAGCT---------------------------
--------ATTCTCCACATA------CCTAGAAGAATCAGACAGGGCTTTGAAAGGGCTTTGCTA---TAA
>AF004885
GCT---GTG---------------------------------------
GAAAACTTGTGGGTTACTGTCTATTATGGGGTACCTGTGTGG---AGAGATGCAGAC---
ACCACCTTATTTTGTGCATCAGATGCTAAAGCATATGAGACAGAA---AAGCATAAT---------------
--------------GTCTGGGCTACA---CATGCTTGTGTACCCACAGAC---
CCCAACCCACAAGAAATACATTTGGAC---------AATGTGACAGAAAAGTTTAAC---------------
------------------------ATGTGGAAAAATAACATGGTAGAGCAGATGCACACAGATATA---
ATCAGTCTATGGGATCAAAGCCTA---AAGCCATGTGTAAAGTTA---
ACCCCTCTCTGCGTTACTTTACATTGTACAAATGTCACAAGTGTCAAC------------------------
----
-----------------------------------ACCACTGGAGACAGGGAAGGCTTAAAA---
AACTGCTCTTTCAATATGACCACAGAACTAAGG-------------------------
GATAAGAGACAGAAAGTATATTCACTT---TTTTATAGACTTGATATAGTACCAATTAATGAA---------
----

AATCAGGGTAGTGAATATAGATTAATAAATTGTAATACCTCAGCCATTACACAG---
GCGTGCCCAAAGGTATCCTTTGAGCCAATTCCTATACATTATTGTACCCCAGCTGGTTTTGCGATCCTAAAGT
GTAAG---GATGAGGGGTTCAAT------GGGACAGGGTTATGCAAGAAT----------------------
-GTCAGC---ACAGTCCAATGCACACATGGAATCAAGCCAGTAGTATCAACT---
CAATTGCTGTTGAATGGCAGTCTAGCAGAAAAAAAT-----------
ATAACAATTAGATCTGAAAATATCACAAACAAT---------GCCAAAATTATAATAGTACAACTTGTC----
-----------CAACCTGTGACA---------ATTAAGTGTATCAGACCTAACAACAATACAAGAAAA-----
-AGTATACGTATA------------GGACCAGGACAAGCA---TTC------TATGCAACAGGTGAC------
---------ATAATAGGGGATATA------------AGACAAGCACATTGT---AATGTCACTAGATCA---
AGATGGAATAAGACTTTACAAGAGGTAGCTGAAAAATTAAGA--------------ACATACTTTGGG----
----------------------------------AACAAAACAATAATATTTGCT------
AACTCCTCAGGAGGGGATCTAGAGATCACA---ACACATAGTTTTAATTGTGGAGGAGAA---
TTTAAACAGCTCCAGGCAAGAGTCCTGGCTGTGGAAAGATACCTAAGGGATCAACAGCTCCTAGGAATTTGGG
GCTGCTCTGGAAAACTCATCTGCACCACTAATGTGCCCTGGAACTCTAGTTGGAGT-----------------
----------------------------AATAAATCTCTGGATGAGATATGG------AAC------
AACATGACCTGGCTGCAATGG---GATAAAGAAATTAACAATTACACACAACTAATATAT---
AGACTAATTGAAGAATCGCAGAACCAGCAGGAA---------------------------------------
----
AAGAATGAAAAGGAATTATTGGAGTTGGACAAGTGGGCAAATCTGTGGAGTTGGTTTGACATATCAAATTGGC
TGTGGTATATAAAAATATTTATAATAATAGTAGGAGGCTTAATAGGATTAAGAATAGTTTTTGCT---
GTGCTTTCTGTAATA---------AATAGAGTTAGGCAGGGATACTCACCT------
TTGTCATTTGCTATTAATCTAGTTGATACCATAGCAATAGCAGTAGCTGGC---
TGGACAGATAGGGTTATAGAAATAGCACAAAGAATTGGTAGAGCT----------------------------
--------ATCCTCCACATA------CCTGTAAGAATCAGACAGGGCTTAGAAAGGGCTTTGCTA---TAA
>AF407151
GCT---GCA---
GAAAACTTGTGGGTTACTGTCTACTATGGGGTACCTGTGTGG---AAAGACGCAGAG---
ACCACCCTATTTTGTGCATCAGATGCTAAAACATATGATACAGAG---GTGCATAAT----------------
--------------ATCTGGGCTACA---CATGCCTGTGTACCTACAGAC---
CCCAACCCACAAGAAATAAATTTGGGA---------AATGTGACAGAAGAGTTTAAC---------------
-------------------------ATGTGGAAAAATAACATGGTAGAGCAGATGCATACAGATATA---
ATCAGTCTATGGGACCAAGGCCTA---AAACCATGTGTAAAGTTA---
ACTCCTCTCTGCGTTACTTTAGATTGCTATGATGTCACTAATTTCACC------------------------
----
--AATGACATGCAAGAAGGAATAAAA---
AACTGCTCTTTCAATATAACCACAGAACTAAGG--------------------------
GATAAGAAACAGAAAGTACATTCACTT---TTTTATAGGCTTGATGTAGTACAAATTGGGGAT----------
----

AATAGTAGTAGTGGTCAGTATAGATTGATAAATTGTAACACCTCAGCCATTACCCAG---
GCTTGTCCAAAAGTAACCTTTGAGCCAATTCCTATACATTATTGTGCCCCAGCTGGTTTTGCGATTCTAAAGT
GTAAT---GATAAGGAGTTCAGT------GGAACAGGGCCATGCAAGAAT----------------------
-GTTAGC---TCAGTACAATGCACACATGGAATCAAGCCAGTAGTATCAACT---
CAACTGCTGTTAAATGGCAGTCTAGCAGAAGAAAAA-----------
GTAAGAATTAGATCTGAGAATATCACAGACAAT---------GGCAAAAACATAATAGTACAACTTAAA----
-----------ACACCTGTAAAC---------ATTAGTTGTACCAGACCTAACAACAATACAAGAAAA-----
-AGTATACGTATA------------GGACCAGGACAAGCA---TTC------TATGCAACAGATGAC------
---------ATAATAGGGAATATA------------AGACAAGCATATTGT---ACTGTTAATAGAACA---
CAATGGGATGACACTTTGCAAGAGGTAGCCAACCAATTAAGA--------------ATATACTTT-------
----------------------------------AACAAAACAATAATTTTTAAT------
AACTCAGCAGGAGGGGATCTAGAAATTACA---ACACATAGTTTTAATTGTGGAGGAGAA---
TTTAAACAGCTCCAGGCAAGAGTCCTGGCTGTGGAAAGATACCTAGAGGATCAACAGCTCCTAGGAATTTGGG
GCTGCTCTGGAAAACTCATCTGCACCACTAATGTGCCCTGGAACTCTAGTTGGAGT-----------------
----------------------------AATAAATCCCAGAGTGAGATATGG------GAA------
AACATGACCTGGCTACAATGG---GATAAAGAAATTAACAATTACACACAACTAATATAT---
AGTCTAATTGAAAAATCGCAGACCCAGCAGGAA---------------------------------------
----
ATAAATGAACAAGAATTATTAGCATTGGACAAGTGGGCAAATCTGTGGAATTGGTTTGACATATCAAACTGGC
TGTGGTATATAAGGATATTTATAATAATAGTAGGAGGCTTAATAGGATTAAGAATAGTTTTTGCA---
GTGCTTTCTGTAATA---------AATAGAGTTAGGCAGGGATACTCACCT------

CTATCATTTGCTATTAATTTGCTTGATACCATAGCAATAGCAACAGCTAAC---
TGGACAGACAGGGTTATAGAACTAGGACAAAGACTTTGTAGAGCT----------------------------
--------ATTTACACATA------CCTAGAAGAATCAGACAGGGCTTCGAAAGGGCTTTGCTA---TA-
>AF407162
GCT---GTA--
GACAACTTGTGGGTTACTGTCTACTATGGGGTACCTGTGTGG---AAAGAGGCAGAA---
ACCACCTTATTTTGTGCATCAGATGCTAAAGCATATGAGACAGAA---AAACATAAT----------------
--------------GTCTGGGCTACA---CATGCCTGTGTACCCACAGAC---
CCCAACCCACAAGAAATACATTTGGAA---------AATGTGACAGAAGAGTTTAAC----------------
--------------------------ATGTGGAAAAATAACATGGTAGAGCAGATGCATACAGATATA---
ATCAGTCTATGGGACCAAAGCCTA---AAGCCATGTGTAAAGTTA---
ACCCCTCTCTGTGTTACTTTAGATTGTAACAATGTCACCAATAATGGC-------------------------
----
-------------------------------------ACCAGTGACATGAGAGAAGAAATAAAA---
AACTGCTCTTTCAATATGACCACAGAACTAAGG---------------------------
GATAAGAGACAGAAAGTATATTCACTT---TTTTATAAACTTGATATAGTACAAATTAATGAAAATCAG----
----

GGTAATAGTAGTAACAATAAGTATAGATTAATAACTTGTAATACCTCAGCCATTACACAA---
GCATGCCCAAAGGTAACCTTTGAGCCAATTCCCATACATTATTGTGCTCCAGCTGGTTTTGCGATCCTAAAGT
GTAAG---GATGAGGAGTTCAAT------GGAACAGGGCCATGCAAGAAT-----------------------
-GTCAGC---ACAGTCCAATGCACACATGGAATCAAGCCAGTAGTATCAACT---
CAACTACTGTTAAATGGCAGTCTAGCAGAAAAAGAG------------
GTAAAAATTAGATGTGAAAATATCACAAACAAT---------GCCAAAACTATAATAGTACAACTTGTC----
-----------AATCCTGTGAAA---------ATTAATTGTACCAGACTTAACAACAATACAAGAAAA-----
-AGTATACATATA------------GGACCAGGACAAGCA---TTC------TATACAACAGGTGAC------
---------ATAATAGGGGATATA------------AGACAAGCACATTGT---AATGTCAACAGAACA---
GAATGGAACAACACTTTGCACCAGGTAGTCGAACAATTAAGA---------------AAACACTTTAAGCAA-
-----------------------------TTTAAGAACAAAACAATAAGCTTTGCT------
AACTCCACAGGAGGGGATCTAGAAATAACA---ACACATAGTTTTAATTGTGGAGGAGAA---
TTTAAACAGCTCCAGGCAAGAGTCCTGGCTGTGGAAAGATACCTAAAAGATCAACAGCTCCTAGGAATTTGGG
GCTGCTCTGGAAAACTCATCTGCACCACTAGTGTGCCCTGGAATTCTAGTTGGAGT-----------------
----------------------------AATAAATCCCAGAATGAGATATGG------GAC------
AACATGACCTGGCTGCAATGG---GATAAAGAAATTAGCAATTACACACAGATAATATAT---
GATCTACTTGAAGAATCGCAGAACCAGCAGGAA--

AAGAATGAACAAGACTTATTGGCATTGGACAAGTGGGCAAATCTGTGGAATTGGTTTGACATATCAAACTGGC
TGTGGTATATAAAAATATTTATAATGATAGTAGGAGGTTTAATAGGATTAAGAATAGTTTTTGCT---
GTGCTTTCTGTAATA---------AATAGAGTTAGGCAGGGATACTCACCT------
TTGTCGTTCGCTACTAATTTGCTTGATATCGTAGCAATAGTAATAGCTGGG---
TGGACAGGTAGGGTTATAGAAATAGGACAGAGACTTTGTAGAGCT----------------------------
--------TTTCTCAACATA------CCTAGAAGAATCAGACAGGGCTTCGAAAGGGCTTTGCTA---TAA
>AF407156
GTT---ACA--GGACAG---
TGGGTCACAGTATACTATGGAGTACCTGTATGG---AAAGATGCAGAG---
ACCACCCTATTTTGTGCATCAGATGCTAAAGCATATGAGACAGAA---AAGCATAAT----------------
--------------GTCTGGGCTACA---CATGCCTGTGTACCCACAGAC---
CCCAACCCACAAGAAATACGTTTGGAA---------AATGTAACAGAAGATTTTAAC----------------
--------------------------ATGTGGAAAAATAGCATGGTGGAGCAGATGAATGAGGATATA---
ATTAGTCTATGGGATCAAAGCCTA---AAACCATGTGTAAAATTA---
ACCCCACTCTGTGTCACTCTAAACTGTACTGACTGGACCAATAATGCCACTAGTACGAAC-------------
----
-------------------------CAAACCACTCCTGCCACTAGTGAGGAGACAGGAGTGAAA---
AACTGCTCTTTCAATATAACCACAGAACTAAGA---------------------------
GATAAGAAACAGAAAGTCTATTCACTG---TTTTATAAACTTGATGTAGTACAAATTAGTGAAAATAATAGT-
----

AGTAACAGTAGTAATTTTACTCAGTATAGACTAATAAATTGTAATACCTCAGCCATCACACAG---
GCTTGCCCAAAGGTATCCTTTGAGCCAATTCCTATACATTATTGTGCCCCAGCTGGATTTGCGATTCTAAAAT
GTAAT---GATAGTGTATTCAAT------GGAACAGGGCCATGCAAGAAT-----------------------

```
-GTCAGC---ACAGTACAATGCACACATGGAATCAAGCCAGTAGTATCAACT---
CAACTGNTGTTAAATGGCAGTTTAGCAGAAAGAAAG------------
GTAATGATTAGATCTGAAAATATTACAAACAAT---------GCCAAAAACATAATAGTACAATTTACC----
-----------AAGCCTGTGAAT---------ATTACTTGTATCAGACCTGGCAACAATACAAGAAAA-----
-AGTGTACGCATA------------GGACCAGGACAAGCA---TTC------TATGCAACAGGTGAC------
---------ATAACAGGGGACATA------------AGAAACGCACATTGT---GTTGTCAATAGAACA---
GAGTGGAATAACACTTTACAAAAGGTAGTTGAACAATTAAGA---------------GAATACTTCCCT----
-----------------------------------AATAAAACAATAATCTTTACT------
AACTCCTCAGGGGGGGACATAGAAATTACA---ACACATAGTTTTAATTGTGGAGGAGAA---
TTTAAACAGCTCCAGGCACGAGTCCTTGCTGTGGAAAGATACCTAAAGGATCAACAGCTCCTGGGAATTTGGG
GCTGCTCTGGAAAACTCATCTGCACCACTTCTGTGCCTTGGAACTCTAGTTGGAGT-----------------
----------------------------AATAAAACTCAGCAGGAGATTTGG------AAC------
AACACGACCTGGTTACAATGG---GATAAAGAAATTAGCAATTACACAGGCACAATATAC---
AGGCTACTTGAAGAATCGCAGAACCAGCAGGAA----------------------------------------
-----------------------------
AAGAATGAACAAGACTTATTGGCATTAGATAAGTGGGCAAATCTGTGGAATTGGTTTGACATATCAAAATGGC
TGTGGTATATAAAAATATTTATCATGGTAGTAGGAGGCTTAATAGGATTAAGAATAGTGTTTGCT---
ATAATTTCTGTAGTG---------AATAGAGTTAGGCAGGGATACTCACCT------
TTGTCATTTGCTACTAATTTGCTTGATACCACAGCAATAGCAGTAGCTGGC---
TGGACAGATAGGGCTATAGAAATAGTACAAAGAATTGTTAGAGCT----------------------------
--------ATTCTCCACATA------CCTAGAAGAATAAGACAGGGATTCGAAAGGGCTTTGCTA---TAA
>KT008652
GCT---GCA------------------------------------------
GACAACTTGTGGGTTACTGTCTATTATGGGGTACCTGTGTGG---AGAGATGCAGAG---
ACCACCTTATTTTGTGCATCAGATGCTAAAGCATATAAGACAGAA---ATGCATAAT----------------
--------------GTCTGGCTACA---CATGCCTGTGTACCCACAGAC---
CCCAACCCACAAGAAATACATTTGGAA---------AACGTGACAGAAGAGTTTAAC----------------
--------------------------ATGTGGAAAAATAACATGGTAGAGCAGATGCATACAGATATA---
ATCAGTCTATGGGACCAAAGCCTA---AAACCATGTGTAAAGCTA---
ACCCCTCTCTGCGTTACTTTAAATTGTGCCAATGTCAATGTCACGGGCGCCTCC-------------------
-------------------------------------------------------------------------
----------------------------AGGAATAACTCCCAGGGGGACGGGGGAGAAATGAAA---
AACTGCTCTTTCAATATGACCACAGAGCTAAGG---------------------------
GATAAGAGACAGAAGGTATATTCACTT---TTTTATAGACTTGATATGGTACAAATTAATGGAAAT-------
-------------------------------------------------------------------------
-------
CAAGATAATAGTAGTAATAGTAGTGAGTATAGATTAATAAATTGTAATACCTCAGCCATTACACAA---
GCCTGCCCAAAGGTATCCTTTGAACCAATTCCCATACATTATTGTGCCCCAGCTGGTTTTGCGATCCTAAAGT
GTAAG---GATGAAGAGTTCAAT------GGAACAGGGCCATGCAAGAAT-----------------------
-GTCAGC---ACAGTACAATGCACACATGGAATCAAGCCAGTAGTATCAACT---
CAGCTGCTATTAAATGGCAGTTTAGCAGAAAAAGAG------------
GTAAAAATTAGATCTGAAAATATCACAAACAAT---------GCCAAAAATATAATAGTACAGCTTAAC----
-----------GAGTCTGTGATA---------ATTAATTGTACCAGACCTAACAACAATACAAGAACA-----
-AGTATACGTATA------------GGACCAGGACAAGCA---TTT------TATGCAACAGGTGGC------
---------ATAATAGGGGATATA------------AGGCAAGCACATTGT---AATGTCAGTAAATCA---
AAATGGAATGAAACTTTACAAAAGGTAGTTACCCAATTAAAG---------------AAATACTTTGGG----
-----------------------------------AACAAAACAATAATATTTAAG------
AGCTCCTCAGGAGGGGATCTAGAAATCACA---ACACATAGTTTTAATTGTGGAGGAGAA---
TTTAAACAGCTCCAGGCAAGAGTCCTGGCTGTGGAAAGATACCTAAGGGATCAACAGCTCCTAGGAATTTGGG
GCTGCTCTGGAAAACTCATCTGCACCACTACGGTGCCCTGGAACTCTAGTTGGAGT-----------------
----------------------------AATAAATCTCTGAGTGACATATGG------GAC------
AACATGACCTGGCTGCAATGG---GATAAAGAAATTAGCAATTATACACAAATAATATAT---
GACCTAATTGAAGAAGCGCAAAACCAGCAGGAA----------------------------------------
-----------------------------
AAGAATGAACAAGACTTATTGGCATTGGACAAGTGGGCAAATCTGTGGACTTGGTTTGACATATCAAACTGGC
TGTGGTATATAAAAATATTTATAATGATAGTAGGAGGCTTAATAGGATTAAGAATAGTTTTTGCT---
GTGCTTTCAGTAATA---------AATAGAGTTAGGCAGGGGTACTCACCT------
TTGTCGTTCGCTATTAGTTTGATCGATACTATAGCAATAGTAGTAGCTGGC---
TGGACAGATAGGATTATAGAAATAGGACAAGCAGTTGGTAGAGCT----------------------------
--------ATTCTCCACATA------CCTAGAAGAATCAGACAGGGCTTCGAAAGAGCTTTGCTA---TAA
```

>AF407157
AGT---GCT---GCA------------------------------------
GGAAACTTGTGGGTTACTGTCTACTATGGGGTACCTGTGTGG---AGAGACGCAGAG---
ACCACCTTATTTTGTGCATCAGATGCGAAAGCATATGATAGAGAA---GCGCATAAT----------------
--------------GTCTGGGCTACA---CATGCCTGTGTACCTACAGAC---
CCTAGCCCACAAGAAGTACCTTTGGGA---------AATGTGACAGAAGAGTTTAAC----------------
--------------------------ATGTGGAAAAATAACATGGTAGAGCAGATGCATACAGATATA---
ATCAGTCTATGGGACCAAAGCCTA---CAGCCATGTGTAAAGTTA---
ACCCCTCTCTGTGTTACTTTAAATTGTAGTAATATTAACAATATCCCCAATGTTAATGCT------------
----
----------------------------AGCAGTATCCCCAAGGACGTGAAAGAAGAAATAAAA---
AACTGCTCTTTCAATATGACCACAGAATTAAAG--------------------------
GATAAGAAACAGAATGTATATTCACTT---TTTTATAGACTGGATGTAGTACCACTTGAGACCAACCTA----
----

AAACAGAATAGCAGCCACAGTCGGTATAGATTAATAAATTGTAATACCTCAGCCATTACACAA---
GCTTGTCCAAAGGTATCCTTTGAACCAATTCCCATACATTATTGTGCTCCAGCTGGTTTTGCGATTCTAAAGT
GTAAT---GATAAGGAGTTCAAT------GGAACAGGGCTATGCAAAAAT----------------------
-GTCAGC---ACAGTACAATGCACACATGGAATCAAGCCAGTAGTGTCAACT---
CAACTACTGTTAAATGGCAGTCTAGCAGAAGGAAAG------------
GTAATGGTCAGATCTGAAAATATCACAAACAAT---------GCCAAGAACATAATAGTACAATTTAAC----
-----------AATTCTGTACAA---------ATTAATTGTATCAGACCTGGCAACAATACAAGAAAA-----
-AGTATACATTTA------------GGACCAGGAAAAGTA---TTC------TATGCAACAAAT---------
---------ATAATAGGGGATATA------------AGAAAAGCACATTGT---AATGTCAATAGACAA---
CAATGGAATAAAGCTTTGCAAGATGTAGCCACACAATTAAGA---------------ACACACTTTAGA----
-----------------------------------AACAGAACAATAATCTTTAAT------
AATTCCTTAGGAGGAGATCTAGAAATAACA---ACACATAGTTTTAATTGTAGAGGAGAA---
TTTAAACAGCTCCAGGCAAGAGTCCTGGCTGTGGAAAGATACCTAAAGGATCAACAGCTCCTAGGAATCTGGG
GCTGCTCTGGAAAACTCATCTGCACCACTACTGTGCCCTGGAACCCTAGTTGGAGT-----------------
----------------------------AATAAATCCCAAAGTGAGATATGG------GAC------
AATATGACCTGGATGCAATGG---GATAAAGAAATTAACAATTACACACAGATAATATAT---
GATCTAATTGAAGAATCGCAGAGACAGCAGGAA---

AAGAATGAACAAGACTTATTGGCATTGGACAAGTGGGCAAATCTGTGGAATTGGTTTGACATATCAAATTGGC
TGTGGTATATAAAAATATTCATAATGATAGTAGGAGGCCTAATAGGATTAAGAATAGCTTTTGCT---
GTGCTTTCTGTAATA---------AATAGAGTTAGGCAGGGATACTCACCT------
CTCTCGTTCGCTATTAATTTGCTTGATACCATAGCGATAGCAGTAGCTGAA---
TGGACAGATAGGATTATAGAAATAGGACAAAGACTTTGTAGAGCT----------------------------
--------ATTATCAACATA------CCTAGAAGAATCAGACAGGGCTTCGAAAGGGCTTTGCTA---TAA
>FJ866112
GCT---GCA---
AACAACTTGTGGGTTACTGTCTACTATGGGGTACCTGTGTGG---AAAGACGCAGAG---
ACCACCTTATTTTGTGCATCAGATGCGAAAGCATATGATACGGAA---GTGCATAAT----------------
--------------GTCTGGGCTACA---CATGCCTGTGTGCCTACAGAC---
CCCAACCCACAAGAAATAAATTTGGAA---------AATGTGACAGAAGAGTTTAAC----------------
--------------------------ATGTGGAAAAATGGCATGGTAGAGCAGATGCATACAGATATA---
ATCGGTCTATGGGACCAAAGCCTA---AAGCCATGTGTAAAGTTA---
ACCCCTCTCTGCGTTACTTTAGATTGTAGTAGCTATAATGTCACCACCACCAAT------------------
----
----------------------------------TTCACCAATGAAATGCCAGGAGAAATACAA---
AACTGCTCTTTCAATATGACCACAGAATTAAGG--------------------------
GATAAGAAACAGAAAGTATATTCACTT---TTTTATAGATCAGATGTAGTACCAATCAACAGC----------
----

AGCAGTAGCAGTAGTCAGTATAGACTAATAAATTGTAATACCTCAGCCATTACACAA---
GCTTGTCCAAAGGTAACCTTTGAGCCAATTCCCATACATTATTGTGCACCAGCTGGTTTTGCGATTCTAAAGT
GTAAG---GATAAAGAGTTCAGT------GGAACAGGGTCGTGCAAGAAT----------------------
-GTCAGC---ACAGTACAATGCACACATGGAATCAAGCCAGTAGTATCAACT---
CAACTGCTGTTAAATGGCAGTCTAGCAGAAGGAAAG------------
GTAATGATTAGATCTGAAAATATCACAAACAAT---------GCCAAAAACATAATAGTACAATTTAAC----

-----------AAGCCTGTAGAA---------ATTAACTGTACCAGACCTAACAACAATACAAGAAAA-----
-AGTGTACGTATA------------GGACCAGGACAAGCA---TTC------TATGCAACAGGTGAC------
---------ATAATAGGGGATATA------------AGGAGAGCATATTGT---AATGTCAGTAGAACA---
AAATGGAATCAAACTTTGCGAGAGGTAGTCAGCCAATTAAGA---------------ACACAATTTGAG----
-----------------------------------AACAAAACAATAGTCTTTGAT------
AAACCCATAGGAGGGGATCTAGAAATAACA---ACACATAGTTTTAATTGTGGGGGAGAA---
TTTAAACAGCTCCAGGCACGAGTCCTGGCTCTGGAAAGATACCTAAAGGATCAACAGCTCCTAGGAATTTGGG
GCTGCTCTGGAAAACTCATCTGCACCACTAATGTGCCCTGGAACTCTAGTTGGAGT-----------------
----------------------------AATAAATCCCAGAGTGAGATATGG------GAA------
AACACGACCTGGATGCAATGG---GATAAAGAAATTAGTAATTACACACAGATAATATAT---
GGTCTAATTGAAGAATCGCAGAACCAGCAGGAA--

AAGAATGAACAAGACTTATTGGCATTGGACAAGTGGGCAAATCTGTGGAATTGGTTTGACATATCAAAATGGC
TGTGGTATATAAAAATATTTATCATGATAGTAGGAGGCTTAATAGGATTAAGAATAGTTTTTGCT---
GTGCTTTCTATAATA---------AATAGAGTTAGGCAGGATACTCACCT------
TTGTCGTTCGCTATTAATTTGCTTGATACCATAGCAATAGTAGTAGCTGGG---
TGGACAGATAGGGTTATAGAACTAGGACAAAGGCTTTGTAGAGCT----------------------------
--------ATTCTCCATATA------CCTAGAAGAATCAGACAGGGCGCCGAAAGGGCTTTGCTA---TAA
>AY322190
ACT---GCA---GAA------------------------AAC---TTA---
GAAAAACGGTGGGTTACTGTCTACTATGGGGTACCTGTGTGG---AAAGATGCAGAT---
ACCACCTTATTTTGTGCATCAGATGCTAAAGCATATGAGACAGAA---AAGCATAAT----------------
--------------GTCTGGGCTACA---CATGCCTGCGTACCCACAGAC---
CCCAACCCACAAGAAATACATTTGGAA---------AATGTGACAGAAGAGTTTAAC----------------
--------------------------ATGTGGAAAAATAACATGGTAGAGCAGATGCATACAGATATA---
ATCAGTCTATGGGACCAAAACCTA---AAACCATGTGTAAAGTTA---
ACCCCTCTCTGTGTTACTTTAAATTGTAGCCAGGTCACCAATGCTAGCATCACCACCAAT-------------
----
-------------------------GGTAGTAGGTTCCATGAGAACATGAAAGGAGAAATACAA---
AACTGCTCTTACAATGTGACCACAGAATTAAGG---------------------------
GATAAGAGAAAGAAAGTATATTCACTT---TTTTATAGCCTTGATGTAGTAGAAATTGATAAAGACAAG----
----

AATAATAGTAGGAATAGTAGTCAGTATAGATTAATAAATTGTAATACCTCAGCCATCACACAA---
GCTTGTCCAAAGGTATCCTTTGAGCCAATTCCCATACATTATTGTGCCCCAGCTGGTTTTGCGATCCTAAAGT
GTAAT---GATGAGAATTTCAAT------GGAATAGGGCTATGCAAGAAT-----------------------
-GTCAGC---ACAGTACAATGCACACATGGAATCAAGCCAGTAGTATCAACT---
CAACTGCTGTTAAATGGCAGTCTAGCAGAAAACGAG------------
GTAAAAATTAGATCTGAAAATATTACAAACAAT---------GCCAAAAACATAATAGTACAATTTGCC----
-----------AGGCCTGTAACA---------ATTAATTGTACCAGACCTAATGACAATATAAGAAAA-----
-AGTGTACATATA------------GGACCAGGACAAGCA---TTC------TATGCAACAGGGGAC------
---------ATAATAGGGGATATA------------AGACGAGCACATTGT---AATGTCAGTAGAGTA---
GACTGGTATAAAACCTTACAACAGGTAGCTACACAATTAGGG---------------AAACACTTTGAG----
-----------------------------------AACAAAACAATAACTTTTACT------
AACTCCTCAGGAGGGGATCTAGAAATTACA---ACACATAGTTTTAATTGTGGAGGAGAA---
TTTAAACAGCTCCAGGCGAGAGTCCTGGCTCTGGAAAGATACCTAAGGGATCAACAGCTCCTAGGACTTTGGG
GCTGCTCTGGAAAACTCATCTGCACCACTAATGTGCTCTGGAACAATAGTTGGAGT-----------------
----------------------------AATAAAACTCAGGATGAAATATGG------GAC------
AACATGACCTGGCTACAATGG---GATAAAGAAATTAGCAATTACACACAAGTAATATAT---
ACCCTACTTGAAGACTCGCAGAACCAGCAGGAA--

AAGAATGAACAAGACTTATTGGCATTGGACAAGTGGGCAAATCTATGGAATTGGTTTGACATATCAAATTGGC
TGTGGTATATAAAAATATTTATAATGATAGTAGGAGGCTTAATAGGATTAAGAATAGTTTTTGCA---
GTGCTTTCTGTAATC---------AATAGAGTTAGGCAGGATACTCACCT------
TTATCGTTTGCTATTAATTTGCTTGATACCATAGCAATAGCAGTAGCTGGC---
TGGACAGATAGGGTTATAGAAATAGGACAAAGACTTGGCAGAGCT----------------------------
--------ATTCTCCACATA------CCTAGAAGAATCAGACAGGGCTTTGAAAGAGCTTTGCTA---TAA
>AY322193
ACT---GCA--
GAAAACTTGTGGGTTACTGTTTACTATGGGGTGCCTGTGTGG---AAAGACGCAGAG---

ACCACCCTATTTTGTGCATCAGATGCTAAAGCATATGATACAGAA---GTGCATAAT----------------
--------------ATCTGGGCCACA---CATGCTTGTGTACCTACAGAC---
CCCGACCCACAAGAAATAAATTTGGAA---------AATGTGACAGAAGAGTTTAAC----------------
-------------------------ATGTGGAAAAATAACATGGTAGAGCAGATGCATACAGATATA---
ATCAGTCTATGGGACCAAGGCCTA---AAGCCATGTGTAAAGTTA---
ACCCCTCTCTGCGTTACTTTAGATTGTAGCTATAATGTCACTACCAATGTC----------------------
----
-------------------------------------TCCAATGACATGCAAGGAGAAATAAAA---
AACTGCTCTTTCAATGTGACCACAGTATTAAGG--------------------------
GATAGGAAACAGCAAGTATATTCACTT---TTTTATAGACTTGATGTAGTACCAATGAGTAAT----------
----

GATAGTCAGTACAGATTAATAAATTGTAACACCTCAGCCATTACACAG---
GCTTGTCCAAAGGTAACCTTTGAGCCAATTCCCATACATTATTGTGCCCCAGCTGGTTTTGCGATCCTAAAGT
GTAAG---GATAAGGATTTCAAT------GGAGCAGGGCTATGCAAGAAT-----------------------
-GTCAGC---ACAGTACAATGCACACATGGAATCAAGCCAGTAGTATCAACT---
CAACTGCTGTTAAATGGCAGTCTAGCAGAAGGAAAT------------
ATAACAATTAGATCTGAAAATATCACAGACAAT---------GGCAAAAACATTATAGTACAATTTACC----
-----------AGCCCTGTAAAA---------ATTAATTGTACCAGACCTAACAACAATACAAGGAAA-----
-AGTGTACATATA------------GGACCAGGACAAGCA---TTC------TATGCAACAGGTGAC------
---------ATAATAGGGAATATA------------AGAAAAGCACATTGT---GATGTTAATAGAACA---
GAATGGAATAAAACTTTGCAAGAGGTAGCCAAACAATTAGCA---------------ACATACTTTGAG----
-----------------------------------AACAAAACAATAATCTTTACT------
AACTCCTCAGGAGGGGATCTAGAAGTTACA---ACTCATAGTTTTAATTGTGGAGGAGAA---
TTTAAACAGCTCCAGGCAAGAGTCCTGGCTGTGGAAAGATACCTAAAGGATCAACAGCTCCTAGGAATTTGGG
GCTGCTCTGGAAAACTCATCTGCACCACTAATGTCCCTTGGAACTCTAGTTGGAGT-----------------
----------------------------AATAAATCCCTGACTGAGATATGG------GAC------
AATATGACCTGGCTACAATGG---GATAAAGAAATTAGCAATTACACACAGCAAATATAT---
GATCTAATTGAAAAATCGCAGAACCAGCAGGAA---

AAAAATGAACAAGACTTATTAGCATTGGACAAGTGGACAAATCTGTGGAATTGGTTTGACATATCAAACTGGC
TGTGGTATATAAAGATATTTATAATAATAGTAGGAGGCTTAATAGGATTAAGAATAGTTTTTGCA---
GTGCTTTCTGTAATA---------AATAGAGTTAGGCAGGGATACTCACCT------
TTGTCATTCGCTATTAGTTTGCTTGATACCATAGCAATAGAAGTAGCTGGC---
TGGACAGATAGGGTTATAGAACTAGGACAAGGACTTTGTAGAGCT----------------------------
--------ATTTTACACATA------CCTAGAAGAATCAGACAGGGCTTAGAAAGGGCTTTGCTA---TAA
>FJ396018
GCT---GCA---
CAAAACTTGTGGGTTACTGTCTACTATGGGGTACCTGTGTGG---CATGACGCAGAT---
ACCACCTTATTTTGTGCATCAGATGCTAAAGCATATGAGACAGAA---AAGCATAAT----------------
--------------GTCTGGGCTACA---CATGCCTGTGTACCCACAGAC---
CCCAACCCACAAGAAATACATTTGGAA---------AATGTGACAGAAAAATTTAAC----------------
-------------------------ATGTGGAAAAATAACATGGTAGAGCAGATGCATACAGATATA---
ATCAGTTTATGGGACCAAAGCCTA---AAGCCATGTGTAAAGTTA---
ACCCCTCTTTGTGTTACTTTACACTGTACCAACGTCAGCAACAATGTCAGCATCAGCAACACG----------
----
----------------------AACATGACAGAAGAAATAAAAAACATGAAAGAAGAAATAAAA---
AACTGCTCTTTCAATATGACAACAGAACTAAGG--------------------------
GATAAGAAACAGGAAGTATATTCACTT---TTTTATAGACTTGATATAGTACAAATTAAGGAAAGTAAT----
----

AATAGTACTAATACCAGTGAGTATAGATTAATAAATTGTAATACCTCAGCCATTACACAG---
GCTTGTCCAAAGGTATCCTTTGAGCCAATTCCCATACATTATTGTGCCCCAGCTGGTTTTGCGATACTAAAGT
GTAAG---GATAAGGAGTTCAAT------GGAACAGGGCTATGTAAGAAT-----------------------
-GTCAGC---ACAGTACAATGCACACATGGAATCAAGCCAGTAGTATCAACT---
CAGTTGCTGTTAAATGGCAGTCTAGCAGAAGAAAAG------------
ATAATGATTAGATCTGAAAATATCACAGACAAT---------GCCAAAAACATAATAGTACAATTTACC----
-----------CAGCCTGTAAAA---------ATTAATTGTACCAGACCTAACAACAATACAAGAACA-----
-AGTATACGTATA------------GGACCAGGACAAACA---TTC------TATGCAACAGGTGAA------
---------ATAATAGGGGATATA------------AGAAAAGCACATTGT---AATGTCAGTAGAGCA---

GAATGGAATAGCACTTTGCAAAATGTAACTGAACAATTAAGA---------------ACATACTTTAAG----
-----------------------------------AACAAAACAATAATATTTAAT------
AGCTCCTCAGGAGGGGATCCAGAAATCACG---ATGCATAGTTTTAATTGTGGAGGAGAA---
TTTAAACAGCTCCAGGCAAGAGTCCTGGCTGTGGAAAGATACCTAAAGGATCAACAGCTCCTAGGAATTTGGG
GCTGCTCTGGAAAGCTCATCTGCACCACTAATGTGCCTTGGAACTCTAGTTGGAGT-----------------
----------------------------AATAAATCACAGAGTGAGATATGG------GAC------
AACATGACCTGGCTGCAATGG---GATAAAGAAATTAGCAATTACACACAACTAATATAT---
ACCCTAATTGAAGAATCGCAGAACCAGCAGGAA--

AAGAATGAACAAGACTTGTTGGCATTGGACAAGTGGGCAAATCTGTGGACTTGGTTTGACATATCAAATTGGC
TGTGGTATATAAAAATATTTATAATGACAGTAGGAGGCTTAATAGGATTAAGAATAGTTTTTGCT---
GTGCTTTCAGTAATA---------AATAGAGTTAGGCAGGGATACTCACCT------
TTGTCGTTTGCTATTAATTTGATTAATACCATAGCAATAGTAGTAGCTGGC---
TGGACAGATAGGATTATAGAAATAGGACAAGGAATTGCTAGAGCT----------------------------
--------ATTCTCCACATA------CCTAGAAGAATCAGACAGGGCTTTGAAAGAGCTTTGCTA---TAA
>AY736811
AAT---GCA--
GAAAATTTGTGGGTTACTGTCTATTATGGGGTACCTGTGTGG---AAAGATGCAGAC---
ACCACCTTATTTTGTGCATCAGATGCTAAAGCATATAAGCGAGAA---GTGCATAAT----------------
--------------GTCTGGGCTACA---CATGCCTGTGTGCCCACAGAC---
CCCAACCCACAAGAAATAGATTTGGAA---------AATGTGACAGAAGACTTTAAC----------------
--------------------------ATGTGGAAAAATAAAATGGTAGAGCAGATGCATGAAGACATA---
ATTAGTCTATGGGACCAAAGCCTA---AAGCCATGTGTAAAGTTA---
ACCCCTCTCTGCGTTACTTTAAACTGTAGCGATGTCGTCAATGGCACT-------------------------
----
-------------------------------------GAYGACAACATGAAAGGAGAAATGAGA---
AACTGCTCTTACAATATGACCACAGAACTAAGG--------------------------
GATAAGAAAAAGAAAGTATACTCACTA---
TTTTATAGACTAGATGTAGTGCAAATTAATAATGGTAATAGTAGT----------------------------
----
AATGGTAATAGTAGCAGTGGTAATAGTAGCAGTAGTGAGTATAGATTAATAAATTGTAATACCTCAGCCATTA
CACAA---
GCTTGTCCAAAAGTAACCTTTGAGCCAATTCCCATACATTATTGTGCCCCAGCTGGTTTTGCGATCCTAAAAT
GTAAG---GACAAGGAGTTCAAT------GGAACAGGACCATGCAGAAAT-----------------------
-GTCAGC---ACAGTACAATGCACACATGGAATCAAGCCAGTAGTATCAACT---
CAACTGCTGTTAAATGGCAGTCTAGCAGAAGGAAAG-----------
GTAAGAATCAGATCTGAAAATATCTCAGATAAT---------GCTAAAACCATAATAGTACAACTTACC----
-----------GAGCCTGTAACA---------ATTAATTGTACCAGACCTAACAACAATACAAGAAAA-----
-GGAATACATATA------------GGACCAGGACAAGCA---TTT------TATGCGACAGGTGAA------
---------ATAATAGGGGATATA------------AGACGAGCACATTGT---AATGTCAGTAGCTCA---
AAATGGAATAAAACCTTGCAACAGGTAGTTACACAATTAAGG--------------AATTACTGG-------
-----------------------------------AACAAAACAATAGTCTTTACT------
CCCTCCTCAGGAGGGGATATAGAAATTACA---ACACATAGTTTTAATTGTGGGGGAGAA---
TTTAAACAGCTCCAGGCAAGAGTCCTGGCTGTGGAAAGATACCTAAGGGATCAACAGCTCCTAGGAATTTGGG
GCTGCTCTGGAAAACTCATCTGCACCACTAATGTGCCCTGGAACTCTAGTTGGAGT-----------------
----------------------------AATAAATCTTATGAGCAGATATGG------AAT------
AACATGACCTGGTTGCAATGG---GATAAAGAAGTCAGCAATTACACACAAATAATATAT---
CAGTTAATTGAAGAATCGCAGAACCAGCAAGAA--

AAGAATGAACAAGATTTATTGGCATTGGACAAATGGGCAAGTCTGTGGAATTGGTTTGACATAACAAAATGGC
TGTGGTATATAAGAATATTTATAATGATAGTAGGAGGCTTAATAGGGTTAAGAATAGTTTTTGCT---
GTGCTTTCTATAATA---------AATAGAGTTAGGCAGGGATATTCACCC------
TTGTCGTTTGCTATTAAYTTGTTTGATACTATAGCAATAGCAGTAGCTGGC---
TGGACAGATAGGGTTATAGAAATAGGACAAAGAATTGGTAGAGCT----------------------------
--------ATTCTCAACATA------CCTAGAAGAATCAGACAGGGCTTGGAAAGAGCTTTGCTA---TAA
>AY736814
GCT---ACA--
GAACAATTGTGGGTTACTGTCTACTATGGGGTGCCTGTGTGG---AAAGATGCAGAG---
ACCACCCTATTTTGTGCATCAGATGCTAAAGCACATGAGACAGAA---GTACATAAT----------------
--------------GTCTGGGCTACG---CATGCCTGTGTACCCACAGAC---

CCCAACCCACAAGAAATAGAGTTGAAA---------AATGTGACAGAAGAGTTTAAC----------------
--------------------------ATGTGGAAAAATAACATGGTAGAACAGATGCATACAGATATA---
ATCAGTCTATGGGACCAAAGCCTA---AAGCCATGTGTAAAGTTA---
ACCCCTCTCTGTGTTACTCTAAATTGTAGTAACTTTAGTGTCACTAATAATAGCACCAATAATAGC-------
----
-------------------ACCAATACTACCACCATTGACAATAATATGCAAGGAGAAATAAAA---
AACTGCTCTTACAATGTGACCACAGTAATAAGG--------------------------
GATGAGAAACAGAAAGTATACTCGCTT---TTTTATAGAACTGATGTAGTAGAAATTGAGAAT----------
----

AATAGTAGTCAGTATAGATTAATAAATTGTAATACCTCAGCCATTACACAG---
GCTTGTCCAAAGGTAACCTTTGAGCCAATTCCCATACATTATTGTGCTCCAGCTGGTTTTGCGATTCTAAAGT
GTAGG---AATGAGACGTTCAAT------GGAACAGGACCATGTAACAAT----------------------
-GTCAGC---ACAGTACAATGCACACATGGAATCAGGCCAGTAGTATCAACT---
CAGCTGCTGTTAAATGGTAGTCTAGCAGAAAAAGAA-----------
ATAATGATTAGATCTGAAAATATCTCAGACAAT---------GCCAAAAGCATACTAGTACAATTGACA----
-----------AGGCCTGTAATA---------ATTAATTGTACTAGACCTAACAACAACACAAGAAAA-----
-AGTATACATATA------------GGACCAGGACAAGCA---TTC------TATGCAACAGGTGAC------
---------ATAATAGGGGATATA------------AGACAAGCATATTGT---ACTGTCAATAGAACA---
GAATGGAATGAAACTTTGCACAATGTGACCACTCAATTAAAA---------------ACATACTTT-------
-----------------------------------AACAAAACAATAGTCTTTACT------
AGCCACTCAGGAGGGGATCTAGAGATTACA---ACACATACTTTTAATTGTGGAGGAGAA---
TTTAAACAGCTCCAGGCAAGAGTCCTGGCTGTGGAAAGATACCTAAGGGATCAACAGCTCCTAGGACTTTGGG
GCTGCTCTGGAAAACTCATCTGCACCACTACTGTGCCCTGGAACTCTAGCTGGAGT-----------------
-----------------------------AATAAATCACAGAATGAGATATGG------AAC------
AACATGACCTGGCTGCAATGG---GATAAAGAAATTAGCAATTACACACAGATAATATAT---
GACCTAATTGAAGAATCGCAGAACCAGCAGGAA--

AAGAATGAACAAGACTTATTGGCATTGGACAAGTGGGCAAATCTGTGGAGTTGGTTTGACATATCAAGTTGGC
TGTGGTATATAAGAATATTTATAATGATAGTAGGAGGCTTAATAGGATTAAGAATAGTTTTTGCA---
GTGCTTTCTATAATA---------AAGAGAGTTAGGCAGGGATACTCACCT------
TTATCATTTGCTATTAATTTGTTTGATACCTTAGCAATAACAGTAGCTGGC---
TGGACAGATAGGGTTATAGAAGTAGGACAAAGACTTTGTAGAGCT---------------------------
--------CTTTTACACATA------CCTAGAAGAATCAGACAGGGCTTAGAAAGGGCTTTGCTA---TAA
>AF457075
GCT---GCA---
GGAAACTTGTGGGTTACTGTCTACTATGGGGTACCTGTGTGG---AAAGATGCAGAG---
ACCACCCTATTTTGTGCATCAGATGCTAAAGCATATGAGACAGAA---AAACATAAT----------------
--------------GTCTGGGCTACA---CATGCCTGTGTACCCACAGAC---
CCCAACCCACAAGAAATACATTTGGAA---------AATGTGACAGAAGAATTTAAC---------------
--------------------------ATGTGGAAAAATAAAATGGTAGAGCAGATGCATACAGATATA---
ATCAGTCTATGGGATCAAAGCCTA---AAGCCATGTGTAAAATTA---
ACCCCTCTCTGCGTTACTTTAAACTGTAGTGATGTCACAACAAACAATGTCACA------------------
----
-------------------------------ACAAATGTCACAGAGGGCGGGGAGGACATGAGA---
AACTGCTCTTTCAATATGACTACAGAATTAAGG--------------------------
GATAAACAGAAGAAGGTATATTCACTT---TTTTATAGACTTGATGTAGTACAAATTAATGGAAGTCAA----
----

AGTAATAGTAATTACAGTGAGTATAGACTAATAAATTGTAATACCTCAGCCATTACACAG---
GCCTGCCCAAAGGTATCCTTTGAGCCAATTCCCATACATTATTGTGCTCCAGCTGGTTTTGCGATCCTAAAGT
GTAAT---GATGAGAATTTCAAT------GGAACAGGGCCATGCAAAAAG----------------------
-GTCAGC---ACAGTACAATGTACACATGGAATCAAGCCAGTAGTATCAACT---
CAACTGCTGTTAAATGGCAGTCTAGCAGCAGGAAAT---------
AAAACAAAAATTAGATCTGAAAATATCACAAACAAT---------GCCAAAAATATAATAGTACAATTTACC-
--------------GAGCCTGTGGAA---------ATTAATTGTATCAGACCTAACAACAATACAAGAAAA--
----AGTATGCGTATA------------GGACCAGGACAAGCA---TTC------TATGCAACAGGGGAT---
------------ATAGTAGGGGATATA------------AGAAAAGCATATTGT---AATATCTCTGAATCA-
--AAATGGAATATAACTTTACAAAAGGTAGGTGAACAATTAAGA---------------ACATACTTTGTG--
------------------------------------AACAAAGCAATAATATTTACT------

AACTCCTCAGGAGGGGATCTAGAAATCACA---ACACATAGTTTTAATTGTGGAGGAGAA---
TTTAAACAGCTCCAGGCAAGAGTCCTGGCTGTGGAAAGATACCTAAGGGATCAACAGCTCCTAGGAATTTGGG
GCTGCTCTGGAAAACTCATCTGCACCACTAATGTGCCTTGGAACTCTAGTTGGAGT-----------------
----------------------------AATAAATCTGAAAAGGAAATATGG------GAT------
AACATGACCTGGCTGCAATGG---GATAAAGAAATTGGCAATTACACAGACATAATATAT---
AGGCTAATTGAAGAATCGCAGAATCAGCAGGAA--

AAGAATGAACAAGACTTATTGGCATTGGACAAGTGGGCAGGCCTGTGGAATTGGTTTAGCATAACAAACTGGC
TGTGGTATATAAGAATATTTATAATGATAGTAGGAGGCTTAATAGGATTAAGAATAGTTTTTGCT---
GTGCTTTCTATAATA---------AATAGAGTTAGGCAGGGGTACTCACCT------
TTGTCATTTGCTGTTAATTTGATTGATACCATAGCAATAGTAGTAGCTGGC---
TGGACAGATAGGGTTATAGAAATAGGACAAGTAGTAGGTAGAGCT----------------------------
--------ATTCTCCACATA------CCTAGAAGAATCAGACAGGGCCTCGAAAGAATTTTGCTA---TAA
>DQ208458
GCA---GCA--
GAAAACTTGTGGGTCACTGTCTACTATGGGGTACCTGTGTGG---AAAGACGCAGAG---
ACCACCTTATTTTGTGCATCAGATGCTAAAGCATATGAGACAGAA---AAGCATAAT----------------
--------------GTCTGGGCTACA---CATGCCTGTGTACCCACAGAC---
CCCAACCCACAAGAAATACATTTGGAA---------AATGTGACTGAAGAGTTTAAC----------------
--------------------------ATGTGGAAAAATAACATGGTAGAGCAGATGCATACAGATATC---
ATCAGTCTATGGGACCAAAGCCTA---AAGCCATGTGTAAAGTTA---
ACCCCTCTCTGCGTTACTCTACAGTGTACCAATGTCACCAATAATATC-------------------------
----
-------------------------------------ACTGATGACATGAGGGGAGAATTAAAA---
AACTGCTCTTTCAATATGACCACAGAGCTAAGG---------------------------
GATAAGAAACAGAAGGTTTATTCACTT---TTTTATAGACTAGATGTAGTACAAATTAACGAGAATCAAGGT-
----

AATAGGAGTAATAATAGTAACAAGGAGTATAGATTAATAAATTGTAATACCTCAGCCATTACACAG---
GCTTGTCCAAAGGTATCCTTTGAGCCAATTCCCATACATTATTGTGCCCCAGCTGGTTTTGCGATCCTAAAGT
GTAAG---GATAAGAAGTTCAAT------GGAACAGGGCCATGCCCAAGT-----------------------
-GTCAGC---ACAGTACAATGCACACATGGAATCAAGCCAGTAGTATCAACT---
CAACTGCTGTTAAATGGCAGTCTAGCAGAAGAAGAG-----------
GTAATGATTAGATCTGAAAATATCACAAACAAT---------GCCAAAAACATACTAGTACAATTTAAC----
-----------ACGCCTGTGCAA---------ATTAATTGTACCAGACCTAACAACAATACAAGGAAA-----
-AGTATACGTATA------------GGACCAGGACAAGCA---TTC------TATGCAACAGGGGAC------
---------ATAATAGGGGATATA------------AGACAAGCACATTGT---ACTGTCAGTAAAGCA---
ACATGGAATGAAACTTTGGGAAAGGTGGTCAAACAATTAAGA---------------AAACACTTTGGG----
--------------------------------AACAACACAATAATAAGATTTGCT------
AATTCCTCAGGAGGGGATCTAGAAGTCACA---ACACATAGTTTTAATTGTGGAGGAGAA---
TTTAAACAGCTCCAGGCAAGGGTCCTGGCTGTGGAAAGATACCTAAGGGATCAACAGCTTCTAGGAATTTGGG
GCTGCTCTGGAAAACTCATCTGCACCACTAATGTGCCCTGGAACTCTAGTTGGAGT-----------------
----------------------------AATAGAAACCTGAGTGAGATATGG------GAC------
AACATGACCTGGCTGCAATGG---GATAAAGAAATTAGCAATTACACACAGATAATATAT---
GGGCTACTTGAAGAATCGCAGAACCAGCAGGAA--

AAGAATGAACAAGACTTATTGGCATTGGATAAGTGGGCAAGTCTGTGGAATTGGTTTGACATATCAAACTGGC
TGTGGTATATAAAAATATTTATAATGATAGTAGGAGGCTTAATAGGATTAAGAATAGTTTTTGCT---
GTGCTTTCTGTAATA---------CATAGAGTTAGGCAGGGATACTCACCT------
TTGTCGTTTGCTATTAATTTGTTTGATACCATAGCAATAGCAGTAGCTGAG---
TGGACAGATAGGGTTATAGAAATAGGACAAAGACTTTGTAGAGCT----------------------------
--------TTTCTCCACATA------CCTAGAAGAATCAGACAGGGCCTCGAAAGGGCTTTGCTA---TAA
>DQ208467
GCT---GCA--
GAACAATTGTGGGTTACTGTCTACTATGGGGTACCTGTGTGG---AAAGATGCAGAG---
ACCACCTTATTTTGTGCATCAGATGCCAAAGCATATGAGACAGAA---AAGCATAAT----------------
--------------GTCTGGGCTACA---CATGCCTGTGTACCCACAGAC---
CCCAACCCACAAGAAATACATTTGGAA---------AATGTGACAGAAGAGTTTAAC----------------
--------------------------ATGTGGAAAAATAACATGGTAGAGCAGATGCATACAGATATA---
ATCAGTCTATGGGACCAAAGCCTA---AAGCCATGTGTAAAGTTA---

ACCCCTCTCTGCGTCACTTTACATTGTAACAATGTCACTGGCACGAATAACAACACG----------------
----
-------------------------------GAGGGAGAAAGGAAGGCCAGGGAAGAACTAAGA---
AATTGCTCTTTCAATATGACCACAGAGCTAAGG---------------------------
GATAAGAAACAGAAGGTATATTCACTT---TTTTATAGACTTGATGTAGTAAAACTTAATGAAAAT-------
----

CAAGATAATAGTAGTGATGAGTATAGATTAATAAATTGTAATACCTCAGCCATTACACAA---
GCTTGTCCAAAGGTAACCTTTGATCCAATTCCCATACATTATTGTGCCCCAGCTGGTTTTGCGATCCTAAAGT
GTAAG---GATGAGGAGTTCAAT------GGAACAGGGCCATGCAAGAAT-----------------------
-GTCAGC---ACAGTACAATGCACACATGGAATCAAGCCAGTAGTATCAACT---
CAACTACTGTTAAATGGCAGTCTAGCAGAAACGAAG------------
GTAAAAATTAGATCTGAAAATATCACAAACAAT---------GCCAAAAGTATAATAGTACAACTTGAC----
-----------AATCCTGTGACA---------ATTAATTGTACCAGACCCAACAACAATACAAGAACA-----
-AGTGTACGTATA------------GGACCAGGACAAGCA---TTC------TATGCAACAGGTGCC------
---------ATAATAGGGAACATA------------AGACAAGCACATTGT---AATGTCAGTAGATCA---
GCATGGAATCAAACTTTACAAAAGGTAGTTACACAATTAAGA---------------ACATACTTTGGG----
-----------------------------------AACAAAACAATAATATTTGCT------
AACTCCTCAGGAGGGGATCTAGAAATCACA---ACACATAGTTTTAATTGTGGAGGAGAA---
TTTAAACAGCTCCAGGCAAGAGTCCTGGCTGTGGAAAGATACCTAAGGGATCAACAGCTCCTAGGAATTTGGG
GCTGCTCTGGAAAACTCATCTGCACCACTAATGTGCCCTGGAACTCTAGTTGGAGT-----------------
----------------------------AATAAATCTCAGAATGAGATCTGG------GAG------
AACATGACCTGGCTACAATGG---GATAAAGAAATTAGCAATTACACAGACATAATATAT---
AAACTAATTGAAGAATCGCAGAACCAGCAGGAA--

AAGAATGAACAAGACTTGTTGGCATTGGACAAGTGGACAAGTCTGTGGACTTGGTTTGACATATCAAATTGGC
TGTGGTATATAAAAATATTTATAATGATAGTAGGGGGCTTGATAGGATTAAGAATAATTTTTGCT---
GTGCTTTCTGTAATA---------AATAGAGTTAGGCAGGGGTACTCACCT------
GTGTCGTTTGCTGTTAATTTGGTTGATACTATAGCAATAGCAGTAGCTGGC---
TGGACAGATAGGATTATAGAAATAGGACAAGGAATTGGTAGAGCT----------------------------
--------GTTCTCCACATA------CCTAGAAGAATCAGACAGGGCTTCGAAAGAGCTTTGCTA---TAA
>DQ208448
ACT---GCA--
GAAAACTTGTGGGTTACTGTCTACTATGGGGTACCTGTATGG---AGAGACGCAGGG---
ACCACCTTATTTTGTGCATCAGATGCGAAAGCATATGATACAGAA---GTACATAAT----------------
--------------GTCTGGCCACA---CATGCTTGTGTACCTACAGAC---
CCCAACCCACAAGAAATAAATTTGGAA---------AATGTGACAGAAAAGTTTAAC----------------
--------------------------ATGTGGAAAAATAACATGGTAGAGCAGATGCATACAGATATA---
ATCAGTCTATGGGACCAAAGCCTA---AAGCCATGTGTAAAGTTA---
ACCCCTCTCTGCGTTACTCTAGATTGTAGCCATAATGTCACCAATAATGTTACCAAGATCGAT----------
----
-------------------------AATAGCACCACTGACAAGGAAATAAAAGGAGAGATAAAA---
AACTGCTCTTTCAATATAACCACAGAATTAAGG---------------------------
GATAAGAGACGGAGAGTATTTTCACTT---TTTTATAAACTGGATGTAGTACAATTTAATGAC----------
----

AGTAAAAATAGTTGGTATAGATTAATAAATTGTAATACCTCAGCCATTACACAG---
GCTTGTCCAAAGGTATCCTTTGAACCAATTCCAATACATTATTGTGCCCCAGCTGGTTTTGCGATTCTAAAGT
GTAAT---GATAGGGAGTTTAAT------GGAACAGGACTATGCAAGAAT-----------------------
-GTCAGC---ACAGTACAATGCACACATGGAATCAAACCAGTAGTATCAACT---
CAACTACTGTTAAATGGCAGTCTAGCAGAAGGAAAG------------
GTAATGATCAGATCTGAAAATCTCACAAACAAT---------GCCAAAAACATAATAGTACAATTTAAC----
-----------GAGTCTGTAAAA---------ATTGATTGTATCAGGCCTAACAACAATACAAGAAAA-----
-AGTATACATATA------------GGACCAGGACAAGCA---TTC------TATGCAACAGGTGCC------
---------ATATTAGGGGATATA------------AGACAAGCACATTGT---AAAGTTAATAAAACA---
GAATGGAATAAAGCTTTGCAAAAGGTAGTCACCCAATTAGGA---------------ACATACTTTAAG----
-----------------------------------GACAAAGCAATAACCTTTGAT------
AACTCCATAGGAGGGGATCTAGAAATAACA---ACACATAGTTTTAATTGTGGGGGAGAA---
TTTAAACAGCTCCAGGCAAGAGTCCTGGCTGTGGAAAGATACCTAAAGGATCAACAGCTCCTAGGAATTTGGG
GCTGCTCTGGAAAACTCATCTGCACCACTAATGTGCCCTGGAACTCTAGTTGGAGT-----------------

----------------------------AATAAGTCCCAGAGAGAGATATGG------GAC------
AACATGACCTGGCTACAATGG---GATAAAGAAATTAGCAATTACACACAGGTAATATAT---
AGTCTAATTGAAGAATCGCAGAATCAGCAGGAA--

AAGAATGAACAAGACTTATTGGCATTGGACAAGTGGACAAATCTGTGGAATTGGTTTGACATATCAAGATGGC
TGTGGTATATAAAAATATTTATAATAATAGTAGGAGGCTTGATAGGATTAAGAATAGTTTTTGCT---
GTGCTTTCTATAATA---------AATAGAGTTAGGCAGGATACTCACCT------
TTGTCGTTCGCTATTAATTTGTTTGATACCATAGCAATAGTAGTAGCTGGG---
TGGACAGATAGGGTTATAGAACTAGTACAAAGACTTTGTAGAGCT----------------------------
--------ATTCTCAACATA------CCTGTAAGAATTAGACAGGGCTTGGAAAGGGCTTTGCTA---TAA
>KU749411
ACT---GCA--
GAAAACTTGTGGGTTACTGTCTACTATGGGGTACCTGTGTGG---AAAGACGCAAAG---
ACCACCCTATTTTGTGCATCAGATGCTAAAGCATATAAGACAGAG---GTGCATAAT----------------
--------------GTCTGGGCCACG---CACGCATGTGTACCCACAGAC---
CCCAACCCACAAGAAATAGATTTAGAA---------AATGTGACAGAAATGTTTAAC----------------
--------------------------ATGTGGAAAAATGACATGGTGGAGCAGATGCATACAGATATA---
ATTAGTCTATGGGACCAAAGCCTA---CAACCATGTGTAAAATTA---
ACCCCTCTCTGCGTCACCCTAAACTGTACCCATAATGTCACTGTCGAGGACAGTACCAATGTCACTGTCAGCA
ACAGTAGCMAYGAGASCAATGKC--
-------------ACTGKCACTARTAGCACTGTCRSTAGCCCTGTCACTGAACAAGAAATGAAA---
AACTGCTCTTTCAATACAACCACAGAAATAAGG---------------------------
GACAAGAAACAGAAAGTATATTCACTT---TTTTATAAACTTGATATAGTACCAATTAATAAA----------
----

AATACTAGTGAGTATATTTTAATAAATTGTAATACCTCAGCCATTACACAG---
GCTTGTCCAAAGGTAAGCTTTGAGCCAATTCCTATACATTATTGTGCCCCAGCTGGTTTTGCGATCCTAAAGT
GTAAG---GATAAGAAGTTCAAT------GGAACAGGGCTGTGCAAGAAT-----------------------
-GTCAGC---ACAGTACAATGCACACATGGAATCAAGCCAGTAGTATCAACT---
CAACTACTGTTAAATGGCAGTCTAGCAGAGGAAGAA------------
ATAATAATTAGATCTGAAAATATCTCAGACAAT---------GCTAAAACCATAATAGTACAGCTTAAC----
-----------AAGCCTGTACAA---------ATCAATTGTACCAGACCTAATAACAATACAAGAAAG-----
-AGTATACATATA------------GGACCAGGACAAGCA---TTT------TATGGAACAGGGACA------
ACAGGGGCTATAATAGGGAATATA------------AGACAAGCGCATTGT---AATGTTAGTAAATCA---
TTATGGAATSAAACTTTAAAACAGGTAGCTGACAAATTAAGA---------------GAACATTGG-------
-----------------------------------AACAAAACAATAATCTTTACT------
AAACCCTCAGGAGGAGATTTAGAAATTACA---ACACATAGTTTTAATTGTGGAGGAGAG---
TTTAAACAGCTCCAGGCAAGAGTCCTGGCTGTGGAAAGTTACCTAAGGGATCAACAGCTCCTGGGAATTTGGG
GTTGCTCTGGAAAACTCATCTGCACCACTACTGTGCCCTGGAACTCTAGTTGGAGT-----------------
----------------------------AATAAATCTTACAGTGAGATATGG------GAT------
AACATGACCTGGCTGCAATGG---GACAGAGAAATTGACAATTACACAGAAGTAATATAT---
AAGTTAATTGAAGAATCACAGAACCAGCAGGAA--

AGAAATGAACAAGACTTATTGGCATTGGACAAGTGGGCAAGCCTGTGGAATTGGTTTAACATATCAAATTGGC
TGTGGTATATAAGAATATTTATAATAATAGTAGGAGGCTTAATAGGATTAAGAATAGTTTTTGCT---
GTGCTCTCTATAATA---------AATAGAGTTAGGCAGGATACTCACCT------
CTGTCGTTTGCTATTAGCTTGTTTGATACCATAGCAATAGTAATAGCTGGC---
TGGACAGATAGGGTTATAGAAGTAGGGCAAAGAATTGGTAGAGCT----------------------------
--------TTTCTCAACATA------CCTAGAAGAATCAGACAGGGTGCCGAAAGGGCTTTAATA---TAG
>KX232594
GCT---GCA--
GGAAAATTGTGGGTTACTGTCTACTATGGGGTACCTGTATGG---AAAGATGCAGAT---
ACCACCCTATTTTGTGCATCAGATGCTAAAGCATATAAGACAGAA---GCGCATAAT----------------
--------------GTATGGGCTACA---CACGCTTGTGTACCCACAGAC---
CCCAACCCACAAGAAATAAATTTAACA---------AATGTGACAGAAAAGTTTAAC----------------
--------------------------ATGTGGAAAAATAACATGGTGGAGCAGATGCATTCAGATGTA---
ATTAGTATATGGGACCAAAGCCTA---AAGCCATGTGTAAAGTTA---
ACCCCTCTCTGCGTCACCCTAAGCTGTAACAATAATGTCACGGTCACTCCCAATAATAGCACTGTTAATGCCA
GT---
---GCCAATGACACTGAAATGAAA---

AACTGCTCTTACAATATAACCACAGAAATAAGG---------------------------
GATAAGAAAAGGAAAGTATATTCACTT---TTTTATAAACTTGATATAGTACCAATTAATGAG----------
----

AATAATAATAGTAATAGTGAGTATATCTTAATAAATTGTAATACCTCAGCCATTACGCAG---
GCTTGTCCAAAGATAAGCTTTGAGCCAATTCCCATACATTATTGTACCCCAGCTGGTTTTGCGATCTTAAAGT
GTAAG---GATAAGGATTTCAAT------GGAACAGGTCTATGTAAGAAT----------------------
-GTCAGC---ACAGTACAATGCACACATGGAATCAAGCCAGTAGTATCAACT---
CAACTGCTGTTAAATGGCAGCCTAGCAGAAGGAAAG------------
ATAATGATTAGATCTGAAAATATCTCAGACAAT---------GCTAAAACCATAATAGTACAGCTTAAC----
-----------AAGACTGTGTCA---------ATTAATTGTACCAGACCTAACAACAATACAAGAAAG-----
-AGTGTACCTATA------------GGACCAGGACAAGCA---TTC------TATGCAACAGGTGAT------
---------ATAATAGGGAATATA------------AGACAAGCACATTGT---AATGTCAGTAGATCA---
GATTGGAATAACACTTTAAGACAGGTAGCTGAAAAATTAAGA---------------GAATATTGG-------
-----------------------------------AACAATACAATAATCTTTACT------
AAACCCTCAGGAGGAGATTTAGAAATTACA---ACACATAGTTTTAATTGTGGAGGAGAA---
TTTAAACAGCTCCAGGCAAGAGTCCTGGCTGTGGAAAGTTACCTAAGGGATCAACAGCTCCTAGGAATTTGGG
GTTGCTCTGGAAAACTCATCTGCACCACTAATGTGCCCTGGAACTCTAGTTGGAGT-----------------
----------------------------AATAAAACTTACAATGATATATGG------GAT------
AACATGACTTGGCTGCAATGG---GATAAAGAAATTGAAAATTACACAGGTGAAATATAT---
AGGTTAATTGAAGAATCGCAGAACCAGCAGGAA--

AAAAATGAACAAGAGTTATTGGCATTGGACAAGTGGGCAAGCCTGTGGAATTGGTTTGACATATCAAACTGGC
TGTGGTATATACGAATATTTATAATGATAGTAGGAGGCTTAATAGGATTAAGAATAGTTTTTGCT---
GTGCTCTCTATAATA---------AAAAGAGTTAGGCAGGGATACTCACCT------
CTGTCATTCGCTGTTGACTTGTATAATACCACAGCAATAGTAGTAGCTGGT---
TGGACAGATTGGGTTATAAACGGAGGGCAAAGAATTGTTAGAGCT---------------------------
--------TTTCTCAACATA------CCTAGAAGAATCAGGCAGGGCTTAGAAAGGGCTTTACTA---TAA
>KX232597
ACT---ACA---
GAACAATTGTGGGTTACTATTTACTATGGGGTACCTGCGTGG---AAAGATGCAGAC---
ACCACCCTATTTTGTGCATCAGATGCTAAAGCATATAAGACGGAA---ATGCATAAT----------------
--------------GTATGGGCTACA---CACGCCTGTGTACCCACAGAC---
CCCAGCCCACAAGAAATAAATTTAGCA---------AATGTGACAGAAAATTTTAAC---------------
--------------------------ATATGGAAAAATAACATGGTGGAGCAGATGCATACAGATATA---
ATTAGTATATGGGACCAAAGCCTA---AAACCATGTGTAACGTTG---
ACCCCTCTCTGCGTCACCCTAAATTGTACCGATGTCAATACCAATAGTACCAATAGTGCC------------
----
--AATGTCACACAAGAAATGAAA---
AACTGCACCTACAATATAACCACAGAAATAAGA---------------------------
GATAAGACAAAGAAAGTATATTCACTT---TTTTATAAACTTGATATAGTACAAATTGATAAAGGGAAT----
----

AATAGTAATAGTAGTGAGTATATTTTAATAAATTGTAATACCTCAGCCATTACACAG---
GCTTGTCCAAAAGTAAGCTTTGAGCCAATTCCCATACATTATTGTGCCCCAGCTGGTTTTGCAATTCTAAAGT
GTAAT---GATAAGAAGTTCAAT------GGAACAGGGCCATGCAGTAAT----------------------
-GTCAGC---ACAGTACAATGCACACATGGAATCAAGCCAGTAGTATCAACT---
CACCTGCTGTTAAATGGCAGTCTAGCAGAAGAAGAG------------
ATAGTGATTAGATCTGAAAATATCTCAGACAAT---------GCTAAAACCATAATAGTGCAGCTTAAC----
-----------CAGTCTGTATCA---------ATTAATTGTACCAGATTTAAC-ACAATACAAGAAAG-----
-AGTGTACCTATA------------GGACCAGGACAAGCA---ATC------TATACAACAGGTGAT------
---------ATAATAGGGGATATA------------AGACAAGCACATTGT---AATATCAGTAGATCA---
GCATGGAATAGAACTTTAAGACAGGTAGCTAACAAATTAAGA---------------AGCTATTGGAGC----
-----------------------------------AACAAAACAATAATCTTTACT------
AAACCCTCAGGAGGTGATTTAGAAATTATG---ACACATAGTTTTAATTGTGGAGGAGAA---
TTTAAACAGCTCCAGGCAAGAGTCCTGGCTGTGGAAAGTTACCTAAAGGATCAACAGCTCCTAGGAATTTGGG
GTTGCTCTGGAAAACTCATCTGCACCACTAGTGTGCCCTGGAACTCTAGTTGGAGT-----------------
----------------------------AATAAATCATATGATAATATATGG------AAT------
AACATGACCTGGCTGCAATGG---GAGAGAGAAATTGATAATTACACAGGTTTAATATAT---
CAATTAATTGAAGAATCACAGAACCAGCAAGAA--

AAAAATGAACAAGAATTATTGTCATTGGACAAGTGGGCAAGTCTGTGGAGTTGGTTTAACATAACACAATGGC
TGTGGTATATAAGAATATTTATAATGATAGTAGGAGGCTTAATAGGATTAAGAATAATTCTTGCT---
GTGCTTTCTATAGTA---------AATAGAGTTAGGCAGGGATACTCACCT------
CTGTCGTTCGCTATTAGCTTGTTTGATACCATAGCAGTAGCAGTAGCTGGC---
TGGACAGATACAGTTATAGAAGCAGCACAAAGATTTGGTAGAGCT----------------------------
--------ATTCTCAACATA------CCTAGAAGAATCAGGCAGGGCTTTGAAATAGCTTTACTA---TAA
>KX232609
ACT---GCA--
GAAAAATTGTGGGCTACTGTCTACTATGGGGTACCTGTGTGG---AAAGATGCAGAG---
ACCACTCTATTTTGTGCATCAGATGCTAAAGCATATGAGACAGAA---GCACATAAT----------------
--------------GTATGGGCTACA---CACGCCTGTGTACCCACAGAC---
CCCAACCCACAAGAAATAGATTTAGAA---------AATGTGACAGAAGAGTTCAAC----------------
--------------------------ATGTGGAAAAATAGCATGGTGGAGCAGATGCATACAGATATA---
ATTAGTATATGGACCAAAGCCTA---AAGCCATGTGTAAAGTTA---
ACCCCTCTCTGCGTCACCCTACACTGTGCCAATATCAATAGCAGCGTCAMACACTGTGCCAAT----------
----
----------------------------ATCAATAGCAGCGTCAATGCCACAAAAGAAATGAAA---
AACTGCTCTTACAATATAACCACAGAGATAAGG---------------------------
GATAAGATACAGAAAGTATATTCACTT---TTCTATAAACTTGATATAGTACCAATTAATCAA----------
----

AATAATGCTAGTAATGTCAGTGAGTATATTTTAATAAATTGTAATACCTCAGCCATTACACAG---
GCTTGTCCAAAGGTAAGCTTTGAGCCAATTCCTATACATTATTGTGCCCCAGCTGGTTTTGCGATCCTAAAAT
GTAAG---GATACAGAGTTCAAT------GGAACAGGGCTATGCAAGAAT-----------------------
-GTCAGC---ACAGTACAATGCACACATGGAATCAAGCCAGTAGTATCAACT---
CAACTGCTGTTAAATGGCAGTCTAGCAGAAAAAGAA------------
ATAATGATTAGATCTGAAAATATCTCAGACAAT---------GCTAAAACCATAATAGTACAGCTTAAC----
-----------CAGTCTGTAACA---------ATTAATTGTACCAGATTTAACAACAATACAAGAAAG-----
-AGTATACATATA------------GGACCAGGACAAGCG---TTC------TATGCAACA---GAT------
---------ATAATAGGGGATATA------------AGACAAGCATATTGT---AATGTCAGCAGATCA---
GCATGGAATAAAACTTTAGGAGAGGTAGCTAAAAGATTAAGA---------------AAATATTGG-------
-----------------------------------AACAGGACAATAATCTTTACT------
AAACCCTCAGGAGGAGATTTAGAAATTACA---ACACATAGTTTTAATTGTGGAGGAGAA---
TTTAAACAGCTCCAGGCAAGAGTCCTGGCTGTGGAGAGTTACCTAAAGGATCAACAGCTCCTAGGAATTTGGG
GTTGCTCTGGAAAACTCATCTGCCCCACTACTGTGCCCTGGAACGCTAGTTGGAGT-----------------
----------------------------AATAAATCTTACAATGAGATATGG------GAT------
AGCATGACCTGGCTGCAATGG---GATAAAGAAATTGACAATTACACAAATGAAATATAT---
AAGTTAATTGAAGAATCACAGAACCAGCAAGAA--

AAAAATGAACAAGATTTATTGGCATTGGACAAGTGGGCAAGTCTGTGGAATTGGTTTAACATATCAAATTGGC
TGTGGTATATAAAAATATTTATAATGATAGTAGGAGGCTTAATAGGATTAAGAATAATTTTTGCT---
GTGCTTGCTATAATA---------AATAGAGTTAGGCAGGGATACTCACCT------
CTGTCGTTCGCTATTAGCTTGCTTGATACCATAGCAATAGTAGTAGCTGGA---
TGGACAGATAGGGCTATAGAACTAGGACAAAGCATTGGTAGAGCT----------------------------
--------ATTCTCAACATA------CCTAGAAGAATCAGGCAAGGTTTAGAAAGGATTTTGCTA---TAA
>KX232613
ACT---ACA--
GAAAAGGTGTGGGTTACTGTTTATTATGGGGTACCTGTGTGG---AAAGAGGCAGAC---
ACCACCTTATTTTGTGCATCAGATGCTAGAGCATATGATACAGAA---GTGCATAAT----------------
--------------GTCTGGGCTACA---CATGCCTGTGTACCCACAGAC---
CCCAACCCACAAGAAATACATTTGGAA---------AATGTAACAGAAAATTTTAAC----------------
--------------------------ATGTGGAAAAATAACATGGTAGAGCAGATGCATGCAGATATA---
ATCAGTCTATGGACCAAAGCCTA---AAGCCTTGTGTAAAGTTA---
ACCCCTCTCTGTGTTACTTTAGAGTGTACCAATGTCAACAGAAATGATAGTAAGACAAATGAC----------
----
----------------------------AGCGGTACGGTGGAAAGCACACAAAGAGAAATAACA---
AACTGTTCTTTCAATATGACCACAGAACTAAGA---------------------------
GACAAGAGACAGAAAGTATATTCACTT---TTTTATAGACTTGATGTAGTACCACTTGATGGC----------
----

AACAGTAGTAGTAACCAGTATAGATTAATAAACTGTAATACCTCAGCCATTACACAG---
GCTTGTCCAAAGGTAACTTTTGAGCCAATTCCCATACATTATTGTGCCCCAGCTGGTTTTGCGATCCTAAAGT
GTAAT---GATAAAGAGTTCAAT------GGAACAGGGCCATGCAAGAAT----------------------
-GTCAGT---ACAGTACAGTGCACACATGGAATCAGGCCAGTAGTATCAACT---
CAACTACTGTTAAATGGCAGTTTAGCAGAACAAGAT-----------
ATAAGAATTAGAGTTGAAGATATCTTAGAGAAT---------ACTAAGACTATAATAGTACAATTTAAT----
-----------GAGTCTGTAGTA---------ATTAATTGTACCAGACCTAACAATAATACAAGAAGA-----
-AGTGTGCGTATA------------GGACCAGGACAAACA---TTC------TATGCA---AGTGAC------
---------ATAATAGGGGATATA------------AGAGAAGCATATTGT---AATGTCAGTAGATCA---
GAATGGAATAAAACTTTACAAGGAGTAGCTAAACAACTAAGA---------------AGACACTGG-------
-----------------------------------AACAAAACAATAATCTTTAGC------
AACTCCTCAGGAGGAGATTTAGAAATTACA---ACACATATGTTTAATTGTCAAGGAGAA---
TTTAAACAGCTCCAGGCAAGAGTCCTGGCTGTGGAAAGATACCTAAAGGATCAACAGCTCCTAGGAATTTGGG
GCTGCTCTGGAAAGCTCATCTGCCCCACTAATGTGCCCTGGAACTCTAGTTGGAGC-----------------
----------------------------AATAAAACTCAGGATGAGATATGG------GGA------
AACATGACTTGGCTGCAGTGG---GATAAAGAAATTAGTAATTACACAAGCTTAATATAT---
CAGCTAATTGAGGAATCACAGAACCAGCAGGAA--

AAGAATGAACAAGACTTATTGGCATTGGACCAGTGGGCAAGTCTGTGGAATTGGTTTAACATATCAAACTGGC
TGTGGTATATAAAGATATTTATAATGATAGTAGGAGGCTTAATAGGATTAAGAATAGTTTTTGCT---
GTGCTTTCTATAATA---------AATAGAGTTAGGCAGGGATACTCACCT------
TTGTCGTTTGCTATTAGTTTATTAGATACCATAGCAATAGCAGTAGCTGGC---
TGGACAGATAGGGTGATAGAATTAGGACAAAGAATTGGTAGAGCT----------------------------
--------ASGCAGAGGGCT------GAAAGAGSTGCTAYGCAGAGGGCTGAAAGAGCTKTGCTA---TAA
>KX232614
ACT---GCA---
GAAAACTTGTGGGTCACTGTCTACTATGGGGTACCTGTGTGG---AAAGACGCAGAG---
ACCACCCTATTTTGTGCATCAGAGGCTAAGGCATATAAGACAGAA---GTACACAAT----------------
--------------GTATGGGCTACA---CACGCTTGTGTACCCACAGAC---
CCCAACCCACAAGAAATAAAATTAGAA---------AATGTGACAGAAATGTTTAAC----------------
--------------------------ATGTGGAAAAATCCCATGGTGGAGCAGATGCATACAGATATA---
ATTAGTCTATGGGACCAAAGTCTA---AAGCCATGTGTAAAGTTA---
ACCCCTCTCTGTGTCACCCTACACTGTACCAATGTCAAAGACAATGTCACTGTCCCCAGTACCAATGCCACT-
----
-------------GTCGCTCCCAATGACACTGCCACTGTCAATGAAACTGACAGTGACAGGAAA---
AACTGCTCTTTCAATATAACCACAGAAATAAGG--------------------------
GATAAGAAACAGAAAGCATATGCACTT---TTTTATAAGCTTGATTTAGTACCCATTAATACT----------
----

AGTAAGAATGACAGTGAGTATATTTTAATAAATTGTAATACCTCAGCCATTACACAG---
GCTTGTCCAAAGGTAAGCTTTGAGCCAATTCCCATACATTATTGTGCCCCAGCTGGCTTTGCAATCCTAAAGT
GTAAT---GATAGGAATTTCAAT------GGGACAGGGCTATGCAGGAAT----------------------
-GTCAGC---ACAGTACAGTGCACACATGGAATCAGGCCAGTAGTATCAACT---
CAACTGCTATTAAATGGCAGTCTAGCAGAAGGAGAA-----------
ACAATGATTAGATCTGAAAATATCTCAGACAAT---------GCTAAGACCATAATAGTACAGCTTAAC----
-----------CAGTCTGTATCA---------ATTAATTGTACCAGACCTAACAACAATACAAGAAAG-----
-AGTATACATATA------------GGACCAGGACAAGCA---TTT------TATGCAACAGGGGAG------
---------GTAATAGGGGACATA------------AGACGAGCATATTGT---AATGTCAGTGGATCA---
GAATGGAATAAAGCTTTACAACAGGTAGTGGCCAAATTAAGA---------------GAACATTGGCCC----
-----------------------------------AACGGAACAATAAACTTTACT------
AAACCCTCAGGAGGAGATTTAGAAATTACA---ACACACAGTTTTAATTGTGGAGGAGAG---
TTTAAACAGCTCCAGGCAAGAGTCCTGGCTATGGAAAGATACCTAAAGGATCAACAGCTCCTAGGAATTTGGG
GATGCTCTGGAAAACTCATCTGCACCACTACTGTGCCCTGGAACTCTAGTTGGAGT-----------------
----------------------------AATAAATCTTACCATGAGATATGG------GAT------
AACATGACCTGGCTGCAATGG---GATAAAGAAATTAACAATTACACAGAAATAATATAT---
CAGTTGATTGAAGAATCGCAGAACCAGCAGGAG--

AAAAATGAAAAAGACTTATTGGAATTGGACAAGTGGGCAAGTCTGTGGAATTGGTTTGACATATCAAACTGGC
TGTGGTATATAAGAATATTTATAATGATAGTAGGAGGCTTAATAGGATTAAGAATAGTTTTTGCT---

GTGCTCTCTATAATA---------AATAGAGTTAGGCAGGGATACTCACCT------
CTGTCGTTCGCTATTAGCTTGGTTGATACCACAGCAATAGCAGTAGCTGGC---
TGGACAGATAGGATTATAGAAATAGGACAAGGAATTGGTAGAGCT----------------------------
--------TTTCTCAACATA------CCTAGAAGAATCAGGCAGGGCCTTGAAAGAGCTCTCCTA---TAA
>KX232618
ACT---GCA--
GAAAACTTGTGGGTTACTGTCTACTATGGGGTACCTGTGTGG---AAAGACGCAGAG---
ACCACCCTATTTTGTGCATCAGAAGCGAAAGCATATAAGACAGAG---GTACATAAT----------------
--------------GTATGGGCTACA---CATGCATGTGTACCCACAGAC---
CCCAACCCACAAGAAATAGATTTAGAA---------AATGTGACAGAAGAGTTTAAC----------------
--------------------------ATGTGGAGAAATAACATGGTGGAGCAGATGCATACAGATATA---
ATTAGTCTATGGGACCAAAGCCTA---AAACCATGTGTAAAATTA---
ACCCCTCTCTGTGTCACCTTACACTGTACCAGTGTCACTGTCAATAACACTGTTACTGAA-------------
----
--CAAGAAATGAAA---
AACTGCTCTTTCAATACAACCACAGGAATAAGG---------------------------
GACAAGAAACAGAGAGTATATTCACTT---
TTTTATAAGCTTGATATAGTACCAATTAATAAAAATAATACTAGTCAT-------------------------
----
AATGACAGTGAGTACATTTTAATAAATTGTAATACCTCAGCCATTACACAA---
GCTTGTCCAAAGGTAAGCTTTGAGCCAATTCCTATACATTATTGTGCCCCAGCTGGTTTTGCGATCCTAAAGT
GTAAG---GATAAGAAGTTCAAT------GGAACAGGGCTATGCAGGAAT-----------------------
-GTCAGC---ACAGTACAATGCACACATGGAATCAAGCCAGTAGTATCAACT---
CAACTGCTGTTAAATGGCAGTCTAGCAGAGGAAGAA------------
ATAATGATTAGATCTGAAAATATCTCAGACAAT---------GCTAAAACCATAATAGTACAGCTTACC----
-----------CGGCCTGTATCA---------ATTAATTGTACCAGACCTAACAACAATACTAGAAAG-----
-AGTATACGTATA------------GGGCCAGGACAAGCA---TTC------TATGCAACAGGGGAT------
---------ATAATAGGGGACATA------------AGACAAGCACATTGT---AATGTCAGTAAATCA---
GAATGGAATACAACTTTAAAACAGGTAGCTGACAAATTAAGA---------------GAACTTTAC-------
-----------------------------------AACAACACAATAATTTTTACT------
GAACCCTCAGGAGGAGATTTAGAAATTACA---ACACATAGTTTTAATTGTGGAGGAGAG---
TTTAAACAGCTCCAGGCAAGAGTCCTGGCTGTGGAAAGATACCTAAAGGATCAACAGCTCCTGGGAATTTGGG
GATGCTCTGGAAAACTCATCTGCACCACTACTGTGCCCTGGAACTCTAGTTGGAGT-----------------
----------------------------AATAAATCCTACAATGAGATATGG------GAT------
AACATGACCTGGCTGCAATGG---GATAAAGAAATTGACAATTACACAGACCTAATATAT---
AAGTTAATTGAAGAATCACAGAACCAGCAGGAA--

AAAAATGAACAAGACTTATTGGCATTAGACAAGTGGGCAAGTCTGTGGAATTGGTTTGACATATCAAATTGGC
TGTGGTATATAAAAATATTTATAATGATAGTAGGAGGCTTAATAGGGTTAAGAATAGTTTTTGCT---
GTGCTCTCTATAATA---------AATAGAGTTAGGCAGGGATACTCACCT------
CTGTCGTTCGCTACTAGCTTGTTTGATACCATAGCAATAGTAATAGCTGGC---
TGGACAGATAGGGTTATAGAAAGAGGACGAAGAATTGGTAGAGCT----------------------------
--------TTTCTCAACATA------CCTAGAAGAATCAGGCAGGGTGCCGAAAGAGCTTTAATA---TAA
>KX232620
ACT---GCA--
GAAAACTTGTGGGTTACTATCTACTATGGGGTACCTGTGTGG---AAAGATGCAGAG---
ACCACCCTATTTTGTGCATCAGATGCTAAAGCATATAAGACAGAA---GTACATAAT----------------
--------------GTATGGGCTACA---CATGCCTGTGTACCCACAGAC---
CCCAACCCACAAGAAATGGATTTAAAA---------AATGTGACAGAAAGGTTTGAC----------------
--------------------------ATGTGGAAAAATAGCATGGTGGAGCAGATGCATGCAGATATA---
ATTAGTTTATGGGACCAAAGCCTA---AAGCCATGTGTAAAATTA---
ACCCCTCTCTGCGTCACCCTAAACTGTACCGATGTCCAGAAGACGACTATCAATATCAGTAACATTGCAGAAG
AAATGAAAAACTGCTCTGCT---
-------GTACCGATGTCCAGAAG-ACGACTATCAATATCAGTAACATTGCAGAAGAAATGAAA---
AACTGCTCTTTCAATATAACCACAGAAATAAGG---------------------------
GATAAGAAACGAAAAGCATATTCACTT---TTTTATAAACTTGATTTAGTACAAATTAATAGT----------
----

AATAATAGTAATAATAACAGTGAGTATATTTTAATAAATTGTAATACCTCAGCCATTACACAG---
GCTTGTCCAAAGGTGAGTTTTGAGCCAATTCCCATACATTATTGTGCTCCAGCTGGTTTTGCGATTCTAAAAT

GTAAG---GATAAGAATTTCAAT------GGAACAGGGCTGTGCAAGAAT----------------------
-GTCAGC---ACAGTGCAATGCACACATGGAATCAGGCCAGTAGTATCAACT---
CAATTGTTGTTAAATGGCAGTCTAGCAGAAGAAGAA------------
ATAATAATTAGATCTGAAAATATCTCAGACAAT---------GCTAAAACCATAATAGTACAGCTTACC----
-----------AAGGCTGTCCAA---------ATTAATTGTTCCAGACTTAACAACAATACAAGACAG-----
-AGTATACGTATA------------GGACCAGGGCAAGCA---TTC------TATACAACAGGTGAT------
---------ATAATAGGGGATATA------------AGAAAAGCACAGTGT---TTTGTCAATGGAACA---
TTATGGAAGGAAACTTTAAAAAAGGTAGCTGACAAACTAAGA---------------GAAAATTGGGCT----
-----------------------------------AACAAAACAATAATCTTTACT------
CAACCTTCAGGAGGAGATTTAGAAATTACA---ACACATAGTTTTAATTGTGGAGGAGAG---
TTTAAACAGCTCCAGGCGAGAGTCCTGGCTGTGGAAAGATACCTACAGGATCAACAGCTCCTAGGAATTTGGG
GTTGCTCTGGAAAACTCATCTGCACCACTAATGTGCCCTGGAACTCTAGTTGGAGT-----------------
----------------------------AATAAATCTTACAATGAGATATGG------AAT------
AATATGACCTGGCTGCAATGG---GATAAAGAAATTGACAATTACACAGAAACAATATAT---
ATGTTAATTGAAGAATCGCAGAAACAGCAGGAA---

AGAAATGAACAAGAACTATTGGCATTGGACAAGTGGGCAAGTCTGTGGAATTGGTTTGACATATCAAAGTGGC
TGTGGTATATAAAGATATTTATAATGATAGTAGGAGGCTTAATAGGATTAAGAATAGTTTTTGCT---
GTGCTTTCTATAATA---------AATAGAGTTAGGCAGGGATACTCACCT------
CTGTCGTTCGCTATTAACTTGTATGATACCATAGCAATAGCAGTAGCTGGC---
TGGACAGATAGGGTTATAGAACTAGGACAACGTATTGGTAGAGCT---------------------------
--------ATTCTCAACATA------CCTAGAAGAATCAGGCAGGGCTTAGAAAGGTCTTTACTA---TAA
>KX232624
ACT---GCA---
GAAAAATTGTGGGTTACTGTTTACTATGGGGTACCTGTGTGG---AAAGATGCAGAG---
ACCACCCTATTTTGTGCATCAGATGCTAAAGCATATAAGACAGAA---GTACATAAT----------------
--------------GTATGGGCTACA---CATGCCTGTGTACCCACAGAC---
CCCAATCCACAAGAAATAGACTTAAAA---------AATGTGACAGAAAAGTTTGAC----------------
--------------------------ATGTGGAAAAATAACATGGTAGAGCAGATGCATACAGATATA---
ATTAGCATATGGGACCAAAGTCTA---AAGCCATGTGTAAAATTA---
ACCCCTCTCTGCGTCACCCTAAACTGTACCGATGTCAACAACAATGTCACTGTCAACAACAAT----------
----
---AGCAATGTACAAGAAATGAAA---
AACTGCTCTTTCAGTACAACCACAGGAATAAGG--------------------------
GATAAGAAACGGAAAGCATATTCACTT---TTTTATAAACTTGATCTAGTATCAATGAATAAT----------
----

AATAATAATAGTAATGAGTATATTCTAATAAATTGTAATACCTCAACCATTACACAG---
GCTTGTCCAAAGGTGAGCTTTGAGCCAATTCCCATACACTATTGTGCTCCAGCTGGTTTTGCGATTCTAAAGT
GTAAG---GATAAGAATTTCACT------GGAACAGGGCTGTGCAAGAAT----------------------
-GTCAGC---ACAGTGCAATGCACACATGGAATCAGGCCAGTAGTATCAACT---
CAACTGCTGTTAAATGGCAGTCTAGCAGAAGAAGAA------------
ATAATAATTAGATCTGAAAATATCTCAAACAAT---------GCTAAAACCATAATAGTACAGCTTACC----
-----------AAGGCCGTCCAA---------ATTAATTGTACCAGACCTAACAACAATACAAGACAG-----
-AGTATACGTATA------------GGACCAGGGCAAGCA---TTC------TATGCAACAGGGGCT------
---------GTAACAGGGGATATA------------AGACAAGCACATTGT---AATGTCAGTAAATCA---
GCATGGAATGCAACTTTACAACAGGTGGCTGCCAAATTAGGA---------------GGGTATTGG-------
-----------------------------------AACAAAACAATAGTCTTTGCT------
AACTCCTCAGGAGGAGATTTAGAAATTACA---ACACATAGTTTTAATTGTGGAGGAGAG---
TTTAAACAGCTCCAGGCAAGAGTCCTGGCTGTAGAAAGCTACCTAAGGGATCAACAGCTCCTGGGAATTTGGG
GTTGCTCTGGAAAACTCATCTGCACCACTACTGTGCCCTGGAACTCTAGTTGGAGT-----------------
----------------------------AATAAATCTTACAATGAGATATGG------GAT------
AACATGACCTGGTTGCAATGG---GACAAAGAAATTGACAATTACACAGAAGAAATATAC---
AGGTTAATTGAAAATTCACAGAACCAGCAGGAG---

AAAAATGAGCAAGACTTATTGGCATTGGACAAGTGGGCAAGTCTGTGGAATTGGTTTGACATATCACATTGGC
TATGGTATATAAAAATATTTATAATGATAGTAGGAGGCTTAATAGGATTAAGAATAGTTTTTGCT---
GTGCTTTCTCTAATA---------AATAGAGTTAGGCAGGGATACTCACCT------
CTGTCGTTCGCTATTAGCTTGTTTGATACCATAGCAATAGTAATAGCTGGC---

TGGACAGATAGGGTTATAGAAGGAGGACAAAGAATTGGTAGAGCT-------------------------
--------ATTCTCAACATA------CCTAGAAGAATCAGGCAGGGTGCTGAAAGAGCTTTTCTA---TAA
>KX232626
ACT---GCA--
GAAAACTTGTGGGTTACTGTCTACTACGGGGTACCTGTGTGG---AGAGATGCAGAG---
ACCACCCTATTTTGTGCATCAGATGCTAAAGCATATGAGACAGAA---ATGCATAAT----------------
--------------GTATGGGCTACA---CACGCCTGTGTACCCACAGAC---
CCCAACCCACAAGAAATAAATTTAGAA---------AATGTGACAGAAGAGTTTAAC----------------
-------------------------ATGTGGAAAAATAGCATGGTGGAGCAGATGCATACAGATATA---
ATTAGCATATGGACCAAAGCCTA---AAGCCATGCGTGAAATTA---
ACCCCTCTCTGCATCACCCTACACTGTACCAATGTCAATGTCACAAACAATAGCAATGTCAGCAACAATACTG
TCACT---
---GACATGAAA---
AACTGCTCTTTCAATACAACCACAGAAATAAGG---------------------------
GACAAGAAACAGAAAGCATATTCACTT---TTTTATAAACTTGATATAGTACCAATTAATAAT----------
----

AATACTAGTGAGTATATTTTAATAAATTGTAATACCTCAGCCATTACTCAG---
GCTTGTCCAAAGGTAAGCTTTGAGCCAATTCCTATACATTATTGTACCCCAGCTGGTTTTGCGATCCTGAAGT
GTAAT---GATAAAAAGTTCAAT------GGAACAGGGCTATGCAGGAAT-----------------------
-GTCAGC---ACAGTACAATGTACACATGGAATCAAGCCAGTAGTATCAACT---
CAACTGCTGTTAAATGGCAGTCTAGCAGAGGAAGGA------------
ATAATGATTAGATCTGAAAATATCTCAGACAAT---------GCTAAAACCATAATAGTACAGCTTAAC----
-----------CAGTCTGTATCA---------ATTAATTGTACCAGACCTAACAACAATACAAGAAAG-----
-AGTATACGTATA------------GGACCAGGACAAGCA---TTC------TATGCAACAGGGGAT------
---------ATAATAGGGGATATA------------AGACAAGCACATTGT---AATGTCAGTAAATCA---
GGATGGAATACAACTTTAAATAGGGTAGCTGCCAAATTAAGA---------------GAACATTGGAAC----
-------------------------------------ATCAGCACAATAATCTTTAAT------
AAATCCTCAGGAGGAGATTTAGAAATTACA---ACACATAGTTTTAATTGTGGAGGAGAG---
TTCAAACAGCTCCGGGCAAGAGTCCTGGCTGTGGAAAGATACCTAAGGGATCAACAGCTCCTGGGAATTTGGG
GTTGCTCTGGAAAACTCATCTGCACCACTACTGTGCCCTGGAACTCTAGTTGGAGT-----------------
----------------------------AATAAATCTTACAATGAAATATGG------AAT------
AACATGACCTGGTTGCAATGG---GACAAAGAAGTTAACAGTTACACAAAAACAATATAT---
CAGTTAATTGAAGAATCACAAAACCAGCAGGAA---

AAAAATGAACAAGACTTATTAGCATTGGACAAGTGGGCAAGTCTGTGGAATTGGTTTAACATATCAAACTGGC
TGTGGTATATAAAGATATTTATAATAATAGTAGGAGGCTTAATAGGATTAAGAATAGTTTTTACT---
GTGCTCTCTATAATA---------AATAGAGTTAGGCAGGGATACTCACCT------
CTGTCGTTCGCTACTAGCTTGTTTGATACCATAGCAATAGTAATAGCTGGC---
TGGACAGATAGGGTTATAGAAGGAGGACAAAGAATTGGTAGGGCT---------------------------
--------TTTCTCAACATA------CCTAGAAGAATCAGACAGGGTGCCGAAAGGGCCTTAATA---TAA
>KU749423
GCT---GCA--
GAAAACTTGTGGGTTACTATCTATTATGGGGTACCTGTGTGG---AAAGACGCAGAG---
ACCACCTTATTCTGTGCATCAGATGCTAAAGCATATGAAACAGAA---AAGCATAAT----------------
--------------GTCTGGGCTACA---CATGCCTGTGTACCCACAGAC---
CCCAACCCACAGGAAGTACATTTGCCT---------AATGTGACAGAAAAGTTTAAT----------------
-------------------------ATGTGGAAAAATAATATGGTAGAACAGATGCACACAGATATA---
ATCAGTCTATGGGACCAAAGCCTA---AAGCCATGTGTACAGTTA---
ACCCCTCTCTGTGTCACCTTAGATTGTCAATATAACGTCACCCATAACATCAAAGATGAC-------------
----
-------------------ATCCTCAGTATCTCAAATCAGACTGACATGAAAGAAGAAATAAAA---
AATTGCTCTTTCGAGGTGACCACAGAATTACAG----------------------------
GATAGGAAACAGAGAGTGCATTCACTT---
TTTTATAGACTTGATATAGTAAAAATGGAAAAAGATAACAGTAGTAAT-------------------------
----
CAAAATACAAGTGATATAAAGGACCCTTATAGATTAATAAATTGTAATACCTCAACCATTACACAA---
GCTTGTCCAAAAGTATCCTTTGAACCAATTCCCATACATTATTGTGCCCCAGCTGGTTTTGCGATTCTAAAAT
GTAGG---GATGAGGAGTTCAAT------GGAACAGGGCCATGCAAGAAT-----------------------
-GTCAGC---TCAGTACAGTGTACACATGGAATCAAGCCAACAGTGTCAACT---

CAACTACTGTTAAATGGCAGTATAGCAAAG---GAG------------
GTAATAATTAGATCTGAAAATATCACAAACAAT---------GCCAAAATCATAATAGTACACCTTAAC----
-----------CAGACTGTAAAC---------ATTACTTGTATCAGACCTAACAACAATACAAGAACC-----
-AGTGTACGTATA------------GGACCAGGGCAAGCA---TTA------TTCCATGCAGGTGAC------
---------ATAATAGGAGATATA------------AGACAAGCACATTGT---AATATCAGTAGAAAA---
GCATGGAATGAAACTTTAGAAAAGGTAGCCATACAATTAAGA---------------AAACACTTTGTG----
-----------------------------------AACAGTACAATAACCTTTGCT------
AACTCTTCAGGAGGGGATATAGAGATTACA---ACACATAGCTTTAATTGTGGAGGAGAA---
TTTAAACAACTCCAGGCAAGAGTCCTGGCTGTGGAAGGATACCTAAGGGATCAACAGCTCCTAGGAATTTGGG
GCTGCTCTGGAAAACTCATCTGCACCACTACTGTGCCTTGGAACTCTAGTTGGAGT-----------------
----------------------------AATAAATCTTATGATGACATATGG------GGG------
AACATGACCTGGATGCAATGG---GATAGAGAAATTAACAACTACACAAAGACAATATAT---
AATCTAATTGAAGAATCGCAGAACCAGCAAGAA--

AAAAATGAACAAGAATTATTGGAACTTGACAAGTGGGCAAGTCTATGGAATTGGTTTAACATAACACAATGGC
TGTGGTATATAAAAATATTTATAATAATAATAGGAGGCTTAATAGGATTAAGAATAGTTTTTGCT---
GTACTATCTATAATA---------AATAGAGTTAGGCAGGGATACTCACCT------
TTGTCATTCGCTATTAGTTTGCTTGATACCTTAGCAATAACAGTAGCTGGC---
TGGACAGATAGGTGCATAGAGATATTACAAAGAATTGGTAGAGCT----------------------------
--------ATCCTTAACATA------CCTAGAAGAATCAGACAGGGCTTAGAAAGGGCTTTGCTA---TAA
>KX983643
GCT---GCA---
GAAAAATTGTGGGTTACTGTCTACTATGGGGTACCTGTATGG---AAAGATGCAGAA---
ACCACCCTATTTTGTGCATCAGATGCTAAAGGATATGAGACAGAA---GTGCATAAT----------------
--------------GTCTGGGCTACA---CATGCCTGTGTACCCACAGAC---
CCCAACCCACAAGAAATGGATTTGAAG---------AATGTAACAGAGGAGTTTAAC----------------
--------------------------ATGTGGAAAAATAACATGGTAGAGCAGATGCATACAGATATA---
ATTAGTCTATGGGACCAAAGCCTA---AAGCCATGCGTAAAGTTA---
ACTCCTCTCTGCGTTACCTTAAATTGTAGTGATGTCACTGTCAACAGAACTGGTGACAGCAACAGCACG----
----
---GGGATACAGGGAGAAATAAAA---
AACTGCACTTACAATGTGACTACAGAACTAAAG--------------------------
GATAAGAGAAAACAAGTATCTTCGCTT---TTTTATAAACTTGATGTAGTACCACTTGGAGCCACTGGCCAT-
----

AGGAACAGCACTTATACTGATTATAGATTAATAAATTGTAATACCTCAGCCATTACACAG---
GCTTGTCCAAAAGTATCCTTTGAGCCAATTCCCATACATTATTGTGCCCCAGCTGGTTTTGGGATCCTAAAGT
GTAAG---GATGAGAAGTTCAAT------GGAACAGGGCCATGCACGAAT----------------------
-GTTAGT---ACAGTACAATGCACACATGGAATCAAGCCAGTAGTATCAACT---
CAACTGCTGTTAAATGGCAGTCTAGCAGAAAAAGAG------------
GTAAGAATTAGATCTGAAAATATCACAAACAAT---------GCCAAAATCATAATAGTACAACTTGTC----
-----------ACGCCTGTACGA---------ATTAATTGTACCAGACCTAACAACAATACAAGAACA-----
-AGCATACATATA------------GGGCCAGGACAAGCA---TTC------TATGCAACAGGTGAC------
---------ATAATTGGGGATATA------------AGACAAGCACATTGT---GAGGTCAACAAATCA---
GAATGGAATGAAACTTTACAACAGGTAGCTATACAATTAGAA---------------AAACACTTTCAG----
-----------------------------------AATAAAACAATAACCTTTAAT------
AACTCCGCAGGAGGGGATCTAGAAATTACA---ACACATAGTTTTAACTGTGGAGGAGAA---
TTTAAACAGCTCCAGGCAAGGGTCCTGGCTGTGGAAAGATACCTACAGGATCAACAGCTCCTAGGAATTTGGG
GCTGCTCTGGAAAACTCATCTGCACCACTAATGTGCCATGGAACTCTAGTTGGAGC-----------------
----------------------------AATAAATCTCAGAGTGAGATATGG------AAT------
AACATGACCTGGCTGCAATGG---GATAAGGAAATTAACAATTATACAAACATAATATAT---
ACTCTACTTGTGGAATCACAGAACCAGCAGGAA--

AAGAATGAACAAGATTTATTGGCATTAGATAAATGGGCAAGTCTGTGGAATTGGTTTGACATATCAAAATGGC
TGTGGTATATAAAAATATTTATAATGATAGTAGGAGGCTTAATAGGATTAAGAATAGTTTTTGCA---
GTGCTTTCTGTAATA---------AATAGAGTTAGGCAGGGATACTCACCT------
TTGTCGTTTGCTAGTAATCTGATTGATACCATAGCAATAGCAGTAGCTGGC---
TGGACAGATAGGGTTATAGAGTTAGCACAAAGAGTTGGTAGAGCT----------------------------
--------ATTCTTAACATA------CCTACAAGAATTAGACAGGGGTTAGAAAGGGCTTTGCTG---TAA
>KX983542

GCT---GAA--
AACGGCTTGTGGGTTACTGTCTACTATGGGGTACCTGTGTGG---AAAGATGCAGAG---
ACCACCTTATTTTGTGCATCAGATGCTAAAGGATATGATACAGAA---GTACATAAT----------------
--------------GTCTGGGCTACA---CATGCCTGTGTACCCACAGAC---
CCCAATCCACAAGAAATACATTTGGAA---------AATGTGACAGAAAAGTTTGAC----------------
--------------------------ATGTGGAAAAATAACATGGTAGAGCAGATGCATACAGATATA---
ATCAGTCTATGGGACCAAAGCCTA---AAGCCATGTGTACAATTA---
ACCCCTCTCTGTGTCACCTTAAATTGTAGTGACGTGCAAAGAATGCCACCAATGCCACCAAT-----------
----
-------------CCCATACAAGGAAATGCCACCAATCAGGATGGCATGCAAGGAGAAATAAAA---
AACTGCTCTTACAATATGACCACAGTATTAAGA---------------------------
GATAAGACGCAGAAAGTGTATTCACTT---TTTTATAAACTTGATGTAGCACAAATTAACAAC----------
----

AGTAGTAGTGAGTATAGATTAATAAATTGTAATACCTCAACCATTACACAG---
GCTTGTCCAAAGGTAACATTTGAGCCAATTCCCATACATTATTGTGCCCCAGCTGGTTTTGCGATTTTAAAGT
GCAGG---GATACAGAGTTCAAT------GGAACAGGGCTATGCAAGAAT-----------------------
-GTCAGC---ACAGTACAATGCACACATGGAATCAAGCCAGTAGTATCAACC---
CAACTACTGTTAAATGGCAGCCTAGCAGAAGGTGGG------------
ATAAGGATTAGATCTGAAAATATCACAGATAAT---------GTCAAAACCATAATAGTACAGCTTGAT----
-----------GAACCTGTAAAT---------ATTACTTGTATTAGACCTAACAATAATACAAGAAAA-----
-AGCGTGCATATA------------GGACCAGGACGAACA---TTC------TATGCAACAGGTGAC------
---------ATAATAGGGGATATA------------AGACAAGCACATTGC---AATGTCAGTCAAGCA---
GCTTGGGGTAACGCTTTATTCAAGGTAGCTCAACAATTAAAC---------------AACTACTTTAAC----
-----------------------------------AACAAAACATTAGACTTCAAT------
AAATCCTCAGGAGGGGACTTAGAAATTACA---AAACACAGTTTTAATTGTGGAGGAGAA---
TTTAAACAGCTCCGGGCAAGAATCCTAGCTGTCGAAAGATACCTAAAGGATCAACAGCTCCTAGGAATTTGGG
GCTGCTCTGGAAAACTCATCTGCACCACTAATGTGCCCTGGAACTCTAGTTGGAGT-----------------
----------------------------AATAAATCTCAGAATGAAATCTGG------GAA------
AACATGACCTGGCTGCAATGG---GATAGAGAAATTAGCAATTACACAAGTAAAATATAT---
TCTCTAATTGAAGAATCGCAGAACCAGCAGGAA--

AAGAATGAACAAGACTTATTGGCATTAGACAAGTGGGCAAGCCTGTGGACTTGGTTTGATATATCAAATTGGC
TGTGGTATATAAAAATATTCATAATGATAGTAGGAGGTTTAATAGGATTAAGAATAGTTTTTGCT---
GTGCTTGCTATAATA---------AATAGAGTTAGGCAGGGATACTCACCT------
TTGTCATTTGCTATTAATTTGTTTGATACCATAGCAATAGCAGTAGCTGGC---
TGGACAGACAGGGTTATAGAAATATTACAAAGGATTTGTAGAGCT----------------------------
--------ATTCTCAACATA------CCTAGAAGAGTTAGACAGGGCTTAGAAAGGGCTTTGCTA---TAA
>KX983566
GCT---GCA---
GACTTGTGGGTTACTGTCTATTATGGGGTACCTGTGTGG---GAAAACGCAGAG---
ACTACCTTATTTTGTGCTTCAGATGCTAAGGCATATGATACAGAA---GTGCATAAT----------------
--------------ATCTGGGCTACA---CATGCCTGTGTACCCACAGAC---
CCCAACCCACGAGAAATACATCTGGAA---------AATGTGACAGAAGAGTTTAAC----------------
--------------------------ATATGGAAAAATAACATGGTAGAGCAGATGCATACAGATATA---
ATCAGTCTATGGGACCAAGGCCTG---AAGCCATGTGTAAAGTTA---
ACCCCTCTCTGTGTTACTCTAGATTGTCATAACCACACCATAAATACTACGAATGAT----------------
----
--GAAGAAAGAAAA---
AACTGCTCTTTCAATGTGACCACAGAATTAAGG---------------------------
GATAAGAAACAGAAAGTGCATGCACTT---TTTTATAGGCTGGACATAGTACAAATTAATAATAAT-------
----

GATAGTCATAGTTATAGATTAATAAATTGTAATACCTCAGCCATTACACAG---
GCTTGTCCAAAGGTATCCTTTGAGCCAATTCCCATACATTATTGTGCTCCAGCTGGTTTTGCGATCCTAAAGT
GTAGG---GATAGTGAATTCAAT------GGAACAGGGCCATGCAAGAAT-----------------------
-GTCAGC---ACAGTACAATGCACACATGGAATCAAGCCAGTAGTATCAACT---
CAGCTGCTGTTAAACGGCAGTCTAGCAGAAGGAAAG------------
GTAATAATTAGATCTGAAAATATCACAAACAAT---------GCTAAAACCATAATAGTACAATTTAAC----
-----------GAGTCTGTAGGA---------ATTAGTTGTATCAGACCTAACAACAATACAAGAAAA-----

-GGTGTACATATA------------GGACCAGGACAAGCA---TTC------TATGCAACAGGTGAA------
---------ATAATAGGGAAAATA------------AGACAAGCCCATTGT---AATGTCAGTACCCGA---
GACTGGAATAACACTTTACAACGGGTAGCTATACGATTAGGA---------------GACTACTTTCAG----
-----------------------------------GGTAAAACAATAATCTTTGAT------
AACTCCACAGGAGGGGATCTAGAGATTGCG---ACACATAGTTTTAATTGTGGAGGAGAA---
TTTAAACAGCTCCAGGCAAGAGTCCTGGCTGTGGAAAGATACCTAAAGGATCAACAGCTCCTAGGAATTTGGG
GCTGCTCTGGAAAACTCATCTGCACCACTAATGTGCCCTGGAACTCTAGTTGGAGT-----------------
----------------------------AATAAATCTCAGAATGAGATATGG------GAT------
AACATGACCTGGCTGCAATGG---GATAAAGAAATTAGCAATTACACACAAATAATATAT---
GGGCTAATAGAAGAATCGCAGAACCAGCAGGAA--

AAGAATGAACAGGACTTATTGGCATTGGACAAGTGGGCAAATCTGTGGAATTGGTTTGACATCTCAAATTGGC
TGTGGTATATAAAAATATTTATAATGATAGTAGGTGGCTTAATAGGATTAAGAATAATTTTTGCT---
GTGCTTGCTGTAATA---------AATAGAGTTAGGCAGGATACTCACCA------
TTGTCATTTGCCATTAATTTGGTTGATACAGTAGCAATAGCAGTAGCGGGC---
TGGACAGATAGGGTTATAGAAATAGGACAGAGCATTGGTAGGGCT----------------------------
--------ATCCTCCACATA------CCTAGAAGAATTAGACAAGGCTTAGAAAGGGCATTGCTA---TAA
>JX236677
GTT---GCA--
GGAAACTTGTGGGTTACTGTATACTATGGAGTACCTGTGTGG---AGAGACGCAGAT---
ACCACCTTATTTTGTGCCTCAGATGCTAAAGCATATGATACAGAA---GTGCATAAT----------------
--------------GTCTGGGCTACA---CATGCCTGTGTACCCACAGAC---
CCCAGCCCACAAGAGATACATCTGGAA---------AATGTGACAGAAAAGTTTAAC----------------
--------------------------ATGTGGAAAAATAACATGGTAGAGCAAATGCATGAAGATATA---
ATCAGTTTATGGGACCAAAGCCTA---AAACCATGTGTAAAGTTA---
ACCCCTCTCTGCGTTACTTTAGAGTGTAAAGCCCCCAACATCACTGCA-------------------------
----
---GACATGCGAGAAGAAATAAAA---
AACTGCTCTTTCAATATGACTACAGAATTAAGG---------------------------
GATAGGAGACGGAAAATGTATTCACTT---TTCTATAGGCTTGATATAGTGCCAATTAATAAA----------
----

AGTAGGGAAAATAATAGTGAGTATAGATTAATAAATTGTAATACCTCAGCCATTACCCAA---
GCTTGTCCAAAGGTATCCTTTGAGCCAATTCCCATACATTATTGTGCTCCAGCTGGTTTTGCGATTCTAAAGT
GTAAA---GATACAGAGTTCAAT------GGAACAGGGCCATGCAAGAAT-----------------------
-GTCAGC---ACAGTACAATGCACACATGGAATTAAGCCAGTAGTATCAACT---
CAACTGTTGTTAAATGGCAGTCTAGCAGAAAATGGG------------
ATAAGGATTAGATCTGAAAATATTACAAACAAT---------GCCAAAACCATAATAGTACAACTTGAC----
-----------AGGCCTGTACAA---------ATTAATTGTACCAGACCTAACAACAACACAAGAAAA-----
-AGTGAACGTATA------------GGACCAGGACAGACA---TTC------TATGCAACAAATGCC------
---------ATAATAGGGGATATA------------AGGAAAGCACATTGT---AATGTTAGCAAAGCA---
GAATGGAATGAAACTGTACGAAGGGTAGCCGAACAATTAGAA---------------AAATATTTCAAA----
-----------------------------------AACAAAACAATAAAATTTACT------
AACTCCTCAGGAGGGGATTTAGAAATTACC---ACACATAGTTTTAATTGTGGAGGAGAA---
TTTAAACAGCTCCAGGCAAGAGTCCTGGCTGTGGAAAGATACCTAAGGGATCAACAGCTCCTAGGACTTTGGG
GCTGCTCTGGAAAACTCATCTGCACCACTAATGTGCCCTGGAACTCTAGTTGGAAT-----------------
----------------------------AATAAATCTTATAGTGAGATATGG------GAT------
AGCATGACCTGGCTGCAATGG---GATAAAGAAATTAACAATTACACAAGTATAATATAT---
AGCCTAATTGAAGAATCACAGAACCAGCAAGAA--

AAAAATGAACAAGACTTATTGGCATTAGACAAGTGGGCAACTTTGTGGAATTGGTTTGACATAACAAATTGGC
TGTGGTATATAAGAATAGTAATAATAATAGTAGGAGGTTTAATAGGATTAAGAATAGTTTTTGCC---
ATGCTATCTATAATA---------AATAGAGTTAGGCAGGATACTCACCT------
CTGTCGTTGGCTATTAATTTGCTTGATACCACAGCAATAGCAGTAGCTAAC---
TGGACAGATAGGATTATAGAAATAGGACAAAGACTTTGTAGAGCT----------------------------
--------ATTATTCATATA------CCTAGAAGAATCAGACAGGGATTCGAAAGGGCTTTGCTA---TAA
>JX236678
GCT---ACA--
GAACAATTGTGGGTTACTGTCTACTATGGGGTACCTGTGTGG---AAAGACGCAGAG---
ACCACCCTATTTTGTGCATCAGATGCTAAAGCATATAAGACAGAA---ATGCATAAT----------------

--------------GTCTGGCTACA---CATGCCTGTGTACCCACAGAC---
CCCAACCCACAAGAAATACCTTTGGAA---------AATGTAACAGAAAATTTTAAC----------------
--------------------------ATGTGGAAAAATAACATGGTAGAGCAGATGAATACAGATATA---
ATCAACCTATGGATCAAAGCCTA---AAGCCATGTGTAAAGTTA---
ACCCCGCTCTGTGTTACTTTAAATTGTAGCAAAGTCAATGGTAACAAGACAGAGGCCAACACC----------
----
--GAGGGGAGTGGGGAGGAAATGAAA---
AACTGCTCTTTCAATATGACAACAGAACTAAGG---------------------------
GATAGAAGAAAGAAAGTATATTCACTT---TTTTATAAACTTGATGTAGTACAACTTAATAAAAATAATAAT-
----

AGTAGGAATAGTACTTACAATGAATATAGATTAATAAATTGTAATACCTCAGCCATTACACAA---
GCTTGTCCAAAGGTAACCTTTGAGCCAATTCCTATACATTATTGTGCCCCAGCTGGTTTTGCGATCCTAAAGT
GTAGG---GATGAGGAGTTCAGT------GGGATAGGGCTATGTAAGAAT-----------------------
-GTCAGC---ACAGTACAATGCACACATGGAATTAAACCAGTAGTATCAACT---
CAACTGCTATTAAATGGTAGTCTAGCAGAAAAAAAG------------
GTAAGAATTAGATCTGAAAATATCACAAACAAT---------GTCAAAACCATATTAGTACAACTTGCC----
-----------CAGCCTGTGATA---------ATTACGTGTATTAGAACTGGCAACAATACAAGAACA-----
-AGTATACGTATA------------GGACCAGGACAAACA---TTC------TATGCAACAGGTGAC------
---------ATAATAGGGAATATA------------AGAAAAGCATATTGT---AATGTCAGTAGAACA---
GAATGGAATGACACTTTACAACAGGTAGTTATACAATTAGGA---------------GAGTACTTTAAA----
-----------------------------------AACAAAACAATAACCTTTAAT------
AGCTCCTCAGGAGGGGATCTAGAAATCACA---ACCCATAGTTTTAATTGTGGAGGAGAA---
TTTAAACAGCTCCAGGCAAGAGTCCTGGCTGTGGAAAGATACCTAAGGGATCAACAGCTCCTAGGAATTTGGG
GCTGCTCTGGAAAACTCATCTGCACCACTAATGTGCCCTGGAATGCTAGTTGGAGT-----------------
----------------------------AATAAATCTCAGAGTGAGATATGG------GAC------
AATATGACCTGGCTGCAATGG---GATAAAGAAATTAGCAATTACACACACATAATATAT---
AAACTAATTGAAGAATCACAGAACCAGCAGGAG--

AAGAATGAACAAGACTTATTGGCATTGGACAAGTGGGCAAATCTGTGGAATTGGTTTGACATATCAAATTGGC
TGTGGTATATAAAAATATTCATAATAATAGTAGGAGGCTTAATAGGTTTAAGAATAGTTTTTGCT---
GTGCTTTCTATAATA---------AATAGAGTTAGGCAGGGATACTCACCG------
TTGTCGTTTGCTATTAATTTAGTAGATACCATAGCAATAGCAGTAGCTGGC---
TGGACAGATAGAATTATAGAAATAGGACAAGGAATTGGTAGAGCT----------------------------
--------ATTCTCCACATA------CCTAGAAGAATTAGACAGGGCTTCGAAAGGGCTTTGCTA---TAA
>KU749424
GCT---GCA--
GATAAAATGTGGGTTACTGTATACTATGGGGTACCTGTGTGG---AAGGATGCAGAG---
ACCACCCTATTTTGTGCATCAGATGCTAAAGCATATGAGAAAGAA---GTGCATAAT----------------
--------------GTCTGGCTACA---CATGCCTGTGTACCCACAGAC---
CCCAATCCACAAGAATTAAAATTGGAG---------AATGTGACAGAAGAATTTAAC----------------
--------------------------ATGTGGAAAAATCCCATGGTAGAGCAGATGCATACAGATATA---
ATCAGTCTATGGACCAAAGCCTA---AAGCCATGTGTACAGTTA---
ACCCCTCTCTGTGTTACCTTAAATTGTAGCAGTGTCAACAGCAGTATCACAGCTAACAGCAGCAGTGGCAATG
GCACAGCTAAC--
----------------------AGTACAAGTAGCTACGACAAGGAGGCAGTCGAAGAAATGAGA---
AACTGCTCTTACAATATGACCACAGAATTAAGG---------------------------
GATAAAAAACAGAAAATATATTCACTT---TTTTATAAACTTGATATAGTACCAATTAATGGTGAG-------
----

GATAATAAGAGTAGTGAATATAGGTTAATAAACTGTAATACCTCAACCATTACACAG---
GCTTGTCCAAAGGTATCCTTTGATCCAATTCCCATACATTATTGTGCTCCAGCTGGTTTTGCGATCCTAAAGT
GCAAT---GATACGAAGTTCAAT------GGAACAGGGCTATGCAAAAAT-----------------------
-GTCAGC---ACAGTTCAATGCACACATGGAATCAGACCAGTAGTATCAACT---
CAACTACTGTTAAATGGCAGTCTAGCAGAAAAAGAG------------
GTAATGATTAGATCTGAAAATATCACAAACAAT---------GTCAAAAACATAATAGTACAATTGGTC----
-----------AACTCTGTGAAT---------ATTACTTGTATCAGACCTGCCAATAATACAAGAAAA-----
-AGTGTACATATA------------GGACCAGGGCAAGCA---TTT------TATGCAATGGGGAT------
---------GTAATAGGGGATATA------------AGAAAAGCACATTGC---AATGTTAGTAGAACA---
GAATGGAATGAAGCTCTAAAACAGGTAGCTAACCAATTAGGA---------------AAACACTTTGGC----

----------------------------------AACAAAACAATAATATTTAAT------
AACTCCTCAGGAGGAGATTTGGAAATCACA---ACACATAGTTTTAATTGTAGAGGAGAA---
TTTAAACAGCTCCAGGCAAGAGTCCTGGCTGTGGAAAGATACCTAAGGGATCAACAGCTCCTAGGAATTTGGG
GTTGCTCTGGAAGACTCATCTGCACCACTAATGTGCCTTGGAATGAAAGTTGGAGT-----------------
----------------------------AATAAATCTTATGATGAAATATGG------GGA------
AACATGACCTGGCTGCAATGG---GAAAGAGAAATTGACAATTACACAGGCATAATATAT---
AACCTAATTGAAGAATCGCAGAACCAGCAGGAA---

AGAAATGAAAAAGACTTATTGGCATTGGATAAGTGGGCAAGTCTGTGGAATTGGTTTAGCATAACAAAATGGC
TGTGGTATATAAAAATATTTATAATGATAGTAGGAGGCTTAATAGGGTTAAGAATAGTTTTTGCA---
GTGCTTTCTATAATA---------AATAGAGTTAGGCAGGGATACTCACCT------
TTGTCGTTCGCTACTAGTTTGCTTGATACCATAGCAATAGCAGTAGCTGGC---
TGGACAGATAGGATTATAGAACTAGGACAAAGCATTGGTAGGGCT-----------------------------
--------ATCCTCCACATA------CCTAGAAGAATCAGACAGGGTTTGGAAAGGGCTTTGCTA---TAA
>MF565934
GCT---GCA---
AACAACTTGTGGGTTACTGTCTACTATGGGGTACCTGTGTGG---AGAGATGCAGAG---
ACCACCTTATTTTGTGCATCAGATGCTAAAGCATATGACACAGAA---GTGCATAAT----------------
--------------GTCTGGGCTACA---CATGCTTGTGTACCCACAGAC---
CCCAGCCCGCAAGAAATACATTTGGCA---------AATGTGACAGAAAAGTTTAAC----------------
-------------------------ATGTGGAAAAACAGCATGGTAGAGCAGATGCATACAGATATA---
ATCAGTCTATGGGACGAAAGCCTA---AAGCCATGTGTAAAATTA---
ACCCCTCTTTGCATTACTTTAAATTGTACTAACATCACAAGGAAGAACGTCACA-------------------
----
-------------------------GGGGGGAACCTCACAGAGGATGGCAAGGAAGAATTAAAA---
AACTGCTCTTTCAATGCAACCACAGAACTAAGG--------------------------
AATAAGAGACAAAAAGTACATTCACTT---TTTTATAGACTTGATATAGTAGAACTTAATGAGGGTAAT----
----

AGTAGTAATAGTAATACTAGTATGTATAGATTAATAAATTGTAATACCTCAGCAATTACACAG---
GCTTGTCCAAAGGTATCCTTTGAGCCAATTCCCATACATTATTGTGCCCCAGCTGGTTTTGCGATTCTAAAGT
GTAGG---GAGGAGGAGTTTAAT------GGAACAGGGCCATGCAAAAAT----------------------
-GTCAGC---ACAGTACAATGCACACATGGGATCAAGCCAGTAGTATCAACT---
CAGCTGCTGTTAAATGGCAGTCTAGCAGAAGGAACG-----------
GTAAAAATTAGATGTGAAAATATCTCAAACAAT---------GCCAAAACCATACTAGTCCAACTTACT----
-----------ACGCCTGTGAGA---------ATTAATTGTACCAGGCCTAACAACAATACAAGAACA-----
-AGTATACGTATA------------GGACCAGGACAATCG---TTC------TATGCAACAGGTGAC------
---------ATAATAGGGGATATA------------AGAAAAGCATATTGT---AATGTTTCAGGATCA---
GAATGGAAGGAAGCTTTAGGGAAGGTAGTTGTACAATTAAGA---------------AGCCACTTT-------
----------------------------------AATAAAACAATAACCTTTGCT------
AGCTCCTCAGGAGGGGATTTAGAAATCACA---ACACATAGTTTTAATTGTGGAGGAGAA---
TTTAAACAGCTCCAGGCAAGAGTCCTGGCTGTGGAAAGATACCTGAGTGATCAACAGCTCCTAGGAATTTGGG
GCTGCTCTGGAAAGCTCATCTGCACCACTACTGTGCCCTGGAACTCTAGTTGGAGT----------------
----------------------------AATAAATCTCAGGATGAGATATGG------AAT------
AACATGACCTGGCTACAATGG---GATAAAGAAATTAGCAATTACACAGACACAATATAT---
TACTTAATTGAAAAATCGCAGAATCAGCAGGAG---

GTAAATGAAAAAGACTTATTGGCATTGGACAAATGGACAAATCTGTGGAATTGGTTTGACATATCAAAGTGGC
TGTGGTATATAAAAATATTTATAATGATAGTAGGAGGCTTAATAGGATTAAGGATAATTTTTGCT---
GTGCTTTCTGTAATA---------AATAGAGTTAGGCAGGGATACTCACCT------
GTGTCATTTGCTGTTAATCTGGTTGATACCATAGCAATAGTAGTAGCTGGC---
TGGACAGATAGAGTTATAGAAGTATTACAAGGAATTGGTAGAGCT---------------------------
--------TTCCTTCACATA------CCTAGAAGAATCAGACAGGGCTTAGAAAGAGCATTGCTA---TAA
>KX983624
GTT---GCA---
GAAAACTTGTGGGTTACTGTCTATTATGGGGTGCCTGTGTGG---AAAGATGCAGAG---
ACCACTTTATTTTGTGCATCAGATGCTAAAGCATATGATACAGAA---GCGCATAAT----------------
--------------GTCTGGGCTACA---CATGCCTGTGTACCCACAGAC---
CCCAACCCACAAGAAATACATTTGGAA---------AATGTGACAGAAGAGTTTAAC---------------
-------------------------ATGTGGAAAAGTAACATGGTAGAGCAGATGCATACAGACATA---

ATCAGTCTATGGGACCAGAGCCTA---AAGCCATGTGTAAAATTA---
ACCCCTCTCTGTGTTACTTTAGATTGTCATAACGTCACC----------------------------------
----
-------------------------AATAATTTCACGGAGGGGGTCCCGGAAGAAGAAATAAAA---
AACTGCACTTTCAATATGACCACAGAGTTAAGG---------------------------
GATAAGAAGCAGTCAGTGTATTCACTT---TTTTATAGACTTGATATAGTACAAATTGGTGGA----------
----

AAGAATAATAGTCAGCATTATAGATTAATAAATTGTAATACCTCAGCCATTACACAG---
GCTTGTCCAAAGGTATCCTTTGAGCCAATCCCTATACATTATTGTGCCCCAGCTGGTTTTGCGATTCTAAAGT
GTAGG---GATACAAAGTTCAAT------GGAACAGGACCATGCAAGAAT-----------------------
-GTCAGC---ACAGTACAATGCACACATGGAATCAGGCCAGTAGTATCAACT---
CAACTGCTGTTAAATGGCAGTCTAGCAGAAGAAGAG------------
GTAGTAGTGAGGTCTGAAAATATCACAAATAAT---------GTCAAACCCATAATAGTGCAATTGGTC----
-----------AGGCCTGTAAAC---------ATTACTTGTGTCAGACCTAACAACAATACAAGAAAA-----
-AGTGTACGTATA------------GGACCAGGACAAGCA---TTC------TATGCAAGGGGTGGC------
---------ATAATAGGGGATATA------------AAACAAGTATATTGT---ATTGTCAATGAAACA---
GAATGGAAGGGAGCTTTAAGAACTGTATCCCTACAATTAGGA---------------AACTACTTTAAG----
-----------------------------------GACAAAGCAATAAACTTTACT------
AACTCCTCAGGAGGGGATGTAGAAATTACA---ACACATAGTTTTAATTGTGGAGGAGAA---
TTTAAACAGCTCCAGGCAAGAGTCCTGGCTGTGGAAAGATACCTAAAGGATCAACAGCTCCTAGGAATTTGGG
GCTGCTCTGGAAAACTCATCTGCACCACTAATGTGCCCTGGAACTCTAGTTGGAGT-----------------
----------------------------AATAAATCTCAGAGTGAAATATGG------AAT------
AACATGACCTGGATGCAATGG---GATAAGGAAATTGACAATTACACACAGGAAATATAT---
AGACTAATTGAAGAATCGCAGAACCAGCAGGAA--

AAGAATGAACAAGACTTATTGGCATTGGACAAGTGGGCAAGTCTGTGGACCTGGTTTGACATATCAAATTGGC
TGTGGTATATAAGAATATTTATAATGATAGTAGGAGGCTTAATAGGATTAAGAATAGTTTTTGCT---
GTGCTTTCTGTAATA---------AATAGAGTTAGGCAGGGATACTCACCT------
CTGTCATTTGCTATTAATTTGGTTGATACCATAGCAATAGCAGTAGCTGGC---
TGGACAGATAGGGCTATAGAAATAGGACAAGGAATTGGTAGAGCT----------------------------
--------ATAATCCACATA------CCAAGAAGAATCAGACAGGGCTTAGAAAGGGCTTTGCTA---TAA
>KF716472
GCT---ACA--
GAAAACTTGTGGGTTACTGTCTACTATGGGGTACCTGTGTGG---AAGAACGCAGAG---
ACCACCTTATTTTGTGCATCAGATGCTAAAGCATATGATAAAGAG---GTGCATAAT----------------
--------------GTCTGGGCTACA---CATGCCTGTGTACCCACAGAC---
CCCAACCCACAAGAGGTATATTTAGAA---------AATGTGACAGAAGAGTTCAAC----------------
--------------------------ATGTGGAAAAATAACATGGTAGAGCAAATGCATACAGATATA---
ATCAGTCTATGGGACCAAAGCTTA---AAGCCATGTGTACAGTTA---
ACCCCTCTCTGCGTTACTTTAAATTGTACTAAT--

-------------AACATCACCGTGAACGGTAACACAAAAGGGACAGAGATAGAAGGAATGACA---
AACTGTTCTTTCAATGCAACCACAGAATTAAGG---------------------------
GATAAAAACCAGAAAATGTATTCACTT---
TTTTATAAGCTTGATATAGTACAAATTAATGAAGATAAGAATAAT----------------------------
----
AGTAGTAAGAATAATAGTAGTTCTCTGAATAGGTTAATAAATTGTAATACCTCAGCCATTACACAG---
GCTTGTCCAAAGGTATCCTTTGAGCCAATTCCCATACATTATTGTGCCCCAGCTGGTTTTGCAATTCTAAAGT
GTAGG---GACCCAAAGTTCAAT------GGAACAGGACCATGCAAGAAT-----------------------
-GTCAGC---TCAGTACAATGCACACATGGAATCAAGCCAGTAGTATCAACT---
CAACTGCTGTTGAATGGCAGTCTAGCAGAAAAGGAG------------
GTGATAATTAGATCTGAAAATATCACCAACAAT---------GCCAAAACCATAATAGTACAACTTGTC----
-----------AAGCCTATAGGA---------ATTAATTGTACCAGGCCTAGCAACAATACAAGAAAA-----
-AGTGTACGTATA------------GGACCAGGACAAACA---TTC------TATGCAACAGGTGAG------
---------ATAATAGGGGATATA------------AGACAAGCACATTGT---AATGTCAGTAGAGAA---
GACTGGTTTGAAGCTTTAAAAAATATATCCAAACAATTAGGA---------------AAACACTTTGGG----
-----------------------------------AACAAAACAATAGTCTATGAT------
AAATCCTCAGGAGGGGATTTAGAAGTTACA---ACACATAGTTTTAATTGTGGAGGAGAA---
TTTAAACAGCTCCAGGCAAGAATCCTGGCTGTGGAGAGATACCTAAAGGATCAACAGCTCCTGGGAATTTGGG

```
GATGCTCTGGAAAACTTATCTGCACCACTAGTGTACCCTGGAACTCTAGCTGGAGT-----------------
----------------------------AATAAATCTCAGGATGAGATATGG------CAG------
AATATGACCTGGATACAATGG---GATAAAGAAATTAACAATTACACAGAATTAATATAT---
AATCTAATTGAACAATCGCAAAACCAGCAGGAA----------------------------------------
-----------------------------
AAGAATGAACAAGAATTATTGGCATTGGACAAGTGGGCAAGTCTGTGGACTTGGTTTGACATCTCAAACTGGC
TGTGGTATATAAAGATATTTATAATGATAGTAGGAGGCTTAATAGGGTTAAGAATAGTTTTTGCT---
GTGCTTTCTATAATA---------AATAGAGTTAGGCAGGGATACTCACCC------
TTGTCATTTGCTATTAGTTTGTTTGATACAATAGCAATAACAGTAGCTGGC---
TGGACAGATAGAGTTATAGAAATAGTGCAAAGAATTTGTAGAGCT----------------------------
--------ATTCTTAACATA------CCAACAAGAATCAGACAGGGCTTCGAAAGGGCTTTGCTA---TAA
>AB253421
GCT---ACA------------------------------------------
GAAAACTTGTGGGTTACTGTTTACTATGGGGTACCTGTGTGG---AAAGATGCAAAG---
ACCACCCTATTTTGTGCATCAGATGCTAAAGCATATGAGACAGAA---AAGCATAAT----------------
--------------GTCTGGGCTACA---CATGCCTGTGTACCCACAGAC---
CCCAACCCACAAGAGATACATTTGGAA---------AATGTGACAGAAGATTTTAAC----------------
--------------------------ATGTGGAAAAATAACATGGTAGAACAGATGCATACAGATATA---
ATCAGTCTATGGGACCAAAGCCTA---AAGCCATGTGTAAAGCTA---
ACCCCTCTCTGTGTCACTTTACATTGTAGCAATGTCACGGGTGCCAACAGC----------------------
-------------------------------------------------------------------------
-------------------------------------ACTGGCACGGGTGGGAAGAAATAAAA---
AACTGCTCTTACAATATTACCACAGAACTAAGG---------------------------
GATAAAAGAAAGAAAGTATATTCACTT---TTTTATAGACTAGACATAGTACAACTTAGTAGCAAT-------
-------------------------------------------------------------------------
----------------
AATAGTAATAGTAATGAGTATAGATTAATAAATTGTAATACCTCAGCCATTACACAA---
GCTTGTCCAAAGGTATCCTTTGAGCCAATTCCTATACATTATTGTGCCCCAGCTGGTTTTGCGATCCTAAAGT
GTAAG---GATGAGGAGTTCAAT------GGAACAGGGCCATGTAAGAAT-----------------------
-GTCAGC---ACAGTACAATGCACACATGGAATCAAGCCAGTAGTGTCAACT---
CAACTGTTGTTAAATGGCAGTCTAGCAAAAGAAAAG------------
GTAATAATTAGATCTGAAAATATCACAAACAAT---------GTCAAAACCATAATAGTACAACTTGTC----
-----------AAGCCTGTGAAA---------ATTAATTGTACCAGACCTAACAACAACACAAGAACA-----
-AGTATACGTATA------------GGACCAGGACAATCA---TTC------CATGCAACAGGTGAC------
---------ATAATAGGGGATATC------------AGACAAGCACATTGC---AATGTCAGTAGATCA---
GAATGGAATGAAGCCTTACGACAGGTAGTTGAACAACTAAGA---------------GGACACTTTGGG----
-----------------------------------AACAAAACAATAATCTTTACT------
AACTCCTCAGGAGGGGATATAGAAATCACA---ACACATAGTTTTAATTGTGGAGGAGAA---
TTTAAACAGCTCCAGGCAAGAGTCCTGGCTGTGGAAAGCTACCTAAGGGATCAACAGCTCCTAGGAATTTGGG
GCTGCTCTGGAAAACTCATCTGCACCACTACTGTGCCCTGGAATGCTAGTTGGAGT-----------------
----------------------------AATAAATCTTACAGTGAGATATGG------GAG------
AATATGACCTGGTTGCAATGG---GATAAAGAAATTAGCAATTACACAAACCTAATATAT---
GGCCTAATTGAAGAATCGCAGAACCAGCAGGAA----------------------------------------
-----------------------------
AAGAATGAACAAGACTTATTGGCATTGGACAAGTGGGCAAATCTGTGGAGTTGGTTTGAAATATCAAATTGGC
TGTGGTATATAAAAATATTTATAATGATAGTAGGAGGCTTAATAGGATTAAGAATAGTTTTTGCT---
GTGCTTTCTATAATA---------AATAGAGTTAGGCAGGGGTACTCACCT------
TTGTCGTTTGCTACCAGTTTGGTTGATACCATAGCAATAGTAGTAGCTGGC---
TGGACAGATAGAGTTATAGAAATAGTACAAGGAATTGGTAGAGCT----------------------------
--------ATTCTCCACATA------CCTAGAAGAATCAGACAGGGTCTTGAAAGGGCTTTGCTA---TAA
>AB287377
ACT---GCA------------------------------------------
GAAAACTTGTGGGTTACTGTCTACTATGGGGTACCTGTGTGG---AAAGATGCAGAA---
ACCACCTTATTTTGTGCATCAGATGCTAAAGCATACGACACAGAG---GCGCATAAT----------------
--------------GTCTGGGCTACA---CATGCCTGTGTACCCACAGAC---
CCCAACCCACAAGAAATATATTTGGAA---------AATGTGACAGAAGAGTTTAAC----------------
--------------------------ATGTGGAAAAATAACATGGTAGAGCAGATGCATACAGATATA---
ATCAGTCTATGGGATCAAAGCCTA---AAGCCATGTGTACAGTTA---
ACCCCTCTCTGCGTTACTTTAAATTGTAATAACATCACCATCAATAACATCACCACC----------------
-------------------------------------------------------------------------
```

-------------------------------AACATCACTGAGGACATGAGAGGAGAAATAAAA---
AACTGCTCGTACAATATGACCACAGTATTAAGG---------------------------
GATAAGAGAAGGAAAGTGTATTCACTT---TTTTATAGACTTGATATAGTACCACTTGATGAGGGGAAT----
----

AATAACTCTGCTGGGAGTAGTGACTATAGATTAATAAATTGTAATACCTCAACCATAACACAA---
GCCTGTCCAAAGGTCTCTTTTGACCCAATTCCTATACATTATTGTGCTCCAGCTGGTTTTGCGATTCTAAAAT
GTAAG---GATCCAGATTTCAAT------GGAACAGGGCCATGCAAGAAT-----------------------
-GTCAGC---ACAGTACAATGCACACATGGAATCAAGCCAGTAGTATCAACT---
CAACTGCTGTTAAATGGCAGTCTAGCAGAAGGAAAG------------
GTAAGAATTAGATCTGAAAATATTACAAACAAT---------GCCAAAAACATAATAGTACAACTTGTC----
-----------AAGCCTGTAAAA---------ATTAATTGTGTCAGACCTAACAACAATACAAGAACA-----
-AGTGTACGTATA------------GGACCAGGACAAACA---TTC------TATGCAACAGGTGAA------
---------ATAATAGGGGATATA------------AGACAAGCATTTTGT---ACTGTCAATGAATCA---
GAATGGAATGAAACTTTACAACAGGTAGCTACGCAATTAAGA---------------GAACACTTTGAG----
-----------------------------------AACAAAACAATAAAATTTACT------
AACTCCTCAGGAGGGGATTTAGAAATTACA---ACACATAGCTTTAATTGTGGAGGAGAA---
TTTAAACAGCTCCAGGCAAGAGTCCTTGCTGTGGAAAGATACCTGCAGGATCAACAGCTCCTAGGAATTTGGG
GCTGCTCTGGAAAACTCATCTGCACCACTAATGTGCCCTGGAACTCTAGTTGGAGT-----------------
----------------------------AATAAATCTCAGAGTGAGATATGG------GAG------
AACATGACCTGGCTGCAATGG---GATAAAGAAATTAGCAGTTACACAGGCATAATATAT---
AAACTAATTGAAGAATCGCAGAACCAGCAGGAA--

AAGAATGAACAAGACTTATTGGCATTGGACAAGTGGGCAAGTCTATGGAATTGGTTTGAAATATCAAAGTGGC
TGTGGTATATAAAAATATTTATAATGATAGTAGGAGGATTAATAGGATTAAGAATAGTTTTTGCT---
GTGCTTTCTATAATC---------AATAGAGTTAGGCAGGGATACTCACCT------
TTGTCATTTGCTGTTAATTTAGTTGATACCATAGCAATAGCAGTAGCTGGC---
TGGACAGATAGGATTATAGAAACAGGACAAAGATTTTGTAGAGCT----------------------------
--------CTTCTCAACGTA------CCTAGAAGAATCAGACAAGGATTTGAAAGGGCTCTGCTA---TAA
>AB287379
GCT---GTA--
GAAAACTTGTGGGTTACTGTCTACTATGGGGTACCTGTGTGG---AAAGACGCAGAG---
ACCACCTTATTTTGTGCATCAGACGCTAAAGCATATGATACAGAA---GTGCATAAT----------------
--------------GTCTGGGCTACA---CATGCTTGTGTACCCACAGAC---
CCCAACCCACAAGAAATACATTTGGAA---------AATGTGACAGAAGAGTTCAAC----------------
--------------------------ATGTGGAAAAATAACATGGTAGAGCAGATGCATACAGATATA---
ATTAGTCTATGGGATCAAAGCCTA---AAACCATGTGTAAAGTTA---
ACCCCTCTCTGTGTTACTTTAGATTGCCATAAGTATAACAGCACCTATAACGGCACC----------------
----
-------------------------------AACAGTACGGTTGACATGCGAGAAGAAATAAAA---
AACTGCTCTTTCAATATAACCACAGAATTAAGG---------------------------
GATAAGAAAAAGAAAGAGCATTCACTT---TTTTATAGACTTGACATAGTACAAATTAATAAT----------
----

AGCAGTAATAGCAGTCAGTATAGATTAATAAATTGTAATACCTCAGCCATTACACAA---
GCTTGTCCAAAGGTAACCTTTGAGCCAATTCCCATACATTATTGTGCCCCAGCTGGTTTTGCGATTCTAAAGT
GTAAA---GAGATAGAGTTCAAT------GGAACAGGACCATGCAAGAAT-----------------------
-GTCAGC---ACAGTACAATGCACACATGGAATCAGGCCAGTAATATCAACT---
CAACTGCTGTTAAATGGCAGTCTAGCAGAACAAAAG------------
ATAAGGATTAGATCTGAAAATATCACAAACAAT---------GCCAAAACCATAATAGTACAACTTGTC----
-----------CAGCCTGTAGAA---------ATTAATTGTACAAGACCTAACAACAATACAAGAAGA-----
-AGTGTACATATA------------GGACCAGGACAAGCA---TTC------TATGCAACAGGTAAA------
---------ATAATAGGGGATATA------------AGACAAGCACATTGT---AATGTCAGTAGAGCA---
AAATGGAATGATACTTTACGAGAGGTGGCCACACAATTAAGA---------------GAATACTTTAAG----
-----------------------------------AACAAAACAATAATCTTTGAT------
AAATCCTCAGGGGGGGATTTGGAAATTACA---ACACATAGTTTTAATTGTGGAGGAGAA---
TTTAAACAGCTCCAGGCACGAGTCCTGGCTGTGGAAAGATACCTAAAGGATCAACAGCTCCTGGGAATTTGGG
GCTGCTCTGGAAAACTCATCTGCACCACTAATGTGCCCTGGAACTCTAGTTGGAGT-----------------
----------------------------AATAAATCTCAGAGTAAGATATGG------GAT------
AACATGACCTGGCTGCAATGG---GATAAGGAAATTAGCAATTACACAGACATAATATAT---

AGTCTAATTGAAGAAGCGCAGAACCAGCAAGAA---------------------------------------
----
AAGAATGAACAAGACTTATTGGCATTCGACAAGTGGGCAAGTCTGTGGAATTGGTTTGACATATCAAAATGGC
TGTGGTATATAAAAATATTTATAATGATAGTAGGAGGCTTAATAGGATTAAGAATAGTTTTTGCT---
GTGCTTTCTATAATA---------AATAGAGTTAGGCAGGGATACTCACCT------
CTGTCGTTTGCTGTTAACTTGTTTGATACCATAGCTATAGCAATAGCTGGC---
TGGACAGATAGGATTATAGAACTAGGACAAAGAATTTGTAGAGCT----------------------------
--------ATTCTCCACATA------CCTAGAAGAATCAGACAAGGCTTCGAAAGGGCTTTACTA---TAA
>AY713406
GCT---GTA---
GAAAACTTGTGGGTTACTGTCTACTATGGGGTACCGGTGTGG---AAAGATGCAGAG---
ACCACCCTATTTTGTGCCTCAGATGCTAAAGCATATGAGACAGAA---GTGCATAAT----------------
--------------GTCTGGGCTACA---CATGCCTGTGTGCCCACAGAC---
CCAAACCCACAAGAAATACCTTTGGAA---------AATGTGACAGAAAAGTTTAAC----------------
--------------------------ATGTGGAAAAATTATATGGTAGAGCAGATGCATACAGATATA---
ATCAGCCTATGGGATGAAAGCCTA---AAGCCATGTGTAAAGTTA---
ACCCCTCTCTGCGTTACTTTAAATTGTAGCGATGCCATTGGCAACTTT------------------------
----
--ACAAATATCAAGGAAGAAATAACA---
AACTGCTCTTTCAATGTGACCACAGAACTAAGG--------------------------
GATAAGACAAAGAAAGTACATTCACTT---
TTTTATAGACTTGATATAGTACCACTTAATGAATATAGTAGTAGC----------------------------
----
AATAAAAGTAGTAACAATAATAATAGTAGCCAGGAATATGTATTAATAAATTGTAATACCTCAGCCATTACAC
AA---
GCCTGTCCAAAGGTAACCTTTGAGCCAATTCCCATACATTATTGTGCCCCAGCTGGTTTTGCGATCCTAAAGT
GTAAG---GACACAGATTACAAT------GGAACAGGGCCATGCAGGAAT----------------------
-GTCAGC---ACAGTACAATGCACACATGGAATCAAGCCAGTAGTATCAACT---
CAACTGCTGTTAAATGGCAGCCTAGCAGAAAGAAAT-----------
GTAACAATTAGATCTGAAAATATCACAAACAAT---------GCCAAAACCATACTAGTACAACTTGTC----
-----------AAGCCTGTGAGA---------ATTAATTGTACCAGACCTTATAACAATACATTCAGA-----
-AGGGTACGTATA------------GGACCAGGACAATCA---TTC------CATGCATCAGATAAC------
---------ATAGTAGGGGATATA-----------AGAAAAGCACATTGT---AATGTTAGTAGATCA---
GAATGGAATGAAACTTTACAACAGGTAGTTACTCAATTAAGA--------------GGGTACTTTGGG----
------------------------------------AACAAGACAATAGTCTTTACT------
AAGCCCTCAGGAGGGGATCTAGAAATCATA---ACACATAGTTTTAATTGTAGAGGAGAA---
TTTAAACAGCTCCAGGCAAGAGTCCTGGCTGTGGAAAGATACCTAAAGGATCAACAGCTCCTAGGAATTTGGG
GTTGCTCTGGGAAACTCATCTGCACCACTAATGTGCCCTGGAATACTAGTTGGAGT-----------------
-------------------------AAAAATAAATCTCTGAGTGAAATATGG------GAT------
AACATGACCTGGCTGCAATGG---GATAAAGAAATTAGCAATTACACAAACCTAATATAT---
AACCTAATTGAAGAATCGCAGAACCAGCAGGAA---------------------------------------
----
AAGAATGAACAAGACTTATTAGCATTGGATAAGTGGGCAAATCTTTGGACTTGGTTTGACATATCAAATTGGC
TGTGGTATATAAAAATATTCATAATGATAGTAGGAGGCTTAATAGGATTAAGAATAGTTTTTGCT---
GTGCTTACTGTAATA---------AATAGAGTTAGGCAGGGATACTCACCT------
TTGTCATTTGCTATTAATTTGTTTGATACCATAGCAATAGCAGTAGCTGGC---
TGGACAGATAGGGTTATAGAAGTAGGACAAAGAGTTGGTAGAGCT----------------------------
--------TTGCTCCACGTA------CCTAGAAGGATCAGACAGGGCTTTGAAAGGGCTTTGCTA---TAA
>L07082
GTT---GCA---
GGAAACTTGTGGGTTACTGTCTACTATGGGGTACCTGTGTGG---AAAGATGCAGAA---
ACCACCTTATTTTGTGCATCAGATGCTAAAGCATATGATACAGAA---GCACATAAT----------------
--------------GTCTGGGCTACA---CATGCCTGTGTACCCACAGAC---
CCCAACCCACAAGAAATACTTTTGAAA---------AATGTGACAGAAGAGTTTAAC----------------
--------------------------ATGTGGAAAAATGGCATGGTAGAGCAGATGCATACAGATATA---
ATCAGTCTATGGGACCAAAGTCTA---AAGCCTTGTGTACAGTTA---
ACCCCTCTCTGCGTTACCTTAGACTGTAATTATAACATCACCATCAATAGCACCAAAACCCCTTCC-------
----
----------------------CCTGTCACCAATATCTCCGAGGAAATGAGAGAAGAAATAAAA---
AACTGCTCTTTCAATATGACCACAGAATTAAGG--------------------------

GATAAGAAACAGAAAGTGCATTCACTT---TTTTATAAACTTGATATAGTACACCTTAATGAC----------
----

AGTAAGCCTAAGCAGTATAGATTAATAAATTGTAATACCTCAGCCATTACACAG---
GCTTGTCCAAAGGTATCCTTTGAGCCAATTCCCATACATTATTGTGCCCCAGCTGGTTTTGCGATTCTAAAGT
GTAAA---CGTGCAAACTTCACT------GGAACAGGGCCATGCAAAAT------------------------
-GTCAGC---TCAGTACAATGCACACATGGAATCAGGCCAGTAATATCAACT---
CAACTGTTGTTAAATGGCAGTCTAGCAGAAGAAAAA---------
GATGTACAGATTAGATCTGAAAATATCACAAACAAT---------GCCAAAACCATAATAGTACAATTTACC-
--------------AAGGCTGTAAAA---------ATTAATTGTACCAGACCTAACAAAACTACAGAAAA--
----GGTGTGCGTATA------------GGACCAGGACAAGCA---TGG------TATGCAAGAGGTAAC---
------------ATGATAGGAGATATA------------AGAAAAGCATATTGT---AATGTCAGTAGAACA-
--GAATGGAATCAAACATTACAAAAGGTAGCCACACAATTAGGA---------------AAACACTTT-----
-------------------------------------AACAAAACAATAACCTTTACT------
AAATCCTCAGGAGGGGATTTAGAAATTACA---ACACATAGTTTTAATTGTGGAGGAGAA---
TTCAAACAGCTCCAGGCAAGAGTCCTGGCTGTGGAAAGATACCTAAAGGATCAACAGCTCCTAGGAATTTGGG
GCTGCTCTGGAAAACTCATCTGCCCCACTACTGTGCCCTGGAACTCTAGTTGGAGT-----------------
----------------------------AATAAAACTCTGAGTGAAATATGG------GAT------
AACATGACCTGGCTGCAATGG---GATAAAGAGATTAGTAATTACACAGATTTAATATAT---
CAGCTAATTGAAGACTCGCAGAACCAGCAGGAA--

AAGAATGAACAAGACTTGTTGGCATTGGACAAGTGGGCAAGTCTGTGGAATTGGTTTGACATATCAAATTGGC
TGTGGTATATAAGAATATTTATAATGATAGTAGGAGGCTTAATAGGATTGAGAATAATTTTTGCT---
GTGCTTTCTATAATA---------AATAGAGTTAGGCAGGATACTCACCT------
CTGTCGTTTGCTATCAATTTGTTTGATACCATAGCAATAGTAGTAGCTGGC---
TGGACAGATAGGGTTATAGAAGTAGGACAAAGAATTGGTAGAGCT----------------------------
--------TTTCTTAACATA------CCTAGAAGAATCAGACAGGGCGCTGAAAGGGCTTTGATA---TAA
>MF373167
GCT---GCA--
GAGAACTTGTGGGTTACTGTCTACTATGGGGTACCTGTGTGG---AAAGATGCAGAG---
ACCACCTTATTTTGTGCATCAGATGCGAAAGCATATAAAACAGAA---GTGCATAAT----------------
--------------GTCTGGGCTACA---CATGCCTGTGTACCTACAGAC---
CCCAACCCACAAGAAATACATTTGGAA---------AATGTGACAGAAGAGTTTAAC----------------
--------------------------ATGTGGAAAAATAACATGGTAGAGCAGATGCAGACAGATATA---
ATCAGTCTATGGGACCAAAGCCTA---AAGCCATGTGTAAAGTTA---
ACCCCTCTCTGTGTTACTTTAGATTGTAGTGACTATAATGGCACCAATATCACCGGT----------------
----
---GACATGACAAGGGAAATAAAA---
AACTGCTCTTTCAATATAACCACAGAATTAAGG---------------------------
GATAAGAAACAGAAAGTACATTCACTT---TTTTATAGACTAGATGTAGTACAAATTAATAAT----------
----

AGCAGTAACAGCAATAACAGTCAGTATAGATTAATAAATTGTAATACCTCAGCCATTACACAA---
GCTTGTCCAAAGGTATCCTTTGAGCCAATTCCCATACATTATTGTGCCCCAGCTGGTTTTGCGATTCTAAAAT
GTAAT---GATAAGGAGTTCAGT------GGAACAGGGCTATGCAGGAAT-----------------------
-GTCAGC---ACAGTACAATGCACACATGGAATCAAGCCAGTAGTATCAACT---
CAACTGCTGTTAAATGGCAGTCTAGCAGAAGGAAAG------------
GCAATGATTAGATCTGAAAATATCACAAACAAT---------GGTAAAAACATAATAGTACAACTTGCC----
-----------AAGCCTGTACAC---------ATTAATTGTACCAGACCTAATAACAATACAAGAAAA-----
-AGTGTACGTATA------------GGACCAGGACAAGCA---TTT------TATGCAACAGGGGAA------
---------ATAATAGGGAACATA------------AGAAAGGCACATTGT---ACGGTCAATAAAACA---
GAATGGACTAGAACTTTGCGAGAGGTAGCCAAACAATTAAGA------------AAAGAACACTTTAAG----
-----------------------------------AATAAAACAATAATTTTTACT------
AACTCCTCAGGAGGGGATCTAGAAATTACA---ACACATAGTTTTAATTGTAGAGGAGAA---
TTTAAACAGCTCCAGGCAAGAGTCCTAGCTGTGGAAAGATACCTAAGGGATCAACAGCTCCTAGGAATTTGGG
GCTGCTCTGGAAAGCTCATCTGCACCACTAATGTGCCCTGGAACTCTACTTGGAGT-----------------
----------------------------AATAAAACCCAGAGTGAGATATGG------GAC------
AACATGACTTGGCTGCAATGG---GATAAAGAAATTAGCAATTATACACAATTAATATAT---
AGGCTAATTGAAGAATCGCAGACCCAGCAGGAA--

ATAAATGAAAAAGACTTATTGGCATTGGACAAGTGGGCAAATCTGTGGAGTTGGTTTGACATCTCAAATTGGC
TGTGGTATATAAAAATATTTGTAATGATAATAGGAGGCTTAATAGGATTAAGAATAGTATTTGCT---
GTGCTTTCAGTAATA---------AATAGAGTTAGGCAGGGGTACTCACCT------
TTGTCGTTTGCTGTTAATTTGTTTAATACCATAGCAATAGCAGTAGCTGGC---
TGGACAGATAGGGTCATAGAGACAGGACACAGACTTTGTAGAGCT----------------------------
--------ATTTTCAACATA------CCCAGAAGAATCAGACAGGGCCTCGAAAGGGCTTTGCTA---TAA
>MF373168
ACT---GCA---GAC---------------------------------
AACAACAACTTGTGGGTTACGGTCTACTATGGGGTACCTGTGTGG---AGAGATGCAGAA---
ACCACCCTATTTTGTGCATCAGATGCTAAAGCTTATAAGACAGAA---ATGCATAAT----------------
--------------GTATGGGCTACA---CATGCCTGTGTACCCACAGAC---
CCCAACCCACAAGAACTGCCTTTGGGA---------AATGTGACAGAAGAGTTTAAT----------------
--------------------------ATGTGGAAAAATAGCATGGTAGACCAGATGCATACAGATATA---
ATTAGCCTATGGGACGAAAGCCTA---AAACCATGTGTAAAGTTA---
ACCCCTCTCTGTGTTACTTTAAATTGTAACAATGTTTCTGTTTCTACCAATAATACCAATGAT----------
----
-------------------------------------ACAGTGGAAGCAAACAAAGAATTGAGA---
AACTGCTCTTTCAATATGACTACAATGCTAAGG---------------------------
GATAAGAAACAGAAAATATATTCACTT---
TTTTATAAGCTTGATATAGTACAAATTAATGGTAATGATAATAAT----------------------------
----
AATGATACTGTTAGTAATAGTAATTATAGGTTAATAAATTGTAACACCTCAGCCATTACACAG---
GCTTGTCCAAAGGTATCCTTTGAGCCAATTCCCATACATTATTGTGCCCCAGCTGGTTTTGCGATCCTAAAAT
GTAGG---GATGAGGAGTTTAAT------GGAACAGGGTCATGCAAAAAT-----------------------
-GTAAGC---ACAGTACAGTGCACACATGGAATCAAGCCCGTAGTATCAACT---
CAATTGCTATTAAATGGTAGTCTAGCAGAAAAAAAG------------
ATAATGATTAGATCTAAAAATATCTCAGATAAT---------ACCAAAACCATAATAGTACAACTTAAG----
-----------GAGCCTGTAAAA---------ATTGATTGTATCAGACCTAACAACAAGACAAGAAAA-----
-AGTATACGTATA------------GGACCAGGACAAGCA---TTC------TATGCAGCAGGTGAC------
---------ATAATAGGGGATATA------------AGACAAGCACATTGT---GTGGTCAATAGATCA---
AGCTGGGATAACACTCTACGAGAGATAGTTACACAATTAAGA---------------GCACACTGG-------
-----------------------------------AATGGAACAATAAGCTTTAAG------
AGCTCCTCAGGAGGGGATTTAGAGATTACA---ACACATAGTTTCGATTGTGGAGGGGAA---
TTCAAACAGCTCCAGGCGAGAGTCCTGGCTCTGGAAAGATACCTAAGGGATCAACAGCTCCTAGGACTTTGGG
GCTGCTCTGGAAAACTCATCTGCACCACTAATGTGCTCTGGAACAATAGTTGGAGT-----------------
----------------------------AATAAAACTCAGGATGAAATATGG------GAC------
AACATGACCTGGCTACAATGG---GATAAAGAAATTAGCAATTACACACAAGTAATATAT---
ACCCTACTTGAAGACTCGCAGAACCAGCAGGAA--

AAGAATGAAAAAGACTTCCTGGAATTGGACAAATGGGCAAGTCTGTGGAATTGGTTTGATATAACAAATTGGC
TGTGGTACATAAAAATATTCATAATGATAGTAGGAGGCTTAATAGGATTAAGAATAGTTTTTGCT---
GTGCTTTCTGTTATA---------AATAAAGTTAGGCAGGGATACTCACCT------
CTGTCGTTTGCTGTTAATTTGTTTGATACCATAGCAATAGCAGTAGCTGGC---
TGGACAGATAGGGTTATAGAAATAGGACAAAGAATTGGTAGAGCT----------------------------
--------ATTCTCAACATA------CCTAGAAGAATTAGACAGGGCTTAGAAAGGGCTTTGCTA---TAA
>MF373181
ACT---GCA--
GAAAATTTGTGGGTTACTGTTTACTATGGGGTACCTGTATGG---AGAGACGCAAAG---
ACCACCCTGTTTTGTGCATCAGATGCTAAAGCATATAAGACAGAA---GCGCATAAT----------------
--------------ATCTGGGCTACA---CATGCCTGTGTACCCACAGAC---
CCCAACCCACAGGAACTAGATTTGGAA---------AATGTGACAGAAGAGTTTAAC----------------
--------------------------ATGTGGAAAAATAACATGGTAGAGCAGATGCATACAGATATA---
ATCAACCTATGGGATCAAAGCCTA---AAGCCATGTGTAAAGTTA---
ACCCCTCTCTGTGTTACTTTGAATTGTACAAATAATGTAACAAGTATTAACAGTACTGCAACAGTTAATAAC-
----
---AGTGCCATAGAAGAAATAAAA---
AACTGTTCTTTCAATGTGACCACAGAAATATGG---------------------------
GATAAGAGACAAAAAGTGTATTCACTT---TTTTATAAACATGATATAGTACAAATTGATGAG----------
----

AATAGTACTGAGTATATATTAATAAATTGTAATACCTCAGCCATTAAACAA---
GCTTGTCCAAAGGTGTCCTTTGAGCCGATTCCCATACATTATTGTGCCCCAGCTGGTTTTGCGATCCTAAAGT
GTAAG---GATAAGGGATACAAT------GGAACAGGGCCATGCAAAAT------------------------
-GTCAGC---TCAGTACAATGTACACATGGATCAAGCCAGTAGTTTCAACT---
CAATTGCTGTTAAATGGCAGTCTAGCAGAAGAAGAG------------
ATAAGAATTAGATCTGAAAATTTCTCAGACAAT---------GCTAAAACCATAATAGTACAACTTGTT----
-----------GATCCTGTAATA---------ATTAATTGCACCAGACCTAACCAGAAGAAGAGCAGA-----
-AGTATGCGTATA------------GGACCAGGACGAGTA---TTT------TATGCAACAGAAAAA------
---------ATAACAGGG------------------AAACAAGCATCTTGT---ACTGTCAATGGGTCA---
GCATGGAATGAAACTTTACAAAAGGTAGCTGCACAATTAAGA---------------AAACACTGG-------
-----------------------------------AACAAAACAATAATCTTTACT------
CGCCCCTCAGGAGGGGATCTAGAAATTACA---ATGCATAGTTTTAATTGTGGAGGAGAA---
TTTAAACAGCTTCAGGCGAGAGTCCTGGCTATAGAAGGATACCTAAAGGACCAACAGCTCCTAGGGATTTGGG
GCTGCTCTGGAAAACTCATCTGCACCACTAATGTGCCTTGGAACTCTAGTTGGAGT-----------------
----------------------------AATAAATCTTACACGGATATATGG------GGC------
AACATGACCTGGCTGCAGTGG---GATAAAGAAATTAGCAATTATACACAAACAATATAT---
GAGTTAATTGAAGAATCGCAGAGCCAGCAGGAA--

AAGAATGAACAAGACTTATTGGCATTGGACAAGTGGGCAAGTCTGTGGACTTGGTTTGACATAACAAAATGGC
TGTGGTATATAAGAATATTTATAATGATAGTAGGAGGCTTAATAGGGTTAAGAATAGTTTTTGCT---
GTGCTTTCTATAATA---------AATAGAGTTAGGCAGGGATACTCACCT------
CTGTCGTTTGCTATTAATCTGTTTGATACCATAGCAATAGCAGTAGCTGGC---
TGGACTGATAGGGTTATAGAAATAGGACAAGGAATTTGTAGAGCT----------------------------
--------ATTCTCAACATA------CCTAGAAGAATCAGACAGGGCTTGGAAAGGATCCTGCTA---TAA
>MF373182
ACT---ACA--
GAGAACTTGTGGGTTACTGTCTATTATGGGGTACCTGTGTGG---AAAGATGCAAAG---
ACCACCCTATTTTGTGCATCAGATGCAAAAGCATATGATAAAGAA---GTGCATAAT----------------
--------------GTCTGGCTACA---CATGCCTGTGTACCCACGGAC---
CCCAACCCACAAGAATTATATTTGAAA---------AATGTAACAGAAGAGTTTGAC----------------
--------------------------ATGTGGAACAATAACATGGTAGAGCAGATGCATGCAGATATA---
ATCAGTCTATGGGATGAAAGCCTA---AAGCCATGTGTACAATTA---
ACCCCTCTCTGTGTTACTCTAAATTGTAGAGATGTCAATTCTTCCAAT-------------------------
----
----------------------------GCTACCTCCCTTGTCCAGGACATAGCAGAAATCAAG---
AACTGCTCTTTCAATATGACCGCTCATTTAAGG---------------------------
GATACCAACAACAAAGTGTACTCACTT---TTTAATGGATCTGACGTAACTCAAATTAATGAA----------
----

ACGCATGGACCTATAAACTGCTGAGTTGTAATACCTCATCCATTAAAAAA---
CCTTGGCCAATGGAATCCTTTGACCCAATCCCCCTACTTAATCGAGCCCCAGCCGGTATTGGGCTCCTAAAGG
GTAAG---GATACAAATTCCAGT------GGGACAAGACCTTGCATGAAG-----------------------
-GTGGCC---ATAGCAAAATGGTCACCTGGAATCAAGGCACCGGTGTCAACT---
CAGCTGCTGTTAAATGGCCCTGTGGTAAAGGGAGGG------------ATT------
AACTTAAGGCATATCTCGGATAAT---------ACCAAAATTATAATAGTACAACTTGAC-------------
--AAGTCTGTAACC---------ATTAACTGCACCAGACCTAACAATAATACAAGAAAG------
ACTATACGTTTT------------GGACCAGGACACTCA---TTC------TATGCAACAGATGGC-------
--------ATAATAGGGAACATA------------AGGAAAGCACATTGT---AATGTCAGTAGATCA---
GAATGGAAGGGAGCCTTACAAAAAGTAGCTACACAATTAAGA---------------AAACACTTTGAG----
-----------------------------------AACAAAACAATAAATTTTACA------
AGCTCCTCAGGAGGGGATCTAGAAATCACA---ACACATAGTTTTAATTGTAGAGGAGAA---
TTTAAACAGCTCCAGGCAAGACTCCTGGCTGTGGAAAGATACCTAAAGGATCAACAGCTCCTAGGAATTTGGG
GCTGCTCTGGAAGACTCATCTGCACCACTAATGTGCCCTGGAACTTTACTTGGAGT-----------------
----------------------------AATAAATCTTATAATGAGATATGG------GAT------
AACATGACCTGGATGCAATGG---GATAAAGAAATTAACAATTACACACAAGCAATATAT---
GATCTAATTGAAAAATCACAAATCCAGCAGGAG--

CAGAATGAACCGGATTTTTTGGCATTGGCCAAGTGGACAAATTTGTGGAATTGGTTTGCCATATCCAATTGGC
TGTGGTATATAAGAATTTTTATAATGATAGTAGGAGGCTTAATGGGATTAGGAATAGTTTTTGCA---
GTGCTTTCTGTAATA---------AATAGAGTTAGGCAGGGTTACTCACCT------

CTGTCGTTTGCCATTAATTTGCTTGATACCATAGCAATAGCAGTAGCTGGC---
TGGACAGATAGGGTTATAGAACTAGGACAAAGGTTTGGTAGAGCT----------------------------
--------ATTCTCAACATA------CCTAGAAGAATCAGACAGGGCTTCGAAAGGCTTTGCTA---TAA
>AF069670
ACT---GCA--
GACAACTTGTGGGTTACTGTCTACTATGGGGTACCTGTGTGG---AAAGATGCAGAG---
ACCACCCTATTTTGTGCATCAGATGCTCAAGCATATAAGACAGAA---ATGCATAAT----------------
--------------GTATGGGCTACA---CATGCCTGTGTACCCACAGAC---
CCCAACCCACAAGAACTGCATTTGAAA---------AATGTGACAGAAGAGTTTAAC----------------
--------------------------ATGTGGAAAAATAGCATGGTAGAGCAGATGCATACAGATATA---
ATCAGTCTATGGGACGAAAGCCTA---AAGCCATGTGTAAAGTTA---
ACCCCTCTCTGCGTTACTTTAAATTGTACCAATGCCAACGGAACCCAGAAT----------------------
----
-------------------------------------GTCAACATCACGAATGTAGGAATGAGA---
AACTGCTCTTTCAATATGACCACAGAGCTAAGG---------------------------
GATAAGAAACAGAAAGGATATTCACTT---TTTTATAAGCTTGATATAGTACAAATTAATGATAATGGT----
----

AATAACAGTAATAACAGTAGTGAGTATAGGTTAATAAATTGTAATACCTCAGCCATTACACAG---
GCTTGTCCAAAGGTATCCTTTGAGCCAATTCCCATACACTATTGTGCCCCAGCTGGTTTTGCGATCCTAAAGT
GTAGG---GATAAGGAGTTCAAT------GGAACAGGGCCATGCAACAAT-----------------------
-GTAAGC---ACAGTACAGTGCACACATGGAATCAAGCCAGTAGTATCAACT---
CAACTGCTGTTAAATGGCAGTCTAGCAGAAGAAAAG------------
ATAATGATTAGATCTGAAAATATCTCAGACAAT---------GCCAAAACCATAATAGTACAACTTACC----
-----------GAGCCTGTAACA---------ATTAATTGTACCAGACCTAGCAACAATACAAGAACA-----
-AGTATACGTATA------------GGACCAGGACAAGCA---TTC------TATGCAACAGGTGAC------
---------ATAACAGGGGATATA------------AGACAAGCACATTGT---AATGTCAGTAGATCA---
AGCTGGAATAAAACTCTACAAGACATAGTTACACAATTAAGA---------------GTATACTGG-------
-----------------------------------AATAGAACAATAATCTTTAAT------
AGCTCCTCAGGAGGGGATTTAGAAATTACA---ACACATAGTTTTAATTGTGGAGGAGAA---
TTCAAACAGCTCCAGGCAAGAGTCCTGGCTGTGGAAAGATACCTAAAGGATCAACAGCTCCTAGGAATTTGGG
GCTGCTCTGGAAAACTCATCTGCACCACTAATGTGCCCTGGAACTCTAGTTGGAGT-----------------
----------------------------AATAAATCTCAGAATGAAATATGG------GAA------
GACATGACCTGGCTACAATGG---GACAGAGAAATTAGCAATTACACAGAAATAATATAT---
AAACTAATTGAAGAATCGCAGAACCAGCAGGAA--

AAGAATGAACAAGACTTACTGGCATTGGACAAATGGGCAAATCTGTGGAATTGGTTTGAGATAACAAAATGGC
TGTGGTATATAAAAATATTTATAATGATAGTAGGAGGCTTAATAGGATTAAGAATAGTTTTTGCT---
GTGTTTTCTGTTATA---------AATAGAGTTAGGCAGGGATACTCACCT------
TTGTCGTTTGCTATTAGTTTGTTTGATACCCCAGCAATAGCAGTAGCTGGC---
TGGACAGATAGGGGTATAGAATTAATACAAAGAATTGGTAGAGCT----------------------------
--------ATTCTCAACATA------CCTAGAAGAATTAGACAGGGCTTTGAAGAGGCTTTGCTA---TAA
>AF069671
GTT---GCA--
GGAAACTTGTGGGTTACTGTCTACTATGGGGTACCTGTGTGG---AAAGATGCAGAG---
ACCACCCTATTTTGTGCATCAGATGCTAAAGCATATGAGACAGAA---AAGCATAAT----------------
--------------GTCTGGGCTACA---CATGCCTGTGTACCCACAGAC---
CCCAACCCACAAGAAATACATTTGAAA---------AATGTGACAGAAAAGTTTAAC----------------
--------------------------ATGTGGAAAAATTACATGGTGGAGCAGATGCATACAGATATA---
ATCAGTCTATGGGACCAAAGCCTA---GAGCCATGTGTAAAGTTA---
ACCCCTCTCTGTGTTACTTTAGAGTGTCACTATAATATCACTGTCAAGAATATCACTGTCAGCAGC-------
----
----------------------AACAATAATATTAGTATCAGCAACAGCACGGAGGACATGAGA---
AACTGCTCTTTCAATATGACCACAGAACTAAGA---------------------------
GATAAGCAACAGAAAGTATATTCACTT---TTTTATAGACTTGATCTAGTAAAAATTGATGAAAATAAG----
----

AGTAATAGTAGTAATAGTAAGTATAGATTAATAAATTGTAATACCTCAGCCATTACACAA---
GCTTGTCCAAAGGTAACCTTTGAACCAATTCCCATACATTATTGTGCCCCAGCTGGTTTTGCGATCCTAAAGT
GTAAT---GATAAAGAGTTCAAT------GGAACAGGGCTGTGCAACAAT-----------------------

-GTCAGC---ACAGTACAATGCACACATGGAATCAAGCCAGTAGTGTCAACT---
CAACTGCTGTTGAATGGCAGTCTAGCAACA---AAG-----------
ATAATGATTAGATCTGAAAATATCACAAACAAT---------GCCAAAACCATAATAGTACAACTTGTC----
-----------GAGCCTGTGGAA---------ATTAATTGTACCAGACCTAACAACAATACAAGAACA-----
-AGTGTACCTATA------------GGACCAGGAAAAGTA---TTC------TATGCAACAGGTGAG------
---------ATAATAGGGGATATA------------AGACAAGCACATTGT---AATGTCAGTAAATCA---
AAATGGAATGCAACTTTACAAAAGGTAGCTATAAAATTAAGA---------------GAATACTTTGAT----
-----------------------------------GACAAAACAATAATCTTTACC------
AAACCCTCAGGAGGGGATCTAGAAATCACA---ACACATAGTTTTAATTGTGGAGGAGAA---
TTTAAACAGCTCCAGGCAAGAGTCCTGGCTGTGGAAAGATACCTAAAGGATCAACAGCTCCTAGGAATTTGGG
GCTGCTCTGGAAAACTCATCTGCACCACTACTGTGCCCTGGAACTCTAGTTGGAGT-----------------
----------------------------AATAAATCTTACGATGAGATATGG------GAT------
AACATGACCTGGCTGCAATGG---GATAAAGAAATTAGTAATTACACAGAAATAATATAT---
CAACTAATTGAAAAATCGCAGAACCAGCAGGAA--

AAGAATGAACAAGACTTAATGGCATTGGACAAATGGACAAATCTGTGGACTTGGTTTAGCATATCAAACTGGC
TGTGGTATATAAGAATATTTATAATGATAGTAGGAGGCTTGATAGGTTTAAGAATAGTTTTTGCT---
GTGCTTGCTATAATA---------AATAGAGTTAGGCAGGGGTACTCACCA------
TTGTCATTTGCTATTAATTTGGTTGATACCATAGCAATAGCAGTAGCTGGC---
TGGACAGACAGGATTATAGAAATAGGACTAAGAATTGGTAGAGCT-----------------------------
--------TTTCTCCACATA------CCTAGAAGAATTAGGCAGGGCTTTGAAAGGGCTTTGCTA---TAA
>AF069673
GCT---GCA---
GAAAACTTGTGGGTTACTGTCTATTATGGGATACCTGTGTGG---AGAGACGCAGAG---
ACCACCTTATTTTGTGCATCAGATGCAAAAGCATATGATACAGAG---GTGCATAAT----------------
--------------GTCTGGGCCACA---CATGCCTGTGTACCTACAGAC---
CCCAACCCACAAGAAATAAATTTGGAA---------AATGTGACAGAAGAGTTTAAC----------------
--------------------------ATGTGGAAAAATAACATGGTAGAGCAGATGCATACAGATATA---
ATCAGTCTATGGGACCAAAGCCTA---AAGCCATGTGTAAAGTTA---
ACCCCTCTCTGCGTTACTTTAAATTGTAGCTCTGTCACCAATAGCTCTGTCACT-------------------
----
-------------------------------AATATCACCAGTGACATGGCAGGAGAAATAAAA---
AACTGCTCTTTCAATATGACCACAGAAATAAGG--------------------------
GATAAGAGGCAGAAAGTACATGCACTC---TTTTATAGACTGGATGTAGTACCAATGGACAAC----------
----

AATAATAGTTTGTATAGATTAATAAATTGTAATACCTCAGCCATTACACAA---
GCTTGTCCAAAGGTCTCCTTTGAGCCAATTCCCATACATTATTGTGCCCCAGCTGGTTTTGCGATCCTAAAGT
GCAAT---GATAAGGAGTTTAAT------GGAACAGGGCCATGTAAGAAT-----------------------
-GTCAGC---TCAGTACAATGCACACATGGAATCAGGCCAGTAGTATCAACT---
CAACTGCTGTTAAATGGCAGTTTAGCAGAAACAGAG-----------
GTAATGATTAGAGCTGAGAATATCACAAACAAT---------ATCAAAAACATAATAGTACAATTTAAC----
-----------AAGTCTGTAGAA---------ATTATTTGTATCAGACCTAACAACAATACAAGAAAA-----
-AGTATACGTATA------------GGACCAGGACAAGCA---TTC------TATGCAACAGGAGAC------
---------ATAATAGGGGATATA------------AGACAAGCATATTGT---GATGTCAATAGAACA---
GAATGGAATGAAGCTTTACAAAAGGTAGTCAACCAATTAAAA---------------ACACACTTTAAG----
-----------------------------------AACAAAACAATAATCTTTAAT------
AGCTCCTCAGGAGGGGATCTAGAAATAACA---ACACATAGTTTTAATTGTGGAGGAGAA---
TTTAAACAGCTCCAGGCACAAGTCCTGGCTGTGGAAAGATACCTAAGGGATCAACAGCTCCTAGGAATTTGGG
GCTGCTCTGGAAAACTCATCTGCACCACTACTGTGCCTTGGAACTCTAGTTGGAGT-----------------
----------------------------GATGTATCCCAGAGTGAGATATGG------GAG------
AACATGACCTGGCTGCAATGG---GATAAAGAAATTAGCAATTACACACAGATAATATAT---
AGTCTAATTGAAGAATCACAGAACCAGCAGGAA--

AAGAATGAACAAGACTTATTGGCATTGGACAAGTGGGCAAATCTGTGGAATTGGTTTGACATATCAAAATGGC
TGTGGTATATAAAAATATTTATAATGATAGTAGGAGGCTTAATAGGATTAAGAATAGTTATTGCT---
GTGATTTCTATAATA---------AATAGAGTTAGGCAGGGATACTCACCT------
TTGTCGTTCGCTATTAATTTGCTTGATACCATAGCAATAGTAATAGCTGGG---
TGGACAGATAGGGTTATAGAAATAGGACAAGGATTTTGTAGAGCT---------------------------
--------ATTTTCC--ATA------CCTAGAAGAAGCAAACAGGGCTTAAAAAGGGCTTTGCAA---TAA

>AF107771
ACT---GCA---
GAAAACTTGTGGGTTACTGTTTACTATGGGGTACCGGTGTGG---AAAGACGCAGAG---
ACCACCCTATTTTGTGCATCAGATGCCAAAGCATATGAGAAAGAA---GTGCATAAT----------------
--------------GTCTGGGCTACA---CATGCCTGTGTACCCACAGAT---
CCCAACCCACAAGAATTATATTTGGAA---------AATGTGACAGAAGATTTTAAC----------------
--------------------------ATGTGGAAAAATAATATGGTAGAGCAGATGCATACAGATATA---
ATCAGTCTATGGGATCAAAGCCTA---AAGCCATGTGTACAGTTA---
ACCCCTCTCTGCGTTACCTTAAATTGTAGCAACAATGTCACTGCCAACACCAACAGCACG------------
----
-------------------------AGTGCCAACCTCACGGATAGCGTGAAAGGAGAAATGAGA---
AACTGCTCTTTCAATATTACCACAGAACTAAGG---------------------------
GATAAGAAAAAGAAAGTATACTCACTT---
TTTTATAAACTTGATATAGTAAAAATTAATAAAAATAAAAGCTTC----------------------------
----
AGAGGTAAAAATAGCAGTGGTAATAGTAGCAGTGATCGATATAGATTAATAAATTGTAATACCTCAGCCATTA
CACAA---
GCTTGTCCAAAGGTATCCTTTGAGCCAATTCCCATACATTATTGTGCCCCAGCTGGTTTTGCGATCCTAAAGT
GTAAT---GAAGATGAGTTCAAT------GGAACTGGGACATGCAGGAAT----------------------
-GTCAGC---ACAGTACAATGCACACATGGAATCAGGCCAGTAGTATCAACT---
CAACTACTGTTAAATGGCAGTCTAGCAAAAGAAGAG-----------
GTAAGAATTAGATCTGAAAATATCTCAGACAAT---------GCTAAAACCATAATAGTACAATTTACC----
-----------AAACCTGTAGAA---------ATTATTTGTACCAGACCTAACAACAATACAAGGAAA-----
-AGTATACGTATA------------GGACCAGGACAAGCA---TTC------TATGGAATGGGTGAC------
---------ATAATAGGGGATATA------------AGAAAAGCACATTGT---AATGTCAGTAGATCA---
AAATGGAATGAAACTTTAAAAAAGGTGGCTATACAATTAAGA---------------AAATACTGG-------
-----------------------------------AACACAACAATAATCTTTACT------
AACTCCTCAGGAGGGGATTTAGAAATTACA---ACACATAGTTTTAATTGTGGAGGAGAA---
TTTAAACAGCTCCAGGCAAGAGTCATGGCTGTGGAAAGATACCTAAAGGATCAACAGCTTCTAGGAATTTGGG
GCTGCTCTGGAAAGATCATCTGCACCACTGCTGTGCCTTGGAACTCTACCTGGAGT-----------------
----------------------------AATAAATCTTACACTCAAATATGG------GAT------
AACATGACCTGGCTGCAATGG---GATAAAGAAATTAGCAATTACACAGACATAATATAT---
CAACTAATTGAAGAATCGCAATACCAGCAGGAA--

AAGAATGAAAAGGAATTATTGGAATTGGACAAGTGGGCAAATCTGTGGAATTGGTTTGACATATCCAACTGGC
TGTGGTATATAAAAATATTTATAATGATAGTAGGAGGCTTAATAGGCTTAAGAATAGTTTTTGCT---
GTGCTTACTGTAATA---------AAAAGAGTTAGGCAGGGATACTCACCT------
CTGTCGTTCGCTATTAGTTTGTTTGACACCATAGCAATAGCAGTAGCTGGC---
TGGACAGATAGGGTTATAGAAATAGGACAAAGAATTGGTAGAGCT----------------------------
--------ATTCTCCATATA------CCTAGAAGAATTAGACAGGGCTTTGAAAGGGCTTTACTA---TAA
>HQ659604
GCT---ACA---
AGCAAATTGTGGGTTACTGTCTATTATGGGGTACCTGTGTGG---AAAGACGCAGAG---
ACCACCTTATTTTGTGCATCAGATGCTAAAGCATATGAGAGAGAA---GTGCATAAT----------------
--------------GTCTGGGCGACA---CATGCCTGTGTACCCACAGAC---
CCCGACCCACAAGAAATATATTTGGAA---------AATGTGACAGAAGGGTTTAAC----------------
--------------------------ATGTGGAAAAATAACATGGTAGAGCAGATGCATACAGATATA---
ATCAGTCTATGGGACCAGAGTCTA---AAGCCATGTGTAAAGTTA---
ACCCCTCTCTGCGTTACTTTAAATTGTAGCCATAACATCACCACCCATAACATCACCACCAAC----------
----
-------------------------AACCCCAATATCACCAAATACATGGAAGGAGAAATAAAG---
AACTGCTCTTACAATATGACCACAGAACTAAGG---------------------------
GATAAGAAACAGAAAGTGTATTCACTT---TTTTATAAACTTGATGTAGTACCAATTGATAAAAATAAT----
----

GGTAATAACAATATAAGGACTCAGTATAGATTAATAAATTGTAATACCTCAGCCATTACACAG---
GCTTGTCCAAAAGTATCCTTTGAGCCAATTCCCATACATTATTGTGCTCCAGCTGGTTTTGCGATTCTAAAGT
GTAAT---GATGCAGAGTTCAAT------GGAACAGGGCCATGCAAGAAT----------------------
-GTCAGC---ACAGTACAATGCACACATGGAATCAGGCCAGTAATATCAACC---
CAACTGCTGTTAAATGGCAGTCTAGCAGAAGGAAGG-----------

GTAAAGATTAGATCTGAAAATATCACAAACAAC---------GCCAAAACCATAATAGTACAACTTAAC----
-----------AAGACTGTAGAA---------ATTAATTGTACCAGACCTAACAACAATACAAGAAAA-----
-AGTGTACGTATA------------GGACCAGGACAAGCA---TTC------TATGCAACAGGTGAC------
---------ATAATAGGGGATATA------------AGACAAGCATATTGT---AATGTCAGTAGAGCA---
GACTGGAATAAAACTTTACAAGGGGTAGCCAACCAATTAAAA---------------AGTTACTTTAGT----
-----------------------------------AACAAAACAATAATCTTTGCT------
AGCTCCTCAGGAGGGGATTTAGAAATTACA---ACACATAGTTTTAATTGTGGAGGAGAA---
TTTAAACAGCTCCAGGCAAGAGTCCTGGCTGTGGAAAGATACCTAAAGGATCAACAGCTCCTAGGAATTTGGG
GCTGCTCTGGAAAACTCATCTGCACCACTAATGTGCCCTGGAACTCTAGTTGGAGT----------------
----------------------------AATAGAACTCAGAGTGAGATATGG------AAT------
AACATGACCTGGCTGCAATGG---GATAAAGAAATTAGCAATTACACAGACATAATATAT---
AATCTAATTGAAGAATCGCAGATCCAGCAGGAA--

AAGAATGAACAAGAATTATTGGCATTGGACAAGTGGGCAAATCTGTGGAATTGGTTTGACATATCAAAGTGGC
TGTGGTATATAAGGATATTTATAATGATAGTAGGAGGCTTAATAGGATTAAGAATAGTTTTTGCT---
GTGCTTTCTATAATA---------AATAGAGTTAGGCAGGGATACTCACCT------
CTGTCATTTGCTATTAATTTGCTTGATACCATAGCAATAGCAGTAGCTGGC---
TGGACAGATAGGGTTATAGAAATAGGACAGGCAATTTGTAGAGCT---------------------------
--------ATTCTCAACATA------CCTAGAAGAATCAGACAGGGCTTCGAAAGGGCTTTGTTA---TAA
>HQ659597
ACT---GCA---
GAAAATTTGTGGGTTACTGTATACTATGGGGTGCCTGTGTGG---AAAGATGCAGAG---
ACCACCCTATTTTGTGCATCAGATGCTCAGGCATATAACCCAGAA---GCACATAAT----------------
--------------GTCTGGGCTACG---CATGCCTGCGTACCCACAGAC---
CCCAACCCACAAGAAATACATTTGGAA---------AATGTGACAGAAAAGTTTGAC----------------
-------------------------ATGTGGAAAAATAACATGGTAGAGCAGATGCATACAGATATA---
ATCAGTCTATGGGATCAAAGCCTA---AAGCCATGTGTAAAGTTA---
ACCCCTCTCTGTGTTACTTTAGAGTGTAACGATAATGTCTCCAGAAATGGCTCCAAT----------------
----
-----------------------------------GTCACCAATGGAATGGTAGGAGAGATAAAA---
AACTGCTCTTACAATGTGACCACAGTAATAAGG--------------------------
GATAAGAAACAGAAAGTATCTTCACTT---TTTTATAAACTTGATGTAGTACAACTTGACAAGAAT-------
----

AGTAGTCAGTATTATAGATTAATAAATTGTAATACCTCAGCCATTACACAG---
GCTTGTCCAAAGGTGTCTTTTGAGCCAATTCCCATACATTATTGTGCTCCAGCTGGTTTTGCGATTCTAAAGT
GTAAT---GATAAGCAGTTCAAT------GGAACAGGGCTATGCAAGAAT----------------------
-GTTAGC---ACAGTACAATGCACACATGGAATCAAACCAGTAGTATCAACC---
CAACTGCTGTTAAATGGCAGTCTAGCAGAAGAACGG-----------
GTAATGATTAGATCTGAAAATCTCACAAACAAT---------GCCAAAAACATAATAGTACAATTTACT----
-----------AAGCCTGTAGAA---------ATTGAGTGTATCAGACCTAACAACAATACAAGACAA-----
-AGTGTACATATA------------GGACCAGGACAAGCA---TTC------TATGCAACAGGTGAC------
---------ATAATAGGGGATATA------------AGGCAGGCACATTGT---AATGTCAGTAGAAAA---
AACTGGACTGACACTTTGGAACAGGTAGCCATACAATTAAAA---------------ACACACTTT-------
-----------------------------------AACAAAACAATAATCTTTGCT------
AACTCCTCAGGAGGGGATATAGAAATTACA---ACACATAGTTTTAATTGTGGAGGGGAA---
TTTAAACAGCTCCAGGCACGAATCCTGGCTGTGGAAAGATACCTAAGGGATCAACAGCTCCTAGGAATTTGGG
GCTGCTCTGGAAAACTCATCTGCACCACTAATGTGCCCTGGAACTCTAGTTGGAGT----------------
----------------------------AATAAATCCCAGGATGAGATATGG------GGC------
AACATGACCTGGCTGGACTGG---GATAAAGAAATTAGCAATTACACACAGATAATATAT---
GATCTAATTGAACAATCACAGAACCAGCAGGAA--

AAGAATGAACAAGATTTATTGGCATTAGACAAGTGGGCAAGTCTGTGGAATTGGTTTGACATCTCAAAATGGC
TGTGGTATATAAAGATATTTATAATGATAGTAGGAGGCTTAATAGGATTAAGAATAGTTTTTGCA---
GTGCTTTCTATAATA---------AATAGAGTTAGGCAGGGTTACTCACCT------
TTGTCATTTGCTATTAGTTTATTGGATACAATAGCAATAGGAGTAGCTGGC---
TGGACAGATAGGATTTTAGAATTAGGACAAAGACTTTGTAGAGCT---------------------------
--------ATTTACAACATA------CCTAGAAGAATCAGACAGGGCTTCGAAAGGGCTTTGCTA---TAA
>HQ697994

GTG---ATG--
GGAAATTTGTGGGTCACAGTATATTATGGGGTGCCTGTGTGG---AAAGACGCAGAG---
ACCACCCTATTTTGTGCATCAGATGCGAAAGCATATCATACAGAA---GTGCATAAT----------------
--------------GTCTGGGCTACG---CATGCCTGTGTACCCACAGAC---
CCCAACCCACAAGAAATAAATTTGGAA---------AATGTGACAGAAGAGTTTAAC----------------
--------------------------ATGTGGAAAAATAAAATGGTAGAGCAGATGCATACAGATATA---
ATCAGTTTATGGGATCAAAGCCTA---AAGCCATGTGTACAGTTA---
ACCCCTCTCTGTGTTACTTTAGATTGTCAATATAATGTCACCAATATCAATAGCACC----------------
----
--AGTGACATGGCAAGAGAAATAAAC---
AACTGCTCTTACAATATAACCACAGAATTAAGG---------------------------
GATAGGGAACAGAAAGTATATTCACTT---TTTTATAGATCTGATATAGTACAAATGAATAGTGAT-------
----

AATAGTAGTAAGTATAGATTAATAAATTGTAATACCTCAGCCATTAAACAG---
GCTTGTCCAAAGGTAACCTTTGAGCCAATTCCCATACATTATTGTGCTCCAGCTGGTTTTGCAATTCTAAAGT
GTAAA---GATAAAGAGTTCAAT------GGAACAGGACCATGCAAGAAT-----------------------
-GTCAGC---ACAGTACAATGCACACATGGAATCAAGCCAGTAGTATCAACT---
CAACTGCTGTTAAATGGCAGTCTAGCAGAAGAGAAG------------
GTAATGATTAGATCTGAAAATATCACAGACAAT---------GCCAAAAACATCATAGTACAATTTAAG----
-----------GAGCCTGTAAAA---------ATTAATTGTACCAGACCTAACAATAATACAAGAAAA-----
-AGTGTACGTATA------------GGACCAGGACAAACA---TTC------TATGCAACAGGTGAA------
---------ATAATAGGAGATATA------------AGACAAGCACACTGC---AATGTCAGTAAAGCA---
CACTGGGAGAACACGTTGCAAGAGGTAGCCAACCAATTAAAA---------------TTAATGATACACTCT-
-----------------------------------AACAAAACAATAATCTTTGCT------
AACTCCTCAGGAGGGGATCTAGAAATTACA---ACACATAGTTTTAATTGTGGAGGAGAG---
TTTAAACAGCTCAAGGCACGAGTCCTGGCTGTGGAAAGATACCTAAAGGATCAACAGCTCCTAGGAATTTGGG
GCTGCTCTGGAAAACTCATATGCACCACTAATGTGCCCTGGAACTCTAGTTGGAGT-----------------
----------------------------AATAAATCCCTGGGGGAAATATGG------GAC------
AACATGACCTGGTTGCAATGG---GATAAAGAAATCGAGAATTACACACAGATAATATAT---
GAGCTAATTGAAGAATCGCAGAACCAGCAGGAA--

AAGAATGAACAAGATTTATTGGCATTGGACAAGTGGGCAAGTCTGTGGAATTGGTTTGAAATATCAAGGTGGC
TGTGGTATATAAAAATATTTATAATGGTAGTAGGAGGCTTAATAGGATTAAGAATAGTCTTTGCA---
GTGCTTTCTATAATA---------AATAGAGTTAGGCAGGGATACTCACCT------
TTATCATTTGCTACTAATTTGTTTGATACCATAGCAATAGTAGTAGCTGGC---
TGGACAGATAGGGTTATAGAAACAGGACAAAGACTTTGTAGGGCT----------------------------
--------ATTGTCAACGTA------CCTGTAAGAATCAGACAGGGCTTAGAAAGGGCTTTGTTA---TAA
>HQ698029
GCT---GCA--
GCAAACTTGTGGGTTACTGTTTACTATGGGGTGCCTGTGTGG---AAAGATGCAGAG---
ACCACCCTATTTTGTGCATCAGATGCTAAAGCATATGAGACAGAA---GCACATAAT----------------
--------------GTCTGGGCTACA---CATGCCTGTGTACCCACAGAC---
CCCAACCCACAAGAAATACAGTTGGAA---------AATGTGACAGAAGAGTTTAAC----------------
--------------------------ATGTGGAAAAATAACATGGTAGAACAGATGCATACAGATATA---
ATCAGTCTATGGGACCAAAGTCTA---AAGCCATGTGTAAAGTTA---
ACCCCTCTCTGCGTTACTTTGGATTGTAGCCATAATGTCACCAATAATATCACCGAT----------------
----
-------------------------------------AAAGATGGAATGAGAGGAGAAATAAAA---
AATTGCTCTTTCAATGTGACCACAGTACTAAGG---------------------------
GATAAGAGACAGCAAGTATATTCACTT---TTTTATAAACTTGATGTAGTACAAATTGGTAATGAG-------
----

AGTAATAATCAGTATAGATTAATAAATTGTAATACCTCAGCCATTACACAA---
GCTTGTCCAAAAGTATCCTTTCAGCCAATTCCCATACATTATTGTGCTCCAGCTGGTTTTGCAATTCTAAAAT
GTAGG---GAGAAGGAGTTCACT------GGAACAGGGCCATGCAAGAAT-----------------------
-GTCAGC---ACAGTACAATGCACACATGGAATCAGGCCAGTGGTATCAACT---
CAACTGTTATTAAATGGCAGTCTAGCAGAAAGTACG------------
GTAATGATTAGGGCTGAAAATATCACACACAAT---------ACCAAGAACATAATAGTTCAACTTAAA----
-----------GAGCCTGTAAGA---------ATTAATTGTACCAGACCTAACAACAATACAAGAAAA-----

```
-AGTGTACCTATA------------GGACCAGGACAAGCA---TTC------TATGCAACAGGGGAC------
---------ATAATAGGAAATATA------------AGACAAGCATATTGT---AATGTTAGTAAAAAA---
GAATGGAATAGAACGTTGCAACAGGTAGTCAACCAACTAAAA---------------CTACACTTTGTG----
-----------------------------------GACAAAACAATAATCTTTACT------
AACTCCTCAGGAGGGGATCTAGAACTTACC---ACCCATAGTTTTAATTGTGGAGGAGAA---
TTTAAACAGCTCCAGGCAAGAGTCCTGGCTGTGGAAAGATACCTAAGGGATCAACAGCTCCTAGGACTTTGGG
GCTGCTCTGGAAAACTCATCTGCACCACTAGTGTGCCCTGGAACTCTAGTTGGAGC-----------------
----------------------------AATAAATCCCAGCATGAGATATGG------GAC------
AACATGACCTGGCTGCAATGG---GATAAAGAAATTAGCAATTACACAGATATAATATAT---
AGGCTAATTGAAGAATCACAGAATCAGCAGGAA----------------------------------------
-----------------------------
AAGAATGAACAAGATTTATTAGCATTGGACAAGTGGGCAAATCTGTGGAATTGGTTTGACATATCAACATGGC
TGTGGTATATAAAGATATTTATAATGATAGTAGGAGGCTTAATAGGATTAAGAATAGTTTTTGCA---
GTGCTTTCTGTAATA---------AATAGAGTTAGGCAGGGATACTCACCT------
TTGTCATTTGCTATTAATTTGATTGATACCATAGCAATAGCAGTAGCTGGC---
TGGACAGATAGGGTTATAGAAATAGGACAAAGGCTTTGTAGAGCT----------------------------
--------ATTCTTAATATA------CCTGTAAGAATCAGACAGGGTTTGGAAAGGGCTTTGCTA---TAA
>AY253305
GCT---GCA------------------------------------------
GACAACTTATGGGTTACAGTCTACTATGGGGTACCAGTGTGG---AAAGATGCAGAG---
ACCACCTTGTTTTGTGCATCAGATGCGAAAGCATATGATGCAGAA---GTGCATAAT----------------
--------------GTCTGGCTACA---CATGCCTGTGTACCTACAGAC---
CCCAACCCACAAGAAATACATTTGGAA---------AATGTGACAGAAGAGTTTAAC----------------
--------------------------ATGTGGAAAAATAACATGGTAGAGCAGATGCATACAGATATA---
ATCAGTCTATGGGACCAAAGCTTA---AAGCCATGTGTAAAGTTA---
ACCCCTCTCTGCGTTACTTTAAATTGTAGCAATGTCAGTAATAATACTGACAGTGACAACAGTGACAAT----
-------------------------------------------------------------------------
-------------------GCCACCAATTCCACCATCACCAGGGAAATGATTGGAGAAATAAAA---
AACTGCTCTTTCAATATAACCACAGAAATAAGG---------------------------
GACAAGAAACAGAAAGTATATTCACTT---TTTTATAAACTTGATGTAGTGGAAATTAGTAAT----------
-------------------------------------------------------------------------
-------------------
AGTAATAGTAGTCAGTATAGATTAATAAACTGTAATACCTCAGCCATTACCCAG---
GCTTGTCCAAAGGTAACCTTTGAGCCAATTCCCATACACTATTGTGCTCCAGCTGGTTTTGCGATTCTAAAGT
GTAAG---AATAAAACGTTCAAT------GGAACAGGGCCATGCAATAAT--------------------------
-GTCAGC---TCAGTACAATGCACACATGGAATCAGGCCAGTAGTATCAACT---
CAACTGCTGTTAAATGGCAGTCTAGCAGAAGGAGAG------------
ATACAAATTAGATCTGAGAATATCACAAACAAT---------GCCAAAAATATAATAGTACAATTTACC----
-----------GAGCCTGTACAA---------ATTATTTGCATCAGACCTAACAACAATACAAGAAAA-----
-AGGGTACATATA------------GGACCAGGACAGACA---TTC------TATGCAACA---GAC------
---------ATAATAGGGAATATA------------AGACAAGCATATTGT---ACTGTCAATAGAACA---
GAATGGAATACAACTTTGCAAAAGGTAGGCAAACAATTAAGA---------------GATTTATACTTT----
--------------------------------AATAACAAAACAATAATTTTTACT------
AGCTCCTCAGGAGGGGATCTAGAAATTACA---ACACATAGTTTTAATTGTGGGGGAGAA---
TTTAAACAGCTCCAGGCAAGAGTCCTGGCTGTGGAAAGATACCTAAAGGATCAACAGCTCCTAGGAATTTGGG
GCTGCTCTGGAAAACTCATCTGCACCACTAATGTGCCCTGGAATTCTAGTTGGAGT-----------------
----------------------------AATAAATCCCATGATGACATATGG------AAT------
AACATGACCTGGCTGCAATGG---GATAAAGAGATTAGCAATTACACACAGCTAATATAT---
AATCTAATTGAAGAATCACAGAACCAGCAGGAA----------------------------------------
-----------------------------
AAGAATGAACAAGAGTTATTGGCATTGGACAAGTGGGCAAGTCTGTGGAATTGGTTTGATATATCAAATTGGC
TGTGGTATATAAGGATATTTATAATGATAGTAGGAGGCTTAATAGGATTAAGAATAATTTTTGCA---
GTGCTTTCTATAATA---------AATAGAGTTAGGCAGGGATACTCACCT------
TTGTCATTTGCTATTAATTTGCTTGATACCATAGCAATAGCAGTAGCTGGC---
TGGACAGATAGGGTTATAGAAATAGGACAAAGAATTTGTAGAGCT----------------------------
--------ATTTACAACATA------CCTAGAAGAATCAGACAGGGCTTAGAAAGGGCTTTGCTA---TAA
>AY253314
ACT---GCA------------------------------------------
GACAACTTGTGGGTTACTGTCTATTATGGGGTACCTGTGTGG---AGAGACGCAGAA---
ACCACCTTATTTTGTGCATCAGATGCTAAAGCATATAGTACAGAA---AAGCATAAT----------------
```

--------------GTCTGGGCTACA---CATGCCTGTGTACCCACAGAC---
CCCAACCCACAAGAAATACCTTTGGAC---------AATGTGACAGAAGAGTTTAAC----------------
--------------------------ATGTGGAAAAATAACATGGTAGATCAGATGCATGAAGATATA---
ATCAGTTTATGGACCAAAGCCTA---AAGCCATGTGTACAGTTA---
ACCCCTCTCTGTGTTACTTTAAATTGTAGTAATGCCAGGGTCAATGCCACTTTT-------------------
----
----------------------------------AACAGTACGGAGGACAGGGAAGGAATGAAA---
AACTGTTCTTTCAATATGACCACAGAACTAAGG---------------------------
GATAAAAAACAACAAGTTTATTCACTT---TTTTATAGACTAGATATAGAAAAGATTAATAGT----------
----

AGTAACAATAATAGTGAGTATAGATTGGTAAATTGTAATACCTCAGCCATTACACAG---
GCTTGCCCAAAGGTAACCTTTGAACCAATTCCCATACATTATTGTGCCCCAGCTGGTTTTGCGATCCTAAAGT
GTAAT---GATACGGAGTTCAAT------GGAACAGGGCCATGCAAGAAT-----------------------
-GTCAGC---ACAGTACAATGCACACATGGAATCAAGCCAGTAGTATCAACT---
CAACTGCTGTTAAATGGCAGTCTAGCAGAAAGAGAG------------
GTAAGAATCAGATCTGAAAATATCGCAAATAAT---------GCCAAAAATATAATAGTACAGTTTGCC----
-----------AGTCCTGTGAAA---------ATTAATTGTATCAGGCCTAACAACAATACAAGGAAA-----
-AGTTACCGAATA------------GGACCAGGACAAACA---TTC------TATGCAACA---GAC------
---------ATAGTAGGGGATATA------------AGGCAAGCACATTGT---AATGTCAGTAGAACA---
GATTGGAATAACACTTTAAGACTGGTAGCAAATCAATTAAGA---------------AAATACTTTAGT----
-----------------------------------AACAAAACAATAATATTCACT------
AACTCCTCAGGAGGGGATCTAGAAATCACA---ACACATAGTTTTAACTGTGGAGGGGAA---
TTTAAACAGCTCCAGGCAAGAGTCCTGGCTGTGGAAAGGTACCTAAGGGATCAACAGCTCCTAGGAATTTGGG
GCTGCTCTGGAAAACTCATCTGCACCACTAATGTGCCCTGGAACTCTAGCTGGAGT-----------------
----------------------------AATAAATCTTACGATGACATATGG------CAG------
AACATGACCTGGCTGCAATGG---GATAAGGAAATTAGCAATTACACAGACATAATATAT---
AGCCTAATTGAAGAATCACAAAACCAGCAGGAA--

AAGAATGAGCAAGACTTATTGGCATTGGACAAGTGGGCAAATCTGTGGAATTGGTTTGACATATCAAAATGGC
TGTGGTATATAAAAATATTTATAATGATAATAGGAGGCTTAATAGGATTAAGAATAGTTTTTGCT---
GTGCTGTCTGTAATA---------AATAGAGTTAGGCAGGGCTACTCACCT------
TTGTCGTTTGCTATTAGTTTGGTGGATACCATAGCAATAGTAGTAGCTGGC---
TGGACAGATAGGGTTATAGAAATAGGACTAAGAATTGGTAGAGCT----------------------------
--------CTTCTCCACATA------CCTAGAAGAATCAGACAGGGCTTCGAAAGGGCTTTGCTA---TAA
>KX907352
ACT---GCA--
GAAAACTTGTGGGTTACTGTCTACTATGGGGTACCTGTGTGG---AAAGAGGCAGAG---
ACCACCTTATTTTGTGCCTCAGATGCTAAAGCATATGAGACAGAA---ATGCATAAT----------------
--------------GTCTGGGCTACA---CATGCTTGTGTACCCACAGAC---
CCCAACCCACAAGAAATATATTTGGA----------AATGTGACAGAAGAGTTTAAC----------------
--------------------------ATGTGGAAAAATAAGATGGTAGAGCAGATGCATACAGATATA---
ATCAGTCTATGGACCAAAGCCTA---AAGCCATGTGTAAAGCTA---
ACCCCTCTCTGCGTTACTTTAAATTGTAGCAATGTCAATGGCACGAACAGTGCCAATGGCACG----------
----
----------------------------GACAATGTCAATGTCACAGGCAGTGAAGAAATGAAA---
AATTGCTCTTTCAATATGACCACAGAGCTAAGG---------------------------
GATAAGAGAAAGAGGGTCTATTCACTA---TTTTATAGACTTGATATAGTAAAAATTGATGAT----------
----

AATAGTAGTAACCATGAGTATAGATTAATAAATTGTAATACCTCAGCCATTACACAG---
GCCTGCCCAAAGATAACTTTTGAGCCAATTCCCATACATTATTGTGCCCCAGCTGGTTTTGCGATCCTAAAGT
GTAAT---GATGAAGAGTTCAAT------GGAACAGGGCCATGCAAAAAT-----------------------
-GTCAGC---ACAGTACAATGCACACATGGAATTAAGCCAGTAGTATCAACT---
CAACTGTTGTTAAATGGTAGTCTAGCACAAACAGAG------------
GTAAAAATTCGATCTGAAAATATCACAAACAAT---------GCCAAAACTATAATAGTACAATTTGTC----
-----------AAGCCTGTGCAA---------ATTAATTGTACCAGACCTAACAACAATACAAGACAA-----
-AGTATACGTATA------------GGACCAGGACAAGCA---TTC------TATGCACACGGTGAG------
---------ATAATAGGGGATATA------------AGACAAGCATATTGT---AATGTCAGTGGATCA---
ACATGGAATGTAACTTTAAAACAGGTAGTTGAACAATTAAGA---------------AAACACTTTGGG----

------------------------------------AACAAAACAATAATATTTACT------
AACTCCTCAGGAGGGGATCTAGAAATCACA---ACACATAGTTTTAATTGTGGAGGAGAA---
TTTAAACAGCTCCAGGCAAGAGTCCTGGCTGTGGAAAGATACCTAAGGGATCAACAGCTCCTAGGAATTTGGG
GCTGCTCTGGAAAACTCATCTGCACCACTAATGTGCCCTGGAACTCTAGTTGGAGT-----------------
-----------------------------AATAAATCTCAGACTGAGATATGG------GAC------
AATATGACCTGGCTGCAATGG---GATAAAGAAATTAGCAATTACACACAATTAATATAT---
AACCTAATTGAAGAATCGCAGAATCAGCAGGAA---

AAGAATGAACTAGACTTATTGGCATTGGACAAGTGGGCAGATCTGTGGAATTGGTTTGACATAACAAATTGGC
TGTGGTATATAAAAATATTTATAATAATAGTAGGAAGCTTAATAGGATTAAGAATAGTTTTTGCT---
GTGCTGTCAGTTATA---------AATAGAGTTAGGCAGGGGTACTCACCT------
CTATCGTTTGCTATTAATTTGGTTGATACTATAGCAATAACAGTAGCTGGC---
TGGACAGATAGGGCTATAGAAATAGGACAAAGAATTGGTAGAGCT-----------------------------
--------ATCCTCCACATA------CCTAGAAGAATTAGACAGGGCTTTGAAAGAGCTTTGCAA---TAA
>KX907348
GCT---
GAAAATTTGTGGGTTACCGTCTACTATGGGGTACCTGTGTGG---AGAGACACAGAG---
ACCACTCTATTTTGTGCATCAGATGCTAAAGCATATAAGGCAGAA---GCGCATAAT----------------
--------------GTCTGGGCTACG---CATGCCTGTGTACCCACAGAC---
CCCAACCCACAAGAAATATATTTGAAA---------AATGTGACAGAAAATTTTAAC----------------
---------------------------ATATGGAAAAATAACATGGTAGACCAGATGCATACAGATATA---
ATCAGTCTATGGGACCAAAGCCTA---AAGCCATGTGTAAAATTA---
ACCCCTCTCTGCGTTACTTTAAATTGTAGCAACTATAATGGCACTAATGGCAATCAGAATATCACCAAT----
----
---GAAATGGTAGGAGAAATAAAA---
AACTGCTCTTACAATATAACCACAGTACTAAAG---------------------------
GATAAGGTACAGAAAGTATATTCACTT---
TTTTATAGACTTGATGTAGTACAAATTGATGACAATAGTAATAAT-----------------------------
----
AGTCAAAATAGTAATGGTAGTGGGCAGTATAGATTAATAAATTGTAATACCTCAGCCATTACACAG---
GCTTGTCCAAAGATATCCTTTGAGCCAATTCCCATACATTATTGTGCTCCAGCTGGCTTTGCGATTCTAAAAT
GTAAT---GATAAGGAGTTCAAT------GGAACAGGGCCATGTAATAAT-----------------------
-GTCAGC---ACAGTACAATGCACACATGGAATCAGGCCAGTAGTATCAACT---
CAATTGCTGTTAAATGGCAGTCTAGCAGAAGAAGGT-----------
GTAATGATTAGATCTGAAAATATCACAAACAAT---------GCCAAAAACATACTAGTACAACTTAAG----
-----------GAGCCTGTAATA---------ATTAATTGTACCAGACCTAACAACAATACAAGAAAA-----
-GGTGTACATCTA------------GGACCAGGACGAGCA---TTC------TATGCAACAGGTAAC------
---------ATAATAGGGAATATA------------AGACAAGCACATTGT---ACAGTCAATAGAACA---
GCATGGAATGCAACTCTGCAAACGGTAGTCGACAAATTAAGA---------------GAACACTTTGGG----
------------------------------------AACAAAACAATAATCTTTGAT------
AAACCCACAGGAGGGGATCTAGAAATTACA---ACGCATAGTTTTAATTGTGGGGGAGAA---
TTTAAACAGCTCCAGGCAAGAGTCCTAGCTGTGGAAAGATACCTAAGAGATCAACAGCTCCTAGGAATTTGGG
GCTGCTCTGGAAAGCTCATCTGCACCACTAATGTGCCCTGGAACTCTAGTTGGAGT----------------
-----------------------------AATAAATCTCAGGATGAGATATGG------AAG------
AACATGACCTGGCTGCAATGG---GATAAAGAAATTAACAATTACACAGAGATAATATAT---
AACCTAATTGAAGAATCGCAGACTCAACAGGAA---

AGAAATGAACATGACTTATTGGCATTGGACAAGTGGGCAAGTCTGTGGAATTGGTTTGACATATCAAACTGGC
TGTGGTATATAAGAATATTTATAATGATAGTAGGAGGCTTAATAGGATTAAGAATAGTTTTTACA---
GTGCTTTCTATAATA---------AATAGAGTTAGGCAGGGATACTCACCT------
TTGTCGTTTGCTATTAATTTGTTTGATACCATAGCAATAGCAACAGCTAGC---
TGGACAGATAGTATTATAGAACTAGTACAAAGATTTTGTAGAGCT----------------------------
--------ATTCTCAACATA------CCTAGAAGGATCAGACAGGGCCTCGAAAGGGCTTTGTTA---TAA
>KX907336
GCT---GCA---------------------------------------
GGAAACTTGTGGGTTACTGTCTACTATGGGGTACCTGTGTGG---AAAGAAGCAGAG---
ACCACCTTATTTTGTGCCTCTGAAGCTAAGGCATATGGGACAGAA---GTGCATAAT----------------
--------------GTTTGGGCTACA---CATGCCTGTGTGCCCACAGAC---
CCCAACCCACGAGAGATATATTTGCCA---------AATGTGACAGAAGAGTTTAAC---------------
--------------------------ATGTGGAAAAATAGCATGGTAGAGCAGATGCATACAGATATA---

ATTAGTCTATGGGACCAAAGCCTA---AAGCCATGTGTAAAGTTA---
ACCCCACTCTGTGTTACTTTAGATTGC---CAGTATAACATCACCACGAACAACACCAAT-------------
----
----------------------------AGGAACAATAGCTTTGACATAGGAGAAGAAATGAAA---
AACTGTTCTTTCAATATGACTACAGAAGTAAGG---------------------------
GATAAGAAACAGAAAGTGTATTCACTT---TTTTATAGACTGGATATAGTACCAATTAATACC----------
----

AATAATAGCTATAGATTAATAAATTGTAATACCTCAGCCATTACACAA---
GCCTGTCCAAAGGTATCCTTTGAGCCAATTCCCATACATTATTGTGCCCCAGCTGGTTTTGCGATTCTGAGGT
GTAAC---GATATAGAGTTCAAT------GGAACAGGACCATGCAAGAAT-----------------------
-GTCAGC---ACAGTACAATGCACACATGGAATCAGGCCAGTAATGTCAACT---
CAGCTGTTGTTAAATGGCAGTTTAGCAGAAAAAGAG------------
GTAAGGATTAGATGTGAAAATATTACAAACAAT---------GCCAAAACCATAATAGTACAACTTAAC----
-----------CAGTCTGTACCA---------ATTAATTGCACCAGGCCTTACAACAACACAAGAAGA-----
-AGTACACGTATA-------------GGACCAGGACAAGCA---TTC------TATGCTACAGGTGAC------
---------ATAATAGGAGATATA------------AGACAAGCACATTGT---AATGTCAGTAGAAAA---
CAATGGAATGACATGTTACACATGGTAGTCACACAATTAAGA---------------AAACACTTT-------
-----------------------------------AACAAAACAATGATCTTTAAT------
GAATCCTCAGGGGGAGATTTAGAAATTACA---ACACATAGTTTTAATTGTGGAGGAGAA---
TTTAAACAGCTCCAGGCACGAGTCCTGGCTGTGGAAAGATACCTAAGGGATCAACAGCTCCTAGGAATTTGGG
GCTGCTCTGGAAAACTCATCTGCACCACTAATGTGCCCTGGAATTCTAGTTGGAAC-----------------
----------------------------AATAAATCTCAGAGTGAGATATGG------GAG------
AACATGACCTGGCTGCAATGG---GATAAAGAAATTAGCAATTACACATATACAATATAT---
AATCTACTTGAAGAAGCACAAAACCAACAGGAA--

AAAAATGAACAAGACTTATTGGCATTGGACAAGTGGGAAAGTCTGTGGAATTGGTTTGACATATCAAAATGGC
TGTGGTATATAAAAATATTTATAATGATAGTAGGAGGCTTAATAGGATTAAGAATAGTTTTTGCT---
ATACTTTCTGTAATA---------AATAGAGTTAGGCAGGGATACTCACCT------
TTGTCGTTTGCTATTAATTTGCTTGATACCATAGCAATAGCAGTAGCTGGT---
TGGACAGATAGGGCTATAGAAATAGGACAAAGAGTTTGTAGAGCT----------------------------
--------ATTCTCAACATA------CCTAGAAGAATCAGACAGGGCTTCGAAAGGGCTTTGCTA---TAA
>KX907372
GCT---GCA--
GAAAACATGTGGGTTACTGTCTATTATGGGGTACCTGTGTGG---AGAGATGCAGAG---
ACAACCTTATTTTGTGCATCAGATGCTAAAGGATATGAGACAGAA---AAGCATAAT----------------
--------------GTCTGGGCTACA---CATGCCTGTGTACCCACAGAC---
CCCAACCCACAAGAAATACATTTGGAA---------AATGTGACAGAAGAGTTTAAC----------------
--------------------------ATGTGGAAAAATAACATGGTAGATCAGATGCATACAGATATA---
ATTAGTCTATGGGACCAAAGCCTA---AAACCATGTGTAAAGTTA---
ACCCCTCTCTGCGTTACTTTACAGTGTAGCGATGTCACTAGCAATGGCACTAACAGCAATGTC----------
----
---ACTAACAGGGAAGAAATGAAA---
AACTGCTCTTTCAATGTAACCACAGAAATAAGG---------------------------
GATAAAAAACAAAAAGTATATTCACTT---TTTTATAGACTTGATATAGTACAAATTAGTAAT----------
----

AGTAGTAATAGTAGTGAGTATATATTAATAAATTGTAACACCTCAACCCTTACACAG---
GCTTGCCCAAAGATATCTTTTGAGCCAATTCCCATACATTATTGTGCCCCAGCTGGTTTTGCGATACTAAAGT
GTAAG---GATGAGGAATTCGAT------GGAACAGGACCTTGTAAGAAT-----------------------
-GTCAGC---ACAGTACAATGCACACATGGAATCAAGCCAGTGGTATCAACT---
CAACTGCTGCTAAATGGCAGTTTAGCAGAAAAAGGA------------
GTAAAAGTTAGATCTGAGAATATCACAAACAAT---------GCCAAAAATATAATAATACAACTTTCC----
-----------AAGCCTGTGGAA---------ATTAACTGTAYCAGACCTAACAACAATACAAGAAAA-----
-AGTATACGTATA-------------GGACCAGGACAATCA---TTC------TATGCAACAGGTGAC------
---------ATAATAGGGGATATA------------AGGGAAGCACATTGT---AATGTCAGTCAAACA---
GAATGGAATAACACTTTACATCAGGTAGCAGAACAATTACAA---------------AAATACTTTAAT----
-----------------------------------AACAAAACAATAATATTTAAT------
GGATCCTCAGGAGGGGATCTAGAAATTACA---ACACATAGTTTTAATTGTGGAGGAGAA---
TTTAAACAGCTCCAGGCAAGAGTCCTGGCTGTGGAAAGATACCTAAGGGATCAACAGCTCCTAGGAATTTGGG

GCTGCTCTGGAAAGCTCATCTGCACCACTAATGTACCCTGGAACTCTAGCTGGAGT-----------------
-----------------------------AATAAATCTTACAGTGAGATATGG------GAC------
AACATGACCTGGCTACAATGG---GATAAGGAAGTTAGCAATTACACACAGATAATATAT---
CAACTAATTGAAGAATCGCAGAACCAGCAGGAA--

AAGAATGAACAAGACTTACTGGCGTTGGACAAGTGGGCAAGTCTGTGGAATTGGTTTAACATATCAAATTGGC
TGTGGTATATAAAAATATTTATAATGATAATAGGAGGCTTAATAGGATTAAGAATAGTTTTTATT---
GTAATCTCTGTAATA---------AATAGAGTTAGGCAGGGCTACTCACCT------
TTGTCATTCGCTATTAATTTGTTTGATACCATAGCAATAGCAGTAGCTGGC---
TGGACAGATAGGATTATAGAAATAGGACAAAGAATTGGTAGAGCT----------------------------
--------ATTCTTAATATA------CCTACAAGAATCAGACAGGGCTTCGAAAGGGCTTTGCTT---TAA
>KX907383
GCT---GCA---
GAAAACTTGTGGGTCACTGTTTATTATGGGGTGCCTGTGTGG---AAAGATGCAGAA---
ACCACCCTATTTTGTGCATCAGATGCTAGAGCATATGAGAAAGAA---GCGCATAAT----------------
--------------ATCTGGGCTACG---CATGCATGTGTACCCACCGAC---
CCCGACCCACAAGAATTATATTTGGAA---------AATGTGACAGAAGAATTTAAC----------------
-------------------------ATGTGGAAAAATAACATGGTAGAGCAGATGCATACAGATATA---
ATTAGTCTATGGGACCAAAGCCTA---AAACCCTGTGTACAGTTA---
ACCCCTCTCTGCGTTACTTTAAATTGTAACGATGTCACCAATAATGGCACCAAT-------------------
----
-------------------------GTTGCTAACATCAGCATTGACATGAAAGGAGAAATAAAA---
AACTGCTCTTTCAACATGACCACAGAATTAAGG---------------------------
GATAAGAAACAGAAAGTATATTCACTT---TTCTATAAACTTGATGTAGTACAAATGAATAGT----------
----

AGTCRGTATAGATTAATAAATTGTAATACCTCAGCCATTAAACAA---
GCTTGTCCAAAGGTATCCTTTGAGCCAATTCCCATACATTATTGTGCTCCAGCTGGTTTTGCGATTCTAAAAT
GTAGG---GATGAGGAGTTCGAT------GGAACAGGGCCATGCACTAAT----------------------
-GTCAGT---ACAGTACAATGCACACATGGAATCAAGCCAGTAGTATCAACT---
CAACTGCTGTTAAATGGCAGTCTAGCAGAAAAAAGG------------
GTAATGGTTAGGTCTGAAAATATCACAAACAAT---------GTCAAAAACATAATAGTACAATTTAAT----
-----------GAGACTGTAAAC---------ATAACTTGTATCAGACCTAACAACAATACAAGAAAA-----
-AGTGTACATATA------------GGGCCAGGACAAGCA---TTC------TATGCAACAGGTGAC------
---------ATAATAGGGAATATA------------AGAAAAGCACATTGT---CATGTTAATAAAACA---
GAATGGAATAGAATGTTGGCAAAGGCAGCCCAACAATTAATT---------------AACCACTTT-------
-------------------------------------AACAAAACAATAACCTTTAAG------
AACTCCTCAGGAGGGGATCTAGAAATTACA---TCACATAGTTTTAATTGTGGAGGAGAA---
TTTAAACAGCTCCAGGCAAGAGTCCTGGCTGTGGAAAGATACCTAAAGGATCAACAGCTCCTAGGAATTTGGG
GCTGCTCTGGAAAACTCATCTGCACCACTAATGTGCCCTGGAACAATAGTTGGAGT----------------
-----------------------------AATAAATCCCTAGAGGATATATGG------GAG------
AATATGACCTGGATGCAATGG---GACAAGGAAATTAACAATTACACACATCTGATATAT---
AATCTAATTGAAGAATCGCAGTACCAGCAGGAA--

AAGAATGAAAAAGAGTTATTAGAATTGGACAAGTGGGCTAATCTGTGGAATTGGTTTGATATATCAAACTGGC
TGTGGTATATAAGGATATTTATAATGATAGTAGGAGGCTTAATAGGCTTAAGAATAGTTTTTGCA---
GTACTTTCTGTAATA---------AATAGAGTTAGGCAGGGGTACTCACCT------
GTGTCATTTGCTGTTAACTTGTTTGATACCATAGCAATAGCAGTAGCTGGC---
TGGACAGATAGGATTATAGAACTAGGACAAAGACTTTGTAGAGCT---------------------------
--------TTTCTTAACATA------CCTAGAAGAATCAGACAGGGATTTGAAAGGGCTTTGCTG---TAA
>KX907389
GCT---GAT--
GAAAAAACGTGGGTTACTGTCTATTATGGGGTGCCTGTGTGG---AAAGACGCGGAT---
ACCACCCTATTTTGCGCATCAGATGCTAAAGCATATGATACAGAG---GTACATAAT----------------
--------------GTCTGGGCTACG---CATGCCTGTGTACCCGCAGAC---
CCCAACCCACAAGAAATACATATGGAA---------AATGTGATAGAAGAGTTTAAC----------------
-------------------------ATGTGGAAAAATAACATGGTAGAACAGATGCATACAGATATA---
ATTAGTTTATGGGACCAAAGCCTA---AAGCCATGTGTACAGTTA---
ACCCCTCTCTGTGTCACTTTAAATTGTAGCACTGTCAATAATAGCACCAGTATCAAT----------------
----

----------------------------AGCACTCTCTATGAAGACATGAAAGGAGAACTAAAA---
AACTGCTCTTTCAATATGACCACAGAACTAAGG---------------------------
GATAAGAAACAGAAAGTATATTCACTT---TTTTATAAGATTGATGTAGTACCAATTAATCAA----------
----

AATAGTGGTAATAGTAGTAGTGATCAGTATAGGTTAATAAATTGTAATACCTCAGCCATTAAACAG---
GCTTGTCCAAAGGTAACCTTTGAGCCAATTCCCATACATTATTGTGCTCCAGCTGGCTTTGCGATTCTAAAGT
GTAAG---GATGATGAGTTCAAT------GGAACAGGGCCATGCAAAAAT-----------------------
-GTCAGC---ACAGTACAATGCACACATGGAATCAAGCCAGTAATATCAACT---
CAACTGCTGTTAAATGGCAGTCTAGCAGAAAAAGAG------------
GTARGAATTAGATCTGAAAATATCACAAACAAT---------GCCAAAAACATACTAGTACAACTTAAA----
-----------GAACCTGTAAAC---------ATTAATTGTACCAGACCTAACAATAATACAAGGAAA-----
-AGTGTACGTATA------------GGACCAGGACAAACA---TTC------TATGCAACAGGTGAC------
---------ATAGTAGGGGATATA------------AGACAAGCATATTGT---AATATCAGTAGATCA---
AAATGGAATGAAGCTTTGTACCATGTAGCCAAACAATTAAGA---------------ACACACTTC-------
-----------------------------------AACAAAACAATAATCTTTAAA------
AATTCCTCAGGGGGAGATCTAGAAATTACA---ACACATAGTTTTAATTGTGGAGGAGAA---
TTTAAACAGCTCCAGGCAAGAGTCCTGGCTGTGGAAAGATACCTAAAAGATCAACAGCTCCTAGGAATTTGGG
GCTGCTCTGGAAAGCTCATCTGCACCACTAATGTGCCCTGGAACACTAGTTGGAGT-----------------
-------------------------AATAAATCCCAGGATGAGATATGGGAA------AGC------
AACATGACCTGGCAGCAATGG---GAGAAAGAAATTAGCAATTACACACAGATAATATAT---
AATCTAATTGAAGACTCGCAGAACCAGCAGGAA--

AAGAATGAACATGATTTATTGGCATTGGACAAGTGGGCAAGTCTGTGGAATTGGTTTGACATATCAAACTGGC
TGTGGTACATAAAAATATTTATAATGATAGTAGGAGGCTTAATAGGATTAAGAATAGTTTTTGCA---
GTGCTTTCTATAATA---------AATAGAGTTAGGCAGGGATACTCACCT------
TTGTCGTTTGCTATTAATTTGCTTGATACCATAGCAATAGCAGTAGCTGGC---
TGGACAGACAGGGTTATAGAAATAGGACAAAGGTTTTGTAGAGCT----------------------------
--------ATTCTCCATATA------CCTAGAAGAATCAGACAGGGCTTAGAAAGGATTTTGCTA---TAA
>KX907341
GCT---GTA--
GGGAACTTGTGGGTTACTGTCTACTACGGGGTGCCTGTGTGG---AAAGATGCAGAG---
ACCACCCTATTTTGTGCATCAGACGCGAAAGCATATGATAAAGAA---GTGCATAAT----------------
--------------GTCTGGGCTACG---CGTGCCTGTGTACCCACAGAC---
CCCAACCCACAAGAAGTATTTTTGGAA---------AATGTGACAGAAGAGTTTAAC----------------
--------------------------ATGTGGAAAAATAACATGGTAGAGCAGATGCATACAGATATA---
ATCAGTCTATGGGATGAAAGCCTA---AAGCCATGTGTACAGTTA---
ACCCCTCTCTGTGTTACTTTAAATTGTACAGACTATAAGGGTAACTATACAAATTATGATGGCACTGGCCACA
ATATCACCAGT--
---GATATGGAAGGAGAAATAAAA---
GACTGTTCTTTCAATGTAACCACAGAATTAAGG---------------------------
GATAAGAGACAGAAAGTACATTCACTT---TTTTATAGACTTGATATAGTACAAATTAATAGTAGTCAAACT-
----

AATAGTAGTGAGTATAGATTAATAAATTGTAATACTTCAGCCATTACACAG---
GCTTGTCCAAAGATTTCCTTTAATCCAATTCCCATACATTATTGTGCTCCAGCTGGTTTTGCGATTCTAAAGT
GTAAT---GATAAAAAGTTCCAT------GGAACAGGGCCATGCAAGAAT-----------------------
-GTCAGC---TCAGTACAATGCACACATGGGATCAAGCCAATAGTATCAACT---
CAACTACTGTTAAATGGCAGTCTAGCAGAAGAAAAA------------
GTAGCGATTAGATCTGAAAATATTACAAACAAT---------GCCAAAAACATATTAGTACAACTTAAA----
-----------AGTCCTGTAAAC---------ATTACTTGTGTCAGACCTAACAACAATACAAGAACA-----
-AGTGTACGTATA------------GGACCAGGACAAACA---TTC------TATGCAACAGGTGCC------
---------ATAACAGGGGACATA------------AGACAAGCACATTGT---AATGTCAGTAAAGCA---
AAATGGAATGAAACTTTAAGAAAGGTAGCTGGACAATTAAAA---------------ACATATTTT-------
-----------------------------------AACAAAACAATAATCTTTAAT------
AGCTCTGCAGGAGGGGATCTAGAAATTACA---ACACATAGTTTTAATTGTGGAGGAGAA---
TTTAAACAGCTCCAAGCAAGAGTCCTGGCTGTGGAAAGATACCTAAGGGATCAACAGCTCCTAGGAATTTGGG
GCTGCTCTGGAAAACTCATCTGCACCACTAATGTGCCCTGGAACTCTAGTTGGAGT-----------------
----------------------------AATAAATCCCAGAATGAGATATGG------GAA------
AACATGACCTGGCTGCAATGG---GAGAAAGAAATTAGCAACTACACAGATGAAATTTAT---

AATCTACTTGAAAAATCACAGAACCAGCAGGAA---------------------------------------
----
AAGAATGAACAAGATTTACTGGCATTGGACAAGTGGGCAAGTCTGTGGAATTGGTTTGACATATCAAATTGGC
TGTGGTATATAAAAATATTTATAATGATAGTAGGAGGTTTAATAGGATTAAGAATAATTTTTGCA---
GTGCTTTCTATAATA---------AATAGAGTTAGGCAGGGATACTCACCT------
TTGTCATTTGCTATTAATCTGCTTGATACCGTAGCAATAGCCACAGCTAAC---
TGGACAGATAGGGTTATAGAAATAGGACAAAGACTTTGTAGAGCT----------------------------
--------TTTCTCAATATA------CCTAGAAGAATCAGACAGGGCTTCGAAAGGGCCTTGCTA---TAA
>KX907347
ACT---GCA---
GACAAGTTGTGGGTTACTGTATACTATGGGGTACCTGTGTGG---AAAGATGCAGAG---
ACCACCTTATTCTGTGCATCAGACGCTAAAGCATATGACAAAGAA---AAGCATAAT----------------
--------------GTCTGGGCTACG---CATGCCTGTGTACCCACAGAC---
CCTAACCCACAAGAAATACATTTGGAA---------AATGTGACAGAAGAGTTTAAC----------------
-------------------------ATGTGGAAAAATAACATGGTAGAGCAGATGCATACAGATATA---
ATCAGTCTATGGGATCAAAGCCTT---AAGCCATGTGTAAAGCTA---
ACCCCTCTCTGCGTTACTCTAAATTGTAGCCACAACATTACTAGGCTCAACACCAGTAGTCCCAACATCACAG
GAAACGACACA--
----------------AACAGTAGTCCCAACATCACAGAAAACGACACAGAAAACGAAAGGAAA---
AACTGCTCTTTCAATATAACCACAGAATTAAGG--------------------------
GATAAGAGCAAACAAGTATATTCACTT---TTTTATAGACTTGATATAGTACCAATTGATGGTAGTGAC----
----

AATAGCAGTGACAATAGCAATAATTATAGATTAATAAATTGTAATACCTCAGCCATTACACAG---
GCTTGCCCAAAAGTGTCCTTTGAGCCAATTCCCATACATTATTGTGCCCCAGCTGGTTTTGCGATTCTAAAAT
GTAAG---GATGGAAATTTCAGT------GGAAAAGGGCCATGCAAGAAT----------------------
-GTCAGC---ACAGTACAATGCACACATGGAATCAAGCCAGTAGTATCAACT---
CAACTGCTGTTAAATGGCAGTCTAGCAGAAGAAAAG-----------
GTAAAAATTAGATCTGAAAATATCAGTGACAAT---------ACCAAAAACATAATAGTACAACTTGTC----
-----------CAACCTGTGAAG---------ATTAACTGTACCAGACCTGACAACAACACAAGAAAG-----
-AGTATACATATA------------GGACCAGGACAAGCA---TTC------TTTGCAACAGGTGCC------
---------ATAATAGGGAAGATA------------AGACAAGCACATTGT---AATGTCAGTAAAACA---
GAATGGAATAAAGCTTTAAAACAGGTAGTTGAACAATTAAGA---------------AGAGACTTTGGA----
-----------------------------------AACAAAACAATAATATTCAAA------
AACTCCTCAGGAGGAGATCTAGAAATCACA---ACACATAGTTTCAATTGTGGAGGAGAA---
TTTAAACAGCTCCAGGCGAGAGTCCTGGCTGTGGAAAGATACCTAAGGGATCAACAGCTCCTAGGAATTTGGG
GCTGCTCTGGAAAACTCATCTGCACCACTACTGTGCCCTGGAACTCTAGCTGGAGC-----------------
----------------------------AATAAATCTTACGATGATATATGG------GAG------
AACATGACCTGGCAGCAATGG---GAGCGGGAAATTAGCAATTACACAGATATAATATAC---
AGATTGATTGAAGACTCGCAGAACCAGCAGGAA---------------------------------------
----
ATGAATGAAAAAGACTTATTGGCATTAGACAAGTGGGCAAGTCTGTGGAATTGGTTTGACATAACAAAGTGGC
TGTGGTATATAAAAATATTTATAATGATAGTAGGAGGCTTAATAGGATTAAGAATAGTTTTTGCT---
GTGCTTGCTATAATA---------AATAGAGTTAGGCAGGGATACTCACCT------
TTGTCGTTCGCTATTAATTTGGTTGATACCATAGCAATAGTAGTAGCTGGC---
TGGACAGATAGGGTTGTAGAAATAGGACAAAGATTTGGTAGGGCT---------------------------
--------ATCCTCTACATA------CCTAGAAGAATAAGACAGGGCTTAGAAGGGGCTTTGCTA---TAA
>KX907364
GCT---ACA---ACT--------------------------------
ACAGACAAGATGTGGGTTACGGTCTACTATGGGGTACCTGTGTGG---AAAGATGCAGAG---
ACCACCCTATTTTGTGCATCAGATGCAAAAGCATATGATCCAGAA---ATGCATAAT----------------
--------------GTCTGGGCTACA---CATGCCTGTGTTCCTACAGAC---
CCTAACCCACAAGAAATAAATTTGGAA---------AATGTGACAGAACAGTTTAAC----------------
-------------------------ATGTGGAAAAATAACATGGTAGAGCAGATGCATACAGATATA---
ATCAGTCTATGGGACCAAAGCCTA---AAACCATGTGTAAAGTTA---
ACCCCTCTCTGTGTTACTCTAGAATGTACCAATGCCAATATCACCAATAATATCACCAATGCCAATGTCACC-
----
--AATGACATGCAAGAAGTAAAA---
AACTGCTCTTTCAATATAACCACAGAACTAAGG--------------------------
GATAAAAGAAAGAAAATATATTCACTT---

TTTTATAGACTTGATATAGTACAAATAAATGAAGGGGAGGATACT----------------------------
----
AATAATAGTAGTAATAGTAGTCAGTATAGATTAATAAACTGTAATACCTCAGCCATCACACAA---
GCTTGTCCAAAGGTAACCTTTCAGCCAATCCCCATACATTATTGTGCCCCAGCTGGTTTTGCGATTCTAAAGT
GTAAT---GATGAGGATTTCAAT------GGGACAGGACTATGTAAGAAT-----------------------
-GTCAGC---ACAGTACAATGCACACATGGAATCAAGCCAGTAGTATCAACG---
CAACTGCTGTTAAATGGAAGTCTAGCAGAAGGAAAT------------
GTAACAATTAGATCTGAAAATTTTACAAACAAT---------GCCAAAAACATACTAGTACAATTTAAA----
-----------AAGCCTGTACAA---------ATTAATTGTACTAGACCTAGCAACAATACAAGGACA-----
-AGTGTACGTATA------------GGACCAGGACAATCA---TTC------TATGCAACAGGTGCC------
---------ATAATAGGGGATATA------------AGACAAGCACATTGC---AATGTCAGTGCAGCA---
GAATGGAATACAACTTTGAAAAGTGTGGTTGACCAATTAAGA---------------AAACACTTTGGA----
--------------------------------GAAAACAAAACAATAATYTTTACT------
AACCACTCAGGAGGGGATCTAGAAATTACA---ACACATAGTTTTAATTGTGGAGGAGAA---
TTTAAACAGCTCCAGGCAAGAGTCCTGGCTGTGGAGAGATACCTAAGGGATCAACAGCTCCTAGGAATTTGGG
GCTGCTCTGGAAAGCTCATCTGCACCACTAATGTGCCCTGGAACTCTAGTTGGAAT-----------------
----------------------------AATAAATCCTTCAATGAAATATGG------GAC------
AACATGACCTGGCTGCAATGG---GATAAAGAAATTAGCAATTACACACAGATAATATAT---
GACCTAATTGAAGAATCGCAGAACCAGCAGGAA--

AAGAATGAACAAGATTTATTGGCATTGGACAAGTGGGCAAATCTGTGGAATTGGTTTGACATATCAAACTGGT
TGTGGTATATAAGAATATTTATAATAATAGTAGGAGGCTTAATAGGATTAAGAATAGTTGTTGCA---
GTGTTTCTATAATA---------AATAGAGTTAGGCAGGGATACTCACCT------
TTGTCATTCGCTATTAATTTGCTTGATACCATAGCAATAGCAATAGCTAAC---
TGGACAGATAGGGGTATAGAAATAGGACAAAGACTTTGTAGAGCG----------------------------
--------CTTTTAAACATA------CCTAGAAGAATCAGACAGGGCTTTGAAAGGGCTTTGCTA---TAA
>KX907412
GCT---GCA--
GAAAACTTGTGGGTTACTGTCTACTATGGGGTACCTGTATGG---AAAGATGCAGAG---
ACCACCCTATTTTGTGCATCAGATGCTAAAACATATGATAAAGAA---GTGCATAAT----------------
--------------GTCTGGGCTACA---CATGCCTGTGTACCTACAGAC---
CCCAACCCACAAGAGATAAATTTGGAA---------AATGTGACAGAAGATTTTAAC----------------
-------------------------ATGTGGAAAAATAACATGGTAGATCAGATGCATGCAGATATA---
ATCAGTCTATGGGATCAAGCCCTA---AAGCCATGTGTAAAGCTA---
ACCCCTCTCTGTGTTACTCTAAATTGTAGTGATAGTGTCACCACTGCTAATAACTCTATC-------------
----
-------------------------------------CAAAATGAAATGAAAGGAGAAATAAAA---
AACTGCTCTTTCAATGTGACCACAGAACTAAGG---------------------------
GATAAGAGACAGAAAGTATATTCACTT---TTTTATAGACTTGATATAGTACAAATTGATAAT----------
----

AATAATAGGAATAGTACACAGTATAGATTAATAAATTGTAATACATCAGCCATTACACAG---
GCTTGTCCAAAGGTATCCTTTGATCCAATTCCCATACATTATTGTGCTCCAGCTGGTTTTGCAATTCTAAAGT
GTAAG---GATGAGAATTTCACA------GGAGTAGGACCATGTAAGAAT-----------------------
-GTCAGC---ACAGTACAATGCACACATGGAATCAAGCCAGTAGTATCAACT---
CAACTACTGTTAAATGGCAGTCTAGCAGAAAAAAAG------------
GTAATGATTAGATCTGAAAATATTTCAAACAAT---------GCTAAAAACATACTAGTACAACTTACC----
-----------ACGCCTGTAAAC---------ATTACGTGTATCAGACCTGGCAACAATACAAGAAAA-----
-AGTGTACATATA------------GCACCAGGACAAGCA---TTC------TATGCAACAGGTGAC------
---------ATAATAGGGGATATA------------AGACAAGCACATTGT---AATGTCAATAGAACA---
CAATGGAATAATACTTTGAAAGAGGTAGCCAATCAATTAAGA---------------ACATACTTTGGG----
--------------------------------AACGAAACAGCAATAATCTTTGCT------
AGCTCCTCAGGAGGAGATCTAGAAATTACA---ACACATAGTTTTAATTGTAGGGGAGAA---
TTTAAACAGCTCCAGGCAAGAGTCCTGGCTGTGGAAAGATACCTAAAGGACCAACAGCTCCTAGGAATTTGGG
GCTGCTCTGGAAAGCTCATCTGCACCACCACTGTGCCCTGGAACTCTAGCTGGAGT-----------------
----------------------------AATAGATCCCAGAGTGAGATATGG------GGC------
AACATGACCTGGCAGCAATGG---GAAAAAGAAATTAGCAATTACACAGAGGAAATATAT---
AGGCTAATTGAAAATTCGCAGAACCAGCAGGAA--

AAAAATGAACAAGACTTATTGGCATTGGACAAGTGGGCAAGTCTGTGGAATTGGTTTGACATATCAAACTGGC

TGTGGTATATAAGGCTATTTGTAATAATAGTAGGAGGCTTAATAGGATTAAGAATAGTTCTTGCA---
GTGTTTTCTATAATA---------AATAGAGTTAGGCAGGGATACTCACCT------
TTGTCATTTGCTATTAATTTGCTTGATACCTTAGCAATAGCAACAGCTAAC---
TGGACAGATAGGGTTATAGAATTATTACAAGCACTTGGTAGAGCT----------------------------
--------TTTCTCAACATA------CCCAGAAGAATCAGACAGGGTGCAGAAAGGGCTTGGCTA---TAA
>KX907414
GCT---GTA--
GGAAACCTGTGGGTTACTGTCTACTATGGGGTACCTGTGTGG---AAAGAAGCAGAG---
ACCACCCTATTTTGTGCATCAGATGCTAAAGCATATGAGACAGAA---AAACATAAT----------------
--------------GTCTGGGCTACA---CATGCCTGTGTGCCTACAGAC---
CCCAGCCCACAAGAGATAAATTTGGTG---------AATGTGACAGAAGATTTTAAC----------------
--------------------------ATGTGGAAAAATTACATGGTAGAGCAGATGCATACAGATATA---
ATCAGTCTATGGGACCAAAGCCTA---AAGCCATGTGTAAAGTTA---
ACCCCCCTCTGTGTTACTTTATACTGCAAGAATGTCACCAGAGATAATAGTACCAAAAATAGCACCGAAAACG
AGAACACCACT--
-------------------------------------CATATCATCAATGACAGGTCAATAACA---
AACTGCTCTTTTAATATGACCACAGTGCTACAG---------------------------
GATAAGAGGCAGCAAGTCTATTCACTT---TTTTATAGGACTGATGTAGTACCAATAGAGGAT----------
----

AGAAAGAATGATAGTCAGTATAGATTAATAAATTGCAATACCTCAGCCATTACACAG---
GCTTGTCCAAAAGTATCCTTTGAGCCAATTCCCATACATTATTGTGCTCCAGCTGGTTTTGCGATTCTAAAGT
GTAAT---GAGGAGGAGTTCAGT------GGAAAAGGGCCATGTAAGAAT-----------------------
-GTCAGC---ACAGTACAATGTACACATGGGATCAAGCCAGTGGTGTCAACC---
CAACTGCTATTAAATGGCAGCCTAGCAGACAAAAAG------------
GTAATGATTAGATCTGAAAATATTACAAATAAT---------GTCAAGAATATAATAGTACAATTTAAT----
-----------AAGACTGTAGCA---------ATTAATTGTATTAGACCTAACAACAATACAAGGAAA-----
-AGTATAAGGATA------------GGGCCAGGACAAAAA---TGG------TATTCATGGAATGAC------
---------ATAATAGGGAATATA------------AGAGAAGCACATTGT---ACTGTTAATGGAACA---
GAATGGAATATGGCTTTGCAAGAGGTAGTCAACCAATTAAGA---------------ACACACTTT-------
-----------------------------------AGTGGAACAATAATCTTTAAT------
AGCTCCTCAGGAGGGGATCTAGAAGTTACA---ACACATAGTTTTAACTGTGGAGGAGAA---
TTTAAACAGCTCCAGGCGAGAATCCTGGCTGTGGAAAGTTACCTAAAGGATCAACAGCTCCTAGGAATTTGGG
GTTGCTCTGGAAAACTCATCTGCACCACTAATGTGCCCTGGAACTCTAGTTGGAGT-----------------
----------------------------AATAAATCCCTGGATGAGATATGG------CAA------
AATATGACCTGGCTGCAATGG---GATAGAGAAATTAGCAATTACACAGATACAATATAC---
AGCTTAATTGAAGAAGCGCAGAACCAACAGGAG--

AGAAATGAAAAAGATTTATTAGCATTTGACAAATGGGCAAGTCTGTGGAATTGGTTTGACATCTCGAACTGGC
TGTGGTATATAAAGATATTTATAATAATAGTAGGAGGCTTAATAGGATTAAGAATAGTTTTTACT---
GTGCTTTCTGTAATA---------AATAGAGTTAGGCAGGGATACTCACCA------
TTGTCATTCGCTATTAGTTTGCTTGATACCATAGCAATAGTAGTAGCTGGC---
TGGACAGATAGGGTTATAGAATTAGGACAACGCTTTTGTAGAGCT----------------------------
--------ATCCTCAACATA------CCTAGAAGAATCAGACAGGGCTTTGAAAGAGCTTTGCTA---TAA
>KX907423
GCT---GCA--
GAAAACTTGTGGGTTACTGTCTATTATGGGGTACCTGTATGG---AAAGACGCAAAG---
ACCACTCTATTTTGTGCATCAGATGCTAAAGCATATGATACAGAA---GTGCATAAT----------------
--------------GTCTGGGCTACA---CATGCCTGTGTACCTACAGAC---
CCCAACCCACAAGAGATAAATTTGGTA---------AATGTGACAGAAGATTTTAAC----------------
--------------------------ATGTGGAAAAATAACATGGTAGATCAGATGCATGAAGATATA---
ATCAGCCTATGGGATCAATCCCTA---AAGCCATGTGTAAAGCTA---
ACCCCTCTTTGTGTTACTCTGAATTGTAGTGATGTCAAGAATGGCAGTAGCAGTGATGGTAAT----------
----
-------------------AACCAGAATATTAGTGTCTCCACGGAAATGCAAGGAGAAATAAAA---
AACTGCTCTTTCAATATGACCACAGAATTAAGG---------------------------
GATAAGAGACAGCAAGTACATTCACTT---TTTTATAGGCTTGATGTAGTACCAATTAATAGT----------
----

GATAATAATAGTAGTTACTATAGATTAATAAATTGTAATACATCAGCCATTACACAG---

GCTTGTCCAAAGGTATCCTTTGAGCCAATTCCCATACATTATTGTGCTCCAGCTGGTTTTGCAATTCTAAAGT
GTAAA---GAGGAAAATTTCACA------GGAAAAGGGCAATGTAAGAAT-----------------------
-GTCAGC---ACAGTACAATGCACACATGGAATCAAGCCAGTAGTATCAACT---
CAACTACTGTTAAATGGCAGTCTGGCAGAAAAAAAG------------
GTGATGATTAGATCTGAAAATATTACAAACAAT---------GCTAAAAACATACTAGTACAACTTACC----
-----------AAGCCTGTAGAA---------ATTAATTGTACCAGACCTAACAACAATACAAGAAAA-----
-AGTATACATATA------------GGACCAGGACAAGCA---TTC------TAT---ACAAGTGGC------
---------ATAATAGGGGATATA------------AGACAAGCATTTTGT---ACTGTCAATAGAACA---
CAATGGAACAGCACTTTGAAAGAGGTAGTCGATCAATTAAGA---------------GCAGACTTTGAG----
-----------------------------------AACGCAACAATAATCTTTACT------
AACTCCTCAGGAGGAGATCTAGAAATTACA---ACACATAGTTTTAATTGTAGGGGAGAA---
TTTAAACAGCTCCGGGCAAGAGTCCTGGCTGTGGAAAGATACCTAAAGGACCAACAGCTCCTAGGAATTTGGG
GCTGCTCTGGAAAGCTCATCTGCACCATTGCTGTGCCCTGGAACTCTAGTTGGAGT-----------------
----------------------------AATAAAACCCAAAATGAGATATGG------GAC------
AACATGACCTGGCAGGACTGG---GAAAAAGAAATTAGTAATTACACAGAGATAATATAT---
AGCCTAATTGAAGAATCGCAGATCCAGCAGGAA--

AAGAATGAACAAGACTTATTGGCATTGGACAAATGGGCAAGTCTGTGGAATTGGTTTGACATATCAAAATGGC
TATGGTACATAAAGATATTTATAATAATAGTAGGAGGCTTAATAGGATTAAGAATAGTTTTTGCA---
GTGTTTTCTATAATA---------AATAGAGTTAGGCAGGGATACTCACCT------
TTGTCATTTGCTATTAATTTGCTTGATACCTTAGCAATAGCAACAGCTAAC---
TGGACAGATAGGTTTATAGAATTAGTACAAACATTTTGTAGAGCT----------------------------
--------ATTCTCCACATA------CCCAGAAGAATCAGACAGGGTGCAGAAAGGCTTTGGCTA---TAA
>KX907431
GCA---GCA--
GACAACTTGTGGGTTACTGTTTACTATGGGGTACCTGTGTGG---AGAAATGCAGAG---
ACCACCTTATTCTGTGCATCAGATGCTAAAGCATATGATACAGAA---GTACATAAT----------------
--------------GTTTGGCTACA---CATGCCTGTGTACCCACAGAC---
CCCAATCCACAAGAGATAAATTTGGAG---------AATGTGACAGAAGATTTTAAC----------------
--------------------------ATGTGGAAAAATCACATGGTAAAGCAGATGCATACAGATATA---
ATCAGTCTATGGGATCAAAGTCTA---AAGCCATGTGTAAAGTTA---
ACCCCTCTCTGTGTTACTTTAAATTGCAATAATAATATCACCACTAACACCACCACTGAA-------------
----
--TGGGGAATGACA---
AACTGCTCTTACAATGTGACCACAGAAATAAGG---------------------------
GATAAGAAAAAGAAAGAATATTCACTT---TTTTATAAGATTGATGTAGTAAAAATTGATAATAAT-------
----

AATAATAGCCAGTATAGGTTGATAAATTGTAATACCTCAGCCATTACACAG---
GCTTGTCCAAAGGTATCTTTTGAGCCAATTCCCATACATTATTGTGCCCCAGCTGGCTTTGCGATTCTAAAGT
GCAAG---GATAAGGAGTTCAAT------GGAACAGGGCCATGTAAAAAT-----------------------
-GTCAGC---ACAGTACAATGCACACATGGAATCAAGCCAGTAGTATCAACT---
CAACTGCTGCTAAATGGCAGTCTAGCAGAAGAAGAG------------
GTAAGAATTAGATCTGAAAACATCACAAATAAT---------GGCAAAAACATAATAGTACAATTTAAC----
-----------GAGCCTGTAAGA---------ATTAATTGTACTAGACCTAACAACAATACAAGAAAA-----
-GGTGTACATATA------------GGACCAGGACAAACA---TTT------TATGCAACAGGTGCA------
---------ATAATAGGAAATATA------------AGACAAGCACATTGT---AATGTCAGTAAAAA---
GACTGGAATGAAGCTTTGAAAAGGGTAGCCAAACAATTAAGG---------------AAACACTTT-------
-----------------------------------AACAAAACAATAATCTTTAGT------
AACTCCTCAGGAGGGGATCTAGAAATCACA---ACACATAGTTTTAATTGTAGAGGAGAA---
TTTAAACAGCTCCAGGCAAGAGTCCTGGCTGTGGAAAGCTACCTAAAGGATCAACAGCTCCTAGGAATTTGGG
GCTGCTCTGGAAAACTCATCTGCACCACTAATGTGCCCTGGAACTCTAGTTGGAGT-----------------
----------------------------AATAAAACACAGGATGAGATATGG------AAC------
AACATGACTTGGCTGCAATGG---GATAAAGAAATTAGCAATTACACAAAAGTAATATAT---
GGTTTAATTGAAGAATCGCAGAATCAGCAGGAA--

AAGAATGAATTAGACTTATTGGCATTGGACAAGTGGGCAGATCTGTGGAATTGGTTTGACATATCAAAATGGC
TGTGGTATATAAAAATATTTGTAATGATGGTAGGAGGCTTAATAGGATTAAGAATAGTTTTTGCA---
GTGCTTTCTATAATA---------AATAGAGTTAGGCAGGGGTACTCACCT------
TTGTCATTTGCTATTAGTTTATATGATACATTAGCAATAGCAGTAGCTGAC---

TGGACAGATTGGGTTATAGAAATAGGACAGAGATTTTGTAGAGCT----------------------------
--------ATTCTCAATATA------CCTAGGAGAATCAGACAGGGTGCTGAAAGGGCTTTGCAA---TAA
>HQ615968
ACT---GCA---
GACAACTTGTGGGTTACTGTCTATTATGGGGTGCCTGTGTGG---AGAGATGCAAAT---
ACCACCCTATTTTGTGCATCAGATGCTAAAACATATGAGAGAGAA---GTGCATAAT----------------
--------------GTCTGGGCTACG---CATGCCTGTGTACCCACAGAC---
CCCAACCCACAAGAAATACATTTGGAA---------AATGTAACAGAAGAGTTTAAC---------------
--------------------------ATGTGGAAAAATAACATGGTAGAGCAGATGCATACAGATATA---
ATCAGTCTATGGGATCAAAGCCTA---AAGCCATGTGTAAAGTTA---
ACCCCTCTCTGTGTTACTTTAGAGTGTGGCGATGCCATCGTCAACAATAGCGATGCCATCGTCAACAATAGCA
ATGCCACC---
-------GTCAGCAATAGCAATGCCACCGTCAATGCCACCGCCACCAATGTAGGAGAAATGACA---
AATTGCTCTTTCAACATGACCACAGTAGTAAGG--------------------------
GATAAGACAAAGAAAGTATATTCGCTT---TTTTATAGACTTGATGTAGTACCAATTGATAATGAT-------
----

AATAGTAGTAAGTATAGATTAATAAATTGTAATACCTCAACCATTACACAG---
GCTTGTCCAAAGGTGTCTTTTGAGCCAATTCCCATACATTATTGTGCTCCAGCTGGTTTTGCGATTCTAAAGT
GTAAT---GATAAGAATTTCACG------GGAATGGGGGAATGCAAGAAT----------------------
-GTCAGC---ACAGTACAGTGCACACATGGAATCAAGCCAGTAATATCAACC---
CAGCTGTTGTTAAATGGCAGTCTAGCAGAAAAAGAG------------
GTAATGATTAGATCTGAAAATCTCACAGACAAT---------GCCAAAAACATAATAGTACAATTTAAC----
-----------GAGTCTGTAAGG---------ATTATTTGTAGCAGACCTAGCAACAATACAAGAACA-----
-AGTGTAAGGATA------------GGACCAGGACAAACA---TTC------TATGCAACAGGTGCC------
---------ATAACAGGGAACATA------------AGACAAGCGCATTGT---AATGTTAGTAGAGCA---
GAATGGAACAAAACTTTGGAAAAGGTAGCCCACCAATTAAGA--------------ACAAACTATAAC----
-----------------------------------AAATCAAAAATAATCTTTACT------
AGTTCCTCAGGAGGGGATCTAGAAATTACA---ACACATAGTTTTAATTGTGGAGGAGAA---
TTTAAACAGCTCCAGGCAAGAGTCCTGGCTGTGGAAAGATACCTAAGGGATCAACAGCTCCTAGGAATTTGGG
GCTGCTCTGGAAAACTCATCTGCACCACTAATGTGCCCTGGAACTCTAGTTGGAGT----------------
----------------------------AATAAATCCCAGAGTGAGATATGG------AAC------
AACATGACCTGGTTGAAATGG---GATAAAGAAATTAATAAGTACACAAAGACAATATAT---
GATTTACTTGAGGAATCGCAGATCCAGCAGGAG--

AAGAATGAACAAGATTTATTGGCATTGGACAAGTGGGCAAGTCTGTGGAATTGGTTTGACATATCAAATTGGC
TATGGTATATAAGAATATTTATAATGATAGTAGGAGGCTTAATAGGGTTAAGAATAGTCTTTGCA---
GTGTTCTCTATAATA---------AATAGAGCTAGGAAGGGATACTCACCT------
TTATCATTTGCTATTAGTTTGCTTGATACTGTAACAATAGCAGTAGCTGGC---
TGGACAGATAGGGTTATAGAAATAGGACAAAGATTTTGTAGAGCT---------------------------
--------ATTCTCCACATA------CCTAGAAGAATCAGACAGGGCTTTGAAAGGGCTTTGCTA---TAA
>HQ615970
GCT---GCA---
GAAAACTTGTGGGTTACTGTCTACTATGGGGTACCTGTGTGG---AAAGATGCAGAA---
ACCACCTTATTTTGTGCATCAGATGCTAAAGCACATGAGGCAGAA---AAGCATAAT----------------
--------------GTCTGGGCTACA---CATGCCTGTGTACCCACAGAC---
CCCAACCCACAAGAAATACCTTTGGTA---------AATGTGACAGAAGAGTTTAAC---------------
--------------------------ATGTGGAAAAATAAAATGGTAGAGCAGATGCATACAGATATA---
ATCAGTCTATGGGACCAAAGTCTA---AAGCCATGTGTACAGTTA---
ACCCCTCTTTGCGTTACTCTACATTGTAACGATGTTAACACGACCAATAACACAACCAAT------------
----
------------------------CACACCACTGTCGCCCCGGACATGGTAGAAGAAAGAAAA---
AATTGCTCTTTCAATATGACCACAGAACTAAGG--------------------------
GATAAGAAACAAAAAGTATATTCAATT---TTTTATAGGCTTGATATAGTACCACTTAATGAG---------
----

AGTCAAGTTAATAGTAGTGAGTATAGATTAATAAATTGTAATACCTCAGCCATTACACAG---
GCCTGCCCAAAGGTATCCTTTGAGCCAATTCCCATACATTATTGTGCCCCAGCTGGTTTTGCAATTCTAAAAT
GTAGG---GATAAGGATTTCAAT------GGAACAGGGCTATGCAAGAAT---------------------
-GTCAGC---ACAGTACAGTGCACACATGGAATCAAGCCAGTAGTATCAACA---

CAGCTGCTGTTAAATGGCAGTTTAGCAGAAGAAGAG------------
GTAGTGATTAGATCTGAAAATATCACAGACAAT---------GTCAAAACTATACTAGTACAACTTGTC----
-----------AAGCCGGTGAAA---------ATTAATTGTACCAGACCTAGTAACAATACAAGGAAA-----
-AGTGTACATATA------------GGACCAGGACAAGCA---TTC------TATGCAACAGGTGAC------
---------ATAATAGGTGATATA------------AGACAAGCACATTGT---ATTGTCAATGGATCA---
GAATGGAATGAAACTTTACACCAGGTAGCTAAACAATTAAAC---------------AAACACTTT-------
-----------------------------------AACAAAACAATAACATTTACT------
AAATCCTCAGGAGGGGATCTAGAAATAACA---ACACATAGCTTTAATTGTGGAGGAGAA---
TTTAAACAGCTCCAGGCAAGAGTCCTGGCTGTGGAAAGTTACCTAAAGGATCAACAGCTCCTAGGAATTTGGG
GCTGCTCTGGAAAACTCATCTGCACCACTAATGTGCCCTGGAACTCTAGTTGGAGT-----------------
----------------------------AATAAATCTTATGATGATATATGG------GAG------
AACATGACTTGGGTAGAATGG---GAGAGAGAAATTAGCAATTACACAGAAAAAATATAT---
GGCCTACTTGAAGAATCACAGAACCAGCAGGAA--

AAGAATGAACAAGACTTGTTGGCATTAGACAAATGGGCAAGTCTGTGGAATTGGTTTAACATATCAAGATGGC
TGTGGTATATAAAAATATTTATAATGATAGTAGGAGGCTTAATAGGGTTAAGAATAGTTCTTACT---
GTGCTTTCTTTAATA---------AATAGAGTTAGGCAGGGATACTCACCC------
TTGTCGTTCGCTGTTAGTCTGCTTGACACCATAGCAATAGCAGTAGCTGGC---
TGGACAGATAGAATTATAGAAATAGGACAAGCAATTGGTAGAGCT----------------------------
--------ATCCTCCACATA------CCTAGAAGAATCAGACAGGGCGCTGAAAGAATTTTGCTA---TAA
>HQ615971
GCT---GCA--
GAAAACTTGTGGGTCACTGTATACTATGGGGTACCTGTGTGG---AGAGACGCAGAA---
ACCACTTTATTTTGTGCATCCGATGCTAAGGCATATGATACAGAA---GTGCATAAT----------------
--------------GTATGGGCCACA---CATGCCTGTGTACCCACAGAC---
CCCAACCCACAAGAAATACATTTGAAA---------AATGTGACAGAAGAGTTTAAC----------------
--------------------------ATGTGGAAAAATAGCATGGTAGAGCAGATGCATACAGATATA---
ATTAGTCTATGGGACCAAAGCCTA---AAACCATGTGTACAGTTA---
ACCCCTCTCTGTGTCACTTTAAATTGCTCCCATAATATCACCAATAACATTACCAAGATGGAC----------
----
----------------------AACAATACGGACAATACAATAGACATGAAAGAAGAAATAAAA---
AACTGCTCTTTCAATATGACTACAGAATTAAGG---------------------------
GATAAGCAACGAAAAGTGCACTCACTT---TTTTATAGACTTGATATAGTACAAATTAATGAAAATGACAAT-
----

GCTACTCAAAATAATAGTGGTAAGTATAGATTAATAAATTGTAATACCTCAGCCATTACACAA---
GCCTGTCCAAAGGTAACCTTTGAGCCAATTCCCATACATTATTGTGCCCCAGCTGGTTTTGCGATTCTAAAGT
GTAAT---GATAAAAGTTTCAAT------GGAACAGGACCATGCGGGAAT-----------------------
-GTCAGC---ACAGTACAATGTACACATGGAATCAAGCCAGTAATATCAACT---
CAGCTGCTGTTAAATGGCAGTCTAGCAGAACAAGAG------------
ATAATGATTAGAAGTGAAAATATCACAAACAAT---------GCCAAAACCATAATAGTACAACTTAAC----
-----------CAGTCTGTAGAA---------ATTACTTGTATCAGACCTAACAACAATACAAGAACA-----
-GGTATACATATA------------GGACCAGGGCAAGCA---TTC------TATGCAACAGGTAAC------
---------ATAATAGGAGATATA------------AGACAAGCACATTGC---AATATCAGTAGAAAA---
AATTGGACTGACACGTTACAAAAGGTAGCCACACAATTAAGA---------------AAATACTTTAAC----
-----------------------------------AACTCAACAATAATCTTTGAT------
GAACCCTCAGGGGGAGATTTAGAAATTACA---ACACATAGCTTTAATTGTGGAGGAGAA---
TTTAAACAGCTCCAGGCACGAGTCCTGGCTGTGGAAAGATACCTAAGGGATCAACAGCTCCTAGGAATTTGGG
GCTGCTCTGGAAAACTCATCTGCAACACTAATGTGCCCTGGAATTCTAGTTGGAGT-----------------
----------------------------AATAAGTCTCAGGATGAGATATGG------AAA------
AACATGACCTGGTTGCAATGG---GATAAAGAGATTAGCAATTACACACAAATAATATAT---
GATCTACTTGGAGAAGCGCAAAACCAACAGGAA--

AACAATGAACAAGACTTATTGGCCTTGGACAAATGGGCAAATCTGTGGAATTGGTTCGACATATCAAACTGGC
TGTGGTATATAAAAATATTTATAATGGTAGTAGGAGGCTTAATAGGATTAAGAATAGTTTTTGCT---
ATATTTTCTCTAATA---------AATAGAGTTAGGCAGGGATACTCACCT------
TTGTCGTTCGCTATTAATTTGTATGATACCATAGCAATAGCAGTAGCTGGC---
TGGACAGATAGGGTTATAGAAGTAGGACAAAGATTTGGTAGAGCT----------------------------
--------ATCCTCAACATA------CCTAGAAGAATCAGACAGGGCTTCGAAAGGCTTTGCTA---TAA
>HM204620

GCT---GCA---
GAAAAATTGTGGGTTAATGTTTACTATGGGGTGCCTGTGTGG---AAAAATGCAGAT---
ACCACTCTATTTTGTGCATCAGATGCTAAAGCATATAACCCAGAA---GCACATAAT----------------
--------------ATCTGGGCTACG---CATGCCTGTGTACCCACGGAC---
CCCAGCCCACAAGAAATATACTTGGAA---------AATGTGACAGAAGATTTTAAC----------------
--------------------------ATGTGGAAAAATAACATGGTAGAGCAGATGCATGCAGATATA---
ATCAGTCTATGGGACCAAAGTCTA---AAGCCATGTGTACAGTTA---
ACCCCTCTCTGTGTTACTTTAGAGTGTAAGAAGATCAATATCACTAATACCACTGGCAATAATGTC-------
----
----------------------ACTGCCAATAATGACACTATCCTTGCCAGAGAAGAAATGAGA---
AACTGCTCTTACAATATGACCACAGAACTAAGG--------------------------
GATAAGAAACAGAAAGTACATTCACTT---
TTTTATAGACTTGATATAGTAGAAATTAATAATAATAATAATGGG----------------------------
----
AGTAGTAATAATAATAGTGATAATGATAGTAGGCCTTATAGATTAATAAATTGTAATACCTCAGCCATTACAC
AG---
GCTTGTCCAAAGATATCCTTTGAGCCAATTCCCATACATTATTGTGCCCCAGCTGGTTTTGCGATTCTAAAGT
GCAGG---GATAAGGAGTTCAAT------GGAACAGGGCCATGCAAGAAT----------------------
-GTCAGT---ACAGTACAATGCACACATGGAATTAAGCCAGTAGTATCAACT---
CAACTACTGTTAAATGGCAGTTTAGCAGAAAAAAGG-----------
GTAATGATTAGAACTGAAAACATTACAAATAAT---------GCCAAAAACATAATAGTACAATTTAAT----
-----------GAGTCTGTAGAA---------ATTAATTGTACCAGACCTAACAACAATACAAGACAA-----
-AGTGTACGTATA------------GGACCAGGACAAGCA---TTC------TAT---ACAACAGAA------
---------ATAATAGGGGATATA------------AGACGAGCATATTGT---ACTGTCAATGAAACA---
AAATGGAATCAAACTTTGTATAGGGTAGCTGAGCAATTAAGA---------------AAATATTTTAAT----
--------------------------------AATGATACAACAATAATCTTTGCT------
AACTCCTCAGGAGGGGACGTAGAAATCACA---ACACATAGTTTTAATTGTGGAGGAGAA---
TTTAAACAGCTCCAGGCAAGACTCCTGGCTGTGGAAAGATACCTAAGGGATCAACAGCTCCTAGGAATTTGGG
GCTGCTCTGGAAAACTCATCTGCGCCACTAATGTGCCCTGGAACTCTAGTTGGAGT-----------------
----------------------------AATAAATCCTATGATGAGATATGG------GGG------
AACATGACCTGGCTGCAATGG---GATAAAGAAATTGACAACTACACACAGCTAATATAT---
CAATTAATTGAAGACTCGCAGAACCAGCAGGAA--

AAGAATGAACAAGAGTTATTGGCATTGGACAAGTGGGCAAATCTGTGGAATTGGTTTGAGATATCAAATTGGC
TGTGGTATATAAGACTATTTGTAATGATAGTAGGAGGCTTAATAGGATTAAGAATAGTTTTGGCA---
GTGCTTTCTGTAATC---------AATAGAGTTAGGCAGGGATACTCACCT------
TTGTCATTTGCTATTAATTTGCTTGATACCATAGCAATAGCAGTAGCTGGC---
TGGACAGATAGGGTTATAGAACTAGGACAAAGACTTTGTAGAGCT----------------------------
--------TTTCTTCACATA------CCTAGAAGAATCAGACAGGGCTTCGAAAGGGCTTTGCTA---TAA
>HM204621
GCT---GCA---
GAAAACTTGTGGGTTAATGTCTACTATGGGGTACCTGTGTGG---AAAGAAGCAGAG---
ACCACTTTATTTTGTGCATCTGATGCTAAAGCATATTCTACAGAA---GCACATAAT----------------
--------------GTTTGGGCTACA---CATGCCTGTGTACCCACAGAC---
CCCAACCCACAAGAAGTATATTTAGAA---------AATGTGACAGAAGAGTTCAAC----------------
--------------------------ATGTGGAGAAATAAAATGGTAGATCAGATGCAGGAAGATATA---
GCCAGTCTATGGGACCAATTCCTA---CAACCATGTGTGAAGTTA---
ACCCCTCTCTGTGTTACCTTAAATTGCAGTAACCCCAAGAACCCCGATAACAGTACAGATAAC----------
----
--------------------------AGTACAGGCATAGGCAGAGAAGACATGAAAGACATGAAA---
AACTGCTCTTTCAATATGACCACAGAATTAAGG--------------------------
GATAAACACCAAAAAATGTATTCTCTT---
TTTTATAGACTTGATATAGAAGAACTCAATGAAAATAGTAATAGC----------------------------
----
AGTAATAGCAGTAGCAGTAATAGCAGTGAGTATAGATTAATAAATTGTAATACCTCAACCATTGCACAA---
GCTTGTCCAAAGGTATCCTTTGAGCCAATCCCCATACATTATTGTGCCCCAGCTGGTTTTGCGATTCTAAAGT
GTAGG---GATAAAAAGTTCAAT------GGAACAGGACCATGCAGGAAT----------------------
-GTCAGC---ACAGTACAATGCACACATGGAATCAAGCCAGTAGTATCAACT---
CAACTGCTGTTAAATGGCAGTCTAGCAGAAAAAGGG-----------
ATAAAGATTAGATCTGAGAATATCTCAGAAAGT---------GCTAAAACTATAATAGTACAACTTGAC----

-----------CAGCCTGTAGTA---------ATTAATTGTACCAGACCTAACAACAATACAAGAACA-----
-AGTATACCTATG------------GGACCAGGACGAGCA---TTA------TATGCAACAGGTGCC------
---------ATAACAGGGGATCCA------------AGACAAGCACATTGT---AATATTAGTAGAGAA---
AAATGGAATGAAACTTTAAGCAAGGTAGCTAAAAAATTAAAA---------------GAATATTTTAAT----
-----------------------------------AATAGAACAATAATCTTTACA------
AACGCCTCAGGGGGGGATGTAGAGGTTACA---ACACATAGTTTTAATTGTGGAGGAGAG---
TTTAAACAGCTCCAGGCAAGAGTCCTGGCTGTGGAAAGATACCTACAGGATCAACAGGTCCTAGGACTTTGGG
GCTGCTCTGGGAAAATCATCTGCGCCACTAATGTGCCCTGGAATTCTAGTTGGAGT----------------
----------------------------AATAAATCCTATGGGGAGATATGG------GAT------
AACATGACCTGGCTGGAATGG---GATAAAGAAGTTAGCAATTACACAGACATAATCTAT---
GATCTAATTGCAAAATCGCAAAATCAACAGGAA--

AAGAATGAACAAGACTTATTGGCATTGGACAAGTGGACAAGTTTGTGGGGTTGGTTTGAAATATCAAGATGGC
TGTGGTATATAAAAATATTCATAATGATAGTAGGAGGCTTAATAGGATTAAGAATAGTTTTTGTT---
ATACTGTCTATAATA---------AATAGAGTTAGGCAGGGATACTCACCT------
TTGTCGTTCGCAATTAAGTTGCTTGATACCATAGCAATAGCAGTAGCTGGC---
TGGACAGATAGGGTTATAGAAGTAGGACAAAGAATTGGTAGAGCT---------------------------
--------ATCCTCAACATA------CCTAGAAGAATCAGACAAGGTTTAGAAAGGCTTCTGCTA---TAA
>KY658695
GCT---ACA---------------------------------------
GATAACTTGTGGGTTACTGTTTACTATGGGGTGCCTGTATGG---AAAGATGCAGAG---
ACCACCTTATTTTGTGCATCAGATGCAAAAGCACATGAGACAGAA---GCGCATAAT----------------
--------------GTCTGGGCTACA---CATGCCTGTGTACCTACAGAC---
CCCAACCCACAAGAAATAAATTTGGGA---------AATGTGACAGAAGAGTTTAAT----------------
--------------------------ATGTGGAAAAATAACATGGTAGAACAGATGCATGAAGATATA---
ATCAGTCTATGGGACCAAAGCCTA---AAGCCATGTGTAAAGTTA---
ACCCCTCTCTGTGTTACTTTAGAGTGTAGCAATGTCAATGGCACCAATAGCACCAATAKCACC----------
----
-------------------------AGTGATAGTAAACTGGAAGAAGAAATAGGAGAAATAAAA---
AACTGCTCTTTCAATATGACCACAGAATTAAGG---------------------------
GATAAGCAAAGGAAAGTATATTCACTT---TTTTATAAACTAGATGTAGTACAAATGGGTGAC----------
----

AACAATAGCAGTAAGTATAGATTAATAAATTGTAATACCTCAGCCATTACACAA---
GCTTGTCCAAAGGTAACCTTTGAGCCAATTCCCATACATTATTGTGCCCCAGCTGGTTTTGCGATTCTAAAGT
GTAAT---GATGAGGAGTTTAAT------GGAACAGGGCCATGCATGAAT-----------------------
-GTCAGC---TCAGTACAATGCACACATGGAATTAAGCCAGTAGTATCAACT---
CAACTGCTGTTAAATGGCAGTCTAGCAGAAGGAACG------------
ATAAAGATTAGATCTGAAAATATCACAAACAAT---------GCCAAAAACATAATAGTACAATTTACT----
-----------GAGTCTGTAAAA---------ATTGATTGTATCAGACCTAACAACAATACAAGGACA-----
-AGTGTACGTATA------------GGACCAGGACAAACA---TTC------TATGCAACAGGTGGC------
---------ATAATAGGGAATATA------------AGAAAAGCACATTGT---GTGGTCAATGGAACA---
AAATGGAATAGAACTTTGTCTAAGGTAGTCAACCAATTAAGA---------------ACATACTTTGGA----
-----------------------------------AATAAAACAATAATCTTTGAT------
AAATCCGCAGGAGGGGATTTAGAAATAACA---ACACATAGTTTTAATTGTGGAGGAGAA---
TTTAAACAGCTCCAGGCAAGAGTCCTGGCTGTGGAAAGATACCTAAAGGATCAACAGCTCCTAGGAATTTGGG
GCTGCTCTGGAAAACTCATCTGCACCACTAATGTGCCCTGGAACGATAGTTGGAGTAATAGTAGT--------
----------------------------AAGATACCCCTGGATCAGATATGG------GAC------
AACATGACCTGGCTGCAATGG---GATAAAGAAATTAGCAATTACACATATGTAATATAT---
AATCTGCTTGAAGAATCGCAGAACCAGCAGGAA--

AAGAATGAACAGGATTTATTGGCATTGGACAAATGGGCAAATCTGTGGAATTGGTTTGACATATCAAACTGGC
TGTGGTATATAAAAATATTTATAATGATAGTAGGAGGCTTAATAGGATTAAGAATAGTTTTTGCT---
GTGCTTTCTATAATA---------AATAGAGTTAGGCAGGGATACTCACCT------
TTGTCGTTCGCTATTAATTTGCTTGATACCATAGCAGTAGCAGTAGCTGGG---
TGGACAGATAGGATTATAGAACTAGTACAAAGATTTTGTAGAGCT---------------------------
--------ATTCTCAACATA------CCTAGAAGAATTAGACAGGGCTTCGAAAGGGCTTTGCTA---TAA
>KY658696
GCT---GTA---------------------------------------
GATAACTTATGGGTTACTGTCTACTATGGGGTACCTGTGTGG---AAAGATGCAGAG---

ACCACCTTATTTTGTGCATCAGATGCTAAAGCATATGATACAGAA---GTGCATAAT----------------
--------------GTCTGGGCTACA---CATGCCTGTGTACCCACAGAC---
CCCAACCCACAAGAAATGCCTTTGGAA---------AATGTGACAGAAGAGTTTAAC----------------
--------------------------ATGTGGAAAAATAACATGGTAGAGCAGATGCAGACAGATATA---
ATCAGTCTATGGGACCAAAGTCTA---AAACCATGTGTAAAGTTA---
ACCCCTCTCTGCGTTACTCTAGAGTGCCAGAACATCAACAGGAGCCACACCACTAACAATACAGATAACACCA
CTACCAAT---
---AACATGCAAGGAGAAATAAAA---
AACTGCTCTTTCAATATGACCACAGAGCTAAGG---------------------------
GATCAGAAAAAGAAAGTGCATTCACTT---TTTTATAAACTTGATGTAGTACAAATTGGTGAC----------
----

AACAGCAGTACGTATAGATTAATAAATTGCAATACCTCAGCCATTACACAG---
GCTTGTCCAAAGGTATCCTTTGAGCCAATTCCCATACATTATTGTGCTCCAGCTGGTTTTGCGATTCTAAAGT
GTAGA---GATATAGAGTTCAAT------GGAACAGGACCATGCAAGAAT-----------------------
-GTCAGC---ACAGTACAATGCACACATGGAATCAAGCCAGTAGTATCAACT---
CAACTGCTGTTAAATGGCAGTCTAGCAGAACAGGGG------------
ATAAGGATTAGATCTGAAAATATCACAGCAAAT---------CACAAAAACATAATAGTACAACTTAAC----
-----------GAGTCTGTAGAA---------ATTTATTGTATCAGACCTAACAACAATACAAGAAAA-----
-AGTGTACATATA------------GGACCAGGACAAGCA---TTC------TATGCAACAGGAGAC------
---------ATAATAGGGGACATA------------AGACAAGCACACTGT---AATATCAGTGAAGAA---
AAGTGGAATAGAACTTTAAGCCAGGTAGCCACACGATTAAGA---------------GAATACTTT-------
-----------------------------------AATAAAACAATAATCTTTACT------
AGCCACTCAGGGGGGGATTTAGAAGTTGCA---ACACATAGTTTTAATTGTAGAGGAGAA---
TTTAAACAGCTCCAGGCGAGAGTCCTGGCTGTGGAAAGATACCTAAAGGATCAACAGCTCCTAGGAATTTGGG
GCTGCTCTGGAAAACTCATCTGCACCACTAATGTGCCTTGGAATTCTAGTTGGAGT-----------------
----------------------------AATAAATCTCAGAGTGAGATATGG------GAA------
AATATGACCTGGTTGCAATGG---GATAAAGAAATTAGCAATTACACAGACATAATATAT---
AAGCTAATTGAAGACTCGCAACTCCAACAGGAA--

AAGAATGAACAAGACTTATTGGCATTGGACAAGTGGGCAAGCCTGTGGAATTGGTTTGACATATCAAACTGGT
TGTGGTATATAAAAATATTTATAATAATAGTAGGAGGCTTAATAGGATTAAGAATAATTTTTGCT---
ATAGTAGCTGTAATA---------AATAGAGTTAGGCAGGATATTCACCT------
TTGTCATTTGCTATTAATTTGTTGGATACCATAGCAATAGCAGTAGCTGGC---
TGGACAGATAGGGTTATAGAAATAGGACAAAGATTTTGTAGAGCT----------------------------
--------ATTCTCAACATT------CCTAGAAGAATCAGACAAGGCCTCGAAAGGGCTTTGTTA---TAA
>AF361872
GCT---GCA--
GAAAACTTGTGGGTTACTGTCTACTATGGGGTACCTGTGTGG---AAAGATGCAAAG---
ACCACCTTATTTTGTGCATCAGATGCTAAAGCATATGAGACAGAA---AAGCATAAT----------------
--------------GTTTGGGCTACA---CATGCCTGTGTACCCACAGAC---
CCCAACCCACAAGAAATACATTTGGAC---------AATGTGACAGAAGAGTTTAAC----------------
--------------------------ATGTGGAAAAATGACATGGTAGAGCAGATGCATACAGATATA---
ATCAGTCTATGGGACCAAAGCCTA---AAGCCATGTGTAAAGTTA---
ACCCCTCTCTGCGTTACTTTACATTGTAACGATGTTAAGGTTAACAGC-------------------------
----
--ACGGGGGAGGACAAGGAAATGACA---
ATCTGCACTTACAATGTAACCACAGAGATAAGG---------------------------
GACAAAAAAGAGCAAGTATATTCACTG---TTTTATAGACTTGATGTAGTACAAATTAATGAAAGTCAA----
----

GGTAGCAGTAGTAACAATAAGTATAGATTAATAAATTGTAATACCTCAGCCATGACACAG---
GCTTGCCCAAAGGTATCCTTTGAGCCAATCCCCATACATTATTGTGCCCCAGCTGGTTTTGCGATCCTCAAGT
GTAAG---GATGAAGAGTTCAGT------GGAACAGGGCCGTGCAAGAAT-----------------------
-GTCAGC---ACAGTACAATGCACACATGGAATCAAGCCAGTAGTGTCAACT---
CAACTGCTGTTAAATGGCAGTCTAGCAGAAAATCAG------------
ATAAAAATTAGATCTGAAAATATCACAAACAAT---------GCCAAAAACATAATAGTACAATTTGTC----
-----------AATCCTGTGCCA---------ATTATTTGTATCAGACCTAACAACAATACAAGAAAG-----
-AGTATACGTATA------------GGACCAGGGCAAACA---TTT------TATGCAACAGGTGAC------
---------ATAATAGGGGATATA------------AGACAAGCCTATTGT---AATGTCAGTAAAACA---

GAGTGGAATAAAACTTTACATCAGGTAGCTACACGATTAGGA--------------CAATACTTTAAT----
-----------------------------------AACAAAACAATAATATTTACT------
AACTCCTCAGGAGGAGATCTAGAAATCACA---ACACATAGTTTTAATTGTGGAGGAGAA---
TTTAAACAGCTCCAGGCAAGAGTCCTGGCTGTGGAAAGATACCTAAGGGATCAACAGCTCCTAGGAATTTGGG
GCTGCTCTGGAAAACTCATCTGCCCCACTAATGTGCCCTGGAACTCTAGTTGGAGT----------------
----------------------------AATAAATCTCAGAGTGAGATATGG------GAC------
AACATGACCTGGCTGCAATGG---GATAGAGAAATTAGCAATTACACAGAAATGATATAT---
AAACTAATTGAAGAATCGCAGAACCAGCAGGAA---

AAGAATGAACAAGACTTATTGGCATTGGACAAGTGGGCAAATCTGTGGAATTGGTTTGACATATCAAAATGGC
TGTGGTATATAAGAATATTTATAATGATAGTAGGAGGCTTAATAGGATTAAGAATAGTTTTTGCT---
GTGCTTTCTATAATA---------AATAGAGTTAGGCAGGGATACTCACCT------
TTGTCGTTCGCTATTAATTTGATTGATACCATAGCAATAGTAGTAGCTGGC---
TGGACAGATAGGGTTATAGAGATAGGACAAGGGATTGGTAGAGCT---------------------------
--------ATTCTCCACATA------CCTAGAAGAATCAGACAGGGCTTAGAAAGAGCTTTGCAA---TAA
>AF361873
GCT---GCA--
AATAATTTGTGGGTTACTGTTTACTATGGGGTACCTGTGTGG---AGAGATGCAGAG---
ACCACCCTCTTTTGTGCATCAGATGCTAAAGCATATAAGACAGAA---AGGCATAAT---------------
--------------GTCTGGGCTACG---CATGCCTGTGTACCCACAGAC---
CCCAACCCACAAGAAATACATTTGAAA---------AATGTGACAGAAAACTTTAAC---------------
--------------------------ATGTGGAAAAATAACATGGTAGAACAAATGCATACAGATATA---
ATCAGTCTATGGGATGAAAGCCTA---AAGCCATGTGTAAAGTTA---
ACCCCTCTCTGTGTTACTTTAACTTGCTCTAATGCCAACTTTACTAATGTCAAC------------------
----
----------------------------------GTTAGCACCACTGACATGCAAGAAGTAAAA---
AACTGCTCTTACAATATAACCACAGAATTAAGG-------------------------
GATAAGAAACAGCAAGTATATTCACTC---TTTTATAGACTTGATATAGTACAACTGAATGAAAATAAGAAG-
----

AGTAGTAGTCAGAATAATAGTCAATATAGATTAATAAATTGTAATACCTCAGCCATTACACAG---
GCTTGTCCAAAGGTATCCTTTGAGCCAATTCCCATACATTATTGTGCTCCAGCTGGTTTTGCGATTCTAAAGT
GTAAG---GATAAAGAGTTCAAT------GGAACAGGGCCATGCAAGAAT----------------------
-GTCAGC---ACAGTACAATGCACACATGGAATCAAGCCAGTAGCATCAACT---
CAACTGCTGTTAAATGGCAGTCTAGCAGAAAGCAAG-----------
ACAATGATTAGATCTGAAAATATCACAAACAAT---------ATAAAAAACATAATAGTACAGCTCGCC----
-----------AACCCTGTAAAA---------ATTAATTGTACCAGACCTAACAACAATACAAGGAAA-----
-AGTGTACATATA------------GGACCAGGACAAGCA---TTC------TATGCAACAGGTGCC------
---------ATAATAGGGGATATA-----------AGACAAGCATATTGT---AATGTTAGTGAAGTA---
GAATGGAATAAAACTTTGCATGAGGTAGTCAAACAATTAAGA--------------ACATACTTTAGG----
-----------------------------------AATAAAACAATAATCTTTGAC------
AAATCAGCAGGAGGGGATCCAGAAATTACA---ACGCATACTTTTAATTGTGGGGGAGAA---
TTTAAACAGCTCCAGGCAAGAGTCCTGGCTGTGGAAAGATACCTAAGGGATCAACAGCTCCTAGGAATTTGGG
GCTGCTCTGGAAGACTCATCTGCACCACTAATGTACCCTGGAACTCTACTTGGAGT----------------
----------------------------AATAAATCCTATCATGACATATGG------GAT------
AACATGACCTGGCTGCAATGG---GATAAAGAAATTAGCAATTACACACAGATAATATAT---
GATCTAATTGAAGAATCGCAGAACCAGCAGGAA---

AAGAATGAACAAGACCTATTGGCATTGGACAAATGGGCAAATCTGTGGAACTGGTTTGACATAACAAACTGGC
TGTGGTATATAAAAATATTTATAATGATAGTAGGAGGCTTAATAGGATTAAGAATAGTTTTTGCA---
GTGCTTTCTATAATA---------AATAGAGTCAGGCAGGGATACTCACCT------
TTGTCATTTGCTATTAATTTGCTTGATACCATAGCAATAGCAGTAGCTGGC---
TGGACAGATAGGGTTATAGAAATAGGACAAAGATTTTGTAGAGCT---------------------------
--------ATTTACAACATA------CCTAGAAGAATCAGACAGGGCTTTGAAAGGGCTTTGCTA---TAA
>HM027846
GCT---GCA--
AACAACTTGTGGGTTACTGTTTACTATGGGGTACCTGTATGG---AAAGATGCAGAA---
ACCACCTTATTTTGTGCATCAGATGCGAAAGCATATGATACAGAA---GTGCATAAT---------------
--------------GTTTGGGCTACA---CATGCCTGTGTACCTACAGAC---
CCCAACCCACAAGAAATGAAGTTGGAA---------AATGTGACAGACAAGTTTAAC---------------

-------------------------ATGTGGAAAAATAACATGGTAGATCAAATGCATACCGACATA---
ATCAGTCTGTGGGACCAAAGTCTA---AAGCCATGTGTACAGTTA---
ACCCCTCTCTGCGTTACTTTAAATTGCTCTAATGTCAACACCACCGAGTCCAAAAGCTCT-------------
----
-------------------------CCTACCATCAAGCCCACTGTCTTCTCGTCTTAAACAAAA---
AACTGCTCTTTCAATATGACCACAGAATTAAGG---------------------------
GATAAAAAACAGCAAGTATATTCACTT---TTTTATAAACTGGATGTAGTACCAATTAATGGCAAC-------
----

GACAGCGATGCCAATGGTCAGTATAGATTAGTGAATTGTAATACCTCAGCCATTACACAA---
ACTTGTCCAAAAGTAACCTTTGAGCCAATTCCCATACATTATTGTGCCCCAGCTGGTTTTGCAATTTTAAAGT
GTAAT---GATAAGACGTTCAAT------GGAACAGGGACATGCAAAAT------------------------
-GTCAGC---ATAGTACAATGCACACATGGAATCAAGCCAGTAGTATCAACT---
CAACTACTGTTAAATGGCAGTCTAGCAGAAGATGAG------------
GTAATGATTAGATCTGAAAATATCACAGACAAT---------ATCAAAAACATAATAGTACAATTTAAC----
-----------AGGTCTGTAGAA---------ATTAATTGTACCAGACCTAACAACAATACAAGAAAA-----
-AGTGTACGTATA------------GGGCCAGGACAAGCA---TTC------TATGCAACAGGTGAC------
---------ATAATAGGGAATATA------------AGACAAGCACATTGT---ACTGTCAATAGGACA---
GAATGGAATGAAGCTTTGAAAGAGGTAGCCCAAAAATTAAGT---------------ACACTCTTTGTG----
-----------------------------------AACAAAACAATAAACTTTACT------
AGCCCCTCAGGAGGGGATGTAGAACTAATA---ACACATAGTTTTAATTGTAGAGGAGAG---
TTTAAACAGCTCCAGGCAAGAGTCCTGGCTGTGGAAAGATACCTAAAGGATCAACAGCTCCTAGGACTTTGGG
GCTGCTCTGGAAAACTCATCTGCACCACTACTGTGCCCTGGAACTCTAGTTGGAGT-----------------
----------------------------AATAAATCCCAGAGTGAGATATGG------GAT------
AACATGACCTGGATGCAATGG---GATAGAGAAATTAGCAATTACACACAGGAAATATAT---
AGTCTAATTGAAAAATCGCAGAACCAGCAGGAA--

AAGAATGAACAAGACTTATTGGCATTGGACAAGTGGACAAATCTGTGGACTTGGTTTGACATATCAAACTGGC
TGTGGTATATAAAAATCTTTATAATGATAGTGGGAGGCTTAATAGGATTAAGAATAGTTTTTGCT---
GTGCTTTCTATAATA---------AATAGAGTTAGGCAGGGATACTCACCT------
TTGTCATTCGCTATTAGTTTGCTTGATACCATAGCAATAGTAGTAGCTGGG---
TGGACAGATAGAGTTATAGAAATAGGACAAAGAATCTGTAGAGCT----------------------------
--------TTTCTCCACATA------CCTAGAAGAATTAGACAGGGCTTCGAAAGGGCTTTGCTA---TAA
>JX877522
GCT---GCA--
GACAAATTGTGGGTTACTGTCTACTATGGGGTACCTGTGTGG---AGAGATGCAGAA---
ACCACCTTATTTTGTGCATCTGATGCAAAAGCATATGAAAGAGAA---GTGCATAAT----------------
--------------ATCTGGGCTACG---CATGCCTGTGTACCTACAGAC---
CCCAACCCACAAGAAATACATTTGAAA---------AATGTGACAGAAGAATTTAAC----------------
--------------------------ATGTGGAAAAATAACATGGTAGAACAGATGCATACAGATATA---
ATCAGTCTATGGGACCAAAGCCTA---AAGCCATGTGTAAAGTTA---
ACCCCTCTCTGCGTTACTTTAAATTGTAGTGATGATGTTAGGGTTAGTAACCCTAATGGTACTGCCAGCAGCT
CTAATGTTAGT--
-------------------AACCAAAATAGTAACCAAAATGACAGTATCACCGGGGACATGAAA---
AACTGCTCTTTCAATGCGACCACAGAATTATGG---------------------------
GATAAGAAACAGAAAGTATATTCACTT---TTTTATAAGATGGATATAATATCAATTGAGAAC----------
----

AACAATAATGACAATAATACACAGTATAGATTAATAAATTGTAATACTTCAACCATTACACAG---
GCTTGTCCAAAGGTAACCTTTGAGCCAATTCCTATACATTATTGTGCCCCCGCTGGTTTTGCGATCTTAAAGT
GTAAT---GATGAGGAGTTCAAT------GGAACAGGGGTATGCAAGAAT-----------------------
-GTCAGC---ACAGTACAATGCACACATGGAATCAAGCCAGTAGTGTCAACT---
CAACTGCTGCTAAATGGAAGTCTAGCAGAGGGAAAG------------
ATAATGATTAGATCTGAAAATATCACAAACAAT---------GCCAAAAACATACTAGTACAACTTAAC----
-----------GAGACTGTAACC---------ATTAATTGTACCAGACCTGGCAACAATACAAGAAAG-----
-AGTGTACGTATA------------GGACCAGGCCAAGCA---TTC------CTTACAACAGGTGAG------
---------ATAATAGGGGATCCA------------AGAAAAGCACATTGT---AATGTCAGTAGAGCA---
GATTGGAATAAAACTTTGGCAAGGGTAGCCCAACAATTAAGA---------------ACACACTTTAAA----
-----------------------------------AACAACACAATAATCTTTAAT------
AGCTCCACAGGAGGGGATCCAGAAATAACA---ACACATAGTTTTAATTGTGGAGGAGAA---

TTTAAACAGCTCCAGGCAAGAGTCCTGGCTGTGGAAAGATACCTAAAGGATCAACAGCTCCTAGGAATTTGGG
GCTGCTCTGGAAAACTCATCTGCACCACTAATGTGCCTTGGAACGATAGTTGGAGT-----------------
----------------------------AATAAAACCCAAAATGAGATATGG------GAC------
AACATGACCTGGCTGCAATGG---GATAAAGAAATTAACAATTACACACAGGTAATATAT---
AATTTACTTGAAGAATCGCAGAACCAGCAGGAC--

AAGAATGAACAAGACTTGTTAGCATTGGACAAGTGGGCAAGCCTGTGGACTTGGTTTGACATATCAAACTGGC
TGTGGTATATAAGAATATTTCTAATGGTAGTAGGAGGCTTAATAGGATTAAGAATAGTTTTTGCT---
GTGCTTGCTATAATA---------AGTAGAGTTAGGCAGGGATACTCACCC------
TTGTCGTTCGCTATTAATTTGCTTGATACCATAGCAATAGTAGTGGCTGGG---
TGGACAGACAGGATCATAGAAGTAGGACTAGGGCTTTGTAGAGCT----------------------------
--------ATTTTACACATA------CCTAGAAGAATCAGACAAGGCTTAGAAAGAGCATTGCTA---TAA
>HM215272
GCT---GCA--
GGAAACTTGTGGGTTACAGTCTACTATGGGGTACCTGTGTGG---AAAGATGCAGAG---
ACCACCTTATTTTGTGCATCAGATGCTAAAGCATATAGTACAGAA---AAGCATAAT----------------
--------------ATCTGGGCTACA---CATGCCTGTGTACCCACAGAC---
CCCAACCCACAAGAAATAGATTTGGAA---------AATGTGACAGAAAAGTTTAAC----------------
--------------------------ATGTGGAAAAATGGCATGGTAGAGCAGATGCATACAGATATA---
ATTAGTATATGGGACCAAAGCCTA---CAGCCATGTGTAAAGTTA---
ACCCCTCTCTGTGTTACTTTAATTTGTATCGATATTGCCAATGACACAAGCCGC-------------------
----
-------------------------------AATGTCACTGTTGAAATGAAAAGAGAAATACAA---
AACTGCTCTTACAATGTGACCACAGAAGTAAGA---------------------------
GATAAAAAACAGAAAGTATATTCACTT---TTTTATAAAGTTGATATAGTGCAAATTAGTAAT----------
----

AATAGCAGTGAGTATAGATTAATAAATTGTAATACCTCAGTCATTAAACAA---
GCTTGTCCAAAGGTAACCTTTGAGCCAATTCCCATACATTATTGTGCTCCAGCTGGTTATGCGATCCTAAAGT
GTAAG---GATGATAAGTTCAAT------GGAACAGGGCTATGCAAAAAT-----------------------
-GTCAGC---ACAGTACAATGCACACATGGAATCAAACCAGTAGTGTCAACC---
CAACTGCTGTTAAATGGCAGCCTAGCAGAAGAAGAG------------
GTAATCATTAGATCTGAAAATATTAACAACAAT---------GCCAAAACGATAATAGTGCAACTATAC----
-----------CAGCCTGTAAAA---------ATTAATTGTACTAGACCTGGCAATAATACAAGGAAA-----
-AGTGTACATATA------------GGAACAGGACAAGCA---TAC------TTTGCAACAGGTGAC------
---------ATAATAGGGGATATA------------AGACAAGCATATTGT---AAGGTTAATAAATCA---
GAATGGAATAACACTTTACAACAGGTAGTTGCACAATTAAGA---------------AAACATTGG-------
-----------------------------------AACAAAACTATAATCTTTAAT------
AGCTCCTCAGGAGGAGATTTAGAAATTACA---ACACATCGCTTTAATTGTGGAGGAGAA---
TTTAAACAGCTCCAGGCAAGAATCCTGGCTGTGGAAAATTACCTAAAGGACCAACAGCTCCTAGGAATTTGGG
GCTGCTCTGGAAGACTCATCTGCACCACTAATGTGAAATGGAACTCTACTTGGAGT-----------------
----------------------------AATAAATCCAAGAGTGAGATATGG------GAT------
AAAATGACCTGGCTGGAATGG---GATAGAGAAATTAGCAATTACACAGACATAATATAT---
CAGCTAATTGAAGAATCGCAAAACCAGCAGGAA--

AAGAATGAACAAGATTTATTGGCATTGGACAAGTGGGCAAATCTATGGAATTGGTTTGACATATCAAATTGGC
TGTGGTATATAAAAATATTTATAATGATAGTAGGAGGCTTAATAGGATTAAGAATAGTTTTTGCA---
GTGCTCTCTGTAATA---------AATAGAGTTAGGCAGGGATACTCACCT------
CTGTCGTTTATTATCAACTTATATGATACCATAGCAATAGCAGTAGCTGGC---
TGGACAGATAGGATTATAGAAATAGGACAAAGAATTGGTAGAGCT----------------------------
--------ATTCTCAACATA------CCAAGGAGAATCAGGCAGGGTTTTGAAAGGGCTTTACTA---TAA
>HM027824
GCT---ACA--
AACAACTTGTGGGTTACTGTCTATTATGGGGTACCTGTATGG---AAAGAAGCAGAG---
ACCACCTTATTTTGTGCATCAGATGCGAAAGCATATGATAAAGAA---GTGCATAAT----------------
--------------GTCTGGGCTACG---CATGCCTGTGTACCTACAGAC---
CCTAACCCACAGGAAATAAAATTAGAA---------AATGTGACAGAAAATTTTAAC----------------
--------------------------ATGTGGAACAATAGCATGGTAGAGCAAATGCATACAGATATA---
ATCAGTCTATGGGACCAAAGCCTA---AAGCCATGTGTAAAGTTA---
ACCCCTCTCTGTGTTACTTTAGAGTGCTCTAATGTCACCAACAAGGGCACCAACAGCACTAACAGT-------

----
-------------------AGCCTCAATACCACCCTTGCCAGTGACATGGTAGGAGAAATAAAA---
AACTGCTCTTTCAATATGACCACAGAATTAAGG---------------------------
GATAAAAATCAGAAAGTACATTCACTT---TTTTATAAACTAGATATAGTATCAATTAATGGC----------
----

AGCAATAGTAGTTATAGATTAGTGAATTGTAATACCTCAACCATTAAACAG---
GCTTGTCCAAAAGTAACCTTTGAGCCAATTCCCATACATTATTGTGCCCCAGCTGGTTTTGCGATTCTAAAGT
GTGGT---GATACAGGGTTCAAT------GGGACAGGATGGTGCCGCAAT-----------------------
-GTCAGC---ACAGTACAATGCACACATGGAATCAAGCCAGTAGTATCAACT---
CAACTGCTGTTAAATGGCAGTCTAGCAGAAGAACAG------------
GTAGTGATTAGATCTGAAAATATCACAAACAAT---------GCCAAAAACATAATAGTACAATTTGCC----
-----------AGGCCTGTACAA---------ATTAATTGCACCAGACCTAACAACAATACAAGAAGA-----
-GGTATACGTATA------------GGACCAGGACAATCA---TTC------TATGCAACAGATGAC------
---------ATAATAGGGGATATA------------AGGCAAGCACATTGT---AATGTCAGTAGGACA---
GAATGGAATAAAACTCTGCAAGAGGTAGCCAGTCAATTAAGT---------------ACACTCTTTGGG----
-----------------------------------AACAGAATAATAAACTTTACT------
AGCCCATCAGGAGGAGATCTAGAAATAACA---ACACATAGTTTTAATTGTAGAGGAGAA---
TTTAAACAGCTCCAGGCAAGAGTCCTGGCTGTGGAAAGATACCTAAAGGATCAACAGCTCCTAGGAATTTGGG
GTTGCTCTGGAAAACTCATCTGCACCACTAATGTGCCCTGGAACTCTAGTTGGAGT-----------------
-------------------------AAGAATAAATCCCTGAGTGAGATATGG------GAC------
AACATGACCTGGATGGACTGG---GATAAAGAAATTGGCCAGTACACACAGGTCATATAT---
AATCTACTTGAGAAATCGCAGAACCAACAGGAA--

ATGAATGAACAAGACTTATTGGCATTGGACAAGTGGGCAAGCCTGTGGAATTGGTTTGACATATCAAACTGGC
TGTGGTATATAAAAATATTTATAATGATAGTAGGAGGCTTAATAGGATTAAGAATAGTTTTTGCT---
GTGCTTTCTATAATA---------AATAGAGTTAGGCAGGGATACTCACCT------
TTATCATTCGCTACTAGTTTGCTTGATACCATAGCAGTAGTAGTAGCTGGG---
TGGACAGACAGGGTTATAGAAATAGGACAAAGACTTGGTAGAGCT----------------------------
--------TTTCTTCACATA------CCTAGAAGAATTAGACAGGGCTTTGAAAGGGCTTTGCTA---TAA
>JX236669
GCT---ACA--
GAAAACTTGTGGGTGACTGTCTACTATGGGGTACCTGTGTGG---AGAGATGCAGAG---
ACCACCTTATTTTGTGCATCAGATGCTAAAGCATACGACACAGAA---ATGCATAAT----------------
--------------GTCTGGGCTACA---CATGCCTGTGTGCCCACAGAC---
CCCAACCCACAAGAAATAGATTTGGAA---------AATGTGACAGAAAAGTTTAAC----------------
--------------------------ATGTGGAAAAATAATATGGTAGAGCAGATGCATACAGATATA---
ATCAGTCTATGGGACCAAAGCCTA---AAGCCATGTGTAAAATTA---
ACCCCTCTCTGTGTTACTTTAAACTGTACTGCCATAACAAATGACACGAGAGGAAATGAAACA----------
----
----------------------------GGAATAAACAGGACAGTAGAAACAACTGAAATGACA---
AACTGCTCTTTCAATATGACCACAGAATTAAGG---------------------------
GATAGGAAAAAGAAAGTAAATGCACTT---TTTTATAAACTTGATATAGTACAAATTGGTGAAAAC-------
----

AGTAGTAGTCAGTATAGATTAATAAATTGTAATACCTCAGTCATTACACAA---
GCTTGTCCAAAGGTAACCTTTGAGCCAATTCCTATACATTATTGTGCCCCAGCTGGTTTTGCGATCCTAAAGT
GTAAG---GATAAGGAGTTCAAT------GGAACAGGGACATGCAGGAAT-----------------------
-GTCAGC---TCAGTACAATGCACACATGGAATCAAGCCAGTAGTATCAACT---
CAACTGCTGTTAAATGGCAGTCTGGCAGAAGGACAG------------
GTAAAAATTAGATCTGAAAATATTTCAGACAAT---------GCTAAAACCATAATAGTACAACTTAAT----
-----------GAGTCTGTACCA---------ATTAATTGTACCAGACCTAACAACAATACAAGACGA-----
-GGTATACATTTA------------GGACCAGGACAGACA---TTC------TTTGCAACA---GAC------
---------ATAATAGGGGATATA------------AGGCAAGCACATTGT---AATGTCAGTGAGTCA---
AAATGGAATAAAGCTTTACAAGAGGTAGTTAAACAATTAAGA---------------CAACACTGG-------
-----------------------------------AACAAAACAATAATCTTTAAA------
AGCTCCTCAGGAGGGGATCTAGAAATTACA---ACACATAGTTTTAATTGTGGAGGAGAA---
TTTAAACAGCTCCAGGCAAGAGTCCTGGCTGTGGAAAGATACCTAAAGGATCAACAGCTCCTAGGAATTTGGG
GCTGCTCTGGAAAACTCATCTGCCCCACTACTGTGCCCTGGAACTCTAGTTGGAGT-----------------
----------------------------AATAAATCTCAGAATGAGATATGG------GAT------

AACATGACCTGGTTGCAATGG---GATAAAGAAATTAGCAATTACACACAATTAATATAT---
AGCCTAATTGAAGAATCACAGAACCAGCAGGAA--

AAGAATGAACAAGAGTTATTGGCATTGGACAAGTGGGCAAGTCTATGGAATTGGTTTGACATATCAAAATGGC
TGTGGTATATAAGGATATTTATAATGATAGTAGGAGGCTTAATAGGATTAAGAATAGTTTTTACT---
GTGCTTTCTATAATA---------AATAGAGTTAGGCAGGGATACTCACCT------
TTGTCGTTTGCTCTTAATTTGCTTGACACTATAGCAATAGCAGTAGCTGGC---
TGGACAGATAGGGTCATAGAAATAGGACAAAGAATTTGCAGAGCT----------------------------
--------ATTCGCAACATA------CCTAGAAGAATTAGACAGGGCGCCGAAAGGCTTTTCTA---TAA
>JX236671
ACT---GCA--
GGAAACTTGTGGGTTACTGTCTATTATGGGGTGCCTGTGTGG---AAAGATGCAGAT---
ACCACCTTATTTTGTGCATCAGATGCCAAAGCATACGAGACAGAA---AAGCATAAT----------------
--------------GTCTGGGCTACA---CATGCCTGTGTGCCCACAGAC---
CCCAACCCACAAGAAACATATTTGGAA---------AATGTGACAGAAGAGTTTAAC----------------
--------------------------ATGTGGAAAAATAACATGGTAGAGCAGATGCATACAGATATA---
ATCAGCTTATGGGATCAAAGCCTA---AAGCCATGTGTAAAGTTA---
ACCCCTCTCTGCGTTACTTTACATTGTACCGATGTCAATGTCACCAACACCAATGTCACCAACACCAATGGC-
----
-------------ACCAGCACCAGCAGCTCCAAGGTCACCATCAACGACAGGGAAGAAATAAAA---
AACTGCTCTTTCAATGTAACCACAGAATTAAGG---------------------------
GATAAGGAAAAGAAAGTATATTCACTT---TTTTATAAGCTTGATGTAGTAAAAATAGATAAG----------
----

AATAAGAGTGACTATATATTAATAAATTGTAATACCTCAACCATTACACAG---
GCTTGCCCAAAGGTATCCTTTGAACCAATTCCCATACATTATTGTGCCCCAGCTGGTTTTGCGATCCTAAAGT
GTAGA---GATATAGACTTCAAT------GGAACAGGGCCATGCAAGAAT-----------------------
-GTTAGC---ACAGTCCAATGCACACATGGAATCAAGCCAGTAGTGTCAACT---
CAACTGCTGTTAAATGGCAGTCTAGCAGAAGGAGGG------------
ATAAGAATTAGATTTGAAAATATCACAAACAAT---------GCCAAAACTATAATAGTACAATTAAAC----
-----------AGTTCTGTGCCA---------ATTAATTGTACCAGACCTAATAACAATACAAGGCAA-----
-AGTGTGCGTATA------------GGACCAGGACAATCA---TTC------TTTGCAACAGGGGAG------
---------ATAATAGGGGATATA------------AGACAAGCACATTGT---AATGTCTCTAAATCA---
GACTGGCATAAGACTTTACAACAGGTGGCTGAACAATTAGAA---------------AAACACTTTAAG----
-----------------------------------AACAAAACAATAATATTTGAT------
CAACCCTCAGGGGGGGATCTAGAAATCACA---ACACATAGTTTTAATTGTGGAGGAGAA---
TTTAAACAGCTCCAGGCAAGAGTCCTGGCTGTGGAAAGATACCTAAGGGATCAACAGCTCCTAGGAATTTGGG
GCTGCTCTGGAAAACTCATCTGCCCCACTAATGTGCCCTGGAACTCTAGTTGGAGT-----------------
----------------------------AATAAAACTATGAGTGAAATATGG------GAC------
AACATGACCTGGCTGCAATGG---GATAAAGAAATTAGCAATTACACAAACATAATATAT---
AATCTAATTGAAGAATCGCAGAACCAGCAAGAA--

AAGAATGAACAAGACTTATTGGCATTGGACAAATGGGCAAGTCTTTGGAATTGGTTTGACATATCAAAGTGGC
TGTGGTATATAAAAATATTTATAATGATAGTAGGAGGCTTAATAGGATTAAGAATAATTTTTGCT---
GTGTTTTCTATAATA---------AATAGAGTTAGGCAGGGATACTCACCT------
CTGTCGTTTGCTATTAGCTTGTTTGATACCCTAGCAATAGTAGTAGCTGGC---
TGGACAGATAGAGTTATAGAAATAGGACAAAGACTTGGTAGAGGT----------------------------
--------TTTCTTCACATA------CCTAGAAGAATTAGACAAGGCTTAGAAAGGGTTTTGCTA---TAA
>JX236676
GCT---GCA--
GACAACTTGTGGGTTACTGTCTACTATGGGGTACCTGTGTGG---AGAGACGCAGAA---
ACCACCTTATTTTGTGCATCAGATGCAAAAGCATATGAAAAAGAA---GTGCATAAT----------------
--------------ATCTGGGCTACG---CATGCCTGTGTACCTACAGAC---
CCCAACCCACAAGAAATACATTTGGAA---------AATGTGACAGAAGAGTTTAAC----------------
--------------------------ATGTGGAAAAATAACATGGTAGAGCAGATGCATACAGATATA---
ATCAGTCTATGGGACCAAAGCCTA---AAGCCATGTGTAAAGTTA---
ACCCCTCTCTGCGTTACTTTAAATTGTAGCCAGTATGTCAGTAGCCATGTCAATAACCATAATAAC-------
----
-------------AGTAGCCATAATGTCAGTAGCCATAGTGGCAATATCACCAGTGACATGAAA---
ATCTGCTCTTTCAATACAACTACAGAAGTAAGG---------------------------

GATAAGAAACAGAAAGTATATTCACTT---TTTTATAAGTTGGATGTAGTACCAATCAGTAAT----------
----

GACAGTAGTCAGTATAGATTAATAAATTGTAATACCTCAGCCATTACACAG---
GCTTGTCCAAAGGTAACCTTTGAGCCAATTCCTATACATTATTGTGCCCCTGCTGGTTTTGCGATTTTAAAGT
GTAAG---GATAAGGAGTTCAAT------GGAACAGGGCTATGCAAGAAT-----------------------
-GTCAGC---ACAGTACAATGCACACATGGAATCAAGCCAGTAGTATCAACT---
CAACTGCTGTTAAATGGCAGTCTAGCAGAAGGAACG------------
GTAAGGATTAGATCTGAAAATATCACAAACAAT---------GCCAAAAACATACTAGTACAACTTACC----
-----------AGGCCTGTAAAC---------ATTAATTGTACCAGACCTAACAACAATACAAGAAAA-----
-AGTGTACATATA------------GGACCAGGACAAGCA---TTC------TATGCAATAGGAGCT------
---------ATAATAGGAAATATA------------AGACAAGCACATTGT---AATGTCAGTAAAGCA---
AGTTGGAATAGCGCTTTGCAAGAGGTAGCCCAACAGTTAAAA---------------ACACACTTTGGG----
-----------------------------------AACAAAACAATAATCTTTGCT------
AACTCCACAGGGGGGGATCCAGAAATAACA---ACACATAGTTTTAATTGTGGAGGAGAA---
TTTAAACAGCTCCAGGCAAGAGTCCTGGCTGTGGAAAGATACCTAAGAGATCAACAGCTCCTAGGAATTTGGG
GCTGCTCTGGAAAACTCATCTGCACCACTAATGTGCCCTGGAACTCTAGTTGGAGT-----------------
----------------------------AATAAAACTCAGAGTGAGATATGG------GAC------
AACATGACCTGGATGCAATGG---GATAAAGAAATTAACAATTACACACAGTTAATATAT---
GGTCTACTTGAAGAGTCGCAGAACCAGCAGGAA--

AAGAATGAACAAGACTTGTTGGCATTGGACAAGTGGGCAGATCTGTGGAATTGGTTTGACATATCAAAATGGC
TGTGGTATATAAAAATATTTATAATGATAGTAGCAGGCTTAATAGGATTAAGAATAGTTTTTGCT---
GTGCTTGCTATAATA---------AATAGAGTTAGGCAGGGATACTCACCC------
TTGTCGTTCGCTATTAACTTGCTTGATACCATAGCAATAGTAGTAGCTGGG---
TGGACAGACAGGGTCATAGAAGTAGGACAAAGACTTTGTAGAGTT----------------------------
--------ATTTTACACATA------CCTAGAAGAATCAGACAGGGCTTAGAAAGGGCTTTGCTA---TAA
>KF716478
GCT---ACA--
GACAAATTGTGGGTTACTGTCTACTATGGGGTACCTGTATGG---AAAGACGCAGAG---
ACCACCTTATTTTGTGCATCAGATGCGAAAGCATATGAGAAAGAA---ATGCACAAT----------------
--------------GTCTGGGCTACA---CATGCCTGTGTACCTACAGAC---
CCCAACCCACAAGAAGTAGCTTTGGAA---------AATGTGACAGAAGAGTTTAAT----------------
--------------------------ATGTGGAAAAATAACATGGTAGAACAGATGCAGACAGATATA---
ATCAGTCTATGGGACCAAAGCCTA---AAGCCATGTGTAGAGTTA---
ACCCCTCTCTGCGTCACTTTGGAGTGTAGCAATATCAACAGCACCAATAACACCGCCACC-------------
----
-------------------------------AATTTCACCAATTTCATGGGAGAAGACATAAAA---
AACTGCTCTTTCAATATGACCACAGAATTAAGG---------------------------
GATAAGGAGAAGAAAGTATATTCACTT---TTCTATAAAGTGGATATAACACCATTGGAAAAC----------
----

AGCAACGAAACTAAGTATAGGTTAATAAATTGTAATACCTCAGCCATTAAACAA---
GCCTGTCCAAAGGTATCCTTTGAGCCAATTCCCATACATTATTGTGCTCCAGCTGGTTTTGCGATCCTAAAGT
GTAGA---GATGAGGAGTTCAAT------GGAACAGGGCCATGCAAAAAT-----------------------
-GTCAGC---ACAGTACAATGCACACATGGAATCAAGCCAGTAGTATCAACT---
CAACTACTATTAAATGGCAGTCTAGCAGAAAAGAAG------------
GTAATGATTAGATCTGAAAATTTCACAAACAAT---------GCTAAAAACATCATAGTACAATTTAAT----
-----------AAGTCAGTAGAA---------ATTAATTGTACCAGACCTAACAACAACACCAGAAAA-----
-AGTGTACATATA------------GGACCAGGACGAGCA---TTC------TATGCAACA---GAT------
---------ATAATAGGGGACATA------------AGATGGGCATATTGT---AATGTCAGTAGATCA---
GAATGGAAGGAAACTTTACAACAGGTAGTCAAACAATTAGGA---------------AACCATTGG-------
-----------------------------------AATAAAACAATACACTTTAAT------
AATTCTGCAGGAGGGGATTTAGAAATTACA---ACACATAGTTTTAATTGTGGAGGAGAA---
TTTAAACAGCTCCGGGCAAGAGTCCTGGCTGTGGAAAGGTACCTAAAGGATCAACAGCTCCTAGGAATTTGGG
GCTGCTCTGGAAAACTCATCTGCCCCACTACTGTGCCCTGGAACTCTAGTTGGAGT-----------------
----------------------------AATAAAAGTTACAGTGAGATATGG------GAT------
AACATGACCTGGCTGCAGTGG---GATAAAGAAATTAGCAATTACACAGACCTAATATAT---
GGGCTAATTGAAGAATCGCAAAACCAGCAGGAA--

AAGAATGAACTAGACTTATTAGCATTAAACAAGTGGGCAGAGCTGTGGAATTGGTTTGACTTATCAAACTGGC
TGTGGTATATAAGAATATTTATAATGATAGTAGGAGGCTTAATAGGATTAAGAATAGTTTTTGCT---
GTGCTTTCTATAATA---------AATAGAGTTAGGCAGGGATACTCACCT------
TTGTCGTTCGCTATTAGTTTGCTTGATACCATAGCAATAGTAGTAGCTGGC---
TGGACAGATAGGGCTATAGAAATAGGACAAGGAATTGGTAGAGCT----------------------------
--------ATCCTCAACATA------CCTAGAAGAATCAGGCAGGGCTTAGAAAGGGCTTTGCTG---TAA
>KP109490
GCT---GTA--
GAAGACTTGTGGGTTACTGTCTATTATGGGGTACCTGTGTGG---AAAGACGCAGAG---
ACCACCTTATTTTGTGCATCAGATGCTAAAGCATATGAGACAGAA---AAGCATAAT----------------
--------------GTCTGGGCTACA---CATGCCTGTGTACCCACAGAC---
CCCAGCCCACAAGAAATACCTTTGGAA---------AATGTGACAGAAGAGTTTAAT----------------
--------------------------ATGTGGAAAAATAACATGGTAGAGCAGATGCATACAGATATA---
ATCAGTCTATGGGACCAAAGTCTA---AAGCCATGTGTAAAGTTA---
ACCCCGCTCTGCGTCACTTTAAATTGTAACAATATCAATGCCACGAGCAGCAATGCCACA-------------
----
----------------------GTCCCGGGCAATGACACAGCTAATAACAAGGAAGAAATAACA---
AACTGCTCTTTCAATATGACTACAGAAGTAAGG---------------------------
GATAAGAAAAAGCAAGTACATTCACTT---TTTTATAAACTTGATGTAGTACAAATAGGAAAT----------
----

AGTAATAGTAGCGAGTATAGATTAATAAATTGTAATACCTCAGTCATTACACAG---
GCGTGCCCAAAGGTAAACTTTGAGCCAATTCCCATACATTACTGTGCTCCAGCTGGTTTTGCGATCCTAAAGT
GTAGG---GATGAACAGTTCAAT------GGAACAGGGCTGTGCAAGAAT-----------------------
-GTCAGC---ACAGTCCAATGCACACATGGAATCAAGCCAGTAGTATCAACT---
CAACTGCTGTTAAATGGCAGTCTAGCACAAAAAGAG------------
GTACAAATTAGATCTGAAAATATCACAAACAAT---------GCCAAAAATATAATAGTACAATTTGCC----
-----------AAGCCTGTGGAA---------ATTAATTGTACCAGACCTAGTAATAATACAAGAAAA-----
-AGTATACATATA------------GGACCAGGACAAGCC---TTC------TATGCAACAGATGCC------
---------ATAATAGGGGATACA------------AGACAAGCACATTGT---AACGTCAGTAAAGAA---
AAATGGAATAAGACTTTAGAAGAAGTAGCTAAACAATTAAAG---------------AAACACTTT-------
-----------------------------------AACAAAACAATAATATTTGCT------
AACTCCGCAGGAGGGGATCTAGAAATCACA---ACACATAGTTTTAATTGCAGAGGAGAA---
TTTAAACAGCTCCAGGCAAGAGTCCTGGCTGTGGAAAGATTTCTAAAGGATCAACAGCTCCTAGGAATTTGGG
GCTGCTCTGGAAAACTCATCTGCACCACTAATGTGCGTTGGAACTCTAGTTGGAGT-----------------
----------------------------AATAAAAGTCAAAGTGAAATATGG------GAC------
AACATGACCTGGCTACAATGG---GATAAAGAAATTAACAATTACACAGAAATAATATAT---
AAACTACTTGAAGAATCGCAAAATCAGCAGGAA--

AAGAATGAACAAGACTTATTGGCATTGGACCAATGGGCGAGTTTGTGGAGTTGGTTTGACATAACAAAATGGC
TGTGGTATATAAAAATATTTATAATGATAGTAGGAGGCTTAATAGGATTAAGAATAGTTTTTGCT---
GTGCTTTCTGTACTA---------AAGAGAGTTAGGCAGGGATACTCACCT------
TTGTCATTTGCTATTAGCTTGTTTGATACCTTAGCAATAGCAGTAGCTGGC---
TGGACAGATAGGGTTATAGAAATAGGACAAAGAATTGGTAGAGCT----------------------------
--------TTTCTCCACATA------CCTAGAAGAATTAGACAGGGCTTTGAAAGGGCTTTGCTA---TAA
>JF680921
ACT---GCA--
GAAGACTTGTGGGTTACTGTCTACTATGGGGTACCTGTATGG---AAAGAAGCAAAT---
GCCACCTTATTTTGTGCATCTGATGCTAAAGCATATGATCAAGAA---GTGCATAAT----------------
--------------GTCTGGGCTACA---CATGCCTGTGTACCCACAGAC---
CCCAACCCACAAGAAATATATTTGCAA---------AATGTGACAGAAGATTTTAAC----------------
--------------------------ATGTGGAAAAATAACATGGTAGAACAGATGCATACAGATATA---
ATTAGTCTATGGGACCAAAGCTTA---AAGCCATGTGTACAATTA---
ACACCTCTCTGCGTTACTTTAGATTGTAGCCATACTATCTCCTATATTAACAACACCCATAATGGCACCAGAA
TC---
-------------AATGATACCACCATGGATAACGATACCAGAAAAATACTAGAAGAAATGAAA---
AACTGCTCTTTCAATATGACCACAGAATTAAGA---------------------------
GATAAGAAACAGAAAGTGTATTCACTT---
TTCTATAGACTTGATATAATAGAAATTGATAAAGATAAGAATAGCAGAGAGAATAAT----------------
----

AGTAGCAGTAATAGTAATAATAGTAGTTATAGATTAATAAATTGTAATACCTCAGCCATTACACAA---
GCTTGTCCAAAGGTAACCTTTGAGCCAATTCCCATACATTATTGTGCCCCAGCTGGTTTTGCAATTTTAAAGT
GTAAT---GAGATAGGGTTCAAT------GGAACAGGACCATGCAAGAAT----------------------
-GTCAGC---ACAGTACAATGTACACATGGAATCAAGCCAGTAGTATCAACT---
CAACTGCTGTTAAATGGCAGTCTAGCAGAACAAAAT-----------
GTAGTAATTAAATCTGAAAATATCACAGACAAT---------GCCAAAAACATCATAGTACAATTTAAT----
-----------GAGTCTGTAGTA---------ATTAATTGTACCAGACCTAACAACAATACAAGAAAG-----
-AGTGTACGTATA------------GGACCAGGACAATCA---TTC------TATGCGACAGGTGAA------
---------ATAATAGGGAATATA------------AGACAAGCACACTGT---ACTGTCAATAGAACA---
AAATGGATAAGAGCTTTACAAAAAGTAGCCACACAATTAGGC---------------AAAAACTTTAAG----
------------------------------AACAAAACAAAAATAATCTTTACT------
AGCCCCTCAGGAGGAGATGTAGAAATTACA---ACACATAGTTTTAATTGTCAAGGAGAA---
TTTAAACAGCTCCAGGCAAGAGTCCTGGCTATGGAAAGATACCTACATGATCAACAGCTCCTAGGAATTTGGG
GCTGCTCTGGAAAACTCATCTGCACCACTACTGTGCCCTGGAACTCTAGTTGGAGC-----------------
------------------------AGTAATAAATCTCAGGATGAGATATGG------AAC------
AAAATGACCGGGCTGCAATGG---GATAAAGAAGTTAGCCAATACACAAACACAATATAT---
AACCTACTTGAAGAATCACAGAACCAGCAGGAA-------------------------------------
----
AAGAATGAACAAGAATTATTGGCATTAGATGAGTGGGCAAATCTATGGAATTGGTTTGACATATCAAAATGGC
TGTGGTATATAAAAATATTTATAATAATAGTAGGAGGCTTAATAGGATTAAGAATAGTTTTTGCT---
GTACTTTCTATAATA---------AATAGAGTTAGGCAGGGATACTCACCT------
CTGTCGTTTGCTATTAGTTTGCTTGATACCATAGCAATAACAGTAGCTGGC---
TGGACAGATAGGATTATAGAAATAGGACAAAGATTTTGTAGAGCT----------------------------
--------ATTCTCAACATA------CCTAGAAGAATCAGACAAGGCTTTGAGAGGGCTCTGCTA---TAA
>KF859745
GTT---GCA--
GGAAACTTGTGGGTTACTGTCTACTATGGGGTACCTGTGTGG---AGAGATGCAGAG---
ACCACCTTATTTTGTGCATCAGATGCAAAAGCATATGATACAGAA---GTGCATAAT----------------
-------------GTCTGGGCCACA---CATGCCTGTGTACCTGCAGAC---
CCCAACCCACAAGAAATAGAGTTGAAA---------AATGTGACAGAAAAGTTTAAC---------------
-------------------------ATGTGGAAAAATAACATGGTAGAGCAGATGCATGCAGACATA---
ATCAGTCTATGGGACCAAAGCCTA---AAGCCATGTGTGAAGTTA---
ACCCCTCTCTGCGTTACTCTAAAGTGTACAGACCTAGACCTTAATGGCACTCAGAGT---------------
----
-------------------CTTAATGGCACTCAGAGGCTAAATGGCACTGAGGTAGGTATGACA---
AACTGCTCTTTCAATACAACCACAGAATTAAGG--------------------------
GATAAGATAAGGAAAGAATATGCACTT---TTTTATAGGCTTGATATAGTACAAATTAATGGA----------
----

AGTGATAACAGTACATATAGATTAATAAATTGTAATACCTCAGCCATTAAACAG---
GCTTGTCCAAAGGTATCCTTTGAGCCAATTCCCATACATTATTGTGCCCCAGCTGGTTTTGCGATTCTAAAGT
GTAAG---GATGCAGAGTTCAAT------GGAACAGGGCCATGCAACAAT----------------------
-GTCAGC---ACAGTACAATGCACACATGGGATCAGGCCAGTAGTATCAACT---
CAACTGCTGTTGAATGGCAGTCTAGCAGAAAGAAAG-----------
GTAATGATTAGATCTGAAAATATCACAAACAAT---------GCCAAAAACATAATAGTACAATTTAAT----
-----------AAGTCTGTAGAA---------ATTAATTGTACCAGACCTAACAATAATACAAGAAAA-----
-AGTGTACGCATA------------GGACCAGGACAAACA---TTC------TATGTAACAGGTGAC------
---------ATAATAGGGGATATA------------AGACAAGCACACTGT---AATGTCAGTAGAAAG---
AAATGGAATGACACTTTGAGAGAGGTAGTCAGCGAATTAAGG---------------AAATACTTTGGG----
---------------------------------AACAAAACAATAATCTTTACT------
AACTCCTCAGGAGGGGATCTAGAGATTACA---ACACATAGTTTTAATTGTGGAGGAGAA---
TTTAAACAGCTCCAGGCAAGAGTCCTGGCTCTGGAAAGTTACCTAAGGGATCAACAGCTCCTAGGAATTTGGG
GCTGCTCTGGAAAACTCATCTGCACCACTAATGTGCCCTGGAACTCTAGTTGGAGT----------------
----------------------------AATAAGTCCCAAGATGAGATATGG------CAA------
AACATGACCTGGTTGCAATGG---GATAGAGAAATTAGCAATTACACAGATCTAATATAC---
ACTCTGATTGAAGAATCGCAGATCCAGCAGGAA-------------------------------------
----
AAGAATGAACAAGACTTATTAGCATTGGACAAGTGGGCAAATCTATGGAATTGGTTTGACATATCAAACTGGC
TGTGGTATATAAAAATATTTATAATGATAGTAGGAGGCTTAATAGGATTAAGAATAATTTGTGCT---
GTGCTTGCTATAATA---------AATAGAGTTAGGCAGGGATACTCACCT------

CTATCGTTCGCCATCAATTTGCTTGATACCATAGCAATAGCAGTAGCTGGC---
TGGACAGATAGGATTATAGAAATAGGACAAAGCATTGGTAGGGCT-------------------------
--------ATTCTTCACATA------CCTAGAAGAATCAGACAGGGTGCTGAAAGGGCTTTGCTA---TAA
>KF716486
GCT---GCT--
CAAAACTTGTGGGTTACTGTCTACTATGGGGTACCTGTGTGG---AAAGACGCAGAG---
ACCACCCTGTTTTGTGCGTCAGATGCTAAAGCATATGAAACAGAG---AAGCATAAT-------------
--------------GTCTGGGCTACA---CATGCCTGTGTACCCACAGAC---
CCCAACCCACAAGAAATACATTTGGAA---------AATGTAACAGAAGAATTTAAC-------------
-------------------------ATGTGGAAAAATAATATGGTAGAGCAGATGCATACAGATATT---
ATCAGTCTATGGGACCAAAGCCTA---AAGCCATGTGTAAGGTTA---
ACTCCTCTCTGTGTTACTTTAAGCTGTACCAATGCTAAAAGCGGCAGCACTGGGAAC-------------
----
---------------------------AGCAGCAGCACGGTGGAAGGGGAGGGAGAAATAAAA---
AATTGTTCTTTCAATATGACCACAGAACTAAGG------------------------
GATAGGAAAAGGAGAATGTATGCACTT---TTTTATAAACTTGATCTACAACAAATTAATGAAACT-------
----

AATAATAGTTACTATAGCTTAATAAATTGTAATACCTCAGCCCTTACACAG---
GCTTGTCCAAAGGTATCCTTTGAGCCAATTCCCATACATTATTGTGCTCCAGCTGGTTTTGCGATCCTAAAGT
GTAGG---GATGAGGAGTTCAAT------GGAACAGGGCCATGCAAGAAT----------------------
-GTCAGC---ACAGTACAATGCACACATGGAATTAAGCCAGTAGTATCAACT---
CAATTGCTGTTAAATGGCAGTCTAGTAAAAGGAGAG-----------
GTAAGAATTAAATCTGAAAATATTTCAGAAAAT---------CATAAAAACATAATTGTACACCTTACA----
-----------AAGCCTGTATCA---------ATTAGGTGTATCAGACCTAACAACAATACACGAACA-----
-AGTGTACGTATA------------GGGCCAGGACAAGAA---TTC------TTTACAAGTAAG---------
---------ATAATAGGGAATATA-----------AGACAAGCATACTGT---AACGTCAGTAAATCA---
GAATGGGAGAACACTTTACAACAGGTAGCTACACAATTAAGA--------------AAATACTGG-------
----------------------------------AACAAAACAATAAAATTTACT---
GGCCCGGTCTCAGGAGGGGATGTAGAAATTACA---ACACATAGTTTTAATTGTGGAGGAGAA---
TTTAAACAGCTCCAGGCAAGAGTCTTAGCTGTGGAAAGATACCTAAAGGATCAACAGCTCCTAGGAATTTGGG
GCTGCTCTGGAAAACTCATCTGCACCACTACTGTGCCCTGGAACTCTAGTTGGAGT----------------
---------------------------AACAAAACTTACAATGAAATATGG------GAC------
AACATGACCTGGCTGCAATGG---GATAAAGAAATTAGCAATTACACAGAGGAGATATAC---
AGGCTAATTGAAGCATCACAGATCCAGCAGGAA-------------------------------------
----
AAGAATGAACAAGACTTATTGGCATTGGACAAGTGGGCAAGTCTGTGGAGTTGGTTTGACATATCAAATTGGC
TGTGGTATATAAAGATATTTATAATGATAGTAGGAGGCTTAATAGGATTAAGAATAGTTCTTGCT---
GTGCTTTCATTAATC---------AATAGAGTTAGGCAGGGATACTCACCT------
CTATCGTTTGCTATTGATTTGTTTAACACCACAGCAATAGCAGTAGCTGGC---
TGGACAGATAGGATTATAGAAATAGGGCAAAGAATTGGTAGAGCT-------------------------
--------ATCCTCAACGTA------CCTACAAGAATCAGGCAGGGCTTAGAAAGGGCTTTACTA---TAA
>M62320
GCA---CAA---
CAATTGTGGGTCACGGTCTACTATGGGGTACCTGTGTGG---AAAGATGCAGTT---
ACCACCCTATTTTGTGCATCAGATGCTAAAGCATATGATGCAGAA---GTGCATAAT-------------
--------------GTCTGGGCTACA---CATGCCTGTGTACCCACAGAC---
CCCAACCCACAAGAAATAGATTTGGTC---------AATGTGACAGAAGAATTTAAC-------------
-------------------------ATGTGGAAAAATAACATGGTAGATCAGATGCATGAAGATATA---
ATCAGTCTATGGGACCAAAGCCTA---AAGCCATGTGTAAAGTTA---
ACCCCTCTCTGCGTTACGTTAGATTGCCATAACATCACCATTAACAACACCAATAACAAC----------
----
---------------------------ACCAATATCACAGATGGAGTGAGGGAAGAAATGAAA---
AATTGCTCTTTCAATATGACCACAGAACTAAGA------------------------
GATAAGAAACAGAAAGTATATTCACTT---TTTTATAGACTTGATATAGTACAAATCAATAAA----------
----

ACTGATAACAATAGTTATAGACTAATAAATTGTAATACCTCAACCATTACACAG---
GCTTGTCCCAAGGTGTCCTTTGAGCCAATTCCCATACATTATTGTGCCCCAGCTGGTTTTGCGATTCTAAAGT
GTAAG---GATCCTGAGTTCAAT------GGAAAAGGGCCATGCAGGAAT----------------------

-GTCAGC---ACAGTACAATGTACACATGGAATCAAGCCAGTGGTATCAACT---
CAACTGCTGTTAAATGGCAGTCTAGCAGAAAGAGAA------------
ATAAGGATTAGATCTGAAAATTTCACAAACAAT---------GCAAAAACCATAATAGTACAGCTTGTC----
-----------AATCCTGTAAAA---------ATCAATTGTTCCAGACCTTACAATACAAGAAAAAAT-----
-ATACGTAGGTATAGT------ATAGGATCAGGACAAGCA---TTC------TATGTAACAGGTAAA------
---------ATAATAGGGGATATA------------AGACAAGCACATTGT---AATGTCAGTAGAAGG---
GACTGGAATAGAACTATACAACAGGTAGCTGAACAATTAAAG---------------AAAAAGTTTAAT----
-----------------------------------AACAAAACAATAATCTTTGCT------
AGCTCCTCAGGAGGGGATATAGAAATTACA---ACACATAGCTTTAATTGTGGAGGAGAA---
TTTAAACAGCTCCAGGCAAGAGTCCTGGCTGTGGAAAGATACCTACAGGATCAACAGCTCCTAGGAATTTGGG
GCTGCTCTGGAAAGCTCATCTGCACCACTACTGTGCCCTGGAACTCTAGTTGGAGT-----------------
----------------------------AATAAATCTCAGGAGGACATATGG------AAT------
AACATGACCTGGCTGCAATGG---GAGAAAGAAATTAGTAGTTACACAGGCATAATATAT---
CAACTAATTGAGGAATCGCAGAACCAGCAGGAA--

AAGAATGAACTAGACTTATTGGCATTGGACAAGTGGGCAAATCTGTGAAATTGGTTTAACATCTCAAATTGGC
TGTGGTATATTAGACTATTTGTAATAATAGTAGGAGGCTTAATAGGATTAAGAATAGTTTTTACT---
GTGCTTTCTATAATC---------AATAGAGTTAGGCAGGGATACTCACCT------
CTGTCGTTTGCTATTACTTTGCTTGACGCTGTAGCAGTAGCAGTAGCTGGG---
TGGATAGATAGGGTTATAGAAATAGGACAAACAATTGGTAGAGCT----------------------------
--------ATTCTCAACATA------CCTAGAAGAATCAGACAAGGCTTAGAAAGGGCTTTGCTA---TAA
>L22957
ACT---GCA--
AAAAACTTGTGGGTTACTGTATACTATGGGGTACCTGTGTGG---AGAGACGCAGAG---
ACCACCCTATTTTGCGCATCAGATGCTAAAGCATATGAGACAGAA---ATGCATAAT----------------
--------------GTCTGGCTACA---CATGCCTGTGTACCCACAGAC---
CCTAACCCACAAGAAATACGTTTGGAA---------AATGTGACAGAAGAGTTTAAC----------------
--------------------------ATATGGAAAAATAACATGGTAGAGCAGATGCATACAGATATA---
ATCAGTCTATGGGACCAAAGCCTA---AAGCCATGTGTAAAGTTA---
ACCCCTCTCTGCGTTACTTTAAATTGTACCAATGCCAACCTCACCAATGCCAATAAAAATGCCAAGGTC----
----
----------------AACAGCGCCACGAATGGCACCATCCAGAGCATGGAAAGAGAAATAAAA---
AACTGCTCTTTCAATATGACCACAGAACTAAGG---------------------------
GATAAGACACGGAGAGTATATTCACTT---TTTTATAAACTTGATGTAGTACAAATTAAAAAT----------
----

AGTAGTGAGTATAGATTAATAAATTGTAATACCTCAGCCATTACACAG---
GCTTGTCCAAAGGTATCCTTTGAGCCAATTCCCATACATTATTGTGCCCCAGCTGGTTTTGCGATCCTAAAAT
GTAAT---GATAAGGAGTTCAAT------GGAACAGGGCTATGCAAGAAT-----------------------
-GTCAGC---TCAGTACAATGCACACATGGAATCAGGCCAGTAGTATCAACT---
CAACTGCTGTTAAATGGCAGTCTAGCAGAAACAGAG------------
GTAAAAATTAAATCTGAAAATATCTCAGACAAT---------GCTAAAACCCTAATAGTACAACTTACC----
-----------ACGCCTGTAAAA---------ATTAATTGTACTAGACCTGGCAACAATACAAGAACA-----
-AGTGTACGTATA------------GGACCAGGACAAGCA---TTC------TATGCAACAGGTGAT------
---------ATAATAGGAGATATA------------AGACAAGCACATTGT---AATGTCAGTAGATCA---
GAATGGAAGGAAACTTTGCAAAAGGTAGTCAAACAATTAAGA---------------ACACACTGG-------
-----------------------------------AACAAAACAATAATCTTTACA------
AACTCCTCAGGAGGGGATTTAGAAATTACA---ACACATAGTTTTAATTGTGGAGGAGAA---
TTTAAACAACTCCAGGCAAGACTCCTGGCTGTGGAAAGATACCTAAGGGATCAACAGCTCCTAGGAATTTGGG
GCTGCTCTGGAAAACTCATCTGCACCACTACTGTGCCCTGGAACTCTAGTTGGAGT-----------------
----------------------------AATAAATCTCAAAGTGAGATATGG------GAT------
AACATGACCTGGCTGCAATGG---GATAAAGAAATTAGTAATTACACAAACATAATATAT---
GATCTAATTGAAGAAGCGCAGAACCAGCAGGAA--

AAGAATGAACAAGACTTATTGGCATTGGACAAGTGGGCAAGTCTGTGGAGTTGGTTTGACATATCAAATTGGC
TGTGGTATATAAGACTATTTATAATGATAGTAGGAGGCTTAATAGGATTAAGAATAGTTCTTGCT---
GTGTTTTCTGTAATA---------AATAGAGTTAGGAAGGGATACTCACCT------
GTGTCATTTGCTATTAATTTGCTTGATACCATAGCAATAGCAGTAGCTGGC---
TGGACAGATAGAGTTATAGAAATAGGACAAAGAATTGGTAGGGCT----------------------------
--------TTTCTTAACATA------CCTAGAAGAATCAGGCAGGGCTTCGAAAGAGCTTTACTA---TAA

>L22951
ACT---GCA---------------------------------------
GAAGACTTGTGGGTTACTGTCTACTATGGGGTACCTGTATGG---AGAGACGCAGAG---
ACCACCTTATTTTGTGCATCAGATGCGAAAGCATATGATACAGAA---ATGCATAAT---------------
-------------GTCTGGGCTACA---CATGCCTGTGTACCTACAGAC---
CCCAACCCACAAGAAATAAATTTGGGA---------AATGTGACAGAAAAATTTAAC---------------
-------------------------ATGTGGAAAAATAACATGGTAGAGCAGATGCATGAAGATATA---
ATCAGTCTATGGGATCAAAGCCTA---AAGCCATGTGTAAAGCTA---
ACCCCTCTCTGCGTTACTTTAAATTGTAGTGATGTCAATAATGTCACTGTCACCAAT---------------
----
---------------------------CATAGTATTAGCAGTGACATGAAAGGAGAAATAAAA---
AACTGCTCTTTCAATATGACCACAGAATTAAGG--------------------------
GATAAGCAACAGAAAATACATCCACTT---TTTTATAGACTGGATGTAGTACCAATTAATGGC----------
----

AGCAACAACAGGCAGTATAGATTAATAAATTGTAATACTTCAGCCATTACACAG---
GCTTGTCCAAAAGTATCCTTTGAGCCAATTCCCATACATTATTGCGCCCCAGCTGGTTTTGCGATTCTAAAGT
GTAAT---GATCAGGAGTTCAAT------GGAACAGGGATATGTAATAAT----------------------
-GTCAGC---ACAGTACAATGCACACATGGAATCAAGCCAGTAGTATCAACT---
CAGCTGCTGTTAAATGGCAGTTTAGCAGAAGGAAAG-----------
GTAAAGATTAGATCTGAAAATATCACAAACAAT---------GCCAAAAATATACTGGTATAACTTACC----
-----------ACGCCTGTAACA---------ATTAATTGTACCAGACCTAACAACAATACAAGAACA-----
-AGTGTACGTATA------------GGACCAGGACAATCA---TTC------TATGCAACAGGTGAC------
---------ATAATAGGGGATATA------------AAACAAGCACATTGT---AATATCAGTAGAGCA---
GAATGGAATGAAACTTTACAAAAGGTAGTCAGCCAATTAAGA---------------ACACACTTTGGC----
-----------------------------------AACAAAACAATAATCTTTGGT------
AACTCCTCAGGAGGGGATATAGAAATAACA---ACACATAGTTTTAATTGTGGAAGAGAA---
TTTAAACAGCTCCAGGCAAGAGTCCTGGCTGTGGAAAGATACCTAAGGGATCAACAGCTCCTAGGAATTTGGG
GCTGCTCTGGAAAACTCATCTGCACCACTAATGTGCCCTGGAACTCTAGTTGGAGT----------------
---------------------------AATAAATCCCAGAGTGATATATGG------GAC------
AACATGACCTGGCTGCAATGG---GATAAGGAAATAAACAATTACACAGAGCTAATATAT---
AGGCTACTTGAAGAATCGCAGAACCAGCAGGAA---------------------------------------
----
AAGAATGAACAAGACTTATTGGCATTGGACAAATGGGCAAATCTGTGGAATTGGTTTGGCATATCAAACTGGC
TGTGGTATATAAGACTATTTATAATGATAGTAGGAGGCTTAATAGGATTAAGAATAGTTTTTGCT---
GTGCTTTCTGTAATA---------AATAGAGTTAGGCAGGGATACTCACCT------
TTGTCGTTCGCTATTAGTTTGTTTGACACCATAGCAGTAGTAATAGCTGGG---
TGGACAGATAGAGTTATAGAATTAGGACAAAGACTTTGTAGAGCT---------------------------
--------ATTTTACACATA------CCTAGAAGAATCAGACAAGGCTTCGAAAGGGCTTTGCTA---TAA
>AB253429
ACT---GCA---------------------------------------
GAAAACTTGTGGGTTACTGTCTACTATGGGGTACCTATATGG---AAGGATGCAAAT---
ACCACCTTATTTTGTGCATCAGATGCGAAAGCATATGATACAGAA---GTGCATAAT---------------
-------------GTCTGGGCTACG---CATGCCTGTGTACCTACAGAC---
CCCAGCCCACAAGAACTAAAAATGGAA---------AATGTGACAGAAGAGTTTAAC---------------
-------------------------ATGTGGAAAAATAACATGGTAGAGCAGATGCATACAGATATA---
ATCAGTCTATGGGACCAAAGCCTA---AAACCATGTGTACAGTTA---
ACCCCTCTCTGCGTTACTTTAGATTGTAGCTATAACATCACCAATAATATCACCAATAGC------------
----
-------------------------ATCACCAATAGCTCAGTTAACATGAGAGAAGAAATAAAA---
AACTGCTCTTTCAATATGACCACAGAATTAAGG--------------------------
GATAAGAATCGGAAGGTATATTCACTT---TTTTATAAACTTGATGTAGTACAAATTAATAATGGT-------
----

AATAACAGTAGTAATCTGTATAGATTAATAAATTGTAATACCTCAGCCCTTACACAG---
GCTTGTCCAAAGGTAACCTTTGAGCCAATTCCCATACATTATTGTGCCCCAGCTGGTTATGCGATTCTAAAAT
GTAAT---GATAAGGAGTTCAAT------GGAACAGGGCTATGCAAGAAT----------------------
-GTCAGC---ACAGTACAATGCACACATGGAATCAGGCCAGTAGTATCAACT---
CAACTGCTGTTAAATGGCAGTTTAGCAGAAGGAAAG-----------
GTAATGATTAGATCTGAAAATATCACAAACAAT---------GTCAAAAACATAATAGTACAACTTAAC----

-----------GAGTCTGTAACA---------ATTAATTGTACCAGACCTAACAATAATACAAGAAGA-----
-AGTGTACGTATA------------GGACCAGGACAAACA---TTC------TATGCAACAGGTGAT------
---------ATAATAGGGGATATA------------AGACAAGCACATTGT---AATGTCAGTGGGTCA---
CAATGGAATAAAACTTTACACCAGGTAGTTGAACAATTAAGA---------------AAATATTGGAAC----
-----------------------------------AACAATACAATAATCTTTAAT------
AGCTCCTCAGGAGGGGATTTAGAAATTACA---ACACATAGTTTTAATTGTGCAGGAGAA---
TTTAAACAGCTCCAGGCAAGAGTCCTGGCTGTGGAAAGATACCTAAGGGATCAACAGCTCCTAGGAATTTGGG
GCTGCTCTGGAAAACTCATCTGCCCCACTAATGTGCCCTGGAACTCTAGTTGGAGT----------------
---------------------------AATAAATCTCTAGATGAAATATGG------GAA------
AACATGACCTGGCTGCAATGG---GATAAAGAAATTAGCAATTACACAATCAAAATATAT---
GAGCTAATTGAAGAATCGCAGATCCAGCAGGAA---------------------------------------
----
AGGAATGAAAAAGACTTACTGGAGTTGGACAAGTGGGCAAGTCTGTGGAATTGGTTTGACATATCAAAATGGC
TGTGGTATATAAAAATATTTATAATGATAGTAGGAGGCCTAATAGGATTAAGAATAGTTTTTGCT---
GTGCTTTCTGTAATA---------AATAGAGTTAGGCAGGGATACTCACCC------
CTATCGTTTGCTATTAATTTGCTTGATACCATAGCAATAGCAGTAGCTGGC---
TGGACAGATAGGGTTATAGAAACAGTACAAAGGCTTGGTAGAGCT---------------------------
--------ATTCTCAACATA------CCTAGAAGAATCAGGCAGGGCTTCGAAAGGGCTTTACTA---TAA
>AY494973
ACT---GCA---
GAGAACTTGTGGGTTACTGTCTACTATGGGGTACCTGTGTGG---AGAGATGCAGAC---
ACCACCTTATTTTGTGCATCAGATGCTAAAGCATATGACACAGAA---GTGCATAAT---------------
--------------GTCTGGGCTACA---CATGCCTGTGTGCCCACAGAC---
CCCAACCCACAAGAAATAAATTTGGAA---------AATGTGACAGAAGATTTTAAC---------------
--------------------------ATGTGGAAAAATAACATGGTAGAGCAGATGCATACAGACATA---
ATCAGTCTATGGGACCAAAGCCTA---AAGCCATGTGTAAGTTTA---
ACCCCTCTCTGTGTTACTTTAAATTGTACCGATGTGAAGAACAGCACGGGTAGAGAC---------------
----
----------------------------AACATAACGGGAATTAGTATGGCAGGAGAAATAAAA---
AACTGCTCTTTCAATATGACCACAGAATTAAGG--------------------------
GATAAGAGACAGCAAGTACATTCACTT---TTTTATAAGCTTGATGTAGTACAAATGGATGGA----------
----

AATAATGGTAGTCTGTATAGATTAATAAAGTGTAATACTTCAGCCATTACACAA---
GCTTGTCCAAAGGTATCCTTTGAACCAATTCCCATACATTATTGTGCCCCAGCTGGTTTTGCGATCCTAAAGT
GTAAG---GATGAGGAGTTCAAT------GGAACAGGGCCATGCAAGAAT----------------------
-GTCAGC---ACAGTACAATGCACACATGGAATCAGGCCAGTAGTATCAACT---
CAATTGCTGTTAAATGGCAGTCTAGCAGAAAAAGAG------------
GTAAGAATTAGATCTGAAAATATCTCAGACAAT---------GCTAAAACCATAATAGTACAACTTACC----
-----------AAGCCTGTAACA---------ATTAATTGTACTAGACCTAACAACAATATAAGCAGA-----
-AGAAGGATACAT---------ATAGGAAGAGGACAAGCA---TTC------TATGCAACAGGTGGC------
---------ATGACAGGGAATATA------------AGACAAGCATATTGT---AATGTCAGTGGATCA---
GATTGGAATACAACTTTACAACAGGTAGTTACACAGTTAAGA---------------AGACACTGGCAG----
-----------------------------------AACACAACAATAGTCTTTAAA------
AGCTCCTCAGGAGGGGATGTAGAAATTACA---ACACATAGTTTTAATTGTGGAGGGGAG---
TTTAAACAGCTCCAGGCAAGAGTCCTGGCTGTGGAAAGATACCTAAAGGACCAACAGCTCCTAGGAATTTGGG
GCTGCTCTGGAAAACTCATCTGCACCACCAATGTGCCCTGGAACTCCAGTTGGAGT----------------
---------------------------AATAGATCTCAGAGTGAGATATGG------GAT------
AACATGACCTGGCTGCAATGG---GATAAAGAAATTAACAATTACACATACACAATCTAT---
AGGCTACTTGAAGAATCGCAAAACCAGCAGGAA---------------------------------------
----
AAGAATGAACGAGACTTATTGGCATTGGACAAGTGGGCAAATCTGTGGAATTGGTTTGACATATCAAGATGGC
TGTGGTATATAAAAATATTTATCATGATAGTAGGAGGCTTAATAGGATTAAGAATAGTTTTTGCT---
GTGCTTTCTATAATA---------AATAGAGTTAGGCAGGGATACTCACCT------
TTGTCATTTGCTATTAGTTTGCTTGATGCTACAGCAATAACAGTAGCTGGC---
TGGACAGATAGGGTTATAGAAATAGGACAGAGGCTTTGTAGAGCT---------------------------
--------ATTCGCAACATA------CCTAGAAGAATTAGACAGGGCTTCGAAAGGGCTTTGCTA---TAA
>EU853062
ACT---ACA---
GAAAGATTGTGGGTTACTGTTTATTATGGGGTACCTGTGTGG---AAAGATGCAGAC---

ACCACCTTATTTTGTGCATCAGATGCTAAAGCATTTGAGAGAGAA---GTGCATAAT----------------
--------------GTCTGGGCTACA---CATGCCTGTGTACCCACAGAC---
CCCAACCCACAAGAACTATCTTTGACA---------AATGTGACAGAAAAGTTTAAC----------------
-------------------------ATATGGAAAAATAACATGGTAGAGCAGATGCATGAAGATATA---
ATCAGTCTATGGGACCAAAGTCTA---AAGCCATGTGTAAAGTTA---
ACCCCTCTCTGTGTCACTTTAAATTGTGCTGAGGTCACAGACAGAACGAATGTCTCC----------------
----
------------------------------GTCGTCCAGGAGAACATGAAAGGAGAAATAAAA---
AATTGCTCTTTCAATATGACCACAGAGCTAAGA--------------------------
GATAAGGTGAAGAAAGTACATTCACTT---TTTTATAAATTTGATGTAGTACAAATTGATAAGAAT-------
----

AGTCCTAATAGTAGTGAGTATACATTAATAAATTGTAATACCTCAACCATTACACAG---
GCTTGTCCAAAGGTATCCTTTGAGCCAATTCCCATACATTATTGTGCCCCAGCTGGTTTTGCGATCCTAAAGT
GTAGA---GATGAAGAGTTCAAT------GGAACAGGGCTATGCCAGAAT----------------------
-GTCAGC---ACAGTACAATGCACACATGGAATCAGGCCAGTAGTATCAACT---
CAACTGCTGTTAAATGGCAGTCTAGCAGAAGAAAAC------------
ATAACAATTAAATCTGAAAATATCACAAATAAT---------GCTAAAACCATAATAGTGCAACTTACC----
-----------AAGCCTGTAATA---------ATTAATTGTACCAGACCTAACAACAATACAAGAAAA-----
-GGTGTACACATA------------GGACCAGGACAAGCA---TTC------TTTGCAGCAGGTGAC------
---------ATAATAGGAAACATA------------AGGCAAGCATATTGT---ACGGTCAATGCATCA---
GAATGGAATAACACTTTACAACAGGTAGCTGAACAATTAAGG---------------AAACATTGG-------
------------------------------------AACACAACAATAATCTTCACT------
AACTCCTCAGGAGGGGATGTAGAAATTACA---ACACATAGTTTTAATTGTGGAGGAGAG---
TTTAAACAGCTCCAGGCAAGAGTCCTGGCTGTGGAAAGATACCTAAGGGATCAACAGCTCCTAGGAATTTGGG
GCTGCTCTGGAAAACTCATCTGCACCACTAATGTGCCTTGGAACTCTAGTTGGAGT-----------------
---------------------------AATAAATCTCAGGAGGAGATATGG------GAG------
AACATGACCTGGCTACAATGG---GATAAAGAAATTGACAATTACACACAAATAATATAT---
GAGCTACTTGAAAAAATCGCAGAACCAGCAGGAA--

AAGAATGAACAAGACTTATTGGCATTGGACAAGTGGGCAAGTCTGTGGACTTGGTTTGACATAACAAAATGGC
TGTGGTATATAAAGATATTTATAATGCTAGTAGGAGGCTTAATAGGATTAAGAATAGTTTTTGCT---
GTAATTTCTATAATA---------AATAGAGTTAGGCAGGGATACTCACCT------
TTGTCGTTTGCCATCAATTTGGTTGATACCATAGCAATAGCAGTAGCTGAC---
TGGGCAGATAGGGTTATAGAAATAGGACAAAGAATTGGTAGAGCT----------------------------
--------ATTCTCCACATA------CCTAGAAGAATCAGACAGGGCTTTGAAAGGGCTTTGCTA---TAA
>EU852958
GCT---GCA--
CAAAACTTGTGGGTTACTGTCTACTATGGGGTACCAGTGTGG---AAAGAAGCAGAC---
ACCACCTTGTTTTGTGCGTCAGATGCGAAAGCATATGATACAGAA---ATGCATAAT----------------
--------------GTCTGGGCTACC---CATGCCTGTGTACCTACAGAC---
CCCAACCCACAAGAAATAAATTTAGAA---------AATGTGACAGAAGAGTTTAAC----------------
-------------------------ATGTGGAAAAATAACATGGTAGAGCAGATGCATACAGATATA---
ATCAGTCTATGGGACCAAAGCCTA---AAGCCATGTGTAAAATTA---
ACCCCTCTCTGCGTTACTTTAAATTGTAGTGACAAGGTCAGCTCTAATAATAATGTCACCTAT----------
----
----------------------ATTGACACTGTCACCCATGTCAATATGACAGGGGAAATAAAA---
AACTGCTCTTTCAATATGACCACAGAATTAAGG--------------------------
GATAAGCAACGGCAAGTACGTTCACTT---TTTTATAAACTGGATATAGTACAAGTTAGCAAC----------
----

AACAGTAACAGTCTGTATAGGTTAATAAATTGTAATACCTCAGCCATTACACAG---
GCTTGTCCAAAGGTATCCTTTGAGCCAATTCCCATACATTATTGTGCCCCAGCTGGTTTTGCGATTCTAAAGT
GTAAG---GATGAGGGGTTCAAT------GGGACAGGGCTATGCAAGAAT----------------------
-GTTAGC---ACAGTACAATGCACACATGGAATCAAGCCAGTAGTGTCAACT---
CAACTGCTGTTAAATGGCAGTCTAGCAGAAGGAGAG------------
GTAATGATTAGATCTGAAAATATCACAGACAAT---------ACCAAAAACATAATAGTACAATTTAAC----
-----------CAGTCTGTACAA---------ATTATTTGTACCAGACCTAACAACAATACAAGAGAA-----
-AGTATACATATA------------GGACCAGGACAGGCA---TTC------TATGCAACAGGTGAA------
---------ATAATAGGGAATATA------------AGACAAGCACATTGC---AATGTCAGTGGAACA---

AGATGGAATAAAACTTTGCAAGAGGTAGCCAACAAATTAAGA---------------ATACACTTTGGG----
-----------------------------------AACACAACAATAAACTTTACT------
AGCCCCGCAGGAGGGGATCCAGAAATAACA---ACACATAGTTTCAATTGTGGAGGAGAA---
TTTAAACAGCTCCAGGCAAGAGTCCTGGCTGTGGAAAGATACCTAAAGGATCAACAGCTCCTAGGAATTTGGG
GCTGCTCTGGAAAACTCATCTGCACCACTAATGTGCCTTGGAATTCTAGTTGGAGT-----------------
----------------------------AATAAATCCCTGAATGAGATATGG------GAC------
AACATGACCTGGCTGCAATGG---GATAAAGAAATTAGCAACTACACACAGATAATATAT---
GGGCTAATTGAAGAATCACAGAACCAGCAGGAA--

AAGAATGAACTAGACTTATTGGCATTGGACAAGTGGGCAAGTCTGTGGAATTGGTTTGACATAACAAAATGGC
TGAGGTATATACAAATATTTATAATGATAGTAGGAGGCTTAATAGGATTAAGAATAGTTTTTGCT---
GTGCTTTCTGTAATA---------AATAGAGTTAGGCAGGGATACTCACCT------
GTGTCGTTCGCTATTAGCTTGCTTGATACCATAACAATAGTAATAGCTGGG---
TGGACAGATAGGGTTATAGAAATAGGACAAAGAATTGGTAGAGCT----------------------------
--------ATCCTCAACGTA------CCTACAAGAATCAGACAGGGCTTTGAAAGGGCTTTGCTA---TAA
>EU852954
GCT---GCA--
CAAAACTTGTGGGTTACTGTCTACTATGGGGTACCTGTGTGG---AAAGACGCAGCC---
ACCACCTTGTTTTGTGCGTCAGATGCAAAAGCATATGAAACAGAA---ATGCATAAT----------------
--------------GTCTGGGCTACC---CATGCCTGTGTACCTACAGAC---
CCCAACCCACAAGAAATAGATTTAGTA---------AATGTGACAGAAGAGTTTAAC----------------
--------------------------ATGTGGAAAAATAACATGGTAGAGCAGATGCATACAGATATA---
ATCAGTCTATGGACCAAAGCCTA---AAGCCATGTGTAAAATTA---
ACCCCTCTCTGCGTTACTTTAGAGTGTTACAACAATGTCACCGCTATTAATGGCACCTTT-------------
----
----------------------------GAGAATACCACCCATAAAATAACAAAGGAAATGATG---
AACTGCTCTTTCAATATGACCACAGAAGTAAGA---------------------------
GATAGGCAAAAGAAAGTATCTTCACTT---
TTCTATAGACTGGATGTAGTACCAGTTGCTGCAAATAGCAACAGC----------------------------
----
AGCAACAACAATAGCAACAGCAGCAACAACAATCTGCATAGATTAATAAATTGTAATACCTCAGCCATTACAC
AG---
GCTTGTCCAAAGGTATCCTTTGAGCCAATTCCCATACATTATTGTACCCCAGCTGGTTTTGCGATTCTAAAGT
GTAAG---GATGAGGAGTTCAAT------GGAACAGGGCTATGCAAGAAT-------------------------
-GTTAGC---ACAGTGCAATGCACACATGGAATCAAGCCAGTAGTGTCAACT---
CAACTGCTGTTAAATGGCAGTCTAGCAGAAGGAGAG------------
GTAATGATTAGATCTGAAAATATCACAGACAAT---------GCCAAAAACATAATAGTACAATTTAAC----
-----------CAGTCTGTACCA---------ATTAATTGTACCAGACCTGGCAACAATACAAGAGAA-----
-AGTATACACATA------------GGACCAGGACAGGCA---TTC------TATACAACAGGTGCC------
---------ATAATAGGGGATATA------------AGACAAGCACATTGC---AATGTCAGCAGAGCA---
AAATGGAATGAAACTTTGCAAAGGGTAGTCAAAAAATTAGGA---------------ACACACTTTAAG----
-----------------------------------AACAAAACAATAATCTTTACT------
AAACCTGCAGGAGGAGATCCAGAAATAACA---GCACATAGTTTTAATTGTGGAGGAGAA---
TTTAAACAACTCCAGGCAAGAGTCCTGGCTGTGGAAAGATACCTAAAGGATCAACAGCTCCTAGGAATTTGGG
GCTGCTCTGGAAAACTCATCTGCACCACTAATGTGCCTTGGAATTCTAGTTGGAGT-----------------
----------------------------AATAAATCCCTGAATGAGATATGG------GAC------
AACATGACCTGGCTGCAATGG---GATAAAGAAATTAGCAACTACACACAGATAATATAT---
GGGCTAATTGAAGAATCACAGAACCAGCAGGAA--

AAGAATGAACTAGACTTATTGGCATTGGACAAGTGGGCAAGTCTGTGGAATTGGTTTGACATAACAAACTGGC
TGAGGTATATACAAATATTTATAATGATAGTAGGAGGCTTAATAGGATTAAGAATAGTTTTTGCT---
GTGCTTTCTGTAATA---------AATAGAGTTAGGCAGGGATACTCACCT------
GTGTCATTCGCTGTTAGTTTGCTTGATACCATAGCAATAGTAGTAGCTGGG---
TGGACAGATAGGGTTATAGAAATAGGACAAAGAATTGGTAGGGCT----------------------------
--------ATCCTTAACATA------CCTACAAGAATCAGACAGGGCTTCGAAAGGGCTTTGCTA---TAA
>AF484507
GCT---GCA--
GAAAACTTGTGGGTTACTGTCTATTATGGGGTACCTGTATGG---AAAGACGCAGGG---
ACCACCTTATTTTGTGCCTCAGATGCTAAAGCATATAAGAAAGAA---GCACATAAT----------------
--------------GTATGGGCTACA---CATGCCTGTGTACCCACAGAC---

CCCAACCCACAAGAAATAGACTTGGAC---------AATGTGACAGAAGATTTTAAC----------------
--------------------------ATGTGGAAAAATAACATGGTAGAGCAGATGCATACAGATATA---
ATCAGTCTATGGACCAAAGCCTA---AAGCCATGTGTAAAGCTA---
ACCCCTCTCTGCGTCACTCTAAATTGTAGCCATACCAATGTCAATGTCGGTAGCATGTAT-------------
----
----------------------------CTCAACATCAAGGAAAGCCTGAAAGAAGAAATAAAA---
AACTGTTCTTTCAATATGACCACAGAAATAAGG---------------------------
GATAAGGAAAAAAAAGTATATTCACTT---
TTTTATAAACTTGATGTAGTAGAAATTAATGATAAAAATAATAGT----------------------------
----
AGTAATAATAACAGTAACAGTAGTGAGTCTAAACTGTATAGATTAATAAATTGTAATACCTCAGCCATCACAC
AA---
GCTTGTCCAAAGGTATCCTTTGAGCCAATTCCTATACATTATTGTGCCCCAGCTGGTTTTGCGATCCTAAAGT
GTAAT---GATGAGGAGTTCAAT------GGAACAGGACCATGCAAGAAG-----------------------
-GTCAGC---ACAGTGCAATGCACACATGGAATCAGGCCAGTGGTTTCAACT---
CAACTGCTATTAAATGGCAGTCTAGCAAAAGGAGAG------------
GTAAAAATTAGATCTGAAAATATCTCAGACAAT---------GCTAAAACCATAATAGTACAACTTACA----
-----------GAGCCTGTAAAC---------ATTAGTTGTATCAGACCTAACAACAATACAAGGACA-----
-AGTATACATATA------------GCACCAGGACAAGCA---TTC------TATGCAACAGGTGGC------
---------ATAATAGGGGATATA------------AGACAAGCACATTGT---AATGTCAGTGGAGCA---
AAATGGAATCAAACATTACAAAAGGTAGTTAAGCAATTAGGA---------------AAGCACTGGAAC----
-----------------------------------ACAACAACAATAATCTTTAAA------
AACTCCTCGGGGGGGGATTTAGAAATTACA---ACACATAGTTTTAATTGTGGAGGAGAA---
TTTAAACAGCTCCAGGCAAGAGTCCTGGCTGTGGAAAAGTACCTAAAGGATCAACAGCTCCTAGGAATTTGGG
GCTGCTCTGGAAAACTCATCTGCACCACTAATGTGCCTTGGAACTCTAGTTGGAGT-----------------
----------------------------AATAAATCCCAGCATGAGATATGG------GAT------
AACCTGACCTGGCTGCAATGG---GATAAAGAGATTAACAATTACACACACGAAATATAT---
AGGCTACTTGAAGAATCGCAGATCCAGCAGGAA--

AAGAATGAACAAGACTTATTGGCATTAGACAAGTGGGCAAGTCTGTGGAATTGGTTTGACATATCAAGATGGC
TGTGGTATATAAGAATATTTATAATGATAGTAGGAGGCTTAGTAGGATTAAGAATAGTTTTTGCA---
GTGCTTTCTATAATA---------AATAGAGTTAGGCAGGGATACTCACCT------
TTGTCGTTTGCTATTAATTTGCTTGATACTATAGCAATAGCAGTAGCTGGC---
TGGACAGATAGGGTTATAGAAATAGGACAAGGAATTGGTAGAGCT----------------------------
--------ATTCTCAACATA------CCTAGAAGAATCAGACAGGGCTTAGAAAGGGCTTTGCTA---TAA
>AF484508
GCT---GCA---
GAAACTTGTGGGTTACTATCTACTATGGGGTACCTGTGTGG---AAAGATGCGGAG---
ACCACCTTATTTTGTGCATCAGATGCGAAGGCATATGAAAAAGAA---GTGCATAAT----------------
--------------GTCTGGCTACA---CATGCCTGTGTACCTACAGAC---
CCCAACCCACAAGAAATAAATTTGGA----------AATGTGACAGAAAAGTTTAAC----------------
--------------------------ATGTGGAAAAATAACATGGTAGACCAGATGCATGCAGATGTA---
ATCAGTCTATGGACGAAAGCCTA---AAGCCATGTGTAAAGTTA---
ACCCCTCTCTGCGTTACTTTAAATTGTAATCAGGTCAATGTCACCGGTGACGAGAAT----------------
----
-------------------------------CATACCCTCAGTAATATCAAAGAGGACCTAAGA---
AACTGCTCTTTCAATATGACCACAGTATTAAGG---------------------------
GATAAGACTCAGAAAGTACATTCACTT---TTTTATAAACTGGATATAGTAGAAATTGGGAAT----------
----

AGCAGTAGTCAGTATAGATTAATAAACTGTAATACCTCAGCCATTACACAA---
GCCTGTCCAAAGGTATCCTTTGAGCCAATTCCCATACATTATTGTGCCCCAGCTGGGTTTGCGATTCTAAAAT
GTAAC---GATAAGAAGTTCAAT------GGAACAGGGCTATGCAAGAAT-----------------------
-GTTAGC---ACAGTACAATGCACACATGGAATCAAGCCAGTAGTATCAACT---
CAACTGCTGTTAAATGGCAGTCTAGCAGAAGGAGAG------------
GTAATGATTAGATCTGAAAATATCACAAACAAT---------GCCAAAAACATAATAGTACAATTTAAA----
-----------GAGGCTGTACAA---------ATTAATTGTAGCAGACCTGGCAACAATACAAGAAAA-----
-AGTGTACCTATG------------GGACCAGGAAAAGCA---TTC------TATGCAACAGGTGAC------
---------ATAATAGGGGATATA------------AGAAAAGCACATTGT---AATGTCAGTAGAGCA---
GCATGGAATAAAACTTTACAGGAGGTAGCCAAACAATTAAGA---------------ACATACTTTAAG----

-----------------------------------AACAAAACAATAATCTTTACT------
AACACCTCAGGAGGGGATCCAGAGATAACA---ACACATAGTTTTAATTGTGGAGGAGAA---
TTTAAACAGCTCCAGGCAAGAATCCTGGCTGTGGAGAGATACCCAAAGGATCAACAGCTCCTAGGAATTTGGG
GCTGCTCTGGAAAAATCATCTGCACCACTAATGTGCCCTGGAACTCTAGTTGGAGT-----------------
-----------------------------AATAAATCCCAGAGTGAGATATGG------GAC------
AACATGACCTGGCTGCAGTGG---GATAAAGAAATTAGCAATTACACAACCATAATATAT---
AAGCTAATTGAAGAATCGCAGACCCAGCAGGAA---

ATAAATGAAAAAGACTTATTGGCATTGGACAAGTGGGCAAATCTGTGGAATTGGTTTGAAATATCAAAATGGC
TGTGGTATATAAAAATATTTATAATGATAATAGGAGGCTTAATAGGATTAAGAATAGTTTTTGCT---
GTGCTTTCTATAATA---------AATAGAGTTAGGCAGGGATACTCACCT------
TTGTCATTCGCTATCAATTTGGTTGATACCATAGCAATAGCAGTAGCTGGG---
TGGACAGATAGGATTATAGAAGTAGGACAAAGAATTTGTAGAGCT----------------------------
--------ATTTTACATATA------CCTAGAAGAATCAGACAAGGCTTCGAAAGGGCTTTGCTA---TAA
>AF484509
GCT---GCA---
GAAAACTTGTGGGTTACTGTCTACTATGGGGTACCTGTGTGG---AGAGATGCAGAG---
ACCACCTTATTTTGTGCATCAGATGCAAAATCATATGATACAGAA---ATGCATAAT----------------
--------------GTCTGGGCCACA---CATGCCTGTGTACCTACAGAC---
CCCAACCCACAAGAAATAAATTTGAAA---------AATGTGACAGAAGAGTTTAAC----------------
--------------------------ATGTGGAAAAATAAAATGGTAGAGCAGATGCATACAGATATA---
ATCAGTCTATGGGACCAAAGCTTA---AAGCCATGTGTAAAGTTA---
ACCCCTCTCTGTGTTACTTTAGAGTGTAGCAACCCATCAACAGGTATCAATAAAACC----------------
----
-------------------------------ACAGACATGAGAAATAACATCACAGACATGAAA---
AACTGCTCTTTCAATATGACCACAGAATTAAGG--------------------------
GATAAGAAACAGAGAGTATACTCACTT---TTTTATAAACTGGATGTAGTACCAATTAATGAC----------
----

AACAGTAGCAATACCTATAGATTAATAAATTGTAATACCTCAGCCATAACACAG---
GCTTGTCCAAAGGTATCCTTTGAGCCAATTCCCATACATTACTGTGCTCCAGCTGGTTTTGCGATTCTAAAGT
GTAAT---GATAAAGAGTTCAAT------GGAACAGGGCTATGCAAGGAT----------------------
-GTCAGC---ACAGTACAATGCACACATGGAATCAAGCCAGTAGTATCAACT---
CAACTGCTGTTAAATGGCAGTCTAGCAAAAGAAAGG-----------
GTAATAATTAGATCTGAAAATATTACAGACAAT---------ATCAAAAACATAATAGTACAACTTAAC----
-----------GAGTCTGTAGAA---------ATTAGTTGTATCAGACCTAACAACAATACAAGAAAA-----
-AGTGTACGTATA------------GGACCAGGACAAGCA---TTC------TATGCAACA---GAC------
---------ATAACAGGGGATATA------------AGACAAGCATATTGT---AATGTTAGTAAAAGG---
GGATGGAATAACACTTTGCAAAAGGTAGTTGAACAATTAAGA---------------GTACACTTTGAG----
-----------------------------------AACAAAACAATAACCTTTGCT------
AACTCCTCAGGAGGGGATCTAGAAATAACA---ACACATAGTTTTAATTGTGGAGGAGAA---
TTTAAACAGCTCCATGCAAGAGTCCTGGCTGTGGAAGGATACCTAAGGGATCAACAGCTCCTAGGAATTTGGG
GCTGCTCTGGAAAACTCATCTGCACCACTAATGTGCCCTGGAACTCTAGTTGGAGT----------------
-----------------------------AATAAATCCCAGAATGAGATATGG------GGC------
AACATGACCTGGCTGCAGTGG---GATAAAGAAATTAGCAATTACACACAGATAATATAT---
AGCCTAATTGAAGAATCACAGAACCAGCAGGAA---

AAGAATGAACAAGACTTATTGGCATTGGATAAATGGGCAAATCTGTGGAATTGGTTTGACATATCAAACTGGC
TGTGGTATATAAAAATATTTATAATGATAGTAGGAGGCTTAATAGGATTAAGAATAGTTTTTGCT---
GTACTTTCTATAATA---------AATAGAGTTAGGCAGGGATACTCACCC------
TTGTCGTTCGCTATTAACCTGTTTGATACCATAGCAATAGCAGTAGCTGAG---
TGGACAGATAGGGTTGTAGAAATAGGACAAAGACTTTGTAGAGCT----------------------------
--------ATTCTCCACATA------CCTAGAAGAATCAGACAAGGCTTAGAAAGGGCTTTGCTA---TAA
>AF484512
AGT---ACA---
GATAACTTGTGGGTTACTGTCTACTATGGGGTACCTGTGTGG---AAAGATGCAGAG---
ACCACCCTCTTTTGTGCATCAGATGCTAAAGCATATGAGAGAGAG---GTGCATAAT----------------
--------------GTCTGGGCTACA---CATGCTTGTGTACCCACAGAC---
CCCAACCCACAAGAAGTACATTTGAAA---------AATGTGACAGAACAATTTAAT----------------
--------------------------ATGTGGAAAAATAACATGGTAGAGCAGATGCATACAGATATA---

ATCAGTCTATGGGATGAAAGCCTA---AAGCCATGTGTAAAGTTA---
ACCCCTCTCTGTGTTACTTTAAAGTGTAGCAATATCAGCAATATCAACAGCACGAGGGGCAGC----------
----
----------------------ACAGAGGGCAACAGCACGTTAGAAAACAGGGAAGAAATAAAA---
AACTGCTCTTTCAATATGACCACAGAACTAAGG---------------------------
GATAAGAAACAAAAAGTACATGCACTT---TTTTATAGACTTGATATAGTAGAAATAGATGACAAGAAG----
----

AGTAATGGTAGTAACAATGAGTATAGATTAATAAATTGTAATACCTCAACCATTACACAG---
GCTTGTCCAAAGGTATCCTTTGAGCCAATTCCCATACATTATTGTGCCCCAGCTGGTTTTGCGATCCTAAAGT
GTAAT---GATAAGAGGTTCAAT------GGAACAGGGCCATGCAAGAAT-----------------------
-GTCAGC---ACAGTACAATGCACACATGGAATCAGGCCAGTAGTATCAACT---
CAACTGCTGTTAAATGGCAGTCTAGCAGAAGAAGAG------------
ATAAGAATTAGTTCTGAAAATATCACAGACAAT---------GCCAAAACCATAATAGTACAATTTATC----
-----------AAGCCTGTAAAC---------ATTACTTGTGTCTGACCTAACAACAATACAAGGACA-----
-AGCATACGTATA------------GGACCAGGACAAACA---TTC------TATACAACAGGTGAT------
---------ATAATAGGAAATATA------------AGACAAGCACATTGT---AATGTCAGCAGATCA---
GAATGGAATGATAACTTAAAAGAGGTAGCTACACGATTAAAA---------------GAATACTTTAAT----
--------------------------------AAAACACAAACAATAATCTTTACT------
AATTCCTCAGGAGGGGATCTAGAAATCACA---ACACATAGTTTTAATTGTGGAGGAGAA---
TTTAAACAGCTCCAGGCACGAGTCCTGGCTCTGGAGAGATACCTAAAGGATCAACAGCTCCTAGGAATTTGGG
GCTGCTCTGGAAAACTCATCTGCACCACTAATGTGCCCTGGAACTCTAGTTGGAGT-----------------
----------------------------AATAGATCTTACAATGAAATATGG------GAT------
AACATGGCCTGGCTGCAATGG---GATAGAGAAATTAACAATTACACAGACATAATATAT---
AAATTAATTGAAGAATCGCAGAACCAGCAGGAA--

AAGAATGAACAAGATTTATTGGCATTGGACAAGTGGGCAAATCTGTGGAATTGGTTTGGCATATCAAATTGGC
TGTGGTATATAAAAATATTTATAATGATAGTAGGAGGCTTAATAGGATTAAGAATAGTGTTTACT---
GTGCTTTCTATAATA---------AATAGGGTTAGGCAGGGATACTCACCT------
TTGTCGTTTGCTATTAGCTTGCTTGATACCACAGCAATAGCAGTAGCTGGC---
TGGACAGATAGGGTTATAGAAATAGGACAAAGAATTGGTAGAGCT----------------------------
--------ATTCTCCACATA------CCTAGAAGAATCAGACAGGGCTTCGAAAGAGCTTTGCTA---TAA
>EU853030
GCT---GCA--
GAAAATATGTGGGTTACTGTCTACTATGGGGTACCTGTGTGG---AGAGATGCAGAG---
ACCACCCTATTTTGTGCATCAGATGCTAAAGCATATGAGACAGAA---AGGCATAAT----------------
--------------GTTTGGGCTACA---CATGCTTGTGTACCCACAGAC---
CCCAACCCACAGGAAATACATTTGGAA---------AATGTGACAGAACAGTTTAAC----------------
--------------------------ATGTGGAAAAATAGCATGGTAGAACAGATGCGTACAGATATA---
ATCAGTCTATGGGACCAAAGCCTA---AAGCCATGTGTAAAGTTA---
ACCCCTCTCTGTGTCACTTTAAATTGTGTCAATGTCACGACGAATGCCACGAAA-------------------
----
-------------------------------AACAACAGCACGGGGGACGAGGGAGAAATGAAA---
AACTGCTCTTTCAATATGACCACAGAACTAAGG---------------------------
GATAAGAAACAAAGAGTATATTCACTT---TTTTATAGACTTGATATAGTAAAAATTAATGAAAGTGAG----
----

AGTGACAGTAGTAATAGTAGTGAGTATAGATTAATAAATTGTAATACCTCAGCCATTACACAA---
GCTTGCCCAAAGGTATCCTTTGAGCCAATTCCTATACATTATTGTGCCCCAGCTGGTTTTGCGATCCTAAAGT
GTAAT---GATAAGGAGTTCAAT------GGAACAGGGCTATGCCAGAAT-----------------------
-GTCAGC---ACAGTACAATGCACACATGGAATCAAGCCAGTAGTATCAACT---
CAACTGCTGCTAAATGGCAGTCTAGCAGAAGGGACG------------
GTAAAAATTAGATCTGAAAACATCACAAACAAT---------GTCAAAACCATAATAGTACACCTTAAC----
-----------GAGTCTGTGGAA---------ATTAATTGTACCAGACCTAACAACAATACAAGAACA-----
-AGTGTACGTATA------------GGACCAGGACAAGCA---TTC------TATGCAACA---GAC------
---------ATAATAGGAGATATA------------AGGAAGGCATATTGT---AAAGTCAATCGCTCA---
GAATGGCATAAAGCTTTACAAAAGGTAGTCGAGCAATTAAGA---------------AAACACTTTAAG----
-----------------------------------AACAAAACAATAATCTTTGCT------
AGCTCCTCAGGAGGGGATCTAGAAATCACA---ACACATAGTTTTAATTGTGGAGGAGAA---
TTCAAACAGCTCCAGGCAAGAGTCCTGGCTGTGGAAAGATACCTAAGGGATCAACAGCTCCTAGGAATTTGGG

GCTGCTCTGGAAAACTCATTTGCACCACTAATGTGCCCTGGAACTCTAGTTGGAGT-----------------
----------------------------AATAAATCTTTTGAAGAGATATGG------GAT------
AACATGACCTGGCTGCAATGG---GATAAAGAAGTTAGCAATTACACACAAATAATATAT---
GATCTAATTGAAGAATCGCAGAACCAGCAGGAA--

AAGAATGAACAGGACTTATTGGCATTAGACAAGTGGGCAAATCTGTGGAATTGGTTTGAAATATCAAAATGGC
TGTGGTATATAAAAATATTTATAATGGTAGTAGGAGGCTTAATAGGATTAAGAATAGTTTTTGCT---
GTGCTTTCTATAATA---------AATAGAGTTAGGCAGGGATACTCACCT------
TTATCATTTGCTACTAATTTGGTTGACACCATAGCAATAGCAGTAGCTGGC---
TGGACAGATAGGGTTATAGAAATAGGACAAAGAATTGGTAGGGCT----------------------------
--------ATCCTACACATA------CCTAGAAGAATCAGACAGGGTTTCGAAAGGGCTTTGCTA---TAA
>AF484478
ACT---GCA--
GAAAACTTGTGGGTTACTGTCTACTATGGGGTACCTGTGTGG---AAAGACGCAGAG---
ACCACCTTATTTTGTGCATCAGATGCGAAAGCGTATGATACAGAA---ATGCATAAT----------------
--------------GTCTGGGCTACG---CATGCCTGTGTACCTACAGAC---
CCCAACCCACAAGAAATAAATTTGAAA---------AATGTGACAGAAGAATTTAAC----------------
--------------------------ATGTGGAAAAATAACATGGTAGAGCAGATGCATGCAGATATA---
CTTAGTCTATGGGACCAAAGCCTA---AAGCCATGTGTAAAGTTA---
ACCCCTCTCTGCGTTACTTTAGAATGTCAAAACTATGAAGGCACCAATTTAACTAATAGTAAC----------
----
----------------------ACCACTGTCAACATCACCGAGGACATGAAAGGAGAAATAAAA---
AACTGCTCTTTCAATATGACCACAGAATTAAGG---------------------------
GATAAGAGGCAGAAAATGCATTCGCTT---TTTTATAGACTGGATATAGTACAAATTAATGGCAAC-------
----

AGAAGCAATAGTAGTGAACAGTATAGATTAATAAATTGTAATACCTCAGCCATTACACAA---
GCTTGTCCAAAGGTATCCTTTGAGCCAATTCCCATACATTATTGTGCCCCAGCTGGTTTTGCGATTCTGAAGT
GTAAG---GATAGAGATTTCAAT------GGAACAGGGCCATGCAAGAAT-------------------------
-GTCAGC---ACAGTACAGTGCACACATGGAATCAAGCCAGTAGCATCAACT---
CAATTGCTGTTAAATGGCAGTCTAGCAGAAGGAGAG------------
GTTATGATTAGATCTGAAAATATCACAAACAAT---------GCCAAAAACATAATAGTACAACTTAAC----
-----------AAGCCTGTAAAC---------ATTACTTGTATCAGACCTAACAACAATACAAGACAA-----
-AGTGTACGTATA------------GGACCAGGACAAGCA---TTC------TATGCAACAGGTAAC------
---------ATAATAGGGGATATA------------AGACAAGCATATTGT---GATGTCAATAGAACA---
GAATGGAATAAAACTTTGCAAGAGGTAGCCTACCAATTAGGA---------------AAACACTTTGGG----
-----------------------------------AACAAAACAATAATCTTTGCT------
AACTCCACAGGAGGGGATCTAGAAATAACA---ACACATAGTTTTAATTGTGGAGGAGAA---
TTTAAACAGCTCCAGGCAAGAGTCCTGGCTGTGGAAAGATACCTAAAGGATCAACAGCTCCTAGGAATTTGGG
GCTGCTCTGGAAAACTCATCTGCACCACTAATGTGCCCTGGAACTCTAGTTGGAGT-----------------
----------------------------AATAAATCCCAGAGTGAGATATGG------GAC------
AACATGACCTGGCTGCAATGG---GATAAAGAAATTAGCAATTACACACAGATAATATAT---
AATCTAATTGAAGAATCGCAGATCCAGCAGAAA--

AAGAATGAACAAGACTTATTGGCATTGGACAAGTGGGCAAACCTGTGGAATTGGTTTGACATATCAAACTGRC
TGTGGTATATAAGAATATTTATAATGATAGTAGGAGGCTTAATAGGATTAAGAATAGTTTTTACT---
GTGCTTTCCATAATA---------AATAGAGTTAGGCAGGGATACTCACCT------
TTGTCGTTCGCTATTAATTTGCTTGATACCATAGCAATAGCAACAGCTGAG---
TGGACAGATAGGGTTATAGAAATAGGACAAAGACTTTGTAGAGCT----------------------------
--------ATTCTCCACATA------CCTAGAAGAATCAGACAGGCCTTAGAAAGGGCTTTGCTA---TAA
>AF484493
GCA---AAC--
CACCCTTTGTGGGTCACGGTCTACTATGGGGTACCTGTGTGG---AAAGAAGCAGCT---
ACCACCCTATTTTGTGCATCAGATGCTAAAGCACATGATACAGAA---GCGCATAAT----------------
--------------GTCTGGGCTACA---CATGCCTGTGTACCCACAGAC---
CCCAACCCACAAGAAATAAATTTGGTC---------AATGTGACAGAAAAGTTTAAC----------------
--------------------------ATGTGGAAAAATAACATGGTAGAGCAGATGCATGAAGATATA---
ATCAGTCTATGGGACCAAAGCCTA---CAGCCATGTGTAAAGTTA---
ACCCCTCTCTGCGTCACGTTAGAGTGTAATCATACCACCAATAACATCACCAATGAT----------------
----

---------------------------ATCAATAGTACAGTTAACATGAAAGAAGAAATAAAA---
AACTGCTCTTTCAATATGACCACAGAACTAAGA--------------------------
GATAAGAAACAGAAAGTATATTCACTT---
TTTTATAAACTTGATATAGTACAAATGAAAGAAGATAATAGTACA----------------------------
----
ATGAAAGAAGATAATAGTAATACCAGTAGGTATAGATTAATAAATTGTAATACCTCAGCCATTACACAA---
GCTTGTCCAAAGGTAACCTTTGAGCCAATTCCCATACATTATTGTGCCCCAGCTGGTTTTGCGATCCTAAAGT
GTAAT---GACAAGGAGTTCAAT------GGAACAGGGCTATGCAAGAAT----------------------
-GTCAGC---ACAGTACAATGCACACATGGAATCAAGCCAGTGGTATCAACT---
CAACTGCTGTTAAATGGCAGTCTAGCAGAGGGTGAG-----------
ATAAGAATTAGATCTGAAAATATCTCAGACAAT---------GCTAAAACCATAATAGTACAACTTGTC----
-----------AAGTCTGTACCA---------ATTAAGTGTATCAGACCTAACAACAATACAAGAAAA-----
-AGTATACGTATA------------GGACCAGGACAAACA---TTC------TATGCA---GGTGAT------
---------GTAATAGGGGATATA------------AGACAAGCACATTGT---AATGTCAGTAGGTCA---
GATTGGAATAAAACTTTACATCAGGTAGCTACACAATTAAGA---------------CAATACTGGAAC----
-----------------------------------AGCAGCATAATAATCTTTACT------
AACTCCTCAGGAGGGGATTTAGAAATTACA---ACACATAGTTTTAATTGTGGAGGAGAA---
TTTAAACAGCTCCAGGCAAGAGTCCTGGCTGTGGAAAGATACCTAAAGGATCAACAGCTCCTAGGAATTTGGG
GCTGCTCTGGAAAACTCATCTGCACCACTAATGTGCCCTGGAACGCTAGCTGGAGT-----------------
---------------------------AATAAATCTTATGGGGAGATATGG------GAT------
AACATGACCTGGCTGCAATGG---GATAAAGAAATTAGCAATTACACAAACATAATATAT---
AAGCTAATTGAAGAATCGCAGAACCAGCAGGAA---------------------------------------
----
AAGAATGAACAAGACTTGTTGGCATTGGACCAGTGGGCAAGTCTGTGGAATTGGTTTGACATAACAAAATGGC
TGTGGTACATAAAAATATTTATAATGATAGTAGGAGGTATAATAGGATTAAGAATAGTTCTTGCT---
GTGCTTTCTGTAATA---------AATAGAGTTAGGCAGGGATACTCACCT------
CTGTCGTTTGCTATTAGTTTGTATGATACCATAGCAATAACAGTAGCTGGC---
TGGACAGATAGGGTTATAGAAAGAGGACAAAGACTTGGTAGAGCT---------------------------
--------CTTATCCACATA------CCTAGAAGAATCAGGCAGGGCTTAGAAAGGGCTTTACTA---TAA
>AB098330
GCT---GCA---
GAAAACTTGTGGGTTACTGTCTACTACGGGGTACCTGTGTGG---AAAGACGCAGAG---
ACCACCTTATTTTGTGCATCAGATGCTAAAGCATATGATACAGAA---GTACATAAT---------------
--------------GTCTGGGCGACA---CATGCCTGTGTACCCACAGAC---
CCCAACCCACAAGAAATACACTTGGAA---------AATGTGACAGAAGATTTTAAC---------------
-------------------------ATGTGGAAAAATAATATGGTAGAGCAGATGCATACAGATATA---
ATCAGTCTATGGGATCAGAGCCTA---AAGCCATGTGTAGAATTA---
ACCCCTCTCTGCGTTACTTTAGATTGTCTAAACGCCACCCTCAATGCCACCGCCCCC----------------
----
------------------------------AATGTCACCAATGACATGGAAGGAGAAATGAAA---
AACTGCTCTTACAATATAACCACAGAATTAAAG-------------------------
GATAAGAAACAGCAAGTGTATTCACTT---TTTTATAAGCTTGATGTAGTACAAATTAATGAA----------
----

AAGAATAAAACAAACAAGTATAGATTAATAAATTGTAATACCTCAGCCATTACACAG---
GCTTGTCCAAAGGTATCCTTTGAGCCAATTCCCATACATTATTGTGCCCCAGCTGGCTTTGCGATTCTAAAGT
GTAAG---GATACAGAGTTCAAT------GGAACAGGGCCATGCAAGAAT----------------------
-GTCAGC---ACAGTACAATGCACACATGGAATCCGGCCAGTAATATCAACT---
CAACTGCTGTTAAATGGTAGTCTAGCAGAAGGAGGG-----------
ATACAGATTAGATCTGAAAATATCACAAACAAT---------GCCAAAACCATAATAGTACAACTTGAT----
-----------AAGGCTGTAAAA---------ATTAATTGTACCAGACCTAACAACAATACAAGAAAA-----
-AGTGTAAGGATA------------GGACCAGGACAAGCA---TTC------TATGCAACAGGTGAT------
---------ATAATAGGGGATATA------------AGACAGGCACATTGT---AATGTCAGTAGAGCA---
AAATGGAACGAAACTTTACGAGGGATAGCCAAAAAACTAAGC---------------GAACACTTTAAA----
-----------------------------------AACAAAATAATAATCTTTGAG------
AAATCCTCAGGAGGGGATATAGAAATTACA---ACACATAGTTTTAATTGTGGAGGAGAA---
TTTAAACAGCTCCAGGCACGAGTCCTGGCTGTGGAAAGATATCTAAAGGATCAACAGCTCCTAGGAATTTGGG
GCTGCTCTGGAAAACTCATCTGCACCACTAATGTGCCCTGGAACTCTAGTTGGAGT-----------------
---------------------------AATAAATCTATGAATGAGATATGG------GAT------
AACATGACTTGGCTGCAATGG---GAAAAAGAAATTAGCAATTACACACAATTAATATAT---

AATCTAATTGAAGAATCGCAGAACCAGCAGGAA-------------------------------------
----
AAGAATGAACAAGACTTGTTGGCATTGGACAAGTGGGCAAGTCTGTGGAATTGGTTTGACATATCAAGGTGGC
TGTGGTATATAAAAATATTTATAATGATAGTAGGAGGCTTAATAGGATTAAGAATAGTTTTTGCT---
GTGCTTTCTGTAATA---------AATAGAGTTAGGCAGGGATACTCACCT------
CTGTCATTCGCTGTCAATTTGGTTGATACCATAGCAATAGCAGTAGCTGGC---
TGGACAGATAGGGTTATAGAAATAGGACAAAGAATTTTTAGAGCT-------------------------
--------ATTCTTAACATA------CCTAGAAGAATCAGACAGGGCCTTGAAAGGGGCTTGCTA---TAA
>GU367412
GCT---ACA--
GAAAATTTGTGGGTCACGGTCTACTATGGGGTACCAGTGTGG---AGAGACGCAGAG---
ACCACCCTATTCTGTGCATCAGATGCTAAAGCATATGATCAAGAA---GCACATAAT-------------
-------------GTTTGGGCTACA---CACGCCTGTGTACCCACAGAC---
CCCAACCCACAAGAAATAGATTTAGAA---------AATGTGACAGAAGAGTTTAAC-------------
-------------------------ATGTGGAAAAATAACATGGTGGAGCAGATGCATACAGATATA---
ATCAGTCTATGGGACCAAAGCTTA---AAGCCATGTGTAAAATTA---
ACCCCTATCTGCGTTACTTTAAACTGTGAAAACGTCACCATCAATGCCAATGCC----------------
----
------------------------------AATGTCACGAATGAAATGGGAGAAGAAATAAAA---
AACTGCTCATACAATACAACTACAGAGCTAAGA------------------------
GATAAGAAACAAAAGGTATATTCACTT---
TTTTATAGACTGGATATAGTACAAATGAATAGTAGTAATAGTGAT-------------------------
----
AAAAATAAGGGTAATAATAATAAAAGTAGTAGTAATAGTGAGTATAGACTAATAAATTGTAATACCTCGGCCA
TTACACAG---
GCTTGTCCAAAGGTATCTTTTGAGCCAATTCCCATACATTATTGTGCCCCAGCTGGTTTTGCGATTCTAAAGT
GTAAG---GATAAGAAATTCAAT------GGAGCAGGGCCATGTAGAAAT--------------------
-GTCAGC---ACAGTACAATGCACACATGGAATCAGGCCAGTAGCATCAACT---
CAACTGCTGTTGAATGGCAGTCTAGCAGAAGAAGAG-----------
GTAATGATTAGATCTGAAAATTTCTCAAACAAT---------GCCAAAACCATAATAGTACAGCTTGTT----
-----------AATCCTGTAGAA---------ATTAATTGTACCAGACCCAACAACAATACAAGAAAA-----
-AGTGTACGCATA------------GGACCAGGACAGGCA---TTC------TATGCAACAGGTGAC------
---------ATAATAGGGAATATA------------AAAAAGTCATCTTGT---ACTGTCAATAAAACA---
GAATGGAATAAAGCTTTACAAGAGGTAGCTAAAAAGTTAAAA---------------CAACACCTTAAT----
-----------------------------------AAAACAACAATAAAATTTGCT------
GAATCCTCAGGAGGGGATTTAGAAATTGCA---ACACATAGCTTTAATTGTGGAGGAGAA---
TTTAAACAGCTCCAGGCAAGAGTCCTGGCTGTGGAAGGGTACCTAAAGGATCAACAGCTCCTAGGAATTTGGG
GTTGCTCTGGAAAACTCATCTGCACCACTAATGTGCCCTGGAACTCTAGTTGGAGT----------------
-----------------------------AATAAATCTCAGAGTGAAATATGG------GAT------
AGTATGACCTGGCTGCAATGG---GATAAAGAAATTGGCAATTACACAAACATAATATAT---
GATCTACTTGAAGAATCGCAGATCCAGCAGGAA-------------------------------------
----
AAGAATGAACAAGACTTATTGGCATTGGACAAATGGGCAAATCTGTGGAATTGGTTTGACATATCAAATTGGT
TATGGTATATAAAAATATTTATAATGATAATAGGGGGCTTAATAGGATTAAGAATAGTTTTTACT---
GTGCTTTCTATAATA---------AATAGAGTTAGGCAGGGATACTCACCT------
CTATCATTTGCTATTTCTTTGCTTGATGCTACAGCAATAGCAGTAGCTGAG---
TGGACAGATAGGGTTATAGAAGTAGGACAAAGGGTTGGTAGAGCT-------------------------
--------CTTCTCCACATA------CCTAGAAGAATCAGACAGGGCTTCGAAAGGGCTTTGATA---TAA
>FJ647148
ACT---GCA--
GACAACTTGTGGGTTACTGTTTACTATGGGGTACCTGTGTGG---AAAGATGCAGAG---
ACCACCCTATTTTGTGCATCAGATGCTAAAGCATATGAGAAAGAA---GTGCATAAT-------------
-------------GTCTGGGCTACA---CATGCCTGTGTACCCACAGAC---
CCCAACCCACAAGAAATACATTTGGTA---------AATGTGACAGAAAATTTTAAT-------------
-------------------------ATGTGGAAAAATAAAATGGTAGAGCAGATGCATGCAGATATA---
ATCAGTCTATGGGACCAAAGCCTA---AAGCCATGTGTAAAGCTA---
ACCCCTCTCTGTGTAACTTTAAATTGTACCAATGCCAATATCACCTATGTCAGTACCAACAGCACG-------
----
----------------------AAGGCCTATGTCACTGTCAACGGCACAACGGAAGAAATAAAA---
AACTGCTCTTATAATATGACCACAGAACTAAGG------------------------

GATAAGAAACAGAAAGTATATTCACTT---TTTTATAGACTTGATGTAGTACAGATTAATAAAAATAATAAT-
----

AGTAGAGATAATGATAGTGGTGAGTATAGATTAATAAATTGTAATACCTCAGCCATTACACAA---
GCTTGTCCAAAGGTCTCCTTTGAGCCAATTCCCATACATTATTGTGCTCCAGCTGGTTTTGCGATCCTAAAAT
GTAAT---GAGGAGGAGTTCAAC------GGAACAGGGCCATGCAAGAAT----------------------
-GTCAGC---TCAGTACAATGCACACATGGAATCAGGCCAGTAGTATCAACT---
CAACTGCTGTTAAATGGCAGTCTAGCCCAAGGAGAG-----------
GTAAAAATTAGATCTGAAAATATCTCAGACAAT---------GCTAAAACCATAATAGTACAATTTAAC----
-----------CAGTCTGTAATA---------ATTAATTGTACCAGACCTAGCAACAATACAAGGAGA-----
-AGTGTACGTATA------------GGACCAGGACAAGCA---TTC------TATGCAACAGGTGAG------
---------ATAATAGGGGACATA------------AGGAAAGCACATTGT---AATGTCAGTGAATCA---
GAATGGAATAAAGCTTTACAACAGGTAGCTACACAATTAGGA---------------AGATACTGGAGT----
-----------------------------------AACAAAACAATAATTTTTAAT------
AGCTCCTCAGGAGGGGATTTAGAAATTACA---ACACATAGTTTTAATTGTGGAGGAGTA---
TTTAAACAGCTCCAGGCAAGAGTCCTGGCTCTAGAGAGATACCTAAGGGATCAACAGCTCCTAGGAATTTGGG
GCTGCTCTGGAAAACTCATTTGCGCCACTAATGTGCCTTGGAACTCTAGTTGGAGT-----------------
----------------------------AATAAATCTTATAATGAAATATGG------GAT------
AACATGACCTGGCTGCAGTGG---GATAAAGAAATTGACAATTACACAGAAACAATATAT---
AGGCTAATTGAAGAATCGCAAAACCAGCAGGAA---

AGGAATGAACAAGACTTATTGGCATTGGACAAGTGGACAAATCTGTGGAGTTGGTTTGACATATCGAACTGGC
TGTGGTATATAAAAATATTTATAATGATAGTAGGAGGCTTAATAGGATTAAGAATAGTTTTTGCT---
GTGCTTTCTATAATA---------AATAGAGTTAGGCAGGGATACTCACCT------
TTGTCATTTGCTATTAATTTGCTTGATACCATAGCAATAGCAGTAGCTGGC---
TGGACAGATAGGGTTATAGAAGTAGGACAAAGATTTGGTAGAGCT----------------------------
--------ATTCTCCACATA------CCTAGAAGGATCAGACAAGGACTTGAAAGAGCTTTACTA---TAA
>KT183312
GCT---GCA---AAT-----------------------------------
GAAAACTTGTGGGTTACTGTTTACTATGGGGTGCCTGTATGG---AAGGAAGCAACT---
ACCACCCTATTTTGTGCTTCAGATGCTAAAGCATATGATACAGAG---AAGCATAAT----------------
--------------GTCTGGGCTACG---CATGCCTGTGTACCCACAGAC---
CCCAACCCACAAGAAATGCATTTGGAA---------AATGTGACAGAAGAATTTAAC---------------
--------------------------ATGTGGAAAAATGACATGGTAGAGCAGATGCATACAGATATA---
ATCAGTCTATGGGACCAAAGCCTA---CAGCCATGTGTAAAGTTA---
ACCCCTCTCTGTGTTACTTTAGATTGCCATGATGTCAGTCTCAGCAGTAACCAGAATGGCAAT----------
----
------------------AGCAGCAATAATGACAGTATCAGTAACATAAAAGAAGAAATAAAA---
AACTGCTCTTTCAATACAACCACAGAACTATGG--------------------------
GATAAGAAACAGAAAGTATATTCACTC---TTTTATAAACTAGATGTAGTAGCAATGAATGAA----------
----

AGCAATAATAGTAGTACTCAGTATAGATTAATAAATTGTAATACCTCAACCATCACACAG---
GCTTGTCCAAAGGTAACCTTTGAGCCAATTCCCATACATTATTGTGCTCCAGCTGGTTTTGCGATTCTAAAAT
GTAAT---GACAAGAAGTTCAAT------GGAACAGGGCCATGCAAGAAT----------------------
-GTCAGC---ACAGTACAATGCACACATGGAATCAAGCCAGTAGTATCAACT---
CAACTGCTGTTAAATGGCAGTCTAGCAGAAGAAGAG-----------
ACAATGGTTAGATCTGAAAATATCACAAACAAT---------ATCAAAAACATAATAATACAATTTAAT----
-----------GATTCTGTAGCA---------ATTAGTTGTATCAGACCTGGCAATAATACAAGAAAA-----
-AGTGTACGTATA------------GGTCCAGGACAAGCA---TTC------TATGCAACAGGTGAC------
---------ATAATAGGGGATATA------------AGAAAAGCATATTGT---AATGTCAGTAGAGTA---
ACATGGAATACAACTTTACAAAAGGTAGCCAAACAATTATCA---------------AAATACTTTGAG----
-----------------------------------AACAAAACAATAACATTTAAT------
AGCACCACAGGAGGGGATCTAGAAATTACA---ATGCATAGCTTTAATTGTGGGGGAGAA---
TTTAAACAGCTCCAGGCAAGAGTCCTAGCTGTGGAAAGATACCTAAGGGATCAACAGCTCCTAGGAATTTGGG
GCTGCTCTGGAAAAATCATCTGCACCACTAATGTGCCCTGGAATTATAGTTGGAGT-----------------
----------------------------AATAAATCCTATCATGAGATATGG------GAC------
AACATGACCTGGCTACAATGG---GATAAAGAAATTGACAATTACACACAGATAATATAT---
AGTCTAATTGAAGAATCACAGATCCAGCAAGAA---

AAAAATGAGCAAGACTTATTGGCATTGGACAAGTGGGCAAGTCTGTGGAATTGGTTTAACATAACAGAATGGC
TGTGGTATATAAGAATATTTATAATGATAGTAGGAGGCTTAATAGGATTAAGAATAGTTTTTGCA---
GTGCTCTCTATAATA---------AATAGAGTTAGGCAGGGATACTCACCT------
TTGTCTTTTGCTATCAATTTGCTTGATACCATAGCAATAGCAGTAGCTGGC---
TGGACAGATAGGGTTATAGAAATAGGACAAAGAGTTGGTAGAGCT---------------------------
--------ATTCTTAACATA------CCTACAAGAATTAGACAGGGCTTTGAAAGGGCTTTGCTA---TAA
>KC863311
GCT---GCA---
AACAACTTGTGGGTTACTGTCTATTATGGGGTACCTGTGTGG---AGAGATGCAGAA---
ACCACCTTATTTTGTGCATCGGATGCTAAAGCATATGATACAGAG---GTACATAAT----------------
--------------GTCTGGGCTACA---CATGCTTGTGTACCCACAGAC---
CCCGACCCACAAGAAATAGATTTAAAA---------AATGTGACAGAAGAGTTTAAC----------------
-------------------------ATGTGGAAAAATAACATGGTAGAGCAAATGCATACAGATATA---
ATTAGTCTATGGGACCAAAGCCTA---AAGCCATGTGTAAAGTTA---
ACCCATCTCTGCGTCACTTTAAATTGTAGCACTGCTAATGTTAATGTCACCGATTAC----------------
----
------------------------------AACATCACGACGGGGGACAAGGAAGAAATAAAA---
AACTGCTCTTTCAATATGACCACAGAACTAAGT--------------------------
GATAAGAAACAGAAAGTACATTCACTT---
TTTTATAGACTAGATGTAGTACCAATTAAACCTGATAATAGTGATGAT-------------------------
----
AATAGAAGTAATAGTAGTTATAGTAATTATAGATTAATAAATTGTAATACCTCAGCCATTACACAA---
GCATGCCCAAAGGTAACCTTTGATCCAATTCCCATACATTATTGTGCTCCAGCTGGTTTTGCGATCCTAAAGT
GTAAG---GATGATGGGTTCAAT------GGAACAGGACCATGCAAGAAT-----------------------
-GTTAGC---TCAGTCCAATGCACACATGGAATCAAGCCAGTAGTATCAACT---
CAATTGTTGTTAAATGGCAGTCTAGCAGGAAAAGGG-----------
ATAAGAATTAGATCTGAAAATATCACAGACAAT---------ACCAAAACTATAATAGTACAACTTGAC----
-----------AAGCCTGTGAGG---------ATTAATTGTACCAGACCTAACAACAATACAAGAAAA-----
-AGTATGCGTATA------------GGACCAGGACAAACA---TTC------TTTGCAACAGGTGAC------
---------ATAATAGGGGATATA------------AGAAAAGCACATTGT---AATATCAGTATATCA---
GAATGGAATGAGACTCTATACAAGGTAGCTGAACAGTTAGGA---------------GGAATTATTGGT----
-----------------------------------AACAAAACAGTAAAATTTGCT------
AACTCCTCAGGAGGGGATCTAGAAATCACA---ACACATAGTTTTAATTGTGGAGGAGAA---
TTTAAACAGCTCCAGGCAAGAGTCCTGGCTGTGGAAAGATACCTAAAGGATCAACAGCTCCTAGGAATTTGGG
GCTGCTCTGGAAAACTCATCTGCACCACTAATGTGCCCTGGAACTCTAGTTGGAGT-----------------
---------------------------AATAAAAATCAGAGTGAGATATGG------GAT------
AACATGACCTGGCTGCAATGG---GATAAAGAAATTAACAATTACACACAAATAATATAT---
GACCTACTTGAAGAATCGCAGAACCAGCAAGAA--

AAGAATGAACAGGAATTATTGGCATTGGACAAGTGGACAAATCTGTGGAATTGGTTTAACATAACAAACTGGC
TGTGGTATATAAAAATATTTATAATGATAGTAGGAGGCTTAATAGGATTAAGAATAGTTTTTGCT---
GTGCTTTCTATAATA---------AATAGAGTTAGGCAGGGATACTCACCT------
TTGTCATTTGCTATTAGTTTATTTGATACCATAGCAATAGCAGTAGCTGGC---
TGGACAGATAGGGTTTTAGAAATAGGACAAGCAATTTGTAGAGCT---------------------------
--------ATCCTTCACATA------CCTAGAAGAATCAGACAGGGCCTTGAAAGGCTTTTGCTA---TAA
>HM623589
GCT---AAA---
GAAGACTTGTGGGTTACAGTTTATTATGGGGTACCTGTGTGG---AAGGATGCAGAG---
ACCACTCTATTTTGTGCATCAGATGCTAAAGCATATGATACAGAA---AAACATAAT----------------
--------------GTCTGGGCAACA---CATGCTTGTGTACCCACAGAC---
CCCAACCCACAAGAAATAGACCTAGAC---------AATGTAACAGAAAATTTTAAC----------------
-------------------------ATGTGGAAAAATGGCATGGTAGATCAGATGCATAAAGATATA---
ATCAACCTATGGGATCAAAGTCTA---AAGCCATGTGTAAAGTTA---
ACCCCTCTCTGCGTTACCTTGAATTGCACTAACATTAACATCAACAACAGCACTGACAAT-------------
----
------------------------GGCACTTCTATCACTAATGAGATAGGAGACGAGATAAAA---
AACTGCTCTTACAATATTACTACAGAATTTAGA--------------------------
GATAAGAGACGGAGAGTGTCTTCACTG---TTTTATAGACATGATATAATGCCAATTAATGAA----------
----

AAGAATGATAGTAGGACTTATATACTAATAAATTGTAATACCACAACCATTACACAG---
GCTTGTCCAAAGGTGTCCTTTGAACCAATTCCTATACACTATTGTGCCCCTGCTGGTTATGCGATTCTAAAAT
GTAAG---GACAAAGATTTCAAT------GGGACAGGACCATGCAAGAAT----------------------
-GTCAGC---ACAGTACAATGTACACATGGAATTAAGCCAGTGGTATCAACT---
CAACTGCTGTTAAATGGCAGTCTAGCAGAAGGAGGG-----------
ATAAAAATTAGATCTGAAAATATTACAAACAAT---------GCCAAAACTATAATAGTACAGCTTGAT----
-----------ACGCCTGTAAAA---------ATTAATTGTAGCAGGCCCAACAACAATACAAGGGAC-----
-AGGATAGGTATA------------GGACCAGGACAAGCA---TTC------TTTGCAAGTAATAGG------
---------ATAATTGGGAATATA------------AGACAAGCACACTGT---AATATCAGTGCCTCA---
ATATGGAATATGATTTTACAAAAGGTAGCTGAACAATTAGCC---------------AAGCAGTTCCCT----
-----------------------------------AATAAAAACATAAGCTTTACA------
AATCACTCAGGAGGAGACCTAGAAATTACA---TCACACACTTTTAATTGTGGAGGAGAG---
TTTAAGCAGCTCCAGGCAAGAGTCCTGGCTCTGGAAAGATACCTAAGGGATCAACAGCTCCTAGGAATTTGGG
GCTGCTCTGGAAAAACTATCTGCACCACTACTGTGCCTTGGAACTCTAGTTGGAGT-----------------
----------------------------AATAAATCTCAGGAGGAGATTTGG------GGA------
AACATGACTTGGTTGCAGTGG---GATAAAGAAATTAGCAATTACACAGACTTAATATAC---
GTGCTGCTTCAAGAGTCACAAAACCAGCAGGAC---------------------------------------
----
AGGAATGAACAAGAGCTATTGGCATTGGACAAGTGGGCAAATCTGTGGAATTGGTTTGACATATCAAATTGGC
TATGGTACATAAGAATATTTATAATGGTAGTAGGAGGCTTAGTAGGCTTAAGAATAGTTTTTGCA---
GTAATTTCTATAGTA---------AATAGAGTTAGGCAGGGATACTCACCT------
TTGTCATTGGCTATTAGTCTATTTGATACCACAGCAATAGCAGTAGCTAAT---
TGGACAGATAGGGCTATAGAATTAGGACAAAGAATCTGTAGAGCT----------------------------
--------ATCCGTAACATA------CCTAGAAGAATAAGACAGGGCTTTGAAACAGCTTTGCTA---TAA
>MH705163
GCT---ACA---
AATTTGTGGGTCACAGTATACTATGGAGTACCTGTATGG---AGAGATGCAGAA---
ACCACCCTATTTTGTGCATCAGATGCCAAAGCATATGATACAGAG---GTGCATAAT----------------
--------------GTCTGGGCCACA---CATGCCTGTGTACCCACAGAC---
CCCAACCCACAAGAAGTAAACCTGGAA---------AATGTAACAGAAGATTTTAAC---------------
-------------------------ATGTGGAAAAATAGCATGGTAGAGCAGATGCATGTAGATATA---
ATCAGTCTATGGGATCAAAGCCTA---AAGCCATGTGTAAAATTA---
ACCCCTCTCTGCGTTACTTTAAATTGTAGCAATGCCACCGTCAATAGCACCAATAGCACCATCACT-------
----
--GAAGAAATAAAA---
AACTGCTCTTACAAGGTTACCCCAGAACTAAAA--------------------------
GATAAGCCACAGAGAGTCTATTCACTG---TTTTATAAACTTGATGTAGTACAAATTAGTAGT----------
----

AGTACACAGAAGGATAATGGTACTCAGTATAGACTAATAAATTGTAATACCTCAGTCCTTACCCAA---
GCTTGTCCAAAGGTATCCTTTGAGCCAATTCCTATACATTATTGTGCTCCAGCTGGTTTTGCGATTCTAAAGT
GTAAG---GATAAGGAATTCAAT------GGAACAGGGTCATGCAAGAAT----------------------
-GTTAGC---TCAGTACAATGCACACATGGAATTAGGCCAGTAGCATCAACT---
CAACTACTGTTGAATGGCAGCCTAGCAGAAGGAAAG-----------
ATAATGATTAGATCTGAAAATATTACAGACAAT---------GCCAAAAACATAATAGTACAGTTTAAT----
-----------GAGTCTGTACCA---------ATTAATTGTAGCAGGCCCAACAACAATACAAGAAAA-----
-AGTATACGCTTT------------GGACCAGGACAAGCC---TTC------TAT---ACAAATGAC------
---------ATAATAGGGGATATA------------AGACAAGCACATTGT---AATATCAGCAAAACA---
AAATGGAATGACACTTTACAAAAGATAGTTGAACAATTAAGA---------------GAACACTTCCCT----
-----------------------------------AATAAAACAATAGTCTTTAAA------
AAACCCTCAGGAGGGGACCTAGAAATTACA---ACACATAGTTTTAATTGTGGGGGAGAA---
TTTAAACAGCTCCAGGCAAGAGTCCTGGCTGTGGAAAGATACCTACAGGATCAACAGCTCCTGGGAATTTGGG
GCTGCTCTGGTAAACTCATCTGCACCACTAATGTACCCTGGAACTCTAGTTGGAGT----------------
----------------------------AATAAGACTCAGAGTGAGATTTGG------GAC------
AACATGACCTGGTTGCAATGG---GATAAAGAAATTAGCAATTACACAGATACAATATAC---
AGACTACTTGAAGACTCGCAGAACCAGCAGGAA---------------------------------------
----
AAAAATGAACAAGACTTATTGGCATTAGACAAATGGGCAAGTTTGTGGAGTTGGTTTGACATAACAAAATGGC
TGTGGTATATAAAAATATTTATAATGATAGTAGGAGGCTTAATAGGATTAAGAATAGTTGTTGCT---
ATAATTTCTGTAGTA---------AGTAGAGTTATGGGGGGATACTCACCT------

TTGTCATTTGCTATTAGTTTATTTGATACCATAGCAGTAGCAGTAGCTGAG---
TGGACAGATAGAGTTATAGAAATAGGACAAAGAATTTGCAGAGCT----------------------------
--------ATTCGCAACATA------CCTAGAAGAATCAGACAGGGCTTCGAAAGGGCTTTACTA---TAA
>AF286241
GCT---ACA---
GATTGGTGGGTCACAGTATACTATGGAGTACCTGTGTGG---AAAGATGCAGAA---
ACCACCTTATTTTGCGCATCAGATGATAAAGCATATGAGACAGAA---GCGCATAAT----------------
--------------GTCTGGGCCACA---CATGCCTGTGTACCCACAGAC---
CCCAACCCACAAGAAGTAAACCTAAAA---------AATGTGACAGAAGATTTTAAC----------------
--------------------------ATGTGGAAAAATAATATGGTAGAGCAGATGCATGAAGATATA---
ATCAGTCTATGGGATCAAAGCCTA---AAGCCATGTGTAAAATTA---
ACCCCTCTCTGTGTCACGTTAAACTGTAGCAATGCCAACACCAATAGCACCAATAGC----------------
----
----------------------------ACTAGCGCCCCTAGCATGGGCCCTGGAGAAATAAAA---
AACTGTTCTTTTAATGTTACCACAGAAGTAAGA---------------------------
GATAAAGAAAAGAAAGTCTATGCACTG---TTTTATAAACTTGATGTAGTACAAATTAATGAAAGTGACAGT-
----

AATAGTACAAAGGATAGTACTCAGTATAGACTAATAAATTGTAATACCTCAGCCATCACACAG---
GCTTGTCCAAAGGTATCCTTTGAGCCAATTCCTATACATTATTGTGCCCCAGCTGGTTTTGCGATTCTAAAGT
GTGAG---GATCCGAGATTCAAT------GGAACAGGACCATGCAATAAT-----------------------
-GTTAGC---TCAGTACAATGTACACATGGAATTATGCCAGTAGCATCAACT---
CAACTGCTGTTGAATGGCAGTCTAGCAGAAAAAGAG------------
GTGATGATTAGATCTGAAAATATTACAAACAAT---------GCCAAAAACATAATAGTACAGTTTAAT----
-----------GAATCGGTACCA---------ATTACTTGTATCAGACCCAACAACAATACGAGAAAA-----
-GGTATACCTATT------------GGACCAGGACAAGTC---TTC------TAT---ACAAGTGAC------
---------ATAATAGGGGATATA------------AGACAAGCATATTGT---AGTATCAACAAAACA---
AAATGGATGCCTCTTTACAAAAGGTAGCTGAACAATTAAGA----------------AAACACTTCCCT----
-----------------------------------AATAAAACAATAAATTTTACC------
AAACCCTCAGGAGGGGATCTAGAAATTACA---ACACATAGTTTTAATTGTGGAGGAGAA---
TTTAAACAGCTCCAGGCAAGAGTCCTGGCTCTGGAGAGATACCTACAAGATCAACAGCTCCTGGGAATTTGGG
GTTGCTCTGGAAAACTCATCTGCACCACTACTGTGCCCTGGAACTCTAGTTGGAGT-----------------
----------------------------AATAAGACTTACGAGGAGATTTGG------AAC------
AACATGACCTGGTTGCAATGG---GATAGAGAAATTGACAATTACACAAATATAATATAC---
AATCTACTTGAAGAATCGCAGAACCAGCAGGAA--

AAGAATGAACAAGACTTACTGGCATTAGATAAATGGGCAAGTTTGTGGAATTGGTTTAGCATAACAAACTGGC
TGTGGTATATAAGAATATTCATAATGATAGTAGGAGGCTTGATAGGATTAAGAATAGTTATGGCT---
ATAATTTCTGTAGTG---------AATAGAGTTAGGCAGGGATACTCACCT------
TTGTCATTTGCTATTAGTTTACTAAATTCCACAGCAATAGCAGTAGCTGAG---
TGGACAGATAGGGTTATAGAAATAGGACAAAGAGCTTGCAGAGCT----------------------------
--------ATTCTCAACATA------CCTAGAAGAATCAGACAGGGCTTCGAAAGAGCTTTACTA---TAA
>AF286238
GCT---GCA---
GATTTGTGGGTCACAGTATACTATGGAGTACCTGTGTGG---AGAGATGCAGAT---
ACCACCCTATTTTGTGCATCAGACGCTAAAGCATATGCTACAGAA---AAGCATAAT----------------
--------------GTCTGGGCCACA---CATGCCTGTGTACCCACAGAC---
CCCAACCCACAAGAAGTAAACCTGGCA---------AATGTAACAGAAGATTTTAAC----------------
--------------------------ATGTGGAAAAATAACATGGTAGAGCAGATGCATGCAGATATA---
ATCAGTCTATGGGATCAAAGCCTA---AAGCCATGTGTAAAATTA---
ACCCCTCTCTGCGTCACTTTAAATTGTAGCAATGCCAACACCACTAAC-------------------------
----
--ACCAATAGCACTGAAGAAATAAAA---
AACTGCTCTTACAATATGCCCACAGAACTAAAA---------------------------
GATAAAACACAGAAAGTCTATTCACTG---TTTTATGAACTTGATGTAGTACTACTTAATAGAAGT-------
----

AAGAATAGTAGTTATAGTACTTATAGACTAATAAGTTGTAATACCTCAGTCATCACACAA---
GCTTGTCCAAAGGTATCCTTTGAGCCAATTCCTATACATTATTGTGCCCCAGCTGGTTATGCGATTCTAAAGT
GTAAG---GATAAGGAATTCAAT------GGAAAAGGGTCATGCAGTAAT-----------------------

-GTTAGC---TCAGTACAATGTGCACATGGAATTAGGCCAGTAGCATCAACT---
CAACTGCTGCTGAATGGCAGTCTAGCAGAAGGAAAG-----------
GTAATGATTAGATCTGAAAATATTACAGACAAT---------GCCAAAAACATAATAGTACAGTTTAAT----
-----------AAACCTGTACCA---------ATTAATTGTACCAGACCCAACAACAATACAAGAAAA-----
-AGTATACGCTTT------------GGACCAGGACAGGCC---TTC------TATACAAATAATAAC------
---------ATAATAGGGGATATA------------AGACAAGCACATTGT---AATATCAGCATAACA---
GAATGGAATGCCACTCTAAAAAAGGTAGTTGAACAATTAAGA---------------GAACACTTCCCT----
-----------------------------------AATAAAACAATAATCTTTAAT------
AGCTCCTCAGGAGGGGACCTAGAAATTACA---ACACATAGTTTTAATTGTGGAGGAGAA---
TTTAAACAGCTCCAGGCAAGAGTCCTGGCTCTGGAAAGATACCTACAGGATCAACAGCTCCTAGGAATTTGGG
GCTGCTCTGGAAAACTCATCTGCGCCACTGATGTGAGATGGAACTCTAGTTGGAGT----------------
----------------------------AATAAGACTCAGGAGCAGATTTGG------AAA------
AACATGACCTGGTTGCAATGG---GATAAGGAAATTAGCACTTACACAGACATAATATAT---
ATGCTACTTGAGGAATCGCAGAACCAGCAGGAA--

AAGAATGAACAAGACTTATTGGCATTAGACAAATGGGCAAATTTGTGGAATTGGTTTGACATAACAAGATGGC
TGTGGTATATAAAAATATTTATAATGATAGTAGGAGGCTTGATAGGATTAAGAATAGTTATTGCT---
ATAATTTCTGTAGTA---------AAAAGAGTTAGGCAGGGATACTCACCA------
TTGTCATTTGCTATTAGATTGCTTGATACCATAGCAGTAGCAGTAGCTGAG---
TGGACAGATAGGGTTATAGAAATAGGACAAAGAGCTTGCAGAGCT----------------------------
--------ATCCGCAACATA------CCTAGAAGAATCAGACAGGGCCTCGAAAGGGCGTTACTA---TAA
>AF286237
GCT---ACA--
GATTTGTGGGTCACAGTATACTATGGAGTACCTGTGTGG---AAAGATGCAGAT---
ACCATCCTATTTTGTGCATCAGATGCTAAAGCATATGATACAGAA---GTGCATAAT----------------
--------------GTATGGGCCACA---CATGCCTGTGTACCCACAGAC---
CCCAACCCACAAGAAATAAACCTGGAA---------AATGTAACAGAAAATTTTAAT---------------
--------------------------ATGTGGAAAAATAACATGGTAGAGCAGATGCAAGAAGATATA---
ATCAGCTTATGGGATCAAAGCCTA---AAGCCATGTGTAAAATTA---
ACCCCGCTCTGCGTCATTTTAAATTGTAGCAATGCCAATACCAGCACCCATAGCAATAGC------------
----
----------------------------AGTAGCACCCAGAGCCCCATTAATGAAGAAATAAAA---
AACTGCTCTTACAATACTACCACAATACTAAGA--------------------------
GATAAGACACAAAAAGTTTATTCACTG---TTTTATAGACTTGATGTAGTACAACTTGATGAAAGTGAAAAT-
----

AAGAATACATCAGGTAGTAATACTCTGTATAGACTAATAAATTGTAATACCTCAACCATCACACAA---
GCGTGTCCAAAGGTAACCTTTGAGCCAATTCCTATACATTATTGTGCCCCAGCTGGTTTTGCGATTCTAAAGT
GTAAG---GATCCGAGATTCAAT------GGAACAGGGTCATGCAAGAAT----------------------
-GTTAGC---TCAGTACAATGTACACATGGAATTAAACCAGTAGCATCAACT---
CAACTGCTGTTGAATGGCAGTCTAGCAGAAGGAGGG---------
AAAATAATGATTAGATCTGAAAATATTACAAACAAT---------GCCAAAAACATAATAGTTCAGTTTACT-
--------------AAGCCTGTACTA---------ATTACTTGTATCAGACCCAACAACAATACAAGAAAA--
----AGTATACGCTTT------------GGACCAGGACAAGCC---TTC------TAT---ACAAATGAA---
------------ATAATAGGGGACATA------------AGACAAGCACATTGT---AATATCAACAAAACA-
--TTATGGAATGACACTTTACAAAAGGTAGCTGAACAATTAAGA---------------GAGAAATTCCCT--
--------------------------------------AAGAAAACCATAATCTTTACT------
AACTCCTCAGGAGGGGACCCAGAAATTACA---ACACTTAGTTTTAATTGTGCAGGAGAA---
TTTAAACAGCTCCAGGCGAGGGTCCTGGCTGTGGAAAGATACCTAAAGGATCAACAGCTCCTGGGAATTTGGG
GCTGCTCTGGAAAACTCATCTGCGCCACTACTGTGCCCTGGAACACTAGTTGGAGT-----------------
----------------------------AATAAGTCTCAGGATGAGATTTGG------GAC------
AACATGACCTGGTTGCAATGG---GATAAAGAAATTAGCAATTACACAAACATAATATAT---
AGGTTACTTGAAGAATCGCAAAACCAGCAGGAA--

AAGAATGAGCAAGACTTATTGGCATTAGACAAATGGGCAGATTTGTGGAGTTGGTTCAACATTTCACACTGGC
TGTGGTATATAAGAATATTTATAATGATAGTAGGAGGCTTGATAGGATTAAGAATAGTTTTTGCT---
ATAATTACTGTAGTA---------AATAGAGTTAGGCAGGGATACTCACCT------
GTGTCATTTGCTATTAGCTTATTTGATACTATAGCAGTAGCAGTAGCTGAG---
TGGACAGATAGGGTTATAGAAATAGGGCAAAGAGCTTTCAGAGCT---------------------------
--------ATTCTCAACATA------CCTAGAAGAATCAGACAGGGCTTAGAAAGGGCTTTACTA---TAA

>AY521629
GCT---ACA--
GAAAACTTGTGGGTCACGGTCTACTATGGGGTACCGGTGTGG---AAAGACGCAGAG---
ACCACCCTATTTTGTGCATCAGATGCTAAAGCATATGATCAAGAA---GTGCATAAT----------------
--------------GTTTGGGCTACA---CACGCCTGTGTACCCACAGAC---
CCCAACCCACAAGAGTTAGATTTGGAA---------AATGTGACAGAAGAGTTTAAC----------------
--------------------------ATGTGGAAAAATAACATGGTGGAGCAGATGCATACAGACATA---
ATCAGTCTATGGGACCAAAGCTTA---AAGCCATGTGTACAGTTA---
ACCCCTCTCTGCGTTACTCTAAACTGTACCGACATCCCCAAGAACTTTACCAATACC----------------
----
-------------------------------GAGAATGTCACCAAGGCCTATGAAGAGATGAAA---
AACTGCTCGTTCAATATAACCACAGAACTAAGA---------------------------
GATAAGAAACAGAAAATATATTCACTT---TTTTATAGACTTGATGTAGTACCAATTGAGAATGGTAGT----
----

AACAGTACTGAGAACAGTACTGAATATAGATTAATAAATTGTAACACCTCAGCCATTACACAG---
GCTTGTCCAAAGGTATCCTTTGAGCCAATTCCCATACATTATTGTGCCCCAGCTGGTTTTGCGATTCTAAAGT
GTAAG---GAGAAGAAATTCAAT------GGAACAGGGCCATGCAGGAAT-----------------------
-GTCAGC---ACGGTACAATGTACACATGGAATCAAGCCAGTAGTATCAACT---
CAACTGCTGTTGAATGGCAGTCTAGCGGAAGAAGAG------------
GTAATGATTAGATCTGAAAATCTCACAAACAAT---------GCCAAAACCATAATAGTACAGCTTGTT----
-----------AATCCTGTGCAA---------ATTAGTTGTACCAGACCCAACAACAATACAAGAACA-----
-AGTGTACGCATA------------GGACCAGGACAAGCA---TTC------TATGCAACAGGTGAC------
---------ATAATAGGGGATATA------------AGGCAAGCACATTGT---AATGTCAGTAGAGAA---
AATTGGACTGAGACTTTACAACAGGTAGCTAAACAATTAAAA---------------AGACACTTT-------
-----------------------------------AACAAAACAATAATCTTTACT------
GGGTCCTCAGGAGGAGATTTAGAAATTACA---ACACATAGCTTTAATTGTGGAGGAGAA---
TTTAAACAGCTCCAGGCAAGAGTCCTGGCTATAGAAAGATACCTAAAGGATCAACAGCTCCTAGGACTTTGGG
GTTGCTCTGGGAAACTCATCTGCACCACTAATGTGCCCTGGAACTCTAATTGGAGT-----------------
----------------------------AGAAAGTCTCAGGAGGAAATATGG------GGT------
AATATGACCTGGCTGCAATGG---GATAAAGAAATTAGCAATTACACAGACATAATATAT---
AGTCTAATTGAAGAATCACAGAACCAGCAGGAA--

AAGAATGAACAAGACTTATTGGCATTGGACAAATGGGCAAGTCTGTGGAGTTGGTTTGACATATCAAAGTGGT
TATGGTATATAAAAATATTTATAATGATAGTAGGGGGCTTAATAGGATTAAGAATAGTTTTTGCT---
GTGCTTTCTATAATA--------AATAGAGTTAGGCAGGGTTACTCACCT------
CTCTCGTTTGCTATCTCTTTGCTTAATGCTACAGCAATAGTAGTAGCTGAG---
TGGACAGATAGGGTTATAGAAATAGGACAAAGAGCTGGTAGAGCT----------------------------
--------CTTCTCCATATA------CCTAGAAGAGTCAGACAGGGCTTAGAAAGGGCTTTGCTA---TAA
>AY521631
GCT---ACA--
GAAAAGTTGTGGGTCACAGTCTACTATGGGGTACCGGTGTGG---AAAGACGCAGAG---
ACCACCCTATTTTGTGCATCAGATGCTAAAGCATATGAGCGAGAA---GTGCATAAT----------------
--------------GTTTGGGCTACA---CACGCCTGTGTGCCCACAGAC---
CCCAACCCACAAGAAATAGATTTGGAA---------AATGTGACAGAAGAGTTTAAC----------------
--------------------------ATGTGGAAAAATAATATGGTGGAGCAGATGCATACAGATATA---
ATCAGTCTATGGGACCAAAGCTTA---AAGCCATGTGTAAAGTTA---
ACCCCTCTCTGCGTTACTTTAAACTGCCACAACGTCACCAAGACCAATTCCAATTCCAAT-------------
----
----------------------------TCCAGTGTCAAAAATGAAATGCCTGGAGAAATAAAA---
AACTGCTCGTACAATGTAACCACAGCAATAAGA---------------------------
GATAAGAGGCAGAATGTATATTCGCTT---TTTTATAGAGTTGATGTAGTACCAATTAATGAAAATAGT----
----

AATAGTAGTGAGAATAGTAGTGAGTATAGACTAATAAATTGTAATACTTCAGCCATTACACAG---
GCTTGCCCAAAGGTAACCTTTGAGCCAATTCCCATACATTATTGTGCCCCAGCTGGTTTTGCGATTCTAAAGT
GTAAG---GATAAGGATTTCAAT------GGAACAGGAACATGCAGGAAT-----------------------
-GTCAGC---ACAGTACAATGCACACATGGAATCAAGCCAGTAGTGACAACT---
CAACTGCTGTTGAATGGCAGTCTAGCAGAAGGAAAG------------
GTAATGATTAGATCTGAAAATCTCACAAACAAT---------GCCAAAACCATAATAGTACAGCTTGAT----

-----------GAGCCTGTATAC---------ATTAATTGTACCAGACCCAACAACAATACAAGGAAA-----
-AGTGTACATATA------------GGACCAGGACAGGCA---TTC------TATGCAACAGGAGAC------
---------ATAATAGGGGATATA------------AGACAAGCACATTGT---AATGTCAGTAGAGAA---
GATTGGAATAAAACTTTATATAAGGTGGTTGAACAATTAAGA---------------AAGCACTTTAAC----
-----------------------------------AACAGAACAATAAACTTTACT------
GCACCCTCAGGAGGGGATTTAGAAATTACA---ACACATAGCTTTAATTGTGGAGGAGAA---
TTTAAACAGCTCCAGGCAAGAGTCCTGGCTGTGGAAAGGTACCTAAAGGATCAACAGCTCCTAGGAATTTGGG
GTTGCTCTGGAAAACTCATCTGCACCACTAATGTGCCTTGGAACTCTAGTTGGAGT----------------
----------------------------AATAAATCTTATAGTGAAATATGG------GAT------
AATATGACTTGGCTGCAATGG---GATAAAGAAATTAGCAATTACACAGACTTAATATAT---
AATCTAATTGAAGAATCGCAGAACCAGCAGGAA---------------------------------------
----
AAGAATGAACAAGACTTATTGGCATTGGACAAGTGGGCAAGTCTATGGAATTGGTTTAACATATCAAATTGGC
TATGGTACATAAGAATATTTATAATGATAGTAGGGGGCTTAATAGGATTAAGAATAGTTTTTACT---
GTACTTTCTATAATA---------AATAGAGTTAGGAAGGGATACTCACCT------
CTGTCATTTGCTATTTCTTTGCTTGACGCTATAGCAATAACAGTAGCGGGG---
TGGACAGATAGGGTTATAGAAGTAGGACAAAGAGCTGGTAGAGCT---------------------------
--------CTTCTCCACATA------CCTAGAAGAATTAGACAGGGCTTGGAAAGGGCTTTGCTA---TAA
>AY521630
GCT---ACA---
GAAAACTTGTGGGTCACGGTCTACTATGGGGTACCGGTGTGG---AAAGACGCAGAG---
ACCACCCTATTTTGTGCATCAGATGCTAAAGCATATGATCAAGAA---GTGCATAAT----------------
--------------GTTTGGGCTACA---CACGCCTGTGTACCCACAGAC---
CCCAACCCACAAGAATTAGATTTGGAA---------AATGTGACAGAAGAATTTAAC---------------
--------------------------ATGTGGAAAAATAACATGGTGGAGCAGATGCATACAGATATA---
ATCAGTCTATGGGACCAAAGCTTA---AAGCCATGTGTAAAGTTA---
ACCCCTCTCTGCGTTACTTTAAACTGTAGCAACATCAACAGCACCAGGTAT---------------------
----
------------------------------------GACACCAAGGACAAGGAAGAGATGAAA---
AACTGCTCGTTCAATATAACCACAGAACTAAGA--------------------------
GATAAGAGACAGAGTGTATATTCACTT---TTTTATAAACTTGATGTAGTACCAATTAATAATAATAGT----
----

AGTAATAATAGTAGTAATGAGTATAGACTAATAAATTGTAACACCTCAGCCATTACACAG---
GCTTGTCCAAAGGTTTCCTTTGAGCCAATTCCCATACATTATTGTGCCCCAGCTGGTTTTGCGATCCTAAAGT
GTAAG---GATAAGAAATTCAAT------GGAACAGGGCCATGCAGGAAT----------------------
-GTCAGC---ACGGTACAATGTACACATGGAATCAAGCCAGTAGTATCAACT---
CAACTGTTGTTGAATGGCAGTCTAGCGGAAGAAGAG-----------
GTAATGATTAGATCTGAAAATCTCACAAACAAT---------GCCAAAACCATAATAGTACAGCTTGTT----
-----------ACTCCTGTACAA---------ATTAATTGTACCAGACCCAACAACAATACAAGAACA-----
-AGTGTACGCATA------------GGACCAGGACAAGCA---TTC------TATGCAACAGGTGAC------
---------ATAATAGGGAAGATA------------AGACAAGCACATTGT---ACTGTCAGAAGAGCA---
CAATGGAATACCACTTTACAACAGGTAGCTGACCAATTAAGA---------------AAACACTTT-------
-----------------------------------AACAAAACAATAAACTTTACT------
AGGCCCTCAGGAGGAGATTTAGAAATTACA---ACACATAGCTTTAATTGTGGAGGAGAA---
TTTAAACAGCTCCAGGCAAGAGTCCTGGCTGTGGAAAGATACCTAAGGGATCAACAGCTCCTAGGACTTTGGG
GTTGCTCTGGGAAACTCATCTGCACCACTGCTGTGCCCTGGAACTCTAGTTGGAGT----------------
----------------------------AATAAATCTCAGAATGAAATATGG------GAG------
AATATGACCTGGCTACAATGG---GATAAAGAAATTAGCAATTACACAGACATAATATAT---
AATCTAATTGAAGAATCGCAGAACCAGCAGGAA---------------------------------------
----
AAGAATGAACAAGACTTATTGGCATTGGACAAATGGGCAAATCTGTGGAATTGGTTTGACATATCAAAATGGT
TATGGTATATAAAAATATTTATAATGATAGTAGGGGGCTTAATAGGATTAAGAATAGTTTTTGCT---
GTGCTTTCTATAATA---------AATAGAGTTAGGCAGGGTTACTCACCT------
CTCTCGTTTGCTATTTCTTTGCTTAATGCTACAGCAATAGTAGTAGCTGAG---
TGGACAGATAGGGTTATAGAAGTAGGACAAAGAGCTGGTAGAGCT---------------------------
--------CTTCTCCACATA------CCTAGAAGAATCAGACAGGGCTTAGAAAGGGCTTTGCTA---TAA
>AM000053
GCT---GCA---
GAAAAATTGTGGGTCACGGTCTACTATGGGGTACCCGTGTGG---AAAGATGCAGAA---

ACCACCCTGTTTTGTGCATCACATGCTAATGCATATGAGAAAGAA---CAACATATT---------------
--------------GTCTGGGCTACC---CATGCCTGTGTTCCCACAGAC---
CCCAACCCACTCGAAATGGATTTAGAT---------AATGTGACAGAAGAGTTTAAC----------------
--------------------------ATGTGGAAAAATAATATGGTAGAGCAGATTCATGATGATATA---
ATCCGTCTATGGGATCGCAGCTTA---CAGCCACGTGTAAAATTA---
ACCCCTCTCTGCGTTACTTTAGACTGCCAGCCAGTTAACAGCACCAACAAT---------------------
----
----------------------------------ACTAAGGTAGAAGTGCCGGGAGAAATGACA---
AACTGCTCTTTCAATATGACCACAGAACTAAGT---------------------------
GATAAGAAACAGAAAGTACGGTCTCTT---TTTTATAGGATTGATTTAGTACAGATTGGTAATAATACAAAT-
----

GACAGTAGTAATAGGAGTCTACAGTATAGACTAATAAATTGTAACACCTCAACCATTACCCAG---
GCTTGTCCCAAGGTATCCTTTGAGCCAGTTCCCATACACTATTGTGCCCCAGCTGGTTTTGCGATTCTGAAGT
GTAAA---GATCAGGAGTTCAAT------GGAACAGGGCCATGCAAAAAT----------------------
-GTCAGC---ACAGTACAATGTACACATGGAATTAAACCTGTAGTATCAACT---
CAACTGCTGTTAAATGGCAGTTTAGCAGAAGGAGGA-----------
ATAAAGATTAGATCTGCAAATATCTCATACAAT---------GCCAAAAACATAATAGTACAGCTTGAC----
-----------ATACCTGTAAAA---------ATTAATTGTAGTAGGCCCAACAACAATACAAGAACA-----
-AGTGTGCGTATA------------GGACCAGGGCAAACA---TTT------TATGCAACAGGTGAT------
---------ATAATAGGGAATATA-----------AGACAAGCACACTGC---AACCTTAGTAGAACA---
GCATGGAACGATACTTTATACAATGTATCTAAAGCCTTAAGA---------------GAACACTTCCCA----
-----------------------------------AATAAAACAATAATCTTTAAT------
AAATCATCAGGAGGGGACCTAGAAGTGACA---CAACATATGTTTATTTGTGGAGGAGAA---
TTTAAACAGCTCCAGGCAAGAGTCCTGGCTGTGGAAAGATACCTAAAGGATCAACAGCTCCTAGGAATTTGGG
GCTGCTCTGGAAAACTCATCTGCACCACTACTGTGCCTTGGAACTCTAGTTGGAGT-----------------
---------------------------AATAAATCTCAGGATGAGATTTGG------AAC------
AACATGACCTGGATGCAATGG---GATAAAGAAATTAATAATTACACAGAAATAATATAC---
GGGCTCATTGAAGAATCGCAGAAACAGCAGGAG---------------------------------------
----
AAAAATGAATTTGAGTTATTGGAATTGGACAAGTGGGCAAATCTGTGGAATTGGTTTGAAATATCAAATTGGC
TATGGTATATAAAAATATTTATAATGATAGTAGGAGGCTTAATAGGATTAAGAATAGTTTTTGCA---
GTGCTTTCTATAGTA---------AATAGAGTTAGGCAGGGATACTCACCT------
TTATCGTTTGCTGTCAGTGCATTTAATGCTTTAGCAATAACAGTTGCTGAG---
TGGACAGATAGGGCTATAGAAGTAGGACAAAGAATTGGTAGAGCT---------------------------
--------TTTCTCAACATA------CCTAGAAGAATCAGACAGGGCTTCGAAAGAGCTTTGCTA---TAA
>AM000054
GCT---GCA--
GAAAACTTGTGGGTCACGGTCTACTATGGGGTACCTGTGTGG---AAAGATGCAGAA---
ACCACCCTATTCTGTGCATCAGACGCCAAAGCATATGAGAGAGAA---GTGCATAAT---------------
--------------GTCTGGGCTACA---CATGCCTGTGTTCCCACAGAC---
CCCAACCCACAAGTAATGCCTCTAGCC---------GATGTGACAGAAGTATTTAAC----------------
--------------------------ATGTGGATGAATGATATGGTAGAGCAGATGCATGAAGATATA---
ATCAGCCTATGGGATCAAAGCTTA---AAACCATGTGTACAGTTG---
ACCCCTCTCTGTGTTACTTTACATTGCCATTATGTCATCAACAACACAGCCCACAAG---------------
----
----------------------------GTTTCTGTTTCTCCAGAAATGCAAGAAAAAGTAGCA---
AACTGCTCTTTCAACGTGACCACAGAACTAAGA--------------------------
GACAGAAAACAGAAAGTATATTCTCTT---
TTTTATAGGGAAGATTTAGTACCTATTGATGAGGATAAAAATAACAGTAATAGTGCTAGCATCAGTACTTCTA
GT-----------------
AACAGCAGTAATTGTAGTATTTCTGATAGCAACAGTACTTGTATTAACATCAGTACTTATAGACTAATAAATT
GTAATACCTCAACCATTACACAG---
GCTTGTCCAAAGGTATCCTTTGAGCCAATTCCCATACACTATTGTGTCCCAGCTGGTTTTGCAATTCTAAAAT
GTAAC---GAGAAGGAGTTCAAT------GGAACAGGGCCATGCAAGAAT----------------------
-GTTAGC---TCAGTACAATGTACACATGGAATCAAGCCAGTAGTATCAACT---
CAACTGCTGCTAAATGGCAGTCTAGCAAAAGAAACA-----------
GTAATAATTAGAACTGAAAATATTTCAGACAAT---------GGCAAAAACATAATAGTACAGTTTGTT----
-----------GATCCTGTAAGA---------ATTAATTGTATCAGACCCAACAACAATACAAGAACA-----
-AGTGTACGTATA------------GGACCAGGACAAACA---TTT------TAT---ACAAGTGAT------

---------ATAATAGGGGATATA------------AGACAAGCCTACTGC---GAAGTCAATAAAACA---
CAATGGAACATGACTTTACAAAAGGTAGCTGACCAATTAAGA--------------AAATTCTTCCCG----
-----------------------------------AACAAAACAATAAACTTTAAT------
CAAACCTCAGGAGGGGACTTAGAAGTTACA---ACACATATTTTTAATTGTAGAGGAGAA---
TTTAAACAGCTCCAGGCAAGAGTCCTGGCTGTGGAAAGATACCTAAAGGATCAACAGCTCCTAGGAATTTGGG
GCTGCTCTGGGAAACTCATCTGCACCACTAATGTGCCTTGGAACTCTAGCTGGAGT----------------
----------------------------AATAAATCTCAGGGTGAAATTTGG------GAT------
AACATGACCTGGATGCAATGG---GATAGAGAAATTAGCAATTACACAGATATAATATAC---
AAGTTAATTGAGGAATCGCAGGAACAGCAGGAG--

AAGAATGAACTTAAGTTATTGGCATTGGACCAGTGGGCAAGTCTGTGGAATTGGTTTGACATATCAAATTGGC
TATGGTATATAAGAATATTTATAATGATAGTAGGAGGCTTAATAGGATTAAGAATAGTTTTTGCA---
GTGCTTTCTGTAGTA---------AATAGAGTTAGGCAGGGATACTCACCT------
TTATCGTTTGCTATTAGTTTATTTGATACTCTAGCAATAGCAGTAGCTGAG---
TGGACAGATAGGATTATAGAAGTAGGACACAGAATTGGTAGAGCT---------------------------
--------TTTCTCAACATA------CCTAGAAGAATCAGACAGGGCTTCGAAAGAGCTTTGCTA---TAA
>FJ388892
GCT---ACA---
GAAAACTTGTGGGTCACGGTATACTATGGGGTACCTGTGTGG---AGAGATGCAGAG---
ACCACCCTATTTTGTGCATCAGATGCTAAAGCATATGAGAAAGAA---GTACACAAT----------------
--------------GTCTGGGCTACA---CATGCCTGTGTACCCACAGAC---
CCTAACCCACAAGAAATAAATTTGGAA---------AATGTGACAGAAAATTTTAAC---------------
--------------------------ATGTGGAAAAACAGTATGGTAGAACAAATGCAAACAGATATA---
ATCAGTCTCTGGGACCAAAGCCTA---AAGCCATGTGTACAGTTA---
ACCCCTCTCTGCGTCACTCTAAATTGTACTGACCCCACACCCAACATCACTAGCTCTAACAACAGT-------
----
-------------------AGCGTTAACAATAACAGCAGCGATAGCCTGTATCAAGAAATGAAA---
AACTGCTCTTTCAACATGACCACAGAACTAAGA--------------------------
GATAAAAAGAAAAAGGTACATTCACTT---TTTTATAAACTTGATATAGTATCAACTGGTAGTAATAAT----
----

GATAGTAGTAATGATAGTCTGTATAGACTAATAAATTGTAACACATCTGCCATGACACAG---
GCTTGTCCTAAGGTAACCTTTGAGCCAATTCCTATACATTATTGTGCCCCAGCTGGTTTTGCGATTCTAAAGT
GCAAG---GATCCAAATTTTAAT------GGAACAGGGCCATGCAAGAAT----------------------
-GTCAGC---ACAGTACAATGCACACATGGAATCAAACCAGTAGTATCAACT---
CAACTGCTGTTAAATGGCAGCCTAGCAGAGAAAGAG-----------
GTAATGATTAGATCTGAAAATATCACAGACAAT---------GGCAAAATCATAATAGTGCAGCTTAAT----
-----------GAGTCTATAAGC---------ATCACTTGTATCAGACCCAACAACAACACAAGAACA-----
-AGTATACGTATA------------GGACCAGGACAAACC---TTC------TATGCAACAGGGGAT------
---------GTAACAGGGGACATA------------AGAAAAGCATATTGT---AATGTCAGCAGAACA---
AACTGGACTAGCACTTTACGAAAGATAAGTACACAATTAAGG--------------AAACACTTTACT----
-----------------------------------AACAAAACAATAATCTTTAAG------
AACTCTGCAGGAGGGGATTTAGAAGTTACA---ACACATAGTTTCAATTGTGGAGGAGAA---
TTTAAACAGCTCCAGGCAAGAGTCCTGGCTGTGGAACAATACCTAAAGGATCAGCAGCTCCTAGGAATTTGGG
GTTGCTCTGGAAAACTCATCTGCACCACTAATGTGCCCTGGAACTTTAGTTGGAGT----------------
----------------------------AATAAATCTCAGAGTGAGATATGG------GAT------
AACATGACCTGGATGCAATGG---GACAAGGAAGTTATCAATTACACAGGCATAATATAT---
GATCTACTTGAAAAAATCGCAAAACCAGCAGGAA---------------------------------------
----
AAGAATGAACAAGATTTATTGGCATTAGATAAGTGGACAAGTCTGTGGAGTTGGTTTGACATATCAAATTGGT
TATGGTATATAAGAATATTTATAATAATAGTAGGAGGCTTAATAGGATTAAGAATAGTTTTTGCT---
GTGCTTTCTATAATA---------AATAGAGTCAGGCAGGGATACTCACCC------
TTGTCATTGGCTATTAATCTGATAGATACCATAGCAATAGCAGTAGCTGGG---
TGGACAGATAGGGTTATAGAATTAGCACAAAGACTTGGTAGAGCT---------------------------
--------ATCCTTAACATA------CCTAGGAGAATCAGACAGGGCGCAGAAAAGGCTTTGCAA---TAA
>JF683763
GCT---ACA---
GACAACTTGTGGGTCACGGTATACTATGGGGTACCTGTGTGG---AGAGATGCAGAG---
ACCACCCTATTTTGTGCATCAGATGCTAAAGCATATGACAAAGAA---GTACATAAT----------------
--------------GTCTGGGCTACA---CATGCCTGTGTTCCCACAGAC---

CCTAACCCACAAGAAATGGCTTTGGAA---------AATGTGACAGAAAATTTTAAC---------------
--------------------------ATGTGGAAAAATAACATGGTAGAACAAATGCAAACAGATATA---
ATCAGTCTCTGGGACCAAAGCCTA---AAGCCATGTGTAAAGTTA---
ACCCCTCTCTGCGTTACTTTAAATTGTACTGCACCCAACAGCACTAGCTCTAACAACAGTAGC---------
----
----------------------ATTAACAGCAACAGAAACGATAGTATGTTTGAAGAAATGAGA---
AACTGCTCTTTCAACATGACCACAGAAATAAGA--------------------------
GATAAAAGGAAAACTGTACATTCACTT---TTTTATAAACTTGATATAGTATCAATTGGTAGT---------
----

AATGATAGTAATGATAGTGGCAGTATAGACTAATAAATTGCAATACATCAGCCATGACACAG---
GCCTGTCCTAAGGTAACTTTTGAGCCAATTCCTATACATTATTGTGCCCCAGCTGGTTTTGCGATTCTAAAGT
GCATA---GATACAACTTTTAAT------GGAACAGGGGCATGCAGGAAT----------------------
-GTCAGC---ACAGTACAATGCACACATGGAATCAGGCCAGTAGTATCAACT---
CAACTGCTGTTAAATGGCAGTCTGGCAGAAAAAGAG-----------
GTAATGATTAGATCTAAAAATATCACAGACAAT---------AGCAAAATCATAATAGTACAGCTTACT----
-----------GAGCCTGTAAAC---------ATCACTTGTATCAGACCTGGCACCAATACAAGAACA-----
-AGTATACGTATA------------GGACCAGGACAAACC---TTC------TATGGAACAGGTGAT------
---------GTAATAGGGGATATA------------AGAAAAGCACATTGT---AATGTCAACAGAACA---
GCTTGGAATAGCACTTTACAAAAAATAAGTATACAATTAAGA---------------AAATACTTTGGT----
-----------------------------------AACAAAACAATAATCTTTAAG------
AACTCCACAGGAGGGGATTTAGAAATTACA---ACACATAGTTTCAATTGTGGAGGAGAA---
TTTAAACAGCTCCAGGCAAGAGTCCTGGCTGTGGAAAGATACCTAAGGGATCAGCAGCTCCTAGGAATTTGGG
GTTGCTCTGGAAAACTCATCTGCACCACTAATGTGCCCTGGAACACTAGTTGGAGT----------------
-----------------------------AACAAATCTCTGGAGGACATATGG------AAT------
AACATGACCTGGATGCAATGG---GACAGGGAAGTCATCAATTACACAAACACAATATAT---
GAACTAATTGAAAAATCGCAAAACCAGCAGGAA---------------------------------------
----
AAGAATGAACAAGAATTATTGGCCTTGGATAAGTGGGCAAGTCTGTGGAGTTGGTTTGACATATCAAATTGGT
TATGGTATATAAAAATATTTATAATAATAGTAGGAGGCTTAATAGGATTAAGAATAGTTTTTGCT---
GTACTTTCTATAATA---------AATAGAGTCAGGCAGGGATACTCACCC------
TTGTCATTGGCTATCAATTTAATAGATACCTTAGCAATAGCCGTAGGTAGG---
TGGACAGATAGGATTATAGAAATAGGACAAAGACTTTGTAGAGCC--------------------------
--------ATTCGTAACATA------CTTAGGAGAATCAGACAGGGCGCAGAAAAGGCTTTGCAA---TAA
>JF683798
GCT---GCA---
GAAAACTTGTGGGTCACGGTATACTATGGGGTACCTGTGTGG---AGAGATGCAGAT---
ACCACCCTATTTTGTGCATCAGATGCTAAAGCATATGAGAAAGAA---GCACACAAT---------------
--------------ATCTGGGCTACA---CATGCCTGTGTACCCACAGAC---
CCTGACCCAAACGAAATAATGTTGGAA---------AATGTGACAGAAAAATTTGAC---------------
--------------------------ATGTGGAAAAATAACATGGTAGAACAAATGCAAACAGATATA---
ATCAGTCTCTGGGACCAAAGCCTA---AAGCCATGTGTAAAGTTA---
ACCCCTCTCTGCGTTACTTTAAATTGTACTGACCCCATCAGCAATAACAACAAT------------------
----
-------------------------------ACACTTAACAACAGTAACGATGGAGAAATGACA---
AACTGCTCTTTCAATGCGACCACAGAACTAAGA--------------------------
GATAAAACGAAAACTGTATATTCACTT---TTTTATAAGCTTGATATAGTATCAACTAATAGT---------
----

AGTGATAATAGGACATACAGACTAATAAATTGTAACACATCAACCACGACACAG---
GCCTGTCCTAAGGTATCCTTTGAGCCAATTCCTATACATTATTGTGCCCCAGCTGGTTTTGCGATTCTTAAGT
GCAAC---GAAAAAAGTTTTAAT------GGAACAGGGCCATGCAAGAAT----------------------
-GTCAGC---ACAGTACAATGCACACATGGAATCAAGCCAGTAGTATCAACT---
CAACTGCTGTTAAATGGCAGTCTAGCAGAGAAAGAG-----------
GTAATGATTAGATCTGAAAATATCACAGACAAT---------GGCAAAATCATAATAGTACAGCTTACT----
-----------GAGCCTGTAAAC---------ATCACTTGTATCAGACCTAACAACAATACAAGAACA-----
-AGTATACGTATA------------GGACCAGGACAAACT---TTC------TATGGGACA---GAT------
---------GTAATAGGGGACATA------------AGAAAAGCACATTGT---AATGTCAACAGAACA---
GCATGGAATCGCACTTTACAACAGATAAGTAAAAAATTGAGA---------------GGATACTTTAAT----
-----------------------------------AACAAAACAATAATCTTTAAG------

AACTCCTCAGGAGGGGATTTAGAAGTTACA---ACACATAGTTTCAATTGTGGAGGAGAG---
TTTAAACAGCTCCAGGCAAGAGTCCTGGCTGTGGGACAATACCTAAAGGATCAGCAGCTCCTAGGAATTTGGG
GTTGCTCTGGAAGACTCATCTGCACCACTAATGTGCCCCGGAACTCTAGTTGGAGT-----------------
----------------------------AATAAATCTCAGAATGAAATATGG------GAT------
AACCTGACCTGGATGCAATGG---GACAAGGAAGTTATCAATTACACAAACATAATATAT---
GAGCTACTTGAAGAATCGCAAAACCAGCAGGAA--

AAGAATGAACAAGATTTATTGGCATTGGATAAGTGGGCAAGTCTGTGGAGTTGGTTTGACATATCAAATTGGT
TATGGTATATAAAAATATTTATAATAATAGTAGGAGGTTTAATAGGATTAAGAATAGTTTTTGCT---
GTGCTTTCTATAATA---------AATAGAGTCAGGCAGGGATACTCACCC------
TTGTCATTGGCTATTAATCTGATAGATACCATAGCAATAGTAGTAGCTGGG---
TGGACAGATAGGGTTATAGAAATAGGACAAAGACTTGGTAGAGCT----------------------------
--------ATTTGTAACATA------CCTAGGAGAATCAGACAGGGCGCAGAAAGGGCTTTGCAA---TAA
>EF545108
GCT---ACA--
GGAAACCTGTGGGTCACGGTATACTATGGGGTACCCGTGTGG---AGAGATGCAGAG---
ACCACCCTATTTTGTGCATCAGATGCTAAAGCATATGATCCAGAA---GTACATAAT----------------
--------------GTCTGGGCTACA---CATGCCTGTGTACCCACAGAC---
CCTGACCCACAAGAAATAACTTTGGAC---------AATGTGACAGAAGAATTTAAT----------------
--------------------------ATGTGGAAAAATAACATGGTAGAACAGATGCAAACAGATATA---
ATTAGTCTATGGACCAAAGCCTA---CAGCCATGTGTAAAGTTA---
ACCCCTCTCTGCGTTACTTTAAATTGTCAAGAGATCAGCAATGTCACTAACTATAACAGT-------------
----
----------------------------AGCATGTCTAACAGCAGCAGGTATGGAGAAATGAAA---
AACTGCTCTTTCAATATGACCACAGAATTAAGA---------------------------
GATAGGAAAAGAAAGGTACATTCACTT---TTTTATAAACTTGATATAGTATCAACTAGTAGT----------
----

AATGAGAGTGGACCGTATAGACTAATAAATTGTAATACATCAGCCATGACACAG---
GCCTGTCCTAAGGTGGTCTTTGAGCCAATTCCTATACATTATTGTGCCCCAGCTGGTTTTGCGATTCTAAAGT
GCAAC---CAAAAAGATTTTAAT------GGAACAGGGCCATGCAGGAAT-----------------------
-GTCAGC---ACAGTACAATGCACACATGGAATCAAGCCAGTAGTATCAACT---
CAACTGCTGTTAAATGGCAGTCTAGCAGAGGGAAAT------------
GTAACGATTAGATCTGAAAATATCACAGACAAT---------ACCAAAACCATAATAGTACAGCTTAGT----
-----------AAGCCTGTAAAC---------ATTACTTGTATCAGACCTGGCAACAATACAAGAACA-----
-AGTATACGTATA------------GGACCAGGACAAACA---TTC------TATGCAACAGGTGAT------
---------ATAATAGGGGACACA------------AGAAAAGCACATTGT---AATGTCAGTAGAACA---
GAATGGGGAAACGCTACAGAAGAGATACGTAAACAATTACAA---------------ACTTACTTTCCA----
-----------------------------------AACAAAACAATAAGCTTTAAG------
AACTCCTCAGGAGGAGATTTAGAAATTACA---ACACATAGTTTTAATTGTGGAGGAGAA---
TTTAAACAGCTCCAGGCAAGAGTCCTGGCTGTGGAAAGATACCTAAAGGATCAACAGCTCCTAGGAATTTGGG
GTTGCTCTGGAAAACTCATATGCACCACTAATGTACCCTGGAACTCTAGTTGGAGT-----------------
----------------------------AATAAATCTCAGAGTGATATATGG------GAT------
AATCTGACCTGGATGCAATGG---GACAGAGAAATTCACAATTATACAGACATAATATAT---
GATCTAATTGAAAAATCGCAAAACCAGCAGGAA--

AAGAATGAGCAAGATTTATTGGCATTGGATAAGTGGGCAAGTCTGTGGAATTGGTTTGACATATCAAATTGGC
TATGGTATATAAAACTATTTATAATGATAGTAGGAGGCTTAATAGGATTAAGAATAGTTTTTGCT---
GTGCTTTCTGTAATA---------AATAGAGTCAGGCAGGGATACTCACCC------
TTGTCATTKGCTATTAATTTGGTAGATACCATAGCAATAGCAGTAGCTGGG---
TGGACAGATAGGGTTATAGAAATAGGACAAAGAGTTTGTAGAGCT----------------------------
--------ATYYTTCACATA------CCTAKAAGAATCAGACAGGGCTTCGAAAAGGCTTTGCAA---TAA
>GU481566
GCT---GCA--
GAAAACTTGTGGGTCACAGTATACTATGGGGTACCTGTGTGG---AGAGATGCAGAG---
ACCACCCTATTTTGTGCATCAGATGCTAAGGCATATGATAAAGAA---GTACACAAT----------------
--------------GTCTGGGCTACA---CATGCCTGCGTACCCACAGAC---
CCTAACCCACAAGAAATAGCTTTGGAA---------AATATGACAGAAAATTTTAAC----------------
--------------------------ATGTGGAAAAATAACATGGTAGAACAAATGCAAACAGATATA---
ATCAGTCTCTGGACCAAAGCCTA---AAGCCATGTGTACAGTTA---

```
ACCCCTCTCTGCGTTACTTTAAACTGTACTGAACCCACCAGCACTAACAGCACCAACAGCACTAGCGGTAACA
GCACTAGC-----------------------------------------------------------------
-------CTTAACAGCACTAGCGGTTACAGCAACAGCAACGATAGTTTGCTTAAAGAAATGAAA---
AACTGCACTTTCAACATGACCACAGAACTAAGA---------------------------
GATAAAAAGAAAAAAGTACATTCACTT---TTTTATAAACTTGATATAGTGCCAACTGATAAT----------
-------------------------------------------------------------------------
----------------
AATGGTAGTAGTGGGGAGTATAGACTAATAAATTGCAATACATCAACCATAACACAG---
GCCTGTCCTAAGGTAACCTTTGAGCCAATTCCTATACATTATTGTGCCCCAGCTGGTTTTGCGATTCTAAAGT
GCAAA---GATACAGAATTTAAT------GGAACAGGGCCATGCAAGAAT-----------------------
-GTCAGC---ACAGTACAATGCACACATGGAATCAAGCCAGTAGTATCAACT---
CAACTACTGTTAAATGGCAGTCTAGCAGAGGGAAAG------------
GTAATAATTAGAGCTGAAAACATCACAGACAAT---------AGCAAAACCATAATAGTACAGCTTACT----
-----------AAACCTGTAAAC---------ATCACTTGTATCAGACCTGGCAACAATACAAGAACA-----
-GGTATACATATA------------GGACCAGGACAAACC---TTC------TTTGCAACAGGTGCT------
---------GTAATAGGGGACATA------------AGAAAAGCACATTGT---AATGTCAGCAGATCA---
GCATGGAATAAAACTTTACAAGAGATAAGTATACAATTAAGA---------------AAATACTTTGAT----
-----------------------------------AACAAAACAATAATCTTTAAT------
AGCTCCACAGGAGGGGATTTAGAAGTTACA---ACACATAGTTTCAATTGTGGAGGAGAA---
TTTAAACAGCTCCAGGCAAGAGTCCTGGCTGTGGAAAGATACCTAAAGGATCAGCAGCTCCTAGGAATTTGGG
GTTGCTCTGGAAAACTCATCTGCACCACTGCTGTGCCCTGGAACTCTAGTTGGAGT-----------------
----------------------------AACAAATCTCTGGGTGAGATATGG------GAT------
AACATGACCTGGATGCAATGG---GACAAGGAAGTTATCAATTACACAAACATAATATAT---
GATCTAATTGAAGAAGCGCAAAACCAGCAGGAA----------------------------------------
-----------------------------
AAGAATGAACAAGATTTATTGGCATTGGATAAGTGGGCAAGTCTGTGGAATTGGTTTGGCATATCAAAGTGGT
TATGGTATATAAAAATATTTATAATAATAGTAGGAGGCTTAATAGGATTAAGAATAGTTTTTGCT---
GTGCTTTCTATAATA---------AATAGAGTCAGGCAGGATACTCACCC------
TTGTCATTGGCTATCAATCTAATAAACACCATAGCAATAGCAGTAGCTGGG---
TGGACAGATAGGGTTATAGAAATAGGACAAAGACTTTGTAGAGCC----------------------------
--------ATTTGTAACATA------CCTAGGAGAATCAGACAGGGCGCAGAAAAGGCTTTGCAA---TAA
>GU481615
GCT---GCA------------------------------------------
GGAAACTTGTGGGTCACGGTATACTATGGGGTACCTGTGTGG---AGAGATGCAGAG---
ACCACCCTATTTTGTGCATCAGATGCTAAAGCTTATGATAAAGAA---GTACACAAT----------------
--------------GTCTGGGCTACA---CATGCCTGTGTACCCACAGAT---
CCTGACCCACAAGAAATAATTTTGGAA---------AATGTGACAGAAAATTATGAC----------------
--------------------------ATGTGGAAAAATAACATGGTAGAACAAATGCAAACCGATATA---
ATCAGTCTCTGGGACCAAAGCCTA---AAGCCATGTGTAGAGTTA---
ACCCCTCTCTGCGTTACTTTAAATTGTACTGCACCCAACAGCAATAGCTCTAGCAAC----------------
-------------------------------------------------------------------------
----------------------------AGTAACAGCAGCGATAACATATATGAAGAAATGAAA---
AACTGCTCTTACAACATGACCACAGAACTAAGA---------------------------
GATAAAAGAAAAATTGTACATTCACTT---TTTTATAAACTTGATATAGTATCAATTGATAGTAATAGTAGT-
-------------------------------------------------------------------------
-------------------
GAACAGTATAGACAGTATAGACTAATAAATTGTAATATGTCAACCATGACACAG---
GCCTGTCCTAAGGTAACCTTTGAGCCAATTCCTATATATTATTGTACCCCAGCTGGTTTTGCGATTCTAAAGT
GCAAG---GATCCAAATTTTAAT------GGAACAGGGCCATGCAAGAAT-----------------------
-GTCAGC---ACAGTACAATGCACACATGGAATCAAGCCAGTAGTATCAACT---
CAACTGCTGTTAAATGGCAGTCTAGCAGAGAAAAAG------------
GTAATGATTAGATCTCAAAATATCACAGACAAT---------ACCAAAATCATAATAGTACAGCTTACT----
-----------GAGCCTGTAAAC---------ATCACTTGTATCAGACCTAGTAGCAAGACAAGAGCA-----
-AGTATACGTATA------------GGACCAGGACGAACC---TTC------TATGCAATGGATGGG------
---------GTAGGAGGGAACCCA------------AGAAAAGCATATTGT---GAGGTCAACAAAACA---
GCATGGAATAGCGCTTTACAAAACATAAGTACACAATTAAGA---------------GAATACTTT-------
-----------------------------------AACAAAACAATAATCTTTAAG------
AACTCCTCAGGAGGGGATTTAGAAGTTACA---ACACATAGTTTCAATTGTGGAGGAGAG---
TTTAAACAGCTCCAGGCAAGAGTCCTGGCTGTGGAAAGATACCTAAGGGACCAGCAGCTCCTAGGAATTTGGG
GTTGCTCTGGAAAACTCATCTGCACCACTAATGTGCCCTGGAACTCTAGTTGGAGT-----------------
```

----------------------------AATAAATCTGAGATTGAGATATGG------GGA------
AACATGACCTGGATGCAATGG---GACAAGGAAGTGTTCAATTACACAAACATAATATAT---
GATTTGCTTGAAAAATCGCAAAACCAGCAGGAA--

AAGAATGAACAAGATTTATTGGCATTGGATAAATGGGGAAGCCTGTGGAGTTGGTTTGACATATCAAATTGGT
TATGGTACATAAAAATATTTATAATAATAGTAGGAGGCTTAATAGGATTAAGAATAGTTTTTGCT---
GTGCTTTCTGTAATA---------AATAGAGCCAGGCAGGGATACTCACCT------
TTGTCATTGGCTATTAATTTGATAGATACCATAGCAGTAGCAGTAGCTGGG---
TGGACAGATAGGGTTATAGAAATAGGACAAAGAGCTTGTAGAGCT----------------------------
--------ATTTGTAACATA------CCTAGGAGAATCAGACAGGGCGCAGAAAAGGCTTTGCAA---TAA
>HQ896489
GCT---ACA--
GAAGACTTGTGGGTCACGGTATACTATGGGGTACCTGTGTGG---AGAGATGCAGAT---
ACCACCCTATTTTGTGCATCAGATGCTAAAGCATATGATAAGGAA---GTACACAAT----------------
--------------GTCTGGGCTACA---CATGCCTGTGTACCCACAGAC---
CCTGACCCACGAGAAATAACTTTGGAA---------AATGTGACAGAAAATTTTAAC----------------
--------------------------ATGTGGAAAAATAACATGGTAGAACAAATGCAAGAAGATATA---
ATCAGTCTCTGGGACCAAAGCCTA---AAGCCATGTGTAAAGTTA---
ACCCCTCTATGCGTTACTTTAAATTGTACTGAACCCAAAACCAACGGAACTAACTCTAACGACAGT-------
----
----------------------AGTGGTAACAACAGCAGCGGTAGGTTGGTTGAAGAAATGAGA---
AACTGCTCTTTCAAAATGACCACAGAACTAAGA---------------------------
GATAAACAGAAAACTGTATATTCACTT---TTTTATAAACTTGATATAGTATCAACTGATAAT----------
----

AGTAATAATGATAGTGGGCAGTATAGATTAATAAATTGCAATACGTCAGCCGTGACACAA---
GCCTGTCCTAAGGTAACCTTTGAGCCAATTCCTATACATTATTGTGCCCCAGCTGGTTTTGCGATTCTAAAGT
GCAAG---GATACAAATTTTACT------GGAACAGGGCCATGCAAGAAT-----------------------
-GTCAGC---ACAGTACAATGCACACATGGAATCAAGCCAGTAGTATCAACT---
CAACTGCTGTTAAATGGCAGCCTAGCAGAGAAAGAT------------
GTAGTGATTAGGTCTGAAAATATCACAGACAAT---------AGCAAAATCATAATAGTACAGCTTAAT----
-----------GAGCCTGTAAAC---------ATCAATTGTACCAGACCTGGCAACAATACAAGAACA-----
-AGTATACGTATA------------GGACCAGGACAAACC---TTC------TATGCAACAGGTGAT------
---------GTAATAGGGGACCCA------------AGAAAAGCACATTGT---AATGTCAGCAGAGCA---
GCATGGAATAGCACTTTACAAAAGATAAGTATACAATTAGGA---------------AAATACTTTAAT----
-----------------------------------AACAAAACAATAATCTTTAAG------
AAATCGTCAGGAGGGGATTTAGAAGTTACA---ACACATAGTTTCAATTGTGGAGGAGAA---
TTTAAGCAGCTCCAGGCAAGAGTCCTGGCTGTGGAAAGATACCTAAAGGATCAGCAGCTCCTAGGAATTTGGG
GTTGCTCTGGAAAACTCATCTGCACCACTAATGTGCCCTGGAACTCTAGTTGGAGT-----------------
----------------------------AATAAATCTCAAAATGAGATATGG------GAT------
AACATGACCTGGATGCAATGG---GACAAGGAAGTTATCAATTACACAAACATAATATAT---
GATCTAATTGAAAAATCGCAAAACCAGCAGGAA--

AAGAATGAACAAGATTTATTGGCATTGGATAAGTGGGCAAGTCTGTGGAGTTGGTTTAACATATCAAATTGGT
TATGGTATATAAGAATATTTATAATAATAGTAGGAGGTTTAATAGGATTAAGAATAGTTTTTGCT---
GTGCTTTCTATAATA---------AATAGAGTCAGGCAGGGATACTCACCC------
TTGTCATTGGCTATCAATCTAGTAGATACCATAGCAATAGCAGTAGCTGGG---
TGGACAGATAGGTTTATAGAGATAGTACAAAGACTTTGTAGAGCC----------------------------
--------ATTTGTAACATA------CCTAGGAGAATCAGACAGGGTGCAGAAAGGGCTTTGCAA---TAA
>JQ292897
GCT---GCA--
GAAAACTTGTGGGTCACGGTATACTATGGGGTACCTGTGTGG---AGAGATGCAGAG---
ACCACCCTATTCTGTGCATCGGATGCTAAAGCATATGATAAAGAA---GTACACAAT----------------
--------------GTCTGGGCCACA---CATGCCTGTGTACCCACAGAC---
CCTAACCCGCAAGAAATAATTTTGGAA---------AATGTGACAGAAGAATTTAAC----------------
--------------------------ATGTGGAAAAATAACATGGTAGAACAAATGCAAACAGATATA---
ATCAGTCTCTGGGACCAAAGCCTA---AAGCCATGTGTACAGTTA---
ACCCCTCTCTGCGTTACTTTAAATTGTACTGAACCCATCAGCACTAACTCTAACAACAGTATCTTTAACTCTA
ACAACAGTATCTTTAAC--
-------------------AACAGTAGCGTTAACAGCAATAATAGCATGGTAGGAGAAATAAAA---

AACTGCTCTTTCAACATGACCACAGAACTAAGA---------------------------
GATAAAAGGAAAACTGTACATTCACTT---TTTTATAAACTTGATATAGTACCAATTGATAATGAT-------
----

AATAATAATAGTAGTCAGTATAGACTAATAAATTGTAATACATCAACCGTGACACAG---
GCCTGTCCTAAGGTAAACTTTGAGCCAATTCCTATACATTATTGTACCCCAGCTGGTTTTGCGATTCTAAAGT
GCAGG---GATCCAAACTTTACG------GGAAAAGGGCCATGCAAGAAT-----------------------
-GTCAGC---ACAGTACAATGCACACATGGAATCAGGCCAGTAGTATCAACT---
CAACTGCTGTTAATGGCAGTCTAGCAGAAGAAGAA-------------
GTAATGATTAGATCTGAAAATATTACAGACAAT---------GGCAAGACCATAATAGTACAGCTTACT----
-----------GAGCCTGTAAAC---------ATCACTTGTATCAGACCTGGCAACAATACAAGAACA-----
-AGTATACGTATA------------GGACCAGGACAAACC---TTC------TATGCAACAGGTGAT------
---------GTAATAGGGGACCCA------------AGAAAAGCACACTGT---GAAGTCAACAAAACA---
GCATGGAATAACACTTTACAAAAGATAAGTATGCAATTAAGA---------------AAATACTTTAAT----
-----------------------------------AACAAAACAATAATCTTTAAG------
AAATCCTCAGGAGGGGATTTAGAAGTTACA---ACACATAGTTTCAATTGTGGAGGAGAA---
TTTAAACAGCTCCAGGCAAGAGTCCTGGCTGTGGAAAGATACCTAAAGGATCAGCAGCTCCTAGGAATTTGGG
GTTGCTCTGGAAAACTCATCTGCACCACTACTGTGCCCTGGAACTCTAGTTGGAGT-----------------
----------------------------AATAAATCTCAGGATGAGATATGG------GAG------
AACATGACCTGGATGCAATGG---GACAAGGAAATTATCAATTACACAAACAAAATATAT---
GATCTAATTGAAAAATCGCAAAACCAGCAGGAA--

AAGAATGAACAAGATTTATTAGCATTGGATAAGTGGGAAGGTCTGTGGAGTTGGTTTAGCATATCAAATTGGT
TATGGTATATAAGAATATTTATAATAATAGTAGGAGGCTTAATAGGATTAAGAATAGTTTTTGCT---
GTGCTTTCTATAATA---------AATAGAGCCAGGCAGGGATACTCACCC------
TTGTCATTGGCTATTAATCTGGTAGATACCATAGCAATAGTAGTAGCTGGG---
TGGACAGATAGGGTTTTGGAAATAGGACAAAGATTTTGTAGAGCT----------------------------
--------ATTTGTAACATA------CCTAGGAGAATCAGACAGGGCGCAGAAAAGGCTTTGCAA---TAA
>JQ292899
GCT---GCA--
GAAAACTTGTGGGTCACGGTATACTATGGGGTACCTGTGTGG---AGAGATGCAGAR---
ACCACYCTATTTTGTGCATCAGATGCTAARGCATATGATAAAGAA---GTACACAAT----------------
--------------GTCTGGGCTACA---CATGCCTGTGTACCCACAGAC---
CCTAACCCGCAAGAAATACCTTTGGAA---------AATGTGACAGAAGAATTTGAC----------------
--------------------------ATGTGGAAAAATAACATGGTAGAACAAATGCAAACAGATATA---
ATCAGTCTCTGGGACCAAAGCCTA---ARGCCATGTGTAAAGTTA---
ACCCCTCTCTGTGTTACTTTAAATTGTGCTGAACTCAACAGCAATACCTCTAACAGT----------------
----
--GAAGAAATGAAA---
AACTGCTCTTTTAACACGAGCACAGAACTAAGA---------------------------
GATAAAAGGAAAACTGCATATTCACTT---TTTTATAAACTTGATGTAGTATCAATTGATAGT----------
----

AATGGTAGTAATGGTAGTGGGCAGTATAGACTAATAAATTGTAATACATCAACCATGACACAG---
GCCTGTCCTAAGGTAACCTTTGAGCCAATTCCTATATATTATTGTACCCCAGCTGGTTTTGCGATTCTAAAGT
GCAAG---GATACAAACTTTACT------GGAACAGGGCCATGCCAGAAT-----------------------
-GTCAGC---ACAGTACAATGCACACATGGAATCAAGCCAGTAGTATCAACT---
CAACTGCTGTTAATGGCAGTCTAGCAGAAAAAGAG-------------
GTAATGATTAGATCTAAAAATATTACAGACAAT---------AGCAAAATCATAATAGTACAGCTTACT----
-----------GAGCCTGTAGAC---------ATCACTTGTATCAGACCTGGCAACAATACAAGAACA-----
-AGTGTACGTATA------------GGACCAGGACAAACC---TTC------TATGCAACAGGT---------
---------GTAATAGGGGACATA------------AGAAAAGCATATTGT---AATGTCAGCAGAGCA---
GCATGGAATAACACTTTACAAAAGATAAGTATACAATTAAGA---------------AAATACTTTAAT----
-----------------------------------AACAAAACAATAATCTTTAAG------
AACTCCTCAGGAGGGGATTTAGAAGTTACA---ACACATAGTTTCAATTGTGGAGGAGAA---
TTTAAACAGCTCCAGGCAAGAGTCCTGGCTGTGGAAAGCTACCTAAAGGATCAGCAGCTCCTAGGACTTTGGG
GTTGCTCTGGAAAACTCATCTGCACCACTAATGTGCCCTGGAACTCTAGTTGGAGT-----------------
----------------------------AATAAATCTCACAATGAGATATGG------GAT------
AACATGACCTGGATGCAATGG---GACAAGGAAATAATCAATTACACAGAAAAAATATAT---
AATCTAATTGAAAAATCGCAAAACCAGCAGGAA--

AAGAATGAACAAGATTTATTAGCATTGGATAAGTGGGCAGGTCTGTGGAATTGGTTTAGCATATCAAAGTGGT
TATGGTATATAAAAATATTTATAATAATAGTAGGAGGCTTGATAGGCTTAAGAATAGTTTTTGCT---
GTGCTTTCTATAATA---------AATAGAGCCAGGCAGGATACTCACCC------
TTGTCATTGGCTATTAATCAGATAGATAACATAGCAATAGCAGTAGCTGGG---
TGGACAGATAGGGTTTTAGAAATAGGACAAAGATTTTGTAGAGCT----------------------------
--------ATTYGTAACATA------CCTAGGAGAATCAGACAGGGCGCAGAAAAGGCTTTGCAA---TAA
>HQ385841
GCT---GCA--
GAAAAACTGTGGGTCACGGTATACTATGGGGTACCTGTGTGG---AGAGATGCAGAG---
ACCACCCTATTTTGTGCCTCAGATGCTAAAGCATATGATAAAGAA---GTACACAAT----------------
--------------GTCTGGGCTACA---CATGCCTGTGTACCCACAGAC---
CCTAACCCACAAGAAATAATTTTGGAA---------AATGTGACAGAAGACTTTAAC----------------
--------------------------ATGTGGAAAAATAACATGGTAGAACAAATGCAAACAGATATA---
ATCAGTCTCTGGACCAAAGCCTA---AAGCCATGTGTACAGTTA---
ACCCCTCTCTGCGTTACTTTAAATTGTACTAAACCCACCAGCAACAGCACTAGCTCTAACAGCAGTAGCACTA
GCTCTAACAGCAGTAGCACTAGCTCTAACAGCAGT--------------------------------------
-------AGCACTAGCTCTAACAACAGTAGCGTTGACAGAGATAAATTGCTTGAAGAAATGAAA---
AACTGCTCTTTCAACATGACCACAGAACTAAGA---------------------------
GATAAAAAGAAAAATGTACATTCACTT---TTTTATAAACTTGATATAGTATCAATTGATAGTAATAGTAGT-
----

GGGCAGTATAGTAATAGTAGTGGGCTGTATAGACTAATAAATTGTAATACATCAACCGTGACACAG---
GCCTGTCCTAAGGTAACCTTTGAGCCAATTCCTATACATTATTGTACCCCAGCTGGTTTTGCGATTCTAAAGT
GCAGG---GATCCAAAATTTAAT------GGAACAGGGCCGTGCAAGAAT-----------------------
-GTCAGC---ACAGTACAATGCACACATGGAATCAAGCCAGTAGTATCAACT---
CAACTGCTGTTAAATGGCAGTCTAGCAAAAGACGAG------------
GTAATGATTAGATCTGAAAATATAACAGACAAT---------GGCAAAATCATAATAGTACAGCTTAAT----
-----------GAGCCTGTAAAC---------ATCACTTGTATGAGACCTGGCAACAATACCAGAACA-----
-AGTATACGTATA------------GGACCAGGACAAACC---TTC------TATGCAACAGGTGAG------
---------GTAATAGGGGACCCA------------AGAAAAGCACATTGT---AATGTCAGCAGCGCA---
GCATGGAATAGCACTTTACAAAAGATAAGTGAAAAATTAAGA---------------AGATACTTTGAT----
-----------------------------------AACAAAACAATAATCTTTAAT------
AGCTCCTCAGGAGGGGATTTAGAAGTTACA---ACACATAGTTTCAATTGTGGAGGAGAA---
TTTAAACAGCTCCAGGCAAGAGTCCTGGCTGTGGAAAGATACCTAAAGGATCAGCAGCTCCTAGGAATTTGGG
GGTGCTCTGGAAAACTCATCTGCACCACTGCTGTGCCCTGGAACTCTAGTTGGAGT-----------------
----------------------------AATAAAACTTATAATGATATATGG------GAT------
AACATGACCTGGATGCAATGG---GACAAGGAAATTATCAATTACACAAACAAAATATAT---
GATCTAATTGAAAAATCGCAAAACCAGCAGGAA--

AAGAATGAACAAGATTTATTAGCATTGGATAAGTGGACAGGTCTGTGGAGTTGGTTTGACATATCAAATTGGT
TATGGTATATAAGAATATTTATAATGATAGTAGGAGGATTAATAGGCCTAAGAATAGTTTTTGCT---
GTGCTTTCTATAATA---------AATAGAGCCAGGCAGGATACTCACCC------
TTGTCATTGGCTATTAATCTGGTAGATACTATAGCAATAGCAGTAGCTGGG---
TGGACAGATAGGGTTTTAGAAATAGGACAAAGAGTTTGTAGAGCT----------------------------
--------ATTCGCAACATA------CCTAGGAGAATCAGACAGGGCGCAGAAAAGGCTTTGCAA---TAA
>HQ385843
GCT---ACA--
GAAAACTTGTGGGTCACGGTATACTATGGGGTACCTGTGTGG---AGAGACGCAGAC---
ACCACCCTATTTTGTGCATCAGATGCTAAAGCATATGATAAAGAA---GCACACAAT----------------
--------------GTCTGGGCTACA---CATGCTTGTGTACCCACAGAC---
CCTAATCCACAAGAAATAGATTTGGAA---------AATGTGACAGAAGAATTTAAC----------------
--------------------------ATGTGGAAAAATAACATGGTAGAACAAATGCAAACAGATATA---
ATCAGTCTCTGGACCAAAGCCTA---AAGCCATGTGTACAGTTA---
ACCCCTCTCTGCGTTACGTTAACTTGTACTGCACCCAACAGCACTAGCTCTAACAGCAGTGCATCCAAC----
----
-------------------AGCACTAGCTCTAACAACAGTAGTGTTAACAGCGAAATAATGAAA---
AACTGCTCTTTCAACATGACCACAGAACTAAGA---------------------------
GATAAAAGGAAAAAGGTATATTCACTT---TTTTATAAACTTGATATAGTATCAATTGATAGT----------
----

AATAGTAGCCAGTATAGACTAATAAATTGTAATACATCAACTGTGACACAG---
GCCTGTCCTAAGACAACCTTTGAGCCAATTCCTATACATTATTGTACCCCAGCTGGTTTTGCGATTCTAAAGT
GCAAG---GATACCAACTTTACA------GGAAAAGGGCCATGCAAGAAT----------------------
-GTCAGC---ACAGTACAATGCACACATGGAATTAAACCAGTAGTATCAACT---
CAACTGCTGTTAAATGGCAGTCTAGCAGAAGAAGAG-----------
GTAATGATTAGATCTGAAAATATTACAGACAAT---------AGCAAAACCATAATAGTACAGCTTAAT----
-----------AAGCCTGTAAAC---------ATCACTTGTATCAGACCTGGCAACAATACAAGAACA-----
-AGCATACATATA------------GGACCAGGACGAACC---TTC------TTTGCAACAGGTGAT------
---------GTAGTAGGGGACATA------------AGAAAAGCACATTGT---AATGTCAGCAGAACA---
GCATGGAATAACACTTTACAAAAGATAAGTGAACAATTAAGA--------------AAATACTTTAAA----
-----------------------------------AACAAAACAATAATTTTTAAG------
AACTCCTCAGGAGGGGATTTAGAAGTTACA---ACACATAGTTTCAATTGTGGAGGAGAA---
TTTAAACAGCTCCAGGCAAGAGTCCTGGCTGTGGAAAGATACCTAAAGGATCAGCAGCTCCTAGGAATTTGGG
GTTGCTCTGGAAAACTCATCTGCACCACTAATGTGCCCTGGAACTCTAGTTGGAGT----------------
----------------------------AATAAATCTCTGAATGACATATGG------GAT------
AACATGACCTGGATGCAATGG---GACAAGGAAATTATTAATTACACAAACAAAATATAT---
GATCTAATTGAAAAATCGCAAAACCAGCAGGAA---------------------------------------
----
AAGAATGAACAAGATTTATTAGCATTGGATAAGTGGGCAAGTCTGTGGAATTGGTTCAACATATCAAATTGGT
TATGGTATATAAAAATATTTATAATAATAGTAGGAGGCTTAATAGGATTAAGAATAGTTTTTGCT---
GTGCTTTCTATAATA---------AATAGAGTCAGGCAGGGATACTCACCC------
TTGTCATTGGCTATTAACCTGATAGATACCATAGCAATAGTAGTAGCTGGG---
TGGACAGATAGGGTTTTAGAAATAGGACAAAGATTTTGTAGAGCT---------------------------
--------ATTTGTAACATA------CCTAGGAGAATTAGACAGGGCGCAGAAAGGGCTTTGCAA---TAA
>HQ385846
GCT---GCA---
GAAAACTTGTGGGTCACGGTATACTATGGGGTACCTGTGTGG---AGAGATGCAGAG---
ACCACCCTATTTTGTGCATCAGATGCTAAAGCATATGATAAAGAA---GTACACAAT----------------
--------------GTCTGGGCTACA---CATGCCTGTGTACCCACAGAC---
CCTGACCCACAAGAAATAGTTTTGGAA---------AATGTAACAGAAAATTTTGAT---------------
--------------------------ATGTGGAAAAATAACATGGTAGAACAAATGCAAACAGATATA---
ATCAGTCTCTGGGATCAAAGCCTA---AAACCATGTGTACAGTTA---
ACCCCTCTCTGCGTTACTTTAAATTGTACTAAGCCCAACAGCACTAGCTCTAACAACAGTAGCGTTAACAGCA
CTAGC---
-------TCTAACAACAGTAGCGTTAACAGCAATAGCAGCAATAGTGTGTATGAAGAAATGAAA---
AACTGCACTTACAACATGACCACAGAACTAAGA--------------------------
GATAAAAGGAAAACTGTACATTCACTT---TTTTATAAACTTGATATAGTATCAACTGATAAT----------
----

AATAGTAGTGGGCAGTATAGACTAATAAATTGCAATACATCAACCATGACACAG---
GCCTGTCCTAAGGTAACCTTTGAGCCAATTCCTATACATTATTGTGCCCCAGCTGGTTTTGCGATTCTAAAGT
GCAAG---GATCCAAATTTTAAT------GGAACAGGGCCATGCAAGAAT----------------------
-GTCAGC---ACAGTACAATGCACACATGGAATCAAGCCAGTAGTATCAACT---
CAATTGCTGTTAAATGGCAGTCTAGCAGAGGAAGAG-----------
GTAATGATTAGATCTGCAAATATCACAGACAAT---------AGCAAAACCATAATAGTACAACTCAAA----
-----------GAGCCTGTAAAA---------ATCACTTGTATCAGACCTAGCAGCAAGATAAGAACA-----
-AGTATACGTATA------------GGACCAGGACAAACC---TTC------TATGCAACAGATGGT------
---------ATAGAAGGAGATATA------------AGAAAAGCATATTGT---AATGTCAGCAGAGCA---
GAATGGAATAGCACTTTATACAATATAAGTATACAATTAGGA--------------AAATACTTTAAT----
-----------------------------------AACAAAACAATAATCTTTAAG------
AACTCCACAGGAGGGGATTTAGAAATTACA---ACACATAGTTTCAATTGTGGAGGAGAA---
TTTAAACAGCTCCAGGCAAGAGTCCTGGCTGTGGAAAGATACCTAAAGGATCAACAGCTCCTAGGAATTTGGG
GTTGCTCTGGAAAACTCATCTGCACCACTAGTGTGCTCTGGAACTCTAGTTGGAGT----------------
----------------------------AACAAATCTCAGAGTGAGATATGG------GAC------
AACATGACCTGGATGCAATGG---GACAAGGAAGTTAGCAATTACACAAAATTAATATAT---
GACCTACTTGAAGAATCGCAAAACCAGCAGGAA---------------------------------------
----
AAGAATGAACAAGATTTATTGGCATTAGATAAGTGGGCAAGTCTGTGGAGTTGGTTTGACATATCACATTGGT
TATGGTATATAAGAATATTTATAATGATAGTAGGAGGCTTAATAGGATTAAGAATAGTTTTTGCT---

GTGCTTTCTATAATA---------AATAGAGTCAGGCAGGATACTCACCA-------
TTGTCATTGGCTATCAATCTACTAGATACCATAGCAATAGCAGTAGCTGGG---
TGGACAGATAGGGTTATAGAAATAGGACAAAGACTTTGTAGAGCC----------------------------
--------ATTAGTAACATA------CCTAGGAGAATCAGACAGGGCGCAGAAAAGGCTTTGCAA---TAA
>GU481434
GCT---GCA--
GAAAACTTGTGGGTCACGGTATACTATGGGGTACCGGTGTGG---AGAGATGCAGAG---
ACCACCCTATTTTGTGCATCAGATGCTAAAGCATATGATAAAGAG---GTACACAAT----------------
--------------GTCTGGGCTACA---CATGCCTGTGTACCTACAGAC---
CCTAACCCACAAGAAATAATTTTGGAA---------AATGTGACAGAAGAATTTGAC----------------
--------------------------ATGTGGAAAAATAACATGGTAGAACAAATGCAAACAGATATA---
ATCAGCCTCTGGGACCAGAGCCTAA--AAGCCATGTGTAAAGTTA---
ACCCCTCTCTGCGTTACTTTAAATTGTACTGAACCCAACAGAACTAGCTTTAACAACAGT-------------
----
-------------------------GACGTTAGCAGCAATAATAGCTTGTATGAAGAAATGAAA---
AACTGCTCTTTCAACATGACCACAGAACTAAGA---------------------------
GATAAGAGGAAACTTGTACATTCACTT---TTTTATAAACTTGATATAGTATCTATGGATAAT----------
----

AATGATAGTGGGCAGTATAGACTAATAAATTGTAATACATCAACCATGACACAG---
GCCTGTCCTAAGGTAACCTTTGAGCCAATTCCTATATATTATTGTACTCCAGCTGGTTTTGCACTTCTAAAGT
GCAAG---GATACAAATTTTACT------GGAACAGGGCCATGCAAGAAT-----------------------
-GTCAGC---ACAGTACAATGCACACATGGAATCAAGCCAGTAGTATCAACT---
CAACTGTTATTAAATGGCAGTCTAGCAGAAAAAGAG------------
GTAATGATTAGATCTCAAAATATTACAGACAAT---------AGCAAAATCATAATAGTACAGCTTACT----
-----------GAGCCTGTAAAC---------ATCACTTGTATCAGACCTGGCAACAATACAAGAACA-----
-AGTGTACGTATA------------GGACCAGGACAAACC---TGG------TATGCAACAGGTGGT------
---------GTAACAGGGGACATA------------AGAAGAGCATATTGT---AATGTCAGCAGAGCA---
GCATGGAATAGCACTTTACATAAGATAAGTACACAATTGAAA---------------AAATACTTTAAT----
-----------------------------------AACAAAACAATAATCTTTAAG------
AACTCCTCAGGAGGGGATTTAGAAGTTACA---ACACATAGTTTCAATTGTGGAGGAGAA---
TTTAAACAGCTCCAGGCAAGAGTCCTGGCTGTGGAAAGATACCTAAGGGATCAGCAGCTCCTAGGAATTTGGG
GTTGCTCTGGAAAACTCATCTGCACCACTAATGTGCCCTGGAACTCTAGTTGGAGT-----------------
----------------------------AATAAATCTAAGGATGAGATATGG------GAG------
AACATGACCTGGATGCAATGG---GATAAGGAAATTATCAATTACACAAACAAAATATAT---
GATCTAATTGAAGAAGCGCAAAGCCAGCAGGAA--

AAGAATGAACAAGATTTATTGGCATTGGATACGTGGACAGGTCTGTGGAATTGGTTTAGCATATCAAATTGGT
TATGGTATATAAAAATATTTATAATAATAGTAGGAGGCTTGATAGGCCTAAGAATAGTTTTTGCT---
GTGCTTTCTATAATA---------AATAGAGCCAGGCAGGATACTCACCC-------
TTGTCATTGGCTATTAGTCTGATAAATACCATAGCAATAGTAGTAGCTGGG---
TGGACAGATAGGGTTTTAGAAGTAGGACAAAGATTTTGCAGGGCT----------------------------
--------ATTCTTAACATA------CCTAGGAGAATCAGACAGGGCGCAGAAAGGGCTTTGCAA---TAA
>GU481486
GCT---ACA--
GAAAACTTGTGGGTCACGGTATACTATGGGGTACCTGTGTGG---AGAGATGCAAAG---
ACCACCCTATTTTGTGCATCAGATGCAAAAGCTTACGATAAAGAA---GTCCACAAT----------------
--------------GTATGGGCTACA---CATGCCTGTGTACCCACAGAC---
CCTAACCCGCAAGAAATAAATTTGGAA---------AATGTCACAGAAGAATTTGAC----------------
--------------------------ATGTGGAAAAATAACATGGTAGAACAAATGCAAACAGATATA---
ATCAGTCTCTGGGACCAAAGCCTA---AAGCCATGTGTAAAGTTA---
ACCCCTCTCTGCGTTACTTTAAATTGTACTAAACCTAGCAGCACTAGCAATAACAACAGT-------------
----
-------------------------AGCGTTAACAGCAATAATAGCTTGTTTGAAGAAATGAAA---
AACTGCTCTTTCAACATGACCACAGAATTAAGA---------------------------
GATAAAAGGGAAAAAGTACATTCACTT---TTTTATAAACTTGATATAGTATCAATTGATAGT----------
----

AATAATAATAGTCAGTATAGACTAATAAATTGTAATACATCAACCATGACACAG---
GCCTGTCCTAAGGTAACCTTTGAGCCAATTCCTATATATTATTGTACCCCAGCTGGTTTTGCGATTCTAAAGT

GCACG---GATCCAAACTTTAAT------GGAACAGGGCCATGCAAGAAT-----------------------
-GTCAGT---ACAGTACAATGCACACATGGAATCAAGCCAGTAGTATCAACT---
CATCTGCTGTTAAATGGCAGTCTAGCAGAAGAAGAG------------
GTAATGATTAGATCTAAAAATATTACAGACAAT---------GGCAAAACCATAATAGTACAGCTTAAG----
-----------GAGCCTGTAAAC---------ATCACTTGTATCAGACCTGGCAACAATACAAGAACA-----
-AGTATACGTATA-------------GGACCAGGACAAACC---TTC------TATGCAACAGGTGAT------
---------GTAATAGGGGACATA------------AGAAGAGCATATTGT---AATGTCAACAGAACA---
GCATGGAATAGAACTTTACAAGAGATAAGTAAACAATTAAGA---------------AAATACTTTAAT----
-----------------------------------AACAAAACAATAATCTTTAAG------
AGCTCCTCAGGAGGGGATTTAGAAGTTACA---ACACATAGTTTCAATTGTGGAGGAGAA---
TTTAAACAGCTCCAGGCAAGAGTCCTGGCTGTGGAAAGATACCTAAAGGATCAGCAGCTCTTAGGAATTTGGG
GTTGCTCTGGAAAACTCATCTGCACCACTAATGTGCCCTGGAACTCTAGTTGGAGT-----------------
----------------------------AATAAATCTCAGACAGAGATATGG------GAT------
AACATGACCTGGATGCAATGG---GACAAAGAAATTATAAATTACACAGACAAAATATAT---
GATCTAATTGAAAAATCGCAAAACCAGCAGAAA--

AAGAATGAACAAGATTTATTAGCATTGGATAAGTGGGCAGGTTTGTGGAATTGGTTTGACATATCAAATTGGT
TATGGTATATAAAAATATTTATAATAATAGTAGGAGGCTTAATAGGATTAAGAATAGTTTTTGCT---
GTGCTTTCTATAATA---------AATAGAGTCAGGCAGGGATACTCACCC------
TTGTCATTGGCTATTAATCTGATAGATAACATAGCAATAGCAGTAGCTGGG---
TGGACAGATAGGGTTTTAGAAATAGGACAAAGATTTTGTAGAGCT----------------------------
--------ATTCGTAACATA------CCTAGGAGAATCAGACAGGGCGCAGAAAGGCTTTGCAA---TAA
>GU481644
GCT---GCA---
GACTTGTGGGTTACGGTATACTATGGGGTACCTGTGTGG---AAAGATGCAGAG---
ACCACCCTATTTTGTGCATCAGATGCTAAAGCATATGATAAAGAA---GTACACAAT----------------
--------------GTCTGGCTACA---CATGCCTGTGTACCCACAGAC---
CCTGACCCACAAGAAATAAATTTGGAA---------AATGTGACAGAAGAATTTAAC----------------
--------------------------ATGTGGAAAAATAACATGGTAGAACAGATGCAAACAGACATA---
ATCAGTCTCTGGACCAAAGCCTA---AAGCCATGTGTACGATTA---
ACCCCTCTCTGCGTTACTTTAGATTGTAATGCACCCAACAGCCCTAGCTCTAACAACAGT-------------
----
-------------------------AGCGTTAGCAACAATGATACCTTGTTTGAAGAAATGAAA---
AACTGCTCTTTCAACATGACCACAGAACTAAGA---------------------------
GATAAAACGAAAAAAGCATATTCACTT---TTTTATAAACTTGATATAGTATCAATTGATAGTAATGGT----
----

AGTAATAGTAATGGTAGTGGGCAATATAGACTAATAAATTGTAATACATCAACCACGACACAG---
GCCTGTCCTAAGGTATCCTTTGAGCCAATTCCTATACATTATTGTACTCCAGCTGGCTTTGCGATTCTAAAGT
GCAAG---GATCCAAAGTTTAAT------GGAACAGGGCCATGCAAGAAT-----------------------
-GTCAGC---ACAGTACAATGCACACATGGAATCAAGCCAGTAGTATCAACT---
CAACTGCTGTTAAATGGCAGTCTAGCAGAAGAAGAG------------
GTAATGATTAGATCTGAAAATATTACAGACAAT---------GGCAAAATCATAATAGTACAGCTTACT----
-----------GAGCCTGTAAAC---------ATCACTTGTATCAGACCTGGCAACAATACAAGAACA-----
-AGTGTACGTATA-------------GGACCAGGACAAACC---TTC------TATGCAACAGGTGCT------
---------GTAATAGGGGACATA------------AGAAGAGCACATTGT---AATGTCAGCAGAACA---
GCATGGAATAGCACTTTACAAAAGATAAGTAAACAATTAAGA---------------AAATACTTT-------
-----------------------------------AACAAAACAATAATCTTTAGG------
AACTCCGCAGGAGGGGATTTAGAAGTTACA---ACACATAGTTTCAATTGTGGAGGAGAA---
TTTAAACAGCTCCAGGCAAGAGTCCTGGCTGTGGAAAGATACCTAAAGGATCAGCAGCTCCTAGGAATTTGGG
GTTGCTCTGGAAAACTCATCTGCACCACTAATGTGCCCTGGAACTCTAGTTGGAGT-----------------
----------------------------AATAAATCTTATAGTGAGATATGG------GAT------
AACATGACCTGGATGCAATGG---GACAGGGAAATTATCAATTACACAAACAAAATATAT---
GAATTAATTGAAGAATCACAAAACCAGCAGGAG--

AAGAATGAACAAGATTTATTGGCATTGGATAAGTGGACAGGTCTGTGGAGTTGGTTTGACATATCAAATTGGT
TATGGTATATAAAAATATTTATAATAATAGTAGGAGGATTAATAGGCCTAAGAATAGTTTTTGCT---
GTGCTTTCTATAATA---------AATAGAGCCAGGCAGGGATACTCACCC------
TTGTCATTGGCTATTAATCTGATAGATACCATAGCAATAGCAGTAGGAGGG---

TGGACAGATAGGGTTTTAGAACTAGGACAAAGATTTTGTAGAGCT----------------------------
--------CTTTGTAACATA------CCTAGGAGAATCAGACAGGGCGCAGAAAAGGCTTTGCAA---TAA
>JQ292891
GCT---GCA--
GATAACTTGTGGGTCACGGTATACTATGGGGTACCTGTGTGG---AGAGATGCAGAG---
ACCACCCTATTTTGTGCATCAGATGCTAAAGCATATGATAAAGAA---GTACACAAT----------------
--------------GTCTGGGCTACA---CATGCCTGTGCACCCACAGAC---
CCTAACCCACAAGAAATAGCTTTGGAA---------AATGTGACAGAAAATTTTGAT----------------
--------------------------ATGTGGAAAAATAACATGGTAGAACAAATGCAAACAGATATA---
ATCAGTCTCTGGGACCAAAGCCTA---CAGCCATGTGTAAAGTTA---
ACCCCTCTCTGCGTTACTTTAAATTGTGTTGACCCCAACAGCAACAGC-------------------------
----
-------------------------AGTAGGAACAACAGTGATAATGTGCTTGAAGAAATGAGA---
AACTGCTCTTACAACATGACCACAGAACTAAGA---------------------------
GATAAAAGGAAAAATGTACATTCACTC---TTTTATAAACTTGATATAGTATCAACTGATAGT----------
----

AATAGTGGGCAGTATAGACTAATAAATTGCAATACATCAGCCATGACACAG---
GCCTGTCCTAAGGTAACCTTTGAGCCAATTCCTATACATTATTGTGCCCCAGCTGGTTTTGCGATTCTAAAGT
GCAGG---GATACAAATTTTACT------GGAAAGGGGCAATGCAACAAT-----------------------
-GTCAGC---ACAGTACAATGCACACATGGAATCAAGCCAGTAGTATCAACT---
CAACTGCTGTTAAATGGCAGTCTAGCAGAGAAAGAG------------
GTAATGATTAGATCTGAAAATATCACAGACAAT---------AGCAAAATCATAATAGTACAGCTTACT----
-----------GAGCCTGTAAAC---------ATCACTTGTATCAGACCTAGCAACAATACAAGAACA-----
-AGTATAAGTATA------------GGACCAGGACGAGCC---TTC------TATGCAACAGGTGAT------
---------GTAACGGGGGACACA------------AGGAAAGCATATTGT---AAGGTCAACAAAACA---
GCATGGAATAGCACTTTACAAAAGGTAAGCACACAATTAAGA---------------AACTACTTTAAG----
-----------------------------------ACCAAAACAATAATCTTTAAG------
AACTCCTCAGGAGGGGATTTAGAAATTACA---ACACATAGTTTCAATTGTGGAGGAGAA---
TTTAAACAGCTCCAGGCAAGAGTCCTGGCTGTGGAAAGATACCTGAAGGATCAGCAGCTCCTAGGAATTTGGG
GTTGCTCTGGAAAACTCATCTGCACCACTAATGTGCCCTGGAACTCTAGTTGGAGT-----------------
----------------------------AACAAATCTCAGAGTGAGATATGG------AAT------
AACATGACCTGGATGCAATGG---GATAAGGAAGTTATCAATTACACAAACATAATATAT---
AATCTAATTGAAGAATCGCAAAACCAGCAGGAA--

AAGAATGAACAAGATTTATTGGCATTGGATAAGTGGGCAAGTCTGTGGAATTGGTTTAACATATCAAATTGGT
TATGGTATATAAAAATATTCATAATGATAGTAGGAGGCTTAATAGGATTAAGAATAGTTTTTGCT---
GTGCTTTCTATAATA---------AATAGAGCCAGGCAGGGATATTCACCC------
TTGTCATTAGCTATCAATCTATTAGATACCATAGCAATAACAGTAGCTGGG---
TGGACAGATAGTATTATAGAAATAGGACAAAGACTTTGTAGAGCC----------------------------
--------ATTCTTAACATA------CCTAGGAGAATCAGACAGGGCGCAGAAAAGGCTTTGCAA---TAA
>HQ385820
GCT---GCA--
GAAAACTTGTGGGTCACGGTATACTATGGGGTACCTGTGTGG---AGAGATGCAGAG---
ACCACCCTATTTTGTGCATCAGATGCTAAAGCATACGATAAAGAA---GTACACAAT----------------
--------------GTCTGGGCTACA---CATGCCTGTGTACCCACAGAC---
CCTGACCCACAAGAAATAGTTTTGGAA---------AATGTGACAGAAAATTTTGAC----------------
--------------------------ATGTGGAAAAATAACATGGTAGAACAAATGCAAACAGATATA---
ATCAGCCTCTGGGACCAAAGCCTA---AAGCCTTGTGTAAAGTTA---
ACCCCTCTCTGCGTTACTTTAAATTGTGCTGAACCCAACAGCACTAGCTCTAACAATAGT-------------
----
-------------------------AGTGTTTACAAAAACGATAGTTTGTATGAAGAAATGAAA---
AACTGCTCTTTCAACATGACCACAGAAATATTA---------------------------
GATAAAAGGAAAACTGTACATTCACTT--CTTTTATAAACTTGATATAGTATCAACTGGTAGT----------
----

AATGATAGTGGACAGTATAGACTAATAAATTGTAATACATCAGCCATGACACAG---
GCCTGTCCTAAGGTAACCTTTGAGCCAATTCCTATACATTATTGTGCCCCAGCTGGTTTTGCGATTTTAAAAT
GCAAA---GATACAAATTTTAAT------GGAACAGGGCCATGCAAGAAT-----------------------
-GTCAGC---ACAGTACAATGCACACATGGAATCAAGCCAGTAGTATCAACT---

CAACTGCTGTTAAATGGCAGTCTAGCAGAGAAAAAG------------
GTAATGATTAGATCTAAAAACATCACAGACAAT---------GGCAAAACCATAATAGTACAGCTTACT----
-----------GAGCCTGTAAAC---------ATCACTTGTATCAGACCTGGCAACAATACAAGAACA-----
-AGCATACGTATA------------GGACCAGGACAAACC---TTC------TATGCAACAGGTGCT------
---------GTAGTAGGGGACATA------------AGAAGAGCACATTGT---AATGTCAGCAGAACG---
GCATGGGATAGAGCTTTACAAAAGATAAGTACACAATTAAGA---------------AAATACTTTAAT----
-----------------------------------AACAAAACAATAATCTTTAAG------
AACAGCTCAGGAGGGGATTTAGAAGTTACA---ACACATAGTTTCATTTGTGGAGGAGAA---
TTTAAACAGCTCCAGGCAAGGGTCCTGGCTGTAGAAAGATACCTAAAGGATCAGCAGCTCCTAGGAATTTGGG
GTTGCTCTGGAAAACTCATCTGCACCACTAATGTGCCCTGGAACTCTAGTTGGAGT-----------------
----------------------------AACAAATCCCAGGAGGAGATATGG------GAT------
AACATGACCTGGATGCAATGG---GACAAGGAAATTAACAATTACACAGACTTAATATAT---
AATCTAATTGAAAAATCGCAAAACCAGCAGGAA--

AAGAATGAACAAGATTTATTGGCATTGGATAAGTGGGCAGGTCTGTGGAATTGGTTTGACATATCAAATTGGT
TATGGTATATAAGAATATTTATAATAATAGTAGGAGGCTTAATAGGATTAAGAATAGTTTTTGCT---
GCGCTTTCTATAATA---------AAAAGAGCCAGGCAGGGATACTCACCC------
TTGTCATTTGCTATCAATCTAATAGATACCATAGCAATAGCAGTAGCTGGG---
TGGACAGATAGGGTTATAGAAATAGGACAAGGATTTTGTAGAGCC----------------------------
--------ATTCGTAACATA------CCTAGGAGAATCAGACAGGGCGCAGAAAGAGCTTTGCAA---TAA
>JF952018
GCT---GCA--
GAAAACTTGTGGGTCACGGTATACTATGGGGTACCTGTGTGG---AGAGATGCAGAG---
ACCACCCTATTTTGTGCCTCAGATGCTAAAGCATATGATAAAGAA---GTACACAAT----------------
--------------GTCTGGGCTACA---CATGCCTGTGTACCCACAGAC---
CCTGACCCACAAGAAATAGCTTTGGGA---------AATGTGACAGAAAATTTTAAC----------------
--------------------------ATGTGGAAAAATAACATGGTAGAACAAATGCAAACAGATATA---
ATCAGTCTCTGGGACCAAAGCCTA---AAGCCATGTGTACAGTTA---
ACCCCTCTCTGCGTTACTTTACTTTGTGATGAATTCAACAGCACTAGCTCTAACAACAGTAGC----------
----
----------------------GTTAACAGCAGCAGCAGCGATAGTTTGGTTACAGAAATGAAA---
AACTGCTCTTTCAACATGACCACAGAACTAAGA---------------------------
GATAAAAAGCAAAATGTATATTCACTT---TTTTATAAACTGGATATAGTACAAACTGATAGTAAT-------
----

GATAGCAGGCAGTATAGACTAATAAATTGTAATACATCAGCCATGACACAG---
GCCTGTCCTAAGGTAACCTTTGAGCCACTTCCTATACATTATTGTGCCCCAGCTGGTTTTGCGATTCTAAAAT
GCAAT---GATACAAAATTTAAT------GGAACAGGGCCATGTAAGAAT-----------------------
-GTCAGC---ACAGTACAATGCACACATGGAATCAAGCCAGTAGTATCAACT---
CAACTGCTGTTAAATGGCAGTCTAGCA-AGGAAGAG------------
GTGATGATTAGATCTGAAAATATCACAGACAAT---------GCCAAAACCATAATAGTACAGCTTACT----
-----------AAGCCTGTAAAC---------ATCACTTGTATCAGACCTGGCAACAATACAAGAACA-----
-AGTATACGTATA------------GGACCAGGACAAACC---TTC------TGGGGAACAGGTGAT------
---------GTAATAGGGGACATA------------AGAAAAGCATATTGT---AATGTCAGCAGAGCA---
GCATGGAATAGCACACTATATGAGATAAGTACCCAATTAAGA---------------AAATACTTTAGT----
-----------------------------------AACAAAACAATAATATTTAAC------
AGCTCCTCAGGAGGGGATTTAGAAGTTACA---ACACATAGTTTCAATTGTGGAGGAGAA---
TTTAAACAGCTCCAGGCAAGAGTCCTGGCTGTGGAAAGATACCTAAAGGATCAGCAGCTCCTAGGAATTTGGG
GTTGCTCTGGAAAACTCATCTGCACCACTAATGTGCCCTGGAACTCTAGTTGGAGT-----------------
----------------------------AACAAATCTCAGAAGGAGATATGG------GAT------
AACATGACCTGGATGCAATGG---GACAAGGAAGTTATCAATTACACAGGCATAATATAT---
GATCTAATTGAAAAATCGCAAAACCAGCAGGAA--

AAGAATGAACAAGATTTATTGGCATTAGATAAGTGGGCAAGTCTGTGGAATTGGTTTGACATATCAAGTTGGT
TATGGTATATAAGAATATTTATAATAATAGTAGGAGGCTTAATAGGATTAAGAATAGTTTTTGTT---
GTGCTTTCTGTAATA---------AATAGAGTCAGGCAGGGATACTCACCA------
TTGTCATTGGCTATCAATCTAATAGATACCATAGCAATAGCAGTAGCTGGG---
TGGACAGATAGGGTTATAGAAATAGGACAAAGATTTTGTAGAGCC----------------------------
--------ATTTGTAACATA------CCTAGGAGAATCAGACAGGGCGCAGAAAAAGCTTTGCAA---TAG
>HQ338112

GCT---ACA---
GAAAACTTGTGGGTCACGGTATACTATGGGGTACCTGTGTGG---AGAGAGGCAGAG---
ACCACCCTATTTTGTGCATCAGATGCTAAAGCATATGATAAAGAA---GTACACAAT----------------
--------------GTCTGGGCTACA---CATGCCTGTGTACCCACAGAC---
CCTAACCCACAAGAAATACCTTTGGAA---------AATGTGACAGAAAATTTTAAC----------------
--------------------------ATGTGGAAAAATAACATGGTAGAACAAATGCAAACAGATATA---
ATCAGTCTCTGGGACCAAAGCCTA---CAGCCATGTGTAAAGTTA---
ACCCCTCTCTGCGTCACTTTAGAGTGTACTAAACCCAACAGCACTAGCTTTAACATCACTAGCTCTAACAAC-
----
----------------AGTAGCGGTAACAGCAACAGCAGTAATAGTTTGTATGAAGAAATGAGC---
AACTGCACTTTCAACATGACAACAGAACTAAGA--------------------------
GATAAAAAGAAAAAGGTACACTCACTT---TTTTATAAACTTGATATAGTACCAATTGGTAATAAT-------
----

AATAGTAGTCAGTATAGACTAATAAATTGCAATACATCAGCCATGACACAG---
GCCTGTCCTAAGGTAACCTTTGAGCCAATTCCTATACATTATTGTGCCCCAGCTGGTTTTGCGATTCTAAAGT
GCAAG---GATCCAACTTTTAAT------GGAACAGGGCCATGCAGGAAT-----------------------
-GTCAGC---ACAGTACAATGCACACATGGAATCAGGCCAGTAGTATCAACT---
CAACTGCTGTTAAATGGCAGTCTAGCAGAGAAAGAT-----------
GTAATGATTAGATCTGAAAATATCACAGACAAT---------GGCAAAATCATAATAGTACAGCTTACT----
-----------GAGCCTGTAAAC---------ATCACTTGTATCAGACCTGGCAACAATACAAGAACA-----
-AGTATACGTATA------------GGACCAGGACAAACC---TTC------TATGCAACAGGTGAG------
---------GTAACAGGGGACATA------------AGGAAAGCACATTGT---AATGTCAGCAGAGCA---
GCATGGAATAACACTTTACAAAAGATAAGTAAACAATTAGGA---------------AAATACTTTAAT----
-----------------------------------AACAAAACAATAATCTTTAAG------
AACTCCTCAGGAGGGGATGTAGAAGTTACA---ACACATAGTTTCAATTGTGGAGGAGAA---
TTTAAACAGCTCCAGGCAAGAGTCCTGGCTGTGGAAAGATACCTGAAGGATCAGCAGCTCCTAGGAATTTGGG
GTTGCTCTGGAAAACTCATCTGCACCACTAATGTGCCCTGGAACTCTAGTTGGAGT-----------------
----------------------------AACAAATCTCATGATGAGATATGG------AAT------
AACATGACCTGGATGCAATGG---GACAGGGAAGTTATCAATTACACAGAAAAAATATAT---
CAGCTAATTGAAGAGTCGCAAAACCAGCAGGAA--

AAGAATGAACAAGATTTATTGGCATTGGACAAGTGGGCAAGTCTGTGGAGTTGGTTTGGCATATCAAATTGGC
TATGGTATATAAGAATATTTATAATAATAGTAGGAGGCTTAATAGGATTAAGAATAGTTTTTGCT---
GTGCTTTCTATAATA---------AATAGAGTCAGGCAGGGATACTCACCC------
CTGTCGTTGGCTATCAATCTACTAGATACCATAGCAATAGCAGTAGCTGGG---
TGGACAGATAGGGTTATAGAAATAGTACAAAGAATTTGTAGAGCC----------------------------
--------ATTCGTAACATA------CCTAGGAGAATCAGACAGGGCGCAGAAAGGGCTTTGCAA---TAA
>GU481277
GCT---GCA---
GATAACTTGTGGGTCACGGTATACTATGGGGTACCTGTGTGG---AGAGATGCAGAA---
ACCACCCTATTTTGTGCATCAGATGCTAAAGCATATGATAGGGAG---GTACACAAT----------------
--------------GTCTGGGCTACA---CATGCCTGTGTACCCACAGAC---
CCTAACCCACAAGAAATAATGTTGGAA---------AATGTGACAGAAGAATTTGAC----------------
--------------------------ATGTGGAAAAATAACATGGTAGAACAAATGCAAGCAGACATA---
ATCAGTCTCTGGGACCAAAGCCTA---AAGCCATGTGTAAAGTTA---
ACCCCTCTCTGCGTTACTTTAAATTGT---GATTACCTCAACAGCACTAGCTCT-------------------
----
------------------------------AACAACAGTAGCGATTTGTTTGAAGAAATGAAA---
AACTGCTCTTTCAACATGACCACAGAACTAAGA--------------------------
GATAAAAGGAAAACTGTGCATTCGCTT---TTTTATAAACTTGATATAGTATCAATTGATAGT----------
----

AATAATAATAGTAGGCAATATAGACTAATAAATTGTAATACATCAACCATGACACAG---
GCCTGTCCTAAGGTAACCTTTGAGCCAATTCCTATATATTATTGTACCCCAGCTGGTTTTGCGATTCTAAAGT
GCAGG---GATCCAAACTTTAAT------GGAACAGGGCCATGCAAGAAT-----------------------
-GTCAGC---ACAGTACAATGCACACATGGAATCAAGCCAGTAGTATCAACT---
CAACTGCTGTTAAATGGCAGTCTAGCAGAAAAAGAG-----------
GTAATGATTAGATCTAAAAATATTACAGACAAT---------AGCAAAATTATAATAGTACAGCTTAAA----
-----------GAGCCTGTAAAC---------ATCACTTGTATTAGACCTAGCAACAATACAAGAACA-----

-GGTATACGTATA------------GGACCAGGACAAACC---TTC------TATGCAACAGGTAAT------
---------GTAATAGGGGACATA------------AGAAAAGCATATTGT---AATGTCAGCAGAGCA---
AACTGGACTAGCACTTTACAAAAGATAAGTAAACAATTAAGA---------------AAATACTTTCAT----
-----------------------------------AACAAAACAATAAGCTTTAAG------
AACTCCTCAGGAGGGGATTTAGAAGTTACA---ACACATAGTTTCAATTGTGGAGGAGAA---
TTTAAACAGCTCCAGGCAAGAGTCCTGGCTGTGGAAAGATACCTAAAGGATCAGCAGCTCCTAGGAATTTGGG
GTTGCTCTGGAAAACTCATCTGCACCACTGATGTGCCCTGGAACTCTAGTTGGAGT----------------
----------------------------AATAAATCTCAAAGTGAGATATGG------GAT------
AACATGACCTGGATGCAATGG---GACAAGGAAATTATCAATTACACAGAAAAAATATAT---
GATCTAATTGAAAAATCGCAAAACCAGCAGGAA--

AAGAATGAACAAGATTTATTAGCATTGGATAAGTGGGCAGGTCTGTGGAGTTGGTTTAGCATATCAAATTGGT
TATGGTATATAAGAATATTTATAATAATAGTAGGAGGCTTAATAGGATTAAGAATAGTTTTTGCG---
GTGCTTTCTATAATA---------AATAGAGTCAGGCAGGGATACTCACCC------
TTGTCATTGGCTGTTAATCTGATAGATACCCTAGCAATAGCAGTAGCTGAG---
TGGACAGATAGGGTTTTAGAAATAGGACAAAGATTTGGTAGAGCT---------------------------
--------ATTTGTAACATA------CCTAGGAGAATCAGACAGGGCGCAGAAAAAGCTTTGCAA---TAA
>GU481544
GCT---GCA---
GAAAACTTGTGGGTCACGGTATACTATGGGGTACCTGTGTGG---AGAGATGCAGAG---
ACCACCCTATTTTGTGCATCAGATGCTAAAGCATATGATAAAGAA---GTACACAAT---------------
--------------GTCTGGGCTACA---CATGCCTGTGTACCCACAGAC---
CCTGACCCACAAGAAATAAATTTGGAA---------AATGTGACAGAAAACTTTAAC---------------
-------------------------ATGTGGAAACATAACATGGTAGAACAAATGCAAACAGATATA---
ATCAGTCTCTGGGACCAAAGCCTA---AAGCCATGTGTAAAGTTA---
ACCCCTCTCTGCGTTACTTTAACTTGTACTGAACCCAACATCACTAGCTCTAACGACAGTAGCATTAACAGG-
----
----------------AACAGCAGTGATAGCTTTAACAGGAACAGCAACGATGAAGAAATGAAA---
AACTGCTCTTTCAACATGACCACAGAACTAAGA--------------------------
GACAAAAGGAAAATTGTACATTCACTT---TTTTATAAACTTGATATAGTATCAATTGATAGA----------
----

AATATTAGTGGGCAGTATAGACTAATAAATTGTAATACATCAACCATGACACAG---
GCCTGTCCTAAGATAACCTTTGAGCCAATTCCTATACATTACTGTGCCCCAGCTGGTTTTGCGATTCTAAAGT
GCAAA---GATACAGAGTTTAGT------GGAACAGGGCCATGCAAGAAT----------------------
-GTCAGC---ACAGTACAATGCACACATGGAATCAAGCCAGTAGTATCAACT---
CAACTGCTGTTAAATGGCAGTCTAGCAGAGGAAAAG------------
GTAATGGTTAGATCTGAAAATATCACAAACAAT---------GGCAAAAACATAATAATACAGCTTACT----
-----------GAGCCTGTAAAA---------ATCACTTGTATCAGACCTAGCAACAATACAAGAACA-----
-AGTATACGTATA------------GGACCAGGACAAACC---TTC------TATGCAACAGGTAAA------
---------GTAGTAGGGAACATA------------AGAAAAGCATATTGT---AATGTCAGCGGAGCA---
AAATGGAATGAAACTCTACAAAAGATAAGTATACAATTAAGA---------------ACATACTTTAAT----
-----------------------------------AACAAAACAATAATCTTTAAG------
AACTCCTCAGGAGGGGATTTAGAAGTTACA---ACACATAGTTTCAATTGTGGAGGAGAA---
TTTAAACAGCTCCAGGCAAGAGTCCTGGCTGTGGAAAGATACCTAAAGGATCAGCAGCTCCTAGGAATTTGGG
GTTGCTCTGGAAAACTCATCTGCACCACTAATGTGCCCTGGAACTCTAGTTGGAGT----------------
----------------------------AATAAATCTCAGAGTGAGATATGG------AAT------
AACATGACCTGGATGCAATGG---GACAAGGAAGTTATCAATTACACAAACATAATATAT---
GATCTAATTGAACAATCGCAAACCCAGCAGGAA--

AAGAATGAACAAGATTTATTGGCATTGGATAAGTGGACAAGTCTGTGGAATTGGTTTGACATATCAAAGTGGT
TATGGTATATAAGAATATTTATAATAATAGTAGGAGGCTTAATAGGATTAAGAATAGTTTTTGCT---
GTGCTTTCTATAATA---------AATAGAGTCAGGCAGGGATACTCACCC------
TTGTCATTGGCTATCAATCTGGTGGATACCATAGCAATAGCAGTAGCTGGA---
TGGACAGATAGGGTTATAGAAATAGGACAAGGAGTTTGTAGAGCT---------------------------
--------ATTTGTAACATA------CCTAGGAGAATCAGACAGGGCGCAGAAAAGGCTTTGCAA---TAA
>GU481661
GCT---GCA---
GAAAACTTGTGGGTCACAGTATACTATGGGGTACCTGTGTGG---AAAAATGCAGAT---
ACCACCTTATTTTGTGCATCAGATGCTAAAGCATTTGATAAAGAA---GTACACAAT---------------

--------------GTCTGGGCTACA---CATGCCTGTGTACCCACAGAC---
CCTGACCCGCAAGAAATACCTTTGGAA---------AATGTGACAGAAGAGTTTAAC----------------
--------------------------ATGTGGAAAAATAACATGGTAGAACAAATGCAAACAGATATA---
ATCAGTCTCTGGGACCAAAGCCTA---AAGCCATGTGTAAAGTTA---
ACCCCTCTCTGTGTTACTTTAAATTGTACTGAACCCAACAAACCCAACAGCACTGTCTCCAACAACACTGTCA
CT---
----------GTCTCTAACAACAGTAGCTTTAACAACAATAGTAGATTGGTTGAAGAAATGAGA---
AACTGCTCTTTCAATGTGACCACAGAACTAAAA---------------------------
GATAAAAAGAGAAAGGTACATTCACTT---TTTTATAAACTTGATATAGTACCAATTGATAGT----------
----

AATAATAGTAGGGAGTATAGACTAATAAATTGTAATACATCAACCGTGACACAG---
GCCTGTCCTAAGGTAACCTTTGAGCCAATTCCTATATATTATTGTACCCCAGCTGGTTTTGCGCTTCTAAAGT
GCAGG---GATCCAAACTTTAAT------GGAACAGGGCCATGCAAAAAT-----------------------
-GTCAGC---ACAGTACAATGCACACATGGAATCAAGCCAGTAGTATCAACT---
CAACTGCTGTTAAATGGCAGTCTAGCAGAAAAAGAG------------
ATAATGATTAGATCTGAAAATATTACAAACAAT---------GGCAAAAACATAATAGTACAGCTTACT----
-----------GAGCCTGTAAAC---------ATCACTTGTATCAGACCTGGCAACAATACAAGAACA-----
-AGTATACGCATA------------GGACCAGGACAAGCC---TTC------TATGCAACAGGTGAG------
---------GTAACAGGGGACATA------------AGAAAAGCACATTGT---ATTGTCAACAGAGTA---
GCATGGGAGAACACTTTAAAAAACATAAGTACACAATTAAGA---------------AAATACTTT-------
-----------------------------------AACAGAACAATAATCTTTGAG------
AAATCCTCAGGAGGGGATTTAGAAGTTACA---ACACATAGTTTTAATTGTGGAGGAGAA---
TTTAAACAGCTCCAGGCAAGAGTCCTGGCTGTGGAAAGATACCTAAAGGATCAGCAGCTCCTAGGAATTTGGG
GTTGCTCTGGAAAACTCATCTGCACCACTACTGTGCCCTGGAACTCTAGTTGGAGT-----------------
----------------------------AATAAATCTTACAGTGACATAGGG------GAT------
AACATGACCTGGATGCAATGG---GACAAAGAAATTATCAATTACACAAACAAAATATAT---
GATCTAATTGAAGAATCGCAAAACCAGCAGGAA--

AAGAATGAACAAGATTTATTGGCATTGGATAAGTGGGCAAGCCTGTGGAATTGGTTTGACATATCAAATTGGT
TATGGTATATAAAAATATTTATAATGATAGTAGGAGGCTTAATAGGATTAAGAATAGTTTTTGCT---
GTGTTTTCTATAATA---------AATAGAGTTAGGCAGGGATACTCACCC------
TTGTCATTGGCTGTTAATCTGGTAGATACCATAGCAATAGCAGTAGGTGGG---
TGGACAGATAGGGTTTTAGAAATAGGACAAAGAATTTGTAGAGCT----------------------------
--------ATTCGCAACATA------CCTAGAAGAATCAGACAGGGCGCAGAAAAAGCTTTGCAA---TAA
>KF716491
GCT---GCA--
GAAAACTTGTGGGTCACGGTATATTATGGGGTACCTGTGTGG---AGAGATGCAGAG---
ACCACTCTATTTTGTGCATCAGATGCTAAGGCATATGATACAGAA---GTACACAAT----------------
--------------GTCTGGGCTACA---CATGCCTGTGTACCCACAGAC---
CCTAACCCACAAGAAATAGCTTTGGAA---------AATGTGACAGAAAATTTTAAC----------------
--------------------------ATGTGGAAAAATGGCATGGTAGAACAAATGCAAACAGATATA---
ATCAGTCTCTGGGACCAGAGCCTA---AAGCCATGTGTAAAGTTA---
ACCCCTCTCTGCGTTACTTTAAATTGTACTGCACCCAACAGCACTAGACCTGACAACAGC-------------
----
---AGCAGGTATGAAGAAATGAAA---
AACTGCTCTTTCAACATGACCACAGAACTAAGA---------------------------
GATAAAAGGACAAAAGTATATTCACTT---TTTTATAAACTTGATATAGTAGCAACTGATAGTAAT-------
----

AATGGTAGTAATAGTAGTGGGCAGTATAGACTAATAAATTGTAATACATCAGCCATGACACAG---
GCCTGTCCTAAGATAACCTTTGAGCCAATTCCTATACATTATTGTGCCCCAGCTGGTTTTGCGATTCTAAAGT
GCAGG---GATCCAAATTTTAAT------GGAACAGGGCCATGCGGGAAT-----------------------
-GTCAGC---ACAGTACAATGCACACATGGAATCAAGCCAGTAATATCAACT---
CAACTGCTGTTAAATGGCAGTCTAGCAGAGAAAGAG------------
ATAATAATTAGATCTGAAAATATCACAGACAAT---------GGCAAAATCATAATAGTACAGCTTAAA----
-----------GAGCCTGTAAAA---------ATCAATTGTACCAGACCTGACAACAATACAAGAACA-----
-AGTTATCGTATA------------GGACCAGGACAAACC---TTC------TATGCAACAGGTGAT------
---------GTAATAGGGGACCCA------------AAGAAAGCATATTGT---AATGTCAGCAGAGCA---
ACATGGAATAACACTTTACGAAACATAAGTGCACAATTAATA---------------AAACACTTT-------

-----------------------------------AACAAAACAATAATCTTTAGG------
AACTCCTCAGGAGGGGATTTAGAAGTTACA---ACACATAGTTTCAATTGTGGAGGAGAA---
TTTAAACAGCTCCAGGCAAGAGTCCTGGCTTTGGAAAGATACCTAAAGGATCAGCAGCTCCTAGGAATTTGGG
GTTGCTCTGGAAAACTCATCTGCACCACTAATGTGCCCTGGAACCTTAGTTGGAGT-----------------
----------------------------AATAGATCTCAGAGTGACATATGG------GAT------
AACATGACCTGGATGCAATGG---GACAGGGAAGTCATCAATTACACAAACATAATATAT---
GATCTACTTGAAGAATCACAAAATCAGCAGGAA--

AAGAATGAACAAGATTTATTGGCATTGGATAAGTGGGCAAGTCTGTGGAATTGGTTTGACATAACAAATTGGT
TATGGTATATAAAAATATTCATAATGATAGTAGGAGGCTTAATAGGATTAAGAATAGTTTTTGCT---
GTGCTTTCTATAATA---------AATAGAGTCAGGCAGGGATACTCACCC------
TTGTCATTTGCTATCAATCTAATAGATACCATAGCAATAGTAGTAGCTGGG---
TGGACAGATAGGGTTATAGAATTAGGACAAAGACTTTGTAGAGCC----------------------------
--------ATTTGTAACATA------CCTAGAAGAATCAGACAGGGCGCAGAAAAAGCTTTGCAA---TAA
>KF716492
GCT---GCA--
GAAAACTTGTGGGTCACGATATACTATGGGGTACCTGTGTGG---AGAGATGCAGAG---
ACCACCCTATTTTGTGCCTCAGATGCTAAAGCATATGATAAAGAA---GTACACAAT----------------
--------------GTCTGGGCTACA---CATGCCTGTGTACCCACAGAC---
CCTGACCCACAAGAAATAATTTTGGAA---------AATGTGACAGAAGATTTCAAC----------------
--------------------------ATGTGGAAAAATAACATGGTAGAACAAATGCAAACAGATATA---
ATCAGTCTCTGGGACCAAAGCCTA---AAGCCATGTGTAAAGTTA---
ACCCCTCTCTGCGTTACTTTAAATTGTACTGAGCCCAACAGCGCTAGCTCTAACAACAATAGCACT-------
----
-------------------------AACAGCAACAGCAGCGATAGCTTGTTTGAAGAAATGAGA---
AATTGCTCTTTCAACATGACCACAGAACTAAGA---------------------------
GATAAGAGAAAAACTGTATATTCACTT---TTTTATAAACTTGATATAGTACCAACTGATAGT----------
----

AGTAATGGTAGTAAGCAGTATAGACTAATAAATTGTAATACATCAGCCATGACACAG---
GCCTGTCCTAAGGTAACCTTTGAGCCAATTCCTATACATTATTGTGCCCCAGCTGGTTTTGCGATTCTAAAGT
GCAAG---GATACAGATTTTATT------GGGAAAGGGCCATGCACGAAT-----------------------
-GTCAGC---ACAGTACAATGCACACATGGAATCAAGCCAGTAGTATCAACT---
CAACTGCTATTAAATGGCAGTCTAGCAGAGGAAAAG------------
GTAATGATTAGGTCTGAAAATATCACAGACAAT---------GGCAAAATCATAATAGTACAGCTTACT----
-----------AAGCCCGTAAGC---------ATCACTTGTATCAGACCTGGCAACAATACAAGAACA-----
-AGTATACGTATA------------GGACCAGGACAAACC---TTC------TATGCAACAGGTGAT------
---------GTAATAGGGGACATA------------AGGAAAGCATATTGT---AATGTCAGCAGAACA---
GAATGGAATAGCACTTTACAAAAGATAAGTACACAATTAAAA---------------GAATACTTTAAT----
-----------------------------------AACAAAACAATAATCTTTCAG------
AACTCCTCAGGAGGGGATTTAGAAGTTACA---ACACATAGTTTCAATTGTGGAGGAGAA---
TTTAAACAGCTCCAGGCAAGAGTCCTGGCTGTGGAAAGATACCTAAAGGATCAGCAGCTCCTAGGAATTTGGG
GTTGCTCTGGAAGACTCATCTGCCCCACTAATGTGCCCTGGAACTCTAGTTGGAGT-----------------
----------------------------AATAAATCTCAGGAGGAGATATGG------GGT------
AACATGACCTGGATGCAATGG---GACAAGGAAGTTATCAATTACACAAACATAATATAT---
GATCTAATTGAACAATCGCAAAACCAGCAGGAA--

AAGAATGAACAAGATTTATTGGCATTAGATAAGTGGGCAGATCTGTGGAATTGGTTTAGCATATCAAATTGGT
TATGGTATATAAGAATATTTATAATAATAGTAGGAGGCTTAATAGGATTAAGAATAGTTTTTGCT---
GTGCTTTCTATAATA---------AATAGAGTCAGGCAGGGATACTCACCC------
TTGTCATTGGCTATTAATCTGTTAGATGTCACAGCAATAGCAGTAGCTGGG---
TGGACAGATAGGATTATAGAAATAGGACAAAGATTTTGTAGAGCT----------------------------
--------ATTTGTAACATA------CCTAGGAGAATCAGACAGGGCGCAGAAAAGGCTTTGCAA---TAA
>FJ864679
GTT---GCA--
GGAAACTTGTGGGTCACGGTATACTATGGGGTACCTGTGTGG---AGAGATGCAAAG---
ACCACACTATTTTGTGCATCAGATGCTAAAGCATATGATAAAGAA---GTACACAAT----------------
--------------GTCTGGGCTACA---CATGCCTGTGTACCCACAGAC---
CCTAACCCACAAGAAATAGTTTTGGAA---------AATGTGACAGAAGACTTTGAC----------------
--------------------------ATATGGAAAAATGACATGGTAGAACAAATGCAAACAGATATA---

ATCAATCTCTGGGACCAGAGCCTA---AAGCCATGTGTAAAGTTA---
ACCCCTCTCTGCGTTACTTTAAATTGTTCTGACCCCACTGAATTCAACAGCACTAGCTATAGC----------
----
----------------------AACATTAGCAGTAGCAGAATCAGAAATGATACTGATATGAAA---
AACTGCTCTTTCCAAATGACCACAGAACTAAGA---------------------------
GATAGAACAAAAACTGTACATTCACTT---TTTTATAGACTTGATATAGTATCAACTAATGAT----------
----

AGTGATAATAGGCTGTATAGACTAATAAGTTGCAATACATCAGAGGTGAAACAG---
GCCTGTCCTAAGGTAACCTTTGAGCCAATTCCTATACATTATTGTGCCCCAGCTGGTTTTGCGATTCTAAAAT
GTAGG---GATGCAAATTTTACA------GGAAAAGGACATGCCAGAAT------------------------
-GTCAGC---ACAGTACAATGCACACATGGAATCAAGCCAGTAGTATCAACT---
CAACTGCTGTTAAATGGCAGTCTAGCAGAGAAAGAG------------
ATAATGATTAGATCTAAAAATCTCTCAAACAAT---------GCCAAAAACATAATAGTACAGCTTAAT----
-----------GAGCCTGTAAAC---------ATCACTTGTATCAGACCTAACATCAAGACAAGAAAA---
GGTATACATGTAGGACAT------ACAGGACCAGGACAAGCC---ATC------TATGTAACAGGTGCT----
-----------GTAACAGGGGACATA------------AGAAAAGCACATTGT---AATGTCAGCAGAGCA--
-AAATGGAATACAACGTTACAAAAACTAAGTGAAAGATTAAGA------------AAATACTTTGTA------
------------------------------------AACAAAACAATAATCTTTAAG------
AACCACTCAGGAGGGGATGCAGAAATTACA---ACACATAGTTTCAATTGTGGAGGAGAA---
TTTAAACAGCTCCAGGCAAGAGTCCTGGCTGTGGAAAGATACCTGAAGGATCAGCAGCTCCTAGGAATTTGGG
GTTGCTCTGGAAAACTCATCTGCACCACTAATGTGCCCTGGAACTCTAGTTGGAGT-----------------
----------------------------AACAAATCTCTGAGGGCGATATGG------GAT------
AACATGACCTGGATGGAATGG---GATAAGGAAGTTCACAATTACACAAACATAATATAT---
CAGCTAATTGAAGAATCGCAAAACCAGCAGGAA--

AAGAATGAACAAGAATTATTGGCATTTGATAAGTGGGCAAGTCTGTGGAATTGGTTTGACATATCAAAGTGGT
TATGGTATATAAGAATATTTATAATGATAGTAGGAGGCTTAATAGGATTAAGAATAGTTTTTACT---
GTGCTTTCTATAATA---------AATAGAGTCAGGCAGGGATACTCACCC------
TTGTCATTGGCTATCAATCTAATAGATACCATAGCAATAGTAGTAGCTGGG---
TGGACAGATAGGGTTATAGAAATAGGACAAAGATTTTGTAGAGCC----------------------------
--------ATTCGTAACATA------CCTAGGAGAATCAGACAAGGAGCAGAAAGGGCTTTGCAG---TAA
>JQ292893
GCT---ACA--
GAAAACTTGTGGGTCACAATATACTATGGGGTACCTGTGTGG---AGAGATGCTGAG---
ACCACCTTATTTTGTGCATCAGATGCTAAGGCATATGATAAAGAA---GTACACAAT----------------
--------------GTCTGGGCTACA---CATGCCTGTGTACCCACAGAC---
CCTAACCCACAAGAAATAGCTTTGGGA---------AATGTGACAGAAAATTTTGAC----------------
--------------------------ATGTGGAAAAATAACATGGTAGAACAAATGCAAACAGATATA---
ATCAGTCTCTGGGACCAAAGCCTA---AAGCCATGTGTAAAGTTA---
ACCCCTCTCTGCGTTACTTTAAATTGTGCTGAACCCAACAGCACTAGATCTAACAGCACTAGCTCTAAC----
----
-------------AACAGTAGCGTTAACAGCAACAGCGGTGATAGTTTATTTGAAGAAGTGAAA---
AACTGCACTTTCAACATGACCACAGAATTAAGA---------------------------
GATAAAAGGAAAACTGTACATTCACTT---TTTTATAAACTTGATATAGTGTCAACTAATAAT----------
----

GGTAGTGGGCAGTATAGACTAATAAACTGCAATACATCAGCCATGACACAG---
GCCTGTCCTAAGGTATCCTTTGAGCCAATTCCTATACATTATTGTGCCCCAGCTGGTTTTGCGATCCTAAAAT
GCAAG---GATATAAATTTTAAT------GGAACAGGGCCATGCAGGAAT-----------------------
-GTCAGC---ACAGTACAATGCACACATGGAATCAAGCCAGTAGTATCAACT---
CAACTGCTGTTAAATGGCAGTCTAGCAGAGAAAGAT------------
GTAATGATTAGGTCTAAGAATATCACAGACAAT---------GGCAAAATCATAATAGTACAGCTTACT----
-----------GAGCCTGTAAAC---------ATCACTTGTATCAGACCTGGCAACAATACAAGAACA-----
-AGTATACGTATA-------------GGACCAGGACAAACC---TTC------TATGCAACAGGTGCT------
---------GTAATAGGGAACATA------------AGAAAAGCATATTGT---TATATGAACAGAACA---
GCATGGAATAGAACTTTACAAAAGATAAGTATACAATTAAGA---------------ACATACTTTAAA----
-----------------------------------AACAAAACAATAATCTTTAAG------
AACTCTTCAGGAGGGGATATAGAAGTTACA---ACACATAGTTTCAATTGTGGAGGAGAA---
TTTAAACAGCTCCAGGCAAGAGTCCTGGCTGTGGAAAGATACCTAAAGGATCAGCAGCTCCTAGGAATTTGGG

GTTGCTCTGGAAAACTCATCTGCACCACTAATGTGCCCTGGAACTCTAGTTGGAGT-----------------
----------------------------AACAAATCTCAGAAGGAGATATGG------GAT------
AACATGACCTGGATGCAATGG---GACAAGGAAGTTATCAATTACACAGAAATAATATAT---
GAGCTAATTGAAGAATCGCAAAACCAGCAGGAA--

AAGAATGAACAAGATTTATTGGCATTGGATAAGTGGGCAAGTCTGTGGAGTTGGTTTGACATATCAAATTGGT
TATGGTACATAAGAATATTTATAATAATAGTAGGAGGCTTAATAGGATTAARAATAGTTTTTGCT---
GTGCTTTCTATAATA---------AATAGAGCCAGGCAGGGATACTCACCT------
TTGTCATTTGCTATCAATCTAGTAGATACCATAGCAATAGCAGTAGCTGGG---
TGGACAGATAGGGTTATAGAAGTAGGACAAAGACTTTGTAGAGCC----------------------------
--------ATTTGTAACATA------CCTAGGAGAATCAGACAGGGCGCAGAAAAGGCTTTGCAA---TAA
>JF430898
GTT---ACA--
GGAAACTTGTGGGTCACGGTATACTATGGGGTACCTGTGTGG---AGAGATGCAGAG---
ACCACCCTATTTTGTGCATCAGATGCTAAAGCATATGATAAAGAG---GTACACAAT----------------
--------------GTCTGGGCTACA---CATGCCTGTGTCCCCACAGAC---
CCTAACCCACAAGAAATAGATTTGGAA---------AATGTGACAGAAAAATTTGAC----------------
--------------------------ATGTGGAAAAATAACATGGTAGAACAAATGCAAACAGATATA---
ATCAGCCTCTGGGACCAAAGCCTA---AAGCCATGTGTAAAGTTA---
ACCCCTCTCTGTGTTACTTTAAATTGTACTGAACCCAACCCCAACAGCACTAGTGTTAACATCACTAGCATGA
ACAACACTAGA--
----------------------GTTAGCATCAACAGCAACGATAGTTTGTATGAAGAAATGAAA---
AACTGCTCTTTCAACATGACCACAGAACTAAGA---------------------------
GATAAAAGGAAAAAGGCATATTCACTT---TTTTATAAACTTGATATAGTATCAATTGATAGT----------
----

AATGGTAGTAATGATAGTAGGCAGTATAGACTAATAAATTGCAATACATCAGCCATGACACAG---
GCCTGTCCTAAGGTAACTTTTGAGCCAATTCCTATACATTATTGTGCCCCAGCTGGTTTTGCGATTCTAAAGT
GCAAG---GATCCATATTTCAAT------GGAACAGGACCATGCAAGAAT-----------------------
-GTCAGC---ACAGTACAATGCACACATGGAATCAAGCCAGTAGTATCAACT---
CAACTGCTGTTAAATGGCAGTCTAGCCGAGAAAGAG------------
ATAATGATTAGATCTAAAAATATCACAGACAAT---------GGCAAGATCATAATAGTACAGCTTACT----
-----------GAGCCTGTAAAA---------ATCACTTGTATCAGACCTGGCAACAATAGAAGAACA-----
-AGTATACATATG------------GGACCGGGACAAGCT---TTC------TATGCAACAGGTGCT------
---------ATAACAGGGGACATA------------AGAAAAGCACATTGT---CAAGTCAACAGAACA---
GCGTGGAATAGCACTTTACAACAGATAAGTATAAAATTAAGA---------------CAATACTTTGCT----
-----------------------------------AACAAAACAATAATCTTTAAG------
AACTCCTCAGGAGGGGATTTAGAAGTTACA---ACACATAGTTTCAATTGTGGAGGAGAA---
TTCAAACAGCTCCAGGCAAGAGTCCTGGCTGTAGAAAGATACCTAAAGGATCAGCAGCTCCTAGGAATTTGGG
GTTGCTCTGGAAAACTCATCTGCACCACTAATGTGCCCTGGAACTCTAGTTGGAGT-----------------
----------------------------AACAAATCTCAGAGTGAAATATGG------AAT------
AACATGACCTGGATGCAATGG---GACAGGGAAGTTATCAATTACACAAACATAATATAT---
GATCTAATTGAAGAATCGCAAAACCAGCAGGAA--

AAGAATGAACAAGATTTATTGGCATTGGATAAGTGGGCAAGTCTGTGGAATTGGTTTGACATATCAAAGTGGC
TATGGTATATAAAAATATTTATAATAATAGTAGGAGGCTTAATAGGATTAAGAATAGTTTTTGCT---
GTGCTTTCTATAATA---------AATAGAGTCAGGCAGGGATACTCACCC------
TTGTCATTGGCTATCAAGCTAATAGATACCATAGCAATAGCAGTAGCTGGG---
TGGACAGATAGGATTATAGAAATAGGACAAAGATTTTGTAGGGCA----------------------------
--------ATCTGTAACATA------CCTAGGAGAATCAGACAGGGCGCAGAAAAGGCTTTGCAA---TAA
>HQ834967
GCT---GCA--
GAAAACTTGTGGGTCACAGTATACTATGGGGTACCTGTGTGG---AGAGATGCAGAG---
ACCACCCTATTTTGTGCATCAGATGCTAAAGCATATGATAAAGAA---GTGCACAAT----------------
--------------GTCTGGGCTACA---CATGCCTGTGTACCCACAGAC---
CCTAGCCCACAAGAAATAGCTTTGGAA---------AATGTGACAGAAAATTTTGAC----------------
--------------------------ATGTGGAAAAATAACATGGTAGAACAAATGCAAACAGATATA---
ATCAGTCTCTGGGACCAAAGCCTA---AAGCCATGTGTAAAGTTA---
ACCCCTCTCTGCGTTACTTTAGATTGTGTTAAACCTACCAGTGACAACGCTAGC-------------------
----

---------------------------------------CTTAACAACAGTAGCGTTATGAAA---
AACTGCTCTTTCAACATGACCACAGAACTAAGA--------------------------
GATAAAAGGAAAAATGTATATTCACTT---TTTTATAAACTTGATATAGTATCAATTAATAGT---------
----

AGTAATAATGGTGATGGGCCTTATAGACTAATAAATTGTAATACATCTACTGTGACACAG---
GCCTGTCCTAAGGTAACATTTGAGCCAATTCCTATACATTATTGTGCCCCAGCTGGTTTTGCGATTCTAAGT
GCAGG---GATCCAAATTTTAAT------GGAACAGGGCCATGCAAGAAT----------------------
-GTCAGC---TCAGTACAATGCACACATGGAATCAAGCCAGTAGTATCAACT---
CAACTACTGTTAAATGGCAGCCTAGCAGAGGAAAAG-----------
GTAATGATTAGATCTCAAAATATCACAGACAAT--------GGCAAAATCATAATAGTACAGCTTACT----
-----------GAGCCTGTAAAC--------ATCACTTGTATCAGACCTGGCAACAATACAAGAAAA-----
-AGTATACGTATA-----------GGACCAGGACAGACC---TTC------TATGCAACAGGTGAT------
---------GTAATAAGTGACATA------------AGAAAAGCACATTGT---AATGTCAGCAAATCA---
AAATGGAATAGCACTTTACAAGAGATAGGTACACAATTAAGA--------------AAATACTTTAAT----
----------------------------------AACAAAACAATAATCTTTAAG------
AGCTCCTCAGGAGGGGATTTAGAAGTTACA---ACACATAGTTTCAATTGTGGAGGAGAA---
TTTAAACAGCTCCAGGCAAGAGTCCTGGCTGTGGAAAGATACCTAAAGGATCAGCAGCTCCTAGGAATTTGGG
GTTGCTCTGGAAAACTCATCTGCACCACTAGTGTGCCCTGGAACTCTAGTTGGAGT----------------
---------------------------AACAAATCTCATAATGAGATATGG------GAA------
AACATGACCTGGATGCAATGG---GACAGGGAAATTATCAATTACACAAACCTAATATAT---
AATCTAATTGAAGAATCGCAAAACCAGCAGGAA---------------------------------------
----
AAGAATGAACAGGATTTATTGGCATTGGATAAGTGGTCAAGTCTGTGGAGTTGGTTTGACATAACAAATTGGT
TATGGTATATAAGAATATTTATAATGATAGTAGGAGGCTTAATAGGATTAAGAATAGTTTTTGCT---
GTACTTTCTATAATA---------AATAGAGTCAGGCAGGGATACTCACCC------
TTGTCATTTGCTATCAATCTAGTAGATACCTTAGCAATAGCAGTAGCTGGG---
TGGACAGATAGGATTATAGAAATAGTACAAAGATTTTGTAGAGCC---------------------------
--------ATTTGGAACATA------CCTAGGAGAATCAGACAGGGCGCAGAAATAGCTTTGCAA---TAA
>JX500696
GCT---GCA---
GAAAACTTGTGGGTCACGGTGTACTATGGAGTACCTGTGTGG---AGAGATGCAGAG---
ACCACCCTATTTTGTGCATCAGATGCTAAAGCATATGATAAAGAA---GTACACAAT---------------
--------------GTCTGGGCTACA---CATGCCTGTGTACCCACAGAC---
CCTAACCCACAAGAAATAGCTTTGGAA---------AATGTGACAGAAGATTTTGAC---------------
-------------------------ATATGGAAAAATAACATGGTAGAACAAATGCAAACAGATATA---
ATTAGTCTCTGGGACCAAAGTCTA---AAGCCATGTGTAAAGCTA---
ACCCCCCTCTGTGTTACTTTAAATTGTACTGACCTCAACAGCACTAGCTCTAACAACATTACC---------
----
---------------------------CGCAACAGCAGCGATAGTTTGTTAGGAGAAATGAAA---
AACTGCTCTTTCAACATGACCACAGAAATCAGA--------------------------
GATAAAAAGAAAACTGTATACTCACTT---TTTTATAAACTTGATATAGTATCAACTGATAGAAAT-------
----

GATAGTAATCAGTATAGACTAATAAATTGCAATACATCAACCATGACACAG---
GCCTGCCCTAAGGTAACCTTTGAGCCAATTCCTATACATTATTGTGCTCCAGCTGGTTTTGCGATTCTAAGT
GCAGG---GATACAAATTTTACG------GGAAAAGGGGCATGCAGGAAT----------------------
-GTCAGC---ACAGTACAATGCACACATGGAATCAAGCCAGTAGTATCAACT---
CAACTGCTGTTAAATGGCAGTCTAGCAGAGAGAGAG-----------
GTAATGATTAGATCAGAAAATATCACAGACAAT--------AGCAAAACCATAATAGTGCAGCTTACT----
-----------AAGCCTGTAAAC--------ATCACTTGTATCAGACCTGGCAACAATACAAGAACA-----
-AGTATACGAATA-----------GGGCCAGGACAAACC---TTC------TATGCAACAGGTGAG------
---------GTAATAGGGGACATA------------AGAAAAGCATATTGT---AATGTCAGCAAAAAA---
GAATGGAATGACACTTTACAAGAGATAAGTGCACAATTAAGA--------------GAATACTTTAAT----
----------------------------------AACAAAACAATAATCTTTAAG------
AACTCCACAGGAGGGGATTTAGAAGTTACA---ACACATAGTTTCAATTGTGGAGGAGAA---
TTTAAACAGCTCCAGGCAAGAGTCCTGGCTGTGGAAAGATACCTAAAGGATCAGCAGCTCCTAGGAATTTGGG
GTTGCTCTGGGAAACTCATCTGCACCACTAATGTGCCCTGGAACTCTAGTTGGAGT----------------
---------------------------AACAAATCTCAGAGTGAGATATGG------GAT------
AACATGACCTGGATGCAATGG---GACAAGGAAATTATCAATTACACAAACATAATATAT---

```
AATCTAATTGAAGAATCGCAGAACCAGCAGGAA---------------------------------------
----------------------------
AAGAATGAACAAGATTTATTGGCATTGGATAAGTGGGCAAGTCTGTGGAGTTGGTTTGACATATCAATTGGT
TATGGTATATAAGAATATTTATCATAATAGTAGGAGGCTTAATAGGATTAAGAATAGTTTTTGCT---
GTGCTCTCTATAATA---------AATAGAGCCAGGCAGGATACTCACCC------
TTGTCATTGGCTATCAATCTAATAGATACCATAGCAGTAGCAGTAGCTGGG---
TGGACAGATAGGGTTATAGAAACAGGACAAAGACTGTGTAAAGCC---------------------------
--------ATTCGTAACATA------CCTAGAAGAATCAGACAGGGCGCAGAAAAAGCTTTGCAA---TAA
>JX500695
GCT---GCA-----------------------------------------
GAAAACTTGTGGGTCACAGTATACTATGGGGTACCTGTGTGG---AGAGATGCAGAG---
ACCACCCTATTTTGTGCATCAGATGCTAAAGCATTTGAAAAAGAA---GTACACAAT---------------
--------------GTCTGGGCTACA---CATGCCTGTGTGCCCACAGAC---
CCTAACCCACAAGAAATAGCTTTGGAA---------AATGTGACAGAAAATTTTAAC---------------
-------------------------ATGTGGAAAAATAACATGGTAGAACAAATGCAAACAGATATA---
ATCAGTCTCTGGGACCAAAGCCTA---AAGCCATGTGTAAAGTTA---
ACCCCTCTCTGCGTTACTTTAAATTGTGCTGAGCCCTCTAACAACAGGAGCGCTAACAAC------------
------------------------------------------------------------------------
------------------------------AACAGCAGTGATAATTTGTTTGAAGAAATGAAA---
AACTGCTCTTTCAACATGACCACAGAACTAAGA--------------------------
GATAGAAGGAAAACTGTGTATTCACTT---TTTTATAAACTTAATATAGTATCAGCTGATAGTAAT------
------------------------------------------------------------------------
----------------------
AGTAGTGGGAAGTATAGACTAATAAATTGCAATACATCAACCATGACACAG---
GCCTGTCCTAAGATAACATTTGAGCCAATTCCTATACATTATTGTGCCCCAGCTGGTTTTGCAATTCTAAAAT
GCAAG---GATCCAAATTTTAAT------GGAACAGGGCCATGCAAGAAT----------------------
-GTCAGC---ACAGTACAATGCACACATGGAATCAAGCCAGTAGTATCAACT---
CAACTGCTGTTAAATGGCAGTCTAGCAGAGAAAGAG-----------
GTAATGATCAGATCTGAAAATATCACAGACAAT---------GGCAAAATCATAATAGTACAGCTTGCT---
-----------GATCCTGTAAAC---------ATCACTTGTATCAGACCTGGCAACAATACAAGAACA----
-AGTATACGTATA------------GGACCAGGACAAACC---TTC------TATGCAACAGGTGAT-----
---------ATAATAGGGGACATA------------AGAAAAGCATATTGT---AATATCAGCAGAGAA---
GCATGGGATAGCGCTTTACAAAAAATAAGTACACAATTAAGA--------------AAATACTTTAAT----
-----------------------------------AACAAAACAATAATCTTTGAG------
AAATCCTCAGGAGGGGATTTAGAAGTTACA---ACACATAGTTTCAATTGTGGAGGAGAG---
TTTAAACAGCTCCAGGCAAGAGTCCTGGCTGTGGAAAGATACCTAAAGGATCAGCAGCTCCTAGGAATTTGGG
GTTGCTCTGGAAAACTCATCTGCACCACTAATGTGCCCTGGAACTCTAGTTGGAGT----------------
----------------------------AACAAATCTCAGAGTGAGATATGG------AAT------
AACATGACTTGGATGCAATGG---GACAGGGAAGTTATCAATTACACAAACATGATATAT---
GATCTACTTGAAGAATCGCAAAACCAGCAGGAA---------------------------------------
----------------------------
AAGAATGAACAAGATTTATTGGCGTTGGATAAGTGGGCAAGTCTGTGGAATTGGTTTGACATATCACATTGGT
TATGGTATATAAAAATATTTATAATGATAGTAGGAAGCTTAATAGGATTAAGAATAGTTTTTGCT---
GTGCTTTCTATAATA---------AATAGAGTCAGGCAGGATACTCACCC------
TTGTCATTGGCTATCAATCTAATAGATACCATAGCAATAGCAGTAGGTAGG---
TGGACAGATAGTGTTATAGAAATAGGACAAAGACTTTGTCGAGCC---------------------------
--------ATTCGTAATATA------CCTAGGAGAATCAGACAGGGCGCAGAAAAAGCTTTGCAA---TAA
>JX500694
GCT---GCA-----------------------------------------
GAACATTTGTGGGTCACGGTATACTATGGAGTACCTGTGTGG---AGAGATGCAGAG---
ACCACCCTATTTTGTGCATCAGATGCTAAAGCATATGATAAAGAA---GTACACAAT---------------
--------------GTCTGGGCTACA---CATGCCTGTGTACCCACAGAC---
CCTGACCCACAAGAAATAATGTTGGAA---------AATGTGACAGAAAATTTTGAC---------------
-------------------------ATGTGGAAAAATAACATGGTAGAACAAATGCAAACAGATATA---
ATCAGTCTCTGGGACCAAAGTCTA---AAGCCATGTGTACAGTTA---
ACCCCTCTCTGCGTTACTTTACATTGTGCTGGATCCAACAGCACTAGCTCTAACAACAGTACTAGCTATAAC-
------------------------------------------------------------------------
-------------------AACAGTAGTAGCAACAGCAGCGATAGCTGGGTTGAAGTAATGAGA---
AACTGCTCTTTCAACGTGACCACAGAGCTAAGA--------------------------
GATAAAAAGAAAAAAGTATATTCACTT---TTTTATAGACTTGATATAGTATCAACTGGTAGTAAT------
```


GATAGTGAGCAGTATAGACTAATTAATTGTAATACATCAGCCGTGACACAG---
GCCTGTCCCAAGGTAACCTTTGAGCCAATTCCTATACATTATTGTGCCCCAGCTGGTTTTGCGATTCTAAAGT
GCAAG---GATACAAATTTTACT------GGAAAAGGGCCATGCAAGAAT-----------------------
-GTCAGC---ACAGTACAATGCACACATGGAATCAAGCCAGTAGTATCAACT---
CAACTGCTGTTAAATGGCAGTCTAGCAGAGAAAGAG-----------
GTAATGATTAGATCTGAAAATATCACAGACAAT---------GGCAAAATCATAATAGTACAGCTTACT----
-----------GAGTCTGTGAGC---------ATCACTTGTACCAGACCTGGCAACAATACAAGAACA-----
-AGTATACGTATA------------GGACCAGGACAAACC---TTC------TATGCAACAGGTGAT------
---------GTAAYAGGGGACATA------------AGAAGAGCATATTGT---AATGTCAGCAGAACA---
GAATGGAATAGCACCTTACAAAAGATAAGTACACAATTAAGA---------------AGATACTTTCAT----
-----------------------------------AACAAAACAATAATCTTTAGG------
AACTCCTCAGGAGGGGATGTAGAAGTTACA---ACACATAGTTTCAATTGTGGAGGAGAA---
TTTAAACAGCTCCAGGCAAGAGTCCTGGCTGTGGAAAGATACCTAAGGGATCAGCAGCTCCTAGGAATTTGGG
GTTGCTCTGGAAAACTCATCTGCACCACTAGTGTGCCCTGGAACTCTAGTTGGAGT-----------------
----------------------------AATAAATCTCGGCAGGAGATATGG------GAT------
AACATGACCTGGATGCAATGG---GACAAGGAAGTTTTCAATTACACAAATCTAATATAT---
GAGCTGCTTGAAGAATCGCAAAACCAGCAGGAA--

AAGAATGAACAAGATTTATTGGCATTGGATAAGTGGACAAGTCTGTGGAATTGGTTTGACATATCAAATTGGT
TATGGTATATAAAAATATTTATAATGATAGTAGGAGGCTTGATAGGATTAAGAATAATTTTTACT---
GTGCTTTCTATAATA---------AATAGAGCCAGGCAGGGATACTCACCC------
TTGTCATTGGCTATTAATCTGGTAGATACCATAGCAATAGTAGTAGCTGGG---
TGGACAGATAGGGTTATAGAAATAGGACAAGGAGCTTGTAGAGCT----------------------------
--------ATTTGTAACATA------CCTAGGAGAATCAGACAGGGCGCAGAAAGGGCTTTGCAA---TAA
>MH603967
GCT---GCA--
GAAAAATTTTGGGTCACAGTATATTATGGGGTACCTGTGTGG---AGAGATGCAGAG---
ACCACTCTGTTTTGTGCATCAGATGCTAAAGCATATGAGAAAGAA---GTACACAAT----------------
--------------GTCTGGGCTACA---CATGCCTGTGTACCCACAGAC---
CCTAACCCACAAGAAATAAGTTTGGGA---------AATGTGATAGAAAATTTTGAC----------------
--------------------------ATGTGGAACAATAACATGGTAGAACAAATGCAAACAGATATA---
ATCAGTCTCTGGGATCAAAGCCTA---AAGCCATGTGTAAAGTTA---
ACCCCTCTCTGTGTTACTTTAAATTGTACTGAACCCAGAAAAAGTAATAGCTCTAAAATTAATAACAGTACAG
ATTACAGTGGC--
----------------AACAGTAACAGTACAGTTAACAGCAGCAGCATATATGAAGAAATGAGA---
AACTGCTCTTTCAACATGACCACAGAACTAAGA--------------------------
GATAAAAAGAAAAATGTCTATTCACTC---TTTTATAAACTTGATGTAGTACCACTTGGTAGTAAT-------
----

AATAGTGGAGAGTATAGATTAATAAATTGTAATACATCAACCATGACACAG---
GCCTGCCCTAAGGTAACCTTTGAGCCAATTCCTATATATTATTGTGCCCCAGCTGGTTTTGCAATTCTAAAAT
GCAAG---GATCCAAAATTTAAT------GGAACAGGGCCATGCAAGAAT-----------------------
-GTCAGC---TCAGTACAATGCACACATGGAATCAAGCCAGTAGTATCAACT---
CAACTGCTGTTAAATGGCAGTCTAGCAGAGGAAAAG-----------
GTAATGATTAGATCTAAAAATATCACAGACAAT---------AGCAAAAATATAATAGTACAGCTTACT----
-----------GAGCCTGTAAAC---------ATCACTTGTATCAGAACTGGCAATAATACAAGGACA-----
-AGTATACGTATA------------GGACCAGGACAAACC---TTC------TATGCAACAGGTGAT------
---------GTAATAAGAGACATA------------AGAAAAGCACATTGT---TATGTCAACAAAACA---
GCATGGAATAGAGCTTTACAAAATATAAGTATACAATTAAGA---------------CAGCACTTT-------
-----------------------------------AATAAAACAATAATCTTTAGG------
AACTCCACAGGAGGGGATTTAGAAATTACA---ACACATAGTTTCAATTGTGGAGGAGAA---
TTTAAACAGCTCCAGGCAAGAGTCCTGGCTGTGGAAAGATTCCTAAGGGATCAGCAGCTCCTAGGAATTTGGG
GTTGCTCTGGAAAACTCATCTGCACCACTAATGTGCCCTGGAACTCTAGTTGGAGT-----------------
----------------------------AATAAATCTCTGAATGAGATATGG------GAT------
AACATGACCTGGTTGCAATGG---GACAGGGAAGTTAGCAATTACACAAACCTAATATAT---
GACTTGCTTGAAAAATCGCAGAACCAGCAGGAA--

AAGAATGAACAAGATTTATTGGAACTGGATAAGTGGGCAAGCCTGTGGAATTGGTTTGACATATCAAATTGGT

TATGGTATATAAAAATATTTATAATGATAGTAGGAGGCTTAATAGGATTAAGAATAGTGTTTGCT---
GTGCTATCTATAATA---------AGGAGAGTCAGGCAGGGATACTCACCT------
GTGTCATTGGCTATTAATCTGCTAGATACCATAGCAATAGCAGTAGCTGGG---
TGGACAGATAGAGTCATAGAAATAGGACAAAGATTTGGTAGAGCT----------------------------
--------ATTTGTAACATA------CCTAGGAGAATCAGACAGGGCGCAGAAAAGGCTTTGCAA---TAA
>MH604120
GCT---GCA--
GGAAACTTGTGGGTCACGGTATACTATGGGGTACCTGTGTGG---AGAGATGCAGAA---
ACCACCCTATTTTGTGCGTCAGATGCTAAAGCATATGACAAAGAA---GTACACAAT----------------
--------------GTCTGGGCTACA---CATGCCTGTGTACCCACAGAC---
CCTGACCCACAAGAAATAGATTTGGAA---------AATGTGACAGAAGAATTTAAC----------------
--------------------------ATGTGGAAAAATAACATGGTAGAACAAATGCAAACAGATATA---
ATCAGTCTCTGGGACCAAAGCCTA---AAGCCATGTGTAAAGTTA---
ACCCCTCTCTGCGTTACTTTAAATTGTACTGCACCCAACAGCACTAGCTCTAGCAACAGT-------------
----
-------------------------AGCGTTAACAGCAATAGTAGCATGTTTGAAGAAATGAAA---
AACTGCTCTTTCAACATGACCACAGAACTAAGA---------------------------
GATAAAAGGAAAACTGTATATTCACTT---TTTTATAAACTTGATATAGTATCAATTGATAGTGAT-------
----

GGTAATAATAATAATAGTAATGGGCAGTATAGACTAATAAATTGTAATACATCAACCACGACACAG---
GCCTGTCCCAAGGTATCCTTTGAGCCAATTCCTATACATTATTGTACTCCAGCTGGTTTTGCGATTCTAAAGT
GCAAG---GATCCAAAATTTAAT------GGAACAGGGCCATGCAAAAAT-----------------------
-GTCAGC---ACAGTACAATGCACACATGGAATCAGGCCAGTAGTATCAACT---
CAACTGCTGTTAAATGGTAGTCTAGCAGAAGAAAAG-----------
GTAATGATTAGATCTGAAAATATTACAGACAAT---------GGCAAAATCATAATAGTACAGCTTAAG----
-----------AACCCTGTAAAC---------ATCACTTGTGCCAGACCTGGCAACAATACAAGAACA-----
-AGTATACGTATA------------GGACCAGGACAAACC---TTC------TATGCAACAGGTGCT------
---------GTAATAGGGGACATA-----------AGAAAAGCACATTGT---AATGTTAGCAGAACA---
GAATGGAATAACACTTTAAAAGAAATAAGTAACCAATTAGGA---------------GAATACTTTAAG----
-----------------------------------AACAAAACAATAATCTTTAAG------
AAATCCTCAGGAGGGGACTTAGAAATTACA---ACACATAGTTTCAATTGTGGAGGAGAA---
TTTAAGCAGCTCCAGGCAAGAGTCCTGGCTGTGGAAAGATACCTAAAGGATCAGCAGCTCCTGGGAATTTGGG
GTTGCTCTGGAAAACTCATCTGCCCCACTAGTGTGCCCTGGAACTCTAGTTGGAGT-----------------
----------------------------AATAAATCTTATGATGAAATATGG------GGT------
AACATGACCTGGATGCAATGG---GACAGGGAAGTTATCAATTACACAAACAAAATATAT---
GAGCTAATTGAACAATCGCAAGACCAACAGGAA--

AAGAATGAACAAGATTTATTGGCATTGGATAAGTGGGAAGGTCTGTGGAATTGGTTTAACATAACAAACTGGT
TATGGTATATAAAAATATTTATAATGATAGTAGGAGGCTTAATAGGATTAAGAATAGTTTTTACT---
GTGCTTTCTATAATA---------AATAGAGCCAGGCAGGGATACTCACCC------
TTGTCATTGGCTATTAATCTAATAGATACCATAGCAATAGCAGTAGCTGGG---
TGGACAGATAGGGTTTTAGAATTAGGACAAAGACTTTGTAGAGCT----------------------------
--------ATCCGTAACATA------CCTAGAAGAATCAGACAGGGCGCAGAAAGGGCGTTGCAG---TAA
>MH604308
GCT---ATG--
GACAACTTGTGGGTCACGGTATACTATGGGGTACCTGTGTGG---AGAGATGCAGAG---
ACCACCCTATTTTGTGCATCAGATGCTAAAGCATATGATAAAGAA---GTACACAAT----------------
--------------GTCTGGGCTACA---CATGCCTGTGTACCCACAGAC---
CCTAACCCGCAAGAAATAAATTTGGAA---------AATGTGACAGAAGGATTTAAC----------------
--------------------------ATGTGGAAAAATAACATGGTAGAACAAATGCAAACAGATATA---
ATCAGTCTCTGGGATCAAAGCCTA---AAGCCATGTGTAAAGTTA---
ACCCCTCTCTGCGTTACTTTAAATTGTGCTGAACCCAACAGCACTAGCTCTAACAGCAGT-------------
----
-------------------------AGCGGTAACAGCAGTAATAGCTTGTTTGAAGAAATGAAA---
AACTGCTCTTTCAACATGACCACAGAACTAAGG--------------------------
GATAAAAGAAAAAATGTACATTCACTC---TTTTATAAACTTGATATAGTATCAATTGATAATAAT-------
----

AGTAGTGGGCAGTATAGACTAATAAATTGCAATACATCAGCCATGACACAG---

GCCTGTCCTAAGGTAACCTTTGAGCCAATTCCTATATATTATTGTACCCCCGCTGGTTTTGCGATTCTAAAGT
GCAAG---GATCCAAACTTTAAT------GGAACAGGGCCATGCAAGAAT----------------------
-GTCAGC---ACAGTACAATGCACACATGGAATCAAGCCAGTAGTATCAACT---
CAACTGCTGTTAAATGGCAGTCTAGCACAAGAACAG------------
GTAATGATTAGATCTAAAAATATTACAGACAAT---------GGCAAAATCATAATAGTGCAGCTTACT----
-----------GAGCCTGTAAAT---------ATCACTTGTATCAGACCTGGCAACAATACAAGAACA-----
-AGTATACGTATA------------GGACCAGGACAAACC---TTC------TATGCAACAGGTGAT------
---------GTAATAGGGGACATA------------AGAAAAGCACATTGT---AATGTCAGCAGAGCA---
GCATGGAGTAGCACTTTACAAAAGATAAGTACACAATTAAGA---------------AAATACTTTAAT----
-----------------------------------AACAAAACAATAATCTTTAAA------
AACTCCTCAGGAGGGGATTTAGAAATTACA---ACACATAGTTTCAATTGTGGAGGAGAA---
TTTAAACAGCTCCAGGCAAGAGTCCTGGCTGTGGAAAGATACCTAAGGGATCAGCAGCTCCTGGGAATTTGGG
GTTGCTCTGGAAAACTCATCTGCACCACTACTGTGCCCTGGAACTCTAGTTGGAGT-----------------
----------------------------AATAAATCTCAGGAAGAGATATGG------AAT------
AACATGACCTGGATGCAATGG---GACAAAGAAGTTATCAATTACACAGAAAAAATATAT---
GATCTACTTGAAAAATCGCAAAATCAGCAGGAA---------------------------------------
----
AAGAATGAACAAGATTTATTAGCATTAGATAAGTGGGCAGGTCTGTGGAATTGGTTTGACATAACAAATTGGT
TATGGTATATAAAAATATTTATAATGATAGTAGGAGGCTTGATAGGCCTAAGAATAGTTTTTGCT---
GTGCTTTCTATAATA---------AATAGAGCCAGGCAGGGATACTCACCC------
TTGTCATTGGCTACCAATCTAATAGATACCATAGCAATAGTAGTAGCTGGG---
TGGACAGATAGGGTTTTAGAAGTAGGACAAAGACTTTGTAGAGCT---------------------------
--------ATTAGTAACATA------CCTAGGAGAATCAGACAGGGCGCAGAAAAGGCGTTGCAA---TAA
>MH604056
GCT---GCA---
GACAATTTGTGGGTCACAGTATACTATGGGGTACCTGTGTGG---AGAGATGCAGAA---
ACCACCCTATTTTGTGCATCAGATGCTAAAGCATATGATAAAGAG---GTACACAAT----------------
--------------GTCTGGGCCACA---CATGCCTGTGTACCCACAGAC---
CCTAACCCGCAAGAAATACCTTTGGGA---------AATGTGACAGAAGATTTTGAC----------------
--------------------------ATGTGGAAAAATAACATGGTAGAACAAATGCAAACAGATATA---
ATTAGTCTCTGGGACCAAAGCCTA---AAGCCATGTGTAAAGTTA---
ACCCCTCTCTGCGTTACTTTAAATTGTACTAAACCCAACAACAGTACAGATAACGGCAATTAT----------
----
---AGCTTGGGTGAAGAAATGAAA---
AACTGCTCTTTCAACATGACCACAGAAATAAGA--------------------------
GATAAAAAGAAAAAAGTACATTCACTT---TTTTATAAGCTTGATATAGTATCAATGGATGGT----------
----

AATAGTAATGATAGTAGTGGGCAGTATAGACTAATAAATTGTAATACATCAACCATGACACAG---
GCCTGTCCTAAGGTAACCTTTGAGCCAATTCCTATATATTATTGTACCCCGGCTGGTTTTGCGATTCTAAAGT
GCAAG---GATCCAGACTTTAAT------GGAACAGGGCCATGCAAGGAT----------------------
-GTCAGC---ACAGTACAATGCACACATGGAATCAAGCCAGTAGTATCAACT---
CAACTGCTGTTAAATGGCAGTCTAGCAGAAAAAAAG------------
GTAATGATTAGATCTGAAAATATTACAGACAAT---------AGCAAAACCATAATAGTACAGCTTACT----
-----------AAGCCTGTAAAC---------ATCACTTGTGTCAGACCTAACAACAATACAAGAACA-----
-GGTATACATATA------------GGACCAGGACAAACC---TTC------TATGCAACAGGTGAT------
---------GTAATAGGGGACATA------------AGACAAGCACATTGT---AATGTCAGCAAAGCA---
GCATGGAATAGCACTTTACAAGAGATAAGTGCACAATTAAGA---------------AAATACTTTGAT----
-----------------------------------AACAGAACAATAATCTTTAAA------
AATTCCTCAGGAGGAGATTTAGAAATTACA---ACACATAGTTTCAATTGTGGAGGAGAA---
TTCAAACAGCTCCAGGCAAGAGTCCTGGCTGTGGAAAACTACCTAAAGGATCAGCAGCTCCTAGGACTTTGGG
GTTGCTCTGGGAAACTCATCTGCACCACTAATGTGCCCTGGAACTCTAGTTGGAAT----------------
----------------------------AATAAATCTTTCAATGAGATATGG------GAT------
AACATGACCTGGATGCAATGG---GACAAAGAAATTATCAATTACACAAACAAAATATAT---
GATCTAATTGAAGAATCGCAAAACCAGCAGGAA---------------------------------------
----
AAGAATGAACAAGATTTATTGGCATTGGATAAGTGGGCAGATCTGTGGAGTTGGTTTAAGATAACAAATTGGT
TATGGTATATAAGAATATTTATAATGATAGTAGGAGGCTTAATAGGATTAAGAATAGTTTTTGCT---
GTGCTTTCTATAATA---------AATAGAGCCAGGCAGGGATACTCACCC------
TTGTCATTAGCTATTAATCTGCTAGATACTATAGCAATAGTAGTAGCTGGG---

TGGACAGATAGGGTTTTAGAACTAGGACAAAGATTTTGTAGAGCT----------------------------
--------ATTTGTAACATA------CCTAGGAGAATCAGACAGGGCGCAGAAAAGGCTTTGCAA---TAA
>MH604408
GCT---GCT--
GACAACTTGTGGGTCACGGTATACTATGGGGTACCTGTATGG---AGAGATGCAGAG---
ACTACCCTATTTTGTGCATCAGATGCTAAAGCATATGATAAAGAA---GTACACAAT----------------
--------------GTTTGGGCTACA---CATGCCTGTGTACCCACAGAC---
CCTGACCCACAAGAAATAAATTTGGGA---------AATGTGACAGAAGAATTTAAT----------------
--------------------------ATGTGGAAAAATAACATGGTAGAACAAATGCAAACAGATATA---
ATCAGTCTCTGGGACCAAAGCTTA---AAGCCATGTGTAAAGTTA---
ACCCCTCTCTGCGTTACTTTAAATTGTACTGCACCCAACAGCACTAGCTCTAACAGCACTAGCTCT-------
----
-------------------------AACAGCAGCAGCAGTGATAGTTTGTTTGAAGAAATGAAA---
AACTGCTCTTTCAACATGACCACAGAACTAAGA---------------------------
GATAAAAGGAAAACTGTATATTCACTT---TTTTATAAACTTGATATAGTATCAATTGATGGTAAT-------
----

AGTAGTGAGCAATATAGACTAATAAATTGTAATACATCAACCATGACACAG---
GCCTGTCCTAAGGTAACCTTTGAGCCAATTCCTATACATTATTGTACCCCAGCTGGTTTTGCGATTCTAAAAT
GCAAG---GATCCAAACTTTAAT------GGAACAGGGCCATGCAAGAAT-----------------------
-GTCAGC---ACAGTACAATGCACACATGGAATCAAGCCAGTAGTATCAACT---
CAACTACTGTTAAATGGCAGTCTAGCAGAAAAAAAG------------
GTAATGATTAGATCTGAAAATATTACAGACAAT---------AGCAAAACCATAATAGTACAGCTTACT----
-----------GAGCCTGTAAAC---------ATCACTTGTACCAGACCTGGCAACAATACAAGAACA-----
-AGTATACGTATA------------GGACCAGGACAAACC---TTC------TATGCAACAGGTGAT------
---------GTAATAGGGGACATA------------AGAAGAGCATATTGT---GAGGTCAACAAAACA---
GCATGGATAGCACTTTACAAAAGATAAGTATACAATTAAGA----------------AGATACTTTAAT----
-----------------------------------AACAAAACAATAATCTTTAGG------
AACTCCTCAGGAGGAGATTTAGAAGTTACA---ACACATAGTTTTAATTGTGGAGGAGAA---
TTTAAGCAGCTCCAGGCAAGAGTCCTGGCTGTGGAAAGATACCTAAAGGATCAGCAGCTCCTAGGAATTTGGG
GTTGCTCTGGAAAACTCATCTGCACCACTACTGTGCCCTGGAACTCTAGTTGGAGT-----------------
----------------------------AACAAATCTCAGAATGAGATATGG------GAG------
AACATGACCTGGATGCAATGG---GACAAGGAAGTTAGAAATTACACAGAAGTAATATAT---
GATCTGATTGAAAAATCGCAAAACCAGCAGGAA--

AAGAATGAACAAGACTTATTAGCATTGGATAAGTGGGCAAGTCTGTGGAATTGGTTTAACATAACAAATTGGC
TATGGTATATAAAAATATTTATAATGATAGTAGGAGGCTTAATAGGATTAAAAATAGTTTTTGCT---
GTGCTTTCTATAATA---------AATAGAGCCAGGCAGGGATACTCACCC------
CTGTCATTGGCTATTAATCTGATAGATAACTTAGCAATAGTAGTAGCTGGG---
TGGACAGATAGGGTTTTAGAAATAGGACAAAGATTTTGTAGAGCT----------------------------
--------ATTAGCAACATA------CCTAGGAGAATCAGACAGGGAGCAGAAAAGGCTTTGCAA---TAA
>MH604109
GCT---GCA--
GGAAACTTGTGGGTCACGGTATACTATGGGGTACCTGTGTGG---AGAGATGCAGAG---
ACCACCTTATTTTGTGCATCAGATGCTAAAGCATATGATAAAGAG---GTACACAAT----------------
--------------GTCTGGGCCACA---CATGCCTGTGTACCCACAGAC---
CCTAACCCACAAGAAATAATTTTGGAA---------AATGTGACAGAAGAATTTAAC----------------
--------------------------ATGTGGAAAAATAACATGGTAGAACAAATGCAAACAGATATA---
ATTAGTCTCTGGGATCAAAGCCTA---AAGCCATGTGTAAAGATA---
ACCCCTCTCTGCGTCACTTTAACTTGTACTGAACCCACCATCAACAGTACTAGCTCTAACAACAGTAGCAACA
GAAACAGTACT--
----AGACCTAACAACAGTAGCAGTAGCATTAGCAGCAATTATAGTTTGGGGGAAGAAATGAAA---
AACTGCTCTTACAACATGACCACAGAAGTAAGA---------------------------
GATAAAAAGAAAAAGGTATATTCACTT---TTTTATAAACTTGATATAGTATCACTTAATGATAATAAT----
----

AATAGTAGTAATGGTAGTGGACAGTATAGACTAATAAATTGTAATACATCAACCACGACACAG---
GCCTGTCCTAAGGTAACCTTTGAGCCAATTCCTATACATTATTGTGCCCCAGCTGGTTTTGCGATTCTAAAAT
GCAGG---GATCCATACTTTAAT------GGAACAGGGCCATGCAATAAG-----------------------
-GTTAGC---ACAGTACAATGCACACATGGAATCAAGCCAGTAGTATCAACT---

CAACTGCTGTTAAATGGCAGTCTAGCAGAAGATGAA------------
ATAATAATTAGATCTGAAAATATTACAGATAAT---------AGCAAAACCATAATAGTACAGCTTACT----
-----------GAACCTGTAAAC---------ATCACTTGTAGCAGACCTAACAACAATACAAGAACA-----
-AGTGTACGTATA------------GGACCAGGACAAACC---TTT------TATGCAACAGATGGT------
---------GTAATAGGGAACATA------------AGAAAAGCATATTGT---GATGTCAACAGAGCA---
GCATGGAATAGAACTTTACAAAAGATAAGTGAACAATTAAGA---------------AAATACTTTAAT----
-----------------------------------AACAAAACAATAGAGTTTAAG------
AACTCCTCAGGAGGGGATTTAGAAATTACA---ACACATAGTTTCAATTGTAGAGGAGAA---
TTTAAACAGCTCCAGGCAAGAGTCCTGGCTCTGGAAAGCTACCTAAAGGATCAGCAGCTCCTAGGAATTTGGG
GTTGCTCTGGAAAGCTCATCTGCACCACTAATGTGCCCTGGAACTCTAGTTGGAGT-----------------
----------------------------AATAAATCTCTGGATGACATATGG------AAG------
AACATGACCTGGATGCAATGG---GACAGGGAAGTTAGCAATTACACAAACCAAATATAT---
AATCTAATTGAAGAAGCGCAAAATCAGCAGGAA---

AAGAATGAACAAGATTTATTAGCATTAGATACGTGGGCAGGCCTGTGGAATTGGTTCACCATATCAAAGTGGT
TATGGTATATAAAAATATTTATAATGATAGTAGGAGGCTTAATAGGATTAAGAATAGTTTTTGCT---
GTGCTTGCTATGATA---------AATAGAGCCAGGCAGGGATACTCACCC------
TTGTCATTGGCTATTAATCTGATAGATACCATAGCAATAGTAGTAGCTGGG---
TGGACAGATAGGGTTTTGGAAATAATACAAAGATTTTGTAGAGCT----------------------------
--------ATTTGTAACATA------CCTAGGAGAATCAGACAGGGCGCAGAAAGGGCTTTGCTA---TAA
>MH604508
GCT---GCA---
GGAAACTTGTGGGTCACAGTATACTATGGGGTACCTGTATGG---AAAGATGCAGAG---
ACCACCCTATTTTGTGCATCAGATGCTAAAGCATATGATAAAGAA---GTACACAAT----------------
--------------GTCTGGGCTACA---CATGCCTGTGTACCCACAGAC---
CCTAACCCACAAGAAATAAATTTGGAA---------AATGTGACAGAAGAATTTGAC----------------
--------------------------ATGTGGAAAAATAACATGGTAGAACAGATGCAAACAGATATA---
ATCAGTCTTTGGGACCAAAGCCTA---AAGCCATGTGTAAAGTTA---
ACCCCTCTCTGTGTTACTTTAAGGTGTACTGAACCCAAAAGTAATAGCTCTAATAATAGT-------------
----
-------------------------AGCGTTACCAACAACAGCAGTATGTTTGAAGAAATGAAA---
AACTGCTCTTTCAACATGACCACAGAACTAAGA--------------------------
GATAAAAAGAAAAATGTATATTCACTT---TTTTATAAACTTGATATAGTATCAACTGGTAGT----------
----

AATATTATTAATGGTAGTGGAGAGTATAGATTAATAAATTGTAATACATCAACTATGACACAG---
GCCTGTCCTAAAGTAAACTTTGAGCCAATTCCTATATATTATTGTGCCCCAGCTGGTTTTGCGATTCTAAAGT
GCAAG---GATGCAAATTTTAAT------GGAACAGGGCCATGCAAGAAT----------------------
-GTCAGC---ACAGTACAATGCACACATGGAATCAAGCCAGTAGTATCAACT---
CAACTGCTGTTAAATGGCAGTCTAGCAGAGAAAGAG------------
GTAATGATTAGATCTAAAAACATCACAGACAAT---------AGCAAAATCATAATAGTACAGCTTACT----
-----------GAGCCGGTAAAC---------ATCACTTGTATCAGACCTGGCAATAATACAAGAACA-----
-AGTATGCATATA------------GGACCAGGACAAACC---TTC------TATGCAACAGGTGAT------
---------GTAATAGGGGACATA------------AGAAAAGCATATTGT---AATGTCAGCAGAGAA---
GCATGGAATAGAACTTTACAAAAAGTAAGTAGACAATTAAGA---------------AAGCACTTTAAT----
-----------------------------------AACAAAACAATAATCTTTAAG------
AACTCCACAGGGGGGGATTTAGAAATTACA---ACACATAGTTTCAATTGTGGAGGAGAA---
TTTAAACAGCTCCAGGCAAGAGTCCTGGCTGTGGAGAGATACCTAAGGGATCAGCAGCTCCTAGGAATTTGGG
GTTGCTCTGGAAAACTCATCTGCACCACTAATGTGCCCTGGAACTCTAGTTGGAGT-----------------
----------------------------AATAAATCTCAGAATGATATATGG------AAT------
AACATGACCTGGATGCAATGG---GACAAGGAAGTTATCAATTACACAGACATAATATAT---
GATCTACTTGAAAAATCACAAAACCAGCAGGAA---

AAGAATGAACAGGAGTTATTGGCATTGGATAAATGGACAAGTCTGTGGAGTTGGTTTGACATATCAAGTTGGT
TATGGTATATAAAAATATTTATAATGATAGTAGGAGGCTTAATAGGATTAAGAATAGTTTTTGCT---
GTACTGTCTATAATA---------AATAGAGCTAGGCAGGGATACTCACCC------
TTGTCATTGGCTATTAATCTGGTAGATACCATAGCAATAGTAGTAGCTGGG---
TGGACAGATAGGGTCATAGAAATAGGACAAAGACTTGGTAGAGCT----------------------------
--------ATTTGGAACATA------CCTAGGAGAATCAGACAGGGAGCAGAAAAGGCTTTGCAA---TAA
>MH604524

GCT---GCA--
GAAAACTTGTGGGTCACGGTATACTATGGGGTACCTGTGTGG---AGAGATGCAGAG---
ACCACCCTATTTTGTGCATCAGATGCTAAAGCATATGATAAAGAA---GTACACAAT----------------
--------------GTCTGGGCTACA---CATGCCTGTGTACCCACAGAC---
CCTAACCCACAAGAAATAGTTTTGGAA---------AATGTGACAGAAAAATTTAAC----------------
--------------------------ATGTGGAAAAATAACATGGTAGAACAAATGCAAACAGATATA---
ATCAGTCTCTGGGACCAAAGCCTA---AAGCCATGTGTAAAGTTA---
ACCCCTCTCTGTGTCACTTTAGATTGTGCTGAACCCAACAGCCATAGCTCTAACAACAAT------------
----
-------------------------AGCGTTAACAGCAGTAATAGCTTGTTTGAAGAAATGAAA---
AACTGCTCTTTCAACACGACTACAGAACTAAGA---------------------------
GATAAAAGGAAAACTGCATATTCACTT---TTTTATAAGCTTGATATAGTACCACTTGATAGT----------
----

AGGAATAATAGTAGACAGTATAGGCTAATAAATTGTAATACATCAACTGTGACACAG---
GCCTGTCCTAAGGTAAACTTTGAGCCAATTCCTATATATTATTGTACCCCAGCTGGTTTTGCGATTTTAAAGT
GTAAG---GAAACAAATTTTACT------GGGAAAGGGTCATGCAAGAAT----------------------
-GTCAGC---ACAGTACAATGCACACATGGAATCAGACCAGTAGTATCAACT---
CAACTGCTGTTAAATGGCAGTCTAGCAGAAAAACAG------------
GTAATGATTAGATCTGAAAATATTACAGACAAT---------AGCAAAATCATAATAGTACAGCTTACT----
-----------GAGCCTGTAAAC---------ATCACTTGTATCAGACCTGGCAACAATACAAGAACA-----
-AGTACACGTATA------------GGACCAGGACAAACC---TTC------TATGCAACAGGTGAT------
---------ATAATAGGGGACATA------------AGAAAAGCATATTGT---AATGTCAACAAAACA---
GCATGGAATAGCACTTTACAAAAGATAAGTATAGAATTAGGA---------------AAATACTTTAAT----
-----------------------------------AACAAAACAATAATCTTTAAG------
AACTCCTCAGGAGGGAATTTAGAAGTTATA---ACACATAGTTTCAATTGTGGAGGAGAA---
TTTAAACAGCTCCAGGCAAGAGTCCTGGCTGTGGAAAGATACCTGAAGGATCAGCAGCTCCTAGGAATTTGGG
GTTGCTCTGGAAAACTCATCTGCACCACTAATGTGCCCTGGAACTCTAGTTGGAGT----------------
----------------------------AATAAATCTCAGAATGAGATATGG------GAT------
AACATGACCTGGATGCAATGG---GACAAGGAAGTTATCAATTACACAGATATAATATTT---
GAGCTAATTGAAAAATCGCAAAACCAGCAGGAA--

AAGAATGAACAAGATTTATTAGCATTGGATAAGTGGACAGGTCTGTGGAATTGGTTTAGCATATCAAATTGGT
TATGGTATATAAGAATATTTATAATAATAGTAGGAGGCTTAATAGGATTAAGAATAGTTTTTGCT---
GTACTTTCTATAATA---------AATAGAGTCAGGCAGGGATACTCACCC------
TTGTCATTGGCTATTAATCTGGTAGATACCATAGCAATAGTAGTAGCTGGG---
TGGACAGATAGGGTTTTAGAAATAGGACAAAGAGTTTGTAGAGCT---------------------------
--------ATTTGTAACATA------CCTAGGAGAATCAGACAGGGCGCAGAAAGGGCTTTGCAA---TAA
>MH604541
GCT---GCA--
GAAAACTTGTGGGTCACCGTATACTATGGGGTACCTGTGTGG---AGAGATGCAGAG---
GCCACCCTATTTTGTGCATCAGATGCTAAAGCATATGATAAAGAA---GTACACAAT----------------
--------------GTCTGGGCTACA---CATGCCTGTGTCCCCACAGAC---
CCTAACCCACAAGAAATAATGTTGGAA---------AATGTGATAGAAGAATTTAAC----------------
--------------------------ATGTGGAAAAATAACATGGTAGAACAAATGCAAACAGATATA---
ATCAGTCTCTGGGACCAAAGCCTA---AAGCCATGTGTAAAGTTA---
ACCCCTCTTTGCGTTACTTTAAATTGTACTGCACCCAACAGCACTAGCTCTAACAACAAT------------
----
-------------------------AGTGTTAACAGCAATAATAGCTTGGTTGAAGAAATGCAA---
AACTGCTCTTTTTACATGACCACAGAACTAAGA---------------------------
GATAAAAAGAAAAAGGTATACTCACTT---TTTTATAAACTTGATATAGTATCAATTGATAGT----------
----

AATAACAGTAGTAATATTACTCAGTATAGACTAATAAATTGTAATACATCAACCGTGACACAG---
GCCTGTCCTAAGGTAACCTTTGAGCCAATCCCTATACATTATTGTACCCCAGCTGGTTTTGCGATTCTAAAGT
GCAAG---GATCCAAACTTTAAT------GGAACAGGGCCATGCAAGAAT----------------------
-GACAGC---ACAGTACAATGCACACATGGCATCAAACCAGTAGTATCAACT---
CAGCTGCTGTTAAATGGCAGTCTAGCAGAAAAAGAG------------
GTAATGATTAGATCTGCAAATATTACAGACAAT---------AGCAAAATCATAATAGTACAGCTTAAT----
-----------GAGTCTGTACCC---------ATCACTTGTATCAGACCTGGCAACAACACAAGAACA-----

-AGTATACGTATA------------GGACCAGGACAAACC---TTC------TATGCAACAGGTGAT------
---------ATAATAGGGGACATA------------AGAAAAGCACATTGT---AATGTCAGCAAAACA---
GCATGGAATAGAACTTTACAAAGGATAAGTACACAATTAAGC---------------AAATACTTTAAA----
-----------------------------------AATAAAACAATAATCTTTAAG------
AACTCCTCAGGGGGGGGATTTAGAAATTACA---ACACATAGTTTTAATTGTGGAGGAGAA---
TTTAAACAGCTCCAGGCAAGAGTCCTGGCTGTGGAAAGCTACCTAAAGGATCAGCAGCTCCTAGGAATTTGGG
GTTGCTCTGGAAAACTCATCTGCACCACTAATGTGCCCTGGAACTCTAGCTGGAGT-----------------
----------------------------AATAAATCTTGGAATGAAATATGG------GAT------
AACATGACCTGGATGCAATGG---GACAGGGAAATTATCAATTACACAAACATAATATGG---
GATCTAATTGAAGAATCGCAAAACCAGCAGGAA--

AAGAATGAACAAGATTTATTAGCATTGGATAAGTGGACAAGTCTGTGGAATTGGTTTAGCATATCAAATTGGT
TATGGTACATAAAAATATTTATAATAATAGTAGGAGGCTTAATAGGATTAAGAATAGTTTTTACT---
GTGCTTTCTATAATA---------AATAGGGTCAGGCAGGGATACTCACCT------
TTGTCATTGGCTATTAATCTGATAGATTACATTGCAGTAGCAGTAGCTGGG---
TGGACAGATAGGGTTTTAGAAATAGGACAAAGATTTTGTAGAGCT---------------------------
--------ATTTGTAACATA------CCTAGGAGAATCAGACAGGGCGCAGAAAGGGCTTTGCAA---TAA
>MH330347
GCA---
GGAAACTTGTGGGTCACGGTATACTATGGGGTACCTGTGTGG---AGAGATGCAGAG---
ACCACCCTATTTTGTGCATCAGATGCTAAAGCATATGATAAAGAA---GTACACAAT----------------
--------------GTCTGGGCTACA---CATGCCTGTGTACCCACAGAC---
CCTGACCCACAAGAAATAATTTTGGAA---------AATGTGACAGAAAACTTTAAC----------------
-------------------------ATGTGGAAAAATAACATGGTAGAACAAATGCAGACAGATATA---
ATCAGTCTCTGGGACCAAAGCCTA---AAGCCATGTGTAAAATTA---
ACCCCTCTCTGCGTTACATTAGATTGTACTACCCCCCAATTTAACAGCACTAGCTCTAACAACAGTAGTGGTA
ACAGCAGCAGC--
-------------------------AGTGATAGCCTGTCTGAAGACCTGTCTGAAGAAATGAAA---
AACTGCTCTTTCAACATGACCACAGAACTAAGA--------------------------
GATAAAAGGAAACAAGTATATTCACTT---TTTTATAAACTTGATATAGTATCAACTGGTAGTAAT-------
----

GGTAGTGGACAGTATAGACTAATAAATTGTAACACATCAGCCGTGACCCAG---
GCTTGTCCTAAGGTAACCTTTGAGCCAATTCCTATACATTATTGTGCCCCAGCTGGTTTTGCGATTGTAAAGT
GCAAG---GATACAGATTTTTCT------GGAACAGGGCTATGCAAGAAT----------------------
-GTCAGC---ACAGTACAATGCACACATGGAATCAAGCCAGTGGTATCAACT---
CAACTGCTGTTAAATGGCAGTTTAGCAGAGAAAGAG------------
GTAGTGATTAGATCTGAAAATATCACAAACCCT---------GGAAAAAACATAATAGTACAGCTTACT----
-----------GAGCCTGTAAAA---------ATCACTTGTATCAGACCTGGCAACAATACAAGAACA-----
-AGTATACATATA------------GGACCAGGACAAGCC---TTC------TTTGCAACAGGTGCT------
---------GTAACAGGGGACATA------------AGAAAAGCATATTGT---ACTGTCAACGAAACA---
GCATGGAATAGCACTTTACAAAAGATAAGTACACAATTAAGA---------------AAATACTTTAAT----
-----------------------------------AACAAAACAATAATCTTTAAA------
AACTCCTCAGGAGGGGATTTAGAAATTACA---ACACATAGTTTCAATTGTGGAGGAGAA---
TTTAAACAGCTCCAGGCAAGAATCCTGGCTGTGGAAAGATACCTAAAGGATCAGCAGCTCCTAGGAATTTGGG
GTTGCTCTGGAAAACTCATCTGCACCACTAATGTGCCCTGGAACTCTAGTTGGAGT----------------
----------------------------AATAAATCTCTGAATGAGATATGG------GAT------
AACATGACCTGGATGCAATGG---GACAAGGAAGTTATTAATTACACAAACACAATATAT---
GAGCTAATTGAACAATCGCAAATCCAGCAGGAA--

AAGAATGAACAAGATTTATTGGCATTGGATAAGTGGGCAAGTCTGTGGAATTGGTTTGACATATCACAGTGGT
TATGGTATATAAAAATATTTATAATAATAGTAGGAGGCTTAATAGGATTAAGAATAGTTTTTGCA---
GTGCTTTCCATAATA---------AATAGAGCCAGGCAGGGATACTCACCC------
TTGTCATTGGCTATTAATCTGATAGATACCCTAGCAGTAGCAGTAGCTGAG---
TGGACAGATAGGGTTATAGAAATAGGACAAAGATTTTGTAGAGCT---------------------------
--------ATTCGTAATATA------CCTAGGAGAATTAGACAGGGCGCAGAAAAAGCTTTGCAA---TAA
>MH330348
GCT---GCA--
GATTTGTGGGTCACGGTATACTATGGGGTACCTGTGTGG---AGAGATGCAGAG---
ACCACCCTATTTTGTGCATCAGATGCTAAAGCATATGATAAAGAA---GTGCACAAT----------------

--------------GTCTGGGCTACA---CATGCCTGTGTACCCACAGAC---
CCTAACCCACAAGAAATAGCTTTGGAA---------AATGTGACAGAAAATTTTGAC----------------
--------------------------ATGTGGAAAAATAACATGGTAGAACAAATGCAAACAGATATA---
ATCAGTCTCTGGGACCAAAGCCTA---AAGCCATGTGTAAGGTTA---
ACCCCTCTCTGCGTTACCTTAGATTGTACTGAACCCAACAACACTAGCTCTAACAACAGTAGTACTAGC----
----
-------------------------TCTAACAACAGTAGTGATAGTATGTATGGAGAAATGAAA---
AACTGCTCTTTCAACATGACCACAGAACTAAGA---------------------------
GATAAAAGGAAAACTGCATATTCACTT---TTTTATAAACTTGATATAGTATCAACTGATACTGAT-------
----

AGTAATAGTAGTAGGCAGTATAGACTAATAAATTGCAATACATCAGCCATGACACAG---
GCCTGTCCTAAGGTCACCTTTGAGCCAATTCCTATACATTATTGTGCCCCAGCTGGTTTTGCAATTCTAAAGT
GCAAG---GATCCAAATTTTAAT------GGAACAGGGCCATGCAATAAT-----------------------
-GTCAGC---ACAGTACAATGCACACATGGAATTAAGCCAGTAGTATCAACT---
CAACTGCTGTTAAATGGCAGTCTAGCAGAGAAAAAG-----------
GTAATAATTAGATCTAAAAATATCACAGACAAT---------AGCAAAATCATAATAGTACAGCTTACT----
-----------AAGCCTGTAAAC---------ATCACTTGTATCAGACCTGGCAACAACACAAGAACA-----
-AGTATACGTATA------------GGACCAGGACAAACC---TTC------TATGCAACAGGTGCG------
---------GTAACAGGAGACACA------------AGAAGAGCACATTGT---AATGTCAACAGAACA---
GAATGGAATAGAACTGTACAAGAGATAAGGGAACAATTAAGA---------------GGATACTTTAAT----
-----------------------------------AATCGAACAATAAATTTTAAG------
AACTCCTCAGGAGGGGATTTAGAAGTTACA---ACACATAGTTTCAATTGTAGAGGAGAA---
TTTAAACAGCTCCAGGCAAGAATCCTGGCTGTGGAAAGATACCTGAAGGATCAGCAGCTCCTAGGAATTTGGG
GTTGCTCTGGAAAACTCATCTGCACCACTAATGTGCCCTGGAACTCTAGTTGGAGT-----------------
----------------------------AACAAATCTCAGAGTGAGATATGG------GAA------
AACATGACCTGGATGCAATGG---GACAAGGAAGTTATCAATTACACAAACCTAATATAT---
AGTTTAATTGAAGAATCGCAAAACCAGCAGGAA--

AAGAATGAACAAGATTTATTGGCATTGGATAAATGGGCAGGTCTGTGGAATTGGTTTGACATATCAAATTGGT
TATGGTATATAAAAATATTTATAATAATAGTAGGAGGCTTAATAGGATTAAGAATAGTTTTTGCT---
GTGCTTTCTATAATG---------AATAGAGTCAGGCAGGGATACTCACCC------
TTGTCATTGGCTATCAGTCTAATAGATACCATAGCAATAGCAGTAGCTGGG---
TGGACAGATAGGGTTATAGAATTAGGACAAAGATTTTGTAGAGCC----------------------------
--------ATTTGTAACATA------CCTAGGAGAATCAGGCAGGGTGCAGAAAGGGCTTTGCAA---TAA
>MH330349
GCT---ACA--
GAAGACTTGTGGGTCACAGTATACTATGGGGTACCTGTGTGG---AGAGATGCAGAG---
ACCACCCTATTTTGTGCATCAGATGCTAAAGCATATGATAAAGAA---GTACACAAT----------------
--------------GTCTGGGCTACA---CATGCCTGTGTACCCACAGAC---
CCTAACCCACAAGAAATAGATTTGGAG---------AATGTGACAGAAGAGTTTGAC----------------
--------------------------ATGTGGAAAAATAACATGGTAGAACAAATGCAAACAGATATA---
ATCAGTCTCTGGGACGAAAGCCTA---AAGCCATGTGTACAGTTA---
ACCCCTCTCTGCGTTACTTTAAATTGTACTGAACCCAACAGCACTAGACCTAACAGCACTAGCTCTAACAACA
GTAACAGCACT--
----ACACCTACCAGCAGTAGTGGTCACAGCAGCAGTAGTGATAGTTTGTATGGAGACATGAAA---
AACTGTTCTTTCAATATGACCACAGAACTAAGA---------------------------
GATAAAAAGAAAAAGGTACATTCACTT---TTTTATAAACTTGATATAGTATCCACTGATAGAAAT-------
----

AATAGTGGGCATTATAGACTAATAAATTGCAATACATCAACCATGACACAG---
GCCTGTCCTAAGGTAACCTTTGAGCCAATTCCTATACATTATTGTGCCCCAGCTGGTTTTGCGATTCTAAAGT
GCAAG---GATCCAAATTTTAAT------GGAACAGGGCCATGTAAGAAT-----------------------
-GTCAGC---ACAGTGCAATGCACACATGGAATCAAGCCAGTAGTATCAACT---
CAACTGCTGTTAAATGGCAGTCTAGCAGAGGAAAAG-----------
GTAATACTTAGATTTGAAAATATCACAAACAAT---------GGCAAAACCATAATAGTACAGCTTAAG----
-----------GAGCCTGTAAAC---------ATCACTTGTATCAGACCTGGCAACAATACCAGAACA-----
-AGTATACATATA------------GGACCAGGAAGAGCC---TTC------TATGCAACAGGTGAG------
---------GTAATAGGGGACATA------------AGAAGAGCACATTGT---AATGTCAACGGAACA---
GAATGGAATAAAACTTTACACGAAATAAGTAAACAATTAGGA---------------AAACACTTT-------

-----------------------------------AACAAAACAATTATCTTTAAG------
AGTTCCTCAGGAGGGGATTTAGAAGTTACA---ACACATAGTTTCAATTGTGGAGGAGAA---
TTTAAACAGCTCCAGGCAAGAGTCCTGGCTGTGGAAAGATACCTGAAGGATCAGCAGCTCCTAGGAATTTGGG
GTTGCTCTGGAAGACTCATCTGCACCACTACTGTGCCCTGGAACACTAGTTGGAGT-----------------
----------------------------AACAAAACTTATACTGAGATATGG------GAT------
AACATGACCTGGATGCAATGG---GACAAGGAAGTTATCAATTACACAAACATAATATAT---
AATCTAATTGAAGAATCGCAAAACCAGCAGGAA--

AAGAATGAACAAGATTTATTGGCATTGGATAAGTGGGCAAGTCTGTGGAATTGGTTTGACATATCAAGTTGGT
TATGGTATATAAGAATATTTATAATGATAGTAGGAGGCTTAATAGGATTAAGAATAGTTTTTGCT---
GTGCTTTCTATAATA---------AATAGAGTCAGGCAGGATACTCACCT------
GTGTCATTGGCTATCAATCTATTAGACACCGTAGCAGTAGCAGTAGCTGGG---
GGGACAGATAGGGTTATAGAAATAGGACAAAGACTTTGTAGAGCC----------------------------
--------ATTTGTAACATA------CCTAGGAGAATCAGACAGGGCGCAGAAAAAGCTTTGCAA---TAA
>MH330350
GCT---GCA--
GAAAACTTGTGGGTCACGGTATACTATGGGGTACCTGTGTGG---AGAGATGCAGAG---
ACCACCCTATTTTGTGCATCAGATGCTAAAGCATATGATAAAGAA---GTACACAAT----------------
--------------GTCTGGGCTACA---CATGCCTGTGTACCCACAGAC---
CCTAACCCGCAAGAAATAATTTTGGAA---------AATGTGACAGAAGAATTTAAC----------------
--------------------------ATGTGGAAAAATAACATGGTAGAACAGATGCAAACAGATATA---
ATCAGTCTCTGGGACCAAAGCCTA---AAGCCATGTGTAAAGTTA---
ACCCCTCTCTGCGTTACTTTAAATTGTACTGCACCCAACATCACTAGCTCTAACAACAGT-------------
----
-------------------------ACCATCAACAGCAATAGTAGCATGGGTGAAGAAATGAAA---
AACTGCTCTTTCAACATGACCACGGAACTAAGT---------------------------
GATAAAAAGAAAAAAGTACATTCACTT---TTTTATAAACTTGACATAGTATCAATTGATAGT----------
----

AATAGTAGTAGTAATAGTAGTGGGCAGTATAGACTAATAAATTGTAATACATCAACCATGACACAG---
GCCTGTCCCAAGGTAACCTTTGAGCCAATTCCTATATATTATTGTACCCCAGCTGGTTTTGCGATTCTAAAGT
GCAAC---AGTCCAGACTTTAAT------GGAACAGGGCCATGCAAGAAT-----------------------
-GTCAGC---ACAGTACAATGCACACATGGAATCAAGCCAGTAGTATCAACT---
CAACTGCTGTTAAATGGCAGTCTAGCAGAAGGAAAG------------
GTAATGATTAGATCTGAAAATATTACAGACAAT---------AGAAAAACCATAATAGTACAGCTTACT----
-----------GAGCCTGTAAAT---------ATCACGTGTATCAGACCTAGCAACAATACAAGAACA-----
-AGTATACGTATA------------GGACCAGGACAAACC---TTC------TATGCAACAGGTGCT------
---------GTAACAGGGGACATA------------AGGAAAGCACATTGT---AATGTCAGCAAAGCA---
GCATGGGACAGAACTTTACAAAACATAAGTACACAATTAAGA---------------AAATACTTTAAT----
-----------------------------------AATAAAACAATAGTCTTTAAG------
AACTCCTCAGGAGGGGATTTAGAAGTTACA---ACACATAGTTTCAATTGTGGAGGAGAA---
TTTAAACAGCTCCAGGCAAGAGTCCTGGCTGTGGAAAGATACCTAAAGGATCAGCAGCTCCTAGGAATTTGGG
GTTGCTCTGGAAAACTCATCTGCACCACTACTGTGCCCTGGAACTCTAGTTGGAGT-----------------
----------------------------AATAAATCTCAGAGTGAGATATGG------GAT------
AACATGACCTGGATGCAATGG---GACAAGGAAATTATCAATTACACAAACAAAATATAT---
GAGCTAATTGAAAAATCGCAAAACCAGCAGGAA--

CAGAATGAACAAGAGTTATTAGCATTGGATAAGTGGGCAAGTCTGTGGAATTGGTTTGACATATCAAGTTGGT
TATGGTATATAAAAATATTTATAATAATAGTAGGAGGCTTAATAGGATTAAGAATAGTTTTTGCT---
GTGCTTTCGATAATA---------AATAGAGCCAGGCAGGATACTCACCC------
TTGTCGTTGGCTATTAATCTGATAGATACCTTAGCAATAGCAGTAGCTGGG---
TGGACAGATAGGGTTTTAGAAATAGGACAAAGATTTTGGAGAGCT----------------------------
--------ATTTGTAACATA------CCTAGAAGAATCAGACAGGGCACAGAAAAGGCTTTGCAA---TAA
>MH330351
GCT---ACA--
GAAAAATTGTGGGTCACAGTATACTATGGGGTACCTGTGTGG---AGAGATGCAGAG---
ACCACCCTATTTTGTGCATCAGATGCTAAAGCATATGATAAAGAA---GTACACAAT----------------
--------------GTCTGGGCTACA---CATGCCTGTGTACCCACAGAC---
CCCGACCCACAAGAAATAGACTTGAAA---------AATGTGACAGAAAATTTTAAC----------------
--------------------------ATGTGGAAAAATAACATGGTAGAACAAATGCAAACAGATATA---

ATCAGTCTCTGGGACCAAAGCCTA---AAGCCATGTGTAAAGTTA---
ACCCCTCTCTGCGTTACTTTAAATTGTACTGAACTCAACAGCACTAGCTCTAACAACAGTAGT---------
----
-------------------------GGTAACAGCAACAGCAGCAGTATGTATGAAGAAATGAGA---
AACTGCTCTTTCAATATGACAGCAGAACTAAGA--------------------------
GATAAAAAGAAAACTGTATATTCACTT---TTTTATAAACTTGATATAGTATCAACTGATAAT---------
----

AGTAGTAGTGGGTATTATAGACTAATAAATTGTAATACATCAGCCATGACACAG---
GCCTGTCCTAAGATAACCTTTGAGCCAATTCCTATACATTATTGTGCCCCAGCTGGTTTTGCGATTCTAAAGT
GCAAG---GATGCAAATTTTAAT------GGAACAGGGCCATGCAAGAAT----------------------
-GTCAGC---ACAGTGCAATGCACACATGGAATCAAGCCAGTAGTATCAACT---
CAACTGCTGTTAAATGGCAGTCTAGCAGAGGAACAG-----------
GTAATGATTAGATCTGAAAATATCACAGACAAT---------GGCAAAATCATAATAGTACAGCTTAAG----
-----------GAGCCTGTAAAC---------ATCACTTGTATCAGACCTGGCAACAATACAAGAACA-----
-AGTATACGTATA------------GGACCAGGACAAACC---TTC------TATGCAACAGGTGGT------
---------GTAATAGGGGATATA------------AGAAAAGCACATTGT---AATGTCAGCAGAACA---
GCATGGAATAACACTTTACAAAAGATAAGTGCACAGTTAAGA--------------CAATACTTTAAT----
----------------------------------AACAAAACAATAATCTTTAAA------
AACTCCTCAGGAGGGGATTTAGAAGTTACA---ACACATAGTTTTAATTGTGGAGGAGAA---
TTTAAACAGCTCCAGGCAAGAGTCCTGGCTGTGGAAAGATACCTAAAGGATCAGCAGCTCCTAGGAATTTGGG
GTTGCTCTGGAAAACTCATCTGCACCACTAGTGTGCCCTGGAACTCTAGTTGGAGT----------------
----------------------------AACAAATCTCAGAGTGAGATATGG------GAT------
AACATGACCTGGATGCAATGG---GACAAGGAAATTATCAATTACACAAGCATAATATAT---
AATCTAATTGAAGAATCGCAAAACCAGCAGGAA---------------------------------------
----
AAGAATGAACAAGAGTTATTGGCATTGGATAAGTGGGCAAGTCTGTGGACTTGGTTTGACATATCAAATTGGT
TATGGTATATAAGAATGTTTATAATAATAGTAGGAGGCTTAATAGGATTAAGAATAGTTTTTGCT---
GTGCTTTCTATAATA---------AATAGAGCCAGGCAGGGATACTCACCC------
TTGTCATTCGCTATCAATCTAGTAGATACCATAGCAATAGCAGTAGCTGGG---
TGGACAGATAGGGTTATAGAGCTAGGACAAAGATTGTGTAGAGCC---------------------------
--------ATTCGTAACGTA------CCTAGGAGAATCAGACAGGGCGCAGAAAAAGCTTTGCAA---TAA
>MH330352
GCT---ACA--
GAAAACTTGTGGGTCACGGTATACTATGGGGTACCGGTGTGG---AGAGATGCAGAG---
ACCACCCTATTTTGTGCATCAGATGCTAATGCACTTGATAAAGAA---GTACACAAT---------------
--------------GTCTGGGCTACA---CATGCCTGTGTACCCACAGAC---
CCTAACCCACAAGAAATAGATTTGGAA---------AATGTGACAGAAGATTTTGAC---------------
--------------------------ATGTGGAAAAATGGCATGGTAGAACAAATGCAAACAGATATA---
ATCAGTCTCTGGGACCAAAGTCTA---AAGCCATGTGTAAAGTTA---
ACCCCTCTCTGTGTTACTTTATATTGTACTGAACCCAACAGCACTAGCTCTAACAGCACTAGCTATAGC----
----
-------------AACAGTAGCAGTAACAGCAACAGCAGCGATAGTTTGCTTAAAGAAATGAGC---
AACTGCACTTACAACATGACCACAGAACTAAGA--------------------------
GATAAAAGGAAAACTGTACATTCACTC---TTTTATAAACTTGATATAGTATCAATTGATGGT---------
----

AATGGTAGTAATGGTAGTGGGCATTATAGACTAATAAATTGTAATACATCAACCATGACACAG---
GCCTGTCCTAAAGTAACCTTTGAGCCAATTCCTATACATTATTGTGCCCCAGCTGGTTTTGCGATTCTAAAGT
GCAAG---GATCCAGATTTTAAT------GGAACAGGGCCATGCAACAAT----------------------
-GTCAGC---ACAGTACAATGCACACATGGAATCAAGCCAGTAGTATCAACT---
CAACTGCTGTTAAATGGCAGTCTAGCAGAGGAAAAG-----------
GTAATAATTAGATCTAAAAATATTACAGACAAT---------AGCAAAATCATAATAGTACAGCTTAAT----
-----------GAGCCTGTAAAC---------ATCACTTGTATCAGACCTGGCAACAATACAAGAACA-----
-AGTATACGTATA------------GGACCAGGACAAACC---TTC------TATGCAACAGGTGAT------
---------ATAATAGGGAACCCA------------AGAAAAGCATATTGT---AATGTCAGCAGAGCA---
AAATGGAATGAAACTTTACAAAAGATAAGTAAACAATTACAG--------------GAACACTTT-------
----------------------------------AACAAAACAATAATCTTTAAA------
AGCTCCTCAGGAGGGGATTTAGAAGTTACA---ACACATAGTTTCAATTGTGGAGGAGAA---
TTTAAACAGCTCCAGGCAAGAGTCCTGGCTGTGGAAAGATACCTGAAGGATCAGCAGCTCCTAGGAATTTGGG

GTTGCTCTGGAAAACTCATCTGCACCACTAATGTGCCTTGGAACTCTAGTTGGAGT-----------------
-----------------------------AACAAATCTCTGGATCAGATATGG------GAT------
AACATGACCTGGATGCAATGG---GACAAGGAAGTTATCAATTACACAAACATAATATAT---
GGTCTAATTGAAGAATCGCAAAACCAGCAGGAA---

AAGAATGAACAAGATTTATTGGCATTGGATAAGTGGGCAAGTCTGTGGAATTGGTTTGACATATCAAATTGGT
TATGGTATATAAGAATATTTATAATGATAATAGGAGGCTTAATAGGATTAAGAATAGTTTTTACT---
GTGCTTTCTATAATA---------AATAGAGCCAGGCAGGGATACTCACCC------
TTGTCATTGGCTATCAATCTACTAGATACCATAGCAATAGCAGTAGCTGGG---
TGGACAGATAGGGTTATAGAATTAGGACAAAGACTTGGTAGAGCT----------------------------
--------ATTTGTAACATA------CCTAGGAGAATCAGACAGGGCGCAGAAAGAGCTTTGCAG---TAA
>MH330353
GCT---ACA---
GAAAACTTGTGGGTCACAGTATACTATGGGGTACCTGTGTGG---AGAGATGCAGAG---
ACCACCCTATTTTGTGCATCAGATGCTAAAGCATATGATAAGGAA---GTACACAAT----------------
--------------GTCTGGGCTACA---CATGCCTGTGTACCCACAGAC---
CCTGACCCACAAGAAATAGATTTGGAA---------AATGTGACAGAAAATTTTAAC----------------
-------------------------ATGTGGAAAAATAACATGGTAGAACAAATGCAAATAGATATA---
ATCAGCCTCTGGGACCAAAGCCTA---AAGCCATGTGTAAAGTTA---
ACCCCTCTCTGCGTAACTTTAAATTGTACTGAACCCATCAGAACCAACAGAACTAGTAACAGCAGTACCAACA
GCACTAGCTCT---
----------------------ACCAGCAGTAGTGTTAACGATAGTTTGTATGAGGAACTGAAA---
AACTGCTCTTTCAACATGACCACAGAACTAAGA---------------------------
GATAAAAGGAAAAAAGTATACTCACTT---TTTTATAAACTTGATATAGTATCAACTGATAGGAAT-------
----

AATAGTGGAGAGTATAGACTAATAAATTGCAATACATCAACCATGACACAG---
GCCTGTCCTAAAGTAACCTTTGAGCCAATTCCTATACATTATTGTGCCCCAGCTGGTTTTGCGATTCTAAAGT
GCAGG---GATCCAAAGTTTAAT------GGAACAGGGCCATGCACGAAT----------------------
-GTCAGC---ACAGTACAATGCACACATGGAATCAAGCCAGTAGTATCAACT---
CAACTGCTGTTAAATGGCAGTCTAGCAGAGAAAGAG------------
ATAATGATTAGATCTGAAAATATCACAGACAAT---------AGTAAAACCATAATAGTACAGCTTACT----
-----------AAGCCTGTAGGT---------ATCACTTGTATCAGACCTAACAACAATACAAGAACA-----
-AGTATACATATA------------GGACCAGGACAAGCC---TTC------TATGCAAGAGGTGAT------
---------GTAATAGGAGACATA------------AGGAAAGCATATTGT---ATTATCAATGGAACA---
GAATGGAATAACACTTTACAACAGATAAGTGAAAAAATTAAGA---------------GGATACTTTGAT----
-----------------------------------AATAAAACAATAATCTTTAAG------
AACTCCTCAGGAGGGGATTTAGAAGTGGCA---ACACATAGTTTCAATTGTGGAGGAGAA---
TTTAAACAGCTCCAGGCAAGAGTCCTGGCTTTGGAAAGATACCTGAAGGATCAGCAGCTCCTAGGAATTTGGG
GTTGCTCTGGAAAACTCATCTGCCCCACTAATGTGCCTTGGAATTCTAGTTGGAGT-----------------
-----------------------------AACAAATCCCTGAATGATATATGG------AAT------
AACATGACCTGGATGCAATGG---GACAAGGAAGTTTTCAATTACACAAACATAATATAT---
GAACTAATTGAAGAATCACAAAACCAGCAGGAA---

AAGAATGAACAAGATTTATTGGCATTGGATAAGTGGGCAAGTCTGTGGAATTGGTTTGACATATCACATTGGT
TATGGTATATAAGAATCTTTATAATAATAGTAGGAGGCTTAATAGGATTAAGAATAGTTTTTACT---
GTGCTTTCTCTAATA---------AATAGAGTCAGGCAGGGATACTCACCC------
TTGTCATTTGCTATCAATCTACTAGATACCATAGCAATAGCAGTAGCTGGG---
TGGACAGATAGGGTTATAGAGGTAGTACAAAGACTTTGTAGAGCC----------------------------
--------ATTTGTAACATA------CCTAGGAGAATCAGACAGGGCGCAGAAAAAGCTTTGCAA---TAA
>MH330354
GCT---GCA---
GAACACTTGTGGGTCACGGTATACTATGGGGTACCGGTGTGG---AGAGATGCAGAG---
ACCACCCTATTTTGTGCATCAGATGCTAAAGCATATGATAAAGAA---GTACACAAT----------------
--------------GTCTGGGCTACA---CATGCCTGTGTACCCACAGAC---
CCTAACCCACAAGAAATAGCTTTGGAA---------AATGTGACAGAAAATTTTGAT----------------
-------------------------ATGTGGAAAAATAACATGGTAGAACAAATGCAAACAGATATA---
ATCAGTCTCTGGGACCAAAGTCTA---AAACCATGTGTACAGTTA---
ACCCCTCTCTGCGTCACTTTAAATTGTACTGACCCCACTTTAAATTGTACTGACCCCCACAATAATAGGAGTG
AAGGAATAAGC---

-------------------------AACAACAGCAATAACAGCTGTATGTATGAAGAAATGAAA---
AACTGCACTTTCAACATGACCACAGAACTAAGA---------------------------
GATAAGAGGAAAACTGTACATTCACTC---TTTTATAAACTTGATATAGTACCAACTGATATTAAT-------
----

AATAGTGGAGAGTATAGACTAATAAATTGCAATACATCAACCATGACACAG---
GCCTGTCCTAAGGTAACCTTTGAGCCAATTCCTATACATTATTGTACCCCAGCTGGTTTTGCGATTCTAAAGT
GCAAG---GATACAGAGTTTAAT------GGAACAGGGCCATGCAAGAAT-----------------------
-GTCAGC---ACAGTACAATGCACACATGGAATCAAGCCAGTAGTATCAACA---
CAACTGCTGTTAAATGGCAGTCTAGCAGAGGAAAAG------------
GTAATGATTAGATCTAAAAATATCACAGACAAT---------AGCAAGATCATAATAGTACAGCTTACT----
-----------AAGCCTGTAAAC---------ATCACTTGTATCAGACCTAACAACAATACAAGAACA-----
-AGTATACGTATA------------GGACCAGGACAAACC---TTC------TATGCAACAGGTGCT------
---------GTAACAGGGGACATA------------AGAAAAGCATATTGT---GAAGTCAACAGAACA---
GAATGGAATAGCACTTTACAAGAGATAAGTTCACAATTAAGA---------------AACTACTTTAAG----
-----------------------------------AACAAAACAATAATCTTTAAA------
AACTCCACAGGAGGGGATTTAGAAATTACA---ACACATAGTTTCAATTGTGGAGGAGAA---
TTTAAACAGCTCCAGGCAAGAGTCCTGGCTGTGGAAAGATACCTGAAGGATCAGCAGCTCCTAGGAATTTGGG
GTTGCTCTGGAAAACTCATCTGCACCACTACTGTGCCCTGGAACTCTAGTTGGAGT-----------------
----------------------------AACAAATCTATGAATGAAATATGG------GAT------
AACATGACCTGGATGCAATGG---GACAAGGAAGTTATCAATTACACAAACATAATATAT---
GATCTAATTGAAGAATCGCAAAACCAGCAGGAA--

AAGAATGAACAAGATTTATTAGCATTGGATAAGTGGGCAAGTCTGTGGAGTTGGTTTGACATATCAAATTGGT
TATGGTATATAAAAATATTCATAATAATAGTAGGAGGCTTAATAGGATTAAGAATAGTTTTTGCT---
GTGCTTTCTATAATA---------AATAGAGTCAGGCAGGGATACTCACCC------
GTGTCATTAGCTATCAATCTAATAGATACCTTAGCAATAGCAGTAGCTGGG---
TGGACAGATAGGGTTATAGAAATAGGACAAAGAATTTGTAGAGCC----------------------------
--------ATTCGTAACATA------CCTAGGAGAATCAGACAGGGCGCAGAAAAGGCTTTGCAA---TAA
>MH330355
GCT---ACA--
GGAAACTTGTGGGTCACGGTATACTATGGGGTACCGGTGTGG---AGAGATGCAGAA---
ACCACCCTATTTTGTGCATCAGATGCTAAAGCATATGATAAAGAA---GTACACAAT----------------
--------------GTCTGGGCTACA---CATGCCTGTGTACCCACAGAC---
CCTAACCCACAAGAAATGCCTTTGGAA---------AATGTGACAGAAAATTTTAAC----------------
--------------------------ATGTGGAAAAATAACATGGTAGAACAAATGCAAACAGATATA---
ATCAGTCTCTGGGACCAAAGTCTA---AAGCCATGTGTACAGTTA---
ACCCCTCTCTGTGTTACTTTAAATTGTACTGAACCCATCTTTAACTCTACCATCACTAACTCTACCAACATTA
ACCGCAACAAC--
--AATGATAGTAGTGATAGTATG------
ATCTGCACTTACAACATGACAACAGAACTAAGA---------------------------
GATAAAAAGAAAAATGTACGTTCACTC---TTTTACAAACTTGATATAGTATCAACTGGTAGGAAT-------
----

AATAGTGGGGAGTATAGACTAATAAATTGCAATACATCAACCATGACACAG---
GCCTGTCCTAAGGTAACCTTTGAGCCAATTCCTATACATTATTGTGCCCCAGCTGGTTTTGCGATTCTAAAGT
GCAGG---GATCCAAATTTTAAT------GGAACAGGGCCATGCAAGAAT-----------------------
-GTCAGC---ACAGTACAATGCACACATGGAATCAAGCCAGTAGTATCAACT---
CAACTGCTGTTAAATGGCAGTCTAGCAGAGAACAAG------------
GTAATGATTAGATCTGAAAATATCACAGACAAT---------AACAAAAACATAATAGTACAGCTTACT----
-----------AAGCCTGTAAAC---------ATCACTTGTATCAGAACTGGCAACAATACCAGAGAA-----
-GGTGTAGGTATA------------GGACCAGGACAAACT---TTC------TATGCAGCAAGTCCT------
---------ATAATAGGGGACATA------------AGAAAAGCATATTGT---AATGTCAGCAGAGCA---
GAATGGAACAACACTTTAAAAGAGGTGAGTGCACAATTAAGA---------------ACATACTTTAAT----
-----------------------------------AACAAAACAATAGTCTTTAAC------
AGCTCCTCAGGAGGGGATGTAGAAATTACA---ACACATAGTTTCAATTGTGGAGGAGAA---
TTTAAACAGCTCCAGGCAAGAGTCCTGGCTGTGGAAAGATACCTGAAGGATCAGCAGCTCCTAGGAATTTGGG
GTTGCTCTGGAAAACTCATCTGCAACACTAATGTGCCTTGGAACTCTAGTTGGAGT-----------------
----------------------------AACAAATCTCTGGATTATATATGG------GGT------
AACATGACCTGGATGCAATGG---GATAAGGAAGTTAGCAATTACACAAACATAATATAT---

AATTTAATTGAAGAATCACAAAACCAGCAGGAA--

AAGAATGAACAAGAATTATTGGCATTGGATCAGTGGGCAAGCCTGTGGAATTGGTTTAACATATCAAATTGGT
TATGGTATATAAGAATATTTATAATGATAGTAGGAGGCTTAATAGGATTAAGAATAGTTTTTGCT---
GTGCTTTCTATAATA---------AATAGAGTCAGGCAGGGATACTCACCC------
TTGTCATTGGCTATCAATCTACTAGATACCATAGCAATAGCAGTAGCTGGG---
TGGACAGATAGGGTTATAGAACTAGGACAAAGATTTTGTAGAGCT----------------------------
--------ATTTGTAACATA------CCTAGAAGAATCAGACAGGGCGCAGAAAGGGCTTTGCAA---TAA
>MH330356
GCT---ACA--
GAAAAATTGTGGGTCACGGTATACTATGGGGTACCTGTATGG---AGAGATGCAGAG---
ACCACCCTATTTTGTGCATCAGATGCTAAAGCATATGATAAAGAA---GTACACAAT----------------
--------------GTCTGGGCTACC---CATGCCTGTGTACCCACAGAC---
CCTAACCCACAGGAAATAGATTTGAAA---------AATGTGACAGAAGATTTTAAC---------------
-------------------------ATGTGGAAAAATAACATGGTAGAACAAATGCAAACAGATATA---
ATCAGTCTCTGGGACCAAAGCCTA---AAGCCATGTGTACAGTTA---
ACCCCTCTCTGCGTTACTTTAGATTGTATTGACTTCAACAGCACTAGCTCTGATGACAGTAGCAATAACAGCA
CTAGCTCTAAC--
----------------AACAGTAGCAGTCACAGCAACAACAGCCATTGGGTTGAAGATATGAAA---
AACTGCTCTTTCAACATGACCACAGAACTAAGA--------------------------
GATAAAAGGAAAACTGTATATTCACTT---TTTTATAAACTTGATATAGTACCAACTGATAGT---------
----

AGTAATAGTAATAATAGTAGTAGGCATTATAGACTAATAAGTTGCAATACATCAACTGTGACACAG---
GCCTGTCCTAAGGTATCCTTTGAGCCAATTCCTATACATTATTGTGCCCCAGCTGGTTTTGCGATTCTAAAGT
GCAAA---GAAAAAAATTTTACT------GGAAAAGGGCAATGCAAGAAT----------------------
-GTCAGC---ACAGTACAATGCACACATGGGATCAAGCCAGTAGTATCAACT---
CAACTGCTGTTAAATGGCAGTCTAGCAGAGGAAGAG-----------
GTAATGATTAGATCTGAAAATATCACAGACAAT---------AGCAAAACCATAATAGTACAGCTTAAA----
-----------GAGCCTGTAAAC---------ATCACTTGCCTCAGACATAACAACAATACAAGAACA-----
-AGTATACGTATA------------GGACCAGGACAAACC---TTC------TATGCAACAGGTGAG------
---------GTAATAGGGGACCCA-----------AGACGAGCACATTGT---AATGTCAGCAGAGCA---
GCTTGGAATAGGACTTTACATGGGATAAGTATACAATTAAAA--------------AAATACTTTCCT----
-----------------------------------AACAAAACAATAAAGTTTCAA------
AACTCCTCAGGAGGGGATTTAGAAATTACA---ACACATAGTTTCAATTGTGGAGGAGAA---
TTTAAACAGCTCCAGGCAAGAGTCCTGGCTGTGGAAAGATACCTGAAGGATCAGCAGCTCCTAGGAATTTGGG
GTTGCTCTGGAAAACTCATCTGCACCACTGCTGTGCCCTGGAACATTAGTTGGAGT----------------
-----------------------------AACAAATCTTATGATGATATATGG------GGG------
AACATGACCTGGATGCAATGG---GACAGGGAAGTTATCAATTACACAAACATAATATAT---
GGTCTAATTGAAGACTCGCAAAACCAGCAGGAA--

AAGAATGAACAAGATTTATTGGCATTGGATAAGTGGGCAAGTCTGTGGAGTTGGTTTGACATAACAAGTTGGT
TATGGTATATAAGAATATTTATAATAATAGTAGGAGGCTTAATAGGATTAAGAATAGTTTTTGTT---
GTGCTTTCTATAATA---------AAAAGAGCCAGGCAGGGATACTCACCA------
TTGTCATTGGCTATCAATCTAGTAGATACCATAGCAATAGCAGTAGGTGGG---
TGGACAGATAGGGTCATAGAAATAGGACAAAGACTTTGTAGAGCC--------------------------
--------ATTTGTAACATA------CCTAGGAGAATCAGACAGGGCGCAGAAAGAGCTTTGCAA---TAA
>MG902950
GCT---GCA--
GAAAACTTGTGGGTCACAGTATATTATGGGGTACCTGTGTGG---AGGGATGCAGAG---
ACCACCCTATTTTGTGCATCAGATGCTCAGGCATATAACAAAGAA---GTACACAAT----------------
--------------GTCTGGGCTACA---CATGCCTGCGTACCCACAGAC---
CCTAACCCACAAGAAATAACTTTGGAA---------AATGTGACAGAAGATTTTAAC---------------
-------------------------ATGTGGAAAAATAACATGGTAGAACAAATGCACACAGATATA---
ATCAGTCTCTGGGACCAAAGCCTA---AAGCCATGTGTACAGTTA---
ACCCCTCTCTGTGTCACTTTAAATTGTGTTGAACAACCCAACAGCACTAGCTCTAACAACAGTAGCGCTAATA
ACAGCAGCAGC--
----------------------GTTACTAACAGCAGCGATAGTAAGTGGGGAGAAGTAATGAGA---
AACTGCTCTTTCAACATGACCACAGAACTAAGA--------------------------
GATAAGAGGAAAACTGTATATTCACTT---TTTTATAAACTTGATATAGTATCAACTGATAGT---------

----

AATAATAATAGTAGGCAGTATAGACTAATAAATTGCAATACATCAACCATGACACAG---
GCCTGTCCTAAGGTGACCTTTGATCCAATTCCTATACATTATTGTGCCCCAGCTGGTTTTGCGATTCTAAAGT
GCAAG---GATACAAATTTTACT------GGAAAAGGGTCATGCAAGAAT----------------------
-GTCAGC---ACAGTACAATGCACACATGGAATTAAGCCAGTAGTATCAACT---
CAACTGCTGTTAAATGGCAGTCTAGCAGAGAAAGAG-----------
GTAATGATTAGATCTGAAAATATCACAGACAAT---------AGCAAAACCATAATAGTGCAGCTTACT----
-----------AAGCCTGTAAAC---------ATTACTTGTATCAGACCTGGTAACAATACAAGAACA-----
-AGTATACGTATA------------GGACCAGGACAAACC---TTC------TATGGAACAGGTGAA------
---------GTAGTAGGGGACATA------------AGAAACGCACATTGT---AATGTCAGCAGAGCA---
GCATGGAATGAAACTCTACAAAAGATAAGTATACAATTAAGA---------------AAACACTTTGGT----
---------------------------------AACAATAAAACAATAAATTTTAAG------
AACTCCTCAGGAGGGGATTTAGAAATAACA---ACACATAGTTTCAATTGTGGAGGAGAA---
TTTAAACAGCTCCAGGCAAGAATCCTGGCTGTGGAAAGATACCTAAAGGATCAGCAGCTCCTAGGAATTTGGG
GTTGCTCTGGAAAACTCATCTGCACCACTAATGTGCCCTGGAACTCTAGTTGGAGT-----------------
----------------------------AACAAATCTCAGGATGAGATATGG------AAT------
AACATGACCTGGATGCAATGG---GACAAGGAAGTTCTCAATTACACAGTCCTAATATAT---
GATCTACTTGAAGAATCGCAAAACCAGCAGGAA---------------------------------------
----
AAGAATGAACAAGATTTATTGGCATTAGATAAGTGGGCAAGTCTGTGGAATTGGTTTAATATATCAAATTGGT
TGTGGTATATAAAAATATTTATAATAATAGTAGGAGGCTTAATAGGATTAAGAATAGTTTTTATT---
GTGCTTTCTATAATA---------AATAGAGTCAGGCAGGGATACTCACCC------
TTGTCATTGGCTATCAATCTGTTAGATACCATAGCAATAGCAGTAGCTGGG---
TGGACGGATAGGATTATAGAAATAGGACGCAGACTTTGTAGAGCC-----------------------------
--------ATCCGTAACATA------CCTAGGAGAATCAGACAGGGCGCAGAAAGGGCTTTGCAA---TAA
>MG902951
GCT---ACA---
GAAAATTTGTGGGTCACGGTATACTATGGGGTACCTGTGTGG---AGAGATGCAGAA---
ACCACACTATTTTGTGCATCAGATGCTAAAGCATATGATAAAGAA---GTACACAAT----------------
--------------GTCTGGGCGACA---CATGCCTGTGTACCCACAGAC---
CCTGACCCAAAAGAAATAAATTTGGAA---------AATGTGACAGAAAATTTTAAT----------------
--------------------------ATGTGGAAAAATAACATGGTAGAACAAATGCAAACAGATATA---
ATCAGTCTCTGGGACCAAAGCCTA---AAGCCATGTGTAAAATTA---
ACCCCTCTCTGTGTTACTTTAAATTGTGTTGAACCCAACAACACTAGCTCTAGCAACAATAGCGTCAACAGTA
GCAGCAGTGAT---
-------AGTTTGAACTGCACTAGCAACAATAGCGTCAGCAGTAGCAGCAGTGATAGTTTG------
AACTGCACTTTCAACATGACCACAGAACTAAGA---------------------------
GATAAAAAGAAAACTGTACATTCACTT---TTTTATAAACTTGATATAGTATCAACTGATGAT----------
----

AATAATAGTAGTGGGCAGTATAGACTAATAAATTGCAATACATCAACCGTGACACAG---
GCTTGTCCTAAGGTAACCTTTGAGCCAATTCCTATACATTATTGTACCCCAGCTGGTTTTGCGATTCTAAAGT
GCAAG---GATCCAAATTTTAAT------GGAACAGGGCCATGCAAGAGT----------------------
-GTCAGC---TCAGTACAATGCACACATGGAATTAAGCCAGTAGTATCAACT---
CAACTGCTGTTAAATGGCAGTCTAGCAGAGAAACAG-----------
GTAATGATTAGATCTGAAAATATCACAGACAAT---------AGCAAAACCATAATAGTACAACTTACT----
-----------GAGCCTGTAAAC---------ATCACTTGTATCAGACCTAACAACAATACAAGAACA-----
-AGTATGCGTATA------------GGACCAGGACAAACC---TTC------TATGCAAGAGGTGAT------
---------GTAATAGGGGACATA------------AGACAAGCACATTGT---AATGTCAGCGGAGAA---
GCATGGAATAAAGCTTTACAAAACATAAGTACACAGTTAGGA---------------AAATACTTTAAT----
------------------------------------AACAAAACAATAATCTTTAAG------
AACTCCTCAGGAGGGGATTTAGAAGTTACA---ACACATAGTTTCAATTGTGGAGGAGAG---
TTTAAACAGCTCCAGGCAAGAGTCCTGGCTGTGGAAAGATACCTAAAGGATCAGCAGCTCCTAGGAATTTGGG
GTTGCTCTGGAAGACTCATCTGCACCACTAATGTGCTCTGGAACACTAGTTGGAGC-----------------
----------------------------AACAAATCTTACAATGAAATATGG------GAT------
AACATGACCTGGATGCAATGG---GACAAGGAAGTTAACAATTACACAGACACAATATAT---
AGGCTAATTGAAAAATCGCAAAACCAGCAGGAA---------------------------------------
----
AAGAATGAACAAGATTTATTGGCATTGGATAAGTGGACAAGTCTGTGGAGTTGGTTTGACATATCACATTGGT

TATGGTATATAAAAATATTTATAATGATAGTAGGGGGCTTAATAGGATTAAGAATAGTTTTTACT---
GTGCTTTCTATAATA---------AATAGAGTCAGGCAGGGATACTCACCC------
TTGTCATTGGCTATCAATCTAATAGATACCATAGCAATAGCAGTAGCTGGG---
TGGACAGATAGGGTTATAGAAATAGTACAAAGATTTTGTAGAGCT----------------------------
--------ATTTGTAACATA------CCTAGGAGAATCAGACAGGGCGCAGAAAGAGCTTTGCAA---TAA
>MH330337
GCT---ACA---
GAAAACTTGTGGGTCACGGTATACTATGGAGTACCTGTGTGG---AGAGATGCAGAC---
ACCACCCTATTTTGTGCATCAGATGCTAAAGCATATGATAAAGAA---GTACACAAT----------------
--------------GTCTGGGCTACA---CATGCCTGTGTACCCACAGAC---
CCTAACCCGCAAGAAATAATTTTGGAA---------AATGTAACAGAAGAATTTGAC----------------
--------------------------ATGTGGAAAAATAACATGGTAGAACAAATGCACACAGATATA---
ATCAGTCTCTGGGACCAAAGCTTA---AAGCCATGTGTAAAGTTA---
ACCCCTCTCTGCGTTACTTTAAATTGTACTAAACCCAGCCACAACAGTACTAACAGCAGTAGT----------
----
---GGCTTGATTGAAGAAATGAAA---
AACTGCTCTTTCAACATGACCACAGAACTAAGA--------------------------
GATAAAAGGAAAACTGTACATTCACTT---TTTTATAAACTTGATATAGTATCAATTGATAGT----------
----

AATGGTAGTGATAGTAATGGTAGTGGGCAGTATAGACTAATAAATTGTAATACATCAACCGTGACACAG---
GCCTGTCCTAAGGTAACCTTTGAGCCAATTCCTATATATTATTGTACCCCAGCTGGTTTTGCGATTCTAAAGT
GCAGG---GATACAAACTTTACT------GGAAAAGGGTCATGCAAGAAT-----------------------
-GTCAGT---ACAGTACAATGCACACATGGAATCAAGCCAGTAGTATCAACT---
CAACTGCTGTTAAATGGCAGTCTAGCAGAAAAAGAG------------
GTAATGATTAGATCTGAAAATATTACAGACAAT---------GGCAAAACCATAATAGTACAGCTTACT----
-----------GAGCCTGTAAAC---------ATCACTTGTACCAGACTTAGCAACAATACAAGAACA-----
-AGTATACGTATA------------GGACCAGGACAAACC---TTC------TATGCAACAGGTGAT------
---------GTAATAGGAGATATA------------AGAAAAGCATCTTGT---GAGGTCAACAGAACA---
GTATGGAATAGCACTTTACAAAAGATAAGTACACAATTAAGA---------------AAATACTTTAAT----
-----------------------------------AACAAAACAATAATCTTTAAG------
AACTCCTCAGGAGGGGATTTAGAAGTTACA---ACACATAGTTTCAATTGTGGAGGAGAA---
TTTAAACAGCTCCAGGCAAGAGTCCTGGCTGTGGAAAGATACCTACAGGATCAGCAGCTCCTAGGAATTTGGG
GTTGCTCTGGAAAACTCATCTGCACCACTAATGTGCCCTGGAACTTTAGTTGGAGT-----------------
-----------------------------AATAAATCTCAGGATGAGATATGG------GGA------
AACATGACCTGGATGCAATGG---GACAAGGAAATTAGCAATTACACAGACAAAATATAT---
GATCTAATTGAAAAAATCGCAAAATCAGCAGGAA---------------------------------------
----
AAAAATGAACAAGAGTTATTAGCATTGGATAAGTGGGCAGGTCTGTGGAATTGGTTTAACATATCAAATTGGT
TATGGTATATACAAATATTTATAATAATAGTAGGAGGCTTAATAGGATTAAGAATAGTTTTTGCT---
GTACTTTCTATAATA---------AATAGAGCCAGGCAGGGATACTCACCC------
TTGTCATTGGCTATTAATCTGATAGATCACATAGCAATAGTAGTAGCTGGG---
TGGACAGATAGGGTTTTAGAAATAGGACAAAGATTTTGTAGAGCT----------------------------
--------ATTCGTAACATA------CCTAGGAGAATCAGACAGGGCGCAGAAAAAGCGTTGCAA---TAA
>MH330338
GCT---GCA---
GAACAATTGTGGGTCACGGTATACTATGGGGTACCTGTGTGG---AGAGATGCAGAG---
ACCACCCTATTTTGTGCATCAGATGCTAAAGCATATGATAAAGAG---GTACACAAT----------------
--------------GTCTGGGCTACA---CATGCCTGTGTACCCACAGAC---
CCTAACCCACTAGAAATGGATTTGGAG---------AATGTGACAGAAGAGTTTAAT----------------
--------------------------ATGTGGAAAAATAACATGGTAGAACAAATGCAAACAGATATA---
ATCAGTCTCTGGGACCAAAGCCTA---AAGCCATGTGTACAGCTA---
ACCCCTCTCTGTGTTACTTTAAATTGTTCTGACTTCAACAACACTAGACCTAACAGCACTAGTTCTAGTACCA
CTAGCGCTCCT--
----------------------AACAGCACTGCTAACGAAACATACAAGAATGAAGAAATGAAA---
AACTGCTCTTTCAACATAACCACAGTACTAAGA--------------------------
GATAAAAAGAAAAATGTATATTCACTT---
TTTTATAAACTTGATATAGTACCAATTGATAGTGATAGTAATGGTAGTAAG----------------------
----
CAGGATAATGGTAGTGGGCAGTATAATGGTAGTGTGCAGTATGGGCAGTATAGACTAATAAATTGTAATACAT

CAACCATGACACAG---
GCCTGTCCTAAGGTATCCTTTGAGCCAATTCCTATACATTATTGTGCCCCAGCTGGTTTTGCGATTCTAAAGT
GCAGG---GAGACAAACTTTACA------GGGAAAGGGCCATGCAAGAAT----------------------
-GTCAGC---ACAGTACAATGCACACATGGAATCAAGCCAGTAGTATCAACT---
CAACTGCTGTTAAATGGCAGTCTAGCAGAAGAACAG-----------
GTAATGGTTAGATCTGAAAATTTCACAGACAAT---------GGCAAAATCATAATAGTACAGCTTAAT----
-----------AAGCCTGTAAAC---------ATCACTTGTATCAGACCTAACAACAATACAAGAACA-----
-AGTGTACGTATA------------GGACCAGGACAAACC---TTC------TATGCAACAGGTGAG------
---------GTAATAGGGGACGTA------------AGGAAAGCATATTGT---AATATCAGCAGAACA---
GAATGGAATAACACTTTACAAAATGTAAGTGCACAATTAAGA---------------CAACACTTTCAG----
-----------------------------------AACAAAACAATAATCTTTAGG------
AACTCCTCAGGAGGGGATTTAGAAATTACA---ACACATAGTTTCAATTGTGGAGGAGAA---
TTTAAACAGCTCCAGGCAAGAGTCCTGGCTGTGGAAAGATACCTAAGGGATCAGCAGCTCCTAGGAATTTGGG
GTTGCTCTGGAAAACTCATCTGCACCACTACTGTGCCTTGGAACTCTAGTTGGAGT-----------------
----------------------------AACAAATCTCATGATGAGATATGG------AAT------
AATATGACCTGGATGGAATGG---GACAAGGAAGTTATCAATTACACAAACACAATATAT---
AAGCTAATTGAAAAAGCGCAAAACCAGCAGGAA--

AAGAATGAACAAGATTTATTGGCATTGGATAAGTGGGCAGGTCTGTGGAATTGGTTTAACATATCAAACTGGT
TATGGTATATAAAAATATTTATAATAATAGTAGGAGGCTTAATAGGATTAAGAATAGTTTTTGCT---
GTGCTTTCTATAATA---------AATAGAGTCAGGCAGGGATACTCACCC------
TTGTCATTTGCTATCAATCTAATAGATACCATAGCAATAGCAGTAGCTGGG---
TGGACAGATAGGGTTATAGAATTAGGACAAAGGCTTTGTAGAGCC----------------------------
--------ATTCTTAACATA------CCTAGAAGAATTAGACAGGGCGCAGAAAGAGCTTTGCAA---TAA
>MH330339
GCT---GCA--
GACAAATTGTGGGTCACAGTATACTATGGGGTACCTGTGTGG---AGAGATGCAAAG---
ACCACCCTATTCTGTGCATCAGATGCTAAAGCATATGATAAGGAA---GTACATAAT----------------
--------------GTCTGGGCTACA---CATGCCTGTGTACCCACAGAC---
CCTAACCCACAAGAAATAAGTTTG------------AATGTGACAGAAAATTTTAAC---------------
--------------------------ATGTGGGAAAATGACATGGTAGAACAAATGCAGACAGATATA---
GTCAGCCTCTGGGATCAAAGCCTA---AAGCCATGTGTAAAGTTA---
ACCCCTCTCTGCGTTACTTTAGATTGTGTTGAGCCCCTTAACAACAGT------------------------
----
-------------------------AATATTACAGGCAATTATAACTTGTCTGAAGACATGAGA---
AACTGCTCTTTCAATATGACCACAGAACTAAGA---------------------------
GATAAAAGGAAAAAATGTACATTCACTT---
TTTTATAAACTTGATATAGTACCAATTGATAATGGTAGTAATAGT----------------------------
----
AGTAGTAATGGTAGTAGGCAGTATAGACTAATAAATTGTAACACATCAACCACGACACAG---
GCCTGTCCTAAGGTAAGCTTTGAGCCAATTCCTATATATTATTGTACCCCAGCTGGTTTTGCGATTCTAAAGT
GCACG---GATCCAAAATTTAAT------GGAACAGGGCCATGCAATAAT----------------------
-GTCAGC---ACAGTACAATGCACACATGGAATCAAGCCAGTAGTATCAACT---
CAACTGCTGTTAAATGGCAGTCTAGCAGAAGAAGAG-----------
GTAATGATTAGATCTGCAAATATTACAGACAAT---------AGCAAAAACATAATAGTACAGCTTACT----
-----------GAGCCTGTAAAC---------ATCACTTGTATCAGACATAACAACAATACAAGAACA-----
-AGTATACGTATA------------GGACCAGGACAAACC---TTC------TATGCAACAGGTGAT------
---------GTAATAGGGGACATA------------AGAAGAGCACAGTGT---ATTGTCAATAGAACA---
GCATGGAATAGCACTTTATATAAGATAAGCACACAATTAAAA---------------AAGTACTTTAAC----
-----------------------------------AACAGAACAATAATCTTTAAT------
AGCTCCTCAGGAGGGGATGTAGAAGTTACA---ACACATAGTTTCAATTGTGGAGGAGAA---
TTTAAACAGCTCCAGGCAAGAGTCCTGGCTGTGGAAAGATACCTAAGGGATCAGCAGCTCCTAGGAATTTGGG
GTTGCTCTGGAAAACTCATCTGCACCACTAATGTGCCCTGGAACTCTAGTTGGAGT----------------
----------------------------AATAAAAATCAGAGTGAGATATGG------GAT------
AACATGACCTGGATACAATGG---GAAAAGGAAATTAGCAATTACACAAACCATATATAT---
GAGCTACTTGAAGAATCACAAAACCAGCAGGAA--

AAGAATGAACAAGATTTATTAGCATTGGATAAGTGGGCAGGTCTGTGGAATTGGTTTGACATCTCAAATTGGT
TATGGTATATAAGAATATTTATAATAATAGTAGGAGGCTTGATAGGCCTAAGAATAGTTTTTGCT---
GTGCTTTCTATAGTA---------AATAGAGTCAGGCAGGGATACTCACCC------

TTGTCATTGGCTATTAATCTGATAGATACATTAGCAATAGCAGTAGCTGGG---
TGGACAGATAGAGTTTTAGAACTAGGACAAAGATTTTGTAGAGCT----------------------------
--------ATTTGTAACATA------CCTAGGAGAATCAGACAGGGCGCAGAAAGGCTTTGCAA---TAA
>MH330340
GCT---ACA--
GGAAACTTGTGGGTCACGGTATACTATGGGGTACCTGTGTGG---AGGGAGGCAAAA---
ACCACCCTATTTTGTGCATCAGATGCCAAAGCATATGATAAAGAA---GTGCACAAT----------------
--------------GTCTGGGCTACA---CATGCCTGTGTGCCCACAGAC---
CCTAGCCCACAAGAAATAATGATGGAA---------AATGTAACAGAAAATTTTAAC----------------
--------------------------ATGTGGAAAAATGATATGGTAGAACAAATGCATACAGATATA---
ATCAGTCTCTGGGACCAAAGCTTA---AAGCCATGTGTAAAGCTA---
ACCCCTCTCTGTGTTACTTTAAATTGTACTGAACCCATCAGCAACAGTAGTATCAGCAACAAT----------
----
---AGTTTGGGGGACGTAATGAAA---
AACTGCTCTTTCAACATGACTACAGAAGTAAGA---------------------------
GATAAAAGGAAAACTGTATATTCACTC---TTTTATAAGCTTGATATAGTATCAACTGATAGTAAT-------
----

AGTAGTGGGCAGTATAGACTAATAAGTTGTAATACATCAACCATGACACAG---
GCCTGTCCTAAGGTAACCTTTGAGCCAATTCCTATACATTATTGTGCCCCAGCTGGTTTTGCGATTCTAAAGT
GCAAT---GAGGCAAAATTTAAT------GGAACAGGGCCATGCAAGAAT-----------------------
-GTCAGC---ACAGTACAATGTACACATGGAATCAAGCCAGTAGTATCAACT---
CAACTGCTGTTAAATGGCAGTCTAGCAGAAGAAGAG------------
GTAATGATTAGATCTGAAAATATCACAGACAAT---------GGCAAAAACATAATAGTACAGCTTAAG----
-----------GAGGCTGTAAAC---------ATCACTTGTATCAGACCTGGCAACAATACAAGAACA-----
-AGTATACGTATA-------------GGACCAGGACAAGCC---TTC------TATGCAACAGGTGCT------
---------GTAATAGGGGACATA------------AGAAGAGCACATTGT---AATGTCAGCAGAGCA---
AAATGGAATAGCACTCTACAACAGATAAGTACACAATTAAAA---------------AGGTACTTTAAT----
-----------------------------------AACAAAACAATAATCTTTAGG------
AATTCCTCAGGAGGGGATTTAGAAATTACA---ACACATAGTTTCAATTGTGGAGGAGAA---
TTTAAGCAGCTCCAGGCAAGAGTCCTGGCTGTGGAAAGATACCTAAGGGATCAGCAGATCCTGGGAATTTGGG
GTTGCTCTGGAAAACTCATCTGCACCACTAATGTGCCCTGGAACTCTAGTTGGAGT-----------------
----------------------------AATAAATCTCAAGAGGAGATATGG------GAG------
AACATGACCTGGATGCAATGG---GACAAGGAAGTTTTCAATTACACAAACCTAATATAT---
AATCTAATTGAAGAATCGCAAAACCAGCAGGAA--

AAGAATGAACAAGATTTATTGGCATTGGATAGGTGGGCAAGTCTGTGGAATTGGTTTGACATAACAAATTGGC
TATGGTATATAAAAATATTTATAATGATAGTAGGAGGCTTAATAGGATTAAGAATAGTTTTTGCT---
GTGCTTTCTATAATA---------AATAGAGCCAGGCAGGGATACTCACCC------
TTGTCATTGGCTATTAATCTAATAGATACCATAGCAATAGCAGTAGCTGGG---
TGGACAGATAGGGTTATAGAAATAGGACAAAGATTTTGTAGAGCT----------------------------
--------ATTTGTAACATA------CCTAGGAGAATCAGACAAGGCGCAGAAAGAGCTTTGCAA---TAA
>MH330341
GGT---GCA--
GGAAACTTGTGGGTCACAGTATACTATGGGGTACCTGTGTGG---AGAGATGCAGAG---
ACCACCCTATTTTGTGCATCAGATGCTAAAGCATATGATAAGGAA---GTACACAAT----------------
--------------GTCTGGGCCACA---CATGCCTGTGTACCCACAGAC---
CCTAACCCGCAAGAAATAATTTTGGAA---------AATGTGACAGAAGAATTTAAC----------------
--------------------------ATGTGGAAAAATAACATGGTGGAACAAATGCAAACAGATATA---
ATCAGTCTTTGGGACCAAAGCTTA---AAGCCATGTGTAAAGTTA---
ACCCCTCTCTGCGTTACTTTAGATTGTGTTGAACCCAACATCACTAGCTCTAACAGCAGT-------------
----
-------------------------AGCACTAACAGCAGTATTGACCAGTATGAAGAAACGAAA---
AACTGCTCTTTCAACATGACCACAGAACTAACA---------------------------
GATAAAAGGAAAACTGTACATTCACTT---TTTTATAAACTTGATATAGTATCAATGGATAGT----------
----

AATAATAATAGTAGTAAGCAGTATAGACTAATAAATTGTAATACATCAACCACGACACAG---
GCCTGTCCTAAGGTAACCTTTGAGCCAATTCCTATACATTATTGTACCCCAGCTGGTTTTGCGATTCTAAAGT
GCAAG---GAGACAAACTTTACT------GGAAAAGGGCCATGCAAGAAT-----------------------

-GTCAGC---ACAGTACAATGCACACATGGAATCAAGCCAGTAGTATCAACT---
CAACTGCTGTTAAATGGCAGTTTAGCAGAAGAAAAG------------
ATAATGATTAGATCTGAAAATATTACAGACAAT---------AGCAAAATTATAATAGTACAGCTTAAG----
-----------GAGCCTGTAAAC---------ATCACTTGTATCAGACCTGGCAACAATACAAGAACA-----
-AGTATACGAATA------------GGACCAGGACAAGCC---TTC------TATGCAGCAGGTGCG------
---------GTAATAGGGAACATA------------AGAAAAGCACATTGT---GAGGTCAACAGAACA---
GCCTGGAATAGCACTTTACAAAAGATAAGTACACAATTAAGA---------------CAATACTTTAAG----
-----------------------------------AACAAAACAATAATCTTTAAG------
AACTCCTCAGGAGGGGATTTAGAAGTTACA---GCACATAGTTTCAATTGTGGAGGAGAA---
TTTAAACAGCTCCAGGCAAGAGTCCTGGCTATAGAAAGATACCTAAAGGATCAGCAGCTCCTAGGAATTTGGG
GTTGCTCTGGAAAACTCATCTGCACCACTAATGTGCCCTGGAACTCTAGTTGGAGT-----------------
----------------------------AATAAATCTCAGAATGAGATATGG------GAT------
AACATGACCTGGATGCAATGG---GACAAGGAAATTATCAATTACACAAACAAAATATAT---
GATTTAATTGAAGAATCGCAAAACCAGCAGGAA---------------------------------------
----
AAGAATGAACAAGATTTACTAGCATTGGATAAGTGGACAGGTCTGTGGAATTGGTTTAACATATCAAATTGGC
TATGGTATATAAAAATATTTATAATAATAGTAGGAGGCTTAATAGGATTAAGAATAGTTTTTGCT---
GTGCTTTCTATAATA---------AATAGAGCCAGGCAGGGATACTCACCC------
TTGTCATTGGCTATTAATCTGGTAAATAGCATAGCAATAGCAGTAGCTGAG---
TGGACAGATAGGGTTATAGAAATAGGACAAATATTTTGTAGAGCT----------------------------
--------ATT-------------------CGAGAACCAGACAGGGCATAGAAAAGGCTTTGCAA---TAA
>MH330342
GCT---ACA--
GAAAACTTGTGGGTCACGGTTTACTATGGGGTACCTGTGTGG---AGAGATGCAGAG---
ACCACCCTATTTTGTGCATCAGATGCTAAAGCATATGATAAAGAA---GTACACAAT----------------
--------------GTCTGGGCTACA---CATGCCTGTGTACCCACAGAC---
CCTAACCCACAAGAAATAAAATTGGAA---------AATGTGACAGAAAACTTTGAC----------------
--------------------------ATATGGAACAATAACATGGTAGAACAAATGCAAACAGATATA---
ATCAGTCTCTGGGACCAAAGCCTA---AAGCCATGTGTAAAATTA---
ACCCCTCTTTGCGTTACTCTAAATTGTACTGAAGTCAACAGCACTAGCTCTATCAAC----------------
----
-------------------------AACTTTAGCAACAGTACCGATATCAAGAGCGAGATGAAA---
AACTGCTCTTTCAACATAACCACAGAACTAAGA---------------------------
GATAAAAGGAAAACTGTACATTCACTT---TTTTATAAACTTGATATAGTATCAACAACTGGT----------
----

AGTAATAATAGTAGTGGTAGTGGGCAGTATAGACTAATAAATTGTAATACATCAACCATGACACAG---
GCCTGTCCTAAGGTAACCTTTGAGCCAATTCCTATACATTATTGTGCCCCAGCTGGTTTTGCGATTCTAAAGT
GCAAT---AATCCAGATTTTAAT------GGAACAGGTCCATGCACGAAT-----------------------
-GTCAGC---ACAGTACAATGCACACATGGAATCAAGCCAGTAGTAACTACT---
CAACTACTGTTAAATGGCAGCCTAGCAGAGAACGAG------------
GTAATGATTAGAGCTAAAAATATCACAGACAAT---------AGCGGAAGCATAATAGTACAGCTTAAT----
-----------GAGACCGTAAAA---------ATCACTTGTATCAGACCTGGCAACAATACAAGAACA-----
-AGTATACGTATA------------GGACCAGGACAAACC---TTC------TATGCAACAGGTGCT------
---------GTAATAAGGGACATA------------AGAAAAGCATATTGT---AATGTCAGCAAAACA---
GCATGGAGCAGCACGTTACAAAAGATAAGAGAAAAATTAAGA---------------AATCACTTT-------
-----------------------------------AACAAAACAATACTCTTTAGG------
CCCTCCTCAGGAGGGGATTTAGAGGTTACA---ACACATAGTTTCAATTGTGGTGGAGAA---
TTTAAACAGCTCCAGGCAAGAGTCCTGGCTGTGGAAAGATACATAAAGGATCAGCAGCTCCTAGGACTTTGGG
GTTGCTCTGGAAAACTCATCTGCACCACTACTGTGCCCTGGAACACTACTTGGAGT-----------------
----------------------------AATAAATCTCTTGATTACATATGG------GGT------
AACATGACCTGGATGCAATGG---GACAAGGAAGTTATCAATTACACAAACAAAATATAT---
GATCTAATTGAAAAATCACAAAACCAGCAGGAA---------------------------------------
----
AAGAATGAACAAGATTTATTGGCATTGGATAAGTGGGCAGGTCTGTGGAATTGGTTTAACATATCAAATTGGT
TATGGTACATAAGAATATTTATAATGATAGTAGGAGGCTTAATAGGATTAAGAATAGTCTTTGCT---
GTGCTTTCTGTAATA---------AATAGAGCCAGGCAGGGATACTCACCC------
TTGTCATTGGCTATTAATCTGCTAGATACTATAGCAATAGCAATAGCTGGG---
TGGACAGATAGGGTTATAGAAGGAGGACAAAGATTGTGTAGAGCT----------------------------
--------ATTCGCAACATA------CCTAGAAGAATCAGACAGGGTGCAGAAAGAGCTTTGCAA---TAA

>MH330344
GCT---GCA--
GAAAACTTGTGGGTCACGGTATACTATGGGGTACCTGTGTGG---AGAGATGCAGAG---
ACCACCCTATTTTGTGCATCAGATGCTAAAGCATATGATAAAGAA---GTACACAAC----------------
--------------GTCTGGGCTACA---CATGCCTGTGTACCCACAGAC---
CCTAACCCACAAGAAATAGCTTTGGAG---------AATGTGACAGAAAATTTTGAC----------------
--------------------------ATGTGGAAAAATAACATGGTAGAACAAATGCAAACTGATATA---
ATCAGTCTCTGGGACCAAAGCCTA---AAGCCATGTGTAAAGCTA---
ACCCCTCTCTGCGTTACTTTAAATTGTACTGAACCCAGCTTTAATAATACCAGCTTTAATAATAAC-------
----
-------------------------AGTAGCGTTAACGTTAGTAATAGTGAAATAGAAATGAAA---
AACTGCTCTTTCAACATGACCACAGAACTAAGA---------------------------
GATAAAAGGAAAACTGTATATTCACTT---TTTTATACACCAGATATAGTATCAATGGATAGT----------
----

AATAGTAGTAATAGTAGTGGGCAGTATAGACTAATAAGTTGTAATACATCAACCATAACACAG---
GCCTGTCCTAAGGTAAGCTTTGAGCCAATTCCTATATATTATTGTACCCCAGCTGGTTTTGCGATTCTAAAGT
GCAAG---GATACAAATTTTAAT------GGAACAGGGCCATGCAAGAAT-----------------------
-GTCAGC---ACAGTACAATGCACACATGGAATCAAGCCAGTAGTATCAACT---
CAACTGCTGTTAAATGGCAGTCTAGCAGAAAAAGAG------------
GTAATGATTAGATCTAAAAATATCACAGACAAT---------AGCAAAACCATAATAGTACAGCTTACT----
-----------AAGCCTGTAAAC---------ATCACTTGTATCAGAACTGGCAACAATACAAGAACA-----
-AGTATACGTATA------------GGACCAGGACAAACC---TTC------TATGCAACAGGCGAT------
---------GTAATAGGGGACATA------------AGAAGAGCATATTGT---AATGTCAGCAGAGCA---
GAATGGAATAGGACTTTACAGGAGATAAGTACACAGTTAAGA---------------AAACACTTTGAG----
-----------------------------------AACAAAACAATAATCTTTAAG------
CACTCCTCAGGAGGGGATTTAGAAGTTGCA---ACACATAGTTTCAATTGTGGAGGAGAA---
TTTAAACAGCTCCAGGCAAGAGTCCTGGCTGTGGAAAGATACCTAAAGGATCAGCAGCTCCTAGGAATTTGGG
GTTGCTCTGGAAAACTCATCTGCACCACTAATGTGCCCTGGAACTCTAGTTGGAGT-----------------
----------------------------AACAAATCTCAGAGTGAGATATGG------GAT------
AACATGACCTGGATGCAATGG---GACAAGGAAGTTATCAATTACACAAACCTAATATAT---
AATCTAATTGAAGAATCACAAAACCAGCAGGAA--

AAGAATGAACAAGATTTATTGGCATTAGATAAGTGGGCAAGTCTGTGGAGTTGGTTTGACATATCAAACTGGT
TATGGTATATAAAAATATTTATAATAATAGTAGGAGGCTTAATAGGATTAAGAATAGTTTTTGCT---
GTGCTTTCTGTCATA---------AATAGAGTCAGGCAGGGATACTCACCC------
TTGTCATTGGCTATCAACCTAATAGATACCCTAGCAATAGCAGTAGCTGGG---
TGGACAGATAGGGTTATAGAAGTAGGACAAAGATTTTGTAGAACC----------------------------
--------ATCCGTAATATA------CCTAGAAGAATCAGACAGGGCGCAGAAAAGGCTTTGCAA---TAA
>MH330345
GCT---ACA--
GAAAACTTGTGGGTCACGGTATACTATGGAGTACCTGTGTGG---AGAGATGCAGAG---
ACCACCCTATTTTGTGCATCAGATGCTAAAGCATATGATAAAGAG---GTACACAAT----------------
--------------GTCTGGGCTACA---CATGCCTGTGTACCCACAGAC---
CCTGAACCACAAGAAATAAATTTGGAA---------AATGTGACAGAAGATTTTAAC----------------
--------------------------ATGTGGAAAAATAACATGGTAGAACAAATGCAAACAGATATA---
ATCAGTCTCTGGGACCAAAGTCTA---AAGCCATGTGTAAAGTTA---
ACCCCTCTCTGCGTTACTTTAAATTGTGTTGAACCCAACATCACTAGCTCTAACAGCAGTAGCGTTAACAGCA
AC---
-------------------------AACAGTAGTGTTAACAACAACAGGCTTGAAGAAATGAAA---
AACTGCTCTTTCAACATGACCACAGAACTAAGA---------------------------
GATAAAAAGGAAAAGGTACATGCACTT---TTTTATAGACTTGATATAGTCTCACTTGGTAAGAAGGAT----
----

AGTAATAATAGTGGTAGTGAGCAGTATAGATTAATAAATTGTAATACATCAGCCATGACACAG---
GCCTGTCCTAAGGTAACCTTTGAGCCAATTCCTATACATTACTGTGCCCCAGCTGGTTTTGCGATTCTAAAGT
GCAAG---GATACAACTTTTAAT------GGAACAGGGCCATGCAAGAAT-----------------------
-GTCAGC---ACAGTACAATGCACACATGGAATAAGGCCAGTAGTATCAACT---
CAACTGCTATTAAATGGCAGTCTAGCAGAGGAACAG------------
GTAATGATTAGATCCCAAAATATCACAGACACT---------AGTAAAACCATAATAGTACAGCTTACT----

-----------GAGCCTGTAAAC---------ATCACTTGTATCAGAAATGGCAGCAATACGAGAACA-----
-AGTATACGTATA------------GGACCAGGACAAGCC---TTC------TTTGCAACAGGAGCT------
---------GTAACAGGGGACATA------------AGAAAAGCATATTGT---ATTGTCAACGGAACA---
GCATGGAATAGCACTTTACAAAAGATAAGTACACAATTAGAA--------------AACCACTTTAAA----
------------------------------------AACAAAACAATAACCTTTAAG------
AACTCCTCAGGAGGGGATTTAGAAGTTACA---ACACATAGTTTCAATTGTGGAGGAGAA---
TTTAAACAGCTCCAGGCAAGAGTCCTGGCTGTGGAAAGATACCTAAAGGATCAGCAGCTCCTAGGACTTTGGG
GTTGCTCTGGAAAACTCATCTGCACCACTACTGTGCCCTGGAACTCTAGTTGGAGT----------------
-----------------------------AATAAATCTTACAGTGATATATGG------GAT------
AACATGACCTGGATGCAATGG---GACAAGGAAGTTATCAATTACACAAACATAATATAT---
GATCTAATTGAAAAATCGCAAACCCAGCAGGAA---

AAGAATGAACAAGATTTATTGGCATTGGATAAGTGGGCAAGTCTGTGGAATTGGTTTACCATATCAAAGTGGT
TATGGTATATAAAAATATTTATAATGATAGTAGGAGGCTTAATAGGATTAAGAATAGTTTTTGCT---
GTGCTTTCTATAATA---------AATAGAGTTAGGCAGGGATACTCACCC------
GTGTCATTGGCTATCAATCTAATAGATACCATAGCAATAGTAGTAGCTGGG---
TGGACAGATAGGGTTATAGAAGTAGGACACAGACTTTGTAGAGCC----------------------------
--------CTTTGTAACATA------CCTAGAAGAATCAGACAGGGTGCAGAAAGAGCTTTGCAA---TAA
>MH330346
GCT---GCA--
GAAAACTTGTGGGTCACAGTATACTATGGGGTACCTGTGTGG---AGAGATGCAGAG---
ACCACCCTATTTTGTGCATCAGATGCTAAAGCATATGATAAAGAA---GTACACAAT----------------
--------------GTCTGGGCTACA---CATGCCTGTGTGCCCACAGAC---
CCTGACCCACAAGAAATAGCTTTGAAA---------AATGTGACAGAAAATTTTAAC---------------
--------------------------ATGTGGAAGAATAACATGGTAGAACAAATGCAAACAGATATA---
ATCAGTCTCTGGGACCAAAGCCTA---AAGCCATGTGTAAAGTTA---
ACCCCTCTCTGTGTTACTTTAAATTGCACTAACCCAAGCAGCACTAGCTTGAACAGCAATAAT----------
----
-------------------------AGCGATAACAGCAATGAGAGTTTGTATGAAGAGATGAAA---
AACTGCTCTTACAACATAGCCACAGAACTAAGA--------------------------
GATAGAAAGAAAACTGTATATTCACTT---TTTTATAAGCTTGATATAGTATTAACTGATAGT----------
----

AATAATAATAATAGTAATAGTGAAAAGTATAGACTAATAAATTGCAATACATCAACCATGACACAG---
GCCTGTCCTAAGGTAACCTTTGAGCCAATTCCTATCCATTATTGTGCCCCAGCTGGTTTTGCGATTCTAAAGT
GCAGG---GAACCAAATTTTAGT------GGAAAAGGGCCATGCAAGAAT----------------------
-GTCAGC---ACAGTACAATGCACACATGGAATCAAGCCAGTAGTATCAACT---
CAACTGCTGTTAAATGGCAGTCTAGCAGAGGGAGAG---------
AATGTAACAATTAGATCTGAAAATATCACAGACAAT---------AGCAAAATCATAATAGTACAGCTTAAG-
--------------AAGCCTGTAAAC---------ATCACTTGTATCAGACCTAGCAACAATACAAGAACA--
----AGTATACGTATA------------GGACCAGGACAAACC---TTC------TATGCAACAGGTGAT---
------------GTAATAGGGAACCCA-----------AGAAAAGCACATTGT---GAGGTCAACAAAACA-
--GAATGGAATAGAGCTTTACGAGAGATAAAGGAACAATTGAGA------------AACAACTACTTTCAT--
--------------------------------------AACAAAACAATAAAATTTGAA------
AAATCCTCAGGAGGAGATTTAGAAGTTATA---ACACATACTTTCAATTGTGGAGGAGAA---
TTTAAACAGCTCCAGGCAAGAGTCCTGGCTGTGGAAAGATACCTAAAGGATCAGCAGATCCTAGGAATTTGGG
GTTGCTCTGGAAGACTCATCTGCGCCACTAATGTGCCCTGGAACTCTAGTTGGAGT----------------
-----------------------------AATAAATCTTATGATGAGATATGG------AAGAGT---
AACATGACCTGGATGCAATGG---GACAAGGAAGTTAACAATTACACAGGCAGAATATAT---
GAGCTAATTGAAAAATCGCAAAACCAGCAGGAA---

AAGAATGAACAAGAGTTATTGGAATTGGATAAGTGGTCAAGTCTGTGGAATTGGTTTGACATATCAAATTGGT
TATGGTATATAAAAATATTTATAATGATAGTAGGAGGCTTAATAGGATTAAGAATAGTTTTTGCT---
GTGCTTTCTATAATA---------AATAGAGTCAGGCAGGGATACTCACCC------
TTGTCATTTGCTATCAATCTACTAGATACCATAGCAATAGCAGTAGGTGGG---
TGGACAGATAGGGTTATAGAAATAGGACAAAGACTTTGTAGAGCC----------------------------
--------ATTCGCAACATA------CCTAGGAGAATCAGACAGGGCGCAGAAAGAGCTTTGCAG---TAA
>HQ616082
GCT---ACA--
GAAAACTTGTGGGTCACGGTATACTATGGGGTACCTGTATGG---AGAGATGCAGAG---

ACCACCCTATTTTGTGCATCAGATGCTAAAGCATATGATAAAGAG---GTACACAAT----------------
--------------GTTTGGGCTACA---CATGCCTGTGTACCCACAGAC---
CCTGACCCACGAGAAATAGATTTAGAA---------AATGTGACAGAAAATTTTAAC----------------
--------------------------ATGTGGAAAAATAACATGGTAGAACAAATGCAAACAGATATA---
ATCAGTCTCTGGGACCAAAGCCTA---AAGCCATGTGTAAAGTTA---
ACCCCTCTCTGTGTTACTTTAAATTGTTCTGACCCCACCAGCAACAGCACTAGCTCTAACAACAGCACTATC-
----
----------------TCTAACAACAGCACTAGCTTTAATGACAGCAGGTATGAAGAAATGAAA---
AACTGCTCTTTCAACATGACCACAGAACTAAGA---------------------------
GATAAAAGAAGAAATGTATATTCACTT---TTTTATAAACTTGATATAATAGCAACTAATAGTAAT-------
----

AATAGTAGGCTGTATAGATTAATAAATTGCAATACATCAGCCATGACACAG---
GCCTGTCCCAAGGTAACATTTGAGCCAATACCTATACATTATTGTGCCCCAGCTGGTTTTGCGATTCTAAAGT
GCAAG---GATCCAAATTTTAAT------GGAACAGGGCCATGCAAGAAT-----------------------
-GTCAGC---ACAGTGCAATGCACACATGGAATCAAGCCAGTAGTATCAACT---
CAACTGCTGTTAAATGGCAGTCTAGCAGAGAAAGAT------------
GTAATGATTAGATCTGAAAATATCACAGACAAT---------AGCAAAACCATAATAGTACAGCTCACT----
-----------GAGCCTGTAAAC---------ATCACTTGTATCAGACCTGGCAACAATACAAGAACA-----
-AGTATACGTATA-------------GGACCAGGACAAGCT---TTC------TATGCAACAGGTGAG------
---------ATAATAGGGGACACA------------AGAAAAGCACATTGT---ACTGTCAACAAAACA---
GCATGGAGAGCACTTTACAAAAGACAAGTATGAAATTAAGA----------------GAATACTTTAAG----
-----------------------------------AACAAAACAATAGTCTTTAAG------
AACTCCTCAGGAGGGGATTTAGAAGTTACA---ACACATAGTTTCAATTGTGGAGGAGAA---
TTTAAACAGCTCCAGGCAAGGGTCGTGGCTGTGGAAAGATACCTAAAGGATCAGCAGCTCCTAGGAATTTGGG
GTTGCTCTGGAAAACTCATCTGCCCCACTAATGTGCCCTGGAACTCTAGTTGGAGT-----------------
----------------------------AACAAATCTCAGAATGAGATATGG------AAT------
AACATGACCTGGATGCAATGG---GACAAGGAAGTTATCAATTACACAAACCTAATATAT---
AATTTAATTGAAGAATCGCAAAACCAGCAGGAA--

AAGAATGAACAAGATTTATTGGCATTGGATAAGTGGGCAAGTCTGTGGAGTTGGTTTGACATATCAAATTGGT
TATGGTATATAAGAATATTTATAATAATAGTAGGAGGATTAATAGGATTAAGAATAGTTTTTGCT---
GTGCTTTCTATAATA---------AATAGAGTCAGGCAGGGATACTCACCC------
TTGTCATTGGCTATCAATCTGCTAGATACCACAGCAATAGTAATAGCGGGG---
TGGACAGATAGGGTTATAGAACTAGGACAAAGACTTTGTAGAGCA----------------------------
--------ATTATTAACATA------CCTAGGAGAATCAGACAGGGCGCAGAAAAAGCTTTGCAA---TAA
>DQ823357
GCT---ACA--
GAAAACTTGTGGGTCACAGTATACTATGGGGTACCTGTGTGG---AGAGATGCAGAG---
ACCACTCTATTTTGTGCATCAGATGCTAAAGCATATGATAAAGAA---GTACATAAT----------------
--------------GTCTGGGCTACA---CATGCCTGTGTACCCACAGAC---
CCTAACCCACAAGAAATACCTTTGGAA---------AATGTGACAGAAAATTTTAAT----------------
--------------------------ATGTGGAAAAATAACATGGTAGAACAAATGCAAACAGATATA---
ATCAGTCTCTGGGACCAAAGCCTA---AAACCATGTGTAAAGCTA---
ACCCCTCTCTGCGTTACTTTAAATTGTACTAAACCCACGCCCCCAACTTTAAATTGTACTGCATCC-------
----
----------------------AACTGCACTATCTCTAACAGCAGCTTGTATGAAGAAATGAGA---
AACTGCTCTTTCAACATGACAACAGAACTAAGA---------------------------
GATAAAGAGAAAAAAGTACATTCACTT---TTTTATAAACTTGATATAGTATCAACTGGTAGT----------
----

AATGATAGTGGGGAGTATAGACTAATAAATTGTAATACATCAGCCATGACACAG---
GCCTGTCCTAAGGTAACCTTTGAGCCAATTCCTATACATTATTGTGCCCCAGCTGGTTTTGCGATTCTAAAGT
GCAAT---GATACAACTTTTAAT------GGAACAGGACCATGCAGGAAT-----------------------
-GTCAGC---ACAGTACAATGCACACATGGAATCAAGCCAGTAGTATCAACT---
CAACTGCTGTTAAATGGCAGTCTAGCAGAAAATGAG------------
GTAATGATTAGATCTGAAAATATTACAGACAAT---------GGCAAAAACATAATAGTACAGCTTACT----
-----------GAGCCTGTAAAC---------ATCACTTGTATCAGACCTGGCAACAATACAAGAACA-----
-AGTATACGTATA-------------GGACCAGGACAAACC---TTC------TATGCAACAGGTGAA------
---------ATAATAGGGAACCCA------------AGAAAAGCACATTGT---AATGTCAGCAGAGTA---

GCATGGAATAACACTTTACAAAAGATAAGTATACAATTAAGA--------------AAATACTTTAAT----
-----------------------------------AACAAAACAATAACCTTTAAG------
AACTCCTCAGGAGGGGATTTAGAAGTTACA---ACACATAGTTTCAATTGTGGAGGAGAA---
TTTAAACAGCTACAGGCAAGAGTCCTGGCTGTGGAAAGATACCTAAAGGATCAGCAGCTCCTAGGAATTTGGG
GTTGCTCTGGAAAACTCATCTGCACCACTAATGTGCCCTGGAACTCTAATTGGAGT----------------
----------------------------AGTAAATCTCATGATGAGATATGG------AAT------
AACATGACCTGGATGCAATGG---GACAAGGAAGTTATCAATTACACAAACATAATATAT---
GGTCTAATTGAAAAATCGCAAAACCAGCAGGAA--

AAGAATGAACAAGATTTATTGGCATTGGATAAATGGGCAAGTCTGTGGAGTTGGTTTGACATATCAAATTGGT
TATGGTATATAAAAATATTTATAATGATAGTAGGAGGCTTAATAGGATTAAGAATAGTTTTTGCT---
GTGCTTTCTATAATA---------AATAGAGTCAGGCAGGGATACTCACCC------
TTGTCATTGGCTATTAATCTGCTAGATACCATAGCAATAGCAGTAGCTGGG---
TGGACAGATAGGGTTATAGAAGTAGGACAAAGATTTTGTAGAGCT---------------------------
--------ATTTGTAACATA------CCTAGGAGAATCAGACAGGGCGCAGAAAAGGCTTTGCAA---TAA
>KU749399
GCT---ACA--
GAAAACTTATGGGTCACAGTATACTATGGGGTACCTGTGTGG---AGAGATGCAGAG---
ACCACCCTATTTTGTGCATCAGATGCCAAAGCATATGATAAAGAA---GTACACAAT---------------
--------------GTCTGGGCTACA---CATGCCTGTGTACCCACAGAC---
CCTAATCCACAAGAAATAAGTTTGGGA---------AATGTGACAGAAAATTTTAAC---------------
--------------------------ATGTGGAAAAATAACATGATAGAACAAATGCAGACAGATATA---
ATCAGTCTCTGGGACCAAAGCCTA---AAGCCATGTGTACAGTTA---
ACCCCTCTCTGCGTTACTCTAAAGTGTACTGACCCCACTAACAACAACTCT---------------------
----
--GAAGTAATGAAA---
AACTGCTCTTTCAACATGACCACAGAACTAAGA--------------------------
GATAAAAAGAAAGCTGTATATTCACTA---TTTTATAAACTTGATATAGTACCAACTGATGGTAAT-------
----

GATAATAGTAGTTATAGACTAATAAATTGTAATACATCAACCGTGACACAG---
GCCTGTCCTAAGGTAACCTTTGAGCCAATTCCTATACATTATTGTGCCCCAGCTGGTTTTGCGATTCTAAAGT
GCAAG---GATAAAAAATTTTAAT------GGAACAGGGCCATGCAAGAAT----------------------
-GTCAGC---ACAGTACAATGCACACATGGGATCAAGCCAGTAGTATCAACT---
CAACTGCTGTTAAATGGCAGTCTAGCAGAGGATGAG-----------
GTAATGATTAGATCTGAAAATATCACAGACAAT---------GGCAAGACCATAATAGTACAGCTTAAG----
-----------GAGCCTGTAAAT---------ATCACTTGTATCAGACCTGGTAATAATACAAGAACA-----
-GGTATACATATA------------GGACCAGGACAAGCC---TTC------TTTGCAACAGGTGAC------
---------GTAATAGGGGACATA-----------AGAAACGCACATTGT---AATGTCAGCAGAGCA---
ACATGGAATAACACTTTACAACAGATAAGTGAACAATTAAGA--------------AAATACTTTCAT----
-----------------------------------AACAAAACAATAATCTTTGAG------
AGCTCCTCAGGTGGGGATTTAGAAGTTACA---ACACATAGTTTCAATTGTGGAGGAGAA---
TTTAAACAGCTCCAGGCAAGAGTCCTGGCTGTGGAAAGATACCTAAAGGATCAGCAGCTCCTAGGAATTTGGG
GTTGCTCTGGAAAACTCATCTGCACCACTAATGTGCCCTGGAACGTTAGTTGGAGT----------------
----------------------------AATAAATCTCAGCAGGAGATATGG------GAT------
AACATGACCTGGATGCAATGG---GACAAGGAAGTTATCAACTATACAGGCATAATATAT---
GATCTACTTGAAAAATCGCAAAACCAGCAGGAA--

AAGAATGAACAAGAATTATTGGCATTGGACAAGTGGGCAAGTCTGTGGAGTTGGTTTGACATAACAAATTGGT
TATGGTATATAAAAATATTTGTAATAATAGTAGGAGGCTTAATAGGATTAAGAATAGTTTTTGCT---
GTGCTTTCTATAATA---------AATAGAGTCAGGCAGGGATACTCACCC------
GTGTCATTGGCTATTAATCTGCTAAATACCACAGCAATAGTAGTAGCTGGG---
TGGACAGATAGGGTTATAGAAATAGGACAAAGATTTTGTAGAGCT---------------------------
--------ATTTGTAACATA------CCTAGGAGAATCAGACAGGGCGCAGAAAACGCTTTGCAA---TAA
>KU749401
GCT---ACA--
GAAAACTTGTGGGTCACAGTATATTATGGGGTACCTGTGTGG---AGAGATGCAGAG---
ACCACCCTATTTTGTGCATCAGATGCTAAGGCTTATGATAAGGAA---GTACACAAT---------------
--------------GTCTGGGCTACA---CATGCCTGTGTACCCACAGAC---
CCTAACCCACAAGAAATAAATTTGAAA---------AATGTGACAGAAAACTTTGAC---------------

--------------------------ATGTGGAATAATAGCATGGTAGAACAAATGCAAACAGATATA---
ATCAGTCTATGGGATCAAAGCCTA---AAGCCATGTGTACAGTTA---
ACCCCGCTCTGTGTTACTTTAAATTGTACTGAACCCAACCTCAACAGCACCAACACTACT-------------
----
-------------ATCCCTAACAATAGTGGTGGTAACAGCAGCGAGGTTAAAGAACTAATGAGA---
AACTGCACTTTCAACGTGACCACAGAACTAAGA---------------------------
GATAAAAAGAAGAGTGTGCATTCACTT---TTTTATAAGCTTGATATAGTATCAATTGATAGT----------
----

AATAGTAATAGTACACAGTATAGACTAATAAATTGTAATACATCAACCATAACACAG---
GCCTGTCCTAAGGTAACCTTTGAGCCAATTCCTATACATTATTGTACCCCAGCTGGTTTTGCGATTCTAGAGT
GCAAG---GAGAAAAATTTTACT------GGAAAAGGGCCATGCAGGAAT-----------------------
-GTCAGC---ACAGTACAATGTACACATGGAATCAAGCCAGTAGTATCAACT---
CAACTGCTGTTAAATGGCAGTCTAGCAGAGGGAGAG------------
GTAATGATTAGATCTAAAAATATCTCAGACAAT---------GGCAAAATCATAATAGTACAGCTTGAT----
-----------GAGCCTGTAAAC---------ATCACTTGTATCAGACCTGGCAACAATACAAGAACA-----
-AGTATAAGTATA------------GGACCAGGACGAGCC---TTC------TATGCAACAGGTGAT------
---------GTAATAGGGGACATA------------AGGAAAGCATATTGT---AATGTCAGCAAAACA---
GCATGGACTAACACTTTACAAAAGATAAGTATGCAATTAAGA---------------AAACACTTTAAT----
-----------------------------------AACAAAACAATAATCTTTAAG------
AACTCCTCAGGAGGGGATTTAGAAGTTACA---ACACATAGTTTCAATTGTGGAGGAGAA---
TTTAAACAGCTCCAGGCCAGAGTCCTGGCTGTGGAAAGATACCTAAGGGATCAGCAGCTCCTAGGAATTTGGG
GTTGCTCTGGAAGACTCATCTGCACCACTAATGTGCCCTGGAACTCTACTTGGAGT-----------------
----------------------------AATAAAAATCTGACTGAAATATGG------GAT------
AACATGACCTGGATGCAATGG---GACAGGGAAGTTAGCAATTACACAGACACAATATAT---
CAGCTAATTGAAAAATCGCAAAACCAGCAGGAA--

AAGAATGAACAAGATTTATTGGCATTAGATAAGTGGGCAAGTCTGTGGAATTGGTTTGACATATCACATTGGT
TATGGTATATAAAAATATTTATAATGATAATAGGAGGCTTAATAGGATTAAGAATAGTTTTTGCT---
GTGCTTTCTATAATA---------AATAGAGTCAGGCAGGGATACTCACCA------
TTGTCGTTGGCTATTAATCTGCTAGATACCACAGCAATAGCAGTAGCTGGG---
TGGACAGATAGGGTTATAGAAGTAGTACAAAGATTTTTTAGAGCT----------------------------
--------ATTTGTAACATA------CCTAGGAGAATCAGACAGGGCGCAGAAATAGCTTTGCTA---TAA
>KY658681
GCT--
GGAGGCTTGTGGGTCACAGTATACTATGGGGTACCTGTGTGG---AGAGATGCAGAG---
ACCACTCTATTTTGTGCATCAGATGCTAAAGCATTTGATAAAGAA---GCACACAAT----------------
--------------GTCTGGGCCACA---CATGCCTGTGTGCCCACAGAC---
CCTGACCCACAAGAAATAACTTTGGAC---------AATGTGACAGAAAAATTTGAC----------------
--------------------------ATGTGGAAAAATAACATGGTAGAACAAATGCAAGCAGACATA---
ATCAGTCTCTGGGACCAAAGCCTA---AAGCCATGTGTAAAGTTA---
ACCCCTCTCTGTGTTACCTTAAATTGTACTGCACCCAACAGCACTAGATGTAACAACAGT-------------
----
-------------------------CGTGTTAACAACAGTGATTGTGTGTTTGAAGAGATGAGA---
AACTGCTCTTACAACATGACCACAGAACTAAGA---------------------------
GATAAAAGGAAAACTGTACATTCACTT---TTTTATGCACTAGATATAATATCAATTGGTAGT----------
----

ACTGGTAGTAATGGTAGTGGGCAGTATAGACTAATAAATTGTAATACATCATCCATGACACAG---
GCCTGTCCTAAAGTATCCTTTGAGCCAATTCCTATACATTATTGTGCCCCAGCTGGTTTTGCAATTCTAAAAT
GCAAG---GATGCAAATTTTACT------GGAAAAGGGCCATGCAAGAAT-----------------------
-GTCAGC---ACAGTACAATGCACACATGGAATCAGGCCAGTAGTATCAACT---
CAACTGCTATTAAATGGCAGTCTAGCAGAGGAGAAG------------
ATAATGATTAGATCTGAAAATATCACAAACAAT---------CACAAAACCATAATAGTACAGCTTACT----
-----------GAGCCTGTAAAC---------ATCACTTGTATCAGACCTAATAACAATACAAGAACA-----
-AGTATACGTATA------------GGACCAGGACAAACC---TTC------TATGCAACAGGTGAT------
---------ATAATAGGGAACCCA------------AGAAAAGCATATTGT---AATATCAGTAGAAAA---
GCATGGAATAGCACTTTAGGAAAAGTAAGTATACAATTAAGA---------------GAATACTTT-------
-----------------------------------AACAAAACAATAATCTTTAAG------
AACTCCACAGGAGGGGATTTAGAAATTACA---ACACATAGTTTCAATTGTGGAGGAGAA---

TTTAAACAGCTCCAGGCAAGAGTCCTGGCTGTGGAAAGATACCTAAAGGATCAGCAGCTCCTAGGAATTTGGG
GTTGCTCTGGAAAACTCATCTGCACCACTAATGTGCCCTGGAACTCTAGTTGGAGT-----------------
----------------------------AATAAATCTCTGCCTGAGATATGG------AAT------
AACATGACCTGGATGGAATGG---GACAAGGAAGTTTTCAATTACACAAACACAATATAT---
GAGCTAATTGAAAAATCGCAAAACCAGCAGGAA--

AAGAATGAACTAGAGTTATTGGAATTGGATAAATGGGCAAGTCTGTGGAGTTGGTTTGACATAACAAATTGGT
TATGGTATATAAAAATATTTATAATAATAGTAGGAGGCTTAATAGGATTAAGAATAGTTTTTGCT---
GTGCTTTCTTTAATA---------AATAGAGTCAGGCAGGGATACTCACCC------
TTGTCATTGGCTATTAATCTGATAGATACCATAGCAATAGCAGTAGCTGAA---
GGAACAGATAGAATTATAGAAGTAGTACAAAGATTTTGTAGAGCT----------------------------
--------ATTTGTAACATA------CCTAGGAGAATCAGACAGGGCGCAGAAAAGGCTTTGCAA---TAA
>KU749402
GCT---ACA--
GAAAACTTGTGGGTCACGGTATACTATGGGGTACCTGTGTGG---AGAGATGCAGAG---
ACCACCCTATTTTGTGCATCAGATGCTAAAGCATATGATAAAGAG---GTACACAAT----------------
--------------GTCTGGGCTACA---CATGCCTGTGTACCCACAGAC---
CCTGACCCACGAGAAATACTTTTGGAA---------AATGTGACAGAAGAATTTAAC----------------
--------------------------ATGTGGAAAAATAACATGGTAGAACAAATGCAAATAGATATA---
ATCAGTCTCTGGGACCAAAGTCTA---AAGCCATGTGTAAAGTTA---
ACCCCTCTCTGTGTTACTTTAAATTGTACTAAACCCAAATTCAACAGTACCTCTAGCAACAATACCTTTTAC-
----
-------------------AACAGTACCTTTTACAGCAACAACAGCATGTTAGAAGAAATGAAA---
AACTGCTCTTTCAACATGACCACAGAACTAAGA---------------------------
GATAAAACAAAAAAAGTACATTCACTT---TTTTATAAGCTTGATATAGTAGCAACTGATGGTGGGCAT----
----

ACTAATGATAGTGGGCAGTATAGATTAATAAATTGTAATACATCAACCATGACACAG---
GCCTGTCCTAAGGTAACCTTTGAGCCAATTCCTATATATTATTGTGCTCCAGCTGGTTTTGCGATTCTAAAGT
GCAAC---GATCCAAATTTTAAK------GGAAAAGGGCCATGCAACAAT-----------------------
-GTCAGC---ACAGTACAATGCACACATGGAATCAAGCCAGTAGTATCAACT---
CAACTGCTGTTAAATGGCAGCCTAGCAAAGGAAAAG------------
GTAATGATTAGATCTGAAAATATCTCAAACAAT---------GCCAAAAACATAATAGTACAGCTTACT----
-----------AAGCCTGTAAAC---------ATCTCTTGTATCAGACCTAGCAACAATACAAGAACA-----
-AGTATACATATA------------GCACCAGGACAAGCC---TTC------TATGCAACAGGTGAG------
---------GTAATAGGGGACATA------------AGAAAAGCACATTGC---AATGTCAGCAGAACA---
TTATGGAATAGCACTTTACAAGAGATAAGTAAACAATTAAGA---------------GAACACTTTAAG----
-----------------------------------AACAAAACAATAATCTTTAAA------
AACCCTATAGGAGGGGATTTAGAAATTACA---ACACATAGTGTCAATTGTGGTGGAGAA---
TTTAAACAGCTCCAGGCAAGAGTCCTTGCTGTGGAAAGATTCCTAAGGGATCAGCAGCTCCTAGGAATTTGGG
GCTGCTCTGGAAGACTCATCTGCAACACTAATGTGCCCTGGAACTCTAGTTGGAGT-----------------
----------------------------AATAAATCTCAAAGTGAAATATGG------GAG------
AACATGACCTGGATGCAATGG---GACAAGGAAGTTATCAATTACACAAGCAAAATATAT---
GAGCTACTTGAACAATCGCAAAATCAGCAGGAA--

AAGAATGAACAAGATTTATTGGCATTGGATGAGTGGACAAGTCTGTGGAGTTGGTTTGACATATCAAAGTGGT
TATGGTATATAAAAATATTTATAATGATAGTAGGAGGCTTAATAGGATTAAGAATAGTTTTTGCT---
GTGCTTTCTATAATA---------AATAGAGTCAGGCAGGGATACTCACCA------
TTGTCATTGGCTATTAATCTGCTAGATACCATAGCAATAGCAGTAGCTGGG---
TGCACAGACAGGGTTATAGAAGTAGTACAAAGATTTTTTAGAGCT----------------------------
--------CTTCGTAATATA------CCTAGGAGAATTAGACAGGGAGCAGAAATAGCTTTGCAA---TAA
>KU749403
GCT---GCA---
GACATGTGGGTTACGGTATACTATGGGGTACCTGTGTGG---AGAGATGCAAAG---
ACCACCCTATTTTGTGCATCAGATGCTAAAGCACATGATACAGAA---GTACACAAT----------------
--------------GTCTGGGCTACA---CATGCCTGTGTACCCACAGAT---
CCTAACCCACAAGAAATATCTTTGGAA---------AATGTGACAGAAGATTTTAAC----------------
--------------------------ATGTGGAAAAATGATATGGTAGAACAAATGCAAACAGATATA---
ATCAGTCTCTGGGATGAAAGCCTA---AAGCCATGTGTACAGTTA---
ACCCCTCTCTGTGTTACTTTAAATTGTACTGATCCCAAACCCAACAGCACTATATCTAACAGCAGT-------

----
----------------------AGTAACAGCTACAGCAATGATAGCTGGGTTAAAGACATGAAA---
AACTGCTCTTACTACATAACCACAGAACTAAGA---------------------------
GATAAAAAGAAAAATGTACACTCACTT---TTTTATAAACTTGATGTAGTATCACTTGGTACT----------
----

AGTAATAATAGTAGTCAGTATAGACAGTATAGACTAATACATTGTAATACATCAACCATAAAGCAG---
GCCTGTCCTAAGGTAACCTTTGAGCCAATTCCTATACATTATTGTGCCCCAGCTGGTTTTGCGATTCTAAAGT
GCAAA---GATCCAATGTTTAAT------GGAACAGGGCCATGCAAGAAT-----------------------
-GTCAGC---ACAGTACAATGCACACATGGAATCAAGCCAGTAGCATCAACT---
CAACTACTGTTAAATGGCAGTCTAGCAGAGAAAGAG-----------
GTAATGATTAGATCTCAAAATATCACAGATAAT---------AGCAAAACCATAATAGTACAGCTTCAG----
-----------GAGCCTGTAAAC---------ATCACTTGTATCAAACCAGGCACCAATACAAGAACA-----
-AGGATACATCAT---------ATAGGACCAGGACGAGCC---TTT------TATACACATGGTGAT------
---------ATAATAGGAAACCCA------------AGAATAGCACATTGT---AATGTCAGCAGTGCA---
AAATGGAATAAAACTTTACAAGGGATAAGGAGACAATTAAGA---------------AACTACTTTACT----
-----------------------------------AACAAAACAATAATCTTTAGG------
AACTCCTCAGGAGGGGATGTAGAAATTACA---ACACATAGTTTCAATTGTGGAGGAGAA---
TTTAAACAGCTCCAGGCAAGAGTCCTGGCTGTGGAAAGATACCTAAAGGATCAGCAGCTCCTAGGAATTTGGG
GTTGCTCTGGAAGACTCATCTGCACCACTGCTGTGCCCTGGAATTCTAGTTGGAGT-----------------
----------------------------AATAAATCTTACAATGACATATGG------AAT------
AACATGACCTGGATGCAATGG---GACAAGGAAGTTAACAATTACACACACATAATATAT---
GATCTAATAGAACAATCGCAAAACCAGCAGGAA--

AAAAATGAACAAGAGTTATTGGCATTGGATAAGTGGGCAAGTCTGTGGAATTGGCTTGACATATCAAATTGGT
TATGGTACATAAGAATATTTATAATGATAGTAGGAGGCTTAGTAGGATTAAGAATAGTTTTTGCT---
GTGCTTTCTATAATA---------AATAGAGTCAGGCAGGGATACTCACCT------
GTGTCATTGGCTATTAATCTCATAGATACCATAGCAATAGTAGTAGCTGGG---
TGGACAGATAGAGTTATAGAAATAGGACAAAGAGTTTGTAGAGCT----------------------------
--------ATTTGCAACATA------CCTAGGAGAATCAGACAGGGCGCAGAAAGGGCTTTGCAG---TAA
>KU749404
GCT---GGA--
GAAAACTTGTGGGTCACAGTATACTATGGGGTACCTGTGTGG---AGAGATGCAGAG---
ACCACCCTATTTTGTGCATCAGATGCTAAAGCATATGATAAAGAA---GTACATAAT----------------
--------------GTCTGGGCTACA---CATGCCTGTGTACCCACAGAC---
CCTGACCCACAAGAAATAGTTTTGGGA---------AATGTGACAGAAGAATTTGAC----------------
--------------------------ATGTGGAAAAATAACATGGTAGAACAAATGCAAACAGATATA---
ATCAGTCTCTGGGACCAAAGCCTA---AAGCCATGTGTAAAGTTA---
ACCCCTCTCTGCGTTACTTTAAATTGTACTGAACCCAGATCCAACAGCAATAGTTCTAGC-------------
----
----------------------AACAATAACAACAGCACTGATAGATTGTATGAGGATATGAGA---
AACTGCTCTTTCAACATGACCACAGAACTAAGA---------------------------
GATAAAAGGAAAAATGTATATTCACTT---TTTTATAAACTTGATATAGTAGCAATTGATAGTGAG-------
----

AATAGTAGTCAGTATAGACTAATAAATTGTAATACATCAGCCATGACACAG---
GCCTGTCCTAAGGTAAACTTTGAGCCAATTCCCATACATTATTGTGCCCCAGCTGGTTTTGCGATTCTAAAGT
GCAAT---GATCCAAAGTTTACT------GGAAAAGGGCCATGCAAGAAT-----------------------
-GTCAGC---ACAGTACAATGCACACATGGAATCAAGCCAGTAGTTTCAACT---
CAACTGCTGTTAAATGGCAGTCTAGCAGAAGACGAG-----------
ATAATGATTAGATCTGAAAATATCACAGACAAT---------AGCAAAAACATAATAGTACAGCTTAAT----
-----------GAGTCTGTAAGC---------ATCACTTGTGTCAGACCTGGCAACAATACAAGAACA-----
-AGTATACGTATA------------GGACCAGGACAAACC---TTC------TATGGAACAGGTGAT------
---------GTAATAGGGGACATA------------AGAAAAGCATATTGT---AATGTCAACGGAACA---
GCATGGGATAGCACTTTACAAAAGATAAGAATACAATTGAGA---------------AAGTACTTTGGT----
-----------------------------------AACAGAACAATAAATTTTAGA------
AACTCCTCAGGAGGGGATTTAGAAGTTAGA---ACACATAGTTTCAATTGTGGAGGAGAA---
TTTAAACAGCTCCAGGCAAGAGTCCTGGCTATAGAAAGATACCTAAAGGATCAGCAGCTCCTAGGAATTTGGG
GATGCTCTGGAAAACTCATCTGCACCACTAATGTGCCTTGGAACTCTAGTTGGAGT-----------------
----------------------------AATAAATCTCTGAATGACATATGG------GAT------

AACATGACCTGGATGCAATGG---GACAAGGAAGTTTTCAATTACACAAACATAATATAT---
GAGCTAATTGAAGAATCGCAAAACCAGCAGGAA--

AAGAATGAACAAGATTTATTGGCATTAGATAAGTGGGCAGGCCTGTGGAATTGGTTTGACATAACAAATTGGT
TATGGTATATAAAAATATTTATAATGATAGTAGGAGGCTTAATAGGATTAAGAATAGTTTTTGCT---
GTACTTTCTATAATA---------AATAGAGTCAGGCAGGGATACTCACCC------
TTATCATTTGCTATTAATCTGTTAGATACCATAGCAATAGCAGTAGCTGGG---
GGAACAGATAGGATTATAGAAACAGTACAGAGAATTTGTAGAGCT----------------------------
--------ATTTGTAACATA------CCTAGAAGAATTAGACAGGGAGCAGAAAAGGCTTTGCAA---TAA
>KU749405
GCT---ACA--
GAAAACTTGTGGGTCACGGTATATTATGGGGTACCTGTGTGG---AGAGAAGCAGAG---
ACCACCCTATTTTGTGCATCAGATGCTAAAGCATATGAGAAAGAA---GTGCACAAT----------------
--------------GTCTGGGCTACA---CATGCCTGTGTACCCACAGAC---
CCTAACCCACAAGAACTGGCTTTGGAA---------AATGTGACAGAAAATTTTAAT----------------
--------------------------ATGTGGAAAAATAACATGGTAGAACAAATGCAAACAGATGTG---
ATCAGTCTCTGGGACCAAAGCCTA---AAGCCATGTGTACAGTTA---
ACCCCTCTCTGCGTTACTTTAAATTGTACTGCACCCAGGTTAGATTGTATTAACAGCACC-------------
----
-------------------------AGCCACTACACCCACGATAGCTTGTATGAAGAAGTGAGA---
AACTGCTCTTATAACATGACCACAGAATTAAGG---------------------------
GATAAAAGGAAAAAGGTATATTCTCTT---TTTTATAAACTTGATATAATACCAACTGATAAT----------
----

AATACTAATAGTGATAGTAGACAGTATAGACTAATAAATTGTAACTCATCAACTGTGACACAG---
GCCTGTCCTAAGGTATCCTTTGAGCCAATTCCTATACATTATTGTGCCCCAGCTGGTTTTGCGATTCTAAAGT
GCAGG---GATCCAAAGTTTAAT------GGAACAGGGCCATGCAAGAAT-----------------------
-GTCAGC---ACAGTACAATGCACACATGGAATCAAGCCAGTAGTATCAACT---
CAACTGCTGTTAAATGGCAGTCTAGCAGAGGGGGAG------------
GTAATGGTTAGATCTGAAAATTTCACAGACAAT---------GCCAAGGTCATAATAGTACAGCTTAAG----
-----------GACCCTGTAAAC---------ATCACTTGTATCAGACCTAACAACAATACAAGGAAA-----
-AGTATACCTATG------------GGACCAGGACGAGCC---TTC------TATGCAACAGGTGAG------
---------GTAATAGGGGACATA------------AGAAAAGCATATTGT---TTTGTCAATAAAACA---
AAATGGAATAACACTTTAGAACAGATAAGTATAAAATTAAGA---------------GAACAATTTCCT----
-----------------------------------GACAAAACAATAGTCTTTAGG------
AACTCCTCAGGAGGGGATGTAGAAATTACA---ACACATAGTTTCAATTGTGGAGGAGAA---
TTTAAACAGCTCCAGGCAAGAGTCCTGGCTGTGGAAAGATATCTAAAGGATCAGCAGGTCCTAGGAATTTGGG
GCTGCTCTGGAAAACTCATCTGCACCACTAATGTGCCTTGGAACTCTAGCTGGAGT-----------------
----------------------------AATAGATCTCTAAATGAGATATGG------GAA------
AACATGACCTGGATGCAATGG---GACAAGGAAGTTAACAATTACACACAAATAATATAT---
GAGCTAATTCAAGAATCGCAAATCCAGCAGGAA--

AAGAATGAACAAGATTTATTGGCATTGGATAAGTGGGCAAGTCTGTGGAATTGGTTTAACATATCAAATTGGT
TATGGTATATAAAAATATTTATAATGATAGTAGGAGGCTTAATAGGATTAAGAATAATTTTTGCT---
GTGCTTTCTATAATA---------AATAGAGTCAGGCAGGGATACTCACCC------
TTGTCATTGGCTATTAATCTGATAGATACCATAGCAATAGCAGTAGCTGAG---
TGGACAGATAGGGTTATAGAAATAGGACAAAGAATTGGTAGAGCT----------------------------
--------ATTTGCAACATA------CCTAGGAGAATCAGACAGGGAGCAGAAAAAGCTTTGCAA---TAA
>KY658682
GCT---GCA---
AACTTGTGGGTCACAGTATACTATGGGGTACCTGTGTGG---AGAGATGCAGAG---
ACCACCCTATTTTGTGCCTCAGATGCCAAGGCTTATGATAAAGAA---GTACACAAT----------------
--------------GTCTGGGCTACA---CATGCCTGTGTACCCACAGAC---
CCTAACCCACAAGAAATAATTTTAGAA---------AATGTGACAGAAAATTTTAAT----------------
--------------------------ATGTGGAAAAATAACATGGTAGAACAAATGCACACAGATATA---
ATCAGTCTCTGGGACCAAAGTCTA---AAGCCATGTGTACAACTA---
ACCCCTCTCTGTGTTACTTTAAATTGTACTGAACCCACTATCGACAGTAACAATAACAGCACT----------
----
----------------------------AACACCAGCTAYAATAGAAAGATTGAAGAAATGAGA---
AACTGCTCTTTCAACATAACCACAGAACTAAAT---------------------------

GATAAACAGAAAAAAGTACATTCACTT---TTTTATAAACTTGATATAGTACCAACTGATAAT----------
----

GGTAGTAGTCAGTATAGACTAATAAATTGTAATACATCAACCATGACACAG---
GCCTGTCCTAAGGTATCCTTTGAGCCAATTCCTATATATTATTGTACCCCAGCTGGTTTTGCGATTATAAAAT
GCAAT---GATGCAAATTTTACA------GGAATAGGGCCATGCAGGAAT----------------------
-GTCAGC---ACAGTACAATGTACACATGGAATCAAGCCAGTAGTATCAACT---
CAATTGCTGTTAAATGGTAGTCTAGCTGAGAAAGAG-----------
ATAATGATTAGATCTGAGAATATCACAGACAAT---------ACTAAAAACATAATAGTACAGCTTACT----
-----------AAGGCTGTAAAC---------ATCACTTGCTCCAGACCTGGCAACAATACAAGAACA-----
-AGTATACCTATA------------GGACCAGGACGAGCC---TGG------TTTGCAACAGGTGAT------
---------GTAACAGGGGATCCA------------AGGAAAGCACATTGT---AATGTCAGCAAAGCA---
GATTGGACTAGCACTTTAAAAAATATAAGTATACAATTAAGA---------------AAACACTTT-------
-----------------------------------AACAAAACAATAATCTTTCAG------
AATTCCTCAGGAGGGGATTTAGAAGTTACA---ACACATAGTTTCAATTGTGGGGGAGAA---
TTTAAACAGCTCCAGGCAAGAGTCCTGGCTGTGGAAAGATACCTAAGAGATCAGCAACTCCTAGGAATTTGGG
GTTGCTCTGGAAAACTCATCTGCACCACTACTGTGCCCTGGAACTCTAGTTGGAGT-----------------
---------------------------AATAGATCTCAGGATGATATATGG------AAT------
AACATGACCTGGATGCAATGG---GACAGGGAAGTTAGCAATTACACAGAATTAATATAT---
AAGCTAATTGAAGAATCGCAAAACCAGCAGGAA---------------------------------------
----
AAGAATGAACAAGATTTATTGGCATTGGATGAGTGGGCAAGTCTGTGGAATTGGTTTAAAATAACACAGTGGT
TATGGTATATAAAAATATTTATAATGATAGTAGGAGGCTTAATAGGATTAAGGATAGTTTTTGCT---
GTGCTTTCTATAGTA---------AATAGAGTCAGGCAGGGATACTCACCC------
TTGTCATTGGCTATAAGTCTGATAGATACCATAGCAATAGTAGTAGCTGGG---
TGGACAGATAGGGTTTTAGAACTAGGACAGAGAATTTGTAGAGCT----------------------------
--------ATTTGTAACATA------CCTAGGAGAATCAGACAGGGCGCAGAAAGAGCTTTGCAA---TAA
>KU749407
GCT---GCA--
GAAAACACGTGGGTCACAGTATACTATGGGGTACCTGTGTGG---AGAGATGCAGAG---
ACCACCCTATTTTGTGCATCAGATGCTAAAGGAGTGGAAAAAGAA---GTACACAAT----------------
--------------GTCTGGGCTACA---CATGCCTGTGTACCCACAGAC---
CCTGACCCACAAGAAATAAATTTGAAA---------AATATGACAGAAGAGTTTAAC---------------
--------------------------ATGTGGAAAAATAACATGGTAGAACAAATGCAAACAGATATA---
ATCAGTCTCTGGGACCAAAGCCTA---AAGCCATGTGTACAGTTA---
ACCCCTCTCTGCGTTACTTTAAATTGTACTGCACCCAAAAACAACAGCAATGATAACTTCATT----------
----
--GGAGAAATGAAA---
AACTGCTCTTTCAACATGACCACAGAAGTAAGA--------------------------
GATAAAAGGAAAATTGTACATTCACTT---TTTTATAAGCTTGATATAGTATCAATTGGTAATAACAAA----
----

AATGATAATAGTAACTATAGTGAGTATAGACTAATAAATTGTAATCAAACAACCATGACACAG---
GCCTGTCCTAAGGTAACATTTGAGCCAATTCCTATACATTATTGTGCCCCAGCTGGTTTTGCGATTCTAAAAT
GCAGG---GATGCAAATTTTACT------GGAAAAGGGCAATGCAATAAT----------------------
-GTCAGC---ACAGTACAATGCACACATGGAATCAAGCCAGTAGTGTCAACT---
CAACTGCTGTTAAATGGTAGTCTAGCAGAGAAAAAG-----------
GTAATGATTAGATCTGAAAATATCACAGACAAT---------AGCAAAACCATAATAGTACAGCTTACT----
-----------GAGCCTGTAAAC---------ATCACTTGTATCAGACCTGGCAACAATACAAGAAAA-----
-AGAATACATATA------------GGACCAGGACGAGCC---TTC------ATTGCAACAGGTGCT------
---------GTACTAGGGGACCCA------------AGAAAAGCATATTGT---AATGTCAGCAAAACA---
GCATGGAATAGCACTTTACAAAAGATAAGTATACAATTAAGA---------------CAATACTTTGAA----
-----------------------------------AACAGAACAATAAYCTTTAAG------
AACTCCTCAGGAGGGGATTTAGAAGTTGCA---ACACATAGTTTCAATTGTGGAGGAGAA---
TTTAAACAGCTCCAGGCAAGAGTCCTGGCTGTGGAAAGATACCTAAGGGATCAGCAGCTCCTAGGAATTTGGG
GTTGCTCTGGAAAACTCATCTGCACCACTAATGTGCCCTGGAACTCTAGTTGGAGT----------------
---------------------------AATAAATCCTTGAGTGAGATATGG------GAT------
AACATGACCTGGATGCAATGG---GATAAGGAAGTTGCCAATTACACAAACATAATATAT---
AATCTAATTGAAGAATCACAAAACCAGCAGGAA---------------------------------------
----

AAGAATGAACAAGACTTGTTGGCATTGGACAAGTGGGCAAGTCTGTGGAATTGGTTTGACATATCAAATTGGT
TATGGTATATAAAAATATTTATAATGATAGTAGGAGGCTTAATAGGATTAAGAATAGTTTTTGCT---
GTGCTTTCTATAATA---------AATAGAGTCAGGCAGGGATACTCACCC------
GTGTCGTTGGCTATTAATCTGCTAGATACCATAGCAATAGCAGTAGCTGGG---
GGAACAGATAGGATTATAGAAATAGTACAGAGAATTGGTAGAGCT----------------------------
--------ATCTGGAACATA------CCTAGGAGAATCAGACAGGGCGCAGAAAGGGCTTTGCTA---TAA
>AF082486
GCT---GCA--
GAAAACTTGTGGGTCACGGTATACTATGGGGTACCTGTGTGG---AGAGATGCAGAG---
ACCACCCTATTTTGTGCATCAGATGCTAAAGCATATGATAAAGAA---GTACACAAT----------------
--------------GTCTGGGCTACA---CATGCCTGTGTACCCACAGAC---
CCTAACCCACAAGAAATAAGTTTGGAA---------AATGTGACAGAAAAATTTGAC----------------
--------------------------ATGTGGAAAAATAACATGGTAGAACAAATGCAAACAGATATA---
ATCAGTCTCTGGGACCAAAGCCTA---AAGCCATGTGTAAAGTTA---
ACCCCTCTCTGCGTTACTTTAAATTGTGCTGAACCCAAAAGCACTAGCTCTAACAACAGTAGC----------
----
----------------------GTTAACAGCAACAGCAGCGATAGCTTGTTTGAAGAAATGAAA---
AACTGCTCTTTCAACATGACCACAGAACTAAGA---------------------------
GATAAAAGGAAAACTGTACATTCACTT---TTTTATAAACTTGATATAGTATCAACTGGTART----------
----

AATGGTAGTGGGCAGTATAGACTAATAAATTGTAATACATCAGCCATGACACAG---
GCCTGTCCTAAGGTAACCTTTGAGCCAATTCCTATATATTATTGTGCCCCAGCTGGTTTTGCGATTCTAAAGT
GCAAG---GATACAAATTTTACT------GGAACAGGGCCATGCAAGAAT-----------------------
-GTCAGC---ACAGTACAATGCACACATGGAATCAAGCCAGTAGTATCAACT---
CAACTGCTGTTAAATGGCAGTCTAGCAGAGAAAGAG------------
GTAATGATTAGATCTGAAAATATCACAGACAAT---------AGCAAAATCATAATAGTACAGCTTACT----
-----------GAGCCTGTAAAC---------ATCACTTGTATCAGACCTGGCAACAATACAAGAACA-----
-AGTATACGTATA------------GGACCAGGACAAACC---TTC------TATGCAACAGGTGAT------
---------GTAATAGGGGACATA------------AGAAAAGCATATTGT---AATGTCAGCAGAGCA---
GCATGGAATAGCACTTTACAAAAGATAAGTACACAATTAAGA---------------AGATACTTTAAT----
-----------------------------------AACAAAACAATAATCTTTAAG------
AACTCCCCAGGAGGGGATTTAGAAGTTACA---ACACATAGTTTCAATTGTGGAGGAGAA---
TTTAAACAGCTCCAGGCAAGAGTCCTGGCTGTGGAAAGATACCTAAAGGATCAGCAGCTCCTAGGAATTTGGG
GTTGCTCTGGAAAACTCATCTGCACCACTAATGTGCCCTGGAACTCTAATTGGAGT-----------------
----------------------------AATAAATCTCAGAGTGAGATATGG------GAT------
AACATGACCTGGATGCAATGG---GACAGGGAAGTTATCTATTACACAGACATAATATAT---
GATCTAATTGAGAAATCGCAAAACCCGCAGGAA--

AAGAATGAACAAGATTTATTGGCCTTGGATAAGTGGGCAAGTCTGTGGAGTTGGTTTGACATATCAAATTGGT
TATGGTATATAAAAATATTTATAATAATAGTAGGAGGCTTAATAGGATTAAGAATAGTTTTTGCT---
GTGCTTTCTATAATA---------AATAGAGCCAGGCAGGGATACTCACCC------
TTGTCATTGGCTATTAATCTGATAGATACCATAGCAATAGCAGTAGCTGGG---
TGGACAGATAGGGTTATAGAAATAGGACAAAGATTTTGTAGAGCT----------------------------
--------ATTCGTAACATA------CCTARGAGAATCAGACAGGGCGCAGAAAAGGCTTTGCAA---TAA
>KY968402
GCT---ACA--
GAAAAATTGTGGGTCACAGTCTATTATGGGGTACCTGTGTGG---AAAGAAACAACA---
ACCACTCTATTTTGTGCATCAAATGCTAAAGCATATGCTACAGAG---GTACATAAT----------------
--------------GTTTGGGCCACA---CATGCCTGTGTACCCACAGAC---
CCCGACCCACAAGAACTAGTGTTATCA---------AATGTGACAGAAAAATTTGAC----------------
--------------------------ATGTGGAAAAATAACATGGTAGAACAGATGCATGAGGATATA---
ATCAGTTTATGGGATCAAAGCCTA---AAACCATGTGTAAAATTA---
ACCCCACTCTGTGTTACTTTAAATTGCACTGCATTGAAGACCAATAGTACTACTAGTGATAGTACC-------
----
-------------------------AGTAATGGTACCGTTAGCCCAGCAAGAGAAGAAATGACA---
AACTGCTCTTTCAATGTCACCACAAACATAAGA---------------------------
AATAAGATACAGAAAGAATATGCACTT---TTTGATAAACTTGATGTAGTTCCAACACAGGAT----------
----

AATAATAACAGCTATATTTTGAGAAATGGTAATGCCTCAGTCCTTACACAG---
GCCTGTCAAAAGGTATCCTTTGAGCCAATTCCCATACATTACTGTACCCCAGCTGGTTTTGCACTTCTAAAAT
GTAAC---GATAAAAAGTTCAAT------GGAACAGGATTATGTACAAAT----------------------
-GTCAGC---ACAGTACAATGTACACATGGAATTAAGCCAGTAGTATCAACT---
CAACTGCTGTTAAATGGCAGTCTAGCAGAAGAAGAG-----------
GTAGTAATTAGATCTCAGAATTTCAGTGACAAT---------GCTAAAATCATAATAGTACAACTGAAT----
-----------GAATCTGTAGTA---------ATTAATTGTACAAGACCCAACAACAATACAAGAAGA-----
-GGTATACATATG------------GGACCAGGAGGAGTA---TTT------TATGCAACAGGAGAC------
---------ATAATAGGAGATATA-----------AGACAAGCACATTGT---AACATTAGTAGCAAA---
GAATGGAATAAAACCTTAGGACAGATAGCTAAAAAATTAAGA--------------GAACAATTTGGG----
-----------------------------------AATAAAACAATAGCCTTTAAT------
CAATCCTCAGGAGGGGACCCAGAAATTGTA---ATGCACAGTTTTAATTGTGGAGGAGAA---
TTTAAACAGCTCCAGGCAAGAGTCCTGGCTGTGGAAAGATACCTAAAGGATCAACAGCTCCTAGGGATTTGGG
GTTGCTCTGGAAAACTCATCTGCACCACTACTGTGCCTTGGAATACTAGCTGGAGT-----------------
----------------------------AATAAATCTCTGGGTGACATTTGG------GAT------
AACATGACCTGGATGGAGTGG---GAAAGACAGATTGAAAATTACACAGATTTAATATAC---
ACCTTACTTGAAGATTCACAGAGACAACAAGAA--

CAGAATGAACTAGAATTATTAAAATTAGATACGTGGGACAGTCTGTGGAGTTGGTTTAGCATATCAAATTGGC
TGGGGTACATAAGAATATTCATAATAGTAGTAGGAGGCTTGATAGGCTTAAGAATAATTTTTGCT---
GTATTTTCTATAGTG---------AATAGAGTTAGGCAGGGATACTCACCA------TTATCATTT-----
TGTCTTGTTCAATGCTATAGCCATAGCAGTAGCAGGG------ACAGAG---
ATTATAGAAATAGTACAAAGAACTTTA---GCT------------------------------------
ATTCTCAACATA------CCA---AGAATAAGACAGGGCTTGGAAAGGGCTTTGCTA---TA-
>KY968404
ACT---GGA---
GAACCATTGTGGGTCACAGTCTATTATGGGGTACCTGTGTGG---AGAGAAGCAACT---
ACCACCCTATTTTGTGCATCAGATGCTAAAGCATATGATACAGAG---GTACACAAT----------------
--------------GTTTGGGCCACA---CATGCCTGTGTACCCACAGAC---
CCCAACCCACAAGAAATAATATTGGCA---------AATGTGACAGAAGAATTTAAC---------------
--------------------------ATGTGGAAAAATAACATGGTGGAACAGATGCAAGAGGATATA---
ATCAGTCTATGGGATCAAAGTCTA---AAGCCATGTGTAAAATTA---
ACCCCACTCTGTGTTACTTTAAATTGTAGTGATGCTAATGTTACTAGGGCCACTAATATT------------
----
----------------------------AGCTGGGGGGAAGCGATAGGGAAAGGAGAAATAAAA---
AACTGCTCTTTCAATGTCACCACAAACATGAGA--------------------------
GATAAGGTGAAAAAAGCATATGCAAAT---TTTTATAATCTTGATATAGTACAAATAGATAAT---------
----

GATAATACCAGCTATATATTAAGAAGTTGTAATACCTCAGTCATTACACAG---
GCCTGTCCAAAGATAACCTTTGAGCCAATTCCCATACATTATTGTGCCCCGGCTGGATATGCGATTCTAAAGT
GTAGA---GATAAGAAGTTTAAT------GGAACAGGAACATGTAGAAAT----------------------
-GTCAGC---TCAGTACAATGTACACATGGAATTAGGCCAGTAGTATCAACT---
CAACTGTTGTTAAATGGCAGTCTAGCAGAAGAAGAG-----------
GTAATAATTAGATCTGAGAATTTCAGTAACAAT---------GCTAAAACCATAATAGTACAGCTAAAC----
-----------GATCCTGTAGTA---------ATTAATTGTACAAGACCCARCAACAATACAAGAAGA-----
-AGTATACCTATA------------GGACCAGGAAGAGCA---TTT------TATGCAACAGGAGAC------
---------ATAATAGGAGATATA-----------AGACAAGCACATTGT---AATGTTAGTAGAACA---
AAATGGAATGAGACTTTAGAAAAGATAGTTAAAAAATTACAA------------GAAATAGAATTTAAG----
-----------------------------------AATAAAACAATAGTATTTAAT------
CACTCCACAGGAGGGGACCCAGAAATTGTA---ATGCACAGTGTTAATTGTGGAGGAGAA---
TTTAAGCAGCTCCAGGCAAGAGTCCTGGCTATAGAAAGATACCTAAAGGATCAACAGCTCCTGGGAATTTGGG
GTTGCTCTGGAAAACTTATTTGCACCACTACTGTGCCTTGGAATACTAGTTGGAGT----------------
----------------------------AATAAATCTCTAAATCAGATTTGG------GAT------
AACATGACCTGGATGCAGTGG---GATAAAGAAATTAACAATTACACAACCTTAATATAC---
ACTTTGATTGAAGAATCGCAGATCCAGCAAGAA--

AAGAATGAACAAGAATTATTAAAATTGGATAGTTGGGCAAGTTTGTGGAATTGGTTTGACATATCAAACTGGC
TGTGGTATATAAAGATATTCATAATGATAGTAGGAGGCTTAGTAGGTTTAAGAATAATTTTTACT---
GTACTTTCTATAGTA---------AATAGAGTTAGGCAGGGATACTCACCA------

TTATCGTTGGCTGTTAGCTTGTTTGATACTATAGCCATAGCAGTAGCTGAG---
GGGACAGATAGGATTATAGAAGTAGGACAAAGAGCTTGTAGAGCT----------------------------
--------ATTATCCACATA------CCA---AGAATAAGAC--------------------ACT---TAA
>KY968408
GCT---ACA--
GAAAATTTGTGGGTCACAGTCTATTATGGGGTACCTGTGTGG---AAAGAAGCAACC---
ACCACTCTATTTTGTGCATCAGATGCTAAAGCAGTTGACAAAGAG---GTACATAAT----------------
--------------GTTTGGGCCACA---CATGCCTGTGTACCCACAGAC---
CCTAATCCACAAGAATTAGTATTGGAA---------AATGTGACAGAAAATTTTAAC----------------
--------------------------ATGTGGAAAAATGACATGGTAGAACAGATGCATGAGGATATA---
ATCAGCTTATGGGATCAAAGCTTA---AAGCCATGTGTAAAATTA---
ACCCCACTCTGTGTTATGTTAAATTGCACTGATTATACTAATAACAGCACTAATAGCACTAAT----------
----
-------------------------------------TTGGAACAGATGAAGGGAGAAATAAAA---
AACTGCTCTTTTAATATCACCACAAACATAAAA---------------------------
AATAAGATACAAAAAGAATATGCACTT---ATGTATAAACTTGATTTAATGCCAATAAGTAAT----------
----

GATAATACAAGCTATACATTAATAAACTGTAACACCTCAGTCATTACACAG---
GCCTGTCCAAAGGTATCCTTTGAACCAATTCCCATACATTATTGTGCCCCGGCTGGTTTTGCGATTCTAAAAT
GTAAT---GATAAGAAGTTCAAT------GGAACAGGACCATGTACAAAT-----------------------
-GTCAGC---ACAGTACAATGCACACATGGAATTAGGCCAGTAGTGTCAACT---
CAACTGCTGTTAAATGGCAGCCTAGCAGAGGGAGAA------------
ATAGTAATTAGATCTGAAAATTTCACGAACAAT---------GTCAAAACCATAATAGTACAGCTGAAC----
-----------GAATCCATAGCA---------ATTAATTGTACAAGACCCAACAACAATACAAGAAAA-----
-GGTATACATATA------------GCATGGGGAAGAACA---TTT------TATGCAACAGGAGAA------
---------ATAATAGGAGATATA------------AGACAAGCACATTGT---AATCTTAGTGGAGCA---
GAATGGGAAAAGACTTTAGAACGGGTAGCTAAAAAATTAAGA---------------GAACAATTTAAT----
-----------------------------------AATAAAACAATAGTCTTTAAT------
CAATCCTCAGGAGGGGACCCAGAAATTGTA---ATGCACAGTTTCAATTGTGGAGGGGAA---
TTTAAGCAGCTCCAGGCAAGAGTCCTGGCCGTGGAAAGATACCTAAAGGATCAACAGCTCCTAGGATTTGGG
GTTGCTCTGGAAAACTCATTTGCACCACTGCTGTGCCTTGGAATATTAGTTGGAGT-----------------
----------------------------AATAAATCTCAGGATGACATTTGG------AAC------
AACCTGACCTGGATGCAGTGG---GAGAAAGAAATTGACAATTACACAGGCTTAATATAC---
ACCTTACTTGAAGAATCGCAGAACCAACAAGAA--

AAAAATGAACTAGAATTATTGGAATTGGATAAGTGGGCAAGTTTGTGGAATTGGTTTGACATATCAAACTGGC
TGTGGTATATAAAATTATTCATAATGATAGTAGGAGGCTTAGTAGGTTTAAGGATAGTTTTTGCT---
GTACTTTCTATAGTG---------AATAGAGTTAGGCAGGGATACTCACCA------TTATCATTT-----
TGTTTTGCTCAACGCTATAGCCATAGCAGTAGCGGGG------ACAGAG---
GTTATAGAATTAGTACAAAGACTTTGA---GGT------------------------------------
ATTCTCCACATA------CCA---AGAATAAGACAGGGATTAGAAAGGGCTTTGCTA---TA-
>DQ383749
GCT---GGA--
GAACAATTGTGGGTTACAGTCTATTATGGGGTACCTGTGTGG---AAAGAAGCAACC---
ACCACTCTATTTTGTGCATCAGATGCTAAAGGATATGATACAGAA---GCACATAAT----------------
--------------GTTTGGGCCACA---CATGCCTGTGTACCCACAGAC---
CCCAACCCACAAGAAATAGTATTGGGA---------AATGTGACAGAAAATTTTAAT----------------
--------------------------ATGTGGAAAAACAACATGGTAGAACAGATGCATGAGGATATA---
ATCAGTTTATGGGAGGAAAGCCTA---AAGCCATGTGTAAAATTA---
ACCCCACTCTGTGTTACTTTACATTGCACTGATGTGAATGATACTAAGGCCAATAGTAATACT----------
----
----------------------ACTGGGAGTAGCTGGGACATGCCGGGGGGAGGAGAAATGACA---
AATTGCTCTTTCAATATCACCTCAAGCATAAGA---------------------------
GATAAGTGGCAGAAAGAATATGCACTT---TTTTATAGACTTGATGTAGTACCAATAGATAATAAT-------
----

AATAATAGTGATAATACCAGCTATAGGTTGATAAGTTGTAATACCTCAGTCGTTACACAA---
GCCTGTCCTAAGACATCTTTTGAGCCAATTCCCATACATTATTGTGCCCCGGCTGGTTTTGCAATTCTAAAGT
GTAAT---GATAAAAAGTTCAAT------GGAACAGGACCATGTACAAAT-----------------------

-GTCAGC---ACAGTACAGTGTACACATGGAATCAGGCCAGTAGTATCAACC---
CAACTGCTGTTAAATGGCAGTCTAGCAGAAGAAGAG------------
ATAGTAATTAGGTCTGAAAATTTCACAAACAAT---------GCTAAAACCATAATAGTACAGCTGAAC----
-----------ACATCTGTAGAA---------ATAAATTGTACAAGACCCAACAACAACACAAGAAAG-----
-AGTATACATATA------------GGACCAGGGAGATCA---TTA------TATACAACA---GAA------
---------ATAATAGGAAATATA------------AGAAAGGCATATTGT---ATCATTAATAGCACA---
AAATGGAATAATACTTTACAACAGATAAGTATAAAACTAAGA---------------GAACAATTTAAG----
-----------------------------------AATAAAACAATAGTCTTTAAT------
CAATCCTCAGGAGGGGACCCAGAAATTATG---ATGCTCACCCTTAATTGTGGAGGGGAA---
TTTAAACAGCTCCAGGCAAGAGTCCTGGCTGTGGAAAGATACCTATGTGATCAACAGCTCCTAGGGATTTGGG
GTTGCTCTGGAAAACTCATCTGCCCCACTACTGTGCCTTGGAATACTAGTTGGAGT-----------------
----------------------------AATAAATCTCTGGATAAGATTTGG------AAT------
AACATGACATGGATGGAGTGG---GAGAGGGAAATTGACAATTACACGAACATAATATAC---
TCCTTACTTGAACAATCACAAATCCAACAAGAA--

ACGAATGAAAAAGAATTATTAGAATTAGATAAATGGGCAAGTTTGTGGAATTGGTTTAGCATAACAAATTGGC
TATGGTATATAAAAATATTCATAATGATAGTAGGAGGCTTGATAGGTTTAAGAATAGTTTTTACT---
GTACTTTCTATAGTG---------AATAGGGTTAGGCAGGATACTCACCA------
TTATCGTTTGCTGTTAGCTTGCTCAACGCCACAGCCATAGCAGTAGCTGAG---
GGGACAGATAGGGTTATAGAAGTAGTGCAAAGAACTTATAGAGCT----------------------------
--------ATTCTCCACATA------CCTACAAGAATAAGACAGGGCTTGGAAAGGCTTTACTA---TAA
>MH078532
GCT---ACA--
AAAGACTTGTGGGTCACAGTCTATTATGGGGTACCTGTGTGG---AAAGAAGCAACC---
ACCACTCTATTTTGTGCATCAGATGCTAAAGCATATGACCCAGAA---GCACATAAT----------------
--------------GTTTGGCCACA---CATGCTTGTGTACCCACAGAC---
CCTAACCCACAAGAAGTAAAATTGGAA---------AATGTGACAGAAAGTTTTAAC----------------
--------------------------ATGTGGAAAAATAAAATGGTAGAACAAATGCATGAGGATATA---
ATCAGTTTATGGGATCAAAGCCTA---AAACCATGTGTAAAATTA---
ACCCCACTCTGTGTTACGTTAAGATGCAGTAATGTCACTGATACCAATGCCACTAATACCAATACCACT----
----
-------------------------AATACCAATGTTACTAGGGGGATGAGGGAAGAAATGCAA---
AACTGCTCTTTCAATGTCTCAGAAGGCATAAAA---------------------------
GATAAGATAAGGAAAGAATATGCACTC---TTTTATAAAATGGATCTAGTATCAATAGATAGT----------
----

AATAGTAGTAGTGCAAAGGATAGTACAGATTATATGTTGATAAATTGTAATACCTCAGTCATTACCCAG---
GCTTGTCCAAAGACAACCTTCGAACCAATTCCCATACATTATTGTGCCCCAGCTGGTTTTGCGATTCTAAAGT
GTACA---GATAAGAAATTCAAT------GGAACAGGACCATGTAAAAT------------------------
-GTTAGC---ACAGTACAGTGTACACATGGAATTAGGCCAGTAGTGTCAACT---
CATTTGTTGTTAAATGGCAGTTTAGCAGAAGAAGAG------------
ATAGTAATTAGATCTGAAAATATCACAAACAAT---------GCTAAAACTATAATAATACAGCTGAAG----
-----------GAAACTGTAAAA---------ATCACTTGTATAAGACCCGGCAACAATACAAGGAAA-----
-AGTATAAGTTTT------------GGACCTGGGAGTACA---TGG------TATGCAACAGGAGAC------
---------ATAATAGGAGATATC------------AGAAAAGCATATTGT---GACATCAATGGGACA---
GAATGGAATAATACATTAAGACAAATAGTTACAAAATTAAGA------------GAACAACACTTTAAT----
-----------------------------------AATAAAACAATAATCTTTGAA---
CAACCTATGGCAGGAGGGGACCCAGAAATTACA---ATGCACACTTTTAATTGTGGAGGAGAA---
TTTAAGCAGCTCCAGGCAAGACTCCTGGCTGTGGAAAGATACCTACGGGATCAACAGCTCCTAGGGATTTGGG
GATGCTCGGGAAAACTCATTTGCACCACTGCTGTGCCTTGGAATGCTAGTTGGAGT-----------------
----------------------------AATAAATCTGTAGATTACATTTGG------AAT------
AACATGACCTGGATGCAGTGG---GAGAAAAAAATTGACAACTACACAGAATTTATATAC---
ACCTTACTTGAGGAATCGCAGAACCAACAAGAA--

AAAAATGAACAGGAATTATTGAAATTGGATAGTTGGGCAAATTTGTGGAGTTGGTTCAGCATATCAAATTGGC
TGTGGTATATAAGAATATTCATAATAATAATAGGAGGCTTAATAGGTTTAAGAATAATTTTTACT---
GTACTTTCTATAGTG---------AATAGAGTTAGGCAGGATACTCACCA------
TTGTCATTCGCTGTTAGTTTGTTTAATGCAATAGCTATAGCAGTAGCTGAG---
GGGACAGATAGGATTATAGAAATAGTACAAAGATTTTGTAGAGGT----------------------------
--------ATCCTTCACATA------CCTACAAGAATAAGACAGGGCTTAGAAAGGCTTTGCTA---TAA

>AF538307
GCA---GCA---
GAAAAATTATGGGTCACAGTCTATTATGGGGTACCTGTGTGG---AAGGAAGCAACC---
ACCACTCTATTTTGTGCATCAGATGCTAAAGCATATGGTACAGAA---GTACATAAT---------------
--------------GTTTGGCCACA---CACGCCTGTGTACCCACAGAC---
CCCAACCCACAAGAAGTAGAATTGAAA---------AATCTGACAGAAAACTTTAAT---------------
--------------------------ATGTGGAAAAATGACATGGTAGAACAAATGCATGAGGATATA---
ATCAGTTTATGGGATCAAAGTCTA---AAGCCATGTGTAAAATTA---
ACCCCACTCTGTGTTACTTTAAATTGCACTGATGTCAATACTAATACCACTACTAATGTCACTGGTATT----
----
-------------------AATAATAGTAGTGTCAAGTTAGAATTGGAGAAAGGAGAAATAAAA---
AACTGCTCTTTCTACACCACCACAAGCATAACA--------------------------
GATAAGTATCAAAAAGTCTATGCGCTT---TTTTATAAACTTGATGTAGTGCCAATAGATGATAGT-------
----

AATAACAATACTAATACTAGCTATAGGTTGATAAGCTGTAACACCTCAGTCATTACCCAG---
GCCTGTCCAAAAGTATCCTTTGAGCCAATTCCCATACATTATTGTGCCCCGGCTGGTTTTGCGATTCTAAAAT
GTAAT---GATAAAAAGTTCAAT------GGAACAGGACCATGTAAAAAT----------------------
-GTCAGC---ACAGTACAATGTACACATGGAATTAGGCCAGTAGTGTCAACT---
CAACTGCTGTTAAATGGCAGCCTAGCAGAAGAAGAG-----------
GTAGTAATTAGATCTAGCAATTTCTCAGACAAT---------ACTAGAAACATAATAGTACAGCTGAAA----
-----------GACGCTGTAAAC---------ATTACTTGTATGAGGCCCAGCAACAATACAAGAAAA-----
-AGTATATCTATA------------GGACCAGGGAGAGCA---TTT------GATGCAACAGGAGAA------
---------ATAATAGGAGATATA------------AGACGAGCACACTGT---AACCTTAGTAGACAA---
GATTGGAATAACACTTTAAAACAGATACATAGCAAATTAAGG--------------GAACAATTTGGG----
-----------------------------------AATAAAACAATAGTCTTTAAT------
CACTCCTCAGGAGGGGACCCAGAAATTGTA---ATGCACAGTTTTAATTGTGGAGGGGAA---
TTTAAACAGCTCCAGGCAAGAGTCCTGGCTGTGGAAAGATACCTAAGGGATCAACAACTCCTAGGGATTTGGG
GTTGCTCTGGAAAACTCATTTGCACCACTAATGTGCCTTGGAATGCTAGTTGGAGT----------------
----------------------------AATAAATCTCTAAATGATATCTGG------GAT------
AACATGACCTGGATGGAGTGG---GAAAGAGAAATTGACAATTACACAAATACCATATAC---
ACCTTACTTGAAGAATCGCAAAACCAACAAGAA---------------------------------------
----
AAGAATGAACAAGAATCATTGGAATTAGATAAATGGGCAAGTTTGTGGAATTGGTTTGACATAACAAACTGGC
TGTGGTATATAAGAATATTCATAATGATAGTAGGAGGCTTGATAGGTTTAAGAATAGTTTTTAGT---
GTACTTTCTATAGTA---------AATAGAGTTAGGCAGGGATATTCACCA------
TTATCGTTTGCTGTTAGCTTGCTCGACGCCATAGCCATAGCAGTAGCTGAG---
GGGACAGATAGGGCTATAGAAATATTACAGAGAATCTTTAGAGCT---------------------------
--------ATTATCCACATA------CCTACAAGAATAAGACAGGGCTTGGAAAGGGCTTTGCTA---TAA
>GU367404
GCT---GCA---
GAAAAGTTGTGGGTCACAGTCTATTATGGGGTACCTGTGTGG---AAGGAAGCAAAT---
ACCACTCTATTTTGTGCATCAGATGCTAGAGCATATGATACAGAG---GTGCATAAT---------------
--------------GTTTGGCCACA---CATGCCTGTGTACCCACAGAC---
CCCAACCCACAAGAAGTATTATTGGAA---------AATGTGACAGAAACTTTTAAC---------------
--------------------------ATGTGGAAAAATAACATGGTAGAACAGATGCATGAAGATATA---
ATCAGTTTATGGGATCAAAGCCTA---AAGCCATGTGTAAAATTA---
ACCCCACTCTGTGTTAGTTTAAATTGCACCGATTTGAAGAATGCCACTAAGGAG------------------
----
-------------------------------ATGAATACCACTAAGGAGACGATGGAAATGAAA---
AACTGCTCTTTTAATATCAGCACAAGAATAAAA--------------------------
GCTAAGCTACAGGAAGAATATGCACTT---TTTTACAAACTTGATATAGTACCATTAGGTAAG---------
----

GACAGTAATGATACCAGCTATAGGTTGAAACACTGTAATACCTCAGTCATTACACAG---
GCCTGTCCAAAGGTATCCTTTGAGCCAATTCCCATACATTATTGTGCCCCGGCTGGTTTTGCGATTCTAAAGT
GTAAA---GATAAGAAGTTCAAT------GGAGAAGGACCATGTACAAAT----------------------
-GTTAGT---ACAGTGCAATGTACACATGGAATTAGACCAGTAGTGTCAACT---
CAACTGCTGTTAAATGGCAGTCTAGCAGAAGAAGAG-----------
ATAGTAATTAGATCTGACAATTTCACGGATAAT---------ACTAAAACCATAATAGTACAGCTGAAG----

-----------GAACCTGTAGAA---------ATTAATTGTACAAGACACAACAACAATACAAGAAAA-----
-AGTATATCTATG------------GGACCAGGGAAAGCA---TTT------TATGCAACAGGAGAC------
---------ATAATAGGAAATATA------------AGAAAAGCAGATTGC---ATCATCAATGAAACA---
AAATGGAATGACACTTTATACCAGATAGCTATGAAATTAAGA---------------GCACAATTTAGG----
-----------------------------------AATAAAACAATAGTCTTTAAT------
CGATCCTCAGGAGGGGACCCAGAAATTGTA---ATGCACAGTTTTAATTGTGGAGGGGAA---
TTTAAGCAGCTCCAGGCAAGACTCCTGGCTGTGGAAAGATACCTAAAGGATCAACAGCTCCTAGGGATTTGGG
GTTGCTCTGGAAAACTCATTTGCACCACTGCTGTGCCTTGGAATTCTAGTTGGAGT-----------------
----------------------------AATAAATCTCTGGATGAGATTTGG------GAT------
AACATGACCTGGATGCAGTGG---GAAAAAGAAATTAGCAATTACACAAACTTAATATAC---
ACCTTAATTGAAAAATCGCAGAACCAGCAAGAA--

AAGAATGAACAAGAATTATTGGAATTAGATAAGTGGGCAAGTTTGTGGAATTGGTTTGACATAACAAACTGGC
TGTGGTATATAAAAATATTCATAATGATAGTAGGAGGCTTGGTAGGTTTAAGAATAGTTTTTGCT---
GTACTTTCTATAGTG---------AATAGAGTTAGGCAGGGATACTCACCA------
TTGTCATTTGCTGTTAGCTTGCTCAATGCCACAGCTATAGCAGTAGCTGAG---
GGAACAGATAGGATTATAGAGGTAGTACAAAGAGTTTTTAGAGTT----------------------------
--------ATTCTCCACATA------CCTAGAAGAATAAGACAGGGCTTGGAAAGAAGTTTGCTA---TAA
>AF146728
GCA---ACA--
GAAAAATTGTGGGTCACAGTCTATTATGGGGTACCTGTGTGG---AAAGAAGCAACC---
ACCACTCTATTTTGTGCATCAGATGCTAAAGCATATGATAAAGAG---GTACATAAT----------------
--------------GTTTGGCCACA---CATGCTTGTGTACCCACAGAC---
CCCAACCCACAAGAAATATTACTAGAA---------AATGTGACAGAAGAGTTTAAC----------------
--------------------------ATGTGGAAAAATAACATGGTAGAACAGATGCATGAGGATATA---
ATCAGTTTATGGGATCAAAGCCTA---AAGCCATGTGTACAATTA---
ACCCCACTCTGTGTTACTTTAAATTGCACTGATGAGTTGACGAATGTTACTTTTACTAATAGTAGGCAT----
----
----------------GTGACTAATAGTAGTTATGTGGGAAGTATGGAAAAAGGAGAAATGAAA---
AACTGCTCTTTCAACATCACCACAAGCATAAGA---------------------------
GATAAGAGGCACAAAGAATTTGCACTT---TTTTATAAACTTGATGTAGTACAAATAGATGGT----------
----

AGTAATACTAGCTATAGATTAATAAATTGTAATACCTCAGTCATTACACAG---
GCCTGTCCAAAGGTATCCTTTGAGCCAATTCCCATACATTATTGTGCCCCGGCTGGTTTTGCGATTCTAAAGT
GTAAC---AATAAGACGTTCAAT------GGAAAAGGACCATGTGCAAAT-----------------------
-ATCAGC---ACAGTACAATGTACACATGGAATTAGGCCAGTAGTGTCAACT---
CAACTGTTGTTAAATGGCAGTCTAGCAGAAAAAGAG------------
ATAGTAATTAGATCTGACAATTTCACGGACAAT---------GCTAAAAGCATAATAGTACAGCTGAAT----
-----------GAATCTGTAGAA---------ATTCATTGTATGAGACCCAACAACAATACAAGAAAA-----
-GGGATATATGTA------------GGACCAGGGAGACAC---ATC------TATGCAACAGAGAAA------
---------ATAGTAGGAGATATA------------AGACAAGCACATTGT---AACATCAGTAGAACA---
AACTGGACTAGCGTGCTAAGACAGATAGCTGTAAAATTAAGA---------------GAACGATTTAAG----
-----------------------------------AATAAAACAATAGTCTTTAAT------
CACTCCTCTGGAGGGGACCCAGAAATTGTA---AGGCACAGTTTTAATTGTGGAGGGGAA---
TTTAAACAGCTCCAAGCAAGAATCCTGGCTGTGGAAAGATACCTAGAGGATCAACAGCTCCTAGGGATTTGGG
GTTGCTCTGGAAAACTCATTTGCACCACTTCTGTGCCTTGGAATGCTAGTTGGAGT-----------------
----------------------------AATAAATCTCTAAGTGACATTTGG------GAT------
AACATGACCTGGATGCAGTGG---GAAAGAGAAATTGGCAATTACACAGGCTTAATATAT---
CATTTACTTGAAGAATCGCAGAACCAACAAGAA--

AAGAATGAACAAGAATTATTGGCATTAGATAAGTGGGCAAGTTTGTGGAATTGGTTTAGCATAACAAAATGGC
TGTGGTATATAAAAATATTCATAATGATAGTAGGAGGCTTAGTAGGTTTAAGAATAGTTTTTGCT---
GTACTTTCTCTAGTG---------AAGAAAGTTAGGGAGGGATACTCACCA------
TTGTCATTGGCTATTAGCTTGCTCAACGCCACAGCTATAGCAGTAGCTGAG---
GGAACAGATAGGGTTATAGAAGTAGTACAAAGAGCTTGTAGAGCT----------------------------
--------ATTCTCCACATA------CCTAGAAGAATCAGACAGGGCTTGGAAAGGCTTTTACTA---TAA
>MN485974
GCT---GCA--
GATCAAAAGTGGGTCACAGTCTATTATGGGGTACCTGTGTGG---AAAGAAGCAAT---

ACCACTCTATTTTGTGCATCAGATGCTAAAGCATATGATACAGAG---GTACATAAT----------------
--------------GTTTGGGCCACA---CATGCCTGTGTACCCACAGAC---
CCCAGCCCACAAGAAGTAGCATTGGAA---------AATGTAACAGAGGATTTTAAC----------------
--------------------------ATGTGGAAAAATAACATGGTAGAACAGATGCATGAAGATATG---
ATCAGTTTATGGATCAAAGCCTA---AAGCCATGTGTAAAATTA---
ACCCCACTCTGTGTTACCTTAAAGTGCACTACGTATAAGAATGCTACTAATACCACTCAGACTCCCACTGACA
GTCCT--
---AGAGAAGAAATAAAA---
AACTGCTCCTTCAATGTTAGCACAAGTGTAAGA---------------------------
GATAAGAGTAAGAGAGAACATGCACTT---TTCTATGACAGTGATATAGTACCAATAGATAAT----------
----

GCCACATATAGGTTGATACATTGTAATACCTCTATCATTACACAG---
GCCTGTCCAAAGGTATCATTTGAACCAATTCCCATACATTTCTGTGCCCCGGCTGGTTTTGCGATTCTAAAGT
GTAAA---GAGACAAAGTTTAAT------GGAACAGGACCATGTAAAAAT-----------------------
-GTCAGC---ACAGTACAATGCACACATGGAATCAGACCAGTAGTGTCAACT---
CAACTACTGTTAAATGGCAGTCTAGCAGAAGAAGAG------------
ATAGTAATTAGATCTGCCAATTTCACGGACAAT---------AATAAAAACATAATAGTACAGTTGAAT----
-----------GAAACTATAAAA---------ATTAATTGTRCAAGACCCAATAACAATACAAGGAAA-----
-AGTATACATATA------------GGACCAGGGAGAGCA---TTT------TATGCAACAGGAGAA------
---------ATAATAGGAAACATA------------AGACAAGCACATTGT---AACCTTAGTAGCACA---
CAGTGGAATAACACTTTAAAACAAATAGCTATAGAATTAAGG---------------AACATATTTCCA----
-----------------------------------AATAAAACAATAGTCTTTAAT------
CATTCCTCAGGAGGAGACCCAGAAGTTGTG---ATGCACATTTTTAATTGTGGAGGGGAA---
TTTAAGCAGCTCCAGGCAAGAGTCCTGGCTGTGGAGAGATACCTARTGGATCAACAGCTCCTAGGGATTTGGG
GTTGCTCTGGAAAACTCATCTGCACCACTGCTGTACCTTGGAATAATAGTTGGAGT-----------------
----------------------------AATAGATCTCTAAATGACATTTGG------GAT------
AACATGACCTGGATGCAGTGG---GAAAAAGAAATTGACAATTACACAGGCTTAATATAC---
AAYTTACTTGAAGAATCGCAGAACCAACAAGAA--

AAGAATGAACAAGACTTATTGGCATTGGATAAGTGGGCAAGTTTGTGGAATTGGTTTRACATAACAAAMTGGC
TGTGGTATATAAAGATATTCATAATGATAGTAGGAGGCTTRATAGGTTTAAGGATAGTTTTTACT---
GTGCTCTCTATAGTG---------AATAGAGTTAGGAAGGATACTCACCA------
YTATCGTTCGCTATTAGCTTGCTCAACGCCACAGCCATAGCAGTAGCTGAG---
GGGACAGATAGAATTATAGAAGTAGTGCAAAGAACTGGTAGAGCT----------------------------
--------ATCCGCCATATA------CCTAGAAGAATAAGRCAGGGCCTCGAAAGGATTTTGCTA---TAA
>MN485990
GCT---CTA--
GAAGAATCGTGGGTCACAGTCTATTATGGAGTACCTGTGTGG---AAGGAAGCAACC---
ACCACTCTATTTTGTGCATCAGATGCTAAAGGGTATAAAACAGAG---GCACATAAT----------------
--------------ATTTGGGCCACG---CATGCCTGTGTACCCACAGAC---
CCCAATCCACAAGAAATAAAATTGGAA---------AATGTGACAGAAAATTTTAAC----------------
--------------------------ATGTGGAAAAATAACATGGTAGACCAGATGCATGAGGACGTA---
ATCAGTTTATGGATCAAAGCCTA---AAGCCATGTGTAAAATTA---
ACTCCACTCTGTGTTATTTTAAATTGCACTGAGTATGTGGGAAACAATRCSCRA-------------------
----
-------------------------GACAATGAMACTCCTAAGGAAGAAAAAGGAGAAATACAA---
AACTGCTCTTTTTATACAAACCCAATAACACAA---------------------------
GATAAGAAGCAGAAAGAATATGCACTT---
TTTTATAAACTTGATGTAGTACCAATAGATAATAGTGCTGGAAATGAT-------------------------
----
AGTAATGATAGTAATATTACTAGCTATAGGTTGATAAGTTGTAATACCTCAGTCATTACACAA---
GCCTGTCCAAAGGTGTCCTTTGAACCAATTCCCATACATTATTGTGCCCCAGCTGGTTTTGCGATTCTGAAGT
GTAAT---AATGAGATGTTCAAT------GGAACAGGACCGTGTAACAAT-----------------------
-GTCAGC---ACAGTACAATGTACACATGGAATTAAACCAGTAGTATCAACT---
CAACTGCTGTTAAATGGCAGCCTAGCAAAA---GAA------------
GTAGTGATTAGATCTGAAAATTTCACAGACAAT---------GCTAAAACCATAATAGTGCAGCTAAAT----
-----------GAATCTGTAGTA---------ATTAAGTGTACAAGACCCAGCAACAATACAAGAAGA-----
-AGTATACATATA------------GGACCAGGACAAACA---TGG------TATGCAACAGGAGCC------
---------ATAATAGGAGATATA------------AGAAAAGCACATTGT---AACATTAGTAGAAAA---

AACTGGAATGACACTCTACAAAAGATAGTTACAAAATTAGGA---------------GAACAATTTGGG----
-----------------------------------AATAAGACAATAATCTTTAAA------
AACTCCTCAGGAGGGGACCCAGAAATTGTA---ATGCACAGTTTTAATTGTGGAGGGGAA---
TTTAAGCAGCTCCAGGCAAGAGTCCTGGCTGTGGAAAGATACCTAAGGGATCAACAGCTCCTAGGGATTTGGG
GTTGCTCTGGAAAACTCATCTGCACCACTACTGTGCCTTGGAATACTAGTTGGAGT-----------------
----------------------------AATAAATCTTTACAAGAAATTTGG------AAT------
AACATGACCTGGATGGAGTGG---GAAAGAGAAATTGAAAATTATACAGACTTAATATAC---
AACTTAATTGAAGGTGCACAGAACCAACAAGAA---

AAAAATGAACAAGAATTATTGCAATTAGATAAGTGGGCAAGTTTGTGGAGTTGGTTTGACATAACAAATTGGC
TGTGGTATATAAAAATCTTCATAATGATAGTAGGAGGCTTGGTAGGTTTAAGAATAGTTTTTACT---
ATACTTTCTCTAGTG---------AATAGAGTTAGGCAGGGTTACTCACCA------
TTGTCATTTGCTATTAGCTTGTTCAATGCTACAGCCATAACAGTAGCTGAA---
GGGACAGATAGGATTATAGAAGCAGCACAAGGAATTGGTAGAGCT----------------------------
--------ATCCTCAACATA------CCTAGAAGAATAAGACAAGGATTAGAAAGGCTTCTACAA---TAA
>MN485995
GCT---ACA---
GGCAACTTGTGGGTCACAGTCTATTATGGGGTACCTGTGTGG---AAAGAAGCAATT---
ACCACTCTATTTTGTGCATCAGATGCTAAGGCATATGATACAGAA---GTACATAAT----------------
--------------GTTTGGGCCACA---CATGCCTGTGTACCCACAGAC---
CCCCAACCACAAGAAGTAGTCATGGCA---------AATGTGACAGAAAATTTTAAT----------------
--------------------------ATGTGGAAAAATAACATGGTAGAACAAATGCAGGAGGATATA---
ATCAGTTTATGGGATCAAAGTCTA---AAGCCATGTGTAAAATTA---
ACCCCACTCTGTGTTACTTTAAACTGCACGAATGCTAGG------------------------------------
----
----------------------------AATGCAGCGAGAAATGAAACAATAGGAGAAATGAAA---
AACTGCTCTTTCAATGTTACCACAGACATAAGG--------------------------
GATAAGGTGCAGAAAAAATATGCACTT---
TTCTATGCACTTGATATAGTACCAATAGATGATGATAATACCACCAGA-------------------------
----
CATAATCCTAGCTCTAATTATAGGTTAACAAGTTGTAACACCTCAATCATTACACAA---
GCCTGTCCAAAGGTATCCTTTGAGCCAATTCCCATACATTATTGTGCCCCGGCTGGTTTTGCGATTTTACAAT
GTAAC---AATAAGACTTTCAAT------GGATCAGGACCATGTACAAAT----------------------
-GTCAGC---ACAGTACAATGTACACATGGAATTAGGCCAGTAGTATCAACT---
CAACTGCTGTTGAATGGCAGTTTAGCAGAAGGAGAG------------
GTAATGATTAGATCTGCCAATTTCACGGACAAT---------ACTAAAACCATAATAGTGCAGCTGAGA----
-----------AAAGCTGTACAA---------ATTAATTGTACAAGGCCCAACAATAATACAAGAAAA-----
-GGTATACATATA------------GGACCAGGGAGAACA---CTC------TATGCAACAGGAGAA------
---------GTAGTAGGAGATATA------------AGACAAGCACATTGT---AATATTAGTAGAGCA---
AATTGGAATACCACTTTAACAGAGATAGTTAAACAATTAAGA---------------AAACAATTTGGA----
-----------------------------------AATGCAACAATAGAATTTAAA------
CACCCTTCAGGAGGGGATCCAGAAATTGTA---ATGCACAGTTTTAATTGTGGAGGAGAA---
TTTAAGCAGCTCCAGGCAAGAGTCCTAGCTCTGGAAAGATACCTAAAGGATCAACAGCTCCTGGGGATTTGGG
GTTGCTCTGGAAAACTCATTTGCACCACTGCTGTGCCTTGGAATACTAGTTGGAGT-----------------
----------------------------AATAAATCCATGGAATACATTTGG------GAC------
AACATGACCTGGATGGAGTGG---GAAAGAGAAATTGACAATTACACAGGCTTGATATAC---
AACTTGCTTGAAAAATCGCAAAACCAACAAGAA---

AAGAATGAACAAGAATTATTGGAATTGGATAAATGGGCAAATTTGTGGAATTGGTTTGACATAACAAATTGGC
TGTGGTATATAAAAATATTCATAATGATAGTAGGAGGCTTGATAGGTTTAAGAATAGTTTTTACT---
GTACTTTCTATAGCA---------AATAGAGTTAGGCAGGGATACTCACCT------
TTGTCATTTGCTGTTAGCTTATTCAACGCCACAGCAATAGCAGTAGCTGAC---
GGGACAGATAGAATTTTAGAAATATTGCAAAGAGCTTGTAGAGCT----------------------------
--------ATACTCAACATA------CCTACTAGAATAAGACAGGGTTTAGAAAGGGCTTTACAG---TAA
>MN486034
GCT---GTA---
GATCAATTATGGGTCACARTCTATTATGGGGTACCTGTGTGG---AAAGAAGCTACC---
ACCACTCTATTTTGTGCATCAGATGCTAAAGCATATAAAACAGAG---GTACATAAT----------------
--------------GTTKGGGCCACA---CATGCCTGTGTACCCACAGAC---
CCCAGCCCACAAGAAGTACTATTGGTA---------AATGTAACAGAAAATTTTAAC----------------

--------------------------ATGTGGACAAATAACATGGTAGAACAGATGCATGAGGATATA---
ATCAGTTTATGGGATCAAAGCCTA---AAGCCATGTGTGAAATTA---
ACCCCACTCTGTGTTACTTTAAATTGCACTGACAATTTAACACCAACACCTAGAAATGGTACT---------
----
-------------------------AATGTCACATTGGACAAAGGGATGGAAGGAGAAATAAAA---
AACTGCTCTTTCAATACTACCACAAACATACAA--------------------------
AATAAGATGCAGAATGCATATGCACTT---TTTTATAAACTTGATTTAGTACCATTAGGTAATGGTAAT----
----

AATAATAATACTAATTATACTACTTATAGGTTGATAAGTTGTAACACTTCAGTCATTACACAG---
GCCTGTCCAAAGGTATCCTTTGAGCCAATTCCCATACATTATTGTGCCCCGGCTGGTTTTGCGCTTCTACAGT
GTAAC---GAGAAGAACTTCAGT------GGAAAAGGAGAATGTAGAAAT----------------------
-GTCAGT---ACAGTACAATGTACACATGGAATTAGGCCAGTAGTATCAACT---
CAACTACTGTTAAATGGCAGCCTAGCAGAAGGAGAG-----------
GTAGTAATTAGATCTGCCAATTTCTCGGACAAT---------AATAAAAACATAATAGTACAACTAAAT----
-----------GAAACTGTAACA---------ATTACTTGTGTAAGACCCAATAACAATACAAAAAGA-----
-AGTATACATATA------------GGACCAGGGAAAGCA---TTT------GAT---ACAGGGGAC------
---------ATAATAGGAGATATA------------AGACAAGCACATTGT---AACATTAGTGAACAA---
AACTGGAATAACACTTTAATTAGGGTAGCCAAAAAATTAAGA---------------GAACAATTT-------
----------------------------------AATAAAACAATAGTCTTTAAC------
CAATCCTCAGGAGGGGACATAGAAATTGTA---ACACACAGTTTTAACTGTGGAGGAGAA---
TTTAAACAGCTCCAGGCAAGAGTCCTGGCTCTAGAAAGGTACCTAAGGGATCAACAGCTCCTGGGAATTTGGG
GTTGCTCTGGAAAACTCATTTGCACCACTACTGTGCCGTGGAATAGTAGTTGGAGT-----------------
-------------------------AAGAATAAAAATTTAACAGACATATGG------GAT------
AAAATGACCTGGAATCAGTGG---GAAAGAGAAATTGAAAATTACACAGAACTAATATAC---
TCCTTAATTGAAGAATCGCAAAACCAGCAAGAA---------------------------------------
----
AAGAATGAAAAAGACCTATTGGAATTAGATAAATGGGCAAGTTTGTGGAATTGGTTTGACATAACAAATTGGC
TGTGGTACATAAAAATATTCATAATGATAGTAGGAGGCTTAATAGGCTTAAGGATAGTTTTTCT---
GTACTTTCCATAGTG---------AATAGAGTTAGGCAGGGTTACTCACCA------
TTGTCATTTGCTGTTAGCTTGCTCAACGCTATAGCCATAGCAGTAGCTGAG---
GGGACAGATAGAATTGTAGAAGTAGTACAAAGAGCTTGTAGAGCT---------------------------
--------ATCCTTCACATA------CCTAGGAGAATAAGACARGGCTTGGAAAGGCTTTTGCTT---TAA
>DQ358805
GCT---ACA--
CGACAATTGTGGGTCACAGTCCATTATGGGGTACCTGTGTGG---AAAGAAGCAAAT---
ACCACTCTATTTTGTGCATCAGATGCTAAAGCATATGACACAGAG---GTACATAAT----------------
--------------GTTTGGGCCACA---CATGCTTGTGTACCCACAGAC---
CCTAGCCCACAAGAAGTACCATTAGTA---------AATGTGACAGAAAATTTTAAC----------------
--------------------------ATGTGGAAAAATAACATGGTGGAACAGATGCATGAGGATGTA---
ATCAGCTTGTGGGATCAAAGCCTA---AAGCCATGTGTAAAATTA---
ACTCCACTCTGTGTGACTTTAAATTGTACTAATTTGAAGAATGATACTAATACCAATAGT------------
----
-------------------------AGTAGCGGGAGAATGATAATGGAGAAAGGAGAAATAAAA---
AACTGCTCTTTTAATATCACCGCAAGCATAAAA--------------------------
AATAAGGTGAAACAGGAATCTGTACTT---TTTAATAGACTTGATGTAGTGCCAATAGATAAT----------
----

GATACTACAAGCTATATATTGATAACTTGTAACACCTCAGTCATTACACAG---
GCCTGTCCAAAGATAACCTTTGAACCAATTCCCATCCATTATTGTGCCCCGGCTGGTTTTGCGATTCTAAAGT
GTAAT---GATAAGAAGTTCAAT------GGAACAGGACCCTGTACAAAT----------------------
-GTCAGT---ACAGTACAATGTACACATGGAATTAGGCCAGTAGTGTCAACT---
CAACTGCTGTTAAATGGCAGTCTAGCAGAAGAAGAG-----------
ATAGTAATTAGATCTGAAAATATCACGGACAAT---------ACTAAAATCATAATAATACAGCTGAAC----
-----------GAAACTGTAGAA---------ATTAATTGTACAAGACCCAACAATAATACAAGAAAA-----
-AGGATAACTATG------------GGACCAGGAAGAGTA---TAT------TATGCAACAGGAGGC------
---------ATAGTAGGAGATATA------------AGACAAGCACATTGT---AACATTAGTGAAACA---
AAATGGAACAGCACTTTGGAAAAGATAGCTAGAAAATTAAGG---------------GAACACTTT-------
----------------------------------AATAGAACAATAGTCTTTAAT------
CGATCCTCAGGAGGGGACCCAGAAATTGTA---ATGCACAGTTTTAATTGTGGAGGGGAA---

TTTAAACAGCTCCAGGCAAGAGTCCTGGCTGTAGAAAGATACCTAAGGGATCAACAGCTCCTAGGGCTTTGGG
GTTGCTCTGGAAAACTCATTTGCAACACTAATGTGCCTTGGAATACTAGTTGGAGT-----------------
----------------------------AATAAATCTTTAGAACAGATTTGG------GGA------
AACATGACCTGGATAGAGTGG---GAGAGAGAAATTGACAATTACACAGGCATAATATAC---
ACCTTACTTGAAGAATCGCAAAACCAACAGGAA--

AAGAATGAACAAGACTTATTAAAATTGGATAAGTGGGCAGATTTGTGGAGTTGGTTTAACATAACAAATTGGC
TGTGGTATATAAGAATATTCATAATGATAGTAGGAGGCTTAGTAGGCTTAAGAATAGTTTTTACT---
GTAATTTCTATAGTG---------AATAGAGTTAGGAAGGGATACTCACCA------
CTATCATTCGCTGTTAGTTTGCTTAATGCCACAGCTATAGCAGTAGCTGAG---
GGGACAGATAGGATTATAGAATTAGCACAAAGAATTGGTAGAGCT----------------------------
--------TTTATCCACGTA------CCTAGAAGAATAAGACAGGGCTTCGAAAGGGCTTTGCTA---TAA
>JN692439
GCT---ACA--
GACAAATTGTGGGTCACAGTCTATTATGGGGTACCTGTGTGG---AGAGAAGCAAAT---
ACCACCTTATTTTGTGCATCAGATGCTAAAGCATATGACACAGAG---GCACATAAT----------------
--------------GTTTGGCCACA---CATGCCTGTGTGCCCACAGAC---
CCTGACCCACAAGAAATACTATTGCCA---------AATGTAACAGAGAATTTTAAT----------------
--------------------------ATGTGGAAAAATGATATGGTAGAACAGATGCATAAGGATATA---
ATCAGTTTATGGGATCAAAGCCTA---AAGCCATGTGTAAAAATA---
ACCCCACTCTGTGTTATTTTACATTGCTCTAATTACACTAGTGCTAACACCACTAGTAATAAT----------
----
-------------------------ACCACTAGTAGATTGCTAAACATGGAGGGAGAAATAAAA---
AACTGTACTTTCAATATCACCACAAACATAAGA---------------------------
AATAAGGTAAGGCAAGAATATGCAATT---TTTGATAAACTTGATATAATACAAATAGATAAGAAT-------
----

GATAGTACAAGCTATATGTTGAGACATTGTAATACCTCAGTCATTACACAG---
GCCTGTCCAAAGACATCCTTTGAACCAATTCCCATACATTTTTGTGCCCCGGCTGGTTTTGCGATTCTGAAGT
GTAAT---GATAAGACATTCAAT------GGATCAGGACCATGTAAAAAT-----------------------
-GTCAGC---ACAGTACAGTGTACACATGGAATTAGGCCAGTAGTATCAACT---
CAATTACTGTTAAATGGCAGTCTAGCAGAAGGAGAT------------
GTAATGATTAGATCTGAAAATTTCACAAACAAT---------ATTAAAACCATAATAGTACAGCTGAAC----
-----------GAAACTGTAAAA---------ATTAATTGTACAAGAACCAACAACCATACAAGAAAG-----
-AGTATACACATG------------GGATGGGGGAGAGCA---CTT------TATACAAACGGAGAA------
---------ATACTAGGAGATATA------------AGACGAGCACATTGT---AACATTAGTAGATCA---
GAATGGGGAAAAGCTGTAAAACAGATAATGGCAAAATTAAAA---------------GAACAAGTGGGG----
-----------------------------GACAACAAGAAGACAATAATCTTTAAT------
CAATCCGCAGGAGGGGACCTAGAAATTGTA---ACACACAGTTTTAATTGTGGAGGAGAA---
TTTAAGCAGCTCCAGGCAAGAGTCCTGGCTGTAGAAAGATACCTACAGGATCAACAGCGCCTAGGGATTTGGG
GTTGCTCAGGAAAACTCATTTGCACCACCTCTGTGCCTTGGAATACTAGTTGGAGT-----------------
----------------------------AATAGATCTCTAGAACACATTTGG------AAT------
AACATGACCTGGATGGAGTGG---GAGAAAGAAATTAACAAACACACAGACACAATATAC---
CGTATACTTGAAGAGTCACAAAACCAACAAGAA--

AAGAATGAACAAGAATTATTAGAATTGGATAAGTGGGCAAATTTGTGGAATTGGTTTAGCATAACAAAATGGC
TGTGGTATATAAAAATATTCATAATGATAATAGGAGGCATAGTAGCTTTAAGAATAGTTTTTACT---
GTATTATCTTTAGTG---------AACAAGGTTAGGCAGGGATACTCACCA------
TTGTCGTTCGCTATTAGTCTGCTTAATGCCACAGCTATTGCAGTAGCTGAG---
GGGACAGATAGGGTTATAGAAGTAGCACAAAGATTTGGTAGAGGT----------------------------
--------GTTATCCATATA------CCTAGAAGAATAAGACAGGGCTTGGAAAGAGCTTTGCAA---TAA
>JN692462
GCT---ACA---GAA---------------------------------
CAAGAAAAATTGTGGGTCACAGTCTATTATGGGGTACCTGTGTGG---AAAGAAGCAAAA---
ACCACTCTATTTTGTGCATCAGATGCTAAAGCAGTTGACCCAGAG---GTACATAAT----------------
--------------GTTTGGCCACA---CATGCCTGTGTACCCACAGAC---
CCTAAACCACAAGAAATAGTATTAGAA---------AATGTAACAGAAAAATTTAAC----------------
--------------------------ATGTGGAAAAATGATATGGTAGAACAGATGCATGAGGATATA---
ATCAGTTTATGGGATCAAAGCCTA---AAGCCATGTGTGAAATTA---
ACCCCACTCTGTGTTACGTTAAATTGTACTGATATTTCTACCAATAATACTAATAGCAGTAGTAAT-------

----
-------------------ACTAATAGCAGTAGTTGGAAAAATATGGAACTGGGAGAAATGAAA---
AATTGCTCTTTCAATATCACCACAAACATAGGA---------------------------
AATAAGATGCAGAAAGAATATGCACTT---TTTTATAAACTTGATGTACAACAAATAGATAAT----------
----

ACAAGCTATACATTGATAAACTGTAACACCTCAGTCATTACACAG---
GCCTGTCCAAAGGTAAGCTTTGATCCAATTCCCATACATTATTGTGCCCCGGCTGGTTTTGCAATTGTAAAGT
GTAAT---GATAAGAAATTTAAT------GGAACAGGGCCATGTAAAAAT-----------------------
-GTCAGC---ACAGTACAATGTACACATGGAATTAAGCCAGTAGTGTCAACT---
CAACTATTGTTAAATGGCAGTCTAGCAGAAGAAGAG------------
ATAGTAATTAGATCTGAAAATTTCACAAACAAT---------GCTAAAATTATAATAGTACAGCTGAAT----
-----------GAATCAGTAGAA---------ATTAATTGTACAAGACCCAACAACAATACAAGAAAA-----
-GGTATACATATG------------GGATGGGGGAGAGCA---TTT------TATGCAACAAATGCA------
---------ATAGTAGGAGATATA------------AGGCAAGCACATTGT---AACCTTAGTAAGGAA---
AATTGGGAAAGAACGTTAAAACAGGTAGCTGAAAAATTACAA---------------AAACAATTT-------
-----------------------------------AATACCACAATAATCTTTAAT------
CAGTCCGCAGGAGGGGACCCAGAAATTGTA---ATGCACAATTTTAATTGTAGAGGGGAA---
TTTAAGCAGCTCCAGGCAAGACTCCTGGCTGTGGAAAGATACCTAGGGGATCAACAGCTCCTAGGGATATGGG
GTTGCTCTGGAAAACTCATCTGCACCACTAATGTGCCTTGGAATGCTAGTTGGAGT-----------------
----------------------------AATAAATCTCTGAATGAGATTTGG------GGA------
AACATGACCTGGATGGAGTGG---GAGAAAGAAATTGACAATTACACAGGCTTAATATAC---
ACCTTACTGCTAGAATCGCAAAACCAACAAGAA--

AAAAATGAGCAAGAATTATTGGCATTAGATAAGTGGGCAAATTTGTGGAATTGGTTTGATATAACAAAATGGC
TGTGGTATATAAAAATATTCATAATGATAGTAGGAGGCTTAGTAGGTTTAAGAATAGTTTTTGCT---
GTACTTTCTTTAGTG---------AATAGAGTTAGGCAGGGGTACTCACCA------
TTGTCATTCGCTGTTAGTTTGCTTGATGCCATAGCTATAGCAGTAGCTGAG---
GGGACAGATAGGATTATAGAAGTAATACAAAGAATTTGTAGAGGT----------------------------
--------ATCTTACACATA------CCTAGAAGAATAAGACAAGGCTTAGAAAGGCTTTTACTA---TAA
>JN692465
GTT---GCA--
GGAAATTGGTGGGTCACAGTCTATTATGGGGTACCTGTGTGG---AAAGAGGCAAAT---
ACCACTCTATTTTGTGCATCAGATGCTAAAGCATATAGTACAGAG---ATGCATAAT----------------
--------------GTTTGGGCTACA---CATGCCTGTGTACCCACAGAC---
CCCAGCCCACACGAAATACCATTGGAA---------AATGTAACAGAAAATTTTAAC----------------
--------------------------ATGTGGAAAAATAACATGGTAGAACAGATGCATGAGGATATA---
ATCAGCTTATGGGATCAAAGCCTA---AAGCCATGTGTCAAACTA---
ACCCCACTCTGTGTTACTATGAATTGCACAGATGCAGAGACTAATGTTACTAAAAGT----------------
----
----------------------------AATAGTAGTGATTTGGAGACCAAAGGAGAAATGAAA---
AACTGCTCTTTCAATGTCACCTCAGGCGTAAGA---------------------------
GACAAAGTAAAGAGAGAACATGCACTT---TTTTATGCACTTGATGTAGTACCAATAGATAAT----------
----

GGTAAGAATAATACTAGCTATATGCTGATAAAATGTAATACCTCAGTCATTACGCAG---
GCCTGTCCAAAGGTATCCTTTGAGCCAATTCCCATACATTACTGCGCCCCAGCTGGTTTTGCGATTTTAAAGT
GTAAC---AATAAGACATTCAAT------GGATCAGGGCCATGTACAAAT-----------------------
-GTCAGC---ACAGTACAGTGTACACATGGAATTAAGCCAGTAGTGTCAACT---
CAACTGCTGTTAAATGGCAGTTTAGCAGAAGAAGAG------------
ATAGTAATTAGGTCTGAAAATTTCACGAACAAT---------GCTAAAAGCATATTAGTACAGCTGAAG----
-----------GACCCCGTACAC---------ATTACTTGTATAAGACCCAACAACAATACAAGAAGA-----
-GGTGTAACTATG------------GGACCAGGGAGAGTA------------TATTTTACAAAG---------
---------ATAATAGGAGATATA------------AGAAAAGCACATTGC---AACATTAGTATGACA---
GACTGGAATAACACTTTAACACAGATAGCTGAAAAATTAAGG---------------AAACAGTATGGG----
--------------------------------AACAACAAAACAATAGTCTTTAAT------
CAATCCTCAGGAGGAGACCCAGAAATTGTA---GAGCACAGTTTTAATTGTGGAGGGGAA---
TTCAAGCAGCTCCAGGCAAGAGTCCTGGCTGTGGAAAGATACCTAAGGGATCAACAGCTCCTAGGGATTTGGG
GCTGCTCTGGAAAACTCATCTGCACCACTGCTGTGCCCTGGAATGCTAGTTGGAGTAAGAGT-----------
-------------------------TGGAATGGATCTCTGGATGACATTTGG------ACT------

AACATGACCTGGATGCAGTGG---GAAAGAGAAATTGACAACTACACACAAACAATATAT---
AGCTTAATTGAAAAATCGCAAAACCAACAAGAA--

AAGAATGAACAAGATTTATTGGCATTAGATAAGTGGCAAGTTTGTGGAATTGGTTTGACATATCAAAATGGC
TGTGGTACATAAAAATATTCATAATGATAGTAGGAGGCTTGATAGGTTTAAGAATATTTTTTGCT---
GTAGTTTCTATAATA---------AATAGAGTTAGGCAGGATATTCACCC------
TTGTCATTTGCTGTCAGCTTGTATAACGCTACAGCTATAGCAGTAGCTGAG---
GGGACAGATAGGATCATAGAAGCAGTGCTAACAATTTGGAGAGCT----------------------------
--------TTTCTACACATA------CCTAGAAGAATAAGACAGGGCTTTGAAAGGGCTTTGCTA---TAA
>JN692473
GCT---GCT--
GAACAATTGTGGGTCACAGTCTATTATGGGGTGCCTGTGTGG---AAAGACGCGACC---
ACCACTCTATTTTGTGCATCTGATGCTAAAGCATATAGCACAGAG---GCACATAAT----------------
--------------GTTTGGCCACA---CACGCCTGTGTACCCACAGAC---
CCTAGCCCACAAGAAGTAGTATTGGCA---------AATGTGACAGAAAATTTTAAC----------------
--------------------------ATGTGGAAAAATGACATGGTAGAGCAGATGCAGGAGGATGTA---
ATCAGTTTATGGGATCAAAGCCTA---AAGCCATGTGTAAAATTA---
ACCCCACTCTGTGTTACTTTACGTTGCACTAATTATGCTGGGGAAAATGATACTATTGGTAATTGTACTGGGG
GGAATAATAATAGTAGTAAT---
TGTACTAGGGGGAATGATACTAGTACTAATAGTACCGAGTGGAGTGAGATGGTGGGAGAAATAAAA---
AACTGCTCTTTCAATATTACCACAAGCATAAAA---------------------------
AATAAGGTGCGACAAGAATATGCACTT---TTTAATAAACTTGATGTGGTACCAATAGATAAT----------
----

GATAATACAAGCTATATGTTGATAAATTGTAACACCTCAGTCATTACACAG---
GCCTGTCCAAAGATAACCTTTGAACCAATTCCCATACATTATTGTGCCCCGGCTGGTTTTGCGATTCTAAAGT
GTAAT---GCTAAGAATTTCAAT------GGAACAGGACCATGTACAAAT-----------------------
-GTCAGT---ACAGTACAATGTACACATGGAATTAAGCCAGTAGTGTCAACT---
CAACTGCTGTTAAATGGCAGTCTAGCAGAAGAAGAG------------
ATAGTAATTAGATCTAAAAATCTCACGGACAAT---------GCTAACAACATAATAGTACAGCTAGAC----
-----------AAACCTGTAGAA---------ATTAATTGTACAAGACCCAACAACAATACAAGCAAA-----
-AGTATACATATG------------GCATGGGGGAGAGCA---TTT------TATGCAACAGAAAGA------
---------ATAATAGGAAAGATA------------AGACAAGCACATTGT---GACGTTAATGCAACA---
GACTGGAACCACACTTTAACAAGGATAAAGGCAAAACTAAAA---------------GAACATTTT-------
-----------------------------------AATAAAACAATATACTTTGAT------
CAGCCCTCAGGAGGGGACCCAGAAATTATA---CTGCACACTTTTAATTGTGGAGGAGAA---
TTTAAGCAGCTCCAGGCAAGAGTCCTGGCTGTGGAAAGATACCTAAGGGATCAACAGCTCCTGGGGATTTGGG
GTTGCTCTGGAAAACTCATCTGCACCACTGCTGTGCCTTGGAATGCCAGTTGGAGC-----------------
----------------------------AATAGATCTCAGGATGACATTTGG------CAG------
AACATGACTTGGATGGAGTGG---GAAAGAGAGATTGACAATTACACAGACATAATATAC---
AGCTTACTTGAAAAATCGCAGACCCAACAAGAA--

AAAAATGAACAAGACTTATTGGCATTGGATAAGTGGACAAATTTGTGGAATTGGTTTAACATAACAAATTGGC
TGTGGTATATAAAAATATTCATAATGATAGTAGGAGGCTTAATAGGTTTAAGAATAATTTTTACT---
GTACTGTCTATAGTG---------AATAGAGTTAGGCAGGATACTCACCA------
TTGTCATTCGCTGTTAGTTTGCTTAATACCATAGCTATAGCAGTAGCTGAG---
GGGACAGATAGGGTTATAGAAGTAGTACAAAGAATTGGTAGAGGT----------------------------
--------ATTCTCCATATA------CCTGTAAGAATAAGACAAGGCTTAGAAAGGGCTTTGCTA---TAA
>JN692474
GCT---ACA--
GACAATTTGTGGGTCACAGTGTATTATGGGGTACCTGTGTGG---AAAGAAGCAACC---
ACCACTCTATTTTGTGCATCAGATGCTAAATCATATGACACAGAG---GTACATAAT----------------
--------------GTTTGGCCACA---CATGCCTGTGTACCCACAGAC---
CCTAACCCACAAGAGGTAGTATTGGAA---------AATGTGACAGAAAATTTTAAC----------------
--------------------------ATGTGGAAAAATAGCATGGTAGATCAGATGCATGAAGATATA---
ATCAGTTTATGGGATCAAAGCCTA---AAGCCATGTGTAAAATTA---
ACTCCACTCTGTGTCACTTTAAATTGCACTGATTGGAAACAGAATAATACCACTAATGGGACCCAA-------
----
-------------------AATAGTAATAAAACTATTGGGCAAAAGATGGAGGGAGAAATAAAA---
AACTGCTCTTTCAATGTCACCACAGGCATAGCA---------------------------

AATAAGTATCACAGAGAATATGCGCTT---TTGTATAAAACTGATGTTGTACCAATAGAAAAG----------
----

GATAATACAAGTTATATGTTAATAAGTTGTAACACCTCAGTCATTACACAG---
GCCTGTCCAAAGATATCCTTTGAACCAATTCCCATACATTATTGTGCCCCGGCTGGTTTTGCGATTCTAAAGT
GTAAT---AATAAGACATTCAAT------GGAACAGGACCATGTACAAAT-----------------------
-GTCAGC---ACAGTACAATGTACACATGGAATTAGGCCAGTAGTGTCAACT---
CAACTACTGTTAAATGGCAGTCTAGCAGAAGAAGAG------------
GTAATAATTAGATCTGAAAACCTTACGGACAAT---------GCTAAAACCATAATAGTACAGCTGAAT----
-----------GAATCTGTAGCA---------ATTAATTGTACAAGGCCCAGCAACAATACAAGAAAA-----
-AGTATACAGTTA------------GGATGGGGAAGAGTA---TTG------TATGCAACAGGAGAC------
---------ATAATAGGGGATATA------------AGACAAGCACATTGT---AACCTGAGTAGAAAA---
GACTGGAACAGAACTTTACATCAAATAGTTATGAAATTAAGA---------------GAACACTTT-------
-----------------------------------AACAAAACAATAATCTTTAAG------
CAATCCTCAGGAGGGGACCCAGAAATTGAA---ATGCACAGTTTTAATTGTGGAGGGGAA---
TTTAAGCAGCTCCAGGCAAGAGTCCTGGCTGTAGAAAGATACCTAAAGGATCAACAGCTCCTAGGGATTTGGG
GTTGCTCTGGAAAACTCATTTGCACCACTACTGTGCCTTGGAATAAGACCTGGAGT-----------------
----------------------------AATAAATCTCTGGATGAAATTTGG------GAT------
AACATGACCTGGATGCAGTGG---GAAAAAGAAATTGACAATTACACAAGCTTAATATAC---
ACCTTAATTGAAGAATCGCAAATCCAACAAGAA--

AAGAATGAACAAGAATTATTGCAATTGGATTCATGGGCAAGTTTGTGGAATTGGTTTAGCATATCAAACTGGC
TGTGGTATATAAAAATATTCATAATGATAGTAGGGGGTTTAGTAGGGTTAAGAATAGTTTTTACT---
GTATTTTCTATAGTG---------AATAGAGTTAGGCAGGGATACTCACCA------
TTATCATTCGCTATTAGTTTGCTTAATGCCACAGCTATAGCAGTAGCTGAG---
GGGACAGATAGAATCATAGAAGCAGCACAAGGAATTGGTAGGGCT----------------------------
--------ATTCTCCACATA------CCTGTAAGAATAAGACAGGGCTTAGAAAGGGCTTTGGTA---TAA
>JN692480
GCT---GCA--
AAGCAATTATGGGTCACAGTCTATTATGGAGTACCTGTGTGG---AAAGAAGCAAAC---
ACCACTCTGTTTTGTGCATCAGATGCTAAAGCATATGATACAGAG---GTACATAAT----------------
--------------GTTTGGGCCACA---CATGCCTGTGTACCCACAGAC---
CCTGACCCACAAGAAGTAGTATTAAAA---------AATGTGACAGAAAATTTTAAC----------------
--------------------------ATGTGGAAAAATGACATGGTAAGACAGATGCATGAGGATGTA---
ATCAGTTTATGGGATCAGAGCTTA---AAGCCATGTGTAAAATTA---
ACCCCACTCTGTGTCACTTTGAATTGCTCCTATATCAATAGTACTTGTGGCAATAGT----------------
----
----------------------------ACTGATGGTTGGCAAGATATGAGGGAAGAAATAAAA---
AACTGCTCTTTCAATAGCACCACAGGCATAGGA---------------------------
AATAAGGTGCGAAAAGAATTTGCTTTC---TTTAATAGGCTTGATATAGTACCAATAGATGAT----------
----

GGTAATTCAAGCTACATGTTGAGAAGTTGTAACACCACAGTCATTACACAG---
GCCTGTCCAAAGGTAACCTTTGAACCAATTCCCATACATTATTGTGCCCCGGCTGGTTTTGCGATTCTAAAGT
GTAAT---GATAAGAAGTTCAAT------GGAACAGGATCATGTAACAAT-----------------------
-GTCAGC---ACAGTACAATGTACACATGGAATTAGGCCAGTAGTGTCAACT---
CAACTGCTGTTAAATGGCAGTCTAGCAGAAAAAGAG------------
ATAATAATTAGATCTGAAAATTTCACGAACAAT---------GCTAAAAACATACTAGTGCAGCTGAAC----
-----------GAATCTGTAGTA---------ATTAATTGTACAAGACCCAACAACAATACAAGGAAA-----
-AGTATACATCTA------------GGACCGGGAAGAGCA---TTT------TATGCAACAGGAGAA------
---------ATAATAGGAAATATA------------AGACAAGCATATTGT---ACCCTTAACGCATCA---
CAATGGAAAAAACTTTAGGAAAGGTAGCTATAAAATTAAGA---------------
GAACAAATTAATAACACATTA------CACTTA---------------AATAACACAAAAATAGCCTTTACA-
-----AACTCCTCAGGAGGGGACCCAGAAATTATA---ATGCACAGCTTTAATTGTGGAGGGGAA---
TTTAAGCAGCTCCAGGCAAGAGTCCTGGCTGTGGAAAGATACCTAAAGGATCAACAGCTCCTAGGGATTTGGG
GTTGCTCTGGAAAACTCATCTGCACCACTAATGTGCCTTGGAATACTAGTTGGAGT-----------------
----------------------------AATAAATCTTTGGGGGAAATTTGG------GAT------
AACATGACTTGGATGCAGTGG---GAAAAAGAAATTGACAAATACACAAACTCAATATAC---
AACTTACTTGAAGATTCGCAAAACCAGCAAGAA--

AAGAATGAACAAGACTTATTGGCATTGGATAAGTGGACAAATTTATGGAATTGGTTTAGCATATCAAACTGGC
TGTGGTATATAAAAATATTCATAATGATAGTAGGAGGCTTAGTAGGATTAAGAATAATTTTTACT---
GTTCTTTCTATAGTA---------GATAGAGTTAGGCAGGGCTACTCACCA------
TTATCTTTCGCTGTTAGCTTGCTAAATGCCACAGCTATAGTAGTAGCTGAG---
GGAACAGATAGAGTTATAGAAGTAGTACAAGGATTTGTTAGAGCT----------------------------
--------ATTCGCAATATA------CCTAGAAGAATAAGACAGGGCCTAGAAAGGAGCTTACTA---TAA
>HQ377423
GCT---ACG--
CAACAGATGTGGGTTACAGTCTATTATGGGGTACCTGTGTGG---AAAGAAGCAACC---
ACTACTCTATTTTGTGCATCAGATGCTAAAGCATATGATACAGAG---GTACATAAT----------------
--------------GTCTGGGCCACA---CATGCTTGTGTACCCACAGAC---
CCCAACCCGCAAGAAGTAGACTTGGTA---------AATGTGACAGAAAATTTTAAT----------------
--------------------------ATGTGGAAAAATAACATGGTAGAACAAATGCATGAGGATATA---
ATTAGTTTATGGGATCAAAGCCTA---AAACCATGTGTAAAATTA---
ACCCCGCTCTGTGTTACCTTAGATTGCAAAGATGTGAATATTACTAAAAGCAATGGTACTGTCAACATTACT-
----
----------------AACGTGAATACTACTAGCTGGCCACAAATGCAAGCAGGAGAAATAAAA---
AATTGCTCTTTCAATATTAGCACAGACATAAGA---------------------------
GATAAGGTGCGGAAAGAATATGCACTT---TTTTATAACCTTGATGTAGTAAAGATAGAAGGGGGG-------
----

AATAGTACTAGCTATAGGTTGGTAAGTTGTAACACCTCAGTCCTTACACAG---
GCCTGTCCAAAGGTATCCTTTGAGCCAATTCCCATACATTATTGTGCCCCGGCTGGCTTTGCGATTCTAAAAT
GTAAA---GATAAGGAGTTCAAT------GGATCAGGACCATGTACAAAT-----------------------
-GTCAGC---ACAGTACAATGTACACATGGAATTAGGCCAGTAGTGTCAACT---
CAACTGCTACTAAATGGCAGTCTAGCAGAAGGAGGG------------
GTAGTAATTAGATCTGAAAATTTCACAAACAAT---------GCTAAAACCATAATAGTACAGCTGAAT----
-----------GAATCTGTAATA---------ATTAATTGCACAAGACCTAACAACAATACAAGAAGG-----
-AGTATAGATATA------------GGACCAGGCAGTGCA---TTT------TATGCAACAGGAGAT------
---------ATAATAGGAGATATA------------AGACAAGCACATTGT---AATATTAGTGGACTA---
AAATGGAACAACACATTAAAACAAATAAGTGGAAAATTAAGA---------------GAACAATTTATT----
-----------------------------------AATAAAACAATAGTCTTTAAT------
CACTCCTCAGGAGGGGACCCAGAAGTTGTA---ATGCACAGTTTTATTTGTGGAGGGGAA---
TTTAAGCAACTCCAGGCAAGAGTCCTGGCTGTGGAAAGATACCTAAAGGATCAACAGCTCCTGGGAATTTGGG
GTTGCTCTGGAAAGCTCATTTGCGCCACTGCTGTGCCTTGGAATACTAGTTGGAGT-----------------
----------------------------AATAAATCCTATGATCAGATTTGG------GGG------
AATATGACATGGATGGAATGG---GAAAGAGAAATTGATAATTACACAGGCTTAATATAC---
AGCTTAATTGAAGAATCGCAAATCCAACAAGAA--

AAGAATGAACAAGACTTATTGTCATTAGGTCAATGGGCAAGTTTGTGGAATTGGTTTGACATATCAAACTGGC
TATCGTATATAAAAATATTCATAATGATAGTAGGGGGCTTAATAGGTTTAAGAATAGTTTTTGCT---
GTACTTTCTATAATA---------AATAGAGTTAGGCAGGGATACTCACCC------
TTATCGTTCGCTATTAGCTTGCTTAATACCACAGCAATAGCAGTGGCTGAG---
GGGACAGACAGAATTATAGAAGCTTTGCAAAGAATTGGAAGAGCT----------------------------
--------ATCCTCAATATA------CCTAGAAGAATAAGACAGGGCTTGGAAAGGGCCTTGCTA---TAA
>KT427681
GCT---ACA--
GAAAAATTGTGGGTCACAGTATATTATGGRGTACCTGTGTGG---AAAGAAGCAAMC---
ACCACTTTATTTTGTGCATCAGATGCTAAAGCATATGACACAGAA---GCACATAAT----------------
--------------GTTTGGGCCACA---CATGCCTGTGTACCYACAGAC---
CCTAACCCACAAGAAGTAGTATTGGAA---------AATGTGACAGAAAATTTTAAC----------------
--------------------------ATGTGGAAAAAYAACATGGTAGAACAGATGCATGAGGATATA---
ATTAGTTTATGGGATCAAAGCCTA---AAGCCATGTGTAAAGTTA---
ACCCCACTCTGTGTTACCTTAAATTGCACTAAGATAAATACTAATAGTACTGGAAATAGTAGTAGT-------
----
----------------------GTTACCAATAGTACCAGCGAGGATGGTGGGGAACCAATGAGA---
AACTGCTCTTTCAATGTC---ACAGATGGAATA------------------------------
AAGAGGAAAAAAGACTATGCACTC---TTTTATAGTGTTGATGTAGTACCAATAGAAGAT-------------
----
-------------------GATAATACAAGCTATATGTTAAGAAGTTGTAACACCTCAGTCATTACACAA---

GCCTGTCCAAAGATAAACTTTGACCCAATTCCCATACATTATTGTACCCCGGCTGGTTTTGCGATTCTAAAAT
GTAAT---GATGAGAAGTTCAGT------GGAATAGGACCATGCACAAAT-----------------------
-GTCAGC---ACAGTACAATGTACACATGGAATTAGGCCAGTAGTGTCAACT---
CAACTGCTGTTAAATGGCAGTTTAGCAAGAAAAGAG------------
ATAATACTCAGATCTGAAAATTTCACGGACAAT---------GCTAGAAACATAATAGTCCAACTGAAT----
-----------GAATCTATAGAA---------ATTAATTGTACAAGACCCAGCAACAATACAAGGAAA-----
-AGTATACATTTT------------GGACCGGGGCGAACA---ATG------TATGCAACAGGAGAA------
---------ATAATAGGAGATATA------------AGAAAAGCATTTTGT---ACCCTGAATGGAATA---
AAATGGAATAACACTTTAAAACAGATAGCTAGAAAATTAAAA---------------GAACAATTT-------
---------------------------------AATAAAAGCATAGTCTTTGAG---
CCAGCCATCAAAGGAGGGGACCCAGAAATTATA---ATGCACAGTTTTAATTGTAGAGGAGAA---
TTTAAGCAGCTCCAGGCAAGAGTCCTGGCTGTGGAAAGATACCTAAAGGATCAACAGCTCCTAGGAATTTGGG
GTTGCTCTGGAAAACTCATCTGCACCACCGCTGTGCCTTGGAATTCTAGTTGGAGT-----------------
---------------------------AATAAAACTTATAATGACATTTGG------AAT------
AACATGACCTGGATGCAGTGG---GAAAAAGAAATTGACAACTACACAGGCTTAATATAC---
ACTTTAATTGAAAAATCGCAAAAGCAGCAGGAA--

AAGAATGAGCAAGAACTATTGGAACTGGATAAATGGGCAAACTTGTGGAATTGGTTTGACATAACAAAATGGC
TGTGGTATATAAAAATATTCATAATGATAGTAGGAGGCTTAATAGGTTTAAAAATAGTT--------------
--------------------------------------TCACCA------C--------
GCTATTAGCTTGCTTAATACCACTGCTATAGCAGTAGCTGAA---
GGGACAGATAGGATTATAGAAATAGTACAAAGATTTTGTAGAGGC----------------------------
--------ATCCTTCACATA------CCTAGAAGAATAAGACAGGGCTTAGAAAGGAGTTTATTA---TAA
>KT427732
GCT---GAA---------------------------------------
GATCAAATGTGGGTCACAGTCTATTATGGGGTACCTGTGTGG---AAAGAAGCAAAA---
ACTACCCTGTTTTGTGCATCAGATGCTAAAGCATATAAGACAGAG---GCACATAAT----------------
--------------ATTTGGGCCACA---CATGCCTGTGTACCCACAGAC---
CCTAATCCACAAGAAGTAGTATTGGGA---------AATGTGACAGAAAATTTTAAC----------------
-------------------------ATGTGGACAAATAACATGGTGGAACAGATGCATGAGGATATA---
ATCAGTTTATGGGATCAAACCCTA---AAGCCATGTGTAAAAATA---
ACCCCACTCTGTGTTATTTTAAACTGCACTGATTGGAAGAGGAACAMTACCMATAGTAMT-------------
----
------------------AGCTATAATAGTACGGTGGAAGGGATGGAGAAGGGAAATTTG-------------
CTCTTTCAATATTACCACAGACATAGA-------------------------
GAATGGTGCAACAAGACTATGCACTTT------GATACACTTGATATAGTACCAATARAAAATAAGACAGAA-
----
-
AATCRGACATAWGTAAKCTATACATWGGTAACCTRTAACAYCATAACTTGTAGCACCACAAAARTTACACAG-
--
ACCTGCCCAAAGATATCCTTTGAACCAATTCCCATACATTATTGCGCCCCGGCTGGTTTTGCGATTCTAAAGT
GTAAT---GATAAAAGGTTCAAT------GGATCAGGACCATGTACAAAT-----------------------
-GTCAGC---ACAGTACAATGTACACATGGAATTAGGCCAGTAGTGTCAACT---
CAACTGCTGTTAAATGGCAGTACTAGCAGAAGAAGA------------
GACAGTAGTAGATCTAAAAATTTCTCAGACAAT---------GCTAAAAATATAATAGTGCAGCTAAAT----
-----------GAAACTGTAAAA---------ATTGATTGTATAAGACCCAGCAACAATACAAGAAGG-----
-AGTATACACATG------------GGATGGGGGAGAGCA---ATT------TATACAACAGGAGAC------
---------ATAATAGGAGACATA------------AGACAAGCACATTGT---AATGTTAGTGCAGGA---
ARATGGAATAACACTTTAAGACTGATAGCTATAAAATTAATG---------------GAACAATTTCCA----
---------------------------------AATAAAACAATAATCTTTAAT------
GAATCCACAGGAGGGGACCCAGAAGTAGAA---CTGCACATGACTAATTGTGGAGGGGAA---
TTTAAGCAGCTCCAGGCAAGAGTCCTGGCTGTGGAAAGATACCTAAGGGATCAACAGCTCCTAGGGATTTGGG
GTTGCTCTGGAAAACTCATTTGCACCACCTCTGTGCCTTGGAATGCTAGTTGGAGT-----------------
---------------------------AATAAATCTTATGATAAGATTTGG------AAT------
AACATGACCTGGATGCAGTGG---GAGAGAGAAATTGACAATTACACAGACTTTATATAC---
AATTTACTTGAAGAATCGCAAAACCAACAAGAA--

AAGAATGAACAAGAATTATTGGAATTAGATAAGTGGGCAAATTTGTGGAATTGGTTTGATATATCAMACTGGC
TGTGGTATATAAAAATATTYATAATGATAGTAGGAGGCTTAATAGGTTTAAGAATAATTTTTACT---
GTACTTTCTATAGTG---------AATAGAGTTAGGCAGGGATACTCACCA------

TTGTCGTTCGCTGTTAATTTGCTTAATTTCATAGCTATAGCAGTAGCTGAG---
GGAACAGATAGGATTATAGAAGTAATACAAGGAATTGGTAGAGCT----------------------------
--------TTCCTCCACATA------CCAAGAAGAATAAGACAGGGCTTAGAAAGGCTTTTGCTA---TAA
>KT427713
GCT---ACA--
AAAAATTTGTGGGTCACAGTCTATTATGGGGTACCTGTGTGG---AAAGATGCAACC---
ACCACCCTATTCTGTGCATCAGATGCTAAAGCATATGATACAGAG---GCACATAAT----------------
--------------GTTTGGGCCACA---CATGCCTGTGTACCCACAGAC---
CCCAGCCCACAAGAAGTAGWRTTARAM---------AATGTGACAGAAAATTTTAAC----------------
-------------------------ATGTRGAAAAATAATATRGTAGAACAGATGCAAGAGGATGTA---
ATTAGTTTATRRGATGAAAGCCTA---AAGCCATGTGTAAAATTA---
ACCCCACTCTGTGTTACCTTAAATTGCACTGATTGGGACAATAATGCCAATAGT-------------------
----
----------------------------AGTAGTGTGAATAAGATAGACAAAGGAGAAATAAAA---
AACTACTCTTTCAATATCTCCACAAATCTGAAA---------------------------
GATAAGAAACAGAAAGAATATGCACTC---TTTTATAAACTTGATGTAGTATCAATAGATAAT----------
----

AGTAATACTAGCTATAGTTTAATAAGTTGTAACACCTCAGTTGTTACACAG---
GCCTGTCCAAAGGTATCTTTTGAGCCAATTCCCATACATTATTGTGCCCCGGCTAGTTTTGCGATTCTAAAGT
GTAAA---GATAAGAACTTCAAT------GGAACARGATTATGTCAAAAT-----------------------
-GTCAGC---ACAGTACAATGTACACATGGAATTAAACCAGTGGTATCAACT---
CAATTGCTGTTAAATGGCAGTCTAGCAGAAGGAGAG------------
ATAGTAATTAGATCTGAAAATTTCACAAACAAT---------GCTAAAACTATAATAGTACAGCTGAAT----
-----------GAAGCTGTAGAA---------ATTAATTGTACAAGACCCAACAACAACACAAGGAAA-----
-AGTATAAATATA------------GGACCAGGGAGAGCC---TTT------TATACAACAGGAGAC------
---------ATAATAGGAAATATA------------AGACAAGCACATTGT---AACATTAATGGAACA---
AAATGGACTAACATGTTAAAACAGTTAGCTACAAAATTAAGA---------------GAACAATTTAAT----
--------------------------------AATACAACAACAATAGTCTTTAAT------
AGCTCCTCAGGAGGGGACCCAGAAATTGTA---ATGCACAGTTTTAATTGTGGAGGGGAA---
TTTAAACAGCTCCAGGCAAGAGTCCTGGCTGTGGAAAGATACCTAGGGGATCAACAGCTCCTGGGAATGTGGG
GTTGCTCTGGAAAACTCATCTGCACCACTGCTGTGCCTTGGAATGCTAGTTGGAGT-----------------
----------------------------AATAAATCTCAGGATGACATTTGG------AAT------
AACATGACATGGATAGAATGG---GAAAGAGAAATTAGCAATTACACAGGCGTCATATAC---
AGCTTACTTGAAGAATCGCAGAACCAACAAGAA--

AAGAATGAACAAGAATTATTGGCATTAGATAAATGGGCAAGTTTATGGAATTGGTTTAACATAACAAACTGGC
TGTGGTATATAAAAATATTCATAATGATAGTAGGAGGCTTGGTAGGTCTAAGAATAGTCTTTTCT---
GTACTTTCTCTAGTG---------AATAGAATTAGGCAGGGATACTCACCA------
TTATCGTTGGCTGTTAGCTTGTTTGATGCCATAGCCATAGCAGTAGCTGAG---
GGGACAGATAGGGTTATAGAAGTATTACAAAGAATTGGTAGAGCT----------------------------
--------ATTCTCCACATA------CCTGTAAGAATAAGACAGGGCCTAGAAAGGGCTTTGCTA---TAA
>KT427870
GCT---TCA--
GAAAAGTTATGGGTCACAGTCTATTATGGGGTACCTGTGTGG---AGAGAAGCATCC---
ACCACTCTATTTTGTGCATCAGATGCTAATGCAGCTAACACAGAG---GTGCATAAT----------------
--------------GTTTGGGCCACA---CATGCCTGTGTACCCACAGAC---
CCTAATCCACAAGAACTAGTATTGGAA---------AATGTGACAGAAAATTTTAAC----------------
-------------------------ATGTGGAAAAATAACATGGTAGAACAAATGCATGAGGATATA---
ATCAGTTTATGGGATGAAAGCCTA---AAGCCATGTGTAAAATTA---
ACCCCACTCTGTGTTACTCTAAATTGCACTAATTTGAATATTACTAATAATAATAGTACA-------------
----
----------------------------AGTAATAGTAGTGTTCAGGAATCGGGAGAAATGAAA---
AATTGCTCTTTCAATATCACCACAAGCCTAAAA---------------------------
GATAAGAAGAAGAAAGAATATGCGCTT---TTTTATAAACTTGATATAGAACAAATAGATAAT----------
----

ACAAGCTATACATTGATAAATTGTAACACCTCAGTCATTACACAG---
GCCTGTCCAAAGGTATCCTTTGAACCAATTCCTATACATTATTGTACCCCAGCTGGTTTTGCGATTCTAAAGT
GTAAT---AATAAGACGTTCAAT------GGAACAGGACCATGTACAAAT-----------------------

-GTCAGC---ACAGTACAATGTACACATGGAATTAAGCCAATAGTGTCAACC---
CAACTGCTGTTAAATGGCAGTCTAGCAGAAGAAAAG------------
ATAGTAATTAGGTCTGAAAGTTTAACAAACAAT---------GCAAAAGTCATAATAGTACAGCTGAAT----
-----------AAATCTGTAGAA---------ATTAATTGTACGAGACCCAACAACAATACAAGAAAA-----
-AGTATACATATG------------GGATGGGGGAGGACA---TTG------TATGCAACAGGAGAA------
---------ATAATAGGAAACATA------------AGACAAGCACATTGT---AACCTTAGCAGGGTA---
AATTGGAACAACACTTTAAGACAGATAGCTGGAGAATTAAGA---------------GCAATATTT-------
-----------------------------------AATAAAACAATAATCTTTAAT------
CAGTCCTCAGGAGGGGACCCAGAAATTGAA---ATGCACACTTTTAATTGTGGAGGGGAA---
TTTAAGCAGCTCCAGGCAAGAGTCCTGGCTGTGGAAAGATACCTACAGGATCAACGGCTCCTAGGAATTTGGG
GTTGCTCTGGAAAACTCATCTGCACCACTGCTGTGCCTTGGAATACTAGTTGGAGT-----------------
----------------------------AATAAATCTCTGAATGCAATTTGG------GAA------
AACATGACCTGGATGGAGTGG---GAGAGAGAAATTGACAATTACACAACCTTAATATAC---
ACCTTAATTGAAGAATCGCAGAGCCAGCAGGAA--

AAGAATGAAAAGGAATTATTGGAATTGGATAAGTGGGCAAGTTTGTGGAATTGGTTTGACATAACACACTGGC
TGTGGTATATAAAAATATTCATAATGATAGTAGCAGGCTTAGTAGGTTTAAGAATAGTTTTTACT---
GTAATTTCCATAGTG---------AATAGAGTTAGGGAGGGATACTCACCA------
TTATCATTCGCTGTTAGTTTGCTTAATACCACAGCTATAGCAGTAGCTGAG---
GGGACAGATAGGATCATAGAAATAGTACAAAGAATTGCTAGAGCT----------------------------
--------ATCCTCCATATA------CCTAGAAGAATAAGACAAGGATTCGAAAGGGCTTTGCTA---TAA
>KT427707
GCT---GAA---GAA------------------------------------
GAAAAATCATGGGTCACAGTCTATTATGGGGTACCTGTGTGG---AAAGAGGCAACC---
ACCACTCTATTTTGTGCATCAGATGCTAAGGCATATGACACAGAG---GTACATAAT----------------
--------------GTTTGGGCCACA---CATGCCTGTGTACCCACAGAC---
CCTAGCCCACAAGAAGTAGTATTGGAA---------AATGTGACAGAAAATTTTAAT----------------
--------------------------ATGTGGAAAAATGACATGGTAGATCAGATGCATGAGGATGTA---
ATCAGTTTATGGGATCAAAGCCTA---AAACCATGTGTAAAATTA---
ACCCCACTCTGTGTYACTCTAAATTGCAYTAATTGTGCCAATACTACTAATACTACTACCTGTACTACTAATA
CTACTGATAGT--
---AAGATGGAGGGAGAAATAAAA---
AACTGCACTTTCAATGTCACCACAAGCATAAAA--------------------------
AATAGGGTGAAAAAAGAATATGCACTT---TTTAATACACTTGATTTAGTACCAATAGATAAA----------
----

GATAATACAAGCTATATGTTGATAAGTTGTAACACCTCAGTCATTACACAG---
TCTTGTCCAAAGGTGACCTTTGAACCAATTCCCATACATTATTGTGCCCCAGCTGGTTTTGCGATTCTACRAT
GTAAT---GATAAGAAATTCAAT------GGAACAGGGCCATGTAGAAAT-----------------------
-GTCAGC---ACAGTACAATGTACACATGGAATTAGGCCAGTAGTGTCAACT---
CAACTGCTGTTAAATGGCAGCCTAGCAGAAGAAGAG------------
GTAGTAATTAAATCTGAGAATTTCTCAAACAAT---------GCTAAAACCATAATAGTACAGCTAAAT----
-----------AGCTCTGTAACA---------ATTAATTGTACAAGACCCAACAACAATACAAGGAAA-----
-AGTATACATATA------------RRACCGGRAAAAACA---TTT------TATGCAACAGAAGCA------
---------ATAATAAAAAATATA------------AGACAAGCACATTGT---AACCTTAGTAAAACA---
GAATGGGACCAAGCTTTAATGCACACAGCTACAAAATTAAAA---------------GAGCAATTT-------
-----------------------------------AATAAAACAATAATCTTTAAT------
GCATCCTCAGGAGGGGACCCAGAGATTGTA---ATGCACACTTTTAATTGTGGAGGGGAA---
TTTAAGCAGCTCCAGGCAAGAGTCCTGGCTGTGGAAAGATACCTAAAGGATCAACAGCTCCTAGGGATTTGGG
GTTGCTCTGGAAAACTCATCTGCACCACTACTGTGCCCTGGAATGCTAGTTGGAGT-----------------
----------------------------AATAAATCTCTGAGTACAATTTGG------GAT------
AACATGACCTGGATGCAGTGG---GAGAAAGAAATTGACAATTACACAGGCTTAATATAC---
ACTTTACTTGAAGAATCGCAAAACCAACAAGAA--

AAGAATGAACAAGAATTACTGGCATTGGATAAGTGGGCAAGTTTGTGGAGTTGGTTCAAAATATCAAACTGGC
TGTGGTATATAAGAATATTCATAATGATAGTAGGGGGCTTAATAGGTTTAAGAATAGTTTTTGCT---
GTGCTTTCTATAATC---------AATAGAGTTAGGCAGGGATACTCACCA------
TTGTCATTCGCTGTTAGTCTGCTTAATGCCACAGCTATTGCAGTAGCTGAG---
GGGACAGATAGGATTATAGAGGTAGGACAAAGATTTGGTAGGGGT---------------------------
--------ATCCTCCACGTA------CCTAGAAGAATAAGACAGGGCTTAGAAAGGGCTTTGCAA---TAA

>KT427873
GCT---ACA--
GAAAAGTTGTGGGTCACAGTCTATTATGGGGTACCTGTGTGG---AAAGACGCAAGC---
ACCACTCTATTTTGTGCCTCAGATGCTAAAGCATATGACAGAGAG---GTACATAAT----------------
--------------GTTTGGGCCACA---CATGCCTGTGTACCCACAGAC---
CCCAACCCACAAGAAGTAGTATTGGAA---------AATGTGACAGAAGAATTTAAC----------------
--------------------------ATGTGGAAGAATAACATGGTAGAACAGATGCATGAGGATATA---
ATCAGTTTATGGGATCAAAGCCTA---AAGCCATGTGTAAAATTA---
ACCCCACTCTGTGTTACTCTAAATTGCACGGATGTGAAGAATACAAGTGTTACGGAAAAT-------------
----
---GTATACAATACAGACATGAAA---
AACTGCTCTTTCAATGTCACCGCAAGCATAAAA---------------------------
GATAGGATTCATCAGGAATATGCATTA---
TTTTATAAGCTTGATATAGTGCCAATAGATAATAGAGATAATAGT----------------------------
----
AATATTACAGAGAATGGTACTAGGGAATATAGGTTGATAAATTGTAACACCTCAGTCATTACACAG---
GCCTGTCCAAAAGTATCATTTGAACCAATTCCCATACATTATTGTACCCCGGCTGGTTTTGCGATTCTAAAGT
GTAAT---GACAAACAGTTCAGT------GGAAAAGGAGACTGTAAAAT------------------------
-GTTAGC---ACAGTACAGTGCACACACGGAATTAGGCCAGTAGTGTCAACT---
CAACTGCTGTTAAATGGCAGTCTAGCAGAAGGAAAT------------
GTGACAATTAGATCTGACAATTTCACAGACAAT---------ACTAAAACCATAATAGTACAACTGAAT----
-----------GAAAGTATAGAA---------ATTAATTGTACAAGACCCAACAATAATACAAGAAGA-----
-AGTATACCTATA------------GGACCAGGGAGAGCA---TTT------TATGCAACAGGAGAT------
---------ATAATAGGAGATATA------------AGACAAGCACATTGT---AACATTAGTAAAGGC---
CAATGGAATGACACTTTAAAAAAGATAGTTGAACAATTAAGA---------------GAAAAATTCCCA----
-----------------------------------AATAAAACAATAGTCTTTAAT------
CAATCTGCAGGAGGGGATCCAGAAATTGTA---ATGCACAGTTTTATCTGTGGAGGGGAA---
TTTAAACAGCTCCAGGCGAGAGTCCTGGCTGTGGAAAGATACCTAAGGGATCAACAGCTCCTAGGGATTTGGG
GTTGCTCTGGAAAACTCATTTGCACCACTACTGTGCCTTGGAATAATAGTTGGAAT-----------------
----------------------------AATAAATCTCTGGAAGAAATTTGG------AAT------
AACAGGACATGGATGCAGTGG---GAAAAAGAAATTGAAAATTACACAGACTTAATATAC---
AACTTACTTGAAGAATCGCAGAACCAGCAAGAA--

AAGAATGAACAAGAATTGTTGGAATTGGATAAGTGGGCAAGTTTGTGGAATTGGTTTGATATAACAAAATGGC
TGTGGTATATAAAAATATTCATAATGATAGTAGGAGGCTTGATAGGTTTAAGAATAGTTTTTGCT---
GTACTTTCTATAGTA---------AATAGAGTTAGGCAGGGATATTCACCA------
TTGTCATTGGCAGTTAGCTTGCTCAATGCTACAGCTATAGCAGTAGCTGAG---
GGAACAGATAGGATTATAGAAGTAGTACAAAGAATTGGTAGAGCT----------------------------
--------ATTCTCCACATA------CCTAGAAGAGTGAGACAGGGCTTAGAAAGAGCTTTGCTA---TAA
>KT427695
GCT---GCA--
GATCAATCATGGGTCACAGTATATTATGGGGTACCAGTGTGG---AAAGAAGCAAGC---
ACCACTTTATTTTGTGCATCAGATGCTAAATCATATGACACAGAG---GCACACAAT----------------
--------------GTTTGGGCCTCA---CATGCCTGTGTACCCACAGAC---
CCTAACCCACAAGAAATAGTATTGAAA---------AATGTGACAGAAAATTTTAAT----------------
--------------------------ATGTGGAAAAATAACATGGTAGAACAGATGCATGAGGATATA---
ATCAGTTTATGGGATCAAAGCTTA---AAGCCATGTGTAAAATTA---
ACCCCAMTTTGTGTTACGTTAAATTGCACAGATGYGAACAACAATGGCACGRACAACAGT-------------
----
----------------------------GGCAATTTTAGCACTGAGGTGAAGGGAGAAGTAAAA---
AATTGCTCTTTCAATGCTACCACAGGAATTGGG---------------------------
AACAAGGTGAAGGGACAATATGCACTT---TTTAATACAATTGATATAAAGCAACTAGGTGAG----------
----

GATAATACAACCTATATGTTAACACATTGTAACACCTCAGTCATTACACAG---
GCATGTCCAAAGGTATCCTTTCAGCCAATTCCCATACATTATTGTACCCCGGCTGGTTTTGCGATTCTAAAGT
GTAAC---GATGGGAAATTCAAT------GGAACAGGACCATGTACAAAT------------------------
-GTCAGC---ACAGTACAATGTACACATGGAATTAAGCCAGTGGTGTCAACT---
CAACTACTGTTAAATGGCAGTCTAGCAGAAGGAGAG------------
GTAGTAATTAGATCTGAAAATTTCACAAACAAT---------GTTAAAACCATAATAGTACAGCTGAAT----

-----------GAATCTGTAGTA---------ATTAATTGTACAAGACCCAACAACAATACAAGAAAA-----
-AGTATACATCTA------------GGATGGGGGAGGGCA---TTT------TATACAACAGGAGAM------
---------ATAATAGGTAATATA------------AGACAGGCACATTGT---AACCTTWCTGRAGYA---
GATTGGGCAAAAACTYTAMAACGGGTAGAAGMAAAATTAAAG---------------GCAAAATTT-------
-----------------------------------AATAAAACAATACACTTTAAC------
AGTTCCTCAGGAGGAGACCCAGAACTTGTA---ATGCACACTTTTAATTGTGGAGGGGAA---
TTTAAGCAGCTCCAGGCAAGACTCCTGGCTGTAGAAAGATACCTAAAGGATCAACAGCTCCTAGGGATTTGGG
GTTGCTCTGGAAAACTCATCTGCACCACTACTGTGCCTTGGAATGCTAGTTGGAGT-----------------
----------------------------AATAAATCTCTGAATGAGATTTGG------AAT------
AACATGACCTGGATAGAGTGG---GAGAGAGAAATTGACAATTACACAGAATTAATATAC---
ACCTTACTTGAAGAATCGCAAAACCAACAAGAA--

AAGAATGAACAAGAATTATTGGCATTGGATAAGTGGGCAAGTTTGTGGAATTGGTTTGACATATCAAAATGGC
TGTGGTATATAAAGTTATTCATAATGATAGTAGGAGGCTTAGTAGGTTTAAGAATAGTTTTTACT---
GTGCTTTCTATAGTG---------AATAGAGTTAGGCAGGGATACTCACCA------
TTGTCATTCGCTATCAGCTTGCTTAATGCTACAGCTATCACAGTAGCTGAG---
GGAACAGATAGGATTATAGAAGTAGTGCAAAGAATTGGTAGAGCT----------------------------
--------ATTCTCAACATA------CCTACAAGAATAAGACAAGGCTTGGAAAGGGCTTTGCTG---TAA
>KT427785
GCT---ACA--
GAACAGTTGTGGGTCACAGTATATTATGGGGTACCTGTGTGG---AAAGAAGCAACC---
ACCACTCTATTCTGTGCATCAGATGCTAAAGCATATAGTCCAAAG---GCACATAAT----------------
--------------GTTTGGGCCACA---CATGCCTGTGTACCCACAGAC---
CCCAACCCACAAGAAATAGTATTGGAA---------AATGTGACAGAAAATTTTAAC----------------
--------------------------ATGTGGAAAAATAACATGGTAAAACAGATGCATAAAAATATA---
ATCAGTTTATGGAATAAAAGCCTA---AAGCCATGTGTAAAATTA---
ACCCCACTCTGTGTTACTTTAGAGTGCAAGAATCCTAAGCCCACTAATACTAATGCCAATAATACTGGTGCCA
CTATTAATAGC--
--ACAGGACAGGGAAAAATAAAA---
AACTGCTCTTTCAATATTACCACAAACATAAGA---------------------------
GATAAGGTGCAGAAACAACGTGCACTT---
TTTTATAACCTAAATATAGTACAAATAAATAATAAAAAAAAAGATAAT-------------------------
----
AGAACTAAGAATAATAATAAAACTAAATATAGGTTAATAAGTTGTAATACCTCAGTCCTTACACAG---
GCCTGTCCAAAGGTATCCTTTAAGCCAATTCCCATACATTTTTGTGCCCCAGCTAGTTTTGCGATTCTAAAGT
GTAAT---AATAAAAAATTTCAAT------GGGAAAGGACCGTGTACAAAT-----------------------
-GTCAGC---ACAGTACAATGTACACATGAAATTAAACCAGTGGTATCAACT---
CAACTACTGTTAAATGGCAGTCTAGCAGAAAAAAAT------------
GTAGTAATTAAATCTGAAAATTTCACAGACAAT---------GCTAAAACTATAATAGTACAGCTGAAA----
-----------AAAAATATAACA---------ATTAACTGTGTAAGACCCAACAATAATACAAGAACA-----
-AGTATACCTATA------------GGACCAGGGAGAGCA---TTC------TATGCAACAGGTGAT------
---------ATAATAGGAGATATA------------AGACAAGCACACTGT---AACCTTAGTAAAAAA---
GCATGGTATAACGCTTCAGTACAAATATTTAAAAAATTACAA---------------AGACAATTTGGG----
--------------------------------AACAAAACAACAATAGTTTTTAAT------
CAATCCTCAGGAGGGGACCCAGAAATTGTA---AAGCTTACTTTTAATTGTGGAGGGGAA---
TTTAAGCAGCTCCAGGCAAGAGTCCTGGCTGTAGAAAGATACCTAAAGGATCAACAGCTCCTGGGAATTTGGG
GTTGCTCTGGAAAACTCATTTGCACCACTGCTGTGCCTTGGAATGCTAGTTGGAGT-----------------
----------------------------AATAAGACTCTACATGAGATTTGG------GAT------
AACATGACCTGGATGCAATGG---GAAAGAGAAATTGACAATTACACAAGCTTAATATAC---
ACTTTAATTGAAGAATCGCAGAACCAACAGGAA--

AAGAATGAACAAGAATTATTAGAATTAGATAAATGGGCAAATTTGTGGAATTGGTTTGATATAACAAACTGGC
TGTGGTATATAAAAATATTCATAATGGTAGTAGGAGGTTTGATAGGGTTAAGAATAGTTTTTACT---
GTACTTTCTATAGTG---------AATAGAGTTAGGCAGGGATACTCACCA------
TTGTCGTTTGCTGTTAGCTTGCTCAACGCCACAGCCATAGCAGTAGCAGAG---
GGAACAGATAGGGTTATAGAGATAATCCAAAGAGCTGTTGGAGCT----------------------------
--------ATCCTCAATATA------CCTAGAAGAATAAGACAGGGCTTCGAAAGGGCTTTACTA---TAA
>KJ849814
GCA---
GAAGACATGTGGGTCACAGTATATTATGGGGTACCTGTGTGG---AAAGAAGCAACC---

ACCACTCTATTTTGTGCATCAGATGCTAAAGCATATGACACAGAA---GCACATAAT----------------
--------------GTTTGGCCACA---CATGCCTGTGTACCCACAGAC---
CCTAGTCCACAAGAAGTAAAATTGGTA---------AATGTGACAGAAAATTTTAAC----------------
--------------------------ATGTGGAAAAATAACATGGTGGACCAAATGCATGAGGATGTA---
ATCAGTTTATGGGATCAAAGCCTA---AAGCCATGTGTAAAATTA---
ACCCCACTCTGTGTCACTTTAAATTGCACTGATTATAAAAAGAATTCAACGAGCAAT----------------
----
----------------------------AATGCCACTAGTAGTCCAACAGACTCAGACATGAAA---
AACTGCTCTTTCAATGTTACTACAAGAATAGGA---------------------------
AATAAGATGCAAAAAGAATATGCACTT---TTTTATAAGCTTGATGTAACACCAATAGATAAT----------
----

AATGGTAATAGTACAGACTACATATTGATAAATTGTAACACCTCAGTCATTACACAG---
GCCTGTCCAAAGGTATCCTTTGAACCAATTCCCATACATTATTGTGCCCCGGCTGGTTTTGCGATTCTAAAGT
GTAAT---GATAAGAACTTCAAT------GGAACAGGACCATGTGAAAAT-----------------------
-GTTAGC---ACAGTACAATGTACACATGGAATTAGGCCAGTAGTGTCAACT---
CAATTGTTGTTAAATGGCAGTCTAGCAGAAACAGAG------------
ATAGTGATCAAATCTGAGAATTTCACAAATAAT---------GCTAAAACCATAATAGTGCAGCTGAAC----
-----------GAATCTGTAGTG---------ATTAATTGTACAAGACCTAGCAACAATACAAGAAAG-----
-AGTATATCTTTA------------GGATTTGGGAGAGCG---CTA------TATGCAACAGGAGAA------
---------ATAATAGGAAATATA------------AGACAAGCACATTGT---AACATTAGTAAAGCA---
AAATGGGAAACTACTTTAAGCAGGACTGCTGAAAAATTAAGA---------------AAACAATTTAGG----
--------------------------------GAGAACACAACAATAATCTTTAAC------
AGTTCTTCAGGAGGGGACCCAGAAATTGTA---ATGCACACTTTTAATTGTGGAGGGGAA---
TTTAAACAGCTCCAGGCAAGAGTCCTGGCTCTGGAAAGATACCTATGGGATCAACAGCTCCTAGGGATTTGGG
GTTGCTCGGGAAAAATCATCTGCAACACTGCTGTGCCCTGGAATGCTAGTTGGAGT-----------------
----------------------------AATAAATCTCTTAACACAATTTGG------GAT------
AACATGACTTGGATGCAGTGG---GAGAAAGAAATTGAAAATTACACAGGCTTAATATAC---
AATTTGCTTGAAGAATCGCAGAACCAGCAAGAA--

AAAAATGAACAAGAATTATTGGAATTGGATAAGTGGGCAAGTTTGTGGAATTGGTTTGACATAACAAATTGGC
TGTGGTATATAAAAATATTCATAATGATAGTAGGAGGCTTAATAGGTTTAAGAATAGTTTTGATT---
GTACTTTCTATAGTG---------AATAGAGTTAGGCAGGGATACTCACCA------
TTGTCATTCGCTGTTAGTTTGCTTGATGCAATAGCTGTAGCAGTAGCTGAA---
GGAACAGATAGGGTTATTGAAATAATACAAAGATTTGTTAGGGGC----------------------------
--------ATTCTCCACATA------CCTACAAGAATCAGACAGGGCTTAGAAAGGGCTTTGCTA---TAA
>KT427771
GCT---AAG--
GAAAATTTGTGGGTCACAGTCTATTATGGGGTGCCTGTGTGG---AGAGACGCAAAC---
ACCACTCTATTTTGTGCATCAGATGCTAAGGCATATGATACAGAG---GTACATAAT----------------
--------------GTTTGGCCACA---CATGCCTGTGTACCCACAGAC---
CCCAACCCACAAGAAGTACTATTGGAA---------AATGTGACAGAAAATTTTAAT----------------
--------------------------ATGTGGAAAAATAACATGGTAGAACAGATGCAGGAGGATGTA---
ATCAGTTTATGGGATCAAAGTCTA---AAACCATGTGTACAATTA---
ACCCCACTCTGTGTTACTTTAAATTGCACTGACAATTGGAGGAATGATACCAATAGTACT-------------
----
-------------------------------------AGCTTGGACATGAAAGGAGAAATAAAA---
AACTGCTCTTTTAATGTCACCACAGACGTAAGA---------------------------
GATAAGATGCACAAAGATTATGCACTT---TTTTATAAGCTTGATGTAGTACAAATAGATACTAAT-------
----

GATAATACTAGCTATAGGTTGATAAGTTGTAACACCTCAGTCCTTACACAG---
GCCTGTCCAAAGGTATCCTTTGAGCCAATTCCCATACATTTTTGTGCCCCGGCTGGTTTTGCGATTCTAAAGT
GTAGA---GACAAAAGATTCAAT------GGAACAGGACTATGCAAAAAT-----------------------
-GTCAGC---ACAGTACAATGCACGCATGGAATTAGGCCAGTGGTGTCAACT---
CAACTGCTGTTAAATGGCAGTCTAGCAGAAGATGAG------------
GTAGTAATTAGGTCTGAAAATTTCACGAACAAT---------GCTAAAACCATAATAGTACAGCTGAAA----
-----------AAGGCTGTAAAC---------ATTACTTGTGTAAGACCCAACAACAATACAAGAAGA-----
-AGTATACCTATA------------GGACCAGGAAGAGCA---TTT------TATACAACAGGGCAA------
---------ATAATAGGAGACATA------------AGACGAGCATATTGT---AACCTTAGTAGAGCA---

GACTGGAATAAGACTTTAGAACAGATAGCTAAAGAATTAAGA---------------GAACAATTTGGG----
--------------------------------AAAAATAAAACAATAGTCTTTACT------
CAATCCTCAGGAGGGGACCTAGAAATTGTA---ACACACAGTTTTAATTGTGGAGGAGAA---
TTTAAGCAGCTCCAGGCAAGAATCCTAGCTGTGGAAAGATACCTAGGGGATCAACAGCTCCTAGGGATGTGGG
GTTGCTCTGGAAAACTCATTTGCACCACTGCTGTGCCTTGGAATGCTAGTTGGAGT----------------
----------------------------AATAAATCTTACAATGAAATTTGG------GAT------
AACATGACCTGGATGCAGTGG---GACAAAGAAATTAACAATTACACACAGTTAATATAC---
ACCTTAATTGAAGTTTCACAAAACCAGCAAGAA--

CAGAATGAACAGAAATTGTTGGAATTGGATAAATGGGACAGTCTGTGGAACTGGTTTAGTATAACAAACTGGC
TGTGGTATATAAAAATATTCATAATGATAGTAGGAGGCTTAATAGGTTTAAGAATAGTTTTATCT---
GTACTTTCTATAGTG---------AACAGAGTTAGGCAGGGATACTCACCA------
CTATCGTTTGCTGTTAGCTTGCTTAATGTCACAGCTATAGCAGTAGCTGAG---
GGGACAGATAGGATTATAGAACTAGCACAAACAATTGTTAGA------------------------------
---GGACGAGAATGGGAA---TAA
>KT427753
GCT---ACA---
GAACAATTGTGGGTCACAGTATATTATGGGGTACCTGTGTGG---AAAGAAGCAACT---
ACCACTCTATTTTGTGCATCAGATGCTAAGTCATATGAGACAGAG---GTACATAAT----------------
--------------GTTTGGGCCACA---CATGCCTGTGTACCCACAGAC---
CCCAACCCACAAGAAATGGACTTGRAA---------AATGTGACAGAAAATTTTAAC---------------
--------------------------ATGTGGAAAAATAACATGGTAGAACAGATGCATGAGGATATA---
ATCAGCTTATGGGATCAAAGCCTA---AAGCCATGTGTAAAATTA---
ACCCCACTATGTGTTACTTTAAATTGCACTGATAATTTGRATGRTACTAATATCAATGGTACG----------
----
-------------------RRCARKGCTAATCTCACAGGAAGGATAGASAAGGGAGAAATAAAA---
AACTGCTCTTTCAATGTCACCACAAGCATAAGA--------------------------
GATAAGGTACAGAAACAATATGCACTT---TTTTATAATATGGATATAGTACAAATAGATGATGATAATAAA-
----

AATATAACTAATAATTATACTAGCTATAGATTAACAAGTTGTAACACCTCAGTCATTACACAG---
GCCTGTCCAAAGGTATCCTTTGAACCAATTCCCATACATTATTGTGCCCCAGCTGGTTTTGCGATTCTAAAGT
GTAAG---GATAAGAAGTTCAAT------GGAACAGGACCGTGTAAAAAT----------------------
-GTCAGT---ACAGTACAATGTACACATGGAATTAGGCCAGTGGTATCAACT---
CAACTATTGTTAAATGGCAGTCTAGCAGAAGAAGAG-----------
GTAGTAATCAGATCTGAGAATATCACAGATAAT---------GCTAAAACTATAATAGTACAGCTGAAC----
-----------AAAACTGTAATA---------ATTAATTGTACAAGACCCAACAATAATACAAGAAAA-----
-AGTATACCTATA------------GGACCAGGGAAAGCA---TTT------TATGCAACA---GAC------
---------ATAATAGGAGATATA------------AGACAAGCACATTGT---AACATTAGTAGAACA---
AACTGGAATAACACTTTAAAGAAAATAGTTGAAAAATTAGGA---------------ACACAATTTGGG----
--------------------------------AAGAATAAAACTATAGTCTTTCAA------
CACTCCTCAGGAGGGGACCCAGAAGTTACA---ATGCACACTTTTATTTGTGGAGGGGAA---
TTTAAACAGCTCCAGGCAAGAGTCCTGGCTGTGGAAAGATACCTAAAAGATCAACAGCTCCTGGGGATTTGGG
GTTGCTCTGGAAAACTCATTTGCACCACCTCTGTGCCTTGGAATGCTAGTTGGAGT----------------
----------------------------AATAAATCTCTAGATTATATTTGG------AGT------
AACATGACGTGGATGCAGTGG---GAAAAAGAAATTGACAATTATACAGGGACAATATAC---
GAATTAATTGAACAATCGCAAAACCAACAAGAA--

AAGAATGAACAAGAATTATTGGAATTAGATAAATGGGCAAGTTTGTGGAATTGGTTTAATATAACACAATGGC
TGTGGTATATAAAAATATTCATAATGATAGTAGGAGGATTAATAGGTTTAAGAATAGTTTTTACT---
GTAATTTCTATAGTG---------AATAGAGTTAGGCAGGGATACTCACCA------
TTATCGTTGGCTGTTAGCTTGCTAAACGCCACAGCTATAGCAGTAGCAGAG---
GGGACAGATAGGGTTATAGAGGCACTCCAAAGAATTGGTAGAGCT---------------------------
--------ATTATCAACATA------CCTACAAGAATAAGACAGGGCTTCGAAAGGGCTTTGCTA---TAA
>KT427749
GCT---ACA---
GAACAACTGTGGGTCACAGTATATTATGGGGTACCTGTGTGG---AAAGAAGCAACT---
ACCACTCTATTTTGTGCATCAGATGCTAAATCATATGATACAGAG---GTACATAAT----------------
--------------GTTTGGGCCACA---CATGCTTGTGTACCCACAGAC---
CCCAACCCACAAGAAATGAAATTGGAA---------AATGTGACAGAAAATTTTAAC---------------

--------------------------ATGTGGCAAAATAACATGGTAGAACAGATGCATGAGGATATA---
ATCAGCTTATGGGATCAAAGCCTA---AAGCCATGTGTAAAATTA---
ACCCCACTATGTGTTACTTTAAATTGCACTGATTGGGGGAATAGTACTGCTGCCAATGTTACG----------
----
-------------------AGCAGTGCTAGTTTCACAGGAAAGATAGAGAAGGGAGAAATAAAA---
AACTGCTCTTTCAATATCTCCACAAACATAGAA---------------------------
GATAAGAGACAGAARCAATATGCACTT---TTTTATAAWATGGATGTAGTACAAATAAATGAT----------
----

GATAATAATACTAATAGCTATAGGTTAATAAGTTGTAACACCTCAGTCATTACACAG---
GCCTGTCCAAAGGTATCCTTTGAACCAATTCCCATACATTATTGTGCCCCAGCTGGTTTTGCGATTCTAAAGT
GTAAA---GATAAGAAGTTCAAT------GGAACAGGACCGTGTAAAAAT-----------------------
-GTCAGC---ACAGTACAATGTACACATGGAATTAGGCCAGTGGTATCAACT---
CAACTACTGTTAAATGGCAGCCTAGCAGAAGAAGAG-----------
GTAGTAATTAGATCTGAGAATATCACAGATAAT---------ACTAAAACTATAATAGTACAGCTGAAT----
-----------GAAACTGTAAGA---------ATTAATTGTACAAGACCCAACAATAATACAAGAAAA-----
-AGTATAACTATA------------GGACCAGGGAGAGCA---TTT------TATGCAACAACAGAC------
---------ATAATAGGAGATATA------------AGACAAGCACATTGT---AACATTAGTAGAACA---
GACTGGAATAACACTCTAAAGAAAATAGTTGAAAAATTAGGA---------------ACACAATTTGGG----
-----------------------------------AATAAAACTATAGTCTTTCGA------
AACTCCTCAGGAGGGGACCCAGAAGTTACA---ATGCATACTTTTAATTGTGGAGGGGAA---
TTTAAACAGCTCCAGGCAAGAGTCCTGGCTGTAGAAAGATACCTACAGGATCAACGGCTCCTGGGGATTTGGG
GTTGCTCTGGAAAACTCATTTGCACCACTTCTGTGCCTTGGAATGCTAGTTGGAGT-----------------
----------------------------AATAAATCCCTAGATTATATTTGG------AGT------
AACATGACCTGGATGCAGTGG---GAAAAAGAAATTGACAATTATACAGACTTAATATAC---
AGTTTAATTGAACAATCGCAGACCCAACAAGAA--

AAGAATGAACAAGAATTATTGGAATTAGATAAATGGGCAAGTTTGTGGARTTGGTTTAGTATAACACACTGGC
TGTGGTATATAAAAATATTCATAATGATAGTAGGAGGATTAATAGGTTTAAGAATAGTTTTTACT---
GTAATTTCTATAGTG---------AATAGAGTTAGGCAGGGATACTCACCA------
TTATCGTTTGCTGTTAGCTTGCTCAACGCTACAGCCATAGCAGTAGCAGAG---
GGGACAGATAGAATTGTAGAAATACTCCAAAGGATTGGTAGAGCT----------------------------
--------ATTATCAACATA------CCTAGAAGAATAAGACAGGGCTTCGAAAGGGCTTTGCTA---TAA
>KT427826
GCT---GAC--
CAACCATTGTGGGTCACAGTTTATTATGGGGTACCTGTGTGG---AAAGATGCAACC---
ACCACTCTATTTTGTGCATCAGATGCTGAAGCACATAGTACAGAG---AAACATAAT----------------
--------------GTTTGGGCCACA---CATGCCTGTGTACCCACAGAC---
CCCAGCCCACAAGAAATGAAATTGGAA---------AATGTGACAGAAAATTTTAAC----------------
--------------------------ATGTGGAAAAATGATATGGTGGAACAGATGCATGAGGACATA---
ATCAGTTTATGGGATGAAAGCCTA---AAGCCATGTGTAAAATTA---
ACCCCACTCTGTGTTACTTTACATTGCACTAATGCCAATGACACTAAGACTACTAATGCCACT----------
----
----------------------------------GGCACTATTACAGAGCCAGGAGAACTGAAA---
AACTGCTCTTTCAATGTCACCTCCAGCAGAAAT---------------------------
GATAAGAAGAAAAACGAACATGCAATT---TTTAATGCACTTGATATAGTTCCAATAAGTGAGGATAAT----
----

ACTAATGATAATTATAGTTATATTTTGAGAAGTTGTAATACCTCAGTCATTACACAG---
GCCTGTCCAAAGATATCCTTTGAGCCAATTCCCATACATTATTGTGCCCCGGCTGGTTTTGCGATTCTAAAGT
GTAAC---AATGAAACGTTCAAT------GGAACAGGACCATGTACAAAT-----------------------
-GTTAGC---ACAATACAATGCACACATGGAATTAAGCCAGTAGTGTCAACT---
CAACTGCTGTTAAATGGCAGCCTAGCAGAAAAAAAG-----------
ATAGTAATTAGATCTGCAAACATATCAGATAAT---------GCTAAAACTATAATAGTACACCTGAAT----
-----------GCATCAGTAGAA---------ATTAATTGCACAAGACCCAACAACAATACAAGGAAA-----
-AGTATAAATATA------------GGACCAGGGAGAGCA---GTG------TATATGACAGGAGAA------
---------ATAATAGGAAATATA------------AGACAAGCACACTGT---AACATTAGTGGAGCA---
ACATGGAAGGACACTTTACAAAAGATAGTTGGAGAACTAAGA---------------AAACTCTTT-------
-----------------------------------AATAAAACAATAATCTTTAAC------
CAATCTACAGGAGGGGACCCAGAAATAACA---ATGCACAGCTTCAATTGTGGAGGGGAA---

TTTAAGCAGCTCCAGGCAAGAGTCCTGGCTTTGGAAAGATTCCTACAGGATCAACAGCTCCTGGGAATTTGGG
GATGCTCTGGGAAAATCATTTGCACCACTAATGTGCCTTGGAATACTAGTTGGAGT--------------
---------------------------AATAGATCTATAAATCATATTTGG------GAT------
AACATGACCTGGATGGAGTGG---GAAAGAGAAATTGATAATCACACAGAATTAATATAC---
AAATTACTTGAAGAATCGCAGAACCAACAAGAA-------------------------------------
----
CAGAATGAACTAGACTTATTGAAATTAGATACATGGGCAAGTTTGTGGAATTGGTTTAGCATAACCAACTGGC
TGTGGTATATAAAAATATTCATAATGATAGTAGGAGGCTTGATAGGTTTAAGAATAGTTTTTACT---
ATACTTTCTATAGTG---------AATAGAGTTAGGCAGGGATACTCACCA------
TTATCGTTTGCTGTTAGCTTGCTCAACGCCACAGCTATAGCAGTAGCTGAA---
GGAACAGATAGGATTATAGAAATAGCACAAACAATTTTTAGAGCT-------------------------
--------ATCCTCAACATA------CCTGTAAGGATAAGACAAGGCTTGGAAAGGATTTTGCTT---TAA
>KJ849821
GCT---GCA--
GATCAATTGTGGGTCACAGTTTATTATGGGGTACCTGTGTGG---AAAGATGCAACC---
ACCACTCTATTTTGTGCATCAGATGCTAAAGCATATGACACAGAG---GTACATAAT---------------
--------------GTTTGGGCCACA---CATGCCTGTATACCCACAGAT---
CCTAGCCCGCAAGAAGTACAATTG------------AATGTGACAGAAAATTTTAAT---------------
-------------------------ATGTGGAAAAATAACATGGTGGAACAAATGCATGAGGATATA---
ATCAGTTTATGGGATCAAAGCTTA---AAGCCATGTGTAAAATTA---
ACCCCACTCTGTGTTACTTTAAGTTGTAGTAATTTGACCAATAGTAATAATAGTACACATAACACTAGT----
----
----------------GATTTGGGAAACAATACTGATTTGGGAAGTATGGCGGGAGAAATGAAA---
AACTGCTCTTTCAATATTACCACACAAATAAAA------------------------
AATAAGAATAGAAAGGAACATGCACTT---TTTAATAAGATTGATTTAGTACCAATAGATAAT----------
----

AATAATACAAATTATATGTTAGTAAATTGTAACACCTCAGTCATTACACAG---
GCCTGCCCAAAGGTAACCTTTGAACCAATTCCAATACATTACTGTACCCCGGCTGGTTTTGCGCTTCTAAAGT
GTAAG---GATAAGATGTTCAAT------GGAACAGGACCATGTAAAAAT----------------------
-GTCAGC---ACAGTACAATGTACACATGGAATTAGGCCAGTAGTGTCAACT---
CAACTGCTGTTGAATGGCAGTCTGGCAGAAGAAGAA-----------
GTAATAATTAGATCTGAAAATTTCACAAACAAT---------GCTAAAACCATAATAGTACAGCTGAAT----
-----------GAAACTGTACAA---------ATTAATTGTACAAGACCCAACAACAATACAAGGAAA-----
-AGTATACACTTT------------GGACCAGGACAAGCA---TTA------TATGCAACAGGAGAA------
---------ATAATAGGAGATATA------------AGAAAAGCCCATTGT---AACATTAGTAAAGAA---
AAATGGAACAACACTTTACAACACATAGTTAGAAAATTAGGA---------------GAACAATTCAAA----
--------------------------------AATCTAACAACAATATCCTTTAAT------
AAATCCTCAGGGGGGGACCCAGAAATTACA---ATGCACAGTTTTAATTGTGGAGGGGAA---
TTTAAGCAGCTCCAGGCTAGAGTCCTGGCTGTGGAAAGATACCTACGGGATCAACAGCTCCTAGGAATTTGGG
GTTGCTCTGGAAGACTCATTTGCACCACTAATGTGCCTTGGAATACTAGTTGGAGT----------------
----------------------------AATAAAACTCTGGAAAAGATTTGG------GAT------
AACATGACCTGGATGGAGTGG---GAGAGAGAAATTGACAATTACACAGGCATAATATAC---
AACTTAATTGAAGAATCACAGAACCAGCAGGAA-------------------------------------
----
AAGAATGAATTGGAATTATTGGAATTGGATAAATGGTCAAGTCTGTGGAGTTGGTTTGACATAACACAATGGC
TGTGGTATATAAGAATATTCATAATAATAGTAGCAGGTTTAATAGGTTTAAGAATAGTTTTATTT---
GTACTTTCTGTAGTA---------AAGAGAGTTAGGCAGGGCTACTCACCA------
TTATCATTTGCTGTTAGTCTGCTTAACGCCATAGCTATAGCAGTAGCTGAG---
GGGACAGATAGGAGTATAGAAATAGTACAAAGAATTGGTAGAGCT-------------------------
--------ATTATCCACATA------CCTAGAAGAATAAGACAGGGTTTAGAAAGGGCTTTGCTA---TAA
>KT427811
GCT---AAT---GAA-----------------------------------
GACAAGTGGTGGGTCACAGTCTATTATGGGGTACCTGTATGG---AAAGAAGCAACC---
ACCACTCTATTCTGTGCATCAGATGCTAAAGCATATAAACCAGAG---ATGCATAAT---------------
--------------GTTTGGGCCACA---CATGCCTGTGTGCCCACAGAC---
CCTAGTCCACAAGAGTTAGTGTTGGAA---------AATGTGACAGAAAATTTTAAC---------------
-------------------------ATGTGGAAAAATAACATGGTAGAGCAGATGCATGAGGATATA---
ATCAGTTTATGGGATCAGAGCCTA---AAGCCATGTGTAAAATTA---
ACCCCACTCTGTGTTACTTTAAATTGCACTAATGCTAATGTTACTAGGAATACCACCAGTAGTAGT-------

----
-------CCCACTGGGGGTAACTGGACAGATAAAATAATACAGGAAGACGAGGGAGAAATAAAA---
AACTGCTCTTTCAATATCACCACAAGCATAAGA---------------------------
GATAAGGTACAAAAACAATATGCACTT---TTTGATAGACTTGATGTAGTACCAATGGATACT----------
----

AATAGTAATAATACAAGTTATATGCTAATAAATTGTAACACCTCAGTCATTACACAG---
GCCTGTCCAAAAATATCCTTTGAACCAATTCCCATACATTATTGCACCCCGGCTGGTTTTGCGATTCTAAAGT
GTAAT---GATAAAAAGTTCAAT------GGAACAGGACCATGTACAAAT-----------------------
-GTCAGT---ATAGTACAGTGTACACATGGAATTAGGCCAGTAGTGACAACT---
CAATTACTGCTAAATGGCAGTCTAGCAGAAGATGAG------------
ATAGTAATTAGGTCTGAAAATATCACGAACAAT---------GCTAAAACCATACTAGTACAGCTGAAT----
-----------AAAACCATAATA---------ATTAATTGTACAAGACCCAACAACAATACAAGAAGA-----
-AGTATATCTGTA------------GCATGGGGGAGAGCA---TTA------TTTACACCAGGAGAA------
---------ATAATAGGAGATATA------------AGAAAAGCATATTGT---ATCCTTAATGGAACA---
GAATGGAACAACACTTTAAGACAGGTAGTTGTAAAATTAAGA---------------GAGAAATTTGGT----
-----------------------------------AATAAAACAATAGTCTTTAAT------
CAATCCTCAGGAGGGGATCCAGAACTTACA---ATGCACACATTTAATTGTGGAGGGGAA---
TTTAAGCAGCTCCAGGCGAGAGTCCTGGCTGTGGAAAGATACCTAAGGGACCAACAGCTCCTGGGAATTTGGG
GTTGCTCTGGAAAACTCATCTGCACCACTAATGTGCCTTGGAATACTAGCTGGAGT-----------------
----------------------------AATACATCTCTGGATAAAATCTGG------AGC------
AACATGACCTGGATGCAGTGG---GAGAGAGAAATTAACAATTACACAGACTTAATATAC---
ACCTTACTTGAAGAATCGCAGAGCCAGCAAGAA--

AAGAATGAAAAGGAATTATTGGAATTGGATAAATGGTCAAATTTGTGGAATTGGTTCACCATATCAAACTGGC
TGTGGTATATAAAAATATTCATAATGATAGTAGGAGGCTTAATAGGTTTAAGAATAGTTTTTGCT---
ATACTTTCTATAGTG---------AATAGAGTTAGGCAGGGATACTCACCA------
TTATCGTTTGCTGTTAGTTTGATTAATGCAATAGCTATAGCAGTAGCTGAG---
GGAACAGATAGGATTATAGAAGGAATACTAACATTTGGTAGAGCT----------------------------
--------GTTCTTCACATA------CCCACAAGAATAAGACAAGGTTTAGAAAGAGCTTTGCTA---TAA
>KT427796
GCT---AAA---GAA------------------------------------
GATAATTTGTGGGTCACAGTCTATTATGGGGTACCTGTGTGG---AAAGATGCAAAC---
ACCACTCTATTTTGTGCATCAGATGCTAAAGGATATAGCACAGAG---ATGCATAAT----------------
--------------ATTTGGGCCACA---CATGCCTGTGTACCCACAGAT---
CCTGACCCACAAGAAATAGTATTGGAA---------AATGTGACAGAAAATTTTAAC----------------
--------------------------ATGTGGAAAAATAACATGGTAGATCAGATGCATGAGGATGTA---
ATCAGTTTATGGGATCAAAGCCTA---AAACCATGTGTAAAATTA---
ACCCCACTCTGTGTTACTTTAAATTGTACTAATTTGTATAGAAATTGTAATAATTCAAATGATACCCAAAGTA
AGAGTTGTAATAAGGGGAAT---
----------------AAGGACACCGTAAAGGTGGAGAAACAGATGGAGGAGGGCGAAATAAAA---
AATTGCTCTTTCAATGTCACCACAAATGTAAAA---------------------------
GATAAGGTTCAAAAGCAATATGGAATT---TTTAATAAATTTGATATAGTACCAATAGATAAT----------
----

GATAATACAAGCTATATGCTGGTAAGTTGTAACACCTCAGTCATTACACAG---
ACTTGTCCAAAGGTAACCTTTGAACCAATTCCCATACATTATTGTACCCCGGCTGGTTTTGCGATTCTAAAGT
GCAAT---GATAAAAAGTTTAAT------GGAACAGGACCATGTAAAAAT-----------------------
-GTCAGC---ACAGTACAATGTACACATGGGATTAAGCCAGTAGTGTCAACT---
CAGTTGCTATTGAATGGCAGTCTAGCAGAAAAAGAG------------
ATAGTAATTAGATCTGAAAATTTTACAAACAAT---------GCTAAAATCATAATAGTACAGCTGAAT----
-----------GAAACTGTAAAA---------ATTAATTGTACAAGACCCAATAACAATACAAGGAAA-----
-AGTATACATATG------------GGATTTGGGAGAGCA---TTT------CATACAACAGGGGAC------
---------ATAATAGGTGATATA------------AGACGAGCACATTGT---AACATTAGTAGAAAG---
GAATGGAACAACACTTTAAAACAGGTAGCTAAAAAATTAAAA---------------GAACAATTTAAT----
-----------------------------------AAAACAGTAATAGAGTTTAAG------
CAATCCTCAGGAGGGGACCCAGAAATTGTA---ATGCACACTTTTAATTGTGCAGGGGAA---
TTCAAACAGCTCCAGGCAAGAGTCCTGGCTGTAGAAAGATACCTAAGGGATCAACAGCTCCTAGGGATTTGGG
GTTGCTCTGGAAAACTCATCTGCACCACTGCTGTGCCTTGGAATACTAGTTGGAGT-----------------
----------------------------AATAAAAATCTGAGTACAATTTGG------GAT------

AACATGACCTGGATACAGTGG---GAGAGAGAAATTGAAAAGTACACAGGCTCAATATAC---
CAATTGCTCGAAGAATCGCAAAACCAACAAGAA--

AAGAATGAACAAGAATTATTGGCATTAGATAAGTGGGCAGGTTTGTGGAATTGGTTTGACATAACAAATTGGC
TGTGGTATATAAAAATATTCATAATGATAGTAGGAGGCTTAATAGGATTAAGAATAGTTTTTGCT---
GTACTTTCTGTAGTG---------AATAGAGTTAGGAAGGGATACTCACCA------
TTGTCATTCGCTATTAGTTTGCTTAATGCAACAGCTATAGCAGTAGCTGAG---
GGAACAGATAGAATTATAGAAGTACTACAAAGATTTGGTAGAGGT----------------------------
--------ATTATCCACATA------CCTAGAAGAATAAGACAGGGCTTAGAAAGAGCTTTGCTA---TAA
>KX181909
GCT---GAA---GAA------------------------------------
GAGAAAACGTGGGTCACAGTCTATTTTGGGGTACCTGTGTGG---AAAGAAGCAAAG---
ACCACTCTATTTTGTGCATCAGATGCTAAGGGACATAGCACAGAG---GCACATAAT----------------
--------------GTTTGGGCCACA---CATGCCTGTGTACCCACAGAC---
CCCGATCCACAAGAAGTAGAATTGGTA---------AATGTAACAGAAAATTTTAAC----------------
--------------------------ATGTGGGACAATGACATGGTAGACCAGATGCAAGAGGATATA---
ATCAGCTTATGGGATCAAAGCCTA---AAGCCATGTGTAAAATTA---
ACCCCACTCTGTGTTGTTTTAAGTTGCACTAATGTGACTGTTACTGAGACCAATAGGACT-------------
----
-------------------GAGACCAATGGTACTATTTGGGAAAATATGAAGGGAGAAATAAAA---
AACTGCTCTTTCAATGTCACAGAAAAGGTAAAA---------------------------
AATACAGTGCGGGAAGAGCATGCACTT---TTTAATAGACTTGATGTAGTACCAATAGATAATAAT-------
----

AATAATGAAAGCTATATGTTGATAGGTTGTAATACCTCAGTCATTACACAG---
GCCTGTCCAAAAACAACCTTTGAACCAATTCCCATACATTATTGTGCCCCGGCTGGTTTTGCAATTCTAAAAT
GTGGA---GATAAGAAGTTCAAT------GGAACAGGACCATGTCAAAAT-----------------------
-GTCAGC---ACAGTACAGTGTACACATGGAATTAAGCCAGTAGTGTCAACT---
CAATTATTGTTAAATGGCAGTCTATCAGAAGGAGAA------------
ATAATAATTAGAAGTGAAAATATCTCAGACAAT---------GCTAAAAACATAATAGTACAGCTAAAC----
-----------CAATCTGTAGTA---------ATTAACTGTACAAGACCCAACAACAATACAAGAAAA-----
-GGGATAACTATA------------GGACCAGGGAGAGCA---TTT------TTTGCAACAGGAAGT------
---------ATAATAGGAGATATA------------AGACAAGCACATTGT---AACCTTAGTAAAAAA---
GATTGGGGAGACACTTTAAAAAAGATAGAAGAAAAATTAAAA---------------GGATACTTT-------
-----------------------------------AATAAAACAATAGCATTTAAT------
CAATCCTCAGGAGGGGACCCAGAGATTGTA---ATGCACACTTTTAATTGTGGAGGGGAA---
TTTAAGCAGCTCCAGGCCAGAGTCCTGGCTGTGGAAAGATACCTAGAGGTTCAACAGCTCCTAGGAATTTGGG
GTTGCTCTGAAAAACTCATCTGCACCACTACTGTGCCTTGGAATATTAGCTGGAGT-----------------
----------------------------AATAGATCTCAGAAAGAAATTTGG------AAA------
AACATAACCTGGATGCAGTGG---AAGAAAAAAATTGATAATTACACAGGCTTGATATAT---
GAATTGCTTGAAAAATCGCAAAACCAGCAGAAA--

AAAAATGAACAAGAATTATTAAAATTGAATAAGTGGGCAAATTTGTGGAATTGGTTTGACATATCGAACTGGC
TGTGGTATATAAAAATATTTATAATGATAGTAGAAGGCTTAATAGGTTTAAGAATAGTTTTTACT---
GTACTTTCCATAGTA---------AATAAAGTTAGGCAGGGATACTCACCA------
TTGTCATTCGCTGTTAGTTTGCTCAATACCATAGCTGTAGTAGTAGCTGAG---
GGAACAGATAGGGTTATAGAAGTAGTACAAGGAATTGGTAGGGGT----------------------------
--------ATCCTTAATATA------CCTAGAAGAATAAGACAGGGCTTAGAAAGAAGTTTGCTA---TAA
>KX181908
GCT---ACA--
AACCAATTGTGGGTCACAGTTTATTATGGGGTACCTGTGTGG---AGAGAAGCAACC---
ACCACTCTGTTTTGTGCATCAGATGCTAAAGCATATGACACAGAG---GTACATAAT----------------
--------------GTTTGGGCCACA---CATGCCTGTGTACCCACAGAC---
CCCAACCCACGAGAAGTAATATTGGTA---------AATGTGACAGAAAACTTTAAC----------------
--------------------------ATGTGGACAAATAACATGGTAGAACAGATGCATCAGGATATA---
ATCAGTTTATGGGATGAAAGTCTA---AAGCCATGTGTAAAATTA---
ACCCCACTCTGTGTTACTTTAAATTGTAGCAGTCCAATTTTTAATAATAGCAATAATGCTAGT----------
----
-------------------------ATCAATGGTAATTGGGTAAATATGACTACAGAAATGAAA---
AACTGCTCATTCAATATCACCACAAGCATAAGA---------------------------

GATAGGAGGCGTAAAGAATATGCACTT---TTTTATAAAACTGATATAGTATCAGCAGATGGT----------
----

GGTAATACAAGCTATACAATGATAAACTGTAACACCTCAGTCATCACACAA---
ACTTGTCCAAAGGTAACCTTTGAACCAATTCCCATACATTATTGTACCCCAGCTGGTTTTGCGATTCTAAAGT
GTAAT---GATAAAAAGTTCAAT------GGAACAGGACCATGTAAAAAT----------------------
-GTCAGC---TCAGTACAATGTACACATGGAATTAGGCCAGTAGTATCAACT---
CAGCTACTGTTAAATGGCAGTCTAGCAGAAGAAGAA-----------
GTAGTAATTAGATCCCAAAACATCACGGACAAT---------GCTAGAACCATAATAGTACACCTGAAT----
-----------GAATCTGTACAA---------ATTAATTGTACAAGACCTGGCAACAATACCAGAAGA-----
-AGCATACACATA------------GGACCAGGAAGCGCT---TGG------TATACAACAGGAGAC------
---------ATAATAGGGGATATA------------AGACAAGCACATTGC---AACATTAGTGAAGCA---
GCCTGGAACAGAACTTTAAAACAGGTAGTTGCAAAATTAAGA---------------GAAAAATTTGGG----
-----------------------------------AACAAAACAATAGTCTTTAAT------
TCATCCTCAGGAGGAGACCCAGAAATTGAA---AAGCACACTTTTAATTGTGGAGGGGAA---
TTCAAGCAGCTCCAGGCAAGAGTCCTAGCTGTGGAAAGTTACCTAAGGGATCAACAGCTCCTGGGGATTTGGG
GTTGCTCTGGAAAACTCATTTGCACCACTGCTGTGCCTTGGAATGCTAGTTGGAGT-----------------
----------------------------AATAAAACTCTGAACCAGATTTGG------GAC------
AACATGACCTGGATAGAATGG---GAGAGAGAAATTGACAATTACACAGACTTAATATAT---
TCCCTAATTGAAGAATCGCAGAACCAACAGGAA---

AAGAATGAACAAAAATTATTGGCACTTGATAAATGGGCAAGTTTGTGGAATTGGTTTGACATATCACGATGGC
TGTGGTATATAAGAATATTCATAATGATAGTAGGAGGCTTAATAGGTTTAAGAATAGTTTTTACT---
GTACTTTCTATAGTG---------AATAGAGTTAGGCAGGGATACTCACCA------
TTATCATTCGCTGTTAGTTTGCTTAATGCCATAGCCATAGCAGTAGGTGAG---
GGGACAGATAGGGTTATAGAAGCAATACAAAGAATTGGTAGAGCT----------------------------
--------TTTCTCAACATA------CCTAGAAGAATAAGACAGGGCTTCGAAAGGGCTTTGCTA---TAA
>KX181898
GCT---ACA---
AAAGAATTGTGGGTCACAGTATATTATGGGGTACCTGTGTGG---AAAGAAGCAACC---
ACCACTCTGTTTTGTGCATCAGATGCTAAAGCATATGACACAGAA---GCACATAAT----------------
--------------GTTTGGGCCACA---CATGCCTGTGTACCCACAGAC---
CCTAACCCACAAGAAGTAGAATTAAAA---------AATGTGACAGAAGATTTCAAT----------------
--------------------------ATGTGGAAAAATAACATGGTAGAACAGATGCATGAGGATATA---
ATCAGTTTATGGGATCAAAGCCTA---AAGCCATGTGTAAAATTA---
ACCCCACTCTGTGTTACTTTAAATTGCAGTAATTGTAATATTACTGCTGACAATACTACCAATACCAATGCCA
ATAGCAGT---
---TGTGAGGAGATGGGAATGAAA---
AACTGCTCTTTCAATGTCACCACAGGCATAGGA--------------------------
AATAAGAGGCAGAAAGAATATGCGCTG---TTTTACAAGTCTGATATAGTACCAATGGATAATGAT-------
----

AATAATAATAGTACAAGCTATAGGTTGACACAGTGTAACACCTCAGTCATCACACGT---
GCCTGTCCAAAGACATCCTTTGAACCAATTCCCATACATTATTGTACTCCAGCTGGTTTTGCGATTCTAAAGT
GTAAT---GATAAGAAGTTTAAT------GGAACAGGAACATGTAAAAAT----------------------
-GTCAGC---ACAGTACAATGTACACATGGAATTAGGCCAGTAGTGTCAACT---
CAACTGCTGTTAAATGGTAGTCTAGCAGAGGAAGAG-----------
ATAATAGTCAGATCTGAAAATTTCACAGACAAT---------GTTAAAACCATAATAGTACAGCTGAAC----
-----------GAGTCGATACAA---------ATTAATTGTACAAGACCCAGCAACAATACAAGCAAA-----
-GGTATACATCTA------------GGATGGGGGAGAGCA---TTT------TATGCAACAGAAAGA------
---------ATAATAGGAGATATA------------AGAGAAGCACATTGT---AACCTTAGTAAAGCA---
AAATGGGAAGACACTTTACAAAAAATAGCTAACAAATTAAAA---------------GAACATTATAAT----
-----------------------------------AATAGAACAATATCCTTTAAT------
AAATCCTCAGGAGGGGACGACGAAATTGTA---AGGCACAGTTTTAATTGTGGAGGGGAA---
TTTAAGCAGCTCCAGGCAAGAGTCCTGGCTGTGGAAAGCTACCTAAGGGATCAACAGCTCCTAGGGATTTGGG
GTTGCTCTGGAAAACTCATTTGCACCACTGCTGTGCCTTGGAATGCTAGTTGGAGT-----------------
----------------------------AATAAATCTAAGGAAGAGATTTGG------GGG------
AACATGACTTGGATGCAGTGG---GAAAGAGAAATTGACAATTACACAGAATATATATAC---
AACTTACTTGAAAAATCGCAAAACCAACAAGAA---

AAAAATGAACAAGAATTATTGGAATTGGATAAGTGGGCAAGTTTGTGGAGTTGGTTTGACATAACACAATGGC
TGTGGTATATAAAAATATTCATAATGATAGTAGGAGGCTTAATAGGTTTAAGAATAGTTTTTACT---
GTGCTTTCTATAGTG---------AATAGAGTTAGGCAGGATACTCACCA------
TTGTCATTCGCTGTTAGTTTGCTTAATGCCACAGCTATAGCAGTAGCTGAA---
GGAACAGATAGGATCATAGAAGTAATACAAAGAATTTGTAGAGCT----------------------------
--------ATTCTCCACATA------CCTACAAGAATAAGACAGGGCTTAGAAAGGGCTTTACTA---TAA
>MK041573
GCT---GCA--
GAAGAATTGTGGGTCACAGTCTATTATGGGGTACCTGTGTGG---AAGGAAGCAAAT---
GCTACCCTATTCTGTGCATCAGATGCTAAAGCATATGATACAGAG---GTACATAAT----------------
--------------GTTTGGCCACA---CATGCCTGTGTACCCACAGAC---
CCCAACCCACAAGAAGTAGATTTGGTA---------AATGTGACAGAAAATTTTAAC----------------
--------------------------ATGTGGAAAAATAACATGGTAGAACAAATGCATGAGGATGTA---
ATCAGTCTATGGGACCAAAGCCTA---AAGCCATGTGTACAATTA---
ACCCCACTCTGTGTTACTTTAAATTGCACTGATTGGAATAATACTGCTACCAATGTTACTAAT----------
----
-------------------------ACCAATGTTACCCTTGCCATGGACAGAGGAGAAATAAAG---
AACTGCTCTTTTAATGTCACCACACCCATAAGA---------------------------
GATAAGAAACAGAAAGAATATGCACTT---TTTTATAAACTTGATGTAGTACTAATAGGTAATGAT-------
----

AATAGTAGCTATAGTAGCTATAGAATGATAAGTTGTAACACTTCAGTCATTACACAG---
GCTTGTCCAAAGATATCCTTTGAGCCAATTCCAATACATTTTTGTGCCCCGGCTGGTTATGCGATTTTAAAGT
GTAAA---GATAAGAAGTTCAAG------GGAACAGGACCATGTACAAAT-----------------------
-GTTAGC---ACGGTACAATGTACACATGGAATTAAGCCAGTAGTATCAACT---
CAACTGCTGTTAAATGGCAGTCTAGCAGAAGAAGAG------------
GTAGTAATTAGATCTCAAAATTTCACGAACAAT---------GCTAGAACCATAATAGTACAGCTAAAA----
-----------GAAGCTGTAAAC---------ATTACTTGTACAAGACCCAGCAACAATACAAGAAAA-----
-GGTATACATATA------------GGACCAGGGAGTGCA---TTT------TATACAACAGGACAA------
---------ATAATAGGGGATATA------------AGACAAGCACATTGT---AACATTAGCAAAAAA---
GACTGGAATAAGACTTTAGAACAGGTAGCTAAAAAATTAAGA---------------GAACAATTTGGG----
-----------------------------------AATAAAACAATAATCTTTAAT------
CAGTCCTCAGGAGGGGACCTAGAAATTGTA---ACACACAGTTTTAATTGTGGAGGGGAA---
TTTAAGCAGCTCCAGGCAAGAGTCCTTGCTGTGGAAAGATACCTAAAGGATCAACAGCTCCTAGGAATTTGGG
GTTGCTCTGGAAAACTCATTTGCACCACTGCTGTGCCCTGGAATGCTAGTTGGAGT-----------------
----------------------------AATAAATCCTACAATGACATTTGG------GAT------
AACATGACCTGGATGCAGTGG---GACAGAGAAATTAACAATTACACAAAGCAAATATAC---
ACCTTGATTGAACAATCGCAGAACCAACAAGAA--

AAGAATGAACAGGAATTATTGGAATTGGATAAATGGGCAAGTTTGTGGAACTGGTTTGACATATCAAATTGGC
TGTGGTATATAAAAATATTCATAATGATAGTAGGAGGCCTGGTAGGTTTAAGAATAATCTTTTCT---
GTACTTTCTATAGTA---------AATAGAGTTAGGCAGGATATTCACCA------
TTATCGTTCGCTGTTAGTTTGCTTAATTGCACAGCTATAGCAGTAGCTGAG---
GGGACAGATAGGCTTATAGAAGTAGCACAAAGAATTGTTAGAGGT----------------------------
--------ATTCTCCATATA------CCTAGAAGAATAAGACAGGGCTTGGAAAGGGCTTTGCAA---TAA
>MK041575
GCT---GCA--
GAAAACTTGTGGGTCACAGTCTATTATGGGGTACCTGTGTGG---AAAGAAGCAACC---
ACCACTCTATTCTGTGCATCAGATGCTAAAGCATATGACACAGAG---GTGCATAAT----------------
--------------GTTTGGCCACA---CATGCCTGTGTACCCACAAAC---
CCTGACCCACAAGAAATAAAATTGGAA---------AATGTGACAGAAAGTTTTAAT----------------
--------------------------ATATGGAAAAATGACATGGTAAAACAGATGCATGAGGATATA---
ATCAGTTTATGGGATCAAAGCCTA---AAACCATGTGTAAAATTA---
ACCCCACTCTGTGTTACTCTAAATTGCACTGAATATACCGGAACGAATGTTAGTGTTACCAATAGT-------
----
-------------------------CCCGCTAATAGTAGTGTGGAAGAAAGGGAAGAAATAAAA---
AACTGCTCTTTTAATATCACCACAGGCATAGGA---------------------------
GATAAGATGAGGAAAGAATATGCTCTC---TTTTATAAACTTGATGTAGAACCAATAGAGAAT----------
----

GATACTCACAGATATACGTTGGTAAGTTGTAATACCTCAGTCATTACACAG---
GCCTGTCCAAAGGTATCCTTTGAACCAATTCCCATACATTATTGTGCTCCAGCTGGTTTTGCGCTTCTAAAGT
GTAGA---GATGCAAAGTTCAAT------GGAACAGGACTATGTAAAAAT-----------------------
-GTCAGT---ACAGTACAATGCACACATGGAATTAGGCCAGTAGTGTCAACT---
CAATTGCTGTTAAATGGCAGTCTAGCAGAAGAAGAG------------
ATAGTAATTCGATCTGAAAATATCACCGACAAT---------GCTAAAACCATAATAGTACAGCTGAAC----
-----------AAAACTGTAGTA---------ATCAATTGTACAAGACCCAACAACAATACAAGAAAA-----
-AGTATACATACA------------GGATTTGGGAGAGCA---ATT------TTTGCAACAGGACAA------
---------ATAATAGGAGACATA------------AGACAAGCACATTGT---AACCTTAGTGGAGCA---
GGCTGGGGAGAAGCTTTAATAATGGTAGCAAGAAAATTAAAA---------------CAACATTTTGTG----
-----------------------------------AATAAAACAATAAGCTTTAGG------
AAAGCCTCAGGAGGGGACCCAGAAATTCTA---ATGCACCATTTTAATTGTGGAGGGGAA---
TTTAAGCAGCTCCAGGCAAGAGTCCTAGCTATAGAAAGATACCTACAGGACCAACAGCTCCTGGGGATTTGGG
GTTGCTCTGGAAAACTTATCTGCACCACTACTGTGCCTTGGAATAATAGTTGGAGT---------AAC-----
----------------------------AATAAAGCTCTAAATGATATTTGG------GAT------
AATATGACCTGGATGCAGTGG---GAAAAAGAAATTAGCAATTACACGGACCAAATATAC---
AAATTAATTGAAGAATCGCAGAACCAGCAAGAA--

AAGAATGAACAAGAATTACTAGAATTGGATAAGTGGGCAAGTTTGTGGACTTGGTTCGAAATATCAAATTGGC
TGTGGTATATAAAAATATTCATAATGATAGTAGGAGGCTTAGTAGGTTTAAGAATAGTTTTTGCT---
GTAATTTCTATAGTA---------AATAGAGTTAGGCAGGGATACTCACCA------
TTGTCATTCGCTGTTAGTTTGCTTAATGCAACAGCTATAGCAGTAGCTGAG---
GGGACAGATAGAGTTATAGAAGCAGTACAAAGGATTGGTAGAGCT----------------------------
--------TTTCTCCACATA------CCTAGAAGAATAAGACAGGGCTTCGAAAGGGCTTTGCTA---TAA
>MG571983
ACT---GCA---
AAGTTGTGGGTCACAGTCTATTATGGGGTACCTGTGTGG---AAAGAAGCAACC---
ACCACTCTATTTTGTGCATCAGATGCTAAAGCAGTTGAGAAAGAG---GTGCATAAT----------------
--------------GTTTGGGCCACA---CATGCCTGTGTACCCACAGAC---
CCTAATCCACAAGAAATACACTTG------------AATGTGACAGAAAATTTTAAC----------------
--------------------------ATGTGGAAAAATGACATGGTAGACCAAATGCATAAGGATATA---
ATCAGTTTATGGGATGAAAGCCTA---AAGCCATGTGTAAAATTA---
ACCCCGCTCTGTGTTACTTTAAATTGTACTAATTATAATGGCACTGCTAATTCAACTACTGCTAATCCAACTA
CT---
-------------------------GATACTAACAGCCTGAAAATGGAGGAGGGAGAAATAAAA---
AATTGTTCTTTCAATATCACCACAAATATAGGA---------------------------
AATAAGTTGCAGAGAGAAGATGCACTT---TTTTATAGACAGGATATAATGGCAATAGATGGA----------
----

AGTAATACAAGCTATACACTAACAAACTGTAACACCTCAGTCATTAAACAG---
GCCTGTCCAAAGGTATCCTTTGAACCAATTCCCATACATTATTGTACCCCGGCTGGTTTTGCGATTCTAAAGT
GTAAT---GATAAAAAGTTCAAT------GGAACAGGACCCTGTAACAAT-----------------------
-GTCAGC---GTAGTACAATGTACACATGGAATTAAGCCAGTAGTGTCAACT---
CAATTGCTGTTAAATGGCAGTCTAGCAGAAGAAGAG------------
ATAGTAATTAGATCTGAAAATTTCACAAATAAT---------GCTAAAATCATAATAGTACAGCTGAAC----
-----------AAATCTATAGCA---------ATTACTTGTATAAGACCTAACAATAATACAAGAAAA-----
-AGTATACATTTG------------GGACCAGGAGGAACA---TTC------TATGCAACAGGAGAA------
---------ATAATAGGAAATATA------------AGACAAGCATATTGT---AGCCTTAATGGAACG---
CAATGGAATAAAACTATGGAACAGATAAGTACAAAATTAAAA---------------GAACAATATAAT----
--------------------------------AATAATAGAACAATATTATTTAAT------
CAATCCTCAGGAGGGGATCCAGAGATTGTC---ATGCACACTTTTAATTGTGGAGGGGAA---
TTTAAGCAGCTCCAGGCAAGAGTCCTGGCTGTGGAAAGATACCTACAGGATCAACGGCTCCTAGGGATTTGGG
GTTGCTCTGGGAAACTCATTTGCGCCACTAATGTGCCTTGGAATATTAGTTGGAGT-----------------
----------------------------AATAAATCTCATGATGAGATTTGG------GGC------
AACATGACCTGGATGCAGTGG---GAAAAAGAAATTGACAATTACACAGGCTTAATATAC---
ACCTTGCTTGAAGAATCGCAAATCCAACAAGAA--

AAAAATGAAAAAGAATTATTGGAATTGGATAAGTGGGCAAGTTTGTGGAATTGGTTTGATATATCAAACTGGC
TGTGGTATATAAGATTATTCATAATAATAGTAGGAGGCTTAGTAGGCTTAAGGATAATTTTTATT---
GTACTTTCTTTAGTG---------AATAGAGTTAGGCAGGATACTCACCA------

CTATCATTCGCTGTTAGTTTGCTTAACGCCATAGCCATAGCAGTAGCTGAG---
GGGACAGATAGGATTTAGAAGGAATACAAGGGATTGGTAGAGCT----------------------------
--------TTTCTCCACATA------CCTAGAAGAATAAGACAGGGCTTCGAGAGGGCTTTACTA---TAA
>MG571987
GCT---GTA---
GACCAATTGTGGGTCACAGTCTATTATGGGGTGCCTGTGTGG---AAAGAAGCAAAT---
ACCACTTTATTTTGTGCATCAGATGCTAAAGCATATGATACAGAG---GCACATAAT---------------
--------------GTTTGGGCCACA---CATGCCTGTGTACCCACAGAC---
CCTAACCCACAAGAAGTAGTATTGGGA---------AATGTGACAGAAAATTTCAAT---------------
--------------------------ATGTGGAAAAATAACATGGTAGAACAAATGCATGAGGATGTA---
ATCAGTTTATGGGATCAAAGCCTA---AAGCCATGTGTAAAATTA---
ACCCCACTCTGTGTTGTTTTAAATTGCAGTGATATTAGTATCACTAGTACTCAGAATGCTACTAAT------
----
----------------------------AATAGTAGTGGGAAGACATGAAGGGAGAACTAAAA---
AACTGCTCTTTCAATATCACTTCAGGCATAAAA--------------------------
AAGAAGGTGCAAACAGAATATGCAGTG---TTTAATAGACTTGATGTAGTGCCAATTGATAATAAT------
----

GATACTACAAGCTTTATGTTAATAAATTGTAACACCTCAGTCATTACACAG---
GCCTGTCCAAAGGTATCCTTTGAACCAATTCCAATACATTACTGTGCCCCGGCTGGTTTTGCGATTCTAAAGT
GTAAT---GATAAAAGTTTTAAT------GGAACAGGACCATGTAAAAT-----------------------
-GTCAGT---ACAGTACAATGTACACATGGAATTAGGCCAGTAGTGTCAACA---
CAACTGCTATTAAATGGCAGTCTAGCAGAAGGAGAG-----------
GTAGTAATTAAATCTCAAAATTTCACAGACAAT---------GCTAAAACCATAATAGTACAGCTAAAT----
-----------GAAACTGTAAAA---------ATTAACTGTACAAGACCAGGCAACAATACAAGAAGA-----
-AGTATAAATATG------------GGACCAGGGAGAGTA---CTT------TATGCAACAGGAGCC------
---------ATAATAGGAGATATA-----------AGAAGAGCACATTGT---AAAATTAGGAGAGAT---
GATTGGAATAACACTTTAAAAAAGATAGCTATAAAATTACAG---------------GAACAATATAAT----
-----------------------------------ACAACAAACATAACCTTTAAT------
CAATCCTCAGGAGGGGACCCAGAAATTGTA---ATGCACAGTTTTATCTGTGGAGGGGAA---
TTTAAGCAGCTCCAGGCAAGAGTCCTAGCTGTGGAAAGATACCTACAGGATCAACAGCTCCTGGGGATTTGGG
GTTGCTCTGGAAAACTCATCTGCACCACTGCTGTGCCTTGGAATAGTAGTTGGAGT-----------------
----------------------------AATAAATCTTATAATCAAATTTGG------GAT------
AACATGACCTGGATGCAGTGG---GAGAGAGAAATTGACAATTACACACAAAGCATATAC---
ACCTTACTTGAGGCATCGCAGAACCAGCAGGAA---------------------------------------
----
AAGAATGAACAAGAACTGTTGGAATTGGATAAGTGGGCAAGTTTATGGAATTGGTTTGACATAACAAAATGGC
TGTGGTATATAAAAATATTCATAATGATAGTAGGAGGCTTAGTAGGTTTAAGAATAGCTTTTACT---
GTACTTTCTGTAGTA---------AATAGAGTTAGGAAGGGATACTCACCA------
TTATCGTTCGCTATTAGTTTGCTTAATACTACAGCTATAGCAGTAGCTGAG---
GGGACAGATAGGATTATAGAAGCAGTACAAAGAGCTGGTAGAGCT----------------------------
--------ATTCTCAACATA------CCTAGAAGAATAAGACAGGGTGCTGAAAGGGCTTTGATA---TAA
>MG571990
GCT---GCA---
GAAGAATTGTGGGTCACAGTCTATTATGGGGTACCTGTGTGG---AAGGAAGCAAAT---
GCTACCCTATTCTGTGCATCAGATGCTAAAGCATATGATACAGAG---GTACATAAT---------------
--------------GTTTGGGCCACA---CATGCCTGTGTACCCACAGAC---
CCCAACCCACAAGAAGTAGATTTGGTA---------AATGTGACAGAAAATTTTAAC---------------
--------------------------ATGTGGAAAAATAACATGGTAGAACAAATGCATGAGGATGTA---
ATCAGTCTATGGGACCAAAGCCTA---AAGCCATGTGTACAATTA---
ACCCCACTCTGTGTTACTTTAAATTGCACTGATTGGAATAATACTGCTACCAATGTTACTAATACC------
----
----------------------------AATGTTACCCTTGCCATGGACAGAGGAGAAATAAAG---
AACTGCTCTTTTAATGTCACCACACCCATAAGA--------------------------
GATAAGAAACAGAAAGAATATGCACTT---TTTTATAAACTTGATGTAGTACTAATAGGTAATGAT------
----

AATAGTAGCTATAGTAGCTATAGAATGATAAGTTGTAACACTTCAGTCATTACACAG---
GCTTGTCCAAAGATATCCTTTGAGCCAATTCCAATACATTTTTGTGCCCCGGCTGGTTATGCGATTTTAAAGT
GTAAA---GATAAGAAGTTCAAG------GGAACAGGACCATGTACAAAT-----------------------

-GTTAGC---ACGGTACAATGTACACATGGAATTAAGCCAGTAGTATCAACT---
CAACTGCTGTTAAATGGCAGTCTAGCAGAAGAAGAG------------
GTAGTAATTAGATCTCAAAATTTCACGAACAAT---------GCTAGAACCATAATAGTACAGCTAAAA----
-----------GAAGCTGTAAAC---------ATTACTTGTACAAGACCCAGCAACAATACAAGAAAA-----
-GGTATACATATA------------GGACCAGGGAGTGCA---TTT------TATACAACAGGACAA------
---------ATAATAGGGGATATA------------AGACAAGCACATTGT---AACATTAGCAAAAAA---
GACTGGAATAAGACTTTAGAACAGGTAGCTAAAAAATTAAGA---------------GAACAATTTGGG----
-----------------------------------AATAAAACAATAATCTTTAAT------
CAGTCCTCAGGAGGGGACCTAGAAATTGTA---ACACACAGTTTTAATTGTGGAGGGGAA---
TTTAAGCAGCTCCAGGCAAGAGTCCTTGCTGTGGAAAGATACCTAAAGGATCAACAGCTCCTAGGAATTTGGG
GTTGCTCTGGAAAACTCATTTGCACCACTGCTGTGCCCTGGAATGCTAGTTGGAGT-----------------
----------------------------AATAAATCCTACAATGACATTTGG------GAT------
AACATGACCTGGATGCAGTGG---GACAGAGAAATTAACAATTACACAAAGCAAATATAC---
ACCTTGATTGAACAATCGCAGAACCAACAAGAA---

AAGAATGAACAGGAATTATTGGAATTGGATAAATGGGCAAGTTTGTGGAACTGGTTTGACATATCAAATTGGC
TGTGGTATATAAAAATATTCATAATGATAGTAGGAGGCCTGGTAGGTTTAAGAATAATCTTTTCT---
GTACTTTCTATAGTA---------AATAGAGTTAGGCAGGGATATTCACCA------
TTATCGTTCGCTGTTAGTTTGCTTAATTGCACAGCTATAGCAGTAGCTGAG---
GGGACAGATAGGCTTATAGAAGTAGCACAAAGAATTGTTAGAGGT----------------------------
--------ATTCTCCATATA------CCTAGAAGAATAAGACAGGGCTTGGAAAGGGCTTTGCAA---TAA
>MG572002
ACT---ACA---
GAACAACTGTGGGTCACAGTCTATTATGGAGTACCAGTGTGG---AAAGAAGCAAAA---
ACCACTTTATTTTGTGCATCAGATGCTAAAGCATATGACACAGAG---GCTCATAAT----------------
--------------GTTTGGGCCACA---CATGCTTGTGTACCCACAGAC---
CCCAATCCACAAGAAATAGTATTGGAA---------AATGTGACAGAAAATTTTAAC---------------
--------------------------ATGGGAAAAAATGACATGGTAGATCAGATGCATGAGGATATA---
ATCAGCTTATGGGACCAAAGCCTA---AAGCCATGTGTAAAATTA---
ACCCCACTCTGTGTTACTTTAAATTGCACTGAGCTGAATAATGATACTATAGCTAGTGCTACTTCTAGCAATA
CTAGCGCTACT---
----------------------------------GCCGCGGAAGAATTAAGGAGAGAAATGACA---
AACTGCTCTTTCAATATCACCACAAAAATAGGA--------------------------
AATAGGATACAGAAAGAATATGCACTT---
TTTTATAAATCTGATGTAGTACTAATAGGTAATAGTACAAACAAT----------------------------
----
GGTACAAATAGTAGTGCACACTATATGTATATGTTGACAACCTGTAACACCTCAACCCTTACACAA---
GCCTGTCCAAAGACATCCTTTGAACCAATTCCCATACATTATTGTGCCCCAGCTGGATTTGCGATTCTAAAGT
GTAAT---GATAAGAAGTTCAAT------GGAACAGGTCCATGTACAAAT-----------------------
-GTCAGC---ACAGTACAATGTACACATGGAATTAGGCCAGTAGTGTCAACC---
CACTTGCTGTTAAATGGCAGTCTATCAGAAGAAGAA------------
GTAGTACTTAGATCTGAAAACTTCACAGACAAT---------GCTAAAACCATAATAGTACAACTGAAT----
-----------GAAACTGTGGTA---------ATTACTTGTATAAGACCCAATAACAATACAAGAAAA-----
-AGTATACATCTA------------GGATGGGGGAGAGCA---TTT------TATGCAACAGGACAA------
---------ATAACAGGAGATATA------------AGAAAAGCACACTGT---ATCCTTAATAGAACA---
AACTGGGAGAACACTCTAGAAAAGGTAGCTAGAAAGTTATCC---------------ACACAATTCAAT----
-----------------------------------AATACAAAAATAATCTTTACA------
CCACCCGCAGGAGGGGACCCAGAAATTGTA---ATGCACACTTTTAATTGTGGAGGAGAA---
TTTAAGCAGCTCCAGGCAAGAGTCCTGGCTCTGGAAAGATACCTAAAGGATCAACAGCTCCTAGGGATTTGGG
GTTGCTCTGGAAGACTCATTTGCACCACTGCTGTGCCTTGGAATACTAGTTGGAGT-----------------
----------------------------AATAGAAATATGACTCAGATCTGG------AAT------
AACATGACATGGATGCAGTGG---GAAAGAGAAATTGACAATTACACAGAAGTAATATAC---
AGTCTACTTGAAGAATCGCAAAACCAACAAGAA---

AAGAATGAACAAGAATTATTAGCATTGGACAAGTGGGCAAGTTTGTGGAATTGGTTCGATATATCAAACTGGC
TGAGGTATATACAAATATTCATAATGATAGTAGGAGGCTTAATAGGATTAAGAATAGTTTTTGCT---
GTACTTTCTATAGTG---------AATAGAGTTAGGCAGGGATACTCACCA------
TTGTCATTCGCTATCAATTTGCTTAATACAGTAGCTGTAGCAGTAGCTGAG---
GGGACAGATAGGATTATAGAAGTAGGACAAGGACTTGGTAGAGCT---------------------------
--------ATTCTCCACATA------CCTAGAAGAATAAGACAGGGATTAGAAAGGGCTTTGGTA---TAA

>JF320427
ACT---GCA--
GAAAAATTGTGGGTCACAGTCTATTATGGGGTACCTGTGTGG---AAAGAAGCAACC---
ACCACTCTATTTTGTGCATCTGATGCTAAAGCATATGATTCAGAG---GTACATAAT----------------
--------------GTTTGGGCCACA---CATGCCTGTGTACCCACAGAC---
CCCAGTCCACAAGAAGTAGTTTTGGGA---------AATGTGACAGAAAAGTTTAAC----------------
--------------------------ATGTGGAAAAATGACATGGTAGAACAGATGCATGAGGATATA---
ATTAGTTTATGGGATCAAAGCTTA---AAGCCATGTGTAAAATTA---
ACCCCACTCTGTGTTACTTTAAATTGCACTGATAAAGTAACGGTGAGACACAATAAT----------------
----
----------------------------ACTGTTTCTTTTAATGATACCAAAGGAGAAATACAA---
AACTGCTCTTTCAATATCACCTCAAATATAAGA---------------------------
GATAAGGTGCAGAAAGCATATGCACTT---TTTTATAAACTTGATGTAGTACCAATAGATAGTAAT-------
----

GATAATACCAGTTATAGGCTGATAAGTTGTAACACCTCAGTCATCACACAG---
GCCTGTCCAAAGATAACCTTTGAACCAATTCCTATACACTATTGTGCTCCTGCTGGTTTTGCGATTCTAAAAT
GTAAT---GATAAAAAGTTCAAT------GGAACAGGACCATGTACAAAT-----------------------
-GTCAGC---ACAGTACAATGTACACATGGAATTAGACCAGTAGTATCAACC---
CAACTACTGTTAAATGGTAGTCTGGCAGAAGAAGAG------------
ATAGTAATTAGATCTGAAAATATCACAAACAAT---------GCTAAAACCATAATAGTGCATCTGAAC----
-----------GAATCTGTAGAA---------ATTAATTGTACAAGACCCAACAACAATACAAGAAAA-----
-GGTATACATCTA------------GGACTAGGGAGGGCA---TTC------TATACAACAGGAGAA------
---------ATAATAGGAGATATA------------AGACAAGCACATTGC---ACCCTTAATGAGGCA---
AAATGGAATGACACTTTAAAAAAGGTAGTTAAAAAATTAAGA---------------
GAACAATTTGAGGAATTT------------------------------AAGAATAAAGCAATAGTTTTTAAT-
-----CAATCTGCAGGAGGGGACCCAGAAATTACA---ATGCTCACTTTTAATTGTGGAGGAGAA---
TTTAAGCAGCTCCAAGCAAGAGTCCTGGCTGTGGAAAGATACCTAAGGGATCAACAGCTCCTGGGAATTTGGG
GTTGCTCTGGAAAACTCATTTGCACCACTGCTGTGCCTTGGAATGACAGTTGGAGT-----------------
-------------------------CATAATAGATCTCTAGAGCAGATTTGG------AAC------
AACATGACCTGGATGGAGTGG---GACAGAGAAATTAGCAATTACACAGGCTTAATATAC---
TCCTTAATTGAACAATCGCAAAGCCAACAGGAA--

AAGAATGAAAAAGAATTATTAGAATTGGATAAATGGGCAAATTTATGGAATTGGTTTGATATAACAAACTGGC
TGTGGTATATAAAAATATTCATAATGATAGTAGGAGGCTTGATAGGTTTAAGAATAGTTTTTACA---
GTACTCTCTCTAGTG---------AATAGAGTTAGGCAGGGATACTCACCA------
TTATCGTTCGCTGTTAGCTTGCTTAACGCTATAGCCATAGCAGTAGCTGAG---
GGAACAGATAGGATTATAGAAATAGTACAAAGAGCTGGTAGAGCT----------------------------
--------ATCCTCAACGTA------CCTAGAAGAATAAGACAGGGCTTTGAAAGGGCTTTGCTG---TAA
>GU562236
GCT---ACA--
GAACGGTTGTGGGTCACAGTCTACTATGGGGTACCGGTGTGG---AAAGATGCAACC---
ACCACTCTATTTTGTGCATCCGATGCTAAAGCATATGATACAGAG---GTACATAAT----------------
--------------ATCTGGGCCACA---CATGCCTGTGTACCCACAGAC---
CCCAACCCACAAGAAATAGTATTGCAT---------AATGTGACAGAAAATTTTAAC----------------
--------------------------ATGTGGAAAAATAACATGGTAGAACAGATGCATGAGGATATA---
CTCAGTTTATGGGATGAAAGCCTA---AAGCCATGTGTAAAATTA---
ACCCCACTCTGTGTTCCTTTAAATTGCACTGATGCAAAGACGAACAGT-------------------------
----
--ACTGTGGGGAGAGAAGAAATGAAA---
AACTGCTCTTTCGATGTTACCACAGGCATAAGA---------------------------
AGTAAAGTGCAGAAAGAATATGCACTT---
TTGTATAAACTTGATATAGTACAAATAGATAAAATTAATGATAGTATAATT----------------------
----
AATGGTAGTAATAGTAACTATAGTAGCTATAGTAACTATAGTAGCTATGCATTGATAAGTTGTAACACCTCAG
TCATTAAACAG---
GCCTGCCCAAAGGTAACCTTTGAGCCAATTCCCATACATTATTGTGCCCCAGCTGGTTATGCGATTGTAAAGT
GTAAT---GATAAGAGGTTCAAT------GGAACAGGACCATGTAAAAAT-----------------------
-GTCAGC---ACAGTACAGTGCACACATGGAATTAGGCCAGTAGTGTCAACT---
CAACTGCTGTTGAATGGCAGTCTAGCAGAAGAAGAG------------

GTAATAATTAGGTCTGAAAATTTCACGGACAAT---------GCTAAAACCATAATAGTACAGCTAAAT----
-----------GAAACTGTAAAA---------ATTGAGTGTATAAGACCCAACAACAATACAAGAAAA-----
-AGTATACGTATA------------GGACCAGGGAGTACA---TTT------TATGCAACAGGAGCC------
---------GTAATAGGAGAGATA------------AGACAAGCACATTGT---AACATTAGTAGAACA---
AACTGGACTAACACTTTACAAAAGGTAGTTACAAAATTAAGA---------------GAACAATTTGGG----
--------------------------------AAGAATAAAACAATAATCTTTAAG------
CCACCAGCAGGAGGAGACCCAGAGATTGTA---ACTCACAGTTTTAATTGTGGAGGGGAG---
TTTAAACAGCTCCAGGCAAGAGTCCTAGCTGTGGAAAGATACCTAAAGGATCAACAGCTCCTAGGGATTTGGG
GCTGCTCTGGAAAACTCATCTGCACCACTGCTGTGCCTTGGAATGCTAGTTGGAGT-----------------
----------------------------AATAAATCTCTGGAGAGTATCTGG------GAA------
AACATGACCTGGATGGAGTGG---GAAAAAGAAATTGACAATTACACAGAACAAATATAC---
CTCTTACTTGAACAATCGCAGAGCCAGCAAGAA--

AAGAATGAACAGGAATTATTGGAATTGGATAAGTGGGCAAGTTTGTGGAATTGGTTTAGCATATCAAACTGGC
TGTGGTATATAAGAATATTCATAATGATAATAGGAGGCTTGGTAGGTTTAAGAATAGTGTTTGCT---
GTACTTTCTGTAGTG---------AATAAAGTTAGGCAGGGATACTCACCA------
TTATCATTTGCAGTTAGCTTGCTTAATGTCACAGCAATAGCAGTAGCTGAG---
GGGACAGATAGGATTATAGAACTAGTACAAAGAATTTTTAGAGCT----------------------------
--------TTTCTCAACATA------CCTAGAAGAATAAGACAGGGCTTAGAAAGAGCTTTGCAA---TAA
>JQ403024
GCT---AAA--
GAACAATTATGGGTCACAGTCTATTATGGGGTACCTGTGTGG---AAAGAAGCAAAC---
ACCACTCTATTTTGTGCATCAGATGCTAAAGCATATGATACAGAG---GTACATAAT----------------
--------------GTTTGGGCCACA---CATGCCTGTGTACCCACAGAC---
CCCAACCCACAAGAAATAGTAATGGAA---------AATATAACAGAAGAGTTTAAC----------------
--------------------------GTGTGGAAAAATAACATGGTAGAACAGATGCATGAGGACATA---
GTCAATTTATGGGATCAAAGTCTA---AAGCCATGTGTAAAATTA---
ACCCCACTCTGTGTTACTTTAAATTGCACTGATTATGTGAGGAATGATAATAATACCAAT-------------
----
-------------------------AATAGTAGTGTGTTAAGGATGGAGCCAGGAGAAATAAAA---
AACTGCTCTTTCAATATTACCACAAACATAAGA---------------------------
GATAAGATACAAAGAACATATGCATTG---TTTTATAAACTTGATGTAGTACCAATAGATAAT----------
----

GAGAATAATAGCTATAGGTTGATAAGTTGTAACACCTCAGTCATTACACAA---
GCTTGTCCAAAGGTATCCTTTCAGCCAATTCCCATACATTATTGTGCCCCGGCTGGTTTTGCGATTCTAAAGT
GTAAC---GAGAAGGGGTTCAGT------GGAAAAGGAGAATGTAAAAAT-----------------------
-GTCAGC---ACAGTACAATGTACACATGGAATTAGGCCAGTAGTGTCAACT---
CAACTGTTGTTAAATGGCAGTCTAGCAGAAGAAGAG------------
GTAGTAATTAGATCTGAAAATTTCACAGACAAT---------ACTAAAACCATAATAGTACAGCTGAAC----
-----------AAAGCTGTAGTA---------ATTAATTGTACAAGACCCAACAACAACACAAGAAAA-----
-GGTATACATATA------------GGACCAGGGAGTGCA---TTT------TATACAACAGGAGAC------
---------ATAATAGGAGATATA------------AGACAAGCACATTGT---AACATTAGTGAAGCA---
GACTGGAATAATACTTTAGGACAGATAGCTGCAAAATTAAAA---------------GAAAAATTT-------
-----------------------------------AATAAAACAATAGTCTTCAAT------
CAATCCTCAGGAGGGGACCCAGAAATTGTA---ATGCACAGTTTTAATTGTGGAGGAGAA---
TTTAAGCAGCTCCAGGCAAGAGTCCTGGCTGTGGAAAGATACCTAAAGGATCAACAGCTCCTAGGGATTTGGG
GTTGCTCTGGAAAACTCATTTGCACCACTGCTGTGCCTTGGAATGCTAGTTGGAGT-----------------
----------------------------AATAAATCTCTAGATGACATCTGG------AAT------
AACATGACCTGGATACAATGG---GAAAGAGAAATTGACAATTACACAGGCTTAATATAC---
ACCTTACTTGAAAAATCGCAGAACCAGCAAGAA--

AAGAATGAACAAGAATTATTAGAATTGGATAAGTGGGCAAGTTTGTGGAATTGGTTTAGCATAACAAAATGGC
TGTGGTATATAAAAATATTCATAATGATAGTAGGAGGCTTAATAGGTTTAAGAATAATTTTTGCT---
GTGCTTTCTATAGTG---------AATAGAGTTAGGCAGGGATATTCACCA------
CTATCATTGGCTATTAGCTTGCTGAACGCCACTGCTATAGTAGTAGCTGAG---
GGGACAGATAGGATTATAGAAGGAGTACAAAGGGCTTTTAGAGCT----------------------------
--------TTTATTCACATA------CCTGTAAGAATAAGACAGGGCTTGGAAAGGGCTTTGCTA---TAA
>JQ403041

GCT---GGA---
CAACAGTTGTGGGTCACAGTCTATTATGGGGTACCTGTGTGG---AAAGAAGCAACT---
ACCACTCTATTCTGTGCATCAGATGCTAGAGCATATCATACAGAG---GTACATAAT---------------
--------------GTTTGGGCCACA---CATGCCTGTGTACCCACAGAC---
CCCAGCCCACAGGAAGTAGTATTGCAA---------AATGTGACAGAAAATTTTAAC---------------
--------------------------ATGTGGAAAAATAACATGGTAGATCAGATGCATGAGGATATA---
ATTAGTTTATGGGATCAAAGCCTA---AAGCCATGTGTAAAGTTA---
ACCCCACTCTGTGTCACTTTAAATTGTGCTGATTGGAAGAATGGTACTAATACCAGGAATGATTCTAAT----
----
-------------------GCCACTAATAGTAGCATGGGAGTGGTGGAAGGAAAAGAAATGAAA---
AATTGCTCTTTCAATATCACCACAAGCATAAGA-------------------------
GATAAGGTGAAGAAGGAATATGCACTT---TTTTATAAACTTGATGTAGTACCCATAGACAAT----------
----

GAGAATGATAGCTATAGGTTGATAAGTTGTAACACCTCAGTCATTACACAG---
GCCTGTCCAAAGGTCTCCTTTGAGCCAATTCCCATACATTATTGTGCCCCGGCTGGTTTTGCGATTATAAAGT
GTAAT---AATAAGACATTCAAT------GGAAGAGGACCATGTACAAAT----------------------
-GTCAGC---ACAGTACAATGTACACATGGAATTAGGCCAGTAGTATCAACT---
CAACTGCTGTTAAATGGCAGTCTAGCAGAAGAGGAG-----------
GTAGTAATCAGATCAGAAAATTTCACGAACAAT---------GCTAAAGTCATAATAGTACAGCTGAAA----
-----------AAACCTGTAGAG---------ATTAATTGTACAAGACCCGGCAACAATACAAGAAAA-----
-AGTATAAATATA------------GGACCAGGCAGAGCA---TTT------TATGCAACAGGAAAC------
---------ATAATAGGAGACATA-----------AGACAAGCACATTGT---AATCTTAGTAGAGCA---
ACATGGGATGAAACTTTAAAAGAGATAGCTACAAAATTAAGA---------------GAACAATTTGTG----
------------------------------------AATAAAACAATAGTCTTTAAA------
GATTCCTCAGGAGGGGATCCAGAAGTTGTG---ATGCACACTTTTAATTGTGGAGGGGAA---
TTTAAGCAGCTCCAGGCGAGAGTCCTGGCTGTGGAAAGATACCTAAAGGATCAACAGCTCCTAGGGATTTGGG
GTTGCTCTGGAAAGCTCATTTGCACCACTAATGTACCTTGGAATAAGACTTGGAGT----------------
-----------------------------AATAAAACTCAGGAGCAGATTTGG------GAT------
AACATGACCTGGATGCAGTGG---GAAAGAGAAATTGATAACTACACAGGTTTAATATAC---
ACCTTACTTGAAGAATCGCAAAACCAACAAGAA---------------------------------------
----
AAGAATGAACAAGAATTATTGGCATTGGATAAATGGGCAAGTTTGTGGAATTGGTTTGACATAACAAAATGGC
TGTGGTATATAAAAATATTCATAATGATAGTAGGAGGTTTGGTAGGTTTAAGAATAGTTTTTGTT---
GTACTTTCTATAGTG---------AATAGAGTTAGGCAGGGATACTCACCA------
TTATCATTCGCTGTTAGCTTGCTCAACGCCCTAGCCATAGCAGTAGCTGAG---
GGGACTGATAGGATTATAGAATTAGTGCAAAGAACTTGTAGAGCT---------------------------
--------ATTATCAACATA------CCCACACGAATAAGACAGGGCTTGGAAAGGGCTTTGCTA---TAA
>KC797225
AGT---GCT---GCA------------------------------------
GATCAATTGTGGGTCACAGTATATTATGGGGTACCTGTGTGG---AAAGAAGCAACT---
ACCACCCTATTTTGTGCATCAGATGCTAAAGCATATGATACAGAG---GTACATAAT---------------
--------------GTTTGGGCCACA---CATGCCTGTGTACCCACAGAC---
CCCAACCCACAAGAAGTAGTATTGGGA---------AATGTGACAGAAAATTTTAAC---------------
--------------------------ATGTGGAAAAATAACATGGTGGAACAGATGCATGAGGATATA---
ATTAGTTTATGGGATCAAAGTCTA---AAGCCATGTGTAAAATTA---
ACCCCACTCTGTGTTACTCTAAATTGCACTGATGACTTGAAAAATAATAATAATAGTAGTAGTAATACCAATA
GTACT--
-------------AGTAATGCCAATAGTACTAGTGAGGCACCAATGGAAAAAGGAGAAATAAAA---
AACTGCTCTTTTGATGTCGACACAGGCATAAGC--------------------------
AAGAAGATACAGAAAGAATATGCACTT---
TTTTATAAACTTGATGTAGTACCAATAGAAGATGATAACAAGACAACT------------------------
----
AATGGTAGCTATAATAATACAAAGTATAGTAACTATAGATTAATAAGTTGTAACACCTCAATCATTACACAG-
--
GCCTGTCCAAAGGTAACCTTCGAGCCAATTCCCATACATTATTGTGCCCCGGCTGGTTTTGCAATTCTCAAGT
GTAAT---GATAAGAAGTTCAAT------GGAACAGGACCATGTACAAAT----------------------
-GTCAGT---ACAGTACAATGTACACATGGAATTAGGCCAGTAGTATCAACT---
CAATTGTTGTTAAATGGCAGTCTATCAGAAGAGGAG-----------
GTAGTAATTAGATCTGAAAATTTTACAAATAAT---------GCTAAAACTATAATAGTACAGCTGAAT----

-----------GAAACCATACAA---------ATTAATTGTACAAGACCCAACAACAATACAAGAAAG-----
-GGTATACATATA------------GGTCCAGGAAGAACA---TTT------TTTGCAACTGGAGAT------
---------ATAATAGGGGATATA------------AGACAAGCACATTGT---ACCATTAATGGAGCA---
CAATGGAATGCCACTTTACAACAGATAGTTGCAAAATTAAGA---------------GAACAATTTAGG----
---------------------------------GGAAATAAAACAATAAAATTTGAG---
CCACCATCCCCAGGAGGGGATCCAGAAATTATG---ATGCACAGTTTTAACTGTGGAGGGGAA---
TTTAAGCAGCTCCAGGCAAGAGTCCTTGCTGTGGAAAGATACCTAAGGGATCAACAGCTCCTAGGGATTTGGG
GTTGCTCTGGAAAACTCATCTGCACCACTGCTGTGCCTTGGAATACTACCTGGAGT----------------
-----------------------------AATAAAACTCTGGAACAAATTTGG------GGT------
AACATGACCTGGATGCAGTGG---GAAAAAGAAATTGACAATTACACTAACGTAATATAC---
ACCTTAATTGAGGATGCGCAAAACCAGCAGGAA--

AAGAATGAACAAGAATTATTGGAATTGGATAAGTGGGCAAATTTGTGGAGTTGGTTCGACATATCAAACTGGC
TGTGGTATATAAAAATATTCATAATGATAGTAGGAGGCTTAATAGTTTTAAGAATAATTTTTGCT---
GTACTTTCTATAGTG---------AGAAGAGTTAGGCAGGGATATTCACCA------
TTGTCATTTGCTACTAACTTGCTTAATACCATAGCTATAGCAGTAGCTGAG---
GGGACAGATAGGATTATAGAATTAGCACAAAGAGGTTTTAGAGCT----------------------------
--------TTTCTCCACATA------CCTAGAAGAATAAGACAGGGCCTTGAAAGGGCTTTGCAA---TAA
>HQ699978
GCT---GCA--
GACAAATTGTGGGTCACAGTCTATTATGGGGTACCTGTATGG---AAAGAAGCAACT---
ACCACTCTATTTTGTGCATCAGATGCTAAAGCATATGAGACAGAG---GTACATAAT----------------
--------------ATTTGGGCTACA---CATGCCTGTGTACCCACAGAC---
CCCAACCCACAAGAAGTAGTCTTGGGA---------AATGTGACAGAGAACTTTGAT----------------
--------------------------ATGTGGGAAAATAACATGGTAGAACAGATGCATGAAGATATA---
ATCAGTTTATGGGATCAAAGCCTA---AAGCCATGTGTAAAATTA---
ACCCCACTCTGTGTTACTTTAAATTGCACTAATTTGAATGATACTACTAAAACCAGTAATACTACCAGTAATA
ATACCAGT---
-------GATACTACTAATACCAGTAGTACTACCAGTAGTATGATAAGGGAAGGAGAAATAAAA---
AACTGCTCTTTCAATATCACCACAAGCATAAGG---------------------------ACTAAGGTA---
AAAGACTATGCATTT---TTTTATAAACTTGATATAGTACAAATAGATAAT---------------------
----
----GATAATAGTAATACCAGCTATAGGTTGATAAATTGTAACACCTCAGTCATTACACAA---
GCTTGTCCAAAGGTATCCTTTGAACCAATTCCCATACATTATTGTGCCCCGGCTGGTTTTGCGATTCTAAAGT
GTAAC---AATAAAACGTTCAAT------GGAACAGGACCATGTACAAAT----------------------
-ATCAGT---ACAGTACAATGTACACATGGAATTAGGCCAGTAGTATCAACT---
CAACTGCTGTTAAATGGCAGTTTAGCAGAAGAAGAG-----------
GTAGTAATTAGATCTAGCAATTTCACGGACAAT---------CTTAAAACCATAATAGTACAGTTGAAT----
-----------AAATCTGTAGAA---------ATTAATTGTACAAGACCCAACAACAATACAAGAAAA-----
-AGTATACATCTA------------GGACCAGGGCAAGCA---TGG------CATACAACAGGAGAA------
---------ATAATAGGAGATATA------------AGACAAGCACATTGT---AACATTAGTAGAGCA---
AAATGGAATAACACTTTAGAACAGATAACTAAAAAAATTAAGA---------------GAAATATAC-------
-----------------------------------AACAAAACAATAAGCTTTAAG------
CAACATGCAGGAGGGGACCCAGAGGTTGTA---ATGCACAGTTTTAATTGTGGAGGGGAA---
TTTAAGCAGCTCCAGGCAAGAGTCTTGGCTGTGGAAAGATACCTAAAGGATCAACAGCTCCTAGGGATTTGGG
GTTGCTCTGGAAAACTCATTTGCACCACTTCTGTGCCTTGGAATGCTAGTTGGAGT----------------
-----------------------------AATAAATCTCTGGATGAGATTTGG------AAT------
AACATGACTTGGATGGAGTGG---GAAAGAGAAATTGACAATTACACAAACGAAATATAC---
AAATTAATTGAAGAATCGCAGAACCAACAAGAA--

AAGAATGAACTAGAACTATTGGAATTGGATAAGTGGGCAAGTTTGTGGAATTGGTTTAGCATAACAAACTGGC
TGTGGTATATCAAAATATTCATAATGATAGTAGGAGGCTTGGTAGGTTTAAGAATAGTTTTTGCT---
GTACTTTCTATAGTA---------AATAGAGTTAGGCAGGGATACTCACCA------
TTGTCATTAGCTATTAGCTTGCTTAACGCCACAGCCATAGCAGTAGCTGAG---
GGGACAGATAGGGTTATAGAAGTAGTACAAAGAGCCTGTAGGGCT----------------------------
--------ATTCTCAACATA------CCTACAAGAATCAGACAGGGCCTCGAAAGGGCTTTGCTA---TAA
>EU131792
GCT---GCA--
AAAAACTTGTGGGTCACAGTCTATTATGGGGTACCTGTATGG---AAAGAAGCAACT---
ACTACTCTATTTTGTGCATCAGATGCTAAAGCATATGAGACAGAG---GTACATAAT----------------

--------------ATTTGGGCCACA---CATGCCTGTGTACCCACAGAC---
CCCAGCCCACAAGAAGTAGTCTTGGAA---------AATGTGACAGAAAACTTTAAC----------------
--------------------------ATGTGGAAAAATAACATGGTAGACCAGATGCATGAAGATATA---
ATCAGTTTATGGGATCAAAGCCTA---AAGCCATGTGTAAGATTA---
ACCCCACTCTGTGTTACTTTAAATTGCACTAATTTGAAAAATACTACTAATACCAGTAAT-------------
----
----------------------------AATATCAGTAGTACTACAGAGGGAGGAAAAATAAAA---
AACTGCTCTTTCAATATCACCACAAGCACAAAA---------------------------ACTAAGGTG---
AGAGACTATGCACTT---TTTTATGAACTTGATATAGTATCAATAAAAAAT----------------------
----
----------GATAGTACCAGCTATAGGTTGATAAATTGTAACACCTCAGTCATTACACAA---
GCTTGTCCAAAGGTATCCTTTGAGCCAATTCCCATACATTATTGTGCCCCGGCTGGTTTTGCGATTCTAAAAT
GTAAC---AATAAAACGTTCAAT------GGAACAGGACCATGTACAAAT-----------------------
-GTCAGT---ACAGTACAATGTACACATGGAATTAGGCCAGTAGTATCAACT---
CAACTGCTGTTAAATGGCAGTTTAGCAGAAGAAGAG------------
GTAGTAATTAGATCTAGCAATTTCACGGACAAT---------ACTAAAGTCATAATAGTACAGCTTAAT----
-----------GAATCTGTAAGA---------ATTGATTGTACAAGGCCCAACAACAATACAAGAAAA-----
-AGTATACATCTA------------GGACCAGGGAGAGCA---TGG------TATACAACAGGACAA------
---------ATAATAGGAGATATA------------AGACAAGCACATTGT---AACATTAGTAGAGAA---
AAATGGAATAACGCCTTAGGACTGATAACTGAAAAATTAAAA---------------GAACAATTTGGG----
-----------------------------------AACAAAACAATAATCTTTGAT------
CAATCTTCAGGAGGGGACCCAGAGATTGTA---ATGCACAGTTTTAATTGTGGAGGGGAA---
TTTAAGCAGCTCCAGGCAAGAGTCTTGGCTGTGGAAAGATACCTAAAGGATCAACAGCTCCTAGGGATTTGGG
GTTGCTCTGGAAAACTCATTTGCACCACTGCTGTGCCTTGGAATGCTAGTTGGAGT-----------------
----------------------------AATAAATCCCTGAGTGAGATTTGG------GGC------
AACATGACTTGGATGGAGTGG---GAAAAAGAAATTGGCAATTACACAAGAGAAATATAC---
ACCTTAATTGAAGATTCGCAGAACCAGCAAGAA--

AAAAATGAACTAGAACTATTGGAATTGGATAAGTGGGCAAGCTTGTGGAATTGGTTTGACATAACACAATGGC
TGTGGTACATCAAAATATTCATAATGGTAGTAGGGGGCTTGGTAGCTTTAAGAATAGTTTTTGCT---
GTACTTTCTATAGTA---------AATAGAGTTAGGCAGGGATACTCACCC------
TTGTCGTTTGCTATTAGCTTGCTTAACGCCACAGCCATAGCAGTAGCTGAG---
GGGACAGATAGGATCATAGAAGTAGCACAAGGAGCTTATAGGGCT----------------------------
--------ATTCTTAACATA------CCTACAAGAATCAGGCAGGGCCTAGAAAGGGCTTTGTTA---TAA
>EU131803
GCT---GAA--
GAAAAGTTGTGGGTCACAGTCTATTATGGGGTACCTGTATGG---AAAGAAGCAACT---
ACCACTCTATTTTGTGCATCAGATGCTAAAGCATATGATACAGAG---GTACATAAT----------------
--------------ATTTGGGCCACA---CATGCCTGTGTACCCACAGAC---
CCCAACCCACAAGAAGTAGTCTTGGGA---------AATGTGACAGAAAACTTTAAC----------------
--------------------------ATGTGGAAAAATAACATGGTAGAACAGATGCATGAAGATATA---
ATCAGTTTATGGGATCAAAGCCTA---AAGCCATGTGTAAAATTA---
ACCCCACTCTGTGTTACTTTAAATTGCACTAATTTGAGGAATATTACTAATACCAGTAGT-------------
----
----------------------------AATACCAGTAGTACGACAGAGGGAGGAGAAATAAAA---
AACTGCTCTTTTAATATCACCACAAAAATAAGA---------------------------ACTAAGGTA---
AAAGACTATGCACTT---TTTTATAAACTTGATGTAGTACCAATAGATAAT----------------------
----
----------GATAATACCAGCTATAGGTTGATAAATTGTAACACCTCAGTCATTACACAA---
GCTTGTCCAAAGGTATCCTTTGAGCCAATTCCCATACATTATTGTACCCCGGCTGGTTTTGCGATTCTAAAGT
GTAAC---AATAAAACATTCAAT------GGAACAGGACCATGTACAAAT-----------------------
-GTCAGT---ACAGTACAATGTACACATGGAATTAGGCCAGTAGTATCAACT---
CAACTGCTGTTAAATGGCAGTTTAGCAGAAGAAGAG------------
ATAGTAATTAGATCTAGCAATTTCACAGACAAT---------GCTAAAGTCATAATAGTACAGTTGAAT----
-----------AAATCTGTAGAA---------ATTAATTGTACAAGACCCAACAACAATACAAGAAAA-----
-GGTATAAATATA------------GGACCAGGGCGAGCA---TGG------TATACAACAGGAGAT------
---------ATAATAGGAGATATA------------AGACAAGCACATTGT---AACCTTAGTAGGACA---
GAATGGAATAACACTTTAAAACAGATAGCTGAAAAATTAAGA---------------GAACAATTTGG----
-----------------------------------AACAAAACAATAATCTTTAAT------
CAATCTTCAGGAGGGGACCCAGAGATTGTA---ATGCGCAGTTTTAATTGTGGAGGGGAA---

TTTAAGCAGCTCCAGGCAAGAGTCCTGGCTGTGGAAAGATACCTAAAGGATCAACAGCTCCTAGGGATTTGGG
GTTGCTCTGGAAAACTCATTTGCACCACTACTGTGCCTTGGAATACTAGTTGGAGT-----------------
----------------------------AATAAATCTCTGGATCAAATTTGG------AAT------
AACATGACTTGGATGGAGTGG---GAAAGAGAAATTGGCAATTACACAAGAGAAATATAC---
ACCTTAATTGAACAATCGCAGAACCAGCAGGAA---

GAGAATGAATTAGAACTATTGGAATTGGATAAGTGGTCAAGTTTGTGGAGTTGGTTTAGCATAACAAATTGGC
TGTGGTATATCAGAATATTCATAATGATAGTAGGAGGCTTGATAGGTTTAAGAATAGTTTTTGCT---
GTACTTTCTATAATA---------AATAGAGTTAGGCAGGGATACTCACCA------
TTGTCATTTGCTATTAGCTTGCTTAACGCCACAGCCATAGCAGTAGCTGAG---
GGAACAGATAGGGTTATAGAAGTAGCACAAAGAGCTTATAGGGCA----------------------------
--------ATTCTCAATATA------CCTACAAGAATCAGACAGGGCCTAGAAAGGGCTTTGCTA---TAA
>EU363829
GCT---GCA---
GAAAACTTGTGGGTCACAGTCTATTATGGGGTACCTGTATGG---AAAGATGCAAAT---
ACCACTCTATTTTGTGCATCAGATGCTAAAGCATATGAGACAGAG---GTACATAAT----------------
--------------ATTTGGGCCACA---CATGCCTGTGTACCCACAAAC---
CCCAACCCACAAGAAGTAGTCTTGGAA---------AATGTGACAGAAAACTTTAAC----------------
--------------------------ATGTGGAAAAATGACATGGCAGAACAAATGCAGGAAGATATA---
ATCAGTTTATGGGATCAAAGCCTA---AAGCCATGTGTAGAATTA---
ACCCCACTCTGTGTTACTTTAAATTGTACTAATGTTACTATTAGTGATAAAAAT------------------
----
-----------------------------------AGTACAATAGAGGGAGGAGGAGAAGTAAGA---
AACTGCTCTTTCAATATCACCACAAGCATAAAT---------------------------ACTCAGGTG---
AAAGACCATGCAATT---TTTTATAAACATGATATAGAAAAAATAGATAAT----------------------
----
----------GAGACTACCAGCTACAGGTTGACAAGATGTAACACCACAATCATTAAACAG---
GCTTGTCCAAAGGTATCCTTTGAGCCAATTCCCATACATTATTGTGCCCCGGCTGGTTTTGCGATTCTAAAGT
GTAAC---AATAAAACGTTCAGT------GGAACAGGACCATGTACAAAT----------------------
-GTCAGT---ACAGTACAATGTACACATGGAATTAGGCCAGTAGTATCAACT---
CAACTGCTGTTAAATGGCAGTTTAGCAGAAAAAGAG-----------
GTAATGATTAGATCTAGCAATTTCATGGATAAT---------GCTAAAATAATAATAGTACAGCTGAAT----
-----------GAATCGGTAGAA---------ATTAATTGTACAAGACCCAACAATAATACAAGAAAA-----
-AGGGTAACTCTA------------GGACCAGGGCGAGTA---TGG------TATACAACAGGACAA------
---------ATAATAGGAGATATA------------AGAAAAGCATATTGT---AACATTAGTAGAATA---
AAATGGAATAACACTTTACATCAGATAACTAAAAAATTAAGA---------------GAACAATTTGGG----
-----------------------------------AACAAAACAATAATCTTTAAT------
CAATCCTCAGGAGGGGACCCAGAGATTCAA---ATGCACAGTTTTAATTGTGGAGGAGAA---
TTTAAGCAGCTCCAGGCAAGAGTCCTGGCTGTGGAAAGATACCTAAAGGATCAACAGCTCCTAGGGATTTGGG
GTTGCTCTGGAAAACTCATTTGCACCACTACTGTGCCTTGGAATGTTAGTTGGAGT----------------
----------------------------AATAAATCTCTGGATGAGATTTGG------AAT------
AACATGACTTGGATGGAGTGG---GAAAAAGAAATTGGCAACTACACAAATATAATATAC---
AACTTAATTGAAGAATCGCAGAACCAACAAGAA---

AAGAATGAACTAGACCTATTGAAATTGGATCAGTGGGCAAGTTTGTGGAATTGGTTTGACATATCAAAATGGC
TGTGGTATATTAAAATATTCATAATGATAGTAGGAGGCTTGGTAGGTTTAAGAATAGTTTTTGCT---
GTACTTTCTATAGTA---------AATAGAGTTAGGCAGGGATACTCACCA------
TTGTCATTAGCTATTAGCTTGCTTAACGTCACAGCCATAGCAGTAGCTGAG---
GGGACAGATAGGATTATAGAAGTAGTACAAAGAACTTATAGGGCA----------------------------
--------ATTCTTAACATA------CCTACAAGAATCAGACAGGGCCTCGAAAGGGCTTTGCTA---TAA
>HM215413
GCT---GAA---
GAAAAATTGTGGGTCACAGTCTATTATGGGGTACCTGTATGG---AAAGAAGCAACC---
ACCACTCTATTTTGTGCATCAGATGCTAAAGCATATGATCCAGAG---GTACATAAT----------------
--------------ATTTGGGCCACA---CATGCCTGTGTACCCACAGAC---
CCCAACCCACAAGAAGTAGTCTTGGGA---------AATGTGACAGAAAACTTTAAC----------------
--------------------------ATGTGGAAAAATGACATGGTAGAACAGATGCAGGAAGATATA---
ATCAGTTTATGGGATCAAAGCTTA---CAGCCATGTGTAAAATTA---
ACCCCACTCTGTGTCAATTTAACTTGCACTAATGCCACAGCTACCAATGCCACAGCTACCAATAGTACGGTGG
CC--

-------------CCAGCTCCCAATAGTACGGCGGCCCGCACGATGACAGACTATGAAATGAAA---
AACTGCTCTTTCAATATCACCACCAGCACAAGC---------------------------ACTAAGATG---
ACAGGCTATGCAGTT---TTTTATAACCTTGATGTAGTACCAATAGGTAAG----------------------
----
----GGTAATGATACTAAAAGCTATAGGTTGCTAAAGTGTAACACCTCAGTCATTACACAA---
GCTTGTCCAAAGGTATCCTTTGAACCAATTCCCCTACATTATTGTGCCCCGGCTGGTTTTGCGATTCTAAAGT
GTAAC---AACAAAACGTTCAGT------GGAACAGGACCATGTACAAAT-----------------------
-GTCAGT---ACAGTACAATGTACACATGGAATTAGGCCAGTAGTATCAACT---
CAACTTCTGTTGAATGGCAGTTTAGCAGAAGAAGAG------------
GTAGTAATTAGATCTAGCAATTTCTCGAACAAT---------GCTAAAACCATAATAGTACAGCTGAAG----
-----------GAACCTGTAGAA---------ATTAATTGTACAAGACCCAACAACAATACAAGAAAA-----
-AGTATGACTCTA------------GGACCAGGGCGAGCA---TGG------TATACAACAGGACAA------
---------ATAATAGGAGATATA------------AGAAAAGCACATTGT---AACCTTAGTAGCACA---
AAATGGCATGACACTTTAAGACAAATAGCTAAAAAATTAAGT---------------AAACAATTTGGG----
-----------------------------------AACAAAACAGTAGTCTTTAAT------
CAATCCTCAGGAGGAGACCCAGAGATTGTA---ATGCACAGTTTTAATTGTGGAGGGGAA---
TTTAAGCAGCTCCAGGCAAGAGTCTTGGCTGTGGAAAGATACCTAAAGGATCAACAGCTCCTAGGGATTTGGG
GTTGCTCCGGAAAACTCATCTGCACCACTGCTGTGCCTTGGAATGATAGTTGGAGT-----------------
-------------------------AGGAATAAAACTCTGAAGGACATTTGG------AAT------
AACATGACTTGGATGCAGTGG---GAAAGAGAAATTGACAATTACACAAGTGAAATATAC---
ACCTTAATTGAGGAATCGCAGAACCAACAAGAA--

AAGAATGAACTAGAATTATTGGAATTGGACAAGTGGGCAAGTTTGTGGAATTGGTTTGACATAACACAATGGC
TGTGGTATATAAAAATATTCATAATGATAGTAGGAGGCTTGGTAGGTTTAAGAATAGTTTTTGCT---
GTACTTTCACTAGTA---------AATAGAGTTAGGCAGGGATACTCACCA------
TTGTCATTAGCTGTTAGCTTGCTCAATGCCACAGCTATAGCAGTAGCTGAG---
GGGACAGATAGGGTTATAGAAGTAGTACAAAGAGCTTGTAGAGCT----------------------------
--------ATTCTCCACATA------CCCACAAGAATAAGACAGGGTCTTGAAAGGGCTTTGCTA---TAA
>EU131802
GCT---GAA--GAAAAG---
TGGGTCACAGTCTATTATGGGGTACCTGTATGG---AGAGATGCAAAT---
ACCACTCTATTTTGTGCATCAGATGCTAAAACATATGATACAGAG---GTACATAAT----------------
--------------GTTTGGCCACA---CATGCCTGTGTACCCACAGAC---
CCCAACCCACAAGAAGTAGTCTTGGGA---------AATGTGACAGAAAACTTTAAC----------------
--------------------------ATGTGGAAAAATAACATGGTAGAACAGATGCATGAAGATATA---
ATCAGTTTATGGGATCAAAGCCTA---AAGCCATGTGTAAAATTA---
ACCCCACTCTGTGTTACTTTAAATTGCACTAATTTGGGGAGTACTACTAATACCAGTAGT-------------
----
----------------------------AATACCAGTAGTACGACAAAGGGAGGAGAAATAAAA---
AACTGCTCTTTCGATATCACCACAAACACAGGA------------------------------AAGGTA---
AGAGACTATGCACTT---CTTTATAAACTTGATGTAGTACCAATGGGTAAA----------------------
----
----------GTTAATACCAGCTTTAGGTTGATACATTGTAACACCTCAGTCATTACACAA---
GCTTGTCCAAAGGTATCCTTTGAGCCAATTCCCATACATTATTGTACCCCGGCTGGTTTTGCGCTTCTAAAGT
GTAAC---AATAAAACATTCAGT------GGAACAGGACCATGTACAAAT-----------------------
-GTCAGT---ACAGTACAGTGTACACATGGAATTAGGCCAGTAGCATCCACT---
CAACTGCTGTTAAATGGCAGTTTAGCAGAAGAAAGG------------
GTAGTAATTAGGTCTAGCAATTTCACGGACAAT---------GCTAAAATCATAATAGTACAGCTAAAT----
-----------GAATCTGTAGAA---------ATTAATTGTACAAGACCCAACAACAATACAAGAAAA-----
-GGTATATATCTA------------GGACAAGGGCGAGCA---TGG------TATACAACAAGAAGA------
---------ATAATAGGAGATATA------------AGACAAGCACATTGT---AACATTAGTAGAGAA---
AAATGGAATAACACTTTAAAACTGATAACTGAAAAATTAAGA---------------GAACAATTTGGG----
-----------------------------------AACAAAACAATAATCTTTAAT------
CGATCTTCAGGAGGGGACCTAGAGGTTGTA---ATGCACAGTTTTAATTGTGGAGGGGAA---
TTTAAGCAGCTCCAGGCAAGAGTCTTGGCTGTGGAAAGATACCTAAAGGATCAACAGCTCATGGGAATTTGGG
GTTGCTCTGGAAAACTCATTTGCACCACTGCTGTGCCTTGGAATGCTAGTTGGAGT-----------------
----------------------------AATAAATCTCTAGATCAGATTTGG------AAT------
AACATGACTTGGATGGAGTGG---GAAAGAGAAATTGACAATTACACAAGAGAAATATAC---
AACTTAATTGAACTATCGCAGAACCAACAAGAA--

CAGAATGAACAAGAACTATTGGAATTGGATAAATGGGCAAGTTTGTGGAATTGGTTTGACATAACAAAATGGC
TGTGGTATATCAAAATATTCATAATGGTAGTAGGAGGCTTGGTAGGTTTAAGAATAGTTTTTGCT---
GTACTTTCTATAGTA---------AATAGAGTTAGGCAGGGATACTCACCA------
TTGTCATTAGCTATTAGCTTGCTTAACACCACAGCCATAGCAGTAGCTGAG---
GGAACAGATAGGGTTATAGAAGTAGTACAAAGAGCTTATAGGGCT----------------------------
--------ATTCTCAATATA------CCTACAAGAATCAGACAGGGCCTAGAAAGGGCTTTGCTA---TAA
>EU131804
GCT---GAA--
GAAAAGTTGTGGGTCACAGTCTATTATGGGGTACCTGTATGG---AAAGAAGCAACT---
ACCACTCTATTTTGTGCATCAGATGCTAAAGCATATGATACAGAA---GTACATAAT----------------
--------------GTTTGGGCCACA---CATGCCTGTGTACCCACAGAC---
CCCAGCCCACAAGAAGTAGTCTTGGAA---------AATGTGACAGAAGACTTTAAC----------------
--------------------------ATGTGGAAAAATAACATGGTAGAACAGATGCATGAAGATATA---
ATTAGTTTATGGGATCAAAGCCTA---AAGCCATGTGTAAAATTA---
ACCCCACTCTGTGTTACTTTAAATTGCGCTAAATTGAATACCACTTTGAGCAATGGTACTACTACC-------
----
----------------------AGTAATAATACCAGTAGTACAGTAGAGGGAGGAGAAATGAAA---
AACTGCTCTTTCAATATCACCACAAACATAGGA---------------------------ACTAAGGTA---
AAAGACTATGCACTT---TTTTATAACCTTGATGTAGTACCAATAGATGAG----------------------
----
-------AAGGATAATACCAGCTATAGGTTGATGAATTGTAACACCTCAGTAATTACACAA---
ACTTGTCCAAAGGTATCCTTTGAGCCAATTCCCATACATTATTGTACCCCGGCTGGTTTTGCGCTTCTAAAGT
GTAAC---AATAAAACGTTCAGT------GGAACAGGACCATGTACAAAT-----------------------
-GTCAGT---ACAGTACAATGCACACATGGAATTAGGCCAGTAGCATCCACT---
CAACTGCTGTTAAATGGCAGTTTATCAGAAGAAGGG------------
GTAGTAATTAGGTCTAGCAATTTCACAGACAAT---------GCTAAAGTCATAATAGTACAGCTGAGT----
-----------GAATCTGTAGCA---------ATTAATTATACAAGACCCAACAACAATACAAGAAAG-----
-AGTATACCTATA------------GGACCAGGGCAAGCA---TGG------TATACGACAGGACAA------
---------GTAATAGGAGATATA------------AGGCAAGCACATTGT---AACCTTAGTAGCACA---
CAATGGAATAACACTTTAAGTCAGATAACTGAAAAATTAAGA---------------GAGCAATTTGAG----
-----------------------------------AACAAAACAATAATCTTTAAT------
CAATCTTCAGGAGGGGACCCAGAAATTGTA---ATGCACAGTTTTAATTGTGGAGGGGAA---
TTTAAGCAGCTCCAGGCAAGAGTCTTGGCTGTGGAAAGATACCTAAAGGATCAACAGCTCCTAGGGATTTGGG
GCTGCTCTGGAAAACTCATTTGCACCACTACTGTGCCTTGGAATGTTAGTTGGAGT-----------------
----------------------------AATAAATCTCTAAGTCAGATTTGG------GAT------
AACATGACTTGGATGCAGTGG---GAAAGAGAAATTGATAATTACACAAAAGAAATATAC---
ACCTTAATTGAACAATCGCAGATCCAACAAGAA--

AAGAATGAACTAGAACTATTGGAATTGGATAAGTGGGCAAGTTTGTGGAATTGGTTTGACATATCACAATGGC
TGTGGTATATCAGAATATTCATAATGATAGTAGGAGGCTTGATAGGTTTAAGAATAGTTTTTGCT---
GTACTTTCTGTAGTA---------AATAGAGTTAGGCAGGGATACTCACCA------
TTGTCATTTGCTACTAGCTTGCTTAACGCCGCAGCCATAGCAGTAGCTGAG---
GGGACAGATAGGGTTATAGAAGTAGTACAAAGAACTTATAGGGCT----------------------------
--------ATTCTCAATATA------CCTACAAGAATCAGACAGGGTCTAGAAAGAGCTTTGCTA---TAA
>JF932472
GCT---GCA--
GAAAAATTGTGGGTCACAGTCTATTATGGGGTACCTGTATGG---AAAGAAGCAACT---
ACTACTCTATTTTGTGCATCAGATGCTAAAGCATATGATACAGAG---GTGCATAAT----------------
--------------ATTTGGGCCACA---CATGCCTGTGTACCCACAGAC---
CCCAGCCCACAAGAAGTAGTCTTGGGA---------AATGTGACAGAAAATTTTAAC----------------
--------------------------ATGTGGAAAAATGACATGGTAGAACAGATGCATGAAGATATA---
ATTAGTTTATGGGATCAAAGCATA---AAGCCATGTGTAAAATTA---
ACCCCACTCTGTGTTACTTTAAATTGCACTAATTTGGAGAATACTAATAATACCAGTAGT-------------
----
----------------------------AATACCAGTAGTGTGACAGAGGGAGGAGAAATAAAA---
AACTGTTCTTTCAATATCACCACAAGCATAAAA---------------------------ACTAAGGTG---
AAAGACTATGCATTT---TTTTATAAACTTGATATAGTACCAATAGATAAT----------------------
----
----------------ACCAGCTATAGGTTGATAAGTTGTAACACCTCAGTCATTACACAG---
GCTTGTCCAAAGGTATCTTTTGAGCCAATTCCTATACATTATTGTGCTCCGGCTGGTTTTGCGATTCTAAAAT

GTAAC---AATAAAACGTTCAGT------GGAACAGGACCATGTACAAAT-----------------------
-GTCAGT---ACAGTGCAATGTACACATGGAATTAGGCCAGTAGTATCAACT---
CAACTGCTGTTAAATGGCAGTTTAGCAGAAGAAGAG------------
GTAATAATTAGATCTAGCAATTTCACGAACAAT---------GCTAAAGTCATAATAGTACAGCTGAAT----
-----------AAAGCTGTAAAA---------ATTAATTGTATAAGACCCAACAACAATACAAGAAAA-----
-AGTATACCTCTA------------GGATCAGGGAAAGCA---TGG------TATACAACAGGACAA------
---------ATAATAGGAAATATA------------AGACAAGCACATTGT---AACCTTAGTAGACCA---
ATATGGAATAACACTTTACAACAGATAACTAAAAAATTAAGA---------------GAACAATTTGGG----
-----------------------------------AACAAAACAATAGTCTTTAAT------
CAATCTTCAGGAGGGGACCCAGAGATTGTA---ATGCACAGTTTTAATTGTGGAGGGGAA---
TTTAAGCAGCTCCAGGCAAGAGTCCTGGCTGTGGAAAGATACCTAAAGGATCAACAGCTCCTAGGGATTTGGG
GTTGCTCTGGAAAACTCATCTGCCCCACTGCTGTGCCTTGGAATGTTAGTTGGAGT-----------------
----------------------------AATAAATCTCTGAGTGAAATTTGG------GAT------
AACATGACTTGGATGGAGTGG---GAAAGAGAAATTGACAATTACACAAGAGAAATATAC---
ACCTTAATTGAAGAATCGCAGAACCAACAAGAA--

AAGAATGAACTAGAGTTGTTGGAATTGGATAAGTGGGCAAGTTTGTGGAATTGGTTTGACATAACGAAATGGC
TGTGGTATATCAAAATATTCATAATGGTAGTAGGAGGCTTGATAGGCTTAAGAATAATTTTTGCT---
GTACTTTCTATAGGA---------TATAGAGTTAGGCAGGGATACTCACCA------
TTGTCATTAGCTATTAGCTTGCTTAACGCCACAGCCGTAGCAGTAGCTGAG---
GGGACAGATAGAGTTATAGAAGTAGCACAAAGAGCTTATAGGGCT----------------------------
--------TTTATTAACATA------CCTACAAGAATCAGACAGGGCCTCGAAAGGGCTTTGCTA---TAA
>JF932474
GCT---GCA--
GAAAACTTGTGGGTCACAGTCTATTATGGGGTACCTGTATGG---AAAGAAGCAAAT---
ACCACCTTATTTTGTGCATCAGATGCTAGAGCATATGATACAGAG---GTACATAAT----------------
--------------ATTTGGGCCACA---CATGCCTGTGTACCCACAGAC---
CCCAACCCACAAGAAGTAGTCTTGGGA---------AATGTGACAGAAAACTTTAAC----------------
--------------------------ATGTGGAACAATAACATGGTAGAACAGATGCATGAAGATATA---
ATCAGTTTATGGGATCAAAGTCTA---AAGCCATGTGTAAAATTA---
ACCCCACTCTGTGTTACTCTAAATTGCACTGATTATTTGGGGAATACTAATACTACCAGTAATAATACTAGTA
CTGATGATACCAGAAGTACT---
-AACAGTAGTACTGATAATGCCAGTAGTAATACCAGTAGTACGATAAAGAGAGGAGAAATAAAA---
GAATGCTCTTTCAATATCACCACAAACATAAGA---------------------------
GATAAGAAGGTGAAAGACTATGCACTT---TTTTATAAACTTGATCTAGTACAATTAAATGATGAT-------
----

ACTAATACCAATACCAGCTATAGGAGCTATAGGTTAATAAGTTGTAACACCTCAGTCATTACACAA---
GCTTGTCCAAAGGTATCCTTTGAGCCAATTCCCATACATTATTGTGCCCCGGCTGGTTTTGCGATTCTAAAGT
GTAAC---AATAAAACATTCAGT------GGAACAGGACCATGTACAAAT-----------------------
-GTCAGT---ACAGTACAATGTACACATGGAATTAGGCCAGTAGTATCAACT---
CAACTGCTGTTAAATGGCAGCTTAGCAGAAGAAAAG------------
ATAGTAATTAGATCTAGCAATTTCACGAACAAT---------GCTAAAGTCATAATAGTACAGCTGAAA----
-----------GAATCTGTACAA---------ATTAATTGTACAAGACCTAACAACAATACAAGAAAA-----
-AGTATATCTCTA------------GGACAAGGGCAAGCA---TGG------TTTACAACAGGACAA------
---------ATAATAGGAGATATA------------AGACAAGCACATTGT---AACCTTAGTAGAACA---
GCATGGAATAACACTTTAAAACAGATAGCTCAAAAATTAAGA---------------GAACAATTTGGG----
--------------------------------AACAACAAAACAATAGACTTTAAG------
CCATCTTCAGGAGGGGACCCAGAAATTGTA---ATGCACAGTTTTAATTGTAGAGGGGAA---
TTTAAGCAGCTCCAGGCAAGAGTCTTGGCTGTGGAAAGATACCTAAAGGATCAACAGCTCCTAGGGATTTGGG
GTTGCTCTGGAAAACTCATTTGCCCCACTGCTGTGCCTTGGAATGATAGTTGGAGT-----------------
----------------------------AGTAAATCTTTGGATGAGATTTGG------AAT------
AACATGACTTGGATGGAGTGG---GAAAGAGAAATTGCCAATTACACAGGACATATATAC---
ACCTTAATTGAAGAATCGCAGAACCTACAAGAA--

AAGAATGAACTAGAACTATTGGAATTGGATAAGTGGGCAAGTTTGTGGAATTGGTTTGACATTTCAAAATGGC
TGTGGTATATCAAAATATTCATAATGATAGTAGGAGGCTTGGTAGGGTTAAGAATAGTTTTTGCT---
GTACTTTCTATAGTA---------AATAGAGTTAGGCAGGGATACTCACCC------
TTGTCATTGGCTATTAGCTTGCTTAACGCTACATCCATAGTAGTAGCTGAA---

GGAACAGATAGGGTTATAGAAGGAGCACAAAGAGCTTATAGGGCT----------------------------
--------ATTCGCAACATA------CCTACAAGAATCAGACAGGGCCTCGAAAGGGCTTTGCTA---TAA
>HQ215554
GCT---GAA--
GACAATTTGTGGGTCACAGTCTATTATGGGGTACCTGTGTGG---AAAGAAGCAACT---
ACCACTTTATTTTGTGCATCAGATGCTAAAGCATATAGCTCAGAG---GCACATAAT----------------
--------------GTTTGGGCTACA---CATGCCTGTGTACCCACAGAT---
CCTAACCCACAAGAAGTAGTATTGGAA---------AATGTGACAGAAAATTTTAAT----------------
--------------------------ATTTGGAAAAATGATATGGTAAGACAGATGCATGAGGATGTA---
ATCAGTTTATGGGATGAAAGCCTA---AAGCCATGTGTAAAATTA---
ACCCCACTCTGTGTTACTTTAAATTGCACTGATATGGGGTGTGCTACCAATGCTACCAATAATAAT-------
----
-------------------AGTAGTAACAATACTAGGTGTGATATGCCACTGGGAGAAATAAAG---
AACTGCTCTTTCAATATCACCACACAAATAGGA---------------------------
AAGAAAAGGAAAAAAGACTATGCAATT---TTTAATAAACTTGATGTAGTACCAATAGATAAT----------
----

GATAACAGTAGCTATATGTTGATAAATTGTAACACCTCAGTCATCAAGCAG---
GCTTGTCCAAAGGTATCCTTTGAGCCAATTCCCATACATTATTGTACCCCAGCTGGTTTTGCAATTATAAAGT
GTAAT---GATAAGGAGTTCAAT------GGAATAGGACCATGTAAAAAT-----------------------
-GTCAGC---ACAGTACAATGTACACATGGAATTAGGCCAGTAGTGTCAACT---
CAATTACTGTTAAATGGCAGTCTAGCAGAAGGAGAG------------
ATAGTAATTAAATCTCAGAATTTCTCAAACAAT---------GCTAAAAACATAATAGTACAGCTAAAT----
-----------GAAACTGTACCA---------ATTAATTGTACAAGACCCAACAACAATACAAGAAAG-----
-GGTATACATATG------------GCAGTAGGGGGAGCA---TTA------TATGCAACAGGGGCC------
---------ATAATAGGAGATATA------------AGACAAGCATATTGT---ACTGTTAATGCAACA---
AAATGGAGTGAAACTTTAAAAAAGGTAGCTATAAAATTAAGA---------------
GAACAATTAGAAAAAATAGGA------CTT------------------CCGAATAAAACAATAGTCTTTAAC-
-----CAATCCGCAGGAGGGGACCTAGAAATTGTA---ATGCACATACAAAATTGTGGAGGGGAA---
TTTAAGCAGCTCCAGGCAAGAGTCCTAGCTGTGGAAAGATTCCTAAGGGATCAACAGCTCCTAGGGATTTGGG
GTTGCTCTGGAAAGCTCATCTGCACCACTGCTGTACCTTGGAATACTAGTTGGAGT-----------------
----------------------------AATAAAACTAGGATCAGATTTGG------GAT------
AACATGACCTGGATACAGTGG---GAGAGAGAAATTGACAACTACACAGGCTTAATATAT---
GACTTACTTGAAAAATCACAGATCCAGCAAGAA--

ATGAATGAACAAGACTTATTGGAATTGGATAAGTGGACAAGTTTGTGGAATTGGTTTAACATATCAAACTGGC
TGTGGTATATAAAAATATTCATAATGATAGTAGGAGGCTTAATAGGTTTAAGAATAATTTTTACT---
GTACTTTCTATAGTG---------AATAGAGTTAGGCAGGGATACTCACCA------
TTGTCATTCGCTGTTAGTTTGTTTAATGCAATAGCTATAGCAGTGGCTGAG---
GGGACAGATAGAATCATAGAAATAGCACAAAGAATTGGAAGGGCT----------------------------
--------ACCCTCCACATA------CCTAGAAGAATAAGACAGGGTGCAGAAAGGGCTTTGCAG---TAA
>JF346917
GCT---GCA--
GAAAACTTGTGGGTCACAGTCTATTATGGGGTACCTGTATGG---AAAGAAGCAACT---
ACCACTCTATTTTGTGCATCAGATGCTAAAGCATATGATACAGAG---GTACATAAT----------------
--------------ATTTGGGCCACA---CATGCCTGTGTACCCACAGAC---
CCCAACCCACAAGAGGTAGTATTGGAA---------AATGTGACAGAAAATTTTAAC----------------
--------------------------ATGTGGAAAAATGACATGGTAGAACAGATGCATGAAGATATA---
ATCAGTTTATGGGATCAAAGCCTA---AAGCCATGTGTAAAATTA---
ACCCCAATATGTGTTACTTTAAATTGCACTGATTTGAAGAATACTACTAATACCAAT----------------
----
-------------------------------ATCAGTAGTACAATAGAGGGAGGAGAAATAAAA---
AACTGCTCTTTCAATATCACCACTAGAACAAGA---------------------------AAAAAGGTG---
GTAGACTATGCATTT---TTTTATAAACTTGATGTAGTACCAATAAATGAG----------------------
----
----GATAATGATACTACCAGCTATAGGATGATACATTGCAACACCTCAGAAATTACACAA---
GCTTGTCCAAAGGTATCCTTTGAGCCAATTCCCATATTTATTGCACCCCGGCTGGTTTTGCGATTCTAAAGT
GTAAC---AATAAAACATTCAGT------GGAACAGGACCATGTACAAAT-----------------------
-GTCAGC---ACAGTACAATGTACACATGGAATTAAGCCAGTAGTATCAACT---
CAACTGCTGCTAAATGGCAGTTTAACAGGAGAAAAA---------

GAGGTAGTAATTAGATCTAGCAATTTCTCAGACAAT---------GCTAAAATTATAATAGTACAGCTGAAT-
--------------GAATCTGTGGTA---------ATTAATTGTACAAGACCCAACAACAACACATTGAAA--
----AGTATACATCTA------------GGACAAGGGCGAGCA---TGG------CATACAACAGGACAA---
------------ATAATAGGAGATATA------------AGACAAGCACATTGT---AATCTTAGTAAATCA-
--GACTGGGATAAAACTTTAGAAAGGATAGCAAAAAAATTAAGA---------------GGACAATTTGGG--
-------------------------------------AACAGAACAATAATCTTTAAT------
CAATCCTCAGGAGGGGACCCAGAGATTACA---ATGCATAGTTTTAATTGTGGAGGGGAA---
TTTAAGCAACTCCAGGCAAGAGTCTTGGCTGTGGAAAGATACCTAAAGGATCAACAGCTCCTAGGGATTTGGG
GTTGCTCTGGAAAACTCATCTGCACCACTGCTGTGCCTTGGAATACTAGTTGGAGT-----------------
----------------------------AATAGAACTCTGAGTGAGATTTGG------GAT------
AACATGACTTGGATGCAGTGG---GAAAGAGAAATTGACAATTACACACAAGAAATATAC---
TCCTTAATTGAAGAATCGCAGAACCAACAGGAA--

AAGAATGAACTAGAACTATTGGAATTGGATAAATGGGCAAGTTTGTGGAGTTGGTTTGACATATCAAACTGGC
TGTGGTATATCAAAATATTCATAATGATAGTAGGAGGCTTGGTAGGCTTAAGAATAGTTTTTGCT---
GTACTTTCTATAATA---------AATAGAGTTAGGCAGGGATACTCACCA------
TTGTCGTTAGCTATTAGCTTGCTTAACGCCACAGCCATAGCAGTAGCTGAG---
GGAACAGATAGGATTATAGAAGTAGTACAAAGAGCTTATAGGGCT----------------------------
--------ATTCTCAACATA------CCTAGAAGAATCAGACAGGGTCTAGAAAGAGCTTTGCTA---TAA
>JX960599
GCT---ACA--
GGACGATTTGGGTTACAGTCTATTATGGGGTACCTGTGTGG---AAAGAAGCAATC---
ACCACTTTATTTTGTGCATCAGATGCTAAAGCATATGACACAGAG---GTACATAAT----------------
--------------GTTTGGGCCACA---CATGCCTGTGTACCCACAGAC---
CCCAACCCACAAGAGATAAAATTGGAA---------AATGTGACAGAAGATTTTAAC----------------
--------------------------ATGTGGGAAAATAACATGGTAGAACAAATGCATGAAGATATA---
ATTAGCTTATGGGATCAAAGCTTA---AAGCCATGTGTAAAGTTA---
ACCCCACTCTGTGTTACTTTAAACTGCACTGATGTTAAGACCAATAGTAGTAGCACTGATGCT----------
----
-------------------------------------AGGACCAATAGTAGTACAGAAATGAAA---
AACTGCACTTTCAATGTCACCACAATATTAAGA---------------------------
GATAAGGTACAGAAAGAATTTGCACTT---TTTTATAGGCTTGATGTAGTACCAATAGATGATGGTAATAAT-
----

AATAATAATACTAATACCAACAGCTATAGGTTAATAAATTGTAACACCTCAACCATTACACAA---
GCTTGTCCAAAGGTATCGTTTGAGCCAATTCCCATACACTATTGTACCCCAGCTGGTTTTGCGCTTCTAAAGT
GTAAT---GATAAGAAGTTCAGT------GGAAACGGATCATGCACCAAT-----------------------
-GTCAGC---TCAGTACAATGTACACATGGAATTAGGCCAACAGTGTCAACT---
CAACTGTTGTTAAATGGCAGTCTAGCAGAAGAAGAG------------
GTAATAATTAGATCTAAAAATTTCACAAATAAT---------GCTGAAACCATAATAGTACAGCTAAAC----
-----------AACCCTGTAGGA---------ATTCATTGTATAAGACCCAACAACAACACAAGAAAA-----
-GGTATCCATATG------------GGCCCAGGACGAGCA---TTC------TATGTAACAGGAGAG------
---------ATAATAGGAGATATA------------AGACAAGCATATTGT---AACATTAGTAGAGGA---
GAATGGAATACCACTTTACAACAGGTAGCTATAAAATTAAGA---------------GAACAATTTAAT----
-----------------------------------GCAACAACAATAGTCTTTAAT------
CAAAGCTCAGGAGGAGACCCAGAAATTGTA---ATGCACAATTTTAATTGTGGAGGGGAA---
TTTAAACAGCTCCAGGCGAGAGTCCTGGCTGTGGAAAGATACCTAAGGGATCAACAGCTCCTAGGGATTTGGG
GCTGCTCTGGAAAACTCATTTGCACCACTGCTGTGCCTTGGAATAGTAGTTGGAGT-----------------
-------------------------GAAAATAAAACTCTAGATGCTATTTGG------AAT------
AACATGACCTGGAACCAATGG---GAAAAAGAAATTGACAATTACACAGACTTAATATAC---
TCCTTACTTGAAAATTCACAGATCCAACAAGAA--

AAGAATGAAGAAGAATTATTGAAATTAGATAGTTGGGCAAGCTTGTGGAATTGGTTTGACATATCAAGGTGGC
TGTGGTATATAAAGATATTCATAATGATAGTAGGGGGCTTAGTAGGTTTAAGGATAATTTTTGCT---
GTACTTTCTATAGTA---------AATAGAGTTAGGCAGGGATACTCACCA------
TTATCATTTGCTATTAGCTTGCTGAATGTCACAGCTGTAGCAGTAGCTGAG---
GGGACAGATAGGATTATAGAAATAGTACAAAGGGCTTGGAGAGCT----------------------------
--------TTTCTTCACATA------CCTGCTAGAATAAGACAGGGCTTAGAAAGGGCTTTGCTA---TAA
>JF932497

GCT---GCA---
GAAAACTTGTGGGTCACAGTCTATTATGGGGTACCTGTATGG---AAAGAAGCAACT---
ACCACTCTATTTTGTGCATCAGATGCTAAAGCATATGATGCAGAG---GTACATAAT---------------
--------------GTTTGGGCCACA---CATGCCTGTGTACCCACAGAC---
CCCAACCCACAAGAAGTAATCTTGGGA------AAAAATGTGACAGAAAACTTTAAC---------------
-------------------------ATGTGGAAAAATAACATGGTAGAACAGATGCATGAAGATATA---
ATCAGTCTATGGGATCAAAGCCTA---AAACCATGTGTAAAATTA---
ACCCCACACTGCATTATTTTAAATTGCACTAATGTGACTATTACTAATACCAGTAATAATACTACTAAT----
----
-------------------ACCAGTAGTACTACCAGTAGTACAATAGAGAGAGAAGAAATAAAA---
AACTGCTCTTTCAATATCACCACAAGCATAAGA--------------------------
ACTAAAACTAAAAAAGAATATGCACTC---
TTTAATAAAATTGATATAGTACCAATAGATGATGATGATGATAAT---------------------------
----
ACCAGTAATACCAGTAATACCAGCTATAGGTTGAGACATTGTAACACCTCAGTCATTACACAA---
GCTTGTCCAAAGGTATCCTTTGAGCCAATTCCCATACATTACTGTGCCCCGGCTGGTTTTGCGATTCTAAAGT
GTAGA---GATAAAAAGTTCAAT------GGAACAGGACTATGTGAAAAT----------------------
-GTCAGT---ACAGTACAATGTACACATGGAATCAGGCCAGTAGTATCAACT---
CAACTGCTGTTAAATGGCAGTTTAGCAGAAGAAGAG------------
GTAGTAATCAGATCTAGCAATTTCTCGAACAAT---------GCTAAAGTCATAATAGTACAGCTGAAG----
-----------GAATCTGTAGAA---------ATTAATTGTACAAGACCCAACAACAATACAAGAAAA-----
-GGGGTAACTCTA------------GGACCAGGGCGAGTA---TGG------TATACAACAGGACAA------
---------ATAATTGGAGATATA------------AGACGAGCACATTGT---AACATTAGTAGAGCA---
AAATGGAATAATACTTTAGATCAGATAACTAAAAAATTAAGA---------------GAACAATTTGAG----
----------------------------------AACAAAACAATAATCTTTAAG------
CAACCTTCAGGAGGGGACTCAGAGATTGTA---ATGCACAGTTTTAATTGTAGAGGAGAA---
TTTAAGCAGCTCCAGGCAAGAGTCCTGGCTGTAGAAAGATACCTGAAGGATCAACAGCTCCTAGGGATTTGGG
GATGCTCTGGAAAACTCATTTGCACCACTGCTGTGCCTTGGAATGTTAGTTGGAGT----------------
---------------------------AATAGGACTCTAGATGAGATTTGG------AAT------
AACATGACTTGGATGAAGTGG---GAAAGAGAAATTGAAAATTACACAAAAGAAATATAC---
ACCTTAATTGAAGAATCGCAAAACCAACAAGAA---------------------------------------
----
AAGAATGAACTAGACCTATTGGAATTGGATAAGTGGGCAAGTTTGTGGAATTGGTTTGACATAACAAAATGGC
TGTGGTATATCAAAATATTCATAATGATAGTAGGAGGCTTGGTAGGTTTAAGAATAGTTTTTGCT---
GTACTTTCTATAGTG---------AATAGAGTTAGGCAGGGATACTCACCA------
TTGTCATTTGCTATTAGCTTGCTTAACGCCACAGCCATAGCAGTAGCTGAG---
GGGACAGATAGGATTATAGAAGTAGCACAAAGAGCTTATAGGGCT---------------------------
--------ATTCTCAACATA------CCTACAAGAATCAGACAGGGACTCGAAAGGGCTTTGCTA---TAA
>KX693642
GCT---GCA---
GAAAACTTGTGGGTCACAGTCTACTATGGGGTACCTGTATGG---AAAGAAGCAACT---
ACCACTCTATTTTGTGCATCAGATGCTAAAGCATATAAGACAGAG---GTACATAAT---------------
--------------GTTTGGGCCACA---CATGCCTGTGTACCCACAGAC---
CCCAACCCACAAGAAGTAGTCTTGGTA---------AATGTGACAGAAAATTTTAAC---------------
-------------------------ATGTGGAAAAATGACATGGTAGAACAGATGCATGAAGATATA---
ATCAGTTTATGGGATCAAAGCCTA---AAGCCATGTGTAAAATTA---
ACTCCACTCTGTGTTACTTTAAATTGCACTGATGTGAGTAATGCTAATAATACCTGTAATAATACCAATATTA
CTAGAAGTAGTAATGCTACCTGTAATAATACCAGT-------------------------------------
------------------------AGTACTAATAGTAGTATGACAGAGGAAGGAGACATGAAA---
AACTGCTCTTTCGATTTCACCACAAGCATGAGT--------------------------ACTAAGGTA---
AAAGACTATGCACTT---TTTTATAAACTTGATGTAGTACCAGTAAATAAT---------------------
----
-------ACCAATAATACCGGTTATAGGTTGATAAGTTGTAATACCTCAGTCATTACACAA---
GCTTGTCCAAAGATATCCTTTGAGCCAATTCCCATACATTATTGTGCCCCGGCTGGTTTTGCGATTCTAAAGT
GTAAC---AATAAAACATTCAAT------GGAACAGGACCATGTAAAAAT----------------------
-GTTAGT---ACAGTACAATGTACACATGGAATTAGGCCAGTAGTGTCAACT---
CAACTACTGTTAAATGGCAGTTTAGCAGAAGAAGGG------------
GTCGTAATTAGATCTAGCAATTTCTCGGACAAT---------GCTCAAGTCATAATAGTACAGCTGACT----
-----------AACTCTGTAAAC---------ATTAGTTGTGAAAGACCCAACAACAATACAAGAAAA-----
-AGTATAAATATA------------GGACCAGGGCGAGCA---TGG------TATGCAACAGGAAAC------

```
---------GTAATAGGAGATATA------------AGACAAGCACATTGT---AACCTTAGTAAAGCA---
GATTGGAATAACACTTTAAGACAGATATCTAAAAAATTAAGA---------------GAACAATTTAAG----
-----------------------------------AACAAAACAATAATCTTTAAG------
CAATCTTCAGGAGGGGACCCAGAGATTACA---ATGCACAGTTTTAATTGTGGAGGGGAA---
TTTAAGCAGCTCCAGGCAAGAGTCTTGGCTGTGGAAAGATACCTAAAGGATCAACAGCTCCTAGGGATTTGGG
GTTGCTCTGGAAAACTCATTTGCACCACTGCTGTGCCTTGGAATGAGAGTTGGAGT-----------------
----------------------------AATAAAACTATGGATCAGATTTGG------AAT------
AACATGACTTGGATGGAGTGG---GAAAGAGAAATTGACAATTACACAAGAGAAATATAC---
ACCTTAATCGAACTATCGCAGACCCAACAAGAA----------------------------------------
-----------------------------
CAGAATGAACTAGAACTATTGGAATTGGATAAATGGGCAAGCTTGTGGAATTGGTTTGACATAACAAATTGGC
TGTGGTATATCAAAATATTCATAATGATAGTAGGAGGCTTGGTAGGTTTAAGAATAGTTTTTGCT---
GTACTGTCTGTAGTA---------AATAGAGTTAGGCAGGGATACTCACCA------
TTGTCATTTGCTATTAGCTTGTTTAACGCCACAGCCATAGCAGTAGCTGAG---
GGGACCGATAGGGTTATAGAAATAGCACACAGAGCTTTTAGGGCT----------------------------
--------ATTCTCAACATA------CCTACAAGAATCAGACAAGGCCTCGAAAGGGCTTTGCTG---TAA
>KX693171
GCT---GCA------------------------------------------
GAAAAATTGTGGGTCACAGTCTATTATGGGGTACCTGTATGG---AAAGAAGCAACT---
ACCACTCTATTTTGTGCATCAGATGCTAAAGCATATGAGACAGAG---GTCCATAAT----------------
--------------GTTTGGCCACA---CATGCCTGTGTACCCACAGAC---
CCCAACCCACAAGAAGTAGTCTTGGAG---------AATGTGACAGAAAATTTTAAC----------------
--------------------------ATGTGGAAAAATGACATGGTAGAACAAATGCATGAAGATATA---
ATCAGTTTATGGGATCAAAGCCTA---AAGCCATGTGTAAAATTA---
ACCCCACTCTGTGTTACTTTAAATTGCACTGATGATTTGACTAATTTGAGGAATACTACTAATAACAGTACTA
CTACTAACAGTAGTACGACAGAG--------------------------------------------------
----------GGGAATGCTACTACTACTAATAACAGTAGTACGATAGAGAGAGGAGAAATAAAA---
AACTGCTCTTTCGATATCACCACAAGCATACAG---------------------------ACTAAGAGG---
AAAGACTATGCACTT---TTTTATAAATATGATATAGTAAAAATAGATAATGATGATATAGATAAT-------
-----------------------------------------------------------------
GGTAATATCAGCCATAAGGATAATACCAGCTATAGGTTGATAAATTGTAACACCTCAGTCATTACACAA---
GCTTGTCCAAAGATATCCTTTGAGCCAATTCCCATACATTATTGTACCCCGGCTGGTTTTGCGATTCTAAAGT
GTAAC---AATAAAAGGTTCAAT------GGAACAGGGCCATGTACAAAT--------------------------
-GTCAGT---ACAGTACAATGTACACATGGAATTAGGCCAGTAGTATCAACT---
CAACTGCTATTAAATGGCAGTTTGGCAGAAGAAGAG------------
GTAGTAATTAGATCTAGCAATTTCACAGACAAT---------GCTAGAATCATAATAGTACAGCTGAAT----
-----------AAATCTGTAGAA---------ATTAATTGTACAAGACCCAACAACAATACAAGAAAA-----
-AGTATAAATATA------------GGACCAGGGCGAGCA---TGG------TATGCAACAGGAGAA------
---------ATAATAGGAGATATA------------AGACAAGCACATTGT---AACCTTAGTAGAACA---
GCATGGAATAACACTTTAAAACAGATAACTGAAAAATTAAGA---------------GAGGAATTTGGG----
-----------------------------------AACAAAACAATAATCTTTAAT------
CAATCTGCAGGAGGGGACCCAGAAATCGTA---ATGCACAGTTTTAATTGTGGAGGGGAA---
TTTAAGCAGCTCCAGGCGAGAGTCTTGGCTGTGGAAAGATACCTAGGGGATCAACAGCTCCTAGGGATTTGGG
GTTGCTCTGGAAAACTCATTTGCACCACTGTTGTGCCTTGGAATGCTAGTTGGAGT-----------------
----------------------------AATAGATCCCTGAATGAGATTTGG------AAT------
AACATGACTTGGATGGAGTGG---GAAAGAGAAATTGACAATTACACAAAAGAAATATAC---
ACCTTAATTGAAATATCGCAGAACCAACAAGAA----------------------------------------
-----------------------------
AAGAATGAACTAGAACTATTGAAATTGGATGAGTGGGCAAGCTTGTGGAATTGGTTTGACATAACAAAATGGC
TGTGGTATATCAAAATATTCATAATGATAGTAGGAGGCTTGGTAGGTTTAAGAATAGTTTTTGCT---
GTACTTTCTATAGTA---------AATAGAGTTAGGCAGGGATATTCACCA------
TTGTCATTAGCTATTAGCCTGCTTAATACCTCAGCTATAGCAGTAGCTGAG---
GGGACAGATAGGGTTATAGAAGTAGCACAAAGAGCTTATAGGGCC----------------------------
--------ATTCTCCACATA------CCTAGAAGAATCAGACAGGGCCTCGAAAGGGCTTTGGTA---TAA
>KX693309
GCT---GCA------------------------------------------
GAAAACTTGTGGGTCACAGTCTATTATGGGGTACCTGTATGG---AAAGAAGCAACT---
ACCACTCTATTTTGTGCATCAGATGCTAAAGCATATGCTACAGAG---GTACATAAT----------------
--------------GTTTGGCCACA---CATGCCTGTGTACCCACAGAC---
CCCAACCCACAAGAAGTAGTCTTGGGA---------AATGTGACAGAAAACTTTAAC----------------
```

-------------------------ATGTGGAAAAATAACATGGTAGACCAGATGCATGAAGATATA---
ATCAGTTTATGGGATCAAAGCCTA---AAGCCATGTGTAAAATTA---
ACCCCACTCTGTGTTACTTTATATTGCACTAATGCTACTATTACCAGTAATAATACCAGTAGTAATACT----
----
------------------ACCAGTAGTAATACTAGTAGTACGATAGAGGGAGGACAAATGAAA---
AACTGCTCTTTCAATGTCACCACAAGCATAGGA-------------------------ACTAAAGTG---
AGCGACTATGCACTT---TTTTATAAACTTGATGTAGAACCAATGAATAAT-------------------
----
----GATACTAATACTACCAGCTATAGGTTGATAAATTGTAACACCTCAGTCATTACACAA---
GCTTGTCCAAAGGTATCCTTTGAGCCAATTCCCATACATTATTGTGCCCCGGCTGGTTTTGCGATTCTAAAGT
GTAAC---AGTAAAAAGTTCAAT------GGAACAGGACCATGTACAAAT--------------------
-GTCAGT---ACCGTACAGTGTACACATGGAATTAGGCCAGTAGTATCAACC---
CAACTGCTGTTAAATGGCAGTTTAGCAGAAGAAGAG-----------
GTAGTGATTAGATCCAGCAATTTCACGGACAAT---------GCTAAAGTCATAATAGTACAGCTAAAT----
-----------GAATCTGTAGAA---------ATTAATTGTACAAGACCCAACAACAATACACTGAAA-----
-AGTATACATCTA------------GGACCAGGGCGAGCA---TGG------TATACAACAGGACAA------
---------ATAATAGGAGATATA-----------AGACAAGCACATTGC---AACATTAGTAGAACA---
AAATGGAATAACACTTTAAGACTGGTAGCTGAAAAATTAAAA--------------GAAATATAC-------
----------------------------------CACAAAACAATAATCTTTAAT------
CGATCTTCAGGAGGGGACCCAGAGATTGTA---ATGCACAGTTTTAATTGTGGAGGGGAA---
TTTAAGCAGCTCCAGGCAAGAGTCTTGGCTGTGGAAAGATACCTAAAGGATCAACAGCTCCTAGGAATTTGGG
GATGCTCTGGAAAACTCATCTGCACCACTGCTGTGCCTTGGAATACTAGTTGGAGT----------------
---------------------------AATAGATCTCTGAGTGAGATTTGG------GAT------
AACATGACTTGGATGGAGTGG---GAAAGAGAGATTGACAATTACACAAAAGAAATATAC---
ACATTAATTGAACAATCGCAGAACCAACAAGAA-------------------------------------
----
AAGAATGAACTAGACCTATTGGAATTGGATAAGTGGGCAAGTTTGTGGAATTGGTTTGACATATCAAACTGGC
TGTGGTATATCAAAATATTCATAATGGTAGTAGGAGGCTTAGTAGGTTTAAGAATAGTTTTTGCT---
GTACTTTCTATAGTA---------AATAGAGTTAGGCAGGGATACTCACCA------
TTGTCATTAGCTATTAGCTTGTTTAACGCCACAGCAGTAGCAGTAGCTGAG---
GGGACAGATAGGGTTATAGAAGTAGCACAAAGAGCTTTTAGGGCT-------------------------
--------GTTCTCAACGTA------CCTAGAAGAATCAGACAGGGCTTCGAAAGGGCTTTGCTA---TAA
>KX693362
GCT---GCA--
GAAAACTTGTGGGTCACAGTCTATTATGGGGTACCTGTATGG---AAAGAAGCAACT---
ACCACTCTATTTTGTGCATCAGATGCTAAAGCATATGATACAGAG---GTACATAAT---------------
------------GTTTGGGCCACG---CATGCCTGTGTACCCACAGAC---
CCCAACCCACAAGAAGTAGTCTTGGGA---------AATGTGACAGAAAACTTTAAC-------------
-------------------------ATGTGGAAAAATGACATGGTAGAACAGATGCATGAAGATATA---
ATCAGTTTATGGGATCAAAGCCTA---AAGCCATGTGTAAAATTA---
ACCCCACTCTGTGTTACTTTAAATTGCACTAATTTGAATACTACTATTACCAGTAGTAATACCAGTAGTACCA
AT--
------------ACCAGTACCAGTAGTAATAACAGTAGTATGATAGAGGGAGGAGAACTAAAA---
AATTGCTCTTTCAATATCACCACAAGCATAAAT-------------------------ACTAGGGTG---
AAAGACTATGCATTT---TTTTATAAAGTTGATATAGAACCAATGAATAAT-------------------
----
----GATACTAATACTACCAGCTATAGGTTGATGCATTGTAACACCTCAGTCATTACACAA---
GCTTGTCCAAAGATATCCTTTGAGCCAATTCCCATACATTATTGTACCCCGGCTGGTTTTGCGATTCTAAAGT
GTAAC---AATAAAACGTTCAAT------GGAACAGGACCATGTACAAAT--------------------
-GTCAGT---ACAGTACAATGCACACATGGAATTAGGCCAGTAGTATCAACT---
CAACTGCTGTTAAATGGCAGCTTAGCAGAAGAAGAG-----------
GTAGTAATTAGATCTAGCAATTTCACAGACAAT---------ACTAAGATCATAATAGTACAGCTGAAT----
-----------GAATCTGTAGCA---------ATTAATTGTACAAGACCCAACAACAATACAAGAAAA-----
-AGAATATCTCTA------------GGACCAGGGCGAGTG---TGG------TATACAACAGGAGAA------
---------ATAATAGGAGATATA-----------AGAAAAGCATATTGT---AACCTTAGTAGAATA---
GCATGGAATAACACTTTAAGACAGATAACTGAAAAATTAAAA--------------GGACAATTTGGG----
----------------------------------AACAAAACAATAATCTTTAAT------
CACTCTTCAGGAGGGGACCAAGAGATTGTA---ATGCACAGTTTTATTTGTGGAGGGGAA---
TTTAAGCAGCTCCAGGCAAGAGTCTTGGCTGTGGAAAGATACCTAAAGGATCAACAGCTCCTAGGGCTTTGGG
GTTGCTCTGGAAAACTCATCTGCACCACTGCTGTGCCTTGGAATGCTAGTTGGAGT----------------

----------------------------AATAAATCTCTGAGTGAGATTTGG------AAT------
AACATGACTTGGATGGAGTGG---GAAAGAGAGATTGACAATTACACAAGAGAAATCTAC---
AACTTAATTGAGGAATCGCAGAACCAACAAGAA--

AAGAATGAACTAGAACTATTGGAATTGGATAAGTGGGCAAGTTTGTGGAATTGGTTTGACATAACACAATGGC
TGTGGTATATCAAAATATTTATAATGATAGTAGGAGGCTTGGTAGGTTTAAGAATAGTTTTTGCT---
GTACTTTCTGTAGTG---------AATAGAGTTAGGCAGGGATACTCACCA------
TTGTCATTAGCTATTAGCTTGCTTAACGCTACAGCCATAGCAGTAGCTGAG---
GGGACAGATAGGGTTATAGAAGTAGCACAAAGAGCTTATAGGGCT----------------------------
--------ATTCTCAACATA------CCTACAAGAATCAGACAGGGCCTCGAAAGGGCTTTGGTA---TAA
>KX693411
GCT---ACA--
GAAAAATTGTGGGTCACAGTCTATTATGGGGTACCTGTATGG---AAAGATGCGACT---
ACCACTCTATTTTGTGCATCAGATGCTAAAGCATATGATACAGAG---GTTCATAAT----------------
--------------ATTTGGGCCACA---CATGCCTGTGTACCCACAGAC---
CCCAACCCACAAGAAGTAGTTTTGGGA---------AATGTGACAGAAAACTTTAAC----------------
--------------------------ATGTGGAAAAATGAGATGGTAGAACAGATGCATGAAGATATA---
ATCAGTTTATGGGATCAAAGCCTA---AAGCCATGTGTAAAATTA---
ACCCCACTCTGTGTCACTTTAAATTGCACTAATGCTACCGGTAGCCATAATGTTAGTACTACCAGTACTCCTA
CCACCAGTAGT--
----ACCGGTGGTAATACCAATAGTACTACCAGTAGTCCGATAGAGGGAGGAGGAGAAATAAAA---
AAATGCTCTTTCAATATCACCACAAGCATAGGA---------------------------ACTAAGGTG---
AAAGACTATGCACTT---TTTAATAGACTTGATATAGTACCAATAAAAGATGATAGTACCAGCAATAAT----
----
AGTACCAGCAATAATAGTACCAACAATGCCAGCTATAGGTTGATAAATTGTAACACCTCAGTCATTACACAA-
--
GCTTGTCCAAAGGTATCCTTTGAGCCAATTCCCATACATTATTGCACCCCGGCTGGTTATGCGATTCTAAAGT
GTAAC---AATAAAACGTTCAGT------GGAACAGGACCATGTACAAAT-----------------------
-GTCAGT---ACAGTACAATGTACACATGGAATCAGGCCAGTAGTATCAACT---
CAACTGCTGTTAAATGGCAGTCTAGCAGAAGAAGAG------------
GTAGTAATTAGATCTAGCAATTTCACGGACAAT---------GCTAAAATCATAATAGTACAGCTGAAT----
-----------GTATCTGTACAA---------ATTAATTGTACAAGACCCAACAACAATACAAGAAAA-----
-AGAGTAACCCTA------------GGACCAGGGCGAGTA---TGG------TATACAACAGGACAA------
---------ATAATAGGAGATATA------------AGACGAGCACATTGT---AACCTTAGTAGTACA---
GCATGGAATAAAACTTTAGAACAGGTAGCTAAAAAATTAGGA---------------GAACAATTTAAG----
-----------------------------------AACAAAACAATAGTCTTTACT------
CAACCTTCAGGAGGGGACCCAGAGATTGTA---ATGCACAGCTTTAATTGTGGAGGGGAA---
TTTAAGCAGCTCCAGGCAAGAGTCCTGGCTGTGGAAAGATACCTAAAGGATCAACAGCTCCTAGGGATTTGGG
GTTGCTCTGGAAAACTCATTTGCACCACTGCTGTGCCTTGGAATGCTAGTTGGAGT-----------------
----------------------------AATAAATCTCTGAATGAGATTTGG------GAT------
AATATGACTTGGATGGAGTGG---GAAAGAGAAATTGAAAAGTACACAGGAGAAATATAC---
AACTTAATTGAAGAATCGCAGAACCAACAGGAA--

AAGAATGAGCAAGAACTATTGGAATTGGATAAGTGGGCAAGCTTGTGGAATTGGTTTAGCATAACAAACTGGC
TGTGGTATATCAGAATATTCATAATGATAGTAGGAGGCTTGGTAGGTTTAAGAATAGTTTTTGCT---
GTACTTTCTATAATA---------AATAGAGTTAGGCAGGGATACTCACCA------
TTGTCATTAGCTATTAGCTTGCTTAACGCCACAGCCATAGCAGTAGCTGAG---
GGGACAGATAGGATTATAGAAATAGTACAAAGAGCTTGTAGGGCT----------------------------
--------ATTCTCAATATA------CCTACAAGAATCAGACAAGGCCTCGAAAGGGCTTTGCTA---TAA
>KR914676
GCT---AAA--
GAACAATTGTGGGTAACAGTCTATTATGGGGTAACTGTGTGG---AAGGGGCTA------
CCTGTGTGGAAAAATGCAACCACCACTTTATTTTGTGATACAGAG---CTACATAAT----------------
--------------GTCAGGGACAAA---TGTGCCTGTGTACCCACAGAC---
CCCAACCCACAAGAAGTAGTAATGGGA---------AATGTGACAGAATATTTTAAC----------------
--------------------------ATGTGGAAAAATGACATGGTAGAGCAGATGCATGAGGATATA---
ATCAGTTTATGGGATCGAAGCTTA---AAGCCATGTGTAATATTC---
ACCCCACTCTGTGTCACTTTAAATTGCAATGATTTGAAGAATGTTACTAATACCAATTGT-------------
----
-------------------------AGTAGCGGGAGAGGGATAGTTGAGAAACTAGATATATCA---

AACTGCTCTGTCAGTATCAGCACATTTATAACA---------------------------
GGTCACGTAGTGATAGCAGATGAAGTT---ATGGATACAGATGACATACTACTAGTGTCTATT----------
----

GATTCTATCAACAATAAGTTGACTCTCTGTAACACCTCACTCATGACACAG---
GCCTGTCGATCAGAACGGTGTAAGCCAATTCCCATACATTATTGTGCCCCGGCCGGTTTTGCGATTCTAAAGT
GTAAC---AATAAGACATTCAAT------GGAAAAGGACCATGTAAAAT------------------------
-GTTAGC---ACAGTACAATGTACACATGGAATTAAGCCAGTAGTTTCAACT---
CAACTGCTATTAAATGGCAGTCTAGCAGAAGAAGAG------------
GTAGTAATAAGATCTGAAAATTTCACAGACAAT---------ACTAAAACCATAATAGTACAGCTGAAT----
-----------GAATCTGTGGTA---------ATTAATTGTACTAGACCCAGCAACAATACAAGAAAA-----
-AGCATACGTATCCAT------AGAGGACCAGGGCAAGCA---TTC------TTTGCAACAGGA---------
---------AAAATAGGAGATATA------------AGACAAGCACATTGT---AACCTTAGTAGAGCA---
GAATGGAATAAAACTTTAGCACAGATAGCTAAAAAATTAGGC---------------AAACAATTTGGA----
--------------------------------GAAAATAAAACAATATCCTTTCAG------
CCATCCTCAGGAGGGGACATAGAAATTGTA---ACACACAGTTTTAATTGTGGAGGGGAA---
TTTAAACAGCTCCAGGCAAGAGTCCTGGCTGTGGAAAGATACCTAAAGGATCAACAGCTCCTGGGAATTTGGG
GGTGCTCTGGAAAACTCATTTGCACCACTGCTGTGCCTTGGAATGCTAGTTGGAGT-----------------
----------------------------AATAAATCCCTGGAGTACATTTGG------GAT------
AATATGACCTGGATGGAGTGG---GAAAGAGAAATTAACAATTACACAAGATTAATATAC---
CACTTAATTGAAGAATCGCAGAACCAACAAGAA--

AAGAATGAAAAGGAATTATTGGAATTGGATAAATGGGCAAGTTTGTGGAATTGGTTTGACATAACAAAATGGC
TGTGGTATATAAAGATATTTATAATGATAGTAGGAGGCTTAATAGGATTAAGAATAGTTTTTGTA---
GTACTTTCTATAATA---------AATAGAGTTAGGCAGGGATATTCACCA------
CTCTCGTTTGCTGTTAGCTTGCTCAACGCCACAGCAGTAGCAGTAGCAGAA---
GGGACAGATAGGATTATAGAAATAGTGCAAAGAGCTTGTAGAGCT----------------------------
--------TTTCTCCACATC------CCTAGAAGAATAAGACAGGGCTTCGAAAGGCTTTTGCTA---TAA
>FJ388899
GCT---ACA--
TAAGACTTGTGGGTCACAGTCTATTATGGGGTACCTGTGTGG---AGAGAAACAAT---
ACCACTCTATTTTGTGCATCAAATGCTAAAGCCTATGATACAGAG---GTACATAAT----------------
--------------GTTTGGGCCACA---CATGCCTGTGTACCCACAGAC---
CCCAACCCAAAAAAAATAAAATTGGAA---------AATGTAACAGAAGACTTTAAC----------------
--------------------------ATGTGGGAAAAGGGCATGGAAAAACAGATGCATAAAGACATA---
ATCAGTTTATGGGATCAAAGCCTA---AAGCCATGTGTAAAATTA---
ACCCCACTCTGTGTTACTTTAAATTGCACTGATTGTAAGTTGAATGATACTAAATCTGGTACGAATAGTACTG
AATCT--
-------AGTTTGAATTGTCCTAATATTACTAATACCACTGATATGAAAAAAGGAGAAATTAAA---
GAATGCTCTTTCAACGTCATCACAGGCATAAGA---------------------------
GACAAGGTGCAGAAAAAATTATGCACTT---TTTTATGACACTGATGTAACACCAATAGATAAC---------
----

ACCAGCTATATACTGAGAAGTTGTAACACCTCAATCATTACACAG---
GCCTGCCCAAAGGTAACTTTTGAGCCAATTCCCATACATTATTGTGCCCCGGCTGGTTTTGCGATTCTAAAGT
GTAGA---GATAAGAAGTTCAAT------GGAACAGGACCGTGTAAAAT------------------------
-GTCAGT---ACAGTACAATGTACACATGGAATTAGGCCAGTAGTATCAACT---
CAACTGCTGTTGAATGGCAGTCTAGCAGAAGAAGAG------------
GTAATAATTAGATCTGAAAATATCTCGAACAAT---------GCTAAAAACATAATAGTACAGCTGAAA----
-----------GAACCTATAGAA---------ATTAATTGTACAAGACCCAGCAACAATACAAGCAAA-----
-AGCATACATCTA------------GGACCAGGGGGAGCA---TTT------TATGCAACAGAAAGA------
---------ATAATAGGAGATATA------------AGACACGCATATTGT---AACATTAATAAAGGA---
AAATGGGAGGAAACTTTAAAGCAGATAGCTGCAAAATTAGGA---------------GAACAATTTGGG----
-----------------------------------AACAGGACAATAAGCTTTAAT------
CAATCCTCAGGAGGGGATCCAGAAATTGTA---CAGCACAGTTTTAATTGTGGAGGGGAG---
TTTAAGCAGCTCCAGGCAAGAGTCCTGGCTGTGGAGAGATACCTAAAGGATCAACAGCTCCTGGGAATTTGGG
GTTGCTCTGGAAAACTCATCTGCACCACTACTGTGCCTTGGAATGCTAGTTGGAGC-----------------
----------------------------AATAAATCTCTAAATGACATTTGG------GAT------
AACATGACCTGGATGCAGTGG---GACAGAGAAATTAACAATTACACAAGCTTAATATAC---
TCCTTAATAGAAGAATCGCAAAACCAACAAGAA--

AAGAATGAACTAGAATTATTAGAACTAGATAAATGGGCAAGTTTGTGGAGTTGGTTTGACATAACAAATTGGC
TGTGGTATATAAAAATATTCATAATGATAGTAGGAGGCTTGGTAGGTTTAAGAATAGTTTTTACT---
GTACTTTCTGTAGTG---------AATAGAGTTAGGCAGGGATACTCACCA------
TTATCGTTTGCTATTACCTTGCTTAACGCCACAGCAATAGCAGTAGCTGAG---
GGGACAGATAGGGTTATAGAAGTATTACAAAGAATTTTTAGGGCT----------------------------
--------ATTATCCACATA------CCTAGAAGAATAAGGCAGGGCTTGGAAAGGGGTTTGCTA---TAA
>FJ388916
GCT---AAA---GAA------------------------------------
GAAAATCTGTGGGTCACAGTCTATTATGGGGTACCTGTGTGG---AAAGAAGCAACT---
ACCACTCTATTTTGTGCATCAGATGCTCAAGCACATAACACAGAG---GCACATAAT----------------
--------------GTTTGGGCCACA---CATGCCTGTGTACCCACAGAT---
CCTAACCCACAAGAAGTAGTAATGGAA---------AATGTAACAGAAAATTTTAAC----------------
--------------------------ATGTGGAAAAATAACATGGTAGAACAGATGCAGGAGGATATA---
ATTAGTTTATGGGATCAAAGCCTA---AAGCCATGTGTAAAATTA---
ACCCCACTTTGTGTTACTTTAAATTGCACAGATGAGTTGAATATTAATGGGACTACTGGTAATGGGAATGGG-
----
-------------ACTACAGCCAGTAATAGTAGTATTGAAGAAGGGATGAAAGGAGAAATAAAA---
AACTGCTCTTTCAATGTCACCAAAAGCATAGGA---------------------------
AATAAGCTGCAGAAAGAATATGCACTT---TTTTATAAACTTGATGTAGTACAAATAGAAGATGATAATACA-
----

AAAGATAATAATACAACTAAAATATATACATTGATAAATTGTAACACCTCAGTCATTACACAG---
GCCTGTCCAAAGGTAACCTTTGAACCAATTCCCATACATTATTGTGCCCCGGCTGGTTTTGCGATTCTAAAGT
GTAAT---GATAAAAAGTTCAAT------GGAACAGGACCATGTAAAAAT-----------------------
-GTCAGC---ACAATACAATGTACACATGGAATTAAGCCAGTAGTGTCAACT---
CAATTACTGTTAAATGGCAGTCTAGCAGAAGAAGAG------------
GTAGTGATCAGATCTGAAAATTTCACAGACAAT---------GCTAAAAACATAATAGTACAGCTGAAG----
-----------GTACCTGTAGAA---------ATTAATTGTACAAGACCCCACAACAATACAAGAAAA-----
-AGTATACCTATA------------GGACCAGGGAGAGCA---TTT------TATGCAACAGGAGAC------
---------ATAATAGGAGATATA------------AGGCAAGCACATTGT---AACATTAGTAGTAAA---
AAATGGAATGAAACTTTAGAACGTGTAGTTACAAAATTAGGA---------------GAACAATTTGGG----
-----------------------------------AACAAAACAATAATTTTTAAA------
CACTCCTCAGGAGGAGACCCAGAAATTGTA---ATGCACAGTTTTAATTGTGGAGGGGAG---
TTTAAGCAGCTCCAGGCAAGAGTCCTGGCTGTAGAAAGATACCTACAGGATCAACAGCTCCTAGGGATTTGGG
GTTGCTCTGGAAAACTCATTTGCACCACTGCTGTGCCTTGGAATGCTAGTTGGAGT-----------------
-------------------------AATAATAAATCTTATAATGACATTTGG------GAT------
AACATGACCTGGATGGAGTGG---GAGAGAGAAATTGACAATTACACAGGCTTAATATAC---
AGCTTACTTGAAAAATCACAGAACCAACAAGAA--

AAGAATGAACAAGAATTATTGGAATTGGATAAGTGGGCAAATTTGTGGAATTGGTTTAGCATAACAAACTGGC
TGTGGTATATAAGAATATTCATAATGATAATAGGAGGTTTAATAGGTTTAAGAATAGTTTTTGCT---
ATACTTTCTGTAGTG---------AATAGAGTTAGGCAGGGATACTCACCA------
TTATCGTTTGCTGTTAGTTTGCTTAATACCATAGCTATAGCAGTAGCTGAG---
GGGACAGATAGGATTATAGAAGTAGCACAAAGAGCTGGTAGAGCT----------------------------
--------ATTCTCCACATA------CCTAGAAGAATAAGACAAGGCTTAGAAAGGTCTTTACTA---TAA
>JF683738
GCT---TCA---
AATCTTTGGGTCACAGTCTATTATGGGGTACCTGTGTGG---AAAGAAGCAACA---
CCCACTTTATTTTGTGCATCTGATGCTAAAGCATATGACACAGAG---GTACATAAT----------------
--------------GTTTGGGCCACA---CATGCCTGTGTACCCACAGAT---
CCTAGCCCACAAGAAATGGTATTAGAA---------AATGTGACAGAAAATTTTAAC----------------
--------------------------ATGTGGAAAAATTATATGGTAGAACAGATGCAAGAGGATATA---
ATCAGTTTATGGGATGAAAGCCTA---AAGCCATGTGTAAAATTA---
ACTCCACTCTGTGTTACTTTAAATTGCACTAACGCGAATCTTACTAGTACCAAT-------------------
----
-------------------------------AGTTCCATGGGGATGGAAGCAGGAGAAATAAAA---
AACTGCTCTTTCAAGATCACCACAAGCATAAAA---------------------------
GATAAGACAAAGAATGAATATGCACTC---TTTTATAAACTTGATGTAACACCAATAGATAAT----------
----

AATAGTACAAATAGTACAAACTATACTTTGATAAATTGTAACACCTCAGCCATTACACAA---
GCCTGTCCAAAGGTATCCTTTGAACCAATTCCCATACATTATTGTGCCCCGGCTGGTTATGCGATTCTAAAGT
GTAAG---GATAAGAAGTTCAAT------GGGACAGGACCATGTAAAAAT----------------------
-GTTAGC---ACAGTACAATGTACACATGGAATTCGGCCAGTGGTGTCAACT---
CAACTGCTGTTAAATGGCAGTCTAGCAGAAGAAGAG-----------
GTAGTAATTAGATCTGAAAACTTCACAAACAAT---------GCCAAAACCATAATAGTACAGCTGAAA----
-----------GAACCTATAAAC---------ATTACTTGTATCAGACCCGGCAACAATACAAGAAAA-----
-AGTATACATATA------------GGACCGGGAAGGGCA---TTT------TATGCAACAGGAGAC------
---------ATAATAGGAAATATA------------AGACAAGCACATTGT---AACCTTAGTGAAACA---
AAATGGAATAACACTTTAAAACAGATAGTTACAAAATTAAGA---------------GAAAAATTTGAA----
---------------------------------AATAAAACAATAGCCTTTAAT------
CAATCCTCAGGAGGGGACCCAGAAATTACA---ATGCACAGTTTTAATTGTGGAGGGGAA---
TTTAAACAGCTCCAGGCAAGAGTCCTGGCTGTGGAAAGATACCTACAGGATCGACAGCTCCTAGGGCTTTGGG
GTTGCTCTGGAAAGCTCATCTGCACCACTTCTGTACCTTGGAATGCTAGTTGGAGT----------------
----------------------------AATAAATCTCAGGAGGAGATTTGG------GGT------
AACATGACCTGGATGCAGTGG---GAGAGAGAAATTGGAAATTACACAGAATTAATATAC---
ACCTTACTTCAAGAATCGCAGTACCAACAAGAA--

AAGAATGAACAAGAATTATTGGAATTAGATAAGTGGGCAAGTTTGTGGAATTGGTTTGACATATCAAAATGGC
TGTGGTATATAAAAATATTCATAATGATAGTAGGAGGTTTAGTAGGTTTAAGAATAATTTTTGCT---
GTACTTTCTATAGTG---------AATAGAGTTAGGAAGGGATACTCACCA------
TTATCATTCGCCATTAGCTTGCTTAATACTACAGCTATAGTAGTAGCTGAG---
GGAACAGATAGAATTATAGAAGTAGTACAAAGATTTTGTAGAGCT---------------------------
--------ATTCTCCACATA------CCTAGAAGAATAAGACAGGGTTTAGAAAGAATTTTGCTA---TAA
>JF683756
GCT---GCA--
GACAAACTGTGGGTCACAGTCTATTATGGAGTACCTGTGTGG---AAAGAAGCAACC---
ACCACTCTATTTTGTGCATCAGATGCTAAAGCATATGACACAGAG---GTACATAAT----------------
--------------GTTTGGGCCACA---CATGCCTGTGTACCCACAGAC---
CCTAGCCCACAAGAAGTAGAATTGAAG---------AATGTGACAGAGAATTTTAAC---------------
--------------------------ATGTGGAAAAATAACATGGTAGAACAGATGCATGAGGATATA---
ATCAGTTTATGGGATCAAAGCCTA---AAGCCATGTGTAAAATTA---
ACCCCACTCTGTGTTACTTTAAATTGCACTGATGTGAATAATACCAATAGTACCAATGTG------------
----
----------------------------AATAGTAGTGACCAGATAGAGGCGGGAGAAATAAAA---
AACTGCACCTTCAATATTACTAATAGCATAGGA-------------------------
AATAAGCTGCAAAAAGAATATGCACTT---TTTTACAAGCTTGATATAATGTCATTAGATAAT----------
----

AATAATGGTACTTATACGTTAAGAAGTTGTAACACCTCAGTCATTACACAG---
GCCTGTCCAAAGGTATCCTTTGAACCAATTCCTATACATTATTGTACCCCGGCTGGTTTTGCGATTCTAAAGT
GTAAT---GATAAACAGTTTAAT------GGATCAGGGCTATGTCAAAAT----------------------
-GTCAGT---ACAGTACAATGTACACATGGAATTAGGCCAGTAGTGTCAACT---
CAACTACTGTTAAATGGCAGTCTAACAGAAGGGGAT-----------
GTAGTAATTAGGTCTCAAAATTTCACAAACAAT---------GCTAGAACCATAATAGTACAGCTGAAC----
-----------GAAACTGTAAAA---------ATTAATTGTACAAGACCCAACAACAATACAAGAAGA-----
-AGTATACATATA------------GGACCAGGGAGAGCA---TTT------GAGACAACAGGAGCC------
---------ATAATAGGAGATATA------------AGACAAGCACATTGT---AACATTAGTGGCACA---
GAATGGAATAACGCTTTAAAACAAGTAGTTAGAAAATTAGGA---------------GAACAATTTAGG----
------------------------------AATGCGCAAAACATAACCTTTCAG---
CAACCATCCCCAGGAGGGGACCCAGAAATTATA---TTGCACAGTTTTAACTGTGGAGGGGAA---
TTTAAGCAGCTCCAGGCAAGAGTCCTGGCTGTGGAAAGATACCTAAAGGATCAACAGCTCCTAGGAATTTGGG
GTTGCTCTGGAAAACTCATCTGCACCACTACTGTGCCTTGGAATACTAGTTGGAGC-----------------
----------------------------AATAAATCTTTCAATGAGATTTGG------AAT------
AACATGACCTGGGTACAGTGG---GAGAGAGAAATTGACAATTACACAAGCACAATATAT---
TCCTTGATTGAACAATCGCAAAACCAACAAGAA--

AAGAATGAACAAGAATTATTGGAATTGGATAAGTGGGACAGTTTATGGTCTTGGTTTAGTATAACACAATGGC
TGTGGTATATAAGAATATTCATAATGATAGTAGGAGGCTTGATAGGTTTAAGAATAGTTTTTGCT---

GTACTTTCTATAATA---------AAAAGAGTTAGGCAGGGATACTCACCA------
TTGTCGTTCGCATTTTAGTTTGCTTAGCT-------ATAGCAGTAACTAAG---
GGAACAGATAGGGTTATAGAAGTAGTACAAAGAGCTTGTAGAGCT----------------------------
--------ATTCTCCACATA------TCTGCAAAAATAAGACAGGGTTCAGAAAGGGCATAGCTA---TAA
>JF683764
GCT---GCA--
GAACAATTGTGGGTCACAGTTTATTATGGGGTACCTGTGTGG---AAAGAAGCAACC---
ACCACTCTATTTTGTGCATCAGATGCTAAGGCATATGATACAGAG---GCCCATAAT----------------
---------------GTTTGGCCACA---CATGCCTGTGTACCCACAGAC---
CCCAACCCACAAGAAATAGAAATGAAA---------AATGTGACAGAAGATTTTAAC----------------
--------------------------ATGTGGAAAAATAACATGGTAGAACAAATGCATGAGGATATA---
ATCAGTTTATGGGATCAAAGCCTA---AAGCCATGTGTAAAATTA---
ACCCCACTCTGTGTTACTCTAAATTGCACCGATTTACGGGAAAATGATGGGAATAGTACAACT----------
----
-------------------------AATGAGAATTGGAGAAAGGAGGAGAAAGGAGAAATGAAA---
AATTTCTCTTTCCATATCAACACAGGAAAGGAAAAA---------------------
ACACAACCGACACGTACAAATTATGCTATT------------
TTTGATGTAATAAATATAAATACAAATGGTAAT--

GGGAATAATAGTAATTATAGTTATTATAGGTTGACAAGTTGTAACACCTCAGTCATTACACAA---
GCCTGTCCAAAGATATCCTTTGAACCAATTCCCATACATTATTGTGCCCCGGCTGGGTTTGCGATTCTAAAGT
GTAAC---AATAAGACATTTAAT------GGAAAAGGACCATGTACAAAT-----------------------
-GTCAGC---ACAGTGCAATGTACACATGGAATTAGGCCAGTAGTATCAACT---
CAACTGCTGTTAAATGGCAGTCTAGCAAAT---GAT------------
GTAGTAATTAAATCTGAAAATCTCACGGACAAT---------ACTAAAAACATAATAGTACAGCTGAAG----
-----------GAAGCTGTAAAA---------ATTACTTGCATAAGACCCAACAACAATACAAGACAA-----
-AGTTTAAATCTA------------GGACCAGGGAGAGCA---CTC------TTTATAACAGGAGAC------
---------ATAATAGGAAATATA------------AGAAAAGCACATTGT---AACATTAATGGCACA---
CAATGGAATAAAACTTTAGAACAGATAGTTGGAAAATTAAGA---------------GAACAATTTAAG----
-----------------------------------AATAAAACAATAAACTTTACT------
CAATCTGCAGGAGGGGACCCAGAAATTGTA---ATGCACAGTTTTAATTGTGGAGGGGAA---
TTTAAGCAGCTCCAGGCAAGACTCCTGGCTGTGGAAAGATACCTAAGGGATCAGCAGATCCTAGGGATTTGGG
GTTGCTCTGGAAAGCTCATCTGCACCACGACTGTGCCTTGGAATGCTAGCTGGAGT-----------------
----------------------------AATAAATCTCTAGAGGAGATTTGG------AAG------
AACATGACCTGGATGGCTTGG---GAAAAAGAAATTGACAATTACACAAACTACATATAT---
GACTTACTTGAAAAATCGCAGATCCAACAAGAA--

AAGAATGAACAAGAATTATTGGAATTGGATAAGTGGGCAAGTTTGTGGAATTGGTTTGACATATCACAATGGC
TGTGGTATATAAGACTATTCATAATGATAGTAGGAGGCTTGATAGGTTTAAGAATAGTTTTTGCT---
GTGCTTTCTGTAGTG---------AATAAAGTTAGGCAGGGATACTCACCA------
CTGTCATTTGCTGTTAGCTTGTTCAATACTACAGCTATAGCAGTAGCTGAG---
GGGACAGATAGGATTATAGAAGTAGTACAAGGAACTTTTAGAGCT----------------------------
--------ATCCTCAACATA------CCTAGAAGAATAAGACAGGGCTTAGAAAGGGCTTTGCTA---TAA
>JF683773
ATT---GCA---GGA---------------------------------
GAGGAAAAAGCGTGGGTCACAGTCTATTATGGAGTACCTGTGTGG---AAAGAAGCAACC---
ACCACTCTATTTTGTGCATCAAATGCTAAAGGCTATGATACAGAG---GTACATAAT----------------
---------------GTTTGGCCACA---CATGCCTGTGTACCCACAGAC---
CCAGACCCACAAGAAATAAAATTGGAA---------AATGTGACAGAAAATTTTGAC----------------
--------------------------ATGTGGAAAAATAACATGGTAGAACAGATGCATGAGGATATA---
ATCAGCTTATGGGATCAAAGCCTA---AAGCCATGTGTAAAATTA---
ACCCCACTCTGTGTTACTTTAAATTGCACTGATGATGTGAAGGTGAATGGTACCGCT----------------
----
-------------------------------GATGTCACTACTGAGGAAAAAGGAGAGATAAAA---
AACTGCTCTTTCAATGTTAGCACAAGCATAAAA---------------------------
GATAAGATACAAAAAGAATATGTTTTT---TTTTATAATGTTGATGTACTACCAATAGAGGGTAAT-------
----

GATAATACTAGTTATAGGTTGATAAGTTGTAATACCTCACTCCTTACACAG---
GCCTGTCCAAAGGTATCCTTTGAGCCAATTCCCATACATTATTGTGCCCCGGCTGGTTTTGCGATTCTAAAGT

GTAAA---GATAACAATTTCAAT------GGAACAGGACCATGTAACAAT-----------------------
-GTCAGC---ACAGTACAATGTACACATGGAATTAGGCCAGTAGTGTCAACT---
CAACTGCTGTTAAATGGCAGTCTAGCAGAAGGGGAA------------
ATAGTACTTAGATCTGCCAATTTCTCGGACAAT---------AATAAAAACATAATAGTACAGCTAAAG----
-----------GAAGGTGTAAAC---------ATTACTTGTATAAGACCCAACAACAATACAAGAAAA-----
-GGTATACCTATA------------GGACCAGGGAGAGCA---TTC------TATGCAACAGGAGAC------
---------ATAATAGGAGATATA------------AGACAGGCACATTGT---AACATCAGTAGAAAA---
TCATGGAATAACACTCTATATCAGATAGCTGAAAAATTAGGA---------------AAACAATATAAG----
-----------------------------------AATAAAACAATAGTTTTTACT------
AAATCCTCAGGAGGGGACCCAGAAATTGTA---ATGCACAGTTTCAATTGTGGAGGGGAA---
TTTATCCAGCTCCTGCCAACAATAGTGGCGGTGGAAAGATTCCTCCGGGATCATCAGCTCCTAGGGATTAGGG
GGGGCTCTTGAAAAATCATCATCTGCACCACTGCTCCTCGGAATAGTAGTAGGAGT-----------------
-------------------------AATAATACATTAGTTGACATCATGAAT------GGT------
AACATGACCTGGATGCAGTGG---GAAAGAGAAATTGACAATTACACACAAACAATATAC---
ACCTTAATTGAAGAATCGCAGAACCAACAAGAA--

AAGAATGAAAAAGAATTATTGGAATTAGATAAGTGGGCAAGTTTGTGGAGTTGGTTTGACCTAACACAGTGGC
TGTGGTATATAAGAATATTCATAATGGTAATAGGAGGCTTAATAGGTTTAAGAATAGTTTTTGCT---
GTACTTTCCATAGTA---------AAGAGAGTTAGGCAGGGATACTCACCT------
TTGTCATTTGCTGTCAGCTTGCTTAATACCACTGCTATAGTAGTAGCTGAG---
GGAACAGATAGGGTTATAGAAGTAGTACAAAGAATTTTTAGAGCT----------------------------
--------ATTCTCCACATA------CCTAGAAGAGTGAGACAGGGCTTGGAATTAAGCTTGCTA---TAA
>JQ416158
GCT---GCA--
GACAACTGGTGGGTTACAGTCTATTATGGGGTACCTGTGTGG---AAAGAAGCAACC---
ACCACTCTATTTTGTGCATCAGATGCTAAAGGATATGTTAAAGAG---GCACATAAT----------------
--------------GTTTGGGCCACA---CATGCCTGTGTACCCACAGAC---
CCTGACCCACAAGAAATACCATTGGAA---------AATGTAACAGAAAATTTTAAC----------------
--------------------------ATGTGGAAAAATAGCATGGTAGAACAGATGCAAGAGGATATA---
ATCAGTTTATGGGATCAAAGTCTA---AAGCCATGTGTAAAACTA---
ACCCCACTCTGTGTTACCTTAAATTGCAGTGATTGGGAAAAGGAAAATGTTACTAGTAGTGCTAGTAATAGTC
CAAAT--
-------------------AACAATAGTAGTAGTGAATTAAGGATGGAGAAAGGAGAATTAAAA---
AACTGCTCTTTCAATGTCACCACAAGCATAAGA---------------------------
GATAAGATACAGAAAGAATATGCACTT---TTCTATAGACTTGATGTAGTACCAATAGAGGGT----------
----

GATAATACTAGCTATAGGTTAATAAATTGTAATACCTCAGTCATTACTCAA---
GCCTGTCCAAAGGTAACCTTTGAGCCAATTCCCATACATTATTGTGCCCCAGCTGGTTTTGCACTTCTAAAAT
GTAAG---GATGAGTTATTCAAT------GGAACAGGACCATGTAAAAAT-----------------------
-GTCAGC---ACAGTACAATGTACACATGGAATTATGCCAGTAGTGTCAACT---
CAACTGCTGTTAAATGGCAGCCTAGCAGAAAAAGAG------------
GTAGTAATTAGATCTGCCAATCTCTCAGACAAT---------ACTAAAAACATAATAGTACATCTGAAC----
-----------GAATCTGTAAAG---------ATTAATTGTACAAGGCCCAACAACAATACAAGAAAA-----
-AGTATAAATATA------------GGACCAGGGAGAGCA---TTT------ATTACAACAGGAGCT------
---------ATAATAGGAGATATA------------AGGCAAGCACATTGT---AACATTAGTAGAGAA---
AAATGGAACAAAACTTTACAAATGGTAGTTGAAAAATTAAGA---------------GAAAAATTTGAA----
-----------------------------------AATAAAACAATAGCCTTTAAA------
CCCCCCTCAGGAGGGGACCCAGAAATTGTA---ATGCATAGTTTCAATTGTGGAGGGGAA---
TTTAAGCAGCTCCAGGCAAGAGTCCTGGCTGTGGAGAGATACCTAAGGGACCAACAGCTCCTAGGAATTTGGG
GTTGCTCGGGAAAACTCATCTGCACCACTAATGTGCCTTGGAATAAAACTTGGAGCAACTAC---AGA-----
----------------------------------AATCTGACTCAGATTTGG------GAT------
AACATGACCTGGATACAGTGG---GAAAGAGAAATTGACAATTACACAGACTTCATATAC---
TCTTTAATTGAACAATCGCAAAACCAACAAGAA--

AAGAATGAACAAGAATTATTGGAATTAGATAAGTGGGCAAATTTGTGGAATTGGTTTGACATAACAAAATGGC
TGTGGTATATAAAAATATTCATAATGATAATAGGAGGCTTGATAGGTTTAAGAATAGTTTTTACT---
ATACTTTCTATAGTG---------AATAGAGTTAGGAAGGGATACTCACCA------
TTATCATTTGCTGTCAGCTTGTTCAACACCACAGCTATAGCAGTAGCTGAG---

GGGACAGATAGAATTATAGAGTTAGCACAAAGATTTTTTAGAGCT-----------------------------
--------ATCCTTCACATA------CCTGTAAGAATAAGACAGGGCTTAGAAAGGGCTTTGCTA---TAA
>KT124803
GCT---GTA--
GAACAATTTTGGGTCACAGTTTATTATGGAGTACCTGTGTGG---AAAGATGCAACC---
ACCACTTTATTTTGTGCATCAGATGCTAAAGCACATAATACAGAG---GTACATAAT----------------
--------------GTTTGGGCCACA---CATGCCTGTGTACCCACAGAT---
CCCAGCCCACAAGAAGTCCTAATGGAA---------AATGTGACAGAAACTTTTGAC----------------
--------------------------ATGTGGAACAATAACATGGTAGAACAGATGCATGAGGATATA---
ATCAGTTTATGGGATCAAAGTCTA---AAGCCATGTGTAAAGTTA---
ACCCCACTCTGTGTTACTTTAAATTGCACTGACATGGAGAATGGGAATACTCCTAATACT-------------
----
----------------------AATAATAGTAGCTGGAAAAAGATGGAAAAAGGAGAATTAAAG---
AACTGCTCTTTCAATATCACCACAAGCTTAAGA--------------------------
GATAAGATACAGAAAGAATATGCACTT---
TTTTATAAACTTGATGTAGTACCAATAGATGATGATAATAGTAGC-----------------------------
----
AATAATAATTATAGTAGCTATAATAACTATAGGTTGATAAGTTGTAATACCTCAGTCATTACACAG---
GCCTGTCCAAAGGTATCCTTTGAGCCAATTCCCATACATTATTGTGCCCCAGCTGGTTTTGCGATTCTAAAGT
GTAAC---AATAAGACATTTAAT------GGAACAGGACCATGTAAAAAT-----------------------
-GTCAGT---ACAGTACAATGTACACATGGTATTAGGCCAGTAGTATCAACT---
CAGCTGTTGTTAAATGGCAGTCTAGCAGAAGAAGAG------------
ATAGTAATTAGATCTGAAAATATCACGAACAAT---------GCTAAAAACATAATAGTACAGCTGAAC----
-----------ACATCAGTAAAG---------ATTAATTGTACAAGACCCAACAACAATACAAGAAAA-----
-AGTATTCATATA------------GCACCAGGGAGAGCA---TTC------TATGCAACAGGAGAA------
---------ATAATAGGAGATATA-----------AGACAAGCACATTGT---AACATTAGTAGAGCA---
AAGTGGAATAACACTTTAGAACAGGTGGCTGGAAAATTAAGA---------------GAACACTTTAGG----
-----------------------------------AATAAAACAATAGTCTTTACT------
CACTCCTCAGGAGGGGATCCAGAAATTGTA---ATGCACAGCTTTAATTGTGGAGGGGAA---
TTTAAGCAGCTCCAGGCAAGAGTCCTGGCTGTGGAAAGATACCTAAAGGATCAACAGCTCCTGGGAATTTGGG
GTTGCTCTGGAAAAATAATTTGCACCACTGCTGTGCCTTGGAATAGTAGTTGGAGTAGTAGA---TGGAAT--
----------------------------AATAAATCTCTGGGAGACATATGG------AAT------
AACATGACATGGATGGAATGG---GAAAAAGAGATTGATAATTACACAGGCTTAATATAC---
AGCTTACTTGAAGAATCACAGAACCAACAAGAA--

AAGAATGAACAAGAATTATTAGAATTGGATAAATGGGCAAATTTGTGGAATTGGTTTGACATAACAAACTGGC
TGTGGTATATAAAAATATTCATAATGATAGTAGGAGGCTTGATAGGTTTAAGAATAGCTTTTACT---
GTGCTATCTATAGTG---------AATAGAGTTAGGCAGGGATACTCACCA------
TTATCGTTTGCTATTAGTTTGCTTAATGCCACAGCTATAGCAGTAGCTGAG---
GGAACAGATAGGGTTATAGAAGTAGTACAAAGAATTGGTAGAGCT---------------------------
--------TTTCTCCATATA------CCTAGAAGAATAAGACAAGGCTTAGAAAGAATTTTGCAA---TAA
>KT124789
GCT--
GGAGATTTGTGGGTCACAGTCTATTATGGAGTGCCTGTGTGG---AAAGAAGCAACC---
ACCACTTTATTTTGTGCATCAGATGCTAAGGCATATGATACAGAG---GCACATAAT----------------
--------------GTTTGGGCCACA---CATGCCTGTGTTCCCACAGAC---
CCTAACCCACAAGAAGTAGTATTGGAA---------AATGTGACAGAAAATTTTAAC----------------
--------------------------ATGTGGAAAAATAACATGGTAGAACAAATGAATGAAGATATA---
ATTAGTTTATGGGATCAAAGCTTA---AAGCCATGTGTAAAATTA---
ACCCCACTCTGTGTCACCTTAAATTGCGTTGATGCCAAGAATTGCAACGACACTGGTGCTACAAATTGTAGTA
GTGGAGGAACC--
----------------AACAGTACTACTGGTATTGGGGGATTGATGGACAAAGGGGAAATAAAA---
AACTGCTCTTTCAATAACACCACAAGCATAGGA--------------------------
AATAAGGTAAAGAAAGAATATGCACTT---TTTTATAGACTTGATGTAGTACAAATGGATAATATG-------
----

GATAATACAAGCTATACATTGATAAATTGTAACACCTCAGTCATTACACAG---
GCCTGTCCAAAGGTATCCTTTGAACCAATTCCCATACATTATTGTGCTCCGGCTGGTTTTGCGATTCTAAAGT
GTAAT---GATAATAAGTTTAAT------GGAACAGGACCATGTAAAAAT-----------------------
-GTCAGC---ACAGTACAATGTACACATGGAATTAAACCAGTGGTGTCAACT---

CAACTGCTGTTGAATGGTAGTCTAGCAGAAGGAGGA---------
GAGGTAATAATTAAATCTGAAAATATCACAGATAAT---------ACTAAAAACATAATAGTACAGCTAAAT-
--------------GCATCTGTAGGA---------ATTGATTGTACAAGACCCAACAACAATACAAGAAAA--
----AGTATACATATA------------GGACCAGGGAGAGCA---TTC------TATGCAACA---GAT---
------------ATAATAGGAGATATA------------AGACAAGCACATTGT---AATATTAGTAGAGCA-
--AAATGGAATGACACATTAAAAAGTATAGCTATAAAATTAAAA---------------GAACAGTTT-----
-------------------------------------AACAAAACAATAATTTTTAAT------
CGATCCTCAGGAGGGGACCCAGAAATTGAA---ATGCACAGCTTTAATTGTGGAGGGGAA---
TTTAAGCAGCTCCAGGCTAGAGTCCTGGCTGTGGAAAGCTACCTAAAGGATCAACAGCTCCTGGGGATTTGGG
GTTGCTCTGGAAAACTCATCTGCACCACTAATGTGCCTTGGAATACTAGTTGGAGT-----------------
----------------------------AATAGAACTCTGAAAGACATTTGG------GAT------
AACATGACCTGGATGGAATGG---GACAGAGAAATTAACAATTACACAAACTTAATATAC---
AACTTAATTGAAATATCACAGATCCAGCAGGAA--

AAGAATGAACAAGAATTGTTGGAATTGGATAAGTGGACAGGCTTGTGGAATTGGTTTGACATATCAAATTGGC
TGTGGTATATAAAAATATTCATAATGATAGTGGGGGGCTTAATAGGTTTAAGAATAGTTTTTACT---
GTACTGTCTATAGTG---------AATAGAGTTAGGCAGGGATATTCACCA------
TTATCATTTGCTGTTAGTCTGCTTAATGCCACAGCTTTAGCAGTAGCTGAG---
GGGACAGATAGGATTATAGAAGTAGGACAAAGACTTTGTAGAGCA----------------------------
--------ATTCTCCACATA------CCTAGAAGAATAAGACAGGGTTTAGAAAGGGCTTTGCTA---TAA
>KT124805
GCT---ACA--
GAAAACTTGTGGGTCACAGTTTATTATGGGGTACCTGTATGG---AGAGAAGCAAAC---
ACCACTTTATTTTGTGCATCAGATGCTAAAGCATATGATACAGAG---GTCCATAAT----------------
--------------GTTTGGCCACA---CATGCCTGTGTACCCACAGAC---
CCTAACCCACAAGAAATAAAATTGGAA---------AATGTGACAGAAAAGTTTGAC----------------
--------------------------ATATGGAAAAATAACATGGTGGAACAGATGCATGAAGATATA---
ATTAGTTTATGGGATCAAAGCCTA---AAACCATGTGTAAAACTA---
ACCCCACTCTGTGTCACTTTAAATTGTACTGATTTGAAGAATAATAGTTGTACTAATTTG-------------
----
-------------GAGTCTAATAATTGTACCAATAGTAGTATAGTGGATAAAGAAGAAATGACA---
AACTGTTCTTTCAATGTAACCACAATCAGGAGA---------------------------
GATAAGATGCAGAAAGAATATGCATAT---TTTCATAAACTTGATGTAGTACCAATAGATAAT----------
----

GATAATACCAGTTATATGTTGAGAAGTTGTAATACCTCAGTCATTACACAG---
GCCTGTCCAAAAGTATCCTTTGAGCCAATTCCCATACATTACTGTGCCCCGGCTGGTTTTGCGATATTAAAGT
GTAAC---AATAAAACATTTAAT------GGAAGAGGGCCATGTAGAAAT-----------------------
-GTCAGC---ACAGTACAATGCACACATGGAATTAAGCCAGTAGTATCAACT---
CAACTGCTGTTAAATGGCAGTTTAGCAGAAGAAGGC------------
ATAGTACTTAGATCTGCCAACTTCTCAGACAAT---------GCTAAAACCATAATAGTACAATTGAAG----
-----------GATCCTGTAAGA---------ATTAATTGCACAAGGCCCAACAACAATACAAGAAGA-----
-AGTATACATATA------------GCACCAGGGAGAGCA---TTC------TATACAACAGGAGAA------
---------ATAATAGGGGACATA------------AGACAAGCACACTGC---AATATTACTAGAGGG---
GATTGGGATAAAACTCTAGGGCAGATAGTTATAAAATTAAGA---------------AAACAATTTGGA----
-----------------------------------AATAAAATAATAGCCTTTAAC------
AAATCCTCAGGAGGGGACCCAGAAATTGTA---ATGCACAGTTTTAATTGTGGAGGGGAA---
TTCAAGCAGCTCCAGGCAAGAGTCCTGGCTATAGAAAGATACCTACGGGATCAACAGCTCCTGGGAATTTGGG
GTTGCTCTGGAAAACTCATCTGCCGCACTGCTGTGCCTTGGAATGCTAGTTGGAGT-----------------
----------------------------AATAAATCTTATAATGACATTTGG------GAT------
AACATGACCTGGATGGAGTGG---GATAGAGAAGTTAACAATTACACAGACCTAATATAC---
AATTTAATTGAACAATCGCAAAACCAGCAAGAA--

AAGAATGAACAAGACTTACTGGAATTGGATAAATGGGCAAGTTTGTGGAATTGGTTTGACATATCAAATTGGC
TGTGGTATATAAGAATATTCATAATGATAATAGGAGGCTTGGTAGGTTTAAGAATAATTTTTGCT---
GTGCTTTCTATAGTA---------AATAGAGTTAGGCAGGGATATTCACCA------
TTATCGTTTGCTGTTAGCTTGCTCAACGCCACAGCCATAGTAGTAGCTGAA---
GGGACAGATAGGATTATAGAAGTAGTACAAAGAACCTTTAGAGCT----------------------------
--------CTCATCCATATA------CCTAGAAGAATAAGACAGGGCTTAGAAAGGGCCTTGATA---TAA
>KT124763

GCT---ACA---------------------------------------
GAAGAGAAGTGGGTCACAGTCTATTATGGAGTACCTGTGTGG---AAAGAAGCAACC---
ACTACTCTATTTTGTGCATCAGATGCTAAAGCATATGATACAGAG---GTGCATAAT----------------
--------------GTTTGGGCCACA---CATGCCTGTGTACCCACAGAC---
CCCAGCCCACAAGAAGTAGCATTGGAA---------AATGTGACAGAAGATTTTAAC----------------
-------------------------ATGTGGAAAAATAGCATGATAGACCAGATGCACGAGGATATA---
ATCAGTTTATGGGATCAAAGCTTA---AAGCCATGTGTAAAATTA---
ACTCCACTCTGTGTTGCTTTAAATTGTACTACTTTTTCTAAGAATTGTACTACTTTTAATAAGAATGTTACCG
GTAACGAGACG--
GTGGAAAATGGAAACTGCACTAATAATACTGAGACACAGACAAAGGAAGTACAAGAACTGCAAGAAATAAAA-
--AACTGTTCTTTCAAAACCTTCACAAGGATAAGA---------------------------
GATAAGACACAGGGAGAAGAATCTGCTCTTTTTTATAGAGATGATCTAGTACTAATAGATAATGATAATAAAA
CTAATAAT---

AGCTATAATAAAACTAATAAGAGCTATAGGTTGGTAAGTTGTAACACCTCAGTCATTACACAG---
GCCTGTCCAAAGGTATCCTTTGAGCCAATTCCCATACATTATTGTGCCCCAGCTGGTTTTGCGATTCTAAAAT
GTAAC---GATAAGAAGTTCGAG------GGAAAAGGATCATGTGAAAAT----------------------
-GTTAGC---ACAGTACAATGTACACATGGAATTAGGCCAGTAGTATCAACT---
CAACTGTTGTTAAATGGCAGCCTAGCAGAAGAGGAG-----------
GTAGTAATTAGATCTGAAAATTTCACAAACAAT---------GCTAAAATCATAATAGTGCAGCTAAAA----
-----------GAAGCTGTAAAA---------ATTAATTGTACAAGACCCAATAACAATACAAGAAAA-----
-AGTATACATATA------------GGACCAGGGAAAGCA---TTT------TATACAACAGGAGAT------
---------ATAATAGGAGATATA------------AGACAAGCACATTGT---AACATTAGTAGAGAA---
CAATGGAATAACACTTTAAAAAAGGTAGTTGAAAAACTAAGG---------------AAAAAATTTAAC----
-----------------------------------AATAAAACAATAAGCTTTAAG------
CAATCCTCAGGAGGAGACCCAGAAATTGTA---ATGCACAGTTTTAATTGTGGAGGGGAA---
TTTAAGCAGCTCCAGGCAAGGCTCCTGGCTGTAGAAAGATATCTAAAGGATCAACAGCTCCTGGGGATTTGGG
GTTGCTCTGGAAAACTCATTTGCACCACTGCTGTGCCTTGGAATGATAGTTGGAGT----------------
-----------------------------AATAAATCTCTGAACCAGATTTGG------AAT------
AACATGACTTGGATGCAGTGG---GAGAAAGAAATCAACAATTACACAAATTTTATATAT---
AAATTAATTGAAGAATCGCAAAACCAACAAGAA---------------------------------------
----
AAGAATGAACAAGAATTATTGGAGTTAGATAAATGGGCAAGTTTGTGGAATTGGTTTTCCATAACAAACTGGC
TGTGGTATATAAGACTATTCATAATGATAGTAGGAGGCTTAATAGGCTTAAGAATAGTTTTTGCT---
GTACTTTCTATAGTG---------AATAGAGTTAGGCAGGGATACTCACCC------
TTGTCGTTTGCTGTTAACTTGCTTGACACTATAGCTATAGCAGTAGCTGAG---
GGAACAGATAGGATTTTAGAAGGCTTACAAGGAATTGGTAGAGCT---------------------------
--------TTTCTCCACATA------CCTAGAAGAATAAGACAGGGCCTCGAAAGAGCTTTGCAA---TAA
>KT124779
ACT---GCA---------------------------------------
GAACAATTGTGGGTAACAATCTATTATGGGGTACCTGTGTGG---AAAGAAGCAAAC---
ACCACTCTATTTTGTGCATCAGATGCTAAAGCATATGATACAGAG---ATACATAAT----------------
--------------GTTTGGGCCACA---CATGCCTGTGTACCCACAGAC---
CCCAACCCACAAGAAGTAGTATTGGGA---------AATGTGACAGAAAATTTCAAC----------------
-------------------------ATGTGGAAAAATAACATGGTAGAACAGATGCATGAAGATATA---
ATCAGTCTATGGGATCAAGGCCTA---AAGCCATGTGTAAAATTA---
ACCCCACTCTGTGTTAAGTTAAATTGCACTAATGTGAGGAATGATACTAATACTAATATGACTGATACTAGT-
----
--ATGACA---
AATTGCACTTTCAATATTACCACAAAATTAGGA---------------------------GGG---
ACTAAGCAAGAAAGGGCGATT---TTTAATAAACTTGATGTAGTACTAATAAGTAATGGGACAAAA-------
----
----ATGGGTAATAGGACATATGAGATATATAGGTTAATACACTGTAACACCTCAGTCATTTCACAG---
GCCTGTCCAAAGATATCATTTGAGCCAATTCCCATACATTATTGCGCCCCGGCTGGTTTTGCGATTCTAAAAT
GTAAT---GATAAGAAGTTCAAT------GGAACAGGACCATGTAAAAAC----------------------
-GTCAGC---ACAGTACAATGTACACATGGAATTAGACCAGTAGTGTCAACT---
CAACTGCTGTTAAATGGCAGTCTAGCAGAAGAAGAG-----------
ATAGTAATTAGATCTGAAAATTTCACAAACAAT---------GCTAAAACCATAATAGTACAGCTGAAT----
-----------GAATCTGTACAG---------ATTAATTGTACAAGACCCAACAACAATACAAGAAAA-----
-AGTATACATATA------------GGACCAGGGAGAGCA---TTT------TATGCAACAGGAGAT------

---------ATAACAGGAAATATA------------AGACAAGCACATTGT---AACCTTTCAAGAACA---
CAATGGAATAATACTTTAAACCGGGTAGCTATAAAATTAAGG--------------GAACAATTTAGG----
-----------------------------------AATAAAACAATAGTCTTTAAT------
CAATCCTCAGGAGGGGACCCAGAAATTGTA---ATGCATAATTTTAATTGTGGAGGGGAA---
TTTAAGCAGCTCCAGGCAAGAGTCCTGGCTGTGGAAAGATACCTAAAGGATCAACAGCTCCTAGGAATTTGGG
GTTGCTCTGGAAAACTCATCTGCACCACTGCTGTGCCTTGGAATGCTAGTTGGAGT----------------
----------------------------AATAAATCTTATTCAGACATTTGG------GAA------
AACATGACATGGATGGAGTGG---GAAAGAGAAATCGATAATTACACAAATTTAATATAC---
ACCTTAATTGAAGAATCGCAGAACCAGCAAGAA--

AAGAATGAACTCGAATTATTGGAATTAGATAAGTGGGCAAGTTTGTGGAATTGGTTTAATATAACAGAATGGC
TGTGGTATATAAAAATATTCATAATGGTAATAGGAGGCTTGATAGGTTTAAAAATAGTTTTTACT---
GTACTTTCTATAGTG---------AACAGAGTTAGGCAGGGATACTCACCA------
TTGTCATTCGCTATTAGCTTGCTTAATGCTACAGCCATAGCAGTAGCTGAG---
GGGACAGATAGGATTATAGAAGTAGCACAAAGAGTTGGTAGAGCT---------------------------
--------TTTCTTCACATA------CCTAGAAGGATAAGACAAGGCGCAGAAAGGTTTTTGCTA---TAA
>KT124787
GCA---ACA---
GAAAAATTGTGGGTCACAGTTTATTATGGGGTACCTGTATGG---AAAGAAGCAGAC---
ACCACTTTATTTTGTGCATCAGATGCTAAGGCACAGGCAACAGAG---GTACATAAT----------------
--------------GTTTGGGCCACA---CATGCCTGTGTGCCCACAGAC---
CCCAACCCACAAGAAGTAGTATTGGTA---------AATGTGACAGAAGATTATAAC---------------
--------------------------ATGTGGAAAAATAACATGGTAGAACAGATGCATGAGGATATA---
ATCAGTTTATGGGATCAAAGCCTA---AAGCCATGTGTAAAATTA---
ACCCCACTCTGTGTTACTTTACATTGCACTGATCTAAATGTTACTAATACCAATAGTAGTAGTGCTAAG----
----
-------------------------AATACCAATAATAGTATGATAGATGGAGGAGAAATGAAA---
AACTGCTCTTTCAATGTCACCACAAACCTAAGG--------------------------
GATAAGATGGAGAGAGAATTTGCACTC---CTGTATAAACTTGATATAGTACCAATAGGTAAT----------
----

GATAGCAACAGTAATAGCTATAGGTTGATAAGTTGTAACACCTCAATCATTACACAG---
GCCTGTCCAAAGGTATCCTTCGAGCCAATTCCCATACATTATTGTGCCCCGGCTGGTTTTGCGATTCTAAAGT
GTAAT---GATAAGGATTTTAAC------GGAACAGGAACATGTACCAAT----------------------
-GTCAGC---ACAGTACAATGTACACATGGAATTAGGCCAGTTGTGTCAACT---
CAACTGCTGCTAAATGGCAGTCTAGCAGAAAAAGAG-----------
GTAGTAATTAGATCTGAAAATTTCACTGACAAT---------ACTAAAAACATAATAGTACAGCTGAAC----
-----------CAATCTATATAC---------ATTAATTGTACAAGACCCAACAACAATACAAGAAAA-----
-AGTATACGTATA------------GGACCAGGACAAGCA---TTT------TATGCAACAGGAGAT------
---------ATAATAGGAGATATA------------AGACAAGCACATTGT---AACATTAGTAAGAAG---
GATTGGTATAACACTTTAACACAGATAGCTGCAAAATTACAA-----------AAACAAGAAGAATTT----
-----------------------------------AACAAAACAATAGTCTTTAAT------
CATTCCTCAGGAGGGGACCCAGAAATAGTA---ATGCATAGTTTTAATTGTGGAGGGGAA---
TTTAAACAGCTCCAGGCGAGAGTCCTGGCTGTGGAAAGATACCTACATGATCAACAGCTCCTAGGGATTTGGG
GTTGCTCTGGAAAGCTCATCTGCACCACTGCTGTGCCTTGGAATACTAGTTGGAGT----------------
----------------------------AACAAATCTATAGATATGATTTGG------CAA------
AACATGACCTGGATGCAATGG---GAAAAGGAAATTGACAATTACACAAGCTTAATATAT---
ACCTTACTCGAAAAAATCGCAGAACCAGCAGGAA--

AAGAATGAACAAGACTTATTGGAATTGGATAAATGGGACAGTTTGTGGAATTGGTTTAGTATATCAAATTGGC
TGTGGTATATAAAAATATTCATAATGATAGTAGGAGGCTTGGTAGGTTTAAGAATAGTTTTTGCT---
GTACTTTCTTTAGTA---------AATAGAGTTAGGCAGGGATATTCACCC------
TTATCATTAGCTGTTAGCTTGTTCAACGCTACAGCTATTGTAGTAGCTGAA---
GGGACAGATAGGGTTATAGAAATAGTACAAAGAGCTTTTAGAGCT---------------------------
--------ATTCTCCACATA------CCTAGGAGAATAAGGCAGGGCGCGGAAAGGGCTTTACAA---TAA
>KC596067
GCT---ACA---
GAAAAATTGTGGGTCACAGTCTATTATGGGGTACCTGTGTGG---AAAGAAGCAACC---
ACCACTCTGTTTTGTGCATCAGATGCTAAAGCATATGACAGAGAG---GTGCATAAT---------------
--------------GTTTGGGCCACA---CATGCCTGTGTACCCACAGAC---

CCCAACCCACAAGAAATAGAATTGAAA---------AATGTGACAGAAAATTTTGAC----------------
--------------------------GTATGGAAAAATAAAATGGTAGAACAGATGCATGAAGACATA---
ATTAGTTTATGGGATCAAAGCCTA---AAACCATGTGTAAAATTA---
ACCCCACTCTGTGTTACTTTAAATTGCACTAATACTAACAATAGTATTGTAAACTCCACT-------------
----
---CAGGAGATGAAA---
AACTGCTCTTTCAATGTCACCACAGGCATAAGA---------------------------
GATAAGGTAAAGAAAGAAAATGCATAC---TTTTATAACATTGATCTAGTACAAATAAAAGAT----------
----

GATAGTGATAATACTAGCTATATATTAACAAGTTGTAACACCTCAGTCATTACACAG---
GCCTGTCCAAAGGTATCCTTTGAGCCAATTCCCATACATTTTTGTGCCCCGGCTGGTTTTGCGATTCTAAAGT
GTAAT---GATAAGAAGTTCAGT------GGAAAAGGATCATGTACAAGT----------------------
-GTCAGC---ACAGTACAATGTACACATGGAATTAGACCAGTAGTATCAACT---
CAACTGTTGTTAAATGGCAGCCTAGCAGAAGAAGAG-----------
GTAGTAATTAGATCTGAAAATATCACAAACAAT---------GCTAAGACCATAATAGTACAGTTGAAA----
-----------GAACCAGTAAAG---------ATTAATTGTACAAGACCCGGCAACAATACAAGAAGA-----
-AGTATATCTATA------------GGACCAGGGAGAGCA---TTT------TATACAATGGGGCAA------
---------ATAATAGGAAACATA------------AGAGAAGCACACTGT---AATATTAGTGGAACA---
GACTGGAATAACACTTTAAAACAGATAGTTGGAAAATTAAGA---------------GAACAATTTGGG----
-----------------------------------AATAAAACAATTGTCTTTAAT------
AAATCCTCAGGAGGGGACCCAGAAATTATA---ATGCACAGTTTTAATTGTGGAGGGGAA---
TTTAAACAGCTCCAGGCAAGAGTCCTGGCTGTGGAAAGATACCTAAGGGATCAACAGCTCCTAGGGATTTGGG
GCTGCTCTGGAAAACTCATCTGTACCACTACTGTGCCTTGGAACACTAGTTGGAGC-----------------
----------------------------AATAGATCTCTGGATAGGATTTGG------AAC------
AACATGACATGGATGGAGTGG---GAAAAAGAAATTGACAATTACACAGGATTAATATAC---
ACATTGCTTGAAGAGTCACAAAACCAGCAGGAA--

AACAATGAGAAAGAATTATTGGAATTGGATAAATGGGCAAATCTATGGAATTGGTTTGATATAACAAATTGGC
TGTGGTATATAAAAATATTCATAATGATAGTAGGAGGCTTAATAGGTTTAAGAATAGTTTTTACT---
GTATTTTCTATAGTG---------AACAGAGTTAGGCAGGGATACTCACCA------
TTATCGTTTGCTATTAGCTTGCTCAACGCCACAGCCATAGCAGTAGCTGAG---
GGGACAGATAGGATTATAGAAATATTACAAAGAATTGGTAGGGGT---------------------------
--------ATCCTCCACATA------CCTACAAGAATAAGACAGGGCTTGGAAAGGGCTTTGCTA---TAA
>EF514700
GCA---GCA---
GAAGAATTGTGGGTCACAGTCTATTATGGGGTACCTGTGTGG---AAAGAAGCAAAC---
ACCACTTTATTTTGTGCATCAGATGCTAAAGCATATAAAACAGAA---GTACATAAT----------------
--------------GTTTGGGCCACA---CATGCCTGTGTACCCACAGAT---
CCCAACCCACAAGAAGTAGTATTGGGA---------AATGTGACAGAATATTTTAAC---------------
--------------------------ATATGGAAAAATAACATGGTAGAACAAATGCATGAGGATATA---
ATCAGTCTATGGGATCAAAGTCTA---AAGCCATGTGTAAAATTA---
ACCCCACTCTGTGTTACTTTAAATTGCAGTAATTACATCCCACCCTGTGTTAATAGCACTAAGAGTACTAATT
GCACTAATAGTAAT---
AGTACTAATAGTAATACTACTGAGGTGACGAATGGTACTGATACCCAAGGGGAGATAGGAATGAAA---
AACTGCTCTTTCAATGTCACCACAAACATAAGA---------------------------
GATAAGATACAGAAAGAATATGCACTT---TTTTATAGACTTGATGTAGTACCAATAGATGAT----------
----

GATAATGATACTAATAGCTATAGGTTAATAAGTTGTAATACCTCAGTCATTACACAA---
GCCTGTCCAAAGGTATCCTTTGAGCCAATTCCCATACATTATTGTACCCCGGCTGGTTTTGCACTTCTAAAGT
GTAAC---AATAAGACATTTGAG------GGAAAAGGAGAATGTACAAAT----------------------
-GTCAGC---ACAGTACAATGTACACATGGAATTAGGCCAGTAGTGTCAACT---
CAACTGCTGTTAAATGGCAGTCTAGCAGAAGAAGAG-----------
GTAGTGATTAGATCTGACAATTTTACAAACAAT---------GCTAAAACCATAATAGTACAGTTTAAT----
-----------GAATCTGTAGAA---------ATTAATTGTACAAGACCCAACAACAATACAAGAAAA-----
-AGTATACATATA------------GGACCAGGAAGAGCA---TTT------TATGCAACAGGAAAT------
---------ATAATAGGAAATATA------------AGACAAGCACATTGC---AATATTAGTAAAGCA---
GGATGGAATAAAGTGTTAAGACAGGTAGCTGCAAAATTAAGA---------------GGACAATTTGGG----
-----------------------------------AATAAAACAATAAACTTTACT------

CAACACTCTGGAGGGGATCCAGAAATTGTA---ATGCACAGTTTTAATTGTGGAGGGGAA---
TTTAAGCAGCTCCAGGCAAGACTCCTGGCTGTGGAAAGATACCTAAGGGATCAACAGCTCCTAGGGATTTGGG
GTTGCTCTGGAAAACTCATTTGCACCACTACTGTGCCTTGGAATAGTAGTTGGAAT-----------------
----------------------------AATAAAACTCAGAAGGAGATTTGG------GAT------
AACATGACCTGGATGCAGTGG---GATAAAGAAATTAACAATTACACAAACTTAATATAC---
AGCTTACTTGAAACATCACAGAACCAACAAGAA--

AAGAATGAACAAGACTTATTGAAATTAGATGAGTGGGCAAGTTTATGGAATTGGTTTGACATAACAAAGTGGC
TGTGGTATATAAAAATATTCATAATGATAGTAGGAGGCTTGGTAGGTTTAAGAATAGTTTTTGCA---
GTACTTTCTATAATA---------AATAGAGTTAGGCAGGGATACTCACCA------
TTGTCATTTGCAATTAGCTTGCTTAACGCCACAGCAATAGCAGTAGCTGAG---
GGGACAGATAGGATTATAGAAGTAGTACAAAGAATTTGCAGAGCT----------------------------
--------TTTCGCCACGTA------CCTAGAAGAATAAGACAGGGCTTGGAAAGGATTTTGCTA---TAA
>EU839598
GCT---AAG---
GAAGATTTGTGGGTCACAGTATATTATGGGGTACCTGTGTGG---AAAGAAGCATCC---
ACCACTCTATTTTGTGCATCAGATGCTAAAGCAGTTGAAAGAGAG---GCACATAAT----------------
--------------GTCTGGGCCACA---CATGCCTGTGTACCCACAGAC---
CCCAACCCACAAGAAGTAAAATTGGAC---------AATGTGACAGAATATTTTAAT----------------
--------------------------GTGTGGAAAAATAACATGGTAGATCAGATGCATGGGGATATA---
ATCAGTTTATGGGACCAAAGTCTA---AAGCCATGTGTAAAATTA---
ACCCCACTCTGTGTTACCTTAAATTGCACCAATTGGGAAAGGGGTAATGTCACTGAAAGGAGTAATGTC----
----
----------------ACTAATAATAACAGTACAGTGACACAGATGGGGGAAGAGGAAGTAAAA---
AACTGCTCTTTCAACATCACCACAGATATGAAA---------------------------
GATAGGTATCAGAGAGAATATGCACTT---TTTTATAACCTTGATGTAGTACCAATAGATAATAATGTT----
----

ACAAATACCACATATACCACATATAGGTTGCTACAGTGTAATACTTCAGTCATTACACAG---
GCCTGTCCAAAGACATCCTTTGAACCAATTCCTATTTATTATTGTGCCCCGGCTGGTTTTACGATTGTGAAGT
GTAAC---AATAAAACGTTCAGT------GGGAAAGGAGAATGTAACAAT----------------------
-GTCAGC---ACAGTACAATGTACACATGGAATTAGGCCAGTAGTGTCAACT---
CAGCTGCTGTTAAATGGCAGTCTAGCAGAAGAAGAG-----------
GTAGTACTTAGATCTGAAAATTTCACAAACAAT---------ATTAAAACCATAATAGTACAGCTGAAT----
-----------AAAACTGTAGTA---------ATTAATTGTACAAGACCCAGCAATAATACAAGAAAA-----
-AGTATAAATATA------------GGGCCAGGAAGAGCA---TTT------TATGCAACAGGAGCA------
---------ATAATAGGAGATATA------------AGACAAGCACATTGT---AACCTTAGTAGAAAA---
GATTGGAATGACACTTTAAAACTGGTAGTTGCAAAATTAAAA---------------GAACAATTCAAT----
-----------------------------------ACATCAAAAATAATCTTTCAG---
CCACCATCCCCAGGAGGGGACCTAGAAATTGTA---ATGCACAGTTTTAATTGTGGAGGGGAG---
TTCAAACAGCTCCAGGCAAGAGTCCTGGCTCTGGAAAGATACCTACAGGATCAACAGCTCCTAGGGATTTGGG
GTTGCACTGGAAAACTCATTTGCACCACTACTGTGCCTTGGAACACTAGTTGGAGT-----------------
----------------------------ACAAAAAATTATAGTCAAATTTGG------AAT------
AACATGACCTGGATGCAGTGG---GAAAAAGAAATTGACAATTACACAGAGTTAATATAC---
TCCTTAATTGAAAAATCGCAGACCCAGCAAGAA--

CTAAATGAACAAGAACTATTGGCATTGGATAAGTGGGCAAATCTGTGGAATTGGTTTGATATAACATACTGGT
TGTGGTATATAAAAATATTTATAATGATAATAGGAGGCTTGGTAGGTTTAAGAATAATTTTTGCT---
GTATTTTCTGTAGTG---------AATAGAGTTAGGCAGGGATACTCACCC------
TTGTCATTTGCTGTTAGCTTGTTTGATACCACAGCAGTAGCAGTAGCTGAG---
GGGACAGATAGAATTATAGAAGGAGTAAGAAGGGCTTTTAGAGCT----------------------------
--------ATTTTACACATA------CCTAGAAGAATAAGACAGGGCTTGGAAAGGGCTTTGGTA---TAA
>KC595194
GCT---GCA---
GAAAAATTGTGGGTTACAGTTTATTATGGGGTACCTGTGTGG---AAAGAAGCGACC---
ACCACTCTATTTTGTGCATCAGATGCTAAAGCATATGATACAGAG---GTACATAAT----------------
--------------GTTTGGGCCACA---CATGCCTGTGTACCCACAGAC---
CCCAACCCACAAGAAGTAGTATTGGGA---------AATGTGACAGAAAATTTTAAC----------------
--------------------------ATGTGGAAAAATAACATGGTAGAACAAATGCATGAGGATATA---
ATCAGTTTATGGGATCAAAGCTTA---AAGCCATGTGTAAAATTA---

ACCCCACTCTGTGTTACTTTAAATTGCACCGATTGGAAGAATGCTACTAATACCACTACTACTAATACC----
----
-------ACTGCTACTAATATCACTGGTAGTAGCGGGAAAATGTGGGAGAAAGGAGAAATAAAA---
AACTGCTCTTTCAATGTCACCACAAACATAAGA--------------------------
GATAAAATACAGAGAGAATATGGACTT---TTTTATAAACTTGATGTAGTACCAATAGGCAAT---------
----

AGTAATAGTAGCTATAGGTTGATAAATTGTAACACCTCAGTCATTACACAA---
GCCTGTCCAAAAGTATCCTTTGATCCAATTCCCATACATTATTGTGCCCCGGCTGGTTTTGCGATTCTAAAGT
GCAAC---GATAAGAAGTTCAAT------GGAACAGGACCATGTACAAAT----------------------
-GTCAGC---ACAGTACAATGTACACATGGAATTAGGCCAGTAGTATCAACC---
CAACTGCTGTTAAATGGAAGCCTAGCAGAAGAAGAG-----------
ATAGTAATTAGATCTGCCAATTTCACGGACAAT---------GCCAAAACCATAATAGTACAGCTGAAA----
-----------GAACCTATAACA---------ATTAATTGTACAAGGCCCAGCAACAATACAAGAAGA-----
-AGTATACATATA------------GGACCAGGAAGAGCA---TTT------TATGCAACAGAAGCA------
---------ATAACAGGAGATATA------------AGGCAAGCATATTGT---ACCCTTAATGGAACA---
AAATGGAATAACACTTTACAACAGATAGCTACAAAATTAAGA---------------GCACAATTAAGT----
-------------------------------AATATAACAACAATAGTCTTTAAC------
AGATCCTCAGGAGGGGACCCAGAAATTGTA---ATGCACAGTTTTAATTGTGGAGGGGAA---
TTTAAGCAGCTCCAAGCAAGAGTCCTGGCTGTGGAAAGATACCTAAAGGATCAACAGCTTCTGGGGATGTGGG
GTTGCTCTGGAAAACTCATTTGCACCACTACTGTGCCTTGGAATGCTAGTTGGAGT----------------
-----------------------------AATAAATCTCTGAATAGTATTTGG------GAT------
AACATGACCTGGATGGAGTGG---GAAAAAGAAATTAACAATTACACAGGCTTAATATAC---
ACCTTAATTGAAGAATCGCAGAACCAACAAGAA--

AAGAATGAACAAGAATTGTTAGAATTGGACAAATGGGCAAGTTTGTGGAATTGGTTTAACATAACAGAATGGC
TGTGGTATATAAAAATATTCATAATGATAGTAGGAGGCTTGGTAGGGTTAAGAATAGTTTTTACT---
GTACTTTCTATAGTA---------AATAGAGTTAGGCAGGGATACTCACCA------
TTATCGTTTGCTATTAGCTTGCTTAATGCCACAGCCATAGCAGTAGCTGAG---
GGGACAGATAGGGTTATAGAATTAGTACGAAGAGCTTGTAGAGCT---------------------------
--------ATTCTCCACATA------CCTACAAGAATAAGGCAGGGCTTGGAAAGAGCTTTGCTA---TAA
>KC595151
GCT---GCA---
GAACAATTGTGGGTCACAGTCTATTATGGGGTACCCGTGTGG---AAAGAAGCAAAC---
ACCACTCTATTTTGTGCATCAGATGCTAAAGGATATGATACAGAG---GTACATAAT----------------
--------------GTTTGGGCCACA---CATGCCTGTGTACCCACAGAC---
CCCAACCCACAAGAAATAGAATTGAAA---------AATGTGACAGAAAATTTTAAC---------------
-------------------------ATGTGGAAAAATAACATGGTAGAACAGATGCATGAGGATATA---
ATCAGTTTATGGGATCAGAGCTTA---AAACCATGTGTAAAATTA---
ACCCCACTTTGTGTTACTTTACATTGCACTAATGCTAGTGCTACTAATGCCACTGGTGATGCTACTGCT----
----
----------------------GCTGGTGCCACTAGTGGTACTAATATTACAGAAGACATGAAA---
AACTGCTCTTTCAATATCACCACAAGCATAAGA-------------------------
GATAAGATGCAGAAAGTATATGCGCTT---TTTTATAAACTTGATGTAGTACCAATAGATGAT---------
----

GCCAATAGTAATGCCAGCTATAGGTTGATAAATTGTAACACCTCAGTCATTACACAG---
GCCTGTCCAAAAGTATCCTTTGAGCCAATTCCCATACATTATTGTACCCCGGCTGGTTTTGCGATTCTAAAAT
GTAAT---GACAAGAAGTTCAAT------GGAACAGGACCATGTGAAAGT----------------------
-GTCAGC---ACAGTACAATGTACACATGGAATTAGGCCAGTAGTGTCAACT---
CAACTGCTGTTAAATGGCAGTCTAGCAGAAGAAGAG-----------
GTAGTAATTAGATCTGAAAATTTCACGGACAAT---------GTTAAAACCATAATAGTACAGCTGAAT----
-----------GTATCTATACAA---------ATTAATTGTACAAGACCCAACAACAATACAAGAAAA-----
-AGTATACCTATA------------GGTCCAGGGAGAGCA---TTT------TATACAACAGGAGAA------
---------ATAATAGGAGATATA------------AGACAAGCACATTGT---AACCTTAGTGCAACA---
AAATGGAATAACACCTTAAAGCTGGTAGTTGCCAAATTAAGA---------------GAACAATTTGGG----
-----------------------------------AATAAAACAATAATCTTTAAT------
CAATCCTCAGGAGGTGACCCAGAAATTGTA---ATGCACAGCTTTAATTGTGGAGGGGAA---
TTTAAGCAGCTCCAGGCAAGAGTCCTGGCTGTGGAAAGATACCTATGGGATCAACAGCTCCTGGGGATTTGGG
GTTGCTCTGGAAAACTCATTTGCCCCACTGCTGTGCCTTGGAATAGTAGTTGGAGT----------------

----------------------------AATAAATCCCTGGAAACGATTTGG------GGA------
AACATGACCTGGATGCAGTGG---GAAAGAGAAATTGACAATTACACAGGCCTAATATAC---
ACCTTAATTGAAGAATCGCAGAACCAACAGGAA--

AAGAATGAACAAGAGTTATTGGCATTGGATAAATGGGCAAGTTTGTGGAATTGGTTTGACATAACAAAATGGC
TGTGGTATATAAAAATATTCATAATGATAGTAGGAGGCTTGGTAGGTTTAAGAATAGTTTTTACT---
GTACTTTCTATAGTA---------AATAGAGTTAGGCAGGATACTCACCA------
TTATCGTTTGCCGTTAGCTTGCTCAACGCCACAGCCATAGCAGTAGCTGAG---
GGGACAGATAGAGTTATAGAAGCATTACAAAGAGCTTTTAGAGCT----------------------------
--------ATTCTCCATATA------CCTACAAGAATAAGACAGGGCTTAGAAAGAGCTTTGCTA---TAA
>EU786679
GCG---GCA--
GAACAACTGTGGGTCACAGTCTATTATGGGGTACCTGTGTGG---AAAGAAGCAACC---
ACCACTCTATTTTGTGCATCAGAGGCTAAAGCATATAAGACAGAG---GTTCATAAT----------------
--------------ATCTGGGCCACA---CATACCTGTGTACCCACAGAC---
CCCAATCCACAAGAAGTAGTATTAGAG---------AATGTGACAGAAGATTTTAAC----------------
--------------------------ATGTGGAAAAATGACATGGTAGAACAGATGCAGGAGGATATA---
ATCAGTTTATGGGATCAAAGCCTA---AAGCCATGTGTAAAATTA---
ACCCCACTCTGTGTTACTTTAAATTGCACTGATTTGAAGAATGTTACTAGT----------------------
----
-------------------------------------AACAGCACTGATAATAGTAGTATGACA---
AATTGCTCTTTCAATATCACCACAAGCATGAGA---------------------------
GATAAGATGCAGAAAGAATATGCAATC---TTTTATAAAACTGATGTAGTAGCAATAGATAAT----------
----

GATACCAACTATAGGTTGATAAGTTGTAATACCTCAGTCATTACACAG---
GCCTGTCCAAAAGTATCCTTTGAGCCAATTCCCATACATTATTGTGCCCCGGCTGGTTTTGCGATTTTAAAAT
GTAAT---AATAAAAAGTTCAAT------GGAACAGGAACATGTAAAAAT-----------------------
-GTCAGC---ACAGTACAATGTACACACGGAATTAGGCCAGTAGTATCAACT---
CAACTGCTGTTAAATGGCAGTCTAGCAGAAGAAGAG------------
GTGGTAATTAGATCTGCCAATTTCTCGGACAAT---------GCTAAAACCATAATAGTACAGCTGAAT----
-----------GAATCTGTAGCA---------ATTAATTGTACAAGACCCAATAACAATACAAGAAGA-----
-AGTATAACTATA------------GGACCAGGGAGAGCA---TTT------TATGCAACAGGAGAA------
---------ATAATAGGAGATATA------------AGAAAAGCATATTGC---AACATTAGTGGAACA---
AAATGGAATAACACTTTAAAACAGATAGCTAAAAAACTAAGA---------------GAACAATTTAAG----
-----------------------------------AATAAAACAATAGTCTTTCAG------
CCGCCCTCAGGAGGGGACCTAGAAATTGTA---ATGCACAGTTTTAATTGTGGAGGGGAA---
TTTAAGCAGCTCCAGGCAAGAGTCCGAGCTGTGGAAAGATACCTACAGGATCAACAGCTCCTGGGAATTTGGG
GTTGCTCTGGAAAACTCATTTGCACCACTGCTGTGCCTTGGAATGCTAGTTGGAGT-----------------
----------------------------AATAAATCTGTAGATGATATTTGG------AGT------
AACATGACCTGGATGCAGTGG---GAAAAAGAAATTGACAATTACACAAATGTAATATAC---
ACCTTACTTGAGCAATCGCAAAACCAACAAGAA--

AAGAATGAACAAGAATTATTGGAATTGGATAAATGGGCAAGTCTGTGGAATTGGTTTGACATAACAAATTGGC
TGTGGTATATAAAAATATTCATAATGGTAGTAGGAGGCGTGATAGGGTTAAGAATAGTCTTTACT---
ATACTTGCTATAGTG---------AATAGAGTTAGGCAGGATACTCACCT------
TTGTCATTTGCTGTTAGCTTGCTCAACGCCACAGCCATAGCAGTAGCTGAG---
GGGACAGATAGGGTTATAGAAGTAGTACAAGGAGCTGGTAGAGCT----------------------------
--------ATTCTCCACATA------CCTAGAAGAATAAGACAAGGCGCAGAGAGGATTTTACAA---TAA
>KC473841
ACT---GCA---GAA------------------------------------
GAAAATTTGTGGGTCACAGTCTATTATGGGGTACCTGTGTGG---AGAGATGCAAAC---
ACCACTCTATTTTGTGCATCAGATGCGAAGGCATATGATACAGAG---GTACATAAT----------------
--------------GTTTGGGCCACG---CATGCCTGTGTACCCACAGAC---
CCCAATCCACAAGAATTAGAATTGGAA---------AATGTGACAGAAAATTTTAAC----------------
--------------------------ATGTGGAAAAATAACATGGTAGAACAGATGCAAGAGGATATA---
ATCAGCTTATGGGATGAAAAATTA---AAGCCATGTGTAAAATTA---
ACACCACTCTGTGTTACTTTAAATTGCATTAATATTAATATCACCAATAATGCCAATCAGACCAATAAAACCA
ATCAG--
-------------------ACCAATGCCAATCAGACCAGTATAGAACAAACAGACGAAGTGAAA---

AACTGCTCTTTCAATACCACCACACACATAAGG---------------------------
GACAAGGTGAAGAAAGAATATGCTCTT---TTTTATAGTTCTGATGTAGTCCCAATAGGCAAT----------
----

AATACATCTATATTGATAAGCTGTAATACCTCAGTCACTACACAC---
GCCTGTCCAAAGGTATCCTTTGAACCAATTCCCATACATTATTGTGCCCCGGCTGGTTTTGCAATTCTAAAGT
GTAAG---GATGAGACATTTAAT------GGAACAGGACCATGTACAAAT-----------------------
-GTCAGT---ACAGTACAATGCACACATGGAATTAGGCCAGTAGTCTCAACT---
CAATTGCTGTTAAATGGCAGCCTAGCAGAAAAAGAG------------
GTAGTAATTAGATCTGAAAACTTCACAGACAAT---------GCTAAAACCATAATAGTACAGCTGAAA----
-----------GAAGCTGTACAA---------ATTAATTGTACGAGACACAGCAATAATACAAGAAGA-----
-AGTATACGTATA------------GGACCAGGGCAAGCA---TTT------TATGCAACAGGAGAA------
---------ATAATAGGAGATATA------------AGGAAAGCACACTGT---AACATTAGTGGAGCA---
CAATGGAATAAAACTTTAGAACAAATAGTAAAGAAATTAAGA---------------GAACAATTTAAT----
-----------------------------------AATAGAACGATAATCTTTAGT------
CAATCTTCAGGAGGAGACCCAGAAATTGTA---ATGCACAGTTTTAATTGTGGAGGGGAA---
TTTAAGCAGCTCCAGGCAAGAGTCCTGGCTGTGGAAAGATACCTAAGGGATCAACAGCTCCTGGGGATTTGGG
GTTGCTCTGGAAAACTCATTTGCACCACTGCTGTACCTTGGAATGCTAGTTGGAGT-----------------
----------------------------AATAAATCTCAGGATCAAATTTGG------AAA------
AATATGACCTGGATGGAGTGG---GAAAGAGAAATTGACAATTACACAAGCTTAATATAC---
ACCTTACTTGAAGAATCGCAATACCAGCAAGAA--

AAGAATGAACAAGAATTATTGGAATTGGACAAATGGGCAAGTTTGTGGAATTGGTTTGACATAACAAAATGGC
TGTGGTATATAAAAATATTCATAATGATAGTAGGAGGCTTGGTAGGTTTAAGAATAGTTTTTACT---
GTACTTTCTATAGTG---------AATAGAGTTAGGCAGGGATACTCACCA------
TTATCGTTGGCTGTTAGCTTGTTTGACACCATAGCCATAGCAGTAGCTGGG---
GGGACAGATAGGGTTCTAGAAGCATTGCGAAGAGCTTGCTTGGCT----------------------------
--------GTTATACATATA------CCTAGAAGAATAAGACAGGGCTTGGAAAGGGCTTTGCTA---TAA
>KT200357
GCT---ACA--
GAACAATTGTGGGTCACAGTCTATTATGGGGTACCTGTGTGG---AAGGAGGCTACC---
ACCACTCTATTCTGTGCATCAGATGCTAAAGCCTATAAAACAGAG---GCACATAAT----------------
--------------GTTTGGGCCACA---CATGCCTGTGTACCCACAGAC---
CCTAACCCACAAGAAGTAGAATTGGTA---------AATGTAACAGAAAATTTTAAC----------------
--------------------------ATGTGGAAAAATAACATGGTAGAACAGATGCATGAGGATATA---
ATTAGTTTATGGGATGAAAGCCTA---AAACCATGTGTGAAATTA---
ACCCCACTCTGTGTTACTTTAAATTGCAGTAATGCTAATTTCAATTGCAATAATACTAAT-------------
----
----------------------------AACGCTAGTTGTCTAGAGATGCAGGCAGAAGTAAAA---
AATTGCTCTTTCAATATCTCCACAAGCATAAAA---------------------------
AACAAAGAAAAGAAAGAACATGCACTT---TTTTATAGTCTTGATGTAATGTCAATAGATAAT----------
----

ACAAGCTATGCATTGAGACAGTGTAACACCTCAGTCATTAAACAG---
GCCTGTCCAAAGGTATCCTTTACACCAATTCCCATACATTATTGTGCCCCAGCTGGTTTTGCGATTCTAAAAT
GTAAG---GATAAGAAATTCAAT------GGAACAGGACCATGTAAAAAT-----------------------
-GTCAGC---ACAGTACAATGTACACATGGAATTAAGCCAGTAGTGTCAACT---
CAACTGCTGTTAAATGGCAGTCTAGCAGAAGAAGAG------------
GTAGTGATTAGATCTGAAAATCTCACAGACAAT---------GCTAAAACCATAATAGTGCAGCTGAAT----
-----------GAAACTGTAAAA---------ATTAATTGTACAAGACCCAGTAACAACACAAGAAAA-----
-GGTATACACATA------------GGACCGGGGAGTGCA---TTT------TATGCAACAGGAGAA------
---------ATAATAGGAGATATA------------AGACAAGCACATTGT---AACGTTAGTAGAACA---
GAATGGAACAAAACTTTAGGATACATAGTTGAAAAATTAAGA---------------GAACAATTTAAG----
-----------------------------------AATACAACAATAATCTTTAAC------
CAGTCCTCAGGAGGGGACCCAGAAATTGAA---ATGCACAGTTTTAATTGTGGAGGGGAA---
TTTAAACAGCTCCGGGCAAGAGTCCTAGCTCTGGAAAGATACCTACAGGATCAACAGCTCCTAGGCATTTGGG
GTTGCTCTGGAAAACTCATTTGCACCACTAATGTGCCTTGGAATGCTAGTTGGAGT-----------------
----------------------------AACAAAACCCGTAGTGAGATTTGG------GAA------
AACATGACCTGGATGCAGTGG---GAAAGAGAAATTAGCAATTACACAGCAACAATATAC---
TCCTTAATTGAAGAATCGCAAATCCAACAAGAA--

AAGAATGAACAAGAATTATTAGAATTAGATAAGTGGGCAAGTTTGTGGAATTGGTTTGACATAACAAAATGGC
TGTGGTATGTAAAAATATTCATAATGATAGTAGCAGGCTTAGTAGGTTTAAGAATAGCATTTACT---
ATATTTTCTTTAGTA---------AATAGAGTTAGGCAGGGATACTCACCA------
TTATCATTCGCAGTTAGTTTATTTAATGCCACAGCTATAACAGTAGCCGAA---
GGGACAGATAGGGCTATAGAATTACTTCAAAGCATTGGTAGAGCT----------------------------
--------ATTCTCCACATA------CCTAGAAGAATAAGACAGGGCTTAGAAAGGGCTTTGCAA---TAA
>KP109518
GCT---ACA--
GAAAAATTATGGGTCACAGTCTATTATGGAGTACCTGTGTGG---AAGGAAGCAACC---
ACCACTCTATTTTGTGCATCAGATGCTAAAGCATATAGTACAGAA---AAACATAAT----------------
--------------GTTTGGGCCACA---CATGCCTGTGTACCCACAGAC---
CCCGATCCACAAGAAATATTATTGGTA---------AATGTGACAGAAAAGTTTAAC----------------
--------------------------ATGTGGAAAAATAACATGGTAGAACAAATGCATGAGGATGTA---
ATCAGTTTATGGGATCAAAGCTTA---AAACCATGTGTAAAATTA---
ACCCCACTCTGTGTTACTTTAAATTGCACTGATTTGAAAACCAAGAATTGCACTAATTTGGGAACCAAGAATT
GCACTGGT---
-------TTGAATACCACTAATACTCAAAACAATATTAGTAGTGAGGAAAAAGGAGATATGAAA---
AACTGCTCTTTCAATATC---ACAAACGTAAGG---------------------------
AATCAGATACAGAAAGAATATGCAGCT---TTTTATAAACTTGATATAACACCAATAGATACGGGTAAG----
----

AATAATAGTAATACCAGCTATATATTGACAAGCTGTAACGCCTCAGTCATTACACAG---
GCCTGTCCAAAGGTAACCTTTGAACCAATTCCCATACATTACTGTGCCCCGGCTGGCTTTGCGATTTTAAAAT
GTAAA---GATAAGAACTTCAAT------GGAACAGGAACATGTACAAAT-----------------------
-GTCAGC---ACAGTACAATGTACACATGGAATTAGGCCAGTAGTATCAACT---
CAACTGCTGTTAAATGGCAGTCTAGCAGAAGAAGAT------------
GTAGTAATTAGATCTGAGAATTTCACAGACAAT---------GCTAAAACCATCATAGTACAGTTGAAC----
-----------AATTCTGTAGAA---------ATTAATTGTACAAGACCCAATAACAATACAAGAAGA-----
-AGTATACGTATA------------GGACCAGGGAGTGTA---TAT------TATGCAACAGGGGAC------
---------ATAATAGGAGAAATA------------AGAAAAGCACAGTGT---ATCCTTAAAGCGCAA---
CAGTGGGAGGAAACTTTAAAAAAGGTAGCTATAAAATTAAGA---------------GAGCAATTTAAT----
-----------------------------------AATAGAACAATAATCTTTAGT------
CACTCCTCAGGAGGGGACCCAGAAATTGTA---ATGCACAGTTTTAATTGTGGAGGGGAA---
TTTAAGCAGCTCCAGGCAAGAGTCCTGGCTGTAGAAAGATACCTAAGGGATCAACAGCTCCTGGGGATTTGGG
GCTGCTCTGGAAAACTCATCTGCACCACTAATGTGCCTTGGAATACTAGTTGGAGT-----------------
----------------------------AACAAATCTCTGGATAGCATTTGG------GAT------
AACATGACTTGGATGGATTGG---GATAAAGAAATTAATAATTACACAAACCTAATATAT---
AATTTACTTGAAGAATCGCAGAACCAGCAAGAA--

AAGAATGAACAAGAATTGTTGGAATTGGATAAATGGGCAAGTTTATGGACATGGTTTGACATTACGAATTGGC
TGTGGTATATAAAAATATTCATAATGATAGTAGGAGGCTTGATAGGTTTAAGAATAGTTTTTACT---
GTGCTTTCTATAGTG---------AATAGGGTTAGGCAGGGATACTCACCC------
TTATCGTTTGCTGTTAGCTTGTTTAATGCAATTGCCATAGCAGTAGCTGGG---
GGGACAGATAGGGTCTTAGAATTAATACAAAGAATTTATAGAGGT----------------------------
--------ATTCTCCACGTA------CCTGTAAGAATAAGACAGGGATTGGAAAGGCTTTTGCTA---TAA
>KY465969
GCT---ATA--
GAAGATTTGTGGGTCACAGTCTATTATGGGGTACCTGTGTGG---AAAGAAGCAACC---
ACCACTCTATTTTGTGCTTCAGATGCTAAAGCATATGACACAGAG---ATACATAAT----------------
--------------ATTTGGGCCACA---CATGCCTGTGTACCCACAGAC---
CCTAACCCACAAGAACTACTTTTGAAA---------AATGTAACAGAAAACTTTAAC----------------
--------------------------ATGTGGAAAAATGACATGGTAGAACAGATGCATAAGGATATA---
ATCAGTTTATGGGATGAAGGCCTA---AAGCCATGTGTAAAATTA---
ACCCCATTATGTGTTACTTTAGAGTGTGCTAATATAACTTGCAACAAAAATAGTACTTGC-------------
----
---GAGGATGGAGGAGGAATGAAA---
AACTGCTCGTTCAATGTCACCACAGGCATAGGA---------------------------
AATAAATGGCATAAAGAATATGCACTT---TTTTATAAGCTTGATATAGTACCAGTGGAAAAT----------
----

AGTAGTACAAGCTATACATTGATAAGTTGTAACACCTCAGTCATTACACAG---
GCCTGTCCAAAGGTAACCTTTGAACCAATTCCCATACATTATTGTGCCCCGGCTGGTTTTGCGATTCTAAAGT
GTAAT---GAAAAGAAATTCAAT------GGATCAGGATCATGTAAAAAT-----------------------
-GTCAGC---ACAGTACAATGTACGCATGGAATTAGGCCAGTAGTGTCAACT---
CAACTGTTGCTAAATGGCAGTCTAGCAGAGGAAGAG-----------
GTAGTAATTAGATCTGAAAATTTCACGGACAAT---------GCTAAAAACATAATAGTACATCTGAAC----
-----------GAATCTATAGTA---------ATTAATTGTACAAGACCCAACAACAATACAAGAAAA-----
-AGTATACAGATG------------GGATGGGGGAGAGCA---TTT------TATGCAACAGGAGAA------
---------ATAATAGGAGATATA------------AGACAAGCACATTGT---AACATTAGTAAAGCA---
CAATGGAACAGAACTTTAAAACAGGTAGCTGCAAAATTAATG---------------AACCAATTCAAT----
-----------------------------------ACTACAACAATAATCTTTAAT------
CCATCCTCAGGAGGGGACCTAGAAATTACA---ACACATAGTTTTAATTGTGGAGGGGAA---
TTTAAGCAGCTCCAGGCAAGAGTCCTGGCTGTGGAAAGATACCTAAAGGATCAGCAGCTCCTAGGAATTTGGG
GTTGCTCTGGAAAACTCATCTGCACCACTGCTGTGCCTTGGAATGCTAGTTGGAGT-----------------
----------------------------AATAAATCTCTAAATACCATTTGG------GAC------
AATATGACCTGGATGGAATGG---GAAAGAGAAATTGACAATTACACAGGCATAATATAC---
AACTTACTTGAAAAATCACAGATACAACAGGAA--

AAGAATGAAAAGGACTTATTGGAATTGGATAGTTGGGCAAGTTTGTGGACCTGGTTTGATATAACAAGGTGGC
TGTGGTATATAAAAATATTCATAATAATAGTAGGAGGCTTAATAGGTTTAAGAATAGTTTTTACT---
GTACTTTCTATAGTG---------AATAGAGTTAGGAAGGGATACTCACCA------
TTATCATTCGCTATCAATTTGTATGATACCATAGCTATAGCAGTAGCTGAA---
GGGACAGATAGAGCTATAGAAACATTACAAAGAATTGGTAGAGCT---------------------------
--------TTCCTCCACATA------CCTAGAAGAATAAGACAGGGCTTTGAAAGAGCTTTGCTA---TAA
>KC595204
GTT---GCA---
GGACAATTGTGGGTCACAGTCTATTATGGGGTACCTGTGTGG---AAAGAAGCAACC---
ACCACTCTATTTTGTGCGTCAGATGCTAAAGCATATGATCCAGAG---GTACATAAT----------------
--------------GTTTGGGCCACA---CATGCCTGTGTACCCACAGAC---
CCCAACCCACAAGAAGTAGTATTGGGA---------AATGTGACAGAAAATTTTAAC---------------
--------------------------ATGTGGAAAAATAACATGGTAGAACAGATGCATGAGGATATA---
GTCAGTTTATGGGATCAAAGCCTA---AAGCCATGTGTAAAATTA---
ACCCCACTCTGTGTTACTTTAAATTGCACTGATTATAATACCACT---------------------------
----
----------------------------AGTAGAGGGAAACAGATGGAGGGAGGAGAAATAAAA---
AACTGCTCTTTCAATATCACCAAAAGCATAAGA--------------------------
GATAAGGTGCAGAAAGAATATGCAATT---TTTTATAAGCTTGATATAGTACCAATAGATAAT----------
----

GATAGCTATAGGTTGATAAGTTGTAACACCTCAGTCATCACACAG---
GCCTGTCCAAAGGTAACCTTTGAGCCAATTCCCATACATTATTGTACCCCGGCTGGTTTTGCGATTCTAAAGT
GTAAC---GATAAGAAGTTCAAT------GGAACGGGACCATGCACAAAT-----------------------
-GTCAGC---ACAGTACAATGTACACATGGAATTAGGCCAGTAGTATCAACT---
CAACTGCTGTTAAATGGCAGTCTAACAGAAGAAGAG-----------
GTAGTAATTAGGTCTGAAAATTTCACGGACAAT---------ACTAAAACCATAATAGTACAACTGAAA----
-----------GAATCTGTAGAA---------ATTAATTGTACAAGACCCAACAACAATACAAGAAAA-----
-AGTATAAATATA------------GGACCAGGGAGAGCA---TTT------TATACAACAGGAGAC------
---------ATAATAGGAGATATA------------AGACAAGCACATTGT---AACCTTAGTAGAGCA---
AAATGGAATGACACCTTAGAACAGATAGTTGTCAAATTAAGA---------------GAACAATTCGAG----
-----------------------------------AATAAAACAATAGTCTTTAAT------
CAATCCTCAGGAGGGGACCTAGAGATTGTA---ATGCACAGTTTTAATTGTGGAGGGGAA---
TTTAAGCAGCTCCAGGCAAGAGTCCTGGCTGTGGAAAGATACCTAAAGGATCAACAGCTCCTGGGGATTTGGG
GTTGCTCTGGAAAACTCATCTGCACCACTGCTGTGCCTTGGAATACTAGTTGGAGT-----------------
----------------------------AATAAATCTCTGGATAAGATTTGG------GAT------
AACATGACCTGGATGGAGTGG---GAAAGAGAAATTAATAATTACACAGACTTAATATAC---
AACTTACTTGAAGAATCGCAAAACCAACAAGAA--

AAGAATGAACAAGAATTATTGGAATTAGATAAATGGGCAAGTTTGTGGAATTGGTTTGACATAACAAGCTGGC
TGTGGTATATAAAAATATTCATAATGATAGTAGGAGGCTTGGTAGGTTTAAGAATAGTTTTTACT---

GTACTTTCTATAGTG---------AATAGAGTTAGGCAGGGATACTCACCA------
TTATCGTTTGCTGTTAGCTTGCTCAATGCCACAGCCATAATAGTAGCTGAG---
GGGACAGATAGGGCTATAGAAGTATTACAAAGAGCTTATAGAGCT----------------------------
--------ATTCTCCACATA------CCTACACGAATAAGACAGGGAGCGGAAAGGGCTTTGCTA---TAA
>DQ141342
GCT---AAA---GAA---------------------------------
CAAGACAAATTGTGGGTCACAGTCTATTATGGGGTACCTGTGTGG---AAAGAAGCAACC---
ACCACTCTATTTTGTGCAGCAGATGCTAAAGCATATGATCCAGAG---GTACATAAT----------------
--------------GTTTGGGCCACA---CATGCCTGTGTACCCACAGAC---
CCCAACCCACAAGAATTAGTATTAGGA---------AATGTGACAGAAAATTTTAAC----------------
--------------------------ATGTGGAAAAATAACATGGTAGAACAGATGCATGAGGATATA---
ATCAGTTTATGGGATGAAGGCCTA---AAGCCATGTGTGAAATTA---
ACCCCACTCTGTGTTACTCTAAATTGCACTGATGCTACTAATTCCAATAGTACAAGGAATAGTACT-------
----
-------------------AATGACAATAATAGTAACATGAAAATGGAGGTAGGAGAAATAAAA---
AATTGCTCTTTCAATATCACCACACACATGGA---------------------------
GATAAGATGCAGAAAGAATATGCAATT---TTTTCTAAACTTGATGTAGTACCAATAGCCAAT----------
----

GCTAGTAAGAGCTACAGGTTGATAAGTTGTAACACCTCAGTCACTACACAG---
GCCTGTCCAAAAGTAACCTTTGAGCCAATTCCCATACATTATTGTGCCCCGGCTGGTTTTGCGATTCTAAAAT
GTAAT---AATAAGACGTTCAAT------GGAACAGGACCATGTAAAAAT-----------------------
-GTCAGC---ACAGTACAATGTACACATGGAATTAGGCCAGTAATATCAACT---
CAACTGCTGTTAAATGGCAGTCTAGCAGAAGAAGAG------------
GTAGTAATTAGATCTGAAAATTTCTCGGATAAT---------GCTAAGACCATAATAGTACAGCTGAAA----
-----------GATCCTATAGAA---------ATCAATTGTACAAGACCCAATAACAATACAAGAAGA-----
-AGTATACATGTA------------GGACCAGGAAGAGCA---TTG------TATACAACA---GAC------
---------ATAATAGGAGACATA------------AGAAAAGCACATTGT---AACATTAGTAGAGAA---
AAATGGAATAACACTTTAAGGCAGATAGTTACAAAATTAAGA---------------GAACAATTAGGG----
--------------------------------AATCAAACAACAATAGTTTTTAAG------
CAATCCTCAGGAGGAGACCCAGAAATTGTA---ATGCACAGTTTTAATTGTGGAGGGGAA---
TTTAAGCAGCTCCAGGCAAGAGTCCTGGCTGTGGAAAGATATCTAAGGGATCAACAGCTCCTGGGGATTTGGG
GTTGCTCTGGAAAACTCATTTGCACCACTGCTGTGCCTTGGAATGCTAGTTGGAGT-----------------
----------------------------AATAAAACTCAGGATGAGATTTGG------AAT------
GAAACGACCTGGATGCAGTGG---GACAAAGAAGTTAGCAATTACACAGAATTAATATAC---
TCCTTAATTGAAAAATCGCAGAACCAGCAGGAA--

CAGAACGAACTAGACTTATTAAAATTGGATGAATGGGCAAGTTTGTGGAATTGGTTTGACATATCAAAATGGC
TGTGGTATATAAGAATATTCATAATGATAGTAGGAGGCTTGGTAGGTTTAAGAATAGTTTTTGCT---
GTACTTTCTATAGTA---------AGTAGAGTTAGGAAGGGATACTCACCA------
TTATCGTTTGCTATTAGCTTGCTCAACGCCACAGCCATAGCAGTAGCTGAA---
GGGACAGATAGGATTATAGAGATATTACAAAGAGCTTGTAGAGCT----------------------------
--------ATTCTCCACATA------CCTAGAAGAATAAGACAGGGCTTGGAAAGAATTTTGCTA---TAA
>DQ141343
GCT---AAA--
GAAGAATTGTGGGTCACAGTCTATTATGGGGTACCTGTGTGG---AAAGAAGCAACC---
ACCACTTTATTTTGTGCATCAGATGCTAAGGCATATGATACAGAG---GTACATAAT----------------
--------------GTTTGGGCCACA---CATGCCTGTGTACCCACAGAC---
CCCAACCCACAAGAAATGAAATTGGGA---------AATGTGACAGAAAATTTTAAC----------------
--------------------------ATGTGGAAAAATAACATGGTAGAACAGATGCATGAGGATATA---
ATCAGTTTATGGGATCAAAGTCTA---AAGCCATGTGTAAAATTA---
ACCCCACTCTGCGTTACTTTAAATTGCACTAATTATTGGAAGAATGATACCAATACCACT-------------
----
-------------------------GTGAATAACGGGGAAATAATGGAGAAAGGAGAGATGAAA---
AACTGCTCTTTCAATATCACCACAAACATGAGA--------------------------
GATAAGATGCAAAAAGAATATGCAGTT---
TTTTATGAATCTGATGTAGAACCAATGAACAATAGCAATGTAGAA----------------------------
----
CCAAAAAACAATAGCAGTAATGAGACTATCTATAGAGAATATAGGTTGACAAGTTGTAACACCTCAATCATTA
CACAG---

GCCTGTCCTAAAGTATCCTTTGAGCCAATTCCCATACATTATTGTGCCCCGGCTGGCTTTGCGATTCTAAAGT
GTAAT---GATAAGAAGTTCAAT------GGAACAGGACCATGTAAAAT------------------------
-GTCAGC---ACAGTACAATGTACACATGGAATTAGGCCAATAGTATCAACT---
CAATTGCTGTTAAATGGCAGTCTAGCAGAA---GAG------------
GTAGTAATTAAATCTGAAAATATCACAAATAAT---------GCTAAAACCATAATAGTACAGCTGAAC----
-----------GAAACTATAACC---------ATTAATTGTACAAGACCCAACAACAATACAAGAAGA-----
-AGTATACCTATA------------GGACCAGGGAGAGCA---TTT------TATGCAACAGGAGAC------
---------ATAATTGGAGATACA------------AGAAAAGCATATTGT---ACCATTAATAAAACA---
AAATGGAATAACACTTTATACCAGATAGCTACAAAGTTAGGA---------------GAACAATTTAAG----
-----------------------------------GATAAAATAATAGTCTTTAAT------
AAATCCTCAGGAGGGGACCCAGAAATTGTA---ATGCACAGTTTTAATTGTGGAGGAGAA---
TTTAAGCAGCTCCAGGCAAGAGTCCTGGCTGTGGAAAGATTCCTACAGGATCAACAGCTCCTGGGGATTTGGG
GTTGCTCTGGAAAACTCATCTGCACCACTACTGTGCCTTGGAATACTACTTGGAGT-----------------
----------------------------AATAAAAATCTGAGTCAGATCTGG------AAT------
AATATGACCTGGATGGAGTGG---GAAAAAGAAATTGACAATTACACAGGCTTAATATAC---
TCCTTAATTGAAGAATCGCAAAACCAGCAAGAA--

AAGAATGAACAAGACTTATTGGCATTGGATAAATGGGCAAGTTTGTGGAATTGGTTTAGCATAACAAAATGGC
TGTGGTATATAAAAATATTCATAATGATAGTAGGAGGCTTGGTAGGTTTAAGAATAATTTTTGCT---
GTACTTTCTATAGTG---------AATAGAGTTAGGCAGGGGTACTCACCA------
TTATCGTTTGCTGTTAGCTTGTTCAACGCCATAGCCATAGCAGTAGCTGAG---
GGAACAGATAGAATTTTAGAAGTATTACAAAGAATTGGTAGAGCT----------------------------
--------ATTCGCCACATA------CCTACAAGAATAAGACAGGGCTTGGAAAGGGCTTTGCTA---TAA
>DQ448819
GCT---AAA---GAA---------------------------------
GATGATAACTTGTGGGTCACAGTCTATTATGGGGTACCTGTGTGG---AAAGAAGCAAAC---
ACCACTCTATTTTGTGCATCAGATGCTAAAGCATATGATAAAGAG---GTACATAAT----------------
--------------GTTTGGGCCACA---CATGCCTGTGTACCCACAGAC---
CCCAACCCACAAGAAGTAGTATTGGGA---------AATGTGACAGAAAATTTTAAC----------------
--------------------------ATGTGGAAAAATGATATGGTAGAACAGATGCATGAGGATATA---
ATCAGTTTATGGGATCAAAGCCTA---AAGCCATGTGTAAAATTA---
ACCCCACTCTGTGTTACTTTAAATTGCACTGATGTGAATATTACTAATACTAATAATAACACTAGTGCT----
----
----------------AATAGCACTAGTGGTAACTGGACAATGATGGAGAGAGGAGAAATAAAA---
AATTGCTCTTTCAATATTACCACAAATATAGGA---------------------------
GATAAGAGGCAGAAAGAACGTGCACTT---TTTTATAACCTTGATATAGTACCAATAGACAAT----------
----

AGTAGTAATTACAGGTTGCTAAGTTGTAATACCTCAGTCATTACACAG---
GCCTGTCCAAAAATAACCTTTGAACCAATTCCCATACATTATTGTGCCCCGGCTGGTTTTGCGATTCTAAAAT
GTAAT---GATAAGAAGTTCAAT------GGAACAGGACCATGTACAAAT-----------------------
-GTCAGT---ACAATACAATGTACACATGGCATTAGGCCAGTAGTATCAACT---
CAACTGCTGTTAAATGGCAGTCTAGCAGAAGAAGAG------------
ATAGTAATTAGATCTGAAAATATCACAGATAAC---------ATTAAAAACATAATAGTACAGCTGAAC----
-----------ACAACTATAAAA---------ATTAATTGTACAAGACCTAACAACAATACAAGAAAA-----
-AGTATACATATA------------GGACCAGGGAGTGCA---TTT------TATGCAACAGGAGAC------
---------ATAATAGGAGATATA------------AGGCAAGCACATTGT---ATCATTAATGGAACA---
GAATGGAATAACACTTTAAAACAGATAGTGACAAAATTAAGA---------------GAAAAATTAGGA----
--------------------------------GAAAATAAAACAATAGTCTTTAAT------
CAATCCTCAGGAGGGGACCCAGAAATTGTA---ATGCACAGTTTTAATTGTGGAGGGGAA---
TTTAAGCAGCTCCAGGCAAGAATCCTGGCTGTAGAAAGATACCTACAGGATCAACAGCTCCTGGGAATTTGGG
GTTGCTCGGGAAAACTCATCTGCACCACTGCTGTGCCCTGGAATGCTAGTTGGAGT-----------------
----------------------------AATAAATCTCAAGATGCAATTTGG------AAT------
AACATGACCTGGATGGAGTGG---GAAAAGGAAATTGATAATTACACAGGCTTAATATAC---
ACCTTAATTGAAAAATCACAGAACCAGCAAGAA--

AAGAATGAACTAGAATTATTGCAATTGGATAAATGGGCAAGTTTGTGGACTTGGTTTGACATAACAAAATGGC
TGTGGTATATAAAAATATTCATAATGATAGTAGGAGGCTTGATAGGTTTAAGAATAGTCTTTACT---
GTACTTTCTATAGTA---------AATAGAGTTAGGAAGGGATACTCACCA------
TTATCGTTTGCTGTTAGCTTGCTCGACGCCATAGCCATAGCAGTAGCTGAG---

GGGACAGATAGAGTTCTAGAAGGGTTACAAAGAGCTTTTAGGGCT----------------------------
--------ATTCTCAATATA------CCTAGAAGAATAAGACAGGGCTTGGAAAGGGCTTTGCTA---TAA
>EF531331
GTT---GCA--
GGAAATTTATGGGTCACCGTCTATTATGGGGTACCTGTGTGG---AAAGAAGCAACC---
ACAACTCTATTCTGTGCATCAGATGCTAAAGCATATGATACAGAG---AAACATAAT----------------
--------------GTCTGGGCCACA---CATGCCTGTGTACCCACAGAC---
CCCAGTCCACAAGAAGTAGTATTGGCA---------AATGTGACAGAAAATTTTGAC---------------
--------------------------GTGTGGAACAATGACATGGTAGAACAGATGCATGAGGATATA---
ATTAGTTTATGGGATCAGAGCCTA---AAGCCATGTGTAAAATTA---
ACCCCACTCTGTGTTACTTTAAATTGCACTAATGTGACCACTACTAATACCAATAGCCCTACTACCACTAGTA
GCACC---
-------AGTAGCCTTACTGCGAGTACTGCTCCTACCACTAGTAGCATCAAGGATGAAATAAAA---
AATTGCTCTTTCAATACCACCACAAGCATAAGC--------------------------
GATAAATGGCAAAAAGCATATGCACTT---TTTTATAAACTTGATGTAGAACCAATAGGAAAT----------
----

GATGATACTAGTTATAGGTTGATAAGTTGTAACACCTCAGTCATTACACAA---
GCCTGTCCAAAGGTAACCTTTGAGCCAATTCCCATACATTTTTGTGCCCCGGCTGGTTTTGCGATTCTAAAGT
GTAAC---AATAAGACGTTCCAG------GGACAAGGACCATGTAAAAAC----------------------
-GTCAGC---ACAGTGCAATGTACACATGGAATTAAGCCAGTAGTATCAACT---
CAACTGCTGTTAAATGGCAGTCTAGCAGAAGAAGAG-----------
ATAGTAATTAGATCCGCCAATTTCACGGACAAT---------GCTAAAGTCATAATAGTACAGCTGAAG----
-----------GAATTTGTAGAA---------ATTAATTGTACAAGACCCAACAACAATACAAAAAGA-----
-AGTATACATATA------------GGACCAGGCAGCGCC---TTG------TATACAAGAAGA---------
---------ATAATAGGAAATATA-----------AGACAAGCATATTGT---ACCATTAATGGAACA---
AAATGGAATAACACTTTAAGACAAGTAGCTATAAAATTAAAA--------------GAACAATTTAAG----
-----------------------------------AATAAAACAATAGTCTTTAAT------
CACTCCTCAGGAGGGGATCCAGAAATTGTA---ATGCACAGCTTTAATTGTGGAGGGGAA---
TTTAAACAGCTCCAGGCCAGAGTCCTGGCTGTGGAAAGATACCTAAAGGATCAACAGCTCCTGGGGATTTGGG
GTTGCTCTGGAAAACTCATTTGCACCACTACTGTGCCTTGGAATACTAGTTGGAGT----------------
----------------------------AATAGATCTCTCGAGCACATTTGG------AAT------
AACATGACCTGGATGGAGTGG---GAAAAAGAAATTGATAATTACACAGACTTAATATAC---
AACTTAATTGAAAAAATCGCAAAACCAGCAAGAA--------------------------------------
----
AAGAATGAGCAAGACTTATTGGCATTAGACACATGGGCAAGTTTGTGGAATTGGTTTGACATATCAAAATGGT
TGTGGTATATAAAAATATTCATAATGATAGTAGGAGGCTTAATAGGTTTAAGAATAATTTTTGCT---
GTACTTTCTATAGTG---------AATAGAGTTAGGCGGGGATACTCACCC------
TTATCGTTCGCTGTTAGTTTGTTCAACGCCACAGCCATAGCAGTAGCTGAG---
GGGACTGATAGGATTATAGAGGCATTGCAAAGAGCTTATAGAGCT--------------------------
--------ATTCTTCACATA------CCTAGAAGAATAAGACAGGGCTTGGAAAGGATTTTGCTA---TAA
>MH000288
GCT---ACA--
GAGCAATTATGGGTCACAGTCTATTATGGGGTACCTGTGTGG---AAAGAAGCAAAT---
ACCACTCTATTTTGTGCATCAGATGCTAGAGCATATGATACAGAG---GTGCATAAT----------------
--------------GTTTGGGCCACA---CATGCCTGTGTACCCACAGAC---
CCCAACCCACAAGAAGTAGTATTGGAA---------AATGTGACAGAAAATTTTAAC---------------
--------------------------ATGTGGAAAAATAACATGGTAGAACAAATGCATAAGGATATA---
ATCAGTTTATGGGATCAAAGCCTA---AAGCCATGTGTAAAGTTA---
ACCCCACTCTGTGTTACTTTAGATTGCAAAGAATTG-------------------------------------
----
--GAAATGAAA---
AACTGCTCTTTCAATGTCACCGCAGGCATAAGA--------------------------
GATAAGATACAGAAAGAACATGCATTA---TTTTATACATATGATTTAGTACCAATAACCAAT----------
----

TACAGGTTGATAAATTGTAATACCTCAGTCATTACACAG---
GCCTGTCCAAAAGTAACATTTGAACCAATCCCCATACATTATTGTGCCCCAGCTGGTTTTGCGATTATAAAGT
GTAAT---GATAAGCAGTTCAAT------GGAACAGGACCATGTAAAAAT----------------------
-GTTAGC---ACAGTACAATGTACACATGGAATTAGGCCAGTAGTATCAACT---

CAATTGCTGTTAAATGGCAGTTTGGCAGAAGAAGAG------------
GTAATAATTAGATCTGAAAATTTCACAAACAAT---------GCCAAAACCATAATAGTACAGCTAAAT----
-----------GAAACTGTAACA---------ATCAATTGTACAAGACCCAACAACAATACAAGAAAA-----
-GGTATACATATA------------GGACCAGGGAGAGCA---TTT------TATGCAACAGGAAGC------
---------ATAATAGGAAACATA------------AGACAGGCATACTGT---AACATTAGTGGAACA---
AAATGGAATAGCACCTTAAAACACATAGCTGCAAAATTAAGA---------------GAACAATTTGGG----
--------------------------------GAGAATAAAGCAATAGTCTTTAAT------
CGCTCGTCAGGAGGGGACCCAGAAATTGTA---ATGCATAGTTTTAATTGTGGAGGGGAA---
TTTAAGCAGCTCCAGGCAAGAGTCCTGGCTGTGGAAAGATACCTAAAGGACCAACAGCTCCTAGGGATTTGGG
GTTGCTCTGGAAAACTCATTTGCACCACTACTGTGCCTTGGAATACTAGTTGGAGT-----------------
----------------------------AATAAATCTTATAATGAGATTTGG------GAT------
AACATGACCTGGATGCAGTGG---GAGAGAGAAATTGATAATTACACATGCTTAATATAC---
TCCTTAATTGAAAAATCGCAGAACCAGCAAGAA--

CAGGATGAACAAGAATTATTGGCATTAGATAAATGGGCAAGTTTGTGGAATTGGTTTGACATAACAAATTGGC
TGTGGTATATAAAAATATTCATAATGATAGTAGGAGGCCTAATAGGTTTAAGAATAGTTTTTACT---
GTATTTTCATTAGTA---------AATAGAGTTAGGCAGGGATATTCACCA------
TTATCGTTGGCTGTCAGCTTGCTTAACGCCACAGCTATAGCAGTAGCTGAG---
GGGACAGATAGGGTTATAGGAGCAGTACAAAGAGTTGGTAGAGCT----------------------------
--------TTTATCCACATA------CCTAGAAGAGTGAGACAGGGCTTGGAAAGGGCTTTGCTA---TAA
>KC699040
GCT---TCA--
GAAAAATTGTGGGTCACAGTCTATTATGGGGTACCTGTGTGG---AAGGAAGCAAAA---
ACCACTCTATTTTGTGCATCAGATGCTAAAGCATATGATACAGAG---GTGCATAAT----------------
--------------GTTTGGGCCACA---CATGCCTGTGTACCCACAGAC---
CCCAACCCACAAGAAGTAACATTGGAA---------AATGTAACAGAAAATTTTGAC----------------
--------------------------ATGTGGAAAAATAACATGGTAGAACAGATGCATGAGGATGTA---
ATCAGTCTATGGGATCAAAGCTTA---AAACCATGTACAAAACTA---
ACCCCACTCTGTGTTACTTTAAATTGCACTAATGTGACTGTTATTAGTACCAATAATGTTACTTATGCC----
----
----------------------------AGTGGTGGTATCAGTAATATCACGGAAAAAATGAAA---
AACTGCACTTTCAATATCACCACAGACGTAAGA---------------------------
GATAAGATGAAAAGGGTATATTCACTT---
TTTTATAAAGAGGATATAGAACTAATAAAGAATCCTGATGGTCCTAATACCAACAATAGTGCT----------
----
AATAATACCAGCAATAGTACTGATAGTCCTAGTACCAGCTATAGGTTGACAAAGTGTAACACCTCAGTCATTA
CACAA---
GCCTGTCCAAAGGTATCCTTTGAGCCAATTCCCATACATTATTGTGCCCCGGCTGGTTTTGCAATTCTAAAAT
GTAAT---GATAAGAAATTCAAT------GGAACAGGACCATGTAAAAAT-----------------------
-GTCAGC---ACAGTACAATGTACACATGGAATTAGGCCAGTAGTATCAACT---
CAACTATTGTTAAATGGTAGTCTAGCAGAAGAAGAG------------
ATAGTAATTAGAACTAAAGACCTTGAAGAAAAT---------ACTAAAAACATAATAGTACAGCTGAAT----
-----------AAAACTGTAAAA---------ATTAACTGTACAAGACCCGGCAACAATACAAGGAAA-----
-AGCCTACATATA------------GGACCAGGGAGAGCA---TTT------TTTGCAACAAATGAT------
---------ATAATAGGAGATATA------------AGACAAGCACATTGT---AACATTAGTGCAGAA---
GAATGGAATGAAACTTTAGCAAAGGTAGCTAGCAAAATAAGA---------------
GAACTATTTAGTGAAAGAAAC------AGCAGTTATGAG------------AATGATACAATAGTCTTTAGT-
--CCACCATCCCCAGGAGGGGACCCAGAAATTATA---ATGCACAGTTTTAATTGCAGAGGGGAA---
TTTAAGCAGCTCCAGGCAAGAGTCCTGGCTCTGGAAAGATACCTAGCGGATCAACAGCTCCTGGGACTTTGGG
GTTGCTCTGGAAAACTCATCTGCACCACTGCTGTGCCTTGGAATGCTAGTTGGAGT-----------------
----------------------------AATAGATCTCTGACAGAAATTTGG------GAT------
AACATGACCTGGATGGAGTGG---GAAAGAGAAATTAATAATTATACAGGTATAATATAC---
TCCTTAATTGAAGGAGCGCAGAACCAGCAAGAA--

AAGAATGAAGAAGACTTATTAAAATTAGATCAATGGGCAAATTTGTGGAATTGGTTTAACATAACACAATGGC
TGTGGTATATAAAAATATTCATAATGATAGTAGGAGGTTTAATAGGTTTAAGAATAGTTTTTGCT---
GTACTTTCTATAGTG---------AATAGAGTTAGGCAGGGATACTCACCC------
TTGTCGTTAGCTGTTAGCTTGCTTAACGCCACAGCCATAACAGTAGCTGAG---
GGAACAGACAGGGTTATAGAAGCATTACTAAGAGCTTGGAGGGCT----------------------------
--------ATTCTCCACATA------CCTAGAAGAATAAGACAAGGCTTAGAAAGGGCTTTGATA---TAA

>KC699032
GCT---GAA---GAA------------------------------------
GGAAAATTGTGGGTCACAGTCTATTATGGGGTACCTGTGTGG---AAAGAAGCAACT---
ACCACTCTATTTTGTGCATCAGATGCTAAGGCATATGAAACAGAA---GCACATAAT----------------
--------------GTTTGGGCCACA---CATGCCTGTGTACCCACAGAT---
CCTAGTCCACAAGAAGTATCATTGGGA---------AATGTGACAGAAAATTTTAAT----------------
--------------------------ATGTGGAAAAATAACATGGTAGAACAAATGCATGAGGATGTA---
ATCAGTTTATGGGATCAAAGTCTA---AAGCCATGTGTAAAATTA---
ACCCCACTTTGTGTTAAGTTAACTTGCACTGATAAGTTGGATAATGATACCGATACTAATACTACT-------
----
-------------------------------------TTGAAAAATTTAGAAGGGGAAGTAAAA---
AACTGCTCTTTCAATGTCAGCACAAACATAGTA---------------------------
AATAAGATGCAGAGACAATATGCACTT---CTTTATAAACTTGATGTAATGCAAATAGATGAAGAT-------
----

GATAATACAAGCTATACATTAACAAGTTGTAACACCTCAGTCATTACACAA---
GCTTGTCCAAAGGTATCCTTTGAACCAATTCCAATACATTATTGTGCCCCGGCTGGTTTTGCGATTCTAAAGT
GTAAA---GATAAGAATTTCAAT------GGAACAGGACCATGTACAAAT-----------------------
-GTCAGT---ACAGTACAATGCACACATGGAATTAAGCCAGTAGTGTCAACT---
CAACTACTGTTAAATGGCAGTCTAGCAGAAGAAGAG------------
ATAGTACTCAGATCTGAGAATTTCACGAACAAT---------GCAAAAAACATAATAGTACAACTGAAG----
-----------GACCCTGTAAAA---------ATTAATTGTACAAGACCCCACAACAATACAAGAAAA-----
-AGTATACATATA------------ACACCAGGGAGAGCA---TTT------TATGCAACAGGAGCT------
---------ATAATAGGAGATATA------------AGACAAGCACATTGT---AACATTAGTAGAGAA---
AAATGGAATAACACTTTAGGGGAGATAGCAAAAAAGTTAGGA---------------GAACAGTTTGGT----
--------------------------------AAGAATAAAACAATAATCTTTACT------
CAGCCCTCAGGAGGGGACCCAGAAATTATA---ATGCACAGTTTTAATTGTGGAGGGGAA---
TTCAAACAGCTCCAGGCAAGAGTCCTGGCTGTAGAAAGATACCTAAAGGATCAGCAGCTCCTAGGAATCTGGG
GTTGCTCTGGAAAGCTCATTTGCACCACTGCTGTGCCTTGGAATATTAGTTGGAGT-----------------
----------------------------AATAGATCTCACGATTATATTTGG------GAT------
AACATGACATGGATGCAGTGG---GAGAAAGAAATTGACAATTACACAGACTTAATATAC---
AATTTACTTGAACAATCGCAGAACCAACAAGAA--

AAGAATGAACAAGACTTATTGGCATTGGATAAATGGGCAAGTTTGTGGAGTTGGTTTAGCATAACAAATTGGC
TGTGGTATATAAAAATATTCATAATGATAGTAGGGGGCTTAGTAGGTTTAAGAATAGTTTTTTCT---
GTATTTTCTATGGTG---------AATAGAGTTAGGCAGGGATACTCACCA------
TTGTCATTTGCTGTTAGTCTGTTTAATGCCATAGCTATAGCAGTAGCTGAG---
GGAACAGATAGGGTTATAGAAGTAATACAAAGATTTTGTAGAGCT----------------------------
--------ATTCTACACATA------CCTAGAAGAATAAGACAGGGCTTAGAAAGGCTTTTGCTA---TAA
>KC699037
GCT---GCA--
GAACAATTGTGGGTCACAGTCTATTATGGGGTACCTGTGTGG---AAAGAAGCAACT---
ACCACCCTATTTTGTGCATCAGATGCTAAAGCATATGAGAAAGAG---GCACATAAT----------------
--------------GTTTGGGCCACG---CATGCCTGTGTACCCACAGAC---
CCCAACCCACAAGAAGTAATATTGGGA---------AATGTGACAGAAGATTTTAAC----------------
--------------------------ATGTGGAAAAATAACATGGTAGAGCAGATGCATGAAGATATA---
ATCAGTTTATGGGATCAGAGCCTA---AAGCCATGTGTAAAATTA---
ACCCCACTCTGTGTTACTTTAAATTGCACTGATGTTGATGGGAATAATACCAAGGTTAATGAGAAT-------
----
----------------------GCCACTTATTATACTGGGAAAATAGAACCAGGAGAAATAAAA---
AACTGCTCTTTTAATGTCACCCCAGGCATAATA---------------------------
GATAAAGTAAAGAAAGAACGTGCACTT---TTTAATAGACTTGATGTAGTGCCAATAGATAAT----------
----

GATAATACTAGTTATAGGCTGATAAATTGTAATACCTCAGTCATTACACAG---
GCCTGTCCAAAGGTATCCTTTGATCCAATTCCTATACATTATTGTGCCCCGGCTGGTTTTGCGATTTTGAAGT
GTAAC---AATAACACATTCAAT------GGAAAAGGACCATGTACAAAT-----------------------
-GTCAGT---ACAGTACAATGTACACATGGAATTAAGCCTGTAGTGTCAACT---
CAACTACTGTTAAATGGAAGCCTAGCAGAAGGAGGG------------
ATAGTAATTAGATCTGACAATTTCTCAGATAAT---------GCTAAAACCATAATAGTACATCTGAAT----

-----------AAAACTGTAAAC---------ATTACCTGTACAAGAGTCAACAACAATACAAGACAA-----
-AGTATACCTATA------------GGACCAGGGAGAGCA---TTT------TATGCGACAGGAGAT------
---------ATAATAGGAGACATA------------AGACAAGCACATTGT---AACATCTCTAGAGCA---
GAATGGAATAGTACTTTAGAACAGATAGTTAAAAAATTAAAA---------------GAACAATATAAT----
-----------------------------------AATAACACAATAATCTTTAAG------
CCACCAGCAGGAGGAGACCCAGAAATTGTA---ATGCACAGTTTCACTTGTGGAGGGGAA---
TTTAAACAGCTCCAGGCAAGAGTCCTGGCTGTGGAAAGATACCTACAGGATCAGCGGCTCCTAGGGATTTGGG
GTTGCTCTGGAAAACTCATCTGTACCACTACTGTGCCTTGGAATGATAGTTGGAGT-----------------
----------------------------AATAGATCTTATGATAGTATTTGG------GAC------
AATATGACCTGGATGCAGTGG---GAAAAAGAAATTGACAATTACACAGGTGTGATATAC---
AACTTACTTGAAAAATCGCAAAACCAACAAGAA--

AAGAATGAACAAGAATTATTAGAATTGGATAAGTGGGCAAGTTTGTGGAATTGGTTTGACATAACAAAATGGC
TGTGGTATATAAAAATATTCATAATGATAATAGGAGGCTTGATAGGTTTAAGAATAGTTTGTGCT---
GTGCTTTCTATAGTG---------AATAGAGTTAGGCAGGGATACTCACCA------
TTGTTGTTTGCTGTTAGCTTGCTTAATGCCACAGCTGTCACAGTAGCTGAG---
GGAACAGATAGGATTATAGAAGCAGTACAAAGAATCTCTAGAGCT----------------------------
--------ATTATCCACATT------CCCACAAGAGTGAGACAGGGCTTAGAAAGGGCTTTGCAA---TAA
>MH000292
GCA---ACC---
CAATTGTGGGTCACAGTCTATTATGGGGTACCTGTGTGG---AAAGATGCTACT---
ACCACTCTATTTTGTGCATCAGATGCCAGAGCATATGATACAGAG---AAGCATAAT----------------
--------------GTCTGGGCCACA---CATGCCTGTGTGCCCACAGAC---
CCTAACCCACAAGAAATAGTATTGGGA---------AATGTGACAGAAAATTTTAAT----------------
--------------------------GCGTGGAAAAATGACATGGTAGACCAGATGCATGAAGATATA---
ATCAGTTTATGGGATCAAAGCCTG---AAGCCATGTGTAAAATTA---
ACCCCACTCTGTGCTACTTTACAATGCACTAATTTG-------------------------------------
----
---GAAATAAGA---
AACTGCTCTTTCAATATCACCACAGATATGAGA---------------------------
GATAAAGTAAAGAAAGAACATGCACTT---TTTTATAGGCTTGATATAGTACCAATAACTAGT----------
----

TATAGGTTGATAAGTTGTAATACTTCAGTCCTTACACAG---
GCCTGTCCAAAGGTATCCTTTGAACCAATTCCCATACATTATTGTGCCCCGGCTGGTTTTGCGATTCTAAAAT
GTAAT---GATAAGAATTTTAAT------GGATCAGGACCATGTAACAAT-----------------------
-GTCAGC---ACAGTACAATGTACACATGGAATTAAGCCAGTGGTGTCAACC---
CAACTGCTGTTAAACGGCAGTCTAGCAGAAGAAGGG------------
GTAGTAATTAGATCTGAAAATTTTACGAACAAT---------GCTAAAACTATAATAGTACAGCTGAAC----
-----------AAAGCTGTAGTA---------ATTAATTGTACAAGACCCAACAACAATACAAGAAGA-----
-GGTATCCATATA------------GCACCAGGAAGAGCA---TTT------TATGCAACAGGAGAC------
---------ATAGTAGGAGATATA------------AGACAAGCACATTGT---AATCTTAGTGAAGGA---
GACTGGAATGATACTTTAAAAGAAGTAGTTAAAAAATTAAGA---------------GACCAATTTGGG----
-----------------------------------AATAAAACAATACTCTTTGCA------
AATTCCTCAGGAGGGGACCCAGAAATTGTG---ACACACAGTTTTAATTGTGGAGGGGAA---
TTTAAGCAACTCCAGGCAAGAGTCCTGGCTGTGGAAAGATACCTAAAGGATCAACAGCTCCTGGGAATTTGGG
GTTGCTCTGGAAAGCTCATCTGCACCACTGCTGTGCCTTGGAATACTAGTTGGAGT-----------------
----------------------------AATAAAAATTACAGTGATATTTGG------GAT------
AACATGACCTGGATGCAGTGG---GAGAAAGAAATTGATAATTACACAGATTTAATATAC---
ACCTTGCTTGAACAATCGCAGAACCAGCAGGAA--

AAGAATGAACAAGAATTATTGGAATTGGATAAATGGGCAAGTTTGTGGAATTGGTTTGACATAACACGATGGC
TGTGGTATATAAAAATATTCATAATGATAGTAGGAGGTTTAATAGGTTTAAGAATAGTTTTTGCT---
GTGCTTTCTATAGCG---------AATAGAGTTAGGCAGGGATACTCACCA------
TTGTCATTCGCTGTTAGTTTGTTTAATGCAATAGCTATAGCAGTAGCTGAG---
GGGACAGATAGGATTATAGAAATAGTACAGAGAGCTGGTAGAGCT----------------------------
--------ATCCTCCACATA------CCTACAAGAATAAGACAGGGCTTAGAAAGGGCTTTGCAA---TAA
>KC699006
GCT---GCA--
GAAAAATTGTGGGTCACAGTCTATTATGGGGTACCTGTGTGG---AAAGAAGCAACC---

```
ACCACTCTATTTTGTGCATCAGATGCTAAAGCATATGACACAGAG---ATACATAAT----------------
--------------GTTTGGGCCACA---CATGCCTGTGTACCCACAGAC---
CCCAACCCACAAGAAGTATTATTGGAA---------AATGTGACAGAAGATTTTAAC----------------
--------------------------ATGTGGAAAAATAACATGGTAGAACAGATGCATGAGGATATA---
ATCAGTTTATGGGATCAAAGCCTA---AAGCCATGTGTAAAATTA---
ACCCCACTCTGTGTTACTTTAAATTGCACTGATTGGAATGCCAATGTCACTAATACCAATGCCAATAAT----
-------------------------------------------------------------------------
-------------------ACCAATAGTAGTAATTTGGAGACGATGGAAAAAGGAGAAATAAAA---
AACTGCTCTTTCAATATCACCTCAAACATAAGA---------------------------
GATAAGATGCAGAAAGAATATGCTCTT---TTTTATAAACTTGATGTAGTACCAATAGATAATGAT-------
-------------------------------------------------------------------------
----------------------
GATAATACTAGCTATAGGATGATACATTGTAACACCTCAGTCATTACACAA---
GCCTGTCCAAAGATATCCTTTGAACCAATTCCCATACACTATTGTGCCCCGGCTGGTTTTGCGATTTTAAAGT
GTAAC---AATAAGACGTTCAAT------GGAACGGGACCATGTAAAAT------------------------
-GTCAGC---ACAGTACAATGTACACATGGAATTAGGCCAGTAGTGTCAACT---
CAACTGCTGTTAAATGGCAGTCTAGCAGAAGAAGAG------------
GTAATAATTAGATCTGCCAATTTCACAGACAAT---------GCTAAAACCATAATAGTGCAGCTGAAC----
-----------AAATCTGTAGTA---------ATTAATTGTACAAGACCCAGCAACAACACAAGAAAA-----
-GGTATACATATA------------GGACCAGGGAGAGCA---TTT------TATACAACAGGAAAC------
---------ATAATAGGAGATATA------------AGACAAGCACATTGT---AACATTAGTAGAGTA---
GAATGGAATAACACTTTAGGACAGGTAGTTGCAAAATTAAGA---------------GAACAATTTAAG----
-----------------------------------AATAAAACAATAGTTTTTAAT------
CAATCCTCAGGAGGGGACCCAGAAATTGTA---ATGCACAGTTTTAATTGTGGAGGGGGAA---
TTCAAGCAGCTCCAGGCAAGAGTCCTGGCTGTGGAAAGATACCTAAAGGATCAACAGCTCCTAGGGATTTGGG
GTTGCTCTGGAAAACTCATCTGCACCACCACTGTGCCTTGGAATACTAGTTGGAGT-----------------
----------------------------AATAAATCTCTAGATACAATTTGG------AAT------
AACATGACCTGGATGGAGTGG---GAAAGAGAAATTGACAATTACACAGGCTTAATATAC---
ACCTTAATTGAACAATCGCAAAACCAACAAGAA----------------------------------------
-----------------------------
AAGAATGAACAAGAATTATTGGAATTGGATAAATGGGCAAGTTTGTGGACTTGGTTTGACATAACAAACTGGC
TGTGGTATATAAAAATATTCATAATGATAGTAGGAGGCTTGATAGGTTTAAGAATAGTCTTTACT---
GTACTTTCTATAGTG---------AATAGAGTTAGGCAGGGATACTCACCA------
TTATCGTTGGCTATTAGCTTGCTCAATGCTACAGCTATAACAGTAGCTGAG---
GGAACAGATAGGGTTGCAGAAGCAGTACAAACAATTTTTAGAGCT----------------------------
--------ATTATCCACATA------CCTAGAAGAATAAGACAGGGCTTGGAAAGGGCTTTGCTA---TAA
>KC699007
GCT---GCA------------------------------------------
GACAAATTGTGGGTCACAGTCTATTATGGGGTACCTGTGTGG---AAAGAAGCAACC---
ACCACTCTATTTTGTGCATCAGATGCTAAAGCATATGATACAGAG---GTACATAAT----------------
--------------GTTTGGGCCACA---CATGCCTGTGTACCCACAGAC---
CCCAACCCACAAGAAGTAGTATTGGGG---------AATGTGACAGAAAATTTTAAC----------------
--------------------------ATGTGGAAAAATAACATGGTAGAACAGATGCATGAGGATATA---
ATTAGTTTATGGGATCAAAGCCTA---AAGCCATGCGTAAAATTA---
ACCCCACTCTGTGTTACTTTAAATTGCACTGATTATTTGAAGAATGCTACTAACACCACT-------------
-------------------------------------------------------------------------
-------------------------AATAGTAGCGGGGGAATGAAGGAGGAAGGAGAAATAAAA---
AATTGCTCTTTCAATATCACCACAAGCATAAGA---------------------------
GATAAGTTGCAGAAAGAATATGCACTT---TTTTATAAACTTGATGTAATACCAATAGATACT----------
-------------------------------------------------------------------------
----------------------
GATAATACTAGCTATAGGTTGATAAGTTGTAACACCTCAGTCATTACACAG---
GCCTGTCCAAAGGTATCCTTTGAGCCAATTCCCATACATTATTGCGCCCCGGCTGGTTTTGCGATTCTAAAGT
GTAAC---GATAAGAAGTTCAAT------GGAACAGGACCATGTACAAAT------------------------
-GTCAGC---ACAGTACAATGTACACATGGAATTAGGCCAGTGGTATCAACT---
CAACTGCTGTTAAATGGCAGTCTAGCAGAAAAAGAG------------
GTAGTAATTAGATCCGCCAATTTCACGAACAAT---------GCTAAAATCATAATAGTACAGCTGAAT----
-----------GAATCTGTAGAA---------ATTAATTGTACAAGACCCAACAACAATACAAGAAAA-----
-AGTATAAATATA------------GGACCAGGCAGAGCA---TTT------CATACAACAGGAGCA------
---------ATAATAGGAGATATA------------AGACAAGCACATTGT---AACATTAGTAGAGCA---
```

AAATGGAATGACACTTTAAAAAAGATAGTTATAAAATTAAGA---------------GAACAATTTAGG----
-----------------------------------AATAAAACAATAGTCTTTAAT------
CAATCCTCAGGAGGGGACCCAGAAATTGTA---ATGCACAGTTTTAATTGTGGAGGGGAA---
TTCAAGCAGCTCCAGGCAAGAGTCCTGGCTGTGGAAAGATACCTAAAGGATCAACAGCTCCTGGGGATTTGGG
GTTGCTCTGGAAAACTCATTTGCACCACTGCTGTGCCTTGGAATGGTAATTGGAGT-----------------
----------------------------AATAAATCTCTGGATCAGATTTGG------AAT------
AACATGACCTGGATGGAGTGG---GAAAGAGAAATTAATAATTACACAGACATAATATAC---
ACCTTAATTGAAGAATCGCAAAATCAACAAGAA--

AAAAATGAAAAAGAATTATTGGAATTAGATAAATGGGCAAGTTTGTGGAATTGGTTTGACATAACAAAATGGC
TGTGGTATATAAAAATATTCATAATGATAGTAGGAGGCTTGGTAGGTTTAAGAATAGTTTTTGCT---
GTGCTTTCTATAGTG---------AATAGAGTTAGGCAGGGATACTCACCA------
CTATCGTTAGCTGTGAACTTGCTTAATACCACAGCCATAGCAGTAGCTGAG---
GGGACTGATAGGGTTATAGAAGCATTACAAAGAGCTTGTAGAGCT----------------------------
--------ATTCTCCACATA------CCTGTACGAATAAGACAGGGCTTGGAAAGGGCTTTGCTA---TAA
>KC699025
GCT---GCA--
GAACAATTGTGGGTCACAATCTATTATGGGGTACCTGTGTGG---AAAGATGCAACT---
ACCACTCTATTTTGTGCATCAGATGCTAAAGCATATGATACAGAG---GTACATAAT----------------
--------------GTCTGGGCCACA---CATGCCTGTGTACCCACAGAC---
CCCAACCCACAAGAAGTAGTAATGGGA---------AATGTAACAGAAGAGTTTAAC----------------
--------------------------ATGTGGAAAAATAACATGGTAGAACAGATGCATGAGGATATA---
ATCAGTTTATGGGATCAAAGCCTA---AAGCCATGTGTAAAATTA---
ACCCCACTCTGTGTTACTTTAAATTGCACTGATTTAGCTAGGAATACCACTAATAGTAATGCCACTAATAATA
ATACCATTAGT--
----------------------------ACTATTGCCTCTACGATGGAACCAGTAGGAATAAAA---
AACTGCTCTTTCAACATCTCCACAAGCTTAGGA---------------------------
GATCAGGTAAAGAAAGAATATGCATTT---TTTTATAATCTTGATATAGTACAACTAGATGAA----------
----

GGTAATAATAAAAATAATTATAGGTTGATAAGCTGTAATACCTCAGCCCTCACACAG---
GCCTGTCCAAAGGTATCCTTTGAACCAATTCCTATACATTATTGTGCCCCGGCTGGTTTTGCGATTCTAAAGT
GTAAC---AATAAAACGTTCAGT------GGAAAAGGACAATGTACAAAT-----------------------
-GTCAGC---ACAGTACAATGTACACATGGAATTAGGCCAGTAGTGTCAACT---
CAGCTGCTGTTGAATGGCAGTTTAGCAGAGAAAGAG------------
ATAGTAATGAGATCTGACAATTTCTCAGATAAT---------GTTAAAACCATAATAGTACAGCTGAAT----
-----------GCATCTGTGCCA---------ATTACTTGTGAAAGACCCAACAACAATACAAGGAAA-----
-AGCATAACTATA------------GGACCAGGGAGAGCA---TTT------TATACAACAGGACAA------
---------ATAATAGGAAATATA-----------AGACAAGCACATTGT---AACATTACTAGAGAG---
AAATGGAATAACACTTTAGAACAGATAGTTAAAAAACTAAAA---------------GGACAATTTAAG----
-----------------------------------AATAAAACAATAGTCTTTAGG------
CCATCCTCAGGAGGGGACCTAGAAATTGTA---ACGCATAGTTTTAATTGTGGAGGAGAA---
TTTAAGCAGCTCCAGGCAAGAGTCCTGGCTGTGGAAAGATACCTACAGGATCAACAGCTCCTAGGGATTTGGG
GTTGCTCGGGAAAACTCATCTGCACCACTGCTGTGCCTTGGAATAGTAGTTGGAGT-----------------
----------------------------AATAAATCCTTGAATCAGATTTGG------GAT------
AACATGACCTGGATGCAGTGG---GAAAGAGAAATTGACAATTACACAACAGAAATATAC---
AACTTACTTGAAAAATCGCAGAACCAACAAGAA--

AAGAATGAACAAGAATTATTGGAATTAGATCATTGGGCAAGTTTATGGAATTGGTTCAACATAACAGAGTGGC
TGTGGTATATAAAAATATTCATAATGATAGTAGGAGGCTTGATAGGTTTAAGAATAGTTTTTGCT---
GTACTTTCTATAATG---------AATAGAGTTAGGCAGGGATACTCACCA------
TTATCATTTGCTGTTAGCTTGCTCAATGCCACAGCTATCGCAGTAGCTGAG---
GGAACAGATAGGATTATAGAAGTAGTACGAAGAGCTTATAGAGCT----------------------------
--------ATCCTTCACATA------CCTACAAGAGTGAGACAGGGCTTGGAAAGGGCTTTGCTA---TAA
>JF706474
GCT---GAA--
GACAATTTGTGGGTCACAGTCTATTATGGGGTACCTGTGTGG---AAAGAAGCAACT---
ACCACTCTATTTTGTGCATCCGATGCTAAAGCATATGATAGAGAG---GTACATAAT----------------
--------------GTTTGGGCCACA---CATGCCTGTGTACCCACAGAC---
CCCAGCCCACAAGAAGTACCATTGACA---------AATGTGACAGAAAATTTTAAC----------------

--------------------------ATGTGGAAAAATAATATGGTAGAACAGATGCATGAAGATATA---
ATCAGTTTATGGGATCAAAGTCTA---AAACCATGTGTAAAGTTG---
ACCCCACTCTGTGTTACTTTAGATTGCACTGATTATAATAATACTAATGGGAATAATGTTACTAATGCCAATG
CTACTAAT---
-------GGGAATAGTAGTACTAATACCAATATTATCTATCCAATGGAGAAAGGAGAAATAAAA---
AACTGCTCTTTCAATATCACCACAAAATTAAGA------------------------
GATGATAAGGTGCAGAAAGAATATGCACTC---TTTAATAGACTTGATATAGTACCAATGGACAGTGGTAAT-
----

GGTACTAATACTACCAGTTACAGGTTGATAAATTGTAATACCTCAGTCATTACACAG---
GCCTGTCCAAAGGTAACCTTTGAACCAATTCCCATACATTATTGTGCCCCGGCTGGTTTTGCGCTTCTAAAGT
GTAAT---GATAAGAAGTTCAAT------GGAACAGGACCATGTAAAAT----------------------
-GTCAGC---ACAGTACAATGTACACATGGAATTAGGCCAGTAGTGTCAACT---
CAACTACTATTGAATGGCAGTCTAGCAGAAGAAGAG-----------
GTAGTAATCAGATCTGAAAATCTCACGGATAAT---------GCTAAAACCATAATAGTGCAGCTGAAG----
-----------GACGCAGTAAAC---------ATTACTTGTACAAGACCCAACAACAATACAAGAAAA-----
-AGTATACATGTA------------GGACCAGGGCAAGCA---ATT------TATGCAGCAGGACAA------
---------ATAATAGGAAATATA------------AGACAAGCATATTGT---GACATTAATAAAGCA---
AAATGGAATGACACTTTAAACCAGATAGTTAAAAAATTAAGA---------------GAACCATTTGGA----
------------------------------------AATAAAACAATAACCTTTAAG------
CCACCCGCAGGAGGAGACCCAGAAGTTGTA---ATGTTCACTACTAATTGTGGAGGAGAA---
TTTAAGCAGCTCCAGGCAAGAGTCCTGGCTGTGGAAAGATACCTACAGGATCAACAGCTCCTAGGGATTTGGG
GTTGCTCTGGAAAACTCATTTGCACCACTGCTGTGCCTTGGAATGCTAGCTGGAGT-----------------
-----------------------------AATAAATCTCAGGATCAGATTTGG------GGC------
AACATGACCTGGATGGAGTGG---GAAAGAGAAATTGACAATTACACAGGCTTAATATAC---
AGCTTACTTGAAAAATCACAGACCCAACAAGAA--

ATGAATGAAAAAGACTTATTGGAATTAGATAAGTGGGCAAGTTTGTGGAATTGGTTTGACATAACCAATTGGC
TGTGGTATATAAAAATATTCATAATGATAGTAGGAGGCTTGATAGGTTTAAGAATAGTTTTTGCT---
GTACTTTCTATAGTA---------AATAGAGTTAGGCAGGGATATTCACCA------
CTATCGTTTGCTGTCAGCTTGCTTAATGCCACAGCTATAGCAGTAGCTGAG---
GGAACAGACAGGGTTATAGAAGCAGTACAAAGAATTTATAGAGCT----------------------------
--------TTTCTTCACATA------CCTAGAAGAATAAGACAGGGCTTTGAAAGGGCTTTGCTA---TAA
>HM204630
GCT---GTA---
GATAAACTGTGGGTCACAGTCTATTATGGAGTACCTGTGTGG---AGAGAAGCCAAC---
ACCACACTGTTTTGTGCATCAGATGCTAAAGCATATGATACAGAG---GTACATAAT----------------
--------------GTTTGGGCCACG---CATGCCTGTGTACCCACAGAC---
CCCAACCCACAAGAAATAGCATTGGAA---------AATGTGACAGAAAATTTTAAC----------------
-------------------------ATGTGGAAAAATAACATGGTAGAACAGATGCAGGAGGATATA---
ATTAGTTTATGGGATCAAAGTCTA---AAGCCATGTGTAAAATTA---
ACCCCACTCTGTGTTACTTTAAATTGCACTGAATTGAGGAATAGTACTGATAATGCCACTATT----------
----
----------------------AATAATGCCACTTTTAGTAACGAGGAGAAAGGAGAAATAAAA---
AACTGCTCTTTCAATGTCACCACAGGCATAAGA--------------------------
GACAGGATAAAGAAAGAATATGCACTT---CTGTATAAACTTGATTTAGTATCAATAGATGAT----------
----

AATGATAATACTAGTTATAGGTTAATAAGTTGTAATACCTCAGTCATCACACAG---
GCCTGTCCAAAAGTAACCTTTGAACCAATTCCCATACATTATTGTGCCCCGGCTGGTTTTGCGATTCTAAAGT
GTAAT---GACAAGAAGTTCAAT------GGAACAGGACCATGTAAAAT----------------------
-GTTAGT---ACAGTACAATGTACACATGGAATTAGGCCAGTAGTATCAACT---
CAACTGCTGTTAAATGGCAGTCTCGCAAAAGAAGAT-----------
GTAGTAATTAGATCTGTTAATTTCACAGACAAT---------GCTAAAGTCATAATAGTACAACTAAAT----
-----------GAATCTGTACAA---------ATTAATTGTACGAGACCCAACAACAATACAAGAAGA-----
-GGTATAAATATA------------GGACCAGGGAGGGCA---TGG------TATGCAATGGGAGAC------
---------ATAGTAGGAGATATA------------AGACAAGCACATTGT---AACATTAGTGCAACA---
GAATGGAATAAAACTTTAGAAAAGGTAGCCAGAAAATTAAAA---------------GAACAATTCGGG----
------------------------------------AATAAAACAATAGTCTTTAAG------
CAATCCTCAGGAGGGGACCCAGAAATTGTA---ATGCACAGTTTTAATTGTAAAGGGGAA---

TTTAAGCAGCTCCAGGCGAGAGTCCTGGCTGTGGAAAGATACCTAAGGGATCAACAGCTCCTGGGAATTTGGG
GTTGCTCTGGAAAACTCATTTGCACCACTGCTGTGCCTTGGAATGCTAGTTGGAGT-----------------
----------------------------AATAGATCTCTAGAATACATTTGG------GGG------
AACATGACCTGGATGGAATGG---GAAAGAGAAATTGACAATTACACAGAAATGATATAC---
AGTTTACTTGAAGAATCGCAGAACCAACAAGAA--

AAGAATGAGAAAGACTTATTGGAATTGGACAAATGGGCAAGTTTGTGGAATTGGTTTGACATAACAAATTGGC
TGTGGTATATAAAAATATTCATAATGATAGTAGGAGGCCTGATAGGTTTAAGAATAGTTTTTGCT---
GTACTTTCATTAGTG---------AATAGAGTTAGGCAGGGATACTCACCT------
TTATCGTTTGCTATTAGCTTGCTTAATACCTCAGCCATAGCAGTAGCTGAG---
GGGACAGATAGGATTATAGAAGTACTACAAAGAGGTTTTAGAGCT----------------------------
--------ATTATCCACATA------CCTAGAAGAATAAGACAGGGCTTGGAAAGGGCTTTGCTA---TAA
>HM204631
GCT---ACA--
GAACAATTGTGGGTCACAGTCTATTATGGGGTACCTGTGTGG---AAAGAAGCAACC---
ACCACTCTATTTTGTGCATCAGATGCTAAAGCATATTCTACAGAG---GCACATAAT----------------
--------------GTTTGGGCCACT---CATGCCTGTGTACCCACAGAC---
CCCAGTCCACAAGAAGTATTCTTGAAA---------AATGTGTCAGAAAATTATAAC----------------
--------------------------ATGTGGGAAAATAACATGGTAGAACAAATGCATGAGGATATA---
ATCAGTTTATGGGATCAAAGCCTA---AAGCCATGTGTAACATTA---
ACCCCACTCTGTGTTACTTTAAACTGCACTGACTGGGGAATAGTACTAATGGTAATACT--------------
----
----------------------------AGTAGGGGAGAAAAGATGGAAAAAGGGGAAATAAAA---
AACTGCTCTTTCTATACCACCCCAAGCACAACA---------------------------
GACAAACAACAGAAAACATTTGCACTT---TTTTATAATCTTGATATAGTGCCAATAGAGGATAATAACAAG-
----
-------TCTTCTAACGGAACTAACCATACTAGCTATAGGTTGATAACTTGTAAC------------------
----
--AAT--------------------
----GTCAGC---ACAGTACAATGTACACATGGAATTAGGCCAGTAGTGTCGACT---
CAATTACTATTAAATGGCAGTCTAGCAGAAGAAGAG------------
GTAGCAATTAGATCTCAAAATTTCACGGACAAT---------GCTAAAACCATAATAGTACAGCTGAAG----
-----------ACATCTGTAAAC---------ATTACTTGTACAAGACCCAACAACAACACAAGAAAA-----
-AGTATACATATA------------GCACCAGGGAGAGCA---TTT------TATGCAACAGGAGAC------
---------ATAATAGGAGATATA------------AGACAAGCACATTGT---AACATTAGCAAAGAA---
GATTGGATGAATGCTTTAAACCAAACAGCTAAAAAATTAAGA---------------GAACAATTTGGG----
--------------------------------GAGAATAAGACAATAAAGTTTGAG------
CCACCCACAGGAGGGGACCCAGAAGTTGTG---ATGTACACTTTTAATTGTGGAGGGGAA---
TTTAAACAGCTCCAGGCAAGAGTCCTGGCTGTGGAAAGATACCTAAGGGATCAACAGCTCCTAGGGCTTTGGG
GTTGCTCTGGAAAACTCATCTGCACCACTGCTGTGCCTTGGAATGCTAGTTGGAGT-----------------
----------------------------AATAGATCTCTGAATGACATTTGG------GAT------
AACATGACCTGGAGGGATTGG---GAAAGAGAAATTGACAATTACACAAATGTAATATAC---
GACTTGCTTCAAGAATCGCAGAACCAACAAGAA--

AAAAATGAAAAAGAATTATTGGAATTGGATCGATGGGCAAATCTGTGGAATTGGTTTGACATATCTAACTGGC
TGTGGTATATAAAAATATTCATAATGATAGTAGCAGGCTTGATAGGTTTAAGAATAGTTTTTACT---
GTGCTTTCTATAGTG---------AATAGAGTCAGGCAGGGATACTCACCA------
TTGTCATTTGCTGTTAACTTGCTCAATACCACAGCTATAGTAGTAGGTGAA---
GGGACAGATAGGGTTATAGAAGTAGTACAAAGATTTTTTAGGGGT----------------------------
--------ATCCTCCATATC------CCTAGAAGAATAAGACAGGGCTTCGAAAGGCTTTTACTA---TAA
>HM204636
GCT---AAA--
GAAAAATTATGGGTCACAGTCTATTATGGGGTACCTGTGTGG---AAAGAAGCAACC---
ACCACTCTATTTTGTGCATCAGATGCTAAAGCATATGACACAGAG---GTCCATAAT----------------
--------------GTTTGGGCCACA---CATGCCTGTGTACCCACAGAC---
CCTAGCCCACAAGAACTACCATTAATA---------AATGTGACAGAAAATTTTACT----------------
--------------------------ATGTGGAAAAATAACATGGTAGAACAGATGCATCAGGATATA---
ATCAGTTTATGGGATGAAAGCCTA---AAACCATGTGTGAAATTA---
ACCCCACTCTGTGTCATTTTAAATTGCACTAATGCTAATATTACCAATAGTACTAGTATCACT----------
----

-------------------------AGGAGTAGTATTGGGGGAAACATAACGGAAGAAATGAAA---
AACTGTTCTTTTAATATCACCACAAGCATAGGA---------------------------
GATAAGATGCAGAAAGAATATGCACTT---TTTTATAAACTTGATATAGTACCAATAAAGGAA----------
----

GATAATATGAGTTATACATTGAAAAATTGTAACACCTCAGTCATTAAACAG---
GCCTGTCCAAAGGTATCCTTTGAACCAATTCCTATACATTATTGTGCCCCAGCTGGTTTTGCGATTCTAAAAT
GTAAG---GATAAGAAGTTTAGT------GGAACGGGTCCATGTAAAAAT-----------------------
-GTCAGC---ACAGTACAATGTACACATGGAATTAGGCCAGTGGTGTCAACC---
CAATTACTGTTAAATGGCAGTCTAGCAGAAGAAGGG------------
GTAGTAATTAGATCTGAAAATTTCACAAACAAT---------GCTAAAACCATAATAGTGCAGCTGAAG----
-----------GACCCTGTAAAA---------ATTAATTGTACAAGACCCGGTAATAATACAAGAAAG-----
-AGTATACATCTA------------GCACAAGGAAGAGCA---TTT------TATGCAATGGGAGAC------
---------ATAATAGGAGATATA------------AGACAGGCACACTGT---AACATTAGTAGAAAA---
CAATGGAATGACACTTTAGGGCAGATAGTTGGAAAATTAAGA---------------GAACAATTTGGA----
-----------------------------------AATAAAACAATAGCCTTTAAT------
CAATCATCAGGAGGGGACCCAGAAATTGTA---ATGCACACTTTTAATTGTGGAGGGGAA---
TTTAAGCAGCTCCAGGCAAGAATCCTGGCTGTGGAGAGATTCCTACAGGATCAACAGCTCCTAGGGATTTGGG
GTTGCTCTGGAAAGCGCATTTGCACCACTAATGTGCCTTGGAATAGTAGTTGGAGT-----------------
-------------------------AATAATAAACCTCTGAAGGACATTTGG------GAT------
AACATGACCTGGATACAGTGG---GAGAGACAAATTGAAAATTACACAAATACAATATAC---
CAATTACTTGGAGAATCGCAAATCCAACAAGAA--

AAGAATGAACAAGAATTATTGGAATTGGATTCATGGGACAGTTTGTGGAGTTGGTTTCAACTATCAAAGTGGA
TGTGGTATATAAAAATATTCATAATAATAGTAGCAGGTTTAGTAGGGTTAAGGATAGTTTTTACT---
GTACTTTCTATAGTG---------AATAGAGTTAGGCAGGGATACTCACCA------
TTGTCATTCGCTGTTAGTTTGCTTGATACCATAGCTATAGCAGTAGCTGAG---
GGGACAGATAGGGTTATAGAAGCAATACAAAGAACTTGTAGAGCC----------------------------
--------ATTTGCCGCGTA------CCTGCAAGAATAAGACAGGGCTTAGAAAGGTTTCTACTA------
>HM204637
GCT---ACA--
GAACAATTGTGGGTCACAGTTTACTATGGAGTACCTGTGTGG---AAAGAAGCAACC---
ACCACTTTATTTTGTGCCTCAGATGCTAAAGCAGTTGAGAGAGAG---GTGCATAAT----------------
--------------GTTTGGCCACA---CATGCCTGTGTACCCACAGAC---
CCTAACCCACAAGAAATAGAATTGAAA---------AATGTGACAGAAAATTTTAAC----------------
--------------------------ATGTGGAAAAATAACATGGTAGAACAGATGCAGGAAGATATA---
ATCAGTTTATGGGATCAAAGCCTA---AAACCATGTGTAAAGATA---
ACCCCACTCTGTGTTACTTTAAATTGCACTAATTTGAACAAGACCAGTAACAATAACACTCATGTC-------
----
----------------------ACTAATAGCAGCGGGAGAACAGTGGAAAGCTGGGAAATGACA---
AACTGCTCTTTTAATGTCACCACAGAGATAAGA---------------------------
GATAAGGTGAAGAAAGAATATGCACTT---TTTTATAGACTTGATGTAGTACCAATAGATAAT----------
----

GATAATACCAGCTATAGGTTGATAAACTGTAATTCCTCAGTCATTACACAA---
GCCTGTCCAAAGGTAACCTTTGAACCAATTCCCATACATTATTGTGCCCCAGCTGGTTTTGCAATTCTAAAGT
GTAAG---GACAAGAAGTTCAAT------GGAACAGGATCATGTAAAAAT-----------------------
-GTCAGT---ACAGTACAATGTACACATGGAATTAGGCCAGTAGTGTCAACT---
CAACTGCTGTTAAATGGCAGTCTAGCAGAAGAAGAG------------
GTAGTAATTAGGTCTGAAAATTTCACAGATAAT---------ACTAAGAACATAATAGTACAGCTAAAT----
-----------GAATCTGTAATA---------ATTAATTGTACAAGACCCAACAACAATACAAGAAAA-----
-AGTATACATATA------------GGACCAGGGAGAGCA---TTC------TATGCAACAGGAGAC------
---------ATAATAGGAGATATA------------AGACAAGCACATTGT---AACCTTAGTGAGGCA---
CAATGGACTAGAACTTTACAACAGGTAGCTACAAAATTAAAA---------------GAACAGTTTAAG----
-----------------------------------AATAAAACAATAACCTTTAAT------
CAAGCCTCAGGAGGGGACCCAGAAATTGTA---ATGCACAGTTTTAATTGTGGGGGGGAA---
TTTAAGCAGCTCCAGGCAAGAGTCCTGGCTGTGGAAAGATACCTACAGGATCAACAGCTCCTGGGGATTTGGG
GCTGCTCTGGAAAACTCATTTGCACCACTACTGTGCCTTGGAATGCTAGTTGGAGT-----------------
----------------------------AATAAATCTCTAAATGACATTTGG------AAT------
AACATGACCTGGATACAGTGG---GAGAAAGAAATTGACAATTACACAAGCTTAATATAC---

ACCTTAATTGAAACATCGCAAAACCAGCAGGAA--

AAGAATGAACAAGAATTATTGGCATTGGATAAATGGAACATTTTGTGGAGTTGGTTTGACATAACAAGCTGGC
TGTGGTATATAAAAATATTCATAATGATAGTAGGAGGTTTAATAGGGTTAAGAATAGTTTTTGCT---
GTACTTTCTGTAGTG---------AATAGAGTTAGGCAGGATACTCACCA------
TTGTCATTCGCTATTAGCTTGTTCAACGCCACAGCCATAGCAGTAGCTGAG---
GGGACAGATAGGGTTATAGAAGGATTTCAAAGAGCTTTTAGGGCT----------------------------
--------ATTCTCCACATA------CCTAGAAGAATAAGACAGGGCTTCGAAAGGGCTTTGCTA---TAA
>HM204644
GCA---AAA---GAA------------------------AAA---CAA---
GAAGAGTTGTGGGTCACAGTCTATTATGGGGTACCTGTGTGG---AAAGAAACAACC---
ACCACTTTATTTTGTGCAACAGATGCTAAAGCATATGAGACAGAA---AAACATAAT----------------
--------------GTTTGGGCCACA---CATGCTTGTGTACCCACAGAC---
CCCAACCCACGAGAATTAAATTTGGTT---------AATGTGACAGAAAATTTTAAC----------------
-------------------------ATGTGGAAAAATGACATGGTAGAACAGATGCATGAGGACATA---
ATCAGTTTATGGGATCAAAGCCTA---AAACCATGTGTAAAAATA---
ACCCCACTCTGTGTTACTTTAAATTGTACCAATGCTACTAATAAGACTGGTAGTACCAATAGT----------
----
----------------------AGTGAGAGTGTTGCTCCTCCCATAGAGCCAGGAGAAATAAAA---
AACTGCTCTTTCAATATCACCACAAGCACAAGA---------------------------
GGTAAGATGCAGAAAGAATATGCAACT---TTTTATAACATTGATATAGTAAAAATAGATAATGCTAAT----
----

AGTGCTAATACTAGCTATAATAACTATAGGTTGGTAAGTTGTAACACCTCAGTCATTACACAA---
GCCTGTCCAAAGGTATCCTTTGAACCAATTCCCATACATTATTGTGCCCCGGCTGGTTATGCGATTATAAAAT
GTAAT---AATAACACATTCAAT------GGAACAGGACCATGTAATAAT-----------------------
-GTCAGC---ACAGTACAATGTACACATGGAATTAGACCAGTGGTATCAACT---
CAACTGCTGCTAAATGGCAGTCTAGCAGAAGAAGAA------------
GTAGTAATTAGATCTGAAAATTTCACAAACAAT---------GCTAAAACTATAATAGTACAGCTGAAA----
-----------GAACCAGTACAA---------ATTAATTGTACAAGACCCAACAACAATACAAGAAAA-----
-GGTATACATATA------------GGACCAGGGAAAGCA---TTC------TATACAACAGGAGAA------
---------ATAATAGGAGATATT------------AGACAAGCATACTGT---AACATTAGTAGGGCA---
AAATGGAATAACACTTTAAGTGAAATAGCTAAAAAACTAGGA---------------GAACAATTTGGG----
-----------------------------------AATAAAACAATAGTCTTTAAT------
CGATCTTCAGGAGGGGACCCAGAGATCACA---ACACACAGTTTTAATTGTGGAGGGGAA---
TTTAAGCAGCTCCAGGCAAGAGTCCTGGCTGTGGAAAGATACCTAAGGGATCAGCAGCTCCTGGGGATCTGGG
GTTGCTCTGGAAAACTCATCTGCACCACTACTGTGCCTTGGAATAATACTTGGAGT-----------------
----------------------------AATAAATCTCTGGACCGCATATGG------GGG------
AATATGACCTGGATGCAGTGG---GAAAGAGAAATTGACAATTACACAGCAGAAATATAC---
TCCTTAATTGAAGAATCGCAAAAACAGCAAGAA--

AAGAATGAACAAGAATTATTGGCATTAGATAAATGGGCAAGTTTGTGGAATTGGTTTGACATATCAAGTTGGC
TGTGGTATATAAAAATATTCATAATGATAGTAGGAGGCTTGGTAGGTTTAAGAATAGTTTTTTCT---
GTACTTTCTATAGTG---------AATAGAGTTAGGCAGGATACTCACCA------
TTATCGTTTGCAGTTAGCTTGTTTAATGCAATAGCCATAGCAGTAGCTGAG---
GGGACTGATAGGATTATAGAAACATTGCAAAGAATTGGTAGAGCT----------------------------
--------GTCCTTCATATA------CCTAGAAGAATAAGACAGGGCTTAGAAAGGGCTTTGCTA---TAA
>JF680923
GCT---ACA--
GAAAAACTGTGGGTCACAGTCTATTATGGGGTACCTGTGTGG---AAAAATGCATCC---
ACCACTCTATTTTGTGCATCAGATGCTAGAGCATATGATACAGAG---GTACATAAT----------------
--------------GTTTGGGCCACA---CATGCTTGTGTCCCCACAGAC---
CCCAACCCACAGGAAATAGTATTAGAA---------AATGTGACAGAATATTTTAAC----------------
-------------------------ATGTGGAAAAATGATATGGTAGACCAGATGCAAGAGGACATA---
GTCAATCTATGGGACCAAAGCCTA---AAGCCATGTGTAAAATTA---
ACCCCACTCTGTGTCACTTTAAATTGCAGTGATGTCAATGTCACTAATGTCAATGTCACTAAT----------
----
-------------------------ATCAATGTCACTGATATCCAGGGAGAAGGAGAAATAAAA---
AACTGCTCTTTCAATATCACCACAGACCTGAAA---------------------------
GATAAACGGCAGAAGGAATATGCACTT---TTTTATAAACTTGATGTAGTAACAATAAGTAAT----------

----

GATAGCAGCAGCTATAGGTTGATAAGTTGTAACACCTCAGTCATTACACAA---
GCCTGTCCAAAAGTATCCTTTGAGCCAATTCCCATACATTATTGTGCCCCGGCTGGTTTTGCGATTCTAAAAT
GTAAT---AATAAGACGTTCAGT------GGAAAAGGATCATGTACAAAT-----------------------
-GTCAGC---ACAGTACAATGCACACATGGAATTAGGCCAGTAGTATCAACT---
CAACTGCTGTTAAATGGCAGTCTAGCAGAAGAAGAG------------
GTAGTAATTAGGTCTGACAATTTCTCGGACAAT---------GCTAAAACCATAATAGTACAGCTGAAT----
-----------GAATCTGTAGAA---------ATTAATTGTACAAGACCCAACAACAATACAAGAAAA-----
-AGTATAAATATA------------GGACCCGGAAGGGCA---TTT------TATACAACAGGACAA------
---------ATAATAGGAAATATA------------AGACAAGCACATTGT---AACATTAGTGAACAC---
AAATGGAATAAAACTTTAAAACAGATAGTTGAGAAATTACAA---------------GAACAATATGGG----
-----------------------------------AATAAAACAATAATCTTTAAG------
CATTCCTCAGGAGGGGACCCAGAAATTGTA---ATGCACAGCTTTAATTGTGAAGGGGAA---
TTTAAGCAGCTCCAGGCAAGAGTCCTGGCTGTGGAAAGATACCTAAGGGATCAACAGCTCCTGGGACTTTGGG
GGTGCTCTGGGAAACTCATCTGCACCACTACTGTGCCTTGGAATGCTAGTTGGAGC-----------------
----------------------------AATAAAAATCTAAGTCAGATCTGG------GAT------
AACATGACCTGGATGCAGTGG---GAAAAAGAAATTGACAATTACACAGAATTAATATAC---
ACCTTAATTGAACAATCGCAGAACCAGCAGGAA--

AAGAATGAACAAGAACTATTGGAATTGGATAAATGGGCAAGTTTGTGGAACTGGTTTGACATAACAAACTGGC
TGTGGTATATAAAAATATTCATAATGATAGTAGGAGGATTGATAGGTTTAAGAATAGTTTTTACA---
GTACTCGCTATAGTA---------AATAGAGTTAGGAAGGGATATTCACCA------
TTGTCATTTGCTATTAGCTTGCTCAATGCCACAGCTATCACAGTAGCTGAA---
GGGACAGATAGGTTTATAGAAACATTACAAAGAATTGGCAGAGCT----------------------------
--------TTTCTTCATATA------CCTAGAAGAGTAAGACAGGGCTTAGAAAGGGCTTTGCTA---TAA
>JF680924
GCT---ACA--
GAAAATTTGTGGGTTACTGTCTATTATGGGGTGCCTGTGTGG---AAAGAAGCAACC---
ACCACTTTATTCTGTGCATCAGATGCTAGAGCATATGATACAGAG---GTACATAAT----------------
--------------GTCTGGGCTACA---CATGCCTGTGTACCCACAGAC---
CCCAACCCACAAGAAATAGACTTGAAG---------AATGTGACAGAAGATTTTAAC----------------
--------------------------ATGTGGAAAAATGACATGGTAGAACAGATGCATGAAGATATA---
ATCAGTTTATGGACCAAAGCCTG---AAGCCCTGTGTAAAATTA---
ACTCCACTCTGTGTAACTTTAAATTGCACTGATTATGAAGGTACAAATACCACTAATCATACC----------
----
----------------------ACTAATCATAATACTAATGTCACGGAGAAAGAAGAAATGAAA---
AATTGCTCTTTCAATATCACCACAAGTATAAGA---------------------------
GATAAGGTGACAAAAGAATATGCACTT---TTCTATAGGCTTGATATAGCACAAATAAATGATGATAGTAAA-
----

AATGATAGTAGCAGTAATAATAGTAGCTATAGATTGATAAATTGTAATACCTCAGTCATTACACAA---
GCATGTCCAAAAGTATCCTTTGAACCAATTCCCATACATTTTTGTGCCCCGGCTGGTTTTGCGATTTTAAAAT
GTAAG---GATAAGACATTCAAT------GGAAAAGGAACATGTACAAAT-----------------------
-GTCAGC---ACAGTACAATGTACACATGGAATTAGACCAGTAGTGTCAACT---
CAACTGCTGTTAAATGGCAGTCTAGCAGAAGAAGAG------------
ATAGTAATTAGATCTGAAAATTTCACAAATAAT---------GTTAAAACCATAATAGTACAGCTAAAT----
-----------AAATCTGTAGCA---------ATTGAATGTACAAGACCCAACAACAATACAATAAAA-----
-GGTATACATATA------------GGACCAGGAAGCGCA---TTT------TATACAACAGGACAA------
---------GTAGTAGGAGATATA------------AGACAAGCATATTGT---AGCTTTAACAGAACA---
GAATGGAATAAAACTTTAGAACAGGTAGTTAAAAAATTAAGA---------------AAACAATTTGAG----
-----------------------------------AATAAAACTATAATCTTTAGG---
CAACCTGCGCGGGGAGGGGACCCAGAACTTGTA---ATGCACAGCTTTAATTGTGGAGGGGAA---
TTTAAGCAGCTCCAGGCAAGAGTCCTGGCTGTGGAAAGATACCTAAAGGATCAACAGCTCCTGGGGATTTGGG
GTTGCTCTGGAAAACTTATCTGCACCACTTCCGTGCCTTGGAACACTAGTTGGAGT-----------------
----------------------------AATAAATCTCAGGAGCAGATTTGG------GAG------
AACATGACCTGGATGCAGTGG---GAAAAAGAGATTGATAATTACACAGACGTCATATAC---
AACTTGCTTGAAAAATCGCAGAACCAGCAGGAG--

AAGAATGAAAAAGAATTATTAGAATTGGATAAATGGGCAAGTTTGTGGAATTGGTTTGACATAACAAGATGGC

TGTGGTATATAAAAATATTCATAATGATAGTAGGAGGCTTGGTAGGTTTAAGAATAATTTTTGCT---
GTACTTTCTATAGTG---------AAAAAAGTTAGGCAGGGATACTCACCC------
TTGTCGTTGGCTATTAGCTTGCTTAATGCCACAGCAATTGCAGTAGCTGAG---
GGGACAGATAGGATTATAGAAATAGTACAAAGAACTGTTAGAGCT----------------------------
--------ATTCTCAACATA------CCTAGAAGAATAAGACAGGGCCTGGAAAGAATTTTGCTA---TAA
>JF680933
GCT---ACA---
GAACAATTATGGGTCACAGTCTATTATGGGGTACCTGTGTGG---AAAGAAGCAACC---
ACCACTCTATTTTGTGCATCAGATGCTAAGGCATATGATACAGAG---GTACATAAT---------------
--------------GTTTGGGCCACA---CATGCCTGTGTACCCACAGAC---
CCCAGCCCACAAGAATTAAAATTGGAA---------AATGTGACAGAAAATTTTAAC---------------
--------------------------ATGTGGAAAAATAACATGGTAGACCAGATGCATGAGGATATA---
ATCAGTTTATGGGATCAAAGCCTA---AAGCCATGTGTAAAGTTA---
ACCCCACTCTGTGTCACTCTAAATTGCAGTAATGCCACTTATGCCAATACCACTTATGCCACTAAAGCC----
----
-------------------AATAATAGTAGCGAGAAAAGGATGATAGAGGTAGAAGAAATAAAA---
AACTGCTCTTTCAATATCACCACAAGCATAGGG--------------------------
AATAAGCTGCAAAACCAATATGCAACT---TTTTCTAAACTTGATATAGTGTCATTAAATAGT---------
----

AATGATACTAAGAATACTAGCTATAGGTTGATAAATTGTAACACCTCAGTCATTACACAG---
GCCTGTCCAAAGGTATCCTTTGAGCCAATTCCCATACATTATTGTGCCCCAGCTGGTTTTGCGATTCTAAAGT
GTAAT---GAGAAGAATTTCAAT------GGAACAGGACCATGTAAAAAT----------------------
-GTCAGT---ACAGTACAATGTACACATGGAATTAGGCCAGTAGTATCAACT---
CAACTGCTGTTAAATGGCAGTCTAGCAGAAAACGAG-----------
GTAGTAATTAAATCTGAGAATATCACAGACAAT---------ACTAAAAACATAATAGTACAGCTGAAT----
-----------GCCTCTATAGCA---------ATTAATTGCACAAGACCCAACAACAATACAAGGAGA-----
-GGTATACATATG------------GGACCAGGAAGAACA---TTC------TTTACAGCAGAT---------
---------ATAATAGGAGATATA-----------AGACAAGCACATTGT---AACCTTAGTGGAGCA---
GACTGGAATAGCACCTTAGGCAAGATAGTTATAAAATTAAGA--------------GAATCATTTGTG----
-----------------------------------AATAAAACAATAATCTTTAAG------
CCACCAGCAGGAGGGGACCCAGAAATTGTA---CTACACAGCTTTAATTGTGGAGGGGAA---
TTTAAGCAGCTCCAGGCAAGAGTCCTGGCTGTGGAAAGATACCTACGGGATCAACAGCTCCTAGGGATTTGGG
GTTGCTCTGGAAAACTCATCTGCCCCACTGCTGTGCCTTGGAATGTTAGTTGGAGC----------------
-----------------------------AATAAATCTCTGGATCAGATTTGG------GGC------
AACATGACATGGATGCAGTGG---GAAAGAGAAATTGACAATTACACAAACATAATATAC---
ACCTTAATTGAAGACTCGCAACTCCAACAAGAA---------------------------------------
----
AAGAATGAACAAGAATTATTGGAATTGGATAAATGGGCAAGTTTGTGGAATTGGTTTGACATATCAAAATGGC
TGTGGTATATAAAAATATTCATAATGATAGTAGGAGGCTTGATAGGTTTAAGAATAATCTTTCTT---
GTACTTTCTATAGTG---------AATAGAGTTAGGCAGGGATACTCACCA------
TTATCGTTGGCTGTTAGCTTGTTCAACGCCATAGCCATAGCAGTAGCTGAG---
GGGACAGATAGAGTTATAGAAATATTACAAAGAGCTTGTAGAGCC---------------------------
--------ATTCGCAACATC------CCTAGAAGAATAAGACAGGGTTTGGAAAGGTCTTTGCTA---TAA
>HQ595797
GCT---GCA---CAA--------------------------------
CAAGAACAATTGTGGGTCACAGTCTATTATGGGGTACCTGTGTGG---AAAGAAGCAACC---
ACCACTTTATTTTGTGCCTCAGATGCTAGAGCATATGATACAGAG---GCACATAAT---------------
--------------GTTTGGGCCACA---CATGCCTGTGTGCCCACAGAC---
CCCAACCCACAAGAAGTAGTATTGGGA---------AATGTGACAGAAAATTTTAAC---------------
--------------------------ATGTGGAAAAATAACATGGTAGAACAGATGAATGATGATATA---
ATCAGTTTATGGGATGAAAGCCTA---AAGCCATGTGTAAAATTA---
ACTCCACTCTGTGTTACTTTAAATTGTACTGATTTGAATATTAATATTACTAGAAGCAATGGT---------
----
---------------------ACTGAGGATAATATTACTAGAATGGACCCAGGAGAAATAAAA---
AACTGTTCTTTCTATGTCAAACCAGTCATAAAG--------------------------
GATAAGGTGCTTAAAGCACATGCATCT---TTTTATAAATATGATGTAGTACAAATAGGCAAT---------
----

AATAATTCTAGCTATAGGTTGATAAGTTGTAACGCCTCAGTCATTACACAG---

GCCTGTCCAAAAACATCCTTTGAGCCAATTCCCATACATTATTGTGCCCCGGCTGGTTTTGCGATTCTAAAAT
GTAAT---GATAATAATTTCAAT------GGAACAGGGCCATGCAAAAAT-----------------------
-GTCAGC---ACAGTCCAATGTACACATGGAATTAGGCCAGTAGTGTCAACT---
CAACTGCTATTAAATGGCAGTCTAGCAGCAGAAAAG------------
GTAGTACTTAGATCTGCCAATTTCTCAGACAAT---------ACTAAAACCATAATAGTACAGCTGAAT----
-----------AAAACAATAAAA---------TTTGATTGTACAAGACCCGGCAACAATACAAGGAGG-----
-AGTATAACTATA------------GGACCAGGGAGAACA---TTT------TTTACAGGAGAA---------
---------ATAATAGGAGATATA------------AGAAAAGCACATTGT---AACATTAGTAGAACC---
CAATGGAATAACACTCTAGAAAAGGTAGTTGCAGAACTAAGA------------GGAATTGAACAATTTAAG-
-----------------------------------AATAAAACAATAAAATTTAGT------
CACTCTACAGGAGGGGACCCAGAAGTTACG---ATGATCAATTTTAATTGTGGAGGGGAA---
TTTAAGCAGCTTCAGGCTAGAGTCCTGGCTCTGGAAAGATATCTAAGGGATCAACAGCTCCTAGGGATTTGGG
GTTGCTCTGGAAAACTCATCTGCACCACTGCTGTGCCTTGGAATACTAGTTGGAGT-----------------
----------------------------AATAAATCTTACACAACGATTTGG------GAA------
AACATGACCTGGATGGAGTGG---GAGAGAGAAATTGACAATTACACAAGTTTAATATAT---
TCCTTAATTGAAGACTCGCAAAACCAGCAAGAA--

AAGAATGAACAAGAATTATTGGAATTAGATAAGTGGGCAAGTTTGTGGAATTGGTTCAGCATAACACACTGGC
TGTGGTATATAAAATTATTCATAATGATAGTAGGAGGCTTGATAGGTTTAAGAATAGTTTTTGCT---
GTATTTTCTATAGTG---------AATAGAGTTAGGCAGGATATTCACCA------
CTATCATTTGCTGTTAGCTTGCTCAACGCCACTGCTATAATAGTAGCTGAG---
GGAACAGATAGGATTATAGAAGTAGTACAAACAATTTATAGGGGT----------------------------
--------ATCCTCCACATA------CCTACAAGAATAAGACAGGGCTTGGAAAAGGCTTTACTA---TAA
>AJ535611
GCT---GCT---GCA------------------------------------
GGACAATTGTGGGTCACAGTCTATTATGGAGTACCTGTGTGG---AAAGAAGCAACC---
ACCACTTTATTTTGTGCATCAGATGCTAAAGCATATGACACAGAG---GCACACAAT----------------
--------------GTGTGGCCACA---CATGCTTGTGTACCCACAGAC---
CCTAACCCACAAGAAATAGTAATGGGA---------AATGTAACAGAAAATTTTAAT----------------
--------------------------ATGTGGAAAAATAATATGGTAGAACAGATGAATGAGGATATA---
ATCAGCTTATGGGATCAAAGCTTA---AAACCATGTGTAAAATTA---
ACCCCACTCTGTGTTGTTTTAAATTGCACTGATGCGAAGGCTAACAATAGTAGTAACAAT-------------
----
-------------------------AGTAGTGATTGGCTAAAGATGGAGAAAGGAGAAATACAA---
AACTGCTCTTTCAAC---ACCACAAGAATAGGA---------------------------
AATAAGATACAGAAAGAATATGCACTT---
TTTTACAAACTTGATGTAGTACCAATAGATCAGGATAATACAAGT----------------------------
-----------------------------CAGCAG---
AATAATACAAGTTATACAAATCAGACAAAGTATACAAGGTATACATTGATAAATTGTAATACCTCAGTCATTA
CACAA---
GCCTGTCCAAAGATATCCTTTGAACCAATTCCCATACATTATTGTGCCCCGGCTGGTTTTGCGCTTCTAAAGT
GTAAC---AATAAGAAGTTCAAT------GGAACAGGACCATGTACAAAT-----------------------
-GTCAGC---ACAGTACAATGTACACATGGAATTAGGCCAGTAGTGCCAACT---
CAATTACTGTTAAATGGCAGTCTAGCAGAAGAAGAG------------
GTAGTAATTAGATCTGAAAATTTCACAGACAAT---------GCAAAAATCATAATAGTACAGCTGAAT----
-----------GCAACTGTAAGA---------ATTAATTGTACAAGACCCAACAACAATACAAGAAAA-----
-AGTATACATATA------------GGACCAGGGAGAGTA---TTT------TATGCAACAGGAGAT------
---------ATAATAGGAGATATA------------AGACAAGCACATTGT---AATATTAGTAAAACA---
GAATGGAATAACACTTTAAGACAGATAGCTACAAAATTAGCA---------------AAACAATTTAAT----
-----------------------------------AATAGAACAATAATCTTTAAG------
CAATCCTCAGGAGGGGACCCAGAAATTGTA---ATGCACAGTTTTAATTGTGGAGGGGAA---
TTTAAGCAGCTCCAGGCAAGAGTCCTAGCTGTGGAAAGATACCTAAGGGATCAACAGCTCCTAGGGATTTGGG
GTTGCTCTGGAAAACTCATTTGCACCACTACAGTGCCTTGGAATGCTAGTTGGAGT-----------------
----------------------------AATAAATCCCTGAATCAGATTTGG------GAA------
AACATGACCTGGATGGAGTGG---GAGAGAGAAATTGACAATTATACAAACACAATATAC---
AACTTAATTGAAAAATCACAAAACCAGCAGGAA--

AAGAATGAACAAGAATTATTGGAATTGGATAAGTGGGCAAGTTTGTGGAATTGGTTTGACATAACAAGTTGGC
TGTGGTATATAAAAATATTCATAATGATAATAGGAGGTTTAGTAGGTTTAAGAATAGTTTTTGCT---
GTGCTTTCTATAATA---------AATAAAGTTAGGCAGGATACTCACCA------

TTATCATTTGCTATTAGTTTGCTTAATACTACAGCTATCGCAGTAGCTGAG---
GGGACAGATAGAGTTATAGAAGTAGTACAAAGAACTTGTAGAGCT----------------------------
--------ATCCTCCATATA------CCTAGAAGAATAAGACAAGGCTTAGAAAGGCTTTTACTG---TAA
>AJ286339
GCT---ACA--
GAACAATTGTGGGTCACAGTCTATTATGGGGTACCTGTGTGG---AAAGAAGCAACT---
ACCACTCTATTCTGTGCCTCAGATGCTAAAGCATATGAAACAGAG---GCACATAAT----------------
--------------GTCTGGCCACA---CATGCCTGTGTACCCACAGAC---
CCTAACCCACAAGAAGTAAAATTGGAA---------AATGTGACAGAAAACTTTAAC----------------
--------------------------ATGTGGAAAAATAACATGGTAGAACAGATGCAGGAGGATATA---
ATCAGTTTATGGGATGAAAGCCTA---AAGCCATGTGTAGACTTA---
ACCCCAATCTGTGTTACTTTAAATTGCACTGATTGTTTAAAGAATGCCACCGATAATAGTACATTAGCTTGCA
CTAAGGCTAATAAC---
-AATAATGCTACTAATATCAATAATAGTGGTTATTGGAAAAAGATGGAAGCAGGAGAAATGAAA---
AGATGCTCTTTCAATATCACCACAAACATAGGA---------------------------
AATAAGGTGAAACAAGAATATGCCCTT---TTCAATAGACTTGATATAGTACCAATAAATACT----------
----

GGTAATGATAATACCAGCTATATGTTGATAAATTGTAATACCTCAGTCATTAAACAA---
GCCTGTCCAAAGATATCTTTTGAACCAATTCCCATACATTATTGTGCCCCGGCTGGTTTTGCGATTCTAAAGT
GTAAG---GATAAAAATTTCAAT------GGAACAGGACCATGTAAAAAT-----------------------
-GTCAGC---ACAGTACAATGTACACATGGAATTAGGCCAGTAGTGTCAACT---
CAACTGCTGTTAAATGGCAGTCTATCAGAAGGAGAT------------
GTAGTAATTAGATCTGAAAATTTCACAAACAAT---------ATTAAAAACATAATAGTACAGCTGAAG----
-----------GAATCTGTAGAA---------ATTACTTGTATGAGACCCAACAACAATACAAGAAAA-----
-GGGATAAATATA------------GGACCAGGGAGAGCA---TGG------TATACAACAGGAGAG------
---------ATAATAGGAGATATA------------AGACAAGCACATTGT---AACATTAGTAAAGGA---
AACTGGACTAAAACTTTAAGACAGATAGTTGCAAAATTAAGA---------------GAACAATATGGG----
--------------------------------GTAAATAAAACAATAATCTTTAAT------
CACTCCTCAGGAGGAGACCCAGAAATTGTA---ACACACAGTTTTAATTGTGGAGGAGAA---
TTTAAGCAGCTCCAGGCAAGAGTCCTGGCTGTGGAAAAATACCTAAGGGATCAACAGCTCCTAGGGATTTGGG
GTTGCTCTGGAAAACTCATCTGCACCACTACTGTGCCTTGGAATGCTAGTTGGAGT-----------------
----------------------------AATAGAACTCTGGGAGAGATTTGG------AAT------
AATATGACTTGGATGCAGTGG---GAAAAAGAAATTGACAATTACACAGGCCTAATATAC---
ACCTTACTTGAAGAATCGCAAACCCAACAAGAA--

AAGAATGAACAAGAATTATTGGAATTGGATAAGTGGGCAAGTTTGTGGGATTGGTTTAGCATAACAAATTGGC
TGTGGTATATAAGAATATTCATAATGATAGTAGGAGGCTTGGTAGGTTTAAAAATAGTTTTTGCT---
GTGTTTTCTATAGTA---------AATAGAGTTAGGCAGGGATACTCACCA------
TTATCATTTGCAGTTAGCTTGTTCAACGCTACAGCTATAGCAGTAGCTGAG---
GGGACAGATAGGATTATAGAAGTAGTACAAAGAACTTGTAGAGCT----------------------------
--------ATTCGCCACATA------CCTAGAAGAATAAGACAGGGCACAGAAAGGGCTTTAGTA---TAA
>DQ207942
GCT---ACA--
GAAAAATTATGGGTCACAGTCTATTATGGGGTACCTGTGTGG---AAAGATGCAACC---
ACCACTCTATTTTGTGCATCAGATGCTAAAGCATATGACACAGAG---ATGCATAAT----------------
--------------GTTTGGCCACA---CATGCATGTGTACCCACAGAC---
CCTGACCCACAAGAGGTAAGATTGGAA---------AATGTGACAGAAAATTTTAAT----------------
--------------------------ATGTGGAAAAATAACATGGTAGATCAAATGCATGAGGATATA---
ATCAGTTTATGGGATCAAAGCTTA---AAGCCATGTGTAAAACTA---
ACCCCACTCTGTATCACTCTAAATTGCACTGATTTGAACAATAGTACAACCAAT-------------------
----
-------------------------------AGTAGTAATAGTTGGAAAGAGGTGGAAATGAAA---
AATTGCTCTTTCAATGTCACCACAAGCATAGGA---------------------------
AATAAGATGCACAAAGAATATGCACTT---TTGTATAAGCTCGATTTAGTACCAATAGAAGAG----------
----

AATAATAATACAATCTATACATTGATAAGTTGTAACACCTCAGTCATTACACAG---
GCTTGTCCAAAGATATCCTTTGAACCAATTCCCATACATTATTGTGCCCCGGCTGGATTTGCAATTGTAAAAT
GTAAT---GATGAGAAGTTCAAT------GGAACAGGACCATGTAAAAAT-----------------------

-GTTAGC---ACAGTACAATGTACACATGGAATTAGGCCAACAGTGTCAACT---
CAACTGCTATTAAATGGCAGTCTAGCAAAAGAAGAA------------
GTAATAATTAGATCTGAAAATTTCACAGACAAT---------GTTAAAACCATAATAGTGCAGCTGAAT----
-----------GAATCTGTACAA---------ATTAATTGTACAAGACCTAACAACAATACAAGAAAA-----
-AGTATACATATA------------GGACCAGGGAGAGCA---TTT------TATGCAACAGGAGAA------
---------ATAATAGGAGATATA------------AGACAAGCACATTGT---AACCTTAGTAGTGAT---
CAATGGAGTAACACTTTAAAACACATAGCTACAAAATTAAGA---------------GAACAATTTAAG----
-----------------------------------AATAAAACAATAGCCTTTAAC------
CAATCCTCAGGAGGGGACCCAGAAATTACA---ATGTTCAATTTTAATTGTGGAGGGGAA---
TTTAAGCAGCTCCAGGCAAGAGTCCTGGCTGTAGAAAGATACCTACAGGATCAACGGCTCCTAGGGCTTTGGG
GTTGCTCTGGAAAACTCATTTGCACCACTACTGTGCCTTGGAATGCTAGTTGGAGT-----------------
----------------------------AATAAATCTCTAAATGAAATTTGG------GAT------
AACATGACCTGGATGCAGTGG---GAGAGAGAAATTGATAATTACACAGGCATAATATAC---
AATTTAATTGAAATATCACAAACCCAACAAGAA--

AAGAATGAACATGAATTATTGGAATTAGACAAGTGGGCAAGTTTATGGAATTGGTTTGACATATCAAACTGGC
TGTGGTATATAAAAATATTCATAATGATAGTAGGAGGTTTAATAGGTTTAAGAATAGTTTTTACT---
GTACTTTCTATAGCA---------AATAGAGTTAGGCAGGGATACTCACCA------
TTGTCATTCGCTGTTAATTTGCTTAATGCTGCAGCCATAGCAGTAGCTGAG---
GGGACAGATAGAATTGTAGAAGTAGTACAAAGAATTGGTAGAGCT----------------------------
--------ATTATCCACATA------CCTAGAAGAATAAGACAGGGCTTTGAAAGGTCTTTGCTA---TAA
>MH078546
GCT---GAA---
GACAAATTGTGGGTCACAGTCTATTATGGGGTACCAGTGTGG---GAAGAAGCAACC---
ACCACTCTGTTTTGTGCATCAGATGCTAAAGGATATGAAACAGAG---AAACACAAT----------------
--------------GTATGGGCCACA---CATGCCTGTGTACCTACAGAC---
CCCAGCCCACAAGAAGTAGTATTGAAA---------AATGTAACAGAAAATTTTAAC---------------
--------------------------ATGTGGAAAAATGACATGGTAGATCAGATGCATGAGGATGTA---
ATCAGTTTATGGGATCAAAGCCTA---AAGCCATGTGTAGAATTG---
ACCCCACTCTGTGTTACTTTAAATTGTACAGATATTACTAGGAATAATACTAAGTCTACT-------------
----
--------------------------------------GGAACAATAGAGAGTGCAGACATGAAA---
AACTGCTCTTTCAATATCACCACAAGCATGAGA--------------------------
GATAAGAGGCAAAAAGAATATGCACTT---TTTTATAAGCTTGATATAGTACCAATAGAAGATAATAAGAAT-
----

GATAGTATTAATAGTAATAGTAGTGAATATAGGTTGATAAGTTGTAACACCTCAGTCATTACACAG---
GCCTGTCCAAAAGTATCCTTTGAGCCAATTCCAATACATTATTGTGCCCCAGCTGGTTTTGCGATTCTAAAAT
GTAAT---AATGATACATTCAAT------GGAACAGGACCATGTACAAAT-----------------------
-GTCAGC---ACAGTACAATGTACACATGGAATTAAGCCAGTGGTATCAACT---
CAGCTGTTGTTAAATGGCAGTCTAGCAAAAGAAGAC------------
ATAGTAATTAGATCTGAGAATTTCACAGACAAT---------GCTAAAACCATCATAGTACATCTGAAT----
-----------GAATCTATAATG---------ATCAATTGTACAAGACCCAACAACAATACAAGGAAG-----
-AGTATCCATATGGGA------CTAGGGAGATGGAGATCA---ATT------TCTGCAACAGGAGAT------
---------ATAATAGGAGATATA------------AGACAAGCACATTGT---AACATTAGTGAAGAA---
CAATGGAAGAAAACATTAAGCAAGGTAGTTATAAAATTAAGA---------------
GAAAAATTAACTAATCAA---------------------------------ACAAGAACAATAGTCTTTAAT-
-----CAATCCTCAGGAGGGGACCCAGAAATTGTA---ATGCACAGTTTTAATTGTGGAGGGGAA---
TTTAAACAGCTCCAGGCAAGAGTCCTGGCTGTAGAAAGATACCTACAGGATCAACAGCTCCTGGGAATTTGGG
GTTGCTCTGGAAAACTCATTTGCACCACTAATGTGCCTTGGAATGCCAGTTGGAGT-----------------
-------------------------AAGAACAGAAATCTGACAGATATATGG------GAT------
AACATGACCTGGATGGAGTGG---GAAAGGGAAATTAACAATTACACAGGAGCAATATAT---
AGACTGATTGAAGATTCGCAAAACCAGCAAGAA--

AAGAATGAACAAGAATTATTAAAATTTGATAAATGGGCAAGTTTGTGGAATTGGTTTGACATCTCACGCTGGC
TGTGGTATATAAGGCTATTCATAATGATAGTAGGAGGCCTGATAGGTTTAAGAATAGTTTTTGCT---
GTGCTTTCTATAGTA---------AATAGAGTTAGGCAGGGATACTCACCA------
TTGTCGTTAGCTGTTAGCTTGTTCAATGCCACAGCCATAGCAGTAGCTGAG---
GGAACAGATAGGGTTATAGAAATAGTACAAAGAGCTTTTAGAGCT---------------------------
--------ATCCTCCACATA------CCTGTAAGAATCAGACAGGGCCTGGAAAGATTGTTGCTA---TAA

>EU839600
GCA---GCA--
GAAAAATTGTGGGTCACAGTATATTATGGGGTACCTGTGTGG---AAAGATGCAACC---
ACCACTCTATTTTGTGCATCAGATGCTAAAGCATATGATACAGAG---GCACATAAT----------------
--------------GTCTGGGCCACA---CATGCCTGTGTACCCACAGAC---
CCCAACCCACAAGAAGTAAAATTG------------AATGTGACAGAAAATTTTAAT----------------
--------------------------ATGTGGAAAAATAACATGGTAGATCAGATGCATGAGGATGTA---
ATCAGTTTATGGGATCAAAGCCTA---AAGCCATGTGTAAAGTTA---
ACCCCACTCTGTGTTACTTTACATTGCATTAGTGTGAATTTGACTGCAAATTATTCT----------------
----
-------------------------------ACTATTGATAACAATACTGATTCAGGGATAACA---
AACTGCTCTTTCAATATTACCACAAACATAAGG---------------------------
GATAAGTTGCAAAAAGAATATGCACTC---
TTTTATAAACCTGATGTAGTACCAATAGATGAAAGTACTGATAAT----------------------------
----
AATACTACTGTTAATAAGGATACTAAATATAGATTGATAAGTTGCAATACTTCAGTCATTACACAG---
GCCTGTCCAAAGATAACCTTTGAGCCAATTCCCATACATTATTGTGCCCCGGCTGGTTTTGCAATTCTGAACT
GTACA---GATAAGATGTTCAAT------GGAACAGGGCCATGTAAAAAT----------------------
-GTCAGC---ATAGTACAATGTACACATGGAATTAAGCCAGTAGTGTCAACT---
CAACTGCTGTTAAATGGCAGTCTAGCAGAAGGTGGG------------
GTAGTAATCAGATCTGAAAATTTCACAGACAAT---------GCTAAGACCATAATAGTACAGTTGGAA----
-----------AGCTCTGTAAAA---------ATTGAGTGTACAAGACCCAATAACAATACAAGAAGA-----
-AGCCTACATATG------------GGACCAGGGAAGGCA---TTT------TATACAACA---GAC------
---------ATAATAGGAGATATA------------AGACAAGCATATTGT---AACATTAGTGGAGAA---
GAGTGGCATAAAGCTTTACAAAATGTAAGTAAAAAATTAAGA---------------GAACAATATAAT----
-----------------------------------GACTCAGGAATAAATTTTGAG---
CAACCCCCGTCAGGAGGGGACCCAGAAATTGTA---ACACATAGTTTTAATTGTGGAGGGGAA---
TTTAAACAGCTCCAGGCAAGACTCCTGGCTGTGGAAAGATACCTAAAGGATCAACAGCTCCTGGGGATCTGGG
GTTGCTCTGGAAAATTCATCTGCACCACTACTGTGCCTTGGAATGTTAGTTGGAAC-----------------
----------------------------AATAGATCTGTAGATGACATTTGG------AAT------
AACCTGACCTGGATGCAGTGG---GAAAGAGAAATTGACAATTACACAAGCATAATATAT---
GGCTTACTTGAGGAATCGCAGATCCAGCAAGAT---

ATAAATGAACAAGAATTATTAGAATTAGATAAGTGGGCAAATTTGTGGAATTGGTTTGATATAAGCAACTGGC
TGTGGTATATAAAAATATTCATAATGATAGTAGGAGGCTTGATAGGTTTAAGAATAGTTTTTGCT---
GTACTTTCTATAGTA---------AATAGAGTTAGGCAGGGGTATTCACCA------
TTGTCATTTGCTGTTAGCTTGCTTAATACTACAGCTATAGCAGTAGCTGAG---
GGAACAGATAGGATTATAGAAGCAGTACAAAGAGTTTGCAGAGCT----------------------------
--------ATCCTTCACATA------CCTAGAAGAGTAAGACAGGGCTTGGAAAGGCTTTTGCTA---TAA
>MK303349
GCT--
GAAGACTTGTGGGTCACAGTCTATTATGGGGTACCTGTGTGG---AGAGAAGCAAAC---
ACCTCTCTATTTTGTGCATCAGATGCTAAAGCATATGATAACGAG---GTGCACAAT----------------
--------------GTTTGGGCCACA---CATGCCTGTGTGCCCACAGAC---
CCCAACCCACACGAAGTAAGATTGGAA---------AATGTGACAGAAAATTTT-------------------
--------------------------ACATGGAAAAATAACATGGTAGATCAAATGCATGAGGATATA---
ATCAGTTTATGGGATCAAAGCCTA---AAGCCATGTGTAAAATTA---
ACCCCACTCTGTGTCACTCTACATTGCAATAATGTTACTAATGATAGGAATACTGCTGGT-------------
----
-------------------------AGCAGTAATGGTACATTGAAAAATATAGGAGAAATAAAA---
AACTGCTCTTTTAATATCACCACAAGCACCAAG---------------------------GAGAAGGTG---
AAAGACTATGCACTC---TTTTATGAACTAGATATACAGCCAATAAGTAAT---------------------
----
----------------GATAGCTATAGATTACTAAAGTGTAATACCTCAGTCATTACACAA---
GCTTGTCCAAAGGTATCCTTTGAGCCAATTCCCATACATTATTGTGCCCCAGCTGGTTTTGCGATTCTAAAAT
GTAAA---GATAAGAATTTCAAT------GGAACAGGACCATGTAGAAAT----------------------
-GTCAGC---ACAGTACAATGCACACATGGAATTAGGCCAGTAGTGTCAACT---
CAGTTGCTGTTAAATGGCAGCTTAGCAGAAGACGAG------------
GTAGTAATTAGATCTGAAAATTTCTCGGACAAT---------GCTAAAACCATAATAGTACATCTGAAT----
-----------AAATCTGTGGCA---------ATTAATTGTACAAGACCCAACAACAATACAATAAAA-----

-AGTATACACATG------------GGACCAGGGAAAGCA---TTT------TGGGCAACAGGAAAT------
---------ATAATAGGAGATATA------------AGACAAGCACATTGT---AATCTTAGCAGAACA---
GATTGGAATGACACTTTAAACAAGGTAGCTATAAAGTTAAGA---------------GAACGATTTGGA----
---------------------------------GAAAACAAAACAATAGTCTTTAAT------
CAATCCTCAGGAGGGGACATAGAAATTGTA---ATGCACAGTTTTATTTGTGGAGGGGAA---
TTTAAGCAGCTCCAGGCAAGAGTCCTGGCTGTGGAAAGATACCTAAAGGATCAACAGCTCCTGGGGCTGTGGG
GCTGCTCTGGAAAACTCATTTGCACCACTGCTGTGCCTTGGAATAGTAGTTGGAGC-----------------
----------------------------AATAAAACTCAGGAGGAGATTTGG------GAG------
AACATGACCTGGATGCAGTGG---GAAAAAGAAATTGATAATTACACAGGCTTAATATAC---
AGCTTAATTGAGCAATCGCAAATCCAACAAGAA--

AAGAATGAGCAAGAATTATTGGAATTAGATAAATGGGCAAGTTTGTGGAATTGGTTTTCAATATCAAACTGGC
TGTGGTATATAAGGTTATTCATAATGATAGTAGGAGGCATAGTAGGTTTAAGAATAGTCATGACT---
GTATTTTCTTTAGTA---------AATAGAGTTAGGAAGGGATACTCACCA------
TTGTCATTTGCTACTAGTTTGCTTAATGCCACAGCTATAGCAGTAGGGGAA---
GGGACAGATAGGATTATAGAAATAGTACAAAGAGGTGTTAGGGCT----------------------------
--------ATTCTCCATATA------CCTAGAAGAATAAGACAGGGCTTAGAAAGAGCTTTGATA---TAA
>EF694037
GCT---GCA--
GGCCAATTGTGGGTCACAGTCTATTATGGGGTACCTGTGTGG---AAGGAAGCAACC---
ACCACGCTATTTTGTGCGTCAGATGCTAAAGCATATGATACAGAG---GTACATAAT----------------
--------------GTTTGGGCCACA---CATGCCTGTGTACCCACAGAC---
CCCAACCCACAAGAAGTAGTATTGGAA---------AATGTGACAGAAAATTTTAAC----------------
-------------------------ATGTGGAAAAATAACATGGTAGAACAGATGCATGAGGATATA---
ATCAGTTTATGGGATCAAAGCCTA---AAGCCATGTGTAAAATTA---
ACCCCACTTTGTGTCACTTTAAATTGCACTGATTGGAAGAATAATACTAATACCAATAGT-------------
----
----------------------------AGTAGCGGGGAAACGGTGGAGCAAGGAGAAATAAAA---
AACTGCTCTTTCAATATCACCACAAACATAAGA--------------------------
GATAGGGTGCAGAGAGAATATGCATTA---TTTTATAAGCTTGATGTAGTACCAATAGAAAAGGAT-------
----

AATACCAGCTATGCCAATTATAGGTTGATAAGTTGTAACACCTCAAGCCTTACACAG---
GCCTGTCCAAAGGTATCCTTTGAGCCAATTCCCATACATTATTGTGCCCCGGCTGGTTTTGCGATTCTAAAGT
GTAAC---GATAAGAAGTTCAAT------GGAACGGGACCATGTAAAAAT-----------------------
-GTCAGC---ACAGTACAATGTACACATGGAATTAGGCCAGTAGTATCAACT---
CAACTGCTGTTAAATGGCAGTCTAGCAGAAGAAGAG------------
GTAGTAATTAGATCTGAAAATTTCACGAACAAT---------GCTAAAATCATAATAGTACAGCTGAAA----
-----------GAAGCTGTAGAA---------ATTAATTGCACTAGACCCAGCAACAATACAAGAAAA-----
-AGTATACATCTA------------GGACCAGGGGCAGCA---TTT------TATGCAACAGGAAAC------
---------ATAATAGGAGACATA------------AGACAAGCACATTGT---AACATTAGTAGAGCA---
AAATGGAATAACACTTTAAGCCAGATAGTTAAAAAATTAAAA---------------GAAATAGTTGGG----
---------------------------------AAGAATCAAACAGTAGTCTTTAAT------
TCATCCTCAGGAGGGGATCCAGAAATTGTA---ATGCACAGTTTTAATTGTGGAGGGGAA---
TTTAAGCAACTCCAGGCAAGAGTCCTGGCTGTGGAAAGGTGCCTAAAGGATCAACAGCTCCTGGGAATTTGGG
GTTGCACGGGAAAACTCATTTGCACCACTGCTGTGCCTTGGAACACTAGTTGGAGT-----------------
----------------------------AATAAATCTCTAGATGCGATTTGG------GAT------
AACATGACCTGGATGGAATGG---GAAAGAGAAATTAGCAATTACACAAGCAGAATATAC---
ACCTTAATTGAAGAATCGCAGAACCAACAAGAA--

AAGAATGAACAAGAATTATTGGAATTAGATAAATGGGCTAGTTTGTGGAATTGGTTTGACGTATCAAATTGGC
TGTGGTATATAAAAATATTCATAATGGTAGTAGGAGGCTTGGTAGGTTTAAGAGTAATTTTTACT---
GTACTTTCTATAGTG---------AATAGAGTTAGGCAGGGATACTCACCA------
TTGTCGTTTGCTATTAGCTTGCTTAACGCAACAGCCATAGTAGTAGCTGAG---
GGAACAGATAGGGCTATAGAAATATTACAAAGAGCTTTTAGA-------------------------------
--GACAGGGCTTAGAAAGGGCTTTGCTA---TAA
>JF680930
ACT---GAA--
GGACAATTGTGGGTCACAGTCTATTATGGGGTACCTGTGTGG---AAAGAAGCAACC---
ACCACTCTATTTTGTGCATCAGATGCTAAAGCATATGATGCAGAG---GTACATAAT----------------

--------------GTTTGGGCCACA---CATGCCTGTGTACCTACAGAC---
CCCAACCCACAAGAACTAGACTTGGAA---------AATGTGACAGAAAATTTTAAC----------------
--------------------------ATGTGGAAAAATAACATGGTAGAACAAATGCAGGAGGATATA---
ATCAGTTTATGGGATCAAAGCCTA---AAGCCATGTGTAAAATTA---
ACCCCACTCTGTGTTACATTAAATTGCACTGATTATAATAGTACCAATAATGTTAATACTACC----------
----
-------------------------AGCAGTAATGTTGCTAATATGGAAAAGGGAGAAATAAAA---
AACTGCTCTTTTAATGTCACCACAAACATAAGA---------------------------
GATAAGGTAATGAAAGAAAATGCACTT---TTTAATAAACTTGATATAGTACCAATAGATGAT----------
----

GATAATGATAATACTAGCTATAGGCTGATAAGTTGTAATACCTCAGTCATTACACAG---
GCCTGTCCAAAGGTAACCTTTGAGCCAATTCCCATACATTATTGTGCCCCGGCTGGTTTTGCGATTCTAAAGT
GTAAA---GATAACAAGTTCAAT------GGAACAGGACCATGTAAAAAT-----------------------
-GTTAGC---ACAGTACAATGTACACATGGAATTAGGCCAGTGGTATCAACT---
CAACTGCTGTTAAATGGCAGTTTAGCAGAAGAAGAG------------
ATAGTAGTTAGGTCTGAAAATTTCACGGACAAT---------GCTAAAACCATAATAGTACAGTTGAAC----
-----------GAAACTGTAGAA---------ATTAATTGTACAAGACCCAACAACAATACAAGAAAA-----
-AGTATACATATA------------GGACCAGGGAGAGCA---TTT------TATGCAACAGGAGAA------
---------ATAATAGGAAATATA------------AGACAAGCACATTGT---AACATTAGTGGATTA---
AAATGGAATAATACCTTAAGACAGGTAGCTAACAAATTAAGA---------------GAACAGTTTAAG----
-----------------------------------AATAAAACAATAGTCTTTAAC------
CAATCCTCAGGGGGGGACCCAGAAATTACA---ATGCACACTTTTAATTGTGGAGGGGAA---
TTTAAGCAGCTCCAGGCAAGAGTCCTGGCTGTGGAAAGTTACCTAAAGGATCAACAGCTCCTGGGGATCTGGG
GTTGCTCTGGAAAACTCATTTGCACCACTGCTGTGCCTTGGAATATTACTTGGAGT-----------------
----------------------------AATAAAACTCTGGAGACAATTTGG------AAT------
AACATGACCTGGATGGAGTGG---GAAAGAGAAATTGATAATTACACAGGTGAAATATAC---
AAATTAATTGAACAATCGCAAAGCCAGCAAGAA--

AAGAATGAACAAGAATTATTGCAATTAGATCAATGGGCAAGTTTGTGGAATTGGTTTGACATAACGAAATGGC
TGTGGTATATAAAAATATTCATAATGATAGTAGGAGGCTTGATAGGTTTAAGAATAGTTTTTACT---
GTACTTTCTATAGTG---------AATAGAGTTAGGCAGGATACTCACCA------
CTATCGTTCGCTGTTAGCTTGCTCAACGCCACAGCCATAGCAGTAGCTGAG---
GGGACTGATAGGGTTCTAGAAGTATTACAAAGGGCTTGTAGAGCT----------------------------
--------ATTCTAAACGTA------CCTACACGCATAAGACAGGGCTTGGAAAGAGCTTTGCTA---TAA
>AB287372
GCT---GCA--
GAACAATTGTGGGTCACAGTCTATTATGGGGTACCTGTGTGG---AGAGATGCAAGC---
ACCACTCTATTTTGTGCATCAGATGCTAAAGCATATGTTACAGAA---GTACACAAT----------------
--------------GTTTGGGCCACA---CATGCCTGTGTACCCATAGAC---
CCTGACCCACAAGAAATAGTATTAGAA---------AATGTGACAGAGAATTTTAAC----------------
--------------------------ATGTGGAAAAATAACATGGTAGAACAAATGCATGAGGATGTA---
ATCAGTTTATGGGATCAAAGCCTA---AAGCCATGTGTAAAATTA---
ACCCCACTCTGTGTCACTTTAAATTGCACTGATTGCACGAATACTTCGTGCGCTAAGAATGCTACTTAT----
----
-------------------ACCAATATTGAGCAGTGGGAAAAGGTGAAGGATGGAGAAATAAAA---
AACTGCTCTTTCAATATCACCACAAACATAAGA---------------------------
GATAAGGTACAGAAAAACTATGCACTG---TTTTATGATCTTGATGTAGTACCAATAGATAAAAATAATAAT-
----

ACAAGTAACAATAATTATACTAGCTATAGGATGATAAGTTGTAATACCTCAATCATTACACAG---
GCCTGTCCAAAGGTATCCTTTGAGCCAATTCCCATACATTATTGTGCCCCGGCTGGTTTTGCGATTCTAAAGT
GTAAA---GATAAGCAGTTCAAT------GGAACAGGACCATGTAAAAAT-----------------------
-GTTAGC---ACAGTACAATGTACACATGGAATTAGGCCAGTAGTGTCAACT---
CAACTACTGTTAAATGGCAGTCTATCAGAAGGAGAG------------
GTAGTAATTAGATCTGAAAATTTCACAAACAAT---------GCTAAAACTATAATAGTACAGCTAAAT----
-----------GAAACCGTAGAA---------ATTAATTGTACAAGACCCAACAACAATACTAGGAAA-----
-GGTATACATATG------------GGACCAGGGAAATTT---TAT------GCAACAGGAGAA---------
---------ATAATAGGAGATATA------------AGACAAGCACATTGT---AACATTACTAGAGAG---
AAATGGAATAACACTTTACAAAGATAGCTGAAAAATTAAGA---------------GAACAATTCAAC----

-----------------------------------AAAACAACAATAGTCTTTACT------
AGTTCCTCAGGAGGGGACCCAGAAATTGTA---ATGCACAATTTTAATTGTGGAGGGGAA---
TTCAAGCAGCTCCAGGCAAGAGTCCTGGCTGTGGAAAGATACCTAAAGGATCAACAGCTCCTAGGGATTTGGG
GTTGCTCTGGAAAACTCATCTGCACCACTTCTGTGCCTTGGAATGATACTTGGAGT-----------------
----------------------------AATAAATCTCTGAATACAATTTGG------GAT------
AACATGACCTGGATGGAGTGG---GACAGAGAAATTGAAAATTACACAAAAGAAATATAT---
ATCTTACTTGAAGAATCGCAAAAACAACAGGAA--

AAGAATGAACAAGAATTATTAGCATTAGATCAGTGGGCAAATTTGTGGAATTGGTTTGACATATCAAATTGGC
TGTGGTATATAAAAATATTCATAATGATAGTAGGAGGCTTAATAGGTTTAAGAATAGTATTTGCT---
GTATTATCTATAGTG---------AATAGAGTCAGGCAGGATATTCACCA------
TTGTCATTTGCTGTTAGCTTGCTCAACGCCACAGCTATAGCAGTAGGTGAG---
GGAACAGATAGGGTTATAGAAATAGCACAAAGAATCCTTAGAGCT----------------------------
--------TTTATCCATATA------CCTAGAAGAATAAGACAGGGCTTTGAAAGGGCTTTGATA---TAA
>AB588264
GCT---GTA--
GAACAATTGTGGGTTACAGTCTATTATGGGGTACCTGTGTGG---AAAGAAGCAACC---
ACCACTTTATTTTGTGCATCAGATGCTAAAGCATATGACACAGAG---GTACATAAT----------------
--------------GTTTGGGCCACA---CATGCCTGTGTACCCACAGAC---
CCCAACCCACAAGAAGTAGTAATGGAA---------AATGTGACAGAAGATTTTAAC----------------
--------------------------ATGTGGAAAAATAACATGGTAGAACAGATGCATGAAGATATA---
ATTAGTTTATGGGATCAAAGCTTA---AAGCCATGTGTAAAGTTA---
ACCCCACTCTGTGTTACTTTAAACTGCACTGATGCTAAACCCAGTAATAGTAGCACTGATGCTAACCCC----
----
----------------AATAATAGTAGTGTTACCAACAATACTGTAATAGAACAAGAAATGAAA---
AACTGCTCTTTCAATGTTACCACAACATTAAGA---------------------------
GATAAGGTGAACAAAGAATATGCACTT---TTTTATAAACTTGATATAGTACCAATAGATAAT----------
----

AATAATACTAGCTATAGGTTAATAAGTTGTAACTCCTCAGTCCTTACACAG---
GCCTGTCCAAAGGTATCCTTTGAGCCAATTCCCATACACTATTGTACCCCGGCTGGTTTTGCGCTTCTAAAGT
GTAAC---AATAAGACGTTCAGT------GGAAAAGGACCATGTACCAAT-----------------------
-GTCAGC---ACAGTACAATGTACACATGGAATTAAGCCAGTAGTGTCAACT---
CAACTGCTATTAAATGGCAGTCTAGCAGAAGAAGAG------------
GTAATAATTAGATCTGAAAATTTCACAAATAAT---------GCTAAAACCATAATAGTACAGCTGAAC----
-----------CAATCTGTAGTA---------ATTAATTGTACAAGACCCAACAACAACACAAGAAAA-----
-GGAATTCCTATA------------GGACCAGGACGAGCA---TTC------TATACAACAGGAGAA------
---------ATAGTAGGAGAAATA------------AGACAAGCACATTGT---AACATTAGTAAAGTA---
GACTGGAATAACACTTTAAGACAGGTAGTTGAAAAATTAAAA---------------GAAACATTTAAT----
-----------------------------------GCAACAACAATAGCCTTTAAG------
CAATCCTCAGGAGGAGACCCAGAAATTGTA---ATGCACAGTTTTAATTGTGGAGGGGAA---
TTTAAGCAGCTCCAGGCAAGAGTCCTAGCTGTGGAAAGATACCTAAGGGATCAACAGCTCCTAGGGATTTGGG
GCTGCTCTGGAAAACTCATCTGCACCACTGCTGTGCCTTGGAATAGTAGTTGGAGT-----------------
----------------------------AATAAATCTCGAGATGAGATTTGG------GAT------
AACATGACCTGGCTACAGTGG---GAAAGAGAAATTGACAATTACACAGACTTAATATAC---
TCCTTACTTGAAGAATCGCAAAACCAGCAGGAA--

AAGAATGAACAAGAGTTATTGAAATTAGATAGTTGGGCAAGCCTGTGGACTTGGTTTAACATATCAAATTGGC
TGTGGTATATAAGGATATTCATAATGATAGTAGGAGGCTTAGTAGGTTTAAGAATAGTTTTTGCT---
GTACTTTCTATAGTA---------AATAGAGTTAGGCAGGATACTCACCA------
ATATCATTGGCTGTTAGCTTGCTTAATGTTACAGCTGTAGCAGTAGCTGAG---
GGGACAGATAGGATTATAGAAACAGTACAAAGAGCTTGGAGAGGT----------------------------
--------TTTCTTCACATA------CCTGTAAGAATAAGACAGGGCTTAGAAAGGGCTTTGCTA---TAA
>AB731669
GCA---GCA--
GATAAATTGTGGGTCACAGTCTATTATGGGGTACCTGTGTGG---AAAGAAGCAACT---
ACCACTCTATTTTGTGCATCAGATGCTAAAGCATATAAAACAGAG---GTGCATAAT----------------
--------------GTTTGGGCCACA---CATGCCTGTGTACCCACAGAC---
CCCAACCCACAAGAAGTATTATTGGAA---------AATGTGACAGAAAATTTTAAC----------------
--------------------------ATGTGGAAAAATGACATGGTAGAACAGATGCAGGAGGATGTA---

ATCAGTTTATGGGATGAAAGCCTA---AAGCCATGTGTAAAACTA---
ACCCCACTCTGTGTTACTTTAAATTGCATTGATTATAATAATACAAAGAGTAATAGTACAAGTAAGACTCCTA
CCAATAATAGT--
-------------------------AGTATGGGGAAGAGGTGATGGAAAAAGGAGAAATAAAA---
AACTGCACTTTCAATATCACCACCAACATAAGA---------------------------GGA---
AAGCATAAAGACTATGCACTC---TTTTATAGAAGTGATGTAGTATCAATAAATAAT----------------
----
----------AGAACTAATAGTACTAGCTATAGGTTGATAAGTTGTAACACCTCAGTCATTACACAA---
GCCTGTCCAAAGGTATCTTTTGAGCCAATTCCCATACATTATTGTACCCCGGCTGGTTTTGCGCTTCTAAAGT
GTAAT---GATAAGAAATTTAAT------GGAACAGGGCCATGTACAAAT-----------------------
-GTCAGC---ACAGTACAATGTACACATGGAATTAGGCCAGTAGTGACAACT---
CAACTGCTGTTAAATGGCAGTCTGGCAAAAGAAGAG------------
ATAATAATTAGGTCTGCCAATTTCTCGGACAAT---------GCTAAAACCATAATTGTACAGCTAAAT----
-----------GAAACTATAGAA---------ATTAATTGTACAAGACCCAACAACAATACAAGAAAA-----
-AGTATACCTCTA------------GGACCAGGGAGAACA---TTT------TATGCAACAGGAGAC------
---------ATAATAGGAGATATA------------AGACAAGCACATTGT---AACATTAGTGTAGCA---
CAATGGACAACACTTTAAAACAAATAGCTGAAAAATTGAGA----------------GAACAATTTGGG----
--------------------------------AAAAACAAAACAATAAAATTTGAG------
CCATCCTCAGGAGGGGACCCAGAAATTGTA---ATGCATACTTTTAATTGTGGAGGGGAA---
TTTAAGCAGCTCCAGGCAAGAGTCCTGGCTGTGGAAAGATACCTAAGGGATCAACAGCTCCTAGGGATTTGGG
GATGCTCTGGAAGACTCATTTGCACCACTAATGTGCCTTGGAATAGTAGTTGGAGT-----------------
----------------------------AATAAAACTTTGGATCAGATTTGG------GAT------
AACATGACTTGGATGCAGTGG---GAAAGAGAAATTGTTAATTACACAAAAGAAATATAC---
AACTTAATTGAAGAAGCGCAGAACCAGCAAGAA--

CAGAATGAACATGACTTATTGGAGTTGGATAAGTGGGACAATTTGTGGAATTGGTTTAACATAACAAACTGGC
TGTGGTACATAAGATTATTCATAATGATAGTAGGAGGCTTGATAGGTTTAAGAATAGTTTTTGCT---
GTACTTTCTATAGTA---------AATAGAGTTAGGCAGGATATTCACCA------
TTGTCATTGGCTGTTAGCTTGCTCAACGCTACAGCTATAACAGTAGCTGGG---
GGACTGATAGAATTATAGAAATAGCACAAAGAATTTATAGAGGC-----------------------------
--------ATTCTCCACATA------CCTAGAAGAATAAGACAGGGCTTGGAAAGGGCTTTACTA---TAA
>AB565478
GCT---GAA--
GAACAATTGTGGGTCACAGTTTATTATGGGGTACCTGTGTGG---AAAGAAGCAACC---
ACCACTCTATTTTGTGCATCAGATGCTAAAGCATATGACACAGAG---GTACATAAT----------------
--------------GTTTGGCTACA---CATGCCTGTGTACCCACAGAC---
CCTAACCCACAAGAAGTAGAATTAAAA---------AATGTGACAGAAAATTTTAAC----------------
--------------------------ATGTGGAAAAATAATATGGTAGAACAAATGCATGAGGATATA---
ATCAGTTTATGGGATCAAAGCCTA---AAGCCATGTGTAAAATTA---
ACCCCACTCTGTGTTACTTTAAATTGCACTAATTTGAGGAATGATACTGCTAATAGCAACAGTACTGCT----
----
-------------------AATGACAATAGTACTAATTGGGAATGGATAGATAAAGAAATGACA---
AACTGCTCTTTCAATGTCACCACAATTATAAGA---------------------------
AATAAGATGAAGAAAGAATATGCACTT---TTTTATAGACATGATGTAGTACAAATAGGTACA----------
----

GATAATACAAGCTATACGTTGACACATTGTAACACCTCAGTCATTACACAG---
GCCTGTCCAAAAGTATCCTTTGAACCAATTCCCATACATTATTGTACCCCAGCTGGTTTTGCGCTTCTAAAGT
GTAAT---GATAAAAAGTTCAAT------GGAAAAGGACCATGTAAAAAT-----------------------
-GTCAGC---ACAGTACAATGTACACATGGAATTAGGCCAGTAGTGTCAACT---
CAGCTACTGTTAAATGGCAGTCTAGCAGAAGAAGAG------------
GTAGTAATTAGAACTGAAAATTCCACAAACAAT---------GCTAAAACCATAATAGTACAGCTGAAT----
-----------CAAACTGTAGAA---------ATTAATTGTACAAGACCCAACAACAATACAAGAAAA-----
-AGTATACATATG------------GGACCAGGGAGGTGG---AGC------GTACTTTATGCAACA------
---GGAGCTATAGTAGGAGATATA------------AGACAAGCACATTGT---AACCTTAGTAGACAA---
GACTGGGAAAAGACTTTAAAAAAGATAGTTATAAAATTAAAA---------------GAACAATTTAAT----
--------------------------------ATAACAAAAAACATTACCTTTAAA------
GAATCCTCAGGAGGGGACCCAGAAATTACA---ATGCACAGTTTTAATTGTGGAGGGGAA---
TTTAAGCAGCTCCAGGCCAGAGTCCTGGCTGTGGAAAGATACCTAAAGGATCAACAGCTCATGGGGATTTGGG
GCTGCTCTGGAAAACTCATCTGCACCACTGCTGTGCCTTGGAATACTAGTTGGAGT-----------------

----------------------------AATAAAACTCTAAATCAGATATGG------GAT------
AACATGACCTGGATGGAGTGG---GAGAGAGAAATTAACAATTACACAGGCTTGATATAC---
AACTTAATTGAAGAATCGCAGAACCAACAAGAA--

AAGAATGAACAAGAGTTATTGGAATTGGATAAGTGGGCAAGTTTGTGGACTTGGTTTGACATATCAAATTGGC
TGTGGTATATAAGAATTTTCATAATAATAGTAGGAGGCTTAATAGGTTTAAGAATAGTTTTTGCT---
GTACTTTCTATAGTG---------AATAGAGTTAGGCAGGGATACTCACCA------
TTATCGTTGGCTGTTAGTCTGCTTAATGCCACAGCTGTAGCAGTAGCTGGG---
GGGACAGATAGGATTCTAGAAGTAGTACAAAGAATTGGGAGGGGT----------------------------
--------ATCCTCCACATA------CCTAGGAGGATAAGACAGGGCTTAGAAAGGGCTTTGCTA---TAA
>JQ316131
GTT---GCA---ACA------------------------------------
GGAGGATTGTGGGTCACGGGCTATTATGGGGAACCTGTGTGG---AAAGAAGCGACC---
ACCACCCTATTTTGTGCATCTGATGCTAAAGCATATGACACAGAG---GTACATAAT----------------
--------------GTTTGGGCCGCA---CGTGCCTGTGTACCCAGAGAC---
CCCAACCCACAAGAAGTAGTATTGGTA---------AATGTGACAGAAAATTTTAAC----------------
--------------------------ATGTGGAAAAATGACATGGTAGAACAGATGCATGAGGATATA---
ATCAGTTTATGGGATCAAAGCCTA---AAGCCATGTGTAAAATTA---
ACTCCACTCTGTGTTACTTTAAATTGTAGTAATTATAATGATAGTAAAGTGAACAATGCCACT----------
----
-------------------------AATAGTAATGGGACAGTGGTAGGGGAAACAGAAGTAAAA---
AACTGCACCTTCAATGTTACCACAAGCATAAGA---------------------------
GATAAGGTAAAAAAAGAATATGCTCTT---TTTTATAGACTGGATGTAAAACAAATAGGTGATACA-------
----

GATAATACAAGCTATACAATGATAAATTGTAACTCCTCAGCCATTACACAG---
GCCTGTCCAAAGGTATCCTTTGAACCAATTCCCATACATTATTGTACCCCGGCTGGTTTTGCGATTCTCAAAT
GTAAT---GATAAGAAGTTCAAT------GGAACAGGACCATGTTCAAAT-----------------------
-GTCAGC---ACAGTACAATGTACACATGGAATTAGGCCAGTAGTAACAACT---
CAACTGCTGTTAAATGGCAGTCTAGCAGAAGAAGAG------------
GTAGTAATTAGATCTGAAAATTTCACGAACAAT---------GCAAAAACCATAATAGTACAGCTCAAT----
-----------GTATCTGTACAA---------ATTAATTGTACAAGACCCCACAACAATACAAGAAAA-----
-GGTATAGGGATA------------GGACCAGGGAGCATG---TTT------TATGCAACAGGAGCC------
---------ATAATAGGAGATATA------------AGACAAGCACATTGT---AACATTAGTACAACA---
AAATGGAATGACACTTTAAAACAGGTAGTTATAAAATTAAGG---------------AAACAATTTGTA----
-----------------------------------AATAAAACAATAGTCTTTGAT---
CAACCATCCCCAGGAGGGGACCCAGAGATTGTA---ATGCACAGTTTTAATTGTGGAGGGGAA---
TTTAAGCAGCTCCAGGCGAGAGTCCTGGCTGTGGAAAGATACCTAAGGGATCAACAGCTCCTGGGGATTTGGG
GATGCTCTGGAAAGCTTATCTGCACCACTAATGTGCCTTGGAATACTAGTTGGAGT-----------------
----------------------------AATAAATCTCAGGAGGAGATCTGG------AAT------
AACATGACATGGATGCAGTGG---GACAGAGAAATTGACAATTACACAAACTTTATATAC---
AACTTACTTGAAGAATCGCAGAACCAACAAGAA--

AAGAATGAACAGGAGTTATTGGAATTGGATAAATGGGCAAGTCTGTGGAGTTGGTTTGAAATATCAAACTGGC
TGTGGTATATAAAAATATTCATAATGATAGTAGGAGGCTTAGTAGGTTTAAGAATAATTTTTTCT---
GTGCTTTCTATAGTG---------AATAGAGTTAGGCAGGGGTACTCACCG------
TTATCGTTTGCTGTTAGTTTGCTTAATGCCACAGCTGTAGCAGTAGCTGAG---
GGGACAGATAGGATTATAGAAATATTACAAAGAGCTTATAGAGCT----------------------------
--------ATTCTCAACATA------CCTAGAAGAATAAGACAGGGCTTGGAAAGGGCTTTGCTA---TAA
>JQ316133
GCC---AAT---
AAATCGTGGGTCACAGTCTATTATGGGGTACCTGTATGG---AAAGAAGCAACC---
ACCACCCTATTTTGTGCATCAGATGCTAAAGCATATGACACAGAG---GTACATAAT----------------
--------------GTTTGGGCCACA---CATGCCTGTGTACCCATAGAC---
CCCAACCCACAAGAAGTAGTATTGGGA---------AATGTGACAGAAAATTTTAAC----------------
--------------------------ATGTGGAAGAATAACATGGTAGACCAGATGCATGAGGATATA---
ATCAGTTTATGGGATCAAAGCCTA---AAGCCATGTGTAAAAATA---
ACTCCACTATGTGTTACTTTGAATTGCAGTAATTTGAAGAATGAGACTGCCAATAGTACTAGTGCCAACAGTA
ATGAGACTGCCAAT--
AGTACTACTGCCAACAGTAATAGTACCAGTAATAGTAGTGGGGAAGTGATGGAGGAAGTAGACATGAAA---

AACTGCTCTTTCACCGTTACCACCGAATTAAAA--------------------------
AATACGGTGAGCAAACAATATGCTCTT---TTTTATAAACTGCAAGCCCTA---------------------
----

--------------CCTAAAGATAAT------GGAACAGGACCATGTTCAAAT-------------------
----GTCAGC---ACAGTACAATGTACTCATGGAATTAGGCCAGTGGTATCAACT---
CAACTACTGCTAAATGGCAGTCTAGCAGAAGAAGAG-----------
GTAGTAATCAGATCTGAAAATTTCACGAACAAT---------GCTAAAAACATAATAGTACAGCTGAAC----
-----------GCATCTGTAGAA---------ATTAATTGTACAAGACCCGAC---AACACAATAAAA-----
-AATATAAGAATAGGA------CCAGGGATAGGACTAGGG---AGT------ACATTACATGCAACA------
---AAAAGCATAACAGGAAGAATA------------AGACCA---CATTGC---AACATTAGTGAAGCA---
AAATGGAACAACACTTTAAAACAGATAGCCATAAAATTAAGA---------------GAACAATTTAGG----
-----------------------------------AATAAAACAATAATCTTTAAT------
CACTCCTCAGGAGGGGACCCAGAGATTGAA---ATGCACAGTTTTAATTGTGGAGGGGAA---
TTTAAGCAGCTCCAGGCAAGAGTCCTGGCTGTAGAAAGATACCTAAAGGATCAACAGCTCCTGGGGATTTGGG
GATGCTCTGGAAAGCTCATCTGCACCACTAATGTGCCTTGGAATACTAGTTGGAGT-----------------
----------------------------AATAAAACTCAGGAGGATATCTGG------ACT------
AACCTGACCTGGATGCAGTGG---GAAAGAGAAATTGACAATTACACAAACGTAATATAC---
TCCTTGCTTGAAGAATCGCAGAATCAGCAAGAA---------------------------------------
----
AAGAATGAACAGGAATTATTGCAATTGGATAAATGGGCAAGTTTGTGGAATTGGTTTGACATAACTAGTTGGC
TGTGGTATATAAAAATATTCATAATGATAGTAGGAGGCTTGATAGGTTTAAGAATAATTTTTACT---
GTACTTTCTATAGTG---------AATAGAGTTAGGCAGGGATACTCACCA------
TTATCGTTTGCTGTTAGTTTGCTTCAAGCCACAGCTAGAGCAGTAGTTGAG---
GGGACAGATAGGGTTATAGAAACATTACAAAGAGCTTATAGAGCT----------------------------
--------ATTCTCCACATA------CCTAGAAGAATAAGACAGGGTTTGGAAAGGGCTTTGCTA---TAA
>DQ295192
GCT---ACA---GCA-----------------------------------
GAAGACTTGTGGGTCACAGTATATTATGGGGTACCTGTGTGG---AAAGAAACAACC---
ACCACTCTATTTTGTGCATCAGATGCTAAAGCATATGACACAGAG---GTACATAAT---------------
--------------GTTTGGGCTACA---CATGCCTGTGTACCCATAGAC---
CCCAACCCACCAGAGGTAGTATTGGTA---------AATGTGACAGAAAATTTTAAC---------------
--------------------------ATGTGGAAAAATAACATGGTAGAACAGATGCATGAGGATATA---
ATCAGTTTATGGGATCAAAGCCTA---AAGCCATGTGTAAAATTA---
ACTCCACTCTGTGTTACTTTGAATTGTGGGGATGCGAATTTTACTAATACCAATAGTAGTAGGACTACC----
----
-------------------AATGATAGTAGAAATGAGACAGTGATGGCGATCGGAGAAATAAAA---
AACTGCTCTTTCAATGTTACCACAGGCATAAGA--------------------------
GATAAGGTGCAAAAAGAATATGCTCTT---TTTCATAAACTTGATGTAGTACAAACGGATAAT---------
----

TCAAGCTATACATTGATAAGTTGTAACACCTCAGTCATTAAACAA---
GCCTGTCCAAAGGTATCCTTTGAACCAATTCCCATACATTATTGTACCCCGGCTGGTTTTGCGCTTCTACAGT
GTAAT---GATAAGAAATTCAAT------GGAACAGGACCATGTTCAAAT----------------------
-GTCAGC---ACAGTACAATGTACACATGGAATTAGGCCAGTAGTATCAACT---
CAACTACTGTTAAATGGCAGTCTAGCAGAAGAAGAG-----------
ATAGTAATTAGATCTGAGAATTTCACGAACAAT---------GCTAAAACCATAATAGTACAGCTGAAT----
-----------GTATCTGTAGAA---------ATTAATTGTACAAGACCCTACAACAATACAAGAAAA-----
-AGTATAAGAATA------------GGACCAGGGAGCGCA---TTT------TATGCAACAGGAGAT------
---------GTAATAGGAGATATA------------AGAAAAGCACATTGT---AACATTAGTGAAACA---
AAATGGAATAACACTTTGAGACAGCTAGTCACAAAATTAGGA---------------GAACAATTTGGG----
-----------------------------------AATAGCACAATAATCTTTAAT------
AAATCCTCAGGAGGGGACCCAGAGATTGTA---ATGCACAGTTTTAATTGTGGAGGGGAA---
TTTAAGCAGCTCCAGGCAAGAGTCCTGGCTGTAGAAAGATACCTAAAGGATCAACAGCTCCTGGGGATTTGGG
GATGCTCTGGAAAGCTCATCTGCACCACTAATGTGCCTTGGAATACTAGTTGGAGT-----------------
----------------------------AATAAATCTCTGAATAGAATCTGG------GAT------
AACATGACCTGGATGGAGTGG---GATAGAGAAATTAACAATTACACAAACTTAATATAT---
CACTTGCTTGAAGAATCGCAGAACCAGCAAGAT---------------------------------------
----

AAAAATGAACAGGAATTATTGGAATTGGATAAATGGGCAAGTTTGTGGAGTTGGTTTGACATAACAAACTGGC
TGTGGTATATAAAAATATTCATAATGATAGTAGGAGGCTTGATAGGTTTAAGAATAGTCTTTTCT---
GTACTTTCTATAGTA---------AATAGAGTTAGGCAGGGATACTCACCC------
TTATCGTTTGCTGTTAGTTTGCTTAATGCCACAACTATAGTAGTAGCTGAG---
GGGACAGATAGGATTATAGAACTATTGCAAAGAACTTATAGAGCT----------------------------
--------ATTATCCACATA------CCTACAAGAATAAGACAGGGCTTGGAAAGGGCTTTGCTA---TAA
>KT878039
GTT---GTC---ACA------------------------------------
AAAGATTTGTGGGTCACAGTCTATTATGGGGTACCTGTGTGG---AGAGATGCAAAC---
ACCACCCTATTTTGTGCATCAGATGCTAAAGCATATGTTGAAGAA---GCACATAAT----------------
--------------ATTTGGCCACA---CATGCCTGTGTACCCATAGAC---
CCCAACCCACAAGAAGTAGCATTGGTA---------AATGTGACAGAAAATTTTAAC----------------
--------------------------ATGTGGAAAAATAACATGGTAGAACAGATGCATGAGGATATA---
ATCAATTTATGGGATCAAAGTCTA---AAGCCATGTGTAAAATTA---
ACTCCACTCTGTGTTACCTTGAATTGTACAGATTTGACTGGTACTAATACTACTAGCCAGAATAATAGT----
----
-------------AGTCCAACCAATAGTAGTAGTGGGGGATGGATGGAGGCATTAGAAATGAAA---
AACTGTTCTTTCAATGTTACCACAAGCATAAGA---------------------------
GATAAGGTGAAGCAAGAATATGCTCTT---TTTTATAAAGTTGATGTAACACCAACAGACAAT----------
----

AGTAATACAAGCTATATATTGATAAATTGTAACTCCTCAACCATTACACAG---
GCCTGTCCAAAGACATCCTTTGAACCAATTCCCATACATTATTGTGCCCCAGCTGGTTTTGCGCTTCTAAAGT
GTAAG---GATAAGAAGTTCAAT------GGAACAGGAACATGTTCAAAT-----------------------
-GTCAGC---ACAGTACAATGTACACATGGAATTAGGCCAGTAGTATCAACT---
CAGCTACTGTTAAATGGCAGTCTAGCAGAAGAAGAG------------
ATAGTAATTAGATCTGAAAATTTCACGAACAAT---------GCTAAAACCATAATAGTGCAGCTGAAT----
-----------GCATCTGTAGAA---------ATCAATTGTACAAGACCCAGCAACAATACAAGAAAA-----
-GGTATACCTATA------------GGACCAGGGAGTGTA---ATG------TATGCAACAGGAGAA------
---------ATAATAGGAGATATA------------AGACAAGCACATTGT---AACATTAGTAGAGTA---
AAATGGAACAACACTTTAGGACAGATAGCAAAAAAATTAGGA---------------GAACAATTTAAA----
-----------------------------------AATAACACAATAGCCTTTAAC------
CAATCCGCAGGAGGGGACCAAGAGATTACA---ATGCACAATTTTATTTGTGGAGGGGAA---
TTTAAGCAGCTCCAGGCAAGAGTCCTGGCTGTGGAAAGATACCTAGGGGATCAACAGCTCCTGGGGATTTGGG
GATGCTCTGGAAAGCTCATCTGCACCACTAATGTACCTTGGAATAATAGCTGGAGT-----------------
----------------------------AATAAATCTATAACATATATCTGG------GAA------
AACATGACCTGGAGGGAGTGG---GACAGAGAAATTAATAATTACACAAACTTAATATAC---
AACTTGCTTGAGAAGTCGCAAAACCAGCAAGAA--

AAGAATGAACAGGAATTATTGGAATTGGATAAGTGGACAAGTTTGTGGAATTGGTTTGACATATCACACTGGC
TGTGGTATATAAGACTATTCATAATGATAGTAGGAGGCTTAATAGGTTTAAGAATAATTTTTTTT---
GTACTTTCTATAGTA---------AATAGAGTTAGGCAGGGATACTCACCA------
TTATCGTTGGCTGTTAGTTTGCTTAACGCCATAGCTATAGCAGTAGCTGAG---
GGGACAGATAGGGCTATAGAAATATTACGAAGAGCTATTAGAGCT----------------------------
--------ATTCTCAACATA------CCTAGAAGAATAAGACAGGGCTTGGAAAGGGCTTTACTA---TAA
>KT878021
GCT---GTA--
GAAAAGTTATGGGTCACAGTCTATTATGGGGTACCTGTATGG---AAAGAAGCAACC---
ACTGTTCTATTTTGTGCATCAGATGCTAAAGCATATGACACAGAG---GCACATAAT----------------
--------------GTTTGGCCACA---CATGCCTGTGTACCCACAGAC---
CCTAACCCACAAGAAGTAGTATTGGAG---------AATGTGACAGAAAATTTTAAC----------------
---------------------------ATGTGGAAAAATGACATGGTAGAACAGATGCATGGGACATA---
ATTAGTTTATGGGATGAGAGTCTA---AAGCCATGTGTAAAGTTA---
ACCCCACTCTGTGTTACTTTAAATTGCACTAATTATAATTATAATAAGACTAATTATAAT-------------
----
----------------------------ACAACTAAACTGGAGGAAGGAGGCACAGACATAAAA---
AATTGCTCTTTCAATATCACAGGAGGAATAGGA---------------------------
AAAGAGAAGAAAAAAGATTATGCAATT---TTTAATGCACTTGATATAATACCAGTAGATAAT----------
----

GATAACACAAGCTATATGTTGGTAGGTTGTAACACCTCAGTCATTACACAA---
GCCTGTCCAAAGATATCCTTTGACCCAATTCCCATACATTATTGTACCCCGGCTGGTTTTGCGATTCTAAAGT
GTAAT---GATAAAAAGTTCAAT------GGAACAGGACCATGTAAAAAT-----------------------
-GTCAGC---ACTGTACAATGCACACATGGAATTAGGCCAGTAGTGTCAACT---
CAACTGCTGTTAAATGGCAGTCTAGCAGAAGAAGAT------------
ATAGTAATTAGATCTGAAAATTTCACGAACAAT---------GCTAAAACCATAATAGTACAGCTAAAT----
-----------GAATCTGTAATA---------ATTAATTGTACAAGACCCAACAACAATACGAGAAAA-----
-AGTATACATATA------------GGCCCAGGGGGAGCA---ATG------TATGCAACAGGGGAC------
---------ATAATAGGAGACATA------------AGAAGAGCACATTGT---AACATTAGTAGTACA---
CAATGGAATAACACTTTACAAAAGGTAGCTACAAAATTAAAG---------------GAACAATTTGGG----
-----------------------------------AATAAAACAATAAGCTTTAAT------
CAATCCTCAGGAGGGGACCCAGAAATTGTA---ATGCATAGTTTTAGTTGTGGAGGGGAA---
TTTAAGCAGCTCCAGGCAAGAGTCCTGGCTGTAGAAAGATACCTAAAGGATCAACAGCTCCTAGGGATTTGGG
GTTGCTCTGGAAAACTCATTTGCACCACTGCTGTGCCTTGGAATGCTAGTTGGAGT-----------------
----------------------------AATAAATCTCTGAATCAAATTTGG------GAT------
AATATGACCTGGATGGAGTGG---GAGAAAGAAATTGACAATTACACAAGCTTAATATAC---
ACTTTAATTGAAGAATCGCAAATCCAACAAGAA--

AAAAATGAACAAGAATTACTAGAATTGGATAAGTGGGCAAGTCTGTGGAATTGGTTTGACATAACAAAATGGC
TGTGGTATATAAAGATCTTCATAATGATAGTAGGAGGCTTAGTAGGTTTAAGAATAATTTTTGCT---
GTGCTTTCTATAGTG---------AATAGAGTTAGGCAGGGCTACTCACCA------
TTATCATTAGCTGTCAGCTTGTATAACACCACAGCTATAGTAGTAGCTGAG---
GGGACAGATAGAATTATAGAAGGAGTACGCAGAATTCTTAGAGCT----------------------------
--------TTATTCCACATA------CCTAGAAGAATAAGACAGGGCTTAGAAAGGGCTTTGCTA---TAA
>KT878029
GCT---GCA---AGA------------------------------------
GAAGACTCATGGGTCACAGTCTATTATGGGGTACCTGTGTGG---AGAGATGCAAAC---
ACCACCCTATTTGTGCATCAGAGGCTAAAGCATATGTCACAGAG---GCACATAAT-----------------
--------------GTTTGGCCACA---CATGCCTGTGTACCCATAGAC---
CCTGACCCACAAGAAGTAGTATTGGTA---------AATGTGACAGAAAATTTTAAC----------------
--------------------------ATGTGGAAAAATAACATGGTAGAACAGATGCATGCGGATATA---
ATCAGTTTATGGGACCAAAGCCTA---AAACCATGTGTAAAATTA---
ACTCCACTCTGTGTTACTTTGAATTGTAGTAATGTGACTGATACTACTAATAGTACCAGT-------------
----
-------------------GTCCATAATAGTAGTGGGAGAGTGATGGGGGGAGGAGAAATGAAA---
AACTGTTCTTTCAATGTTACCACAGACATAAGA---------------------------
AAGAAGCAAAAGAAAGAATATGCTCTT---TTTTATGACCTTGATGTAGAACCAATAGATAAT----------
----

ACAAGCTATATATTGAAACATTGTAATGCCTCAGTCATTACACAG---
GCCTGTCCAAAGGTATCTTTTGAACCAATTCCAATACATTATTGTACCCCAGCTGGTTTTGCGATTCTAAAAT
GTAAT---GATAAGAAGTTCAAT------GGATCAGGACCATGTTCAAAT-----------------------
-GTCAGC---ACAGTACAATGTACACATGGAATTAAGCCAGTAGTATCAACT---
CAACTGCTGTTAAATGGCAGTCTAGCAGAAGAAGAG------------
ATAGTAATTAGATCTGAAAATTTCTCGGATAAT---------GCTAAAATCATAATAGTACAGCTGAAT----
-----------GCATCTGTAGAA---------ATTAATTGTACAAGACCCAACAACAATACAAGAAAA-----
-GGTATACATATA------------GGACCAGGGAGGACA---TTT------TATGCAACAGGAGAC------
---------ATAATAGGAGATATA------------AGACAAGCACATTGT---AACATTAGTATAGAA---
GAATGGAACAACACTTTAAGACAGCTAGTTATAAAATTAAGA---------------GAACAATATGGG----
--------------------------------AATAATAAAACAATAGTCTTTAAT------
CAATCCTCAGGAGGGGACCCAGAGATTGTA---AGGCACAGTGTTAATTGTGGAGGGGAA---
TTTAAGCAGCTCCAGGCAAGAATCCTGGCTGTGGAAAGATACCTAAAGGATCAACAGCTCCTGGGGATTTGGG
GATGCTCTGGAAGACTCATCTGCACCACCAATGTGCCTTGGAATGCTAGTTGGAGT-----------------
----------------------------AATAAATCTATAGATGATATCTGG------GAT------
AACATGACCTGGATGGAGTGG---GACAGAGAAATTAACAATTACACAAGCTTAATATAT---
ACATTGCTTGAAGAATCGCAGAACCAACAAGAA--

AAGAATGAACAGGACTTATTGGCATTGGATGACTGGGCAAGTTTGTGGAATTGGTTTGACATATCAAACTGGC
TGTGGTATATAAAAATATTCATAATAATAGTAGGAGGCTTGGTAGGTTTAAGAATAATTTTGGCT---
GTACTTTCTATAGTG---------AATAGAGTTAGGCAGGGATACTCACCA------

TTATCGTTGGCTGTTAGTTTGCTTGATGCAACAGCTAGAGCAGTAGCTGAG---
GGGACAGATAGGGTTATAGAATTATTGCAAAGAGCTTTTAGAGCT---------------------------
--------ATTCTCAACATA------CCTAGAAGAATAAGACAGGGCTTGGAAAGGGCTTTGCTA---TAA
>KF561441
GCT---GCA---ACA-----------------------------------
GGAGGATTGTGGGTCACAGTCTATTATGGGGTACCTGTGTGG---AGAGAAGCAATC---
ACCACCCTATTTTGTGCATCAGATGCTAAAGCATATGAAACAGAG---ATGCATAAT---------------
--------------GTTTGGGCCACA---CATGCCTGTGTACCCATAGAC---
CCCAACCCACTAGAAGTAGACTTGCCA---------AATGTGACAGAAAACTTTAAC---------------
--------------------------ATGTGGAAAAACAACATGGTAGAACAGATGCATGAGGATATA---
ATCAGTTTATGGGATCAAAGCCTA---AAGCCATGTGTAAAAATA---
ACTCCACTCTGTGTTGTTCTAAATTGTAGTGATGCAAAGAATAGTACTAGTACCAATAAT------------
----
---------------------------AGTACGGAGAAAGTAATGGAGAAAGGAGAAATGAAA---
AACTGCTCTTTCAATGTCACCACAAACATAGAA--------------------------
AATAAGGTGAAGGAAGCACGTGCTATT---
TTTGATAAACTTGATGTAGTCCAAATAGATGATAATGATACTAATACTATGATAGGGATACAA---------
----
----------TACTACAAATGTTACTCCTCGAGCATTACACAG---
GCCTGTCCAATGGTATCCTTTGAACCAATTCCCATACATTATTGTACCCCGGCTGGTTTTGCGATTCTACAGT
GTAAA---GATAAGAAGTTCAAT------GGAACAGGACCATGTACAAAT----------------------
-GTCAGC---ACAGTACAATGTACACATGGAATTAGGCCAGTAGTATCAACT---
CAACTGCTGTTAAATGGCAGTCTAGCAGAAGAAGAA------------
ATAGTAATTAGATCTGAAAATTTCACGAACAAT---------GCTAAAACCATAATAGTACAGCTGAAC----
-----------GCATCTGTAGAA---------ATTAATTGTACAAGACCCAACAACAATACAAGGAAA-----
-AGTATACCTATA------------GGACCAGGGAGAGCA---TTT------TATGCAACAGGAGAT------
---------ATAATAGGAGATATA-----------AGACGAGCACATTGT---AACATTAGTAAAGCA---
ACATGGAACAACACTTTAAGACAGCTAGCTATAAAATTAAGA--------------GAACAACTTGAAGAT-
--------------------------------AATACAACAACAATAATCTTTAAG------
AATTCCTCAGGGGGGGACCCAGAGATTGAA---GCACACAGCTTTAATTGTAGAGAGGAA---
TTTAAACAGCTCCAGGCAAGAGTCCTGGCTGTAGAAAGATACCTACAGGATCAACGGCTCCTGGGGATTTGGG
GATGCTCTGGGAAGCTCATCTGCACCACTAGTGTGCCTTGGAATACTAGTTGGAGT-----------------
---------------------------AATAAATCTCATGATGAAATCTGG------AAT------
AACATGACCTGGATGGAGTGG---GATAGAGAAATTAACAATTACACACAGGTGATATAC---
GACCTGCTTGAGGAATCACAGATCCAGCAAGAA---------------------------------------
----
AAGAATGAGCAGGAATTATTGAAATTGGATTCATGGGCAAGTCTGTGGAAGTGGTTGGACATATCAAACTGGC
TGTGGTATATACAAATATTCATGATGATGGTAGGAGGCTTGGTAGGTTTAAGAATAATTCTTTGT---
GTACTTTCTGTAGTG---------AATAGAGTTAGGCAGGGATACTCACCA------
TTACCGTTTGCTGTTAGTTTGCTTAATGCCACAGCTATAGCAGTAGCTGAG---
GGGACAGATAGAGTTATAGAAATATTACAAAGAATTTATAGAGCT---------------------------
--------ATTCTCCACATA------CCTACAAGAATAAGACAGGGCTTAGAAAGGGCTTTGCTA---TAA
>MN043577
GCT---GCA---------------------------------------
GCAGGAGGATTGTGGGTCACAGTCTATTATGGGGTACCTGTGTGG---AAAGAAGCAACC---
ACCACCCTATTTTGTGCATCAGATGCTAAAGCATATGACACAGAG---GTACATAAT---------------
--------------GTTTGGGCCACA---CATGCCTGTGTACCCATAGAC---
CCCAACCCACAAGAAGTAGTATTGGAA---------AATGTGACAGAAAATTTTAAC---------------
--------------------------ATGTGGAAAAATAACATGGTAGAACAGATGCATGAGGATATA---
ATCAGTTTATGGGATCAAAGCCTA---AAGCCATGTGTAAAATTA---
ACTCCACTCTGTGTTACTTTAAATTGTAGTAATTATTTCAAGAATAATACTAATACCACTACCACT-------
----
-------------ACTAATAACAATACTAGTAGTGGGAAAGTGATGGAGGAAACAGAAATGAAA---
AACTGCTCTTTCAATGTTACCACAAACATAAGA--------------------------
GATAAGGTGAAAAAAGAATATGCTCTT---TTTTATAAACTTGATGTACAACAAATAGATAAT---------
----

ACAAGCTATACATTGATAAATTGTAACTCCTCAGTCATTACACAG---
GCCTGTCCAAAGGTATCCTTTGAACCAATTCCCATACATTATTGTACCCCGGCTGGTTTTGCGATTCTACAAT
GTAAT---GATAAGAAGTTCAAT------GGAACAGGACCATGTTCAAAT----------------------

-GTCAGC---ACAGTACAATGTACACATGGAATTAGGCCAGTAGTATCAACT---
CAACTGCTGTTAAATGGCAGTCTAGCAGAAGAAGAG-----------
GTAGTAATTAGATCTGAAAATTTCACGAACAAT---------GCTAAAACCATAATAGTACAGCTGAAT----
-----------GCATCTGTAGAA---------ATTAATTGTACAAGACCCAACAACAATACAAGAAAA-----
-GGTATAGGGATA-----------GGACCAGGGAGCATG---TTT------TATGCAACAGGAGAA------
---------ATAATAGGAGATATA-----------AGACAAGCACATTGT---AACATTAGTACAGCA---
AAATGGAATGACACTTTAAAACAGCTAGTTATAAAATTAAGA--------------GAACAATTTGGG----
----------------------------------AATAAAACAATAGTCTTTAAT------
CAATCCTCAGGAGGGGACCCAGAGATTGTA---ATGCACAGTTTTAATTGTGGAGGGGAA---
TTTAAGCAGCTCCAGGCAAGAGTCCTGGCTGTGGAAAGATACCTAAAGGATCAACAGCTCCTGGGGATTTGGG
GATGCTCTGGAAAGCTCATCTGCACCACTAATGTGCCTTGGAATACTAGTTGGAGT----------------
----------------------------AATAAATCTCAGAAGGAGATCTGG------GAT------
AACATGACCTGGATGGAGTGG---GACAGAGAAATTAACAATTACACAAACTTAATATAC---
ACCTTACTTGAAGAATCGCAGAACCAACAAGAA--

AAGAATGAACAGGAATTATTGGAATTGGATAAATGGGCAACTTTGTGGAATTGGTTTGAAATATCACAATGGC
TGTGGTATATAAAAATATTCATAATGATAGTACGAGGCTTAGTAGGTTTAAGAATAATTTTTTCT---
GTACTTTCTATAGTG---------AATAGAGTTAGGCAGGGATACTCACCA------
TTATCGTTTGCTGTTAGTGTGCTTAATGCCACAGCTGTAGCAGTAGCTGAG---
GGGACAGATAGGGTTATAGAAATATTACAAAGAGCTTATAGAGCT---------------------------
--------ATTCTCCACATA------CCTAGAAGAATAAGACAGGGCTTGGAAAGGGCTTTGCTA---TAA
>MN043581
GCT---GCA---------------------------------------
GCAGGAGGATTGTGGGTTACAGTCTATTATGGGGTACCTGTGTGG---AAAGAAGCAATC---
ACCACCCTATTTTGTGCATCAGATGCTAAAGCATATGACACAGAG---GTACATAAT---------------
--------------GTTTGGGCCACA---CATGCCTGTGTACCCATAGAC---
CCCAACCCACAAGAAGTAGTATTGGAA---------AATGTGACAGAAAATTTTAAC---------------
--------------------------ATGTGGAAAAATAACATGGTAGAACAGATGCATGAGGATATA---
ATCAGTTTATGGGATCAAAGCCTA---AAGCCATGTGTAAAATTA---
ACTCCACTCTGTGTTACTTTAAATTGTAGTGATTTGAAAAATGATACTAATACCAATAAT------------
----
----------------------------AGTAGTGAGAAAGTGATGGAGAAAGGAGAAATGAAA---
AACTGCTCTTTCAATGTTACCACAAACATAAAA-------------------------
AATAAGGTGCAGAAAGAATATGCTCTT---TTTTATAAACTTGATGTAATGCAAATAGATAAT---------
----

ACAAGCTATACGTTGATAAGTTGTAACTCCTCAGTCATTACACAG---
GCCTGTCCAAAGGTATCCTTTGAACCAATTCCCATACATTATTGTACCCCGGCTGGTTTTGCGATTCTACAGT
GTAAT---GATAAGAAGTTCAAT------GGAGCAGGACCATGTTCAAAT----------------------
-GTCAGC---ACAGTACAATGCACACATGGAATTAGGCCAGTAGTATCAACT---
CAACTGCTGTTAAATGGCAGTCTAGCAGAAGAAGAG-----------
GTAGTAATTAGATCTGAAAATTTCACGAACAAT---------GCTAAAACCATAATAGTACAGCTGAAT----
-----------GCATCTGTAGAA---------ATTAATTGTACAAGACCCAACAACAATACAAGAAAA-----
-AGTATAAGTATA-----------GGACCAGGGAGAACT---TTT------TATGCAACAGGAGAC------
---------ATAATAGGAGATATA-----------AGACAAGCACATTGT---AACATTAGTGCAGCA---
AAATGGAATAACACTTTAAGACAGATAGTTATAAAATTAAGA--------------GAACAATTTGGG----
----------------------------------AATAAAACAATAGTCTTTAAT------
CAATCCTCAGGAGGGGACCCAGAGATTGTA---ATGCACAGTTTTAATTGTGGAGGGGAA---
TTTAAGCAGCTCCAGGCAAGAGTCCTGGCTGTGGAAAGATACCTAAAGGATCAACAGCTCCTGGGGATTTGGG
GATGCTCTGGAAAGCTCATCTGCACCACTAATGTGCCTTGGAATACTAGTTGGAGT----------------
----------------------------AATAAATCTCAGAATGATATCTGG------CAT------
AACATGACCTGGATGGAGTGG---GACAGAGAAATTAACAATTACACAAGCTTAATATAC---
ACCTTACTTGAAGAATCGCAGAACCAGCAAGAA--

AAGAATGAACAGGAATTATTGGAATTGGATAAATGGGCAAGTTTGTGGAGTTGGTTTGAAATATCAAACTGGC
TGTGGTATATAAAAATATTCATAATGATAGTAGGAGGCTTGGTAGGATTAAGAATAATTTTTTCT---
GTACTTTCTATAGTA---------AATAGAGTTAGGCAGGGATACTCACCA------
TTATCGTTTGCTGTTAGTTTGCTTAATGCCACAGCTATAGCAGTAGCTGAG---
GGGACAGATAGGGTTATAGAAATATTACAAAGAGCTTATAGGGCT---------------------------
--------ATTCTCCACGTA------CCTACAAGAATAAGACAGGGCTTGGAAAGGGCTTTGCTA---TAA

>HQ386180
GCT---GTA--
GGAAACTTGTGGGTCACAGTTTATTATGGGGTACCTGTGTGG---AAAGAAGCAACT---
ACCACTCTATTTTGTGCATCAGATGCTAAAGCATATGATACAGAA---GTACATAAT----------------
--------------GTTTGGGCCACA---CATGCCTGTGTACCCACAGAC---
CCCAACCCACAAGAAATAATATTGGAA---------AATGTGACAGAAGAATTTAAC----------------
--------------------------ATGTGGAAAAATAACATGGTAGACCAGATGCATGAGGATATA---
ATCAGTTTATGGGATCAAAGCCTG---AAGCCATGTGTAAAATTA---
ACCCCACTATGTGTCACTTTAAATTGCACTGATGATTGGAAGAATATTACTACTGCCAATGTTACTAAT----
----
-------------------CCCAATACTACTAATACCGCTGGTTGGAAAAAAGGGGAAATAAAA---
AGTTGCTCTTTCAATGTCACCACAGGTGTAAGA---------------------------
GATAAGATAAAGAGAGAAAATGCACTA---TTTTATAAGCTTGATCTAGTACCAATAAATGAT----------
----

AAAAATACTAGCAATAATACTAGCTATAGATTGATAAGTTGTAACTCCTCAATCATTACACAG---
GCCTGTCCAAAGGTAACCTTTGAACCAATTCCCATACATTATTGTGCCCCGGCTGGGTTTGCGATTCTAAGT
GTAAC---GATAAGAAGTTCAAT------GGAACAGGACAATGTAAAAAT-----------------------
-GTCAGC---ACAGTACAATGTACACATGGAATTAGGCCAGTAGTGTCAACT---
CAACTGCTGTTAAATGGCAGTCTAGCAGAAGAAGAG------------
GTAATAATTAGATCTGCCAATATTACAGACAAT---------GCTAAAACCATAATAGTACAGCTAAAT----
-----------GAATCTGTCAAA---------ATTGATTGCATAAGACCCAACAACAATACAAAGAAA-----
-AGTATATATATA------------GGACCAGGGAGAGCA---TGG------CATACAGCAGGAAGA------
---------ATAGTAGGAGATATA------------AGACAAGCACATTGT---AACCTTAGTAGAACA---
AACTGGACTAACACTTTAAAACAGGTAGTTGCAAAATTAAGA---------------GAACACTTTAAG----
-----------------------------------AACAAAACAATAGTCTTTAAT------
TCATCCTCAGGAGGGGACCTAGAAATTGTA---ACGCACAGTTTTAATTGTGGAGGGGAA---
TTTAAGCAGCTCCAGGCAAGACTCCTGGCTGTAGAAAGATACCTACAGGATCAACAGCTCCTAGGGATTTGGG
GTTGCTCTGGAAAACTCATTTGCACCACTGCTGTGCCTTGGAATACTAGTTGGAGT-----------------
----------------------------AATAGATCTCTAAATTATATTTGG------GAA------
AACATGACCTGGATGGCGTGG---GAAAGAGAAATTGACAATTACACAAGCACAATATAC---
ACTTTAATTGAAGAATCGCAGATCCAACAGGAA--

AAGAATGAAAAGGAGTTATTAGAATTAGATGAGTGGGCAAGTTTGTGGAATTGGTTCACTATATCAAAATGGC
TGTGGTATATAAAGATATTCATAATGATAGTAGGAGGATTGATAGGTTTAAGAATAGTTTTTTCT---
GTGCTTTCTATAGTG---------AATAGAGTTAGGAAGGGGTACTCACCA------
TTATCATTCGCAGTTAACTTGCTCAATACCACAGCTATAGCAGTAGCTGAG---
GGGACAGATAGGGTTATAGAAGTAGTACAAAGAGCTTGTAGAGCT----------------------------
--------ATTCTCCATATA------CCTAGAAGAATAAGACAGGGCTTGGAAAGAATTTTGCTA---TAA
>EU744081
GCT---ACA--
GACCAATTGTGGGTCACAGTCTATTATGGAGTACCTGTGTGG---AAAGATACAACC---
ACCACTCTATTTTGTGCATCAGATGCCAAAGCATATGCTACAGAG---GTACATAAT----------------
--------------GTTTGGGCCACA---CATGCCTGTGTACCCACAGAC---
CCCAACCCACAAGAAATAGTATTGAAA---------AATGTAACAGAAGATTTTAAC----------------
--------------------------ATGTGGAAAAATAACATGGTAGAACAGATGCATGAAGATATA---
ATCAGTTTATGGGATCAAAGCCTA---AAGCCATGTGTAAAACTA---
ACCCCACTCTGTGTTACTTTAAATTGTACTGAGTTACAGACTAATGATACTAGTGTCACTAGTGGTAATAAGA
CTGATACT---
-------AATAATAGCACTAGTGGTAATAGGACTATCGAGGATAGTATGAGGGGAGAAATAAAA---
AACTGCTCTTTTAATATCACCACAAGCATAAAT---------------------------GATAAGAAG---
AAAGACTATGCTCTT---TTTAATAGACTTGATATAGTACCAATAAATAAT----------------------
----
----------GATAATACTAGCTATAGGTTGATAAGTTGTAACACCTCAGTCATTACACAA---
GCCTGTCCAAAGGTAACCTTTGAGCCAATTCCCATACATTATTGTACCCCGGCTGGTTTTGCACTTCTAAGT
GTAAC---AATAAGACGTTCAGT------GGAAAAGGAAAATGTACAAAT-----------------------
-GTCAGC---ACAGTACAATGTACACATGGAATTAGGCCAGTAGTGTCAACT---
CAACTGCTGTTAAATGGAAGTCTAGCAGAAGAAGAG------------
ATAGTAATTAGATCTGAAAATTTCACAAACAAT---------GCTAAAACCATAATAGTACAGCTGAAT----
-----------GAATCTGTAGCA---------ATTAATTGTACAAGACTCAACAACAATACAAGAAAA-----

-AGTATAACTTTA------------GGACCAGGGAGAGCA---TTT------TATGCAACAGGAGAC------
---------ATAATAGGAAATATA------------AGGCAAGCACAGTGT---GAACTTAATAGAACA---
AAATGGAATAACACTTTAAAAAAGATAGTTATAAAATTAGGA---------------GAACAATTTGGG----
-----------------------------------AATAGAACAATAGTTTTTAAT------
CACTCCTCAGGAGGGGACCCAGAAATTGTA---ATGCACAGTTTTAATTGTGGAGGGGAA---
TTTAAGCAGCTCCAAGCAAGAGTCCTGGCTGTGGAAAGATACCTAAGGGATCAACAGCTCCTAGGGATTTGGG
GTTGCTCTGGAAAACTCATTTGCACCACTGCTGTGCCTTGGAATGCTAGTTGGAGT-----------------
----------------------------AATAAATCTCTAGATCAGATTTGG------GAG------
AATATGACCTGGATGGATTGG---GAAAGAGAAATTAACAATTACACAGGCTTAATATAC---
ACCTTAATTGAAGAATCGCAGAACCAGCAGGAA--

AAGAATGAACAAGAATTATTGGAATTGGACAAGTGGTCAAATTTGTGGACCTGGTTTGATATAACAAACTGGC
TGTGGTATATAAAAATATTCATAATGATAGTAGGAGGATTGGTAGGTTTAAGAATAATTTTTTCT---
GTACTTTCTATAGTG---------AAAAGAGTTAGGCAGGGATACTCACCA------
TTATCGTTTGCTGTTAGCTTGTACAATACAATAGCCATAGCAGTAGCTGAG---
GGGACAGATAGGATTATAGAAGGATTACAAAGAGCCTTTAGAGCT----------------------------
--------TTTCTCCACATA------CCTAGAAGAATAAGACAGGGCGCAGAAAGAGCTTTGCAA---TAA
>JF320244
GCT---ACA---
GAAAACTTGTGGGTCACAGTCTATTATGGGGTACCTGTGTGG---AAAGAAGCAACC---
ACCACTCTATTTTGTGCATCAGATGCTAAAGCATATGATACAGAA---GTGCATAAT----------------
--------------GTTTGGGCCACA---CATGCCTGTGTACCCACAGAC---
CCCAACCCACAAGAAATAGAATTGGAA---------AATGTGACAGAAGATTTTAAC----------------
--------------------------ATGTGGAAAAATAACATGGTAGAGCAAATGCAGGAAGATGTA---
ATCAGTTTATGGGATCAAAGCCTA---AAGCCATGTGTAAAATTA---
ACCCCACTCTGTGTTACTTTGGATTGCAATGATTTTGATAAGAATAGTACTAATAGC----------------
----
----------------------------ACTACTGAGGAAAAGATGGACAAAGGAGAAATAAAA---
AATTGCTCTTTCAATAGCACCACAGACATGAGA---------------------------
GACAAGGTACGGAAAGAACATGCACTT---
TTTTATAGCCTTGATATAGTACCAATAGATAGTAGTGATAGTAATAAT-------------------------
----
AGTTCTAATAGTAGTAGTTCTAATAATTATAGGTTAATAAGTTGTAACACCTCAATCATTACACAA---
GCCTGTCCAAAAGTAACCTTTGAGCCAATTCCCATACATTATTGTGCCCCGGCTGGTTTTGCGATTCTAAAGT
GTAAA---GATAAGGATTCAAT------GGAACAGGGACATGTAAAAT-------------------------
-GTCAGC---ACAGTACAGTGCACACATGGAATTAGGCCAGTAGTGTCAACT---
CAACTATTGTTGAATGGCAGTCTAGCAGAGGGAGAG------------
GTAGTAATTAAATCTGCCAATTTCTCAGACAAT---------GCTAAAAACATAATAGTACAGTTAAAT----
-----------GAGTCTGTACCA---------ATAACTTGTATAAGACCCAACAATAATACAAGAAGA-----
-AGTATACCTATA------------GGGCCAGGAAGAGCA---TTT------TATACCACAGGAGAT------
---------ATAATAGGAGATATA------------AGAAAAGCACATTGT---ACCATTAGTAAAAAA---
GCATGGAATAATACTTTATACCAGTTAAGTAGAAAATTAAAA---------------GACCAGTATAAT----
-----------------------------------ACGACAACAATAGTCTTTAAA------
CCATCCTCAGGAGGGGACCCAGAAATTGTG---ATGCACAGTTTTAATTGTGGAGGAGAA---
TTTAAGCAGCTCCGGGCGAGAGTCCTGGCTGTGGAAAGATACCTAAGGGATCAACAGCTCCTAGGGATTTGGG
GTTGCTCTGGAAAACTCATTTGCACCACTGCTGTGCCTTGGAATGCTAGTTGGAGT-----------------
----------------------------AATAAATCTCTAGACTATATTTGG------GAT------
AACATGACCTGGATGCAGTGG---GAAAAGGAAATTGACAATTACACAGAACTTATATAC---
TCCTTGCTTGAAGAATCGCAGAACCAGCAGGAA--

AAGAATGAACAAGAATTATTGGAATTGGATAAGTGGGCAAGTCTGTGGAATTGGTTTAACATATCAAATTGGC
TGTGGTATATAAGAATATTCATAATAATAGTAGGAGGCTTGATAGGTTTAAGAATAGTTTTTACT---
GTACTTTCTGTAGTG---------AATAGAGTTAGGCAGGATACTCACCT------
GTGTCTTTTGCTGTCAGCTTGTTCAACGCAACAGCTATAGCAGTAGCTGAG---
GGAACAGATAGAATTATAGAAGTAGGACAAAGAATTGGTAGAGCT----------------------------
--------ATTCTCCACATA------CCTAGAAGAATAAGACAAGGCTTGGAAAGGTCTTTGCTA---TAA
>JF320226
GCT---GAA---
GAAAAATCGTGGGTCACAGTCTATTATGGGGTACCTGTGTGG---AAAGAAGCAACC---
ACCACTCTATTTTGTGCATCAGATGCTAAAGCATATGATACAGAG---GTACATAAT----------------

--------------GTTTGGGCTACA---CATGCCTGTGTACCCACAGAC---
CCCAACCCACGAGAAATAGTATTGGAA---------AATGTGACAGAAGAGTTTAAC----------------
--------------------------ATGTGGAAAAATAATATGGTGGAACAGATGCATGAGGATATA---
ATCAGTTTATGGGATCAAAGCCTA---AAGCCATGTGTAAAATTA---
ACCCCACTCTGTGTCACTTTAAATTGCACTGATGCTAGGCATAATACCACTGATGCTAGGAATAGTAAT----
----
----------------GGTACTAGTACTACCACTAATAGTTCAATAGACATAGGAGAGATGAAG---
AATTGCTCTTTCAATATCACCTCAAACATAAGA---------------------------
GACAAGATGAGGAAGGAAAATGCACTG---TTTTACAGACTTGATGTAGTACCAATAGGAAAT----------
----

AGTACTTCTGACTATAGGTTGATAAATTGTAACACCTCAGTCATTACACAG---
GCCTGTCCAAAGATATCCTTTGAGCCAATTCCCATACATTATTGTACCCCGGCTGGTTTTGCGATCTTAAAGT
GCAAT---GATAAGAAGTTCAAT------GGAACAGGAACATGTACAAAG-----------------------
-GTCAGC---ACAGTACAATGCACACATGGAATTAGGCCAGTAGTGTCCACT---
CAACTGCTGTTAAATGGCAGTCTAGCAAAAGAAGAG------------
GTAGTAATCAGATCTGCCAATTTCACAGACAAT---------GCTAAAACCATAATAGTGCAGTTAAAC----
-----------CACTCTGTACAA---------ATAGATTGTGAAAGACCCAACAATAATACAAGAAAA-----
-GGTATACATATA------------GGGCCAGGGAGAACA---TTT------TATGCAACAGGAGAT------
---------ATAATAGGAGACATA------------AGACAAGCACACTGT---AATGTTAGTAAAGCA---
GACTGGAATTACACTTTACAACTCATAGGTAAAAAATTAGCA--------------GACCAATTT-------
-----------------------------------AATAAAACAATAGTCTTTAAC------
CACTCCTCAGGAGGAGACCCTGAAGTTGTG---ATGCACACTTTTAATTGTGGAGGAGAA---
TTTAAGCAACTCCAGGCAAGAGTCCTGGCTGTAGAAAGATACCTACAGGATCAACAGCTCCTAGGAATTTGGG
GTTGCTCTGGAAAACTCATTTGCACCACTGCTGTGCCTTGGAATGCTAGTTGGAGT-----------------
----------------------------AATAAATCTCTGGACCAAATTTGG------CAT------
AACATGACCTGGATGGAGTGG---GAAAAAGAAATTGACAATTACACAGGGTTAATATAC---
ACCTTACTTGAAACATCGCAGAACCAGCAAGAA--

AAGAATGAACAAGAATTATTGGAATTGGATAAGTGGGCAAGTCTGTGGAACTGGTTTGACATAACACAATGGC
TGTGGTATATAAAAATATTCATAATGATAGTAGGAGGCTTGATAGGTTTAAGAATAGTTTTTGCT---
GTACTTTCTGTAGTA---------AATAGAGTTAGGAAGGGATACTCACCA------
TTGTCATTCGCTATTAGCTTGTTCAACGCCACAGCTATCGCAGTAGCCGAA---
GGAACAGATAGGGTTATAGAAACAGGGCAAAGATTAGGTAGAGCT----------------------------
--------CTTCTCCACATA------CCTAGAAGAATAAGACAGGGCTTGGAAAGGATTTTGCTT---TAA
>JF320189
GCC---GCA--
GATCAAATGTGGGTCACAGTCTATTATGGGGTACCTGTATGG---AAAGAAGCAACC---
ACCACTCTATTTTGTGCATCAGATGCTAAAGCATATGATAGAGAG---GTACATAAT----------------
--------------GTTTGGGCCACA---CATGCCTGTGTACCCACAGAC---
CCCAACCCACAAGAATTAGTATTGGGA---------AATGTAACAGAAGAATTTAAC----------------
--------------------------ATGTGGAAAAATAGCATGGTAGACCAGATGCATGAGGATATA---
ATAGAGTTATGGGATCAAAGCCTA---AAACCATGTGTAGAATTA---
ACCCCACTCTGTGTTATTTTAAATTGCGTTGATTATAATGGTACTGCTAATAATACCACTCAGAAT-------
----
-------------------GCCACTGAAAGTAGCAAGATAAAAATGGAGCAAGGAGAAATACAA---
AACTGCTCTTTCAACATCTCCACAAGCATAAGA---------------------------
GATAAGGTGCAGAAAGAACATGCACTT---TTCTATAAAATTGATATAGTACCAATAGATAAT----------
----

GATGGTAATAATAGTAGCTATAGGTTATTAAGTTGTAACACCTCAGTCATTAAACAG---
GCCTGTCCAAAAGTAACCTTTGAGCCAATTCCCATACATTATTGTGCCCCAGCTGGTTTTGCGATTCTACAAT
GTAAG---GATAAAAAGTTCAAT------GGAACAGGACAATGTAAAAAT-----------------------
-GTCAGC---ACAGTACAATGTACACATGGGATTAGGCCAGTAGTATCAACT---
CAACTGCTGTTAAATGGCAGTCTAGCAGAAGAAGAG------------
ATAATAATTAGATCTGAGAATCTCACAAATAAT---------GCTAAAACCATAATAGTACATCTGAAT----
-----------GAATCTGTAGAA---------ATTAATTGTACAAGACCCAATAACAATACAAGAAGA-----
-AGTATACATATA------------GGACCAGGGAAAGCA---TTT------TATGCAACAGGAGGT------
---------ATAATAGGAGACATA------------AGAAAAGCACATTGT---AACATTAGTGGAACA---
AAATGGAAGAACATTTTAAAACAGATAGTTACAAAATTAAAA---------------GACAAATTTGGG----

-----------------------------------AACAAAACAATAATCTTTAAG------
CCATCCTCAGGAGGGGACCAAGAAGTTATG---ATGCACACTTTTAATTGTGGAGGAGAA---
TTTAAGCAGCTCCAGGCAAGAGTCCTTGCTATAGAGAGATACCTAAAGGATCAACAGCTCCTGGGGATTTGGG
GTTGCTCTGGAAAACTCATTTGCACCACTTCTGTACCTTGGAATAATAGTTGGAGT-----------------
-----------------------------AATAAATCTTTCAATGATATTTGG------GAT------
AACATGACCTGGATGGAGTGG---GAAAAAGAAATTGACAATTACACAAGAATAATATAC---
ACCCTAATTGAAGAATCGCAGTACCAGCAAGAA--

AAGAATGAACAAGAATTATTGGCATTGGATAAATGGGCAAGTTTGTGGAATTGGTTTGACATAACAAAATGGC
TATGGTATATAAAAATATTCATAATGATAGTAGGAGGCTTAATAGGTTTAAGAATAGTTTTTGCT---
GTACTTTATCTAGTA---------AGAAGAGTTAGGCAGGGGTACTCACCA------
TTGTCATTTGCTGTTAGTTTGCTCAATACCACAGCCATAGTAGTAGCTGAG---
GGGACAGATAGGATTGTAGAAGTAGTACAAAGAGCTTTTAGGGCT----------------------------
--------ATTATCCACATA------CCTACAAGAATAAGACAGGGCTTAGAAAGACTTCGGCTA---TAA
>EF210727
ACT---GCA---
GGAAAATTGTGGGTAACAGTCTATTATGGGGTACCTGTGTGG---AAAGAAGCAACC---
ACTACTCTATTTTGTGCATCAGATGCTAAAGCATATGATACAGAG---AAACATAAT----------------
--------------GTTTGGGCCACA---CATGCCTGTGTACCTACAGAC---
CCCAACCCACAAGAAATGGTATTGGAA---------AATGTGACAGAAGATTTTAAC----------------
--------------------------GTGTGGAAAAATAACATGGTAGAACAGATGCATGAGGATATA---
ATCAGTTTGTGGGATGAAAGCCTA---AAGCCATGTGTAAAGTTA---
ACCCCACTCTGTGTTAATTTAAATTGCACTGATGTTAAAATTAATAGTACTTCCCCTAATAATACT-------
----
----------------------GTTGGTTCTTCCATTAATAGTAGCATGATAGAAGAAATGAAA---
AACTGCTCTTTCAATATCTCCACAAAAATAAAA--------------------------
GATAAGGCGCAAAAAGAATATGCACTT---TTTTATAAACTTGATATAGCACAAATAGATAAT----------
----

AGTACTGGATATAGGTTGATAAGTTGTAACACCTCAGTCATTACACAG---
GCCTGTCCAAAAGTATCTTTTGAGCCAATTCCAATACATTATTGCACCCCGGCTGGTTTTGCGCTTCTGAAAT
GTAAT---GATAAGAAGTTCAAT------GGAACGGGACCATGTACAAAT----------------------
-GTCAGC---ACAGTACAATGTACACATGGAATTAGACCAGTGGTATCAACT---
CAATTGCTGGTAAATGGCAGTCTAGCAGAAGAAGAG-----------
GTAGTAATTAGATCTGAAAATATCACGGACAAT---------ATTAAAACCATAATAGTACAGCTGAAT----
-----------GAATCTGTAGAA---------ATTAATTGTACAAGACCCAGCAACAATACAAGAAAA-----
-AGTATACAATTA------------GGACTAGGGAGAGCA---TGG------TATGCAACAGGAGCA------
---------ATAATAGGAGATATA------------AGAAAAGCATATTGT---GAGGTTAATGCAGGA---
AAATGGAACAAAACCTTAAAAATGGTAGCTAGAAAATTAAAC---------------GAACTTATT-------
-----------------------------------AACAAAACAATAGTCTTCAAT------
CAATCTTCAGGAGGGGATCCAGAAATTGTA---ATGTACAGTTTTAATTGTGGAGGGGAA---
TTTAAGCAGCTCCAGGCAAGAGTCCTGGCTGTGGAAAGATACCTAAAGGATCAACAGCTCCTAGGAATTTGGG
GTTGCTCTGGAAAGCTCATCTGCCCCACTGCTGTGCCTTGGAATACTAGTTGGAGC----------------
-----------------------------AATAAATCTCAGGATGAGATTTGG------AAG------
AACATGACCTGGATGCAGTGG---GAAAGAGAAATTGATAACTACACAAATGAAATATAT---
AGCTTAATTGCAGAGTCGCAAAACCAACAAGAC--

AAGAATGAACAAGAATTATTGGAATTGGATAAATGGGCAGGTTTGTGGGATTGGTTTAGCATAACAAACTGGC
TGTGGTATATAAAAATATTCATAATAATAGTAGGAGGTTTAATAGGTTTAAGAATAGTTTTTACT---
GTACTTTCTATAGTG---------AATAGAGTTAGGCAGGGATACTCACCA------
TTGTCATTCGCTATTAGCTTGCTCAATGCCACAGCCATAACAGTAGCCGAG---
GGGACAGATAGGGTTATAGAAGTAGCACAAAGAATTGTTAGGGCT--------------------------
--------ATTCTCCACATA------CCTAGGAGAATAAGACAGGGCTTGGAAAGGGCTTTGCTA---TAA
>EF210729
GCT---GAA---
GACAAATTGTGGGTCACAGTCTATTATGGGGTACCTGTGTGG---AAAGAAGCAACT---
ACCACTCTATTTTGTGCATCAGATGCTAAAGCATATAACACAGAG---GTACATAAT----------------
--------------GTTTGGGCCACA---CATGCCTGTGTACCCACAGAC---
CCTAACCCACAAGAAGTAAAATTGGAA---------AATGTGACAGAAAATTTTAAT---------------
--------------------------ATGTGGAAAAATAACATGGTAGAACAAATGCATGAGGATATA---

ATCAGTTTATGGGATCAAAGCCTA---AAGCCATGTGTAAAATTA---
ACCCCACTCTGTGTTGCTTTAAACTGCAGTGCGCTTGCGCCAAGTCTTTGCACTAATAATGTTACTAATGCCA
ATAGTACTACA--
----TCGAATAGTACTACTAATGCCACTAATACAGATTGTGAAAACCTGAGGCGAGAAATGACA---
AACTGTTCTTTTAATGCTACCACAGGCATAACA--------------------------
AATAAGATGAAAAAAGAATATGCACTT---TTTGATAGACTTGATGTAATACCAATAGATAAT---------
----

AGTTCAATATATGCATTGATAAATTGTAATACCTCAGTCATTAAACAG---
GCCTGTCCAAAGGTAACCTTTGAACCAATTCCCATACATTATTGTGCCCCGGCTGGTTTTGCGATTCTAAAGT
GTAAT---GATAAGAAGTTCAAT------GGAACAGGCATGTGTACAAAT----------------------
-GTTAGT---ACAGTACAATGTACACATGGAATTAGGCCAGTAGTATCAACT---
CAATTGCTGCTAAATGGCAGTCTAGCAGAAGGAGAT-----------
GTAGTACTTAAATCTGAAAATTTCACAAACAAT---------GCTAAAACCATACTAGTACAGCTGAAT----
-----------AATTCTGTAACA---------ATTAATTGTACAAGACCCTACAACAATACGAGAAGA-----
-GGTGTACATATG------------GGACCGGGGCAAGCA---CTT------TTTGCAACAGGGGCA------
---------ATAATAGGAGACATA------------AGACAAGCACATTGT---AACATTAGTAGAAAA---
GATTGGAATGACACTTTAAAACAGATAGTTACAAAATTAGGA--------------GAACAATTTGGG----
-------------------------------AAAAGTAAAACAATAGTCTTTAAT------
CAATCCTCAGGAGGAGACCCAGAGATTGTA---ATGCACAGCTTTAATTGTGGAGGGGAA---
TTCAAACAGCTCCAGGCAAGAGTCCTGGCTGTGGAAAGATACCTAAAGGATCAACAGCTCCTAGGGATTTGGG
GCTGCTCTGGAAAACTCATCTGCACCACTGCTGTGCCTTGGAATGCTAGTTGGAGT-----------------
---------------------------AATAAATCTATAGAAAAAATTTGG------AAT------
AACATGACCTGGATGCAGTGG---GAGAGAGAAATTGACAATTACACAGACTTAATATAC---
AATTTACTTGAACAATCGCAGAACCAGCAAGAA---------------------------------------
----
AAGAATGAACAAGAATTATTGGAATTGGATAAATGGGCAAGTTTGTGGAATTGGTTTGATATATCACACTGGC
TGTGGTATATAAAAATATTCATAATGATAGTAGGAGGCTTGGTAGGTTTAAGAATAGTTTGTGCT---
GTAATTTCTATAGCA---------AATAGAGTTAGGCAGGGATACTCACCA------
TTATCATTGGCTGTTAGTTTGCTTAATGCCACAGCTATAGCAGTAGCTGAG---
GGAACAGATAGGGTTATAGAAACATTACAAGGAATTGGTAGAGCT---------------------------
--------ATTCTAAACGTA------CCTAGAAGAATAAGACAGGGCTTTGAGAGGGCTTTACTA---TAA
>EF210731
GCT---GCA--
AAAGAATTGTGGGTCACAGTCTATTATGGGGTACCTGTGTGG---AAAGATGCAACC---
ACTACTCTATTTTGTGCATCAGATGCTAAAGGATATGATACAGAG---GTACACAAT----------------
--------------GTTTGGGCCACA---CATGCCTGTGTACCCACAGAC---
CCCAACCCACAAGAAGTACTATTGGAA---------AATGTGACAGAATACTTTAAC----------------
-------------------------ATGTGGAAAAATGACATGGTAGAACAGATGCAGGAGGATATA---
ATCAGCTTATGGGATCAAAGTCTA---AAACCATGTGTAAAACTA---
ACCCCACTCTGTGTTACTTTAAACTGTACTGATTTGAATGTTACTAAGACTGATTTGAATGTTACTAAG----
----
------------------ACTAATAATAGTAGTGAAACAACAATAGAAAAGGGAGAAATGAAA---
AACTGCTCTTTCAATGTCACCAAAGACTGGAAT--------------------------
GGTAGAGGGCAGAAAGAGTATGCAGTT---TTTCAAAAACTTGATGTAGTACCAATAGAGAATAAT-------
----

AATGGTAGTAATAATACCTATAGGTTGATAAGTTGTAACACCTCAGTTGTTACGCAA---
GCCTGTCCAAAGATATCTTTTGATCCAATTCCCATACATTATTGTGTTCCTGCTGGTTTTGCGATTGTAAAGT
GTAAT---GATAAAAAGTTCAAT------GGAACAGGACCATGTAAAAAT----------------------
-GTCAGC---ACAGTACAATGTACTCATGGAATTAAGCCAGTAGTATCAACT---
CAACTGTTGTTAAATGGCAGCCTAGCAGAAGAAGAG-----------
GTAGTAATTAAATCTGCCAATTTCACAAACAAT---------GCTAAAATCATAATAGTACAACTGAAA----
-----------GAAGCTGTAACC---------ATTACTTGTACAAGACCCAACAATAATACAAGAAAA-----
-AGTGTACATATG------------GGACCCGGGAAAGCA---TTT------TATTTAACA---GAT------
---------GTAATAGGAGATATA------------AGACAAGCATATTGT---AACATTAGCGGTAAA---
AAATGGAATGACACTTTAAGACAGGTAGCTGAAAAATTAAGC---------------AAACAATTTAAG----
----------------------------------AATAAAACAATAACCTTTAAT------
CAATCTTCAGGAGGGGACCCAGAAATTGTA---ATGCACAGTTTCAATTGTAGAGGGGAA---
TTTAAGCAGCTCCAGGCAAGGGTCCTGGCTGTGGAAAGATACCTATATGATCAACAGCTCCTGGGACTGTGGG

GTTGCTCTGGAAAACTCATTTGCACCACTACTGTGCCTTGGAATACTAGTTGGAGT-----------------
----------------------------AATAAATCTCGGTGGGAAATTTGG------GAG------
AACATGACCTGGATGGAATGG---GATAAAGAAATTAGCAATTACACAGAATTAATATAT---
AACTTACTTGAACAATCGCAAAACCAACAAGAA--

AAGAATGAACAGGAATTATTGGCACTCGATAAATGGTCAAATTTGTGGAATTGGTTTGACATATCAAATTGGC
TATGGTATATAAAAATATTCATAATGGTAGTAGGAGGCTTGATAGGCTTAAGAATAATTTTTACT---
GTGCTATCTATAGTG---------AATAGAGTTAGGCAGGGGTACTCACCA------
TTATCATTTGCTGTTAGCTTGCTTAATGCCACAGCCATAGCAGTAGCTGAG---
GGGACAGATAGGATTATAGAAGGCTTACAACGAATTGGTAGAGCT----------------------------
--------ATCCTCCACATA------CCTAGAAGAATAAGACAGGGCTTAGAAAGGGCTTTGCAA---TAA
>EF210735
GCT---GCA--
GAAAACTTGTGGGTTACAGTCTATTATGGGGTACCTGTGTGG---AAAGAAGCAAC---
ACCACTTTATTTTGTGCATCAGATGCTAGAGCATATGATACAGAG---GTACATAAT----------------
--------------GTTTGGGCCACA---CATGCCTGTGTACCCACAGAC---
CCCAACCCACAAGAAATAGCATTGGGA---------AATGTGACAGAAGATTTTAAC----------------
--------------------------ATGTGGAAAAATAACATGGTAGAACAAATGCATGAGGATGTA---
ATCAGTTTATGGGATCAAAGCCTA---AAGCCATGTGTAAAACTA---
ACCCCACTCTGTGTAACTTTAAGTTGTACTGATGTCAATAGTACTGATACTAAGGGCAATAATACTGGT----
----
-------------------AGTAATAGTACTAGTGGGAAATGATGGAGAAAGGAGAAATAAAA---
AATTGCTCTTTCAATGCTACAACAAGCATGCAA---------------------------
GACAAGATAAAGAAAAACTATGCACTG---TTTTATAGCCTTGATATAGTACCAATAGACAAT----------
----

ACTAGCTATAGGTTAATCAGTTGTAACACCTCAATCATTACACAG---
GCCTGTCCAAAGGTATCCTTTGAGCCAATTCCCATACATTATTGTGCTCCGGCTGGTTTTGCGATTCTAAAAT
GTAGT---GATAAGGGATTCAAT------GGAACAGGAAAATGTACAAAT-----------------------
-GTCAGC---ACAGTACAATGCACACATGGGATTAGGCCAGTAGTGTCAACC---
CAACTGCTGTTAAATGGCAGTCTAGCAGAAGAAGAG------------
GTAGTACTTAGATCTGCCAATTTCTCAGACAAT---------ACTAAAACCATAATAGTACAGCTAAAG----
-----------GAGCCTGTAGAA---------ATAAATTGTACAAGACCCAACAATAATACAAGAAAA-----
-AGTATACCTATA-------------GGGCCAGGGCGAGCA---TTT------TATGCAACAGGAGAA------
---------ATAATAGGAGATATA------------AGAAAAGCACATTGT---AACATTAGTAGAGCA---
AAATGGAATAAAACTTTACACCAGATAGCTACCAAATTAAGA---------------GCCCAATAT-------
-----------------------------------AATAAAACAATAGCCTTTAAT------
AAATCCTCAGGAGGGGACCTAGAAATTGTG---ATGCATACTTTTAATTGTGGAGGAGAA---
TTCAAACAGCTCCAGGCAAGAGTCCTGGCTGTGGAAAGATACCTACAGGATCAACAGCTCCTAGGGATCTGGG
GTTGCTCTGGGAAACTCATTTGCGCCACTAATGTGCCCTGGAACACTAGTTGGAGTGAGAAT---ATGACT--
-------------------------AGTAATATATCTCTGAACCAAATTTGG------GAT------
ACAATGACCTGGATGCAGTGG---GAAAAAGAAATAGAAAGGTACACAGAAGACATATAC---
ACCTTACTTGAAAAATCGCAGAACCAGCAGGAA--

AAGAATGAACAAGAATTATTGAAATTGGATGAGTGGGCAAGCCTGTGGAACTGGTTTGACATATCAAAATGGC
TGTGGTATATAAAAATATTCATAATGATAGTAGGAGGTTTGGTAGGTTTAAGAATAATTTTTGCT---
GTACTTTCTATAGTG---------AATAGAGTTAGGCAGGGATACTCACCA------
ATATCATTGGCTGTCAGCTTGCTCAATGTCACAGCAATAGCAGTAGCCGAG---
GGGACAGATAGGATTATAGAAGTAGTACAAATAGCTGGTAGAGCT----------------------------
--------ATTCTCCACATA------CCTAGAAGAATAAGACAAGGCTTGGAAAGGTGTCTGCTA---TAA
>MH327746
GCT---ACA--
GAAAAATTGTGGGTCACAGTCTATTATGGAGTACCTGTGTGG---AAAGAAGCAACT---
ACCACTCTATTTTGTGCTTCAGATGCTAAAGCATATGATACAGAG---GTACATAAT----------------
--------------GTGTGGGCTACA---CATGCCTGTGTACCCACAGAC---
CCCGATCCAAAAGAGGTAGTATTAGAA---------AATGTGACAGAAAATTTTAAT----------------
--------------------------GTATGGAAAAATGACATGGTAGAGCAGATGCATGAGGATATA---
ATCAATTTATGGGATCAAAGCCTA---AAGCCATGTGTAAAATTA---
ACCCCACTCTGTGTTATTTTAAATTGCACTAATGCCACGATTAACAGTACGACCCCGGAG-------------
----

-------------------------------ACAGAATTAAGGACCCTGAAGGAAGAAGTAAGG---
AACTGCTCTTTCAATGTCACCCCAAGCATAAAA---------------------------
AACAAGATGCAGAAGGAATATGCACTT---TTGTATAAACTTGATCTAGTAGCAATAAATAAG----------
----

ACTGATAATACTAGCTATAGGTTAATAAGTTGTAACACTTCGACCCTTACACAG---
GCCTGTCCAAAGGTATCCTTTGAGCCAATTCCCATACATTATTGTGCCCCGGCTGGTTTTGCGATTATAAAGT
GTAAT---GATAAAAAGTTCAGT------GGAAAAGGATCATGTACAAAT-----------------------
-GTCAGC---ACAGTACAATGTACACATGGGATTAGGCCAGTAGTATCAACT---
CAACTGCTGTTGAATGGCAGTCTAGCAGAAGATGAG------------
ATAGTAATTAGATCTGAAAATATCACAGACAAT---------GCCAAAACCATCATAGTGCACCTTAGT----
-----------GAAGCTGTACAA---------ATCAATTGTACCAGACCCAATAACAATACAAGGAAA-----
-AGTATAACTATA------------GGACCAGGGAGAGCA---TTT------TATGCAACAGGAGAA------
---------GTAACAGGAGATATA------------AGACAAGCACATTGT---AACATTAGTGGAACA---
GAATGGAATAACACTTTAAGGCAGGTGAGTGAAAAATTAAGA---------------GATAAATTTAAG----
-----------------------------------AATAAAACAATAGTCTTTAAG------
CCATCCTCAGGAGGGGACCTAGAAATTGAA---ATGCACAGTTTTAATTGTGGAGGGGAA---
TTTAAGCAGCTCCAGGCAAGAGTCCTGGCTATGGAAAGATACCTAAAGGATCAACAGCTCCTAGGGATTTGGG
GTTGCTCTGGAAAACTCATTTGCACCACTGCTGTGCCTTGGAATGCTAGTTGGAGT-----------------
----------------------------AATAAGACTCTAAATGAGATTTGG------GAT------
AACATGACCTGGATGCAGTGG---GAAAAAGAAATTGACAATTACACAGCAGTAATATAC---
TCCTTACTTGAAGAATCGCAGAACCAGCAAGAC--

AAGAATGAACAAGAATTATTGGCATTGGATAAGTGGGCAAATCTGTGGAATTGGTTTGACATAACAAATTGGC
TGTGGTATATAAGAATATTCATAATGATAGTAGGAGGCTTGATAGGTTTAAGAATAGTTTTTACT---
GTGCTTTCTTTAATA---------AATAGGGTTAGGCAGGGATACTCACCA------
TTGTCATTCGCTATTAGCTTGCTCAATGCCACAGCCATCGCAGTAGCTGAG---
GGGACAGATAGGATTATAGAAATAGTGCAAAGAATTGGTAGAGCT----------------------------
--------ATTCTTAATATA------CCTAGAAGAATAAGACAAGGCTTGGAAAGAAGTTTGCTA---TAA
>KY658690
GCT---ACA--
GAAAAATTGTGGGTCACAGTCTATTATGGAGTACCTGTGTGG---AAAGAAGCAACT---
ACCACTCTATTTTGTGCTTCAGATGCTAAAGCATATGATACAGAG---GTACATAAT----------------
--------------GTATGGGCTACA---CATGCCTGTGTACCCACAGAC---
CCCGATCCAAAAGAGGTAGTATTAGAA---------AATGTGACAGAAAATTTTAAT----------------
--------------------------GTATGGAAAAATGACATGGTAGAGCAGATGCATGAGGATATA---
ATCAATTTATGGGATCAAAGCCTA---AAGCCATGTGTAAAATTA---
ACCCCACTCTGTGTTATTTTAAATTGCACTAATGCCACGATTAACAGTACGACCCCGGAG-------------
----
-------------------------------ACAGAATTAAGGACCCTGAAGGAAGAAGTAAGG---
AACTGCTCTTTCAATGTCACCCCAAGCATAAAA---------------------------
AACAAGATGCAGAAGGAATATGCACTT---TTGTATAAACTTGATCTAGTAGCAATAAATAAG----------
----

ACTGATAATACTAGCTATAGGTTAATAAGTTGTAACACTTCGACCCTTACACAG---
GCCTGTCCAAAGGTATCCTTTGAGCCAATTCCCATACATTATTGTGCCCCGGCTGGTTTTGCGATTATAAAGT
GTAAT---GATAAAAAGTTCAGT------GGAAAAGGATCATGTACAAAT-----------------------
-GTCAGC---ACAGTACAATGTACACATGGGATTAGGCCAGTAGTATCAACT---
CAACTGCTGTTGAATGGCAGTCTAGCAGAAGATGAG------------
ATAGTAATTAGATCTGAAAATATCACAGACAAT---------GCCAAAACCATCATAGTGCACCTTAGT----
-----------GAAGCTGTACAA---------ATCAATTGTACCAGACCCAATAACAATACAAGGAAA-----
-AGTATAACTATA------------GGACCAGGGAGAGCA---TTT------TATGCAACAGGAGAA------
---------GTAACAGGAGATATA------------AGACAAGCACATTGT---AACATTAGTGGAACA---
GAATGGAATAACACTTTAAGGCAGGTGAGTGAAAAATTAAGA---------------GATAAATTTAAG----
-----------------------------------AATAAAACAATAGTCTTTAAG------
CCATCCTCAGGAGGGGACCTAGAAATTGAA---ATGCACAGTTTTAATTGTGGAGGGGAA---
TTTAAGCAGCTCCAGGCAAGAGTCCTGGCTATGGAAAGATACCTAAAGGATCAACAGCTCCTAGGGATTTGGG
GTTGCTCTGGAAAACTCATTTGCACCACTGCTGTGCCTTGGAATGCTAGTTGGAGT-----------------
----------------------------AATAAGACTCTAAATGAGATTTGG------GAT------
AACATGACCTGGATGCAGTGG---GAAAAAGAAATTGACAATTACACAGCAGTAATATAC---

TCCTTACTTGAAGAATCGCAGAACCAGCAAGAC--

AAGAATGAACAAGAATTATTGGCATTGGATAAGTGGGCAAATCTGTGGAATTGGTTTGACATAACAAATTGGC
TGTGGTATATAAGAATATTCATAATGATAGTAGGAGGCTTGATAGGTTTAAGAATAGTTTTTACT---
GTGCTTTCTTTAATA---------AATAGGGTTAGGCAGGGATACTCACCA------
TTGTCATTCGCTATTAGCTTGCTCAATGCCACAGCCATCGCAGTAGCTGAG---
GGGACAGATAGGATTATAGAAATAGTGCAAAGAATTGGTAGAGCT----------------------------
--------ATTCTTAATATA------CCTAGAAGAATAAGACAAGGCTTGGAAAGAAGTTTGCTA---TAA
>MF373127
GCT---TCA--
GATCAATTGTGGGTCACAGTCTATTATGGGGTACCTGTGTGG---AAAGAAGCAAAC---
ACCACTCTATTTTGTGCATCAGATGCTAAAGCATATGATACAGAG---GTACATAAT----------------
--------------GTTTGGGCCACA---CATGCCTGTGTACCCACAGAC---
CCCAACCCACAAGAAATAGGATTGGGA---------AATGTGACAGAAAATTTTAAC----------------
--------------------------ATGTGGAAAAATAACATGGTAGAACAAATGCATGAGGATATA---
ATTAGTTTATGGGATCAAAGTCTA---AAGCCATGTGTAAAATTA---
ACCCCACTCTGTGTTACTTTAAATTGCACTGATTGTAAAAAGGATAATGATACTTTACAATGTGATTGTAATA
AT---
----GAGAGACTGATAATAATACTATCACTAGTGACTTATACATGGGAGAGAGGAGAAATAAGA---
AACTGCTCTGTCACTGCTACCACAGACATAGAA---------------------------
GATAGGAGGAAAAATGAAGATGCTTCT---TATCATAATCGTGGTATAATACAAATTAATAAT----------
----

ATTAATAATACCTATACCTTGATGTTGAGTAGTTGTACCACTTCTATCATTGCCGGT---
CCCTGTCCATCCGTATCCCTTGATCCTATTCCCAATTGTTATTGTGCCGCGGCTGGTTTTCCTATTCGGAATG
GTAAT---GATAACAACGTCGCT------GGACCGGGACCAAGTGATATT-----------------------
-GTCACC---CCAGGACCATGTGCACTTGGGATTATGCCAGTGGCACCACCT---
CTGCTGCTGGTAAGTGGCCCTCTAGCAGAGGAAGAA------------
GTAATAACTACGTCTGACATTTTCACTAACAAT---------GCTAAAACCATTATAGTACAGCTGAAT----
-----------GAAACTGTAAAA---------ATTAACTGTACAAGGCCCAGCAACAATACAAGGAAA-----
-AGTATAACTATA------------GGACCAGGGAGAGCA---TTT------TATGCAACAGGAGAC------
---------ATAATAGGAGATATA------------AGACAAGCACATTGC---AACATTAGTCAAGCA---
AAATGGAATAACACTTTAAAACAGGTAGTTGTCAAATTAAGA---------------GAGCAATTTGGG----
--------------------------------ATGAATAAAACAATAATCTTTAAT------
CAATCCTCAGGAGGGGACCCAGAAATTGTA---ATGCACAGTTTTAATTGTGGAGGGGAA---
TTTAAGCAGCTCCAGGCAAGAATCCTGGCTGTAGAAAGATACCTACAGGATCAGCAGTTCCTAGGGTTTTGGG
GATGCTCTGGAAAACGCATCTGCACCACTGCTGTGCCTTGGAATGCTAGTTGGAGT-----------------
----------------------------AATAAATCTCTAGATGAGATTTGG------AAT------
AATATGACCTGGATGGAGTGG---GAAAAAGAAATTGACAATTACACAGGCTTAATATAT---
AACTTACTTATAGAATCGCAGAACCAACAAGAA--

AAGAATGAACAAGAATTATTGGCATTGGATAAATGGGCAAGTTTGTGGAATTGGTTTGACATATCAAATTGGC
TGTGGTATATAAGAATATTCATAATGATAGTAGGAGGCTTAGTAGGTTTAAGAATAGTTTTTACT---
GTACTTTCTATAGTG---------AGAAGAGTTAGGCAGAGATATTCACCA------
ATATCGGTAGCTGTTAGTTTGTTCAATGCCACAGCTATTGTAGTTGCTGAA---
GGGACAGATAGGGTTATAGAAGCAATACAAAGAGCTTTTAGAGCT----------------------------
--------ATTCTCCACATA------CCTAGAAGAATCAGACAGGGAGCGGAAAGGGCTTTGATA---TGA
>MF373129
GCT---TCA--
GGTCAATTGTGGGTCACAGTCTATTATGGGGTACCTGTGTGG---AAAGAAGCAACC---
ACCACTCTATTTTGTGCATCAGATGCTAAAGCATATGATACAGAG---GTACATAAT----------------
--------------GTTTGGGCCACA---CATGCCTGTGTACCCACAGAC---
CCCAACCCACAAGAAGTACTATTGGAA---------AATGTGACAGAAAATTTTGAT----------------
--------------------------ATGTGGAAAAATGACATGGTAGAACAGATGCATGAAGATATA---
ATCAGTTTATGGGATCAAAGTCTA---AAGCCATGTGTAAAATTA---
ACCCCACTCTGTGTTACTTTAAATTGCACTGATTATAGTAGAAAAAATACCACTAGTAAT-------------
----
-------------------------AACAGTACCATTGGTAACAACATGGAAGGAGAAATAAAA---
AACTGCTCTTTCAATATCACCACAAACAGACAA---------------------------
GATAAATGGCAGAAAGAATATGCACTT---TTTTATTAATATGATATAGTACCAGTAGATGGAGAT-------

----

ACTAATAATACTAATAATAATACCTATAGGTTGACAAGTTGTAACACCTCAGTCATCACACAG---
GCCTGTCCAAAAACATCCTTTGAGCCAATTCCCATACATTATTGTGCCCCGGCTGGTTTTGCGATTCTAAAGT
GTAAT---GATAAGAAGTTCAAT------GGAACAGGAAAATGTATAAAT--------------------
-GTTAGC---TCAGTACAATGTACACATGGAATTAGGCCAGTAGTGTCAACT---
CAACTGCTGTTAAATGGCAGTCTAGCAGAAGAAGAG-----------
GTAGTAATTAGGTCTGAAAATTTCACGAACAAT---------ATTAAAACCATTATAGTACAGTTGAAT----
-----------GAATCTGTAGAA---------ATTAATTGTACAAGACCCAACAACAATACAAGGAAA-----
-AGTATACATATG------------GGACAAGGGAGAGCA---TTT------TATGCAACAGGAGAC------
---------ATAATAGGAGATATA-----------AGACAAGCACATTGT---AACATTAGTAGAGCA---
AAATGGAATAACACTTTAAAACAGATAGTTGCCAAATTAAGA---------------GAGCAATTTGGG----
-----------------------------------AATAAAACAATAATCTTTAAT------
CAATCTTCAGGAGGGGACCCAGAAATTGTA---ATGCACAGTTTTAATTGTGGAGGGGAA---
TTTAAGCAGCTCCAGGCAAGACTCCTGGCTGTGGAACGATACCTAAGGGATCAACAGCTCCAGGAACTTTGGG
GGTGCTCTGGAAAACTCATTTGCACCACTGCTGTGCCTTGGAATGCTAGTTGGAGT-----------------
---------------------------AATAAATCTCTAGATATGATTTGG------CGT------
AACATGACCTGGAGGGAGTGG---GAAAGAGAAATTGACAATTACACAGACTTAATATAC---
AACTTACTTGAAGAATCGCAGAACCAGCAAGAA-------------------------------------
----
AAGAATGAACAAGACTTATTGGAATTGGATAAGTGGGCAAGTTTATGGAATTGGTTTACCATAACAAACTGGC
TGTGGTATATAAAAATATTCATAATGATAGTAGGAGGCTTGGTAGGTTTAAGAATAGTTTTTACT---
GTACTTTCTATAGTA---------AATAGAGTTAGGCAGGGATACTCACCA------
ATATCGTTAGCTGTTAGTTTGCTCAACGCCACAGCTATAGCAGTTGCTGAG---
GGGACAGATAGGATTATAGAAGCAATCCAAAGAGCTTATAGAGCT---------------------------
--------ATTATCCACATA------CCTACAAGAATAAGACAGGGCGCGGAAAGGGCTTTGCTA---TAA
>KP411829
GCT---TTA--
GATCAATTGTGGGTCACAGTCTATTATGGGGTACCTGTGTGG---AAAGAAGCAACC---
ACCACTCTATTTTGTGCATCAGATGCTAAAGCATATGATACAGAG---GTACATAAT----------------
--------------GTTTGGGCCACA---CATGCCTGTGTACCCACAGAC---
CCCAGCCCACAAGAAGTAGTACTGGGA---------AATGTGACAGAAAATTTTAAC----------------
-------------------------ATGTGGAAAAATGACATGGTAGAACAGATGCATGAGGATATA---
ATCAGTTTATGGGATGAAAGTCTA---AAGCCATGTGTAAAATTA---
ACCCCGTTCTTTGTTACTTTAATGTGCACTGATTTGGAAAATGAAACTAATAGTACTTTAAAAAATGAAACTA
ATAGTAATAATGCGACTATG--
--GAAGATGGAGAGAGGAGAAATAAA---
AAACTGCTCTTTCAATCTCACAAGCCTAGGAAA-----------------------------
AAGATGCAGAAAGAATATGCAACT---TTTTATAAGCTTGATATAGTATCCATAGATAAT-------------
----
-------------------GATAATACTAGCTATAAGTTGATATTTTGTATCACCTCATTCATTACTAAG---
GCCTGCCCTAAGATCTCCTTTGAGTCAGTTTCAATACATTATACTGCCCCGGCGGGTTTTGCGATTATAAAGT
GTAGA---GATACAAAGTTCAAT------GGAACAGGGCCATGTAAAAAT--------------------
-GTCAGC---ACAGTACAATGTACACATGGAATTAAGCCAGTGGTATCAACT---
CAACTGCTGTTGAATGGCAGTGTAGCAGAAGAAGAT-----------
ATAGTCATTAGGTCTGAAAATTTCACGGACAAT---------GCTAAAACTATAATAGTACAGCTGAAC----
-----------AAGTCTGTAGAA---------ATTCATTGTATAAGACCCAACAACAATACAAGAAAA-----
-GGTATACATATG------------GGACTAGGGAGAGCA---TTT------TATACAACAGGAGAA------
---------ATAATAGGAGATATA-----------AGGCAAGCACATTGT---ATCCTTATTAGCACA---
AATTGGACTAACACTTTAAGACAGATATCTGTCAAATTAAAA---------------GAACTTTTTAAG----
-----------------------------------AATAAAACAATATACTTTACT---
CGACTCTCTGATGGGGCTACTCTAGAAATGACC---ACCCATATCTTTGTTTTGGGAGGAGAA---
TTCAAGCAGCTCCAGGCAAGAGTCCTGGCTGTGGAAAGATACCTAAAGGATCAACAGCTCCTGGGGATTTGGG
GTTGCTCTGGAAAACTCATTTGCACCACTACTGTGCCTTGGAATAAGACTTGGAGT-----------------
---------------------------AATAAATCTCTGGATGACATTTGG------AAA------
AACATGACCTGGATGCAGTGG---GAAAGAGAAATTGATAATTACACAGACTTAATATAC---
ACCTTACTTGCAGACTCACAGCGCCAACAAGAA-------------------------------------
----
AAGAATGAACAAGAATTATTGGAATTAGATAAATGGGCAAGTTTGTGGAATTGGTTTGACATAACAAACTGGC
TGTGGTATATAAAAATATTCATAATGATAATAGGAGGCTTGGTAGGTTTAAGAATAGTTTTTACT---

GTACTTTCTATAGTG---------AATAGAGTTAGGCAGGGATACTCACCA------
TTATCGTTTGCTGTTAGCTTGTTTAATGCCATAGCCATAGTAGTAGCTGAG---
GGAACAGATAGGGTTATAGAAGTATTACAAAGAGCTTTTAGGGCT----------------------------
--------ATCCGCCATATA------CCTACAAGAATAAGACAGGGCGCGGAAAGGGCTTTGCTA---TAA
>MF373151
GCT---AAA------GAA---------------CAA---GCT---AAA---
GAACAATTGTGGGTCACAGTCTATTATGGGGTGCCTGTGTGG---AAAGAAGCAACC---
ACCACTCTATTTTGTGCATCAGATGCTAAAGCATATGACACAGAG---GTGCATAAT----------------
--------------GTTTGGGCCACA---CATGCCTGTGTACCCACAGAC---
CCCAACCCACAAGAGATACATTTGGAA---------AATGTGACAGAAAATTTTAAC----------------
--------------------------ATGTGGAAAAATAACATGGTAGAACAAATGCATGAGGATATA---
ATTAATTTATGGGATGAAAGTCTA---AAACCATGTGTAAAATTA---
ACCCCACTCTGTGTTACTTTAAATTGTGAGCGAGGTAATAGCACTAGTATCCAGGCAATCGTTAGTAGAAGTG
CCCGCAGTAGT--
-------GTTATTCAAGTAATAGCCATATTACGGGCAATAGGAGTTTGGAATCGGGAGGAATCA---
ATTTGCTCTTTCAATGTCACCACAGGTGTAAGG---------------------------
GACAAAGTAATGAAAGAATATGCTCTT---TTTTATAAACTTGATGTAGTACAAATAGATAAT----------
----

AGTAATACTAGCTATAGGTTGATAAATTGTAATACCTCAGTCATTACACAG---
GCCTGTCCAAAGGTGTCTTTTGAGCCAATTCCAATACATTATTGTACCCCGGCTGGTTATGCGCTTTTAAAGT
GTAAT---AACAAAAAGTTCAAT------GGAACAGGACCATGTAAAAAT-----------------------
-GTCAGC---ACAGTACAATGTACACATGGAATTAGACCAGTAGTATCAACT---
CAATTGCTGTTAAATGGCAGTCTAGCAGAAGAAGAG------------
GTAGTAATTAGGTATGCTAATTTCTCGGACAAT---------GCTAAAACCATAATAGTACAGCTGAAA----
-----------GCATCTGTAAAA---------ATTGATTGTACAAGACCCAACAATAATACAAGAAAA-----
-AGTATACATATA------------GGACCAGGGAGGGCA---TTT------TATGCAACAGGAGAT------
---------ATAATAGGAAATATA------------AGAGAAGCACATTGT---AACATTAGTAGAGCA---
AAATGGAATGACACTTTAAGCCAAGTAGCTAGAAAATTAAAA---------------GAACAATTT-------
-----------------------------------AATAAAACAATAGTCTTCCAG------
CCACCCTCAGGAGGGGACCCAGAAGTTATG---ATGCTCAGCTTTAATTGTGGAGGGGAA---
TTTAAGCAGCTCCGGGCAAGAGTCCTGGCTGTGGAGAGATACCTACAGGATCAACAGCTCCTAGGGATTTGGG
GTTGCTCTGGAAAACTCATTTGCACCACTACTGTGCCTTGGAATTCTAGTTGGAGT-----------------
----------------------------AATAGATCTCTGGATAAGATTTGG------AAT------
AACATGACCTGGATGGAGTGG---GAAAGAGAGATTGACAATTACACAGATATAATATAC---
AGCTTAATTGAACAATCGCAAAACCAACAAGAA--

AAGAATGAACAAGACTTATTGGCATTAGATAAATGGGCAAGTTTGTGGAATTGGTTTAACATAACAAATTGGC
TGTGGTATATAAAAATATTCATAATGATAGTAGGAGGCTTGATAGGTTTAAGAATAGTTTTTACA---
GTAATTTCTATAGTA---------AATAGAGTTAGGCAGGGGTACTCACCT------
TTATCGTTTGCTGTTAGCTTGCTAGATACCACAGCTGTAATAGTAGCTGAG---
GGACAGATAGGGTTATAGAATTTGTACAAAGGGCTTTTAGAGCT-----------------------------
--------ATCCTCCACATA------CCTGTAAGAATAAGACAGGGCTTGGAAAGGGCTTTGCTG---TAA
>KP411823
GCT---TTA--
GATCAATTGTGGGTCACAGTCTATTATGGGGTACCTGTGTGG---AAAGAAGCAACC---
ACCACTCTATTTTGTGCCTCTGATGCTAAAGCATATGATACAGAG---GTACATAAT----------------
--------------GTTTGGGCCACA---CATGCCTGTGTACCCACAGAC---
CCCAACCCACAAGAAGTAGTATTGGAA---------AATGTGACAGAAAATTTTAAC----------------
--------------------------ATGTGGAAAAATAACATGGTAGACCAGATGCATGAGGATATA---
ATCAGTTTATGGGATCAAAGTCTA---AAGCCATGTGTAAAATTA---
ACCCCACTCTGTGTTACTTTGAATTGTACTGATTTGAGAAATAATACTGAGAGCAATGATACT----------
----
----------------ACCAGTGGAATGATACTGGGGAAAGACAAGATAAAAATGATTCTATTC---
AACTGCTCTTTCAATATCACCACAAGCAGAAGA---------------------------
GATAAATGGCAGCAAGAATATGCATTT---TTTTATAAGCTTGATATAATGCCAATAGATGAAGAGAAT----
----

AATACTAATACCTATACGTTGATAAGTTGTAATACCTCAGTCATTACACAG---
GCCTGTCCAAAGGTATCCTTTGAGCCAATTCCCATACATTATTGTACCCCAGCTGGTTTTGCGCTTCTAAAGT

GTAAT---GATAAGAAGTTCAAT------GGAACAGGACTATGTAAAAAT-----------------------
-GTTAGC---ACAGTACAGTGTACACATGGAATTAGGCCAGTAGTATCAACT---
CAACTGCTGTTAAATGGCAGTCTAGCAGAAGAAGAG------------
GTAGTAATTAGGTCTGAAAATTTCACAGACAAT---------ATTAAAACCATTATAGTACAGCTGAAT----
-----------GAATCTGTAGAA---------ATTAATTGCACAAGACCCAACAACAATACAAGGAGA-----
-AGTATCCGTAACCAT------AGAGGACCAGGGAGAGCA---TTT------CATACAACAGGAGAA------
---------ATAATAGGGAATATA------------AGACAAGCACATTGT---AACATTAGTAGAGCA---
AAATGGAATAACACTTTAAAACAGATAGTTGCCAAATTAAGA---------------GAGCAATTTGGG----
--------------------------------AAGAATAAAACAATAGTCTTTAAT------
CACTCCTCAGGAGGGGACCCAGAAATTGTG---ATGCACAGTTTTAATTGTGGAGGGGAA---
TTTAAGCAGCTCCAGGCAAGAGTCCTGGCTATAGAAGGATACCTAAAGGATCAACAGCTCCTGGGACTTTGGG
GATGCTCTGGAAAACTCATTTGTACCACTGCTGTGCCTTGGAATGTTAGTTGGAGT-----------------
----------------------------AATAAATCTCTAGATAAGATTTGG------AAT------
AACATGACCTGGAGGGAGTGG---GAAAGAGAAATTGACAATTACACAGGCTTAATATAC---
AACTTACTTGAAACATCGCAGAACCAGCAAGAA--

AAGAATGAACAAGAATTATTGGAATTGGATAAATGGGCAAGTTTGTGGAATTGGTTTGACATAACAAACTGGC
TGTGGTATATAAAAATATTCATAATGATAGTAGGAGGCTTAGTAGGTTTAAGAATAGTTTTTACT---
GTACTTTCTATAGTG---------AATAGAGTTAGGCAGGGATACTCACCA------
ATATCGTTAGCTAATGAGGTTTTCAATTGCCCCGTTAATGCCACCGGTGAA---
AGCACCGGTAGGGGTATAGAAACCTTTCAAAGAACCTTTAAAAGTATTTTTCAA-------------------
--------ATTCTTTCCCAA------ATTACCCCGGGACAAACGGGCGCCAAAAAGGGTTGGGTA---TAA
>MF373162
ACT---GTA--
GCACAATGGTGGGTCACAGTCTATTATGGGGTACCTGTGTGG---AAAGATGCAACT---
ACCACTCTATTTTGTGCGTCAGATGCTAAAGCATATGAAACAGAG---GTACATAAT----------------
--------------GTTTGGCCACA---CATGCTTGTGTACCCACAGAC---
CCTAACCCACAAGAAATAAGATTGGAA---------AATGTGACAGAAAATTTTAAT----------------
--------------------------ATGTGGAAAAATAACATGGTAGACCAAATGCATGAGGATATA---
ATCAGTTTATGGGATCAAAGTCTA---AAGCCATGTGTAAAATTA---
ACCCCACTCTGTGTTACTTTGAATTGCTCTAACTTAAGAAATAATACTAATACCAATAGTACT----------
----
-------------------------AGTACCAATCATACTATTGGAGGAGAGGGTGATATGAAA---
AACTGCTCTTTCAATACCACCACAGGGATAGAT---------------------------
AATAAGATACAAAAACAATATGCACTT---TTTTATACACTTGATGTAGTACAAATTAAAAT----------
----

GATACTAAAAGCTATACATTGGTAAATTGTAATACCACAGTCATTTCACAG---
GCCTGTCCAAAGGTAACCTTCGAACCAATTCCCATACATTATTGTGCCCCGGCTGGTTTTGCGATTCTAAAAT
GCAAG---GATGAAAAGTTCAAT------GGTTCAGGACCATGTAACAAT-----------------------
-GTCAGC---ACAGTACAATGTACACATGGAATTAGGCCAGTAGTGTCAACT---
CAACTGCTATTAAATGGCAGTCTAGCAGAAGGAGGA---------
GAAGTAGTAATTAGATCTGAAAATTTCACAAACAAT---------GCTAAAACCATAATAGTACAGCTGGAC-
--------------ACACGTGTAGAA---------ATTGATTGTATAAGACCCAACAACAACACAAGGAGA--
----AGTATACATATA-------------GCACCAGGGAGTGCA---TTT------TATGCAACAGGAGAC---
------------ATAATAGGAGATATA------------AGACAAGCACATTGT---AACATTAGTAGAGCA-
--GAATGGAATAGTACATTAGTCAAGATAGCTAAAAAATTACGA---------------GAACAATTT-----
-------------------------------------AATACAACAATAGCCTTTAAT------
AAATCCTCAGGAGGGGATCTAGAAATTGTA---ATGCACAGTTTTAATTGTGGAGGAGAA---
TTTAAGCAGCTCCAGGCTAGAGTCCTGGCTGTAGAAAGATACCTAAGGGATCAACAGCTCCTTGGGATTTGGG
GTTGCTCTGGAAAACTCATCTGCCACACTGCTGTGCCTTGGAATAAGAGTTGGAGTGAGAAT---
ATATCTAGTAAG------------------------AATATATCTATAGACCAGATTTGG------CAA----
--AACATGACCTGGATGCAGTGG---GAGAGAGAAATTGATAATCACACAGAATTAATATAC---
AATTTACTTGAACAATCGCAGATCCAGCAGGAA--

AAGAATAAACAAGAATTACTAGAATTGGATAAGTGGACAAATTTGTTGGATTGGTTTGACATAACAAAATGGC
TGTGGTATATAAAAATATTTATAATGATAGTAGGAGGCTTAGTAGGCTTAAGAATAGTTTTTACT---
GTACTTTCTATAGTA---------AATAGAGTTAGGCAGGGATATTCACCA------
TTATCATTTGCTGTTAGCTTGCTTAATACCACTGCTATAGTAGTAGCTGAG---

GGGACAGATAGGATTATACAAGCAGCACAAATAGTTGGCAGAGCT----------------------------
--------ATTCTCCATATA------CCTAGAAGAATAAGACAGGGTCTTGAAAGGGCTTTGCAA---TAA
>KP411824
ACT---GCA--
CCAAGCTTGTGGGTCACAGTCTATTATGGAGTGCCTGTGTGG---AAAGAAGCAACT---
ACCACCCTATTTTGTGCATCAGATGCTAAGGCATATGAGAAAGAG---GCACATAAT----------------
--------------ATTTGGGCCACA---CATGCCTGTGTACCCACAGAC---
CCCAATCCACAAGAAGTAGTATTGGAA---------AATGTGACAGAGGATTTTAAC---------------
--------------------------ATGTGGAAAAATAACATGGTAGAACAGATGCATGAGGATATA---
ATCAGTTTATGGGATCAAAGCTTA---AAGCCATGTGTAAAATTA---
ACCCCACTCTGTGTCACTTTAAATTGCACTGATCCTTGGTATGATAATACTGCTAATAGGACT----------
----
----------------------------ACAGAGAGAATGATAATGGAGAAAGGAGAGATAAAA---
AAATGCTCGATCGAGGTCAACACATGCTTGATA--------------------------
GATGTGTGGATGGAGCATTGTGCACTT---CTTTCTAAACTACATTTAGTACGACTAGAGAAC----------
----

ACTACATTCATCTATATGCAGACAAGTCGTCACTCCTCGAAACGTACAAAA---
GGTTGTCCAAGGTATTCCATAGAACCGATTCCACTACAATATTCTGCCCCGGCAGGTTCTGCCATTCTAATTC
GTAAA---AATGATATGGTTCAT------GGTACCGGGACATGTACTCAT----------------------
-GTCCCC---CCAGTACAATGTTCGAACGGAATCAGGCCAGTTGTATCGAGT---
CAACATCCCTCGCATGACATATCAGGAGCAGCG--------------GTGCTAACTAAA---
GACAATTTCAAGGACACA---------GTTAGAACCATAATAGTACAGCTCAAT---------------
GATTCTGTACCA---------ATTAATTGTACAAGACCCAATAACAATACAAGAAAA------
AGTATACATATA------------GGACCAGGGAGAGCA---TTT------TATGCAACAGGAGAC-------
--------ATAATAGGAGATATA------------AGACAAGCACATTGT---AACATTAGTCTAGTA---
AAATGGAATAACACTTTAGAAAAAATAGTTACAAAATTGAGA---------------GAACAATTTAAG----
-----------------------------------AACAAGACAATAGTCTTTTCT------
CCCCCCTCAGGAGGGGATCCAGAAATTGTA---TCACACAGTTTTAATTGTGGAGGAGAG---
TTTAAGCAGCTCCAGGCAAGAGTCCTGGCTGTGGAAAGATACCTAAAGGATCAGCAGCTCCTAGGGATTTGGG
GCTGTTCTGGAAAACTCATTTGCACCACCACTGTGCCTTGGAATATTAGTTGGAGT----------------
----------------------------AATAAAACTCTAGAGCAGATTTGG------GAT------
AACATGACCTGGATGCAGTGG---GACAAAGAAATTCACAATTACACAGAATTAATATAC---
ACCTTAATTGAAGAATCGCAGATCCAGCAGGAA--

AAGAACGAACAAGAATTATTAGAGTTAGATAAATGGTCAAATTTGTGGAATTGGTTTGACATAACAAACTGGC
TGTGGTATATAAAAATATTCATAATGATAGTAGGAGGCTTGATAGGTTTAAGAATAGTTTTTGTT---
GTGCTTTCTATAATA---------AATAGAGTTAGGAAGGGATACTCACCA------
TTATCGTTCGCTATTAGTATTATTAATGTTACAGCCACAGCCGTAGCCGTA---
GCAGCAGATACGGTTATAGATACAGTACCAGCAGCAGGTAGAGGT---------------------------
--------ATCCTCCTCATA------CTTAGACGAATAAGACAAGGACTGGAAAGGGATTTGCTA---TAA
>MF373185
GCT---GCA--
GAACAATTGTGGGTCACAGTCTATTATGGGGTACCTGTGTGG---AAAGAAGCAACC---
ACCACTCTATTTTGTGCATCAGATGCTAAAGCATATGATACAGAG---GTACATAAT----------------
--------------GTTTGGGCCACA---CATGCCTGTGTACCCACAGAC---
CCCAACCCACAAGAAGTAAAATTGGAA---------AATGTGACAGAAAATTTTAAT---------------
--------------------------ATGTGGAAAAATAACATGGTAGAACAGATGCATGAAGATATA---
ATCAGCTTATGGGATCAGAGTCTA---AAACCATGTGTAAAATTA---
ACCCCACTCTGTGTTACCTTAAATTGCACTAATATTACTAGTATTAATGCCACCAGAGTGATACCAATAATAG
CATGGGGAATGGAATGAAGACATGAAAAA--

TGCTCTTTCAATATCACCACAAACATGAGA--------------------------
GATAAGCTAAAGAGAGAATATACACTT---
TTGTATAAAATGGATATAGTACCATTAGATGATAGTGAAAATAGC---------------------------
----
AATAGTAGTAATGGCAATTATAGCAGTTATAGATTGATAAGTTGTAACACCTCAGTCATTACACAG---
GCCTGTCCAAAGGTCTCTTTTGAGCCAATTCCCATACATTATTGTGCCCCGGCTGGTTTTGCGATTCTAAAAT
GTAAT---GATAAGGAGTTCAAT------GGAACAGGACCATGTAATAAT----------------------
-GTCAGC---ACAGTACATTGTACACATGGAATTAGGCCAGTAGTATCAACT---

CAGCTGCTGTTAAATGGCAGTCTAGCAGAAAATGAT-----------
ACAGTAATTAGGTCTGAAAATTTCACAAACAAT---------GCTAAAACTATAATAGTACAGCTGAAA----
-----------GAACCTGTAAGC---------ATTACTTGTGTAAGACCCAACAACAACACAAGAAAA-----
-GGTATACCTATA------------GGACCAGGGAGAGCA---TGG------TATACAACAGGACAA------
---------ATAGTAGGAAATATA------------AGAGCAGCATATTGT---GATGTTAATAGAGTA---
GCGTGGAATAACACTTTAAAACAGATAGCTAGCAAACTCAGA---------------GAGCAATTTAGG----
-----------------------------------AATAAGACAATAGTCTTTACT------
AACTCCTCAGGAGGGGACCCAGAAATTGTA---ATGCTCAGTGTTAATTGTGGAGGGGAA---
TTTAAGCAGCTCCAGGCAAGAGTCCTGGCTCAGGAAAGATACCTAAAAGATCAACAGCTCCTAGGGATTTGGG
GTTGCTCTGGAAAAATCATCTGCACCACTAATGTGCCTTGGAATGCTAGTTGGAGT-----------------
----------------------------AATAAATCTTATAATGACATTTGG------GAT------
AACATGACCTGGAGGGATTGG---GAAAGAGAAATTGATAGTTACACAAACACAATATAC---
AACTTACTTGAACTCTCGCAAATCCAACAAGAA--

AAGAATGAACAAGAATTATTACAATTAGATAAATGGGCAAATTTGTGGAATTGGTTTGACATAACAAAATGGC
TGTGGTACATAAAAATATTCATAATGATAGTAGGAGGCTTAATAGGTTTAAGAATAGTTTTTGCT---
GTACTTTCTATAGTG---------AATAGAGTTAGGCAGGGATACTCACCA------
ATATCGTTGGCTTATAGCTTGCCTAACGCTACGGCTATAGCAGTACCTGAG---CACCGA---
TTGACTTATGAAGCATCACGAAAGTCGATA---GGT-----------------------------------
ATTCCTCCATAC------CTAGTAAGAATAAGACAGGGCTTGGAAAGGGCTTTGCTA---TAA
>MF373186
GCT---GAA--GAAAAG---
TGGGTTACAGTCTATTATGGGGTACCTGTGTGG---AGAGAAGCAACC---
ACCACTTTATTCTGTGCATCAAATGCTAAAGGATATGATACAGAG---GCACATAAT----------------
--------------GTTTGGGCCACA---CATGCCTGTGTGCCCATAGAC---
CCCAACCCACAAGAGTTAGTATTGGAA---------AATGTAACAGAAAATTTTAAC----------------
--------------------------ATGTGGAAAAATAACATGGTAGAACAGATGCATGAGAATATA---
ATTGAATTATGGGATCAAAGTCTA---AAGCCATGTGTAGAATTA---
ACCCCACTCTGTGTTACTATAAACTGCACCACCATGGGGGCAAATAATACCACCTCTAGCCCC----------
----
----------------------------CTTACTAATAGTACAATAGATGAAGGGATAATGACA---
AACTGCTCTTTCAATATCACCACAGACATAAGA---------------------------
GATAGGAAGAAGGAGGACTATGCATTG---TTTTATAGAACTGATGTAGAAAGGATAAGTGAA----------
----

GATAGTGCTAATACCACCTATAGGTTGAGAAGTTGTAATACCTCAGTCATTAGACAG---
GCCTGTCCAAAGGTATCCTTTGAGCCAATTCCCATACATTTTTGTGCCCCAGCTGGTTTTGCGATTCTAAAAT
GTAAG---GATGAGGATTTCAAT------GGAACAGGACCATGTAAAAAT-----------------------
-GTCAGC---ACAGTACAATGTACACATGGAATTAGGCCAGTAGTATCAACT---
CAATTGCTGTTAAATGGCAGTCTAGCAGAAAAAGAG-----------
ATAGTAATTAGATCTCAAAATTTCTCGGATAAT---------GCTAAAACCATCCTAGTACAGCTAAAT----
-----------GCATCTATAGAA---------ATTAATTGTACAAGACCTAACAACAATACAAGAAAA-----
-AGCATACATATA------------GCACCAGGGAAAGCA---TTT------TATGCAACAGGAGAA------
---------ATAATAGGAAATATA------------AGACAAGCACATTGT---AATCTTAGTGGAGCA---
CAATGGAATAACACTTTAAAACATATAGCTATAAAATTAAGA---------------GAACAATATCCT----
-----------------------------------AATAAAACAATAAACTTTACC------
AAGTCCTCAGGAGGGGACCCAGAAATTGTA---CTGCATATCTTTAATTGTGGAGGGGAA---
TTTAAGCAGCTCCAGGCGAGAGTCCTGGCTGTAGAAAGATACCTAAAGGATCAACAGCTCCTGGGAATTTGGG
GTTGCTCTGGAAAACAAATCTGCACCACTGCTGTGTCTTGGAATGATAGTTGGAGT-----------------
----------------------------AATAAATCTCTAGATTATATTTGG------AAC------
AACATGACCTGGATGCAGTGG---GATAAAGAAATTCACAATTACACAAATTTCATATAT---
GACTTACTTGAAGATTCGCAAAAACTACAAAAC--

AAGAATGAAAAAGAATTGTTGGAATTGGATAACTGGGCAAGTTTGTGGAATTGGTTTACCATATCAAATTGGC
TGTGGTATATAAGAATATTCATAATGATAGTAGGAGGCTTGATAGGTTTAAGAATAGTTTTTACT---
GTACTGTCTATAGTG---------AATAGAGTTAGGCAGGGGTACTCACCC------
TTATCGTTTGCTGTTAGCCTGCTCAATGCCACGGCCATAGCAGTAGCTGAG---
GGGACAGATAGGGTTATAGAAATAATACAAAGACCTTGTAGA-------------------------------
--------------------------------AGAATAAGACAAGGCCTGGAAAGAGGTTTGCTA---TAA
>KY213743

GCA---GAA--
GACAATTGGTGGGTCACAGTCTATTATGGAGTACCTGTATGG---AAAAATGCAACC---
ACCACTCTATTTTGTGCATCAGATGCTAAAGCATATGATACAGAG---GTACACAAT----------------
--------------GTTTGGGCCACA---CATGCCTGTGTACCCACAGAC---
CCCAACCCACAAGAAGTAGTATTGGGA---------AATGTGACAGAATATTTTAAC----------------
--------------------------ATGTGGGAAAATGATATGGTAGAACAGATGCAGGAGGATGTA---
ATCAGTTTATGGGATCAAAGCTTA---AAGCCATGTGTGAAATTA---
ACCCCACTCTGTGTCACTTTAAATTGCACTGAGTTAAATACTACTAGTAATGCTACTGGG-------------
----
-------TTAAATACTACTAGTAATACTAGCACTGTGTCACATAATAGTAAGGATGAAATGAAA---
AACTGCTCTTTCAATGTCACCTCAAACATAAGA---------------------------
GATAAGTTGCAGAGAGAATATGCACTT---TTCTATAAACTTGATATAGTACCAATAGATAAT----------
----

GATAATAGCAGCTATAGATTGATAAGTTGTAACACCTCAGTCATTACACAG---
GCGTGTCCAAAGGTATCTTTTACGCCAATTCCTATACATTATTGTACTCCAGCTGGTTTTGCGCTTCTAAAGT
GTAAC---AATAAGACATTCAAT------GGAAAAGGACCATGTACAAAT-----------------------
-GTCAGC---ACTGTACAGTGTACTCATGGGATTAGGCCAGTAGTATCAACT---
CAGCTGTTGTTAAATGGCAGTCTAGCAGAAGAAGAA------------
GTAATAATTAGAGCTGACAATTTCACTGATAAC---------GCCAAAACCATAATAGTACAGCTGAAG----
-----------GAACCTGTAAAA---------ATTAATTGTACAAGACCCCACAACAATACAAGAAAG-----
-AGTATACATATA------------GGACCAGGGAGAGCA---TTC------TATGCAACAACAGAT------
---------ATAATAGGAGATATA------------AGACAAGCACATTGT---AACATTAGTGCAAAA---
GACTGGGGAAAAACTTTACAACAGGTAGCTGAAAAATTAAAA---------------GAACAATTTAAG----
-----------------------------------AACAAAACAATATACTTTAAT------
CAATCCTCAGGAGGGGACCCAGAAATTGTA---ATGCATAGTGTTAATTGTGGAGGGGAA---
TTTAAGCAGCTCCAGGCAAGAGTCCTGGCTATAGAAAGATACCTAAGGGATCAACAGCTCCTGGGGATTTGGG
GCTGCTCTGGAAAACTCATTTGCACCACTAATGTGCCTTGGAATGCTAGTTGGAGT-----------------
-------------------------AAAAATAAAAGTATAGATGAGGTTTGG------AAT------
AACATGACCTGGATGCAGTGG---GAAAATGAGATTGACAATTACACAGGCTTAATATAT---
ACTTTACTTGAAGAATCGCAAAACCAGCAAGAA--

AAGAATGAGCAAGAATTATTGGAATTGGATAAATGGGCAAGTTTGTGGAATTGGTTTGACATAACAAATTGGC
TGTGGTATATAAAAATATTCATAATGATAGTAGGAGGCTTGATAGGCTTAAGAATAGTTTTTGCT---
GTACTTTCTATAGTG---------AATAGAGTTAGGAAGGGATACTCACCA------
TTGTCATTCGCTGTTAGTTTGCTTAATACCATAGCTATAGCTGTAGCTGAG---
GGAACAGATAGGGTTATAGAAATAGTACAAAGAGTTTGTAGAGCT----------------------------
--------ATTCGCAACATA------CCTAGAAGAATAAGACAGGGCTTAGAAAGGGCTTTGCAG---TAA
>KY213745
GCT---GCA--
GAACAATTGTGGGTTACAGTATATTATGGGGTACCTGTGTGG---AAAGAAGCAATC---
ACCACTTTATTTTGTGCATCAGATGCTAAAGCATATGACACAGAG---GTACATAAT----------------
--------------GTTTGGGCCACA---CATGCCTGTGTACCCACAGAC---
CCCAACCCACAAGAAGTAAGGATGGAA---------AATGTGACAGAAGATTTTAAC----------------
--------------------------ATGTGGGAAAATAATATGGTAGAACAGATGCATGAAGACATA---
ATCAGTTTATGGGATCAAAGTTTA---AAGCCATGTGTAAAATTA---
ACCCCACTCTGTGTTACTTTAGAGTGCGATGATTATACCACCAATAGTACTGTTACCAAT-------------
----
-------------------------AGTAGTAGTGAGAAAACAGGGGAAAAAGGAGAAATAAAA---
AACTGCTCTTTCAATGTCACCACAACAATAAGA---------------------------
GATAAGGTGGAAAAAGAACAAGCACTG---TTTTATAAACTTGATGTAGTACCAATAGAAAAT----------
----

AGTAGTAATAGTACCAAATATAGGTTAAGAAATTGTAATACCTCAGTCATTACACAG---
ACTTGTCCAAAGGTATCCTTTGAGCCAATTCCCATACACTATTGTGCCCCGGCTGGTTTTGCGATTGTAAAGT
GTAAC---AATAAGACATTCAGT------GGAAAAGGACCATGTACCAAT-----------------------
-GTCAGC---ACAGTACAATGTACACATGGAATTAGGCCAGTAGTGTCAACT---
CAGCTGTTGTTAAATGGCAGTCTAGCAGAAGAAGAC------------
ATAGTAATTAGATCTAAGAATTTCACAGATAAT---------TCAAAAACCATAATAGTACAACTGAAC----
-----------AAATCCATAGCA---------ATTAATTGTACAAGACCCAACAACAACACAAGAAAA-----

-AGTATACATTTA------------GGACCAGGGCAAGCA---TGG------TATACAACAGGAGCC------
---------ATAATAGGAGATATA------------AGACAAGCACATTGT---AACATTAGTAAAATA---
GAATGGAATAATACTTTAAGACAGGTAGTTAAAAAATTAAAA---------------GAAAAATTTAAT----
-----------------------------------GCAACAACAATATTCTTTAAT------
CAATCCTCAGGGGGAGACCCAGAAATTGTA---ATGCACAGCTTTAATTGTGGAGGGGAA---
TTTAAGCAGCTCCAGGCAAGGGTCCTGGCTGTGGAAAGATACCTAAGGGATCAACAGCTCCTAGGGATTTGGG
GCTGCTCTGGAAAACTCATTTGCAACACTGCTGTGCCTTGGAATACTAGTTGGAGT-----------------
----------------------------AATAAATCTCTTAATGAAATTTGG------AAT------
AACATGACCTGGTTAGAGTGG---GAAAGAGAAATTGACAATTACACAGGTTTAATATAC---
ACCTTACTTGAAAAAGCACAGAACCAGCAAGAA--

AAGAATGAACAAGAATTATTGAAATTGGATAGTTGGGCAAGTCTGTGGAATTGGTTTAGCATATCAAACTGGC
TGTGGTATATAAGGATATTCATAATAGTAGTAGGAGGCTTAGTAGGTTTAAGAATAGTTTTTGCT---
GTACTTTCTATAGTA---------AATAGAGTTAGGCAGGGATATTCACCA------
TTATCATTGGCTATTAGCTTGCTAAATGTCACAGCTGTAGCAGTAGCTGAA---
GGGACAGATAGAATTATAACAGTAGTACAAAGAGCTTATAGAGCT----------------------------
--------TTTCTTCACATA------CCTGTAAGAATAAGACAAGGCTTGGAAAGGGCTCTGCTA---TAA
>JN248346
ATG---GCA--
CAACAATTGTGGGTCACAGTCTATTATGGGGTACCTGTGTGG---AAAGAAGCAAAA---
ACCACTCTATTTTGTGCTTCAGATGCTAAAGCATATGATACAGAG---GTACATAAT----------------
--------------GTCTGGCCACA---CATGCCTGTGTACCCACAGAT---
CCCAACCCACAAGAAGTAGAATTGGTA---------AATGTGACAGAAGATTTTAAC----------------
--------------------------ATGTGGAAAAATAACATGGTAGAACAAATGCATGAGGATGTA---
ATCAGTTTATGGGATCAAAGCCTA---AAACCATGTGTCAAATTA---
ACCCCACTCTGTGTTACTTTAAATTGCAGTGATTGTGATACTGCAAATTGCAGTATTGGGAATAACAATACGA
AT---
-------------GCCACTATTAAAAATAACTCTGCTACTCATAACGCTAGTAGAGAAATAAAA---
ATCTGCTCTTTTAATGTCACAACAGATATAAGA---------------------------
GATAAGGTGAAGAAAGAATATGCGCTT---TTTCATAGTCTTGATGTAATATCAATAGATAAG----------
----

AATAATACTAGCTATAGGTTGATAAGTTGTAATACCTCAACCCTTACACAG---
GCCTGTCCAAAAGTATCATTCCAGCCAATTCCTATACATTATTGTACCCCAGCTGGCTATGCGCTTCTGAAGT
GTAAA---GATAAGAATTTCAAT------GGAACAGGAAAATGTCATAAT-----------------------
-GTTAGC---ACAGTACAATGTACACATGGAATTAGACCAGTAGTATCAACT---
CAACTGCTGTTAAATGGCAGTCTATCAGAAGGAGAT------------
GTAATAATTAGATCTGAAAATTTCACAAACAAT---------GCCAAAACCATAATAGTACAGCTGAAT----
-----------GAAACTGTAAAA---------ATTAATTGTACAAGACCCAATAATAATACAAGAAAA-----
-AGCATACATATA------------GCACCAGGGAGGACA---TTC------TTTGCAACAGGAGAG------
---------ATAATAGGAGATATA------------AGACAAGCACATTGC---AACATTAGTAGAAAA---
GAATGGAATGACACTTTAAAACTGATAGCTACAAAATTAAGG---------------GAACAATATAAA----
-----------------------------------AACAAAACAATAGTTTTTCAG------
CCACCCTCAGGAGGGGACCCAGAAATTGTA---AGGCACAGTTTTAATTGTGGGGGGGAA---
TTTAAGCAGCTCCAGGCAAGAGTCCTGGCTGTGGAAAGATACCTAAGGGATCAACAGCTCCTGGGGATTTGGG
GTTGCTCTGGAAAACTCATCTGCACCACTGCTGTGCCTTGGAATAATAGTTGGAGT-----------------
-------------------------AAGAATAAATCTCTGAAGGAGATTTGG------AAT------
AACATGACCTGGATGCAGTGG---GAAAAAGAAATTGACAATTACACAAGCTTAATATAC---
AGCTTAATTGAAGAATCGCAGAACCAACAAGAA--

AAGAATGAACAAGAATTATTAGAATTAGATAAATGGGCAAATCTGTGGAGTTGGTTTGACATATCAAATTGGC
TGTGGTATATAAGAATATTCATAATGATAGTAGGAGGCTTGATAGGTTTAAAAATAGTTTTTGCT---
GTACTTTCTGTAGTG---------AATAGAGTTAGGCAGGGATACTCACCA------
TTATCGTTTGCTGTTAGCTTGCTCAACGCCACCTCCATAGCAGTAGCTGAA---
GGGACAGATAGGATTATAGAAGTGTTACAAAGAGTTTTTAGAGCT----------------------------
--------ATTCTCCACATA------CCTACTAGAATAAGACAAGGCTTCGAAAGGGCTTTGCTA---TAA
>KJ769147
GCT---GCA--
AAAAACTTGTGGGTCACAGTCTATTATGGGGTACCTGTATGG---AAAGAAACAACC---
ACCACTCTATTTTGTGCATCAGATGCTAAAGCATACGATACAGAA---GCACATAAT----------------

--------------GTTTGGGCCACA---CATGCCTGTGTACCCACAGAC---
CCCAACCCACAAGAAGTAGAATTGGAA---------AATGTGACAGAAAACTTTAAC---------------
--------------------------ATGTGGAAAAATAACATGGTAGAACAGATGCATGAAGATATA---
ATCAGTTTATGGGATCAAAGTCTA---AAGCCATGTGTAAAATTA---
ACCCCTCTCTGTGTTACTCTAAATTGCACTGATGTGAAAAATGCTACG------------------------
----
-------------------------------------ATGGAGGGAAAAGGAGGAGAACTAAAA---
AACTGCTCTTTCAATATCACCACAAGCATAAGA--------------------------ACTGAGGTG---
AAAGACTATGCACTC---
TTTAATAAACTTGATGTAGAACCAATGACTAATGATAATACCAGTAATACCAGACCAAGCAGTACC------
----
AATAATACCAGTAATACCAACTATACCAGCTATAGGCTGTTGCATTGTAACACCTCAGTCATTACACAA---
GCTTGTCCAAAGATATCCTTTGAGCCAATTCCCATACATTATTGTACCCCAGCTGGTTTTGTGCTTCTAAGT
GTAAA---GATAAAAAGTTCAAT------GGAACGGGACCATGTAAAAAT----------------------
-GTCAGT---ACAGTACAATGTACACATGGAATTAGGCCAGTAGTATCAACT---
CAACTGCTGTTAAATGGCAGTTTAGCAGAAGAAGAG------------
GTAGTAATTAGATCTAGCAATTTCTCGGACAAT--------GCTAGAATCATAATAGTACAACTGAAT----
-----------GAATCTGTAGAA---------ATTAATTGTACAAGACCCAATAACAATACAAGAAAA----
-CGTGTATCTCTA------------GGACCAGGGCGAGTA---TGG------TATACAACAGGACAA-----
---------ATAGTAGGAGATATA------------AGACAAGCACATTGT---ACCATTAATAGCACA---
AAATGGAATAGAACTTTAGAACTGCTAACTAAAAAATTAAGA--------------GAACAATTTGGG----
-----------------------------------AACAAAACAATAGTCTTTAAT------
CAATCCTCAGGAGGGGACCCAGAGATTGTA---ATGCACAATGTTAATTGTGGAGGGGAA---
TTTAAGCAGCTCCAGGCAAGAGTCCTGGCTGTGGAAAGATACCTAAAGGATCAACAGCTCCTAGGGATTTGGG
GTTGCTCTGGAAAACTCATTTGCACCACTGCTGTGCCTTGGAATGCTAGTTGGAGT----------------
----------------------------AATAAATCTCTGGAGGAGATTTGG------GAT------
AACATGACTTGGATGCAGTGG---GAAAGAGAGATTAACAATTACACAAAAGAAATATAC---
ACCTTAATTGAAGAATCACAAAACCAACAAGAA---------------------------------------
----
AAAAATGAACTAGAATTATTAGAATTGGATAAGTGGGCAAGCTTGTGGAATTGGTTTGACATAACAAAATGGC
TGTGGTATATAAAAATATTCATAATGATAGTAGGAGGCTTGATAGGTTTAAGAATAATTTTTGCT---
GTACTTTCTATAGTA---------AATAGAGTTAGGCAGGGATACTCACCC------
TTGTCATTGGCTGTTAGCTTGCTCAACGCCACAGCTATAACAGTAGCTGAA---
AGGACAGATAGGGTTATAGAAATAGCACAAAGAGCTTATAGAGCA---------------------------
--------ATTCTCCACATA------CCTACAAGAATAAGACAGGGCTTAGAAAGGGCTTTGCTA---TAA
>KP109514
GCT---GAG--
GAATTGTGGGTCACAGTCTATTATGGGGTACCTGTGTGG---AAAGAAGCAGTC---
ACCACTCTATTTTGTGCATCAGATGCTAAAGCATATGACAAAGAG---GTACATAAT---------------
--------------GTTTGGGCCACA---CATGCCTGTGTACCCACAGAC---
CCCAACCCACAAGAAATAGAATTGGAA---------AATGTGACAGAAAATTTTGAC---------------
--------------------------ATGTGGAACAATAACATGGTAGACCAGATGCATGAGGATATA---
ATTAGTTTATGGGATCAAAGCCTA---AAACCATGTGTAAAATTA---
ACCCCAGTCTGTGTTACTTTACATTGCTGTAAGTTGAATGATACTAAGTGCCATGATTTGAATGTCACTAATA
GTACTACCCCT---
-------------GAAGGTAACAATACCACTAATATTACTAACAATGGTGGTGATGATTTAAGA---
AATTGCTCTTTCAATATGACCACAATAATGACA--------------------------
GAGAAGATGCAGAAAAATTATGCACTT---TTCTATACATCTGATATAGTACCTATAGATAAT---------
----

ACAGATAATACTAGTGACTATAGGTTGATAAGTTGTAACACCTCAATCCTTACACAA---
GCCTGTCCAAAGGTATCCTTTGAGCCAATTCCAATACATTATTGTGCCCCGGCTGGTTTTGCAATCTTAAAGT
GTAAA---GACAAGACATTCAAT------GGAACAGGACCATGTACAAAT----------------------
-GTCAGC---ACAGTACAATGTACACATGGAATTAGGCCAGTAGTATCAACT---
CAATTGTTGTTAAATGGCAGTCTAGCAGAGGAAGAC------------
ATAGTAATTAGATCTAAAAATTTCTCGGACAAT--------GCTAAAACCATAATAGTACAGCTGAAT----
-----------GAATCTATACCA---------ATTGTGTGTGTAAGACCCAACAACAATACAAGAAAA----
-AGTATATCTATA------------GGACCAGGGAGAACA---TGG------TATGCAACAGGAGAC-----
---------ATAATAGGAGACATA------------AGACAAGCATATTGT---AACATTACTGTTGGG---
AATTGGAGTAACACTTTACAACGGATAGATAAAAAATTAAAA--------------GAACAATTTAGA----

-----------------------------------AATAAAACAATAAACTTTAAT------
CAATCCTCAGGAGGGGACCCAGAAATTGTA---ATGCACAGCTTTAATTGTGGAGGGGAA---
TTTAAGCAGCTCCAGGCAAGAATCCTGGCTGTGGAAAGATACCTACAGGATCAACAGCTCCTGGGGATTTGGG
GTTGCTCTGGAAAACTCATTTGCACCACTACTGTGCCTTGGAATGCTAGTTGGAGT-----------------
----------------------------AATAAATCTCTGGATACCATTTGG------AAT------
AACATGACCTGGATGCAGTGG---GATAGAGAAATTAACAATTACACAAGCTTAATATAC---
ACCTTACTTGAAAAATCGCAGAACCAGCAAGAA---

AAGAATGAACAAGAATTATTGGAATTGGATAAGTGGGCAAATCTGTGGAATTGGTTTGACATAACAAATTGGC
TGTGGTATATAAAAATATTCATAATGATAGTAGGAGGCTTGATAGGTTTAAGAATAGTTTTTACT---
GTACTTTCTATAATA---------AATGGAGTTAGGCAGGGATACTCACCA------
TTATCGTTTGCAATTAGCTTGATCAACGCCGCAGCAGTAGCAGTAGCTGAG---GGGACAGGTAGG-------
----
CCACGCA------CCTAGAAGAATAAGACAGGAATTAGAAAGGGCTTTGCAA---TAA
>EU578539
GCT---GCA--
GAACAATTGTGGGTCACCGTCTATTATGGGGTACCTGTGTGG---AGAGAAGCAACC---
ACCACTTTATTTTGTGCATCGGATGCTAAAGCATATGATGCAGAG---GCACATAAT----------------
--------------GTCTGGGCCACA---CATGCCTGTGTACCCACAGAC---
CCCAACCCACAAGAAGTATTATTGGAA---------AATGTGACAGAAAATTTTAAC----------------
--------------------------ATGTGGAAAAATAACATGGTAGAACAGATGCATGAGGATATA---
ATCAGTTTATGGGATCAAAGCCTA---AAGCCATGTGTAAAATTA---
ACCCCACTCTGTGTTACTTTAAATTGCACTGATGAGTTGAGAAATAATACTAGTGGCACT-------------
----
-------------------------AGCAATAGCAGCTTGGAATTAGGGAGAGGAGAAATAAAA---
AATTGCTCTTTCAATGTTACCACAGGCATAAGA---------------------------
GACCAGAGAAAGAAAGAATATGCACTT---TTCTATAAACTTGATGTGATACAAATAAATAGTGGGAAT----
----

AATAATAGTAATTATAGTAGCTATAGATTGATAAATTGTAATACCTCAGTCATTACACAG---
GCCTGTCCAAAGGTATCCTTTGAACCAATTCCCATACATTTTTGTGCCCCGGCTGGTTTTGCGATTCTAAAGT
GTAAT---GATAAAAAGTTCAAT------GGAACAGGACCATGTACAAAT----------------------
-GTCAGC---ACAGTACAATGTACACATGGAATTAGACCAGTAGTGTCAACT---
CAACTGCTGTTAAATGGCAGTCTAGCAGAAGAAGAA-----------
GTAGTAATTAGATCTGAAAATTTCACAAACAAT---------GCTAAAACCATAATAGTACAGCTGAAT----
-----------GAATCTGTAGTG---------ATTAATTGTACAAGACCCAACAACAATACAAGAAGA-----
-AGCATACATCTG------------GGACCAGGGGCAGCA---TTT------ATTACAGGAGAA---------
---------ATAATAGGAGATATA------------AGACAAGCCTACTGT---ACCCTTAATGGAACA---
CAATGGAATAACACTTTAAAACAGGTAGTTATAAAATTAAGA---------------GAACAATTTGGG----
-----------------------------------AATAAAACAATAGTCTTTAAT------
CGATCCTCAGGGGGGGACATAGAAATTGTA---ACGCACAGTTTTAATTGTGGAGGGGAA---
TTTAAACAGCTCCAGGCAAGACTCCTGGCTGTGGAAAGATACCTAAGGGATCAACAGCTCCTAGGGATGTGGG
GTTGCTCTGGAAAACTCATTTGCACCACTAGTTTGCCTTGGAACGCTAGTTGGAGT----------------
----------------------------AATAAATCTCTAAATGATATTTGG------GAT------
AACATGACCTGGATGCAGTGG---GATAGAGAGATTAGCAATTACACAGACTTCATATAT---
ACCTTACTTGAAGACTCGCAGAACCAGCAGGAA---

AAGAATGAACAAGAATTATTAGAATTGGACAAATGGGCAAGTTTATGGAATTGGTTTGACATATCAAACTGGC
TGTGGTATATAAAAATATTCATAATGATAGTAGGCGGCTTGATAGGTTTAAGAATAGTTTTTGCT---
GTACTTTCTATAGTG---------AATAGAGTTAGGCAGGGATACTCACCA------
TTGTCGTTTGCTGTTGGCTTGCTTGATACAATAGCTATAGCAGTAGCTGAG---
GGAACAGATAGGATTATAGAATCAGTAAGAAGAATTTTTAGAGCT--------------------------
--------ATTCTCCACATA------CCTACAAGAATAAGACAGGGCTTGGAAAGGGCTTTACTA---TAA
>HQ217760
ACT---ACA--
CAACAATTGTGGGTCACAGTCTATTATGGGGTACCTGTGTGG---AAAGAAGCAACC---
ACCACTCTATTTTGTGCATCAGATGCTAAAGCATATGATACAGAG---GTACATAAT----------------
--------------GTATGGGCCACA---CATGCCTGTGTACCCACAGAC---
CCCAACCCACAAGAAGTAGTATTGGGA---------AATGTGACAGAAAATTTTAAC---------------
--------------------------ATGTGGAAAAATAATATGGTAGATCAGATGCATGAGGATGTA---

ATCAGTTTATGGGATCAAAGTCTA---AAGCCGTGTGTAAAATTA---
ACCCCACTCTGTGTTACTTTAAATTGCACTGATGTGTGGACCAATAGCACTAACAATACCACT---------
----
----------------------AACAGTAGCACTTGGGGCGGGGAGGAGAGAGGAGAAATAAAA---
AACTGCTCTTTTAATATCACCACAGGCATAAGA--------------------------GGT---
AATAGGCAAGAGTATTCACCT---TTTTATAAACTTGATGTAATACCAATAGATAATAGCAATAAT-------
----
-TCTAGCAATAATTCTAATAGTGGTAGCTATATATTGATAAATTGTAATACCTCAGTCATTACACAG---
GCCTGTCCAAAAGTGTCCTTCGACCCAATTCCCATACATTATTGTGCCCCGGCTGGTTTTGCGATTCTAAAGT
GTAAT---GCTAAAGAGTTCAAT------GGAACAGGACCATGCACAAAT----------------------
-GTCAGC---ACAGTACAATGTACACATGGAATTAAACCAGTAGTGTCAACT---
CAACTGCTGTTAAATGGCAGTCTAGCAGAAGAAGAG-----------
GTAGTAATTAGATCTGAAAATCTCACGGACAAT---------GCTAAAACCATAATAGTACAGCTGAAT----
-----------CAGTCTGTAAAG---------ATTACTTGTGTAAGACCCAATAACAATACAAGAAGA-----
-AGTATACCTATA------------GGACCAGGGAGAGCA---TTC------TATACAGGAGAA---------
---------ATAATAGGAGATATA------------AGGAAAGCACATTGT---AACCTTAGTAGAGCA---
GACTGGAATAACACTTTACAACGGATAGTTATAAAATTAAGA---------------AAACAATTTGAG----
-----------------------------------AATAAAACAATAGTCTTTAAT------
CAATCTTCAGGAGGGGACGTAGAAATTGTA---ATGCACAGTTTTAATTGTGGAGGGGAA---
TTTAAACAGCTCCAGGCAAGAGTCCTGGCTGTGGAGAGATACCTAAGGGATCAACAGCTCCTAGGGATTTGGG
GTTGCTCTGGAAGACTCATTTGCACCACTAATGTGCCTTGGAACGCGAGTTGGAGT----------------
-----------------------------AATAAATCTTACAATGAGATTTGG------GAC------
AACATGACCTGGATGCAGTGG---GAGAGAGAGATTGACAATTACACAAGAACCATATAT---
ACCTTACTTGAAGAATCGCAGAACCAGCAAGAA---------------------------------------
----
AAGAATGAACAAGAATTATTAGAATTGGATAAGTGGGCAAGTCTGTGGAATTGGTTTGACATAACAAAATGGC
TGTGGTATATAAAAATATTCATAATGATAGTAGGTGGCTTGATAGGTTTAAGAATAGTTTTTGCT---
GTACTTTCTATAGTG---------AATAGAGTTAGGCAGGGATACTCACCA------
TTGTCATTTGCTGTTAGCTTGTTTGATACCATAGCTATAGCAGTAGCTGAG---
GGAACAGATAGGGTTCTGCAGGTAGTACGAAGCGCTTTTAGAGCT---------------------------
--------ATTCTCCACATA------CCTAGAAGAATAAGACAAGGCTTGGAAAGGGCTTTACTA---TAA
>AF277071
TGT---AGT---ACT--------------------------------
ACAGAAAAAGGGTGGGTCACAGTCTATTATGGGGTACCTGTGTGG---AGAGAAGCAACC---
ACCACTTTATTTTGTGCATCAGATGCTAAAGCACATAATACAGAG---GTACATAAT----------------
--------------GTCTGGGCCACA---CATGCCTGTGTACCCACAGAC---
CCCAACCCACAAGAAGTAGTAATGGGA---------AATGTGACAGAAAAATTTAAC---------------
--------------------------ATGTGGAAAAATAACATGGTAGACCAGATGCATGAGGATATA---
ATCAGTTTATGGGATCAAAGCCTG---AAGCCCTGTGTAAAATTA---
ACTCCACTCTGTGTTACTTTAAATTGCATTGATTATGTGAAGAATGATACTAATACCAACAATAGT-------
----
-------------------ACTAATACCAACAATAGTAGTGTGATGGAGACAGGAGAAATGAAA---
AACTGCTCTTTCAATATTACCACAAGTGTAAGA-------------------------
GATAAGGTACGGAAAGAATATGCACTT---TTTTATAAACTTGATGTAACACCAATAGATGAT---------
----

AATAATGATAATAGTGGCTATAGATTGATAAATTGTAATACATCAGTCATTACACAA---
GCCTGTCCAAAGGTATCCTTTGAACCAATTCCCATACATTATTGTACCCCGGCTGGTTTTGCGATTTTAAAAT
GTAAT---GATAAGAAATTCAAT------GGAACAGGACCCTGTACAAAT----------------------
-GTCAGC---ACAGTACAATGTACACATGGAATTAGACCAGTAGTGTCAACT---
CAACTGTTATTAAATGGCAGTCTAGCAGAAGAAGAG-----------
GTAGTAATTAGATCTGAAAATTTCACAAACAAT---------GCTAAAACCATAATAGTACAACTGAAT----
-----------AAATCTGTAGTA---------ATCAATTGTACAAGACCCAACAACAATACAAGAAAA-----
-AGTATACCTATA------------GGACCAGGGAGAGCA---TTT------TATGCAACAGGAGAA------
---------ATAATAGGAAATATA------------AGACAAGCACATTGT---AATCTTAGTAGAGCA---
GATTGGGATAACACTTTAACTCACATAGTTAGCAAATTAAGA---------------GAACAATTTGGG----
-----------------------------------AATAAAACAATAGTCTTTAAC------
CAATCCTCTGGAGGGGACCCAGAAATTGTA---ATGCACAGTTTTAATTGTGGAGGGGAA---
TTTAAGCAGCTCCAGGCAAGAGTCCTGGCTGTGGAAAGATACCTAAAGGATCAACAGCTCCTGGGGATTTGGG
GTTGCTCTGGAAAACTCATTTGCACCACTAATGTGCCTTGGAATGTTAGTTGGAGT----------------

----------------------------AATAGATCTATGGATGATATTTGG------AAT------
AACATGACCTGGATGGAGTGG---GATAGAGAAGTTAACAATTACACAAGCCTCATATAC---
ACCTTACTTGAAGAATCGCAAAACCAGCAAGAA--

AAGAATGAACAAGAATTATTAGAATTGGATAAATGGGCAAGTTTATGGAATTGGTTTAACATAACACAATGGC
TGTGGTATATAAAAATATTCATAATGATAGTAGGAGGCTTGATAGGTTTAAGAATAGTTTTTGCT---
GTACTTTCTGTAGTG---------AATAGAGTTAGGCAGGGATACTTACCA------
TTACCGTTCGCTGTTAGCTTGCTTAATTCCACAGCCATCGCAGTAGCTGAG---
GGGACAGATAGGGTTATAGAAGTAGTACAAAGAGCTTTTAGAGCT----------------------------
--------ATTCTCCACATA------CCTAGAAGAATAAGACAGGGCTTGGAAAGGGCTTTGCTA---TAA
>EF593267
GCT---GCA--
GAAGAATTGTGGGTCACAGTCTATTATGGGGTACCTGTGTGG---AAAGAAGCAAAC---
ACCACTCTATTTTGTGCATCAGATGCTAAAGCATATAAGACAGAG---GTACATAAT----------------
--------------GTTTGGGCCACA---CACGCCTGTGTACCCACAGAC---
CCCAACCCACAAGAGGTAGTATTGGGA---------AATGTAACAGAAAATTTTAAC----------------
--------------------------ATGTGGAAAAATAACATGGTAGATCAGATGCATGAGGATATA---
ATCAGCTTATGGGATCAAAGCCTA---AAGCCATGTGTAAAATTA---
ACCCCACTCTGTGTTACCTTAAATTGCACTGATGTGTGGGGAAATAGTAATAGTAGCACTAAC----------
----
-------------------------AGTAGCAGCTGGGATAAGGTAGAGAAAGGAGAAATAAAA---
AACTGCTCTTTCAATATCTCCACAATTACAAGA---------------------------GGCAAGGTG---
AATGAATATTCACTG---TTTTATAAACTTGATGTAATACCAATAGGGAATACT-------------------
----
-TCTAATAGTAATTTTAGTAACTATAGATTGATAAATTGTAATACCTCAGTCATTAAACAG---
GCCTGTCCAAAGGTATCCTTTGAACCAGTTCCCATACATTATTGTGCCCCGGCTGGTTTTGCGATTCTAAAGT
GTAAT---GATAAAAAGTTCAAT------GGAACAGGACCATGTACAAAT-----------------------
-GTCAGT---ACAGTACAATGTACACATGGAATTAAACCAGTAGTGTCAACA---
CAACTGCTGTTAAATGGCAGTCTAGCAGAAGAAGAG------------
GTAGTAATTAGATCTGAAAACTTCACGAACAAT---------GCTAGAACCATAATAGTACAGTTGAAT----
-----------GAATCTGTAGCA---------ATTAATTGTACAAGACCCAACAACAATACAAGAAGA-----
-GGTATACATGTG------------GGACCAGGGCAAGCA---TTA------TATACAGGAGAC---------
---------ATAATAGGAGATATA------------AGACAAGCACATTGT---AACATTAGTGGAGTA---
CAATGGAATAACACTTTAAAACTGACCGTTACAAAATTAGGA---------------GAACAATTTGGG----
-----------------------------------AATAGAACAATAGTCTTTAAT------
CAATCCTCAGGAGGGGACGTAGAAATTGTA---ATGCACAGTTTTAATTGTGGAGGGGAA---
TTCAAACAGCTCCAGGCAAGAATCCTGGCTGTAGAGAGATACCTAAGGGATCAACAGCTCCTAGGGATTTGGG
GTTGCTCTGGAAAACTCATTTGCACCACTAATGTGCCTTGGAACGCTAGTTGGAGT-----------------
----------------------------AATAAATCTCTAAATAACATTTGG------GAA------
AACATGACCTGGATGGAGTGG---GAGAAAGAAATTGACAATTACACAAGCCTCATATAT---
ACCTTACTTGAGACATCGCAGAACCAGCAAGAA--

AAGAATGAACAAGAATTATTAGAGTTGGATAAGTGGGCAAATTTGTGGAATTGGTTTGACATAACAAACTGGC
TGTGGTATATAAAAATATTCATAATGATAGTAGGTGGCTTGATAGGTTTAAGAATAGTTTTTGCT---
GTACTTTCTATAGTG---------AATAGGGTTAGGCAGGGATACTCACCA------
TTGTCATTTGCTGTTAGCTTGTTTAATGCCATAGCTATAGCAGTAGCTGAG---
GGAACAGATAGGATTATAGAAGCGGCACAAAGAATTTTTAGAGCT----------------------------
--------TTTATCCACATA------CCAAGAAGAGTAAGACAAGGCTTGGAAAGGGCTCTGCTA---TAA
>DQ853436
GCT---GCA--
GATAACTTGTGGGTCACAGTCTATTATGGGGTACCTGTGTGG---AAAGAAGCAACC---
ACCACTCTATTTTGTGCCTCAGATGCTAAAGCTTATGATACAGAG---GCACATAAT----------------
--------------GTTTGGGCCACA---CATGCCTGTGTACCCACAGAC---
CCCAGCCCACAAGAAGTAGTATTGGAG---------AGTGTGACAGAAGATTTTGAC----------------
--------------------------ATGTGGAAAAATAACATGGTAGAACAGATGCATGAGGATATA---
ATCAGTTTATGGGATCAAAGCCTA---AAGCCATGTGTAAAATTA---
ACCCCACTCTGTGTTACTTTAAATTGTACTAATTTGAGAAATACTACTAAT----------------------
----
----------------------------------ACCACTAAAATAGACAAAGAAGAAATAAAA---
AACTGCTCTTTCCAGGTCACCACAGAAATAAGA---------------------------

GATAAGACAAGGAAAGAATATGCACTT---TTT-
ATAGGCTTGATGTAGTACCAATAGATAATAAAGATAATGCTGGAAATAATACT--------------------
----
AATAATACTAGCTATAGGTTAATAAATTGTAACTCCTCAGTCATTACACAG---
GCTTGTCCAAAGATATCCTTTGAGCCAATTCCCATACATTATTGTGCCCCGGCTGGTTTTGCGATTATAAAGT
GTAAT---GATAAGATGTTCAAT------GGAACAGGACCATGCACAAAT----------------------
-GTCAGC---ACAGTACAATGTACACATGGAATTAGGCCAGTAGTGTCAACT---
CAACTGCTGTTGAACGGCAGTCTAGCAGAAGAAGAT-----------
GTAATGATTAGATCACAGAATTTCACGGACAAT---------GCTAAAACCATATTAGTACAGCTGAAT----
-----------GAAACTGTACGA---------ATTAATTGTACAAGACCCAACAACAATACAAGAAAA-----
-AGCATACATATA------------GCACCAGGGAGAGCA---TTT------TATGCAACAGGAGAA------
---------ATAATAGGAGATATA------------AGACAAGCACATTGT---AACATTAGTAAAGAA---
AAATGGAATGACACTTTAAGCAAGATAGTTACAAAATTAAGA---------------GAACAATTTGAA----
--------------------------------GGTAATAAAACAATAGTCTTTAAT------
TCATCCTCAGGAGGGGACCCAGAAATTGTA---ATGCATAGTTTTAATTGTGGAGGGGAA---
TTTAAGCAGCTCCAGGCAAGAGTCCTGGCTGTGGAAAGATACCTAAGGGATCAACAGCTCCTGGGAATTTGGG
GTTGCTCTGGAGAACTCATCTGCACCACTGCTGTGCCTTGGAATAGTAGTTGGAGC-----------------
----------------------------AATAAATCTCTAGAAAAAATTTGG------GAT------
AACATGACCTGGATGCAGTGG---GAAAGAGAAATTGACAATTACACAGGCAAAATATAT---
GACCTACTTGAAAAATCGCAGAACCAGCAAGAA---

AAAAATGAACAAGAATTATTGGAGTTGGATAAATGGGCAAGTTTGTGGAATTGGTTTGACATAACAAAATGGC
TGTGGTATATAAAAATATTCATAATGATAGTAGGAGGCTTAATAGGTTTAAGAATAGTCTTTGCT---
GTATTTTCTATAGTG---------AATAAAGTTAGGCAGGGATACTCACCA------
TTGTCATTGGCCATTAATTTGCTCAACACCACAGCTATAGTAGTAGCTGAG---
GGGACAGATAGGATTATAGAAGTAGTGCTAAGAGCTTATAGAGCT----------------------------
--------ATTCTTCACATA------CCTAGAAGAATAAGACAGGGCTTGGAAAGGCTTTTGCTG---TAA
>KT124806
ACT---GCA---
GAACAGTTGTGGGTTACAGTCTATTATGGGGTACCTGTGTGG---AAAGAAGCAAAC---
ACCACTCTATTTTGTGCATCAGATGCTAAAGCATATGATACAGAG---GTACATAAT----------------
--------------GTTTGGGCCACT---CATGCCTGTGTGCCCACAGAC---
CCCAACCCACAAGAAGTAGTATTGAAA---------AATGTGACAGAAGATTTTAAC----------------
--------------------------ATGTGGAAGAATAACATGGTAGAACAGATGCATGAGGATATA---
ATCAGTTTATGGGATCAAAGCCTA---AAGCCATGTGTAAAATTA---
ACCCCACTCTGTGTCACTTTAAATTGCACTGATTATTGTAAGAATAATACTAACAATGGTAATACTACTGAAA
ATGGTACTGGT--
----------------------AACTGTAATACTACTATTAATAGCACTGATAGTAATATGAAA---
AACTGCTCTTTCAATGTCACCACAAGTGTAAGA--------------------------
GATAAGTTGCAGAAAGAATATGCACTT---TTTTATAAACTTGATGTAGTATCAATAGATGGT----------
----

AATAATACTAGCTATAGGTTGATAAATTGTAACACCTCAGTCATTACACAG---
GCCTGTCCAAAGATATCCTTTGAACCAATTCCCATACATTATTGTACCCCAGCTGGTTATGCGCTTCTAAAGT
GTAAC---AATAAGACATTCAAT------GGATCAGGACCATGTAACAAT----------------------
-GTCAGC---ACAGTACAATGTACACATGGAATTAGGCCAGTAGTTTCAACT---
CAACTGCTGCTAAATGGCAGTCTAGCAGAGGAA--------------
GTAGTAATTAGATCTGAAAATTTCTCGGACAAT---------GCTAAAACCATAATAGTACAGCTGAAC----
-----------GAATCTGTAGTA---------ATTAATTGCACAAGACCTAACAACAATACAAGAAAA-----
-AGTATACATATA------------GGACCAGGGAGAGCA---TTT------TATGCAACAGGAGAA------
---------ATAATAGGAAATATA------------AGACAAGCACATTGT---AACCTTAGTAGAGCA---
GCCTGGAATAACACTTTAGGAAAGGTAGTTAGAAAATTAAGA---------------GAAAAGTTT-------
-------------------------------------AATAACACAATAATCTTCAAT------
CACTCCTCAGGAGGGGACCCAGAAATTGTA---ATGCACAGTTTTAATTGTGGAGGGGAA---
TTTAAGCAGCTCCAGGCAAGAGTCCTGGCTGTGGAAAGATACCTAAGGGATCAACAGCTCCTAGGGATTTGGG
GTTGCTCTGGAAAACTCATTTGCACCACTACTGTGCCTTGGAATACTAGTTGGAGT-----------------
----------------------------AATAAAAATCTGACAGCAATTTGG------GAT------
AACATGACCTGGATGCAGTGG---GAAAAAGAAATTAGCAATTACACAGATATAATATAC---
AACTTAATTGAAGAATCACAGAACCAACAAGAA---

AAGAATGAACAAGAATTATTGGAATTAGATAAGTGGGCAAATTTGTGGAATTGGTTTAGCATATCAAACTGGC
TGTGGTATATAAAAATATTCATAATGATAGTAGGAGGCTTAATAGGTTTAAGAATAGTTTTTGCT---
GTGCTTTCTATAATA---------AATAGAGTTAGGCAGGGATACTCACCT------
TTGTCATTCGCTATTAGCTTGTTCAATGCTACAGCTATCGCAGTAGGTGAG---
GGGACAGATAGGGTTATAGAAGTAGTACAAAGAGCTTGGAGAGCT---------------------------
--------ATCCTCCACATA------CCTAGAAGAATAAGACAGGGCTTGGAAAGGATTTTGGTG---TAA
>EF593290
GCA---GAA---
AAATTGTGGGTCACAGTTTATTATGGGGTACCTGTGTGG---AAAGAAGCAACC---
ACCACTCTATTTTGTGCATCAGATGCTAAGGCATATAGTACAGAG---GTACATAAT---------------
--------------GTTTGGGCCACG---CATGCCTGTGTACCCACAGAC---
CCCAACCCACGGGAAGTAGAATTGACA---------AATGTGACAGAACCTTTTAAC---------------
--------------------------ATGTGGAAAAATAACATGGTAGAACAGATGCATGAGGATATA---
ATCAGTCTATGGGATCAAAGCCTA---AAGCCATGTGTAAAACTA---
ACCCCACTCTGTGTTACTTTAAACTGCACTAACGCTACTTCACACCATGTGAATGAGACTGCTTGTAATGGTA
CT--
----------AAGAATAATAATGTTAGTATGAAAAACTGCTCTTTCATAAGTGAAGAAATGAAA---
AACTGCTCTTTCAATATCACCTCAGGCATAAGA--------------------------
GATAAGGTGCGGAAAGAATATGCAGTT---TTCTATAACCTTGATATAGTACAAATAGATAAA----------
----

AATGATACTCACAGTTATAGGTTGGTAAATTGTAATACCTCAGTCATTACACAG---
GCCTGTCCAAAGGTATCCTTTGAGCCAATTCCCATACATTATTGTACCCCGGCTGGTTTTGCACTCCTAAAAT
GTAAT---GATAAGAAGTTCAAT------GGAACAGGGCCATGTAAAAAT-----------------------
-GTCAGC---ACAGTACAATGCACACATGGAATTAAGCCAGTAGTGTCAACT---
CAATTGCTGTTAAATGGCAGTCTAGCAGAAGAGGGG-----------
ATAGTAATTAGATCTGAGAATTTCTCGAACAAT---------GCTAAAACTATAATAGTACAGCTGAAT----
-----------CAATCTGTAGCA---------ATCAACTGTACAAGACCCAACAACAATACAAGAAAA-----
-AGTATACATATA------------GGACCAGGGAGAGCA---TTT------TATGCAACAGGAGAC------
---------ATAATAGGGAATATA------------AGACAAGCACATTGT---AACCTTAGTAGAGCA---
GCCTGGAATAACACTTTAGGACAGATAGTTATAAAATTAAGA---------------GAACAATTTGGG----
---------------------------------GAGAATAAAACAATAGTCTTTAAT------
CAATCCTCAGGGGGAGACCCAGAAATTGTG---AGGCACAGCTTCAATTGCGGAGGGGAA---
TTTAAGCAGCTCCAGGCAAGAGTCCTGGCTATAGAAAGATACCTACAGGATCAACAGCTCCTAGGAATTTGGG
GTTGCTCTGGAAAACTCATTTGTACCACTGCTGTGCCTTGGAATGCTACTTGGAGT-----------------
----------------------------AATAAATCTCTGGATAGAATTTGG------GAT------
AACATGACCTGGATGGAGTGG---GAGAGAGAAATTGACAATTACACAGGGGTAATATAC---
AACTTACTTGAAAAATCGCAAAACCAGCAAGAA---

AAGAATGAACAAGAATTATTGGAATTGGACAAGTGGTCAAGTCTGTGGACTTGGTTTGACATATCAAATTGGC
TGTGGTATATAAAAATATTCATAATGATAGTAGGAGGCTTGGTAGGTTTAAGAATAATTTTTGCT---
GTACTTTCTATAGTG---------AATAGAGTCAGGCAGGGATACTCACCA------
TTGTCGTTCGCTGTTAGCTTGCTCAACGCCACAGCAATAGCAGTAGCTGAG---
GGGACAGATAGGGTTATAGAAGTAGTACTAAGAACTCTTAGAGCT---------------------------
--------ATACTCCACATA------CCTAGAAGAATAAGACAGGGCTTGGAAAGGGCTTTGCTA---TAA
>JX863967
GCT---GCA---
GAAAACTTGTGGGTCACAGTCTATTATGGGGTACCTGTGTGG---AAAGAAGCAACC---
ACCACTCTATTTTGTGCATCAGATGCTAAAGCATATGAGAGAGAA---GTACATAAT---------------
--------------GTTTGGGCCACA---CATGCCTGTGTACCCACAGAC---
CCCAACCCACAAGAAGTAGTATTGGAA---------AATGTGACAGAAAATTTTAAT---------------
--------------------------ATGTGGAAAAATGACATGGTAGAACAGATGCATGAGGATATA---
ATCAGTTTATGGGATCAAAGCCTA---AAGCCATGTGTAAAATTA---
ACCCCACTCTGTGTTACTTTAAATTGCCCTGATAATTGGAGGAATGCTACTAATACCACT------------
----
-------------------------------------AGTATTGAAGCCATTAGAGAAATAACA---
AACTGCTCTTTCAACACTACCACAGGCATAAGA--------------------------
GATAAGATGCAGAAGGAATATGCACTT---TTTTATAAACTTGATATAGTACAATTAAAAGAT----------
----

AGGGACAATACTAGCTATAGGTTGATAAATTGTAACACCTCAGTCATTACACAG---
GCCTGTCCAAAGGTAACTTTTGAACCAATTCCCATACATTATTGTACCCCAGCTGGTTTTGCGATTCTAAAGT
GTAAT---GAGGAGAATTTCACG------GGAAAAGGACAATGTAAAAAT----------------------
-GTCAGC---ACAGTACAATGCACACATGGAATTAAGCCAGTAGTATCAACT---
CAACTGCTGTTAAATGGCAGTCTAGCAAAAGAAGAG-----------
GTAATAATTAGATCTGACAATTTCACGAACAAT---------GCTAAAATCATAATAGTACAGCTGAAT----
-----------GAATCTGTAGAG---------ATTAACTGTACAAGACCCAACAACAATACAAGGAAA-----
-AGTATACATATA------------GGGCCAGGAAGAGCA---TTT------TATACTACAAGAGAC------
---------ATAATAGGAGATATA------------AGACAAGCACATTGT---GAAATTAGTGAAAAA---
AAATGGAGGAACACTTTAAAACAGGTAGCCAGAAAGTTAAGA---------------GAACAATTTAAG----
-----------------------------------AATAAAACAATAGCCTTTAAT------
CAATCCTCAGGAGGGGATCCAGAAATTGTA---ATGCACACTTTTAATTGTGGAGGGGAA---
TTTAAACAGCTCCAGGCAAGAGTCCTGGCTATGGAAAGATACCTAAAGGATCAACAGCTCCTAGGAATTTGGG
GATGCTCTGGAAAACTCATCTGCACCACCTCTGTGCCTTGGAATGCTAGTTGGAGT-----------------
-------------------------AACAACAAATCACTAGAACATATTTGG------AAT------
AACATGACCTGGATGGAATGG---GAAAAAGAAATTGACAATTACACACACATAATATAC---
AACCTAATTGAAGAGTCGCAAAACCAACAAGAA---------------------------------------
----
AAGAATGAACAAGATTTATTGGAATTGGATAAGTGGGCAAATTTGTGGAATTGGTTTAGCATATCAAACTGGC
TGTGGTATATAAAAATATTCATAATGATAGTAGGAGGCTTAATAGGTTTAAGAATAATTTTTGCT---
GTGCTTTCTATAGTG---------AATAGAGTTAGGCAGGGATACTCACCA------
TTATCATTTGCAGTTAGCTTGCTCAACGCCACAGCTATAGCAGTAGCTGAG---
GGGACAGATAGGATAATAGAAATAGTACAGATAATTTTTAGAGCT----------------------------
--------ATCCTCAACATA------CCTAGAAGAATAAGACAGGGCTTGGAAAGGGCTTTGCTA---TAA
>KT124764
GCT---GTA---
GAACAAACGTGGGTCACAGTCTATTATGGGGTACCTGTGTGG---AGAGAAGCAAAC---
ACCACTCTATTTTGTGCATCAGATGCTCAAGCATATAATACAGAG---GTACATAAT----------------
--------------GTCTGGGCCACA---CATGCCTGTGTACCCACAGAC---
CCCAACCCACAAGAAGTAGAATTGGAA---------AATGTGACAGAAAATTTTAAT---------------
-------------------------ATGTGGAAAAACAACATGGTAGATCAGATGCATGAGGATATA---
ATCAATTTATGGGATCAAAGCCTA---AAGCCATGTGTAAAATTA---
ACTCCACTCTGTGTTACTTTAAATTGCACTGATATGGGGAATGATACTAGTACCAATACT------------
----
----------------------------AGTAGCTGGGATAAAATGGAAAAAGGAGAAATAAAG---
AACTGCTCTTTCAATATCACCACAAACATGAGA--------------------------
AATAAGATGCAGAAACAATATGCACTT---TTCTATAAACTTGATGTAGTACCAATAGAGGAGAAT-------
----

ACCACCACTACCTATAGGTTGGTAAGTTGTAACACCTCAGTCATTACACAG---
GCCTGTCCAAAGGTAACCTTTGAACCAATTCCCATACATTATTGTGCCCCAGCTGGTTTTGCGCTTCTAAAAT
GTAAG---GATAAGAAATTCAAT------GGAACAGGACCATGTAAAAAT----------------------
-GTTAGC---ACAGTACAATGCACACATGGAATTAAGCCAGTAGTATCAACT---
CAGCTGCTATTAAATGGCAGTCTAGCAGAAGAAGAG-----------
GTAATAATAAGATCTGAGAATTTCTCAAATAAT---------GCTAGAACCATAATAGTACAACTGAAT----
-----------AAATCTGTAGAA---------ATAAATTGTATAAGACCCAACAACAATACAAGAAAA-----
-AGTATACCTATA------------GGACCAGGGAGAGCA---TTT------TATACAACAGGGGAT------
---------ATAATAGGAGATATA------------AGACAAGCACATTGT---AACATTAGTAGACAA---
AATTGGAATGACACTTTAAAACAGATAGTGGAAAAGTTAAGA---------------GAACAATTTGGG----
-----------------------------------AATAAAACAATAGTCTTTAAT------
AAATCCTCAGGAGGGGACCCAGAAATTGTA---ATGCACACTTTTAATTGTGCAGGGGAA---
TTTAAGCAGCTCCAGGCAAGAGTCCTGGCTGTGGAAAGATACCTACGTGATCAACAGCTCCTGGGAATTTGGG
GTTGCTCTGGAAAACTCATCTGCACCACTGCTGTGCCTTGGAATAGCAGTTGGAAC----------------
----------------------------AATAGATCTGTGGATGACATTTGG------GGA------
AACATGACATGGATGCAGTGG---GATAGAGAAATTAGTAATTACACAAGTTACATATAC---
ACCTTAATTGAAGAATCGCAGAACCAGCAAGAA---------------------------------------
----
AAGAATGAACAAGAATTATTGGCATTAGATAAATGGGCAAATTTGTGGAATTGGTTCGACATAACAAAATGGC
TGTGGTATATAAAAATATTCATAATGATAGTAGGAGGCTTGATAGGTTTAAGAATAGTTTTTGCT---
GTGCTTTCAATAGTG---------AATAGAGTTAGGCAGGGATACTCACCA------

TTATCGTTTGCTGTTAGCTTGCTCAACGCCATAGCCATAGCAGTAGGTGAC---
GGGACAGATAGGATTATAGAAGCATTTAGAATAATTTTTAGAGCT---------------------------
--------ATTCGCCACATA------CCCACAAGAATAAGACAGGGCTTGGAAAGGTCTTTGCTA---TAA
>KT124814
GCA---GAC---
AACAACTTGTGGGTCACAGTCTATTATGGGGTACCTGTGTGG---AGAGAAGCAGAC---
ACCACTCTATTTTGTGCATCAGATGCTAAAGGATATAGTGCAGAG---GTACATAAT---------------
--------------GTTTGGGCCACA---CATGCCTGTGTACCCACAGAC---
CCCAACCCACAAGAAGTATTATTGGCA---------AATGTAACAGAAAATTTTAAC---------------
-------------------------ATGTGGACAAATAACATGGTAAAACAGATGCAGGAGAATATA---
ATCAGTTTATGGGATAAAAGCCTA---AAGCCATGTGTAAAATTA---
ACCCCACTCTGTGTTACTTTAAATTGCACTGATAGTATTACTACTATTGCCACTGCTAATACTACCACT---
----
-------------GCTCCTAATATCAATGGTAGCAGTGGGAGGATGGAGATAGGAGAAATAAAA---
AATTGCTCTTTCTATGTCACCTCAAACATAAGA--------------------------
GATAAGAAACAGAGAAAATTTGCACTT---TTCTATAAACTTGATGTAGTACAAATAGATAAT---------
----

AATACCAGTGAGAATAGGACCAGCTATAGGTTGACAAGTTGTAACACCTCAGTCATTACACAA---
GCCTGTCCAAAGATATCCTTTGAGCCAATTCCCATACATTATTGTGCCCCGGCTGGTTTTGCGATTCTAAAGT
GTAAT---GATAAAAGGTTCAAT------GGAACAGGACCATGTACAAAT----------------------
-GTCAGC---ACAGTACAATGTACACATGGAATCAGGCCAGTAGTATCAACT---
CAACTGCTGTTAAATGGCAGTCTAGCAGAAGAAGAG------------
GTAATAATTAGATCTGAAAATTTCACAAACAAT---------GCTAAAATCATAATAGTACAGCTGAAC---
-----------GAAACTGTAAAA---------ATTAATTGTATGAGACCCAACAACAATACAAGAAAA----
-AGTATACATATA------------GCACCAGGAAAAGCA---TTT------TATGCAACAGGAGAC-----
---------ATAATAGGAAATATA------------AGACAAGCACATTGT---AACATTAGTATGAAA---
GAATGGAGGAACACTTTAAATCAGACAGTTAAAAAATTAAGA--------------GAACAATTTGGG----
-----------------------------------AATAAAACAATAAAGTTTCAG------
CCATCCTCAGGAGGAGACCCAGAAATTGTA---ATGCACAGTTTTAATTGTGGAGGGGAA---
TTTAAGCAGCTCCAGGCAAGAGTCCTGGCTGTGAAAAGATACCTAAGGGATCAACAGCTCCTGGGAATTTGGG
GCTGCTCTGGAAGACTCATCTGCACCACTAATGTGCCTTGGAATGCTAGCTGGAGT----------------
---------------------------AATAAATCTTATAATGATATTTGG------AAC------
AACATGACCTGGATGCAGTGG---GATAGAGAAATTAGCAATTACACAGGCTTAATACAC---
AGATTACTTGAAGACTCGCAAAACCAACAAGAA---------------------------------------
----
AAGAATGAACAAGAATTATTGGAATTAGATAAATGGGCAAGTTTGTGGAGTTGGTTTGATATAACAAAATGGC
TGTGGTATATAAAAATATTCATAATAATAGTAGGAGGCTTGATAGGCTTAAAAATAATTTTTACA---
GTACTTTCTATAGTA---------AATAAAGTTAGGCAGGATACTCACCA------
ATATCGTTTGCTGTTAGCTTGTTCAACGCCATAGCTATAGCAGTAGCTGAG---
GGGACAGATAGGATTATAGAGATAATACAAGCAACTTTTAGAGCT---------------------------
--------ATTCTCCACATA------CCTACAAGAATAAGACAGGGCTTAGAAAGGGCTTTGCTA---TAA
>EU578104
GCT---ACA---
GAACAATTGTGGGTCACAGTCTATTATGGGGTACCTGTGTGG---AAAGAAGCAACC---
ACCACTCTATTTTGTGCATCAGATGCTAAAGCATATCACGCAGAG---AAGCACAAT---------------
--------------GTTTGGGCCACA---CATGCCTGTGTACCCACAGAC---
CCTAACCCACAAGAAGTAGTATTGGGA---------AATGTAACAGAAAATTTTAAT---------------
-------------------------ATGTGGAAAAATAACATGGTAGACCAGATGCATGAGGATATA---
ATTAGTTTATGGGATCAAAGCCTA---AAGCCATGTGTAAAAATA---
ACCCCACTCTGTGTTACTCTAAATTGCACTAATTTCAATGGTACTAAGACCAATAGTACTACTAGTCCCACTA
AT--
-------------AGTCCCAACAATAATAGTAGTTGGGAAAGGATGGAGGTAGGAGAAGTAAAA---
AACTGCTCTTTCAATGTTACCACTAGCATACAA--------------------------
GATAAGATAACTAAACAATATGCACTT---TTTTATAGACTTGATGTAGTACCAATAGATAAG---------
----

GATAATACAAGCTATACATTGATAAATTGTAACACCTCAGTCATTACACAG---
GCCTGTCCAAAGGTATCCTTTGAACCAATTCCCATACATTATTGTGCCCCGGCTGGTTTTGCGATTCTAAAAT
GTAAT---AATAAGACATTCAAT------GGAAAGGACCATGTACAAAT----------------------

-GTCAGC---ACAGTACAATGTACACATGGAATTAGGCCAGTAGTGTCAACT---
CAACTACTGTTAAATGGCAGCTTAGCAGAAGAAGGG------------
GTAATGATTAGATCTGAAAATTTCTCGGACAAT---------GCTAAAACCATAATAGTACAGCTGAAT----
-----------GAATCTGTAACA---------ATTAATTGTACAAGACCCAACAACAATACAAGGAGA-----
-AGTATACCTATA------------GGACCAGGGAGAGCA---TTT------TATGCAACAGGAGAT------
---------ATAATAGGAGATATA------------AGAAAAGCATATTGT---ACCCTTAGTGGAAAA---
GCATGGCATGACACTCTAAAACAGATAGTTGTAAAATTAAAA---------------GAACAATTTGGT----
--------------------------------GAGAATAAAACAATAGTCTTTACT------
AAATCTTCAGGAGGAGATCCAGAAATTGTA---ATGCACACTTTTAATTGTGGAGGGGAA---
TTTAAGCAGCTCCAGGCAAGAGTCCTGGCTGTAGAAAGATACCTAAAGGATCAACAGCTCCTGGGAATTTGGG
GATGCTCTGGAAAGCTCATCTGCACCACTAATGTGCCTTGGAATACTAGTTGGAGC-----------------
----------------------------AATAAATCTCTAAATCAGATTTGG------GAT------
AACATGACCTGGATGGAGTGG---GAGAGAGAGATTGACAATTACACAAGCTTGATATAC---
TCTTTAATTGAAGAATCGCAAAACCAACAGGAA--

AAGAATGAACAAGAGTTATTGGAATTGGATAAGTGGGCAAGTCTATGGAATTGGTTTAGCATATCACAATGGC
TGTGGTACATAAAAATATTCATAATGATAGTAGGAGGTTTAATAGGTTTAAGAATAGTTTTTACT---
GTGCTTTCTATAGTG---------AATAGAGTTAGGCAGGGATACTCACCA------
TTGTCATTGGCTGTTAGTTTATATAACGCCATAGCTATAGCAGTAGCTGAG---
GGAACAGATAGGGTTATAGAACTAGTACAAAGAGCTGGTAGAGCT----------------------------
--------GTTCTCCACATA------CCAAGAAGAATAAGACAGGGCTTAGAAAGGGCTTTGCTA---TAA
>JQ403105
GCT---GTA--
GAACAAACGTGGGTCACAGTCTATTATGGGGTACCTGTGTGG---AAAGAAGCAAAT---
ACCACTTTATTTTGTGCATCAGATGCTCAAGCATATAATACAGAG---GTACATAAT----------------
--------------GTTTGGGCCACA---CATGCCTGTGTACCCACAGAC---
CCCAACCCACAAGAAGTACCATTGGAA---------AATGTGACAGAAAATTTTAAC----------------
--------------------------ATGTGGAAAAACAACATGGTAGATCAGATGCATGAGGATATA---
ATCAGTTTATGGGATCAAAGCCTA---AAGCCATGTGTAAAATTA---
ACTCCACTCTGTGTTACTTTAAATTGCAATGATACGGTGGGGAATGATACTAATACCAAT-------------
----
-------------------------AATAGTAGCTGGGATAGAATGGAAAAGGGAGAAATAAAG---
AACTGCTCTTTCAATATCACCACAAACATGAGA---------------------------
GATAAGATGCAGAAACAATATGCACTT---TTTTATAAACTTGATGTAGTACCAATAGAGGAAGATGAA----
----

AATAGTACCAACTATAGATTGATAAGTTGTAACACCTCAGTCATTACACAG---
GCCTGTCCAAAGGTAACCTTTGAGCCAATTCCCATACATTATTGTGCCCCAGCTGGTTTTGCGCTTCTAAAAT
GTAAG---GATAAGAAATTCAAT------GGAACAGGACCATGTAAAAAT-----------------------
-GTTAGC---ACAGTACAATGTACACATGGAATTAAGCCAGTAGTATCAACT---
CAGCTGTTATTAAATGGCAGTCTAGCAGAAAAAGAG------------
GTAGTAATAAGATCTGAGAATTTCTCGAATAAT---------GCTAAAACCATAATAGTACAGCTGAAT----
-----------ACATCTGTAGCA---------ATAAATTGTACAAGACCCAACAACAATACAAGAAAA-----
-AGTATACATATA------------GGACCAGGGAGAGCA---TTT------TATACAACAGGAGAT------
---------ATAATAGGAAATATA------------AGACAAGCACATTGT---AACATTAGTAAAAGA---
GATTGGAATAACACTTTAAAACAGATAGCTGAAAAGTTAAGA---------------GAACAATTTGAG----
-----------------------------------AATAAAACAATAGTCTTTAAA------
AATTCCTCAGGAGGGGATCCAGAAATTGTC---ATGCACACTTTTAATTGTGCAGGGGAA---
TTTAAGCAGCTCCAGGCAAGAGTCCTGGCTGTGGAAAGATACCTACGTGATCAACAGCTCCTGGGAATTTGGG
GTTGCTCTGGAAAACTCATCTGCACCACTGCTGTGCCTTGGAATAGTAGTTGGAAC-----------------
----------------------------AATAGATCTGTGGATGACATTTGG------GGA------
AACATGACATGGATGCAGTGG---GATAGGGAAATTAGTAATTACACAAGTTTAATATAC---
ACCTTAATTGAGGAATCGCAACACCAACAAGAA--

AAGAATGAACAAGAATTACTGGCATTAGATAAATGGGCAAATTTGTGGAATTGGTTCGACATAACAAAATGGC
TGTGGTATATAAGAATATTCATAATGATAGTAGGAGGCTTGATAGGATTAAGAATAGTTTTTGCT---
GTGCTTTCTATAGTG---------AATAGAGTTAGGCAGGGATACTCACCA------
TTATCGTTTGCTGTTAGCTTGCTCAACACCCTAGCCATAGCAGTAGGTGAA---
GGGACAGATAGGATTATAGAAGTATTTAGACTAATTTTTAGAGCT----------------------------
--------ATTCTCCGCATA------CCTACAAGAATAAGACAGGGCTTGGAAAGGTCTTTGCTA---TAA

>EU578230
GCT---GCA---CAA------------------------------------
CAACAAATGTGGGTCACAGTCTATTATGGGGTACCTGTGTGG---AAAGAAGCAAAC---
ACCACTTTATTTTGTGCATCAGATGCTAAAGCATATGATACAGAG---GCACATAAT----------------
--------------GTTTGGGCCACA---CATGCCTGTGTACCCACAGAC---
CCCAACCCACAAGAAGTAGTATTA------------AATGTGACAGAAAATTTTAAC----------------
--------------------------ATGTGGAAAAATAACATGGTAGAACAGATGCATGAGGATATA---
ATCAGTTTATGGGATCAAAGCCTA---AAGCCATGTGTGAAATTA---
ACCCCACTCTGTGTCACTTTAAATTGTACTAATGCCACTGGTGGT---------------------------
----
---------------------------------------AATACCACCTCTCCAGAGATAAAA---
AACTGCACTTTCAATACCACCACAAGCATGACA---------------------------
AATAAGATGCAGAAACAAAATGCACTT---TTTAATAGGCTTGATATAGTGCCAATAGATAATGATAGTGCT-
----

AATCATACTAATAATAATACTAATGAATATAGGTTGATAAGTTGTAACACCTCAGTCATTACACAG---
GCCTGTCCAAAGGTATCCTTTGAGCCAATTCCCATACATTATTGTGCTCCTGCTGGTTTTGCGATTCTAAAAT
GTAAT---GATAAGAAATTCAAT------GGAAAAGGGCCATGTACAAAT-----------------------
-GTCAGC---ACAGTACAATGTACACATGGAATTAAGCCAGTAGTATCAACT---
CAATTGTTGTTAAATGGCAGTCTAGCAGAAGAAGAG-----------
ATAATAATTAGATCTGAGAATTTCTCAAACAAT---------GCTAAAACCATAATAGTACAGCTGAAT----
-----------GCATCAGTAGAA---------ATTAATTGTACAAGACCCAATAACAATACAAGAAGA-----
-AGTATTCATATG------------GGACCAGGGAAAACA---TAT------TATGCAACAGGAGAC------
---------ATAATAGGAGATATA------------AGACAAGCACATTGT---AACATCAGTAGAGAA---
AAATGGAATAAAACTTTAGAACAGGTAGGTATAAAACTAAGA---------------GAACAATTTAAT----
-----------------------------------AATAAAACAATAGTCTTTACT------
CACTCCTCAGGAGGGGACCTAGAAATTGTA---ATGCACAGTTTTAATTGTGGAGGGGAA---
TTCAAACAGCTCCAGGCAAGAGTCCTGGCTGTGGAAAGATACCTAAGGGATCAACAGCTCCTAGGAATTTGGG
GCTGCTCTGGAAAACTCATCTGCCCCACTGCTGTGCCTTGGAATATTAGTTGGAGT-----------------
----------------------------AATAAAACTTATGATCAGATTTGG------GAT------
AACATGACCTGGATGCAGTGG---GAAAAAGAAATTGACAGTTACACAAACACAATATAC---
AATTTAATTGAAGAATCGCAGAATCAGCAGGAA--

AAGAATGAACAAGACTTATTACAATTGGATAAATGGTCAAATTTGTGGAATTGGTTTAGCATATCATATTGGC
TATGGTATATAAAAATATTCATAATGATAGTAGGAGGCTTGATAGGTTTAAGAATAGTTTTTGCT---
GTGCTTTCTATAGTG---------AATAGAGTTAGGCAGGGATACTCACCC------
TTATCGTTTGCTATTAGCTTGCTCAATACCACTGCTATAGTAGTAGCTGAG---
GGGACAGATAGGGTTATAGAAATAGTACAAAGAGTTTTTAGAGCT----------------------------
--------ATCTTACATATA------CCTAGAAGAGTAAGACAAGGCCTGGAAAGAGCTTTGCTA---TAA
>FJ469754
GCT---GCA---------------------------------------
GATAATTTGTGGGTCACAGTCTATTATGGGGTACCTGTGTGG---AAAGAAGCAAAC---
ACCACCCTATTTTGTGCATCAGATGCTAAAGCACAGGATAAAGAG---GTACATAAT----------------
--------------GTTTGGGCAACA---CATGCCTGTGTACCCACAGAC---
CCCGACCCACAAGAAATAAAATTGAAA---------AATGTGACAGAATATTTTAAC----------------
--------------------------ATATGGAAAAATAACATGGTAGACCAGATGCAGGAGGATATA---
ATCAGTTTATGGGATCAAAGCTTA---AAGCCATGTGTAAAGCTA---
ACTCCACTCTGTGTTGCTTTAAATTGCAGTAATTGCAATACTACAAGTTGCAATTTGAATGACGCCAATACTC
CCACTAATGCTACT---
ACTAGCAGTACTACTAGTACTCCTAGTAGTCTAGTCTACCCTACTATGAGTACAGGAGAAATAAAA---
AACTGCTCTTTCAATATCACCACAGGAATAGGA---------------------------AAAATGGAG---
AAAAACTATGAATTA---TTATATTGTATTGATATAGTACCAATAGATAGG---------------------
----
-------GATGATACTACCAGCTATAGGCTGATAAGTTGTAACACCTCAATCATTACACAG---
GCCTGTCCAAAGGTATCCTTTGAACCAATTCCCATACATTATTGTGCCCCGGCTGGTTTTGCGATTCTAAAGT
GTAAC---GATACGAAATTCAGT------GGAAATGGCACATGTAGCAAA-----------------------
-GTCAGC---ACAGTACAATGTACACATGGAATTAGGCCAGTAGTATCAACT---
CAACTGCTGTTAAATGGCAGTCTAGCAGAAGAAGAG-----------
GTAGTAATTAGATCTGCCAATTTCTCAGACAAT---------GCTAAAACCATAATAGTGCAGCTGAAA----
-----------GAACCTGTAGAA---------ATTAATTGTACAAGACCCAATAACAATACAAGAAGA-----

-AGTATAAATATA------------GGACCAGGGCGAGCA---TTT------TATGCAGCAGGACAA------
---------ATAATAGGAGATATA------------AGACAAGCATATTGT---AACATTAGTGAAGCA---
AGATGGGAGAACACTTTAAAACAGGTAGCTAGAAAATTAGGA---------------AAACAATTTGGG----
---------------------------------GAGAACAAAACAATGATTTTTACT------
AAATCCTCAGGAGGGGACCCAGAAATTGTG---ATGCATAGTTTTAATTGTGGAGGGGAA---
TTTAAGCAGCTCCAGGCGAGAGTCCTGGCTATAGAAAGATACCTAAAGGATCAACAGCTCCTGGGATTTGGG
GTTGCTCTGGAAAGCTCATCTGCCCCACTGCTGTGCCTTGGAATACTAGTTGGAGT-----------------
-----------------------------AATAAATCTTGGGAAACAATTTGG------GAT------
AACATGACCTGGATGCAGTGG---GATAAAGAAATTAACAATTACACAGGCTTAATATAC---
ACTTTACTTGAAGAATCGCAAAACCAACAAGAA--

AAGAATGAACAAGAATTATTGGCATTGGATAAATGGGCAAATTTGTGGAATTGGTTTGACATATCAAGATGGC
TGTGGTATATTAGAATATTCATAATGATAGTAGGGGGCTTAATAGGCTTAAGAATAGTTTTTGCT---
GTACTTTCTATAGTG---------AATAGAGTCAGGCAGGGATACTCACCA------
TTGTCATTCGCTGTTAGCTTGCTCAATACAACAGCCATAGTAGTAGCTGAG---
GGGACAGATAGGATTATAGAAGCATCACAAAGAGCTGGTAGAGCT----------------------------
--------ATTCTCCACATA------CCTAGAAGAATAAGACAGGGCTTGGAAAGGGCTTTGCTA---TAA
>FJ469757
GCT---ACA---
GAACAATTGTGGGTCACAGTATATTATGGGGTACCTGTGTGG---AAAGAAGCAACT---
ACCACTCTATTTTGTGCATCAGATGCTAAAGCATATGATACAGAA---GTACATAAT----------------
--------------GTTTGGGCCACA---CATGCCTGTGTACCCACAGAC---
CCCAACCCACAAGAAGTAGTATTGGCA---------AATGTGACAGAAAATTTTAAT----------------
--------------------------GTGTGGAAGAATGACATGGTAGATCAGATGCATGAGGATGTA---
ATCAGTTTATGGGATCAAAGCCTG---AAGCCATGTGTAAAATTA---
ACCCCACTCTGTGTTACCTTGAATTGCAGTGATTATGAGGGGAATACTACTAGACAG----------------
----
-----------------------------AATATAACAATGAAGGAGGAAAAAGGAGAAATAAAA---
AATTGCACTTTCAAGATCACCACAGACATAAGA---------------------------
GATAAGATGCGAAAAGAATATGCACTT---
TTTTATAAACTTGATATAACACCAATAGAGGAAGATAATAATAGT----------------------------
----
AATAATAGTAGTAGTGCTAATAGTAGTAATAGTAATGCTAATTATACTAACTATAGGTTGATAAGTTGTAACA
CCTCAGTCATTACACAG---
GCCTGTCCAAAGGTATCCTTTGAACCAATTCCCATACATTATTGTGCCCCAGCTGGTTTTGCGATTCTAAAGT
GTAAT---AATAAAACATTCAAT------GGAAAAGGACCATGTACAAAT-----------------------
-GTCAGC---ACAGTACAATGTACACATGGCATTAGGCCAGTAGTATCAACT---
CAATTGCTGTTAAATGGCAGTCTAGCAGAAGGAGAT------------
GTAGTAATTAGATCTGTCAATTTCTCGAACAAT---------GCTAAAACCATAATAGTACAGCTGAAT----
-----------CAGTCTTTAGAA---------ATTAATTGTACAAGACCCAACAACAATACAAGAAGA-----
-AGTATACATATG------------GGACTAGGGAGAGCA---TGG------TATGCAGCAGGAGGA------
---------ATAGTAGGAGATATA------------AGAAAAGCACATTGT---AACATTAGTGGGACA---
AAATGGAAGAACACCCTAAAACAGATAGTTAAAAAATTAGGA---------------GACCAATTTAAG----
---------------------------------AATAAAACAACAATAACCTTTAAT------
CACTCCTCAGGAGGGGACCCAGAAATTGTA---ATGCACATGTTTATTTGTGGAGGGGAA---
TTTAAGCAGCTCCGAGCGAGAGTCCTGGCTGTGGAAAGATACCTAAAGGATCAACAGCTCCTGGGATTTGGG
GTTGCTCTGGAAAACTCATTTGCACCACTACTGTGCCTTGGAATACTAGTTGGAGT-----------------
-----------------------------AATAAAACTATGACAGAGATTTGG------GAG------
AACATGACCTGGATGCAGTGG---GAGAGAGAAATTGACAACTACACAGGCATAATATAC---
ACCCTAATTGAAGAAGCGCAAAACCAACAAGAA--

CAGAATGAACAAGAATTACTAGCATTAGATAAATGGGCAAGTTTGTGGAATTGGTTCAGCATAACAAACTGGC
TGTGGTATATAAAAATATTCATAATGATAGTAGGAGGATTAATAGGTTTAAGAATAGTTTTTACT---
GTACTTTCTATAGTG---------AATAGAGTTAGGCAGGGATACTCACCA------
TTATCGTTTGCTGTTAGCTTGCTCAACGCCATAGCCATAGCAGTAGCTGAG---
GGGACAGATAGGGTCATAGAAGCAGTCCAAAGAGCTTTTAGAGCT----------------------------
--------ATCCTTCACATA------CCTACAAGAATAAGACAGGGCTTAGAAAGGGCTTTGCTA---TAA
>GU330538
GCT---GCA---
GAACAATTGTGGGTCACAGTCTATTATGGAGTACCTGTGTGG---AAAGATGCAAAC---

ACCACTCTATTTTGCGCATCAGATGCTAAGGCATATGATACAGAG---GTACATAAT----------------
--------------GTTTGGGCCACA---CATGCCTGTGTACCCACAGAC---
CCCAACCCACAAGCAATAGAATTAAAA---------AATGTGACAGAAGATTTTAAC----------------
-------------------------ATGTGGAAAAATAACATGGTAGAACAGATGCATGAGGATATA---
ATCAGTTTATGGGATCAAAGTCTA---AAGCCATGTGTAAAATTA---
ACCCCACTCTGTGTCACTTTAGATTGCAATAATACTGTGACTCCCACTAGGACCAATAGTAGTAATAATGCCA
CTAGTTCCACTAACAGTCCTACCACTAGTACT---
----ACCACTAGTCCCACTACCAGTACTACCAATAATAGTAGTTGGGAAAAAGAAGAAATAAAA---
AACTGCTCTTTCAATATCACCACAAGCATAAGA-------------------------
GATAAAGTGCAGAGGGAATATGCACTT--TTTTTATAGCCTTGATGTAGTACAAATAGATAAG----------
----

ACTAGCTATAGGTTAATAAATTGTAACACCTCAGTCATTACACAG---
GCCTGTCCAAAGGTATCCTTTGAACCAATTCCCATACATTATTGTGCCCCGGCTGGTTTTGCGATTCTAAGGT
GTAAT---GATAAGAAGTTCAGT------GGAAAAGGAAAATGTGACAAT----------------------
-GTCAGC---ACAGTACAGTGTACACATGGAATTAAGCCAGTAGTGTCAACT---
CAACTGCTGTTAAATGGCAGTCTAGCAGAAGGAGAT-----------
GTAGTGCTTAAATCTGACAATTTTTCAAACAAT---------GCTAAAACCATAATAGTACAGCTAAAC----
-----------GAAACTGTAAGA---------ATTAATTGTTCAAGACCCAACAACAACACAAGAAAA-----
-GGTATACATATA------------GGACCAGGGAGAGCA---TTT------TATACAACAGGAGAA------
---------ATAATAGGAGACATA------------AGAAAGGCACATTGT---AACATTAGTAGAACA---
AACTGGACTAACACTTTAGGGCAGATAGCAAAAAAATTAGGA------------GAACAGTTTAAC-------
-----------------------------------ACTAGCACAGTAAACTTTAGA------
CCATCCTCAGGAGGGGACCCAGAAATTGTA---ATGCACAGTTTTAATTGTAGAGGGGAA---
TTTAAGCAGCTCCAGGCAAGAGTCCTTGCTGTGGAGAGATACCTACGGGATCAACAGCTCCTAGGGATGTGGG
GTTGCTCTGGAAAACTCATTTGCACCACTACTGTGCCTTGGAATACTAGTTGGAGT-----------------
----------------------------GGTAAAAATCTAACTGACATTTGG------GAT------
AACATGACCTGGATGGAGTGG---GAAAAAGAAATTGACAATTACACAGACCTCATATAC---
ACCTTACTTGAAGCATCGCAAACCCAACAAGAG--

ATAAATGAACAAGAATTATTGGCACTAGATAAGTGGGCAAGCTTGTGGAATTGGTTTGACATAACAAACTGGC
TGTGGTATATAAGATTATTCATAATGATAGTAGGAGGCTTGATAGGTTTAAGAATTATTTTTGCT---
GTGCTTTCTATAGTG---------AATAGAGTTAGGCAGGGATACTCACCA------
TTATCATTTGCTGTTAACTTGCTTAATGCCACAGCTATCGCAGTAGCTGAG---
GGGACAGATAGGATTATAGAAGTAGTACAAAGAGCTTATAGAGCT----------------------------
--------TTTATCCACATA------CCTAGAAGAATAAGACAGGGTTTAGAAAGAGCTTTGCAA---TAA
>GU330550
GCT---ACA---AAAAAG---
TGGGTCACAGTCTATTATGGGGTACCTGTGTGG---AAAGAAGCAAGC---
ACCGTTCTGTTTTGTGCATCAGATGCTAAAGCATATGATACAGAG---GCACATAAT----------------
--------------GTTTGGGCCACA---CATGCCTGTGTGCCCACAGAC---
CCTAACCCACAAGAAGTATTAATGGAA---------AATGTGACAGAATATTTCAAT----------------
-------------------------GTGTGGAAAAATGACATGGTAGAACAAATGCATAAGGATATA---
ATCAGTTTATGGGATCAAAGCCTA---AAACCATGTGTAAAATTA---
ACCCCACTCTGTGTTACTTTAAATTGCACTGATTATGAGGCTAATAATACTACTAAT----------------
----
----------------------------GCCAATGATACCGCTGATAGTAAACAGGAAATAAAG---
AACTGCTCTTTCAATGTCACCACAGTCGTAAAA-------------------------
GATAAGGTGAAAAAAGAATATGCACTT---TTTTATAGCCTTGATGTAGTACCAATAGATGAT----------
----

GATAATACTAGCTATAGGTTAATAAGTTGTAACACCTCAGTCATTACACAG---
GCCTGTCCAAAAGTAACCTTTGAACCAATTCCTATACATTATTGTGCCCCAGCTGGTTTTGCGATTCTAAAAT
GTAAC---AATAAGACATTCAGT------GGAGAAGGAGATTGTAAAAAT----------------------
-GTCAGT---ACAGTACAATGTACACATGGAATTAGGCCAGTAGTATCCACT---
CAACTGCTGTTAAATGGCAGCCTAGCAGAAGAAGAG-----------
ATAATAATTAGATCTGAGGATTTCACAAACAAT---------GCTAAAACCATAATAGTACAGCTGAAA----
-----------GACCCTGTAAAA---------ATTAATTGTACAAGACCCAACAACAATACAAGAAAA-----
-GGCATACATATA------------GGACCAGGGAGAGCA---TTT------TATACAACA---GAA------
---------ATAGTAGGAGATATA------------AGACAAGCATATTGT---AACATTAGTAAAGAA---

GCATGGAATAAGACATTAGAACAGGTAGTTCAAAAATTAAGA--------------GAACAATTTGGA----
----------------------------------AATAAAACAATAACCTTTAAT------
CAATCCTCAGGAGGGGACCCAGAAATTGTA---CTGCACAGTTTTAATTGTGGAGGGGAA---
TTTAAGCAGCTCCAGGCAAGAGTCCTGGCTGTGGAAAGATACCTAAAGGATCAACAGCTCCTGGGAATTTGGG
GTTGCTCTGGAAAACTCATCTGCACCACTGCTGTGCCTTGGAACGATAGTTGGAGT----------------
---------------------------AATAGATCTCTGGATGAGATTTGG------GAA------
AACATGACCTGGATGCAGTGG---GAAAAAGAAATTGACAATTACACAAACGTAATATAC---
ACCCTAATTGAAAAATCGCAGAACCAGCAGGAA---------------------------------------
----
ATGAATGAAAAAGAATTATTGGAATTGGATAAATGGGCAAGTTTGTGGACTTGGTTTGACATAACAAACTGGC
TGTGGTACATAAAAATATTCATAATGATAGTAGGAGGCTTGATAGGTTTAAGAATAGTTTTTGCT---
GTATTGTCTATAGTG---------AATAGAGTTAGGCAGGGATACTCACCA------
TTATCGTTGGCTGTTAGCTTACTCAATGCCACAGCCATAGCAGTAGCTGAG---
GGGACAGATAGGATTATAGAAGCATTACAAAGAATTTATAGAGCT---------------------------
--------TTTTTTCACATA------CCTAGAAGAATTAGACAGGGCTTAGAAAGGGCTTTACTA---TAA
>KT124781
------ACA---
GAAAAATTGTGGGTCACAGTCTATTATGGGGTACCTGTGTGG---AAAGAAGCAACC---
ACCACTCTATTTTGTGCATCAGATGCTAAAGCATATGATACAGAG---ATGCATAAT----------------
--------------GTTTGGGCCACA---CATGCCTGTGTACCCACAGAC---
CCCAACCCACAAGAAGTAGTATTGGAA---------AATGTGACAGAAAATTTTAAC---------------
--------------------------ATGTGGAAAAATAACATGGTAGAACAGATGCATGAGGATATA---
ATCAGTTTATGGGATCAAAGCCTA---AAGCCATGTGTAAAATTA---
ACCCCACTCTGTGTAACTTTAAATTGCACTGATGTTAACCCGGAGATTACTAAGACCTATAATAGTAGC----
----
--CAGGAAATGAAA---
AACTGCTCTTTCAAGGTCAACCCAGGCATAAAA--------------------------
GACAAGACGCAGAGAGAACGGGCACTT---TTCTATGCACTTGATTTAGTACCAATAAATAAT---------
----

ACAAATGGTAATGATACCAAATATATATTAATAAGTTGTAATACCTCAGTCATTACACAG---
GCCTGTCCAAAGATATCCTTTGAGCCAATTCCCATACATTATTGTGCCCCGGCTGGTTTTGCGATTCTAAAGT
GTAAT---GATAAGACATTCAAT------GGAACAGGACCCTGTACAAAT----------------------
-GTCAGC---ACAGTACAATGTACACATGGAATTAGGCCAGTAGTATCAACT---
CAATTGCTGTTAAATGGCAGTCTAGCAGAAGAAGAG-----------
ATAGTAATTAGATCTGAAAACTTCACAGACCAT---------TTTAAAAACATAATAGTACAGCTGAAC----
-----------GATACTGTAAAC---------ATTACTTGTATAAGACCAAATAACAATACAATAAAA-----
-AGTATACATATA------------GGACCAGGGAAAGCA---TTT------TATGCAACAGGAAAG------
---------ATAATAGGAGATATA------------AGACAAGCATATTGT---AGCATTAATAGAGCA---
AAATGGAATAACACTTTAAGACAGATAGCTATAAAATTAAGA--------------AAACAATTAGAA----
-----------------CAATTTTGGAAT---AAAACAAAAACAATAGTCTTTAAT------
CCCTCCTCAGGAGGGGACTCAGAAATTGTA---ATGCACACCTTTAATTGTGGAGGGGAA---
TTTAAGCAGCTCCAGGCAAGAGTCCTGGCTCTGGAAAGATATCTAAGGGATCAACAGCTCCTGGGGATTTGGG
GTTGCTCTGGAAAACTCATTTGCACCACTGCAGTACCTTGGAATGATAGTTGGAGT----------------
---------------------------AATAAATCTTTGAATGAGATTTGG------AAT------
AACATGACCTGGATGGAGTGG---GAAAGAGAAATTGACAATTACACAGGCTTGATATAC---
AACTTACTTGAACAATCGCAAAACCAACAGGAC---------------------------------------
----
AAGAATGAACAAGACTTATTGGAATTAAGTAAATGGGACAGTTTGTGGAATTGGTTTGACATAACAAATTGGC
TGTGGTATATAAAAATATTCATAATGATAGTAGGAGGCTTGATAGGTTTAAGAATAGTTTTTACT---
GTACTTTCTATAGTG---------AATAGAGTTAGGAAGGGATACTCACCA------
TTATCGTTTGCTATTAGCTTGTTTGATGCTACAGCCATAGCAGTAGCTGAG---
GGGACAGATAGGGTTATAGAATTTTTACAAAGAGCTTGTAGAGCT---------------------------
--------ATTCTCCACATA------CCTACTAGAATAAGACAGGGCTTGGAAAGGCTTTTGCTA---TAA
>MH060965
GCT---GCA---GAA-----------------------------------
GAGAAAACGTGGGTCACAGTCTACTATGGGGTACCTGTGTGG---AAAGAAGCAAAC---
ACCACTCTATTTTGTGCATCAGATGCTAAAGCATATATTACAGAG---GTACATAAT----------------
--------------GTTTGGGCCACA---CATGCCTGTGTACCCACAGAC---
CCCAGCCCACAAGAAATAGAATTGAAA---------AATGTGACAGAAAATTTTAAC---------------

---------------------------ATGTGGAAAAATAACATGGTAGAACAGATGCAGGAAGATATA---
ATCAGTTTATGGGATCAAAGCTTA---AAGCCATGTGTAAAATTA---
ACCCCACTCTGTGTTACTTTAAATTGCACTAATTATAATAACACCAGTGATAATAAGACCATTAATGAT----
----
--GACCCTAAAAGGGAAATAAAA---
AATTGCTCTTTCAATGTCGCCACAGATATAAGT----------------------------
GGTAAGATGAAGAAAGAATATGCATAT---TTTAATAGACTTGATGTAGTACCAATAGATAGT------------
----

AATAATAATACTGATACCAGCTATATGTTGAGGGGTTGTAACACCTCAGTCATTACACAG---
GCCTGTCCAAAGGTATCCTTTGAGCCAATTCCTATACATTATTGTGCCCCGGCTGGTTTTGCGATTCTAAAGT
GTAGA---GATAAGAAGTTCAAT------GGAACAGGACCATGTGGAAAT-------------------------
-GTCAGC---ACAGTACAATGTACACATGGAATTAGGCCAGTAGTATCAACT---
CAACTACTGTTAAATGGCAGTCTAGCAGAAGAAGAG------------
GTAGTAATTAGATCTGAAAATTTCACAAATAAT---------GCTAAAAACATATTAGTACAGCTGAAG----
-----------GAGCCTGTAGAA---------ATTAATTGTACAAGACCCAACAACAATACAAGAAAA-----
-AGTATACGTATA------------GGACCAGGGAGTGCA---TTT------TTTGCAACAGGAGAA------
---------ATAATAGGAGATATA------------AGACGAGCCTATTGT---AACATTAGTAGAGCA---
AAATGGAATGACACTTTAGAACAGGTAGTGAGAAAATTAAGA---------------GAACAATTTGTG----
------------------------------------AATAGAACAATAATATTTAAT------
TCATCCTCAGGAGGGGACCCAGAAATTGTA---ATGCACAGTTTTAATTGTGGAGGGGAA---
TTTAAACAGCTCCAGGCAAGAGTCCTGGCTGTGGAAAGATACCTAAGGGATCAACAGCTCCTGGGAATTTGGG
GTTGCTCTGGAAAACTCATCTGCCACACTGCTGTGCCTTGGAATACTAGTTGGAGT-----------------
-----------------------------AATAAATCTCAAAGTGAAATTTGG------GAC------
AACATGACTTGGATGCAGTGG---GAGAAAGAAATTAGCAACTACACAGGAATAATATAC---
AGCTTAATTGAAGAATCGCAAAACCAGCAAGAA--

AAGAATGAACAAGATTTATTGGCATTAGATAAGTGGGCAAGTTTGTGGAGTTGGTTTGACATAACCAATTGGC
TGTGGTACATAAGAATATTCATAATGATAGTAGGAGGCTTGGTAGGTTTAAGAATAGTTTTTGCT---
GTGCTTTCCATAGTG---------AATAGAGTTAGGCAGGGATACTCACCA------
TTGTCATTGGCTATTAGCTTATACAACGCCATAGCCATAGCAGTAGCTGAG---
GGGACAGATAGGGCTATAGAAGTATTACAAAGAGTTTTTAGAGCT-----------------------------
--------ATCCTTCACATA------CCCAGAAGAATAAGACAGGGCTTAGAAAGGTGTTTGCTG---TAA
>FJ469731
GCT---GTT---
GAAAAGACGTGGGTCACAGTTTATTATGGGGTACCTGTGTGG---AAAGAAGCAACC---
ACCACTCTATTTTGTGCCTCAGATGCTAAATCATATGATACAGAG---GCACATAAT----------------
--------------GTTTGGGCCACA---CATGCCTGTGTACCCACAGAC---
CCCAACCCACAAGAAGTAGTATTGGGA---------AATGTGACAGAAAATTTTAAC----------------
---------------------------ATGTGGAAAAATAACATGGTAGATCAAATGCATGAGGATATA---
ATCAGTTTATGGGATCAAAGTCTA---AAGCCATGTGTGAAATTA---
ACCCCACTCTGTGTTACTTTAAATTGCACTGATTTGAATAAAAATGCCACTAACAATAGTACCACT-------
----
----------------------GCTTCTACTAACAATAATAGGAAATGGAAAGCGAAAAAGAAA---
ATCAGATCTTTCAATATCACCACCACCATAAGA----------------------------
GATAAGATGCAGAGAACATATGCACAT---TTTTATAAACATGATATTGATACACTACATGCTAAT-------
----

AATAGTAATAGTACCACATATAGGTTGATAAGTTGTAACACCTCAGTCATTACACAG---
GCCTGTCCAAAAGTATCCTTTGAGCCAATTCCCATACATTACTGTGCCCCGGCTGGTTTTGCGATTCTAAAGT
GTAAT---GATAAGAAATTCAAT------GGAACAGGACCATGTAAAAAT------------------------
-GTCAGC---ACAGTACAATGCACACATGGAATTAGGCCAGTAGTGTCAACT---
CAATTGCTGTTGAATGGCAGTCTAGCAGAAGAAGAG------------
ATAGTAATTAGATCTGAGAATATCACAGACAAT---------GCTAAAACCATAATAGTACAGCTGAAC----
-----------ACATCTGTAGTA---------ATTAATTGTACAAGACCCAACAACAATACAAGGAAA-----
-AGTATACATATA------------GCACCAGGGAGAGCA---TTT------TATGCAACAGGAGAC------
---------ATAATAGGAAAGATA------------AGACAAGCACATTGT---AATGTTAGTAGAAGC---
AAATGGGAAGACACTTTAAGAAAGGTAGCTATAAAATTAAAA---------------GAACAATTT-------
------------------------------------AATAACACAATAATCTTTACA------
AACTCCTCAGGAGGGGACCCAGAAATTGTA---ATGCACAATTTCATTGTGGAGGGGAA---

TTTAAGCAGCTCCAGGCAAGAATCCTGGCTGTGGAAAGATACCTAAAGGATCAACAGCTCCTAGGGATTTGGG
GTTGCTCTGGAAAACTCATCTGCACCACTACTGTGCCTTGGAATGCTAGTTGGAGT-----------------
----------------------------AATAGATCTCTGGATAATATCTGG------CAT------
AACATGACTTGGATGCAATGG---GAAAGAGAAATTGACAATTACACAGAATACATATAC---
TCATTGATTGAAGAATCGCAGAACCAGCAAGAA--

AAGAATGAAAAAGAATTATTGGAATTGGATAAGTGGGACAATCTGTGGAATTGGTTTACAATAACACACTGGC
TGTGGTATATAAAAATATTCATAATGATAGTAGGAGGCTTGATAGGTTTAAGAATAGTTTTTGCT---
GTACTTTCTATAGTG---------AATAGAGTTAGGCAGGGATATTCACCA------
TTGTCGTTTGCTGTTAGCTTGCTCAACGCCTTTGCCATAGCAGTAGCTGAG---
GGGACAGATAGGATTATAGAAGCAGTACAAAGAGCTTGTAGAGCT----------------------------
--------ATTCTCCACATA------CCTAGAAGAATAAGACAGGGCTTGGAAAGGGCTTTGCTA---TAA
>HQ216397
GCT---GCA--
GAACAATTGTGGGTCACAGTCTATTATGGGGTACCTGTGTGG---AAAGAAGCAACC---
ACCACTCTATTTTGTGCATCAGATGCTAAAGCATATCACACAGAG---GTACATAAT----------------
--------------GTTTGGGCCACA---CATGCCTGTGTACCCACAGAC---
CCCAACCCACAAGAAGTAGTAATGGAA---------AATGTGACAGAAAATTTTAAC----------------
--------------------------ATGTGGAAAAACAACATGGTAGAACAGATGCAGGAGGATATA---
ATCAGTCTGTGGGATCAAAGCCTA---AAGCCATGTGTAAAATTA---
ACCCCACTCTGTGTTACTTTAAATTGCACTGACTTGGAGAATAATAGTACTAATACTACT-------------
----
-------------------------AATGGTAACTGGGAAACGCTGGAGAGAGGAGAAGTAAAA---
AACTGCTCTTTCAATGTCACAATAAACAGAGGA---------------------------
GAGGAGCTGAGGAAAGAATATGCACTT---CTTTATAAACTTGATATAGTACCAATGGGAGAG----------
----

AATAGTACCAGCTATAGGTTGATGAGTTGTAACACCTCAGTCATTACACAG---
GCCTGTCCAAAGGTATCCTTTGAACCAATTCCCATACATTATTGTGCCCCGGCTGGTTTTGCGATTCTAAAGT
GTAAT---AGTAAGACATTCAAT------GGAACAGGACCATGTAAAAT------------------------
-GTCAGC---ACAGTACAATGCACACATGGAATTAGGCCAGTAGTATCAACT---
CAACTACTGCTAAATGGCAGTCTAGCAGAAGAAGAG------------
GTAGTAATTAGATCTGAAAATTTCACAAACAAT---------GCTAAAACCATAATAGTTCAGCTGAAT----
-----------AAAACTGTAACA---------ATTAATTGTACAAGACCCAACAACAATACAAGGAAA-----
-AGTATACATATG------------GGACCAGGGAAAGCA---TTT------TATGCAACAGGAGAC------
---------ATAATAGGAAATATA------------AGAAGAGCACATTGT---AATATTAGTAGAGCA---
GAATGGAATAGCACTTTACAAAAGGTAGTTACAAAATTACAA---------------AAACAATTTGGG----
--------------------------------GAGAATAAAACAATAGTCTTTAAT------
CACTCCTCAGGAGGGGACCCAGAAATTGTA---ATGCACAGTTTTAATTGTGGAGGGGAA---
TTTAAGCAGCTCCAAGCAAGACTCCTAGCTGTGGAAAGTTACCTAAGGGATCAACAGCTCCTAGGGATTTGGG
GTTGCTCTGGAAAACTCATTTGCACCACTAATGTGCCTTGGAATACTAGTTGGAGT-----------------
----------------------------AACAGATCACTGAATAGTATTTGG------GAT------
AACATGACCTGGATGGAGTGG---GATAGAGAAATTAACAATTACACAAACATAATATAC---
AACTTACTTGAACAATCGCAAAACCAACAAGAA--

AAGAATGAAAAAGACTTATTGGAATTGGATAAGTGGGCAAGTTTGTGGAATTGGTTTGACATCTCAAACTGGC
TGTGGTATATAAAAATATTCATAATGATAGTAGGAGGTTTAGTAGGATTAAGAATAGTTATGGCT---
GTGCTTTCTATAGTG---------AATAGAGTTAGGCAGGGATACTCACCA------
TTGTCATTTGCTGTTAGTTTGCTTAACGCCATCGCTATAGCAGTAGCTGAG---
GGAACAGATAGGGTTATAGAAGCAGTACAAAGAATTTGTAGAGCT----------------------------
--------ATCCTCCACATA------CCTAGAAGAATAAGACAGGGCTTGGAAAGGCTTTTACTA---TAA
>KT124758
GCT---GTA--GAAAAG---
TGGGTTACAGTCTATTATGGGGTACCTGTGTGG---AGAGAAGCAAGC---
ACCACTTTATTCTGTGCATCAAATGCTAAAGGATATAATACAGAG---GCACATAAT----------------
--------------GTTTGGGCCACA---CATGCCTGTGTGCCCATAGAC---
CCCAACCCACAAGAAGTAGTAATGGAA---------AATGTAACAGAAAATTTTAAC----------------
--------------------------ATGTGGAAAAATAACATGGTAGAACAGATGCATGAGAATATA---
ATCGATTTATGGGATCAAAGCCTA---AAGCCATGTGTAGAATTA---
ACCCCACTCTGTGTTACCTTAAATTGCACTGATTTGACTAATATCAATACCACCACGGGGACAAAT-------

----
-------------ACCACTGTCGCTGGCAATACTAGTGGTACAATAAATGAAGGGATAATGACA---
AACTGCTCTTTCAATATCACCACAAACATAAGA--------------------------
GATAAGATAAAGAAAGACTATGCATTG---TTTTATAGAACTGATGTAGAAAGGATAATTGAAGAAAAT----
----

GATAATACCAGCTATAGGTTGAGAAGTTGTAATACCTCAGTCATTAGACAG---
GCCTGTCCAAAGGTATCCTTTGAGCCAATTCCCATACATTTTTGTGCCCCAGCTGGTTTTGCGATTCTAAAAT
GTAAT---GATAAGAAGTTCAAT------GGAACAGGACCATGTAAAAAT----------------------
-GTCAGC---ACAGTACAATGTACACATGGAATTAGGCCAGTAGTATCAACT---
CAATTGCTGTTAAATGGCAGTCTAGCAGAAGAAGAG-----------
ATAGTAATTAGATCTGAAAATTTCTCGGATAAT---------GCTAAAACCATCATAGTACAGCTGAAT----
-----------GCATCTGTAGAA---------ATTAATTGTACAAGACCCAACAACAATACAAGAAAA-----
-AGCATACATATA------------GCACCAGGGAGAGCA---TTT------TATGCAACAGGAGAA------
---------ATAATAGGAAATATA------------AGACAAGCACATTGT---AATCTTAGTGGAGCA---
CAATGGAATAACACTTTAAAACATATAGCTATAAAATTAAGA---------------GAACAATATCCT----
-----------------------------------AATAAAACAATAAATTTTACC------
CAATCCTCAGGAGGGGACCCAGAAATTGTA---ATGCATATCTTTAATTGTGGAGGGGAA---
TTTAAGCAGCTCCAGGCGAGAGTCCTGGCTGTAGAAAGATACCTAAAGGATCAACAGCTCCTGGGAATTTGGG
GTTGCTCTGGAAAACTCATCTGCACCACTGCTGTGCCTTGGAATGATAGTTGGAGT-----------------
----------------------------AACAAATCTCTGAGTGATATTTGG------GAC------
AACATGACCTGGTTGCAGTGG---GATAAAGAAATTCACAATTACACAACTTTAATATAC---
ACCTTACTTGAAGAATCGCAGAACCTACAAGAC---------------------------------------
----
AAGAATGAAAAAGAATTATTGGAATTGGATAACTGGGCAAGTTTGTGGAGTTGGTTTGACATATCAAATTGGC
TGTGGTATATAAGAATATTCATAATGATAGTAGGAGGCTTGATAGGTTTAAGAATAGTTTTTGCT---
GTACTGTCTATAGTG---------AATAGAGTTAGGCAGGGATACTCACCC------
TTATCGTTTGCTGTTAGCTTGCTTAATGCCACGGCCATAGCAGTAGCTGAG---
GGGACAGATAGGGTTATAGAAGTAATACAAAGAACTTGTAGAGCT---------------------------
--------ATCCGCCATATA------CCTAGAAGAATAAGACAGGGCCTGGAAAGAATTTTGTTA---TAA
>JX863968
ACA---GAA---AAG---
TGGGTCACAGTCTATTATGGGGTACCTGTGTGG---AAAGAAGCAACC---
ACCACTCTATTTTGTGCATCAGATGCTAAAGCATATGATACAGAG---ATGCATAAT---------------
--------------GTTTGGGCCACA---CATGCCTGCGTACCCACAGAC---
CCAAACCCACAAGAAATAGGATTAGAA---------AATGTGACAGAAAATTTTAAC---------------
--------------------------ATGTGGAAAAATAACATGGTAGAACAGATGCATGAGGATATA---
ATTAGTTTATGGGATCAAAGCCTA---AAGCCATGTGTAAAATTA---
ACCCCACTCTGTGTTACTTTAAATTGCACTGATTATACTGGTAATGGAACTGTGAAAAATGCTACTGATACT-
----
----------------------GTTAATAGAACTGTGGAAGGGACGATGAGGGGAGAAATAAAA---
AACTGCTCTTTCAATATCACCACAAACATAAGA--------------------------
GATAAGATAAAGAAAGAACATGCATTA---TTTTATAAACTTGATGTAGTGCAAATAGATGAGAAT-------
----

AATACTAACAGCTATAGGTTGATAAGTTGTAACACCTCAGCCCTTACCCAG---
GCCTGTCCAAAGGTATCCTTTGAGCCAATTCCCATACATTATTGTGCCCCGGCTGGTTTTGCGATTCTAAAAT
GTAAT---GATACAAAGTTCAAT------GGATCAGGACCATGTACAAAT----------------------
-GTCAGC---ACAGTACAGTGTACACATGGAATTAGGCCAGTAGTATCAACT---
CAACTGCTGTTAAATGGCAGTCTAGCAAAAGAAGAG-----------
GTAGTAATTAGATCTGAAAATTTCACGAACAAT---------GCTAAGACCATAATAGTACAGCTGAAT----
-----------GAATCTGTAGTA---------ATTACTTGTACAAGACCCAACAACAATACAAGAAGA-----
-AGTATACATATA------------GCACCAGGGAGAGCA---TTT------TATGCAACAGGAAAT------
---------ATAGTAGGAGATATA------------AGACAAGCACATTGT---AACATTAGTAAAGCA---
AACTGGACTAACACTTTAAAACAGGTAGTTGAAAAATTAAGA---------------GAACAATTTGGG----
-----------------------------------AATAAAACAATAGTCTTTAAT------
CAATCCTCAGGAGGGGACCCAGAAATTGTA---AGTCACAGTTTTAATTGTGGAGGAGAA---
TTTAAGCAGCTCCAGGCAAGAGTCCTGGCTGTGGAAAGATACCTAAGAGATCAACAGCTCCTGGGGATTTGGG
GTTGCTCTGGAAAACTCATTTGCACCACTGCTGTGCCTTGGAATGATACTTGGAGT----------------
----------------------------AATAAATCCCTAGATGATATTTGG------GAT------

AACATGACCTGGATGCAGTGG---GAAAGAGAGATTGACAATTACACAGCTACAATATAC---
AACCTACTTGAAAAATCGCAGAACCAGCAGGAA--

AAGAATGAACAAGAATTATTGGAATTGGATAAATGGGCAAGCTTGTGGAATTGGTTTGACATAACAAATTGGC
TATGGTATATAAAGATATTCATAATGATAGTAGGGGGTTTGATAGGTTTAAGACTAGTTTTTACT---
GTACTTTCTATAGTG---------AATAGAGTTAGGCAGGGATACTCACCA------
TTATCGTTTGCTGTTAGCTTGCTCAACGCCACAGCCATAGCAGTAGCTGAG---
GGGACAGATAGGGTTATAGAAGTATTACAAAGAATTGTTAGGGCT----------------------------
--------ATCCTTCACATA------CCTACAAGAATAAGACAGGGTTTGGAAAGGCTTTGCTA---TAA
>EF593190
GCT---GGA--
GAACAATTGTGGGTAACAGTCTATTATGGGGTACCTGTATGG---AAAGAAGCAACC---
ACCACTCTATTTTGTGCATCAGATACTAAAGCATATCATACAGAG---GTACATAAT----------------
--------------GTTTGGGCTACA---CATGCCTGTGTACCCACAGAC---
CCCAGCCCACAAGAAGTAGTATTGGGA---------AATGTAACAGAAAATTTTAAC----------------
--------------------------ATGTGGAAAAATAACATGGTAGAACAGATGCATGAGGATATA---
ATCAGTTTATGGGATCAAAGCCTA---AAGCCATGTGTAAAATTA---
ACCCCACTCTGTGTTACTTTAAATTGCACTGATAATTTGAGCAATTTGAACAATGTTACTCATAAACATAAC-
----
----------------AATAGTAATAGCGGGGAAGATCTGAAAGGAGAAATAGGAGAAATAAAA---
AACTGCTCTTTCAATGTCACCACAATCATAAAG---------------------------
GATAAGATACAGAAAGAATATGCACTT---TTTTATAAATCTGATGTAGTACCAATAGAGAGTGAGAAT----
----

AATACTAGTAACTATACTAGCTATAGGTTGATACATTGTAACACCTCAGTCATTACACAA---
GCCTGTCCAAAGGTATCCTTTGAACCAATTCCCATACATTTTTGCGCCCCGGCTGGTTTTGCGATTCTAAAAT
GTAAC---AATAAGACATTCGCA------GGAAAAGGGCCATGCACAAAT-----------------------
-GTCAGT---ACAGTACAATGTACACATGGAATCAGGCCAGTAGTATCAACT---
CAACTGCTGTTAAATGGTAGTCTAGCAGAGAAAGAG------------
GTAGTAATCAGAACTGAAAATTTCACAGACAAT---------GCTAAAACCATAATAGTACACCTGAAT----
-----------GAATCGGTAACC---------ATTAATTGTACAAGACCTAACAACAATACAAGGAAA-----
-GGTATACATATA------------GGACCAGGGAGAGCA---TTT------TATGCAACAGGACAA------
---------ATAATAGGAGATATA------------AGACAAGCACATTGT---ACTCTTAATATAACA---
CAATGGAATAACACTTTAAATCAGATAGTTACAAAATTAAGA---------------GAAAATTTTAATTTG-
--------------------------------GAAAATAAAACAATAATCTTTAAT------
CACTCCTCAGGAGGGGACCCAGAAATTATA---ATGCACAGTTTTAATTGTGCAGGAGAA---
TTTAAGCAGCTCCAGGCAAGAGTCCTGGCTGTGGAAAGATACCTGAAGGATCAACAGCTCCTGGGAATTTGGG
GTTGCTCTGGAAAACTCATTTGCACCACTAATGTGCCTTGGAATGCTAGTTGGAGT-----------------
----------------------------AATAAATCTCTGAATGACATCTGG------GAC------
AACATGACCTGGATGCAGTGG---GAAAAAGAAATTGACAATTACACAGGCTTAATATAC---
TCCTTAATTGAAGAATCGCAAAACCAACAAGAA--

AAGAATGAACAAGAATTATTGGCATTGGATAGATGGGCGAGTTTGTGGAATTGGTTTGACATAACAAGATGGC
TGTGGTATATAAAAATATTCATAATGATAGTAGGGGGCTTAATAGGGTTAAGAATAGTTTTTACT---
GTACTTTCTATAGTG---------AATAGAGTTAGGCAGGGCTACTCACCA------
TTATCATTTGCTGTTAGCTTGTTCAACGCCATAGCCATAGCAGTAGCTGAG---
GGGACAGATAGGGTTATAGAAATAGTACAGAGAGCTGTTAGAATT----------------------------
--------ATTCTCAACATA------CCTACACGAATAAGACAGGGCTTGGAAATTGCCTTGCTA---TAA
>MH060681
GGT---AAA--
GAAGGCCTGTGGGTCACAGTCTATTATGGGGTACCTGTGTGG---AGAGAAGCAAAC---
ACCACTCTATTTTGTGCATCAGATGCTAAAGCATATGAGACAGAG---GTGCATAAT----------------
--------------GTCTGGGCCACA---CACGCCTGTGTACCCACAGAC---
CCTAGCCCACAAGAAGTAGTATTGGAA---------AATGTAACAGAAAATTTTAAC----------------
--------------------------ATGTGGAAAAATAACATGGTAGAACAGATGCATGAGGATATA---
ATCAGTTTATGGGATGAAAGCCTA---AAGCCATGTGTGAAATTA---
ACCCCACTCTGTGTTACTCTAAATTGCACTGATATCACTAGTGCTGCCACTAATGCCACTGCCAACTCT----
----
-------------------------AATGCCACTAGTTGGACGGATGGAAGAGAAGAAATGAGA---
AACTGCTCTTTTAATATCACCACAAACGTAAGC---------------------------

GATAAGAAGCAGAAAGAATATGCAATC---TTTTATAAATATGATATAGTATCAACAGATAAT----------
----
----------------------GGTACTACCAGTTAC---
TTAACACATTGTAACACCTCAATAATTAGACAG---
GCCTGTCCAAAAGTATCCTTTCAGCCAATTCCCATACATTATTGTGCCCCGGCTGGTTTTGCGATTCTGAAGT
GTAAT---GATAAGAAGTTCAAT------GGATCAGGGCCATGTACAAAT----------------------
-GTCAGC---ACAGTACAATGTACACATGGAATTAAGCCAGTAGTGTCAACT---
CAATTGCTGTTAAATGGCAGTCTAGCAGAAGAAGAC-----------
ATAGTAATCAGATCTGAAAATATCTCGGACAAT---------ACTAAAACTATAATAGTACATTTGAAT----
-----------GAAACTGTACAA---------ATTAATTGTATAAGACCCAATAACAATACAAGAAAA-----
-AGTATACATATA------------GGACCAGGGAGAGCA---TTT------TATGCAACAGGAAAC------
---------ATAATAGGAGACATA------------AGACGAGCACATTGT---AATGTTAGTAGAGCA---
AAATGGAATAGCACTTTAGAAAGGATAGCTGCAAAATTAAGA---------------GAAAAATTTGGG----
--------------------------------AATAACAAAACAATAGTCTTTAAT------
TCATCCTCAGGAGGGGACCCAGAAATTGAA---ATGCACACTTTTAATTGTGGAGGGGAA---
TTTAAGCAGCTCCAGGCAAGAGTCCTGGCTTTGGAAAGATACCTAAGGGATCAACAGCTCCTAGGGATTTGGG
GTTGCTCTGGAAAACTCATTTGCCCCACTGCTGTGCCTTGGAATGCTAGTTGGAGT-----------------
----------------------------AATAGATCTCAGGAGACAATTTGG------AAC------
AACATGACCTGGATGCAGTGG---GAAAAAGAGATTGACAATTACACAGGCATAATATAC---
AACTTACTTCAGGAATCGCAAAACCAGCAAGAA---

AAGAATGAACAAGAATTATTGGAATTAGATAAATGGGCAAGTTTGTGGAATTGGTTTAGCATAACACAGTGGC
TGTGGTATATTAAATTATTCATAATGATAGTAGGAGGCCTGATAGGTTTAAGAATAGTTTTTAGT---
GTACTTTCTATAGTA---------AAGAGAGTTAGGCAGGGATATTCACCA------
TTATCGTTTGCTGTTAGCTTGCTTAATGCCATAGCCAGAGCAATAGCTGAA---
GGGACAGATAGGGTTATAGAATTAGCACAAGGAGTTTTTAGAGCT----------------------------
--------ATCATTCACATA------CCTAGAAGAATAAGACAGGGACTTGAAATAGCTTTGCTG---TAA
>MH060990
GCT---ACA---
GAAAAATTGTGGGTCACAGTCTATTATGGGGTACCTGTGTGG---AAAGAAGCAACT---
ACCACTCTATTTTGTGCATCAGATGCTAAAGCATATGATACAGAG---GTACATAAT----------------
--------------GTTTGGGCTACA---CATGCCTGTGTGCCCACAGAC---
CCCAACCCACAAGAAGTAGTATTGGGA---------AATGTGACAGAAAACTTTAAC---------------
--------------------------ATGTGGAGAAATGACATGGTAGAACAGATGCATGAGGATATA---
ATTAGTTTATGGGATCAAAGCCTA---AAGCCATGTGTAAAATTA---
ACCCCACTCTGTGTTACTTTAAAGTGCACTGATTATGGGAACAATGCTACCAATGCCAATACTACCAATACC-
----
-------------------------AATAGTAGTAGTAAAGTGGAAATGATAGGAGAAATAAAA---
AATTGCTCTTTCAACATCACCACAAACATAAGA--------------------------
GATAAGATAAAGAAAGAATATGCATTG---TTTTATAAACTTGATGTAGTACCAATAGATAAT----------
----

ACTAGCTATAGGTTGATAAGTTGTAATACCTCAGTCCTTACACAG---
GCCTGCCCAAAGGTATCCTTTGAGCCAATTCCCATACATTATTGTGCCCCGGCTGGTTTTGCGATTCTAAAAT
GTAAC---AATAAAAAGTTCAAT------GGGACAGGATTATGTAAAAAT----------------------
-GTCAGC---ACAGTACAATGTACTCATGGAATTAGACCAGTAGTATCAACT---
CAACTGTTGTTAAATGGCAGTCTAGCAGAAGAGGAG-----------
GTAGTAATCAGATCTGAAAATTTCACGAACAAT---------GCTAAAATCATAATAGTACAGCTGAAC----
-----------GAAGCTGTAGAA---------ATTAATTGTACAAGACCCAACAACAATACAAGAAAA-----
-GATATACATATA------------GGACCAGGGAGAGCA---TTT------TATACAACAGGAGAA------
---------ATAATAGGAGATATA------------AGACAAGCACATTGT---AACATTAGTAGAGAA---
AAATGGAATAACACTTTAGGACAGATAGTTAAAAAATTAAGA---------------GAACAATTTGGG----
-----------------------------------AATAAAACAATAATCTTTAAT------
CAATCCTCAGGAGGGGACCCAGAAGTTGTA---ATGCACACTTTTAACTGTGGAGGGGAA---
TTTAAGCAGCTCCAGGCAAGAGTCCTGGCTRTAGAAAGATACCTAAGGGATCAACAGCTCCTGGGAATTTGGG
GTTGCTCTGGAAAACTCATCTGCACCACTGCTGTGCCCTGGAATTCTAGTTGGAGT----------------
----------------------------AATAAATCTCTGGATCAGATTTGG------AAT------
AACATGACCTGGATGGAGTGG---GAAAGAGAAATTGACAATTACACAGGCACAATATAC---
AGGTTAATTGAAGAATCGCAAAACCAACAAGAA---

AAGAATGAAAAAGAATTATTGGAATTAGATAAATGGGCAAGTTTATGGAATTGGTTTGACATAACAAGCTGGC
TGTGGTATATAAAAATATTCATAATGATAGTAGGAGGCATAGTAGGTTTAAGAATAATTTTTTCT---
ATACTTTCTATAATA---------AATAGAGTTAGGCAGGGATACTCACCA------
TTATCGTTTGCTATTAGCTTGTTCAATGCCACAGCTATAGTAGTAGCTGAA---
GGGACAGATAGAATTTTAGAAGCATTACAAAGAACTTTTAGAGCT----------------------------
--------ATCCTCAACATA------CCTAGAAGAATAAGACAGGGCTTAGAAAGAATTTTGCTA---TAA
>DQ410526
GCT---ACA--
GAGCAATTGTGGGTCACAGTATATTATGGGGTATCTGTGTGG---AAAGAAGCAAAC---
ACCACTCTATTTTGTGCATCAGATGCTAAAGGATATGATACAGAA---AAACATAAT----------------
--------------GTCTGGCCACA---CATGCCTGTGTACCCACAGAC---
CCCAGCCCACAAGAATTAGTATTGGCA---------AATGTGACAGAAAACTTTAAC----------------
--------------------------ATGTGGAAAAATAACATGGTAGAACAGATGCATGAGGATATA---
ATCAGTTTATGGGACCAAAGCCTA---AAGCCATGTGTAAAATTA---
ACTCCACTCTGTGTTACTTTAACTTGCAAAAATGTGACTAAAACCAATGTGACTAATAAAGTCAATGTG----
----
----------------ACTAATACCACTAATAGTAGCGAGAAAACAATGAGAGAAGAAATGAAA---
AATTGTTCTTTCAATATTACCACAAACATAAGA---------------------------
GATAAGATACAGAAGCAATATGCGCTT---TTTTATAAACTTGATATAGTACAAATAGATGAG----------
----

AATGATAATACTAGCTATAGGTTGATAAGTTGTAACACCTCAGTCATTACACAG---
GCCTGTCCAAAGGTATCCTTTGAGCCAATTCCCATTCATTATTGTGCCCCGGCTGGTTTTGCGATTCTAAAGT
GTAAT---AATGAGACTTTCAAT------GGAACAGGACCATGTAAAAT------------------------
-GTCAGT---ACAGTACAATGCACACATGGAATTAGGCCAGTAGTATCAACT---
CAACTACTGTTAAATGGCAGTCTAGCAAAAGGAGAG------------
GTAAGAATTAGGTCCGCCAATTTCTCGGACAAT---------GCTAAAACCATAATAGTACAGCTGAAT----
-----------GAATCTGTAATA---------ATTAATTGTACAAGACCCAACAACAATACAAGAAAA-----
-AGTATACCCATA------------GGACCAGGCAGAGCA---TTT------TATGCAACAGGGGAC------
---------ATAATAGGAGACATA------------AGACAAGCACATTGT---AATGTTAGTAGAAAA---
CATTGGAATAACACTTTAAAGTGGGTAGCTGCAAAATTAAGA---------------GAACAATTT-------
-----------------------------------AATAAAACAATAGTCTTTAGG------
AACTCCTCAGGAGGGGACCCAGAAATTGTA---ATGCACAGCTTTAATTGTGGAGGGGAA---
TTTAAGCAGCTCCAGGCAAGAATCCTGGCTGTGGAAAGATACCTAAAGGATCAACAGCTCCTGGGGATTTGGG
GATGCTCTGGAAAACTCATTTGCCCCACTACAGTGCCTTGGAATAATAGTTGGAGT-----------------
----------------------------AATAAAAATCTGAGTGTTATTTGG------GGA------
AACATGACCTGGATGCAGTGG---GAAAGAGAAATTGAGAATTACACAGGCTTAATATAC---
AGCTTAATTGAAGAATCGCAAAACCAACAAGAA--

ATGAATGAAAAAGAATTATTGGAATTAGATAAATGGGCAAGTTTGTGGAATTGGTTTGACATAACAAATTGGC
TGTGGTATATAAGATTATTCATAATGATAGTAGGAGGCTTAGTAGGTTTAAGAATAATTTTTGCT---
GTCCTTTCTATAGTG---------AATAGAGTTAGGCAGGGATACTCACCA------
TTATCGTTTGCTGTTAGCTTGCTCAACGCCATAGCCATAGCAGTAGCTGAG---
GGGACAGATAGGATTTTAGAAATATTGCAAAGAGCTTTTAGAGCT----------------------------
--------ATTCTCCACATA------CCTACAAGAATAAGACAGGGCTTGGAAAGAGCTTTGCTA---TAA
>EF363127
GCT---GCA--
AACAATTTGTGGGTCACAGACTATTATGGGGTACCTGTGTGG---AAAGAAGCAAAC---
ACCACTCTATTTTGTGCCTCAGATGCTAAAGCATATAGTACAGAG---GTACATAAT----------------
--------------GTTTGGCCACA---CATGCCTGTGTACCCACAGAC---
CCCAACCCACAAGAAGTAGCATTGAAA---------AATGTGACAGAAAATTTTAAC----------------
--------------------------ATGTGGAAAAATAACATGGTAGAACAAATGCAGGAAGATATA---
ATCAGTTTATGGATCAAAGCCTA---AAGCCATGTGTAAAATTA---
ACTCCACTCTGTGTTACTTTAAATTGCACTGATTGGGATAATGCTACT-------------------------
----
-------------------------------------CTTGCAAATATGACGGGAGAAATAAAA---
AACTGCACTTTCAATATCACCACAACCATGAGA---------------------------
GATAAGGTGCAAAAAGCATATGCACTC---TTTTATAGCACTGATGTAGTAAAAATAAATAAT----------
----

AATGATAATAATAGTAGCTATAGGTTGATAAGTTGTAACACCTCAGTCATTACACAA---
GCCTGTCCAAAAGTATCCTTTGAGCCAATTCCCATACATTATTGTGCCCCGGCTGGTTTTGCGATTCTGAAGT
GTAGA---GATAAAAAGTTCAAT------GGAACAGGACCATGTACAAAT----------------------
-GTTAGC---ACTGTACAATGTACTCATGGAATTAGGCCAGTAGTATCAACT---
CAACTGCTGTTAAATGGTAGTCTAGCAGAAGAAGAG-----------
GTAATAATTAGATCTGCCAATATCACAGACAAT---------ACTAAAACCATAATAGTACATCTGAAC----
-----------AACTCTGTAGGA---------ATTACATGTACAAGACCCGGCAACAATACAAGACAA-----
-AGTATACATATA------------GCACCAGGAAGAGCA---TTT------TATGCAACAGGTGCA------
---------ATAACAGGAAATATA------------AGAAAAGCACATTGT---AACATTAGTATAGTA---
AGTTGGAATGACACCTTAAAACAGGTAGTTGAAAAATTACAA--------------GAACACTTCCCA----
----------------------------------AATAAAACAATAGCCTTTAAA------
AATTCCTCAGGAGGAGACCCAGAAATTGTA---ATGCACAGGTTTAATTGCGGAGGGGAA---
TTTAAACAGCTCCAGGCAAGAGTCCTGGCTGTGGAGAGATACCTAAGGGATCAACAGCTCCTGGGAATTTGGG
GTTGCTCTGGAAAACTCATCTGCACCACTAATGTGCCTTGGAATATTAGTTGGAGT-----------------
----------------------------AATAAAAATCTATCTCAGATTTGG------GAT------
AACATGACCTGGATGGAGTGG---GAAAGAGAAATTGAGAATTACACACAAACAATATAC---
AACTTAATTGAAGAATCGCAGAACCAGCAAGAA---------------------------------------
----
AAGAATGAAAAAGAATTATTAGAATTAGATAAATGGGCAAGTTTGTGGAATTGGTTTGACATAACAAACTGGC
TGTGGTATATAAAAATATTCATAATGATAGTAGGAGGCTTGATAGGTTTAAGAATAGTTTTTACT---
GTGCTTTCTATAGTG---------AATAGAGTTAGGCAGGGATACTCACCA------
TTATCGTTCGCTATCAGTTTGTTTAATGCCATAGCTATAGCAGTAGCTGAG---
GGGACAGATAGGGTTATAGAAATAGTACAAAGAGTTTGCAGAGCT----------------------------
--------ATTCGCCACATA------CCTAGAAGAATAAGACAAGGCTTGGAAAGGAGTTTGCTA---TAA
>DQ410205
GCT---GCA--
GAGAAATTGTGGGTCACAGTCTATTATGGGGTACCTGTGTGG---AAAGAAGCAACC---
ACCACTCTATTTTGTGCGTCAGATGCTAGAGCATATGATACAGAG---GTACATAAT----------------
--------------GTTTGGGCCACA---CATGCCTGTGTACCCACAGAC---
CCCGACCCACAAGAAATAGTATTGGAG---------AATGTGACAGAAAATTTTAAC---------------
--------------------------ATGTGGAAAAATAACATGGTAGAACAGATGCATGAGGATATA---
ATCAGTCTATGGGATCAAAGTCTA---AAGCCATGTGTAAAATTA---
ACCCCACTCTGTGTTACTTTAAATTGCACTGAGTTGAGGAATGCTACTAATACCAATGCTACTAAT-------
----
-------------------ACCACTAGTAGTAGTGGGGGAATGATGGAGGGAGGAGAAATAAAA---
AATTGCTCTTTCAATATCACCACAAGCATAAAA--------------------------
GAAAAGATGAAGAAAGAATATGCACTT---TTTTATAGTCTTGATGTAATACCAATAGATGAT---------
----

AATAATACTAGTTATAGGTTGAGAAGTTGTAACACCTCAGTCATTACACAG---
GCCTGTCCAAAGGTATCCTTTGAGCCAATTCCCATACATTATTGTGCCCCGGCTGGTTTTGCGATTCTAAAGT
GTAAA---GATAAAAAGTTCAAT------GGAATAGGACCATGTACAAAT----------------------
-GTCAGC---ACAGTACAATGTACACATGGGATTAAGCCAGTAGTATCAACT---
CAACTGCTGTTAAATGGCAGTCTAGCAGAAGGAGAG-----------
GTAGTAATTAGATCCTATAATTTCACGGACAAT---------GCTAAAATCATAATAGTACAGCTGAAT----
-----------GAGTCTGTAGGA---------ATTAACTGTACAAGACCCAACAACAATACAAGAAAA-----
-AGTATACATATA------------GGACAAGGCAGGGCA---CTT------TATGCAACAGGAGAC------
---------ATAATAGGAGATATA------------AGACAAGCACATTGT---AATCTTAGTATGACA---
AAATGGGATAACACTTTAAAACGGATAGTTGTAAAATTAAGA--------------GAACAATTTAAG----
----------------------------------AATAACACAATAGTCTTTAAT------
CAATCCTCAGGAGGGGACCTAGAAATTGTA---ATGCACAGTTTTAATTGTGGAGGGGAA---
TTTAAGCAGCTCCAGGCAAGAGTCCTGGCTGTGGAAAGATACCTAAAGGATCAACAGCTCCTGGGGATTTGGG
GTTGCTCTGGAAAACTCATTTGTACCACTGCGGTGCCTTGGAACACTAGTTGGAGT----------------
----------------------------AATAAATCTCTGAATCAGATTTGG------AAT------
AACATGACCTGGATGGAGTGG---GAAAGAGAAATTGATAATTACACAGGCTTAATATAC---
AACCTAATTGAAGAATCGCAGAACCAACAAGAA---------------------------------------
----
AAGAATGAACAAGAATTATTGGAATTAGATAAATGGGCAAGTTTGTGGAATTGGTTTGACATAACAAAATGGC
TGTGGTATATAAAAATATTCATAATGATAGTAGGGGGCTTGGTAGGTTTAAGAATAGTTTTTACT---
GTACTTTCTATAGTG---------AATAGAGTTAGGCAGGGATACTCACCA------

TTATCGTTTGCTGTTAGCTTGCTCAATGCCACAGCTATAGCAGTAGCTGAG---
GGGACAGATAGGATTATAGAAGTATTACAAAGAGTTTATAGAGCT---------------------------
--------GTTCTCCACATA------CCTACAAGAATAAGACAGGGCTTAGAAAGGGCTTTGCTA---TAA
>FJ469733
GCT---TCA--
GAAAAGTTGTGGGTCACAGTATATTATGGGGTACCTGTGTGG---AAAGATGCAAAG---
ACCACTCTATTTTGTGCATCAGATGCTAAAGGATATGAGACAGAG---ATGCACAAT----------------
--------------GTTTGGGCCACA---CATGCCTGTGTACCCACAGAC---
CCCAACCCACAAGAAATAGTATTGGAA---------AATGTGACAGAAGATTTTAAC----------------
--------------------------ATGTGGAAAAATAACATGGTAGAACAGATGCAGGAGGATATA---
ATTAGTTTATGGGATCAAAGCCTC---AAGCCATGTGTACAACTA---
ACCCCACTCTGTGTTACTTTAGATTGCACTGATTATAAGGGGAGTAATACAACCAAGAATACTACAACAGCC-
----
----------------AATAGTACAAGTAATAATAGTAGCTTAAGGGAGGATACAGGAATGAAA---
AACTGCTCTTTCAATGTCACCACAAACATAAGA--------------------------
GATAAGAGGCAGAAAGAATATGCACTT---TTATATAAACTTGATTTAGTACCAATAAATGATGCT-------
----

AATGCTAGTAATACTAGCTATAGGTTGATAAGTTGTAACACCTCAGTCATTACACAG---
GCTTGTCCAAAGGTATCCTTTGAGCCAATTCCAATACATTATTGTGCCCCGGCTGGTTTTGCGATTCTAAAAT
GTAAT---AGCAAGACATTTAGT------GGAAAAGGACCATGTACAAAT----------------------
-GTCAGT---ACAGTACAATGTACACATGGAATTAGACCAGTGGTATCAACT---
CAACTGCTATTAAATGGCAGTCTAGCAGAAAAAGAG------------
GTAGTAATTAGATCTGTCAATTTCTCGGACAAT---------GCTAAAACCATAATAGTACAGCTGAAC----
-----------AAATCTGTAGAA---------ATTAATTGTACAAGACCTAATAACAATACAAGAAAG-----
-AGTATACATATA------------GGACCAGGGAGGGCA---TTC------TATGCAACAGGAGAC------
---------ATAATAGGAAACATA-----------AGACAAGCACATTGT---AACCTTAGTGGAGTA---
GATTGGAATACCACTTTAAGCCTAATAGTTAAAAAATTAAGA---------------GAACAATTTGGG----
--------------------------------GAAAATAAAACAATAAAATTTGAG------
CGATCCTCAGGAGGAGACCCAGAAGTTGAA---ATGCACAGTTTTAATTGTGGAGGGGAA---
TTTAAGCAGCTCCAGGCTAGAGTCCTGGCTGTAGAAAGATACCTAAAGGATCAACAGCTCCTGGGAATTTGGG
GGTGCACTGGAAAACTCATTTGCACCACTGCTGTGCCTTGGAATGCTAGTTGGAGT-----------------
---------------------------AATAAAACTATGGAAAGGATTTGG------GAT------
AATATGACCTGGATGCAGTGG---GAAAAAGAAATTGACAATTACACAGGAGAGATATAC---
AGACTGCTTGAAGATGCGCAGAACCAGCAAGAA---------------------------------------
----
AAGAATGAACAAGAATTATTGGAATTGGATAAATGGGCAAGTTTGTGGAGTTGGTTTGACATAACACAATGGC
TGTGGTATATAAAAATATTCATAATGATAGTAGGAGGCTTGATAGGTTTAAGAATAGTTTTTACT---
GTACTTTCTTTAGTA---------AATAGAGTTAGGCAGGGATATTCACCA------
TTATCGTTTGCTGTTAGCTTGCTTAATGTCACAGCTATAACAGTAGCTGAA---
GGGACAGATAGGATTATAGAAGCAATACAAAGAGTTTTTAGAGCT---------------------------
--------TTTCTCAACATA------CCTAGAAGAATAAGACAGGGCTTGGAAAGGGCTTTGCTA---TAA
>JQ403107
GCT---ACA--
GAACAATTGTGGGTCACAGTCTATTATGGGGTACCTGTGTGG---AAAGAGGCAACC---
ACCACTCTGTTTTGTGCATCAGATGCTAAAGCATATGATACAGAG---GTGCATAAT----------------
--------------GTTTGGGCCACA---CATGCCTGTGTACCCACAGAC---
CCCAACCCACAAGAAGTAGTAATGGAA---------AATGTGACAGAAAATTTTAAC----------------
--------------------------ATGTGGAAAAATGACATGGCAGAGCAAATGCATGAGGATATA---
ATCAGTTTATGGGATCAAAGCCTA---AAGCCATGTGTAAAATTA---
ACCCCACTCTGTGTCACTTTACATTGCACTGATTATAATGGTACCCATATGAATTCTACT-------------
----
-------------GGGAATAACACTGATGGTTATGGGGAACGGATGAAATCAGGAGAAATGAAA---
AACTGCTCTTTCAATGTCACCACAGAAATAAGA--------------------------
GATAGAGTGAAGAAACAATCTGCACTT---
TTTTATAAACTTGATGTAGTATCAATAGATGATGATAAAATTAGGAATACCAGTAAT----------------
----
AGTAGTACCAGTAATACTAACTATACTAGCTATAGGTTGATAAATTGTAACACCTCAGCCATTACACAA---
GCCTGTCCAAAGGTATCCTTTGAGCCAATTCCCATACATTATTGTGCCCCAGCTGGTTTTGCGATTGTAAAGT
GTAAT---GATAAGAAGTTCAAT------GGAACAGGATCATGTAAAAAT----------------------

-GTCAGC---ACAGTACAATGTACACATGGAATCAGGCCAGTAGTGTCAACT---
CAACTGCTGTTAAATGGCAGTTTAGCAGAAGAAGAT------------
ATAGTAATTAGATCTGAAAATATCTCAGACAAT---------GCTAAAACCATAATAGTACATCTGAAT----
-----------AGATCTGTAGCA---------ATTAATTGTATAAGACCCAATAACAATACAAGAAAA-----
-AGTATACCTATG------------GGACCAGGGAGAGCC---TTT------TTTGCAACAGGAGAC------
---------GTAATAGGAGATATA------------AGACAAGCATATTGT---ACCATTAATAGAACA---
GAATGGAATACAACTTTAAAACAGATAGCTATAAAATTAGGA---------------GAACAATTTGAG----
-----------------------------------AATAAAAAAATAATCTTTAAT------
CACTCCTCAGGAGGGGACCCAGAAATTGTA---ATGCACAGTTTTAATTGTGGAGGGGAG---
TTTAAGCAACTCCAGGCAAGAATCCTGGCTGTGGAAAGATACCTAAAGGATCAACAGCTCCTAGGAATTTGGG
GATGCTCTGGAAAACTCATCTGCACCACTGCTGTGCCTTGGAATACTAGCTGGAGT-----------------
-------------------------AATAATAGAACTTATGATGACATTTGG------AAT------
AACCTGACCTGGATGCAGTGG---GAAAAACAAATTGACAATTACACAGACATAATATAT---
GACTTACTTGAAAAATCGCAAACCCAACAAGAA--

ATAAATGAAAAGGAATTATTGGAATTGGACAAGTGGGCAAGTTTGTGGAATTGGTTTGACATCTCAAATTGGC
TGTGGTATATAAAAATATTCATAATGATAGTAGGAGGCTTGATAGGTTTAAGAATAGTTTTTGCT---
GTACTTTCTATAGTG---------AATAGGGTTAGGCAGGATACTCACCA------
TTGTCATTTGCTATTAGCTTGCTCAATGCCACAGCCATAGCAGTAGCTGAG---
GGAACAGATAGGGTTATAGAAGTACTACAAAGAGTTTTTAGAGCT----------------------------
--------ATCCTCCACATA------CCTAGAAGAATAAGACAGGGCTTGGAAAGGTCTTTGCTA---TAA
>JN024100
GCT---ACA--
GAAAAATTGTGGGTCACAGTCTATTATGGGGTACCTGTGTGG---AAAGAAGCAACT---
ACCACTCTATTTTGTGCATCAGATGCTAAAGCATATGATACAGAG---GTACATAAT----------------
--------------GTTTGGCCACA---CATGCCTGTGTGCCCACAGAC---
CCCAACCCACAAGAAGTAGTATTGGGA---------AATGTGACAGAAAACTTTAAC----------------
--------------------------ATGTGGAGAAATGACATGGTAGAACAGATGCATGAGGATATA---
ATTAGTTTATGGGATCAAAGTCTA---AAGCCATGTGTAAAATTA---
ACCCCACTCTGTGTTACTTTAAATTGCAATGATTATAATGGTACCAATACCAATGGTACCAATACC-------
----
-------------------------AAGAATAGTAGTAACGTGGAAATGATAGGAGAAATAAAG---
AATTGCTCTTTCAACATCACCACAAACATAAGA---------------------------
GATAAGATAAAGAAAGAATATGCATTG---TTTTATAAACTTGATGTAGTACCAATAGATAAT----------
----

GATAATACTAGCTATAGGTTGATAAGTTGTAATACCTCAGTCATTACACAG---
GCCTGCCCAAAGATATCCTTTGAGCCAATTCCCATACATTATTGTGCCCCGGCTGGTTTTGCGATTCTAAAAT
GTAAC---AATAAAAAGTTCAAT------GGGACAGGACCATGTACAAAT-----------------------
-GTCAGC---ACAGTACAATGTACTCATGGAATTAGGCCAGTAGTATCAACT---
CAACTGTTGTTAAATGGCAGTTTAGCAGAGGAAGAG------------
GTAGTAATCAGATCTAAAAATTTCACGGACAAT---------GCTAAAATCATAATAGTACAGCTGAAC----
-----------GAAGCTGTAGAA---------ATTAATTGTACAAGACCCAACAACAATACAAGAAAA-----
-GGTATACATATA------------GGACCAGGAAGAGCA---TTT------TATACAACAGGAGAA------
---------ATAATAGGAGATATA------------AGACAAGCACATTGT---AACATTAGTAAAGTA---
AAATGGAATAACACTTTAGGACAGATAGTTAAAAAATTAAAA---------------GAACAATTTGAG----
-----------------------------------GATAAAACAATAATTTTTAAT------
CAATCCTCAGGAAGGGACCCAGAAGTTGTA---ATGCACACTTTTAATTGTGGAGGGGAA---
TTTAAGCAGCTCCAGGCAAGAGTCCTGGCTATAGAAAGATACCTAAAGGATCAACAGCTCCTGGGAATTTGGG
GTTGCTCTGGAAAACTCATCTGCACCACTGCTGCGCCCTGGAATTCTAGTTGGAGT-----------------
----------------------------AATAAATCTCTGAATCAGATATGG------GAT------
AACATGACCTGGATGGAGTGG---GAAAGAGAAATTGACAATTACACAAGCACAATATAC---
AGGTTAATTGAAGAATCGCAAAACCAACAAGAA--

AAGAATGAACAAGAATTATTGGAATTAGATAAATGGGCAAGTTTATGGAATTGGTTTGACATAACAAAATGGC
TGTGGTATATAAAAATATTCATAATGATAGTAGCAGGCATAGTAGGTTTAAGAATAACTTTTTCT---
ATATTTTCTATAATA---------AATAGAGTTAGGCAGGATACTCACCA------
TTGTCGTTTGCTGTTAGCTTGCTCGACGCCGCAGCCATAGTAGTAGCTGAG---
GGGACAGATAGGGTTATAGAAATATTACAAAGAATTTTCAGGGCT----------------------------
--------ATTATCCACATA------CCTGTTAGAATAAGACAGGGCTTAGAAAGGGCTTTGCTG---TAA

>HQ216601
GCT---ACA---------------------------------------
GAAAAATTGTGGGTCACAGTCTATTATGGGGTACCTGTGTGG---AAAGAAGCAACT---
ACCACTTTATTTTGTGCATCAGATGCTAAAGCATATGATACAGAG---GCACATAAC---------------
--------------GTCTGGGCCACA---TATGCCTGTGTGCCCACAGAC---
CCCAACCCACAAGAAGTATTATTGGAA---------AATGTAACAGAAAACTTTAAC---------------
-------------------------ATGTGGAAAAATAACATGGTAGAACAAATGCATGAGGATATA---
ATCAGTTTATGGGATCAAAGCCTA---AAGCCATGTGTGAAATTA---
ACCCCACTCTGTGTTACTTTAAATTGCACTGATTGGAATAATGCTACCAATACCAATGGT------------
----
---------------------------AGTGACTGGGAAAGGGTGGAGAAAGGAGAAATAAAA---
AACTGCTCTTTCAACATCACCACAAACATAAGA--------------------------
GATAAGATGCAGAAAGAATATGCATTG---TTTTATAAACTTGATATAGTACCAATAGAGAAGGATAATGAT-
----

AATAGGAATGATAATACTAGCTATAGGTTGATAAGTTGTAATACCTCAGTCATTACACAA---
GCCTGTCCAAAGGTATCCTTCGAGCCAATTCCCATACATTATTGTGCCCCGGCTGGTTTTGCGATTCTAAAGT
GTAAA---AATAAGAAGTTCAAT------GGAACAGGACCATGTAAGAAT----------------------
-GTCAGC---ACAGTACAATGTACACATGGAATTAGGCCAGTAGTATCAACT---
CAATTGTTGTTAAATGGCAGTCTAGCAGAAGAGGAA-----------
GTAGTGATCAGATCTGAAAATTTCATGAACAAT---------GCTAAAATCATAATAGTACAGCTGAAT----
-----------GAATCTGTAGAA---------ATTAATTGTACAAGACCCAACAACAATACAAGAAAA-----
-AGTATACATATA------------GGACCAGGGAGAGCA---TTT------TATGCAACAGGAGAA------
---------ATAATAGGAAATATA------------AGACAAGCACATTGT---AACATTAGTAGAGAA---
AAATGGAATAACACTTTAAGACAGATAGTTGGAAAATTAAGA--------------CAACAATTTGGG----
-----------------------------------AATAAAGCAATAATCTTTAAT------
CAATCCTCAGGAGGGGACCCAGAAGTTGTA---AAACACAGTTTTAATTGTGGAGGGGAG---
TTCAAGCAGCTCCAGGCAAGAGTCCTGGCTCTAGAAAGATACCTAGCGGATCAACAGCTCCTGGGAATTTGGG
GCTGCTCTGGAAAACTCATTTGCACCACTGCTGTGCCTTGGAATTCTAGTTGGAGT----------------
---------------------------AATAAATCTCTGAATCAGATTTGG------GAT------
AACATGACCTGGATGGAGTGG---GAAAGAGAAATTGACAATTACACAGACACAATATAC---
AGATTAATTGAAGACTCGCAAAACCAACAAGAA---------------------------------------
----
AAGAATGAACAAGAATTATTGAAATTAGATCACTGGGCAAGTTTGTGGAATTGGTTTGACATAACAAACTGGC
TGTGGTATATAAAAATATTCATAATGATAGTAGGAGGCTTAGTAGGTTTAAGAATAATTTGTTCT---
ATACTTTCTATGGTA---------AATAGAGTTAGGCAGGGATACTCACCA------
TTATCGTTTGCTGTTAGCTTGCTTAACGCCACAGCCATAGCAGTAGCTGAG---
GGGACAGATAGGGTTATAGAAATATTACAAAGAATTCTTAGGGCT---------------------------
--------ATTATCCACATA------CCTGTTAGAATAAGACAGGGCTTAGAAAGGGCTTTGCTG---TAA
>HQ216795
GTT---GCA---------------------------------------
GGACAATTGTGGGTCACAGTCTATTATGGGGTACCTGTGTGG---AAAGAAGCAACC---
ACCACTCTATTTTGTGCATCAGATGCTAAAGCATATGACACAGAG---GCACATAAT---------------
--------------GTTTGGGCCACA---CATGCCTGTGTACCCACAGAC---
CCTAACCCACAAGAAGTAGTATTGCAA---------AATGTGACAGAAAATTTTAAC---------------
-------------------------ATGTGGAAAAATGACATGGTAGAACAGATGCATCAGGATGTA---
ATCAGTTTATGGGATGAAAGCTTA---AAGCCATGTGTAAAATTA---
ACCCCACTCTGTGTTACTTTAAGTTGCACTGATGTAAATACTACTACTACCAATAATGTAAATACTACTACTA
CC--
-------------AGTAATAATAGTACCAATGATCCAATGTTGGAACAGACAGGAGAAATAAAA---
AACTGCTCTTTCAATGTCACCACAAGCATAAGA--------------------------
GATAAGCAAAAGAAAGAATATGCGCTT---TTTTATAGACTTGATGTAGTACCAATAGATGGG---------
----

AAACAAAATAGTACAGACTATACATTGATAAATTGTAACACCTCAGTTATCACACAG---
GCTTGTCCAAAGGTGTCCTTTGAACCAATTCCTATACATTATTGTACCCCGGCTGGTTTTGCGCTTCTAAAGT
GTAAA---GATAAGAAGTTCAAT------GGAACAGGACCTTGTAAAAAT----------------------
-GTCAGT---ACAGTACAATGTACACATGGAATTAGGCCAGTAGTGTCAACT---
CAATTGCTGTTAAATGGCAGTCTAGCAGAAGAAGAA-----------
GTAGTACTTAGATCTGAAAATTTTACAAACAAT---------GCTAAAACCATAATAGTACAGCTGAAG----

-----------GAAGCTGTAGTA---------ATGAATTGTACAAGACCCAACAACAATACAAGAAGA-----
-AGTATACATATA------------GCACCAGGGAGAGCA---TTT------TATGCAACAGGAGAA------
---------ATAATAGGAGATATA------------AGACAAGCCCATTGT---AACATTAGTATAGAA---
AAGTGGAATAAAACTTTAAGAGGGATAGCTACAAAATTAAAA---------------GAACAATTTAAG----
-----------------------------------AATAAAACAATATACTTTAAT------
CAATCCTCAGGAGGGGACGCAGAAGTTGTA---ATGCACAGTTTTAATTGTGGAGGAGAA---
TTTAAGCAGCTCCGAGCAAGAGTCCTGGCTGTGGAAAGATACCTAAAGGATCAACAGCTCCTAGGAATTTGGG
GTTGCTCTGGAAAACTCATTTGCACCACTACTGTGCCTTGGAATGCTAGTTGGAGT-----------------
----------------------------AATAAATCTCTAGATTATATTTGG------AAT------
AACATGACCTGGATGCAGTGG---GAGAGAGAAATTGACAATTATACAGGATTAATATAC---
ACCTTACTTGAAGAATCGCAAAACCAACAAGAA--

AAGAATGAACAAGAATTATTGGAATTGGATAAGTGGACAAGTTTGTTGAATTGGTTCTCTATAACAAGCTGGC
TGTGGTACATAAAAATATTCATAATGATAGTAGGAGGTTTAATAGGTTTAAGAATAGTTTTTTCT---
GTGCTTTCCATAGTG---------AATAGAGTTAGGCAGGGATACTCACCA------
TTATCATTTGCTGTTAGTTTGCTTAATACCATAGCTATAGTAGTAGCTGAA---
GGGACAGATAGGGTTATAGAAATAGCACAAAGATTAGTTAGAGCT----------------------------
--------ATTATCCACATA------CCTAGAAGAATAAGACAAGGCTTAGAAAGGGCTTTGCTA---TAA
>JX863965
GTT---GCA--
GGAAATTTGTGGGTCACAGTTTATTATGGAGTACCTGTGTGG---AAAGAAGCAACC---
ACCACTCTATTTTGTGCATCAGATGCTAGAGCATATGATACAGAG---GCACATAAT----------------
--------------GTTTGGGCCACA---CATGCCTGTGTACCCACAGAC---
CCCAATCCACAAGAAATATTATTGGAA---------AATGTGACAGAAAATTTTAAC----------------
--------------------------ATGTGGAAAAATAACATGGTAGAACAGATGCATGAGGATATA---
ATCAGCTTATGGGATCAAAGCCTA---AAGCCATGTGTAAAATTA---
ACCCCACTCTGTGTTACTTTAAATTGCACTAATGTGAGGAATATTACTGCCACTAATAATACTACT-------
----
----------------------------AATAATGTGACAACGATGGAAAAAGGAGAAATAAAA---
AACTGCTCTTTTAATATCACCACACACAGAAAT---------------------------GAGAGGCTA---
AAAGACTATGCATTG---TTTTATAAACTTGATGTAGTACCAATGGATGAG----------------------
----
-AATAATACTACTAGTACTCGCTATATGTTGATAAGTTGTAACACCTCAGTCATTAAACAG---
GCCTGTCCAAAAGTAACCTTTGAGCCAATTCCCATACATTATTGTGCCCCGGCTGGTTTTGCGATTCTAAAGT
GTAGA---GACAAGAAGTTCAAT------GGAACAGGACCATGTAAAAAT-----------------------
-GTCAGC---ACAGTACAATGTACACATGGGATTAGGCCAGTAGTATCAACC---
CAACTGCTGTTAAATGGCAGTCTAGCAGAAGAAGAG------------
GTAGTAATTAGGTCTGCCAATTTCACGGACAAT---------GCTAAAACCATAATAGTACAGCTGAAA----
-----------GACCCTGTAGTA---------ATTAATTGTACAAGACCCAACAACAATACAAGAAAA-----
-AGTATACCTATG------------GGACCAGGGAGAGCC---TTT------TATGCAACAGGAGAT------
---------ATAACAGGAGATATA------------AGACAAGCACATCGT---AACCTTAGTAGAACA---
GCATGGAATAACACTTTAAGACAGATAGTTAGCAAATTAAGA---------------GGACAATTTAAG----
-----------------------------------AATAGAACAATAGTCTTTAAT------
CAATCCTCAGGAGGGGACCCAGAAATTGTA---ACACACAGTTTTAATTGTGGAGGGGAA---
TTTAAGCAGCTCCAGGCAAGAGTCCTGGCTGTGGAAAGCTACCTAAAGGATCAACAGCTCCTAGGGATCTGGG
GTTGCTCTGGAAAACTCATCTGCACCACTGCTGTGCCTTGGAATACTAGTTGGAGT-----------------
----------------------------AATAAATCTCTAGATGATATTTGG------AAT------
AACATGACCTGGATGGAGTGG---GACAGAGAAATTAGCAATTACACAAACATAATATAC---
ACCTTAATTGAAGAATCGCAGAACCAACAGGAA--

AAGAATGAACAAGAATTATTGGCCTTAGATAAATGGGCAAGTTTGTGGAATTGGTTTGACATAACAAAGTGGC
TGTGGTATATAAAAATATTCATAATGATAGTAGGAGGCTTGGTAGGTTTAAGAATAGTTTTTGCT---
GTACTTTCTATAGTG---------AATAGAGTTAGGCAGGGATACTCACCT------
TTATCATTCGCTATTAGCTTGCTTAATTTCATAGCCATAGCAGTAGCTGAG---
GGGACAGATAGGATTATAGAAGTATTACAAAGAACTTGTAGAGCT----------------------------
--------ATTTGCAACATA------CCTAGAAGAATAAGACAGGGCTTGGAAAGGGCTTTGCTA---TAA
>JF320043
GCT---ACA---GAC------------------------------------
AAAGATAAGTGGGTCACAGTCTATTATGGGGTACCTGTGTGG---AAAGAAGCAAAC---
ACTACTCTATTTTGTGCATCAGATGCTAAATCATATAGCACAGAG---GCACATAAT----------------

--------------GTTTGGGCCACA---CATGCCTGTGTACCCACAGAC---
CCCAACCCACAAGAGGTAGAATTGAAA---------AATGTAACAGAAAAATTTAAC---------------
-------------------------ATGTGGAAAAATAACATGGTTGAACAAATGCATGAGGATATA---
ATCAGTTTATGGGATCAAAGCCTA---AAGCCATGTGTAAAATTA---
ACCCCACTCTGTGTTACTTTAAATTGCACTGACGCGGAGAGGAATAAGACTAATGCC---------------
----
---------------------------ACAGGTGTAAATCTGATAGAGAAAGGAGAAATAAAA---
AATTGCTCTTTCAATGTCACCACAGGAGTAAGA-------------------------
GATAAGATAAAGAGAGATTATGCACTT---TTTTATAGCCTTGATGTGATACAAATAAATGATACAGATGCA-
----

GTAAGTAATACCAGCAATAATTATACCAGCTATATGTTGAGAAGTTGTAACACCTCAATCATTACACAA---
GCCTGTCCAAAGGTAACCTTTGAGCCAATTCCCATACATTTTTGTACCCCGGCTGGTTTTGCGCTTCTACAAT
GTAAT---GAGAAGAATTTCAAT------GGATCAGGACTATGTACAAAT----------------------
-GTCAGC---ACAGTACAATGTACACATGGAATTAGGCCAGTAGTATCAACT---
CAACTGCTGTTAAATGGTAGTCTAGCAAAAGATGAG-----------
GTAGTAATTAGATCTCAAAATTTCTCAGACAAT---------GCTAAAACCATATTAGTACAGCTGAAC----
-----------AAAACTATAGAA---------ATTAATTGTACAAGACCCAACAACAATACAAGAAGA-----
-AGTATAACTATA------------GGACCAGGGAGGGTC---TTC------TATCCAACA---GAC------
---------ATAATAGGAGATATA-----------AGACAAGCACATTGT---AACATTAGTGCATCA---
AAATGGAACGACACTTTAAGACTGGTAGTTGCAAAATTAAGA--------------GAACAATATGGG----
---------------------------------AGTAATAAAACAATAGTCTTTAAG------
AATTCCTCAGGAGGGGACCCAGAAATTGTG---ATGCATAGTTTTAATTGTGGAGGGGAA---
TTTAAGCAGCTCCAGGCAAGAGTCCTGGCTGTGGAAAGATACCTAAAGGATCAACAGCTCCTGGGAATTTGGG
GATGCTCTGGAAAACTCATCTGCACCACTGCTGTGCCTTGGAATAATAGTTGGAGT----------------
---------------------------AATAGATCTCTAGATGACATTTGG------AAC------
AACATGACCTGGATGCAGTGG---GAAAAAGAAATTGAAAATTACACAGAGTATATATAC---
AATTTAATTGAAATATCGCAAAACCAGCAAGAA---------------------------------------
----
AAGAATGAACAGGAATTATTGGAATTGGACAAATGGGCAAACTTGTGGAGTTGGTTTGACATAACAAACTGGC
TGTGGTACATAAGAATATTCATAATGATAGTAGGAGGCTTGATAGGTTTAAGGATAGTGTTTGCT---
GTACTTTCTATAGTA---------AATAGAGTTAGGAAGGGATACTCACCA------
TTGTCGTTTGCTGTTAGCTTGTTCAACGCCACAGCAATAGCAGTAGCTGAG---
GGGACAGATAGGATTATAGAATTAGCACAAGGAATTGGTGGAGCT--------------------------
--------ATTCTCAATATA------CCTAGAAGAATTAGACAGGGCTTGGAAAGGGCTTTACTA---TAA
>EU577873
ACT---GAC--
GAAAAGATGTGGGTCACAGTCTATTATGGGGTACCTGTGTGG---AAAGAAGCAACC---
ACCACTCTATTTTGTGCATCAGATGCTAAAGCATATCATACAGAG---GTACATAAT----------------
--------------GTATGGGCCACA---CATGCCTGTGTACCCACAGAT---
CCCAACCCACAGGAAATGAAATTGGAA---------AATGTAACAGAAGAGTTTAAC---------------
-------------------------ATGTGGAAAAATAACATGGTAGAACAGATGCATGAGGATATA---
ATAAGTTTATGGGATCAAAGCCTA---AAGCCATGTGTAAAATTA---
ACTCCACTCTGTGTTACTTTAAATTGCTCTGATGCTCATACTGGGAATGATACTAACTCCAACAGT-------
----
---------------------ACTGCAAGCAACAGGACAGTAGGGGAGAAAGGAGAAATGAAA---
AACTGTTCTTTCAATGTCACATCAGGCATAAGA-------------------------
GATAAGATGCAACAGGTGTATGCACTT---TTTTATAAACTTGATGTAGTACCAATAGATAATGATAGT----
----

AGTAGTAATACTAGTGCCAGTTATAGGTTGATAAATTGTAATACCTCAGTCATTACACAG---
GCCTGTCCAAAGGTATCCTTTGAGCCAATTCCCATACATTATTGTGCCCCGGCTGGTTTTGCGATTCTAAAAT
GTAAT---GATAAGACATTTGAG------GGAAAAGGAGAATGTAAAAAT----------------------
-ATCAGC---ACAGTACAATGTACACATGGCATTAGGCCAGTAGTATCAACA---
CAACTACTGTTAAATGGCAGTCTAGCAGAAGAAGAG-----------
GTAGTAATTAGATCTGACAATTTCACAGACAAT---------GCTAAAACCATAATAGTACAGTTGAAT----
-----------GAAACTGTACAG---------ATTAACTGCACAAGACCCGGTAACAATACAAGAAAA-----
-AGTATACATATA------------GGACCAGGGCAAGCA---TTT------TATGCAACAGGAGAC------
---------ATAATAGGAGATATA-----------AGACAAGCACATTGT---AACATTAGTAAAACA---
CAATGGGAGAGCACTTTAAAAAAGGTAGCTGAAAAATTAAGA--------------GGAAAATTTAAG----

-----------------------------------AATAAAACTATAGTCTTCAAC------
CACTCCTCAGGAGGGGACCCAGAGATTGTA---ATGCACAGTTTCAATTGTGGAGGGGAA---
TTTAAGCAGCTCCAGGCAAGAGTCCTGGCTGTGGAAAGATACCTAAGGGATCAACAGCTCCTGGGGATTTGGG
GATGCTCTGGAAAGCTCATCTGCCCCACTGCTGTGCCTTGGAATGCTAGTTGGAGT-----------------
----------------------------AATAAATCTTTCAATCAGATTTGG------GAT------
AACATGACCTGGATGCAGTGG---GAGAAAGAAATTGACAATTACACGGAAGTGATATAT---
AACTTAATTGAAAAATCGCAAAACCAACAAGAA--

AAGAATGAACAGGAATTATTGGAATTGGATAAATGGGCAAGTTTGTGGAATTGGTTTGACATAACACAATGGC
TGTGGTATATAAAAATATTCATAATGATAGTAGGAGGCTTGATAGGCTTAAGAATAGTTTTTACT---
GTACTTTCCATAGTG---------AATAGAGTTAGGCAGGGATACTCACCA------
TTATCATTTGCCATTAGCTTGTTCAACGCCACAGCCATAGCAGTAGCTGAG---
GGGACAGATAGGGTTATAGAAGTATTACAAAGAGTTGGTAGAGCT----------------------------
--------ATTCTCCACATA------CCTACAAGAATAAGACAAGGCTTGGAAAGGCTTTTGCTA---TAA
>KT124778
------GAA--
GACAAGTTGTGGGTCACAGTCTATTATGGGGTACCTGTGTGG---AAAGATGCAAAC---
ACCACTTTATTTTGTGCATCAGATGCTAAAGCATATGATACAGAG---GTACATAAT----------------
--------------GTTTGGGCCACA---CATGCCTGTGTACCCACAGAC---
CCCAACCCACAAGAAGTAGTATTGGGA---------AATGTGACAGAAAAATTTAAC----------------
--------------------------ATGTGGAAAAATAACATGGTAGAACAGATGCATGAGGATATA---
ATTAGTTTATGGGATCAAAGTCTA---AAGCCATGTGTAAAATTA---
ACCCCACTCTGTGTTACTTTAAATTGCATTGATACCAATACTACTAAAACCAAT-------------------
----
-------------------------AGTAGTATGGGGGAAATGATGGAGAAAGGAGAAATAAAA---
AACTGCTCTTTCAATATCACCATAAACAAATGG---------------------------
GACAAGATGCAGAAAACATATGCACTT---TTTTATAAATCTGATGTAGTACCAATAAATGATACA-------
----

GGTAATTCTAGCTATAGGTTGATACAATGTAACACCTCAGTCATTACACAG---
GCCTGTCCAAAGGTATCCTTTGAGCCAATTCCCATACATTATTGTGCCCCGGCTGGTTATGCGATTATAAAGT
GTAAC---AATAAGCAGTTCAAT------GGAACAGGACAATGTACAAAT-----------------------
-GTCAGC---ACAATACAATGTACACATGGAATTAGGCCAGTAGTGTCAACT---
CAACTGCTGTTAAATGGCAGTCTAGCAGAAGAAGAG------------
ATAGTAATTAGATCTGAAAATTTCTCGAACAAT---------GCTAAAACCATAATAGTACAGCTGAAG----
-----------GAACCTGTACAA---------ATTAATTGTACAAGACCCAACAATAATACAAGAAGG-----
-AGTATACATATA------------GGACCAGGGAGAGCA---TTT------TATGCAACAGGAGAT------
---------ATAATAGGACCCATA------------AGAAAAGCATATTGT---AACGTTAGTAAAACA---
AGATGGAATGACACTTTACAACAGGTAGCTACAAAATTAAGA---------------GCACAATTTGGG----
-----------------------------------AATAAAACAATAGTCTTTAAG------
CACTCCTCAGGAGGGGACCCAGAAGTGGTA---ATGCACAGTTTTAATTGTGGAGGGGAA---
TTTAAACAGCTCCAGGCAAGAGTCCTGGCTGTGGAAAGATACCTAAAGGATCAACAGCTTCTAGGAATTTGGG
GTTGCTCTGGAAAACTCATTTGCACCACTGCTGTGCCTTGGAATGCTAGTTGGAGT-----------------
----------------------------AATAAATCTATGGATGACATTTGG------GGT------
AACATGACCTGGATGGAGTGG---GAAAGAGAAATTGACAATTACACAGGCTTAATATAC---
ACCTTAATTGAAGAATCGCAGAACCAGCAAGAA--

AAGAATGAACTAGAATTATTGGAATTAGATAAGTGGGCAAATTTGTGGAATTGGTTTGACATAACAAATTGGC
TGTGGTATATAAAAATATTCATAATGATAGTAGGAGGCTTGATAGGTTTAAGAATAGTTTTTACT---
GTGCTTTCTATAGTA---------AATAGAGTTAGGAAGGGATACTCACCA------
TTATCATTTGCTGTTAGCTTGCTTAATGCTACAGCTATAGCAGTAGCTGAG---
GGAACAGATAGGGTTATAGAAGTAGCACAAGGAGCTTTTAGAGCT----------------------------
--------ATTCTCCACATA------CCTACACGAATAAGACAGGGCTTGGAAAGAGCTTTGCTA---TAA
>DQ410562
GCT---GCA--
GAACAATTGTGGGTCACAGTCTATTATGGGGTACCTGTGTGG---AAAGAAGCAACC---
ACCACTCTATTTTGTGCATCAGATGCTAAAGCATATGATACAGAG---ATGCATAAT----------------
--------------ATTTGGGCCACA---CATGCCTGTGTACCCACAGAC---
CCCAACCCACAAGAATTAGTATTGGAA---------AATGTGACAGAAGATTTTAAC----------------
--------------------------ATGTGGAAAAATAACATGGTAGAACAGATGCATGAGGATATA---

ATCAGTTTATGGGATCAAAGTCTA---AAGCCATGTGTAAAATTA---
ACCCCACTCTGTGTTACTTTGAATTGTAGTGATGTGGAGAATAGTACTATGACTAATAGTACTGGGACTAATG
CT---
-------------ACTAGTGCTAATAATATTACTATTAGTAAGGAGATGGAGGGGGAAATAAAA---
AACTGCTCTTTCAATATCACCACAAACATAAGG---------------------------
GATAAGGTACAAAAAGGATATGCATTC---TTTTATAACCTTGATGTAGTAAGAATAGATGATGAT-------
----

AGTAGTCAGGATAATACTAGTTATAGGTTGATAAGTTGTAATACCTCAGTCATTACACAA---
GCCTGTCCAAAGGTATCCTTTGAGCCAATTCCCATACATTATTGTGCCCCGGCTGGTTTTGCAATTCTAAAGT
GTAAT---GATAAGAAATTCAAT------GGAACAGGACAATGTAAAAAT-----------------------
-GTCAGT---ACAGTACAATGTACACATGGAATTAGGCCAGTAGTGTCAACT---
CAACTGCTGCTAAATGGCAGTCTAGCAGAAGATGAG------------
GTAGTAATTAGATCTGAAAATTTTACAGACAAT---------GCTAAAACCATAATAGTGCAGCTAAAT----
-----------GAATCTGTAGTA---------ATTAATTGCATAAGACCCAACAACAATACAAGAAAA-----
-AGTATACATATG------------GGACCAGGGGGAGCA---TTT------TATGCAACAGGACAA------
---------ATAATAGGAAATATA------------AGACAAGCACATTGT---AATATTAATGAGACA---
ACATGGAATAACACTTTAAAAAAGGTAGCTGAAAAATTAAAA---------------GAACAATTTAAT----
-----------------------------------GCAACAAAAGTTGTCTTTAAT------
AAATCCTCAGGAGGGGACCCAGAAATTGTA---ATGCACAGTTTTAATTGTGGAGGAGAA---
TTTAAGCAGCTCCAGGCAAGAGTCCTGGCTGTGGAGAAATACCTAAAGGATCAACAGCTCCTAGGGATTTGGG
GTTGCTCTGGAAAACTCATTTGCACCACTGCTGTGCCTTGGAATACTAGTTGGAGT-----------------
----------------------------AATAAAAGTTTGAATCAAATTTGG------GAT------
AACATGACCTGGATGCAGTGG---GAAAAAGAGATTGACAATTATACAGACACAATATAT---
AGATTAATTGAAATATCGCAAAACCAACAAGAA--

AAGAATGAACAAGAATTATTGGAATTGGATCAATGGGCAAGTCTGTGGAATTGGTTTGACATAACAAAGTGGC
TGTGGTATATAAAAATATTCATAATGATAGTAGGAGGCTTGATAGGTTTAAGAATAGTTTTTGCT---
GTGCTTTCTATAGTA---------AATAGAGTTAGGCAGGGATACTCACCA------
TTATCGTTTGCTGTTAGCTTGCTTAACGCCACAGCCATAGCAGTAGCTGAG---
GGGACAGATAGGATCATAGAAATAGTACAAAGAATTTTTAGAGGT----------------------------
--------ATCCTCCATATA------CCTACAAGAATAAGACAGGGCTTAGAAAGGGCTTTGCTA---TAA
>FJ469717
ACT---GCA--
GGACAATTGTGGGTCACAGTCTATTATGGGGTACCTGTGTGG---AAAGAAGCAACT---
ACCACTCTATTCTGTGCCTCAGATGCTAAAGCATATGATACAGAG---GTACATAAT----------------
--------------GTTTGGGCCACA---CATGCATGTGTGCCCACAGAC---
CCCAACCCACAAGAAATAGAATTGAAA---------AATGTGACAGAGAATTTTAAC----------------
--------------------------ATGTGGAAAAATGACATGGTGGAACAGATGCAGGAGGATATA---
ATTAGTTTATGGGACCAAAGCCTA---AATCCATGTGTAAAATTA---
ACCCCACTCTGTGTTACTTTACATTGCACTAATTATACCAGGAATCATAATAATAGCACTAGT----------
----
-------------------------GCTAATAGCACTAGGGTAGAAAATAAAGGAGAAATAAAA---
AACTGCTCTTTCAATACCACCACAGGCATAAGA---------------------------
GATAAGATAAAGAAAGAACATGCACTT---TTTTATACAGATGATGTAGTACCAATAGAGGAT----------
----

AATAATGATAGTATTAGCTATAGGTTAATAAATTGTAATACCTCAGTCATTACACAA---
GCCTGTCCAAAGGTGTCCTTTGAACCAATTCCCATACATTATTGTGCTCCAGCTGGTTTTGCGATGCTAAAAT
GTAAG---GATAAAAAATTCAAT------GGAACAGGACCATGTAAAAAT-----------------------
-GTCAGC---ACAGTACAATGTACACATGGAATTAGACCAGTAGTGTCAACT---
CAACTGTTATTAAATGGCAGTCTAGCAGAAAAAGAT------------
GTAGTAATTAGATCTGAAAATTTTACTGATAAT---------ACTAAAAACATAATAGTACAGTTGAAC----
-----------AAAACTGTACAA---------ATTACCTGTATAAGACCCAACAACAACACAAGAAAA-----
-AGTATAACTATA------------GGACCAGGGAGAACA---TTC------TTTGCAGCAGGAGAC------
---------ATAATAGGAGATATA------------AGACAAGCATATTGT---AACATTAGTGGAACA---
AAATGGAATGACACTTTAAAACAGATAGTTATAAAATTAAAA---------------
GAACAATTGGAGAAATTG------------------GGAAAT------AAGACTAATAAGATTGCCTTTAAG-
-----CAATCCTCAGGAGGGGACCCAGAAATTGTA---ATACACAGTTTTAATTGTGGAGGGGAA---
TTTAAGCAGCTCCAGGCAAGAGTCCTGGCTGTGAAAAGATACCTAAGGGATCAACAGCTCCTAGGAATTTGGG

GTTGCTCTGGAAAGCTCATCTGCACCACTGCTGTGCCTTGGAATGCTAATTGGAGT-----------------
----------------------------GATAGATCTGTAGATGACATTTAG------AAT------
AACATAACCTGGATGCAGTGG---GAGAGAGAAATTGACAATTACACAGAAATAATATAC---
AACTTAATTAAGAAATCGCAAAACCAACAAGAA--

CAGAATAAAAAAGATTTATTAGAGTTAGATTCATGGGCAAATTTGTGGAATTGGTTTCACATATCAAACTGGC
TGTGGTATATAAGAATATTCATAATGATAGTAGGAGGTTTAATAGGTTTAAAAATAGTTTTTGCT---
GTACTTTCTATAGTA---------AATAGAGTTAGGCAGGATACTCACCA------
TTATCGTTTGCCATTAGCTTGTTCAATGCCACAGCTATAGCAGTAGCTGAA---
GGGACAGATAGGGTTATAGAAATAGCAAGCAGAATTTTTAGAGCT----------------------------
--------TTTCTCCACATA------CCTAGAAGAATAAGACAGGGCTTAGAAAGG---------------
>FJ469724
GCT---GCA--
AATGATTTGTGGGTCACAGTCTATTATGGGGTACCTGTGTGG---AAAGAAGCAACC---
ACCACTCTATTTTGTGCATCAGATGCTAAAGCATATGATAAAGAG---GCACATAAT----------------
--------------GTTTGGGCCACA---CATGCCTGTGTACCCACAGAC---
CCCAACCCACAAGAAATACTATTGGAA---------AATGTGACAGAAAATTTTAAC----------------
--------------------------ATGTGGAAAAATAACATGGTAGAACAAATGCATGAGGATATA---
ATCAGTTTATGGGATGAAAGCCTA---AAACCATGTGTAAAATTA---
ACCCCACTCTGTGTTACTTTAAATTGTACTGATGCCACTATTAGAAATGAAACTATTGAT-------------
----
-------------------------AATGCTAGAGAGAACGGGACGAAGGTAGAAGAAATAAAA---
AATTGCTCTTTCTATGTCCCCACAAACATAAAA---------------------------
GATAGGATGCGAAAGGTATATGCAACT---
TTTTATAAACTTGATCTAGAACCAATAGGTGATGATAGTGCTAGTAATACTAATAGT----------------
----
AGTAATAGTACCAGTAATAATAGTAGTAATTATACATTGATAAGTTGTAACACCTCAGTCATCACACAG---
GCCTGTCCAAAGATATCCTTTACGCCAATTCCCATACATTATTGTGCCCCGGCTGGTTTTGCGCTTCTAAAGT
GTAAT---GATAAGAAGTTCAAT------GGAACAGGACCATGTAAAAAT-----------------------
-GTCAGC---ACAGTACAATGTACACATGGAATTAGGCCAGTAGTATCAACT---
CAACTACTGTTAAATGGCAGTCTAGCAGAA---GAG-----------
GTAGTAATTAGATCTGAAAATCTCACGAAAAAT---------ACTAAAACCATAATAGTACAGCTGAAA----
-----------GACCCTGTAAAA---------ATTAATTGCATAAGACCCGGCAACAATACAAGAAAA-----
-AGTATACCTATG------------GGACCAGGGAGAGCA---TGG------TTTGCAACAGGAGAC------
---------ATAATAGGAGATATA------------AGACAAGCACATTGT---AACATTAGTGAAGCA---
GAATGGAATGACACTTTGAAACAGGTAGCTAAAAAATTAGGA---------------GAACAATTGAAT----
-----------------------------------ATTAAGACAATAATCTTTAAT------
AACCACTCAGGAGGGGACCCAGAAATTGCA---ATGCACAGCTTTAATTGTGGAGGGGAA---
TTTAAGCAGCTCCAGGCAAGAGTCCTGGCTGTGGAAAGATACCTAAAGGATCAACAGCTCCTGGGAATTTGGG
GTTGCTCTGGAAAACTCATCTGCACCACTGCTGTGCCTTGGAATACTAGTTGGAGT-----------------
----------------------------AATAAATCTGTGCATGACATTTGG------GAT------
AACATGACCTGGATGGAGTGG---GAAAGAGAAATTAGCAATTACACAGACACAATATAT---
GACTTGATTGCAAAATCGCAGATTCAACAAGAA--

AAAAATGAACAAGAATTATTGGAATTAGATAAATGGGCAAACTTGTGGAATTGGTTTGACATATCAAATTGGC
TGTGGTATATAAGAATATTCATAATGATAGTAGGAGGCTTGATAGGTTTAAGAATAGTTTTTTCT---
GTGCTTTCTATAGTA---------AAGAGAGTTAGGCAGGGATACTCACCA------
TTATCGTTTGCTGTTAGCTTGTACAACGCAATAGCCATCGCAGTAGCTGAG---
GGGACAGATAGGGTTATAGAAGTAGTACAAAGAGCTTTTAGGGCT----------------------------
--------ATTCTCCATATA------CCTACAAGAATAAGACAGGGTTTGGAAAGAGCTTTGCTA---TAA
>FJ653159
GCT---AAG---AAA------------------GAA---GAA---GAG---
GAACAGTTGTGGGTTACAGTCTATTATGGGGTACCTGTGTGG---AAAGAAGCAACC---
ACCACTCTATTCTGCGCATCAGATGCTAAAGCATATAAGACAGAG---GTGCATAAT----------------
--------------GTATGGGCCACG---CATGCCTGTGTACCCACAGAC---
CCCAACCCACAGGAAGTGAACTTGGCA---------AATGTGACAGAGAATTTTAAC----------------
--------------------------GTGTGGAAAAATAACATGGTTGAACAGATGCATGAGGATATA---
ATCAGTTTATGGGATCAAAGCCTA---AAACCATGTGTAAAGCTA---
ACCCCACTCTGTGTTACTTTAAATTGCATTAATGTTAATGTTACAAAGGGTAATTCCACAAATGGTACT----
----

----------------GATCCCACTATATCCACTATACTGACAGAAACAAAAGGAGAAATAAAA---
AACTGCTCTTTCAATGTCACCGCAGGTATAAGG---------------------------
GATAAGGTGCAGAAACAACATGCACTT---TTTTATAGCCTTGATGTAGTACCAATAGATAAT----------
----

AGCAACGACACCTATAGGTTGGTAAGTTGTAATACCTCAGTCATTACACAG---
GCCTGTCCAAAGATATCCTTTGAGCCAATTCCCATACATTTTTGTGCCCCAGCTGGTTTTGCAATTCTAAGGT
GTAAT---AATGAGACGTTCAAT------GGAACAGGACCATGTACAAAT-----------------------
-GTCAGC---ACAGTACAATGTACCCATGGGATTAGGCCAGTAGTGTCAACT---
CAACTACTGTTAAATGGTAGTCTATCAGAAGGGGAG------------
GTAATGATTAGATCTGAAAATTTCACAAACAAT---------GCTAAAACCATAATAGTGCAGCTAAAT----
-----------GAAATTGTACAG---------ATTAATTGTAGCAGACCCAACAACAATACGAGAAAA-----
-GGCATACATATA------------GGACCAGGGAGAGCA---TTT------TATACCACAGGAGAC------
---------ATAATAGGAGACATA------------AGAAAAGCACATTGT---AATATTAGTAGAGGA---
CAATGGAATAACACTCTAAAAAAGATAGTTATAAAATTAAGA---------------GAGCAATTTGGG----
-----------------------------------AATAAGACAATAAGCTTTAAT------
CAATCATCAGGAGGGGACCCAGAAATTGTA---ATGCACAGTTTCAATTGTGGAGGGGAG---
TTCAAGCAGCTCCAGGCAAGAGTCCTGGCTGTGGAAAGATACCTAAAGGATCAACAGCTCCTAGGGATTTGGG
GTTGCTCTGGAAAACTCATTTGCACCACTACTGTGCCTTGGAATAAAACTTGGAGC-----------------
----------------------------AAGAAATCTTATAATGAGATTTGG------GAT------
AACATGACCTGGATGCAGTGG---GAAAAAGAAATTGACAATTACACAGACTTCATATAC---
TCCTTAATTGAAGATGCACAGAACCAACAAGAA--

AAGAATGAGCAAGACTTATTAGCATTAGATAAGTGGGCAAGTCTGTGGAATTGGTTTAACATAACAAACTGGC
TGTGGTATATAAAAATATTCATAATAATAGTAGGGGGTTTGATAGGTTTAAGAATAGTTTTTACT---
GTATTTGCTATAATA---------AATAGAGTTAGGCAGGGATACTCACCC------
TTGTCATTTGCTATTAGCTTGCTCAACGCCACAGCCATAACAGTAGCTGAA---
GGGACAGATAGGATTATAGAAGTATTACAAAGATTTGGTAGAGCT----------------------------
--------ATTATTCACATA------CCTAGAAGACTAAGACAGGGCTTAGAAAGGGCTTTGCTA---TAA
>FJ653573
GCT---GCA--
GAAAACTTGTGGGTCACAGTATATTATGGAGTACCTGTGTGG---AAAGAAGCAAAC---
ACCACTCTCTTTTGTGCATCAGATGCTAAAGCATATGAGACAGAG---AAGCATAAT----------------
--------------GTCTGGCCACA---CATGCCTGTGTACCCACAGAT---
CCCAACCCACAAGAAATAGTAATGGGA---------AATGTGACAGAAAATTTTAAC----------------
--------------------------ATATGGAAAAATAACATGGTAGATCAGATGCATGAGGATATA---
ATTAGCTTATGGGATCAAAGCTTA---AAGCCATGTGTAAAATTA---
ACCCCACTATGTGTTACTCTAAATTGCACTGATAAGTTGAGTACTACTGGGAATAATACT-------------
----
----------------------------GTGGAAAAGAATGATACTAATATGGGAGAAATAAAA---
AACTGCTCTTTCAATATTACCACAGGCATAAGA---------------------------
GATAAGGTGCAGAAAGAATATGCTCTT---TTATATAAACTTGATGTAGTACCAATAGAGGATAGTGGAAAC-
----

AATAGTGATAGTAACTATAGTAGCTATAGGTTGATAAACTGTAATACCTCAGTCATTACACAA---
GCCTGTCCAAAGGTATCTTTTGAGCCAATTCCCATACATTATTGTGCCCCGGCTGGTTTTGCGATTCTAAAGT
GTAAT---GATAAGATGTTCAAT------GGATCAGGACCATGTACAAAT-----------------------
-GTCAGC---ACAGTACAATGTACACATGGAATTAGGCCAGTAGTGTCAACT---
CAATTGCTGCTAAATGGCAGCCTAGCAGAAGAAGAT------------
GTAGTAATTAGATCTGAAAATATCACAAACAAT---------GCTAAAACCATAATAGTACAGCTGAAA----
-----------GAAACTGTAGAA---------ATTAATTGCACAAGACCCAACAACAATACAAGAAAA-----
-GGTATACATATA------------GGACCAGGGAGAGCA---TTT------TTTGCAACAGGAGAT------
---------ATAATAGGAGATATA------------AGACAAGCATATTGT---AACATTAGTAGAGCA---
GAGTGGAATGACACTTTAAAACAGGTAGTGAACAAATTAAGA---------------GAGAAATTTAAG----
-----------------------------------AATAAAACAATAGCCTTTAGA------
CCATCCTCAGGAGGGGACATAGAAATTGTG---ACTCACAGTTTTAATTGTGGAGGGGAA---
TTCAAACAGCTCCAGGCAAGAGTCCTGGCTGTGGAAAGATACCTAAGGGATCAACAGCTCCTAGGGATTTGGG
GTTGCTCTGGAAAACTCATCTGCACCACTAATGTGCCTTGGAACTCTAGTTGGAGT-----------------
----------------------------AATAGATCTCTGAGTGAGATTTGG------GAT------
AACATGACCTGGATGGAGTGG---GAAAGAGAAATTGACAAAGATCTGCAGGTAATATAT---

AACTTACTTGAAGTATCACAAAACCAGCAAGAA---

AAGAATGAACAAGAATTATTAGAATTGGATAAGTGGGCAAGTTTGTGGAATTGGTTCAGCATAACAAATTGGC
TGTGGTATATAAAAATATTCATAATGATAGTAGGAGGCTTGATAGGTTTAAGAATAGTTTTTGCT---
GTGCTTTCCATAGTA---------AATAGAGTTAGGCAGGGATATTCACCA------
TTGTCGTTTGCTGTTAGCTTGCTTGATACCACAGCAATAGCAGTAGCTGAG---
GGGACAGATAGGATTATAGAAGGATTAAGAACAATTGTTAGAGCT----------------------------
--------TTTCTCCACATA------CCTAGAAGAATAAGACAGGGTTTGGAAAGGGCTTTGCTA---TAA
>EU578629
GCT---GTA---
GAACAAACGTGGGTCACAGTCTATTATGGGGTACCTGTGTGG---AAAGAAGCAAGC---
ACCACTCTATTTTGTGCCTCAGATGCTAAAGCATATGACACAGAG---GCGCATAAT----------------
--------------GTCTGGGCCACA---CATGCCTGTGTGCCCACAGAC---
CCCAGCCCACAAGAAGTAAAATTGGAA---------AATGTGACAGAAAATTTTAAT----------------
--------------------------ATGTGGAAAAATGACATGGTAGAACAGATGCATGAGGATATA---
ATCAGTTTATGGGATCAAAGCCTA---AAGCCATGTGTGAAAATA---
ACCCCACTCTGTGTTACTCTAAATTGCACTCACAAAATTAGCGTTAAAAATGTAACTTAC-------------
----
----------------------------AGCACTAATCTGAAAAATATTACTGAAGATATGAAA---
AACTGCTCTTTCAATACCACCACAATCTTAAGA--------------------------
GATAAGGTGACAAAAGAATATGCGCTA---TTTTATAAATCTGATGTAGTACCATTAGATAAT----------
----

GATAATACCAGCTATAGATTGATAAATTGTAACACCTCAACCATTACACAG---
GCCTGTCCAAAAATATCCTTTGAGCCAATTCCCATACATTATTGTGCCCCGGCTGGTTTTGCGATTCTAAGAT
GTAAC---AATAAGACATTCAGT------GGAAAGGGACCATGTACAAAT-----------------------
-GTCAGC---ACAGTACAATGTACACATGGAATTAGGCCAGTAGTATCAACT---
CAACTGCTGCTAAATGGCAGTCTAACAGGAGAAGAG-----------
ATAGTAATTAGATCTGACAATTTTACAGACAAT---------GCTAAACCCATAATAGTACATCTGAAT----
-----------GAATCTGTAGAA---------ATTAATTGTACAAGACTCAGCAACAATACAAGAAAA-----
-GGTGTACATTTG------------GGACCAGGGAGCGCA---ATG------TATGCAACAGGAGAA------
---------ATAATAGGAGATATA------------AGACAAGCACATTGT---AACATTAGTGAAGCA---
AAATGGAATAACACTTTAAGACAGATAGCTAAAAAATTAGGA---------------GAACAATATGGG----
--------------------------------AATAATAAAACAATAATCTTTACT------
AATTCCTCAGGAGGGGACCCAGAAATTGTA---ATGCACACTTTTAATTGTGGAGGGGAG---
TTTAAGCAGCTCCGGGCAAGAGTCCTGGCTGTGGAAAGATACCTAAAGGATCAACAGCTCTTAGGGATTTGGG
GTTGCTCTGGAAAATCTATTTGCACCACTTCTGTGCCTTGGAATGCTAGTTGGAGT-----------------
----------------------------AATAAATCTTTGAATGACATTTGG------GAG------
AACATGACCTGGATGGAGTGG---GACAGAGAAATTAGCAATTACACAGGTTTAATATAC---
TCCTTAATTGGAGAAGCACAAATCCAGCAAGAA---

AAGAATGAACAAGAATTATTGGAATTGGATAAATGGGCAAGTCTGTGGAATTGGTTTGACATATCAAACTGGC
TGTGGTATATAAAAATATTCATAATGATAGTAGGAGGCTTGATAGGTTTAAGAATAATTTTTACT---
GTGCTTGCTATAGTG---------AATAGAGTTAGGAAGGGATACTCACCA------
TTATCATTGGCTGTTAGCTTGCTTAATGCCATTGCCATAGCAGTAGCTGAG---
GGGACAGATAGGGTTATAGAATTAGGACAAAGAGCTGGTAGAGCT----------------------------
--------GTCCTCCACATA------CCTAGAAGAATAAGACAGGGCGCGGAAAGGGCTTTGGTA---TAA
>HQ217860
GCT---GAT---
GAAAAATTGTGGGTCACAGTCTATTATGGGGTGCCTGTGTGG---AAAGACGCAGCC---
ACCACTCTATTTTGTGCATCAGATGCTAAATCATATGATACAGAG---GTACATAAT----------------
--------------GTTTGGGCCACA---CATGCATGTGTACCCACAGAC---
CCCAACCCACAAGAAGTAGTATTGGAG---------AATGTGACAGAACCTTTTAAC----------------
--------------------------ATGTGGAAAAATAATATGGTAGATCAGATGCATGAAGATATA---
ATCAGTTTATGGGATCAAAGCCTA---AAGCCATGTGTAAAATTA---
ACCCCACTCTGTGTTACTTTAAATTGTACTAATTACAATAATACGAGGGATGGTACTACGACCACTAGTCCTG
CT---
----------AATAATACCACTAGTTATATCTGGGGGGAAGAGATGAAAGAAGGAGAAATAAAG---
AATTGTTCTTTCAATGTCACCACAAACATAGTA--------------------------
GATAAGATAGAGAAACAATATGCACTT---TTTTATGAAGTTGATGTGGTGCCAATAGAAGAT----------

----

AGTAATAATAGTACCCATACCAGCTATAGGTTAATAAGTTGTAACACCTCTGTCATTACACAA---
GCTTGTCCAAAGGTATCCTTTGAGCCAATTCCCATACATTATTGTGCCCCGGCTGGTTTTGCGATTCTAAAGT
GTAAT---AACAAAACATTCAAT------GGAAAAGGACCATGTAAAAAT----------------------
-GTCAGC---ACAGTACAATGTACACATGGAATTAAGCCAGTAGTATCAACT---
CAACTGTTGTTAAATGGCAGTCTAGCAGAAGAAGAG-----------
GTAGTAATTAGATCTGACAATTTCACAGACAAT---------GCTAAAACCATAATAGTACAGCTGAAT----
-----------GAAACTGTACAA---------ATTCAGTGTGTAAGACCCAACAACAATACAAGAAAA-----
-GGTATACATATA------------GGACCAGGGAGAGCA---TTT------TATGCAGCAGGAGAA------
---------ATAATAGGAAATATA------------AGACGAGCATATTGT---AACATTAGTGAAGCA---
AGTTGGAATAACACCTTACACAAGATAGCTAAAAAACTAAAT---------------GAACAATTT-------
-----------------------------------AATAAAACAATAGTCTTTAAT------
CAATCCTCAGGGGGGGACCCAGAAGTTGTA---ATGCACATTTTTAATTGTGGAGGGGAA---
TTTAAACAGCTCCAGGCAAGAGTCCTGGCTGTAGAAAGATACTTAAAGGATCAACAGCTCCTGGGGATTTGGG
GTTGCTCAGGAAAACTCATTTGCACCACCTCTGTGCCTTGGAACTCTAGTTGGAGT----------------
----------------------------AATAGATCTCTGAATTACATTTGG------CAG------
AACATGACCTGGATGCAGTGG---GAAAAAGAAATTGACAATTACACAAACATAATATAC---
AACTTAATTGAAGAATCGCAAAACCGACAAGAA---------------------------------------
----
AGCAATGAAAAGGAATTATTAGAGTTAGATAAATGGGCAAGTTTGTGGAATTGGTTTGACATAACAAACTGGC
TGTGGTATATAAAAATATTCATAATGATAGTAGGAGGCTTGATAGGTTTAAGAATAGTTTTTACT---
GTACTTTCTATAGTG---------AATAGAGTTAGGCAGGGATACTCACCA------
TTATCGTTTGCTGTTAGCTTGCTCAACGCTATAGCCATAGCAGTAGCTGAG---
GGAACAGATAGGGTTATAGAAGTATTACAAAGAGCTCTTAGGGCT---------------------------
--------ATTCTCCACATA------CCTAGAAGAATAAGACAGGGCTTAGAAAGGGCTTTGCTA---TAA
>JF689865
GCA---GAA--
CAATTGTGGGTCACAGTCTATTATGGGGTACCTGTGTGG---AAAGAAGCAAAT---
ACTACTCTATTTTGTGCATCAGATGCTAAAGCATATGATACAGAG---GTACATAAT---------------
--------------GTTTGGGCCACA---CATGCCTGTGTACCCACAGAC---
CCCAACCCACAAGAAGTAGTATTGGGA---------AATGTGACAGAAAATTTTAAT---------------
-------------------------ATGTGGAAAAATAACATGGTAGAACAAATGCATGAGGATATA---
ATTAGTTTATGGGATCAAAGCCTA---AAGCCATGTGTAAAATTA---
ACCCCACTCTGTGTTACTTTAGATTGCATTGATCCAACGAATAATGTTACTAAT------------------
----
-------------------------------ATCACGGGTGTTATAGAGAGGGGGGAAATAAAA---
AATTGCTCTTTCAATGTCACTACAAGCATAAGA--------------------------
GATAAGATGCAGAAAGCATTTGCAACT---TTTAATAGACTTGATATAGTACCAATAGAAGGT----------
----

AGCAATAATAGCTATAGGTTGATAAGTTGTAATACCTCAGTCATTACCCAG---
GCCTGTCCAAAGGTATCCTTTGAGCCAATTCCCATACATCTTTGTGCCCCGGCTGGTTTTGCGATTCTAAAGT
GTAAT---GATAAGAAATTCAAT------GGAACAGGACCATGTAAAAAT----------------------
-GTCAGC---ACAGTACAATGCACACATGGAATTAGGCCAGTGGTATCAACT---
CAACTGCTGTTAAATGGCAGTCTAGCAGAAAAAGAA-----------
ATAGTAATTAGGTCCGCCAATCTCACGGACAAT---------ACTAAAACCATAATAGTACAGCTAAAT----
-----------AAGTCTGTAGTA---------ATAAATTGTACAAGACCCAATAACAATACAAGGAAG-----
-AGTATACATATA------------GGACCAGGCAGAGCA---TTT------TATGCAACAGGAGAC------
---------ATAATAGGAGATATA------------AGACAGGCACATTGT---AATCTGAGTAGAGCA---
AATTGGAATAACACTTTAGGAAAGATAGCTATAAAATTAAGA---------------GAACAATTC-------
-----------------------------------AATAAAACAATAGTCTTTAAT------
CATTCCTCAGGAGGGGACCCAGAGATTGTA---ATGCTCAATTTAAATTGTGGAGGGGAA---
TTTAAGCAGCTCCAGGCGAGAGTCCTGGCTGTGGAAAGATACCTACAGGATCAACAGCTCCTGGGAATTTGGG
GTTGCTCTGGAAAACTCATTTGCACCACTACTGTGCCTTGGAATGCTAGTTGGAGT----------------
----------------------------AATAAAACTATAAAGTATATTTGG------GAT------
AACATGACCTGGATGCAGTGG---GAAAAAGAAATTGAGAATTACACAGGCATAATATAC---
AACTTAATTGAAGAATCGCAAAACCAACAAGAA---------------------------------------
----
AAGAATGAACATGAATTATTGGAATTAGATAAATGGGCAAGTTTGTGGAATTGGTTCAACATAACAAACTGGC

TGTGGTATATAAAAATATTCATAATGATAGTAGGAGGCTTGATAGGTTTAAGAGTAGTTTTTACT---
GTACTTTCTATAGTG---------AATAGAGTTAGGCAGGGATACTCACCA------
TTATCGTTCGCTGTTAGCTTGCTCAATACCATAGCCATAGCAGTAGCTGAG---
GGAACAGATAGGGTTATAGAAGCAATACAAAGAGTTTTTAGAGCT---------------------------
--------ATTCTCCACATA------CCTACAAGAATAAGACAGGGCTTAGAACGGGCTTTGCTA---TAA
>JF689874
GCT---AAA--
GAAAATTTGTGGGTCACAGTCTATTATGGGGTACCTGTGTGG---ARGGAAGCAACT---
ACCACTCTATTTTGTGCATCAGATGCTAAAGCATATGATACAGAG---GTACATAAT---------------
--------------GTTTGGGCTACA---CATGCCTGTGTACCCACAGAC---
CCCAACCCACAAGAAATAGTAATGGAA---------AATGTGACAGAAAATTTTAAC---------------
--------------------------ATGTGGAAAAATCCTATGGTAGAACAGATGCATGAGGATGTA---
ATCAGTTTATGGGATCAAAGCCTA---AAGCCATGTGTAAAATTA---
ACTCCACTATGTGTTACTTTAAATTGCACTGATTTGAAGACTAATACCAGTAAATTGAAGAGTAATACT----
----
----------------ACTAACACTAATAGTAGCGGGGGAACGGTAGAGGAAGAAGAAATGAAA---
AATTGCTCTTTCAATGTCACCACAAATGTAAAA-------------------------
GATAAGGTGAGAAAAGAATATGCACTT---TTGTATAAACTTGATATAACACCAATGGATGAT---------
----

GATAATACTAGCTATAGGTTGATAAGTTGTAACACCTCAGTCCTTACACAG---
GCTTGTCCAAAAATATCCTTCGAGCCAATTCCCATACATTATTGTGCCCCGGCTGGTTTTGCGATTCTAAAGT
GTAAT---GATAAGAAGTTCAAT------GGAACAGGATTATGTAAAAAT----------------------
-GTTAGC---ACAATACAATGCACACATGGAATTAGGCCAGTGGTGTCGACT---
CAACTGCTGTTGAATGGCAGTCTGGCAGAAGAAGGG-----------
GTAGTAATTAGATCTGAAAATATCACGGACAAT---------GCTAAAACCATAATAGTACACTTGAAT----
-----------GAATCTGTAGAA---------ATTAATTGTACAAGACCTAACAACAATACAAGAAGA-----
-AGTATATCTATA------------GGACCAGGGAGAGCA---TTT------TTCGCAACAGGAGAA------
---------ATAATAGGAGATATA------------AGGCAAGCACATTGC---AATATTAGTAAAGCA---
CAATGGAATAAAACTTTGACACAGGTAGCTAGCAAATTAAAA---------------GAACTATTTGGA----
----------------------------------AATAAAACAATAATCTTTAAT------
CACTCCTCAGGAGGGGACCCAGAAATTACA---ATGCACAATTTTAATTGTGGAGGGGAA---
TTTAAACAGCTCCAGGCAAGAGTCCTGGCTGTGGAAAGATACCTACGGGACCAACAGCTCCTGGGGATTTGGG
GTTGCTCTGGAAAACTCATCTGCACCACTAATGTGCCTTGGAATGCTAGTTGGAGC----------------
----------------------------AATAAATCACAGGATATGATTTGG------AAC------
AGCACGACCTGGATGCAGTGG---GAAGAAGAAATTGACAATTACACAGGCTTAATATAC---
TCCCTACTTGAAGAATCGCAAACCCAGCAAGAA---------------------------------------
----
AAGAATGAACAAGAATTATTGGGATTAGATAAATGGGCAAGTTTGTGGAGTTGGTTTGACATAACAAATTGGC
TGTGGTATATAAAGATATTCATAATGATAGTAGGAGGCTTGATAGGTTTAAGAATAGTTTTTATG---
GTACTTTCTATAGTA---------AATAGAGTTAGGCAGGGATRCTCACCT------
TTATCGTTTGCTGTTAGCTTGCTTAGTGCCACAGCCATAGCAGTAGCTGAA---
GGGACAGATAGGATTATAGAAGCAGTACAAAGAGCTTGGAGAGCT---------------------------
--------TTTCTCCACATA------CCTAGAAGAGTCAGACAGGGCTTGGAAAGGGCTTTGCTA---TAA
>JF689875
GCT---GCA--
GAAGATTTGTGGGTCACAGTCTATTATGGGGTACCTGTATGG---AAAGAAGCAAAA---
ACCACTCTATTTTGTGCATCAGATGCTAAAGCATATGATACTGAA---GCACATAAT---------------
--------------GTTTGGGCCACA---CATGCTTGTGTACCCACAGAC---
CCCAACCCACAAGAAGTAGTATTGGAA---------AATGTGACAGAAAATTTTAAT---------------
--------------------------ATGTGGAAAAATAACATGGTAGAACAGATGCATGAGGATATA---
ATCAGTTTATGGGATCAAAGCCTA---AAGCCATGTGTAAAATTA---
ACCCCACTCTGTGTCACTTTAAATTGCACTGATTGGAATGCTACCACTAATAAT------------------
----
-------------------------------AGCAAGGAAAAATTAAAGGGAAAAGAAATGAAA---
AATTGCTCTTTTGATGTCACCCCAAGCATAAAA-------------------------
GATAAAAAGCAGAGAGAATATGCACTT---TTTTATAAACTTGATGTAACACAAATAGGAGGA---------
----

GATAATACTAGCTATAGGTTAATAAGTTGTAACACCTCAGTCCTTACACAG---

```
GCCTGTCCAAAGATATCCTTTGAGCCAATTCCTATACATTATTGTGCCCCGGCTGGTTTTGCGATTCTAAAGT
GTAAT---GATAAGAAGTTCAAT------GGAACAGGACCATGTACAAAT-----------------------
-GTCAGC---ACTGTACAATGTACACATGGATTAGGCCAGTGGTGTCAACT---
CAACTACTGTTAAATGGCAGCCTAGCAGAAGAAGAG------------
ATAGTAATTAGATCTGAAAATTTCACGGATAAT---------GTTAAAAGCATAATAGTACAGCTGAAT----
-----------AAATCTATAGAA---------ATTAATTGTACAAGACCTGGCAACAATACAAGAAAA-----
-AGTATACATATA------------GGACCAGGGAGAGCA---TTT------TATGCAACAGGAGGA------
---------ATAATAGGAGATACA------------AGGCAGGCACATTGT---ACCATTAGTGGGGCA---
GATTGGAATAATACTTTAAAACAGATAGTTGACAAATTAAGA---------------GAACAAAAACAATTT-
--------------------------------GAGAATAAAACAATAAATTTTAAT------
TCATCCTCAGGAGGGGACCCAGAAATTGTA---ATGCACAGTTTTAATTGTGGAGGGGA----
TTTAAGCAGCTCCAAGCAAGAGTCCTAGCTGTAGAAAGATACCTACAGGATCAACAGCTCCTTGGGATTTGGG
GTTGCTCTGGAAAACTCATCTGCACCACAACTGTGCCTTGGAACACTAGTTGGAGT-----------------
----------------------------AATAAATCTCTGGATAAAATTTGG------CAT------
AACATGACCTGGATGGAGTGG---GAAAGAGAAATTGACAATTACACAGACTACATATAC---
ATCTTACTTGAAAAATCGCAGAACCAGCAGGAA----------------------------------------
-----------------------------
AAGAATGAACAGGAATTATTGGAATTGGATAAATGGGCAAGTTTGTGGAATTGGTTTGACATAACACACTGGC
TGTGGTATATAAAAATATTCATAATGATAGTAGGAGGCTTGGTAGGTTTAAGAATAGTTTTTATT---
GTACTTTCTGTAGTG---------AATAGAGTTAGGCAGGATACTCACCA------
TTATCGTTCGCTGTTAGCTTGCTCAATGCCACAGCCATAGCAGTAGCTGAG---
GGGACAGATAGGGTTATAGAAGTATTACAAAGAGCTTGTAGAGCT----------------------------
--------ATTCTCCATATA------CCTAGAAGAATAAGACAGGGCTTGGAAAGGGCTTTGCTA---TAA
>JX863990
GCT---ACA------------------------------------------
GAACAATTGTGGGTCACAGTCTATTATGGGGTACCTGTGTGG---AAAGAAGCAACC---
ACCACTCTATTTTGTGCATCAGATGCTAAGGCATATGATACAGAG---ATACACAAT----------------
--------------GTTTGGCCACA---CATGCCTGTGTACCCACAGAC---
CCCAACCCACAAGAAGTAGTATTGGAA---------AATGTGACAGAAAATTTTAAC----------------
--------------------------ATGTGGAAAAATGACATGGTAGAACAGATGCATGAGGATATA---
ATCAGTTTATGGGACCAAAGCCTA---AAGCCATGTGTGAAATTA---
ACCCCACTCTGTGTTACTTTAAATTGCACTAATGTCTTGAGGAATGCTACTAATACCAATAGTACCAGTGAGC
AGAATATTACT--------------------------------------------------------------
-------------------------AGTGCCRCTAATAATAGTTGGATAGGGACAGAAATGAAA---
AATTGCTCTTTCAATGTCACCACAGAAATAAGA---------------------------
GATAAGATGAAGAAAGAATATGCACTT---TTTTATAAACTTGACATAGTATCAATGAATAATGGTGAG----
-------------------------------------------------------------------------
----------------
AATAATAATAGTAATAGTTATAGGTTGATAAATTGTAACACCTCAAGCCTTACACAA---
GCCTGTCCAAAGGTATCCTTTGAGCCAATTCCCATACATTATTGTGCCCCGGCTGGTTTTGCGATTCTAAAGT
GTAAT---GATAAGACGTTCAAT------GGAACAGGATCATGTCACAAT-----------------------
-GTCAGC---ACAGTACAATGTACACATGGAATTAAGCCAGTAGTGTCAACT---
CAACTGCTGTTAAATGGAAGCCTAGCAGAAAATGAA------------
ACAGTAATTAGATCTGCCAATTTCTCAGACAAT---------GCTAAAACCATAATAGTACAGCTGAAG----
-----------ACACCTGTAAAA---------ATTAATTGTACAAGACCCGGCAACAATACAAGAAAA-----
-AGTATACATATA------------GCACCAGGGAGAGCA---TTT------TATGCAACAGGAGAT------
---------ATAATAGGAGATATA------------AGACAAGCACATTGT---AACATTAGTAAAACA---
GAATGGGAGAAAACTTTACAAGAGGTAGCTAAAAAATTAAAA---------------GAACAATATGGG----
--------------------------------GAAAATAAAACAATAGCCTTTAAT------
CAATCCACAGGAGGGGACCCAGAAATTGTA---ATGCACAGTTTTAATTGTGGAGGGGAG---
TTTAAGCAGCTCCAGGCAAGAGTCCTGGCTGTGGAAAAATACCTAAAGGATCAACAGCTCCTAGGGATTTGGG
GTTGCTCTGGAAAACTCATTTGCACCACCTCTGTGCCTTGGAATGCTAGTTGGAGT-----------------
----------------------------AATAAAACTCAGGAGGAGATTTGG------GGA------
AACATGACCTGGATGCAATGG---GAAAGAGAAATTGACAATTACACAAGTGAAATATAC---
AACTTAATTGAGATAGCACAAAACCAGCAAGAA----------------------------------------
-----------------------------
AAGAATGAACAAGAATTGTTGGCATTAGATCAATGGGCAAATTTGTGGAATTGGTTTGACATAACAAAGTGGC
TGTGGTATATAAAAATATTCATAATGATAATGGGAGGCTTGATAGGTTTAAGAATATTTTTTGCT---
GTAATTTCTATAGTA---------AATAGAGTTAGGCAGGATACTCACCA------
TTATCGTTTGCTGTTAGCTTGCTCAACGCCACAGCCATAGCAGTAGCTGAG---
```

GGAACAGATAGGATCATAGAAATAGTACAAAGATTTTTAGGGCT---------------------------
--------ATCCTTCATATA------CCTACAAGAGTGAGACAAGGCTTAGAAAGGGCTTTACTA---TAA
>JF320363
GCT---GCA---GAGAAA---
TGGGTCACAGTCTATTATGGGGTACCTGTGTGG---AAAGAAGCGACC---
ACCACTCTCTTTTGTGCATCAGATGCTAAAGCATATGTTGCAGAG---GCACATAAT---------------
--------------GTTTGGGCCACT---CATGCCTGTGTACCCACAGAC---
CCCAACCCACAAGAAGTAGAATTGGTA---------AATGTGACAGAAAATTTTAAC---------------
--------------------------ATGTGGAGAAATGACATGGTAGAACAGATGCATGAGGATATA---
ATCAGTTTATGGGATCAAAGCCTG---AAGCCATGTGTAAAATTA---
ACCCCACTCTGTGTTACTTTAAATTGCACTAATGTGACTGTGACTACTAATGAGACTGCTAATACC-------
----
----------------------ACTATTAGTCCAGAGGAAATGATGGCAAGAGAAGAAATAAAA---
AACTGCTCTTTCAATATCACCACAAGCATAAAA--------------------------ACTAAGGTG---
CAAGAATATGCATTA---TTTTATAAAATTGATATAGCACCAATAAATAATAACAGCTCT------------
----
----AATACCAACTATACCAACTATAGGTTGATAAGTTGTAACACCTCAGTCATTACACAG---
GCCTGTCCAAAGGTATCCTTCGAGCCAATTCCCATACATTATTGTGCCCCGGCTGGATTTGCGATTCTAAAGT
GTAAT---GAAAAGGGATTCAAG------GGAACAGGACCATGTAAAAAT----------------------
-GTCAGT---ACAGTACAGTGTACACATGGAATTAGGCCAGTAGTATCAACT---
CAACTGATGTTAAATGGCAGTCTAGCAGAAGGAGAG-----------
GTAGTAATTAGATCTGAAAATTTCACAAACAAT---------GCTAAAACCATAATAGTACAGCTGAAC----
-----------GAAACTGTACAA---------ATTAATTGTACAAGACCCAACAACAATACAAGGAAA-----
-GGTATACATATG------------GGACCAGGGAAAACA---CTT------TATGCAACAGGAGCA------
---------ATAATAGGAAATATA------------AGACGAGCACATTGT---AATATTAGTAAAACA---
AAATGGAATAACACTTTACAACAGGTAATTGCAAAATTAAAA---------------GAACAATATAAT----
----------------------------------AATAAAACTATACTCTTTAAT------
CAATCCTCAGGGGGAGACCCAGAAATTGAA---ATGCACAGTTTTAATTGTGGAGGGGAA---
TTTAAGCAGCTCCAAGCAAGAATCCTGGCTGTGGAAAGATACCTACAGGATCAACAGCTCCTGGGAATTTGGG
GTTGCTCTGGAAAACTCATTTGCACCACTGCTGTGCCTTGGAATGATAGTTGGAGT----------------
----------------------------AATAAATCTCTGGATTATATTTGG------AAT------
AACATGACCTGGATGGAGTGG---GAAAGAGAAGTTAATAATTACACAGGCTTAATATAC---
ACCTTAATTGAAGACTCGCAAATCCAACAAGAA---------------------------------------
----
AGGAATGAAAAAGATTTATTGGAATTAGATAAATGGGCAAGTTTGTGGAATTGGTTCGACATATCAAACTGGC
TGTGGTATATAAAAATATTCATAATGATAGTAGGAGGCTTAGTAGGTTTAAGAATAGTTTTTCTT---
GTACTTTCTATAGTA---------AATAGAGTTAGGCAGGGATATTCACCA------
TTATCGTTGGCTGTTAGTTTGCTTAATTTCACAGCTATAGCAGTAGCTGAG---
GGGACAGATAGGGTTATAGAAATAGTACGAAGAAGTTTTAGGGCT---------------------------
--------ATCCTCCACATA------CCTAGAAGAATAAGACAGGGCTTAGAAAGGATTCTGATA---TAA
>JF320036
GCT---ACA---
GAAAAATTGTGGGTCACAGTCTATTATGGGGTACCTGTATGG---AAAGAAGCCACT---
ACCACTCTATTTTGTGCATCAGAGGCTAAAGCATATGATACAGAG---GTACATAAT---------------
--------------GTTTGGGCTACA---CATGCCTGTGTGCCCACAGAT---
CCCAACCCACAAGAAGTAGTATTGGAA---------AATGTGACAGAAAACTTTAAC---------------
--------------------------ATGTGGAAAAATAACATGGTAGACCAGATGCATGAGGATATA---
ATCAGTTTATGGGATCAAAGCCTA---AAGCCATGTGTAAAATTA---
ACCCCACTCTGTGTTACTTTAAATTGCACTGATTATTTGAATAATACTACTAAGACTACCAATAATACTGAG-
----
----------------GCTACCAATACTAGTAGCGGGGAAAAGATGGAGCAAGGGGAAATAAAA---
AACTGCTCTTTCAATATCACCACAAACATAGGA--------------------------
GATAAGAGGCAGAAAGAATATGCATTG---TTTTATAAACTTGATGTAGTACCAATAGATAAGGTT-------
----

GATAATACTAGCTATAGGTTGATAAATTGTAATACCTCAGTCATTACACAG---
GCCTGTCCAAAGGTATCCTTTGAGCCAATTCCCATACATTATTGTGCCCCGGCTGGTTTTGCGATTCTAAAGT
GTAAT---AATAAGACATTCAAT------GGGACAGGGCCATGTCACAAT----------------------
-GTCAGC---ACAGTACAATGTACACATGGAATTAGGCCAGTAGTATCAACT---
CAACTGTTGTTAAATGGCAGTCTAGCAGAAGAAGAT-----------

GTAGTAATTAGATCTGAAAATTTCACGAACAAT---------GCTAAGATCATAATAGTACAGCTAAAT----
-----------GAATCTGTACAA---------ATTGATTGTATAAGACCCAACAACAATACAAGAAAA-----
-GGTATACATATA------------GGACCAGGGAGTGCA---TTT------TATACAACAGGAGAA------
---------ATAATAGGAAATATA------------AGACAAGCACATTGT---AACATTAGTGAAGGA---
AAATGGAATAATACTTTAAAACAGATAGCAGAAAAATTAAGA---------------GAACAATTTGGG----
--------------------------------AAGAATAAAACAATAGTCTTTAAT------
CAATCCTCAGGAGGGGACCTAGAAATTGTA---ACACACAGTTTTAATTGTGGAGGAGAA---
TTTAAACAGCTCCAGGCAAGAGTCCTGGCTGTGGAAAGATACCTACAGGATCAACAGCTCCTAGGAATTTGGG
GTTGCTCTGGAAAACTCATTTGCACCACTGCTGTGCCCTGGAATTCTAGTTGGAGT-----------------
----------------------------AATAAATCTCTGGATCAGATTTGG------AAT------
AACATGACCTGGATGGATTGG---GAAAAAGAAATTGACAATTACACAGGCATAATATAC---
AACTTACTTGAAAACTCGCAAAACCAACAAGAA--

AAGAATGAACAAGAATTATTGGAATTAGATAAATGGGCAAGTTTGTGGAATTGGTTTGACATAACACAATGGC
TGTGGTATATAAAAATATTCATAATGATAGTAGGAGGCCTGATAGGTCTAAGAATAGTTTTTTCT---
ATACTTTCTATAGTA---------AATAGAGTTAGGCAGGGATATTCACCA------
TTGTCGTTTGCTGTTAGTTTGCTTAATTCTACAGCTATAGCAGTAGCAGAG---
GGGACAGATAGGCTTATAGAAGTAGCACAAAGACTTTTTAGAGGT----------------------------
--------ATTCTCCACATA------CCTAGAAGAATAAGACAGGGCTTGGAAAGGGCTTTACAA---TAA
>JF320502
GCT---AAA--
GAAAAATCATGGGTCACAGTCTATTATGGAGTACCTGTGTGG---AAAGAAGCAACC---
ACCACTCTATTTTGTGCATCAGATGCTAAAGCATATGATACCGAG---GCACATAAT----------------
--------------GTTTGGGCCACA---CATGCCTGTGTACCCACAGAC---
CCCAGTCCACAAGAAGTATTATTGGCA---------AATGTGACAGAAAATTTTAAC----------------
--------------------------ATGTGGAAAAATAACATGGTGGAACAGATGCATGAGGATATA---
ATCAGTCTATGGGATCAAAGCCTA---AAGCCATGTGTAAAATTA---
ACTCCACTTTGTGTTACTTTAAATTGCACTAATGTCACTAGTAATGTCACTAGTAGTGCTAATAGCACT----
----
-------------------AGCACTAATAATACCACCAGTGATATCAAGGACGGAGGAATGAAA---
AACTGCTCTTTCAATATCACCATCACAGGCAGA------------------------
AGAGATAAGATGAAAAAAGAATATGCTCTT---TTTTATAGCCTTGATATAGTGCCAATAGATAATGAT----
----

AATAATACAAGCTATATGTTGAGAAGTTGTAACACCTCGGTCATTACACAG---
GCCTGTCCAAAGATATCATTTGAGCCAATTCCCATACATTATTGTGCCCCGGCTGGTTTTGCGATTCTAAAGT
GTAAT---GATAAAAAGTTCAAT------GGATCAGGACTATGCAGGAAT-----------------------
-GTTAGC---ACAGTACAATGTACACATGGGATTAAGCCAGTAGTATCAACT---
CAACTGCTGTTAAATGGCAGTCTAGCAGAAGAAGAG------------
GTAGTAATTAGATCTGAAAATTTCACAGACAAT---------GCTAAAAATATAATAGTACAGCTGACA----
-----------GAAGCTGTAAAC---------ATTACTTGTATAAGACCCAACAACAATACAAGAAGA-----
-AGAATATCTATG------------GGACCAGGGAGAGTA---CTT------TATACAACAGGAGAA------
---------ATAATAGGAGATATA------------AGAAAAGCACACTGT---AACCTTAGTAGAACA---
CAGTGGAATAACACTTTAAAACAGATAGCTAAAAAATTAACA---------------GAACAATTTGGG----
-----------------------------------AATAAAACAATAATCTTTAAA------
CCCCCCTCAGGAGGGGACCTGGAAATTGTA---ACGCACAGTGTTAATTGTGGAGGAGAA---
TTTAAACAACTCCAGGCAAGAGTCCTGTCTGTGGAAAGATACCTAAGGGATCAACAGCTCCTGGGGATTTGGG
GTTGCTCTGGAAAACTCATCTGCACCACTGCTGTGCCTTGGAACACTAGTTGGAGT-----------------
----------------------------AATAAATCTGTAGATGACATTTGG------GGT------
AACATGACCTGGAGGCAGTGG---GAAAGAGAAATTGACAATTACACAAGCTTAATATAC---
ACCCTACTTGAAGAATCGCAGAACCAGCAAGAA--

AAGAATGAACAAGAATTATTGGAATTGGATAAATGGGCAAATTTGTGGAATTGGTTTAGTATAACAAACTGGC
TGTGGTATATAAAATTATTCATAATGATAGTAGGAGGCTTGATAGGTGTAAGAATAGTTTTTACT---
GTACTTTCTATAGTG---------AATAGAGTTAGGCAGGGATACTCACCT------
TTGTCGTTTGCTATTAGCTTGCTCAACGCCACAGCCATAGCAGTAGCTGAG---
GGAACAGATAGGGTTATAGAAGTATTACAAAGAATTTTTAGAGCT----------------------------
--------ATTTGCCACATA------CCTACACGAATAAGACAGGGCTTGGAAAGGCTTTTGCTA---TAA
>JF320151

GCT---ACA---
GAACAATTGTGGGTCACAGTCTATTATGGGGTACCTGTGTGG---AAAGAAGCAACC---
ACCACTCTATTTTGTGCATCAGATGCTAAAGCATATGATACAGAG---GCACATAAT---------------
--------------GTTTGGGCCACA---CATGCCTGTGTACCCACAGAC---
CCTAACCCACAAGAAGTAGTATTGGGA---------AATGTGACAGAATATTTTAAT---------------
--------------------------GCATGGAAAAATGACATGGTAGAACAGATGCATGAGGATATA---
ATTAGTTTATGGGATCAAAGCCTA---AAGCCATGTGTCAAATTA---
ACCCCACTTTGTGTTACTCTAAATTGCACTGATTTGAAGAATACTACTAGTACCAATGCTACTAGG-------
----
-------------------CCCAATACTAGTCCCAATAATACTATGGAAGAAGGAGACATGAAA---
AACTGCTCTTTCAATGTCACCACAAGCATAAGA---------------------------
AATAAATTGCAAAAAGAATATGCACTT---TTCTATAAACTTGATATAGTACCAATAGATGGT----------
----

GATAATGAAAGCTATGCTTTAATAAGTTGTAACACCTCAGTCATTACACAG---
GCCTGTCCAAAGGTATCCTTTGAACCAATTCCCATACATTATTGTGCCCCGGCTGGTTTTGCGATTCTAAAGT
GTAAT---GAGGAGAATTTCAAT------GGAACAGGACCATGTACAAAT-----------------------
-GTCAGC---ACAGTACAATGTACACATGGAATTAAGCCAGTAGTGTCAACT---
CAGTTGTTATTAAATGGCAGCCTAGCAGAAAAGGAG-----------
GTAGTAATTAGGTCTGAAAATATCACAGACAAT---------GCTAAAACCATAATGGTGCATCTGAAT----
-----------AAATCTGTAATA---------ATTAATTGTACAAGATACAATAACAACACAAGAAGA-----
-AGTATAAATATA------------GGACCGGGGAGAGCA---TTT------TATGCAACAGGGGAT------
---------ATAATAGGAGATATA-----------AGAAAATCACATTGT---AATGTTAGTAAAGCA---
GATTGGATGAGAACTTTAAAAGATGTGGCTCAAAAATTAAGA---------------GAAAAATTTGGG----
--------------------------------AATGATACAAAAATAGCCTTTAAT------
AAAACCTCAGGAGGAGACCCAGAAATTGTA---ATGCACAGTTTTAATTGCGGAGGGGAA---
TTTAAGCAGCTCCAGGCAAGAGTCCTGGCTGTGGAAAGATACCTAAAGGATCAACAGCTCCTAGGAATTTGGG
GTTGCTCTGGAAAACTCATCTGCACCACTAATGTGCCTTGGAATGTTACTTGGAGT----------------
----------------------------AATAAATCTCTAGATAACATTTGG------AAT------
AACATGACCTGGATGGAGTGG---GAGAGAGAAATTGACAATTACACAAGCCTAATATAC---
TCCTTAATTGAAGAATCGCAGAACCAACAGGAA--

AAGAATGAACAAGAATTATTGGCATTGGATAAGTGGGCAAGTTTGTGGAATTGGTTTAACATATCAAATTGGC
TGTGGTACATAAAAAATATTCATAATGATAGTAGGAGGCTTGATAGGGTTAAGAATAGTTTTTACT---
GTGCTTTCTATAGTG---------AATAGAGTTAGGCAGGGGTACTCACCA------
TTATCGTTTGCTGTTAGTTTGCTTAATGCTACAGCTATAGCAGTAGCTGAG---
GGGACAGATAGGATTATAGAAATATTACAAAGATTTTGTAGGGGT---------------------------
--------ATCCTTCACATA------CCTACAAGAATAAGACAGGGCTTTGAAAGAGCTTTGCTT---TGA
>JF320145
GCT---GAA---
GAAAAATTGTGGGTCACAGTCTATTATGGGGTACCTGTGTGG---AAAGAAGCAACC---
ACCACTTTATTTTGTGCATCAGATGCTAAAGGATATGATACAGAG---GTACATAAT---------------
--------------GTTTGGGCCACA---CATGCCTGTGTACCCACAGAC---
CCCAACCCACAAGAAGTAGAATTGAAA---------AATGTGACAGAATATTTTAAC---------------
--------------------------ATATGGAAAAATAACATGGTAGAACAGATGCATGAGGATATA---
ATCAGTTTATGGGATCAAAGCCTA---AAGCCATGTGTAAAATTG---
ACCCCACTCTGTGTTACTCTAAATTGCACTGATAATTGGAGGAAGGCTACTGATTGGAAGAATAATAGTAAT-
----
-------------GCTATTGCCAATAATGGTAGTTGGGAAAAATTAGAGAAGGGAGAAATAAAA---
AACTGCTCTTTCAATGTCACCACAAGCATAAAA---------------------------
GATAAGATAACAAAAGAACATGCACTT---TTTTATAGACTTGATATAGTACCAATAGATAGTGAT-------
----

AGTAAGAGTGATAGTAGTTATACCAGCTATAGGTTGATAAATTGTAATACCTCAGTCATTACACAG---
GCCTGTCCAAAAGTAACCTTTGAACCAATTCCCATACATTATTGTGCCCCGGCTGGGTTTGCGATTCTAAAGT
GCAAT---AATAAGACATTCAGT------GGAAAGGGAGCATGTAACAAT-----------------------
-GTCAGC---ACAGTACAATGTACACATGGAATTAGGCCAGTAGTATCAACT---
CAGCTGCTATTGAATGGCAGTCTAGCAGAAGAAGAG-----------
ATAGTAATTAGGTCTGAGAATTTCTCAGACAAT---------GCTAAAATCATAATAGTACACCTGAAT----
-----------GAATCTGTAGAG---------ATTAATTGCACAAGACCCAACAATAATACAAGAAAA-----

-GGTATACATATA------------GGACCAGGGAGGGCA---TGG------TTTGCAACAGAAAAT------
---------ATAATAGGAAACATA------------AGACAAGCACATTGT---AACATTAGTAGTAGA---
AAATGGTATAGCACTTTAGAAAAGGTAGTTGAAAAATTAAGA---------------GAGCAATATGGG----
-----------------------------------AATAAAACAATAGTCTTTAAA------
AATTCCTCAGGAGGGGACCCAGAAATGGTA---ACACACAGCTTTAATTGTGGAGGAGAA---
TTTAAGCAGCTCCAGGCAAGAGTCCTGGCTGTAGAAAGATACCTAAGGGATCAACAGCTCCTTGGGATTTGGG
GTTGCTCTGGAAAACTCATCTGCACCACTAATGTGCCTTGGAATGCTAGTTGGAGT-----------------
----------------------------AATAAAAATCTGAGCCAGATTTGG------GAC------
AACATGACCTGGATGCAGTGG---GAAAAGGAAATTGACAATTACACAAGCTTAATATAC---
ACCTTAATTGAAGAATCGCAAAACCAACAAGAA--

AAAAATGAACTAGATTTATTGGAATTGGATAAATGGGCAAGTTTGTGGAATTGGTTTGACATATCAAACTGGC
TGTGGTATATAAGAATATTCATAATGATAGTAGGAGGCTTAATAGGTTTAAGAATAGTTTTTGCT---
ATGCTTTCTGTAGTG---------AATAGAGTTAGGCAGGATACTCACCA------
TTGTCATTTGCTGTTAGCTTGCTCAACGCCATAGCCATAGCAATAGCTGAG---
GGGACAGATAGGATTATAGAAATAGTACAAAGAGCTTGTAGAGCT----------------------------
--------GTTCTCAACATA------CCTAGAAGAATAAGACAGGGCTTCGAAAGGGCTTTGCTA---TAA
>HQ908232
GCT---ACA--
AACAACTTGTGGGTCACAGTCTATTATGGGGTACCTGTATGG---AAAGATGCAACC---
ACCACTCTATTTTGTGCATCAGATGCTAAAGCATATGACACAGAG---GCACATAAT----------------
--------------ATTTGGGCCACA---CATGCCTGTGTACCCACAGAC---
CCTAACCCACAAGAAGTAGTACTGGGA---------AATGTGACAGAAGACTTTAAT----------------
--------------------------GTTTGGAAAAATGACATGGTAGAACAGATGCAAGAGGATATA---
ATCAGTTTATGGGATCAAAGCCTA---AAACCATGTGTAAAATTA---
ACCCCACTTTGTGTCACTTTTAATTGCACTAATGTAGTTAATGGTACCACCAATACCACT-------------
----
-------------------------AGGGATAGTTGGCAAATGATGAAGGAAGGAGAAATAAAA---
AACTGTTCTTTCAATGTCACCACAAACATAATA---------------------------
GATAAGATGCAGAAAGAATATGCATTA---TTTTATAAATCTGATATAGTACCAATAGATAAT----------
----

AGTAGTAATGGCTATAGGCTAATAAGTTGTAACACCTCAGTCATTACACAG---
GCCTGTCCAAAGACATCCTTTGATCCAATTCCCATACATTATTGTACCCCGGCTGGTTATGCGATTCTAAAGT
GCAAC---AATAAGACGTTCGAG------GGGAAAGGACACTGCAAAAAT-----------------------
-GTTAGC---ACAGTACAGTGTACACATGGAATTAGGCCAGTAGTGTCAACT---
CAACTGCTGCTAAATGGCAGTCTAGCAGAGGAAGAT------------
GTAGTAATTAGATCTGACAATTTCTCAGATAAT---------GCAAAAACTATAATAGTACAGCTGAAG----
-----------GAAGCTGTAGAA---------ATTAATTGTACAAGACCCAACAACAATACAAGAAAA-----
-AGTATACATATA------------GCACCAGGGAGAGCA---TTT------TATGCAACAGGAGAA------
---------ATAATAGGGGATATA------------AGACAAGCACATTGT---AACATTAGTAAGGAA---
GCCTGGAATAACACTTTAAAACAGATAGTTGAAAAATTAAGA---------------GTTAAATTTGGG----
-----------------------------------AATAAAACAATAGTCTTTAAT------
CAATCCTCAGGAGGGGACCCAGAAATTGTA---ATGCACAGTTTTAATTGTGGAGGGGAA---
TTCAAGCAGCTCCAGGCAAGAGTTCTGGCTGTGGAAAGATACCTAAAGGATCAACAGCTCCTGGGAATTTGGG
GTTGCTCTGGAAAACTCATCTGCACCACTACTGTGCCTTGGAATAATAGTTGGAGT-----------------
----------------------------AATAAAAATCTGAGTACAATTTGG------GAT------
AACATGACCTGGATGGAATGG---GAAAGAGAAATTGACAATTATACAGACTTAATATAC---
AGGTTACTTGAAGAATCGCAGAACCAGCAAGAA--

AAGAATGAACAAGACTTATTAGCATTAGATAAGTGGGCAAGTTTGTGGAATTGGTTTAACATAACACAATGGC
TGTGGTATATAAAAATATTCATAATGATAGTAGGAGGCTTAGTAGGTTTAAGAATAGTTTTTACT---
GTACTTTCTATAGTG---------AATAGAGTTAGGCAGGATACTCACCA------
TTATCGTTTGCTGTTAGTTTGTTTAATGCCACGGCCATAGCAGTAGCTGAG---
GGGACAGATAGGATTATAGAAGTGATACTAAGAGTTTATAGAGCT----------------------------
--------ATCCTCCACATA------CCTACAAGAATAAGACAGGGCTTGGAACGCGCTTTGCTA---TAA
>KM353665
ACT---GCA--
GAACAATTGTGGGTCACAGTCTATTATGGGGTGCCTGTGTGG---AAAGAAGCAACT---
ACCACTCTATTTTGTGCATCAGATGCTAAAGCATATGATACAGAG---ATGCATAAT----------------

```
--------------GTTTGGGCCACA---CATGCCTGTGTCCCCACAGAC---
CCCAGCCCACAAGAAGTAGTATTAGCA---------AATGTGACAGAAAATTTTAAC----------------
--------------------------ATGTGGAAAAATAACATGGTAGACCAGATGCATGAAGATATA---
ATTAGTTTATGGGAGCAAAGCCTA---AAGCCATGTGTACAGTTA---
ACCCCACTCTGTGTTACTTTAAATTGTACTGAATATTTAGGGAATAATGCTACCAGTAAGACTAATACC----
-------------------------------------------------------------------------
----------------ACCAATAATACTACCAGTAGTCCTGATATCGATAAAGATGCCATGAAA---
AGCTGCTCTTTCAATAGCACCACAAGCATGAGA---------------------------
GATAAGATTAAGAAAGAACATGCACTT---TTTTATACACTTGATTTAGTACCACTAGATAAT----------
-------------------------------------------------------------------------
----------------
AATAGTAATCAAACCACCTATAAGTTGACAAGTTGTAATACCTCAGTCATTACACAG---
GCCTGTCCAAAGATATCATTTGAACCAATTCCCATACATTATTGTGCCCCAGCTGGTTTTGCGATTCTAAAAT
GTAAC---AATAAGACATTCAAT------GGAACAGGACCATGTACAAAT-----------------------
-GTCAGC---ACAGTACAATGTACACATGGAATTAGGCCAGCGGTGTCAACT---
CAGCTGCTGTTAAATGGCAGTCTAGCAGAAGAAGAG------------
GTAGTAATTAGATCTGCCAATTTCACGGACAAT---------GCTGGAAACATAATAGTGCAGCTGAAT----
-----------GAAACTGTACCA---------ATTACTTGTATAAGACCCAACAATAATACAAGAAAA-----
-AGTATAAATATG------------GGACCAGGGAGAGCA---TTT------TATGCAACAGACAAA------
---------GTAATAGGAGATATA------------AGACAGGCACACTGT---ACACTTAATGGAACA---
CAATGGCATAAAACCTTAGAAATGATAGTTGAAAAATTAAAA---------------GGACAATTTGGG----
-----------------------------------AATAAGACAATAGTCTTTAAC------
AAATCCTCAGGAGGGGATCCAGAAATTGAA---ATGCACAGTTTTAATTGTGGAGGGGAA---
TTTAAACAACTCCAGGCAAGAGTCCTGGCTGTGGAAAGATACCTACAGGATCAACAGCTCCTAGGGATCTGGG
GTTGCTCTGGAAAACTCATCTGCACCACTGCTGTGCCTTGGAATAGTAGTTGGAGT-----------------
----------------------------AATAAATCTTACAATGAAATTTGG------AAT------
AACATGACCTGGATGGAATGG---GAAAGAGAAATTGACAATTACACAGGCTTAATATAC---
AACCTACTTGAAACAACGCAGAACCAACAAGAA----------------------------------------
-----------------------------
AAGAATGAACAAGAATTATTAGAGTTAGAAAAGTGGGCAAACCTATGGAATTGGTTTGATATATCAAACTGGC
TGTGGTATATAAAAATATTCATAATGGTAGTAGGAGGCTTGATAGGTTTAAGGATAATTTTTGCT---
GTACTTTCTGTAGTG---------AATAGAGTTAGGCAGGGATACTCACCA------
TTGTCATTTGCTGTTAGCTTGCTTAATGCCACAGCTATAGCAGTAGCTGAG---
GGAACAGATAGGATTATAGAAGTAGCACAAAGAGCTTATAGAGCT----------------------------
--------ATTCTCCACATA------CCTAGAAGAATAAGACAGGGCGCGGAAAGGGCTTTGGTA---TAA
>JN687750
TCT---GCA------------------------------------------
GAAAACTTGTGGGTCACAGTCTATTATGGGGTACCTGTGTGG---AAAGAAGCAACC---
ACCACTCTATTTTGTGCATCAGATGCTAAAGCTTATGATACAGAG---GTACATAAT----------------
--------------GTATGGGCCACA---CATGCCTGTGTACCCACAGAC---
CCCAACCCACAAGAAGTAGTATTGGGA---------AATGTAACAGAAGATTTTAAC----------------
--------------------------ATGTGGAAAAATAACATGGTAGAACAGATGCATGAGGATATA---
ATCAGTTTATGGGATCAAAGTCTA---AAGCCATGTGTAAAATTA---
ACCCCACTCTGTGTTACTCTACATTGCACTAATTATTCCGGGAATGTTATCAATGTCACCAGAAGTGGTAATA
AT-----------------------------------------------------------------------
-------------ACCAATAATGCCACTATTAAGATAGATGAAGAAATGAAGGGAGAAATAAAA---
AACTGCTCTTTTAATATCACCACACACCTAAGA---------------------------
GATAAGGTGCAGAAAGCACGTGCATTC---TTTAATAGACTTGATGTAGTACCAATAGATAAT----------
-------------------------------------------------------------------------
----------------
GATAATGATAATACTAGCTATAGGTTGATAAGTTGTAACACCTCAAGCATTACACAA---
GCCTGTCCAAAAATAACCTTTGAGCCAATTCCCATACATTTTTGTGCCCCGGCTGGTTTTGCGATTCTAAAGT
GTAAT---AATAAAACATTTGAT------GGAAAGGGATCATGTACAAAT-----------------------
-GTCAGT---ACAGTACAATGTACACATGGAATTAAGCCAGTAGTGTCAACT---
CAACTGCTGCTAAATGGCAGTCTAGCAGAAGAAGAG------------
ATAGTAATTAGATCTGAAAATTTCACAAACAAT---------GCTAAAACCATAATAGTACAGCTGACG----
-----------GAACCTATAAAT---------ATTACTTGTGTCAGACCCAACAACAATACAAGAACA-----
-AGCATACATATG------------GGACCAGGGAAAGCA---TTT------TATGCAGCAGGAGAA------
---------GTAATAGGAGATATA------------AGGAGAGCATATTGT---AAGATTAATATAACA---
GAATGGAATAACACTTTAGAAAAAATAGTTGGAAAATTCAGA---------------GAACAATTTGGG----
```

-----------------------------------AATAAAACAATAGTCTTTAAT------
CAATCCTCAGGAGGGGACCCAGAAATTGTA---ACTCACAGTTTTAATTGTAGAGGGGAA---
TTTAAACAGCTCCAGGCAAGAGTCCTGGCTGTGGAAAGATACCTAAGGGATCAACAGCTCCTAGGGATTTGGG
GTTGCTCTGGAAAACTCATTTGCACCACTACTGTGCCTTGGAACAATAGCTGGAGT-----------------
-------------------------AAAAATAGATCTTGGGAGGAAATTTGG------GAA------
AACATGACCTGGATGCAATGG---GAAAAAGAAATTGACAATTACACAGGCTTAATATAC---
AACTTAATTGAAATATCGCAAAACCAGCAAGAA--

AAGAATGAACAAGACTTATTGGCATTGGATAAATGGGAAAGTTTGTGGAATTGGTTTGACATATCAAATTGGC
TGTGGTACATAAAAATATTCATAATGATAGTAGGAGGCTTAGTAGGTTTAAGAATAGTTTTTACT---
GTACTTTCTATAGTG---------AATAGAGTTAGGCAGGGATACTCACCA------
TTGTCGTTTGCTGTTAGCTTGCTTAATGCTTTTGCAATAACAGTAGCTGAA---
GGGACAGATAGGATTATAGAACTAGCAAGAAGAATTTTTAGAGCT----------------------------
--------ATCCTTCATATA------CCTACAAGAATAAGACAAGGCTTGGAGAGGGCTTTACTA---TAA
>HM204582
GCT---GCA--
GAAAATTTGTGGGTCACAGTCTATTATGGGGTACCTGTGTGG---AAAGAAGCAAAC---
ACCACTCTGTTTTGTGCATCAGATGCTAAAGGATATGAGACAGAG---GTACATAAT----------------
--------------GTCTGGCCACA---CATGCCTGTGTACCCACAGAC---
CCCAACCCACAAGAAATAGTATTGGAA---------AATGTAACAGAAAATTTTAAC----------------
--------------------------ATGTGGAAAAATAACATGGTAGAACAGATGCATGAGGATATA---
ATCAGTTTATGGGATCAAAGCCTA---AAGCCATGTGTAAAATTA---
ACTCCACTCTGTGTTACTTTAAATTGCACTAATTTAAGGAATGATACTAATACTACCAGT-------------
----
----------------------------AATAATGAGACAATGCAAGCGAGAGAAGAAATAAGA---
AATTGCTCTTTCAATGTCACTGCAGGCATAAGA---------------------------
GATAAGATACAAAAAGAATATGCTCTC---TTCTATAAAGTTGATGTAGTACCAATAAAGGAT----------
----

AATAATGATTCTACTAGCTTTAGATTGATAAGTTGTAATACCTCAGTCATTACACAG---
GCCTGTCCAAAGGTAACCTTTGAGCCAATTCCCATACATTATTGTGCCCCAGCTGGTTTTGCGATTCTAAAGT
GTAAC---AATAAAACATTCAGT------GGAACAGGGCCATGTACAAAT-----------------------
-GTTAGC---ACAGTACAATGTACACATGGAATTAAACCAGTAGTGTCAACT---
CAACTGCTGTTGAATGGCAGTCTAGCAGAAGAAGAG------------
ATAGTAATTAGGTCTGAAAATTTCACAAACAAT---------GCTAAAACCATAATAATACAGCTGAAG----
-----------GAGCCTGTACAA---------ATTAATTGTTCAAGACCCAACAACAATACAAGAAAA-----
-AGTATACATATA------------GGACCAGGGAGAGCA---TTT------TATACAACAGGAGAA------
---------ATAATAGGGGATATA------------AGAAAAGCACATTGT---AATATTAGTGGAACA---
GCCTGGAATAATACGTTAAGCCAGATAGCTACAAAATTAAGA---------------GGAAACTTAAC----
--------------------------------AATAACACCACAATAATCTTTAAG------
CCATCCTCAGGAGGGGACCCAGAAATTGTA---GCTCACAGTTTTAATTGTGGAGGAGAA---
TTTAAACAGCTCCAAGCAAGAGTCCTGGCTGTGGAAAGATACCTAAAGGATCAACAGCTCCTAGGGATTTGGG
GTTGCTCTGGAAAACTCATTTGCCCCACTACTGTGCCTTGGAACACTAGCTGGAGT-----------------
----------------------------AACAAATCTCTGAGTGAGATTTGG------GAT------
AACATGACCTGGATGGAGTGG---GAAAGAGAAATTGACAATTACACAAGCTTAATATAC---
AACTTAATTGAACAATCGCAGAACCAGCAGGAG--

AAAAATGAACAAGAATTATTAGAATGGGATAAGTGGGCAAGTTTGTGGAATTGGTTTAGCATAACAAAGTGGC
TGTGGTATATAAAAATATTCATAATGATAGTAGGAGGCTTAATAGGTTTAAGAATAGTTTTTACT---
GTACTTTCTATAATA---------AATAGAGTTAGGCAGGGATATTCACCA------
ATATCATTTGCTGTTAGCTTGTTTAATGCCATAGCAATAGCAGTAGCTGAA---
GGAACAGATAGGATTATAGAACTAGCACAAAGAATTCTTAGAGCT----------------------------
--------ATCCTCAACATA------CCTACAAGAATAAGACAGGGCTTAGAAAGAGCTTTAGTA---TAA
>HM204589
ACT---GCA--
GAAAACTTGTGGGTCACAGTCTATTATGGGGTACCTGTGTGG---AAAGAAGCAACC---
ACCACTCTATTTTGTGCCTCAGATGCTAAAGCACATGATACAGAG---GCACATAAT----------------
--------------GTTTGGCCACA---CATGCCTGTGTACCCACAGAC---
CCCAACCCACAAGAAATAAAATTG------------AATGTTACAGAAGATTTTAAC----------------
--------------------------ATGTGGAAAAATAGCATGGTAGAACAGATGCATGAGGATGTA---

ATCAGTTTATGGGATCAAAGCCTA---AAGCCATGTGTAAAATTA---
ACCCCACTCTGTGTCACTTTAAATTGCACTGATGAGTTGACCAATAAGACTTTTACTAATAGT----------
----
----------------------ACTCCTACCGCTATTGGTAAGATAGAGAAAGGAGAAATAAAA---
AACTGCTCTTTCAATATCACCACAAACTTAAGA---------------------------
GATAAGATGCAAAGAGTATATGCACTT---ATGTATAAACTTGATGTAGTACCAATAGATGAG----------
----

AATAATAGTAGCTATAGGTTGATAAGTTGTAACACCTCAGTCATTACACAA---
GCCTGTCCAAAAATATCCTTTGAACCAATTCCAATACATTATTGTACCCCGCCTGGTTTTGCACTTTTAAAAT
GTAGA---GACAAGAAGTTCAAT------GGAACAGGAGAATGTAAAAT------------------------
-GTCAGC---ACAGTACAATGTACACATGGGATTAGGCCAGTAGTATCAACT---
CAACTGCTGTTAAATGGTAGTCTAGCAGAAGAAGAG------------
GTAGTGATTAGGTCTGAAAATTTCACAAACAAT---------GTTAAAACCATAATAGTACACCTGAAT----
-----------GTATCTGTACAA---------ATGAATTGTACAAGACCCAACAACAATACAAGAAAA-----
-GGTATACATATA------------GGACCAGGGAGAGCA---TTT------TATGCAACAGGAGAC------
---------ATAATTGGAGATATA------------AGACAAGCACATTGT---AATGTGAGCAGAGGA---
GATTGGAATAACACTTTAAAATGGGTAGTTGAAAAATTAAGA---------------GAACAATTTGTG----
-----------------------------------AATAAAACAATAGTCTTTAAT------
CAATCTTCAGGAGGGGACCCAGAAATTGTA---ATGATCAATTTTAATTGTGGAGGGGAA---
TTCAAACAGCTCCAGGCAAGAGTCCTGGCTCTGGAAAGATACCTAAGGGATCAACAGCTCCTGGGAATTTGGG
GATGCTCTGGAAAACTCATCTGCACCACTGCTGTGCCTTGGAATGCTAGTTGGAGT-----------------
-------------------------AAAAATAAATCTTTAAATGATATTTGG------GAT------
AACATGACCTGGATGCAGTGG---GAAAAAGAAATTGACAATTACACAGAGGTAATATAC---
AGCTTACTTGAAAAGGCACAAAACCAACAAGAA--

AAGAATGAACAAGAATTATTAGAATTAGATAAATGGGCAAGTTTGTGGAGTTGGTTTGACATAACAAACTGGC
TGTGGTACATACAAATATTCATAATGATAGTAGGAGGCTTGATAGGTCTAAGAATAGTTTTTGCT---
GTGCTTTCTGTAGTA---------AATAGAGTTAGGCAGGGATACTCACCA------
TTATCGTTTGCTGTTAGCTTGCTTAATGTCACAGCCATAGTAGTAGCTGAG---
GGGACAGATAGGGTTATAGAAGTAGTACAAAGAACTTTTAGAGCT----------------------------
--------TTTCTCCATATA------CCTAGAAGAATAAGACAGGGCTTAGAAAGGATTTACTA---TAA
>KY112061
GCT---GCA--
GACAACTTGTGGGTCACAGTCTATTATGGGGTACCTGTGTGG---AGAGAAGCAACC---
ACCACTCTATTTTGTGCATCAGATGCTAAGGCATATGATACAGAG---GCACATAAT----------------
--------------GTTTGGGCCACA---CATGCCTGTGTACCCACAGAC---
CCCAACCCACAAGAAGTAGAATTGGAA---------AATGTGACAGAAAATTTTAAC----------------
--------------------------ATGTGGGAAAATAACATGGTAGAACAGATGCATGAGGATATA---
ATCAGTTTATGGGATCAAAGCTTA---AAACCATGTGTAAAATTA---
ACACCACTCTGTGTTACTTTAGCTTGCACTAATGCTAATTGGACAGATAGAAATCAGACT-------------
----
----------------------ATCGATAATAGTAGCCTAAAAATGGAGGAAGGAGAAGTAAAG---
AACTGCTCTTTTAATATCACCACAGAAATAAGA---------------------------
GATAAGATAAGGAAAGAATATGCACTT---TTTTATAAATCTGACATAGTATCAGTGAATAAGGAT-------
----

AATAATACTAGGTATAGGTTGATAAGTTGTAACACCTCAGTCCTTACACAG---
GCCTGTCCAAAAGTATCCTTTGAGCCAATTCCTATACATTATTGTGCCCCGGCTGGTTTTGCGATTCTAAAGT
GTAAT---GATAAGCAATTTATT------GGAACAGGACCATGTACAAAT-----------------------
-GTCAGC---ACAGTACAATGTACACATGGAATTAGGCCAGTAGTATCAACT---
CAACTGCTGTTAAATGGCAGTTTAGCAGAAGAAGAG------------
GTAGTAATTAGATCAGTTAATTTCAGTGACAAT---------GCTAAAACCATAATAGTACAACTGAAT----
-----------AAATCTGTAGAA---------ATTACTTGCACAAGACCCAACAACAATACAAGAAAA-----
-AGTATACCCATG------------GGACCAGGGAAAGCA---TTT------TATGCAAGAGGAGAC------
---------ATAATAGGAGACATA------------AGAAAAGCATATTGT---GAAATTAATGGAACA---
GAATGGCATAACACTTTAAAACTGGTAGTTGAAAAATTAAGA---------------GAACAATAT-------
-----------------------------------AATAAAACAATAGTCTTTAAT------
CGCTCCTCAGGAGGGGACCCAGAAATTGTA---ATGTACAGTTTCAATTGCGGAGGGGAA---
TTTAAGCAGCTCCAGGCAAGAGTCCTGGCTGTGGAAAGATACCTAAAGGATCAACAGCTCCTGGGGATTTGGG

GTTGCTCTGGAAAACTCATCTGCACCACTACTGTGCCTTGGAATACTAGTTGGAGT----------------
----------------------------AATAAATCTCTGGAACAGATTTGG------GAT------
AACATGACTTGGATGGAGTGG---GAAAGAGAAATTGACAATTATACAGGCTTAATATAC---
CAATTAATTGAAGAATCGCAGAACCAACAAGAA---------------------------------------
----
AAGAATGAACAAGAATTATTGGCATTGGATAAATGGGCAAGTTTGTGGAATTGGTTTGACATAACAAACTGGC
TGTGGTATATAAGACTATTCATAATGATAGTAGGAGGCTTAATAGGTTTAAGAATAGTTTTTACT---
GTGCTTTCTATAGTG---------AATAGAGTTAGGCAGGGATACTCACCA------
TTATCATTTGCTGTTAGCTTGCTCAACGCCACAGCCATAGCAGTAGCTGAG---
GGGACAGATAGGGTTATAGAAGTAGTACAAAGAGCTTGTAGAGCT---------------------------
--------ATTCTCCACATA------CCTAGAAGAATAAGACAGGGCTTGGAAAGGGCTTTGCTA---TAA
>EU576345
GCA---GCA---GAA--------------------------------
ACAGGACAATTGTGGGTCACAGTCTATTATGGGGTACCTGTGTGG---AAAGAAGCAACC---
ACCACTCTATTCTGTGCATCAGATGCTAAAGCATATTCAACAGAA---AAACATAAT---------------
--------------GTTTGGGCCACA---CATGCCTGTGTACCCACAGAC---
CCCAACCCACAAGAAATAAAATTGGAA---------AATGTGACAGAAAATTTTAAC---------------
--------------------------ATGTGGAAAAATGACATGGTAGAACAGATGCATGAAGATATA---
GTTAGTTTATGGGATCAAAGTCTA---AAACCATGTGTAAAATTA---
ACCCCACTCTGTGTTACCTTAGATTGCTCTCATAATGTCACTAGTAAAAATGCTACTAAT------------
----
----------------------------ACCAATAGTAGTAGGGTGGAGGGAGGAGACATGAAA---
AACTGCTCTTTCAATGTCACCACAAGCATAAGA--------------------------
GATAAGGTGAAAAACGAATATGCACTA---TTTTATAAACTTGATGTAGTACCAATAGATGGG---------
----

AGTAATACTAGCTATAGGTTAATAAATTGTAATACCTCAGTCATTACCCAG---
GCTTGTCCAAAAGTAACCTTTGAACCAATTCCCATACATTATTGTACCCCGGCTGGTTTTGCGATTCTAAAGT
GTGGA---GATAAGAAGTTCAAT------GGAACAGGACCATGTAAAAAT----------------------
-GTCAGT---ACAGTACAATGTACACATGGAATTAAACCAGTAGTGTCAACT---
CAACTGCTGTTAAATGGCAGTCTAGCAGAAGAAGAG-----------
GTAGTAATTAGATCTGCCAATCTCTCAGACAAT--------ACTAAAACCATAATAGTACAGCTGAAA----
-----------GATTCTGTAGAA---------ATTAATTGTATAAGACCCAACAACAATACAAGAAAA----
-AGTATAAGTATA-----------GGACCAGGGAGAGCA---TTT------TATGCAACAGGAGAT------
---------ATAATAGGAGATATA------------AGACAAGCACATTGT---AACATTAGTAGAAAA---
GCATGGAATGAAACGCTAAGACAGATAGCTATAAAATTAAGA---------------GAAAAATTTAAA----
-----------------------------------AATAAAACAATAGTCTTTAAT------
CAATCCTCTGGAGGGGATCCAGAAATTATA---ATGCACAGTTTTAATTGTAACGGGGAA---
TTTAAGCAGCTCCGAGCAAGAGTCCTGGCTGTGGAAAGATACCTACAGGATCAACAGCTCCTGGGAATTTGGG
GCTGCTCTGGAAAACTCATTTGCACCACTACTGTGCCTTGGAATGCTAGTTGGAGT----------------
----------------------------AATAAATCTCTGAATGATATTTGG------GAT------
AACATGACCTGGATGGAGTGG---GAAAGAGAAATTAGCAATTATACAAGCATAATATAC---
ACCTTACTTGAAAAATCGCAAAACCAACAGGAA---------------------------------------
----
AAGAATGAACAAGAATTATTGGAATTGGACAAGTGGGCAAATTTGTGGAATTGGTTTGATATATCAAACTGGC
TGTGGTATATAAAAATATTCATAATGATAGTAGGAGGCTTGGTAGGTTTAAGAATAGTTTTTGCT---
GTACTTTCTATAGTG---------AATAGAGTTAGGCAGGGATACTCACCA------
TTGTCATTTGCTGTTAGCTTGCTCAACGCCACAGCAATAGCAGTAGCTGAG---
GGGACAGATAGAATTATAGAAGTAGTACAAAGAACTTTTAGAGCT---------------------------
--------ATTCTCCACATA------CCTACAAGAATAAGACAGGGTTTGGAAAGGGCTTTGCTA---TAA
>EU576383
GCT---TCA---
AACAATTTGTGGGTCACAGTCTATTATGGGGTGCCTGTGTGG---AGAGAAGCAACC---
ACCACTCTATTTTGTGCATCAGATGCTAAAGCATATGATACAGAG---GTACATAAT---------------
--------------GTGTGGGCCACA---CATGCCTGTGTACCCACAGAC---
CCCAACCCACAAGAAGTAAGATTGGAA---------AATGTGACAGAAAATTTCAAC---------------
--------------------------ATGTGGACAAATAACATGGTAGAGCAGATGCATGAGGACATA---
ATCAGTTTATGGGATCAAAGCCTA---AAGCCATGTGTAAAATTA---
ACCCCAATCTGTGTCACTTTAAATTGCACCGACTATAATAATACTAACACTAACTCGAATACTACTAGA----
----

-------------------AGCAATAGTACTCATGAGAAATTGATGGTCAAAGGAGAAATACAG---
AACTGCTCTTTCAATATTGCCACAGACATAGGA---------------------------
GATAAGAGGCAGAAAGCATATGCACTG---
TTTTATAAAATTGATATAGTACCAATAAGTAGTAATAATAATACT----------------------------
----
AATTATACCAGCTCTAATGATAATAGGACCAGCTCTAATTATACCAGCTATAGATTGATAAGTTGTAATACCT
CAGTCATTACACAG---
GCCTGTCCAAAGGTATCCTTTGAGCCAATTCCCATACATTATTGTGCCCCGGCTGGTTTTGCGATTCTAAAGT
GTAAT---GATAAGAAGTTCAAT------GGAAGTGGACCATGTGCAAAT-----------------------
-GTCAGC---ACAGTACAGTGTACACATGGAATTAGGCCAGTAGTATCAACT---
CAACTGCTGTTAAATGGCAGTTTAGCAGAAGAAGAA------------
GTAGTAGTTAGATCTGCCAATTTCTCAGACAAT---------GCTAAATCCATAATAGTACAGCTGAAA----
-----------GAATCTGTTACA---------ATTAATTGTACAAGACCCAACAACAATACAAGAAAA-----
-AGTATACATATA------------GCACCAGGGAGAGCA---TTT------TATGCAACAGGAGAA------
---------ATAATAGGAGATATA------------AGACAAGCATATTGC---AACATTAGTGGAACA---
CAATGGAATAAAACTTTATACCAAATAGTTGAAAAATTAAGA---------------GAAAAAATTGGG----
-----------------------------------AATAAAACAATAATCTTTAAT------
CAATCAGCAGGAGGGGACCCAGAAATTGTA---ACACACAGTTTTAATTGTGGAGGGGAA---
TTTAAGCAGCTCCAGGCAAGAGTCCTGGCTGTGGAAAGATACCTACAGGATCAACAGCTCCTAGGGATGTGGG
GTTGCTCTGGAAAACTCATCTGCACCACTACTGTGCCTTGGAATACTAGTTGGAGT-----------------
----------------------------AATAAAACCCAGGAAGAAATTTGG------AAT------
AACATGACCTGGATGCAGTGG---GATAGAGAAATTAACAATTACACAGGCTTAATATAC---
ACCTTAATTACAGAATCGCACATCCAGCAAGAA--

AAGAATGAAAAAGAATTATTGGCACTAGATAAATGGGCAAGTTTGTGGAATTGGTTTGACATATCAAAATGGC
TGTGGTATATAAGAATATTCATAATGATAATAGGAGGCTTGATAGGTTTAAGAATAGTTTTTACT---
GTACTTTCTATAGTG---------AATAGAGTTAGGCAGGGATACTCACCA------
TTGTCGTTGGCTGTTAGCTTGCTTAATGCCACGGCCATAGCAGTAGCTGAA---
GGAACAGATAGGGTTATAGAAGGATTGCAAAGAGCTTTTAGGGCT----------------------------
--------ATTCTCCACATA------CCTACAAGAATAAGACAGGGCTTAGAAAGGGCTTTGCTA---TAA
>KX129209
ACT---ACA--
GAGCAATTGTGGGTCACGGTCTATTATGGGGTACCTGTGTGG---AAAGAAGCAAAA---
ACCACTCTATTTTGTGCATCAGATGCTAAAGCATATGAGACAGAG---GCCCATAAT----------------
--------------ATTTGGGCCACA---CATGCCTGTGTACCTACAGAC---
CCCAACCCACAGGAAGTAGAATTAAAA---------AATGTGACAGAACAATTTGAC----------------
--------------------------ATGTGGAAAAATAACATGGTAGAACAGATGCATGAGGATATA---
ATCAGTTTATGGGATCAAAGCCTA---AAACCATGTGTAAAATTA---
ACTCCACTCTGTGTTACTTTAAATTGCACTGACGCTAAGAATAATGGTACTAATACCACTGCT----------
----
-------------------------------------AGTGGGATGGATACAGGAGAAGTAAAA---
AACTGCTCTTTCAATGTCACCACAGAACTAAGA---------------------------
GATAAGATGAAGAACGAGTATGCACTT---TTTTATAGACTTGATGTAGAACCAATAGATAAG----------
----

AGTAATAATAATAGCTATAAGTTGAGAAGTTGTAATACCTCAGCCATTACACAG---
GCCTGTCCAAAGATATCCTTTGAGCCAATTCCCATACATTATTGTGCCCCGGCTGGTTTTGCGATTCTAAAGT
GTAAT---GATAAGGATTACAAT------GGAACAGGAACGTGTACAAAT-----------------------
-GTCAGC---ACAGTACAATGTACACATGGAATTAGGCCAGTAGTATCAACT---
CAATTACTGTTAAATGGTAGTCTAGCAGAAAAAGGG------------
ATAGTACTTAGATCTGAAAATTTCACAAACAAT---------GCTAAAACCATAATAGTACAGTTGAAA----
-----------GAAGCTGTAAAC---------ATTACTTGTGTAAGACCCAACAACAATACAAGAAAA-----
-AGTATACACATA------------GCACCAGGGAGCGCA---TTT------TATGCGACAGGAGAC------
---------ATAATAGGAAACATA------------AGACAAGCACATTGT---AACATTAGTAGAGAA---
CAATGGAATAATACTTTACAACAGATAGCTGAAAAATTACAA---------------CAACAATTTGGG----
-----------------------------------CATAACAGAACAGTCTTTAAT------
CAATCCTCGGGAGGAGACCCAGAAATTGTA---ATGTTCAATTTTAATTGTGGAGGGGAA---
TTTAAGCAGCTCCAGGCAAGAGTCCTGGCTGTGGAAAGATACCTAAAGGATCAACAGCTCCTGGGAATTTGGG
GTTGCTCTGGAAAACTCATTTGCACCACTGCTGTGCCTTGGAATACTAGTTGGAGT-----------------
----------------------------AATAAATCTCTAGATAAGATTTGG------AAT------

AACATGACCTGGATGCAGTGG---GAAAGGGAAATTGACAATTACACAAACTTAATATAC---
AGCTTAATTGAAGACTCGCAGAACCAGCAAGAA---------------------------------------
----
AAGAATGAAAAAGATTTATTAGAATTGGATAACTGGCAAGTTTGTGGAATTGGTTTGAAATAACAAACTGGC
TGTGGTATATATAAAAATATTCATAATGATAGTAGGAGGCTTGATAGGTTTAAGAATAGTTTTTACT---
GTACTTTCTATAATA---------AAGAGAGTTAGGCAGGGATACTCACCA------
TTGTCGTTTGCTGTTAGCTTGCTCAATGCCACAGCTATAGCAGTAGCTGAA---
GGGACAGATAGGATTATAGAAGGATTACAAAGAACTGCCAGAGCT---------------------------
--------ATTTGCAACATA------CCTAGACGAATAAGACAGGGCTGGGAAAGGCTTGGCTGCGA---
>KM259534
GCT---ACA--
CAAAAATTATGGGTCACAGTCTATTATGGGGTACCTGTGTGG---AAAGAAGCAACC---
ACCACTCTATTTTGTGCATCAGATGCTAAAGCCTATGAGACAGAG---GTACATAAT---------------
--------------GTTTGGGCCACA---CATGCCTGTGTACCCACAGAC---
CCTAGCCCACAAGAAGTAGTATTGGAA---------AATGTGACAGAAAATTTTAAC---------------
--------------------------ATGTGGAAAAATAACATGGTTGAACAAATGCATAAGGATATA---
ATCAGTCTATGGGATCAAAGCCTA---AAGCCATGTGTAAAATTA---
ACCCCACTTTGTGTTACCTTAAATTGCACTAATTATGAGGCTAATAGCACCAAGAATTCTACTAATAGCACCA
ACAATTCTACTCAGGCC---
-TCGGGAAGTAGCAATTCTACTCAGGCCTCGGGAAGTAGCGAAGAAATGATAGGGGAAATGAAA---
AACTGCTCTTTCAATGTCACCACAAGCATGAGA--------------------------
GATAAGATACAGAAAAACTATGCACTT---TTTTATGCACTTGATCTAGTACCAATAGATGGT---------
----

AGTAATAGTACTGAATATAGGTTGATAAGTTGTAATACCTCAGTCATTACACAG---
GCCTGTCCAAAGGTATCCTTTGAGCCAATTCCCATACATTATTGTGCCCCAGCTGGTTTTGCGATTCTAAAGT
GTAGA---GATAATAAGTTCAAT------GGAACAGGACTATGTAAGAAT----------------------
-GTCAGC---ACAGTACAATGTACACATGGAATTAGGCCAGTAGTATCAACT---
CAACTGCTGTTAAATGGCAGTCTAGCAGAAGAAGAG-----------
GTAGTAATTAGATCTGAAAATCTCAGGAACAAT---------GCTAAAAACATCATAGTACAGCTGAAT----
-----------ACAAGTGTAAAA---------ATTAATTGTACAAGACCTAACAACAATACAAGAAGA-----
-GGTATACATATA------------GGACCAGGGCAGGTT---TTT------TAT---ACAGGAGAG------
---------ATAATAGGAGATATA-----------AGACAAGCACATTGT---AACATTAGCAGTAAA---
GAATGGAATACCACTCTATACCAGGTAGCTATGAAATTAAGA--------------GAACAATTTAAC----
-----------------------------------AATAAAACAATAATTTTTAAT------
CAATCCTCAGGAGGGGACCTAGAAATTCAA---ATGCACAGTTTTAATTGTGGAGGGGAA---
TTTAAGCAGCTCCAGGCAAGAGTCCTGGCTGTGGAAAGATACCTACGGGATCAACAGCTCCTGGGGATTTGGG
GTTGCTCTGGAAAACTCATCTGCACCACTGCTGTACCTTGGAATACTAGTTGGAGT----------------
----------------------------AATAAATCGCTAAATGAAATATGG------GAT------
AACATGACCTGGATGGAGTGG---GAAAAGGAGATTGATAATTACACAGGCTTGATATAC---
AACTTACTCGAGAAATCGCAAAACCAACAGGAA---------------------------------------
----
AACAATGAAAAAGAATTATTGGCATTAGATAAATGGGCAAATTTGTGGACTTGGTTTGACATAACAAATTGGC
TGTGGTACATAAAAATATTTATAATGATAGTAGGAGGCTTAATAGGTTTAAGAATAATTTTTGCT---
GTGCTTTCTATAGCG---------AATAGAGTTAGGCAGGGATACTCACCA------
ATCTCGTTGGCTGTCAGCTTGCTCAACGCCACCGCTATAGCAGTAGCTGAG---
GGAACAGATAGGATTATAGAAGTAGTACAAAGAGTTGGCAGAGGT---------------------------
--------ATCCGTCACATA------CCTAGACGAATAAGACAGGGCTTGGAAAGGCTTTGCTA---TAA
>HQ217957
GGT---ACA---GAA------------------------AAC---AAA---
GAAAATTTGTGGGTCACAGTCTATTATGGGGTACCTGTGTGG---AAAGAAGCAACC---
ACCACTCTATTTTGTGCATCAGATGCTAAAGCATACAAACCAGAG---GCACATAAT---------------
--------------ATCTGGGCCACA---CATGCCTGTGTACCCACAGAC---
CCCAACCCACAAGAAGTATTACTGGAA---------AATGTGACAGAATATTTTAAT---------------
--------------------------ATGTGGAAAAATGACATGGTAGACCAGATGCATGAGGATATA---
ATCAGTTTATGGGATCAGAGCTTA---AAGCCATGTGTACAATTA---
ACCCCACTCTGTGTTACTTTAAATTGCACTGATATTAATTTGAATAATGATACTTCTGCTAATTTG------
----
----------------------ACGGATGCTAATATGACGATAGAATCGGTAAAAGAAATGAAA---
AACTGTTCTTTCAATACCACCACAGAGGTAATA--------------------------

GATAAGAGGCAGAAAACCTATGCTTTA---TTCCATAAACTTGATGTAACACAAACAGATGAA----------
----

ACTAGTAGTAATACTAGCTATAGATTGATAAGTTGTAATACCTCAGTCATTACACAA---
GCCTGTCCAAAGACAAGCTTTGAGCCAATTCCTATACATTATTGTACCCCGGCTGGTTTTGCAATTCTAAAGT
GTAAC---GATAAACAGTTCAAT------GGAACAGGAGACTGTAAAAAT-----------------------
-GTCAGC---ACAGTACAATGCACACATGGAATCAGGCCAGTAGTGTCAACT---
CAACTGCTATTAAATGGCAGTCTAGCAGAAGGAGAG------------
ATAATAATTAGATCTGAAAATTTCTCGAACAAT---------GCTAAAACCATAATAGTACAGCTGAAC----
-----------CACTCTGTAGTA---------ATTAATTGTACAAGACTCAATAACAATACAAGAAAA-----
-AGTATACATATA------------GGACCAGGAAGAGCA---TTT------TATACAACAGGAGAC------
---------ATAATAGGAGATATA------------AGAAAAGCACACTGT---AACATTAGTAGAGAA---
GACTGGAATAAAACTTTAAGACAGATAGCTATAAAATTAAAA---------------GAACAATTTGTG----
--------------------------------AATAGTACAACAATAAGTTTTAAT------
TCATCCTCAGGAGGGGACCCAGAGATTGTA---CTTCACAGTTTTAATTGTGGAGGGGAA---
TTTAAACAGCTCCAAGCAAGAGTCCTGGCTGTGGAAAGATACCTAAGGGATCAACAGCTCCTAGGGATCTGGG
GTTGCTCTGGAAAACTCATTTGCACCACTAATGTGCCTTGGAACACTACTTGGAGT-----------------
----------------------------AATAAAACTATGAGGGACATTTGG------AAT------
AACCTGACCTGGGTGCAGTGG---GAAAAAGAAATTGACAATTACACAGAAGTAATATAC---
CACTTACTTGAGATGGCGCAAAACCAGCAAGAA--

AACAATGAACAAGACTTATTGAAATTGGAGCAATGGGACAGTTTATGGAATTGGTTTAACATAACAAACTGGC
TGTGGTATATAAAAATATTCATAATGATAGTAGCAGGCTTAGTAGGTTTAAGAATAGTTTTTACT---
GTACTTTCTATAGTG---------AATAGAGTTAGGCAGGGATACTCACCT------
ATATCATTGGCTGTTAGCTTGTTTAATGCCATAGCAATAGTAGTAGCTGAG---
GGAACAGATAGGATTATAGAAATAGCACAAAGAATTTTTAGAGCT----------------------------
--------ATTATCCACATA------CCTACAAGAATAAGACAGGGCTTTGAAAGGGCTTTACTA---TAA
>HQ217984
GCC---ACA--
GAAAATAAATGGGTCACAGTTTATTATGGGGTACCTGTGTGG---AAAGAAGCAAAC---
ACCACTCTATTTTGTGCATCAGATGCTAAAGGATATGAAACAGAG---GTACATAAT----------------
--------------GTATGGGCTACA---CATGCTTGTGTACCCACAGAC---
CCCAACCCACAAGAAGTGAAATTAGTC---------AATGTGACAGAAAAGTTTAAC----------------
--------------------------ATGTGGAAAAATAACATGGTAGAACAGATGCATGAGGACATA---
ATCAGTTTATGGGATCAAAGCCTA---AAGCCATGTGTAAAATTA---
ACCCCACTCTGTGTTACTTTAACTTGCACTAATGTTACTAATGCTACTACACCTAAT----------------
----
----------------------------------ATCACTATGAGTGCTGCTGATGAAATAAAA---
AATTGCTCTTTCAATGTCACCACTGATGTAAGA---------------------------
GATAAGATGAAGAAAGAATATGCACTT---TTTTATAGCCTTGATATAGTAAAACTGGATACT----------
----

AATAATGATAGTACTGATTATAGGTTGACAAGTTGTAATACCTCAGTCATTACACAG---
GCCTGTCCAAAGGTATCCTTTGAGCCAATTCCCATACATTTCTGTGCCCCGGCTGGTTTTGCGATTCTAAAGT
GTAACAATAAGACATTTAGT------GGAAAAGGACCATGTAACAAT-----------------------
-GTCAGC---ACAGTACAATGCACACATGGAATTAAGCCAGTAGTATCAACT---
CAACTGCTGTTAAATGGCAGTCTAGCAGAAGAAGAA------------
ATAGTAATTAGATCTGACAATTTCTCAGACAAT---------GCTAAAACCATAATAGTACAGCTAGAA----
-----------ACCCCTGTAAAC---------ATTACTTGTACAAGACCCAACAACAATACAAGAAAA-----
-AGCATACATATA------------GCACCAGGGAGAGCA---TTT------TATGCAACAGGAGAC------
---------ATAATAGGAGATATA------------AGAAAAGCATACTGT---AACATTAGTAGGGCT---
CTGTGGAATAAAACTTTACAAAAGATAGCTAACAAATTAGGA---------------GAACAATTTAAT----
--------------------------------AATATAACAAAACTATCCTTTAAA------
GAATCCTCAGGGGGGGATCCAGAAATTGTA---AGGTTCAACGTTAATTGTGGAGGGGAA---
TTTAAGCAGCTCCAGGCAAGAGTCCTGGCTGTGGAAAGATACCTAAAGGATCAACAGCTCCTGGGGATTTGGG
GTTGCTCTGGCAAACTCATTTGCACCACTAATGTACCTTGGAATAACAGTTGGAGT-----------------
----------------------------AACACATCTCTGGATGTGATTTGG------AAT------
AACATGACCTGGATGCAGTGG---GAAAAAGAAGTTGACAATTACACAAACATAATATAC---
AATTTACTTGAAGAATCGCAGAACCAACAAGAA--

AAGAATGAACAAGAATTATTAGAATTGGACAAATGGGCAAATTTGTGGAATTGGTTTAGCATAACAAATTGGC
TGTGGTATATAAAAATATTCATAATGATAGTAGGAGGTTTGATAGGTTTAAGAATAGTTTTTGCT---
GTGCTTTCTATAGTA---------AATAGAGTTAGGCAGGATACTCACCA------
TTGTCATTTGCTGTTAGCTTGCTTAACGCCACAGCCATAGCAGTAGCTGAG---
GGAACAGATAGGATCATAGAAATAGTACAAAGACTTTGCAGAGCT----------------------------
--------ATCCGTAACATA------CCTACAAGAATAAGACAGGGCGCAGAAAGGGCTTTGCTA---TAA
>JF689886
GCA---GAT---
CAATTGTGGGTCACAGTCTATTATGGGGTACCTGTGTGG---AAGGAAGCAACC---
ACCACTTTATTTTGTGCATCAGATGCTAAAGCATATGTTAAAGAG---GTACATAAT----------------
--------------GTCTGGGCCACA---CATGCCTGTGTACCCACAGAT---
CCCAACCCACAAGAAGTGGTATTGAAA---------AATGTAACAGAAAATTTTAAC----------------
--------------------------ATGTGGAAAAATAACATGGTAGATCAGATGCATGAGGATATA---
ATCAGCTTATGGGATGAGAGCCTA---AAGCCATGTGTAAAATTA---
ACCCCACTCTGTGTTACTTTAAATTGCACTAGTAATATCACTACGGCCACT----------------------
----
----------------------------------GTTAGTCCAGAGATGACAGGAGAAATAAAA---
AACTGCTCTTTCAATATCACCACAAGCAGAAGA---------------------------
GATAAGGTGCGGAGAGAATATGCACTC---TTTTATAGCCTTGATATACTAGCAATAGGTGAT----------
----

AGTAATGATAGCTATATAATAAGAAGTTGTAATACCTCAGTCATTACACAG---
GCCTGTCCAAAGGTATCCTTTGAGCCAATTCCCATACATTATTGTACCCCGGCTGGTTTTGCACTTCTAAAGT
GTAAT---GATAAGAATTTCAAT------GGAACAGGACCATGTAAAAAT-----------------------
-GTCAGC---ACAGTACAATGTACACATGGAATTAAGCCAGTAGTATCAACT---
CAACTGTTGTTAAATGGCAGTCTAGCAGAAGAAGAC------------
ATAATAATTAGATCTGAAAATTTCTCTAACAAT---------GCTAAAACCATAATAGTACAGCTGAAT----
-----------GAAACTATAGAA---------ATTAATTGTACAAGACCCAACAACAATACCAGAAAA-----
-AGTATAAATATG------------GGTCCAGGGAGGGCA---ATT------TATGCAGCAGGACAA------
---------ATAATAGGAAATATA------------AGACAAGCATATTGT---ACCATTAATGGTACA---
AAATGGAAGGATACTTTAAAAAGGATAGTTGAAAAATTAGGA---------------GAACAATTTAAG----
-----------------------------------AATAATGAAACAATCTTTAGG------
AGTTCCTCAGGAGGGGACCCAGAAATTACA---ATGCACACTGTTAATTGTGGAGGGGAA---
TTTAAACAGCTCCAGGCAAGAGTCCTGGCTGTGGAAAGATACCTAAAGGATCAACAGCTCCTAGGGATCTGGG
GTTGCTCTGGAAAACTCATCTGCTCCACTAATGTGCCTTGGAATACTAGTTGGAGT-----------------
----------------------------AATAAATCTTTGAATGAAATTTGG------GAT------
AACATGACCTGGATGCAGTGG---GATAAAGAAGTTAGCAATTACACAGATTTAATATAC---
AACCTACTTGAAAAATCACAGACCCAACAGGAA--

AAGAATGAACAAGAATTATTAGAGTTAGACAAATGGTCAAGTCTGTGGAGTTGGTTTAGCATATCAAATTGGC
TGTGGTATATAAGAATATTCATAATGATAGTAGGAGGCTTGATAGGTTTAAGAATAGTCTTTGCT---
GTGCTTTCTATAGTG---------AATAGAGTTAGGCAGGATACTCACCA------
TTATCGTTTGCTGTCAGCTTGCTTAATGCTACAGCAATAGCAGTAGCTGAA---
GGGACAGATAGGATTATAGAAATAGCAAGGGGAATTTTTAGAGCT----------------------------
--------ATTTGCAACATA------CCTAGAAGAATCAGACAGGGCTTGGAAAGGCTTTTGCTA---TAA
>JF320385
GCT---GCA---GAA------------------------------------
CAACAATTGTGAGTCACAGTCTATTATGGGGTACCTGTATGG---AAAGAAGCTAAC---
ACCATTCTATTTTGTGCATCAGATGCTAAAGCATATGATACAGAG---GTGCATAAT----------------
--------------GTTTGGGCCACA---CATGCCTGTGTACCCACAGAC---
CCCAACCCACAAGAAGTAGTATTGGGA---------AATGTGACAGAAAATTTTAAC----------------
--------------------------ATGTGGAAAAATAACATGGTAGAACAGATGCATGAGGATATA---
ATCAGTTTATGGGATGAAAGCCTA---AAGCCATGTGTAAAATTA---
ACCCCACTTTGTGTTACTTTAAATTGCTCTGATTGGGAGAATGCTAATAATACCATTAATAGTACTGCTACTC
CT---
----------ACTACTAGTATCAATAGTAGTAGGGAGTTGGCGTTGGGGAAAGGAGAAATAAAA---
AACTGCTCTTTCTATGTCACCTCAGAG---------------------------------
GATAAGAAGCACAAAGAACGTGCACTG---TTTTATAAACTTGATGTAGTATCAATGGATGAT----------
----

AATAATACTACCTATAGGCTGATAAGTTGTAATACCTCAGTCATTAAACAG---
GCCTGTCCAAAGGTATCCTTTGAGCCAATTCCTATACATTATTGTGCCCCTGCTGGTTTTGCGATTCTAAAGT
GTAAC---AATAAGACATTCAAT------GGATCAGGACCATGTACAAAT-----------------------
-GTTAGT---ACAGTACAATGTACACATGGAATTAAGCCAGTAGTATCAACT---
CAACTGCTGTTAAATGGCAGTCTAGCAGAAGAAGAA------------
ATAGTAATTAGATCTGAAAATTTCTCAGATAAT---------GCTAAAACCATAATAGTGCAGCTAAAT----
-----------GAAACTGTAGAG---------ATTAATTGTACAAGACCCAGCAACAATACAAGAAAG-----
-AGTATACATCTA------------GGACCAGGGAGCACA---TTT------TTTGCAACAGGAGAA------
---------ATAATAGGAGATATA------------AGACAAGCACATTGT---AACATTAGTGGAGTA---
AAATGGAATAACACTTTAGAAAAGGTAGTTAAAAAATTAGGA---------------GGACAATTTAAT----
-----------------------------------ACTAGCAAAATAATCTTTAAT------
CAATCCTCAGGAGGGGACCCAGAAATTGTA---ATGCACAGTTTTAATTGTGGAGGAGAA---
TTTAAACAGCTCCAGGCAAGAATCCTGGCTGTAGAAAGATACCTAAAGGATCAACAGCTCCTGGGGATTTGGG
GTTGCTCTGGAAAACTCATTTGCACCACTGCTGTGCCTTGGAATACTAGTTGGAGT-----------------
-------------------------AGTAAAACAAATCTGAGTACAATTTGG------GAT------
AACATGACCTGGATGCAGTGG---GAAAAAGAAATTAGCAATTACACAGACTTAATATAC---
ACCCTAATTGAAGAATCGCAGAACCAACAGGAA--

AAGAATGAACAAGACTTACTGGCATTAGATAAATGGGCAAGTTTGTGGAATTGGTTTGACATAACAAAATGGC
TGTGGTATATAAAAATATTCATAATGATAGTAGGAGGCTTGATAGGTTTAAGAATAGTTTTTACT---
GTATTTCTTTAGTA----------AATAGAGTTAGGCAGGGATATTCACCA------
TTATCGTTTGCTGTTAGCTTGTTCAACGCCATAGCCATAGCAGTAGCTGAG---
GGAACAGATAGGGTTATAGAAATATTACAAAGAGCTTTTAGAGCT----------------------------
--------ATCCTCCACATA------CCTAGAAGAATTAGACAGGGCTTGGAAAGGCTTTGTTG---TAA
>JF320539
GCA---GAA---
CAATTGTGGGTCACAGTCTATTATGGGGTACCTGTGTGG---AAAGAAGCAACC---
ACCACTCTATTTTGTGCATCAGATGCTAAAGCATATGATACAGAG---GTACATAAT----------------
--------------GTTTGGCCACA---CACGCCTGTGTACCCACTGAC---
CCCAGCCCACAAGAAATAAATTTGAAA---------AATGTGACAGAAGATTTTAAC----------------
--------------------------ATGTGGAAAAATGACATGGTAGATCAGATGCATGAGGATATA---
ATCAGTTTATGGGATCAAAGCCTG---AAGCCATGTGTGAAATTG---
ACCCCACTCTGTGTCACTTTAAACTGCAGTAACTTAAATAATAGCACTAGGAATGAG----------------
----
----------------------------AGTAATACTAACTTGGAGGGGAAAGAAGAAATGAAA---
AACTGCTCTTTCAATATCACCACAAGCATAAAA---------------------------
GATAAGGTGCAGGACAATATGCATTG---TTTTATAGTCTTGATATAGTACAAATAGATAAT-----------
----

GATAATACTAGTTATAGGTTGAGAAGTTGTAACACCTCAGTCATCACACAA---
GCCTGCCCAAAGATGTCATTTGAGCCAATTCCCATACATTATTGTGCCCCGGCTGGTTTTGCGATTCTAAAGT
GTAAT---GATAAGAAGTTCAAT------GGATCAGGACCATGTACAAAT-----------------------
-GTCAGC---ACAGTACAATGTACACATGGGATTAGGCCAGTAGTATCAACT---
CAACTGCTGTTAAATGGCAGTCTAGCAGAAGAGGAG------------
GTAGTAATTAGATCTGAAAACTTTACGAACAAT---------GCTAAAACCATAATGGTCCAGTTGAAA----
-----------GATTCTGTAAAC---------ATTACTTGTGCAAGACCCAATAACAATACAAGGAAA-----
-AGTATAAACATA------------GGACCAGGGAGAGCA---TTT------TATGCAACAGGAGAC------
---------ATAATAGGAAATATA------------AGACAAGCACATTGT---AACATTAGTGGAACA---
AAATGGAATAACACGTTAATGCAGGTAGTTAAAAAATTAAGA---------------GAACAATTT-------
-----------------------------------AATAAAACAATAACTTTTAAG------
CCCTCCTCAGGAGGGGACCCAGAAATTGTA---ATGCACACTTTTAATTGTGGAGGAGAA---
TTTAAGCAGCTCCAGGCAAGAGTCCTGGCTGTGGAAAGATACCTAAAGGATCAACAGCTCCTGGGGATTTGGG
GTTGCTCTGGAAAACTCATCTGCACCACTGCTGTGCCTTGGAATGCTAGTTGGAGT-----------------
----------------------------AATAAATCTATGAATGAGATTTGG------AAT------
AACATGACCTGGATACAGTGG---GAAAGAGAAATTGACAATTACACAGGCTTAATATAC---
AGCTTAATTGAACAATCGCAAAACCAGCAAGAA--

AAGAATGAACTAGATTTATTGGCATTAGATAAATGGGCAAGTTTGTGGGATTGGTTTGATATATCAAATTGGC
TGTGGTATATAAAAATATTCATAATGATAGTAGGAGGCTTGATAGGTTTAAGAATAGTCTTTACT---
ATACTTTCTATAGTG---------AATAGAGTTAGGCAGGGGTACTCACCA------

CTGTCGTTTGCTGTTAGCTTGCTCAATACCATAGCCATAGTAGTAGCTGAG---
GGAACAGATAGGGTTCTAGAAATATTGCAAAGAGCTGGTAGAGCT---------------------------
--------ATCCTTAATATA------CCCACAAGAATAAGACAGGGCTTCGAAAGGGCTTTGCTA---TAA
>JF320131
GCT---CAA---
CAATTGTGGGTCACAGTCTATTATGGGGTACCTGTGTGG---AAAGAAGCAACC---
ACCACTCTATTTTGTGCATCAGATGCTAAGACATATGATACAGAG---GTACATAAT----------------
--------------GTTTGGGCCACA---CATGCCTGTGTACCCACAGAC---
CCCAGCCCACAAGAAGTAGAATTGGAA---------AATGTGACAGAAGATTTTAAT----------------
-------------------------ATGTGGAAAAATGATATGGTAGAACAAATGCATGAGGATATA---
ATCAGTTTATGGGATGAAAGCCTA---AAGCCATGTGTAAAATTA---
ACTCCACTCTGTGTTACTTTAAATTGTACTGATTATCAGGGAAATCAGACTAATGCC----------------
----
---ACTGTTACTACTAATATGAAA---
AACTGCTCTTTCAATGTCACCACAGACAGAAGA--------------------------
GATAAGATGAAAAAAGAAAATGCACTT---TTTTATAATCTTGATATAGTACAAGTAGAAAAG----------
----

AACAGTACTAGCTATAGGTTGGTAAGTTGTAATACCTCAGTCATTACACAG---
GCCTGTCCAAAAGTATCCTTTGATCCAATTCCCATACATTACTGTACCCCGGCTGGTTTTGCTATTCTAAGGT
GTAAT---AATAAGACATTCAAT------GGAAAAGGACCATGTACAAAT----------------------
-GTCAGT---ACAGTACAATGTACACATGGAATTAAGCCAGTAGTATCAACT---
CAACTGCTGTTAAATGGCAGCCTAGCAGAAGAAGAG-----------
GTAGTAATTAGATCTGAGAATCTCACAGACAAT---------GGTAAAACCATAATAGTACAACTGAAA----
-----------GATCCTGTAAAT---------ATTACTTGTGTAAGGCCCAATAACAATACAAGAAAA-----
-GGTATACATATA------------GGACCAGGGAGAGCA---ATG------TTTACAACAGGAGAA------
---------ATAGTAGGAGATATA-----------AGACAAGCACATTGT---AACCTTAGTGTGGAA---
AAATGGGATGCCGCTTTAGAACAGATAGTTGGAAAATTAGGA---------------GCACAACTTAAT----
-----------------------------------ATTACATCAATAAGATTTGCT------
AATTCCTCAGGAGGGGACCCAGAAATTGTA---ATGCATAGTTTTAATTGTAGAGGGGAA---
TTTAAACAGCTCCAGGCAAGAGTCCTGGCTGTGGAAAGATACCTAAGAGACCAACAGCTCCTGGGAATTTGGG
GTTGCTCTGGAAAACTCATCTGCACCACTGCTGTGCGTTGGAATAATAGTTGGAGT-----------------
---------------------------AATAAATCTCAGAATGACATTTGG------AAC------
AATATGACCTGGATGCAGTGG---GAAAGAGAAATTGACAATTATACAGGCTTAATATAC---
AACTTACTTGAAGAAGCGCAAAATAAACAAGAA--

AAGAATGAACAAGAATTATTGGAATTGGATAAATGGGCAAGTTTGTGGAGTTGGTTTGATATAACAAACTGGC
TGTGGTATATAAGAATAGTCATAATGATAGTAGGAGGCTTGATAGGTTTAAGAATAGTTTTTACT---
GTACTTTCTATAGTG---------AATAGAGTTAGGCAGGGATACTCACCC------
TTATCGTTTGCTGTCAGCTTGTTTAACGCCATAGCCATCGCAGTAGCTGAA---
GGGACAGATAGGATTATAGAAGTAGTACAAAGGGCTGGTGGAGCT---------------------------
--------GTTCTCCACATA------CCTAGAAGAATAAGACAGGGCTTGGAAAGGGCTTTGCTA---TAA
>HM638503
GCT---GCA---
GGGAAATTTTGGGTCACAGTTTATTATGGGGTACCTGTGTGG---AAAGAAGCAAAC---
ACCACCTTATTTTGTGCATCAGATGCTAAAGCATATGATACAGAG---ATGCATAAT----------------
--------------GTTTGGGCCACA---CATGCCTGTGTACCCACAGAC---
CCCAACCCACAAGAAATAGACTTAGAG---------AATGTGACAGAAGAATTTAAC----------------
-------------------------ATGTGGAAAAATGACATGGTAGAACAAATGCATGAGGATGTA---
ATCAATTTATGGGATCAAAGCCTA---AAACCATGTGTAAAATTA---
ACTCCACTCTGTGTTACTTTAAATTGCACTAAGTTAAATGCTACTATCTTAGCAAATCTTACTAGT-------
----
----------------------AATGGCAACCATAGTATGGGGAACATGACAGAGGAAATGAAA---
AACTGCACTTTTAATATCTCCACAAGTGTAAAA--------------------------
GATAAGATGGAGAAAAAATATGCATTT---
TTTTATAACCTTGATATAGTACAAATAATAGATAGTAGTAACAGACAAATAAATAGTAGTAAC----------
----
AGACAAATAAACGATAGTAATAACAGCACTAATGACAGCACTAGCTATAGGTTGATAAGTTGTAATACCTCAG
TCCTTACGCAG---
GCCTGTCCAAAGGTATCCTTTGAACCAATTCCCATACATTATTGTGCCCCGGCTGGTTTTGCGATTCTAAAAT

GTAAG---GATGTAACATTCCAG------GGACAAGGAAATTGTTCAAAT-----------------------
-GTCAGC---ACAGTACAATGTACACATGGAATTAGGCCAGTAGTATCCACT---
CAATTGCTGTTAAATGGCAGTCTAGCAGAAAAGGAG------------
GTAGTAATTAGATCTGAAAACTTCTCGGACAAT---------GCTAAAACCATAATAGTACAGCTGAAT----
-----------AAAACTGTAGTA---------ATTAACTGTACAAGACCTAACAACAATACAAGAACA-----
-GGTATACATATG------------GGACCAGGGAAAGTA---TTT------TATGCAACAGATAAT------
---------ATAATAGGAGATATA------------AGAAAAGCACACTGT---ACCATTAATAGAACA---
GAATGGAATAACACTTTAAGACAGGTAGCTACAAAATTAAGG---------------GAACAATTTAAT----
-----------------------------------AAAAGTGAAATAGCCTTTAAG------
CCCTCCTCTGGAGGGGACCCAGAAATTGTA---ATGCACACATTTAATTGTGGAGGGGAA---
TTTAAGCAGCTCCAGGCAAGAGTCCTAGCTGTGGAAAGATACCTAAAGGATCAACAGCTCCTAGGGATGTGGG
GTTGCTCTGGAAAACTCATCTGCACCACTGCTGTGCCTTGGAATACTAGTTGGAGT-----------------
----------------AAGAATACG---AATACATCTTTAGATAAGATTTGG------GAT------
ACCATGACCTGGATGCAGTGG---GAAAAAGAAATTGATAATTACACAGGTTTAATATAC---
ACCTTACTTGAAGACTCGCAACTCCAACAAGAC--

AAAAATGAACAAGAATTATTAGAATTAGATAAATGGGCAAGTTTGTGGAATTGGTTTAACATAACAAACTGGC
TGTGGTATATAAAAATATTCATAATGATAGTAGGAGGCTTGATAGGTTTAAGAATAGTTTTTTCT---
ATACTTTCTATAGTG---------AATAGAGTTAGGCAGGGATACTCACCA------
TTATCGTTTGCTGTTAGCTTGTTTGATGCCATAGCCATAGCAGTAGCTGAA---
GGGACAGATAGGATTATAGAAGGATTACGAAGAGTTTTTAGAGCA----------------------------
--------GTTCTCCACGTA------CCTACAAGAATAAGACAGGGCTTGGAAAGGGCTTTGCTA---TAA
>KT124801
GCT---GCA--
GATCAAATGTGGGTCACAGTCTATTATGGGGTACCTGTGTGG---AGAAATGCAACC---
ACCGTTCTCTTCTGTGCATCAGATGCTAAAGCATATGAGACAGAG---GTACATAAT----------------
--------------GTTTGGCCACA---CATGCCTGTGTACCCACAGAT---
CCCAGCCCACAAGAAATGGTATTGGAA---------AATGTGACAGAATATTATGAT----------------
--------------------------ATGTGGAAAAATAACATGGTAGATCAGATGCATGAGGATATA---
ATTAGTTTATGGGATCAAAGCCTA---AAGCCATGTGTAAAATTA---
ACCCCACTCTGTGTTACCTTAAACTGCACTGATAATATTAACAATACCACTAATAATAAC-------------
----
----------------------------------AGGACCATTATAGAGAAAGGAGAAATAAAA---
AATTGCTCTTTCAATGTCACCACAAATATAAGA---------------------------
GATAAGATGCAAAAAGAATATGCACTG---TTTTATAAACTTGATGTAGTACCAATAGATGGGAATAAT----
----

AATAATATTAGTAATTATACAAACTATAGGTTAGGAAGTTGTAACACCTCAGTCATTAGGCAG---
GCCTGCCCAAAAATATCCTTTGAACCAATTCCCATACATTATTGTGCCCCGGCTGGTTTTGCGATTCTAAAAT
GTAAT---GATAAAAAATTCAGT------GGAAAAGGAGAATGTAACAAT-----------------------
-GTCAGC---ACAGTTCAATGTACACATGGAATTAGGCCAGTAGTATCAACT---
CAACTGCTGTTAAATGGCAGCCTAGCAGAAGAGGAG------------
GTAGTAATTAGATCTGACAATTTCTCGAACAAT---------GCTAAAACCATAATAGTACAGTTGAAT----
-----------CAATCTGTAGCA---------ATTAATTGTACAAGACCCAACAACAATACAAGAAAA-----
-AGCATACATATA------------GCACCAGGGAGTGCA---TTT------TATGCAACAGGAGAT------
---------ATAATAGGAGATATA------------AGGCAAGCACATTGT---AACCTTAGTAGAAAA---
GCATGGAACAACACATTAAGACAGGTAGCTGAAAAATTAGGA---------------GAGGAATTTCAG----
-----------------------------------AATAAAACAATAATCTTTACG------
AAATCCTCAGGAGGGGACCCAGAAATTGTA---ATGCACAGTTTTAATTGTGGAGGGGAA---
TTTAAGCAGCTCCAGGCAAGAGTCCTGGCTGTAGAAAGATACCTAAGGGATCAACAGCTCCTGGGGATTTGGG
GTTGCTCTGGAAAACTCATCTGCACCACTGCTGTGCCTTGGAATGTTAGTTGGAGT-----------------
----------------------------AATAAAACTCTGGAACAGATTTGG------GAT------
AATATGACCTGGATGCAGTGG---GAAAAAGAAATTGACAATTACACAGGAATAATATAC---
ACTTTGCTTGAAAAATCGCAAAACCAACAAGAA--

AAGAATGAACAAGAGTTATTGGAATTGGACAAATGGGCAAGTCTGTGGGATTGGTTTGACATAACAAATTGGC
TGTGGTATATAAGGATATTCATAATTATAGTAGGAGGCTTGATAGGTTTAAGAATAGTTTTTGCT---
GTGCTTTCCATAGTG---------AATAGAATTAGGCAGGGTTACTCACCA------
TTATCGTTGGCTGTTAGCTTGCTCAACGCCACAGCAATAGCAGTAGCTGAG---

GGAACAGATAGGATTATAGAATTAGCACAAAGAATTTTTAGAGCT---------------------------
--------TTTCTCCACATC------CCTAGAAGAATAAGACAGGGCTTCGAAAGGGCTTTGCTA---TAA
>JN687763
GCT---GCA---
AACCAATCGTGGGTCACAGTCTATTATGGAGTGCCTGTGTGG---AAAGAAGCAGTC---
ACAACTCTATTCTGTGCATCAGATGCTAAAGCATATGAGACAGAA---GTGCATAAT---------------
--------------GTTTGGGCCACA---CATGCCTGTGTACCCACAGAT---
CCCAATCCACAAGAAGTAATGTTAGAA---------AATGTGACAGAAAATTTTAAC---------------
--------------------------ATGTGGAAAAATAATATGGTAGATCAGATGCATGAGGATATA---
ATCAGTTTATGGGATCAAAGCCTA---AAACCATGTGTAAAATTA---
ACCCCACTCTGTGTTACTTTGCATTGCACTAATTACAATGGTACTAAACCTAAT------------------
----
---------------------------------ATTAGTGAAGAAATGAAAGGAGAAATAAAA---
AACTGCTCTTTCAATGTCTCCACAGATGTAAGA--------------------------
GATAAGGTAAAGAAACAAAATGCATTG---TTTTATAATCTTGATATAATACAAATAAGTGATGATACAACA-
----

CAAGGAAATAATAGTAATAATATTTATAGGTTGACAAGTTGTAATACCTCAGTCATTACACAG---
GCTTGTCCAAAGGTAACCTTTGAGCCAATTCCCATACATTATTGTGCCCCGGCTGGTTTTGCAATTCTAAAGT
GTAAC---AATGAAACATTCAAT------GGAACAGGACCGTGTACAAAT----------------------
-GTCAGC---ACAGTACAATGTACACATGGAATTAAGCCAGTAGTATCAACG---
CAACTACTGTTAAATGGTAGTCTATCAAAAGCAGGG-----------
GTAATACTTAGATCTGAAAATTTCACGGACAAT--------GTTAAAAGCATAATAGTACAGTTGAAT----
-----------GAATCTGTAACA---------ATTAATTGCACAAGACCCAACAATAATACAAGAAAA----
-AGTATAAATATA-----------GGACCAGGGAGGGCA---TGG------TATGCAACAGGAGAA------
---------ATAATAGGAAACATA-----------AGACAAGCACATTGT---AACATTAATAAAGAA---
AAGTGGAATAAAACTTTAGAAAAGATAGTTGAAAAATTAAGA--------------GAACAATATGGG----
--------------------------------AATAATAAAACAATAATCTTTAAG---
CCACCACCTCCAGGAGGGGACCCAGAAATTGTA---ACACATAGTTTCAATTGTGCAGGGGAA---
TTTAAACAGCTCCAGGCAAGAGTCCTGGCTGTGGAAAGATACCTAAAGGACCAACAGCTCCTGGGAATTTGGG
GTTGCTCTGGAAAACTCATTTGCACCACTACTGTGCCTTGGAATACTAGTTGGAGT----------------
----------------------------AATAGATCTCTAGATTACATTTGG------GGT------
AACATGACCTGGATGGAGTGG---GAAAAAGAAATTGAAAGTTACACAAGCGAAATATAC---
ACCTTAATTGAAGCATCGCAGAACCAACAAGAA---------------------------------------
----
AAGAATGAGCAAGAATTATTGGAATTAGATAAATGGGCAAGTTTGTGGAATTGGTTTAACATATCAAATTGGC
TGTGGTATATAAGAATATTCATAATGATAGTAGGAGGCTTGATAGGTTTAAGAATAGTTTTTACT---
GTACTTTCTATAGTG---------AATAGAGTCAGGCAGGGATACTCACCC------
TTATCGTTTGCTGTTAGCTTGCTCAATACAGTAGCCATAGTAGTAGCTGAG---
GGAACAGATAGGATTATAGAAGTGTTACAAAGAATTTTTAGAGCT---------------------------
--------GTTATCCACATA------CCTGTAAGAATAAGACAGGGTTTAGAAAGGGCTTTGCTA---TAA
>JQ403073
GCT---GTA---
GAACAGTTGTGGGTCACAGTCTATTATGGGGTACCTGTGTGG---AAAGAAGCAACC---
ACCACTCTATTTTGTGCATCAGATGCTAAATCATATGACACAGAG---GTACATAAT---------------
--------------GTTTGGGCCACA---CATGCCTGTGTACCCACAGAC---
CCCAGCCCACAAGAAGTATTCCTACCA---------AATGTGACAGAAAACTTTAAC---------------
--------------------------ATGTGGAAAAATAACATGGTAGACCAAATGCATGAGGATATA---
ATCAGTTTATGGGATCAAAGCCTA---AAGCCATGTGTAAAATTA---
ACCCCACTCTGTGTCACTTTAAATTGCACTGATTTTGGGAATGCTACTAATACTGGGAATGTTACTAATGGTG
GGGAAAATGGT---
-------------------ACTAATACTAATGACACATTAAGAATGGAGAAAGGAGAAATAAAA---
AACTGCTCTTTCAATATCACCTCAAACATAAGA--------------------------
GATAAAATGCAGAAAGAAAATGCACTT---
TTTTATAGAGATGATCTAGTGCCAATAGCTAATGATACTGCCAGCCAGACCAATATTAGCGATAGT------
----
AATATTACCAGCAATAATAATACTACCAGATATAAGAAATATATATTGATAAGTTGTAACACCTCAGTCATCA
CACAG---
GCTTGTCCAAAGGTATCCTTTGAGCCAATTCCAATACATTATTGTGCCCCGGCTGGTTTTGCGATTCTAAAGT
GTAAC---GAAGAAGAGTTCAAT------GGAACAGGACCATGTAAAAAT----------------------

-GTCAGC---ACAGTACAATGTACACATGGAATCAGGCCAGTAGTATCAACT---
CAACTACTGTTAAATGGCAGTCTAGCAAAAGGAGAG------------
GTAGTAATTAGATCTGCCAACATCTCAGACAAT---------GCTAAAACCATAATAGTACAGCTGAAC----
-----------GTATCTGTAATA---------ATTAATTGTACAAGACCCAACAACAATACAAGAAAA-----
-AGTATACTTATA------------TCACCAGGGAGAGCA---TTT------TATGCAAGAGAAGGG------
---------ATAATAGGAGATATA------------AGAAAAGCACATTGT---AACATTAGTAGAACA---
GAATGGAATAGGACTCTAAACCAGATAGCTGAAAAATTAAAA------------TTAAGCAAAGAGTTTAAG-
-----------------------------------AATAAAACAATAGTCTTTAAA------
CCATCCTCAGGAGGGGACCCAGAGATTGTG---ATGCACAGTTTTAATTGTGGAGGGGAA---
TTTAAGCAGCTCCAGGCAAGAATCCTGGCTGTGGAAAGATACCTACAGGATCAACAGCTCCTGGGGATTTGGG
GCTGCTCTGGAAGACTCATTTGCACCACTGCTGTGCCTTGGAATACTAGTTGGAGT-----------------
----------------------------AATAAATCTCTGAATAAGATTTGG------GAT------
AACATGACCTGGATGGAATGG---GAAAGAGAAATTGACAATTACACAGGCTTAATATAC---
AACCTACTTGAAGAATCGCAGAACCAACAAGAA--

AAGAATGAACAAGAATTATTGGAATTGGATAAATGGGCAAGTTTGTGGACTTGGTTTAACATAACAAATTGGC
TGTGGTATATAAAATTATTCATAATGATAGTAGGAGGCTTGATAGGTTTAAGAATAATTTTTGCT---
GTACTTTCTATAGTG---------AATAGAGTTAGGCAGGGATACTCACCA------
TTATCGTTTGCTGTTAGCTTGCTCAATACCACAGCAATAGCAGTAGCTGAG---
GGAACAGATAGGATTATAGAAATAGTACAAAGGGCTTTTAGAGCT----------------------------
--------ATTCTCCACATA------CCTACAAGAATAAGACAGGGTTTGGAAAGGGCTTTGCTA---TAA
>JQ403078
GCT---ACA--
GAAAAATTGTGGGTCACAGTCTATTATGGGGTACCTGTGTGG---AAAGAAGCAACC---
ACCACTCTATTTTGTGCATCAGATGCTAAGGCATATGCTGCAGAG---GCACATAAT----------------
--------------GTTTGGGCTACA---CATGCCTGTGTACCCACAGAC---
CCCAACCCACAAGAAGTAGTATTGGGA---------AATGTGACAGAAAATTTTAAT----------------
--------------------------ATGTGGGAAAACAACATGGTAGAACAGATGCATGAGGATATA---
ATCAGCTTATGGGATCAAAGCCTA---AAGCCATGTGTAAAATTA---
ACCCCACTCTGTGTTACTTTACATTGCACTAATTATAATAATACTAATAATACTGTTACCAATACTAATAATA
CT---
-------------GTTACCAATACTAGCAATAGTGAAATACCAATGGAGGTAGGAGAAATAAAA---
AACTGCTCTTTCAATATTTCCTCATCAATAAAT---------------------------
AAAAAGGTAAAGAAAGAATATGCACTT---TTTTATAGCATTGATGTAGTACCATCAGAAGAA----------
----

AATAATACAAGCTATACATTGAGAAGTTGTAACACCTCAGTCATTACACAA---
GCTTGTCCAAAGGTATCCTTTGAACCAATTCCCATACATTATTGTGCCCCGGCTGGTTTTGCGATTCTAAAAT
GTAAT---GCTGAGGATTTCAAT------GGAACAGGACCATGTAAAAAT-----------------------
-GTCAGT---ACTGTACAATGTACACATGGAATTAAGCCAGTAGTGTCAACT---
CAACTGCTGTTAAATGGAAGTCTAGCAGAAAAAGAG------------
GTAGTAATCAGGTCTGAAAATTTCACGAACAAT---------GCTAAAACCATAATAGTACAGCTGAAG----
-----------GACCCTATAAAA---------ATTAATTGTACAAGACCCAACAACAATACAAGAAGA-----
-GGTATACATATA------------GGACCAGGGGGAGCA---TTG------TATGCAACAGGACAA------
---------ATAATAGGAGATATA------------AGACAAGCATATTGT---AACATTAGTAGAATA---
CAATGGAATAATACTTTAATACAGATAGTTAAAGTATTACAA---------------GAACAATTTAAA----
-----------------------------------AATAAAACAATAGTCTTTAAT------
CGATCATCAGGAGGGGACCCAGAAATTGTA---ATGCACAGTTTTAATTGTGGAGGGGAA---
TTTAAACAGCTCCAGGCAAGAGTCCTGGCTGTGGAGAGATACCTAAAGGATCAACAGCTCCTGGGAATTTGGG
GTTGCTCTGGAAAACTCATCTGCACCACTGCTGTGCCTTGGAATACTAGTTGGAGT-----------------
----------------------------AATAAATCTCTGGATGAGATTTGG------AAT------
AACATGACCTGGATGCAGTGG---GAGAAAGAAATTGACAATTACACAGACTTAATATAC---
AATTTAATTGAACAATCGCAAAACCAGCAAGAA--

AAGAATGAACAAGAATTATTGGAATTGGATAAGTGGGCAAGTTTGTGGAATTGGTTTAGCATAACAAAATGGC
TGTGGTATATAAAAATATTCATAATGATAGTAGGAGGTTTAATAGGTTTAAGAATAGTTTTTGCT---
GTGCTTTCTATAGTA---------AATAGAGTTAGGCAGGGATATTCACCA------
TTATCGTTGGCTGTTAGTTTGCTTAATGCCACAGCTATAGCAGTAGCTGAG---
GGGACAGATAGGATTCTAGAAGTAGTACAGAGAATTGGTAGAGCT----------------------------
--------ATTATACACATA------CCTACAAGAATAAGACAAGGCTTTGAAAGGGCTTTGCTG---TAA

>JQ610064
GCT---GCA--
GAAAAATTGTGGGTCACAGTCTATTATGGGGTACCTGTGTGG---AGAGATGCAAAT---
GCTACCCTATTTTGCGCATCTGATGCTAAAGCATATGTTACAGAG---GTACATAAT----------------
--------------GTTTGGGCCACA---CATGCCTGTGTACCCACAGAC---
CCCAACCCACAAGAATTAGTATTAGAA---------AATGTGACAGAAGATTTTAAC----------------
--------------------------ATGTGGAAAAATAATATGGTAGACCAGATGCATGAGGATATA---
ATCAGTCTGTGGGAACAAAGCCTA---AAACCATGTGTAAAATTA---
ACCCCACTCTGTGTTACTTTAAATTGCACTGATCCAAAACCTTTAAATTACACTGATCTAAATAATTCAAGTT
GCACTGATCTACAAGGCAACGAAACTAGCAATAAT------------------------------
GGCACTGGTTCGGCTAGCAACATTAGTAATAGTTATAATAGCAGCTGGGAAGACATGAAAGGAGAAATAAAA-
--AACTGCTCTTTCAATATCACCACAAGCATG------------------------------
AATAAGGTGCAACAAAAATATTCAATG---TTTAATAGACTTGATATAGTGCCAATAGATAAT----------
----

GATAGTAATAGTACTAGCTATATGTTGATAAGTTGTAATACCTCAGTCATTACACAG---
GCCTGTCCAAAGGTATCCTTTGAGCCAATTCCCATACATTATTGTGCCCCGGCTGGTTTTGCGATTCTAAAGT
GTAAT---AATAAGACATTCAAT------GGAACAGGGAAATGTACAAAT-----------------------
-GTCAGC---ACAGTACAATGTACACATGGAATTAGACCAGTAGTATCAACT---
CAACTGCTGTTAAATGGCAGTCTAGCAGAAGAGGAG------------
GTAATAATTAGATCTGCCAACTTCTCGGACAAT---------GCTAAAACCATAATAGTACAGCTGAAG----
-----------GACCCTGTACAA---------ATTAATTGCACAAGACCCAACAACAATACAAGGAAA-----
-AGTATACATATA------------GGACCAGGAAGAGCA---TTT------TATACAACA---GAC------
---------ATAATAGGAGATATA------------AGACGAGCACATTGT---AACATTAGTAAAGCA---
AGATGGAGGGAAGCTTTAAAACAGACAAGTAAAAAATTAATA---------------GAACAATATAAT----
-----------------------------------ACATCAAAAATAATCTTTAAA------
CCATCCTCAGGAGGGGACCCAGAAATTGTA---ATGCACACCTTTAATTGTGGAGGGGAA---
TTTAAACAGCTCCAGGCAAGAGTCCTGGCTATAGAAAGATACCTAAGGGATCAACAGATCCTAGGGATTTGGG
GTTGCTCTGGAAAACTCATTTGCACCACTACTGTGCCTTGGAATGCTAGTTGGAGT-----------------
-------------------------AAAAATAGATCTCTAGATGACATTTGG------CAT------
AACATGACCTGGATGCAGTGG---GAAAAAGAAATTGAAAATTACACAAGTACAATATAC---
AACTTAATTGAAATATCGCAAAACCAGCAGGAA--

AAGAATGAACAAGAATTATTGGAATTGGATAAGTGGGACAGTTTGTGGAATTGGTTTGACATAACAAACTGGC
TGTGGTATATAAAAATATTCATAATGATAGTAGGAGGCTTAATAGGTTTAAGAATAGTTCTTGCT---
GTAATTTCTATAGTA---------AATAGAGTTAGGCAGGGATACTTACCA------
CTATCATTTGCTGTTAACTTGCTTAATACCATAGCTATAGCAGTAGGTGAG---
GGGACAGATAGGGTTATAGAGGTAGTACAAAGAACTTGTAGAGCT----------------------------
--------ATTATCCACATA------CCTAGAAGAATAAGACAGGGCTTGGAAAGGGCTTTGCAA---TAG
>HQ217713
GCT---GCA--
GAACAATCGTGGGTCACAGTCTATTATGGGGTACCTGTGTGG---AGAGAAGCAACT---
ACCACTCTATTCTGTGCATCAGATGCTAAAGCATATGACACAGAA---AAACATAAT----------------
--------------GTTTGGGCCACA---CATGCCTGTGTACCCACAGAC---
CCCAACCCACAAGAAGTACAATTA------------AATGTGACAGAAAATTTTAAC----------------
--------------------------ATGTGGAAAAATAACATGGTAGACCAGATGCATGAGGATATA---
ATCAGTCTATGGGATCAAAGCCTA---AAGCCATGTGTACAATTG---
ACCCCACTCTGTGTTACTTTAAATTGCTCTGATACTATCAATGCCACTACTGCCAATAATACTATCAATGCCA
CTACTACC---
-------ACTCCTACTATCAATGCCACTACTGCCAATAAGAGTATGGAAATAGGAGAAATGAGA---
AACTGCTCTTTCAATATCACCAACATGGGA---------------------------------
AAGAAAATGAAAGAATATGCGCTT---TTTTATAACCTTGATGTAGTGTCAATAGATGAAGAT----------
----
-------------AATAATAATAAAACAAGCTATAGGTTGAAGAGTTGTAACACCTCAGTCATTACACAA---
GCCTGTCCAAAGGTATCCTTTAAACCAATTCCCATACATTATTGTGCCCCGGCTGGTTTTGCGATTCTAAAGT
GTAAC---GATAAGAAGTTCAAT------GGAACAGGACCATGTGGAAAT-----------------------
-GTCAGC---ACAGTACAATGTACACATGGAATTAAGCCAGTAGTATCAACT---
CAACTGCTGTTAAATGGCAGTCTAGCAGAAGAAGAG------------
GTAGTAATTAGATCTGAAAATTTCACGAACAAT---------GTTAAAACCATAATAGTACAGCTGAAA----
-----------AACCCTGTAATG---------ATTAATTGTACAAGACCCAACAACAATACAAGAAAA-----

-AGTATACATATG------------GGACCAGGGCAAGCA---TTT------TATGCAACAGGAGCA------
---------ATAATAGGAGATATA------------AGACAAGCACATTGT---AATATTAGTGAAAAA---
GAATGAAATAACACTTTAGAATTGATAGTTGGGAAATTAAGA---------------GAACAATTTAG----
-----------------------------------AATAAAACAATAGTCTTCAAT------
CAATCCGCAGGAGGAGACCTAGAAATTGTA---ACGCACAGTTTTAATTGTGGAGGGGAA---
TTTAAGCAGCTCCAAGCAAGAGTCCTGGCTGTGGAAAGATACCTAAAGGATCAACAGCTCCTGGGGATTTGGG
GTTGCTCTGGAAAACTCATTTGCACCACTACTGTGCCTTGGAATACTAGTTGGAGT-----------------
----------------------------AATAAATCTGTAGATTACATTTGG------GCG------
AACATGACCTGGATGCAGTGG---GAAAAAGAAATTGACAATTACACAAACGAAATATAC---
AGGTTAATTGAACAATCGCAAAACCAACAAGAA--

AAGAATGAACAAGAATTATTGGAATTGGATAAATGGGCAAGTTTGTGGAATTGGTTTGACATAACAAACTGGC
TGTGGTATATAAAAATATTCATAATGATAATAGGAGGCTTGGTAGGTTTAAGAATAGTTTTTACT---
GTACTTTCTATAGTA---------AATAGAGTTAGGCAGGATATTCACCA------
ATATCGTTTGCTGTTAGCTTGCTCAACACCACAGCCATAGCAGTAGCAGAG---
GGGACAGATAGAGTTATAGAAATAGTTCAAAGAGCTTGCAGAGCT----------------------------
--------GTGCTACACATA------CCTAGAAGAATAAGACAGGGCTTGGAAAGGATTTTGCTA---TAA
>MK383397
GCT---GCA--
GAAAATTTGTGGGTCACAGTATATTATGGGGTACCTGTGTGG---AAAGATGCGAAC---
ACCACTCTATTTTGTGCATCAGATGCTAAAGCATATGATAAAGAG---GCCCATAAT----------------
--------------GTTTGGCCACA---CATGCCTGTGTACCCACAGAC---
CCCAACCCACAAGAAGTAAAATTGGAA---------AATGTAACAGAAAATTTTAAC----------------
--------------------------ATGTGGAAAAATAACATGGTAGAACAAATGCATGAAGATGTA---
ATTAGTTTATGGGATCAAAGTCTA---AAACCATGTGTAAAATTA---
ACCCCACTCTGTGTTACTTTAAACTGCACTGATTATAATACTACTAGTACCAATAGCACTGGTGTTGCTAGTA
ATGCTACTACC--
----ACTAGCGCTCCTGTTACTAGTACCACTAGTAGTCTAAAAATGGAGAGAGGAGAAATAAAA---
AACTGCTCTTTCAATGTCACCTCAAACATGAGA---------------------------
GATAAGGTACAGAAAGAATATGCACTT---TTTTATAGACTTGATGTAGAGCAAATAGGGAATAAT-------
----

GATAATAATAGTTATAGGTTGATAAGTTGCAATACCTCACGCCTTACACAG---
GCCTGCCCAAAAATATCCTTTGAACCAATTCCCATACATTATTGTGCCCCGGCTGGTTTTGCGATTCTAAAAT
GTAAT---AATAAGGAGTTCAAT------GGAACAGGACCATGTAGCAAT-----------------------
-GTCAGC---ACAGTACAATGTACACATGGAATTAAGCCAGTAGTATCAACT---
CAGCTGCTGTTAAATGGCAGTCTAGCAGAAAGAGAG------------
GTAGTGATTAGATCTCAAAATTTCTCAGACAAT---------ACTAAAACCATAATAGTACAGCTGAAC----
-----------GAAACTGTACAA---------ATTAATTGTACAAGACCCGGCAACAACACAAGAAAA-----
-AGTATATCTCTA------------GGACCAGGGAGTGCA---ATT------TATGCAACAGGACAA------
---------ATAATAGGAGATATA------------AGACAAGCACATTGT---AACATTAGTACAGAA---
AAATGGAATAACACTTTAAAACAGGTAGTTAAAAAATTAAGG---------------GGACAATATGGG----
--------------------------------GAAAATAAAACAATAATCTTTAGT------
AGTTCCTCAGGAGGGGACCCAGAAATTGTA---ATGCACAGTTTTACTTGTGGAGGAGAA---
TTTAAACAGCTCCAGGCAAGAATCCTGGCTGTGGAAAGATACCTAAGGGATCAACAGCTCCTGGGGATTTGGG
GCTGCTCTGGAAAACTCATCTGCACCACTGCTGTGCCTTGGAATAATAGTTGGAGT-----------------
----------------------------AATAGATCTCAGGAGGACATTTGG------CAG------
AACATGACATGGATGCAGTGG---GAAAGAGAAATTGACAATTACACAGGCTTAATATAT---
GACTTACTTGCAAAATCGCAAGACCAACAAGAA--

AAGAATGAAAAAGAATTATTGGAATTAGATAAATGGGCAAGTTTGTGGAGTTGGTTTGACATAACACACTGGC
TGTGGTATATAAAAATATTCATAATGATAGTAGGAGGCTTGGTAGGTTTAAGAATAGTTTTCCTT---
ATACTTTCTGTAGTA---------AATAGAGTCAGGCAGGATACTCACCA------
TTATCGTTTGCTATTAACTTGTTCAATACCACTGCTATAGCAGTAGCTGAG---
GGGACAGATAGGGTTATAGAAGTAGTACTAAGAATTTATAGGGCT----------------------------
--------ATCCTCCACATA------CCTAGAAGAATAAGACAGGGCTTTGAAAGAATTTTACTA---TAA
>KT124761
GCT---ACA--
GAACAATGGTGGGTCACAGTATACTATGGGGTACCTGTGTGG---AAAGAAGCAACC---
ACCACTTTATTTTGTGCATCAGATGCTAAGGCATATGACAAAGAG---GCACATAAT----------------

--------------GTTTGGGCCACA---CATGCATGTGTACCCACAGAC---
CCTGACCCACAAGAAATACCAATGCCA---------AATGTGACAGAAAATTTTAAC----------------
--------------------------ATGTGGAAAAATGACATGGTAGAACAAATGCATGAGGATATA---
ATCAGCTTATGGGATGAAAGCCTA---AAACCCTGTGTAAAATTA---
ACCCCACTCTGTGTTAATTTAAGTTGTACTAATGATATTAACATTAATACTACCAATATAAATAATACTGGTA
CCTCTACTAAT--
-------------GTTACTACCAATAATACTAGTGATCTGGGAAAAATGTCAGAAGGAATGAAA---
AACTGCTCTTTCAATATC---ACAAGCATAGGA---------------------------
AATGAGGTAAAGAAAGAATATGCACTC---TTTCATACACTTGATATAAAATCACAACATAGT----------
----

GGAGGCTATATGTTGACAAGTTGTAATGCCTCAGTCATTAAACAG---
GCCTGTCCAAAGACCTCCTTTGAACCAATTCCGATACATTATTGTGCCCCGGCTGGTTTTGCGATTTTAAAGT
GTAAG---GATAAGAAGTTCAAT------GGAACAGGACCATGTAAAAAT-----------------------
-GTCAGC---ACAGTACAATGTACACATGGAATTAAGCCAGTGGTGTCAACT---
CAACTGCTGTTAAATGGCAGTCTAGCAGAAGAAGAG------------
ATAGTAATTAGATCTGAAAATATTACAAACAAT---------GCTAAAAACATAATAGTACAGCTAAAA----
-----------ACATCTGTAGAA---------ATAAATTGTACAAGACCCGGCAACAATACAAGAAGA-----
-AGTATACGTATA------------GGACCTGGGAGTGCA---TAT------TTCAACACAGGAGAC------
---------ATAATAGGAAATCCA------------AAACAAGCACATTGT---AACGTTAGTGGAGCA---
GCATGGAAAGAAACTTTAAAACAGGTAGCTACAAAATTAAGA---------------GAACAATTTATT----
-----------------------------------AATAAAACAATTACCTTTACT------
AACCACTCAGGAGGAGACCCAGAAATCACA---ATGCACAGTTTTACTTGTGGAGGGGAA---
TTTAAGCAGCTCCAGGCGAGAGTCCTGGCTGTAGAAAGATACCTAAGGGATCAACAGCTCCTAGGAATTTGGG
GTTGCTCTGGAAAACTCATATGCCCCACCGCTGTGCCTTGGAATGTTAGTTGGAGT-----------------
-------------------------AATAGAAGTAATCTAAGTGCTATTTGG------GAA------
AACATGACCTGGATGGAGTGG---GAGAGAGAAATTGACAATCATACAGAATTAATATAC---
ACACTATTAGAAAAATCGCAAAACCAGCAAGAA--

AAGAATGAACAAGAATTATTGGCATTGGATAAGTGGGCAGATTTGTGGAGTTGGTTTGATATAACAAAATGGC
TGTGGTATATAAGAATATTCATAATGATAGTAGGAGGCTTAATAGGCTTAAGAATAGTTTTTACT---
GTACTTTCTATAGTG---------AATAGAGTTAGGCAGGGATACTCACCA------
TTGTCATTCGCTATTAGCTTGCTTAATGCCACAGCTATAACAGTAGCAGAG---
GGGACAGATAGGGTTATAGAAGTAATACAAAGAGCTGGGAGAGCT----------------------------
--------ATTCTCCACATA------CCTAGAAGAATAAGACAGGGCTTAGAAAGGGCTTTGCAA---TAA
>KM353846
GCT---GCA--
GAAAATTTGTGGGTCACAGTCTATTATGGGGTACCTGTGTGG---AAAGAAACAACC---
ACCACTCTATTCTGTGCATCAGATGCTAAAGCATATGACACAGAG---GTACATAAT----------------
--------------GTTTGGGCCACA---CATGCCTGTGTACCCACCGAC---
CCCAACCCACAAGAAGTGGAATTAAAA---------AATGTGACAGAAAATTTTAAT----------------
--------------------------ATGTGGAAAAATGACATGGTAGACCAGATGCATGAGGATATA---
ATCAGTTTATGGGATGAAAGCCTA---AAGCCATGTGTAAAATTA---
ACCCCACTTTGTGTTACTTTAAATTGCAGTGATGTAAGGAATGATACTACTACTAATACCAAT----------
----
-------------------------AATAGCTGGGGAAATAAAATGGAGAGGGGAGAAATGAAA---
AATTGCTCTTTCAATGTCACCACAAGCATAAGA---------------------------
GATAAGGCACAGAAAGAAGCGGCACTT---TTTTATAGACTTGATATAGTACCAATAGAAGATAATAATGAT-
----

AATAGTAGCTCTAGAAATTATAGTAGCTATAGATTGATAAATTGTAATACCTCAGTCATTACACAG---
GCCTGTCCAAAGATATCCTTTGAGCCAATTCCCATACATTATTGTGCCCCGGCTGGTTTTGCGATTCTAAAAT
GTACT---AATAAGACGTTCCAG------GGAAAAGGACCATGCACAAAT-----------------------
-GTCAGC---ACAGTACAATGCACACATGGAATTAGGCCAGTAGTGTCAACT---
CAACTGCTATTAAATGGCAGTCTAGCAGAAGAAGAG------------
GTAGTAATTAGATCTGATAATTTCACAGACAAT---------GCTAAAATCATAATAGTGCAGCTGAAT----
-----------GAATCTGTAGTA---------ATTAATTGTACAAGACCCAACAACAATACAAGAAAA-----
-AGTATATCTCTA------------GGACCAGGGAGCGCA---TGG------TATGCAACAGGAGAT------
---------ATAATAGGAGATATA------------AGACAAGCACATTGT---AACATTAGTGGAGCA---
AACTGGACTAGAGCTTTAAAACTAATAAGTGAAAAATTAAAA---------------GAACAATTTGTC----

-----------------------------------AATAAAACAATAACCTTTAAA------
CCATCTTCAGGAGGGGACCCAGAAATTGTT---ATGCACAGCTTCAATTGTGGAGGGGAA---
TTTAAGCAGCTCCAGGCAAGAGTCCTGGCTGTAGAAAGATACCTAAGGGATCAACAGCTCCTAGGAATTTGGG
GTTGCTCTGGAAAACTCATCTGCACCACTACTGTGCCTTGGAATACTAGTTGGAGC-----------------
----------------------------AATAAATCCCTGGATTACATTTGG------CAA------
AATATGACTTGGATGCAGTGG---GAAAGTGAAATTGAAAATTACACAGACCTAATATAT---
GACTTAATTGAAAAATCGCAGAACCAGCAAGAA--

AAGAATGAACAAGAATTATTGGAATTGGACAAATGGGCAAATTTGTGGTCTTGGTTTGACATAACAAACTGGC
TGTGGTATATAAAAATATTCATAATGATAGTAGGAGGCTTGATAGGTTTAAGAATAATTTTTACT---
GTGCTTTCTATAGTA---------AATAGAGTTAGGAAGGGATACTCACCA------
TTGTCATTCGCTGTTAACTTGTTTAATACCACAGCAATAGTAGTAGCTGAA---
GGGACAGATAGGATTATAGAAACAGTAAGAAGAATTTTTAGAGCT----------------------------
--------TTTCTACATATA------CCTACAAGAGTAAGACAGGGCTTAGAAAGGGCTTTGCTA---TAA
>KY112135
GCT--
GAAGATATGTGGGTCACAGTCTATTATGGGGTACCTGTGTGG---AAGGAAGCAACT---
ACTACTCTATTTTGTGCATCAGATGCTAAAGCATATGTTACAGAG---AAACATAAT----------------
--------------GTTTGGGCCACA---CATGCCTGTGTACCCACAGAC---
CCCAACCCACGAGAAGTAGTATTAGGA---------AATGTGACAGAAGAATTTAAC----------------
--------------------------ATGTGGAAAAATAACATGGTAGAACAGATGCATGAGGATGTA---
ATCAGTTTATGGGAACAAAGCCTG---AAGCCATGTGTAAAATTA---
ACCCCACTCTGTGTTACTTTAAATTGCACTGATAATGTTGCTAGTACCACCACCAATAATAGTACTAATGCCA
CTGTTACTAGT--
---AAGGGAGAAATAAAA---
AACTGCTCTTTCAATGTTACCACTAGCATAAGA---------------------------
GATAAAGTTAAGAAAGAATATGCACTC---TTTTATAAACTTGATGTAGTACCAATAGATAAT----------
----

ACTACTGTTAATAATTATACCAATTATAGATTGATAAATTGTAACACCTCAGTCATTACACAG---
GCCTGTCCAAAGGTTACATTTGAGCCAATTCCTATACATTATTGTGCCCCGGCTGGTTTTGCAATACTAAAGT
GCAAT---GATAAGAAGTTCAAG------GGAAAAGGACCATGTGCAAAT-----------------------
-GTCAGC---ACAGTACAATGTACACATGGAATTAGGCCAGTAGTGTCAACC---
CAGCTGCTGTTAAATGGCAGTCTAGCAGAAGACGAT------------
GTAGTAATTAGATCTGACAATTTCTCGGACAAT---------GCTAAAATCATAATAGTACATCTGAAC----
-----------GAATCTATAAAA---------ATTAATTGTACAAGACCCAATAACAATACAAGAAAA-----
-AGTATACCTATA------------GGACCAGGGAGAGCA---TTT------TATGCAACAGGAGAC------
---------ATAATAGGAGATATA------------AGACAAGCACATTGT---AACATAAGTAAAAAA---
GACTGGAACAAAACTTTACAACTAGTAGTGGAAAAGTTACGA---------------GAAGAATTTAAT----
-----------------------------------AAAACAACAATAATCTTTAAA------
AACTCCTCAGGAGGGGACCCAGAAATTGTA---ATGCACAGTTTTAATTGTGGAGGGGAA---
TTTAAGCAGCTCCAGGCAAGACTCCTGGCTGTGGAAAGATACCTAAGGGATCAACAGCTCCTAGGGATTTGGG
GTTGCTCTGGAAGAATCATTTGCACCACTACTGTGCCTTGGAATGCTAGTTGGAGT-----------------
----------------------------AATAAAACTTACAATGAGATTTGG------GAG------
AATATGACCTGGATGCAGTGG---GAAAGAGAAATTGACAATTACACAAGCTTAATATAC---
ACCTTAATTGAGGAATCACAGAACCAACAAGAA--

AAGAATGAGCAGGATTTATTGGCATTAGATCAGTGGGCAAGTTTGTGGAATTGGTTTAGCATAACACAGTGGC
TGTGGTATATAAAAATATTCATAATGATAGTAGGAGGCTTGGTAGGTTTAAGAATAGTTTTTGCT---
GTGCTTTCCATAGTG---------AATAGAGTTAGGCAGGGATACTCACCA------
TTATCATTTGCTGTCAGCTTGCTTAATGCCACAGCTATAGCAGTAGCTGAG---
GGGACAGATAGGGTTATAGAAGCAGCACGAACAATATTTAGAGCT----------------------------
--------TTTATACACATA------CCTAGAAGAATAAGACAGGGCTTGGAAAGGGCTTTGCTA---TAA
>JQ403070
GCT---AAT---AAT------------------------GCT---AAA---
GAACAATTGTGGGTCACAGTCTATTATGGGGTACCTGTGTGG---AAAGAAGCGACC---
ACCACTCTATTTTGTGCATCAGATGCTAAGGCATATGACACTGAA---GTACATAAT----------------
--------------GTTTGGGCCACA---CATGCCTGTGTACCCACAGAC---
CCTAATCCACAAGAGATAGAATTGAAA---------AATGTGACAGAAAATTTTAAC----------------
--------------------------ATGTGGAAAAATAATATGGTAGAACAGATGCATGAGGATATA---

ATCAGTTTATGGGATCAAAGCCTA---AAGCCATGTGTAAAATTA---
ACCCCAGTCTGTGTTAAGTTAAATTGCACGAATGTCAATTTTACGAAGACTGATGATACAAAT----------
----
----------------------ATCTATAATGGTACCTTGAAGGTAGAGGTAGGAGAAGTAAAA---
AACTGCTCTTTCAATGTCACCACAATGATAAAA---------------------------
AATGAGGCGCGGAAAGAATATGCACTT---TTTTATAAACTTGATATAGTACCAATAGAGGGT----------
----

GATAATACAAGCTATACTTTGATAAATTGTAATACCTCAGTCATTACACAA---
GCCTGTCCAAAGGTATCCTTTGAACCAATTCCCATACATTACTGTGCCCCAGCTGGCTTTGCGATTCTAAAGT
GTAGG---GATAAGAAGTTCAAT------GGAACAGGACCATGTAAAGAG-----------------------
-GTCAGC---ACAGTACAATGTACACATGGAATTAGGCCAGTAGTGTCAACT---
CAACTGCTGTTAAATGGCAGTCTAGCAGAAGAAGAG------------
GTAGTACTTAGATCTGAAAATTTCACGGACAAT---------GTTAAAACCATAATAATACAGCTGAAT----
-----------AAATCTATACAA---------ATTAATTGTACAAGACCCAACAACAATACAAGCAAG-----
-AGCATACCTATG------------GGGCCGGGGAGAGCA---TTT------TATGCAACAGAAAGA------
---------ATAATAGGAGATATA------------AGACAAGCACATTGT---AACATTAGTAGAAAA---
GATTGGAATAACACTTTAAAAATGGTAGTTGCAAAATTAAGA---------------GAACAATATAAT----
-----------------------------------AATAAAACAATAATATTTAAC------
CAATCCTCAGGAGGGGATCCAGAAATTGTA---ATGCACAGTTTTAATTGTGGAGGGGAA---
TTTAAACAGCTCCAGGCGAGACTCCTGGCTGTGGAAAGATACCTAAAGGATCAACAGCTCCTAGGGATTTGGG
GTTGCTCTGGGAAACTCATTTGCACCACTAATGTGCCTTGGAATATTAGTTGGAGT-----------------
----------------------------AATAAATCTCTTGATCAGATTTGG------GAT------
AACATGACCTGGATGCAGTGG---GAGAGAGAGATTGACAATTACACAGGTTTAATATAC---
AGCTTAATTGAAGAATCGCAAACCCAACAAGAA--

AAGAATGAACAAGAATTATTGCAATTGGATACATGGGCAAGTTTGTGGACTTGGTTTTCCATAACAAATTGGC
TGTGGTATATAAGAATATTCATAATAATAGTAGGAGGTTTAATAGGTTTAAGAATTGTTTTTACT---
GTACTTTCTATAGTG---------AATAGAGTTAGGCAGGGATACTCACCT------
CTGTCATTTGCTGTTAGTTTGCTTAATGCCACAGCTATAGCAGTAGCTGAG---
GGGACAGATAGGATTATAGGAGTAATACAAAGCATTGGCAGAGCT----------------------------
--------ATTCTCCATATA------CCTAGAAGAATCAGACAGGGCTTAGAAAGGGCTTTGCTA---TAA
>JQ403031
GCT---GCA--
GAACAATTGTGGGTTGCAGTATATTATGGGGTACCTGTGTGG---AAAGATGCAAAC---
ACCACTCTATTTTGTGCATCAGATGCTAAAGCATATTCCACAGAG---GCACATAAT----------------
--------------GTTTGGCCACA---CATGCCTGTGTACCCACAGAC---
CCCAACCCACAAGAAGTAATGATGGAA---------AATGTGACAGAAAATTTTAAC----------------
--------------------------ATGTGGAAAATAATATGGTAGAACAGATGCATGAAGATATA---
ATCAGTTTATGGGATCAAAGCTTA---AAGCCATGTGTAAAATTA---
ACCCCACTCTGTGTTACTTTAAACTGCAGTGATTATAATCCTCCTCCTATCAATAGTAGTAGTGTTGACAATA
ATAGTGTTGCC--
----------------------------AGCAATATTAGTGAGGAAATGAAAAGAGAAATAAAA---
AACTGCTCTTTCAATATCACCACAACAATAAGA---------------------------
GATAAGGTGACAAAAGTACAAGCTCTT---TTTTATAGACTTGATGTAGTACCAATAGATGAT----------
----

AATGGTAATAATAGTAACATATATAGGTTAATAAATTGTAATACCTCAGCCATTACACAG---
GCTTGTCCAAAGATATCCTTTGAGCCAATTCCCATACACTATTGTGCCCCGGCTGGTTTTGCGATTTTAAAGT
GTAAC---AATAAGACATTCAGT------GGAGTAGGACCATGTACCAAT-----------------------
-GTCAGC---ACAGTACAATGTACACATGGAATTAGGCCAGTAGTGTCAACT---
CAGCTGTTGTTGAATGGCAGTCTAGCAGAAAAAGAG------------
ATAGTAATTAGATCTAAAAATTTCACAGATAAT---------GCTAAAACTATAATAGTACAACTGAAT----
-----------GAATCCGTAGTA---------ATTAATTGTACAAGACCTCACAACAACACAAGAAAA-----
-GGTATACATATA------------GGACCAGGGCAAGCA---TGG------TATGCAACAGGAGAA------
---------ATAATAGGAGATATA------------AGACAAGCACACTGT---AACATTAGTAAAACA---
GCATGGAATAATACTTTAAGAAAGGTAGCTGCAAAATTAAGA---------------GAAATAATTAAT----
-----------------------------------GCAACAACAATAATCTTTAAT------
CACTCCTCAGGGGGAGACCCAGAAGTTGTA---TTGCACAGCTTTAATTGTGGAGGGGAA---
TTTAAGCAGCTCCAGGCAAGGATCCTGGCTGTGGAAAGATACCTAAGGATCAACAGCTCCTAGGGATTTGGG

GCTGCTCTGGGAAAATCATCTGCAACACTGCTGTGCCTTGGAATACTAGTTGGAGT-----------------
-----------------------------AATAAATCTCTTGATGACATTTGG------ACT------
AACATGACCTGGATAGAGTGG---GAAAAAGAAATTGGCAATTACACAAGCTTAATATAC---
ACCTTACTTGAAACAGCGCAGAACCAGCAAGAA---

AAGAATGAACAAGAGTTATTGGAATTGGATAGTTGGGCAAGTCTGTGGAGTTGGTTTGACATAACAAAATGGC
TGTGGTATATAAAGATATTCATAATGATAGTAGGAGGCTTAATAGGTTTTAGAATAGTTTTTGCT---
GTATTTTCTATAGTA---------AATAGAGTTAGGCAGGGATATTCACCA------
TTATCATTGGCTATTAGCTTGCTGAATGCCACAGCTGTAGCAGTAGCTGAA---
GGGACAGATAGGATTATAGAAGTAGTACAAAGAGCTTATAGAGCT----------------------------
--------TTCATTCACATA------CCTGTAAGAGTAAGACAAGGTTTAGAAAGGGCTTTGCTA---TAA
>JQ403085
GGT---ACA---
GAAAAATTGTGGGTCACAGTCTATTATGGGGTACCTGTGTGG---AAGGAGGCAACC---
ACCACTCTATTTTGTGCATCAGATGCTAAGGCATATGATACAGAG---GTACATAAT----------------
--------------GTTTGGGCCACA---CATGCCTGTGTACCCACAGAC---
CCCAACCCACAAGAAATAGAATTG------------AATGTGACAGAAAATTTTAAC----------------
-------------------------ATGTGGAAAAATGACATGGTAGAACAGATGCAGGAGGATATA---
ATCAGTCTATGGGATCAAAGCCTA---AAGCCATGTGTAAAATTG---
ACCCCACTTTGTGTTACTTTGAATTGCAGTGATGTTGGTGGCACTAATGCTACTAATACCACTGGTGCC----
----
-------------ACTAACACCACTGATAGTAGCATGGGAATAATAGAGAAGGGAGAAATGAAA---
AACTGTTCTTTCAATGTCACTAGTACCCTAAGA---------------------------
GATAAGGTGCAGACAGAATATGCACTG---TTTTATAAACCTGATGTAGTATCAATAAAAGAT----------
----

AATAGTACTAGTTATAGGTTGATAAGTTGTAACACCTCAACCATTACACAA---
GCCTGTCCAAAAGTATCTTTTGAGCCAATTCCCATACATTATTGTGCCCCGGCTGGTTTTGCACTTCTAAAGT
GTAAG---GATAAGAAGTTCAAT------GGAACAGGGCCATGTAGCAAT-----------------------
-GTCAGC---ACAGTACAGTGTACACATGGAATTAAGCCAGTAGTATCAACT---
CAACTACTGTTAAATGGCAGTCTAGCAGAAGAAGAG------------
ATAGTAATTAGATCTGAAAATTTCACAAACAAT---------GCTAAAACCATAATAGTACAGCTGAAG----
-----------GAACCTGTAAAA---------ATTACTTGTATAAGACCCGGTAACAATACAAGAAAA-----
-AGTATACCTATA------------GGACCAGGGAGAGCA---TTT------TATACAACAGGACAA------
---------ATAATAGGAGATATA------------AGAAAAGCATATTGT---AACATTAGTAGAGCA---
GATTGGAATAAAACTTTACAAAGGGTAGCTATCAAATTAAAG---------------GAACAATTTGAA----
-----------------------------------AATAAAACAATAATCTTTAAT------
CAATCCTCAGGAGGGGACCCAGAAATTGTA---ATGCACAGTTTTAATTGTAGAGGGGAA---
TTTAAGCAGCTCCAGGCAAGAGTCCTGGCTGTAGAAAGATACCTAAAGGATCAACAGCTCTTGGGAATTTGGG
GTTGCTCTGGAAAACTCATCTGCACCACTGCTGTGCCTTGGAATACTAGTTGGAATAATAAT-----------
-----------------------------AAAACAAATTTGAGTCAGATTTGG------GAC------
AAAATGACCTGGATGCAGTGG---GAAAGGGAAATTGACAATTACACAGACTTAATATAC---
AGCTTAATTGAAGAATCGCAGAACCAGCAGGAA---

AAGAATGAACAAGAATTATTGGAATTGGATAAATGGGACAGCTTGTGGAGTTGGTTTAGTATAACAAATTGGC
TGTGGTATATAAAAATATTCATAATGATAGTAGGAGGCTTGATAGGTTTAAGAATAGTTTTTGCT---
GTGCTTTCTATAGTA---------AATAGAGTTAGGCAGGGATACTCACCT------
CTGTCGTTTGCTGTTAGCTTATTCAATGCCATAGCCATAACAGTAGCTGAG---
GGGACAGATAGGGCTATAGAAATATTACAAAGAATTTTTAGAGCT----------------------------
--------TTTCTTCATATA------CCTAGAAGAGTAAGACAGGGCTTAGAAAGGGCTTTGCTA---TAA
>JQ403094
GCT---GCA---
GAAGATTTGTGGGTCACAGTCTATTATGGGGTACCTGTGTGG---AAAGAAGCAAAC---
ACCACTCTATTTTGTGCATCAGATGCTAAAGCATATGATACAGAG---GTACATAAT----------------
--------------GTTTGGGCCACA---CATGCCTGTGTACCCACAGAC---
CCTAGCCCACAAGAAATAAAATTGGAA---------AATGTGACAGAAGAATTTGAC----------------
-------------------------ATGTGGAAAAATAACATGGTAGAACAAATGCATGAAGATATA---
ATTAGTATATGGGATCAAAGTTTA---AAGCCATGTGTAAAATTA---
ACTCCACTCTGTGTCACTTTACACTGCAATGATTCAAGAAATGAGACTAGTTCCAATAGTACTAGTACT----
----

----------------AGATCCAATATTACTAGCGAGGAAAAGATGGATCCAGGAGAAATGAAA---
AACTGCTCTTTCAACATCACCTCAGGCATAAGA---------------------------
GATAAGGTAAAGGGAGCACGTGCAGTT---TTCTATTCAATTGATCTAGTCCCAATAGATGATGATGAACAT-
----

AATAGTACCAGCAACATTACCAGCTATAGGTTGAGAAGTTGTAATACCTCAGTCATTACACAG---
GCCTGTCCAAAGGTAACCTTTGAGCCAATTCCCATACATTATTGTGCCCCGGCTGGTTATGCGATTCTACAAT
GTAAA---GATAAAAAGTTCAAT------GGATCGGGACTATGTCAAAAT-----------------------
-GTCAGC---ACAGTACAATGTACACATGGAATTAGGCCAGTAGTATCAACT---
CAACTGCTGTTAAATGGCAGTCTGGCAGAAGAAGAT------------
ATAGTAATTAGATCTCAAAATTTCTCAAACAAT---------GCAAAAACCATAATGGTACAGCTGAAC----
-----------GAATCTGTAGAA---------ATTAATTGTACAAGGCCCAACAATAATACAAGAACA-----
-AGCATACCTATA------------GGACCAGGGAGAGCA---TTT------TATGCAACAGGAGAC------
---------GTACTAGGAGACATA------------AGAAAAGCATATTGT---AACATTAGTAGAGCA---
GCATGGAATGAAACTTTAAGAAAGATAGCTGGGAAATTACAA---------------GAACACTTTAAG----
-----------------------------------AATAAAACTATAATCTTTAAC------
CAATCCTCAGGAGGGGACCTAGAGATTGTA---ATGCACAGTTTTAATTGTGCAGGGGAA---
TTTAAGCAGCTCCAGGCAAGAGTCCTGGCTGTGGAAAGATACCTAAAGGATCAACAGCTCCTGGGGCTTTGGG
GTTGCTCTGGAAAACTCATTTGCACCACTACTGTGCCTTGGAATGCTAGTTGGAGT-----------------
----------------------------AATAGATCTCTAGACAGGATTTGG------AAC------
AACATGACCTGGATGCAGTGG---GAAAAAGAAATTGACAATTACACAGGCTTAATATAC---
ACCTTAATTGAAGAATCGCAGAACCAACAAGAA--

AAGAACGAACAAGAATTATTGGAATTAGATAAATGGGCAAGTTTGTGGAATTGGTTTGACATAACAAGTTGGC
TGTGGTATATAAAAATATTCATAATGATAGTAGGAGGCTTAATAGGTTTAAGAATAGTTTTTGTT---
GTACTTTCTATAGTG---------AATAGAGTTAGGCAGGGATACTCACCA------
TTGTCGTTCGCTGTTAGCTTGCTCAACGCCATAGCCATAGCAGTAGCTGAG---
GGGACAGATAGGATTATAGAAATAGCACAAAGAATTGGTAGAGCT----------------------------
--------ATTATCCACATA------CCTACAAGAATAAGACAGGGCTTGGAGAGGCTTTTGCTA---TAA
>KR423482
GTA---GCA--
GAACAATTGTGGGTCACAGTCTATTATGGGGTACCTGTGTGG---AAAGAAGCAAAC---
ACCACTCTATTTTGTGCATCAGATGCTAAAGCATATGATACAGAG---GTCCATAAT----------------
--------------GTTTGGGCCACA---CATGCCTGTGTACCCACAGAC---
CCCAACCCACAAGAAATAGCATTGCCA---------AATGTAACAGAAAATTTTAAC----------------
--------------------------ATGTGGAAAAATAACATGGTAGAGCAGATTCATGAGGATGTA---
ATTAGTCTATGGGATCAAAGCCTA---AAGCCATGTGTAAAACTA---
ACCCCACTCTGTGTTACTTTAAATTGCACTGAGGCTAATAATACCACAAGTAATAGTACCACA----------
----
-------------------------AGTAATAGCACTACCACAAGGGAAGAAGGAGAAATGAAA---
AACTGCTCTTTCAACATTACCACAAACATAAAC---------------------------
GATAAGTACCAGAAAACATATGCACTC---
TTTTATAAACTTGATATAGTACCAATAAGTGAGGAAAGTCAGAAT----------------------------
----
AGTAGTACTAGTTATAGGTTAATAAGTTGTAATACCTCAGTCCTTACCCAA---
GCCTGTCCAAAGGTAACCTTCGAACCAATTCCCATACATTATTGTGCCCCGGCTGGTTTTGCGATTCTAAAAT
GCAGA---GATAAGAAATTCAAT------GGAACAGGACCATGTAAGAAT-----------------------
-GTCAGC---ACAGTACAATGTACACATGGAATTAAGCCAGTAGTATCAACT---
CAATTACTGTTAAATGGCAGTCTAGCAGAAGAAGAG------------
ATAGTAATTAGATCTGCCAACTTCACAGACAAT---------ACTAAAGTCATAATAGTACAGCTAACA----
-----------GAAGCTGTGGAA---------ATTAATTGTACAAGGCCCAACAACAATACAAGAAAG-----
-AGTATACACATA------------GGACCAGGGAGAGCA---TTT------TATGCAACAGGAGAC------
---------ATAATAGGAGATATA------------AGACAAGCACATTGT---AACATTAGTAAAGCA---
AAATGGAATAACACTTTAAAACAGATAGCTAAAAAATTGATA---------------GAACAATTT-------
-----------------------------------AATAAAACAATAAATTTTAAT------
CAATCCTCAGGAGGGGATCTGGAAATTGTA---ACACATAGTTTTAATTGTGGAGGGGAA---
TTTAAGCAGCTCCAGGCAAGAGTCCTGGCTGTGGAAAGATACCTAAGGGATCAACAGCTCCTAGGGATTTGGG
GTTGCTCTGGAAAACTCATTTGCACCACTGCTGTGCCTTGGAACACTAGTTGGAGT-----------------
----------------------------AATAAAAATTACACTCAGATTTGG------GAT------
AACATGACCTGGATGCAGTGG---GAGAAAGAAATTGACAATTACACAGACTTAATATAC---

ACCTTACTTGAAGAATCGCAAAACCAACAAGAA--

AAGAATGAACAAGAATTATTAGCATTAGATAAGTGGGCAAGTTTGTGGAATTGGTTTGATATAACAAATTGGC
TGTGGTATATAAAAATATTCATAATGATAGTAGGAGGCTTGGTAGGCTTAAGAATAGTTTTTACT---
GTATTTTCTATAGTG---------AATAGAGTTAGGCAGGATACTCACCC------
TTATCGTTTGCTGTCAGCTTGCTCAACACCACAGCCATAGCAGTAGCTGAG---
GGAACAGATAGGGTTATAGAAGTGTTACAAAGAATTTGTAGAGCT----------------------------
--------TTGCGCCACATA------CCTAGAAGAATAAGACAGGGCCTCGAAAGGATTTTGCTA---TAA
>GU733713
GCT---ACA--
GAACAATTGTGGGTCACAGTCTATTATGGGGTACCAGTATGG---AAAGAAGCAACC---
ACCACTCTATTTTGTGCATCAGATGCTAGAGCATATAAGACAGAG---GTACATAAT----------------
--------------GTTTGGGCCACA---CATGCCTGTGTACCCACAGAC---
CCTAGCCCACAAGAAATACCATTGATA---------AATGTGACAGAAAATTTTAAC----------------
--------------------------ATGTGGAAAATAAAATGGTAGACCAAATGCATGAGGATATA---
ATCAGTTTATGGGATCAAAGCCTA---AAGCCATGTGTAAAATTA---
ACCCCACTCTGTGTTACTTTAAATTGCATTGATGTGAATATTACTAATACCAATAGCACTAAT----------
----
----------------------CCCACTAGTAGCTGGGAAACGATGGAGAAAGGAGAAATAAAA---
AACTGCTCTTTCAATATCACCACAAACATAAGA---------------------------
GATAAGATACAGAAAGAATATGCATTG---TTTAATAGACTTGATGTAGTACCAATAAATAAT----------
----

AGTAATACCTACTATAGGTTGATAAGTTGTAACACCTCAGTCCTTACACAA---
GCCTGTCCAAAGGTATCCTTTGAGCCAATTCCCATACACTACTGTGCCCCGGCTGGTTTTGCAATTCTAAAGT
GTAAC---GAGACAAAGTTCAAT------GGATCAGGACCATGTAAAAAT-----------------------
-GTCACC---ACAGTACAATGTACACATGGAATTAGGCCAGTAGTATCAACT---
CAATTGCTGTTAAATGGCAGTCTAGCAGTAGAGGAG------------
GTAGTAATTAGATCTGAAAATTTCTCAGACAAT---------GCTAAAACTATAATAATACAGCTGAAC----
-----------ACCTCTGTAGAA---------ATTAATTGTACAAGACCCAACAACAATACAAGAAAA-----
-GGTATACATATA------------GGGCCAGGGAGAGCA---TTT------TATGCAACAGGAGAC------
---------ATAATAGGAGATATA------------AGACAAGCACATTGT---AACATTAGTACAGCA---
AAATGGAATGACACTTTAAAACACATAGTTGGAAAATTGAAA---------------GAACAATTTAGG----
--------------------------------AATGCAACAAAAATAGTCTTTAAT------
CACTCCTCAGGAGGGGACCCAGAAATTGTA---ATGCACAGTTTTAATTGTGGAGGAGAA---
TTTAAGCAGCTCCAGGCAAGAGTCCTGGCTGTGGAAAGATACCTAAGGGATCAACAGCTCCTGGGAATTTGGG
GATGCTCTGGAAAACTCATCTGCACCACTACTGTACCTTGGAATGCTAGTTGGAGC-----------------
----------------------------AATAAAAATCTAACTACAATTTGG------AAT------
AACATGACCTGGATGCAGTGG---GAAAAAGAAATTGACAATTACACAGAAATAATATAT---
GATTTAATTGAACAATCACAGAACCAGCAAGAA--

AAGAATGAACAAGAATTATTGGAATTGGATAAATGGGCAAGTTTGTGGAGTTGGTTTGATATAACAAATTGGC
TGTGGTATATAAAAATATTCATAATGATAGTAGGAGGCTTAATAGGTTTAAGAATAATTTTTGCT---
GTGCTTTCTATAGTG---------AATAGAGTTAGGCAGGATACTCACCA------
TTATCGTTTGCTGTTAGCTTGCTTGATGCCACAGCTGTAACAGTAGCTGGG---
GGGACAGATAGAATTTTAGAAGTATTACGAAGAATTTGGAGAGCT----------------------------
--------TTTCTCCACATC------CCTAGAAGAATAAGACAGGGCTTAGAAAGGGCTTTGCTA---TAA
>MK164668
GCT---ACA--
GAAAAATTGTGGGTCACAGTCTATTATGGGGTACCTGTGTGG---AAAGAAGCAGAC---
ACCACTCTATTTTGTGCATCAAATGCTAAAGCATATGATACAGAG---GTACATAAT----------------
--------------GTTTGGGCCACA---CATGCCTGTGTACCCACAGAC---
CCCAACCCACAAGAGATAGCATTGGAA---------AATGTGACAGAAAATTTTAAC----------------
--------------------------ATGTGGAAAAACAACATGGTAGAACAGATGCAGGAGGATATA---
ATCAGTTTATGGGATCAAAGCCTA---AAGCCATGTGTAAAATTA---
ACCCCGCTCTGTGTTACTTTAAATTGTACTGATTATATGGGGAATGGCACTAGTGGGAGTGCCACTAATAGGG
CT---
-------------------------GTCACTAAAAATAATACTATTGACAAGGGAGAAATAAAA---
AACTGCTCTTTCAATGTCACCACAAATAGAAGA---------------------------
AATAAAGCACAGAAAGAATATGCACTT---TTTTATAAGCTTGATGTAGTACCAATAGAGAAT----------

----

GATAATACTAGCTATAGGTTAATAAGTTGTAACACCTCAGTCATTACACAG---
GCCTGTCCAAAGGTATCCTTTGAACCAATTCCCATACATTATTGTGCCCCGGCTGGTTTTGCTATTCTAAAGT
GTAAA---GATAAGAATTTCAAT------GGAAAAGGGTCATGTGAAAAT-------------------------
-GTCAGC---ACAGTACAATGTACACATGGAATTAGACCAGTAGTATCAACC---
CAACTGCTGTTAAATGGTAGCCTAGCAGAAGAAGAC------------
GTAGTAATTAAATCTGACAATTTCTCGGACAAT---------GCTAAAAATATAATAGTACAGCTGAAT----
-----------GAAACAGTAGAA---------ATTGATTGTGCAAGACCCAATAACAATATGAGAAAA-----
-AGTATACCTATA------------GGACCAGGGAGAGCA---TTT------TATGCAACAGGAGAA------
---------ATAATAGGAGATATA------------AGACAAGCACATTGT---AACCTTAGTAAAGCA---
AATTGGACTCAAACTTTAAGTAGGATAGCAGAAAAATTAAAA---------------GAACAATTTAAT----
------------------------------------ACAACAACAATAAAATTTAAT------
CGATCCGCAGGAGGGGACCCAGAAGTTGAA---ATGCACAGTTTCAATTGTGGAGGGGAA---
TTTAAGCAGCTCCAGGCGAGAGTCCTGGCTGTGGAAAGATACCTACAGGATCAACAGCTCCTGGGAATTTGGG
GTTGCTCTGGGAAACTCATCTGCACCACTACTGTGCCTTGGAATGCTAGTTGGAGT----------------
-----------------------------AATAAATCTCTGGATTATATCTGG------AAT------
AACATGACCTGGATGAAGTGG---GAAAGAGAAATTGACAATTACACAAGTTTAATATAC---
ACCTTAATTGCAGAATCGCAGAACCAACAAGAA--

AAGAATGAACAAGACTTATTGACATTAGACAAATGGGATAGTTTGTGGAATTGGTTTAGCATAACAAAATGGC
TGTGGTATATAAAAGTATTCATAATGATAGTAGGAGGCTTGATAGGTTTAAGAATAGTTTTTACT---
GTACTTTCTATAGTG---------AATAGAGTTAGGCAGGGATACTCACCA------
TTATCGTTTGCTGTTAGCTTGTTCAATACTACAGCTATAGTAGTAGCTGAG---
GGAACAGATAGAATTATAGAAGCATTCCAAATAGCTTGTAGAGCT------------------------------
--------ATTAGCCACATA------CCTAGAAGAATAAGACAGGGCTTAGAAAGACTCTTACTA---TAA
>KC935959
ACT---GCA---
GAGGAATTGTGGGTCACAGTTTATTATGGGGTACCTGTGTGG---AAAGAAGCAAAC---
ACCACTCTATTTTGTGCATCAGATGCTAAATCATATGATACAGAG---GTACATAAT----------------
--------------GTTTGGGCCACA---CATGCCTGTGTACCCACAGAC---
CCCAGCCCACAAGAATTACTATTGGTA---------AATGTGACAGAAAATTTTAAC----------------
---------------------------ATGTGGAAAAATAACATGGTAGAACAGATGCATGAGGATGTA---
ATTAGTTTATGGGATCAAAGTCTA---AAGCCATGTGTAAAATTA---
ACCCCTCTCTGTGTTACTTTAAATTGCACAGATGTTACTAATACCACTGGCAATGCTACTCATAGTCAGAGTA
AGGTAATGAAA--
----------------------CAAAGGG--AGAAATGAAAATGAAACAGTGGGAGAAATGAAA---
AACTGCACTTTCAATATCACCACAAGTATGAGC----------------------------
AATAAGGTGAAGCGAGAATATGCACTT---TTTTATACACTTGATATAATACCAACAGAAGAA----------
----

AGTGGGAATACTAGTTATAGGTTGATAAGTTGCAACACCTCAGTCGTTACACAG---
GCCTGTCCAAAGGTAACCTTTGAACCAATTCCCATACATTATTGTGCCCCGGCTGGTTTTGCAATTCTAAAGT
GTAAT---AATAAGACATTCAAT------GGAACAGGACCATGTACAAAT-------------------------
-GTTAGC---ACAGTACAATGTACACATGGAATTAGACCAGTAGTATCAACT---
CAGCTGCTGTTAAATGGCAGTCTATCAGAAGAAGAG------------
ATAATAATTAGATCCGCCGATCTCTCAGACAAT---------CTTAAAACCATAATAGTACAGCTGAAT----
-----------CAATCTGTACAA---------ATGACTTGTATAAGACCCAACAACAATACAAGAAAA-----
-GGCATACATCTA------------GGACCAGGCAGAGCA---TTT------GATGCAATAGGAGGA------
---------ATAATAGGAGATATA------------AGACAAGCACATTGT---AACATTAGTAAAAGC---
AAGTGGAATACTACTTTACAAAATTTAGCTGCAAAATTGAGA---------------GAACAATTT-------
------------------------------------AATAAAACAATAATTTTTAAT------
CAATCCGCAGGAGGGGACCCAGAAATTACA---ATGCTCAATTTTAATTGTGGAGGGGAA---
TTCAAGCAGCTCCAGGCAAGAGTCCTGGCTGTAGAAAGATACCTAAAGGATCAACAGCTCCTAGGGATTTGGG
GTTGCTCTGGAAAACTCATTTGCACCACTACTGTGCCTTGGAACAATAGTTGGAGT----------------
-------------------------GGAAACAAAAGTCTGAGTCATATTTGG------GAC------
AACATGACCTGGATGCAGTGG---GAAAAAGAGATTGAGAATTACACAGACACAATATAC---
ACGTTAATTGCAGAATCGCAAAGCCAACAAGAA--

AAGAATGAAAAAGAATTATTGGAATGGGATAAATGGGCAAGTTTGTGGAATTGGTTTAACATAACAAACTGGC

TGTGGTATATAAAAATATTCATAATGATAGTAGGAGGCCTAATAGGTTTAAGAATAGTTTTTACT---
GTATTTTCTATAGTG---------AATAGAGTTAGGCAGGGATACTCACCA------
TTATCGTTCACTATTAGCTTGTACAATACCATAGCCATAGCAGTAGCTGAG---
GGGACAGATAGGATTATAGAAGCATTGCAAAGAGCTGGTAGAGCT----------------------------
--------ATTCTCCACATA------CCTAGAAGAATAAGACAGGGCTTGGAAAGGATTTTGATA---TAA
>KM082006
GTT---GCA--
GGACAATTGTGGGTCACAGTCTATTATGGGGTACCTGTATGG---AAAGAAGCAGAC---
ACCACTCTATTCTGTGCATCAAATGCTAAAGCATATGACACAGAG---ACACATAAT----------------
--------------GTTTGGGCCACA---CATGCCTGTGTACCCACAGAC---
CCTAACCCACAAGAAGTAGAATTGGAA---------AATGTGACAGAGAATTTTAAC----------------
--------------------------ATGTGGAAAAATAACATGGTAGAACAGATGCATGCGGATATA---
ATCAGTTTATGGGATGAAAGTCTA---AAGCCATGTGTAAAATTA---
ACCCCACTCTGTGTTACTTTACAGTGCACTGATAATATTACTATTACTAATGGCACTAATGTTACTAGTACCA
CT---
----------------------AGTGGCAATAGCACTTTGGGAAGTATGAAAGAAGAGATGAAA---
AACTGCTCTTTCAATGTCACCACAAGCATAAGA---------------------------
AATAAGATGCAGAAAGAATATGCACTT---TTTTATAAACTTGATGTAGTACCAATAGATAAT----------
----

GATAATACAAGCTATACATTGATAAATTGTAACACCTCAGTCATTACACAG---
GCCTGTCCAAAGGTATCCTTTGAACCAATTCCAATACATTATTGTACCCCAGCTGGTTTTGCGCTTCTAAAGT
GTAGG---GATAAGAAGTTCAAT------GGAAAAGGACCATGTAGAAAT------------------------
-GTCAGC---ACAGTACAATGTACACATGGAATTAGGCCAGTGGTGTCAACC---
CAATTGCTGCTAAATGGCAGTCTAGCAGAAGGAGAG-----------
GTAGTAATTAGATCTGAAAATTTCACAAACAAT---------GCCAAAGTCATAATAGTACAGCTAAAT----
-----------GAATCTATAGAA---------ATTAATTGTACAAGACCCAACAACAATACAAGAAGA-----
-AGTATCCATATA------------GCACCAGGAAGAGCA---TTC------TATGCAACAGGAGAA------
---------ATAATAGGAGATATA------------AGACAAGCATATTGT---AGCATTAGTAATAGA---
AAATGGAATGACACTTTAAGCAAGATAGCTATAAAATTAGGA---------------GAACAATTT-------
-----------------------------------AATAAAACCATAATCTTTAAT------
CAATCCTCAGGAGGGGACCCAGAAATTGTA---ATGCACAGTTTTAATTGTGGAGGGGAA---
TTTAAGCAGCTCCAGGCAAGAGTCCTGGCTTTGGAAAGATACCTAAGGGATCAACAGCTCCTAGGGATTTGGG
GATGCTCTGGAAAACTCATTTGCACCACTGCTGTGCCTTGGAATGCTAGTTGGAGT-----------------
-----------------------------AATAAATCTCATGATCAGATTTGG------CAA------
AACATGACCTGGTTACAGTGG---GAGAGAGAGATTGACAATTACACAGGCTTAATATAC---
ACCTTGCTTGAAGAATCGCAAAACCAGCAAGAA--

AAGAATGAACAAGACCTATTGGCATTGGATAACTGGACAAGTTTGTGGAGTTGGTTTAGCATATCAAATTGGC
TATGGTATATAAGAATATTCATAATGATAGTAGGAGGTTTAGTAGGTTTAAGAATAGTTTTTGCT---
GTGTTTTCTATAGTG---------AATAGAGTTAGGCAGGGATACTCACCA------
TTGTCGTTCGCTATTAGTTTGCTTAATGCCACAGCTATAGCAGTAGCTGAG---
GGGACAGATAGGATTATAGAAACAGTACAAAGAATTTGTAGAGCT---------------------------
--------ATTCTCAACATA------CCTAGAAGAACAAGACAGGGCTTAGAAAGGGCTTTGCTA---TAA
>MG196775
GCT---AAA---
GAACAGTTGTGGGTCACAGTCTATTATGGGGTACCTGTGTGG---AAAGAAGCAACT---
ACCACTCTATTTTGTGCATCAGATGCTAAAGCATATAAGCCAGAG---GCACATAAT----------------
--------------ATTTGGGCCACA---CATGCCTGTGTGCCCACAGAC---
CCCAACCCACAAGAAGTAGCATTGGAA---------AATGTGACAGAGAATTTTAAC----------------
--------------------------ATGTGGAAAAATAACATGGTAGAACAGATGCATGAAGATATA---
ATTAGTTTATGGGATGAAAGCCTA---AAGCCATGTGTAAAATTA---
ACCCCACTCTGTGTTACTTTAAATTGCACTAACGTAACAGTCATTAATGCCACTACTACTACT----------
----
-------------------------AGTAATGACAATAGTAGTTATTGGAAAGATGATATGAAA---
AACTGCTCTTTCAATACCACCACAATCTTAAGA---------------------------
GATAAGGCTAAGAAAGAATATGCACTT---
TTTTATAAGCTTGATATAGTACCAATAGATGAGGATAAGAATACTAAG-------------------------
----
GGTAATAATAGTAATAGTGGTAGTAGTAGCTATAGGTTGATAAATTGTAATTCCTCAACCATCACACAA---

GCCTGTCCAAAAATAGCATTTGAACCAATTCCCATACATTATTGTGCCCCGGCTGGTTTTGCGATTCTAAAGT
GCAGA---GATAAAAAATTTGAG------GGAAAAGGACCATGTAAAAAT-----------------------
-GTCAGC---ACAGTACAATGTACACATGGAATTAGACCAGTAGTGTCAACT---
CAACTACTGTTAAATGGCAGTCTAGCAGAAGAAGAA------------
GTAGTAGTTAGATCTGAAAATTTCTCGGACAAT---------GTTAAAAACATAATAGTACAGCTGAAC----
-----------GCATCTGTACAA---------ATTAACTGTACAAGACCCAACAACAATACAAGAAAA-----
-AGTATACATATG------------GGACCAGGGGGAGCA---ATG------TATGCAACAGGAGCC------
---------ATAATAGGAGATATA------------AGACAAGCACACTGT---AACCTTAGCATAAAA---
CAGTGGTATGACACTTTAAGACAGGTAGTTATAAAATTAGGA---------------
GAACAATTTAATAAAACAAAT------AATTTGAATAAAACA------GAAAATTTTACAATAGCCTTTAAG-
-----CCATCCTCAGGAGGGGACCCAGAAGTTGAA---ATGCACAGCCTTAATTGTGGAGGGGAA---
TTTAAGCAGCTCCAGGCAAGAGTCCTGGCTGTAGAAAGTTACCTAAGGGATCAACAGCTCCTAGGGATTTGGG
GTTGCTCTGGAAGACTCATCTGCACCACTACTGTGCCTTGGAATAATAGTTGGAGT-----------------
----------------------------AGTAAAAGTTATGACGACATTTGG------GGT------
AACATGACCTGGATGGAGTGG---GAAAAAGAAATTAGCAATTACACAGGATTAATATAC---
ACCTTAATTGAAGACTCACAAAACCAGCAAGAA--

AAGAATGAACAAGAGTTATTATCACTGGATAAGTGGGCAAGTTTGTGGAATTGGTTTGACATAACAAAGTGGC
TGTGGTATATAAAGATATTCATAATGATAGTAGGAGGCTTGATAGGTTTAAGAATAATTTGTGCT---
GTGCTTTCTATAGTG---------AATAGAGTTAGGCAGGGATACTCACCA------
TTGTCGTTCGCTGTTAGCTTGTACAACGCCATAGCTATAGCAGTAGCTGAA---
GGGACAGATAGCATTATAGAAGCAATACTAAGAGTTGGTAGAGCT---------------------------
--------ATCCTCCACATA------CCTAGAAGAGTAAGACAGGGCTTGGAAAGGGCTTTGCAA---TAA
>JQ182826
GCT---AAA--
GAACAATTGTGGGTCACAGTCTATTATGGGGTACCTGTGTGG---AAAGAAGCAACT---
ACCACTCTATTTTGTGCATCAGATGCTAGAGCATATGGTAAAGAA---GTACATAAT----------------
--------------ATTTGGGCCACA---CATGCCTGTGTACCCACAGAC---
CCCAACCCACAAGAAGTAATATTGGAA---------AATGTAACAGAAAATTTTAAC----------------
---------------------------ATGTGGAAAAATAACATGGTAGAACAGATGCATGAGGATATA---
ATCAGTTTATGGGATCAAAGTCTA---AAGCCATGTGTGAAATTA---
ACCCCACTTTGTGTTACTTTAAATTGCACTGATTATTTTAAGAATACTACTAATACTAGTAATAAAACTAATA
GCACT--
----------AGTCCTAACACCAACAATAGTAGCTTGGGAATAATGGATAAAGGAGAAATAAAA---
AACTGCTCTTTCAATATTACCTCAAGCATAAGA---------------------------
GATAAAATGCAGAAAGAATATGCACTT---TTGTATAAACTTGATATAGTACCAATAGGGGGT----------
----

GATAATACTAGTTATAGGTTGATAAGTTGTAACACCTCAGTCATTACACAG---
GCTTGTCCAAAAGTATCCTTTGAGCCAATTCCTATACATTACTGTGCTCCAGCTGGTTTTGCGATTCTAAGGT
GTAAT---AATGAGACGTTCAAT------GGAACAGGACCATGTACAAAT-----------------------
-GTTAGC---ACAGTACAATGTACACATGGAATTAAGCCAGTAGTATCAACT---
CAACTGCTGTTGAATGGCAGTCTAGCAGAAAAAGAG------------
GTAGTAATTAGATCTGAAAATATTACAGACAAT---------GGTAAAATCATAATAGTACAGCTGAAT----
-----------GCATCTGTAGAA---------ATTAATTGTACAAGACCCAACAACAATACAAGAAGA-----
-GGTATAAACATA------------GGACCAGGAAGAGCA---TGG------TATACAACAGGAGAA------
---------ATAATAGGAGATATA------------AGACAAGCACATTGT---ACCATTAGTAGAGAA---
CAATGGAATAAAACTCTACAACAGGTAGTTGAAAAATTAGGG---------------GAACATTTTAAG----
-----------------------------------AATAAAACAATAGTTTTTAAT------
CACTCCTCAGGAGGAGACCCAGAAGTTGTA---ATGCACAGTTTTAATTGTGGAGGGGAA---
TTTAAACAGCTCCAGGCAAGAGTCCTAGCTGTAGAAAGATACCTAAAGGATCAACAGCTCCTGGGAATTTGGG
GTTGTTCTGGAAAACTCATCTGCACCACTACTGTGCCTTGGAATACTAGTTGGAGT-----------------
----------------------------AATAAAAATCTGAGTCAAATTTGG------GAT------
AACATGACCTGGATGCAATGG---GAGAAAGAAATTGATAATTACACAAATGCAATATAC---
TATTTACTTGAAAAAGCGCAGAACCAGCAAGAA--

AAGAATGAACAAGAATTATTGGAATTGGATAAATGGGCAAGTTTGTGGAATTGGTTTGACATATCAAACTGGC
TGTGGTATATAAAAATATTCATAATGATAGTAGGAGGCCTAGTAGGTTTAAGAATAGTTTTTGCT---
GTGCTTTCTATAGTG---------AATAGAGTTAGGCAGGGATACTCACCA------
TTATCGTTTGCTGTTAGCTTGTTCAATTCTACTGCCATAGTAGTAGCTGAG---

GGAACAGATCGGGTTATAGAAATATTACAGAGAGCTCTTAGAGTT----------------------------
--------GTTCTCCATATA------CCTACAAGAATAAGACAGGGTTTAGAAAGGGCTTTGCTA---TAA
>KM353922
GCT---ACA---
GAAAAATTGTGGGTCACAGTCTATTATGGGGTACCAGTGTGG---AAAGAAGCAACC---
ACCACTTTATTTTGTGCCTCAGATGCTAAAGCATATGATACAGAG---GTACATAAT----------------
--------------GTTTGGGCCACA---CATGCCTGTGTACCCACAGAC---
CCTAACCCACAAGAAGTAGTATTGGGA---------AATGTAACAGAAAATTTTAAC---------------
--------------------------ATGTGGAAAAATAACATGGTAGAACAGATGCATGAGGATATA---
ATCAGTCTATGGGATCAAAGCCTA---AAACCATGTGTAAAATTA---
ACCCCACTCTGTGTTACTTTAAATTGTAGTAATAATATAATTACTGTTAAT---------------------
----
---------------------------------GCCACTAATGATGGGAAAGAAGACTTAAGA---
AACTGTTCTTTCAAAATCACCACAAGCATAAGA--------------------------
GATAAGATGCAGAAAGAATATGCACTC---TTTTATAAACTAGATGTAGTACAAATAAATGAT----------
----

AATGATACTGATAGTTATAGGTTAATAAGTTGTAATACCTCAGTCATTACTCAG---
GCCTGTCCAAAGATATCCTTTGAACCAATTCCCATACATTATTGTGCCCCGGCTGGTTTTGCGATTCTAAAGT
GTAAT---AATAAGACATTCAAT------GGAACAGGACCATGTACAAAT-----------------------
-GTCAGC---ACAGTACAATGTACACATGGAATTAAGCCAGTAGTATCAACT---
CAACTACTGTTAAATGGCAGTCTAGCAGAAGAAGAG------------
ATAGTACTTAGATCTGAAAATTTCTCAAACAAT---------GCTAAAACCATAATAGTACATTTAAAT----
-----------GAATCTGTAGTA---------ATTAATTGTACAAGACCCAACAACAATACGAGAAAA-----
-AGTATAAATATA------------GGGCCAGGGAGAGCA---TTT------TATGCAGCAGGAGAA------
---------ATAATAGGAGATATA------------AGACGAGCATATTGT---AACATTAGTAGAACA---
AAATGGAATAACACTTTAAAACACATAGTTGTAAAATTAAAG---------------GAACAATTTGCA----
-----------------------------------AATAGAACAATAATCTTTAAT------
CACTCCTCAGGAGGGGATCCGGAAATTGTA---ATGCACAGTTTTAATTGTGGAGGAGAA---
TTTAAGCAGCTCCAGGCACGAGTCCTGGCTGTGGAAAGATACCTAAAGGATCAACAGCTCCTGGGAATTTGGG
GTTGCTCTGGAAAACTCATTTGCACCACTGCTGTGCCTTGGAATGCTAGTTGGAGT----------------
----------------------------AATAAATCTTTTGGTCAGATTTGG------GAT------
AACATGACCTGGATGGAGTGG---GAAAAAGAAATTGACAATTACACAGGCTTGATATAC---
ACTTTAATTGAAGAATCGCAAAACCAACAAGAA--

AAGAATGAACAAGAATTATTGGCATTAGATAAATGGGCAAGTTTGTGGAATTGGTTTGACATAACAAATTGGC
TGTGGTATATAAAAATATTCATAATGATAGTAGGAGGTTTGGTAGGTTTAAGAATAGTTTTTACT---
GTGCTTTCTATAGTG---------AATAGAGTTAGGAAGGGATACTCACCA------
TTATCGTTCGCTGTTAGCTTGTTTAATGCCACAGCTATAGCAGTAGCTGAG---
GGAACAGATAGGATTATAGAAGGATTACAAAGAGCTTATAGAGCT--------------------------
--------ATTATCCACATA------CCTAGAAGAATAAGACAGGGGGCGGAAAAGGCTTTGCAA---TAA
>KM354862
GCT---ACA---
GGAAACTTGTGGGTCACAGTCTATTATGGGGTACCTGTGTGG---AGAGAAGCAACC---
ACCACTTTATTTTGTGCATCAGATGCTAAAGCACATGATACAGAG---GTACACAAT----------------
--------------GTTTGGGCCACA---CATGCCTGTGTACCCACAGAC---
CCCAATCCACAAGAAGTACAATTG------------AATGTAACAGAAAATTTTAAC---------------
--------------------------ATGTGGAAAAATAACATGGTAGAACAAATGCATGAGGATATA---
ATTAATTTATGGGATGAAAGCTTA---AAGCCATGTGTAAAATTA---
ACCCCACTCTGTGTTAAGTTAAATTGCACTAATGTGAGGAATGATACTATCAATTTGAAGAAT----------
----
---------------------AATAATTGGACTGTAGATCACCAAATGAAAGGAGAAATAAAA---
AACTGCTCTTTCAATGTCACCACAGATCTGAAA--------------------------
GATAAGATAAAGAAAGAACATGCACTT---TTTTATAAACTTGATATAGTACCAATAGAGAATGATAATGAT-
----

AATAATACTAGTAATAATACTAGCTATAGGTTGATAAGTTGTAACGCCACAACCATTACACAG---
GCCTGTCCAAAAGTATCCTTTGAGCCAATTCCTATATATTATTGTGCCCCGGCTGGTTTTGCGATTCTAAAAT
GTAAT---AATAAGAAGTTCAAT------GGAACAGGGCCATGTACAAAT----------------------
-GTCAGC---ACAGTACAATGTACACATGGAATTAAGCCAGTAGTATCAACT---

CAACTGCTATTAAATGGCAGCATAGCAGAAGAAGAG------------
GTAGTAATTAGATCTGAAAATTTCACAAACAAT---------GCTAAAATCATAATAGTACAGCTGAAT----
-----------GACACTGTACAA---------ATTAATTGTACAAGACCCAACAATAATACAAGGAAA-----
-AGTATAAATATA------------GGACCAGGGAGAGCA---TTT------TATGCAGCAGGAGAT------
---------ATAATAGGAGATATA------------AGACAAGCACATTGT---AACATTAGTGGAGCA---
GAATGGAATAACACTTTAAAGCAGATAGCTAACAAATTAAGA---------------GAACAATATGGG----
--------------------------------AAGAATAAAACAATAGAATTTAGG------
CCATCCTCAGGAGGGGACCCAGAAATTGTA---ATGCACAATTTTAATTGTGGTGGGGAA---
TTTAAGCAGCTCCAGGCAAGAGTCCTGGCTCTGGAAAGATACCTAAGGGATCAACAGCTCCTGGGAATTTGGG
GTTGCTCTGGAAAACTCATCTGCCCCACTGCTGTGCCTTGGAATACTAGTTGGAGT-----------------
----------------------------AATAAGTCTTATAATGAAATTTGG------AAT------
AACATGACCTGGATGGAATGG---GATAGAGAAATTAACAATTACACAAACTTAATATAT---
AGCTTAATTGAAGAATCGCAGAACCAACAAGAA--

AAAAATGAACAAGACTTATTGGCATTGGATAAATGGGCAAATTTGTGGAGTTGGTTTGACATATCAAATTGGT
TGTGGTATATAAGAATATTCATAATAATAGTAGGAGGCTTGATAGGATTAAGAATAGTTTTTACT---
GTACTTTCTATAGTA---------AATAGAGTTAGGCAGGGATACTCACCA------
TTGTCATTAGCTGTTAGCTTGCTTAACGCCACAGCCATAGTAGTAGGTGAG---
GGGACAGATAGGGTTTTAGAAGGATTACAAAGAGCTTTTAGAGCT----------------------------
--------ATTCTCCACATA------CCTAGAAGAATAAGACAGGGCGCAGAAAGGGCTTTGCTA---TAA
>KX587241
GCT---GCA--
GAACAATTGTGGGTCACAGTCTATTATGGAGTACCTGTGTGG---AAAGAAGCAACC---
ACCACACTATTTTGTGCATCAGATGCTAAAGGATATGATACAGAG---GCACATAAT----------------
--------------GTTTGGGCCACA---CATGCTTGTGTACCCACAGAC---
CCCAATCCACAAGAAGTAGTATTGGGA---------AATGTGACAGAAGATTTTGAT----------------
-------------------------GTGTGGAAAAATAACATGGTGGAACAAATGCATGAGGATATA---
ATCAGTTTGTGGGATCAAAGCCTA---AAGCCATGTGTAAAACTA---
ACTCCACTCTGTGTTACTTTAAATTGTCATAACATTACAGACAATAGTACTACAACCTCT-------------
----
----------------------------AATACAAGCATCAATGCGGCAAGCAGAGAAATGAAA---
AACTGCTCTTTCAACATCACCACAAATATAAGA--------------------------
GATAAGATAAAGCAAGAATATGCACTT---TTTTATAGCCTTGATGTAGTACCAATAAATAAT----------
----

GATAAGAATAATGATACTTATAGGTTGCTAAGTTGTAACACCTCAGTCATTACACAA---
GCCTGTCCAAAGATATCATTTGAACCAATTCCCATACATTATTGTACCCCAGCTGGTTTTACGATTCTAAAAT
GTAAT---AATGAGAGTTTCAAG------GGAAAAGGACCATGCACAAAT-----------------------
-GTCAGC---ACAGTACAATGTACACATGGAATTAAGCCAGTAGTGTCAACC---
CAACTGCTGTTAAATGGCAGTCTAGCAAAAGAAAAT------------
ATTGTACTTAGATCTGAGAATTTCTCGGACAAT---------GCTAAAAACATAATAGTACAGCTGAAA----
-----------GAACCTGTAAAA---------ATTAATTGTACAAGACCCAATAATAATACAAGAAAA-----
-AGTATACCTATG------------GGTCCAGGGAAAGCA---TTT------TATGCAACAGGAGAC------
---------ATAATAGGAGATATA------------AGACAAGCACATTGT---AACATTAGTAAAACA---
GAATGGAATGATACTTTAACATATATAGCTATAAAATTAAAA---------------GAACAATTTAAT----
-----------------------------------AAGGAAACAATAGTCTTTAAT------
CAATCCTCAGGAGGGGACCTAGAAAGTGTA---ATGCACACCTTTAATTGTGGAGGGGAA---
TTTAAGCAGCTCCAGGCAAGAGTCCTGGCTGTGGAAGGATACCTAAAGGATCAACAGCTCCTGGGAATTTGGG
GTTGCTCTGGAAAACTCATCTGCACCACTGCGGTGCCTTGGAATGCTAGTTGGAGT-----------------
----------------------------AATAAATCTCTAGATAAGATTTGG------AAT------
AACATGACCTGGATGCAGTGG---GAAAAAGAAATTGACAATTACACAGGCTTAATATAC---
AACTTACTTGAGACATCGCAAAACCAGCAAGAG--

AAAAATGAACAAGAATTATTAGAATTGGATAAATGGGACAGTTTGTGGAGTTGGTTCAACATAACAAATTGGC
TGTGGTATATAAAAATATTCATAATGATAGTAGGAGGCTTAATAGGTTTAAGAATAATTTTTACT---
GTACTTTCTATAGTA---------AATAGAGTTAGGCAGGGATATTCACCA------
TTATCGTTTGCTATTAGCTTGTTCAACGCCACAGCCATAGCAATAGCTGAG---
GGAACAGATAGGATTATAGAAGTAGTACTAAGAATTAGTAGAGCT----------------------------
--------ATCCTCCACATA------CCTACAAGAATAAGACAGGGCTTCGAAAGGGCTTTGCAA---TAA
>KX587299

GCT---AAA--
GAAGATTGGTGGGTAACAGTTTACTATGGGGTACCTGTGTGG---AAAGAAGCTACC---
ACCACTTTATTTTGTGCATCAGATGCTAAAGCATATGATACAGAG---GCACATAAT----------------
--------------GTCTGGCCACA---CATGCCTGTGTACCTACAGAC---
CCCAACCCACAAGAAGTAAAATTGGAA---------AATGTGACAGAAAATTTTAAT----------------
--------------------------ATGTGGAAAAATAACATGGTGGAACAAATGCATGAGGATGTA---
ATCAGTTTATGGGATCAAAGCCTA---AAGCCATGTGTAAAATTA---
ACCCCACTCTGTGTTACTTTAAATTGCACTGATTATGTGGGAAATGCTACTGCTGCACCACCAACTGTTACTA
CTACCAATAATAGTAACTCCACTACAACATCA---
----------------ACTACTACCAAAAGTAGTCCTACTGCTAATGCCACAATTGAAATGAAA---
AACTGCTCTTTTTATACTACCCCAAACATAAGA---------------------------
GATAAGGTAAGGAAAGAATATGCACTT---
TTTTATAGCCTTGATGTAGTTCCAATAGATAAGAAAGATAATACTAGTAAT----------------------
----
GAAAATAATACTGTTAGAAATAATACTAGCTATATGTTGATAAGTTGTAACACCTCAGTCATTACACAG---
GCCTGTCCAAAGATATCCTTTGAACCAATTCCCATACATTATTGTGCCCCGGCTGGTTTTGCGATTCTAAAAT
GTAAT---GATACGAAGTTCAAT------GGAACAGGACCATGTAAAAAT-----------------------
-GTCAGC---ACAGTACAATGTACACATGGAATTAAGCCAGTAGTATCAACT---
CAACTGCTATTAAATGGCAGTCTAGCAGAAGAAGAG------------
GTAATAATTAGATCTGCCAATCTCTCAGATAAT---------ACCAAAAACATAATAGTACATCTGAAT----
-----------GAAAGTGTAACC---------ATTAACTGTACAAGACACAACAACAATACAAGAAAA-----
-AGTATACCTATG------------GGACCAGGGAGAGCA---TTT------TATGCATATGGAGAA------
---------ATAATAGGAGATATA------------AGACAAGCATATTGT---AACATTAGTAGAGAA---
GAGTGGAATAAAACTTTAAGACTGATAGTTGAGAAGTTAAGA---------------GAAAAGTTTAAT-----
-----------------------------------AAAACAACAATAATCTTTAGG------
AACCACTCAGGAGGGGACCTAGAAGTTGCA---ATGCACACTTTTAATTGTGGAGGGGAA---
TTTAAGCAGCTCCAGGCAAGAGTCCTGGCCATAGAAAGATTCCTAGGGGATCAACAGCTCCTGGGAATTTGGG
GTTGCTCTGGAAAACTCATTTGCACCACTACTGTGCCTTGGAATCATAGTTGGAGT-----------------
-------------------------AATAATACAAATCTAACTAAGGTTTGG------GAA------
AACATGACCTGGATACAGTGG---GAAAAAGAAATTGACAATTACACAGACTTAATATAC---
AACTTAATTGAACACTCGCAGATCCAGCAAGAA--

AAGAATGAACAAGAACTATTGGAATTTGATAAATGGGCAAATTTGTGGAATTGGTTTAACATATCTAATTGGC
TGTGGTATATAAAAATCTTCATAATGATAGTAGGAGGCTTGATAGGTTTAAGAATAGTTTTTAGT---
GTACTTTCTATAGTG---------AATAGAGTTAGGCAGGGATACTCACCA------
CTATCGTTTGCTATTAGCTTGCTCAACGCCACAGCCATAGCAGTAGCTGAG---
GGAACAGATAGGGTTATAGAAGCAGTACAAAGAGTTGGTAGAGCT----------------------------
--------ATCCTTCACATA------CCTAGAAGAATAAGACAGGGCTTTGAAAGGGCTTTACTG---TAA
>MG900412
GCT---GCA--
GACAACTTGTGGGTCACAGTCTATTATGGGGTACCTGTGTGG---AGAGAAGCAACC---
ACTACTCTATTTTGTGCATCAGATGCTAAAGCATATGATACAGAG---GCACATAAT----------------
--------------GTTTGGCCACA---CACGCCTGTGTACCCACAGAC---
CCCAACCCACAAGAAGTAGAATTAAAA---------AATGTGACAGAAAATTTTAAT----------------
--------------------------ATGGGGAAAAATAACATGGTAGAACAGATGCATGAGGATATA---
ATCAGTTTATGGGATCAAAGCTTA---AAACCATGTGTAAAATTA---
ACTCCACTCTGTGTTACTTTAAATTGCACTGACAATGTTACTACTACCACTAATAGGAACGGGACAAATTGCA
CTGATTTAAGG--
-------AATGTTACTACTACCACTAATAGTAACGAGACATTGATGGAGAAGGGAGAAGTAAAA---
AACTGTTCTTTCATGATCACCACAGACGTAAAA---------------------------
GATAGAATGCGAAAGGAATATGCACTT---TTTTATAAACTTGATGTAGTACCAATAAATGAT----------
----

ACCAGATATAGGTTAGTAAGCTGTAACACCTCAGTCATTACACAG---
GCCTGTCCAAAGGTATCCTTTGAGCCAATTCCAATACATTATTGTGCCCCGGCTGGTTTTGCGATTCTAAAGT
GTAAT---GATAAGCAATTCAAT------GGAACAGGACCATGTACAAAT-----------------------
-GTCAGC---ACAGTACAATGTACACATGGAATTAGGCCAGTAGTATCAACT---
CAACTGCTGTTAAATGGCAGTTTAGCAGAAGAAGAG------------
GTAGTAATTAGATCAGTCAATTTCAGTGACAAT---------GCTAAAACAATAATAGTACAACTGAAT----
-----------AAATCTGTAGAA---------ATTAATTGCACAAGACCCAACAACAATACAAGAAAA-----

-AGTATACCCATG------------GGACCAGGGAAAGCA---TTT------TATGCAAGAGGAGCC------
---------ATAATAGGAGACATA------------AGAAAAGCACATTGT---AACATTAGTAGAGCA---
GAATGGCATAGCACTTTAAAACTGGTAGCTGAAAAATTAAGG---------------GAACAATAT-------
-----------------------------------AATAAAACAATAGTCTTTAAT------
CACTCCTCAGGAGGGGACCCAGAAATTGTA---ATGTACAGTTTCAATTGCGGAGGGGAA---
TTTAAGCAGCTCCAGGCAAGAGTCCTGGCTGTGGAAAGATACCTAAAGGATCAACAGCTCCTGGGGATTTGGG
GTTGCTCTGGAAAACTCATCTGCACCACTACTGTGCCTTGGAATACTAGTTGGAGT-----------------
----------------------------AATAAATCTCTGGAACAGATTTGG------GAT------
AACATGACATGGATGGAGTGG---GAAAAAGAAATTAACAATTATACAGGCTACATATAC---
CAATTAATTGAAGAATCGCAGAACCAACAAGAA--

AAGAATGAACAAGAATTATTGGCATGGGATAAATGGGCAAGTTTGTGGAATTGGTTTGACATAACAAACTGGC
TGTGGTATATAAAAATATTCATAATGATAGTAGGAGGCTTAATAGGTTTAAGAATAGTTTTTACT---
GTACTTTCTATAGTG---------AATAGAGTTAGGCAGGGATACTCACCA------
TTATCATTTGCTGTTAGCTTGCTCAACGCCACAGCCATAGCAGTAGCTGAG---
GGGACAGATAGAGTTATAGAAGTAGTACAAAGAGCTGGTAGAGCT----------------------------
--------ATTCGCCACATA------CCTAGAAGAATAAGACAGGGCTTGGAAAGGGCTTTGCTA---TAA
>KC473826
GCT---GAA--GAAAAG---
TGGGTCACAGTCTATTATGGGGTACCTGTGTGG---AAAGAAGCAAAC---
ACCACTCTATTTTGTGCATCAGATGCTAAAGCATATAAGAGAGAG---GTACATAAT----------------
--------------GTGTGGCTACA---CATGCCTGCGTACCCACAGAC---
CCCAGTCCACAAGAAGTGGAATTAAAG---------AATGTGACAGAAGAGTTTAAC----------------
--------------------------ATGTGGAAAAATAACATGGTAGAACAGATGCATGAGGACATA---
ATCAGTTTATGGGATCAAAGCTTA---AAGCCATGTGTAAAATTA---
ACACCACTCTGTATTACTTTAAATTGCACTGATGTGAAGAATACTACTAATACTACCACTGCT----------
----
---AGCAGCGGGGATGAAATGAAA---
AATTGCTCTTTCAATATCTCCACAGACGTAAGA---------------------------
GATAAGATGAAGAAAGAATATGCACTT---TTTTATAGCCTTGATATAGTAAAACTGAATGGTAAT-------
----

GATAGTAACAGATATAGGTTGACAAGTTGTAATACCTCAGTCATTACACAG---
GCCTGTCCAAAGGTATCCTTTGAGCCAATTCCCATACATTATTGTGCCCCGGCTGGTTTTGCGATTATAAAAT
GTAAC---AATAAGACATTCAGT------GGAAAAGGATCATGTAACAAT-----------------------
-GTCAGC---ACAGTACAATGCACACATGGAATTAAGCCAGTAGTATCAACT---
CAATTGCTGTTAAATGGCAGTCTAGCAGAAGGAGAC---------
AACATAGTAATTAGGTCTGACAATTTCTCGAACAAT---------GCTAGGACCATAATAGTACAGCTAACA-
--------------GAACCTGTAAAT---------ATTACTTGTACAAGACCCAACAACAATACAAGAAAA--
----AGCATACATATA------------GCACCAGGGAAGGCA---TTT------TATGCAACAGGAGAC---
------------ATAATAGGAAATATA------------AGACAAGCACACTGT---AACATTAGTAAAGCA-
--GAGTGGAATAACACTTTACAAAAGATAGTTAAAAAACTAGGA---------------GAACAATATAAT--
-------------------------------------ACAACAAATTTATCCTTTAAA------
AACTCCTCAGGAGGGGATCCAGAAATTGTG---ATGCACAGCTTTAATTGTAGAGGGGAA---
TTCAAGCAGCTCCAGGCAAGAGTCCTGGCTGTGGAAAGATACCTAAAGGATCAGCAGCTCCTGGGGATTTGGG
GTTGCTCTGGAAAACTCATTTGCACCACTAATGTACGTTGGAATAATAGTTGGAGT-----------------
----------------------------AATAAATCTTATGAAACGATTTGG------GAT------
AACATGACCTGGATGCAGTGG---GAAAAAGAAATTGACAGATACACAAACACAATATAC---
ACTTTACTTGAAGAATCGCAGAACCAGCAGGAA--

AAGAATGAACAAGAATTATTGGAATGGGACAAATGGGCAAATCTGTGGAATTGGTTTGACATAACAAACTGGC
TGTGGTATATAAAAATATTCATAATGATAGTAGGAGGTTTGATAGGTTTAAGAATAATTTTTGCT---
ATGCTTTCTATAGTG---------AATAGAGTTAGGCAGGGATACTCACCA------
TTATCGTTTGCTGTTAGCTTGCTCAACGCCACAGCCATAGCAGTAGCTGAG---
GGGACAGATAGGATTATAGAAGTAGTACAAAGAAGTTGTAGAGCT----------------------------
--------ATCCTTAACATA------CCTAGGAGAATAAGACAGGGCGCGGAAAGGGCTTTGCTA---TAA
>KC473828
GCT---GCA--
AATCAAACGTGGGTCACAGTCTATTATGGGGTACCTGTGTGG---AAAGATGCAATC---
ACCACTCTATTTTGTGCATCAGATGCTAAATCATATACTACAGAG---GCACATAAT----------------

--------------GTTTGGGCCACA---CATGCCTGTGTTCCTACAGAC---
CCCAACCCACAAGAAGTAGTATTGAAA---------AATGTGACAGAAGATTTTAAC----------------
--------------------------ATGTGGAAAAATAACATGGTGGAACAGATGCATGAAGATATA---
ATCAGTTTATGGGATCAAAGTCTA---AAGCCATGTGTAAAATTA---
ACCCCACTCTGTGTTACTTTAAAGTGCACTGATGTGAATACCACTAGTGCCAATAACGCTAGT----------
----
-------------GCCACTAACAGTAGTAGTAGCGGGGAATTAATGGAGCCAGGAGAAATAAAA---
AACTGCTCTTTCAATGTCACAACAGGCCTAAGA---------------------------
GATAAAAGGAAGAAAGAATATGCACTT---TTTTATAACCTTGATATAATACACTTAAGTGAT----------
----

AATGAAAATGATAATACTAGCTATAGGTTAACAAGTTGTAATACCTCAGTCATTACACAG---
GCCTGTCCAAAAATATCCTTTGAACCAATTCCCATACATTATTGTGCCCCGGCTGGTTTTGCGATTCTAAAAT
GTAAT---GAAAAGGATTCAAT------GGAACTGGACCATGCAAAAAT-----------------------
-GTCAGC---ACAGTACAATGTACACATGGAATTAGGCCAGTAGTCTCAACT---
CAACTGCTGTTAAATGGCAGTCTAGCAGAAGAAGAG------------
ATAGTAATTAGATCTGAAAATATCACAGACAAT---------GCAAAAACCATGATAGTACAGTTGAAT----
-----------GAGTCTGTAGCA---------ATTAATTGCACAAGACCCAACAACAATACAAGAAAA-----
-AGCATACATATA------------GCACCAGGAGGCGCA---TTT------TTTGCAACAGGAGAC------
---------ATAATAGGAGATATA------------AGAAAAGCACATTGT---AGCATTAGCAAAAAA---
GATTGGAATAACACTTTATATAAGGTAGCAATAAAATTAAGG---------------GAACAGTTT-------
-----------------------------------AATAAAACAATAATCTTTAAA------
AACTCCTCAGGAGGGGACCCAGAAATTGTA---ATGCACACTTTCAATTGTGGAGGGGAG---
TTTAAGCAGCTCCAGGCAAGAGTCCTAGCTGTAGAAAGATACCTAAGAGATCAACAGCTCCTGGGAATTTGGG
GTTGCTCTGGAAAACTCATCTGCACCACTGCTGTGCCTTGGAATAGTAGTTGGAGT-----------------
----------------------------AATAGATCTCTGGATGACATTTGG------GAT------
AACATGACCTGGATGATGTGG---GAAAAGGAAATTGAAAATTACACAGACTTAATATAC---
AACTTAATTGAGAAATCGCAAGACCAACAAGAA--

AAGAATGAACAAGAATTATTGGCATTGGATAAATGGGCAAGTTTGTGGAATTGGTTTGACATAACAAACTGGC
TGTGGTATATAAAAATATTCATAATGATAGTAGGGGGCTTAGTAGGCTTAAGAATAGTGTGTACT---
GTACTTTCCATAGCA---------AATAGAGTTAGGCAGGGATACTCACCT------
TTATCATTTGCTGTTAGCTTGTTTGATGCCATAGCTATTGCAGTAGCTGAG---
GGAACAGATAGGATTATAGAAATATTACAAAGAGGCTTTAGAGCT----------------------------
--------ATCCTCCATATA------CCTACAAGAATAAGACAGGGTTTCGAAAGGGCTTTGCTA---TAA
>KC473829
GCT---GCA--
GAAGAGACGTGGGTCACAGTCTATTATGGGGTACCTGTGTGG---AAAGATGCAGAT---
ACCACTCTATTTTGTGCATCAGATGCTAAAGCATATGAAACAGAG---GTACATAAT----------------
--------------GTTTGGGCCACA---CATGCCTGTGTACCCACAGAC---
CCGAACCCACAAGAAGTACAATTGGAA---------AATGTGACAGAAAATTTTAAC----------------
--------------------------ATGTGGAAAAATAACATGGTAGAACAGATGCATAATGATATA---
ATCAGCTTATGGGATCAAAGCCTG---AAGCCATGTGTAAAATTA---
ACCCCACTCTGTGTTACTTTAAATTGCACTGATGTGAAGATCAATACTACCAGCACCGAAATTAGT-------
----
-------------------ACCAAGACCGAATATAATACCATAGAGGAAAAAGGAGAAATAAAA---
AACTGCTCTTTCAATATCACCACAGGTATAAGA---------------------------
GATAAGGTAGAGAAACAATATGCAATT---
TTTTATAAATCTGATGTAGTGCAAATAGAGAAAAATGATATAGGA----------------------------
----
AGTAATAGTAGTGATAACAAGACTAGCTATAGGTTGATAAGTTGTAACACCTCAGTCATTACACAG---
GCCTGTCCAAAGGTAACATTTGAGCCAATTCCAATACATTATTGTACCCCAGCTGGTTTTGCGCTTCTAAAGT
GTAAT---AATGAGACGTTCAAT------GGAACAGGACCATGTAACAAT-----------------------
-GTTAGT---ACAGTACAGTGTACACATGGAATTAAGCCAGTGGTATCAACT---
CAATTGCTGTTAAATGGCAGTCTAGCAGAAAAGGAC------------
ATAGTAATTAGATCCCAAAATATCTCAGACAAT---------ACTAAAACCATAATAGTACATCTAAAG----
-----------GAACCTGTAAAT---------ATCACTTGTATAAGACCCAACAATAATACAAGAAAA-----
-GGTATACATATA------------GGACCAGGGAGAGCA---TTT------TATGCAACAGGAGAC------
---------ATAATAGGAAATATA------------AGAAAGGCACATTGT---AACATTACTAGAGCA---
AATTGGAATGTAACCTTAGGAAAGGTAGTTGAAAAATTAAAA---------------GAAAAGTTTAGG----

---------------------------------AATAAAACAATAAGATTTAGG------
CCACCCTCAGGAGGGGATCAAGAAATTACA---ATGCATATTTTTAATTGTAAAGGAGAA---
TTTAAACAGCTCCAGGCAAGAGTCCTAGCTGTGGAAAGATACCTAAGGGATCAACAGCTCCTGGGGATCTGGG
GTTGCTCTGGAAAACTCATTTGTACCACTGCTGTGCCTTGGAATGCTAGCTGGAGT-----------------
---------------------------AATAGATCTGTGAATGACATTTGG------GAT------
AACATGACCTGGATGGAGTGG---GAAAAGGAAATTGACAATTACACAGAGATAATATAC---
AACTTACTTGAGAAGTCACAGAACCAGCAAGAA-------------------------------------
----
AAGAATGAACAAGAATTATTGGAATTGGACAAATGGGCAAGTTTGTGGAATTGGTTTGACATATCAAAATGGC
TGTGGTATATAAAAATATTTATAATGATAGTAGGAGGCTTAATAGGTTTAAGAATAGTCTTTACT---
GTGCTTTCTATAGTG---------AATAGAGTTAGGCAGGGATACTCACCA------
TTGTCGTTGGCTGTTAGCTTGTTCAACGCCACAGCAGTAGCAGTAGCTGAG---
GGGACAGATAGAATTATAGACCTATTTCAAAGAATTTTTAGAGCT-------------------------
--------ATTCTTCATATC------CCTACAAGGATAAGACAGGGCTTGGAAAGGGCTTTGCTA---TAA
>KC473830
GCT---ATA--
GAAATGTGGGTCACAGTCTATTATGGGGTACCTGTATGG---AGAGAAGCAAAC---
ACCACTCTATTTTGTGCATCAGATGCTAAAGGATATGAGACAGAG---GTACATAAT-------------
--------------GTTTGGGCTACA---CATGCCTGTGTACCCACAGAC---
CCCAGTCCACAAGAAGTGGAATTAAAA---------AATGTGACAGAAGAATTTAAT-------------
-------------------------ATGTGGAAAAATAACATGGTAGAACAGATGCATGAGGACATA---
ATTAGCTTATGGGATCAAAGCTTA---AAGCCATGTGTAAAATTA---
ACACCACTCTGTGTCACTTTAACTTGCACTAATATAACTGAAACGAATACTACTAATGCCACTGCT-------
----
---AGCAACCGGAATGAAATGAGA---
AATTGCTCTTTCAATGTCACCACAGATGTAAGA--------------------------
GATAAGGTGAAGAAAGAATATGCACTT---TTTTATAGCCTTGATATAGTAAAACTGGATGGT----------
----

AATACCAGCTATAGGTTAACAAGTTGTAACACCTCAGTCATTACACAG---
GCCTGTCCAAAAAATATCCTTTGAACCAATTCCCATACATTATTGTGCCCCGGCTGGTTTTGCGATTTTAAAGT
GCAAC---AATAAGACATTCAGT------GGAAAAGGACCATGTAACAAT----------------------
-GTCAGC---ACAGTACAATGCACACATGGAATTAGGCCAGTAGTATCAACT---
CAATTGCTGTTAAATGGTAGTCTAGCAGAAGAAGAC-----------
ATAGTAATTAGGTCTGACAATTTCTCGAACAAT---------GCTAGAACCATAATAGTACAGCTAAAA----
-----------GACCCTGTAAAT---------ATTAATTGTACAAGACCCAACAACAATACAAGAAAA-----
-AGCATACATATA------------GCACCAGGGAGGGCA---TTT------TATGCAACAGGAGCC------
---------ATAACAGGAGATATA------------AGACAAGCACACTGT---AACATTAGTAGAGCA---
CAGTGGAATAACACTTTACAAAAGATAGCTACCAAATTAGGA---------------GAACAATATACA----
---------------------------------GCAAACAACATATCCTTTAAA------
AACTCCTCAGGAGGAGATCCAGAAATTGTA---ATGCACAGCTTTAATTGTGGAGGGGAA---
TTCAAGCAGCTCCAGGCAAGAGTCCTGGCTGTGGAAAGATACCTAAGGGATCAACAGCTCCTGGGGATTTGGG
GTTGCTCTGGTAAACTCATTTGCACCACTAATGTACGTTGGAATAATAGTTGGAGTAGTAGT---TGG-----
------------------------AGTAGTAAAAATCTGAGTGATATTTGG------AAC------
AACATGACCTGGATGCAGTGG---GAAAAAGAAATTGACAATTACACAAGCATAATATAC---
ACTTTACTTGAAGAATCGCAGAACCAGCAGGAA-------------------------------------
----
AAGAATGAACAAGAATTATTGGAATTGGACAAATGGGCAAATTTGTGGAATTGGTTTGACATAACACACTGGC
TGTGGTATATAAAAATATTCATAATGATAGTAGGAGGTTTGATAGGTTTAAGAATAGTTTTTGCT---
GTGCTTTCTATAGTG---------AATAGAGTTAGGCAGGGATACTCACCC------
TTATCGTTTGCTATTAGCTTGCTCAACGCCACAGCCATAGCAGTAGCTGAG---
GGGACAGATAGGATTATAGAATTAGTACAAAGAATTTGTAGAGCT-------------------------
--------ATCTGTAATATA------CCTAGGAGAATAAGACAGGGCGCGGAAAGGGCTTTGGTA---TAA
>KU677990
GCT---GCA--
GAAAACTTGTGGGTCACAGTCTATTATGGGGTACCTGTGTGG---AAAGAAGCAACT---
ACCACTCTCTTTTGTGCATCAGATGCTAAAGCATATGATACAGAG---GTACATAAT-------------
--------------GTTTGGGCCACA---CATGCCTGTGTACCCACAGAC---
CCCAACCCACAAGAAGTACAATTGAAA---------AATGTGACAGAAAATTTTAAC-------------
-------------------------GTGTGGAAAAATGATATGGTAGAACAGATGCATGAGGATATA---

ATCAGTTTATGGGATCAAAGCCTA---AAGCCATGTGTAAAATTA---
ACCCCACTCTGTGTAACTTTAAATTGCACTGATTTGAAGAATGTTACTAATACCAATAGTACTAATGCCACTA
GCACTATTGGCAAAGGTAGTAATACT---
-------GCTGGTAATAATGCCAATAATAGTAGCGGGGAAATACTGGGGGGAGGAGAAATAAAA---
AACTGCTCTTTCAATATCACCACAAGCATAAGA---------------------------
GATAAGGTGAAGAAAGAATATGCACTT---TTTTATACACTTGATATAGTGCCAATAGAGGAGGATAATACT-
----

GCTAATGATAGTACCAGTTATAGGTTGACAAGTTGTAATACCTCAGTCATTACACAG---
GCCTGTCCAAAGGTATCCTTTGAGCCAATTCCAATACATTATTGTGCCCCTGCTGGTTTTGCGATTCTAAAGT
GTAAT---GATAAGAATTTCACT------GGCTCAGGGCCATGCAGAAAT-----------------------
-GTCAGC---TCAGTACAATGTACACATGGAATTAGACCAGTAGTATCAACT---
CAACTACTGTTAAATGGCAGTCTAGCAGAAGATGAA---------
AAGGTAGTAATTAGATCTGAGAATTTCACAAACAAT---------GCCAAAACCATAATAGTACAGCTGAGA-
--------------GAAGCAGTAGTA---------ATTAATTGTACAAGGCCCAACAATAATACAAGAAAA--
----GGTATACATATA------------GGACCAGGGAGAGCA---TTT------TATGCAACAGGAGCA---
------------ATAATAGGAGACATA------------AGACAAGCACATTGT---AACCTCAGTGCAACA-
--GATTGGAATACTACTTTAAAAGAGGTAGCTGAAAAATTAAGA---------------GGACAATTTGGG--
----------------------------------AAAAATAAAACAATAGTCTTTAAT------
CAATCCTCAGGAGGGGACCCAGAAATTGTA---ATGCACACTTTTAATTGTGGAGGGGAA---
TTTAAGCAGCTCCAGGCAAGAGTCCTGGCTGTGGAAAGATACCTAAAGGATCAACAGCTCCTAGGAATTTGGG
GTTGCTCTGGAAAACTCATCTGCACCACTGCTGTGCCCTGGAATACTAGTTGGAGT-----------------
----------------------------AATAAATCTTATGATGACATTTGG------TAC------
AACATGACCTGGCTGGAGTGG---GAAAGAGAAATTGACAATTACACAGGCATAATATAC---
AACTTAATTGAAAAATCGCAAAACCAACAGGAA--

AAGAATGAACAAGAATTATTGGCATTAGATAAATGGGCAAGTTTGTGGACTTGGTTTGACATATCAAACTGGC
TGTGGTATATAAAAATATTCATAATGATAGTAGGAGGCTTGATAGGTTTAAGAATAATTTTTACT---
GTGCTTTCTATAGTG---------AATAAAGTTAGGCAGGGATACTCACCA------
TTATCGTTTGCTGTTAGCTTGCTCAACGCCACAGCCATAGCAGTAGGTGAG---
GGGACAGATAGGATTCTAGAAATATTACAAAGAGCTTTTAGAGGT----------------------------
--------GTCCTTCACATA------CCTAGAAGAATAAGACAGGGCTTTGAAAGGGCTTTGCTT---TGA
>KF384800
GGT---ACA--
GAAAAATTGTGGGTCACGGTCTATTATGGGGTACCAGTGTGG---AAGGAAGCAACC---
ACCACTCTATTTTGTGCATCAGATGCTAAAGCATATGAGAAAGAG---GTACATAAT----------------
--------------GTTTGGGCCACA---CATGCCTGTGTACCCACAGAC---
CCCAACCCACAAGAAATAGAATTGAAA---------AATGTGACAGAAAATTTTAAC----------------
--------------------------ATGTGGAAAAATAACATGGTAGAACAGATGCAAGAGGATATA---
ATCAGTTTATGGGATGAAAGCCTA---AAGCCATGTGTAAAATTA---
ACCCCACTCTGTGTTACTTTAAATTGCACTGATAATATTACTGCTACTAATACCACTAATGGTACTACT----
----
-------------ACTACTGTCAGCAATGGTAATGGTACAGCGGAGGAAAAGGGGGAAATGAAA---
AACTGCTCTTTCAATATCACCACAGCCATAAGA---------------------------
AATAAGATACAGAAAGAATATGCACTT---TTCTATAAACTTGATTTAACACCAATAGATAATGCT-------
----

GACAATACTAGCTATAGGTTGATAAGTTGTAACACCTCAGTCCTTACACAG---
GCCTGTCCAAAGGTGTCCTTTGAGCCAATTCCCATACATTATTGTGTCCCGGCTGGTTTTGCGATGCTAAAGT
GTAAA---GATAGAAATTTTAAT------GGAACAGGACAGTGTACAAGT-----------------------
-GTTAGC---ACAGTACAATGTACACATGGAATTAGGCCAGTGGTATCAACT---
CAACTACTGTTAAATGGCAGTCTAGCAGAAGGAGAG------------
GTAGTAATTAGATCTGAAAATTTCACAAACAAT---------GCTAAAATAATAATAGTACAGCTGAAC----
-----------AATTCTGTAAAA---------ATTAATTGTACAAGACCCTACCGAGTTATAACGAAA-----
-AGAATAATGCAT---------ATAGGACCAGGGAGAACA---TTC------CATACAACAGGA---------
---------ACAATAGGGAATATA-------------AGACACGCGTATTGT---AACATTAGTAGAAAA---
GATTGGAATAACACTTTAGCACAGATAGTTGAAAAATTAAAA---------------GAACAATTTGGG----
--------------------------------AAAAATAAAACAATAATCTTTAAG------
CCATCCTCAGGAGGGGACCCAGAAATTGTA---ACGCACAGTTTTAATTGTGGAGGGGAA---
TTTAAGCAGCTCCAGGCAAGAGTCCTGGCTCTGGAAAGATACCTAAGGGATCAACAGCTCCTGGGAATTTGGG

GTTGCTCTGGAAAACTCATTTGCACCACTACTGTGCCTTGGAATGCTAGTTGGAGT-----------------
----------------------------AATAGATCTCTAGATGAGATTTGG------GAT------
AACATGACCTGGATGAAATGG---GAAAGAGAAATTGACAATTACACAGGCTTAATATAC---
AACTTACTTGAACAATCGCAGAACCAGCAAGAA--

AAGAATGAACAAGAATTGTTGGAATTGGATCAATGGGCAAGTTTGTGGAATTGGTTCCACATAACAAATTGGC
TGTGGTATATAAAAATATTCATAATGATAGTAGGAGGCTTGGTAGGCTTAAGAATAGTTTTTGCT---
ATACTTTCTGTAGTG---------AATAGAGTTAGGCAGGGATACTCACCT------
TTATCGTTTGCTGTTAGCTTGCTCAACGCCACAGCTATAGCAGTAGCTGAG---
TGGACAGATAGGGTTATAGAAGTATTACAAAGAGCTGGTAGAGCT----------------------------
--------ATTATCCACATA------CCTACAAGAATAAGACAGGGCTTGGAAAGGGCTTTGCAA---TAA
>KF384801
GCT---GCA--
AAACAATTGTGGGTCACAGTCTATTATGGGGTACCCGTGTGG---AAAGAGGCCCCC---
CCCACTCTATTTTGTGCATCAGATGCTAGAGGATATGATACAGAG---GCACATAAT----------------
--------------GTCTGGGCCACA---CATGCCTGTGTACCCACAGAC---
CCCAGCCCACAAGAACTAGTATTGGCA---------AATGTGACAGAAAATTTTAAC----------------
-------------------------ATGTGGAAAAATAACATGGTAGAACAGATGCATGAGGATATA---
ATCAGTTTATGGGATCAAAGTCTA---AAACCATGTGTGAAATTA---
ACCCCACTCTGTGTTACTTTAAATTGCACTGATGCTAATAGAACCTGCGATAATGTCACTAATACTAGT----
----
----------------------AGTACTACTATTGACTGTAAGGACATGCGAAGAGAAATAAAA---
AACTGCTCTTTCAATATCACCTCAGGCATAAGA--------------------------
GATAAGATGAAAAAGGAATATGCACTT---TTTTATGCACTTGATGTAATACCAATAGATAAT----------
----

AGTAATACCAGTTATAGGTTGTTAAGATGTAACACCTCAGTCATTACACAG---
GCCTGTCCAAAGGTATCCTTTGAGCCAATTCCCATACATTACTGTGCCCCGGCTGGTTTTGCGATTCTAAAGT
GTAAC---AATAAGACATTCAAT------GGAACAGGGCCATGTACAAAT----------------------
-GTCAGT---ACAGTACAATGTACACATGGAATTAGACCAGTAGTGTCAACT---
CAACTGCTGTTAAATGGCAGCCTAGCAGAAGAAGAG-----------
GTAGTCATTAGGTCTGAGAATTTCACAGACAAT---------GCTAAAACTATAATAGTACAGCTGAAT----
-----------GAATCTGTACAA---------ATTAACTGTACAAGACCCAACAACAATATAAGAAAA-----
-AGAATAATACAT---------ATAGGACCAGGGAGACCA---TTT------TATGCAACAGGAGAC------
------------ATAGGAAATATA------------AGACGAGCACAGTGT---ACCCTCAATGGAACA---
GCTTGGAATAACACTTTAGAAAAGATAGTTGGAAAATTAAGA---------------GAACAATTTGGG----
----------------------------------AATAAGACAATAGTCTTTAAT------
TCATCCTCAGGAGGGGACCCAGAAATTGTA---ATGCACACTTTTAATTGTGGAGGAGAA---
TTTAAGCAGCTCCAGGCAAGAGTCCTGGCTCTGGAAAGATACCTAAGGGATCAACAGCTCCTAGGGATTTGGG
GTTGCTCTGGAAAACTCATCTGCACCACTAATGTGCCTTGGAATACTAGTTGGAGT----------------
----------------------------AACAAATCCCTAAGTTATATATGG------GAC------
AACATGACATGGATGGAGTGG---GAAAGAGAAATTGACAATTATACAGGCTTAATATAT---
AACTTAATTGAAGAATCGCAAAACCAACAAGAA--

AAGAATGAACTAGAATTATTAAAATTAGATTCATGGGCAAGTCTGTGGAATTGGTTTGACATATCCAAATGGC
TGTGGTACATAAAAATATTCATAATGATAGTAGGAGGCTTGATAGGTTTAAGAATAATCTTTACT---
GTGCTTTCTATAGTG---------AATAGAGTTAGGCAGGGATACTCACCA------
TTATCATTGGCAGTTAGCTTGCTCAACGCCACAGCAATAGCAGTAGGTGAG---
GGAACAGATAGGGTTATAGAGCTATTACAAAGAATTTATAGAGCT----------------------------
--------ATTCTTCACATA------CCTACAAGAATAAGACAGGGCTTAGAAAGGGCTTTGCTA---TAA
>KF384803
GCT---GCA--
GAACAATTGTGGGTCACAGTCTATTATGGGGTACCTGTGTGG---AAAGAAGCAAAT---
ACTACACTATTTTGTGCATCAGATGCTAAATCATATGAGGCAGAG---GCACATAAT----------------
--------------GTTTGGGCCACA---CATGCCTGTGTGCCCACAGAC---
CCTAACCCACAAGAAGTATTATTGGGA---------AATGTGACAGAAAATTTTAAC----------------
-------------------------ATGTGGAAAAATAACATGGTAGAACAGATGCATGAGGATGTA---
ATCAGTTTATGGGATCAAAGCCTA---AAGCCATGTGTAAAATTA---
ACCCCACTCTGCGTTACTTTAAACTGCACTGATTATGTGAATAATGCTACTAATATCTCTAGC----------
----

----------------------------AGCGGGGGAGAAGCGATGGAAAAAGGCGAAATGAAA---
AACTGCTCTTTCAATATCACCACAAGCCTAAGA--------------------------
GATAAGGTGCAGAAAGAATATACACTT---
TTTTATAAACTTGATTTAGTACCAATAGAGGATGAGAATAACAAA-----------------------------
----
ACCAATACCAGCTATAGGTTGATAAGCTGTAACACCTCAGTCCTTACACAG---
GCCTGTCCAAAGGTATCCTTTGAGCCAATTCCCATACATTATTGTGCCCCAGCTGGTTTTGCGATTCTAAAGT
GTAAA---GATGAGAAGTTCAAT------GGAACAGGACCGTGTACAAAT----------------------
-GTCAGC---ACCGTACAATGTACACATGGAATTAGGCCAGTAGTATCAACC---
CAACTGCTGTTGAATGGCAGTCTAGCAGAAAAAGAG------------
GTAGTAATTAGATCTAGCAACTTCACGGACAAT---------GCTAAAACCATAATAGTACAGCTGAAA----
-----------ACACCTGTAAAA---------ATTAATTGTACAAGACCCTACAACAAGATAATAAAA-----
-AGAAGAATACAT---------ATAGGACCAGGGAGAGCA---TTT------TCTACAACAGGAGGA------
---------ATAGGAGGAGAGATA------------AGAAAAGCACATTGT---AACATTAGTAGAACA---
GAGTGGAATAACACTCTAAAAGAGATAGTTAACAAATTAAGA---------------GAACAATTTGGG----
-----------------------------------AATAAAACAATAATCTTTAAT------
AAATCTGCAGGAGGGGACCCAGAAATTGTA---ATGCACAGTTTTAATTGTGGAGGGGAA---
TTTAAACAGCTCCAGGCAAGAGTCCTGGCTGTGGAAAGATACCTAAGGGATCAACAGCTCCTGGGGATTTGGG
GTTGCTCTGGAAAACTCATTTGCACCACTACTGTGCCTTGGAATGCTAGCTGGAGT-----------------
----------------------------AATAAATCTCTGGAAAAGATTTGG------GAT------
AACATGACCTGGATGGAGTGG---GAAAGAGAAATTGACAATTACACAAATTTAATATAC---
ACTTTACTTGAAGAATCGCAGAACCAACAAGAA---------------------------------------
----
AAGAATGAACAAGAATTATTGGAATTAGATAAATGGGCAAATTTGTGGAATTGGTTTGATATAACAAAATGGC
TGTGGTACATAAAAATATTTATAATGGTAGTAGGAGGCTTGGTAGGTTTAAGAATAGTTTTTGCT---
GTATTTTCTATAGTG---------AATAGAGTTAGGCAGGGATATTCACCA------
CTCTCGTTTGCTGTTAGCTTGTTCAATGCCATAGCCATAGCAGTAGCTGAG---
GGGACAGATAGGGTTTTAGAAGTATTACAAAGAGCTTTTAGAGCT---------------------------
--------ATTCTACACATA------CCTAGAAGAATAAGACAGGGCTTTGAAAGGGCTTTGCTA---TAA
>KF384804
GCT---ACA---
AAACCATTGTGGGTCACAGTCTATTATGGGGTACCTGTGTGG---AAAGAAGCAACT---
ACCACTTTATTTTGTGCATCAGATGCTAAAGCATATAATACAGAG---AAGCACAAT----------------
--------------GTTTGGGCCACA---CATGCCTGTGTACCCACAGAC---
CCCAACCCACAAGAAGTAGTAATGGAA---------AATGTGACAGAAAATTTTAAC----------------
--------------------------ATGTGGAAAAATAACATGGTAGAACAGATGCATGAGGATATA---
ATTAGTTTATGGGATCAAAGCTTA---AAGCCATGTGTGAAATTA---
ACCCCACTCTGTGTTACTTTAAATTGCACTGATGCTACTAAGACCAAT-------------------------
----
-------------------------AGTAGTAGAGGGGAAATGAAGGATGGACAAGAAATGAAA---
AATTGCTCTTTCAATATCACCACAAGCATAAGA--------------------------
GGTAAGATGCAGAAAGAATATGCGCTT---TTTTATAAGCTTGATGTAGTACCAGTAGAAAAG----------
----

GATAATGCTAGCTATAGGTTGATAAGTTGTAACACCTCAGTCATTACACAG---
GCCTGTCCAAAGGTATCCTTTGAACCAATTCCCATACATTATTGTACCCCGGCTGGTTTTGCGATTATAAAAT
GTAAT---AATAAGAAGTTCAAT------GGAACAGGACCATGTAAAAAT----------------------
-GTCAGC---ACAGTACAGTGTACACATGGAATTAAGCCAGTAGTATCAACT---
CAACTGCTGTTAAATGGCAGTCTAGCAGAAGAAGAG------------
GTAGTAATTAGATCTGAAAACTTCACAAACAAT---------GCTAAAGTCATAATAGTACAATTGAAT----
-----------GAGTCTGTGTCA---------ATTAATTGTACAAGACCTGGCAACAATACAAGCAAA-----
-GGTATACATATA------------GGACCAGGCAGAGCA---TTG------CATGTAACAAGAACA------
AGAGGAAAAATAATAGGAGATATA------------AGACAAGCACATTGT---AACATTAGTAAAGCA---
GATTGGAATAACACTCTAAAGAAGATAGTGGTAAAATTAAGA---------------GAACAATTTGGG----
-----------------------------------AATAAAACAATAGTCTTTAAT------
CAATCCTCAGGAGGGGACATAGAAATTGTA---ACACACAGTTTTAATTGTGGAGGGGAA---
TTTAAGCAGCTCCAGGCAAGAGTCCTGGCTGTAGAAAGATACCTAAAGGATCAACAGCTCCTGGGAATTTGGG
GTTGCTCTGGAAAACTCATCTGCACCACTAATGTGCCTTGGAACACTAGCTGGAGT----------------
---------------------------AATAAATCTCTAAAACAGATTTGG------GAC------
AACATGACCTGGATGGAGTGG---GAAAGAGAAATTGACAACTACACAAGTGTAATATAC---

ACCTTAATTGAACAATCGCAAAACCAGCAAGAA---------------------------------------
----
AAGAATGAAGAAGAGTTATTATCTTTAGATAAATGGACAATTTATGGAATTGGTTTAACATAACAAACTGGC
TGTGGTATATAAAAATATTCATAATGATAGTAGGAGGCTTGATAGGTTTAAGAATAGTTTTTACT---
GTACTTTCTATAGTA---------AATAGAGTTAGGCAGGATATTCACCA------
TTATCGTTCGCTGTAAGCTTGCTCAATTTCATAGCTATAGCAGTAGCTGAG---
GGGACAGATAGGATCATAGAAGTATTACAAAGAATTGGTAGAGGC---------------------------
--------ATCCTCCACATA------CCTACAAGAATAAGACAGGGCTTAGAAAGGCTTTTGCTA---TAA
>KF384807
GCT---GCA---
GAGGATATGTGGGTCACAATATATTATGGGGTACCTGTGTGG---AAAGAAGCAAAT---
ACCACTCTATTTTGTGCATCAGATGCTAAAGCATATAAAACAGAG---GCACATAAT---------------
--------------GTTTGGGCTACA---CATGCCTGTGTACCCACAGAC---
CCCAACCCACAAGAAGTAGTATTGGAA---------AATGTGATAGAAAATTTTAAC---------------
-------------------------ATGTGGAAAAATAACATGGTAGAACAGATGCATGAGGATGTA---
ATTAGTTTATGGGATCAAAGTCTA---AAACCATGTGTAAAATTA---
ACCCCACTCTGTGTTACTTTAACTTGCACTGATGCTAATGTCACTGATACTAATGCCACG------------
----
------------------------AATACTAACAAGACAGGGCTGGAGGGAGAAGGAATGAAA---
AATTGCTCTTTTAATGTCACCACAGACGTAAGT--------------------------
GATAAAAGAAAGAAAGAATATGCACTT---TTTTATAACCTTGATGTAGTACAAATAGATGGG---------
----

AATAGGGATAATACTAGCTACAAGTTAATAAGTTGTAACACCTCAGTCGTTACACAA---
GCCTGTCCAAAGATATCCTTTGAGCCAATTCCCATACATTATTGTGCCCCGGCTGGTTTTGCGATTCTAAAGT
GTAAT---AATAAGACATTCAAT------GGAACAGGACCATGTACAAAT----------------------
-GTCAGC---ACAGTACAATGTACACATGGATTAGGCCAGTAGTATCAACT---
CAACTACTGTTAAATGGCAGTTTGGCAGAAGAAAAG-----------
GTAGTCCTTAGATCTGAAAATTTCACAGACAAT---------AGTAAGACCATAATAGTACAACTGAAG----
-----------GAACCAGTACAA---------ATTAATTGTACAAGACCCAGTAACAATACAATAAAA-----
-GGTATACATATG------------GGACCAGGAAGAGCA---TTT------TATGCAACAGAACAG------
---------GTAATAGGAGATATA-----------AGGCAGGCACATTGT---AACCTTAGTAGAGAA---
CAATGGAATAACACTTTAAAACAGATAGCTAGCAAATTAGGA--------------GAACAATATGGG----
--------------------------------AGGAATAAAACAATAATCTTTAAA------
CACTCCTCAGGAGGGGACCTAGAAATTGTA---ACGCACACTTTTAATTGTGGGGGAGAA---
TTTAAGCAGCTCCAGGCAAGGGTTCTGGCTGTGGAAAGATACCTAAGGGATCAACAGCTCCTGGGGATCTGGG
GTTGCTCTGGAAAACTCATTTGCACCACTACTGTGCCTTGGAATGCTAGTTGGAGC----------------
----------------------------AATAAATCTTTTAATGAAATTTGG------GAT------
AACCTGACCTGGATGCAATGG---GAAAGAGAAATTGACAATTACACAGCAATAATATAC---
TCCTTACTTGAAGAATCGCAGAACCAGCAAGAA---------------------------------------
----
AAGAATGAACAAGACTTATTGGCATTAGATAAATGGTCAAATTTGTGGAATTGGTTTAACATAACAAACTGGC
TGTGGTATATAAAAATATTCATAATGATAGTAGGGGGCATGATAGGTTTAAGGATAGTTTTTATT---
ATATTTTCTATAGTG---------AATAGAGTTAGGCAGGATACTCACCA------
TTGTCGTTTGCCATTAGCTTGCTTAACGCTACAGCCATAACAGTAGCTGAG---
GGGACAGATAGGGTTATAGAAATAGTACAAAGAGCTTGGGGAGCT---------------------------
--------TTTCTCCACATA------CCTACTAGAATAAGACAGGGCTTGGAAAGGGCTTTGCTA---TAA
>KF384811
GCT---GCA---
GAAAATTTGTGGGTCACAGTCTATTATGGAGTACCTGTGTGG---AAAGAAGCAACT---
ACTACTCTATTTTGTGCATCAGATGCTAAAGCATACGACACAGAG---ATGCATAAT---------------
--------------GTTTGGGCCACA---CATGCCTGTGTACCCACAGAC---
CCCAACCCACAAGAAGTAGTACTGGAA---------AATGTGACAGAGAATTTTAAC---------------
-------------------------ATGTGGAAAAATGACATGGTAGAACAGATGCATGAGGATATA---
ATTAGTTTATGGGATCAAAGCCTA---AAGCCATGTGTAAAATTA---
ACCCCACTCTGTGTTACTTTAAATTGCGCTGATGTTAATAGTACCACCAATAAT------------------
----
---------------------------AGTAGTACGGGAGAGATGCAGGAAGGAGAAATAAAA---
AACTGCTCTTTCAATACCACCACAAACATAAAA--------------------------
GATAGGATACGGAAGGAATATGCACTT---

TTTTATAAACTTGATGTAGTACCAATAGTGGATGATGGAACCAAC----------------------------
----
AATACTAATGCCACTAGTACCCACTATAGGTTGATAAGTTGTAATACCTCAGTCATTACACAG---
GCCTGTCCAAAGATATCCTTTGAGCCAATTCCCATACATTATTGTGCCCCGGCTGGTTTTGCGATTCTAAAGT
GTAAT---GATAAGAAGTTTAAT------GGAACAGGACCATGTAAAAT-----------------------
-GTCAGC---ACAGTACAATGTACACATGGAATTAAGCCAGTAGTATCAACT---
CAGCTACTGTTAAATGGCAGTCTAGCAGAAGGAGAG------------
GTAATAATTAGATCTGAGAATCTCACAAACAAT---------GCTAAGACTATACTAGTACCCCTGAAT----
-----------AAATCTGTAGAA---------ATTATTTGTGAAAGACCCAACAACAATACAAGAAGG-----
-AGTATACCTATA------------GGACCAGGGAGAGTC---TTT------TTTACATCAGAA---------
---------ATAATAGGAGATATA------------AGACAAGCATATTGT---ACAATTAATGGAACA---
ACCTGGAGTGAAACTTTAAAGCAGGTAGCTATCAAATTAGGT---------------GAAAAATTTCCG----
-----------------------------------AACAAAACAATAGCCTTTAAT------
CGGTCCTCAGGAGGGGACCCAGAAATTGTA---ATGTACAGTTTTAATTGTGGAGGGGAA---
TTTAAGCAGCTCCAGGCAAGAGTCCTGGCTGTGGAAAGATACCTAAGAGATCAACAGCTCCTAGGAATTTGGG
GTTGCTCTGGAAAACTCATTTGCACCACTACTGTGCCTTGGAATAATAGTTGGAGT-----------------
----------------------------AATAAATCTTATGACCACATTTGG------GGT------
AACATGACCTGGATGGAGTGG---GAAAGAGAAATTGATAATTACACACACCTAATATAC---
AACTTAATTGAAGACTCGCAAAACCAACAAGAA--

AAGAATGAACTAGACTTATTAGAATTGGATAAGTGGGCAAGTCTGTGGAATTGGTTTGACATAACAAAATGGC
TGTGGTATATAAAAATATTCATAATGATAGTAGGAGGCTTAATAGGATTAAGACTAGTTTTTACT---
GTACTTTCTATAGTG---------AATAAAGTTAGGAAGGGATACTCACCA------
TTGTCGTTCGCTATTAGCTTGCTCAATACCACTGCTGTAGCAGTAGCTGAG---
GGGACAGATAGGGTTATAGAATTTTTACAAGGAATTGGTAGAGGC----------------------------
--------ATTCTCCACATA------CCTAGAAGAATAAGACAGGGCTTGGAAAGGGCTTTGCTA---TAA
>KX156409
GTT---ACA--
GGAAATTTGTGGGTCACAGTCTATTATGGAGTACCTGTGTGG---AAAGAAGCAACC---
ACCACTCTATTTTGTGCATCAGATGCTAAAGCATATGATACAGAG---GTACATAAT----------------
--------------GTTTGGGCCACA---CATGCCTGTGTACCCACAGAT---
CCCAATCCACACGAAGTACCATTGGTA---------AATGTGACAGAAAAATTTAAC----------------
--------------------------ATGTGGAAAAATAACATGGTAGAACAGATGCATGAAGATATA---
ATCAGTTTATGGGATCAAAGCCTA---AAGCCATGTGCAAAATTA---
ACCCCACTCTGTGTTACTTTAGATTGCAAGGATGTTAATGCCACTAGAGATAATGCCAATACTAATAATACCA
CTAGTGCCCCT--
-------------------------------AGTGGTAATAGTAGTATGATAGGAGAAATGAGA---
AACTGCTCTTTCAATGTCACCACAAACATAAGA---------------------------
GATAAGATACGGAAAGAATATGCTCTT---
TTTTATAGATATGATGTAGTACCAATAGAAGAGGAGAAAAGGCAA----------------------------
----
AACAAAACCACCTATAGACTAATAAATTGTAACACCTCAGAAATTACACAG---
GCCTGTCCAAAGGTATCCTTTGAGCCCATTCCCATACATTACTGTGCCCCGGCTGGTTTTGCGCTTCTAAAGT
GTGGA---GAGAAGAACTTTAAT------GGAACAGGAAAATGTAGAAAT-----------------------
-GTCAGC---ACAGTACAATGTACACATGGAATTAAGCCAGTAGTGTCAACT---
CAACTGTTGTTAAATGGTAGTCTAGCAGAAGAAGAT------------
GTAGTACTTAAATCTGAGAACTTCACGAACAAT---------GCTAAAGTCATAATAGTACAGCTAAAT----
-----------GAAACTATAGAA---------ATAAATTGTACAAGACCCAACAACAATACAAGAAGG-----
-AGTATACATATA------------GCACCAGGGAGAGCA---TTC------TATGCAACAGGAGAA------
---------ATAACAGGAGACATA------------AGAAAAGCATATTGT---AATGTCAGTAAAGAA---
AATTGGAATAAAGCATTACAAAGGATAGCTATGAAATTAAGA---------------GAACAATTTGGG----
--------------------------------GAAAACAAAACAATAAATTTTACT------
CAATCCTCAGGGGGGGACTCAGAAATTGTA---ATGCATAGTTTTAATTGTGGAGGGGAA---
TTTAAGCAGCTCCAGGCAAGAGTCCTGGCTCTGGAAAGATACCTACATGATCAACAGCTCCTAGGAATTTGGG
GTTGCTCTGGAAAACTCATCTGCACCACTGCTGTGCCTTGGAATACTAGTTGGAGT-----------------
----------------------------AATAAAACTCTAGAAAGGATTTGG------AAT------
AACATGACCTGGATGCAGTGG---GAGAAAGAAATTGACAATTATACAGAAACAATATAC---
TCCTTACTTGAAAAATCGCAAAACCAGCAAGAA--

AAGAATGAACAAGAATTATTAGAATTAGATAAATGGGCAAGTCTGTGGAATTGGTTTGATATAACAAAGTGGC

TGTGGTATATACAAATATTCATAATAATAGTAGGAAGCTTAGTAGGTTTAAGAATAGTTTTTGCT---
GTACTTTCTATAGTG---------AATAGAGTTAGGCAGGGATACTCACCA------
TTATCGTTTGCTGTTAGCTTGCTAAGTGCTATTGCTACTGCTATAGCTGGG---
GGGACAGATAGGATTATAGAAATAGCACAAAGAATCTTTAGAGCT----------------------------
--------ATCCTTAACGTA------CCTACTAGAATAAGACAGGGCTTAGAAAGGGCTTTGCTA---TAA
>KY778615
GCT---GCA---
GAAAATTTGTGGGTCACAGTCTATTATGGGGTACCTGTGTGG---AAAGAAGCAACC---
ACCACTCTATTCTGTGCATCAGATGCTAAAGCATATGACACAGAG---GTACATAAT----------------
--------------GTTTGGGCCACA---CATGCCTGTGTACCCACCGAC---
CCCAACCCACAAGAAGTGGAATTAAAA---------AATGTGACAGAAAATTTTAAT----------------
--------------------------ATGTGGAAAAATGACATGGTAGACCAGATGCATGAGGATATA---
ATCAGTTTATGGGATGAAAGCCTA---AAGCCATGTGCAAAATTA---
ACCCCACTTTGTGTTACTTTAAATTGCAGTGATGTAAGGAATGATACTACTACTAATACC-------------
----
----------------------AATAGTAGCTGGGGAAATACAATGGAGAGAGGAGAAATGAAA---
AATTGCTCTTTCAATGTCACCACAAGCATAAGA---------------------------
GATAAGACACAGAAAGAAGCGGCACTT---
TTTTATAGACTTGATATAGTACCAATAGAAGATAATAATGATAAT----------------------------
----
AGTAGCTCTAGAAATTATGGTAGCTATAGATTGATAAATTGTAATACCTCAGTCATTACACAG---
GCCTGTCCAAAGATATCCTTTGAGCCAATTCCCATACATTATTGTGCCCCGGCTGGTTTTGCGATTCTAAAAT
GTACT---AATAAGACGTTCCAG------GGAAAAGGACCATGCACAAAT-----------------------
-GTCAGC---ACAGTACAATGCACACATGGAATTAGGCCAGTAGTGTCAACT---
CAACTGCTATTAAATGGCAGTCTAGCAGAAGAAGAG-----------
GTAGTAATTAGATCTGATAATTTCACAGACAAT---------GCTAAAATCATAATAGTGCAGCTGAAT----
-----------GAATCTGTAGTA---------ATTAATTGTACAAGACCCAACAACAATACAAGAAAA-----
-AGTATATCTATA------------GGACAAGGGAGGGCA---TGG------TATGCAACAGGAGAT------
---------ATAATAGGAGATATA------------AGACAAGCACATTGT---AACATTAGTGGAGCA---
AACTGGGCTAGAGCTTTAAAACTAATAAGGGAAAAATTAAAA---------------GAACAATTTGTC----
-----------------------------------AATAGAACAATAACCTTTAAA------
CCATCTTCAGGAGGGGACCCAGAAATTGTT---ATGCACAGTTTCAATTGTGGAGGGGAA---
TTTAAGCAGCTCCAGGCAAGAGTCCTGGCTGTAGAAAGATACCTAAGGGATCAACAGCTCCTAGGAATTTGGG
GTTGCTCTGGAAAACTCATCTGCACCACTACTGTGCCTTGGAATACTAGTTGGAGC-----------------
-----------------------------AATAAATCCCTGGATTACATTTGG------CAG------
AATATGACTTGGATGCAGTGG---GAAAGTGAAATTGAAAATTACACAGACCTAATATAT---
GACTTAATTGAAAAATCGCAAAACCAGCAAGAA--

AGGAATGAACAAGAATTATTGGAATTGGACAAATGGGCAAATTTGTGGTCTTGGTTTGACATAACAAACTGGC
TGTGGTATATAAAAATATTCATAATGATAGTAGGAGGCTTGATAGGTTTAAGAATAATTTTTACT---
GTGCTTTCTATAGTA---------AATAGAGTTAGGAAGGGATACTCACCA------
TTGTCATTTGCTGTTAACTTGTTTAATACCACAGCAATAGTAGTAGCTGAA---
GGGACAGATAGGATTATAGAAGCAGGAAGAAGAATTTTTAGAGCT---------------------------
--------TTTCTACATATA------CCTAGAAGAATAAGACAGGGCTTAGAAAGGGCTTTGCTA---TAA
>KT124768
GCT---ACA---
AATCAATTATGGGTAACAGTCTATTATGGGGTACCTGTGTGG---AAAGAAGCAAGA---
ACCACTCTATTTTGTGCATCAGATGCTAAAGGATATGAGACAGAG---GCACATAAT----------------
--------------GTTTGGGCCACA---CATGCCTGTGTACCCACAGAC---
CCCAACCCACAAGAAGTAGAATTGGAA---------AATGTGACAGAAAATTTTAAC----------------
--------------------------ATGTGGAAAAATGACATGGCAGAACAGATGCATGAGGATATA---
ATCAGTTTATGGGATCAAAGTTTA---AAGCCATGTGTAAAATTA---
ACCCCACTCTGTGTTACTTTAGAGTGCAGTGATAATGTTATTATAAATGGCACTACTAATAAGACCGCTAGTA
CTATCAGTCCA--
-------------------------------AAGAGCACTAATATACCAGAAGGGGAAATAAAA---
AACTGCTCTTTCAATATCACCACAAACATGAGA---------------------------
GATAAATATCAAAGAGAATACGCACTT---TTTTATAAACTTGATTTAGAAGAACTAATAGAT----------
----

AACAATAAGACTACCTATAGGTTAATAAGTTGTAACACCTCAGTCATTACACAA---

GCCTGTCCTAAGGTATCCTTTGAGCCAATTCCTATACATTATTGTGCCCCGGCTGGTTTTGCGATTCTAAAGT
GTAAA---GATAATATGTTTAAT------GGAACAGGACCATGTTACAAT-----------------------
-GTTAGC---ACAGTACAATGTACACATGGAATTAGACCAGTAGTATCAACT---
CAACTGTTGTTAAATGGCAGCCTAGCAGAAGAAGAT------------
GTAGTAATTAGATCTGAAAATATCACGGACAAT---------ACTAAGAACATAATAGTACAGCTAAAA----
-----------GATCCTGTAGAA---------ATTAACTGCACAAGACCCAACAACAATACAAGAAGA-----
-AGTATACATATA------------GCACCAGGGAGAGCA---TTC------TATGCATCAGGAGGA------
---------ATAATAGGAGATATA------------AGACAAGCACATTGT---AACATTAGTAGAATA---
AAATGGAATAACACTTTAGAACAAATAGTTAAAAAACTGAGA---------------GCACAATTTGAA----
-----------------GAATTTAAACAT------AATAAAACAATAGCCTTTAGA---
CAACCACCTCCAGGTGGGGACCCAGAAATTACA---ATGCATACTTTTAATTGTGGAGGGGAA---
TTTAAGCAGCTCCAGGCAAGAGTCCTGGCTGTGGAAAGATACCTAAAGGATCAACAGCTCCTGGGGATTTGGG
GTTGCTCTGGAAAACTCATCTGCCCCACTGCTGTGCCTTGGAATAGCACTTGGAGT-----------------
----------------------------AATAGATCTCAGAGTGAAATTTGG------GAT------
AACATGACCTGGATGCAGTGG---GAGAAAGAAATTGGCAATTACACAGACTTAATATAC---
AACTTAATTGAAGAATCGCAAATCCAACAAGAA--

AAGAATGAAAAAGACTTATTGGAATTAAGTAACTGGGCAAGTTTGTGGAATTGGTTTGACATATCAAAATGGC
TGTGGTATATAAAAATATTCATAATGATAGTAGGAGGCTTGATAGGTTTACGAATAGTTTTTGCT---
GTAATTTCTATAGTG---------AATAGAGCTAGGCAGGGATACTCACTA------
CTGTCGTTTGCTGTTAGCTTGCTTGACGCCATCGCCATAGCAGTAGCTGGG---GGAACCGATAGG---
ACAGAGAATACAGAGAATTGGTAGAGCTAT---
-----------------------TCTCAACACATCTAAAGAGCTTTACTA---TAA
>MG196879
GCT---GCA--
GATAATTTGTGGGTCACAGTCTATTATGGGGTACCTGTGTGG---AAAGAAGCAACC---
ACTACTTTATTTTGTGCATCAGATAGTAAAGCATATGATACAGAA---GTACATAAT----------------
--------------GTTTGGGCCACA---CATGCCTGTGTACCCACAGAC---
CCCAACCCACAAGAAGTAGTATTGGAA---------AATGTAACAGAAAATTTTAAC----------------
--------------------------ATGTGGGAAAATAACATGGTAGAACAGATGCATGGGGATATA---
ATCAATTTATGGGATCAAAGCCTA---AAGCCATGTGTAAAATTA---
ACCCCACTTTGTGTTACTTTAAATTGCACTGATTATGTAGGGAATAGTGCTAAAAATGGTACTGATTCG----
----
-------------------GGGAATAGTACTGTGGCGAATAATGCTACTATTGGGGAAATAAAA---
AACTGCTCTTTCAATATCACCACCAGCATAAAA---------------------------
GATAAGGTGCAGAAAGAAAAAGCATTG---TTTTATAAATATGATTTAGTACAAATAACTGAAGATAAA----
----

AATAGTAATACGAGCTATAGTAGCTATAGGTTGGTAGGTTGCAACACCTCAGTCATTACACAA---
GCCTGTCCAAAAATATCCTTTGAGCCAATTCCCATACATTATTGTGCCCCGGCTGGTTTTGCGATTCTAAAGT
GTAAT---GATAAAAGGTTCAAT------GGAAAAGGACCATGTAAAAAT-----------------------
-GTCAGC---ACAGTACAATGTACACATGGAATAAGACCAGTAGTATCAACT---
CAACTACTGTTAAATGGCAGCTTAGCAGAGAAAGAA------------
ATAGTAATTAGATCTGAAAATTTCACGAGCAAT---------ACTAAAAACATAATAGTACAGTTAAAT----
-----------GAATCTGTAGAG---------ATTAAGTGTACAAGACCCAACAACAATACAAGAAAA-----
-GGTATACATTTA------------GGACCAGGAAAAGCC---TTT------TATGCAACAACAGAC------
---------ATAATAGGAGATATA------------AGACAAGCACATTGT---AACATTAGTGGAACA---
AAATGGAATAACACATTAAAACAGATAGCTGAAAAATTAAAA---------------GAACAATATAAT----
--------------------------------AATAATAAAACAATAGTCTTCAAT------
CAATCCTCAGGAGGGGATCCAGAAATTGTA---ATGCACAGTTTTATCTGTGGAGGGGAA---
TTTAAACAGCTCCAGGCAAGAGTCATGGCTGTGGAAAGATACCTGCAGGATCAACAGCTCCTGGGAATTTGGG
GTTGCTCTGGAAAGCTCATTTGCACCACTTCTGTGCCTTGGAATGCGAGCTGGAGT-----------------
----------------------------AACAAATCTCTGGAGAAAATTTGG------GAT------
AACATGACCTGGATGGAGTGG---GAAAAAGAGATTGACAATTACACAAACGAAATATAC---
ACCTTAATTGAAATAGCGCAAAACCAACAAGAA--

AAGAATGAACAAGAATTATTGGAATTAGATAAATGGGCAAGTTTGTGGAATTGGTTTGACATAACAAACTGGC
TGTGGTATGTAAAAATATTCATAATGATAGTAGGAGGCTTGATAGGTTTAAGAATAGTTTTTACT---
GTACTTTCTATAGTG---------AATAGAGTTAGGCAGGGATACTCACCA------
TTATCGTTTGCTACTAGCTTGCTCAACGCCACAGCCCTAGCAGTAGCCGAG---

GGAACAGATAGGGTTATAGAAGTAATACAAAGAATTGGTAGAGCT----------------------------
--------TTTCTCCATATA------CCTAGAAGAATAAGACAGGGCTTCGAAAGGGCTTTGCAA---TAA
>MG197013
GCT---GCA--
GAACAACTGTGGGTCACAGTCTATTATGGGGTACCTGTGTGG---AAAGACGCAAAC---
ACCACTCTATTTTGTGCATCAGATGCTAAAGGATACCATACAGAG---AAGCATAAT---------------
--------------GTTTGGGCCACA---CATGCCTGTGTACCCACAGAC---
CCCAACCCACAAGAAGTAGTATTGGAA---------AATGTGACAGAAAAATTTAAC---------------
--------------------------ATGTGGAAAAATAACATGGTAGAACAGATGCATGAGGATATA---
ATCAATTTGTGGGATCAAAGTCTA---AAGCCATGTGTAAAACTA---
ACCCCACTCTGTGTTACTTTACATTGCATTAATGTGACTAATACCACTGGTAATAATACCAAT---------
----
---------------------GGTGTTAGGCAGGGAGAGATGATTAATAAAGGAGAAATAAAA---
AACTGCTCTTTCAATATCACCGCAGGCATAAGA--------------------------
GATAAAGTGCAAAAAGAATATGCTCTT---
TTTTATAAAATTGATTTAGTACCAATAGAGAATGATAATACAAGC----------------------------
----
AGCTATGATAATACAAGCTATAGGTTGATAAGTTGTAACACCTCAGTCATTACACAA---
GCCTGTCCAAAGGTATCCTTTGAGCCAATTCCCATACATTATTGTGCCCCGGCTGGTTTTGCGATTCTAAAGT
GCAAT---GATAAGAAGTTCAGT------GGAAAAGGAAACTGTAAAAAT----------------------
-GTCAGC---ACAGTACAATGTACACATGGAATTAGACCAGTAGTGTCAACT---
CAACTGCTGTTAAATGGCAGTTTAGCAGAAGAAGAG------------
GTAGTGGTTAGATCTGACAATTTCACAGACAAT---------GTTAAAACCATAATAGTGAATCTGAAT----
-----------GAATCTGTAACA---------ATTAATTGTACAAGACCCGGCAACAACACAAGAAAA-----
-AGTGTATCTATA------------GGACCAGGAAGAGCA---TTT------TATGCAACAGGAGAC------
---------ATAATAGGAGATATA-----------AGACAAGCACACTGT---AACATTAGTAAAGGA---
GACTGGGATAAAATGTTAAAAAGGGTAGCTACAAAATTAAGA--------------GAAAAATTT-------
-----------------------------------AATAAAACAATAGCCTTTAAT------
AAATCCTCAGGAGGAGACCCAGAAATTGTA---ATGCACAATTTTAATTGTGGAGGGGAA---
TTTAAGCAGCTCCAGGCAAGAGTCCTGGCTGTGGAAAAATACCTAAGGGATCAACAGCTCCTAGGGATTTGGG
GTTGCTCTGGAAAACTCATTTGCACCACTGCTGTGCCTTGGAATTCTAGTTGGAGT----------------
----------------------------AATAAATCTCTAGATCAAATTTGG------GAT------
AACATGACCTGGAATGATTGG---GAAAAAGAAATTAGCAATTACACACAAAAAATATAC---
ACCTTAATTGAAAAATCGCAGAACCAACAAGAA---------------------------------------
----
AAGAATGAAAAAGAATTATTGGAATTGGATCAATGGGCAAATTTGTGGACTTGGTTTGATATAACAAACTGGC
TGTGGTATATAAAAATATTCATAATGATAGTAGGAGGCTTGATAGGTTTAAGAATAGTTTTTGCT---
GTATTTTCTTTAGTG---------AATAGAGTTAGGCAGGGATATTCACCA------
CTATCATTTGCTGTCAGCTTGTTCAACGCCACAGCTATAGCAGTAGCTGAG---
GGAACAGATAGGATTATAAAAGTAGCACAAAGAGTTGGTAGAGCT---------------------------
--------TTTCTCAACATA------CCTAGAAGAATAAGACAGGGTTTGGAAAGGGCTTTGCTA---TAA
>MG197046
GCA---ACA--
GAAGGCTTGTGGGTCACAGTCTATTATGGGGTACCTGTGTGG---AAAGAGGCAAAG---
ACCACTCTATTTTGTGCCTCAGATGCTAAAGGATATGATACAGAG---GCACATAAT---------------
--------------ATCTGGGCCACA---CATGCCTGTGTACCCACAGAC---
CCCAACCCACAAGAAATAGTATTGGGA---------AATGTGACAGAAAATTTTAAC---------------
--------------------------GCGTGGAAAAATGACATGATAGACCAGATGCATGAGGATATA---
ATCAATTTATGGGATGAAAGCCTA---AAACCATGTGCAAAACTA---
ACCCCACTCTGTGTTACTTTAGATTGCAAGGAACTCAATAAAACTAGGAATGCT------------------
----
--ACTATAGAAGGAGAAGTAAAA---
AATTGTACTTTCAATACCACCACACCCGTAAGG--------------------------
AATGAGGTGCAGAAGCAATATGCCCTT---TTTTATAGCACGGATATAATACCAATAGACAGT---------
----

AATAATAACAATACCATATATAGATTGATAAGGTGTAACACCTCAGTCATTACACAG---
GCCTGCCCAAAGATATCCTTTGAGCCAATTCCCATACATTATTGTGCCCCGGCTGGTTTTGCAATTCTAAAAT
GTAAC---AATAAGACATTCAGT------GGAAAAGGACCATGCACAAAT----------------------
-GTCAGC---ACAGTACAATGCACACATGGAATTAAGCCAGTAGTATCAACC---

CAACTACTGTTAAATGGCAGTCTAGCAGAAGAAGAT------------
GTAGTAATTAGATCTGAAAATTTCACGGACAAT---------GCAAAAACCATAATAGTACATCTAAAT----
-----------AAATCTGTAACA---------ATTAATTGTACAAGGCCCAACAATAATACAAGAAAA-----
-AGTATACATATA------------GCACCAGGGAGAACA---TTC------TATGCAACAGGAGAC------
---------ATAATAGGAGATATA------------AGACAAGCACATTGT---AACATTAGTGGTACA---
GAATGGGAACAAGCTTTAACAAATGTGGTTGGAAAACTAAGA---------------GAACAATATGGG----
---------------------------------GGAAATAAAACAATAATCTTTAAT------
CAATCCTCAGGAGGGGACCCAGAGATTGTA---ATGCACAGTTTTAATTGTAGGGGGGAA---
TTTAAACAGCTCCAGGCAAGAGTCCTGGCTGTGGAAAGATACCTAAGGGATCAACAGCTCCTGGGGATTTGGG
GTTGCTCTGGAAAACTCATTTGCCCCACTGCTGTGTCTTGGAATGCTACTTGGAGT-----------------
-----------------------------AATAAATCTCTGGAGGAAATTTGG------AAT------
AACATGACCTGGATGGAGTGG---GATAGAGAGATTAACAATTACACACAACACATATAC---
ACGTTGCTTGAAGAATCACAAAACCAGCAAGAA---

AAGAATGAAAAGGAATTATTGGAATTGGATAAATGGGCAGGTTTGTGGAATTGGTTTGACATATCAAATTGGC
TGTGGTATATAAAAATATTCATAATGATAGTAGGAGGCTTGATAGGTTTAAAAATAGCTTTTACT---
GTACTTTCTATAGTG---------AATAGAGTTAGGCAGGGATATTCACCA------
CTATCGTTTGCTGTTAGCTTGCTCAACGCTTTAGCCATAGCAGTAGCTGAG---
AGGACAGATAGGATCTTAGAAGTATTACAAAGAATTGGTAGGGCT----------------------------
--------ATCCTTCATATA------CCCACAAGAATAAGACAAGGCCTGGAAAGAGCTTTGCTA---TAA
>MG197064
GCT---ACA---
GAAAAATTGTGGGTCACAGTCTATTATGGAGTACCTGTGTGG---AGAGAAGCAGAC---
ACCACTCTATTTTGTGCATCAGATGCTAAAGCATATGATACAGAG---GCCCATAAT----------------
--------------GTTTGGGCTACA---TATGCCTGTGTACCCACAGAC---
CCCAGTCCACAAGAAGTAGAATTAAAA---------AATGTGACAGAAAATTTTAAC----------------
-------------------------ATGTGGAAAAATAACATGGTAGATCAGATGCATCAGGATATA---
ATCAGTTTATGGGATCAAAGCCTA---AAGCCATGTGTAAAATTA---
ACCCCACTCTGTGTTACTTTAAATTGCACTAATGCTACTGCTATCGTCAATAATACTATT-------------
----
-----------------------------GCCAATGCCAGTAGCGAGGGGAGAGAAGAAATAAAA---
AACTGCTCGTTCAATATCACCACAGGGGTAAGA---------------------------
GATAAGATGCAGAAAGCATATGCAACT---TTTTATAAACTTGATATAGTACCAATAGAGGAGGAC-------
----
ATAAAACCAACTAGTAGTGCTGGCAATGCCAGTCATACTAACTCCAACTATACTAACTATAGGATAATAAATT
GTAACACCTCAGTCATTACACAA---
GCCTGTCCAAAGGTATCCTTTGAGCCAATTCCCATACATTATTGTGCCCCGGCTGGTTTTGCGATTCTAAAAT
GTAAT---GACAAGGAGTTCAAT------GGAACAGGATTATGTAAAAAT-----------------------
-GTCAGC---ACAGTACAGTGTACACATGGAATTAAGCCAGTAGTATCAACT---
CAACTGCTATTAAATGGCAGCCTAGCAAAGGGAGAG------------
ATAGTAATTAGATCTGAAAATCTCACGGACAAT---------GCTAAAACCATAATAGTACACTTGAAT----
-----------GAAACTGTAGAC---------ATTACTTGTGTAAGGCCCAACAACAATACAAGAAAA-----
-AGCATACATATA------------GGACCAGGGAGAACA---TTT------TACGCAACAGGAGAC------
---------ATAATAGGAGATATA------------AGAAAAGCACATTGT---AGCATTACTGCAGCA---
ATGTGGAATAAAACTTTACAACAGGTAGCCACAAAATTAAGT---------------GAAAAATTTGAG----
------------------------------------GGTAAAAATATAACCTTTACA------
CAACACTCAGGAGGGGATCTAGAAATTGTA---ATGCACACTTTTAACTGTGGAGGGGAA---
TTTAAGCAGCTCCAGGCAAGAGTCTTGGCTGTGGAAAGATACCTAAGGGATCAACAGCTCCTGGGAATTTGGG
GTTGTTCTGGAAAACTCATCTGCACCACTACTGTGCCTTGGAATACTAGTTGGAGT-----------------
-----------------------------AATAAATCTCTGGAGCAGATTTGG------GAT------
AACATGACCTGGATGGATTGG---GAAAGAGAAATTGACAATTACACAAGTACAATATAC---
AGATTAATTGAAGAATCGCAAAACCAACAAGAA---

AAAAATGAACAAGACTTATTGGCACTTGATAAATGGGACAGTCTGTGGAGTTGGTTTAATATAACAAATTGGC
TGTGGTATATACAAATATTCATAATGATAGTAGGAGGATTGATAGGTTTAAGAATAGTTTGTGCT---
GTAATTTCTGTAGTG---------AATAAAGTTAGGCAGGGATACTCACCA------
TTATCGTTCGCTATTAGCTTGTTCAATGCCACAGCAATAACAGTAGCTGAG---
GGGACAGATAGGGTTATAGAAATATTGCAAAGAGCTTGTAGGGCT----------------------------
--------ATCCTCCATATA------CCTAGAAGAGTAAGACAGGGCTTGGAAAGGGCTTTGCTA---TAA
>MG197113

GCA--
GAACAGTTGTGGGTCACAGTCTATTATGGGGTACCTGTGTGG---AAAGAAGCAACT---
ACCACTCTATTTTGTGCATCAGATGCCAAAGCCTATGACACTGAG---GCACATAAT----------------
--------------GTTTGGGCCACA---CATGCCTGTGTGCCCACAGAC---
CCTCACCCACAAGAAGTAGTATTGGGA---------AATGTGACAGAAAATTTTAAC----------------
--------------------------ATGTGGAAAAATTACATGGTAGATCAGATGCATGAGGATATA---
ATCAGTTTATGGGATGAAAGCCTA---AAGCCATGTGTAAAATTA---
ACTCCACTATGTGTTACTTTAACTTGCACCAACGTGAATCATACCAATGGCAATCAAACTAAGCTTACCAAGA
GTAATCAAACT--
----------------------AATAATAGTAGTGAGTTGGAAGAGATGAGGGGAGAAATAAAA---
AACTGCTCTTTCAATATTACCACAACCATAAGT---------------------------
GATAAGAGGAAGAAAGAATATGCTCTT---TTTTATAGACTTGATGTAGTACCAATAGCTGAT----------
----

GATAATACAAGCTATACGTTGATAAATTGTAACACCTCAGTCATTACACAG---
GCCTGTCCAAAGATATCCTTTGAACCAATTCCCATACATTATTGTGCTCCGGCTGGTTTTGCGATTCTAAAGT
GTAAT---AATAAGAAATTCAAT------GGAACAGGACCATGCACAAAT-----------------------
-GTCAGC---ACCGTACAATGTACACATGGAATTAAACCAGTAGTATCAACT---
CAATTGCTGTTAAATGGCAGTCTAGCAGAAGAAGAC------------
ATAGTAATTAGATCTGAGAATTTTACGAACAAT---------GCTAAAACTATAATAGTAAATCTGAAT----
-----------GAATCTGTAGTA---------ATTAATTGTACAAGACCCAACAACAATACAAGAAAA-----
-AGTATACATATA-----------GGACCGGGGGGAGCA---TTT------TATGCAACAGGACAA-------
---------ATAATAGGAGATATA------------AGACAAGCATACTGT---ACTGTTAATAAAACA---
GGATGGGAAAACACTTTAAGACAGGTGGCTAGAAAATTAAGT---------------GAGCAATTT-------
-----------------------------------AATAAAACAATAGCTTTTAAT------
CAATCCACAGGAGGGGATCCAGAAGTTGTA---ATGCACACTTTTAATTGTGGAGGGGAA---
TTTAAGCAGCTCCAAGCAAGAGTCCTGGCTGTGGAAAGATACCTAAAGGATCAACAGCTCCTAGGAATTTGGG
GCTGCTCTGGAAAACTCATATGCACCACTACTGTGCCTTGGAATAATAGTTGGAGC-----------------
----------------------------AATAAATCTTACAATGAGATTTGG------GAC------
AACATGACCTGGATGCAGTGG---GAAAGGGAAATAGACAACTACACAAACTTAATATAAC--
AACTTAATTGAAGAATCGCAGAACCAACAAGCA--

AAGAATGAACTAGAATTATTGGAGTTGGACTCTTGGGCAAATTTGTGGAATTGGTTTAGCATATCAAACTGGC
TGTGGTATATAAAAATCTTCATACTAGTAGTAGGAGGCTTAGTAGGTTTAAGAATAGTTTTTGCT---
GTACTTTCTATAGTA---------AATAAGGTTAGGAAGGGATACTCACCA------
TTATCGTTCGCTATCAGTTTGCTTAATGCCACAGCTATAGCAGTAGCTGAG---
GGGACAGATAGGGTTATAGAAGTAGTACAAAGAATTGGTAGAGGT----------------------------
--------ATCCTCCATATA------CCTGTAAGAATAAGACAGGGCTTGGAAAGGGCTTTACTA---TAA
>KM355159
GCT---ACA--
CAAAATTTGTGGGTCACAGTCTATTATGGGGTACCTGTGTGG---AAAGAAGCAACC---
ACCACTCTATTTTGTGCATCAGATGCTAAAGCATATAAGACAGAG---GCGCATAAT----------------
--------------GTTTGGGCCACA---CATGCCTGTGTACCCACAGAC---
CCCAACCCACAAGAAGTGTTTTTAAAA---------AATGTGTCAGAATATTTTAAC----------------
--------------------------ATGTGGAAAAATAACATGGTAGAACAGATGCATGAGGATATA---
ATCAGTTTATGGGATGAGAGCTTA---AAGCCATGTGTAAAATTA---
ACCCCGCTCTGTGTTACTTTAAATTGCACTGATGTGAGAAATGGTACTGGGAATAGTACTGGG----------
----
----------------------AATAGTAATATTACTGACAGGATAGAGAAAGGAGAAATGAAA---
AACTGCTCTTTCAATATCACCACAATTCTAGGA---------------------------
AATAAGAAGCAGAAAAAAATATACACTT---TTTGATAGGCTTGATCTAGTACCAATAGATGGT---------
----

GGTGATAATACAACCAACTATAGATTGATAAGTTGTAACACCTCAACTCTTACACAG---
GCCTGTCCAAAGACATCCTTTGAACCAATTCCAATACATTATTGTGCTCCGGCTGGTTTTGCGATTCTAAAAT
GTAAT---GATAAAAGGTTTAAT------GGATCAGAACAATGTAAAAAT-----------------------
-GTCAGC---ACAGTACAATGTACACATGGGATCAAACCAGTAGTATCAACT---
CAGCTGTTGTTAAATGGCAGTCTAGCAGAAGAAGAT------------
GTAGTGCTTAGATCTAAAAATTTCTCGGACAAT---------AATAATATCATAATAGTACAGTTAAAA----
-----------GAACCTGTAGAA---------ATTCGGTGTGAAAGACCCAACAACAATACAAGAAAA-----

-AGTATAACTATA------------GGACCAGGAAAAGCA---ATG------TTTACAACA---GAC------
---------ATAATAGGAGATATA------------AGAAAGGCACATTGT---AACATTAGTAGAACA---
AGATGGAATAACACTCTACATAAAATAGTGGAAAAATTAAAA---------------ACACAATTTAAC----
-----------------------------------AATAAAACAATAGTCTTTAAT------
CAATCCTCAGGAGGGGATCCAGAAGTGGTA---ATGCACATGTTTAACTGTGGAGGGGAA---
TTTAAGCAGCTCCAGGCAAGAGTCCTGGCTGTGGAAAGATACCTAAAGGATCAACAGCTCCTGGGAATTTGGG
GTTGCTCTGGAAAACTCATCTGCACCACTGCTGTGCCTTGGAATGCTAGTTGGAGT-----------------
----------------------------AATAAAACTTATAATGACATTTGG------GAT------
AACATGACCTGGATGCAGTGG---GAAAAAGAAATTGACAATTACACAGGCTTAATATAC---
ACATTGCTTGAAGAATCGCAGACCCAACAGGAA--

AAGAATGAACAAGAATTATTGGAATTGGATAAATGGACAAGTTTGTGGAGTTGGTTTGACATAACAAATTGGC
TGTGGTATATAAAACTGTTCATAATGATAGTAGGAGGATTAGTAGGTTTAAGAATAGTTTTTGCT---
GTGCTTTCTATAGTG---------AATAGAGTTAGGCAGGGATACTCACCA------
TTACCGTTTGCTATTAGCTTGTTCAACGCCACCGCCATAGCAGTAGCTGAG---
GGGACAGATAGGATTATAGAAGTATTACAAAGAATTGGTAGGGCT----------------------------
--------ATCATCCACATA------CCTCGAAGAGTAAGACAGGGCTTAGAAAGGGCTTTGCTA---TAA
>MG196745
ACT---AAT--
CAACAATTGTGGGTCACAGTATATTATGGGGTACCTGTATGG---AAGGAAGCAACC---
ACAGTCCTCTTTTGTGCATCAGATGCTAAAGCACATGGTACAGAG---GCACATAAT----------------
--------------GTTTGGGCCACA---CATGCCTGTGTACCCACAGAC---
CCCAACCCACAAGAAATAATATTGGAA---------AATATGACAGAAAATTTTAAC----------------
--------------------------ATGTGGAAAAATAACATGGTAGAACAAATGCAAGAGGATATA---
ATCAGTTTATGGGATCAAAGCCTA---AAGCCATGTGTAGAACTA---
ACTCCACTCTGTGTCACTTTAAATTGCACTAAATTAAATCACAATGCCACTCAG-------------------
----
----------------------------AGTAACGAGACAGCAGAGGAAGGAGGAGGAATGAAA---
AACTGCTCTTTCAATATCACCACAAGCATGAGA---------------------------
GATAAACAGCAAAAAGAATATGGACTT---TTCTATGACCTTGATATAGTACAAATAGATAAT----------
----

AGCACCTATAGGTTGAAAAGTTGTAACACCTCAGTTATTAAACAA---
GCCTGTCCAAAGGTATCCTTTGAACCAATTCCCATACATTATTGTGCCCCGGCTGGTTTTGCGATTCTAAAAT
GTAAT---GATAAAACTTTTAAT------GGATCAGGACCATGTACAAAT-----------------------
-GTCAGT---ACAGTACAATGTACACATGGAATTAGGCCAGTAGTATCAACT---
CACCTACTGTTAAATGGCAGTCTAGCAGAAGGAGAA---------
GATATAGTAATTAGATCTGAGAATTTCACAAACAAT---------GCTAAAATCATAATAGTACAGCTGAAC-
--------------GAATCTGTAGTA---------ATTAATTGTACAAGACCTAACAACAATACAAGAAGA--
----GGTATAAATATA------------GGACCAGGGAGAGCA---TTT------TATACAACAGGAGAA---
------------ATAATAGGAGATATA------------AGACAAGCACATTGT---AACGTTAGTATAGTA-
--AAATGGAACAAAACTTTAAATCTAGTAAAGGAAAAATTAAAA---------------AAGCAGTTTAAT--
-------------------------------------AATACAACAATACTTTTTGAT------
GAACCCTCAGGAGGGGACCCAGAAATCACA---ATGCACAGTTTTAATTGTGGAGGGGAA---
TTCAAGCAGCTCCAGGCAAGAGTCCTGGCTGTGGAAAGATACCTAAAGGATCAACRGCTCCTGGGGATTTGGG
GTTGCTCTGGAAAACTCATTTGCACCACTGCTGTGCCTTGGAATACTAGCTGGAGT-----------------
----------------------------AATAAATCTTACAATGACATCTGG------GAT------
AATATGACATGGATGCAGTGG---GAGAGAGAAATTGACAATTACACAGAAGAAATATAC---
AACTTACTAGAAAAATCGCAAAACCAGCAAGAA--

AAGAATGAGCAAGAATTATTGGAATTGGATAAATGGACAAGTTTGTGGAGTTGGTTTGACATAACAAACTGGC
TGTGGTACATAAGAATATTCATAATGGTAGTAGGAGGCTTAATAGGTTTAAGAATAGCTTTTGCT---
GTACTTTCTATAGCA---------AATAGAGTTAGGCAGGGATACTCACCC------
CTGTCGTTTGCTGTTAGCTTGCTAAACGCCACAGCTATAGCAGTAGCTGAA---
GGGACAGATAGGGTTATAGAAGTAGGAAGAAGAGCTTTTAGAGCT----------------------------
--------ATACTCCACATA------CCTACAAGAATAAGACAGGGCTTTGAAAGGGCTTTGCTA---TAA
>MG196964
ACT---GCA--
GATAAGTTGTGGGTTACAGTCTATTATGGGGTACCTGTGTGG---AAGGAGGCAACC---
ACCACTCTATTTTGTGCATCAGATGCTAAAGCATATGATACAGAG---GTACATAAT----------------

--------------GTTTGGGCCACA---CATGCCTGTGTACCCACAGAC---
CCCAGCCCACAAGAGATAGTGTTGGTA---------AATGTGACAGAAAATTTTAAC----------------
--------------------------ATGTGGAAAAATAACATGGTGGAACAGATGCATGAAGATATA---
ATCAGTTTATGGATCAAAGCCTA---AAGCCATGTGTACAACTC---
ACCCCAATCTGTGTTACCTTAAATTGCACGGATTATAGTGGGAATACCACTAATCAGGCC-------------
----
----------------------AATAACAGTAGTGAGAAGGGGGTAGTGCAAGGAGAAATAAAA---
AATTGCACTTTCAATGTCACAGCAGCAGCACAA------------------------------
AAAAGGAAGAAGGAATATGCACTT---TTTTATAGACATGATATAGTACAAGATGATAAT-------------
----
-------------------GATAATACCAGCTATAGGTTAATAAGTTGTAACACCTCAGTCATTACACAG---
GCTTGTCCAAAGATATCCTTTGAGCCAATTCCCATACATTATTGTGCCCCGGCTGGTTTTGCGATTCTAAAGT
GTAAC---GATAAAAGGTTCAAT------GGAACAGGACCATGTAAGAAT-----------------------
-GTCAGC---ACAGTACAATGTACACATGGAATTAGACCAGTAGTATCAACT---
CAACTGCTGTTAAATGGCAGTCTAGCAGAAGAAGAG------------
GTAGTGATTAGATCTGAAAATATCACGGACAAT---------GCTAAAACCATAATAGTACAGCTAAAA----
-----------GAACCTGTATGG---------ATTAATTGTACAAGACCCAACAACAATACAAGAAAA-----
-GGTATACATCTA------------GGACCAGGGAGTGTA---TTT------TTTGCAGCAGGCGAA------
---------ATAATAGGAGATATA------------AGGCAAGCACATTGT---AACATTAGTAGAGCA---
GAATGGAATCACACTTTAAGGCAGGTAGTTAATAAATTAAGA---------------GAACAATTTAAC----
-----------------------------------AACAAAACAATAATCTTTAAT------CAATCCTCA--
-GGAGACCCAGAAACTATA---ATGCACACTTTTAATTGTGGAGGGGAA---
TTTAAGCAGCTCCAGGCAAGAGTCCTGGCTGTGGAAAGATACCTAAAGGATCAACAGCTCCTGGGAATCTGGG
GTTGCTCTGGAAAACTCATTTGCACCACTAATGTGCCTTGGAATGCTAGTTGGAGT-----------------
----------------------------AATAAATCCCTGCAAGATATTTGG------GAT------
AACATGACCTGGATGGAGTGG---GAGAGAGAAATTGACAATTACACAAGGTTAATATAC---
ACCTTAATTGAAGAATCGCAGAACCAGCAGGAA--

AAGAATGAACAAGAATTATTGGAATTGGATAAATGGGCAAGTTTGTGGAATTGGTTTAGCATAACAAACTGGC
TGTGGTATATAAGATTATTCATAATGATAGTAGGAGGCCTGATAGGGCTCAGAATAGTTTTTACT---
GTACTTTCTATAGTA---------AATAGAGTTAGGCAGGATACTCACCA------
TTATCGTTTGCTATTAGCTTGCTCAATACCACAGCCATAGCAGTAGCTGGG---
GGAACAGATAGGATTACAGAAATATTACAAGCAGCTTGTAGAGCT----------------------------
--------ATTCTCAACATA------CCTAGAAGAATAAGACAGGGCTTGGAAAGAATTTTGCTA---TAA
>MG197035
ATG---GTG---------------------ATCTGTAGTGAT---GAA---
GAAAAAACGTGGGTCACAGTCTATTATGGGGTACCTGTGTGG---AAAGAAGCAACC---
ACCACCTTGTTTTGTGCATCAGATGCTAAAGCTTATAGTAAAGAG---GTACATAAT----------------
--------------GTCTGGGCCACA---CATGCCTGTGTACCCACAGAC---
CCCAGCCCACATGAACAAAAATTGGAA---------AATGTGACAGAAAATTTTAAC----------------
--------------------------ATGTGGAAAAATGACATGGTAGAACAAATGCAGGAAGATATA---
ATCAGTCTATGGATCAAAGCCTA---AAGCCATGTGTAAAACTA---
ACCCCACTCTGTGTTACTTTAGAATGCACTAATAATATTACTTATCATCGTGTCAATAGTACT----------
----
-------------------AAGGTTCATGCTACTCTTAATGACACTGAGAAAATAGGCATGAAA---
AACTGCTCTTTCAATGCCACCACAGACATAAGA---------------------------
GATAAGATGCGAAAAGAATATGCACTT---TTTTATACCCTTGATATAGTACCAATAGATAATAAT-------
----

GATACTAGTAGCTATAGATTGATAAGTTGTAACACCTCAGTCCTTACACAG---
GCCTGTCCAAAGGTATCTTTTGAGCCAATTCCCATACATTATTGTGCCCCGGCTGGTTTTGCGATTCTAAAGT
GTAAC---GATACGGCATTCAAT------GGAAATGGAACATGTAAAAAT-----------------------
-GTCAGC---ACAGTACAATGTACACATGGAATTAGGCCAGTAATATCAACT---
CAATTACTGCTAAATGGCAGTATAGCAGAAGAAGAG------------
ATAGTAATTAGATCTGCAAATTTCTCAGACACT---------GGTAAAACTATAATAGTGCAGCTGAAC----
-----------GAAGCTGTAAAA---------ATTAATTGCACAAGACCCAACAACAATACAAGAAGA-----
-GGTGTACATATA------------GGTCCAGGGGGAGCA---TTT------TACACAACAGGAGAC------
---------ATAATAGGAGATATA------------AGGCAAGCACATTGT---AACATTAGTAGAGAA---
CACTGGTATAATACTACCAAATGGGTAGCTGCAAAATTAAGA---------------GAACAATTTGGA----
-----------------------------------AATAAAACAATAGTCTTTAAT------

CACTCCTCAGGAGGGGACCCAGAAGTAGAA---ATGCACACTTTTAATTGTGGAGGGGAA---
TTTAAGCRGCTCCAGGCAAGAGTCCTGGCCATAGAAAGATACCTAAAGGATCAACAGCTCCTAGGGATTTGGG
GTTGCTCTGGAAAACTCATTTGCACCACTTCTGTGCCTTGGAATACTAGTTGGAGT-----------------
----------------------------AATAAAACACAGGAGACAATTTGG------AAC------
AACATGACCTGGATGCAATGG---GAAAAAGAAATTGACCAATACACAAATGAAATATAT---
GATTTACTTGAAAAATCGCAGAACCAGCAGGAA--

CAAAATGAACAAGAATTGTTGGAATTGGATAAATGGGCAAGTTTGTGGAATTGGTTTGATATAACAAGATGGC
TGTGGTATATAAAAATATTCATAATGATAATAGGAGGCTTGATAGGATTAAGAATAGTTTTTGCT---
GTGCTTAATATAGTG---------TATAGAGTTAGGCAGGGATATTCACCC------
TTATCGTTTGCTGTTAGCTTGCTTAATGCCATAGCCATAGCAGTAGCTGAG---
GGAACAGATAGGATTATAGAAGTATTACAAGGAGCTTTTAGAGCT----------------------------
--------ATTCTCCACATA------CCTAGAAGAATAAGACAAGGCTTAGAAAGGCTTTTACTA---TAA
>MK214316
GCT---ACA--
GGAACATTATGGGTCACAGTCTATTATGGGGTACCTGTGTGG---AGAGAAGCAACC---
ACCACTTTATTTTGTGCATCAGATGCTAAAGCACAGGAAACAGAG---GCACACAAT----------------
--------------GTTTGGGCCACA---CATGCCTGTGTACCCACAGAC---
CCCAATCCACAAGAAGTACAATTG------------AATGTAACAGAAAATTTTAAC----------------
-------------------------ATGTGGAAAAATAACATGGTAGAACAGATGCATGAGGATATA---
ATTAATTTATGGGATGAAAGCTTA---AAGCCATGTGTAAAATTA---
ACCCCACTCTGTGTTGAGTTAAATTGCACGGATTTAAGGAATGAGATTCAGACTAGCAATGAGACTCAGACTA
GCAATAGGAAT--
----------------CAGACTAGCAATAGGACTATAGAGCCACAAATGCAAGGAGAAATAAAA---
AACTGCTCCTTCAACGTCACCACAGATTGGAAG--------------------------
GATAAGAAAAAGAAAGAACATGCACTT---TTCTATGCATTTGATTTGACAGCAATAGAGAAT----------
----

GATAATACTAGCTATAGGTTGACAAGTTGTAATGCCACATCCATTACACAG---
GCCTGTCCAAAAGTATCCTTTGAGCCAATTCCTATATATTATTGTGCCCCGGCTGGTTTTGCGATTCTAAAAT
GTAAT---AATAAAAAGTTCAAT------GGAACAGGGCCATGTACAAAT-----------------------
-GTCAGC---ACAGTGCAATGTACACATGGAATTAAGCCAGTAGTATCAACT---
CAACTGTTATTAAATGGCAGCATAGCAGAAGAAGAA-----------
GTAGTAATTAGATCTGAAAATTTCACAAACAAT---------GCTAAAATCATAATAGTACAGCTGAAT----
-----------GTCTCTGTAGAA---------ATTAATTGTACAAGACCCAACAATAATACAAGAAGA-----
-AGTATACCTATA------------GGACCAGGGAGAGCA---TTT------TAT---ACAGGAGAG------
---------ATAATAGGAGATATA------------AGACAAGCACATTGT---AACATTAGTAAAGCA---
GAATGGAATAACACTTTAAGGCTGATAGCTGACAAATTAAAA---------------GATCAATATGGA----
--------------------------------AAGAATAAAACAATACAATTTAAT------
TCATCCTCAGGAGGGGACCCAGAAATTGTA---ATGCACACTTTTAATTGTGGTGGGGAA---
TTTAAGCAGCTCCAGGCAAGAGTCCTGGCTCTGGAAAGATACCTAAGGGATCAACAGCTCCTGGGAATTTGGG
GCTGCTCTGGAAAACTCATCTGCCCCACTGCTGTGCCTTGGAATACTAGTTGGAGT-----------------
----------------------------AATAAATCTTATAATGACATTTGG------AAT------
AACATGACCTGGATGGAATGG---GATAGAGAAATTAACAATTACACAAACTTAATATAT---
AGCTTAATTGAAGAATCGCAGAACCAACAGGAA--

AAAAATGAACAAGACTTATTGGCATTGGATAAATGGGCAAATTTGTGGAATTGGTTTGGCATATCAAATTGGT
TGTGGTATATAAAAATATTCATAATGATAGTAGGAGGCTTGATAGGATTAAGAATAGTTTTTACT---
GTACTTTCTATAGTA---------AATAGAGTTAGGCAGGGATACTCACCA------
TTGTCATTAGCTGTTAGCTTGCTTAACGCCACAGCCATAGCAGTAGGTGAA---
GGAACAGATAGGATTATAGCAATAACTCAAAGAGCTTTTAGAGCT----------------------------
--------GTTCTTAACATA------CCTAGAAGAATAAGACAGGGCTTAGAAAGGGCTTTGCTG---TAA
>KX505616
GCT---TCA--
GAAAACTTGTGGGTCACAGTCTATTATGGGGTACCTGTGTGG---AAAGAAGCAACC---
ACCACTCTATTCTGTGCATCAGATGCTAAAGCATATGATACAGAG---ATGCATAAT----------------
--------------GTTTGGGCCACA---CATGCCTGTGTACCCACAGAC---
CCCAACCCACAAGAAGTAGCATTGGAA---------AATGTGACAGAAAATTTTAAC----------------
-------------------------ATGTGGAAAAATAACATGGTAGAACAGATGCATGAGGATATA---
ATTAGTTTATGGGATCAAAGCCTA---AAACCATGCGTAAAATTA---

ACCCCACTCTGTGTTACTTTAAATTGCACTGATTGGGAGAGGAAGAATGATACTACTAATACCACT-------
----
-------------------------AGTAGTAGCGGGGAAAATTTGGAGGGAGGAGAAATAAAA---
AACTGCTCTTTCAAGGTCACCACGAGCATAAAA---------------------------
GATAAGACGCAGAGAGAGTATGCACTT---
TTTTATAAGCTTGATGTAGTACCAATAGATGAGGATAATAACAGGACC-------------------------
----
AATAGTAGTAATACCAATTATAGGTTGATAAGTTGTAACACCTCAGTCCTTACACAA---
GCCTGTCCAAAGGTATCCTTTGAGCCAATTCCCATACATTATTGTGCCCCGGCTGGTTTTGCGATTCTAAAGT
GTAAT---GATAAACAGTTCAGT------GGAAAAGGATCATGTAACAAT-----------------------
-GTCAGC---ACAGTACAATGTACACATGGAATTAGGCCAGTAGTATCAACT---
CAACTGCTGTTAAATGGCAGTCTAGCAGAAAAAGAG------------
GTAGTAATTAGATCTGACAATTTCACGGACAAT---------GCTAAAGTCATAATAGTGCAGCTGAAC----
-----------AAATCTGTAGAA---------ATTAATTGTACAAGACCCAACAACAATACAAGAAAA-----
-GGTATACATATA------------GGACCAGGGAGAGCA---TTT------TATGCAACAGGTGAA------
---------ATAACAGGAGATATA------------AGACAAGCACATTGT---AACATTAGTAGAGCA---
GAATGGAATAACACTTTGGAACAGATAGCTAAAAAATTAAGA---------------GAACAATTTAAG----
--------------------------------AATACAACAACAATATCCTTTAAT------
CGGTCCTCAGGAGGGGACCTAGAAATTGTG---ATGCACACTTTTAATTGTGGAGGGGAA---
TTTAAGCAGCTCCAGGCAAGAGTCCTGGCTATAGAAAGATACCTAAGGGATCAACAGCTCCTGGGGATTTGGG
GTTGCTCTGGAAAACTCATCTGCACCACTACAGTGCCTTGGAATGCTAGTTGGAGT-----------------
----------------------------AATAAATCTCTGAATCAGATTTGG------GAT------
AACATGACCTGGATGGAGTGG---GAAAGAGAAATTGACAATTACACAAGCGTAATATAC---
ACCTTACTTGAAGAATCGCAGAACCAACAGGAA--

AAGAATGAACAAGAATTATTGGAATTGGATAAATGGGCAAATTTGTGGAATTGGTTTGACATAACAAATTGGC
TGTGGTATATAAAAATGTTCATAATGATAGTAGGAGGCTTGATAGGTTTAAGAATAGTTTTTACT---
GTACTTTCTATAGTG---------AATAGAGTTAGGCAGGGCTACTCACCA------
TTATCATTTGCTGTTAGCTTGCTCAACGCCACAGCCATAGCAGTAGCTGAG---
GGGACAGATAGAATTATAGAAGTAGTACAAAGAGCTGGTAGGGCT----------------------------
--------ATTCTCCACATA------CCTGTAAGAATAAGACAGGGCTTGGAAAGGGCTTTGCTA---TAA
>KR182192
GCT---ACA--
GAACAATTGTGGGTCACAGTCTATTATGGGGTACCTGTATGG---AAAGAAGCAACC---
ACCACTCTATTTTGTGCATCAGATGCCAAAGCATATGACACAGAG---GCGCATAAT----------------
---------------GTTTGGCCACA---CATGCCTGTGTACCCACAGAC---
CCTAACCCACAAGAAGTAGAATTGGGA---------AATGTGACAGAAAATTTTAAC----------------
--------------------------ATGTGGAAAAATAACATGGTGGAACAGATGCATGAGGATATA---
ATCAGTCTATGGGATCAAAGCCTA---AAGCCATGTGTAAAATTA---
ACCCCACTCTGTGTTACTTTAAATTGCACTAATTTGAGTGTCACTAAGACCAATGGTAGT-------------
----
----------------------------GGTATTGGGGAAAAGATGGAGGAAGGAGAAGTGAAA---
AATTGCTCTTTCAATATCACCACACGCATAAAA---------------------------
AATAAGCTGCAGAGAGAATATGCACTC---TTTTATAAACTTGATGTAGTACCAATAGATGATGAT-------
----

GATAATACAAGCTATATGTTGATAAATTGTAACACCTCAGTCATTACACAG---
GCCTGTCCAAAGGTATCCTTTGAACCAATTCCCATACATTATTGTACCCCTGCTGGTTTTGCGATTCTAAAGT
GTAAA---GATAAGAATTTCAAT------GGAACAGGACCATGTAAAAAT-----------------------
-GTCAGC---ACAGTACAATGTACACATGGAATCAGGCCAGTAGTATCAACT---
CAACTACTGTTGAATGGCAGTCTAGCAGAAGAAGAG------------
GTAGTAATTAGATCTGAAAATATCACAAACAAT---------GTTAAATCCATAATAGTACAGCTGAAT----
-----------GAATCTGTAGAA---------ATTAATTGTACAAGACCCAACAACAATACAAGAAAA-----
-AGTATCCACATA------------GGACCGGGGAGAGCA---TTT------TATGCAACAGGAGAA------
---------ATAATAGGAGACATA------------AGACAAGCATATTGT---AACGTTAGTGAAGCA---
AAATGGAATAAAACTCTAAAATGGATAGTTATAAAATTACGA---------------GAACAATTTAAG----
-----------------------------------AATACAACAATAGTCTTTAAT------
CAATCCTCAGGAGGGGACCCAGAAATTGTA---ATGCACAGTTTTAATTGTGGAGGGGAA---
TTTAAACAGCTCCAGGCAAGAGTCCTGGCTGTGGAAAGATACCTAAAGGATCAACAGCTCCTAGGGATTTGGG
GTTGCTCTGGAAAACTCATTTGCACCACTTCTGTGCCTTGGAATGCTAGTTGGAGT-----------------

----------------------------AATAAATCTCTGGATGACATTTGG------AAT------
AACATGACCTGGATGGAGTGG---GAAAGAGAAATTAACAATTACACAGACTTAATATAC---
ACCCTAATTGAAAAATCGCAGAACCAGCAGGAA--

AAGAATGAACAAGAATTATTGGAATTGGATAAGTGGGCAAGTTTGTGGAATTGGTTTGACATAACAAAATGGC
TGTGGTATATAAAGATATTCATAATGATAGTAGGAGGTTTAATAGGTTTAAGAATAGTTTTTGCT---
GTGCTTTCTATAGTG---------AATAGAGTTAGGCAGGATACTCACCA------
TTATCATTCGCTGTTAGTTTGCTTAATGCTACAGCTATAGCAGTAGCTGAG---
GGGACAGATAGGATTATAGAAGTAGTACAAAGACTTTGTAGGGCT----------------------------
--------ATCCTACACATA------CCTGTAAGAATAAGACAGGGCTTAGAAAGGGCTTTGCTA---TAA
>KR182242
ACA---GAA--
GAAAATACGTGGGTCACAGTCTATTATGGGGTACCTGTGTGG---AAAGAAGCAACC---
ACCACTCTATTTTGTGCATCAGATGCTAAAGCATATGATACAGAA---GCACATAAT----------------
--------------GTTTGGGCCACA---CATGCCTGTGTACCCACAGAC---
CCCAGCCCACAAGAAGTAAAATTGGCT---------AATGTAACAGAAAACTTTAAT----------------
--------------------------ATGTGGAAAAATGACATGGTAGACCAGATGCATGAGGATGTA---
ATCAGCTTATGGGATCAAAGCTTA---AAGCCATGTGTAAAATTA---
ACTCCACTCTGTGTTACTTTAAATTGCACTGATTGGAAGAATGACACTACCAACACTGCTAACAAA-------
----
-------------------ACCACTAGTAGCAGCGAGGGAACGTTGGAGGGAGGAGAAGTAAAA---
AATTGCTCTTTCAACATAACCACAAACATAAGA---------------------------
GATAGGTATCAGAAAGAATATGCACTG---TTTTATAAACTTGATGTAGTACCAATAGATAAT----------
----

AGTAGTAATAATAGTACTAACTATACTAACTATAGGTTGATAAATTGTAACACCTCAGTCATTAGACAG---
GCTTGTCCAAAGGTATCCTTCGAGCCAATTCCCATACATTATTGTGCCCCGGCTGGTTATGCGATTCTAAAGT
GTAAC---AATAATACATTCAAT------GGAACAGGACCATGTACAAAT-----------------------
-GTTAGC---ACAGTACAGTGTACACATGGAATTAAGCCAGTAGTATCAACT---
CAACTGCTGTTAAATGGCAGTCTAGCAGAAGGAGAA------------
GTAGCAATTAGATCTGCCAATTTCTCAGACAAT---------GCTAAAATCATAATAGTACAACTAAAG----
-----------AAAGCTGTAAAC---------ATTACTTGCGCAAGACCCAGCAATAATACAAGAAAA-----
-GGTATACATATG------------GGACCAGGCAGTGCA---TGG------TTT---ACGGGAAAA------
---------ATAACAGGAGATATA------------AGACAAGCACATTGT---AACCTTAGCAGGGCA---
GACTGGAATGACACTTTAAGACAGATAGCTATAAAATTAAAA---------------GAACAATTT-------
-----------------------------------AATAAAACAATAGCCTTTAAT------
CAATCCTCAGGAGGGGACCCAGAAATTGTA---ATGCATATGCAGAATTGTGGAGGGGAA---
TTTAAGCAGCTCCAGGCAAGAATCCTGGCTGTAGAAAGATACCTAAGGGATCAGCAGCTCCTGGGGATTTGGG
GTTGCTCTGGAAAACTCATCTGCACCACTACTGTGCCTTGGAATAATAGTTGGAGT-----------------
----------------------------AGTAGATCTGTGGATGACATTTGG------GAA------
AACATGACCTGGATGCAGTGG---GAAAAAGAAATTGAAAATTACACAGGCGTAATATAC---
AACTTGCTTGAAGAATCGCAAAACCAACAAGAA--

AAGAATGAACATGAATTATTGGCATTAGATAAATGGGCAAGTTTGTGGAATTGGTTTGACATAACAAATTGGC
TGTGGTATATAAAAATATTCATAATGATAGTAGGAGGCTTAATAGGTTTAAGAATAGTTTTTACT---
GTGCTTTCTATAGTG---------AATAGAGTTAGGCAGGATACTCACCC------
TTATCGTTCGCTGTTAGCTTGCTCAACGCCATAGCCATAGCAGTAGCTGAG---
GGAACAGATAGGGCTATAGAAGTATTACAAAGAATTTGCAGAGCT----------------------------
--------GTTCTTCACATA------CCTACAAGAATAAGACAGGGCTTAGAAAGGGCTTTGCTA---TAA
>KR182343
GCT---GCA---GAA---------------------------------
GAAGAAAAAGCGTGGGTCACAGTTTATTATGGGGTACCTGTGTGG---AAAGAAGCAACC---
ACCACACTATTTTGTGCATCAGATGCTAAAGCATATGATACAGAG---GCACATAAT----------------
--------------GTTTGGGCCACA---CATGCCTGTGTACCCACAGAC---
CCCAACCCACAAGAAATAGTAATGGAA---------AATGTGACAGAAAATTTTAAT----------------
--------------------------ATGTGGAAAAATAACATGGTAGAACAGATGCATGAGGATGTA---
ATCAGTTTATGGGATCAAAGCCTA---AAGCCATGTGTAAAATTA---
ACCCCACTCTGTGTCACTCTAGAGTGCACTAAATTGAGGAATAAAACTTGCACTAGTAATGACACT-------
----
----------------------GGTTGTAATACCACTGCTGGCATCGAGGGAGGAGGAATGAAA---

AATTGCTCTTTCAATTTCACCAACAGCATA------------------------------
AATGAGGTTAAAAAAGGACGTGCACTT---TTTTATGAACGTGATATAATACAGATAAATAGT----------
----

ACAAAAAATGGTAGTACTAATCTTGGCTATAGGTTGACAAGCTGTAATACCTCAGTCATTACACAG---
GCCTGTCCAAAAATATCCTTTGAGCCAATTCCCATACATTATTGTGCCCCGGCTGGTTTTGCAATTCTAAAGT
GTAAC---AATGAGAGTTTCAAT------GGAACAGGACCATGTACAAAT----------------------
-GTAAGC---ACAGTACAATGTACACATGGAATTAGACCAGTAGTATCAACT---
CAATTGCTGTTAAATGGCAGTCTAGCAGAAAAAGAG-----------
GTAGTAATTAGATCTGAAAATTTCACAAACAAT---------GCTTACAACATAATAGTACAACTGAAT----
-----------GAGTCTGTATCA---------ATTAATTGTACAAGACCCAACAACAATACAAGAACA-----
-AGTATATCTTTT------------GGACCAGGCAAAGCA---TTT------TATGCAACAGGAGAT------
---------ATAATAGGGGATATA------------AGACAAGCATATTGT---ACCCTTAGTGAAGCA---
AAATGGAATAACACTTTGAAAAAGATAGTTGGAAAACTAAGA---------------GAACAATTTCCA----
-----------------------------------AATAAAACAATAATCTTTAAT------
CACAGCACAGGAGGAGATCCAGAGGTTATA---ATGCACACTTTTAATTGTAGAGGGGAA---
TTTAAGCAGCTCCAGGCAAGAGTCCTGGCTGTGGAAAGATACCTAAGGGATCAGCAGCTCCTAGGGATTTGGG
GTTGCTCTGGAAAACTCATCTGCACCACTTCTGTGCCTTGGAATGCTAGCTGGAGC-----------------
----------------------------AATAGATCTGTGGATGACATTTGG------GAG------
AACATGACCTGGATGCAGTGG---GAAAAAGAAATAGATAATTACACAGGCTTAATATAT---
AGTTTACTTGAAAGTTCACAAATACAGCAAGAA--

AACAATGAAAAAGAACTATTAGAGTTAGATAAATGGGCAAGTTTGTGGAATTGGTTTGACATATCAAATTGGC
TATGGTATATAAAAATATTCATAATGATAGTAGGAGGCCTGATAGGTTTAAGAATAGTTTTTACT---
GTACTTTCTATAGTG---------AATAGAGTTAGGCAGGGATACTCACCA------
TTGTCGTTCGCTATTAGCTTGCTCAACGCCACAGCAGTAGCAGTAGCTGAG---
GGGACAGATAGGATCTTAGAAATATTACAAGCAATTTATAGAGTT----------------------------
--------GTTCTTCACATA------CCTGTAAGAATAAGACAAGGCTTGGAAAGGGCTTTGCTT---TAA
>KT986555
GCA---GGA--
CAACAATTGTGGGTCACAGTCTATTATGGGGTACCTGTGTGG---AAAGAAGCAACC---
ACCACTCTATTTTGTGCATCAGATGCTAAAGCATATGATACAGAG---GCACATAAT----------------
--------------GTTTGGGCCACA---CATGCCTGTGTGCCCACAGAC---
CCCAACCCACAAGAAGTAGTATTGGGA---------AATGTGACAGAAAATTTTAAT----------------
--------------------------ATGTGGAAAAATAACATGGTGGAACAGATGCATGAGGATATA---
ATCAGCTTATGGGATCAAAGCTTA---AAGCCATGTGTAAAGTTA---
ACCCCACTCTGTGTTACTTTAAATTGCACTGATGCAATAATTACTGGTAATAGTAGTGCTAGCAATAAT----
----
----------------------AGTGGTGCTAGTAATAGTAGTGCTAGTGATAGTGGAATGAAA---
AACTGCACTTTCAATGTTTCCACAGAAGTAAAC-------------------------
GATAAGTATCAGAAAGTATTTGCACTC---TTTTATAAAATGGATGTAGTGTCACTAGATAAC----------
----

AATTCTAATTCTACTAAGTATAGGTTGATAAATTGTAACACCTCAGTCATTACACAG---
GCCTGTCCAAAGATATCCTTTGAGCCAATTCCCATACATTATTGTGCCCCGGCTGGGTTTGCGATTCTAAAAT
GTAAT---GAAAAGAATTTCACT------GGAAAAGGACCATGTACAAAT----------------------
-GTCAGC---ACAGTACAATGTACACATGGGATTAAGCCAGTAGTATCAACT---
CAATTGCTGTTAAATGGCAGTATAGCTGAAAGAGAG-----------
CCAGTACTTAGATCTGACAATTTCTCAGACAAT---------GCTAAAACCATAATAGTGCAGCTGAAT----
-----------GAAACTGTAGAA---------ATTAATTGTACAAGACCCAACAACAATACAAGAAAA-----
-AGTATACATATA------------GCACCAGGGAGAGCA---TTT------TATGCAACAGGAGAC------
---------ATAATAGGAGATATA------------AGACAAGCACATTGT---AACATTACCAGAGCA---
AAATGGAATAACACTTTAAAACAGATAGTTAACAAATTATCT---------------GAACACTTTGAA----
--------------------------------AACAAAACAAAGATAGTCTTTGAG------
CCACCCACAGGAGGGGACCCAGAAATTGTA---ATGCACATGTTTAATTGTGGAGGGGAA---
TTTAAACAGCTCCAGGCAAGAATCCTGGCTGTAGAAAGGTACCTAAGGGATCAACAGCTCCTGGGGATTTGGG
GTTGCTCTGGAAAGCTCATCTGCACCACTACTGTGCCTTGGAATGCTAGTTGGAGT-----------------
----------------------------AATAGATCTCTTGATTACATTTGG------GAT------
AACATGACCTGGATGGAGTGG---GAAAGAGAAATTGGCAATTACACAGATTTAATATAC---
ACCTTAATTGAAAATTCGCAAGTTCAGCAAGAA--

```
-----------------------------
AAGAATGAACAAGACCTATTAGCATTGGACAAATGGGCAAGTTTGTGGAATTGGTTTGACATATCAAACTGGC
TGTGGTATATAAAAATATTCATAATGATAGTAGGAGGCTTGATAGGTTTAAGAATAGTTTTTGCT---
GTACTTTCTATAGTA---------AATAGAGTTAGGCAGGGATATTCACCA------
CTCTCGTTGGCTGTTAGCTTGCTCGATACCATAGCTATAGCAGTAGCTGAG---
GGAACAGATAGGATTATAGAAGCAGTAAGAAGATTTTGCAGGGCT----------------------------
--------ATCCTACACACA------CCTAGAAGAATAAGACAGGGCTTAGAAATAGCTTTACTA---TAA
>KX505508
GCT---ACA------------------------------------------
CAAAATTTGTGGGTCACAGTCTATTATGGGGTACCTGTGTGG---AAAGAAGCAACC---
ACCACTCTATTTTGTGCATCAGATGCTAAAGCATATAAGACAGAG---GCGCATAAT----------------
--------------GTTTGGCCACA---CATGCCTGTGTACCCACAGAC---
CCCAACCCACAAGAAGTGTTTTTAAAA---------AATGTGTCAGAATATTTTAAC----------------
--------------------------ATGTGGAAAAATAACATGGTAGAACAGATGCATGAGGATATA---
ATCAGTTTATGGGATGAGAGCTTA---AAGCCATGTGTAAAATTA---
ACCCCGCTCTGTGTTACTTTAAATTGCACTGATGTGAGAAATGGTACTGGGAATAGTACT-------------
-------------------------------------------------------------------------
-------------------GGGAATAGTAATATTACTGACAGGATAGAGAAAGGAGAAATGAAA---
AACTGCTCTTTCAATATCACCACAATTCTAGGA---------------------------
AATAAGAAGCAGAAAAAATATACACTT---TTTGATAGGCTTGATCTAGTACCAATAGATGGT----------
-------------------------------------------------------------------------
----------------
GGTGATAATACAACCAACTATAGATTGATAAGTTGTAACACCTCAACTCTTACACAG---
GCCTGTCCAAAGACATCCTTTGAACCAATTCCAATACATTATTGTGCTCCGGCTGGTTTTGCGATTCTAAAAT
GTAAT---GATAAAAGGTTTAAT------GGATCAGAACAATGTAAAAAT-----------------------
-GTCAGC---ACAGTACAATGTACACATGGGATCAAACCAGTAGTATCAACT---
CAGCTGTTGTTAAATGGCAGTCTAGCAGAAGAAGAT------------
GTAGTGCTTAGATCTAAAAATTTCTCGGACAAT---------AATAATATCATAATAGTACAGTTAAAA----
-----------GAACCTGTAGAA---------ATTCGGTGTGAAAGACCCAACAACAATACAAGAAAA-----
-AGTATAACTATA------------GGACCAGGAAAAGCA---ATG------TTTACAACA---GAC------
---------ATAATAGGAGATATA------------AGAAAGGCACATTGT---AACATTAGTAGAACA---
AGATGGAATAACACTCTACATAAAATAGTGGAAAAATTAAAA---------------ACACAATTTAAC----
-----------------------------------AATAAAACAATAGTCTTTAAT------
CAATCCTCAGGAGGGGATCCAGAAGTGGTA---ATGCACATGTTTAACTGTGGAGGGGAA---
TTTAAGCAGCTCCAGGCAAGAGTCCTGGCTGTGGAAAGATACCTAAAGGATCAACAGCTCCTGGGAATTTGGG
GTTGCTCTGGAAAACTCATCTGCACCACTGCTGTGCCTTGGAATGCTAGTTGGAGT-----------------
----------------------------AATAAAACTTATAATGACATTTGG------GAT------
AACATGACCTGGATGCAGTGG---GAAAAAGAAATTGACAATTACACAGGCTTAATATAC---
ACATTGCTTGAAGAATCGCAGACCCAACAAGAA----------------------------------------
-----------------------------
AAGAATGAACAAGAATTATTGGAATTGGATAAATGGACAAGTTTGTGGAGTTGGTTTGACATAACAAATTGGC
TGTGGTATATAAAACTGTTCATAATGATAGTAGGAGGATTAGTAGGTTTAAGAATAGTTTTTGCT---
GTGCTTTCTATAGTG---------AATAGAGTTAGGCAGGGATACTCACCA------
TTACCGTTTGCTATTAGCTTGTTCAACGCCACCGCCATAGCAGTAGCTGAG---
GGGACAGATAGGATTATAGAAGTATTACAAAGAATTGGTAGGGCT----------------------------
--------ATCATCCACATA------CCTCGAAGAGTAAGACAGGGCTTAGAAAGGCTTTGCTA---TAA
>KX027971
GCT---GCA------------------------------------------
GAAGATTTGTGGGTCACAGTCTATTATGGGGTACCTGTGTGG---AAAGAAGCAGAC---
ACCACTCTATTCTGTGCATCAGATGCTAGAGCATATGATACAGAA---GCACATAAT----------------
--------------GTCTGGCCACA---CATGCCTGTGTACCCACAGAC---
CCCAACCCACAAGAAGTAGTATTGGGA---------AATGTGACAGAAAATTTTAAC----------------
--------------------------ATGTGGAAAAATAACATGGTAGAACAGATGCATGAGGATATA---
ATCAGCTTATGGGATGAAAGCCTA---AAGCCATGTGTAAAAATA---
ACCCCACTCTGTGTTACTTTAGATTGCACTGATGTAAGCAATAGCACTGATCTGAATACCAAT----------
-------------------------------------------------------------------------
----------------------CAGACCCTTGATGATAGTGAAAAAATGAGGAAAGAAATGACA---
AATTGCTCTTTCAATGTCACCACAATTATACAA---------------------------
AATAAAGTAAAGAAAGAATATGCACTT---
TTTTATAAAATTGATGTAGTACCAATAGACAATGATAATAAAAACACATCACAA-------------------
```

AATAATAAAACCACACCACGAAATAATAGTACCATATATAGGTTAATAAATTGTAACTCCTCAGTCATTACAC
AG---
ACCTGTCCAAAGGTATCCTTTGAACCAATTCCCATACATTATTGTGCCCCGGCTGGTTTTGCGATTCTAAAGT
GTAAT---GATAAGAAGTTTAAT------GGAACAGGACCATGCACAAAT-----------------------
-GTTAGC---ACAGTACAATGTACACATGGAATTAAGCCAGTAGTGTCAACT---
CAACTACTGTTAAATGGCAGCCTAGCAGAAGAAGAG------------
ATAGTAATTAGATTTGAGAATATCACAAACAAT---------GCTAAAACCATAATAGTACAGTTAAAT----
-----------GAGTCAGTAACA---------ATTAATTGTACAAGACCCAACAATAATACAAGAAAG-----
-AGTATACATCTA------------GGACCAGGGAAAGCA---TTT------TATGCAACAGGAGAC------
---------ATAACAGGAGATATA------------AGAAAAGCATATTGT---AACATTAGTAGAGCA---
AAATGGAATAACACTTTACAACAGGTAAGTAAAAAATTAAGA---------------GAACACTTTGAG----
--------------------------------AATACAACAACAATAACCTTTACC------
AACCACTCAGGAGGAGACCCAGAAATTGAA---ATGCACAGTTTTAATTGTGGAGGGGAA---
TTTAAGCAGCTCCAGGCAAGGCTCCTGGCTGTGGAAAGATACCTAAGGGATCAACAGCTCCTGGGGATTTGGG
GTTGCTCTGGAAAACTCATCTGCACCACTACTGTGCCTTGGAATACTAGTTGGAGT-----------------
----------------------------AATAAATCTCTAGAACAGATTTGG------AAT------
AACATGACATGGATGGAGTGG---GAAAATGAAATTAGCAAATACACAGGCTTAATATAC---
ACACTAATTGAAGACTCGCAAAACCAGCAAGAA--

AAGAATGAACTAGAATTATTGGAATTAGATAAATGGGCAAATCTGTGGAATTGGTTTGACATAACAAGCTGGC
TATGGTACATAAAAATATTCATAATAATAATAGGAAGCTTGATAGGTTTAAGAATAGTTTTTGCT---
GTACTTTCTATAGTG---------AATAGAGTTAGGCAGGGATACTCACCA------
TTATCATTGGCTGTCAGCTTGCTTAACGCTACAGCCATAGCAGTAGGTGAG---
GGAACAGATAGGATTATAGAAGGGTTACAAAGAATTTGTAGAGCT----------------------------
--------ATCTGCAACATA------CCTAGAAGAATAAGACAGGGCTTAGAAAGGGCTTTGCTA---TAA
>KX505446
GCT---GCA--
AAACAGAAGTGGGTCACAGTCTATTATGGGGTACCTGTGTGG---AAAGAAGCAACC---
ACCACTCTCTTCTGTGCATCAGATGCTAAATCATATGAGACAGAG---AAACATAAT----------------
--------------GTTTGGCCACA---CATGCTTGTGTACCCACAGAC---
CCCAACCCACAAGAAATAGTGATGGAC---------AATGTGACAGAAGACTTTAAT----------------
--------------------------GTGTGGAAAAATGACATGGTAGACCAGATGCATGAGGATGTA---
ATTAATTTATGGGATCAAAGCCTA---AAGCCATGTGTAAAATTA---
ACCCCACTCTGTGTTACTTTAAATTGTACTAATGCCACTGCTATTAATGCCACTAGTAGTAGTAAAACT----
----
---AATATCACTAGTGAAATAAAG---
AAATGCTCTTTCGATGTTACCACAGACAGAAGA---------------------------
GATAAAGTAAAAAAGGAGTATGCACTT---
TTTTATAAACTTGATATAGTACAAATAGATGAGAATAACAGTAACAAG-------------------------
----
AATAAGAGTAGCAATGACACAGTCGAACAATATAGGTTGATAAGTTGTAACACCTCAGTCCTTACACAA---
GCTTGTCCAAAGGTATCCTTTCAGCCAATTCCCATACATTATTGTGCCCCGGCTGGTTTTGCGATTCTAAAGT
GTAAT---AACAAGAATTTTACT------GGGTCAGGATCATGTACAAAT-----------------------
-GTCAGC---ACAGTACAATGTACACATGGGATTAAGCCAGTAGTGTCAACT---
CAACTGCTGTTAAATGGCAGCCTAGCAGAGGAAGGG------------
GTAGTAATTAGATCTGAAAACTTCTCAAACAAT---------GCTAAAACTATAATAGTACAGCTGAAA----
-----------ACATCTGTATCA---------ATTAATTGTACAAGACCCAATAACAATACAAGAAAA-----
-GGTATACATATA------------GGACCAGGAAGAACA---TTT------TATGCAGCAGGAGAA------
---------ATAATAGGAGATATA------------AGACAAGCACATTGT---AACATTAGTAGAGAA---
GATTGGAATAATACTCTAAAACAGGTAGTGGAAAAATTGGGG---------------GACCAATATAAT----
-----------------------------------ATAACAAAAATAATCTTCAAT------
CACTCATCAGGAGGAGACCCAGAAATTGTA---ATGCACAGTTTTAATTGTGGAGGGGAA---
TTTAAGCAGCTCCAGGCAAGAGTCCTGGCTCTGGAAAGATACCTAAGGGATCAACAGCTCCTAGGAATTTGGG
GTTGCTCTGGAAAACTCATTTGCACCACTAATGTGCCGTGGAATGATAGTTGGAGT-----------------
----------------------------AATACATCAGTGAAAGACATTTGG------AAC------
AGCATGACCTGGATGCAGTGG---GAGAAAGAAATTGACAATTACACAGATGTAATATAC---
CAATTACTTGAAGAATCGCAAAACCAACAAGAA--

AAGAATGAACAAGAATTATTAGAATTAGATAAATGGGCAAGTTTGTGGAGTTGGTTTGACATAACACAGTGGC

TGTGGTATATAAAAATATTCATAATGATAGTAGGAGGCTTGGTAGGTTTAAGAATAGTTTTTTCT---
GTACTTTCTATAGTA---------AATAGAGTTAGGCAGGGGTATTCACCA------
TTATCGTTTGCTGTTAGCTTGCTAAATACCACTGCTATAGTAGTAGCTGAA---
GGGACAGATAGGGTTATAGAAGCAGCACGAAGAGTTTGTAGAGCT----------------------------
--------ATCCGCAATATA------CCTACAAGAATAAGACAGGGTTTGGAAAGGGCTTTGCTA---TAA
>KT986947
GCT---GCA--
GAACAGTTGTGGGTCACAGTCTATTATGGGGTACCTGTGTGG---AAAGAAGCAAAC---
ACCACTCTATTTTGTGCATCAGATGCTAAAGCCTATGAGACAGAA---GTACATAAT----------------
--------------GTTTGGGCCACA---CATGCTTGTGTACCCACAGAC---
CCCGACCCACAAGAAGTAGTATTGGGA---------AATGTGACAGAAAACTTTAAT----------------
-------------------------GCATGGAAAAATGATATGGTAGACCAGATGCATGAGGATATA---
ATCAATTTATGGGATCAAAGCCTA---AAGCCATGTGTGAAATTA---
ACCCCACTCTGTGTTACTTTAAAGTGCACTGATGACATTAACAGCACTGAGGTCAATAACAGGACTAACAGCA
CTGGGTCCAAT--
----AGCGGGACCACGAATAATGTGAACGGGACCACGAATGTGAGCGGGGTATTAGAAATGAAA---
AATTGCTCTTTCAATATCAGCACAGACATAAGA--------------------------
GATAAGATAAAGACAGAGCATGCACTT---TTTTATAAACTTGATGTAGTGCCAATAGATAAT----------
----

AATAGTTCTAGCTATAGATTAATAAGTTGTAATACCTCAACCCTTACACAG---
GCTTGTCCAAAGGTTACCTTTGAACCAATTCCCATACATTATTGTGCCCCGGCTGGTTTTGCGATTCTAAAGT
GTAAT---AATGAAACATTTAAT------GGAAAAGGACCATGTACAAAT-----------------------
-GTCAGC---ACAGTACAATGTACACATGGAATTAAGCCAATAGTGTCAACC---
CAACTGCTGTTAAATGGTAGCCTAGCAAAAGATGGG-----------
GTAGTAATCAGATCTGAAAATTTCACAGACAAT---------GCTAAAACCATAATAGTACAGCTGAAA----
-----------GACCCTGTAAAA---------ATTAATTGTACAAGACCCTATAATAATACAAGAAGA-----
-AGTATAACTATG------------GGACCAGGGAGAGCA---TTT------TATGCATCAGACAAG------
---------ATAATAGGAAACATA------------AGACAAGCACATTGT---AACATTAGTAAAGCA---
GAATGGAATGACACTTTGAAGCAGGTAGCTATAAAATTACGA---------------GAGCAATATGGG----
--------------------------------GAAAATAAAACAATAGTCTTTAAT------
CAATCTTCAGGAGGGGATCCAGAAATTGTA---ATGCACAGTTTTATTTGTGCAGGAGAA---
TTCAAACAGCTCCAGGCAAGAATCCTAGCTGTGGAAAGATACCTAAGGGATCAACAGCTCCTGGGACTTTGGG
GTTGCTCTGGAAAACTCATCTGCACCACTGCTGTGCCTTGGAATACTAGTTGGAGT----------------
------------------------------AATAAATCTCTGGAGGACATTTGG------GAC------
AACATGACCTGGATGCAGTGG---GAAAAAGAAATTGACAATTACACAGGCTTAATATAC---
ACCTTAATTGAAAATTCGCAGAACCAACAAGAA--

AAGAATGAACAGGAATTATTGGAACTAGATAAATGGGCAAGTTTATGGAATTGGTTTGATATAACAAAATGGT
TGTGGTATATAAAAATATTCATAATGATAGTAGGAGGCTTGATAGGTTTAAGAATAGTCTTTTTT---
GTACTTTCTATAGTA---------AATAGAGTTAGGCAGGGATACTTACCA------
TTGTCGTTCGCTGTTAGCTTGCTTAATACCATAGCCATAGCAGTAGCTGAG---
GGGACAGATAGGATTATAGAAATATTACAAGCAGGCTTTAGAGCT---------------------------
--------ATCCTCCACATA------CCTGTAAGAATAAGACAAGGCTTGGAAAGGCTTCTGCTA---TAA
>MH264238
GCT---ACA--
GAAAATTTGTGGGTAACAGTCTACTATGGGGTACCTGTGTGG---AAAGAAGCTATC---
ACCACTTTATTTTGTGCATCAGATGCTAAAGCATATGATACAGAG---GTACATAAT----------------
--------------GTCTGGGCCACA---CATGCCTGTGTACCTACAGAC---
CCCAGCCCACAAGAAGTAGTATTGAAA---------AATGTGACAGAAAATTTTAAT----------------
-------------------------ATGTGGAAAAATAACATGGTAGAACAGATGCATGAGGATATA---
ATCAGTTTATGGGATCAAAGCCTA---AAGCCATGTGTAAAATTA---
ACCCCACTCTGTGTTACTTTAAATTGCACTGCTTATGTGGGAGATACTACTACTACCAATAGTAGTACTACT-
----
-----------------------------ACTAATACCAACATTAGTATGGAGGGAGAAATGAAA---
AACTGCTCTTTTTATACTACCCCAAACATAAGA--------------------------
GATAAGGAACAGAAAGAATATGCACTT---TTTTATAGCCTTGATGYAGTTCCAATAGATAAT----------
----

ACTAGCTATAGGTTGATAAGTTGTAACACCTCAGTCATTACACAG---

GCCTGTCCAAAGGTATCCTTTGAACCAATTCCCATACATTATTGTGCCCCGGCTGGTTTTGCGATTCTAAAAT
GTAAT---GATAAGAAGTTCAAT------GGAACAGGACCATGTAAAAT------------------------
-GTCAGC---ACAGTACAATGTACACATGGAATTAAGCCAGTAGTATCAACT---
CAACTGCTATTAAATGGCAGTCTAGCAGAAGAAGAG------------
GTAATAATTAGATCTGCCAATCTCTCGGATAAT---------GCTAAAACCATAATAGTACAGCTGAAT----
-----------AAAACTGTAGAA---------ATTAATTGTGTAAGACCCAACAACAATACAAGAAAA-----
-AGTATACATATG------------GGACCAGGGAAAGCA---TTT------TATGCATATGGAGAA------
---------ATAATAGGAAATATA------------AGACAAGCACATTGT---AACATTAGTAAAGGA---
GATTGGAATAAAACTTTAGGACGGATAGTTAAGAAATTAAGA---------------GAAAAGTTT-------
-----------------------------------AATAAAACAATAACCTTTAAG------
CAACACTCAGGAGGGGACCCAGAAATTGTA---ATGCACACTTTTAATTGTGGAGGGGAA---
TTTAAGCAGCTCCAGGCAAGAGTCCTGGCCATAGAAAGATTCCTAAAGGATCAACAGCTCCTGGGAATTTGGG
GTTGCTCTGGAAAACTCATTTGCACCACTACTGTGCCTTGGAATGCTAGTTGGAGT-----------------
----------------------------AATAGATCTCTAGATTATATTTGG------GGT------
AACATGACCTGGATGCAGTGG---GAAAAAGAAATTGACAATTACACAGAATTAATATAC---
AGCTTAATTGAAAAATCGCAGAACCAGCAAGAA--

AAGAATGAACAAGAACTATTGGAAATGGATAAATGGGCAAATTTGTGGAATTGGTTTGACATATCTCATTGGC
TGTGGTATATAAAAATCTTCATAATGATAGTAGGAGGCTTGGTAGGTTTAAGAATAGTTTTTAGT---
GTACTTTCTATAGTG---------AATAGAGTTAGGCAGGGATACTCACCA------
CTATCGTTTGCTGTTAGCTTGCTCAACGCCACAGCCATAGCAGTAGCTGAG---
GGGACAGATAGGGCTATAGAAGCAGTACAAAGAGCTTGTAGAGCT----------------------------
--------ATCCTCCACATA------CCTAGAAGAATAAGACAGGGCTTTGAAAGGGCTTTACTG---TAA
>KX587339
GCT---ACA--
AAACAATTGTGGGTCACAGTCTATTATGGGGTACCTGTGTGG---AAAGAAGCAACC---
ACCACTCTATTTTGTGCCTCAGATGCTAAATCATATGATACAGAG---GTACATAAT----------------
--------------GTTTGGGCCACA---CATGCCTGTGTACCCACAGAC---
CCCAACCCACAAGAAGTAGTAATGGGA---------AATGTGACAGAAGAGTTTAAC----------------
--------------------------ATGTGGAAAAATGACATGGTAGAACAGATGCATGAAGATATA---
ATCAGTTTATGGGATCAAAGCCTA---AAGCCATGTGTAAAATTA---
ACCCCACTCTGTGTTACTTTAAATTGCACTGATGCTATTGACAATTGCACTATTGTGAATGGTACTACCAAGT
GCACTAAGTATAATAATAGTAGCACTAATACT---
-------------------------ACCAGTAAGAGTAGCGTGGAAATGATGGGAGAAATAAAG---
AATTGCTCTTTCAATGTTACCACAGGATTAAGT---------------------------
AATAAGTTGCAGCGGGAATATGCACTT---
TTTTATAAACTTGATGTAGTACCAATAATAGAGAGTAATGGTGGG----------------------------
----
AATAATAGCAGCAATAGCAGCTATAGGTTGATAAGTTGTAACACCTCAGTTATTACACAA---
GCCTGTCCAAAGGTAACCTTTGACCCAATTCCCATACATTATTGTGCCCCGGCTGGTTTTGCGATTCTAAAGT
GTAAT---AATGAGACATTCAAT------GGGAAAGGATCATGCACAAAT------------------------
-GTCAGC---ACAGTTCAATGTACACATGGAATTAAGCCAGTAGTATCCACT---
CAACTGCTGTTAAATGGCAGTCTAGCAGAAAAGGAG------------
ATAATAATTAGATCTGAAAATTTCACGGACAAT---------GCTAAAACCATAATAGTACATCTGAAT----
-----------GAATCTGTACAA---------ATTAATTGTACAAGACCCAACAACAATACAAGAAAA-----
-GGTATACATTTA------------GCAGCAGGGAGAGCA---ATT------TATACAACAGGA---------
---------ATAATAGGAGACATA------------AGACAAGCACATTGT---AACATTAGTAGAGAA---
AAATGGAAAAAACTTTAAGACAGGTAGCTACAAAATTAAGC---------------CAACAATTTAATAAG-
--------------------------------GATAATACAACAATAGCATTTAAA------
CCATCCTCAGGAGGGGACCCAGAAATTGCA---ACACACATGTTTATTTGTAGAGGAGAA---
TTTAAGCAGCTCCAGGCAAGAATCCTGGCTGTGGAAAGATACCTAAAGGATCAACAGCTCCTGGGGATTTGGG
GTTGCTCTGGAAAACTCATCTGCACCACTGCTGTGCCTTGGAATGCTAGTTGGAGT-----------------
----------------------------AATAAAACTCTGGGGGAAATTTGG------AAT------
AACATGACCTGGATGCAGTGG---GAAAAAGAAATTGACAATTACACTGACACAATATAC---
AGATTAATTGAGGAATCGCAAAACCAACAGGAA--

AAGAATGAACAAGACTTATTGGCATTAGACAAATGGGCAAATTTGTGGAATTGGTTTGACATAACAAACTGGC
TGTGGTATATAAAAATATTCATAATGATAGTAGGAGGCTTGATAGGTTTAAGAATAGTTTTTACT---
GTGCTTTCTATAGTG---------AATAGAGTTAGGCAGGGATACTCACCA------
TTGTCATTTGCTATTAGCTTGTACAATACCACCGCCATAGTAGTAGCTGAG---

GGGACAGATAGGGTTATAGAAATAGTACAAAGAGCTTGTAGAGCT----------------------------
--------ATTTGCCACATA------CCTAGAAGAATAAGACAGGGCTTGGAAAGGAGCTTGCTA---TAA
>KX505536
GCT---ACA--
GAACAATTGTGGGTCACAGTCTATTATGGGGTACCTGTGTGG---AAAGAGGCAACC---
ACTACTCTATTTTGTGCCTCAGATGCTAAAGGATATGAAACAGAG---GCACATAAT---------------
--------------GTTTGGGCCACA---CATGCCTGTGTGCCCACAGAC---
CCCAGCCCACAAGAAATAAAAATGGAA---------AATGTGACAGAACATTTTAAC---------------
--------------------------ATGTGGAAAAATTCCATGGTAGAACAAATGCATGAGGATATA---
ATCAGTTTATGGGATCAAAGTCTA---AAGCCATGTGTAAAACTA---
ACCCCACTCTGTGTTACTTTAAATTGCATTAAT--

----------------ACTACTAGTAGTGGTGTGAACGGGACAATAGAGGATGGGGACATGAAA---
AACTGCTCTTTCAATATAACCACAGACATGAAA--------------------------
GATAAGGTGCAGAGAGAATATGCATTT---TTCAACAAACTTGATATAGTTCCAATGGATAGT----------
----

AGTGGTAGTACTAATGAAACTAACTATAGGTTGATAAGTTGTAACACCTCAGCCATTACACAG---
GCCTGTCCAAAGATATCCTTTGAGCCAATTCCAATACATTATTGTGCCCCAGCAGGTTTTGCAATTATAAAGT
GTAAT---AATAAGACATTCAAT------GGAACAGGACCATGTACAAAT----------------------
-GTCAGC---ACAGTACAATGCACACATGGAATTAAGCCAGTAGTATCAACT---
CAACTACTGTTAAATGGCAGTCTAGCAGAAGAAGAT------------
ATAATAATTAGATCTGAAAATCTCACAAATAAT---------GCTAAAACCATAATAGTGCAGCTGAAG----
-----------GACCCTGTAGAA---------ATTACTTGTATAAGACCCAACAACAATACAAGAAAG-----
-AGTATAACTATG------------GGACCAGGAAGAGCA---TTC------TATACAACA---GAC------
---------ATAATAGGAAATATA-----------AGGCAAGCACATTGT---AACATTAGTAGAGCA---
AATTGGACTGACACTCTAAAAAAGATAGCTAGCAAATTAAGA---------------GAAAAATTT-------
-----------------------------------AATAAAACAATAATCTTTGAT------
CAGCCTTCAGGAGGAGACCTAGAAATTGTA---ATGCACAGCTTTAATTGTGGAGGGGAA---
TTTAAACAGCTCCAGGCAAGAGTCCTGGCTATGGAAAGATACCTAAGGGACCAACAGCTCCTGGGGATTTGGG
GTTGCTCTGGAAAACTCATCTGCACCACTGCCGTGCCTTGGAATATTACTTGGAGT----------------
-----------------------------AATAAAAATCTGACTACAATTTGG------GAA------
AACATGACCTGGATGGAGTGG---GAAAAAGAAATTGACAATTACACAGAGTTGATATAC---
AGCTTACTTGAAAATTCGCAAATACAACAAGAA--

AAGAATGAACAAGAATTATTGGCATTGGACCAATGGGCAAATTTGTGGAGTTGGTTTGACATAACAAAATGGC
TGTGGTATATAAAAATATTCATGATGATAGTAGGAGGCTTGATAGGTTTAAAAATAGTTTTTGCT---
GTACTTTCTATAGTA---------AATAGAGTTAGGCAGGGATACTCACCA------
TTATCGTTGGCTACTAGCTTGCTTAACGCTACAGCCATAGCAGTAGGTGAG---
GGAACAGATAGGATCATAGAGGTATTACAAAGATTTGGTAGGGGC--------------------------
--------ATTCTCCACATA------CCTAGAAGAATAAGACAGGGCTTGGAAAGGGCCTTGCTG---TAA
>MG196412
ACT---GCA--
GGAAACTGGTGGGTCACAGTCTATTATGGGGTACCTGTGTGG---AAAGAAGCAACC---
ACCACTTTATTTTGTGCATCAGATGCTAAAGCATATGATACAGAG---GCACATAAT---------------
--------------GTTTGGGCCACA---CATGCCTGTGTACCCACAGAC---
CCCAACCCACAAGAAGTATTCCTGGTA---------AATGTGACAGAAAATTTTAAT---------------
--------------------------ATGTGGAAAAATAATATGGTAGACCAGATGCATGAGGATATA---
ATCAGTTTATGGGATCAAAGTCTA---AAGCCATGTGTAGAATTA---
ACCCCACTCTGTGTTACTTTAAATTGCACTAATGCCACTAAGCCTAATGCCACTAATTCTAAT----------
----
----------GATACTAATGATACTAGTTATAATGAGACAATAATAGAGAAGGGAGAAATAAAA---
AACTGCTCTTTCTATATCAACACAAACATAGGA--------------------------
GATAAGACACATAAAAATTATGCACTT---TTTTATAGCATTGATATAGAACCAATAGATAAT----------
----

GATAGTACCAGTTATAGGTTGACGAAGTGTAACACCTCAATCATTACACAA---
GCTTGTCCAAAGGTATCTTTTGAGCCAATTCCTATACATTACTGTACCCCGGCTGGTTTTGCGATTCTAAAGT
GTAGA---GAGAAGGATTTTAAT------GGAACAGGACCATGTAAAAAT----------------------
-GTTAGC---ACAGTGCAATGTACACATGGAATTAAGCCAGTAGTGTCAACT---

CAACTGCTGTTAAATGGAAGTTTAGCGGAAAACGAG------------
ACAGTAATTAGATCTGAGAATTTCACAGACAAT---------GCTAAAACTATAATAATACAGTTAAAG----
-----------GACCCTGTAGAA---------ATTAATTGTACAAGACCCCACAACAATACAAGAAAA-----
-AGTATAAATACG------------GGACCAGGGAGAATA---CTT------TATGCAACAGGAGAA------
---------ATAATAGGAGACATA------------AGACAAGCACATTGT---AACATTAGTGGAGAA---
CAATGGAATAAAACTTTAAGTCAGATAGTTAAAAAATTAAGA---------------GAACAATTTAAT----
-----------------------------------AATAGCGCAATAATCTTTAAG------
CAATCCTCAGGAGGGGACCCAGAAATTGTA---ATGCACAGTTTTAATTGTGGAGGGGAA---
TTTAAGCAGCTCCAGGCAAGAGTCCTGGCTGTGGAAAGATACCTAAAGGATCAACAGCTCCTGGGGATTTGGG
GTTGCTCTGGAAAACTCATCTGCACCACTGCTGTGCCTTGGAATAGTAGTTGGAGT-----------------
----------------------------AGTAGCAATGTAAATGATATTTGG------GAC------
AACATGACCTGGATGCAATGG---GAAAAAGAAATTGACAATTACACAGAGATAATATAC---
ACCTTACTTGAACAATCGCAGAACCAACAAGAA--

AAGAATGAACAAGAATTATTGGAATTGGATAAATGGGCAAGTCTGTGGAATTGGTTTGACATATCAAGTTGGC
TGTGGTATATAAGAACATTCATAATGATAATAGGAGGCTTAATAGGTTTAAGAATAATTTTTGCT---
GTAATTTCTATAGTG---------AAAAGAGTTAGGCAGGGATACTCACCA------
TTATCGTTAGCTATTAGCTTGTTCAACGCCACAGCCATAGCAGTAGCTGAG---
GGAACAGATAGGATTATAGAAATAGTACAAAGAACTTGTAGAGCT----------------------------
--------ATTCTCAACATA------CCTAGAAGAATAAGACAGGGCTTAGAAAGGGCTTTGCTA---TAA
>MK169417
GCT---ACA--
ACAAACTTGTGGGTCACAGTCTATTATGGGGTACCTGTGTGG---AAAGATGCAAGC---
ACCACTCTATTCTGTGCATCAGATGCTAAAGCATATGATACAGAG---GTACATAAT----------------
--------------GTTTGGGCTACA---CACGCCTGTGTACCCACAGAC---
CCCAACCCACAAGAAGTAGTATTGGAA---------AATGTGACAGAACATTTTAAC----------------
--------------------------ATGTGGAAAAATAACATGGTAGAACAGATGCATGAGGATATA---
ATCAGTTTATGGATCAAAGCCTA---AAGCCATGTGTGAAATTA---
ACCCCACTCTGTGTTACTTTAGATTGCACTAATGTTAATGCTACTAATGTTAATGCTACTAATCCCACT----
----
-------------------------AATAATAGCAAGGAACTGATGGCCAAAGAAGAAATGAAA---
AACTGCTCTTTCAATGTTACTGCAGGCATAAGA---------------------------
GATAAGATGCAAAAAGAATTTGCGCTT---TTTTATAAACTTGATATAGTGCCAATAAATAAT----------
----

ACTAATAATAATAGTAAAGACTATAGGCTGATAAGTTGTAACACCTCAGTCATTACACAA---
GCCTGTCCAAAGGTAACCTTTGAGCCAATTCCCATACATTATTGTACCCCGGCTGGTTTTGCAATTATAAAGT
GTAAC---AATAAAAAGTTCAAT------GGAAAAGGACCATGTAAAAAT-----------------------
-GTCAGC---ACAGTACAATGTACACATGGAATTAGGCCAATAATATCAACT---
CAACTGTTGTTAAATGGCAGTCTAGCAGAAGAAGAA------------
GTAGTAATTAGATCTGACAATTTCAGTGACAAT---------GTTAAAACCATAATAGTACAGCTGAAT----
-----------GAGTCCATACAA---------ATTAACTGTACAAGACCCAACAATAATACAAGAAGA-----
-AGTATAAATATA------------GGACCAGGAAGAGCA---TTC------TTTGCAACAGGACAC------
---------ATAATAGGAGATATA------------AGGAAAGCACATTGT---AACCTTACTAGAACA---
GAATGGAATAACACTTTAAAAAGGATAGTTATAAAATTAGGA---------------GAACAATTTAAT----
-----------------------------------AATAGCAAAACAGTCTTTAAA------
CCACCTGCAGGTGGAGACCTAGAAATTGTA---ACGCACAGTTTTAATTGTGGAGGGGAA---
TTTAAGCAGCTCCAGGCAAGAGTCCTGGCTGTGGAAAGATACCTAAAGGATCAACAGCTCCTGGGGATTTGGG
GTTGCACAGGAAAACTCATTTGCACCACTGCAGTGCCTTGGAATGCTAGTTGGAGT-----------------
----------------------------AATAAATCTCTGGATCAAATTTGG------AAT------
AACATGACCTGGATGCAGTGG---GAAAGAGAAATTAACAATTACACAGGCTTAATATAC---
ACCTTACTTGAGGAATCGCAAAACCAACAAGAA--

AAAAATGAACAAGAATTATTGGAATTGGATAAATGGGCAGATTTGTGGAATTGGTTTGACATATCAAACTGGC
TGTGGTATATAAGAATATTCATAATAATAGTAGGAGGCTTGGTAGGTTTAAAAATAGCTTTTACT---
GTGCTTGCTATAGTG---------AATAGAGTTAGGAAGGGATACTCACCA------
TTATCGTTTGCTGTTAGCTTGTTTAATACCATAGCCATAGCAGTAGCTGAG---
GGGACAGATAGGATTATAGAAATATTGCAAAGAGCTGGTAGAGCC----------------------------
--------ATTCTGCACATA------CCTAGAAGAATAAGACAGGGTTTGGAAAGAATTTTGGTA---TAA
>MK169834

GCT---GCA--
GCACAACTTTGGGTCACAGTTTATTATGGGGTACCTGTGTGG---AGAGAAGCAAAC---
CCCACTCTATTTTGTGCATCAGACGCTAAAGCATATGATACAGAA---GTACACAAT----------------
--------------GTTTGGGCCACA---CATGCCTGTGTACCCACAGAC---
CCCAACCCACAAGAGGTAAAATTGGAC---------AATGTAACAGAATATTTTAAC----------------
--------------------------ATGTGGAATAATAGCATGGTAGAACAAATGCATGAGGATATA---
ATCAGTTTATGGGATCAAAGCTTA---AAGCCTTGTGTAAAATTA---
ACTCCACTCTGTGTCACTCTAAATTGCACTGATGTGAATAATAGGACA------------------------
----
----------------------GAGAATAATAATAGAACTGTGGAGAATCCAGGGGAAATAAAA---
AACTGCTCTTTCAATATCACTGCAGGAGTTCGA---------------------------
GATAAGGTGAATAAAGAATATGCATTC---TTTTATAAATATGATGTAGTACCCTTACAAAAT----------
----

GATAATACTAGCTATAGGTTGACAAGTTGTAACACCTCAGTCATTACACAG---
ACCTGTCCAAAGGTAACCTTTGAGCCAATTCCTATACATTATTGTGCCCCGGCTGGCTATGCAATACTAAAAT
GTAAT---GATAAGAGATTCAAT------GGAACAGGACCATGTACCAAT----------------------
-GTTAGC---ACAGTACAATGTACACATGGGATTAAGCCAGTAGTGTCAACT---
CAATTGCTGTTAAATGGCAGTCTCGCAGAAGAAGAG-----------
GTAGTAATTAGATCTGAAAATTTCACAAACAAT---------GCTAAAAACATAATAGTACAGCTAAAT----
-----------GAATCTGTAGTA---------ATTAATTGTACAAGACCCAACAACAATACAAGAAAA-----
-AGTATACATATA------------GGACCAGGGAGGGCA---TTT------TATGCAGCAGGAGAT------
---------ATAATAGGAGATATA------------AGGCAAGCACATTGT---AACATTACTGCAAAC---
GAATGGAATAAAACTTTAGAACGGATAAAGGAAAAATTGGGA---------------GAACAATTTAAG----
-----------------------------------AATAAAACAATAAAATTTAAT------
CAGTCCTCAGGAGGGGACCCAGAAATTACA---ATGCACAGTGTTAATTGTGGAGGGGAA---
TTTAAACAGCTCCAGGCAAGAGTCCTGGCTGTGGAAAGATACCTAAAGGATCAACAGCTCCTGGGGATTTGGG
GTTGCTCTGGAAAACTCATCTGCACCACCTCTGTGCCTTGGAATAGTAGTTGGAGT-----------------
-----------------------------AATAAATCTCTGGATATGATTTGG------GAT------
AACATGACCTGGATGCAGTGG---GATAGAGAAATTAGCAAGTATTCAAAGACAATATAC---
AACTTACTTGAAGAATCACAAAACCAACAGGAA--

AAGAATGAACAAGAATTATTAGCATTAGACAAGTGGGACAGTTTGTGGAGTTGGTTTGACATATCAAATTGGC
TGTGGTATATAAGAATATTCATAATGATAATAGGAGGCTTAATAGGCTTAAGAATAGTTTTTACT---
GTACTTTCTATAGTG---------AATAGAGTTAGGCAGGGATACTCACCT------
TTATCATTTGCTGTTAGCTTGCTTAACGCCACCGCCATAGTAGTAGCTGAG---
GGGACAGACAGGATTATAGAAGGACTACAAAGAGCTGGTAGAGCT---------------------------
--------ATCCTCCGCATA------CCAACAAGAATAAGACAGGGCTTAGAAAGGGCTTTGGTG---TAA
>AF025753
GCT---ACA--
GAAAAATTGTGGGTCACAGTCTATTATGGGGTACCCGTGTGG---AAAGAAGCAACC---
ACCACTCTATTTTGTGCATCAGATGCTAAAGCATATGATACAGAG---GTACATAAT----------------
--------------GTTTGGGCCACA---CATGCCTGTGTACCCACAGAC---
CCCAGCCCACAAGAAATGATATTGAAA---------AATGTGACAGAAAATTTTAAC----------------
--------------------------ATGTGGAAAAATAACATGGTAGAACAGATGCATGAAGATATA---
ATCAGTTTATGGGATCAAAGCCTA---AAGCCATGTGTAAAAATA---
ACCCCACTCTGTGTCACTTTAAATTGCACTGATTTGAGGAATACTAATAATACCACT----------------
----
-------------------------------AGTAGTAGCGAGGGACTGAGAGGGGAAATGAAA---
AACTGCTCTTTCAATATCACCACAACACTAAGA---------------------------
GACAAGATGCAGGAAGATTATGCACTT---TTTGATAACCTTGATATAGTACCAATAGATAAT----------
----

GATAATACCAGCTATAGGTTGATAAATTGTAACACCTCAGTCATTACACAG---
GCCTGTCCAAAGGTATCCTTTGAGCCAATTCCCATACATTTTTGTGCCCCGGCTGGTTTTGCGATTCTAAAGT
GTAAT---GATAAGAAGTTCAAT------GGAACAGGACCATGTAAAAAT----------------------
-GTCAGC---ACAGTACAATGTACACATGGAATTAGGCCAGTAGTATCAACT---
CAACTGCTGTTAAATGGCAGTCTAGCAGAAGAAGAG-----------
GTAGTAATTAGATCTGAAGATTTCACGAACAAT---------GCTAAAACCATAATAATACAGCTGAAT----
-----------GAAACTGTAGAA---------ATTAATTGTACAAGACCCAACAACAATACAAGAAGA-----

-AGTATAAATATA------------GGACCAGGGAGAGCA---TTT------TATGCAACAGGG---------
---------ATAATAGGGGATATA------------AGAGAAGCACATTGT---AACATTAGTGGAACA---
AAATGGAATAATACTTTAAAACAGATAGCTCAAAAATTAAGA--------------GAACAATTTGGG----
----------------------------------AATAAAACAATAGTCTTTAAC------
CAATCCTTAGGAGGAGACCCAGAAATTGTA---ACGCACAGTTTTAATTGTGCAGGGGAA---
TTTAAGCAGCTCCAGGCGAGAGTCCTAGCTGTGGAAAGATACCTAAAGGATCAACAGCTCCTGGGGATTTGGG
GTTGCTCTGGGAAACTCATTTGCACCACTACTGTGCCTTGGAATACTAGTTGGAGT-----------------
---------------------------AATAAATCTCGGGATGAAATTTGG------GAT------
AACATGACCTGGATGCAGTGG---GAAAAAGAAATTAGCAATTACACAAGCCAAATATAC---
ACCTTAATTGGAGAATCGCAGATCCAACAAGAA---------------------------------------
----
AAGAATGAACAAGAATTATTGGAATTGGATAAATAGGCAAGTTTGTGGAATTGGTTTGACATAACACATTGGC
TGTGGTATATAAAAATATTCATAATGATAGTAGGAGGCTTGGTAGGTTTAAGAATAGTTTTTACT---
GTACTTTCTATAGTG---------AATAGAGTTAGGCAGGGATACTCACCA------
TTATCGTTCGCTGTTAGCTTGCTCAATGCCACAGCAATCGCAGTAGCTGAG---
GGGACAGATAGGGTTATAGAAGGATTACAAAGAGCTTATAGGGCT---------------------------
--------ATTCTCCACATA------CCTACAAGAATAAGACAGGGCTTGGAAAGGGCTTTGCTA---TAA
>MH012806
GCT---GCA---
GACCAATTGTGGGTCACAGTCTATTATGGGGTACCTGTGTGG---AAAGAAGCTACC---
ACCACTCTATTTTGTGCATCAGATGCTAAGGCATATGATACAGAG---ATGCATAAT----------------
--------------GTTTGGGCCACA---CATGCTTGTGTACCCACAGAC---
CCCAGCCCACAAGAAATAGTATTGGAA---------AATGTGACAGAAAATTTTAAC----------------
-------------------------ATGTGGAAAAATAACATGGTAGAACAGATGCATGAGGATATA---
ATCAGTTTATGGGATCAAAGCCTA---AAGCCATGTGTAAAGTTA---
ACCCCACTCTGTGTCACTTTAAATTGCACCAATTTGAAGTTGAATGCTACTGATACCACTAGTAATAATACCA
AT---
---AGTATAGCAACAGAAATGAAA---
AACTGCTCTTTCAAGATCACCACAAGCTTAAGA-------------------------
GATAAGACACGAAAAGAATATGCACTT---TTTTATAAACTTGATGTAGTACCAATAGAAGGAAAT-------
----

GATAATACTAGCTATAGGTTGATAAGTTGTAACACCTCAGTCATTACACAG---
GCCTGTCCAAAGGTATCCTTTGAGCCAATTCCCATACATTATTGTACCCCGGCTGGTTTTGTGATCCTCAAGT
GTAAT---GATAAGAAGTTCAAT------GGAACAGGAGCATGTAAAAAT----------------------
-GTCAGC---ACAGTACAATGTACACATGGAATTAGGCCAGTAGTATCAACT---
CAACTGCTGTTAAATGGCAGTCTAGCAGAAGATGAG-----------
GTAGTAATTAGATCTGCCAATTTCACGAACAAT---------GCTAAAATCATAATAGTACAGCTGAAT----
-----------GAATCTGTAGAA---------ATTAATTGTACAAGACCCAACAACAATACAAGAAAA-----
-AGTATAAGTATA------------GGACCAGGGAGAACA---TTT------TTTACAACAGGACAA------
---------ATAATAGGAGATATA------------AGACAAGCACATTGT---AACATTAGTAGTACA---
AAATGGAACAACACTTTAAGACTGATAGTTGGAAAATTAAAA--------------GAACAATTTGGG----
----------------------------------AATAAAACAATAGTCTTTAAT------
CACTCCTCAGGAGGGGACCCAGAAATTGTA---ATGCACAGTTTTAATTGTGGAGGAGAA---
TTTAAGCAGCTCCAGGCAAGAGTCCTGGCTGTGGAAAGATACCTAAAGGATCAACAGCTCCTGGGGATTTGGG
GTTGCTCTGGAAAACTCATTTGCACCACTGCTGTGCCTTGGAATACTAGTTGGAGT----------------
----------------------------AATAGATCTCTGGATCAGATTTGG------AAT------
AACATGACCTGGATGGAGTGG---GAAAGAGAAATTGATAATTACACAGAATTAATATAC---
ACTTTAATTGAAGGAGCGCAAAACCAGCAAGAA---------------------------------------
----
AAGAATGAACAAGAATTAATGGAATTAGATAAATGGGCAAGTCTGTGGAATTGGTTTGACATAACAAATTGGC
TGTGGTATATAAAAATGTTCATAATGATAGTAGGAGGCTTGGTAGGTTTAAGAATAGTTTTTACT---
GTACTTTCCCTAATA---------AATAGAGTTAGGCAGGGATACTCACCG------
TTATCGTTCGCTGTTAGCTTGCTCAATGTCACAGCCATAGCAGTAGCTGAG---
AGGACAGATAGGGTTATAGAAGTATTACAAAGAGCTTTTAGAGCT---------------------------
--------ATTCTCCATATA------CCTAGAAGAATAAGACAGGGCTTGGAAAGGGCTTTGCTA---TAA
>MH012971
GCT---GCA---
GAGCAATTGTGGGTCACAGTCTATTATGGGGTACCTGTGTGG---AAAGAAGCAACC---
ACCACTCTATTTTGTGCATCAGATGCTAAATCATATGATACAGAG---GTACATAAT----------------

--------------GTATGGGCCACA---CATGCTTGTGTACCCACAGAC---
CCCAACCCACAAGAAGTAGTATTGGAA---------AATGTGACAGAAAATTTTAAC----------------
--------------------------ATGTGGAAAAATAACATGGTAGAACAGATGCATGAGGATATA---
ATTAGTTTATGGGATCAAAGCCTA---AAGCCTTGTGTAAAATTA---
ACTCCACTCTGTGTTACTTTAAATTGCTCTGATGTGGAGAATCCTAAAGGGGGGAATACTACTAATACCACTA
GTGGAAGGAAT--
----------AATACTAATACCAATAATAGTAGCGGAGAAATAATGGAGAAAGGAGAATTAAAA---
AACTGTTCTTTCAATATCACCACAAACATAAGA---------------------------
GATAAGGTGCAAAGAGAATTTGCATAT---TTTTATAAACTTGATGTAGCACCAATAGATAAT----------
----

ACTACTAACAATACTGAATATAGGTTGATAAGTTGTAACACCTCAGTCATTACACAG---
GCCTGTCCAAAGGTATCCTTTGAGCCAATTCCCATACATTATTGTGCCCCGGCTGGTTTTGCGATTCTAAAGT
GTAAT---GATAAGAAGTTCAAT------GGAACAGGAACATGTACAAAT-----------------------
-GTCAGC---ACAGTACAATGCACACATGGAATTAGGCCAGTAGTATCAACT---
CAGCTGCTGTTAAATGGCAGTCTAGCAGAAAAAGAG------------
GTAGTAATTAGATCTGAAAATTTCACAAACAAT---------GTTAAAACCATAATAGTACAGCTAAAT----
-----------AAGTCTGTAGAA---------ATTAATTGTATAAGACCCAACAACAATACAAGGAAA-----
-AGTATACCTATA------------GGACCAGGGAGAGCA---TTT------TATGCAACAGGACAA------
---------ATAATAGGAGATATA------------AGACAAGCACATTGT---AACATTAGTAAAGCA---
GAATGGAATAACACTTTAAGACAAGTAGCTGAAAAATTAAGA---------------GAACAATTT-------
-----------------------------------AATAAAACAATAATCTTTGAT------
CAGTCCTCAGGAGGGGACCCAGAAATTGTA---ATGCACAGTTTTAATTGTGGAGGGGAA---
TTTAAGCAGCTCCAGGCAAGAATCCTGGCTGTGGAAAGATACCTAAGGGATCAACAGCTCCTGGGGATTTGGG
GTTGCTCTGGAAAGCTCATCTGCACCACTGCTGTACCTTGGAATGCTAGTTGGAGC-----------------
----------------------------AATAAATCTCTGAATGAGATTTGG------GAT------
AACATGACCTGGATGGAGTGG---GAAAGAGAAATTGACAATTACACAGGCTTAATATAC---
ACCTTAATTGAAGAATCGCAAATCCAACAAGAA--

AAGAATGAACAAGAATTATTGAAATTAGATAAATGGACAAGTTTGTGGAATTGGTTTAACATAACAAACTGGC
TGTGGTATATAAAAATATTCATAATGATAGTAGGAGGCTTGATAGGTTTAAGAATAGTTTTTACT---
GTATTTGCTATAGTG---------AATAGAGTTAGGCAGGGATACTCACCA------
TTATCGTTTGCTGTTAGCTTGCTCAACGCCACAGCAATAGCAGTAGCTGAG---
GGAACAGATAGGGTTATAGAAATATTACAAAGAATTTTTAGAGCT----------------------------
--------ATTCTCCACATA------CCTACAAGAATAAGACAGGGCTTGGAAAGGGCTTTGCAA---TAA
>MH012542
GCT---AAA--
GAAAAATTGTGGGTCACAGTCTATTATGGGGTACCTGTGTGG---AAAGAAGCAACC---
ACCACTCTATTTTGTGCATCAGATGCTAAAGCATATGATACAGAG---GTACATAAT----------------
--------------GTTTGGGCCACA---CATGCCTGTGTACCCACAGAC---
CCCAACCCACAAGAAATAGTATTGAAA---------AATGTGACAGAAAATTTTAAC----------------
--------------------------ATGTGGAAAAATAACATGGTAGAACAGATGCATGAGGATATA---
ATCAGTTTATGGGATCAAAGCCTA---AAGCCATGTGTAAAATTA---
ACTCCACTCTGTGTTACTTTAAATTGCACTGATTTGGGGAATGCTACTAATACCCATAGAAGT----------
----
-------------GAGGCTACCACTAGTAGTAGCAAGGGAACGATAGAGGAAGGAGAAATAAAA---
AACTGCTCTTTCAATATCACCACAAACATAAGA---------------------------
GGTAAGGTGCAGACAGCATCTGCACTT---TTTTATAGACTTGATGTAGTACCAATAGAAAAT----------
----

GAAAATACAAGCTATAGGTTGATAAGTTGTAACACCTCAGTCATTACACAG---
GCCTGCCCAAAGGTATCTTTTGAGCCAATTCCCATACATTATTGTGCCCCGGCTGGTTTTGCGATTCTAAAGT
GTAAA---GATAAGAAGTTCAAT------GGAACAGGACCATGTACAAAT-----------------------
-GTCAGC---ACAGTACAATGTACACATGAAATTAAACCAGTAGTATCAACT---
CAACTGCTGTTAAATGGCAGTCTAGCAGAAGAAGGG------------
GTAGTAATTAGATCTGAGAATTTCACAGACAAT---------GCTAAAACCATAATAGTACAGCTGAAA----
-----------GAACATGTAGAA---------ATTAATTGTACAAGACCCAACAACAATACAAGAAAA-----
-GATATACATATA------------GGACCAGGGAGAGCA---TTT------CATGCAACAGGAGAA------
---------ATAATAGGAGATATA------------AGACAAGCACATTGT---AATATTAGTAAAGCA---
AAATGGAATGACACTTTAGGACAGATAGTTACAAAATTAAGA---------------GAACAATTTAAG----

-----------------------------------AATAAAACAATAGTCTTTAAT------
CACTCCTCAGGAGGGGACCTAGAAATTGTA---ATGCACAGTTTTAATTGTGGAGGGGAA---
TTCAAGCAGCTCCAGGCAAGAGTCCTGGCTGTGGAAAGATACCTAAAGGATCAACAGCTCCTGGGGATTTGGG
GTTGCTCTGGAAAACTCATTTGCACCACTGCTGTGCCTTGGAATGCTAGTTGGAGT-----------------
----------------------------AATAAATCTCTGAATAACATTTGG------GAT------
AACATGACCTGGATGGAGTGG---GACAGAGAAATTAGCAATTACACAAACTCAATATAC---
AACTTAATTGAACAATCGCAGAACCAACAAGAA--

AAGAATGAACAAGAATTATTAGAGTTAGATAAATGGGACAGTTTGTGGAATTGGTTTAACATAACAAAGTGGC
TGTGGTATATAAAAATATTCATAATGATAGTAGGAGGCTTGGTAGGTTTAAGAATAGTTTTTACT---
GTACTTTCTATAGCG---------AATAGAGTTAGGCAGGGATACTCACCA------
TTATCGTTTGCTGTTAGCTTGCTCAACGCCACAGCCATAGCAGTAGCTGAA---
GGGACAGATAGGGTTATAGAACTAGTACAAAGAGCTTGTAGAGCT----------------------------
--------ATTCTCCACATA------CCTAGAAGAATAAGACAGGGTTTGGAAAGGGCTTTGCTA---TAA
>AY835435
GCT---GCA--
GACAAATTGTGGGTCACAGTCTATTATGGGGTACCTGTGTGG---AAAGAAACAACC---
ACCACTCTATTTTGTGCATCAGATGCTAGAGCATATGATACAGAG---GTGCATAAT----------------
--------------GTTTGGGCCACA---CATGCCTGTGTACCCACAGAC---
CCCAACCCACAAGAAGTAGTATTGGGA---------AATGTGACAGAAAATTTTAAC----------------
--------------------------ATGTGGAAAAATAACATGGTAGAACAGATGCATGAGGATATA---
ATCAGTTTATGGGATCAAAGCCTA---AAGCCATGTGTAAGACTA---
ACCCCACTCTGTGTTACTTTAAATTGCATTGATTATTATGGAAATACTACTAATAGCAAT-------------
----
-------------------------AATAGTAGTGAGACAATGATGGAAAAAGGAGAAATAAAA---
AATTGCTCTTTCAATATCACCACAAGACTAAAA---------------------------
GATAAGATGCAGAAAGAATATGCACTT---TTTTATAAATATGATATAGTACCAATAGATAATAGGGTAGGA-
----

AATGATACCAGTAATGCTACCAGCTATAGGTTAACAAGTTGTAACACCTCAGTCATTACCCAG---
GCCTGTCCAAAGGTATCCTTTGAGCCAATTCCCATACATTATTGTGCCCCGGCTGGTTTTGCGATTCTAAAGT
GTAAT---GATAAGAAGTTCAAT------GGAACAGGACCCTGTAAAAAT-----------------------
-GTCAGC---ACAGTACAATGTACACATGGAATTAAGCCAGTAGTATCAACT---
CAACTGCTGTTAAATGGCAGTCTAGCAGAAGAAGAG------------
GTAATGATTAGATCTGAAAATTTCACAGACAAT---------GCTAAAACCATAATAGTACAGCTGAAT----
-----------GAAACTGTAGAA---------ATTAATTGTACAAGACCCAACAATAATACAAGAAAA-----
-AGTATACATATG------------GGACCAGGGAAAGTA---TTT------TACACAACAGGAGAA------
---------ATAATAGGAGATATA------------AGACAAGCACATTGT---AACATTAATAGAGCA---
AAATGGAATAACACTTTAATAAAGATAGTTGAAAAATTAAGA---------------GTAAAATTT-------
-----------------------------------AATAAAACAATAAGCTTTAAG------
CAATCCTCAGGAGGGGACCCAGAAATTGAA---ATGCACAGTTTTAATTGTGGAGGGGAA---
TTTAAACAGCTCCAGGCAAGAGTCCTAGCTGTGGAAAGATACCTAAAGGATCAACAGCTCCTGGGGATTTGGG
GTTGCTCTGGAAAACTCATCTGCACCACTGCTGTGCCTTGGAATGCTAGTTGGAGT-----------------
----------------------------AATAAATCTCTGAATGAAATTTGG------GAT------
AACATGACCTGGATGGAGTGG---GAAAAAGAAATTGACAATTACACAAGCTTAATATAC---
ACCTTAATTGAAGAATCGCAAAACCAACAAGAA--

AAGAATGAACAAGAATTATTGGAATTAGATAAATGGGCAAGTTTGTGGAATTGGTTTAGCATAACAAACTGGC
TGTGGTATATAAAAATATTTATAATGATAGTAGGAGGCTCGATAGGTTTAAGAATAGTTTTTGCT---
GTGCTTTCTATAGTG---------AACAGAGTTAGGCAGGGATACTCACCA------
CTATCGTTTGCCATTAGCTTGCTCAATGCAACAGCCATAGCAGTAGCTGAG---
GGGACAGATAGAGTTATAGAAGTAGTACAAAGAACTTGTAGAGCT----------------------------
--------ATTATCCACATA------CCTAGAAGAATAAGACAGGGCTTGGAAAGGCTTTTGCTA---TAA
>AY331292
GCT---GAA--
AAAGACTCGTGGGTCACAGTCTATTATGGGGTACCTGTGTGG---AAAGAAGCAATC---
ACCACTCTATTTTGTGCTTCAGATGCTAAAGCATATAAGACAGAG---GTACATAAT----------------
--------------GTTTGGGCCACA---CATGCCTGTGTACCCACAGAC---
CCCAACCCACAAGAAGTAGTATTGGCA---------AATGTGACAGAACATTTTAAT----------------
--------------------------ATATGGGAAAATAACATGGTAGAACAAATGCATGAGGATATA---

ATCAGTTTATGGGATGAAAGCCTA---AAGCCATGTGTAAAATTA---
ACCCCACTCTGTGTTACTTTAAATTGCACTGACTATATTAATAACAGTACTGAAAGTAAG-------------
----
-------------------------AAAACCAATGAAGGCTTGAACAGTACTATAGAAATGAAA---
AACTGCTCTTTCAAAATAACCACAGGCATGAGA---------------------------
GATAAGATGCAGAAAGCATATGCACTT---
TTTTATAAGCTTGATATAGTACCAATAGATGATGATAATATTAAT----------------------------
----
AAGACTAGCACCAACCATAATTATACCAGCTATAGGTTAATAAGTTGTAACACCTCAGTCATTACACAG---
GCCTGTCCAAAGGTATCCTTTGAGCCCATTCCCATACATTTTTGTGCCCCGGCTGGTTTTGCGATTCTAAAGT
GTAAC---AACAAGAGTTACACA------GGAAAAGGACCATGTACAAAT-----------------------
-GTCAGC---ACAGTACAATGTACACATGGAATTAGGCCAGTAGTATCAACT---
CAACTGCTGTTAAATGGCAGTCTAGCAGAAGAAGAG------------
ATAGTRATTAGATCTGCCAATTTCTCTAACAAT---------GCTAAAACCATAATAGTACAGCTAAAT----
-----------AAAACTGTAGTA---------ATTAATTGTATAAGACCTAACAACAATACAAGAAAA-----
-AGTATACATATA------------GGACCAGGGAGAGCA---TTT------TATGCAACAGGAGAA------
---------ATAATAGGAGATATA------------AGACARGCACATTGT---ACCCTTAATGGAACA---
CAATGGAATAACACTTTAAGACAGATAGCTTTAAAATTAAAA---------------GAACAATTTAAG----
-----------------------------------GGTAAAACAATAGTCTTTAAT------
CACTCCTCAGGAGGGGACCCAGAAATTGTA---AAGCACTCTTTTAATTGTGCAGGGGAA---
TTTAAGCAGCTCCAAGCAAGAGTCCTGGCTGTGGAAAGATACCTAAAGGATCAACAGCTCCTGGGGATTTGGG
GTTGCTCTGGAAAACTCATTTGCACCACTGATGTGCCTTGGAATGATAGCTGGAGT-----------------
----------------------------AATAAATCTCTGGATAAGATTTGG------AAT------
AACATGACCTGGATGCAGTGG---GAAAGAGAAATTGACAATTACACAGATGTAATATAC---
AACTTACTTGAAGAATTGCAAAACCAACAAGAA--

AAGAATGAACAAGAATTATTGCAATTAGATAAATGGGCAAGTTTGTGGACTTGGTTTGACATAACAAAATGGC
TGTGGTATATAAAAATAGTCATAATGATAATAGGAGGCTTAATAGGATTAAGAATAGTTTTTGTT---
GTACTTTCTATAGTG---------AATAGAGTTAGGCAGGATACTCACCA------
TTGTCGTTTGCTGTTAGCTTGCTTAATTCCACTGCCATAGTAGTAGCTGAG---
GGGACAGATAGGATTATAGAAGTAGTACTAAGAGCTTTTAGAGCT----------------------------
--------GTTCTTCACATA------CCTACAAGAATAAGACAGGGCTTAGAAAGGGCTTTGCTA---TAA
>EU289191
GCT---GAA--
GAAGATACGTGGGTCACAGTCTATTATGGGGTACCTGTGTGG---AAAGAAGCAACC---
ACCACTCTATTTTGTGCATCAGATGCTAAAGCATATGATACAGAG---GTACATAAT----------------
--------------GTTTGGGCTACA---CATGCCTGTGTACCCACAGAC---
CCCAACCCACAAGAAGTAGTATTGGGA---------AATGTGACAGAAAATTTTAAC----------------
--------------------------ATGTGGAAAAATAACATGGTAGAACAGATGCATGAGGATATA---
ATCAGTTTATGGATCAAAGCCTA---AAGCCATGTGTGAAACTA---
ACTCCACTCTGTGTTACCTTAAATTGTACTGATTATTGGAATACTAATAACACCAATACTACTGCCAGTACTA
CTACT--
-------ACCAGTACTCCTACTTCCAGTAATATAGGAGGAATGATGAAAAAAGGGGAAATACAA---
CAATGCTCTTTCAATATTACCACAACCATAAAA---------------------------
GATAAAGTGCAGAAAGAATATGCTCTT---
TTTTATAGAACAGATGTAGTACCAATAAATGATGATGTTAAAAAT----------------------------
----
AATAATAATGATAGTGTTAAGAATAGTACCAAATATACCAACTATAGGTTGATAAATTGTAACACCTCAGTCA
TTACACAG---
GCCTGTCCAAAAATAACCTTCGAGCCAATTCCCATACATTATTGTGCCCCGGCTGGTTTTGCGATTCTAAAGT
GTAAC---AATAAGACGTTCGAG------GGAAAAGGACCATGCACGAAT-----------------------
-GTCAGC---ACAGTACAGTGTACACATGGAATTAGACCAGTAGTATCAACT---
CAACTGCTGTTAAATGGCAGTCTAGCAGAGGAAGAA------------
ATAGTAATTAGATCTGACAATTTCTCGAACAAT---------GCTAAAATCATAATAGTACAGTTGAAT----
-----------GAATCTGTAGTG---------ATTGATTGTACAAGACCCAGCAACAATACAAGGAAA-----
-AGTATACATATG------------GGACCAGGGGGAGCA---TTT------TATGCAACAGGGAGC------
---------ATAATAGGAGATATA------------AGACAAGCACATTGT---AACATTAGTAGAGCA---
AAATGGAATGACACTTTAAATCAGATAGCTATAAAATTAAGA---------------AAACAATTTAAT----
-----------------------------------AAAACAACAATAATATTTAAG------
CAATCCTCAGGAGGGGACCCAGAAATTGTA---ATGCACAGCTTTAATTGTGGAGGAGAA---

TTTAAGCAGCTCCAGGCAAGAGTCCTGGCTGTGGAAAGATACCTAAGAGATCAACAGCTCCTGGGAATTTGGG
GTTGCTCTGGAAAACTCATTTGCACCACTACTGTGCCTTGGAATGCTAGCTGGAGT-----------------
----------------------------AATAGATCTATAGATCAAATTTGG------GAT------
AACATGACCTGGATGCAATGG---GAAAACGAAATTAGCAACTACACTGACTTAATATAT---
TCCTTAATTGAAAAATCGCAAAACCAACAGGAA--

CAGAATGAACAAGACTTATTGAAATTGGATGAATGGGCAAGTTTGTGGAATTGGTTTGACATATCAAAATGGC
TGTGGTATATAAAAATATTCATAATGATAGTAGGAGGCTTGATAGGTTTAAGAATAATTTTTACT---
GTAGTTAATATAGTG---------TATAGAGCTAGGCAGGGATACTCACCA------
TTATCATTTGCTGTTAGTTTGCTCAACGCCACAGCAGTAGCAGTAGCTGAG---
GGAACAGATAGGGTTATAGAAGTAGTACAAAGATTCTGTAGAGGT----------------------------
--------GTTCTCCACATA------CCTAGAAGAATAAGACAGGGCTTGGAAAGGGCTTTACTA---TAA
>KT124770
GCT---GTA--
GATCAATTGTGGGTCACAGTTTATTATGGGGTACCTGTGTGG---AAAGAAGCAACT---
ACCACTCTATTTTGTGCATCAGATGCTAAAGCATATGATACAGAG---GTACATAAT----------------
--------------GTTTGGGCCACA---CATGCCTGTGTACCCACAGAC---
CCCAACCCACAAGAAATAGTAATGGGA---------AATGTGACAGAAAATTTTAAC----------------
--------------------------ATGTGGAAAAATGACATGGTAGAGCAGATGCATGAGGATATA---
ATCAGTTTATGGGATCAAAGCCTA---AAGCCATGTGTAAAATTA---
ACCCCACTCTGTGTCACTTTAAATTGCACTGCTGTTAATACCACTAGTGGTAACACTACTGATACCACTAGGG
GTAATACTAGT--
-------ACCATCCCCACTACTAGTAGTCTTACTACTACCCCTGGTATTAGAGAGGACATGAAA---
AACTGCTCTTTCAATATCACCACAGACCTAAGA---------------------------
GATAAAGTGCAAAGAGAATATGCATTA---CTTTATAAGCTTGATATAGCACCAATAGATAAT----------
----

GATAATATTAGCTATAGATTGATAAGTTGTAACACCTCAAGCATTACACAG---
GCCTGTCCAAAGGTATCCTTTGAGCCAATTCCCATACATTATTGTGCCCCAGCTGGTTTTGCGATTCTAAAGT
GTAAT---AATAAGACGTTCAAT------GGAAGTGGACCATGTACAAAT-----------------------
-GTTAGC---ACAGTACAATGTACTCATGGAATTAGGCCAGTAGTATCAACT---
CAACTGCTGTTAAATGGCAGTCTAGCAGAAGAAGAG------------
GTAGTAATTAGATCTGAAAATTTCACAAACAAT---------GCTAAAACCATAATAGTACAGCTGAAT----
-----------GAAACTGTAAAA---------ATTGAATGTATGAGACCCAGCAACAATACAAGAAAA-----
-AGCATACATATA------------GGACCAGGGAGAGCA---TTT------TATACAACAGGAGAT------
---------ATAATAGGAAATACA------------AGACAAGCACATTGT---AACGTTACTAGAACA---
GAATGGAATAAGGCTTTAAAACGGATAGTTATAAAATTAGGA---------------GAACAATTTGGA----
-----------------------------------AATAAAACAATAAAATTTCAG------
CAACCCTCAGGAGGGGACCCAGAGATTGTA---ATGCACAGTTTTAATTGTGGAGGAGAA---
TTTAAGCAGCTCCAGGCAAGAGTCCTGGCTGTGGAAAGATACCTAAAGGATCAACAGCTCATGGGGATTTGGG
GCTGCTCTGGAAAACTCATTTGCACCACTACTGTGCCTTGGAATACTAGTTGGAGT-----------------
----------------------------AATAAATCTCTGAATCAGATTTGG------GAT------
AACATGACTTGGATGGAGTGG---GAAAAAGAAATTGACAGTTACACAAATGAGATATAT---
ATCTTACTTCAAGAATCGCAGATCCAACAAGAA--

AAGAATGAACAAGAATTATTGGCATTAGATAAGTGGGCAAGCTTGTGGAATTGGTTTGATATAACACATTGGC
TGTGGTATATAAGAATATTCATAATGATAGTAGGAGGCCTGATAGGTTTAAGAATAGTTTTTGCT---
GTACTTTCTATAGTG---------AAAAGGGTTAGGCAGGGATATTCACCA------
TTATCGTTTGCTGTTAGCTTGCTCAATGCCACGGCCATAACAGTAGCTGAG---
GGAACAGATAGGATTATAGAAGTAGCACAAAGAGCTTGGAGAGCT----------------------------
--------TTCCTACACATA------CCTAGAAGAATAAGACAGGGCTTGGAAAGGGCTTTGCTA---TAA
>EU575670
GCT---ACA--
GAAAATTTGTGGGTCACAGTCTATTATGGGGTACCTGTGTGG---AAAGAAGCAAAC---
ACCACTCTGTTTTGTGCATCAGACGCTAAAGCATATGACACAGAG---GTACATAAT----------------
--------------GTTTGGGCCACA---CATGCCTGTGTACCCACAGAC---
CCCAACCCACAAGAAGTAGAATTAAAA---------AATGTGACAGAAAATTTTAAC----------------
--------------------------ATGTGGAAAAATCACATGGTAGAACAGATGCATGAGGATATA---
ATCAGTTTATGGGATCAAAGCCTA---AAGCCATGTGTAAAATTA---
ACCCCACTCTGTGTCACTTTAAATTGCACTGATTTGAAGAATAATACTCTTAATGTC----------------

----
---------------------------AATAGTAGTACCAAGGGAATAGGGGGAGAAATGACA---
AACTGCTCTTTCAACATCACCACAAGCATAAGA--------------------------
GATAAGGTGACAAGAGAATATGCACTT---TTTTATAGAGTTGATGTAGTACCAATAGAAAAT---------
----

GAAGATAATAGCTATAGGTTGATAAATTGTAACACCTCAGTCATTACACAA---
GCCTGTCCAAAGACATCCTTTGAGCCAATTCCCATACATTATTGTGCCCCAGCTGGTTTTGCGATTCTAAAGT
GTAGA---GATAATAAGTTCAAT------GGAACAGGACCATGTACAAAT----------------------
-GTTAGC---ACAGTACAATGTACACATGGAATTAAGCCAGTAGTATCAACT---
CAACTGCTGTTAAATGGCAGTCTAGCAGAAGGAGAG-----------
GTAATGATAAGATCTGAAAATCTCACGAACAAT--------GCTAAAACCATAATAATACAGCTAAAA----
-----------GAATCTGTAGAA--------ATTAATTGTACAAGACCCAACAACAATACAAGAAAA-----
-AGTATACATATA------------GGACCAGGGAGAGCA---TTT-----TATGCAACAGGAGAA------
---------ATAATAGGAGATATA------------AGACAAGCACATTGT---AACATTAGTAGAAAA---
AAGTGGAATGACACTTTAGCACAGATAGCTATAAAATTAGGA--------------GAACAATTTAAA----
----------------------------------AATAAAACAATAGTATTTAAT------
CAATCCTCAGGAGGGGACCCAGAAATTGTA---CTGCATAGTTTTAATTGTGGAGGGGAA---
TTTAAGCAGCTCCAGGCAAGAGTCCTGGCTGTGGAAAGATACCTAAAGGATCAACAGCTACTAGGGATTTGGG
GCTGCTCTGGAAAACTCATTTGCACCACTACTGTACCTTGGAATACTAGTTGGAGT----------------
---------------------------AATAAAAATCTGAGCACGATTTGG------GAA------
AGCATGACCTGGATGGATTGG---GAAAGAGAAATTGACAATTACACAGGCTTAATATAT---
TCCTTAATTGAAGAATCGCAAAACCAACAAGAA---------------------------------------
----
AAGAATGAACAAGAATTATTGGCATTAGATAAATGGGCAAGTTTGTGGAATTGGTTTGACATAACAAATTGGC
TGTGGTATATAAGAATATTCATAATGATAGTAGGAGGCTTGGTAGGTTTAAGAATAGTTCTTTCT---
GTAATTGCTATAGTG---------AATAGAGTTAGGAAGGATACTCACCA------
TTATCGTTCGCTGTTAGCTTGTTCAATGCCATAGCAGTAGCAGTAGCTGAG---
GGAACAGATAGGGTTATAGGAGTAGTACAAAGAATTATTAGAGGT---------------------------
--------ATTATCCACATA------CCTAGAAGGATAAGACAGGGCTTTGAAAGAGCTTTGCT------
>EU575927
ACA---GAA--
AAATTGTGGGTCACAGTCTATTATGGGGTACCTGTGTGG---AAAGAAGCAACC---
ACCACTTTATTTTGTGCATCAGATGCTAAAGGATATGATACAGAG---GTACATAAT---------------
--------------GTTTGGCCACA---CATGCCTGTGTACCCACAGAC---
CCCAACCCACAAGAAGTAGTATTGGGA---------AATGTGACAGAAAACTTTAAC---------------
-------------------------ATGTGGAAAAATAACATGGTAGACCAGATGCATGAAGATATA---
ATCAGTTTATGGGATCAAAGCCTA---AAACCATGTGTAAAATTA---
ACTCCACTCTGTGTTACTTTAGATTGCACTGATGCTAATAGTACTAGTAATGCAACCCAGGGCAATGGGACT-
----
------------ACTAAGGCCCCTGATAGTAGCTGGGGAGAGATAGAGAGAGGAGAGATAAAA---
AACTGCTCTTTCAATATCACCACAAGCATAAGA--------------------------
GATAAGGTGCAGAAAGAACATGCAAAT---TTTTATAAACTTGATTTAGTATCAATAGGTAAT---------
----

TCTAATACTAGCACTAGCTATAGGTTGATAAGTTGTAACACCTCAGTCATTACACAA---
GCCTGTCCAAAGGTATCCTTTGAGCCAATTCCTATACATTATTGTGCCCCGGCTGGTTATGCGATTCTAAAAT
GTAAT---AATAAGACATTCAAT------GGATCAGGACCATGTACAAAT----------------------
-GTTAGC---ACAGTACAATGTACACATGGAATAAGGCCAGTAGTATCAACT---
CAACTGCTGTTAAATGGCAGTCTAGCAGAAGAAGAT-----------
GTAATAATTAGATCTGCCAATTTCTCAGACAAT--------ACTAAGACAATAATAGTTCAGCTGAAT----
-----------GAATCTGTAACA--------ATTAATTGTACAAGACTCGGCAACAATACAAGAAAA-----
-AGCATACCTATA------------GGACCAGGGAGAGCA---TTT-----TATACAACA---GAC------
---------ATAATAGGAGACATA------------AGAAAAGCACATTGT---AACATTAGTAGAACA---
CAATGGAATAACACTTTAGAACAGGTAGTTATAAAATTAGGA--------------GAACAATATAAG----
----------------------------------AATAAAACAATAGTCTTTAAT------
CACTCCTCAGGAGGGGACCCAGAAATTGTA---ATGCACAGTTTTAATTGTGGAGGGGAA---
TTTAAGCAGCTCCAGGCAAGACTCCTAGCTGTGGAAAGATACCTAAAGGATCAACAGCTCCTGGGGATTTGGG
GATGCTCTGGAAAGCTCATTTGCACCACTGCTGTGCCTTGGAATAATAGTTGGAGT----------------
---------------------------AATAAATCTCTAGATGATATTTGG------GAT------

CACATGACCTGGATGGAGTGG---GACAGAGAAATTAACAATTACACAAGTTTAATATAC---
ACCTTAATTGAAGAATCGCAGCACCAGCAGGAA---------------------------------------
----
AAGAATGAAAAAGAATTATTAGAATTGGATAAATGGACAAGTTTGTGGAATTGGTTTACCATAACAAACTGGC
TGTGGTATGTAAAAATATTCATAATGATAGTAGGAGGCTTGGTAGGTTTAAGAATAGTTTTTGCT---
GTACTTTCTATAGTG---------AATAGAGTTAGGCAGGGATACTCACCA------
TTATCGTTTGCTGTTAGCTTGTTTAACGCCACAGCCATAGCAGTAGCTGAG---
GGAACAGATAGAATTATAGAAATAGTGCAAAGAGCTTTTAGGGCT---------------------------
--------ATTCTCCACATA------CCTAGAAGAATAAGACAGGGCTTTGAAAGGCTTTACTA---TAA
>AY835442
GCT---GAA---
GACAATTTGTGGGTCACAGTCTATTATGGGGTACCTGTGTGG---AAGGAAGCAACC---
ACCACTCTATTTTGTGCATCAGATGCTAAAGCATATGATACAGAA---AAGCATAAT---------------
--------------GTTTGGGCCACA---CATGCCTGTGTACCCACAGAC---
CCCAACCCACAAGAAATAGTATTGAAA---------AATGTGACAGAAAATTTTAAC---------------
-------------------------ATGTGGAAAAATAACATGGTAGAACAGATGCATGAGGATATA---
ATCAGTTTATGGGATCAAAGCCTA---AAGCCATGTGTAAAATTA---
ACCCCACTCTGTGTTACTCTAAATTGTACTGATTATTTTGGAAATACTACC---------------------
----
---------------------------------AATAATAGTAGCAGGGAAGCGATGATGAAA---
AAATGCTCTTTCAATATCACCACAAATATAAGA--------------------------
GATAAGGGGCAGAAAGAATATGCTCTT---TTTTATAAAACTGATGTAGTGCCAATAGATAAT---------
----

GATAATACCAGCTATAGGATGATAAGTTGTAACACCTCAGTCATTACACAG---
GCCTGTCCAAAGATATCCTTTGAACCAATTCCCATACATTTTTGTGCCCCGGCTGGTTTTGCGATTCTAAAAT
GTTTT---AATAAGACATTCAAT------GGAACAGGACCATGTACAAAT----------------------
-GTCAGC---ACAGTACAATGTACACATGGAATTAGACCAGTAGTATCAACT---
CAACTGCTGTTAAATGGCAGTTTGGCAGAAGAAGAG-----------
GTAGTAATTAGATCTGAAAATCTCACAGACAAT---------ACTAAAATCATAATAGTACAGCTGAAC----
-----------AAATCTGTAGTA---------ATTAATTGTACAAGACCCAACAACAATACAAGAAAA-----
-AGTATACATATA------------GGACCAGGGAGAGCA---TTT------TATGCAACAGGAGGA------
---------GTAATAGGAGATATA-----------AGACAAGCACATTGT---AACATTAGTAGAAAA---
GACTGGTATGAAGCTTTACAACAGATAGCTATCAAATTAAGA--------------GAGCAATTTGAG----
-----------------------------------AATAGAACAATAGCCTTTAAT------
CGATCCTCGGGAGGAGATCCAGAAATTGTA---ACACACAGTTTTAATTGTGCAGGGGAA---
TTTAAGCAGCTCCAGGCAAGAGTCCTGGCTCTGGAAAGATACCTAAGGGATCAACAGCTCCTGGGAATTTGGG
GTTGCTCTGGAAAACTCATTTGCACCACTTCTGTGCCTTGGAATACTAGTTGGAGT----------------
----------------------------AATAAATCCTATAAAGAGATATGG------GAG------
AACATGACCTGGATACAATGG---GAAAGAGAAATTGACAATTACACAGGCATAATATAT---
GAATTAATTGAAGAATCGCAGAACCAGCAAGAA---------------------------------------
----
AAGAATGAACAAGAATTATTGGAATTGGATAAATGGGAAAATTTGTGGAATTGGTTTAGCATAACAAATTGGC
TGTGGTATATAAAAATATTTATAATGGTAGTAGGAGGCTTGGTAGGTTTAAGAATAGTTTTTTCT---
GTACTTTCTATAGTG---------AAGAGAGTTAGGCAGGGATACTCACCC------
TTATCGTTTGCTGTTAGCTTGCTCAATGCCACAGCAATAACAGTAGCTGAG---
GGGACAGATAGGATTTTAGAACTATTACAAAGAGTTTGTAGAGCT---------------------------
--------ATTCGCCACATA------CCTAGAAGAATAAGACAGGGCTTGGAAAGGATTTTGCTA---TAA
>DQ853463
GCT---GCA---
GAAAACTTGTGGGTAACAGTCTATTATGGGGTACCTGTGTGG---AAAGAAGCAACC---
ACTACTCTATTTTGTGCATCAGATGCTAAAGCTTATGAGACAGAG---GTACATAAT---------------
--------------GTTTGGGCCACA---CATGCCTGTGTACCCACAGAC---
CCCAACCCACAAGAGATAGTATTGGAG---------AATGTGACAGAAAATTTTAAC---------------
-------------------------ATGTGGAAAAATAACATGGTAGAACAAATGCATGAAGATATA---
ATCAGTTTATGGGATCAAAGCCTA---AAACCATGTGTAAAATTA---
ACCCCACTCTGTGTTACTTTAAATTGTACTGATTATTTGGGGAATACTACTAAAACCAATACTACTAGTGCCC
CT--
-------------ACTACTAGTACCACTACTACTAATACCACTAATAATAAGGGGGAATTAAAA---
AACTGCTCTTTCCAGGTCACCACAGGCATAGGA--------------------------

```
GATAGAACAAAGAAAGAATATGCACTT---TTTTATAAGCATGATGTAGTACCAATAGATAATGATAAT----
-------------------------------------------------------------------------
-------------
AATAAGACTAATAATAGTAACTTTATATTAATACATTGTAATAGCTCAGTCATTACACAG---
GCTTGTCCAAAGGTGTCCTTTGAGCCAATCCCCATACATTATTGTGCCCCGGCTGGTTTCGCGATTCTAAAGT
GTAAA---GATAAGAAGTTCAAT------GGAACAGGACCATGCAAAAT------------------------
-GTCAGC---ACAGTACAATGTACACATGGAATTAGGCCAGTAGTGTCAACT---
CAACTGCTGTTGAATGGCAGTCTAGCAGAAGAAGAG------------
ATAGTAATTAGATCACAAAATTTTACGGACAAT---------GTTAAATCCATAATAGTACAGCTGAAT----
-----------GAAACTGTAAAA---------ATTAATTGTACAAGACCCAACAACAATACAAGAAAA-----
-GCTATACGTATA------------GGACGAGGGAGAGCA---ATT------TATGCAACAGATAGA------
---------ATAATAGGAGATATA------------AGACAAGCATATTGT---AACATTAGTAGAACA---
AAATGGAATGACACTTTAGGCCAGATAGCTACAAAATTAAGA---------------GAACAATTTGGG----
-----------------------------------AACAAAACAATAGTCTTTAAT------
TCATCCTCAGGAGGGGACCCAGAAATTGTT---ATGCACAGTTTTAATTGTGGAGGGGAA---
TTTAAGCAGCTCCAGGCAAGAATCCTGGCTGTGGAAAGATACCTAAGGGATCAACAGCTCCTGGGGATTTGGG
GTTGCTCTGGAAAACTCATCTGCCCCACTGCTGTGCCTTGGAATGCTAGTTGGAGT-----------------
----------------------------AATAAATCTCTAGAAGAAATTTGG------GAG------
AACATGACCTGGAGGGAGTGG---GAAAGAGAAATTGACAATTACACAGGCAAAATATAT---
GACCTACTTGCAAAATCGCAGAACCAGCGAGAA----------------------------------------
-----------------------------
ATGAATGAACAAGAATTATTAAAATTAGATAAATGGGCAGATTTGTGGAATTGGTTTGACATAACACAATGGC
TGTGGTATATAAAAATATTTATAATGATAGTAGGAGGCTTGATAGGTTTAAGAATAATTTTTGCT---
GTAATTTCTATAGTG---------AATAGAGTTAGGCAGGGATACTCACCA------
TTGTCATTGGCCATTAATTTGCTCAACACCACAGCTATAGTAGTAGCTGAG---
GGGACAGATAGGGTTATAGAAGTAGTGCTAAGAGCTTATAGAGCT----------------------------
--------ATTCTCCACATA------CCTAGAAGAATAAGACAGGGCTTGGAAAGGCTTTTGCTA---TAA
>KT124769
GCT---GTA------------------------------------------
GAACAAACGTGGGTCACAGTCTATTATGGGGTACCTGTGTGG---AAAGATGCAAAC---
ACCACTCTATTTTGTGCATCAGATGCTAAAGCATATGATACAGAG---GTACATAAT----------------
--------------GTTTGGGCCACA---CATGCCTGTGTACCCACAGAC---
CCCAACCCACAAGAAGTAGAATTGGCA---------AATGTGACAGAAAATTTTAAC----------------
--------------------------ATGTGGAAAAACAACATGGTAGATCAGATGCATGAGGATATA---
ATCAGTTTATGGGATCAAAGCCTA---AAGCCATGTGTAAAATTA---
ACTCCACTCTGTGTTACTCTAAATTGCACTGATAATGTGGGGAATAATACTAGTACCAAT-------------
-------------------------------------------------------------------------
-------------------------AATAGTAGATGGGATAAAATGGAAAAAGGAGAAATAAAG---
AACTGCTCTTTCAATATCACCACAAACATGAGA---------------------------
GATAAGATGCAGAAACAATATGCACTT---TTTTATAAACTTGATGTAGTACCAATAAAGGAAGATGAG----
-------------------------------------------------------------------------
----------
AATAATACCAGTAATTATACCGACTATAGGTTGATAAGTTGTAACACCTCAGTCATTACACAG---
GCCTGTCCAAAGGTAACCTTTGAGCCAATTCCCATACATTATTGTGCCCCAGCTGGTTTTGCGCTTCTAAAAT
GTAAG---GATAAGAAATTCAAT------GGAACAGGACCATGTAAAAT------------------------
-GTTAGC---ACAGTACAATGTACACATGGAATTAAGCCAGTAGTATCAACT---
CAGCTGCTACTAAATGGCAGTCTAGCAGAAGAAGAG------------
GTAGTAATAAGATCTGAGAATTTCTCGAACAAT---------GCTAGAACCATAATAGTACAGCTGAAT----
-----------ACATCTGTAGAA---------ATAAAGTGTATAAGACCCAACAACAATACAAGAAAA-----
-GGTATACATATA------------GGACCAGGGAGAGCA---TTT------TATACAACAGGAGAT------
---------ATAATAGGAGATATA------------AGACAAGCACATTGT---AACATTAGTAGACAA---
AATTGGACTGACACTTTAAAACAGATAGCTGAAAAGTTAAGA---------------GAACAATTTGGG----
-----------------------------------AAAAAAACAATAGTCTTTAGT------
AACTCCTCAGGAGGGGATCCAGAAATTGTA---ATGCACACTTTTAATTGTGCAGGGGAA---
TTTAAGCAGCTCCAGGCAAGAGTCCTGGCTGTGGAAAGATACCTACGGGATCAACAGCTCCTGGGGATTTGGG
GTTGCTCTGGAAAACTCATCTGCACCACTGCTGTGCCTTGGAATGTTAGTTGGAAT-----------------
----------------------------AATAGATCTGTGGATGACATTTGG------GAA------
AACATGACCTGGATGCAGTGG---GATAGAGAAATCAGTAATTACACAAGTTTAATATAC---
ACCTTAATTGAAGAATCGCAAAACCAACAAGAA----------------------------------------
-----------------------------
```

AAGAATGAACAAGAATTATTGGCATTAGATAAATGGGCAGATTTGTGGAATTGGTTCAGCATAACAAAATGGC
TGTGGTATATAAAAATATTCATAATGATAGTAGGAGGCTTGGTAGGTTTAAGAATAGTTTTTGCT---
GTGCTTTCTATAGTG---------AATAGAGTTAGGCAGGATACTCACCA------
TTATCGTTTGCTGTTAGCTTGCTCAACGCCATAGCCATAGCAGTAGGTGAG---
GGGACAGATAGGATTATAGAAGCATTTAGAAGAATTTTTAGAGCT----------------------------
--------ATTCTCCACATA------CCTACAAGAATAAGACAGGGCTTGGAAAGGCTTTGCTA---TAA
>EU289196
GCT---GCA--
GAAAACTTGTGGGTCACAGTCTATTATGGGGTACCTGTGTGG---AAAGAAGCAACC---
ACCACTCTATTTTGTGCATCAGATGCTAAAGCATATGATACAGAG---GTACACAAT----------------
--------------GTTTGGCCACA---CATGCCTGTGTACCCACAGAC---
CCCAACCCACAAGAAATGGTATTAGAA---------AATGTGACAGAATATTTTAAC----------------
--------------------------ATGTGGAAAAATAACATGGTAGAACAGATGCATGAGGATATA---
ATCAGTTTATGGGATCAAAGCCTA---AAGCCATGTGTAAAATTA---
ACCCCACTCTGTGTTACTTTAACTTGCACTGATTATGAATGGAATTGTACTGGTATCAGAAATAGTATCTGTA
AATATAAT---
----AATATGACTAATAATAGTAGCTCAGGGAATTATACTGGATGGGAAAGGGGAGAAATAAAA---
AACTGCTCTTTCAATAGCACCATCTCGGGCATA------------------------
AGAGATAAGGTGCGGAAAGAGTATGCACTT---TTGTATAAAATAGATCTAGTATCAATAGATGGA-------
----

AGTAATACAAGCTATAGGATGATAAGTTGTAACACCTCAGTCATTACTCAG---
TCCTGTCCAAAGATATCCTTCGAGCCAATTCCCTTACATTATTGTACCCCGGCTGGTTTTGCGCTTCTAAAGT
GTAAT---GATAAGAAGTTCAAT------GGAACAGGGCTATGTCACAAT-------------------------
-GTCAGC---ACAGTACAATGTACACATGGAATTAAGCCAGTAGTATCAACT---
CAACTACTGTTAAATGGCAGCCTAGCAGAAGAAGAG------------
GTAGTAATCAGATCTAAAAATTTCACGGACAAT---------GCTAAAATCATAATAGTACAGCTAAAT----
-----------GAAACTGTAGAA---------ATTAATTGTACAAGACCCGGCAACAACACAAGAAAA-----
-AGTATACATATA------------GCACCAGGGAGAACA---TTC------TATGCAACAGGAGAA------
---------ATAATAGGAGATATA-----------AGACGAGCACATTGT---AACATTAGTAGAGAA---
AAATGGAATACCACTTTACATAGGATAGCTACAAAATTAAGA---------------GAACAATAT-------
-----------------------------------AATAAAACAATAGTCTTTAAT------
CAATCCTCAGGAGGGGACCCAGAAATTGTA---ATGCACAGTGTCAATTGTGGAGGGGAA---
TTTAAGCAGCTCCAGGCAAGAGTCCTGGCTCTGGAAAGATACCTAAGGGATCAACAGCTCATGGGGATTTGGG
GTTGCTCTGGAAAACTCATTTGCACCACTGCTGTGCCTTGGAATGCTAGTTGGAGT-----------------
----------------------------AATAAATCTCTGAATGATATTTGG------AAT------
AACATGACCTGGATGCAATGG---GAAAGAGAAATTGACAATTACACAGGACTAATATAC---
AGCTTACTTGAGGAATCGCAAAACCAACAAGAA--

AAGAATGAACAAGATTTATTGGCATTAGATAAATGGGCAAATTTGTGGACTTGGTTTGATATATCAAATTGGC
TGTGGTATATAAAAATATTCATAATGATAGTAGGAGGCTTGGTAGGTTTAAGAATAGTTTGTACT---
GTATTTTCTATAGTG---------AATAGAGTTAGGCAGGATACTCACCA------
CTATCGTTGGCTATTAGCTTGCTCAATGCCACAGCCATAGCAGTAGCTGAG---
GGGACAGATAGGATTATAGAAGTATTACAAAGACTTTTTAGAGCT----------------------------
--------TTTCTCAACATA------CCTAGAAGAATAAGACAGGGCTTAGAAAGGCTCTTCTA---TAA
>EU576141
GCT---GCA--AAAAG---
TGGGTCACAGTCTATTATGGGGTACCTGTGTGG---AAAGAAGCAACC---
ACCACTCTATTTTGTGCATCAGATGCTAAAGCATATGATACAGAG---GTACATAAT----------------
--------------GTTTGGCCACA---CATGCCTGTGTACCCACAGAC---
CCTAACCCACAAGAAATAGTATTGGGA---------AATGTGACAGAAAATTTTAAC----------------
--------------------------ATGTGGAAAAATAACATGGTAGAACAGATGCATGAGGATATA---
ATCAGTTTATGGGATCAAAGCCTA---AAGCCATGTGTAAAATTA---
ACCCCACTCTGTGTTACTCTAAATTGTAATAATGTTAATACTAATAATACCAATAATAGTACTAAC-------
----
-------------------GCCACTATTAGTGACTGGGAAAAGATGGAGACAGGAGAAATGAAA---
AATTGCTCTTTCAATGTCACCACAAGCATAAGA---------------------------
GATAAGATAAAAAAAGGAATATGCACT---
GTTTTATAAGCTTGATGTAGTACCACTAGAAAATAAAAATAATAT----------------------------
---TAATAATACT--

AATATTACTAACTATAGGTTGATAAATTGTAACACCTCAGTCATTACACAG---
GCCTGTCCAAAGGTATCCTTTGAACCAATTCCCATACATTATTGTGCCCCGGCTGGTTTTGCGATTCTAAAAT
GTAAC---AGTAAGACATTCAAT------GGATCAGGACCATGTACAAAT----------------------
-GTCAGT---ACAGTACAATGCACACATGGAATCAGGCCAGTGGTATCAACT---
CAACTGCTGTTAAATGGCAGTTTAGCAGAAGAAGAG-----------
ATAGTTATTAGATCTGAAAATATCACAGACAAT---------GCTAAAACCATAATAGTACAGCTGAAT----
-----------GAAGCTGTAGAA---------ATTAATTGTACAAGACCCAACAACAATACAAGAAAA-----
-AGTATACATATA------------GGGCCAGGGAGAGCA---TTT------TATGCAACAGGAGAT------
---------ATAATAGGAAATATA------------AGACAAGCACATTGT---AACATTAGTAAAGCA---
AGATGGAATGAAACTTTAGGACAGATAGTTGCAAAATTAGAG---------------GAACAATTTCCA----
-----------------------------------AATAAAACAATAATCTTTAAT------
CATTCCTCAGGAGGGGACCCAGAAATTGTG---ACACACAGTTTTAATTGTGGAGGAGAA---
TTTAAACAGCTCCAGGCAAGAGTCCTGGCTGTGGAAAGATACCTAAGGGATCAACAGCTCCTGGGAATTTGGG
GTTGCTCTGGAAAAATCATTTGCACCACTAATGTGCCTTGGAATGATAGTTGGAGT-----------------
----------------------------AATAAAACTATAAATGAAATTTGG------GAC------
AACATGACTTGGATGCAGTGG---GAAAAAGAAATTGACAATTACACACAACACATATAC---
ACCTTACTTGAAGTATCGCAAATCCAGCAAGAA---------------------------------------
----
AAGAATGAACAAGAATTATTGGAATTGGATAAATGGGATAGTTTGTGGAATTGGTTCAGCATATCAAATTGGC
TGTGGTATATAAAAATATTCATAATGATAGTAGGAGGCTTGATAGGTTTAAGAATAGTTTTTACT---
GTGCTTTCTATAATA---------AGTAGAGTTAGGCAGGGATACTCACCA------
TTATCGTTTGCTGTTAGCTTGCTCAACGCCACAGCCATAGCAGTAGCTGAG---
GGAACAGATAGGGTTATAGAAGTAGTACAAAGGATTGTTAGGGGT----------------------------
--------ATCCTTCACATA------CCTACAAGAATAAGACAGGGCTTGGAAAGGGCTTTGCTA---TAA
>AY835446
GCT---GTA---
GAACAAACGTGGGTCACAGTCTATTATGGGGTACCTGTGTGG---AAAGAAGCAAAC---
ACCACTTTATTTTGTGCATCAGATGCTAAAGCATATAATACAGAG---GTACATAAT----------------
--------------GTTTGGGCCACA---CATGCCTGTGTACCCACAGAC---
CCCAACCCACAAGAAGTAGAATTGGAA---------AATGTGACAGAAAATTTTAAC---------------
-------------------------ATGTGGAAAAACAACATGGTAGATCAGATGCATGAGGATATA---
ATCAGTTTATGGGATCAAAGCCTA---AAGCCATGTGTAAAATTA---
ACTCCACTCTGTGTTACTTTAAGTTGCACTGATAATGTGGGAAATGATACTAGTACCAAT------------
----
-------------------------AATAGTAGATGGGATAAAATGGAAAAAGGAGAAATAAAG---
AACTGCTCTTTCAATATCACCACAAACATGAGA--------------------------
GATAAGATGCAGAAACAATATGCACTT---TTTTATAAACTTGATGTAGTACCAATAGAGGAAGGTAAG----
----

AATAATAACAGTAGTTTTACCGACTATAGGTTGATAAGTTGTAACACCTCAGTCATTACACAG---
GCCTGTCCAAAGGTAACCTTTGAGCCAATTCCCATACATTATTGTGCCCCAGCTGGTTTTGCGCTTCTAAAAT
GTAAG---GATAAGAAATTCAAT------GGAACAGGACCATGTAAAAAT----------------------
-GTTAGC---ACAGTACAATGTACACATGGAATTAAGCCAGTAGTATCAACT---
CAGCTGCTATTAAATGGCAGTCTAGCAGAAGAAGAG-----------
GTAGTAATAAGATCTGAGAATTTCTCGAACAAT---------GCTAGAACCATAATAGTACAGCTGAAT----
-----------ACATCTGTAGAA---------ATAAAGTGTATAAGACCCAACAACAATACAAGAAAA-----
-GGTATACATATA------------GGACCAGGGAGAGCA---TTT------TATACAACAGGAGAT------
---------ATAATAGGAGATATA------------AGACAAGCACATTGT---AACATTAGTAGACAA---
AATTGGAATAACACTTTAAAACAGATAGCTGAAAAGTTAAGA---------------GAACAATTTGGG----
-----------------------------------AATAAAACAATAGTCTTTAGA------
AACTCCTCTGGAGGGGATCCAGAAATTGTA---ATGCACACTTTTAATTGTGCAGGGGAA---
TTTAAGCAGCTCCAGGCAAGAGTCCTGGCTGTGGAAAGATACCTACGTGATCAACAGCTCCTGGGAATTTGGG
GTTGCTCTGGAAAACTCATCTGCACCACTGCTGTGCCTTGGAATGTTAGTTGGAAT----------------
----------------------------AATAGATCTGTGGATGACATTTGG------GAA------
AACATGACCTGGATGCAGTGG---GATAGAGAAATTAGTAATTACACAAGTTTAATATAC---
ACCTTAATTGAAGAATCGCAGAATCAGCAAGAA---------------------------------------
----
AAGAATGAACAAGAATTATTGGCATTAGATAAATGGGCAAATTTGTGGAATTGGTTCAACATAACAGAATGGC
TGTGGTATATAAAAATATTCATAATGATAGTAGGAGGCTTGGTAGGTTTAAGAATAGTTTTTGCT---
GTGCTTTCTATAGTG---------AATAGAGTTAGGCAGGGATACTCACCA------

TTATCGTTTGCTGTTAGCTTGCTCAACGCCATAGCCATAGCAGTAGGTGAG---
GGGAAAGATAGGATTATAGAAGCATTTAGAAGCATTTTTAGAGCT---------------------------
--------ATTCTCCACATA------CCTACAAGAATAAGACAGGGCTTGGAAAGGTCTTTGCTA---TAA
>EF593282
GCT---ACA---
GAAAAATTGTGGGTCACAGTCTATTATGGGGTACCTGTGTGG---AAAGAAGCAACC---
ACCACTCTATTTTGTGCATCAGATGCTAAAGCATATGATACAGAG---GTACATAAT----------------
--------------GTTTGGGCCACA---CATGCCTGTGTACCCACAGAC---
CCCAACCCACAAGAAGTGTTATTGGGA---------AATGTGACAGAGAATTTTAAC----------------
--------------------------ATGTGGAAAAATAACATGGTAGAACAGATGCATGAAGATATA---
ATTAGTTTATGGGATCAAAGCCTA---AAGCCATGTGTAAAATTA---
ACCCCACTCTGTGTTACCTTACATTGCACTAATGCTAATACCACTGGTACTAATAGC----------------
----
-------------------------------ACTAGTGGTAGCGGGATGGAGAGTGAAATAAAA---
AACTGCTCTTTCAATGTCACCACACCCATAAGA--------------------------
GATAAGGTGCAGAAAGAATTTGCATTA---TTTTATAAACTTGATGTAGTACCAATAGATAATGGTAATAAT-
----

AGTAATGGTAATACTAGTGCTAGATATAGGTTAATAAGTTGTAACACCTCAATTATTACACAG---
GCCTGTCCAAAGGTAACCTTTGAGCCAATTCCCATACATTATTGTGCCCCGGCTGGTTTTGCGATTCTAAAGT
GTAAT---GATAAGAAGTTCAAT------GGAACAGGACCATGTAAAAAT----------------------
-GTCAGC---ACAGTACAATGTACGCATGGTATTAAGCCAGTAGTATCAACT---
CAACTGCTGTTAAATGGCAGTCTAGCAGAAAAAGAG------------
GTAGTAATTAGATCTAGAAATTTCTCAAACAAT---------GCTAATATCATAATAGTACAGCTGAAT----
-----------GAACCTGTAAAA---------ATGAATTGTACAAGACCCAACAACAATACAAGAAAA-----
-AGTATACATATA------------GGACCAGGGAGAGCA---TTT------TATGCAACAGGAGAA------
---------ATAATGGGAGATATA-----------AGGCAAGCACATTGT---AACATTAGTAGAGCA---
GAATGGAATAAAACTTTAGAAAAGGTAGTTGAAAAATTAAGA---------------GAACAATTTAAG----
-----------------------------------AATAAAACAATAGCCTTTAAT------
CCATCCTCAGGAGGGGACCCAGAAATTGTA---ATGCACAGTCTTAATTGTGGAGGGGAA---
TTTAAGCAGCTCCAGGCAAGAGTCCTGGCTGTGGAAAGATACCTAAAGGATCAACAGCTCCTAGGGATTTGGG
GTTGCTCTGGGAAACTCATTTGCACCACTTCTGTGCCTTGGAATGCTAGTTGGAGT-----------------
---------------------------AATAAATCTCTGGATGCAATTTGG------AAT------
AACATGACCTGGATGGAGTGG---GAAAGAGAAATTGACAATTACACAGGTTTAATATAC---
ACCTTAATTGAAGAATCGCAAAACCAACAAGAA--

AAGAATGAACAAGAGTTATTGGAATTGGATAAATGGGCAAGTTTGTGGAATTGGTTTGATATAACAAAATGGC
TGTGGTATATAAAAATATTCATAATGATAGTAGGAGGCTTGATAGGTTTAAGAATAGTTTTTACT---
GTACTTTCTATAGTG---------AATAGAGTTAGGCAGGGATACTCACCC------
TTATCGTTTGCTGTTAGCTTGCTTAATGTCACAGCCATAGCAGTAGCTGAG---
GGGACAGATAGAGTTATAGAACTAATACAAGGAGCTTTTAGAGCT---------------------------
--------ATTCTCAACATA------CCTACAAGAATAAGACAGGGCTTGGAAAGGGCTTTGCTA---TAA
>JQ610043
GCT---GCA---
GAAAATTTGTGGGTCACAGTCTATTATGGGGTACCTGTGTGG---AAAGAAGCAACC---
ACCACTCTATTCTGTGCATCAGATGCTAAAGCATATGACACAGAG---GTACATAAT----------------
--------------GTTTGGGCCACA---CATGCCTGTGTACCCACTGAC---
CCAAACCCACAAGAAGTAGTATTGGAA---------AATGTGACAGAAAATTTTAAC----------------
--------------------------ATGTGGAAAAATAACATGGTAGAACAAATGCATGAAGATGTA---
ATCAGCTTATGGGATCAAAGCCTA---AAGCCATGTGTAAAATTA---
ACCCCACTCTGTGTTACTTTAAATTGCACTGACTGTGCTACTGCAAATTGCACTGTCTGGAATTCTACTAGTA
CCAAT--
-------------AATACTACCCATAATACTAGGGAAAGAAAGATGGAGGGCTCAGAAATAAAA---
AAATGCTCTTTCAATGTCACCACAGACATAGGA--------------------------
AATAAGGTAAAGGAAGAATATGCATTG---TTTTATGAATCTGATATAATACAAATAGATAGG----------
----

AGTACTAATACAAGCTATATGTTAAAAAATTGTAATACCACAGTCATTACCCAG---
GCCTGCCCAAAAGTATCCTGGGAACCAATTCCCATACATTATTGTACTCCAGCTGGTTTTGCGATTCTAAAGT
GTAAC---GATAAAAAGTTCAAT------GGAACAGGACCATGTACAAAT----------------------

-GTCAGC---ACCGTACAGTGTACACATGGAATTAGGCCAGTAGTGTCAACT---
CAACTGCTGTTAAATGGCAGTCTAGCAGAAGAAGAG------------
ATAGTAATTAGATCTGAAAATTTCACGAGCAAT---------GTTAAAACCATAATAGTACATCTGAAT----
-----------GAATCTGTAAAA---------ATTAATTGTACAAGACCCAACAACAATACAAGAAAA-----
-GGTATACATATA------------GGGCCAGGGAGAGCA---TTT------TATGCAGCAGGAGAA------
---------ATAGTAGGAAATATA------------AGACAAGCCCATTGT---AACCTTAGTAGAGCA---
GCATGGAATAAGACTTTAGGACACATAGTTGTAAAATTAAGA---------------
GAACAAGCAGGTTTAGG----------------------------------AATAAAACAATAATCTTTAAT-
-----CAACACTCAGGAGGAGACCCAGAAATTGTA---AGCCACAGTTTTAATTGTGGAGGGGAA---
TTTAAGCAGCTCCAGGCAAGAGTCCTGGCTGTGGAAAGATACCTAAAGGATCAACAGCTCCTAGGAATTTGGG
GTTGCTCTGGAAAACTCATTTGCACCACTGCTGTACCTTGGAATACTAGTTGGAGT-----------------
----------------------------AATAAAACTTATAGTGAGATTTGG------GAA------
AACATGACCTGGATGGAATGG---GAGAGAGAGATTGACAATTACACAAGCTTAATATAC---
ACCTTAATTGAAGAATCGCAGAACCAACAGGAA--

AAGAATGAACAAGAATTATTGGCATTGGATGATTGGGCAGGTTTGTTGAGTTGGCTTAACCTATCAAAATGGC
TGGGGTATGTAAAAATATTCATAATAGTAATAGGAGGTTTAGTAGGTTTAAGGATAATATTTTTT---
GTACTTTCTATAGTG---------AATAGAGTTAGGCAGGGATACTCACCA------
TTATCATTTGCTGTTAATTTGCTTAATACCATAGCTATAGCAGTAGCTGAG---
GGGACAGATAGGGTTATAGAAGCAGTACAAAGAGTTTGTAGAGCC----------------------------
--------ATTCGCCACATA------CCTAGAAGAATAAGACAGGGTGCAGAAAGGTTTTTGCTT---TAA
>EF593212
GCT---GCA--
GATCAATTGTGGGTCACAGTCTATTATGGGGTACCTGTGTGG---AAAGAAACAACC---
ACCACTCTATTCTGTGCATCAGATGCTAAAGCCTATGACACAGAG---GTACATAAT----------------
--------------GTTTGGCCACA---CATGCCTGTGTACCCACAGAC---
CCCAGCCCACAGGAAGTAGAATTGGGA---------AATGTGACAGAAAATTTTAAT----------------
--------------------------ATGTGGAAAAATAACATGGTGGACCAGATGCATGAGGATATA---
ATCAGTTTATGGGATCAAAGCCTA---AAGCCATGTGTAAAATTA---
ACCCCACTCTGTGTTACTTTAAATTGCACTGATTGGACGAATGCTAATAATACCAGTGCTGATAATACCACT-
----
-------------GCTACTAATAATACCACTCCTAGTACCAAAATAGAGGAAGTAGAAATAAAA---
AACTGCTCTTTCAATATTACCACAAACATAAGA---------------------------
GATAAGGTGAAGAAAGAATATGCACTT---TTTTATAAACTTGATGTAGTACCAATAGAGGAT----------
----

GATAGTAGGAATAATAGCTATAGGTTAATAAGTTGTAACACCTCAGTCATTACACAG---
GCCTGTCCAAAGGTATCCTTTGAGCCAATTCCCATACATTATTGTGCCCCGGCTGGTTTTGCGATTCTAAAGT
GTAAT---AATAAGACATTCAAT------GGATCAGGGCCATGTAAAAAT-----------------------
-GTCAGC---ACAGTACAATGTACACATGGAATTAGGCCAGTAGTATCAACA---
CAACTGCTGTTAAATGGCAGTCTAGCAGAAGAAGAT------------
ATAGTAATTAGATCTGAAAATTTCACTAACAAT---------GCTAAAACCATAATAGTACAGTTGAAG----
-----------GAACCTGTAATA---------ATCAATTGTACCAGACCCAACAACAATACAAGAAGA-----
-AGTATACCCATG------------GGACCAGGGAAAGTA---TTT------TATACAGCAGAC---------
---------ATAATAGGAGATATA------------AGACAAGCACATTGT---AACATTAGTGGAGAA---
CAATGGAATAACACTTTAAGTGGGGTAGTTGGAAAATTAAGA---------------GAACAATTTAAG----
-----------------------------------GATAAAACAATAATCTTTAAT------
CAATCCTCAGGAGGGGACCCAGAAATTGTA---ATGCACACTTTTAATTGTAGAGGGGAA---
TTTAAGCAGCTCCAGGCAAGAGTCCTGGCTGTGGAAAGATACCTAAAGGATCAACAGCTCCTAGGGATTTGGG
GTTGCTCTGGAAAACTCATCTGCACCACTACTGTGCCTTGGAATACTAGTTGGAGT-----------------
----------------------------AACAAATCTGTAGATGAGATTTGG------GGT------
AACATGACCTGGATGCAATGG---GAAAAAGAAATTGACAATTACACAGACTTTATATAC---
ACTTTAATTGAACAATCGCAGATCCAGCAAGAA--

AAGAATGAACAAGAATTATTGGAATTGGATAAATGGGCAAGTTTGTGGAATTGGTTCACCATATCAAATTGGC
TGTGGTACATAAAAATATTCATAATGATAGTAGGAGGTTTGATAGGTTTAAGAATAGTTTTTTCT---
GTACTTTCTGTAGTG---------AATAGAGTTAGGCAGGGATACTCACCA------
TTATCATTTGCTGTTAGCTTGTTCAATACCACAGCCATAGCAGTAGCTGAG---
GGAACAGATAGGATTATAGCAGTATTACGCAGGGCTTTTAGAGCT----------------------------
--------GTTCTTCATATA------CCTACAAGAATAAGACAGGGCTTGGAAAGGCTTTGCTA---TAA

>EF593300
GCT---ACA--
GATCAATTGTGGGTCACAGTCTATTATGGGGTACCTGTATGG---AAAGAAGCAACC---
ACCACTCTATTTTGTGCATCAGATGCTAAAGCTTATGAGACAGAG---GTACATAAT----------------
--------------GTTTGGGCCACA---CATGCCTGTGTACCCACAGAC---
CCTGACCCACAAGAATTAAAATTGGTA---------AATGTGACAGAAAATTTTAAC----------------
--------------------------ATGTGGAAAAATAACATGGTAGAACAGATGCATCAGGATATA---
ATCAGTTTATGGGATCAAAGCTTA---AAGCCATGTGTAAAACTA---
ACCCCACTCTGTGTCACTTTAAATTGCACTGATTTGAGGAATGCTACTGGCACTAATACTACTAAA-------
----
-------------------ACCTATAATATTAGCGGGGAACTAATGGAAAAAGGAGAAATGAAA---
AACTGCTCTTTCAATGTCACCACAAGCACAGGA---------------------------
GAAAAAAGGCAAAAAGAACGTGCACTG---TTTTATAAACTTGATCTAATGCAAATAGATAGTGAT-------
----

AATAATAGTAGCTATAATAGTTATAGGTTGACAAGTTGTAATGCCTCAGTCATTACACAG---
GCCTGCCCAAAAGTATCCTTTGAGCCAATTCCAATACATTTCTGTGCCCCGGCTGGTTTTGCAATTCTAAAGT
GTAAT---GATAAAAAGTTCAAT------GGATCAGGACCATGTAAAGAT-----------------------
-GTCAGC---ACAGTACAATGTACCCATGGAATCAGGCCAGTAGTATCAACT---
CAATTGCTGTTAAATGGCAGTCTAGCAGAAGAAGAG------------
GTAGTTCTTAGGTCTGAAAATTTCACAGACAAT---------GCTAAAAACATAATAGTACAACTGAAA----
-----------ACAGCTGTAAAC---------ATTACTTGTATAAGACCCAACAACAATACAAGAACA-----
-AGTATATCTATG------------GGACCAGGGAGAGCA---TTT------TTTGCAACAGGAGAT------
---------ATAATAGGAGATATA------------AGACAAGCACATTGT---AACATTAGTAGAAAA---
GACTGGAATAATACTTTACACGAGATAGTTATAAAATTAAGA---------------GAACAATTTCAA----
-----------------------------------AATAAAACAATAATCTTTAAT------
CAATCCTCAGGAGGGGACCCAGAGATTGTA---ACACACAGTTTTAATTGTGGAGGGGAA---
TTTAAGCAGCTCCAGGCAAGAGTCCTGGCTGTGGAAACATACCTAAAGGATCAACAGCTCCTGGGAATTTGGG
GTTGCTCTGGAAGACGCATCTGCACCACTGCTGTGCCTTGGAATACTAGTTGGAGT-----------------
----------------------------AATAAATCCCTGGGAGAGATTTGG------GAT------
AACATGACCTGGATGCAGTGG---GAAAAAGAAATTCACAATCACACAGAAGCAATATAT---
GCCTTACTTGAAATAGCGCAAGGCCAGCAAGAA--

AAGAATGAAAAGGAATTATTGGAATTGGATAAATGGGCAAGTTTGTGGACTTGGTTTGACATATCAAATTGGA
TGAGGTATATACAAATATTCATAATGATAGTAGGAGGCTTAATAGGATTAAAAATAGTTTTTGCT---
GTACTTTCTATAGTG---------AAAAGAGTTAGGCAGGGATACTCACCA------
CTATCATTTGCTGTTAGCTTGCTCAACGCCACAGCCATAGCAGTAGCTGAG---
GGGACAGATAGGATTATAGAAGTGGGACAAACAATTTTTAGGGCT----------------------------
--------ATCATTCACATA------CCCAGAAGAATAAGACAGGGCTTGGAAAGGGCTTTACTA---TAA
>GQ153937
GCT---GCA--
GAAAAAATGTGGGTCACAGTCTATTATGGGGTACCTGTGTGG---AAAGAAGCAACT---
ACCACTCTATTTTGTGCATCAGATGCTAAATCTTATGATACAGAG---GTACATAAT----------------
--------------GTTTGGGCCACA---CATGCCTGTGTACCCACAGAC---
CCCAACCCACAAGAAGTAGTATTGGGA---------AATGTGACAGAAAATTTTAAT----------------
--------------------------ATGTGGAAAAATAACATGGTAGAACAGATGCATGAGGATGTA---
ATCAGTTTATGGGATCAAAGCCTA---AAGCCATGTGTAAAATTA---
ACCCCTCTCTGTGTTACTTTAAATTGCACTGATAAGTTGAGAAATGACGCTTTT-------------------
----
-------------------------------GGAGTAAATAATACAATGGAAGGGGAAATGAAA---
AACTGCTCTTTCAATACCACCACAAGCCTAAGA---------------------------
GATAAGATACAGAAAGAATATGCACTT---TTTTACAAGCTTGATGTAGTACAAATAAAAAATAAT-------
----

AATAATAGTAATTATACTAGCTATAGGTTAATAAATTGTAACACCTCAGTCATTACACAG---
GCCTGTCCAAAGGTAACCTTTGAGCCAATTCCCATACATTATTGTACCCCAGCTGGTTTTGCGATTCTTAAAT
GTAAT---AATAAGACTTTCTCG------GGGAAAGGAACATGTACAAAT-----------------------
-GTCAGC---ACAGTACAATGCACTCATGGAATTAGGCCAGTAGTATCAACT---
CAACTGCTGTTAAATGGCAGTCTAGCAGAAGAAGAT------------
GTAGTAATTAGATCAGACAATTTCTCGCAAAAT---------GCCAAAATCATAATAGTACAGCTAAAT----

-----------GAAGCTGTAGTA---------ATTAATTGTACAAGACCCGGCAACAATACAAGGAAA-----
-AGTATACCTATA------------GGACCAGGGAGAGCA---TTT------TATGCAACAGGAGAC------
---------ATAATAGGAAGTATA------------AGACAAGCACATTGT---AACGTTAGTAGTACA---
AAATGGAATAATACTTTACAAAAGATAGTTGAAAAATTAAGA---------------GAACAATTTGGG----
-----------------------------------AATAAAACAATAAAATTTACA---
TCACCCTCCCCAGGAGGGGACCCAGAAATTGTA---ATGCACAGTTTTAATTGTGGAGGAGAA---
TTTAAACAGCTCCAGGCAAGAGTCCTGGCTGTGGAAAGATACCTAAGAGATCAACAGCTCCTGGGATTTGGG
GATGCTCTGGAAAACTCATCTGCACCACTGCTGTGCCCTGGAATGCTAGTTGGAGT----------------
----------------------------AATAAATCTTACAGACATTTGG------GAT------
AACATGACATGGATGCAGTGG---GAAGAAGAAATAGAAAATTACACAAGTTTAATATAC---
ACCCTAATTGAAGACTCGCAAAACCAACGGGAA---------------------------------------
----
AAGAATGAACAAGAATTATTAGAATTGGATAAGTGGGCAAGCCTGTGGAATTGGTTTGACATAACAAGCTGGC
TGTGGTATATAAAAATATTCATAATGATAGTAGGAGGCTTGATAGGTTTAAGAATAGTTTTTGCT---
GTACTTTCTATAGTA---------AATAGAGTCAGGCAGGGATACTCACCA------
TTATCGTTGGCAGTTAGCTTGCTCAATACAATAGCTATAGCAGTAGCTGAG---
GGGACAGATAGGACTATAGAATTAGCACATAGAATTTGTAGAGCT---------------------------
--------ATTCTCCACATA------CCTAGAAGAATAAGACAGGGCTTTGAAAGGGCTTTGCTA---TAA
>MH263520
GCA---ACA---
ACAAATTTGTGGGTCACAGTCTATTATGGGGTACCTGTGTGG---AAAGAAGCAAAC---
ACCACTCTATTTTGTGCATCAGATGCTAAAGCATATGAAACAGAG---GCACATAAT----------------
--------------GTTTGGGCCACA---CATGCCTGTGTACCCACAGAC---
CCCAGCCCACAAGAAATACCATTGGAA---------AATGTGACAGAAAATTTTAAC---------------
--------------------------ATGTGGAAAAATAACATGGTAGAACAGATGCATGAGGATATA---
ATCAGTTTATGGGATCAAAGCCTA---AAACCATGTGTAAAATTA---
ACCCCACTCTGTGTTACCCTAGATTGCACTAATTTGAATGCTACTAATTCAAATACTACTAATTCAAATGCTA
CTAATTCAAAT---
----------------ACTACCAATCATAGTAGCTGGGAAAGGATGGATAAAGAAGAAATAAAA---
AACTGCTCTTTTAATGTCACCACAAACATAGGA---------------------------
GATAAAATGCAGAAAGCATATGCACTT---TTCTATAGACCTGATATAGTACAATATATACCAATA-------
----

AATACGACCAAATATAGGTTGATAAGTTGTAACACCTCAGTCATTACACAA---
GCCTGTCCAAAGACATCCTTTGAACCAATTCCCATACATTATTGTGCCCCGGCTGGTTTTGCGATTCTAAAGT
GTAGA---GATAAGAAGTTTAAT------GGAACAGGACCGTGTAAAAAT----------------------
-GTCAGC---ACAGTACAATGCACACATGGAATTAGGCCAGTAGTATCAACT---
CAACTGCTGCTAAATGGCAGTCTAGCAGAAGAAGAG------------
GTAGTAGTTAGATCTGCCAATTTCACGGACAAT---------GCTAAAACCATAATAGTACAGCTGAAA----
-----------GAACCTGTAGAG---------ATCAATTGTACAAGACCCAACAACAATACAAGAAAA-----
-AGTATATATATA------------GGACCAGGGAGAGCA---TTT------CATGTAACAGGAAAA------
---------ATAATAGGAGATATA------------AGACAAGCACATTGT---AACATCACTAGAGCA---
AAATGGAATGAAACGTTAAGACAGATAGTTGAAAAATTAAGA---------------GAAAAATTTAAT----
-----------------------------------AATAGAACAATAGTTTTTAAC------
CAATCTTCAGGAGGGGACCTAGAAATTGTA---ATGCACAGTTTTATTTGTGGAGGGGAA---
TTTAAGCAGCTCCAGGCAAGAGTCCTAGCTGTGGAAAGATACCTAAAGGATCAACAGCTCCTGGGAATTTGGG
GTTGCTCTGGAAAACTCATCTGCACCACTACTGTGCCTTGGAATGCTAGTTGGAGT----------------
----------------------------AATAAATCTCTGGAGACAATTTGG------AAT------
AACATGACCTGGATGCAGTGG---GAGAAAGAAATTGACAATTACACAAGCTTAATATAC---
ACCTTAATTGAAGAATCGCAAAACCAGCAAGAA---------------------------------------
----
AAGAATGAACTAGAATTATTGGAATTAGATAAATGGGCAAGTTTGTGGAATTGGTTTGACATATCAAACTGGC
TGTGGTATATAAAAATATTCATAATGATAGTAGGAGGCTTGGTAGGTTTAAGAATAATTTTTACT---
TTGCTTTCTATAGTA---------AATAGAGTTAGGCAGGGATATTCACCA------
TTGTCATTTGCTGTTAGCTTGTTCAACGCATTCGCCATAGCAGTAGCTGAG---
GGGACAGATAGGGTTATAAACTTAGTACAAAGAGCTGGTAGAGCT---------------------------
--------ATTCTTCACATC------CCTAGAAGAATAAGACAGGGCTTGGAAAGGGCTTTGCTA---TAA
>KX984437
GCT---GAA---GAA-----------------------------------
GAAAAATTGTGGGTCACAGTCTATTATGGGGTACCTGTGTGG---AAAGACGCAACC---

ACCACTCTATTTTGTGCATCAGATGCTAAAGCATACAAGACAGAG---GCACATAAT---------------
--------------GTCTGGGCCACA---CATGCCTGTGTACCCACAGAC---
CCCAACCCACAAGAAGTAGTATTGGAA---------AATGTGACAGAAAATTTCAAC---------------
--------------------------GCATGGAAAAATGACATGGTAGAACAGATGCATGAGGATATA---
ATCAGTTTATGGGATCAAAGCCTA---AAGCCATGTGTAAAATTA---
ACCCCACTCTGTGTTACTTTAAATTGCACTAATGTCACTACTAGTGCTACTAATAGTAGTAGCACTAAT----
----
------------------AATAATGCTACTGCTACTAACAGTAGTAGCGAGGGAGAAATGAAA---
AACTGCACTTTCAATATCACCACATTCATGAGT--------------------------
GATAAAAAGCAGACAGTATATGCAAAT---TTTTATAACCTTGATTTAGTACCAATAAATGATGGTAAG----
----

AATGATACTAAAAATAGTTATAGGTTGATAAGTTGTAATACCTCAGTCATTACACAG---
GCCTGTCCAAAGATATCCTTTGAGCCAATTCCCATACATTATTGTGCCCCGGCTGGTTTTGCGATTTTAAAAT
GTAAT---GATAAGAAGTTCAAT------GGAACAGGACCATGTACAAAT----------------------
-GTCAGC---ACAGTACAATGTACACATGGAATTATGCCAGTGGTATCAACT---
CAACTGCTGTTAAATGGCAGCCTAGCAGAAGAAGAG-----------
GTAGTAATTAGTTCTGCCAATATCTCGGATAAT---------GCTAAAACCATAATAGTGCATCTGAAC----
-----------GAATCTGTAGAA---------ATTAATTGTACAAGGCCCAACAACAATACAAGAAAA-----
-AGTATAAATATA------------GGACCAGGGAGAGCA---TTT------TATGCAACAGGAGAC------
---------ATAATAGGAGATATA-----------AGACAAGCACATTGT---AACATCAGTAGTACA---
AAATGGAATAACACTTTAAAAAAGATAGTTGAAAAACTAGGA---------------GAACAATTTAAT----
-----------------------------------ACTCAAACAATAATCTTTGAT------
CAACCCTCAGGAGGGGACCCAGAAATTGTA---ATGCACAGTTTTAATTGTGGAGGAGAA---
TTTAAGCAGCTCCAGGCAAGAGTCCTGGCTGTGGAAAGATACCTACAGGATCAACAGCTCCTAGGAATTTGGG
GTTGCTCTGGAAAACTCATTTGCACCACCGCTGTGCCTTGGAATGCTAGTTGGAGT-----------------
------------------------AAGAATAGATCTCTGAAGGATATTTGG------AAT------
AACATGACCTGGATGGAATGG---GAGAGAGAAATTGACAATTACACAGGCTTAATATAC---
AACTTAATTGAACAATCGCAAAACCAGCAAGAA---------------------------------------
----
AAGAATGAACAAGACTTATTGGCACTAGATAAATGGGAAAGTTTATGGAATTGGTTTAACATAACAAAATGGC
TGTGGTATATAAAAATATTCATAATGATAGTAGGAGGCTTGGTAGGTTTAAGAATAGTGTTTTCT---
GTACTTGCTTTAGTG---------AATAGAGTTAGGAAGGGATACTCACCA------
TTATCGTTTGCTGTTAGCTTGCTCGACGCCACAGCCATAGCAGTAGCTGAG---
GGGACAGATAGGATTATAGCAGTAATACAAAGAGCTTTTAGAGCT---------------------------
--------GTTCTTAATATA------CCTACAAGAGTAAGACAGGGCTTGGAAAGGGCTTTGCTA---TAA
>KX984699
GCT---ACA---GAA--------------------------------
AAAGAAAACTTGTGGGTCACTGTCTATTATGGGGTACCTGTGTGG---AAAGATGCAAAT---
ACCACCCTATTTTGTGCATCAGATGCTAAAGCATATAAGACAGAG---GTACATAAT---------------
--------------GTTTGGGCCACA---CACGCTTGTGTACCCACAGAC---
CCCGACCCACAAGAAGTAGGACTGGGA---------AATGTGACAGAAAATTTCAAC---------------
--------------------------ATGTGGAAAAATAACATGGTAGAACAGATGCATGAGGATATA---
ATCAGTTTATGGGATCAAAGCCTA---AAGCCATGTGTAAAATTA---
ACCCCACTCTGTGTTACTTTAAAATGCTTTGATTTTAATGCCACTAGTAATAGTACTAATGGGACTACCCCTA
GTCCTAGCCCC--
------------------ACTAGGCCTAGCACTGATTTGAATGATACTAGTAATGGGATGAAA---
AACTGCTCTTTCAATATCACCACAAGCATAAGA--------------------------
GATAAGGTGAAACAAGAATATGCACTT---TTATATAAACTTGATATAGTACAAATAGATAGT---------
----

AATAACACCTATAGGTTAATAAGTTGTAATACCTCAGTCCTTACACAG---
GCCTGTCCAAAGGTAACCTTTGAGCCAATTCCCATACATTATTGTGCCCCGGCTGGTTTTGCGATTCTAAAGT
GTAGA---GATAAAAAGTTCAAT------GGGACAGGATCATGCAAAAAT----------------------
-GTCAGC---ACAGTACAATGTACACATGGAATTAAGCCAGTAGTGTCAACT---
CAACTGCTGTTAAATGGTAGTCTATCAGAAGCAGGA-----------
ATAGTAATTAGATCTGAAAATTTCACAAATAAT---------GCTAAAACCATAATAGTACAGCTGGAC----
-----------AAATATGTAAAA---------ATTAATTGTACAAGACCCAACAACAATACAAGGAAA-----
-AGTATAACTATA------------GGACCAGGGAGAGCA---TTT------TATACAACAGGAGAA------
---------ATAATAGGAAATATA-----------AGACAAGCATATTGT---ATAGTTAATGGAACA---

CAATGGAATAACACTTTAACACAGGTAGTTCATGAATTAAGA---------------AAACAATTCCCA----
-----------------------------------AATAAAACAATAGTCTTTAAT------
CAATCCTCAGGAGGGGACCCAGAAATTGTA---ATGCACAGTTTTAATTGTAGAGGGGAA---
TTTAAGCAGCTCCAGGCAAGAGTCCTGGCTATAGAGAGATACCTACAGGATCAACAGCTCCTGGGAATTTGGG
GTTGCTCTGGAAAGCTCATTTGCACCACTGCTGTGCCTTGGAATACTAGTTGGAGT-----------------
----------------------------AATAGATCTCTGGATGCGATTTGG------CAA------
AACATGACCTGGATGCAGTGG---GAAAAAGAAATTGAAAAGTACACAGGCACAATATAC---
ACATTACTTGAAGAATCGCAGAACCAGCAAGAA--

AAAAATGAACACGAGTTATTGGAATTGGATAAATGGGCAAATTTGTGGACTTGGTTTAGCATAACAAACTGGC
TGTGGTATATAAAAATATTCATAATGATAATAGGAGGCTTGATAGGTTTAAGAATAGTTTTTGCT---
GTACTTTCTATAGTG---------AATAGAGTTAGGCAGGGATACTCACCA------
TTATCGTTTGCTGTTAGCTTGTTTAATGCCATAGCCATAGCAGTAGCTGAG---
GGGACAGATAGGGTTATAGAGGTATTACAAAGAGCTTGTAGAGCT----------------------------
--------ATTCTCCATATA------CCTACAAGAATAAGACAGGGCTTGGAAAGGGCTTTGCTA---TAA
>MH632763
GCT---GAA---AAA---------------------------------
CAAGAGCGAGGGTGGGTCACAGTTTATTATGGGGTACCTGTGTGG---AGAGAAGCAAAC---
ACCACTCTATTTTGTGCATCAGATGCTAGAGCATATAATAAAGAG---GTACATAAT----------------
--------------GTTTGGGCCACA---CATGCCTGTGTACCCACAGAC---
CCCAACCCACAAGAGGTAATATTGGAA---------AATGTAACAGAACAGTTTAAC----------------
--------------------------ATATGGAAAAATAACATGGTAGAACAGATGCATGAGGATATA---
ATCAGTTTATGGGATCAAAGCTTA---AAACCATGTGTAAAACTA---
ACCCCACTCTGTGTTACTTTAAAATGCACTAATTTGACTAATGTCACTGAGAGT-------------------
----
-------------------------------------GACCAGATGAAGGAAGGAGGAATGCAA---
AACTGCTCTTTCAATGTCACAGCAGGCATAAAA---------------------------
GATAGGATGCAGAAAGAATATGCACTT---TTTTATAAACTTGATATAGTACCAATAGCTGATGATAAGAAT-
----

AGTAGTGAGACTAACTATACCAACTATAGGTTGATAAGTTGTAACACCTCAGTCATTACACAG---
GCCTGTCCAAAAGTATCCTTTGAGCCAATTCCCATACATTATTGTGCCCCGGCTGGTTTTGCGATTCTAAAAT
GTAAT---GATAAGAATTTCAAT------GGATCAGGGTCATGTACAAAT-----------------------
-GTCAGC---ACAGTACAATGTACACATGGAATTAAGCCAGTAGTATCCACT---
CAACTGCTGCTAAATGGCAGTCTAGCAGAAGATGAG------------
ATAGTAGTTAGATCTGCCAACTTCTCGAACAAT---------GCTAAAATCATAATAGTACAGCTGAAT----
-----------GAATCTGTAACA---------ATTAATTGCACAAGACCCAGCAACAATACAAGAAAA-----
-AGTATAACTATA------------GGACCAGGGAGAGCA---TTC------TATGCAACAGGACAA------
---------ATAATAGGAGATATA------------AGAGCAGCACATTGT---AACATTAGTGAGAAA---
CAATGGAATAAAACTTTAAAACACGTAGCTGACAAATTAAGA---------------GAACAATTT-------
-----------------------------------AACAAGAGCATAGTCTTTAAG------
AACTCCTCAGGAGGGGACCCAGAAATTGTA---ATGCATACTTTTAATTGTGGAGGGGAA---
TTTAAACAGCTCCAGGCAAGAGTCCTGGCTGTGGAAAGATACCTAAGGGATCAACAGCTCCTAGGGATTTGGG
GGTGCTCTGGAAAACTCATCTGCACCACTAATGTGCCTTGGAATGCTAGTTGGAGT-----------------
----------------------------AATAAATCTATAGAGGATATCTGG------GAA------
AACATGACCTGGATGCAATGG---GAAAGAGAAATTGACAATTACACAAACATAATATAC---
TCCTTGATTGAAGAATCGCAGAACCAGCAGGAA--

AAGAATGAACAAGAATTATTAGCATTAGATAAATGGGCAAATTTGTGGAATTGGTTTAGCATAACAAATTGGC
TGTGGTATATAAAAATATTCATAATGATAATAGGAGGCCTGATAGGCTTAAGAATAATTTTTGCT---
GTACTTACTGTAGTG---------AATAGAGTTAGGCAGGGATACTCACCT------
TTGTCATTCGCTGTTAGCTTGTTCAACGCCATAGTCATAGCAGTAGCTGGG---
GGGACAGATAGGATTATAGAAGTATTACAAAGAATTGGTAGAGCT----------------------------
--------ATTCTTCACATA------CCTACAAGAATAAGACAGGGCTTGGAAAGAGCTTTGCAA---TAG
>MH576107
GCT---GCA--
GACAATTTGTGGGTCACAGTCTATTATGGGGTACCTGTGTGG---AGAGAAGCAAAC---
ACCACTCTATTTTGTGCATCAGATGCTAAAGCATATGCTAAAGAG---GTACATAAT----------------
--------------GTTTGGGCCACA---CATGCCTGTGTACCTACAGAC---
CCCAACCCACAAGAAGTAGCATTAGAA---------AATGTGACAGAAAATTTTAAC----------------

--------------------------ATGTGGAAAAATAGCATGGTAGAACAAATGCATGAGGATATA---
ATCAGTTTGTGGGATCAAAGCCTA---AAGCCATGTGTAAAATTA---
ACCCCACTTTGTGTTACCTTAAATTGCACTGATGTGAATGCTACTGCTACTATGGGGAATGAT----------
----
-------------ACTAATGACAATAGTAGCAAAGGGAAATTGATGGAGATAGGAGAAATGAAA---
AATTGCTCTTTCAATATCACCACAAGCATAGGA---------------------------GAGAAGAAG---
ACAGAAAATGCACTT---TTTTATACACTTGATGTAGCACCACTAGACAGG---------------------
----
----------GATAACGACAGCTATAGGTTGATAAGTTGTAATACCTCAGTCATTACACAA---
GCCTGTCCAAAGGTATCCTTTGAGCCAATTCCTATACATTATTGTGCCCCGGCTGGTTTTGCGATTCTAAAAT
GTAAT---GATAAAAAGTTCAAT------GGAACAGGGCCATGTAAAAAT----------------------
-GTCAGC---ACAGTACAATGTACACATGGAATCAGGCCAGTAGCATCAACT---
CAATTGCTGTTAAATGGCAGTCTAGCAGAAGGAAAG-----------
GTAGTAATTAGATCTGCCAACTTCACAGACAAT---------ACTAAAATCATGATAGTGCAGCTGAAC----
-----------GAACCTATAAAT---------ATTACTTGTATAAGACCCAATAATAATACAAGAAAA-----
-AGTATATCCATA------------GGACCAGGGGGAGCA---ATT------TATGCAACAGGAGAC------
---------ATAATAGGAGATATA------------AGACAAGCATATTGT---ACCCTTGATGGAACA---
AAATGGAATGAAACTTTAAAAAACATATCTGACATATTACAA--------------AAGCAATTTGAG----
-----------------------------------AATAAAACAATACTCTTTAGG------
CCACCCTCAGGAGGGGACCCAGAAGTTGTA---ACACACAGTTTTAATTGTGGAGGGGAA---
TTTAAGCAGCTCCAGGCAAGAGTCCTGGCTGTGGAAAGATACCTAAAGGATCAACAGCTCCTGGGGATTTGGG
GATGCTCTGGAAAACTCATTTGCACCACTAATGTACCTTGGAATAGTAGTTGGAGT-----------------
-----------------------------AATAAATCTCTAGATATGATTTGG------AAT------
AACATGACCTGGATGGAGTGG---GAAAAAGAAATTAGCAATTACACAAGCTTAATATAC---
AACTTAATTGAAGAATCGCAGAACCAACAAGAA---------------------------------------
----
AAAAATGAACAAGAATTATTGGCATTGGATAAATGGGCAAGTTTGTGGAATTGGTTTGACATATCAAATTGGC
TGTGGTATATAAAAATATTCATAATGATAGTAGGAGGCTTGATAGGTTTAAGAATAGTTTTTACT---
GTACTTTCTATAGTG---------AATAGAGTTAGGCAGGGATACTCACCA------
TTGTCATTTGCTGTTAGCTTGCTCAACGCCACAGCCATAGCAGTAGCTGAG---
GGGACAGATAGGATTATAGAAGTAGTACAAAGGGCTGGTAGAGCT---------------------------
--------ATTCTCCACATA------CCTAGAAGAATAAGACAGGGCTTTGAAAGGGCTTTGCTA---TAA
>EU616649
GCT---ACA---
GAAAAGTTGTGGGTCACAGTCTATTATGGGGTACCTGTATGG---AAAGAAGCAACC---
ACCACTCTATTTTGTGCATCAGATGCTAAAGCATATGCTACAGAG---ATGCACAAT----------------
--------------GTTTGGGCCACA---CATGCCTGTGTACCCACAGAC---
CCTAACCCACAAGAAGTAGTATTGACA---------AATGTGACAGAAAATTTTAAC----------------
--------------------------ATGTGGAAAAATAACATGGTGGAACAGATGCAGGAGGATATA---
ATCAGTTTATGGGACCAAAGCCTA---AAGCCATGTGTCAAATTG---
ACCCCACTATGTGTTACTTTAAATTGCACTGACTTGAAGAATGGTACTAATGGGAAT----------------
----
----------------------------GTTACACTGGAAGTGGGAGCGGAAGGAGAAATAAAA---
AACTGCTCTTTCAATATCACCACCAACCTAAAA---------------------------
GATAAGGTACAGACAGCATATGCAAAT---TTTTATAAGCTTGATGTAGTACAAATAGATAATGATAAT----
----

ACCAGCCATGAAAATTATACCAGCTATAGGTTGATAAGTTGTAACACCTCAGTCATTACACAA---
GCCTGTCCAAAGGTTTCCTTTGAACCAATTCCTATACATTATTGTACCCCAGCTGGTTTTGTGATCATAAAGT
GTAAT---GATAAGAAGTTCAAT------GGAACAGGACCATGTAAAAAT----------------------
-GTCAGC---ACAGTACAGTGTACACATGGAATTAGGCCAGTAGTAACAACT---
CAACTGCTATTAAATGGCAGTCTAGCAGAAGAAGAG-----------
GTAGTAATTAGATCCGCCAATTTCACAGACAAT---------GCTAAAATCATAATAGTACATCTGAAT----
-----------GAATCAGTAGAA---------ATTAACTGTACAAGACCCAACAACAATACAAGGAAA-----
-GGTATACATATA------------GGACCAGGGAGAGCA---TTT------TATGCAGCAGGAGAA------
---------ATAATAGGAAATATA------------AGAAAAGCATATTGT---ACCATTAATAGAACA---
AAATGGAATACCACTTTACAACGGGTAGTTGAAAAACTAAGA--------------GGACAATTTGGT----
-----------------------------------AATAAAACAATAGTCTTTAAC------
CAATCCTCAGGAGGGGACCCAGAAATTGTA---ATGCACAGTTTTAATTGTAGAGGGGAA---
TTTAAGCAGCTCCAGGCCAGAGTCCTGGCTGTGGAAAGATACCTAAAGGATCAACAGCTCCTGGGGATTTGGG

GTTGCTCTGGAAAACTCATCTGCACCACTGCTGTGCCTTGGAATACTAGTTGGAGT-----------------
----------------------------AACAAAAGTTATACAGACATTTGG------GAC------
AACATGACCTGGATGCAGTGG---GAAAAAGAAATTGATAATTACACAGAAACAATATAC---
ACATTACTTCAAGAATCACAAAACCAGCAAGAA--

AAGAATGAACAAGAATTATTAGAATTAGATAAATGGGCAAGTTTGTGGAATTGGTTTACCATAACAAAGTGGC
TGTGGTATATAAAAATATTCATAATGATAGTAGGAGGCTTAATAGGGTTAAGAATAGTTTTTACT---
GTACTTTCTATAGTG---------AATAGGGTTAGGAAGGGATACTCACCA------
TTATCGTTCGCTATTAGCTTGCTCAACGCCACCGCCATAGCAGTAGCTGAG---
GGGACAGATAGGATTATAGAAGTATTACAAAGAATTTGTAGAGCT----------------------------
--------ATTATCCACATA------CCTGTACGAATAAGACAGGGCTTGGAAAGGGCTTTGCTA---TAA
>FJ469704
GCT---ACA---
GAAAATTTGTGGGTCACAGTCTATTATGGGGTACCTGTGTGG---AAAGAAGCAACC---
ACCACTCTATTTTGTGCATCAGATGCTAAGGCATATGATACAGAG---GTACATAAT----------------
--------------GTTTGGGCCACA---CATGCCTGTGTACCCACAGAC---
CCCAACCCACAAGAAGTAGAATTGGAA---------AATGTGACAGAAAATTTTAAC----------------
-------------------------ATGTGGAAAAATAGCATGGTAGAACAGATGCATGAGGATATA---
ATCAGTTTATGGGATCAAAGCCTA---AAACCATGTGTAAAACTA---
ACACCACTCTGTGTTACTTTAAACTGCACTGATGATTTGAGGAATGATACCATGAGTAGTAATGCC-------
----
----------------------AGGAATAGTAGCTGGGGAATGATGGAGAAAGGAGAAATAAAA---
AACTGCTCTTTCAATATCACCACAAGCATAAGA--------------------------
GATAAGGTTCAGAAAGAATATGCACTT---TTTTATAAACTTGATGTAGTACCAATAGATAGTGATAAT----
----

ACCAGCTATAGTAATAATACCAGCTATAGGTTGATAAGTTGTAACACCTCAGTCATTACACAG---
GCCTGTCCAAAGGTATCATTTGAGCCAATTCCCATACATTATTGTGCCCCGGCTGGTTTTGCGATTCTAAAGT
GTAAA---GACAAGAAGTTCAAT------GGAACAGGACCATGTAAAAAT-----------------------
-GTCAGC---ACAGTACAATGTACACATGGAATTAAACCAGTAGTGTCAACT---
CAACTGCTGTTAAATGGCAGTCTAGCAGAAGAAGAG-----------
GTAGTAATTAGATCTGAAAATTTCACAAATAAT---------GCTAAAATCATAATAGTTCATTTGAAT----
-----------GAATCTATAGAA---------ATTAATTGTACAAGACCCAACAATAATACAAGAAAA-----
-GGTATACATATA------------GGACCAGGGAGAGCA---TTT------TTTGGAACA---GAA------
---------ATAATAGGAAATATA------------AGACAAGCACATTGT---AACATTAGTGAGACA---
AAATGGAATAATACTTTAAAAAAGATAGTTACAAAATTAAGA---------------GAACAATTTAAG----
----------------------------------AATAAAACAATAGTCTTTAAT------
CAATCTTCAGGAGGGGACCCAGAAATTGTA---ATGCACAGTTTTAATTGTGGAGGGGAA---
TTTAAGCAGCTCCAGGCAAGAGTCCTGGCTGTGGAAAGATACCTAAGGGATCAGCAGCTCCTAGGGATTTGGG
GTTGCTCTGGAAAACTCATCTGCACCACTTCTGTGCCTTGGAATGCTAGTTGGAGT-----------------
----------------------------AACAAATCTCAGGAGGCGATTTGG------GGG------
AACATGACCTGGATGGAGTGG---GAAAGAGAAATTAACAATTATACAAACTTAATATAC---
AACTTACTTGAAGATTCGCAAAACCAACAAGAA--

AAGAATGAACAGGAATTATTGGAATTGGATAAGTGGGCAAATTTGTGGAATTGGTTTGACATAACACACTGGC
TGTGGTATATAAAAATATTCATAATGATAGTAGGAGGTTTGGTAGGTTTAAGGATAGTTTTTGCT---
ATGCTTGCTATAGTG---------AATAAAGTTAGGCAGGGATACTCACCA------
TTATCGTTTGCTGTTAGCCTGCTCAATACTACAGCCATAGCAGTAGCTGAG---
GGGACAGATAGGGTTATAGAAGTAGCACAAAGAATTGGTAGAGGT----------------------------
--------ATCCTCCACATA------CCTGTAAGAATAAGACAAGGCTTAGAAAGGGCTTTGCTA---TAA
>MN515543
GCT---ACA---
GACAAGTTGTGGGTCACAGTCTATTATGGAGTACCTGTGTGG---AAAGAAGCAACC---
ACCACTCTATTTTGTGCATCAGATGCTAGAGCATATGCTACAGAG---GTACATAAT----------------
--------------GTTTGGGCCACA---CATGCCTGTGTACCCACAGAT---
CCCAGCCCACTAGAAACAGAATTGGGA---------AATGTGACAGAAGATTTTAAT----------------
-------------------------ATGTGGAAAAATAACATGGTAGAACAGATGCATGAGGATATA---
ATCAGTTTATGGGATCAAAGCCTA---AAGCCATGTGTAAAATTA---
ACCCCACTCTGTGTTACCTTAAACTGCACAGATTGGGAAAGTGCTAAGAATAATACTAATGCCACTGATGCTA
AGAATAATACT--

----------GATGCCACTAATGGTACTGCTGAGTGGAACACGATAGAGAAAGGAGAAATAAAA---
AACTGCTCTTTCAATGTCACCTCAGGTATAAGA---------------------------
GATAAGATGCAGAAAGAATATGCACTT---TTCTATAAATTTGATATAGTACCAATAGATAAT----------
----

GCTACCAATGCTACCAGCTATAGGTTGTTAAGTTGTAATACCTCAGTCATTACACAG---
AAATGTCCAAAGGTAACCTTTGAGCCAATTCCCATACATTATTGTGCCCCGGCTGGTTTTGCGATTCTAAAGT
GTAAC---AATAAGACATTTAAT------GGAAAAGGATCATGTACAAAT-----------------------
-GTCAGC---ACAGTACAATGTACACATGGAATTAAGCCAGTAGTATCTACT---
CAATTGCTGTTAAATGGCAGTCTAGCAGAAGAAGAT------------
GTAGTAATTAGATCTGACAATTTCTCGGACAAT---------GCTAAAACCATAATAGTACAGCTGAAG----
-----------GATCCTGTAGTT---------ATTAATTGTACAAGACCCCACAACAATACAAGAAAA-----
-GGTATACATATA------------GGACCAGGGAGAGCA---TTT------TATGCAACAGGAAGC------
---------ATAATAGGAGATATA------------AGACAAGCACATTGT---AACATTAGTAGAAAA---
GCCTGGAATAACACCTTAAAACAAATAGTAGGAAAATTAAGA---------------GAACAATTTAGG----
-----------------------------------AACAAAACAATAGTCTTTAAG------
CACTCCGCAGGAGGGGACCCAGAAATTACA---ATGCACAGTTTTAATTGTGCAGGGGAA---
TTTAAGCAGCTCCAGGCAAGAGTCCTGGCTGTAGAAAGATACCTACGGGATCAACAGCTCCTGGGGATCTGGG
GTTGCTCTGGAAAACTCATCTGCACCACTGCTGTGCCTTGGAATGCTAGTTGGAGT-----------------
----------------------------AATAAATCTCTAGATGACATTTGG------AAT------
AACATGACCTGGATGCAGTGG---GAAAAAGAAATTGACAATTATACAGAATCAATATAC---
AAATTACTTGAAAAATCGCAGAACCAACAAGAA--

AAGAATGAACAAGAATTATTGGAATTGGATAAATGGGCAAATTTGTGGAATTGGTTTGACATAACGAACTGGC
TGTGGTATATAAAAATATTCATAATGATAGTAGGAGGCTTGATAGGTTTAAGAATAGTTTTTACT---
GTACTTTCTATAGTA---------AATAGAGTTAGGCAGGATATTCACCA------
TTATCGTTTGCTGTTAGCTTGCTTAATGCCACAGCCATAGCAGTAGCTGAG---
GGACAGATAGGATTATAGAAATATTACAAAGAACTGTTAGAGCT-----------------------------
--------ATCCTCCACATA------CCTACAAGAATAAGACAGGGCTTGGAGAGGGCTTTGCTA---TAA
>MN515731
GCT---ACA--
GAACAGTTGTGGGTCACAGTCTATTATGGGGTACCTGTGTGG---AGAGACGCAAAC---
ACCACTCTATTTTGTGCATCAGATGCTAAAGCATATGAGACAGAG---GTACACAAT----------------
--------------GTTTGGCCACA---CATGCCTGTGTACCCACAGAC---
CCCAACCCACAAGAAATACCATTGGAA---------AATGTGACAGAATATTTTAAC----------------
--------------------------ATGTGGAAGAATAACATGGTAGAACAGATGCATGAGGATATA---
ATCAGTTTATGGATGAAAGCTTA---AAACCATGTGTAAAATTA---
ACCCCACTCTGTGTTACTTTAAATTGTACTAATGTGAATGTTACTAATACTTGTAGGAATCATACTAAGAATA
GTACTGCTGAGAATGATAATAATAATACTTGTAGCAATACT--------------------------------
----------CATAATACTACTAGTTATGATAATAGTACTTTGGAAACTCCAGGAGAAATAAAA---
AACTGCTCTTTCAATATCACCACAAGCATAAGA---------------------------
GATAAGGTGCAGAAAGAGTATGCGCTT---TTTTATAGACTTGATGTAGTACAAATAAATAAT----------
----

GATAATACTAGCTATAGATTGATACATTGTAATTCCTCAGTCATTACACAG---
GCCTGTCCAAAGGTATCCTTTGATCCAATTCCCATACATTACTGTGCCCCGGCTGGTTTTGCGATTCTAAAAT
GTAAT---GATAAAAGGTTTAAT------GGATCAGACATATGTAGAAAT-----------------------
-GTCAGC---ACAGTACAATGTACACATGGAATTAGACCAGTAGTGTCAACT---
CAACTGTTGTTAAATGGCAGTCTAGCAGAAGAAGAG------------
ATAGTAATTAGATCTGCAAATTTCAGTGACAAT---------GATGAAACCATAATAGTACAGCTGAAC----
-----------GAATCTGTGGTA---------ATTAATTGTACAAGACCCAACAACAATACAAGAAAA-----
-AGTATAACTAGG------------ACACCAGGGAGAGTC---ATT------TATACAACAGGAGCC------
---------ATAGTAGGAGACATA------------AGGAAAGCACATTGT---ACCATTAATGGAACA---
AAATGGAATAACACTTTAGCAAAGATAGTTACAAAATTAAGA---------------GAACAATTTGGG----
-----------------------------------AATAAAACAATAGTCTTTAAT------
CATTCCTCAGGAGGGGACCCAGAAATTGTA---ACGCACAGTTTTAATTGTGGAGGGGAA---
TTCAAGCAGCTCCAGGCAAGAATCCTGGCTGTAGAAAGATACCTAAAGGATCAACAGCTCCTAGGGATTTGGG
GTTGCTCTGGAAAACTCATCTGCACCACTACTGTGCCTTGGAACAATAGTTGGAGT-----------------
-------------------------AAAAATAAATCTCTGGATGAAATTTGG------GAT------
AACATGACTTGGATACAGTGG---GAAAAAGAAATTAACAAATACACAGGGGAAATATAC---

AATTTAATTGAAAAATCGCAGAACCAGCAGGAA--

CAAAATGAACTAGACTTATTGAAATTGGATGAGTGGGCAAGTCTGTGGAATTGGTTTGACATAACAAAATGGC
TGTGGTATATAAAAATATTCATAATGATAGTAGGAGGCTTGATAGGTTTAAGAATAGTTTTTACT---
GTACTTTCTATAGTG---------AATAGAGTTAGGCAGGGATACTCACCA------
TTGTCATTTGCTGTTAGCTTGCTCAACGCCACAGCCATAGCAGTAGCTGAG---
TGGACAGATAGGGTTATAGAAGTATTACAAAGAGCTGGTAGAGCT----------------------------
--------ATTCTCAACATA------CCTAGAAGAATAAGACAGGGCTTGGAAAGGGCTTTGCTA---TAA
>GU728412
GCT---ACA---
GAAAAATTGTGGGTCACAGTCTATTATGGGGTACCTGTGTGG---AAAGAAGCAACT---
ACCACTCTATTTTGTGCATCAGATGCTAAAGCATATGATACAGAG---GTACATAAT----------------
--------------GTTTGGGCCACA---CATGCCTGTGTGCCCACAGAC---
CCCAACCCACAAGAAGTAGTATTGGGA---------AATGTAACAGAAAACTTTAAC----------------
-------------------------ATGTGGAAAAATAACATGGTAGAACAGATGCATGAGGATATA---
ATCAGTTTATGGGATCAAAGCCTA---AAGCCATGTGTAAAATTA---
ACCCCACTCTGTGTTACTTTAAATTGCACTGATTTGAATACTACCAGTACCAATAGT----------------
----
------------------------------AGTTGGGGAAAAATAGACCCAGGAGAAATAAAA---
AACTGCTCTTTCAATGTCACCACAAACATAAGA--------------------------
GATAAGATGCAGAAAGAATATGCATTG---TTGTATAAACTGGATATAGTACCAATAGATGAT----------
----

GATAATAGTAGCTATAGGTTGATAAGTTGTAATACCTCAGTCATTACACAG---
GCCTGTCCAAAGGTATCCTTTGAGCCAATTCCTATACATTATTGTGCCCCGGCTGGTTTTGCGATTATAAAGT
GTAAT---AATAAGACATTCAAT------GGGACAGGACCATGTCACAAT----------------------
-GTCAGC---ACAGTACAATGCACACATGGAATTAGACCAGTAGTATCAACT---
CAACTATTGTTAAATGGCAGTCTAGCAGAAGAAGAG-----------
GTAGTAATCAGATCTGAAAATTTCACGAACAAT---------GCTAAAATCATAATAGTACAGCTGAAA----
-----------GAACCTGTGAAA---------ATTAATTGTACAAGACCCAGCAACAATACAAGAAAA-----
-AGTATAAATATA------------GGACCAGGGAGAGCA---TTT------TATACAACAGGAGAA------
---------ATAATAGGAGATATA------------AGACAAGCACATTGT---AACATTAGTAGAGAA---
GAGTGGAATAAAACTTTAGGACAGATAGTTGAAAAATTAAGA---------------GAACAATTTGGT----
--------------------------------AGGAATAAAACAATAATTTTTAAT------
CAATCCTCAGGAGGGGACCCAGAAATTGTA---ACGCACAGCTTTAATTGTGGAGGGGAA---
TTTAAGCAGCTCCAAGCAAGAGTCCTGGCTGTAGAAAGATACCTACGGGATCAACAGCTCCTGGGAATTTGGG
GTTGCTCTGGAAAACTCATTTGCACCACTGCTGTGCCCTGGAATTCTAGTTGGAGT-----------------
----------------------------AATAAATCTCTAGATCAGATCTGG------AAT------
AACATGACCTGGATGGATTGG---GAAAAAGAAATTGACAATTACACAGGCATAATATAC---
AGATTACTTGAAAACTCGCAAAACCAGCAAGAA--

AAGAATGAACAAGAATTATTGGAATTAGATAAATGGGCAAGTTTGTGGAATTGGTTTGACATAACAAACTGGC
TGTGGTATATAAAAATATTCATAATGATAGTAGGAGGCATAGTAGGTTTAAGAATAATTTTGTCT---
ATACTTGCTATAGTA---------AATAGAGTTAGGAAGGGATATTCACCA------
TTATCGTTTGCTGTTAGCTTGCTCAACGCCACAGCCATAGCAGTAGCTGAN---
GGGACAGATAGGATTATAGAAATACTACGAAKAGCTTTTAGGGCT----------------------------
--------ATTATCCACATA------CCTGTTAGAATAAGACAGGGCTTGGAAAGAGCTTTGCTA---TAA
>GU727880
GCT---ACA---
CAAAACTTGTGGGTTACAGTCTATTATGGGGTACCTGTGTGG---AAAGAAGCAAAC---
ACCACTCTATTTTGTGCATCAGATGCTAAATCATATGAGACAGAG---GTACATAAT----------------
--------------GTCTGGGCCACA---CATGCCTGTGTACCCACAGAC---
CCCAACCCACAAGAAGTAGTATTGGGA---------AATGTAACAGAAAATTATAAC----------------
-------------------------ATGTGGAAAAATCACATGGTAGAACAGATGCATGAGGATATA---
ATCAGTTTATGGGATCAAAGCCTA---AAGCCATGTGTAAAATTA---
ACCCCACTTTGTGTTACYTTAAATTGCACTGATGTGAGAAATGAGACTAAGGGG------------------
----
------------------------------AATAATAGTATTGGGGAAAATGCTGACATGAAA---
AACTGCTCTTTCRATATCACCACAAGCATGAAA--------------------------
GATAAGTATCAGAACGTATATGCACTT---TTTTATAAACTTGATGTAGTTCCAATAGATGATAAT-------

----

AATAATACTAATACTATCTATAGGTTGGTAAGTTGTAACACCTCAGTCATTACACAG---
GCCTGTCCAAAGGTATCCTTTCAGCCAATTCCCATACATTATTGTGCCCCGGCTGGTTTTGCGATTTAAAAT
GTAAT---GATAAGAAATTCAAT------GGAAAAGGACCATGTACAAAT----------------------
-GTCAGC---ACAGTACAATGTACACATGGAATTAGACCAGTAGTATCAACT---
CAGCTACTGTTAAATGGCAGTCTAGCAGAAGAAGAG------------
ATAATGATTAGATCTGAGAATTTCACAAACAAT--------GCTAAAACCATAATAGTGCAGCTGAAT----
-----------GAATCTGTAATA--------ATTAATTGTACAAGACCCAACAACAATACAAGAAAG-----
-AGGATAACTATG-----------GGACCAGGAAGAGTA---TAT------TACACAACAGGACAA------
---------ATAATAGGTAAGATA-----------AGACAAGCACATTGT---AACATTAGTAGAGCA---
GCATGGAATGATACTTTAAAGAAGGTAGCTGAAAAATTAAGA--------------GGACAATTTGGA----
----------------------------------AATAAAACAATAGTCTTTAAT------
CAATCTTCAGGAGGAGACCTAGAAATTGTA---ATGCACAGTTTTGTTTGTGGAGGGGAG---
TTTAAGCAGCTCCAGGCAAGAATCCTGGCTGTAGAAAGATACCTAAAGGATCAACAGCTCCTAGGGATTTGGG
GCTGCTCTGGAAAACTCATTTGCACCACTGCTGTGCCTTGGAATGCCAGTTGGAGT----------------
----------------------------AWTAAATCTCTGGATAGTATTTGG------GGA------
AACATGACCTGGATGGAGTGG---GAAAAAGAAATTGACAATTACACAGTTTTAATATAC---
AATTTACTTGAAAAATCGCAAATCCAACAAGAA---------------------------------------
----
AAGAATGAACAAGAATTATTGGAATTAGATAAGTGGGCAAGTCTGTGGAATTGGTTTGACATAACAAACTGGC
TGTGGTATATAAAAATATTCATAATGATAGTAGGAGGCCTAGTAGGTTTAAGAATAGTTTTTACT---
GTACTTTCTGTAGTA---------AATAGAGTCAGGCAGGGGTACTCACCA------
TTATCGTTTGCTATTAGCTTGCTTAATACCACTGCTGTAGTAGTAGCTGAG---
GGGACAGATAGGGTTATAGAAGTAGTACAAAGAACTGGTAGAGCT---------------------------
--------ATCTACCACATA------CCTGTTAGAATAAGACAGGGCTTGGAAAGGCTTTTGCTA---TAA
>DQ886031
GCT---GCA---
GAAAAATTGTGGGTCACAGTCTATTATGGGGTACCAGTGTGG---AAAGAAGCAAAC---
ACCACTCTATTTTGTGCATCAGATGCTAAAGCAACTAAGGCAGAG---GTACATAAT---------------
--------------GTTTGGGCTACA---CATGCCTGTGTACCCACGGAC---
CCCAACCCACAAGAACTAGCATTGGAA---------AATGTGACAGAAAGTTTGAC----------------
--------------------------ATGTGGAAAAATAACATGGTAGAACAGATGCATGAGGATATA---
ATCAGTTTATGGGATCAAAGCCTA---AAGCCATGTGTAAAATTA---
ACCCCACTCTGTGTTACCTTAACTTGTACTGATTATTCGGGGAATAATAATACTAATATCACT---------
----
------------------------GGGAAGGCTAATACCACTATTGATGATATAGAAATGAAA---
AATTGCTCTTTCAATATTACCACAGGCATAAGA--------------------------
GATAAGGTGAAGAGAGAATATGCACTG---TTTTATAAAGTTGATGTAGTACCAATAGAGAAG---------
----

AATACCAGTTATAGGTTGATACATTGTAACACCTCAACCATCACACAG---
GCCTGTCCAAAGGTATCCTTTGAGCCAATTCCCATACATTACTGTGCCCCGGCTGGTTTTGCGATTCTAAAGT
GTAAA---GATAAGAAGTTCAAT------GGAACAGGACTATGTACAAAT----------------------
-GTCAGC---ACAGTACAGTGTACACATGGAATTAGGCCAGTAGTGTCAACT---
CAACTGCTGTTAAATGGTAGTCTAGCAGAAGAAGAG------------
GTAGTAATTAGATCTCAGAATTTCACAGACAAT--------GTCAAAACCATAATAGTACAGCTGAAT----
-----------GAATCTGTAGTG--------ATTAATTGTACAAGACCCAACAATAATACAAGAAAA-----
-AGTATACATATA-----------GGACCAGGGGGAGCA---TTC------TATACAACAGGACAA------
---------GTAATAGGAAATATA-----------AGACAGGCATATTGC---AACATTAGTGAAGAA---
CAATGGAATAAAACTTTAAAATACATAGTTGAAAAATTAAGA--------------GAACAATTTGGA----
----------------------------------AATAAAACAATAATCTTTAAT------
CAATCCTCAGGAGGGGATCCAGAAATTGTA---ACGCACAGTTTTAATTGTGGAGGAGAA---
TTCAAGCAACTCCAGGCAAGAGTCCTGGCTGTGGAAAGATACCTAAAGGATCAACAGCTCCTAGGGATTTGGG
GATGCTCTGGAAAACTCATCTGCACCACTAATGTGCCTTGGAATACTAGTTGGAGT----------------
----------------------------AATAGATCTCTGGACTATATTTGG------GGG------
AACATGACCTGGATACAGTGG---GAAAGGAAATTGACAATTACACAGACTTAATATAC---
ACCTTAATTGAAGAATCGCAAGACCAACAAGAA---------------------------------------
----
AAGAATGAACTAGAATTAATGAAACTAGATAAGTGGGACAGTCTGTGGAGTTGGTTTAGCATAACAAACTGGC

TGTGGTATATAAGATTATTCATAATTATAGTAGGAGGCTTAGTAGGTTTAAGAATAGTTTTTACT---
GTACTTTCTATAGTG---------AATAGAGTTAGGCAGGATACTCACCA------
TTGTCATTTGCTGTTAGTTTGCTCAATGCTACAGCTATAACAGTAGCTGAG---
GGGACAGATAGGGCTATAGAACTAATACAAAGAGCTTTTAGGGCT----------------------------
--------TTTCTTCGCATA------CCTACAAGAATAAGACAGGGCTTGGAAAGGGCTTTGCTA---TAA
>DQ886032
GCT---GCA--
GAAAATATGTGGGTCACAGTCTATTATGGGGTACCTGTGTGG---AAGGAAACAACC---
ACCACTTTATTTTGTGCATCAGATGCTAAAGCACATGATACAGAA---GTACATAAT----------------
--------------GTTTGGCCACA---CATGCCTGTGTACCCACAGAT---
CCCAACCCACAAGAAATAGTATTG------------AATGTGACAGAAAATTTTAAC----------------
--------------------------ATGTGGAAAAATAACATGGTTGAACAGATGCATGAGGATGTA---
ATCAGTTTATGGGATCAAAGCCTA---AAGCCATGTGTAAGATTA---
ACCCCACTCTGTGTTACTCTAAATTGCACTGATGTGAAGAATGTTACTAATACCAAGACAAAG----------
----
-------------------------GATACTAATACCAATATTAGTAGTTGGGAAGACATGAAA---
AACTGCTCTTTCAATGTCACCTCAAACATAAGA---------------------------
GATAAAGTACAGAAAGAATATGCACTT---TTTTATAAGCTTGATGTAGTACCAACAGATAAG----------
----

GATAATACTAGCTATAGGATGATAAATTGTAATACCACAGTCATTACACAG---
GCCTGTCCAAAAGTATCCTTTGAGCCAATTCCTATACATTACTGTGCCCCGGCTGGTTTTGCGATTCTAAAGT
GTAAT---GAGGAGAAGTTCAAT------GGATCAGGACCATGTGGAAAT-----------------------
-GTCAGC---ACAGTACAATGTACGCATGGGATTAGGCCAGTAGTGTCAACT---
CAGCTACTGTTAAATGGCAGTCTAGCAGAAAAAGAG------------
GTAGTAATTAGATCTGAGAATTGGACAAACAAT---------GCTAAAATCATAATAGTACAGCTGAAT----
-----------GAATCTGTAGAG---------ATTAATTGTACAAGACCCAACAACAATACAGTAAGA-----
-GGTATACATATA------------GGACCAGGGAGAGCA---TTT------TATGCAACAGGATCA------
---------GTAATAGGAGATATA------------AGACAGGCACATTGT---AACATTAGTGCAGCA---
AAATGGAATAACACTTTAAGACTAATAGCTGATAAGTTAAGA---------------GAACAATTTAGG----
-----------------------------------AATAAAACAATAGTCTTTAAT------
CAATCCTCAGGAGGGGACCCAGAAATTGTA---ATGCATAGTTTTAATTGTGGAGGGGAA---
TTTAAGCAGCTCCAGGCAAGAGTCCTAGCTGTAGAAAGATACCTAAAGGATCAACAGCTTCTAGGGATTTGGG
GTTGCTCTGGAAAACTCATATGCACCACTGCTGTGCCTTGGAATGTTAGTTGGAGT-----------------
----------------------------AATAAATCTTATGATCAAATTTGG------GAC------
AACATGACCTGGATGCAGTGG---GAAAGAGAAATTGACAATTACACAGGCTTGATATAC---
ACCCTAATTGAAGAATCGCAGAACCAACAGGAA--

AAGAATGAACTAGAACTATTGGAATTGGATAAGTGGGGAAGTCTGTGGAATTGGTTTGACATATCAAATTGGC
TGTGGTATATAAGAATATTCATAATGATAGTAGGGGGCTTAATAGGTTTAAGAATAGTTTTTACT---
GTAATTTCTATAGTG---------AATAGAGTTAGGCAGGATATTCACCA------
TTGTCGTTTGCTGTCAGCTTGCTCAACGCCACAGCTATAGCAGTAGCTGAG---
GGGACAGATAGGATTATAGAAGCTTTGCAAAGAGTCTTTAGAGCG----------------------------
--------------------------------TGAATAAGACAGGGCTTGGAAAGGGCTTTGCTA---TAA
>GU367399
GCT---ATA--
GAACAATTGTGGGTCACAGTCTATTATGGGGTACCTGTGTGG---AAAGAAGCAAAC---
ACCACTTTATTCTGTGCATCAGATGCTAAAGCATATAATACAGAG---GTACATAAT----------------
--------------GTTTGGCCACA---CATGCCTGTGTACCCACAGAC---
CCCGACCCACAAGAAGTAGTATTGGAA---------AATGTGACAGAAAATTTTAAC----------------
--------------------------GCGTGGAAAAATAACATGGTAGAGCAAATGCATGAGGATATA---
ATCAGTTTATGGGATCAAAGCCTA---AAACCATGTGTAAAATTA---
ACTCCACTATGTGTTACCTTACATTGCACTAATTACAGTGGCACTAATGACACTGGCACTGGGACT-------
----
-------------------ACTACTGTACCTACTACTGTACTCACCCCTAACGAAGAAGTAAAG---
AACTGCTCTTTCAATGTTACCACAGGCATGAGA---------------------------
GATAAGATAAAGAAAGAATATGCGCTT---TTTTATAGATATGATATAGAAAAAATAGATGAT----------
----

AATACTAATAGTACCAGCTATAGGTTGATAAGGTGCAACACCTCAGTCATTACACAA---

GCCTGTCCAAAGGTATCCTTTGAGCCAATTCCCATACATTTTTGTGCCCCGGCTGGTTTTGCGATTCTAAAAT
GTAAG---GATAAAAGGTTCAAT------GGAACAGGACCATGTACAAAT-----------------------
-GTCAGC---ACAGTACAATGTACACATGGAATTAGGCCAGTAGTCTCAACT---
CAACTGCTGTTAAATGGCAGTCTAGCAGAAGAAGAG------------
ATAGTAATTAGATCTGAAAATATCTCGGACAAT---------GCTAAAACCATAATAGTACAGCTGAAG----
-----------GATTCTGTAAAA---------ATTAATTGTATAAGACCCAACAACAATACAAGAAAA-----
-AGTATACATATG------------GGACCAGGGGCCGCA---CTT------TATACAACAGGAGCA------
---------ATAATAGGAGATATA------------AGACAAGCATATTGT---AAGCTTAATGGAACA---
CAATGGAACAACACTTTAAGACAGATAGCAAGAAAATTAAGT---------------AAACTTGTGAAT----
-----------------------------------AAAACAACAATAGTCTTTAAT------
CAATCCTCAGGAGGGGACCAAGAAATTGTA---ATGCACAGTTTTAATTGTGGAGGGGAA---
TTTAAGCAGCTCCAGGCAAGAGTCCTGGCTGTGGAAAGATACCTAAGGGATCAACAGCTCCTGGGAATTTGGG
GTTGCTCTGGGAAAATCATTTGCAACACTGCTGTGCCTTGGAATACTACTTGGAGT-----------------
----------------------------AATAAATCTCTAGATAATATTTGG------AAT------
AACATGACCTGGATGGAATGG---GAAAGAGAAATTGACAATTACACAGGAGTAATATAT---
GAGTTAATTGAAAAATCGCAAAACCAGCAAGAA--

AAGAATGAACAAGAATTATTGGAATTAGATAAATGGGCAAGTTTGTGGAATTGGTTTAGCATAACAGAATGGC
TGTGGTACATAAAAATATTCATAATGATAGTAGGAGGCTTAGTAGGTTTAAGAATAATTTTTACT---
GTACTTTCTATAGTG---------AATAGAGTTAGGCAGGGATACTCACCA------
TTATCGTTTGCTGTTAGCTTGCTCAATGCCACAGCAATAGCAGTAGCTGAG---
GGAACAGATAGGGTTATAGAAGTATTACAAAGAGCATATAGAGCA----------------------------
--------TTTATCCACATA------CCTAGAAGAATAAGACAGGGCTTGGAAAGGGCTTTGCTA---TAA
>GU367403
GCT---AAA--
GACAAAATGTGGGTCACAGTCTATTATGGGGTACCTGTGTGG---AAAGAAGCAACT---
ACTACTCTATTTTGTGCATCAGATGCTAAAGCATATGATACAGAG---GGTCATAAT----------------
--------------GTTTGGGCCACA---CATGCCTGTGTACCCACAGAC---
CCCAACCCTCAAGAAATAGAATTG------------AATGTGTCAGAAGAGTTCAAC----------------
--------------------------ATGTGGAAAAATAACATGGTAGAACAGATGCAGGAGGATATA---
ATCAGCTTATGGGATCAAAGCCTA---AAGCCATGTGTAGAATTA---
ACACCACTCTGTGTTACTTTATACTGCAGTAATTATTCAGGGAATGGTACTAATGCCACTGGTACTAATACC-
----
----------------ACTACCCTGAATACTACTAGTGACACTGTTGGTATGAAAGAAATGAAA---
AACTGCACTTTCAATATCTCCACAAGCGTAAGA---------------------------
GATAAGATGAAGAAAGAATATGCACTT---TTTTATAAGCTTGATATAGTACCAATAGATAAT----------
----

AATAATAGCTATAGATTGGTAAGTTGTAACACCTCAGTCATTAAACAA---
GCCTGTCCCAAGGTATCCTTTGAGCCAATTCCCATACATTATTGTGCCCCGGCTGGTTTTGCAATTCTCAAGT
GTAGA---GACAAGAAGTTCAAT------GGAACAGGACAGTGTAAAAAT-----------------------
-GTCAGC---ACAGTGCAATGTACACATGGAATTAGACCAGTAGTATCAACT---
CAATTGCTGCTAAATGGCAGTCTAGCAGAAGAAGAG------------
GTAGTACTTAGATCTGTCAATTTCTCAGACAAT---------GCTAAAACCATAATAGTACAACTGAAT----
-----------GAATCTGTAGTA---------ATTAACTGTACAAGACCCGGCAATAATACAAGAAGA-----
-AGTATACATATA------------GCACCAGGTAGAGCA---TTT------TATGCAACAGGAGAG------
---------GTAATAGGGGATATA------------AGACAAGCATCTTGT---ACCATTAATGGGCA---
AAATGGAATAACACTTTAAAACAGATAGTTCAAAAATTAAGA---------------GAAGAATTTAAG----
-----------------------------------AATAAAACAATAGTCTTTAAT------
CAGTCCTCAGGAGGGGATCTAGAAATTGTA---ATGCACAGTTTTAATTGTAGAGGAGAG---
TTTAAACAGCTCCAGGCAAGAGTCCTGGCTGTGGAAAGATACCTGCACGATCAACAGCTCCTGGGGCTTTGGG
GTTGCTCTGGAAAACTCATTTGCACCACTGCTGTGCCTTGGAATGCTAGTTGGAGT-----------------
----------------------------AATAAAAATCTAACTCAGATTTGG------GAT------
AACATGACCTGGATGCAGTGG---GAAAAAGAAATTGACAATTACACAAACGTAATATAT---
ACCTTACTTGAAGAATCGCAGAACCAACAAGAA--

AAGAATGAACAAGAATTATTAGAATTAGATAAATGGGCAAGTTTGTGGAATTGGTTCAGCATAACACAATGGC
TGTGGTATATAAAAATATTCATAATGATAGTAGGAGGCTTGATAGGATTAAGAATAATTTTTGCT---
GTGCTTTCTATAGTA---------AATAGAGTTAGGCAGGGATACTCACCA------
TTATCGTTTGCTGTTAGCTTGTTCAATTTCATAGCCATAGCAGTAGCTGAG---

GGGACAGATAGGGTTATAGAAGTAGTACAAGGAGCTTGTAGAGCT----------------------------
--------ATTCTCAGCATA------CCTAGAAGAATAAGACAGGGCTTAGAAAGGCTTTTGCTA---TAA
>KP754469
------ACA--
GAAAACTTGTGGGTCACAGTCTATTATGGGGTACCTGTGTGG---AAAGAAGCAACC---
ACCACTCTATTTTGTGCATCAGATGCTAAAGCATATGATAAAGAG---GTACATAAT---------------
--------------GTTTGGGCCACA---CATGCCTGTGTACCTACAGAC---
CCTAACCCACAAGAAGTATTATTGGAA---------AATGTGACAGAACCTTTTAAC---------------
--------------------------GCATGGAAAAATGACATGGTAGAACAAATGCATGAGGATATA---
ATCAGTTTATGGGATCAAAGCCTA---AAACCATGTGTAAAATTA---
ACCCCACTCTGTGTCACTTTAAATTGCAGTGATTATAATAGCACTGGTGTTGCTTCTACTACTGTTGCTACTA
GCACTAGTACA--
----------------------CCTCATACTACTAGTAGTATTATAGAGAGAGAACAGATAAAA---
AACTGCTCTTTTAATGTTACCACAAGTATGAGA--------------------------
GACAAGGTGAAAAAAGAACATGCATTG---TTTTATTCAATTGATGTAGTACCGATAGAAGAAAAA-------
----

AATAGTAGTAGCTATATAATGAGAAGTTGTAATACCTCAGTCATTACACAA---
GCCTGTCCAAAAGTATCCTTTGAGCCAATTCCCATACATTTTTGTGCCCCGGCTGGTTTTGCGATTCTAAAAT
GTAAC---AATAAGACATTCAGT------GGAAAAGGAGAATGTAAAAAT-----------------------
-GTCAGC---ACAGTACAATGTACACATGGAATTAGACCAGTAGTATCAACT---
CAATTGCTGTTAAATGGCAGTCTAGCAGAAGAAGAG------------
GTGGTACTTAGATCTGACAATTTCACAAACAAT---------GCTAAAAACATAATAGTACAGCTGAAA----
-----------GAGCCTGTAAAA---------ATTAACTGTACAAGGCCCGGCAACAATACAAGAAAA-----
-AGTATACATATG------------GGACCAGGGAAAGCA---TTT------TATGCAACAGGAGAA------
---------ATAACAGGAGATATA------------AGAAGGGCATATTGT---ACACTTAATGGAACA---
CAATGGAACCAGACCTTAAAACAGGTAGTTGGAAAATTAAGA---------------GAAAAATTTAAT----
-----------------------------------AGCACTAAAATAGCCTTTAAT------
CGATCCTCAGGAGGGGACCCAGAAATTACA---ATGCACATTTTTAATTGTAGAGGGGAA---
TTTAAGCAGCTCCAGGCAAGAGTCCTGGCTCTGGAAAGATACCTAAAGGATCAACAGCTCCTGGGGATTTGGG
GCTGCTCTGGAAAACTCATCTGCACCACTACTGTGCCTTGGAATGCTAGCTGGAGT-----------------
----------------------------AATAAATCTTTCAATGATATTTGG------GAC------
AACATGACTTGGATGCAGTGG---GAAAAAGAAATTGACAATTACACAAACACAATATAC---
ACCTTACTTGAAAAATCGCAGAACCAACAAGAA--

GAGAATGAACAAAAAATTGTTGGAATTGGATAAATGGGACAGTTTGTGGAATTGGTTTGACATAACAAATTGGC
TGTGGTATATAAAAATATTCATAATGATAGTAGGAGGCTTAATAGGTTTAAGAATAGTTTTTTCT---
GTACTTGCTATAGTG---------AATAGAGTTAGGCAGGGATACTCACCA------
TTATCGTTTGCTGTTAGCTTGCTCAACGCCACAGCCATAACAGTAGCTGGG---
GGGACAGACAGAATTCTGGAAGTATTGCAAAGGGCTTATAGAGCT----------------------------
--------CTTTGTAACATT------CCTAGAAGAATAAGACAGGGCTTGGAACGGGCTTTGCTA---TAA
>KP754471
GCT---ACA---AAG------------------------------------
GAGGAAAAGTGGGTCACAGTCTATTATGGGGTACCTGTGTGG---AAAGAGGCAACC---
ACCACTCTATTTTGTGCATCAGATGCTAAAGCATATGATACAGAG---GCACATAAT---------------
--------------GTTTGGGCCACA---CATGCCTGTGTACCCACAGAC---
CCCAACCCACAAGAAATAAGATTGGAA---------AATGTGACAGAAAATTTTAAC---------------
--------------------------ATGTGGAAAAATAACATGGTAGAACAGATGCATGAGGATATA---
ATTAGTTTATGGGATCAAAGCCTA---AAGCCATGTGTAAAATTA---
ACTCCACTCTGTGTTACTTTAAATTGCACTAAGTTGAGTAGCAAAAATAGTACTAGCACCACCAATGGACCTA
GA---
----------------------CCCACTTTTGATAATGAGACAATAGCAAAAGAGGCACTAAGA---
AACTGCTCTTTTAATGTTACCACAAACATAAGA--------------------------
GATAAGGTAAAGAAAGAATATGCATTG---
TTCTATAGCCTTGATATAGTACCAATAAATGAAGATAATAATAAGACT------------------------
----
AATACTAGTTATGCTAGTTATAGGTTGACAAGTTGTAACACTTCAATCATTACGCAG---
GCCTGTCCAAAGACATCCTTTGAACCTATTCCTATACATTTTTGTGCTCCGGCTGGATTTGCGATTATAAAGT
GTAAC---AATAAGACATTCAGC------GTAAAAGGACCATGTAAAAAT----------------------
-GTCAGC---ACAGTACAGTGTACACATGGGATTAGGCCAGTAGTATCAACT---

CAACTGCTGCTAAATGGCAGTCTAGCAGAAGAAGAA-----------
ATAGTAATTAGATCTGACAATTTCTCGGATAAT---------ACTAAAACTATAATAGTACACCTGAAT----
-----------GAATCTATAGTA---------ATTAATTGTACAAGACCCAACAACAACACAAGAAAA-----
-AGTATACATATA------------GGACCAGGGAGAGCA---TTT------TATACAACAGGAGAC------
---------ATAATAGGAGATATA------------AGACAAGCACATTGT---AACATTAGTGAAGAA---
AAATGGAATAAAACTCTAAAACAGATAGCTAAAAAATTAAAA---------------GAACAATTT-------
-----------------------------------AATAAGACAATAATCTTTAAT------
CAATCCTCAGGAGGGGACCCAGAAATTGTA---ATGTACACTTTTAACTGTGGAGGGGAA---
TTTAAGCAGCTTCAGGCAAGGCTCCTGGCTGTGGAAAGATACCTAAATGATCAACAGCTCCTGGGGATTTGGG
GTTGCTCTGGAAAACTCATCTGCACCACTGCTGTGCCTTGGAATAATACTTGGAGT-----------------
-----------------------------AATAAAAGTCTGAGTACAATTTGG------AAT------
AACATGACCTGGATGGATTGG---GAAAGAGAAATTGACAATTACACAGACGAAATATAC---
AAATTGATTGAACAATCGCAGATCCAGCAGGAA--

AAGAATGAACAAGATTTATTGGCATTGGATAATTGGGCAAGTTTGTGGAATTGGTTTAGCATATCAAAATGGT
TGTGGTATATAAAAATATTCATAATGATAATAGGAGGCTTGATAGGTTTAAGAATAGTTTTTACT---
GTACTTTCTATAGTG---------AATAGGGTTAGGCAGGGATATTCACCA------
TTATCGTTTGCTGTCAGCTTGTTTAATACCACAGCAATAGTAGTAGCTGAG---
GGGACAGATAGGATTATAGAAGTAGGACAAAGAATTTTTAGAGCT----------------------------
--------GTTCTTAATATA------CCTAGAAGAATAAGACAGGGCTTTGAAAGGGCTTTGCTA---TAA
>FJ469744
GCT---ACA---
GACAAGTTGTGGGTCACAGTTTATTATGGGGTACCTGTGTGG---AAAGAAGCAAGC---
ACTCCTCTATTTTGTGCATCAGATGCTAAAGCATATGATACAGAG---GCACATAAT----------------
--------------GTTTGGGCCACA---CATGCCTGTGTACCCACAGAC---
CCCAACCCACAAGAAATAGTATTGGAA---------AATGTGACAGAAAATTTTAAC----------------
--------------------------ATGTGGAAAAATAACATGGTAGAACAGATGCATGAGGATATA---
ATCAGTTTATGGGATCAAAGCCTA---AAGCCATGTGTAAAGCTA---
ACCCCACTCTGTGTTACTATGAATTGCATTGATTATAATAATAATAGTACCAATACTACCAAT----------
----
-------------------------AGTGCCAATACCACTAGTAGTCCCCTAGGAGAAATAAAA---
AACTGCTCTTTCAATATCACCACAAACATAAGA--------------------------
GACAAGGTGCAGAAAGAACATGCACTT---TTTTATAGCCTTGATATAGTACCAATAAATAAT----------
----

GAGGGAACTAATACAAGCTATAGGTTGGTAAGTTGTAACACCTCAGTCATTACACAA---
GCCTGTCCAAAGGTATCTTTTGAACCAATTCCCATACATTTGTGTGCCCCGGCTGGTTTTGCGATTCTAAAAT
GTAAC---AATAAGGAGTTCAAT------GGAACAGGACCATGTAAAAAT----------------------
-GTAAGC---ACAGTACAGTGTACGCATGGAATTAGACCGGTAGTATCAACC---
CAACTACTGTTAAATGGCAGTCTAGCAGAGAAAGAG-----------
GTGGTAATTAGGTCTGCCAATTTCTCGGACAAT---------GCTAAGACCATAATAGTACAGCTGAAT----
-----------GCATCAGTACAA---------ATTAATTGTACAAGACCAAACAACAATACAAGAAAA-----
-GGTATACACATA------------GGACCAGGCAGAACA---TGG------TATGCAACAGGAGGA------
---------ATAATAGGAGATATA------------AGACAAGCACATTGT---AACCTTAGTAGAGAA---
GCATGGGAGAACACTTTAAAACAGGTAGCTATAAAACTAGGC---------------GAACAATTTAAG----
-----------------------------------AATAAAAAAATAGCCTTTAAT------
CGATCCTCAGGAGGGGACCCAGAAATTGTA---ATGCACAGTTTTAATTGTGGAGGGGAA---
TTTAAACAGCTCCAGGCAAGAGTCCTGGCTCTGGAAAGATACCTAAAGGATCAGCAGCTCCTGGGGATTTGGG
GTTGCTCTGGAAAACACATCTGCACCACTGCTGTGCCTTGGAACACTAGTTGGAGT-----------------
-----------------------------AATAAATCTTTGAATGCAATTTGG------GAT------
AACATGACCTGGATGGAGTGG---GACAGAGAAATTAGCAATTACACAAACTTAATATAC---
TCCTTAATTGAAGAATCGCAGATCCAACAAGAT--

AAGAATGAAAAAGAACTATTAGAATTAGATAAATGGGCAAGTTTGTGGAATTGGTTTAGTATAACAAATTGGC
TGTGGTACATAAGATTATTCATAATGATAGTAGGGGGCTTGATAGGTTTAAGAATAGTTTTTACT---
GTACTTTCTATAGTG---------AATAGAGTTAGGCAGGGATACTCACCA------
TTATCGTTTGCTGTTAGTTTGCTTAATGCCACAGCCATAGCAGTAGCTGAA---
GGGACAGATAGGATTATAGAAGTAGCACAAAGAGCTTTTAGAGCT----------------------------
--------ATTATCCACATA------CCTACAAGAATAAGACAGGGCTTGGAAAGAGCTTTGCTA---TAA
>FJ687543

GCT---ACA---
GAAAAATTGTGGGTCACAGTCTATTATGGGGTACCTGTGTGG---AAGGAAGCTAAC---
ACCACTCTATTTTGTGCATCAGATGCTAAAACATATGATACAGAG---GCACATAAT------------------
--------------GTTTGGGCCACA---CATGCCTGTGTACCCACAGAC---
CCCAACCCACAAGAAGTAGTAATGACC---------AATGTGACAGAAAATTTTAAC------------------
---------------------------ATGTGGAAAAATAACATGGTAGAACAAATGCATGAGCATATA---
ATCAATTTATGGGATCAAAGCCTA---AAGCCATGTGTAAAATTA---
ACCCCACTCTGTGTTACTTTAAATTGCAGTGATGTAAATGGCACTAGTAGTAATACCGCTAAA------------
----
-----------------------AACATAAGAAACGAGACAATAATGGAGAAAGGAGAAATCAAA---
AACTGCTCTTTCAATATCACCACAGGCATAAGA----------------------------
GATAGGGTGCAGAAACAATATGCAACA---TTTTATAGACTTGATGTAGTACAAATAGATGAT------------
----

AAGCATAAGACTAGATATAGGATGACAAGTTGTAATGCCTCAGTCATTACACAG---
GCCTGTCCAAAGATATCCTTTGAGCCAATTCCCATACATTTTTGTGCCCCGGCTGGTTTTGCGATTCTAAAGT
GTAAA---GAAAAGGGGTTCAAT------GGAACAGGAAAATGTAAAAT----------------------------
-GTCAGC---ACAGTACAATGCACACATGGAATTAGGCCAGTAGTATCAACT---
CAATTGCTGTTAAATGGCAGTCTAGCAGAAGAAGGG------------
GTAGTAATTAGATCTCAAAACTTCACAGACAAT---------ACTAAAACCATAATAGTACAGCTGGAC----
-----------GCAGCTATAAAA---------ATTAATTGTACAAGACCCAACAATAATACAAGAAAA-----
-AGGATGAGTCTA------------GGTCCAGGGAAAGTA---TTT------TATACAACAGGACAA------
---------AAAATGGAGATATA------------AGAAAAGCACATTGT---AACCTTAGTGCAGGA---
GAGTGGTATAGCGCTTTAAAAAGGATAGCCATAAAATTAAGA---------------GCACAATTTAAC----
------------------------------------AATAAAACAATAGTCTTTAAG------
AACTCCTCAGGAGGGGACTCAGAAATTGTA---ACCCACAGTTTTAATTGTGGAGGGGAA---
TTTAAGCAGCTCCAGGCGAGAGTCCTGGCTGTGGAAAGATACCTAAGGGATCAACAGCTCCTGGGGCTCTGGG
GTTGCTCTGGAAAGCTCATTTGCACCACTTCTGTGCCTTGGAATACTAGTTGGAGT-----------------
----------------------------AATAAATCTCTGAATGAGATTTGG------GAT------
AACATGACCTGGAAGGAATGG---GACAGAGAAATTAACAAGTACACAAATATAATATAC---
TCCTTAATTGAAGAATCGCAGAACCAGCAAGAA--

AAGAATGAACAAGAATTATTGGCATTAGATAAGTGGGCAAGTTTATGGAATTGGTTTGACATAACAAAATGGC
TGTGGTATATAAAAATATTCATAATAATAGTAGCAGGCTGGATAGGTTTAAGAATAGTTTTTAGT---
GTACTTTCTATAGTG---------AATAGAGTTAGGCAGGGATACTCACCT------
TTATCGTTTGCTGTTAGCTTGCTCAATGCCACAGCCATAGCAGTAGCTGAG---
GGGACAGATAGGGTTATAGAAGTAGTACAAGGAGCTTGTAGAGCT-----------------------------
--------ATTCGCCACATA------CCTAGAAGAATAAGACAGGGCTTGGAAAGGATTTTGCTA---TAA
>KF725982
GCA---GAC--
CAATTGTGGGTCACAGTCTATTATGGGGTACCTGTGTGG---AAAGAAGCAACC---
ACCACTCTCTTTTGTGCATCAGATGCTAAAGCATATGATACAGAG---GCACATAAT------------------
--------------GTTTGGGCCACA---CATGCCTGCGTACCCACAGAC---
CCCAACCCACAAGAAATAAGACTGGAC---------AATGTGACAGAAAATTTTAAC------------------
---------------------------ATGTGGAAAAATAACATGGTAGAACAGATGCATGAGGATATA---
ATCAGTTTATGGGATCAAAGCTTA---AAGCCATGTGTAAAATTA---
ACCCCACTCTGTGTTACTTTAAACTGTATTAATGCTACTCTCGATTGCAATAATACTGCCAAT------------
----
-------------GTCACTAATTTCAATAAAACTCTTTGCAAGGAGATGGGGACGGAAATAAGA---
AACTGCTCTTTCAATGCCAGCACAGAAATAAGA----------------------------
GATAAGATAAGGAAGGAATATGCACTT---TTTTATAAAACTGATTTAGTATCAATAGATAACAAT-------
----

GACAATACTAGCTTTAGGTTGATAAGTTGTAACACCTCAGTCATTACACAG---
GCCTGTCCAAAGACATCCTTTGAGCCAATTCCCATACATTATTGTGCCCCGGCTGGTTTTGCGATTCTAAAAT
GTAGA---GATAAGAAGTTCAAT------GGAACAGGACCATGTACAAAT----------------------------
-GTCAGC---ACAGTACAATGTACACATGGAATTAAGCCAGTAGTATCAACT---
CAGCTGCTGTTAAATGGCAGTTTAGCAGAAGAAGAG------------
GTAGTAATTAGATCTAGCAATTTCACAGACAAT---------GCTAAAATCATAATAGTACAGCTGAAA----
-----------GAAGCTGTAAAA---------ATTAATTGTACAAGACCCAGTAACAATACAAGAAGG-----

-AGTATACATATA------------GGACCAGGGAGAGCA---TTC------TATGCAACAGGAGCC------
---------ATAATAGGAGATATA------------AGACAAGCACATTGT---AACCTTAGTATAGGG---
GAATGGAAGAAAGCTTTAAATCAAACAGAGGAAAAATTAAAA---------------GAACAATTTAAT----
----------------------------------AATAAAACAATACTCTTTAAA------
CCATCCTCAGGAGGGGACCTAGAAATTGTA---ACACACAGTTTTAATTGTGGAGGGGAA---
TTTAAGCAGCTCCAGGCAAGAGTCCTAGCTGTGGAAAGATACCTAAAGGATCAACAGCTCCTGGGGATTTGGG
GTTGCTCTGGAAAACTCATTTGCACCACTGCTGTGCCTTGGAATGCTAGTTGGAGT-----------------
----------------------------AATAAATCTCATGATCAGATTTGG------AAT------
AACATGACCTGGATGGAGTGG---GAAAGAGAAATTGACAATTACACAAGCTTAATATAC---
TCCTTAATTGAAGAATCGCAGAACCAGCAAGAA--

AAGAATGAACAAGAATTATTGGAATTAGATAAATGGGCAAGTTTGTGGAATTGGTTTGATATATCAAACTGGC
TGTGGTATATAAGAATATTCATAATGATAGTAGGAGGCTTGATAGGCTTAAGAATAATTTTTGCT---
GTACTTTCTTTAGTG---------AATAGAGTTAGGCAGGGATACTCACCA------
TTATCGTTTGCTGTTAGTTTGCTCAACGCCACAGCTATAGCAGTAGCTGAG---
GGGACAGATAGAATTATAGAATTCTTACAAAGAGTTTTTAGAGCT----------------------------
--------TTTATCCACATA------CCTAGAAGAATCAGACAGGGCTTTGAAAGGGCTTTGCTA---TAA
>KF725986
GCT---GCA--
GAAAAATTTTGGGTCACAGTCTATTATGGGGTACCTGTGTGG---AAAGAAGCTAAC---
ACCACTCTATTCTGTGCATCAGATGCTAAAGCATATGACACAGAG---GCCCATAAT----------------
--------------GTTTGGGCCACA---CATGCCTGTGTACCCACAGAC---
CCCGACCCACAAGAAATGGTATTGGCA---------AATGTAACAGAAAATTTTAAC----------------
-------------------------GTGTGGAAGAATGACATGGTAGAACAGATGCATGAGGATATA---
ATCAATTTATGGGATCAAAGCCTA---AAGCCATGTGTAAAATTA---
ACCCCACTCTGTGTCACTTTAAACTGCACTAATATATTGAATAAGACTGATACTAATAAGAATACT-------
----
----------------------AGTAGTAGCAATAACTCGAGAATAGATTATGAAGAAATGAAA---
AATTGTTCTTTCAACATCACCACAGGCATAAAA--------------------------
GATAAAATGAGGAAGGAATATGCACTT---TTTTATAAACTTGACCTAGAACAAACAGGAGAT----------
----

GATAATACAAGTTATACATTAATAAAGTGTAACACCTCAGTTATTACACAG---
GCCTGTCCAAAGATATCCTTTGAACCAATTCCCATACACTTTTGTGCCCCAGCTGGTTTTGCGATTCTAAAAT
GTAAC---AATAATACATTCAAT------GGATCAGGACCATGTACAAAT----------------------
-GTCAGC---ACTGTGCAATGTACACATGGAATCAGGCCAGTAGTGTCAACT---
CAACTGCTGTTAAATGGCAGTCTATCAAAAGGAGAC------------
GTGGTAATTAGATCTGAAAATTTCACAGACAAT---------GCAAAAACCATAATAGTACAGCTGAAT----
-----------GCAACTGTAAAA---------ATTAATTGTACAAGACCCAACAACAATACAAGAAAA-----
-GGTATACATATG------------GGATGGGGAAGAACA---TTT------TATGCAACAGGAGAA------
---------ATAATAGGAGACATA------------AGACAAGCACATTGT---AACATTAGTAAGACA---
GAATGGAATAACACTTTAAGATGGGTAGCAGAAAAAATTAAGA---------------GGACAGCTTAAT----
----------------------------------ATAACAACAATAATCTTTAAT------
CAATCCTCAGGAGGGGACCCAGAAATTGTA---ATGCACCATTTTATTTGTGGAGGGGAA---
TTTAAACAGCTCCAGGCAAGAGTCCTGGCTGTGGAAAGATTCCTAAGGGATCAACAGCTCCTAGGGATTTGGG
GTTGCTCTGGAAAACTCATTTGCACCACTAATGTGCCTTGGAATACTAGTTGGAGT-----------------
-------------------AGGAGT---AGTGATAATCTGAATGCAATTTGG------AAT------
AACATGACCTGGATGGAGTGG---GAGAGAGAAATTGACAATTACACACAAACAATATAT---
ACCTTACTTGAAGAATCGCAGAACCAACAAGAA--

AAGAATGAAAAAGATTTATTAGAATTGGATAAGTGGGACAGTCTGTGGAATTGGTTCAACATAACAAACTGGC
TGTGGTATATAAAAATATTCATAATGATAGTAGGGGGATTAATAGGTTTAAGGATAGTTTTTACT---
ATAATTTCTATAGTG---------AATAGAGTTAGGCAGGGGTACTCACCA------
CTGTCATTCGCTGTTAGTTTGTTTAATGCCATAGCTATAGCAGTAGCTGAA---
GGGACAGATAGAGTAATAGAAGTAGTACTAAGAGTTGGGAGAGGC----------------------------
--------ATTCTCCACATA------CCTACAAGAATAAGACAGGGTTTAGAAAGGCTTTTGCTA---TAA
>KF725988
GCT---GCA---AAA------------------CAA---TCT---GCA---
GAGCAGTTGTGGGTCACAGTCTATTATGGGGTACCTGTGTGG---AAAGACGCCAAC---
ACTACTCTATTCTGTGCATCAGATGCTAAAGCATATGATACAGAG---GCACATAAT----------------

--------------GTTTGGGCCACA---CATGCCTGTGTACCTACAGAC---
CCCAACCCACAAGAAGTAGATTTGGTA---------AATGTGACAGAAGATTTTAAC------------------
---------------------------ATGTGGAAAAATAACATGGTAGAACAGATGCATGAGGATATA---
ATTAGTTTATGGGATCAAAGCCTA---AAGCCATGTGTAAGATTA---
ACCCCACTCTGTGTTACTTTAAAGTGTGATAATACTACTAATAGTACCACTAGTACC------------------
----
--CCTTTCAAAGGAGGAATGACA---
AACTGCTCTTTCAATATCACCACAAGCATGGA-----------------------------
GATAAGATGCAAAGAGAATATGCATTG---TTTTATGAACTTGATGTAGTACCAATAGATAAT----------
----

AGTACCTATAGATTAATTAGTTGTAATACCTCAGTCGTTACACAA---
GCCTGTCAAAAAATATCTTTTGATCCAATTCCCATACATTATTGTGCCCCGGCTGGTTTTGCAATTCTAAAAT
GTAAA---GATAAGAGGTTCAAT------GGAACAGGATCATGTAAAAAT-------------------------
-GTCAGT---ACAGTACAATGTACACATGGAATTAGACCAGTAGTGTCAACT---
CAACTGTTGCTAAATGGCAGTCTAGCAGAAGAACAA------------
GTAGTAATCAGATCTGAAAATTTCTCGGACAAT---------GCTAAAAACATAATAGTACAGCTGAAG----
-----------GAAGCTGTACAA---------ATTAATTGTACGAGACCCAACAACAACACAAGGAAA-----
-AGTATAAATATG------------GGACCAGGGAGGGCA---TTT------TATACAACAGGACAA------
---------ATAATAGGAGATATA------------AGACAAGCACATTGT---AACATTAGTGAGAAA---
AAATGGAATAGCACTTTAAAACAGATAGCTGAAAGATTAAGA---------------GAACAATTTAAG----
------------------------------------AATAAAACAATAGTCTTTACG------
AATTCCTCAGGAGGGGATCCAGAATTG-TA---ACGCACAGTTTTAATTGTGGAGGGGAG---
TTTAAACAGCTCCAGGCAAGAATCCTGGCTGTGGAAAGATACCTCAGGGATCAACAGCTCCTGGGGATTTGGG
GATGCTCTGGGAAACTCATTTGCACCACTGCTGTGCCTTGGAATAATAGTTGGAGT-----------------
-----------------------------AATAGAACTCTAGAGGACATTTGG------GGG------
AACATGACCTGGAAGCAGTGG---GAAACAGAAATTGATAATTACACAAGTTTAATATAC---
ACCTTAATTGAAGAATCGCAGAACCAGCAAGAA--

AAGAATGAACAAGAATTATTGGCATTAGATAAATGGGCAAGTTTGTGGAATTGGTTTGACATAACAAACTGGC
TGTGGTATATAAAAATATTCATAATGATAGTAGGAGGCTTGATAGGTTTAAGAATAGTTTTTACT---
GTACTTTCTATAGTG---------AATAGAGTTAGGCAGGGATACTCACCA------
TTGTCGTTTGCTGTTAGTTTGCTCAATACCATAGCTATAGCAGTAGCCGAG---
GGGACAGATAGGATTATAGAAGTAATACAAGGAATTTTTAGAGCT------------------------------
--------ATTCGCCACATA------CCTAGAAGAATAAGACAGGGCTTGGAAAGGATTTTGCTA---TAA
>KU168260
GCT---GCA---
GATAAATTGTGGGTCACAGTCTATTATGGGGTACCTGTATGG---AAAGAAGCCAAT---
ACCACTCTATTTTGTGCATCAGATGCTAAAGCACATGATACAGAG---GTACATAAT------------------
--------------GTTTGGGCCACG---CATGCCTGCGTACCCACAGAC---
CCCAACCCACAAGAAGTGTTATTGGAA---------AATGTGACTGAAGAGTTTAAC------------------
---------------------------ATGTGGAAAAATAACATGGTAGACCAGATGCATGAGGATATA---
ATCAGTTTATGGGATGAAAGCTTA---AAGCCATGTGTAAAATTA---
ACCCCGCTCTGTGTTACTTTAAATTGCACTGATTTGACAAATGCTACTTTGAGGAATGCTACT----------
----
--GAAATGAAA---
AACTGCTCTTTCAAAACCACCACAAGCTTAAGA----------------------------
GATAAGGCTCAGAAAGAATATGCACTT---TTTTATAAACTTGATGTAGTACCAATAGATGAG----------
----

GCTAAGAATACTACTAACTTTAGATTGATACATTGTAACACCTCAGTCATTACACAG---
GCCTGTCCAAAGATATCATTTGAACCAATTCCCATATATTATTGTGCCCCGGCTGGTTTTGCGATTCTAAAGT
GTAGA---GATAGGAAGTTCAAT------GGAACAGGAATATGTAAAAAT-------------------------
-GTCAGC---ACAGTACAATGTACACATGGAATTAGGCCAGTAGTGTCAACT---
CAACTGCTGTTAAATGGCAGTCTAGCAGAAGAAGAA------------
GTAGTAATTAAATCTGAAAATATCTCGGACAAT---------ACTAAAACCATAATAGTACAGCTGAAA----
-----------AACCCTGTAAAC---------ATTACTTGTACCAGACCCAGCAACAATACAAGAAAA-----
-CGTATAGCGATA------------GGGCCAGGAAGAGCA---GTT------TATACAACAGAACAA------
---------ATAATAGGAGATATA------------AGACAAGCACATTGT---AACCTTAGTGAATCA---
AAATGGAATAAAACTTTACAACAGATAGTTGAAAAATTAAGA---------------GTACACTTTAAG----

-----------------------------------AATAAAACAATAGCCTTTAAT------
AAATCCTCAGGAGGGGACCCAGAAATTGTA---ATGCACAGTTTTAATTGTGGAGGGGAA---
TTTAAGCAGCTCCAGGCAAGAGTCCTAGCTGTGGAAAGATACCTAAGGGATCAACAGCTCCTAGGGATTTGGG
GATGCTCTGGGAGACTCATCTGCACCACTGCTGTGCCTTGGAATGCTAGTTGGAGT-----------------
----------------------------AATAGAAATCTGACTCAGATTTGG------GAT------
AACATGACCTGGATGCAGTGG---GAAAAAGAAATTGACAATTACACAGACTTAATATAC---
TCCTTACTTGAAGAATCGCAAAACCAACAAGAA--

AAGAATGAACAAGAATTATTAGAATTGGATAAGTGGGCAAGTTTGTGGAATTGGTTTGACATATCAAATTGGC
TGTGGTATATAAAAATATTCATAATGATAGTAGGAGGCTTGATAGGCTTAAGAATAGTTTTTGCT---
GTATTTTCTATAGTG---------AATAGAGTTAGGCAGGGATACTCACCA------
TTGTCATTTGCTGTCAGCTTGTTCAACGCCACCGCTATAGCAGTAGCTGAG---
GGAACAGATAGGGTTATAGAAGTATTACTAAGAATTTTTAGAGGA----------------------------
--------ATCCTCCACATA------CCTAGAAGAATAAGACAGGGCTTTGAAAGGTCTTTGCTA---TAA
>KT452481
GCA---GGG--
GAAGATTCGTGGGTCACAGTCTATTATGGAGTACCTGTGTGG---AAAGAAACAACC---
ACCACTTTGTTTTGTGCATCAGATGCTAAGGCATATGACACAGAA---GTACATAAT----------------
--------------GTTTGGGCCACA---CATGCCTGTGTACCCACAGAC---
CCCAGTCCACAAGAAGTATCCTTGCCA---------AATGTGACAGAAAATTTTAAC----------------
--------------------------ATGTGGAAAAATAACATGGTAGAACAGATGCATGAGGATATA---
ATCAGTTTATGGGATCAAAGCTTA---ACGCCATGTGTAAAATTA---
ACCCCACTCTGTGTTACTTTAAATTGCACTGATGTGAATACCACTAGGAATGCTAATACT-------------
----
----------------------AATAATACATGGGGAACGGTAATGGAGAAAGGAGAAGTAAAA---
AATTGTTCTTTCAATATCACCACAAGCATAAGA---------------------------
GATAAAAGGCAGAAAGAATTTGCATTG---TTTTATAGACCTGATGTAGTACCAATAGATGGA----------
----

GATAATAATACCTATAGGTTGATAAGTTGTGACACCTCAGTCATTACTCAA---
GCCTGTCCAAAGATATCCTTTACACCAATTCCCATACATTATTGTGCTCCAGCTGGTTTTGCGATTCTAAAAT
GTAAT---GATAAGAAGTTCAAT------GGAACAGGACCATGTAGAAAT-----------------------
-GTTAGC---ACAGTACAATGTACACATGGAATTAAGCCAGTGGTATCAACT---
CAACTGCTGTTAAATGGCAGTCTAGCAGAAAAGGAG------------
ATAGTAATTAGATCTGCCAATTTCTCAGACAAT---------GCTAAAATCATAATAGTACAGTTGAAT----
-----------GAATCTGTCCAA---------ATTGATTGTATAAGACCCAACAACAATACAAGAAAA-----
-AGTATAAACATA------------GGACCAGGGAGAGCA---TGG------TATACAACAGGAGAC------
---------ATAATAGGAGATATA------------AGACAAGCACACTGT---AACATTAGTGCAGCA---
AAATGGAATCAGGCATTACAAAGGATAGTTGTAAAATTAAGA---------------GAACAATTTGGG----
--------------------------------GAAAATACAACAATAGCTTTTAAT------
CAATCCTCAGGAGGAGACCCAGAAATTGTA---ACACACAGTTTTAATTGTGGAGGGGAA---
TTTAAGCAGCTCCAGGCAAGAGTCCTGGCTGTGGAAAGATACCTAAAGGATCAACAGCTCCTGGGAATTTGGG
GTTGCTCTGGAAAGCTCATTTGCACCACTACTGTGCCTTGGAATAGTAGTTGGAGT-----------------
----------------------------AATAAACCCTTGGATCAAATTTGG------GAT------
AACATGACCTGGATGGAATGG---GAAAGAGAAATTGACAATTACACAGACATAATATAT---
AACTTACTTGAAAAAGCACAAAACCAGCAGGAT--

AAAAATGAACAAGAATTATTGGAACTAGATAAATGGGCAAATCTTTGGAGTTGGTTTGACATAACAAACTGGC
TATGGTATATAAAAATATTCATAATGATAGTAGGAGGCTTGGTAGGTTTAAAAATAGTTTTTACT---
GTGCTTTCTATAGTG---------AATAGAGTTAGGCAGGGATACTCACCA------
TTATCGTTTGCTATTAGCTTGTTCAACGCCACAGCCATAGCAGTAGCTGAG---
GGAACAGATAGGATTATAGAAATAATACAAAGAACTTTTAGAGCT----------------------------
--------ATTATCCACATA------CCTAGAAGAATTAGACAGGGCTTGGAAAGGCATTGCTA---TAA
>KT452508
ACT---GCA--
GGAAATTTGTGGGTCACAGTTTATTATGGGGTACCTGTGTGG---AAAGAAGCAACT---
ACCACTCTATTCTGTGCATCAAATGCTAAAGCATATGAGAAAGAG---GCACATAAT----------------
--------------GTTTGGGCCACA---CATGCCTGTGTACCCACAGAC---
CCCGACCCACAAGAAATGTTCTTG------------AATGTGACAGAAAATTTTAAC----------------
--------------------------ATGTGGGAAAATAACATGGTAGACCAGATGCATGAGGATATA---

ATTAGTTTATGGGATCAAAGCCTA---AAGCCATGTGTAAAATTA---
ACCCCACTCTGTGTTTCTTTAGAATGCACTAATGCAACTAATATAAATATTACTAAGAAT------------
----
---ATGGAAATGAAA---
AATTGCTCTTTCAATATGACCACAAGCATAAGG--------------------------
AATAAGCCGCAGAGAAACTATGCACTT---TTTTATAGCTATGATATAATACCAATAGAAAATGATACA----
----

GATGATGGTAATATTAGCTATAGGTTGAGAAGTTGTAACACCTCAATCATTACACAA---
GCCTGTCCAAAGATATCCTTTGAGCCAATTCCCATACATTATTGTGCCCCGGCTGGTTTTGCGATTCTAAAGT
GTAAT---AATAAGAAGTTCAAT------GGATCAGGGCCATGTACAAAT----------------------
-GTCAGC---ACAGTACAATGTACACATGGAATTAAGCCAGTAGTATCAACC---
CAACTGCTGTTAAATGGCAGTCTAGCAGAAGAAGGG-----------
GTAGTAATTAGATCCGCCAATTTCTCGGACAAT---------AAACATAACATAATAGTACAGCTGAAT----
-----------AAACCTGTAGAA---------ATTAATTGTACAAGACCCAGCAACAATACAATAAGA-----
-AGTATACACATG------------GGACCAGGCAGAGCA---TTT------TCTACAACAGGAGAT------
---------ATAATAGGAGATATA------------AGGAAAGCATATTGT---AACATTAGTGAGGCA---
AAATGGAATAACACTTTAAAACAGATAGCTGACAAATTAATA---------------GAACAATTTAAT----
-------------------------------ACTACAGGTCCAATAGTCTTTAAT------
CAATCCTCAGGAGGAGACCCAGAAATTGTA---ATGCACACTTTTAATTGTGGAGGGGAA---
TTTAAGCAGCTCCAGGCAAGAGTCCTTGCTCTGGAAAGATACCTAAAGGATCAACAGCTCCTGGGAATTTGGG
GTTGCTCTGGAAAACTCATCTGCACCACTAATGTGCCTTGGAATACTAGTTGGAGT-----------------
---------------------------AATAAATCTTATGAGGATATTTGG------GGG------
AACATGACCTGGATGCAGTGG---GAAAGGGAAATTGATAATTACACAGACAAAATATAC---
ACATTGCTTACAGAATCGCAAATCCAACAAGAA---------------------------------------
----
AAGAATGAGCAAGACTTATTGGCATTAGATAAATGGGACAGTCTATGGAGTTGGTTTAACATATCAAATTGGC
TGTGGTACATAAGAATATTCATAATGATAGTAGGAGGCTTGATAGGTTTAAGAATAGTTTTTACT---
GTACTTTCTATAGTG---------AATAGAGTTAGGCAGGGATACTCACCA------
TTATCGTTCGCTGTTAGCTTGTACAACGCTATAGCCATAGCAGTAGCTGAA---
GGGACTGATAGGATTTTAGAAGTATTCCAGAGAGCTTATAGAGCT---------------------------
--------ATTCTCCACATA------CCTAGAAGAATAAGACAGGGCTTAGAAAGGGCTTTGCTA---TAA
>KT452155
GCT---GCA--
GAACAAACGTGGGTCACAGTTTATTATGGGGTACCTGTGTGG---AAAGAAGCAAAC---
ACCACTCTATTTTGTGCATCAGATGCTAAAGCATATGTTACAGAG---AAACATAAT----------------
--------------GTTTGGGCCACA---CATGCCTGTGTACCCACAGAC---
CCCAACCCACGAGAAATACAATTAGAA---------AATGTGACAGAATACTTTAAC----------------
--------------------------ATGTGGAAAAATAACATGGTAGAACAGATGCATGAGGATATA---
ATCAGTTTATGGGATCAAAGCCTA---AAGCCATGTGTAAAACTA---
ACCCCACTTTGTGTTACTCTACAATGCGCAAATATTGTTTGCAATACCACTAATAATACCACTAATAATACCA
CTGATAGTAATAAGATACATTGCAAGTCTGTTAATAAT------------------------------------
-ACCAGTAGTGTTAAGGGGACAAATACCAGTAGTGTTACTGGGACAATAGTGGAGGGAATGAAA---
AACTGCTCTTTCAACATTACCACAGACTTAAGA--------------------------
GATAAGGTGAGGAAAGAATATGCACTG---TTTTATAGCCTTGATATAGTACCAATAGAAAATGAC-------
----

AATAATAACAGTGATAGCTATAGGTTGACAAGTTGTAACACCTCAATCATTACACAG---
GCCTGTCCAAAGGTAACCTTTGAGCCAATTCCTATACATTATTGTACTCCGGCTGGTTTTGCACTCCTAAAAT
GTAAT---GATAAGAATTTCAAT------GGGAATGGAACATGTAACAAT----------------------
-GTCAGC---TCCGTACAATGTACACATGGAATTAGACCAGTAGTGTCAACT---
CAGCTGCTGTTAAATGGTAGTCTAGCAGAGGAAGAG-----------
GTAGTAATTAGGTCTGCAAATTTCACAGACAAT---------GCTAAAACCATAATAGTACAGCTAAAT----
-----------GAATCTGTAGTA---------ATTAATTGCACAAGACCCAACAACAATACAAGAAAA-----
-AGTATTTCCATA---------GGAGGACCAGGGAGAGCA---TTC------TATGCAACAGGAGAC------
---------GTAATAGGAGATATA------------AGGCAAGCATATTGT---AACATTAGTAGAACA---
CAATGGAATAGGACTTTAGGAATGGTAGCTGCAAAATTAGGA---------------AAACATTTT-------
-----------------------------------AATAAAACAATAATCTTTACT------
CAATCCTCAGGAGGGGACCCAGAAATTGTA---ATGCACAGTGTTAACTGTGGAGGGGAA---
TTTAAGCAGCTCCAGGCAAGACTCCTGGCTGTGGAAAGATACCTAACGGACCAACAGTTCCTAGGGCTTTGGG

GATGCTCTGGAAAACGCATTTGCACCACTAATGTGCCTTGGAATCTTAGTTGGAGT-----------------
-----------------------------AATAAAACTCAAGATCAAATTTGG------CAG------
AACATGACCTGGATGCAGTGG---GAAAAAGAAATTGAAAATTACACAGGGGTAATATAC---
ACCTTACTTGAAAACTCGCAAAACCAGCAGGAG---

CAGAATGAAAAGGAATTGTTGGAATTGGATAAGTGGGCAAGTTTGTGGAGTTGGTTTAACATAACACAATGGC
TGTGGTATATAAAAATATTCATAATGATAGTAGGAGGCTTGATAGGTTTAAGAATAGTTTTTGCT---
GTGCTTTCTATAGTG---------AATAGAGTTAGGCAGGGTTACTCACCA------
TTGTCATTTGCTGTCAGCTTGCTTAACGCCACAGCTATAGCAGTAGCTGAA---
GGGACAGATAGGATTATAGAAGTAGCACAAAGAATTTTTACAGCT----------------------------
--------ATTCGCAATATA------CCTAGGAGAATAAGACAGGGCTTGGAAAGGGCTTTATTA---TAA
>U39243
GTG---ATG--
GGGAACTTGTGGGTCACAGTATATTATGGGGTACCTGTGTGG---AAAGAAGCAAAA---
ACTACTCTATTTTGTGCATCAGATGCTAAAGCATATGATACAGAA---GTGCATAAT----------------
--------------GTCTGGGCTACA---CATGCCTGTGTACCCACAGAC---
CCCAGCCCACAAGAAATGCATTTGGAA---------AATGTAACAGAAAATTTTAAC----------------
--------------------------ATGTGGGAAAATGACATGGTGGAACAGATGCATCAGGATATA---
ATCAGTTTATGGGATCAAAGCCTA---AAGCCATGTGTAAAGTTG---
ACCCCACTCTGTGTTACTTTAAACTGTAGCAATGCTAAGAAAGATAATACTACC-------------------
----
----------------------------------ATTGATAATGAAATGAAGGGAGAAATAAAA---
AATTGCTCTTTCAATATAACCACAGAAATAAGA---------------------------
GATAAGAGGCAGAAAGTACATGCACTT---
TTTTATAAACTTGATATAGTACCACCTAATGAGGGGAATAACAAA----------------------------
----
TCTAATGGGGGTAATGACAGTTCTAATGAGTATATATTAATAAATTGTAATACCTCAGCCGTAACACAA---
GCCTGTCCAAAGGTCTCTTTTGACCCAATTCCTATACATTATTGTGCTCCAGCGGGTTATGCGATTCTAAAAT
GTAAT---AATAAGACATTCAAT------GGAACAGGACCATGCAATAAT----------------------
-GTCAGC---ACAGTACTATGTACACATGGAATTAAGCCAGTGGTATCAACT---
CAACTATTGTTAAATGGTAGTTTAGCAGAA---GAG-----------
ATCATAATTAGATCCAAAAATTTGTCAGACAAT---------GCTAAAGTAATAATAGTACATCTTAAG----
-----------GAACCTGTAGAA---------ACTGAGTGTACGAGACCCAACAATAATACAAGAGAA-----
-AGTATAAGGATA------------GGACCAGGACAAGCA---TTC------TATGCAACAGGAGAC------
---------ATAATAGGAGATATA------------AGACAAGCATATTGT---ACCATTAATGGAACA---
AAATGGAATAAGACTTTACAAGAGGTAGGTAAAAAACTAGCA---------------GAGCAATTCAAC----
-----------------------------------AGTTCAAAAATAAAATTTGCA------
CCATCCTCAGGAGGGGACCTAGAAGTTACA---ACACATAGCTTTAATTGCAGAGGAGAA---
TTTAAGCAGCTTCAGACACGAGTCCTGGCTATAGAAAGATACCTAAGGGATCAACGGCTCCTAGGGATTTGGG
GCTGCTCTGGAAAACTCATCTGCACCACTGATGTGCCTTGGAACGCTAATTGGAGT----------------
-----------------------------AATAAATCTCAAGAGGATATTTGG------CAT------
AACATGACCTGGATGCAATGG---GATAGAGAAATTAGTAATTACAAAGGCATAATATAC---
GGATTGCTTGAAGACTCGCAGACCCAGCAGGAA---

AACAATGAAAAAGAGTTACTAGAATTGGACAAGTGGCAAAATCTGTGGAGTTGGTTTGACATAACAAATTGGC
TGTGGTATATAAAAATATTTATAATGATAGTAGGAGGCTTGATAGGTTTAAGAATAATTTTTGCT---
GTGCTCTCTATAGTG---------AATAGAGTTAGGCAGGGATACTCACCC------
TTATCGTTTGCTATTAGTCTGCTTGACGCCATAGCAATAGCAGTAGGTGAA---
GGAACAGATAGGATTATAGAATTAATACAGAGAATTTGTAGAGCT----------------------------
--------ATCTACAACATA------CCTAGAAGAATAAGACAGGGCTTGGAAGCAGCTTTGCAA---TAA
>U39240
GTA---ATG--
GGGAACTTGTGGGTCACAGTCTATTATGGGGTACCTGTGTGGAAAGAAGCACAACCTATACCTACTCTATTTT
GTGCATCAGATGCTAAAGCATATGAGACAGAA---ATGCACAAT-----------------------------
-GTCTGGGCTACA---CATGCCTGTGTACCCACAGAC---CCCAACCCAAACGAATTGGTTTTGGGA------
---AATGTAACAGAAAATTTTAAC--
ATGTGGAAAAATGACATGGTGGAACAGATGCATCAGGATATA---ATCAGTTTATGGGATCAAAGCCTA---
AAGCCATGTGTAAAGTTG---
ACCCCACTCTGTGTTACTTTAAACTGTACGAATGCTAATGCTACCCACAATGGT------------------
----

-------------------------------ACCATAAAGGACGGTGCCCAGGGGAGAATAAAA---
AACTGTACTTTCAATATGACCACAGAACTAAGA---------------------------
GATAAGAGACAGGCAGTACATGCACTT---TTTTATAGAATTGATCTAGTACCACTTGATAAGGGTAAC----
----

AATGCTAGTAATTATAGTGATTATAGATTAATAAATTGTAATACCTCAGCCATAACACAA---
GCCTGTCCAAAGGTCTCTTTTGAACCAATTCCTATACATTATTGTGCTCCTGCTGGTTATGCGATTCTAAAGT
GTAAT---AATGAGACATTCAAT------GGAACAGGACCATGCAGTAAT-----------------------
-GTCAGC---ACAGTACAATGTACACATGGAATTAAACCAGTGGTATCAACT---
CAACTATTGTTAAATGGTAGTCTAGCAGAAGGAGAG------------
ATAATGATTAGATCTAAAAATCTGACAGACAAT---------GCTAAAATAATAATAGTACATCTTAAT----
-----------GAATCTGTACAA---------ATTGATTGTATGAGACCCAACAATAATACAAGAAGA-----
-AGTATAAGGATA------------GGACCAGGACAAGCA---TTC------TATGCAACAGGAGAC------
---------ATAATAGGAGATATA------------AGACAAGCACATTGT---AACATTAGTGGAGAA---
AAATGGAATGAAACTTTAAAACGGGTGAAGGAAAAATTAGAA---------------AAGCACTTCCCT----
-----------------------------------AATAAAACAATACACTTTGCA------
AATTCCTCAGGGGGGGGCCTAGAAATTACA---ACACACAGCTTTAATTGTAGAGGAGAA---
TTTAAGCAGCTCCAGGCAAGAATCCTGGCTATAGAAAGATACCTACAGGATCAACAGCTCCTAGGGATGTGGG
GCTGCTCTGGAAAATCCATCTGCACCACTGCTGTGCCTTGGAACTCTAGTTGGAGT-----------------
----------------------------AATAAATCTCAAGAGGATATTTGG------AAT------
AGAACGACCTGGATGCAGTGG---GAGAAAGAAATTAGTAATTATACACAAACAATATAC---
TGGTTGCTTGAAGACTCGCAAAACCAGCAGGAA--

AAGAATGAACAAGAGTTACTAGCATTAGACAAATGGCAAAATCTGTGGAATTGGTTTGACATATCAAAGTGGC
TGTGGTATATAAGAATATTCATAATGATAGTAGGAGGCTTGATAGGTTTAAAAATAATTTTTGCT---
GTGCTTTCTATAGCG---------AACAGAGTTAGGCAGGGATACTCACCA------
TTGTCGTTTGCTATTAGTCTGCTTGATACCACAGCAATAGCAGTAGCTGAG---
GGAACAGATAGGATTTTAGAACTATTACAGAGAATTGGGAGAGCT----------------------------
--------ATCTACAACATA------CCTAGAAGAATAAGACAGGGCTTTGAAGCAGCTTTGCAA---TAA
>AF443090
GGG---GTG--
GGGGAAATGTGAGTCACAGTCTATTATGGGGTACCTGTGTGG---AAAGAAGCAAAA---
ACTACTCTATTCTGTGCATCAGATGCTAAGGCATATGAGAGAGAA---GTGCATAAT----------------
--------------GTTTGGGCGACA---CATGCCTGTGTACCCACAGAC---
CCCGACCCACAAGAAATGGTTTTGGAA---------AATGTAACAGAGAATTTTAAC----------------
--------------------------ATGTGGAAAAATGACATGGTAGATCAGATGCATGAGGATATA---
ATCAGTTTATGGGATCAAAGCCTA---AAGCCATGTGTAAAGTTG---
ACCCCACTCTGTGTCACTTTAAATTGTACAAACTATAATAATACTAATACTACCCAT----------------
----
----------------------------GGAGAAGGCAATACTACCTATGGAGGAGAAATGAGA---
AATTGCTCTTTCAATATAACCACAGAATTAAGA---------------------------
GATAAGAAAAGGCAAGAGAGTGCACTT---TTTTATAGACTTGATATAGTACCACTTAATGGGAGTGAATCT-
----

AGTGATAGGAACAAATCTAGTGAGTATAGACTAATAAATTGTAATACCTCAGCCATAACACAA---
GCCTGTCCAAAGGTCTCTTTTGACCCGATTCCTATACATTATTGTGCTCCAGCTGGTTATGCGATTCTTAAGT
GTAAT---AACAAGACATTCAAT------GGAACAGGACCATGCAACAAT-----------------------
-GTCAGC---ACAGTACAATGTACACATGGAATTAAGCCAGTGGTATCAACT---
CAACTACTGTTAAATGGTAGCCTAGCAGAAGAAGAG------------
ATAATAATCAGATCCGAAAATCTGGCAAAAAAT---------GACAAAACAATAATAGTACATCTTAAT----
-----------GAATCTGTAGAG---------ATTGTGTGTACAAGACCCTTCAATAATACAAGAAAA-----
-AGTATCAGAATA------------GGACCAGGACAAACC---TTC------TATGCAACAGGAGAC------
---------GTAATAGGAGATATA------------AGACAAGCACATTGT---AACACTAGTGAACAG---
AAATGGAACAAGACTTTAGAACAGGTAGGAAAGAAATTAGCA---------------GAACACTTCCCT----
-----------------------------------AATAAAACGATAATATTTAAT------
TCATCCTCAGGAGGGGACCTAGAAATCACA---ACACATAGCTTTAATTGTGGAGGAGAA---
TTTAAGCAGCTCCAGGCAAGAGTCCTGGCTATAGAAAGATACCTAAAGGATCAACAGCTCCTAGGGATTTGGG
GCTGCTCTGGAAAACTCATCTGCACCACTGCTGTGCCTCGGAACAACAGTTGGAGT-----------------
----------------------------AATAAAACTCTAGGAGATATTTGG------GAG------
AACATGACCTGGATGCAATGG---GATAGAGAAATAAGTAATTACACAGACACAATATAC---

AGGTTGCTTGAAGTCTCGCAAAACCAGCAGGAA---------------------------------------
----
AAAAATGAAAAAGATTTATTAGCACTGGACAGTTGGAAAAATCTGTGGAGTTGGTTTGACATATCAAATTGAC
TGTGGTATATAAGAATATTCATAATGATAGTAGGAGGCTTGATAGGTTTAAGAATAGTTTTTGCT---
GTGCTCTCTTTAGTG---------AATAGAGTTAGGCAGGGATACTCACCT------
TTGTCATTTGCTATTAGTCTGCTTGATACCATAGCAATAGCAGTAGCTGAG---
GGAACAGATAGGATTATAGAATTAATACAAAGAATTTGTAGAGCT----------------------------
--------ATCTACAACATA------CCGAGAAGAATAAGACAGGGCTTTGAAGCAGCTTTGCAA---TAA
>AF443097
GTA---ATG--
GGGAACATGTGGGTCACAGTCTATTATGGGGTACCTGTGTGG---AGAGAAGCAAAA---
GCTCCTCTATTTTGTGCATCAGATGCTAAAGCATATGATAGAGAA---GTGCATAAT----------------
--------------GTCTGGGCTACA---CATGCCTGTGTACCCACAGAC---
CCCAACCCACAAGAAATGGTTTTAAAA---------AATGTAACAGAAAATTTTAAC----------------
-------------------------ATGTGGAAAAATGACATGGTGGAGCAAATGCATGAGGATATA---
ATCAGTTTATGGGATCAAAGCCTA---AAGCCATGTGTAAAGTTG---
ACCCCACTCTGTGTCACTTTAGATTGTACGAATGCTACCATAACTGATAATAAA-------------------
----
-------------------------------ACAATAGACGAAGGAATGGGAAATGAAATAAAA---
AATTGCTCTTTCAATACAACCACAGAACTGAGG--------------------------
GATAAGAAAAAACGTGAATATGCACTT---TTTTATAGACTTGATATAGTATCACTTGATAATGAGAAT----
----

AATAACAAGACGAGTGCAGAGTATAGATTAATAAATTGTAATACCTCAACCATAACACAA---
GCCTGTCCAAAGGTCTCTTTTGACCCAATTCCTATACATTATTGTGCTCCAGCTGGTTATGCGATTCTAAAGT
GTAAT---AATAAAACATTCAAT------GGAAAAGGACCATGCAATAAT-----------------------
-GTCAGC---ACTGTACAATGTACACATGGAATTAAGCCAGTAGTATCAACT---
CAACTACTGTTAAATGGTAGCCTAGCAGAAGGAGGA---------
GAGATAATAATTAGATCTAAAAACCTGACAGATAAT---------GCCAGAACAATAATAGTACATCTTAAT-
--------------GAATCTGTACAA---------ATTGTGTGTACAAGACCCAACAATAATACAAGAAAA--
----AGTGTGAGGCTA------------GGACCAGGACAAGCA---TTC------TATGCAACAGGAGAT---
------------ATAATAGGAGATATA------------AGAAAAGCATATTGT---ACCATTAATGAAAGT-
--AAATGGATAACAACTTTACACAGGGTAAGTGAAAAATTAAAA---------------GAACACTTCCCT--
-------------------------------------AATAAAGCAATAAACTTTACC------
CAACCCAAAGGAGGGGACTTAGAAATTACA---ACACATAGCTTTACTTGTAGAGGAGAG---
TTTAAGCAGCTCCAGACAAGAGTCCTGGCTATAGAAAGATACCTAAAGGATCAACAGCTCCTAGGAATTTGGG
GATGCTCTGGGAAACTCATCTGCACCACTGCTGTGCCTTGGAATGTTAGTTGGAGT-----------------
-----------------------------AATAAATCTTATAAGGCTATTTGG------GAC------
AACATGACCTGGATGCAGTGG---GATAGAGAGATTAGTAATTACACAGACACAATATAC---
AGTTTGCTTGAAGACTCGCAAAACCAGCAGGAA--

AAGAATGAAAAGGATTTATTAGCATTGGACAGTTGGAAAAATCTGTGGAATTGGTTTGACATAACAAATTGGC
TGTGGTATATAAAAATATTCATAATGATAGTAGGAGGCTTGATAGGCTTAAGAATAATTTTTGCT---
GTGCTGTCTATAGTA---------AATAGAGTTAGGCAGGGATACTCACCT------
TTGTCATTTGCTATTAGTCTGCTTGATACCGTAGCAATAGCAGTAGCTGAA---
GGAACAGATAGGATTATAGAATTAATACAAAGAGGTTATAGAGCT----------------------------
--------ATCTGCAACATA------CCTACAAGAATAAGACAGGGATTTGAAGCGGCTTTGCAA---TAA
>AF443109
GTG---GCA--
GGGAACTTGTGGGTCACAGTATATTATGGGGTACCTGTGTGG---AAAGAAGCAAAA---
ACTACTCTATTCTGTGCATCAGATGCTAAAGGATATGAGACAGAA---GTGCATAAT----------------
--------------GTTTGGGCTACA---CATGCCTGTGTACCCACAGAC---
CCCGACCCACAAGAAATGGTCCTGGGA---------AATGTAACAGAAAATTTTAAC----------------
-------------------------ATGTGGGAAAATGACATGGTGGATCAGATGCATGAGGATGTA---
ATCAGTTTATGGGATGAAAGCCTA---AAGCCATGTGTAAAGTTG---
ACCCCACTCTGTGTCACTTTAAACTGTACAAACTATAATGGGACCAAGAACAATGATACCAAAAACAATGAT-
----
----------------ACCAGGAGTGATACTATCTACAATGATACCATGTATGGAGAAATAAAA---
AATTGCTCTTTCAATATGACCACAGAACTAAGA-------------------------
GATAAGAAAGAGAAAATGCATGCACTT---

TTTTATAGACTTGATATAGTACCACTTGAGGACAACTCTAGTAAGAAT-------------------------
----
AGCAGGGGCAACTCTAGTAATTATAGTAATTATAGATTAATAAATTGTAATACCTCAGCCATAACGCAA---
GCCTGTCCAAAGGTCTCTTTTGACCCAATTCCTATACATTATTGTGCTCCAGCTGGTTATGCGATTATAAAGT
GTAAT---AATAAGACATTCAAT------GGGATAGGACCATGCCAGAAT-----------------------
-ATCAGC---ATAGTGCAATGTACACATGGAATTAAGCCAGTGGTATCAACT---
CAACTACTGTTAAATGGTAGCCTAGCAGAAGGAGGA---------
GAGATAATAATTAGGTCTAAAAACCTGTCAGATAAT---------GCCAAAATAATAATAGTACATCTTAAT-
--------------GAATCTGTAGGA---------ATTGTGTGTACAAGACCCAACAATAATACAAGAAAA--
----AGTATAAGGATA------------GGACCAGGACAAACA---TTC------TATGCAGCAGGAGAC---
------------ATAATAGGAAACATA------------AGACAAGCACATTGT---AACATTAGTGAAGGT-
--AACTGGACTAAAACTTTACAAAGGGTAAGTAGAAAGTTAAGA---------------CAAATCTTCAAT--
-------------------------------------AAAAGCAACATAGAATTTAAA------
CCACACTCAGGAGGGGATCCAGAAATTACA---ACACATAGCTTTAATTGTAGAGGAGAA---
TTTAAGCAGCTCCAGGCAAGAGTCTTGGCTATCGAAAGATACCTAAAGGATCAACAGCTCCTAGGTATTTGGG
GATGCTCTGGAAAACTCATCTGCACCACTAATGTGCCTTGGAACTCCAGTTGGAGT-----------------
----------------------------AATAAATCTCTAGAAGATATTTGG------GAT------
AACATGACCTGGATGCAATGG---GATAGAGAAATTAGTAATTACACAAGAGAAATATAC---
AAGTTGCTTGAAGAATCGCAAAACCAGCAGGAA--

ATAAATGAAAAGGATTTATTAGCATTGGACAGTTGGAACAGTCTGTGGAATTGGTTTGACATAACAAAATGGC
TGTGGTATATAAAAATATTCATAATGGTAGTAGGAGGCTTGATAGGCTTGAGAATAATTTTTGCT---
GTGCTTTCTATAGTA---------AATAGAGTTAGGCAGGATACTCACCT------
TTGTCGTTGGCTATTAGTCTGCTTGATGCCATAGCAATAGCAGTAGGTGAA---
GGGACAGATAGGATTCTAGAATTACTACAAAGAATTGGTAGAGGT----------------------------
--------ATCTGCAACATA------CCCAGAAGAATAAGACAGGGCTTTGAAGCAGCTTTGCAA---TAA
>AF443114
GGG---GAA--
GGAAACTTGTGGGTCACAGTCTATTATGGGGTACCTGTGTGG---AAAGAAGCAAAA---
ACCACTCTATTCTGTGCATCAGATGCTAAAGGATATGAGAGGGAG---GTACATAAT----------------
--------------ATCTGGCTACA---CATGCCTGTGTACCCACAGAC---
CCCAACCCACAAGAAATGTTTTTGCAT---------AATGTAACAGAAAATTTTAAC----------------
--------------------------ATGTGGAAAAATGACATGGTGGATCAGATGCATGAGGATATA---
ATTAGTTTATGGGATGAAAGCCTA---AAGCCATGTGTAAAATTG---
ACCCCACTCTGTGTCACTCTAGAATGTAAAAATGTTACCACAAATGTTACCATAAATAATGCC----------
----
-------------------------ACAAGTGTTACCGCGAATAATAATACTAGTGACATGAAA---
AATTGCTCCTTCAATGCAACCACAGAAGTAACA---------------------------
GATAAAATAAGAAAAGAGAATGCACTT---TTTTATACACTTGATATAGTACCACTTGATGAG----------
----

AATCAGAACAATAGTAACTATAGATTAATAAATTGTAATACCTCCAAGGTGACACAA---
GCCTGTCCAAAGGTCTCTTTTGACCCAATTCCTCTACATTATTGTGCTCCAGCTGGTTATGCGATTCTAAAGT
GTAAT---AACAACACATTCAAT------GGGACAGGACCATGCAATAAT-----------------------
-GTCAGT---ACAATACAATGTACACATGGAATCAAGCCAGTAGTATCAACT---
CAACTATTGTTAAATGGTAGTCGAGCAGAAAAAGAG------------
ATAATAATTAGATCTGAAAATATGACAAACAAT---------GCTAAAACAATAATAGTACACCTTAAT----
-----------GAATCCATAGAA---------ATTGAGTGTATAAGACCCAACAACAATACAAGAAAA-----
-AGTATAAGGATA------------GGACCAGGACAAACA---TTC------TATGCAACAAATGGC------
---------ATGATAGGAGACATA------------AGACAAGCACATTGT---AACATTAGTGGAGCT---
GACTGGAATAGAACTTTACAAGGGGTAGGTAGAAAATTAGCA---------------GGGTACTTCCCT----
-----------------------------------AATAAAACCATAAGCTTCCAA------
CCATCCTCAGGAGGGGACCTAGAAATTACA---ACACATAGCTTTAATTGTGGAGGAGAA---
TTCAAGCAGCTCCAGGCAAGAGTCCTGGCTATAGAAAGATACCTAAAGGATCAACAGCTCCTAGGGATTTGGG
GCTGCTCTGGAAAACTCATCTGCACCACTGCTGTGCCTTGGAACTCCAGTTGGAGT-----------------
----------------------------AATAAATCTCAAGCAGACATTTGG------GAT------
AACATGACCTGGATGCAGTGG---GATAGAGAAATTAGCAATTACACAGACACAATATAT---
AGGTTGCTTGAAGTCTCGCAAACCCAGCAGGAA--

CAAAATGAACAAGATTTATTAGCATTGAACAAGTGGCAACATCTGTGGAATTGGTTTGACATAACAAAGTGGC

TGTGGTATATAAAAATATTCATAATGATAGTAGGAGGCTTGATAGGTTTGAGAATAATTTTTGCT---
GTGCTATCTATAGTA---------AATAGAGTTAGGCAGGGATACTCACCT------
TTATCATTGGCTATTAGTCTGCTTGATAGTATAGCAATAGCAGTAGCTGAG---
GGGACAGATAGGATTATAGCATTCATACAAAGACTTTTTAGAGCT----------------------------
--------ATCTGCAACCTA------CCTAGAAGAATAAGACAGGGCTTTGAAGCATCTTTGCTA---TA-
>KR861314
GTG---GTA---
GGAAACTTGTGGGTCACAGTCTATTATGGGGTACCTGTGTGG---AAAGAAGCAAAA---
ACTACTCTATTCTGTGCATCAGATGCTAAAGCATATGAGAAGGAA---GTGCATAAT---------------
--------------GTCTGGGCTACA---CATGCCTGTGTACCCACAGAC---
CCCAGCCCACAAGAAGTGGTTTTGAAA---------AATGTAACTGAAAATTTTAAT---------------
--------------------------ATGTGGAAAAATGACATGGTGGATCAGATGCATGAGGATATA---
ATCAGTTTATGGGATCAAAGCCTA---AAGCCATGTGTAAAGTTG---
ACCCCACTATGTGTTACTTTAAACTGTATAAATGCTACTGCTAGTATGAATGCTACTACATAC---------
----
---AATGAAACCATGGAAATAAAA---
AATTGCTCTTTCAATATGACCACAGAACTAAGA--------------------------
GATAAAAAACAGAACGTGTATGCACTT---TTTTATAAACTTGATATAGTAAAACTTAATGGC---------
----

AATTCGGATGAGTATAGATTAATAAATTGTAATACCTCAGCCATAACACAA---
GCCTGTCCAAAGGTCTCTTTTGACCCAATTCCTATACATTATTGTGCTCCAGCTGGGTATGCGATTCTAAAGT
GTAAT---GATAAGACATTCAAT------GGAACAGGACCATGTAATAAT----------------------
-GTTAGC---ACAGTACAATGTACACATGGAATTAAGCCAGTGGTCTCAACT---
CAATTACTGTTAAATGGTAGTCTAGCAGAAGAAGAC-----------
ATAGTAATTAGATCTGAAAATTTGAAAGACAAT---------ATCAAAACAATAATAGTACATCTTAAT----
-----------GAATCTGTAGAA---------ATTAATTGTACAAGACCTAACAATAATACAAGGAAA-----
-AGTATAAGGATA------------GGACCAGGACAGACC---TTC------TATGCAACA---GAA------
---------ATAATAGGAGACATA------------AGAGAAGCACATTGT---AACATTAATGCAGGA---
AAATGGAATGAAACTTTAGAAAAGGTAAAAGGAAAGTTAAAA---------------GAACATCACAAT----
-----------------------------------GGAAGCGAGATAATATTTAAA------
CCACCTTCAGGAGGGGACCTAGAAATTACA---ACACATAGTTTTAATTGTAGAGGAGAA---
TTTAAGCAGCTCCAGGCAAGAGTCCTGGCTATAGAAAGATACCTAAAGGATCAACAGCTCCTAGGGATTTGGG
GTTGCTCTGGAAAGCTCATCTGCACCACTAATGTGCCTTGGAACTCCAGTTGGAGT----------------
-----------------------------AATAGATCTCAAGGAGATATTTGG------GAG------
AGCATGACCTGGATGCAGTGG---GACAGAGAAATTAATAATTACACAAGCACAATATAC---
AGTTTGCTTGAAGAATCACAAACCCAGCAGGAA---------------------------------------
----
AAAAATGAAAAGGATTTATTAGCATTGGACAGTTGGCAAAGTCTGTGGAGTTGGTTTAGCATAACAAACTGGC
TGTGGTATATAAAAATATTCATAATAATAGTAGGAGGCTTGATAGGCTTAAGAATAATTTTTGCT---
GTGCTTTCTATAGTG---------AATAGAGTTAGGCAGGGATACTCACCT------
TTGTCGTTCGCTATTAATCTGCTTGATACCACAGCAATAGCAGTAGCTGAA---
GGAACAGATAGGATTATAGAATTAATACAAAGATTTTGTAGAGCT---------------------------
--------ATTCGCAATATA------CCTACAAGAATAAGACAAGGCTTTGAAGCAGCTTTACAA---TAA
>KR861341
GTG---TGG---
GGGCAATTGTGGGTCACAGTCTACTATGGGGTACCTGTGTGG---AAAGAAGCAAAA---
ACTACTCTATTCTGTGCATCAGATGCTAAGGCATATGAGAAAGAG---GTGCATAAT---------------
--------------GTCTGGGCTACA---CATGCCTGTGTACCCACAGAC---
CCCAACCCACAAGAAATAGTCTTGGGC---------AATGTAACAGAAAATTTTGAC---------------
--------------------------ATGTGGGAAAATGACATGGTAGATCAGATGCATGAGGATATA---
AGCAATTTATGGGATCAAAGCATA---AAGCCATGTGTAAAGTTG---
ACCCCACTCTGTGTCACTTTAAATTGTACTGATGCTAAGACTAGTACTACAAATACTACTGCTGCAAATACTA
CT---
--AATGAGATAAAA---
AATTGCACTTTTAATGCAACCACAGAAATAAAA--------------------------
GATAAGAAAAGGAAAGTGCAAGCACTT---
TTTTATAAACTTGATATAATACCACTTGATGAGGATAACAACAGT---------------------------
----
TCTACTAGAAACAGCAGCTATGTGAAGTATAGATTAATAAATTGTAATACCTCAGCCATAACACAA---

GCCTGTCCAAAGATCACTTTTGACCCAATTCCTATACATTATTGTGCGCCAGCTGGTTATGCGATTCTAAAGT
GTAAT---AACAAGACATTCAAG------GGGACAGGACCATGCAGAAAT-----------------------
-GTCAGC---ACAGTACAATGTACACATGGAATTAAGCCAATGGTAACAACT---
CAACTACTGTTAAATGGTAGCCTAGCAGAAGAAGGG------------
ATAATAATTAGATCTAAAAATCTGAGTGACAAT---------GGCAAAAATATAATAGTACAACTTAAG----
-----------GATGCTGTAAAA---------ATTGAGTGCATAAGACCCAACAATAATACAAGAAAA-----
-AGTGTAAGGATA------------GGACCAGGACAGACA---TTC------TTTGCAACAAAT---------
---------ATAATAGGAGACATA------------AGAGAAGCACATTGT---AACATTAGTAGAAAG---
GAATGGAACAAAACTTTACAATCTGTAGGAAGAAAATTAGCA---------------GAATATTTCCCT----
--------------------------------AATAATACAACAATAGAATTTGCA------
CCACACTCAGGAGGGGATCTAGAAATTACC---ACACATTTCTTTAACTGTAGAGGAGAA---
TTTAAGCAGCTCCAGGCAAGAGTCCTGGCTATAGAAAGATACCTAAAGGATCAACAGCTCCTAGGGATGTGGG
GCTGCTCTGGAAAACTCATCTGCACCACTTCTGTGCCTTGGAATTCCAGTTGGAGT-----------------
----------------------------AATAAATCTCATACAGAAATTTGG------AAT------
AACATGACCTGGATGCAATGG---GAGAAAGAAATTAGTAATTACACAGAAATAATATAC---
AAGATGCTTGGAGACTCGCAAAACCAGCAGGAA--

CAAAATGAAAAAGAGTTACTAGCATTGGACAAGTGGCAAAATCTGTGGAGTTGGTTTGACATATCAAATTGGC
TGTGGTATATAAAAATATTCATAATGATAGTAGGAGGCTTGATAGGGTTAAGAATAATTTTTGCT---
GTGCTTTCTATAGTA---------AATAGAGTTAGGCAGGGATACTCACCT------
TTGTCGTTTGCCATTAATCTATTGGATACCACAGCAATAGCAGTAGCTGAA---
GGAACAGATAGGATTATAGAATTAATACAAACACTTTGTAGAGCT----------------------------
--------ATCCGCAACATA------CCTAGAAGAATAAGACAGGGCTTGGAAGCAGCGTTGCTA---TAA
>KR861270
GGG--
AATAAATTGTGGGTCACAGTCTATTATGGGGTACCTGTGTGG---AAAGAAGCAAAA---
ACTACTCTATTTTGTGCATCAGATGCTAAAGGATATGAGAAAGAA---GTGCATAAT----------------
--------------GTTTGGGCCACA---CATGCCTGTGTACCCACAGAT---
CCCAACCCACAAGAAATGTTTTTAGAA---------AACGTAACAGAAAATTTTAAT----------------
--------------------------ATGTGGAAAAATGACATGGCAGATCAGATGCATGAGGATGTA---
ATCAGTTTATGGGATCAAAGTTTA---AAGCCATGTGTAAAGTTG---
ACCCCACTCTGTGTCACTTTAAACTGTACAGATGCTACTGTAAATGCTACAGATGCTACT-------------
----
--GTAAATGATACCATAGAAATGAAA---
AATTGTACTTTCAATACGACCACAGAAATAAGA---------------------------
GATAAGAAAAAGCAAGAAAGGGCACTC---TTTTATAAACTTGATATAGTACAACTTGATGGC----------
----

AACTCTAGTAAGTATATATTAATAAATTGTAATACCTCAGTCATAACACAA---
GCCTGTCCAAAGGTCTCTTTTGACCCTATTCCTATACATTATTGTGCTCCAGCTGGTTATGCGATCCTAAAGT
GTAAT---GATAAGACATTCAAT------GGAACAGGACCATGCAATAAT-----------------------
-GTTAGC---ACAGTACAATGTACACATGGAATTAAGCCAGTGGTATCAACT---
CAATTACTGTTAAATGGTAGCCTAGCAGAAGACAGA------------
ATAATAATTAGATCTGAAAATCTGACAAACAAT---------GCCAAAACAATAATAGTACAGTTTAAT----
-----------AAATCTGTTGAG---------ATTACGTGTATAAGACCCAACAACAATACAAGGAAA-----
-AGTGTGAGGATA------------GGACCAGGGCAAACA---TTC------TATGCAACAGGAGAC------
---------ATAATAGGAGATATA------------AGACAAGCACATTGT---AACATTAGTAAC------
ACATGGTGGGCAACTTTACAAAAGATAAGGGAAGAATTAAGG------------AAGAAACACTTCCCT----
-----------------------------------AATAAAACCATAAAATTTGAA------
CCACACTCAGGGGGGGACATAGAAATTACA---ACACATAGCTTTAATTGTAGAGGAGAA---
TTTAAACAGCTTCAGGCAAGAGTTTTGGCTCTAGAAAGATACCTAAAGGATCAACAGCTCCTAGGGATTTGGG
GCTGCTCTGGAAAACTCATTTGCAACACTAATGTGCCCTGGAACTCTAGTTGGAGT-----------------
----------------------------AATAAAACTCAAGAGGATATTTGG------GGG------
AACATGACCTGGATGCAGTGG---GAGAAAGAAATTGACAATTACACAAACACAATATAC---
AGGTTGCTTGAAGAATCACAAAACCAGCAGGAA--

CAAAATGAAAAGGATTTACTAGCATTGGACAGTTGGAACAATCTGTGGAATTGGTTTCACATAACAAAATGGC
TGTGGTATATAAAAATATTCATAATGATAGTAGGAGGCTTGATAGGTTTAAGAATAATTTTTGCT---
GTGCTCTCTATAGTG---------AATAGAGTTAGGCAGGGGTACTCACCT------
CTGTCGTTTGCTATTAATCTGCTTGATACCATAGCAATAAGAGTAGCTGAA---GGAACAGATAGGATTATA-

-----------GGATTTTGTAGAGCT------------------------------------
ATCTGCCGCATA------CCTAGAAGAATAAGACAGGGCTTTGAAGCAATTTTGCAA---TAA
>KR861273
GTG---ATG--
GGAAACATGTGGGTCACAGTCTATTATGGGGTACCTGTGTGG---AAAGAAGCAAAA---
ACTACTCTATTCTGTGCATCAGATGCTAAAGCATATGAGAAGGAG---GTGCATAAT----------------
--------------GTCTGGGCAACA---CATGCCTGTGTACCCACAGAC---
CCCAACCCACAAGAAATAGAATTAAAA---------AATGTAACAGAAAATTTTAAC----------------
--------------------------ATGTGGAAAAATAATATGGTGGATCAAATGCATGAGGATATA---
ATCAGTTTATGGGATCAAAGCCTA---AAGCCATGTGTAAAGTTA---
ACCCCACTCTGTGTCACTTTGAAGTGCAGTGATGCTACTGTTACCGGCGGTAATAATAAC-------------
----
-------------------------CAAATTAGCACCTTAGGTAACATAACGGAAGAAATAAAA---
AATTGCACTTTCAATATGACCACAGATGTAAGG---------------------------
GATAAAAGAAAGAATGTAGCTGCACTT---TTTTATACACTTGATATAGTACCACTTAATGAG----------
----

AACTCCAGTGAGTATAGATTAATAAATTGTAATACCTCAGCCATGACACAA---
GCCTGTCCAAAGGTCTCCTTTGACCCAATTCCTATACATTATTGTGCTCCAGCTGGTTATGCGATTCTAAAGT
GTAAT---AATAAGACATACAAT------GGAACAGGACCATGCAATAAT-----------------------
-GTCAGC---ACAGTGCAATGTACACATGGAATTAAGCCAGTGGTATCAACT---
CAATTACTGTTAAATGGTAGTCTAGCAGAAGAGGGC------------
ATAATAGTTAGATCTGAAAATATTACAGACAAT---------GCCAAAACAATAATAGTACAGCTTAAA----
-----------AATGCTGTAGGA---------ATTAATTGTACAAGGCCTGGCAATAATACAAGGAAA-----
-AGTATGAGGATA------------GGACCAGGACAAGCA---TTC------TTTGCAACAGGAGAT------
---------ATAATAGGAGATATA------------AGACAAGCACATTGT---AACATTAGTGAAGGA---
GATTGGAATAAAACTTTAAAAGAGGTAGCTAACAAATTACGA---------------GACCAATTCCCT----
-----------------------------------AATAAAACAATAGTATTTGAA------
CCAACCTCAGGAGGGGACCTAGAGATTACA---ACACATAGCTTTAATTGTGGAGGAGAA---
TTTAAGCAGCTCCAAGCAAGAATCCTGGCTATAGAAAGATACCTACAGGATCAACAGCTCCTAGGGCTTTGGG
GCTGCTCTGGAAAACTCATCTGCACCACTGCCGTGCCTTGGAATGCTAGTTGGAGT-----------------
----------------------------AATAAAAGTCACAATGACATTTGG------CAA------
AACATGACCTGGATGCAGTGG---GAAAAAGAAATTAGTAATTACACAAACACAATATAC---
AATTTGCTTGAAAAATCACAGAACCAGCAGGAG--

AAAAATGAACAGGATTTACTAGCATTGGACAGTTGGAAAAATCTGTGGAATTGGTTTGACATAACACATTGGC
TGTGGTACATAAAAATATTCATAATGATAGTAGGAGGACTGATAGGGTTAAGAATAGTTTTTGCT---
GTGCTATCCATAGTG---------AATAGAGTTAGGCAGGGATACTCACCT------
TTGTCATTTGCTATTAGTTTGCTTGATGCCACAGCAATAGTAGTAGCTGAG---
GGAACAGATAGAATTATAGAAGGGCTACAAAGAGGTGGTAGAGCT----------------------------
--------ATCCGCAACATA------CCTAGAAGAATAAGACAGGGCTTTGAAATAGCTTTGCAA---TAA
>KY658708
GTG---GTG--
GGAAACTTGTGGGTCACAGTTTATTATGGGGTACCTGTGTGG---AAAGATGCAAAA---
ACTACTCTATTCTGTGCATCAGATGCTAAAGCATATGAAAAAGAA---GTGCATAAT----------------
--------------GTCTGGGCTACA---CATGCCTGTGTACCCACAGAC---
CCCAACCCACAAGAAATAGTTTTGGAA---------AATGTAACAGAAAATTTTAAC----------------
--------------------------ATGTGGAAAAATGACATGGTGGATCAGATGCATGAGGATATA---
ATCAGTTTATGGGACCAAAGCCTA---AAGCCATGTGTAAAGTTG---
ACCCCACTCTGTGTCACTTTAAAATGTGTAAATGTTAGTAATAAATTTAACCTA-------------------
----
-------------------------CAAAATAGCATGAATGAAGATCCGAAGGAAGAAATAAGA---
AATTGCTCTTTCAATGCAACCACAGAATTAAGA---------------------------
GATAAGAAACAGAAAGTGCATGCACTC---TTTTATAAACTTGATATAGTACCACTTCATGGC----------
----

AACAACTCTAGTGAGTATAGATTAATAAATTGTAATACCTCAACCATAACACAA---
GCCTGTCCAAAGGTCTCTTTTGATCCAATTCCTATACATTATTGTACTCCAGCTGGTTATGCGATTTTAAAGT
GTAAT---AATCAGACATTCAAT------GGGACAGGACCATGCAATAAT-----------------------
-GTCAGC---TCAGTACAATGTACACATGGAATTAGGCCAGTAGTATCAACT---

CAGTTACTGTTAAATGGTAGCCTAGCAAAAGGAGAG------------
ATAATAATTAGATCTGAAAATCTGACAAACAAT---------GCCAAAATAATAATAGTACATCTTAAT----
-----------AAACCTGTAAAA---------ATTGTGTGTGTAAGGCCCAACAATAATACAAGAAAA-----
-AGTGTAAGGATA------------GGACCAGGACAAACA---TTC------TATGCAACAGGTGAA------
---------ATAATAGGAGACATA------------AGACAAGCATATTGT---ATCATTAATAAAACT---
GAATGGAATAACACTTTACAAGGGGTAAGTAAAAAATTAGGA---------------GAACACTTCTCT----
------------------------------------AATAAAACAATAAAATTTGAA------
CCGTCATCAGGAGGGGACCTAGAAATTACA---ACACATAGCTTTAATTGTAGAGGAGAA---
TTTAAGCAGCTCCAGACAAGAGTCTTGGCTATGGAAAGATACCTAAAGGATCAACAGCTCCTAGGGATTTGGG
GCTGCTCTGGAAAACTCATCTGCACCACTGCCGTGCCTTGGAACTCCAGTTGGAGT-----------------
-----------------------------AATAGATCTCATGATGAGATTTGG------GAT------
AACATGACCTGGATGCAGTGG---GATAGAGAAATTAATAATTACACAGACACAATATAC---
AGGTTGCTTGAAGAATCACAAAACCAGCAGGAG--

AAAAATGAAAAGGATTTATTAGCATTGGACAGTTGGCAAAATCTGTGGAATTGGTTTAGCATAACAAATTGGC
TGTGGTATATAAAAATATTCATAATGATAGTAGGAGGCTTGATAGGTTTAAGAATAATTTTTGCT---
GTGCTTTCTATAGTG---------AATAGAGTTAGGCAGGGATACTCACCT------
CTGTCGTTTGCTATTAGTCTGTTTGATACCATAGCAATAGCAGTAGCTGAA---
GGAACAGATAGGATTATAGAATTAATACAAAGAATTTGTAGAGCT----------------------------
--------ATTCGCAACATA------CCTAGAAGAATAAGACAGGGCTTTGAAGCAGCTTTGCAA---TAA
>AF443084
GTG---GTA---
GGGAACTTGTGGGTTACAGTCTATTATGGGGTACCTGTGTGG---AAAGAAGCAAAA---
ACTACTCTATTCTGTGCATCAGATGCTAAAGCATATGATAAAGAA---GTGCATAAT----------------
--------------GTCTGGGCTACA---CATGCCTGTGTACCCACAGAC---
CCCAACCCACAAGAAATAGTTTTGGAA---------AATGTAACAGAAAATTTTAAC----------------
--------------------------ATGTGGAAAAATGACATGGTAGATCAGATGCATGAGGATATA---
ATCAGTTTATGGGATCAAAGCCTA---AAGCCATGTGTAAAGTTG---
ACCCCACTCTGTGTCACTTTAAAATGTACAAATGTTAACGCTACAAATGTTAACGCT----------------
----
-----------------------------ACCTACAGTAATAGGACGATGACTGAGGAAATAAAA---
AATTGCTCTTTCAATATAACCATGGAACTAAGA---------------------------
GATAGGAAACAGAAGGTGAATGCACTT---TTTTATAAACTTGATATAGTACCACTTAATGAGAAGGCT----
----

AACAATAACAGCTATAGCTACTATAGATTAATAAATTGTAATACCTCAGCCATAACACAA---
GCCTGTCCAAAGGTCTCTTTTGACCCAATTCCTATACATTATTGTGCTCCAGCTGGTTATGCGATTCTAAAGT
GTAAT---AATAAGACATTTAAT------GGGACAGGACCATGCCAGAAT----------------------
-GTCAGC---ACAGTACAGTGCACACATGGAATTAAGCCAGTGGTATCAACT---
CAACTACTGTTAAATGGTAGCCTAGCAGAAGGAGAG------------
ATAATAGTTAGATCTGAAAATCTGACAGACAAT---------GTCAAAACAATAATAGTACATCTTGAT----
-----------AAACCTGTAGGA---------ATTGTGTGTATAAGACCCAACAATAATACAAGGAAA-----
-AGTATAAGGATA------------GGGCCAGGACAAACA---TTT------TATGCAACAGGAGAC------
---------ATAATAGGAAACATA------------AAAGAAGCATATTGT---AACATTAAGGAATCT---
GAATGGAATAGAACTTTACAAAGGGTAGGAGAAAAACTGGGA---------------AAATACTTCCCT----
------------------------------------AGTAAAACAATAAAATTTAAC------
TCATCCTCAGGAGGGGACCTAGAAATTACA---ACACATAGCTTTAATTGTAGAGGAGAA---
TTCAAGCAGCTTCAGGCAAGAGTCCTGGCTATAGAAAGATACCTAAAGGATCAACAGCTCCTAGGGCTTTGGG
GCTGCTCTGGAAAACTCATCTGCACCACTGCTGTGCCTTGGAACTCCAGTTGGAGT-----------------
-----------------------------AATAAATCTAAGGAAGATATTTGG------AAT------
AACATGACCTGGATGCAGTGG---GATAGAGAAATTAGTAATTACACAAACACAATATAC---
AGGTTGCTTGAAGACTCGCAAAGCCAGCAGGAA--

AAAAATGAAAAAGATTTATTAGCATTGGACAGTTGGAAAAATCTGTGGACTTGGTTTGACATATCAAATTGGC
TGTGGTATATAAGAATATTCATAATGATAGTAGGAGGCTTAATAGGCTTGAGAATAATTTTTGCT---
GTACTCTCTATAGTG---------AATAGAGTTAGGCAGGGATACTCACCT------
TTATCGTTTGCTACTAGCCTGATTGATGCCATAGCAATAGCAGTAGCTGAA---
GGAACAGATAGAATTATAGACTTAATACAAAGAATTTGTAGAGCT----------------------------
--------ATCAGAAATATA------CCTAGAAGAATAAGACAGGGCTTTGAAGCAGCTTTGCAA---TAA
>KC899004

GTG---GGA---
GGAAACTTATGGGTCACAGTTTATTATGGGGTACCTGTATGG---AGAGAAGCAAAA---
ACTACTCTATTTTGTGCATCAGATGCTAGAGCATATGATACAGAG---GTACATAAT----------------
--------------GTCTGGGCTACA---CATGCCTGTGTACCCACAGAC---
CCCAACCCACAAGAAATAGAGTTGAAA---------AATGTAACAGAAAATTTTAAC----------------
--------------------------ATGTGGAAAAATGAAATGGTGAATCAGATGCATGAGGATGTA---
ATCAGCTTATGGGATCAAAGCCTA---AAGCCATGTGTAAAATTG---
ACCCCACTCTGTGTCACTTTAGAATGTGGAAATGTTAACCGCACTAGCAATACTACCCACAGC----------
----
----------------------------AATGATACCTACAATGACAGTGGGAAGGAAATAAAA---
AATTGCTCTTTTAATGCAACCACAGAAGTAAGA---------------------------
GATAAAAAGCAGGCAGTGTATGCACTT---TTTTATAGACTTGATATAGTGCCACTTAATGAA----------
----

AGAAGCGGTAGGAACTCTACTGAGTATTATAGATTAATAAATTGTAATACCTCAGCCATAACACAA---
GCCTGTCCAAAGGTCACTTTTGATCCAATTCCTATACACTATTGTGCTCCAGCTGGTTTTGCAATTCTAAAGT
GTAAT---GATAAGACATTCAAT------GGGACAGGACCATGCCTTAAT----------------------
-GTTAGT---ACAGTGCAATGTACACATGGAATTAAGCCAGTGGTATCAACT---
CAACTACTGTTAAATGGTAGCCTAGCAGAAGGAGAA------------
ATAATAATTAGATCTGAAAATCTGACAGACAAT---------GTCAAAACAATAATAGTACATCTTAAT----
-----------CAATCTGTAGAA---------ATTGTATGTACAAGACCCAACAATAATACAAGAAAA-----
-AGTATAAGGATA------------GGACCAGGACAAACA---TTC------TATGCAACAGGAGAC------
---------ATAATAGGGGACATA------------AGACAAGCATATTGT---AACATTAATAAAAAA---
GTATGGAATGAAACTTTACAAAGGGTAGGGAAAAAATTAGCA---------------AAACACTTCCCT----
-----------------------------------AATAAAACAATAAATTTCAAT------
TCATCCTCAGGAGGAGACCTAGAAATTACA---ACACATAGCTTTAATTGTGGAGGAGAA---
TTTAAGCAGCTCCAGACAAGAGTCCTGGCTATAGAAAGATACCTACAGGATCAACAGCTCCTAGGGATTTGGG
GCTGCTCTGGAAAACTCATCTGCACCACTAATGTACCTTGGAACTCCAGTTGGAGT----------------
----------------------------AACAGATCTCAAGAAAAGATTTGG------AAA------
AACATGACCTGGATGCAGTGG---GATAAAGAAATTAGTAATTACACAGACACAATATAC---
GGATTGCTTGAAAAATCGCAAAACCAGCAGGAA--

AGAAATGAAAAAGATTTATTAGCATTGGACAGTTGGAAAAATCTATGGAATTGGTTTGACATAACAAACTGGC
TGTGGTATATAAAAATATTCATAATGATAGTAGGAGGCTTGATAGGCTTAAGAATAATTTTTGCT---
GTGCTCTCTATAGTA---------AATAGAGTTAGGCAGGGGTACTCACCT------
TTGTCGTTTGCTACCAGTCTGCTTGATATCACAGCCATAGCAGTAGCTAAC---
GGAACAGATAGGATTATAGAATTAGTACTCAGCATTTGTAGAGCT---------------------------
--------ATCCGCAACATA------CCTACAAGAATAAGACAGGGCCTTGAAATAGCTTTGCAA---TAA
>KF835522
GTG---GGA---
GGAAACTTGTGGGTCACAGTCTATTATGGGGTACCTGTGTGG---AAAGAAGCAAAA---
ACTACTCTATTCTGTGCATCAGAGGCTAAAGCATATGAGCAAGAA---GTGCATAAT----------------
--------------GTTTGGGCTACA---CATGCATGTGTACCCACAGAC---
CCTGACCCACAAGAAATATTTTTGAAA---------AATGTAACAGAAAATTTTAAC----------------
--------------------------ATGTGGAAAAATGACATGGTGAATCAGATGCATGAGGATGTA---
ATCAGTTTATGGGATCAAAGCCTA---AAGCCATGTGTAAAGTTG---
ACCCCACTCTGTGTCACTTTAGAATGTGGAAAGGTTAGTAATACCTAT-------------------------
----
----------------------------ATTAAGATCTTCAATGAGAGTGAGGAGGAAATAAAA---
AACTGCACTTTCAATGCAACCACAGTAGTAAGA---------------------------
GATAGGAAGCAGTCAGCGTATGCCCTT---TTTTATAGACTTGATATAGTACCACTTAATGAG----------
----

TCTAGTAATTCTAGTTATTATAGATTAATAAATTGTAATACCTCAGCCATAACACAA---
GCCTGTCCAAAGGTCACTTTTGATCCAATTCCTATACACTATTGTACTCCAGCTGGTTATGCGATTCTAAAGT
GTAAT---GATGAGACATTCAGT------GGGACAGGACCATGCTCTAAT----------------------
-GTTAGC---ACAGTACAATGTACACATGGAATTAAGCCAGTAGTATCAACT---
CAACTACTGTTAAATGGTAGCCTAGCAGAAGGAGAT------------
ATAATCATTAGATCTGAAAACCTGACAAATAAT---------GTCAAAACAATAATAGTGCATCTTAAC----
-----------GAATCTGTAGAA---------ATTGTATGTACAAGACCCAACAATAATACAAGAAAA-----

-AGTATAAGGATA------------GGACCAGGACAAACA---TTC------TATGCAACAGGAGGC------
---------ATAATAGGAAACATA------------AGAGAAGCACATTGT---AACATTAGTGGAAAT---
AAATGGAATGAAACTTTACAAAAGGTAGGTAAAAAATTAGCA--------------GAACACTTCCCT----
-----------------------------------AATAAAACAATAAAATTTAAC------
TCATCCTCAGGAGGGGACCTAGAAATTACA---ACACATAGCTTTAATTGTGGAGGAGAA---
TTTAAGCAGCTCCAGACAAGAGTCCTGGCTATAGAAAGATACCTAAAGGATCAACAGCTCCTAGGGATTTGGG
GCTGCTCTGGAAAACTCATCTGCACTACTGCTGTACCTTGGAACTCCAGTTGGAGT----------------
----------------------------AACAAATCTCAACAAGAGATTTGG------GAT------
AACATGACCTGGATGCAGTGG---GATAGAGAAATTAGTAATTACACAGACACAATATAC---
AGATTGCTTGAGGATTCGCAAAACCAGCAGGAA--

AAGAATGAAAAAGATTTATTAGCATTGGACAGTTGGAAAGATCTATGGAGTTGGTTTGACATATCAAGTTGGC
TGTGGTATATAAGAATATTCATAATGATAGTAGGAGGCTTGATAGGTTTAAGAATAATTTTTGCT---
GTGCTTTCTATAGTG---------AATAGAGTTAGGCAGGGATACTCACCC------
TTGTCGTTTGCTATTAGTCTGTTTGATACCATAGCAATAGTAGTAGCTGAA---
GGAACAGATAGGATTATATACTTAGTAGTAAGAATTTGTAGAGCT---------------------------
--------ATCTGCAACATA------CCTAACAGAATAAGACAGGGCTTTGAAGCAGCTTTGCAA---TAA
>HM215403
GTG---GGA--
GGAAACTTGTGGGTCACAGTTTATTATGGGGTACCTGTGTGG---AAAGAAGCAAAA---
ACTACTCTATTTTGTGCATCAGATGCTAAAGCATATGAGACAGAG---GTACATAAT---------------
--------------GTCTGGGCTACA---CATGCCTGTGTACCCACAGAC---
CCCAACCCACAAGAAATGGTTTTGGGA---------AATGTAACAGAAAATTTTAAT---------------
-------------------------ATGTGGAAAAATGACATGGTGAATCAGATGCATGAGGATGTA---
ATTAGTTTATGGGATCAAAGCCTA---AAGCCATGTGTAAAGTTG---
ACCCCACTCTGTGTCACTTTAGAATGTACAGATGTAAATGTTACTAATGTAAATGTTACTATGAGCAATGGTA
CTATGAGCAATGATACT---
ATGAGCAATAGTACACAAGTAAATGTTACTATGAACAATGGGAGTTGGATTACGGATGGGATGCAA---
AATTGCTCTTTCAATGCAACCACAGAATTAAGA--------------------------
GATAAGAAGAATAAAGTGTTTGCACTT---TTTTATAAACTTGATATAGTGCCACTTAGTGAG---------
----

ACAAACTCTAGTAACTATTATAGATTAATAAATTGTAATACCTCAGCCATAACACAA---
GCCTGTCCAAAAGTCACTTTTGATCCAATTCCTATACACTATTGCACTCCAGCTGGTTATGCGCTTCTAAAGT
GTAAT---GCAAAAGATTTCAAT------GGGACAGGACCATGCAGTAAT----------------------
-GTTAGC---ACAGTACAATGTACACATGGAATTAAGCCAGTAGTATCAACT---
CAGCTACTGTTAAATGGTAGCCTAGCAGAAGAAGAC-----------
ATAATAATTAGATCTGAAAACCTGACAGACAAT---------GCCAAAACAATAATAGTACATCTCAAT----
-----------AAATCTGTAGAA---------ATTGTATGTACAAGACCCAGCAATAATACAAGGAAA-----
-AGTATAAGGATA------------GGACCAGGACAAACA---TTC------TATGCAACAGGAGAC------
---------ATAATAGGGGACATA------------AGACAAGCACATTGT---AACATTAGTAAAGGA---
AAATGGAATGACACTTTACAAAGGGTAGGTATAAAATTAGCA--------------GAATACTTCCCG----
-----------------------------------AATAAAACAATAAATTTTACC------
TCATCCTCAGGAGGGGACCTAGAAATTACA---ACACATAGCTTTAATTGTGGAGGAGAA---
TTTAAGCAGCTCCAGACAAGAGTCCTGGCTATAGAAAGATACCTAAAGGATCAACAGCTCCTAGGGATTTGGG
GCTGCTCTGGAAAACTCATCTGCACTACTGCTGTACCTTGGAACTCCAGTTGGAGT----------------
----------------------------AACAGAACTCAAGATGAGATTTGG------GAT------
AACATGACCTGGATGCAGTGG---GATAAAGAAATTAATAACTACACAGACACAATATAC---
AGACTGCTTGAAGAATCGCAAAACCAGCAGGAG--

AAAAATGAAAAAGATTTATTAGCATTGGACAGTTGGAAAAATCTATGGAATTGGTTTGGCATAACAAAGTGGC
TGTGGTATATAAAAAATATTCATAATGATAGTAGGAGGCTTGATAGGCTTAAGAATAATTTTTGCT---
GTGCTTTCTATAGTA---------AATAGAGTTAGGAAGGGATACTCACCT------
TTGTCATTTGCTATTAGTCTGTTTGATACCATAGCCATAGCAGTAGCTGAA---
GGAACAGATAGGATTATAGAATTAGTACTCAGCATTTGTAGAGCT---------------------------
--------ATCCGCAACATA------CCTAGAAGAATAAGACAGGGCTTTGAATTAGCTTTGCAA---TAA
>HM215408
GGG---GAA--
GGAAACTTGTGGGTCACAGTTTATTATGGGGTACCTGTGTGG---AAAGAAGCAAAA---
ACTACTCTATTCTGTGCGTCAGATGCTAAAGCACATGAGACAGAA---GTGCATAAT---------------

--------------GTCTGGGCTACA---CATGCCTGTGTACCCACAGAC---
CCCAACCCACAAGAAATAGTTCTGGGA---------AATGTAACAGAAAATTTTAAT----------------
--------------------------ATGTGGAATAATGATATGGCAGATCAGATGCATGAGGATGTA---
ATCAGTTTATGGGATCAAAGCCTA---AAGCCATGTGTAAAGTTG---
ACCCCACTCTGTGTCACTTTAAATTGTACAGATGTTAACAATAGTAGCCTTGCTAATAACAATGGTAGC----
----
-------------------ATTGCTAATAGAACTATTGATGAGGAGGCCTTCAAAGAGATAAAA---
AACTGCTCTTTCAATGCAACCACATTATTAAGA---------------------------
GATAAGAAGAAGACAGAGTATGCACTT---TTTTATAGAGTTGATATAGTACCACTTAATGAAGGGAACTCT-
----

GGTAAGAACTTTAGTAACTTTAGTAAGTATAGATTAATAAATTGTAATACCTCAGCCATAACACAA---
GCCTGTCCAAAGGTTAATTTTGATCCAATTCCTATACACTATTGTACTCCAGCTGGTTATGCGATTCTAAAAT
GTAAG---GATAAGACATTCAAT------GGGACAGGGCCATGCCATAAT-----------------------
-GTTAGC---ACAGTACAATGTACTCATGGAATTAAGCCAGTGGTATCAACT---
CAACTACTGTTAAATGGTAGCCTAGCAGAAGAAGAG------------
ATACTAATTAAATCTGAAAATCTGACAGACAAT---------GCCAAAACAATAATAGTACATCTTAAT----
-----------GAATCTGTAGAA---------ATTGTATGCACAAGACCCAACAATAATACAAGGAAA-----
-AGTATAAGGATA------------GGACCAGGGCAAACA---TTC------TATGCAACAGGAGAC------
---------ATCATAGGAGACATA------------AGACAAGCACATTGT---AACATTAGTAAACAT---
AAATGGAAAGAAGCTTTACAGAACATAAGTAAAAAATTAGCA---------------GAACACTTCCCT----
-----------------------------------AATAAAACAATAAACTTTACA------
TCATCCTCAGGAGGGGACCTAGAAATCACA---ACACATAGCTTTAATTGTAGAGGAGAA---
TTTAAGCAGCTACAGACAAGAGTTCTGGCTATAGAAAGATACCTAAAGGATCAACAGCTCCTAGGGATTTGGG
GCTGCTCTGGAAAACTCATCTGCACTACTGCTGTACCTTGGAACTCCAGTTGGAGC-----------------
----------------------------AACAAGTCTCAAACAGAGATTTGG------GAT------
AACATGACCTGGATGCAGTGG---GATAAGGAAATTAGTAATTACACAAACACAATATAC---
AGTTTGCTTGAGGAATCGCAAAACCAGCAGGAA--

AGAAATGAAAAGGATTTATTAGCATTGGACAGCTGGAAAAATCTATGGAGTTGGTTTGACATTTCAAATTGGC
TGTGGTATATAAAAATATTCATAATGATAGTAGGAGGCTTGATAGGTTTAAGAATAATTTTTGCT---
GTGCTTTCTGTAGTG---------AATAGAGTTAGGCAGGGATACTCACCT------
TTGTCGTTCGCTATTAGTCTGCTTGATACCATAGCAATAGCAGTAGCTGAA---
GGAACAGATAGTATTATAAACATAGTGCAAAGAATTTGTAAAGCT----------------------------
--------ATCCGCAACATA------CCTAGAAGAATAAGACAGGGCTTAGAAGCAGCTTTGCAA---TAA
>HQ699950
GTG---GGA--
GGAAACTTATGGGTCACAGTTTATTATGGGGTACCTGTGTGG---AAAGAAGCAAAA---
ACTACTCTATTTTGTGCATCAGATGCTAAAGCATATGATACAGAG---GTACATAAT----------------
--------------GTCTGGGCTACA---CATGCCTGTGTACCCACAGAC---
CCCAACCCACAAGAAATATTTTTGGAA---------AATGTAACAGAAAACTTTAAC----------------
--------------------------ATGTGGAAAAATGAAATGGTGAATCAGATGCATGAAGATGTA---
ATCAGTTTATGGGATCAAAGCCTA---AAGCCATGTGTAGAGTTG---
ACCCCCCTCTGTGTCACTTTAGAATGTAGAAATGTTACCCACAATGTTACCCACAAT----------------
----
----------------------------------GAGAGTGTGAAGGAAATAAAGGAAATAAAA---
AATTGCTCTTTTAATGCAACCACAGAATTAAGA---------------------------
GATAGGAAGCAGGCAGTGCATGCACTT---TTTTATAGACTTGATATAGTGCCACTTGATGAGAAGAACGTT-
----

AGTAAAAACTCTAGTGAGTATTATAGATTAATAAATTGTAATACCTCAGCCATAACACAA---
GCCTGTCCAAAGGTCACTTTTGATCCAATTCCTATACACTATTGCACTCCAGCTGGTTATGCGATTCTAAAGT
GTAAT---GATAAGACATTCAAT------GGGACAGGACCATGCCGTAAT-----------------------
-GTTAGC---ACAGTACAATGTACACATGGAATTAAGCCAGTGGTATCAACT---
CAACTACTGTTGAATGGTAGCCTAGCAGAAGAAGAA------------
ATAATAATTAGATCTGAAAATCTGACAGACAAT---------GTCAAAACAATAATAGTACATCTTAAT----
-----------CAACCTGTAGAA---------ATTGTATGTACAAGACCCAACAATAATACAAGAAAA-----
-AGTATAAGGATA------------GGACCAGGACAAACA---TTC------TATGCAACAGGAGAC------
---------ATAATAGGGGACATA------------AGACAAGCACATTGT---AACATTAGTAAAGCT---
AGATGGAATGAAACCTTACAAGGGGTAGGAAGAAAATTAGCA---------------GAATACTTCCCT----

-----------------------------------AATAAAAAAATAAACTTCACA------
TCATCCTCAGGAGGAGACCTAGAAATTGCA---ACACATAGCTTTAATTGTGGAGGAGAA---
TTTAAGCAGCTCCAGACAAGAGTCCTGGCTATAGAAAGATACCTAAAGGATCAACAGCTCCTAGGGATTTGGG
GCTGCTCTGGAAAACTCGTCTGCACCACTAATGTACCTTGGAACTCCAGTTGGAGT-----------------
----------------------------AACAAAAATCAAGAAGAGATTTGG------GGT------
AACATGACCTGGATGCAGTGG---GATAGAGAAATTAGTAATTTCACAGACACAATATAC---
AGATTGCTTGAAGACTCGCAAAACCAGCAGGAA--

AGAAATGAAAAAGATTTATTAGCATTAGACAGTTGGAAAAATCTATGGAATTGGTTTGACATAACAAACTGGC
TGTGGTATATAAAAATATTCATAATGATAGTAGGAGGCTTGATAGGCTTAAGAATAATTTTTGCT---
GTGCTCTCTATAGTA---------AATAGAGTTAGGCAGGGATACTCACCT------
TTGTCGTTTGCTACTAGTCTGCTTGATACCATAGCCATAGCAGTAGCTAAC---
GGAACAGATAGGATTATAGAATTAGTACTCAGCATTTGTAGAGCT----------------------------
--------ATCCGCAACATA------CCTACAAGAATAAGACAGGGCCTTGAAATAGCTTTGCAA---TAA
>HQ699981
GGG---GGA--
GGAGACTTGTGGGTCACAGTTTATTATGGGGTACCTGTGTGG---AAAGAAGCAAAA---
ACTACTCTATTTTGTGCATCAGATGCTAAAGCATATGATACAGAG---GTACATAAT----------------
--------------GTATGGGCTACA---CATGCCTGTGTACCCACAGAC---
CCCAACCCACAAGAAATAGTTTTGAAA---------AATGTAACAGAACAGTTTAAC----------------
--------------------------ATGTGGGAAAATGAAATGGTGAATCAGATGCATGAGGATGTA---
ATCAGTTTATGGGATCAAAGCCTA---AAGCCATGCGTAAAGTTG---
ACCCCACTCTGTGTCACTTTAGAATGTAGAAATGCTAGTAGAAATGATAGTGAA-------------------
----
-------------------------------ACCCACAATATTACCTACACGGAGGAAATAAAA---
AATTGCTCTTTCAATACAACCACAGAAATAAGA---------------------------
GATAAGGTGCAGAAAGTACATGCACTT---TTTTATAGACTTGATGTAGTGCCACTTGGTAAGGAGAAC----
----

CCTAGTACCAACTCTAGTGAGTATTATAGATTAATAAATTGTAATACCTCAGCCATAACACAA---
GCCTGCCCAAAGGTCAATTTTGATCCAATTCCTATACATTATTGCACTCCTGCTGGTTATGCGATTCTAAAGT
GTAAT---AATAAGACATTCAAT------GGGACAGGACTATGCAATAAT-----------------------
-GTTAGC---ACAGTACAATGTACACATGGAATTAAGCCAGTGGTGTCAACT---
CAACTACTGTTAAATGGTAGCCTAGCAGAAGAAGAA------------
ATAATAATTAGTTCTAAAAATGTGACAGACAAT---------GGCAAAATAATAATAGTACATCTTAAT----
-----------AAACCTGTAAAA---------ATTGAATGTACAAGACCCAACAATAATACAAGAAAA-----
-AGTGTAAGGATA------------GGACCAGGACAAGCA---TTC------TATGCAACAGGAGAC------
---------ATAATAGGGAACATA------------AGAGAAGCACATTGT---AACATTAGTAAAAGT---
AAATGGAATGACACTTTACAAAGGGTAGGTGAAAAATTAGCA---------------AAACACTTCCCT----
-----------------------------------AATAAAACAATAAAATTTAAT------
TCATCCTCAGGAGGGGACCTAGAAATTACA---ACACATAGCTTTAATTGTAGAGGAGAA---
TTTAAGCAGCTCCAGACAAGAGTCCTGGCTATGGAAAGATACCTACAGGATCAACAGCTCCTAGGGTTGTGGG
GCTGCTCTGGAAAACTCATCTGCACTACTGCTGTACATTGGAACTCCAGTTGGAGT-----------------
----------------------------AGCAAATCTCATGATGACATTTGG------AAA------
AACCTGACCTGGATGCAGTGG---GATAAGGAAATTAGTAATTACACAGACACAATATAC---
AGATTGCTTGAAGACTCGCAAAACCAGCAGGAA--

GAAAATGAAAAAGATTTATTAGCATTGGACAGTTGGAAAAATCTATGGAGTTGGTTTGACATAACAAAGTGGC
TGTGGTATATAAAAATATTCATAATGATAGTAGGAGGCTTGATAGGCTTAAGAATAATTTTTGCT---
GTGCTTTCTATAGTA---------AATAGAGTTAGGCAGGGATACTCACCT------
TTGTCGTTTGCTACTAGTCTGCTTGATAGCATAGCCATAGCAGTAGCTGAA---
GGAACAGATAGGATTATAGAATTAATACGCAGCATTTGGAGAGCT----------------------------
--------ATCCGCAACATA------CCTACAAAGATAAGACAGGGCTTTGAACTAGCTTTGCAA---TAA
>KC807928
ATG---GAA--
GAAAAATTGTGGGTCACAGTCTATTATGGGGTACCTGTGTGG---AAAGATGCAAGA---
ACTACTCTATTCTGTGCATCAGAGGCTAAAGCATATGAGACAGAA---GTGCATAAT----------------
--------------GTATGGGCTACA---CATGCTTGTGTACCCACAGAC---
CCCAACCCACAAGAGATGGTTATGGGA---------AATGTAACAGAAAATTTTAAC----------------
--------------------------ATGTGGGATAATGATATGGTGGATCAGATGCATGAGGATGTA---

ATCAGTTTATGGGATCAAAGCCTA---AAGCCATGTGTAAAGTTG---
ACCCCACTCTGTGTCACTTTAGACTGTAGAAGTGTTAATATGAGAAGTGTTAATATGACC-------------
----
-------------------------AACAATGTGACCGTCAATGGAACCTACGATGAGATAAAA---
AATTGCTCTTTCAATACAACCACATTATTAACA---------------------------
GATAGGAAAAAGACAGAGTATGCACTT---
TTTTACAAACTTGATATAGTACCCCTTGATAATGAGAATCCTAGTAAG-------------------------
----
AACTCTAGTAACAACTCTAGTGATTATTATAGATTAATAAATTGTAATACCTCAGCCATAAGACAA---
GCCTGTCCAAAGGTCACTTTTGATCCAATTCCTATACACTATTGCACTCCAGCTGGTTTTGTGATTCTAAAGT
GTAAT---AATAAGACATTCAAT------GGGACAGGACCATGCCATAAT-----------------------
-GTTAGC---ACAGTACAATGTACACATGGAATTAAGCCAGTGGTATCAACT---
CAACTACTGTTGAATGGTAGCCTAGCAGAAGAAGAG------------
ATAATAATTAGATCTAAAAATCTGTCAGACAAT---------GCCAAAACAATAATAGTACATCTTAAT----
-----------GAATCTGTAGAA---------ATTGTATGTACAAGACCCAACAATAATACAAGAAAA-----
-AGTATAAGGATA------------GGACCAGGACAAACA---TTC------TATGCAACAGGAGAT------
---------ATCATAGGAGACATA------------AGACAAGCATATTGT---AACATTACTAAAAT---
AAATGGGTTGAAACTTTACAGAAAGTAAGTAAAAAATTAGCA---------------GAACGCTTCCCT----
-----------------------------------AATAGAACAATAACATTTAAC------
TCATCCTCAGGAGGGGACCTAGAGATTACA---ACACATAGCTTTAATTGTAGGAGGAGAA---
TTTAAACAGCTACAGACAAGAGTCCTGGCTCTAGAAAGATACCTAAGGGATCAACAGCTCCTAGGGATTTGGG
GCTGCTCTGGAAGACTTATCTGCACTACTGCTGTACCTTGGAACTCCAGTTGGAGT-----------------
----------------------------AACAAATCTCTAAATGAGATTTGG------GAG------
AACATGACCTGGATGCAGTGG---GAAAAGGAAATTGATAATTACACAGACTACATATAC---
ACATTGCTTGAAGTCTCGCAAAACCAGCAGGAA--

AAGAATGAAAAAGATTTATTAGCATTGGACAGTTGGAAAAACCTATGGAGTTGGTTTAACATATCAAATTGGC
TGTGGTATATAAAAATATTCATAATGATAGTAGGAGGCTTGATAGGCTTAAGAATAATTTTTGCT---
GTGCTTTCTATAGTG---------AATAGAGTTAGGCAGGGATACTCACCT------
TTGTCGTTTGCTATTAGTCTGTTTGATACCATAGCAATAACAGTAGCTGAA---
GGAACAGATAGGGTTATAGCCATAGTACAAAGAATTTGTAGAGCT----------------------------
--------ATCCGCAACGGA------CCTAGAAGAGTAAGACAGGGCTTGGAAGCATCTTTGCAA---TAA
>JF683757
GTG---GTG---GGG---------------------------------
AACAAGGACTTGTGGGTCACAGTCTATTATGGGGTACCTGTGTGG---AAAGAAGCAAAA---
ACTACTCTATTCTGTGCATCAAATGCTAAAGCATATGAGAGGGAG---GTGCATAAT----------------
--------------GTTTGGGCTACA---CATGCCTGTGTACCCACAGAC---
CCCAACCCACAGAAAATAGATTTGGAA---------AATGTAACAGAAAATTTTAAC----------------
--------------------------ATGTGGAAAAATGACATGGTGGATCAAATGCATGCAGATGTA---
ATCAGTTTATGGAAGGAAAGCCTA---AAGCCATGTGTAAAGCTG---
ACCCCACTCTGTGTCACTTTAAATTGTACAGAGGTTACTGTTATCAAAAATACAGGGGTTAATATGACCAATA
ATAGT--
-------ACTAGTACCAGTATTGACACTAGTATCCTTAATGATACCAGTAATGCAGACTTGAAG---
AATTGCTCTTTCAATACAACCACAAAAATAAGT---------------------------
GATAATGAAATTTTTGAGTTTGCTGTT---AATAATACTCTTGATGTAGAACCAATTGATGGA----------
----

ACATACACCTCCATAACCTTTAGATTAACAAATTCCAATTCCTCCTCCATACCACAA---
GCTTGTCCAAAGGTTTCTTTTGACCCACTTCCTATTCCTTATTGTGCTCCAGCTGGGTATACGGGTCTAAGGT
GTAGT---AATAATACATTCACT------GGAACAGGACCATGCCGTAAT-----------------------
-GTCAGC---ACAGTACAATGTACACATGGAATTAAGCCAGTGGTATCAACT---
CAGCTACTGTTAAATGGTAGCCTAGCAGAAGGAGAG------------
ATAATGATTAGATCTGAAAATCTGACAAATAAT---------GCCAAAACAATAATAGTACATCTTAAT----
-----------GAATCTGTAAAC---------ATTGTATGTACAAGACCCAACAACAATACCAGAAAA-----
-GGTGTAGGGATA------------GGACCAGGACAAGCA---ATC------TATGCAACAGGAGCA------
---------ATAATAGGAGACATA------------AGACAAGCACATTGT---AACATTACTAAAGG---
GGATGGAATGGACTCCCACAAAAGGTAAAGGGAAAGTTAAGG---------------GAACACCTCCCC----
-----------------------------------GGTAAATTAATAATATTTCAA------
CCACACTCAGGAGGGGACCTACAAATTACA---ATGCATACCTTTAATTGGGGAGGAGAA---
TTTAAACAGCTCCAGGCAAGAGTCTTGGCTCTGGAAAGATACCTAAAGGATCAACAGCTCCTAGGGATTTGGG

GCTGCTCTGGAAAACTCATCTGCACCACTACTGTACCTTGGAACACCAGTTGGAGT-----------------
----------------------------AATAGATCCAAAGAGGAGATTTGG------AAT------
AACATGACCTGGATGCAGTGG---GAGAGAGAAATTGATAATTACACAGAAATAATATAC---
CAGTTACTTGGAGAATCGCAGATTCAGCAGGAA--

CAAAATGAAAAGGATTTATTAGCATTGGACAGCTGGAAAAATCTGTGGAGTTGGTTTTCCATATCAAATTGGC
TGTGGTATATAAGAATATTCATAATGATAGTAGGAGGCTTGATAGGCTTAAGAATAATTTTTGCT---
GTACTTTCTATAATA---------CATAGAGTTAGGCAGGGATACTCACCT------
TTGTCATTGGCTATTAGTCTGCTTGATACTGTAGCAATAGCAGTAGCTGGG---
AGGACAGATAGGATTATAGAAGTAATACAAGAAATTTGTAGAGCT----------------------------
--------ATCTACCACATA------CCTAGAAGAATAAGACAGGGCTTTGAAGCAGCTTTGCTA---TAA
>L23065
GGG---ATG--
GGGAACTTGTGGGTCACAGTCTATTATGGGGTACCTGTGTGG---CAAGAAGCAAAC---
CCTACTCTATTTTGTGCATCAGATGCTAAAGCATATGAGACAGAA---GTGCATAAT----------------
--------------GTCTGGGCTACA---CATGCCTGTGTACCCACAGAC---
CCCAGCCCACAGGAACTAGTTATGGAA---------AATGTAACAGAAAATTTTAAC----------------
--------------------------ATGTGGAAAAATGGCATGGTGGATCAGATGCATCAGGATATA---
ATCAGTTTATGGGATGAAGGCCTA---AAGCCATGTGTAAAGTTG---
ACCCCACTCTGTGTTACTTTAAACTGTAGTAATAAGGTTACTGCTACCGGTAATGGT----------------
----
----------------------------AGTGTTACCTACAATAGCACAGAAAAAGACATGAGA---
AATTGCTCTTTCAACATAACCACAGAACTAAGA---------------------------
GATAAGAAACAGAAAAAATATGCACTT---TTTTATAAACTTGATATAGTACCACTTGGGAAT----------
----

GGGAATGAGGAATATCGATTAATAAATTGTAATACCTCATCCATAACACAA---
GCCTGTCCAAAGGTCTCTTTTGAACCAATTCCTATACATTATTGTGCTCCAGCTGGTTATGCGATTCTAAAGT
GTAGG---GATACAAAATTCAAT------GGAACAGGACCATGCAAGAAT-----------------------
-GTCAGT---ACAGTACAATGTACACATGGAATTAAACCAGTGGTATCAACT---
CAACTATTGTTAAATGGTAGTACAGCAGAGGAGGAT------------
ATAATAATTAGATCTGAAAATCTGACAAACAAT---------GCCAAAATAATAATAGTACAGCTTAAT----
-----------CAATCTGTAGAA---------ATTAATTGTACAAGACCCAACAATAATACAAGGCAA-----
-AGCATAAGGATA------------GGACCAGGACAAACA---TTC------TATGCAACAGGAGAT------
---------ATAATAGGAGATATA------------AGACAAGCACATTGT---AACATTAGTAGACAA---
AAGTGGAAAGAAACTTTACAACAGGTAAAAGGAAAATTAAAA---------------GAACACTTC-------
-----------------------------------AATAAAACAATAAAATTTGCA------
CCATCCTCAGGAGGAGACATAGAAATTACA---ACACATAGCTTTAATTGTGGAGGAGAA---
TTTAAGCAGCTCCAGACAAGAGTCCTGGCTATAGAAAGATACCTGAAGGATCAACAGCTCCTAGGGATTTGGG
GCTGCTCTGGAAAACTCATCTGCACCACTGCTGTGCCTTGGAACTCTAGTTGGAGT-----------------
----------------------------AATAAATCTCAAGAGGAGATTTGG------GAC------
AACATGACCTGGATGCAGTGG---GATAGAGAAATTAATAATTATACAGAAACAATATAC---
AGGTTGCTTGAAGTCTCGCAAACCCAGCAGGAG--

CAAAATGAAAAAGATTTACTAGCATTGGACAAATGGCAAAATCTGTGGAGTTGGTTTGACATAACAAAGTGGC
TGTGGTATATAAAAATATTCATAATGATAGTAGGGGGCTTGATAGGTTTAAGAATAATTTTTGCT---
GTGCTTTCTATAGTG---------AATAGAGTTAGGCAGGGATACTCACCA------
TTATCATTTGCTGTTAGTCTGCTTGATACCACAGCAATAGCAGTAGCTGAA---
GGAACAGATAGGATTATAGAAATAATACAAAGAATTTGGAGAGCT----------------------------
--------TTCTGCAACATA------CCTAGAAGAATAAGACAGGGCTTTGAAGCAGCTTTGCAA---TAA
>EF531335
GTA---AAG---
GGCTTATGGGTCACCGTCTATTATGGGGTACCTGTGTGG---AGAGAAGCAAAT---
ACTACTCTATTTTGTGCATCAGATGCTAAAGCATATGACAAAGAA---GTGCATAAT----------------
--------------GTCTGGGCTACA---CATGCCTGTGTACCCACAGAC---
CCCAAACCACAAGAACAATGGTTGAAA---------AATGTAACAGAAGATTTTAAC----------------
--------------------------ATGTGGAAAAATAACATGGTGGAACAAATGCATGAGGATATA---
ATCAGTTTATGGGATCAGAGCCTA---AAGCCATGTGTAAAGTTG---
ACCCCACTCTGTGTCACTTTAAACTGTACTAATGCTACCAGTATTGTTAATATGACTGATGTTGCCAGTAATA
GGAGTAGGAGTAATGATACCAGTGTTGAAAATGTT--------------------------------------

----------AATGGTACCAGTGTTGAAAATATGACTATTGTTAACAGTACTGATGAAATAAAA---
AATTGCTCTTTCAATATAACCACAGGAATAGGA------------------------
GATAAGCCAAAGAAAGAAAGTGCACTT---TTTTATAGCCTTGATATAGTGCCACTTAACAAA---------
----

AGTGCTAATAATAGTGAGTATATATTAATAACTTGTAAAACCTCCAAGATAACACAA---
GCCTGTCCAAAGGTCTCTTTTGAGCCAATTCCTATACATTATTGTGCTCCAGCTGGTTATGCGATTCTAAGGT
GTGAT---AATAAGACATTCAAT------GGAACAGGACCATGCAATAAT---------------------
-GTCAGC---TCAGTACAATGTACACATGGAATTAGGCCAGTGGTAACAACT---
CAATTACTGTTAAATGGTAGCTTAGCAGAAGAAGAT-----------
ATAATAATTAGATCTGTAAATATGTCAAACAAT---------GCCAAAACAATAATAGTACATCTTAAT----
-----------GAATCGGTAGAG---------ATTATATGTACAAGACCCAATAATAATACAAGAAAA-----
-AGTGTGGGGATG------------GGACCAGGACAAGCA---ATC------TATACAACAGGAGCC------
---------ATAATAGGAAATATA-----------AGACAAGCACATTGT---AACATCAGTGGAAGG---
AACCGGAGTAGAACTTTACAAAGGGTAAGTGAAAAATTACAA--------------AAATATTTTTAT----
-----------------------------------AATAAAACAATAAAATTCCAA------
CCACACTCAGGAGGGGACCCAGAAATTACA---ACACATAGTTTTAATTGTGGAGGAGAA---
TTTAAGCAGCTCCAGGCGGGAGTCCTGGCTCTAGAAAGATACCTACAGGATCAACAGATCCTAGGGATTTGGG
GCTGCTCGGGAAAACGCATCTGTACCACTGATGTGCCTTGGAACTCTAGTTGGAGT----------------
----------------------------AATAAAACTAAAGAATACATTTGG------GGA------
AATATGACCTGGATGCAGTGG---GATAAAGAAATTAGTAATTACACAGGCATAATATAC---
GACTTGCTTGAAGAATCGCAAAACCAGCAGGAA-------------------------------------
----
AAAAATGAACAGGAATTACTAGCATTGGACAGTTGGAAAAACCTGTGGAATTGGTTTAACATAACAAACTGGC
TGTGGTATATAAAAATATTCATAATGATAATAGGAGGCTTGATAGGTTTAAGAATAATATTTGCT---
GTGCTATCTATAGTG---------CATAGAGTTAGGCAGGGATACTCACCC------
TTGTCGTTTGCTATTAGTCTGCTTGATACCATAGCAATAGCAGTAGCTGAA---
GGAACAGATAGGATTATAGAGTTTATACAAAGAATTTGCAGAGCT---------------------------
--------ATCTGCAACATA------CCTAGAAGAATAAGACAGGGCTTGGAAAGGATTTTGCTA---TAA
>KU319529
GTG---GGG---
GGGAACTTGTGGGTCACAGTTTATTATGGGGTACCTGTGTGG---AGAGAAGCAAAA---
ACTACTCTATTTTGTGCATCAGATGCTAAAGGATATGAGAGAGAA---GTGCATAAT---------------
--------------GTCTGGGCTACA---CATGCCTGTGTACCCACAAAC---
CCCGACCCACAAGAATTGGTTTTGGGA---------AATGTAACAGAAAATTTTAAC---------------
-------------------------ATGTGGAAAAATGATATGGTGGATCAGATGCATGAGGATGTA---
ATCAGTTTATGGGATCAAGGCCTA---AAGCCATGTGTAAAATTG---
ACCCCACTCTGTGTCACTTTAACCTGTATTAATGCTACTATCTTCAATGGTAGCGAAATT------------
----
--GACGAAGCAAAA---
AATTGCTCTTTCAGTATGACCGCGGAAGTAAGA------------------------
GATAGCACTATCAATGTATCCTCAGTT---TATTATAGACTTGATATTGTACCCCTTGATAATAATAAGACC-
----

TCTAGCAGTGATGGTGAATATAGATTAATAAATTGTAATACCTCAACTGTAACACAA---
GCCTGTCCAAAGGTCTCTTTTGACCCATTTCCTATACATTATTGTGCTCCAGCTGGTTATGCGATTCTAAAGT
GTAAT---AACAAGACATTCAAT------GGAACAGGACCATGCACTAAT---------------------
-GTCAGT---ACAGTACAATGTACACATGGAATTAAACCAGTAGTATCAACT---
CAACTACTGTTAAATGGGAGTCTAGCAGAAAAAGAA-----------
ATAGTAATTAGGTCTAAAAATCTGACAGACAAT---------GCCAAAATAATAATAGTCCATCTTAAA----
-----------AAATCTGTAGAA---------ATAATATGTATAAGGACTGGCAATAATACAAGGAAG-----
-AGTATAAGGATA------------GGACCTGGACAAACA---TTT------TATGCAACAGGAGAA------
---------ATAATAGGAGACATA-----------AGACAAGCATATTGT---AACATTAATGAAAAG---
ACATGGAATGACACTTTACAAGAGGTAGCGAAAAAATTACAT--------------GAACACTTCCCT----
--------------------------------AATAGCACAAACATAAGATTTGCA------
CCACCCTCAGGAGGAGACCTAGAAATTACA---ACACATAGCTTTAATTGTAGAGGAGAA---
TTTAAGCAGCTCCAGGCAAGAGTCCTGGCTATAGAAAGATACCTAAAGGATCAACAGCTCCTGGGGATTTGGG
GCTGCTCTGGAAAGCTCATCTGCACCACTAATGTGCCTTGGAACTCTAGTTGGAGT----------------
----------------------------AACAAATCTCTAGAGGATATTTGG------AGT------
AATATGACTTGGATGCAGTGG---GATAGAGAAATTAATAATTACACAAACACAATATAC---

AGTTTGCTTGAAGAATCGCAAATCCAACAGGAG---------------------------------------
----
AAAAATGAAAAAGATTTACTAGAATTGGACAAGTGGGATAATCTGTGGAATTGGTTTAGCATAACAAATTGGC
TGTGGTACATAAAGGTATTCATAATGGTAGTAGGAGGCTTGATAGGTTTAAGAATAATTTTTACT---
GTGCTTTCTATAGTA---------AATAGAGTTAGGCAGGGATACTCACCT------
TTGTCATTGGCTATTAGTCTGCTTAATACCACAGCAATAGTAGTAGCTGAA---
GGAACAGATAGGATTATAGAATTAGTACAAAGAACTTGGAGAGCT---------------------------
--------ATCTGCAATATA------CCAAGAAGGACAAGACAGGGCTTGGAAGCAGCTTTGCAG---TAA
>KU319530
GTG---GGG---
GGGAACTTGTGGGTCACAGTTTATTACGGGGTACCAGTGTGG---AAAGAGGCAAAA---
GCTCCTTTATTTTGTGCATCAGATGCTAAAGCACATGAGAACGAA---GTGCATAAT----------------
--------------GTCTGGGCTACA---TATGCCTGTGTACCCACAGAC---
CCCAACCCACAAGAAATGGTGTTGGAA---------AATGTAACAGAGAAGTTTAAC---------------
-------------------------ATGTGGAAAAATGACATGGTTGAACAGATGCATGAGGATATA---
ATCAGTTTATGGGATCAAAGCCTA---AAGCCATGTGTAAAGTTG---
ACCCCACTCTGTGTCACTTTAAACTGCGGTCCTGTTAGTAATAGTACTGGTGATAATGTTACT----------
----
----------------------------ATTACCATCAATAATACCACGAAGGAGGACATGAAA---
AATTGCTCTTTCAATGTAACCACAGAATTAAGA--------------------------
GATAAAAGAAAGAAGGAATATGCACTT---TTTTATAAACTCGATATAGTGCAACTTAATAGAGAGAAG----
----

AACCCCAGTGAGTATAGATTAATAAATTGTAATACCTCAGCCGTAACACAA---
GCCTGTCCAAAGGTCTCTTTTGACCCAATTCCTATACATTATTGTGCTCCAGCTGGTTATGCGATTCTAAAGT
GTAAT---AACAAGACATTCAAT------GGAACAGGACCGTGCAATAAT----------------------
-GTCAGC---ACAGTACAATGTACACATGGAATTAAACCAGTAGTATCAACT---
CAACTACTATTAAATGGTAGTCTAGCAGAAGAAGAA-----------
ATAGTAATTAGATCTGAAAATCTGACAAACAAT---------GTCAAAATAATAATAGTACATCTCCAA----
-----------CAACCTGTAGAA---------ATTGTATGTACAAGGCCAGGCAATAATACAAGGAAA-----
-AGTGTGAGGATA------------GGACCAGGACAAACA---TTT------TATGCAACAGGAGAC------
---------ATAATAGGAGAAATA------------AGACGAGCACATTGT---AACATTAAGGAAAAG---
ACATGGAATGAAACTTTACAAAATGTAAGTAAAAAATTACAA---------------GCACACTTCCCT----
-----------------------------------AATAAAACAATATACTTTGAA------
CCATCCTCAGGAGGGGACCTAGAAATTACA---ACACATAGTTTTAATTGTAGAGGAGAA---
TTTAAACAGCTCCAGACAAGAGTCCTGGCTATAGAAAGATACCTAAAGGATCAACAGCTCCTAGGGATTTGGG
GCTGCTCTGGAAAGCTCATCTGCACCACTGCAGTACCTTGGAACACTAGTTGGAGT----------------
----------------------------AATAAATCACAAGGGGATATTTGG------GAT------
AACAGGACTTGGATGCAGTGG---GATAAAGAAATTAGTAATTACACAGGTACAATATAC---
AGGTTGCTTGAAGACTCGCAAAACCAGCAGGAA---------------------------------------
----
AAAAATGAAAAAGAACTACTCGCATTAGACAGTTGGAAAAAATTTATGGACTTGGTTTGACATATCAAGGTGGC
TGTGGTATATAAAGATATTCATAATGATAGTAGGAGGCTTGATAGGTTTAAGAATAATTTTTGCT---
GTACTTTCTATAGTA---------AATAGAGTTAGGCAGGGATACTCACCT------
TTGTCATTTGCTATTAGTCTGTTTGATACCATAGCAATAGCAGTAGCTGAA---
GGAACAGATAGAATACTAGAAATAATACAAAGAGTTTGTAGAGCT---------------------------
--------ATCTACAACATA------CCTAGAAGAATAAGACAGGGCTTTGAAGCAGCTTTGCAA---TAA
>KU319539
ATA---ATA---
AGAAACGTGCTTTTCACAATTTATTATGGGGGACCTGATTGG---AAAGAAACAAAA---
ACTACTCTATTTTTTTAACAAATGCTAAAACATATAAAAGAGAG---GTACATAAT----------------
--------------GTCTGGGCTACT---CACGCCTGTGGACCCGACACT---
CCCAACGCCCCAGAGATAATTTTGGTT---------CATGGCGCACAAAATTTTACC---------------
-------------------------TTGCGAAAAATTGACATGGTGGAACAAACGCCTGATGATATA---
CTCAGTTTGTGTGATGAGATCCTA---AAGTCGTGTGTAAAGATG---
ACCCCAGTCTGTGTCACTTTAAACTGTAGCAATGCAACTGCTAATACAACTGCTAATAGAAAT----------
----
---ATCGGTAGAAGTGTAGAGCAT---
TATTGTTATTTCAATAACACTACTAGAATTCACGGC-----------------
GTAATGATAGGTATGCAGGAATATTGTAAATTT---GTTTGTCTAGTAGATATGATGCCGATAGATAAT----

----

GGCACTACTAGATATAGGTTGATAAGTTGTAGATCCTCAGTCAGTAAACAA---
ACCTGTCTAATGGTCTCTTTTGCCCCAATTCCTATACATTATTGTGCTCCAGCTGGTTTTGCGATTATAAAGT
GTAAT---GACAAGACATTCAAT------GGAGTAGGACCATGTAATAAT-----------------------
-GTCAGC---ACAGTACAATGTACACATGGAATTAAGCCAGTGGTATCAACT---
CAACTACTGTTAAATGGTAGTCTAGCTGAAGAAGGG------------
ATAGTAATTAGATCTGAAAATCTGAAAGACAAT---------GCCAAAATAATAATAGTGCATCTTAAA----
-----------AACCCTGTGAAA---------ATTGTGTGTACAAGGCCAAGCAATAATACAAGGAGA-----
-AATATAGGGATA------------GGACCAGGACAAACA---TTC------TTTGCACCAGGAACC------
---------ATAATAGGAGATATA------------AGACGAGCATATTGT---ACCATTAGGAAAAGG---
AAATGGAATAACACTTTACAAGAGGTAACTAAAGAATTACTA---------------AAACTATTCCGA----
--------------------------------AATAGCAGCACAATAAAATTTGCA------
CCATCTTCAGGAGGGGACCTAGAAATTACA---ACACATAGCTTTAATTGTAGAGGAGAA---
TTTAAGCAGCTCCAGGCAAGAGTCCTGGCTATCGAAAGATACCTAAAGGATCAACAGCTCCTAGGGATTTGGG
GCTGCTCTGGAAAACTCATCTGCACCACTGCTGTGCCTTGGAACTCTAGTTGGAGT-----------------
----------------------------AATAAATCTAAAGATGATATCTGG------CAT------
AACATGACTTGGATGGAGTGG---GATAGAGAAATTAGTAATTACACAGGCACAATATAC---
AGGTTGCTTGAAGAATCGCAAACCCAGCAGGAA--

AAAAATGAAAAAGATTTACTCGAATTGGACAGTTGGGAATATTTGTGGAATTGGTTTTATTTTTCAAATTGGC
TGCGGTATATAATGCTGTTCATAATGATAATAGGAGGCTTGATAGGTTTAAGAATAATTTTTGCT---
GAGATATCTATAACA---------AATAAAATTATGCAAGGATACTCAACT------
ATATCATTGGCTATTAGTATAATAGATGCTTTAGCAATAACAGTAGCAGAA---
GGAACAGATAGAATTATAGAAGTAGTACAAAGATTTTGTAGAACT----------------------------
--------ATCCTTAACATC------CCTAGAAGAATAAGACAGGGCTTTGAAGCAGCATTGCAA---TAA
>KU319542
GTG---GGA--
GGGGACTTGTGGGTCACAGTGTATTATGGGGTACCTGTGTGG---AGAGAAGCAAAA---
GCCCCTCTATTTTGTGCATCAGATGCTAAAACATATGAGAGAGAA---GTGCATAAT----------------
--------------GTTTGGGCTACA---CATGCCTGTGTACCCACAGAC---
CCCGACCCACAAGAAATAGCTTTGAAA---------AATGTAACAGAAAGTTTTAAC----------------
--------------------------ATGTGGAAAAATGACATGGTAGATCAGATGCATGAGGATATA---
ATCAGTTTATGGGATCAAAGCCTG---AAGCCATGTGTAAAGTTG---
ACCCCACTCTGTGTCACTTTAAACTGTAATGATACTTATAATACTACTAATAATGATAAC-------------
----
-------------------------------GCAAGAATACTAACGCAACTAAGAGAAATGACA---
CAGTGCTCTTTCAATATAACCACAGAATTGAGA---------------------------
GATAAGAAAAAGACTGAATATGCACTT---TTTTATAAACTTGATGTAGTACCACTTAATAGT----------
----

AGTAATAGTAATAGTAGTGAGTATAGATTAATAAATTGTAATACCTCAGCCGTAACACAA---
GCCTGTCCAAAGGTTTCTTTTGACCCAATTCCTATACATTATTGTGCTCCAGCTGGTTATGCGATTTTAAAGT
GTAAT---AACAAGACATTCAAT------GGAACAGGACCATGCGAAAAT-----------------------
-GTCAGC---ACAGTACAATGTACACATGGAATTAAACCAGTAGTATCAACT---
CAGCTACTGTTAAATGGTAGTCTAGCAGAA---GAG------------
ATAGTAATCAAATCTGAAAATCTGACAGACAAT---------GCCAAAACAATAATAGTACATCTTAAT----
-----------GAATCTGTAGAT---------ATTGTGTGTACAAGGCCTAACAATAATACAAGGAAA-----
-AGTATAAGAATA------------GGACCAGGACAAACA---TTC------TATGCAACAGGAGAT------
---------ATAATAGGAAATATA------------AGAGAAGCACATTGT---AACATTAGTGAAAGG---
AAATGGAATAATACTTTACAAAAGGTAGGTAAAAAATTAAAA---------------GAACTCTTCCCT----
-----------------------------------AATAAAACAATAGCATTTGCA------
CCATCCTCAGGAGGGGACCTAGAAATTACA---ACACATAGCTTTAATTGTAGGGGAGAA---
TTTAAGCAGCTCCAGACAAGAGTCCTGGCTATAGAAAGATACCTAAAGGATCAACAGCTCCTAGGGATTTGGG
GATGCTCTGGAAAAATCATCTGCACCACTGCTGTGCTTTGGAACACTAGTTGGAGT-----------------
----------------------------AATAAATCTAAAGAGGAAATTTGG------GAA------
AACATGACTTGGATGCAGTGG---GATAGAGAAATTAGTAATTACACAGACACAATATAC---
AGGTTGCTTGAAATCTCGCAAAACCAGCAGGAA--

GAAAATGAAAAAGATTTACTAGCATTGAACAAGTGGGACAATCTGTGGTCTTGGTTTGACATATCACATTGGC

TGTGGTATATAAGGATATTCATAATGATAGTAGGAGGCTTGATAGGTTTAAGAATAATTTTTGCT---
GTGCTTTCTATAGTA---------AATAGAGTCAGGCAGGGATACTCACCT------
TTGTCATTGGCCATTAGTCTGTTTGATACCATAGCAATAGCAGTAGCTGAG---
GGAACAGATAGAATTATAGAAGTAGCACAAAGATTTGGTAGAGGT----------------------------
--------ATCAGGAATATA------CCTAGAAGAATAAGACAGGGCTTTGAAGCAGCTTTGCAG---TAA
>KU319543
GTG---TGG--
GGGAACTTGTGGGTCACAGTCTATTATGGGGTACCTGTGTGG---AAAGAAGCAAAA---
ACTACTCTATTTTGTGCATCAGATGCTAAAGCATATGATAAAGAA---GTGCACAAT----------------
--------------GTCTGGGCTACA---CATGCCTGTGTACCCACTGAC---
CCCAACCCACTAGAAATGGTTTTGAAA---------AATGTAACAGAAAATTTTAAC----------------
--------------------------ATGTGGAAAAATGATATGGTGGATCAGATGCATGAGGACATA---
ATCAGTTTATGGGATGAAAGCCTA---AAGCCATGTGTAAAGTTG---
ACCCCACTTTGTGTTACTTTAAACTGTAGTCATGAGATTAATGTTAACAGTAGTAACAATTTT----------
----
-------------------GACTTTGATAGTATGCTACCTACCGTGATATTACAGATACATGGA---
AATTGTTCTTTCAATATAACCACAGAAATAAGA---------------------------
GATAAGAAAAGGAAAGCATATGCACTT---TTTTATAAACTTGATGTTTTACCACTTAATAAG----------
----

AGAAGAAATAATGGGGGTGGACTGTTAAATATAGATTCATAAGTTGTAATACCACAGCTGTAACACCA---
GCCTGTCCAGAGGCCTCATTTGCTCCTGTTCCTATTCATTATTGTGCTCCTGCTGGTTATGCTATTCTAAGTT
GTAAT---AACAATACCTTCAAT------GGACTAGGACCATGCAATAAT-----------------------
-GTCACC---GCAGTACAATGTACACGTGTAATTAAACCTGTGGTATCAACT---
CAACTACTGTTAAATGGTAGTCTAGCAGAAGAGGAG------------
ATAGTAATTAAAACTGAAATTATGACAAACAAT---------GCCAAAATAATAATAGTACAGCTTAAA----
-----------CAACCAGTAAAT---------ATTGTGTGTACGAGGACTGGCAGTACTACAAGGAGA-----
-AGTGTGAGGATA------------GGACCAGGGCAAGCA---TTC------TATACAACAGGAGAA------
---------ATAATAGGAAATATA------------AGAGAAGCACATTGT---AATATTAATGAACAT---
AAATGGAACCAAACTTTAAAGAAGGTAAGGGAACAGTTACTA---------------AAACACTTCCCT----
-----------------------------------AATAAGTCAATAATATTTGCC------
AATTCCTCAGGAGGGGACCTAGAAATTACA---ACACATAGCTTTAATTGTAGAGGAGAA---
TTTAAGCAGCTCCAGGCAAGAGTCCTGGCTATAGAAAGATACCTAAAGGATCAACAGCTCCTAGGGATTTGGG
GCTGTTCTGGAAAACTCATCTGCACCACTGCTGTGCCTTGGAACTCTAGTTGGAGT-----------------
----------------------------AATAGGTCTCAAGATGATATTTGG------AAT------
AACATGACTTGGATGCAGTGG---GATAGAGAAATTAATAAGCACACAAACACAATATAC---
AGGTTGCTTGAAGACTCGCAAAACCAGCAGGAG--

AAAAATGAAAAAGATTTACTAGCATTGGACAGTTGGGAAAGTTTGTGGAATTGGTTTAGCATAACAAAATGGC
TGTGGTATATAAAAATATTCATAATGATAGTAGGAGGCTTGATAGGTTTAAGAATAGTTTTTGCT---
GTGCTTTCTATAGTA---------AATAGAGTTAGGCAGGGATACTCACCT------
TTGTCGTTTGCTATTAGTCTGCTTGATACAGTAGCAATAGCAGTAGTTGAA---
GGAACAGATTGGATTATAATCTTAGTACAAAGAATTTGGAGAGCT----------------------------
--------TTCTGCAACATA------CCTAGAAGAATAAGACAGGGCTTTGAAGCAGCTTTGCAG---TAA
>KU319548
GGG---GGG--
GAAGACTTGTGGGTTACAGTCTATTATGGGGGACCTGTGTGG---AAAGAAGCAAAA---
ACTACTCTATTTTGTGCATCAGATGCTAAAGCATATGAGAAAGAT---GTGCATAAT----------------
--------------GTTTGGGCTACA---CATGCCTGTGTACCCACAGAC---
CCCAACCCACTTGAAATGTTTTTGGAA---------GATGTAACAGACAATTTTAAC----------------
--------------------------ATGTGGAAAAATGACAGGGTGGATCACATGCATGCGGATATA---
ATCAGGTTATGGGTTCAAATCATA---AAGACATGCGTCAAATTG---
ACTACGCTCAGTATTACTATCAATAGCAGTAATGATAATCTTACC----------------------------
----
----------------------------AGTAGAGGTAATGTGACCATGAATTGGGAAATAGTA---
AACTGTGGTTTCAGTGTTACGACTGAGATAAGA---------------------------
GATACGAGAAAGCATGAATATGCTCTA---ATTTCTAGTAATGATGTCGTTACATTTAATGAGGGC-------
----

AATTCCAGTGAGTATAGATTAATAAATTGTAATACCTCAGCTGTAACACAA---

GCCTGTCCAAAGGTCTCTTTTGATCCAATTCCTATACATTATTGTGCTCCTGCTGGTTATGCGATTCTAAAGT
GTAAT---AATGGAACATTCAAT------GGAACAGGACCATGCAATAAT-----------------------
-GTCAGC---ACAGTACAATGTACACATGGAATTAAACCAGTGGTATCAACT---
CAACTACTGCTAAATGGTAGTCTAGCAGAAAAAGAG------------
ATAGTAATTAGATCTGAAAATCTGACAAACAAT---------GGCAAAATAATAATAGTACATCTTAAA----
-----------GAATCTGTACAA---------ATTAAGTGTACAAGGCCCGGTAATAATACAAGGAAA-----
-AGTATGAGGATA------------GGACCAGGACAAACA---TTC------TATGCAACAGGAGAT------
---------ATAACAGGAGACATA------------AGACAAGCACATTGT---AACATTAGTGGAAAG---
GACTGGAATGCCACTTTACAAAAGGTAAGTGATAAATTAAAA---------------CAACACTTCCCT----
-----------------------------------AGTATATCAATAATCTTTGCA------
CCAGCTTCAGGAGGGGACCTAGAAATTACC---ACACTCAGCTTTAATTGTAGAGGAGAA---
TTCAAGCAGCTCCAGACAAGAGTCCTGGCTATAGAAAGATACCTAAAGGATCAACAGCTCCTAGGGCTTTGGG
GCTGCTCTGGAAAACTCATCTGCACCACTGCTGTGCCTTGGAACGACAGCTGGAGT-----------------
----------------------------AATAAATCTCAAAAGGATATTTGG------GAT------
AACATGACTTGGATGCAGTGG---GATAAGGAAATTAATAATTACACAAGCACAATATAC---
AGGTTGCTTGAAGACTCACAAAGCCAGCAGGAG--

AAAAATGAAAAAGATTTGCTAGCATTGGACAGTTGGAAAAAGCTGTGGAGTTGGTTTGACATATCAAATTGGC
TGTGGTATATACAAATATTCATAATGATAGTAGGAGGCTTGATAGGTGTAAGAATAATTTTTGCT---
GTACTATCTATAATA---------AATAGAGTTAGGCAGGATACTCACCT------
TTGTCGCTTGCTGTTAGTCTGCTTGATACTATAGCAATAGTAGTAGCTGAA---
GGAACAGATAGACTTATAGAAGTAGTACAAAGATTTTGTAGAGGT----------------------------
--------ATCTACAACATA------CCTAGAAGAATAAGACAGGGCTTTGAAGCAGCATTGCAA---TAG
>KU319549
GGA---ACA--
GGAAACTTGTGGGTCACAGTCTATTATGGGGTACCTGTGTGGAAAGAAGCAAACCCTACTCCACCTTTATTTT
GTGCATCAGATGCTAAAGCATATGAGACAGAG---GTGCATAAT-----------------------------
-GTTTGGGCTACA---CATGCCTGTGTACCCACAGAC---CCCGACCCACAAGAGCTAGAGTTGAAA------
---AATGTAACAGAAGATTTTAAC--
ATGTGGAAAAATGGTATGGTGGATCAGATGCATCAGGATGTA---ATCAGCTTATGGGAGCAAAGCCTA---
AAGCCATGTGTAAAAATG---
ACCCCACTCTGTGTTACCTTAAATTGTACGTATGTCACCAGTAAAAATACCACCGATAATAGTACTAGTACA-
----
-------------------------ATGCTACTATCTGTTAATGCCACTGATAGTGACATGAAA---
AATTGCTCTTTCAATGTAACCACAGAACTAAAA---------------------------
GATAAGCAAAAGAAGGTATATGCTCTT---
TTTTATTTACTTGATATTAGACAACTTAATGACAGTTCTAACACACATACTGGAAATTCT-------------
----
AGTAATACTGGAAATTATAGTGATTATATATTAATAAATTGCAATACCTCAACCATAGCACAA---
GCCTGTCCAAAGGTCTCTTTTGACCCAATTCCTATACCTTATTGTGCTCCTGCTGGTTATGCTATTCTTAAGT
GTAAT---GATAAGAATTTTAAT------GGAACAGGAACATGTACAAAT-----------------------
-GTCAGC---ACAGTTCAATGTACACGTGGAATTAAACCAGTGGTATCAACT---
CCACTATTATTAAATGGTAGTATATCAGAAGGTGAT------------
ATAGTAATTATATCTAAAAATCTGACAGACAAT---------GCCAAAACAATAATAGTACAGCTTAAC----
-----------CAAACTGTAGCA---------GTTAATTGTACGAGACCCACCAATAATACTAGAACA-----
-GGTATAAGGATA------------GGACCAGGACGAGCC---TTC------TATGCAACAGGAGAC------
---------ATAATAAGAGATATA------------AGACAGGCACATTGT---AACATTAGTGGAGGA---
GATTGTAATAGAACTCTAGAAGAGGTAGAGAAAAAATTAAAT---------------GAGTCCTTCTCT----
--------------------------------GGTAATGAAACAATACAATTTGAA------
CCAACCTCAGGAGGGGATCCAGAAATTACA---ACTCGTAGTTTTAATTGTGGAGGGGAA---
TTTAAGCAGCTCCAGACAAGAGTCCTGGCTCTAGAAAGATACCTAAGGGATCAACAACTCCTAGGGATTTGGG
GCTGCTCTGGAAAAATCATCTGCACCACTGCTGTGCCTTGGAACTCTAGTTGGAGT-----------------
----------------------------AATAAAACTCATGATCAGATTTGG------AAG------
AACATGACTTGGATGCAGTGG---GAAAGAGAAATTGATAATTATACAGGCATAATATAT---
AATTTGCTTGAAGTCTCGCAAATCCAGCAGGAG--

CAAAATGAGCAAGATTTATTGGCATTGGACAAATGGCAAAATCTGTGGTCTTGGTTTGATATAACAAACTGGC
TGTGGTATATAAAAATATTCATAATGATAGTAGGAGGCTTGATAGGTATAAGAATAATTTTTGCT---
GTGCTATCTATAGTG---------AATAGAGTTAGGCAGGATACTCACCA------
TTATCGTTTGCTGTTAGTCTGCTTGATACTGTAGCAATAGCAGTAGCTGAA---

GGAACAGATAGGATTATAGAAGTAATACAAAGAGCTTGGAGAGCT----------------------------
--------TTCTGCAATATA------CCCACAAGAATAAGACAGGGCTTTGAAATAGCTTTGCTA---TAA
>AF219266
GTG---AGA--
GGGAACTTGTGGGTTACAGTCTATTATGGGGTACCTGTGTGG---AAAGAAGCAAAA---
ACTACTCTATTCTGTGCATCAGATGCTAAAGCATATGAGAGAGAG---GTGCACAAT---------------
--------------GTCTGGGCTACA---CATGCCTGTGTGCCCACGGAC---
CCCAACCCACAAGAAATGGTTCTGGAA---------AATGTAACAGAAAAATTTAAC---------------
-------------------------ATGTGGAAAAATGACATGGTGGATCAGATGCATGAGGATATA---
ATTAGTTTATGGGAGCAAAGCCTA---AAGCCATGTGTAAAGTTG---
ACCCCACTCTGTGTCACTTTAAATTGTGGTAATGCTATCTATAAT---------------------------
----
---AAAAGCATAAATGAAGAAATAAAA---
AATTGCTCTTTCAATGCAACCACAGAACTAAAG--------------------------
GATAGGAAACAAAAAGTGCGTGCACTT---TTTTATAGACTTGATGTAGTACCGCTTAATAATGAA-------
----

ACTGGAAACAATACTCTCTATAGGTTAATAAATTGTAATACCTCAGCCATAACACAA---
GCCTGTCCAAAGGTCACTTTTGACCCAATTCCTATACGTTATTGTGCTCCCGCTGGTTATGCGATTCTAAAGT
GTAAT---AATAAGACATTCAAT------GGGACAGGACCATGCCATAAT----------------------
-GTCAGC---ACAGTACAATGTACACATGGAATTAAGCCAGTGGTATCAACT---
CAACTACTGTTAAATGGTAGCCTAGCAGAAGAAGAA-----------
ATAATAATTAGATCTGAGAATCTGACAAACAAT---------GCCAAAACAATAATAGTACATCTTAAG----
-----------GAAGCTGTAGAA---------ATTGTGTGTACAAGACCCAACAATAATACAAGAAAA-----
-AGTGTAAGGATA------------GGACCAGGACAAACA---TTC------TATGCAACAGGAGAC------
---------ATAATAGGAAACATA-----------AGACAAGCATATTGT---AACATTGATGAAGGT---
AAATGGAATAAAACTTTACAAAATGTAAGTAAAAAATTAAAA--------------GAACTCTTCCCT----
--------------------------------GATAAAACTGAAATAAAATTTGCA------
CCATCCTCAGGAGGGGACCTAGAAATTACA---ACACATAGCTTTAATTGTAGGGGAGAG---
TTTAAACAGCTTCAGACTAGAGTCCTGGCTATAGAAAGGTACCTAAAGGATCAACAGCTCCTAGGGCTTTGGG
GCTGCTCTGGGAAACTCATCTGCACCACTGCTGTGCCTTGGAACTCCAGTTGGAGT----------------
----------------------------AATAAATCTCAAACAGATATTTGG------GAT------
AACATGACCTGGATGCAGTGG---GATAGAGAAATTAGTAATTACACAAACACAATATAC---
AGGTTGCTTGAAGACTCGCAAATCCAGCAGGAA---------------------------------------
----
AAAAATGAAAAAGATTTATTAGCATTGGACAGTTGGGAAAATCTGTGGAGTTGGTTTAACATAACAAATTGGC
TGTGGTATATAAGAATATTCATAATAATAGTAGGAGGCTTGATAGGTTTGAGAATAATTTTTGCT---
GTGTTCTCTATAGTG---------AATAGAGTTAGGCAGGGATACTCACCT------
TTGTCGTTTGCCATTAGTCTGCTTGATACCATAGCAATAGCAGTAGCTGAA---
GGAACAGATAGGATTATAGAAGTAATACAAAGAATTTGTAGAGCT---------------------------
--------ATCCGCAACATA------CCTACAAGACTAAGACAGGGCTTTGAAGCAGCTTTGCTA---TAA
>FJ977095
GTG---ATA--
GGGAATTTGTGGGTCACAGTCTATTATGGGGTACCTGTGTGG---AAAGAAGCAAAA---
ACTACTCTATTTTGTGCATCAGATGCTAAAGCATATGTTACAGAA---GTGCACAAT---------------
--------------GTCTGGGCTACA---CATGCCTGTGTACCCACAGAC---
CCCAACCCACAAGAAATGGTTTTGGAA---------AATGTAACAGAAAATTTTAAC---------------
-------------------------ATGTGGAAAAATGACATGGTGGATCAGATGCATCAGGATATA---
ATCAGTTTATGGGATCAAAGCCTA---AAGCCATGTGTACAGTTG---
ACCCCACTCTGTGTTACTTTAAATTGTACAAATGCTAATGTGACCAGAACCAATGCC---------------
----
-------------------------------ACAATGGAAGAAGGAATGAAAAATGAAATAAAA---
AATTGTTCTTTCAATATCACCACAGAAATGAGA--------------------------
GATAAGAGACAGAAAGTACATGCACTT---TTTTATAGACTTGATGTAGTACTAATAAATGAA---------
----

ACAATAGATAATACTAGTTATAGATTAATAAATTGTAATACCTCAGCCATAACACAA---
GCCTGTCCAAAGGTCTCTTTTGACCCAATTCCTATACATTATTGTGCTCCAGCTGGTTATGCGATTCTAAAAT
GTAAT---AATAAGACATTCAAT------GGAACAGGACCATGCAATAAT----------------------
-GTCAGC---ACAGTGCAGTGTACACATGGAATTAAACCAGTGGTATCAACT---

CAACTATTGTTAAATGGTAGTCTAGCAGAAAAAGAG------------
ATAGTAATTAGATCTGAAAATCTGACAAACAAT---------GCCAAAATAATAATAGTACATCTTAAT----
-----------GAATCTGTAGAA---------ATTAATTGTACAAGACCCAACAACAATACAAGACAA-----
-AGTATAAGGATA------------GGACCAGGGCAAGCA---TTT------TATGCAACAGGAGAT------
---------ATAATAGGAGATATA------------AGACAAGCACATTGT---AACATTAGTGCAAAA---
CGATGGAACGAAACTTTAGAAAGGGTAAGACTAAAATTAAAG---------------GAGCACTTCCCT----
-----------------------------------GATAAAGCAATAACCTTTAAT------
CACTCCTCAGGAGGGGATCTAGAAATTACA---ACACATAGCTTTAATTGTAGAGGAGAA---
TTTAAGCAGCTCCAGACAAGAGTCCTGGCTATAGAAAGGTACCTAAAGGATCAACAGCTCCTAGGGATTTGGG
GCTGCTCTGGAAAACTCATCTGCACCACTGCTGTGCCTTGGAACTCTAGTTGGAGT-----------------
----------------------------AATAAATCTCAAGATGAGATTTGG------AAT------
AACATGACCTGGATGCAGTGG---GATAGAGAAATTAGTAACTATACAAACACAATATAC---
ACGTTGCTTGCAGACTCGCAAAACCAGCAGGAC--

AAAAATGAAAAGGAGTTACTAGCATTAGATAGCTGGAAAAATCTGTGGAATTGGTTTAACATAACAAATTGGC
TGTGGTATATAAAAATATTCATAATGATAGTAGGGGGCTTAATAGGTTTAAGAATAATTTTTGCT---
GTGATCTCTATAGTG---------AACAGAGTTAGGCAGGATACTCACCA------
TTGTCGTTCGCTATTAGCTTGCTTGATACCACAGCAATAGCAGTAGCTGGA---
GGGACAGATAGGATTATAGAAGTAGTATTAAGAATTGGTAGAGCT----------------------------
--------ATCTACAACATA------CCTAGAAGAATCAGACAGGGCTTAGAAAGGGCTTTGCTA---TAA
>AY255823
GTG---ATG---GGG---------------------------------
AACAGGAACCTGTGGGTCACAGTCTATTATGGGGTACCTGTGTGG---AAAGAAGCAAAC---
CCTACTCTATTTTGTGCATCAGATGCTAAAGCATATAAGACAGAA---ATACATAAT----------------
--------------GTCTGGCTACA---CATGCCTGTGTACCCACAGAC---
CCCAGCCCACAGGAAATAGCTTTGAAA---------AATGTAACAGAAAATTTTAAC----------------
--------------------------ATGTGGAAAAATGGCATGGTGGAGCAGATGCATCAGGATGTA---
ATCAGTTTATGGATCAAGGCCTA---AAGCCATGTGTAAAGTTG---
ACCCCACTCTGTGTTACTTTAAACTGTAGCAAGGCTATCAATAGTAGTGCGGAT-------------------
----
-------------------------------GTTGATACCTACAATGAAACCATGAAAGTGAAA---
AATTGTTCTTTCGATGTAACCACAGAACTAAGA---------------------------
GATAAACAACGGAAAGCATATGCACTT---TTTTATAAACTTGATATAGTACCACTTAATGAGACT-------
----

GAGGGTAATTATAGTGATTATAGATTAATAAACTGTAATGCTTCAACCATAACACAA---
GCCTGTCCAAAGGTCTCTTTTGACCCAATTCCTATACATTATTGTGCTCCAGCTGGTTATGCGATTCTAAAGT
GTAAT---GATACAAAATTCAAT------GGAACAGGACCATGCAATAAT-----------------------
-GTCACC---ACAGTACAATGTACACATGGAATTAAACCAGTGGTATCAACA---
CAATTATTGTTAAATGGTAGTACAGCAGAAGAA---------------
GTGATAATTAGATCTGAAAATCTGACAAACAAT---------GCCAAGATAATAATAGTACAGCTTAGG----
-----------AAATCTGTATAC---------ATTAATTGTACGAGACACAACAATAATACAAGAAAA-----
-GGTATAGGGATA------------GGACCAGGACAAACA---TTC------TATGCAACAGAAGCC------
---------ATAATAGGAAATATA------------AGACAAGCACATTGT---AACATTAGTGGAGAA---
GAATGGAATACAACTGTACACGAGGTAAAAGGAAAATTAGGA---------------AAGCTCTTCCAT----
-----------------------------------AATAAAACAATAGTATTTGCA------
CCATCCTCAGGAGGGGACCTAGAAATTACA---ACACATAGCTTTAATTGTAGAGGAGAA---
TTTAAGCAGCTCCAGGCAAGAGTCCTGGCTATAGAAAGATACCTAGGGGATCAACAGCTCCTAGGGATTTGGG
GCTGCTCTGGAAAACTCATCTGCACCACCACTGTGCCTTGGAACTCTAGTTGGAGT-----------------
----------------------------AATAAAACTCAAAAGGAAATTTGG------GAT------
AATATGACCTGGATGCAGTGG---GATAGAGAAATTAATAATTATACAAACATAATATAC---
AGGTTGCTTGAAGACTCGCAAAACCAGCAGGAG--

AAAAATGAAAAAGATTTACTAGCATTGGACAAATGGCAAAATCTGTGGGGTTGGTTTCAAATATCAAGATGGC
TGTGGTATATAAGAATATTCATAATGATAGTAGGAGGCTTGATAGGCTTAAGAATAGTTTTTGCT---
GTGCTCTCTATAGTG---------AATAGAGTTAGGCAGGATACTCACCG------
TTATCGTTTGCTATTAGTCTGCTTGATACCACAGCAGTAGCAGTAGCTGAA---
GGAACAGATAGGATATTAGAATTAATACAAAGAGTTTGGAGAGCT----------------------------
--------ATCTACAACATA------CCTAGAAGAATAAGACAGGGCTTTGAAGCAGCTTTGCTA---TAA
>KP109480

GGG---GGA---AGA------------------------------------
GAAGACTTGTGGGTCACAGTCTATTATGGGGTACCTGTGTGG---AAAGAAGCAAAA---
ACTACTCTATTCTGTGCATCAGATGCTAAAGCACATGAAAGAGAA---GTGCATAAT----------------
--------------GTCTGGGCTACA---TATGCCTGTGTACCCACAGAC---
CCCAACCCACAAGAAATAGTTTTGAAA---------AATGTAACAGAAAATTTTAAC----------------
--------------------------ATGTGGGAAAACGACATGGTGGATCAGATGCAGGAGGATGTA---
ATCAGTTTATGGGATCAAAGCCTA---AAGCCATGTATAAAGTTG---
ACCCCACTCTGTGTCACTTTAGAATGTACAAATGTTAATATTATAAAT-------------------------
----
----------------------------GGTACTATCCACAATGAGACCTACGAGAGCATGAAA---
AATTGCTCTTTCAATACAACCACAGAACTAAAA---------------------------
GATAAGAAGCAGTCAGTGTATGCACTT---TTTTATAGATTAGATATAGTGCCACTTAACAAC----------
----

TCTAATGAGTATTATAGATTAATAAATTGTAATACCTCAGCCATAAAACAA---
GCCTGTCCAAAGGTCACTTTTGACCCAATTCCTATACACTATTGTGCTCCAGCTGGTTATGCGATTCTAAAGT
GTAAT---GATAAGACATTCAGT------GGGACAGGACCATGCCATAAT-----------------------
-GTCAGC---ACAGTACAATGTACACATGGAATTAAGCCAGTGGTATCAACT---
CAACTACTGTTAAATGGTAGCCTAGCAGAAGGAGAG------------
ATAATAATTAGATCTAAAAAACTGGACGACAAC---------GCCAACACAATAATAGTACATCTTGAT----
-----------GAACCTGTAAAA---------ATTGAGTGTACAAGACCCAACAATAATACAAGAAAA-----
-AGTATAAGGATA------------GGACCAGGACAAACA---TTC------TATGCAACAGGAGAA------
---------ATAATAGGAAACATA------------AGGCAAGCACATTGT---AACATTAGTGAAGAT---
CAGTGGAATGAAACTTTGCAAAGGGTAGGTAAAAAATTAGCA---------------GAACTCTTCCCT----
-----------------------------------AATAAAACAATAACCTTTAAT------
TCATCCTCAGGAGGGGATCTAGAAATCACA---ACACATAGCTTTAATTGTAGAGGAGAA---
TTTAAGCAGCTCCAGACAAGAGTCCTGGCTATAGAAAGATACCTAAAGGATCAACAGCTCCTAGGGATTTGGG
GCTGCTCTGGAAAACTCATCTGCACCACTTCTGTACCTTGGAACTCTAGTTGGAGT-----------------
-------------------------AACAACAGATCTCAAGACGATATTTGG------GAG------
AACATGACCTGGATGCAGTGG---GATAGAGAAATTAGTAATTACACAAACACAATATAC---
AGGTTGCTTGAAGACTCGCAAAACCAGCAGGAA--

AATAATGAAAAAGATTTATTAGCATTGGACAGTTGGAAAAATCTGTGGAGTTGGTTTGATATAACAAATTGGC
TGTGGTATATAAGAATATTCATAATAATAGTAGGAGGCTTGATAGGGTTAAGAATAATTTTTGCT---
GTGCTTTCTATAGTG---------AATAGAGTTAGGCAGGGATACTCACCT------
TTGTCGTTTGCTATTAATCTGCTTGATATCACAGCAATAGCAGTAGCTGAA---
GGAACAGATAGGATTATAGAATTAGTGCAAAGAATTTATAGAGCT----------------------------
--------ATCCGCAACATA------CCTAGAAGAATAAGACAGGGCTTTGAAGCAGCTTTGCAA---TAA
>KP109483
GTG---GGA--
GGAAACTTGTGGGTCACAGTTTATTATGGGGTACCTGTGTGG---AGAGAAGCAAAA---
ACTACTCTATTCTGTGCATCAGATGCTAAAGCATATGAGAAAGAA---GTGCATAAT----------------
--------------GTCTGGGCTACA---CATGCCTGTGTACCCACAGAC---
CCCAACCCACAAGAAATAGATTTG------------AATGTAACAGAAAATTTTAAC----------------
--------------------------ATGTGGAAAAATGACATGGTGGAGCAGATGCATGAGGATGTA---
ATCAGTTTATGGGATCAAAGCCTA---AAGCCATGTGTAAAGTTG---
ACCCCAATTTGTGTCACTTTAGAATGTACAGATGCTAATATTACCTGCAATAGTACTACTAGCAGTAATAATT
GTACC--
-------------------------AGCTATGAGATCAACAAGGAGGACATGGGGGAAATAAAA---
AATTGCTCTTTCAATACAACCACAGAATTAATA---------------------------
GATAAGCAGAAGAAAGTGCATGCACTC---
TTTTATAGACTTGATATAGTATCACTAGAAAAGGACAACTCTAGTAAG-------------------------
----
AAGAACGACTCTAATGAGTATTATAGATTAATAAATTGTAATACCTCAGCCATAACACAA---
GCCTGTCCAAAGGTCACTTTTGACCCAATTCCTATACACTATTGTGCTCCGGCTGGTTATGCGATTCTAAAGT
GTAAA---AATAAGACATTCAAT------GGGACGGGACCATGCAATAAT-----------------------
-GTTAGC---ACAGTACAATGTACACATGGAATTAAGCCAGTGGTATCAACT---
CAACTACTGTTAAATGGTAGCCTAGCAGAAGAAGAG------------
ATAATAATTAGATCTGAAAATCTGACAAACAAT---------GCCAAAATAATAATAGTACATCTTAAT----
-----------CAAGCTGTAGAA---------ATTGTATGTACAAGACCTGGCAATAATACAAGAAAA-----

```
-AGTATAAGGATA------------GGGCCAGGACAAACA---TTC------TATGCAACAGGAGAC------
---------ATAATAGGAGACATA------------AGACAAGCACATTGT---AACATTAGTGAAGCT---
AAGTGGAATAAAACCTTGCGAGAGGTAAGTAAAAAATTAGCA---------------GAACACTTCCCT----
-----------------------------------AATAAAACAATAATATTTAAC------
TCATCCTCAGGAGGGGACCTAGAAATTACA---ACACATAGCTTTAATTGTGGAGGAGAA---
TTTAAACAGCTCCAGACAAGAGTCCTGGCTATAGAAAGATACCTAAAGGATCAACAGCTCCTAGGGATTTGGG
GCTGCTCTGGAAAACTCATCTGCACTACTGCTGTACCTTGGAACTCCAGTTGGAGT-----------------
----------------------------AACAAATCTCAAGACCAGATTTGG------AAT------
GATACGACCTGGATGCAGTGG---GATAAAGAAATTAGTAATTACACAGACATAATATAC---
ACGTTGCTTGAAAAATCGCAAAACCAGCAGGAA----------------------------------------
-----------------------------
ATAAATGAAAAGGATTTATTAGCATTGGACAGTTGGAACAGTCTATGGAATTGGTTTGACATAACAAAGTGGC
TGTGGTATATAAAAATATTCATAATGATAGTAGGAGGCTTGATAGGTTTAAGAATAATTTTTGCT---
GTGCTTTCTATAGTG---------AATAGAGTTAGGCAGGGATACTCACCT------
TTGTCGTTTGCTATTAGTCTGTTTGATACCATAGCAATAGCAGTAGCTGAA---
GGAACAGATAGGCTTATAGAATTCATACAAAGAACTGGTAGAGCT----------------------------
--------ATCTGCAACATA------CCTAGAAGAATAAGACAGGGTTTTGAAGCAGCTTTGCAA---TAA
>KY929363
GTG---TTA------------------------------------------
GGAAACCTGTGGGTCACAGTCTATTATGGGGTACCTGTGTGG---AAAGAAGCAAAA---
ACTACTCTATTCTGTGCATCAGATGCTAAAGCTTATGAAACAGAA---GTGCATAAT----------------
--------------GTCTGGGCAACA---CATGCCTGTGTACCCACAGAC---
CCCAACCCACAAGAAATGTTTTTGGAA---------AATGTAACAGAAAATTTTAAC----------------
--------------------------ATGTGGGAAAATGACATGGTGGATCAGATGCATGAGGATGTA---
ATCAGTTTATGGGATCAAAGCCTA---AAGCCATGTGTAAAGTTG---
ACCCCACTCTGTGTCACATTAAATTGTACACATGATATTAAAATTAATAGTACCGTTAATAATAGTACT----
-------------------------------------------------------------------------
-------------------------------------------------------GACATGAGA---
AATTGCTCTTTCAATTCAACCACAGAACTAAGA---------------------------
GATAAGAGACAGAAAGTATATGCACTT---TTTTATAAACTTGATGTAGTACCAATTGATGGT----------
-------------------------------------------------------------------------
----------------------
GGTAACTCTAGTTATAGATTAATAAATTGTAATACCTCAGCCATAACACAA---
GCCTGTCCAAAGGTCTCATTTGACCCAATTCCTATACATTATTGTGCTCCAGCTGGTTATGCGATTTTAAAGT
GTAAT---AATAAGACATTCGAT------GGGAAAGGACCATGCAATAAT-----------------------
-GTCAGC---ACAGTACAATGTACACATGGAATTAAGCCAGTAGTATCAACT---
CAACTACTGTTAAATGGTAGCCTAGCAGAAGAAGAC------------
ATAATAATTAGAACTGAGAATCTGACAAAGAAT---------AGCAAAATAATAATAGTACATCTTAAT----
-----------AAGTCTGTAGAA---------ATTAAGTGTATAAGACCTAACAATAATACAAGAAAA-----
-AGTATAAGAATA------------GGACCAGGACAAACA---TTC------TATGCAACTGGAGAC------
---------ATAATAGGAGACATA------------AGAGAAGCACATTGT---ACCATTAGTAAC-------
--TGGACTGAAATTTTACAAAATGTAAGTAAAAAATTAGCA---------------GGACTCTTCCCT-----
----------------------------------AATAAAACAATAAAATTTGCA------
CCATCCTCAGGAGGGGACCTAGAAGTTACA---ACACATATCTTTAATTGTAGAGGAGAA---
TTTAAGCAGCTCCAGACAAGAGTCCTGGCTATAGAAAGATACCTAAAGGAACAACAGCTCCTAGGGATTTGGG
GCTGCTCTGGAAAACTCATCTGCACCACCAATGTACCTTGGAACTCCAGTTGGAGT-----------------
----------------------------AACAAAACTGAAGAAGACATTTGG------AAT------
AACATGACTTGGATGCAGTGG---GATAGAGAAATTAGTAATTACACAGATACAATATAC---
AGGTTGCTTGAGGAATCGCAAAACCAGCAGGAA----------------------------------------
-----------------------------
AGAAATGAAAAAGATTTGTTAGCATTGGACAGTTGGAAAAATCTATGGAGTTGGTTTAGTATAACAAACTGGC
TGTGGTATATAAGAATCTTCATAATAATAGTAGGAGGCTTGATAGGCTTGAGACTAATCTTTGCT---
GTGCTTTCCATAGTG---------AATAGAGTTAGGCAGGGTTACTCACCT------
TTGTCGTTCGCTATTAGCCTGTTGGATATTGTAGCAATAGCAGTAGCTGAG---
GGAACAGATAGAATTATAGAATTAATAAGAAGAATTTGTAGAGCT----------------------------
--------ATCTGCAACATA------CCTAGAAGAATAAGACAGGGTTTTGAAGCAGCTTTGCAG---TAA
>KY929365
AAG---GAA------------------------------------------
GAAGAATTGTGGGTCACAGTCTATTATGGGGTACCTGTGTGG---AAAGAAGCTAAA---
ACTACTCTATTCTGTGCATCAGATGCTAAAGCATACGAGCAAGAA---GTGCATAAT----------------
```

--------------GTTTGGGCTACA---CGTGCCTGTGTACCCACAGAC---
CCCAACCCACAAGAAGTGGTTTTGGAA---------AATGTAACAGAAAATTTTAAT---------------
--------------------------ATGTGGAAAAATGACATGGTGGATCAGATGCATGATGATATA---
ATTAGTTTATGGGATGAAAGCTTA---AAGCCATGTGTAAAGTTG---
ACCCCACTTTGTGTCACTTTAGATTGTACAAATGTTAATGTTACAGGTAATGATACAACCACTGGTAATGCT-
----
--ATTGCTGAAGTAAAA---
AATTGTACTTTCAATGCAAGCACAGAAATAAGA-------------------------
GATAAGAAGCAGACAGTATATGCACTA---TTTTATAAACCTGACATAGTACAACTTGGAGAG---------
----

AATAATAGCACATATAGATTAATAAGTTGCAATACCTCAACCATAACACAA---
GCCTGTCCAAAGATCAGTTTTGATCCAATTCCTATACACTATTGTGCTCCAGCTGGTTATGCGATTCTAAAGT
GTAAT---AACAAAACATTTAAT------GGGACGGGACCATGCCAAAAT----------------------
-GTCAGC---ACAGTACAATGTACACATGGAATTAAGCCAGTGGTATCAACT---
CAATTACTGTTAAATGGTAGCCTAGCAGAAAAAGAG------------
ATAGTAGTTAGATCTGAAAATCTGACAAACAAT---------GTCAAAACAATAATAGAACAGCTTAAT----
-----------CAATCTGTAACG---------ATTGTGTGTACAAGACCCAATAATAATACAAGAAAA-----
-AGTATAAGGATA------------GGACCAGGACAAACA---TTT------TATGCAGCAGGAGCC------
---------ATAATAGGAGACATA------------AGACAAGCACATTGT---
AACATTAGTAACACTAGTGACTGGAATACAACTTTGCTAAATGTAGCTAAAAAATTAGCA------------
--GAACACTTTCCT---------------------------------------
AATAAAACAATAAAATTTGAA------CCACCCTCAGGAGGAGACCTAGAAATTACA---
ACACATAGTTTTAATTGTAGGGGAGAA---
TTTAAGCAGCTCCAGGCAAGAATCCTGGCTATAGAAAGATACCTACAGGATCAACAGCTCCTAGGGATGTGGG
GCTGCTCTGGAAAACTTATCTGCACCACTGCTGTACGTTGGAACACCAGTTGGAGT-----------------
----------------------------AACAAATCTGAATCAGACATATGG------AAT------
AACATGACCTGGATGGAGTGG---GATAGAGAAATCAATAATTACACAACAACAATATAC---
AGGTTGCTTGAAAAATCACAAAACCAGCAGGAA---------------------------------------
----
AGAAATGAAAAAGATTTATTAGAATTGGACAGTTGGAAAAATCTGTGGACTTGGTTTGACATAACAAACTGGC
TGTGGTATATAAAAATATTCATAATGATAGTAGGAGGCTTGATAGGCTTAAGAATAATTTTTGCT---
GTGCTCTCTATAGTG---------AATAGAGTTAGGCAGGGATACTCACCT------
CTGTCGTTTGCTGTTAGTCTGTTAGATACCACAGCAATAGTAGTAGCCGAA---
GGAACAGATAGGATTTTAGAAGCAATACGAAGAATTTGTAGAGCT---------------------------
--------ATCTACAACATA------CCTAGAAGAATAAGACAGGGCTTTGAAGCAGCTTTGCTA---TAA
>KY929369
GGG---GGA---
AACAACTTATGGGTCACAGTTTATTATGGGGTACCTGTGTGG---AAAGAAGCAAAT---
CCTACTCTATTCTGTGCATCAGATGCTAAAGCATATGAGAGAGAA---GTGCATAAT---------------
--------------GTTTGGGCTACA---CATGCCTGTGTACCCACAGAC---
CCTAGCCCACAAGAAGTAAATTTGGGA---------AATGTAACAGAAAATTTTAAT---------------
--------------------------GTGTGGAAAAATGGCATGGTGGACCAAATGCATGAGGATGTA---
ATCAGTTTATGGGATCAAAGCCTA---AAGCCATGTGTAAAGTTG---
ACCCCACTCTGTGTCACTTTAGAATGTAAAGATATCAGTAATAGTAGTATCAGTAATAGTAGTGATAGCGGCA
GTAATAGTAGT---
----------AATAGTATTATTAAT------------------------------GAGATAACA---
AATTGCTCTTTCAATGCGTCCACAGAGATAAAA-------------------------
GATAGGAAGCAGACAGTGTATGCACTT---TTTTATAAACTAGATATAGTACCACTTGATGATAATAATGAA-
----

AATATAACCAAGTATAGGTTAATAAAGTGTAATACCTCAGTCATAACACAA---
GCCTGTCCAAAGGTCTCTTTTGATCCAATTCCTATACACTATTGTACTCCAGCTGGTTATGCGATTCTTAGGT
GTAAT---GATAAGACATTCAAT------GGGACAGGACCATGCTCTAAT----------------------
-GTCAGC---ACAGTACAATGTACACATGGAATTAAGCCAGTGGTATCAACT---
CAACTACTGTTGAATGGTAGCCTAGCAGAAGAGGAG------------
ATAATAATTAGATCTAAAAATCTGACAAACAAT---------GTCAACACAATAATAGTACATCTTAAT----
-----------GAATCTGTAGAA---------ATTAAGTGTATAAGACCTGGCAATAATACAAGAAAA-----
-AGTATAAGGATA------------GGACCAGGACAAACA---TTC------TATGCCACAGGAGCT------
---------ATAATAGGAAACATA------------AGACAAGCACATTGT---AACATTAGTAGAGAG---

AAGTGGAATGCAACTTTAGAAAGGGTAAGGAAAAAATTAGAA---------------
GAGCACTTCCACTTCCTT---------------------------------AATAAAACAATAGAATTTGCA-
-----CCATCCTCAGGAGGGGACTTAGAAGTTACA---TCACATTACTTCAATTGTAGAGGAGAA---
TTTAAGCAGCTTCAGACAAGGGTCCTGGCTATAGAAAGATACCTAAAGGAACAACAGCTCCTAGGGATTTGGG
GCTGCTCTGGAAAACTCATCTGCACCACTGCTGTACCTTGGAACTCCAGTTGGAGT-----------------
----------------------------AACAAAAATCTAACAGATATTTGG------GAT------
AACATGACCTGGATGCAATGG---GATAGAGAAATTAGTAATTACACAGATACCATATAC---
AGGTTGCTTGAAGAATCACAGAACCAGCAGGAA--

CAAAATGAAAAGGATTTATTAGCATTGGACAGTTGGAAAAATCTGTGGAATTGGTTTGACATAACAAATTGGC
TGTGGTATATAAAGATATTCATAATGATAGTAGGAGGCTTGATAGGTTTAAGAATAGTTTTTACT---
GTGCTATCTATAGTG---------AATAGAGTTAGGCAGGGATACTCACCT------
TTGTCGTTTGCTATTAGTCTGCTTGATGCATTAGCAATATCAGTAGCTGAA---
GGAACAGATAGGATTATAGAGTTAGTACAAAGATTTTTTAGAGCT----------------------------
--------ATCTACAATATA------CCTACAAGAATAAGACAGGGCCTTGAAGCAGCTTTGCAA---TAA
>KY929370
GGG---GGA--
AACAACTTATGGGTCACAGTTTATTATGGGGTACCTGTGTGG---AAAGAAGCAAAT---
GCTACTCTATTCTGTGCATCAGATGCTAAAGCATATGAGAAAGAA---GTGCATAAT----------------
--------------GTTTGGGCTACA---CATGCCTGTGTACCCACAGAC---
CCCAACCCACAAGAAATGTTATTGAAA---------AATGTAACGGAAAATTTTAAT----------------
--------------------------ATGTGGGAAAATGGCATGGTGGACCAAATGCAGGAGGATGTA---
ATCAGTTTATGGGATCAAAGCCTA---AAGCCATGTGTAAAGTTG---
ACCCCACTCTGTGTCACTTTAGAATGTAAAAATATTAGCAGTAATAGTAGCAGTAATAGTAAT----------
----
-------------------------AATAGTGCTAGTGACCAGGAACTCTACAATGAGATAAAA---
AATTGCTCTTTCAATGCAACCACAGAGATAAGA---------------------------
GATAGGAATCAGGCAGTGGATGCACTT---
TTTTATAAACTAGATATAGTACCACTTGAGAATAACAATGGAAGT----------------------------
----
AATGGCAATAGAAGTAACACTTATAGGTTAATAAATTGTAATACCTCAGTCATAACACAA---
GCCTGTCCAAAAGTCTCTTTTGATCCAATTCCTATACACTATTGTGCTCCAGCTGGTTATGCGATTCTTAAGT
GTAAT---AATAAGACATTCAAT------GGGACAGGACCATGCTCTAAT-----------------------
-GTCAGC---ACAGTACAATGTACACATGGAATTAAGCCAGTGGTATCAACT---
CAACTACTGTTGAATGGTAGCCTAGCCGAAAAGGAG------------
ATAATAATTAGATCTAAAAATCTGACAGACAAT---------ACCAAAATAATAATAGTACATCTTAAT----
-----------GAATCTGTAGAA---------ATTAATTGTACAAGACCCAACAATAATACAAGAAAA-----
-AGTATAAGGATA------------GGACCAGGACAAACA---TTC------TATGCAACAGGAGAC------
---------ATAATAGGAGACATA------------AGACAAGCACATTGT---AACATTAGTAGAAAG---
AAGTGGGAAGAAACTTTACAAAGGGTAAGTAAAAAATTAGCA---------------GAGCACTTCCTT----
-----------------------------------AATAAAACAATAAAATTTGCA------
TCATCCTCAGGAGGGGACCCAGAAATTACA---ACACATAGCTTCAATTGTAGAGGAGAA---
TTTAAGCAGCTCCAGACAAGAGTCCTGGCTATAGAAAGATACCTAAAGGAACAACAGCTCCTAGGGATTTGGG
GCTGCTCTGGAAAACTCATCTGCACCACTGCTGTACCTTGGAACTCCAGTTGGAGT-----------------
----------------------------AACAAAAATCTAACAGATATTTGG------GAT------
AACATGACCTGGATGCAGTGG---GATAGAGAAATTAGTAATTACACAGATACCATATAC---
AGGTTGCTTGAAGAATCACAGAACCAGCAGGAA--

CAAAATGAAAAGGATTTATTAGCATTGGACAGTTGGAAAAATCTGTGGAATTGGTTTGACATAACAAATTGGC
TGTGGTATATAAAGATATTCATAATGGTAGTAGGAGGCTTGATAGGTTTAAGAATAGTTTTTGCT---
GTGATATCTATAGTG---------AATAGAGTTAGGCAGGGATACTCACCT------
TTGTCGTTTGCTATTAGTCTGCTTGATGCCTTAGCAATATCAGTAGCTGAA---
GGAACAGATAGGATTATAGAATTAGTACAAAGATTTTGTAGAGCT----------------------------
--------ATCCGCAATATA------CCTACAAGACTTAGACAGGGCCTTGAAGCAGCTTTGCAA---TAA
>KX756609
GTG---GCA--
GGAAACTTGTGGGTCACAGTGTATTATGGGGTACCTGTGTGG---AGAGAAGCAAAA---
ACTACTCTATTTTGTGCATCAGATGCTAAAGGATATGAAAAGGAA---GTGCATAAT----------------
--------------ATCTGGGCAACA---CATGCCTGTGTACCCACAGAC---
CCCAGACCACAAGAAATAGAGTTGGAA---------AATGTAACAGAAAATTTTAAC----------------

--------------------------ATGTGGGAAAATGACATGGTGGATCAGATGCATGAGGATGTA---
ATCAGTATATGGGATCAAGGCCTA---AAGCCATGTGTAAAGTTA---
ACCCCACTCTGTGTCACTTTARACTGTAGAAATGTTACT------------------------------------
----
-------------------------AATAACAGTACGGGAATAGATAACAAGGAGGAGGTAAAA---
AATTGCTCTTTCAACACAACCACAGAAATCAAA---------------------------
GATAGGAAGCAGAAGGTGCATGCACTT---
TTTTATAGGCTTGATATAGTACCACTTGATAACAATATTGAAGATTCT-------------------------
----
AATAAAACTRAAAATTCTAAGRGGCGGTATAGATTAATAAATTGTAATACCTCAGCCATAACACAA---
GCCTGTCCAAAAGTCAATTTTGATCCAATTCCTATACACTATTGTGCTCCAGCTGGTTATGCGATTCTAAAGT
GTAGG---GATAAGAAATTCAAT------GGGACAGGACCATGCAAGAAC-----------------------
-GTTAGT---ACAGTACAATGTACACATGGAATTAAGCCAGTAGTATCAACT---
CAACTGCTGTTAAATGGTACCCTAGCAGAAGATGAT-----------
ATAATAATTAGCTTTGAAAATAAGACAGACAAT---------GCTAAATCAATCATAGTACATTTTAAT----
-----------GAATCTGTAAAA---------ATTGTGTGTACAAGACCCAGCAATAACACAAGAAAA-----
-AGTATAAGGATA------------GGACCAGGACAAACG---TTC------TATGCAACAGGAGAA------
---------ATAATAGGAGACATA------------AGACAAGCACACTGT---AACATTACTAAC-------
--TGGAATAACACTTTACAAAGGGTAAAGAGAAAATTAGGA---------------GAACACTTCCCT-----
----------------------------------AATGCAACAATAAAATTTGCA------
CCACATTCAGGAGGGGACCTAGAAATTACA---ACACATAGTTTTAATTGTAGAGGAGAA---
TTTAAGCAGCTCCAGGCAAGAGTCCTGGCTATAGAAAGATACCTACAGGGACAACAGCTCCTAGGGATTTGGG
GCTGCTCTGGAAAACTCGTCTGCACCACTAATGTACCTTKGAACTCCAGTTGGAGT-----------------
---------------------------AACAAATCTCAAGACAAAATTTGG------GGA------
AACATGACCTGGATGGAATGG---GATAGGGAAATTAGTAATTACACAGAAGAAATATAC---
AAGTTACTTGAGGAATCGCAGAACCAGCAGGAA--

ATAAATGAAAAAGATTTATTAGCATTGGACAGTTGGAAAAATCTGTGGAATTGGTTTGATATATCAAATTGGC
TGTGGTATATAAAAATATTCATAATGATAGTAGGAGGCTTAATAGGTTTGAGAATAATTTTTGCT---
GTGCTATCTATAGTG---------AATAGAGTTAGGCAGGGATACTCACCC------
TTGTCGTTTGCTATTAGTCTGCTTGATACCTTAGCAATAGCAGTAGCTGAA---
GGAACAGATAGGATTATAGAAGTAGTACTAACAATTTGTAGAGCT----------------------------
--------ATCCGCAACATA------CCTAGAAGAATAAGACAGGGTCTTGAAGCAGCTTTGCAA---TAA
>MK076687
GTA---GAA--
GGAGACTCGTGGGTCACAGTCTATTATGGGGTAGCTGTGTGG---AAAGAAGCAAAA---
ACTACACTATTCTGTGCATCAGATGCTAAAGCATATGAGAAAGAA---GTGCATAAT----------------
--------------GTCTGGGCTACA---CACGCCTGTGTACCCACAGAC---
CCCAGCCCACAAGAAATAAAATTGGAA---------AATGTAACAGAAAATTTTAAC----------------
--------------------------ATGTGGAAAAGTGACATGGTGGATCAGATGCAGGAGGATGTA---
ATTAGCTTATGGGATGAAAGCCTA---AAGCCATGTGTAAAGTTG---
ACTCCACTCTGTGTCACTTTAAATTGCACAAAT--

----------------ACTACTACCCTCAATAGGACCCTTGATGGGAGTGAACGTGAACTAAGA---
AATTGCTCTTTCAATACAACCACAGAAATACAT---------------------------
GATAAACAGCAGAAAGTGCATGCACTT---
TTCTATAGACTTGATCTACAACCATTGGGTAATGAGACAGATTCCTCTAAAAATTCC----------------
----
TCTACAAATTCCTCTACAAACTCCTCTGAGTATATATTAATAAATTGTAATACCTCAGCCATAGCACAA---
GCCTGTCCAAAGGTCTCTTTTGATCCAATTCCTATACACTATTGTGCTCCAGCTGGTTATGCGATTCTAAAGT
GTAAT---GATAAGAAATTCAAT------GGGACAGGACCATGCCATAAT-----------------------
-GTTAGC---ACAGTACAATGTACACATGGAATTAAGCCAGTGGTGTCAACT---
CAACTACTGTTAAATGGTAGCCTAGCAGAAGAAGAG-----------
ATAATAATTAGATTTGAAAATATAACAGACAAT---------GCCAAGACAATAATAGTACATCTTAAT----
-----------CGATCTGTAAGA---------ATTGAGTGTACAAGACCCAACAATAATACAAGAAAA-----
-AGTTATAGGATA------------GGACCAGGACAAACA---TTC------TATGCAACAGGCGAC------
---------ATAATAGGGGACATA------------AGACAAGCACATTGT---AACATTAGTGAAAAG---
GACTGGTATGGAACTTTAAAAAATATAAGTAAAAAATTAGCA---------------
GAACACTTCCCTAATCCT--------------------------------AATAAAACAATAAACTTTGAA-
-----GAACACTCAGGAGGGGACCCAGAAATCACA---ATGCATAGCTTTAATTGCAGAGGAGAA---

TTTAAGCAGCTTCAGACAAGAGTTCTGGCTATAGAAAAATACCTGACGGATCAACAGCTCCTAGGGGTTTGGG
GTTGCTCTGGAAAAACCATCTGCACCACTGCTGTGCCTTGGAACTCCAGTTGGAGT-----------------
----------------------------AACAAATCTCAAGATGAAATTTGG------AAA------
AACATGACCTGGATGCAGTGG---GATAGAGAAATTAGTAATTACACAGACACAATCTAC---
TGGTTGCTTGAAAAATCGCAAAACCAACAGGAG--

GAAAATGAAAAGGATCTATTAGCATTGGACAGTTGGAAAAATCTGTGGAGTTGGTTTGATATAACAAAATGGC
TGTGGTATATAAAAATATTCATAATGATAGTAGGAGGCTTAATAGGTTTAAGAATAATTTTTGCT---
GTGCTTTCTATAGTG---------AATAGAGTTAGGCAGGATACTCACCT------
TTGTCGTTCGCTATTAGTTTGTTTGATACCATAGCATTAGCAGTAGCAGAA---
GGAACAGATAGAATTATAGAATTAATATTAGGAATTGGTAGAGCT----------------------------
--------ATCTGCAACATA------CCTAGAAGAATAAGACAGGGCTTTGAAGCAGCTTTGCAA---TAA
>KY713231
GTG---GTA--
GGAAACTTGTGGGTCACAGTCTATTACGGGGTACCTGTGTGG---ACAGAAGCAAAA---
ACTACTCTATTCTGTGCATCAGATGCTAAAGCATATGAGACAGAA---GTGCATAAT----------------
--------------GTATGGCTACA---CATGCCTGTGTACCCACAGAC---
CCCAACCCACAAGAATTGGTTTTGGGA---------AATGTAACAGAAAATTTTAAC----------------
--------------------------ATGTGGAAAATAACATGGTGGATCAGATGCATGAGGATGTA---
ATCAGTATATGGGATCAAAGCCTA---AAGCCATGTGTAAAGTTG---
ACCCCACTCTGTGTCACTTTAGACTGTAAAAATGTTACCTATAATGTTACCAATAGTACAGCT----------
----
-------------------------GGTGGAAATGTTAACATTAGTACTTGGGAAGAAATGAAA---
AATTGCTCTTTTAATACAACCGCAGAACTAAAA---------------------------
GACAAGAAGCAGAAGGCGTATGCACTT---TTTTATAGCCTTGATATAACACCACTTGATGATGGG-------
----

AACAGCTCTAGGTATAGATTAATAAAATGTGATACCTCAGCCATAACACAA---
GCCTGTCCAAAGGTTACTTTTGATCCAATACCTATACATTATTGCGCTCCAGCTGGTTATGCGATTCTAAAGT
GTAAT---AATAAGACATTCAAT------GGGACAGGACCATGCCAGAAT-----------------------
-GTTAGC---ACAGTACAATGTACACATGGAATTAAGCCAGTGGTATCAACT---
CAACTACTGTTGAATGGTAGCCTAGCAGAAGAAGAG------------
ATAATAATTAGATCTGAAAATCTGACAAAGAAT---------GACAGAACAATAATAGTACATCTTAAT----
-----------GAATCTGTAGAA---------ATTGAGTGTACAAGACCCAACAATAATACAAGGAAA-----
-AGTATAAGGATA------------GGACCAGGACAAACA---TTC------TATGCAACAGGAGAT------
---------ATAATAGGAGACATA------------AGACAAGCACATTGT---AACATTAGTAATGAA---
AAATGGAATGAAACTTTACGAAGGGTACGTAGAAAATTAGAA---------------GAATACTTTCCT----
-----------------------------------GGTAAAAACATAACTTTTAAC------
CCATCCTCAGGAGGGGACCTAGAAATTGCA---ACTCATAGCTTTAATTGTAGAGGAGAA---
TTTAAGCAACTCCAGACAAGAGTCCTGGCTATAGAAAGATACCTAAAGGATCAACAGCTCCTAGGGATTTGGG
GCTGCTCTGGAAAGCTCATCTGCACCACTGCAGTACCTTGGAACTCCAGTTGGAGT-----------------
----------------------------AATAGATCTCAAACAGATATTTGG------GAG------
AACATGACCTGGATGCAGTGG---GATAAGGAAATTAGTAATTACACAAGCACAATATAC---
AGGTTACTTGAGGAATCGCAAAACCAGCAGGAA--

AGAAATGAAAAAGATTTATTAGCATTGGACAGTTGGAAAAATCTGTGGAGTTGGTTTGACATATCAAATTGGC
TGTGGTATATAAGAATATTCATAATGATAGTGGGAGGCTTGATAGGTTTAAGAATAATTTTTGCT---
GTGCTCGCTATAGTG---------AATAGAGTTAGGCAGGATACTCACCT------
TTGTCGTTCGCTATTAGTCTGTTTGATACTATAGCAATAGCAGTAGCTGAA---
GGAACAGATAGGATTATAGAAATAGTACAAAGAGGTTATAGAGCT----------------------------
--------ATCTGTAACATA------CCTAGAAGAATAAGACAGGGCTTTGAAGCAGCTCTTTGCAA-TAA
>KY713235
GTG---GGA--
GGAAACTTGTGGGTCACAGTCTATTATGGGGTACCTGTGTGG---AAAGAAGCAAAA---
GCTACTCTATTCTGTGCATCAGATGCTAAAGCATATGATAAAGAA---ATACATAAT----------------
--------------GTATGGCTACA---CATGCCTGTGTACCCACAGAC---
CCCAACCCACAAGAACTGGTTTTGGAA---------AATGTAACAGAAAATTTTAAC----------------
--------------------------ATGTGGAGAAATGATATGGCGGAACAGATGCATGAGGATGTA---
ATCAGTTTATGGGATCAAGGCCTA---AAGCCATGTGTGAAATTG---
ACCCCACTCTGTGTCACTTTAAATTGTACAAATGCTATTGCACATAATAGTACAAATGTTACTAGTACTGCAA

AGCCAACCACT--
---GATAGTGTGGGGAAATAAAA---
AATTGCTCTTTCAATGCAACCACAGAAATAACA---------------------------
GATAAAAGGAGGAAAGTGCATGCACTC---
TTTTATAGACTTGATATAGTACCACTTGATATAGAAAACAAATCTGCA-------------------------
----
AGCAACTCTAGTGAGTTTTATAGATTAATAAATTGTAATACCTCAGCCATAACACAA---
GCCTGTCCAAAGGTCACTTTTGATCCAATTCCTATACATTATTGTGCTCCAGCTGGTTATGCAATTCTAAAGT
GTAAT---AATAAGACATTCAAT------GGGACAGGGCCATGTCAGAAT-----------------------
-GTTAGC---ACAGTACAATGTACACATGGAATTAAGCCAGTGGTATCAACT---
CAATTCCTGTTAAATGGTAGCCTAGCAGAAGAAGAG------------
ATAATAATTAGATCTGAAAATCTGACAGATAAT---------GCCAAAACAATAATAGTACATCTTAAT----
-----------AAATCTGTAGAA---------ATTACGTGTATAAGACCCAACAATAATACAAGGACA-----
-AGTATAAGGATA------------GGACCAGGACAAATG---TTC------TATACAACAGGAGAC------
---------ATCATAGGAGACATA------------AGACAAGCATATTGT---ACCATTAATAGAACA---
AGTTGGAATGACACATTACAAAGTGTAAGAAATAAATTAGCA---------------GAACACTTCAAT----
-----------------------------------AATACAATAATAAAATTTGAA------
CCACCCTCAGGAGGGGACCTAGAAATTACA---
ACACATAGCTTTAATTGTAGAGGAGAATTAGATAAGCAGCTCCAAACAAGAGTCCTGGCTATAGAAAGATACC
TAAAGGATCAACAGCTCCTAGGGATTTGGGGCTGCTCTGGAAAACTCATCTGCACTACTAATGTACCTTGGAA
CTCCACTTGGAGT---
AACAAATCTCTAACAGAGATTTGG------AGT------AACATGACCTGGATGGAGTGG---
GAGAGAGAAATTGATAATTACACAAACACAATCTAC---AGGCTACTTGAAGACTCGCAAAACCAGCAGGAA-
----
ATAAATGAAAAAGATTTATTAGCATTGGATAAGTGGAACAGTCTATGGAATTGGTTTAACATAACAAACTGGC
TGTGGTATATAAAAATATTCATAATGATAGTAGGAGGATTAATAGGTTTAAGAATAATTTTTGCT---
GTGCTTTCTGTAGTA---------AATAGAGTTAGGCAGGGATACTCACCT------
TTGTCATTCGCTATTAGTTTGTATGATACCATAGCAATAGCAGTAGCTGAA---
GGAACAGATAGAATTATAGCAGTGATACAAGGAATTTGTAGAGCT----------------------------
--------ATCTGCAACATA------CCTAGAAGAATAAGACAGGGCTTGGAAGCAGCTTTGCAA---TAA
>MN703343
GGG---GGA--
GGAAACTTGTGGGTCACAGTCTATTATGGGGTACCTGTGTGG---AAAGAAGCAAAA---
ACTACTCTATTCTGTGCATCAGATGCCAAAGCATATGAGAAAGAA---GTGCATAAT----------------
--------------GTCTGGGCTACA---CATGCCTGTGTACCCACAGAC---
CCCAACCCACAAGAATTAGGGTTGACA---------AATGTAACAGAAAATTTTGAC----------------
--------------------------ATGTGGAAGAATGACATGGTGGATCAAATGCATGAGGATGTG---
ATTAGTTTATGGGATCAAAGCCTG---AAGCCATGTGTAAAGTTG---
ACCCCACTCTGTGCCACTTTAAACTGTAGTATCGTTAAGGTTAATAATACTGGTAGCAATGGTACT-------
----
-------------------------GTTAGTAATGAGACCCAAATCGACGATGAAATCATGACA---
AATTGCACTTTCAATACAACCACAGAATTAAGA---------------------------
GATAAAAAGCAGAGTAGGCGTGCACTT---TTTTATAAGCTTGATGTAGTACCAATTAATGAG----------
----

AACTCTAGTATGTATAGATTAATAAATTGTAATACCTCAGCCATAACACAA---
GCCTGTCCAAAGATCTCTTTTGATCCAATTCCTATACACTATTGTGCTCCAGCTGGTTATGCGATTCTAAAGT
GTAAC---AATAAGACATTCAAT------GGGACAGGACCATGCAATAAT-----------------------
-GTTAGC---ACAGTACAATGTACACATGGGATTAAGCCAGTGGTATCAACT---
CAACTACTGTTGAATGGTAGTCTAGCAGAAAAAGAG------------
ATAATAATTAGATCTGAAAATCTGACAAACAAT---------GCCAAAACAATAATAGTTCATCTTAAT----
-----------GAATCTGTACGA---------ATTGAGTGTACAAGACCCAACAACAATACAAGAGAA-----
-AGTATAAGGATA------------GGACCAGGACAAACA---TTC------TATGCAACAGGAGAC------
---------ATAATAGGAGACATA------------AGACAAGCACATTGT---AACATTAGTGAAAAA---
AACTGGACTATAGCTTTACACAGGGTAAGTAAAAAATTAGAA---------------GAACACTCCCCT----
-----------------------------------AATAAAACAATAAGATTTGAC------
CAATCCTCAGGAGGGGACCTAGAAATTAGC---ACACATAGCTTTAATTGTGGAGGAGAA---
TTTAAGCAGCTCCAGGCAAGAGTCCTGGCTATAGAAAGATACCTAAAGGATCAACAGCTCCTGGGAATTTGGG
GCTGCTCTGGAAAACTCATCTGCACTACTGCTGTACCTTGGAACTCCAGTTGGAGT-----------------
----------------------------AACAAATCTAAAGAGGAGATTTGG------GAT------

AACATGACCTGGATGCAGTGG---GATAGAGAAATTAATATTTACACAGACATAATATAC---
AGTTTGCTTGAAAAATCACAAATCCAGCAGGAG---

GAAAATGAAAAAGAGTTATTAGCATTGGACAGTTGGAATAGTTTATGGAATTGGTTTGACATAACAAATTGGC
TGTGGTATGTAAAAATATTCATAATGATAGTAGGAGGCTTGATAGGTTTAAGAATAATTTTTGCT---
GTGCTTTCTATAGTA---------AATAGAGTTAGGCAGGGATACTCACCT------
TTGTCGTTGGCTATTAACTTGCTTGACACCACAGCAATAGCAGTAGCTGAA---
GGGACAGATAGGATTATAGAATTAGCACTAACAATTATTAGAGCT---------------------------
--------ATAGGCAACATA------CCTAGAAGAATAAGACAGGGCTTGGAAGGAGCTTTGCTA---TAA
>MN703361
GTG---GTA---
GGGAATTTGTGGGTCACAGTTTATTATGGGGTACCTGTGTGG---AGAGAAGCAAAA---
ACTAATTTATTCTGTGCATCAGATGCTAAAACATATGAGGCGGAA---GTGCATAAT----------------
--------------GTCTGGGCAACA---TATGCCTGTGTACCCACAAAC---
CCCGAACCACACGAAACATTTTTGGAA---------AATGTAACAGAAAATTTTAAT----------------
--------------------------ATGTGGAAAAATGATATGGTGGAGCAGATGCATGAGGATGTA---
ATCAGTATATGGGACCAAGGCCTA---AAGCCGTGTGTAAAGTTG---
ACCCCACTCTGTGTCACTTTAAACTGTACAAATGCTACAAGTGTTGGCACAAATGTTACAAGT----------
----
--GAGGAAATGAAA---
AATTGCTCTTTCAATGCAGCCACAACAGTAAAA--------------------------
GACAAGAGGCAGAAAGAGTATGCAACT---
TTTTATAACCTGGATATAGTACAACTTAATGAGGGAACGTCTAACGAG-------------------------
----
AGCTCTAGCTATTATAGATTAATCAGTTGTAATACCTCAGCCATAACACAA---
GCCTGTCCAAAAGTCAATTTTGATCCAATTCCTATACATTATTGTGCTCCAGCTGGTTTTGCGATTCTAAAGT
GTAAT---AACAAGACATTCAAT------GGGACAGGACCATGCACGAAT----------------------
-GTTAGC---ACAGTACAATGTACACATGGGATTAGGCCAGTAGTATCAACT---
CACCTACTGTTAAATGGTAGCCTAGCAGAAGAAAAG-----------
ATAATGATTAGGTCTGAAAATCTGACAAACAAT---------GCTAAAACAATAATAGTGCAGTTTAAT----
-----------AAATCTGTAGAG---------ATTGTCTGTACAAGACCCAACAATAATACAAGAAAA-----
-AGTATAAGAATA------------GGACCAGGACAAACA---TTC------TATGCAACAGGAGAA------
---------ATAATAGGAGACATA-----------AGACGAGCCCATTGT---ACAGTTAATGCTAGT---
GATTGGACTAGCACCTTAGAAAAGGTAAGAAAAGAATTAGGA---------------ACACACTTC-------
-------------------------------------AATCGTACAATAATATTTAAC------
CAATCCTCAGGAGGGGACTTAGAAGTCACA---ACACATAGCTTTAATTGTAGAGGAGAA---
TTTAAGCAGCTCCAGACGAGAGTCCTGGCTATAGAAAGATACCTAAGGGATCAACAGCTCCTAGGGATTTGGG
GCTGCTCTGGAAAACTCATCTGCACTACTGCTGTACCTTGGAACTCCAGTCGGAGT-----------------
-----------------------------AACAGATCTGAAACAGAAATTTGG------GGC------
AACATGACCTGGATGCAGTGG---GATAGAGAAATCAGTAATTACACAGTTGAAATATAC---
AAATTGCTTGAAGAGTCGCAAAGCCAGCAGGAA---

AGAAATGAAAAAGATTTATTAGCATTGGACAGTTGGAACAATTTATGGAATTGGTTTGGCATAACAAAATGGT
TGTGGTATATAAAAATATTCATAATGATAGTAGGAGGCTTAATAGGTTTAAGAATAATTTTTGCT---
GTGCTATCTATAGTA---------AATAGAGTTAGGCAGGGATACTCACCC------
TTGTCGTTTGCTATTAGTCTGCTTGATACCACAGCAATAGTAGTAGCTGAA---
GGAACAGATAGGATCATAGAAGTAGCACAAAGAATTGGTAGAGCT---------------------------
--------ATTTGCAACATA------CCTAGAAGAATAAGACAGGGCTTTGAAGCAGCTTTGCAA---TAA
>AF067159
GGG---GGA---
AAAGACTTGTGGGTCACAGTCTATTATGGGGTACCTGTGTGG---AAAGAAGCAAAA---
ACTACTCTATTTTGTGCATCAGATGCTAAAGCATATGGGAAAGAA---GTGCATAAT----------------
--------------GTATGGGCTACA---CATGCCTGTGTACCCACAGAC---
CCCAACCCACAAGAAATATCTTTGGAA---------AATGTAACAGAAAATTTTAAC----------------
-------------------------ATGTGGAAAAGTGATATGGTGAATCAGATGCATGAGGATGTA---
ATCAGTTTATGGGATCAAAGCCTA---AAGCCATGTGTAAAGTTG---
ACCCCACTCTGTGTCACGTTAGAATGTGGAAATGTTACCCAAAATGGTACC----------------------
----
----------------------------------TACAATGATGAGAGCAATAAGGAAATAACA---
AATTGCACTTTCAATACAACCACAGAAATAAGA--------------------------

GGTAGGAAGCAGAAAGTGTATGCACTC---TTTTATAAACTTGATATAGTACCAATTAGTGAG----------
----

ACTAGTAATCAGTCTAGATTAATAAGTTGTAATACCTCAGTCATAACACAA---
GCCTGTCCAAAGGTCAGTTTTGATCCAATTCCTATACACTATTGTACTCCGGCTGGTTATGCGATTCTAAAGT
GTAAT---GATAAGACATTCAAT------GGGACAGGACCATGCCGTAAT-----------------------
-GTTAGC---ACAGTACAATGTACACATGGAATTAAGCCAGTAGTATCAACT---
CAACTACTGTTAAATGGTAGCCTATCAGAAGGAGAG------------
ATAATAATTAGATCTGAAAATCTGACAAACAAT---------GTCAAAACAATAATAGTACATCTTAAT----
-----------AAATCTGTAGAA---------ATTGTGTGTACAAGACCCAACAATAATACAAGAAAA-----
-AGTATAAGGATA------------GGACCAGGACAAACA---TTC------TATGCAACAGGAGAA------
---------ATAGTAGGAAACATA------------AGACAAGCACATTGT---AACATTAGTAAAAGG---
GATTGGAATGAAACTTTACAAAGGGTAAGTGAAAAATTAGCA---------------AAACACTTCCCT----
-----------------------------------AATAAAACAATAAAATTTGCA------
CCATCCTCAGGAGGGGACCTAGAAATTACA---ACACATAGCTTTAATTGTAGAGGAGAA---
TTTAAGCAGCTACAGACAAGAGTCCTGGCTATAGAAAGATACCTAAAGGATCAACAGCTCCTAGGGATTTGGG
GCTGCTCTGGAAAACTCATCTGCACTACTGCTGTACCTTGGAACTCCAGCTGGAGT-----------------
----------------------------AACAGAACTCAAGAAGAGATTTGG------AAT------
AACATGACCTGGATGCAGTGG---GATAGAGAAATTAATAATTACACAAATACAATATAC---
AGGTTACTTGAAGACTCACAAAACCAGCAGGAG--

AAAAATGAAAAAGATTTATTAGCATTGGACAGTTGGAAAAATCTATGGAGTTGGTTTGACATAACAAATTGGC
TGTGGTATATAAAAATATTCATAATGATAGTAGGAGGCTTGATAGGTTTAAGAATAATTTTTGCT---
GTGCTCTCTATAGTG---------AATAGAGTTAGGCAGGGATACTCACCT------
TTGTCGTTTGCTATCAGGCTGCTTGATATCATAGCAATAGCAGTAGCTGAA---
GGAACAGATAGGATTATAGAAATAATACAAGGAACTTGTAGAGCT----------------------------
--------ATCCGCAACATA------CCTAGAAGAATAAGACAGGGCTTGGAAGCAGCTTTGCAA---TAA
>FJ866133
GGG---GTG--
GGGAACTTGTGGGTTACAGTATATTATGGGGTACCTGTGTGG---AGAGATGCAAAA---
ACCACTCTATTCTGTGCATCAGAAGCTAAAGCATATGATCCAGAA---GTGCATAAT----------------
--------------GTCTGGCTACA---CATGCCTGTGTACCCACAGAC---
CCCAACCCACAAGAAATAGTTTTGGGA---------AATGTAACAGAAAATTTTAAC----------------
--------------------------ATATGGGAAAATGACATGGTGGATCAAATGCATCAGGATATA---
ATCAGTTTATGGACCAAAGCCTA---AAGCCATGTGTAAAGTTG---
ACCCCACTCTGTGTTACCTTAAACTGTAGGGATGCTGATTTTACTAGTATCCAC-------------------
----
-------------------------------AATAATACCATGGATATGAATGGAGAAATAAAA---
AATTGTTCTTTTAATATGACCACAGTACTAAAA---------------------------
GATAAGAAACAGAAGGTACAGGCACTT---TTCTATAGACTTGATGTAGTACAACTTAGTAAT----------
----

AACAATAGTAGTAGTGAGTATAGATTAATAAATTGTAATACCTCAGCCATTACACAA---
GCTTGTCCAAAGGTCTCTTTTGACCCAATTCCTATATATTATTGTGCTCCAGCTGGTTATGCGATTCTAAAGT
GTAAT---AACAAGACATTCAAT------GGAACAGGACCATGCAGAAAT-----------------------
-GTCGGC---ACAGTACAATGTACACATGGAATTAAGCCAGTGGTATCAACT---
CAATTGCTGTTAAATGGTAGTCTAGCAGAAGAGAAA------------
ATAATAATTAGATCTAAGAATCTGACAAACAAT---------GCCAAAACAATAATAGTACATCTTAAG----
-----------GAACCTGTATCA---------ATTAATTGTACGAGACCTAATAACAATACAAGAGAA-----
-AGTATAGGGATA------------GGACCAGGACAAATG---TTC------TATGCAATGGGAGCG------
---------ATAATAGGAGATATA------------AGACAAGCACATTGT---AACATTAGTGGAGAA---
AAATGGAATAACACTTTACAAAAGGTAAAAGAAGAATTAGAA---------------AAGCACTTTCCT----
-----------------------------------AATAAAACAATACAATTTAAC------
AATTCAGCAGGAGGGGACCTAGAAGTTACA---ACACACAGCTTTAATTGTAGAGGAGAA---
TTTAAGCAGCTCCAGGCAAGAGTCCTGGCTATAGAAAGATACCTACAGGATCAACAGCTCCTAGGGATTTGGG
GCTGCTCTGGAAAACTCATCTGCACCACTGCTGTGCCTTGGAACTCTAGTTGGAGT-----------------
----------------------------AATAAATCTCAGGAAGATATTTGG------AAT------
AACATGACCTGGATGCAGTGG---GACAGAGAAATTAGTAATTATACAAACACAATATAC---
AGGTTGCTTGAGGTCTCGCAGAACCAGCAGGAG--

CAGAATGAAAAAGATTTACTAGCATTAGACAAATGGCAAAATCTGTGGAATTGGTTTGACATATCAAAGTGGC
TGTGGTATATAAAAATATTCATAATAATAGTAGGAGGCTTGATAGGTTTAAGAATAATTTTTGCT---
GTGCTCTCTATAGTG---------AACAGAGTTAGGCAGGGATACTCACCA------
TTATCGTTTGCTATTAATTTGTTAGATACAGTAGCAATAGCAACAGCTAAC---
TGGACAGATAGGGTTATAGAAGTAGCACAAAATATTGGTAGAGCT----------------------------
--------ATCTACAACATA------CCTAGAAGAATAAGACAGGGCTTTGAAGCAGCTTTGCAA---TAA
>AB097871
GTG---GGA--
GGAAACTTGTGGGTCACAGTCTATTATGGGGTACCTGTGTGG---AGAGAAGCAAAA---
ACTACTCTATTCTGTGCATCAAATGCTAAAGCATATGAGACAGAA---GTGCATAAT----------------
--------------GTCTGGGCTACA---CATGCCTGTGTACCCACAGAC---
CCCAACCCACAAGAAATGGATTTGGAA---------AATGTAACAGAAAATTTTAAC----------------
--------------------------ATGTGGACAATGATATGGTGGATCAGATGCATGAGGATGTA---
GTCAGTTTATGGATCAAAGCCTA---AAGCCATGTGTAAAGTTG---
ACCCCACTCTGTGTCACTTTAGAATGTAAAAATGTTAAAATTGTTAACAGTACTGGTAGAAATGTTAGCAGT-
----
----------------AGTAGTGGTAGTAATGGTACCTACATTGGGAACTACACTGAGATAAAA---
AATTGCTCTTTCAATACAACCACAGTTTTAAAA---------------------------
GATAGGAAGCAGTCAGTGTATGCACTT---TTTTATAGACTTGATATAGTACCACTTAATGAGAAGAAA----
----

TCTAGTGAAAACTCTAGTGAGTATTATAGATTAATAAATTGGAATACCTCAGCCATAACACAA---
GCCTGTCCAAAGGTCACTTTTGACCCAATTCCTATACACTATTGCGCTCCAGCTGGTTATGCGATTCTAAAGT
GTAAG---GATAAGAAATTCAAT------GGGACAGGACCACGCCGTAAT-----------------------
-GTTAGC---ACAGTACAATGTACACATGGAATTAAGCCAGTGGTATCAACC---
CAACTACTGTTAAATGGTAGCCTAGCAGAAGAAGAG------------
ATAATAATTAGATCTGAAAATCTGACAGACCAT---------TTCAAAACAATAATAGTACAACTTAAT----
-----------CAACCTGTAGAA---------ATTGAATGTACAAGACCCAACAATAATACAAGAAAA-----
-AGTATAAGGATA------------GGACCAGGACAAACA---TTC------TATGCAACGGGAGAA------
---------ATAATAGGAGACATA------------AGACAAGCACATTGT---AACATTAGTAAACAA---
AAATGGAATGAAACTTTACAAAGGGTAGGTAGAAAATTAGCA---------------GAACTCTTCCCT----
-----------------------------------AATAGAACAATAGTATTTAAC------
TCATCCCCAGGAGGGGACCTAGAAATTGCA---ACACATAGCTTTAATTGTAGAGGAGAA---
TTTAAGCAGCTCCAGGCAAGAGTCCTGGCTATAGAAAGATACCTAAAGGATCAACAGCTCCTAGGGATTTGGG
GCTGCTCTGGAAAACTCATCTGCACTACTGCTGTACCTTGGAACTCCAGTTGGAGT-----------------
----------------------------AACAAATCTCAAGAAGAGATTTGG------AAC------
AACATGACCTGGATGGAGTGG---GATAAAGAAATTAATAATTACACAGACACAATATAC---
AGGTTGCTTGAAGACTCGCAAAACCAGCAGGAA--

AGAAATGAAAAAGATTTATTAGCATTGGACAGTTGGAAAAATCTATGGAATTGGTTTAACATAACAAATTGGC
TGTGGTATATAAAAATATTCATAATGATAGTAGGAGGCTTGATAGGTTTAAGAATAATTTTTGCT---
GTACTCTCTGTAGTG---------AATAGAGTTAGGCAGGATACTCACCT------
TTGTCGTTTGCTATTAGTCTGCTTGATAGCATAGCCATAACAGTAGCTGAA---
GGAACAGATAGGATTATAGCAGTAGTACACAGAATTTGTAGAGCT----------------------------
--------ATCCGCAACATA------CCTAGAAGAATAAGACAGGGCTTTGAAGCAGCTTTGCAG---TAA
>KC894073
GTG---GTG--
GGAAACTTGTGGGTCACAGTCTATTATGGGGTACCTGTGTGG---AAAGAAGCAAAA---
ACTACTCTATTCTGTGCATCAGATGCTAGAGCATATGAGAGAGAA---GTGCATAAT----------------
--------------GTCTGGGCTACA---CATGCCTGTGTACCCACAGAC---
CCCAACCCACAAGAAATGGTTTTGGTC---------AATGTAACAGAAAATTTTAAC----------------
--------------------------ATGTGGAAAAATGATATGGTGGATCAGATGCATGAGGATATA---
ATCAGTTTATGGACCAAAGCCTA---AAGCCATGTGTAAAATTG---
ACCCCACTCTGTGTCATTTTAGAGTGTAATAATGCTAATGGTACCACCAATAATGGTAGT-------------
----
-------------------GTTATTGTTGTTAATGAAAATAGTACCATGTATGGAGAAATACAA---
AATTGCTCTTTCAAGGTAAATTCAGAAATAAAA---------------------------
GGTAAGAAACAAGACGTGTATGCACTT---TTTAATAGCCTTGATATAGTAAAACTTTATAATAAT-------
----

GGAACTAGTCAGTATAGATTAATAAATTGTAATACCTCAACCTTAACACAA---
GCCTGTCCAAAGGTCTCTTTTGATCCAATTCCTATACATTATTGTGCTCCAGCTGGTTATGCGATTCTCAAGT
GTAAT---AATAAGACATTCAAT------GGGACAGGACCATGCAATAAT-----------------------
-GTCAGC---ACAGTACAATGTACACATGGAATTAAGCCAGTGGTGTCAACT---
CAATTACTGTTAAATGGTAGCCTAGCAGAGGGAGAG------------
ATAATAATTAGATCTAAAAATCTGACAGACAAT---------ACCAAAACAATAATAGTACATCTTAAT----
-----------GAGTCTATAAAA---------ATTAATTGCATAAGACCCAACAATAATACAAGAAGA-----
-AGTATAAGGATA------------GGACCAGGACAAGCA---TTC------TATGCAGCAAATGGC------
---------ATAGTAGGAAATATA------------AGACAAGCACATTGT---AACATTAGCGAAGGT---
GAATGGAATAAAACTTTATACAGGGTAAGTAGAAAATTAGCA---------------GAACACTTCCCT----
-----------------------------------GGTAAAGAAATAAAATTTAAA------
CCACATTCAGGAGGGGACTTAGAAATCACA---ACACATAGCTTTAATTGTAGAGGAGAA---
TTTAAACAGCTCCAGGCAAGAGTCCTGGCTATGGAAAGATACCTACAGGATCAACAGCTCCTAGGGATTTGGG
GCTGCTCTGGAAAACTCATCTGCACCACTGCTGTGCCTTGGAACTCTAGTTGGAGT-----------------
----------------------------AACAAAACTCTAGAGTATATTTGG------GGA------
AACATGACTTGGATGCAGTGG---GATAGAGAAATTGACAATTACACAGGCATAATATAC---
GATTTACTTGAAGACTCGCAAATCCAGCAGGAA--

AAAAATGAAAAAGATCTACTAGCATTGGACAGTTGGAAAAATCTATGGAGTTGGTTTAGCATAACAAATTGGT
TGTGGTATATAAAGATATTCATAATGATAGTAGGAGGCTTGATAGGTTTAAGAATAATTTTTGCT---
GTGCTTTCTATAGTG---------AATAGAGTTAGGCAGGGATACTCACCT------
TTGTCGTTTGCTATTAGTCTGCTTGATACCATAGCAATAGCAGTAGCTGAA---
GGAACAGATAGGATTATAGAAGTAGTACAAAGAATTTGTAGAGCT----------------------------
--------ATCTATAACATA------CCTAGAAGAATAAGACAGGGCTTTGAAGCAGCTTTGCAA---TAG
>FJ444207
AGT---GGA---
TCCTTGTGGGTCACAGTCTATTATGGGGTACCTGTGTGG---AGGGAAGCAAAA---
ACTACCCTATTCTGTGCATCAGATGCTAAAGCATATGAGAAGGAA---GCGCATAAT----------------
--------------GTTTGGGCTACA---CATGCCTGTGTGCCCACAGAC---
CCCAACCCACAAGAAGTAGTTTTGGAA---------AATGTAACAGAAAATTTTAAC----------------
--------------------------ATGTGGAAAAATGACATGGTGGATCAGATGCATGAGGATATA---
ATCAGTTTATGGGATCAAAGCCTA---AAGCCATGTGTAAAGTTG---
ACCCCACTCTGTGTCACTTTAATTTGTAGCAATGTTAATGTTAACAATAGCAGCAAT----------------
----
-------------------------------GTTAATGTTAACAATAGCACTAATGAAATGAAA---
AATTGCTCTTTCAATACAACCACAGAACTAAGA---------------------------
GATAAGAGAAAGAAGGAATATGCACTC---TTTTATAGACCTGATGTAGTACCACTTAATGAG----------
----

GGGAATAATGACAGTTATAGATTAATAAATTGCAATACCTCAACCATAACACAA---
GCCTGTCCAAAGGTCTCTTTTGACCCAATTCCTATACATTATTGTGCTCCAGCTGGTTATGCGATTCTAAAGT
GTAAT---AATAAGACATTCAAT------GGAACAGGACCATGCAATAAT-----------------------
-GTTAGC---ACAGTGCAATGTACACATGGAATTAAGCCAGTGGTATCAACT---
CAACTACTGTTAAATGGTAGTCTAGCAGAAGGAGAG------------
ATAATAATTAGATCTGAAAATCTGACAAACGAT---------GTCAAAACAATAATAGTACACCTTAAT----
-----------GAATCTGTAAGA---------ATTGTGTGTACAAGACCCCACAATAATACAAGAAAA-----
-AGTGTGAGGATA------------GGACCAGGACAAGTA---TTC------TATGCAACAGAAAGA------
---------ATAATAGGAGACATA------------AGAGAAGCACATTGT---AACATTAGTGAAGAA---
AAATGGAACAAAACTTTAAATGATGTAAAGAAAAAATTATTA---------------GAACACTTCCCT----
-----------------------------------AATAAAACTATAAGGTTTCAA------
CCACATTCAGGAGGGGACCTAGAAATTACA---ACACATAGTTTTAATTGTAGAGGAGAA---
TTTAAGCAGCTCCAGGCAAGAGTCCTGGCTCTAGAAAGATACCTAAAGGATCAACAGCTCCTAGGGATTTGGG
GCTGCTCTGGAAAACTCATCTGCACCACCAATGTGCCTTGGAACGGGAGTTGGAGT-----------------
----------------------------AATAGATCTCAAGAGGAAATTTGG------GGT------
AACATGACTTGGATGCAGTGG---GATAGAGAAATTAACAATTACACAAACACGATATAC---
AGGTTGCTTGAAGACTCGCAAAACCAGCAGGAA--

CAAAATGAAAAAGATTTACTAGCATTGGACAGTTGGAATAGTTTATGGAGTTGGTTTAACATAACAAATTGGC
TGTGGTATATAAAAATATTCATAATGATAGTAGGAGGTTTAATAGGCTTAAGAATAATTTTTGCT---
GTACTATCCATAGTA---------AATAGAGTTAGGAAGGGATACTCACCA------

TTATCGTTTGCTATTAATCTGCTTGATACCATAGCAATAGCAGTAGCTGAA---
GGAACAGATAGGATTATAGAAGTAATACAAAGAGGTTGTAGAGCT----------------------------
--------ATCCTCAACATA------CCTAGAAGAATAAGACAGGGCTTTGAAGCAGCTTTGCAA---TAA
>KC894078
GTG---ATG--
GGAAACTTGTGGGTCACAGTCTATTATGGGGTACCAGTTTGG---AAAGAAGCAAAA---
ACTACTCTATTTTGTGCATCAGATGCTAAAGCATATGAGAGAGAA---GTGCATAAT----------------
--------------GTCTGGCTACA---CATGCCTGTGTACCCACAGAC---
CCCAGCCCACAAGAAATGGAGTTAAAA---------AATGTAACAGAAAATTTTAAC----------------
--------------------------ATGTGGGAAAATGACATGGTGGATCAGATGCATGAGGATGTA---
ATCAGTTTATGGGATCAAAGCCTA---AAGCCATGTGTAAAGTTG---
ACCCCACTCTGTGTCACTTTAAACTGTAGTGATGTTAAGAATAATGTTACTACTAATGGTACCTCT-------
----
----------------------GATAGTAGTAATATTACCTCTAATATAAAGGATGAAATGAAA---
AATTGCTCTTTCAATATAACCACAGAAATAAGA---------------------------
GATAAGAGAAAGAAGGAATATGCACTT---TTTTATAAACTTGATATAGTACCACTTGAGACAAAT-------
----

TCCAGTAGTGAATACAGATTAATAAATTGTAATACCTCAGCCGTAACACAA---
GCTTGTCCAAAGGTCTCTTTTGATCCAATTCCTATACATTATTGTGCTCCAGCTGGTTATGCGATTCTAAGGT
GTAAT---AATAAGACATTCAAT------GGAACAGGACCATGCAATAAT-----------------------
-GTCAGT---ACAGTACAATGTACACATGGAATTAAGCCAGTGGTATCAACT---
CAGCTACTGTTAAATGGTAGCCTGGCAGAAAAAGAC------------
ATAATAATTAGCTCTAAAAATATATCAGACAAT---------GCCAAAACAATAATAGTACATCTTAAT----
-----------GAATCTGTACAA---------ATTGTGTGTATAAGGCCCGGCAATAATACAAGGAAA-----
-AGTGTGTGGATA------------GGACCAGGACAAGCA---TTC------TATGCAACAGGAGAC------
---------ATAATAGGAGATATA------------AGACAAGCACATTGT---AACATTACTGCAGGC---
AAATGGAATAGAACTTTAGAAAAGGTAAGGAAAAAATTAGAA---------------GAACACTTCCCT----
--------------------------------AATAAAACTATAAACTTTGAACCA---
GTACCAGCCTCAGGAGGGGACCTAGAAATTACA---ACACATAGCTTTAATTGTAGAGGAGAA---
TTTAAGCAGCTCCGGGCAAGAGTCCTGGCTATAGAAAGATACCTACAGGATCAACAGCTCCTAGGGATTTGGG
GCTGCTCTGGAAAACTCATCTGCACCACTAATGTGCCTTGGAACTCCAGTTGGAGT-----------------
----------------------------AATAAAACTCAAAACGAGATTTGG------GAG------
AACATGACCTGGATGCAGTGG---GATAGAGAAATTAGTAATTACACAGACACAATCTAT---
AGGTTGCTTGAAGACTCGCAAAACCAGCAGGAA--

ATTAATGAAAAGGATTTATTAGCATTGGACAGTTGGAAAAATCTGTGGAGTTGGTTTAGCATATCAAATTGGC
TGTGGTATATAAGAATATTCATAATGATAGTAGGAGGCTTGATAGGTTTAAGAATAATTTTTGCT---
GTGCTCTCTATAATA---------AATAGAGTTAGGCAGGATACTCACCT------
TTGTCATTTGCTATTAGTCTGCTTGATACCATAGCAATAATAGTAGCTGAA---
GGAACAGATAGAATTATAGAATTAGTACAAGGAATTTGTAGAGCT----------------------------
--------ATCCGCAACATA------CCTAGAAGAATAAGACAGGGCTTTGAAGCAGCTTTGCTA---TAA
>KC894079
ATG---GAG---
GGCTCGTGGGTCACAGTTTACTATGGAGTGCCTGTGTGG---AAAGAAGCAAAA---
ACTACTCTATTCTGTGCATCAGATGCTAAAGCATATGAGAAAGAA---GTGCATAAT----------------
--------------GTCTGGCTACA---CATGCCTGTGTGCCCACAGAT---
CCCAACCCACAAGAAATGGTTTTGGCA---------AATGTAACAGAAAATTTTAAC----------------
--------------------------ATGTGGAAAAATGATATGGTAGAGCAGATGCATGAGGATATA---
ATTAGTTTGTGGGATGAAAGCCTG---AAGCCATGTGTGAAGTTG---
ACCCCACTCTGTGTCACTTTAAATTGTACAAATGTTAAAGGGAATGAGAGTGACACCAGT-------------
----
--GAAGTAATGAAA---
AATTGCTCTTTCAAGGCAACCACGGAACTAAAG---------------------------
GATAAAAAACATAAGGTGCATGCGCTT---TTTTATAAACTTGATGTAGTACCACTTAATGGA----------
----

AACAGCAGCAGCTCTGGAGAGTATAGATTAATAAATTGCAATACCTCAGCCATAACACAA---
GCCTGTCCAAAGGTCTCTTTTGACCCAATTCCTTTACATTACTGTGCACCAGCTGGTTTTGCGATTCTAAAGT
GTAAT---AATAAGACATTCAAT------GGGACAGGACCATGTCGTAAT-----------------------

-GTCAGC---ACAGTACAATGTACACATGGAATTAAGCCAGTGGTATCAACT---
CAACTACTGTTAAATGGTAGCCTAGCAGAAGAAGAG------------
ATAATAATTAGATCTGAAAATCTGACAAACAAT---------GCCAAAACAATAATAGTACACCTCAAT----
-----------GAATCTGTAAAC---------ATTGTGTGTACAAGACCCAATAATAATACAAGAAAA-----
-AGTATAAGGATA------------GGACCAGGACAAACA---TTC------TATGCAACAGGTGAC------
---------ATAATAGGAAACATA------------AGACAGGCACATTGT---AACATTAATGAAAGT---
AAATGGAACAACACTTTACAAAAGGTAGGAGAAGAATTAGCA---------------AAACACTTCCCT----
-----------------------------------AGTAAAACAATAAAGTTTGAA------
CCATCCTCAGGAGGGGATCTAGAAATTACA---ACACATAGCTTTAATTGTAGAGGAGAG---
TTTAAACAGCTCCAGGCAAGAGTCCTGGCTATAGAAAGATACCTAAAGGATCAACAGCTCCTAGGGATGTGGG
GCTGCTCTGGAAAACTCATCTGCACCACTGCTGTGCCTTGGAACTCCAGTTGGAGT-----------------
----------------------------AACAAATCTCAAAATGAAATTTGG------GGG------
AACATGACCTGGATGCAGTGG---GACAGAGAAATTAATAATTACACAAACACAATATAT---
AGGTTACTTGAAGACTCACAAAACCAGCAGGAA--

AAAAATGAGAAAGATTTGTTAGCATTGGACAGTTGGAAAAATCTGTGGAATTGGTTTGACATATCAAAGTGGC
TGTGGTATATAAAAATATTCATAATGATAATAGGAGGCTTGATAGGTTTGAGGATAATTTTTGCT---
GTGCTTTCTATAGTA---------AAAAGAGTTAGGCAGGATACTCACCT------
TTGTCATTTGCTATTAGTCTAATTGATACCATAGCCATAGCAGTAGCTGAA---
AGGACAGATAGGATTATAGAAATAGTACAAAGAATTTGTAGAGCT----------------------------
--------ATCCGCAACATA------CCTAGAAGAATAAGACAGGGCTTTGAAACAGCTTTGCTA---TAA
>KC894081
GTG---GTG--
GGAAACTTGTGGGTCACAGTTTATTATGGGGTACCTGTGTGG---AAAGAAGCAAAA---
ACTACTCTATTTTGTGCATCAGATGCTAAAGCATATGAGAAGGAA---GTGCATAAT----------------
--------------GTCTGGGCTACA---CATGCCTGTGTACCCACAGAC---
CCCAACCCACAAGAAATGGTTTTGGAA---------AATGTAACAGAAAATTTTAAC----------------
--------------------------ATGTGGAAAAATGATATGGTAGATCAGATGCATGAGGATGTA---
ATCAGTTTATGGGATCAAAGCCTA---AAGCCATGTGTAAAGTTG---
ACCCCACTCTGTGTCACTTTAACTTGTACAAATACTACTGTTAGTAATGGTAGCAGTAATAGTAATGCTAACT
TT---
--GAAGAAATGAAA---
AATTGCTCTTTCAATGCAACCACAGAAATAAAA---------------------------
GATAAGAAAAAGAACGAGTATGCACTT---TTTTATAAACTGGATATAGTACCACTTAATAAC----------
----

AGCTCAGGCAAGTATAGATTAATAAATTGCAATACCTCAGCCATAGCACAA---
GCCTGTCCAAAGGTCACTTTTGAGCCAATTCCTATACATTATTGTGCTCCAGCTGGTTATGCGATTCTAAAGT
GTAAT---AATAAGACATTCAAT------GGAACAGGACCATGCAATAAT-----------------------
-GTCAGC---ACAGTACAATGTACACACGGAATTAAGCCAGTGGTATCAACT---
CAATTGCTGTTAAATGGTAGCCTAGCAGAAAAAGAA------------
ATAATAATTAGATCTGAAAACCTGACAAACAAT---------GCCAAAACAATAATAATACACTTTAAT----
-----------GAGTCTGTAGGG---------ATTGTGTGTACAAGACCCAGCAATAATACAAGGAAA-----
-AGTATAAGGATA------------GGACCAGGACAAACA---TTT------TATGCAACAGGTGAC------
---------ATAATAGGAGACATA------------AGACAAGCACATTGT---AACGTTAGTAAACAG---
AATTGGAACAGAACTTTACAACAGGTAGGAAGAAAATTAGCA---------------GAACATTTCCCT----
-----------------------------------AATAGAAACATAACATTTAAC------
CATTCCTCAGGAGGGGATCTAGAAATTACA---ACACATAGCTTTAATTGTAGAGGAGAA---
TTTAAGCAGCTCCAGGCAAGAGTCTTGGCCATAGAAAGATACCTAAAGGATCAACAGCTCCTAGGGATTTGGG
GCTGTTCTGGAAAACTCATCTGCACCACTAATGTGCCTTGGAACTCCAGTTGGAGT-----------------
----------------------------AATAGATCTCAAGAGGATATTTGG------AAT------
AACATGACCTGGATGGAGTGG---GAAAGGGAAATTGATAATTACACACACACAATATAC---
AGCTTGCTTGAAGAATCACAAATCCAGCAGGAA--

AAAAATGAAAAAGATTTATTGGCATTGGACAGTTGGCAAAATCTGTGGAGTTGGTTTAGCATAACCAAATGGC
TGTGGTATATAAAGATATTCATAATGATAGTAGGAGGCTTGATAGGTTTAAGAATAATTTTTGCT---
GTGCTCTCTATAGTG---------AATAGAGTTAGGCAGGATACTCACCT------
TTATCATTGGCTACCAGTCTGCTTGATACCACAGCAATAGTAGTAGTTGAA---
GGAACAGATAGAATTATAGGAATAATACAAGCAATTTGTAGAGCT----------------------------
--------CTCCTCAACCTA------CCTAGAAGAATAAGACAGGGCTTTGAAGCAGCTTTGCTA---TAA

>FJ444522
GTG---GTG--
GGGAACTTGTGGGTCACAGTCTATTATGGGGTACCTGTGTGG---AAAGAAGCAAAA---
ACTACTCTATTCTGTGCATCAGATGCTAAAGCATATGAGAGAGAA---GCACATAAT----------------
--------------GTCTGGGCTACA---CATGCCTGTGTACCCACAGAC---
CCCAACCCACAAGAAATAGTTTTGGAA---------AATGTAACAGAAAATTTTAAT----------------
--------------------------ATGTGGAAAAATGACATGGTAGATCAGATGCATGAGGATATA---
ATCAGTTTATGGGATCAAAGCCTG---AAGCCATGTGTAAAGTTG---
ACCCCACTCTGTGTCACTTTAAAATGTACAAATACAAATGGTACCAGTAGT----------------------
----
----------------------------------AGTAATATTACCAAAGAAGGAGACATGAAA---
AATTGCTCTTTCAATACAACCACAGAAATAAGA---------------------------
GATAAAAAACAGAAAGTGTATGCACTT---TTTTATAAACTTGATGTAGTACCACTTAATGAG----------
----

AATAACAACAGCTATATATTAATAAACTGCAATACCTCAGCCATAACACAA---
GCCTGTCCAAAGGTCTCTTTTGACCCAATTCCTATACATTATTGTGCACCAGCTGGTTATGCGATTCTAAAGT
GTAAC---AATAAGACATTCAAT------GGGACAGGACCATGCCATAAT-----------------------
-GTCAGC---ACAGTACAATGTACACATGGAATTAGGCCAGTGGTATCAACT---
CAACTACTGTTAAATGGCAGCCTAGCAGAAGAAGAG------------
ATAATAATTAGATTTGAAAACCTGACAAACAAT---------GCCAAAACAATAATAGTACATTTTAAT----
-----------GAATCTGTACAG---------ATTAATTGTACAAGACCCAACAATAATACAAGAAAA-----
-AGTATAAGGATA-----------GGACCAGGACAAACA---TTC------TATGCAACAAATGGC------
---------ATAATAGGAGACATA------------AGAGAAGCGCATTGT---AACATTAGTGCAAAG---
CAATGGAACAAAACATTACAGCAGGTAGGGAAAAAGTTAGCA---------------GAACGCTTCCCT----
-----------------------------------AATAAAACAATAATTTTTAAC------
CAATCCTCAGGAGGGGATCTAGAAATTACA---ATGCATAGCTTTACTTGTAGAGGAGAA---
TTTAAGCAGCTCCAGGCAAGAGTCCTGGCTATCGAAAGATACCTAAAGGATCAACAGCTCCTAGGGATTTGGG
GCTGCTCTGGAAAACTCATCTGCACCACTGCTGTGCCCTGGAACTCCAGTTGGAGT-----------------
----------------------------AATAAAACTCAGGACGCTATTTGG------AAG------
AACATGACCTGGATGCAGTGG---GATAGAGAAATTAGTAATTATACAGACACAATATAC---
AGATTGCTTGAAGACTCACAAAACCAGCAGGAA--

AGCAATGAAAAGGATTTACTAGCATTGGACAGTTGGAAAAATCTATGGACTTGGTTTGACATAACAAACTGGC
TGTGGTATATAAAAATATTCATAATGATAGTAGGAGGCTTGATAGGTTTGAGAATAATTTTTGCT---
GTGCTTTCTATAGTA---------AATAGAGTCAGGCAGGGATACTCACCT------
TTGTCTTTTGCTATCAGTCTAATTGATACCCTAGCCATAGCAGTAGCTGAA---
GGAACAGATAGGATTATAGAAGTAATACAAAGATTTGTAGAGCA-----------------------------
--------ATCCGCAACGTA------CCTATAAGAATAAGACAGGGCTTTGAAGCAGCTTTGCTA---TAA
>KC894089
GGG---AAG---GAT------------------------------------
GAGGATTTGTGGGTCACAGTCTACTATGGGGTACCTGTGTGG---AAAGAAGCAAAA---
ACTACTCTATTCTGTGCATCAGATGCTAAAGCATATAAGAAAGAA---GTACATAAT----------------
--------------GTCTGGGCCACA---CATGCCTGTGTACCCACAGAC---
CCCAACCCACAAGAAATAGTTTTGGGA---------AATGTAACAGAAAATTTTAAC----------------
--------------------------ATGTGGGAAAATGACATGGTAGATCAGATGCATGAGGATGTA---
ATCAGTTTATGGGATCAAAGCCTA---AAGCCATGTGTAAAGTTG---
ACCCCCCTCTGTGTTACTTTAAATTGTACAAAGGCTACTCCTGAGAATAGGACTAATACTAATCCT-------
----
-------------AGTACTAATGGTACTAATATTACTGTTCCTGGGTTATTGGACGACATAAAA---
AATTGCTCTTTCATTGCAACCACAGAAATAAGA---------------------------
GATAAGAAAAAGAAAGAGTATGCACTT---TTTTATAGACTTGATATAGTACCACTTGATGGGGAT-------
----

AAGAATGACAGCACCGAGTATAGATTAATAAATTGCAATACCTCAACCATAACACAA---
GCCTGTCCAAAGGTCTCTTTTGACCCAATTCCTATACATTATTGTGCTCCAGCTGGTTATGCGATCTTAAAGT
GTAAT---AATGAGACATTCAAT------GGGACAGGACCATGCAATAAT-----------------------
-GTCAGC---ACAGTACAATGTACACATGGAATTAAGCCAGTGGTATCAACT---
CAACTACTGTTAAATGGTAGCCTAGCAGAAAAAGAA------------
GTAATAATTAGATCTAAAAATATATCAGACAAT---------GCCAAAACAATAATAGTACAACTTAAT----

-----------GAATCTGTAGCG---------ATTGTGTGTACAAGACCCGGCAATAATACAAGAAGA-----
-AGTGTAAGGATA------------GGACCAGGATATGCA---TTC------CTTACAAATAAA---------
---------ATAATAGGAGACCCA------------AGACAAGCACATTGT---AATATTAGTGCAAAC---
AAATGGCACGAAGCTTTACGAAGAGTAGGACAAAAATTAAGC---------------AAACACTTCCCT----
-----------------------------------AATAAGACAATAGCATTTAAT------
TCATCCTCAGGAGGGGACCCAGAAATTACA---ACACATAGCTTCAATTGCAGAGGAGAG---
TTTAAGCAGCTCCAGGCAAGAGTCCTGGCCATAGAAAGATACCTACAGGATCAACAGCTCCTAGGGCTGTGGG
GCTGCTCTGGAAAACTCATATGCACCACTAATGTGCCTTGGAACTCCAGTTGGAGT-----------------
----------------------------AATAAATCTCGAACAGATATTTGG------GAT------
AACATGACCTGGATGCAGTGG---GATAAAGAAATTAGTAATTATACAGGCATAATATAC---
CATTTGCTTGAAAACTCGCAAAACCAGCAAGAA--

AATAATGAAAAGGAGTTATTAGAATTGGACAGGTGGAGCAGTCTGTGGACTTGGTTTGACATAACAAAATGGC
TGTGGTATATAAAAATATTCATAATGATAGTAGGGGGCTTGATAGGTTTAAGAATAATTTTTACT---
GTAATCTCTCTAGTG---------AATAGAGTTAGGCAGGGATACTCACCT------
TTATCGTTTGCTGTTAGTTTATTTGATACCTTAGCCATAGCAGTAGCTGAA---
GGAACAGATAGAATTTTAGAAATAGTACAAAGAGTCTGTAGAGCT----------------------------
--------ATCCTCAATATA------CCTACAAGAATAAGACAGGGCTTTGAAGCAGCTTTACTA---TAA
>KC894084
GTA---GGG------------------------GGG---AAC---TTG------
AACTTGTGGGTTACAGTCTATTATGGGGTACCTGTGTGG---AAAGAAGCAAAA---
ACTACTCTATTCTGTGCATCAGATGCTAAAGCATATGAGAGAGAA---GTGCATAAT----------------
--------------GTTTGGCTACA---CATGCCTGTGTACCCACAGAC---
CCCAACCCACTAGAAATGGTTTTGGAA---------AATGTAACAGAAAATTTTAAC----------------
--------------------------ATGTGGAAAAATGACATGGTAGATCAGATGCATGAGGATATA---
ATCAGTTTATGGGATCAAAGCCTA---AAGCCATGTGTAAAGTTG---
ACTCCACTCTGTGTCACTTTAAACTGTAGCAATATTACCATAAATGGTGGTGCTACCAATAAT----------
----
-------------GCCATAGCTGGTAATGCTACCATAAATAGTAATATAACTGAAGAAATGAAA---
AATTGCTCTTTCAATACAACCACAGAGATAAGA---------------------------
GATAAGAAGAAGAAAGAGTATGCACTT---TTTTATACACTTGATATAGTACCACTTGATAAG----------
----

AATGCCAAGGAGTATAGATTAATAAGTTGTAATACCTCAGCCATAACACAA---
GCCTGTCCAAAGGTCTCTTTTGACCCAATTCCTATACATTATTGTGCTCCAGCTGGTTATGCAATTCTAAAGT
GTAAT---AATAAGACATTCAAT------GGAACAGGACCATGCAGTAAT-----------------------
-GTTAGC---ACAGTACAATGCACACATGGAATTAAGCCAGTAGTAACAACT---
CAACTGCTGTTAAATGGTAGTTTAGCAGAAGAAGAG------------
ATAATAATTAGATCTGAAAATCTGACAGACAAT---------GCCAAAACAATAATAGTACACCTTAAT----
-----------AAGTCTGTAGAA---------ATTGTGTGTACAAGGCCCAACAATAATACAAGGAAA-----
-AGTGTGAGAATA------------GGACCAGGACAAACA---TTC------TATGCGACAGGAGAA------
---------GTAATAGGAAATATA------------AGACAAGCATATTGT---AGCATTAACAAAACA---
ACATGGAATGAAGCTTTACAAAAGGTAGGTAAAAAATTAGCA---------------GAACTCTTCCCT----
-----------------------------------AATAAAAAAATAGTATTTAAC------
CAATCTTCAGGAGGGGACCTAGAAATTACA---ACACATAGCTTTAATTGCAGAGGAGAA---
TTTAAGCAGCTCCAGACAAGAGTCCTGGCTCTAGAAAGATACCTAAGGGATCAACAGCTCCTAGGGCTTTGGG
GCTGCTCTGGAAAACTCATCTGCACCACTACAGTGCCTTGGAACACCAGTTGGAGT-----------------
----------------------------AATAAATCTCTAGACGATATTTGG------AAA------
AACATGACCTGGATGCAGTGG---GATAGAGAAATTGATAATTACACAGACACAATATAC---
AGGTTGCTTGAAACCTCACAAAACCAGCAGGAA--

CAAAATGAAAAAGATTTACTAGCATTGGACAGTTGGAAAAATTTGTGGAATTGGTTTGACATAACAAACTGGC
TGTGGTATATAAAAATATTCATAATGATAGTAGGGGGCTTGATAGGTTTGAGAATAATTTTTGCT---
GTGCTTTCTATAGTG---------AATAGAGTTAGGCAGGGATACTCACCT------
TTGTCATTTGCTATTAGTCTGCTTGATACCATAGCAATAGCAGTAGCTGAA---
GGAACAGATAGAATTATAGAATTAATACAAAGAATTGGTAGAGCT----------------------------
--------ATCTTCAACGTA------CCTAGAAGAATAAGACAGGGCTTTGAAGTAGCTTTGCTA---TAA
>KC156114
GGG---ATG--
GGGCAATTGTGGGTCACAGTCTATTATGGGGTACCTGTATGG---AAGGAAGCAAAA---

ACTACTCTATTTTGTGCATCAGATGCTAAAGCATATTCCCAAGAA---GCACATAAT----------------
--------------ATCTGGGCTACA---CATGCCTGCGTACCTACAGAC---
CCCAACCCACAAGAAATGGTTTTAGAA---------AATGTAACAGAAAATTTTAAC----------------
--------------------------ATGTGGAAAAATGACATGGTAGATCAGATGCATGAGGATATA---
ATCAGTTTATGGGATCAAAGCCTA---AAGCCATGTGTAAAGTTG---
ACCCCACTCTGTGTCACTTTAAACTGTACTACTGTTAATATTACCCAGGAAAAT-------------------
----
----------------------------AATCCCCTTAATGATAGTGATAGTAATTACATGAAA---
AACTGCTCTTTCAATATGACCACAGAACTAAAA---------------------------
GATAAAAAGAAGGAAGAGAGGGCATTG---TTTCATAGACTTGATCTAGTACCACTTAATGAGAAT-------
----

AGTAGCAAGTATGAGAATAGTAACAAGTATATATTAACAAATTGTAGGACCTCAACCATAGCACAG---
GCCTGTCCAAAGATCTCGTTTGATCCGATTCCTATACATTATTGTGCTCCAGCTGGTTATGCGATTCTAAAAT
GTAAT---AATAAGGCATTCAAT------GGATCAGGACCATGCACTAAT-----------------------
-GTCAGC---ACAGTACAATGTACACATGGAATTAAGCCAGTGGTATCAACT---
CAGCTACTGTTAAATGGTAGCCTAGCAGAA---GAG------------
ATAATAATCAGATTTGAAAATCTGACAAACAAT---------GTCAAAACAATAATAGTACACCTTAAT----
-----------GAATCTGTAGAA---------ATTTCGTGTACAAGACCCAACAATAATACAAGAAAA-----
-AGTGTGAGGATA------------GGACCAGGACAAACA---TTC------TATGCAACAGGAGAA------
---------ATAATAGGAGATATA------------AGACAAGCATATTGT---ACCATTAATGAAGGA---
AAATGGAATAAAACTTTAGAAAGGATAAGGAACAAATTAGCA---------------GAGTACTTCCCT----
-----------------------------------AATAAAACAATACAATTTCAA------
AGCGCCTCAGGAGGGGACCTAGAAATTACA---ACACATAGCTTTAATTGTGGAGGAGAA---
TTTAAACAGCTCCAGGCAAGAGTCCTGGCTATAGAAAGATACCTAAAGGATCAACAGCTCCTAGGAATTTGGG
GCTGCTCTGGAAAACTCATCTGCACCACTAATGTGCCTTGGAACTCTAGTTGGAGT-----------------
----------------------------AATAAACCTGAAGAAGAAATTTGG------GGG------
AACATGACCTGGATGCAATGG---GAGAGAGAAATTGATAATTATACAGACACAATATAC---
AGTTTGCTTGAAAAATCGCAAAACCAGCAGGAA--

CAAAATGAACAAGATTTACTAGCATTGGACAGTTGGAAAAACCTGTGGAATTGGTTTAGCATAACAAAATGGT
TGTGGTATATAAAAATATTCATAATGATAGTAGGAGGCTTAATAGGTTTAAGAATAATTTTTGCT---
GTGCTTTCTATAGTG---------AATAGAGTTAGGCAGGATACTCACCT------
TTATCGTTCGCTATTAACCTGCTTGATACCATAGCAATAGCAGTAGCTGAA---
GGAACAGATAGGATCATAGAAGTAATACAAAGAATTTGTAGAGCT----------------------------
--------ATCTGCAATATA------CCTACAAGAATAAGACAGGGCTTTGAAGCAGCTTTGCAA---TAA
>KC156120
GTG---ATG--
GGAAACTTGTGGGTCACAGTCTATTATGGGGTACCTGTGTGG---AAAGAAGCAAAA---
GCTACTCTCTTCTGTGCATCAGATGCTAAAGCATATGATACAGAA---GTGCATAAT----------------
--------------GTCTGGGCTACA---CATGCCTGTGTGCCCACAGAC---
CCCAACCCACAAGAAATAGTTTTGCCA---------AATGTAACAGAAAATTTTAAC----------------
--------------------------ATGTGGAAGAATGACATGGTAGATCAGATGCATAAGGACATA---
ATCAGTTTATGGACCAAAGCCTA---AAACCATGTGTAAAGTTG---
ACCCCACTCTGTGTCACTTTAAATTGTACAAATGCTACAAGCACTAATGCTAATAGTACA-------------
----
-------------------GTGAATGGAGAGAGTACAAATACTAGTATTAGTAATGAAATGAAA---
AATTGTTCTTTCAATATGACCACAGAGTTAAGA---------------------------
GATAAAAAAAGGCAAGTGTATGCACTT---TTTTATAAACTTGATATAGTGCCAATTGATGGGGAAAAT----
----

AATGAGAGGAATGATAGCAACTCCTATAGATTAATAAATTGCAATACCTCAGCCATAACACAA---
GCCTGTCCAAAGGTCTCTTTTGACCCAATCCCTATACATTATTGTGCACCAGCTGGTTATGCGATTCTAAAGT
GTAAT---AATAAGACATTCAAT------GGAACAGGACCATGCAATAAT-----------------------
-GTCAGC---ACAGTACAATGTACCCATGGGATTAAGCCAGTGGTATCAACT---
CAATTACTGTTAAATGGGAGCTTAGCAGAAGAAGAG------------
ATAATAATTAGATCTGAAAATCTGACAGATAAT---------ACTAAAACAATAATAGTACAGCTTAAC----
-----------AAAGCTGTAGAA---------ATTGTGTGTACAAGACCCAATAATAATACAAGAAAA-----
-AGTATAAGAATA------------GGACCAGGACAAGCA---TTC------TTTGCAACAGGTGAC------
---------ATAATAGGAGACATA------------AGAGAAGCACATTGT---AACATTAATAGA-------

--TGGAATGAGACTTTAGAAGGGGTAAGAGAAAAGTTAAAA---------------GGACTCTTT--------
----------------------------------AACAAAACAATAAAATTTAAC------
TCATCTTCAGGAGGGGACCTAGAAATTACA---ACACATAGCTTTAATTGTGGAGGAGAA---
TTTAAGCAGCTCCAGACAAGAATCCTGGCTGTGGAAAGATACCTAAAGGATCAACAGCTCCTAGGGATTTGGG
GCTGCTCTGGAAAACTCATCTGCACCACTAATGTGCCTTGGAACTCCAGTTGGAGC-----------------
----------------------------AATAGATCTCAACAGGATATTTGG------GAT------
AACCTGACCTGGATGCAGTGG---GATAGAGAAATAAGCAATTACACAAGCACAATATAT---
AAATTACTTGAAGAATCACAAAACCAGCAGGAG--

AGAAATGAAAAGGATTTATTAGCATTGGACAGTTGGCAAAATCTGTGGAATTGGTTTGACATATCAAACTGGC
TGTGGTATATAAAAATATTCATAATGATAGTGGAGGCTTGATAGGTTTAAGAATAATTTTTTGCT---
GTACTCTCCATAGTG---------AATAGAGTTAGGCAGGGATACTCACCT------
TTGTCATTTGCTATTAGTCTGCTTGATGCTACAGCTATAGCAGTAGCTGAA---
GGAACAGATAGAATTATAGAAGTTATACAAAGATTTTGTAGAGCT----------------------------
--------GTCCGCAACATA------CCTAGAAGATTAAGACAGGGCTTTGAAACAGCTTTGCTA---TAA
>HQ615984
GTG---GTG--
GGGGACTTGTGGGTCACAGTCTATTATGGAGTACCTGTATGG---AAAGAAGCAAAA---
ACTACTCTATTTTGTGCATCAGATGCTAAAGCATATACAACAGAA---GTGCATAAT----------------
--------------GTCTGGCTACA---CATGCCTGTGTACCTACAGAC---
CCCAACCCACAAGAAATGGTTTTGGAA---------AATGTAACAGAGAATTTTAAT----------------
--------------------------ATGTGGAAAAATGACATGGTGGATCAGATGCATGAGGATATA---
ATCAGTTTATGGGACGAAAGTCTG---AAGCCATGTGTAAAGTTG---
ACCCCACTCTGTGTCACTTTAAATTGTACAGCTACTAATGGTACTGCTACCATAAAT----------------
----
----------------------------ACTGCCAATAATGCCACCGATAATAGTACCATAAAA---
AATTGCTCTTTTGAGGTAACCACAGAAGTAAAA---------------------------
GATAAGAAAAAGCAAGTGTATGCACTT---TTTTATAAACTTGATATAGTAGAACTTAATCAG----------
----

ACTGAGTCTAATCAGACTATGTATAGATTAATAAATTGTAATACCTCAACCATGACACAA---
GCCTGTCCAAAGGTCTCCTTTGATCCAATTCCGATACATTATTGTGCTCCAGCTGGTTATGCGATTCTAAAGT
GTAAT---AATAAGACATTCAAT------GGGACAGGACCATGCAATAAT-----------------------
-GTCAGT---ACAGTACAATGCACACATGGGATTAAGCCAGTGGTATCAACT---
CAATTACTGTTAAATGGTAGCCTATCAGAAGAAGGG------------
ATAATAATTAGATCTGAAAATCTAACAAACAAT---------GCCAAAACAATAATAGTACATCTTAAT----
-----------GAATCTGTAGAC---------ATTGTGTGTACAAGACCCAACAATAATACAAGAAAG-----
-AGTATAAGGATA------------GGGCCAGGACAAACA---TTT------TATGCAACAGGAGAA------
---------ATAATAGGGAACATA------------AGACAAGCACATTGT---AACATTAGTGGAGAT---
AAATGGAATAGAACTTTGCAAAGGGTAGGGGAAGAATTAAGA---------------AAACATTTCCAT----
-----------------------------------AAGAAAACAATAGTATTCAAA------
CCACCTGCAGGAGGGGACTTAGAAATTACA---ACACATAGCTTTAATTGTAGAGGGGAA---
TTTAAGCAGCTCCAGGCAAGAGTCTTGGCTATAGAAAGATACCTAAAGGATCAACAGCTCCTAGGAATTTGGG
GCTGCTCTGGAAAACTCATCTGCACCACTGCTGTGCCATGGAATTCCAGTTGGAGT-----------------
----------------------------AATAAGACTCAAGGAGAGATTTGG------GGA------
AACATGACCTGGATGCAGTGG---GAGAGAGAAATTAATACTTACACAGACACAATATAC---
AAGTTGCTTGAAGAATCGCAAATCCAGCAGGAA--

ACAAATGAAAAAGATTTATTAGCCTTGGACAGTTGGCAAAATCTGTGGAGTTGGTTTAGCATAACAAAATGGC
TGTGGTATATAAAAATATTCATAATGGTAGTAGGAGGCTTGATAGGCTTAAGAATAATTTTTTGCT---
GTGCTTTCTATAGTA---------AATAGAGTTAGGCAGGGATACTCACCT------
TTGTCGTTTGCTATTAGTCTGCTTGATACCATAGCAATAGCAGTAGCTGAA---
GGAACAGATAGGATTATAGAATTAATACAGAGAATTTGTAGAGCT----------------------------
--------ATCCGCAACATA------CCTACAAGAATAAGACAGGGCTTTGAAACAGCTTTGCTA---TAA
>KC156216
GTG---AGG--
GGAAACTTGTGGGTCACAGTCTATTATGGGGTACCTGTGTGG---AAAGAAGCAAAA---
ACTACTCTATTTTGTGCATCAGATGCTAAAGCATATGAGAGAGAA---GTGCATAAT----------------
--------------GTCTGGCTACA---CATGCCTGTGTACCCACAGAC---
CCCAGCCCACAAGAATTGGTTTTGGAA---------AATGTAACAGAAAATTTTAAC----------------

--------------------------ATGTGGAAAAATGATATGGTGGATCAGATGCATGAAGATATA---
ATCAGTTTATGGGATCAAAGCCTA---AAGCCCTGTGTAAAATTG---
ACCCCACTCTGCGTCACGCTAAACTGTATTAATGTCAATATTACCAACAATGCTAGTAGTGCCAATAATACTG
CTGAA--
-------AGTGATAATACTACTGAGGCCACACAGGAAGGGGTAGAGCTAAAAAATGAGCTAAAA---
AATTGCTCTTTCAATATAACCACAGAACTAAAA---------------------------
GATAAGAAAAGGAAAGAATATGCACTT---TTTTATAGACTTGATATAGTAGAACTTAATGGGGAG-------
----

AATAATAATGATACCAGTTATAGATTAATAAATTGTAATACCTCAGCCATAACACAA---
GCCTGTCCAAAGGTCTCTTTTGACCCAATTCCTATACATTATTGTGCTCCAGCTGGTTATGCGATTCTAAAGT
GTAAT---AATAAGACATTCAAT------GGAACAGGACCATGCAATAAT-----------------------
-GTCAGC---ACAGTACAATGTACACATGGAATTAAGCCAGTGGTATCAACT---
CAATTACTGTTAAATGGTAGTCTAGCAGAAGAAGAG-----------
ATAATAATTAGATCTGAAAATCTGACAGACAAT---------GCCAAAACAATAATAGTACATCTTAAC----
-----------GAATCCGTAGGA----------ATTACGTGTACAAGGCCCAACAATAATATAAGGAAA-----
-AGTATGAGGATA------------GGACCAGGACAAACA---TTC------TATGCAACAGGAGGC------
---------ATAATAGGAGACATA------------AGACAAGCACATTGT---AACATTAATGAAGAA---
AGTTGGAATAGAACTCTAAGACAAGTAGTGGAAAAATTAAGA---------------GAACACTTCCCT----
-----------------------------------AATAAAACAATACAATTTGAA------
CCACCCTCAGGAGGGGACCTAGAAGTTACA---ACACATAGTTTTAATTGTATGGGGGAA---
TTTAAGCAGCTCCAGGCAAGAGTCTTGGCTATAGAAAGATACCTAAAGGATCAACAGCTCCTAGGAATGTGGG
GCTGCTCTGGAAAACTCATTTGCACCACTAATGTGCCTTGGAACTCTAGTTGGAGT-----------------
----------------------------AATAAATCTCAAGAGGAAATTTGG------GAC------
AACATGACTTGGATGCAGTGG---GATAGAGAAATTAGTAATTACACAAACACAATATAC---
CAGTTGCTTGGAGACTCGCAGATCCAGCAGGAG--

AAAAATGAAAAAGATTTATTAGCATTGGACAGTTGGAAAAACTTGTGGAGTTGGTTTAGCATAACAAACTGGC
TGTGGTATATAAGAATATTCATAATGATAGTAGGAGGCTTGATAGGCTTAAGAATAATCTTTGCT---
GTGCTTTCTATAGTG---------AATAGAGTTAGGCAGGGATACTCACCT------
TTATCATTTGCTATTAGTCTGTTTGATAGCTTAGCAATAACAGTAGCTGAA---
GGGACAGATAGAATTCTAGAAGTATTACAAAGAATTGGTAGAGCT----------------------------
--------ATCTGCAACATA------CCTAGAAGAATAAGGCAGGGCTTTGAAGCAGCTTTGCAA---TAA
>KC156219
GGG---GTG--
GAAAACTTGTGGGTCACAGTCTATTATGGGGTACCTGTATGG---AAAGAAGCAAAA---
ACTACTTTATTCTGTGCATCAGATGCCAAAGCATATGATAGAGAA---GTGCATAAT----------------
--------------GTCTGGGCTACA---CATGCCTGTGTACCCACAGAC---
CCCAACCCACAAGAAATGGTTTTGGCA---------AATGTAACAGAAAACTTTAAT----------------
--------------------------ATGTGGAAAAATGACATGGTGGATCAGATGCATGAGGATATA---
ATCAGTTTATGGGACCAAAGCCTA---AAGCCATGTGTAAAGTTG---
ACCCCACTCTGTGTCACTTTAAATTGTAGTAATACTATCAATGGTACTGTCCTCTATGAAAAT----------
----
--GATACCATGTATGGAGAAGTAAAA---
AATTGCTCTTTCAATATGACCACAGAAATAAGA---------------------------
GATAAGAAAAAGGGAGTGTATGCACTT---TTTTATAAACTTGATATAGTACCAATTGATAAC----------
----

AGCACGTATAGATTAATAAATTGTAATACCTCAACCATAACACAA---
GCCTGTCCAAAGGTCTCTTGGGACCCAATTCCTATACATTATTGTGCTCCAGCTGGTTATGCGATTCTAAAGT
GTAAT---AATAAGACATTCAAT------GGAACAGGACCATGCACTAAT-----------------------
-GTTAGC---ACAGTACAATGTACACATGGAATAAAGCCTGTGGTATCAACT---
CAACTACTGTTAAATGGCAGCATAGCAGAAGAAGAG-----------
ATAATAATTAGATCTGAAAATCTGACAGACAAT---------ACCAAAACAATAATAGTACATCTCAAT----
-----------AAATCTGTATGG---------ATTAATTGTACAAGACCCAACAATAATACAAGAAAA-----
-AGTATGAGGATA------------GGACCAGGACAAACA---TTC------TATGCAACAGGAGCC------
---------ATAATAGGAAATATA------------AGACAAGCATATTGT---AGCATTAGTAGACAG---
GAATGGAAAGAAACTTTAGGAAAGGTACAGGGAAAATTAGAA---------------GAACACTTTAAG----
-----------------------------------AATAAAAGTATAGTTTTTAAT------
CAATCCTCAGGAGGGGACCTAGAAATCACA---ACACATAGCTTTAATTGTGGGGGAGAA---

TTTAAGCAGCTCCAGGCAAGAGTCCTGGCCATAGAAAGATACCTAAAGGATCAACAGCTCCTAGGGATTTGGG
GCTGCTCTGGAAAACTCATCTGCACCACTGCTGTGCCTTGGAACAATAGTTGGAGT-----------------
----------------------------AATAAAACTCAAGAGCAGATTTGG------GAA------
AACATGACTTGGATGCAGTGG---GAAAAAGAAATTAGCAATTACACAGGCATAATATAC---
GGGTTGCTTGAAGACTCGCAGAACCAGCAGGAA--

ATAAATGAACAAGAGTTACTAGCATTGGACAGTTGGAAAAATTTGTGGAGTTGGTTTGACATATCAAATTGGT
TGTGGTATATAAGAATATTCATAATGGTAGTAGGAGGCTTGATAGGCTTAAGAATAATTTTTGCT---
GTGCTATCTATAGTG---------AATAGAGTTAGGCAGGGATACTCACCT------
TTATCGTTTGCTATTAGTCTGCTTGATATCATAGCAATAGCAGTAGCTGAA---
GGAACAGATAGGATTATAGAAGTAGTACAAAGAATTTGTAGAGCT----------------------------
--------ATCTGCAACATA------CCTAGAAGAATAAGACAGGGCTTTGAAGCAGCTTTGCAA---TAA
>HQ615963
GGA--
ATGTGGGTCACAGTCTACTATGGGGTACCTGTGTGG---AAAGAAGCAAAA---
ACTACTCTATTTTGTGCATCAGATGCTAAGGGATATGAGAAAGAA---GTGCATAAT----------------
--------------GTCTGGGCCACA---CATGCCTGTGTACCCACAGAC---
CCCAATCCACAAGAAATGTTCTTAAAA---------AATGTAACAGAAAATTTTAAC----------------
--------------------------ATGTGGAAAAATGACATGGTGGATCAGATGCATGAAGATGTA---
ATCAGTTTATGGGATCAAAGCCTC---AAGCCATGTGTAAAGTTG---
ACCCCACTCTGTGTCACTCTAATCTGTAGCAATGTTACCCTCAATGAAAGCAAGGCCTCTAAT----------
----
----------------------GAAAACATGACCCTCAATGAAAGCAGAGCAGAGGAAATGAGA---
AATTGTTCTTTCAATGTAACCACAGAATTAAGA---------------------------
GATAAGCAAGAGAAAAAGTATGCACTT---TTTTATAAACTTGATACAGTACCACTAGACAGC----------
----

AACTCTAACAGCTCTAGTCAGTATAGATTAATAAATTGTAATACCTCAACCATAACACAA---
GCCTGTCCAAAGGTCTCTTTTGACCCAATTCCTATACATTATTGTGCTCCAGCTGGTTATGCGATTCTAAAGT
GTAAT---AATAAGACATTCAAT------GGAACAGGACCATGCAATAAT-----------------------
-GTCAGC---ACAGTACAATGTACACATGGAATTAAGCCAGTGGTTTCAACT---
CAATTATTGTTAAATGGTAGCCTAGCAGAAGGAGAG------------
ATAATAATTAGATCTGAAAATATAACAAACAAT---------GTCAAAACAATAATAGTACATCTCAAT----
-----------GAATCTGTAAAG---------ATTGAGTGTGCGAGACCCGGCAATAATACAAGAACA-----
-AGTATAAGAATA------------GGACCAGGACAAGCA---TTT------TATGCAACAGGAGAT------
---------GTAATAGGAGACCCA------------AGAAAAGCACATTGT---AACATT------ACG---
GGATGGTTTAAAACTTTATTTAGGGTAAGTAAAAAATTAAGA---------------GAACACTTCCCT----
-----------------------------------AATAAGACTATAATATTTAAC------
TCATCCTCAGGAGGGGACCTAGAAATTACA---ACACATAGCTTTAATTGTGGAGGAGAA---
TTTAAACAGCTCCAGGCAAGAGTCCTGGCCTTGGAAAGATACCTAAAGGATCAACAGCTCCTAGGGATGTGGG
GCTGCTCTGGAAAACTCATCTGCACCACTAATGTGTTTTGGAACTCTAGTTGGAGT-----------------
----------------------------AATAAAACTTATGGTGATATTTGG------GAT------
AACATGACCTGGATGCAGTGG---GAGAGAGAAATTAGCAATTATACAGGAATAATATAT---
GAATTGCTTGAAGAATCACAAAACCAGCAGGAA--

AAGAATGAACAAGATTTACTAGCATTGGACAGATGGAACAGTCTGTGGAATTGGTTTAACATAACAAATTGGC
TGTGGTATATAAAAATATTCATAATGATAGTAGGAGGCTTGATAGGTTTAAGAATAATTTTTACT---
GTGCTTTCTTTAGTA---------AATAGAGTTAGGCAGGGATACTCACCT------
CTGTCGTTGGCTATTAGTCTATTGGATACCTTAGCAATAGTAGTAGGTGAA---
GGAACAGATAGGATTATAGGATTTATATTAGGAATTTGTAGAGCT----------------------------
--------ATCCGCAACATA------CCTACAAGAATAAGACAGGGCTTTGAAGCAGCTTTGCTA---TAA
>HQ615964
GTG---ATG--
GGGAGCTTGTGGGTTACAGTATATTATGGGGTACCTGTGTGG---AAAGAAGCAAAA---
ACTACTCTATTCTGTGCATCAGATGCTAAAGCACATGAGAAAGAA---GTACATAAT----------------
--------------GTCTGGGCTACA---CATGCCTGTGTACCCACAGAC---
CCCAGCCCACAAGAAATGGTTTTGGGA---------AATGTAACAGAAAATTTTAAC----------------
--------------------------ATGTGGAAAAATGACATGGTAGATCAGATGCATGAGGATATA---
ATCAGTTTATGGGATGAAAGCCTA---AAGCCATGTGTAAAGTTG---
ACCCCACTCTGTGTCACTTTAGAGTGTACAAATGTTACTAAAAATGGTAGTAGTGCAAATACC----------

----
-------------------------ACTAGTAGTAATGGTGCAAATACCGAGGGAGACATGAAA---
AATTGCTCTTTCAATGCAACCACAGAAATAAGA---------------------------
GATAAAAAACAGAAAATGCAGGCACTT---TTTTATAAACTTGATATAGTACCACTTAATGAGAAT-------
----

AGCACTGGGGAGTATAGATTAATAAATTGCAATACCTCAGCTGTAAAACAA---
GCCTGTCCAAAGGTCACTTTTGACCCAATTCCAATACATTATTGTGCTCCAGCTGGTTATGCGATTCTAAAGT
GCAAC---AATAAGACATTCAAT------GGAAAAGGACCATGCAATAAT-----------------------
-GTCAGC---ACAGTACAATGTACACATGGAATTAAGCCAGTGGTATCAACT---
CAACTACTGTTAAATGGTAGTCTAGCAGAAGAAGAG------------
ATAATAGTTAGATCTGAAAATCTGACAAACAAT---------GCCAAAACAATAATAGTGCACCTTAAT----
-----------GAATCTGTAGAA---------ATTATGTGTACAAGACCATCCAATAATACAAGGAAA-----
-AGTATAAGGATA------------GGACCAGGACAAACA---ATC------TATGCAACAGGAGAC------
---------ATAATAGGAGATATA------------AGACAAGCACATTGT---AACATAACTGAGAGT---
AAATGGAATAAAACTCTACAACAGGTGGTTAAAAAATTAAAA---------------GAACACTTCCCT----
-----------------------------------AATAAAACAATAGCATTTCAA------
CCATCCTCAGGAGGGGATCTAGAAATTACA---ACACATAGCTTTAATTGTAGAGGAGAA---
TTTAAGCAGCTCCAGGCAAGAGTCCTGGCTATCGAAAGATACCTAAAGGATCAACAGCTCCTAGGGATTTGGG
GCTGCTCTGGAAAACTCATCTGCACCACTAATGTGCCTTGGAACTCCAGTTGGAGT-----------------
----------------------------AATAGATCAGAATCAGATATTTGG------GAG------
AACATGACCTGGATGCAGTGG---GATAGAGAAATTAGTAATTACACAGACACAATATAC---
AGGTTGCTTGAAGTCTCACAAAACCAGCAGGAA--

CAAAATGAAAAAGATTTATTAGCATTGGACAGTTGGAACAATCTGTGGAGTTGGTTTAGCATAACAAAATGGC
TGTGGTATATAAAAATATTTATAATAATAGTAGGAGGCTTGATAGGCTTAAGAATAATCTTTGCT---
GTGCTCTCTATAGTG---------AATAGAGTTAGGCAGGGATACTCACCT------
TTGTCATTTGCTATTAGTCTGTTTGATACCATAGCAATAAGAGTAGCTGAA---
GGAACAGATAGAATTATAGAAGTAGTACAAAGATTTTGTAGAGCT----------------------------
--------ATCTGCAGCATA------CCTAGAAGAATAAGACAGGGCTTTGAAGCAGCTTTGCAA---TAA
>HM204613
GTG---GTG--
GGAAACTTGTGGGTCACAGTCTATTATGGGGTACCTGTGTGG---AAGGATGCAAAG---
ACCACTTTATTCTGTGCATCAGATGCTAAAGCATATGAGAGAGAG---GTGCATAAT----------------
--------------GTCTGGGCTACA---CATGCCTGTGTACCCACAGAC---
CCCAACCCACAAGAAATGTTTTTGGAA---------AATGTAACAGAAAATTTCAAC----------------
--------------------------ATGTGGGAAAATGACATGGTAGACCAGATGCATGAGGATGTA---
ATCAGTTTATGGGACCAATTCCTA---AAGCCATGTGTAAGGTTG---
ACCCCACTCTGTGTCACTTTAAACTGTAGTGATGCTAGG----------------------------------
----
--GATGATACAGAAAGAAAA---
AATTGCACTTTCAGAGTAACCACAGAAATAAAA---------------------------
GACAAAAAAAAAGAAAGAGACCGCACT---
TTTTTATAGAGTTGATATAGTACCACTTGAGGAGGGTGAGAGTGATAA-------------------------
--------------------------------------CACCACTAGGGAAGAGAGTGATAAC--
ACCACTAGGGAATACAGATTAATAAATTGTAATACCTCAGCCATAACACAA---
GCCTGTCCAAAGGTTTCTTTTGATCCAATTCCTATACATTATTGTACTCCAGCTGGTTATGCGATTCTAAAGT
GTAAT---AATAATACATTCAAT------GGGACAGGACCATGTAAGAAT-----------------------
-GTCAGC---ACAGTACAATGCACACATGGAATTAAGCCAGTGGTATCAACG---
CAATTACTGTTAAATGGTAGCCTAGCAGGAGGAGAA------------
ATAATAATTAGATCTAAAAATCTGACAGACAAT---------ACCAAAACAATAATAGTACATCTTAAG----
-----------GACCCTGTAAAA---------ATTGAGTGTATGAGACCCAACAATAATACAAGAAAA-----
-AGTATAAGGATA------------GGACCAGGACAAGCA---TTC------TTTGCAACAACAGGC------
---------ATAATAGGAAATATA------------AGGCAAGCATCTTGT---AATATTAGCAAAGAG---
GACTGGAATAAGACCTTACAAGAGGTAGTTCAAAAATTAAAA---------------GTATACTTCCCT----
-----------------------------------AATAAAATAATAATATTTAAT------
CACTCTTCAGGAGGGGACCTAGAAATTACA---ACACATAGCTTTAATTGTGGAGGAGAA---
TTTAAGCAGCTCCAGGCAAGAGTCTTGGCCCTAGAAAGATACCTACAGGATCAACAGCTCCTAGGGATTTGGG
GTTGCTCTGGAAAACTCATCTGCACCACTACTGTGCCTTGGAACTCCAGTTGGAGT-----------------
----------------------------AATAGATCTGAAACAGATATTTGG------GGC------

AACATGACCTGGATGCAGTGG---GATAGAGAAATTAGTAATTACACAGGCATAATATAC---
GACTTGCTTGAACAATCGCAAAACCAGCAGGAG---------------------------------------
----
AAAAATGAACAGGATTTATTAGCATTAAACAGTTGGCAAAATCTGTGGACTTGGTTTGACATAACAAAGTGGC
TGTGGTATATAAAAATATTTATAATGATAGTAGGAGGCTTGATAGGTTTGAGAATAATTTTTGCT---
GTGCTCTCTATAGTG---------AATAGAGTTAGGCAGGGATACTCACCC------
TTGTCGTTTGCTATTAGTCTGCTTGATGCCACAGCAATAACAGTAGCTGAA---
GGAACAGATAGAATTATAGAATTAGTACAAAGAATTTGTAGGGCT---------------------------
--------ATCAGCAATATA------CCTAGAAGAATAAGACAGGGCTTTGAAACAGCTTTGCTA---TAA
>KC187578
GTA---GGG---
GGGAACTTGTGGGTCACAGTCTATTATGGGGTACCTGTGTGG---AAAGAAGCAAAA---
ACTACTCTATTCTGTGCATCAGATGCTAAAGCATATGAGAAAGAA---GTGCATAAT---------------
--------------GTTTGGCTACA---CATGCCTGTGTACCCACAGAC---
CCCAACCCACAAGAAATAACTTTGGAA---------AATGTAACAGAAAATTTTAAC---------------
--------------------------ATGTGGGAAAATGACATGGTGGATCAGATGCATGAGGATATA---
ATTAGTTTATGGGACCAAAGCCTA---AAACCATGTGTAAAGTTG---
ACCCCGCTCTGTGTCACTTTAAAATGTGGAAATGTTAATGCTACTAGTAATGGTAAT---------------
----
----------------------------GTTACCTATAATAATACCATGAATGGAGAAATAAAA---
AATTGCTCTTTCAATGCAACCACAGAATTAAGA--------------------------
GATAAGAAAAAGGAAAGGTATGCACTT---TTTTATAAACTTGATATATTACCACTTAAGGGG---------
----

GATAATGACACCTATGTATTAATAAATTGTAATACCTCAACCATAACACAA---
GCTTGTCCAAAGGTCTCTTTTGAGCCAATTCCTATACATTATTGTGCTCCAGCTGGTTATGCGATTCTAAAGT
GTAAT---AATAAGACATTCAAT------GGGACAGGGCCATGCAATAAT----------------------
-GTCAGC---ACAGTACAATGTACACATGGAATTAGGCCAGTGATATCAACT---
CAATTATTATTAAATGGTAGCCTAGCAGAAGAGAAA-----------
ATAATAATTAGATCTAAAAATCTGGCAGACAGT---------GCTAAAACAATAATAGTACAATTTAAC---
-----------AAGTCTGTAGAC---------ATTGTGTGTACAAGGCCCAACAATAATACAAGGACA----
-AGTGTGAGAATA------------GGACCAGGACAAACA---TTC------TATGCAACAGGAGGC-----
---------ATAATAGGAAATATA------------AGACAAGCACATTGT---AATATTAGTGAAAAG---
CTATGGAATGAAACTCTATATGAGGTAGGGAAAAGGTTAGCA---------------GAATACTTCCCT---
-----------------------------------AATAGAACAATAAAATTTGAA------
CCACATTCAGGAGGGGACCTAGAAATTACA---ACACATAGTTTTAATTGTGGAGGAGAA---
TTTAAGCAGCTTCAGGCAAGAGTCCTGGCTATAGAAAGATACCTAAAGGATCAACAGCTCCTAGGGATTTGGG
GCTGCTCTGGAAAACTCATCTGCACCACTGCTGTGCCTTGGAATGCCAGTTGGAGT----------------
----------------------------AATAAATCTCAAGAGGAGATTTGG------GAA------
AACATGACTTGGATGCAGTGG---GAGAGAGAAATTAGCAATTACACAGGCATAATATAC---
AATCTGCTTGAAGTCTCGCAAACCCAGCAGGAG---------------------------------------
----
AAAAATGAAAAAGACTTACTAGCATTGGACAGTTGGAAGAATTTGTGGAATTGGTTTAACATAACAAATTGGC
TGTGGTATATTAAAATATTCATCATGATAGTAGGAGGCTTGATAGGTTTAAGAATAATTTTTGCT---
GTGCTTTCTATAGTA---------AATAGAGTTAGGCAGGGATACTCACCT------
TTGTCGTTTGCTATTAGTCTGCTTGATGCAACAGCAATAACAGTAGCTGAA---
GGAACAGATAGACTTATAGAGGTAGTACAAAGAATTTGTAGGGCC---------------------------
--------ATCCGCAACGTA------CCTACCAGAGTAAGACAGGGTTTTGAAGCAGCATTGCAA---TAA
>KC187323
GTG---GTA---
GGGAACTTGTGGGTCACAGTCTATTATGGGGTACCTGTGTGG---AGAGAAGCAAAG---
ACTACTCTATTCTGTGCATCAGATGCTAAAGCATATGATAGAGAA---GTGCATAAT---------------
--------------GTCTGGCTACA---CATGCCTGTGTACCCACAGAC---
CCCAACCCACAAGAAATGGTTTTGGTA---------AATGTAACAGAAAATTTTAAC---------------
--------------------------ATGTGGGAAAATGACATGGTGGAACAGATGCATGAGGATGTA---
ATCAGTCTATGGGATCAGAGCCTA---AAGCCATGTGTAAAGTTG---
ACCCCACTCTGTGTTACTTTAAACTGTTCCCATAATATTACTGTTAATGGTACCTCCAAT------------
----
----------------------------TTTAATACTACAGGTACCATAGCTGAGGGAATGAAA---
AATTGCTCTTTCAAAGTAATCACAGATCTAAGA--------------------------

GATAAGCCCAAGAAAGAAAATGCACTT---TTTTATAGCCTTGATATAATAAAACTTAAGGAGAAT-------
----

TCCAATTCCAGTTCCAATTCCAGTAAGTATAGATTAAGAAGGTGTAATGCCTCAACCATAACACAA---
GCCTGTCCAAAAGTCTCTTTTGATCCAATTCCTATACATTATTGTGCTCCAGCTGGTTATGCGATTCTAAAAT
GCAAT---AATAAGACATTCAAT------GGAACAGGACCATGCAATAAT-----------------------
-GTCAGC---ACAGTACAATGTACACATGGAATTAAGCCAGTTGTGTCAACT---
CAACTACTATTAAATGGTAGCCTAGCAGAAGGAGAG------------
ATAATAATTAGATCTGAAAATCTGACAGATAAT---------GTCAAAACAATAATAGTACATCTTAAT----
-----------GAAACTGTAGAG---------ATTACGTGTACAAGGCCCAACAATAATACAAGAAGA-----
-AGTGTGAGGATA------------GGACCAGGACAAGCA---TTC------TATACAACAGGAGAA------
---------ATAATAGGAGATACA------------AGACAAGCACATTGT---AACATTAGTGGGAGT---
AACTGGACTACAACATTACACAGGGTAAGTAAAAAAATTAAGA--------------GAACAATTCCCT----
-----------------------------------AATAAAACAATAATATTTGAA------
TCATCCTCAGGAGGGGACTTAGAAATTCAA---ACCCATAGCTTTAATTGTGGAGGAGAA---
TTTAAACAACTCCAGGCAAGAGTCTTGGCTGTAGAAAGATACCTACAGGATCAACAGCTCCTAGGGATTTGGG
GCTGCTCTGGAAAACTCATCTGCACCACTAACGTGCCGTGGAACAGTAGTTGGAGT-----------------
----------------------------AATAGATCTCAAGATGATATTTGG------GGA------
AACATGACCTGGATGCAGTGG---GATAAAGAAATTAATAATTACACAAACACAATATAT---
AGATTGCTTGAAGAATCGCAAAATCAGCAGGAA--

AAAAATGAAAAAGATTTGTTAGCATTGGACAGTTGGAACAGTCTGTGGAATTGGTTTAACATATCAAATTGGC
TGTGGTATATAAAAATATTCATAATGATAGTAGGAGGCTTAATAGGTTTAAGAATAATTTTTACT---
GTACTTTCTATAGTA---------AATAGAGTCAGGCAGGGATACTCACCT------
TTATCGTTTGCTATTAGTCTGCTTGATACCATAGCAATAACAGTAGCTGAA---
GGAACAGATAGAATTATAGAACTGGTACAAAGAATTTGTAGAGCT----------------------------
--------ATCCTTAATATA------CCTAGAAGAATAAGACAGGGCTTGGAAGCAGCTTTGCAA---TAA
>KC186955
GTA---ATG--
GGAAGCTTGTGGGTCACAGTCTATTATGGGGTACCAGTGTGG---AAAGAAGCAAAA---
ACTACTCTATTCTGTGCATCAGATGCTAAAGCATATGAAAAAGAA---GTGCATAAT----------------
--------------ATATGGGCTACA---CATGCCTGTGTACCCACAGAC---
CCCAACCCACAAGAAATGGCTTTGGAA---------AATGTAACAGAAAATTTTAAC----------------
--------------------------ATGTGGAAAAATGACATGGTGGATCAGATGCATGAGGATATA---
ATCAGTTTATGGGATCAAAGCCTA---AAGCCATGTGTAAAGTTA---
ACCCCACTCTGTGTCACTTTAAAATGTAATGAGACCTATGCGAATGGGACCTATGCGAAT-------------
----
--GGGACCGTGAGTAGTGACATGAAA---
AATTGCTCTTTTAATGCAACCACAGAACTAGGA---------------------------
GACAAGAGGAGGCAAGAGTATGCACTT---TTTTATAGACTTGATATAGTACCACTTGATGAGGGGAACAAC-
----

TCTAGCAACTCTACGGAGTATAGATTAACAAATTGTAATACCTCAACCATAACACAA---
GCCTGTCCAAAGGTCTCTTTTGATCCAATTCCTATACATTATTGTACTCCAGCTGGTTATGCGATTCTAAAGT
GTAAT---AATAGGACATTTAAT------GGGAAAGGACCATGCAAGAAT-----------------------
-GTCAGC---ACAGTACAATGTACACATGGAATTAAACCAGTGGTATCAACT---
CAGTTACTGTTAAATGGTAGCCTAGCAGAAGAAGGG------------
ATAATAATTAGATCTGAAAATCTGACAAACAAT---------GTCAAAACAATAATAGTACATCTTAAT----
-----------GAATCTGTAGCA---------ATTAATTGTATAAGACCAGGCAATAATACAAGAAAA-----
-AGTGTAAGAATA------------GGACCAGGACAAACA---TTC------TATGCAACAGGAGAC------
---------ATAATAGGAAATATA------------AGACAAGCACATTGT---AACATTAGCACAAAG---
GACTGGAATAAAACTTTAATAAGGATAAGTAAAAAAATTAAGA--------------GAATACTTCCCT----
-----------------------------------AATAAGACAATAAAATTTGGG---
CCACCTAATAAAGGAGGGGACCTAGAAATTACA---ATGCACAGCTTTAATTGTGGAGGAGAA---
TTTAAGCAGCTCCAGACAAGAGTCCTGGCTATAGAAAAGTACCTAAGGGATCAACAGCTCCTAGGGATGTGGG
GCTGCTCTGGAAAACTCATCTGCACCACTAATGTGCCTTGGAACTCCAGTTGGAGT-----------------
----------------------------AATAAATCTTATAAAGCTATTTGG------GAT------
AACTTGACCTGGATGCAGTGG---GATAGAGAAATTGAGAATTACACAAACACAATATAC---
TATCTGCTTGAAGACTCGCAAATCCAGCAGGAG--

AAAAATGAAAAGGATTTATTTGCACTAAACAGTTGGCAAAACCTGTGGACCTGGTTTGACATATCAAATTGGC
TGTGGTATATAAGAATATTCATAATGATAGTAGGAGGCTTAATAGGGTTAAGGATAATTTTTGCT---
GTGCTTTCCATAGTG---------AATAGAGTTAGGCAGGATACTCACCT------
TTGTCGTTTGCTATTAGTCTGCTTGATACCACAGCAATAGCAGTAGCTGAA---
GGAACAGATAGGATTATAGAAATAGTACAAAGTATTTGTAGAGCT----------------------------
--------ATCCGTAACATA------CCTAGAAGAATAAGACAGGGCTTTGAAGCAGCTTTGCAA---TAA
>KC187164
GTG---GTG------------------------GGA---AAC---TTA------
AACTTGTGGGTCACAGTCTATTATGGGGTACCTGTGTGG---AAAGAAGCAAAT---
ACTACTCTATTCTGTGCATCAGATGCTAAGGCACATGATGAAGAA---GTGCATAAT----------------
--------------ATCTGGGCTACA---CATGCCTGTGTACCCACAGAC---
CCCAACCCACAAGAAATGGTTTTGGTA---------AATGTAACAGAAAATTTTAAT----------------
--------------------------ATGTGGAAAAATGACATGGTGGAACAAATGCATGAGGATATA---
ATCAGTTTATGGACCAAAGCCTA---AAGCCATGTGTACAGTTG---
ACCCCACTCTGTGTCACTTTAAACTGTAGCAATGCTAACAGAAATTTTAGCTTTGTTAATGGA----------
----
----------------------------TCTATTGATAACAATGACAGGGATAGAGAAATAAAA---
AATTGCTCTTTCAATATGACCACAGAATTGAGA---------------------------
GATCAGACAAAGAGAATGTATTCCCTT---
TTTTATAAACTTGATGTAGTACCACTTGATGAAAAGAACAAATCT----------------------------
----
AGTAATGGCTCTGGTATGTATAGATTAATAAATTGCAATACCTCAACCATAACGCAA---
GCCTGTCCAAAGGTTTCTTTTGATCCAATTCCTATACATTATTGTGCTCCAGCTGGGTTTGCGATTTTAAAGT
GTAAT---AATAAGACATTCAAT------GGAACAGGACCATGCAATAAT-----------------------
-GTTAGC---ACAGTACAATGTACACATGGAATCAAGCCAGTGGTATCAACT---
CAATTACTGTTAAATGGTAGCCTAGCAGAAGAAGAC------------
ATAATAATTAGATCTAAGAATCTGACAGACAAT---------ACCAAAACAATAATAGTGCAGCTTAAT----
-----------AAATCTGTACCA---------ATTAATTGTACAAGACCCAACAATAATACAAGACAA-----
-GGTGTAGGGATA------------GGACCAGGACAAATG---TTC------TATACAACA---GAC------
---------ATAATAGGCGATATA------------AGACAAGCACATTGT---AACATTTCCTATAGA---
GAATGGAATAAAACTTTACAAATGGTAGGAAAGAAATTAAGC---------------AAGCACTTCCTT----
-----------------------------------AATAAAACAATAAAATTTGAA------
TCACCTGCAGGAGGGGACCTAGAAATTACA---ATGCATAACTTTAATTGTAGAGGAGAA---
TTTAAGCAGCTCCAGGCAAGAGTCCTGGCCATAGAAAGATACCTAAAGGATCAACAGCTCCTAGGGATTTGGG
GCTGCTCTGGAAAACTCATCTGCACCACTGCTGTGCCTTGGAACTCCAGTTGGAGT-----------------
----------------------------AATAGAACTGCAGACGATATTTGG------CAG------
AACATGACCTGGATGCAGTGG---GAAAAGGAAATTAGTAATTACACAGACACAATATAC---
AATTTGCTTGTAATCTCGCAAACCCAGCAGGAG--

GAAAATGAAAAGGAATTATTAACATTAAACAGTTGGAACAATCTATGGAATTGGTTTGACATAACAAAGTGGT
TGTGGTATATAAAGATATTCATAATGATAGTAGGAGGCTTGATAGGTTTAAGAATAATTTTTGCT---
GTGCTCTCTTTAGTA---------AATAGAGTTAGGCAGGATACTCACCT------
TTGTCGTTTGCAATTAGTCTGCTTGATACCATAGCAATAGCAGTAGCTGAA---
GGAACAGATAGAATTATAGAAATAATACAAAGAAGTTGTAGAGCA----------------------------
--------ATCTGTAACATA------CCTAGAAGAATAAGACAGGGCTTTGAAGCAGCTTTACAA---TAA
>KC187620
GTG---TGG--
GGAAACTTGTGGGTCACAGTCTATTATGGAGTACCTGTGTGG---AAAGAAGCAAAA---
ACTACTCTATTCTGTGCATCAGATGCTAAGGCATATGACCAAGAG---GTGCACAAT----------------
--------------GTCTGGGCTACA---CATGCCTGTGTACCCACAGAC---
CCCAACCCACAAGAATTAGTTTTGGAA---------AATGTAACAGAAAATTTTAAC----------------
--------------------------ATGTGGAAAAATGACATGGTGGATCAGATGCATGAAGATATA---
ATCAGTTTATGGATGAAAGCCTA---AAGCCATGTGTAAAGTTG---
ACCCCACTCTGTGTCACTTTAAATTGTACAGGTGCTAGGATTACAAATGCTACTAGTGATGGTAATGATGAGA
GTACA--
-------------------------AATACTACTGGTAATGTTACCTATAGTGAAGTAATGAAA---
AATTGCTCTTTCAATGCCACTGCAGAACTAAGA---------------------------
GATAAGACAAAGAAAGAATATGCACTT---
TTTTATAAAGTTGATATAGTACCACTTAAAAGGGTAATTAATGAG----------------------------
----

AGCAATAATGATAGTTATGAGTATAGATTAATTAATTGTAATACCTCAACCATAACACAA---
GCCTGTCCAAAGGTCTCTTTTGATCCAATTCCTATACATTATTGTGCTCCAGCTGGTTTTGCGATTCTAAAAT
GTAAT---AATAAGACATTCAAT------GGAACAGGACCATGCAATAAT-----------------------
-GTCAGC---ACAGTACTATGTACACATGGAATTAAGCCAGTGGTATCAACT---
CAACTACTGTTAAATGGTAGCCTAGCAGAAGAAGAG------------
ATAGTAATTAGATCTAAAAATCTGTCAGACAAT---------ACTAAGACAATAATAGTACATCTCAAG----
-----------GAATCTGTAGAG---------ATTGTATGTACAAGACCCAGCAATAATACAAGAAAA-----
-GGTGTGAGGATA------------GGACCAGGACAAACA---TTC------TTTGCAACAGGAGCC------
---------ATAATAGGAGATATA------------AGAAAAGCATATTGT---AACATTAGCAAAACT---
AAATGGAAGAAACTTTATACAATGTAAGTAAAAAAATTAAAA---------------GAACACTTCCCT----
-----------------------------------AGTAAAGAAATAAATTTTACA------
TCATCTGCAGGAGGGGACCTAGAAATTACA---ACACATAGCTTTAATTGTAGAGGAGAA---
TTTAAACAGCTCCAGGCAAGAGTCCTGGCTATAGAAAGATACCTAAAGGATCAACAGCTCCTAGGACTTTGGG
GCTGCTCTGGAAAACTCATCTGCACCACTACCGTGCCGTGGAACACTAGTTGGAGT-----------------
----------------------------AATAAGTCTGTAGAAGATATTTGG------GAC------
AACATGACCTGGATGCAGTGG---GATAGAGAAATTAATAATTACACAAACACAATATAC---
AGTTTGCTTGAAGAATCGCAAAGACAGCAGGAG--

AAGAATGAAAAAGATTTATTGGCATTGGACAGTTGGAACAGTCTGTGGAGTTGGTTTGACATAACAAATTGGT
TGTGGTACATAAGAATATTCATAATGATAGTAGGAGGCTTGATAGGTTTAAGAGTAATTTTTGCT---
GTGCTTTCTATAGTA---------AATAGAGTTAGGCAGGGATACTCACCT------
TTGTCATTGGCTGTTAGTTTGTTTGATGCCATAGCAATAGCAGTAGCTGAA---
GGAACAGATAGGATTATAGAATTAGTACAAAGGCTTTGGAGAGCT----------------------------
--------ATCTGCAATATA------CCTAGAAGAATAAGACAGGGCTTTGAAGTAGCTTTGATA---TAA
>KY111994
GGG---AAA--
GGAAACTTGTGGGTCACAGTCTATTATGGGGTACCAGTATGG---AAAGAAGCAAAA---
ACTACTCTGTTTTGTGCATCAGATGCCAAAGCATATGAGAAAGAA---GTGCATAAT----------------
--------------GTCTGGGCTACA---CATGCCTGTGTACCCACAGAC---
CCCAGCCCACAAGAATTGGTTTTGGAA---------AATGTAACAGAAAATTTTAAC----------------
--------------------------ATGTGGAAAAATGATATGGTAGATCAGATGCATGAGGATATA---
ATCAGTCTATGGGATCAAAGCCTA---AAGCCATGTGTAAAGTTG---
ACCCCACTCTGTGTCACTTTAAATTGTAGTAATGCTACTGTTGACAGTAGTAAAGTTTATGATACTAGG----
----
-------------AGTAATGTTAATGTTACCAGTATCAATAATAACATAATGGGAGAAATGAAA---
AATTGCTCTTTCAATACAACCACAGAAATAAGA---------------------------
GATAAGGTAAAGAAGGAATATGCACTT---TTTTATAGACCTGATGTAGTACCACTTGATGAA----------
----

ACAAGCAATACCAGTGAGTATAGATTAATAAATTGTAATACCTCAGCCGTAACACAA---
GCCTGTCCAAAGGTCACTTTTGAACCAATTCCTATACATTATTGTGCTCCAGCTGGTTATGCGATTCTAAAGT
GTAAT---AATAAGACATTCAAT------GGAACAGGACCATGCAGTAAT-----------------------
-GTCAGC---ACAGTACAATGTACACATGGAATTAGGCCAGTGGTATCAACC---
CAACTACTGTTAAATGGTAGTCTGGCAGAAAAAGAG------------
ATAGTAATTAGATCTGAAAACCTGACAAACAAT---------GCCAAAATAATAATAGTCCATCTTAAC----
-----------ACCTCTGTAGAA---------ATTGTGTGTACAAGGCCCGGCAATAATACAAGGAAA-----
-AGTGTGAGAATA------------GGACCAGGACAAACA---TTC------TATGCAACAGGAGAC------
---------ATAATAGGAGATATA------------AGACAAGCACATTGT---AACATTAGTGAAAAG---
CAATGGAATAACACTTTACAAGAGGTAGGTAAAGAATTGCAA---------------AAACACTTCCCT----
-----------------------------------AATAAGACAATAAAGTATGAA------
CGATCCGCAGGAGGAGACATGGAAATTGCA---ACACATAGCTTTAATTGTGGAGGAGAA---
TTTAAGCAGCTCCAGGCAAGAGTCCTGGCTCTGGAAAGATACCTAAAGGATCAACAGCTCCTAGGGATGTGGG
GCTGCTCTGGAAAACTCATCTGCACCACTACTGTGCCTTGGAACACTAGTTGGAGT-----------------
----------------------------AATAAATCTGAAAAGGATATTTGG------GAT------
AACATGACATGGATGCAGTGG---GAGAGAGAAATTAGCAATTACACAGAGACAATATAC---
ATGTTGCTTGAAGACTCGCAACACCAGCAGGAA--

AGAAATGAAAAAGATTTACTAGCATTGGACAGTTGGAACAGTCTGTGGAATTGGTTTAACATAACAAACTGGC
TGTGGTATATAAGAATATTCATGATGATAGTAGGGGGCTTGATAGGTTTAAGAATAATTTTTGCT---
GTGCTATCTATAGTG---------AATAGAGTCAGGCAGGGATACTCACCT------

TTGTCGTTGGCTATTAGTCTTTTAGATACCATAGCAATAGCAGTAGCTGAA---
GGAACAGATAGGATTATAGAAGTAATACAAAGATTTTGTAGAGCT---------------------------
--------ATCCGCAACATA------CCTACAAGAATAAGACAAGGCCTTGAAGCATCTTTGCTA---TAA
>KC187280
GTG---TTG--
GGGAGCTCATGGGTCACAGTCTATTATGGGGTACCTGTGTGG---AGAGAAGCAAAA---
ACTACTCTATTCTGTGCATCAGATGCTAAAGCATATGAGAAAGAA---GCACATAAT---------------
--------------ATTTGGGCTACA---TATGCCTGTGTACCCACAGAC---
CCCAACCCACAAGAATTAAAGTTGGAA---------AATGTAACAGAAAATTTTAAC---------------
-------------------------ATGTGGAAAAATGATATGGTAGACCAGATGCAGGAGGATATA---
ATTAGCTTATGGGAACAAAGCCTA---AAGCCATGTGTAAGGATG---
ACCCCACTCTGTGTCACTTTGGAGTGTAGTGAAAACATTACTGTTAATGATATCAATAGTACTTCTACT----
----
-------TCCTTTGGTATCAATAGTACTTCTGCTCCAATGGGTAACATTACTGATAATATGAGA---
AATTGCTCTTTCAATGCAACCACAGAAATAAAA-------------------------
GATAAAAAACGGAAGGAGTATGCACTT---TTTTATAGACTTGATATAGTATCACTTGGGAAG---------
----

AATAACTATTATAGACTAATAAATTGCAATACCTCAACCATAACACAA---
GCCTGTCCAAAGATCAATTTTGACCCAATTCCTATACATTATTGTGCGCCAGCTGGTTTTGCGATTCTAAAGT
GTAAT---AATAAGACATTCAAT------GGGACAGGACCATGCAATAAT----------------------
-GTCAGT---ACAGTACAATGTACACATGGAATTAAGCCAGTGGTATCAACT---
CATCTACTATTAAATGGTAGCCTAGCAGAAGGAGAG-----------
ATAATAATTAGGTCTGAAAACCTGACAGTCACT---------CACAAAACAATAATAGTACACCTTAAT----
-----------GAATCTGTAGAA---------ATTGTGTGTACAAGACCTGGCAATAATACAAGAAAA-----
-AGTGTAAGAATA------------GGACCAGGACAAACA---TTC------TATGCAACAGGTGGA------
---------ATAATAGGAGACATA-----------AGACAAGCACATTGT---AACATTAGTGAAACT---
AAGTGGAACAGAACTCTACAACGGGTAGCGAAAAAATTAATA--------------GACTATTTCCCT----
-----------------------------------AATGCAACAATAAAATTTAAA---
CAACCACCCGCAGGAGGGGATCTAGAAATTATG---ATGCATACCTTTAATTGTGGAGGAGAA---
TTTAAGCAGCTCCAGACAAGAGTCCTTGCTATAGAAAGATACCTAAAGGATCAACAGCTCCTAGGGATTTGGG
GTTGCTCTGGAAAACTCATCTGCACCACTAATGTGCCTTGGAACTCCAGTTGGAGT-----------------
---------------------------AATAAATCTATAGACGATATTTGG------GGG------
AGCATGACATGGATGCAGTGG---GATAAAGAAATTAGTAATTACACAGGCATAATATAC---
AGTTTGCTTTCAGACTCGCAAATCCAGCAAGAA---------------------------------------
----
AAAAATGAAAAAGATTTATTAGCCTTGGACAGTTGGCAAAATCTGTGGAATTGGTTTGACATAACAAAGTGGT
TGTGGTATATAAAATTATTCATAATGATAGTAGGAGGCTTAATAGGTTTAAGAATAATTTTTACA---
GTGCTTTCTATAGTA---------AATAGAGTTAGGCAGGGATACTCACCT------
TTATCGTTGGCTATTAGTCTACTAGATACCATAGCCATAGCAGTAGCTGAA---
GGAACAGATAGGATTATAGAGATAATACAAAGAAGTTGTAGAGCT---------------------------
--------ATCCTCAACATA------CCTAGAAGAATAAGACAGGGCCTTGAAGCAGCTTTACTA---TAA
>KC187249
GGG---AGG---GGG---------------------------------
GAAGAGAACTTGTGGGTTACTGTTTATTATGGGGTACCTGTGTGG---AAAGAAGCAAAA---
ACTACTCTATTCTGTGCATCAGATGCTAAAGCCTATGAGAAGGAA---GTGCATAAT---------------
--------------GTCTGGGCTACA---CATGCCTGTGTACCCACAGAC---
CCCAACCCACAAGAAATGGAGTTAAAA---------AATGTAACAGAAAATTTTAAC---------------
-------------------------ATGTGGAAAAATGATATGGTGGATCAGATGCATGAGGATATA---
ATTAGTTTATGGGACCAAAGCCTA---AAGCCATGTGTAAAGTTG---
ACCCCACTCTGTGTCACTTTAAATTGTAGTAATAATATTACTATCAATAATGGTAATAATGGTACTGCTAACT
ATAGTACTACC---
---ACAGAAATGAGA---
AATTGCTCTTTCAATGCAACCACAGAATTAAGA--------------------------
GACAAGAAAAAGAAAGAGCATGCACTT---TTTTATAAGCTTGATTTAGAACCACTTGGTAACAGTAACTCT-
----

AGTGAGAACTATGGAGAGTATATATTAATAAATTGTAATACCTCAACCATAGCACAA---
GCCTGTCCAAAGATCTCTTTTGAACCAATTCCTATACATTATTGTGCTCCAGCTGGTTATGCGATTCTAAAGT
GTAAT---AATAAGACATTCAAT------GGAACAGGACCATGCAATAAT----------------------

-GTCAGC---ACAGTACAATGTACACATGGAATTAAGCCAGTGGTATCAACT---
CAACTACTGTTAAATGGTAGCCTAGCAGAACAGGAG------------
ATAGTAATTAGATCTGAAAATCTGACAGACAAT---------GCCAAAACAATAATAGTACATCTAAAT----
-----------GAATCTGTAAAC---------ATTGTGTGTACAAGACCCAACAATAACACAAGAAAA-----
-AGTATAAGGATA------------GGACCAGGACAAGCA---TTC------TTTGCAGCAGGAGAC------
---------ATAATAGGAGATATA------------AGACAAGCACATTGT---AACATTAGCAAAGTA---
GGTTGGAATAGAACTTTACAAATGGTAGGAAAAAAATTAGCA---------------GAACGCTTTAAT----
-----------------------------------AAGACAAAAGTAGTATTTGCA------
CCACATTCAGGAGGGGACCCAGAAATTACA---ATGCATAGCTTTAATTGTGGAGGAGAA---
TTTAAGCAGCTCCAGGCAAGAGTCTTGGCTATAGAAAGATACCTAAAGGATCAACGGCTCTTAGGGCTTTGGG
GCTGCTCTGGAAAACTCATCTGCACCACTAATGTACCTTGGAACTCTAGTTGGAGT-----------------
----------------------------AACAAAACTCAAGAGGAGATTTGG------GAA------
AACATGACCTGGATGCAGTGG---GAGAGAGAAATTAATAATTACACATACACAATATAC---
AGTTTGCTTGAAGACTCACAAAGCCAGCAGGAG--

AAAAATGAGAAGGATTTATTAGCATTGGACAGTTGGCAAAGTCTGTGGAATTGGTTCAGCATATCAAATTGGC
TGTGGTATATAAGAATATTCATAATGATAGTAGGAGGCTTGATAGGTTTGAGAATAATTTTTGCT---
GTGCTTTCTATAGTA---------AATAGAGTTAGGCAGGGATACTCACCT------
TTGTCCTTTGCTATTAATCTGCTTGATACCATAGCAATAACAGTAGCTGAA---
GGAACAGATAGAATAATAGAAGTAGTACAAAGAATTTGTAGAGCT----------------------------
--------ATTCGCAACATA------CCTAGAAGAATAAGACAGGGCTTTGAAGCAGCTTTGCTA---TAA
>MF502153
GTG---CTT--
GGAAACTTGTGGGTTACAGTCTATTATGGGGTACCTGTGTGG---AGAGAAGCAAAA---
GCTACTCTATTCTGTGCATCAGATGCTAAAGCATATGAGAAAGAA---GTGCATAAT----------------
--------------GTCTGGGCTACA---CATGCCTGTGTACCCACAGAC---
CCCAACCCACAAGAATTGGTTTTGGGA---------AATGTAATAGAAAATTTTAAT----------------
--------------------------ATGTGGAAAAATGACATGGTGGATCAGATGCATGAGGATATA---
ATCAGTCTATGGGATCAAAGCCTA---AAGCCATGTGTAAAGTTG---
ACCCCACTCTGCGTCACTTTAAAATGTATGAATGTGAATGTTACAAGAGAAAATGCTACTGCTGAAAATAATA
CGAATGTTACA--
-------------------AGAGAAAATGCTACTGCTGAAAATAATACCCAAAGTGAAATGAAA---
AACTGCTCTTTCAATATAACCACAGAAATAAGG--------------------------
GATAAGAAAAAGACGGAATATGCACTT---
TTTTACAAACTTGATATAGTACCAATTAAGAATAATAATGATAAT----------------------------
----
AGTAATAATAGTAATAATAGTAATGAGTATATGTTAATAAATTGTAATACCTCAACTGTAACACAA---
GCCTGTCCAAAGGTCTCGTTTGACCCAATTCCTATACATTATTGTGCTCCAGCTGGTTTTGCGATTCTAAAGT
GTAAT---GATAAGACATTTAAT------GGAACAGGACCATGCAATAAT-----------------------
-GTCAGC---ACAGTACAATGTACACATGGAATTAAGCCAGTGGTATCAACT---
CAACTACTGTTGAATGGTAGTTTAACAGAA---GAC------------
ATAGTAATTAGCTCTGAAAATATAACAGACAAT---------GGCAAAACAATAATAGTACATCTTAAT----
-----------GAATCTGTACAA---------ATTAATTGTACAAGGCCCAACAATAATACAAGGAAA-----
-AGTATGAGGATA------------GGACCAGGACAAACA---TTT------TATGCAACAGGAGAA------
---------ATAATAGGAAATATA------------AGAGAAGCACATTGT---AATATTAGTAGACAA---
AATTGGACTAACACTTTAAAAAGGGTGGAGACAAAATTGAAA---------------CAATACTTT-------
-----------------------------------AATGAAACAATAAAATTCGAA------
CCACCTTCAGGAGGGGACCTAGAAATTACA---ACACATAGTTTTAATTGTAGAGGAGAA---
TTTAAGCAGCTCCAGACAAGAGTCCTGGCTATAGAAAGATACCTAAAGGATCAACAGCTCCTAGGGCTTTGGG
GCTGCTCTGGAAAACTCATCTGCACCACTAATGTGCCTTGGAACTCCAGTTGGAGT-----------------
----------------------------AATAAATCTGAAAAAGATATTTGG------GAG------
AACATGACCTGGATGCAGTGG---GATAGAGAAATTACTAATTACACAAACACAATATAT---
GATTTGCTTGAAAAATCACAAAATCAGCAGGAA--

CAAAATGAAAAAGATTTATTAGCATTGGACAGCTGGAAAAATCTATGGAATTGGTTTAGCATAACAAACTGGC
TGTGGTATATAAAATTATTCATAATGATAGTAGGAGGACTAATAGGTTTAAGAATAATTTTTGCT---
GTGCTTTCTATAATA---------AATAGAGTTAGGCAGGGATACTCACCT------
CTATCGTTTGCTATTAACCTGTTTGATACCATAGCAATAGTAGTAGCTGAA---
GGAAGTGACAGAATTATAGAATTAGTACAAAGAATTTGTAGAGCT----------------------------
--------ATCATTAACATA------CCTAGAAGAATAAGACAGGGCTTTGAAGCAGCGTTGCTA---TAA

>MF353063
GGG---ATG---
TGGGTCACAGTCTATTATGGGGTACCTGTGTGG---AAAGAAGCAAAA---
ACTACTCTATTTTGTGCATCAGATGCTAAAGGATATGAGAAGGAA---GTGCATAAT----------------
--------------GTCTGGGCTACA---CATGCCTGTGTACCCACAGAC---
CCCAATCCACAAGAAATGGTTTTAGAA---------AATGTAACAGAAAATTTCAAC----------------
--------------------------ATGTGGAAAAATGACATGGCAGATCAGATGCATGAAGATGTA---
ATTAGTTTATGGGATCAAAGCCTC---AAGCCATGTGTAAAGTTA---
ACCCCACTCTGTGTCACTCTAAACTGTACCGATGCTAAGGCTACTGCCAACAATACCAATGCTACTAATGCTA
CT---
----------------------------GTCAGCAAGAATGACAGTATAACAGAGGAAATGAAA---
AATTGCTCTTTCAATACAACCACAGAATTAAGA---------------------------
GATAAGATAGAGAAAAAGTATGCACTT---TTTTATAAACTTGATATAGTACAACTAGAAGAC----------
----

AGCTCTACTCATTATAGATTCATAAATTGTAATACCTCAGCCATAACACAA---
GCCTGTCCAAAGGTCTCTTTTGACCCAATTCCTATACATTATTGTGCTCCAGCTGGTTATGCGATTCTAAAGT
GTAAT---AATAAGACATTCAAT------GGAACAGGACCGTGTAATAAT-----------------------
-GTCAGC---ACAGTACAATGTACACATGGAATTAAGCCAGTGGTTTCAACT---
CAACTATTGTTAAATGGTAGCCTAGCAGAAGGAGGG------------
ATAATAATTAGATCTGAAAATATAACAGATAAT---------ACCAAAACAATAATAGTACATCTCAAT----
-----------GAATCTGTAGAG---------ATTGTGTGTACGAGACCCGGTAATAACACAAGAAAA-----
-AGTATAGGAATA------------GGACCAGGACAAGCA---TTT------TATGCAACAGGAGAA------
---------ATAATAGGAGACATA------------AGAAAAGCACATTGT---AACATTAGTGAAAGC---
AAATGGAATGAAACTTTACAAAGGGTAAGTGAAAAATTAAAA---------------GAATACTTCCCT----
-----------------------------------GGGAAGAATATAACATTTACA------
CCATCCTCAGGAGGGGACCCAGAAATTGCA---ACACATAGCTTTAATTGTGGAGGAGAA---
TTTAAACAGCTCCAGGCAAGAGTCCTGGCCCTGGAAAGATACCTAAAGGATCAACAGCTCCTAGGGATGTGGG
GCTGCTCTGGAAGATTCATCTGCACCACTAATGTACATTGGAACTCTAGTTGGAGT-----------------
----------------------------AATAAAAATTATAGTGATATTTGG------GAT------
AACATGACCTGGATGCAGTGG---GAGAGAGAAATTAGCAATTATACAGATATAATATAT---
GGATTGCTTGAAGAATCACAAAACCAGCAGGAA--

AAGAATGAACAAGATTTACTAGCATTGGACAGATGGAACAATTTGTGGAATTGGTTTAACATAACAAAATGGC
TGTGGTATATAAAAATATTCATAATGATAGTAGGAGGCTTGATAGGTTTAAGAATAATTTTTGCT---
GTACTTTCTTTAGTA---------AATAGAGTTAGGCAGGATACTCACCT------
CTGTCGTTGGCTATTAGTCTATTGGATACCCTAGCAATAGCAGTAGGTGAA---
GGAACAGATAGGATCCTAGAATTTGTATTAAGAATTTGTAGAGCT----------------------------
--------ATCCGCAACATA------CCTACAAGAATAAGACAGGGCTTTGAAACAGCTTTGCTA---TAA
>HM638803
GTA---TTG--
GGGAACAGGTGGGTCACAGTCTATTATGGGGTACCTGTGTGG---ACAGAAGCAAAA---
ACTACTCTATTCTGTGCATCAGATGCTAAAGCATATGAGAGAGAA---GTGCATAAT----------------
--------------GTCTGGGCTACA---CATGCCTGTGTGCCCACAGAC---
CCCAACCCACAAGAAATGATCTTGGAA---------AATGTAACAGAAAATTTTAAC----------------
--------------------------ATGTGGGAAAATGACATGGTAGATCAGATGCATGAGGATATA---
ATCAGTTTATGGGATCAAAGCCTA---AAGCCATGTGTAAAGTTG---
ACCCCACTTTGTGTCACTTTAAATTGTACAGATGCTAGGGTTAATACTACCAGTAATGGTAATACTACC----
----
----------------ACAACTGGTAATGCTACCCTCAAGGATAACATGAAGGGAGAAATAAAA---
AATTGCTCTTTCAATGCAACCACAGAAATAAGT---------------------------
GATAAGAAAAAGAATGAATATGCACTT---TTTTATAGACTTGATATAGTACCACTTAATGAGGAG-------
----

AATAACAGTTCCAGTGAGTATAGGTTAATAAATTGTAATACCTCAACCGTAACACAA---
GCCTGTCCAAAGGTCTCATTTGACCCAATTCCTATACATTATTGTGCTCCAGCTGGTTATGCGATTCTAAAGT
GTAAT---AATAAGACATTCAAT------GGAACAGGACCATGCAACAAT-----------------------
-GTCAGC---ACAGTACAATGTACACATGGAATTAAGCCAGTGGTATCAACC---
CAACTACTGTTAAATGGTAGCCTAGCAGAAGAAGAG------------
ATAATAATTAGATCTGAAAATCTGACAGACAAT---------GCCAGAACAATAATAGTACATCTTAAT----

-----------CAATCTGTAGAA---------ATTGTATGTACAAGGCCCGGCAATAATACAAGGAAA-----
-AGTGTGAGAATA------------GGGCCAGGACAAGCA---TTC------TATGCAACAGGAGAA------
---------ATAATAGGAAATATA------------AGAGAAGCACATTGT---AACATTAGTAAAGGG---
TTATGGAACAACACTTTACAACAGGTAGGGAAGAAATTAGCA---------------GAACGCTTCCCT----
-----------------------------------AATAAAATAATAGTATTTAAC------
CACTCCTCAGGAGGGGATCCAGAAATTACA---ACACATAGCTTTAATTGTAGGGGAGAA---
TTTAAGCAGCTCCAGGCAAGAGTCCTGGCTATAGAAAGATACCTAAAGGATCAACAGCTCCTAGGACTTTGGG
GCTGCTCTGGAAAACTCATCTGCACCACTAATGTGCCTTGGAACGCCAGTTGGAGT----------------
----------------------------AATAAATCTCAAACAGACATTTGG------GAT------
AACATGACCTGGATGCAGTGG---GATAAGGAAATTAGTAATTACACAGAAACAATATAC---
TGGTTGCTTGAAAAATCACAAAACCAGCAGGAA---------------------------------------
----
CAAAATGAACAGGATTTATTAGCATTGGACAGTTGGAAAAATCTGTGGGATTGGTTTAGCATATCAAATTGGC
TGTGGTATATAAAAATATTCATAATGATAGTAGGAGGCTTGATAGGTTTAAGAATAATTTTTGTT---
GTGCTTTCTATAGTG---------AATAGAGTTAGGCAGGGATACTCACCT------
CTATCATTTGCTATTAGTCTGCTTGATACCATAGCAATAGCAGTAGCTGAA---
GGAACAGATAGAATTATAGAAGTAGTACAAAGAATTTGTAGAGCT---------------------------
--------ATCCGCAACGTA------CCTACAAGAATAAGACAGGGCTTGGAAGCAGCTTTGCTA---TAA
>KC894127
GTG---GTG---
GGAAACTTGTGGGTCACAGTCTATTATGGGGTACCTGTGTGG---AAAGAAGCAAAG---
ACTACTCTATTCTGTGCATCAGATGCTAAAGCATATGACAGAGAA---GTACATAAT----------------
--------------GTCTGGGCTACA---CATGCCTGTGTACCCACAGAC---
CCCAACCCACAAGAAATGGTTTTGGAA---------AATGTAACAGAAAATTTTAAC---------------
--------------------------ATGTGGAAAAATGACATGGTAGATCAGATGCATGAGGATATA---
ATCAGTTTATGGGATCAAAGCCTA---AAGCCATGTGTAAAGTTG---
ACCCCACTCTGTGTCACTTTAAATTGTACAGATGTTACCACAAATGGTACCGCTAGTCAAAAT----------
----
----------------------GTTAGTGATAATGATAGTGTTAATGTTACTGAACAAATGAGA---
AATTGCTCTTTCAATGCAACCACAGAACTAAGA--------------------------
GATAAGAAACAGAAAGTGTATGCACTT---TTTTATAAACTTGATACAGTACCACTTGATGGG----------
----

AATAATAACAGCTATATATTAATAAATTGTAATTCCTCAACCATAACACAA---
GCCTGTCCAAAGGTCTCTTTTGACCCAATTCCTATACATTATTGTGCGCCTGCTGGTTATGCGATTCTAAAGT
GTAAT---AATAAGACATTCAAT------GGGACAGGACCATGCAATAAT-----------------------
-GTCAGC---ACAGTACAATGTACACATGGAATTAAGCCAGTGGTATCAACT---
CAACTACTGTTAAATGGTAGCCTAGCAGAAGGAGAG------------
ATAATAATTAGATCTGAAAATCTGACAAACAAT---------GCCAAAACAATAATAGTACACCTTAAT----
-----------AAGTCTGTAGAA---------ATTGTGTGTACAAGACCCAACAATAACACAAGAAAA-----
-AGTATAAGAATA------------GGACCAGGACAAGCG---TTC------TATGCAACAAATGAA------
---------ATAATAGGAGACATA------------AGACAAGCACATTGT---AACGTGAGTAAAAGT---
GACTGGAACAAAACTTTAGAACAGGTAAAACAAAAATTAGGG--------------ACATACTTCCCT----
--------------------------------AAGAATAAAACAATAATATTTAAC------
ATATCCTCAGGAGGGGATCTAGAAATTACA---ACACATAGCTTTAATTGTAGAGGAGAA---
TTTAAGCAGCTCCAGGCAAGAGTCTTGGCTATAGAAAGATACCTAAAGGATCAACAGCTCCTAGGGATGTGGG
GCTGCTCTGGAAAACTCATCTGCACCACTGCTGTACTTTGGAACTCCACTTGGAGT----------------
----------------------------AACAAATCTTACGAAGATATTTGG------GGT------
AACATGACCTGGATGCAATGG---GACATAGAAATTAGTAATTACACAAACACAATATAC---
AATTTGCTTGAAGAATCACAAAACCAGCAGGAA---------------------------------------
----
AAGAATGAAAAAGATTTATTAGCATTGGACAGTTGGAAAAATCTGTGGAATTGGTTTAGCATATCAAACTGGC
TGTGGTATATAAAAATATTCATAATGATAGTAGGAGGCTTGATAGGTTTGAGAATAATTTTTGCT---
GTGCTATCTATAGTG---------AATAGAGTTAGGCAGGGATACTCACCT------
TTGTCATTTGCTATTAGTCTAATTGATGCCATAGCCATAGCAGTAGCTGAA---
GGAACAGATAGGATTGTAGAATTAATACAAAGAATTTGTAGAGCT---------------------------
--------ATCTGCAACATA------CCTAGAAGAATAAGACAGGGCTTTGAAACAGCTTTGCTA---TAA
>HM639260
GTG---TTG---
GGGAACTTGTGGGTCACAGTCTATTATGGGGTACCTGTGTGG---AGAGAAGCAAAG---

ACCACTCTATTCTGTGCATCAGATGCTAAAGCATATGAGAAAGAA---GTGCATAAT----------------
--------------GCCTGGGCTACA---TATGCCTGTGTACCCACAGAC---
CCCAACCCACAAGAAATAGTTTTGGAA---------AATGTAACAGAGAATTTTAAC----------------
--------------------------ATGTGGAAAAATGACATGGTAGATCAGATGCATGAGGATATA---
ATCAGTTTATGGATCAAAGCCTA---AAGCCATGTGTAAAGTTG---
ACCCCACTCTGTGTCACTCTAAAGTGTACAAATGCTACTTCTAGTGTTAATGTTACTTCTGGTGGTACAAAT-
----
-------------GCTACTAGTGGTAATGTTACCATTAAAAACATTACTAGCGATGAAATGAAA---
AATTGCTCTTTCAATGCAACCACAGAAATAAGA---------------------------
GATAAGAAACAGCAAGTGTATGCACTT---TTTTATAAGCTTGATATAGTACCACTTAATGAGAAT-------
----

AACAGCAGCTCTGGGGAGTATAGATTAATAAATTGCAATACCTCAGCCATAACACAA---
GCCTGTCCAAAGGTCTCTTTTGACCCAATTCCTATACATTATTGTACACCAGCTGGTTATGCGATTCTAAAAT
GTAAT---AATAAGACATTCAAT------GGAACAGGACCATGCCATAAT-----------------------
-GTCAGC---ACAGTACAATGTACACATGGAATTAAGCCAGTGGTATCAACT---
CAACTACTGTTAAATGGTAGCCTAGCAGAAGGAGAG------------
ATAATGATTAGATCTGAAAATCTGACAAACAAT---------GCCAAAACAATAATAGTACACCTTAAT----
-----------GAATCTGTAGAA---------ATTGTGTGTACAAGACCCAACAATAATACAAGAAAA-----
-AGTATAAGGATA------------GGACCAGGACAAGCA---TTC------TATGCAACAGGTGAC------
---------ATAATAGGAGACATA------------AGACAAGCATATTGT---AACATTAGTGAAAGT---
AAATGGAACAAAACTTTATACCAGGTAAGGAAAAAATTAGAA---------------GAACACTTCCTTAAT-
-----------------------------AACAATATAACAAATATAACATTTGCA---
CAACCAAACCCAGGAGGGGATCTAGAAATTACA---ACACATAGCTTTAATTGTAGAGGAGAA---
TTTAAGCAGCTCCAAACAAGAGTCCTGGCTATAGAAAGATACCTAAAGGATCAACAGCTCCTAGGGATATGGG
GCTGCTCTGGAAAACTCATCTGCACCACTGCTGTGCCTTGGAACACTAGTTGGAGT-----------------
----------------------------AATAAGTCTCAAACAGATATTTGG------GAT------
AACATGACCTGGATGCAGTGG---GATAAAGAAATTAGTAATTACACAAACACAATATAC---
AGGTTGCTTGAAGAATCACAAAACCAGCAGGAA--

AAGAATGAAAATGATTTATTAGCATTGGACAGTTGGAACAATCTGTGGAATTGGTTTAACATAACAAACTGGC
TGTGGTATATAAAAATATTCATAATAATAGTAGGAGGCTTGATAGGCTTAAGAATAATTTTTGCT---
GTGCTTTCTATAGTG---------AATAGAGTTAGGCAGGGATACTCACCT------
TTGTCATTTGCTATTAGTTTGCTTGATACCGTAGCAGTAATAGTAGCTGAA---
GGAACAGATAGGATTATAGAAGTAGCACAAAGAATTTGCAGAGCT----------------------------
--------ATTTGCAACATA------CCTAGAAGAATAAGACAGGGTTTTGAAACAGCTTTGCTA---TAA
>KJ541837
GGG---GGA--
AGAGACAGGTGGGTCACAGTCTATTATGGGGTACCTGTGTGG---AAAGAAGCAAAA---
ACTACTCTATTCTGTGCATCAGATGCTAGAGCATATGAGAGAGAA---GTGCATAAT----------------
--------------GTCTGGGCTACA---CATGCCTGTGTACCCACAGAC---
CCTAACCCACAAGAAATACTTATGGAA---------AATGTAACAGAAAATTTTAAC----------------
--------------------------ATGTTTAAAAATGACATGGTGAATCAGATGCATGAGGATGTA---
ATCAGTTTATGGATCAAAGCCTA---AAGCCATGTGTAAAGTTG---
ACCCCACTCTGTGGTGCAAATGTTAGCAATAATGTTACCTGCAATAATAGTAAT-------------------
----
----------------------ACTTGCAATAATACATACAATGAAATAAAAAATGAAATAACA---
AATTGCTCTTTCAATGCAACCACAGAAATAAGA---------------------------
GATAAGAAGCGGCAAGTGTATGCACTC---TTTTATAAACTTGATATAGTACCACTTTTTAATGAGAAAAAC-
----

TCTAGTGACGGAAAACTTTACAGCCAGTATAGATTAATAAATTGTAATACCTCAACCATAACACAA---
GCCTGTCCAAAGGTCACTTTTGATCCAATTCCTATACACTATTGTGCTCCAGCTGGTTATGCGATTCTAAAGT
GTAAT---AACAAAACATTCAAT------GGGACAGGACCATGCAACAAT-----------------------
-GTTAGC---ACAGTACAATGTACACATGGAATTAAACCAGTAGTATCAACT---
CAACTACTGTTAAATGGTAGCCTAGCAGAAGAGGGG------------
ATAATAATTAGATCTAAAAATATGACAGACAAT---------GCCAAAATAATAATAGTACATCTTAAG----
-----------GAACCTGTAGAA---------ATTGTGTGTACAAGACCCAACAATAATACAAGAAAA-----
-AGTGTAAGGATA------------GGACCAGGACAAACA---TTC------TATGCAACAGGAGAC------
---------ATAATAGGAGACATA------------AGACAAGCGTATTGC---AACATTAGTGAAGCT---

AACTGGACTAACACTTTACAAAAGGTAGGTAAAAGATTACAA--------------AAATACTTCCCT----
----------------------------------AATAAAACAATACACTTTGCA------
TCATCCTCAGGAGGGGACCTGGAAGTTACA---ACACATAGCTTTAATTGCAGAGGAGAA---
TTTAAGCAGCTCCAGACAAGAGTCCTGGCTGTAGAAAGATACCTAAAGGATCAACAGCTCCTAGGGATTTGGG
GCTGCTCTGGAAAACTCATCTGCACCACTGATGTACCTTGGAACTCCAGTTGGAGT----------------
---------------------------AATAGATCTCAGGAAGAGATTTGG------GAG------
AACATGACCTGGATGCAGTGG---GATAGAGAAATTAGTAATTACACAAACATAATATAC---
CACTTGCTTGAAGACTCACAAAACCAGCAGGAA---------------------------------------
----
AAAAATGAAAAAGATTTATTGGCATTGGACAGCTGGAAAAATTTATGGAATTGGTTTAGCATATCAAACTGGC
TATGGTATATAAAAATATTCATAATGATAGTAGGAGGCTTGATAGGCTTAAGAATAATTTTTGCT---
GTGCTTTCTATAGTA---------AATAGAGTTAGGCAGGGATACTCACCT------
TTGTCGTTCGCTATTAGTCTGTTTGATGCCATAGCAATAGCAGTAGCTGAA---
GGAACAGATAGGATTATAGAATTAGTACAAAGAGTTTGTAGAGGT---------------------------
--------ATCTACAACATC------CCTAGAAGAATAAGACAGGGCTTTGAAGCAGCTTTGCAA---TAA
>KJ541839
GGG---GGA---
GGAAACTTGTGGGTTACAGTCTATTATGGGGCCCCTGTGGGA---AAAAAACCAAAC---
ACTACTCTTTTTTGTGCATCAAATGCTAAAGCACAGGAAAAAAAA---GTACATAAG---------------
--------------GTCGGGGCTACA---CATGCCTGTGTACCCCCAAAC---
CCCAGCCCACTAAAAACGTTTTTAAAA---------AATGTAACAAAAAATTTTAAC---------------
-------------------------ATGTGGAAAAATGATAGGGGGGAGCAAATGCATGAGGATGTA---
ATCAGTTTATGGAATCAAACCCTA---AAGCCATGTGTAAAGTTG---
ACCCCCCTCTGTGTCACTTTAAACTGTACCAGGGCTAACAATAATTTTACTATTTTCCTC------------
----
---------------------------AAGGGGACCCCCCATGAGAGCATGAAAAAAAAAAAA---
ATTTGCTCTTTCATTGCACCCACCAAATTAAAT-------------------------
GAAAAAAACCAAAAGGTGTATGCCCTC---
TTTTATAAAGTTGATGCCCCCCCCCTTGGGGGGAAAAAACCCCT---------------------------
----
AGTTATAGGTTAATAAATTGTAATGTTTCGGTAATAAAATGTAATACCTCAGCCATCACACAA---
GCCTGTCCTAAGGTCTCTTTTGATCCAATTCCTATACACTATTGTGCTCCAGCTGGTTTTGCAATTCTAAAGT
GTAAC---AATAAGACATTCAAT------GGGACAGGACCATGTAATAAT----------------------
-GTTAGC---ACAGTACAATGTACACATGGAATTAAACCAGTGGTATCAACT---
CAACTACTGTTAAATGGTATCCTAGCAGAAGAAGAG-----------
ATAATAATTAGATCTAAAAATATAACAGATAAT---------GCCAAAACAATAATAGTACAGCTTAAT----
-----------CAATCTGTAGAA---------ATTGTGTGTACAAGACCTAACAATAATACAAGAAAA-----
-AGTATAAGGATA------------GGACCAGGACAAGCA---TTC------TATGCAGCAGAAAAC------
---------ATAATAGGAGATATA------------AGACAAGCACATTGT---AACATTAGTGAAAAG---
AATTGGACTAAAACTTTACAAGAAGTAAGTAAAAAATTAGCA--------------AAACACTTCCCT----
----------------------------------AATAAAACAATAAAATTTGAA------
TCAACATCAGGAGGGGACCTAGAAATTACA---ACACATAGCTTTAATTGTGGAGGAGAA---
TTTAAACAGCTCCAGACAAGAGTCCTGGCCATCGAAAGATACCTAAGGGATCAACAGCTCCTAGGGATTTGGG
GATGCTCTGGAAAACTCTTCTGCGCCACTGCTGAACCTTGGAACTCCAGTTGGAGT----------------
---------------------------AACAGAACTGAAATTGAGATTTGG------GAA------
AACCTGACCTGGATGGAGTGG---GATAAAGAAATTAATAACTACACAAACATAATATAC---
CCGCTGCTTGAACAATCGCGAAACCAGGAGGAA---------------------------------------
----
AAAAATGAAAAGGATTTATTAGAATTGGACAGTTGGAACAATTTATGGAGTTGGTTTGGCATATCAAAATGGC
TGGATTATATAAAAATATTCATAATGGTAGTAGGAGGCTTGATAGGTTTAAGAATAATTTTTGCT---
GTGCTTTCTGTAGTG---------AATAGAGTTAGGCAGGGATACTCACCT------
TTGTCGTTGGCTATTAGTTTGTTTGATACCTTTGCAATAACAGTAGCTGAA---
GGAACAGATAGGATTATAGAATTAGGACTCAAAATTTGTAGAGCT---------------------------
--------ATCTTGAGCATA------CCTAGAAAAATAAGACAGGGCTTTGAAGCATCTTTGCTA---TAA
>KJ541841
GTG---GTA---
GACAACTTGTGGGTTACAGTTTATTATGGGGTTCCTGTGTGG---AAAGATGCAAAT---
ACCACCCTATTTTGTGCATCAGATGCCAAAGCACATAATACAGAA---GTGCATAAT---------------
--------------GTCTGGGCCACT---CATGCCTGTGTACCCACAGAC---
CCCAACCCACAAGAACTACATTTGAAA---------AATGTAACAGAAAATTTTAAC---------------

--------------------------ATGTGGAAAAATGACATGGTGAATCAGATGGATGAGGATGTA---
ATCAGTTTATGGGATCAAAGCCTA---AAGCCATGTGTAAAGTTG---
ACCCCACTCTGTGTCACTTTAGAATGTAGAGATGTTAGCAATAAAACAGCTGATAATAAA-------------
----
----------------------------ACAAGAACAGTTGATAAAAGTATTAAGGAAATAAGT---
AATTGCTCTTTCTATGCTACCACAGAATTAAAC---------------------------
GATAAGCAACAGAAAATGCATGCACTT---TTTTATAGACTTGATATAGTACCACTTGATAATGAGGATAAT-
----

GAGAATAAGAGTAACGCAAGTATCAACTATAGATTAATAAATTGTAATACCTCAACCATAACACAA---
GCCTGTCCAAAGGTCACTTTTGATCCAATTCCTATACACTATTGTGCTCCAGCTGGTTATGCGATTCTAAAGT
GTAAC---AATGAGACATTCAAT------GGGACAGGACCATGTAATAAT-----------------------
-GTCAGC---ACAGTACAATGTACACATGGAATTAAGCCAGTGGTATCAACT---
CAATTACTGTTAAATGGTAGCCTAGCAGAAGAAGAT------------
ATAATAATTAGATCTAAAAATTTAACCGACAAT---------GCCAAAACAATAATAGTACAGCTTAAT----
-----------CAATCTGTAGAA---------ATTGTGTGTACAAGACCCAACAATAATACAAGGAAA-----
-AGTATAAGGATA------------GGACCAGGACAAGCA---TTT------TATGCAGCAGGAAAA------
---------ATAATAGGAGATATA------------AGACAAGCACATTGT---AACATTAGTAAAAG---
AATTGGACTGACACTTTACAAGAAGTAAGTAAAAAATTAGGA---------------AAACACTTCCCT----
-----------------------------------AATAAAACAATAAAATTTGAA------
TCATCCTCAGGAGGGGACCTAGAAATTACA---ACACATAGCTTTAATTGTGGAGGAGAA---
TTTAAACAGCTCCAGGCAAGAGTCCTGGCCATCGAAAGATACCTAAGGGATCAACAGCTCCTAGGGATTTGGG
GATGCTCTGGAAAACTCATCTGCACCACTGCTGTACCTTGGAACTCCAGTTGGAGT-----------------
----------------------------AACAGAACTGAAAGTGAGATTTGG------GAA------
AACATGACCTGGATGGAGTGG---GATAAAGAAATTAATAATTACACAGACATAATATAC---
CAGCTGCTTGAAGAATCGCAAAACCAGCAGGAA--

AAAAATGAAAAGGATTTATTAGCATTGGATAGTTGGAACAATTTATGGAATTGGTTTGGCATATCAAAATGGC
TGGGGTATATAAAAATATTCATAATGATAGTAGGAGGCTTGATAGGTTTAAGAATAATTTTTGCT---
GTGCTTTCTGTAGTA---------AATAGAGTTAGGCAGGGATACTCACCT------
TTGTCGTTGGCTATTAATTTGTTTGATACCTTTGCAATAACAGTAGCTGAA---
GGAACAGATAGGATTATAGAATTAGGACTCAGAATTTGTAGAGCT----------------------------
--------ATCCTCAGCATA------CCTAGAAGAATAAGACAGGGCTTTGAAGGCGCTTTGCTA---TAA
>KJ541848
GGG---GTA--
GGAAACTTGTGGGTCACAGTCTATTATGGGGTACCTGTGTGG---AGAGAAGCAAAA---
ACTACTCTATTCTGTGCATCAGAAGCTAAATCATATGAAAAAGAA---GTGCATAAT----------------
--------------GTCTGGGCTACA---CATGCCTGTGTACCCACAGAC---
CCCAACCCACAAGAAAATGTTATGGGT---------AATGTAACAGAAAATTTTAAC----------------
--------------------------ATGTGGAAAAATGACATGGTGGATCAGATGCATGAGGATGTA---
ATCAGTTTATGGGATCAAAGCCTA---AAGCCATGTGTAAAGATG---
ACCCCACTCTGTGTCACTTTAAACTGTACAGATGTTAGCAATAAAACAGCTGATACATAC-------------
----
----------------------------AACAAGACAGTTGATAATAGTATTAAGGAAATAAGT---
AATTGCTCTTTTTATGGTACCACAGAATTAAAC---------------------------
GATAAGCAACAGAAAATGCATGCACTT---
TTTTATAGACTTGATATAGTACCACTTGATAATGAGAATAAGATTAAT-------------------------
----
GAGAATAAGAGTAACAGCAACGATATCAACTATAGATTAATAAATTGTAATACCTCAACCATAACACAA---
GCATGTCCAAAAGTCAATTTTGATCCAATTCCTATACACTATTGTGCTCCAGCTGGTTATGCAATTCTAAGGT
GTAAT---AATGAGACATTCAAT------GGGACAGGACCATGCAATAAT-----------------------
-GTCAGC---ACAGTACAATGTACACATGGAATTAAGCCAGTGGTATCAACT---
CAATTACTGTTAAATGGTAGCCTAGCAGAAAAAGAT------------
ATAATAATTAGATTTGAAAATTTGGCCAACAAT---------GTCAAAACAATAATAGTACATCTTAAT----
-----------CAATCTGTAGAA---------ATTGTGTGTACAAGACCCAACAATAATACAAGGAAA-----
-AGTATAAGGATA------------GGACCAGGACAAGCA---TTT------TATGCAACAGGAGAC------
---------ATAATAGGAGACATA------------AGACAAGCATATTGT---AACATTAATGAGGAC---
AAATGGAATAAAACTTTACAGATGGTGGGTAAAAAGTTAAAA---------------GAACACTTCCCT----
-----------------------------------AATAAAACAATACCCTTTAAG------
CCACCCTCAGGAGGGGACCTAGAAATTACA---ATGCATAGCTTTAATTGTAGAGGAGAA---

TTTAAGCAGCTCCAGGCAAGAGTCCTGGCTCTAGAAAGATACCTACAGGATCAACAGCTCCTAGGGCTTTGGG
GCTGCTCTGGAAAACTCATCTGCACCACTAATGTACCTTGGAACTCCAGTTGGAGC-----------------
----------------------------AACAGAAATCAAACAGAAATTTGG------GAA------
AAATTGACCTGGATGCAGTGG---GATAAAGAAGTTAGTAATTACACAGACACAATATAT---
AGGTTGCTTGAAGACTCGCAAAACCAGCAGGAA---

AAGAATGAAAAAGATTTATTAGCATTGGACAGTTGGAAAAATCTGTGGAATTGGTTTGACATAACAAATTGGC
TGCGGTATATACAACTATTCATAATGATAGTAGGAGGCTTGATAGGTTTAAGAATATTTTTTGCT---
GTGATCTCTATAGTG---------AATAGAGTTAGGCAGGGATACTCACCT------
TTGTCATTGGCTATTAGTCTGCTTGATACCATAGCAATAACAGTAGCTGAA---
GGCACAGATAGGATTATAGAATTCTTCCTAAGATTTTGTAGAGCT----------------------------
--------ATCTGCAGCATA------CCTAGAAGAATAAGACAGGGCTTTGAAGCAGCTTTGCAG---TAA
>MF373132
GGG---AAG-----------------------GGG---GAC---AAG---
GAGGACTTGTGGGTCACAGTATATTATGGGGTACCTGTGTGG---AGAGATGCACAA---
ACCTCTCTGTTTTGTGCATCAGATGCTAAAGCATATGATACAGAG---GTGCATAAT----------------
--------------GTCTGGGCTACA---CATGCCTGTGTACCCACAGAC---
CCCAACCCACAAGAAATGGTTTTAAAA---------AATGTAACAGAAAATTTTGAC----------------
-------------------------ATGTGGAAAAATAACATGGTGGATCAGATGCATCAGGATATA---
ATCAGTTTATGGGATCAAAGCCTA---AAGCCATGTGTAAAGATG---
ACCCCACTCTGTGTTACTTTAAACTGTACAAATGCTACAACTGCTATGAATGCTACAATTGATAAT-------
----
---AACATGCAGGGAGAAATAAAA---
AATTGTACTTTCAATGTAACCACAGAAATAAGA--------------------------
GATAAGGTAAAGAAGGTACATGCACTT---
TTTTATAAACTTGATATAGCACCACTTGGTAACAGTGCTAGTAGTGATAACAGTTCTAGTGGTGGTAAAGGT-
----
ACTAGGAATAATGAGTATAATGATTATATATTAATAAATTGTAATACCTCAGCCATAGCTCAA---
GCCTGTCCAAAGGTCTCTTTTGACCCAATTCCTATACATTATTGTGCTCCAGCGGGTTATGCGATTCTAAAGT
GTAAT---GATAAGACATTCAAT------GGAACAGGACCATGCAATAAT----------------------
-GTCAGC---ACAGTACAATGTACACATGGAATTAAGCCAGTGGTATCAACT---
CAATTACTGTTAAATGGTAGCCTAGCAGAAGGAGAC-----------
ATAATAATTAGATCTGAGAATATGACAAGCAAT---------ACTAAAACAATAATAGTACATCTTAAT----
-----------GAATCTGTATCA---------ATTAATTGTACAAGACCCAACAATAATACAAGAGAA-----
-AGTTTAAGGATA------------GGACCAGGACAAGCA---TTA------TATGCAACAGGAGAT------
---------ATAATAGGAGATATA-----------AGACAAGCACATTGT---AATATTAGTAAAGAA---
AGGTGGAATAAAACTTTAGAAAGAGTAAAGAAAAGATTAGCA---------------GAGTACTTCCCT----
----------------------------------AATACAACAATACTCTTTGCA------
CCACCCCCAGGAGGGGACCTAGAAGTTACA---ACACATAGCTTTAATTGTAGAGGAGAA---
TTTAAGCAGCTTCAGGCAAGAGTCCTGGCTATAGAAAGATACCTAAAGGATCAACAGCTCCTAGGGATTTGGG
GCTGCTCTGGAAAACTCATCTGCACCACTGCTGTGCCTTGGAATGAAAGTTGGAGT-----------------
----------------------------AATAAGTCTCAAAATGAGATTTGG------GAT------
AACATGACCTGGATGCAGTGG---GATAGGGAAATCAATAATTACACAAATGAAATATAC---
AGATTGCTTGGGGAATCGCAAATCCAGCAGGAG---

AAAAATGAACAGGATTTACTAGCGCTGGACAAGTGGCAAAATCTGTGGAGTTGGTTTGACATAACAAACTGGC
TGTGGTATATAAAAATGTTCATAATGATAGTAGGAGGCTTGATAGGTTTAAGAATAGTTTTTGCT---
GTGCTTTCTATAGTA---------AATAGAATTAGGCAGGGATACTCACCC------
TTATCGTTGGCTATTAGTCTGCTTGATACCACAGCAATAGCAGTAGGTGAA---
GGAACAGATAGGATTGTAGAATTTATACTACGAGTTTGTAGAGCT----------------------------
--------ATCTACAACATA------CCTCGAAGAATAAGACAGGGCTTTGAAGCAGCTTTGCAA---TAA
>MF373137
GAA---ACG---
GGAAATTTGGGGGCCACAGGCTATTATGGGGAACCTGTGTGG---AAAGAACCAAAC---
CCTACTCTATTTTGGGCATCAGATGTTAAAGGATATGAGACTGAA---GGGCACAAT----------------
--------------GTTTGGGCCACA---CATTCCTGGGTACCCACAGAC---
CCCAGTCCACAAGAAATGGTTTTGGAA---------AATGTACCTGAGAATTTTGAC----------------
-------------------------ATGTGGAAAAATAATATGGTGGAGCATATGCATCAGGATGTA---
ATTAGTTTAGGGGATCAAAGTCTA---AAGCCATGTGTAAAGCTG---
ACCTCATTCTGTGTTACCTTAAACTGTACGGATGCTAATGTTAATATTACTGCTATCCACAATAAC-------

----
--ACCATGAAGGGAGAAGTAAAA---
AATTGTACTTTCAATGCTACCACAGAAATAAGA----------------------------
GATAAGAAAAGGAAGGAATATGCACTC---
TTTTATAGACTGGATATAGTACCACTTGATGAGGCGGAGGATAACAAA--------------------------
----
ACTGATGATAGCAAAAACTTTAGTGATTATAGATTAATAAATTGTAATACCTCAGCCATAACACAA---
GCCTGTCCAAAGGTCTCTTTTAACCCAATTCCTATACATTATTGTGCTCCAGCTGGTTTTGCGATTCTAAAGT
GTAAT---AATAAGACATTTAAT------GGAACAGGATCATGCAATAAT------------------------
-GTCAGC---ACAGTACAATGTACACATGGAATTAGACCAGTGGTATCAACT---
CAACTATTGTTAAATGGTAGTCTAGCAGAAGAAGAG-------------
ATAATAATTAGATCAGAAAATCTGACAGACAAT---------ACCAAAACAATAATAGTACATCTTAAT----
-----------GAATCTGTAGAA---------ATTAATTGCACAAGACCCGGCAATAACACAAGAAGG-----
-AGTGTAAGGATA------------GGACCAGGACAAGCA---ATC------TATGCAACAGGAGCC------
---------ATAATAGGAGATATA------------AGACAAGCACATTGC---AACATTAGTGAAGCA---
AAATGGAATAAAACTTTACAACAGGTAGCGGCAAAATTAAAA----------------GAACACTTC-------
------------------------------------AATAAAACAATAATCTTTAGA------
CCATCCTCAGGAGGGGACCCAGAAATTACA---ATGCATAGCTTTAATTGTGGGGGAGAA---
TTTAAGCAGCTCCAGGCAAGAGTCCTGGCTATAGAAAGATACCTAAAGGATCAACAGCTCCTAGGGATTTGGG
GCTGCTCTGGAAGACTCATCTGCACCACTAATGTGCCTTGGAACTCTAGCTGGAGT------------------
----------------------------AATAAATCTAAAGAGGAGATTTGG------GAG------
AACATGACCTGGATGCAGTGG---GATAGAGAAATTAATAATTATACAGGCATAATATAC---
AGGTTGCTTGAACAATCGCAAAACCAGCAGGAA---

AAGAATGAACAAGATTTACTAGCATTGGACAAATGGGAAAATCTGTGGAGTTGGTTTAGCATATCAAAATGGC
TGTGGTATATAAAAATATTCATAATGATAGTAGGAGGATTAATAGGTTTAAGAATAATTTTTGCT---
GTGCTTTCTATAGTA---------AACAGAGTTAGGCAGGGATACTCACCA------
TTGTCATTTGCTATTAATCTATTTGATACTGTAGCAATAGCAGTAGCTGAA---
GGAACAGATAGGATTTTAGAATTGTTACAAGGAATTGGAAGAGCT-----------------------------
--------ATCTATAACATA------CCTAGAAGAATAAGACAGGGCTTTGAAGCAGCTTTGCAA---TAA
>MF373139
GTG---ATG---
GGGAACTTGTGGGTCACAGTCTATTATGGGGTACCTGTGTGG---AAAGAAGCAAAT---
CCTACTCTATTTTGTGCATCAGATGCTAAAGCATATGAGACAGAG---GTGCATAAT-----------------
--------------GTCTGGGCTACA---CATGCCTGTGTACCCACAGAC---
CCCAACCCACAAGAAATGGTCTTGGAA---------AATGTAACAGAAAATTTTAAC-----------------
--------------------------ATGTGGAAAAATGCCATGGTGGATCAGATGCATCAGGATGTA---
ATCAGTTTATGGGATCAGAGCCTA---AAGCCATGTGTAAAGTTG---
ACCCCACTTTGTGTTACTTTAAACTGTCAGAATATTACTATTACCAATACTAACAATACTGTTACCAATACTA
CTAATACTCCC---
--------------------------------CGCCTCAATGACACCACAAGTGGAGAAATAAAA---
AATTGCTCTTTCAATATAACCACAGAAGTAAGA----------------------------
GATAAGGAACGGAAAGCATATGCTCTT---TTTTATAGGCTTGATATAATACCACTTAATGAGGGGAGTAAT-
----

AGCAATTCTAGTAATTCTAGGGATTATAGATTAATAAATTGTAATACCTCAGCCATAACACAA---
GCCTGTCCAAAGGTCTCTTTTGACCCAATTCCTATACATTATTGTGCTCCAGCTGGTTATGCGATTCTAAAGT
GTAAT---AATAAAACATTCAAT------GGAACAGGACCATGCCATAAT------------------------
-GTCAGC---ACAGTACAATGTACACATGGAATCAAACCAGTGGTATCAACT---
CAGCTATTGTTAAATGGCAGTCTATCAGAAGGAGGT-------------
ATAATAATTACATCTCAAAACCTGACAAACAAT---------GCCAAAATAATAATAGTACAGCTTAAC----
-----------CAATCTGTACAA---------ATTAATTGTACAAGACCCAACAATAATACAAGAAAA-----
-AGTATAAGGATA------------GGACCAGGACAAACA---TTC------TATGCAGCAGGAGAG------
---------CTAATAGGAGATATA------------AGACAAGCACATTGT---AACATTAGTAGAACG---
AAGTGGAATCAAACTTTAGCAGAGGTAAAGGAAAAATTAGAA----------------AAGTACTTCCCT----
------------------------------------AATAAAACAATAACATTTAAA------
CCATCCTCAGGAGGGGACCTAGAAATTACA---ACACATAGCTTTAATTGTAGAGGAGAA---
TTTAAGCAGCTCCAGGCAAGAGTCCTGGCCATAGAAAGATACCTACAGGATCAACAGCTCCTAGGGATTTGGG
GCTGCTCTGGAAAACTCATCTGCACCACTGCTGTGCCTTGGAACAATAGTTGGAGT------------------
----------------------------AATAAAAATCAAACGGAAATTTGG------GAT------

AACATGACCTGGATGCAGTGG---GATAGAGAAATTGATAATTATACAAACACAATATAC---
AGGTTGCTTGAAGACTCGCAAAACCAGCAGGAG--

AAAAATGAAAAAGAGTTACTAGAATTGGACAAATGGCAAAATCTGTGGAGTTGGTTTGAAATAACAAGATGGC
TATGGTATATAAAAATATTCATAATGATAGTAGGAGGCTTGATAGGTTTAAGAATAATTTTTGCT---
GTGCTCTCTGTAGTG---------AATAGAGTTAGGCAGGGATACTCACCA------
TTATCGTTTGCTATTAGTCTGCTTGATACTACAGCAATAGCAGTAGCTGAA---
GGAACAGATAGGATTATAGAATTAATACAAAGAATTTGGAGAGCT----------------------------
--------ATCCGC---ATA------CCTAACCAAATAAGACAGGGCTTTGAAGTAGCTTTACTA---TAA
>KP411839
GGG---GAA--
GGAAACTTGTGGTTCACAGTTTATTATGGGTTACCTGTGTGG---ACAGAACCAAAA---
CCTACTCTATTTGGTGCTTCAAATGCTAAAGGATATAAAACAGAA---GTGCATATG----------------
--------------GTCTGGGCTACA---CATGCTTGTGTACCCACAAAC---
CCCAACCAACAGGACCTAGTTTTGGAA---------AATGTAACAGAAAATTTTAGC----------------
--------------------------ATGTGGAAAAATGACATGCTGGATCAGATGCATGAGCATGTA---
GTCATATTCAGTGATCAAGGCCTA---AAACCATATGCAAAAGTG---
ACCTCACTCACAGTCACTGTCACCTGTACTGATTTGAAAAATAGTACTTTCACTAGCACC-------------
----
-------------------AGCAGTAATGATACCTCAATGGTAACGGAGAGTGAGGAAATGAAA---
AATTGTTCTTTCAATATAACCACAGAAATAAGG---------------------------
GATAAGAAAAAGAAGGAATATGCACTC---TTTTATAGACTTGATATAGTACCACTTAAGGAGAAGGAGGAC-
----

AATAACAGTGATTATAGATTAATAAATTGTAATACCTCAGCCGTAACACAA---
GCTTGTCCAAAAGTTTCTTTTGACCCAATTCCTATACATTATTGTGCTCCAGCTGGTTATGCGATTCTAAAGT
GTAAT---AACAAGACATTCAAT------GGAACAGGACCATGCAACAAT-----------------------
-GTCAGC---ACGGTACAATGTACACATGGAATTAAGCCAGTGGTATCAACT---
CAATTACTGTTAAATGGTAGTCTAGCAGAAAAAGAG------------
ATAGTAATTAGATCTGAAAATCTGACAAACAAT---------GCCAAAATAATAATAGTACATCTTAAC----
-----------GAATCTGTAGAA---------ATTGTGTGTACAAGGCCCAACAATAATACCAGGAAA-----
-AGTATGAGGATA------------GGACCAGGACAAACA---TTC------TATGCAACAGGAGAC------
---------ATAATAGGAAATATA------------AGACAAGCATATTGT---AACATTAGTGAAAAA---
AGATGGAATGAAACTTTACAAAAGGTAAGTAAAAAAATTAAGA--------------GAACACTTCCCT----
-----------------------------------AATAAAACAATAAAATTTAAA------
CCATCCTCAGGAGGGGACCTAGAAATTACA---ACACATAGCTTTAATTGTGGAGGAGAG---
TTTAAGCAGCTCCAGACCAGAGTCCTGGCTGTAGAAGGATACCTAAAGGATCAACAGCTCCTACGGATTTGGG
CTGCCTCTGGAAAACTCATCTGCACCACTAACGTGCCTTGGACTCCTAGTTGGAGT-----------------
----------------------------ATTAAGTCCCAGGAAGAGATCTGG------AAT------
AATATGACCTGGATGCAGTGC---GATAGAGAGATTACATATACCGAAAACTTAATACAG---TGCCTAGCT-
-----CCGCAACCGCGAAAATGGAAA---

GATACTACTTGCAATGGCGACTTGGATTTGGACAAATGGCAAAATCTGTGGAAGTGGTTTAACATACCAAAGT
GGCTGTGGTATATAGAATTATTCATAATGATAGTAGGAGGCTTGATAGGTTTAAGAATAATTTTTGCT---
GTGCTTTCTATAGTA---------AATAGAGTTAGGCAGGGATACTCACCT------
TTGTCATTTGCTATTAGTCTGTTTGATACCTTAGCAATAGTAGTAGCTGAA---
GGAACAGATAGAATTATAGAAGTAGTACGAGGATTTTTTAGAGCT----------------------------
--------ATCTACAACATA------CCTAGAAGAATAAGACAAGGCTTAGAAGCAGCATTGCAA---TAA
>MF373148
GTG---GGA--
GAGGACTTGTGGGTCACAGTTTATTATGGGGTACCTGTGTGG---AAAGAAGCAAAA---
ACTACTCTATTTTGTGCATCAGATGCTAGAGCATATGAGAAAGAA---GTACATAAT----------------
--------------GTCTGGGCTACA---CATGCCTGTGTACCCACAGAC---
CCCAACCCACAAGAAATGATTTTGAAA---------AATGTAACAGAAAATTTTAAC----------------
--------------------------ATATGGAAAAATGACATGGTAGATCAGATGCATGAGGACATA---
ATCAGTTTATGGGACCAAGGCCTA---AAGCCATGTGTAAAATTG---
ACCCCGCTATGTGTCACTTTAAATTGTAGTAATGTTAATAGTAGTAGTGTTGCTAATAGTACCAATAGTACCA
ATAGTGGCAAT--
-------------ATTACCAATGCCAATACTACCAATGTCAATATTACCGATAGTGATATGAAG---
AATTGCTCTTTCAATATAACCACAGAACTAAGA---------------------------

GATAAGACAAGGAAAGAATATGCACTT---TTTTATAGACCTGATATAGTACCATTATCCAAT----------
----

TCCAATTCCAGTGAGTATATATTAATAAATTGTAATAGCTCAACAATAACACAA---
GCCTGTCCAAAAGTCTCTTTTGAACCAATTCCTTTGCATTATTGTGCTCCTGCTGGGTATGCGCTTTTAAAGT
GTAGG---GATACAAAATTCAAT------GGAACAGGACCATGCAAGAAT----------------------
-GTCAGC---ACAGTACAATGTACACATGGAATTAAACCAGTGGTATCAACT---
CAATTGTTGCTAAATGGTAGTTTAGCAGAAGATGAG-----------
ATAGTAATTAGATCTGAAAATATAACAAATAAT---------GCTAAAACAATAATAGTACAGCTTAAA----
-----------GAGCCTGTAACA---------ATTATGTGTACAAGGCCCAACAATAATACAAGGAAA-----
-AGTATGAGGATA------------GGACCAGGACAAACA---TTC------TATGCAACAGGAGCC------
---------ATAATAGGAAATATA------------AGACAAGCACATTGT---AACATTAGTAGAACT---
AATTGGACTAAAACTCTACAAGAGGTAGGTGAAAAATTACAA------------GAACAAGAACTCTTTAAG-
-----------------------------------AATAAAACAATAAAATTTACA------
GAACACTCAGGAGGGGACATAGAGATTACA---ACACATAGCTTTAATTGTGGAGGAGAA---
TTTAAGCAGCTTCAGGCAAGAGTCCTGGCTCTAGAGAGATACCTAAGGGATCAACAGCTCCTAGGAATTTGGG
GCTGCTCTGGAAAACTCATCTGCACCACTGCTGTACCTTGGAACTCTAGTTGGAGT-----------------
----------------------------AATAAATCTTATGATGCTATTTGG------GAT------
AACATGACTTGGATGCAATGG---GAGAAAGAAATTGATAATTACACAGACACAATATAC---
AGGTTGCTTGAAGTCTCGCAAACCCAGCAGGAA---------------------------------------
----
CAAAATGAAAGGGATTTACTAGCATTGGACAAGTGGAACAATCTGTGGAGTTGGTTTAACATATCAAATTGGC
TATGGTATATAAAGATATTCATAATGATAGTAGGAGGCTTAATAGGTTTAAGAATAATTTTTGCT---
GTGCTCTCTATAGTG---------AATAGAGTTAGGCAGGGATACTCACCA------
TTATCGTTGGCAATTAGCCTGTTTGATACCCTAGCAATAACAGTAGCTGAA---
GGAACAGATAGAATTATAGAAATAGTACAAAGAACTTGGAGAGCT----------------------------
--------ATCTGCAACATA------CCTAGAAGAATAAGACAAGGCTTTGAGGCAGCTTTGCAA---TAG
>KP411836
GTG---ATG---
GGGGGAATGTGGGTCACAGTCTATTATGGGGTACCTGTGTGG---AAGGAAGCAAAA---
ACTACTCTATTTTGTGCATCAGATGCTAATGCACAGGAGAAAGAA---GTGCATAAT---------------
--------------GTCTGGGCCACA---CATGCCTGTGTTCCCACAGAC---
CCCGACCCAAAAGAAGTAAGGTTGGAA---------AATGTAACAGAAAATTTTAAC---------------
--------------------------ATGTGGAAAAATCACATGGTGGATCAGATGCATGAGGATATA---
ATCAGTTTATGGGATCAAAGTCTA---AAGCCATGTGTAAAGTTG---
ACCCCACTCTGGGTCACTTTAAACTGCAGTGATGTTACTGCCAATGCTACTGTCAATAAT------------
----
---GACAGGGCAAAGGAAATGAAA---
AACTGCTCTTTCAATATAACTACAGAAATAAGA--------------------------
GATAAAAAAAGGAGTGAATATGCACTT---TTTTATAAACTTGATATAGTGCCACTTGATGAG---------
----

AATAAGGAGAATAATGGCAGTGAATATAGATTAATAAATTGTAATACCTCAGCTGTAACACAA---
GCCTGTCCAAAGGTCACTTTTGATCCAATTCCTATACATTATTGTGCTCCTGCTGGTTATGCGATTCTAAAGT
GTAAT---AACAGGACATTCAAT------GGAACAGGACCATGCAATAAT----------------------
-GTCAGC---ACAGTACAATGTACACATGGAATTAAACCAGTGGTATCAACT---
CAACTACTGTTAAAATGTAGTCTAGCAGAAGAAGAG-----------
ATAGTAATCAGATCTGAAAATCTGACAAACAAT---------GTCAAAATAATAATAGTACATCTTAAA----
-----------GAACCTGTACCA---------ATTGAGTGTATAAGACCCGGCAATAATACAAGGAAA-----
-AGTATGAGGATA------------GGACCAGGACAAACA---TTC------TATGCAACAGGAGCC------
---------ATAATAGGAAATATA------------AGAAAAGCATATTGT---AACATTAATGAAAGT---
AAGTGGAATGATACTTTACAAAAGGTAGGTAAGAAATTAGAA--------------GAACACTTCCCT----
-----------------------------------AATAAAACAATAAAGTTTGAA------
CCATCCTCAGGAGGGGACCTAGAAATTACA---ACACATAGCTTTAATTGTAGAGGAGAA---
TTTAAGCAGCTCCAGGCAAGAGTCCTGGCTTTAGAAAGATACCTAAGGGATCAACAGCTCCTAGGAATTTGGG
GCTGCTCTGGAAAACTCATCTGCACCACTAATGTGCCTTGGAATCAGAGTTGGAGT----------------
----------------------------AATAGAACTTATGATGAAATTTGG------GAC------
AACATGACATGGATGCAGTGG---GATAAAGAAGTTAGTAATTACACAGACACAATATAC---
AGGTTGCTTGAAGTCTCGCAAAACCAGCAGGAG--------------------------------------
----

AAAAATGAAGAGGATTTATTAGCATTGGACAGCTGGAAAAATCTGTGGAGTTGGTTCACTATATCAAACTGGC
TGTGGTACATAAAAATATTCATAATGATAGTAGGGGGCTTGATAGGTTTAAGAATAATTTTTGCT---
GTGATCTCTATAGTG---------AATAGAGTTAGGCAGGGATACTCACCT------
TTGTCATTGGCTATTAGTCTGCTTGATACCGTAGCAATAATAGTAGCTGAG---
GGAACAGATAGGATTATAGAATTAATACAAAGAATTTGTAGAGCT---------------------------
--------ATCTGCAACATA------CCTACAAGAATAAGACAGGGCTTAGAAGCAGCTTTGCAA---TAA
>MF373159
GTG---AAG--
GGGAACCTGTGGGTCACAGTCTATTATGGGGTACCTGTGTGG---AAAGAAGCAAAC---
CCTCCTCTATTTTGTGCATCAGATGCTAAAGGATATGAAACAGAA---GTGCATAAT---------------
--------------GTCTGGGCTACA---CATGCCTGTGTACCCACAGAC---
CCCAGCCCACAGGAACTAAAATTGGAA---------AATGTAACAGAAAATTTTAAC---------------
-------------------------ATGTGGAAAAATGGCATGGTGGATCAGATGCATCAGGATGTA---
ATCAGTTTATGGGATCAAAGCCTA---AAGCCATGTGTAAAGTTG---
ACCCCACTCTGTGTTACTTTAAACTGTAGTGCTGTTAAGGTTACCAATAATAGTAGCACTGATAATGACACTT
ACACAGCCACA---
--GAAGAAATGAGA---
AATTGTTCTTTCAATATGACCACAGAGCTAAGA--------------------------
GATAAGAGACAGAGGAGATATGCACTC---TTTTATAAACTTGATATAGTACAACTTAATAATAATGATTCT-
----

GGTAATGGATATAAAGAATATAGATTAATAAATTGCAATACCTCAACCATAACACAA---
GCCTGTCCAAAGGTCTCTTTTGCTCCAATTCCTATACATTATTGTGCTCCAGCTGGTTATGCGATTCTAAAAT
GTAAT---GATGAGAAATTCAAT------GGAATAGGACCATGCAATAAA----------------------
-GTCAGT---ACAGTACAATGTACACATGGAATTAAGCCAGTGGTATCAACT---
CAATTCTTGTTAAATGGTAGTTTAGCAAAAGAGGAA-----------
ATAATGATTAGATCTGAAAATCTAACCGACAAT---------GCCAAAATAATAATAGTACAGCTTAAT----
-----------AAATCTGTAGAA---------ATTAATTGTATGAGACCCAGCAATAATACAAGAGAA-----
-AGTGTAAGGATA-----------GGACCAGGACAAATG---TTC------TATGCAACAGGTGAC------
---------ATAATAGGGGATATA-----------AGACAAGCACATTGT---AACATTAGTAAAGTA---
CAGTGGAGAGAAACTTTACAGAAGGTAGCAGGACAATTAAAA--------------AGTCACTTCATA----
------------------------------------AATAAAACAATAAAATTTGAC------
CAATCCTCAGGAGGGGACCTAGAAATTACA---ACACATAGCTTTAATTGTAGAGGAGAA---
TTTAAGCAGCTCCAGACAAGAGTCCTGGCCATAGAAAGATACCTAAAGGATCAGCAGCTCCTAGGGATTTGGG
GCTGCTCTGGAAAACTCATCTGCACCACTAATGTACCTTGGAACTCTAGTTGGAGT----------------
----------------------------AATAAATCTCACGAGGAGATTTGG------AAT------
AACATGACCTGGGTACAGTGG---GAAAGAGAAATAGATAAATACACAGAAACAATATAT---
CAGTTGCTTGAGATCTCGCAAAACCAGCAGGAG---------------------------------------
----
AAAAATGAACAGGATTTACTAGCATTGGACAAATGGAATAATCTGTGGAATTGGTTTGGCATAACAAAGTGGT
TGGAGTATATAAGAATATTCATAATGGTAGTAGGAGGCTTAATAGGGTTAAGAATAATTTTTGCT---
GTAATTTCTATAGTG---------AATAGAGTTAGGCAGGGATACTCACCA------
TTGTCGTTTGCTATTAGTCTGCTTGATACCACAGCAATAGCAGTAGCTGAA---
GGAACAGATAGGATTATAGAAGTAGTGCTAGCATTTGGAAGAGCT---------------------------
--------ATCTACAACATA------CCTAGAAGAATAAGACAGGGCTTTGAAGCAGCTTTGCAA---TAA
>MF373175
GTG---ACG--
GGAAACTTGTGGGTCACAGTCTATTATGGGGTACCTGTGTGG---AAAGAACCAAGC---
ACTACTCTATTTTGTGCATCAAATGCTAAAGGATATGACACAAAA---TTACATAAT---------------
--------------GTCTGGGCTACA---CATGCCTGTGTACCCACAGAC---
CCCAGCCCACAAGAAATGGTTTTGGAA---------AATGTAACAGAAAATTTAATC---------------
-------------------------ATGTGGAAAAATGATATGGTGGAGCAGATGCATCAGGATATA---
ATCAGTTTATGGGATCAAAGCCTA---AAGCCATGTGTAAAGTTG---
ACCCCACTCTGTGTTACTCTAGACTGT---AAGTATAATATTACTACTACCAGTGATAGTAAT---------
----
-------------------------------------AAGAACTATAATAATACAGATATGAGA---
AATTGTTCTTTCAATATAACCACAGATCTAAGA--------------------------
TATAAGAAAGAGAACACATATGCTCTT---TTTTATAATCGTGATATAGAAAAAGGGGGTGAT---------
----

GGTAATGATTCTTATATATTAACATGTTGTATCACCTCCGCTATAACAAAA---
GCCTGTCCAAAGGTCAATTTTGACCCAATTCTTATACATTATTGTGCTCCAGCTGGTTATGCGATTTTAAAGT
GTAGG---GATGCAAACTTCAGT------GGGAAAGGATTATGCAGGAAT-----------------------
-GTCAGC---ATAGTACAATGTACACAAGGAATTAAACCAGTAGTGTCAAAT---
CAACTGTTGTTAAATGGCAGCACAGCAGAGGAGGGG-----------
ATAATAATTAGATATGAAAATCCGACAAACAAT---------GTCAAAACAATAATAGTACAGATTAAG----
-----------GAACCTGTAAAC---------ATTACCTGTATGAGACCCAACAATAATACAAGAAAA-----
-AGTGTAAGAATA------------GGAGCAGGACAAACA---ATT------TATGCAACAGGAGAT------
---------ATAATAGGAGATATA------------AGACAAGCACATTGT---AACATTAGTAAAAAA---
ATGAGGAATCAAACTTTATTTGAGGTAAAGAAAAAGTTAGAA---------------GAACCGAACATT----
-------------------------------AAGAATAAAACAATACAATTGCAC------
CACTCCTCAGGAGGGGACCTTGGAATTCCA---ACCCATAGCTTTAATTGTAGAGGGGAA---
TTTAAACAGCTCCAGACAAGAGTCCTGGCTATAGAAAGATACCTAAGGGATCAACGGCTCCTAGGGATTTGGG
GCTGCTCTGGAAAAGTCATCTGCACCACTGCTGTGCCTTGGAACTCTAGTTGGAGT-----------------
---------------------------AACAAATCTCAAGAGGAAATTTGG------AAT------
AACATGACCTGGATGCAGTGG---GAGAAAGAAATTGATAATTATACAGAAATAATATAT---
GGGTTGCTTGAAATATCGCAAAACCAGCAGGAG--

AAAAATGAAAAAGATTTGCTAGAATTGGACAAATGGCAGAACTTGTGGAGTTGGTTTGATATAACACATTGGT
TGTGGTATATAAGAATGTTCATCATGATAGTAGGAGGCTTGATAGGTTTAAGAATAATTTTTGCT---
GTGCTTTCTATAGTG---------AATAGAGTTAGGCAGGGATACTCACCA------
TTATCGTTTGCTATTAGTCTGCTTAATACCACAGCAATAGTAGTAGCAGAG---
GGAACAGATAGGATTATAGAATTAGCACAAAGAATTTGGAGAACT----------------------------
--------ATCTGTAACATA------CCTAGAAGAATAAGACAGGGCTTTGAAGCAGCTTTGCAA---TAA
>MF373179
GTG---ATG---
GGGAACTTGTGGGTCACAGTCTATTATGGGGTACCTGTGTGG---AAAGAAGCAAAT---
CCTACTCTATTTTGTGCATCAGATGCTAAAGGATATGAGAGAGAA---GTGCATAAT----------------
--------------GTCTGGGCTACA---CATGCCTGTGTACCCACAGAC---
CCCAACCCACAAGAGCTATTTTTGGAA---------AATATAACAGAAAATTTTAAC----------------
-------------------------ATGTGGAAAAATGAAATGGTGGAGCAGATGCATCAGGATATA---
ATCAGTTTATGGGATCAAAGCCTA---AAGCCATGTGTAAAGTTG---
ACCCCACTCTGTGTTACTCTAAGATGTAGCAACGTTACCTACAAT----------------------------
----
---GAAACCATAAGAGAAATGACA---
AATTGCTCTTTCAATGTAACCACAGAGCTAAGA-------------------------
GATAGGAGACAGGAAGTATATGCACTT---TTTTATAAACTTGATGTAGTATCACTTAATAAA----------
----

AGTGGTGATTATAGATTAATAAATTGTAATACCTCAACCATAACACAA---
GCCTGTCCAAAGGTCTCTTTTGACCCAATTCCTATACATTATTGTGCTCCCGCTGGTTATGCGATTCTAAAGT
GTAAT---GATGAGGAATTTAAT------GGAACAGGACCATGCGGTAAT-----------------------
-GTCAGC---ACAGTACAATGTTCACATGGAATTAAGCCAGTGGTATCAACT---
CAACTATTGTTAAATGGGAGTTTAGCAGAA---AAT-----------
GTGACAATTAGATCTGAAAATCTGACAAACAAT---------GCCAAAACAATAATAGTACAGCTTAGG----
-----------GAGCCTGTATAC---------ATTAATTGTACGAGACCCAACAATAATACAAGAAAA-----
-AGTATAAGGATA------------GGACCAGGACAAGCA---TTG------TATGCAACAGGAGAA------
---------GTAATAGGAGATATA------------AGACATGCACATTGT---AACATCAGTAAAGAA---
AAGTGGAAGACAGCGTTAACAGGGGTAAGAGAAAGATTAAGA---------------GAGCTCTTT-------
-----------------------------------AATAGAACAATACTATTTAAC------
AAGTCATCAGGAGGGGACCTAGAAGTTACA---ACACATAGCTCTAATTGTAGAGGAGAG---
TTTAAGCAGCTCCAGACAAGAGTCCTGGCTCTAGAAAGATACCTAAGGGATCAACAGCTCCTAGGGATTTGGG
GCTGCTCTGGAAAACTCATCTGCACCACTAATGTGCCTTGGAACGGTAGTTGGAGT-----------------
---------------------------AATAGATCTCAACAGGAAATTTGG------GAG------
AACATGACCTGGATGCAATGG---GAAAGAGAAATAGATAATTACACAAACATAATATAT---
GAGTTGCTTGAAGTCTCGCAAAACCAGCAGGAA--

ACAAATGAAAGAGACCTACTAGCTTTGGACAAATGGAACAATCTGTGGAGTTGGTTTAACATATCAAATTGGC
TGTGGTATATAAGACTATTCATAATGATAGTAGGAGGCTTGATAGGTTTAAGAATAATTTTTGCT---
GTGCTTTCTATAGTG---------AATAGAGTTAGGCAGGGATACTCACCA------

TTATCGTTTGCTACTAATATGCTTGATACCACAGCAATAGCAGTAGATGAA---
GTAACAGATAGCATAATAGGATTCGCACAAAGAATTTGGAGAGGT----------------------------
--------ATATGCAACATC------CGTGCAAGGATAAGACAGGGCTATGAAGCAGGGGGGCAA---TAG
>MF373192
GTG---ATG--
GGAAGCTTGTGGGTCACAGTCTATTATGGGGTACCTGTGTGG---AAAGAAGCAAAT---
CCTACTCTATTTTGTGCATCAGATGCTAAAGCATATGAGACAGAA---GTGCATAAT----------------
--------------GTCTGGGCTACA---CATGCCTGTGTACCCACAGAC---
CCCGACCCACAGGAACTAGTTTTAGAA---------AATGTAACAGAAAATTTTAAT----------------
--------------------------ATGTGGAAAAATGACATGGTGGAACAAATGCATCAGGATATA---
ATTAGTTTATGGGATGAAAGCCTA---AAGCCATGTGTAAAGTTG---
ACCCCACTCTGTGTTACTCTAAACTGTAGTAACTATGTTGCCAACAATAGAACC-------------------
----
-------------------------AGTAATGCCACAGTTGCCCCCAAAGATATCGAAATGAAA---
AATTGTTCTTTCAATACAACCGCAGACCTAAGA---------------------------
GATAAGAGACAAACAGCCCATGCAGTT---TTTTCTACACTAGATATAACACCACTTGAAGCT----------
----

AATGGGAGCAATTATAGAATAACAAGTTGTAATACCTCAACCATAACACAA---
GCCTGTCCAAAGGTCTCTTTTGACCCAATTCCTATACATTTTTGTGCTCCAGCTGGGTATGCGATTCTAAAGT
GCAAT---AATAAAACATTCAAT------GGAACAGGACCATGCACTAAT-----------------------
-GTCAGC---ACAGTACAGTGTACACATGGAATCAAACCAGTGGTATCTACT---
CAACTATTATTAAATGGTAGTTTAGCAGAAGGAGAT------------
GTGATAATCAGATCTGAAAATCTGACAAACAAT---------GCCAAAACAATAATAGTACAGCTTAAT----
-----------GAAACTGTACAA---------ATTAATTGTACAAGACCCAACAATAATACAAGAAAA-----
-AGTATAGGGATA------------GGACCAGGACAAACA---TTC------TATGCAGCAGGAGCC------
---------ATAATAGGAGATATA------------AGACAGGCACATTGT---AACATTAGTGGAGAA---
CAATGGAATAAAACTTTAGAAAGAGTAATAGGAAAATTAAAA---------------GAGCACTTTTCC----
-----------------------------------AATAAAACAATACTGTTTAAT------
CAATCCTCAGGAGGGGACCCAGAAATTACA---ACACATAGCTTTAATTGTAGAGGAGAA---
TTTAAGCAGCTCCAGGCAAGAGTCCTGGCTCTGGAAAGATACCTAAGGGATCAACAACTCCTAGGGATTTGGG
GCTGCTCTGGAAAACTCATCTGCACCACTGCTGTGCCTTGGAATTCTAGTTGGAGT-----------------
----------------------------AATAGATCTCAAGATGAGATTTGG------CAA------
AACATGACATGGATGCAATGG---GAGAGAGAAATTGACAATTATACAGACACAATATAC---
AGGTTGCTTGAAGTCTCGCAAACCCAGCAGGAA--

ACAAATGAAAAGGATTTATTAGCTCTTGATAAATGGCAGAATCTGTGGAATTGGTTTGGCATAACAAAATGGC
TGTGGTATATAAAAATATTCATAATGGTAGTAGGAGGCTTGATAGGTTTAAGAATAATTTTTGCT---
GTGCTTTCTATAATA---------AATAGAGTTAGGAAGGATACTCACCA------
TTATCGTTTGCTATTAATCTGTTTGATACCGCAGCAATAGCAGTAGGTGCA---
GGAACAGATAGGATAATAGCCTTTGCACAAGCAATTTGGAGAGCT----------------------------
--------ATCCGTCACATA------CCTACAAGGATAAGACAGGGCTTTGAAGCAGCTTTGCAA---TAA
>KP411835
GGG---
AACTTGTGGGTCACAGTCTATTATGGGGTACCTGTATGG---AAAGAAGCCAAC---
CCTACTCTATTTTGTGCATCAGATGCTAAAGCATATAAGACAGAA---ATGCATAAT----------------
--------------GTCTGGGCTACA---CATGCCTGTGTACCTACAGAC---
CCTAGCCCACAGGAAATAGTTTTGAAA---------AATGTATCAGAAAATTTTAAT----------------
--------------------------ATGTGGAAAAATGGCATGGTGGAACAGATGCATCAGGATATA---
ATCAGTTTATGGGATCAAAGTCTA---AAGCCATGTGTAAAGTTG---
ACCCCACTCTGTGTTACCTTAGACTGTAGTAATAATATTACTAATAATGTTACTACTAACAGT----------
----
----------------------AATGTTATTATCCCCTACAATGAGACAATAGGAGACATGAAA---
AATTGTTCTTTCACTACAACCACAGTGATAAGA---------------------------
GATAAGAAGCAGACAGCATATGCATTT---TATTATAGGCTTGATATAGTCCCCATTAAGGAT----------
----

AAGAATGAGAGTAATAGTGAATATAGATTAATAAATTGTAATATCTCAACCATGACACAA---
GCTTGTCCAAAGGTCTCTTTTGAGCCAATTCCTATACATTATTGTGCTCCAGCTGGTTATGCGATTCTAAAGT
GTAAT---AATAAGACATTCAAT------GGAACAGGACTATGCAATAAT-----------------------

-GTCAGC---ACAGTACAGTGTACACATGGAATTAAACCAGTGGTATCAACT---
CAGCTATTGTTAAATGGGAGTATAGCAGAAAGTGAC------------
ATAATAATTAGATCTGAGAATCTGACAAACAAT---------GCCAAAATAATAATAGTACAGCTTAAT----
-----------GAATCTGTAGAA---------ATTAATTGTACAAGACCCAACAATAATACAAGAAGA-----
-AGTGTAAGGCTA------------GGACCAGGACAAACA---TTC------TATGCAACAGGAGAC------
---------ATAATAGGAGATATA------------AGACAAGCACATTGT---AACATTAGTAGAGAC---
AAATGGAATACAACTTTAAACCAGGTAAGGGGAAAATTAAAA---------------GAGCACTTT-------
-----------------------------------AATAAAACAATAACATTTGAA------
CCTTCCTCAGGAGGGGACCTAGAAATTACA---ACACATAGCTTTAATTGTGGAGGAGAA---
TTTAAGCAGCTCCAGACAAGAGTCCTGGCTATAGAAAGATACCTAAGAGATCAACAGCTCCTAGGGATTTGGG
GCTGCTCTGGAAAACTCATCTGCCCCACTGCTGTGCCTTGGAACAGTAGTTGGAGT-----------------
----------------------------AATAAGTCTCAAGATGAAATTTGG------AAG------
AACATGACCTGGATGCAGTGG---GATAAAGAAATTGATAATTATACATCTGTTAAATGC---
CAGTTTAGCGGAAAGTGCCATAATAATCAGGTC--
--------------------------AGAAAAATACACAAACAATTACCAAATAAC---
AATAGGACAAATCTGTGGAATTGGTTTGGCATATCAAATTGGCTGTGGTATATAAAAATATTCATAATGATAG
TAGGAGGCTTAATAGGTTTAAGAATAATTTTTGCT---GTGCTTTCTATAGTG---------
AACAAAGTTAGGCAGGGATACTCACCA------
TTGTCGTTTGTTATTATTTTGTTTAATACCCCACCAATAGTAGTAGTGAAA---
GAACCAGATAGGTTTATGAAATTATTCCAAAGAGTTGGAAGAGTT----------------------------
--------ATTTCCAACATC------CTTAGAGAATTAAGCCAGGGTTTGGAAATAGTTTTCCAA---TAA
>MF373198
GTG---ATG--
GGGAACTTGTGGGTTACAGTCTATTATGGGGTACCTGTGTGG---AGAGAAGCAAAT---
CCTCCTCTATTTTGTGCATCAGATGCTAAGGCATATGAGACAGAA---AAACATAAT----------------
--------------GTCTGGGCTACA---CATGCCTGTGTACCCACAGAC---
CCCAAACCACAGGAATTAGTTTTGGAA---------AATGTAACAGAAAATTTTAAC----------------
--------------------------ATGTGGAAAAATGGCATGGTGGATCAGATGCATCAGGATATA---
ATCAGTTTATGGGATCAAAGCCTA---CAACCATGTGTAAAGTTG---
ACCCCACTCTGTGTCACTTTAAACTGTACTAATGATATTAGTAATGATATTAGTAAT----------------
----
----------------------------GGTACGTCAATAATAAGGTACCACGGAGACATGAAA---
AATTGCTCTTTCAATATAACCACAGAACTAAGA---------------------------
GATAAGCAAAAGAAAGAATATGCACTT---TTTTATAAACTTGATATAGTATCACTTGGGAATAACAAT----
----

GGTAATAACAATAAGACTGTGGAGGAGTATCGATTAATAAACTGTAATGCCTCAACCATAAAACAA---
GCCTGTCCAAAGGTCTCTTTTGACCCAATTCCTTTACATTATTGTGCTCCAGCTGGTTATGCGATTCTAAAGT
GTAGG---GATGCAAACTTCACT------GGAAAGGGACCATGCAAAAAT-----------------------
-GTCAGC---ACAGTACAATGTACGCATGGAATTAAACCAGTGGTATCAACC---
CAACTATTGTTAAATGGTAGTGTAGCAGAAAATGAG------------
ACAATAATTAGATCTGAGAATCTGACAAACAAT---------GCCAAAATAATAATAGTACAGCTTAAG----
-----------CAACCTGTACAA---------ATTAACTGTACAAGACCCAACAATAATACAAGAAAA-----
-GGCATAGGACTA------------GGAGCAGGACACGCA---TTC------TATGCAACAGATAGG------
---------ATATTAGGAAATATC------------AGACAAGCACATTGT---AACATTAGTGGACAA---
GACTGGAATAAAACTTTAGATGATGTAAAAACACAATTACAA---------------AGACACTTCCAG----
-----------------------------------AATAAAACAATAGCATTTCAA------
CCAGCCTCAGGAGGGGACCTAGAAATTACA---ACACATAGTTTTAACTGTAGAGGAGAA---
TTTAAGCAGCTCCAGACAAGAGTCCTGGCTATAGAAAGATACCTACAGGATCAACAGCTCCTAGGGATTTGGG
GTTGCTCTGGAAAACTCATCTGCACCACTAATGTGCCTTGGAACTCTAGTTGGAGT-----------------
----------------------------AATAAATCTGAAGAGGAGATTTGG------GGT------
AACATGACCTGGATGCAGTGG---GATAGAGAAATTAATAATTATACAGGATATATATAC---
CAGTTACTTGAAGTCTCGCAACACCAGCAGGAG--

AAAAATGAAAAAGATTTACTAGCATTGGACAAATGGCAAGACCTGTGGAGTTGGTTTAGCATAACAAAATGGT
TGTGGTATATAAAAATATTCATAATGATAATAGGAGGCTTGATAGGTTTAAAAATAATTTTTGCT---
GTGCTTTCTATAGTG---------AATAGAGTTAGGCAGGGATACTCACCA------
TTGTCGTTCGCTATTAATCTGCTTGATACCACAGCAATAGCAGCAGCTGAA---
GGAACAGATAGGATAATAGCCGTAGCAAGAAGAATTTGGAGAACT----------------------------
--------TTCTGTAACATA------CCTACAAGAATAAGACAGGGCATAGAAGCAGCTTTGCTA---TAA

>HM215318
GGG---GTG--
GGAAACTTGTGGGTCACAGTTTATTATGGGGTACCTGTGTGG---AGGGAAGCAAAA---
ACTACTCTATTCTGTGCATCAGATGCTAAAGCATATGATAAGGAA---GTGCATAAT----------------
--------------GTCTGGGCTACA---CATGCCTGTGTTCCCACAGAC---
CCCAACCCACAAGAAATGGTTATGGAA---------AATGTAACAGAAAATTTTAAC----------------
--------------------------ATGTGGAAAAATGACATGGTGGATCAGATGCATGAGGATATA---
ATCAGTTTATGGGATCAAAGCCTA---AAGCCATGTGTAAAGTTG---
ACCCCACTCTGTGTTACCTTACGCTGTACTAATGTTACCAGGAATGATAATATT-------------------
----
-------------------------------ACCCACAATGATACCGCGGATGCAGAAATGAAA---
GATTGCTCTTTCAATGTAACCACAGAAATAAGG---------------------------
GATAAAACAAAGAAAGTGCGTTCACTT---TTTTATAGAGTAGATTTAGTATCACTTGATAATAGTAATAGT-
----

AATGATAATAGTAATAGTAGTGAATATATATTAATAAATTGTAATACCTCAACCATAACACAA---
GCCTGTCCAAAGGTCACTTTTGACCCAATTCCTATACATTATTGTGCTCCAGCTGGGTATGCGATTCTAAAGT
GCAAC---AATAAGACATTCAAT------GGGACAGGACCATGCAATAAT-----------------------
-GTCAGC---ACAGTACAATGTACACATGGAATTAAGCCAGTGGTATCAACT---
CAACTACTGTTAAATGGTAGTCTAGCAGAAAGAGAG------------
ATAATAATTAGATCTGAAAATCTGACCAACAAT---------GCCAAAACAATAATAGTACAGTTTAAT----
-----------CAATCTGTAGAA---------ATTGTGTGTATAAGACCTGCCAATAATACAAGGAAG-----
-AGTACCAGAATA------------GGACCAGGACAAGTA---TTC------TATACAAATGAT---------
---------ATAATAGGAGATATA------------AGACGAGCACATTGT---AACATTAGTAGAGGG---
AATTGGAGTAAAACTTTACAAGAGGTAGGTAAGAAATTAAAA---------------GAATATTTC-------
-----------------------------------CCTAAAAATATAACATTTGCA------
CCACACTCAGGAGGGGACCTAGAAATTACA---ACACATAGCTTTAATTGTGGAGGAGAA---
TTTAAGCAGCTCCAGGCAAGAGTCCTGGCTATAGAAAGATACCTAAAGGATCAACAGCTCCTAGGGATTTGGG
GCTGCTCTGGAAAACACATCTGCACCACTGCTGTGCCTTGGAACACCACTTGGAGT-----------------
----------------------------AATAAGACTGAAGAAGAAATTTGG------GGT------
AACATGACCTGGATGCAGTGG---GATAGAGAAATTAGTAATCACACACACACAATCTAC---
AGGTTGCTTAAAGATTCGCAAACCCAGCAAGAA--

CAAAATGAGAAAGATTTATTAGCATTGGACAGTTGGCAAAGTCTGTGGAATTGGTTTAGCATAACAAAATGGC
TGTGGTATATAAAAATATTCATAATGATAGTAGGAGGCTTGATAGGGTTAAGAATAATTTTTGCT---
ATGCTATCTATAGTA---------AATAGAGTTAGGCAGGGATACTCACCT------
TTGTCATTTGCTATTAGTCTATTTGATACCATAGCAATAGCAGTAGCTGAA---
GGAACAGATAGGATTATAGAAGTAATACAAAGAATTTGTAGAGCT----------------------------
--------ATCTACAACATA------CCTAGAAGAATAAGACAGGGCTTTGAAGCAGCTTTGCTA---TAA
>KX907415
GGG---ATG---GGG------------------------------------
AGGGACTTGTGGGTCACAGTCTATTATGGGGTACCTGTGTGG---AAAGAAGCAAAG---
ACTACTCTATTCTGTGCATCAGATGCTAAAGCATATGAGAGAGAA---GTGCATAAT----------------
--------------GTCTGGGCTACA---CATGCCTGTGTACCCACAGAC---
CCCAACCCACAAGAAATGGTTTTGAAA---------AATGTAACAGAAAATTTT-------------------
--------------------------AGGTGGGAAAATGACATGGTAGATCAGATGCATGAAAATATA---
ATCGATTTATGGGATCAAAGCCTA---AAGCCATGTGTAAAGTTG---
ACTCCACTCTGTGTCACTTTAAATTGTAGCAATGTTAATAGCAATGTTAATGTTACCAGTAATGGTACTGCTA
GCCAAGGTAAT--
----------------------------GCTACCTACACTAATAGAATGGAGGAAGAAATAAAA---
AACTGCTCTTTCAATATAACCACAGAAATAAGA---------------------------
GGTAAGAAACAGAAAGTACATGCACTT---TTTTATAAACTTGATGTAGTACCACTTAATGGGGAT-------
----

AGTAGCAACTCTAGTGAGTATAGATTAATAAATTGTAATACCTCAGCCATAACACAA---
GCCTGTCCAAAGGTCTCTTTTGACCCAATTCCTATACATTTTTGTGCTCCAGCTGGTTATGCGATTCTAAAGT
GTAAT---GATAAGACATTCAAT------GGAACAGGACCATGCAATAAT-----------------------
-GTCAGC---ACAGTACAATGTACACATGGAATTAAGCCAGTGATATCAACT---
CAACTACTGTTGAATGGTAGCCTAGCAGAAGGAGAG------------
ATAATAATTAGATCTAAAAATCTGACAGACAAT---------GTCCAAACAATAACGGTACATCTTAAT----

-----------GAATCTATAAGC---------ATTAATTGTACAAGACCCAATAATAATACAAGAAAA-----
-AGTATAAGGATA------------GGTCCAGGACAAATA---TTC------TATGCAACAGGAGAC------
---------ATAATAGGAGACATA------------AGAGAGGCACATTGT---AACATTAGTGCAAGC---
CAATGGAATGCAGCTTTAAAAGGTGTAAAGGCAAAATTAAAA---------------GAACACTTCTCT----
-----------------------------------AATAAAACAATAAACTTTACA------
ACATCCTCAGGAGGGGACCTAGAAATTACA---ACACATAGCTTCAATTGTAGGGGAGAA---
TTTAAGCAGCTCCAAGCAAGAGTCCTGGCTATAGAAAGATACCTAAAGGATCAACAGCTCCTAGGGCTTTGGG
GCTGCTCTGGAAAACTCATCTGCACCACTGCTGTACATTGGAACGACAGTTGGAGT----------------
----------------------------AATAAATCTGTAATAGATATTTGG------GAT------
AACATGACCTGGATGCAGTGG---GATAAAGAAATTAGGAATTACACAAACACAATATAC---
AGGTTGCTTGAAGACTCGCAAACCCAGCAGGAG---------------------------------------
----
AAAAATGAAAAAGATTTATTAGCATTGGACAGTTGGAAAAGTTTGTGGAATTGGTTTGCCATATCAAAGTGGC
TGTGGTATGTAAAAATATTCATAATAATAATAGGAAGCTTGATAGGTTTAAGAATAATTTTTGCT---
ATGCTCTCTATAGTG---------AATAGAGTTAGGCAGGGATACTCACCT------
TTGTCGTTTGCTGTTAGTCTGTTTGATACCATAGCAATAGCAGTAGCTGAA---
GGAACAGATAGGATTATAGAGTTAATACAAAGATTTTGTAGAGCT---------------------------
--------ATCCGCCACATA------CCTACAAGAATAAGACAAGGCTTTGAAGCAGCTTTGCAA---TAA
>KX907398
GGG--
AACTTGTAGGTCACAGTCTATTATGGTGTACCTGTGTGG---AAAGAAGCAAAA---
ACTACTCTATTCTGTGCATCAGATGCTAAAGGATATGAGAAAGAA---GTGCATAAT---------------
--------------GTCTGGGCTACA---CATGCCTGTGTACCCACAGAC---
CCCAACCCACAAGAAATAGTTATGGGA---------AATGTAACAGAAAATTTTAAC---------------
--------------------------ATGTGGAAAAATGACATGGTGGATCAGATGCATGAAGATATA---
ATCAGTTTATGGGATCAAAGCCTA---AAGCCATGTGTAAAGTTG---
ACTCCACTCTGTGTCACTTTAAATTGTAGTAATATTAATAATACTAGTAGTGATAGTAATGGA---------
----
----------------------CAGGATACCTACAATGATAGCCTGGGGAGGGAAGAAATGAAA---
AAATGCTCTTTCAATATAACCACAGAAGTAAGA--------------------------
GACAAGAAAAAGAGCGTGTATGCACCT---TTTTATAAACTTGATATAGTACCACTTAATGAG---------
----

AGTAACAGCAACTCTAGTGAGTATAGATTAATAAATTGTAATACCTCAGTCATAACACAA---
GCCTGTCCAAAGGTCTCTTTTGACCCAATTCCTATACATTATTGTGCTCCAGCTGGTTATGCGATTCTAAAGT
GTAAT---AATAAGACATTCAAT------GGGACAGGACCATGCCATAAT----------------------
-GTCAGC---ACAGTACAATGTACACATGGAATTAAGCCAGTGATATCAACT---
CAACTACTGTTAAATGGTAGCCTATCAGAAGAAGGG-----------
ATAATAATTAGATCTGAAAATCTGGCAAATAAC---------GTCAAGACAATAATAGTACATCTTAAT----
-----------AAATCTATAGAA---------ATTGTGTGTACAAGACCCAACAATAATACAAGAAAA-----
-AGTATAAGGATA------------GGACCAGGACAAACA---TTC------TTTGCAACAGGTGAT------
---------ATAATAGGAGACATA------------AGACTAGCATATTGT---AACATTAATAGAAAA---
AAATGGAATGAAACTTTACACAAGGTAGAAGAAAAATTAAAA---------------GAACACTTCAGG----
-----------------------------------GGTAAAACTATAAGATTTCTA------
CCACACTCAGGAGGGGACCTAGAAATTACA---ACACATAGCTTTAATTGTAGGGGAGAA---
TTTAAGCAGCTCCAGACAAGAGTCCTGGCTATAGAAAGATACCTAAAGGATCAACAGCTCCTAGGGATTTGGG
GCTGCTCTGGAAAACTCATCTGCACCACTAATGTGCCTTGGAACTCCAGCTGGAGT----------------
----------------------------AATAAATCTGTAGGAGATATTTGG------GAT------
AATATGACCTGGATGCAGTGG---GATAAAGAAATTAGTAATTACACAGACACAATATAC---
GGTTTGCTTGAAGAATCACAAAACCAGCAGGAA---------------------------------------
----
AGAAATGAACAAGATTTATTGGCATTGGACAGTTGGAAAAATCTATGGAATTGGTTTGACATAACAAATTGGC
TATGGTATATAAAAATATTCATAATGATAGTAGGAGGCTTGATAGGTTTAAGAATAATTTTTGCT---
ATACTTTCTATAGTG---------AATAGAGTTAGGCAGGGATACTCACCT------
TTGTCGTTTGCTGTCAGCTTGCTTGATACCATAGCAATAACAGTAGCTGAA---
GGAACAGATAGGATTATAGAAATAATACAAAGAATTTGTAGAGCC---------------------------
--------ATCTGCAACGTA------CCTAGAAGAATAAGACAGGGCTTTGAAGCAGCTTTGCAA---TAA
>KX907425
GTG---GTG---
GGGAACTTGTGGGTCACAGTCTATTATGGGGTACCTGTGTGG---AAAGAAGCAAAG---

ACTACTCTATTCTGTGCATCAGATGCTAAAGCATATGAGAGAGAA---GTGCATAAT----------------
--------------GTCTGGGCTACA---CATGCCTGTGTACCCACAGAC---
CCCAACCCACAAGAAATAGTTTTGGAA---------AATGTAACAGAAAATTTT-------------------
-------------------------AGGTGGGAAAATGACATGGTAGATCAGATGCATGAAAATATA---
ATCAGTTTATGGGATCAAAGCTTA---AAGCCATGTGTAAAGTTG---
ACTCCACTCTGTGTCACTTTAAATTGTAGCAATGTTACCCGTGATGAT------------------------
----
---GGCATGGAGAGAGAAATAAAA---
AACTGCTCTTTCAATATAACCACAGAAATAAGA--------------------------
GATAAGAAACAGAAAGTGTATGCACTT---TTTTATAAACTTGATGTAGTACCACTTAATGGAGAT-------
----

AATAGCAACTCTAGTGAGTATAGATTCATAAATTGTAATACCTCAGCCATAACACAA---
GCCTGTCCAAAGGTCTCTTTTAACCCAATTCCTCTACATTTTTGTGCTCCAGCTGGTTATGCGATTCTAAAGT
GTAAT---AATAAGACATTCAAT------GGAACAGGACCATGCAGTAAT----------------------
-GTCAGC---ACAGTACAATGTACACATGGAATTAAGCCAGTGATATCAACT---
CAGCTACTTTTAAATGGTAGCCTAGCAGAG---GAG-----------
ATAATAATTAGATCTAAAAATCTGACAGACAAT---------GCCCAAACAATAACAGTACATCTTAAG----
-----------GAATCTATAAGC---------ATTATTTGTACAAGACCCAATAATAATACAAGAAAA-----
-AGTATAAGGATA------------GGTCCAGGACAAACA---TTC------TATGCAACAGGAGAC------
---------ATAATAGGAGACATA------------AGAGAGGCACATTGT---AACATTAGTACAAAC---
GAATGGAATACAACTTTAGAAAGTGTAACGGCAAAACTAGAA---------------AAACACTTCCCT----
-----------------------------------AATAAAACAATAAAATTTCAA------
CCATCCTCAGGAGGGGACCTAGAGATTACA---ACACATAGCTTTAATTGTAGGGGAGAA---
TTTAAGCAGCTCCAGGCAAGAGTCTTGGCTATAGAAAGATACCTAAAGGATCAACAGCTCCTAGGGCTTTGGG
GCTGCTCTGGAAAACTCGTCTGCACCTCTGCTGTGCCTTGGAACGAAAGTTGGAGT-----------------
---------------------------AATAAATCTGTAACAGATATTTGG------GGT------
AACATGACCTGGATGCAGTGG---GATAAAGAAATTAGTAATTACACACACACAATATAC---
AGGTTGCTTGAAGACTCGCAAACCCGGCAGGAG---------------------------------------
----
AAAAATGAAAAAGATTTATTAGCATTGGACAGTTGGAAAAATCTGTGGAATTGGTTTAGCATATCAAATTGGC
TGTGGTATATAAAAATATTCATAATAATAGTAGGAAGCTTAATAGGTTTAAGAATAATTTTCGCT---
ATGCTCTCTATAGTG---------AATAGAGTTAGGCAGGGATACTCACCT------
TTGTCGTTTGCTGTTAATCGGTTTGATACCATAGCAATAGCAGTAGCTGAA---
GGAACAGATAGGATCATAGAATTAATACAAAGAAGTTGTAGAGCT----------------------------
--------ATCTGCAACATA------CCTACAAGAATAAGACAAGGCTTTGAAGCAGCTTTGCAA---TAA
>KX907428
GTG---TTG---
GGGAACTTGTGGGTCACAGTCTATTATGGGGTACCTGTGTGG---AAAGAAGCAAAG---
ACTACTCTATTCTGTGCATCAGATGCTAAAGGATATGAAAAAGAA---GTACATAAT----------------
--------------GTCTGGGCTACA---CATGCCTGTGTACCCACAGAC---
CCCAACCCACAAGAAATGGTTTTAGAA---------AATGTAACAGAAAATTTT-------------------
-------------------------AGGTGGGAAAATGACATGGTAGATCAGATGCATGAAAATATA---
ATTAATTTATGGGATCAAAGCCTA---AAGCCATGTGTAAAGTTG---
ACTCCACTCTGTGTCACTTTAAATTGTAACAATGTTAATGTTACCAGTAATGGTACTGCTAACCATGGTAATG
GTACTGCTAGC---
-------------------TATGGTAATGATACCTACACTAAGAGCATGGAGGGAGAAATAAAA---
AATTGCTCTTTTAACATAACCACAGAAATAAGA--------------------------
GACAAAAAACGAAAAGTGCATGCACTC---TTTTATAAACTTGATGTAATACCACTTAATGGGGAG-------
----

AATTCTAGTGAGTATAGATTCATAAATTGTAATACTTCAGCCATAACACAA---
GCTTGTCCAAAGGTCTCTTTTGAACCAATTCCTATACATTTTTGTGCTCCAGCTGGTTATGCGATTCTAAAGT
GTAAT---AATAAGACATTTAAT------GGAACAGGACCATGCAATAAT----------------------
-GTCAGC---ACAGTACAATGTACACATGGAATTAAGCCAGTGGTATCAACC---
CAACTACTGTTGAATGGTAGCCTAGCAGAAGGAGAG-----------
ATAATAATTAGGACTAAAAATCTGACAGACAAT---------GCTCAAACAATAACAGTACATCTTAAT----
-----------AAATCTATAAGC---------ATTAATTGTACAAGACCCAATAATAATACAAGACGA-----
-AGTATAAGGATA------------GGTCCAGGACAAACA---TTC------TATGCAACAGGAGAC------
---------ATAATAGGAGACATA------------AGACAGGCACATTGT---AACATTAGCATGAAA---

GAATGGAATGCAACTTTAGAAAGTGTAAAGGCAAAACTAAAC---------------GAATACTTCCAT----
-----------------------------------GGTAACACAATAGAATTTAGA------
GAACACTCAGGAGGGGACATAGAAATTACA---ACACATAGCTTTAATTGTATGGGAGAA---
TTTAAGCAGCTCCAGGCAAGAGTCTTGGCTATAGAAAGATACCTAAAGGATCAACAGCTCCTAGGGCTTTGGG
GCTGCTCTGGAAAACTCATCTGCCCCACCGCTGTGCCTTGGAACAACAGTTGGAGT-----------------
----------------------------AATAAATCTGTAGAAGATATTTGG------GAT------
AACATGACCTGGATGCAGTGG---GATACAGAAATTAGTAATTACACACAGACAATATAC---
AAGTTGCTTGAAGACTCGCAAACCCAGCAGGAG--

AAAAATGAAAAAGATTTATTAGCATTGGACAGTTGGAAAAGTCTGTGGACTTGGTTTGACATATCAAACTGGC
TGTGGTATATAAGAATATTCATAATAATAATAGGAGGCTTGATAAGTTTAAGAATAATTTTTGCT---
GTGCTTTCTATAGTG---------AATAGAGTTAGGCAGGGATACTCACCT------
CTGTCGTTTGCTGTTAGTCTGCTTGATACCATAGCAATAGCAGTAGCTGAA---
GGAACAGATAGGATTATAGGAGTAATACAAAGAGTTGGTAGAGCT----------------------------
--------ATCTGCAACATA------CCTAGAAGAATAAGACAAGGCTTTGAAGCAGCTTTGCAA---TAA
>HQ615976
GGG---
AACTTGTGGGTCACAGTATATTATGGGGTACCTGTGTGG---GAAGAAGCAAAT---
CCTGATCTATTTTGTGCATCAGATGCTAAAGCATATGATACAGAA---GTGCATAAT----------------
--------------GTCTGGCTACA---CATGCCTGTGTACCCACAGAC---
CCCAACCCACAAGAAATGATTTTGGAA---------AATGTAACAGAAAAGTTTGAC----------------
--------------------------ATGTGGAAGAATGATATGGTGGAACAGATGCATCAGGATATA---
ATCAGTTTATGGGATCAAAGTCTA---AAGCCATGTGTAAAGTTG---
ACCCCACTCTGTGTTATTTTAAACTGTATGGATGTTAATATTACCGGCAATAGT-------------------
----
---ACCTGGAGGGAAGAAATAAAA---
AATTGCTCTTTCAATGTAACCACAGAAATATAT---------------------------
GATAAGAAACAGAGAACACATGCACTT---TTTTATAAACTTGATATAGTATCACTCAATGATAATAAG----
----

AGTAGTGATAATAAGAGTAGTGACTATAGATTAATAAATTGTAATACTTCAGCCATAGCACAA---
GCCTGTCCAAAGATCTCTTTTGACCCAATTCCTATACACTATTGTGCTCCAGCGGGTTATGCGATTCTCAAGT
GTAAC---AATAAAACCTTTAAT------GGAACAGGACCATGCAATAAT-----------------------
-GTCAGC---ACAGTACAGTGTACACATGGAATTAAGCCAGTGGTATCAACT---
CAGCTACTGTTAAATGGTAGTTTAGCAGAAGAGGAG------------
ATAATAATTAGATCTGAAAACATATCAGACAAT---------GGCAAAACAATAATAGTACAGCTTAAG----
-----------GAACCTGTAGAA---------ATTGAATGTATAAGACCCAACAATAATACAAGAAAT-----
-AGTATAAGGATA------------GGGCCAGGACAAGCA---TTC------TATGCAACAGGAGAC------
---------ATAATAGGAGATATA------------AGACAGGCACATTGT---AACATTAATAAAACA---
AAATGGAATGAAACTTTACAAGGGGTAGGAGAAAAATTAGCA---------------AAGTACTTCCCT----
-----------------------------------AATAAAACAATACAATTTGCA------
CCATCCTCAGGAGGAGACCTAGAAATTACA---ACACATAGCTTTAATTGTAGAGGAGAA---
TTTAAGCAGCTTCAGGCAAGAGTCCTGGCTATAGAAAGATACCTAAAGGATCAACAGCTCCTAGGGATTTGGG
GCTGCTCTGGAAAACTCATCTGCACCACTTCTGTGCCTTGGAACTCTAGTTGGAGT-----------------
----------------------------AACAAATCTCAGGAGGAAATTTGG------AAT------
AACATGACCTGGATGCAGTGG---GAGAAAGAAATTAGTAATTACACAGACACAATATAC---
AGATTGCTTGAAGACTCGCAAACCCAGCAGGAG--

AAAAATGAAAAGGATTTACTAGCATTGGACAAATGGCAAAATTTATGGAGTTGGTTTAGTATAACAAATTGGC
TGTGGTATATAAAAATATTCATAATGATAGTAGGAGGCTTAATAGGTTTAAGAATAATTTTTGCT---
GTGCTCTCTATAGTG---------AATAGAGTTAGGCAGGGATACTCACCC------
TTATCGTTTGCTATTAATCTGCTGGACATCACAGCAATAGCAACAGCTGAA---
GGAACAGATAGGATTATAGAATTAATACAAAGAATTTGTAGAGCT----------------------------
--------ATCTGCAACATA------CCTAGAAGAATAAGACAGGGTTTAGAAGCAGCTTTGCAA---TAA
>KF526226
GTG---AAG--
GGGAACTTGTGGGTCACAGTCTATTATGGGGTACCTGTGTGG---AAAGAAGCAAAC---
CCTTCTCTATTTTGTGCATCAGATGCTAAAGCATATGAGGCAGAA---GCGCATAAT----------------
--------------GTCTGGCTACA---CATGCCTGTGTACCCACAGAC---
CCCAACCCACAAGAATTGGCTTTGGAA---------AATGTAACAGAAAATTTTAAC----------------

--------------------------ATGTGGAAAAATGGCATGGTGGATCAGATGCATCAGGATATA---
ATCAGTTTATGGGATCAAAGCCTA---AAGCCATGTGTAAAGTTA---
ACCCCACTCTGTGTTACTTTAGACTGT---AACTACAATATCACCAGTAATAGTACTAATACCCACAAT----
----
----------------------GGTACCAATATCCACAATAGTACCACAGAGGGAGATATGAAA---
AATTGTTCTTTCAATGCAACCACAGAAATAAGC---------------------------
GATAAGAAAAGGAAGGAACATGCACTT---TTTTATAAACTTGATATAGTACCACTTAAAAAC----------
----

AATTCTAGTGATTATAAATTAATAAATTGTAATACCTCAGCCATAACACAA---
GCATGTCCAAAGATCTCTTTTGACCCAATTCCTATACATTATTGTGCTCCAGCTGGTTATGCGATTCTCAAGT
GTAAT---GATAAGGCATTCAAT------GGAACAGGACCATGCAAGAAT-----------------------
-GTCAGC---ACAGTACAATGCACACACGGAATTAAACCAGTGGTATCAACT---
CAACTATTGTTAAATGGTAGTATAGCAGAAAACGGG------------
ACAATAATTAGATCTGAAAATCTGACAAACAAT---------GCCAAAACAATAATAGTACAGCTTGAT----
-----------GAACCTGTAGAA---------ATTAACTGTACGAGACCCAACAATAATACAAGAAAA-----
-AGTATAAGGATA------------GGACCAGGACAAACA---TTA------TATGCAACAGGAGAA------
---------ATAATAGGAGATATA------------AGACAAGCACATTGT---AACATTAGTGGAGGA---
AGATGGAATGCAACTTTAGAAAAGATAAAGAAAAAATTAGAA---------------GAGCACTTCCCT----
-----------------------------------AATAAAACAATAAAATTTGCA------
CCATCCTCAGGAGGGGACCCAGAAATTACA---ATGCATAGCTTTAATTGTGGAGGAGAA---
TTTAAGCAGCTCCAGACAAGAGTCCTGGCTATAGAAAGATACCTACAGGATCAACAGCTCCTAGGGATTTGGG
GCTGCTCTGGAAAACTCATCTGCACCACTGCTGTGCCTTGGAACTCTAGTTGGAGT-----------------
----------------------------AATAAGACTCAAGAGGAGATTTGG------AAT------
AACATGACCTGGATGCAGTGG---GAGAGAGAAATTGATAATTATACAGGAACAATATAC---
AGGTTGCTTGAAATCTCGCAAAACCAACAGGAA--
---------------------------A-
AAAAATGAAAAAGATTTACTAGCATTGGACAAATGGCAAAATCTGTGGAATTGGTTTGACATAACACAATGGC
TGTGGTATATAAAAATATTCATAATGATAGTAGAAGGCTTGATAGGTTTAAGAATAATTTTTGCT---
GTGCTCTCTATAGTG---------AATAGAGTTAGGCAGGGATACTCACCA------
TTATCGTTTGCGATTAATCTGCTTAATACCTTAGCAATAGCAGTAGCTGAA---
GGAACAGATAGAATTATAGAATTTCTACAAAGAACTTGGAGAGCT----------------------------
--------ATCTGTAACATA------CCTAGAAGAATAAGACAGGGCTTTGAAGCAGCTTTGCTA---TAA
>AY463229
GTG---ATG--
GGAAACTTGTGGGTCACAGTATATTATGGGGTACCTGTGTGG---AAAGAAGCAAAA---
ACTACTCTATTCTGTGCATCAGATGCTAAAGCATATGAGAGGGAG---GTGCATAAT----------------
--------------GTCTGGGCTACG---CATGCCTGTGTACCCACAAAC---
CCCAACCCACAAGAAATAGTCTTGGGA---------AATGTAACAGAAAATTTTAAC----------------
--------------------------ATGTGGGAAAATGACATGGTGGATCAGATGCATGAGGATATA---
ATCAGTTTATGGGATCAAAGTCTA---AAACCATGTGTAAAGTTG---
ACCCCACTCTGTGTCACTTTAAACTGTACAAAGGCAAATTTTACAAATGTAAATTAT----------------
----
----------------------------ACCAATATAAATGAGACAAATATTGAAGAAATAAGA---
AATTGCACTTTTAATACAACCACAGTAATAAGA---------------------------
GATAAGAAACAGGAAGCGTATGCACTT---TTCTATAGACCTGATATAGTACCACTTAGTAAG----------
----

AATAGCTCTGAGTATATATTAATAAATTGCAATACCTCAACCATAACACAA---
GCCTGTCCAAAGGTCACTTTTGACCCAATTCCTATACATTATTGTGCTCCAGCTGGTTATGCGATTCTAAAGT
GTAAT---AATAAGACATTCAAT------GGGACAGGACCATGCCAGAAT-----------------------
-GTCAGC---ACAGTACAATGTACACATGGAATTAAACCAGTGGTATCAACT---
CAACTACTGTTAAATGGTAGCCTAGCAGAAGGGGAG------------
ATAATAATTAGATCTGAAAATCTGACAAACAAT---------GCCAAAACAATAATAGTACAGCTTAAT----
-----------AAATCCATAGAC---------ATTGTGTGCACAAGACCCGGCAATAATACAAGAAAA-----
-AGTATAAGGATA------------GGACCAGGACAAACA---TTC------TATGCAACAAATGAC------
---------ATAATAGGAAACATA------------AGACAAGCACATTGT---AACATTAGTGCAAAT---
GAATGGAATAGAACTTTACAATGGGTAGGTGAAAAATTGGAA---------------GAACACTTCCCT----
-----------------------------------AATAAAACAATAGAATTTAAA------
CCATCCTCAGGAGGAGATCTAGAAATTACA---ACACATAGTTTTAATTGTAGAGGAGAA---

TTTAAGCAGCTCCAGACAAGAGTCCTGGCTATAGAAAGATACCTAAAGGATCAACAGCTCCTAGGGCTTTGGG
GCTGCTCTGGAAAACTCATCTGCACCACTAATGTACCTTGGAACTCCAGTTGGGGT-----------------
-----------------------------AATAAATCTCAAACAGATATTTGG------GAT------
AACATGACCTGGATGCAGTGG---GATAAAGAAATTAGTAATTACACAGACACAATATAC---
AAGTTGCTTGAAGACTCGCAAAACCAGCAGGAG---

AAAAATGAAAAAGATTTGTTAGCATTGGACAGTTGGAAAAATCTGTGGAATTGGTTTGACATAACAAAATGGT
TATGGTATATAAAAATATTCATAATGATAGTAGGAGGCTTGATAGGTTTGAGAATAATTTTTGCT---
GTGCTATCTATAATA---------AATAGAGTTAGGCAGGGATACTCACCT------
CTATCATTTGCTATTAGTCTGCTTGATACCATAGCGATAGCAGTAGCTGAA---
GGAACAGATAGGATTCTAAAATTCACACAAGGGCTTGGTAGAGCT---------------------------
--------ATCCGCAACCTA------CCTAGAAGATTAAGACAAGGTTTTGAAGCAGCTTTGCTA---TAA
>JX845587
------AGG---
GGAAATTTTTGGGTCACAGTCTATTATGGGGTACCTGTGTGG---AAAGAGGCAAAA---
ACTACTCTATTCTGTGCATCAGATGCTAAAGCATATGAGAAAGAA---GTGCATAAT----------------
--------------GTATGGGCTACA---CATGCCTGTGTACCCACAGAC---
CCCAACCCACAAGAAATAGTTATGGGA---------AATGTAACAGAAAATTTTAAC----------------
--------------------------ATGTGGAAAAATGACATGGTAGATCAGATGCATGAGGATATA---
ATCAGTTTATGGGATCAAAGTCTA---AAACCATGTGTAAAGTTG---
ACCCCACTCTGTGTTACTTTAAATTGTACAAATGCTAATGTTACAAATGCTAACAGTGCTAATGTTACA----
----
-------------AGTGCTAACAGTACTGCTAATTACAGTAATAGCATGTATGGAGAAATAAAA---
AATTGCTCTTTCAATACAACCACAGAAATAAGG--------------------------
GATAAGCAACAGAAAGCGTATGCACTT---TTTTATAGACCTGATTTAGTACCACTTAAAGAG----------
----

AATAACTCTAGTGGAGAGTATATATTAATAAATTGCAATTCCTCAACCATAACACAA---
GCCTGTCCAAAGGTCTCTTTTGACCCAATTCCTATACATTATTGTGCTCCAGCTGGTTATGCGATTCTAAAGT
GTAAT---AATAAGACATTCAAT------GGGACAGGACCATGCCAAAAT----------------------
-GTCAGC---ACAGTACAATGTACACATGGAATTAAGCCAGTGGTATCAACT---
CAACTACTGTTAAATGGTAGCCTAGCAGAAGGGGAT-----------
ATAATAATTAGATCTGAAAATCTGACAGACAAT---------GTCAAAACAATAATAGTCCATCTTAAT----
-----------GAATCTGTAGCA---------ATTAATTGTACAAGACCCAACAATAATACAAGAAAA-----
-AGTATAAGGATA------------GGACCAGGACAAACA---TTC------TATGCAAATGAC---------
---------ATAATAGGAGACATA------------AGACAAGCATATTGT---AGCATTAGTGGTGGA---
AAATGGAACACAACCTTAGAGCAGGTAAAGAAAAAATTACAA---------------GAACACTACCCT----
-----------------------------------AATAAAACAATACAATTTAAA------
GAACCCGTAGGAGGGGATCTAGAAATTACA---ACACATAGCTTTAATTGTAGAGGAGAA---
TTTAAGCAGCTCCAGGCAAGAGTCTTGGCTATAGAAAGATACCTAAAGGATCAACAGCTCCTAGGACTTTGGG
GCTGCTCTGGAAAGCTCATCTGCACCACTAATGTACCTTGGAACTCCAGTTGGAGT-----------------
-----------------------------AATAAATCTCAAGAAGACATTTGG------GAT------
AACATGACCTGGATGCAGTGG---GATAGAGAAATTAGTAATTACACAGGCACAATATAC---
AGGTTGCTTGAGGACTCGCAAAGCCAGCAGGAG---

AAAAATGAAAAAGATTTATTAGCATTGGACAAGTGGAAAGACCTGTGGACTTGGTTTGACATATCCAATTGGC
TGTGGTATATAAGAATATTCATCATGATAGTAGGGGGCTTGATAGGCTTGAGAATAATTTTTGGT---
GTACTCGCTATAGTG---------AAAAGAGTTAGGCAGGGATACTCACCT------
TTGTCGTTTGCTATTAGTCTGCTTGATACCATAGCAATAGCAGTAGCTGAG---
GGAACAGATAGGATTCTTGAAGTACTACAAAGAATTGGTAGAGCT---------------------------
--------ATCTGCAACATA------CCTAGAAGAATAAGACAGGGCTTTGAAGCAGCTTTGCTA---TAA
>AY463235
GTG---ATG---
GGGAACATGTGGGTCACAGTCTACTATGGGGTACCTGTGTGG---AAAGAGGCAAAA---
ACTACTCTATTCTGTGCATCAGATGCTAAGGCATATGAGAAAGAA---GTGCATAAT----------------
--------------GTCTGGGCCACA---CATGCTTGTGTACCCACAGAC---
CCCAACCCACAAGAAATGGAGTTGGAA---------AATGTAACAGAAAATTTTAAC----------------
--------------------------ATGTGGAAAAATGACATGGTGGATCAGATGCATGGGGATATA---
ATCAGTTTATGGGATCAAAGTCTA---AAGCCCTGTGTAAAGTTG---
ACCCCACTCTGTGTTACTTTAAACTGTGTCAATGTTAGCCGTACTGTTAATAGTAACAGTACTGGTAGTAGTA

AC---
----------AGTACTAGTAATAGTACCTACAATGATAACATGGATGGAGCTGGAGAAATAAAA---
AATTGTTCTTTCAATATAACCACAGAACTAAGA---------------------------
GATAAGAAAAAGAGGATACATGCACTT---TTTTATAGACTTGATATAGTACCACTTGATGGG----------
----

AATAATAGTAGTAGTGAATATAGATTAATAAATTGTAATACCTCAGCCATAACACAA---
GCCTGTCCAAAAGTCACTTTTGACCCAATTCCTATACATTATTGTGCTCCAGCGGGTTATGCGATTCTAAAGT
GTAAT---GATAAAACATTCAAT------GGAACAGGACCATGCAGTAAT-----------------------
-ATCAGC---ACAGTACAATGTACACATGGAATTAAGCCAGTGGTATCAACT---
CAACTACTATTAAATGGTAGTCTAGCAGAAAAGGAG------------
ATAATAATTAAATCTGAAAATATAACAGACAAT---------GTCAAAACAATAATAGTACATCTTACG----
-----------AAACCTGTAGAA---------ATTAATTGTACAAGACCCAACAATAATACAAGAAAA-----
-GGTATAGGGATA------------GGGCCAGGACAAACA---TTC------TTTGCAACAGGAGCT------
---------ATAATAGGAGATATA------------AGACAAGCACATTGT---AACATTAGTAAAGGG---
GCATGGAATAAAACTTTACAAGAGGTAGGGGAAAAGTTAAAA---------------AAGTGCTTCCCT----
-----------------------------------GGTAAAGGAATACAATTTGCA------
AATCACTCAGGAGGGGACCTAGAAATTACA---ACACATAGCTTTAATTGTGGAGGAGAA---
TTTAAGCAGCTTCAGGCAAGAGTCCTGGCTATAGAAAGATACCTAAAGGATCAACAGCTCCTAGGGATTTGGG
GCTGCTCTGGAAAACTCATCTGCACCACTAGTGTGCCTTGGAACTCTAGTTGGAGT-----------------
----------------------------AATAAATCTTATAATGCTATTTGG------GAC------
AACATGACCTGGATGCAGTGG---GATATAGAAATTAACAATTACACAGACACAATATAC---
AGGTTGCTTGAAGAATCGCAAAACCAGCAGGAA--

AGAAATGAACAGGAGTTATTAGCATTGGACAAATGGCAAAATCTGTTCAATTGGTTTAATATAACACAGTGGC
TGTGGTATATAAAAATATTCATAATGATAATAGGAGGATTGATAGGTTTAAGAATAATTTTTGCT---
GTACTCTCTATAGTG---------AATAGAGTTAGGCAGGGATACTCACCC------
TTATCGTTTGCTATTAGTCTGCTTAATACCATAGCAATAGCAGTAGCTGAG---
GGAACAGATAGGATTATTGAAGTATTACAAAGAACTTGTAGAGCT----------------------------
--------ATTCGCAATATA------CCTAGAAGAGTAAGACAGGGCTTTGAAACAGCTTTGCTA---TAA
>DQ369995
GTG---GTG--
GGAAACTTGTGGGTCACAGTCTATTATGGGGTACCTGTGTGG---AGAGAAGCAAAA---
GCTACTCTATTCTGTGCATCAGATGCTAAAGCATATGAGAAGGAG---GTACATAAT----------------
--------------GTCTGGCTACA---CATGCCTGTGTACCCACAGAT---
CCCAACCCACTAGAAAGGGTTTTGGAA---------AATGTAACAGAAAATTTTAAT----------------
--------------------------ATGTGGAAAAATGATATGGTGGATCAGATGCATGAGGATATA---
ATCAGTTTATGGGATGAAAGCCTA---AAGCCATGTGTAAAGTTG---
ATCCCACTCTGTGTCACTTTAAATTGTACTACTGCCAAAAAT-------------------------------
----
---AAGTCTGAAGAAATGAA---
AATTGCTCTTTCAAAGTAACCACAGAATTAAGA---------------------------
GATAAGAAGCGCAAAGAGTATGCACTT---
TTTTATAGACTTGATGTAGTACCACTTAATGAGACTAATGAAAGCTCTAATGAT-------------------
----
ACTAATGGAGACTTTAGGGAATATAGATTAATAAATTGTAATACCTCAACCTTAACACAA---
GCCTGTCCAAAGGTCACTTTTGACCCAATTCCTATACATTATTGTGCTCCAGCTGGTTATGCGATTCTAAAGT
GTAAT---AATAAGACATTCAAT------GGGACAGGGCCATGCAATAAT-----------------------
-GTCAGT---ACAGTACAATGTACACATGGAATTAAACCAGTGGTATCAACT---
CAACTACTGTTAAATGGTAGCTTAGCAGAAGAGGAG------------
ATAATAATTAAATCTGAAAATATAACAGACAAT---------GCTAAAACAATAATAGTACAGCTTAAT----
-----------AAAGCCATAGAA---------ATTGAGTGCATAAGACCCAGCAATAACACAGGAAGA-----
-AGTGTAAGGATA------------GGACCAGGACAAACA---TTC------TATACAACTGGAGAA------
---------ATAATAGGAGACATA------------AGACAAGCACATTGT---AACATTAGTGGG------
AATTGGGATAGAACTTTATACAATATAAGTAAAAAATTAGGA---------------GAACACTTCCCT----
-----------------------------------AATAAAAATATAAGCTTTAAA------
CCATCCTCAGGAGGGGACCCAGAGATTACA---ACACATAGTTTTAATTGTGGAGGAGAA---
TTCAAGCAGCTCCAGGCAAGAGTCCTGGCTATAGAAAGATACCTAAAGGATCAACAGCTCCTAGGAATTTGGG
GCTGCTCTGGAAGACAAATCTGCACCACTAATGTGCCTTGGAACACCAGTTGGAGT-----------------
----------------------------AATAAATCCCTAAATGATATTTGG------AAT------

AACATGACCTGGATGCAGTGG---GATAAAGAAATTAGTAATTACACAAACATAATATAC---
CAGTTGCTTGAACTATCGCAAAACCAGCAGGAA-------------------------------------
----
CAAAATGAAAAGGATTTATTAGCATTGGACAAGTGGCAGAATCTGTGGAGTTGGTTTGACATAACACATTGGC
TATGGTATATAAAGATATTCATAATGATAATAGGAGGCTTGATAGGTTTAAGAATAATTTTTGCT---
GTGCTATCCATAGTA---------AATAGAGTTAGGCAGGGATACTCACCT------
TTGTCGTTTGCTATTAGTCTGCTTGATACCATAGCAATAGTAGTAGCTGAG---
GGAACAGATAGGATTATAGAACTCATATTAGGAATTTGTAGAGCT---------------------------
--------ATCCGCAACCTA------CCTAGAAGAATAAGACAGGGTTTTGAAGCAGCGTTGCTA---TAA
>DQ351223
GGG---GTG---
GGGAACTTGTGGGTCACAGTCTACTATGGGGTACCTGTGTGG---AGAGAAGCAAAA---
ACTACTCTATTCTGTGCATCAGATGCTAAAGCATATGAAAGAGAA---GTGCATAAT----------------
--------------GTCTGGGCTACA---CATGCCTGTGTACCGACAGAC---
CCCAACCCACAAGAACTAGTTTTGGGA---------AATGTAACAGAAAATTTTAAT----------------
--------------------------ATGTGGAAAAATGACATGGTAGATCAGATGCATGAGGATATA---
ATCAGTATATGGGATCAAAGTCTA---AAACCATGTGTAAAGTTG---
ACCCCACTATGTGTCACTTTAAACTGTATAGAGGCAAAAGCTAATAGTACC---------------------
----
----------------------------------AGTACTGGTGGTGTGGCTGGAGAAATAACA---
AATTGCTCTTTCAATACAACCACAGAAATAAGA--------------------------
GATAAGAAACAGAACGTGTATGCACTT---TTTTATAGAACTGATGTAGTACCACTCAAGAAG---------
----

AACTCTAGTGAGTATATATTGATAAATTGCAATACCTCAACCATAACACAA---
GCCTGTCCAACGGTCACCTTTGATCCGATTCCTATACATTATTGTGCTCCAGCTGGATATGCGATTCTAAAGT
GTAAT---AATAACACATTTAAT------GGGACAGGGCCATGTTACAAT----------------------
-GTCAGT---ACAGTACAATGTACGCATGGAATTAAGCCAGTGGTATCAACC---
CAATTACTGTTAAATGGTAGCCTAGCAGAAGGGGAG-----------
ATAATCATTAGATCTGAAAATCTGACAAACAGT---------ATCAAAACAATAATAGTACATCTTAAT----
-----------GAATCTTTAGAA---------ATTGTGTGTACAAGACCCAACAATAATACAAGAAAA-----
-GGTATAAGGATA------------GGACCAGGACAAGTA---TTC------TATGCAACTGACGAT------
---------GTAATAGGAAACATA-----------AGGCAAGCATATTGT---ACCATTAATGAAACA---
AAATGGAACGAAACTTTAGAAAGGGTAAAGGAGAAATTAGGA---------------GACCACTTCCCT----
------------------------------------AATAAAACAATAACCTTTGCA------
CCATCCTCAGGAGGGGACCTAGAAGTTACA---ACACATAGTTTCAACTGTAGAGGAGAA---
TTTAAACAGCTCCAAGCAAGAGTCTTGGCTGTAGAAAGATACCTAAAGGATCAACAGCTCCTAGGGCTTTGGG
GATGCTCTGGAAAACTCATCTGCACCACTACTGTGCCTTGGAACTCCAGTTGGAGT-----------------
----------------------------AATAAATCTGAAGCAGATATTTGG------GAT------
AACATGACCTGGATGCAGTGG---GACAGAGAAATTAGTAATTACACAGGCACAATATAC---
AGGTTGCTTGAAGAATCGCAAAACCAGCAGGAG-------------------------------------
----
AAAAATGAAAAAGATTTATTAGCCTTGGACAGTTGGAAAAACCTTTGGAATTGGTTTGACATAACAAATTGGC
TGTGGTATATAAAAATATTTATCATGATAGTAGGAGGCTTGATAGGTTTAAGAATAATTTTTGGT---
GTGCTCGCTATAGTG---------AAAAGAGTTAGGCAGGGATACTCACCT------
TAGTCGTTTGCTATTAATCTGCTTAATACCATAGCAATAGCAGTAGCTGAG---
GGAACAGATAGGATTATAGAATTCGTACAAAGAATCTATAGAACT---------------------------
--------ATCCGCAACGTA------CCTACAAGAATAAGACAGGGCTTTGAAGCAGCTTTGCTA---TAA
>DQ396388
GTG---GTG---
GGGAACTTGTGGGTCACAGTCTACTATGGGGTACCTGTGTGG---AAAGAAGCAAAA---
ACTACTCTATTCTGTGCATCAGATGCTAAAGCATATGAGAAAGAA---GTACATAAT----------------
--------------GTCTGGGCTACA---CATGCCTGTGTACCCACAGAC---
CCCAACCCACAAGAATTGGTTTTGGAA---------AATGTAACAGAAAATTTTAAC----------------
--------------------------ATGTGGAAAAATGACATGGTGGACCAGATGCATGAGGATATA---
ATCAGTTTATGGGATCAAAGCCTA---AAGCCATGTGTAAAGTTG---
ACCCCACTCTGTGTCACTTTAGACTGCAGAAATATTACCTACAATAATGTTACC-------------------
----
-------------------------------ATTAAGGGTAATGATACCATTGAAGAAATGAAA---
AGTTGCTCTTTCAATACAACCACAGAAATAATA--------------------------

GATAAGAAAAACAAAGAGTATGCACTT---TTCTATAAACTTGATATAGTACAACTTAATAAT----------
----

GACAACTCTAGTGAGTATAGATTAATAAATTGTAATAGCTCAACTATGACACAA---
GCCTGTCCAAAGGTCTCTTTTGACCCAATTCCTATACATTATTGTGCTCCAGCTGGTTATGCGATTCTAAAGT
GTAAT---AATAAGACATTCAAT------GGGACAGGACCATGCCATAAT----------------------
-GTCAGC---ACAGTACAATGTACACATGGAATTAAGCCAGTAGTATCAACT---
CAACTACTGTTAAATGGTAGCCTAGCAGAAGGAGAG-----------
ATAATAATTAGATCTGAAAATCTGACAAACAAT---------GCCAAGACAATAATAGTACAGCTTAAT----
-----------CAATCTGTAGAA---------ATTGTGTGTATAAGACCCAACAATAATACAAGAAAA-----
-AGTATAAGGATA------------GGACCAGGACAAGCA---TTC------TATGCAACAGGAGAC------
---------ATAATAGGAAACATA------------AGACAAGCACATTGT---AACATTAATACAGGA---
AACTGGAGTAAAACTTTACAAAGGGTAGCTAAAAAATTAAAA---------------GAACACTTCCCT----
-----------------------------------AATAAAACAATAGAATTTAAA------
CCATCCTCAGGAGGGGACCCAGAAATTACA---ACACATAGCTTTAATTGTGGAGGAGAA---
TTTAAGCAGCTCCAGGCGAGAGTCCTGGCTATAGAAAGATACCTAAGGGATCAACAGCTCCTAGGGATTTGGG
GCTGCTCTGGAAAACTCATCTGCACCACTACTGTGCCTTGGAACTCCAGTTGGAGT-----------------
----------------------------AATAAATCTGAAGAAGAGATTTGG------GGT------
AACATGACCTGGATGCAGTGG---GAGAGAGAAATTGATAATTACACAGAAACAATATAC---
CAGTTGCTTGAAGTCTCGCAAAGCCAGCAGGAG--

CAAAATGAAAAGGAGTTATTAGCATTGGACAAGTGGCAAAATCTGTGGAGTTGGTTTAGCATAACAAACTGGC
TGTGGTATATAAAAATATTTATAATGATAGTAGGAGGCTTGATAGGTTTAAGAATAATTTTTGCT---
GTGCTTTCTATAGTG---------AATAGGGTTAGGCAGGGATACTCACCT------
TTGTCGTTTGCTATTAGCCTGCTTGATACCATAGCAATAGTAGTAGCTGAA---
GGAACAGATAGGATTATACTAGTTATACAAGGAATTTGTAGAGCT----------------------------
--------ATCCGCAACATA------CCTAGAAGAATAAGACAGGGCTTTGAAACAGCGTTGCTA---TAA
>DQ351228
GTG---GGG---
GGAAACTTGTGGGTCACAGTCTATTATGGGGTACCTGTGTGG---AGAGAAGCAAAA---
ACTACTCTATTCTGTGCATCAGATGCTAAAGGATATGAGAGGGAG---GTGCATAAT----------------
--------------GTCTGGGCTACA---CATGCCTGTGTACCCACAGAC---
CCCAACCCACTAGAAATAGTTTTGGGA---------AATGTAACAGAAAATTTTAAC---------------
--------------------------ATGTGGAAAAATGACATGGTAGATCAGATGCATGAGGATATA---
ATCAGTTTATGGGATCAAAGCCTA---AAGCCATGTGTAAAGTTG---
ACCCCACTCTGCGTCACTTTAGCATGTGTTGATACTAATGATACCAATAATGTTAATGCTAATGCT-------
----
----------------------ACCAAAAATGTTACCTACGATAATACCATGCCAGACATAAAA---
AATTGCTCTTTCAATACAACCACAGAAATAAGA--------------------------
GATAAGAAACAGAAAGCGTATGCACTT---TTTTATAGACTGGATATAGTGCCACTTAATGAGAAGAACAAG-
----

AATGAGGAGAGCAACAGTACAAAGTATATATTAATAAATTGTAATACCTCAACCATAACACAA---
GCCTGTCCAAAGGTCTCTTTTGACCCAATTCCTATACATTATTGTGCTCCAGCTGGTTATGCGATTCTGAAGT
GTAAT---AATAAGACATTCAAG------GGGACAGGACCATGCCATAAT----------------------
-GTCAGC---ACAGTACAATGTACACATGGAATTAAACCAGTGGTATCAACT---
CAACTACTGTTAAATGGTAGCATAGCAGAAGAAGAG-----------
ATAATAATTAGATCTGAAAATCTGACAAACAAT---------GCTAAAACAATAATAGTACATCTTAAT----
-----------GAATCTGTGGAA---------ATTGTGTGCACTAGACCCAACAATAACACAAGAAAA-----
-AGTGTAAGGATA------------GGACCAGGACAAACA---TTC------TATGCAACAGGTGCC------
---------ATAATAGGAGACATA------------AGAGAAGCACATTGT---AACATTAGTGAAGAA---
AAATGGAATAGAACCTTACAAAGGGTAAGTGAAAAATTAAAA---------------GAACACTTCCCT----
-----------------------------------AACAAAACAATAAGATTTAAC------
TCATCCTCAGGAGGGGACCTAGAAATTACA---ACACATAGCTTTAATTGTAGAGGAGAA---
TTTAAGCAGCTCCAGACAAGAGTCCTGGCTATAGAAAGATACCTAAAGGATCAACAGCTCCTAGGACTTTGGG
GCTGCTCTGGAAAACTCATCTGCACCACTGCTGTGCCTTGGAACTCCAGTTGGAGT----------------
----------------------------AATAAATCCCAAGAAGATATTTGG------AAT------
AACATGACCTGGATGCAGTGG---GATAGAGAAATTAATAATTACACAAACACAATATAC---
AGGTTGCTTGAAGACTCGCAAAACCAGCAGGAG--

AAAAATGAAAAGGATTTATTAGCATTGGACAGTTGGAAGAGCCTGTGGAGTTGGTTTGACATAACAAATTGGC
TGTGGTATATAAAAATATTCATCATGATAGTAGGAGGCTTGATAGGTTTAAGAATAGTTTTTGCT---
GTGCTATCTATAATA---------AATAGAGTTAGGCAGGGATACTCACCC------
TTGTCGTTTGCCATTAATCTGCTTGATACCTTAGCAATAGCAGTAGGTGAA---
GGAACAGATAGGATTATTGAAATAATACAAAGAGTTTGTAGAGTT----------------------------
--------ATCAGCAACATA------CCTAGAAGAATAAGACAGGGCTTTGAAGCAGCTTTGCTA---TAA
>DQ011175
GGG---GTG--
GGAAACTTGTGGGTCACAGTCTATTATGGGGTACCTGTGTGG---AAAGAAGCAACA---
ACTACTCTATTCTGTGCATCAGATGCTAAAGCATATGAGAGGGAG---GTGCATAAT----------------
--------------GTCTGGGCTACA---CATGCCTGTGTACCCACAGAC---
CCCAACCCACTAGAAATGGTTTTGGAA---------AATGTAACAGAAAATTTTAAC----------------
--------------------------ATGTGGAAAAATGATATGGTAGATCAGATGCATGAGGATATA---
ATCAGTTTATGGGATCAAAGCCTA---AAGCCATGTGTAAAATTG---
ACCCCACTCTGTGTCACTTTAAATTGTACAGAGTTTCATAGGAATGTTACCTGCATTAATAGTACTACC----
----
-------------------TGCGATACAAAAATGAAAAATTGCTCCTGCAATACAGAAATGAAA---
AATTGCTCTTTCAATGTAACCACAGAATTAAGA---------------------------
GATAAGAAGCACAACCAGTATGCACTT---
TTTTATAGACTTGATATAATACCACTTAATGAGGATAGTTCTAAGAATAATTCT-------------------
----
ACTAAGTCTAAGAGTAATTCTACTGAGGAGTGGGAGGAGTATAGATTAATAAATTGTAATACCTCAACCATAA
CACAA---
GCCTGTCCAAAGGTTAATTTTGACCCAATTCCTATACATTATTGTGCCCCAGCTGGCTATGCGATTCTAAAGT
GTAAT---AATAAGACATTCAAT------GGGACAGGACCATGCCAGAAT-----------------------
-GTCAGT---ACAGTACAATGTACACATGGAATTAAACCAGTGGTATCAACT---
CAACCGCTGTTAAATGGTAGCCTAGCAGAAGAAGAG------------
ATAATAATTAGATCTGAAAATCTGACAGACAAT---------GCTAAAACAATAATAGTACAGTTTAAG----
-----------GAATCCATAAAC---------ATTACGTGCACAAGACCCAACAACAACACAAGAACA-----
-AGTGTAAGGATA------------GGACCAGGACAAGCG---TTC------TATGCAACAAATGAT------
---------ATAATAGGAGACATA------------AGACAAGCACATTGT---AACATTAGCGCAAAG---
GACTGGAATAGAACTTTACAAGAGGTAGGTAAAAAACTAGCA---------------AATCACTTTAAT----
-----------------------------------CATACAACAATACACTTTAGA------
CCATCCTCAGGAGGGGATCTAGAAATTGTA---ACACATAGCTTTAATTGTAGAGGAGAA---
TTTAAGCAGCTCCGGACAAGAGTCCTGGCTATAGAAAGATACCTAAAGGATCAACAGCTCCTAGGAATTTGGG
GCTGCTCTGGAAAACTCATCTGCACCACTGCTGTGCCTTGGAACTCCAGTTGGAGT-----------------
----------------------------AATAAATCTCTACATGACATTTGG------GAT------
AACATGACCTGGATGCAGTGG---GATAAAGAAATTAGTAATTACACACAAACAATATAT---
CAGTTGCTTGAAGAATCGCAAATCCAGCAGGAG--

AAAAATGAAAAAGATTTATTAGAATTGGACAGTTGGCAAAATCTGTGGAATTGGTTCAGTATAACAAATTGGC
TATGGTATATAAAAATATTCATAATGATAGTAGGAGGCTTGATAGGTTTAAGAATAATTTTTGCT---
GTACTATCTATAGTA---------AATAGAGTTAGGCAGGGATACTCACCT------
TTGTCGTTTGCTATTAGTCTGCTTGATACCATAGCAATAGCAGTAGCTGGG---
GGAACAGATAGGATTATAGAATTCATACAAGGAATTGGTAGAGCT----------------------------
--------ATCCGCAACCTA------CCCAGAAGAATAAGACAGGGTTTTGAAGCAGCATTGCTA---TAA
>AY901971
GTG---GTG--
GGAAACTTGTGGGCCACAGTTTATTATGGGGTACCTGTGTGG---AGAGAAGCAAAA---
ACTACTCTATTCTGTGCATCAGATGCTAAAGCATATGAGAGAGAA---GTGCATAAT----------------
--------------GTCTGGGCTACA---CATGCCTGTGTACCCACAGAT---
CCCAACCCACAAGAACTAGTTTTGGGA---------AATGTAACAGAAAATTTTAAC----------------
--------------------------ATGTGGGAAAATGACATGGTGGATCAGATGCATGAAGATATA---
ATCAGTCTATGGGATGAAAGCCTA---AAGCCATGTGTAAAGTTG---
ACCCCACTCTGTGTCACTTTAAATTGTAATAATGTTAATACTACCCGAAGTGGTAAT----------------
----
----------------------------AGTACCTACAATAATACCATGGATGCAGAAATGAAA---
AATTGCTCTTTCAATGCAACCACAGAAGTAAGA---------------------------
GATAAGAAGCGCAGAGAGTATGCGCTT---
TTTTATAGGCTAGATATAGTACCACTTGATGGGAATAAGAACAAC----------------------------

----
TCTGGTAATGAGAGCAACTCTATTGGGGAGTATAGATTAATAAATTGTAATGCCTCAACCACAACACAA---
GCCTGTCCAAAGGTCACCTTTGACCCGATTCCCATACATTATTGTGCTCCAGCTGGTTATGCGATTCTAAAGT
GTAAT---AATAAGACATTCAAT------GGGACAGGACCATGCACTAAT------------------------
-GTCAGC---ACAGTACAATGCACACATGGAATTAAGCCAGTGGTATCAACT---
CAACTACTGTTAAATGGTAGTCTAGCAGAAGAGGAG-----------
ATAGTCATTAGATCTAAAAAGCTGACAGAAAGT---------GCCAACACAATAATAGTACATCTTAAT----
-----------GAATCTGTAAAA---------ATTGAGTGTACAAGACCCAGCAATAATACAAGAAAA-----
-AGTATAAGGGTA------------GGACCAGGACAAACA---TTC------TATGCAACAGGAGAA------
---------GTAATAGGAGACATA------------AGACAAGCACATTGT---AACATAAGTAAAAGT---
AAATGGGATAGCACCTTACAAAGGGTAGGTAAAAAATTAAGA---------------GAACACTTCCCT----
------------------------------AATAAAACAGTAATAATCTTTGAC------
GCATCCTCAGGAGGGGATCTAGAAATTGTA---ACACATAGCTTTAATTGTGGAGGAGAA---
TTTAAGCAGCTCCAGGCAAGAGTCCTGGCTGTGGAAAGATACCTACAGGATCAACGGCTCCTAGGAATTTGGG
GCTGCTCTGGAAAACACATCTGCACCACTGCTGTGCCTTGGAACTCCAGTTGGAGC-----------------
---------------------------AATAAAACTTACAATGATATTTGG------GAT------
AACATGACCTGGATGCAGTGG---GATAAAGAAATTAGTAATTACACAGACACAATATAC---
AAGTTGCTTGGGGAATCGCAAATCCAGCAGGAA---

CAAAATGAAAAAGATTTATTAGCATTGGACAAGTGGCAAAATCTGTGGAGTTGGTTTGACATAACAAATTGGC
TATGGTATATAAAAATATTCATAATGATAGTAGGAGGCTTAATAGGTTTGAGAATAATTTTTGCT---
GTGCTATCTATAGTA---------AATAGAGTTAGGCAGGGATACTCACCT------
TTGTCGTTTGCTATTAGTCTGCTTGATACCATAGCAATAGCTGTAGCTGAA---
GGAACAGACAGGATTATAGATTTAATACAAATAATTTGTAGAGCT----------------------------
--------ATCTACCACATA------CCTAGAAGAATAAGACAGGGCTTTGAAGCATCTTTGCTA---TAA
>DQ093597
GTG---GTG--
GGAAACTTGTGGGTCACAGTCTATTATGGGGTACCTGTGTGG---AAAGAAGCAAAG---
ACTACTCTATTCTGTGCATCAGATGCTAAAGCATATGAAAGGGAG---GTACATAAT----------------
--------------GTATGGGCTACC---CATGCCTGTGTACCCACAGAC---
CCCAACCCACTAGAAATAGCTTTGGAA---------AATGTAACAGAAAATTTTAAC----------------
-------------------------ATGTGGAAAAATGACATGGTGGATCAGATGCATGAGGATGTA---
ATCAGTTTATGGGATCAAAGCCTA---AAGCCCTGTGTAAAGTTG---
ACCCCACTCTGTGTCACTTTAAACTGTATTAATGTTAATTTAACATGTAAAAACGACACTGTA----------
----
------------------------GGATGTGGTGTAAATGATACAATGGGGACAGTAATGAAA---
AATTGCTCTTTCAATGCAACTACAGAGATAAAA---------------------------
GACAAGAAGCGCCAAGAGTATGCACTT---
TTTTATAAAATTGATATAGTACCACTTAATGGGGCTGACAAATCT----------------------------
----
AATAAGAGTGATAACAACTATAATGAGTATAGATTAATAAATTGTAATACCTCAACCATAACACAA---
GCCTGTCCAAAGGTCTCTTTTGACCCAATTCCTATACATTATTGTGCTCCAGCTGGATATGCGATTTTAAGGT
GTAAT---AATGATACATTCAAT------GGAACAGGACCATGCAAAAAT----------------------
-GTCAGT---ACAGTACAATGTACACATGGAATTAAACCAGTGGTATCAACT---
CAACTACTGTTAAATGGTAGCCTAGCAGAAAAAGAG-----------
ATAATAATTAGATCTGAAAATATAACAAACAAT---------GTTAAAACAATAATAGTACAGCTTAAT----
-----------GAATCTATAGCA---------ATTGTGTGCGCAAGACCCGGCAATAACACAAGGAGA-----
-AGTGTAAGGATA------------GGACCAGGACAAACA---TTC------TATGCAACAGGAGAT------
---------ATAATAGGAGACATA------------AGACAAGCACATTGT---AACATTAGTAAAGAT---
GATTGGAATAAAACCTTACAAAGGGTAAAAGAAAAATTAGGA---------------GAACACTTCCCT----
------------------------------------AATAAAACCATAATCTTTAAC------
TCATCCTCAGGAGGGGACCTAGAAATTACA---ACACATAGCTTTAATTGTAGAGGAGAA---
TTTAAACAGCTCCAGACAAGAGTCCTGGCTGTGGAAAGATACCTACAGGATCAACAGCTCCTAGGAATTTGGG
GCTGCTCTGGAAAACTCATCTGCACCACTAATGTACCTTGGAACTCCAGTTGGAGT-----------------
---------------------------AATAAATCTCAAAATGATATTTGG------GGT------
AACATGACCTGGATGCAGTGG---GATAAAGAAATTAGTAATTACACATACACAATATAC---
AGTTTGCTTGAAGAATCACAAATCCAGCAGGAA---

CAAAATGAAAAAGATTTATTAGCATTGGACAAATGGCAAAGTCTGTGGACTTGGTTTGACATAACAAAGTGGC
TGTGGTATATAAAAATATTCATAATGATAGTAGGAGGCTTGATAGGTTTGAGGATAATTTTTGCT---

```
GTGCTATCTATAGTA---------AATAGAGTTAGGCAGGATACTCACCT------
TTGTCGTTTGCTATTAATCTGCTTGATACCATAGCAATAGCAGTAGCTGAG---
GGAACAGATAGGATTATAGACTTCCTACAAAGAATTTGTAGAGCT---------------------------
--------ATCCGCAATATA------CCTAGAAGAATAAGACAAGGTTTTGAAGCAGCTTTGCTA---TAA
>AY772692
GTG---GTG-----------------------------------------
GGGAACTTGTGGGTCACAGTTTATTATGGGGTACCTGTATGG---AGAGAAGCAAAA---
ACTACTCTATTCTGTGCATCAGATGCCAAGGCATATGAGAAAGAA---GTGCATAAT----------------
--------------GTCTGGCTACA---CATGCCTGTGTACCCACAGAC---
CCCGACCCACAAGAAATAATCTTAGAA---------AATGTAACAGAAAATTTTAAC---------------
--------------------------ATGTGGAAAAATGACATGGTGGATCAGATGCATGAGGATATA---
ATCAGTTTATGGGATCAAAGTCTA---AAACCATGTGTAAAGTTG---
ACCCCACTCTGTGTCACTTTAAATTGTACAAAGGTTAATGCTACTACTGCTAAAGTTACCAGTATT-------
------------------------------------------------------------------------
----------------------AATGGTACCAGAATTAATGATTCCATGATTGAAGAAATGAGA---
AATTGCTCTTTCAATACAACCACAGAAATAAGA--------------------------
GATAAGCCACAGAAAGCGTATGCACTT---TTTTATAAACTTGATCTAGTACCACTTGGTGGG---------
------------------------------------------------------------------------
------------------
AACAACAAGAATGACTATATATTAATACATTGCAATACCTCAACCATAACACAA---
GCCTGTCCAAAGGTCTCTTTTGACCCAATTCCTATACATTATTGTGCTCCAGCTGGTTATGCGATTCTAAGT
GTAAT---AATGAGACATTCAAT------GGGACAGGACCATGCCGGAAT----------------------
-GTCAGC---ACAGTGCAATGTACACATGGAATTAAACCTGTGGTATCAACT---
CAACTACTGTTAAATGGCAGCCTAACAGAAAAGGAG-----------
ATAGTAATCAGTTTTGAAAATATGACAGACAAT---------GCCAAAACAATAATAGTACACCTTAAT----
-----------GAATCTATAAAC---------ATTGTATGTACAAGACCTGGCAATAATACAAGGAAA---
AATGTAAGGATTATAAGA------ATAGGACCAGGACGAGCA---TTC------CATGCAAATAAC-------
-----------ATAATAGGAGACATA------------AGACAAGCACATTGT---AACATCAGTAAAGCA--
-GACTGGAACAAGACTATAGGAAGGATAAAGACAAAACTAAAA---------------GAACACTTCCCT---
-----------------------------------GGTAAAGCAATAGAATTTAAA------
CCACACTCAGGAGGGGATCTAGAAATTACA---ACACATAGTTTTAATTGTAGAGGAGAA---
TTTAAGCAGCTCCAGACAAGAGTCCTGGCTATAGAAAGATACCTAAAGGATCAACAGCTCCTAGGGCTTTGGG
GCTGCTCTGGAAAACTCATCTGCACCACTAATGTGCCTTGGAACTCCAGTTGGAGT----------------
---------------------------AATAAATCTGAGTCAGAGATTTGG------AAT------
AACATGACCTGGATGGAGTGG---GATAGAGAAATTAATAAGTACACAAACACAATATAC---
AATTTGCTTGAAGTCTCGCAAAACCAGCAGGAG---------------------------------------
----------------------------
AAAAATGAAAAAGATTTATTAGCATTGGACAGTTGGAACAATCTGTGGAGTTGGTTTGACATATCAAATTGGT
TGTGGTATATAAAAATATTCATAATGATAGTAGGAGGCTTAATAGGTTTAAGAATAATTTTTGCT---
GTGCTCTCTATAGTA---------AATAGAGTTAGGCAGGATACTCACCT------
TTGTCGTTTGCTATTAATCTGCTTGATACCATAGCAATAGCAGTAGCTGAA---
GGAACGGATAGGATCATAGAAGTAATACAAAGAGGTTGTAGAGCT---------------------------
--------ATTCGCAACATA------CCTACAAGAATAAGACAGGGCTTTGAATCAGCTTTGCTA---TAA
>DQ164107
GTG---ATG-----------------------------------------
GGAAACTTGTGGGTCACAGTATATTATGGGGTACCTGTGTGG---AGGGAAGCAAAT---
ACTACTCTATTTTGTGCATCAGATGCTAAAGCATATGAGAGAGAG---GTACATAAT----------------
--------------GTCTGGCTACA---CATGCCTGTGTACCCACAGAC---
CCCAACCCACAAGAAATAATCTTGGAA---------AATGTAACAGAAAATTTTAAC---------------
--------------------------ATGTGGAAAAATGGTATGGTGGATCAGATGCATGAGGATATA---
ATCAGTTTATGGGATCAAAGTCTA---AAACCATGCGTAAGGTTG---
ACCCCACTCTGTGTCACTTTAAAGTGTACAACTGTTAACGTTAATGATACCATGAAGGAAGAAATA-------
------------------------------------------------------------------------
----------------------AAAACTGTTAATGTTACTGATACCATGAAGGAAGAAATAAAA---
AATTGCTCTTTCAATACAACCACAGAGCTAAGA--------------------------
GATAAGAAACAGAAAGTGTATGCACTT---TTTTATAGACCTGATATAGTACCACTTAATGAGAATAGC----
------------------------------------------------------------------------
----------
TTTAGTGAGAATAGCTCTAATGATTATATATTAATAAATTGCAATACCTCGACCATAGCACAA---
GCCTGTCCAAAGATCACTTTTGACCCAATTCCTATACATTATTGTGCTCCAGCTGGTTATGCGATTCTAAGT
```

GCAAT---GATAAGACATTCAAT------GGAACAGGACCATGCCATAAT----------------------
-GTCAGC---ACAGTACAATGTACACATGGAATTAAACCAGTGGTATCAACT---
CAATTACTGTTAAATGGTAGCCTAGCAGAAGGGGAG-----------
ATAATAATTAGATCTAAAAATCTGACAGACAAT---------GTCGGAACAATAATAGTACACCTTAAT----
-----------GAATCTATAGAG---------ATTGTGTGTACAAGACCCAACAATAATACAAGAAAA-----
-AGTATACGGATA------------GGACCAGGACAAGCA---TTC------TATGCAACAAATGGC------
---------ATAATAGGAAAAATA-----------AGACAAGCACATTGT---AATATTAGTAAAGAG---
GGCTGGAGTAGAACCCTACAATGGGTAAGAAAAAAATTAGAA--------------AAACACTTCCCT----
----------------------------------AATAAAACAATAAACTTTAAT------
TCATCCTCAGGAGGGGACCTAGAAATTACA---ACACATAGCTTTAATTGTGGAGGAGAA---
TTTAAGCAGCTCCAGGCAAGAGTCCTGGCTATAGAAAGATACCTACAGGATCAACAGCTCCTAGGAATTTGGG
GCTGCTCTGGAAAACTCATCTGCACCACTGCTGTGCCTTGGAACCTTAGTTGGAGT----------------
---------------------------AATAAATCTGAAAATGATATTTGG------GGT------
AACATGACCTGGATGCAGTGG---GATAGGGAAATTAGCAATTACACAAACACAATATAC---
AGGTTACTTGAAGAATCGCAATCCCAGCGGGAA---------------------------------------
----
AAAAATGAAAAGGATTTATTAGCATTGGACAAGTGGCAAAACCTGTGGAGTTGGTTTAACATAACAAATTGGC
TATGGTACATAAGAATATTCATAATGATAGTAGGAGGCTTGATAGGTTTGAGAATAATATTTGCT---
GTGCTATCTATAGTG---------AATAGAGTTAGGCAGGGATACTCACCT------
TTGTCGTTTGCTATTAGTCTGCTTGATAGCACAGCAGTAGCAGTAGCTGAG---
GGAACAGATAGGATTATAGAATTTATACAAAGAATTTGTAGGGCT--------------------------
--------ATCCGCAACCTA------CCTAGAAGAATAAGACAAGGTTTTGAAGCAGCTTTGCTA---TAA
>DQ093600
GTA---ATG--
GGGGACTTGTGGGTCACAGTCTACTATGGGGTACCTGTGTGG---AAAGAAGCAAAA---
GCTACTCTATTCTGTGCATCAGATGCTAAAGCATATGAGAAAGAA---GTGCATAAT---------------
--------------GTCTGGGCTACA---CATGCCTGTGTACCCACAGAC---
CCCAACCCACAAGAATTAGTTTTGGGA---------AATGTAACAGAAAACTTTAAC---------------
-------------------------ATGTGGAAAAATGATATGGTGGATCAGATGCATGAGGATATA---
ATCAGTTTATGGGATGAAAGCCTA---AAACCATGTGTAAAGTTG---
ACCCCACTCTGTGTCACTTTAAATTGTACAAGTGTTAAGGATACCAAGAATAATACT---------------
----
---------------------------ACCAAGCACAATGATAGCATGATTCAAGAAATGAGT---
AATTGCTCTTTCTATACAACCACAGGAATAAGA-------------------------
GATAAGCAACAGCAAGAGTATGCACTT---TTTCATAAACCTGATATAATACCAATTAATAAT---------
----

AATGATAGTGAATATATATTAATCCATTGCAATACCTCAACCATAACACAA---
GCCTGTCCAAAGGTCTCTTTTGACCCAATTCCTATACATTATTGTGCTCCTGCTGGTTATGCGATTCTAAAAT
GCAAT---AATAGGACATTCAAT------GGGACAGGACCATGCAAAAAT----------------------
-GTCAGC---ACAGTGCAATGTACACATGGAATTAAACCAGTGGTATCAACT---
CAACTACTGTTAAACGGTAGCCTAGCAGAAGAGGAT-----------
ATAATAATTAAATCTGAAAATATGACAAACAAT---------ATCAAAACAATAATAGTCCACTTTAAT----
-----------GAATCTGTGCAG---------ATTATGTGTGCAAGACCTGGTAATAAGACAAGCAGA-----
-AGTATAAGGATA------------GGACCAGGACGAGTA---TTC------TATACAAATAAC---------
---------ATAATAGGAGACATA-----------AGACGAGCACATTGT---AATATTACTAGAAAG---
CAATGGAACCAAACTTTAGAACGGGTAAAGGAAAAATTAAGA--------------AAACATTACCCC----
----------------------------------AATAAAACAATAAAATTTGAC------
AAACCTTCAGGAGGGGATCTAGAGATTACA---ACACATAGCTTTAATTGTAGAGGAGAA---
TTTAAGCAGCTCCAGGCAAGAGTCCTGGCTATAGAAAGATACCTAAAGGATCAACAGCTCCTAGGGCTTTGGG
GCTGCTCTGGAAAACTCATCTGCACCACTAATGTGCCTTGGAACTCTAGTTGGAGC----------------
---------------------------AATAAATCTCTAACAGATATTTGG------GAT------
AAAATGACCTGGATGCAGTGG---GAGAAGGAAATTGATAATTACTCACACACAATATAC---
TGGTTGCTTGAAGAATCGCAAAGCCAACAGGAG---------------------------------------
----
AAAAATGAAAAAGATTTATTAGCATTGGACAGTTGGAATAACCTGTGGAGTTGGTTTAGCATATCAAATTGGT
TGTGGTATATAAGAATATTCATCATGATAGTAGGAGGCTTGATAGGTTTAAGAATAATTTTTGGT---
ATACTCTCTATAGTA---------AGAAGAGTTAGGCAGGGATACTCACCT------
TTGTCGTTTGCTATTAATCTGCTTGATACCATAGCAATAGCAGTAGCTGAA---

GGAACAGATAGGATTATTGAAATAGTACAAAGAATTTGTAGAGCT---------------------------
--------ATCAGAAACATA------CCTACAAGAATAAGACAGGGCTTTGAAGCAGCTTTGCTA---TAA
>DQ011166
GGG---GTG--
GGAAACTTGTGGGTCACAGTTTATTATGGGGTACCTGTGTGG---CAAGAAGCAAAA---
ACTACTCTATTCTGTGCATCAGATGCTAAAGCATATGAGAAAGAA---GCACATAAT---------------
--------------ATCTGGGCTACA---CATGCCTGTGTACCCACAGAC---
CCCAACCCACAAGAAGTAAGGTTAGGC---------AATGTAACAGAAAATTTTAAC---------------
-------------------------ATGTGGAAAAATGATATGGTGGATCAAATGCATGAGGATATA---
ATCAGTTTATGGGATGAAAGCCTA---AAGCCATGTGTAAAGTTG---
ACCCCACTCTGTGTTACTTTAAGATGCAATAATATTACCCTTGAGAATGCTACCTGGGGTAATACCAAC----
----
-------------------AATACTACCACTGACTACAATGGTACCAGAAGTGAAGAAGTAAAA---
AATTGCTCTTTCAATACAACCGCAGAGATCAGA--------------------------
GATAAGAAACAGAGAGTGCATGCACTT---TTTTACAAACTAGATTTAGTACCACTTCATGAGAAGAGTGAT-
----

AACCTTAGTGAGAACTTTGATGAGTATATATTAATAAATTGTAATACCTCAACCATAACACAA---
GCCTGTCCAAAGGTCTCTTTTGACCCAATTCCTATACATTATTGTGCTCCAGCTGGTTATGCGATTCTAAAGT
GTAAT---AATAAGACATTTAAT------GGGACAGGACCATGTAATAAT----------------------
-GTCAGC---ACAGTACAATGTACACATGGGATTAAGCCAGTGGTATCAACT---
CAACTACTGTTAAATGGTAGCCTAGCAGAAGAAGAG------------
ATAGTAATTAGAACTGAAAATCTGACAAATAAT---------GCCAAAACAATAATAGTACAGCTTAAA----
-----------GAACCTATAAAC---------ATTACGTGTATCAGACCCAGCAATAATACAAGAAAA-----
-AGCATAAGGATA------------GGACCAGGACAAACA---TTC------TTTGCAACAGGAGAC------
---------ATAATAGGAGATATA-----------AGAAAAGCACATTGT---AACATTAGCAAAAAT---
GATTGGAATAAAACTTTACAAGAGGTAGGTAAAAAATTAGCA--------------GAATACTTCCCT----
-----------------------------------AATAGTACAATAAAATTTGCA------
CCATCTTCAGGAGGGGACCCAGAAATTACA---ACACATAGTTTTAATTGTGGAGGAGAA---
TTTAAGCAGCTCCAGACAAGAGTCCTGGCTATAGAAAGATACCTAAAGGATCAACAGCTCCTAGGACTTTGGG
GCTGCTCTGGAAAACTCATCTGCACCACTGCTGTGTCTTGGAACTCCAGTTGGAGT----------------
----------------------------AATAAATCTAAATCAGATATCTGG------GAT------
AACATGACCTGGATGGAGTGG---GATAGAGAAATTAGTAATTACACAAACATAATATAC---
GGGCTGCTTGAAGAATCACAAAACCAGCAGGAG---------------------------------------
----
AAAAATGAAAAAGATTTATTAGCATTGGACAGTTGGAAAAATCTGTGGAGTTGGTTTAGCATATCAAGTTGGC
TGTGGTATATAAAAATATTCATAATGATAGTAGGAGGCTTAATAGGTTTAAGAATAATTTTTGCT---
GTGCTTTCTATAGTG---------AATAGAGTCAGGCAGGGATACTCACCT------
TTGTCGTTTGCTATTAGTCTGCTTGATACCATAGCAATAGCAGTAGCTGAG---
GGAACAGATAGGATTATAGAACTAATACAAAGAATTTGTAGAGCT---------------------------
--------ATCTGCAACATA------CCTAGGAGAATAAGACAGGGCTTTGAAGCAGCTTTGCTA---TAA
>DQ011167
GTA---ATG--
GGGAATTTGTGGGTCACAGTCTATTATGGAGTACCTGTGTGG---AAAGAAGCAAAA---
ACTACTCTATTCTGTGCATCAGATGCTAAAGCATATGAGAAAGAA---GTACATAAT---------------
--------------GTTTGGGCTACA---CATGCCTGTGTACCCACAGAC---
CCCAACCCACAAGAAATAGTTTTGGAA---------AATGTAACAGAAAATTTTAAC---------------
-------------------------ATGTGG-AAAATGACATGGTGGATCAGATGCATGAAGATATA---
ATCAGTTTATGGGATCAAAGCCTA---AAGCCATGTGTAAAGTTG---
ACCCCACTCTGTGTCACTCTATATTGCACAAATGTTACCAGTAATAGTACCAGT------------------
----
------------------------------AATATTACTGGTGACATGAGTGGAGAAATGAAG---
AATTGCTCTTTCAATATAACCACAGAAATAAGA--------------------------
GATAGGAAAAAGCAAGTGTATGCACTT---TTTTATAAACTGGATATAGTATCACTTGGGGAG---------
----

GGGGAGAATAGTACCTATAGATTAATAAACTGTAATACCTCAGCCATAACACAA---
GCCTGTCCAAAGGTCTCTTTTGATCCAATTCCTATACATTATTGTGCTCCAGCTGGTTATGCGATTCTAAAAT
GTAAT---AATAAGACATTCAAT------GGGACAGGACCATGTCAGAAT---------------------
-GTCAGT---ACAGTACAATGTACACATGGAATTAAGCCAGTGGTATCAACT---

CAACTACTGTTAAATGGTAGCCTAGCAGAAGAAGAG------------
ATAATAATCAGATCTGAGAATCTGACAAACAAT---------GCCAAAACAATAATAGTACATCTTAAT----
-----------AGCTCTGTAGGA---------ATTGTATGTACAAGACCCAACAATAATACAAGAAAA-----
-AGTATAAGGATA------------GGACCAGGGCAAACA---TTC------TATGCAACAGGAGAC------
---------ATAATAGGAGACATA------------AGACAAGCACACTGT---AACATTAGTAGAAGG---
AAATGGAATGAAACCTTAGAAAGGGTAAAAGAAAAATTAAGA---------------AAACACTTCCTT----
-----------------------------------AATAAGACAATTGAATTTAAA------
CCATCCTCAGGAGGGGACCTAGAAATTACA---ACACATACCTTTAATTGTAGAGGAGAA---
TTTAAGCAACTCCAGACAAGAGTCCTGGCTATAGAAAGATACCTAAAGGATCAACAGCTCCTAGGGATTTGGG
GCTGCTCTGGGAAACTCATCTGCACCACTGCTGTGCCTTGGAACCTTAGCTGGAGT-----------------
----------------------------AATAAATCTTATGAAGATATTTGG------GAT------
AACATGACCTGGATGCAGTGG---GATAGAGAAATCAGTAATTACACAAAAACAATATAC---
AGGTTGCTTGAAGACTCGCAAAACCAGCAGGAA---

AAAAATGAAAAAGATTTATTAGCATTGGACAGTTGGAAAAATCTGTGGAGTTGGTTTACCATAACAAATTGGC
TGTGGTATATAAGAATATTTGTAATGATAGTAGGAGGCTTAATAGGCTTAAGAATAATTTTTGCT---
GTGCTCTCTATAGTG---------AATAGAGTTAGGCAGGGATACTCACCT------
TTGTCGTTGGCTATTAGTCTGCTTGATATCATAGCAATAAGAGTAGCTGAG---
GGAACAGATAGAATTATAGAAGTAGTACAAATATTTGGTGGAGCT----------------------------
--------ATCCGCAACATA------CCTAGAAGATA-AGACAGGGCCTTGAAGCAGCTTTGCAA---TAA
>DQ093590
GTG---GTG---
GGAAACTTGTGGGTCACAGTCTATTATGGAGTACCTGTGTGG---AGAGATGCAAAA---
GCTACTCTATTCTGTGCATCAGATGCTAAAGCATATGAGAAAGAA---GTGCATAAT----------------
--------------GTCTGGGCCACA---CATGCCTGTGTACCCACAGAC---
CCCAACCCACAAGAATTAGTTTTGGAA---------AATGTAACAGAAAATCTTAAT----------------
-------------------------ATGTGGAAAAATGATATGGTGGAACAGATGCATGAGGATGTA---
ATTAGTTTATGGGACCAAAGCCTA---AAGCCATGTGTAAAGTTG---
ACTCCACTTTGTGTCACTTTACACTGCACAAATGCTAATATTACCAAGAATGGTAAT----------------
----
----------------------------GCTACCTATAATGATACCATGAAGGGAGAAATACAA---
AATTGCTCTTTCAATACAACCACAGAAATAAGA--------------------------
GATAAGATACAAAAGGTGTATGCACTT---TTTTATAGACCTGATTTAGTACCACTTGATGGG----------
----

GATAACTCTAGTTATATATTAATAAATTGCAATACCTCAACCATAACACAA---
GCCTGTCCAAAGGTCTCTTTTGACCCGATTCCTATACATTATTGTGCTCCAGCTGGTTATGCGATTCTAAAGT
GTAAT---AATGAGACATTCAAT------GGGACAGGACCATGCACTAAT-----------------------
-GTAAGC---ACAGTACAATGTACACATGGGATTAAGCCAGTGGTATCCACT---
CAACTCCTGTTAAATGGTAGCCTAGCAAAAGAGGAG------------
ATAATAATTAGATCTGAAAACCTGACAAACAAT---------GCCAAAACAATAATAGTACACCTTAAT----
-----------GAATCTGTAGAA---------ATTGTATGTACAAGACCCGGCAATAATACAAGAAGA-----
-AGTATAAGGATA------------GGACCAGGACAAACG---TTC------TATGCAACAGGAGAC------
---------ATAATAGGAGACATA------------AGACAAGCACATTGT---AACATTAGCGCAGAT---
AAATGGAATAAAACTTTACAAGAGGTAGGAAAAAAATTAGCA---------------GGACACTTCCCT----
-----------------------------------AATAGAACAATAAAATTTGAA------
CCACATTCAGGCGGGGACCTAGAAATTACA---ACACATAGTTTTAATTGTAGAGGGGAA---
TTTAAGCAGCTCCAGACAGGAGTATTGGCTATAGAAAGATACCTAAAGGATCAACAGCTCCTAGGGGTTTGGG
GCTGCTCTGGAAAGCTCATCTGCACCACTGCTGTGCCTTGGAACTCCAGTTGGAGT-----------------
----------------------------AATAGATCTGAAACAGATATTTGG------AAT------
AACATGACCTGGATGCAGTGG---GATAGAGAAATTAGTAATTACACAGGCATAATATAC---
AGGTTGCTTGAAGACTCACAAAACCAGCAGGAG---

AAAAATGAAAAGGATTTATTTGCATTGGACAGTTGAAAAAATCTGTGGAATTGGTTTAACATCACAAATTGGC
TGTGGTATATAAAAATATTTATAATGATAGTAGGAGGCTTGGTAGGTTTGAGAATAATTTTTGCT---
GTGCTCTCTATAGTG---------AATAGAGTCAGGCAGGGATACTCACCT------
TTGTCGTTTGCTATTAGTCTGTTTGATACCATAGCAATAGCAGTAGCTGAA---
GGAACAGATAGGATTATAGAATTAATACAAAGATTTTGTAGAGCT----------------------------
--------ATCTACCACATA------CCTACAAGAATAAGACAGGGCTTTGAAGCAGCTTTGCAA---TAA
>DQ011178

GTG---GTG--
GGGAACTTGTGGGTCACAGTCTATTATGGAGTACCTGTGTGG---AAAGACGCAAGT---
ACTCCTCTATTCTGTGCATCAGAAGCTAAAGCATATGAGAAAGAA---GTGCATAAT----------------
--------------GTCTGGGCTACA---CATGCCTGTGTACCCACAGAC---
CCCAACCCACAAGAAATGGTTTTGGAA---------AATGTAACAGAAAATTTTAAT----------------
--------------------------ATGTGGAAAAATGACATGGTAGATCAGATGCAGGAAGACATA---
ATCAGTTTATGGGATGAGAGCCTA---AAGCCATGTGTAAAGTTG---
ACCCCACTCTGTGTCACTTTAAACTGTACAACAGTTAATAGTACAACAGCTAATAGTACAACAGCT-------
----
----------------------AATAGTACTTTCTACAATAACACAGAGAATGAACAACTAAAA---
AATTGCTCTTTCAATATAACCACAGAAATAAGA--------------------------
GATAAAAAAACAGCAAGTATATGCACTC---TTTTATAAACCTGATGTAGTACCACTTAATGGG----------
----

AATTCTAGTGAGTATATATTAATTAATTGCAATACCTCAACCACAACACAA---
GCCTGTCCAAAGGTCTCTTTTGACCCAATTCCTATACACTATTGTGTTCCAGCTGGTTATGCGATTCTAAAGT
GTAAT---AATAAGACATTTAAT------GGAACAGGACCATGCCAGAAT-----------------------
-GTCAGT---ACAGTACAATGTACACATGGAATTAAGCCAGTGGTATCAACT---
CAACTACTGTTAAATGGTAGCCTATCAGAAGAAGGG-----------
GTAATAATCAGATCTGAAAATCTGACAGACAAT---------GTCAAAACAATAATAGTACATCTCAAC----
-----------AGATCTGTAGAA---------ATTAATTGTACAAGACCCAACAATAATACAAGAAAA-----
-AGTATAAGGATA------------GGACCAGGGCAAACA---TTC------TATGCAACAGGAGAT------
---------ATAATAGGAGACATA------------AGACAAGCACACTGT---GACGTTAATAGAACA---
AAATGGAAGGAAACTTTACAATGGGTAAAGAATAAATTAGGA---------------GAACATTTCCCT----
-----------------------------------AATAAAACAATAGAATTTGCA------
CAACCCTCAGGAGGGGATCTGGAAATTACA---ACACATACCTTTAATTGTAGAGGAGAA---
TTTAAGCAGCTCCAGGCAAGAGTCCTGGCTATAGAAAGATACCTAAAGGATCAACAGCTCCTAGGGCTTTGGG
GCTGCTCTGGAAAACTCATCTGCACCACTGCTGTGCCTTGGAACTCCAGTTGGAGT-----------------
-----------------------------AATAAATCTGACACAGATATTTGG------GAT------
AACATGACCTGGATGCAGTGG---GATAGAGAAATTAATAATTACACAAGCACAATATAC---
AAGTTGCTTGAAGAATCGCAAAGCCAGCAGGAA---------------------------------------
----
AAAAATGAAAAAGATTTATTAGCATTGGACAGTTGGAACAATCTATGGAGTTGGTTTAGCATAACAAATTGGC
TGTGGTATATAAGAATATTTATAATGATAGTAGGAGGCTTAATAGGCTTGAGAATAATTTTTGCT---
ATACTCTCTATAGTG---------AAAAAAGTTAGGCAGGGATACTCACCT------
TTGTCGTTTGCTGTTAGTCTGTTTGATACCATAGCAATAGCAGTAGCTGGA---
GGAACAGATAGGATTCTAGAAGTATTACAAAGAATTTGTAGAGCT---------------------------
--------ATCTGCAACATA------CCTAGAAGAATAAGACAGGGCTTCGAAACAGCTTTGCAA---TAA
>AY901980
GTG---GTG--
GGAAACTTGTGGGTTACAGTATATTATGGGGTACCTGTGTGG---AGAGAAGCAAAA---
ACTACTCTATTCTGTGCATCAGATGCTAAAGCATATAAGGAGGAG---GTGCATAAT----------------
--------------GTCTGGGCTACA---CGTGCCTGTGTACCCACAGAC---
CCCAACCCACAAGAAATAGTCATGGAA---------AATATAACAGAAAATTTTAAC----------------
--------------------------ATATGGAAAAATGACATGGTGGATCAAATGCATGAGGATATA---
ATCAGTTTATGGGATCAAAGTCTA---AAGCCATGTGTAAAATTG---
ACCCCACTCTGTGTCACTTTAAAATGTATAGATGTAAATGTTACCAGTAATGCTAATAATACCAGTACAAATG
AT---
----------ACCAGTATAAATGCAAATGCTACCAGTGAGAATAGCATGCGGGGAGAAGTAAAA---
AATTGCTCTTTCAACATAACCACAGAAATAAGA--------------------------
GATAAGAAACAGAAAGCATTTGCACTT---TTTTATAGAACTGATATAGTACCACTCAATGAAAATAAC----
----

TCTAGTAAGAATAACCTTAGTGAATATATACTAATAAATTGCAATACCTCGACCATAACACAA---
GCCTGTCCAAAGGTCTCATTTGACCCCATTCCTATACATTATTGTGCTCCAGCTGGCTTTGCGATTCTAAAAT
ACAAT---AATAAGACATTCAAT------GGGACAGGACCATGCTATAAT-----------------------
-GTCAGC---ACAGTACAATGTACACATGGAATTAAACCAGTGGTATCAACT---
CAATTACTGTTAAATGGTAGCCTAGCAGAAGAGGAG-----------
ATAATAATCAAATCTGAAAATCTGACAGACAAT---------GCCAAAACAATAATAATACACCTTAAT----
-----------AAATCTATAGCA---------ATTGTGTGTACAAGACCCAACAATAATACAAGAAAA-----

-AGTATAAGGATA------------GGGCCAGGACAAACA---TTC------TATGCAAGAGGTGAC------
---------ATAATAGGAGACATA------------AGACAAGCACATTGT---AACATTAGTAGGGAT---
GAATGGAATAGAACTCTACAATTGGTAGGAAAAAAATTAAAA---------------GAACACTTCCCT----
-----------------------------------AATAAAACAATAAAGTTTAAT------
GCATCCTCAGGAGGGGACTTAGAAATTACA---ACACATAGCTTTAATTGTAGAGGAGAA---
TTTAAGCAGCTCCAGGCAAGAGTCCTGGCCATAGAAAGATACCTACAGGATCAACAGCTCCTAGGAATTTGGG
GCTGCTCTGGAAAACTCATCTGCACCACTGCTGTGCCTTGGAACTCTAGTTGGAGT-----------------
----------------------------AATAAATCTAAAACTGATATTTGG------GAT------
AACATGACCTGGATGCAGTGG---GATAGAGAAATTAGCAATTACACAGACACAATATAC---
AGGTTGCTTGAAGAATCGCAAGCCCAGCAGGAC--

CAAAATGAAAAAGATTTATTAGCATTGGACAAATGGCAAAATCTGTGGAATTGGTTTAGCATAACAAATTGGC
TATGGTACATAAGAATATTCATAATGATAGTAGGGGGCTTAATAGGTTTAAGAATAATTTTTGCT---
GCGCTATCTATAGTA---------AATAGAGTTAGGCAGGGATACTCACCT------
TTGTCATTTGCTGTTAGTCTGTTTGATACCATAGCAATAGCAGTAGCAGAG---
GGAACAGATAGAATTATAACATTCATACAAAGAATTTGTAGAGCT----------------------------
--------ATCCTCAACCTA------CCCAGAAGAATAAGACAGGGTTTTGAAGCAGCTTTGCTA---TAA
>AY878059
GTA---TGG--
GGGAACTTGTGGGTCACAGTCTATTATGGGGTACCTGTGTGG---GAAGAAGCAAAA---
GCTCCTCTATTCTGTGCATCAGATGCTAAAGCATATGAGAGAGAA---GTGCATAAT----------------
--------------ATCTGGGCTACA---CATGCCTGTGTACCCACAGAC---
CCCAACCCACAAGAAATATGGTTGAAA---------AATGTAACAGAAAATTTTAAC----------------
--------------------------ATGTGGAAAAATGACATGGTGGATCAAATGCATGAGGATATA---
ATTAGTTTATGGGATCAAAGCCTA---AAACCCTGTGTAAAGTTG---
ACCCCACTCTGTGTCACCTTACAGTGTAACGATACTCGCAGTAATGGT-------------------------
----
-------------------------------------ACTGCTGATAACCAGGAAAGAATGAAA---
AATTGCTCTTTCAATACAACCACAGAAATAAGA---------------------------
GATAAGAAACAGAAAGTGTATGCACTT---TTTTATAAATCTGATGTAGTACAATTTGATAAT----------
----

AGTAGTTATATATTAATAAATTGCAATACCTCAACCATAACACAA---
GCCTGCCCAAAGGTCTCTTTTGACCCAATTCCTATACATTATTGTGCTCCAGCTGGATATGCGATTCTAAAGT
GTAAT---GATGTGACATTCAAT------GGGACAGGACCATGCCATAAT-----------------------
-GTCCGC---ACAGTACAATGTACACATGGAATTAAGCCAGTGGTATCAACT---
CAACTTCTGTTAAATGGTAGCCTAGCAGAACAGGAG------------
ATAATAATTAAATCTGAAAATCTGACAGACAAT---------ATCAAAACAATAATAGTCCACCTTAAT----
-----------GAATCTGTAGAA---------ATTAATTGTACAAGACCCAACAATAATACAAGGAAA-----
-AGTATAAGGATA------------GGACCAGGACAAACA---TTC------TATACAAATGAG---------
---------ATAATAGGAGACATA------------AGACAAGCATATTGT---AACATTAATGGAAGT---
AAATGGAACAAAACTTTAGAGCAGGTAAAGAAAAGATTAGAA---------------GAACACTACCCT----
-----------------------------------AATAAAACAATAGAATTTAAA------
CAACACTCAGGAGGGGATCTAGAAATTACA---ACACATAGCTTTAATTGTAGAGGAGAG---
TTTAAGCAGCTCCAGACAAGAGTCTTGGCTATAGAAAGATACCTAAAAGATCAACAGCTCCTAGGACCTTGGG
GCTGCTCTGGAAAAATCATCTGCACCACCGCTGTGCCTTGGAACTCCAGTTGGAGT-----------------
----------------------------AATAAATCTGAAACAGAGATTTGG------AAT------
AACATGACCTGGATGCAGTGG---GATAGAGAAATTAATAGTCACACAGGCACAATATAC---
AGGTTACTTGAAGACTCGCAAAGCCAGCAGGAG--

AAAAATGAAAAAGATTTATTAGCATTGGACAGTTGGAACAACCTGTGGAATTGGTTTGGCATAACAAAATGGC
TGTGGTATATAAAAATATTCATCATAATAGTAGGAGGCTTGATAGGTTTAAGAATAGTTTTAGGT---
GTACTCTCTATAGTG---------AAAAGAGTTAGGCAGGGATACTCACCT------
TTGTCGTTTGCTATTAATCTGCTTGATACCATAGCAATAGCAGTAGCTGAA---
GGAACAGATAGGATTATTGAAGTAATACAAAGAATTTGTAGAGCT----------------------------
--------ATCCTTAACATA------CCTAGAAGAATAAGACAGGGCTTTGAAGCAGCTTTGCTA---TAA
>DQ011174
GTG---GTG--
GGGAACTTGTGGGTCACAGTCTATTATGGGGTACCTGTGTGG---ACAGAAGCAAAA---
GCTACTCTATTCTGTGCATCAGACGCTAAAGCATATGACACAGAA---GTGCATAAT----------------

--------------GTCTGGGCTACA---CATGCCTGTGTACCCACAGAC---
CCCAACCCACAAGAAATAGTTTTGGAA---------AATGTAACAGAAAATTTTAAC----------------
--------------------------ATGTGGAAAAATGATATGGTGGATCAGATGCATGAGGATGTA---
ATCAGTATATGGGATCAAAGTCTA---AAACCATGTATAAAGTTG---
ACCCCACTCTGTGTCACTTTAACCTGTACAAATGCTACTGTTAGTGGTGCCAGAAATAGT-------------
----
-------------------------AGTGTTGCCTACAATAATAGCATAGTAGGAGAAATAAAA---
AATTGCTCTTTCAATACAACCACAGAACTAAGA---------------------------
GATAAGAAGCAGAAAGCGTATGCACTT---TTTTATAAACCTGATATAGTACCACTTAAGGAG----------
----

GATAATTCTAGTTATATATTAATAAATTGTAATACCTCAACCATAGCACAA---
GCCTGTCCAAAGGTCACCTTTGACCCGATTCCTATACATTATTGTGCTCCAGCTGGTTATGCGATTCTAAAGT
GTAAT---AATAAGACATTCAAT------GGGACAGGACCATGCCATAAT-----------------------
-GTCAGC---ACAGTACAATGTACACATGGGATTAAGCCAGTGGTATCAACT---
CGACTACTGTTAAATGGTAGCTTAGCTGAAGGGGA-------------
ATAATAATTAGATCTGAAAATCTGACAAACAAT---------GCCAAAATAATAATAGTACACCTTAAT----
-----------GAATCTGTAGAC---------ATTGTGTGTACAAGACCTGGCAATAATACAAGAAGA-----
-AGCATAAGGATA------------GGACCAGGACAAGCA---ATC------TATGCAACAAATGAC------
---------ATAATAGGAGACATA------------AGACAAGCACATTGT---AACATTAATAGAACA---
AATTGGAACAAAACTTTAGAAAGGGTAAAGGAGAAATTAAGA---------------GAACACTTCCCT----
-----------------------------------AATAAAACAATAACGTTTGCA------
CAACCCGCAGGAGGGGACCTAGAAGTTACA---ACACATAGTTTTAATTGTAGAGGAGAA---
TTTAAGCAGCTCCAGGCAAGAGTCCTGGCTATCGAAAGATACCTAAAGGATCAACAGCTCCTAGGGCTTTGGG
GCTGCTCTGGAAAACTCACCTGCACCACTGCTGTGCCTTGGAACTCCAGTTGGAGT-----------------
----------------------------AATAAATCTCTAGAAGATATTTGG------ACT------
AACATGACCTGGATGCAGTGG---GAGAGAGAAATTAGTAATTACACAGACACAATATAC---
AGGCTACTTGAAGACTCGCAAAGCCAGCAGGAG--

AAAAATGAAAAGGATTTATTAGCATTGGACAGTTGGCAAAACTTGTGGAATTGGTTTAGCATAACAAAATGGC
TGTGGTACATAAAAATATTCATCATGATAGTAGGAGGCTTGATAGGTTTAAGAATAATTTTTGCT---
GTGCTCTCTATAGTG---------AAAAGAGTTAGGCAGGATACTCGCCT------
TTGTCGTTTGCTATTAGTCTGCTTGATACCATAGCAATAGCAGTAGCTGAA---
GGAACAGATAGGATTATAGAATTTATACAAAGAATTTGTAGAGCT----------------------------
--------ATCTGGAACATA------CCTACAAGAATAAGACAGGGCTTTGAAGCAACTTTGCTA---TAA
>GQ999986
GTG---GTA---GGA------------------------------------
CAAAACTTGTGGGTCACAGTCTATTATGGGGTACCTGTATGG---AAAGAAGCAAAA---
ACTACTCTATTCTGTGCATCAAATGCTAAAGCATATGAGAAAGAA---GTGCATAAT----------------
--------------GTCTGGGCTACA---CATGCCTGTGTACCCACAGAC---
CCCAACCCACAAGAAATGGTTTTGGGA---------AATGTAACAGAAAATTTTAAT----------------
--------------------------ATGTGGAAAAATGATATGGTGGATCAGATGCATGAGGATATA---
ATCAGTTTATGGGATGAGAGCCTA---AAGCCATGTGTAAAGTTG---
ACCCCACTCTGTGTCACTTTAATTTGTACAAATGTTAATCATAGTAGTACAAATGTTAGTCATAGTAGTACAA
ATGTTAGTCAG--
----AGTAGTAATAGCAGTCAGGATAATACTACCATTGATGAGAGCATGCGTGAAGAAATAAAA---
AATTGCTCGTACAACTCAACCACAGAGCTATGG---------------------------
GATAAGAAACAAAAGGAGTATGCACTT---TTTTATAAACTTGATATAGTACCAATTAATGGG----------
----

AATGCTAGTGAATATAGATTAATAAATTGTAATACCTCAACCATAAAACAA---
GCCTGCCCAAAGGTCACTTTTGAACCAATTCCTATACATTATTGTGCTCCAGCTGGTTATGCGATTCTAAAGT
GTAAT---AATCAGACATTCGAT------GGGACAGGACCATGTACTAAT-----------------------
-GTCAGC---ACAGTACAATGTACACATGGAATTAAGCCAGTGGTATCAACT---
CAACTACTGTTAAATGGTAGCCTAGCAGAAAAAGAG------------
ATAATAATTAGCTCTGAAAATCTGACAAACAAT---------GCCAAAATAATAATAGTACACCTTAAG----
-----------GATCCTGTACGG---------ATTGTGTGTACAAGACCCAATAATAATACAAGAAAG-----
-AGTATAAGGATA------------GGACCAGGACAAACA---TTC------TATGCAACAGGAGAT------
---------ATAATAGGAGATATA------------AGACAAGCATATTGT---AACATTGATAAAGGG---
GCCTGGAATAAAACTTTACAACAGGTAGGGAAAAAATTACAA---------------GAACATTTCCCT----

-----------------------------------GGTAAAACAATAAAATTTAAC------
TCATCCTCAGGAGGGGATCTAGAAATTACA---ACACATAGCTTTAATTGTAGAGGAGAA---
TTTAAACAGCTCCGGGCAAGAGTCGTGGCTCTAGAAAGATACCTACAGGATCAACAGCTCCTAGGGATTTGGG
GTTGCTCTGGAAAACTCATCTGCACCACTAATGTGCCTTGGAACTCCAGTTGGAGT-----------------
-------------------------------AATAGATCCGCTGACATATGG------GAT------
AACTTGACATGGATGCAGTGG---GAGAAAGAAATCAATAACTACACAGATACAATATAC---
CAGTTGCTTGAAGAATCGCAAATCCAGCAGGAA--

AAAAATGAACAAGATTTATTGGCATTGAACAGTTGGGACAGTCTGTGGAGTTGGTTTAGCATAACAAAATGGC
TGTGGTATATAAAAATATTCATAATGATAATAGGAGGCTTGATAGGTTTAAGAATAATTTTTGCT---
GTGCTTTCTATAGTA---------AATAGAGTTAGGCAGGGATACTCACCT------
TTGTCGTTTGCTGTTAGTCTGCTTGATACCATAGCAATAGCAGTAGCTGAA---
GGAACAGATAGGATTATAGAATTAGTACAAGCAATTGGTAGAGCT----------------------------
--------ATTCGCAACATA------CCTACAAGAATAAGACAGGGCTTTGAAGCAGCTTTGCTA---TAA
>GQ999987
AGG---GGG---
AACTTGTGGGTCACAGTCTATTATGGGGTACCTGTGTGG---AAAGAAGCAAAA---
GCTACTCTATTCTGTGCATCAGATGCTAAAGCATATGAGAGAGAA---GTGCATAAT----------------
--------------GTCTGGGCTACA---CATGCCTGTGTACCGACAGAC---
CCCAACCCACAAGAATTAGTTTTGGGA---------AATGTAACAGAAAATTTTAAC----------------
--------------------------ATGTGGAAAAATGACATGGTGGATCAGATGCATGAGGATATA---
ACCAGTATATGGGATCAAAGCCTA---AAACCATGTGTAAAGTTG---
ACCCCACTCTGTGTCACTTTAAACTGTACAGATATAAAAAACAATAGTACCAGGGGG----------------
----
----------------------------AATAATGGTAGTGATAGCATCAAGGAAAGCATGACA---
AATTGCTCTTTCAATACAACCACAGAATTAAGA---------------------------
GATAAGGAAAAGCAAGCGTATGCACTG---TTTTATAGATCTGATGTAGTACCACTTGGGAACTCT-------
----

AATAAGAACTATAGTGAGTATGTATTAATAAATTGCAATACCTCAACCATAACACAA---
GCCTGTCCAAAGGTCTCCTTTGATCCAATTCCTATACATTATTGTGCTCCAGCTGGTTATGCGATTCTAAAGT
GTAAT---GATAAGACATTCAAT------GGGACAGGACCATGCACTAAT-----------------------
-GTCAGC---ACAGTACAATGTACACATGGAATTAAGCCAGTGGTATCAACT---
CAACTACTGTTAAATGGTAGCCTAGCAGAAAAGGAG------------
GTAGTCATTAGATCTGAAAATCTGACAGACAAT---------GTCAAAACAATAATAGTACATCTTAAT----
-----------GAATCTATAGAA---------ATTGTGTGTACAAGACCCAACAATAATGCAAGAAAA-----
-AGCATAAGAATA------------GGACCAGGACAAACA---TTC------TATGCAACAGGTGAC------
---------ATAATAGGAAACATA------------AGAGAAGCACATTGT---AACGTCAGTGCAGCA---
AAATGGAACAGAACTTTAGGACTGGTAGCGGAAAAATTAAAA---------------GAACACTTCCAT----
-----------------------------------AATAAAACCATATCCTTTAAA------
CCACCCTCAGGAGGGGACTTAGAAATTACA---ACACATAGTTTTAATTGTGGAGGAGAA---
TTTAAGCAGCTCCAAGCAAGAGTCCTGGCTATAGAAAGATACCTAAAGGATCAACAGCTCCTAGGGCTTTGGG
GCTGCTCTGGAAAACTCATCTGCACCACTAATGTGCCTTGGAACTCCAGTTGGAGT-----------------
-----------------------------AATAAATCTCAAACAGATATTGG------GAT------
AACATGACTTGGATGCAGTGG---GATAGAGAAATTAATAATTACACAGGCATAATATAC---
AGATTGCTTGAAGACTCGCAAAACCAGCAGGAG--

AAAAATGAAAAAGATTTATTAGCATTGGACAGTTGGAACAGCTTGTGGAGTTGGTTTAGCATAACAAAGTGGT
TGTGGTACATAAAAATATTCATCATGATAGTAGGAGGCTTAATAGGTTTAAGAATAATTTTTGGT---
GTGCTCTCTATAGTG---------AACAGAGTTAGGCAGGGATACTCACCT------
TTGTCGTTTCTATTAATCTGCTTGATACCANTAGCAATAGTAGTAGCTGAG---
GGGACAGATAGGATTATAGAATTA---
-----------------------------ATAAGAATAAGATAAGGCTTTGAAACAGCTTTGCTA---TAA
>GQ999977
GTG---AGG--
GGAAACTTGTGGGTTACAGTATATTATGGGGTACCTGTGTGG---AGAGAAGCAAAA---
GCTACTTTATTCTGTGTATCAGATGCTAAAGCATATGAGAGGGAG---GTGCATAAT----------------
--------------ATCTGGGCTACA---CATGCCTGTGTACCCACAGAC---
CCCAACCCACAAGAATTAGTTCTGGAA---------AATGTAACAGAAAATTTTAAT----------------
--------------------------ATGTGGAAAAATGACATGGTGGATCAGATGCATGAGGATATA---

ATCAATTTATGGGATCAAAGCCTA---AAGCCATGTGTAAAGTTG---
ACCCCACTCTGTATCACTTTAACATGCACAAATGCTATATACAAG---------------------------
----
---------------------------------------AATACTAGCACGGAGGAAATGAAA---
AATTGCTCTTTCAATGTATCCACAGAAATAAAA--------------------------
GATAAAAAAACATAAAGAGTATGCCACT---TTTTATAGACTTGATATGGTACCACTTAATGAA----------
----

TCTAGTGATAACCAGACTTATAGATTAATAAATTGTAATGCTTCAAAAGTAGCACAA---
GCCTGTCCAAAGGTCAATTTTGACCCAATTCCTATACATTATTGTGCTCCAGCTGGTTATGCGATTCTAAAGT
GTAAT---AATAAGACATTCAAT------GGGACAGGACCATGCTATAAT----------------------
-GTCAGT---ACAGTACAATGTACACATGGAATTAAACCAGTGGTATCAACT---
CAACTACTATTAAATGGTAGCATAGCAGAAGGAGAG-----------
ATAATAATCAGGTCTGAAAATCTGACAGACAAT---------GTTAAAACAATAATAGTGCAGCTTAAT----
-----------GAAACTGTAGAC---------ATTGTGTGTACAAGACCCAACAATAATACAAGGAAA-----
-AGTATAAGGATA------------GGACCAGGACAAACA---TTC------TATGCAACAGGTGAA------
---------ATAATAGGAAACATA------------AGAGAAGCACATTGC---AATATTAGTAAAGGT---
AACTGGTCTAGAACTTTACACAGGGTAAGTGAAAAATTAAAC---------------GAACACCTCCCT----
----------------------------------AATAAAACAATAAAATTTAGT------
CCATCCTCAGGAGGGGACCTAGAAATTACA---ACACATAGCTTTAATTGTAGAGGAGAA---
TTTAAGCAGCTCCAGACAAGAGTCCTGGCTATAGAAAGATACCTACAGGATCAACAGCTCCTAGGAATTTGGG
GCTGCTCTGGAAAACTCATCTGCACCACTGCTGTGCCCTGGAACTCCAGTTGGAGT-----------------
----------------------------AATAAATCTTATGATGAGATTTGG------AAA------
AACATGACATGGATGCAGTGG---GGTAGAGAAATTAGTAATCACACAAATACAATATAC---
CAGTTGCTTGAAGACTCGCAAATCCAGCAGGAG---------------------------------------
----
AAAAATGAAAAGGATTTATTAGCATTGGACAAATGGCAAAATCTGTGGAATTGGTTTAGTATATCAAATTGGC
TATGGTATATAAAAATGTTCATAATGATAGTAGGAGGCTTGATAGGTTTAAGGATAATTTTTGCT---
GTGATATCTATAGTA---------AACAGAGTTAGGCAGGGATACTCACCT------
CTATCGTTTGCTATTAGTCTGCTTGATACCATAGCAATAGCAGTAGCTGAG---
GGAACAGATAGAATTTTAGAATTCATACAAAGACTTTGTAGGGCT---------------------------
--------ATCCTCAATACA------CCTAGAAGAGTAAGACAGGGTTTTGAAGCAGCTTTGCTA---TAA
>JN687821
GGA---AAT---
AACTTGTGGGTCACTGTCTATTATGGGGTACCTGTGTGG---AAAGAAGCAAAA---
ACTACTCTATTCTGTGCATCAGATGCTAAAGCATATGATAAAGAA---GTGCATAAT----------------
--------------GTATGGGCTACA---CATGCCTGTGTACCCACAGAC---
CCCAACCCACAAGAAATAGTTTTGGAA---------AATGTAACAGAAAATTTTAAC----------------
-------------------------ATGTGGAAAAATGACATGGTGGATCAGATGCATGAGGATATA---
ATCAGTTTATGGGATCAAAGTCTA---AAACCMTGTGTAAAGTTG---
ACCCCACTCTGTGTCACTTTAAAATGTACCAATGCTACATTTAATGCT-------------------------
----
------------------------------------ACAGTTAATAGTATGGAARAAATAAAA---
AATTGCTCTTTCAATATAACCACAGAAATAAGA--------------------------
GATAAAAAGAAGAATGAATATGCACTT---TTTTATAGACTTGATATAGTACCACTTGATAAGAATGAA----
----

AATAGCTCTAGTAATTATAGTGAGTATGTATTAATAAATTGTAATACCTCAGCCGTAACACAA---
GCCTGTCCAAAGGTCTCTTTTGAACCAATTCCTATACATTATTGTGCTCCAGCTGGTTATGCGATTCTAAAAT
GTAAT---AATAAGACATTCAAT------GGGACAGGACCATGCAATAAT----------------------
-GTCAGC---ACAGTACAATGCACACATGGAATTAAGCCAGTGGTATCAACT---
CAACTATTGTTAAATGGTAGCCTAGCAGAAGAGGAG-----------
ATAATAATTAGATCTGAAAATCTGACAAATAAT---------ATCAAAACAATAATAGTCCACCTTAAT----
-----------AAATCTGTAGAA---------ATTAACTGTATAAGACCCGGCAATAATACAAGGAAA-----
-AGTATGAGGATA------------GGACCAGGACAAACA---TTC------TATGCAACAGGAGCC------
---------ATAATAGGAAACATA------------AGACAAGCACATTGT---AACATTAGTGGGAAG---
GATTGGAACAACACTTTAGAACAGGTGAAGAAAAAATTACAA---------------GAACAGTAC-------
----------------------------------AATAAAACAATAATATTTGAA------
CAATCCTCAGGAGGGGACCTAGAAATTACA---ACACATAGCTTTAATTGTAGAGGAGAA---
TTTAAGCAGCTCCAGACAAGAGTCCTGGCTATAGAAAGATACCTAAAGGATCAACAGCTCCTAGGGCTTTGGG

```
GCTGCTCTGGAAAACTCATCTGCACCACTGCTGTGCCTTGGAACTCCAGTTGGAGT-----------------
----------------------------AATAAAACTCAAGGGGACATTTGG------GAC------
AACATGACCTGGATGCAGTGG---GATAGAGAAATTAATAATTACACAAATACCATATAC---
AGGTTGCTTGAAGAATCACAGAACCAGCAGGAG----------------------------------------
-----------------------------
AAAAATRAAAAAGATTTATTATCATTGGAAAGCTGGAACAATCTGTGGAATTGGTTTAGCATATCAAATTGGC
TGAGGTATATACAAATATTTCTAATGATAGTAGGAGGCTTGATAGGTTTAAGAATAATTTTTGCT---
GTGATTTCTATAGTR---------AATAGAGTTAGGCAGGGATACTCACCT------
TTGTCGTTTGCTACTAGTCTAGTTGATACCATAGCCATAGCAGTAGCTGAA---
GGAACAGATAGGATTATAGAATTAATACAAAGAATTTGGAGAACT----------------------------
--------ATCCGTAACATA------CCTACAAGAATAAGACAGGGCTTTGAAGCAGCTTTACTA---TAA
>KC863367
GGG---ACA------------------------------------------
GGAAACTTGTGGGTCACAGTCTATTATGGGGTACCTGTGTGG---AAAGAAGCAAAA---
ACTACTCTATTCTGTGCATCAGATGCTAAAGCATATGAGAAAGAA---GTGCATAAT----------------
--------------GTCTGGGCTACA---CATGCCTGTGTACCCACAGAC---
CCCAGCCCAAATGAAATGGATTTGATA---------AATGTAACAGAAGATTTTAAC----------------
--------------------------ATGTGGAAAAATGATATGGTAGATCAGATGCATGAGGATATA---
ATCAGTTTATGGGATCAAAGCCTA---AAACCATGTGTAAAGTTA---
ACCCCACTCTGTGTCACTTTAAACTGTACAAACCTACGCTGTGACAATAGTACAAACCCACGCTGTGTCAATG
ATACAACAAAA--------------------------------------------------------------
----------------------GGCCCACTCTGTGGCAATAGTACAATAAGAGAAGAAATGAAG---
AATTGCTCTTTCAATGCAACCACAGTAATAAAA---------------------------
GATAAGAAACAGAAGGTGTTTGCACTT---
TTTTATAAACTTGATATAGTACCACTTAATAAGGAAAACAATAACAGCTCT----------------------
--------------------------------------------------
GAGGAAAATAATAACAGCTCTGGGGAATATAGATTAATAAATTGTAATACCTCAGCCATAACACAA---
GCCTGTCCAAAGGTCTCTTTTGACCCAATCCCTATACATTATTGTGCTCCAGCTGGTTATGCAATTCTCAAGT
GTAAA---AATAAGACATTCAAT------GGGACAGGACCATGCAACAAT-----------------------
-GTCAGC---ATAGTACAATGTACACATGGAATTAAGCCAGTGGTATCAACT---
CAACTACTGTTAAACGGTAGCCTAGCGGAAGAGGAG------------
ATAATAGTTAGTTCTGAAAATCTGTCAAACAAT---------GCTAAAACAATAATAATACATTTTAAT----
-----------AAATCTGTAGAA---------ATTGTATGTACAAGACCCCACAATAATACAAGAAAA-----
-GGTATAGGGATA------------GGACCAGGACAAACA---TTC------TATGCAACAGATGCA------
---------ATAATAGGAGACATA------------AGACAAGCACATTGT---AACATTAGTAAGGA---
AATTGGAGCAGGACTATAAGAGAGGTAGCGGGAAAATTAAAA---------------GAACACTTCCCC----
-----------------------------------AATAAGACAATAAGATTTAGC------
TCATCCTCAGGAGGGGATCTAGAAATTACA---ACACATAGTTTAATTGTAGAGGAGAA---
TTTAAGCAGCTCCAGGCAAGAGTCCTGGCTATAGAAAGATACCTACAGGATCAACAGCTCCTAGGGATTTGGG
GCTGCTCTGGAAAACTCATCTGCACCACTGCCGTGCCTTGGAACTCCAGTTGGAGT-----------------
----------------------------AATAGAAATCTAACAGAAATTTGG------GAT------
AACATGACCTGGATGCAATGG---GATAGAGAAATTAGTAATTACACAAACACAATATAT---
CAGTTGCTTGAAGAATCGCAAATCCAGCAGGAA----------------------------------------
-----------------------------
AAGAATGAAAAAGATTTATTATCATTGGACAAATGGAACAATCTATTTAATTGGTTTGGCATAACAAAGTGGC
TGTGGTATATAAAAATATTCATAATGATAGTAGGAGGCTTGATAGGGTTAAGAATAATTTTTGCT---
GTGCTATCTATAGTA---------AATAGAGTTAGGCAGGGATACTCACCT------
CTGTCGTTTGCTATTAGCCTGCTTGATACCATAGCAATAGCAGTAGCTGAG---
GGAACAGATAGGATTCTAGAATTAGTACAAAGAGTTGTTGGAGCT----------------------------
--------ATCCTCCACATA------CCTAGAAGAATAAGACAGGGCTTTGAAACAGCTTTGCTA---TAA
>KC894137
GTG---TGG------------------------------------------
GGAAACTTGTGGGTCACAGTCTATTATGGGGTACCTGTGTGG---AAGGAAGCAAAA---
ACTACTCTATTTTGTGCATCAGATGCTAAAGCATATGTGAAAGAA---GTGCATAAT----------------
--------------GTCTGGGCTACA---TATGCCTGTGTACCCACAGAC---
CCCAATCCACAAGAATTGGTTTTGGAA---------AATGTAACAGAAAATTTTAAC----------------
--------------------------ATGTGGAAAAATGACATGGTGGATCAGATGCATGAGGATGTA---
ATCAGCATATGGGATCAAAGCCTA---AAGCCATGTGTAAAGTTG---
ACCCCACTCTGTGTTACTTTACATTGTAGTAATAATATAACAATCAGGGATAGAAACATCACTAGAAATATCA
CT-----------------------------------------------------------------------
```

---------------------AGCACGACGGGAGAACTAAAAAATATCACGGAAGAAGTAAGA---
AATTGCTCTTTCAATGCAACCACAGAAATAAAG------------------------
GATAGGAAACAAAAGGTGTATGCACTC---TTTTATAGGATTGATATAGTACCACTTAATGAG---------
----

AATACCAATGCCAGTGGTGAGTATAGATTAATAAATTGTAATACCTCCACTATAACACAA---
GCCTGTCCAAAGGTCACTTTTGACCCAATTCCTATACATTATTGTGCTCCAGCTGGTTATGCGATTCTAAAGT
GTAAT---AATAAGACATTCAGT------GGAACAGGACCATGTACTAAT----------------------
-GTCAGC---ACAGTACAATGTACACATGGAATTAAGCCAGTAGTATCAACT---
CAACTACTGTTAAATGGTAGCTTAGCAGAAAAAGAG-----------
ATAATAATTAGATCTGAAAATCTGACAGATAAT---------GTCAAAACAATAATAGTACATCTTAAT----
-----------GAATCTGTAGAA---------ATCATGTGCACAAGACCAGGCAATAATACAAGAAAA-----
-AGTGTAAGGATA------------GGACCAGGACAAACA---TTC------TATGCAACAGGAGAA------
---------ATAATAGGAGACATA-----------AGACAAGCACATTGT---AAGATTAATGGAACT---
AAATGGAATGAAACTTTAGAAAAGGTAAAGAAAAAATTAAAA--------------GAACATTTCCCT----
----------------------------------AATAAC---ATAACCTTTGCA------
CCACACTCAGGAGGGGATCTAGAAATTACA---ACACATAGCTTTAATTGTGGAGGAGAA---
TTTAAACAGCTCCAGGCAAGAGTCCTGGCTCTAGAAAGATATCTAAAGGATCAACAGCTCCTAGGACTTTGGG
GCTGCTCTGGAAGACTCATCTGCCCCACTGCTGTGCCTTGGAACATCAGTTGGAGT----------------
---------------------------AATAAAACTTATGAAGATATTTGG------GAT------
AACATGACCTGGATGCAGTGG---GAAAGAGAAATTAGTAATTACACAGGCACAATATAC---
AGGTTGCTTGAAGACTCGCAAAACCAGCAGGAA-------------------------------------
----
AGAAATGAAAAAGATTTATTAGCATTGGACAGTTGGAAAAAATCTGTGGAGTTGGTTTGACATATCAAACTGGC
TGTGGTATATAAGAATATTCATAATGATAGTAGGAGGCTTGATAGGTTTAAGAATAATTTTTGCT---
GTGCTTTCTATAGTA---------AATAGAGTTAGGCAGGGATACTCACCT------
TTGTCGTTTGCTATTAGTCTATTTGATACCGTAGCAATAGTAGTAGCTGAA---
GGAACAGATAGGATTATAGAAGCTGTACAAAGAATTGGTAGAGCT--------------------------
--------ATCCTCAATCTT------CCCAGAAGAATAAGACAGGGCTTCGAGGCAGCTTTGCTA---TAA
>FJ443911
GTG---GGG--
GGAAACATGTGGGTCACAGTATATTATGGGGTACCTGTGTGG---AAAGAAGCAAAA---
ACTACTCTATTCTGTGCATCAGATGCTAAAGCATATGAGAGGGAG---GCGCATAAT---------------
--------------GTCTGGGCTACA---CATGCCTGTGTACCCACAGAC---
CCCAACCCACAAGAAATGGTGATAAATGGAACAAAAAATTTTACAGAAAATTTTAAC---------------
-------------------------ATGTGGAAAAATGACATGGTGGATCAGATGCATGAGGATATA---
ATCAGTTTATGGGATCAAAGCCTA---AAGCCATGTGTAAAGTTG---
ACTCCACTCTGTGTCACTTTAAATTGTACAGAGGCCTACAAGAATGCTAGTACAAAGGCCTGCAATAATGCTA
GTACCAATGAATCTAGCACAAAAAGCACGACTGCTAGCCCGACAAATAATAACACAGAGACCTGCAATAAAAA
TGCTAATACTGTTGCCAGCAAGACAAATAATACTGCTAACATGACAGACCATGCTAGCATGAAA---
AATTGCTCTTTCAATGTAACCACAGAAATAAGA-------------------------
GATAAGATACGCAAAGAGTATGCACTT---TTTTATAAGCTTGATATAGTACCACTTGACAATTCT-------
----

ACTGACGACTATAAGGAGTATGTATTAATAAATTGTAATACCTCAACTGTAACACAA---
GCCTGTCCAAAGGTCTCTTTTGAACCAATTCCTATACATTATTGTGCTCCAGCTGGTTTTGCGATTCTAAAGT
GTAAT---GATAAGACATTCAAT------GGGACAGGACCATGCAAGAAT----------------------
-GTCAGT---ATAGTACAATGTACACATGGAATTAAACCAGTGGTATCAACT---
CAACTACTGTTAAATGGTAGCCTAGCAGAACAAAAG-----------
ATAATAATTAGATCTGAAAATCTGACAAACAAT---------GCTAAAACAATAATAGTACAGCTTAAT----
-----------AAATCCATAGAA---------ATTACGTGCACAAGACACGCCAATAATACAAGAAAT-----
-AGTATAAGGATA------------GGACCAGGACAAGTA---CTC------TTTACAAATGAG---------
---------GTAACAGGAGACATA-----------AGAAAAGCATATTGT---AACATTACTAAAGAG---
AAATGGAATGAAACTTTACAAATGGTATCTAAAGAATTACAA--------------ACATACTTCCCC----
----------------------------------AATAAAACAATAGGGTTTAAA------
CCAGCCTCAGGAGGGGACTTAGAAATTACA---ACACATAGTTTTAATTGTGGAGGAGAA---
TTTAAGCAGCTCCGGGCAAGAGTCCTGGCTATAGAAAGATACCTAAAGGATCAACAGCTCCTAGGACTTTGGG
GCTGCTCTGGAAAACTCATCTGCACCACTAATGTGCCTTGGGAATCCAGGTGGAGT----------------
---------------------------AATAAAACTGAAGACCAGATTTGG------AAT------
CACACGACCTGGATGCAGTGG---GATAAAGAAATTAGCAATTACACAAACACAATATAT---

ACGTTGCTTGAAGAAGCGCAAATCCAGCAGGAA--

AACAATGAAAAAGATTTATTAGCATTGGACAAGTGGCAAAATCTGTGGAATTGGTTTAACATAACACAATGGC
TATGGTATATAAAAATATTCATAATGATAGTAGGGGGCTTGATAGGTTTAAGAATAATTTTTGCT---
GTGCTATCTATAGTG---------AATAGAGTTAGGCAGGGATACTCACCT------
TTGTCGTTTACTATTAGTCTGCTTGATTCTATCGCAATAGCAGTAGCTGAG---
GGAACAGATAGGATTATAGACTTAACACAGAGAATTTGTAGAGCT----------------------------
--------ATCAGAAACCTA------CCTAGAAGAATAAGACAGGGTTTTGAAGCAGCGTTGCTA---TAA
>KT183265
GTG---ATG--
GGAAACTTGTGGGTCACAGTCTATTATGGGGTGCCTGTGTGG---AAAGAAGCAAAA---
ACTACTCTATTCTGTGCATCAGATGCTAAAGCATATGAGCGCGAA---GTACATAAT----------------
--------------GTTTGGGCTACA---CATGCCTGTGTACCCACAGAC---
CCCAACCCACAAGAATTGGTTTTGGAA---------AATGTAACAGAAAACTTTAAC----------------
--------------------------ATGTGGAAAAATGACATGGTAGATCAGATGCATGAGGATATA---
ATCAGTTTATGGGATCAAAGCCTA---AAGCCATGTGTAAAGTTG---
ACCCCACTCTGTGTTACGTTAACCTGTACAAATGTTAGTAGTACAAATGTTAGTGCTAATAGT----------
----
--AATGAGATGACC---
AATTGCTCTTTCAATGCAACCACAGAAGTAAGA---------------------------
GATAAGAAAAAGCAAGTGTTTGCACTT---TTTTATAAACTTGATATAGTGTCACTTGAGGGG----------
----

AATAACAACAGCCAGGGTACATATAGATTAATAAATTGTAATATCTCAACCATAACACAA---
GCCTGTCCAAAGGTCTCTTTTGACCCAATTCCTATACATTATTGTGCACCAGCTGGTTATGCGATTCTAAAGT
GTAAT---AATAAGACATTTAAT------GGGACAGGACCATGCAATAAT-----------------------
-GTCAGC---TCAGTACAATGCACACATGGAATTAAGCCAGTGGTATCAACT---
CAACTATTGTTAAATGGTAGCCTAGCAGAAGGAGAG------------
ATAGTAATTAGATCTAAAAATCTGACAGACAAT---------GCTAAAACAATAATAGTACAACTTAAT----
-----------GAGTCTGTACCA---------ATTGTGTGTATAAGACCCCAAAATAACACAAGAAAA-----
-AGTATAAGGATA------------GGACCAGGACAAACA---CTC------TATGCAACAGGTGAA------
---------ATAATAGGAGACATC------------AGACAAGCACATTGT---AACATTAGTAAGAAT---
GGATGGAACCGTACTTTACAACAGGTAGGAAGAAAATTAGCA---------------GAACACTTCCCT----
-----------------------------------AATAAAACAATAAAATTTGAG------
GGACACTCAGGAGGGGATCTAGAAATCACA---ATGCATAGCTTTAATTGTAGAGGAGAA---
TTTAAGCAGCTCCAGGCAAGAATCCTGGCTATAGAAAGATACCTAAAGGATCAACAACTCCTAGGACTTTGGG
GCTGCTCTGGAAGACTCATCTGCACCACTAATGTGCCTTGGAACTCCAGTTGGAGT-----------------
----------------------------AATAAATCTCATGAAGCTATATGG------GAT------
AACATGACCTGGATGCAGTGG---GATAGGGAAATTAGTAATTACACAAACACAATATAT---
AAGTTGCTTGAAGAATCACAAAACCAGCAAGAA--

AAAAATGAAAAAGATTTATTAGCATTGGACAGTTGGAATAATCTATGGGACTGGTTTAACATAACAAAGTGGC
TGTGGTATATAAAAATATTTATAATGATAGTAGGAGGTTTGATAGGTTTAAGAATAATTTTTGCT---
GTGCTCTCTATAGTA---------AATAGAGTTAGGCAGGGATACTCACCT------
TTGTCGTTTGCTGTTAGTCTACTTGATACCTTAGCAATAGCAGTAGCTGAG---
GGAACAGATAGAATTATAGAATACATACAAAGAATTTGTAGAGCT----------------------------
--------ATCCGCAACCTA------CCTAGAAGAATAAGACAGGGTTTTGAAGCAGCTTTGCTA---TAA
>KC894114
GTG---GCG--
GGGAACAAGTGGGTCACAGTTTATTATGGGGTACCTGTGTGG---AAAGAAGCAAAA---
ACCACCCTATTCTGTGCATCAGATGCTAAAGGATATGAGAGAGAA---GTGCATAAT----------------
--------------ATCTGGGCTACA---CATGCCTGTGTACCCACAGAC---
CCCAGCCCACAAGAAATGTTTTTGGTA---------AATGTAACAGAGAATTTTAAT----------------
--------------------------ATGTGGAAAAATGACATGGTAGATCAGATGCATGAGGATATA---
ATCAGTTTATGGGACCAAAGCCTA---AAGCCATGTGTAAAGTTG---
ACACCACTCTGTGTCACTTTAAATTGTATAAATGCTAATAAGACT----------------------------
----
-------------------------------AGACTAAATGGTACAATGGAGGGAGAAATAAAA---
AATTGCTCTTTTAATGCAACCACAGAGTTAAGA---------------------------
GATAAGAAAAGCAAAGAGTATGCACTT---TTTTATAGGCTTGATATAGTACCACTTGAGGAC----------

----

AATAGCAACTCTAGTACTTATAGATTGATAAATTGTAATACCTCAACCATAACACAA---
GCCTGTCCAAAGGTCTCTTTTGAGCCGATTCCTATACATTATTGTGCTCCAGCTGGTTATGCGATTCTAAAGT
GTAAT---AATAAGACATTCAAT------GGGACAGGACCATGCAATAAT-----------------------
-GTAAGT---ACAGTACAATGTACACATGGAATTAAGCCAGTGGTATCAACT---
CAATTACTGTTAAATGGTAGCCTAGCAGAAGAAGAG------------
ATAATAATTAGATCTGAAAATCTGACAGACAAT---------GCTAAAACAATAATAGTGCATCTTAAT----
-----------GAATCTATAGAA---------ATTGAGTGTGTAAGACCCAACAATAATACAAGAAAA-----
-AGTGTAAGGATA------------GGACCAGGGCAAACA---TTC------TATGCAACAGGAGAC------
---------ATAATAGGAGATATA------------AGAGCTGCATATTGT---GACATTAATAAGAT---
AAATGGAATAGAGCTTTACAAAGGGTAAGTAAAAAATTAAGA---------------GAACTTTACCCT----
--------------------------------AATACAACAACAATACACTTTAAT------
TCATCCTCAGGAGGGGACCCAGAAATTACA---ATGCATAGTTTCAATTGTAGAGGAGAA---
TTTAAGCAGCTCCAGACAAGAGTCCTGGCTCTGGAAAGATACCTAAAGGATCAACAGCTCCTAGGGATTTGGG
GCTGCTCTGGAAAACTCATCTGCACCACTAATGTGCCTTGGAACTCCAGTTGGAGT-----------------
----------------------------AATAAATCTCTAGAAGAAATTTGG------GAA------
AACATGACCTGGATGCAGTGG---GATAAAGAAATTAATAATTACACAAACACAATATAC---
AATTTGCTTGAACTATCGCAAAACCAGCAGGAA--

CAAAATGAAAAAGATTTATTATCATTGGACAAGTGGCAAACTCTGTGGAATTGGTTTGACATAACAAATTGGC
TGTGGTATATAAAAATATTCATAATGATAGTAGGAGGCTTGATAGGTTTGAGAATAATTTTTGCT---
GTACTATCTATAATA---------AATAGAGTTAGGCAGGGGTACTCACCT------
TTGTCATTGGCTGTTAGTCTGTTTGATGCAATAGCAATAGCAGTAGCTGAG---
GGAACAGATAGGATTATAGAACTCATACAGAGAATTTGTAGAGCT----------------------------
--------ATCCGCAACCTA------CCTAGAAGAATAAGACAGGGTTTTGAAGCATCTTTGTTA---TAA
>KC894120
GTG---ACA---
AGCTTGTGGGTCACAGTCTACTATGGGGTACCTGTGTGG---AAAGAAGCAAAA---
ACTACTCTATTCTGTGCATCAGATGCTAAGGCATATGAAAAAGAA---GTGCATAAT----------------
--------------GTATGGGCTACA---CATGCCTGTGTACCCACAGAC---
CCCAATCCACAGGAAATAGTATTGGAA---------AATGTAACAGAAAATTTTAAC----------------
--------------------------ATGTGGAAAATGACATGGTGGATCAGATGCATGAAGATATA---
ATCAGTTTATGGATGAAAGTCTC---AAGCCATGTGTAAAGATG---
ACCCCACTCTGTGTCACTTTAAATTGTGCAAATGCTAATATTAGTAGTACAACGTTTAAT-------------
----
----------------------------GTTAGTGGTGAGATTAACATGAAGGAAGAAGTAAAA---
AATTGCTCTTTCAATGCAACCACAGAAATAAGA---------------------------
GATAAGAAAAAGAAAGTTTATGCACTT---TTTTATAAACCTGATGTAGTACCACTCAATGGGAATGACTCT-
----

AGTGGAAATGGCTCTAGTGAGTATATATTAATAAATTGTAATACCTCAACCATAGCACAA---
GCCTGTCCAAAGGTCTCTTTTGACCCAATTCCTCTACATTATTGTGCTCCAGCTGGCTATGCGATTCTAAAGT
GTAAT---AATAAGACATTTAAT------GGGACAGGACCATGCCAGAAT-----------------------
-GTCAGC---ACAGTACAATGTACACATGGAATTAAGCCAGTGGTATCAACT---
CAACTACTGTTAAATGGCAGCCTAGCAGAAGAAGGG------------
ATAATAATCAGATCTAAAAACCTGACAGACAAT---------ACCAAAACAATAATAGTACACCTTAAT----
-----------GAATCTGTAGGA---------ATTAATTGTACAAGAACTGGTAATAATACAAGGAAA-----
-AGTATAAGGATA------------GGACCAGGACAAACC---TTC------TATGCAACAGGAGAC------
---------ATAATAGGAAATATA------------AGACAAGCACATTGT---AACATTAGTAAAGAA---
AGATGGAATACAACTATGCAAAGGGTAAGACAAAAATTAAAA---------------GGATACTTC-------
-----------------------------------AATAAGACAATAGAGTTTAAG------
TCATCCTCAGGAGGGGATCTAGAAGTTACA---ACACATATCTTTAATTGTAGAGGAGAA---
TTTAAGCAGCTCCAGGCAAGAGTCCTGGCCATAGAAAGATACCTAAAGGATCAACAGCTCCTAGGACTTTGGG
GCTGCTCTGGAAAACTCATCTGCACCACTAATGTGCCTTGGAACTCCAGTTGGAGT-----------------
----------------------------AATAAATCTTATACAGATATTTGG------GAT------
AACATGACCTGGATGCAGTGG---GATAGAGAAATTAATAATTACACAAACACAATATAC---
AAGTTGCTTGAAGACTCGCAAAGCCAGCAGGAG--

AAAAATGAAAGGATTTGTTAGCATTGGACAGTTGGAACAATCTGTGGAATTGGTTTAGCATATCAAATTGGC

TGTGGTATATAAAAATATTCATCATGATAGTAGGAGGCTTAATAGGCTTAAGAATAATTTTTGCT---
GTACTATCTATAGTG---------AATAGAGTTAGGCAGGGATACTCACCT------
CTGTCGTTTGCTATTAGTCTGCTTGATACCATAGCAATAGTAGTAGCTGAA---
GGGACAGATAGGATTATAGAATTAGCACAAAGAATTTGGAGAGCT----------------------------
--------ATCCTCAACATA------CCTACAAGAATAAGACAGGGCTTTGAAGCAGCTTTGCTA---TAA
>KC156115
GTG---CTG--
GAGGACTTGTGGGTCACAGTCTATTATGGAGTACCTGTGTGG---AGAGAAGCAAAA---
ACTACTCTATTCTGTGCATCAGATGCTAAGGCATATGAGAGAGAA---ATACATAAT----------------
---------------GTCTGGCTACA---CATGCCTGTGTACCCACAGAC---
CCCAACCCACAAGAAATAGTTTTGGGA---------AATGTAACAGAAAATTTTAAC----------------
--------------------------ATGTGGAAAAATGACATGGTGGATCAGATGCAGGAAGATATA---
GTCAGTCTATGGGATCAAGGCCTA---AAGCCGTGTGTAAAGTTG---
ACCCCACTCTGTGTCACTCTAAGTTGTACAAATGTTACCAGTAGTAGTAATGTTACCAGTAATAGTAATGATA
CCAGTATT---
--CAGGACATGAGA---
AATTGCTCTTTCAATGCAAGCACAGAAAGATTA---------------------------
GATAAGAGACAGAAAGTGAATGCCCTT---TTTTATAAACTTGATATAGTACAACTTAATGAGAAT-------
----

GACAGCTCCAGCTATAGATTAATAAATTGTAATACCTCAACCATATCACAA---
GCCTGTCCAAAGGTCTCTTTTGACCCAATTCCTATACATTATTGTGCTCCAGCTGGTTATGCGATTCTAAAGT
GTAAT---AATAAGACATTCAAT------GGGACAGGACCATGCCAGAAT-----------------------
-GTCAGC---ACAGTACAATGCACACATGGAATTAAGCCAGTGATATCAACT---
CAACTACTGTTAAATGGTAGCCTAGCAGAAGGAAAC------------
ATAACAATCAGATCTGAAAATCTGACAAACAAT---------GCCAAGACAATAATAGTACACCTTAAT----
-----------GTATCTATAGAA---------ATCATGTGTACAAGACCTAACAATAATACAAGAAAA-----
-AGTATAAGGATA------------GGACCAGGACAAACA---TTC------TATGCAACAGGAGAC------
---------ATAATAGGAGACATA------------AGACAAGCACACTGT---AACATTAGTGGAGAG---
AGATGGAATGAAACTTTACTACAGGTAGGGAAAAAATTATAT---------------GAACACTTCCCT----
-----------------------------------AATAGAACAATAAAATTTGCA------
AAACCCTCAGGAGGGGACCCAGAAGTTACA---ATGCATACCTTTAATTGTAGAGGAGAA---
TTTAAGCAACTCCAGACAAGAGTCCTGGCTATAGAAAGATACCTAAAGGATCAACAGCTCCTAGGGTTGTGGG
GCTGCTCTGGAAAACTCATCTGCACCACTAATGTGCCTTGGAACTCCAGTTGGAGT-----------------
----------------------------AATAAAACTCGAGAAGAAATTTGG------GAT------
AACATGACCTGGATGCAGTGG---GATAGAGAAATTAGTAATTACACATACACAATATAC---
AGGTTGCTTGAAGACTCGCAAACCCAGCAGGAA--

GCAAATGAAAAAGATTTATTAGCATTGGACAGTTGGAAAAATCTATGGAATTGGTTTGACATAACAAAATGGC
TGTGGTATATAAAAATATTTATAATGATAGTAGGAGGCTTGATAGGCTTGAGAATAATTTTTGCT---
GTGCTCTCTATAGTG---------AATAGAGTCAGGCAGGGATACTCACCT------
CTGTCGTTGGCTATTAGTCTGCTTGATACTATAGCAATAACAGTAGCTGAG---
GGGACAGATAGGATTATAGAAGCAATACAAAGAATTGGTAGAGCT----------------------------
--------ATCCTCAGCATA------CCTAGAAGAATAAGACAGGGCTTTGAAGCAGCTTTGCAA---TAA
>KC894124

GGGAACCTGTGGGTCACAGTCTATTATGGGGTACCTGTGTGG---AAAGAAGCAAAA---
ACTACTCTATTCTGTGCATCAGATGCTAAAGCATATGAGACAGAA---GTGCATAAT----------------
---------------GCCTGGGCTACA---CATGCCTGTGTACCCACAGAC---
CCCAACCCACAAGAAATGGTTTTGGAA---------AATGTAACAGAAGATTTTAAT----------------
--------------------------ATGTGGAAAAATGACATGGTGGATCAGATGCATGATGATATA---
ATCAGTTTATGGGACCAAAGCCTA---AAGCCATGTGTAAAGTTG---
ACACCACTCTGTGTCACTTTAAAATGTGTAGAAGTTAACAACACGACA-------------------------
----
-------------------------AATACAACAATTGATGATAATTACAAGGAAGAAGTAAAA---
AATTGCTCTTTCAATACAACCACAGCGATAAGA---------------------------
GATAAGAAACAGCAAGGGTATGCATTT---TTTTATAGATCTGATATAGAACTACTTCCTAAC----------
----

TCTAGTGAGTATATATTAATAAGTTGTAATACCTCAACCATAACACAG---

GCCTGTCCAAAGGTCTCTTTTGACCCGATTCCTATACATTATTGTGCTCCAGCTGGTTATGCGATTCTAAAGT
GTAAT---AATAAGACATTTAAT------GGGACAGGACCATGCAAAAAT-----------------------
-GTCAGC---ACAGTACAATGTACACATGGAATTAAGCCAGTGGTATCAACT---
CAACTACTGTTAAATGGTAGCCTGGCAGAAGAGGAC------------
ATAATAATTAGATCTGAAAATCTGACAAATGAT---------GCTAAAACAATAATAGTACACCTTAAT----
-----------AAATCTGTAGCA---------ATTGTGTGTATAAGACCTGGCAATAATACAAGAAGA-----
-AGTATAAGGATA------------GGACCAGGACAGACA---TAC------TATGCAAATGAC---------
---------ATAATAGGAGACATA------------AGACAAGCATATTGT---AACATTAGTAAAAGT---
CTATGGAATACAACTTTAGAAGGGGTAAAGAAGAAATTAGAA---------------AAACACTTCCCT----
--------------------------------AATAAAACGATAGAGTTTGAACCA---
CAACCACGCTCAGGAGGGGACCTAGAAATTACA---ACACATAGTTTTAATTGTAGAGGAGAA---
TTTAAGCAGCTCCAGGCAAGAGTCCTGGCTATAGAAAGATACCTAAAGGATCAACAGCTCCTAGGGCTTTGGG
GCTGCTCTGGAAAAGTCATCTGCCCCACTGCTGTGCCGTGGAACTCCAGTTGGAGT-----------------
----------------------------AATAAATCTAAAGCAGATATTTGG------GAT------
AACATGACCTGGATGCAGTGG---GATAGAGAAATTGATAACTACACAAACACAATATAC---
AAGTTGCTTGAAGACTCGCAAATCCAGCAGGAG--

AACAATGAAAGAGATTTATTAGCATTAGACAGTTGGAACAGCCTGTGGAATTGGTTTAGCATAACAAAATGGT
TGTGGTATATAAAAATATTCATAATGATAGTAGGAGGCTTGATAGGTTTAAGAATAATTTTTGGT---
GTGCTTTCTATAGTG---------AAAAGAGTTAGGCAGGGATACTCACCT------
TTGTCGTTTGCTGTTAGTTTGCTTGATACTGTAGCAATAGCAGTAGCTGAA---
GGAACAGATAGGATTATAGAATTAGCACAAAGAATTTGTAGAGCT----------------------------
--------ATCTGCAACATA------CCTAGAAGAATAAGACAAGGCTTTGAATCAGCTTTGCTA---TAA
>HM204615
AAT---GTG---GGG------------------------------------
GGGAACTTATGGGTCACAGTCTATTATGGGGTACCTGTGTGG---AAGGAAGCAGAA---
ACTACTCTATTCTGTGCATCAGATGCTAAAGCATATGAGAGAGAA---GTACACAAT----------------
--------------GTCTGGGCTACA---CATGCCTGTGTACCCACAGAC---
CCCAACCCACAAGAAATGGTTTTGGAA---------AATGTAACAGAAGATTTTAAT----------------
--------------------------ATGTGGAAAAATAACATGGTGGATCAGATGCATGAGGATATA---
ATCAGTTTATGGGATCAAAGCCTA---AAGCCATGTGTAAAGTTG---
ACCCCACTCTGTGTAACTTTAAACTGTACTAATGTTAATAGTACTGCTACT----------------------
----
----------------------------------TACAATAATGTTAGCAGGCAGGAAATGAGA---
AATTGCTCTTTCAATATAACTACAGAATTAAGA---------------------------
GATAAGAAAAAACAAGAAAGAGCACTT---TTTTATATACATGATGTAGTACAAATTGGTAAC----------
----

TCTAACTCTAGTGAATATAGATTAATAAATTGTAATACCTCAACCATAACACAA---
GCTTGTCCAAAAGTCTCTTTTGATCCAATTCCTATACATTATTGTGCTCCAGCTGGTTATGCGATTCTAAAAT
GTAAT---AATAAGACATTTAAT------GGAATAGGACCATGCAAGAAT-----------------------
-GTCAGC---ACAGTACAATGTACACATGGAATTAAGCCAGTGGTATCAACT---
CAACTACTGTTAAATGGTAGCCTAGCAGAAGAAGAG------------
ATAATAATTAGATCTGAAAATCTGACAGACAAT---------GTCAAAACAATAATAGTACATCTTAAT----
-----------GAATCTGTAGAA---------ATTGAGTGTGTAAGACCCAACAATAATACAAGGAAA-----
-AGTGTGAGAATA------------GGACCAGGACAAGCA---TTT------TATGCAACA---GAT------
---------ATAATAGGAGACATA------------AGGCAAGCACATTGT---AACATCAGTGCATCT---
ACATGGAACAAAACTTTACAACAGGTAGAAAAAAAATTAAAA---------------GAATACTAC-------
-----------------------------------AATAAAACAATAAGCTTTCAA------
CCATCCTCAGGAGGGGATCTAGAAATTACA---ACACATAGCTTTAATTGTAGAGGAGAA---
TTTAAGCAGCTCCAGGCAAGGGTCCTGGCTATAGAAAGATACCTAAGGGATCAACAGCTCCTAGGGATGTGGG
GTTGCTCTGGAAAACTCATCTGCCCCACTGCTGTGCCTTGGAACACTAGTTGGAGT-----------------
----------------------------AATAGAACTCAAGATGATATTTGG------AAT------
AACATGACCTGGATGCAGTGG---GATAAAGAAATTAGTAATTACACAGGCATAGTATAC---
AGATTGCTTGAAGACTCGCAAAACCAGCAGGAA--

AAAAATGAAAAAGATTTATTAGCACTGGACAGTTGGAACAATCTGTGGAATTGGTTTAACATAACAAATTGGC
TGTGGTATATAAGAATATTCATAATGATAGTAGGAGGCTTAATAGGTTTAAGAATAATTTTTGCT---
GTGCTTTCTATAGTG---------AATAGAGTTAGGCAGGGATACTCACCT------
TTGTCGTTCGCTATTAGTTTACTTGATGCCTTAGCCATATCAGTAGCTGAA---

GGAACAGATAGAGTTATAGAATTGCTACAAAGAATTTGTAGAGCT----------------------------
--------ATCCGCAACATA------CCTGTAAGAATAAGACAGGGCTTTGAAGCAGCTTTGCTA---TAA
>FJ443547
GTA---GAG--
GAGAACTTGTGGGTAACAGTTTATTATGGGGTGCCTGTGTGG---ACAGAAGCAAAA---
GCTACTCTATTCTGTGCATCAGATGCTAAAGCATATGAGAAAGAA---GTGCATAAT---------------
--------------GTCTGGGCTACA---CATGCCTGTGTACCCACAGAC---
CCCAACCCACAAGAATTATATTTGGAA---------AATGTAATAGAAAGTTTCAAC---------------
-------------------------ATGTGGAAAAATGACATGGTGGATCAGATGCATGAGGATGTA---
ATCAGTATGTGGGATCAAAGTCTA---AAGCCATGTGTAAAGCTG---
ACCCCACTCTGTGTCACTTTAAACTGTACAGATGTAAATGTTACCACAAATGCAAATGTT------------
----
------------------------ACCACTAATGGCACTGCTATTGGCGAGGGAGAGATAAAA---
AATTGCTCTTTCAATGCAACCACAGAAATAAGA--------------------------
GATAAAAAACAGAAAGTGTATGCACTT---TTTTATAGACCTGATGTAGTACCACTTGAAAAG---------
----

AACTCTAGTGATTATATATTAATAAACTGCAATACCTCAACCATAGCACAA---
GCCTGTCCAAAGGTCACCTTTGATCCGATTCCTATATATTATTGTGCTCCAGCTGGTTATGCGATTCTAAAAT
GTAAT---AATAAGACATTCAAT------GGGACAGGACCATGCAATAAT----------------------
-GTCAGC---ACAGTACAATGCACACATGGAATTAAGCCAGTGGTCTCAACA---
CAATTACTGTTAAACGGTAGCCTGGCAGAACAAGAG-----------
ATAGTCATTAGATCTGAAAATATAACAGACAAT---------GCCAAAACAATAATAGTACACCTTAAT----
-----------GGAACTATAAAC---------ATTACGTGTACAAGACCCAATAATAATACAAGAAAA-----
-AGTATAAGGATA------------GGACCAGGACAAATG---TTC------TATGCAACAGGTGAA------
---------ATAATAGGAGACATA-----------AGACAAGCATATTGT---AACATCAATATCACA---
AAGTGGAACGCAACTTTAGAGCAGGTAAAGAGAAAATTAGAA--------------AAATACTTCCTT----
-----------------------------------AATAAAACAATAAGATTTGCA------
CCATCTTCAGGAGGGGATCTAGAAGTTACA---ACACATATGTTTAATTGTGGAGGAGAA---
TTTAAACAGCTCCAGACAAGAGTCCTGGCTATCGAAAGATACCTAAAGGATCAACAGCTCCTAGGGCTTTGGG
GCTGCTCTGGAAAACTCATCTGCACCACTGCTGTGCCTTGGAACTCCAGTTGGAGT----------------
----------------------------AATAAATCTCAAAACGATATTTGG------GAT------
AACATGACCTGGATGCAGTGG---GATAGAGAAATTAATAATTACACAGACACAATATAC---
AAGTTGCTTGAAGACTCGCAAAGCCAGCAGGAG---------------------------------------
----
AAAAATGAAAAGGACCTATTAGCATTGGACAGTTGGAAAAACCTGTGGAATTGGTTTAACATAACAAATTGGT
TGTGGTATATAAAATTATTCATCATGATAGTAGGAGGCTTGATAGGTTTAAGAATAATTTTTGGT---
GTACTCTCTATAGTG---------AAAAGAGTTAGGCAGGGATACTCACCT------
TTGTCGTTTGCTATTAGTCTATTTGATACCACAGCAATAGCAGTAGCTGAA---
GGAACAGATAGGATTATAAACTTAGTACAAAGAATCTATAGAGCT--------------------------
--------ATCCGCAACATA------CCTGTAAGAATAAGACAGGGCCTTGAAGCAGCTTTACTA---TAA
>FJ443838
GTG---AGG---
GGCTGGTGGGTCACAGTCTACTATGGGGTGCCTGTGTGG---AAAGAAGCAAAA---
ACTACTCTATTTTGTGCATCAGATGCTAAAGGATATGAGAAAGAA---GCGCATAAT---------------
--------------GTCTGGGCTACA---CATGCCTGTGTGCCCACAGAC---
CCCAACCCACAAGAAATAGTTTTGGTA---------AATGTAACAGAAAATTTTAAC---------------
-------------------------ATGTGGAAAAATGACATGGTAGATCAGATGCATGAAGATATA---
ATCAATTTATGGGATCAAAGCCTT---AAGCCCTGTGTAAAGTTG---
ACCCCACTCTGTGTCACTTTAAATTGCATAAATGCAACTATATTAAATGATACACAAACAAATGCA-------
----
------------------ACTATATTAAATGGTACACAAAAGATAGAAATTGAAGAAATGAAA---
AATTGCTCTTTCAATGTGACCACAGAATTAAGA--------------------------
GATATTAAAAGGAAAGAAAGGACAATT---TTTCATAGCCTTGATATAGTACCAATTGATGGG---------
----

AGGAAGGGTGAATATAGATTAATACATTGTAATACCTCAGTCATAACACAA---
GCCTGTCCAAAAATCTCGTTTGAACCAATTCCTATACATTTTTGTGCTCCAGCTGGTTATGCGATTCTAAAGT
GTAAT---AATAAGACATTCAAT------GGAACAGGACCATGCAATAAT----------------------
-GTCAGC---ACAGTACAATGTACACATGGAATTAAGCCGGTGGTATCAACT---

```
CAACTACTGTTAAATGGTAGCCTAGCAGAAGAAGAT------------
ATAATAATTAGATCTGAAAATCTGACAAACAAT---------GTCAAAACAATAATAGTACATCTTAAT----
-----------GACCCTGTAAAG---------ATTGAGTGTATAAGATTTGCCAATAAGACAAGAAAA-----
-AGTGTGAGAATA------------GGACCAGGACAAGCA---TTC------TATGCAACAGGAGAC------
---------ATAATAGGAGATATC------------AGAGAAGCACATTGT---AACGTTAATGAAAGC---
AAATGGAACCAAACTTTACAGAGGGTAAGTAAGAAATTAAAA---------------GAATTCTTC-------
-----------------------------------AATAAACCAATAGTATTTAAT------
TCATCTTCAGGAGGGGACCTAGAAATTACT---ACACATAGCTTTAATTGTGGAGGAGAA---
TTTAAGCAGCTCCAGGCAAGAGTCCTGGCTATAGAAAGATACCTAAAGGATCAACAGCTCCTAGGGATATGGG
GCTGCTCTGGAAAACTCATCTGCACCACTGATGTACTTTGGAACTCTAGTTGGAGT-----------------
----------------------------AATAAAACTAAAACTGAGATTTGG------GAA------
```
[truncated: 5,278,270 more chars]
