# Supplementary material for: Extending the Coding Potential of Viral Genomes with Overlapping Antisense ORFs: A Case for the De Novo Creation of the Gene Encoding the Antisense Protein ASP of HIV-1
Source: Viruses. 2022 Jan 14;14(1):146. doi: 10.3390/v14010146 (PMC8781085; doi:10.3390/v14010146)
Supplement: Supplementary file 1 [file viruses-14-00146-s001.zip › Supplementary File S3.pdf]

Supplementary File S3

Supplementary File S3 contains a sample set of 637 additional *env/asp* overlaps, all belonging to the group M of HIV-1. For details, see Materials and methods, paragraph *2.4: Assembly of an enlarged sample set of 4362 env/asp overlaps*. This dataset is an extension of the sample set in Supplementary File S1. Briefly, it contains the *env/asp* in which the anti-sense ORF is not delimited by ATG and stop codons at canonical nucleotide positions. However, the anti-sense ORF retains the ability to encode a functional anti-sense protein asp. The nucleotide sequence of each *env*/*asp* overlap is reported both in the alignment format with gaps and in the format without gaps.

```
>MH078531
TCTCAGCTGTTTAATGGGACTGCAAAACAGATGAGAAATAACACAGAAATA----------------------
-------------------------------------------------------------------------
-------------
TATAACAAAACAATCATACTCCCATGCAGAATAAAACAAATTGTAAACAGGTGGCAGGAAGTAGGAAAAGCAA
TGTATGCCCCTCCCATCCAAGGAATAATTAGCTGTTCATCAAAT---
ATTACAGGGCTACTATTAACAAGAGATGGTGGGAACAGCAATACAAGT-------------------------
-------------------------------------------------------------------------
----------
AGAAACAATGAGACCTTCAGACCTGGGGGAGGAGATATGAGGGACAATTGGAGAAGTGAATTATATAAATATA
AAGTAGTGAGAATTGAGCCATTA---GGAATAGCACCCACCAAGGCAAAGAAGAGTGGTG-------------
----------------------------CAGAGAGAAAAAAGAGCA---GTGGGAACACTAGGAGCT------
---ATGTTCCTTGGGTTTCTGGGAACA---GCAGGAAGCACTATG---GGCGCAGCGGCA---
GTGACGCTGACGGTACAA---GCCAGGCAA---TTATTG---TCTGGTATAGTGCAACAGCAGAAC---
AACCTG---CTGAGA---GCTATTGAGGCGCAACAGCATCTGTTGCAACTCACAGTCTGGGGCATC
>MN486020
ACTCAAGGAAATAGTACTGGGAGTAATAAT-------------------------------------------
--------------------------------------------------
AACAATGAGACCATCACACTCCCATGCAGAATAAAACAAATTGTAAACATGTGGCAGGAAGTGGGAAAAGCGA
TGTATGCCCCTCCCATCGGAGGACTAATTAGGTGTACATCAAAT---
ATTACAGGGTTACTATTAACAAGAGATGGTGGTGGAACAGATAACACCACTGAG-------------------
-------------------------------------------------------------------------
-------------
ACATCTGAGACATTCAGACCAGGAGGGGGAAATATGAAGGACAATTGGAGAAGTGAATTATATAAATACAAAG
TAGTAAAAATTGAACCATTA---GGAGTAGCACCCACCAAGGCAAGGAGAAGAGTGGTG--------------
-------------------------CAGAGAGAAAAAAGA------ATTGGAACACTAGGAGCC---------
ATGTTCCTTGGATTCTTGGGAGCA---GCAGGAAGCACTATG---GGCGCAGCGTCA---
ATAACGGTGACGGTACAG---GCCAGACAA---TTACTG---TCTGGGATAGTGCAGCAGCAGAAC---
AATCTG---CTGAGG---GCTATTGAGGCGCAACAGCATATGTTGCAACTCACAGTCTGGGGCATC
>EF637053
ACTCAGAACAGTACTTGGCACAAGAATGAAACT---------------------------------------
ACTTGGCAA---ATGAATGGAAATACTGGAGAG---------------
AGGACTAATGATACTATCACACTCCCATGCAGAATAAAACAAATTATAAACAGGTGGCAGGAAGTAGGAAAAG
CAATGTATGCCCCTCCCATCAGAGGACTAATTAGATGTTCATCAAAT---
ATCACAGGGCTGCTATTAACAAGAGATGGTGGTGGGGACAACGGG----------------------------
-------------------------------------------------------------------------
----------
TCTGAGTCTGAGACCTTCAGACCTGGAGGAGGAAATATGAGGGATAATTGGAGAAGTGAATTATATAAATATA
AAGTAGTAAGAATTGAGCCATTA---GGAGTAGCACCCACCAAGGCAAAGAAGAGTGGTG-------------
----------------------------CAGAGAGAAAAAAGAGCA---GTGGGAACAATAGGAGCT------
---ATGTTCCTTGGGTTCTTGGGAGCA---GCAGGAAGCACTATG---GGCGCAGCGTCA---
CTGACGCTGACGGTACAG---GCCAGACAA---TTATTG---TCTGGTATAGTGCAACAGCAAAAC---
AATTTG---CTGAGA---GCTATTGAGGCGCAACAACATCTGTTGCAACTCACAGTCTGGGGCATC
>JN692454
ACTCAGCTGTTTAATAATACTTGGAATACAACACAGCTTGATGATGATGAGCAGGAT----------------
-------------------------------------------------------------------------
-------------
GACAATGGCACCCTCACCCTCCCATGCAGAATAAAACAAATCATAAACAAGTGGCAGGAAGTAGGAAAAGCAA
```

TATATGCCCCTCCCATCAAAGGCTTAATTAGCTGTAACTCAACA---
ATTACAGGGCTGATATTAACAAGAGATGGTAATGGAACCGACCATGAC-------------------------
----

AATGAGTCTGAAATTTTCAGACCTGGAGGAGGAAACATGAAGGACAATTGGAGAAGTGAATTATATAAATATA
AAGTAGTAAGAATTGAGCCATTA---GGAATAGCACCCTCCAAGGCAAAGAGAAGAGTGGTG-----------
----------------------------CAAAGAGAGAAAAGAGCA---GTGGGAATGATAGGAGCT------
---ATGTTCCTTGGGTTCTTGGGAGCA---GCAGGAAGCACTATG---GGCGCAGCGTCA---
GTAACGCTGACGGTACAG---GCCAGACTA---TTATTG---TCTGGTATAGTGCAACAGCAAAAC---
AACTTG---CTGAGA---GCTATTGAGGCGCAACAGCATATGTTGCAACTCACAGTCTGGGGCATT
>JN692455
AATTATACTTTGGAAAATGATACTTTCAAAAATGATACTAGGCAT----------------------------
----
-
AAAACTGAGAATATCACACTCCAATGCAGAATAAAACAAATTATAAACAGGTGGCAGAAAGCAGGAAAGGCAA
TGTATGCCCCTCCCATCAAAGGACAGATTAGATGTTCATCAAAC---
ATTACAGGGATATTATTAACAAGAGATGGTGGCATACACAACAACAGTAGT----------------------
----

GACAACAATACAGAGATCTTCAGACCTGGAGGAGGAGACATGAGGGACAATTGGAGAAGTGAATTATATAAAT
ATAAAGTAGTAAAAATTGAGCCATTA---GGAGTAGCACCCACCAAGGCAAGGAGAAGAGTGGTG--------
-------------------------------CAGAGAGAAAAAAGAGCA---GTGGGAACGATAGGAGCT---
------ATGTTCCTTGGGTTCTTGGGAGCA---GCAGGAAGCACTATG---GGCGCAGCATCA---
CTGACGCTGACGGTACAG---GCCAGACAA---TTATTG---TCTGGTATAGTGCAACAGCAGAAC---
AATCTG---CTGAGG---GCCATTGAGGCGCAACAGCATCTGTTGCAGCTTACAGTCTGGGGCATC
>FJ195086
GGTCAGTGGGATGGTACTATTGGGCCA--

GATGAGAATGGCAATATCACACTCCCATGCAGAATAAAACAAATTATAAACAGGTGGCAGGAAGTAGGAAAAG
CAATATATGCCCCTCCCATCAGAGGACAAATTAGCTGTTCCTCAAAT---
ATTACAGGGCTGCTACTCACAAGAGATGGTGGTAGTAATAACACG----------------------------
----

AGCAATAATGAGACCTTCAGACCGGGAGGAGGAAATATGAAGGACAATTGGAGAAGTGAATTATATAAATATA
AAGTAGTACAAATTGAGCCATTA---GGAATAGCACCCACCAAGGCAAAAAGAAGAGTGGTG-----------
----------------------------CAGAGAGAGAAAAGAGCA---GTAATA------GGAGCT------
---ATGTTCCTTGGGTTCTTGGGAGCA---GCAGGAAGCACTATG---GGCGCAGCATCA---
CTGACGCTGACGGTACAG---GCCAGACTA---TTATTG---TCTGGTATAGTGCAACAGCAGAGC---
AATCTG---CTGAGG---GCTATTGAGGCGCAACAGCATCTGTTGCAACTCACAGTCTGGGGCATC
>JN692468
AGTTACAACAACACTAGCAAAACTTGGAATAAAACTGAAGAGAAC----------------------------
---CATACT------
GGAGTAAATAACACAATCACACTCCCATGTAGAATAAAGCAAATTATAAACAGGTGGCAGGAAGTAGGAAAAG
CAATGTATGCCCCTCCCATTAGGGGACTAATTAAATGTACATCAAAT---
ATTACAGGGATAATATTAACAAGAGATGGTGGTAATGAGACTGGG----------------------------
----

ACCGAGACCTTCAGACCTGGAGGAGGAGATATGAGGGACAACTGGAGAAGTGAATTATATAAATATAAAGTAG
TAAAAATTGAGCCATTA---GGAATAGCACCCACCAAGGCAAGGAGAAGAGTGGTG-----------------
----------------------CAGAGAGAAAAAAGAGCA---GTGGGAACAATAGGAGCT---------
ATGTTCCTTGGGTTCTTGGGAACA---GCAGGAAGCACTATG---GGCGCAGCGTCA---
ATCACGCTGACGGTACAG---GCCAGACAA---TTATTG---TCTGGTATAGTGCAGCAGCAGAAC---
AATTTG---CTGAAG---GCTATTGAGGCGCAACAACATCTGTTGCAACTCACAGTCTGGGGCATC
>KT427737
ACTCATAGTACTTGGAATACGAATACAACT---

GGAAATGACAATATCACACTCCAGTGCAGAATAAAACAAATTATAAACAGGTGGCAAGAAGTAGGAAAAGCAA
TATATGCCCCTCCCATCAAAGGAAAAATTAATTGTCTATCAAAT---
ATTACAGGGCTGCTGTTAGTAAGAGATGGTGGTAGGAACACAAGT----------------------------
----

GGGTCCGAAACCTTCAGACCTGGAGGAGGAAATATGAGGGACAATTGGAGAAGTGAATTATATAAATATAAAG
TAGTAAAAATTGAACCACTA---GGAATAGCACCCACCAAGGCAAAGAGAAGAGCGGTG--------------
-------------------------CAGAGAGAGAGAAGAGCA---GTGGGAACACTGGGAGCT---------
ATGTTCCTTGGGTTCTTGGGAGCA---GCAGGAAGCACTATG---GGCGCAGCGGCA---
ACAACGCTGACGGTACAG---GCCAGACTA---TTATTG---TCTGGTATAGTGCAACAGCAAAAT---
AATTTG---CTGAGG---GCTATTGAGGCGCAACAGCACATGTTGCAACTCACAGTCTGGGGCATC
>KT427704
AATTATACTCCCAAT--

ACTACAGGAAATATTACACTCCCATGCAGAATAAAACAAATTATAAACAGATGGCAGGAAGTAGGAAAAGCAA
TGTATGCCCCTCCCATCGGAGGACAAATTAAATGTTCATCAAAT---
ATTACAGGGATGATATTAACATGGGATGGGGGGAACGAAACCAGG----------------------------
----

GACAATGAGACCTTCAGACCTGGAGGAGGAGATATGAGGGACAATTGGAGAAGTGAATTATATAAATATAAAG
TAGTAAAAATTGAGCCAATA---GGAGTAGCACCCACCAAGGCAAAGAGAAGAGTGGTG--------------
-------------------------CAGAGAGAAAAAAGAGCG---GTAACG---CTAGGAGCC---------
ATGTTCCTTGGGTTCTTAGGAGCA---GCAGGAAGCACTATG---GGCGCAGCGTCA---
ATGACGCTGACGGTACAG---GCCAGACAA---TTATTG---TCTGGTATAGTGCAACAGCAAAGC---
AATTTG---CTGAGG---GCTATTGAGGCGCAACAGCATCTGTTGCAACTCACAGTCTGGGGCATT
>KT427872
ACTCACTGGTCATTAAATATAACT---

GGCACTGGCAATATCACACTTCCATGCAGAATAAAACAAATTATAAACAAGTGGCAGGAAGTAGGAAGAGCAA
TATACGCCCCTCCCATCCAAGGATTAATTAGATGCTCATCAAAT---
ATTACAGGGCTGCTACTGACACGGGATGGTGGTATCACAAACTCT----------------------------
----

AGCAACCCTGAGGTCTTCAGACCTGGGGGAGGAGATATGAGAGACAATTGGAGAAGTGAACTATATAAATATA
AGGTAGTAAAAATTGAGCCAGTA---GGAATAGCACCCACCAAGGCAAGGAGAAGAGTGGTG-----------
----------------------------CAAAGAGAAAAAAGAGCA---GTGGGGACATTAGGAGCT------
---ATGTTCCTTGGGTTCTTGGGAGCA---GCAGGAAGCACTATG---GGCGCAGCGTCA---
TTGACGCTGACGGTACAG---GCCAGACTA---TTATTG---TCTGGTATAGTGCAACAGCAAAAC---
AATTTG---CTGAGA---GCTATTGAGGCGCAACAGCATATGTTGCAACTCACAGTCTGGGGCATC
>KJ849818
AGTTACCTT--
---AATGAA------
ACTAACGAAACAAATATCACACTCCCATGCAGAATTAAACAAATTATAAACATGTGGCAGAGAGTAGGAAAAG
CAATGTATGCCCCTCCCATTCCAGGACCAATTAACTGTTCATCAAAT---
ATTACAGGGCTGCTCTTAGTAAGAGACGGTGGT--

AATAATAATACAAATGAGACCTTCAGACCTGGAGGAGGAAATATGAAGGACAATTGGAGAAGTGAATTATATC
GATATAAAGTAGTAAAAATTGAACCACTA---GGACTAGCACCTACCACGGCAAAGAGAAGAGTGGTG-----
----------------------------------CAGAGAGAAAGAAGAGCA---GTGGGA---
CTAGGAGCG---------TTGTTCCTTGGGTTCTTAGGAGCA---GCAGGAAGCACTATG---
GGCGCAGCGTCA---CTGACGCTGACGGTACAA---GCCAGACAA---TTATTG---
TCTGGTATAGTGCAACAGCAGAAC---AACCTG---CTGATG---
GCTATTGAGGCGCAACAACATATGTTGCAACTCACAGTCTGGGGCATC
>KT427751
TTTCATTGGCCTGTCAATGACACTAATAGTAATAGT-------------------------------------
----

ACAAATGATATAGTTACCATCCCATGCAGAATAAAACAAATTATAAACAGATGGCAGGAAGTAGGAAAAGCAA
TGTATGCCCCTCCCATCAGAGGGCAGATTAAATGTTCATCAAAT---
ATTACAGGGCTGCTATTGACAAGGGATGGTGGTAACAACAACACG----------------------------
----

GAGAACAATACAGAGGTCTTCAGACCTGAAGGAGGAGATATGAGGGACAATTGGAGAAGTGAACTATATAAAT

ATAAAGTAGTAAAAATTGAGCCATTA---GGAGTAGCACCCACCAAAGCAAAGAGAAGAGTGGTG--------
-------------------------------CAGAGGGAAAAAAGAGCA---GTGGGAGTGATAGGAGCT---
------ATGTTCCTTGGGTTCTTGGGAGCA---GCAGGAAGCACTATG---GGCGCAGCGTCA---
CTGACGCTGACGGTACAG---GCTAGACAA---TTATTG---TCTGGTATAGTGCAACAGCAAAAC---
AATTTG---CTGAGA---GCTATTGAGGCGCAACAGCATCTGTTGCAACTCACAGTCTGGGGCATC
>KT427803
TTTCACAGTTCTTGGAATGCAAGTAATTTGAATACAACACAGAATGGGACT----------------------
----

GATAATAGTAATATCACACTCCCATGCAGAATAAAACAAATTATAAATAGGTGGCAGGAAGTAGGAAAATCAA
TGTATGCCCCTCCCGTCCAAGGACTAATTAAATGTTCATCAAAT---
ATTACAGGGCTGCTATTAACAAGAGATGGTGGTAGCAACACCACG----------------------------
----

GAGAAGGAAGTGTTCAGACCTGGGGGAGGAAATATGAAGGACAATTGGAGAAGTGAATTATATAAATATAAAG
TAGTAAAAATTGAGCCATTA---GGAGTAGCACCCACCAAGGCAAGGAGAAGAGTGGTG--------------
-------------------------CAGAGAGAAAAAAGAGCA---GTGACA---CTAGGAGCT---------
ATGTTCCTTGGGTTCTTGGGAGCA---GCAGGAAGCACTATG---GGCGCAGCGTCA---
CTAACGCTGACGGTACAG---GCCAGACAA---TTATTG---TCTGGTATAGTGCAGCAGCAGAAC---
AATTTG---CTGAGG---GCTATTGAGGCGCAACAACATATGTTGCAACTCACAGTCTGGGGCATC
>KT427797
TTTTATAGTAACTGGACTACAAATTCAACTAACGAAGGAACTGGACTACAAATTCA-----------------
--ACTAAC-----
-
AATGGGAATAACACCATCACACTCCCATGCAGAATAAAACAAATTGTAAACATGTGGCAGGAAGTAGGAAAAG
CAATGTATGCCCCTCCCATCAGTGGAATAATTAAATGTTCATCAAAT---
ATTACAGGGATACTGTTAACAAGAGATGGTGGTAATAATAGTACG----------------------------
----

AACGAGACCTTCAGACCAGCAGGAGGAGATATGAGGGACAATTGGAGAAGTGAATTATATAAATATAAAGTAG
TAAAAATTGAACCATTA---GGATTAGCACCTACCAAGGCAAGGAGAAGAGTGGTG-----------------
----------------------CAGAGAGAAAAAAGAGCA---GTGGGAACACTAGGAGCT---------
ATGTTCCTTGGGTTCTTGGGAGCA---GCGGGAAGCACTATG---GGCGCAGCGTCA---
ATGGCGCTGACGGTACAG---GCCAGACTA---TTATTG---TCTGGTATAGTGCAACAGCAAAAT---
AATTTG---CTGAAG---GCTATTGAGGCGCAACAACACCTGTTGCAACTCACGGTCTGGGGCATT
>KX181894
AGTTATTGGCTGAATAATACTTGGGTGAATACGACTCTGGAA-------------------------------
----
ATCAATGGTACTATCACACTCCCATGCAGAATAAAACAGATTATAAACAGGTGGCAAGAAGTAGGAAAAGCAA
TGTATGCCCCCCCCATCAGAGGACACATTAATTGTTTATCAAAC---
ATTACAGGGCTGATATTAGCAAGGGATGGTGGTATTAATGTGACC----------------------------
----

AACGATAGCGAGACCTTCAGACCTGGAGGAGGAGACATGAGGGACAATTGGAGAAGTGAATTATACAAATATA
AAGTAGTAAAAATTGAGCCATTA---GGAGTAGCACCCACCAAGGCAAAGAGAAGAGTGGTG-----------
----------------------------CAAAGAGAAAAAAGAGCA---GTGGGAACGCTAGGAGCC------
---ATGTTCCTTGGGTTCTTGGGAGCA---GCAGGAAGCACTATG---GGCGCAGCGGCA---
ACAACGCTGACGGTACAG---GCCAGACTA---TTGTTG---TCTGGTATAGTGCAACAGCAGAAC---
AATCTG---CTGAGG---GCTATTGAGGCGCAACAGCATATGTTGCAACTCACAGTCTGGGGCATC
>KX181903
ATTTACGGATGGAATGGAACA--

ATCCCCGCTAACATCACACTTCCATGTAGAATAAAACAAATTATAAACAGGTGGCAGGAAGTAGGAAAAGCAA
TGTATGCCCCTCCCGTCAGCGGGCTAATTAACTGTACATCAAAT---
ATTACAGGACTACTATTAACAAGAGATGGTGGTATCAATGAAAAT----------------------------
----

AATATATCTGAAACCTTCAGACCTGGAGGAGGAGATATGAGGGACAATTGGAGAAGTGAATTATATAAATATA
AAGTAGTAAAAATTGAACCATTA---GGAATAGCACCCTCCAAGGCACAGAGAAGAGTGGTG-----------
----------------------------CAGAGAGAAAAAAGAGCA---GTGGGAACGCTGGGAGCC------

---ATGTTCCTTGGGTTCTTGGGAGCA---GCAGGAAGCACTATG---GGCGCAGCGGCA---
GCAACGCTGACGGTACAA---GCCAGACTA---TTGTTG---TCTGGTATAGTGCAGCAGCAGAAC---
AATCTG---CTGAGG---GCTATTGAGGCGCAACAGCATATGTTGCAACTCACAGTCTGGGGCATC
>KX181914
AATCAGACGTCTAAT--

GGGAATAGGACCATTACACTCCCATGCAGAATAAAACAAATCATAAACAGATGGCAAGAAGTAGGACAAGCAA
TGTATGCCCCTCCCATTAGAGGACAGATTAGATGTACATCAAAT---
ATTACAGGGTTACTATTAACAAGAGATGGTGGTAACAGGAACACCACAGAC----------------------
----

AACAATACTGAGGTTTTCAGACCTGGAGGAGGAGATATGAGGGACAATTGGAGAAGTGAATTATATAAATATA
AAGTAGTAAAAATTGAGCCATTA---GGAATAGCACCCACTAGGGCAAAGAGGAGAGTGGTG-----------
----------------------------CAAAGAGAGAAAAGAGCA---GCGGGA---CTAGGAGCT------
---TTGTTCATTGGGTTCTTGGGAGCA---GCAGGAAGCACTATG---GGCGCAGCGTCA---
GTGACGCTGACGGTACAG---GCCAGACAA---TTATTG---TCTGGAATAGTGCAACAGCAGAAC---
AATTTG---CTGAGA---GCTATTGAGGCGCAACAACATCTGTTGCAACTCACAGTCTGGGGCATC
>MG571982
ACTCATAAAACTTGGGAAAATGGTAAT--

GATAATGAAACTATCACACTCCCATGCAGAATAAAACAAATTATAAACAGGTGGCAGGAAGTAGGAAGAGCAA
TGTATGCCCCTCCCATTGCAGGACAAATTAACTGTACATCAAAT---
ATAACAGGGATGATATTAACAAGAGATGGGGGTAATGAGAGCGCA----------------------------
----

CCTGAAGAAATCTTCAGGCCTGGGGGAGGAGATATGAGGGACAATTGGAGAAGTGAATTATATAAATATAAGG
TAGTAAAAATTGAGCCATTA---GGAGTAGCACCCACCAAGGCAAAGAGAAGAGTGGTG--------------
-------------------------CAGAGAGAAAAAAGAGCG---ATAACG---CTGGGAGCT---------
ATGTTCCTTGGGTTCTTGGGAGCA---GCAGGAAGCACTATG---GGCGCAGCGTCG---
CTGACGCTGACGGTACAG---GCCAGACTA---TTATTG---TCTGGTATAGTGCAGCAGCAGAAC---
AATCTG---CTGAGA---GCTATTGAGGCGCAACAGCATATATTGCAACTCACAGTCTGGGGCATT
>MG571991
AATTATACAGGG---

TCTAATGACACCATCACACTCCCATGCAAAATAAAACAAATTGTAAACAAGTGGCAGGAAGTAGGAAAAGCAA
TATACGCCCCTCCCATCAGTGGAAACATTAGCTGTTCATCAAAT---
ATTACAGGGCTGATATTAACAAGAGATGGAGGCGAT-------------------------------------
----

AATGAGTCTGAAACTTTCAGACCTGGAGGAGGAAATATGAAAGACAATTGGAGAAGTGAATTATATAAATATA
AAGTAGTAAAAATTGAGCCATTA---GGAGTAGCACCCACCAAGGCAAAGAGAAGAGTGGTG-----------
----------------------------CAGAGAGAAAAAAGAGCA---GTGGGAACAATAGGAGCT------
---ATGTTCCTTGGGTTCTTGGGAGCA---GCAGGAAGCACTATG---GGCGCAGCGGCA---
GTAACGCTGACGGTACAG---GCCAGACAA---TTATTG---TCTGGTATAGTGCAACAGCAGAAC---
AACCTG---CTGAGG---GCTATTGAGGCGCAGCAGCATATGTTGCAGCTCACAGTCTGGGGCATC
>FJ653506
GATCAAAGTAATAGTACTTGGCATACTAATAATACT-------------------------------------
---TGGAAT------
AATACTGAAGGCGTTATCACACTCCACTGCAGAATAAAACAAGTTATAAACCTGTGGCAGGAAGTAGGAAAAG
CAATGTATGCCCCTCCCATCCAAGGACAAATTAACTGTACATCAAAT---
ATTACAGGACTGCTTTTAACAAGAGATGGTGGGAATAACAATGGTAGTAAT----------------------
----

AACAGTGAGACCGAGACCTTTAGACCTGGAGGAGGAGATATGAGGGACAATTGGAGAAGTGAATTATATAAAT
ATAAAGTAGTAAGAATTGAACCATTA---GGAATAGCACCCACCAAGGCAAAAAGAAGAGTGGTG--------
-------------------------------CAAAGAGAGAAAAGAGCA---GTGGGA---CTGGGAGCT---
------ATATTCCTTGGGTTCTTGGGAGCA---GCCGGAAGCACTATG---GGCGCAGCGTCG---
ATAACGCTGACGGTACAG---GCCAGACAA---GTATTG---TCTGGTATAGTGCAACAGCAGAAC---
AATTTG---CTGAAG---GCTATTGAGGCGCAACAGCATTTGCTGCAACTCACAGTCTGGGGCATT
>EU023932

ACTCATTTGAGTAATACTACGGAGTCAAAT---

GAAAATGACACAATCACACTCCAATGTAGAATAAAACAAATTATAAACATGTGGCAGGAAGTAGGAAAAGCAA
TGTATGCCCCTCCCATTGCAGGACAAATTAACTGTTCATCAAAA---
ATTACAGGGCTACTTTTAACAAGAGATGGTAGTAGCACAAATGAGACA-------------------------
----

AATGGGACTGAGATCTTCAGACCTGGAGGAGGAGATATGAGGGACAATTGGAGAAGTGAATTATATAAATATA
AAGTAGTAGGAATTGAACCAATA---GGAATAGCACCCACCAAGGCAAAGAGAAGAGTGGTG-----------
----------------------------CAGAGAGAAAAAAGAGCA---GTGGGAACATTAGGAGCT------
---ATGTTCCTTGGGTTCTTGGGAGCG---GCAGGAAGCACTATG---GGCGCAGCGTCA---
ATGACGCTGACGGTACAG---GCCAGACAA---TTATTG---TCTGGTATAGTGCAGCAGCAAAAT---
AATTTG---CTGAGG---GCTATTGAGGCGCAACAACATGTGTTGCAACTCACAGTCTGGGGCATC
>JF932469
AATTATACTTGGGAAGTCACTAATACTTGGAATGATACTGCA-------------------------------
----
-
GGAAATGACAATATCACGCTCCCTTGCAGAATAAAACAAATTGTAAATATGTGGCAGGAAGTAGGGAGAGCAA
TGTATGCCCCCCCCATTGAAGGACAAATTAGATGTTCATCAAAT---
ATTACAGGGTTGCTATTAACAAGAGATGGTGGTAATACTACGAAC----------------------------
----

GGGACCGAGATCTTCAGACCTGGAGGAGGAAATATGAGGGACAATTGGAGAAGTGAATTATATAAATATAAAG
TAGTAAAAATTGAACCATTA---GGAGTAGCACCCACCAAGGCAAAGAGAAGAGTGGTG--------------
-------------------------CAGAGAGAAAAAAGAGCA---GTGGGAACGATAGGAGCT---------
ATGTTCCTTGGGTTCTTGGGAGCA---GCAGGAAGCACTATG---GGCGCAGCGTCA---
ATAACGCTGACGGTACAG---GCCAGACAA---TTGTTG---TCTGGTATAGTGCAACAGCAGAGA---
AATTTG---CTGAGG---GCTATTGAGGCGCAACAGCAGCTGTTGCAACTCACAGTCTGGGGCATC
>KM218138
AATTATACTTGGAATAATGGCACCTGGCTAGCGCCAAATATC-------------------------------
----
-
ACTGGAAATAACATCACCATCCATTGCAGAATAAAACAAATTATAAACAGGTGGCAGGAAGTAGGAAAAGCAA
TGTATGCCCCTCCCATCGCGGGACAACTTAATTGCACATCAAAT---
ATTACAGGGCTGCTATTAGTAAGAGATGGTGGTAACACCACCAACACCATCGAG-------------------
----

AACAACACCGAAATCTTCAGACCTGGAGGAGGAGATATGAGGGACAATTGGAGAAGTGAACTATATAAATATA
AAGTAGTAAAAATTGAGCCATTA---GGACTAGCACCCACCAAGGCAAAGAGAAGAGTGGTG-----------
----------------------------CAGAGAGAAAAAAGAGCA---GTGGGAACAATAGGAGCT------
---ATGTTCCTTGGGTTCTTGGGAGCA---GCAGGAAGCACTATG---GGCGCAGCGTCA---
CTCACGCTGACGGTACAG---GCCAGACTA---TTACTG---TCTGGGATAGTGCAACAGCAAAAC---
AATTTG---CTGAAG---GCTATTGAGGCGCAACAGCACCTGTTGCAACTCACAGTCTGGGGCATC
>KM218195
AATCATACTTGGCTGGCTAATAATACTTGGCAAGAGTCTTGGCAA----------------------------
----
-
GGGTCAGATACTATTACTCTCCAATGCAGAATAAAACAAATTATAAACAGATGGCAGGAAGTAGGAAAAGCAA
TGTATAACCCTCCCATCAGGGGACAAATTAGATGTTCATCAAAT---
ATTACAGGGCTTCTGTTAACAAGAGATGGTGGTAACACGAATGGGTCCAGA----------------------
----

AATGGGCCTGAAACCTTCAGACCTGGAGGAGGAGATATGAGAGACAATTGGAGAAGTGAATTATATAAATATA
AAGTAGTAAAAATTGAGCCACTA---GGAATAGCACCCACCAAGGCAAGGAGAAGAGTGGTG-----------
----------------------------CAGAGAGAAAAAAGAGCA---GTGGGAACAATAGGAGCT------
---ATGTTCCTTGGGTTCTTGGGAGCA---GCAGGAAGCACTATG---GGCGCAGCGTCA---
GTGACGCTGACGGTACAG---GCCAGACTA---TTACTG---TCTGGTATAGTGCAACAGCAGAAC---
AATCTG---CTGAAA---GCTATTGAGGCGCAACAGCACCTGTTGCAACTCACAGTCTGGGGCATC
>JF932478

```
ACTTATAATGGTACTTGGGAAGGTACTACA-------------------------------------------
--------------------------------------------------------------------
GGAAATGACACTATCACGCTCCCTTGCAGAATAAAACAAATTATAAACATGTGGCAGGAAGTAGGGAAAGCAA
TGTATGCCCCTCCCATTAGAGGACAAATTAGATGTTCATCAAAT---
ATTACAGGGTTACTATTAACAAGAGATGGTGGTACTAATGAGACC----------------------------
-------------------------------------------------------------------------
----------
AATACCAACGAGACCTTCAGACCTGGAGGAGGAGATATGAGAGACAATTGGAGAAGTGAATTATATAAATATA
AAGTAGTACAAATTGAACCATTA---GGAGTAGCACCCACCAAGGCAAAAAGGAGAGTGGTG-----------
----------------------------CAGAGAGAAAAAAGAGCA---GTGGGAACAATAGGAGCT------
---ATGTTCCTTGGGTTCTTGGGAGCA---GCAGGAAGCACAATG---GGCGCAGCGTCA---
ATAACGCTGACGGTACAG---GCCAGACAA---TTGTTG---TCTGGCATAGTGCAACAGCAGAGA---
AATTTG---CTGAGG---GCTATTGAGGCGCAACAGCATCTGTTGCAACTCACAGTCTGGGGCATC
>JF346918
ACTTATGCTTGGAATGATACTAGG-------------------------------------------------
-----------------------------------AAT---
ACAGTAAATGACACTATCACGCTCCCTTGCAGAATAAAGCAAATTATAAACATGTGGCAGGGAGTAGGGAAAG
CAATGTATGCCCCTCCCATTAGAGGACAAATTAGGTGTGTATCAAAT---
ATTACAGGGTTGCTATTAACAAGAGATGGTGGTATTAATAAGAAC----------------------------
-------------------------------------------------------------------------
----------
AGCACCCCCGAGACCTTCAGACCTGGAGGAGGAGATATGAGGGACAATTGGAGAAGTGAATTATATAAATATA
AAGTAGTAAGAATTGAACCATTA---GGAGTAGCACCCACCAAGGCAAAGAGAAGAGTGGTG-----------
----------------------------CAGAGAGAAAAAAGAGCA---GTGGGAACGTTCGGAGCT------
---ATGTTCCTTGGGTTCTTGGGAGCA---GCAGGAAGCACTATG---GGCGCAGCGTCA---
CTAACGCTGACGGTACAG---GCCAGACAA---TTGTTG---TCTGGTATAGTGCAACAGCAGAGA---
AATCTG---CTGAGG---GCTATTGAGGCGCAACAGCACATGTTGCAACTCACAGTCTGGGGCATC
>KC596066
ACTTATCTGGCTAATGATACTAGATCAGAGAAGAAT-------------------------------------
--------------------------------------------------------------------
ACTACTGGTGTCATCACACTCCCATGCAGAATAAAACAAATTATAAACAGGTGGCAAGAAGTAGGGAGAGCAA
TGTATGCCCCTCCCATCATGGGAAAAATTAACTGTTCATCAAAT---
ATTACAGGGATACTATTAACAAGAGATGGTGGTGAAACAAATGAGTCC-------------------------
-------------------------------------------------------------------------
-------
AGAAATGGGTCCGAAACCTTCAGACCGGGAGGAGGAAATATGAAAGATAATTGGAGAAGTGAATTATATAAAT
ATAAAGTAGTAAAAATTGAGCCACTA---GGAATAGCACCCACCAAGGCAAGGAGAAGAGTGGTG--------
-------------------------------CAGAGAGAAAAAAGAGCA---GTAACA---CTAGGAGCT---
------GTATTTCTCGGGTTCTTGGGAGCA---GCAGGAAGCACTATG---GGCGCAGCGTCA---
ATGACGCTGACGGTACAG---GCCAGACTA---TTACTG---TCTGGTATAGTGCAACAGCAGAAC---
AATCTG---CTGAGA---GCTATTGAGGCGCAACAGCACATGTTGCAACTCACAGTCTGGGGCATC
>KX693556
CCTCAGATT----------------------------------------------------------------
--------------------GCTACT------
ACAGGAAATGACACTATCACGCTCCCTTGCAGAATAAGACAAATTGTAAACATGTGGCAGCAAGTAGGGAAAG
CAATGTATGCCCCTCCCATAGCAGGACAAATTAGATGTTCATCAAAT---
ATTACAGGGGTGCTATTAACAAGAGATGGTGGTAATAATGAGAGC----------------------------
-------------------------------------------------------------------------
----
AAGGCCAACGCCAACGAGACCTTCAGACCTGCGGGAGGAGATATGAGAGACAATTGGAGAAGTGAATTATATA
AATATAAAGTAGTAAAAATTGAACCATTA---GGAGTAGCACCCACCAAGGCAAAGAGAAGAGTGGTG-----
----------------------------------CAGAGAGAAAAAAGAGCA---
GTGGGAACACTAGGAGCT---------ATGTTCCTTGGGTTCTTGGGAGCA---GCAGGAAGCACTATG---
GGCGCAGCGTCA---ATAACGCTGACGGTACAG---GCCAGACAA---TTGTTG---
TCTGGTATAGTGCAGCAGCAGAGA---AATTTG---CTGAGA---
GCTATTGAGGCGCAACAGCATCTGTTGCAACTCACAGTCTGGGGCATC
>KX693136
ACTCAGACAGGTACTACA-------------------------------------------------------
--------------------------------------------------
GGAAACAGCACTATCACGCTCCCTTGCAGAATAAAACAAATTGTAAACATGTGGCAGGAAGTAGGGAAAGCAA
```

TGTATGCCCCTCCCATTGCAGGAAAAATTAGATGTTCATCAAAT---
ATTACAGGGCTGCTATTAACAAGAGATGGTGGTAATGAGAGCAAGACC-------------------------
----

ACCAACACAGAAACCTTCAGACCTGGAGGAGGAGATATGAGAGACAATTGGAGAAGTGAATTATATAAATATA
AAGTAGTAAAAATTGAACCATTA---GGAGTAGCACCCACCAAGGCAAAGAGAAGAGTGGTG-----------
----------------------------CAGAGAGAAAAAAGAGCA---GTGGGAACAATAGGAGCT------
---ATGTTCCTTGGGTTCTTGGGAGCA---GCAGGAAGCACTATG---GGCGCAGCGTCA---
ATAACGCTGACGGTACAG---GCCAGACAA---TTGTTG---TCTGGTATCGTGCAACAGCAGAGA---
AATTTG---CTGAGA---GCTATTGAGGCGCAACAGCATCTGTTGCAACTCACAGTCTGGGGCATC
>KX693292
AATTATACTAAA---

GAAGATGACAATCTCACGCTCCCTTGCAGAATAAAACAAATTGTAAACATGTGGCAGGGAGTAGGGAGAGCAA
TGTATGCCCCTCCCATTAGAGGACAAATTAGATGTTCATCAAAT---
ATTACAGGGTTGATATTAACAAGAGATGGTGGCAAAAATGGTAAAAATGAGAGC-------------------
----

AACACCAATGAGACCTTCAGACCTATAGGAGGAAATATGAAAGACAATTGGAGAAGTGAATTATATAAATATA
AAATAGTAAAAATTGAACCATTA---GGAGTAGCACCCACCAAGGCAAAGAGAAGAGTGGTG-----------
----------------------------CAGAGAGAAAAAAGAGCA---GTGGGAACGCTGGGAGCT------
---ATGTTCCTTGGGTTCTTGGGAACA---GCAGGAAGCACTATG---GGCGCAGCGTCA---
CTAACGCTGACGGTACAG---GCCAGACAA---TTGTTG---TCTGGTATAGTGCAACAGCAGAGA---
AATCTG---CTGAGG---GCTATTGAGGCGCAACAGCATATGTTGCAACTCACAGTCTGGGGCATC
>KP109511
AATTATACTCTCCTGGCTAATGGTACTTGGCAAGGGCCAAACAGTACTAAT----------------------
----
-
GGAAATGGCACACTCACACTCCCATGCAGAATAAAACAAATCATAAACAGGTGGCAGGAAGTAGGAAAAGCAA
TGTATGCCCCTCCCATCGCGGGAAACATTAGCTGTTTATCAAAT---
ATTACAGGGCTGCTATTAACAAGAGATGGTGGTATCAACATGACGAAT-------------------------
----

AATGGGTCCGAAATCTTCAGACCTGAAGGAGGAGATATGAGGAACAATTGGAGAAGTGAATTATATAAATATA
AAGTAGTAAAAGTTGAGCCACTA---GGAATAGCACCCACCAGGGCAAAGAGAAGAGTGGTG-----------
----------------------------CAGAGAGATAAAAGAGCA---GTGGGAACAATAGGAGCT------
---ATGTTCCTCGGGTTCTTGGGAGCA---GCAGGAAGCACTATG---GGCGCAGCGTCA---
GTGACGCTGACGGTACAG---GCCAGACTA---TTACTG---TCTGGTATAGTGCAACAGCAGAAC---
AATCTG---CTGAGA---GCTATTGAGGCGCAACAGCACATGTTGCAACTCACAGTCTGGGGCATC
>KU724105
AATTATACTTTGTTTAATAATGGTACTTGGAAAGAGGGTACT-------------------------------
----
-
AACAAAAGTGTCATCACACTCCCATGCAGAATAAAACAAATTATAAACAGGTGGCAGGAAGTAGGAAAAGCAA
TGTATGCCCCTCCCATCAGGGGACAAATTAACTGTACATCAAAT---
ATTACAGGGCTGCTATTAACAAGAGATGGTGGTAATAGTGGTACTGGGTCCAAA-------------------
----

AATGGGAACGAAACCTTCAGACCTGGAGGGGGAAATATGAGAGACAATTGGAGAAGTGAATTATATAAATATA
AAGTAGTAAAAATTGAGCCACTA---GGAATAGCCCCCACCACGGCAAGGAGAAGAGTGGTG-----------
----------------------------CAGAGAGAAAGAAGAGCA---GTGGGAACATTAGGAGCT------
---ATGTTCCTCGGGTTCTTGGGAGCA---GCAGGAAGCACTATG---GGCGCAGCGTCG---
CTGACGCTGACGGTACAG---GCCAGACTA---TTACTG---TCTGGTATAGTGCAACAGCAGAAC---
AATCTG---CTGAGA---GCTATTGAGGCGCAACAGCATATGTTGCAACTCACAGTCTGGGGCATC
>AY561238
ACTCAGTGGAAAGGAAATAGCACA---

CTCCCAGAAAATGTTACACTCCCATGCAGAATAAAACAATTTGTAAACATGTGGCAGGAAGTAGGAAAAGCAA
TGTATGCCCCTCCCATCAGCGGACAAATTAGATGCTCATCAAAT---
ATTACAGGGCTGCTATTAACAAGAGATGGTGGTGCCAATGGTACCACT-------------------------

----

GATATAGACGAGATCTTCAGGCCTGGAGGAGGAGATATGAGGGACAATTGGAGAAGTGAATTATATAAATATA
AAGTAGTAAAAATTGAACCATTA---GGAATAGCACCCACCAAGGCAAAGAGAAAAGTGGTG-----------
----------------------------CAAAGAGAAAAAAGAGCA---GTGGGA---CTAGGAGCT------
---TTGTTCCTTAGGTTCTTAGGAGCA---GCAGGAAGCACTATG---GGCGCAGCGTCA---
GTGACGCTGACGGTACAG---GCCAGACAA---TTATTG---TCTGGTATAGTGCAACAGCAGAAC---
AATCTG---CTGAGA---GCTATTGATGCGCAACAGCATCTGTTGCAACTCACAGTCTGGGGCATT
>FJ388940
TTTCAATATAATAGTACTTGGAATGATACTGGAGAG-------------------------------------
----

ATGAATGGCACTATCATGCTCCCATGCAGAATAAGACAAATCATAAACCTGTGGCAGGAAGTAGGAAAAGCAA
TGTATGCCCCTCCCATCAAAGGACTAATCAGATGTTCATCAAAT---
ATTACAGGACTGCTATTACTAAGAGATGGTGGTAATAATAGCAGTGGG-------------------------
----

AGTAATAACACCGAGATCTTCAGACCTGGAGGAGGAGATATGAGGGACAATTGGAGAAGTGAATTATATAAAT
ATAAAGTAGTAAAAATTGAACCATTA---GGAATAGCACCTACCAAGGCAAAGAGAAGAGTGGTG--------
-------------------------------CAGAGAGAAAAAAGAGCA---GTGGGA---TTCGGAGCC---
------GTGTTCCTTGGGTTCTTGGGAGCA---GCAGGAAGCACTATG---GGCGCAGCGTCA---
ATAACGCTGACGGTACAG---GCCAGACAA---TTATTG---TCTGGTATAGTGCAGCAGCAAAAT---
AACTTG---CTGAGG---GCTATTGAGGCGCAGCAGCATCTGTTACAACTCACAGTTTGGGGCATT
>JF683749
AATCACACGGGAAATAAT---

GATAATGATACAATCACACTCCAATGCAGAATAAAGCAAATTATAAACATGTGGCAGGAAGTAGGAAAAGCAA
TGTATGCCCCTCCCATCCAAGGAATAATTAGCTGTTCATCAAAC---
ATTACAGGGCTGCTATTAACAAGAGATGGTGGTGATGACAATAGC----------------------------
----

AGCAGTACAGAGATCTTCAGACCTGGAGGAGGGGATATGAGGGACAATTGGAGAAGCGAATTATATAAATATA
AAGTAGTAAGAATTGAACCATTA---GGGGTAGCACCCACCAAGGCAAAGAGAAGAGTGGTG-----------
----------------------------CAGAGAGAAAAAAGAGCA---ATAGGA---ATAGGAGCT------
---TTGTTCCTCGGGTTCTTGGGAACA---GCAGGAAGCACTATG---GGCGCAGCGTCA---
TTGACGCTGACGGTACAG---GCCAGACAA---TTATTG---TCTGGTATAGTGCAACAGCAGAAC---
AATTTG---CTGAGG---GCTATTGAGGCGCAACAGCATATGTTGCAACTCACAGTCTGGGGCATC
>JF683765
AATCATACTTGGAATGGTACTGCAATGACTGCTTGGAATGGTACTGTAGTGACTACT----------------
--------------------------TGGAAT---GGTACTGCAGTGATTAATAGT---------------
ACTACGGAGTCAAATATCACACTCCAATGCAGGATAAAACAAATAGTAAACATGTGGCAGGAAGTAGGAAAAG
CAATGTATGCCCCTCCCATCAGAGGACAGATTAGATGTTCATCAAAT---
ATTACAGGCCTATTATTAACAAGAGATGGTGGTCTTAACAGCAGC----------------------------
----

AATATCAACGAGACTTTCAGACCAGGAGGAGGAGATATGAGGGACAATTGGAGAAGTGAATTATATAAATATA
AAGTAGTAAAAATTGAACCATTA---GGGGTAGCACCCACCAGGGCAAGGAGAAGAGTGGTG-----------
----------------------------CAGAGAGAAAAAAGAGCA---ATAGGA---CTAGGAGCT------
---GTGTTCCTTGGGTTCTTGGGAGCA---GCAGGAAGCACTATG---GGCGCAGCGTCA---
ATAACGCTGACGGCACAG---GCCAGGCAA---TTGATG---TCTGGTATAGTGCAACAGCAGAAC---
AATCTG---CTGAGA---GCTATTGAGGCGCAACAGCATCTGTTGCAACTCACAGTCTGGGGCATC
>JF683784
ACTCATAATGATACT--

AAAGGAATATATATCACACTCCCATGCAGAATAAGACAAATTGTAAATCTGTGGCAGGAAGTAGGGAAAGCAA
TGTATGCCCCTCCCATCAGGGGACAAATTAACTGTTCCTCAAAT---
ATTACAGGGCTGATATTAACAAGAGATGGTGGTCTGAATAATGAT----------------------------
----

ACTAATGAGATCTTCAGACCTGGAGGAGGAGATATGAGGATAATTGGAGAAGTGAATTATATAAATATAAAG

TAGTAAAAATTGAACCATTA---GGAGTAGCACCCACCAAGGCAAAGAGAAGAGTGGTG--------------
-------------------------CAAAGAGAGAGAAGAGCA---GTGGGA---ATAGGAGCT---------
CTGTTCCTTGGGTTCTTGGGAGCA---GCAGGAAGCACTATG---GGCGCAGCATCA---
ATAACGCTGACGGTACAG---GCCAGACAA---TTACTG---TCTGGTATAGTGCAACAGCAGAAC---
AATCTG---CTGAGG---GCTATTGAGGCGCAACAAAATCTGTTGCAACTCACAGTCTGGGGCATT
>JF683793
AATCATACTGGT---

GCTGAAAGCAATATCACACTCCCATGCAGAATAAGACAAATTATAAATATGTGGCAGAGAGTAGGAAAAGCAA
TGTATGCCCCTCCCATCGAAGGAGAAATTAGATGCTCATCAAAA---
ATTACAGGGCTGCTATTAACAAGAGATGGTGGTAGTAGCGGCAACAAC-------------------------
----

ACCAACGACACAGAGATCTTCAGACCTGCAGGAGGAGATATGAGGGACAATTGGAGAAGTGAATTATATAAAT
ATAAAGTAGTAAGAATTGAACCTTTA---GGAATAGCACCCACTAAGGCAAAGAGAAGAGTGGTG--------
-------------------------------CAAAAAGAGAAAAGAGCA---GTGGGA---ATAGGAGCT---
------CTATTCCTTGGGTTCTTGGGAGCA---GCAGGAAGCACTATG---GGCGCAGCGTCA---
TTGACGCTGACGGTACAG---GCCAGACAG---TTAATG---TCTGGTATAGTGCAACAGCAGAAC---
AATTTG---CTGAGA---GCTATTGAGGCGCAACAACATATGTTGCAACTCACAGTCTGGGGCATC
>JF683796
ACTCAC---

AATAGCAATAACATCACACTCTCATGCAGAATAAGACTAGTTATAAACAGGTGCCGGGAACTAGGAATAGCAA
TGTCTGCCCCTCCCCTCAAAGGCATAGTCAGCTGCTCATCCAAT---
ATTACAGGTCTAGTATTAACAAGGGCTGGTGGCAGTAACAGTGAGTCC-------------------------
----

GACTCCAACGAGACCTTCAGACCTGGAGGGGGAGACATGAGAGATAATTGGAGAAGTGAACTATATAAATATA
AAGTAGTAAAAATTGAGCCATTA---GGAATAGCACCCACCACGGCAAAGAGAAGAGTGGTG-----------
----------------------------CAGAGAGAAAAAAGAGCA---ATAACG---CTAGGAGCT------
---ATGTTCCTTGGGTTCTTGGGAACA---GCAGGAAGCACTATG---GGCGCAGCGTCA---
CTGACGCTGACGGTACAG---GCCAGACAA---TTATTG---TCTGGTATAGTGCAGCAGCAGAGC---
AATCTG---CTGAGG---GCTATTGAGGCGCAACAGCATATGTTGCAACTCACAGTCTGGGGCATC
>JF683805
ACT--

CAAGAAGAGACCATCATACTCCCATGTAGAATAAGGCAAATTGTAAACTTGTGGCAGGAAGTAGGAAAGGCAA
TGTATGCCCCTCCCATAAAGGGAAACATTAGCTGTCCATCAAAC---
ATTACAGGGCTGCTATTAATAAGAGATGGTGAAGATGAGAGTAAT----------------------------
----

AATAGCGAGATCTTCAGACCTGGAGGAGGAGATATGAGGGATAATTGGAGAAGTGAATTATATAAATATAAAG
TAGTAAAAATTGAACCATTA---GGGATAGCACCCACCAAGGCAAAGAGAAGAGTGGTG--------------
-------------------------CAGAGAGAAAAAAGAGCA---GTGGGAGCACTAGGAGCT---------
ATGTTCCTTGGGTTCTTGGGAGCA---GCAGGAAGCACTATG---GGCGCAGCGTCA---
ATGACACTGACGGTACAG---GCCAGACAA---TTATTG---TCTGGTATAGTGCAACAGCAGAAC---
AATTTG---CTGAAG---GCTATTGAGGCGCAACAGCATCTGTTGCAACTCACAGTCTGGGGCATC
>KT124762
ACTCACAACAATGAGAGT---

ACCCCTGAGAATATCACACTCCAATGCAGAATAAAACAAATTATAAACATGTGGCAGGGAGTAGGAAAAGCAA
TGTATGCCCCTCCCATCAGCGGGCTAATTAGATGTTCATCAAAT---
ATTACAGGGCTGTTACTAACAAGGGATGGTGGTACGAATAGCACA----------------------------
----

GATGAGATCTTCAGGCCCGGAGGAGGGGATATGAAGGATAATTGGAGAAGTGAATTATACAAATATAAAGTAG
TAAAAATTGAGCCATTA---GGAGTAGCACCCACCAAGGCAAAGAGAAGAGTGGTG-----------------
----------------------CAGAGAGAAAAAAGAGCA---GTGGGA---TTCGGAGCT---------
TTGTTCCTTGGGTTCTTGGGAGCA---GCAGGAAGCACTATG---GGCGCAGCGTCA---

```
TTGACGCTGACGGTACAG---GCCAGACAA---TTATTG---TCTGGTATAGTGCAACAGCAGAAC---
AATTTG---CTGAGA---GCTATTGATGCGCAACAGCATCTGTTGCAGCTCACAGTCTGGGGCATC
>KT124788
AGTTATAATAGTACTGAG-------------------------------------------------------
-----------------------------------------
AATAATGGTACAATCACACTCCCATGCAGAATAAAACAAATTATAAACATGTGGCAGGAAGTAGGAAAAGCAA
TGTACGCCCCTCCCATCAGAGGACAGATTAGTTGTTCATCAAAT---
ATCACAGGGCTGTTATTAACAAGAGATGGCGGTAAAGGAATAAT-----------------------------
-------------------------------------------------------------------------
-------
GGCAGCAATACTGAGATCTTCAGACCTGGAGGAGGAAATATAAAGGACAATTGGAGGAGTGAATTATATAAAT
ATAAAGTAGTAAAAATTGAACCATTA---GGAGTAGCACCCACCAAGGCAAAGAGAAGAGTGGTG--------
-------------------------------CAGAGAGAAAAAAGAGCA---ATAACA---TTAGGAGCT---
------GTGTTCCTGGGGTTCTTGGGAGCA---GCAGGAAGCACTATG---GGCGCAGCGTCA---
ATAACGCTGACGGTACAG---GCCAGACAA---TTATTG---TCTGGTATAGTGCAACAGCAAAAC---
AACTTG---CTGAAG---GCTATTGAGGCACAACAACATCTGTTGCAACTCACAGTCTGGGGCATT
>KT124782
AATTATACTAAT-------------------------------------------------------------
--------------------------------------------
AACAGCGAAAATATCACACTCCCATGCAGAATAAAGCAAATTATAAACCTATGGCAGGGAGTAGGAAAAGCAA
TGTATGCCAATCCAGTCAAAGGACTAATTAACTGTCTATCAAAT---
ATTACAGGACTGATATTAACAAGAGATGGTGGTGTTAATAATAGC----------------------------
-------------------------------------------------------------------------
-------------
AACAATGAGACCTTTAGGCCTGGGGGAGGAGATATGAGGGACAATTGGAGGAGTGAATTATATAAATATAAAG
TAGTAAAAATTGAACCATTA---GGATTGGCACCCACTACGGCAAAGAGAAGAGTGGTG--------------
-------------------------CAAAGAGAAAAAAGAGCA---GTGGGA---ATAGGAGCT---------
GTGTTCCTTGGGTTCTTGGGAGCA---GCAGGAAGCACTATG---GGCGCAGCGTCA---
ATGACGCTGACGGTACAG---GCCAGACTA---TTACTG---TCTGGTATAGTGCAGCAGCAAAAC---
AATTTG---CTGAGG---GCTATTGAGGCGCAACAGCATCTGTTGCAACTCACAGTCTGGGGCATC
>KT124760
AAT----------------------------------------------------------------------
-----------------------------------
TATACTAGCACAACTAGGCTCCCATGCAGGATAAAACAAATTGTAAACATGTGGCAGGAAGTAGGAAAAGCAA
TGTATGCCCCTCCCATCAGAGGGCGAATCAATTGTTTATCAAAT---
ATCACCGGGTTGCTATTAACAAGAGATGGTGGTAACAGTAACAGC----------------------------
-------------------------------------------------------------------------
-------------
AGCAACGAGACTTTCAGACCAGGAGGAGGAAATATGAAGGATAATTGGAGAAGTGAGTTATATAAATACAAAG
TAGTAAAACTTGAACCATTA---GGAATAGCACCCACCACGGCAAAAAGAAGAGTGGTG--------------
-------------------------CAGAGAGAAAAAAGAGCA---GTGGGA---ATAGGAGCC---------
ATGCTCTTTGGGTTCTTGGGAGCA---GCAGGAAGCACTATG---GGCGCAGCATCA---
ATATCGCTGACGGTACAA---GCCAGGCAA---TTATTG---TCTGGTATAGTGCAACAGCAGAGT---
AATCTG---CTGAGG---GCTATTGAGGCGCAACAACATATGTTGCAACTCACAGTCTGGGGCATC
>KT124780
TATCATAATAGTACTTGGAATAATACTAGGAATAATACTGAA-------------------------------
-----------------------------------------------------------
GGGTCAAATGATATAATCACACTCCCATGCAGAATAAAACAAATTATAAACATGTGGCAGGAAGTAGGAAAAG
CAATGTATGCCCCTCCTATCAAAGGACGAATTAGATGTTCATCAAAT---
ATTACAGGGATGATATTAACAAGAGATGGTGGTAATAATGGGACT----------------------------
-------------------------------------------------------------------------
-------
GACACAGAGATAGAGATCTTCAGACCCCAAGGAGGAAATATGAAGGACAATTGGAGAAGTGAATTGTTTAGAT
ATAAAGTAGTAAGAGTTGAACCATTA---GGAGTAGCTCCCACTAAGGCAAAGAGAAGGGTGGTG--------
-------------------------------CAGAGAGAAAAAAGAGCA---GTAGGA---ATGGGAGCC---
------TTGTTCCTTGGGTTCTTGGGAGCA---GCAGGAAGCACTATG---GGCGCAGCGTCA---
ATGACGCTGACGGTACAG---GCCAGGCAA---TTATTG---TCTGGTATAGTGCAACAGCAGAAC---
AATCTG---CTGAGG---GCTATTGAGGCGCAGCAGCACCTGTTGCAACTCACAGTCTGGGGCATC
>AY173959
```

AATTACAATACA---

AGTGGAGTAAATATCACACTCCCATGTAGAATAAAACAAATCATAAACATGTGGCAGGAAGTAGGAAAAGCAA
TGTATGCCCCTCCCATTAGAGGACAAATTAGATGCATATCAAAT---
ATTACAGGACTGCTATTAACAAGAGATGGTGGTGCTAACCAGACC----------------------------
----

AATGGGACCGAAATCTTCAGACCTGCAGGGGGAGATATGAGGGACAATTGGAGAAGTGAACTATATAAATATA
AAGTAGTAAAAATTGAACCATTA---GGAGTAGCACCCACCAAGGCAAAAAGAAGAGTGGTG-----------
----------------------------CAGAGAGAAAAAAGAGCA---GTGGGG---TTAGGAGCT------
---GTGTTCCTTGGGTTCTTGTCAGCA---GCAGGAAGCACTATG---GGCGCAGCGTCA---
ATGACGCTGACGGTACAG---GCCAGGCAA---TTATTG---TCTGGTATAGTGCAACAGCAGAAC---
AATCTG---CTGAGG---GCTATTGAGGCGCAACAGCATCTGTTGCAACTCACAGTCTGGGGCATT
>KC595166
ACTCAAGAA--

AGTGACACATTAATCACACTCCCATGCAGAATAAAACAAATTGTAAGAATGTGGCAGGAAGTAGGAAAAGCAA
TGTATGCCCCTCCCATCAGAGGACAAATTAGATGTTCATCAAAT---
ATTACAGGGCTGATATTAACAAGAGATGGAGGTAATGACACGGAC----------------------------
----

GAGAACGGGACCGAGATCTTTAGACCTGGAGGAGGAGATATGAGGGACAATTGGAGAAGTGAATTATATAAAT
ATAAAGTAATACAAATTGAACCATTA---GGAGTAGCACCCACCAAGGCAAAGAGAAGAGTGGTG--------
-------------------------------CAAAGAGAAAAAAGAGCA---GCAATA---ATAGGAACT---
------GTGTTCCTTGGGTTCTTGGGAGCA---GCAGGAAGCACTATG---GGCGCAGCGTCA---
ATAACGCTGACGGCACAG---GCCAGACAA---TTATTG---TCTGGTATAGTGCAACAGCAGAGC---
AATTTG---CTGAAG---GCCATTGAGGCGCAACAGCATCTGTTGCAACTCACAGTCTGGGGCATC
>JN054257
CCCTATACTAATGCAAGTATA--

GAAAATATCACACTCACACTCCCATGTAGAATAAAACAAATCATAAACATGTGGCAGGAAGTAGGAAAAGCAA
TGTATGCCCCTCCCATCCCGGGAATAATCCAATGTACATCAAAT---
ATTACAGGACTACTATTAACAAGAGATGGGGGTAACCAAACCAAT----------------------------
----

GGGATTGAGATCTTCAGACCTGCAGGGGGAGATATGAAGGACAATTGGAGAAGTGAATTATATAAATATAAAG
TAGTAAAAATTGAACCATTA---GGAATAGCACCCACCAAGGCAAAAAGAAGAGCGGTG--------------
-------------------------CAAAGAGAAAAAAGAGGA------------ATAGGAGCT---------
GTGATCTTTGGGTTCTTGGGAGCA---GCAGGAAGCACTATG---GGCGCAGCGTCG---
GTAACGCTGACGGTACAG---GCCAGACAA---TTATTG---TCTGGTATAGTGCAACAGCAGAGC---
AACTTG---CTGAGA---GCTATTGAGGCGCAACAGAATCTGTTGCAGCTCACAGTCTGGGGCATT
>KT200348
AGTCATGATGCTTGGAATTGGACTTGGAGTAATGGTACT----------------------------------
--TGGGGGGGTAGT---ACTAAAGAAACAAATAAAACT---------------
GAAGGAAATGTCACCCTCACCCTCCAATGTAGAATAAAACAAATTATAAACATGTGGCAGGAAGTAGGGAAAG
CAATGTATGCCCCTCCCATCAGTGGACCAATTAGATGTAAATCAAAC---
ATTACAGGGATGATATTAACAAGAGATGGGGGTAATGATGACGGTACT-------------------------
----

GACAACAGCAGTGAGACCTTCAGACCTGGGGGAGGAGATATGAGGGATAATTGGAGAAGTGAATTATACAAAT
ATAAAGTAGTAAAAATTGAACCATTA---GGAGTAGCACCCACCAAGGCAAAAAGAAGAGTGGTG--------
-------------------------------CAGAGAGAAAAAAGAGAT---TTAGGA---TTTGGAGCT---
------TTGTTCCTTGGGTTCTTGGGAGCA---GCAGGAAGCACTATG---GGCGCAGCGTCA---
ATAACGCTGACGGCACAG---GCCAGACAA---TTATTG---TCTGGTATAGTGCAACAGCAAAGT---
AATTTG---CTGATG---GCTGTTGAGGCGCAACAGAAGCTGTTGCAACTCACAGTCTGGGGCATC
>KC699038
ACTCAGAATAAC---

ATTACAGAAAATATCACACTCCCATGCAGAATAAAACAGTTTATAAACATGTGGCAGGAAGTAGGAAAAGCAA
TGTATGCCCCTCCCATCAAAGGACAAATTAGATGTTCATCAAAT---

ATTACAGGGCTGATATTAACAAGAGATGGTGGTAATAATGATAAC----------------------------
----

ACAACAGAGACCTTCAGACCTGGAGGAGGAAATATGAAGGACAATTGGAGAAGTGAATTATATAAATATAAAG
TAGTAAAAATTGAACCATTA---GGAGTAGCACCCACCAAGGCAAAGAGAAGAGTGGTG--------------
-------------------------CAGAGAGAAAAAAGAGCA---GCG------ATAGGAGCT---------
GTGTTCCTTGGGTTCTTGGGAGCA---GCAGGAAGCACTATG---GGCGCAGCGTCA---
GTGACGCTGACGGTACAG---GCCAGACAA---TTATTG---TCTGGTATAGTGCAACAGCAAAAC---
AACTTG---CTGAAG---GCTATTGATGCGCAACAGCATATGTTGCAACTCACAGTCTGGGGCATC
>HQ595780
TCTCATAATAACACTGAAGGGGTAAGAAATAATAAC-------------------------------------
--ACT---
GAAGGGGAAAAAAATATCATCCTCCCATGCAGAATAAAACAAGTTATAAACATGTGGCAGGAAGTAGGAAAAG
CAATGTATGCCCCTCCCATCAGAGGACAAATCAGATGTTCATCAAAT---
ATCACAGGGCTGGTATTAACAAGAGATGGTGGCACTAATAATGGCACA-------------------------
----

AACCAGGCCGAGGAGGTCTTCAGACCTGCAGGAGGAAATATGAGGGACAATTGGAGAAGTGAATTATATAGAT
ATAAGGTAGTCAAAATTGAACCATTA---GGAGTAGCACCCACCAAGGCAAAGAGAAGAGTGGTG--------
-------------------------------CAGAGAGAAAAAAGAGCA---GTGGGA---ATAGGAGCT---
------GTATTCCTTGGGTTCTTGGGAGCA---GCAGGAAGCACTATG---GGCGCAGCGTCA---
ATAACGCTGACGGTACAG---GCCAGACAA---TTACTG---TCTGGTATAGTGCAACAGCAGAAC---
AATTTG---CTAAGG---GCTATTGACGCGCAACAGCATCTGTTGCAGCTCACAGTCTGGGGCATC
>HQ595781
TTTCATAGTACTTGGGTTAATGGTAATTGGAGTAATGGCACTTGGAATGGT----------------------
----

ACTGAAGAAATTATCACACTTCCATGCAGAATAAAACAAATTATAAACATGTGGCAGGAAGTAGGAAAAGCAA
TGTATGCTCCTCCCATCAAAGGACAAATTAACTGTACATCAAAT---
ATTACAGGGCTGCTATTAACAAGAGATGGTGGTAACATAACAAACAAC-------------------------
----

AACAACACCGAGACCTTCAGACCTGGAGGAGGAGATATGAGGGACAATTGGAGAAGTGAATTATATAAATATA
AAGTAGTAAAAATTGAACCACTA---GGAATAGCACCCAATAAGGCACAGAGAAGAGTGGTG-----------
----------------------------CAGAGAGGAAAAAGAGCA---GTGGGA---TTAGGAGCT------
---ATGTTCCTTGGGTTCTTGGGAGCA---GCAGGAAGCACTATG---GGCGCAGCGTCA---
ATAACGCTGACGGTACAG---GCCAGGCGA---TTATTG---TCTGGTATAGTGCAACAGCAGAAC---
AATTTG---CTGAAA---GCTATTGAGGCGCAACAGCATCTGTTGCAACTCACAGTCTGGGGCATC
>HQ595805
AATCATACTTGGACAGGTCCAAATAGA--

ACTGACACAAATATCACACTCCAATGCAAAATAAAGCAAATTATAAATATGTGGCAGAAAGTAGGAAAAGCAA
TGTATGCCCCTCCCATCAGAGGACAAATTAAATGTTCATCAAAT---
ATTACAGGGCTGCTATTAACAAGAGATGGTGGAAATGGTGATAATAGT-------------------------
----

ACAAACGGGACTGAGACCTTCAGACCTGGAGGAGGAGATATGAGGGACAATTGGAGAAGTGAATTATACAAAT
ACAAAGTAGTAAAAAATTGAACCATTA---GGAGTAGCACCCACCGAGGCAAAAAGAAGAGTGGTG-------
-------------------------------CAGAGAGAAAAAAGAGCA---GTGGGA---TTTGGAGCT---
------CTGTTCCTTGGTTTCTTGGGAGCA---GCAGGAAGCACTATG---GGCGCAGCATCA---
ATAACGCTGACGGTACAG---GCCAGACAA---TTATTG---TCTGGTATAGTGCAACAGCAAAAC---
AATTTG---CTGAGG---GCTATTGAGGCGCAACAGCATCTGTTGCAACTCACAGTCTGGGGCATT
>AJ535618
TTTCATAGTACTTGGGTGTTTAATAGAACTGGGGGGAATGATACTGAATAC----------------------
----

ACTGAAAAAAATATCACACTTCCATGCGGAATAAAACAAATTATAAACATGTGGCAGGGAGTAGGAAAAGCAA
TGTATGCCCCTCCCATCCAAGGACAAATTAATTGCACATCAAAT---
ATTACAGGGCTGTTATTAACAAGAGATGGTGGTAATGGTGATAAC----------------------------
----

ACAACTGAGATCTTCAGACCTGGAGGAGGAGATATGAGGGACAATTGGAGAAGTGAATTATATAAATATAAAG
TAGTAAAAATTGAGCCATTA---GGAGTAGCACCCACCAAGGCAAAGAGAAGAGTGGTG--------------
-------------------------CAGAGGGAAAAAAGAGAA---GTGGGC---CTAGGAGCT---------
TTGTTCATTGGGTTCTTGGGAGCA---GCAGGAAGCACTATG---GGCGCAGCGTCA---
GTGACGCTGACGGTACAG---GCCAGACAA---TTATTG---TCTGGTATAGTGCAACAGCAGAAC---
AATTTG---CTGAGA---GCTATTGATGCGCAACAGCATCTGTTGCAACTCACAGTCTGGGGCATC
>U36877
AGTCAGGACGGTACTGAA---

GGATATAACATTATCACACTCCCATGCAGAATAAAACAAATTATAAACATGTGGCAGGAAGTAGGAAAAGCAA
TGTATGCCCCTCCCATCAAAGGACAAATTAGATGTTCATCAAAT---
ATTACAGGACTGCTATTAACAAGAGATGGTGGTAATAGTAGTAGC----------------------------
----

ACCACCGAGACCTTCAGACCTGGAGGAGGAGACATGAGGGACAATTGGAGAAGTGAATTATATAAATATAAAG
TAGTAAAAATTGAACCATTA---GGAGTAGCACCCACCAAGGCAAAGAGAAGAGTGGTG--------------
-------------------------CAAAGAGAAAAAAGAGCA---GTGGGA---ATAGGAGCT---------
TTGTTCCTTGGGTTCTTGGGAGCA---GCAGGAAGCACTATG---GGCGCAGCATCA---
ATGACGCTGACGGTACAG---GCCAGACTA---TTATTG---TCTGGTATAGTGCAACAGCAGAAC---
AATTTG---CTGAGA---GCTATTGAGGCGCAACAGCATCTGTTGCAACTCACAGTCTGGGGCATC
>FJ460501
AGTTATATTTTGAATGGTACTAGCTGGAGTAACAATACTAAT-------------------------------
----
GGGGTAAATGATATAATCACACTCCCATGCAGAATAAGACAAATTATAAACATGTGGCAAGAAGTAGGAAAAG
CAATGTATGCCCCGCCCATCATGGGACAAATTAACTGTTCATCAAAT---
ATTACAGGGCTGCTATTAACAAGAGATGGTGGTAATAACACAGACACA-------------------------
----

AACCAAACAGAGATCTTCAGACCTGGAGGAGGAGATATGAGGGACAATTGGAGAAGTGAATTATATAAATATA
AAGTAGTAAGAATTGAACCATTA---GGAATAGCACCCACCAAGGCAAGAAGAAGAGTGGTG-----------
----------------------------CAGAGAGAGAAAAGAGCA---GTGGGA---ATAGGAGCT------
---CTGTTCCTTGGATTCTTGGGAGCA---GCAGGAAGCACTATG---GGCGCAGCGTCA---
CTGACACTGACGGTACAA---GCCAGACAA---TTATTG---TCTGGTATAGTGCAACAGCAGAAT---
AATCTG---CTGAGA---GCTATTGAGGCGCAACAGCATATGTTGCAACTCACAGTCTGGGGCATT
>EU839602
ATTTACCCTTGGAAT--

AAGACTGAAAATATCACACTCCCATGCAGAATAAAACAAATTATCAATATGTGGCAGGGAGTAGGAAAAGCAA
TGTATGCCCCTCCCATCAGCGGACCAATAAGATGTGAATCAAAT---
ATTACAGGGCTGCTATTAACAAGAGATGGTGGTGACCAGAACGGGACG-------------------------
----

GACGCTACCAATGAGACCTTTAGACCTGGAGGAGGAGATATGAGGGACAATTGGAGAAGTGAACTATATAAAT
ATAAAGTAGTAAGAATTGAACCATTA---GGAGTAGCACCCACCGAGGCAAAGAGAAGAGTGGTG--------
-------------------------------CAGAGAGAAAAAAGAGCA---GTGGGA---CTAGGAGCT---
------ATGTTCCTTGGGTTCTTAGGAGCA---GCAGGAAGCACTATG---GGCGCAGCGTCA---
ATAGCGCTGACGGCACAG---GCCAGGCAA---TTGTTG---TCTGGTATAGTGCAGCAGCAGAAC---
AATCTG---CTGAGG---GCTATTGAGGCGCAACAGCATCTGTTGCAACTCACGGTCTGGGGCATC
>EU839604
GAT--

TATAATGACACTATCATACTCCAATGTCGGATAAGACAAATTATAAACATGTGGCAGAAAGTAGGAAAAGCAA
TGTATGCCCCTCCCATAAGTGGAATAATTAAATGTACATCAAAT---
ATTACAGGGCTACTATTAACAAGAGATGGTGGTAATAATACAGGA----------------------------
----

GGGAATGAGACCTTCAGACCTACAGGAGGAAATATGAAGGACAATTGGAGAAGTGAATTATATAAATATAAAG
TAGTACAAATTGACCCAATA---GGAGTAGCACCCACCAAGGCAAAAAGGAGAGTGGTG--------------
-------------------------CAGAGAGAAAAAAGAGCA---GTGGGAGTAGTAGGAGCT---------

ATGTTCCTTGGTTTCTTGGGAGCA---GCAGGAAGCACTATG---GGCGCAGCGTCA---
GTGACGCTGACGGTACAG---GCCAGACAA---TTATTG---TCTGGTATAGTGCAACAGCAGAAT---
AATCTG---CTGAGA---GCTATTGAGGCGCAACAGCATCTGTTGCAACTCACAGTCTGGGGCATC
>HM030560
ACTCAGGAT--

GATACTAATGACACTATCACACTCCCATGCAGAATAAAACAAATTGTAAACATGTGGCAGGAAGTAGGCAAAG
CAATATATGCCCCTCCCATTAGTGGACTAATTAGATGTAACTCAAGT---
ATTACAGGGCTAATGTTAACAAGAGATGGTGGTGGGAATACT-------------------------------
----

AGTAATAATGAGACCTTCAGACCGGGAGGAGGAAATATGAAGGACAATTGGAGAAGTGAATTATATAAATATA
AAGTAGTGAAAATTGAACCATTA---GGAGTAGCACCCACCAGGGCAAGGAGAAGGGTGGTG-----------
----------------------------CAAAGAGAAAAAAGAGCA---GTGGGAGTAATAGGAGCT------
---ATGTTCCTTGGGTTCTTGGGAACA---GCAGGAAGCACTATG---GGCGCAGCGTCA---
GTGACGCTGACGGTACAG---GCCAGACAA---TTATTG---TCTGGTATAGTGCAACAGCAAAAC---
AATTTG---CTGAGG---GCTATTGAGGCGCAACAGCATCTGTTGCAACTCACAGTCTGGGGCATC
>AB588243
AATCAGTCTGCTTGGAAGAGAAATGATACTGAAGGG-------------------------------------
---TTAAAT------
AATACTGAGGACAAAATTACACTCCCATGCAAAATAAAACAAATCATAAACATGTGGCAGAAAGTAGGAAAAG
CAATATATGCCCCTCCCATCAGAGGAGTAATTAGATGTGCATCAAAT---
ATTACAGGGCTGCTATTAACAAGAGATGGTGGTGATGAGAAC-------------------------------
----

AGAACCACCGAGACCTTCAGACCTGGAGGAGGGGATATGAGGGACAATTGGAGAAGTGAATTATATAAATATA
AAGTAGTAAAAATTGAACCATTA---GGAGTAGCACCCACTAAGGCAAAGAGAAGAGTGGTG-----------
----------------------------CAGAGAGAAAAAAGAGCA---GTGGGA---TTTGGAGCT------
---GTATTCCTTGGGTTCTTGGGAGCA---GCAGGAAGCACTATG---GGCGCAGCGTCA---
GTGACGCTGACGGTACAA---GCCAGACAA---TTGCTG---TCTGGTATAGTGCAACAGCAGAGC---
AATCTG---CTGAGA---GCTATTGAGGCGCAGCAACAAATGTTGAAACTCACAGTCTGGGGCATC
>AB588255
AATCATGGA--

GAGTCAAATGACACTATCACACTCCCATGTAGAATAAAACAAATTGTAAACATGTGGCAGGAAGTAGGAAAAG
CAATGTATAACCCTCCCATCGAAGGACAAATTAGATGTTCATCAAAT---
ATTACAGGACTATTATTAATAAGAGATGGTGGTAACAGTAACAACACA-------------------------
----

AACACCACCGAAATCTTCAGACCCGGGGGAGGAAATATGAAGGACAATTGGAGGAGTGAATTATATAAATATA
AAATAGTAAAAATTGAACCATTA---GGAGTAGCACCCACCAAGGCAAAGAGAAGAGTGGTG-----------
----------------------------CAAAGAGAAAAAAGAGCA---GTGGGA---ATAGGAGCT------
---GTGCTCTTTGGATTCTTGGGAGCA---GCAGGAAGCACTATG---GGCGCAGCGTCA---
ATAGCGCTGACGGCACAG---GCCAGACAA---TTATTG---TCTGGTATAGTGCAACAGCAGAAC---
AATATG---CTGAGA---GCCATTGAGGCGCAACAGCACCTGTTGCAACTCACAGTCTGGGGCATC
>AB588271
CTTTATACTTTGAATAGCTCT--

GAAGATAAAAATATCACACTCCAATGCAGAATAAAACAGATTATAAACATGTGGCAGGAAGTAGGAAAAGCAA
CGTATGCCCCTCCTATCAAAGGACAGATTAGATGTTCATCAAAC---
ATTACAGGGCTGCTATTAACAAGAGATGGTGGTACTAATAAAACA----------------------------
----

AGCGATCCCGAGATCTTCAGACCTACAGGAGGTAATATGAAGGACAATTGGAGAAGTGAATTATATAAATATA
AAGTAGTAAAAATTGAACCATTA---GGAGTAGCACCTACCAGGGCAAAGAGAAGAGTGGTG-----------
----------------------------CAGAGAGAAAAAAGAGCA---GTGGGA---CTAGGAGCT------
---GTGTTCCTTGGGTTCTTGGGAGCA---GCAGGAAGCACTATG---GGCGCAGCGTCA---
TTAACGCTGACGGTACAG---GCCAGACAA---TTGTTG---TCTGGTATAGTGCAACAGCAAAAC---
AATTTG---CTGAGA---GCTATTGAGGCGCAACAGAATCTGTTGCAACTCACAGTCTGGGGCATC
>AB262961

AGTTATACTTGGAATGGTACAGGACTGTCTAATAATACTGAGAATAGT-------------------------
----
-
TCAAATAATACTATCACACTCCAATGCAGAATAAAGCAAATTATAAACATGTGGCAGGAAGTAGGAAAAGCAA
TGTATGCCCCTCCCATCAGCGGACAAATTAGATGTTCATCAAAT---
ATTACAGGGCTGCTATTAACAAGAGATGGTGGTAATGGCACGAAC----------------------------
----

GGGACCGAGACCTTCAGACCTGGAGGAGGAGATATGAGGGACAATTGGAGAAGTGAATTATATAAATATAAAG
TAGTAAAAATTGAACCATTA---GGAGTAGCACCCACTAGGGCAAGGAGAAGAGTGGTG--------------
-------------------------CAGAGAGAAAAAAGAGCA---GTGGGA---ATAGGAGCT---------
TTGTTCCTTGGGTTCTTGGGAGCA---GCAGGAAGCACTATG---GGCGCAGCGTCA---
ATGACGCTGACGGTACAG---GCCAGACAA---TTATTG---TCTGGTATAGTGCAACAGCAAAAC---
AATTTG---CTGAGG---GCTATTGAGGCGCAACAGCATCTGTTGCAACTCACAGTCTGGGGCATT
>KJ140264
TGTTATACAACAAAACTGTTTAATAGTACTTGGAATGATACAACACTGTTT----------------------
----
AATAGTACCTGGAATAGT---------
AGTAGTAAAGGAACTATCACACTCCGATGCAGAATACAACATATTATCAACAGGTGGCAGGAAGTAGGAAAAG
CGATGTATGCCCCTCCCATTGGAGGAAAAATTAGATGTTCATCAAAT---
ATTACAGGGCTGCTAATAACAAGAGATGGGGGG--

-
ACTGAGACTGGGACTACTGAGACCTTCAGACCTGGGGGAGGAAATATGAAGGACAATTGGAGAAGTGAATTAT
ATAAATATAAAGTAGTAAGAATTGAGCCATTA---GGGATAGCACCCACCAAGGCAAAGAGAAGAGTGGTG--
-------------------------------------CAGAGAGAAAAAAGAGCA---
GTGGGGACGATAGGAGCT---------GTGTTCCTTGGGTTCTTGGGAGCA---GCAGGAAGCACTATG---
GGCGCAGCGTCA---TTGACGCTGACGGTACAG---GCCAGACAA---TTATTG---
TCTGGTATAGTGCAACAGCAGAAT---AATTTG---CTGAAA---
GCTATTGATGCGCAACAGCATATGTTGCAACTCACAGTCTGGGGCATC
>KJ140265
ACT--
--------------------CATACTGGTACTGAAGGA---------
GTAAATGGAACATATTTCACACTCCCATGCAGAATAAGACAAATTGTAAACAGGTGGCAGGAAGTAGGAAAAG
CAATGTATGCCCCTCCCATCGGAGGACGAATTAGATGTTCATCAAAT---
ATTACAGGGCTGCTATTAACAAGAGATGGGGGG--

AATGAGAATAACGAGACTGAGACGACTGAGACCTTCAGACCTGGGGGAGGAAATATGAAGGACAATTGGAGAA
GTGAATTATATAAATATAAAGTAGTAAAAATTGAGCCATTA---
GGGATAGCACCCACCGAGGCAAGGAGAAGAGTGGTG-------------------------------------
--CAGAGAGAAAAAAGAGCA---GTGGGAGTGATAGGAGCT---------
ATGTTCCTTGGGTTCTTGGGAGCA---GCAGGAAGCACTATG---GGCGCAGCGTCA---
ATGGCGCTGACGGTACAG---ACCAGACAA---TTATTG---TCTGGTATAGTGCAACAGCAGAAT---
AATTTG---CTGAGA---GCCATTGAGGCGCAACAGCATATATTGCAACTCACAGTCTGGGGCATC
>MN043600
TTTCATAGTACTTGGAATGCTACGTTGAATGATACTGAAAGGGTAAATAATAGT-------------------
----

GCAACAAAAAACATTACACTCCAATGCAGAATAAAACAAATTATAAACAGGTGGCAGGAAGTGGGACAAGCAA
TGTATGCCCCTCCCATCAGCGGACTAATTAGATGTTCATCAAAT---
ATTACAGGGCTGATATTAACAAGAGACGGGGGGGTTAACGAGACT----------------------------
----

ACTGAGACCTTCAGACCTGGAGGAGGCGATATGAGGGACAATTGGAGAAGTGAATTATATAAATATAAAGTAG
TAAGGATTGAGCCATTA---GGGATAGCACCCACCAAGGCAAGGAGAAGAGTGGTG-----------------
----------------------CAGAGAGAAAGAAGAGCA---GTGGGAATGTTAGGAGCT---------
ATGTTCCTTGGGTTCTTGGGAGCA---GCAGGAAGCACTATG---GGCGCAGCGTCA---
ATGACGCTGACGGTACAG---GCCAGACAA---TTATTG---TCTGGTATAGTGCAACAGCAGAAT---
AATCTG---CTGAAA---GCTATTGAGGCGCAACAGCATCTGTTGCAACTCACAGTCTGGGGCATC
>KJ140260

AATCATACTAGAGCG--
-----------------------------------AACATTCGGAATAAT---------
CCTGGAAATGGAACTATTATCCTCCCATGCAGAATAAAACAAATTATAAACAGGTGGCAGGAAGTAGGAAAAG
CAATGTATGCCCCTCCCATCGAAGGACAAATTAGATGTACATCAAAT---
ATTACAGGGTTGCTAATAACAAGAGATGGGGGG--

ATAAATGAGACCAAC---
GGGACTGAGACCTTCAGACCTGGGGGAGGAGATATGAGGGACAATTGGAGAAGTGAATTATATAAATATAAAG
TAATAAAAATTGAGCCATTA---GGGATAGCACCCACCAAGGCAAAGAGAAGAGTGGTG--------------
-------------------------CAGAGAGAAAAAAGAGCA---GTGGGAGCGATAGGAGCT---------
ATGTTCCTTGGGTTCTTGGGAGCA---GCAGGAAGCACTATG---GGCGCAGCGTCA---
TTGACGCTGACGGTACAG---GCCAGACAA---TTATTG---TCTGGTATAGTGCAACAGCAGAAT---
AATTTG---CTGAGA---GCTATTGAGGCGCAACAGCATCTATTGCAACTCACAGTCTGGGGCATC
>KT878035
ACTTACAATGACACTGGA---

GTTAATGAAACTATCACCCTCCCTTGCAGAATAAGACAAATTGTAAACAGGTGGCAGGAAGTGGGAAAGGCAA
TGTATGCCACTCCCATCAGTGGACTAATTAGATGTTCATCAAAT---
ATTACAGGGTTACTATTAACAAGAGATGGGGGGACAGGGAACAATAAAACTGGG-------------------
----

AATGGAACTGAGACCTTCAGACCTGGGGGAGGAGACATGAAGGACAATTGGAGGAGTGAATTATACAAATATA
AAGTAGTAAAGATTGAGCCATTA---GGAGTAGCACCCACCAAGGCAAGGAGAAGAGTGGTG-----------
----------------------------CAGAGAGAAAAAAGAGCA---GTGGGAACAATAGGAGCT------
---ATGTTCCTTGGGTTCTTGGGAGCA---GCAGGAAGCACTATG---GGCGCAGCGTCA---
CTGACGCTGACGGTACAG---GCCAGACTA---TTATTG---TCTGGTATAGTGCAACAGCAACAG---
AACTTG---CTGAGA---GCTATTGAGGCGCAACAGCATCTGTTGCAACTCACAGTCTGGGGCATC
>KT878026
ACTCATTGGAATGAGACTTGG--

GATAATGAAACAATCATACTCCCATGCAGAATAAAACAAATTATAAACAGATGGCAGGAAGTAGGAAAGGCAA
TGTATGCCCCTCCCATCAGAGGAAATATTAGTTGTACATCAAAT---
ATTACAGGGCTGCTATTATCAAGAAATGGAGGTGGCAACGGGACT----------------------------
----

AACGGGACTGAGACCTTTAGACCTGGGGGAGGAGATATGAGGGACAATTGGAGAAGTGAATTATATAAATATA
AGGTAATAAGAATTGAGCCATTA---GGATTAGCACCCACCAGGGCAAGGAGAAGAGTGGTG-----------
----------------------------CAGAGAGAAAAAAGAGCA---GTAACG---ATAGGAGCT------
---ATGTTCCTTGGGTTCTTGGGAGCA---GCAGGAAGCACTATG---GGCGCAGCATCA---
CTGACGCTGACGGTACAG---GCCAGGCTA---CTATTG---TCTGGTATAGTGCAACAGCAGAAT---
AATTTG---CTAAGA---GCTATTGAGGCGCAACAGCATCTGTTGCGACTCACAGTCTGGGGCATT
>KT878028
AATCAAAATGGTACCAGGAATGATACTAGT---

ACTAATGCAACTATCACACTTCCATGCAGAATAAAACAATTTATAAACAGGTGGCAGGAAGTAGGAAGAGCAA
TGTATGCCCCTCCCATCGAAGGACAAATCAGATGCTCATCAAAT---
ATTACAGGGCTGCTATTAACAAGAGATGGGGGGAAT-------------------------------------
----

AATGAAACTGAGACCTTCAGACCTGGGGGAGGAAATATGAAAGATAATTGGAGAAGTGAATTATATAAATATA
AAGTAGTAAAAATTGAGCCATTA---GGGATAGCACCCACCAAGGCAAGGAGAAGAGTGGTG-----------
----------------------------CAGAGAGAAAAAAGAGCA---ATAGGAACAATAGGAGCT------
---CTGTTCCTTGGGTTCTTGGGAGCA---GCAGGAAGCACTATG---GGCGCAGCGGCA---
ATGACGCTGACGGTACAG---ACCAGACAA---TTGTTG---TCTGGTATAGTGCAACAGCAGAAT---
AATCTG---CTGAGA---GCTGTTGAGGCGCAACAGCATCTGTTGCAACTCACAGTCTGGGGCATC
>AJ417422
ACTCAACTGTTTAATAGTACTTGGGGGGAACATGATGTTGAAGCAAGTAACACTGAA----------------
----

AATAATGAAAATATCACACTCCCATGCAGAATAAAGCAAATTGTAACCATGTGGCAGAAAGTAGGAAAAGCAA

TGTATGCCCCTCCCATCGAAGGAAAAATTAGCTGTTTATCAAAT---
ATTACAGGGCTACTGTTAATGAGAGATGGTGGTAATATAAACAACAAAACAAAT------------------
----

GGGACCACCCCCCTTGAGATCTTCAGACCTGGAGGAGGAAATATGAAGGATAATTGGAGAAGTGAATTATATA
AATATAAAGTAATAGAAATTGAACCATTA---GGAGTAGCACCCACCAGAGCAAAGAGAAGAGTGGTG-----
----------------------------------CAAAGAGAAAAAAGAGCG------GGA---
ATAGGAGCT---------GTGCTCGTTGGGTTCTTGGGAGCA---GCAGGAAGCACTATG---
GGCGCAGTGTCA---GTGGCGCTGACGGTACAG---GCCAGAAAG---TTAATG---
TCTGGTATAGTGCAACAGCAAAAC---AACTTG---CTGATG---
GCTATTGAGGCGCAACAGCATATGTTGCAACTCACTGTCTGGGGCATC
>JQ316129
ACTCAAAGGTTAGATGAAATTAATAAA---

AATAGCACAATTATCACACTTCCATGCAGAATAAAACAAATTATAAACAGGTGGCAGGAAGTAGGAAAAGCAA
CGTATGCCCCTCCCATCAGAGGACAAATTACATGTTCATCAAAT---
ATTACAGGGTTGCTATTAACAAGAGATGGAGGGAATGAGAGCAGC---------------------------
----

AGTACTGAGACCTTCAGACCTGGGGGAGGAGACATGAGGGACAATTGGAGAAGTGAATTATATAAATATAAAG
TAGTAAAAATTGAGCCATTA---GGGGTAGCACCCACCAAGGCAAGGAGAAGAGTGGTG-------------
-------------------------CAGAGAGAAAAAAGAGCA---GTGGGAACAATAGGAGCT--------
ATGTTCCTTGGGTTCTTGGGAGCA---GCAGGAAGCACTATG---GGCGCAGCGTCA---
CTGACGCTGACGGTACAG---GCCAGACAA---TTGTTG---TCTGGTATAGTGCAACAGCAGAAT---
AATCTG---CTGAGA---GCTATTGAGGCGCAACAGCATCTGTTGCAACTCACAGTCTGGGGCATC
>HQ386198
AATCATAATGGGACTTGGAGTACAAATAGTACTGGGAATGAT------------------------------
---ACCAAC------
AACACAACTGACACTATCACACTCCCATGCAGAATAAAGCAAATTATAAACATGTGGCAGGAAGTAGGGAAAG
CAATGTATGCCCCTCCCATCAGTGGACAAATTAACTGTTCATCAAAT---
ATTACAGGGCTGATATTAACAAGAGATGGTGGTAATAAGACCGAGAAT------------------------
----

GAGAATGGGACCGAGACCTTCAGACCTGGAGGAGGAGATATGAGGGATAATTGGAGAAGTGAATTATATAAAT
ATAAAGTAGTAAAAATTGAACCATTA---GGACTAGCACCCACCAGGGCAAAGAGAAGAGTGGTG-------
-------------------------------CAAAGAGAAAAAAGAGCA---GTGGGAATGCTAGGAGCT---
------ATGTTCCTTGGGTTCTTGGGAGCA---GCAGGAAGCACTATG---GGCGCAGCGTCG---
ATGGCGCTGACGGTACAG---ACCAGACAA---TTAATG---TCTGGGATAGTGCAACAGCAGGAC---
AGTCTG---CTGAGG---GCTATTGAGGCGCAACAGCATCTGTTGCAACTCACAGTCTGGGGCATC
>JF320191
GATCAGGAT---

AAAAATGATACTATTACCCTTCCATGCAGGATAAAACAAATTATAAACATGTGGCAGGAAGTAGGAAAAGCAA
TGTATGCCCCTCCCATCCAAGGACAAATTAGTTGTTCATCAAAT---
ATTACAGGGCTACTCTTAACAAGAGATGGTGGTAATAGCAGTAACACT------------------------
----

AATGGAACTGAGATCTTCAGACCTGGAGGAGGAGATATGAGAGACAATTGGAGAAGTGAATTATATAAGTACA
AAGTAGTAAAAATTGAACCATTA---GGAATAGCACCCACCAAGGCAAAGAGAAGAGTGGTA----------
----------------------------CAAAGAGAAAAAAGAGCA---GTGGGAACGCTGGGAGCT------
---GTGTTCCTTGGGTTCTTGGGAGCA---GCAGGAAGCACTATG---GGCGCAGCGTCA---
ATAACGCTGACGGCACAG---GCCAGACAA---TTACTG---TCTGGTATAGTGCAACAGCAGAAC---
AATATG---CTGAAG---GCTATTGAGGCGCAACAGCATCTGTTGCAACTCACAGTCTGGGGCATT
>MH078554
AATTATAATTGGAATAGTAATAAT--
--------------------------------AGC---
ACTGATAGCAGAAATATCACGCTCCCATGTAGAATAAGACAAATTATAAACATGTGGCAGAGAGTAGGAAAAG
CTATGTATGCCTCTCCCATCAAAGGACCAATTAACTGTACATCAAAC---
ATTACAGGGCTGCTATTGACAAGAGATGGGGGTAATGGCAAAAGCAGCAGT---------------------
----

AATGAGACTGAGACCTTCAGACCTATAGGAGGAGACATGAAGGACAATTGGAGAAGTGAATTATATAAATATA
AAGTAGTACAAATCAAACCGTTA---GGAATAGCACCCACCAGGGCAAAAAGAAGAGTGGTG----------
---------------------------CAGAGAGAAAAAAGAGCA---GTAACA---CTAGGAGCT------
---ATGTTCCTTGGGTTCTTGGGAGCA---GCAGGAAGCACTATG---GGCGCAGCGTCA---
ATAGCGCTGACGGCACAG---GCCAGACAA---TTATTG---TCTGGTATAGTGCAGCAGCAGAAC---
AATCTG---CTGAGG---GCTATTGAGGCGCAACAACATCTGTTGCAACTCACAGTCTGGGGCATC
>EF210725
AACTATACA---

ACAAATGGAACTATCACTCTCCCATGTAGAATAAAACAAGTTATAAACATGTGGCAGGAAGTAGGAAAAGCAA
TGTATGCCCCTCCCATCAAAGGACAAATTAGTTGTTTGTCAAAT---
ATCACAGGGTTGCTATTAACAAGAGATGGAGGTAAGCGCAATGACAGT------------------------
----

AATGAGACAGAGGTCTTTAGACCTGGAGGAGGAGATATGAGGGACAATTGGAGAAGTGAATTATATAAATATA
AAGTAGTAAAAATTGAACCAATA---GGAATAGCACCCACCAAGGCAAAGAGAAGAGTGGTG----------
---------------------------CAAAGAGAAAAAAGAGAG---CTGGGAACGCTAGGAGCT------
---ATGTTCATTGGGTTCTTGGGAGCA---GCAGGAAGCACTATG---GGCGCAGCGTCA---
GTGACGCTGACGGTACAA---GCCAGACAA---TTATTG---TCTGGTATAGTGCAACAGCAGAAC---
AATCTG---CTGAAG---GCTATTGATGCGCAACAGCATCTGTTGCAACTCACAGTCTGGGGCATC
>EF210734
ACTCAA--

GAAAATGAAAATATCACACTCCCATGTAGAATAAAACAAATTATAAACATGTGGCAGGAGGTAGGAAAAGCAA
TGTATGCCCCTCCCATCAAAGGAATAATTAAATGTTCATCAAAC---
ATTACAGGACTGCTCCTAATAAGAGATGGTGGTAATAGTAATGGTAATGGA---------------------
----

AGTAACAATAGCGAGACCTTCAGACCTGGAGGAGGAGATATGAAGGACAATTGGAGAAGTGAATTATATAAAT
ATAAAGTAGTAAAAGTTGAACCAGTA---GGACTAGCACCCACCAAGGCAAAGAGAAGAGTGGTG-------
------------------------------CAGAGAGAAAAAAGAGCA---GTGACA---CTAGGAGCT---
------GTGTTCCTTGGGTTCTTGGGAGCA---GCAGGAAGCACTATG---GGCGCAGCGTCA---
GTGACGCTGACGGTACAG---GCCAGACAA---TTACTG---TCTGGTATAGTGCAACAGCAGAGC---
AATATG---CTGAAG---GCTATTGAGGCGCAACAACATCTGTTGCAACTCACAGTCTGGGGCATC
>KY658689
AATTAC--

ACAAATGGAACTATCACGCTCCCATGCAGAATAAAACAAATTATAAACATGTGGCAGAGAGTAGGAAAAGCAA
TGTATGCCCCTCCCATTGCAGGACAACTTAACTGTTCATCAAAT---
ATTACGGGGATACTATTAACAAGAGATGGGGGTAATGGTAATGTGACA------------------------
----

AATGACACAGAGATCTTCAGACCTGCAGGAGGAGATATGAGGGACAATTGGAGAAATGAACTATACAAATATA
AAGTAGTAAAGATTGAACCATTA---GGAATAGCACCCACCAAGGCAAGGAGAAGAGTGGTG----------
---------------------------CAGAGAGAAAAAAGAGCA---GTGACA---CTGGGAGCT------
---CTGTTCCTTGGGTTCTTGGGAGCT---GCAGGAAGCACTATG---GGCGCAGCGTCA---
GTGACGCTGACGGTACAG---GCCAGGCTA---TTATTG---GCTGGTATAGTGCAACAGCAGAAC---
AATTTG---CTGAGA---GCTATTGAGGTGCAACAGCACATGTTGCAACTCACAGTCTGGGGCATT
>KP411827
ACTCAAAAGGCAAGTAACACA---

GAAAATGAAAATATCACTCTCCCATGCAAAATAAAACAAGTTATAAATATGTGGCAGGAAGTAGGAAAAGCAA
TGTATGCCCCTCCCATCGAAGGACAGATTAAATGTTCATCAAAT---
ATTACAGGGCTACTATTAACAAGAGATGGTGGTAAACATAACAGTAGCGAT---------------------
----

ACTAACAAAACTGAGATCTTCAGACCTGGAGGAGGAGATATGAGGGACAATTGGAGAAGTGAATTATATAAAT
ATAAGGTAGTAAAAATTGAACCATTA---GGAATAGCACCCACCAGGGCAAAGAGAAGAGTGGTG-------
------------------------------CAGAGAGAAAAAAGAGCA---GTGGGA---TTAGGAGCT---

```
------ATGTTCCTTGGGTTCTTGGGAGCA---GCAGGAAGCACTATG---GGCGCAGCATCA---
TTGACGCTGACGGTACAG---GCCAGACAA---TTAATG---TCTGGTATAGTGCAACAGCAAAAC---
AACTTG---CTAAGA---GCTGTTGAGGCGCAACAGCATATGTTGCAACTCACAGTCTGGGGCATC
>MF373174
AATCAAACAACTACC----------------------------------------------------------
--------------------------------------------------
GGTAATGATACCATCCCACTCCAATGCAGGATAAAGCAAATTATAAACATGTGGCAGGAAGTGGGAAAAGCAA
TGTATGCCCCTCCCATTAAAGGACCAATTAATTGTTTATCAAAT---
ATTACAGGGTTGTTATTAACAAGAGATGGTGGAAATAATAATACC----------------------------
-------------------------------------------------------------------------
----------
AATGAAACAGAGACCTTCAGACCTGGAGGAGGAGACATGAGAGACAATTGGAGAAGTGAATTATATAAATACA
AAGTAGTAAAAATTGAACCATTA---GGAGTAGCACCCACCAAGGCAAGGAGAAGAGTGGTG-----------
----------------------------CAGAGAGAAAAAAGAGCA---GTGGGA---CTAGGAGCT------
---TTGTTCCTTGGGTTCTTAGGAGCA---GCAGGAAGCACTATG---GGCGCAGCGTCA---
ATGACGCTGACGGTACAG---GCCAGACAA---TTATTG---TCTGGTATAGTGCAACAGCAGAAC---
AACTTG---CTGAGG---GCTATTGAGGCGCAACAGCACCTGTTGAAACTCACGGTCTGGGGCATT
>JN860769
AATCATACGTTTAATAATACTGAAGGCGAAAATATAACTGAAAGCATA-------------------------
-------------------------------------------------------------------------
-
AATACAACAATTATCAAGCTCCCATGCAGAATAAAACAAATTATAAACATGTGGCAGGAAGTAGGAAAAGCAA
TGTATGCCCCTCCCATCAGTGGACAAATTAGCTGTTCATCAAAT---
ATTACAGGAATACTATTAACAAGAGATGGTGGTGGTAATAATACG----------------------------
-------------------------------------------------------------------------
-------------
GGAAATGAGACCTTCAGACCTGGGGGAGGAAATATGAAAGACAATTGGAGAAGTGAATTATATAAATATAAAG
TAGTAAAAATTGAGCCATTA---GGAATAGCACCCACCAAGGCAAAGAGAAGAGTGGTG--------------
-------------------------CAGAGAGAAAAAAGAGCA---GTGGGAGTGATAGGAGCT---------
ATGTTCCTTGGGTTCTTGGGAGCA---GCAGGAAGCACTATG---GGCGCAGCGTCA---
CTGACGCTGACGGTACAG---GCCAGACAA---TTGTTG---TCTGGTATAGTGCAACAGCAGAAC---
AATTTG---CTGAGA---GCTATTGAGGCGCAACAGCATCTGTTGCAACTCACAGTCTGGGGCATC
>MG989583
AATTATAATATTGAAAAG-------------------------------------------------------
-----------------------------------------------
TCAAATGACACTATTGTACTCCCGTGCAGAATAAAACAAATTATAAACAGGTGGCAGGAAGTAGGAAGAGCAA
TGTATGCCCCTCCCATCAGAGGACAACTTAACTGTACATCAAAT---
ATTACAGGGCTACTATTATTAAGAGATGGTGGGAATAACATTACG----------------------------
-------------------------------------------------------------------------
----------------
AACGAAACCTTCAGACCTGGAGGAGGAAATATGAAGGACAATTGGAGAAGTGAATTATATAAATATAAGGTAG
TAAAAATAGAGCCATTA---GGACTAGCACCCACCAAGGCAAAGAGAAGAGTGGTG-----------------
----------------------CAGAGAGAAAAAAGAGCA---GCA------CTCGGAGCT---------
ATGTTCCTTGGGTTCTTGGCAACA---GCAGGAAGCACTATG---GGCGCAGCATCG---
GTGACGCTGACGGTACAG---GCCAGACAA---TTGTTG---TCTGGTATAGTGCAACAGCAAAAC---
AACTTG---CTGAGG---GCTATTGAGGCGCAACAGCATATGTTGCAACTCACAGTCTGGGGCATC
>AF277065
TTTCATGGTAATTGGACAAGGTCAAATTTCACAGAA-------------------------------------
--------------------------------------------------------
TCAAATAGCACAACTATCACACTCCCATGTAGAATAAAACAAATTGTAAACATGTGGCAGGAAGTAGGCAAAG
CAATGTATGCCCCTCCCATCAGAGGACAAATTAGATGTTCATCAAAT---
ATCACGGGGCTGCTATTAACAAGAGATGGTGGCGTGAACGGGACC----------------------------
-------------------------------------------------------------------------
----------------
CGCGAGACCTTTAGACCTGGAGGAGGAGATATGAGGGACAATTGGAGAAGTGAATTATATAAATATAAAGTGG
TAAAAATTGAGCCATTA---GGAGTAGCACCCACCAAGGCAAAGAGAAGAGTGGTG-----------------
----------------------CAGAGAGAAAAAAGAGCA---GTGGGAACAATAGGAGCT---------
ATGTTCCTTGGGTTCTTGGGAGCA---GCAGGAAGCACTATG---GGCGCAGCGTCA---
ATAACGCTGACGGTACAG---GCCAGACAA---TTATTG---TCTGGTATAGTGCAACAGCAAAAC---
AATTTG---CTGAGA---GCTATTGAGGCGCAACAGCATATGTTGCAACTCACGGTCTGGGGCATC
```

>AF277067
AATCATACTGGGAGTAATAATATAAGATCA---

GAAGACAATGACAATATCACACTTCCATGCAGAATAAAACAAATAGTAAACATGTGGCAGGAAGTAGGCAAAG
CAATGTATGCCCCTCCCATTAGAGGACAAATTAGATGTTCATCAAAT---
ATTACAGGATTACTATTAACAAGAGATGGTGGTAATGACACGAGC----------------------------
----

GACACCGAGATCTTCAGACCTGGAGGAGGAAATATGAGGGACAATTGGAGAAGTGAATTATATAAATATAAAG
TAGTAAGAATTGAACCATTA---GGAGTAGCACCCACCAAGGCAAAGAGAAGAGTGGTG--------------
-------------------------CAGAGAGAAAAAAGAGCA---GTGGGAACATTAGGAGCT---------
ATGTTCCTTGGGTTCTTGGGAACA---GCAGGAAGCACTATG---GGCGCAGCGTCA---
CTGACGCTGACGGTACAG---GCCAGACAA---TTATTG---TCTGGTATAGTGCAACAGCAAAAC---
AATTTG---CTGAGA---GCTATTGAGGCGCAACAGCATCTGTTGCAACTCACGGTCTGGGGCATC
>JN786692
ACTCAGAATGTTACT--

GGAGGAAATGAGACAATCATACTCCCGTGCAGAATAAAACAAATTATAAACATGTGGCAGCAAGTAGGAAAAG
CAATGTATGCCCCTCCCATCAGTGGACTACTTAATTGTACATCAAAT---
ATTACAGGACTACTAATAACAAGAGATGGTGGTACTGGGAACAGG----------------------------
----

ACCAATGAGACCTTCAGACCTGGAGGAGGAGATATGAGGGACAATTGGAGAAGTGAATTATATAAATATAAAG
TAGTAAGAATTGAACCAATA---GGCATAGCACCCACCGAGGCAAAGAGAAGAGTGGTG--------------
-------------------------CAGAGAGAAAAAAGAGCA---GTGGGA---ATAGGAGCT---------
TTGTTCCTTGGGTTCTTGGGAGCA---GCAGGAAGCACTATG---GGCGCAGCGTCG---
ATAACGCTGACGGTACAG---GCCAGACAA---TTATTG---TCTGGTATAGTGCAACAGCAGAAC---
AACTTG---CTGAGG---GCTATTGAGGCGCAACAGCATCTGTTGCAACTCACAGTCTGGGGCATC
>DQ869028
ACTTAT---

ACTAATGGAACTATCACAATCCCATGCAGAATAAGACAGATTGTAAACAGGTGGCAGGAAGTAGGAAAAGCAA
TGTATGCCCCTCCCATCAGAGGATACATTAAATGTTCATCAAAT---
ATTACAGGGCTGCTATTAACGAGAGATGGTGGTAGTAATGCCACG----------------------------
----

AATGAGACCTTCAGACCTGGAGGAGGAGATATGAGGGACAATTGGAGAAGTGAATTATATAAATATAAAGTAG
TAAAAATTGAACCATTA---GGAGTAGCACCCACCAAGGCAAAGAGAAGAGTGGTG-----------------
----------------------CAGAGAGAAAAAAGAGCA---ATAGGGGGATTAGGAGCT---------
TTGTTCCTTGGGTTCTTGGGAGCA---GCAGGAAGCACTATG---GGCGCAGCGTCA---
TTGACGCTGACGGTACAG---GCCAGACAA---TTATTG---TCTGGTATAGTGCAACAGCAGAAC---
AATTTG---CTGAGA---GCTATTGAGGCGCAACAGCATCTGTTGCAACTCACAGTCTGGGGCATC
>JN944911
ACTCAAGGGCCAAGT--

GAAAATAACACAATCATACTCCCATGCAGAATAAAACAAATTATAAACATGTGGCAGAGAGTAGGAAAAGCAA
TATATGCCCCTCCCATCAGTGGACAAATTAGATGTTTATCAAAT---
ATTACAGGGCTGATATTAACAAGAGATGGTGGTAATAGTAGCCTA----------------------------
----

AGCAGCCCCGAGATCTTCAGACCTGGAGGAGGAGATATGAGGGACAACTGGAGAAGTGAATTATATAAATATA
AAGTAGTACAAATTGAACCATTA---GGAATAGCACCCACCAGGGCAAAGAGAAGAGCGGTG-----------
----------------------------CAGAGGGAAAAAAGAGCA---GTGGGA---ATAGGAGCT------
---CTGTTCCTTGGGTTTCTGGGAGCA---GCAGGAAGCACTATG---GGCGCAGCGTCA---
GTGACGCTGACGGTACAG---GCCAGACAG---TTGTTG---TCTGGTATAGTGCAACAGCAGAGC---
AATCTG---CTGAGA---GCTATTGAGGCGCAACAGCATCTGTTGCAACTCACAGTCTGGGGCATT
>FJ469751
AGTCATAATACTGCTATGAATGTT---
-----------------------------------ACT---
GCAGGGAATGATAATATCACACTCCCGTGCAGAATAAAACAAATTATAAACAGGTGGCAGGAAGTAGGAAAAG

CAATGTATGCCCCTCCCATCAGTGGATTCATTAACTGCTCATCAAAT---
ATTACAGGGATAATGTTAATTAGAGATGGT---ATTAATGAAACT----------------------------
----

GATGGGAACGAGACCTTCAGACCAGAAGGAGGAAATATGAAGGACAATTGGAGAAATGAATTATACAAATATA
AAGTAGTAAAAATAGAGCCCTTA---GGAGTAGCACCCACCAAGGCAAAGAGAAGAGTGGTG----------
----------------------------CAGAGAGAAAAAAGAGCA---GTGGGA---ATAGGAGCT------
---CTGTTCCTTGGGTTCTTGGGAGCA---GCAGGAAGCACTATG---GGCGCAGCGTCG---
ATCACGCTGACGGTACAG---GCCAGACAA---TTATTG---TCTGGTATAGTGCAACAGCAGAGC---
AATTTG---CTGAGA---GCTATTGAGGCGCAACAGCATCTGTTGCAACTCACAGTCTGGGGCATC
>DQ869032
ACTTATAACACAACAGATAATAGTACTGAT---

AATGGGAATGAAATTATCACAATCCCATGCAGAATAAAACAGATTATAAACATGTGGCAGGAGGTAGGAAAAG
CAATGTATGCCCCTCCCATTAGAGGACTAATTAGATGTACATCAAAT---
ATTACAGGGCTACTATTAGTAAGAGATGGTGGTACTACTAATACT----------------------------
----

AGTACTAATGAGACCTTCAGACCTGGAGGAGGAGATATGAGGGACAATTGGAGAAGTGAATTATATAAATATA
AAGTAGTAAAAATTGAACCATTA---GGAGTAGCACCCACCAAGGCAAAGAGAAGAGTGGTG----------
----------------------------CAGAGAGAAAAAAGAGCA---ATAGGA---TTTGGAGCT------
---ATGTTCCTTGGGTTCTTGGGAGCA---GCCGGAAGCACTATG---GGCGCAGCGTCA---
CTGACGCTGACGGTACAG---GCCAGACAA---TTATTG---ACTGGTATAGTGCAACAGCAAAAC---
AATTTG---CTGAGG---GCTATTGAGGCGCAACAGCACTTGTTGCAACTCACAGTCTGGGGCATC
>FJ469742
ACTCAGAATGGTACTACATGGTCAAATGAC---

AGTGACCCAATCATCATACTCCCATGCAGAATAAAACAAATTGTAAATAGGTGGCAGGAAGTAGGAAAAGCAA
TGTATGCCCCTCCCATCAGTGGACAAATTAGATGCTCATCAAAT---
ATAACAGGACTGCTATTAACAAGAGATGGTGGTAACACAAATAGC----------------------------
----

ACTATTGAAACCTTCAGACCTGGAGGAGGAGATATGAGGGACAATTGGAGAAGTGAACTATATAAATATAAGG
TAGTACAAATTGAACCATTA---GGAGTAGCACCCACCAAGGCAAAGAGAAGGGTGGTG--------------
-------------------------CAGAGAGAAAAAAGAGCA---GTAGTAGGACTAGGAGCT---------
TTGGTCCTTGGGTTCCTGGGAGCA---GCAGGAAGCACTATG---GGCGCAGCGTCA---
CTGGCGTTGACGGTACAG---ACCAGACAA---TTATTG---TCTGGTATAGTGCAACAGCAGAAT---
AATATG---CTGAGA---GCTATTGAGGCGCAACAACACCTGTTGCAACTCACAGTCTGGGGCATC
>FJ469764
AATTACACA--
-----------------------GAA---
AATGGAAATGACACACTCACAATCCCATGCAGAATAAAACAAATTATAAACATGTGGCAGGAAGTAGGAAAGG
CAATGTATGCCCCTCCCATCAGAGGACAAATTAAATGTTCATCAAAT---
ATTACAGGGCTGCTATTAACAAGAGATGGTGGTAATAGCAACAAC----------------------------
----

ACTAGCAATGAGACCTTCAGACCTGTAGGGGGAGATATGAGGGACAATTGGAGAAGTGAATTATATAAATATA
AAGTGGTAAAAATTGAACCATTA---GGAGTAGCGCCCACCAGGGCAAAGAGAAGAGTGGTG----------
----------------------------CAAAGAGAAAAAAGAGCA---GTGGGA---ATAGGAGCT------
---CTGTTCCTTGGGTTCTTGGGAGCA---GCAGGAAGCACTATG---GGCGCAGCATCA---
CTGACGCTGACGGTACAA---GCCAGACAA---TTATTG---TCTGGTATAGTGCAACAGCAGAAC---
AATCTG---CTGAGG---GCTATTGAGGCGCAACAGCATCTGTTGCAACTCACAGTCTGGGGCATC
>EF593298
TATTACAATGGTACCTGGAATAATACTGAAGAG--

TCAAATGAAACTATCACACTCCCATGCAGAATAAAACAAATTATAAACATGTGGCAGGAAGTAGGAAGAGCAA
TGTATGCCCCTCCCATCAGAGGAATAATTAGATGTTCATCAAAT---
ATAACAGGGCTGCTATTAACAAGAGATGGTACTCCTACTAATAGCACAGAA----------------------
----

AGCAATGACACAGAAATCTTCAGACCGGGAGGAGGAAATATGAGAGACAATTGGAGAAGTGAATTATATAAAT
ATAAAGTAGTACAAATTGAACCAATA---GGACTAGCACCCACAAAGGCAAAGAGAAGAGTGGTG--------
-------------------------------CAGAGAGAAAAAAGAGCA---GTGGGAACAATAGGAGCT---
------ATGTTCCTTGGGTTCTTGGGAACA---GCAGGAAGCACTATG---GGCGCAGCGTCA---
ATAACGCTGACGGTACAG---GCCAGACAA---TTATTG---TCTGGTATAGTGCAACAGCAGAAC---
AATCTG---CTGAAG---GCTATTGAGGCGCAACAACATCTGTTGCAACTCACAGTCTGGGGCATC
>HQ216485
CATTACAAGGAA---

AAAAATGACACAATCACACTCCCATGCAGAATAAAACAAATTATAAACAGGTGGCAGGAGGTAGGAAAAGCAA
TGTATGCCCCTCCCATCAGAGGACAAATTAACTGTTTATCAAAT---
ATTACAGGGCTTCTATTATTAAGAGATGGTGGACATGATAGTGGAAATAATAGT-------------------
----

ACAACGAACCAGAACGAGACCTTCAGGCCTGGGGGGGGAAATATGAAGGACAATTGGAGAAGTGAATTATATA
AATATAAAGTAGTACAAATTGAACCACTA---GGAGTAGCACCCACCAAGGCAAAGAGAAGAGTGGTG-----
----------------------------------CAGAGAGAAAAAAGAGCA---
ATAGGGGAACTAGGAGCT---------GTGTTCCTTGGGTTCTTGGGAGCA---GCAGGAAGCACTATG---
GGCGCAGCATCA---GTGATGCTGACGGTACAG---GCCAGACAA---TTACTG---
TCTGGAATAGTGCAACAGCAGAAC---AATTTG---CTGAGG---
GCTATTGAGGCGCAACAACATTTGTTGCAACTCACAGTCTGGGGCATC
>JX863970
CACTACACCTGGGATGGTACTAATGGTACTTGGGGGAATAATGCA----------------------------
----
-
TCGAAGGGCAATATCACACTCCACTGCAGAATAAAACAAATTGTAAACATGTGGCAGAGAGTAGGAAGAGCAA
TGTATGCCCCCCCTATCAGTGGACCAATTAGCTGTTCATCAAAT---
ATTACAGGGTTACTATTAACAAGAGATGGGGGTGGTATCAACGAGACC-------------------------
----

AACACCACTGAGACCTTTAGACCTGGAGGAGGAGATATGAAGGACAATTGGAGAAGTGAATTATATAAATATA
AAGTAGTACAAATTGAACCAATA---GGAATAGCACCCACCAAGGCAAAAAGAAGAGTGGTG-----------
----------------------------CAGAGAGAGAAAAGAGCA---GTGGGAGTATTTGGAGCT------
---ATGTTCCTTGGGTTCTTGGGAGCA---GCAGGAAGCACTATG---GGCGCAGCATCA---
ATAACGCTGACGGCACAG---GCCAGACAA---TTATTG---TCTGGTATAGTGCAACAGCAGAGC---
AATCTG---CTGAGG---GCTATTGAGGCGCAACAACACATGTTGCAACTCACAGTCTGGGGCATC
>EU578047
AATTACACAGAA---

GGAAATGGCACAATCACACTCCCATGCAGAATAAAACAAATTATAAACATGTGGCAGGAAGTAGGAAAAGCAA
TGTATGCCCCTCCCATCAGAGGACCAATTAATTGTACATCAAAT---
ATTACAGGGTTACTATTAACAAGAGATGGTGGTATACAGAATGGCACT-------------------------
----

GAGAACACCACTGAGACCTTCAGACCTGGAGGAGGAGATATGAGGGACAATTGGAGAAGTGAATTATATAAAT
ATAAAGTAGTAAGAATTGAACCATTA---GGAGTAGCACCCACCAAGGCAATGAGAAGAGTGGTG--------
-------------------------------CAAAGAGAAAAAAGAGCA---GTGGGA---ATAGGAGCT---
------TTGTTCCTTGGGTTCTTAGGAGCA---GCAGGAAGCACTATG---GGCGCAGCGTCG---
CTGACGCTGACGGTACAA---GCCAGACAA---TTATTG---TCTGGTATAGTGCAACAGCAGAAC---
AATTTG---CTGAGA---GCTATTGAGGCGCAACAGCATCTGTTGCAACTCACAGTCTGGGGCATC
>MH060746
CCTCAGAATGAAAGTAGGCCA--

GAAAATGACACTATCACACTCCCATGCAAAATAAGACAAATTGTAAACCTGTGGCAGGAAGTAGGAAAAGCAA
TGTATGCCCCTCCCATCAGAGGACAAATTAGATGTTCATCAAAC---
ATTACAGGGCTGTTATTAACAAGGGATGGTGGTATGAACGTGAGC----------------------------
----

AACACCAACGAGACCTTCAGACCTGGAGGGGGGGATATGAGGGACAATTGGAGAAGTGAATTATATAAATATA
AAGTAGTAAAAATAGAACCATTA---GGAGTAGCACCCACCAAGGCAAAGAGAAGAGTGGTG-----------

----------------------------CAGAGAGAAAAAAGAGCA---GTGACA---CTAGGAGCT------
---ATGTTCCTTGGGTTCTTGGGAGCA---GCAGGAAGCACTATG---GGCGCAGCGTCA---
ATGGCGCTGACGGTACAG---GCCAGACAA---TTATTG---TCTGGTATAGTGCAACAGCAGAAC---
AATTTG---CTGAGA---GCTATTGAGGCGCAACAGCATCTGTTGCAACTCACAGTCTGGGGCATC
>FJ469695
ACTCAGAATAAT---

ACTGACACAATTATCACACTCCCATGCAGAATAAAACAGATAATAAACATGTGGCAAGAAGTAGGAAAAGCAA
TGTATGCCCCTCCCATCCAAGGACAAATTAGATGTTCATCAAAT---
ATTACAGGGTTACTATTAACAAGAGATGGTGCTGGTGGTAATAAA----------------------------
----

AGCGAGAATGAGACCTTCAGACCTGGAGGAGGAAATATGAGGGACAATTGGAGAAGTGAATTATATAAATATA
AAGTAGTAAAAATTGAACCATTA---GGAGTAGCACCCACCAAGGCAAGAAGAAGAGTGGTG-----------
----------------------------CAGAGAGAAAAAAGAGCA---CTAGTAGGACTAGGAGCT------
---GTGTTCCTTGGGTTCTTGGGAGCA---GCAGGAAGCACTATG---GGCGCAGCGTCA---
ATGGCGCTGACGGTACAG---GCCAGACAA---TTATTG---TCTGGTATAGTGCAACAGCAGAAC---
AATTTG---CTGAGA---GCTATTGAGGCGCAACAGCATATGTTGCAACTCACAGTCTGGGGCATC
>FJ469732
AATCAAACTACTAATGAATGGAATGGTACTAAAGGGTTA----------------------------------
--------------------------------------GATGACAGG------
GGAGAAAATGACACAATCACACTCCCATGCAGAATAAAACAAATTGTAAACATGTGGCAGGGAGTAGGAAGAG
CAATGTATGCCCCTCCCATCAGTGGACAAATTAGATGCTTATCAAAT---
ATTACAGGGCTGCTATTAACAAGAGATGGTGGTAATACAAATGGCACG-------------------------
----

AATGGGACTGAAACCTTCAGACCTGGAGGAGGAGATATGAGGGACAATTGGAGAAGTGAATTATATAAATATA
AAGTAGTAAAAATTAAACCATTA---GGAGTAGCACCCACCAAGGCAAAGAGAAGAGTGGTG-----------
----------------------------CAGAGAGAAAAAAGAGCA---GTGGGA---ATAGGAGCT------
---GTGTTCCTTGGATTCTTGGGAGCA---GCAGGAAGCACTATG---GGCGCAGCGTCA---
GTGACGCTGACGGTACAG---GCCAGACAA---TTATTG---TCTGGTATAGTGCAACAGCAGAAC---
AATTTG---CTGAGG---GCTATTGAGGCGCAACAACATCTGTTGCAACTCACAGTCTGGGGCATA
>DQ410403
AAT--

TACACTAGCACTATCACACTCCCATGCAGAATAAAACAAGTTATAAACATGTGGCAGGAAGTAGGAAAAGCAA
TGTATGCCCCTCCCATCAGAGGACAAATTAAATGTTCATCAAAT---
ATTACAGGGCTGATACTAACAAGAGATGGTGGTACAAACAACACC----------------------------
----

AACACCACCGAGACCTTTAGACCTGGAGGAGGAGATATGAGGGACAATTGGAGAAGTGAATTATATAAATACA
AAGTAGTAAAAATTGAACCAATA---GGAATAGCACCCACCAAGGCAAAGAGAAGAACGGTG-----------
----------------------------CAGAGAGAAAGAAGAGCA---ATAGGA---ATAGGAGCT------
---GTGTTCCTTGGGTTCTTGGGAACA---GCTGGAAGCACTATG---GGCGCAGCGTCA---
CTGACGCTGACGGTACAG---GCCAGACAA---TTATTG---TCTGGTATAGTGCAACAGCAAAAC---
AATTTG---CTGAAG---GCTATTGAGGCGCAACAGCATCTGTTGCGACTCACAGTCTGGGGCATC
>EU547186
AGTCAACTTTTTAATAGTACTTGGAATGGTACCGACGGGTCAGATAACACA----------------------
----

ATGGACAAAGATATCTCACTTCCATGCAGAATAAGACAAATTGTAAACATGTGGCAGGAAGTAGGAAAAGCAA
TGTATGCCCCTCCTATCCAAGGACAAATTAGATGTTCATCAAAT---
ATTACAGGGCTACTATTAGTAAGAGATGGTGGTAGTAATACAACC----------------------------
----

AATAATGAGACCTTCAGACCTGGAGGAGGAGACATGAGGGACAACTGGAGAAGTGAATTATATAAATATAAAG
TAGTAAAAATTGAACCAGTA---GGAATAGCACCCACCAAGGCAAAGAGAAGAGTGGTG--------------
-------------------------CAGAGAGAAAAAAGAGCA---GTGGGAACACTAGGAGCT---------
ATGTTCCTTGGGTTCTTGGGAACA---GCAGGAAGCACTATG---GGCGCAGCGTCA---

GTGACGCTGACGGTACAG---GCCAGACAA---TTATTG---TCTGGTATAGTGCAACAGCAAAGC---
AATATG---CTGAGG---GCTATTGAGGCGCAACAGCATCTGTTGCAACTCACAGTCTGGGGCATC
>JF689852
AATCACACT--

AAAGGAAATGGCACAATCACAATTCCATGCAGAATAAAACAAATTATAAACATGTGGCAGGAAGTAGGAAAAG
CAATGTATGCCCCTCCCATCAGAGGACAAATTAGATGTTCATCAAAT---
ATTACAGGGCTGTTATTAACAAGAGATGGTGGCAATGACGGGAC-----------------------------
----

AATACCACCGAAACCTTCAGACCTGGAGGAGGAGATATGAGGGACAATTGGAGAAGTGAATTATATAAATATA
AAGTAGTAAAAATTGAACCATTA---GGAGTAGCACCTACCAAGGCAAAGAGAAGAGTGGTG-----------
----------------------------CAGAGAGGAAAAAGAGCA---GTAGGA---ATAGGAGCT------
---GTGTTCCTTGGGTTCTTGGGAGCA---GCAGGAAGCACTATG---GGCGCAGCGTCA---
TTAACGCTGACGGTACAG---GCCAGACTA---TTATTG---TCTGGTATAGTGCAACAGCAGAAC---
AATTTG---CTGAGG---GCTATTGAGGCACAACAGCATCTGTTGCAACTCACAGTCTGGGGCATC
>HQ216634
GATTACACTACAGCAGAGAAT--
-----------------------------------GAT---
ACTAAAGAAGAAAATATCACACTCCCATGCAGAATAAAACAAATTATAAACATGTGGCAGAAAGTAGGAAAAG
CAATGTATGCCCCTCCCATCAGAGGACAGATTAGATGTTCATCAAAT---
ATTACAGGGCTGCTATTAACAAGAGATGGTGGTAATGGTGATACC----------------------------
----

ACAAACAATGAGACCTTCAGACCTGGAGGAGGAGATATGAGGGATAATTGGAGAAGTGAATTATATAGATATA
AAGTAGTAAGAATTGAACCATTA---GGAATAGCACCCACTAAGGCAAAGAGAAGAGTGGTG-----------
----------------------------CAGAGAGAAAAAAGAGCA---ATAGGGGGAATAGGAGCT------
---ATGTTCCTTGGGATCTTAGGAGTA---GCAGGAAGCACTATG---GGCGCAGCGTCA---
TTGACGCTGACGGTACAG---GCCAGACAA---TTATTG---TCTGGTATAGTGCAACAGCAGAAC---
AATTTG---CTGAGG---GCTATTGAGGCGCAACAGCATCTGTTGCAACTCACAGTCTGGGGCATC
>HQ216874
GTTCATAATAATAATACTGAAAGGTTA--
--------------------------------------AAT---
ACCACTAACAGTACTATCATACTCCCATGCAAAATAAAACAAATTATAAACATGTGGCAGGGAGTAGGAAAAG
CAATGTATGCCCCTCCCATTGCAGGAATAATTAATTGTGTATCAAAT---
ATTACAGGGCTGCTATTAACAAGAGATGGTGGTGGTAGTGGTAATGGCACA----------------------
----

AGAAGGGATAACGAAACCTTCAGACCTGAAGGGGGAAATATGAAGGATAATTGGAGAAGTGAATTATACAAAT
ACAAAGTAGTAAAAATTGAACCATTA---GGAGTAGCACCCACTAGGGCAAAGAGAAGAGTGGTG--------
-------------------------------CAGAGAGAAAAAAGAGCG---ATAGGAGGACTAGGAGCT---
------TTGTTCCTTGGGTTCTTGGGAATG---GCAGGAAGCACTATG---GGCGCAGCGTCG---
CTGACGCTGACGGTACAG---GCCAGGCTG---TTATTG---TCTGGTATAGTGCAACAGCAAAAC---
AATTTG---CTGAGG---GCTATTGAGGCGCAACAGCACCTGTTGCAACTCACAGTCTGGGGCATC
>FJ653571
TCTCATTGGTCTGATAGTACTTGGAATGATACTACAGGG----------------------------------
---CCAAAT------
AACACTACAGAAACAATCATACTCCCATGTAGACTAAAACAAATTATAAACATGTGGCAAGGAGTAGGAAGAG
CAATGTATGCCCCTCCCATCAGAGGACAAATTAGATGTTCATCAAAT---
ATTACAGGGCTGTTATTAACAAGAGATGGTGGTGGTAGTAATAGCAGC-------------------------
----

GGCAGTAACGAGACCTTCAGACCAGAAGGAGGAAATATGAAGGACAATTGGAGAAGTGAACTATATAAATATA
AAGTAGTAAAAATTGAACCATTA---GGAATAGCACCCACTAAGGCAAAAAGAAGAGTGGTG-----------
----------------------------CAAAGAGAAAAAAGAGCA---GTGGGA---CTAGGAGCT------
---CTGTTCCTTGGGTTCTTGGGAGCA---GCAGGAAGCACTATG---GGCGCAGCGGCA---
GTAACGCTGACGGTACAG---GCCAGACAA---TTATTG---TCTGGTATAGTGCAACAGCAAAAC---
AATTTG---CTGAGA---GCTATTGAGGCGCAACAGCATATGTTGCAACTCACAGTCTGGGGCATC
>KC312435

GATCATATTACTGAG--

GAGAACACAACTATCACACTCCAATGCAGAATAAAACAAATTATAAACATGTGGCAAAAAGTAGGAAAAGCAA
TGTATGCCCCTCCCATCAAAGGACAAATTAGCTGTGTATCAAAT---
ATTACAGGGCTGCTATTAACAAGAGATGGTGGTCTAAACCAGTCAGGT-------------------------
----

AACAGTTCCGAGACCTTCAGACCTGGAGGAGGAGATATGAGGGACAACTGGAGAAGTGAATTATATAAATATA
AAGTAGTAAGAATTGAACCATTA---GGAGTAGCACCCACCAAGGCAAAGAGAAGAGTGGTG-----------
----------------------------CAGAGAGAAAAAAGAGCA---GTGGGAGTTCTAGGAGCT------
---ATGTTCCTTGGGTTCTTGGGAGCA---GCAGGAAGCACTATG---GGCGCAGCGTCA---
ATGACGCTGACGGTACAA---GCCAGACAA---TTATTG---TCTGGTATAGTGCAACAGCAGAAC---
AATCTG---CTGATG---GCTATTGAGGCGCAACAGCATATATTGCGACTCACAGTCTGGGGCATC
>HQ216949
AATTAC---

ACTGATGGAACTATCACACTCCCATGCAGAATAAGACAAATTATAAACATGTGGCAGGAAGTAGGAAAAGCAA
TGTATGCCCCTCCCATCGGAGGGCAAATTAGATGTTCATCAGAT---
ATTACAGGGCTACTCTTAACAAGAGATGGTGGCAGAGGAAATAACAAT-------------------------
----

AATGAAACTGAGATCTTCAGACCTGGAGGAGGAGATATGAGGGACAATTGGAGAAGTGAATTATATAAATACA
AAATAGTAAAAATTGAACCATTA---GGAATAGCACCCACCAAGGCGAAGAGAAGAGTGGTG-----------
----------------------------CAGAGAAAGAAAAGAGCA---GTGGGA---ATAGGAGCT------
---GTGTTCCTTGGGTTCTTGGGAGCA---GCAGGAAGCACGATG---GGCGCAGCGTCA---
GTGACGCTGACGGTACAG---GCCAGACAA---TTACTG---TCTGGTATAGTGCAACAGCAGAAC---
AATCTG---CTGAGG---GCTATTGAGGCGCAACAACATCTGTTGCAACTCACAGTCTGGGGCATC
>JX863989
CATCAGAATAATACTAAT---
-----------------ATTACTACA------
GAGTCAAATGGCATCATCACACTCCCATGCAGAATAAAACAAATTATAAACAGGTGGCAGGAAGTAGGAAAAG
CAATGTATGCCCCTCCCATCAGAGGACCAATCAGGTGTAGATCAAAT---
ATTACAGGGGTGCTATTAACAAGAGATGGTGGTAGTACTAATGCC----------------------------
----

ACCAACGAGACCTTCAGACCTGGAGGAGGAGATATGAGGGATAATTGGAGAAGTGAATTATATAAATATAAAG
TAGTAAAAATTGAGCCATTA---GGAATAGCACCCACCAAGGCACAGAGAAGAGTGGTG--------------
-------------------------CAGAGAGAAAAAAGAGCA---GCA------ATTGGAGCT---------
ATGCTCATTGGGTTCTTGGGAATG---GCAGGAAGCACTATG---GGCGCAGCGTCA---
GTGACGCTGACGGTACAA---GCCAGGCTA---TTACTG---TCTGGTATAGTGCAGCAGCAGAGA---
AATTTG---CTGAGA---GCTATTGAGGTGCAACAACATATGTTGCAACTCACAGTCTGGGGCATC
>JX863993
AATTACAAC--

ATTACAGGAAATATCACACTCCCATGCAGAATAAAACAAATTATAAACATGTGGCAGGAAGTAGGAAAAGCAA
TGTATGCTCCTCCCATCGCAGGACTACTTAGATGTTCATCAAAT---
ATTACAGGGCTGCTATTAACACGAGATGGAGGTCTACACCCGGAC----------------------------
----

AATGGGACCGAGATTTTCAGACCTGGAGGAGGAGATATGAGGGACAATTGGAGAAGTGAATTATATAAATATA
AAGTAGTAAAAATTGAACCATTA---GGAATAGCACCCACCAAGGCAAAGAGAAGAGTGGTG-----------
----------------------------CAGAGAGAAAAAAGAGCA---ATAGGA---CTAGGAGCT------
---TTGTTCTTTGGGTTCTTGGGAGCA---GCAGGAAGCACTATG---GGCGCAGCGTCA---
GTGACGCTGACGGTACAG---GCCAGACAG---TTACTG---TCTGGTATAGTGCAACAGCAGAAC---
AATTTG---CTGAGA---GCTATTGAGGCGCAACAACATATGTTGCAACTCACAGTCTGGGGCATC
>JF320263
AATTATACTGATCCTTTAAATGATACTTTGAATAAT-------------------------------------
----
GATACTGAAGAAAATATCACACTCCAATGCAGAATAAAACAATTTATAAACATGTGGCAGAAAGTAGGAAAAG
CAATGTATGCCCCTCCCATTAGTGGACAAATTAACTGTTCATCAAAT---

ATTACAGGGCTGCTATTAATGAGAGATGGTGGTATTAACCAGAGC----------------------------
----

GAGAGCAGCGAGACCTTCAGACCTGGAGGAGGAGATATGAGGGACAATTGGAGAAGTGAATTATATAAATATA
AAGTAGTAAAAATTGAACCTTTA---GGAGTAGCACCCACTAAAGCAAAGAGAAGAGTGGTG-----------
----------------------------CAGAGAGAAAAAAGAGCA---GCA------ATAGGAGCT------
---CTGGTCCTTGGGTTCTTGGGAGCA---GCAGGAAGCACTATG---GGCGCAGCGTCA---
CTGACGCTGACGGTACAG---GCCAGACTA---TTATTG---TCTGGTATAGTGCAGCAGCAAAAC---
AATTTG---CTGAGA---GCTATTGAGGCGCAACAGCATATGTTGCAACTCACAGTCTGGGGCATC
>JF320011
AATCATACTTGGAATAGTACTAATGATACTTGGAAT-------------------------------------
----
-
GGTACTGAGACCATAACACTTCCATGCAGAATAAAACAAATTATAAACATGTGGCAAGAAGTAGGAAAAGCAA
TGTATGCCCCTCCCATCAGAGGACAGATTAACTGCTCGTCAAAT---
ATTACAGGGCTGCTATTAACAAGAGATGGTGGTAACACCACAAAC----------------------------
----

ACCACCGAGATCTTCAGACCTGGAGGGGGAAATATGAAGGACAATTGGAGAAGTGAATTATATAAATACAAAG
TAGTAAAAATTGAACCATTA---GGAGTAGCACCCACCAAGGCAAAGAGGAGAGTGGTG--------------
-------------------------CAGAGAGAAAAAAGAGCA---GCA------ATAGGAGCT---------
TTGTTCCTTGGGTTCTTGGGAGCA---GCAGGAAGCACTATG---GGCGCAGCGTCA---
GTGACGCTGACGGTACAG---GCCAGACTA---TTATTG---TCTGGTATAGTGCAACAGCAGAGC---
AATCTG---CTGAGG---GCTATTGAGGCGCAACAGCATCTGTTGCAACTCACAGTCTGGGGCATC
>JF320126
AGTCATAATGATACCAGGCATAATAGTACTAAA--

GGGACAGATAATATCATACTCCCATGTAAAATAAGACAAATTGTAAACATGTGGCAGGAAGTAGGAAAGGCAA
TGTATGCCCCTCCCATTGCAGGACAAATTAAATGTTCATCAAAT---
ATTACAGGGCTGATATTAACAAGAGATGGTGGTAATAATAATAGCACA-------------------------
----

AACGAGACTGAGACATTCAGACCTGGAGGAGGAAATATGAGGGACAATTGGAGAAGTGAATTATATAAATACA
AAGTAGTAGAAATTGAACCATTA---GGAATAGCACCCACCAAGGCAAAGAGGAGAGTGGTG-----------
----------------------------CAAAGAGAAAAAAGAGCA---GTGGGAACGATAGGAGCT------
---ATGTTCCTTGGGTTCTTGGGAGCA---GCAGGAAGCACTATG---GGCGCAGCGTCG---
ATAACGCTGACGGTACAG---GCCAGACAA---TTATTG---TCTGGTATAGTGCAGCAGCAGAAC---
AATCTG---CTGAGG---GCTATTGAGGCGCAACAACATCTGTTGCAACTCACGGTCTGGGGCATC
>FJ469723
ACTCAGCTGTTTAATAGTACTAATAATAAT---

ACTGCAGAAAACATCACACTCCCATGCAGAATAAGACAAATTATAAACAGGTGGCAAGAAGTAGGAAAAGCAA
TGTATGCCCCTCCCATCAGCGGACTAATTAACTGCTTATCAAAT---
ATTACAGGGCTGCTATTAACAAGAGATGGAGGGAACAATACAGAG----------------------------
----

AGTAAGACTGAGACCTTCAGACCTGGAGGAGGGGATATGAGGGACAATTGGAGAAGTGAATTATACAAGTATA
AAGTAGTAAAAATTGAACCATTA---GGAGTAGCACCCACCAGGGCAAGGAGAAGAGTGGTG-----------
----------------------------CAAAGAGAAAAAAGAGCA---GTGGGAGTGATAGGAGCT------
---ATGTTCCTTGGATTCTTGGGAGCA---GCAGGAAGCACTATG---GGCGCAGCGGCA---
GTGACGCTGACGGTACAG---GCCAGACAA---TTATTG---TCTGGTATAGTGCAACAGCAAAAC---
AATTTG---CTGCAG---GCTATTGAGGCGCAACAACATCTGTTGCAACTCACAGTCTGGGGCATT
>JQ403097
ACTCATTGGAATGATACTAAT--
--------------------------------GAC---
ACCAATAATGACACCATTACACTCCCATGCAGAATAAAACAAATTATAAACTTGTGGCAGGAAGTAGGAAAAG
CAATGTATGCCCCTCCCATCAAAGGACAAATTAGATGTTCATCAAAT---
ATTACAGGGCTGCTATTAACAAGAGATGGTGGTTGTAACACGACC----------------------------
----

AATGGGACTGAGGTCTTCAGACCTGGAGGAGGAGATATGAGGGACAATTGGAGAAGTGAATTATATAAATATA
AAGTAGTAAAAATTGAACCATTA---GGAGTAGCACCCACCAAGGCAAAGAGAAGAGTGGTG-----------
----------------------------CAGAGAGAAAAAAGAGCA---GTGGGAGTAATAGGAGCT------
---ATGTTCCTTGGGTTCTTGGGAGCA---GCAGGAAGCACTATG---GGCGCAGCGTCA---
GTGACGCTGACGGTACAG---GCCAGACAA---TTATTG---TCTGGTATAGTGCAACAGCAGAAC---
AATTTG---CTGAGA---GCTATTGAGGCGCAACAGCATCTGTTGCAACTCACAGTCTGGGGCATC
>EU578223
AATTACACA--

GGAGAAAATGACACAATTACACTCCCATGCAGAATAAAACAAATCATAAATATGTGGCAGGAAGTAGGAAAAG
CAATGTATGCCCCTCCCATCAGAGGAATAATTAGGTGTTCATCTAAT---
ATTACAGGGTTGTTATTAACAAGAGATGGGGGTAGCGGTAACGAG----------------------------
----

AGCGACGCCGAGATCTTCAGACCTGGGGGAGGGAATATGAAGGATAATTGGAGAAGTGAATTATATAAATATA
AAGTAGTAAAAATTAAACCATTA---GGGATAGCACCCACCAAGGCAAAAAGAAGAGTGGTG-----------
----------------------------CAGAGAGAAAAAAGAGCA---GTGGGA---CTAGGAGCT------
---TTGTTCCTTGGGTTCTTGGGAGCA---GCGGGAAGCACTATG---GGCGCAGCGTCA---
GTGACGCTGACGGTACAG---GCCAGAAAC---TTATTG---TCTGGTATAGTGCAACAGCAGAAC---
AATCTG---CTGAGG---GCTATTGAGGCGCAACAGCACCTGTTGCAACTCACAGTCTGGGGCATA
>JF689892
AATTACACTGAA---

GGAAATGATACAATCACACTCCCATGCAAAATAAAACAAATTGTAAACATGTGGCGGGAGGTAGGAAAGGCAA
TGTATGCCCCTCCTATCAGAGGACTAATTAGATGCTCATCAAAT---
ATTACAGGACTGATCTTAACAAGAGATGGTGGTAATATTAATAATGGTACC----------------------
----

ACGGGGAACAGGACTGAGATCTTTAGACCTGGAGGAGGAGATATGAGGGACAATTGGAGAAGTGAATTATATA
AATATAAGGTAGTAAGAATTGAACCATTA---GGAATAGCACCCACCAGGGCAAAGAGAAGAGTGGTG-----
----------------------------------CAAAGAGAAAAACGAGCA---GTGGGA---
GTAGGAGCT---------CTGTTCCTTGGGTTCTTGGGAGCA---GCAGGAAGCACTATG---
GGCGCAGCGTCA---ATAACGCTGACGGTACAG---GCCAGACAA---TTATTG---
TCTGGTATAGTGCAACAGCAGAAC---AATCTG---CTGAAG---
GCAATTGATGCGCAACAGCATCTGTTGCAACTCACAGTCTGGGGCATT
>JF320315
ACTCAAGAAAGT---
--------------------AACACT------
GAAGGAAATGACACAATTATACTACCATGCAGAATAAAACAAATTGTAAACATGTGGCAGGAAGTAGGAAAAG
CAATGTATGCCCCTCCCATTAGTGGACAAATTAACTGTTCATCAAAT---
ATTACAGGGCTAATATTAGCAAGAGATGGTGGTAATGAGACATAC----------------------------
----

AGGAACGAGACCTTCAGACCTATAGGAGGAGATATGAGGGACAATTGGAGAAGTGAATTATATAAATATAAAG
TAGTAAAAATTGAACCATTA---GGAGTAGCACCCACCAAGGCAAAGAGAAGAGTGGTG--------------
-------------------------CAAAGAGAAAAAAGAGCA---GTGGGA---TTAGGAGCT---------
ATGTTCCTTGGGTTCTTGGGAGCA---GCAGGAAGCACTATG---GGCGCGGCGTCA---
GTGACGCTGACGGTACAA---GCCAGACAA---TTATTG---TCTGGTATAGTGCAACAGCAGAAC---
AATCTG---CTGAGA---GCTATTGAGGCGCAACAGCATCTGTTGCAACTCACAGTCTGGGGCATC
>JF320394
ACTCAGAAGATTAAAGGGTTTGAT---

AATAATGGCAATATCACACTCCCCTGTAGAATAAAACAAATTATAAACAGGTGGCAGGAAGTAGGAAAAGCAA
TGTATGCCCCTCCCATTGCAGGACAAATTAAATGTACATCAAAT---
ATTACAGGGCTGCTATTAACATGGGATGGTGGTAATGATGGTGGAAACGGCACT------------GGA----
----
-
ACAAAAAATAACGAAACAGAGGTTTTTAGACCATCAGGAGGAAATATGAGGGACAATTGGAGAAGTGAATTAT
ATAAGTATAAAGTAGTAAAAGTTGAACCATTA---GGAATAGCACCCACCAAGGCAAAGAGAAGAGTGGTG--
-------------------------------------CAGAGAGAAAGAAGAGCA---GTGGGA---

ATAGGAGCT---------GTGTTCCTTGGGTTCTTGGGAGCA---GCAGGAAGCACTATG---
GGCGCAGCGTCA---TTAACGCTGACGGTACAA---GCCAGACTA---TTGTTG---
TCTGGTATAGTGCAACAGCAGAAC---AATCTG---CTGAGA---
GCTATTGAGGCGCAACAGCATCTGCTGCAACTCACAGTCTGGGGCATC
>JF320279
ACTCAATTATTCAATAGTACTTGGCTACTCAATGGATCAAGTAACACTGAA----------------------
----

GGAAATGACACAATCACGATCCCATGCAGAATAAAACAAATTGTAAACATGTGGCAAAAAGTAGGAAAAGCAA
TGTATGCCCCTCCCATCAGAGGAAATATTAGTTGTATATCAAAT---
ATTACAGGAATACTATTAATAAGAGATGGGGGTGTTAGCAACGAGAGTGCT----------------------
----

AACGAGACCAGGAACGAGACCTTCAGACCAGCAGGAGGAGATATGAGGGACAATTGGAGAAGTGAATTATATA
AATATAAAGTAGTAAAAATTGAACCATTA---GGAATAGCACCCACTAAGGCAAAGAGAAGAGTGGTG-----
----------------------------------CAGAGAGAAAAAAGAGCA---GTGGGA---
CTAGGAGCT---------GTGTTCCTTGGGTTCTTGGGAGCA---GCAGGAAGCACTATG---
GGCGCAGCGTCA---GTGACGCTGACGGTACAA---GCCAGACAA---TTATTG---
TCTGGTATAGTGCAACAGCAGAAC---AATTTG---CTGAAG---
GCTATTGAGGCGCAACAGCACCTGTTGCAACTCACAGTCTGGGGCATC
>HQ908109
ACTCAAAATGATACT--

GCTAACAGTACCATTATGCTCCCATGCAGAATAAAACAAGTTATAAACAGGTGGCAGGAAGTAGGAAAAGCAA
TGTATGCCCCTCCCATCAGAGGACCAATTAGCTGTTCATCAAAT---
ATTACAGGGCTGATATTAACAAGGGATGGTGGTATTATTAATGCC----------------------------
----

ACCAATGAGACCTTCAGACCTGGAGGAGGAGATATGAGGGACAATTGGAGAAGTGAATTATACAATATAAAG
TAGTAAAAATTGAACCATTA---GGAGTAGCACCCACCAGGGCAAAGAGAAGAGTGGTG--------------
-------------------------CAAAGAGAAAAAAGAGCA---GTGGGA---ATAGGAGCT---------
CTGTTCCTTGGGTTCTTGGGAGCA---GCAGGAAGCACTATG---GGCGCAGCGTCA---
GTGACGCTGACGGTACAG---GCCAGACAA---TTATTG---TCTGGTATAGTGCAACAGCAGAGC---
AATTTG---CTGAGG---GCTATTGAGGCGCAACAGCATCTGTTGCAACTCACAGTCTGGGGCATC
>FJ469713
TGTTATACATCACGACTGTTTAATAGAACTTTTGGTAATGAGACTATAAATCCTACTAAGATTAATGAA----
----

AACACTACAGAAGTCATCACACTCCCATGTAGAATAAGACAAATTATAAATATGTGGCAGGAAGTAGGAAAAG
CAATGTATGCCCCTCCCATCAGAGGACAAATTAACTGTATATCAAAC---
ATTACAGGACTGTTATTAACAAGAGATGGTGGCGATGACGATACC----------------------------
----

ACCAACACTGAGACCTTCAGACCTACAGGGGGAAATATGAAAGACAATTGGAGAAGTGAATTATATAAATATA
AAGTAGTAAGAATTGAACCATTG---GGACTGGCACCCTCCAAGGCAAAGAGAAGAGTGGTG-----------
----------------------------CAGAGAGAAAAAAGAGCA---GTGGGA---CTAGGAGCT------
---TTGTTCATTGGGTTCTTGGGAGCA---GCAGGAAGCACTATG---GGCGCAGCGTCG---
ATGACGCTGACGGTACAG---GCCAGACTA---TTATTG---TCTGGTATAGTGCAACAGCAGAAC---
AATCTG---CTGAGA---GCTATTGAGGCGCAACAACATCTGTTGCAACTCACAGTCTGGGGCATC
>JQ403071
AGTCATATAGAGTCA--
--------------------------AAT---
GACAATGAAGAAAATATCACACTCCCCTGTAGAATAAGACAAATTGTAAACATGTGGCAGGAAGTAGGAAAAG
CAATGTATGCCCCTCCCATCAGTGGACAAATTAGATGTGTATCAACT---
ATTACAGGGCTGCTATTAACAAGAGATGGTGGGAACGGGACTAAC----------------------------
----

AATACTGAGACCTTTAGACCTGGAGGAGGAGATATGAGGGACAACTGGAGAAGTGAATTATATAAATATAAAG
TAGTAAGAATTGAACCATTA---GGAGTAGCACCCACCAAGGCACAGAGAAGAGTGGTG--------------
-------------------------CAGAGAGAAAAAAGAGCA---GTGGGAATGTTAGGAGCT---------

ATGTTCCTTGGGTTCTTGGGAGCA---GCAGGAAGCACTATG---GGCGCAGCGTCG---
ATAACGCTGACGGTACAA---GCCAGACAA---TTAATG---TCTGGTATAGTGCAACAGCAGAAC---
AATCTG---CTGAAG---GCTATTGAGGCGCAACAACATATGTTGCAACTCACAGTCTGGGGCATC
>KM259279
AGTTATTGGAAT---
--------ACT---ACTGAAGGGTCCAATAACACT---------------
GAAGGAAATGACACCATCACAATACCATGCAGAATAAAACAAATTATAAACATGTGGCAGGAAGTAGGAAAAG
CAATGTATGCCCCTCCCATCAAAGGAATAATTAATTGTTCATCAAAT---
ATCACAGGGCTGTTATTAACAAGAGATGGTGGT--

AACACAACCAACAACAGCACA---------
GAGACCGAGACCTTCAGACCTGGAGGAGGAGATATGAGGGACAATTGGAGAAGTGAATTATATAAGTATAAAG
TAGTAAAAATTGAACCATTA---GGACTAGCACCCACCAAGGCAAAGAGAAGAGTGGTG--------------
-------------------------CAGAGAGAAAAAAGAGCA---ATAGGA---ATAGGAGCT---------
ATGTTCCTTGGGTTCTTGGGAGCA---GCAGGAAGCACTATG---GGCGCAGCATCA---
TTGACGCTGACGGTACAG---GCCAGACAA---TTATTG---TCTGGTATAGTGCAACAGCAGAAC---
AATTTG---CTGAGA---GCTATTGAGGCGCAACAGCATATGTTGCAACTCACAGTCTGGGGCATC
>MK383753
AGTCAC---

ACTGAAGAAAATATCACACTCCCATGCAGAATAAAACAAATTATAAACATGTGGCAGGAAGTAGGAAAAGCAA
TGTATGCCCCTCCCATTAGAGGAAACATTAGCTGTTCATCAAAT---
ATTACAGGGCTGCTATTAACAAGAGATGGTGGTAATAACGAGAGC----------------------------
----

CAGACCGAGACCTTCAGACCTGGAGGAGGAAATATGAAGGATAATTGGAGAAGTGAATTATATAAATATAAAG
TAGTAAAAATTGAACCATTA---GGAGTAGCACCCACCAGGGCAAAGAGAAGAGTGGTG--------------
-------------------------CAGAGAGAAAAAAGAGCA---GTGGGA---ATAGGAGCT---------
GTGTTCCTTGGGTTCTTGGGAGCA---GCAGGAAGCACTATG---GGCGCAGCGTCA---
ATGACGCTGACGGTACAG---GCCAGACTA---TTACTG---TCTGGTATAGTGCAACAGCAGAGC---
AATTTG---CTGAGG---GCTATTGAGGCGCAACAGCGTTTGTTGCAACTCACAGTCTGGGGCATC
>HQ615981
ACTCAGGAGACTAAC--

AGGGACAATGACACTATCACGCTCCCATGCAGAATAAAACAAATTATAAACATGTGGCAGGAAGTAGGAAGAG
CAATGTATGCCCCTCCCATCAAAGGACTAATTAGGTGTTCATCAAAT---
ATCACAGGGCTGCTACTACTAAGAGATGGTGGAAATACCAGTAAC----------------------------
----

AATAATGAGACCTTCAGACCTGGAGGAGGAGATATGAGGGACAATTGGAGAAGTGAATTATATAAATATAAAG
TAGTAAAAGTTGAACCATTA---GGAATAGCACCCACCAAGGCAAAGAGAAGAGTGGTG--------------
-------------------------CAGAGAGAAAAAAGAGCA---GTGGGT---TTTGGAGCT---------
ATGTTCCTTGGGTTCTTGGGAGCA---GCAGGAAGCACTATG---GGCGCAGCATCA---
CTAACGCTGACGGTACAG---GCCAGACAA---TTGTTG---TCTGGTATAGTGCAACAGCAAAAC---
AATTTG---CTGAGA---GCTATTGAGGCGCAACAACATATGTTGCAACTCACAGTCTGGGGCATC
>JQ403080
ACTCAACTGTTTAATAGTACTTGGGTGGTTAATAGTAGTACTGGGGAAGTTAATAGTAGTACTGGG-------
----
GGGTTT------
ACAGGAAATGGCATAATCACACTTCCATGCAGGATAAAACAAATTATAAACATGTGGCAGGAAGTAGGAAAAG
CCATGTATGCCCCTCCCATCGCAGGACAAATTAACTGTTCATCAAAT---
ATTACAGGACTACTACTAATAAGAGATGGTGGTAAAAATGTCAGC----------------------------
----

AACACCACCGAGACCTTTAGACCTGGAGGAGGAAATATGAGGGACAATTGGAGAAGTGAATTATATAAATATA
AGGTAGTAAGACTTGAGCCATTA---GGAATAGCACCCACCAAGGCAAGGAGAAGAGTGGTG-----------
----------------------------CAAAGAGAAAAAAGAGCA---GTGGGAACAATAGGAGTT------
---ATGTTCCTTGGGTTCTTGGGAGCA---GCAGGAAGCACTATG---GGCGCAGCGTCA---
GTGACGCTGATGGTACAG---GCCAGACAA---TTACTG---TCTGGTATAGTGCAGCAGCAAAGC---
AATTTG---CTGAAG---GCTATTGAAGCGCAACACCATCTGTTGCAGCTCACAGTCTGGGGCATC

>KM082128
CATTATAATAATGATACTGGTACTTGGAATACTACTTGGATT-------------------------------
--------------------------------GGGAATGGTACTGAAGGG---------
AATGAAAATAGCACCATCACCCTCCCATGCAGAATAAAACAAATAATAAACATGTGGCAGGAAGTAGGAAAGG
CAATGTATGCCCCTCCCATTAAAGGAATAATTAGCTGTTCATCAAAT---
ATTACAGGGCTGCTATTAACAAGAGATGGTGGTAACAGTAATACC----------------------------
----

ACCGAGAATGAGACCTTCAGACCTGGAGGAGGAGATATGAGGGACAATTGGAGAAGTGAATTATATAAATATA
AAGTAGTAAGAATTGAACCATTA---GGAGTAGCACCCACCCATGCAAAGAGAAGAGTGGTG-----------
----------------------------CAGAGAGCAGCAAGAGCA---GTGGGA---TTCGGAGCT------
---TTGTTCCTTGGGTTCTTGGGAGCA---GCAGGAAGCACTATG---GGCGCAGCGTCA---
ATAACGCTGACGGCACAG---GCCAGACAA---CTGTTG---TCTGGTATAGTGCAACAGCAGAAC---
AATCTG---CTGAGG---GCTATTGAGGCGCAACAACACCTGTTGCAACTCACAGTCTGGGGCATT
>MH666207
TTTCATAGTAATTGGACTAAAACAGGGAACGTTACATACATTCCAGAAAAT----------------------
----

AACTCCACTAACATCACACTCCAATGTAGAATAAAACAAATTATAAACATGTGGCAGAAAGTAGGCAAAGCAA
TGTATGCCCCTCCCATCAGTGGACAAATTAGATGTTCATCAAAC---
ATTACAGGGCTGCTATTAACAAGAGATGGTGGTAAGAAGAACGAATCT-------------------------
----

GAAGGGAACGAGACTGAGGTCTTCAGACCTGGAGGAGGGGATATGAGGGACAATTGGAGAAGTGAATTATATA
AATATAAAGTAGTGAAAATTGAGCCATTA---GGAGTAGCACCCACCAAGGCAAAGAGAAGAGTGGTG-----
----------------------------------CAAAGAGAAAAAAGAGCA---GTGGGA---
CTAGGAGCT---------TTGTTCCTTGGATTCTTGGGAGCA---GCAGGAAGCACTATG---
GGCGCAGCGTCA---ATAACGCTGACGGTACAG---GCCAGACAA---GTATTG---
TCTGGTATAGTGCAGCAGCAACAC---AATTTG---CTGAGG---
GCTATTGAGGCGCAACAGCATCTGTTGCAACTCACAGTCTGGGGCATC
>KR423423
ACTTATACTTGGAATAATACAGAT---

ATCAATGGTACTATCACACTCCAATGCAGAATAAAACAAATTGTAAATAGGTGGCAGGAAGTAGGAAAAGCAA
TGTATGCCCCTCCCATCAGTGGAAAGATTAGTTGCTCATCAAAT---
ATTACAGGGCTGCTATTAATGAGAGATGGTGGAAGCAGCAATGAGACT-------------------------
----

AAAAATAATACTGAGATCTTCAGACCTGGAGGAGGAAATATGAGGGACAATTGGAGAAGTGAATTATATAAAT
ATAAAGTAGTGAGAATTGAACCATTA---GGAATAGCACCCACCAAGGCAAAGAGAAGAGTGGTG--------
-------------------------------CAGAGAGAAAAAAGAGCA---GTGGGA---TTAGGAGCC---
------ATGTTCCTTGGGTTCTTAGGAGCA---GCAGGAAGCACTATG---GGCGCAGCATCA---
ATAACGCTGACGGTACAA---GCCAGACTA---TTATTG---TCTGGTATAGTGCAACAGCAAAAC---
AATCTG---CTGAGA---GCTATTGAGGCGCAACAGCATCTGTTGCAACTCACAGTCTGGGGCATC
>KR423436
AATTACACTGATACTTGGAATAATACTGAAGAG--

ACAAATGAAACTATCACACTCCCATGCAGAATAAAACAAATTATAAACATGTGGCAGGAAGTAGGAAAAGCAA
TGTATGCCCCTCCCATTAGAGGAGCAATTAACTGCTCATCAAAC---
ATAACGGGGCTGCTATTAACAAGAGATGGTAGTACTGATGGCCCA----------------------------
----

GACAATACCACTGAAATCTTCAGACCGGGAGGAGGAGATATGAGGGACAATTGGAGAAGCGAATTATATAAAT
ATAAAGTAGTACAAATTGAACCAATA---GGACTAGCACCCACAAAGGCAAGGAGAAGAGTGGTG--------
-------------------------------CAGAGAGAAAAAAGAGCA---GTAACA---ATGGGAGCT---
------ATGTTCCTTGGGTTCTTGGGAGCA---GCAGGAAGCACTATG---GGCGCAGCGTCA---
ATAACGCTGACGGTACAG---GCCAGACAA---TTATTG---TCTGGTATAGTGCAACAGCAGAAC---
AATCTG---CTGAAG---GCTATTGAGGCGCAACAGCATCTGTTGCAACTCACAGTCTGGGGCATC
>KR423444

TTTCAGGGTACTTGGAATGGTACTACTTGGAATACTAATGATACTTGGAGGCAT-------------------
--TGGAGGCATAGTAAT--------
-
TCATCGAATGACACTGTCACACTCCAATGCAGAATAAGACAAATTGTAAACATGTGGCAGAGAGTAGGAAAAG
CAATATATGCTCCTCCTATCCAAGGAAAAATTAGATGCTCATCAAAT---
ATTACAGGGTTGCTATTAACAAGAGATGGTGGTAATAGCAACGATACC-------------------------
----

AACAGCACTGAGACCTTCAGACCTGGAGGAGGAAATATGAAGGACAATTGGAGAAGTGAATTATATAAATATA
AAGTAGTACAAATTGAACCAGTA---GGAATAGCACCCACCAGGGCAAAAAGAAGAGTGGTG-----------
----------------------------CAGAGAGAGAAAAGAGCA---GTGGGA---ATAGGAGCT------
---CTGTTCCTTGGGTTCTTGGGAGCA---GCAGGAAGCACTATG---GGCGCAGCATCA---
ATAACGCTGACGGTACAG---GCCAGACAA---TTATTG---TCTGGTATAGTGCAACAGCAGAAC---
AATCTG---CTGAGA---GCTATTGAGGCGCAACAGCATATGTTGCAACTCACAGTTTGGGGCATC
>MG197136
ACTTACAGTACTTGGCAGTTTAAAAGTACT---------------------------------
TGGAATGGTACTAACACT---TGGAATAGTACTGTT---------------------
ACAGGAAATGACACTATTACACTCCAATGCAGAATAAAACAAATTATAAACTTGTGGCAGGAAGTAGGAAAAG
CAATGTATGCCCCTCCCATCAGAGGACCAATTAGATGTTCATCAAAT---
ATTACAGGACTACTCTTAACAAGAGATGGTGGTATTACAAATGGGAATAATACT------
AATACTACTGAGAAC---AGTGAT---

AATAATACTGAGATCTTCAGACCTGGAGGAGGAAATATGAAGGACAATTGGAGAAGTGAATTATATAAATATA
AAGTAGTACAAGTTGAACCATTA---GGAATAGCACCCACCAAGGCAAAGAGAAGAGTGGTG-----------
----------------------------CAAAGAGAAAAAAGAGCA---GTGGGAACACTAGGAGCT------
---ATGTTCCTTGGGTTCTTGGGAGCA---GCAGGAAGCACTATG---GGCGCAGCGTCA---
TTGACGCTGACGGTACAA---GCCAGACTA---CTATTG---TCTGGTATAGTGCAACAGCAAAAC---
AACTTG---CTGAGA---GCTATTGAGGCGCAACAACATCTGTTGCAACTCACAGTCTGGGGCATT
>KM354740
ACTCAGCAG--
-----------------------------GTGCAGTTCAACAAT---------
ACTGGGAATGATACTATCATACTCCCATGCAGAATAAAACAGATTATAAACCAGTGGCAGGGAGTAGGAAAAG
CAATGTATGCCCCTCCTATCAGAGGACCAATTAACTGCACATCAAAC---
ATTACAGGGCTGATATTAACAAGAGATGGTGGT--

AATGGTAATGGGAGTGAC---
ACGGAAGAGATCTTCAGACCTGGAGGAGGAGATATGAGGGACAATTGGAGAAGTGAACTATATAAATATAAAG
TAGTACAAATTGAACCAATA---GGAGTAGCACCCACCAAGGCAAAGAGAAGAGTGGTG--------------
-------------------------CAAAGAGAAAAAAGAGCA---GCG------TTGGGAGCT---------
ATGTTCCTTGGGTTCTTGGGAGCA---GCAGGAAGCACTATG---GGCGCAGCGTCA---
GTGGCGCTGACGGTACAG---GCCAGACAA---TTGTTG---TCTGGTATAGTGCAACAGCAGAAC---
AATCTG---CTGAGG---GCTATTGAGGCGCAACAACATTTGTTGCAACTCACAGTCTGGGGCATC
>KM354821
ACTCATTGG--
--------------------------AATGATACTGAAGACTCA---------
AAAACCAATGACACAATCACACTCCCATGCAGAATAAAACAAATTATAAACCTGTGGCAGGAAGTAGGAAAAG
CAATGTATGCCCCTCCCATCAGAGGACAGATTAAATGTTCATCAAAC---
ATTACAGGGCTGCTATTAACAAGGGATGGTGGT--

-
CCTAATAGCACGACCAACGAAACCTTTAGACCTGCAGGAGGAGATATGAGGGATAATTGGAGAAGTGAATTGT
ATAAATATAAAGTAGTAGAAATTGAACCATTA---GGAGTAGCACCCACCAAGGCAAAAAGAAGAGTGGTG--
-------------------------------------CAGAGAGAAAAAAGAGCA---GTGGGA---
CTAGGAGCT---------TTGTTCCTTGGGTTCTTGGGAGCA---GCAGGAAGCACTATG---
GGCGCAGCATCA---ATGGTGCTGACGGTACAG---GCCAGGCAA---TTATTG---
TCTGGTATAGTGCAACAGCAGAAC---AATTTG---CTGAGA---
GCTATTGAGGCGCAACAGCATCTGTTGCAACTCACAGTCTGGGGCATC
>KM082106
CATCAGAATAGTACTAGGAATGAGAGT--

AGTAATAACCTTATCGAACTCCCATGCAGAATAAAACAAATCATAAACATGTGGCAGGAAGTAGGAAAAGCAA
TGTATGCCCCTCCCATCAGAGGAGAAATTAAATGTGTATCAAAT---
ATTACAGGGCTGCTATTAACAAGAGATGGTGGCCAGTCTAATGACACC-------------------------
----

AAAAACGACACAGAGATCTTCAGACCTGGAGGAGGAGATATGAGGGACAATTGGAGAAGTGAATTATACAAAT
ATAAAGTAGTAAAGATTGAACCATTA---GGAATAGCACCCCACAGGGCAAAGAGAAGAGTGGTG--------
-------------------------------CAAAGAGAAAAAAGAGCA---GTGGGA---ATAGGAGCT---
------ATGATCTTTGGGTTCTTGGGAGCA---GCAGGAAGCACTATG---GGCGCAACGTCA---
CTGACGTTGACGGTACAA---GCCAGACTA---TTATTG---TCTGGTATAGTGCAACAGCAGAAC---
AATTTG---CTGAGG---GCTATTGAGGCGCAACAGCATATGTTGCAACTCACAGTCTGGGGCATA
>MK384040
AATCACACTGGAAGA--

AATATCACACTCATCACACTCCCATGCAGAATAAAACAAATTATAAACATGTGGCAGGAAGTAGGAAAAGCAA
TGTATGCCCCTCCCATTAGAGGAAACATTAGCTGTTCATCAAAT---
ATTACAGGGCTGCTATTAACAAGAGACGGTGGTGATAACCAGAGC----------------------------
----

CAGACTGAGATCTTCAGACCTGGAGGAGGAAATATGAAGGATAATTGGAGAAGTGAATTATATAAATATAAAG
TAGTAAAAATTGAACCATTA---GGAGTAGCACCCACCAGGGCAAGGAGAAGAGTGGTG--------------
-------------------------CAGAGAGAAAAAAGAGCA---GTGGGA---ATAGGAGCT---------
GTGTTCCTTGGGTTCTTGGGAGCA---GCAGGAAGCACTATG---GGCGCAGCGTCA---
ATGACGCTGACGGTACAG---GCCAGACTA---TTACTG---TCTGGTATAGTGCAACAGCAGAAC---
AATTTG---CTGAGG---GCTATTGAGGCGCAACAGCGTCTGTTGCAACTCACAGTCTGGGGCATC
>MG196642
TCCTATAATGATACCGGGGAACATGAGGTAGAAAATGGC----------------------------------
----
ACAAGTAAAAACATCACACTTCCATGCAGAATAAAACAATTTATAAACATGTGGCAGGAAGTAGGAAAAGCAA
TGTATGCCCCTCCTATTAGAGGACAAATTAAATGTAAATCAAAT---
ATTACAGGGCTGCTATTAACAAGAGATGGCGGTAAGAATACCACA----------------------------
----

AAACATAATGAGACCTTCAGACCTGGAGGAGGAAATATGAAGGACAATTGGAGAAGTGAATTATATAAATATA
AAGTAGTAAGAATTGAACCATTA---GGAGTGGCGCCCACCAACGCTAAGAGAAGAGTGGTG-----------
----------------------------CAGAGAGAGAAAAGAGCA---GTGACA---CTAGGAGCT------
---CTGTTCCTTGGGTTCTTGGGAGCA---GCAGGAAGCACTATG---GGCGCAGCGTCA---
ATGACGCTGACGGTACAG---GCCAGACTA---TTATTG---TCTGGTATAGTGCAGCAGCAGAAC---
AATCTG---CTGAGA---GCTATTGAGGCGCAACAGCACCTGTTGCAACTCACGGTCTGGGGCATT
>MG196653
AATTATACTTTCAATAGTACTGCATGGTCAAATAATACTAAT-------------------------------
----
-
AGCACTGAAAAAATCACACTCCAATGTAGAATAAAGCAATTTATAAACATGTGGCAGGAAGTAGGAAAGGCAA
TATATGCCCCTCCCATCAAAGGACAAATTAGCTGCTCATCAAAT---
ATTACAGGGCTACTACTAACAAGAGATGGTGGTAACAACAATGACACCAATGGC-------------------
----

AACGATACTGAGGTCTTCAGACCTGCAGGGGGAGATATGAGGGACAATTGGAGAAGTGAGCTATATAAATATA
AAGTAGTAAAAATTGAACCATTA---GGAATAGCACCCACCAAGGCAAAGAGAAGAGTGGTG-----------
----------------------------CAAAGAGAAAAAAGAGCA---GTGGGA---TTAGGAGCT------
---ATGTTCCTTGGGTTCTTGGGAGCA---GCAGGAAGCACTATG---GGCGCAGCGGCA---
GTGACGCTGACGGTACAG---GCCAGGCAA---TTATTG---TCTGGTATAGTGCAACAGCAGAAC---
AATCTG---CTGAGG---GCTATTGAGGCGCAACAGCATATGTTGCAACTCACAGTCTGGGGCATC
>MG196831
AATTATACTACAGGGAATTTTACTACAGAGTCAAATAGC----------------------------------
----
TCAATAGGAAACATCACACTCCCATGCAGAATAAAACAAATTATAAACATGTGGCAGGAAGTAGGAAAAGCAA
TGTATGCCCCTCCCATCAAAGGACAAATCAGCTGCACATCAAAT---
ATTACAGGGCTGCTGATAACAAGAGATGGTGGTAATGATACCGGG----------------------------

----

AGCAAGGAGACCTTCAGACCTGGAGGAGGAGATATGAGGGACAATTGGAGAAGTGAATTATATAAATATAAAG
TAGTAAAAATTGAACCATTA---GGAGTAGCACCCACCAAGGCAAGGAGAAGAGTGGTG--------------
-------------------------CAGAGAGAAAAAAGAGCA---GTGGGA---ATAGGAGCT---------
GTGTTCCTTGGGTTCTTGGGAGCA---GCAGGAAGCACTATG---GGCGCAGCGTCG---
CTAACGCTGACGGTACAG---GCCAGACTA---CTATTG---TCTGGTATAGTGCAACAGCAGAAC---
AACTTG---CTGAGG---GCTATTGAGGCGCAACAGCGTATGTTGCAACTCACAGTCTGGGGCATC
>MG197199
TTTCATAGTGCTTGGCTGGTTAATGGTACTTGGAATAACACTTGGAATGAC----------------------
--ACCATT-----
-
GAAACAAATGGGACAATTACACTCCCATGTAGAATAAAACAAATTATAAACATGTGGCAGGAAGTAGGAAAAG
CAATGTATGCCCCTCCCATCAGAGGAAGAATTAGATGTGAATCAAAT---
ATTACAGGGCTGCTACTAATAAGAGATGGGGGTGTTAACGGGACC----------------------------
----

AATGATACAGAGACCTTCAGACCTGGAGGAGGAAATATGAGGGACAATTGGAGAAGTGAATTATATAAATATA
AAGTAGTAAAAATTGAACCATTA---GGAGTAGCACCTACCAAGGCAAAGAGAAGAGTGGTG-----------
----------------------------CAGAGAGAAAAAAGAGCA---GTGGGA---CTAGGAGCT------
---TTGTTCATTGGGTTCTTGGGAGCA---GCAGGAAGCACTATG---GGCGCAGCGTCA---
ATGACGCTGACGGTACAA---GCCAGACAA---TTATTG---TCTGGTATAGTGCAACAGCAAAAT---
AACTTG---CTGAGA---GCTATTGAGGCGCAACAACATCTGTTGCAACTCACAGTCTGGGGCATC
>KT124784
AACTATACTGAGCTAAACACCACT---

GAAAGGGAAAACATCACACTCCAATGCAGAATAAAACAAATTATAAACATGTGGCAAGAAGTAGGAAAAGCAA
TATATGCCCCTCCCATCGAAGGAATAATTAGATGCACATCAAAT---
ATTACAGGGCTGCTAATAACAAGAGATGGTGGTGGGAACGGAACAGGG-------------------------
----

AACAATGAAACCTTCAGACTTGCAGGAGGGGAGATGAGGGACAATTGGAGAAGTGAGTTATATAAATACAAAG
TAGTAAAAGTTGAACCATTA---GGAATAGCACCCACCAAGGCAAAGAGAAGAGTGGTG--------------
-------------------------CAGAGAGAAAAGAGAGCA---GTGGGA---ATAGGAGCT---------
GTGTTCCTTGGGTTCTTGGGAGCA---GCAGGAAGCACTATG---GGCGCAGCGTCA---
ATGACGCTGACGGTACAG---GCCAGACTA---TTATTG---TCTGGTATAGTGCAACAGCAAAAC---
AATTTG---CTGAGG---GCTATTGAGGCGCAGCAGCATCTGTTGCAACTCACAGTCTGGGGCATT
>KU901858
ACTCACGTGTCAAGC--

GACAATGAAAATATCACACTCCCATGCAGAATAAAACAATTTATAAACATGTGGCAGGAAGTAGGAAAAGCAA
TGTATGCTCCTCCCATCAGCGGGCCAATTGAGTGTTCATCAGAT---
ATCACAGGGCTGCTATTGGTAAGAGATGGTGGTGATAATAATGAAGGTAGG----------------------
----

AACGAAACCGAAATCTTCAGACCTGGAGGAGGAGATATGAGGGACAATTGGAGAAGTGAATTATATAAATATA
AAGTAGTAAAAATTGAACCATTA---GGAATAACACCCACCAAGGCAAGGAGAAGAGTGGTG-----------
----------------------------CAGAGAGAAAAAAGAGCA---GTGACT---CTAGGAGCT------
---ATGTTCCTTGGGTTCTTGGGAGCA---GCAGGAAGCACTATG---GGCGCAGCATCA---
ATAACGCTGACGGTACAG---GCCAGACAA---TTATTG---TCTGGAATAGTGCAACAGCAAAAC---
AATTTA---CTGAGA---GCTATTGAGGCGCAACAGCATATGTTGCAACTCACAGTCTGGGGCATC
>MH843742
AGTCATGATAGTACTACTGAG--

TCAAATGATAATATCACACTCATATGCAAAATAAGACAAATTATAAACAGGTGGCAGGAAGTAGGAAAAGCAA
TGTATGCCCCTCCCATCCAAGGACAAATTAGATGTTCATCAAAT---
ATTACAGGACTGATATTAACAAGAGATGGTGGTGGTAATAGCACG----------------------------
----

GACGGGAACGAGACCTTCAGACCTGGAGGAGGAAATATGAAGGACAATTGGAGAAGTGAATTATATAAATATA

AAGTAGTAAGAATTGAGCCATTA---GGAGTAGCACCCACCAAGGCAAAGAGAAGAGTGGTG-----------
----------------------------CAGAGAGAAAAAAGAGCA---CTAGGAACGATAGGAGCT------
---ATGTTCCTTGGGTTCTTGGGAGCA---GCCGGAAGCACTATG---GGCGCAGCGTCA---
ATGGCGCTGACGGTACAG---GCCAGACAA---TTATTG---TCTGGTATAGTGCAACAGCAGAAC---
AATTTG---CTGAGG---GCTATTGAGGCGCAACAGCATCTGTTGCAACTCACAGTCTGGGGCATT
>KX505589
ACTCAGTCAAATAAT--

GGAAGTAAAACAATCACACTCCCATGCAGAATAAAACAAGTTATTAACATGTGGCAGGGAATAGGAAGAGCAA
TGTATGCCCCTCCCATCGAAGGACACATTAGATGTTCATCAAAT---
ATTACTGGGCTGCTATTAACAAGCGATGAGGGTGACAAAGGGAAC----------------------------
----

AGTTCCAATGTGACCTTCAGACCTGGAGGAGGAAATATGAAGGACAATTGGAGAAGTGAATTATATAAATATA
AAGTAGTACAAGTTGAACCATTA---GGAATAGCACCCACCAAGGCAAAAAGAAGAGTGGTG-----------
----------------------------CAAAAAGAGAAAAGAGCATTAGTGGGA---TTCGGAGCT------
---TTGTTCCTTGGGTTCTTGGGAGCA---GCAGGAAGCACTATG---GGCGCAGCGTCA---
ATAACGCTGACGGTACAG---GCCAGACAA---TTATTG---TCTGGTATAGTGCAACAGCAGAAC---
AACCTG---CTGAAG---GCTATTGAAGCGCAACAGCATATATTGCAACTCACAGTCTGGGGCATC
>KX505695
AGTTATGATTTTAATAATAGTAGTAATTCTAGTAGTAGTGTT-------------------------------
----
TCAAATGGCACTATCATACTCCCATGCAGAATAAAGCAAATTATAAACATGTGGCAGAAGGTGGGAAGAGCAA
TATATGCCCCTCCCATTAGAGGACAACTTAATTGTTCATCAAAT---
ATTACAGGGCTGATATTAACAAGAGATGGTGATAAGCCTAATAGC----------------------------
----

ACCAACGAGACCTTCAGACCTATAGGAGGAAATATGAAGGACAATTGGAGAAGTGAATTATATAAATATAAAG
TAGTAAAAATTGAACCATTA---GGACTAGCACCCACCAAGGCAAAGAGAAGAGTGGTG--------------
-------------------------CAAAGAGAAAAAAGAGCA---GTGGGA---ATAGGAGCT---------
CTGTTCCTTGGGTTCTTGGGAGCA---GCAGGAAGCACTATG---GGCGCAGCATCA---
ATGACGCTGACGGTACAG---GCCAGACTA---TTATTG---TCTGGTATAGTGCAACAGCAGAAC---
AATCTG---CTGAGG---GCTATTGAGGCGCAACAACATCTGTTGCAACTCACAGTCTGGGGCATC
>KX027840
TTTTATGGGACCTTGAATGATACTGGGTGGACAAGTAAAATTGATAAC-------------------------
----

AGTAACGAAAATATTACACTCCAATGCAGAATAAAGCAAATTATAAACATGTGGCAGAAAGTAGGAAAAGCAA
TGTATGCCCCTCCCATCAGCGGACAAATTAGATGTGTATCAAAT---
ATCACAGGGCTAATATTAACAAGAGATGGGGGTAATGGTACAAAC----------------------------
----

AACACTGAGATCTTCAGACCTGAAGGAGGAGATATGAGAGACAATTGGAGAAGTGAATTATATAAATATAAAG
TAGTAAAAATTGAACCATTA---GGAATAGCACCCACCAAGGCAAGGAGAAGAGTGGTG--------------
-------------------------CAGAGAGAAAAAAGAGCA---GTGGGA---ATGGGAGCT---------
TTGTTCCTTGGGTTCTTGGGAGCA---GCAGGAAGCACTATG---GGCGCAGCGTCA---
GTGACGCTGACGGTACAG---GCCAGACAA---TTATTG---TCTGGTATAGTGCAACAGCAGAAC---
AACTTG---CTGAAG---GCTATTGAGGCGCAACAACATCTGTTGCAACTCACTGTCTGGGGCATT
>KY658693
AATTACACTGAGACAACAAAT--
--------------------------AGCACT------
AATGGAACTGACACAATCATACTCCCATGCAGAATAAAACAAATTATAAACATGTGGCAGAAAGTAGGAAAAG
CAATGTATGCCCCTCCCATCAAAGGAATAATTAACTGTAATTCAAGT---
ATTACAGGATTGCTATTAACAAGAGATGGTGGTATTACCAACGATAGT-------------------------
----

AACAGCAATGAGACTTTTAGACCTGGAGGAGGGAATATGAAAGATAATTGGAGAAGTGAATTATATAAATACA
AAGTAGTAAGAATTGAACCATTA---GGAATAGCCCCCACCAGGGCAAAAAGAAGAGTGGTG-----------
----------------------------CAGAGAGAAAAAAGAGCA---ATAGGA---ATGGGAGCT------
---GTGTTCATTGGGTTCTTGGCAACA---GCAGGAAGCGCTATG---GGCGCAGCGTCA---

TTGACGCTGACGGTACAA---GCCAGACAA---TTATTG---TCTGGTATAGTGCAACAGCAGAAC---
AATCTG---CTGAGG---GCTATTGAGGCGCAACAGCATTTGTTGCAACTCACAGTCTGGGGCATT
>KU678026
TTTTATAGTAATTGGACTAAAGAAAATGCTACTGGAGAA----------------------------------
----
AATGACACTGACACAATCATACTCCCATGCAGAATAAAACAAATTATAAACATGTGGCAGGAAGTAGGAAAAG
CAATGTATGCCCCTCCTATCAAAGGACAAATTAGATGTTCATCAAAT---
ATCACAGGGCTGCTGATAACAAGGGATGGTGGTATGAACAACACC----------------------------
----

AACAACGACACTGAGATCTTCAGACCAGGAGGAGGAGATATGAGGGACAATTGGAGAAGTGAATTATATAAAT
ATAAAGTAGTACAAATTGAACCATTA---GGAGTAGCACCCACCAAGGCAAAGAGAAGAGTGGTG--------
-------------------------------CAGAGAGAAAAAAGAGCAGCACTAGGGGGACTAGGAGCT---
------TTGTTCCTTGGGTTCTTGGGAGCA---GCAGGAAGCACTATG---GGCGCAGCGGCA---
GTGACGCTGACGGTACAG---GCCAGACAA---TTATTG---ACTGGTATAGTGCAACAGCAGAAC---
AATTTG---CTGAGG---GCTATTGAGGCGCAACAGCACCTGTTGCAACTCACAGTCTGGGGCATC
>KY323815
GCTTATGAAGAG---

TCAAATGACACTATCACTCTCCCATGCAGAATAAAACAAATTATAAACATGTGGCAGGAAGTAGGAAAAGCAA
TGTATGCCCCTCCCATCAGAGGACAAATTAGCTGTAAATCAAAT---
ATTACAGGGCTGCTATTAACAAGAGATGGTGGTAATGGAACTGAC----------------------------
----

ATGAAAGAGACCTTCAGGCCTGGAGGAGGAGATATGAGGGACAATTGGAGAAGTGAATTATATAAATATAAAG
TAGTTAGAATTGAACCATTA---GGAATAGCACCCACCAAGGCACAGAGAAGAGTGGTG---------------
-------------------------CAGAGAGAAAAAAGAGCA---GTGGGAGTGCTAGGAGCT---------
ATGTTCCTCGGGTTCTTGGGAGCA---GCAGGAAGCACTATG---GGCGCAGCGTCA---
ATGGCGCTGACGGTACAG---GCCAGACAA---TTATTG---GCTGGTATAGTGCAACAACAGAAC---
AATCTG---TTGAGA---GCTATTGAGGCGCAACAGCATATGTTGCAACTCACAGTCTGGGGCATC
>KY324245
CATCAAAAAACA---

TCAAATGGTACATACATACTTCCATGCAGAATAAAACAAATTATAAACATGTGGCAGGAAGTAGGAAAAGCAA
TGTATGCCCCTCCCATCAGAGGAAACATTAGCTGTTCATCAAAT---
ATTACAGGACTGCTATTGGCAAGAGATGGGGGTAGTGGTAGTGATAATAATGGC-------------------
----
ACAAATAATAACACA---
AATGAAGAGATCTTCAGACCTGCAGGAGGAGATATGAGGGACAATTGGAGAAGTGAATTATATAAATACAAAG
TAGTAAAAATAGAGCCATTA---GGATTAGCACCCACCTCGGCAAAGAGAAGAGTGGTG---------------
-------------------------CAGAGAGAAAAAAGAGCA---GTGGGA---ATAGGAGCT---------
CTGTTCCTTGGGTTCTTGGGAGCA---GCAGGAAGCACTATG---GGCGCAGCGTCA---
ATGACGCTGACGGTACAG---GCCAGACTA---TTACTG---TCTGGTATAGTGCAACAGCAAAAC---
AATTTG---CTGAGA---GCTATTGAGGCGCAACAACATCTGTTGCAACTCACAGTCTGGGGCATT
>KY113882
TATCACAATGAGACTACGAGTGTACCGGAG---

GGAAATGAAACAATCACACTTCCATGCAGAATAAAACAAATTATAAACATGTGGCAGGAAGTAGGGAGAGCAA
CGTATGCCCCTCCCATCAAAGGACAAATTAGATGTTCATCAAAT---
ATTACAGGGCTGCTACTAACAAGGGATGGAGGTATTGGGAATGAGACTGAT----------------------
----

AATAATACTGAGATCTTCAGACCTGGAGGAGGAGATATGAGGGACAATTGGAGAAGTGAATTATATAAATATA
AAGTAGTAAGAATTGAACCATTG---GGAGTAGCACCCACCAAGGCAAAGAGGAGAGTGGTG------------
----------------------------CAAAGAGAAAAAAGAGCA---GTGGGA---ATAGGAGCT------
---ATGTTCCTTGGGATCTTAGGAGCA---GCAGGAAGCACTATG---GGCGCAGCGTCA---
TTGACGCTGACGGTACAG---GCCAGACTA---TTATTG---TCTGGTATAGTGCAACAGCAGAAC---
AACTTG---CTGAGG---GCTATTGAGGCGCAACAGCATCTGTTGCAACTCACTGTCTGGGGCATC
>K02007

```
AATCACACTGAAGGAACTAAA----------------------------------------------------
--------------------------------------------------------
GGAAATGACACAATCATACTCCCATGTAGAATAAAACAAATTATAAACATGTGGCAGGAAGTAGGAAAAGCAA
TGTATGCCCCTCCCATTGGAGGACAAATTAGTTGTTCATCAAAT---
ATTACAGGGCTGCTATTAACAAGAGATGGTGGTACAAATGTAACT----------------------------
-------------------------------------------------------------------------
----------
AATGACACCGAGGTCTTCAGACCTGGAGGAGGAGATATGAGGGACAATTGGAGAAGTGAATTATATAAATATA
AAGTAATAAAAATTGAACCATTA---GGAATAGCACCCACCAAGGCAAAGAGAAGAGTGGTG-----------
----------------------------CAGAGAGAAAAAAGAGCA---GTGGGAATAGTAGGAGCT------
---ATGTTCCTTGGGTTCTTGGGAGCA---GCAGGAAGCACTATG---GGCGCAGTGTCA---
TTGACGCTGACGGTACAG---GCCAGACAA---TTATTG---TCTGGTATAGTGCAACAGCAGAAC---
AATTTG---CTGAGG---GCTATTGAGGCGCAACAACATCTGTTGCAACTCACAGTCTGGGGCATC
>HQ110679
ACTCAGGGGTTAAATAAC-------------------------------------------------------
-----------------------------ACT---
GGAAGAAATGACACAATCACACTCCCATGCAGAATAAAACAAATTGTAAACATGTGGCAGGAAGTAGGAAAAG
CAATGTATGCCCCTCCCATCAAAGGAAATATTAGCTGTTCATCAAAT---
ATTACAGGGCTGCTATTAACAAGAGATGGTGGTAGTGATGGTGGTAATAAC----------------------
-------------------------------------------------------------------------
-------
ACGAACGGGAATGAGACCTTCAGACCTGGAGGGGGAGATATGAGGGACAATTGGAGAAGTGAATTATATAAAT
ATAAAGTAGTAAGAATTGAACCATTA---GGAGTAGCACCCACCAAGGCAAAGAGAAGAGTGGTG--------
-------------------------------CAAAGAGAAAAAAGAGCG---GTGGGA---CTAGGAGCT---
------ATGTTCATTGGGTTCTTGGGAGCA---GCAGGAAGCACTATG---GGCGCAGCGTCA---
GTGACGCTGACGGTACAG---GCCAGGCAA---TTATTG---TCTGGTATAGTGCAACAGCAGAGC---
AATTTG---CTGAGA---GCTATTGAGGCGCAACAGCATCTGTTGCAACTCACAGTCTGGGGCATC
>AY713410
ACTCAAGGG----------------------------------------------------------------
--------------------------------------
AACACTGGAAATATCACACTCCCATGCAGAATAAAACAAATTATAAACAGGTGGCAGGAAGTAGGAAAAGCAA
TGTATGCCCCTCCCATCGAAGGAAAAATTAGATGTTCATCAAAT---
ATTACAGGGTTACTATTAACAAGAGATGGTGGTAAGAATAACGAGACC-------------------------
-------------------------------------------------------------------------
----------
AATAACACCGAGACCTTCAGACCTGGAGGAGGAGATATGAAGGACAATTGGAGAAGTGAATTATATAAATATA
AAGTAGTAAAAATTGAGCCATTA---GGAGTAGCACCCACCAAGGCAAAGAGAAGAGTGGTG-----------
----------------------------CAGAGAGAAAAAAGAGCA---GTGACA---CTAGGAGCT------
---ATGTTCCTTGGGTTCTTGGGAGCA---GCAGGAAGCACTATG---GGCGCAGCGTCA---
GTGACGCTGACGGTACAG---TCCAGACAA---TTATTG---TCTGGTATAGTGCAACAGCAAAAC---
AATTTG---CTGAGA---GCTATTGAGGCGCAACAGCATCTGTTGCAACTCACAGTCTGGGGCATT
>EU575593
AATTATTTTGATAACACTAAAGATGAT----------------------------------------------
--------------GGGGCTAATGGGACTGCT---------
AATGGGACTGGAGTTATCATACTTCCATGCAGAATAAAACAAATTGTAAACATGTGGCAGGAAGTAGGAAAAG
CAATGTATGCCCCTCCCATCAGGGGACGAATTAAATGTTTATCAAAT---
ATTACAGGACTGATGTTAACAAGAGATGGTGGTAATAATGGTGGTAACACAACT-------------------
-------------------------------------------------------------------------
-
GAAAACGCCACCGAGACCGAGACCTTCAGACCTGGAGGAGGAGATATGAGGGACAATTGGAGAAGTGAATTAT
ATAAATATAAAGTAGTAAAAATTGAACCATTA---GGAGTAGCACCTACCAAGGCAAAGAGAAGAGTGGTG--
-------------------------------------CAAAGAGAAAAAAGAGCA---GTAGGA---
ATAGGAGCT---------TTGTTCCTTGGGTTCTTGGGAGCA---GCAGGAAGCACTATG---
GGCGCAGCGTCA---GTGACGCTGACGGTACAA---GCCAGACAG---TTATTG---
TCTGGTATAGTGCAACAGCAGAGC---AATCTG---CTGAGG---
GCTATTGAGGCGCAACAGCATCTGTTGCAACTCACAGTCTGGGGCATC
>AY331289
ACTCAGACTAAT-------------------------------------------------------------
--------------------------AGC---
TGGCCAAATGCCACTATCACACTTTCATGCAGAATAAAACAGTTTATAAACATGTGGCAGGAAGTAGGAAAAG
```

CAATGTATGCCCCTCCCATTAGGGGACCAATAAGTTGTTCATCAAAT---
ATTACAGGGTTGCTATTAACAAGAGATGGTGGTAACGGGAACGGCACC-------------------------
----

AACAACAACACCGAGGTCTTCAGACCTACAGGAGGAGATATGAGGGACAATTGGAGAAGTGAATTATATAAAT
ATAAAGTAGTAAGAATTGAACCAATA---GGAGTAGCACCCACCAAGGCAAAGAGAAGAGTGGTG--------
-------------------------------CAGAGAGAAAAAAGAGCA---GTGGGAACAATAGGAGCT---
------ATGTTCCTTGGGTTCTTGGGAGCA---GCAGGAAGCACTATG---GGCGCAGCGTCA---
ATAACGCTGACGGTACAG---GCCAGACTA---TTACTG---TCTGGTATAGTGCAACAGCAGAAC---
AATCTG---CTGAGA---GCTATTGAGGCACAACAACATCTGTTGCAACTCACAGTCTGGGGCATC
>EU575697
ACTCAACTGTTTAATAGTACTTGGAATGGTAATGATACTGAAGGGTCAAATACC-------------------
----

ACTGAAGAAAATATCACACTCCCATGCAGAATAAAACAAATCATAAACATGTGGCAGGAAGTAGGAAAAGCAA
TGTATGCCCCTCCCATCAGAGGACAAATTAGCTGTTCATCAAAT---
ATTACAGGGCTGTTGTTAACAAGAGATGGTGGCGCAGATAGTACA----------------------------
----

AACGAGACCTTCAGACCTGGAGGAGGAGATATGAGGGACAATTGGAGAAGTGAATTATATAAATATAAAGTAG
TAAGAATTGAACCATTA---GGAATAGCACCCTCCAAGGCAAAGAGAAGAGTGGTG-----------------
----------------------CAGAGAGAAAAAAGAGCA---GTGGGA---ATAGGAGCT---------
GTGTTCCTTGGGTTCTTGGGAGCA---GCAGGAAGCACTATG---GGCGCAGCGTCA---
ATGGCGCTGACGGTACAG---GCCAGACAA---TTATTG---TCTGGTATAGTGCAACAGCAGAAC---
AATTTG---CTGAGG---GCTATTGAGGCGCAACAGCATATGTTGCAACTCACAGTCTGGGGCATC
>EU577469
ACTCAA---

GGAAATGACACAATCATACTCCCATGCAGAATAAAACAAATTATAAACATGTGGCAGAGAGTAGGGAAAGCAA
TGTATGCCCCTCCTATCGGAGGACACCTTAATTGTTCATCAAAT---
ATTACAGGGCTGCTATTCACAAGAGATGGTGGCAACAGTAGTGAT----------------------------
----

AACAGTGAGACCTTCAGACCTGGAGGAGGAGATATGAGGGATAATTGGAGAAGTGAATTATATAAATATAAAG
TAGTAAAACTTGAACCCTTA---GGAGTAGCCCCCACCAAGGCCAAGAGAAGAGTGGTG--------------
-------------------------CAAAGAGAAAGAAGAGCA---GTGGGT---TTAGGAGCT---------
ATGTTCCTTGGGTTCTTGGGAGCC---GCAGGAAGCACTATG---GGCGCAGCGTCA---
ATAACGCTGACGGTACAG---GCCAGACAA---TTATTG---TCTGGTATAGTGCAACAGCAGAAC---
AATCTG---CTGAGG---GCTATTGAGGCGCAACAGCATATGTTGCAACTCACAGTCTGGGGCATC
>MN516274
GATCAAAAATATACTGCATGGGCAAATAGCACTGAGTCAAAT-------------------------------
----
GACACTGAAGTAATCACACTCCCATGCAGAATAAAACAAATTATAAACCTGTGGCAGGAAGTAGGGAAAGCAA
TGTATGCCCCTCCCATCAAAGGACAGATTAGATGTTCATCAAAT---
ATTACAGGGCTGCTACTAACAAGAGATGGTGGTAAGAACGAGAGTAGA-------------------------
----

GAGAATGAGACCTTCAGACCTGGAGGAGGAGATATGAGGGACAATTGGAGAAGTGAATTATATAAATATAAAG
TAGTAAAAATTGAACCATTG---GGAGTAGCACCCACCAAGGCAAAGAGAAGAGTGGTG--------------
-------------------------CAAAGAGAAAAAAGAGCA---GTGGGA---ATAGGAGCT---------
TTGTTCCTTGGGTTCTTGGGAGCA---GCAGGAAGCACTATG---GGCGCAGCGTCA---
GTGACGCTGACGGTACAG---GCCAGACTA---TTATTG---TCTGGTATAGTGCAACAGCAGAAC---
AACTTG---CTGAGA---GCTATTGAGGCGCAACAGCATCTGTTGCAACTCACAGTCTGGGGCATC
>GU728005
AATCAAACTGGG---

AGCAATGATACAATTATACTCCCATGTAGAATAAAGCAAATTATAAACCTGTGGCAGGAAGTAGGAAAAGCAA
TGTATGCCCCTCCCATCAGTGGAGTAATTAATTGCACATCAAAT---
ATTACAGGGCTACTATTGACAAGAGATGGTGGTAATAATATTACT----------------------------
----

AACGGGAGCGAGATCTTCAGACCTGGAGGAGGAGATATGAGGGATAATTGGAGAAGTGAATTATATAAATATA
AAGTAGTAAGACTTGAACCATTA---GGAGTAGCACCCACCAGGGCAAAGAGAAGAGTGGTG----------
---------------------------CAGAGAGAAAAAAGAGCA---GTGGGA---CTAGGAGCT------
---TTGTTCCTTGGGTTCTTGGGAGCA---GCAGGAAGCACTATG---GGCGCAGCGTCA---
CTGACGCTGACGGTACAG---GCCAGACTA---CTACTG---TCTGGTATAGTGCAACAGCAGAAC---
AATCTG---CTGAGG---GCTGTTGAAGCGCAACAACATCTGTTGCAACTCACAGTCTGGGGCATC
>GU367405
ACTCAAGGA---

AATGATACTACTATCACACTCCAATGCAAAATAAAACAAATTATAAATATGTGGCAGGAAGTAGGAAAAGCAA
TGTATGCCCCTCCCATCAGAGGATCAATTAAATGCCTATCAAAT---
ATTACAGGGCTGATATTAACAAGAGATGGT---AACGGACCGAGAGC-------------------------
----

ACAAACGAGACCGAGATCTTCAGGCCTGGAGGAGGAGATATGAGGGACAATTGGAGGAGTGAATTATATAAAT
ATAAAGTAGTAAGAATTGAACCATTA---GGAGTAGCACCCACCAAGGCAAAGAGAAGAGTGGTG-------
------------------------------CAGAGAGAAAAAAGAGCA---GTGGGA---CTAGGAGCT---
------TTGTTCCTTGGGTTCTTGGGAGCA---GCAGGAAGCACTATG---GGCGCAGCGTCG---
GTGACGCTGACGGTACAG---GCCAGACTA---TTGCTG---TCTGGTATAGTGCAACAGCAAAAT---
AATTTG---CTGAGG---GCTATTGAGGCGCAACAGCATCTGTTGCAACTCACAGTCTGGGGCATC
>AY795904
ACTCAAATGTTA--

GATAATATCACACTCACACTCCCATGCAGAATAAAACAAATCATAAACATGTGGCAAGAAGTAGGAAAAGCAA
TGTATGCCCCTCCCATTGCAGGAAAAATTAGCTGTTCATCAAAT---
ATTACAGGGATACTATTAACAAGGGATGGTGGTAACGAGAACCAG---------------------------
----

ACCACCCCCGAGGTCTTCAGACCTGCAGGAGGAGATATGAGGGACAATTGGAGAAGTGAATTATATAAATATA
AAGTAGTAAGAGTTGAGCCATTA---GGAATAGCACCCACCAAGGCAAAGAGAAGAGTGGTG----------
---------------------------CAGAGAGAAAAAAGAGCA---ATAGGA---ATGGGAGCT------
---GTGTTCCTTGGGTTCTTGGGAGCA---GCAGGAAGCACTATG---GGCGCAGCGTCA---
ATAACGCTGACGGTACAG---GCCAGACAA---TTATTG---TCTGGTATAGTGCAACAGCAAAGT---
AATTTG---CTGAGA---GCTGTTGAGGCGCAACAACATCTGTTGCAACTCACAGTCTGGGGCATC
>KF725981
AATTATACTTGGAATAATACTACTGAGAGT--

GATAACGGTACTATCATACTCCAGTGCAGAATAAAGCAAATTGTAAACAGGTGGCAGGAAGTAGGAAAAGCAA
TGTATGCCCCTCCCATCAGTGGACAAATTAATTGTTCATCAAAT---
ATTACAGGGCTACTACTAACAAGAGATGGTGGTATCAGTAACACAAGTAGT---------------------
----

GATAACACAAGTGAGACCTTTAGACCTGCAGGAGGAAATATGAAGGACAATTGGAGAAGTGAATTATATAAAT
ATAAAGTAGTAAAAATTGAGCCATTA---GGAATAGCACCCACCAAGGCAAAGAGAAGAGTGGTG-------
------------------------------CAGAGAGAAAAAAGAGCA---GTGGGAACATTAGGAGCT---
------ATGTTCCTTGGGTTCTTGGGAGCA---GCAGGAAGCACTATG---GGCGCAGCGTCA---
TTGACGTTGACGGTACAA---GCCAGACTA---CTATTG---TCTGGTATAGTGCAACAGCAAAAC---
AATTTG---CTGAGA---GCTATTGAGGCGCAACAGCATATGTTGCAACTCACAGTCTGGGGCATC
>MH234639
AATCATACTAAA--

GGAGAAGATATCATCACACTCCCATGTAGAATAAAGCAAATTATAAACATGTGGCAGCAAGTGGGAAAAGCAA
TGTATGCCCCTCCCATCAGTGGACAAATTAGATGTTCATCAAAT---
ATTACAGGGCTGCTATTAACAAGAGATGGTGGTAATAACAATGAC---------------------------
----

ACCACCGAGGTCTTCAGACCTGGAGGAGGAGATATGAGGGACAATTGGAGAAGTGAATTATATAAATATAAAA
TAGTAAAAATTGAACCATTA---GGAGTAGCACCCACCAGGGCAAAGAGAAGAGTGGTG-------------
-------------------------CAGAGAGAAAAAAGAGCA---GTAACA---TTAGGAGCT---------

ATGTTCCTTGGGTTCTTGGGAGCA---GCAGGAAGCACTATG---GGCGCAGCGTCA---
ATGACGTTGACGGTACAG---GCCAGACGA---CTATTG---TCTGGTATAGTGCAACAGCAGAAC---
AATTTG---CTGAGG---GCTGTTGAGGCGCAACAGCATATGTTGCAACTCACAGTCTGGGGCATC
>MH746247
AAT--

CATAATAATACTATCATACTCCCATGCAGAATAAAACAATTTATAAACATGTGGCAGAGAGTAGGACAAGCAA
TGTATGCCCCTCCCATTGAAGGAAACATAACATGTGAATCAAAT---
ATCACAGGACTACTCTTGGTACGTGATGGAGGCAACACAACAGAAACT-------------------------
----

AACAAAGAGACATTCAGACCGGGAGGAGGAGATATGAGGGACAATTGGAGAAGTGAACTATATAAATATAAAG
TGGTAGAAATTAAGCCATTG---GGAATAGCACCCACCAAGGCAAAAAGGAGAGTGGTG--------------
-------------------------GAGAGAGAAAAAAGAGCA---GTGACA---CTAGGAGCT---------
ATGTTCCTTGGGTTCTTGGGAACA---GCAGGAAGCACTATG---GGCGCAGCGTCA---
ATAACGCTGACGGTACAG---GCCAGACAA---TTGCTG---TCTGGGATAGTGCAACAGCAAAGC---
AATTTG---CTGAGA---GCTATAGAGGCGCAACAGCATATGTTGCAACTCACGGTCTGGGGCATT
>KX181928
ACTTACTGTCCTAATAATCCTAATGCAACATATAATGCAACA-------------------------------
----
GAAAATTCAACCATCACAATCCAATGCAAAATAAAACAAATTGTAAACATGTGGCAGGAGGTAGGACGAGCAA
TGTATCCCCCTCCCATTAAAGGACCAATAACATGTGCATCAAAT---
ATCACAGGACTATTGCTGACACGTGATGGAGGAGCAAACTCAAAT----------------------------
----

AACACAGAGACATTCAGACCTGGAGGAGGAGATATGAAGGACAATTGGAGAAGTGAATTATATAAATATAAAG
TGGTAGAAATTAAACCATTA---GGAATAGCACCCACCAAGGCAAAAAGGAGAGTGGTG--------------
-------------------------GAGAGAGAAAAAAGAGCA---GTGGGA---ATAGGAGCT---------
GTGTTCCTTGGGTTTTTGGGAGCA---GCCGGAAGCACTATG---GGCGCAGCGTCA---
ATAACGCTGACGGTACAG---GCCAGGCAA---TTATTG---TTTGGGATAGTGCAACAGCAAAGC---
AATTTG---TTGAGG---GCTATAGAGGCGCAACAGCATATGTTGCAACTCACGGTCTGGGGCATT
>KX181936
AATTATCCAAACATAGGAAACAGCACATATAATGATACAGAAAACAGAAACAGC-------------------
---ACATAT------
AATGATACAGAAAACATCACAATCCCATGCAAAATAAAACAATTTATAAACATGTGGCAGGAGGTAGGGCGAG
CCATGTATGCCTCTCCCATTGAAGGAGTGATAACATGTAAATCAAAT---
ATCACAGGATTTCTACTGACCCGGGATGGGGGCAAAACCACAAAG----------------------------
----

AACGAGACATTCAGGCCCGAAGGAGGAAATATGAGGGACAATTGGAGAAGTGAATTATATAAGTATAAAGTGG
TAGAAATTAAACCATTA---GGAATAGCACCCACTAAAGCAAAAAGGAGAGTGGTG-----------------
----------------------GAGAGAGAAAAAAGAGCA------GGA---ATAGGAGCT---------
GTGTTCCTTGGGTTCTTGGGAGCA---GCAGGAAGCACTATG---GGCGCAGCATCA---
ATAACGCTGACGGTACAG---GCCAGACAA---TTATTG---TCTGGGATAGTGCAACAGCAAAGC---
AATTTG---CTGAGG---GCTATAGAGGCGCAACAGCATATGTTGCAACTCACGGTCTGGGGCATT
>KX181937
CCTCATAGTACAAGTACAAGCAGTACTCCTGGA--

GCAGAAAACGACACACTCACAATCCAATGCAGAATAAAACAAATTGTAAACATGTGGCAGGAGGTAGGACGAG
CAATGTATGCCCCTCCCATTGCAGGAAACATAACATGTAACTCAAGT---
ATCACAGGGCTATTACTGACACGTGATGGGGGAGAAAATAGAACA----------------------------
----
-
GAGAAAGGCACAAAGGAGGAGGAATTCAGGCCTGGGGGGGGAGATATGAGGGACAATTGGAGAAGTGAATTAT
ATAAATACAAAGTAGTAGAAATTAAACCCTTT---GGAATAGCCCCCCCCAAGGCAAAAAGGAGGGTGGTG--
-------------------------------------GAGAGAGAAAAAAAAGCA---GCA------
TTAGGAGCT---------GTGTTCCTTGGGTTTTTGGGAGCA---GCAGGAAGCAATATG---
GGGGCAGCGTCA---GTAGCGCTGACGGTACAG---GCCAGACAA---TTATTG---
TCTGGGATAGTGCAACAGCAAAGC---AATTTG---CTGAAG---
GCTATAGAGGCGCAACAGCATATGTTGCAACTCACAGTCTGGGGCATT

>KX181941
AATTACAAGGTTAATAGTGCAAACAAGGATACAAAA------------------------------------
----
AGTAATGGAACAATCACAATCCCATGCAGAATAAAACAAATTATAAATATGTGGCAGGGGGTGGACGGGCAA
TGTATGCCCCTCCCATTGCAGGAATAATATCATGTAACTCAAGT---
ATCACAGGATTACTACTGACCCGGGATGGCGGCAACAATAACAACACA------------------------
----

GACACAGAGATATTCAGGCCTGGAGGAGGAAATATGAGGGACAATTGGAGAAGTGAGTTATATAAATATAAAG
TAGTAGAAATTAAGCCATTA---GGAATAGCACCCACTAAGGCAAAAAGGAGAGTGGTG-------------
------------------------GGGAGAGAAAAAAGAGCA---GCGGGA---ATAGGAGCT---------
GTGTTCCTTGGGTTCTTGGGAGCA---GCAGGAAGCACTATG---GGCGCAGCATCA---
ATGATGCTGACGGTACAG---GCCAGACAA---TTATTG---TCTGGGATAGTGCAACAGCAAAGC---
AATTTG---CTGAGG---GCTATAGAGGCGCAACAGCATATGTTGCAACTCACGGTCTGGGGCATA
>MK041572
TGCTATACATCAGGCCTGTTTAATAGTACATACAATAGTAATAGTGCAAAAAACAGTAATGAGACA-------
----

GGCAATTCAACCATCACAATCCCATGCAGAATAAAACAAATTATAAACATGTGGCAGGAGGTAGGACGAGCAA
TGTATGCCCCCCCCATTAGTGGAAACATAACATGTAGATCAAAT---
ATCACAGGATTACTGCTGACACGGGATGGAGGTAATACTGGCTCG---------------------------
----

AATGAGACAGAGATATTCAGGCCTGGAGGAGGAGATATGAGGGAGAATTGGAGAAGTGAATTATATAAGTATA
AAGTGGTAGAAATTAAACCATTA---GGAATAGCACCCACTGAGGCAAAAAGGAGAGTGGTG----------
---------------------------GAGAGAGAAAAAAGAGCA---GTGGGA---ATAGGAGCT------
---GTGTTCCTTGGGTTCTTGGGAGCA---GCAGGAAGCACTATG---GGCGCAGCATCA---
ATAACGCTGACGGTACAG---GCCAGACAA---TTATTG---TCTGGGATAGTGCAACAGCAAAGC---
AATTTG---CTGAGG---GCTATAGAGGCGCAACAGCATATGTTGCAACTCACGGTCTGGGGCATT
>AF443091
TGCTATACAACAAAACTGTTTAATAGTACATACAATAGTACATACAATGATACAGGA---------------
----

AGTGAGTCAAACATCACGATTCCATGCAGAATAAAACAAATTATAAACATGTGGCAGGGGGTAGGACAAGCAA
TGTATGCCCCTCCCATTGCAGGAAACATAACATGTAGATCAAAC---
ATCACAGGACTACTATTGACACGTGATGGAGGAATAGGATTAAATGAAGAT---------------------
----

GATAATAACACAGAGATATTCAGACCTGGAGGAGGAAATATGAGGGACAATTGGAGAAGTAAATTATATAAAT
ATAAAGTAGTAGAAATTAAACCATTA---GGAGTAGCACCCAATAAGGCAAAGAGGAGAGTGGTG-------
------------------------------GAGAGAGAAAAAAGAGCA---GTGGGA---CTAGGAGCT---
------GTGTTCCTTGGATTCTTAGGAGCA---GCAGGAAGCACTATG---GGCGCAGCATCA---
ATAACGCTGACGGTACAG---GCCAGACAA---TTATTA---TCTGGTATAGTGCAACAGCAAAAC---
AATCTG---ATGAGA---GCTATAGAGGCGCAACAACATATGTTGCAACTCACAGTCTGGGGCATT
>AF443102
AGTTAT--

AATAGTACAGCAAACATCACACTCCCATGCAGACTAAAACAAATTATAAACATGTGGCAGAAGGTAGGACGAG
GAATATATGCCCCTCCCATTGAGGGAAGCATAACATGTAACTCAAAT---
ATCACAGGACTACTCTTGGTACGGGATGGAGGCATAAATACGAGT---------------------------
----

ACAGTGGAGACATTCAGACCAGAAGGAGGAAATATGAGGGACAATTGGAGAAGTGAACTATATAAATATAAAG
TGGTAGAAATTAAGCCATTG---GGAGTAGCGCCCACTGAGGCACAAAGGAGAGTGGTG-------------
------------------------GAGAAACAAAAAAGAGCA---GTGGGA---ATGGGAGCT---------
GTGTTCCTTGGGTTCTTGGGAGCA---GCAGGAAGCACTATG---GGCGCGGCGTCA---
ATAACGCTGACGGTACAG---GCCAGACAA---CTGTTG---TCTGGAATAGTGCAACAGCAAAGC---
AATTTG---CTGAGA---GCTATAGAGGCGCAACAGCATATGTTGCAACTCACAGTCTGGGGCATT
>AF443103

AGCTATTCAGAACGGCATAATAACACA--

TCAAATTCAACCATCACACTCCCATGCAATATAAAACAAATTATAAACATGTGGCAGGGGGTAGGACGAGCAA
TGTACGCCCCTCCCATTGCAGGAAACATAACATGTACATCAAAT---
ATCACAGGACTAATATTGACACGTGATGGAGGAGGAGGAAATGAAACT-------------------------
----

AATGAAACAGAGACATTCAGACCTGCAGGAGGAGATATGAGGGACAATTGGAGAAGTGAATTATATAAATATA
AAGTGGTAGAAATTAAGCCATTA---GGACTAGCACCCACTAAGGCAAAAAGGAGAGTGGTG-----------
----------------------------GAGAGAGAAAAAAGAGCA---GTAGGA---ATAGGAGCT------
---GTGTTCCTTGGGTTCTTGGGAGCA---GCAGGAAGCACTATG---GGCGCGGCGTCA---
ATAACGCTGACGGTACAG---GCCAGACAA---CTGTTG---TCTGGTATAGTGCAACAGCAAAGC---
AATTTG---CTGAGG---GCTATAGAGGCGCAACAGCATATGTTGCAACTCACGGTCTGGGGCATT
>AF443105
TACTATCCTAATGCTACAAAAAGCGAT--

ACTACAGAAACCATCACACTCCCATGCAGAATAAAACAAATTGTAAACATGTGGCAGGGGGTAGGACGGGCAA
TATATGCCCCTCCCATTGCAGGAAACATAACATGTAACTCAAGT---
ATCACAGGATTACTATTGCTACGTGATGGAGGGACAGATAACGAGACAGAG----------------------
----

AACAACAGGACAGAGACATTCAGACCTGGAGGAGGGGATATGAGAGATAATTGGAGAAGTGAATTATATAAAT
ATAAAGTGGTAGAAATTAAGCCATTA---GGAGTAGCACCCACTAAGGCAAAAAGGAGAGTGGTG--------
-------------------------------GAGAGAGAAAAAAGAGCA---GTAGGA---ATGGGAGCT---
------GTGATCCTTGGGTTCTTGGGAGCA---GCAGGAAGCACTATG---GGCGCGGCGTCA---
ATAACGCTGACGGTACAG---GCCAGACAA---CTGTTG---TTTGGTATAGTGCAACAGCAAAAC---
AATTTG---CTGAGG---GCTATAAAAGCGCAACAGCATCTGTTGCAGCTCACGGTCTGGGGCATT
>AF443107
AACTACAATACAAGCTCAAACTCATCC--

AACAACTCAAACATCACACTCCCATGCAGAATAAAACAAATTATAAATATGTGGCAGGGGGTAGGAAGAGCAA
TATATGCCTCTCCCATTGAAGGAAGCATAACATGTAAATCAAAT---
ATCACAGGACTACTGTTGGTACATGATGGAGGTAACAGTAACACG----------------------------
----

AGTACAGAGATATTCAGACCTGGAGGAGGAGATATGAGGGACAATTGGAGAAGTGAATTATATAAATATAAAG
TGGTAGAAGTTAAGCCATTG---GGAATAGCACCCACTGAGGCAAAAAGGAGAGTGGTG--------------
-------------------------GAGAGAGAAAAAAGAGCA---GTGGGA---ATAGGAGCT---------
GTGTTCCTTGGGTTCTTGGGAGCA---GCAGGAAGCACTATG---GGCGCGGCGTCA---
ATAACGCTGACGGTACAG---GCCAGACAA---CTGTTG---TTTGGTATAGTGCAACAGCAAAGC---
AATTTG---CTGAGG---GCTATAGAGGCGCAACAGCATATGTTGCAACTCACGGTCTGGGGCATT
>AF443108
CCTTACAATGATAATGAT---

ACAGACTCAGACATCACACTTCCATGTAAAATAAAACAAATTATAAACATGTGGCAGGAGGTAGGACGGGCAA
TATATGCCCCTCCCATTGCAGGAAACATAACATGCACATCAAAC---
ATTACAGGACTACTATTGACACGTGATGGAGAGCCAAACAACTCC----------------------------
----

ACAGAGACATTCAGACCTGGAGGAGGAGATATGAGAGACAATTGGAGAAGTGAATTATATAAATATAAAGTGG
TAGAAGTTAAACCATTG---GGAATAGCACCCACTGGAGCAAAAAGGAGAGTGGTG-----------------
----------------------GAGAGAGAGAAAAGAGCA---GTAGGA---ATAGGAGCG---------
GTGTTCCTTGGGTTCTTGGGAGCA---GCAGGAAGCACTATG---GGCGCGGCGTCA---
ATAACGCTGACGATACAG---GCCAGACAA---CTGTTG---TCTGGTATAGTGCAACAGCAAAGC---
AATTTG---CTGAGG---GCTATAGAGGCGCAACAGCATCTGTTGCAACTCACGGTCTGGGGCATT
>KC154012
TGCTATACAACAAACTTGTTTAATACTAGTCTGATTAATACTAATAACACT----------------------
----

GATAATGGCACAATCATACTCCCATGCAAAATAAAACAGATTATAAACATGTGGCAAGAGGTAGGACGAGCAA

TGTATGCCCCTCCCATTGAAGGAAACATAACATGCAACTCAACT---
ATCACAGGACTACTATTGCTACGTGATGGAGGCAATGGCAATGAGACC-------------------------
----

AAAGGGAATGAGACATTCAGACCTGGAGGAGGAAATATGAAAGATAATTGGAGAAGTGAATTATATAAATATA
AAGTGGTAGAAATTAAACCATTG---GGAATAGCACCCACTGAGGCAAAAAGGAGAGTGGTG-----------
----------------------------GAGAGAGAAAAAAGAGCA---GCTACA---ATAGGAGCT------
---GTGCTCCTTGGGTTCTTGGGAGCG---GCAGGAAGCACTATG---GGCGCGGCGTCA---
ATAACGCTGACGGTACAG---GCCAGACAA---TTGTTG---TCTGGTATAGTGCAACAGCAAAGC---
AATTTG---CTGAGA---GCCATAGAGGCGCAACAGCACATGTTGCAACTCACAGTCTGGGGCATT
>KC154014
AATCAGATTAACAGTACAGGAAATACT--

TCAGATTCAAACATCACACTCCCATGCAGAATAAAACAAATTATAAACATGTGGCAGGGAGTAGGACGAGCAA
TGTATGCCCCTCCCATTGCAGGAAACATAACATGTAAATCAAAT---
ATCACAGGACTAATATTGACACGTGATGGAGGAACCCATAACACTAATGATACT-------------------
----

GATACCCCAGAGATATTCAGGCCTGCAGGAGGAGATATGAGGGACAATTGGAGAAGTGAATTATATAAATATA
AAGTGGTAGAAATTAAGCCATTA---GGACTAGCACCCACTAAGGCAAAAAGGAGAGTGGTG-----------
----------------------------GAGAGAGAAAAAAGAGCA---GTGGGA---ATAGGAGCT------
---GTGTTCCTTGGGTTCTTGGGAGCA---GCAGGAAGCACTATG---GGCGCGGCGTCA---
ATAACGCTGACGGTACAA---GCCAGACAA---CTGTTG---TCTGGTATAGTGCAACAGCAAAGC---
AATTTG---CTGAGG---GCTATAGAGGCGCAACAGCATATGTTGCAACTCACGGTCTGGGGCATT
>KF114881
AGTTACCCTAACATTGGTGATACAGAAAAGAAT--

TCAAGCTCAATCATCACACTCCCATGCAGAATAAAACAAATTATAAACATGTGGCAGGGGGTAGGACGAGCAA
TATATGCACCTCCCATTGCAGGAAAAATAACATGTAAATCAAAT---
ATCACAGGACTCCTGTTGACACGTGATGGAGGAAATAGTAATGAGACA-------------------------
----

AATACTACAGAGATATTCAGACCTGGAGGAGGAAATATGAAAGATAATTGGAGGAGTGAATTATATAAATATA
AAGTGGTAGAAATTAAACCATTA---GGATTAGCACCCAATAAGGCAAAAAGGAGAGTGGTG-----------
----------------------------GAGAGACAAAAAAGAGCA---GTAGGA---ATAGGAGCT------
---GTGTTCCTTGGGTTCTTGGGAGCA---GCAGGAAGCACTATG---GGCGCGGCGTCA---
ATAACGCTGACGGTACAG---GCCAGACAA---TTATTG---TCTGGTATAGTGCAACAGCAAAGC---
AATTTG---CTGAGA---GCTATAGAGGCGCAACAGCATCTGTTGCAACTCACAGTCTGGGGCATT
>KR861329
CCTCAGGCAAATGTAAGTGAG--

TCAAATTCAACCATCACACTCCCCTGCAGAATAAAGCAAATTATAAACATGTGGCAGAGGGTGGGAGGAGCAA
TATATGCCCCTCCCATTGCAGGAAACATAACATGTAAATCAAAT---
ATCACGGGACTGCTTTTGGTACGTGATGGAGGTACAAATAAGACA----------------------------
----

GAGCCAGAGACATTCAGACCTGGAGGAGGAGATATGAGGGACAATTGGAGAAGTGAATTGTATAAATATAAAG
TGGTAGAAATTAAGCCATTA---GGAGTAGCACCCACTAAGGCAAGAAGGAGAGTGGTG--------------
-------------------------GAAAGAGAAAAAAGAGCA---GTGGGA---ATAGGAGCT---------
GTGTTCCTTGGGTTCTTGGGAGCA---GCAGGAAGCACTATG---GGCGCGGCGTCA---
ATAACGCTGACGGTACAA---GCCAGACAG---TTATTG---TCTGGTATAGTGCAACAGCAAAGC---
AATTTG---CTGAGG---GCTATAGAGGCGCAACAGCATTTGTTGCAACTCACAGTCTGGGGCATT
>KR861340
AGTCATAACAGTACAAACAGTAATGAATATAATGTAGATAATGGT----------------------------
----
ACAGACAGAAACATCACACTCCCATGCAGAATAAAACAAATTATAAACATGTGGCAGAAGGTAGGAAGAGCAA
TGTATGCCCCTCCTATTAGTGGACTAATAACATGTACCTCAAAT---
ATCACAGGACTACTCTTGGTACGTGATGGG---AACACAAGTGTC----------------------------
----

ACAAATGAGACATTCAGACCAGGAGGAGGAGATATGAGGGACAATTGGAGAAGTGAACTATATAAATATAAAG
TGGTAGAAATTAAGCCATTG---GGAGTAGCGCCCACTGGAGCTAAAAGGAGAGTGGTGGAA-----------
-------------------------AGAAGAGAAAAAAGAGCA---GTGGGA---ATAGGAGCT---------
GTACTCTTTGGGTTCTTGGGAGCA---GCAGGAAGCACTATG---GGCGCAGCGTCA---
ATAGCGCTGACGGCACAG---GCCAGACAA---GTGCTG---TCTGGAATAGTGCAACAGCAAAAC---
AATTTG---CTGAGG---GCTATAGAGGCGCAACAGCATATGTTGCAACTCACAGTCTGGGGCATT
>KR861320
GACTACTGGATAATGGTACCATATCTGGTAAC---

AGTTCAAACATCACCATAATCTCATGCAGAATAAAGCAAATTATAAACATGTGGCAGGGGGTAGGACGAGCAA
TGTATGCCCCTCCCATTGCAGGAAAAATAACATGTAAATCAAAT---
ATCACAGGACTACTATTGTCACGTGATGGGGGTCCAAATGGCACA----------------------------
----

GAAGAAGTATTCAGACCTGGAGGAGGAGATATGAGGGACAATTGGAGAAGTGAATTATATAAATATAAAGTGG
TAGAAATTAAGCCATTA---GGAATAGCACCCACTACTGCAAAAAGGAGAGTGGTG-----------------
----------------------GAAAGAGAAAAAAGAGCA---GTGGGA---ATAGGAGCT---------
GTGCTCCTTGGGTTCTTGGGAGCA---GCAGGAAGCACTATG---GGCGCGGCGTCA---
ATAACGCTGACGGTACAG---GCCAGGCAA---TTGCTG---TCTGGCATAGTGCAACAGCAAAGC---
AATTTG---CTGAAA---GCTATAGAGGCGCAACAGCATCTGTTGCAACTCACGGTCTGGGGCATT
>KR861321
AATTATACACACAATGCAACTGAAAGTGCTATAAAT-------------------------------------
----
-
TCAACAAACATCACCACACTCCATTGCAGAATAAAACAAATTATAAACATGTGGCAGGGGGTAGGGCGGGCAA
TGTATGCCCCCCCCATTGCAGGAAACATAACATGTAGGTCAAAT---
ATCACAGGACTAATATTGACACGTGATGGAGGTAATAAGAATAATAATGAA----------------------
----

ACAACTGGGACAGAGATATTCAGACCTGGAGGAGGAAATATGAGGGACAATTGGAGAAGTGAATTATATAAAT
ATAAAGTGGTGGAGGTTAATCCATTA---GGAGTGGCACCCACTACGGCAAAAAGGAGAGTGGTG--------
-------------------------------GAGAGAGAGAAAAGAGCA---GTGGGA---ATAGGAGCT---
------GTGCTCCTTGGGTTCTTGGGAGCA---GCAGGAAGCACTATG---GGCGCGGCATCA---
ATAACGCTGACGGTACAG---GCCAGACAA---TTATTG---TCTGGTATAGTGCAACAGCAAAAC---
AATTTG---CTGAAG---GCTATAGAGGCGCAACAACATATGTTGCAACTCACAGTCTGGGGCATT
>KR861323
GGTCATAATAGTACAGATAATAGTACAGATAATGGA-------------------------------------
----
AAGTCAAACAAAACCATCACAATACAATGCAAAATAAAACAATTCATAAACCTGTGGCAGGAGGTAGGGCGAG
CAATATATGCCCCTCCCATTGAAGGAAACATAACATGTAAATCAAAT---
ATCACAGGATTACTCTTGACATGGGATGGAGGAGATGATCCAAAA----------------------------
----

GAGTCAAATGACACAGAGATATTCAGACCAGGAGGAGGAGATATGAAGGACAATTGGAGAAGTGAACTATATA
AATATAAAGTGGTAGAAATTAAGCCCTTG---GGAATAGCACCCACTGAGGCAAAAAGGAGAGTGGTG-----
----------------------------------CAGAGAGAAAAAAGAGCA---GTGGGA---
ATGGGAGCT---------GTGCTCCTTGGGTTCTTGGGAGCA---GCTGGAAGCACTATG---
GGCGCAGCGTCA---ATAGCGCTGACGGCACAG---GCCAGACAA---CTGTTG---
TCTGGAATAGTGCAACAGCAAAGC---AATTTG---CTGAGG---
GCTATAGAGGCGCAACAGCATATGTTGCAACTCACGGTCTGGGGCATT
>KR861332
ACCTACCAGGTAAATATTACAAAAGATACTTCAAGTTCA----------------------------------
----
GACAGTAAAAACATCACACTCCAATGCAGAATAAAACAGATTATAAACATGTGGCAGGGAGTAGGACGAGCAA
TGTATGCTCCTCCCATTGAAGGAACCATAACATGTAAATCAAAC---
ATCACAGGACTATTATTGACACGTGATGGAGGAAAACCTAATAAT----------------------------
----

AGTGAAATATTCAGACCTGGAGGAGGGGATATGAGAGATAATTGGAGAAGTGAATTATATAAATATAAAGTGG
TAGAAATTAAGCCATTA---GGGGTAGCACCCACTAAGGCAAAAAGGAGAGTGGTG-----------------

----------------------GAGAGAGAAAAAAGAGCA---GTGGGA---ATAGGAGCT---------
GTGTTCCTTGGGTTCTTGGGAGCA---GCAGGAAGCACTATG---GGCGCGGCGTCA---
ATAACGCTGACGGTACAA---GCCAGACAA---CTGCTG---TCTGGTATAGTGCAGCAGCAAAGC---
AATTTG---CTGAGG---GCTATAGAGGCGCAACAGCATTTGTTGCAACTCACAGTCTGGGGCATT
>KR861334
ACTTACACGCAGCCTAACAGTACAGAAAATACT--

ACAGAGCCAACCATCACACTCAAATGCAGAATAAAACAAATTATAAACATGTGGCAGGAGGTAGGACGAGCAA
TGTATGCCCCTCCCATTGCAGGAAACATAACATGTAGATCAAAT---
ATCACAGGATTAATATTGACACGTGATGGAGGAATTAGTAATGAAGGAAAT----------------------
----

AGGACTAATGAGACATTCAGACCTATAGGAGGAGATATGAGGGACAATTGGAGAAGTGAATTATATAAATATA
AAGTAGTAGAAATTAAGCCATTA---GGACTAGCACCCACTACGGCAAAAAGGAGAGTGGTG-----------
----------------------------GAAAGAGAAAAAAGAGCA---GTGGGA---ATAGGAGCT------
---GTGTTCCTTGGGTTCTTGGGAGCA---GCAGGAAGCACTATG---GGCGCGGCGTCA---
ATAACGCTGACGGTACAG---GCCAGACAA---CTGTTG---TCTGGTATAGTGCAACAGCAAAGT---
AATTTG---CTGAGG---GCTATAGAGGCGCAACAGCATATGTTGCAACTCACGGTCTGGGGCATT
>KR861339
TCCTAC---

CCTAATGACACCATCACCCTCCCATGCAGGATAAAACAAATTATTAACCTGTGGCAGAAGGTAGGACGGGCAA
TGTATGCCCCACCCATTGCAGGAAACATAACATGTAAATCAAAT---
ATCACAGGACTACTATTGACAAGTGATGGAGGTGTAAAAGACAATAAT-------------------------
----

ACTAATAATATTAATACATTCAGACCTGGAGGAGGAAATATGAGGGACAATTGGAGAAGTGAATTATATAAAT
ATAAAGTGGTGGAAGTTAAGCCATTA---GGAATAGCACCCACTGAGTCAAAAAGGAGAGTGGTG--------
-------------------------------GAGAGAGCAAAAAGAGCA---GTGGGA---CTGGGAGCT---
------GTGTTCTTTGGGTTCTTGGGAGCA---GCAGGAAGCACTATG---GGCGCGGCGTCA---
ATAACGCTGACGGTACAG---GCCAGACAA---CTATTG---TCTGGTATAGTGCAACAGCAAAGC---
AATTTG---CTGAGA---GCTATAGAGGCGCAACAACATATGTTGCAACTCACAGTCTGGGGCATT
>KR861342
AGTTATAATGCAACA--

AGCAATAGAACCATCACACTCCCATGCAGAATAAAACAAATTATAAACATGTGGCAGAGGGTAGGACAAGCAA
TATATGCCCCTCCCATTGCAGGAAGAATAACATGTAACTCAAAT---
ATCACAGGAATACTCTTGACACGTGATGGAGGAACAACTAATGACACAGGG----------------------
----

AATGGCACAGAGATATTCAGACCAGGAGGAGGAGACATGAGGGATAATTGGAGAAGTGAACTATATAAATATA
AAGTAGTAGAAATTAAGCCATTG---GGAGTAGCACCCACCAAGGCAAAAAGGAGAGTGGTG-----------
----------------------------GAGAGAGAAAAAAGAGCA---GTAGGA---ATAGGAGCT------
---GTGCTCTTTGGGTTCTTGGGAGCA---GCAGGAAGCACTATG---GGCGCAGCGTCA---
ATAGCGCTGACGGCACAG---GCCAGACAA---GTGCTG---TCTGGAATAGTGCAACAGCAAAGC---
AATTTG---CTGAGG---GCTATAGAGGCGCAACAGCATATGTTGCAACTCACAGTCTGGGGCATT
>KR861328
AGTTATAAT--

GATACAAGAAACATCACACTCCCATGCAGAATAAAACAAATTGTAAATATGTGGCAGGGGGTAGGAAGGGCAA
TATATGCCGCTCCCATTGCAGGACAAATAATGTGCACATCAAAT---
ATTACAGGACTAATATTGACACGTGATGGAGGAAATGGAAATTCT----------------------------
----

ACCCATGAGACATTCAGACCTGAAGGAGGAAATATGAGGGACAATTGGAGAAGTGAATTATATAAATATAAAG
TGGTAGAAATTAAACCATTA---GGAATAGCACCCACTGAGGCAAAAAGGAGAGTGGTG--------------
-------------------------GAGAGAGAAAAAAGAGCA---GTAGGC---CTAGGAGCT---------
GTGTTACTTGGGTTCTTGGGAGCA---GCAGGAAGCACTATG---GGCGCGGCGTCC---
ATGACGCTGACGGTACAG---GCCAGACAA---CTGTTG---TCTGGTATAGTGCAACAGCAAAGC---
AATTTG---CTGAGA---GCTATAGAGGCGCAACAGCATATGTTGCAACTCACAGTCTGGGGCATT

```
>KR861257
AATCATACG----------------------------------------------------------------
-----------------------------------------------
TCAAATTCAAACATCACGCTCCCATGCAGAATAAAACAAATTATAAACATGTGGCAGGAGGTAGGACGAGCAA
TGTATGCCCCGCCCATTGCAGGAAACATAACATGTAAATCAAAT---
ATCACAGGACTACTATTGGTATATGATGGAGGCATGCATAATAGA----------------------------
-------------------------------------------------------------------------
-------------
AGCAACGAGACATTCAGACCGGGAGGAGGAAATATGAAGGACAATTGGCGAAGTGAACTATATAAATATAAAG
TGGTAGAAATTAAACCATTA---GGAATAGCACCCACGAAAGCAAAAAGAAGAGTGGTG--------------
-------------------------GAGAGAGAAAAAAGAGCA---GTGGGA---GTAGGAGCT---------
ATGTTCCTTGGGTTCTTGGGAGCA---GCAGGAAGCACTATG---GGCGCGGCGTCA---
ATAACGCTGACGGTACAG---GCCAGACAA---CTATTG---TCTGGTATAGTGCAACAGCAAAGC---
AATTTG---CTGAGA---GCTATAGAGGCGCAACAACATATGTTGCAACTCACAGTCTGGGGCATT
>KR861259
AATTATACAAATAGCACTACA----------------------------------------------------
-----------------------------------------------------------
GATAATTCAACCATCACAATCCAATGCAGAATAAAACAAATCATAAACATGTGGCAGGAGGTAGGACGAGCAA
TGTATGCCCCTCCCATTGCAGGAAACATAACATGTAGATCAAAT---
ATCACAGGACTACTATTAGTACGTGATGGAGGAAACGGAAACGACACC-------------------------
-------------------------------------------------------------------------
----------
AATAATACAGAGACATTCAGACCTGGAGGAGGAAATATGAAGGACAATTGGAGAAGTGAATTATACAAATATA
AAGTGGTAGAAGTAAAACCATTA---GGAATAGCACCCACTGGGGCAAAAAGGAGAGTGGTG-----------
----------------------------GAGAGAGAAAAAAGAGCA---GTGGGA---ATAGGAGCT------
---GTGTTCCTTGGATTCTTGGGAGCA---GCAGGAAGCACTATG---GGCGCGGCGTCA---
ATAACGCTGACGGTACAG---GCCAGACAA---TTATTG---TCTGGTATAGTGCAACAGCAAAGC---
AATTTG---CTGAGG---GCTATAGAGGCGCAACAGCATCTGTTGCAACTCACAGTCTGGGGCATT
>KR861264
GATTATAATGATACAGAA-------------------------------------------------------
--------------------------------------------------------------
AGTAATGAGACCATCATACTCCAATGCAGAATAAAACAAATTATAAACATGTGGCAGAAGGTAGGAGGAGCAA
TATATGCCCCTCCCATTGCAGGAAACATAACATGTAAATCAAAT---
ATCACAGGACTACTCTTGGTACGGGATGGAGGCAATACCACTAAC----------------------------
-------------------------------------------------------------------------
-------------
ACATCAGAGATATTCAGACCAGGAGGAGGAAATATGAAGGACAATTGGAGAAGTGAACTATATAAATATAAAG
TGGTAGAAATTAAGCCATTG---GGACTAGCGCCCACCAAGGCAAGAAGGAGAGTGGTG--------------
-------------------------GAGAGAGAAAAAAGAGCA---GTGGGA---ATAGGAGCT---------
GTGTTCCTTGGGTTCTTGGGAGCA---GCAGGAAGCACTATG---GGCGCAGCGTCA---
ATAACGCTGACGGTACAG---GCCAGACAA---TTGTTG---TCCGGTATAGTGCAACAGCAAAGT---
AATTTG---CTGAGA---GCTATAGAGGCGCAGCAGCATATGTTGCAACTCACAGTCTGGGGCATT
>KR861269
TGCTATACATCAAACCTGTTTAATGATAGCAATAGGGCAAACATGTCTGAT----------------------
-------------------------------------------------------------------------
----------------------
AACAGTACAGTCATCACACTCTCATGCAGGATAAAACAAATTATAAATATGTGGCAGAAGGTAGGACGAGCAA
TGTATGCCCCTCCCATTGCAGGGAACATAACATGTAAATCAAAT---
ATCACAGGACTACTATTGGTACGTGATGGAGGAACAGGTAACAAA----------------------------
-------------------------------------------------------------------------
----------------
ACAGAGATATTCAGACCTGGAGGGGGAGATATGAGGGACAATTGGAGAAGTGAATTATATAAATATAAAGTGG
TAGAAATAAAGCCATTG---GGAATAGCACCCACTGAGGCAAAAAGGAGAGTGGTG-----------------
----------------------GAGAGAGAAAAAAGAGCA---GTGGGA---ATAGGAGCT---------
GTGTTCCTTGGGTTCTTGGGAGCA---GCAGGAAGCACTATG---GGCGCAGCGTCA---
ATAACGCTGACGGTACAG---GCCAGACAA---TTGTTG---TCTGGAATAGTGCAACAGCAAAGC---
AATTTG---CTGAGG---GCTATAGAGGCGCAACAGCATATGTTGCAACTCACGGTCTGGGGCATT
>KR861276
ACTTAC-------------------------------------------------------------------
--------------------------------------------------
```

ATCACACTCCCATGCAGAATAAAGCAAATTATAAACATGTGGCAGGGGGTAGGACAAGCAATGTATGCCCCTC
CCATTGCAGGGAACATAACATGTACCTCAAAT---
ATCACAGGAATACTGTTGACACGTGATGGAGGAAATGAGGAAAAG----------------------------
----

GAAGAGGAGATATTCAGACCTGGAGGAGGAGATATGAGGGACAATTGGAGAAGTGAGTTGTATAAATATAAAG
TGGTAGAAGTTAAACCATTA---GGAATAGCACCCACTGAGGCAAAAAGAAGAGTAGTG--------------
-------------------------GAGAGACAAAAAAGAGCA---GCGGGA---CTAGGAGCT---------
GTGTTCCTAGGGTTCTTGGGAGCA---GCAGGAAGCACTATG---GGCGCGGCGTCA---
ATAACGCTGACGGTACAG---GCCAGACAA---TTATTG---TCTGGTATAGTGCAACAGCAAAGC---
AATTTG---CTGAGA---GCTATAGAGGCGCAACAACATATGTTGCAACTCACAGTCTGGGGCATT
>KR861281
AATTACATCAAT---

TCATCAGATCTAATCACCCTCCAATGCAGAATAAAACAAATTATAAACTTGTGGCAGAGGGTAGGACGAGCAA
TGTATGCCTCCCCCATTAACGGAATCATAAAATGTAGATCAAAT---
ATCACAGGACTACTATTGACACGTGATGGAGGTAATACAACAAAC----------------------------
----

AGTAGTGAAGAAATATTCAGACCTGAAGGAGGAAATATGAGGGACAATTGGAGAAGTGAATTATATAAATATA
AAGTGGTAGAAATTAAGCCATTA---GGGATAGCACCCACTAGGGCAAAAAGAAGAGTGGTG-----------
----------------------------GAGAGAGAAAAAAGAGCA---GTGGGA---ATAGGAGCT------
---GTGTTCCTTGGGTTCTTGGGAGCA---GCAGGAAGCACTATG---GGCGCGGCGTCA---
ATCACGCTGACGGTACAG---GCCAGACAA---TTATTG---TCTGGTATAGTGCAACAGCAAAGC---
AATTTG---CTGAGA---GCTATAGAGGCGCAACAACATATGTTGCAACTCACAGTCTGGGGCATT
>AF110973
AGTTATAGGGGTACAGAAAGT--

AATAGTTCAATCATCACACTCCCATGCAGAATAAAACAAATTATAAACATGTGGCAGAAGGTAGGACGAGCAA
TATATGCCCCTCCCATCGAAGGGAACATAACATGTAGCTCGAGT---
ATCACAGGACTACTCTTGGCACGTGATGGAGGCTTAGATAATGTC----------------------------
----

ACCACAGAGATATTCAGACCACAAGGAGGAGATATGAAGGACAATTGGAGAAATGAATTATATAAATATAAAG
TAGTAGAAATTAAGCCATTG---GGAGTAGCGCCCACTGAGGCAAAAAGGAGAGTGGTG--------------
-------------------------GAGAGAGAAAAACGAGCA---GTGGGA---ATAGGAGCT---------
GTGATCTTTGGGTTCTTGGGAGCA---GCAGGAAGCACTATG---GGCGCAGCGTCA---
ATAACGCTGACGGCACAG---GCCAGACAA---TTGTTG---TCTGGAATAGTGCAACAGCAAAGC---
AATTTG---CTGAGG---GCTATAGAGGCGCAACAGCATATGTTGCAACTCACAGTCTGGGGCATT
>KY658707
ACCTACAGGTCTAATAGT---

ACAAATGCAACCATCACACTCCCATGCAGAATAAAACAAATTATAAACATGTGGCAAGGGGTAGGACGAGCAA
TGTATGCCCCTCCCATTGCAGGAAACATAACATGTAAATCAAAT---
ATCACAGGACTACTATTGACACGTGACGGAGGAGTTGGTGAGGATAGTGAT----------------------
----

AATGAAACAGAAATATTCAGACCTGGAGGAGGAGATATGAGAGACAATTGGAGAAGTGAATTATATAAATATA
AAGTGGTAGAAATTAAACCATTA---GGAATAGCACCCACTGAGGCAAGAAGGAGAGTGGTG-----------
----------------------------GAGAGAGAAAAAAGAGCA---GTGGGA---ATAGGAGCT------
---GTGTTCCTTGGGTTCTTGGGAGCA---GCAGGAAGCACTATG---GGCGCAGCGTCA---
ATAACGCTGACGGTACAA---GCCAGGCAA---TTATTG---TCTGGTATAGTGCAACAGCAAAGC---
AATTTG---CTGAGA---GCTATAGAGGCGCAACAGCATATGTTGCAACTCACGGTCTGGGGCATT
>AF443077
AGTCAGCCTAACAGTACAGGAAACACT--

CCACATTCAAACATCACACTCCCATGCAAAATAAAACAAATTATAAACATGTGGCAGGGGGTAGGGCGAGCAA
TGTATGCCCCTCCCATTGCAGGAAACATAACATGTATATCAAAT---
ATCACAGGACTAATATTGACACGGGATGGAGGAGTAAATAGAAGT----------------------------
----

GACACAGAAATATTCAGACCTGCAGGAGGAGATATGAGGGATAATTGGAGAAGTGAATTATATAAATATAAAG
TGGTAGAAATTAAACCATTA---GGACTAGCACCCACTAAGGCAAAAAGGAGAGTGGTG--------------
-------------------------GAGAGAGAAAAAAGAGCG---GTGGGA---ATAGGAGCT---------
GTGTTCCTTGGGTTCTTGGGAGCA---GCAGGAAGCACTATG---GGCGCGGCGTCA---
ATAACGCTGACGGTACAG---GTCAGACAA---CTGTTG---TCTGGTATAGTGCAACAGCAAAGC---
AATTTG---CTGAGG---GCTATAGAGGCGCAACAGCATATGTTGCAACTCACGGTATGGGGCATT
>AF443081
TATTATAGTAACAAGACAAGCAGTAATATGACT--

ACAAATGAAATCATCACAATTCCATGCAAAATAAAACAAATTATAAACATGTGGCAGGAGGTAGGACGAGCAA
TGTATGCCCCTCCCATTGCAGGAAACATAACATGTAAATCAAAT---
ATCACAGGACTACTATTAGTACGTGATGGAGGCAACAACAATACC----------------------------
----

ACAGAAACATTCAGACCTGGAGGAGGAAATATGAAGGACAATTGGAGAAGTGAATTATATAAATATAGAGTGG
TAGAAATTAAGCCATTA---GGAATAGCACCCACTGGAGCAAAAAGGAGAGTGGTG-----------------
----------------------GAGAGAGAAAAAAGAGCA---GTGACA---CTAGGAGCT---------
ATGTTCCTTGGGTTCTTGGGAGCA---GCCGGAAGCACTATG---GGCGCGGCGTCA---
ATAACGCTGACGGTACAG---GCCAGACAA---CTGTTG---TCTGGTATAGTGCAACAGCAAAGC---
AATTTG---CTGAGG---GCTATAGAGGCGCAACAGCATATGTTGCAACTCACGGTCTGGGGCATT
>AF443087
TACTATCCAAACATGACCAATGTCACTGATACAAAG-------------------------------------
----
AGTAATTTAACCATCACACTGCCATGCAGAATAAAACAAATTATAAACAGGTGGCAGGAGGTAGGACGAGCAA
TGTATGCCCCTCCCATTGCAGGAAACATAACATGTACGTCAAAT---
ATCACAGGACTACTATTGGTACGTGATGGAGGCCGTACAAGTGAC----------------------------
----

TCTACAAAAGAGATATTCAGACCTGGAGGAGGAGATATGAGGGACAATTGGAGAAGTGAATTATATAAATATA
AAGTGGTAGAAATTAAACCATTG---GGAATAGCACCCACTGAAGCAAAAAGGAGAGTGGTG-----------
----------------------------GAGAGAGAAAAAAGAGCA---GTGACA---ATAGGAGCT------
---ATGTTCCTTGGGTTCTTATCAGCA---GCAGGAAGCACTATG---GGCGCAGCATCA---
ATAACGCTGACGGTACAG---GCCAGACAA---TTATTG---TCTGGTATAGTGCAACAGCAAAGC---
AATTTG---CTGAGA---GCTATAGAGGCGCAACAGCATATGTTGCAACTCACAGTTTGGGGCATT
>KR861318
GACTATAATGTA---

AGCAACATCAACATCACACTCCCATGCAGAATAAAACAATTTATAAACATGTGGCAGGGGGTAGGACGAGCAA
TGTATGCCCCTCCCATTGCAGGAAACATAACATGTATATCAAGT---
ATCACAGGATTAATATTGACACGTGATGGAGGAAGAGGGAATGAGACTGAG----------------------
----

AATAATACTGAGATATTCAGACCTGCAGGAGGAGATATGAGGGACAATTGGAGAAGTGAATTATATAAATATA
AAGTAGTAGAAATTAAGCCATTA---GGACTAGCACCCACTATGGCAAAAAGGAGAGTGGTG-----------
----------------------------GAGAGAGAAAAAAGAGCA---GTGGGA---ATAGGAGCT------
---GTACTCCTTGGGTTCTTGGGAGCA---GCAGGAAGCACTATG---GGCGCGGCGTCA---
ATCACGCTGACGGTACAG---GCCAGACAA---TTGCTG---TCTGGTATAGTGCAACAGCAAAGC---
AATTTG---CTGAGA---GCTATAGAGGCGCAACAGCATATGTTGCAACTCACAGTCTGGGGCATT
>HQ699999
TACTATCCTAATGGTACACTAGTTAAT--

TCAACCTTAAACATCACACTCCAATGCAGAATAAAGCAAATTATAAACATGTGGCAGGAGGTAGGAAAAGCAA
TGTATGCCCCTCCCATTGCAGGAAACATAACATGTAGATCAAAT---
ATCACAGGACTACTATTGGTACGTGATGGAGGGATAACAAATAATACAAACCCA------------AGT----
----
-
GAGATCAAGAATGATACAGAGATATTCAGACCTGGAGGAGGAAACATGAAAGACAATTGTAGAAGTGAATTAT
ATAAATATAAAGTGGTAGAAATTAAGCCATTG---GGAGTAGCACCCACTCCAGCAAAAAGGAGAGTAGTG--
-------------------------------------GAGAGAGAAAAAAGAGCA---GCA------

ATAGGAGCT---------GTGTTCCTTGGGTTCTTGGGAGCA---GCAGGAAGCACTATG---
GGCGCGGCGTCA---CTAACGCTGACGGTACAG---GCCAGACAA---TTGTTG---
TCTGGTATAGTGCAGCAGCAAAGC---AATTTG---CTGAGA---
GCTATAGAGGCGCAACAGCATCTGTTGCAACTCACGGTCTGGGGCATT
>EF514713
TCTCAGTCTAATAATATAAGCATA---

CTTACTAATGAAACCATCACGCTCCCATGCAGAATAAAACAAATTATAAACATGTGGCAGGAGGTAGGACGAG
CAATGTATGCCCCTCCCATTGCAGGAAACATAACATGTAAATCAAAT---
ATCACAGGACTACTATTGGTACGGGATGGAGGAAACCAGGAAAGG----------------------------
----

AACAACGAGACATTTAGACCTGAGGGAGGAAATATGAAGGACAATTGGAGAAGTGAATTATATAAATATAAAG
TGGTAGAAATTAAGCCATTG---GGAATAGCACCCACTGAGGCAAAAAGGAGAGTGGTG--------------
-------------------------GAGAGAGAGAAAAGAGCA---GTGGGA---ATAGGAGCT---------
GTACTCCTTGGGTTCTTGGGAGCA---GCCGGAAGCACTATG---GGCGCGGCGTCA---
ATAACGCTGACGGTACAG---GCCAGACAA---GTATTG---TCTGGTATAGTGCAACAGCAAAGC---
AATTTG---CTGAGA---GCCATAGAGGCGCAACAGCATATGTTGCAACTCACGGTCTGGGGCATT
>KX228820
AGTTATGATTCTAAT--

AGTACAGCAAACATCACACTCCCATGCAAAATAAAACAGATTATAAACTTGTGGCAGGAGGTAGGACGAGCGA
TGTACGCTCCTCCCATTGCAGGAAACATAACATGTAAGTCAACC---
ATCACAGGATTACTATTGGTGCGTGATGGAGGACAAGGAAACAAC----------------------------
----

TCAAATGAGACATTCAGACCTGGAGGAGGAGATATGAGGGATAATTGGAGAAGTGAATTGTATAAGTATAAAG
TGGTAGAAATTAAGCCATTG---GGAGTAGCACCCACTGAGGCAAAAAGGAGAGTGGTG--------------
-------------------------GAGAGAGAAAAAAGAGCA---GTGGGA---CTAGGAGCT---------
GTGATCTTTGGGTTCTTAGGAGCA---GCAGGAAGCACTATG---GGCGCGGCGTCA---
ATAACGCTGACGGTACAG---GCCAGACAA---CTGTTG---TCTGGTATAGTGCAACAGCAAAGC---
AATTTG---CTGAAG---GCTATAGAGGCACAACAGCATATGTTGCAACTCACGGTCTGGGGCATT
>KU319533
ACCTATAAAGGTACAAACATGTCTGAT--

GGTCCTGAATTCATCACACTCCAATGCAGAATAAAACAAATTATAAATATGTGGCAAGGGGTAGGACGAGCAA
TGTATGCCCCTCCCATTGCAGGAAACATAACATGTATATCAAAT---
ATCACAGGAATACTACTGACACGTGATGGA---AAAAATGATGGT----------------------------
----

GCAGATACATTCAGACCTGGAGGAGGAGATATGAGGGATAATTGGAGAAGTGAATTATATAAATACAAAGTAG
TAGAAATTAAACCATTA---GGAATAGCACTCACTAAGGCAAAGAGGAGAGTGGTG-----------------
----------------------GAGAGAGAAAAAAGAGCAGTATTGGGAGGACTAGGAGCT---------
GTGTTCCTTGGGTTCTTGGGGGCA---GCAGGAAGCACTATG---GGCGCAGCATCA---
ATGACGCTGACGGTACAG---GCCAGAAAA---TTGTTG---TCTGGTATAGTGCAACAGCAAGGC---
AATTTG---CTGAGG---GCTATAGAGGCGCAACAGCATATGTTGCAACTCACAGTCTGGGGCATT
>KU319536
AATCATAGTTTTACAAACTCAACCCTGTTTAATAGTAGTAATAAT----------------------------
----
-
GCAAACTCAACTATCACACTCCCATGCAAAATAAAACAAATTATAAACATGTGGCAGAGGGTAGGGCGAGCAA
TATATGCCCCTCCCATTGAAGGAAATATAACATGTAAATCAGGT---
ATCACAGGGCTACTATTGACACGTGATGGAGGAAATGACACAGAGATA-------------------------
----

AATGGCACAGAGACATTCAGACCTGGAGGAAGAGATATGAGGGACAATTGGAGAAGTGAATTATATAGATATA
GGGTGGTAGAAATTAAGCCATTG---GGAATAGCACCCACCAAGGCAAAAAGGAGAGTGGTG-----------
----------------------------GAGAGAGAAAAAAGAGCA---GTGGGA---ATAGGAGCT------
---GTGTTCCTTGGGTTCTTGGGAGCA---GCAGGAAGCACTATG---GGCGCGGCGTCA---

ATAACGCTGACGGTACAG---GCCAGACAA---TTGCTG---TCTGGTATAGTGCAACAGCAAAGC---
AATTTG---CTGAGG---GCTATAGAGGCGCAACAGCATATGTTGCAACTCACAGTTTGGGGCATT
>FJ653288
AGCTACATGTCTAATACTACAAACAGTACAATCAGT-------------------------------------
----
GAAAATTCAACTATCACACTTTCATGCAGAATAAAACAAATTATAAACATGTGGCAGGGGGTAGGACAAGCAA
TATATGCCCCGCCCATTGCAGGAAACATAACATGTATATCAAAT---
ATCACAGGACTACTGTTAACACGTGATGGAGGAAATGAGGGTAAT----------------------------
----

GATACAACAGAGACATTCAGACCTGGAGGGGGAGATATGAGAGACAATTGGAGAAGTGAATTATATAAATATA
AAGTGGTAGAAGTTAAACCATTA---GGAATAGCACCCAATAAGGCAAAAAGGAGAGTGGTG-----------
----------------------------GAGAGAGAAAAAAGAGCA---GTGGGA---ATAGGAGCT------
---GTGCTCCTTGGGTTCTTGGGAGCA---GCAGGAAGCACTATG---GGCGCGGCGTCA---
ATAACGCTGACGGTACAG---GCCAGACAA---TTATTG---TCTGGTATAGTGCAACAGCAAAGC---
AATTTG---CTGAGA---GCTATAGAGGCGCAACAGCATTTGTTGCAACTCACAGTCTGGGGCATT
>DQ207941
AACTACACTGAG---

GCAAATTCAAGCATCACACTCCCATGCAGAATAAAACAAATTATAAACATGTGGCAGGAGGTAGGACGAGCAA
TGTATGCCCCTCCCATTGCAGGATACATAACATGTAATTCAAAT---
ATCACAGGACTACTCTTGACACGTGATGGAGGCAATGGTAGTAGT----------------------------
----

AATTGCACAGAGACATTCAGACCGGGAGGAGGAGACATGAGGGACAATTGGAGAAGTGAATTATATAAATACA
AAGTGGTAGAAATTAAGCCATTA---GGAGTAGCACCCACCAAGGCAAAAAGGAGAGTGGTG-----------
----------------------------GAGAAAGAAAAAAGAGCA---GTGGGA---ATAGGAGCT------
---GTACTCCTTGGGTTCTTGGGAGCA---GCAGGAAGCACTATG---GGCGCAGCGTCA---
ATAACGCTGACGGTACAG---GCCAGACAA---GTGTTG---TCTGGAATAGTGCAACAGCAAAAT---
AATTTG---CTGAGG---GCTATAGAGGCGCAACAGCATATGTTGCAACTCACAGTCTGGGGCATT
>KF766541
AATTATACATACATGCTTAATGGTACACTTACAGGAAGTAATTCA----------------------------
----
-
AGCTCAGACATTATCACACTCCCATGTAGAATAAAACAATTTATAAACATGTGGCAGGAGGTAGGACGAGCAA
TGTATGCTCCTCCCATTGCAGGAAACATAACATGTTCATCAAAA---
ATCACAGGACTACTATTGGTACGTGATGGAGGAAATACAAATAGT----------------------------
----

ACTACAGAGATATTTAGACCTGGAGGAGGAGACATGAGGAACAATTGGAGAAGTGAATTATATAAATACAAAG
TAGTAGAAATTAGACCATTG---GGAGTAGCACCCACTACAGCAAAAAGGAGAGTGGTG--------------
-------------------------GAGAGAGAAAAAAGAGCA---GTGGGA---ATAGGAGCT---------
GTGTTCCTTGGGTTCTTGGGAGCA---GCCGGAAGCACTATG---GGCGCGGCGTCA---
ATGACGCTGACGGTACAG---GCCAGACAA---TTGTTG---TCTGGTATAGTGCAACAGCAAAGC---
AATTTG---CTGAGG---GCTATAGAGGCGCAACAGCATCTGTTGCAACTCACGGTCTGGGGCATT
>MH899155
AGTCATGATGGT---

TTAAACTCAACCATCACACTCCCATGCAAAATAAAGCAGATTATAAATATGTGGCAGGAGGTAGGAAGAGCAA
TGTATGCCCCTCCCATTGAAGGAAACATAACATGTAAATCAAAT---
ATCACAGGCATACTATTGGTACGTGATGGAGGGAATGATAACAGCTCA-------------------------
----

AATGATACAGAAACAGAGACATTCAGACCTGGAGGGGGAGATATGAGAGATAATTGGAGAAGTGAATTATACA
AATATAAAGTGGTAGAAATTAAGCCACTG---GGAGTAGCACCCACTACAGCAAAAAGGAGAGTGGTG-----
----------------------------------GAGAGAGGAAAAAGAGCA---GTGGGC---
ATAGGAGCT---------GTGTTCCTTGGGTTCTTGGGAGCA---GCAGGAAGCACTATG---
GGCGCGGCGTCA---ATAACGCTGACGGTACAG---GCCAGACAA---TTGTTG---
TCTGGTATAGTGCAACAGCAAAGC---AATTTG---CTGAGA---
GCTATAGAGGCGCAACAACATCTGTTGCAACTCACGGTCTGGGGCATT

>KX069220
ACCTACGTGAATGATACCACCAACGGG---

GATAACTCAAACATCACTTTACCATGCCGAATAAAACAAATTATAAACATGTGGCAGGAGGTAGGACGAGCAA
TGTATGCCCCTCCCATTGCAGGAAACATAACATGTAAATCAAAT---
ATTACAGGACTACTATTGGTACGTGATGGAGGGAAAGAGACAAAT-------------------------
----

GGCACAGAGACATTCAGACCTGGAGGAGGAGATATGAGAGACAATTGGAGAAGTGAATTATATAAATATAAAG
TGGTAGAAATTAAGCCATTG---GGAGTAGCACCCACTAAAGCAAAAAGAAGAGTAGTG-----------
------------------------GAGAGAGAAAAAAGAGCA---GCA------CTAGGAGCT--------
GTGTTCCTTGGGTTCTTAGGAGCA---GCAGGAAGCACTATG---GGCGCGGCGTCA---
ATAGCGCTGACGGCACAG---GCCAGACAA---TTGTTG---TCTGGTATAGTGCAACAGCAAAGC---
AATTTG---CTGAGG---GCTATAGAGGCGCAACAGCATCTGTTGCAACTCACAGTCTGGGGCATT
>KY713228
TACTACTTTAATAGTAGTAAAAACAGAGGGGGCACA----------------------------------
----
GAAAGTGATGAAACCATCATCCCATGCAAAATAAAGCAAATCATAAACATGTGGCAGGAGGTAGGAAGAGCAA
TGTATGCCCCTCCCATTGCAGGAAACATAACATGTAAATCAAAC---
ATCACAGGGCTACTATTGGTACGTGATGGAGGAATAAATACTACA-------------------------
----

GAGGCTACAGAGACATTTAGACCAGGAGGAGGAGATATGAGGGACAATTGGAGAAGTGAATTATATAAATATA
AAGTGGTAGAAATTAAGCCACTG---GGAGTAGCACCAACTGGAGCAAAAAGGAGAGTGGTG----------
---------------------------GAGAGAGAAAAAAGAGCA---GTGGGG---CTAGGAGCT------
---GTGTTCCTTGGGTTCTTGGGAGCA---GCAGGAAGCACTATG---GGCGCGGCGTCA---
ATAACGCTGACGGTACAG---GCCAGACAA---CTGTTG---TCTGGTATAGTGCAACAGCAAAGT---
AATTTG---CTGAGA---GCTATAGAGGCGCAACAGCATCTGTTGCAACTCACAGTCTGGGGCATT
>KY713233
AATTACATGGGTACATACAATGAGACAAATGGTACATACAAT----------------------------
----
TCAAACTCGACCATCACAATCCCATGCAGAATAAAACAAATTATAAACATGTGGCAGGAGGTAGGACGAGCAA
TATATGCCCCTCCCATTGCAGGAAACATAACATGTAAATCAAAT---
ATCACAGGACTACTATTGGTACGTGATGGAGGAAGAAACAATACA-------------------------
----

CATAAAAATGAGACATTTAGACCTGGAGGAGGAGATATGAAAGACAATTGGAGAAGTGAATTATATAAATACA
AGGTGGTGGAAATTAAGCCATTA---GGAATAGCACCTACTACAGCAAAAAGGAGAGTGGTG----------
---------------------------GAGAGAGAAAAAAGAGCA---GTGGGA---CTAGGAGCT------
---ATGTTCCTTGGGTTCTTGGGAGCA---GCCGGGAGCACTATG---GGCGCGGCGTCA---
ATAACGCTGACGGTACAG---GCCAGACAA---TTGTTG---TTTGGTATAGTGCAGCAGCAAAGC---
AATTTG---CTGAGA---GCTGTAGAGGCGCAACACCATCTGTTGCAACTCACGGTCTGGGGCATT
>KY713248
AATTACACTGAGACAAATAGTACATACAATGAG-------------------------------------
----
GCAAACTTGACCATCACAATCCCATGCAGAATAAAACAAATTATAAACATGTGGCAGAAGGTAGGAAAAGCAA
TATATGCCCCTCCCATTGCAGGAAACATAACATGTAAATCAAAT---
ATCACAGGACTACTACTGGTACGTGATCGTGGTGGAAACAATACA-------------------------
----

AATAAGAATGAGACATTTAGACCTGGAGGAGGAAATATGAAAGACAATTGGAGAAGTGAATTATATAAATATA
AAGTGGTGGAAATTAAGCCATTA---GGAATAGCACCTACTACAGCAAAAAGGAGAGTGGTG----------
---------------------------GAGAGAGAAAAAAGAGCA---GTGGGA---CTAGGAGCT------
---ATGTTCCTTGGGTTCTTGGGAGCA---GCTGGGAGCACTATG---GGCGCGGCGTCA---
ATAACGCTGACGGTACAA---GCCAGACAA---CTGTTG---TCTGGTATAGTGCAGCAGCAAAGC---
AATTTG---CTGAAA---GCTGTAGAGGCGCAACAGCATCTGTTGCAACTCACTGTCTGGGGCATT
>MK076659
AGTCATGATAGTTTT---
----------------CTAGACTCAACCAACAAC------
TCAACTTCAAAAAAGATCACACTCCCATGCAGAATAAAGCAGATTATAAATATGTGGCAGGAGGTAGGAAGAG

CAATGTATGCCCCTCCCATTGCAGGAAAAATAACATGTAAATCAAAT---
ATCACAGGCATACTATTGACACGTGATGGAGGGCATGATAACAAT----------------------------
----

GTTACAGAGACATTCAGACCTGGAGGGGGAGATATGAGAGATAATTGGAGAAGTGAATTATACAAATATAAAG
TGGTAGAAGTTAAGCCACTG---GGAGTAGCACCCACTGCAGCAAAAAGGAGATTGGTG--------------
-------------------------GAAAGAAACAAAAGAGAC---GTGGGC---CTAGGAGCT---------
GTGTTACTTGGGTTCTTGGGAGCA---GCAGGAAGCACTATG---GGCGCGGCGTCA---
ATAACGCTGACGGTACAG---GCCAGACAA---TTGTTG---TCTGGTATAGTGCAACAGCAAAGC---
AATTTG---CTGAGA---GCTATAGATGCGCAACAACATATGTTGCAACTCACGGTCTGGGGCATT
>AY049708
AGTTATAATGGT---

CCAAGCCCAAATATCACAATCCCATGCAGAATAAAACAAATTATAAACATGTGGCAGGAGGTAGGACGAGCAA
TGTATGCCCCTCCCATTGCAGGAAACATAACATGTAAATCAAAT---
ATCACAGGAATACTATTGGTACGTGATGGGGGAAAAACAAAGGAT----------------------------
----

AATGACACAGAGATATTCAGACCTGGAGGAGGAGATATGAGGGACAATTGGAGAAGTGAATTATATAAATATA
AAGTGGTAGAAATTAAGCCATTG---GGAGTAGCACCCACTGAAGCAAAAAGGAGAGTGGTG-----------
----------------------------GAGAGAGAAAAAAGAGCA---GCG------CTAGGAGCT------
---ATGATCCTTGGGTTCTTGGGAGCA---GCAGGAAGCACTATG---GGCGCAGCGTCA---
ATAACGCTGACGGTACAG---GCCAGACAA---TTGTTG---TCTGGTATAGTGCAACAGCAAAGC---
AATTTG---CTGAAG---GCTATAAATGCGCAACAGCATATGTTGCAACTCACGGTCTGGGGCATT
>EF694036
TTTTATAGGCCTGATGGTAAT--

TCAAGCACAGACATCATAATCCTATGCAGAATAAAACAAATTATAAACATGTGGCAGGAGGTAGGACAAGCAA
TGTATAACCCTCCCATTGCAGGAAACATAACATGTAGATCAAAT---
ATCACAGGACTACTATTGTTACGTGATGGAGGAACAGGAAATACTACA-------------------------
----

GATAATAAAACAGAAATATTCAGACCTGGAGGAGGAGATATGAGGGACAATTGGAGAAGTGAATTATATAAAT
ATAAAGTGGTAGAGATTAAACCATTG---GGGATAGCACCCACTACAGCAAAAAGGAGAGTGGTG--------
-------------------------------GAGAGAGAAAAAAGAGCA---GTGGGA---ATAGGAGCT---
------GTGTTCCTTGGGTTCTTGGGAGCA---GCAGGAAGCACTATG---GGCGCGGCGTCA---
ATAACGCTGACGGTACAG---GCCAGACAA---CTGTTG---TCTGGTATAGTGCAACAGCAAAGC---
AATTTG---CTGAGG---GCTATAGAGGCGCAACAGCATCTGTTGCAACTCACGGTCTGGGGCATT
>AY945738
AACTATACAAAC---

AATACATCAGAGAACATCACACTCCCATGCAGAATAAAACAAATTATAAATATGTGGCAGGGGGTAGGACAAG
CAATATATGCCCCTCCCATTAAAGGGAAGATAATATGTAAATCAAAT---
ATCACAGGGCTACTGCTGACACGTGATGGAGGCAATACAAATAACACA-------------------------
----

AATAACACAGAAATATTCAGACCTGCAGGAGGAGATATGAAGGACAATTGGAGAAGTGAATTATATAAATACA
AAGTGGTAGAAATTAAACCATTA---GGAGTAGCACCCACCAAGGCAAAAAGGAGAGTGGTG-----------
----------------------------GAGAGAGAAAAAAGAGCA---GTGGGA---ATAGGAGCT------
---GCGTTCCTTGGGTTCTTGAGTGCA---GCAGGAAGCACTATG---GGCGCAGCGTCA---
ATAACGCTGACGGTACAG---GCCAGACAA---TTATTG---TCTGGTATAGTGCAACAGCAAAGC---
AATTTG---CTGAGG---GCTATAGAGGCGCAACAGCATATGTTGCAACTCACGGTCTGGGGCATT
>KC894074
TTTTACAAAACAGAGTTTAATAGTACA--

ACAAATTCAACCATCACACTCCAATGTAGAATAAAACAAATTATAAACATGTGGCAAGGGGTAGGGCGGGCAA
TGTATGCCCCTCCCATTGAAGGAAACATAACATGTAAATCAAAC---
ATCACAGGACTACTATTGACACGTGATGGAGGAACAAATGACAGT----------------------------
----

ATGACAGAGACATTCAGACCTGGAGGAGGAGATATGAGGGATAATTGGAGAAGTGAATTATATAAATATAAAG
TGGTAGAAATTAAGCCATTA---GGAGTAGCACCCACTGAGGCAAAAAGGAGAGTGGTG--------------
-------------------------GAGAGAGAAAAAAGAGCA---TTGACA---CTAGGAGCT---------
TTGTTCCTTGGGTTCTTGGGAACA---GCAGGAAGCACTATG---GGCGCGGCGTCA---
ATAACGCTGACGGTACAG---GCCAGACAA---TTGTTG---TCTGGTATAGTGCAACAGCAGAGC---
AATCTG---CTGAAG---GCTATAGAGGCGCAACAGCATCTGTTGCAACTCACGGTCTGGGGCATA
>KC894086
AGCTACATGATTAATGATACAGAAAGCACTAATAAAAGT----------------------------------
----
GCAAATTCAACCATCATACTCCCATGCAGAATAAAACAAATTATAAACATGTGGCAGGGGGTAGGACGAGCAA
TGTATGCCCCTCCCATTGCAGGAACCATAACATGCAACTCAAAT---
ATCACAGGATTACTATTGACCCATGATGGAGACCCTAATAGCAAT----------------------------
----

GGCACAGAGATATTCAGACCTGGAGGAGGAAATATGAAGGACAATTGGAGAAGTGAATTATATAAATATAAGG
TGGTAGAAATTAAGCCATTA---GGAATAGCACCCACTAATGCAAGAAGGAGAGTGGTG--------------
-------------------------GAGAGAGGAAAAAGAGCA---GTGGGA---ATAGGAGCT---------
GTGTTCCTTGGGTTCTTGGGAGCA---GCAGGAAGCACTATG---GGCGCGGCGTCA---
CTAACGCTGACGGTACAA---GCCAGACAA---CTGTTG---TCTGGTATAGTGCAACAGCAAAGC---
AATTTG---CTGAGG---GCTATAGAGGCGCAACAGCATATGTTGCAACTCACGGTCTGGGGCATT
>KC894092
AATTATACAGCAAGTACAAATAAT---

TCAAGTGAGACCATCACACTCCCATGCAGAATCAAACAAATTATAAGAATGTGGCAGGGGGTAGGGCAAGCAA
TGTATGCCCCTCCCATTGCAGGAAACATAACATGTACATCAAAC---
ATCACAGGAATATTATTGGTACGTGATGGAGGAGACAATAATAGC----------------------------
----

ACAAAAGAGATATTCAGACCTGGAGGAGGAGATATGAGGGACAATTGGAGAAGTGAATTATACAAATATAAAG
TAGTAGAAATTAAGCCATTG---GGAATAGCTCCCACTGGAGCAAAAAGGAGAGTGGTG--------------
-------------------------GAGAGAGAAAAAAGAGCA---GTAGGA---ATAGGAGCT---------
GTGCTCCTTGGGTTCTTGGGAGCA---GCAGGAAGCACTATG---GGCGCAGCGTCA---
ATAACGCTGACGGTACAG---GCCAGACAA---TTGTTG---TCTGGTATAGTGCAACAGCAAAGC---
AATTTG---CTGAGG---GCTATAGAGGCGCAACAGCACATGTTGCAACTCACTGTCTGGGGCATT
>KC862923
AATTATACCTTTAATGGTACAGAGCTGTTGAATAGTACCTCAAAT----------------------------
----
-
ACCACAGAGATCATCACACTCCCATGCAGAATAAAACAAATTATAAACATGTGGCAGAGGGTAGGACAAGCAA
TGTATGCCCCTCCCATTAAAGGAAACATAACATGTATATCCAAT---
ATCACAGGACTACTGCTGACACGTGATGGAGGAGGGGACAAAAATGGC-------------------------
----

AAAGAGAATAAAACAGAGACATTCAGACCTGGAGGAGGAAATATGAGGGATAATTGGAGAAGTGAACTATACA
AATATAAAGTGGTAGAAATTAAGCCATTA---GGAATAGCACCCACTAAGGCAAAAAGGAGAGTGGTG-----
----------------------------------GAGAGAGAAAAAAGAGCA---GTGGGA---
ATAGGAGCT---------GTGTTCCTTGGGTTCTTGGGGGCA---GCAGGAAGCACTATG---
GGCGCAGCATCA---ATAACGCTGACGGTACAG---GCCAGACAA---TTATTG---
TCTGGTATAGTGCAACAGCAAAGC---AATTTG---CTGAGG---
GCTATAGAGGCGCAACAGCATATGTTGCAACTCACAGTCTGGGGCATA
>KC894103
TACTATCCTAATAATACAGGAAGTAATTCA---

ACAGACAACATCATCACACTTCCATGCAGAATAAAACAAATTATAAATATGTGGCAGGAAGTAGGAAGAGCAA
TATATGCCCCTCCCATTGAAGGAAGCATAACATGCACATCAAAT---
ATCACAGGACTACTATTGGTACGTGATGGAGGCACTGATAATAGCACA-------------------------
----

AATAACACAGAGACATTCAGACCTGGAGGAGGAGATATGAGGGACAATTGGAGGAGTGAATTATATAAATATA

AAGTGGTAGAGATTAAGCCATTA---GGAGTAGCACCCACTGAGTCAAAAAGGAGAGTGGTG-----------
----------------------------GAGAGACAAAAAAGAGCA---GTGGGA---ATGGGAGCT------
---GTGATCTTTGGGTTCTTGGGAGCA---GCAGGAAGCACTATG---GGCGCGGCGTCA---
ATAACGCTGACGGTACAG---GCCAGACAA---CTGTTG---TCTGGTATAGTGCAACAGCAAAGT---
AATTTG---CTGAAG---GCTATAGAGGCGCAACAGCATCTGTTGCAACTCACGGTCTGGGGCATT
>KC894106
AACTATAATGATACAGGAGGGAAT---

TCAAGCTCAAACATCAGACTCTCATGCAGAATAAAACAATTTATAAACATGTGGCAGAAAGTAGGAAGAGCAA
TGTATGCCCCTCCCATTGAAGGAAACATAACATGTAGATCAAAT---
ATCACAGGACTGCTATTGGTACGAGATGGAGGTATCAGTAGCACC----------------------------
----

ACCGAGACATTCAGACCTGGAGGAGGAAATATGAAGGACAATTGGAGAAGTGAATTATATAAATATAAAGTAG
TAGAAATTAAGCCATTA---GGAATAGCACCCACTAAAGCAAAAAGGAGAGTGGTG-----------------
----------------------GAGAGAGAAAAAAGAGCA---GTGGGA---ATAGGAGCT---------
GTGTTCCTTGGGTTCTTGGGAGCA---GCCGGAAGCACTATG---GGCGCGGCGTCA---
ATAACGCTGACGGTACAG---GCCAGACAA---CTGTTG---TCTGGTATAGTGCAACAGCAAAGC---
AATTTG---CTGAGG---GCCATAGAGGCGCAACAGCATATGTTGCAACTCACAGTCTGGGGCATT
>HM070477
AATTACAGT--

GGGAATGGTACCATCACCATACCATGCAGAATAAAACAAATTATAAATATGTGGCAGGAGGTAGGAAAGGCAA
TATATGCCCCTCCCATTGCAGGAAACATAACATGTAAATCAAAT---
ATCACAGGACTACTACTGACACGTGATGGAGGACAAGGACCAGATAAA-------------------------
----

GATAACACAACAGAGACATTCAGACCTGGAGGAGGAGACATGAGGGACAATTGGAGAAGTGAATTATATAAAT
ATAAAGTAGTAGAAATTAAGCCATTG---GGAATAGCACCCACTACGGCAAAAAGAAGAGTGGTG--------
-------------------------------GAGAGAGAAAAAAGAGCA---GTGGGA---ATAGGAGCT---
------GTGTTCCTTGGGTTCTTGGGAGCA---GCAGGAAGCACTATG---GGCGCAGCGTCA---
ATAACGCTGACGGTACAG---GCCAGACAA---CTGTTG---TTTGGCATAGTGCAACAGCAAAGC---
AATTTG---CTGAGA---GCCATAGAGGCGCAACAGCATATGTTGCAACTCACAGTCTGGGGCATT
>HM070526
TTTCAGAAATCAACAGAAGATAAT---

TCAACCAACATCATCACACTCCCATGCAAAATAAAACAAATTATAAGAATGTGGCAGAGGGTAGGGCAAGCAA
TGTATGCCCCTCCCATTGAAGGAAACATAACATGTAATTCACAA---
ATCACAGGAATACTGTTGACAAGGGATGGAGGGAATAATGGGAATAAT-------------------------
----

GAAGAAAGTGAGACATTCAGGCCTGGAGGAGGAGATATGAGGGACAATTGGAGAAGTGAATTATATAAGTACA
AAGTGGTAGAAGTTAAGCCATTG---GGAATAGCACCCACTGGGGCAAAAAGGAGAGTGGTGGAC--------
-------------------------AGAGACAGAGAAAAAAGAGCA---GTAGGA---ATGGGAGCT------
---GTGTTCCTTGGGTTCTTGGGAGCA---GCAGGAAGCACTATG---GGCGCGGCGTCA---
ATAACGCTGACGGTACAG---GCCAGACAA---CTGTTG---TCTGGCATAGTGCAACAGCAAAGC---
AATTTG---CTGAAG---GCTATAGAGGCGCAACAGCATCTGTTGCAACTCACAGTCTGGGGCATT
>HQ615982
TACTATCCTAATAGTACTGATACAAACAATACAGGAAGT----------------------------------
----
AACTCAAGCTTAATGATCACAATCCCATGCAGAATAAAACAAATTATAAACATGTGGCAGGGGGTAGGAAGAG
CAATATATGCCTCTCCCGTTGCAGGAAACATAACGTGTGTATCAAAA---
ATCACAGGACTCCTATTGACACGTGATGGAGGTACAGATAATAAT----------------------------
----

ATCACAGAGATATTCAGACCTGGAGGAGGAGATATGAAGGACAATTGGAGGAGTGAATTATATAAATATAAAG
TGGTAGAAATTAAGCCATTA---GGAATAGCACCCACTCCGGCAAAAAGGAGAGTGGTG--------------
-------------------------GAGAGAGAAAAAAGAGCA---GTGGGAGTAGTAGGAGCT---------
ATGATCCTTGGGTTCTTGGGAACA---GCAGGAAGCACTATG---GGCGCGGCGGCC---

GTGACGCTGACGGTACAG---GCCAGACAA---CTGTTG---TCTGGTATAGTGCAACAGCAAAGC---
AATTTG---CTGAGG---GCTATAGAGGCGCAACAACACATGTTGCAACTCACGGTCTGGGGCATT
>KC149493
AATTACACATTTAATAATATAAGT---

AAGAATGCAACCATCAAACTTCCATGCAGAATAAAACAAATTATTAACATGTGGCAGGAGGTAGGACGAGCAA
TGTATGCTCCTCCCATTGAAGGAAACATAACATGTAAATCAAAT---
ATCACAGGACTACTACTGGTACGTGATGGAGGCCCAAGTACTGAGAATGGCACA------------AAC----
----

ACAACTGCTGAGGAGATATTCAGGCCTGGAGGAGGAAATATGAAAGACAATTGGAGAAGTGAATTATATAAAT
ATAAAGTGGTAGAAATTAAGCCATTG---GGAATAGCACCCACTAGAGCAAAAAGGAGAGTGGTG--------
-------------------------------GAGAGAGAAAAAAGAGCA---GTGGGA---ATAGGAGCT---
------GTGTTCTTTGGGTTCTTGGGAGCA---GCAGGAAGCACTATG---GGCGCGGCGTCA---
ATAACGCTGACGGTACAG---GCCAGACAA---TTGTTG---TCTGGTATAGTGCAACAGCAAAGC---
AATTTG---CTGAGG---GCTATAGAGGCGCAACAGCATATGTTGCAGCTCACGGTCTGGGGCATT
>KY112254
ACCTACAATATTACTAATAGTACTAGTGCAGAT--

TTAAATTCAACCATCACACTCCCATGCAGAATAAAGCAAATTATAAACATGTGGCAGGAAGTAGGACGAGCAA
TGTATGCCCCTCCCATTGAAGGAAACATAACATGTAAATCAAAT---
ATCACAGGAATACTATTGGTACGTGATGGAGGA--

GACAACACATCTGAGACATTCAGACCTGGAGGAGGAGATATGAGAGATAATTGGAGAAGTGAATTATATAAAT
ATAAAGTGGTAGAAATTAAGCCATTA---GGAATAGCACCCACTAAGGCAAAAAGAAGAGTGGTGGAG-----
-------------------------------AGGAGGGAAAAAAGAGCA---GTGGGA---ATAGGAGCT---
------GTGTTCCTTGGGTTCTTGGGGGCA---GCAGGAAGCACTATG---GGCGCGGCGTCA---
ATAACGCTGACGGTACAG---GCCAGACAA---TTATTG---TCTGGTATAGTGCAACAGCAAAAC---
AATTTG---CTGAGG---GCTATAGAGGCGCAACAGCATATGTTGCAACTCACAGTCTGGGGCATT
>KY112386
ACCTATAATAGTACAGAAAGTGAGTTAGATGCAAACAGC----------------------------------
----
ACAAAGGAAAACATCACACTCCCATGCAGAATAAAACAAATTATAAACATGTGGCAGGAAGTAGGAAAAGCAA
TGTATGCCCCTCCCATTGCAGGAAACATAACATGTAAATCAAAC---
ATCACAGGAATACTATTGGTACGTGATGGAGGAAACACAGATAAC---------------------------
----

ACATCTGAGACATTCAGACCTGGAGGAGGAGATATGAAAGACAATTGGAGAAGTGAATTATATAAATATAAAG
TGGTAGAAATTAAACCATTA---GGACTAGCACCCACCAAGGCAAAAAGAAGAGTGGTGGAG-----------
-------------------------GGGAGAGAAAAAAGAGCA---GTGGGA---ATAGGAGCT---------
GTGTTCCTTGGGTTCTTGGGGGCT---GCAGGAAGCACTATG---GGCGCAGCGTCA---
ATAACGCTGACGGTACAG---GCCAGACAA---TTATTG---TCTGGTATAGTGCAACAGCAAAGC---
AATTTG---CTGAGA---GCTATAGAGGCGCAACAGCATATGTTGCAACTCACGGTCTGGGGCATT
>HQ595814
AAT--

CATACAGACATCATCACACTCCAATGCAGAATAAAACAATTTATAAACATGTGGCAGAAGGTAGGAAGAGCAA
TGTATGCCAATCCCATTGCAGGAAACATAACATGTAGATCAAAT---
ATCACAGGACTACTATTGACACGTGATGGAGGTGTTAGAGGGAATACAACAAAT-------------------
----

AATGAGACTGAGACAGAGACATTCAGACCTGGAGGAGGAGATATGAGGGATAATTGGAGAAGTGAATTGTATA
AATATAAAGTGGTGGAAATTAAGCCATTG---GGAATAGCACCCACTACGGCAAAAAGGAGAGTGGTG-----
----------------------------------GAGAGAGAGAAAAGAGCA---GTGGGA---
ATAGGAGCT---------GTATTCCTTGGGTTCTTAGGAGCA---GCAGGAAGCACTATG---
GGCGCGGCGTCA---ATAACGCTGACGGTACAG---GCCAGACAA---CTACTG---
TCTGGTATAGTGCAACAGCAAAGC---AATTTG---CTGAGA---
GCTATAGAGGCGCAACAGCATATGTTGCAACTCACGGTCTGGGGCATT
>KC186976

ACCTATGGGGATAATGATACAAATGATAATGCAAATACAACTAAT----------------------------
----
GCAAATAGAACTATCATACTCCAATGCAGAATAAAACAAATTATAAACATGTGGCAAGAGGTAGGAAGGGCAA
TGTATGCCCCTCCCATTGCAGGAAACATAACATGTAAATCAAAT---
ATCACAGGACTACTATTGACATGGGATGGAGGAATTAATAATAGG----------------------------
----

ACAAATGAGACATTCAGACCTGGAGGAGGAGATATGAGGGACAATTGGAGAAGTGAATTATATAAATATAAAG
TGGTAGAAATTAAACCATTA---GGGATAGCACCCAATAAGACAAAAAGGAGAGTGGTG--------------
-------------------------GAGAGAGAAAAAAGAGCA---GTGGGA---ATAGGAGCT---------
GTGTTCCTTGGGTTCTTGGGAGTA---GCCGGAAGCACTATG---GGCGCAGCGTCA---
ATAGCGCTGACGGGACAA---GCCAGACAA---TTACTG---TCTGGTATAGTGCAACAGCAAAGC---
AATTTG---CTGAGA---GCTATAGAGGCGCAACAGCATATGTTGCAACTCACGGTCTGGGGCATT
>KC187128
AATTACACGGCTAATAGTACAGAAAGTACTTCA--

AATTCAACCAACATCACAATCCAATGTAGAATAAAACAGATTATAAACCTGTGGCAGGGGGTAGGACGAGCAA
TGTATGCCCCTCCCATTGCAGGAAATATAACATGTAACTCAAAC---
ATCACAGGACTACTATTGACACGTGATGGAGGAAAAGAAGGAGAAAAT-------------------------
----

AATAATACAGAGATATTCAGGCCTGCAGGAGGAAATATGAAGGACAATTGGAGAAGTGAATTATATAAATATA
AAGTGGTAGAAATTAAGCCATTA---GGAATAGCACCCACTAGTGCATCAAGGAGAGTGGTG-----------
----------------------------CAGAGAGAAAAAAGAGCA---GTGGGA---CTGGGAGCC------
---ATGTTCCTTGGGTTCTTGGGAGCA---GCAGGAAGCACTATG---GGCGCAGCATCA---
ATAACGCTGACGGTACAG---GCCAGACAA---TTATTG---TCTGGTATAGTGCAACAGCAAAGC---
AATTTG---CTGAGG---GCCATAGAGGCGCAACAGCATATGTTGCAACTCACGGTCTGGGGCATA
>KC187665
TACTATCCTAATGGTACAAGAAGTAATTCTTCAAAC-------------------------------------
----
TTAAACCCAAACATCACGCTTCCATGCAGAATAAAACAAATTATAAACAGGTGGCAGGAGGTAGGAAGAGCAA
TGTATGCTCCTCCCATTGCAGGAAACATAACATGTAACTCAAGT---
ATCACAGGATTATTATTGACACGTGATGGAGGAAAAAATAATAATGGA-------------------------
----

ACAAATGATACAGAGACATTCAGACCTGGAGGAGGAAATATGAGGGACAATTGGAGAAGTGAATTATATAAAT
ATAAAGTGGTAGAAATCAGGCCATTA---GGAATAGCACCCACTGGTGCAAAAAGGAGAGTGGTG--------
-------------------------------GAGAGAGAGAAAAGAGCA---GTGGGA---ATAGGAGCT---
------GTGTTCCTTGGGTTCTTGGGGACA---GCAGGAAGCACTATG---GGCGCAGCGGCA---
GTGACGCTGACGGTACAG---GTCAGACAA---CTATTG---TCTGGTATAGTGCAACAGCAAAGC---
AATTTG---CTGAGA---GCTATAGAGGCGCAACAGCATATGTTGCAACTCACAGTCTGGGGCATT
>KC186377
AACTAC---

ACCAACACCACACTCACACTCCAATGCAGAATAAGACAAATTGTAAACATGTGGCAAGGGGTAGGACGAGCAA
TGTATGCCCCTCCCATTGCAGGAAACATAACATGTAAATCAAAT---
ATCACAGGACTACTATTGACACGTGATGGAGGAAAAAATAATGGTACT-------------------------
----

AATAACACAGAGATATTCAGACCTGGAGGAGGAGATATGAGGAACAATTGGAGAAGTGAATTATATAAATATA
AAGTGGTAGAAATTAAGCCATTG---GGAATAGCACCCACTAAAGCAAAAAGGAGAGTGGTG-----------
----------------------------GAGAGAGAAAAAAGAGCA---GTGGGA---ATAGGAGCT------
---GTGTTCCTTGGGTTCTTGGGAGCA---GCAGGAAGCACTATG---GGCGCGGCGTCA---
ATAACGCTGACGGTACAA---GCCAGACAA---CTATTG---TCTGGTATAGTGCAACAGCAAAGC---
AATTTG---CTGAGG---GCTATAGAGGCGCAACAGCATATGTTGCAACTCACGGTCTGGGGCATT
>KC186388
AATTATACA--

GATAATTCAACCATCACACTCCAATGCAGAATAAAGCAAATTATAAACATGTGGCAGGAGGTAGGAAAAGCAA
TGTATGCCCCTCCCATTGCAGGAAACATAACATGTGTATCAAGT---

```
ATTACAGGAATACTATTGACACGTGATGGAGGAGATATAAATAGT----------------------------
-------------------------------------------------------------------------
-------------
AGCATAGAGACATTCAGGCCTGGAGGAGGAGATATGAGAGACAATTGGAGAAGTGAATTATATAAATATAAAG
TGGTAGAAGTTAAGCCATTG---GGAATAGCACCCACGGGAGCAAAAAGGCGAGTGGTG--------------
-------------------------GAAAGAGAGAGAAGAGCA---GTGGGA---ATAGGAGCT---------
GTGTTCCTTGGAATCTTGGGAGCG---GCAGGAAGCACTATG---GGCGCGGCGTCA---
ATAACGCTGACGGTACAG---GCCAGACAA---TTGTTG---TCTGGTATAGTGCAACAGCAAAGT---
AATTTG---CTGAGA---GCTATAGAGGCGCAACAGCATATGTTGCAACTCACAGTCTGGGGCATT
>KP109525
TTTCATTCCAATGATACATTAACAGGAGGTAAC----------------------------------------
-----------------------------------------------------------------
TTAACCGGGAACATCACACTCCCATGTAGAATAAAACAAATTATAAACATGTGGCAGGAGGTAGGAAGAGCAA
TGTATGCCCCTCCTATTGCAGGAAACATAACATGTAAATCAAAT---
ATCACAGGACTACTCTTGACACGTGATGGAGGAAATGGAAATGGC----------------------------
-------------------------------------------------------------------------
----------
AGCAATAACGAGACATTTAGGCCTGGAGGAGGAGACATGAGGGACAATTGGAGAAGTGAATTATACAAATACA
AAGTGGTAGAAATTAAACCATTA---GGAATAGCACCCACTAAGGCAAAAAGGAGAGTGGTG-----------
----------------------------GAGAGAGAAAAAAGAGCA---GTGGGT---CTAGGAGCT------
---ATGTTCCTTGGGTTCTTGGGAGCG---GCAGGAAGCACTATG---GGCGCGGCGTCA---
CTAACGCTGACGGTACAG---GCCAGACAA---TTATTG---TCTGGTATAGTGCAACAGCAAAGC---
AATTTG---CTGAGG---GCTATAGAGGCGCAACAGCATTTGTTGCAACTCACAGTCTGGGGCATT
>KC186502
AATTATACATTTAACAAG-------------------------------------------------------
--------------------------------------------------------
ACAGAAGGTAACATCACACTCCCATGCAAAATAAGACAAATTATTAACCTGTGGCAGGAGGTAGGACGAGCAA
TGTATGCCCCTCCCATTGAAGGAAACATAACATGTAGATCAGAC---
ATCACAGGATTACTATTGACACGTGATGGAGGAGAAAATAGTACA----------------------------
-------------------------------------------------------------------------
-------------
AGGAATGAGACATTCAGACCTGGAGGAGGAGATATGAGGGACAATTGGAGAAGTGAATTATATAAATATAAAG
TAGTAGAAATTAAGCCATTA---GGAATAGCACCCACTGAGGCAAGAAGGAGAGTGGTG--------------
-------------------------GAGAGAGAAAAAAGAGCA---ATTGGA---ATAGGAGCT---------
GTGTTCCTTGGGTTCTTGGGAGCA---GCAGGAAGCACTATG---GGCGCAGCATCA---
ATAACGCTGACGGTACAG---GCCAGACAA---TTATTG---TCTGGTATAGTGCAACAGCAAAGC---
AATTTG---CTGAGG---GCTATAGAGGCGCAACAGCATATGTTGCAACTCACAGTCTGGGGCATT
>MF500628
AATCATACAAACAGTAATAGTACAAGTAATAGT----------------------------------------
-----------------------------------------ACAAGT------
AATCCAAATGCAACCATCACCATCCAATGCAGAATAAAACAAATTATAAACATGTGGCAGGGGGTAGGACAAG
CAATATATGCCCCTCCCATTAAAGGAAACATTACATGTAGATCAAAT---
ATCACAGGACTACTATTGACACGTGATGGAGGTAATAATAATACT----------------------------
-------------------------------------------------------------------------
----------------
AATGAGACATTCAGACCTGGAGGAGGAGATATGAGAGACAATTGGAGAAGTGAATTATATAAGTATAAAGTAG
TAGAAATTAAGCCACTA---GGAGTAGCACCCACCAAGGCGAAAAGACGAGTGGTG-----------------
----------------------GAGAGAGAAAAAAGAGCA---GTGGGA---ATAGGAGCT---------
GTACTCCTTGGGTTCTTGGGCGCA---GCAGGAAGCACTATG---GGCGCGGCGTCG---
CTGACGCTGACGGTACAA---GCCAGACAA---TTATTG---TCTGGTATAGTGCAACAGCAAAAC---
AATTTG---CTGAGG---GCTATAGAGGCGCAACAGCACATGTTGCAACTCACAGTCTGGGGCATT
>KC863183
TGCTATACATCAACACTGTTTAATAGTACATACAATGCTGAGTATAATTCAAACTCAACCAAT----------
-------------------------------------------------------------------------
----------------------
TCAAGCTCAACCATCACACTCCAATGCAGAATAAAGCAAATTATAAACATGTGGCAGGGGGTAGGACGAGCAA
TATATGCCCCTCCCATTGAAGGAAACATAACATGTGAGTCAAAT---
ATCACAGGACTATTATTGACACGGGATGGAGGAAAAGATGGAAAT----------------------------
-------------------------------------------------------------------------
-------------
```

AACACCGAGATATTCAGACCTGGAGGAGGAGATATGAGGGACAATTGGAGAAGTGAATTATACAAATATAAAG
TGGTAGAAATTAAGCCATTA---GGAATAGCACCCACTAAGGCCAAAAGGAGAGTGGTG--------------
-------------------------GAGAGAGAAAAAAGAGCA---GTAGGA---ATAGGAGCT---------
GTGTTCCTTGGGTTCTTGGGAGCA---GCAGGAAGCACTATG---GGCGCAGCGTCA---
ATAACGCTGACGGTACAG---GCCAGACAA---TTGTTG---TCTGGTATAGTGCAACAGCAAAGC---
AATTTG---CTGAGA---GCTATAGAGGCGCAACAGCATATGTTGCAACTCACGGTCTGGGGCATT
>KJ158421
AATTATACA--
--------------------------AGAAATAGTACAGGAAAT---------
GATTCAAAAGTAGTCATTACGATCCCATGCAGAATAAAACAAATTATAAACATGTGGCAGGGAGTAGGGCGAG
CAATGTATGCCCCTCCCATTGCAGGAAACATAACTTGTAAATCCAAT---
ATCACAGGACTACTATTGGTACCTGATGTAGGAGAA-------------------------------------
----
GATGGAGGGAACACATTAAATACT---------
ACAGAAGAGACATTCAGACCTGTAGGAGGAAATATGAGGGACAATTGGAGAAGTGAATTATACAAATATAAAG
TGGTAGAAATTAAGCCATTG---GGGGTAGCACCCACTGAAGCAAGAAGGAGAGTGGTG--------------
-------------------------GAAAGAGAAAAAAGAGCA---GTGGGA---GTAGGAGCT---------
ATGTTCCTTGGGTTCTTGGGAGCA---GCAGGAAGCACTATG---GGCGCGGCGTCA---
CTAATGCTGACGGTACAG---GCCAGACAA---TTGTTG---TCTGGTATAGTGCAACAGCAAAGC---
AATTTG---CTGAGA---GCTATAGAGGCGCAACAGCATCTGTTGCAACTCACAGTCTGGGGCATT
>KJ158424
TGCTATACATCAAGACTGTTTAATGGAACATATAAGGATACTGATACC-------------------------
----
CTAAATAATACAAGCAGT---------
AACGCAATATCAAACATCACAATCCCATGCAGAATAAAACAGTGTGTGAACATGTGGCAACAGGTAGGACAGG
GGGGGGATGCCCCTCCCATTGAAGGAGCACGTGAAGCTGCTATCAAA---
TACAAAGGACTACGATTGGTACGTGATGGAGGAGAA-------------------------------------
----
GACAAAAATAATACAGACAGCAAT---------
GAGACAGAGACATTCAGACCTGGGGGAGGAGATATGAGGGACAATTGGAGAAGTGAATTATATAAATATAAAG
TGGTGGAAATTAAGCCATTG---GGAGTAGCACCCACTGCAGCAAGAAGGAGAGTGGTG--------------
-------------------------GAAAGAGAAAAAAGAGCA---GTGGGA---ATAGGAGCT---------
GTGTTCCTTGGGTTCTTGGGAGCA---GCAGGAAGCACTATG---GGCGCGGCATCA---
ATAACGCTGACGGTACAG---GCCAGACAA---TTGTTG---TCTGGTATAGTGCAACAGCAAAAC---
AATTTG---CTGAGG---GCTATAGAGGCGCAACAGCATCTGTTGCAACTCACGGTCTGGGGCATT
>KJ158434
AATTATACA--
--------------------------AGAAATAGTACAGGAAAT---------
GATTCAAACGTAGTCATTACGATCCCATGCAGAATAAAACAAATTATAAACATGTGGCAGGGAGTAGGGCGAG
CAATGTATGCCCCTCCCATTGCAGGAGACATAACATGTATCTCTAAG---
ATCACAGGATTACTATTGGTACGTGATGGGGGAGAA-------------------------------------
----
AACATACAAAGAAATAATGATGCA---------
GGGACAGAGACATTCAGACCTGGAGGAGGAGATATGAGGAACAATTGGAGAAGTGAATTATATAAATATAAAG
TGGTAGAAATTAAGCCATTG---GGGGTAGCACCCACTAAAGCAAAAAGGAGAGTGGTG--------------
-------------------------GAGAGAGAAAAAAGAGCA---ATGGGA---ATAGGAGCT---------
TTGTTCCTTGGGTTCTTGGGAACA---GCAGGAAGCACTATG---GGCGCGGCGTCA---
ATAACGCTGACGGTACAG---GCCAGACAA---TTGTTG---TCTGGTATAGTGCAACAGCAAAAT---
AATTTG---CTGAAG---GCTATAGAGGCGCAACAGCATCTGTTGCAACTCACGGTCTGGGGCATT
>KJ158438
GTTTATAATGGTACA--
--------------------------------GAAAGTAATAGTAACAGT---------
AATAGTAATGCAACCATCACCCTCCCATGCAAAATAAAACAAATTATAAACATGTGGCAGGAGGTAGGACGGG
CAATGTATGCCCCTCCTATTGAAGGAAACATAACATGTAACTCAAAT---
ATCACAGGATTACTCTTGGTACGTGATGGAGGCAAT-------------------------------------
----
GAAAATAATAACAATAATAATAAC---------
TTCGCAGAGACATTCAGACCAGGAGGAGGAGATATGAGGGACAATTGGAGAAGTGAATTATATAAATATAAAG
TAGTAGAAATTAAGCCATTG---GGAGTAGCACCCACTGCAGCAAAAAGGAGAGTGGTG--------------
-------------------------GAGAGAGAAAAAAGAGCGCAGCGG------CTAGGAGCT---------

GTGTTCCTTGGGTTCTTGGGAGCA---GCAGGAAGCACTATG---GGCGCGGCGTCA---
ATAACGCTGACGGTACAA---GCTAGACAA---TTGTTG---TCTGGTATAGTGCAACAGCAAAGC---
AATTTG---CTGAGG---GCTATCGAGGCGCAACAGCATCTGTTGCAACTCACAGTCTGGGGCATT
>KJ158439
ACC---
--------------------TACATTAATGAAACAAAT---------
AGTAATGCTACATCCACCACCCCACCCTGCAGAATAAAACAAATTATAAACATGTGGCAAGAGGTAGGACGAG
CAATGTATGCCCCTCCCATTGCAGGAAATATAACATGTAAATCAAAT---
ATCACAGGAATACTATTGGTACGGGATGGAGGAAAC-------------------------------------
----
AATGATAATGATGATAATGATAAT---------
GATACAGAGACATTCAGACCTGGAGGAGGAGACATGAAGGACAATTGGAGAAGTGAATTATATAAATATAAAG
TGGTAGAAATTAAGCCATTG---GGATTAGCACCCACTACAGCAAAAAGGAGAGTGGTG-------------
-------------------------GAGAGAGAAAAAAGAGCA---GTAGGAACCTTAGGAGCT---------
GTGTTCCTTGGGTTCTTGTCAGCA---GCCGGAAGCACTATG---GGCGCGGCGTCA---
ATAACGCTGACGGTACAG---GCCAGACAA---TTGTTG---TCTGGTATAGTGCAACAGCAAAGC---
AATTTG---CTGAGG---GCTATAGAGGCGCAACAGCATCTGTTGCAACTCACGGTCTGGGGCATT
>KJ541846
TTTTATGATAGT---
-----------------------------ACGAGAAAGGCAAACATC---------
ACAGGAAATTCAACCATCACACTCCCATGCAGAATAAAGCAAATTATAAACATGTGGCAGGAGGTAGGACGAG
CAATGTATGCTCCTCCCATTGCAGGAAACATAACATGTAGATCAAAT---
ATCACAGGACTACTATTGGTACGTGATGGAGGAGAA-------------------------------------
----
GATAACACCAAATCAGAGACCTGG---------
AAGACTGAAACATTTAGACCGGGAGGAGGAGATATGAGGGACAATTGGAGAAGTGAATTATATAAATATAAAG
TGGTAGAAGTTGAGCCAGTG---GGAATAGCACCCACTAAAGCAAAAAGGAGAGTGGTG-------------
-------------------------GAGAGAGAAAAAAGAGCA---GTAGGA---ATGGGAGCT---------
TTGTTCCTTGGGTTCTTGGGAGCA---GCAGGAAGCACTATG---GGCGCGGCGTCA---
ATAACGCTGACGGTACAG---GCCAGACAA---TTGCTG---TCTGGTATAGTGCAACAGCAAAGC---
AATTTG---CTGAGA---GCTATAGAGGCGCAACAGCATCTGTTGCAACTCACAGTCTGGGGCATT
>KP411831
TCTTATAATAGTACCTCTAATACTACTTGCTCGACTAATAATACT---------------------------
----
ACAACTTCAACCTTTACCCTCCAATGCAGAAGAATACATATTATAATCATGTGCCTTGAACCAGGACTAGCAA
TAGATGCCTCTCCCACTGCCGGAGAAATAATCAGTAAATCCAAT---
ACCACTGACCTACTATTGACACGTGCAGGAGGA--

TCGAATAGAACAGAGATATTCAGACCTGGAGGAGGAGATATGAGGGATAATTGGAGAAGTGAGTTATATAAAT
ATAAAGTGGTAGAAATTAAACCATTA---GGGATAGCACCCACTAAGGCAAAAAGGAGAGTGGTG--------
-------------------------------GAGAGAGAAAAAAGAGCA---GTGGGA---ATAGGAGCT---
------GTGTTCCTTGGGTTCTTGGGAGTA---GCAGGAAGCACTATG---GGCGCAGCATCA---
ATAACGCTGACGGTACAG---GCCAGGCAA---TTGTTG---TCTGGTATAGTGCAACAGCAAAGC---
AATTTG---CTGAGG---GCTATAGAGGCGCAACAGCATATGTTGCAACTCACGGTCTGGGGCATT
>MF373158
ACCTACATGTCTAACAGTACTAACAGTATTAAT--

AGTACTGAATCCATCACACTCAAATGCAAAATAAAACAAATTATAAACATGTGGCAAGGGGTAGGACGAGCAA
TGTATGCCCCTCCCGTTGAAGGAAACATAACATGTACCTCAAAT---
ATTACAGGACTACTATTGACACGTGATGGAGGAGGAAATGGCACA----------------------------
----

GAGGAGATATTCAGACCGGGAGGAGGAAATATGAGGGACAATTGGAGAAGTGAATTATATAAATATAAAGTGG
TAGAAGTTAAACCATTA---GGAGTAGCACCCACTAAAGCAAAAAGGAGAGTGGTG----------------
----------------------GAAAGAGAAAAAAGAACA---GTA------CTAGGAGCT---------
GCATTCCTTGGGTTCTTGGGAACA---GCAGGAAGCACTATG---GGCGCAGCGTCA---
CTAACGCTGACGGTACAG---GCCAGAAAA---TTGTTG---TCTGGTATAGTGCAACAGCAAAGT---
AATTTG---CTGAGG---GCTATAGAGGCGCAACAGCATATGTTGCAACTCACGGTCTGGGGCATT
>MF373164

```
AACTAC-------------------------------------------------------------------
--------------------------------------------------------
ACAATAATCCCATGTAGAATAAAACAAATTATAAATATGTGGCAGGCGGTAGGACGAGCAATGTATGCCCCTC
CCATTGCAGGAAACATAACGTGTGCATCAAAC---
ATCACAGGACTACTATTGTCACGTGATGGAGGAAACACAAATACAAATGGCACA-------------------
-------------------------------------------------------------------------
----------
AGTAACACAGAGACTTTCAGACCTGGAGGAGGAGATATGAGAGACAATTGGAGAAGTGAATTATATAAATATA
AGGTGGTAGAAATTAAACCATTA---GGAATAGCACCCACTGGGGCAAAAAGGAGAGTGGTG-----------
----------------------------GAGAGAGAAAAAAGAGCA---GTGGGA---ATAGGAGCT------
---GTGTTCCTTGGGTTCTTGGGAGCA---GCAGGAAGCACTATG---GGCGCGGCGTCA---
GTGACGCTGACGGTACAG---GCCAGACAG---CTGTTG---TCTGGTATAGTGCAACAGCAAAAC---
AACTTG---CTGAAA---GCTATAGAGGCGCAACAGCATATGTTGCAACTCACGGTCTGGGGCATT
>MF373197
AGTTATTCCGCCAATGGC-------------------------------------------------------
--------------------------------------------------
AGCAATGGAAGCATTCCCCTCATATGCCGAATTATACAAATTTTTAACATGTGGCAGCGGGTGGTACGAGCAA
TATATGCTGCTCGTATTGTAGGAAGTATAACATGTATCTCATGT---
ATCACAGGACTACTGTTGACACGTGCTGGAGGAACAAATAATGAT----------------------------
-------------------------------------------------------------------------
----------
ACCACAAGAGAAATATTCAGACCTGGAGGAGGAAATATGAAGGACAATTGGAGAAGTGAATTATACAAATATA
AAGTGGTAGAAGTTAAACCATTA---GGAATAGCACCTACTGAGGCAAAAAGGAGAGTGGTG-----------
----------------------------GGGAGAGACAAAAGAGCA---GTGGGA---ATAGGAGCT------
---GTGTTCCTTGGGTTCTTGGGAGCA---GCAGGAAGCACTATG---GGCGCGGCGTCA---
ATAACGCTGACGGTACAG---GCCAGACAA---TTACTA---TCTGGTATAGTGCAGCAGCAAAGC---
AATTTG---CTGAGA---GCTATAGAGGCGCAACAGCATATGTTGCAACTCACAGTCTGGGGCATT
>AY734556
AGCTATACA----------------------------------------------------------------
--------------------------------------------GGAAAAGATTCAAACTCAATC---
ATCAGAATAAGACAATTTATAAACACATGGCAGAAGGTAGGACGAGCAATGTATGCCCCTCCCATAGCAGGAA
ACATAACATGTAACTCAAGC---ATTACAGGACTACTATTGACATGGGATGGAGGCAGTAGTAAAGAAACA--
-------------------------------------------------------------------------
------------------------------
AGCAACACCACAGAGACATTCAGACCTTTAGGAGGAGACATGAGGGATAATTGGAGACGTGAATTATATAAAT
ATAAAGTGGTAGAAATTAAGCCATTG---GGAATAGCACCCACTGGGGCAAAAAGGAGAGTGGTG--------
-------------------------------GAGAGAGAAAAAAGAGCA---GTGGGA---CTAGGAGCT---
------ATGTTCCTTGGGTTCTTGGGAGCA---GCAGGAAGCACTATG---GGCGCAGCGTCG---
ATAACGCTGACGGCACAG---GCCAGACAA---GTGCTG---TCTGGTATAGTGCAACAGCAAAGC---
AATTTG---CTGAGG---GCCATAGAGGCGCAACAGCATCTGTTGCAACTCACGGTCTGGGGCATT
>KX907339
AATTATACT----------------------------------------------------------------
-----------------------------------------------
ATAAATTCAACCATCACACTCCCATGCAGAATAAAACAAATTATAAATATGTGGCAGGGGGTAGGACGAGCAA
TGTATGCCCCTCCCATTGCTGGAAACATAACATGCAGATCAAAT---
ATCACAGGAATACTATTGACACGTGATGGTGGTGAAACAAATGATGGTAATACAAATGAAACAGGGACAAAGA
CATTCAAATCTAAT-----------------------------------------------------------
-------
GAAACAGAGACAGAGATATTCAGACCTGGAGGAGGAGATATGAGGGACAATTGGAGAAGTGAATTATATAAAT
ATAAAGTGGTAGAAATTAAGCCATTA---GGAATAGCACCCACCAAGGCAAAAAGGAGAGTGGTG--------
-------------------------------GAGAGAGAGAAAAGAGCA---GTGGGA---ATAGGAGCT---
------ATGTTCCTTGGGATCTTGGGAGCA---GCAGGAAGCACTATG---GGCGCGGCGTCA---
ATAACGCTGACGGTACAG---GCCAGACAA---TTATTG---TCTGGTATAGTGCAACAGCAAAGC---
AATTTG---CTGAGA---GCTATAGAGGCGCAACAGCATATGTTGCAACTCACGGTCTGGGGCATT
>KX907433
TACTATCCTAATGGT----------------------------------------------------------
-----------------------------------------------
ACAAATTCAACCCTCATCATCCCATGTAGAATTAAACAAATTATAAACATGTGGCAGGGAGTAGGAAAAGCAA
TATATGCCTCTCCCATTGCAGGAAACATAACATGTAGATCAAAT---
ATCACAGGACTACTATTGACACGTGATGGAGGAGACACAAATGAT----------------------------
```


ACAGAGATATTCAGACCGGCAGGAGGAGATATGAGGGATAATTGGAGAAGTGAATTATATAAATATAAAATAG
TAGAAATTAAACCATTA---GGAGTAGCACCCACTGAGGCAAAAAGGAGAGTGGTG-----------------
----------------------AAGAGAGAAAAAAGAGCA---GTGACA---ATAGGAGCT---------
GTATTCCTTGGGTTCTTGGGAGCA---GCAGGAAGCACTATG---GGCGCGGCGTCA---
ATAACGCTGACGGTACAG---GCCAGACAA---TTACTG---TCTGGTATAGTGCAGCAGCAAAGT---
AATTTG---CTGAGA---GCTATAGAGGCGCAACAGCATATGTTGCAACTCACAGTCTGGGGCATT
>KX907382
ATT--

CAAAATGGAACCACCATACTCCCATGCAGAATAAAACAAATTATAAACATGTGGCAGGAGGTAGGACGAGCAA
TGTATGCCCCTCCCATTGCAGGAAACATAACATGTACATCAAAC---
ATCACAGGACTACTATTGACACGAGATGGAGGCAAAAATAACACA----------------------------
----
GGAGGCAAAAATAACACAAAT---------
GGCATAGAGATATTCAGACCTGAAGGAGGAAATATGAGGGATAATTGGAGAAGTGAATTATATAAATACAAGG
TGGTAGAAATTAAGCCATTG---GGAATAGCACCCACTGAGGCAAAAAGGAGAGTGGTG--------------
-------------------------GAGAGAGAGAAAAGAGCA---GTGGGA---ATAGGAGCT---------
GTGTTCCTTGGGTTCTTGGGAGCA---GCAGGAAGCACTATG---GGCGCGGCGTCA---
ATAACGCTGACGGTACAG---GCCAGACAA---CTGTTG---TCTGGTATAGTGCAACAGCAAAGC---
AATTTG---CTAAGA---GCTATAGAGGCGCAACAGCATCTGTTGCAACTCACGGTTTGGGGCATT
>KX907385
AACTATAATGGTACA--

TCAAATGACACCATCACACTCCGGTGTAGAATAAAACAAATTATAAACATGTGGCAGGGGGTAGGACAAGCAA
TGTATGCCCCTCCCATTGCAGGAAACATAATATGTGCATCAAAT---
ATCACAGGACTACTATTGACACGTGATGGAGGAGACGAAAATAGG----------------------------
----

ACAGATGAGATATTCAGGCCGGAAGGAGGAAATATGAGAGATAATTGGAGAAGTGAATTATATAAATATAAAG
TGGTAGAAATTAAGCCATTA---GGGATAGCACCCACTAAGGCAAAAAGGAGAGTGGTG--------------
-------------------------GAGAGAGAAAAAAGAGCA---GTAGGA---ATAGGAGCT---------
GTGTTCCTTGGGTTCTTAGGAGCA---GCAGGAAGCACTATG---GGCGCAGCATCA---
ATAACGCTGACGGTACAG---GCCAGACAA---TTATTG---TCTGGTATAGTGCAACAGCAAAGC---
AATTTG---CTGAGG---GCTATAGAGGCGCAACAGCATCTGTTGCAACTCACAGTCTGGGGCATT
>KX907368
AACTATACAGAGTTTAAAAATATCTCAACAAACGGTACT----------------------------------
----
-
TCGAATTCAAACATCACACTCCCATGCAGAATAAAACAAATTATAAACATGTGGCAAGGGGTAGGACGAGCAA
TGTATGCCCCTCCCAGGGAAGGAAACATAACATGTGAATCGAAC---
ATCACAGGACTACTATTGACACGAGATGGAGGAAATAGTACAAAA----------------------------
----

AAGGAGATATTCAGACCTGGAGGAGGAGATATGAGGGATAATTGGAGAAGTGAATTATATAAGTATAAAGTGG
TAGAAATTAAGCCATTA---GGAATAGCACCCACTGAGGCAAAAAGGCGAGTGGTG-----------------
----------------------GAGAGAGAGAAAAGAGCA---GTGGGA---ATAGGAGCT---------
GTGTTCCTTGGGTTCTTAGGAGCA---GCAGGAAGCACTATG---GGCGCAGCGTCA---
ATAACGCTGACGGTACAG---GCCAGACAA---TTATTG---TCTGGTATAGTGCAACAGCAAAGC---
AATTTG---CTGAGG---GCTATAGAGGCGCAACAGCATATGTTGCAACTCACAGTCTGGGGCATT
>KT982201
ACTCATGATACAGGAAGTAAT--

TCAACCTCAAACATCACCCTCCAATGCAAAATAAAACAAATTATCAACATGTGGCAGGAGGTAGGACGAGCAA
TGTATGCCCCTCCCATTGCAGGAAACATAACATGTAGATCAAAT---
ATCACAGGACTACTATTGGTACGGGATGGAGGCACAGATAATAGCACAAAT----------------------
----

AAGAGTGACCCAGAGGAGACATTTAGACCTGGAGGAGGAGATATGAGGGACAATTGGAGAAGTGAATTATATA

AATATAAAGTGGTAGAAATTAAGCCATTA---GGGATAGCACCAACTGAGGCAAGGAGGAGAGTGGTG-----
----------------------------------CAGAGAGAAAAAAGAGCA---GTGGGA---
ATAGGAGCT---------CTGTTCCTTGGGTTCTTGGGAGCT---GCAGGAAGCACTATG---
GGCGCGGCGTCA---ATAACGCTGACGGCACAG---GCCAGACAA---GTATTG---
TCTGGTATAGTGCAACAGCAAAGC---AATTTG---CTGAGA---
GCTATAGAGGCGCAACAGCATATGTTGCAACTCACAGTCTGGGGCATT
>AY585266
GAT--

TACAACACAACACTAACACTCCCATGCAGGATAAACCAGATTATAAACATGTGGCAGGAGGTAGGACGAGCAA
TGTATGCTCCTCCCATTGCAGGAAACATAACATGTAACTCAAAT---
ATCACAGGACTACTATTGACACGTGATGGAGGAAACAATGGAGGA----------------------------
----

AACAATGAAACATTCAGACCTGGAGGAGGAAATATGAAAGACAATTGGAGAAGTGAATTATACAAATATAAAG
TGGTAGAGATTAAGCCATTG---GGAATAGCACCCAATAAGGCAAAAAGGAGAGTGGTG--------------
-------------------------GAGAGAGAAAAAAGAGCA---GTGGGA---ATAGGAGCT---------
GTGTTCCTTGGGTTCTTGGGAGCA---GCAGGAAGCACTATG---GGCGCGGCGTCA---
ATAACGCTGACGGCACAG---GCCAGACAA---CTGTTG---TCTGGTATAGTGCAACAGCAAAGT---
AATTTG---CTGAGA---GCTATAGAGGCGCAACAGCATTTGTTGCAACTCACAGTCTGGGGCATT
>AY463224
GTTCAA---

AGCAACGGAAGCATCATACTCCCATGCAAGATAAAACAAATTATAAACATGTGGCAGGGGGTAGGACGAGCAA
TGTATGCCCCTCCCATCGCAGGAAACATAACATGTAACTCAAGT---
ATCACAGGACTACTATTAACGAGGGATGGAGGCATAAGTGAGAAC----------------------------
----

ATGACAGAGACATTCAGACCTGGAGGGGGAAATATGAAGGACAATTGGAGAAGTGAATTATATAAATATAAAG
TAGTAGAAATTAAGCCATTG---GGAATAGCACCCACTAAGGCAAAAAGGAGAGTGGTG--------------
-------------------------GAGAGAGAAAAAAGAGCA---GCA------ATAGGAGCT---------
ATGTTCCTTGGGTTCTTGGGAGCA---GCAGGAAGCACTATG---GGCGTGGCGTCA---
ATAACGCTGACGGTACAG---GCCAGAAAT---CTGTTG---TCTGGTATAGTGCAACAGCAAAGC---
AATTTG---CTGAGA---GCTGTAGAAGCGCAACAACATATGTTGCAACTCACGGTCTGGGGCATT
>JX845593
GATCAAAAT--

GATTCAACAAACATCACACTCCCATGCAGAATAAAACAAATTATAAACATGTGGCAGGAGGTGGGACGAGCAA
TGTATGCCCCTCCCATTGCAGGAAACATAACATGTACATCAAAT---
ATTACAGGACTATTATTGACACGTGATGGAGGAAACGCAAACACAACGGATGGT-------------------
----

AAGAATGACACAGAGACATTCAGACCTGGAGGAGGAAATATGAAGGACAATTGGAGAAGTGAATTATATAAAT
ATAAAGTGGTAGAAATTAAACCATTG---GGAGTAGCACCCACAAAGGCAAAAAGGAGAGTGGTG--------
-------------------------------GAGAGAGAAAAAAGAGCA---GTGGGA---ATGGGAGCT---
------GTGTTCCTTGGGCTCTTGGGAGCA---GCAGGAAGCACTATG---GGCGCGGCGTCA---
ATAACGCTGACGGTACAG---GCCAGACAA---CTGCTG---TCTGGTATAGTGCAACAGCAAAGC---
AATTTG---CTGAGA---GCTATAGAGGCGCAACAGCATATGTTGCAACTCACAGTCTGGGGCATT
>JX845600
AAT--

CACACTCTCAATCTCACTCTCCCATGCAGAATAAAACAAATTATAAACATGTGGCAGGAGGTAGGAAGAGCAA
TGTATGCCCCTCCCATTGCAGGAAACATAACATGTAAATCAAAT---
ATCACAGGACTACTATTGACACGTGATGGAGGAATAAATATTACA----------------------------
----

ACAGAAATATTCAGACCTGCAGGAGGAAATATGAAGGACAATTGGAGAAGTGAATTATATAAATATAAAGTGG
TAGAAATTAAGCCATTA---GGAATAGCACCTACTAAGGCAAAAAGGAGAGTGGTG-----------------
----------------------GACAAAGAAAAAAGAGCA---GTAGGA---ATAGGAGCT---------
GTGTTCCTTGGGTTCTTGGGAGCA---GCAGGAAGCACTATG---GGCGCGGCGTCA---

ATAACGCTGACGGTACAG---GCCAGACAA---CTGTTG---TTTGGTATAGTGCAACAGCAAAAC---
AATTTG---CTGAGA---GCTATAGAGGCGCAACAGCATATGTTGCAACTCACGGTCTGGGGCATT
>AY228557
ACTTATAATAATACAGATAATACAGAT--

AATCCAACCCAAATCACACTCCCATGCAGAATAAAACAAATTATCAACATGTGGCAGGAGGTAGGACGAGCAA
TATATGCCCCTCCCATTGCAGGAAACATAACATGTAAATCGAAT---
ATCACAGGACTACTATTAACATGGGATGGAGGTAGTGGTGAAAAC----------------------------
----

AATACAGAGACATTTAGACCAGGAGGAGGAGATATGAGGGACAATTGGAGAAGTGAATTATATAAATATAAAG
TGGTAGAAATTAAGCCATTA---GGAATAGCACCCACTGAGGCAAAAAGGAGAGTGGTG--------------
-------------------------GAGAGAGAAAAAAGAGCA---ATAGGA---ATAGGAGCT---------
GTGTTCCTTGGGTTCTTGGGAGCA---GCAGGAAGCACTATG---GGCGCAGCGTCA---
ATAACGCTGACGGTACAG---GCCAGACAA---TTGTTG---TCTGGTATAGTGCAACAGCAAAGC---
AATTTG---CTGAGG---GCTATAGAGGCGCAACAGCATCTGTTGCAGCTCACTGTCTGGGGCATT
>DQ275647
CTTCAAAATAATAGTACA---

GGAAATTCAACCATCAAAATCAAATGCAGAATAAAACAAATTATAAACATGTGGCAGGAGGTAGGACGAGCAA
TGTATGCCCCTCCCATTGCAGGAAACATAACATGTAGATCAAAC---
ATCACAGGACTACTATTAGTACGTGATGGAGGCATAAACAACACA----------------------------
----

GAAGAAATATTCAGACCTGGAGGAGGAAATATGAAGGACAATTGGAGAAGTGAATTATATAAATATAAAGTAG
TAGAAATTAAGCCATTG---GGAATAGCACCCACTCCAGCAAAAAGGAGAGTGGTG-----------------
----------------------GAGAGAGAAAAAAGAGCA---GTGGGA---ATAGGAGCT---------
TTGTTCCTTGGGTTCTTGGGAGCA---GCAGGAAGCACTATG---GGCGCAGCGTCA---
ATGACGCTGACGGTACAG---GCCAGACAA---GTGTTG---TCTGGTATAGTGCAACAGCAAAAT---
AATTTG---CTGAAG---GCTATAGAGGCGCAACAGCGTATGTTGCAACTCACAGTCTGGGGCATT
>DQ351224
TCTTATAATGCTACAGGA---

GATAATACAACCATCACACTGCCATGCAAAATAAAACAAATTATAAACATGTGGCAGACGGTAGGAAAAGCGA
TGTATGCCCCTCCCATTGCAGGAAACATAACATGTAAATCAAAT---
ATCACAGGACTACTATTAACACGTGATGGAGGCGAAACTAACAGTACGAAT----------------------
----

GACACAGAGACAGAGATATTCAGACCTGGAGGAGGAAATATGAAGGACAATTGGAGAAGTGAATTATATAAAT
ACAAAGTGGTAGAAATTAAGCCATTG---GGAATAGCACCCACTGAAGCAAAAAGGCGAGTGGTG--------
-------------------------------GAGAGAGAAAAAAGAGCA---GTGGGA---ATAGGAGCT---
------GTGCTCCTTGGGTTCTTGGGAGCA---GCAGGAAGCACTATG---GGCGCAGCGTCA---
ATAACGCTGACGGTACAG---GCCAGACAA---CTGTTG---TCTGGTATAGTGCAACAGCAAAGT---
AATTTG---CTGAGA---GCTATAGAGGCGCAACAGCATCTGTTGCAACTCACGGTCTGGGGCATT
>DQ369977
AGTTATAATAATAGTAAG---

GATAATGAGACCATCACACTCCAATGCAGAATAAAACAAATTATAAACATGTGGCAGAAAGTAGGACGAGCAA
TGTATGCACCTCCCATTGAAGGAAACATAACATGTAACTCAAAT---
ATCACAGGACTACTATTGCTACGTGATGGAGGAAAAGAGAACAAT----------------------------
----

TCCACAGAAATATTCAGACCTGGAGGAGGAAATATGAAGGACAATTGGAGAAGTGAATTATACAAATATAAAG
TGGTAGAAATTAAGCCATTG---GGAATAGCACCCACGGAGGCAAAAAGGAGAGTGGTG--------------
-------------------------GAGAGAGAAAAAAGAGCA---GTAGGA---ATAGGAGCT---------
GTGTTCCTTGGGTTCTTGGGAGCA---GCAGGAAGCACTATG---GGCGCGGCGTCA---
ATAACGCTGACGGTACAG---GCCAGACAA---CTGTTG---TCTGGTATAGTGCAACAGCAAAGC---
AATTTG---CTGAGA---GCTATAGAGGCGCAACAACATATGTTGCAACTCACGGTCTGGGGCATT
>DQ275649

ACTCAGAGTACAAACAGT--

ACAAATTCAACCATCACACTCCAATGCAGAATAAAACAAATTATAAACATGTGGCAGGAGGTGGGACGAGCAA
TGTATGCCCCTCCCATTGCAGGAAACATAACATGTAAATCAAAT---
ATCACAGGACTACTATTGACACGTGATGGAGGAGGAAGAGAAGGAAATAATACA------------------
----

GAAGGAAATAATACAGAGACATTCAGACCTGGAGGAGGAGATATGAGGGACAATTGGAGAAGTGAATTATATA
AATATAAAGTGGTAGAAATTAAGCCATTG---GGAATAGCACCCACTGAGGCAAGAAGGAGAGTGGTG-----
----------------------------------CAGAGAGAGAAAAGAGCA---
ATAATGGGACTAGGAGCT---------GCGTTCCTTGGGTTCTTGGGAGCA---GCAGGAAGCACGATG---
GGCGCAGCGTCA---ATAACGCTGACGGTACAG---GCCAGACAA---TTATTG---
TCTGGTATAGTGCAACAGCAAAGC---AATTTG---CTGAAG---
GCTATAGAGGCGCAACAGCATATGTTGCAACTCACAGTCTGGGGCATT
>DQ396377
ATTCATAATGGGACAGGA--

AGTAATTCAACCATCACACTTAATTGTAAAATAAAGCAAATTATAAACATGTGGCAGGAGGTAGGACGAGCAA
TGTACGCTCCTCCCATTGCAGGAAGCATAACATGTAACTCAAGT---
ATCACAGGACTACTATTGACACGTGATGGAGGACAAGGCCAGGAAAAG------------------------
----

GATAATGACACAGAGGTATTCAGGCCTGGAGGAGGAAACATGAAGGACAATTGGAGAAGTGAATTATATAAAT
ATAAAGTGGTAGAAGTTAAACCATTG---GGAATAGCACCCACTAAAGCAAAAAGGAGAGTGGTG--------
-------------------------------GAGAGAGAAAAAAGAGCA---GTGGGA---ATAGGAGCT---
------GTGTTCCTTGGGTTCTTGGGAGTA---GCAGGAAGCACTATG---GGCGCGGCGTCA---
ATGACGCTGACGGTACAG---GCCAGACAA---CTGTTG---TCTGGTATAGTGCAACAGCAAAAT---
AATTTG---CTGAGA---GCTATAGAGGCGCAACAGCATATGTTGCAACTCACGGTCTGGGGCATT
>DQ369990
ATTCAGAATAGTTCAACCATCACG--

GATAATTCAACCATCACCATCCCATGCAGGATAAAACAAATTATAAACATGTGGCAGGAGGTGGGACGAGCAA
TGTATGCCCCTCCCATTGCAGGAAACATAACATGTAACTCAAGT---
ATCACAGGAATACTATTGACACGTGATGGTGGCGATAACGACACA---------------------------
----

AAAGAGAATGAGACATTCAGACCGGGAGGAGGAAATATGAAGGACAATTGGAGAAGTGAATTATATAAATATA
AAGTGGTAGAAATTAAGCCATTG---GGAGTAGCACCCACGAAGGCAAAAAGGAGAGTGGTG-----------
----------------------------GAGAGAGAAAAAAGAGCA---GTGGGA---ATAGGAGCT------
---GTGTTCCTTGGGTTCTTGGGAGCA---GCAGGAAGCACTATG---GGCGCGGCGTCA---
ATAACGCTGACGGTACAG---GCCAGACAG---CTGTTG---TCTGGTATAGTGCAACAGCAGAGC---
AATTTG---CTGAGA---GCTATAGAGGCGCAACAGCATCTGTTGCAACTCACGGTCTGGGGCATT
>DQ275658
AATTACAGTAAGTCAAACATCACCAAGGGCAGT-------------------------------------
----
-
GATCCAAACATCACCACCATCCAATGCAGGATAAAACAATTTATAAACATGTGGCAGGGGGTAGGACGAGCAA
TGTATGCCCCTCCCATTGCAGGAAACATAACATGTAACTCAAGT---
ATCACAGGACTACTATTGACACGTGATGGTGGGAATGAAACGAAT--------------------------
----

GGCACAGAGACATTCAGACCTGGAGGAGGAAATATGAAGGACAACTGGAGAAGTGAATTATATAAATATAAAG
TGCTAGAAATTAAGCCATTA---GGAGTAGCACCCACTGGAGCAAAAAGGAGAGTGGTG-------------
------------------------GAGAGAGAAAAAAGAGCA---GTGGGA---ATAGGAGCT---------
GTGCTCCTTGGGTTCTTGGGAGCA---GCAGGAAGCACTATG---GGCGCGGCGTCA---
ATAACGCTGACGGTACAG---GCCAGACAA---CTGTTG---TCTGGTATAGTGCAACAGCAAAGC---
AATTTG---CTGAGA---GCTATAGAGGCGCAACAGCATATGTTGCAACTCACGGTCTGGGGCATT
>DQ396391
AGTTATTTAAACATAACTGATGCAAACACAACAAAG----------------------------------
----

```
GCAAACGAAACCATCACACTCCCATGCAGAATAAAACAAATTATAAACATGTGGCAGGAGGTAGGACGAGCAA
TGTATGCCCCTCCCATTGCAGGAAACATAGAATGTAAATCAAAT---
ATCGCAGGACTACTATTGGTACGTGATGGAGGACTAAATAGAACAGAT------------------------
----------------------------------------------------------------------
---------
AATAACACAGAGATATTCAAACCTGAAGGAGGAAATATGAAGGACAATTGGAGAAGTGAATTATATAAATATA
AAGTGGTGGAAATTAACCCATTG---GGAGTAGCACCCACGAAGGCAAAAAGGAGAGTGGTG-----------
---------------------------GAGAGAGAAAAAAGAGCA---GTGGGA---ATAGGAGCT------
---GTGTTCCTTGGGTTCTTGGGAGCA---GCAGGAAGCACTATG---GGCGCGGCGTCA---
TTAACGCTGACGGTACAG---GCCAGACAA---CTGTTG---TCTGGTATAGTGCAGCAGCAAAGC---
AATTTG---CTGAGA---GCTATAGAGGCGCAACAGCATATGTTGCAACTCACGGTCTGGGGCATT
>AY878056
ACTTATAATGCTAGTGAG-----------------------------------------------------
-----------------------------------------------------------
GGAAACAGGACCATCACCATCCTATGCAGGATAAAACAAATTATAAACATGTGGCAGGGGGTAGGACAAGCAA
TGTACGCCCCTCCCATTGCAGGAAACATAACATGTAACTCAAAT---
ATCACAGGACTACTATTGACACGTGATGGTGGTACCAACAACAAC---------------------------
----------------------------------------------------------------------
------------
AACACAGAGACATTCAGACCTGGAGGAGGAAATATGAAGGACAGTTGGAGAAGTGAATTATATAAATATAAAG
TGGTAGAAATTAAGCCATTG---GGAGTAGCACCCACCAAGGCAAAAAGGAGAGTGGTG-------------
-------------------------GAGAGAGAAAAAAGAGCA---GCGGGA---ATAGGAGCT---------
GTGATCTTTGGGTTCTTGGGAGCA---GCAGGAAGCACTATG---GGCGCGGCGTCA---
TTAACGCTGACGGTACAG---GCCAGACAA---CTGTTG---TCTGGTATAGTGCAACAGCAAAGC---
AATTTG---CTGAGA---GCTATAGAGGCGCAACAGCATATGTTGCAACTCACGGTCTGGGGCATT
>DQ164104
TTTTATAATGAAAGTAAAGATAATAGA--------------------------------------------
----------------------------------------------------------------------
----
AGTGAGAACATCACAACAATCCCATGTAAGATAAAACAAATTATAAACATGTGGCAGGGGGTAGGACGAGCAA
TGTATGCCCCTCCCATTGAAGGAAACATAACATGTAACTCAAGT---
ATCACAGGACTACTATTGACACGTGATGGAGGATCCTTAGACAAC---------------------------
----------------------------------------------------------------------
------------
AAAACAGAGATATTTAGACCTGGAGGAGGAAATATGAAGGACAATTGGAGAAGTGAATTATATAAATATAAAG
TGGTAGAAATTAAGCCATTG---GGAGTAGCACCCACTGAGGCAAAAAGGAGAGTGGTG-------------
-------------------------GAGAGAGAAAAAAGAGCA---GTGGGA---ATAGGAGCT---------
GTGCTCCTTGGGTTCTTGGGAGCA---GCAGGAAGCACTATG---GGCGCGGCGTCA---
ATAACGCTGACGGTACAG---GCCAGACAA---CTGTTG---TCTGGTATAGCGCAACAGCAAAGC---
AATTTG---CTGAGA---GCTATAGAGGCGCAACAGCATATGTTGCAACTCACAGTCTGGGGCATT
>DQ011165
AATTACAGTTCTGAATATAATAGTACAGGAAGTAATTCAACC------------------------------
--------------------------------------------------------------
AACAGTAGTTCAACCATCACACTTCCATGCAGAATAAAACAAATTATAAACATGTGGCAGGAGGTAGGACGAG
CAGTGTATGCCCCTCCCATTGCAGGAAACATAACATGTAATTCAAAT---
GTCACAGGATTACTATTAGTACGTGATGGAGGAAACGACAGCACA---------------------------
----------------------------------------------------------------------
------------
CCAACAGAAACATTCAGACCTGAAGGAGGAAATATGAAGGATAATTGGAGAAGTGAATTATATAAATATAAAG
TGGTAGAAGTTAAGCCACTA---GGAATAGCTCCCACTGGAGCAAAAAGGAGAGTGGTG-------------
-------------------------GAGAGAGAAAAAAGAGCA---GTAGGA---ATAGGAGCT---------
GTGTTCCTTGGGTTCTTGGGATTA---GCAGGAAGCACTATG---GGCGCAGCGTCA---
ATAACGCTGACGGCACAG---GCCAGACAA---GTGTTG---TCTGGTATAGTGCAACAGCAAAGC---
GATTTG---CTGAAG---GCTATAGAGGCGCAACAGCATATGTTGCAACTCACGGTCTGGGGCATT
>AY901966
ACCTACAATCCTACATACAATGGTACAAGGAATGAT-----------------------------------
----------------------------------------------------------------------
-
GAGGACACAACCATCACACTCCCATGCAAAATAAAACAAATTATAAATATGTGGCAGAAGGTAGGAAGAGCAA
TGTATGCCCCTCCCATTGCAGGAAACATAACATGTAAGTCAAAT---
```

ATCACAGGACTACTATTAGTACGTGATGGAGGCAACACAAATAAC----------------------------
----

ATAACAGAGATATTCAGACCTGGAGGAGGAAATATGAAAGACAATTGGAGAAGTGAATTATATAAATACAAAG
TGGTAGAAATTAAGCCATTA---GGAATAGCACCCACTGAAGCAAAAAGGAGAGTGGTG--------------
-------------------------AAGAAAGAGAAAAGAGCA---GTGGGA---ATAGGAGCT---------
GTGTTCCTTGGGTTCTTGGGAGCA---GCAGGAAGCACTATG---GGCGCGGCGTCA---
ATGACGCTGACGGTACAG---GCCAGACAA---GTATTG---TCTGGTATAGTGCAACAGCAAAGC---
AATTTG---CTGAGA---GCTATAGAGGCGCAACAGCATATGTTGCAACTCACGGTCTGGGGCATT
>AY901975
AATTAT---

ACAAATGGAATCATCACACTCCCATGCAAGATAAAACAATTTATAAACATGTGGCAAGAGGTAGGACGAGCAA
TGTATGCCCCTCCCATTGCAGGAAACATAACATGCAAATCAAAT---
ATCACAGGACTACTATTGACATGGGATGGAGGAGACACGAATGGGACA-------------------------
----

AAAAATAATAATACATTTAGACCTGGAGGAGGAAATATGAAAGACAATTGGAGAAATGAATTATATAAATATA
AAGTGGTAGAAATTAAGCCATTG---GGAATAGCACCCACTGATTCAAAAAGGAGAGTGGTG-----------
----------------------------GAGAGAGAAAAAAGAGCA---GTGGGA---CTGGGAGCT------
---ATGCTCGTTGGGTTCTTGGGAGCA---GCAGGAAGCACTATG---GGCGCGGCGTCA---
ACGACGCTGACGGTACAG---GCCAGACAA---CTGTTG---TCTGGTATAGTGCAACAGCAAAGC---
AATTTG---CTGAGA---GCTATAGAGGCGCAACAGCATATGTTGCAACTCACAGTCTGGGGCATT
>AY878070
AATTATAGTAGCAGCCACAAG--

CAAAATACAACCATCACAATCCCATGTAAAATAAAACAAATTATAAATATGTGGCAAGGGGTAGGACGGGCAA
TGTATGCCCCTCCCATTGCAGGAAACATAACATGTAAATCAAAT---
ATCACAGGATTACTATTGACACGTGATGGAGGACCCGATAGCAAAAAG-------------------------
----

CCTAATGGGACAGAGACATTCAGACCTGGAGGAGGAGATATGAGGGACAATTGGAGGAGTGAATTATATAAAT
ATAAAGTGGTAGAAATCAGGCCATTA---GGGATAGCACCCACCAAGGCAAAAAGAAGAGTGGTG--------
-------------------------------GAGAGAGAAAAAAGAGCA---GTGACA---ATGGGAGCT---
------ATGTTCCTTGGGTTCTTGGGAGCA---GCAGGAAGCACTATG---GGCGCAGCGTCA---
ATAACGCTGACGGTACAG---GCCAGACAA---CTGTTG---TCTGGTATAGTGCAACAGCAGAAC---
AATTTG---CTGAGA---GCTATAGAGGTGCAACAGCATATGTTGCAGCTCACAGTCTGGGGCATT
>DQ275664
TACTATCCTAATGGTACAAACAGTACTTCAAAGGGT-------------------------------------
----
ACAAATGTAACCATCACACTCCAATGCAGAATAAAACAAATTATAAACATGTGGCAGGGGATAGGACAGGCAA
TGTATGCCCCTCCCATTAAGGGAAACATAACATGTAAGTCAAAT---
ATCACAGGACTACTATTGACACGTGATGGGGGAGAAAATACAAAT----------------------------
----

GGAACAGAAATCTTCAGACCTGGAGGAGGAGATATGAGAGACAATTGGAGAAGTGAATTATATAAATATAAAG
TGGTGGAAATTAAGCCATTA---GGAGTAGCACCCACTACGGCAAAAAGGAGAGTGGTG--------------
-------------------------GAGAGAGAAAAAAGAGCA---GTAGGA---ATAGGAGCT---------
GTGTTCCTTGGGTTCTTGGGAGCA---GCAGGAAGCACTATG---GGCGCAGCATCA---
ATAACGCTGACGGTACAG---GCCAGACAA---TTGTTG---TCTGGTATAGTGCAACAGCAAAGC---
AATTTG---CTGAGG---GCTATAGAGGCGCAACAGCATATGTTGCAACTCACAGTCTGGGGCATT
>DQ093589
AGTCACCTG--

GAAAATACAACCATCACACTTCCATGCAGAATAAAACAAATTATAAACATGTGGCAAGGGGTAGGACGAGCAA
TGTATGCCCCTCCCATTGCAGGAAACATAGAATGTAAATCAAAT---
ATCACAGGAATACTATTGGCACATGATGGAGGAGGAGAGCAGAAT----------------------------
----

GGTACAAATGAGATATTCAGACCTGGAGGAGGAGATATGAGGGACAATTGGAGAAGTGAGTTATATAAATATA

AAGTGGTAGAAATTAAACCATTA---GGAATAGCACCTACTAAGGCAAAAAGGAGAGTGGTG-----------
----------------------------GAGAGAGAAAAAAGAGCA---GCGGGA---ATAGGAGCT------
---GTGTTCCTTGGGTTCTTAGGAGCA---GCAGGAAGCACTATG---GGCGCGGCGTCA---
ATAGCGCTGACGGTACAG---GCCAGACAA---TTACTG---TCTGGTATAGTGCAACAGCAAAGC---
AATTTG---CTGAGA---GCCATAGAGGCGCAACAGCATATGTTGCAACTCACAGTCTGGGGCATT
>KC863250
AATTATCCTAATGGTACAGAAAGTCTTAATAGTACAGAAAGTAAT----------------------------
----
-
TCAAGCTTACCAATCACGATCCCATGCAGAATAAAACAAATTATAAACATGTGGCAGGGGGTAGGACGAGCAA
TGTATGCCCCGCCCATTGCAGGAAACATAACATGTAACTCAAGT---
ATCACAGGACTACTATTGACACGGGATGGAGGAGTGAATACACAG----------------------------
----
GAGGGAGGAGTGAATACAACA---------
AGGACAGAGATATTCAGACCTGGAGGAGGAAATATGAAGGACAATTGGAGAAGTGAATTATATAAATATAAAG
TGGTAGAAATTAAGCCATTA---GGAATAGCACCCACTGAGGCAAAAAGGAGAGTGGTG--------------
-------------------------GAGAGAGAAAAAAGAGCA---GTGACA---CTAGGAGCT---------
GTGTTCCTTGGGTTCTTGGGAGCA---GCAGGAAGCACTATG---GGCGCGGCGTCA---
ATCACGCTGACGGTACAG---GCCAGACAA---CTGCTG---TCTGGTATAGTGCAACAGCAAAGC---
AATTTG---CTGAGA---GCTATAGAGGCGCAACAGCATATGTTGCAACTCACGGTCTGGGGCATT
>DQ275659
TTTCAGAATAATGGTACAAACAGTGAA--

AGTAATTCAACCATCACACTCCCATGCAGAATAAGACAAATTGTAAACATGTGGCAGGAGGTAGGACGAGCAA
TGTATGCCCCTCCCATTGAAGGAAACATAACATGTAAATCAAAT---
ATCACAGGGCTACTATTGCAACGTGATGGAGGCACGAGTAACAATAGC-------------------------
----

AGCAATAACATAGAGATATTCAGGCCTGGAGGAGGAAATATGAAGGACAATTGGAGAAGTGAATTATATAAAT
ATAAAGTGGTAGAAATTAAACCATTG---GGAATAGCACCCACAACGGCAAAAAGGAGAGTGGTG--------
-------------------------------GAGAGAGAAAAAAGAGCA---GTGGGAATCATAGGAGCT---
------ACGATCCTTGGGATCTTGGGAGCA---GCAGGAAGCACTATG---GGCGCAGCATCA---
ATAACGCTGACGGTACAG---GCCAGACAC---CTGCTG---TCTGGTATAGTGCAACAGCAAAGC---
AACTTG---CTGAGA---GCTATAGAGGCGCAACAGCATATGTTGCAACTCACGGTTTGGGGCATT
>DQ093602
TTTCATGATAATTCAAATTTT--

AATAATTCAACCATCACACTCCCATGCAGAATAAAACAATTTATAAACATGTGGCAGGGGGTAGGACGAGCAA
TGTATGCCCCGCCCATTGAAGGAAACATAACATGTACATCAAAT---
ATCACAGGACTACTATTGACACGTGATGGAGGAGTAGGTAATGGGACA-------------------------
----

GATAATACGATAGAAATATTCAGACCTGAAGGAGGAGACATGAGGGACAATTGGAGAAGTGAATTATATAAAT
ATAAAGTGGTGGAAATTAAGCCATTG---GGAGTAGCACCCACTGGGGCAAAAAGGAGAGTGGTG--------
-------------------------------CAGAGAGAAAAAAGAGCA---GTGGGA---ATGGGAGCT---
------ATGATCCTTGGGTTCTTGGCAACA---GCAGGAAGCACTATG---GGCGCGGCGTCA---
ATAACGCTGACGGTACAG---GCCAGACAA---GTGTTG---TCTGGTATAGTGCAACAGCAAAGC---
AATTTG---CTGAGG---GCTATAGAGGCGCAACAGCATATGTTGCAACTCACGGTCTGGGGCATT
>AY901974
ACCTACAAGGAGAAG--

ATAAATAGTACTATCACACTCCAATGCAGAATAAAACAAATTATAAACATGTGGCAGGGGGTAGGACAAGCAA
TGTATGCCCCTCCCATTGAAGGAAACATAACATGTAGATCAAAT---
ATCACAGGACTACTATTAACACGTGATGGAGGAAATGATAAAGGAAAA-------------------------
----

GATAATGACACAGAGATATTCAGACCTGGAGGAGGAGATATGAAGGACAATTGGAGAAGTGAATTGTATAAAT
ATAAAGTGGTAGAAATTAAACCATTA---GGAGTAGCACCCACCAAGGCAAAAAGGAGAGTGGTG--------
-------------------------------GAGAGAGAAAAAAGGACA---GTGGGA---ATGGGAGCT---

------GTGTTCCTTGGGTTCTTGGGAGCA---GCAGGAAGCACTATG---GGCGCAGCATCA---
ATAACGCTGACGGTACAG---GCCAGACAA---TTGCTG---TCTGGTATAGTGCAACAGCAAAGC---
AATTTG---CTGAGA---GCTATAGAGGCGCAACAGCATATGTTGCAACTCACGGTCTGGGGCATT
>DQ011172
AGTTATTCAAGCACA--

GCCAACACAACCATCACGCTCCCATGCAGAATCAGACAGATCATAAACATGTGGCAGCAGGTAGGACGAGCAA
TGTATGCCCCTCCCATTGCAGGAAACATAACATGTAAATCAAT---
ATCACAGGACTACTATTGCTACGTGATGGAGGAAATGAAGACAAT----------------------------
----

AGAACAGAGACATTCAGACCTGGAGGAGGAAATATGAAGGACAATTGGAGAAGTGAATTATATAAATATAAAG
TGGTAGAACTTAAGCCTTTG---GGAGTAGCACCCACTAAGGCAAAAAGGAGAGTGGTG--------------
-------------------------GAGAGAGAAAAAAGAGCA---GTGGGA---ATAGGAGTT---------
GCGCTCCTTGGGTTCTTGGGAGCA---GCAGGAAGCACTATG---GGCGCGGCGTCA---
ATAACGCTGACGGTACAG---GCCAGACAA---CTGTTG---TCTGGTATAGTGCAACAGCAAAGC---
AATTTG---CTGAGA---GCTATAGAGGCGCAACAGCATATGTTGCAACTCACAGTCTGGGGCATT
>DQ093594
CCTCAGAATGATACAAAA---

GATGATTTAACCATCACACTTCCATGCAAAATAAAACAAATTATAAACATGTGGCAGGGGGTAGGACGAGCAA
TGTATGCCCCCCCTATTGCAGGAAACATAACATGTAACTCAAGT---
ATCACAGGACTATTATTGACACGTGATGGAGGAAATAACACAACA----------------------------
----

GGGACAGAGATATTCAGACCTGGAGGAGGAAACATGAAAGACAATTGGAGAAGTGAATTATATAAGTATAAAG
TGGTAGAAATTAAGCCATTG---GGAGTAGCTCCCACTGAAGCAAAAGGGAGAGTGGTG--------------
-------------------------GAGAGAGAAAAAAGAGCA---GTGGGA---ATAGGAGCT---------
GTACTCCTTGGGTTCTTGGGAGCA---GCAGGAAGCACTATG---GGCGCGGCGTCA---
ATAACGCTGACGGTACAG---GCCAGACAA---CTGTTG---TCTGGTATAGTGCAACAGCAAAAC---
AATTTG---CTGAGG---GCTATAGAGGCGCAACAGCATATGTTGCAACTCACGGTCTGGGGCATT
>DQ351217
AACTACACATGTACTGCTCCAGAA---

GGGAATGGAACCATCATACTTCCATGCAGAATAAAACAAATTGTAAACATGTGGCAGGAGGTAGGACGAGCAA
TGTATGCCCCTCCCATTAAAGGAAACATAACATGTAACTCAAAC---
ATCACAGGACTACTATTAGTACGGGATGGAGGCGAGAATATCACG----------------------------
----

GAGGAGACATTCAGGCCTGGAGGAGGAAATATGAAGGACAATTGGAGAAGTGAATTATATAAATATAAAGTGG
TAGAAATTAAGCCATTG---GGAATAGCACCCACTACGGCAAAAAGGAGAGTGGTG-----------------
----------------------GAGAGAGAAAAAAGAGCA---GTGGGA---ATAGGAGCT---------
GTACTCCTTGGGTTCTTGGGAGCG---GCAGGAAGCACTATG---GGCGCGGCGTCA---
ATAACGCTGACGGTACAG---GCCAGACAA---CTGTTG---TCTGGTATAGTGCAACAGCAAAGC---
AATTTG---CTGAAA---GCCATAGAGGCGCAACAGCACATGTTGCAACTCACGGTCTGGGGCATT
>DQ093604
AGTTACAAT--

AGTACAGGAGACATCACACTCCCATGCAGAATAAAACAAATTATAAACATGTGGCAGAAGGTAGGACGAGCAA
TATATGCCCCTCCCATTGCAGGAAACATAACATGTAATTCAACT---
ATCACTGGACTACTCTTGGTAAGAGATGGAGGCAACTCAAATAAC----------------------------
----

CTAAGTGAGACATTCAGACCAGCAGGAGGAAATATGAAGGATAATTGGAGAAGTGAACTATATAAATATAAAG
TGGTAGAAATTAAGCCATTG---GGAATAGCACCCACTAAGGCAAAAAGGCGAGTGGCG--------------
-------------------------GAGAGAGAAAAAAGGGCA---GTAGGA---ATAGGAGCT---------
GTGTTCCTTGGGTTCTTGGGAGCA---GCAGGAAGCACTATG---GGCGCAGCGTCA---
ATAGCGCTGACGGCACAG---GCCAGACAA---GTGTTG---TCTGGAATAGTGCAACAGCAAAGC---
AATTTG---CTGAGG---GCTATAGAGGCGCAACAGCATATGTTGCAACTCACAGTCTGGGGCATT
>AY901973

```
TGTTATACAACACAGCTGTTTAATAGTACATACAATAGTACATACAGGCCTAATGGTACAGAA----------
-------------------------------------------------------------------------
----------------------
AGTAATTCAAACATCACACTCCCATGCAGAATAAAGCAAGTTATAAACATGTGGCAGCAGGTAGGACGAGCAA
TGTATGCTCCTCCCATTGCAGGAAACATAACATGTAAATCAAAT---
ATCACAGGATTACTATTGGTGCGTGATGGAGGAATAAATAACAGT----------------------------
-------------------------------------------------------------------------
-------------
ACCAAAGAGACATTCAGACCTGGAGGAGGAGATATGAAGGACAATTGGAGAAGTGAATTATACAAATATAAAG
TAGTAGAAATTAAACCATTG---GGAATAGCACCTACTGAGGCAAAAAGGAGAGTGGTG--------------
-------------------------GAGAGGGAAAAAAGAGCA---GTGGGA---ATAGGAGCT---------
GTGTTCCTTGGGTTCTTGGGAGCA---GCAGGAAGCACTATG---GGCGCGGCGTCA---
ATAACGCTGACGGTACAG---GCCAGACAA---TTGTTG---TCTGGTATAGTGCAACAGCAAAGC---
AATTTG---CTGAGG---GCCATAGAGGCGCAACAGCATATGTTGCAACTCACGGTCTGGGGCATT
>DQ382374
ACTCATCTGCCTAATGGTACACAGGTTAATGGG----------------------------------------
--------------------------------------------------------------------
ACTGATGCAAATATCACACTTCCATGCAGAATAAAACAATTTATAAACATGTGGCAGAAGGTAAGACTAGCAA
TGTATGCCCCTCCCATTGCAGGAGACATAACATGTGAATCAGAT---
ATTACAGGACTACTGTTAACGCGGGATGGAGGAGATAGTACAAATAGT-------------------------
-------------------------------------------------------------------------
-------
AATAATAATACAGAGACATTCAGACCTGAAGGAGGAAACATGAAAGACAATTGGAGAAGCGAATTATATAAAT
ATAAAGTGGTAGAAATTAAGCCATTA---GGAATAGCACCCACTACTGCAAAAAGGAGAGTGGTG--------
-------------------------------GAAAGAGAGAAAAGAGCA---GTGGGA---CTAGGAGCT---
------GTGTTCCTTGGGTTCTTGGGAGCA---GCAGGAAGCACTATG---GGCGCAGCATCA---
ATAACGCTGACGGTACAG---GCCAGACAA---TTATTG---TCTGGTATAGTGCAACAGCAAAGT---
AATTTG---CTGAGA---GCTATAGAGGCGCAACAGCATATGTTGCAACTCACGGTCTGGGGCATT
>DQ382376
TCCTATCCTCCTTCCTGCCCTCCTAGTACTGCA----------------------------------------
-----------------------------------------------------------------
GATAATTCTTCCATCATCCTTCCTTGCTGCATAATACAACTTATTAACCTGTGGCGGGAGGTAGGACGACCAG
TGTTGGCCGCTCCCCTTGTTGGAAGCATCATATGTTGCTCCTCT---
ATCACCGGAATACTATTATCAACGGATGGAGGCCCCGACGATGAC----------------------------
-------------------------------------------------------------------------
----------------
GCAGAGACATTCAGACCTGGAGGAGGAAATATGAAGGACAATTGGAGAAGTGAATTATATAAATATAAAGTGG
TAGAAGTTAAGCCATTG---GGAATAGCACCCACTAAAGCAAAAAGGAGAGTGGTG-----------------
----------------------GAGAGAGAAAAAAGAGCA---GTGGGA---ATAGGAGCT---------
GTGTTCCTTGGGTTCTTGGGAACA---GCAGGAAGCACTATG---GGCGCAGCGTCA---
ATAACGCTGACGGTACTG---GCCAGACAA---TTATTG---TCTGGTATAGTGCAACAGCAAAGC---
AATTTG---CTGAGG---GCTATAGAGGCGCAACAACATCTGTTGCAACTCACGGTCTGGGGCATT
>DQ382379
TGCTATACAAATGAAACAAGCAAT-------------------------------------------------
-----------------------------------------------------
CCAAATAATACCATCACACTCCCATGCAGAATAAGACAAATTATAAACATGTGGCAGAAGGTAGGACGAGCAA
TGTATGCCCCTCCCATTGCAGGAAACATAAATTGTAAATCAAAT---
ATCACAGGAATACTGTTGACACGTGATGGAGGTAACGAAACAGAGGGTGAC----------------------
-------------------------------------------------------------------------
-------
GGAACAAAGGTAGAGATATTCAGACCTGGAGGAGGAAATATGAGGGACAATTGGAGAAGTGAATTATATAAAT
ATAAAGTGGTAGAAATTAAGCCATTA---GGAGTAGCACCCACTAAGGCAAAAAGGAATGTGGTG--------
-------------------------------CAGAGAGAAAAAAGAGCA---GTGGGA---ATAGGAGTT---
------GTGCTCCTTGGGTTCTTGGGAGCA---GCAGGAAGCACTATG---GGCGCGGCGTCA---
ATAACGCTGACGGTACAG---GCCAGACAA---CTGTTG---TCTGGTATAGTGCAACAGCAAAGC---
AATTTG---CTGAGA---GCCATAGAGGCGCAACAACATATGTTGCAACTCACGGTCTGGGGCATT
>DQ382363
GATTATAATGGTGCAGGAAAT----------------------------------------------------
--------------------------------AGT---
TCATCAGACTCCTTCATCACCATCCTCTGCAGAATAAAACAATTTATAATCATGTGGCAGAGGGTAGGACGAG
```

CAATGTATGCCCCTCCCATTGTTGGAAGCGTCACATGTTGTTCATTT---
ATCACAGGGCTACTATTGACGCGTGATGGAGGAAGTAACACAACA---------------------------
----

GAGACATTCAGACCTGTAGGAGGAGATATGAGGGACAATTGGAGAAGTGAATTATATAAATATAAAGTGGTAG
AAGTTAGGCCATTA---GGAATAGCACCCACTAGGGCACAAAGGAGAGTGGTG-------------------
------------------GAGAGAGTAAAAAGAGCA---GTGGGA---ATAGGAGCT---------
GTGTTCCTTGGGTTCTTGGGAGCA---GCAGGAAGCACTATG---GGCGCAGCATCA---
ATAACGCTGACGGTACAG---GCCAGACAA---TTGCTG---TCTGGTATAGTGCAACAGCAAAGC---
AATTTG---CTGAAG---GCTATAGAGGCGCAACAGCACCTGTTGCAACTCACAGTCTGGGGCATT
>HQ615953
TTTCATAATAATACAAATGAGACAACCATC--

ACAAATGAGACCATCATGCTCCCATGCAGGATAAAACAAATTATAAACATGTGGCAGAAGGTAGGACGAGCAA
TGTATGCTCCTCCCATTGAGGGAAACATAACATGTAATTCAAAT---
ATCACAGGACTACTATTACTACGTGATGGAGGCCTCTTCAATGGGACA------------------------
----

GCAGAAGTAGAGACATTCAGACCTGGAGGGGGAGATATGAGAGACAATTGGAGAAGTGAATTATACAAATATA
AAGTGGTAGAAGTTAAGCCATTG---GGAATAGCACCCACTGGGGCAAGAAGGATAGTGGTG----------
---------------------------AAGAGAGAAAAAAGAGCA---GTGGGA---ATAGGAGCT------
---GTGTTCCTTGGGTTCTTGGGAGCA---GCAGGAAGCACTATG---GGCGCGGCGTCA---
ATGACGCTGACGGTACAG---GCTAGACAA---CTGTTG---TCTGGTATAGTGCAACAGCAAAGC---
AATTTG---CTGAAG---GCTATAGAGGCGCAGCAACATCTGCTGCAACTCACGGTCTGGGGCATC
>GQ999982
AATTATACACTGAGCAATAAC---

AATAATGAAACAATCATACTCCCATGTAGAATGAAACAAATTATAAATATGTGGCAGGGGGTAGGACGAGCAA
TGTATGCCCCTCCCATTGCAGGAAACATAACATGTAACTCAAGT---
ATCACAGGACTGTTATTGACGCGTGACAAAGACCCAGGGCAGAAGAAC------------------------
----

AGCAACACAGAGACATTCAGACCTACAGGAGGAAATATGAAGGACAATTGGAGAAATGAATTATACAAATGTA
AAGTGGTAGAAATTAAACCATTA---GGAGTAGCACCCACTAATGCAAAAAGGAGAGTGGTG----------
---------------------------GAGAGAGAAAAAAGAGCA---GTAGGA---ATAGGAGCT------
---GTGCTCCTTGGGTTCTTGGGAGCA---GCAGGAAGCACTATG---GGCGCGGCGTCA---
ATAACGCTGACGGTACAG---GCCAGACAA---CTGTTA---TCTGGTATAGTGCAACAGCAAAGT---
AATTTG---CTGAGA---GCTATAGAGGCGCAACAGCACATGTTGCAACTCACAGTCTGGGGCATT
>GQ999988
TTTTATGAACCT--

TTAAATTTAACCATCACACTCCAATGCAGAATAAAACAAATTATAAACATGTGGCAGGAGGTGGGACGAGCAA
TGTATGCCCCTCCCATTGCAGGAAACATAACATGTGAATCAAAG---
ATCACAGGGCTAATATTGACACGTGATGGAGGAAGTGACAATGGG---------------------------
----

ACAGAGACATTCAGACCTGGAGGAGGAGATATGAGGGACAACTGGAGAAGTGAATTATATAAATATAAAGTGG
TAGAAATTAAGCCATTG---GGAATAGCACCCACTAATGGAAGAAGGAGAGTGGTG----------------
---------------------CAGAGAGAGAAAAGAGCA---GTGGGA---ATAGGAGCT---------
GTATTCCTTGGGTTCTTGGGAGCA---GCAGGAAGCACTATG---GGCGCGGCATCA---
ATAACGCTGACGGTACAG---GCCAGACAA---TTATTG---TCTGGTATAGTGCAACAGCAAAAC---
AATTTG---CTGAGG---GCTATAGAGGCGCAACAGCATATGTTGCAACTCACGGTCTGGGGCATT
>HQ615952
AATCAGACTACAAAT---

-
AATTCTACTAACATCACACTCCCATGCAGAATAAAACAAATTATCAACATGTGGCAGGAGGTAGGACGAGCAA
TGTATGCCCCTCCTATTGCAGGAAACGTAACATGTAACTCGAGT---
ATCACAGGACTACTACTGACCCGTGATGGGGGAAAAGGAAATGACACA------------------------

----

GGAAATGGCACAGAGATATTCAGACCTGGAGGAGGAAATATGAAAGACAATTGGAGAAGTGAATTATTTAAAT
ATAAAGTGGTAGAAGTTAGGCCATTG---GGAATAGCACCCACTACGGCAAAAAGGAGAGTGGTG--------
-------------------------------GAGAGAGAAAAAAGAGCA---GTGGGA---ATAGGAGCT---
------GTACTCCTTGGGTTCTTGGGAGCA---GCAGGAAGCACTATG---GGCGCGGCGTCA---
ATAACGCTGACGGTACAG---GCCAGACAA---CTGTTG---TCTGGTATAGTGCAACAGCAAAGC---
AATTTG---CTGAGG---GCTATAGAGGCGCAACAGCATATGTTGCAACTCACGGTCTGGGGCATT
>HQ595762
GCTTATAATACACCAAAT---

GCTTCAGAAAACATAACCCTCCCATGTAGAATAAAACAAATTATAAACATGTGGCAGAGGGTAGGACGAGCAA
TGTATGCCCCTCCCATTGCAGGAAACATAACATGTAAATCAAAT---
ATCACAGGACTACTCTTGACACGTGATGGAGGGAGCAATAGTACA----------------------------
----

GGGACAGAAATATTCAGACCTGGAGGAGGAGATATGAGGGATAATTGGAGAAGTGAATTATATAAATATAAAG
TAGTAGAAGTTAAACCATTA---GGAATAGCACCCACTACTGCAAGAAGGAGAGTGGTG--------------
-------------------------CAGAGAGAAAAAAGAGCA---GCA------TTGGGAGCT---------
GTGCTCTTGGGGTTCTTAGGAGCA---GCAGGAAGCACTATG---GGCGCGGCGTCG---
GTGGCGCTGACGGTACAG---GCCAGGCAA---TTATTG---TCTGGTATAGTGCAACAGCAAAGC---
AATTTG---CTGAGA---GCTATAGAGGCACAACACCATTTGTTGCAACTCACAGTCTGGGGCATT
>HQ595758
AATTATAATGATACA--

GAAAGTAATTCAACCATCACACTTCCATGCAGGATAAAACAAATTATAAACATGTGGCAGGAGGTAGGACGAG
CAATGTATGCTCCTCCCATTGCAGGAAACATAACATGTAAATCAAAT---
ATCACAGGATTACTATTGACGCGTGATGGAGGCACAAATGACACA----------------------------
----

AATAACACAGAAATATTCAGACCTGGAGGAGGAAATATGAAGGACAATTGGAGAAGTGAATTGTATAAATATA
AAGTGGTAGAAATTAAACCATTA---GGGATAGCTCCCACTGGAGCAAAAAGGAGAGTGGTG-----------
----------------------------GAGAGAGAGAAAAGAGCA---GTAGGA---ATAGGAGCT------
---ATGATCCTTGGGTTCTTGGGAGCA---GCAGGAAGCACTATG---GGCGCAGCGTCA---
ATAACGCTGACGGTACAC---GCTAGACAA---TTGTTG---TCTGGTATAGTGCAACAGCAAAGC---
AATTTG---CTGAGA---GCTATAGAGGCGCAACAGCATATGTTGCAACTCACGGTCTGGGGCATT
>KT183064
AATCATACAGACAGGTCTAATGATACAGGA---

AGTAATAAAACCATCACACTCCCATGTAAAATAAAACAAATTATAAACATGTGGCAGGAGGTAGGACGAGCAA
TGTATGCCCCTCCCATTGCAGGAAACATAACATGTAAATCAAAT---
ATCACAGGTCTACTATTAGTACGTGATGGAGGAGAAAGTAACTCA----------------------------
----

ACAACGGAGATATTCAGACCTGGAGGAGGAAATATGAAGGACAATTGGAGAAGTGAATTATATAAATATAAAG
TGGTAGAAATTAAACCACTG---GGAATAGCACCCACTGAGGCAAAAAGGAGAGTGGTG--------------
-------------------------GAGAGAGAAAAAAGAGCA---GTTGGC---ATAGGAGCT---------
GTGTTCCTTGGGTTCTTGGGAGCA---GCAGGAAGCACTATG---GGCGCGGCGGCA---
GTGACGCTGACGGTACAG---GCCAGACAA---TTGTTG---TCTGGTATAGTGCAACAGCAAAGC---
AATTTG---CTGAGG---GCTATAGAGGCGCAACAGCATATGTTGCAACTCACAGTCTGGGGCATT
>KT183094
TACTATCCTAATGGTACTGTT--

ACAACAAGTAATATTACACTCCCATGCAAAATAAAACAAATTATAAACATGTGGCAAGGGGTGGGACGAGCAA
TGTATGCCCCTCCCATTGCAGGAAACATAACATGTAAATCAAAC---
ATCACAGGATTACTATTGCTACGTGATGGGGGAAACATGAATAAC----------------------------
----

ACAACAGAAATATTCAGACCTGGAGGAGGAGATATGAAGGACAATTGGAGAAGTGAATTATATAAATATAAAG
TAGTAGAAATTAAACCATTA---GGAATAGCACCCACTGGGGCAAAAAGGAGAGTGGTG--------------

-------------------------GAAAGAGAAAAAAGAGCA---GTGGGA---ATAGGAGCT---------
GTGATCTTTGGGTTCTTGGGAGCA---GCAGGAAGCACTATG---GGCGCAGCGTCA---
ATAGCGCTGACGGTACAG---GCCAGACAA---CTGTTG---TCTGGTATAGTGCAACAGCAAAGC---
AATTTG---CTGAGA---GCTATAGAGGCGCAACAGCATATGTTGCAACTCACGGTCTGGGGCATT
>KT183148
ACTTATAATGGTCCAGAA---

GGAAACTCAAACATCACACTCCCATGCAGAATAAAACAAATTATAAACATGTGGCAGAAAGTAGGACGAGCAA
TATATGCCCCTCCCATTAGAGGAAACATAACATGTAACTCAAGT---
ATCACAGGACTAGTCTTGGTACGTGATGGAGGTATATATAATAATACCAAAAAT------
GAAAATGAGACATTCAGA---CCT---

GAAAATGAGACATTCAGACCGGGAGGAGGAGATATGAGGGACAATTGGAGAAGTGAACTATATAAATATAAAG
TGGTAGAAATTAAACCATTG---GGAATAGCGCCCACTGAAGCAAAAAGGAGAGTGGTG--------------
-------------------------CAGAGAGAAAAAAGAGCA---GTGGGA---ATAGGAGCT---------
TTGTTCCTTGGGTTCTTGGGAGCA---GCAGGAAGCACTATG---GGCGCAGCGTCA---
ATAACGCTGACGGTACAA---GTCAGACAA---TTGTTG---TCTGGAATAGTGCAACAGCAAAGC---
AATTTG---CTGAGA---GCTATAGAGGCGCAACAGCATATGTTGCAACTCACAGTCTGGGGCATT
>KT183196
ATTCAA---

AACAGCACAAATATCACACTCCCATGCAAAATAAAACAATTTGTAAATATGTGGCAGGAGGTAGGACGAGCAA
TGTATGCCCCTCCCATTGCAGGAAACATAACATGTAGATCAAAT---
ATCACAGGACTACTATTGGTGCGTGATGGGGGCAACCAAAGCGGA----------------------------
----

AATGATATAGAGACATTCAGACCTGGAGGAGGAAATATGAAAGACAATTGGAGAAGTGAATTATATAAATATA
AAGTGGTAGAAATTAAACCATTG---GGAATAGCACCCACTAAGGCAAAAAGGAGAGTGGTG-----------
----------------------------GAGAGAGAAAAAAGAGCA---GTGGGA---ATAGGAGCT------
---GTGTTCCTTGGGTTCTTGGGAACA---GCAGGAAGCACTATG---GGCGCGGCGTCA---
ATAGCGCTGACGGTACAG---GCCAGACAA---GTATTG---GCTGGTATAGTGCAACAGCAAAGC---
AATTTG---CTGAGG---GCTATAGAGGCGCAACAGCATATGTTGCAACTCACGGTTTGGGGCATT
>KC156123
GCCTACAATAAAACAGGTGATAATTCA--

ACCATCACAACCATCACACTCCAATGCAGAATAAAACAAGTTATAAACATGTGGCAGGAGGTAGGACGAGCAA
TATATGCCCCTCCCATTGCAGGAAACATAACATGTAGCTCAAAT---
ATCACAGGACTACTGTTGACACGTGATGGAGGAAATAATAATAGTAGT-------------------------
----

AATGAGACAGAGACATTCAGGCCTGGAGGAGGAGATATGAGAGACAATTGGAGAAGTGAATTATATAAATATA
AAACAGTAGAAATTAAACCATTA---GGAATAGCACCCACTACGGCAAAAAGGAGAGTGGTG-----------
----------------------------GAGAGAGAAAAAAGAGCA---GTGGGA---ATAGGAGCT------
---GTGTTCCTTGGGTTCTTGGGAGCA---GCAGGAAGCACTATG---GGCGCGGCGTCA---
ATAACGCTGACGGTACAG---GCCAGACAA---TTGTTG---TCTGGTATAGTGCAACAGCAAAGC---
AATTTG---CTGAGA---GCTATAGAGGCGCAACAACATCTGTTGCAACTCACGGTCTGGGGCATA
>KC156125
TTTTATGCTAATAGCAGT---

-
GATCCCAATGCAACTATCACACTCCCATGCAAAATAAAACAATTTATAAACATGTGGCAGGAGGTAGGACGAG
CAATGTATGCCCCTCCCATTGAAGGAAACATAACATGTAAATCAAAT---
ATCACAGGGCTACTATTGGAACGTGATGGAGACATAAAAGAAAAC----------------------------
----

AATACAGAGACATTCAGACCTATAGGAGGAAATATGAAGAACAATTGGAGAAGTGAATTATATAAATATAAAG
TGGTAGAGATTAAACCATTG---GGAGTAGCACCCACAGAATCAAAAAGGAGAGTGGTG--------------
-------------------------GAGAGAGAAAAAAGAGCA---GTGGGATTAGGCGGAGCT---------
TTGTTCCTTGGGTTCTTGGCAACA---GCAGGAAGCACTATG---GGCGCGGCGTCA---

ATAACGCTGACGGTACAG---GCCAGACAA---CTGTTG---TCTGGTATAGTGCAACAGCAAAAC---
AATTTG---CTGAGA---GCTATAGAGGCGCAACAGCATCTGTTGCAACTCACAGTCTGGGGCATT
>KC156127
AGTCAT---

AATGGTACGAACATCACACTCTCATGCAGAATAAAACAAATTATAAACATGTGGCAGGAGGTAGGAAGGGCAA
TATATAACCCTCCCATTGCAGGAAACATAACATGTAAATCAAAC---
ATCACAGGACTACTATTGGTACGTGATGGAGGTATCACAAATAAC----------------------------
----

AACACAGAGACATTCAGACCTGGAGGAGGAGATATGAGGGACAATTGGAGAAGCGAATTATATAAATATAAAG
TGGTAGAAATTAATCCCTTA---GGAGTAGCACCCACTAGGCAAAAAGGAGAGTGGTG---------------
-------------------------GAGAGAGAAAAAAGAGCA---GTGGGA---GTAGGAGCT---------
ATGTTCCTTGGGTTCTTGAGTGCA---GCAGGAAGCACTATG---GGCGCGGCGTCA---
ATAACGCTGACGGTACAG---GCCAGACAA---CTGTTG---TCTGGCATAGTGCAACAGCAAAGC---
AATTTG---CTGAGG---GCTATAGAGGCGCAACAGCATATGTTGCAACTCACAGTCTGGGGCATT
>JN687717
ATTCATAAGAATGAAAGTGGT--

TCAAATGACACCATTACAATCCCATGCAGAATAAAACAAATTATAAACATGTGGCAGGAGGTAGGAAAAGCAA
TGTATGCCCCTCCCATTGCAGGAAACATAACATGTGAGTCAAAT---
ATCACAGGACTACTATTAGAACGTGATGGAGGCACAAACATTAACACAGGCAATAACACAGGCAATAACACCA
CAGGC--
CATAACACAGGCAATAGCACAGATAACAGAAATGAAACATTCAGACCTGGAGGAGGAAATATGAAGGATAATT
GGAGAAGTGAACTATATAAATATAAAGTGGTAGAAATTAAGCCATTG---
GGAGTAGCACCCACGAAGGCAAGAAGGAGAGTAGTG------------------------------------
ACGGGGAAAGAAAAAAGAGCA---GTGGGA---CTAGGAGCT---------
GTGATCTTTGGGTTCTTGGGAGCA---GCAGGAAGCACTATG---GGCGCGGCGTCA---
GTGGCGCTGACGGTACAG---GCCAGACAA---CTGTTG---TCTGGTATAGTGCAGCAGCAAAGC---
AATTTG---CTGAGA---GCTATAGAAGCGCAACAGCATATGTTGCAACTCACAGTCTGGGGCATT
>KC863629
AATCAT---

TCAAATTCAAACATCACACTCAAATGCAGAATAAAACAAATTATAAACATGTGGCAGGGGGTAGGACAAGCAA
TGTATGCCCCTCCTATTGCAGGAAACATAACATGTAACTCAACC---
ATCACAGGACTACTATTGACACGTGATGGAGGAAGCACAACAAAT----------------------------
----

AGCTCGACAGAAATATTCAGACCTGGAGGAGGAGATATGAGGGACAATTGGAGAAGTGAATTATATAAATATA
AAGTGGTAGAAGTTAAGCCATTA---GGGATAGCACCCACTGAGGCAAAAAGGAGAGTGGTG-----------
----------------------------GAGAGAGAAAAAAGAGCA---GTGGGA---ATAGGAGCT------
---GTGTTCCTTGGGATCTTGGGAGCA---GCAGGAAGCACTATG---GGCGCAGCATCA---
ATAACGCTGACGGTACAG---GCCAGACAA---TTGTTG---TCTGGTATAGTGCAACAGCAAAGC---
AATTTG---CTGAGG---GCTATAGAGGCGCAACAGCATATGTTGCAACTCACGGTCTGGGGCATT
>KC863647
ACCTACAACCATAATGACACATCAAATGCAACCAGCAACAAC-------------------------------
----
ACATCAGATATCATCACCCTCCAATGCAGAATAAAACAAATCATAAACATGTGGCAGGGGGTAGGAAAAGCAA
TATATGCCCCTCCCATTGAAGGAAATATAACCTGTAGATCAGAG---
ATCACAGGACTACTATTGACAAGGGATGGAGGAGATGAAGGTAAA----------------------------
----

AATGATACAGAGATATTCAGACCTGGAGGAGGAGATATGAGAGACAATTGGAGAAGTGAATTATATAAATATA
AAGTAGTAGAAATTAAACCATTA---GGAATAGCACCCACCAAGGCAAAAAGGAGAGTGGTGGAG--------
----------------------------AGGAGAGGAAAAAGAGCA---GTAGGA---ATAGGAGCT------
---ATGTTCCTTGGGTTCTTAGGAGCA---GCAGGAAGCACTATG---GGCGCAGCGTCA---
ATAACGCTGACGGTACAG---GCCAAACAA---TTATTG---TCTGGTATAGTGCAACAGCAAAGC---
AATTTG---CTGAGG---GCTATAGAGGCGCAACAACATATGTTGCAACTCACGGTCTGGGGCATT
>HM204598

GATTATAAACCAACAAATGAT--

ACTGGTGATACCATAATACTTCCATGCAGAATAAAACAAATTATAAACATGTGGCAGGAGGTAGGACGAGCAA
TGTATGCCCCTCCCATTGCAGGAAACATAACATGTAATTCAAGT---
ATCACAGGACTACTATTAGAACATGATGGAGAAACAGATGGAGAT----------------------------
----

AATGTGACATTCAGACCTATAGGAGGAAATATGAAGGACAATTGGAGAAGTGAATTATATAAATATAAAGTGG
TAGAAATTAAGCCATTG---GGAATAGCACCCACTGAGGCAAAAAGGAGAGTGGTG-----------------
----------------------GAGAGAGAGAAAAGAGCA---GCAGTGGGAATAGGAGCT---------
GTGTTCCTTGGGTTCTTGAGCGCA---GCAGGAAGCACTATG---GGCGCGGCGTCA---
ATAACGCTGACGGTACAG---GCCAGACAA---CTGTTG---TTTGGTATAGTGCAACAGCAAAGC---
AATTTG---CTGAAG---GCTATAGAGGCGCAACAGCATATGTTGCAACTCACAGTCTGGGGCATT
>HM204601
AGCTAT---

TCAAACAACACCATCACACTCCCATGCAGGATAAAACAAATTGTAAATATGTGGCAGAAGGTAGGACAAGCAA
TGTATGCCCCTCCCATTGCAGGAAACATAACATGTAACTCAAGT---
ATCACAGGACTAATATTGACACGTGATGGGGGGAACACAGAGAATAACACA----------------------
----

GGGAATAACACAGAGGTATTCAGACCTGGAGGAGGAGATATGAAGAATAATTGGAGAAGTGAATTATATAAAT
ATAAAGTGGTAGAAATTAAACCATTG---GGAATAGCACCCACCACGGCAAAAAGGAGAGTGGTG--------
-------------------------------GAGAGAGAAAAAAGAGCA---GCA------ATAGGAGCT---
------GTGCTCCTTGGGTTCTTGGGAACA---GCAGGAAGCACTATG---GGCGCGGCGTCG---
GTAGCGCTGACGGTACAG---ACCAGACAA---CTGCTG---TCTGGTATAGTGCAACAGCAAAGC---
AATTTG---CTGAGA---GCTATAGAGGCGCAACAGCATATGTTGCAACTCACAGTCTGGGGCATT
>HM204605
AATTACTCGTTTAATGAGACAGACAATGTGACAACAAGCGAG-------------------------------
----
-
CAAGATGCAAACATCACACTCCAATGCAGAATAAAACAAATTATAAACATGTGGCAGGAGGTGGGGCGAGCAA
TGTATGCCTCCCCCATTGCAGGAGAAATAACATGTAACTCAAGT---
ATCACAGGACTACTATTGACACGTGATGGAGGAAGAAAAGAGAATAATACA----------------------
----

GAGAGTAATACAGAAATATTCAGACCTGGAGGAGGAAATATGAGGGACAATTGGAGAAGTGAATTATATAAAT
ATAAAGTGGTAGAAGTTAATCCATTA---GGAATAGCACCCACTAAGGCACGAAGGAGAGTGGTG--------
-------------------------------CAGAAAGAGAAAAGAGCA---ATAACA---CTGGGAGCT---
------ATGTTCCTTGGGTTCTTGGGGGCA---GCAGGAAGCACTATG---GGCGCAGCATCA---
ATAACGCTGACGGTACAG---GCCAGACAA---TTATTG---TCTGGTATAGTGCAACAGCAAAGC---
AATTTG---CTGAGG---GCTATAGAGGCGCAACAGCATATGTTGCAACTCACAGTCTGGGGCATT
>HM204610
CCTCAGGGTTCTAAT--

AGTACAAAAAACATCACAATATATTGCAGAATAAAACAAATTATAAACATGTGGCAGAGGGTAGGACGAGCAA
TGTATGCCCCTCCCATTGCAGGAAACATAACATGTATATCAAAT---
ATCACAGGACTACTATTGGTACGTGATGATCCATCAAATGCT-------------------------------
----

ACATTCAGACCGGGAGGAGGAGATATGAGGGATAACTGGAGAAGTGAATTATATAAATATAAAGTGGTAGAAA
TTAAGCCATTG---GGAATAGCACCCTCTAAGGCAAAAAGGAGAGTGGTG-----------------------
----------------GAGAGAGAAAAAAGAGCA---GCGGGA---CTAGGAGCT---------
GTGTTCCTTGGGTTCTTGGGAGCA---GCAGGAAGCACTATG---GGCGCGGCGTCA---
ATAACGCTGACGGTACAG---GCCAGACAA---TTGCTG---TCTGGTATAGTGCAACAGCAAAAC---
AACTTG---CTGAGG---GCTATAGAGGCGCAACAGCATTTGTTGCAACTCACAGTCTGGGGCATT
>FJ444091
AACTATAATGGTACAGAA---

AGTAATTCAAGCATCATCACACTCCCATGCAGAATAAAACAAATTGTAAACATGTGGCAGGAGGTAGGAAGAG

CAATATATGCCCCTCCTATTGCAGGAAACATAACATGTAACTCAAGT---
ATCACAGGATTACTATTGCTGCGTGATGGAGGCTTAGGCTTGAAT---------------------------
----

GAAACAGGAGAGACATTTAGACCTATAGGAGGAGATATGAGGAACAATTGGAGAAGTGAATTATATAAATATA
AGGTGGTAGAAATTAAGCCATTG---GGAGTAGCACCCACTAAGGCAAAAAGGAGAGTGGTG----------
----------------------------GAGAGAGAAAAAAGAGCA---GTGGGA---CTGGGAGCT-----
---GTGTTCCTTGGGTTCTTGGGAGCA---GCAGGAAGCACTATG---GGCGCGGCGTCA---
ATAACGCTGACGGTACAG---GCCAGACAA---CTGTTG---TCTGGTATAGTGCAACAGCAAAGC---
AATTTG---CTGAGG---GCTATAGAGGCGCAACAGCATATGTTGCAACTCACAGTCTGGGGCATT
>JX140664
AATTATACTTCTAATGATACA---

GAAAACATCACACTCACACTCCCATGCAAAATAAAACAGATTATAAACTTGTGGCAGGAGGTAGGACGAGCGA
TGTACGCTCCTCCCATTGCAGGAAACATAACATGTAAGTCAACC---
ATCACAGGATTACTATTGGTGCGTGATGGAGGACAAGGAAACAGC---------------------------
----

TCAAATGAGACATTCAGACCTGGAGGAGGAGATATGAGGGATAATTGGAGAAGTGAATTGTATAAGTATAAAG
TGGTAGAAATTAAGCCATTG---GGAGTAGCACCCACTGAGGCAAAAAGGAGAGTGGTG-------------
-------------------------GAGAGAGAAAAAAGAGCA---GTGGGA---CTAGGAGCT--------
GTGATCTTTGGGTTCTTGGGAGCA---GCAGGAAGCACTATG---GGCGCGGCGTCA---
ATAACGCTGACGGTACAG---GCCAGACAA---CTGTTG---TCTGGTATAGTGCAACAGCAAAGC---
AATTTG---CTGAAG---GCTATAGAGGCACAACAGAATATGTTGCAACTCACGGTCTGGGGCATT
>KT183083
ACTTATGTAAATATAAGTGAA---

ACAAACAAAACCTTCATACTCCCATGCAGAATAAAACAAATTATAAACATGTGGCAGGGGGTAGGACGAGCAA
TGTATAACCCTCCCATTGCAGGAACCATAACATGTAACTCAAGT---
ATCACAGGACTACTATTGGTACGTGATGGAGGAAACGGGGATACCACAAGT---------------------
----

AATAACAGTAATAACGAGACATTCAGACCTGGAGGGGGAAATATGAAGGACAATTGGAGAAGTGAATTATATA
AATACAAGGTGGTAGAAGTTAAGCCATTG---GGAATAGCACCCACTGAGGCAAAAAGGAGAGTGGTG----
----------------------------------GAGAGAGAAAAAAGAGCA---GCAGGA---
ATAGGAGCT---------GTACTCCTTGGGTTCTTGGGAGCA---GCAGGAAGCACTATG---
GGCGCAGCATCA---ATAACGCTGACGGTACAG---GCCAGGCAA---TTATTG---
TCTGGTATAGTGCAACAGCAAAGC---AATTTG---CTGAGG---
GCTATAGAGGCGCAACAGCATATGTTGCAACTCACGGTCTGGGGCATT
>KT183155
GAT---

TATAATGAAATCATCACACTCCAATGCAGGATAAAACAAATTATAAACATGTGGCAGGGGGTAGGACGAGCAA
TGTATGCCCCTCCCATTGAAGGAATCATAACATGTAACTCAAAT---
ATCACAGGACTACTATTGACACGAGATGGAGGCAACAGCAACAAC---------------------------
----

AACGACACAGAGGTATTCAGACCTGGAGGAGGAAATATGAAAGACAATTGGAGAAGTGAATTATATAAATATA
AGGTGGTAGAAATTAAGCCATTG---GGAATAGCTCCCACTGAAGCAAAAAGGAGAGTGGTG----------
----------------------------GAGAGAGAAAAAAGAGCA---GTGGGA---ATAGGAGCT-----
---GTGCTCCTTGGGTTCTTGGGAGCA---GCAGGAAGCACTATG---GGCGTGGCGTCA---
ATAACGCTAACGGTACAG---GCCAGACAA---TTGTTG---TCTGGTATAGTGCAACAGCAAAGC---
AATTTG---CTGAGA---GCTATAGAGGCGCAACAACATATGTTGCAACTCACGGTCTGGGGCATT
>KT183168
ACTTATAATGATACAAAGCATAAT--

GGAACCAAAGTCATCACACTCCCATGCAGAATAAAACAAATTATAAACATGTGGCAGGAGGTAGGACGAGCAA
TGTATGCTCCTCCCATTGCAGGCAACATAACATGTACGTCAAAT---
ATCACAGGACTACTATTGACAAGGGATGGAGGGAATAATAGCACA---------------------------
----

GAGACAGAGACATTCAGACCTGGAGGAGGAGATATGAGGGACAATTGGAGAAGTGAATTATATAAATACAAAG
TAGTAGAAATTAAGCCATTG---GGAATAGCACCCACTGGAGCAAAAAGGAGAGTGGTG--------------
-------------------------GAGAGAGAAAAAAGAGCA---GTGGGA---ATAGGAGCT---------
GTGTTCCTTGGGTTCTTGGGAGCA---GCAGGAAGCACTATG---GGCGCGGCGTCA---
ATAACGCTGACGGTACAG---GCCAGACAA---CTGTTG---TCTGGTATAGTGCAACAGCAAAGC---
AATTTG---CTGAAG---GCTATAGAGGCGCAACAACACCTGTTGCAACTCACAGTCTGGGGCATT
>KT183188
TCTCATGAGGGGTCAGGC---

AACATCACAAACATCACAATCCCATGCAGGATAAAACAAATCATAAACATGTGGCAGAAGGTAGGACGAGCAA
TGTATGCCCCTCCCATTGCAGGAAGCATAACATGTAACTCAAGT---
ATCACAGGAATACTGTTGACACGTGATGGAGGAAACATAACAAAT----------------------------
----

AGCACAAAGGAGATATTCAGACCTGGAGGAGGAGATATGAAGGACAATTGGAGAAGTGAATTATATAAATATA
AAGTGGTAGAAATTAAGCCATTG---GGAATAGCACCCACTGAATCAAAAAGGAGAGTGGTG-----------
----------------------------GAGAGAGAAAAAAGAGCA---GTGGGA---ATAGGAGCT------
---GTGCTCCTTGGGTTCTTGGGAGCA---GCAGGAAGCACTATG---GGCGCGGCGTCA---
ATAACGCTGACGGCACAG---GCCAGACAA---CTGTTG---TCTGGTATAGTGCAACAGCAAAGC---
AATTTG---CTGAGA---GCCATAGAGGCGCAACAGCATATGTTGCAACTCACGGTCTGGGGCATT
>HM623550
AATCAGACA--

GGAAATGATTCATTCATCATACTTCCATGTAAAATAAAACAAATTATAAATATGTGGCAGAAGGTAGGACGAG
CAATGTATGCTCCTCCCATTGCAGGAAATATAACATGTAAGTCAAAT---
ATCACAGGAATACTATTAGTACGTGATGGAGGCCCAGATAACAGT----------------------------
----

ACAGAAACATTTAGACCTGGAGGAGGAAATATGAAGGACAATTGGAGAAGTGAATTATATAAATATAAAGTGG
TAGAAATTAAGCCATTG---GGAATAGCACCCACTGAAGCAAAAAGGAGAGTGGTG-----------------
----------------------GAGAGAGAAAAAAGAGCA---GTGGGA---ATAGGAGCT---------
GTGTTCCTTGGGTTCTTGGGAGCT---GCAGGAAGCACTATG---GGCGCGGCGTCA---
ATAACGCTGACGGTACAG---GCCAGACAA---CTGTTG---TCTGGTATAGTGCAACAGCAAAGC---
AATTTG---CTGAGA---GCTATAGAGGCGCAACAGCATATGTTGCAACTCACAGTCTGGGGCATT
>HM623567
TGCTATACATCAAGACTGTTTAATAGTACATACAATGGTACACTTAGTATACATAATACACATAATGATACA-
----

GGAAACGAAACACACATCACACTTCCATGCAGAATAAAACAAATTATAAACATGTGGCAGGGGGTAGGGCGAG
CAATGTATGCCCCTCCCATTGCAGGAAACATAACATGTAATTCAAGT---
ATCACAGGTCTACTATTGACACGGGATGGAGGAAACACAACACAT----------------------------
----

AATGACACAGAGACATTCAGACCTGGAGGAGGAAATATGAAGGACAATTGGAGAAGTGAATTATATAAATATA
AAGTAATAGAAATTAAGCCATTG---GGAATAGCTCCCACTGGGGCAAAAAGGAGAGTGGTG-----------
----------------------------GAGAGAGAAAAAAGAGCA---GTGGGA---ATAGGAGCT------
---GTGTTCCTTGGGTTCTTGGGAGCA---GCAGGAAGCACTATG---GGCGCAGCGTCA---
ATAACGCTGACGGTACAG---GCCAGACAA---CTGTTG---TCTGGTATAGTGCAACAGCAAAGT---
AATTTG---CTGAGG---GCTATAGAGGCGCAACAGCATATGTTGCAACTCACAGTCTGGGGCATT
>HM623573
AATTAT---

ACTAATAACACCATCACACTCCCATGCAGAATAAAACAAATTATAAACATGTGGCAGGAGGTAGGAAGAGCAA
TGTATGCCCCTCCCATTGCAGGAAACATAACATGTAACTCAAGT---
ATCACAGGAATACTATTGACACGTGATGGAGGACAAGATAATAATACA-------------------------
----

AAGAATAATAAAGAGATATTCAGACCTGGAGGAGGAGATATGAGAGACAATTGGAGAAGTGAATTATATAAAT
ACAAAGTGGTAGAAATTAAGCCATTG---GGAGTAGCACCCACTGATGCAAAAAGGAGAGTGGTG--------

```
-------------------------------GAGAGAGAAAAAAGAGCA---GTAGGA---CTAGGAGCT---
------GTATTCCTTGGGTTCTTGGGAGCA---GCAGGAAGCACTATG---GGCGCGGCGTCA---
ATAGCGCTGACGGTACAG---GCCAGACAA---CTGTTG---TCTGGTATAGTGCAACAGCAAAGC---
AATTTG---CTGAGG---GCTATAGAGGCGCAACAGCATATGTTGCAACTCACAGTCTGGGGCATT
>KC154017
TTTCATCCAGACAACTCAACCAAAGGCAAA-------------------------------------------
-------------------------------------------------------------------------
----
GACAATGAAACCATCACACTCCCATGCAGGATAAAACAATTTATAAACATGTGGCAGGGAGTAGGACGAGCAA
TGTATGCCTCTCCCATTGAAGGAAACATAACATGTAACTCAAGT---
ATCACAGGGATAATATTGACACGTGATGGAGGAAACACCACAAAC----------------------------
-------------------------------------------------------------------------
-------------
AAAACAGAGGTATTCAGACCTGAAGGAGGAAATATGAAGGACAATTGGAGAAGTGAATTATATAAATATAAAG
TGGTAGAAATTAAGCCATTG---GGAGTAGCTCCAACTGAGGCAAAAAGGAGAGTGGTG--------------
-------------------------GAGAGAGGAAAAAGAGCA---GTGGGA---ATAGGAGCT---------
GTACTCCTTGGGTTCTTGGGAGCA---GCAGGAAGCACTATG---GGCGCAGCATCA---
ATAACGCTGACGGTACAG---GCCAGACAA---CTGTTG---TCTGGTATAGTGCAACAGCAAAGC---
AATTTG---CTAAGA---GCTATAGAGGCGCAACAACATATGTTGCAACTCACAGTCTGGGGCATT
>KC154019
GGTCATAATGGTACAGGA-------------------------------------------------------
-----------------------------------------------------
GATAATTCAACCATCACAATTTCATGCAGAATAAAACAAATTATAAACATGTGGCAGGGGGTAGGGCGAGCAA
TGTATGCCCCTCCCATTGCAGGAAACATAACATGTAACTCAAGT---
ATTACAGGACTACTATTAGTACGTGATGGAGGTGGAAATAGCACA----------------------------
-------------------------------------------------------------------------
----------------
GAAGAGATATTCAGACCTGGAGGAGGAGATATGAGAGACAATTGGAGAAGTGAATTATACAAATATAAAGTAG
TAGAAATTAAGCCCTTA---GGAGTAGCACCCACTAAGGCAAAAAGGAGAGTGGTG-----------------
----------------------GAGAGAGAAAAAAGAGCA---GTGGGA---ATAGGAGCT---------
GTGTTCCTTGGGTTCTTGGGAGCA---GCAGGAAGCACTATG---GGCGCAGCGTCA---
ATAACGCTGACGGTACAG---GCCAGACAA---TTATTG---TCTGGTATAGTGCAACAGCAAAGC---
AATTTG---CTGAGG---GCTATAGAGGCGCAACAGCATATGTTGCAACTCACGGTCTGGGGCATT
>KC154020
GACTATAAT----------------------------------------------------------------
-----------------------------------------------
TCAACTAAGAACATCACACTCCCATGCAGGATAAAACAAATTATAAACATATGGCAGGGGGTAGGACAAGCAA
TGTATGCCCCTCCCGTTGCAGGAAAAATAATATGTACATCAAAT---
ATCACAGGACTACTATTGACACGTGATGGAGGCGACGACACAAAT----------------------------
-------------------------------------------------------------------------
-------------
GACACAGAGATATTCAGACCTGGAGGAGGAAATATGAAGGACAATTGGAGAAGTGAATTATATAAATATAAAG
TAGTAGAAATTAAGCCATTA---GGACTAGCACCCACTGCAGCAAGAAGGAGAGTGGTG--------------
-------------------------CAGAGAGAAAAAAGAGCA---ATAGGA---ATAGGAGCT---------
GTGTTCCTTGGGTTCTTGGGAGCA---GCAGGAAGCACTATG---GGCGCAGCATCA---
ATAACGCTGACGGTACAG---GCCAGACAA---TTGTTG---TCTGGTATAGTGCAACAGCAAAGC---
AATTTG---CTGAGG---GCTATAGAGGCGCAACAGCATATGTTGCAACTCACAGTCTGGGGCATT
>HM204617
TTTCATGGA----------------------------------------------------------------
-----------------------------------------------------------------
AATGAGACAGAAATCATACTCCAATGCAGACTAAAACAATTTATAAATATGTGGCAGGGAGTAGGACAAGCAA
TGTATGCCCCTCCTATTGAAGGAAACATAACATGTACATCAAAT---
ATCACAGGACTATTATTGACACGTGATGGAGGCAAAGATGATAAC----------------------------
-------------------------------------------------------------------------
-------------
ACAACAGAGATATTCAGACCTATAGGAGGAGACATGAAGAACAATTGGAGAAGTGAATTATATAAATATAAAG
TGGTAGAAATTAAGCCATTG---GGAATAGCACCCACTGCTGCAAAAAGGAGAGTGGTG--------------
-------------------------GACAGAGAAAAAAGAGCA---GTGGGA---ATGGGAGCT---------
GTGTTCCTTGGGTTCTTGGGAGCA---GCAGGAAGCACTATG---GGCGCAGCGTCA---
```

ATAACGCTGACGGTACAG---GCCAGACAA---CTGTTG---TCTGGTATAGTGCAGCAGCAAAGC---
AATTTG---CTGAGA---GCTATAGAGGCGCAACAGCATCTGTTGCAACTCACGGTCTGGGGCATT
>KF114894
AAT--

CATAATGGCACCATCACACTCCCATGTAGAATAAAACAAATTATAAACATGTGGCAGAGGGTAGGACGAGCAA
TGTATGCCCCTCCCATTGCAGGAAACATAACATGTAAATCTAAT---
ATCACAGGATTACTATTGGTACGTGATGGAGGGAATGGAATAAT-----------------------------
----

AGCAGAAATGAGACATTCAGACTTGGAGGGGGAGATATGAGAGACAATTGGAGAAGTGAATTATATAAATATA
AAGTGGTAGAAATTAAGCCATTA---GGAGTAGCACCCACCGAGGCAAAAAGGAGAGTGGTG-----------
----------------------------GAGAGAGAAAAAAGAGCA---GTGGGA---ATAGGAGCT------
---GTGTTCCTTGGGTTCTTGGGAGCA---GCAGGAAGCACTATG---GGCGCGGCGTCA---
ATAACGCTGACGGTACAG---GCCAGACAA---TTGTTG---TCTGGTATAGTGCAACAGCAAAAC---
AATTTG---CTGAGG---GCTATAGAGGCGCAACAGCATCTGTTGCAACTCACAGTCTGGGGCATT
>HQ595756
TATCATGCTAATGGTACAGCAAATTCTACAGATTCAAAA----------------------------------
----
AATGATACATACATCACACTCCCATGCAGAATAAAACAAATTATAAACATGTGGCAAGGGGTGGGACAAGCAA
TGTATGCCCCTCCCATTGCAGGAAACATAACATGTAACTCAAAT---
ATCACAGGACTGCTATTGCTACGTGATGGAGGTCCAGAAAACAAT----------------------------
----

ACAGAGACATTCAGACCTGGAGGAGGAAATATGAAGGACAATTGGAGAAGTGAATTGTATAAATATAAAGTAG
TAGAAATTAATCCATTA---GGAATAGCACCCACTAAGGCAAAAAGGAGAGTGGTG-----------------
----------------------GAGAGAGAAAAAAGAGCA---GTGGGA---CTAGGCGCT---------
ATGTTCCTTGGGTTCTTGGGAGCA---GCAGGAAGCACTATG---GGCGCAGCATCA---
ATGACGCTGACGGTACAA---GCCAGACAA---TTGCTG---TCTGGTATAGTGCAACAGCAAAGC---
AATTTG---CTGAAG---GCTATAGAGGCGCAACAGCATATGTTGCAACTCACGGTCTGGGGCATT
>HQ615941
GACTATATTAAAAACAGTACATTAAAC--
-----------------------CTGGATAATAAT------
ACAAACAGTACAGACATCATACTCCAATGCAGAATAAAACAAATTATAAACATGTGGCAGGGGGTAGGAAAAG
CAATGTATGCCCCTCCCATTGCAGGAAATATAACATGTAACTCAACT---
ATCACAGGACTACTATTGACACGTGATGGAGGCAATGCTAAAGGCAATGAT----------------------
----

ACAGACAATAAGACAGAGACATTCAGACCTGCAGGAGGAGATATGAGGGACAATTGGAGAAGTGAATTATATA
AATACAAGGTGGTAGAAATTAAGCCATTA---GGAGTAGCACCCACTGAGGCAAAAAGGAGAGTGGTG-----
----------------------------------GAGAGAGAAAAAAGAGCA---GTAGGA---
CTAGGAGCT---------ATGTTCCTTGGGTTCTTGGGAGCA---GCAGGAAGCACTATG---
GGCGCAGCATCA---ATAACGCTGACGGTACAA---GCCAGACAA---TTACTG---
TCTGGTATAGTGCAACAGCAAAGC---AATTTG---CTGAGG---
GCTATAGAGGCGCAACAACATATGTTGCAACTCACGGTCTGGGGCATT
>GU080194
ACCTACACGCCTAATGCTACAGAAAGTATT---

TCCAATAAAACCATCCCACTTTCATGCAGAATAAAACAAATAATAAATATGTGGCAGGAAGTAGGACGGGCAA
TGTATGCCCCTCCCATTGCAGGAAACATAACATGTAGATCAAAT---
ATCACAGGACTATTATTAGTACGTGATGGAGGCAGCAATAACAAT----------------------------
----

ACCAACGAGACATTCAGACCTGGAGGAGGAAATATGAAGGACAATTGGAGAAGTGAATTATACAAATATAAAG
TGGTAGAAATTAAGCCATTG---GGAATAGCACCCACGAAGGCAAAAAGGAGAGTGGTG--------------
-------------------------GAGAGAGAAAAAAGAGCA---GTGGGA---ATAGGAGCT---------
GTGTTCCTTGGGTTCTTGGGAGCA---GCAGGAAGCACTATG---GGCGCGGCGTCA---
ATAACGCTGACGGTACAG---GCCAGACAA---TTGTTG---TCTGGTATAGTGCAACAGCAAAGC---
AATTTG---CTGAGG---GCTATAGAGGCGCAACAGCATATGTTGCAACTCACGGTCTGGGGCATT
>GU080199

AGTTATACAAGTATTAATAAGACAGGTGAAAACAGCACAAGT-------------------------------
----
GATTCAAACAGCACACTCACACTCCCATGCAGGATAAAACAAATTATAAACATGTGGCAGGAGGTAGGACGAG
CGATGTACGCTCCTCCCATTGCAGGAAATATAACATGTAGATCAAAT---
ATCACAGGACTACTATTGACACGTGATGGAGGAAACACAACAAACAAC-------------------------
----

ACAAACAAAGAGATATTCAGACCTGGAGGAGGAAATATGAAAGACAATTGGAGAAGTGAATTATATAAATATA
AAGTGGTAGAAATTAAGCCATTG---GGAGTAGCACCCACTAAGGCAAAAAGGAGAGTGGTG-----------
----------------------------GAGAGAGAAAAAAGAGCAGTACTGGA---ATGGGAGCT-------
---GTGTTCCTTGGGTTCTTGGGAGCA---GCAGGAAGCACTATG---GGCGCAGCGTCA---
CTAACGCTGACGGTACAG---GCCAGACAA---CTGTTG---TCTGGCATAGTGCAACAGCAAAGC---
AATTTG---CTGAGA---GCTATAGAGGCGCAACAGCATCTGTTGCAACTCACAGTCTGGGGCATT
>HM623585
AACTATACAGATAACAAC---

CAAACAGGTACCATCACACTTCCATGCAAAATAAAACAAATTATAAACATGTGGCAGGGGGTGGGACAAGCAA
TGTATGCCTCTCCCATTGAAGGAAACATAACATGTAGATCAAAT---
ATCACAGGACTACTATTAGTACGTGATGGAGGCAATACAAGTGAGACA-------------------------
----

AATGGAACAGAGACATTCAGACCTGCAGGAGGAGATATGAGGGACAATTGGAGAAGTGAATTATATAAGTATA
AAGTGGTAGAAATTAAGCCATTG---GGAATAGCACCCACTAAAGCAAAAAGGAGAGTGGTG-----------
----------------------------GAGAGAGAAAAAAGAGCA---GTGGGA---ATAGGAGCT------
---GTGTTCCTTGGGTTCTTGGGAGCA---GCAGGAAGCACTATG---GGCGCGGCGTCA---
ATAACGCTGACGGTACAG---GCCAGACAA---CTGTTG---TCTGGTATAGTGCAACAGCAAAAT---
AATTTG---CTGAGG---GCTATAGAGGCGCAACAGCATATGTTGCAACTCACAGTCTGGGGCATT
>KF725903
AATCAC---

TCAACCGATATAACCACCATCCAATGCAGGATAAAACAATTTATAAACATGTGGCAGGAGGTAGGACGAGCAA
TGTATGCCCCTCCCATAGCAGGAAACATAACATGTAAATCAAAT---
ATCACAGGACTACTATTGACAAGTGATTCTGGAAATAGAACTAAT----------------------------
----

GGAGCAGAGATATTCAGACCTGGAGGAGGAAATATGAAGGACAATTGGAGAAGTGAATTATATAAATATAAAG
TGGTAGAAATTAAGCCATTA---GGAGTAGCACCCACTGGGGCAAAAAGGAGAGAGGTG--------------
-------------------------GGGAGAGGAAAAAGAGCA---GCAGGA---ATAGGAGCT---------
ATGATCTTTGGGTTCTTGGGAGCA---GCAGGAAGCACTATG---GGCGCGGCGTCA---
ATAACGCTGACGGTACAG---GCCAGACAA---CTATTG---TCTGGGATAGTGCAACAGCAAAAC---
AATTTG---CTGAGA---GCTATAGATGCGCAACAGCATATGTTGCAGCTCACAGTCTGGGGCATT
>KF725921
GATCAT---

TCAAACTTAACCATCACACTCCCATGCAGAATAAAACAAATTATAAACCTGTGGCAGGAGGTAGGAAGGGCAA
TATATGCCCCTCCCATTGCAGGAAACATAACATGTACATCAAAT---
ATCACAGGATTACTATTGCTACGAGATGGAGGTCTCTTAAACAGC----------------------------
----

ACAAACGAAACATTCAGACCTGGAGGAGGAGACATGAGGAACAATTGGAGAAGTGAATTATATAAATATAAAG
TGGTAGAAATTAAGCCATTG---GGGGTAGCACCCACTAATGCAAAAAGGAGAGTGGTG--------------
-------------------------GAGAGAGAAAAAAGAGCA---GTAGGA---ATAGGAGCT---------
GTGTTCCTTGGGTTCTTGGGAGCA---GCAGGAAGCACTATG---GGCGCGGCGTCA---
ATGACGCTGACGGTACAG---GCCAGACAA---CTGTTG---TCTGGTATAGTGCAACAGCAAAAC---
AATTTG---CTGAGG---GCTATAGAGGCGCAACAGCATATGTTGCAACTCACGGTCTGGGGCATT
>JX140669
AATTATACTGAGACAACA---

GGTAATGGGACTATCATACTTCCATGCAGAATAAAACAAATCATAAACATGTGGCAGAAAGTAGGACGAGCAA
TGTATGCCCCTCCCATTGCAGGAAACATAACATGTAGTTCAAAT---

```
ATCACAGGACTACTATTGACACGTGATGGAGGAAATACAACAGATAATACA----------------------
-------------------------------------------------------------------------
----
ACAAATAATAACACAGAGGTATTCAGACCTGGAGGAGGAAATATAAAAGACAATTGGAGAAGTGAATTATATA
AATATAAAGTGGTAGAAATTAAACCATTA---GGAATAGCTCCAACTAAAGCAAAAAGGAGAGTGGTG-----
----------------------------------GAGAGAGAAAAAAGAGCA---GTGGGA---
ATAGGAGCT---------ATGTTCCTTGGATTCTTGGGAGCA---GCAGGAAGCACTATG---
GGCGCGGCGTCA---ATAACGCTGACGGTACAG---GCCAGACAA---CTGCTG---
TCTGGTATAGTGCAACAGCAAAGT---AATTTG---CTGAGA---
GCTATAGAGGCGCAACAGCATATGTTGCAACTCACAGTCTGGGGCATT
>KF725937
AGCTATACATACAAT----------------------------------------------------------
--------------------------------------------------
GGTAACTCAACCATCACACTTCCATGCAGAATAAAGCAAATTATAAACATGTGGCAGGAGGTAGGACGAGCAA
TGTATGCCCCTCCCATTGCAGGAAACATAACATGTGAATCAAAC---
ATCACAGGATTACTGTTGACACGTGATGGAGGAAACAATAGTACA----------------------------
-------------------------------------------------------------------------
----------
AATGGAACAGAGATATTCAGACCTGAAGGAGGAGATATGAGGGACAATTGGAGAAGTGAATTGTATAAATATA
AAGTGGTGGAAATTAAGCCATTG---GGAGTAGCACCCACTAAGGCAAAAAGGAGAGTGGTG-----------
----------------------------GAGAGAGAAAAAAGAACA---GTGGGA---ATAGGAGCT------
---GTGTTCCTTGGGTTCTTGGGAGCA---GCAGGAAGCACTATG---GGCGCGGCGTCA---
ATAACGCTGACGGTACAG---GCCAGACAA---TTGTTG---TCTGGTATAGTGCAACAGCAAAGC---
AATTTG---CTGAGG---GCTATAGAGGCGCAACAGCATATGTTGCAACTCACGGTCTGGGGCATA
>KF725945
AACTACAATATAAGTAAT-------------------------------------------------------
-----------------------------ATA---
AATTCAACCTCAAATATCACACTCCAATGCAGAATAAAACAAATTGTAAACATGTGGCAAGGGGTAGGACGAG
CAATGTATGCTCCTCCCATTGCAGGAAACATAACATGTAGATCAAAT---
ATCACAGGACTACTATTGACACGTGATGGAGGAAGCAATATTACT----------------------------
-------------------------------------------------------------------------
----------
AATACTACAGAGATATTCAGACCTGCAGGAGGAAATATGAAGGATAATTGGAGAAGTGAGTTATATAAATATA
AAGTGGTAGAAGTTAAACCATTA---GGAATAGCACCCACTGGGGCAAAAAGGAGAGTGGTG-----------
----------------------------GAGAGAGAAAAAAGAGCA---GTGGGA---ATAGGAGCT------
---GTGTTCCTTGGGTTCTTGGGAGCA---GCAGGAAGCACTATG---GGCGCGGCGTCA---
ATAACGCTGACGGTACAG---GCCAGACAA---TTACTG---TCTGGTATAGTGCAACAGCAAAGC---
AATTTG---CTGAGG---GCCATAGAGGCGCAACAGCATATGTTGCAACTCACGGTCTGGGGCATT
>KF725958
AATCATACAGACCTGTTTAATGATACAGAAAGTAATAGCACAAAAGAG-------------------------
-------------------------------------------------------------------------
-
GATAACGCAAACATCACACTCCCATGCAGAATAAAACAAATTATAAACCTGTGGCAGCAAGTGGGACGAGCAA
TGTATGCCCCTCCCATTGCAGGAAACATAACATGTACATCAAAT---
ATTACAGGGATATTATTGACACGTGATGGAGGAGAGAATACAACAGAGGGG----------------------
-------------------------------------------------------------------------
----------
AAGGACACAGAGACATTCAGACCTGCAGGAGGAGACATGAGGGACAATTGGAGAAGTGAATTATATAAATATA
AAGTGGTAGAAATCAGGCCATTG---GGACTAGCACCCACTGGGGCAAAAAGGAGAGTGGTG-----------
----------------------------GGGAGAGAAAAAAGAGCA---GTAGGA---ATAGGAGCT------
---GTGTTCCTTGGATTCTTGGGAGCT---GCAGGAAGCACTATG---GGCGCAGCGTCA---
TTAACGCTGACGGTACAA---GCCAGACAA---TTGTTG---TCTGGTATAGTGCAACAGCAAAGC---
AATTTG---CTGAAG---GCTATAGAGGCGCAACAGCATCTGTTGCAACTCACAGTCTGGGGCATT
>KF725962
TTTTATAGTCCTAATACTACAAATGGTACAGAAGAAAAT----------------------------------
-------------------------------------------------------------------------
----
ACAAACTCCTCCATCACGATACCATGCAGAATAAAACAGATAATAAACATGTGGCAGGAAATAGGACGGGCAA
TGTATGCCCCTCCCGTGGAAGGAAACATAACATGCAACTCAAAC---
ATCACAGGACTGCTACTGGTACGTGATGGAGGAGATAATAATAAAACAGAT----------------------
```


AATAATAAAACAGAGATATTTAGACCTGGAGGAGGAGATATGAGGGATAATTGGAGAAGTAAATTATATAAAT
ATAAAGTGGTAGAAATTAAACCATTG---GGAATAGCACCCACTAAGGCAAAAGGAGAGTGGTG---------
-------------------------------GAGAGAGAAAAAAGAGCA---GTGGGA---ATAGGAGCT---
------GTGTTCCTTGGGTTCTTGGGAGCA---GCAGGAAGCACTATG---GGCGCAGCGTCA---
GTGACGCTGACGGTACAG---GCCAGACAA---TTGTTG---TCTGGTATAGTGCAACAGCAAAGC---
AATTTG---CTGAGG---GCTATAGAGGCGCAACAGCACATGTTGCAGCTCACAGTCTGGGGCATT
>KF726010
AATTATACAAGAAATGCTTCAGATACAACT---

ACAAATAAAACCATCACACTTCCATGTAGAATAAAACAAATTATAAATATGTGGCAGGGGGTAGGAAGAGCAA
TGTATGCCCCTCCCATTGCAGGAAAGATAGCATGTAACTCAAGT---
ATCACAGGATTACTATTATTACGTGATGGAGGTATAAGCAATGAC----------------------------
----

AGTGAGACCGAAATATTCAGACCTGGAGGAGGAAATATGAAAGACAATTGGAGGAGTGAACTATATAAATATA
AAGTGGTAGAAATTAAGCCATTA---GGAATAGCACCCACTGAGGCAAAAGGAGAGTGGTG------------
----------------------------GAGAGACAAAAAAGAGCA---GTAGGA---CTAGGAGCT------
---GTGTTCCTTGGGTTCTTGGGAGCA---GCAGGAAGCACTATG---GGCGCAGCGTCA---
ATAACGCTGACGGTACAG---GCCAGACAA---TTATTG---TCTGGTATAGTGCAACAGCAAAGC---
AATTTG---CTGAGG---GCTATAGAGGCGCAACAGCATATGTTGCAACTCACGGTCTGGGGCATT
>MG901120
GGCTACCAGCAAAATAGTACAGCA---

AATGAGGCAAACATCACACTTCCATGCAGGATAAAACAAATTATAAACATGTGGCAAGGGGTAGGACGAGCAA
TGTATGCCCCTCCTATTGCAGGAACCATACAATGTACATCCAAT---
ATTACAGGACTACTATTAGAACGTGATGGAGGTCCTGATAATAATGGAACTGAAAAT---AATGGAACT----
----

GAAAATAATGGAACTGAAATATTCAGACCTACAGGAGGAAATATGAGGGACAATTGGAGAAGTGAATTATATA
AATATAAAGTGGTGGAAATTAAGCCATTA---GGAATAGCACCCACTGGAGCAAAAGGAGAGTGGTG------
----------------------------------GAGAGACAAAAAAGAGCA---TTGGGA---
ATAGGAGCT---------ATGTTCCTTGGGTTCTTGGGAGCA---GCAGGAAGCACTATG---
GGCGCAGCGTCA---ATAGCGCTGACGGCACAG---ACCAGACAA---CTGATG---
GCTGGTATAGTGCAACAGCAAAGC---AATTTG---CTGAGG---
GCTATAGAGGCGCAACAGCATATGTTGCAACTCACGGTCTGGGGCATT
>KP109516
TACTACCTGTTTAATGGTACGGTTGTTAATACCACA-------------------------------------
----
GATAGTAACACAAATATCACACTACCATGCAGAATAAAACAAATTGTAAACATGTGGCAGGGGGTAGGACGAA
CAATGTATGCTCCTCCCATTGCAGGAGTCATAAACTGTACATCAAAC---
ATAACAGGATTACTATTGACACGTGATGGAGGAATAAATAGCACA----------------------------
----

GGCAATGAGACATTCAGACCTGGAGGAGGAGATATGAGGGACAATTGGAGAAGTGAGTTATATAAATATAAAG
TGGTAGAAATTAAGCCATTA---GGGATAGCACCCACTGAGGCAAAAGGAGAGTGGTG---------------
-------------------------GAGAGAGAGAAAAGAGCA---GCG------ATAGGAGCT---------
GTACTCCTTGGGTTCTTGGGAGTG---GCCGGAAGCACTATG---GGCGCAGCATCA---
ATAACGCTGACGGCACAG---GCCAGACAA---TTGCTG---TCTGGTATAGTGCAACAGCAAAGC---
AATTTG---CTGAGG---GCTATAGAGGCGCAACAGCATATGTTGCAGCTTACAGTCTGGGGCATT
>MN611466
TTTTATAACACTAGCAGGTTTAATAGCAGCATGACAAAC----------------------------------
----

AGTACAAACAACATCACACTCCCATGCAGAATAAAACAAATTATAAACATGTGGCAGGAGGTAGGACGAGCTA
TGTATGCCCCTCCCATTGCAGGAAACATAACATGTATATCAAAT---
ATCACAGGACTACTATTGACACGTGATGGAGGAATAAACAATGGGACA-------------------------
----

AATGAGACAGGTAATGAGACATTCAGACCTGCAGGAGGGAATATGAAGGACAATTGGAGAAGTGAATTATATA
AATATAAAGTGGTGGAGATCAAGCCATTA---GGAGTAGCACCCACTAAAGCAAAAAGGAGAGTGGTG-----
----------------------------------GAGAGAGAAAAAAGAGCA---GTGGGA---
ATAGGAGCT---------GTGTTCCTTGGGTTCTTGGGAGCA---GCAGGAAGCACTATG---
GGCGCGGCGTCA---ATAACGCTGACGGTACAG---GCCAGACAA---CTGTTG---
TCTGGTATAGTGCAACAGCAAAGC---AATTTG---CTGAGG---
GCTATAGAGGCGCAACAGCATATGTTGCAACTCACAGTCTGGGGCATT
>MN611467
ACTTATGCTAATAGAACA---

GGCAATGGTACCTTCACACTCCCATGCAGAATAAAACAAATTATAAACATGTGGCAGGAGGTAGGAAGAGCAA
TGTATGCCCCTCCCATTGCAGGAAACGTAACATGTAGATCAAAT---
ATCACAGGACTACTATTGACACGTGATGGAGGAAAAAACAATACT----------------------------
----

GAAGAGATATTTAGACCTGGAGGAGGAAATATGAGGGACAATTGGAGAAGTGAATTATATAAATATAAAGTGG
TAGAAATTAAGCCATTA---GGAGTAGCACCCACTAATGCACAAAGGAGAGTGGTG-----------------
----------------------AAGAGAGAAAGAAGAGCA---GTGGGA---ATAGGAGCT---------
GTGTTCCTTGGGTTATTGGGAGCA---GCAGGAAGCACTATG---GGCGCAGCGTCA---
ATAACGCTGACGGTACAG---GCCAGACAA---CTGTTG---TCTGGTATAGTGCAACAGCAAAAC---
AATTTG---CTGAGA---GCTATAGAGGCGCAACAGCATATGTTGCAACTCACAGTCTGGGGCATT
>DQ411853
TGCTATACATCAGACTTGTTTAATAGTACATACATGTCTAATAATACA-------------------------
----

GGAGGAGCAAATATCACACTCCAATGCAGAATAAAACAAATTATAAGAATGTGGCAGGGGGTAGGACAAGCAA
TGTATGCCCCTCCCATTGCAGGAAACATAACATGCAAATCAAAT---
ATCACAGGACTACTATTGACACGTGATGGAGGAAAGGAAAAAAAT----------------------------
----

GACACAGAAACATTTAGACCTGGAGGGGGAGATATGAGAGACAATTGGAGAAGTGAATTATATAAATACAAGG
TGGTAGAAATTAAGCCATTA---GGAATAGCACCCGATAAGGCAAAAAGGAGAGTGGTG--------------
-------------------------GAGAGAGAAAAAAGAGCA---GTGGGA---ATAGGAGCT---------
GTGTTCCTTGGGTTCTTGGGAGCA---GCAGGAAGCACCATG---GGCGCAGCATCA---
ATGACGCTGACGGTACAG---GCCAGACAA---TTGTTG---TCTGGTATAGTGCAACAGCAAAGC---
AATTTG---CTGAGG---GCTATAGAGGCGCAACAGCATATGTTGCAACTCACGGTCTGGGGCATT
>AY162223
TACTACCCTAAGAATGGTACATACAAATACAATGGTAAT----------------------------------
----
TCAAGCTTACCCATCACACTCCAATGCAAAATAAAACAAATTGTACGCATGTGGCAAGGGGTAGGACAAGCAA
TGTATGCCCCTCCCATTGCAGGAAACATAACATGTAGATCAAAC---
ATCACAGGAATACTATTGACACGTGATGGGGGATTTAACAACACAAAC-------------------------
----

AACGACACAGAGGAGACATTCAGACCTGGAGGAGGAGATATGAGGGATAACTGGAGAAGTGAATTATATAAAT
ATAAAGTGGTAGAAATTAAGCCATTG---GGAATAGCACCCACTAAGGCAAAAAGAAGAGTGGTG--------
-------------------------------CAGAGAAAAAAAAGAGCA---GTGGGA---ATAGGAGCT---
------GTGTTCCTTGGGTTCTTGGGAGCA---GCAGGAAGCACTATG---GGCGCAGCGTCA---
ATAACGCTGACGGTACAG---GCCAGACAA---CTGTTG---TCTGGTATAGTGCAACAGCAAAGC---
AATTTG---CTGAAG---GCTATAGAGGCGCAACAGCATATGTTGCAACTCACAGTCTGGGGCATT
>AF391235
AGTTATAATACATCAGACCTGTTTAATAGTAAT--

AATGGTTCAGCCATCACACTCCCATGCAGAATAAAACAAATTGTAAACATGTGGCAGGGGGTAGGACGAGCAA
TATATGCCCCTCCCATTGCAGGAAACATAACATGTAACTCAAGT---
ATCACAGGACTACTCTTGGTACGTGATGGAGGAAACACAACCAAC----------------------------
----

TCAACTGAGATATTCAGACCAGAAGGAGGAAATATGAGGGACAATTGGAGAAGTGAATCATATAAATACAAAG
TGGTAGAAATTAAGCCCTTG---GGAATAGCGCCCACTAATGCAAAAAGGAGAGTGGTG--------------

-------------------------GAGAGAGAAAAAAGAGCA---GTGACA---CTAGGAGCT---------
ATGTTCCTTGGGTTCTTGGGAGCA---GCAGGAAGCACTATG---GGCGCAGCGTCA---
ATAACGCTGACGGCACAG---GCCAGACAG---TTGTTG---TCTGGAATAGTGCAACAGCAAAGC---
AATTTG---CTGAGA---GCTATAGAGACGCAACAGCATATGTTGCAACTCACAGTTTGGGGCATT
>AF391240
AATCATACATACCTGTTTAATGGTACAGGCGTGCCTAATAATACCACA-------------------------
----
CCTTCTAATGAGACCATCATACTTCCATGCAGAATAAAACAAATTATAAACATGTGGCAGGAGGTAGGGCGAG
CAATGTATGCCCCTCCCATTGCAGGAAACATCACATGTACATCAAAC---
ATCACAGGACTACTATTAGTACGTGATGGAGGCAACAGTGGCAAAAAT-------------------------
----

ACCACAGAAGAGATATTCAGACCTGGGGGAGGAAATATGAAGGACAATTGGAGAAGTGAATTATATAAATATA
AAGTGGTAGAAATTAAGCCATTA---GGAATAGCTCCCACTGCGGCAAAAAGGAGAGTGGTG-----------
----------------------------GAGAGAGAAAAAAGAGCA---GTGGGA---ATAGGGGCT------
---GTGTTCCTTGGGTTCTTGGGAGCA---GCAGGAAGCACTATG---GGCGCGGCGTCA---
ATAACGCTGACGGTACAG---GCCAGACAA---TTGTTG---TCTGGTATAGTGCAACAGCAAAGC---
AATTTG---CTGAGG---GCTATAGAGGCGCAACAGCATCTGTTGCAACTCACAGTCTGGGGCATT
>AY529668
AATTATATAGCAACAAATACTTCA---

GAGACTTCACCCATCATGATCCAGTGCAGAATAAAGCAAATTATAAACATGTGGCAGAGGGTAGGACGAGCAA
TGTATGCCCCTCCCATTGCTGGAAGCCTAACATGTAACTCAAGT---
ATCACAGGACTAATATTGACACGTGATGGAGGAGAAAATACAAGT----------------------------
----

ATAGAGACATTCAGACCTGGAGGAGGAAATATGAAGGACAATTGGAGAAGTGAATTATATAAATATAAAGTGG
TAGAAGTTAAGCCATTA---GGAATAGCACCCACTGATGCAAAAAGGAGAGTGGTG-----------------
----------------------GAGAGAGAGAAAAGAGCA---GTGGGA---ATAGGAGCT---------
GTGTTCCTTGGGTTCTTGGGAGCA---GCAGGAAGCACTATG---GGCGCGGCGTCA---
ATAACGCTGACGGTACAG---GCCAGACAA---TTATTG---TCTGGTATAGTGCAACAGCAAAGC---
AATTTG---CTGAGG---GCTATAGAGGCGCAACAGCATATGTTGCAACTCACGGTCTGGGGCATT
>AY529670
GATTACACGCCTAATAAGACAGAAAATGATTCA--

AATGCAACTTCAAATATCACACTCCAATGTAGAATAAAGCAAATTGTAAACATGTGGCAGGGGGTAGGACAAG
CAATGTATGCCCCTCCCATTAAAGGATACATAGAATGTATATCAAAT---
ATCACAGGACTACTATTGACACGTGATGGAGGAAAAGAAAAGAAT----------------------------
----

AACACAGAGACATTCAGACCTGGAGGAGGAGATATGAGGGACAATTGGAGAAGTGAATTATATAAATATAAAG
TAGTAGAAGTTGAACCATTA---GGAATAGCACCCACTAAGGCAAAAAGGAGAGTGGTG--------------
-------------------------GAGAGAGAAAAAAGAGCA---GTGGGA---ATAGGAGCT---------
GTGTTCCTTGGGTTCTTGGGAGCA---GCAGGAAGCACTATG---GGCGCGGCATCA---
ATAACGCTGACGGTACAG---GCCAGACAA---TTGTTG---TCTGGTATAGTGCAACAGCAAAGC---
AATTTG---CTGAGG---GCTATAGAGGCGCAACAGCATATGTTGCAACTCACGGTCTGGGGCATT
>DQ447272
AATTACAATGGTACAAAAAAT--

AGTACAGAAAACATCACACTCCCATGCAGAATAAAACAAATTATAAACATGTGGCAGAAGGTAGGACGAGCAA
TGTATGCTCCTCCCATTGAAGGAAAAATAACATGTTCCTCAAAC---
ATCACAGGATTACTAGTGACATATGATGGAGGAACATCAGACAACGAGACA----------------------
----

ACAAAAAACACAGAGACATTCAGACCTGGAGGAGGAGATATGAGGGACAATTGGAGAAGTGAATTATATAAAT
ATAAAGTGGTAGAAATTAAGCCATTG---GGAATAGCACCCACTAAGGCAAAAAGGAGAGTGGTG--------
-------------------------------GAGAGAGAAAAAAGAGCA---GTGGGA---ATAGGAGCT---
------GTGTTCCTTGGGTTCTTGGGAGCA---GCAGGAAGCACTATG---GGCGCGGCGTCA---
ATAACGCTGACGGTACAG---GCCAGACAA---TTGTTG---TCTGGTATAGTGCAACAGCAAAGC---
AATTTG---CTGAGG---GCTATAGAGGCGCAACAGCATATGTTGCAACTCACGGTTTGGGGCATT

>AY522726
AATTACACATCAAAACAGGATAATAGTACAAAAAGAAAT-------------------------------
----
-
TCAAGCTCAATCATCACAATCCCATGCAAAATAAAACAATTTATAAACATGTGGCAGGAGGTAGGACGAGCAA
TGTATGCCCCTCCCATTGAAGGAATCATAAAATGTACATCAAAC---
ATCACAGGATTACTATTACTACGTGATGGAGGGACCAATAATGATACT----------------------
----

AATGACACAGAGATATTCAGACCTGGAGGAGGGGACATGAGGGACAATTGGAGAAGTGAATTATATAAATATA
AAGTAGTAGAAATTAAGCCATTG---GGAATAGCTCCCACTGAAGCAAAAAGGAGAGTGGTG----------
---------------------------GAGAGAGAAAAAAGAGCA---GTAGGA---ATAGGAGCT------
---GTGCTCCTTGGGTTCTTGGGAGCA---GCAGGAAGCACTATG---GGCGCGGCGTCA---
ATAACGCTGACGGTACAG---GCCAGACAA---GTGTTG---TCTGGTATAGTGCAACAGCAAAGC---
AATTTG---CTGAGG---GCCATAGAGGCGCAACAGCATATGTTGCAACTCACGGTCTGGGGCATT
>AY522728
ACTCATTTGCCTAATGATACCAAT--
-----------------------------------AGT---
GAGTCCAATATAAACCTCACGATCCAATGCAGAATAAAACAAATTATAAATATGTGGCAGGAGGTGGGAAGGG
CAATGTATGCCCCTCCCATTGAAGGAAACATAACATGTAAATCAAAT---
ATCACAGGACTACTATTGACGCGTGATGGAGGAAGAAGTAATAAC-------------------------
----

ACAGAAGAGATATTTAGACCTGGAGGAGGAAATATGAAGGATAATTGGAGAAGTGAATTATATAAATATAAAG
TGGTAGAAATTAAGCCATTG---GGAGTAGCACCCACTGAGGCAAAAAGGAGATTGGTG-------------
------------------------GGGAAAGAAAAAAGAGCA---GTGGGA---ATAGGAGCT---------
GTGCTCTTTGGGTTCTTGGGAGCA---GCAGGAAGCACTATG---GGCGCGGCGTCA---
ATAGCGCTGACGGTACAG---GCCAGACAA---TTGCTG---TCTGGTATAGTGCAACAGCAAAAC---
AATTTG---CTGAGG---GCTATAGAGGCGCAACAGCATCTGTTGCAACTCACGGTCTGGGGCATT
>AY522731
AGTTATACAAAACTAAATGGAAGTGAG---

TTAAACAAAACCATCACACTCCCATGCAGGATAAAACAAATTATAAACATGTGGCAGGAGGTAGGACGAGCAA
TGTATGCCCCTCCCATTGCAGGGAAAATAACATGTAACTCAAGT---
ATCACAGGACTACTATTGACACGTGATGGAGGAGGAAACAAAACG-------------------------
----

AATGGCACAGAGGTATTCAGACCTGGAGGAGGAAATATGAAGGACAATTGGAGAAGTGAATTATATAAATATA
AAGTAGTAGAAATTAAGCCATTG---GGAATAGCACCCACTGAGGCAAAAAGGAGAGTGGTG----------
---------------------------GAGAGAGAAAAAAGAGCA---GTGGGA---ATAGGAGCT------
---GTACTCCTTGGGTTCTTGGGAGCA---GCAGGAAGCACTATG---GGCGCGGCGTCA---
ATAACGCTGACGGTACAG---GCCAGACAA---CTGTTG---TCTGGTATAGTGCAACAGCAAAGC---
AATTTG---CTGAGA---GCTATAGAGGCGCAACAGCATATGTTGCAACTCACAGTCTGGGGCATT
>AY522733
GCTTACAGCAACACTGACAACAACAATAGTACTGGA----------------------------------
----
AACGCAACCACCATCACACTGCCATGCAGAATAAAACAAATTATAAACATGTGGCAGGAGGTAGGACGAGCAA
TGTATGCCCCTCCCATTGCAGGAAACATAACATGTAACTCAAGT---
ATCACAGGACTAATATTGACACGTGATGGAGGAAGCAATATGACAGAA----------------------
----

GTAAATACCACAGAGATATTCAGACCTGGAGGAGGAAATATGAAGGACAATTGGAGAAGTGAATTATATAAAT
ATAAAGTGGTAGAAATTAAGCCATTG---GGAATAGCACCCACTGAGGCAAAAAGGAGAGTGGTG-------
------------------------------GAGAGAGAAAGAAGAGCA---GTGGGA---CTAGGAGCT---
------ATGTTCCTTGGGATCTTGGGAGCG---GCAGGAAGCACTATG---GGCGCGGCGTCA---
ATAACGCTGACGGTACAG---ACCAGACAA---CTGTTG---TCTGGTATAGTGCAACAGCAAAGT---
AATTTG---CTGAAG---GCTATAGAGGCGCAACAGCATCTGTTGCAACTCACGGTCTGGGGCATT
>AY522735
TGCTATACCTCAAAGCTGTTTAATAGAACATACAGGCCAAATTCAACCAGTTCAAATTCAAATTCAAATTCA-
----

-------------ACC---
AGTTTAAATTCAACCATCACAATTCCATGCAGAATAAAACAAATTATAAACATGTGGCAGAGGGTAGGACGAG
CAATGTATGCCCCTCCCATTGCAGGAAACATAACATGTAATTCAAGT---
ATCACAGGACTACTATTAGTACGTGATGGAGGAGAAAACAATAGT----------------------------
----

ACAGAAGAGATATTCAGACCTGGAGGAGGAGATATGAGGGACAATTGGAGAAGTGAATTATATAAATATAAAG
TGGTAGAGATTAAGCCATTG---GGAATAGCACCCACTAAGGCAAAAAGGAGAGTGGTG--------------
-------------------------GAGAGAGAAAAAAGAGCAGTAGTGGGA---CTAGGAGCT---------
GTGTTCTTTGGGTTCTTGGGAGCA---GCAGGAAGCACTATG---GGCGCGGCGTCA---
ATAACGCTGACGGTACAG---GCCAGACAA---TTGCTG---TCTGGTATAGTGCAACAGCAAAGC---
AATTTG---CTGAGG---GCTATAGAGGCGCAACAGCATATGTTGCAACTCACGGTCTGGGGCATT
>DQ447268
AATCATAATAATACATACAATACAAGT--

GACAACTCCAACATCACACTCCCATGCAGAATAAAACAAATTATAAACATGTGGCAGGAGGTAGGAAAAGCAA
TATATGCCCCTCCCATCAGAGGAAACATAACATGTAACTCAAGT---
ATCACAGGACTACTATTGGTACGTGATGGGGGTAACTCAAATAATAACTCA----------------------
----

AATGAGACCACAGAGATATTCAGACCTGGAGGAGGAGATATGAGAGATAATTGGAGAAGTGAATTATATAAAT
ATAAAGTGGTAGAAATTAAGCCATTG---GGAGTAGCACCCACTGGGGCAAAAAGGAGAGTGGTG--------
-------------------------------GAGAGAGAAAAAAGAGCA---GTGGGA---ATAGGAGCT---
------GTGTTCCTTGGGTTCTTGGGAGCA---GCTGGAAGCACTATG---GGCGCGGCGTCA---
ATGACGCTGACGGTACAG---GCCAGACAA---CTGTTG---TCTGGCATAGTGCAACAGCAAAGC---
AATTTG---CTGAGG---GCTATAGAGGCGCAACAACATATGTTGCAACTCACAGTCTGGGGCATT
>GU216739
AATTATACTGGCAGTGGA---

AAAAACATAACCACCACCATCCAATGCAAGATAAAACAAATTGTAAATCTGTGGCAAGAGGTAGGACGAGCAA
TGTATGCCCCTCCCATTGCAGGAAACATAACGTGTAACTCAAGT---
ATTACAGGACTACTATTGACACGTGATGGCGGTAGCAACAGCAGC----------------------------
----

AGCCCAGAGACATTCAGACCTGGAGGAGGAAATATGAAGGACAATTGGAGAAGTGAATTATATAAATATAAAG
TGGTAGAAATTAAGCCATTG---GGAGTAGCACCCACTGAGGCAAAAAGGAGAGTGGTG--------------
-------------------------GAGAGAGAAAAAAGGGCA---GTGGGA---ATAGGAGCT---------
GTGTTCCTTGGGTTCTTGGGAGCT---GCAGGAAGCACTATG---GGCGCGGCGTCA---
ATAACGCTGACGGTACAG---GCCAGACAA---CTGTTG---TCTGGTATAGTGCAACAGCAAAGC---
AATTTG---CTGAGA---GCCATAGAGGCGCAACAGCATATGTTGCAACTCACGGTCTGGGGCATT
>GU216844
TCTTATGATACAGAA--

AGTAATGGAAATATCATACTCCCATGCAGAATAAAACAAATTATAAACCTGTGGCAGGGGGTAGGACGAGCAA
TATATGCACCTCCCATTGCAGGAAATATAAAATGTACATCAGAT---
ATCACAGGACTACTATTGACACGTGATGGAGGGAATAACACAATT----------------------------
----

AATGAGACATTCAGACCTATAGGAGGAAATATGAGGGACAATTGGAGAAGTGAATTGTATAAATATAAAGTGG
TAGAAATCAGGCCATTG---GGAGTAGCACCCACTGCAGCAAAAAGGAGAGTGGTG-----------------
----------------------GAGAGAGAAAAAAGAGCA---GTGGGC---ATAGGAGCT---------
GTGTTCCTTGGGTTCTTGGGAGCA---GCAGGAAGCACTATG---GGCGCAGCGTCA---
ATGACGCTGACGGTACAG---GCCAGACAA---CTGTTG---TCTGGTATAGTGCAACAGCAAAGC---
AATTTG---CTGAGG---GCTATAGAGGCGCAACAGCATATGTTGCAACTCACGGTCTGGGGCATT
>HQ625580
ACCTATAATGTTACCAGAACAAGTAATACAACT--

AATAGTAGTATAATTACACTCCAATGCAGAATAAAACAGATTATAAACATGTGGCAAGGGGTAGGACAAGCAA
TGTATGTCCCTCCCATTGCAGGAAACATAATATGCAAATCAAAT---
ATCACAGGACTACTATTGACACGTGATGGAGGCAATAATACAGAAAAC-------------------------

----

AATAATACAGAGATATTCAGACCTGCAGGAGGAGACATGAGGGACAATTGGAGAAGTGAATTATATAGATATA
AAGTGGTAGAAATTAAGCCATTA---GGAATAGCACCCACAAAAGCAAGAAGGAAAGTGGTG-----------
----------------------------CAGAGAGAAAAAAGAGCA---GTGGGA---ATAGGAGCT------
---GTACTCCTTGGGTTCTTGGGAGCA---GCAGGAAGCACTATG---GGCGCGGCGTCA---
ATAACGCTGACGGTACAG---GCCAGACAA---CTGTTG---TCTGGTATAGTGCAGCAGCAAAAC---
AATTTG---CTGAGA---GCTATAGAGGCGCAACAGCATATGTTGCAACTCACAGTCTGGGGCATT
>HQ625591
ACCTATTTAAACACAACCAATGTA---

AACGAAACCGACATCACACTCCAATGCAGGATAAAGCAAATTATAAACATGTGGCAGGGGGTGGACGAGCAA
TGTATGCCCCTCCCATTGCAGGAAACATAACATGTAACTCAAAT---
ATCACAGGACTACTGCTGACACGTGATGGAGGCTTGAATAACACG----------------------------
----

AATAACGAGACATTCAGACCTGGAGGGGGAAATATGAAGGACAATTGGAGAAATGAATTATATAAATACAAAG
TGGTAGAAATTAAGCCATTG---GGAGTAGCACCCACTGCTGCAAAAAGGAGAGTGGTG--------------
-------------------------GAGAGAGAAAAAAGGGGA------------CTAGGAGCT---------
GTGTTCCTTGGGTTCTTGGGAGCA---GCAGGAAGCACTATG---GGCGCGGCGTCA---
ATAACGCTGACGGTACAG---GCCAGACAA---CTGTTG---TCTGGTATAGTGCAACAGCAAAGC---
AATTTG---CTGAGA---GCTATAGAGGCGCAACAGCATATGTTGCAACTCACAGTCTGGGGCATT
>HQ625582
CATCATAAAGGAAGTAATGAA--

AATGAAAGCAGCATCATACTTCCATGCAGAATAAAACAAATTATAAACATGTGGCAGGAGGTAGGACGAGCAA
TGTATGCATCTCCCATTGCAGGAAGGATAACATGTACATCAAAT---
ATCACAGGACTATTATTAGTACGAGATGGAGGCACAGGCAATACC----------------------------
----

AGTAAACCAGAAATATTCAGACCTGGAGGAGGAAACATGAAGGACAATTGGAGAAGTGAATTATATAAATATA
AAGTAGTAGAAATTAAGCCATTA---GGAATAGCACCCACTGGAGCAAAAAGGAGAGTGGTG-----------
----------------------------GAGAGAGAAAAAAGAGCA---GTGGGA---ATAGGAGCT------
---GTACTCCTTGGGTTCTTGGGAGCA---GCAGGAAGCACTATG---GGCGCGGCGTCA---
ATAACGCTGACGGTACAG---GCCAGACAA---CTGTTG---TCTGGTATAGTGCAACAGCAAAGC---
AATTTG---CTGAGG---GCTATAGAGGCGCAACAGCAGATGTTGCAACTCACAGTCTGGGGCATT
>GU939049
TTTTATAAGTTCAATAGGACAGAAAATGGTTCAAATACAGAAAAT----------------------------
----

AGTTCAGGCATCATCACACTCCCATGCAAAATAAAACAAATTATAAACATGTGGCAGGAGGTAGGACGAGCAA
TGTATGCCCCTCCCATTGAAGGAAACATAACATGTAAATCAGAG---
ATTACAGGACTCCTATTGACACGTGACGGA---AAAAATGGAACA----------------------------
----

GAGGAGATAGTTAGACCTATAGGAGGAAATATGAAGGATAATTGGAGAAGTGAATTATATAAATATAAAGTGG
TAGAAATTAAGCCATTA---GGAATAGCACCCACTGAGGCAAAAAGGAGAGTGGTG-----------------
----------------------GAGAGAGAAAAAAGAGCA---GTAGGA---ATAGGAGCT---------
GTGCTCCTTGGGTTCTTGGGAGCA---GCAGGAAGCACTATG---GGCGCGGCGTCA---
ATAACGCTGACGGTACAG---GCCAGACAA---TTGTTG---TCTGGTATAGTGCAACAGCAAAGC---
AATTTG---CTGAGG---GCTATAGAGGCGCAACAGCATCTGTTGCAACTCACAGTCTGGGGCATT
>AB254146
TACTATTGGAACAGTACAACAGGGAAT--

TCAAGCTCAAACATCACAATCCCATGCAAAATAAAACAAATTATAAACATGTGGCAGGAGGTAGGACGAGCAA
TGTATGCCCCTCCCATTGCAGGGAAAATAACATGTGCATCAAAT---
ATCACAGGACTACTATTGACACGTGATGGAGGAGTATCAAATAAGACAGAT----------------------
----

GCAAATAAGACAGAGACATTCAGACCTATAGGAGGAAATATGAGGGACAATTGGAGAAGTGAATTATATAAAT

ACAAAGTGGTAGAAATTAATCCATTA---GGACTGGCACCCACTAAGGCAAAAAGGAGAGTGGTG--------
-------------------------------GAGAGAGAAAAAAGAGCA---GTGGGA---CTAGGAGCT---
------GTGCTCTTTGGGTTCTTGGGAGCA---GCAGGAAGCACTATG---GGCGCAGCATCA---
CTAACGCTGACGGTACAG---GCCAGACAA---TTATTG---TCTGGTATAGTGCAACAGCAAAGC---
AATTTG---CTGAGA---GCTATAGAGGCGCAACAGCATATGTTGCAACTCACGGTCTGGGGCATT
>KY229291
AATTATACAGTA---

GACAATTCTACCATCACACTCCAATGCAGAATAAAACAAATTATAAACACATGGCAGGAGGTAGGACGAGCAA
TATACGCCCCTCCCATTGAAGGAAACATAAAATGTACATCAAAA---
ATCACAGGACTACTCTTGGTACGTGATGGAGGGGGGAATGACACA----------------------------
----

AGGGAAGAGATATTCAGACCTGGAGGAGGAAATATGAGGGACAATTGGAGAAGTGAATTATATAAATATAAAG
TGGTAGAAATTAAACCATTG---GGAATAGCACCCACTAAGGCAAAACGGAGAGTGGTG--------------
-------------------------GAGAGAGAAAAAAGAGCA---GTCGGA---ATGGGAGCT---------
GTGATCCTTGGGTTCTTGGGAGCA---GCAGGAAGCACTATG---GGCGCGGCGTCA---
ATAACGCTGACGGCACAG---GCCAGACAA---GTATTG---TCTGGTATAGTGCAACAGCAAAGC---
AATTTG---CTGAGA---GCTATAGAGGCGCAACAGCATATGTTGCAACTCACAGTCTGGGGCATT
>KY229453
AATCACACAAATAAT--

AGTACATATAATATCACACTCCCATGCAGAATAAGACAGATTGTAAGGATGTGGCAGGGAGTAGGACAAGCAA
TGTATGCCCCTCCCATTGCAGGAAACATAACATGTAGATCAAAT---
ATCACAGGACTACTATTGACACGTGATGGAGGAAAGGAGATAAC-----------------------------
----

AACACAACAGAAACATTCAGACCTGGAGGAGGAGATATGAGGGACAATTGGAGAAGTGAATTATATAAATATA
AAGTGGTAGAGATTAAGCCATTA---GGAATAGCACCCACCAAGGCAAAAAGGAGAGTGGTG-----------
----------------------------GAGAGAGAAAAAAGAGCA---GTGGGA---ATGGGAGCT------
---GTACTCCTTGGGTTCTTGGGAGCT---GCAGGAAGCACTATG---GGCGCGGCGTCA---
ATAACGCTGACGGTACAG---GCCAGACAA---TTGTTG---TCTGGTATAGTGCAACAGCAAAGC---
AATTTG---CTGAGA---GCTATAGAGGCGCAACAGCATATGTTGCAACTCACAGTCTGGGGCATT
>EU166359
TTTCATGGTACATACCTGCCTAATAGTACAGTCATGCTTAATGATACT-------------------------
----

TCAAACAAAACCATCACACTGCTATGCAGAATAAAACAAATTATAAATATGTGGCAGGAGGTAGGGCGAGCAA
TGTATGCCCCACCCATTGCAGGAAGCATAATATGCAACTCAAGT---
ATTACAGGGCTACTATTGACACGTGATGGAGGAGATGGAAATGGAAAGAATACT-------------------
----

ACAGGTAAGGATACAGAGACATTCAGACCTGCAGGAGGAGATATGAGGGACAATTGGAGAAGTGAATTATATA
AATATAAGGTGGTAGAAATTAACCCATTA---GGAATAGCACCCACTAAAGCAAAAAGGAGAGTGGTG-----
----------------------------------GAGAGAGAGAAAAGAGCA---GTGGGA---
ATAGGAGCT---------GTACTCCTTGGGTTCTTGGGAGCA---GCAGGAAGCACTATG---
GGCGCAGCATCA---ATAACGCTGACGGTACAG---GCCAGACAA---TTATTG---
TCTGGTATAGTGCAACAGCAAAGC---AATTTG---CTGAGG---
GCTATAGAGGCGCAACAACATATGTTGCAACTCACGGTCTGGGGCATT
>EU166432
AATTATAACATTACAAACAATAATGTTACAAACATTACAGAC-------------------------------
----
-
GAGAATGTAAACATCACACTCAAATGCAGAATAAAACAGATTATAAACATGTGGCAGGGGGTAGGACAAGCAA
TATATGCCCCTCCCATTGCAGGAAACATAACATGTAAATCAAAT---
ATCACAGGAATACTATTGACACGCGATGGAGGCGACGAAACAAAG----------------------------
----

AATAACACAGAAATATTCAGACCTGGAGGAGGAAATATGAAGAACAATTGGAGAAGTGAATTATATAAATATA
AAGTGGTAGAAATTAAGCCATTA---GGAATAGCACCCACTGAGGCAAAAAGGAGAGTGGTG-----------

---------------------------GAGAGAGAAAAAAGAGCA---GTGGGA---ATAGGAGCT------
---GTGTTCCTTGGGTTCTTGGGAGCA---GCAGGAAGCACTATG---GGCGCGGCATCA---
ATAACGCTGACGGTACAG---GCCAGACAA---TTGTTG---TCTGGTATAGTGCAACAGCAAAGC---
AATTTG---CTGAGA---GCTATAGAGGCGCAACAGCATATGTTGCAACTCACTGTCTGGGGCATT
>DQ388516
ACCTACACGAATGGAAGTAATTCAAATGTAAACATC------------------------------------
----
ACAAGTGCAACCATCACCCTCCCATGCAGAATAAAACAAATTATAAACATGTGGCAAGAAGTAGGACGGGCAA
TGTATGCCCCTCCCATTGCAGGAAACATAACATGTATATCAAAT---
ATCACAGGACTACTCCTGACAAGGGATGGAGGAAATGGAAATGATACA------------------------
----

AATGACACAGAGACATTTAGACCTGCAGGAGGAGATATGAGGGACAATTGGAGAAGTGAATTATATAAATATA
AAGTGGTAGAAATTAAACCATTA---GGAATAGCACCCACTAAAGCAAAAAGGAGAGTGGTG----------
---------------------------GGGAGAGAAAAAAGAGCA---GTGGGA---ATAGGAGCT------
---GTGTTCCTTGGGTTCTTGGGAGCA---GCAGGAAGCACTATG---GGCGCGGCGTCA---
ATAACGCTGACGGTACAG---GCCAGACAA---TTATTG---TCTGGTATAGTGCAACAGCAAAAC---
AATTTG---CTGAGA---GCTATAGAGGCGCAACAGCATCTGTTGCAACTCACGGTCTGGGGCATT
>GU939124
AATTATACATACATGAATGAGACAAGC---

-
ACTAACTCAACGATCAGACTCCCATGTAAGATAAGACAAATTATAAACATGTGGCAGGAGGTAGGACGAGCAA
TATATGCCCCTCCCATTGAAGGAAACATAACATGCAACTCAAGT---
ATCACAGGACTACTATTGGTACGGGATGGAGGCAACACTAGCAGTACC------------------------
----

GGCACAGAGATAGAGATATTTAGACCTGGAGGGGGAGATATGAGGGACAATTGGAGAAGTGAATTATATAAAT
ATAAAGTGGTAGAAATTAAGCCATTG---GGAGTAGCTCCCACTAAGGCAAAAAGAAGAGTGGTG-------
------------------------------GAAAGAGAAAAAAGAGCA---GTGGGA---ATAGGAGCT---
------GTACTCCTTGGGTTCTTGGGAGCA---GCAGGAAGCACTATG---GGCGCAGCGTCA---
ATAACGCTGACGGCACAG---GCCAGACAA---GTGCTG---TCTGGTATAGTGCAACAGCAAAGC---
AATTTG---CTGAGG---GCTATAGAGGCGCAACAGCACCTGTTGCAACTCACAGTCTGGGGCATC
>JX213407
AATTATACATTTAATGATACATTTAATGATATAGGAAGTAAGTCA---------------------------
----
-
AATACAGGAAATATCACACTCCAATGCAGAATAAAACAAATTGTAAACATGTGGCAGAGGGTAGGACGAGCAA
TATATACCCCTCCCATTAAGGGACAGATAACATGTGTATCAAAT---
ATCACAGGAATACTATTGGTACGTGATGGAGGAAACAACAATGGATCAGAG---------------------
----

AATAACTCAGAGATATTCAGACCTGGAGGAGGAGATATGAGGGACAATTGGAGAAATGAATTGTATAAATATA
AAGTAGTAGAAATTAAGCCATTG---GGAATAGCACCCACTGGGGCAAAAAGGAGAGTGGTG----------
---------------------------GAGAGAGAAAAAAGAGCA---GTGGGA---ATAGGAGCT------
---GTGTTCTTTGGGTTCTTGGGAGCA---GCAGGAAGCACTATG---GGCGCGGCGTCA---
ATAACGCTGACGGTACAG---GCCAGACAA---TTATTG---TCTGGTATAGTGCAACAGCAAAGC---
AATTTG---CTGAGG---GCTATAGAGGCGCAACAGCATATGTTGCAACTCACGGTCTGGGGCATT
>JX213435
AATCATACACTGGATGTTAATGGTACATTATCTCCAAATTCA------------------------------
----
-
TCTTCTTTAAACATCACACTCCCATGCAAAATAAAACAAATCATAAACATGTGGCAGGGGGTGGGACGAGCAA
TGTATGCCCCTCCCATTGCAGGAAACATAACATGTAGATCAAAT---
ATCACGGGAATAATATTGACACGTGATGGTGGAAGTGGAAGTGGA---------------------------
----

TCATCTAATGACACAGAGATATTCAGACCTGGAGGAGGAGATATGAGAGACAATTGGAGAAGTGAATTATATA
AATACAAAGTGGTAGAAATTAAGCCATTA---GGAATAGCACCCAACAAGGCAAAAAGGAGAGTGGTG----
---------------------------------GAAAGAGAAAAAAGAGCA---GTGGGA---

ATAGGAGCT---------GTGTTCCTTGGGTTCTTAGGAGCA---GCAGGAAGCACTATG---
GGCGCGGCGTCA---ATGGCGCTGACGGTACAG---GCCAGACAA---GTGTTG---
TCTGGTATAGTGCAACAGCAAAGC---AATTTG---CTGAGA---
GCTATAGAGGCGCAACAGCATATGTTGCAACTCACAGTCTGGGGCATT
>JX213454
AGTTACAAATGCATGCCTAATATGCCTAATGACACATGTAATAGCACAATCACA-------------------
---GAT---
AACAGCACAGGATTCATCATACTCCCATGCAGAATAAAACAAATTGTAAACATGTGGCAGGGGGTAGGACGAG
CAATGTATGCCGCTCCCATTGCCGGGAACATAACATGTAGATCAAAC---
ATCACAGGACTACTATTGACACGTGATGGAGGTACCCCAAGTAAAAATGAG----------------------
----

ACCAAGGAGACATTCAGACCTGGAGGAGGGAATATGAAGGACAATTGGAGAAGTGAATTATATAAGTATAAAG
TGGTAGAAATTAAGCCACTG---GGACTAGCACCCACTGGAGCAAAAAGGAGAGTGGTG--------------
-------------------------GAGAGAGAAAAAAGAGCA---GTGGGA---ATAGGAGCT---------
GTGTTCCTTGGGTTCTTGGGAGCA---GCAGGAAGCACTATG---GGCGCGGCGTCA---
ATGACGCTGACGGTACAG---GCTAGACAA---CTGTTG---TCTGGTATAGTGCAACAGCAAAGC---
AATTTG---CTGAGG---GCCATAGAGGCGCAACAGCATATGTTGCAGCTCACAGTCTGGGGCATT
>GU329195
AGTTACTCAAATGAG--

ACAAACTCAACCATCACACTCCAGTGTAAAATAAAACAAATTATAAACATGTGGCAGGGGGTAGGACGAGCAA
TGTATGCCGCCCCCATTGCAGGAAACATAACATGTATATCAAAC---
ATCACAGGAATACTACTGACACGGGATGGAGGAAACAGTAATAGTAAT-------------------------
----

GAGAGTACAGAAACATTTAGACCAGGAGGGGAAATATGAAAGACAATTGGAGAAGTGAATTATATAAATATA
AAGTGGTAGAGGTTAAGCCATTG---GGAATAGCACCCACTGAGGCAAAAAGGAGAGTGGTG-----------
----------------------------GAGAGAGAAAAAAGAGCA---GTGGGA---CTAGGAGCT------
---GTGTTCCTTGGGTTCTTGGGAGCA---GCAGGAAGCACTATG---GGCGCAGCATCA---
ATAACGCTGACGGTACAG---GCCAGAAAT---CTGTTG---TCTGGCATAGTGCAACAGCAAAAC---
AATTTG---CTGAGG---GCTATAGAGGCGCAACAGCATATGTTGCAACTCACAGTCTGGGGCATT
>GU329205
AATCACACAGACATGTCTAACATGACTAAT---

AATAACATCACACTCACACTCCCATGCAGAATAAAACAAATTATAAACATGTGGCAGGAGGTAGGACGAGCAA
TATATGCACCTCCCATTGTAGGAAACATAATCTGTACATCAAAT---
ATCACAGGACTACTATTGACACGTGATGGAGGAACAGATAACAAC----------------------------
----

AGAACAGAGGAGACGTTCAGACCTGGGGGAGGAGATATGAGAGACAATTGGAGAAGTGAACTATATAAATATA
AGGTGGTAGAAATTAAGCCATTG---GGCATAGCACCCACTAAGGCAAAAAGGAGAGTGGTG-----------
----------------------------GAGAGAGAGAAAAGAGCA---GTGGGA---ATAGGAGCT------
---GTGTTCCTTGGGTTCTTGGGAGCA---GCAGGAAGCACTATG---GGCGCGGCGTCA---
GTGACGCTGACGGTACAG---GCCAGACAA---CTATTG---TCTGGTATAGTGCAACAGCAAAGC---
AATTTG---CTGAAG---GCTATAGAGGCGCAACAGCATATGTTGCAACTCACGGTCTGGGGCATC
>GU329282
CACTATAGTAATGGTACAGACATATTTAATGATAAT-------------------------------------
----
AGTACAAGCAACATCACACTCCCATGCAAAATAAAACAAATTATAAACATGTGGCAGAAAGTAGGACAAGCAA
TGTATGCCCCTCCCATTGCAGGAAACATAAAATGTGAATCAAAT---
ATCACAGGGCTACTATTGACACGTGATGGAGGAGGAGGCTCAGATAATACA----------------------
----

ACAGATAATACAACAGAGACATTCAGACCTGGAGGAGGAGATATGAGGGACAATTGGAGAAGTGAATTATATA
AATATAAAGTGGTAGAAGTTAAGCCATTA---GGAATAGCACCCACTGAGGCAAGAAGGAGAGTGGTG-----
----------------------------------CAGAGAGAAAAAAGAGCA---GTGGGA---
ATAGGAGCA---------ATGTTCCTTGGGTTCTTGGGAGTA---GCAGGAAGCACTATG---
GGCGCAGCGTCA---ATGGCGCTGACGGTACAG---GCCAGACAA---TTGTTG---

TCTGGTATAGTGCAACAGCAAAGT---AATTTG---CTGAGA---
GCCATAGAGGCGCAACAGCATATGTTGCAACTCACGGTCTGGGGCATT
>GU329289
CATTATACAAGAGAGAAT---

GTCTCAAACATCGAGCTGCTCCCATGTAAAATAAAACAGATTATAAACATGTGGCAGGAAGTAGGACGAGCAA
TGTATGCCCCTCCCATTGCAGGAAGCATAACATGTAAATCAAGT---
ATCACAGGACTACTATTGACACGTGATGGAGGAAACAATAGCACC----------------------------
----

GAGACAGAAATATTCAGACCTGGAGGAGGAGATATGAGGGACAATTGGAGAAGTGAATTATATAAATATAAGG
TGGTAGAAATTAAGCCATTA---GGAGTAGCACCCACTACTGCAAAAAGGAGAGTGGTG--------------
-------------------------GAGAGAGAGAAAAGAGCA---GTGGGA---ATAGGAGCT---------
GTGTTCCTTGGGTTCTTAGGAGCA---GCAGGAAGCACTATG---GGCGCAGCATCA---
ATAACGCTGACGGTACAG---GCCAGACAA---CTATTG---TCTGGTATAGTGCAACAGCAAAGC---
AATTTG---CTGAGG---GCTATCGAGGCGCAACAGCATATGTTGCAACTCACAGTCTGGGGCATT
>GU329439
AACTACACGAATGGTGAATACAAGCATAACAGGAATGGTACAGAG----------------------------
-----------------------------------GTGCATATTAATGAG---------
ACTAGCAAACAGAATATCACAATCCAATGCAGAATAAAACAAATTATAAACATGTGGCAGGAGGTAGGGCGAG
CAATGTATGCCCCGCCCATTGCAGGAAACATAACATGTGCATCAAAT---
ATCACAGGACTACTATTGACATGGGATGGAGGAAAAAATAGAAGC----------------------------
----

GAAACAGAGGAGATATTCAGGCCTGGAGGAGGAGATATGAGGGACAATTGGAGAAGTGAATTATACAAATATA
AAGTGGTAGAAATTAAGCCATTA---GGAGTAGCACCCTCTAAGGCAAAAAGGAGAGTGGTG-----------
----------------------------GAGAGAGAAAAAAGAGCA---GTGGGA---ATAGGAGCT------
---GTACTCCTTGGGTTCTTGGGAGCA---GCAGGAAGCACTATG---GGCGCAGCATCA---
TTGACGCTGACGGTACAG---GCCAGACAA---TTATTG---TCTGGTATAGTGCAACAGCAAAGC---
AATTTG---CTGAGG---GCTATAGAGGCGCAACAGCATATGTTGCAACTCACGGTCTGGGGCATT
>KX983735
CCTCAGGATAACAAT--
--------------------ACAAAA------
AATGATGAGACGACCATCAGCCTGCCATGCAGAATAAAACAAATCATAAACATGTGGCAGGAGGTAGGACGAG
CAATATATGCCCCTCCCATTGAAGGAAGCATAACATGTGAATCAAAC---
ATCACAGGACTACTATTGACAAGTGATGGTGGAGGAAAAGGTAAA----------------------------
----

AATGAGACATTCAGACCTGCAGGAGGAGATATGAGGGACAATTGGAGAAGTGAATTATATAAATATAAAGTGG
TAGAAATTAAACCATTA---GGAGTAGCACCCACTAGAGCAAAAAGGAGAGTGGTG-----------------
----------------------GAGAGAGAAAAAAGAGCA---GTGGGA---ATAGGAGCT---------
GTGTTCCTTGGGTTCTTGGGAGCA---GCAGGAAGCACTATG---GGCGCGGCGTCA---
ATAACGCTGACGGCACAG---GCCAGACAA---GTGCTG---TCTGGTATAGTGCAACAGCAAAGC---
AATTTG---CTGAGG---GCTATAGAGGCGCAACAGCATATGTTGCAACTCACGGTTTGGGGCATT
>KX983891
TGCTATACATCGAAACTGTTTAATAGAACACATAATGGTACAGATGATAATGAAACA----------------
----

AGTAGAAACATCACCACTATCCCATGCAGAATAAAACAAATTATTAACATGTGGCAGACAGTGGGACGAGCAA
TGTATGCCCCTCCCATTGAAGGAAACATAACATGTAAATCAAAT---
ATCACAGGAATAATATTAACACGTGATGGAGGAATTACAGAGGAGTCA-------------------------
----

AATAACACAGAAATATTCAGACCTGGAGGAGGAGATATGAGAGATAATTGGAGAAGTGAATTATATAAATATA
AAGTGGTAGAGATTAAACCATTG---GGAATAGCACCCTCCAAGGCAAAAAGGCGAGTGGTG-----------
----------------------------GAGAGAGAAAAAAGAGCA---GTGGGA---ATAGGAGCT------
---GTGTTCCTTGGGTTCTTGGGAGCA---GCAGGAAGCACTATG---GGCGCGGCGTCA---
CTCACGCTGACGGTACAG---GCCAGACAA---TTGTTG---TCTGGTATAGTGCAACAGCAAAAC---
AATTTG---CTGAGA---GCTATTGAGGCGCAACAACATCTGTTGCAACTCACGGTCTGGGGCATT
>AF286224

AATTATACAGAAAATAATACA--

GATGGTACACCCATCACACTCCCATGCAGAATAAGACAAATTATAAATATGTGGCAGGAAGTAGGACGAGCAA
TGTACGCCCCTCCCATTGAAGGAAACATAGCATGTAAATCAGAT---
ATCACAGGGCTACTATTGGTTCGGGATGGAGGAAGCACAAATGATAGCACA----------------------
----

AATAATAACACAGAGATATTCAGACCTGCAGGAGGAGATATGAGGGACAATTGGAGGAGTGAATTGTATAAGT
ATAAAGTGGTAGAAATTAAGCCATTG---GGAATAGCACCCACTGAGGCAAAAAGGAGAGTGGTG--------
-------------------------------GAGAGAGAAAAAAGAGCA---GTGGGA---ATAGGAGCT---
------GTGTTCCTTGGGTTCTTGGGAGCA---GCAGGAAGCACTATG---GGCGCAGCGTCA---
ATAACGCTGACGGCACAG---GCCAGACAA---GTGTTG---TCTGGTATAGTGCAACAGCAAAGC---
AATTTG---CTGAGG---GCTATAGAGGCGCAACAGCATCTGTTGCAACTCACGGTCTGGGGCATT
>KU168262
TTTCATAGTACATACAATAGTACATACAATAGTACAGACAAGGGTAACAGC----------------------
----

ACAGACGATACCATCACACTCCCATGCAGAATAAAACAAATTATAAACATGTGGCAGGAGGTAGGAAAAGCAA
TATATGCCCCTCCTATTCCAGGAAAAATAACATGTAACTCAAGC---
ATCACGGGGCTACTACTGACACGTGATGGAGGGCAAAATAATACA----------------------------
----

AATGAGACATTCAGGCCTGGAGGAGGAGATATGAGAGATAATTGGAGAAGTGAATTATACAAATATAAGGTGG
TAGAAATTAGACCATTA---GGAGTAGCACCCACTGATGCAAAAAGGAGAGTGGTG-----------------
----------------------GAGAGAGAAAAAAGAGCA---GTGGGA---ATAGGAGCT---------
GTGCTCTTAGGGTTCTTGGGAGCA---GCAGGAAGCACTATG---GGCGCAGCGTCA---
GTGGCGCTGACGGTACAG---GCCAGACAA---TTATTG---TCGGGTATAGTGCAACAGCAAAGC---
AATTTG---CTGAGG---GCTATAGAGGCGCAACAGCATATGTTGCAACTCACAGTCTGGGGCATT
>JQ361079
ACTTATAATGCTTCAATAGAA--

AATAATACTATAATCACGCTCCCGTGCAGAATAAAACAAATTATAAACATGTGGCAAGGAGTAGGAAAAGCAA
TATATGCCCCTCCCATTGAAGGACTAATTAAGTGTACATCAAAT---
ATTACAGGAATATTATTAACAAGAGATGGTGGTAGAGATAATAGT----------------------------
----

AGTAGTAACGAGACCTTCAGACCTGGAGGAGGAGATATGAGAGATAATTGGAGAAGTGAATTATACAAATATA
AAGTAGTAAAAATTGAACCAATA---GGTCTAGCACCCACCAAGGCAAAGAGAAGAGTGGTG-----------
----------------------------GAAAGAGAAAAAAGAGCA---ATAGGA---CTAGGAGCT------
---ATGTTCCTTGGGTTCTTGGGAGCA---GCAGGAAGCACGATG---GGCGCAGCGTCA---
GTGACGCTGACGGTACAG---GCCAGACAG---TTATTG---TCTGGTATAGTGCAACAGCAAAAT---
AATTTG---CTGAGG---GCTATAGAGGCGCAACAGCATCTGTTGCAACTCACAGTCTGGGGCATT
>HM215270
AATTATACTGGA---

AATGAGAGTGTAATCAACCTACCATGCAGAATAAAACAAATTATAAACATGTGGCAGGGAGTAGGAAAAGCAA
TGTATGCCCCTCCCATTGAAGGGCTAATCAACTGTTCATCAAAT---
ATTACAGGACTATTGCTGACAAGAGATGGTGGTATAAATAGTAATACG-------------------------
----

AGTAGTTCTGAGACCTTCAGACCTGGAGGAGGAGATATGAGAGACAATTGGAGAAGTGAATTATACAAATATA
AAGTAGTAAGACTTGAACCACTA---GGTCTAGCACCCACCAAGGCAAAAAGAAGAGTGGTG-----------
----------------------------GAAAGAGAAAAAAGAGCA---ATAGGA---CTAGGAGCT------
---GTGTTCCTTGGGTTCTTGGGAGCA---GCAGGAAGCACGATG---GGCGCAGCGTCA---
CTGACGCTGACGGTACAG---GCCAGACAG---TTACTG---TCTGGTATAGTGCAACAGCAAAAT---
AATTTG---CTGAAG---GCTATAGAGGCGCAACAGCATCTGTTGCAACTCACAGTCTGGGGCATT
>L22949
AGTTATAATACATCAGCATTAAATAGTACACAGGGAAAT----------------------------------
----
GAGAGTGAAAACATCACACTCCCATGCAGAATAAAACAAATTATAAACATGTGGCAGGAAGTAGGAAAAGCAA

TGTATGCCCCTCCCATTGAAGGGATAATCAGATGTTCATCAAAT---
ATTACAGGACTATTGTTGACAAGAGATGGTGGTATAACTAACAAT----------------------------
----

AGTAATGAGACCTTCAGACCTGGAGGAGGAAATATGAGAGACAATTGGAGAAGTGAATTATACAAATATAAAG
TAGTAAGAATTGAACCACTA---GGTCTAGCACCCACCACGGCAAAGAGAAGAGTGGTG--------------
-------------------------GAAAGAGAAAAAAGAGCA---ATAGGA---CTGGGAGCT---------
CTGTTCCTTGGGTTCTTGGGAGCA---GCAGGAAGCACAATG---GGCGCAGCGTCA---
CTGACGCTGACGGTACAG---GCCAGACAA---GTATTG---TCCGGCATAGTGCAACAGCAAAAC---
AATTTG---CTGAGG---GCTATAGAGGCGCAACAACATCTGTTGCAACTCACAGTCTGGGGCATT
>L22950
AATTATAATATATCAAATAGTACAGGG--

GGAAATGAGACAATCAGACTCCAATGCAGAATAAAACAAATTATAAACATGTGGCAGGAAGTAGGAAAAGCAA
TGTATGCCCCTCCCATTATAGGACAAATCAAATGTTCATCAAAT---
ATCACAGGACTATTGTTAACAAGAGATGGTGGTGTAAATAATAGTAGTAATGAG-------------------
----

ACTAGCAGTAAAAATGAGATCTTCAGACCTGGAGGAGGAGATATGAGAGACAATTGGAGAAGTGAATTATACA
AATATAAAGTAGTAAGAATTGAACCACTA---GGTCGGGCACCCACCAGGGCAAAAGGAGAGTGGTG------
----------------------------------GAAAGAGAAAAAAGAGCA---ATAGGA---
CTGGGAGCT---------TTGTTCCTTGGGTTCTTGGGAACA---GCAGGAAGCACGATG---
GGCGCAGCGTCA---ATGACGCTGACGGTACAG---GCCAGACAG---GTATTG---
TCTGGTATAGTGCAACAGCAAAAC---AATTTG---CTGAGG---
GCTATAAAGGCGCAACAGCATCTGTTGCAACTCACAGTCTGGGGCATT
>AY623599
ACTTATAATACATCAGGTAGTACAGGGGTA---

AATAACAGTACAATCAAACTCCCATGCAGAATAAAACAAATTATAAACATGTGGCAGGGAGTAGGAAAAGCAA
TGTATGCCCCTCCCATTGAAGGACTAATCAAATGTTCATCAAAC---
ATTACAGGACTATTGTTGACAAGAGATGGGGGAAATAATACTAGG----------------------------
----

CAGAATGAAACCTTCAGGCCTGGAGGAGGGGATATGAGAGACAATTGGAGAAGTGAATTATACAAATATAAAG
TAGTAAGAATTGAACCACTA---GGTCTAGCACCCACTGAGGCAAAGAGAAGAGTGGTG--------------
-------------------------GAAAGAGAAAAAAGAGCA---ATAGGA---CTAGGAGCT---------
ATGTTCCTTGGGTTCTTGGGAGCA---GCAGGAAGCACGATG---GGCGCAGCGTCA---
ATGACGCTGACGGTACAG---GCCAGACAG---TTAATG---TCTGGTATAGTGCAACAGCAAAAC---
AATTTG---CTGAGG---GCTATAGAGGCGCAACAGCATCTGTTGCAACTCACAGTCTGGGGCATT
>AY713418
AATTATAATATATCA--

AATACAGAGGTAATCAGACTCCCATGCAGAATAAAACAAATTATAAACATGTGGCAGGGAGTAGGCAAAGCAA
TGTATGCCCCTCCCATTGAAGGAATAATCAAATGTTCATCAAAT---
ATTACAGGACTATTGTTAACAAGGGATGGTGGTGGTAATAACACT----------------------------
----

CGGAAGAATGAGACCTTCAGGCCTGGAGGGGGAGATATGAGAGACAATTGGAGAAGTGAATTATACAAATATA
AAGTAGTAAGAATTGAACCATTA---GGTCTAGCACCCACCAAGGCAAAGAGAAGAGTGGTG-----------
----------------------------GAAAGAGAAAAAAGAGCA---ATAGGA---CTGGGAGCT------
---CTGTTCCTTGGGTTCTTGGGAGCA---GCAGGAAGCACGATG---GGCGCAGCGTCA---
ATAACGCTGACGGTACAG---GCCAGACAA---GTATTG---TCTGGTATAGTGCAACAGCAAAAC---
AATTTG---CTGAGG---GCTATAGAGGCGCAACAACACTTGTTGCAACTCACAGTCTGGGGCATT
>EU853046
AATTATAGTAGTATGTTTAATAGTAGTACATCAAATAGTAGTATAGGG-------------------------
----
-
GAAAATGAAACAATCACACTCCAATGCAGAATAAAGCAAATTATAAACATGTGGCAGGGAGTAGGAAAAGCAA
TGTATGCACCTCCCGTTGCAGGACAAATCAGATGTTCATCAAAT---
ATTACAGGCCTATTGTTGACAAGAGATGGAGGTGAAAATACTAGT----------------------------

----

GAGGAGATCTTCAGACCTGCAGGAGGAGATATGAGAGACAATTGGAGAAGTGAATTATACAAATATAAAGTAG
TAAGAATTGAACCACTA---GGTCTAGCACCCACCAAGGCAAAGAGAAGAGTGGTG-----------------
----------------------GAAAGAGAAAAAAGAGCA---ATAGGA---CTAGGAGCT---------
GTGTTCCTTGGGTTCTTGGGAGCA---GCAGGAAGCACGATG---GGCGCAGCGTCA---
CTGACGCTGACGGTACAG---GCCAGACAA---TTATTG---TCTGGTATAGTGCAACAGCAAAAC---
AATTTG---CTGAGG---GCTATAGAGGCGCAGCAGCATCTGTTGCAACTCACAGTCTGGGGCATT
>AF484489
ACTTATGATACATCGAATGGTACA---

GGGAGTGAAAATATCACACTCCAATGCAGAATAAAACAAATGATAAACATGTGGCAAGGAGTAGGCAAAGCAA
TGTATGCCCCTCCCATTGAAGGAACAATCAACTGTTTATCAAAT---
ATTACAGGACTATTGTTGACAAGAGATGGTGGTGTAAAAAATAGT----------------------------
----

ACTAATGAGACCTTCAGACCTGGAGGAGGAGATATGAGAGACAATTGGAGAAGTGAATTATATAAGTATAAAG
TAGTAAGACTTGAACCACTA---GGCCTAGCGCCCACAGAGGCAAGAAGAAGAGTGGTG--------------
-------------------------GAAAGAGAAAAAAGAGCA---ATAGGCCTACTAGGAGCT---------
ATGTTCCTTGGGTTCTTGGGAGCA---GCAGGAAGCACGATG---GGCGCAGCGTCA---
CTGACGCTGACGGTACAG---GCCAGACAG---TTAATG---TCTGGTATAGTGCAACAGCAAAAC---
AATCTG---CTGAGG---GCTATAGAGGCGCAACAGCATATGTTGCAACTCACAGTCTGGGGCATT
>JX140673
AGTCAGTCTGAGAAGGCGGGA--

AATAATGGCAGTATCTTCCTTCCATGCAGAATAAGACAAATTGTAAACATGTGGCAAAAGGTAGGACGAGCAA
TGTATGCCCCTCCCATTGCTGGAGTAATTCGGTGTAACTCAAGT---
ATCACAGGTCTGTTATTAACAAGAGATGGTGGGGAAGATAATAGT----------------------------
----

AGCACTGACACCCTCAGACCTGGAGGGGGAGATATGAAAGATAATTGGAGAAGTGAACTGTATAAATATAAGG
TAGTAAAAATTGAACCACTA---GGTGTGGCACCCACCAGGGCAAAAAGACAAGTGGTG--------------
-------------------------AACAGAGAAAAAAGAGCA---GTGGGA---ATAGGAGCT---------
GTGTTCCTTGGATTCTTGGGAACA---GCAGGAAGCACTATG---GGCGCGGCGTCA---
ATAACGCTGACGGTACAG---GTCAGACAA---TTATTG---TCTGGTATAGTGCAACAGCAAAGC---
AATTTG---CTGAGG---GCTATAGAAGCGCAACAGCATATGTTGCAGCTCACAGTCTGGGGCATT
>AJ249236
TACTACAACACATCAGGACTGTTTAATGAAACAGAGGTGGCTAATAAC-------------------------
----

ACAAACGAAAATATCACTCTCCCATGTAGAATAAGACAATTTGTAAACATGTGGCAAAGAATAGGAAGAGCAA
TGTATGCTCCTCCCATTGAAGGAGAAATCCAGTGTACCTCAAAT---
ATCACAGGTCTACTATTGACAAGAGATGGTAGTAAGGATATTGAC----------------------------
----

GGGAAAGAGATTCTCAGACCTATAGGAGGAGATATGAGAGACAATTGGAGAAGTGAACTATATAAATATAAGG
TAGTAAGAATTGAACCAGTA---GGAGTAGCACCCACCAAGGCAAAAAGACGAGTGGTG--------------
-------------------------CAGAGAGCAAAAAGAGCA---GTGGGA---ATGGGAGCT---------
GTGCTCTTTGGGTTCTTGGGAGCA---GCAGGAAGCACTATG---GGCGCGGCGGCA---
ATCACGCTGACGGCACAG---GCCAGACAA---TTATTG---TCTGGTATAGTGCAACAGCAAAGC---
AATCTG---CTGAAG---GCTATAGAAGCGCAACAGCATCTGTTGCAGCTCACAGTCTGGGGCATT
>MH746240
GCTCAGTCTAATAATAGTAGT--

GGAAATGAGACCATCAGACTCTCATGTAAAATAAAACAAATTGTGAGAATGTGGCAGAAAGTGGGACAAGCAA
TGTATGCCCCTCCCATTGCAGGAGAAATTACATGTAGATCAAAC---
ATTACAGGACTACTGCTAACAAGAGATGGTGGAAAGAATATTAGT----------------------------
----

AAAGGAGATGAGATCTTCAGGCCTATAGGAGGAGATATGAGGAACAATTGGAGAAGTGAATTATATAAGTATA

AAGTAGTAAAAATCAAACCACTA---GGGGTAGCACCCACCAAAGCAAGGAGAAGAGTGGTG----------
----------------------------GAGAGAGAAAAAAGAGCA---GTTGGA---CTGGGAGCT------
---GCCTTCCTTGGGTTCTTAGGAGCA---GCAGGAAGCACTATG---GGCGCGGCGTCA---
ATAACGCTGACGGTACAA---GCCAGACAA---TTACTG---TCTGGGATAGTGCAACAGCAAAGC---
AATTTG---CTGAGA---GCTATAGAAGCGCAGCAGCATCTGTTGCAGCTCACAGTCTGGGGCATT
>KR051445
CTTTATGAA--

CTCCCATGTAGAATAAAGCAAATTGTGAGAATGTGGCAGAGAGTGGGACAAGCAATGTATGCCCCTCCCATCA
AAGGAAACATTACTTGTAGATCAAAC---
ATTACAGGACTACTATTAACAAGAGATGGTGGGAGAAATAGGAGT----------------------------
----

AATGACAGAGAGATCTTCAGACCTACAGGAGGAGACATGAGGAACAATTGGAGAAGTGAATTATATAAGTATA
AAATAGTAAAAATAAAACCATTA---GGAGTAGCACCCACCAAGGCAAGGAGAAGAGTGGTG----------
----------------------------GGGAGAGAAAAAAGAGCA---GCTGGC---CTGGGAGCT------
---GTTTTCCTTGGGTTCTTAGGAGCA---GCAGGAAGCACTATG---GGCGCGGCGTCA---
ATAACGCTGACGGTACAG---GTCAGACAA---CTATTG---TCTGGCATAGTGCAACAGCAAAGC---
AATTTG---CTGAGA---GCTATAGAGGCACAGCAACATCTGCTGCAACTCACAGTCTGGGGCATT
>KR051437
AGTCAAGAA--

AATAAAACAGTTATCACAATCCCATGTAGGATAAAACAAATTGTAAGAATGTGGCAGAGAACGGGACAAGCAA
TATATGCCCCTCCCATTGCAGGAAACATTACATGTATATCAAAC---
ATTACAGGACTATTATTAACAAGAGATGGTGGGAATGACAATAATAACAAT----------------------
----
AATACCAATGACAAT---
GACACTGAGGTCTTCAGACCTACAGGAGGAGATATGAGGGACAATTGGAGAAGTGAATTATATAAGTACAAAG
TAGTAAAGATTAAATCACTA---GGAGTAGCACCCACCAAGGCAAGGAGAAGAGTAGTG--------------
-------------------------GGAAGAGAAAAAAGAGCA---GTTGGA---ATAGGAGCT---------
GTCTTTCTTGGGTTCTTAGGAGCA---GCCGGGAGCACTATG---GGCGCGGCGTCA---
CTAACGCTGACGGTACAG---GTCAGACAA---TTATTG---TCTGGCATAGTGCAACAGCAAAGC---
AATTTG---CTGAGA---GCTATAGAGGCGCAACAGCATATGTTGCAACTCACAGTCTGGGGCATT
>KY658701
AGTTATATTAATAGGACATCAACT---

TCTAATGGGACTATCACACTCCCATGTAGAATAAAACAAATTGTAAGGATGTGGCAGAGAGTGGCACAAGCAA
TGTACGCCCCTCCCATTGCAGGAAAACTTGAATGTAACTCAAGC---
ATTACAGGATTAATATTAACAAGAGATGGTGGGAACAATGACAATAACACA----------------------
----

CAGAACGAGACCTTCAGACCTGGAGGAGGAAATATAAGGGACAATTGGAGAAGTGAATTATATAAATATAAAA
CAGTAAAAATCAAATCATTA---GGAATAGCACCCACCAGGGCAAGGAGAAGAGTGGTG--------------
-------------------------GAAAGACAAAAAAGAGCA---GTGGGA---ATGGGAGCT---------
GTCTTCCTTGGGTTCTTAGGAGCA---GCAGGAAGCACTATG---GGCGCGGCGTCA---
ATAACGCTGACGGTACAG---GTCAGACAA---TTATTG---TCTGGCATAGTGCAACAGCAAAGC---
AATTTG---CTGAGA---GCTATAGAGGCGCAACAGCATATGTTGCAACTCACAGTCTGGGGCATT
>FJ817370
AGTCATCTGTTTAATGGTAGTACTCTGTCT---

ACTAATGGTACTATCACACTTCCATGTAGGATAAAACAGATTGTAAGAATGTGGCAGAGAGTGGGACAAGCAA
TGTATGCCCCTCCTATCGCAGGAAACATTACATGTAGGTCAAAC---
ATTACAGGACTACTATTAACAAGAGATGGTGGAACAAATAAAGATACA-------------------------
----

AATGAGGCTGAGACTTTCAGACCTGGAGGAGGAGATATGAGGGACAATTGGAGAAGTGAATTATATAAGTATA
AAGTAGTAAAAATTAAACCATTA---GGAGTAGCACCCACCAGGGCAAGGAGAAGAGTGGTG----------
----------------------------GAGAGAGAAAAAAGAGCA---ATTGGA---CTGGGAACT------
---GTCCTCCTTGGGTTCTTAGGAACA---GCAGGAAGCACTATG---GGCGCAGCGTCA---

ATAACGCTGACGGTACAG---GTCAGACAA---TTATTG---TCTGGCATAGTGCAACAGCAAAGC---
AATTTG---CTGAGG---GCTATAGAGGCGCAGCAGCATCTGTTGCAACTCACAGTCTGGGGCATT
>EU885764
AATCATACAGAT---

ACAAATGAAAATATCACACTCCAGTGTAGGATAAAACAAATTGTGAGAATGTGGCAGAGAGTGGGACAAGCAA
TGTATGCCCCTCCCATCGCTGGAAACATTACATGTATCTCAAAC---
ATTACAGGACTGCTATTAACAAGAGATGGTGTGAATGATACCCATGAC-------------------------
----

AAGGAGAATGAGACCTTCAGACCTACAGGAGGAGATATGAGGGATAATTGGAGAAGTGAATTATATAAGTATA
AAGTAATAAAACTTAAACCACTA---GGAGTAGCACCCACCAGGGCAAGGAGAAGAGTGGTG-----------
----------------------------GAGAGAGGAAAAAGAGAA---GTCACA---CTAGGAGCT------
---TTGTTCCTTACGTTCTTAGGAGCA---GCAGGAAGCACTATG---GGCGCGGCGTCA---
TTAACGCTGACGGTACAG---GCCAGACAA---TTATTG---TCTGGCATAGTGCAACAGCAAAGC---
AATTTG---CTGAGA---GCTATAGAAGCGCAGCAGCATCTGTTGCAGCTCACAGTCTGGGGCATT
>EU885765
AGTTATGGGCCT---

AATGAGACACACATCATACTCCCATGTAGGATAAGACAAATTGTGAGAATGTGGCAGAGAGTAGGACAAGCAA
TGTATGCCCCTCCCATCGCAGGAAACATAACATGTAAATCAAAT---
ATTACAGGACTACTATTAACAAGAGATGGTGGAGAGGATGGCAAT----------------------------
----

AATAGCACTGAGACCTTCAGACCTGCAGGAGGAGACATGAGGGACAATTGGAGAAGTGAATTATATAAGTATA
AAGTAGTAAAAATTAAACCATTA---GGAGTAGCACCCACCAGGGCAAGAAGAAGAGTGGTG-----------
----------------------------AGGAGAGAAAAAAGAGCA---GTTGGA---CTGGGAGCT------
---GTCTTCCTTGGGTTCTTAGGAGCA---GCAGGAAGCACTATG---GGCGCGGCGTCA---
TTAACGCTGACGGTACAG---GCCAGACAA---TTATTG---TCTGGCATAGTGCAACAGCAAAGC---
AATTTG---CTGAGG---GCTATAGAGGCGCAGCAGCATCTGCTGCAACTCACAGTCTGGGGCATT
>HQ236608
AGCTATAATAGTGGCAGTAATAATAATACCACACATAATAGT-------------------------------
----
GGTAATGAGACTATCACAATTCCATGTAGGATAAAACAAATAGTGAGAATGTGGCAGAGAGTGGGACAAGCAA
TGTATGCCCCTCCCGTCGCAGGAAACATTACATGTAGGTCAGAA---
ATTACAGGACTACTATTAACAAGAGATGGT---AATGCAAGTGAA----------------------------
----

AATGAAATTGTCAGACCTGGAGGAGGAGACATGAGGGACAATTGGAGAAGTGAATTATATAAGTATAAAGTAG
TAAAAATTAAACCACTG---GGAGTAGCACCCACCAGGGCAAGGAGAAGAGTGGTG-----------------
----------------------GGGAGAGAAAAAAGAGCA---GTTGGA---CTGGGAGCT---------
GTCCTCCTTGGGTTCTTAGGAACA---GCAGGAAGCACTATG---GGCGCAGCGTCA---
ATAACGCTGACGGTACAG---GTCAGACAA---TTATTG---TCTGGCATAGTGCAACAGCAAAGC---
AATTTG---CTGAGG---GCTATAGAGGCGCAGCAGCATATGTTGCAACTCACAGTCTGGGGCATT
>KX389641
GAT--

TATGAGAGTAATATCACACTCCCATGTAAAATAAAACAAATTGTGAGAATGTGGCAGAGAGTAGGACAAGCAA
TGTATGCCCCTCCCATCGCAGGAAATATTACATGTAGATCAAAC---
ATTACAGGGCTATTATTAACAAGAGATGGTGGTGGGAACAATAAT----------------------------
----

AGCAATGAGACCTTCAGACCTGGAGGAGGAGATATGAGGGACAACTGGAGAAGTGAGTTATATAAGTATAAAA
TAGTAAAAATTAAACCACTA---GGAATAGCACCCACCAAGGCAAAGAGAAGAGTGGTG--------------
-------------------------CAGAGAGAGAAAAGGGCAGTAGTTGGA---CTGGGAGCT---------
GTCTTCCTTGGGTTCTTAGGAGCA---GCAGGAAGCACTATG---GGCGCGGCGTCA---
ATAACGCTGACGGTACAG---GCCAGACAA---TTATTG---TCTGGCATAGTGCAACAGCAAAGC---
AATTTG---CTGAGG---GCTATAGAGGCACAGCAACATCTGTTGCAACTCACAGTCTGGGGCATT
>KY953202

AGTTATATAAATGCTACAAAGGATAATACA---

ACAGGGGATAACATCACGCTCCCATGTAGGATAAAACAGATTGTAAGAATGTGGCAGAGAGTAGGACAAGCAA
TGTATGCCCCTCCCATCGCAGGAAACATTACATGTATATCAAAC---
ATTACAGGGATAATATTAACAAGAGATGGTGGAAATAATAATAAATCA-------------------------
----

AATAATGAAACCTTCAGACCTGGAGGAGGAGATATGAGAGACAATTGGAGAAGTGAGTTATATAAGTATAAAA
TAGTTAAAATTAAACCACTA---GGAATAGCACCCACCAGGGCAAGGAGAAGAGTGGTG--------------
-------------------------CAGAGAGAAAAAAGGGCA---GTTGGA---CTGGGAGCT---------
TTCTTCTTTGGGTTCTTAGGAGCA---GCAGGAAGCACTATG---GGCGCGGCGTCA---
ATAACGCTGACGGTACAG---GTCAGACAA---TTATTG---TCTGGCATAGTGCAACAGCAAAGC---
AATTTG---CTGAGG---GCTATAGAGGCGCAACAACATATGTTGCAACTCACAGTCTGGGGCATT
>FR846409
AGTTAT---

GAAAATGATACTATCACACTTCCATGTAGGATAAAACAAATTGTGAGAATGTGGCAGAGAGTGGGACAAGCAA
TGTATGCCCCTCCCATAGCAGGAAACATTACATGTGTGTCAAAC---
ATTACAGGATTACTATTAGAAAGAGATGGTGGGAATCTTAATAATAGTATAAAT------ACAACTGAG---
CCT--

AAAAATGAAATTTTCAGACCTGTAGGAGGAGACATGAAGAACAATTGGAGAAGTGAATTATATAAATATAAAG
TAGTAAGAATTAAACCACTA---GGAGTAGCACCTACCAGGGCAAGGAGAAGAGTGGTG--------------
-------------------------GGAAGAGAAAAAAGAGCA------GGA---ATAGGAGCT---------
GTCATCTTTGGGTTCTTAGGAGCA---GCAGGAAGCACTATG---GGCGCGGCGTCA---
ATAACGCTGACGGTACAG---GTCAGACAA---TTATTG---TCTGGCATAGTGCAACAGCAAAGC---
AATTTG---CTGAGA---GCAATAGAGGCGCAGCAGCATCTGTTGCAACTCACAGTCTGGGGCATT
>HQ236615
AATTATACTGAGAGTAGA---

AATGATACTATCATCTCAATTCCATGTAGGATAAAACAAATTGTGAGAATGTGGCAGAGAGTGGGACAAGCAA
TGTATGCCCCTCCCATCGCAGGAAACATTACATGTAGGTCAAAT---
ATTACAGGACTATTATTAGTAAGAGATGGTGGCATAAATAATGAG----------------------------
----

ACAACTGAGATTTTCAGACCTGCAGGAGGAGATATGAAGGACAATTGGAGAAGTGAATTATATAAGTATAAAG
TAGTAAAAATTAAACCACTA---GGAGTAGCACCCACCAGGGCAGAG------ATGGTG--------------
-------------------------GGGAGAGAA---AGAGCA---GTTGGA---CTGGGAGCT---------
GCCATCCTTGGGTTCTTAGGAACA---GCAGGAAGCACTATG---GGCGCAGCGTCA---
ATAGCGCTGACGGTACAG---GTCAGACAA---TTATTG---TCTGGCATAGTGCAACAGCAAGGC---
AATTTG---CTGAGG---GCTATAGAGGCGCAGCAGCATCTGTTGCAACTCACAGTCTGGGGCATT
>MH746234
ACTTACACAGGGAGGGGGAATGACACACAGGAG--

AATAACAGCACTATCATACTTCCATGCAGGATAAAACAAATTATAAACATGTGGCAAGGAGCAGGACAAGCAA
TGTATGCTCCTCCCATCAGTGGAAAAATTAATTGTGTATCAAAT---
ATTACAGGAATACTATTGACAAGAGATGGTGGTAATAATGCTACT----------------------------
----

AATGAGACCTTCAGACCTGGAGGAGGAAATATAAAGGACAATTGGAGAAGTGAATTATATAAATATAAAGTAG
TACAAATTGAACCACTG---GGAATAGCACCCACCAAGGCAAAGAGAAGAGTGGTG-----------------
----------------------GAGAGAGAAAAAAGAGCA---GTGGGA---ATAGGAGCT---------
ATGATCTTTGGGTTCTTAGGAGCA---GCAGGAAGCACTATG---GGCGCGGCGTCA---
ATAACGCTGACGGTACAA---GCCAGACAA---TTATTG---TCTGGTATAGTGCAACAGCAAAGC---
AATTTG---CTGAGG---GCTATAGAGGCGCAGCAGCATCTGTTGCAACTCACAGTCTGGGGCATT
>KU600817
ACTTACACAGGAAATGGAACATGGGAGAATAATAGCACT----------------------------------
----
ATTAATGGCACTATCATACTTCCATGCAAGATAAAGCAAATCATAAACATGTGGCAGGGAGCAGGACAAGCAA
TGTATGCTCCTCCCATCAGTGGAATAATTAATTGTGCGTCAAAT---

ATTACAGGACTACTATTGACAAGAGATGGTGGTTTTAGTAATGCAAGTGGT----------------------
----
GGTGGTAGTAATGCA---
AATGAAGAGATCTTCAGACCTGGAGGAGGAAATATAAAAGACAATTGGAGAAGTGAATTATATAAATATAAAG
TAGTACAAATTGAACCATTA---GGAATAGCACCCACCAGGGCAAAGAGAAGAGTGGTG--------------
-------------------------GAGAGAGAAAAAAGAGCA---GTGGGA---ATAGGAGCT---------
ATGATCTTTGGGTTCCTAGGAGCA---GCAGGAAGCACTATG---GGCGCGGCATCA---
ATAACGCTGACGGTACAG---GCCAGACAA---TTATTG---TCTGGTATAGTGCAACAGCAAAGC---
AATTTG---CTGAAA---GCTATAGAGGCGCAACAGCATTTGTTGCAACTCACAGTCTGGGGCATT
>JX112866
ACTTACATGGGAAATGAAACCAAGGGGAAG---

TATAATGGCACTATTATACTTCCATGCAGGATAAAGCAAATTATAAACATGTGGCAGGAAACAGGACAAGCAA
TGTATGCTCCTCCCGTCAGTGGAAGAATTAAGTGTAGATCAAAT---
ATTACAGGAATACTATTGACAAGAGATGGTGGTAATACAACTAAC----------------------------
----

GGGGAAGAGACCTTCAAACCTGGAGGAGGAGATATGAGGGACAATTGGAGAAATAAATTATATAAATATAAAG
TAGTACAAATTGAACCACTA---GGAATAGCACCCACCAGGGCAAAGAGAAGAATGGTG--------------
-------------------------GAGAGAGAAAAAAGAGCA---GTGGGA---ATAGGAGCT---------
ATGATCTTTGGGTTCTTAGGAGCA---GCAGGAAGCACTATG---GGCGCGGCGTCA---
ATAACGCTGACGGTACAG---GCCAGACAA---TTATTG---TCTGGTATAGTGCAACAGCAAAGC---
AATTTG---CTGAGG---GCTATAGAGGCGCAGCAGCATCTGTTGCAACTCACAGTCTGGGGCATT
>GU564225
ACTTACAATGGAAATGAAACCATA---

GGTAATAAAACTATCATACTTCCATGCAGGATAAAGCAAATTATAAACATGTGGCAGGGAGTAGGACAAGCAA
TGTATGCTCCTCCCGTCAGTGGAAGAATTAATTGTGTATCAAAT---
ATTACAGGAATACTATTAACAAGAGATGGTGGTAGTAATAGTACT----------------------------
----

AATGAGACCTTCAGACCTGAAGGAGGAAATATAAAGGACAATTGGAGAAGTGAATTACATAAATATAAAGTAG
TACAAATTGAACCACTA---GGAATAGCACCCACCAGGGCAAAAAGAAGAGTGGTG-----------------
----------------------GAGAGAGAAAAAAGAGCA---GTGGGA---ATAGGAGCT---------
ATGATCTTTGGGTTCTTAGGAGCA---GCAGGAAGCACTATG---GGCGCGGCGTCA---
ATAACGCTGACGGTACAG---GCCAGACAA---TTATTG---TCTGGTATAGTGCAACAGCAAAGC---
AATTTG---CTGAGG---GCTATAGAGGCGCAGCAGCATCTGTTGCAACTCACAGTCTGGGGCATT
>GQ845124
ACTTACACAGAAAATGGAACCATAGAG--

GGGAATAAAACTATCATACTTCCATGTAGGATAAAGCAAATTATAAACATGTGGCAGGGAGTAGGACAAGCAA
TGTATGCTCCTCCCATCAGGGGAAGAATTAATTGTATATCAAAT---
ATTACAGGAATACTATTAACAAGAGATGGTGGTAATAGTAATGCTACCGAT----------------------
----

AGTAATACTAACGAAATCTTCAGACCTGGAGGAGGAAATATAAAGGACAATTGGAGAAGTGAATTATATAAAT
ATAAAGTAGTACAAATTGAACCACTA---GGAGTAGCACCCACCAGGGCAAGGAGAAGAGTGGTG--------
-------------------------------CAGAAAGAAAAAAGAGCA---GTGGGA---ATAGGAGCT---
------ATGATCTTTGGGTTCTTAGGAGCA---GCAGGAAGCACTATG---GGCGCGGCGTCA---
ATAACGCTGACGGTACAG---GCCAGACAA---TTATTG---TCTGGTATAGTGCAACAGCAAAGC---
AATTTG---CTGAGG---GCTATAGAGGCGCAGCAGCATCTGTTGCAACTCACAGTCTGGGGCATT
>GQ845126
ACTCACAAGGAAAATGAAACCATAGGGAGG---

AGAAATGACACTATCATACTTCCATGCCGGATAAAGCAAATTATAAACATGTGGCAGGAAGTAGGACAAGCAA
TGTATGCTCCTCCCATCAGTGGAATAATTAATTGTGTATCAAAT---
ATTACAGGAATACTATTAACAAGAGATGGTGGTAATAATGATACT----------------------------
----

AACGAGACCTTCAGACCTGGAGGAGGAAATATAAAGGACAATTGGAGAAGTGAATTATATAAATATAAAGTAG

TACAAATTGAACCACTA---GGAATAGCACCCACCAGGGCAAAGAGAAGAGTGGTG-----------------
----------------------GAGAGAGAAAAAAGAGCA---GTGGGA---ATAGGAGCT---------
ATGATCTTTGGGTTCTTAGGAGCA---GCAGGAAGCACTATG---GGCGCAGCGTCA---
ATAACGCTGACGGTACAG---GCCAGACAA---TTATTG---TCTGGTATAGTGCAACAGCAAAGC---
AATTTG---CTGAGG---GCTATAGAGGCGCAGCAGCATCTGTTGCAACTCACAGTCTGGGGCATT
>GU564229
ACTCAAACCACAGAG--

GGGAAAGACAATATCACACTTCCATGCAGAATAAAGCAAGTTATAAAAATGTGGCAGAGAGTAGGACAAGCAA
TGTATGCTCCTCCCATCAGTGGAATAATTAATTGTGTATCAAAT---
ATTACAGGTATACTATTAACAAGAGATGGTGGTGATAATGATAGT----------------------------
----

ACTAACGAGACCTTCAGACCTGGAGGAGGAGATATGAGGGACAATTGGAGAAGTGAATTATATAAATATAAAG
TAGTACAAATTGAACCACTA---GGAATAGCACCCACCAAGGCAAAGAGAAGAGTGGTG--------------
-------------------------GAGAGAGAAAAAAGAGCA---GTGGGA---ATAGGAGCT---------
TTGATCTTTGGGTTCTTAGGAGCA---GCCGGAAGCACTATG---GGCGCGGCGTCA---
ATAACGCTGACGGTACAG---GCCAGACAA---TTGTTG---TCCGGTATAGTGCAACAGCAAAGC---
AATTTG---CTGAGG---GCTATAGAGGCGCAGCAGCATATGTTGCAACTCACAGTCTGGGGCATT
>EF036531
ACTTACAATGGAACCCTGAACGAGGAG--

TACAATGGCACTATCATACTTCCATGCAGAATAAAGCAAATTATAAACATGTGGCAGGAAGTAGGACAAGCAA
TGTATGCTCCTCCCATCAGTGGAAGAATTAATTGTGTATCAAAT---
ATTACAGGGATACTATTGACAAGAGATGGTGGTACGAATGAGACT----------------------------
----

AACGAGACCTTCAGACCTGGAGGAGGAAATATAAAGGACAATTGGAGAAATGAATTATATAAATATAAAGTAG
TACAAATTGAACCACTA---GGAATAGCACCCACCAGGGCAAAGAGAAGAGTGGTG-----------------
----------------------GAAAGAGAAAAAAGAGCA---GTGGGA---ATAGGAGCT---------
ATGATCTTTGGGTTCTTAGGAGCA---GCAGGAAGCACTATG---GGCGCGGCGTCA---
ATAACGCTGACGGTACAG---GCCAGACAA---TTATTG---TCTGGTATAGTGCAACAGCAAAAC---
AATTTG---CTGAGG---GCTATAGAGGCGCAGCAGCCTCTGTTTCTTCTCACAGTCTGGGGCATT
>KF835499
AATTACACAGGAAATGGAACA--

-
ACGAGTGGCACTATCATACTTCCATGTAGGATAAAGCAAATTATAAACATGTGGCAGGGAGTAGGACAAGCAA
TGTATGCTCCTCCCATCAGTGGAAGCATTAATTGTGTATCAGAT---
ATCACAGGAATACTATTGACAAGAGATGGTGGTAATAATACAGGTAATAATAAT-------------------
----

AGTGCTAATACAAATGAGATCTTCAGACCTGGAGGAGGAAATATGAAAGACAATTGGAGAAGTGAATTATATA
AATATAAAGTAGTAGAAATTGACCCACTA---GGAGTAGCACCCACCAGGGCAAAAAGAAGAGTGGTG-----
----------------------------------GAGAGAGAAAAAAGAGCA---GTGGGA---
ATAGGAGCT---------ATGATCTTTGGGTTCTTAGGAGCA---GCCGGGAGCACTATG---
GGCGCAGCGTCA---ATAACGCTGACGGTACAG---GCCAGACAA---TTATTG---
TCTGGTATAGTGCAACAGCAAAGC---AATTTG---CTGAGG---
GCTATAGAGGCGCAACAACATATGTTGCAACTCACAGTCTGGGGCATT
>KF835523
ACTCACACGGAAAATGACACC--

ACAAAGGAAAGGATCATACTTCCATGCAGGATAAAGCAAATTATAAACATGTGGCAGAGAGTAGGACAAGCAA
TATACAATCCTCCCATCAGTGGAGCAATTAATTGTCTATCAAAT---
ATTACAGGAATAATATTGACAAGAGATGGTGGTGCTAGTACTAATAATGATACT-------------------
----

AGTACTAACAATGAAACCTTCAGACCTGGAGGAGGAAATATAAAGGACAATTGGAGAAGTGAATTATATAAAT
ATAAAGTAGTACAAATTGAGCCACTA---GGGGTAGCACCCACCAGGGCAAAGAGAAGAGTGGTG--------
-------------------------------GAGAGAGAGAAAAGAGCA---GTGGGA---ATAGGAGCT---

```
------ATGATCTTTGGGTTCTTGGGAGCA---GCAGGAAGCACTATG---GGCGCGGCGTCA---
ATAACGCTGACGGTACAG---GCCAGACAA---TTATTG---TCTGGTATAGTGCAACAGCAAAGC---
AATTTG---CTGAGG---GCTATAGAGGCGCAGCAGCACATGCTGCAACTCACAGTCTGGGGCATT
>GU475020
ACTTATAAAGGAAATGAAACCATA-------------------------------------------------
--------------------------------------------------------------------
GGGAATGGAACTATCATAATTCCATGCAGGATAAAGCAAATTATAAACATGTGGCAGGGAGTAGGACAAGCAA
TGTATGCTCCTCCCATCAGTGGAATAATTAATTGTATATCAAAT---
ATTACAGGAATACTATTAACAAGAGATGGTGGTAGTACTAATAGC----------------------------
-------------------------------------------------------------------------
-------------
ACTAATGAGACCTTCAGACCTGGAGGAGGAAATATAAAGGACAATTGGAGAAGTGAATTATATAAATATAAAG
TAGTACAAATTGAACCACTA---GGAGTAGCACCCACCAGGGCAAAGAGAAGAGTGGTG--------------
-------------------------GAGAGAGAAAAAAGAGCA---GTGGGA---ATAGGAGCT---------
ATGATCTTTGGGTTCTTAGGAGCA---GCAGGAAGCACTATG---GGCGCAGCGTCA---
ATAACGCTGACGGTACAG---GCCAGACAA---TTATTG---TCTGGTATAGTGCAACAGCAAAGC---
AATTTG---CTGAGG---GCTATAGAGGCGCAGCAGCATATGTTGCAACTCACAGTCTGGGGCATT
>HQ699988
ACTTATAATGGAAATGGAACTGTGACCAAG-------------------------------------------
--------------------------------------------------------------------
TATAATGACACTATTATACTTCCATGCAGGATAAAGCAAATTATAAACATGTGGCAGGAAACAGGACAAGCAA
TGTATGCTCCTCCCGTCAGTGGAGTAATAAATTGTAGATCAAAT---
ATTACAGGAATACTATTGACAAGAGATGGGGGTAATAATGGGGGTAATACA----------------------
-------------------------------------------------------------------------
-------
ACTAACGGGGAAGAGACCTTCAGACCTGGAGGAGGAGATATGAGGGACAATTGGAGAAATGAATTATATAAAT
ATAAAGTAGTACAAATTGAACCACTA---GGAATAGCACCCACCAAGGCAAAGAGAAGAGTGGTG--------
-------------------------------CAGAGAGAAAAAAGAGCA---GTGGGA---ATAGGAGCT---
------ATGATCTTTGGGTTCTTAGGAGCA---GCAGGAAGCACTATG---GGCGCGGCGTCA---
ATAACGCTGACGGTACAG---GCCAGACAA---TTATTG---TCTGGTATAGTGCAACAGCAAAGC---
AATTTG---CTGAGA---GCTATTGAGGCGCAGCAGCATCTGTTGCAACTCACAGTCTGGGGCATT
>JX112811
ACTTACTCAGGAAATGAAACCACAGGAAATGAAACCACAGGG-------------------------------
--------------------------------------------------------------------
AAGAATACAAGTATCATACTTCCATGCAAGATAAAGCAAATTATAAACATGTGGCAGGGAGTAGGACAAGCAA
TGTATGCTCCTCCCATCAGTGGAAAAGTTAATTGTATATCAAAT---
ATTACAGGAATACTATTAACAAGAGATGGTGGTGGTAATAATAGT----------------------------
-------------------------------------------------------------------------
-------------
GCTAATGAGACTTTCAGACCGGGAGGAGGAAATATAAAAGACAATTGGAGAAGTGAATTATATAAATATAAAG
TAGTACAAATTGAACCACTA---GGAATAGCACCCACCAGGGCAAAGAGAAGAGTGGTG--------------
-------------------------GAGAGAGAAAAAAGAGCA---GTGGGA---ATAGGAGCT---------
ATGATCTTTGGGTTCTTAGGAGCA---GCAGGAAGCACTATG---GGCGCGGCGTCA---
ATAACGCTGACGGTACAG---GCCAGACAA---TTATTG---TCTGGTATAGTGCAACAGCAAAGC---
AATTTG---CTGATG---GCTATAGAGGCGCAGCAGCATCTGCTGCAACTCACAGTCTGGGGCATT
>JX112817
ACTTACATCAGTAAAATTAACACTACAGGGGGG----------------------------------------
--------------------------------------------------------------------
AGTAATGGCACTATCATACTTCCATGCAAGATAAAGCAAATTATAAACATGTGGCAGGGAGTAGGACAAGCAA
TGTATGCTCCTCCCATCAGTGGAATAATTAATTGTGTAACAAAT---
ATTACAGGAATACTATTAACAAGAGATGGTGGTAATGATAATAGC----------------------------
-------------------------------------------------------------------------
-------------
ATTAATGAGACCTTTAGACCTGGAGGAGGAAATATAAAGGACAATTGGAGAAATGAATTATATAAATATAAAG
TAGTACAAATTGAACCACTA---GGACTAGCACCCACCAGGGCAAAGAGAAGAGTGGTG--------------
-------------------------GAGAGAGAAAAAAGAGCA---GTGGGA---ATAGGAGCT---------
ATGATCTTTGGGTTCTTAGGAGCA---GCAGGAAGCACTATG---GGCGCGGCGTCA---
ATAACGCTGACGGTACAG---GCCAGACAA---TTATTG---TCTGGTATAGTGCAACAGCAAAGC---
AATTTG---CTGAGG---GCTATAGAGGCGCAGCAGCATCTGTTGCAACTCACAGTCTGGGGCATT
>JX112824
```

ACTTATTTAAATAAAACCACAAGGGAG--

AGTAATGGCACTATCATACTTCCATGCAGGATAAAGCAAATTATAAACATGTGGCAGGGAGTAGGACAAGCAA
TGTATGCTCCTCCCATCAGTGGAAGCATTAATTGTGTATCAAAT---
ATTACAGGAATACTATTAACAAGAGATGGTGGTAATAATGGTACT----------------------------
----

AGTGATAAAGAGACCTTCAGACCTGGAGGAGGAAATATAAAGGACAATTGGAGAAGTGAATTATATAAATATA
AAGTAGTAGAAATTGAACCACTA---GGAATAGCACCCACCAGGGCAAAGAGAAGAGTGGTG-----------
----------------------------GAGAGAGAAAAAAGAGCA---GTGGGA---ATAGGAGCT------
---ATGATCTTTGGGTTCTTAGGAGCA---GCAGGAAGCACTATG---GGCGCGGCGTCA---
ATAACGCTGACGGTACAG---GCCAGACAA---TTATTG---TCTGGTATAGTGCAACAGCAAAGC---
AATTTG---CTGAGG---GCTATAGAGGCGCAGCAGCATCTGTTGCAACTCACAGTCTGGGGCATT
>JX112825
ACTTACAATGAAACTGGAACCACAGGGGAGAATAATGGC----------------------------------
----
ACTAATGGCAATATCACACTTCCATGCAGGATAAAGCAAATTATAAACATGTGGCAGGAAGTAGGACAAGCAA
TGTATGCTCCTCCCATCAATGGAATAATTAATTGTGTATCAAAT---
ATTACAGGAATACTATTAACAAGAGATGGTAGTAATGAAACTAGC----------------------------
----

CCTAACGAGACCTTCAGACCTGGAGGAGGAAATATAAAGGACAATTGGAGAAGTGAATTATATAAATATAAAG
TAATACAAATTGAACCACTA---GGAATAGCACCCACCAAGGCAAAGAGAAGAGTGGTG--------------
-------------------------GAGAGAGAAAAAAGAGCA---GTGGGA---ATAGGAGCT---------
TTGATCTTTGGGTTCTTAGGAGCA---GCAGGAAGCACTATG---GGCGCGGCGTCA---
ATAACGCTGACGGTACAG---GCCAGACAA---TTATTG---TCTGGTATAGTGCAACAGCAAAGC---
AATTTG---CTGAGG---GCTATAGAGGCGCAGCAGCATATGTTGCAACTCACAGTCTGGGGCATT
>JX112831
AATTACACAGGAAATAAAACAGGGGGGAATAAT--

AACACTGAAACTATCACACTTCCATGCAACATAAAGCAAATTATAAACATGTGGCAGGGAGTAGGACAAGCAA
TGTATGCTCCTCCCGTCAGTGGAAGAATTAATTGTGTAAGTAAT---
ATTACAGGAATACTATTAACAAGAGATGGTGGTAAAACTAGTGCT----------------------------
----

GACGAGACCTTCAGACCTGGAGGAGGAAATATAAAGGACAATTGGAGAAGTGAATTATATAAATATAAAGTAG
TACAAATTGAACCACTA---GGAATAGCACCCACCAGGGCAAAGAGAAGAGTGGTG-----------------
----------------------GAGAGAGAAAAAAGAGCA---GTGGGA---ATAGGAGCT---------
ATGATCTTTGGGTTCTTAGGAGCA---GCCGGAAGCACTATG---GGCGCGGCGTCA---
ATAACGCTGACGGCACAG---GCTAGACAA---TTATTG---TCTGGTATAGTGCAACAGCAAAGC---
AATTTG---CTGAGG---GCTATAGAGGCGCAACAGCATCTGTTGCAACTCACAGTCTGGGGCATT
>JX112836
ACTTACACAGAAAATGGAACCATAGGGGAA---

AGAAATGACACTATCACACTTCCATGCAAAATAAAGCAAATTATAAACATGTGGCAGGAAATAGGACAAGCAA
TGTATGCTCCCCCCGTCAGTGGAATAATTAATTGTGTATCAAAT---
ATTACAGGAATACTATTAACAAGAGATGGTGGTAATAATGGTACT----------------------------
----

AGTGCTAACGAGACCTTCAGACCTGGAGGAGGAAATATAAAGGACAATTGGAGAAGTGAATTATATAAATATA
AAGTAGTACAAATTGAACCACTA---GGAATAGCACCCACCAGGGCAAAGAGAAGAGTGGTG-----------
----------------------------GACAGAGAAAAAAGAGCA---GTGGGA---ATAGGAGCT------
---ATGATCTTTGGGTTCTTAGGAGCA---GCAGGAAGCACTATG---GGCGCGGCGTCA---
ATAACGCTGACGGTACAG---GCCAGACAA---TTATTG---TCTGGTATAGTGCAACAGCAAAGC---
AATTTG---CTGAGG---GCTATAGAGGCGCAGCAGCATCTGTTGCAACTCACAGTCTGGGGCATT
>JX112837
TATTATGAAAATGGAACCATAGGGGGG--

AGTAATGGCACTATCATACTTCCATGTAGGATAAAGCAAATTATAAACATGTGGCAGGGAGTAGGACAAGCAA
TGTATGCTCCTCCCATCAGTGGAAGCATTAATTGTGTATCAAAT---

ATTACAGGAATACTATTAACAAGAGATGGTGGTGGTAATAATAGA----------------------------
----

ACTAACGAGACCTTCAGACCTGGAGGAGGAAATATAAAGGACAATTGGAGAAGTGAATTATATAAATATAAAG
TAGTAGAAATTGAGCCACTA---GGAGTAGCACCCACCAAGGCAAAGAGAAGAGTGGTG--------------
-------------------------GAGAGAGAAAAAAGAGCA---GTGGGA---ATAGGAGCT---------
ATGATCTTTGGGTTCTTAGGAGCA---GCAGGAAGCACTATG---GGCGCGGCGTCA---
ATAACGCTGACGGTACAG---GCCAGACAA---TTATTG---TCTGGTATAGTGCAACAGCAAAGC---
AATTTG---CTGAGG---GCTATAGAGGCGCAGCAGCATCTGTTGCAACTCACAGTCTGGGGCATT
>JX112838
ATTTACAATGGAAATGAAACCAATATTCCT---

GACAATAAAACTATCATACTTCCATGCAAGATAAAGCAAATTGTAAACTTGTGGCAGGAAGTAGGACAAGCAA
TGTATGCTCCTCCCATCAGGGGAAAAATTAATTGTGTATCAAAC---
ATTACAGGAATACTATTAACAAGAGATGGTGGTAATATTAGTAAT----------------------------
----

ACTAGTACCGAGACCTTCAGACCTGCAGGAGGAGATATGAGGGACAATTGGAGAAATGAATTATATAAATATA
AAGTAGTACAAATTGAACCACTA---GGAATAGCACCCACCAGGGCAAAAAGAAGAGTGGTG-----------
----------------------------AAGAGAGAAAAAAGAGCA---GTGGGA---ATAGGAGCT------
---ATGATCTTTGGGTTCTTAGGAGCA---GCAGGAAGCACTATG---GGCGCGGCGTCA---
ATAACGCTGACGGTACAG---GCCAGACAA---TTATTG---TCTGGTATAGTGCAACAGCAAAGC---
AATTTG---CTGAGG---GCTATAGAGGCGCAGCAGCATATGTTGCAACTCACAGTCTGGGGCATT
>JX112839
CATTACGAAAATGGAACCATAAAGGGGGGTAAT--

AACACTAGCGAGATCACACTTCCATGCAGGATAAAGCAAATTATAAACATGTGGCAGGAAGTAGGACAAGCAA
TGTATGCTCCTCCCGTCAGTGGAAGAATTAATTGTGTATCAAAT---
ATCACAGGAATACTATTAACAAGAGATGGTGGTAATAATAATAGT----------------------------
----

ACTAATGAGACCTTCAGACCTGGAGGAGGAGATATAAAGGACAATTGGAGAAGTGAATTATATAAATATAAAG
TAGTACAAATTGAACCACTA---GGAATAGCACCCACCAGGGCAAAGAGAAGAGTGGTG--------------
-------------------------GAGAGAGAAAAAAGAGCA---GTGGGA---ATAGGAGTT---------
ATGATCTTTGGGTTCTTAGGAGCA---GCTGGAAGCACTATG---GGCGCGGCGTCA---
ATAACGCTGACGGTACAG---GCCAGACAA---TTATTG---TCTGGTATAGTGCAGCAGCAAAGC---
AATTTG---CTGAGA---GCTATTGAGGCGCAACAGCATCTGTTGCAACTCACAGTTTGGGGCATT
>GU475031
ACTTACAAAGGAAATGAAACCATAGGGGAG---

AGGAATGACACTATCATACTTCCATGCAGGATAAAGCAGATTGTAAACATGTGGCAGGAAGTAGGACAAGCAA
TGTATGCTCCTCCCGTCAGTGGAAGAATTAATTGTGTATCAAAT---
ATTACAGGAATACTATTAACAAGAGATGGTGGTAATAATAATACT----------------------------
----

AACGAGACCTTCAGACCTGGAGGAGGAAATATAAAGGACAATTGGAGAAGTGAATTATATAAATATAAAGTAG
TACAAATTGAACCACTA---GGAATAGCACCCACCAGGGCAAAGAGAAGAGTGGTG-----------------
----------------------GAGAGAGAAAAAAGAGCA---GTGGGA---ATAGGAGCT---------
ATGATCTTTGGGTTCTTAGGAGCA---GCAGGAAGCACTATG---GGCGCGGCGTCA---
ATAACGCTGACGGTACAG---GCCAGACAA---TTATTG---TCTGGTATAGTGCAACAGCAAAGC---
AATTTG---CTGAGA---GCTATAGAGGCGCAGCAGCACATGTTGCAACTCACAGTCTGGGGCATT
>GU475030
ACTTACCAAGGAAATGAAACAGGGGGGAAT---

ACTAGCAGCACTATCACACTTCCATGCAAGATAAAGCAAATTATAAACATGTGGCAGGGAGTAGGACAAGCAA
TGTATGCTCCTCCCATCAGTGGAATAATTAATTGTGTATCAAAT---
ATTACAGGAATACTATTAACAAGAGATGGTGGTAATAATAATGCT----------------------------
----

ACTAACGAGACCTTCAGACCTGGAGGAGGAAATATAAAGGACAATTGGAGAAATGAATTATATAAGTATAAAG

TAGTACAAATTGAACCACTA---GGAATAGCACCCACCAGGGCAAAGAGAAGAGTGGTG--------------
-------------------------GAGAGAGAAAAAAGAGCA---GTGGGA---ATAGGAGCT---------
ATGATCTTTGGGTTCTTAGGAGCA---GCTGGAAGCACTATG---GGCGCGGCGTCA---
ATAACGCTGACGGTACAG---GCCAGACAA---TTATTG---TCTGGTATAGTGCAACAGCAAAGC---
AATTTG---CTGAGG---GCTATAGAGGCGCAGCAGCATCTGTTGCAACTCACAGTCTGGGGCATT
>JX112840
ACTTACACAAGAAATGGAACCATAGGGGGG---

AGTAATGGCACTATCATACTGCCATGCAAGATAAAGCAAATTATAAACATGTGGCAGGGAGTAGGACAAGCAA
TGTATGCTCCTCCCATCAGTGGAAGAATTAATTGTGTATCAAAT---
ATCACAGGAATACTATTAACAAGAGATGGTGGTAATAGTAGTGCT----------------------------
----

AACGAGACCTTCAGACCTGGAGGAGGAAATATAAAGGACAATTGGAGAAGTGAATTATATAAATATAAAGTAG
TACAAATTGAACCACTA---GGAATAGCACCCACCAGGGCAAAGAGAAGAGTGGTG-----------------
----------------------GAGAGAGAAAAAAGAGCA---GTGGGA---ATAGGAGCT---------
ATGATCTTTGGGTTCTTAGGAGCA---GCAGGAAGCACTATG---GGCGCGGCGTCA---
ATAACGCTGACGGTACAG---GCCAGACAA---TTATTG---TCTGGTATAGTGCAACAGCAAAGC---
AATTTG---CTGAGG---GCTATAGAGGCGCAGCAGCATCTGTTGCAACTCACAGTCTGGGGCATT
>JX112845
AATTACACAAGAAATGAAACCATGAGTAAG---

GACAAGGACACTCTCATACTTCCATGCAGGATAAAGCAAATTATAAACATGTGGCAGGGAGTAGGACAAGCAA
TGTATGCTCCTCCCATCAGGGGAGTAATTAATTGTTTATCAAAT---
ATTACAGGAATACTATTAACAAGAGATGGTGGTAATAATAGGACT----------------------------
----

AACGAGACCTTCAGACCTGAAGGAGGAAATATAAAGGACAACTGGAGAAATGAATTATATAAATATAAAGTAG
TGCAAATTGAACCACTA---GGAATAGCACCTACCAGGGCAAAGAGAAGAGTGGTG-----------------
----------------------GAGAGAGAAAAAAGAGCA---GTGGGA---ATAGGAGCT---------
ATGATCTTTGGGTTCTTAGGAGCA---GCAGGAAGCACTATG---GGCGCGGCGTCA---
ATAACGCTGACGGTACAG---GCCAGACAA---TTATTG---TCTGGTATAGTGCAACAGCAAAGC---
AATTTG---CTGAGG---GCTATAGAGGCGCAGCAGCATATGTTGCAACTCACAGTCTGGGGCATT
>JX112851
ACTTATGGAAATGAAACCATAGGGGAG--

AGAAATGACAGTATCATACTTCCATGCAAGATAAAGCAAATTATAAACATGTGGCAGGGAGTAGGACAAGCAA
TGTATGCTCCTCCAATCAGTGGAAGAATTAATTGTGTATCAAAT---
ATTACAGGGATACTCTTAACAAGAGATGGTGGTAATAATAATAAT----------------------------
----

ACTAATGAGATCTTCAGACCTGGAGGAGGAAATATAAAGGACAATTGGAGAAGTGAATTATATAAATATAAAG
TAGTACAAATTGAACCACTA---GGAATAGCACCCACCAAGGCAAAGAGAAGAGTGGTG--------------
-------------------------GAGAGAGAAAAAAGAGCA---GTGGGA---ATAGGAGCT---------
ATGATCTTTGGGTTCTTAGGAGCA---GCTGGAAGCACTATG---GGCGCGGCGTCA---
ATAACGCTGACGGTACAG---GCCAGACAA---TTATTG---TCTGGTATAGTGCAACAGCAAAGC---
AATTTG---CTGAGG---GCTATAGAGGCGCAGCAGCATCTGTTGCAACTCACAGTCTGGGGCATT
>GU475023
ACTTACTCAGGAAATGAAAGCACAGGGGGG---

AATGAGAGCACTATCATACTTCCATGCAGGATAAAGCAAATTATAAACATGTGGCAGGGAGTGGGACAAGCAA
TGTATGCTCCTCCCATCAGGGGAAGAATTAATTGTGTAACAAAC---
ATTACAGGAATACTATTAACAAGAGATGGTGGTAATAATACTAAC----------------------------
----

GAGACCTTCAGACCTGGAGGAGGAAATATAAAGGACAATTGGAGAAGTGAATTATATAAATATAAAGTAGTAC
AAATTGAACCACTA---GGAGTAGCACCCACCAGGGCAAAGAGAAGAGTGGTG--------------------
-------------------CAGAGAGAAAAAAGAGCA---GTGGGA---ATAGGAGCT---------
ATGATCTTTGGGTTCTTAGGAGCA---GCAGGAAGCACTATG---GGCGCGGCGTCA---

ATAACGCTGACGGTACAG---GCCAGACAA---TTATTG---TCTGGTATAGTGCAACAGCAAAGC---
AATTTG---CTGAGG---GCTATAGAGGCGCAGCAGCATATGTTGCAACTCACAGTCTGGGGCATT
>GU475027
ACTTACGGAAATGGAACCACAGGGGAGAAG---

AATAGCAGCACTATCATACTTCCATGCAGGATAAAGCAAATTATAAACATGTGGCAGGGAGTAGGACAAGCCA
TGTATGCTCCTCCCATCAGAGGAAACATTAATTGTGTATCAAAT---
ATTACAGGAATACTATTAACAAGAGATGGTGGTGATAATAGT-------------------------------
----

ACTAACGAGATCTTCAGACCTGGAGGAGGAAATATGAAGGACAATTGGAGAAATGAATTATATAAATATAAAG
TAGTACAAATTGAACCACTA---GGAATAGCACCCACCAGGGCAAAGAGAAGAGTGGTG--------------
-------------------------GAGAGACAAAAAAGAGCA---GTGGGA---ATAGGAGCT---------
ATGATCTTTGGGTTCTTAGGAGCA---GCAGGAAGCACTATG---GGCGCGGCGTCA---
ATAACGCTGACGGTACAT---GCCAGACAA---TTATTG---TCTGGTATAGTGCAACAGCAAAGC---
AATTTG---CTGAGG---GCTATAGAGGCGCAGCAGCATCTGTTGCAACTCACAGTCTGGGGCATT
>MG655196
ACTTATACAGAAGGAAATGAAACCATAGAGGAA--

AGTAATGGCAATATCACACTTCCATGCAGGATAAAGCAAATTATAAACATGTGGCAGGGAGTAGGACAAGCAA
TGTATGCTCCTCCCGTCAGTGGAAAAATTAATTGTGTATCAAAT---
ATTACAGGAATACTATTAACAAGAGATGGTGGTGCTGGTACTGGG----------------------------
----

GATAACAATACTGAGACCTTCAGACCTGCAGGAGGAAATATGAAGGACAATTGGAGAAGTGAATTATATAAAT
ATAAAGTAGTGCAAATTGAACCACTA---GGACTAGCACCCACCAGGGCAAAGAGAAGAGTGGTG--------
-------------------------------GAGAGAGAAAAAAGAGCA---GTGGGA---ATAGGAGCT---
------ATGATCTTTGGGTTCTTAGGAGCA---GCCGGAAGCACTATG---GGCGCGGCGTCA---
ATAACGCTGACGGTACAG---GCCAGACAA---TTATTG---TCTGGTATAGTGCAACAGCAAAGC---
AATTTG---CTGAGG---GCTATAGAGGCGCAGCAGCATATGTTGCAACTCACAGTCTGGGGCATT
>MG655197
ACTTACAATGAAAGTGAAACCACAGGGGAA---

AGTAATGGCACTATCATACTTCCATGCAGGATAAAGCAAATTATAAACATGTGGCAGAGAGTAGGACAAGCAA
TGTATGCTCCTCCCATCAGTGGAAGAATTAATTGTATATCAAAT---
ATTACAGGAATACTATTAACAAGAGATGGTGGTAATAATAATAAT----------------------------
----

GATACTGAGATCTTCAGACCTGGAGGAGGAAATATGAAGGACAATTGGAGAAGTGAATTATATAAATATAAAG
TAGTAGAAATTGAACCACTA---GGAATAGCACCCACCAGGGCAAAGAGAAGAGTGGTG--------------
-------------------------GAGAGAGAAAAAAGAGCA---GTGGGA---ATAGGAGCT---------
CTGATCTTTGGGTTCTTAGGAGCA---GCAGGAAGCACTATG---GGCGCAGCGTCA---
ATAACGCTGACGGTACAG---GCCAGACAA---TTATTG---TCTGGTATAGTGCAACAGCAAAGC---
AATTTG---CTGAGA---GCTATAGAGGCGCAGCAGCATATGTTGCAACTCACAGTCTGGGGCATT
>MG655200
ACTTACGGAAATGAAACCATAGGAAATAAAACCGAAGGGGAG-------------------------------
----
AGGAATGGTACTATCACACTTCCATGCAAGATAAAGCAAATTGTAAGAATGTGGCAGGAAGTAGGACAAGCAA
TGTATGCTCCTCCCATTAGTGGAAGCATTAATTGTGTATCAAAT---
ATTACAGGAATACTATTAACAAGAGATGGTGGTAATGGTACTAAC----------------------------
----

GAGACCTTCGAGACCTTCAGACCTGCAGGAGGAGATATAAAGGACAATTGGAGAAGTGAATTATATAAATATA
AAGTAGTACAAATTGAACCACTA---GGAATAGCACCCACCAGGGCAAAGAGAAGAGTGGTG-----------
----------------------------GAGAGAGAAAAAAGAGCA---GTGGGA---ATAGGAGCT------
---ATGATCTTTGGGTTCTTAGGAGCA---GCAGGAAGCACTATG---GGCGCGGCGTCA---
ATAACGCTGACGGTACAG---GCCAGACAA---TTATTG---TCTGGTATAGTGCAACAGCAAAGT---
AATTTG---CTGAGG---GCTATAGAGGCGCAGCAGCATCTGTTGCAACTCACAGTCTGGGGCATT
>JX960603

ACTTACCAAAATGGAACCATAAATGGAGAGAAG--

TATAAGGAAAATATTATACTTCCATGCAGGATCAAGCAAATTATAAACATGTGGCAGAGAGCAGGACAAGCAA
TGTATGCTCCTCCCATCAAGGGAAGAATTAATTGTGTATCAAAT---
ATTACAGGAATACTATTAACAAGAGATGGTGGTGATAATAGCACA----------------------------
----

GCTAACGAGACCTTCAGACCGGGAGGAGGAAATATAAAAGACAATTGGAGAAATGAATTATATAAATATAAGG
TAGTACAAATTGAACCACTA---GGAATAGCACCCACCAGGGCAAAGAGAAGAGTGGTG--------------
-------------------------CAGAGAGAAAAAAGAGCA---GTGGGA---ATAGGAGCT---------
ATGATCTTTGGGTTCTTAGGAGCA---GCAGGAAGCACTATG---GGCGCAGCATCA---
ATAACGCTGACGGTACAG---GCCAGACAG---TTATTG---TCTGGTATAGTGCAACAGCAAAGC---
AATTTG---CTGAGG---GCTATAGAGGCGCAGCAGCATCTGTTGCAACTCACAGTCTGGGGCATT
>KP109508
GATCAT---

GGTAATGGCACTATCACACTTCCATGCAGGATAAAGCAAATTATAAACATGTGGCAAAGAGCAGGACAAGCAA
TGTATGCTCCTCCCATCAGTGGAGTAATTAATTGTGTATCAAAT---
ATTACAGGAATACTATTGACAAGAGATGGTGGTAATACTAGTAGT----------------------------
----

AGTAACGAGACTTTCAGACCTGGAGGAGGAAATCTAAAGGACAATTGGAAAAATGAATTATATAAATATAAAG
TAGTACAAATAGAACCACTA---GGAGTAGCACCCACCAAGGCAAAGCGAAGAGTGGTG--------------
-------------------------GATAGAGAAAAAAGAGCA---GTGGGA---ATAGGAGCT---------
ATGATCTTTGGGTTCTTGGGAGCA---GCAGGAAGCACTATG---GGCGCGGCGTCA---
ATAACGCTGACGGTACAG---GCCAGGCAA---TTATTG---TCTGGTATAGTGCAACAGCAAAGC---
AATTTG---CTGAGA---GCTATAGAGGCGCAGCAGCATATGTTGCAACTCACAGTCTGGGGCATT
>MH327760
AATCACACCGAGGGTAAT---

GGCACTGGCAATATCATACTTCCATGCAAGATAAAGCAAATTATAAACATGTGGCAGGGAGTAGGACAAGCAA
TGTATGCTCCTCCCGTCAGGGGAGTAATTAATTGTGTATCAAAC---
ATTACAGGAATAATATTGACAAGGGATGGTGGGACAAATAGCACG----------------------------
----

AACGATACCGAGATCTTCAGACCTGAAGGAGGAAATATAAAAGACAATTGGAGAAGTGAATTATATAAATATA
AAGTAGTACAAATTGAACCACTA---GGAGTGGCACCCACCAGGGCAAGGAGAAGAGTGGTG-----------
----------------------------GAAAGAGAAAAAAGGGCA---GTGGGA---ATAGGAGCT------
---ATGATCTTTGGGTTCTTAGGAGCA---GCAGGAAGCACTATG---GGCGCGGCGTCA---
ATAACGCTGACGGTACAG---GCCAGACAA---TTATTG---TCTGGTATAGTGCAACAGCAAAGC---
AATTTG---CTGAGG---GCTATAGAGGCGCAACAGCATATGTTGCAACTCACAGTCTGGGGCATT
>KY213720
ACTTATAATAACACTGAGTTAACTGTAGGTAATAATACT----------------------------------
----
GATAATGATACTATCACGCTTCCATGCAGGATAAAGCAAATTATAAACATGTGGCAGGGAGCAGGACAAGCAA
TGTATGCTCCTCCCATCAGTGGAAAAATTAATTGTATATCAAAT---
ATTACAGGAATATTATTGACAAGAGATGGTGGTAATAATGCAACA----------------------------
----

AATGAAGAGATCTTCAGACCTGAAGGAGGAAATATAAAGGACAATTGGAGAAGTGAATTATATAAATATAAAG
TAGTACAAATTGAACCACTA---GGAATAGCACCCACCAGGGCAAAGAGAAGAGTGGTG--------------
-------------------------GAGAGAGAAAAAAGAGCA---GTGGGA---ATAGGAGCT---------
ATGATCTTTGGGTTCTTAGGAGCA---GCCGGAAGCACTATG---GGCGCAGCGTCA---
ATAACGCTGACGGTACAG---GCCAGACAA---TTATTG---TCTGGTATAGTGCAACAGCAAAAC---
AATTTG---CTGAGG---GCTATAGAGGCGCAGCAGCATATGTTGCAACTCACAGTCTGGGGCATT
>KY213739
AATTATACAGAAAATGAAGCCACGAAGGGG---

-
GATAATACAACTATCAGACTTCCATGTAAGATAAAGCAAATTATAAACATGTGGCAGGGAGTAGGACAAGCAA

TGTATGCTCCTCCCATCAGTGGAAGCATTAGTTGTATATCAAAT---
ATTACAGGAATACTATTGACAAGAGATGGTGGTGATAATAATGTT----------------------------
----

AATGAGACCTTCAGACCTGGAGGAGGAAATATAAAGGACAATTGGAGAAGTGAATTATATAAATATAAAGTAG
TACAAATTGAACCACTA---GGAATAGCACCCACCAAGGCAAAGAGAAGAGTGGTG-----------------
----------------------GAGAGAGAGAAAAGAGCA---GTGGGA---ATAGGAGCT---------
ATGATCTTTGGGTTCTTAGGAGCA---GCCGGAAGCACTATG---GGCGCGGCGTCA---
ATAACGCTGACGGTACAA---GCCAGACAA---TTATTG---TCTGGGATAGTGCAACAGCAAAGC---
AATTTG---CTGAGG---GCTATAGAGGCGCAGCAGCATCTGTTGCAACTCACAGTCTGGGGCATC
>KY213717
AATTACACAGCAAATGAAACTGTG---

-
TGGAATGACACTATCATACTTCCATGCAGGATAAAACAAATTGTAAACATGTGGCAGGGAGTAGGACAAGCAA
TGTATGCTCCTCCCGTCAGGGGAATAATTAGTTGTGTATCAAAG---
ATTACAGGATTACTATTGACAAGAGATGGTGGTAATAATAATACT----------------------------
----

AACGAGACCTTCAGACCTGGGGGAGGAAATATGAAGGACAATTGGAGAAGTGAATTATATAAATATAAAGTAG
TACAAATTGAACCACTA---GGAGTAGCACCCACCAGGGCAAGGAGAAGAGTGGTG-----------------
----------------------CAGAGAGAAAAGAGAGCA---GTGGGA---ATAGGAGCT---------
ATGATCTTTGGGTTCTTAGGAGCA---GCAGGAAGCACTATG---GGCGCAGCGTCA---
ATAACGCTGACGGTACAG---GCCAGACAA---TTGTTG---TCTGGTATAGTGCAACAGCAAAGC---
AATTTG---CTGAGA---GCTATTGAGGCGCAGCAGCATATGTTGCAACTCACAGTCTGGGGCATT
>AY945721
ACTTACAGAAAAAATGAAACCAGGGAGAGG---

AATGATAGCACTATCATAATTCCATGCAAGATAAAACAAATTATAAACATGTGGCAGGGAACAGGACAAGCAA
TGTATGCTCCTCCCATCAGTGGAAACATTAATTGTGTATCAAAT---
ATTACAGGAATACTATTGACAAGAGATGGTGGTAATAATAGTGCG----------------------------
----

GATAACGAGACTTTCAGACCTGGAGGAGGAAATATAAAGGACAATTGGAGAAGTGAATTATATAAATATAAAG
TAGTAGAAATTGAGCCACTA---GGAATAGCACCCACCAGGGCAAAGAGAAGAGTGGTG--------------
-------------------------GAGAGAGAAAAAAGAGCA---GTGGGA---ATAGGAGCT---------
ATGATCTTTGGGTTCTTAGGAGCA---GCAGGAAGCACTATG---GGCGTGGCGTCA---
ATAACGCTGACGGTACAG---GCCAGACAA---TTATTG---TCTGGTATAGTGCAACAGCAAAGC---
AATTTG---CTGAGG---GCTATAGAGGCGCAACAGCATATGTTGCAACTCACAGTCTGGGGCATT
>AY945722
AATCACAAGGAG---

TGTAATGGCACTATCACACTTCCATGCAGGATAAAGCAAATTATAAACATGTGGCAGGGAGCAGGACAAGCAA
TGTATGCTCCTCCCATCAGTGGAAAAAATTAATTGTGTATCAAAT---
ATTACAGGAATACTATTGACAAGAGATGGTGGTACTAATAATAATACA-------------------------
----

AATAGCAGCGAGATATTCAGACCTGGAGGAGGAAATATAAAAGATAATTGGAGAAGTAAATTATATAAATATA
AAGTAGTACAAATTGAACCACTA---GGAATAGCACCCACCAGGGCAAAAAGAAGAGTGGTG-----------
----------------------------GAGAGAGAAAAAAGAGCA---GTGGGA---ATAGGAGCT------
---ATGATCTTTGGGTTCTTAGGAGCA---GCAGGAAGCACTATG---GGCGCGGCGTCA---
CTAACGCTGACGGTACAG---GCCAGACAA---TTATTG---TCTGGTATAGTGCAACAGCAAAGC---
AATTTG---CTGAGG---GCTATAGAGGCGCAGCAGCATCTGTTGCAACTCACAGTCTGGGGCATT
>KJ952852
GCTTACAGTGGAAATGAAACCATGAATGAAACCATGGATAGG-------------------------------
----
TATAATGACACGATCATACTTCCATGCAGGATAAAGCAAATTATAAACATGTGGCAGGGAGTAGGACAAGCAA
TGTATGCTCCTCCCATCAGTGGAAGCATTAATTGTGTATCAAAT---
ATTACAGGAATACTATTGACAAGAGATGGTGGTAATAATCTGAAT----------------------------
----

AACAGTAACGAGACCTTCAGACCTGGAGGAGGAAATATAAAGGATAATTGGAGAAGTGAATTATATAAATATA
AAGTAGTACAAATTGAACCACTA---GGAATAGCACCCACCAGGGCAAAGAGAAGAGTGGTG-----------
----------------------------GAGAGAGAAAAAAGAGCA---GTGGGA---ATAGGAGCT------
---ATGATCTTTGGGTTTTTAGGAGCA---GCAGGAAGCACTATG---GGCGCGGCGTCA---
ATAACGCTGACGGTACAG---GCCAGACAA---TTATTG---TCTGGTATAGTGCAACAGCAAAGC---
AATTTG---CTGAGG---GCTATAGAGGCGCAGCAGCATCTGTTGCAACTCACAGTCTGGGGCATT
>KJ953555
ACTTACAGGGAAAATGAAACCATAGGG--

GGTAATGGCACTATCATACTTCCATGCAAGATAAAGCAAATTATAAACATGTGGCAGGGAGTAGGACAAGCAA
TGTATGCTCCTACCGTCCGTGGAACAATTAATTGTGTATCAAAC---
ATTACAGGAATGTTATTAACAAGAGATGGTGGTGTTAATAAGGAT----------------------------
----

GATAATACTAGCGAGACCTTCAGACCTGGAGGAGGAAATATAAAGGACAATTGGAGAAGTGAATTATATAAAT
ATAAAGTAGTACAAATTGAACCACTA---GGAATAGCACCCACCAGGGCAAAGAGAAGAGTGGTG--------
-------------------------------GAGAGAGAAAAAAGAGCA---GTGGGA---ATAGGAGCT---
------ATGATCTTTGGGTTCTTAGGAGCA---GCAGGAAGCACTATG---GGCGCGGCGGCA---
ATAACGCTGACGGTACAG---GCCAGACAA---TTATTG---TCTGGTATAGTGCAACAGCAAAGC---
AATTTG---CTGAGG---GCTATAGAGGCGCAGCAGCATATGTTGCAACTCACAGTCTGGGGCATT
>KJ953646
AGTTACACAAAAGAAAAGGGA--

TGTAATGGCACTATCATACTTCCATGCAAGATAAAGCAAATTATAAACATGTGGCAGGGAGTAGGACAAGCAA
TGTATGCTCCTCCCGTCAGAGGAGTAATTAATTGTGTATCAAAT---
ATTACAGGAATACTATTGACAAGAGATGGTGGTGAGGAGAATAAT----------------------------
----

GGGAGTGAAACCTTCAGACCTGGAGGAGGAAATATAAAGGACAATTGGAGAAGTGAATTATATAAATATAAAG
TAGTACAAATTGAACCACTA---GGAGTAGCACCCACCAAGGCAAAGAGAAGAGTGGTG--------------
-------------------------GAAAGACAAAAAAGAGCA---GTGGGA---ATAGGAGCT---------
ATGATCTTTGGGTTCTTAGGAGCA---GCAGGAAGCACTATG---GGCGCGGCGTCA---
ATAACGCTGACGGTACAG---GCCAGACAA---TTATTG---TCTGGTATAGTGCAACAGCAAAGT---
AATTTG---CTGAGG---GCTATAGAGGCGCAGCAACATCTGCTGCAACTCACAGTCTGGGGCATT
>AY358052
ACTTACACAGGAAATGAAACCATGGAAGGG---

TGTAATGGCACTATCATACTTCCATGCAAGATAAAGCAAATTATAAACATGTGGCAGGGAGTAGGACAAGCAA
TGTATGCTCCTCCCATCAGTGGAAATATTAGTTGTGTATCAAAT---
ATTACAGGAATACTATTGACAAGAGATGGTGGTATTAATGAGGAT----------------------------
----

AACAAGACCGAGATCTTCAGACCTGGAGGAGGAAATATAAAGGACAATTGGAGAAGTGAATTATATAAATATA
AAGTAGTACAAATTGAACCACTA---GGAATAGCACCCACCAGGGCAAAGAGAAGAGTGGTG-----------
----------------------------GAGAGAGAAAAAAGAGCA---GTGGGA---ATAGGAGCT------
---ATGATCTTTGGGTTCTTGGGAGCA---GCAGGAAGCACTATG---GGCGCGGCGTCA---
ATAACGCTGACGGTACAT---GCCAGACAA---TTATTG---TCTGGTATAGTGCAACAGCAAAGC---
AATTTG---CTGAGG---GCTATAGAGGCGCAGCAGCATCTGTTGCAACTCACAGTCTGGGGCATT
>AY945719
AATCATACTTTCAAAAATAATGAAACCACGGAGGGG-------------------------------------
----
-
TATAATGGCACTATCACAATTCCATGCAAGATAAAGCAAATTATAAACATGTGGCAAGGAGTGGGACAAGCAA
TGTATGCTCCTCCCATCAGGGGAAACATTAGTTGTGTATCAAAT---
ATTACAGGCATACTGTTGACAAGAGATGGTGGTAGTAGAGATGCG----------------------------
----

CCTAATGAGACCTTCAGACCTGGAGGAGGAAATATAAAGGACAATTGGAGAAATGAATTATATAAATATAAAG
TAGTACAAATTGAACCACTA---GGAATAGCACCCACCAGGGCAAAGAGAAGAGTGGTG--------------

```
-------------------------GAGAGAGAAAAAAGAGCA---GTGGGA---ATAGGAGCT---------
ATGATCTTTGGGTTCTTAGGAGCA---GCAGGAAGCACTATG---GGCGCAGCGTCA---
ATAACGCTGACGGTACAT---GCCAGACAA---TTATTG---TCTGGTATAGTGCAACAGCAAAGC---
AATTTG---CTGAGG---GCTATAGAGGCGCAGCAGCATCTGTTGCAACTCACAGTCTGGGGCATT
>AY358038
AGTTACCTAAAAAATAAAACCCTTGAC----------------------------------------------
--------------------------------------------------------------------
GGGAATGGCACTATCATAATCCCATGCAGAATAAAGCAAATTGTAAACATGTGGCAGGGAGTAGGACAAGCAA
TGTATGCTCCTCCCATCAATGGAATAATTAATTGTGTATCAAAT---
ATTACAGGAATATTATTAACAAGAGATGGTGGTAACAGTACGAAT----------------------------
-------------------------------------------------------------------------
----------------
AACGAGACTTTCAGACCTGGAGGAGGAAATATAAAGGATAATTGGAGAAGTGAATTATATAAATATAAAGTAG
TACAAATTGAACCACTA---GGAATAGCACCCACCAGGGCAAAGAGAAGAGTGGTG-----------------
----------------------GAGAGAGAAAAAAGAGCA---GTGGGA---ATAGGAGCT---------
ATGCTCTTTGGGTTCTTAGGAGCA---GCCGGAAGCACTATG---GGCGCGGCGTCA---
ATAACGCTGACGGTACAG---GCCAGACAA---TTATTG---TCTGGTATAGTGCAACAGCAAAGC---
AATTTG---CTGAGG---GCTATAGAGGCGCAGCAGCATCTGTTGCAACTCACAGTCTGGGGCATT
>AY358064
ACTTATGGAGAAAATGAAACCATGGAGGGTAAT----------------------------------------
--------------------------------------------------------------------
AACAATAGCACTATCATACTTCCATGCAAGATAAAACAAATTGTAAACATGTGGCAGGGAGCAGGACAAGCAA
TGTATGCTCCTCCCATCAGTGGAACAATTAATTGTGTATCAAAT---
ATTACAGGAATACTATTAACAAGAGATGGTGGTAATAATAATAGT----------------------------
-------------------------------------------------------------------------
-------------
ACTAATGAGACCTTCAGACCTGGAGGAGGAAATATAAAAGACAATTGGAGAAGTGAATTATATAAATATAAAG
TAGTACAAATTGAACCACTA---GGACTAGCACCCACCAGGGCAAGGAGAAGAGTGGTG--------------
-------------------------GAGAGAGAAAAAAGAGCA---GTGGGA---ATAGGAGCT---------
ATGATCTTTGGGTTCTTAGGAGCA---GCAGGAAGCACTATG---GGCGCGGCGTCA---
ATAACGCTGACGGTACAG---GCCAGACAA---TTATTG---TCTGGTATAGTGCAACAGCAAAGC---
AATTTG---CTGAGG---GCTATTGAGGCGCAGCAGCATCTGATGCAACTCACAGTCTGGGGCATT
>JN248324
ACTTACCTAAAAAATGAGACAACGTGTAATGGCACA-------------------------------------
--------------------------------------------------------------------
TGTAATGGCACTATCATACTTCCATGCAAGATAAAACAAATTATAAACATGTGGCAGGGAACAGGACAAGCAA
TGTATGCTCCTCCCATCAGTGGAAGAATTAATTGTGTATCAAAT---
ATTACAGGAATACTATTGACAAGAGATGGCGGTAATGAAAGTAGT----------------------------
-------------------------------------------------------------------------
-------------
GATACTGAGATCTTCAGACCTGAAGGAGGAAATATAAAGGACAATTGGAGAAGTGAATTATATAAATATAAAG
TAGTACAAATTGAACCACTA---GGAGTAGCACCCACCAGGGCAAAGAGAAGAGTGGTG--------------
-------------------------GAGAAAGAAAAAAGAGCA---GTGGGA---ATAGGAGCT---------
GTGATCTTTGGGTTCTTAGGAGCA---GCAGGAAGCACTATG---GGCGCGGCATCA---
ATAACGCTGACGGTACAG---GCCAGACAA---TTATTG---TCTGGTATAGTGCAACAGCAAAGC---
AATTTG---CTGAGG---GCTATAGAGGCGCAACAGCATCTGTTGCAACTCACAGTCTGGGGCATT
>JN248330
ACTTACATGAGAAATGGAACTGTAATAAATGAAGCT-------------------------------------
--------------------------------------------------------------------
GTGAATGGCACTATCTTTCTTCCATGCAGGATAAAGCAAATTATAAACATGTGGCAGGGAGCAGGACAAGCAA
TGTATGCTCCTCCCATCAGGGGAACAATTAATTGTGTCTCAAAT---
ATTACAGGAATACTATTGACAAGAGATGGTGGTACTAATACAACT----------------------------
-------------------------------------------------------------------------
----------------
AATGAGACCTTCAGACCTGAGGGAGGAGATATGAAGGACAATTGGAGAAGTGAATTATATAAATATAAAGTAG
TAGAAATTGAACCACTA---GGAATAGCACCCACCAAGGCAAAGAGAAGAGTGGTG-----------------
----------------------GAAAGAGAAAAAAGAGCA---GTGGGA---ATAGGAGCT---------
ATGATCTTTGGGTTCTTAGGAGCA---GCAGGAAGCACTATG---GGCGCAGCGTCA---
ATAACGCTGACGGTACAG---GCCAGACAA---TTATTG---TCTGGTATAGTGCAACAGCAAAGC---
AATTTG---CTGAGA---GCTATAGAGGCGCAACAGCATATGTTGCAACTCACAGTCTGGGGCATT
```

```
>JN248334
ACTTACTCA----------------------------------------------------------------
--------------------------------------------------
GGAAATGGAACCATCATACTTCCATGCCGGATAAAGCAAATTATAAACATGTGGCAGGGAGTAGGACAGGCAA
TGTATGCTCCTCCCATCAGTGGAGCAATTAAGTGTGTATCAAAT---
ATTACAGGAATACTATTGACAAGAGATGGTGGTACTAATAGGACT----------------------------
-------------------------------------------------------------------------
----------------
AACGAGACTTTCAGACCTGGAGGAGGAAATATAAAGGACAACTGGAGAAGTGAATTATATAAATATAAGGTAG
TAGAAATTGAACCACTA---GGAATAGCACCCACCAGGGCAAAGAGAAGAGTGGTG-----------------
----------------------GAAAGAGAAAAAAGAGCA---GTGGGA---ATAGGAGCT---------
ATGATCTTTGGGTTCTTAGGAGCA---GCAGGAAGCACTATG---GGCGCGGCGTCG---
ATAACGCTGACGGTACAG---GCCAGACAA---TTATTG---TCTGGTATAGTGCAACAGCAAAGC---
AATTTG---CTGAGA---GCTATAGAGGCGCAGCAGCATCTGTTGCAACTCACAGTCTGGGGCATT
>JX447529
GGTCAGGAG----------------------------------------------------------------
-----------------------------------------------------
CATAATGGCACTATCACACTTCCATGCAGGATAAAGCAAATTATAAACATGTGGCAGGGAGTAGGACAAGCAA
TGTATGCTCCTCCCATCAGTGGACAAATTAATTGTGTATCGAAT---
ATTACAGGAATACTATTGACAAGAGATGGTGGTAAGGATAATGAAACG-------------------------
-------------------------------------------------------------------------
----------
AATGGCACAGAGACCTTCAGACCTGGAGGAGGAAACATGAAGGACAATTGGAGGAGTGAATTATATAAATATA
AAGTAATACAAATTGAACCACTA---GGAGTAGCACCCACCAGGGCAAGGAGAAGAGTGGTG-----------
----------------------------GCGAGAGAAAAAAGAGCA---GTGGGA---ATAGGAGCT------
---ATGATCTTTGGGTTCTTAGGAGCA---GCAGGAAGCACTATG---GGCGCGGCGTCA---
ATAACGCTGACGGTACAG---GCCAGACAA---TTATTG---ACTGGTATAGTGCAACAGCAAAGC---
AATTTG---CTGAGA---GCTATAGAGGCGCAACAGCATCTGTTGCAACTCACAGTCTGGGGCATT
>JN248341
ACTTACACAGGAAATGAAACCGGAGAGGGGGGTAAT-------------------------------------
--------------------------------------------------------------------
GGTAATGGCACTATCATAATTCCATGCAGAATAAAGCAAATTATAAACATGTGGCAGGGAGTAGGACAAGCAA
TGTATGCTCCTCCCATCAGTGGAAGCATTAATTGTGTATCAAAT---
ATTACAGGAATACTATTGACAAGAGATGGTGGTAATAACAACATGACT-------------------------
-------------------------------------------------------------------------
----------
AACGATAACGAGACCTTCAGACCTGGAGGAGGAGATATAAGGGACAATTGGAGAAGTGAATTGTATAAATATA
AAGTAGTACAAATTGAACCACTA---GGAGTAGCACCCACCAAGGCAAAGAGAAGAGTGGTG-----------
----------------------------GAGAGAGAAAAAAGAGCA---GTGGGA---ATAGGAGCT------
---ATGATCTTTGGGTTCTTAGGAGCA---GCAGGAAGCACTATG---GGCGCAGCGTCA---
ATAACGCTGACGGTACAG---GCCAGACAA---TTATTG---TCTGGTATAGTGCAACAGCAAAGC---
AATTTG---CTGAGG---GCTATAGAGGCGCAGCAGCATATGTTGCAACTCACTGTCTGGGGCATT
>JN248342
AATTATAATGGCACAAGAAATGAAACG----------------------------------------------
-------------------------------------------------------------------------
-
TGTAATGACACTATCATACTACCATGCAGGATAAAGCAAATTATAAACATGTGGCAGGGAGTAGGACAAGCAA
TATATGCTCCTCCAATCAGAGGAACACTTAAGTGTGTATCAAAT---
ATTACAGGAATACTATTGACAAGAGATGGTGGTAGTAATGAGACTAAA-------------------------
-------------------------------------------------------------------------
----------
AATGGGACCGAGATATTCAGACCTGGAGGGGGAAATATAAAGGACAATTGGAGAAATGAATTATATAAATATA
AAGTAGTACAAATTGAACCACTA---GGAATAGCACCCACCAGGGCAAAGAGAAGAGTGGTG-----------
----------------------------GAGAGAGAAAAAAGAGCA---GTGGGA---ATAGGAGCT------
---ATGATCTTTGGGTTCTTAGGAGCA---GCAGGAAGCACTATG---GGCGCGGCGTCA---
ATAACGCTGACGGTACAG---GCCAGACAA---TTATTG---TCTGGTATAGTGCAACAGCAAAGC---
AATTTG---CTGAGG---GCTATAGAGGCGCAGCAGCATCTGTTGCAACTCACAGTCTGGGGCATT
>JN944654
AATCAAATCATGGGGCAG-------------------------------------------------------
-----------------------------------------------------------
```

CATGATGACACTATGGTACTTCCATGCAAGATAAAGCAAATTATAAATATGTGGCAGGGAGCAGGACAAGCAA
TGTATGCTCCTCCCGTCAGTGGAATAATTAATTGTAGGTCAAAT---
ATTACAGGAATACTATTGACAAGAGATGGTGGTGGTGGTGCTAAC----------------------------
----

AATACAAATGAGACCTTCAGACCTGGGGGAGGAAATATAAAGGATAATTGGAGAAGTGAATTATATAAATATA
AAGTAGTAAAAATTGAACCACTA---GGATTAGCACCCACCAGGGCAAAGAGAAGAGTGGTG-----------
----------------------------GAGAAAGAAAAAAGAGCA---GCGGGA---ATAGGAGCT------
---ATGATCTTTGGGTTCTTAGGAGCA---GCAGGAAGCACTATG---GGCGCGGCGTCA---
ATAACGCTGACGGTACAG---GCCAGACAA---TTATTG---TCTGGTATAGTGCAACAGCAAAGC---
AATTTG---CTGAGA---GCTATAGAGGCGCAACAGCATCTGTTGCAACTCACAGTCTGGGGCATT
>JX447268
ACTTACATAGGAAATGAAACCAGAGAGATATTTAATGGAACTGATGAA-------------------------
----
ACTAATGGAACTATCATACTTCCATGCAAAATAAAGCAAATTATAAACATGTGGCAGGGAGTAGGACAAGCAA
TGTATGCTCCTCCCATCAGTGGAAAAATTAATTGTGTATCAAAT---
ATTACAGGAATACTATTGACAAGAGATGGTAGTTCTAGAAATAAT----------------------------
----

ACGAACGAAACCTTCAGACCTGGAGGGGGAAATATAAAGGATAATTGGAGAAGTGAATTATATAAATATAAAG
TAGTACAAATTGAACCACTA---GGAATAGCACCCACCAGGGCAAAGAGAAGAGTGGTG--------------
-------------------------GAGAGAGAAAAAAGAGCA---GTGGGA---ATAGGAGCT---------
ATGATCTTTGGGTTCTTAGGAGCA---GCAGGAAGCACTATG---GGCGCGGCGTCA---
ATAACGCTGACGGTACAG---GCCAGACAA---TTATTG---TCTGGTATAGTGCAACAGCAAAGC---
AATTTG---CTGAGG---GCTATAGAGGCGCAGCAGCATCTGTTGCAACTCACAGTCTGGGGCATT
>JX447579
GTTTATAATAATGACACAAAGAATAGAACCAGGGGG-------------------------------------
----
AGTAATGACACTATCACAATTCCATGCAAAATAAAGCAAATTATAAACATGTGGCAGGGGATAGGACAAGCAA
TGTATGCTCCTCCCATCAGTGGAACAATTAATTGTGTATCAAAT---
ATTACAGGAATACTATTGACAAGAGATGGTGGTGATAGTACTAAC----------------------------
----

GAGACCTTCAGACCTGGAGGAGGAAATATAAAGGACAATTGGAGAAGTGAATTATATAAATACAAAGTAGTAC
AAATTGAACCACTA---GGAATAGCACCCACCAGGGCAAAGAGAAGAGTGGTG--------------------
-------------------GAAAGAGAAAAAAGAGCA---GTGGGA---ATAGGAGCT---------
ATGATCTTTGGGTTCTTAGGAGCA---GCAGGAAGCACTATG---GGCGCGGCGTCA---
ATAACGCTGACGGTACAG---GCCAGACAA---TTATTG---TCTGGTATAGTGCAACAGCAAAGC---
AATTTG---CTGAGG---GCTATAGAGGCGCAGCAGCATATGTTGCAACTCACAGTCTGGGGCATT
>JX447592
ACTCACATGATAAATGGAACCATGAAGGGGAGTAATGGCACTAATGGC-------------------------
----
ACTAATGGCACTTTCATACTTCCATGCAGGATAAAGCAAATTATAAACATGTGGCAGGGAGTAGGACAAGCAA
TGTATGCTCCTCCCATCAAGGGAATAATTAATTGTGTATCAAAC---
ATTACAGGAATATTATTGACAAGGGATGGTGGTAATAACAACACA----------------------------
----

AATAACGAGACCTTCAGACCTATAGGAGGAAATATAAAGGACAATTGGAGAAGTGAATTATATAAATATAAAG
TAGTACAAATTGAACCACTA---GGAATAGCACCCACCAAGGCAAAGAGAAGAGTGGTG--------------
-------------------------GAGAGAGAAAAAAGAGCA---GTGGGA---ATAGGAGCT---------
ATGATCTTTGGGTTCTTGGGAGCA---GCAGGAAGCACTATG---GGCGCGGCGTCA---
ATAACGCTGACGGTACAG---GCCAGACAA---TTATTG---TCTGGTATAGTGCAACAGCAAAGC---
AATTTG---CTGAGG---GCTATAGAGGCGCAGCAGCATATGTTGCAACTCACAGTCTGGGGCATT
>JX448256
TCTTACCCAGAAAACGAGACTGAGGGGAATACT--

ACCACTACCACTATCATACTTCCATGCAAGATAAAGCGAATTATAAACATGTGGCAGGGAGTAGGACAAGCAA
TGTATGCTCCTCCCATTAGGGGAAAAATTAAGTGTGTATCAAAT---
ATTACAGGAATACTATTGACAAGTGATGGTGGTGGTAAGGGTAATAAT-------------------------
----

AGTAATAGCGAGACCTTCAGACCTGGAGGAGGAAATATAAGGGACAATTGGAGAAGTGAATTATATAAATATA
AAGTAGTGCAAATTGAACCACTA---GGAATAGCACCCACCAAAGCAAAGAGAAGAGTGGTG-----------
----------------------------GAGAGAGAAAAAAGAGCA---GTGGGA---ATAGGAGCT------
---ATGATCTTTGGGTTCTTAGGAGCA---GCAGGAAGCACTATG---GGCGCGGCGTCA---
ATAACGCTGACGGTACAG---GCCAGGCAA---TTATTG---TCTGGTATAGTGCAACAGCAAAGC---
AATTTG---CTGAGG---GCTATAGAGGCGCAGCAGCATATGTTGCAACTCACAGTCTGGGGCATT
>EU743792
GGC--

TATATCATACTTCCATGCAGAATAAAGCAAATTATAAACATGTGGCAGGGAGTAGGACAAGCAATATATGCCC
CTCCTATCAGTGGAAGAATTAGTTGTGTATCAAAT---
ATTACAGGAATCCTATTGACAAGAGATGGTGGTAATAATAATACC----------------------------
----

GAGACCGAGATCTTCAGACCTGGAGGAGGAAATATAAAGGACAATTGGAGAAGTGAATTATATAAATATAAAG
TAGTACAAATTGAACCACTG---GGAATAGCACCCACCAAGGCAAGGAGAAGAGTGGTG--------------
-------------------------GACAGAGAAAAAAGAGCA---GTGGGA---ATAGGAGCT---------
ATGATCTTTGGGTTCTTGGGAGCA---GCAGGAAGCACTATG---GGCGCAGCGTCA---
CTAACGCTGACGGTACAG---GCCAGACAA---TTATTG---TCTGGTATAGTGCAACAGCAAAGC---
AATTTG---CTGAGA---GCTATAGAGGCGCAGCAGCATCTGTTGCAACTCACAGTCTGGGGCATT
>EU743759
CATCAAAACACGACAGGG---

TGTAATGGAACTATCACACTCCCATGCAAGATAAAGCAAATTATAAACATGTGGCAGGAAGTAGGACAAGCAA
TGTATGCTCCTCCCATCAGTGGAAAAATTAAGTGTGTATCAAAT---
ATTACAGGAATACTATTGACAAGAGATGGT---AATAATGAGACT----------------------------
----

GGGGCTAATGAGACCTTCAGACCTGAAGGAGGAAATATGAAGGACAATTGGAGAAATGAATTATATAAATATA
AAGTAGTGCAAATTGAACCACTA---GGAATAGCACCCTCCAGGGCAAAAAGAAGAGTGGTG-----------
----------------------------GAGAGACAAAAAAGAGCA---GTGGGA---ATAGGAGCT------
---ATGATCTTTGGGTTCTTAGGAGCA---GCTGGAAGCACTATG---GGCGCGGCGTCA---
ATAACGCTGACGGTACAG---GCCAGACAA---TTATTG---TCTGGTATAGTGCAACAGCAAAGC---
AATTTG---CTGAGG---GCTATAGAGGCGCAGCAGCATCTGTTGCAACTCACAGTCTGGGGCATT
>EU743768
ACTTACATAAATGGAACCATAAATGAAACCATGGAGGGG----------------------------------
----
CTTAATGACACTATCATACTTCCATGCAAAATAAAGCAAATTATAAACATGTGGCAGAGAGCAGGACAAGCAA
TGTATGCTCCTCCCATCAGGGGAAGCATTAAATGTGTATCAAAT---
ATTACAGGAATACTACTAACAAGGGATAGTGGTGATAATGATACT----------------------------
----

GAGACCTTCAGACCTGAAGGAGGAAATATGAAGGACAATTGGAGAAGTGAATTATATAAATATAAAATAGTAC
AAATTGAACCACTA---GGAATAGCACCCACCATGGCAAAGAGAAGAGTGGTG--------------------
-------------------GAGAGAGAAAAAAGAGCA---GTGGGA---ATAGGAGCT---------
ATGATCTTTGGGTTCTTGGGAGCA---GCAGGAAGCACTATG---GGCGCGGCGTCA---
ATAACGCTGACGGTACAG---GCCAGACAA---TTATTG---TCTGGTATAGTGCAACAGCAAAGC---
AATTTG---CTGAGA---GCTATAGAGGCGCAGCAGCATCTGTTGCAACTCACAGTCTGGGGCATT
>EU743770
AACTACACGGAAAGG--

GTTAATGAAACTATCATACTTCCATGCAAGATAAAGCAAATTATAAATATGTGGCAGAGAGTAGGACAAGCAA
TGTATGCTCCTCCCATCAGGGGAAGAATTGGGTGTATATCAAAT---
ATTACAGGAATACTACTGACAAGGGATAATGGTGGTACCAATAGG----------------------------
----

AGTAGTGAGACCTTCAGACCTGGAGGAGGAGATATAAGGGACAATTGGAGAAGTGAATTATATAAATATAAAG
TAGTAGAAATTGAACCACTA---GGAATAGCACCCACCAGGGCAAAGAGAAGAGTGGTG--------------
-------------------------GAGAGAGAAAAAAGAGCA---GTGGGA---ATAGGAGCT---------

ATGATCTTTGGGTTCTTAGGAGCA---GCAGGAAGCACTATG---GGCGCGGCGTCA---
ATAACGCTGACGGTACAG---GTCAGACAA---TTATTG---TCTGGTATAGTGCAACAGCAAAAC---
AATTTG---CTGAGG---GCTATAGAGGCGCAGCAGCATCTGTTGCAACTCACAGTCTGGGGCATT
>EU743777
AATCAAACCATGCTT--

AATAAAACTACCATACTTCCATGCAGAATAAAGCAAATTATAAACATGTGGCAGGGAACAGGACAAGCAATGT
ATGCTCCTCCCATCCAGGGAAGAATTAATTGTGTATCAAAT---
ATTACAGGAATACTATTGACAAGAGATGGAGGTAATACTAATGAGAAT-------------------------
----

AATAAAACTGAAATCTTCAGACCTGGAGGAGGAAATATGAAGGATAATTGGAGAAGTGAATTATATAAATATA
AAGTAGTACAAATTGAACCATTA---GGAATAGCACCCACCAGGGCAAAGAGAAGAGTGGTG-----------
----------------------------GAGAGAGAAAAAAGAGCA---GTGGGA---ATAGGAGCT------
---ATGATCTTTGGGTTCTTGGGAGCA---GCAGGAAGCACTATG---GGCGCGGCGTCA---
ATAACGCTGACGGTACAG---GCCAGACAA---TTATTG---TCTGGTATAGTGCAACAGCAAAGC---
AATTTG---CTGAGG---GCTATAGAGGCGCAGCAGCATCTGTTGCAACTCACAGTCTGGGGCATT
>EU743780
AATCAAACCCAG---

TGTAAGGGCAATATCACACTTCCATGCAGGATAAAGCAAATTATAAACATGTGGCAGGAAGTAGGAAAAGCAA
TATATGCTCCTCCCGTCAGTGGAAGTATTAATTGTTTATCAAAT---
ATTACAGGGATAATACTGACAAGAGATGGTGGTACTCCTGGTAATGTTACTAAT-------------------
----

AATGGTACTGAAACCGAGATCTTCAGACCTACAGGAGGAAATATGGAGGACAATTGGAGAAGTGAATTATATA
AATATAAAGTAGTACAAATTGAACCACTA---GGAATAGCACCCACCAAGGCAAAGAGAAGAGTGGTG-----
---------------------------------GAGAGAGAAAAAAGAGCA---GTGGGA---
ATAGGAGCT---------ATGATCTTTGGGTTCTTAGGAGCA---GCTGGAAGCACTATG---
GGCGCGGCGGCA---GTAACGCTGACGGTACAG---GCCAGACAA---TTATTG---
TCTGGTATAGTGCAACAGCAAAGC---AATTTG---CTGAGA---
GCTATAGAGGCGCAACAGCATCTGTTGCAACTCACAGTCTGGGGCATT
>JX446961
ACTTACAGAAATGAAACC---

AGTAATAGCACTATCATACTTCCATGCAGAATAAAGCAAATTATAAACATGTGGCAGGAAGTAGGACAAGCAA
TGTATGCTCCTCCCATCAGGGGAAGAATAAATTGTGTATCAAAT---
ATTACAGGAATACTATTGACAAGAGATGGTGGTAATAGTACAAGT----------------------------
----

AATAATGAGACCTTCAGACCTGGAGGAGGAAATATGAAGGACAATTGGAGAAGTGAATTATATAAATATAAAG
TAGTACAAATTGAACCATTA---GGAATAGCACCCACCAGGGCAAAGAGAAGAGTGGTG--------------
-------------------------GACAGAGAAAAAAGAGCA---GTGGGA---ATAGGAGCT---------
ATGATCTTTGGGTTCTTAGGAGCA---GCAGGAAGCACTATG---GGCGCGGCGTCA---
ATAACGCTGACGGTACAG---GCCAGACAA---TTATTG---TCTGGTATAGTGCAACAGCAAAGC---
AATTTG---CTGAGG---GCTATAGAGGCGCAACAGCATCTGTTGCAACTCACAGTCTGGGGCATT
>JX447515
ACTTACACAAATACAACCATACAG---

GGTAATGGTACTATTATACTTCCATGCAAGATAAAGCAAATTATAAATATGTGGCAGGAAGTAGGACGAGCAA
TGTATGCTCCTCCTGTCAGTGGAAGAATTAATTGTATATCAAAT---
ATTACAGGAATACTATTAATAAGAGATGGTGGTAATACTAGTGAG----------------------------
----

TCTAACGAGATCTTCAGACCTGGAGGAGGAAATATGAAAGACAATTGGAGAAATGAATTATATAAATATAAAG
TAGTAGAAATTGAACCACTA---GGAATAGCACCCACCAAGGCAAAAAGAAGAGTGGTG--------------
-------------------------GAGAGAGAAAAAAGAGCA---GTGGGA---ATAGGAGCT---------
TTGATCTTTGGGTTCTTGGGAGCA---GCAGGAAGCACTATG---GGCGCAGCGTCA---

ATAACGCTGACGGTACAG---GCCAGACAA---TTATTG---TCTGGTATAGTGCAACAGCAAAGC---
AATTTG---CTGAGG---GCTATAGAGGCGCAGCAGCATATGTTGCAACTCACAGTCTGGGGCATT
>JX448039
AATTATACTTGCATAAATAATACGTGCATAAATAATGCAACCATGGAT-------------------------
----
-
GATAATGGCACTATCATACTTTCATGCAAAATAAAGCAAATTATAAACATGTGGCAGGGAGTAGGACAAGCAA
TGTATGCTCCTCCCATCAGTGGAAACATTAGTTGTGTATCAAAT---
ATTACTGGAATACTATTGACAAGAGATGGTGGTAATAATAGGACTACG-------------------------
----

ACTACCGAGACCTTCAGACCTGCAGGAGGAGATATGAAGGACAATTGGAGAAGTGAATTATATAAATATAAAG
TAGTACAAATTGAACCACTA---GGAATAGCACCCACCAGGGCAAAGAGAAGAGTGGTG--------------
-------------------------GAGAGAGAAAAAAGGGCA---GTGGGA---ATAGGAGCT---------
ATGATCTTTGGGTTCTTAGGAGCA---GCAGGAAGCACTATG---GGCGCGGCGTCA---
ATAACGCTGACGGTACAG---GCCAGACAA---TTATTG---TCTGGTATAGTGCAACAGCAAAGC---
AATTTG---CTGAGG---GCTATAGAGGCGCAGCAGCATCTGTTGCAACTCACAGTCTGGGGCATT
>JX448198
ACTTACAATGGAACCATGGGGGGATGGAATGGCACTATCAATGGCACT-------------------------
----
-
ATCAATGACACTATCATAATTCCATGCAGAATAAAGCAAATTATAAATATGTGGCAGGGAGTAGGACAAGCAA
TATATGCTCCTCCCATCAGAGGAATAATTAATTGTGTATCAAAT---
ATCACAGGAATACTATTGACAAGAGATGGTGGTGATAATAATGCTACT-------------------------
----

AAAGATACCGAGATCTTCAGACCTGGAGGTGGAAATATAAAGGACAATTGGAGAAGTGAATTATATAAATATA
AAGTAGTACAAATTGAACCACTA---GGAATAGCACCCACCAGGGCAAAGAGAAGAGTGGTG-----------
----------------------------GAAAGAGAAAAAAGAGCA---GTGGGA---ATAGGAGCT------
---ATGATCTTTGGGTTCTTAGGAGCA---GCAGGAAGCACTATG---GGCGCGGCGTCA---
CTAACGCTGACGGTACAG---GCCAGACAA---TTATTG---TCTGGTATAGTGCAACAGCAAAGC---
AATTTG---CTGAGG---GCTATAGAGGCGCAGCAGCATCTGTTGCAACTCACAGTCTGGGGCATT
>JX446877
ACTTACAAAGATAAAGCCATAGAGGGG--

AATAATGGCACTATCATACTTCCATGCAGGATAAAACAAATTATAAACATGTGGCAGGGAGTAGGACAAGCAA
TGTATGCTCCTCCCATCAGGGGAAACATTAGTTGTGTCTCAAAT---
ATTACAGGAATACTATTGACAAGAGATGGTGGTTCTGGTAATGATACAAGA----------------------
----

AATGATACAGAGACCTTCAGACCTGGAGGAGGGAATATAAAGGACAATTGGAGAAGTGAATTATATAAATATA
AAGTAGTACAAATTGAACCACTA---GGACTAGCACCCACCAAGGCAAAGAGAAGAGTGGTG-----------
----------------------------GAGAGACAAAAAAGAGCA---GTGGGA---ATAGGAGCT------
---ATGATCTTTGGGTTCTTAGGAGCA---GCAGGAAGCACTATG---GGCGCGGCGTCA---
ATAACGCTGACGGCACAG---GCCAGACAA---TTATTG---TCTGGTATAGTGCAACAGCAAAGC---
AATTTG---CTGAGA---GCTATAGAGGCGCAGCAGCATCTGTTGCAACTCACAGTCTGGGGCATT
>JX446918
ACTTACGTAAGAAATGAAACCAAG---

GAAAATGATACTATCATACTTCCATGCAAGATAAAGCAAATTATAAACATGTGGCAGGGAGTAGGACAAGCAA
TGTATGCTCCTCCCATCAGTGGAAACATTAGTTGTGTATCAAAT---
ATTACAGGAATAATATTGACAAGAGATGGTGGTCTTGGTAATAAT----------------------------
----

AGTGGAGAGACCTTCAGACCTGGAGGAGGAAATATAAAGGACATTTGGAGAAGTGAATTATATAAATATAAAG
TAGTACAAATAGAACCACTA---GGAATAGCACCCACCAGGGCAAAGAGAAGAGTGGTG--------------
-------------------------GAGAGAGAAAAAAGAGCA---GTGGGA---ATAGGAGCT---------
ATGATCTTTGGGTTCTTAGGAGCA---GCAGGAAGCACTATG---GGCGCGGCGTCA---
ATAACGCTGACGGTACAG---GCCAGACAA---TTATTG---TCTGGTATAGTGCAACAGCAAAGC---
AATTTG---CTGAGG---GCTATAGAGGCGCAGCAGCATATGTTGCAACTCACAGTCTGGGGCATT

>JX447381
ACTTACCTAGGAAATGAAACCACAGGGAGG---

TATAATGACACTATCATAATTCCATGCAAGATAAAGCAAATTATAAGAATGTGGCAGGGGGTAGGACAAGCAA
TGTATGCTCCTCCCATCAGGGGAGTCATTAAGTGTAGATCAAAT---
ATTACAGGAATATTATTGACAAGAGATGGTGATAAAATTAATGCT----------------------------
----

ACTGAAGAGATTTTCAGACCTGGAGGAGGAAATATAAAGGATAATTGGAGAAGTGAATTATATAAATATAAAG
TAGTACAAATTGAACCACTA---GGAATAGCACCCACCAGGGCAAAGAGAAGAGTGGTG--------------
-------------------------GAGAGAGAAAAAAGAGCA---GTGGGA---ATAGGAGCT---------
ATGATCTTTGGGTTCTTAGGAGCA---GCAGGAAGCACTATG---GGCGCGGCGTCA---
ATAACGCTGACGGTACAG---GCCAGACAA---TTATTG---TCTGGTATAGTGCAACAGCAAAGC---
AATTTG---CTGAGG---GCTATAGAGGCGCAGCAGCATCTGTTGCAACTCACAGTCTGGGGCATT
>JX447773
ACTCACATGCCTAATGGTACTATCATA--

GGAAAAGACAATATCACACTTCCATGCAGGATAAAGCAAATCATAAACATGTGGCAGGGAGCAGGACAAGCAA
TGTATGCTCCTCCCATCAGTGGAATAATTAATTGTGTATCAAAT---
ATTACAGGAATACTATTGACAAGAGATGGTGGTGGTAATAGTAGT----------------------------
----

AATGAGAACGAGACCTTCAGACCTGGAGGAGGAAATATAAAGGACAATTGGAGGAGTGAATTATATAAATATA
AAGTAGTACAAATTGAACCACTA---GGAATAGCACCCACCAAGGCACAGAGAAGAGTGGTG-----------
----------------------------CGGATAGAAAAAAGAGCA---GTGGGA---TTAGGAGCT------
---ATGATCTTTGGGTTCTTAGGAGCA---GCAGGAAGCACTATG---GGCGCGGCGTCA---
ATAACGCTGACGGTACAG---GCCAGACAA---TTATTG---TCTGGTATAGTGCAACAGCAAAGC---
AATTTG---CTGAGA---GCTATAGAGGCGCAGCAGCATCTGTTGCAACTCACAGTCTGGGGCATT
>HQ690985
ACTTACCCAGGAAATGAAACC--

ATGGAGGACCATATCATACTTCCATGCAGGATAAAGCAAATTATAAACATGTGGCAGGGAGCAGGACAAGCAA
TGTATGCTCCTCCCATCAGTGGGAGAATTAATTGTGTATCAAAT---
ATTACAGGCATACTACTGACAAGAGATGGTGGTAATAATACTAAT----------------------------
----

GAGACTAAAGAAATCTTCAGACCTGGAGGAGGAAATATGAAGGACAATTGGAGAAGTGAATTATATAAATATA
AAGTAGTACAAATTGAACCACTA---GGAATAGCACCCACCAGGGCAAGGAGAAGAGTGGTG-----------
----------------------------CAGAGAGAAAAAAGAGCA---GTGGGA---ATAGGAGCT------
---ATGATCTTTGGGTTCTTAGGAGCA---GCAGGAAGCACTATG---GGCGCAGCGTCA---
ATAACGCTGACGGTACAG---GCCAGACAA---TTATTG---TCTGGTATAGTGCAACAGCAAAGC---
AATTTG---CTGAGG---GCTATAGAGGCGCAGCAACATCTGTTGCAACTCACAGTCTGGGGCATT
>HQ691052
TGCTACACAACAAAACTGTTTAATGATACTTGCACAGGAAATGAAACCAGCACAGGAAATGAAACCAATAAGG
AATGT--
----AAACTGTCT------
AATGGTAATGGCACTATCATACTTCCATGCAAGATAAAGCAAATTATAAACATGTGGCAGGGAGTAGGACAAG
CAATGTATGCTCCTCCCATCAGTGGAAGAATTAATTGTGTATCAAAT---
ATTACAGGAATACTATTGACAAGAGATGGAGGTACTAACACGATC----------------------------
----

AACAATGAGACTTTCAGACCTGGAGGGGGAAATATAAAGGACAATTGGAGAAGTGAATTATATAAATATAAAG
TAGTACAAATTGAACCACTA---GGAATAGCACCCTCCAGGGCAAGGAGAAGAGTGGTG--------------
-------------------------GAAAGAGAAAAAAGAGCA---GTGGGA---ATAGGAGCT---------
ATGATCTTTGGGTTCTTGGGAGCA---GCAGGAAGCACTATG---GGCGCGGCGTCA---
ATAACGCTGACGGTACAG---GCCAGACAA---TTATTG---TCTGGTATAGTGCAACAGCAAAGC---
AATTTG---CTGAAG---GCTATAGAGGCGCAGCAGCATATGTTGCAACTCACAGTCTGGGGCATT
>HQ691073
ACCTATAATGAC---

```
ACTAATGACCCTATCATACTTCCATGCAGGATAAAGCAAATTATAAACATGTGGCAGGGAGTAGGACAAGCAA
TGTATGCTCCTCCCATCCGTGGAAGCGTTAATTGTTCATCAAAT---
ATTACAGGAATACTATTGACAAGAGATGGGGGTAATAATAGTAAT----------------------------
-------------------------------------------------------------------------
-------------
ACGCAAGAGACTTTCAGACCTGGAGGAGGAAATATAATGGATAATTGGAGAAATGAATTATATAAATATAAAG
TAGTACAAATTGAACCATTA---GGAATAGCACCCACCAGGGCAAAGAGAAGAGTGGTG--------------
-------------------------GAGAGAGAAAAAAGAGCA---GTGGGA---ATAGGAGCT---------
ATGATCTTTGGGTTCTTAGGAGCA---GCAGGAAGCACTATG---GGCGCGGCGTCA---
ATAACGCTGACGGTACAG---GCCAGACAA---TTATTG---TCTGGTATAGTGCAACAGCAAAGC---
AATTTG---CTGAGG---GCTATAGAGGCGCAGCAGCATCTGTTGCAACTCACGGTCTGGGGCATT
>GQ916574
ACTTACACAGGAAATGAAACCACAGGAGAT-------------------------------------------
--------------------------------------------------------------------
GAAACCAAGAATATCACACTTCCATGCAGGATAAAGCAAATTATAAACATGTGGCAGGAAGTAGGACAAGCAA
TGTATGCTCCTCCCATCAGTGGACAAATTAAGTGTGTATCAAAT---
ATTACAGGAATACTATTGACAAGAGATGGTGGTGGTAATGCTAATGAT-------------------------
-------------------------------------------------------------------------
-------
ACTAATAATACCGAGACCTTCAGACCTGGAGGAGGAAATATAAAGGACAACTGGAGAAGTGAATTATATAAAT
ATAAAGTAGTACAAATTGAACCACTA---GGAATAGCACCCACCAAGGCAAAGAGAAGAGTGGTG--------
-------------------------------GAGAGACAGAAAAGAGCA---GTGGGA---ATAGGAGCT---
------ATGATCTTTGGGTTCTTAGGAGCA---GCAGGAAGCACTATG---GGCGCGGCGTCA---
ATAACGCTGACGGTACAG---GCCAGACAA---TTATTG---TCTGGTATAGTGCAGCAGCAAAGA---
AATTTG---CTGAGG---GCTATAGAGGCGCAGCAGCATCTGTTGCAACTCACAGTCTGGGGCATT
>JX446888
GCTTACACAGGAAATGAAACCATGAAA----------------------------------------------
--------------------------------------------------------------------
GGGAATAAAACCATCATACTTCCATGCAGGATAAAGCAAATTATAAACATGTGGCAGGGAGTAGGACAAGCAA
TATATGCTCCTCCCGTCAGGGGAATGATTAATTGTGTATCAAAT---
ATTACAGGAATATTATTGACAAGGGATGGTGGTAATGGTACGACC----------------------------
-------------------------------------------------------------------------
----------------
GAGGAGATTTTCAGACCAGGAGGAGGAAATATAAAAGACAATTGGAGAAGTGAATTATATAAATATAAAGTAG
TACAAATTGAACCACTA---GGCATAGCACCCACCAGGGCAAAGAGAAGAGTGGTG-----------------
----------------------GAGAGAAACAAAAGAGCA---GTTGGA---ATAGGAGCT---------
ATGATCTTTGGGTTCTTAGGAGCA---GCAGGAAGCACTATG---GGCGCGGCGTCA---
ATAACGCTGACGGTACAG---GCCAGACAA---TTATTG---TCTGGTATAGTGCAACAGCAAAGC---
AATTTG---CTGAGG---GCTATAGAGGCGCAGCAGCATCTGTTGCAACTCACAGTCTGGGGCATT
>JX448072
ACTTACATGGAAAATGGAACCATGCAGAATAGTACGCAGAATAACACG-------------------------
--------------------------------------------------------------------
CAAAATGACACTATCATACTCCCATGCAGGATAAAGCAAATTATAAACATGTGGCAGGGAGCAGGACAAGCAA
TGTATGCTCCTCCAGTTAGGGGAAGCATTAAGTGTGTATCAAAT---
ATTACAGGAATACTATTGACAAGAGATGGTGGTGCTAATAATACC----------------------------
-------------------------------------------------------------------------
-------------------
GAGATCTTCAGACCGGCAGGAGGAAATATAAAGGATAATTGGAGAAGTGAATTATATAAATATAAAGTAGTAC
AAATTGAACCACTA---GGAATAGCACCCACCAGGGCAAAAAGAAGAGTGGTG--------------------
-------------------GAGAGACAGAAAAGAGCA---GTGGGA---ATAGGAGCT---------
ATGATCTTTGGGTTCTTAGGAGCA---GCAGGAAGCACTATG---GGCGCGGCGTCA---
ATAACGCTGACGGTACAG---GCCAGACAA---TTATTG---TCTGGTATAGTGCAACAGCAAAGC---
AATTTG---CTGAGG---GCTATAGAGGCGCAGCAGCATATGTTGCAACTCACAGTCTGGGGCATT
>JX448086
AATTACACAAATGGTACTTTAACAAACACCACGGAGGGGGGT-------------------------------
--------------------------------------------------------------------
AGCGACACTACTATCATCCTTCCATGCAAGATAAAGCAAATTATAAACATGTGGCAGGGAATAGGACAAGCAA
TGTATGCTCCTCCCATCAGGGGATATATTAATTGTACATCAAAT---
ATTACAGGAATACTATTGACAAGAGATGGTGACGGTAATATTAATATT-------------------------
-------------------------------------------------------------------------
```

AATGACAGCGAGATCTTCAGACCTGGAGGAGGAAATATGAAGGACAATTGGAGAAGTGAATTATATAAATATA
AAGTAGTACAAATTGAACCACTA---GGAATAGCACCTACCAGGGCAAAGAGAAGAGTGGTG-----------
----------------------------GAGAGAGAAAAAAGAGCA---GTGGGA---ATAGGAGCT------
---ATGATCTTTGGGTTCTTAGGAACA---GCAGGAAGCACTATG---GGCGCAGCGTCA---
ATAACGCTGACGGTACAG---GCCAGACAA---TTATTG---TCTGGTATAGTGCAACAGCAAAGC---
AATTTG---CTGAGG---GCTATAGAGGCGCAGCAGCATCTGTTGCAACTCACAGTCTGGGGCATT
>JX448312
AATTATACTTGCTCGGACAATGGGTGTAATGGCATT-------------------------------------
----
-
ATCAATGGCACTATCATACTTCCATGCAGGATAAAGCAAATTATAAACATGTGGCAAGGAGCAGGACAAGCAA
TGTATGCTCCTCCCATCAGGGGAAGCATTAATTGTGTATCAAAT---
ATTACAGGAATATTATTGACAAGAGATGGTGGTAATGGTGATGATACT-------------------------
----

AATAATACTGAGATCTTCAGACCTGGAGGAGGAAATATAAAGGACAATTGGAGAAGTGAATTATATAAATATA
AAGTAGTACAAATTGAACCACTA---GGAATAGCACCCTCCAGGGCAAAGAGAAGAGTGGTG-----------
----------------------------GAGAGAGAAAAAAGAGCA---GTGGGA---ATAGGAGCT------
---ATGATCTTTGGGTTCTTAGGAGCA---GCAGGAAGCACTATG---GGCGCGGCGTCA---
ATAACGCTGACGGTACAG---GCCAGACAA---TTATTG---TCTGGTATAGTGCAACAGCAAAGC---
AATTTG---CTGAGG---GCTATAGAGGCGCAACAGCATATGTTGCAACTCACAGTCTGGGGCATT
>JN388191
ACTCATACTACT---

TTTAATAAAACTATCAGACTTCCATGCAAGATAAAGCAAATTATAAACATGTGGCAGAGAACAGGACAAGCAA
TGTATGCTCCTCCCATCAGTGGAAACATTAACTGTGTATCAAAT---
ATTACAGGAATACTATTGACAAGGGATGGTGGTAATAATACTAAT----------------------------
----

GAAACCTTTAGACCTGGAGGAGGAAACATAAAAGACAATTGGAGAAGTGAATTATATAAATATAAAGTAGTAG
AAATTGAACCACTA---GGAATAGCGCCCACCAGGGCAAAGAGAAGAGTGGTG--------------------
-------------------GAGAGAGAAAAAAGAGCA---GTGGGA---ATAGGAGCT---------
ATGATCTTTGGGTTCTTAGGAGCA---GCAGGAAGCACTATG---GGCGCGGCGTCA---
ATAACGCTGACGGTACAG---GCCAGACAA---TTATTG---TCTGGTATAGTGCAACAGCAAAGC---
AATTTG---CTGAGG---GCTATAGAGGCGCAGCAGCATATGTTGCAACTCACAGTCTGGGGCATC
>JN388201
ACTTACAATGGAACCCTACATGATGATAAT---

AACAGTACCAATATCACATTTCCATGCAGGATAAAGCAGATTATAAACATGTGGCAGGGAGTAGGACAAGCCA
TGTATGCTCCTCCCATCAGTGGAAGAATTAATTGTGTATCAAAT---
ATTACAGGAATACTATTGACAAGAGATGGTGGTGATAGTAATAAG----------------------------
----

AAAAACGAGACCTTCAGACCTGGAGGAGGAAATATAAAGGACAATTGGAGAAATGAATTATATAAATATAAAG
TAGTAGAAATTGAACCATTA---GGAATAGCACCCACCAGGGCAAAGAGAAGAGTGGTG--------------
-------------------------GAGAGAGAAAAAAGAGCA---GTGGGA---ATAGGAGCT---------
ATGATCTTTGGGTTCTTAGGAGCA---GCAGGAAGCACTATG---GGCGCGGCGTCA---
ATAACGCTGACGGTACAG---GCCAGACAA---TTATTG---TCTGGTATAGTGCAACAGCAAAGC---
AATTTG---CTGAGG---GCTATAGAGGCGCAGCAGCATATGTTGCAACTCACAGTCTGGGGCATT
>GQ916585
TGCTATACAACAAAACTGTTTAATAATACTTGCATGGAAAATGGAACCAAG----------------------
----

TGTAATGACACTATCACACTTCCATGCAAGATAAAGCAAATTATAAACATGTGGCAGGGAATAGGACAAGCAA
TGTATGCTCCTCCCATCAGTGGAACAATTAATTGTGCATCAAAT---
ATTACAGGAATACTATTGACAAGAGATGGTGGTAAGAATACTTCAGTT-------------------------
----

AACGGGACCGAGATCTTCAGACCTGGAGGAGGAAATATGAAGGACAATTGGAGAAGTGAATTATATAAATATA

AAGTAGTACAAATTGAACCACTA---GGAATAGCACCCACCAAGGCAAAGAGAAGAGTGGTG-----------
----------------------------AAGAGACAAAAAAGAGCAGTAGTAGGA---ATAGGAGCT------
---ATGATCTTTGGGTTCTTAGGAGCA---GCAGGAAGCACTATG---GGCGCAGCGTCA---
ATAACGCTGACGGTACAG---GCCAGACAA---TTATTG---TCTGGTATAGTGCAACAGCAAAGC---
AATTTG---CTGAGG---GCTATAGAGGCGCAGCAGAATATGTTGCAACTCACAGTCTGGGGCATT
>GQ916588
TGC--

TATAATAACACTCTCATACTTCCATGTAAGATAAAACAAATTATAAACATGTGGCAGGGAGTAGGACAAGCAA
TGTATGCTCCTCCCATCAATGGAAGAATTAATTGTGTATCAAAT---
ATTACAGGAATACTATTGACAAGAGATGGTGGTGTTAATAATGGGACT-------------------------
----

AACGACACCGAGATCTTCAGACCTGGAGGAGGAAATATAAAGGACAATTGGAGAAGTGAATTATATAAATATA
AAGTAGTACAAATTGAACCACTA---GGAATAGCACCCACCAGGGCAAAGAGAAGAGTGGTG-----------
----------------------------GCGAGAGAAAAAAGAGCA---GTGGGA---ATAGGAGCT------
---GTGATCTTTGGGTTCTTAGGAGCA---GCAGGAAGCACTATG---GGCGCGGCGTCA---
ATAACGCTGACGGTACAG---ACCAGACAA---TTATTG---TCTGGTATAGTGCAACAGCAAAGC---
AATTTG---CTGAGA---GCTATAGAGGCGCAGCAGCATCTGTTGCAACTCACAGTCTGGGGCATT
>GQ916592
AGTTACTTAGAAAATGGAACCATGTGG--

GAAAATAACAGTATCATACTTCTATGCAGGATAAAGCAAATTATAAATATGTGGCAGGGAGTAGGACAAGCAA
TGTATGCTCCTCCCATCAGTGGCTTAATTAAGTGTGTATCGAAT---
ATTACAGGAATACTATTGACAAGAGATGGTGGTAATAGTAATGAGACTAAT----------------------
----

AGTACTGATACTGAGATCTTCAGACCTACAGGAGGAAATATAAAGGACAATTGGAGAAGTGAATTATATAAAT
ACAAAGTAGTACAAATTGACCCACTA---GGAATAGCACCCACCAAGGCAAAGAGAAGAGTGGTG--------
-------------------------------GAAAGAGAAAAAAGAGCA---GTGGGA---CTAGGAGCT---
------ATGATCTTTGGGTTCTTAGGAGCA---GCAGGGAGCACTATG---GGCGCGGCGTCA---
ATAGCGCTGACGGGACAG---GCCAGACAA---TTATTG---TCTGGTATAGTGCAACAGCAAAGC---
AATTTG---CTGAGG---GCTATAGAGGCGCAGCAGCATATGTTGCAACTCACTGTCTGGGGCATT
>GQ916580
ACCTACAGAAATGAA--

AACACGGGCAGTATCATACTCCCATGCAAAATAAAGCAAATTATAAACATGTGGCAGGGAGTAGGACGAGCAA
TGTATGCTCCTCCCATCAGTGGAACAATTAATTGTGTATCAAAT---
ATTACAGGAATACTATTGACAAGAGATGGGGGTGGTGGTGATGAGGTG-------------------------
----

AATAACGAGACCGAGATCTTTAGACCTGGAGGAGGAAACATAAAGGACAATTGGAGAAATGAATTATATAAAT
ATAAAGTAGTACAAATTGAACCACTA---GGAATAGCACCCACCAGGGCAAAGAGAAGAGTGGTG--------
-------------------------------GAGAGAGAAAAAAGAGCA---GTGGGA---ATAGGAGCT---
------ATGATCTTTGGGTTCTTAGGAGCA---GCAGGAAGCACTATG---GGCGCGGCGTCA---
ATAACGCTGACGGTACAG---GCCAGACAA---TTATTG---TCTGGTATAGTGCAACAGCAAAGC---
AATTTG---CTGAGG---GCTATAGAGGCGCAGCAGCATCTGTTGCAACTCACAGTCTGGGGCATT
>KT185994
AATTACACAGAGAATAATACCACGGAGGGG---

-
TGGAATGGCACTATCATACTTCCATGCAGGATAAAGCAAATTGTAAACATGTGGCAGGGAGCAGGACAAGCAA
TGTATAATCCTCCCATCAGTGGAAAAATTAATTGTGTATCAAAT---
ATTACAGGAATACTATTGACAAGAGATGGTGGTAATAATAATGAGACT-------------------------
----

AATGGGACTGAGACTTTCAGACCTGGAGGAGGAAATATAAAGGACAATTGGAGAAATGAATTATATAAGTATA
AAGTAGTACAAATTGAACCACTA---GGAATAGCACCCACCAGGGCAAAGAGAAGAGTGGTG-----------
----------------------------GAGAGAGAGAAAAGAGCA---GTGGGA---ATAGGAGCT------
---ATGATCTTTGGGTTCTTGGGAGCA---GCAGGAAGCACTATG---GGCGCGGCGTCA---

ATAACGCTGACGGTACAG---GCCAGACAA---TTATTG---TCTGGTATAGTGCAGCAGCAAAGC---
AATTTG---CTGAGG---GCTATAGAGGCGCAGCAGCATATGTTGCAACTCACAGTCTGGGGCATT
>KY580553
AATCATACTTGCGAAGGAAATGAAGGGTGTAATGGCACTAGC-------------------------------
----
-
AGTAATGGCACTATCATACTTCCATGCAGGATAAAGCAAATTATAAACATGTGGCAGGGAGCAGGACAAGCAA
TGTATGCTCCTCCCATCAGAGGAACAATTAATTGTGTATCAAAT---
ATTACAGGACTACTATTGACAAGAGATGGTGGTAAAGATAATAGTACT-------------------------
----

AGTGGTAACGAGACCTTCAGACCTGGAGGAGGAAATATAAAAGACAATTGGAGAAGTGAATTATATAAATATA
AAGTAGTACAGATTGAACCACTA---GGAATAGCACCTACCAGGGCAAAGAGAAGAGTGGTG-----------
----------------------------CAGAGAGAAAAAAGAGCA---GTGGGA---ATAGGAGCT------
---ATGCTCTTTGGGTTCTTAGGAGCA---GCAGGAAGCACTATG---GGCGCGGCGTCA---
ATAACGCTGACGGTACAG---GCCAGACAA---TTATTG---TCTGGTATAGTGCAACAGCAAAGC---
AATTTG---CTGAGG---GCTATAGAGGCGCAGCAGCATCTGTTGCAACTCACAGTCTGGGGCATT
>JX447238
ACTTACACAGGAAAAGAAAATGACACCAGGGAAGGGAAG----------------------------------
----
AATGACAATGAAATCATTCTTCCATGCAGAATAAAGCAAATTATAAACATGTGGCAGAGAGTAGGACAAGCAA
TGTATGCTCCTCCCATTAGAGGAAGAATTAATTGTGTATCAAAT---
ATTACAGGAATACTATTGACAAGAGATGGTGGTAATAATGTAAATATG-------------------------
----

ACTAATGAGACCTTCAGGCCTGGAGGAGGACATATAAAGGACAATTGGAGAAGTGAATTGTACAAATATAAAG
TAGTAGAAATTGAACCATTA---GGAATAGCACCCACCAAGGCAAAGAGAAGAGTGGTG--------------
-------------------------GAAAGACAAAAAAGAGCA---GTGGGA---ATAGGAGCT---------
ATGATCTTTGGGTTCTTAGGAGCA---GCAGGAAGCACTATG---GGCGCAGCGTCA---
ATAACGCTGACGGTACAG---GCCAGACAA---TTATTG---TCTGGTATAGTGCAACAGCAAAGC---
AATTTG---CTGAGG---GCTATAGAGGCGCAACAGCACATGTTGCAACTCACAGTCTGGGGCATT
>JN388224
TTTCAGACCAATGTCTGCAATGGGACG--

TGTAATGGCAATATCACACTTCCATGCAGGATAAAGCAAATTATAAACATGTGGCAGGAAGTAGGACAAGCAA
TGTATAACCCTACCAAAAGAGGAGAAATTAAGTGTGTATCAAAT---
ATTACAGGAATACTATTGACAAGAGATGGTGGTAGTAATAATAGG----------------------------
----

TCCAACGAGACCTTCAGACCTGGAGGAGGAAATATGAAGGACAATTGGAGAAGTGAATTATATAAATATAAAG
TAGTAGAAATTGAACCACTA---GGAATAGCACCCACCAGGGCAAAGAGAAGAGTGGTG--------------
-------------------------GAGAGAGAAAAAAGAGCA---ATAGCA---ATAGGAACT---------
ATGCTCTTTGGGTTCTTAGGAGCA---GCCGGAAGCACTATG---GGCGCGGCGTCA---
CTAACGCTGACGGTACAG---GCCAGACAA---TTATTG---TCTGGTATAGTGCAACAGCAAAGC---
AATTTG---CTGAGG---GCTATTGAGGCGCAGCAGCATCTGTTGCAACTCACAGTCTGGGGCATT
>JX512901
ACTTACACAGGAAATGAACCTATGGGGAATAGGACCGAGGGG-------------------------------
----
AATAATACCAATATCATACTTCCATGTAAGATAAAGCAAATTATAAACATGTGGCAGGGAGTAGGACAAGCAA
TGTATGCTCCTCCCATCAGAGGAGCTATTACATGTGTATCAAAT---
ATTACAGGAATACTATTGACAAGAGATGGTGGTAATAGTCCTAAT----------------------------
----

GAGACCTTCAGACCTGAAGGAGGAAATATAAAGGACAATTGGAGAAGTGAATTATATAAATATAAAGTAGTAC
AAATTGAACCACTA---GGAGTAGCACCTACCAGGGCAAAGAGAAGAGTGGTG--------------------
-------------------GAGAGAGAAAAAAGAGCA---GTGGGA---ATAGGAGCT---------
ATGATCTTTGGGTTCTTAGGAGCA---GCAGGAAGCACTATG---GGCGCAGCGTCA---
ATAACGCTGACGGTACAG---GCCAGACAA---TTGTTG---TCTGGTATAGTGCAACAGCAAAGC---
AATTTG---CTGAGG---GCTATAGAGGCGCAGCAGCATCTGTTGCAACTCACAGTCTGGGGCATT

>KU230434
TCTTACATAAATGAAACCACACCTGGG--

TATAATGGCACTATCATACTCCCATGCAGGATAAAACAAATTATAAACATGTGGCAGGGAGTAGGACAAGCAA
TGTATGCTCCTCCCATCAGGGGAATCATTAGTTGTGTATCAAAT---
ATTACAGGAATATTATTGACAAGAGATGGTGGTAATAATAGCACG----------------------------
----

AATGAGACTGAGACCTTCAGACCTGGAGGAGGAAATATAAAGGACAATTGGAGAAGTGAATTATATAAATATA
AGGTAGTACAAATTGAACCACTA---GGACTAGCACCCACCAAGGCAAAGAGAAGAGTGGTG-----------
----------------------------GAGAGAAAAAAAAGAGCA---GTGGGA---ATAGGAGCT------
---ATGATCTTTGGGTTCTTAGGAGCA---GCAGGAAGCACTATG---GGCGCGGCGTCA---
ATAACGCTGACGGTACAG---GCCAGACAA---TTATTG---TCTGGTATAGTGCAACAGCAAAGC---
AATTTG---CTGAGA---GCTATAGAGGCGCAACAGCATATGTTGCAACTCACAGTCTGGGGCATT
>KU230435
AATCATAATTGCACCGAGGAGAGGTGTAATAGCACTACCAATAGCACT-------------------------
----
-
AGCACTGGCAATATCACACTTCCATGTAGAATAAAGCAGATAATAAACATGTGGCAGGGAGTAGGACAAGCAA
TGTACGCTCCTCCCATTAAGGGAAATGTTAGTTGTCTATCAAAT---
ATCACAGGAATACTATTGACAAGAGATGGTGATAATGCCAGTGAT----------------------------
----

AGTGAGACCTTCAGACCGGGAGGAGGAAATATAAAAGACAATTGGAGAAGTGAATTATATAAATATAAAGTAG
TACAAATTGAACCACTA---GGAATAGCACCCACCAGGGCAAAGAGAAGAGTGGTG-----------------
----------------------GAGAGAGAAAAAAGAGCA---GTGGGA---ATAGGAGCT---------
ATGATCTTTGGGTTCTTGGGAGCA---GCAGGAAGCACTATG---GGCGCGGCGTCA---
ATAACGCTGACGGTACAG---GCCAGACAA---TTATTG---TCTGGTATAGTGCAACAGCAAAGC---
AATTTG---CTGAGA---GCTATAGAGGCGCAACAACATATGTTGCAACTCACAGTCTGGGGCATT
>KU230437
ACTTACACAGACGTGAATAATAAAACCAAT---

AAGAATGACACAATCATAATTCCATGCAGAATAAAGCAAATTATAAACATGTGGCAGAGAGTAGGACAAGCAA
TGTATGCTCCTCCCATCAAGGGAAGGATTAGTTGTGTATCAAAT---
ATTACAGGAATACTCTTGACAAGAGATGGTGGTAATAATACTGAT----------------------------
----

GCTAATGAGACCTTCAGACCTGAAGGAGGAAATATGAAGGACAATTGGAGAAATGAATTATATAAATATAAAG
TAGTACAAATTGAACCACTA---GGAATAGCACCCACCAGGGCAAGGAGAAGAGTGGTG--------------
-------------------------GACAGAGAAAAAAGAGCA---GTGGGA---ATAGGAGCT---------
TTGATCTTTGGGTTCTTAGGAGCA---GCCGGAAGCACTATG---GGCGCGGCGTCA---
ATAACGCTGACGGTACAG---GCCAGACAA---TTATTG---TCTGGTATAGTGCAACAGCAAAGC---
AATTTG---CTGAGG---GCTATAGAGGCACAGCAGCATCTGCTGCAACTCACAGTCTGGGGCATT
>MG989575
ACTTACCTAAATGAAACAAATGAA---

TATAATGACACTATCATACTTCCATGCAGGATAAAGCAAATGATAAACATGTGGCAGGGAGCAGGACAAGCAA
TGTATGCTCCTCCCATCAGTGGAATAATTAATTGTACATCAAAT---
ATTACAGGAATACTATTGACAAGAGATGGTGGTGTTAATGAAAGT----------------------------
----

AATGGGACCGAGACCTTCAGACCTGGAGGAGGAAATATAAAGGACAATTGGAGAAATGAATTATATAAATATA
AAGTAGTACAAATTGAACCACTA---GGAATAGCACCCACCAGGGCAAAGAGAAGAGTGGTG-----------
----------------------------GAGAGAGAAAAAAGAGCA---GTGGGA---ATAGGAGCT------
---ATGCTCTTTGGGTTCTTAGGAGCA---GCAGGAAGCACTATG---GGCGCGGCGTCA---
ATAACGCTGACGGTACAA---GCCAGACAA---TTATTG---TCTGGTATAGTGCAACAGCAAAAC---
AATTTG---CTGAGA---GCTATAGAGGCGCAGCAGCATCTGCTGCAACTCACAGTCTGGGGCATT
>MG989515
AATTACACCAAGGAAGGG---

TGTAATGAAACTATCACACTTCCATGCAAGATAAAGCAAATAGTACGCATGTGGCAGGAAGCAGGACAGGCAA
TGTATGCTCCTCCCATCAGGGGAATGATTAATTGTGTATCAAAT---
ATTACAGGAATACTACTGACAAGAGATGGTGGTAGAAATAGTAGC----------------------------
----

AATGAGACCTTCAGACCTGAGGGAGGAAATATAAAGGACAATTGGAGAAATGAATTGTATAAATATAAGGTAG
TACAAATTGAACCACTA---GGAATAGCACCCACCAGGGCAAAGAGAAGAGTGGTG-----------------
----------------------GAGAGAGAAAAAAGAGCA---GTGGGA---ATAGGAGCT---------
ATGATCTTTGGGTTCTTAGGAGCA---GCAGGAAGCACTATG---GGCGCGGCGTCA---
ATAACGCTGACGGTACAA---GCCAGACAA---TTATTG---TCTGGTATAGTGCAACAGCAAAGC---
AATTTG---CTGAGA---GCTATAGAGGCGCAGCAACATCTGCTGCAACTCACAGTCTGGGGCATC
>MG989597
AATTATACAGGAAATGAAACCGAGGGGGAGTATAATGGCACT-------------------------------
----
-
AATAATGGCACTATCACACTTCCATGCAGGATAAAGCAAATAATAAACATGTGGCAGAAAATAGGACAAGCAA
TGTATGCTCCTCCCATCAGGGGAAGTATTAATTGTTTATCAAAT---
ATCACAGGAATACTATTGACAAGAGATGGTGGTGATGGTGGTGCT----------------------------
----

AATGAAAATGATACAGAGACCTTCAGACCTGGAGGAGGAAATATAAAGGACAATTGGAAAAATGAATTATATA
AATATAAAGTAGTACAAATTGAACCACTA---GGAATAGCACCCACCAGGGCAAAGAGAAGAGTGGTG-----
----------------------------------GAGAGAGAAAAAAGAGCA---GTGGGA---
ATAGGAGCC---------ATGATCTTTGGGTTCTTAGGAGCA---GCAGGAAGCACTATG---
GGCGCGGCGTCA---ATAACGCTGACGGTACAG---GCCAGACAA---TTATTG---
TCTGGCATAGTGCAACAGCAAGGT---AACTTG---CTGAGG---
GCTATAGAGGCGCAGCAGCATCTGTTGCAACTCACAGTCTGGGGCATT
>MG989530
ATTTACGTAAGAAATGAAACG--

TATAATGACACTATCATACTCCCATGCAAGATAAAGCAAATTATAAACATGTGGCAGGGAGTAGGACAAGCAA
TGTATGCTCCTCCCATTAGAGGAAGCATTAACTGTGCATCAAAT---
ATTACAGGAATATTATTGACAAGAGATGGTGGTGACGAAAATAGA----------------------------
----

AGCAAAGAGATCTTCAGGCCTGAAGGAGGAAACATAAAGGACAATTGGAGAAATGAATTATATAAATATAAAG
TAGTACAAATTGAACCACTA---GGGGTAGCACCCACCAGGGCAAAGAGAAGAGTGGTG--------------
-------------------------GAGAGAGAAAAAAGAGCA---GTGGGA---ATAGGAGCT---------
ATGATCTTTGGGTTCTTAGGAGCA---GCAGGAAGCACTATG---GGCGCAGCGTCA---
CTAACGCTGACGGTACAG---GCTAGACAA---TTATTG---TCTGGTATAGTGCAACAGCAAAGC---
AATTTG---CTGAGG---GCTATAGAGGCGCAGCAGCATCTGTTGCAACTCACAGTCTGGGGCATT
>MG989559
AATCATAATTGCACCGAGGAGAGGTGTAATAGCACTACCAATAGCACT-------------------------
----
-
AGCACTGGCAATATCACACTTCCATGTAGAATAAAGCAGATAATAAACATGTGGCAGGGAGTAGGACAAGCAA
TGTACGCTCCTCCCATTAAGGGAAATGTTAGTTGTCTATCAAAT---
ATCACAGGAATACTATTGACAAGAGATGGTGATAATGCCAGTGAT----------------------------
----

AGTGAGACCTTCAGACCGGGAGGAGGAAATATAAAAGACAATTGGAGAAGTGAATTATATAAATATAAAGTAG
TACAAATTGAACCACTA---GGAATAGCACCCACCAGGGCAAAGAGAAGAGTGGTG-----------------
----------------------GAGAGAGAAAAAAGAGCA---GTGGGA---ATAGGAGCT---------
ATGATCTTTGGGTTCTTGGGAGCA---GCAGGAAGCACTATG---GGCGCGGCGTCA---
ATAACGCTGACGGTACAG---GCCAGACAA---TTATTG---TCTGGTATAGTGCAACAGCAAAGC---
AATTTG---CTGAGA---GCTATAGAGGCGCAACAACATATGTTGCAACTCACAGTCTGGGGCATT
>MG989562
TCTTACATAAATGAAACCACACCTGGG--

TATAATGGCACTATCATACTCCCATGCAGGATAAAACAAATTATAAACATGTGGCAGGGAGTAGGACAAGCAA

TGTATGCTCCTCCCATCAGGGGAATCATTAGTTGTGTATCAAAT---
ATTACAGGAATATTATTGACAAGAGATGGTGGTAATAATAGCACG----------------------------
----

AATGAGACTGAGACCTTCAGACCTGGAGGAGGAAATATAAAGGACAATTGGAGAAGTGAATTATATAAATATA
AGGTAGTACAAATTGAACCACTA---GGACTAGCACCCACCAAGGCAAAGAGAAGAGTGGTG-----------
----------------------------GAGAGAAAAAAAAGAGCA---GTGGGA---ATAGGAGCT------
---ATGATCTTTGGGTTCTTAGGAGCA---GCAGGAAGCACTATG---GGCGCGGCGTCA---
ATAACGCTGACGGTACAG---GCCAGACAA---TTATTG---TCTGGTATAGTGCAACAGCAAAGC---
AATTTG---CTGAGA---GCTATAGAGGCGCAACAGCATATGTTGCAACTCACAGTCTGGGGCATT
>KT185904
ACTTACATAAATAACAATGGCACT---

ATCAATGGCACTATCATACTTCCATGCAGGATAAAGCAAATTATAAACATGTGGCAGGAAGTAGGACAAGCAA
TGTATGCTCCCCCCATCAGGGGAAGCATTAAATGTAGCTCAAAT---
ATTACAGGAATATTATTAACAAGAGATGGTGGTAGCAGTAATGGC----------------------------
----

AGTAATGAGATCTTCAGACCTACAGGAGGAAATATAAAAGACAATTGGAGAAATGAACTATATAAATATAAAG
TAGTACAAATTGAACCACTA---GGAGTAGCACCCACCAAGGCAAAAAGAAGAGTGGTG--------------
-------------------------GAGAGAGAAAAAAGAGCA---GTGGGA---ATAGGAGCT---------
ATGATCTTTGGGTTCTTAGGAGCA---GCAGGAAGCACTATG---GGCGCGGCGTCA---
ATAACGCTGACGGTACAA---GCCAGACAA---TTATTG---TCTGGTATAGTGCAACAGCAAAGC---
AATTTG---CTGAGG---GCTATAGAGGCGCAGCAGCATATGTTGCAACTCACAGTCTGGGGCATT
>MG989527
ACTTACACCCAGGAG--

AATAATGGCACTATCATACTTCCATGCAAGATAAAACAAATTGTAAGAATGTGGCAAGGAGCAGGACAAGCAA
TGTATGCTCCTCCCATCAAGGGAATAATTAATTGTGTATCAAAT---
ATTACAGGAATACTATTGACAAGAGATGGTGGGAGGGGTAACCATACTGCT----------------------
----

GATAACGAGACCTTCAGACCTGAAGGAGGAGACATGAAGGACAATTGGAGAAGTGAATTATATAAATATAAAG
TAGTACAGATTGAACCACTA---GGAATAGCACCCACCAGGGCAAGGAGAAGAGTGGTG--------------
-------------------------GAGAGAGAAAAAAGAGCA---GTGGGA---ATAGGAGCT---------
ATGATCTTTGGGTTCTTAGGAGCA---GCAGGAAGCACTATG---GGCGCGGCGTCG---
ATAACGCTGACGGTACAG---GCCAGACAA---TTATTG---TCTGGTATAGTGCAACAGCAAAGC---
AATTTG---CTGAGA---GCTATAGAGGCGCAGCAGCATCTGTTGCAACTCACAGTCTGGGGCATT
>MG989617
ACTCACAGAGGAAATGAAACCATGGAG--

GGGAATGGCAATATCACAATTCCATGCAGGATAAAACAAATCATAAACATGTGGCAGGAAACAGGACAAGCAA
TGTATGCTCCTCCCGTCAGGGGAGTAATTAGGTGTGTATCAAAT---
ATTACAGGAATACTATTGACAAGAGATGGTGGTGGTTCTAATAATGCC-------------------------
----

ACTAATGAGACCTTCAGACCTGGGGGAGGAAATATAAAAGACAACTGGAGAAGTGAATTATATAAATATAAAG
TAGTACAAATTGAACCATTA---GGAATAGCACCCACCAAGGCAAAGAGGAGAGTGGTG--------------
-------------------------GAGAGAGAAAAAAGAGCA---GTGGGA---ATAGGAGCT---------
ATGATCTTTGGGTTCTTAGGAGCA---GCAGGAAGCACTATG---GGCGCGGCGTCA---
ATAACGCTGACGGTACAG---GCCAGACAA---TTATTG---TCTGGTATAGTGCAACAGCAAAGC---
AATTTG---CTGAGA---GCTATAGAGGCGCAACAGCATATGTTGCAACTCACAGTCTGGGGCATT
>MF957454
AATCATAGTTGCACAGAGGGACATGGGTGTAACAGTACTACCAAAGAT-------------------------
----
-
GCTAACGGTACTATCACACTTCCATGCAGGATAAAGCAAATTATAAAAATGTGGCAGGGAGTAGGACAAGCAA
TGTATGCTCCTCCCATCAGTGGAAGTATTAATTGTAGATCAAAT---
ATTACAGGAATATTATTGACAAGAGATGGTGGTAAT-------------------------------------
----

AGTTCAAATGAGACCTTCAGACCTGAAGGAGGAAATATAAAAGACAACTGGAGGAGTGAATTATATAAATATA
AAGTAGTGCAAATTGAACCACTA---GGAGTAGCACCCACCAAGGCAAAAAGAAGAGTGGTG----------
----------------------------GACAGACAAAAAAGAGCA---GTGGGA---CTAGGAGCT------
---CTGATCTTTGGATTCTTAGGAGCA---GCAGGAAGCACTATG---GGCGCGGCGTCA---
ATAACGCTGACGGTACAG---GCCAGACAA---TTATTG---TCTGGTATAGTGCAACAGCAAAGC---
AATTTG---CTGAGG---GCTATAGAGGCGCAGCAGCATATGTTGCAACTCACAGTCTGGGGCATT
>MG989509
AATTATAATTGCTCAAAGAATAATACTTGCGAGGGAAATGAGGGAAATAAT---------------------
----
-
CCTAATGGAACCATCATACTTCCATGCAAGATAAAGCAAATTATAAACATGTGGCAGAGAACAGGACAAGCAA
TGTATGCTCCTCCCATCGGGGGATGGATTAATTGTACATCAAAT---
ATTACAGGAATATTACTGACAAGAGATGGGGGTGAGAATAACAAGACTAAC---------------------
----

GAAACCGAGACCCCCGAGACCTTCAGACCTGGAGGAGGGAATATAAAGGACAATTGGAGAAGTGAATTATATA
AATATAAAGTAGTACAAATTGAACCACTA---GGAATAGCACCCACCAGGGCAAAGCGAAGAGTGGTG-----
----------------------------------GACAGAGAAAAAAGAGCA---GTGGGA---
ATAGGAGCT---------TTGATCTTTGGGTTCTTAGGAGCA---GCAGGAAGCACTATG---
GGCGCGGCGTCA---ATAACGCTGACGGTACAG---GCCAGACAA---TTATTG---
TCTGGTATAGTGCAACAGCAAAGC---AATTTG---CTGAGA---
GCTATAGAGGCGCAGCAGCATATGTTGCAACTCACAGTCTGGGGCATT
>MG989603
AATTATACAAATGGAACCATACAGGGG---

-
GGTAATGATACTATCACACTTCCATGCAGAATAAAACAAATAATAAGAATGTGGCAGAGAACAGGACAAGCAA
TATATGCTCCTCCTGTCAGTGGAATAATTAATTGTGTATCAAAT---
ATTACAGGAATACTATTGACAAGAGATGGTGGTGGT-------------------------------------
----

AACGAGACCGAGACCTTCAGACCAGGAGGAGGAAATATGAAGGATAATTGGAGAAATGAATTATATAAATATA
AAGTAGTACGAATTGAACCACTA---GGGGTAGCACCCACAAGGGCAAAGAGAAGAGTGGTG----------
----------------------------GAGAGAGAAAAAAGAGCA---GTGGGA---CTAGGAGCT------
---ATGATCTTTGGGTTCTTAGGAGCA---GCAGGAAGCACTATG---GGCGCAGCGTCA---
ATAACGCTGACGGTACAG---GCCAGACAA---TTATTG---TCTGGTATAGTGCAACAGCAAAGC---
AATTTG---CTGAGG---GCTATAGAGGCGCAACAGCGTCTGTTGCAACTCACAGTCTGGGGCATT
>MG989564
ACTTATGGGGTTAATAATACAAATGAAACCAACAAG-------------------------------------
----
TATAATGACACTATCACACTCCCATGCAGGATAAAGCAAATTATAAACATGTGGCAGAGAACAGGACAGGCAA
TGTATGCTCCTCCCATCAGGGGAGCAATAAATTGTGTATCAAAT---
ATTACAGGAATACTATTGGTAAGAGATGGTGGTCCTAACAGGACT----------------------------
----

AACGAGACTTTCAGACCTGGAGGAGGAAACATGAAGGACAATTGGAGGAGTGAATTATATAAATATAAAGTAG
TACAAATTGAACCACTA---GGAATAGCACCCACCAGGGCAAAGAGAAGAGTGGTG----------------
----------------------GACAGAGAAAAAAGAGCA---GTGGGA---ATAGGAGCT---------
ATGATCTTTGGGTTCTTAGGAGCA---GCAGGAAGCACTATG---GGCGCGGCGTCA---
ATAACGCTGACGGTACAG---GCCAGACAA---TTATTG---TCTGGTATAGTGCAACAGCAAAGC---
AATTTG---CTGAGA---GCTATAGAGGCGCAACAGCATATGTTGCAACTCACAGTCTGGGGCATT
>MG989572
ACTTACCCGTTAAATAGTACTAACATAGAAAATAATACCACGAAGGGG------------------------
----
TATAATGACCCTATCATACTTCCATGCAGAATAAAGCAAATCATAAACTTGTGGCAGGGAGTAGGACAAGCAA
TGTATGCTCCTCCCATCAGGGGAAGCATTAATTGTGTATCAAAT---
ATTACAGGACTACTATTGATAAGGGATGGTGGTATGCATAATGGGACCGAT---------------------
----

AATACCAAGGAGATCTTCAGACCTGGAGGAGGAAATATGAAGGACAATTGGAGAAATAAATTATATAAATATA
AAATAGTACAAATTGAACCGCTA---GGAGTAGCACCCACCAGGGCAAAGAGAAGAGTGGTG-----------
----------------------------GGGAGAGAAAAAAGAGCA---GTGGGA---ATAGGAGCT------
---TTGATCTTTGGGTTCTTGGGAGCA---GCAGGAAGCACTATG---GGCGCGGCGTCA---
ATAACGCTGACGGTACAG---GCCAGACAA---TTACTG---TCTGGTATAGTGCAACAGCAAAGC---
AATTTG---CTGAGA---GCTATAGAGGCGCAGCAGCATCTGTTGCAACTCACAGTCTGGGGCATT
>MG989538
AATTATACGTGGGCTAATAATACATTTAATGGAACCACG----------------------------------
----
-
GAAAATGGGACTATCATACTTCCATGCAGGATCAAGCAAATTGTAAACATGTGGCAGGGAGCAGGACAAGCAA
TATATGCTCCTCCCATCAGGGGAAATATTAGTTGTGCATCAAAT---
ATTACAGGAATACTATTAACAAGAGATGGTGGTAATAATAATGAAACA-------------------------
----

AACGGGACAGAGATCTTCAGACCTGGAGGAGGAAATATAAAGGACAATTGGAGAAGGGAATTATATAAATATA
AAGTAGTACAAATTGAACCACTA---GGAGTAGCACCCACCAAGGCAAAGAGAAGAGTGGTG-----------
----------------------------GAGAGAGAAAAAAGAGCA---GTGGGA---CTAGGAACT------
---ATGATCTTTGGGTTCTTAGGAGCA---GCAGGAAGCACTATG---GGCGCAGCGTCA---
ATAACGCTGACGGTACAA---GCCAGACAA---TTATTG---TCTGGTATAGTGCAACAGCAAAGC---
AATTTG---CTGAGG---GCTATAGAGGCGCAGCAGCATATGTTGCAACTCACAGTCTGGGGCATT
>MG989614
ACTTACAATGGAACCCTGGTGAAAAACAATACT--

GGAAGTGGAAATATCACACTTCCATGTAGGATAAAGCAAATTATAAACATGTGGCAAGGAGCAGGACAAGCAA
TGTATGCTCCTCCCGTTAGTGGAAATATTAATTGTACATCAAAT---
ATTACAGGAATACTATTAACAAGAGATGGTGGTAATAATAATACA----------------------------
----

GCTAACGAGACCTTCAGACCTGGAGGAGGAAATATAAAGGACAATTGGAGAAGTGAATTATATAAATATAAAG
TAGTACAAATTGAACCACTA---GGAATAGCACCCACCAGGGCAAGGAGAAGAGTGGTG--------------
-------------------------GAGAGAGAAAAAAGAGCA---GTGGGA---ATAGGAACT---------
ATGATCTTTGGGTTCTTGGGAGCA---GCAGGAAGCACTATG---GGCGCGGCGTCA---
ATAACGCTGACGGTACAA---GCCAGACAA---TTATTG---TCTGGTATAGTGCAACAGCAAAGC---
AATTTG---CTGAGA---GCTATAGAGGCGCAGCAGCATATGTTGCAACTCACAGTCTGGGGCATT
>MG989496
AGCTACTTAAATAGAAACTTAAGTGAAATCGCAGTA-------------------------------------
----
GAGAACAAAACACTTATACTTCCATGTAGAATAAAGCAAATTATAAACATGTGGCAGGGAGTAGGACAAGCAA
TGTATGCTCCTCCCATCAGTGGGCCAATTAATTGTAAATCAAAT---
ATTACAGGAATACTGTTAACAAGAGATGGTGGTAATGTTACTGGG----------------------------
----

AATAATGAGACCTTCAGACCTACAGGAGGAAACATAAAGGACAATTGGAGAAGTGAATTATATAAATACAAAG
TAGTACAAATTGAACCACTA---GGAATAGCACCCACCAGGGCAAAGAGAAGAGTGGTG--------------
-------------------------GAGAGAGAAAAAAGAGCA---GTGGGA---ATAGGAGCT---------
ATGATCTTTGGGTTCTTAGGAGCA---GCAGGAAGCACTATG---GGCGCGGCGTCA---
ATAACGCTGACGGTACAG---GCCAGACAA---TTATTG---TCTGGTATAGTGCAACAGCAAAGC---
AATTTG---CTGAGA---GCTATAGAGGCGCAACAGCATATGTTGCAACTCACAGTCTGGGGCATT
>MG989539
AATTATAATTGCACAGATGGAAAATGTAATGTCACTAAGGAC-------------------------------
----
-
AATAATGGCACTATCATACTTCCATGCAGAATAAAACAAATTATAAACATGTGGCAAGGAGTAGGACAAGCAA
TGTATGCTCCTCCCATCAGTGGAATAATTAATTGTGTATCAGAC---
ATTACAGGAATACTATTGACAAGAGATGGTGGTAATAATAATAATAATACTAAT-------------------
----

AGTTCGGAAGAGATCTTCAGACCTGAAGGAGGAAACATAAAGGACAATTGGAGAAGTGAATTATATAAATATA
AAGTAGTACAAATTGAACCACTG---GGAATAGCACCCACCAAGGCAAAGAGAAGAGTGGTG-----------

----------------------------GAGAGAGAAAAAAGAGCA---GTGGGA---ATAGGAGCT------
---ATGATCTTTGGGTTCTTAGGAGCA---GCAGGAAGCACTATG---GGCGCGGCGTCA---
ATAACGCTGACGGTACAG---GCCAGACAA---TTATTG---TCTGGTATAGTGCAACAGCAAAGC---
AATTTG---CTGAGA---GCTATAGAGGCGCAGCAGCATATGTTGCAACTCACGGTCTGGGGCATT
>AY713424
ACTTACAGAGGAAATGAAACCATGGAGAGG---

AGTAATGGCACTATCATACTTCCATGCAAGATAAAGCAAATTATAAACATGTGGCAGGAAACAGGACAAGCAA
TGTATGCTCCTCCCATCAATGGAGAAATTAACTGTATATCAAAT---
ATTACAGGAATACTATTGACAAGAGATGGTGGTATTAATAGAACG----------------------------
----

TATAACGAGACCTTCAGACCGGGAGGAGGAAATATGAAGGACAATTGGAGAAATGAATTATATAAATATAAAG
TAGTACAAATTGAACCACTA---GGAGTAGCACCCACCAGGGCAAAGAGAAGAGTGGTG--------------
-------------------------GAGAGAGAAAAAAGAGCA---GTGGGA---ATAGGAATT---------
ATGATCTTTGGGTTCTTAGGAGCA---GCAGGAAGCACTATG---GGCGCGGCGTCA---
ATAACGCTGACGGTACAG---GCCAGACAA---TTATTG---TCTGGTATAGTGCAACAGCAAAGC---
AATTTG---CTGAGG---GCTATAGAGGCGCAGCAGCATCTGTTGCAACTCACAGTCTGGGGCATT
>JN944664
ACTTACAAAGGAAATGAAACCATAGAGGAG---

GGTGATAGCACTATTACAATTCCATGCAAGATAAAGCAAATTATAAACATGTGGCAGGGAACAGGACAAGCAA
TGTATGCTCCTCCCATCAGTGGAAGTATTAATTGTGTATCAAAT---
ATCACAGGAATACTATTGACAAGAGATGGTGGTAATAGTAATGAT----------------------------
----

AACAAGACCGAGACCTTCAGACCTGGAGGAGGAAATATAAAAGACAATTGGAGAAGTGAATTATATAAATATA
AAGTAGTACAAATTGAACCACTA---GGAGTAGCACCCACCAAGGCAAAGAGAAGAGTGGTG-----------
----------------------------GAGAGAGAAAAAAGAGCA---GTGGGA---ATAGGAGCT------
---ATGATCTTTGGGTTCTTAGGAGCA---GCAGGAAGCACTATG---GGCGCGGCGTCA---
ATAACGCTGACGGTACAG---GCCAGACAA---TTATTG---TCTGGTATAGTGCAACAGCAAAGC---
AATTTG---CTGAGG---GCTATAGAGGCGCAGCAGCATCTGTTGCAACTCACAGTCTGGGGCATT
>KJ953503
ACTTACAATGGCACT--

ATCATACTTCCATGCAAGATAAAGCAAATTATAAACATGTGGCAGGGAGTAGGACAAGCAATGTATGCTCCTC
CCATCAGGGGAAGAATTAATTGTGTATCAAAT---
ATTACAGGAATACTATTGACAAGAGATGGTGGTAAGACTAATGAGACT-------------------------
----

AACGTGACTGAGATTTTCAGACCTGGAGGAGGAAATATGAAGGATAATTGGAGAAATGAATTATATAAATATA
AAGTAGTACAAATTGAACCACTA---GGAATAGCACCCACCAGGGCAAAGAGAAGAGTGGTG-----------
----------------------------GAGAGAGAAAAAAGAGCA---GTGGGA---ATAGGAGCT------
---ATGATCTTTGGGTTCTTAGGAGCA---GCAGGAAGCACTATG---GGCGCGGCGTCA---
ATAACGCTGACGGTACAG---GCCAGACAA---TTATTG---TCTGGTATAGTGCAACAGCAAAGC---
AATTTG---CTGAGG---GCTATAGAGGCGCAACAGCATCTGTTGCAACTCACAGTCTGGGGCATT
>KJ952288
AATCACACCATGGAG--

GGGTGTAACAATATCACACTTCCATGCAGGATAAAGCAGATTGTAAACATGTGGCAAGGAGTAGGACAAGCAA
TGTATGCTCCTCCCATCAGTGGACTCATTAATTGTTCATCAAAT---
ATTACAGGAATACTATTGACAAGAGATGGTGGTATTAATAACGAGACTGAA----------------------
----

GTGACTGAGACCTTTAGACCTGGAGGAGGAGATATAAGGGCCAATTGGAGAAGTGAATTATATAAATATAAAG
TAGTACAAATTGAACCACTA---GGCATAGCACCCACCAGGGCAAAGAGAAGAGTGGTG--------------
-------------------------GAGAGAGAAAAAAGAGCA---GTGGGA---ATAGGAGCT---------
ATGATCTTTGGGTTCTTAGGAGCA---GCAGGAAGCACTATG---GGCGCGGCGTCA---

ATAACGCTGACGGTACAG---GCCAGACAA---TTATTG---TCTGGTATAGTGCAACAGCAAAGC---
AATTTG---CTGAGA---GCTATAGAGGCGCAGCAGCATATGTTGCAACTCACAGTCTGGGGCATT
>KJ952992
GATCATACTGGCATAGAAAATGAAACCATAGGG--

-
AATGACACTACTATCATACTCCCATGCAAGATAAAGCAAATTATAAACATGTGGCAGGGAGTAGGACAAGCAA
TGTATGCTCCTCCCATCAGTGGAATAATTAATTGTGTATCAAAT---
ATTACAGGAATACTATTGACAAGAGATGGTGGAGATGGTGATACTAAT-------------------------
----

AACGGGACTGAGACCTTCAGACCTGGAGGAGGAAATATGAAGGACAATTGGAGAAGTGAATTATATAAATATA
AAGTAGTAGAAATTGAACCACTA---GGAATAGCACCCACCAGGGCAAAGAGAAGAGTGGTG-----------
----------------------------GAGAGACAAAAAAGAGCA---GTGGGA---ATAGGAGCT------
---ATGATCTTTGGGTTCTTAGGAGCA---GCAGGAAGCACTATG---GGCGCGGCGTCA---
ATAACGCTGACGGTACAG---GCCAGACAA---TTATTG---TCTGGTATAGTGCAACAGCAGAGC---
AATCTG---CTGAGG---GCTATAGAGGCGCAGCAGCATCTGCTGCAACTCACAGTCTGGGGCATT
>KJ953319
TCTTATGACCCAGAAAATGAAACCATGAAGGAGTATAATGGC-------------------------------
----
ACTAATGGCAATATCACACTTCCATGCAGGATAAAGCAAATTATAAACATGTGGCAGAGAACAGGACAAGCAA
TGTACGCTCCTCCCATCAGTGGAAGTATTAATTGTGTATCAAAT---
ATTACAGGAATACTATTGACAAGAGATGGTGGGGATAATGATGAT----------------------------
----

AACGAGACCTTCAGACCTGGAGGAGGAAACATAAAGGACAATTGGAGAAGTGAATTATATAAATATAAAGTAG
TACAAATTGAACCACTA---GGAATAGCACCCACCAGGGCAAAGAGAAGAGTGGTG-----------------
----------------------GAGAGAGAAAAAAGAGCA---GTGGGA---ATAGGAGCT---------
ATGATCTTTGGGTTCTTAGGAGCA---GCAGGAAGCACTATG---GGCGCGGCGTCA---
ATAACGCTGACGGTACAG---GCCAGACAA---TTATTG---TCTGGTATAGTGCAACAGCAAAGC---
AATTTG---CTGAGG---GCTATAGAGGCGCAGCAGCATATGTTGCAACTCACAGTCTGGGGCATT
>FJ185244
ACTTACCCAATAAATGGAACCATGGAGGGGTATAATGGCACT-------------------------------
----
ATTAATGACACTATCATACTTCCATGCAGGATAAAGCAAATTATAAACATGTGGCAGGAAGTAGGACAAGCAA
TGTATGCTCCTCCCATCAGGGGAAGAATTAATTGTGTATCAAAT---
ATTACAGGAATACTATTGACAAGAGATGGTGGTGCTAATAATACG----------------------------
----

AACGAGACCTTCAGACCTGGAGGAGGAAATATAAAGGACAATTGGAGAAGTGAATTATATAAATATAAAGTAG
TACAAATTGAGCCACTA---GGAATAGCACCCACCAGGGCAAAAAGAAGAGTGGTG-----------------
----------------------GAGAGAGAAAAAAGAGCA---GTGGGA---ATAGGAGCT---------
GTGATCTTTGGGTTCTTAGGAGCA---GCAGGAAGCACTATG---GGCGCGGCGTCA---
ATAACGCTGACGGTACAG---GCCAGACAA---TTATTG---TCTGGTATAGTGCAACAGCAAAGC---
AATTTG---CTGAGG---GCTATAGAGGCGCAGCAGCATATGTTGCAACTCACAGTCTGGGGCATT
>KX389609
AATCAATCA--

CAGGATGGAAATATCACACTCCCATGTAGGATAAAACAAATTGTAAGAATGTGGCAGAGAGTGGGACAGGCAA
TGTATGCCCCTCCCATTGCAGGAAACATTACATGTACACCAAAC---
ATTACAGGATTAATATTAACAAGAGATGGGGGGAACAAGAACAAT----------------------------
----

GAGACTGAGGTCATCAGACCAGGAGGAGGAGATATGAGGGACAACTGGAGAAGTGAACTATATAAGTATAAAG
TAGTAAAAATTAAACCACTA---GGAATAGCACCCACCAAGGCAAGGAGACGAGTAGTG--------------
-------------------------GGGAGAGAAAAAAGAGCA---GTTGGA---ATGGGAGCT---------
GTTCTCCTTGGGTTCTTGGGAACG---GCAGGAAGCACTATG---GGCGCAGCGTCA---
ATAACGCTGACGGTACAG---GTCAGACAA---TTATTG---TCTGGCATAGTGCAACAGCAAGGC---
AACTTG---CTGAGA---GCTATAGAGGCGCAGCAGAATCTGCTGAAACTCACAGTCTGGGGCATT
>KY346462

ACCTACAATGGTACAAGTAAT---

TCAAACACAACCATCACGATCCCATGTAGAATAAAGCAAATTATAAATATGTGGCAGCAGGTAGGACGAGCAA
TGTATGCCCCTCCCATTGCAGGAAACATAACATGTAAATCAAAT---
ATCACAGGACTACTATTGGTACGTGATGGAGGAAAGGGTGAAAAT-------------------------
----

GGTACAGAGATATTCAGACCTGGAGGAGGAGATATGAGGGACAATTGGAGAAGTGAATTATATAAATATAAAG
TGGTAGAAATTAAGCCATTG---GGAATAGCACCCAGTAAAGCAAAAAGGAGAGTGGTG-------------
------------------------GAGAGAGAAAAAAGAGCA---GTGGGA---ATAGGAGCT---------
GTGTTCCTTGGGTTCTTGGGAGCA---GCAGGAAGCACTATG---GGCGCGGCGTCA---
CTGACGCTGACGGTACAG---GCCAGACAA---TTGTTG---TCTGGTATAGTGCAACAGCAGAGC---
AATTTG---CTGAAG---GCTATAGAGGCGCAACAGCATTTGTTGCAACTCACAGTCTGGGGCATT
>HQ326137
TTTCATAGTACACAA---

AATAATTCAACCATCACAATCCCATGCAGAATAAAGCAAATTATAAATATGTGGCAGGAGGTAGGAAGAGCAA
TGTATGCCCCTCCCATTGCAGGAAATATAACATGTAGATCAAAT---
ATCACAGGACTACTATTGGCACGTGATGGAGGAGAAGGCAATGAGACA-----------------------
----

GAGAATGATACAGAGATATTCAGACCTGGAGGAGGAAATATGAGGGACAATTGGAGAAGTGAATTATATAAAT
ATAAAGTGGTAGAAATTAAACCATTG---GGAATAGCACCCACTAAAGCAAAAAGGAGAGTGGTG--------
------------------------------GAGAGAGAGAAAAGAGCA---GTGGGA---ATAGGAGCT---
------GTGTTCCTTGGGTTCTTGGGAGCA---GCAGGAAGCACTATG---GGCGCGGCGTCA---
ATAACGCTGACGGTACAG---GCCAGACAA---TTGCTG---TCTGGTATAGTGCAACAGCAAAGC---
AATTTG---CTGAGG---GCTATAGAGGCGCAACAGCATCTGTTGCAACTCACAGTCTGGGGCATT
>KP718937
AATTAC--

ACAGAGTACAATATAACTCTCCCATGCAAAATAAAGCAAATTGTAAGGATGTGGCAGAGAGTAGGACAAGCAA
TGTACGCCCCTCCTATCCAAGGAGAAATAAGGTGTGACTCAAAC---
ATTACAGGACTACTATTAACAAGAGATGGTGGGAATAATAGTACA-------------------------
----

AATGAAACCTTCAGACCTACAGGAGGAGATATGAAGGATAATTGGAGAAGTGAGTTATATAAGTATAAAGTGG
TGGAAATTAAACCACTA---GGAGTAGCACCCACCAAGGCAAAAAGAAGAGTGGTG----------------
---------------------GAGAGAGAAAAAAGAGCA---GTGGGA---ATAGGAGCT---------
GTGCTCTTTGGGTTCTTGGGAGCA---GCAGGAAGCACTATG---GGCGCAGCGTCA---
ATAACGCTGACGGTACAG---GCCAGACAA---TTATTG---TCTGGCATAGTGCAACAGCAAAGC---
AATTTG---CTGAAG---GCTATCGAGGCACAACAACATATGTTGAAACTCACTGTCTGGGGCATT
>AJ277823
AATTACCCAGGGTCA---

AATGACAATGACACTATAACTCTCCCATGCAGAATAAAGCAAATTGTACATATGTGGCAGAGAGTAGGACAAG
CAATGTATGCCCCTCCCATCCAAGGAAAAATAGAGTGTAAATCAAAC---
ATTACAGGACTACTATTAACAAGAGATGGTGGGAATAAAACACAA-------------------------
----

AATGAAACCTTCAGACCTACAGGAGGAGACATGAGGGACAATTGGAGAAGTGAATTATATAAGTATAAAGTAG
TGGAAATTAGACCACTA---GGAGTAGCACCCACCAAGGCAAAAAGAAGAGTGGTG----------------
---------------------CAGAGAGAAAAAAGAGCA---GTGGGA---ATAGGAGCT---------
GTGCTCTTTGGGTTCTTGGGAGCA---GCAGGAAGCACTATG---GGCGCGGCGTCA---
ATAACGCTGACGGTACAA---GCCAGACAA---TTATTG---TCTGGCATAGTGCAACAGCAAAGC---
AATTTG---CTGAAG---GCTATCGAGGCGCAACAGCAACTGTTGAAACTCACTGTCTGGGGCATT
>JF683802
AATTACACTGACATATATAATTCAACAGATAACAGC----------------------------------
----
ACAAATGACGCAAATCTCACTCTCCCATGCAGAATAAAGCAGATTGTACACATGTGGCAGAGAGTAGGGCAAG
CAATATATGCCCCTCCCATCCAGGGAGTAATAAGGTGTGAGTCAAAC---

ATTACAGGGCTACTGTTAACAAGAGATGGTGGGAATAATGTCACT----------------------------
----

AATGAGACCTTTAGACCCACAGGAGGAAATATGAAGGACAATTGGAGAAGTGAATTATATAAGTATAAGGTGG
TGGAGATTAAACCACTA---GGAGTAGCACCCACCAAGGCAAAAAGAAGAGTGGTG-----------------
----------------------GAGAGAGAAAAAAGAGCA---GTGGGA---ATAGGAGCT---------
GTGCTCTTTGGGTTCTTGGGAGCA---GCAGGAAGCACTATG---GGCGCAGCGTCA---
ATAACGCTGACGGTACAG---GCCAGACAA---TTATTG---TCTGGTATAGTGCAACAGCAAAGC---
AATTTG---CTGAAA---GCTATCGAGGCACAACAGCATCTGTTGAAACTCCCTGTCTGGGGCATT
>KU168270
AATTACACAGAAACAAATAGCACA---

AATAATGAGACTATAACTCTCTCATGCAGAATAAAGCAAATTGTAAATATGTGGCAGAGAGTAGGACGAGCAA
TGTATGCCCCTCCCATCCCAGGAGAAATAAGGTGTGACTCAAAC---
ATTACAGGACTATTATTAACAAGAGATGGTGGGAACAAGACCAAT----------------------------
----

GACGGTGAGGTCTTCAGACCTATAGGAGGAAATATAAGGGACAATTGGAGAAGTGAATTGTATAGGTATAAAG
TGGTGGCAATTGAACCACTA---GGAGTAGCACCCACCAGGGCAAAAAGAAGAGTGGTG--------------
-------------------------GAGAGAGAAAAAAGAGCA---GTGGGA---ATAGGAGCT---------
GTGCTCCTTGGGTTCTTGGGAGCA---GCAGGAAGCACTATG---GGCGCGGCGTCA---
ATAACGCTGACGGTACAA---GCCAGACAA---TTATTG---TCTGGCATAGTGCAACAGCAAGGC---
AATTTG---CTGAAG---GCTATTGAGGCACAACAGCAACTGTTGAGACTCACTGTCTGGGGCATT
>AF516184
TTTCAGAATGGTAATAGTACTTGGAATGATACTGCA-------------------------------------
----
GGAAATGGCACTATCACGCTCCCTTGCAGAATAAGACAAATTGTAAACATGTGGCAGGAAGTAGGAAGAGCAA
TGTATGCCCCTCCCATCAAAGGACAAATTAGATGTTCATCAAAT---
ATTACAGGGTTGCTATTAACAAGAGATGGTGGTAAAAATGAGAATACC-------------------------
----

ACGAATACCACCGAGATCTTCAGACCTGCAGGAGGAGATATGAGGGACAATTGGAGAAGTGAATTATATAAAT
ATAAAATAGTAAAAAATTGAACCATTA---GGAGTAGCACCCACCAAGGCAAAGAGAAGAGTGGTG-------
-------------------------------CAGAGAGAAAAAAGAGCA---GTGGGAACGATAGGAGCT---
------ATGTTCCTTGGGTTCTTGGGAACA---GCAGGAAGCACTATG---GGCGCAGCGTCA---
ATAACGCTGACGGTACAG---GCCAGGCAA---TTGTTG---TCTGGTATAGTGCAACAGCAGAGA---
AATCTG---CTGAGG---GCTATTGAGGCACAACAGCATCTGTTGCAACTCACAGTCTGGGGCATC
>AF457072
AGTCAAACTAATAGTACAGAGTCAGAA--

AATGATACAATAATCACACTCCCATGCAGAATAAAACAAATTATAAATATGTGGCAAGAAGTAGGAAAAGCAA
TGTATGCCCCTCCCATCGCAGGATTCATTAATTGTGCATCAAAT---
ATTACAGGGCTACTATTGACAAGAGATGGTGGGATAACTAATAGG----------------------------
----

TCTAACGAGACCTTCAGACCTGGAGGAGGAGATATGAGGGACAATTGGAGAAGTGAATTATATAAGTATAAGG
TAGTAAGAATTGAACCACTG---GGAGTAGCACCCACCAAGGCAAGGAGAAGAGTGGTG--------------
-------------------------GAAAGAGAAAAAAGAGCA---ATAGGA---CTAGGAGCT---------
ATGTTCCTTGGGTTCTTGGGAGCA---GCAGGAAGCACGATG---GGCGCAGCGTCA---
GTGACGCTGGCGGTACAG---GCCAGACAG---TTATTG---TCTGGTATAGTGCAACAGCAAAAT---
AATTTG---CTGAGG---GCTATAGAGGCGCAACAACATCTGTTGCAACTCACGGTCTGGGGCATT
>JF804812
ATCTACAATGATACTAAGACT--

GAGAATGGTACTATCACACTCCCATGCAGAATAAAACAATTTATAAACAGGTGGCAGGAAGTAGGAAAAGCAA
TGTATGCCCCTCCCATCAGAGGACTAATTAGATGTTCATCAAAC---
ATTACAGGGCTATTAATAACAAGAGATGGTGGTAATAATGAGAGC----------------------------
----

CATCCCCTGAACGAGACCTTCAGACCTGGGGGAGGAGATATGAGGGACAATTGGAGAAGTGAATTATATAAAT

ATAAAGTAGTAAAAATTGAGCCATTA---GGAGTAGCACCCACCAAGGCAAAGAGAAGAGTGGTG--------
-------------------------------CAGAGAGAAAAAAGAGCA---GTGGGAACAATAGGAGCT---
------ATGTTCCTTGGGTTCTTGGGAGCA---GCAGGAAGCACTATG---GGCGCAGCGTCA---
TTGACGCTGACGGTACAA---GCCAGACAA---TTATTG---TCTGGTATAGTGCAACAGCAAAGC---
AATTTG---CTGAGA---GCTATTGAGGCGCAACAGCATCTGTTGCAGCTCACAGTCTGGGGCATC
>AY727526
AATTATACATATAATAGTACAGAAAACATTAATGAA-------------------------------------
----
-
ACAGGAGCAAACATTACAATCCCATGCAGAATAAAACAATTTATAAATATGTGGCAGGAGGTAGGACGAGCAA
TGTATGCCCCTCCCATTAAAGGAATAATAACATGTAAATCAAAT---
ATCACAGGAATATTACTGACACGGGATGGAGGCAGGACTGATACT----------------------------
----

AACACAGAAACATTCAGGCCTGGAGGAGGAGATATGAGGGACAATTGGAGAAGTGAATTATATAAATATAAAG
TGGTAGAAATTAAACCATTA---GGAATAGCACCCACCAAGGCAAAAAGGAGAGTGGTG--------------
-------------------------GAGAGAGAAAAAAGAGCA---GTGGGA---ATAGGAGCT---------
GTGTTCCTTGGGTTCTTGGGAGCA---GCAGGAAGCACTATG---GGCGCAGCGTCA---
ATAACGCTGACGGTACAG---GCCAGACAA---TTATTG---TCTGGGATAGTGCAACAGCAAAGC---
AATTTG---CTGAGG---GCTATAGAGGCGCAACAGCATATGTTGCAACTCACAGTCTGGGGCATT
>AY727527
TACTATAGTACATACAATAGTACAGTTAGTAATACAACA----------------------------------
----
GCAGATGTAAACATAACAATCCCATGCAGAATAAAACAAATTATAAATATGTGGCAGGAGGTAGGAAGAGCAA
TGTATGCCCCTCCCATTAAAGGAGCAATAACATGTATATCAAAC---
ATCACAGGATTATTACTGACACGGGATGGA---GACAAGAATGAC----------------------------
----

ACAGAGACATTCAGGCCTGGAGGAGGAGATATGAGGGACAATTGGAGAAGTGAATTATATAAATATAAAGTGG
TAGAAATTAAACCATTA---GGAATAGCACCCACTAAGGCAAAAAGGAGAGTGGTG-----------------
----------------------GAGAGAGAAAAAAGAGCA---GTGGGA---CTAGGAGCT---------
TTGTTCTTTGGGTTCTTGGGAGCA---GCAGGAAGCACTATG---GGCGCAGCATCA---
ATAACGCTGACGGTACAG---GCCCGACAA---TTGTTG---TCTGGGATAGTGCAACAGCAAAGC---
AATTTG---CTGAGA---GCTATAGAGGCGCAACAGCATATGTTGCAACTCACGGTCTGGGGCATT
>DQ366662
ACTCACAGAGAAAATGAAACCGAGGGAAAG---

GATAATGACACTATCATACTTCCATGCAAAATAAAGCAAATTATAAACATGTGGCAGAGAACAGGACAAGCAA
TGTATGCTCCTCCCATTGAGGGAAAAATTAATTGTGTATCAAAT---
ATTACAGGAATATTATTGACAAGAGATGGTGGTGGTATTAATGATACT-------------------------
----

AGGGAAAACGAGACCTTCAGACCTGGAGGAGGAAATATGGAGGACAATTGGAGAAGTGAATTATATAAATATA
AAGTAGTACAAATTGAACCACTA---GGAATAGCACCCACCAGGGCAAAGAGAAGAGTGGTG-----------
----------------------------GAGAGGGAAAAAAGAGCA---GTGGGA---ATAGGAGCT------
---TTGATCTTTGGGTTCTTAGGAGCA---GCAGGAAGCACTATG---GGCGCGGCGTCC---
ATAACGCTGACGGTACAG---ACCAGACAA---TTATTG---TCTGGGATAGTGCAACAGCAAAGC---
AATTTG---CTGAGG---GCTATAGAGGCGCAGCAGCATCTGTTGCAACTCACAGTCTGGGGCATT
>AB845349
ACTTACTGGATAAATGGAAGTAGGGAGGAGAGTAATGGCACT-------------------------------
----
ACCAATGGCAATATCACTATCCCATGCAAAATAAAACAAATCATAAACATGTGGCAGGGAGTAGGACAAGCAA
TGTATGCTCCCCCCATCAAAGGAAGACTTAATTGTATATCAAAT---
ATTACAGGACTACTATTAACAAGAGATGGTGGTGATGGTAAAGAT----------------------------
----

AACGAGACCTTCAGACCTGCAGGAGGAGAGATGAAGGACAATTGGAGAAGTGAATTATATAAATATAAAGTAG
TACAAATTGAACCACTA---GGAATAGCACCCACCAGGGCAAAGAGAAGAGTGGTG-----------------
----------------------GAGAGAGAAAAAAGAGCA---GTGGGA---ATAGGAGCT---------
ATGATCTTTGGGTTCTTGGGAGCA---GCAGGAAGCACTATG---GGCGCGGCGTCA---

ATAACGCTGACGGTACAG---GCCAGACAA---TTATTG---TCTGGTATAGTGCAACAGCAAAGC---
AATTTG---CTGAGG---GCTATAGAGGCGCAGCAGCATCTGTTGCAACTCACAGTCTGGGGCATT
>KJ671533
AACTACTCAGAACAAAAATTAAAT---
---AATAAAACGTCC------
TCTCCCAATGACACTATCACTATCCCATGTCGAATAAAACAAATTGTAAATATGTGGCAGGAAGTAGGACGAG
CAATGTATACCGCTCCCATTGCAGGAAACATTACCTGTAACTCAAAT---
ATTACAGGTATACTGTTGACAAGAGATGGTGGT--

-
AATATAACTAATGGGACTGAGACATTCAGACCTGGGGGAGGAAATATGAAAGACAATTGGAGAAGTGAATTAT
ATAAATATAAAGTAGTAGAAATTAAACCACTA---GGAGTAGCACCCACCAGGGCAAAAAGACAAGTGGTG--
-------------------------------------AAGAGAGAAAAAAGAGCA---GCAGGA---
ATAGGAGCT---------TTGTTCCTTGGGTTCTTGGGGGCA---GCAGGAAGCACTATG---
GGCGCAGCGTCA---GTGACGCTGACGGTACAG---GCCAGACAA---TTATTG---
TCTGGTATAGTGCAACAGCAGAAC---AATCTG---CTGAGA---
GCTATTGAGGCGCAACAGCATATGTTGCAACTCACAGTCTGGGGCATT
>KU161145
TATTAT---

AATGACACTATCAGACTTCCATGCAGGATAAAGCAGATTATAAACATGTGGCAAGGAGTAGGACAAGCAATGT
ATGCCCCTCCCGTCAGGGGAAGGATTAATTGTGTATCAAAC---
ATTACAGGAATACTATTGACAAGAGATGGTGGTAATACTACTGCA----------------------------
----

GATGCTAGTGAGACCTTCAGACCTGCAGGAGGAAATATGAAGGACAATTGGAGAAGTGAATTATATAAATATA
AAGTAGTAGAAATTGAACCACTA---GGAATAGCACCCACCAAGGCAAAAAGAAGAGTGGTG-----------
----------------------------CAGAGAGAAAAAAGAGCA---GTGGGA---ATAGGAGCT------
---ATGATCTTTGGGTTCTTAGGAGCA---GCCGGAAGCACTATG---GGCGCGGCGTCA---
ATAACGCTGACGGTACAA---GCCAGACAG---TTACTG---TCTGGTATAGTGCAACAGCAAAGC---
AATTTG---CTGAGG---GCTATAGAGGCGCAGCAGCATATGTTGCAACTCACAGTCTGGGGCATT
>KY216147
AATTATACATACAAGACTAATGATAGTAGGACTAATAATACAGCTGAGGATACT-------------------
----
-
AATTCGACCGTCATCACAATCCCATGCAAAATAAAGCAGATTATAAATATGTGGCATGAGGTGGGACGAGCAA
TGTATGCTCCTCCCATTGCAGGAAACATAACATGTAAGTCAAAC---
ATCACAGGAATACTATTAGTACGTGATGGAGGAACAAAGTCAAAT----------------------------
----

AGTACAGAGACATTCAGGCCTGGAGGAGGAGATATGAGGAATAATTGGAGAAGTGAATTATATAAATATAAAG
TGGTAGAAATTAAGCCAGTG---GGAGTAGCACCCACTGAAGCAAAAAGGAGAGTGGTG--------------
-------------------------GAGAGAGAAAAAAGAGCA---GTGGGA---ATAGGAGCT---------
GTGTTCCTTGGGTTCTTGGGAGTA---GCAGGAAGCACTATG---GGCGCGGCGTCA---
GTGACGCTGACGGTACAG---GTCAGACAA---TTGCTG---TCTGGTATAGTGCAACAGCAAAAC---
AATTTG---CTGAGG---GCTATAGAGGCGCAACAGCATATGTTGCAACTCACGGTCTGGGGCATT
>KU820831
TACTATCTTAATAGTACATACAATCCTAATGATACA-------------------------------------
----
GCAAACTCAAACATCACCATCCCATGCAGAATAAAACAGATTGTAAACATGTGGCAGGAGGTAGGACGAGCAA
TGTATGCTCCTCCCATTGCAGGAAAAATAACATGTAAATCAAAT---
ATCACAGGACTACTATTGGTACGTGATGGGGGAATTAATCTAAATACA-------------------------
----

ACAAATAATACCGAAACATTCAGGCCTGGAGGAGGAGATATGAGGGACAATTGGAGAAGTGAATTATATAAAT
ATAAAGTGATAGAAATTAGGCCATTG---GGAATAGCACCCACTAAAGCAAAAAGGAGAGTGGTG--------
-------------------------------GAGAGAGAAAAAAGAGCA---GTGGGA---CTAGGAGCT---
------GTGTTCCTTGGGGTCTTGGGAGCA---GCAGGAAGCACTATG---GGCGCGGCGTCA---
ATAACGCTGACGGTACAA---GCCAGACAA---TTGTTG---TCTGGTATAGTGCAACAGCAAAGT---
AATTTG---CTGAGG---GCTATAGAGGCGCAACAGCATATGTTGCAACTCACGGTCTGGGGCATC

>KU992937
TTCTATACTAATGATACAGGAGGCAGT--

TTAAAATCAAACATCACGATTCCATGTAGAATAAAGCAAATTATAAATATGTGGCAGGAGGTAGGGCGAGCAA
TATATGCCCCTCCCATTGAAGGAAATATAACATGTAAATCAAAG---
ATCACAGGACTACTATTGGTACGTGATGGAGGAAAAGGAAATAAT----------------------------
----

AATACAGAGACATTTAGACCTGGAGGAGGAGATATGAGGGACAATTGGAGAAGTGAATTATATAAATATAAAG
TGGTAGAAGTTAAGCCATTG---GGAGTAGCACCTACTAAAGCAAAAAGGAGAGTGGTG--------------
-------------------------GAGAGAGAAAAAAGAGCA---GTGGGA---ATAGGAGCT---------
GTGTTCCTTGGGTTCTTGGGAGCA---GCAGGAAGCACTATG---GGCGCGGCGTCA---
ATAACGCTGACGGTACAA---GCCAGACAA---TTGTTG---TCTGGTATAGTGCAACAGCAAAGC---
AATTTG---CTGAGA---GCCATAGAGGCGCAACAGCATATGTTGCAACTCACGGTCTGGGGCATT
>KC899012
ACCTATAATGCTACTAGTACAAAAGGTAAT---

TCAAGCTCAGTTATCACACTCCCATGCAGAATAAAGCAAATTATAAACATGTGGCAGGAGGTAGGGCGAGCAA
TGTATGCCCCTCCCATTGCAGGAAACATAACATGTAAATCCAAT---
ATCACAGGAATACTATTGGAATGGGATGGAGGAAATTCAAATAGT----------------------------
----

TCTAATGCTACATTCAGACCTGGAGGAGGAGATATGAAAAACAATTGGAGAAGTGAATTATATAAATATAAAG
TGGTAGAAATAAAGCCATTA---GGAATAGCACCCACTGCAGCAAAAAGGAGAGTGGTG--------------
-------------------------GAGAGAGAAAAAAGAGCA---GTGGGA---ATAGGAGCT---------
GTGTTCCTTGGGTTCTTAGGCGCG---GCAGGAAGCACTATG---GGCGCGGCGTCA---
ATAACGCTGACGGTACAG---GCCAGACAA---TTGCTG---TCTGGTATAGTGCAACAGCAAAGC---
AATTTG---CTGAGG---GCCATAGAGGCGCAACAGCATATGTTGCAACTCACGGTCTGGGGCATT
>MN044632
ACCTACATGCCTAATGGTACATTCAAGTTTAATGATACAGACCTGAATGTT----------------------
---ACAAGT------
AATTCAGATTCACCCATCACAATACCATGCAGAATAAAGCAAATTATAAACATGTGGCAGGAGGTAGGACGAG
CAATGTATGCTCCTCCCATTGAAGGAAACATAACATGTAAATCAAAT---
ATCACAGGACTACTATTGGTACGTGATGGAGGAGAGACAGAGACA----------------------------
----

AACATGACAGAGACATTCAGACCTGGAGGAGGGGATATGAGAGACAATTGGAGAAATGAATTATATAAATATA
AAGTGGTAGAAATTAAGCCATTA---GGAGTAGCACCCACTGAAGCGAAAAGGAGAGTGGTG-----------
----------------------------CAGAGAGAAAAAAGAGCA---GTGGGA---ATAGGAGCT------
---GTGTTCCTTGGGTTCTTGGGAGCA---GCAGGAAGCACTATG---GGCGTGGCGTCA---
ATAACGCTGACGGTACAG---GCCAGGCAA---CTGCTG---TCTGGTATAGTGCAACAGCAAAGC---
AATTTG---CTGAGA---GCTATAGAGGCGCAACAACATGTGTTGCAACTCACTGTCTGGGGCATT
>MK397789
ACTTACCAAAGTGGAACCATAAATGGG--

GATAATGGAACTATTATAATTCCATGTAAAATCAAACAAATTATAAACATGTGGCAGAGAACAGGACAAGCAA
TATATGCTCCTCCCATCAAGGGAAAAATTAATTGTATATCAAAT---
ATTACAGGAATACTATTAACAAGAGATGGTGGTGTTAATAGCACA----------------------------
----

GTTAATGAGACCTTCAGACCGGGAGGAGGAAATATAAAAGACAACTGGAGAAGTGAATTATATAAATATAAAG
TAGTACAAATTGAACCACTA---GGAGTAGCACCCACCAGGGCAAGAAGAAGAGTGGTG--------------
-------------------------CAGAGAGAAAAAAGAGCA---GTGGGA---ATAGGAGCT---------
ATGATCTTTGGGTTCTTAGGAGCA---GCAGGAAGCACTATG---GGCGCGGCGTCA---
ATAACGCTGACGGTACAG---GCCAGACAA---TTATTG---TCTGGTATAGTGCAACAGCAAAGC---
AATTTG---CTGAGA---GCTATAGAGGCGCAGCAGCATCTGTTGCAGCTAACAGTCTGGGGCATT
>EU131799
AATCATAATTTGACTGAGAATAATACTTGGCAAGAGAATGCT-------------------------------
----
-

```
AGCGAAAGTACCATCACACTCCCATGCAGAATAAAACAAATTATAAACAGGTGGCAGGAAGTAGGAAGAGCAA
TGTATGCCCCTCCCATCAGTGGACAAATTAAATGCTCATCAAAT---
ATTACAGGGCTGCTATTAACAAGAGATGGTGGTAAAACGAATGGGTCC-------------------------
-------------------------------------------------------------------------
-------
GAAAATGGGTCCGAAATCTTCAGACCTGGAGGAGGAGATATGAGAGACAATTGGAGAAGTGAATTATATAAAT
ATAAAGTAGTAAAAATTGAGCCACTA---GGAATAGCACCCACCAAGGCAAAAAGAAGAGTGGTG--------
-------------------------------CAGAGAGAAAAAAGAGCA---GTGGGAACAATAGGAGCT---
------ATGTTCCTCGGGTTCTTGGGAGCA---GCAGGAAGCACTATG---GGCGCAGCGTCA---
ATGACGCTGACGGTACAG---GCCAGACTA---TTACTG---TCTGGTATAGTGCAACAGCAGAAC---
AATCTG---CTGAGA---GCTATTGAGGCGCAACAGCACATGTTGCAACTCACAGTCTGGGGCATC
>KM111555
ACTTACAGAGAAAAT----------------------------------------------------------
-----------------------------------------------GGAACCATGGAA------
GGGAGTAATGGCACTATCATACTTCCATGCAAAATAAAGCAAATAATAAACATGTGGCAGGGAGTAGGACAAG
CAATGTATGCTCCTCCCGTCAGTGGAACAATTAATTGTGTATCAAAT---
ATTACAGGAATACTATTAACAAGAGATGGTGGT----------------------------------------
--------------------------------------------------------
GGTAGTACCAATGCGACTAAC---------
GAGACCGAGATCTTCAGACCTGGAGGAGGAAATATAAAGGACAATTGGAGAAGTGAATTATATAAATATAAAG
TAGTAGAACTTGAACCACTA---GGAATAGCACCCACCAGGGCAAAGAGAAGAGTGGTG--------------
-------------------------GCGAGAGAAAAAAGAGCA---GTGGGA---ATAGGAGCT---------
ATGATCTTTGGGTTCTTAGGAGCA---GCAGGAAGCACTATG---GGCGCAGCGTCC---
ATAACGCTGACGGTACAG---GCCAGACAA---TTATTG---TCTGGTATAGTGCAACAGCAAAGC---
AATTTG---CTGAGG---GCTATAGATGCGCAGCAGCATCTGTTGCAACTCACAGTCTGGGGCATT
>KP418805
AATTATACTTTCCAGGCTAATGATACTAGGCAAAGG-------------------------------------
-------------------------------------------------------------------------
-
TCAAACAAAACTATCACACTCCCATGCAGAATAAAACAAATTATAAACAGGTGGCAGGAAGTAGGAAGAGCAA
TGTATGCCCCTCCCATCAGGGGACAAATTAAATGTTCATCAAAT---
ATTACAGGAATACTATTAACAAGAGATGGTGGTGACAAGAGTAGTACTGCA----------------------
-------------------------------------------------------------------------
-------------
ACTAATGAAACCTTCAGACCGGGAGGAGGAGATATGAGGGACAATTGGAGAAGTGAATTGTATAAATATAAAG
TAGTAAAGATTGAACCACTA---GGAATAGCACCCACCAAGGCAAAGAGAAGAGTGGTG--------------
-------------------------CAGAGAGAAAGAAGAGCA---GTGGGA---GTAGGAGCT---------
ATGTTCCTCGGGTTCTTGGGAGCA---GCAGGAAGCACTATG---GGCGCAGCATCA---
ATGACGCTGACGGTACAG---GCCAGACTA---TTACTG---TCTGGTATAGTGCAACAGCAGAAC---
AATCTG---CTGAGA---GCTATTGAGGCGCAACAACACATGTTGCAACTCACAGTCTGGGGCATC
>AB097867
ACTCATAGTATTTGGAATGGTACTATTTGGAATGGT-------------------------------------
-----------------------------------------------ACT---
CGGAATGATACTAATATCACGCTCCCTTGCAGAATAAAACAAATTGTAAACATGTGGCAGGAAGTAGGAAAAG
CAATGTATGCCCCTCCCATCAGAGGACAAATTAGATGTTCATCAAAT---
ATTACAGGGTTGCTATTAACAAGAGATGGTGGTAATGATAAGAAC----------------------------
-------------------------------------------------------------------------
----------
GAGACCACCGAGACCTTCAGACCAGGAGGAGGAGATATGAGGGACAATTGGAGAAGCGAATTATATAAATATA
AAATAGTAAAAATTGAACCATTA---GGAGTAGCACCCACCAAGGCAAAGAGAAGAGTGGTG-----------
----------------------------CAGAGAGAAAAAAGAGCA---GTGGGAACGATAGGAGCT------
---ATGTTCCTTGGGTTCTTGGGAGCA---GCAGGAAGCACTATG---GGCGCAGCGTCA---
ATAACGCTGACGGTACAG---GCCAGACAA---TTGTTG---TCTGGTATAGTGCAGCAGCAGAGA---
AATTTG---CTGATG---GCTATCGAGGCGCAACAGCATCTGTTGCAACTCACAGTCTGGGGCATC
>KT438784
AGTTACAAA----------------------------------------------------------------
--------------------------------------------------------------------
GGAGATGAAACCATCATACTTCCATGTAAAATAAAGCAAATTATAAATATGTGGCAGAGAGTAGGACAAGCAA
TGTATGCTCCTACCATCAGGGGAAAAATTAATTGTGTATCAAAT---
ATTACAGGAATACTATTGACAAGAGATGGTGGAAATGGTAGTGGC----------------------------
```


GAGACCTTCAGACCTGGAGGAGGAGATATGAAGGACAATTGGAGAAGTGAATTATATAAATATAAAGTAGTAC
AAATTGAACCACTA---GGATTAGCACCCACCAAGGCAAAGAGAAGAGTGGTG--------------------
-------------------GAAAGAGAAAAAAGAGCA---GTGGGA---ATAGGAGCT---------
ATGATCTTTGGGTTCTTAGGAGCA---GCTGGAAGCACTATG---GGCGCGGCGTCC---
ATAACGCTGACGGTACAG---GCCAGGCAA---TTATTG---TCTGGTATAGTGCAACAGCAAAGC---
AATTTG---CTGAGG---GCTATAGAGGCGCAGCAGCATATGTTGCAACTCACAGTCTGGGGCATT
>JN248316
ACTTACCTAGGAAATGAAACCTTGGAGAGGTATACTGGCAATAGC----------------------------
----
ACTAATGGCACTATCATACTTCCATGCAGGATAAAGCAAATTGTAAGAATGTGGCAGGGAGTAGGACAAGCAA
TGTATGCTCCTCCCGTCAAGGGAATAATTAATTGTGTATCAAAT---
ATTACAGGAATACTATTGACAAGAGATGGTGGTAATAGAACGAAA----------------------------
----

AATGAGACCTTCAGACCTGAAGGAGGAAATATGAAGGACAATTGGAGAAATGAATTATATAAATATAAAGTAG
TACAAATTGAACCACTA---GGAATAGCACCCACCAAGGCAAAAAGAAGAGTGGTG-----------------
----------------------GAGAGAGAAAAAAGAGCA---GTGGGA---ATAGGAGCT---------
ATGATCTTTGGGTTCTTAGGAGCA---GCAGGAAGCACTATG---GGCGCGGCGTCA---
ATAACGCTGACGGTACAG---GCCAGACAA---TTATTG---TCTGGTATAGTGCAACAGCAAAGC---
AATTTG---CTGAGG---GCTATAGAGGCGCAGCAGCATCTGTTGCAACTCACAGTCTGGGGCATT
>JN248320
ACTTATGACATAGGAAATGAAACCACAGGGGAGGGTAATGGC-------------------------------
----
ACTATTGGCAATATCACACTTCCATGCAAGATAAAGCAAATTATAAACATGTGGCAGGGAGCAGGGCAAGCAA
TGTATGCTCCTCCCATCAGTGGAGTAATTAATTGTGTATCAAAT---
ATTACAGGTATACTATTGACAAGAGATGGTGGTGTTAATAATACGAGTGAG----------------------
----

GAGACCTTCAGACCTGGAGGAGGAAATATAAAGGACAATTGGAGGAGTGAATTATATAAATATAAAGTAGTAC
AAATTGAACCACTA---GGAGTAGCACCCACCAGGGCAAAGAGAAGAGTGGTG--------------------
-------------------GAGAGAGAAAAAAGAGCA---GTGGGA---ATAGGAGCT---------
ATGATCTTTGGGTTCTTGGGAGCA---GCAGGAAGCACTATG---GGCGCAGCGTCA---
ATAACGCTGACGGTACAG---GCCAGACAA---TTATTG---TCTGGTATAGTGCAACAGCAAAGC---
AATTTG---CTGAGG---GCTATAGAGGCGCAACAGCATCTGTTGCAACTCACAGTCTGGGGCATT
>JN248349
AATTATAATGGTACTTGGAATAATAATAATCCTTGGAAT----------------------------------
---GATACT------
ACAGAAAATGGCACTATCATACTCCCTTGCAGAATAAGACAAATAGTAAACATGTGGCAGCAAGTAGGAAGAG
CAATGTATGCCCCTCCCATTGCAGGACAAATTACATGTGCATCAAAT---
ATTACAGGAATACTATTGACAAGAGATGGTGGTGCTAAGAATGAT----------------------------
----

AGTGGCACCGAGACCTTCAGACCTGCAGGAGGAGATATGAGGGACAATTGGAGAAGTGAATTATATAAATATA
AGGTAGTAAAAATTGAACCATTA---GGAGTAGCACCCACCAAGGCAAAGAGAAGAGTGGTG-----------
----------------------------CAGAGAGAAAAAAGAGCA---GTGGGA---ATGGGAGCT------
---ATGTTCCTTGGGTTCTTGGGAGCA---GCAGGAAGCACTATG---GGCGCAGCGTCA---
ATAACGCTGACGGTACAG---GCCAGACAA---TTGTTG---TCTGGTATAGTGCAACAGCAGAGA---
AATTTG---CTGAGG---GCTATTGAGGCGCAACAGCATCTGTTGCAACTCACAGTCTGGGGCATT
>EU743760
ACTCACATCAATGGCACTAGCAACAGCACTGCGAGGGAG----------------------------------
----
TATAATGGCACTATCACACTTCCATGCAGGATAAAGCAAATTATAAATATGTGGCAGGAAGCAGGACAAGCAA
TGTATACTCCTCCCATCAGTGGAATAATTAACTGTATATCAAAT---
ATTACAGGAATACTATTGACAAGAGATGGTGGTGCTAATAATGGG----------------------------
----

ACTATCAATGAGACCTTTAGACCTGGAGGAGGAGATATGAGGGACAATTGGAGAAGTGAATTATATAAATATA
AAGTAGTAAAAATTGAACCATTA---GGAGTAGCACCCACCAAGGCAAGGAGAAGAGTGGTG-----------

```
----------------------------CAGAGAGAAAAAAGAGCA---ATAGGA---ATAGGAGCT------
---GTGTTCCTTGGGTTCTTGGGAGCA---GCAGGAAGCACTATG---GGCGCGGCGTCA---
ATAACGCTGACGGTACAA---GCCAGACAA---TTATTG---TCTGGTATAGTGCAACAGCAGAAC---
AACTTG---CTGAGA---GCTATTGAGGCGCAACAGCATATGTTGCAACTCACTGTCTGGGGCATC
>JN860765
ACTTATAAC----------------------------------------------------------------
--------------------------------------------------------------------
AGTACTGGCAATATCACAATTCCATGTAGGATAAAGCAGATTATAAACATGTGGCAGGAAACAGGACAAGCAA
TGTATGCTCCTCCCATTAGTGGAAAAATTAATTGTGTATCAAAT---
ATTACAGGAATACTATTGACAAGAGATGGAGGTACTAATAATAATAAT-------------------------
-------------------------------------------------------------------------
----------
AATGGAACTGAGACCTTCAGACCTGGAGGAGGAAATATAAAGGACAATTGGAGAAGTGAATTATATAAATATA
AAGTAGTACAAATTGAACCACTA---GGAGTAGCACCCACCAAGGCAAAGAGAAGAGTGGTG-----------
----------------------------GAGAGAGAAAAAAGAGCA---GTAGGA---ATAGGAGCT------
---ATGATCTTTGGGTTCTTAGGAGCA---GCCGGAAGCACTATG---GGCGCGGCGTCA---
ATAACGCTGACGGCACAG---GCCAGACAA---TTACTG---TCTGGTATAGTGCAACAGCAAAGC---
AATTTG---CTGAGG---GCTATAGAGGCGCAGCAGCATATGTTGCAACTCACAGTCTGGGGCATT
>MG989494
AATCATACCAAGGGTGAG-------------------------------------------------------
--------------------------------------------------------
TGTAACAACACTATCATACTTCCATGTAAGATAAAGCAAATTATAAACATGTGGCAGAGAGTAGGACAAGCAA
TGTATGCTCCTCCCATCAGGGGAAACATTACTTGTGTATCAAAT---
ATTACAGGAATATTATTGACAAGAGATGGTGGTAATGGGACTAAC----------------------------
-------------------------------------------------------------------------
-------------
GGGACTGAAACCTTCAGACCTGGAGGAGGAGATATAAAGGACAATTGGAGAAATGAATTATATAAATATAAAG
TAGTACAAATTGATCCACTA---GGAATAGCACCCACCAGGGCAAGAAGAAGAGTGGTG--------------
-------------------------GAGAGGGAAAAAAGAGCA---GTGGGA---ATAGGAGCT---------
ATGATCTTTGGGTTCTTAGGAGCA---GCAGGAAGCACTATG---GGCGCGGCGTCA---
ATAACGCTGACGGTACAG---GCCAGACAA---TTATTG---TCTGGGATAGTGCAACAGCAAAGT---
AATTTG---CTGAGG---GCTATAGAGGCGCAGCAGCATCTGTTGCAACTCACAGTCTGGGGCATT
>MG989490
AATTATACCAAG-------------------------------------------------------------
--------------------------------------------------------
GATAAGGAAAATATCACACTTCCATGCAGGATAAAGCAAATTATAAACATGTGGCAGAGAGTAGGACAAGCAA
TGTATGCTCCTCCCATCAAGGGAAACATTACTTGTGCATCAAAT---
ATTACAGGAATAGTATTGACAAGAGATGGTGGTGGTAATAATGAGAAT-------------------------
-------------------------------------------------------------------------
----------
AGAAACAACGAGACCTTCAGACCAACAGGAGGAAATATAAAGGACAATTGGAGAAATGAATTATATAAATATA
AAGTAGTACAAATTGATCCACTA---GGAATAGCACCCACCAAGGCAAAAAGAAGAGTGGTG-----------
----------------------------GAGAGAGAAAAAAGAGCA---GTGGGA---ATAGGAGCT------
---ATGATCTTTGGGTTCTTAGGAGCA---GCAGGAAGCACTATG---GGCGCGGCGTCA---
ATAACGCTGACGGTACAG---GCCAGACAA---TTATTG---TCTGGGATAGTGCAACAGCAAAGT---
AATTTG---CTGAGG---GCTATAGAGGCGCAGCAGCATATGCTGCAACTCACAGTCTGGGGCATT
>MG989520
TTTCATAATAATGGCACA-------------------------------------------------------
--------------------------------------------------------------------AAT--
-
GGCACAAGTGGAATCATCACAATCCCATGCAGGATAAAGCAAATTATAAACATGTGGCAGAGAGTAGGACAAG
CAATGTATGCTCCTCCCATCAGTGGGGCAATCAGTTGTGTCTCAAAT---
ATTACAGGAATACTATTGACAAGAGATGGTGGTGATGCAAATAACACAAATAACTCTAATACA---ACT----
-------------------------------------------------------------------------
----------
GATCCTAAAGAAATCTTCAGACCTGCAGGAGGAGATACAAGGGACAATTGGAGAAGTGAATTATATAAATATA
AAGTAGTACAAATTGAACCACTA---GGAATAGCACCCACCAAGGCAAAGAGAAGAGTGGTG-----------
----------------------------GACAGAGAAAAAAGAGCA---GTGGGA---ATAGGAGCT------
---ATGATCTTTGGGTTCTTAGGAGCA---GCAGGAAGCACTATG---GGCGCAGCGTCA---
```

```
ATAACGCTGACGGTACAG---GCCAGACAA---TTATTG---TCTGGTATAGTGCAACAGCAAAGC---
AATTTG---CTGAGG---GCTATAGAGGCGCAGCAACATCTGTTGCAACTCACAGTCTGGGGCATT
>MG989573
AAT----------------------------------------------------------------------
-----------------------------------------------
TATAATGGCACCATCATACTTCCATGCAGGATAAAGCAAATTATAAACATGTGGCAGAGAGTAGGACAAGCAA
TGTATGCTCCTCCCATTAGGGGAAACATTACATGTACATCAAAT---
ATTACAGGAATATTATTGACAAGAGATGGCAAAGGTGAAAACGATACA-------------------------
-------------------------------------------------------------------------
-------------
GGGAAAGAGATCTTCAGACCTGGAGGAGGAGATATGAAGGACAATTGGAGAAATGAATTATATAAATATAAAG
TAGTACAAATTGAGCCACTA---GGAATAGCACCCACCAGGGCAAGGAGAAGAGTGGTG--------------
-------------------------CAGAGAGAAAAAAGAGCA---GTAGGA---ATAGGAGCT---------
ATGATCTTTGGGTTCTTAGGAGCA---GCAGGAAGCACTATG---GGCGCGGCGTCA---
ATAACGCTGACGGTACAG---GCCAGACAA---TTATTG---TCTGGTATAGTGCAACAGCAAAGC---
AATTTG---CTGAGG---GCTATAGAGGCGCAACAGCATATGTTGCAACTCACAGTCTGGGGCATT
>MG989599
ACTTATGAC----------------------------------------------------------------
--------------------------------------------------------------------
AGTAATGACACTATCATAATTCCATGTAGGATAAAGCAAATTATAAACATGTGGCAGGAAACAGGACAAGCAA
TGTATGCTCCTCCTGTCAGTGGAGCAATTAATTGTGTATCAAAT---
ATTACAGGAATACTATTGACAAGAGATGGTGGTAATAATAGAACT----------------------------
-------------------------------------------------------------------------
----------------
AATGAGACCTTCAGACCTGGAGGAGGAAATATAAAGGACAATTGGAGAAGTGAATTATATAAATATAAAGTAG
TACAAATTGAACCATTA---GGAATAGCACCCACCAGGGCAAGGAGAAGAGTGGTG-----------------
----------------------GAGAGACAAAAAAGAGCG---GTGGGA---ATAGGAACT---------
CTGATCTTTGGGTTCTTAGGAGCA---GCCGGAAGCACTATG---GGCGTGGCGTCA---
ATGACGCTAACGGTACAG---GCCAGACAA---TTATTG---TCTGGTATAGTGCAACAGCAAAGC---
AATTTG---CTGAGA---GCTATAGAGGCACAGCAGCATATGTTGCAACTCACAGTCTGGGGCATT
>MK697709
TTTTACTTTAATGGTACAAAAAGA-------------------------------------------------
--------------------AATATATTTAATGATACA------
GAAAGTAATTCGACCATCACAATCCCATGCAGGATAAAGCAAATTATAAACATGTGGCAGGAAGTGGGACGAG
CAATGTATGCTCCTCCCATTGCAGGAAACATAACATGTAAGTCAAAT---
ATCACAGGACTGATATTAGTACGTGATGGAGGAAATAGGGCCACA----------------------------
-------------------------------------------------------------------------
----------
AATGATACAGAAATATTCAGGCCTGGAGGAGGAAATATGAGGGATAATTGGAGAAGTGAATTATATAAATATA
AAGTGGTAGAAATTAAGCCATTG---GGAATAGCACCCACTACATCAAAAAGGAGAGTGGTG-----------
----------------------------GAGAGAGAAAAAAGAGCA---GTGGGA---ATAGGAGCT------
---GTGTTCCTTGGGTTCTTGGGAGTA---GCAGGAAGCACTATG---GGCGCGGCGTCA---
GTGGCGCTGACGGTACAG---GCCAGACAA---TTATTG---TCTGGTATAGTGCAGCAGCAAAGC---
AATTTG---CTGAGG---GCTATAGAGGCGCAGCAGCATATGTTGCAACTCACAGTCTGGGGCATT
>MN336527
TATCATACAGAA-------------------------------------------------------------
-----------------------------------------
GACAATTCGACCATCACACTCCCATGCAAAATAAAGCAAATTATAAATATGTGGCAGGAGGTGGGAAGAGCAA
TGTATGCTCCTCCCATTGCAGGAAACATAACATGTAGGTCAAGT---
ATCACAGGACTACTATTAGTACGTGATGGAGGAAATGCAAATGATACA-------------------------
-------------------------------------------------------------------------
----------
AATAATACAGAGATATTCAGGCCTGGAGGAGGAAATATGAGGGATAATTGGAGAAGTGAATTATATAAATATA
AAGTGGTAGAAATTAAGCCATTG---GGAGTAGCACCAACTGGGGCAAAAAGGAGAGTGGTG-----------
----------------------------GAAAGAGAAAAAAGAGCA---GTAGGA---ATAGGAGCT------
---GTGTTCCTTGGGTTCTTGGGAGCA---GCAGGAAGCACTATG---GGCGCGGCGTCA---
GTGACGCTGACGGTACAG---GCCAGACAA---TTGCTG---TCTGGTATAGTGCAACAGCAAAAC---
AATTTG---CTGAGG---GCTATAGAGGCGCAACAGCATATGTTGCAACTCACGGTCTGGGGCATC
>JN864056
```

```
GAC---------------------------------------------------------------------
----------------------------------------------------
TATATACTTCCATGTAAGATAAAACAAATTGTGAGAATGTGGCAGAAAGTGGGACAAGCAATGTATGCCCCTC
CCATCGAAGGAAACATTACATGTACGTCAAAC---
ATCACAGGACTAATATTAACAAGAGATGGTGGGAATAAGAACAGT---------------------------
------------------------------------------------------------------------
-------------
ACAAATGAGACCTTTAGACCTGGGGGAGGAGATATGAGGGACAATTGGAGAAGTGAATTATATAAATATAAAG
TAGTAAAAATTGAGCCAGTA---GGAATAGCTCCCACTAAGGCAAGACGAAGAGTGGTG-------------
-------------------------CAGAGAGAAAAAAGAGCA---GTTGGA---CTAGGAGCT--------
GTCCTCCTTGGGTTCTTAGGAGCA---GCAGGAAGCACTATG---GGCGCGGCGTCA---
ATAACGCTGACGGTACAG---GTCAGACAA---TTATTG---TCTGGCATAGTGCAACAGCAAAGC---
AATTTG---CTGAGG---GCTATAGAGGCGCAGCAGCATCTGTTGCAACTCACAGTCTGGGGCATT
>KT276254
TATCAGAATGATACTGGGAATAGTGTGAATAAC---------------------------------------
-------------------------------------------------------------
ACTGAAGACCTAATTACACTCCCATGTAAAATAAGACAAATTGTAAACATGTGGCAGGAAGTAGGAAAAGCAA
TGTATGCCCCTCCCATCAGAGGGCTGATTAATTGTTCATCAAAT---
ATTACAGGGTTGCTATTAACAAGAGATGGTGGTAATGATACATCT---------------------------
------------------------------------------------------------------------
-------------
GATAAGGAGACCTTCAGACCTGGAGGAGGAGATATGAGAGACAATTGGAGAAGTGAATTATACAAATATAAAG
TAGTAAAAATTGAACCATTA---GGAGTAGCACCCACCAAGGCAAAGAGAAGAGTGGTG-------------
-------------------------CAGAGAGAAAAAAGAGCA---ATAGGA---ATGGGAGCT--------
CTGTTCCTTGGGTTCTTGGGAGCA---GCAGGAAGCACTATG---GGCGCAGCGTCG---
GTAACGCTGACGGTACAG---GCCAGACAA---TTATTG---TCGGGTATAGTGCAACAGCAGAAC---
AACTTG---CTGAGA---GCTATTGAGGCGCAACAGCACCTGTTGCAACTCACAGTCTGGGGCATT
>JN882652
AACTACTCAGGG------------------------------------------------------------
----------------------------------------
GAAAATGACACTATAACTCTCCAATGCAAAATAAAGCAAATTATAAAAATGTGGCAGAAAGTAGGACAAGCAA
TGTATGCCCCTCCCATCCAAGGAGAAATAAGGTGTAACTCAAAC---
ATTACTGGAATACTATTAACAAGAGATGGTGGCAATAATAATGCCACAAACACG------------------
------------------------------------------------------------------------
----------
AGCAAGGCCGAGATCTTCAGACCTGGAGGAGGAGATATGAGGGACAATTGGAGAAGTGAATTATATAAATATA
AAGTAGTAAAAATTGAGCCATTA---GGAATAGCACCCACCAAGGCAAAGAGAAGAGTGGTG----------
----------------------------CAGAGAGAAAAAAGAGCA---GTGGGAGTAATAGGAGCT------
---ATGTTCCTTGGGTTCTTAGGAGCA---GCAGGAAGCACTATG---GGCGCGGCGTCA---
ATAACGCTGACGGTACAG---GCCAGACAA---TTATTG---TCTGGTATAGTGCAACAGCAGAAC---
AATTTG---CTGAGA---GCTATTGAGGCGCAACAGCATCTGTTGCAACTCACAGTCTGGGGCATC
>AJ276595
AGCTATAATAGTACAGAAAGTGTT------------------------------------------------
-------------------------------------------------------
AATGAAACAACCATCATACTCCCATGTAAAATAAAACAAATTATAAACATGTGGCAAGAGGTAGGACGAGCAA
TGTATGCCAATCCCATTGCAGGAAACATAACATGTAACTCAAAT---
ATCACAGGATTACTACTGACACGTGATGGAGGAGTAAATGAGACA---------------------------
------------------------------------------------------------------------
----------
ACAGAGACAGAGATATTCAGGCCTGGAGGAGGAAATATGAAAGACAATTGGAGAAGTGAATTATATAAATATA
AAGTGGTAGAAATTAAACCATTG---GGAGTAGCACCCACTAAAGCAAAAAGAAGAGTGGTG----------
----------------------------GAGAGAGAAAAAAGAGCA---GTGGGA---ATAGGAGCT------
---GTGTTCCTTGGGTTCTTGGGAGCA---GCAGGAAGCACTATG---GGCGCAGCGTCA---
ATAACGCTGACGGTACAA---GCCAGACAA---CTATTG---TCGGGTATAGTGCAACAGCAAAGC---
AATTTG---CTGAGG---GCTATAGAGGCGCAACAGCATATGTTGCAACTCACGGTCTGGGGCATT
>HQ225812
AATTATACATACATGCCTAATGGTACCATGCATAATGATACAACAGAT------------------------
------------------------------------------------------------------------
-
AATTCAACCTTAATCACAATCCCATGCAGAATAAAACAAATTATAAATATGTGGCAGGAGGTAGGACGAGCAA
```

TGTATGCCCCTCCCATTGAAGGAAACATAACATGTAAATCAAAG---
ATCACAGGACTACTATTGGTACGTGATGGAGGCCCCAATAGGACA----------------------------
----

GAAGAGATATTCAGACCTGGAGGAGGAGATATGAAAAACAATTGGGGAAATGAATTATATAAATATAAAGTGG
TAGAAATTAAGCCATTG---GGAGTAGCACCAACTGAAGCAAAAAGGAGAGTAGTG-----------------
----------------------GAGAGAGAAAAAAGAGCA---GTGGGA---ATAGGAGCT---------
GTGTTCCTTGGGTTCTTGGGAGCA---GCAGGAAGCACTATG---GGCGCGGCGTCA---
ATGACGCTGACGGTACAG---GCCAGACAG---TTGTTG---TCTGGTATAGTGCAACAGCAAAGC---
AATTTG---CTGAGA---GCTATAGAGGCGCAACAGCATATGTTGCAACTCACAGTCTGGGGCATT
>KY557336
ACTTACAGTAATTGGAAGTCTAATAGTACT---

GGGAATGATAATATCACACTCCCATGTAGAATAAGACAAATTGTAAACATGTGGCAAGAAGTAGGAAAAGCAA
TGTATGCCCCTCCTATCAGCGGACAAATTAGATGCTCATCAAAT---
ATTACAGGGTTACTATTGACAAGAGATGGTGGTAATAATAAGGAAAGT-------------------------
----

AATAATGCTGAGACCTTCAGACCTGGAGGAGGAAACATGAAGGACAATTGGAGGAGTGAATTGTATAAATATA
AAGTAGTAAAAATTGAACCATTA---GGAGTAGCACCCACCAAGGCAAAGAGAAGAGTGGTG-----------
----------------------------CAGAGAGAAAAGAGAGCA---GTGGGA---ATAGGAGCT------
---TTGCTCGTTGGGTTCTTGGGAGCA---GCAGGAAGCACTATG---GGCGCAGCGTCA---
ATGACGTTGACGGTACAG---GCCAGACAA---CTACTG---TCTGGTATAGTGCAACAGCAGAAC---
AATCTG---CTGAGA---GCTATTGAGGCGCAACAGCATATGTTGCAACTCACAGTCTGGGGCATA
>KM606633
AACTATAGCTCATCAAGC---

AATAAGGAGCCTATCACACTCCCATGTAGAATAAGACAAATTGTAAGAATGTGGCAAAAGGTAGCACAAGCAA
TGTATGCCCCTCCCATTGCAGGAAACATTACATGTAGAACAAAC---
ATTACAGGACTACTATTAACAAGAGATGGGCCTACTACGGAGGAT----------------------------
----

AATAATACTGAGGTTTTCAGACCTGGAGGAGGAAATATGAAGGACAATTGGAGAAGTGAATTATATAAGTATA
AGGTAGTAAAAATTAAACCACTA---GGCATAGCACCCTCCAAGGCAAGGAGAAGAGTAGTG-----------
----------------------------GGCAGAGAGAAAAGAGGA---ATTGGA---CTAGGAGCT------
---GTTTTCCTTGGTTTCTTAGGAACG---GCAGGAAGCACGATG---GGCGCAGCGTCA---
ATAACGCTGACGGTACAG---GTCAGACAA---TTATTG---TCTGGCATCGTGCAACAGCAAAGC---
AATTTG---CTGAGA---GCTATAGAGGCGCAACAGCATTTGTTGCAACTCACAGTCTGGGGCATT
>KJ541851
AATTATACA--
--------------------------AGAAATAGTACAGGAAAT---------
GATTCAAACGTAGTCATTACGATCCCATGCAGAATAAAACAAATTATAAACATGTGGCAGGGAGTAGGGCGAG
CAATGTATGCCCCTCCCATTGCAGGAGAAACCGCATCTATCTCTAAT---
ATCACAGGATTACTATTGGTACGTGATGGAGGAGAA-------------------------------------
----
GACAAAAATAATGAGAATGATAAT---------
GATACAGAGACATTCAGACCTGGAGGAGGAGACATGAAGGACAATTGGAGAAGTGAATTATATAAATATAAAG
TGGTAGAAATTAAGCCATTG---GGATTAGCACCCACTACAGCAAAAAGGAGAGTGGTG--------------
-------------------------GAGAGAGAAAAAAGAGCA---GTAGGA---ATAGGAGCT---------
GTGTTCCTTGGGTTCTTGTCAGCA---GCCGGAAGCACTATG---GGCGCGGCGTCA---
ATAACGCTGACGGTACAG---GCCAGACAA---TTGTTG---TCTGGTATAGTGCAACAGCAAAGC---
AATTTG---CTGAGG---GCTATAGAGGCGCAACAGCATCTGTTGCAACTCACGGTCTGGGGCATT
>AF484510
AATTATAGTATGTCAAATAGTACA---
-----------------------------GGGGTA------
AATGAGAATGCAACCATCATACTCCCATGCAGAATAAAACAAATTATAAACATGTGGCAGGGAGTAGGAAAAG
CAATGTATGCCCCTCCCATTGAAGGACTGATCAATTGTACATCAAAT---
ATTACAGGACTATTGTTGACAAGAGATGGTGGTATAACTAATGAT----------------------------
----

AGTAGGAATGGGACCTTCAGACCTGGAGGGGGAGATATGAGAGATAATTGGAGAAGTGAATTATACAAGTATA
AAGTAGTAAGAGTTGAACCACTA---GGTCTAGCACCCACCAGGGCAAAGAGAAGAGTGGTG-----------
----------------------------GAAAGAGAAAAAAGAGCA---ATAGGA---CTGGGAGCT------
---CTGTTCCTTGGGTTCTTGGGAGCA---GCAGGAAGCACGATG---GGCGCAGCGTCA---
CTGACGCTGACGGTACAG---GCCAGACAA---GTATTG---TCTGGTATAGTGCAACAGCAAAAC---
AATTTG---CTGAGG---GCTATAGAGGCGCAACAGCATCTGTTGCAACTCACAGTCTGGGGCATT
>DQ208433
AATCACACTGACAGCACGTCGAGGTCAGATAGCAAT-------------------------------------
----
ATGACTGGCAATATAACTCTCCTATGCAAAATAAAACAAATTATAAACATGTGGCAGGAAGCAGGAAAAGCAA
TGTATGCCCCTCCCATTGCAGGACTAATCAATTGTTCATCAAAT---
ATTACAGGACTATTACTAACAAGAGATGGTGGGGATAATAGCAGT----------------------------
----

ACAAATGAAACCTTCAGACCTGGAGGAGGAGATATGAGAGACAATTGGAGAAGTGAATTATACAAGTATAAAG
TAGTAAGAATTGAACCACTA---GGTCTAGCACCCACCAGGGCAAAGAGAAGAGTGGTG--------------
-------------------------GAAAGAGAAAAAAGAGCA---ATAGGA---CTGGGAGCT---------
ATGTTCCTTGGGTTCTTGGGAGCA---GCAGGAAGCACGATG---GGCGCAGCGTCA---
CTGGCGCTGACGGTACAG---GCCAGACAG---TTATTG---TCTGGTATAGTGCAACAGCAAAAC---
AATTTG---CTGAGG---GCTATAGAGGCGCAACAACATCTGTTGCAACTCACAGTCTGGGGCATT
>GQ365651
TACTACTTTAATGGTAACAGTACATACAATGATACCAAT----------------------------------
----
ACTGGACCAAATATCACACTCCCATGCAGAATAAAACAAATTATAAACATGTGGCAGGAAGTAGGAAAAGCAA
TGTATGCCCCTCCCATCAGAGGACAAATTAGATGTTCATCAAAT---
ATTACAGGGCTGATATTAACGAGAGATGGTGGTGTGAACAACGCTGAG-------------------------
----

AACAACACTGAGACCTTCAGACCTGGAGGAGGAGATATGAGGGACAATTGGAGAAGTGAACTATATAAATATA
AAGTGGTAGAAATAAAACCATTA---GGAATAGCACCAACTAAGGCAAAAAGGAGAGTGGTG-----------
----------------------------GAGAGAGAAAAAAGAGCA---GCA------CTAGGAGCT------
---ATGTTCCTTGGGTTCTTGGGAGCA---GCAGGAAGCACTATG---GGCGCAGCGTCA---
ATAACGCTGACGGTACAG---GCCAGACAA---TTACTG---TCTGGGATAGTGCAACAGCAAAGC---
AATTTG---CTGAGA---GCTATAGAGGCGCAACAGCATATGTTGCAACTCACAGTCTGGGGCATT
>KF835524
AATTACAATGAG---

AATTCAAGCCTCATCACAATTCCATGCAAAATTAAGCAAATTATAAACATGTGGCAGGAGGTAGGACGAGCAA
TGTATGCCCCTCCCATTGCAGGAAATATAACATGTAAATCAAAT---
ATCACAGGACTACTATTGGTGCGTGATGGAGGAGTGACAAATACA----------------------------
----

AATGAGACAGAGATATTCAGACCTGGAGGAGGAGATATGAGGAATAATTGGAGAAGTGAATTATATAAATATA
AAGTAGTAGAAATTAAGCCATTG---GGAATAGCACCCACTGAAGCAAAAAGGAGAGTGGTG-----------
----------------------------GAGAGAGAAAAAAGAGCA---TTAGGGGGAATAGGAGCT------
---GCGTTCCTTGGGTTCTTGAGTTTA---GCCGGAAGCACTATG---GGCGCGGCGTCA---
CTAACGCTGACGGTACAG---GCCAGACAA---TTGCTG---TCTGGTATAGTGCAACAGCAAAGC---
AATTTG---CTGAGG---GCTATAGAGGCGCAACAGCATATGTTGCAACTCACGGTCTGGGGCATT
>KC898982
GATCAAGATGAG---

TCAAACCCAACCATCATAATCCCATGCAGAATAAAGCAAATTATAAACATGTGGCAGGAGGTAGGACGAGCAA
TGTATGCCCCCCCCATTGCAGGAAACATAACATGTAAATCAAAT---
ATCACAGGACTACTATTGGTACGTGATGGAGGAGACAGAAATGACACA-------------------------
----

AATAATACAGAGAAATTCAGACCTGGAGGAGGAGATATGAGGGACAATTGGAGAAGTGAATTATATAAATATA
AAGTGGTAGAAATTAAGCCATTG---GGAATAGCACCCACTGAAGCAAAGAGGAGAGTAGTG-----------
----------------------------GAGAGAGAAAAAAGAGCA---GTGGGA---ATAGGAGCT------
---GTGTTCCTTGGGTTCTTGGGAGCA---GCAGGAAGCACTATG---GGCGCGGCGTCA---

ATAACGCTGACGGTACAA---GCCAGACAA---TTGTTG---TCTGGTATAGTGCAACAGCAAAGC---
AATTTG---CTGAGG---GCTATAGAGGCGCAACAGCATATGTTGCAACTCACAGTCTGGGGCATT
>HM215402
AGTCAT---

TCAAATTCAATCATCAGGCTCCCATGCAGAATAAAACAAATTATAAACATGTGGCAGAAGGTAGGCCGAGCAA
TGTATGCCCCTCCCATTGAAGGAAACATAACATGTAAATCAAAC---
ATCACAGGACTACTATTGGTACGTGATGGAGGAGAACCTAATAAT----------------------------
----

ATAGAGATATTCAGACCTGGAGGAGGAGATATGAGGGACAATTGGAGAAGTGAATTATATAAATATAAAGTAG
TGAAAATTGAACCATTA---GGAGTAGCACCCACCAAGGCAAAAAGAAGAGTGATG-----------------
----------------------CAGAAAGAAAAAAGAGCA---GTGGGA---ATAGGAGCT---------
GTGTTCCTTGGGTTCTTGGGAGCA---GCAGGGAGCACTATG---GGCGCGGCGTCA---
GTAACGCTGACGGTACAA---GCCAGACAA---TTGTTG---TCTGGTATAGTGCAACAGCAAAGC---
AATTTG---CTGAGG---GCTATAGAGGCGCAACAGCATATGTTGCAACTCACAGTCTGGGGCATC
>KC899013
ACCTACAATGGTACAAGAAGTAAT---

TCAAGCTCAATCATCACTATCCCATGCAGAATAAAGCAAATTATAAACATGTGGCAGGGGGTAGGACGAGCAA
TGTATGCCCCTCCCATTGAAGGAAACATAACATGTAAATCAAAT---
ATCACAGGACTACTCTTGGTACGTGATGGAGGAATAGGGACACAT----------------------------
----

GGTAAAGAGACATTCAGACCTGGAGGAGGAAATATGAGGGACAATTGGAGAAGTGAATTATATAAATACAAAG
TAGTAGAAATTAAGCCATTG---GGAATAGCGCCCACTACAGCAAAAAGGAGAGTAGTG--------------
-------------------------GAGAGAGAAAAAAGAGCA---GTGGGA---ATAGGAGCT---------
GTGTTCCTTGGGTTCTTGGGAGCA---GCAGGAAGCACTATG---GGCGCAGCGTCA---
ATAACGCTGACGGTACAG---GCCAGGCAA---TTGTTG---TCTGGTATAGTGCAACAGCAAAGC---
AATTTG---CTGAAG---GCTATAGAGGCGCAACAGCATATGTTGCAACTCACGGTCTGGGGCATT
>KC899014
AATCATAAGACTAATAGTACAGAAAGTAAT---

TCAAGCCCAGACATCATAATCCCATGCAGAATAAAACAAATTATAAACATGTGGCAGGAGGTAGGACGAGCCA
TGTATGCCCCTCCTATTGCAGGAAACATAACATGTAAATCAAAT---
ATCACAGGACTACTATTAGTACGGGATGGAGGACTATATCCAAAT----------------------------
----

AAGACTGAGACATTCAGACCTGGAGGAGGAGATATGAGGGACAATTGGAGAAGTGAATTATATAAATATAAAG
TGGTAGAAATTAAGCCATTG---GGAGTAGCACCCACTAAAGCAAAAAGGAGAGTGGTG--------------
-------------------------GAGAGAGAAAAAAGGGGA------------ATAGGAGCT---------
GTGTTCCTTGGGTTCTTGGGAGTA---GCAGGAAGCACTATG---GGCGCGGCGTCA---
ATAACGCTGACGGTACAG---GCCAGACAA---TTGTTG---TCTGGTATAGTGCAACAGCAAAGC---
AATTTG---CTGAGG---GCTATAGAGGCGCAACAGCATATGTTGCAACTCACGGTCTGGGGCATT
>KC807958
TACTACCGCCTTAATGGTACATACGAGCCTAATGATATAAGC-------------------------------
----
AATGAGTCAGACATCACACTCCCATGCAGAATAAAGCAAATTATAAATATGTGGCAGGAGGTAGGACGAGCAA
TGTATGCCCCTCCCATTGCAGGAAACATAACATGTAGATCAAAT---
ATCACAGGACTACTATTGGTACGGGATGGAGGAAATGTTACAGGA----------------------------
----

GATACAGAGATATTCAGACCGGGAGGAGGAGATATGAGGGACAATTGGAGAAGTGAATTATATAAATATAAAG
TAGTAGAAATTAAGCCATTG---GGAGTAGCACCCACTGCAGCAAAAAGGAGAGTGGTGGAG-----------
----------------------AGAGAGAGACAAAAAAGAGCA---GTGGGA---CTAGGAGCT---------
GTGTTCCTTGGGATCTTGGGAGTA---GCAGGAAGCACTATG---GGCGCGGCGTCA---
GTGACGCTGACGGTACAG---GCCAGAAAA---TTGCTG---TCTGGTATAGTGCAACAGCAAAGC---
AATTTG---CTGAGG---GCTATAGAGGCGCAACAGCATCTGTTGCAACTCACGGTCTGGGGCATT
>MK792289

TACTACAATAGTACATACCATACATACATAAATGATACAAAA------------------------------
----
AGTAATGAGACCATCACGATCCCATGCAAAATAAAGCAAATTATAAATATGTGGCAGGAGGTGGGACGAGCAA
TGTATGCTCCTCCCATTGCCGGAAACATAACATGTAGGTCAGAT---
ATCACAGGACTGCTATTAGTACGTGATGGAGGAAATGAGCCAAATAGT------------------------
----

ACAAATGGTGCAGAAGAAATATTCAGGCCTGGAGGAGGAAATATGAAGGATAATTGGAGAAGTGAACTATATA
AATATAAAGTAGTAAAAATTGAACCATTA---GGAGTAGCACCCACCAAGGCAAGGAGGAGAGTGGTG-----
---------------------------------CAGAGAGAAAAAAGAGCA---
GTGGGAACGATAGGAGCT---------ATGTTCCTTGGGTTCTTGGGAGCA---GCAGGAAGCACTATG---
GGCGCAGCATCA---ATAACGCTGACGGTACAG---GCCAGACAA---TTGTTG---
TCTGGTATAGTGCAACAGCAGAGA---AATCTG---CTGAGA---
GCTATTGAGGCGCAACAGCATCTGTTGCAACTCACAGTCTGGGGCATC
>FJ388961
AATCATACTAGGCCAATTAACACAGGTGACACTGAA------------------------------------
----
GGAGGAATTATCACCACACCTACATGCAGAATAAAACAAATTATAAACCTGTGGCAGGAAGTAGGAAAAGCAA
TGTATGCCCCTCCCATCGAAGGACAAATTAGTTGTTCATCAAAT---
ATTACCGGGTTGCTATTAACAACAGATGGGGGTACTAACGGGAGC---------------------------
----

AACGAGACCGAGGTCTTCAGACCTGCAGGAGGAAATATGAAGGACAATTGGAGAAGTGAATTATACAAATATA
AAGTGGTACAAATTGAACCATTA---GGAATAGCACCCACCACGGCAAAGAGAAGAGTGGTG----------
---------------------------CAGAGAGAAAAAAGAGCA---GTGGGA---ATAGGAGCT------
---ATGTTCCTTGGGTTCTTGGGAGCA---GCAGGAAGCACTATG---GGCGCAGCATCA---
GTGATGCTGACGGTACAG---GCCAGACAA---TTATTG---TCTGGCATAGTGCAACAGCAAAAC---
AATTTG---CTGAGG---GCTATTGAGGCGCAACAACATCTGTTGCAACTCACAGTCTGGGGCATC
>KU820850
AATTATACAACAGGTAAT--

TCAAGCTCAGTCATCACACTCCCATGCAGAATAAAGCAAATTATAAACATGTGGCAGGAGGTAGGACGAGCAA
TGTATGCCCCTCCCATTGCAGGAAACATAACATGTAAATCAAAT---
ATCACAGGATTACTATTGGTACGTGATGGAGGAGACACAAGTAAT---------------------------
----

ACAACTGAAACATTCAGACCTGGAGGAGGAAATATGAAGGACAATTGGAGAAATGAATTATATAAATACAAAG
TAGTAAAAATTGAACCATTA---GGAGTAGCACCCACCAAGGCAAAGAGAAGCGTGGTG-------------
------------------------CAGAGAGAAAAAAGAGCA---GTGGGA---ATGGGAGCT---------
GTGTTCCTTGGGTTCTTAGGAGCA---GCAGGAAGCACTATG---GGCGCGGCGTCA---
ATAACGCTGACGGTACAG---GCCAGACAA---TTGCTG---TCTGGTATAGTGCAACAGCAAAGT---
AATTTA---CTGAGG---GCTATAGATGCGCAACAGCATATGTTGCAACTCACAGTCTGGGGCATC
>KF725906
AGTTATAAT---

TCAAATGAGACCATCACACTCCCATGCAGAATAAAACAAATTATAAACAGGTGGCAGGAAGTAGGAAAAGCAA
TGTACGCCCCTCCCATCCAAGGAGTAATTAGGTGTGCATCAAAT---
ATTACAGGGCTGCTATTAACAAGAGATGGGGGCAGTAATATTAAG---------------------------
----

AACAACGGTACTGAAACCTTCAGACCTGGAGGAGGAAATATGAGGGACAATTGGAGGAGTGAATTATATAAAT
ATAAAGTAGTAAGAATTGAACCATTA---GGAGTAGCACCCACCAAGGCAAAGAGAAGAGTGGTG--------
------------------------------CAGAGAGAAAAAAGAGCA---GCG------ATAGGAGCT---
------ATGATCCTTGGGTTCTTGGGAACA---GCAGGAAGCACTATG---GGCGCAGCGTCA---
GTGACGCTGACGGTACAG---GCCAGACTA---TTATTG---TCTGGTATAGTGCAACAGCAAAAT---
AATTTG---CTGAGA---GCTATTGAGGCGCAACAACATCTGTTGCAACTCACAGTCTGGGGCATC
>KY496623
AGTCATCTGTTTAATAGTAGTCTGTCTAATAGTAGTAAT---------------------------------
----
AGTAGTAAGTATATCACAATTCCATGTAAGATAAAACAAATTGTAAGAATGTGGCAGAGAGTGGGACAAGCAC

TGTATGCCCCTCCCATCGAAGGAAACATTACATGTAGCTCAAAC---
ATTACAGGACTACTATTAGTTAGAGATGGAGGAAGCAATATAACA----------------------------
----

ATCAATGAGACTTTCAGACCTGCAGGAGGAGACATGAGGGATAATTGGAGAAGTGAATTATATAAATATAAAG
TAGTAAAAATTAAACCACTA---GGAGTAGCACCCACCAGGGCAAGGAGAAGAGTGGTG--------------
-------------------------GGGAGAGAAAAAAGAGCA---GTTGGA---CTGGGAGCT---------
GTCCTCCTTGGGTTCTTAGGAACA---GCAGGAAGCACTATG---GGCGCAGCGGCA---
GTAACGCTGACGGTACAG---GTCAGACAA---CTATTG---TCTGGCATAGTGCAACAGCAAAGC---
AATTTG---CTGAGG---GCTATAGAGGCGCAGCAGCATATGTTGCAACTCACCGTCTGGGGCATT
>DQ383755
AACTACACG--

AAAGAAAATGACACAATCAGACTCCCATGTAGAATAAAACAGATTATAAACATGTGGCAGGAAGTAGGAAAAG
CCATGTATGCCCCTCCCATCAGAGGACTAATTAGGTGTTCATCAAAT---
ATTACAGGGCTGCTATTAACAAGAGATGGTGGTAATGATAACACCACG-------------------------
----

AACGAGTCTGAAATTTTCAGACCTGGAGGAGGAGATATGAGGGACAATTGGAGAAGTGAATTATATAAATACA
AGGTAGTAAGAATTGAGCCAGTA---GGAGTAGCACCCACCAAGGCAAAGAGAAGAGTGGTG-----------
----------------------------CAGAGAGAAAAAAGAGCA---GTGGGAGTGATAGGAGCT------
---ATGTTCCTTGGGTTCTTGGGAGCA---GCAGGAAGCACTATG---GGCGCAGCGTCA---
ATGACGCTGACGGTACAG---GCCAGACAA---TTATTG---TCTGGTATAGTGCAACAGCAAAAC---
AATTTG---CTGAGA---GCTATTGAGGCGCAACAGCATCTGTTGCAACTCACAGTCTGGGGCATC
>KT427679
GGTCAGTGGAATAGCACTGGGAAT---
--------------------------AATACTGAA------
GGGTCAAATGGAACCATCACAATCCCATGCAGAATAAAACAAATTATAAACAGATGGCAGGAAGTAGGAAGAG
CAATGTACGCCCCTCCCATCGCAGGACAAATTAACTGTTCATCACAA---
ATTACAGGGTTACTGTTAACAAGAGATGGTGGTACCAATACTACC----------------------------
----

AGTCCTGAAGAAATCTTCAGACCTGCAGGAGGAGATATGAGGGACAATTGGAGAAGTAAATTATATAAATATA
AAGTAGTAAAAATTGAGCCATTA---GGAATAGCACCCACCAAGGCAAAGAGAAGAGTGGTG-----------
----------------------------CAGAGAGAAAAAAGAGCA---ATGGGAGTACTAGGAGCT------
---GCGGTCCTTGGGTTCCTGGGAGCA---GCAGGAAGCACTATG---GGCGCAGCGTCA---
CTGACGCTGACGGTACAG---GCCAGACTA---TTATTG---TCTGGTATAGTGCAACAGCAGAGC---
AATCTG---TTGAGA---GCTATTGAGGCGCAGCAGCATATGTTGCAACTCACAGTCTGGGGCATC
>KT427801
AATTACAAT--

AGCACTGAGAATATCACTCTCCCATGTCGAATAAAACAAATTGTAAACATGTGGCAGGAAGTGGGGCGAGCAA
TGTATGCCGCTCCCCTTGCAGGAAACATTAACTGTAGTTCAAGT---
ATTACAGGGCTACTATTAACAAGAGATGGTGGTAACAAGAAAGACAATGAAACA-------------------
----

GAAAAAGAGACCTTCAGACCTGGAGGAGGAAATATGAAAGATAATTGGAGAAGTGAATTATATAAATATAAAG
TAGTAGAAATTAAGCCATTA---GGAATAGCACCCACCAAGGCACAGAGAAGAGTGGTG--------------
-------------------------CAGAGGGAAAAAAGAGCA---GTGACA---TTCGGAGCT---------
ATGTTCCTTGGGTTCTTGGGAGCA---GCAGGAAGCACTATG---GGCGCAGTGTCA---
ATGACGCTGACGGTACAG---GCCAGACAA---TTATTG---TCCGGTATAGTGCAACAGCAGAAC---
AATCTG---CTGAGG---GCTATTGAGGCGCAACAGCATATGTTGCAACTCACAGTCTGGGGCATC
>MG365765
ACT--

CATAATGACACTATCACTCTCCCATGTCGAATAAAACAAATTGTAAACCTGTGGCAGGAAGTAGGACGGGCTA
TGTATGCCAATCCCATTGCAGGACAAATTAACTGTACATCAAAT---
ATTACAGGTCTACTATTGACAAGAGATGGTGGTAATAGTAGTAAG----------------------------
----

ACTGAAATCTTCAGACCTGAGGGAGGAAATATGAAAGACAATTGGAGAAGTGAATTATATAAATACAAAGTAG
TAGAAATTGAACCACTA---GGAATAGCACCCACCAGGGCAAAAGGCGAGTGGTG------------------
----------------------GAGAGAGAAAAAAGAGCA---GTGGGA---CTAGGAGCT---------
TTGTTCCTTGGGTTCTTGGGAACA---GCAGGAAGCACTATG---GGCGCAGCGTCA---
ATAACGCTGACGGTACAA---GCCAGACAA---TTATTG---TCTGGAATAGTGCAACAGCAGAGC---
AATTTG---CTGAGG---GCTATTGAAGCGCAACAGCATCTGTTGCAGCTCACAGTCTGGGGCATT
>DQ085870
GCTCACACTTGGAATAGAAATGGG---
--------------------------------------AAT---
GGTACTAATAGTACTATCACACTCCCATGCAGAATAAAACAAATTATAAACAGGTGGCAGGAAGTAGGAAAAG
CAATGTATGCCCCTCCCATCCAAGGACAAATTAGATGTTCATCAAAT---
ATTACAGGGTTGTTATTAACAAGAGATGGAGGTAACAACAACGGGACC-------------------------
----

GAGAACAATACCGAGATCTTCAGACCTGGAGGAGGGAATATGAGGGATAATTGGAGAAGTGAATTATACAAAT
ATAAAGTAGTAAGAATTGAGCCATTA---GGAGTAGCACCCACCAAGGCAAAGAGGAGAGTGGTG--------
-------------------------------CAGAGAGAAAAGAGAGCA---GTGGGA---ATAGGAGCT---
------GTGTTCCTTGGGTTCTTGGGAGCA---GCAGGAAGCACTATG---GGCGCAGCGTCA---
ATAACGCTGACGGTACAG---GCCAGACAA---TTATTG---TCTGGTATAGTGCAACAGCAGAAC---
AATCTG---CTGAGA---GCTATTGAGGCGCAACAACATATGTTGCAACTCACAGTCTGGGGCATC
>AB253430
AATTATAACAATGGGAT--

AATACTGGTGGTAATATCACACTCCCATGCAGAATAAAACAAATTGTAAACAGGTGGCAGGAAGTAGGAAAAG
CAATGTATGCCCCTCCCATCAGAGGAAACATTACCTGTTCATCAAAT---
ATCACAGGGCTGCTATTAACAAGAGATGGTGGTAACAACGTTACC----------------------------
----

AACGAGTCCGAGATTTTCAGACCTGGAGGAGGAAATATGAAGGACAATTGGAGAAGTGAATTATATAAATATA
AAGTAGTAAAAATTGAACCATTA---GGAGTAGCACCCACCAGGGCAAAGAGAAGAGTGGTG-----------
----------------------------CAGAGAGAAAAAAGAGCA---GTGGGAACAATAGGAGCT------
---ATGTTCCTTGGGTTCTTGGGAGCA---GCAGGAAGCACTATG---GGCGCAGCGTCT---
TTGACGCTGACGGTACAA---GCCAGACAA---TTATTG---TCTGGTATAGTGCAACAGCAAAAC---
AATTTG---CTGAGA---GCTATTGAGGCGCAACAGCATCTGTTGCAACTCACAGTCTGGGGCATC
>KC699033
AATCAT---

ACTGAGCAAAATATCACACTACAATGTAGAATAAAACAAATTGTAAACCTGTGGCAGACAGTAGGAAGAGCAA
TATATGCCCCTCCCATCAAAGGACAAATTAGGTGTTTATCAAAC---
ATTACAGGGCTGCTATTAACAAGAGATGGTGGTAACGAGAACTCCACC-------------------------
----

GACAATGACACCGAGACCTTCAGACCTATAGGAGGGAATATAAGGGACAATTGGAGAAGTGAATTATATAAAT
ATAAAGTAGTAAAAATTGAACCATTA---GGAGTGGCACCCACCAGGGCAAGGAGAAGAGTGGTG--------
-------------------------------CAGAGAGAAAAAAGAGCA---GCAGGA---CTAGGAGCT---
------GTGTTCATTGGGTTCTTAGGAGCA---GCAGGAAGCACTATG---GGCGCAGCATCA---
ATAACGCTGACGGTACAG---GCCAGACAA---TTATTG---TCTGGTATAGTGCAACAGCAGAAC---
AATCTG---CTGAGA---GCTATTGAGGCGCAACAGCATCTGTTGCAACTCACAGTCTGGGGCATC
>KX907421
GACTATCCT--

AATGGTACAATCATCACACTTCCATGCAAAATAAAACAAATTATAAACATGTGGCAGGCGATAGGAAAAGCAA
TATATGCCCCTCCCATTGCAGGAAACATAACATGTGTATCAAAT---
ATCACAGGACTATTATTGACACGAGATGGAGGAAGTGGAAATGGTACG-------------------------
----

GACCCAGAGATATTCAGACCTGCAGGAGGAGACATGAGGGATAATTGGAGAAGTGAATTATATAAATATAAAG
TAGTAGAAATTAAGCCATTA---GGAGTAGCACCCACTAAGGCACAAAGGAGAGTGGTG--------------
-------------------------AAGAGAGAGAGAAGAGCA---GTGGGA---ATAGGAGCT---------
GTGCTCTTTGGGTTCTTGGGAGCA---GCAGGAAGCACTATG---GGCGCAGCGTCA---

ATAACGCTGACGGTACAG---GCCAGACAA---TTATTG---TCTGGTATAGTGCAACAGCAAAGC---
AATTTG---CTGAGA---GCTATAGAGGCGCAACAGCATATGTTGCAACTCACAGTCTGGGGCATT
>KX907359
AATTATACATGGAATAATAGTACAGGGCTAGGTAAT-------------------------------------
----
-
ATCACAAGTAATATCACACTACCATGTAGAATAAAACAAATTATAAACATGTGGCAGGGAGTAGGAAAAGCAA
TGTATGCCCCTCCCATTGAAGGACAAATTAAATGTTCATCAAAT---
ATTACAGGGCTAATATTGACAAGAGATGGTGGTAATACAAGTGAT----------------------------
----

GATCCTGAGACCTTCAGACCTGGAGGAGGAAATATGAAGGACAATTGGAGAAGTGAATTATATAAATATAAGG
TAGTAAGAATTGAACCAATA---GGCCTAGCACCCACCAAGGCAAAGAGAAGAGTGGTG--------------
-------------------------GAAAGAGAAAAAAGAGCA---ATAGGA---CTAGGAGCT---------
ATGTTCCTTGGGTTCTTGGGAGCA---GCAGGAAGCACGATG---GGCGCAGCGTCA---
GTGGCGCTGACGGCACAG---GCCAGACAG---TTATTG---TCTGGTATAGTGCAACAGCAAAAC---
AATTTG---CTGAGG---GCTATAGAGGCGCAACAGCATCTGTTGCAACTCACGGTCTGGGGCATT
>DQ054367
AATCAGACAGGGTTA--

AATAATAGTACAATCACACTCCAATGCAGAATAAAACAAATTATAAACATGTGGCAAGAAGTAGGAAGAGCAA
TGTATGCCCCTCCCATTGAAGGAGTAATCAAATGTTCATCAAAC---
ATTACAGGGCTAATATTGACAAGAGATGGTGCAAATAATAATACG----------------------------
----

AATGAGACTTTCAGACCTGGAGGAGGAAATATGAAAGACAATTGGAGAAGTGAACTATATAAATATAAAGTAG
TACAAGTTGAACCAATA---GGACTAGCACCTACCAAGGCAAAGAGAAGAGTGGTG-----------------
----------------------GAAAGGGAAAAAAGAGCA---ATAGGG---CTAGGAGCT---------
ATGTTCCTTGGGTTCTTGGGAGCA---GCAGGAAGCACAATG---GGCGCAGCGTCA---
GTGACGCTGACGGTACAG---GCCAGACAG---TTATTG---TCTGGTATAGTGCAACAGCAAAAC---
AATTTG---CTGAGG---GCTATAGAGGCGCAACAGCATCTGTTGCAACTCACGGTCTGGGGCATT
>AY352654
AGCTATGACATAAGTAACACA--

ACAGATAAGAATATTACACTGCCATGTAGAATAAAACAAATTGTACACATGTGGCAGAGAGTAGGACAAGCAA
TGTATGCCCCTCCCATTGAAGGAAAGATTACATGTACATCAAAT---
ATTACAGGACTAATATTGACAATTGACCATGTTAATCTGACAGGAAAC-------------------------
----

AATGATAATGTTACCCTCAGACCTGGAGGAGGAGATATGAGGGACAATTGGAGAAGTGAACTATATAAATATA
AAGTAGTAAAAATTGAACCACTA---GGAATAGCACCCTCCGAGGCAAGGAGAAGAGTGGTG-----------
----------------------------CAGAGAGAAAAAAGAGCA---GTGGCA---CTGGGAGCT------
---ATGATCTTTGGGTTCTTGGCAGCG---GCAGGAAGCACTATG---GGCGCGGCGTCA---
TTAACGCTGACGGTACAG---GCCAGACAA---TTATTG---TCTGGTATAGTGCAACAGCAAAGC---
AATTTG---CTGAGA---GCTATACAAGCGCAACAGCATATGTTGCAGCTTACTGTCTGGGGCATT
>AY231154
AATTATAGGAGTTTG--

-
ATTAAGGCTAATATCATACTCTCATGTAGAATAAAACAAATTGTAAGAATGTGGCAGAGGGTAGGACAAGCAA
TATATGCCCCTCCCATCGCAGGGAACCTTACATGTACATCAAAT---
ATTACAGGAATGATACTAACATGGGATGGTGGTAGTAATAATAGTAGT-------------------------
----

AAAAATGAATCTGAGACCTTCAGACCTGGAGGAGGAGACATGAAGGACAATTGGAGAAGTGAATTGTATAAAT
ATAAAGTAGTAAAGATTGAACCACTA---GGAATAGCACCCACCAGGGCAAGAAGAAGAGTGGTG--------
-------------------------------CAGAGAGAAAAAAGAGCA---GTGGGA---ATAGGAGCT---
------TTGTTCCTTGGGTTCTTGGGAACA---GCAGGAAGCACTATG---GGCGCGGCGTCA---
ATAACGCTGACGGTACAA---GCCAGACAA---TTATTG---TCTGGCATAGTGCAACAGCAAAGC---
AATTTG---CTGAGA---GCTATAGAAGCGCAACAACAACTGTTGCAGCTCACGGTCTGGGGCATT

```
>MN617383
TTTCATAAGAGAACTTGGTTGTCTAATAATACTGGGCAA----------------------------------
--------------------------------------------------------------
GATACAGAGAACATCACACTCCAATGTAGAATAAAACAAGTTATAAACATGTGGCAGGAAGTAGGAAAAGCAA
TGTATGCCCCTCCCATCAGTGGACAAATTAGCTGTTCATCAAAT---
ATTACAGGGCTGCTATTAACAAGAGATGGTGGTAACAAGAACGAGTCC-------------------------
-------------------------------------------------------------------------
----------
AATGGAACTGAAACCTTCAGACCTGGAGGAGGAGATATGAGGGACAATTGGAGAAGTGAATTATATAAATATA
AAATAGTAAAAATTGAGCCATTA---GGACTAGCACCCACCAAGGCAAGGAGAAGAGTGGTG-----------
----------------------------CAGAGGGAAAAAAGAGCA---GTGGGAGCATTAGGAGCT------
---ATGTTCCTTGGGTTCTTGGGAACA---GCAGGAAGCACTATG---GGCGCAGCGTCA---
CTCACGCTGACGGTACAA---GCCAGAATA---CTACTG---TCTGGTATAGTGCAACAACAGAAC---
AATTTG---CTGAAA---GCTATTGAGGCGCAACAGCGTCTGTTGCAACTCACAGTCTGGGGCATC
>MN617384
AATCAACTGAATAGCAGTGAAACTGAA----------------------------------------------
--------------------------------------------------------
GGCAATGACACAATCACACTCCCATGTAGAATAAAGCAAATTGTAAACATGTGGCAGGAAGTAGGAAAAGCAA
TGTATGCCCCTCCCATCAAAGGACAACTTAACTGCTCATCACAG---
ATTACAGGGCTAATATTAGTAAGAGATGGTGGTAGTAGTGGCAAT----------------------------
-------------------------------------------------------------------------
----------
AATACCACTGAGATCTTCAGACCTGGAGGAGGAGACATGAGGGACAATTGGAGAAGTGAATTATATAAATATA
AAGTAGTAAAAATTGAACCGTTA---GGAATAGCACCCACCAAGGCAAAAAGAAGAGTGGTG-----------
----------------------------CAAAGAGAAAAAAGAGCA---GCGGGA---ATAGGAGCT------
---CTGTTCCTTGGGTTCTTGGGAGCA---GCAGGAAGCACTATG---GGCGCAGCATCG---
ATGACGCTGACGGTACAG---GCCAGACAA---TTATTG---TCTGGTATAGTGCAACAGCAAAAC---
AACTTG---CTGAGG---GCTATTGAGGCGCAACAACATATGTTGCAACTCACAGTCTGGGGCATC
>MN617386
AATCAGACCGTCAAT----------------------------------------------------------
--------------------------------------------
GGAACTGATGTCATCACACTCCCATGCAGAATAAAACAAATTATAAACAGATGGCAGGAAGTAGGAAAAGCAA
TGTATGCTCCTCCCATCAGGGGACAAATTAACTGTACATCAAAT---
ATTACAGGGCTGCTATTAACAAGAGATGGTGGTAACGGTAACGAGACCGGCTCC-------------------
-------------------------------------------------------------------------
----------
AACAGGTCCGAAATCTTCAGACCTGGAGGAGGAGATATGAGGGACAATTGGAGAAGTGAATTATATAAATATA
AAGTAGTAAAAATTGAGCCATTA---GGAATAGCACCCACCAAGGCTAAGAGAAGAGTGGTG-----------
----------------------------CAGAGAGAAAAAAGAGCA---GTGGGAACTATAGGAGCT------
---ATGTTCCTTGGGTTCTTGGGAGCA---GCAGGAAGCACTATG---GGCGCAGCGTCA---
CTCACGCTGACGGTACAG---GCCAGACTA---TTACTG---TCTGGTATAGTGCAACAGCAGAAC---
AATTTG---CTGAAA---GCTATTGAGGCGCAACAGCACCTGTTGCAACTCACAGTCTGGGGCATT
>MN617388
AATTATAATTTGACTAATGGTACTTGGCAATGGTCAAAT----------------------------------
-------------------------------------------------------------------------
-
AACGAAAGTGTCATCACACTCCCATGCAGAATAAAACAAATTATAAACAGGTGGCAGGAAGTAGGAAAAGCAA
TGTATGCCCCTCCCATCAGGGGACAAATTAACTGTACATCAAAT---
ATTACAGGGCTGCTATTAACAAGAGATGGTGGTGGTAATGAGTCC----------------------------
-------------------------------------------------------------------------
----------
AGTGAGTTCGAAATCTTCAGACCTGGAGGGGGAGATATGAGAGACAATTGGAGAAGTGAATTATATAAATATA
AAGTAGTAAAAATTGAGCCACTA---GGGATAGCCCCCACCAAGGCAAAGAGAAGAGTGGTG-----------
----------------------------CAGAGAGAAAGAAGAGCA---GTGGGAACAATAGGAGCT------
---ATGTTCCTCGGGTTCTTGGGAGCA---GCAGGAAGCACTATG---GGCGCAGCGTCG---
ATGACGCTGACGGTACAG---GCCAGACTA---TTACTG---TCTGGTATAGTGCAACAGCAGAAC---
AATCTG---CTGAGA---GCTATTGAGGCGCAACAGCAACTGTTGCAACTCACAGTCTGGGGCATC
>AJ401043
GATCACTCTGGAAAGTCAAATGAAACAAT--------------------------------------------
-------------------------------------------------------------------------
```

-
TCAAATGACACTATTGTACTCCCATGTAAAATAAAACAAATTGTAAGAATGTGGCAGGGAGTAGGACAAGCAA
TGTATGCCCCTCCAATTAAAGGAAAGATTTCCTGTAACTCAAAT---
ATTACAGGACTAATATTGACAAGGGATGGTGGTAATACTACTAAT----------------------------
----

ACTATTGAGACCTTTAGACCTGGAGGAGGAGATATGAGGGACAATTGGAGAAGTGAACTATATAAATATAAGG
TAGTAAGAATTGAACCACTA---GGAATAGCACCCACTAAGGCAAGGAGACGAGTGGTGGAGAGA--------
-------------------GAAAAAATTAGACAAAAAAGAGCA---GTGGGA---ATGGGAGCC---------
TTGTTCCTTGGGTTCTTGGGAGCA---GCAGGAAGCACTATG---GGCGCGGCGTCA---
CTGACGCTGACGGTACAT---GATAGACAA---GTATTG---TCTGGTATAGTGCAACAGCAAAAC---
AATTTG---CTGAGA---GCTATAGAGGCGCAACAGCATCTGTTGCAACTCACAGTCTGGGGCATT
>HQ595767
TTCTATTGTAACACATCAGCTCTGTTTAATGGCACTTGGGGGGACAATACCACCATGCAGGGAAATAGCACAG
AG---

TTAAATGGAACTATAACTCTCCCATGCAGAATAAAGCAAATCATAAATATGTGGCAGAAAACAGGAAAAGCAA
TGTATGCCCCTCCCATCCAAGGAGTAATAAGGTGTGAGTCAAAC---
ATTACAGGACTTATATTAACAAGAGATGGTGGGGATACTAACAGA----------------------------
----

ACAAATGAAACCTTTAGACCTGAAGGAGGAAATATGAAGGACAATTGGAGAAGTGAATTATATAAGTATAAAG
TAGTAAAAATTGAACCACTA---GGAGTAGCACCTACCGAGGCAAGGAGGAGAGTGGTG--------------
-------------------------CAGAGAGAAAAAAGAGCA---TTTGGA---CTGGGAGCT---------
GTTTTCATTGGGTTCTTAGGAGCA---GCAGGAAGCACTATG---GGCGCGGCGTCA---
ATGACGCTGACGGTACAG---GCTAGACAA---TTATTG---TCTGGCATA
>MF373125
TTCTACTGTAATACATCAAAACTGTTTAACAATACTTGGTTTAATGGTACTTGGGTTAATGATACTTGGGATA
CT---

GAGGGTGATAATATCATACTCCCTTGTAGAATAAAACAAATTATAAACATGTGGCAGGAAGTAGGAAAAGCAA
TGTACGCCCCTCCCATTAGAGGACAAATTAGTTGTTCATCAAAT---
ATTACAGGGCTACTATTAACAAGAGATGGTGGTAACCACAGCGAGAGTAAT----------------------
----

AATACCTCAGAGATCTTCAGACCTGGAGGAGGAGATATGAGGGACAATTGGAGAAGTGAATTATATAAATATA
AAGTAGTAAAAATTGAACCATTA---GGAATAGCACCCACCAAGGCAAAGAGAAGAGTGGTG-----------
----------------------------CAGAGAGAAAAAAGAGCG---GTGACA---CTAGGAGCT------
---ATGTTCCTTGGGTTCTTGGGAGCA---GCAGGAAGCACTATG---GGCGCAGCGTCG---
ATGACGCTGACGGTACAG---GCCAGACTA---TTATTG---TCTGGTATAGTGCAACAGCAAAAC---
AATTTG---CTGAGA---GCTATTGAGGCGCAGCAACACATG
>JN024344
TTCTACTGTAATGCATCACAACTGTTTAATAGTAGTTGGAGTGGGAATGACTCTACTTGGAATATAGAG----
----

GCAAATGAAACTATCACACTCCGATGTAATATAAGACAAATTATAAACATGTGGCAGGAAGTAGGGAAAGCAA
TGTATGCCCCTCCCATCAGAGGACTAATTAGATGTTCATCAAAC---
ATTACAGGGCTGTTATTAACAAGAGATGGTGGTAATGGTAATAACACCAAT----------------------
----

AATGACACCGAAATCTTTAGACCTGGAGGAGGAGATATGAGGGACAATTGGAGAAGTGAATTATATAAATATA
AAGTAGTAAGAATTGAACCATTA---GGAATAGCACCCACTAAGGCAAAGAGAAGAGTGGTG-----------
----------------------------CAGAGAGAAAAAAGAGCA---GTGGGA---CTAGGAGCT------
---ATGTTCCTTGGGTTCTTGGGAGCA---GCAGGAAGCACTATG---GGCGCAGCATCA---
ATAACGCTGACGGTACAG---GCCAGACTA---TTATTGTCTGGTATAGTGCAACAGCAGAACAAT---
CTGCTG---AGGGCT---ACTGAGGCGCAACAGCATCTGTTGCAACTCACAGTCTGGGGCATC
>KC894082
TTCTATTGCAATACATCAGAACTGTTTAATGGTACATACAATGGTACATCAGAAAATAAT-------------
----

ACTAACGAGGTGATCACACTCCAATGCAAAATAAAACAATTTATAAACATGTGGCAGGAGATAGGAAGAGCAA
TGTATGCCCCTCCCATTGAAGGAAACATAACATGTAAATCAAAC---
ATCACAGGGCTACTATTGACACGTGATGGAGGAGGGGACACAAAC----------------------------
----

GAGACTGAGACATTCAGACCTGGAGGAGGAGATATGAGGGACAATTGGAGAAGTGAATTATATAAATATAAAG
TGGTAGAAATTAAGCCATTA---GGAGTAGCACCCACTAATGCAAAAAGGAGAGTGGTA--------------
-------------------------CAGAGAGAGAAAAGAGCA---GTGGGA---TTAGGAGCT---------
GTGTTCCTTGGGTTCTTGGGAGTG---GCAGGAAGCACTATG---GGCGCGGCGTCA---
ATAACGCTGACGGTACAG---GCCAGACAA---TTATTG---TCTGGTATAGTGCAACAGCAAAGT---
AATTTG---CTGAGG---GCCATA
>FJ444500
TTCTATTGCAATACATCAAGACTGTTTAATGGTAATAGCACATCCATGAATGAGACATACACAAAAAGTGTGG
AC---

AAAAGTGAGGACATCACACTCCAATGCAGAATAAAACAAATTATCAATATGTGGCAGGGGGTAGGACGAGCAA
TGTATGCCCCTCCCATTGAAGGAAACATAATATGTAACTCAAGT---
ATCACAGGACTATTATTGACACGTGATGGAGGAATAGCAGAAAAG----------------------------
----

AATGGCACAGAGACATTTAGACCTGGAGGAGGAGATATGAGGGATAATTGGAGAAGTGAATTATATAAATATA
AAGTGGTAGAAATTAAGCCATTG---GGAATAGCACCCACTAGGGCAAAAAGGAGAGTGGTG-----------
----------------------------GAGAGAGAGAAAAGAGCA---GTGGGA---ATAGGAGCT------
---GTGTTCCTTGGGTTCTTGGGAGCA---GCAGGAAGCACTATG---GGCGCAGCGTCA---
CTAGCGCTGACGGGACAG---GCCAGACAA---CTGTTG---TCTGGTATAGTGCAACAGCAAAGC---
AACTTG---CTGAAG---GCCATA
>KC894101
TTCTATTGCAATACAACAAAGCTGTTTAATAGTCCTAATGATACTAGTCCTAATGGT----------------
----

ACTAACTCCACCATCACACTCCCATGCAGAATAAAACAAATTATAAACATGTGGCAGGAGGTGGGAAGAGCAA
TGTATGCCCCTCCCATTGCAGGAAACATAACATGCACGTCAAAT---
ATTACAGGACTGCTATTGACACGTGATGGAGGTTTACAGGTCACA----------------------------
----

AATGACTCAGAGACATTCAGACCTGGAGGAGGAAATATGAAGGACAATTGGAGAAGTGAATTATATAAATATA
AAGTGGTGGAAATTAAGCCATTG---GGGATAGCACCCACTGAGGCAAAAAGGAGAGTGGTG-----------
----------------------------GAGAGAGAAAAAAGAGCA---GTGGGA---ATAGGAGCT------
---GTGTTCCTTGGGTTCTTGGGAGCG---GCAGGAAGCACTATG---GGCGCGGCGTCA---
ATGACGCTGACGGTACAG---GCCAGACAA---CTGTTG---TCTGGCATA
>KP411834
TTCTATTGTTGTACATCATACCTGTTGAATAATACACTAGACAATAATACTATAAATGATACTACTAATAATG
GCGGC--

ACAAATGATAGCATCATACTGCAATGCAGAATAAAGCAAATTATCAACGTGGGGCAGGAAGTAGGACGAGCAA
TGTATGCCCCTCCAATAAAAGGCAACATAACATGTAGATCAAAT---
ATCACAGGAATATTGCTGGCAGAAGATGGAGGACCTATCAACGAA----------------------------
----

ACGGAAACATTCAGACGTGGAGGAGGAGATATGAGAGACAATTGGAGAAGTGAATTATATAAATATAAAGTGG
TAGAAATTAAGCCATTA---GGAATAGCACCCACCAAGGCAAAAAGGAGAGTGGTG-----------------
----------------------GGGAGAGAAAAAAGAGCG---GCA------CTAGGAGCT---------
ATGTTCCTTGGGTTCTTGGGAGCA---GCCGGAAGCACTATG---GGCGCAGCGTCA---
ATAACGCTGACGGTACAG---GCCAGACAA---TTATTG---TCTGGTATAGTGCAACAGCAAAGC---
AATTTG---CTGAGG---GCCATA
>HM215317
TTCTACTGCAATACATCAAACCTGTTTACATACAATGGTACATACAATGGTACATACAATGGTACT-------
----

AAAAATTCAACCATCACACTCCAATGCAGAATAAAACAAATTATAAACATGTGGCAGGAGGTAGGGCGAGCAA

TGTATGCCCCTCCCATTGCAGGAAACATAACATGTAAATCAAAT---
ATCACAGGACTACTATTGCTACGTGATGGAGGAAACACAACAGGAGGAAAC----------------------
----

GAAACAGAAGAAAAAGAGATATTCAGACCTGGAGGAGGAAATATGAGGGACAATTGGAGAAGTGAATTATATA
AATATAAAGTGGTGGAGATTAAGCCATTG---GGACTAGCACCCACTAAGGCAAAAAGGAGAGTGGTG-----
----------------------------------GAGAGAGAGAAAAGAGCA---GTGGGA---
ATAGGAGCT---------GTGTTCCTTGGGTTCTTGGGAGCA---GCAGGAAGCACTATG---
GGCGCAGCGTCA---ATAACGCTGACGGTACAG---GCCAGACAA---CTGTTG---
TCTGGTATAGTGCAACAGCAAAGC---AATTTG---CTGAGA---GCCATA
>AY253321
TTCTATTGCAATACATCAAAACTGTTTAATGGTACATACAATGGTACAGACACGACAGGAAGTACT-------
----

CCAAATTCAAACATCACACTCCAATGCAGAATAAAACAAATTATAAACAGGTGGCAGGGGGTAGGGCAAGCAA
TGTATGCCCCTCCCATTGCAGGAAACATAACATGTAAGTCAAAT---
ATCACAGGACTCCTATTGACGCGTGATAGAGAAAACACAACCGGC----------------------------
----

AACGAGACATTCAGACCTGGAGGAGGAGATATGAGGGACAATTGGAGAAGTGAATTATATAAATATAAAGTGG
TAGAAATTAAGCCATTG---GGACTAGCACCCACAGAGGCAAAAAGGAGAGTGGTG-----------------
----------------------GAGAGAGAGAAAAGAGCA---GTGGGA---ATAGGAGCT---------
GTGTTCCTTGGGTTCTTGGGAGCA---GCAGGAAGCACTATG---GGCGCAGCGTCA---
ATAACGCTGACGGTACAG---GCCAGACAA---CTGTTG---TCTGGTATAGTGCAACAGCAAAGC---
AATTTG---CTGAGG---GCCATA
>HQ615958
TTCTATTGCAATACATCAAGCCTGTTTAATGGTACATATAATAACACAGGAAGTAAT----------------
----

GCAAATTCAAACATCACACTCCAATGCAGAATAAAACAAATTATAAACATGTGGCAGGAGGTGGGACGAGCAA
TGTATGCTCCGCCCATTGCAGGAGAGATAACATGTAAATCAAAT---
ATCACAGGACTACTATTGACACGGGATGGGGGAGAAAAAGACAATGAG-------------------------
----

ACAAATAATGCAGAGGTATTCAGACCTGCAGGAGGAGATATGAGGGACAATTGGAGAAGTGAATTATATAAAT
ATAAAGTAGTAGAAATTAAACCATTA---GGATTAGCACCCACTAAAGCAAGAAGGAGAGTGGTG--------
-------------------------------CAGAGGGAGAAAAGAGCA---GTGGGA---CTAGGAGCT---
------GTGCTCTTTGGGTTCTTGGGAGCA---GCAGGAAGCACTATG---GGCGCAGCATCA---
ATAACGCTGACGGTACAG---GCCAGACAA---TTACTG---TCTGGTATAGTGCAACAGCAGAGC---
AATTTG---CTGAGG---GCTATAGAGGCGCAACAGCACATG
>KT183336
TTCTATTGCAATACATCAGGGCTGTTTAATGGGACATACAATCCTAATGCTACAAACAGTAATTCAAGTACT-
----

GTAAATTCAACCATCACACTCCAGTGCAGAATAAAACAAATTATAAACATGTGGCAGGGAGTAGGACAAGCAA
TGTATGCCCCTCCCGTTGCAGGTAACATAACATGTAAATCAAAT---
ATCACAGGAATACTATTGGTAAGGGATGGGGGTAATGACAATAAC----------------------------
----

AGTACCACAGAGACATTCAGACCTGGAGGAGGAGATATGAGGGACAATTGGAGAAGTGAATTGTATAAATATA
AAGTGGTAGAGATTAGGCCATTA---GGAGTAGCACCCACCAAGGCAAAAAGGAGAGTGGTG-----------
----------------------------GAGAGAGAAAAAAGAGCA---GTGGGA---ATAGGAGCT------
---GTACTCCTTGGGTTCTTGGGAGCA---GCCGGAAGCACTATG---GGCGCAGCATCA---
ATAACGCTGACGGTACAG---GCCAGACAA---TTGTTG---TCTGGTATAGTGCAGCAGCAAAGC---
AATTTG---CTGAGG---GCTATAGAGGCGCAGCAACACATG
>JN687734
TTCTATTGCAATACAACAAAACTGTTTAATGAAACACTGTTTAAGTCAGAC----------------------
----

AATGGTTCAGACATCATACTCCCATGCAGAATAAAACAAATCATAAACATGTGGCAGGGGGTAGGACGAGCAA

TGTATGCCCCTCCCATTGCAGGAAACATAACATGCAACTCAAAT---
ATCACAGGACTATTATTGACACGTGATGGAGGAGGTGGCAACAGC----------------------------
----

ACAGAAACATTCAGACCTGGAGGAGGAAATATGAAAGATAATTGGAGAAGTGAATTATATAAATATAAAGTGG
TAGAAATTAAGCCATTG---GGAGTAGCACCCACCAAGGCAAAAAGGAGAGTGGTG-----------------
----------------------GAGAGAGAAAAAAGAGCA---GTGGGA---ATAGGAGCT---------
GTACTCCTTGGGTTCTTGGGAGCA---GCAGGAAGCACTATG---GGCGCGGCGTCA---
ATAACGCTGACGGTACAG---GCCAGACAA---CTGTTG---TCTGGTATAGTGCAACAGCAAAGT---
AATTTG---CTGAGA---GCTATAGAGGCGCAACAGCACATG
>AY529669
TTCTATTGCAATACATCAAAGCTGTTCAATGATACAAGCCTGCTTAATTATACAGAAGGGAATGCAAAC----
----

AATAATGAGACCATCATACTCCCATGCAAAATAAAACAAATTATAAACAGGTGGCAAGGGGTAGGACAAGCAA
TATATGCCCCTCCCATTGCAGGAAACATAACATGTAAATCAAAG---
ATCACAGGACTACTATTGACACGTGATGGAGGTAATACCACAAAT----------------------------
----

AACACAGAGACATTCAGACCAGGAGGAGGCGATATGAGGGACAATTGGAGAAGTGAATTATATAAATATAAAG
TGGTAGAAATTAAGCCATTG---GGAGTAGCACCCACTGAGGCAAAAAGGAGAGTGGTG--------------
-------------------------GAGAGAGAAAAAAGAGCA---GTAGGA---ATAGGAGCT---------
GTGTTCCTTGGGATCTTGGGAGCA---GCAGGAAGCACTATG---GGCGCAGCATCA---
ATAACGCTGACGGTACAG---GCCAGACAA---TTGCTG---TCTGGAATAGTGCAACAGCAAAGT---
AATTTG---CTGAGG---GCCATA
>JX213443
TTCTATTGCAATACATCAAAACTGTTTAATGGTACATACGAGCAGCCTAACAGTAGTGTAGGA----------
----

TCAGATTCAACCATCACACTCCCATGCAGAATAAAACAAATTATAAACTTGTGGCAGGAGGTAGGACGAGCCA
TGTATGCCCCTCCCATTGCAGGAACCATAACATGTAACTCAAGT---
ATCACAGGACTAATATTGACACGTGATGGAGGAAACGAAACTAAGCGC-------------------------
----

AAGGACACAGAGATATTCAGACCTATAGGAGGAGATATGAGGATTAATTGGAGAAGTGAATTATATAAATATA
AAGTGGTAGAAATTAAACCATTA---GGATTAGCACCCACTACGGCAAAAAGGAGAGTGGTG-----------
----------------------------GAGAGAGAAAAAAGAGCA---GTGGGA---ATAGGAGCT------
---GTGTTCCTTGGGTTCTTAGGAGCA---GCAGGAAGCACTATG---GGCGCAGCATCA---
ATAACGCTGACGGTACAG---GCCAGACAA---CTGTTG---TCTGGTATAGTGCAACAGCAAAGT---
AATTTG---CTGAGA---GCCATA
>KF716480
TTCTACTGCAATACATCAGAACTGTTTAATAGTATATGGCTTAATAATACTGGTAACTGGACAGGGAATACAA
CAAACAGT---
-------------ACAGAG------
AAAAATAATGGTACAATCACACTTCCATGCAGAATAAAACAAATTATAAACATGTGGCAGGGAGTAGGAAAAG
CAATGTATGCCCCTCCCATTGAAGGAGAAATCAAATGTATATCAAAT---
ATTACAGGACTATTGTTGACAAGGGATGGTGGTAATAATAATGCT----------------------------
----

ACCAATGAGACCTTCAGACCTGGAGGAGGAGATATGAGAGACAATTGGAGAAGTGAATTATACAAATATAAAG
TAGTAACAATTGAACCACTG---GGTATAGCACCCACCAAGGCAAAGAGAGGAGTGGTG--------------
-------------------------GAAAGAGAAAAAAGAGCA---ATAGGA---CTAGGAGCT---------
ATGTTCCTTGGGTTCTTGGGAGCA---GCAGGAAGCACGATG---GGCGCAGTGTCA---
TTGACGCTGACGGTACAG---GTCAGACAG---CTATTG---TCTGGCATA
>KP411843
TTCTATTGTAATACTTCAGGCCTGTTTAACAGCACTTGGAATGGCACGAAATCTAAAGCGTCAAATGACAATG
ACACTATCCTT--
----------------------------------CGC---------CCGAGA---
AAAAAAAATAAAATTATGTGGAAGAGACAAAGACTAGGACTACAAGCACCTGCCCCTCCTAGCCCAAGAACGA
GAAGGTGTAGCACTACC---AGTACAGGATTACTATTAGATGGAGATGGT---ACGAATACTAAT--------

----

AATGAGACCTTCACGCCGGGACGAGGAGATATAAATATGAATTGCAGAAGTGAACTAGATCTATATAAATTAG
TAGTAATTGAACTTGTACCAGTA---GGAGCCACCCCCTCCCGATCAAGA------GTGGTG-----------
----------------------------CTGATGCAAAAAAAAGCA---GTTGCA---ATGGGAGCT------
---TCGCTCGTTTTCTTCTTATCAGCA---GCAGCAGGAAGTAAT---GACGCGGCGTCG---
ACGACACTGACGGTGCAG---GTCAGGCCA---TTATTG---TCTGGTACAGTGCTACTGCAGCAG---
CATCTG---CTGATG---CCCATA
>HM215265
TTCTATTGTAATACATCAGGACTGTTTAATGGTACTATATCA-------------------------------
----

AATAGTACTAATATCACACTCCCATGTAAGATAAAACAAATTGTAAGAATGTGGCAAAAAGTGGGACAAGCAA
TGTATGCCCCTCCCATTGCAGGAAACATTACATGTACATCAAAC---
ATTACAGGACTACTATTAACAAGAGATGGGGGGAGTAATGGAACC----------------------------
----

CATGATGATGAGGTCTTCAGACCTGGAGGAGGAGATATGAGGGACAATTGGAGAAGTGAATTATATAAGTATA
AAGTAGTAAAAATTAAACCACTA---GGAATAGCACCCACCAAGGCAAGGAGAAGAGTAGTG-----------
----------------------------GGAAGAGAGAAAAGAGCA---GTCGGA---TTTGGAGCT------
---GTTCTCCTTGGGTTCTTAGGAACA---GCAGGAAGCACTATG---GGCGCGGCGTCA---
ATAACGCTGACGGTACAG---GTCAGACAA---TTATTG---TCTGGCATA
>KT022403
TTCTATTGTAATACAACAAGCCTGTTTAATAGCACGTGGGGAAATGACACTGTTAACACACCAAAGAATGCCA
CGGGT--

TCAAATGACACTATAACAATCCTATGCAGAATAAAACAAATTGTAAATATGTGGCAAAGAGTAGGAAGAGCAA
TGTATGCCCCTCCCATTGAAGGAAAAATACAGTGTATATCAAAC---
ATTACAGGTATGATATTAACAAGAGATGGTGGGAATGAGAATATC----------------------------
----

AGTGAGATCTTCAGACCTGGAGGAGGAAATATGAGGGACAATTGGAGAAGTGAATTATATAAGTATAAAGTAG
TAAAAATTGAACCACTA---GGAGTAGCACCCACCCAGGCAAGGAGAAGAGTGGTG-----------------
----------------------GAGAGAGGAAAAAGGGCA---GTTGGA---CTGGGAGCT---------
GTTTTCCTTGGGTTCTTGGGAGCA---GCAGGAAGCACTATG---GGCGCGGCGTCA---
CTGACGCTGACGGTACAG---GCCAGAAAC---TTATTG---TCTGGCATA
>DQ020274
TTCTATTGTAATACATCAGGACTGTTTAATAGTAGTAGTGAAAATAAGACTAGTAGTGAA-------------
----

AATAAGACTAATATCACACTCCCATGCAGAATCAAACAGATTGTGAGAATGTGGCAGAGAGTAGGACAAGCAA
TGTATGCCCCTCCCATCGCAGGAAAAATTACATGCATATCAAAC---
ATTACAGGACTAATACTAACAAGAGATGGTGGGACTAATAGTACT----------------------------
----

AATGAGACCTTCAGACCTGCAGGGGGAGACATGAGGGACAATTGGAGAAGTGAATTATATAAGTATAAAGTAG
TAAAGATTAAACCACTA---GGAGTAGCACCCACCAAGGCAAGGAGAAGAGTGGTG-----------------
----------------------CAGAGAGAGAAAAGAGCA---GTTGGA---ATAGGAGCT---------
GTCCTCCTTGGGTTCTTAGGAGCA---GCAGGAAGCACTATG---GGCGCGGCGTCA---
ATAACGCTGACGGTACAG---GCCAGGCAA---TTATTG---TCTGGCATA
>FM877777
TTCTATTGTAATACAACAAAACTGTTTAATAGCACTTGGCTTAATAGCACTTGGAATGACACGACCCAGGCAA
AT---

AATACAAATGAAAACATAACACTCCCATGCAGGATAAAACAAATTGTAAACATGTGGCAGGGAGTAGGACAAG
CAATGTATGCCCCTCCTATCGCAGGAATGATACAGTGTGTATCAAAT---
ATTACAGGACTGATATTGACAAGAGATGGGGGAGACAATAGTAGT----------------------------
----

AATAATGAGACCTTCAGGCCTGGAGGAGGAAATATGAAGGACAACTGGAGAAGTGAATTATATAAATATAAAG

TAGTACAAATTGAACCACTA---GGGGTAGCACCCAACAAGGCAAAGAGAAGAGTGGTG--------------
-------------------------GAGAGAGAAAAAAGAGCA---GTTGGA---ATAGGAGCT---------
ATGTTCCTTGGGTTCTTAGGAACA---GCAGGAAGCACTATG---GGCGCGGCGTCA---
ATAACGCTGACGGTACAG---GCCAGGCAA---TTATTG---TCTGGCATA
>KJ849769
TTCTACTGTAACTCAACACAACTGTTTCATAGTACTTGGGGGAATAATCAGACT-------------------
----
AAATCAGATAGCAATGGA---------
ACAAGTAATGGAACAATAACACTCCCATGCAGAATAAAACAAATTATAAATATGTGGCAGGGAGTAGGAAAAG
CAATGTATGCCCCTCCCATCAAAGGACAAATTAGCTGTTCATCAAAC---
ATTACAGGACTGTTATTAGTAAGAGATGGTGGT--

-
AATAGTACCAGCAATGAAGAGATCTTCAGACCTACAGGAGGAAATATGAAGGACAATTGGAGAAGTGAATTAT
ATAAATATAAAGTAGTAAAAATTGAACCCTTA---GGAGTAGCACCCACCAAGGCAAAGAGAAGAGTGGTG--
-------------------------------------CAGAGAGAAAAAAGAGCA---GTAGGA---
ATAGGAACT---------ATGTTCCTTGGGTTCTTGGGAGCA---GCAGGAAGCACTATG---
GGCGCAGCGTCA---ATGACGCTGACGGTACAG---GCCAGACAA---TTATTG---
TCTGGTATAGTGCAACAGCAAAAT---AATTTG---CTGAGG---GCCATT
>MG989549
TTCTATTGTAATACAACAAAACTGTTTAATAGTACTTGGACAGGAAATGAAACCAGTGAAGGG----------
----

AATAATAGCACTATCATACTTCCCTGTAAGATAAAGCAAATCATAAACATGTGGCAGGAAGTAGGACAAGCAA
TGTATGCTCCTCCCATCAGTGGAAGCATTAATTGTGTATCAAAT---
ATTACAGGAATACTATTGACAAGAGATGGTGGTGTTAACAATACG----------------------------
----

GGAAATGAGACCTTCAGACCTGGAGGAGGAAATACAAAAGACAATTGGAGAAGTGAATTATATAAATATAAAG
TAGTAGAAATTGAACCACTA---GGAGTAGCACCCACCAGGGCAAAGAGGAGAGTGGTG--------------
-------------------------GAAAGAGAAAAAAGAGCA---GTGGGA---ATAGGAGCT---------
ATGATCTTTGGGTTCTTAGGAGCA---GCAGGAAGCACTATG---GGCGCGGCGTCA---
ATAACGCTGACGGTACAG---GCCAGACAA---TTATTG---TCTGGTATAGTGCAACAGCAAAGC---
AATTTG---CTGAGG---GCTATAGAGGCGCAGCAGCACATG
>MF373150
TTCTATTGCAATACATCAGCACTGTTTAATAGAACTTGGAATAGCAATAGCACTTGGAATAGCACTGGGGAG-
----

AATATCACTGATATACATCTCCCATGCAGAATAAAGCAAATTGTAAATATGTGGCAGAGAGTAGGACAAGCCA
TGTATGCCCCTCCTATCCCAGGAATAATAAGGTGTGATTCAAAC---
ATTACAGGAATACTATTAACAAGAGATGGAGGGAAAAACAATAGT----------------------------
----

AGTAATGAAACCTTCAGACCTGGAGGAGGAGACATGAGGGATAATTGGAGAAGTGAATTATATAAGTATAAAG
TAGTAGAAATTGAACCACTA---GGTGTAGCACCCGCCAGGGCAAGATCAACAGTGGTG--------------
-------------------------GAGAGAGAGACCATAGCA---GTCGGA---CTGGTAGCT---------
GTCGTCCTTGGGGTCTTAGGAGCA---GCAGGAAGAACTATG---GGCGCAGCATCA---
CTAACGCTGACGGTACGA---GCCATACTA---TCAGTG---TCTGGGATACTGCAGCAGCAGATC---
>KU921769
TTCTATTGCAATACAACAGGCCTGTTTAATCACACGTTTAATAGCACGTGGGGAAATGCCACTATTACAGAGA
AT---

GCAAATGAAACTATAATTCTTCCATGCAGAATAAAACAAATTATAAATATGTGGCAGGAAGTAGGACAAGCAA
TGTATGCCCCTCCCATTGAAGGAGTAATAAAGTGTATATCAAAC---
ATTACAGGAATAATATTGACAAGAGATGGTGGGCACATGAGCAATGGCAGC----------------------
----

AATGACAGTATTGAGACCTTCAGACCTGGGGGAGGAAATATGAGGGACAATTGGAGAAGTGAATTATATAAGT
ATAAAGTAGTAAAACTTGAACCACTA---GGAGTAGCACCAACCAGGGCAAGGAGAAGAGTGGTA--------
-------------------------------GAGAGAGAAAAAAGAGCA---GTTGGA---CTGGGAGCT---

------GTCTTCCTTGGGTTCTTGGGAGCA---GCAGGAAGCACTATG---GGCGCAGCGTCA---
ATAACGCTGACGGTACAG---GCCAGACAA---TTATTG---TCTGGCATA
>KF725943
TTCTATTGCGATACATCAGACTTGTTTAATAGTACATACGATCTTACTAATAATTCTACTAATATAAGCAAAG
AG---

TCAAACGAAACAATCACGCTCAAATGCAGAATAAAACAGATCATAAACATGTGGCAGGAGGTAGGAAGAGCAA
TATATGCCCCTCCCATTGCAGGAAACATAACATGTAAATCAAAC---
ATCACAGGACTACTATTAGTACGTGATGGAGGTAATAGCCATGGCACAAATGAC------ACAAATAGC---
ACA--

AATGAAGAGATATTCAGACCTGAAGGAGGAGATATGAGGGACAATTGGAGAAGTGAATTATATAAATACAAAG
TAGTAAAACTTGAACCACTA---GGATTAGCACCCACCAGGGCAAGGAGAAGAGTGGTG--------------
-------------------------GAGAGAGAAAAACGAGCA---GTTGGA---CTGGGAGCT---------
GTCTTCCTTGGGTTCTTGGGAACA---GCAGGAAGCACTATG---GGCGCGGCGTCA---
GTGGCGCTGACGGTACAG---GCCAGACAA---TTATTG---TCTGGCATA
>JX236675
TTCTATTGTAATACATCAGACCTGTTTAATAACATTTGGATAGGAAATGAGACTACAGAAACAAATGAGACTG
AAACAAAT---

TCAAGTAAGCCTATAACGCTCCAGTGCAGAATAAAGCAAATTATAAACATGTGGCAGAGAGTAGGGCAAGCAA
TATATGCCCCTCCCATCCAAGGAAAATTAGAATGTAATTCAAAC---
ATTACAGGACTGCTATTAACAAGAGATGGTGGAAAAAATAATGAC----------------------------
----

AGTAAAGAAATCTTCAGACCTGGAGGAGGAGATATGAGGGATAATTGGAGAAGTGAATTATATAAGTACAAAG
TAGTAAAAATTGAACCACTA---GGAGTAGCACCCACCAGGGCACAGAGAAGAGTGGTA--------------
-------------------------AAGAGAGAAAAAAGAGCAGTCATTGGA---ATGGGAGCG---------
ATGTTCCTTGGGTTCTTAAGTGCA---GCAGGAAGCACTATG---GGCGCAGCGTCA---
CTAACGCTGACGGTACAG---GCCAGACAA---TTATTG---TCTGGCATA
>JN054260
TTCTATTGTAATACATCAGGACTGTTTAATAATAGTGATAATAGTAGTGGTAGTAATAATAGTAGT-------
----

AGGAATGGGAATATCATACTCCCATGCAAGCTCAAACAGATTGTGAGAATGTGGCAGAGAGTAGGACAAGCAA
TGTATGCCCCTCCCATCGCAGGAAAACTCACATGTATATCAAAC---
ATTACAGGACTATTAATAACAAGAGATGGTGGAACTAACAGTACT----------------------------
----

AATGGGACTGAGATCTTCAGACCTGCAGGGGGAGACATGAGGGACAATTGGAGAAGTGAATTATATAAGTATA
AAGTAGTAAAAATTAAACCACTA---GGAGTAGCACCCACCAGGGCAAGGAGAAGAGTGGTG-----------
----------------------------GAGAGAGAAAAAAGAGCA---GTTGGA---ATAGGAGCT------
---GTTTTCTTTGGGTTCTTAGCAGCA---GCAGGAAGCACTATG---GGCGCGGCGTCA---
ATAACGCTGACGGTACAG---GTCAGGCAA---TTATTG---TCTGGCATA
>KT124783
AGTTACTCAAATGAAACG---

GAAGACGATAATATCACACTCCCATGCAGAATAAAACAAATTATAAACATGTGGCAGGAAGTAGGAAAAGCAA
TGTATGCTCCTCCCATCAGAGGACAAATTAGATGTTCATCAAAT---
ATTACAGGGATACTATTAACAAGGGATGGTGGTAAGAACGAGAAC----------------------------
----

AACAACACTGAGATCTTCAGACCTGGAGGAGGAAATATGAGGGACAATTGGAGAAGTGAATTATATAAATATA
AAGTAGTAAAGATTGAGCCATTA---GGAGTAGCACCCACCAAGGCAAAACGAAGAGTGGTG-----------
----------------------------CAGAGAGAAAAAAGAGCA---GTAGGA---CTAGGAGCT------
---GTGTTCCTTGGGTTCTTGGGAGCA---GCAGGAAGCACTATG---GGCGCAGCGGCA---
GTGACGCTGACGGCACAG---GCCAGACAA---TTATTG---TCTGGGATAGTGCAACAGCAGAAC---
AATCTG---CTAAGA---GCTATTGAGGCGCAACAGCATCTGTTGCAACTCACAGTCTGGGGCATC
>KJ698288

AATTATAAT--
--------------AGTACTGAGAATAATACTGAA------------
ACGTCAAATGAGCCTATTATACTCCCATGCAGAATAAAACAAATTATAAACATGTGGCAGGAAGTAGGAAAAG
CAATGTATGCCCCTCCCATTAAAGGACAAATTAGATGCTCATCAAAT---
ATTACAGGGCTGCTATTAACAAGAGATGGTGGA--

AACAACAACACGAACGAGACCTTCAGACCTGGAGGAGGAGATATGAGGGACAATTGGAGAAGTGAATTATATA
AATACAAAATAGTAAAAATAGAACCATTA---GGTGTAGCACCCACCAGGGCAAAGAGAAGAGTGGTG-----
----------------------------------CAGAGAGAAAAAAGAGCA---GTGGGA---
ATAGGAGCT---------GTGCTCTTTGGGTTCTTGGGGGCA---GCAGGAAGCACTATG---
GGCGCAGCGTCA---ATGACGTTGACGGTACAG---GCCAGACAA---TTAATG---
TCTGGTATAGTGCAACAGCAGCAG---AATTTG---CTGAGG---
GCTATTGAGGCGCAGCAGCATCTGTTGCAACTCACAGTCTGGGGCATC
>KU901765
TACTATAGTAGT---
-----------AATAGTAGTGGAGTGGT----------
ACAAATAATAACACAATCATACTCCAATGCAGAATAAAACAAATTATAAACATGTGGCAGACAGTAGGAAAAG
CAATGTATGCCCCCCCCATCAGAGGAAAGATTAGCTGTGTATCAAAT---
ATCACAGGGCTATTATTAGTCAGAGATGGAGGTGATAACAGGAGT----------------------------
----

AACAAAGAGATCTTCAGACCTGCAGGAGGAGATATGAGAGACAATTGGAGAAGTGAATTATATAAATATAAAG
TAGTAAAAATTGAACCATTA---GGAATAGCACCCACTAAGGCAAGGAGAAGAGTGGTG--------------
-------------------------CAAAGAGAAAAAAGAGCA---GTGGGA---ATAGGAGCT---------
TTGTTCCTTGGGTTCTTGGGAGCA---GCAGGAAGCACTATG---GGCGCAGCGTCA---
ATGGCGCTGACGGTACAG---GCTAGACAA---TTATTG---TCTGGTATAGTGCAACAGCAGAAT---
AATTTG---CTGAGA---GCTATTGAGGCGCAACAGCATATGTTGCAACTCACAGTCTGGGGCATC
>EU576268
TATCAGAATAATACT--
--GAAGGGTCAAATAACACT---------
AAAGCAAATAGCACAATCCTACTCCTATGCAGAATAAAACAAATTATAAACATGTGGCAGGAAGTAGGAAAAG
CAATGTATGCCCCTCCCATCAGAGGCAAAATCAGATGTTTATCAAAT---
ATTACAGGGCTGCTACTAACAAGAGATGGTGGTGAGCCTAATAAC----------------------------
----

AAAACCAACGATACCTTCAGACCAGGAGGAGGAGATATGAGGGACAATTGGAGAAGTGAATTATATAAATATA
AAGTAGTACAAATTGAGCCAATA---GGACTAGCACCCACCAAGGCAAAGAGAAGAGTGGTG-----------
----------------------------CAAAGAGAAAAAAGAGCA---GTGGGC---ATAGGAGCT------
---TTGTTCCTTGGGTTCTTGGGAGCA---GCAGGAAGCACTATG---GGCGCAGCGTCA---
TTGACGCTGACGGTACAG---GCCAGACAA---TTATTG---TCTGGTATAGTGCAACAGCAGAAC---
AACTTG---CTGAGA---GCTATTGAGGCGCAACAGCATCTGTTGCAACTCACAGTCTGGGGCATC
>HQ699991
GACTACATGCCTAATGGTACAGCAAGTAAT---

TCAAGCTCAAACATCACACTCCCATGCAGAATAAAGCAAATTATAAACATGTGGCAGGAGGTAGGACGAGCAA
TGTATGCCCCTCCCATTGAAGGAAACATAACATGTAAATCAAAT---
ATCACAGGTCTACTATTGGTCCGTGATGGAGGAAGTACAAATAAT----------------------------
----

ACAGAAGAGACATTCAGACCTATAGGAGGAAATATGAAGGACAATTGGAGAAATGAATTATATAAGTATAAAG
TAGTGGAAATTAAGCCATTG---GGACTAGCACCCACTGGAGCAAAAAGAAGAGTAGTG--------------
-------------------------GAGAGAGAAAAAAGAGCG------GGA---ATAGGAGCT---------
GTGTTCCTTGGGTTCTTAGGAGCA---GCCGGAAGCACTATG---GGCGCGGCGTCA---
ATAACGCTGACGGTACAG---GCCAGACAA---TTGTTG---TCTGGTATAGTGCAACAGCAAAGC---
AATTTG---CTGAAG---GCTATAGAGGCGCAACAGCATATGTTGCAACTCACGGTCTGGGGCATT
>KU319545
TGTCATAATAATCCAACATCCGAACCGTCTAAAAATCGT----------------------------------
----
GCTGCAAATCCAACATTCATAATCCCATGCAGGGTGAAGCAAGTTCTAAGCATGCGCCTCGTATTGCGGGAAG

CAATGTATGCCCATCCTATATCAGGAGACGTACTATTGACATCGGAT---
GTCGGAGGACTGATATCCACACGA---------GATAACGCTAAT----------------------------
----

AGCGCGAACGAGATATTTAGACCTGGAGGAGGAGATATGAGGGACAATTGGAGAAGTGAATTATACAAATATA
AAGTGGTAGAAATTAAACCATTA---GGAATAGCACCCACCAAGGCAAAAAGGAGAGTGGTG-----------
----------------------------GAGAGAGAAAAAAGAGCA---GTGGGA---ATAGGAGCT------
---GTATTCCTTGGGTTCTTGGGAACA---GCCGGAAGCACTATG---GGCGCAGCATCA---
ATAACGCTGACGGTACAG---GCCAGGCAA---TTGTTG---TCTGGTATAGTGCAACAGCAAAGC---
AATTTG---CTGAAG---GCTATAGAGGCGCAACAGCATATGTTGCAACTCACGGTCTGGGGCATT
>KJ158423
TGTTACCTGTTTAATAGATTATACACTGCAGCCAATACA----------------------------------
----
AAGAGTAATTCATGGATT---------
GATATCAACTGCACAGTTGCAATCCCATGCAGAATAAAGCAAATTATAAACATGTGGCAGGAGGTAGGGCGAG
CAATGTATGCCAATCCCATTGAAGGAAATATAACATGTAAATCAGAT---
ATCACAGGGATGCTATTAGAACGTGATTGTGGAAAA-------------------------------------
----
GAACGTGATAAAACGATATCAACT---------
ACGAAAGAGATATTCAGACCTATAGGAGGAAATATGAGGGACAATTGGAGAAGTGAATTATACAAATATAAAG
TGGTAGAAATTAAGCCATTA---GGAGTAGCACCCACGGAAGCAAAAAGGAGAGTGGTG--------------
-------------------------GAGAGAGAAAAAAGAGTA---GCGGGA---CTAGGAGCT---------
GTGTTCCTTGGGTTCTTGGGAGCA---GCAGGAAGCACTATG---GGCGCGGCGTCA---
ATAACGCTGACGGTACAG---GCCAGACAA---TTGTTG---TCTGGTATAGTGCAACAGCAAAGC---
AATTTG---CTGAGA---GCTATAGAGGCGCAACAGCATCTGTTGCAACTCACGGTCTGGGGCATT
>MF373165
GCTCAAGATAAAACGACTGGGGGTAGTGGT---

AATAATGAAACCATTCCCCTCCCCTGCAGGATAAAACAAATTGTCAACATGTGGCAAGTAGTAGGACCAGCAA
TGTATGCTCCCCTCATTGCAGGAAACATAACAAGTAACTCATCT---
ATCACAGGACTACTATTGACACGTGATGGAGGAATCGTAACCAAT----------------------------
----

GCCACCGACACAGAGACATTTAGACCTGGAGGAGGAAATATGAAAGACAATTGGAGAAGTGAATTATATAAAT
ATAAAGTGGTAGAAATTAAGCCATTA---GGAGTAGCACCCACTAAGGCACAGAGGAAAGTGGTGAAG-----
----------------------------AGAGAAAGAGAAAAAAGAGCA---GTGGGA---ATGGGAGCT---
------GTTCTCCTTGGGTTCTTGGGAGCA---GCAGGAAGCACTATG---GGCGCAGCGTCA---
ATAACGCTGACGGTACAG---GCCAGACAA---CTGTTG---TCTGGTATAGTGCAACAGCAAAGC---
AATTTG---CTGAAA---GCTATAGAGGCGCAACAACATCTGTTGCAACTCACAGTCTGGGGCATT
>GU080195
AATCATGATAGTGGGACAGAA--

GATAATTCAACCATCACACTTCCATGCAGAATAAAACAAATTGTAAACATGTGGCAGGAGGTAGGACGAGCAA
TGTACGCTCCTCCCATTGAAGGAAACATAACATGTATCTCAAAT---
ATCACAGGATTACTATTGACACGTGATGGGGGAGAAACAAACAGA----------------------------
----

ACAAATGACACATTCAGACCTGGAGGAGGAAATATGAAGGACAATTGGAGAAGTGAATTATATAAATATAAAG
TGGTAGAAATTAAGCCATTG---GGAATAGCACCCACTGGAGCAAAAAGGAGAGTGGTG--------------
-------------------------GAGAGAGAAAAAAGAGCA---GTGGGA---CTAGGAGCT---------
GTGATCTTTGGGTTCTTGGGAGCA---GCAGGAAGCACTATG---GGCGCAGCGTCA---
ATAACGCCGACGGTACAG---GCCAGACAA---CTGTTG---TCTGGTATAGTGCAACAGCAAAAT---
AATTTG---CTGAGG---GCTATAGAGGCGCAACAGTATATGTTGCAACTCACGGTCTGGGGCATT
>MF285011
AATTATACAGATAATAGTACAACTGATGAT---

ACCCAGACAAACATCACACTCCAATGTAGAATAAAACAAATTATAAACATGTGGCAGGAGGTAGGACGAGCAA
TGTATGCTCCTCCCATTGCAGGAAACATAACATGCAACTCAAAC---
ATCACAGGATTACTATTGACATATGATGGAGGTGAAACAGGTAAT----------------------------
----

GGTACACCAGAGATATTCAGGCCTGGAGGAGGAAATATGAAGGACAATTGGAGAAGTGAATTATATAAATATA
AAGTGGTAGAAATTAAGCCATTG---GGAATAGCACCCACTGGAGCAAAAAGGAGAGTGGTG----------
----------------------------GAGAGAGAAAAAAGAGCA---GTAGGA---ATAGGAGCT------
---GTGTTCCTTGGGTTCTTGGGAGCA---GCAGGAAGCACTATG---GGCGCGGCGTCA---
ATAACGCTGACGGTACAG---GCCAGACAA---CTGTTG---TCTGGTATAGTGCAACAGCAAAGC---
AATTTG---CTGAGA---GCTATAGAGGCGCAACAGCATATGTTGCAACTCACGGTCTGGGGCATT
>KF725969
ACCTATCTT---

AGTAATGAAACCTTTAGACTCCAATGCAAAATAAAACAAATTATAAACATGTGGCAGACGGTAGGACGAGCAA
TGTATGCCCCTCCCATTGCAGGAAACATAACATGTAATTCAAGC---
ATCACAGGATTACTATTGACACGGGATGGAGGTTCCACAGATAAT----------------------------
----

AATAAAACAGAAACGTTCAGACCTGGAGGAGGAGATATGAGAGACAATTGGAGAAGTGAATTATATAAATATA
AAGTGGTGGAAATTAAGCCATTG---GGAATAGCACCCACTAAAGCAAAAAGGAGAGTGGTG----------
----------------------------CAGAGAGAAAAAAGAGCA---GCA------ATAGGAGCT------
---ATGATCCTTGGGTTCTTGGGAGCA---GCAGGAAGCACTATG---GGCGCGGCGTCA---
ATGGCGCTGACGGTACAG---GTCAGACAA---TTGTTG---TCTGGTATAGTGCAACAGCAAAGT---
AATTTG---CTGAGG---GCCATAGAGGCGCAACAGCATATGTTGCAACTCACGGTCTGGGGCATT
>AY322187
TATTATACTGGATGCACCACCAACGTACAACAG--
---AAA---
ACAATGCTTCCCATCAGACTACAACTCATAATAAGTAATGTATGCCCTTGTCCATGGCAAGGAGTGTCAGCAG
CAACATCTGCTGCTACCATCCTA---------------TTATCAACA---
ATAACAGGAGGATCATTGACAACAAGAGGAGTGTTCACAACTGGT----------------------------
----

GGAGGTGATACCTTCAGACCTGGAGGAGGAGATATGAGGGACAATTGGAGAAGTGAATTATACAAATATAAAG
TAGTAAGAATTGAACCACTA---GGTTTAGCACCCACCAGGGCAAAGAGAAGAGTGGTG-------------
-------------------------GAAAGAGAGAAAAGAGCA---ATAGGA---CTAGGAGCT---------
ATGTTCCTTGGGTTCTTGGGAGCA---GCAGGAAGCACGATG---GGCGCAGCGTCA---
CTGACGCTGACGGTACAG---GCCAGACAG---TTATTG---TCTGGTATAGTGCAACAGCAAAAC---
AATTTG---CTGAGG---GCTATAGAGGCGCAACAGCATCTGTTGCAACTCACAGTCTGGGGCATT
>KX587434
TTCTAATCAAACTGAGAGTAACACTACTGAAGGT---------------------------------------
----
AATGACACAGTAATTACACTCCCATGCAGAATAAAGCAAATTATAAACATGTGGCAGCAAGTAGGAAAAGCAA
TGTATGCCCCTCCCATCGCAGGGCAACTTAATTGCTCATCAAAC---
ATTACAGGGATATTATTAACAAGAGATGGTGGTAGCGAGAACCATACC------------------------
----

AAGGGTAATGAGACCTTCAGACCAGTAGGAGGAGACATGAGGGACAATTGGAGAAGTGAATTATATAAATATA
AAATAGTAAAAATTGAACCCTTA---GGAATAGCACCCACCAATGCAAAAAGAAGAGTGGTG----------
----------------------------CAGAGAGAAAAAAGAGCA---GTGGGA---CTAGGAGCC------
---TTGTTCCTTGGGTTCTTGGGAATG---GCAGGAAGCACTATG---GGCGCAGCGTCA---
GTGACGCTGACGGTACAG---GCCAGACAA---TTACTGTCTGGTATAGTGCAGCAGCAAAACAAC---
TTGCTG---AGGGCT---ATTGAGGCGCAACAACACATGTTGCAGCTCACAGTCTGGGGCATC
>MH078531
TCTCAGCTGTTTAATGGGACTGCAAAACAGATGAGAAATAACACAGAAATATATAACAAAACAATCATACTCC
CATGCAGAATAAAACAAATTGTAAACAGGTGGCAGGAAGTAGGAAAAGCAATGTATGCCCCTCCCATCCAAGG
AATAATTAGCTGTTCATCAAATATTACAGGGCTACTATTAACAAGAGATGGTGGGAACAGCAATACAAGTAGA
AACAATGAGACCTTCAGACCTGGGGGAGGAGATATGAGGGACAATTGGAGAAGTGAATTATATAAATATAAAG
TAGTGAGAATTGAGCCATTAGGAATAGCACCCACCAAGGCAAAGAGAAGAGTGGTGCAGAGAGAAAAAAGAGC
AGTGGGAACACTAGGAGCTATGTTCCTTGGGTTTCTGGGAACAGCAGGAAGCACTATGGGCGCAGCGGCAGTG
ACGCTGACGGTACAAGCCAGGCAATTATTGTCTGGTATAGTGCAACAGCAGAACAACCTGCTGAGAGCTATTG
AGGCGCAACAGCATCTGTTGCAACTCACAGTCTGGGGCATC
>MN486020

ACTCAAGGAAATAGTACTGGGAGTAATAATAACAATGAGACCATCACACTCCCATGCAGAATAAAACAAATTG
TAAACATGTGGCAGGAAGTGGGAAAAGCGATGTATGCCCCTCCCATCGGAGGACTAATTAGGTGTACATCAAA
TATTACAGGGTTACTATTAACAAGAGATGGTGGTGGAACAGATAACACCACTGAGACATCTGAGACATTCAGA
CCAGGAGGGGGAAATATGAAGGACAATTGGAGAAGTGAATTATATAAATACAAAGTAGTAAAAATTGAACCAT
TAGGAGTAGCACCCACCAAGGCAAGGAGAAGAGTGGTGCAGAGAGAAAAAAGAATTGGAACACTAGGAGCCAT
GTTCCTTGGATTCTTGGGAGCAGCAGGAAGCACTATGGGCGCAGCGTCAATAACGGTGACGGTACAGGCCAGA
CAATTACTGTCTGGGATAGTGCAGCAGCAGAACAATCTGCTGAGGGCTATTGAGGCGCAACAGCATATGTTGC
AACTCACAGTCTGGGGCATC
>EF637053
ACTCAGAACAGTACTTGGCACAAGAATGAAACTACTTGGCAAATGAATGGAAATACTGGAGAGAGGACTAATG
ATACTATCACACTCCCATGCAGAATAAAACAAATTATAAACAGGTGGCAGGAAGTAGGAAAAGCAATGTATGC
CCCTCCCATCAGAGGACTAATTAGATGTTCATCAAATATCACAGGGCTGCTATTAACAAGAGATGGTGGTGGG
GACAACGGGTCTGAGTCTGAGACCTTCAGACCTGGAGGAGGAAATATGAGGGATAATTGGAGAAGTGAATTAT
ATAAATATAAAGTAGTAAGAATTGAGCCATTAGGAGTAGCACCCACCAAGGCAAAGAGAAGAGTGGTGCAGAG
AGAAAAAAGAGCAGTGGGAACAATAGGAGCTATGTTCCTTGGGTTCTTGGGAGCAGCAGGAAGCACTATGGGC
GCAGCGTCACTGACGCTGACGGTACAGGCCAGACAATTATTGTCTGGTATAGTGCAACAGCAAAACAATTTGC
TGAGAGCTATTGAGGCGCAACAACATCTGTTGCAACTCACAGTCTGGGGCATC
>JN692454
ACTCAGCTGTTTAATAATACTTGGAATACAACACAGCTTGATGATGATGAGCAGGATGACAATGGCACCCTCA
CCCTCCCATGCAGAATAAAACAAATCATAAACAAGTGGCAGGAAGTAGGAAAAGCAATATATGCCCCTCCCAT
CAAAGGCTTAATTAGCTGTAACTCAACAATTACAGGGCTGATATTAACAAGAGATGGTAATGGAACCGACCAT
GACAATGAGTCTGAAATTTTCAGACCTGGAGGAGGAAACATGAAGGACAATTGGAGAAGTGAATTATATAAAT
ATAAAGTAGTAAGAATTGAGCCATTAGGAATAGCACCCTCCAAGGCAAAGAGAAGAGTGGTGCAAAGAGAGAA
AAGAGCAGTGGGAATGATAGGAGCTATGTTCCTTGGGTTCTTGGGAGCAGCAGGAAGCACTATGGGCGCAGCG
TCAGTAACGCTGACGGTACAGGCCAGACTATTATTGTCTGGTATAGTGCAACAGCAAAACAACTTGCTGAGAG
CTATTGAGGCGCAACAGCATATGTTGCAACTCACAGTCTGGGGCATT
>JN692455
AATTATACTTTGGAAAATGATACTTTCAAAAATGATACTAGGCATAAAACTGAGAATATCACACTCCAATGCA
GAATAAAACAAATTATAAACAGGTGGCAGAAAGCAGGAAAGGCAATGTATGCCCCTCCCATCAAAGGACAGAT
TAGATGTTCATCAAACATTACAGGGATATTATTAACAAGAGATGGTGGCATACACAACAACAGTAGTGACAAC
AATACAGAGATCTTCAGACCTGGAGGAGGAGACATGAGGGACAATTGGAGAAGTGAATTATATAAATATAAAG
TAGTAAAAATTGAGCCATTAGGAGTAGCACCCACCAAGGCAAGGAGAAGAGTGGTGCAGAGAGAAAAAAGAGC
AGTGGGAACGATAGGAGCTATGTTCCTTGGGTTCTTGGGAGCAGCAGGAAGCACTATGGGCGCAGCATCACTG
ACGCTGACGGTACAGGCCAGACAATTATTGTCTGGTATAGTGCAACAGCAGAACAATCTGCTGAGGGCCATTG
AGGCGCAACAGCATCTGTTGCAGCTTACAGTCTGGGGCATC
>FJ195086
GGTCAGTGGGATGGTACTATTGGGCCAGATGAGAATGGCAATATCACACTCCCATGCAGAATAAAACAAATTA
TAAACAGGTGGCAGGAAGTAGGAAAAGCAATATATGCCCCTCCCATCAGAGGACAAATTAGCTGTTCCTCAAA
TATTACAGGGCTGCTACTCACAAGAGATGGTGGTAGTAATAACACGAGCAATAATGAGACCTTCAGACCGGGA
GGAGGAAATATGAAGGACAATTGGAGAAGTGAATTATATAAATATAAAGTAGTACAAATTGAGCCATTAGGAA
TAGCACCCACCAAGGCAAAAAGAAGAGTGGTGCAGAGAGAGAAAAGAGCAGTAATAGGAGCTATGTTCCTTGG
GTTCTTGGGAGCAGCAGGAAGCACTATGGGCGCAGCATCACTGACGCTGACGGTACAGGCCAGACTATTATTG
TCTGGTATAGTGCAACAGCAGAGCAATCTGCTGAGGGCTATTGAGGCGCAACAGCATCTGTTGCAACTCACAG
TCTGGGGCATC
>JN692468
AGTTACAACAACACTAGCAAAACTTGGAATAAAACTGAAGAGAACCATACTGGAGTAAATAACACAATCACAC
TCCCATGTAGAATAAAGCAAATTATAAACAGGTGGCAGGAAGTAGGAAAAGCAATGTATGCCCCTCCCATTAG
GGGACTAATTAAATGTACATCAAATATTACAGGGATAATATTAACAAGAGATGGTGGTAATGAGACTGGGACC
GAGACCTTCAGACCTGGAGGAGGAGATATGAGGGACAACTGGAGAAGTGAATTATATAAATATAAAGTAGTAA
AAATTGAGCCATTAGGAATAGCACCCACCAAGGCAAGGAGAAGAGTGGTGCAGAGAGAAAAAAGAGCAGTGGG
AACAATAGGAGCTATGTTCCTTGGGTTCTTGGGAACAGCAGGAAGCACTATGGGCGCAGCGTCAATCACGCTG
ACGGTACAGGCCAGACAATTATTGTCTGGTATAGTGCAGCAGCAGAACAATTTGCTGAAGGCTATTGAGGCGC
AACAACATCTGTTGCAACTCACAGTCTGGGGCATC
>KT427737
ACTCATAGTACTTGGAATACGAATACAACTGGAAATGACAATATCACACTCCAGTGCAGAATAAAACAAATTA
TAAACAGGTGGCAAGAAGTAGGAAAAGCAATATATGCCCCTCCCATCAAAGGAAAAATTAATTGTCTATCAAA
TATTACAGGGCTGCTGTTAGTAAGAGATGGTGGTAGGAACACAAGTGGGTCCGAAACCTTCAGACCTGGAGGA
GGAAATATGAGGGACAATTGGAGAAGTGAATTATATAAATATAAAGTAGTAAAAATTGAACCACTAGGAATAG
CACCCACCAAGGCAAAGAGAAGAGCGGTGCAGAGAGAGAGAAGAGCAGTGGGAACACTGGGAGCTATGTTCCT
TGGGTTCTTGGGAGCAGCAGGAAGCACTATGGGCGCAGCGGCAACAACGCTGACGGTACAGGCCAGACTATTA

TTGTCTGGTATAGTGCAACAGCAAAATAATTTGCTGAGGGCTATTGAGGCGCAACAGCACATGTTGCAACTCA
CAGTCTGGGGCATC
>KT427704
AATTATACTCCCAATACTACAGGAAATATTACACTCCCATGCAGAATAAAACAAATTATAAACAGATGGCAGG
AAGTAGGAAAAGCAATGTATGCCCCTCCCATCGGAGGACAAATTAAATGTTCATCAAATATTACAGGGATGAT
ATTAACATGGGATGGGGGAACGAAACCAGGGACAATGAGACCTTCAGACCTGGAGGAGGAGATATGAGGGAC
AATTGGAGAAGTGAATTATATAAATATAAAGTAGTAAAAATTGAGCCAATAGGAGTAGCACCCACCAAGGCAA
AGAGAAGAGTGGTGCAGAGAGAAAAAAGAGCGGTAACGCTAGGAGCCATGTTCCTTGGGTTCTTAGGAGCAGC
AGGAAGCACTATGGGCGCAGCGTCAATGACGCTGACGGTACAGGCCAGACAATTATTGTCTGGTATAGTGCAA
CAGCAAAGCAATTTGCTGAGGGCTATTGAGGCGCAACAGCATCTGTTGCAACTCACAGTCTGGGGCATT
>KT427872
ACTCACTGGTCATTAAATATAACTGGCACTGGCAATATCACACTTCCATGCAGAATAAAACAAATTATAAACA
AGTGGCAGGAAGTAGGAAGAGCAATATACGCCCCTCCCATCCAAGGATTAATTAGATGCTCATCAAATATTAC
AGGGCTGCTACTGACACGGGATGGTGGTATCACAAACTCTAGCAACCCTGAGGTCTTCAGACCTGGGGGAGGA
GATATGAGAGACAATTGGAGAAGTGAACTATATAAATATAAGGTAGTAAAAATTGAGCCAGTAGGAATAGCAC
CCACCAAGGCAAGGAGAAGAGTGGTGCAAAGAGAAAAAAGAGCAGTGGGGACATTAGGAGCTATGTTCCTTGG
GTTCTTGGGAGCAGCAGGAAGCACTATGGGCGCAGCGTCATTGACGCTGACGGTACAGGCCAGACTATTATTG
TCTGGTATAGTGCAACAGCAAAACAATTTGCTGAGAGCTATTGAGGCGCAACAGCATATGTTGCAACTCACAG
TCTGGGGCATC
>KJ849818
AGTTACCTTAATGAAACTAACGAAACAAATATCACACTCCCATGCAGAATTAAACAAATTATAAACATGTGGC
AGAGAGTAGGAAAAGCAATGTATGCCCCTCCCATTCCAGGACCAATTAACTGTTCATCAAATATTACAGGGCT
GCTCTTAGTAAGAGACGGTGGTAATAATAATACAAATGAGACCTTCAGACCTGGAGGAGGAAATATGAAGGAC
AATTGGAGAAGTGAATTATATCGATATAAAGTAGTAAAAATTGAACCACTAGGACTAGCACCTACCACGGCAA
AGAGAAGAGTGGTGCAGAGAGAAAGAAGAGCAGTGGGACTAGGAGCGTTGTTCCTTGGGTTCTTAGGAGCAGC
AGGAAGCACTATGGGCGCAGCGTCACTGACGCTGACGGTACAAGCCAGACAATTATTGTCTGGTATAGTGCAA
CAGCAGAACAACCTGCTGATGGCTATTGAGGCGCAACAACATATGTTGCAACTCACAGTCTGGGGCATC
>KT427751
TTTCATTGGCCTGTCAATGACACTAATAGTAATAGTACAAATGATATAGTTACCATCCCATGCAGAATAAAAC
AAATTATAAACAGATGGCAGGAAGTAGGAAAAGCAATGTATGCCCCTCCCATCAGAGGGCAGATTAAATGTTC
ATCAAATATTACAGGGCTGCTATTGACAAGGGATGGTGGTAACAACAACACGGAGAACAATACAGAGGTCTTC
AGACCTGAAGGAGGAGATATGAGGGACAATTGGAGAAGTGAACTATATAAATATAAAGTAGTAAAAATTGAGC
CATTAGGAGTAGCACCCACCAAAGCAAAGAGAAGAGTGGTGCAGAGGGAAAAAAGAGCAGTGGGAGTGATAGG
AGCTATGTTCCTTGGGTTCTTGGGAGCAGCAGGAAGCACTATGGGCGCAGCGTCACTGACGCTGACGGTACAG
GCTAGACAATTATTGTCTGGTATAGTGCAACAGCAAAACAATTTGCTGAGAGCTATTGAGGCGCAACAGCATC
TGTTGCAACTCACAGTCTGGGGCATC
>KT427803
TTTCACAGTTCTTGGAATGCAAGTAATTTGAATACAACACAGAATGGGACTGATAATAGTAATATCACACTCC
CATGCAGAATAAAACAAATTATAAATAGGTGGCAGGAAGTAGGAAAATCAATGTATGCCCCTCCCGTCCAAGG
ACTAATTAAATGTTCATCAAATATTACAGGGCTGCTATTAACAAGAGATGGTGGTAGCAACACCACGGAGAAG
GAAGTGTTCAGACCTGGGGGAGGAAATATGAAGGACAATTGGAGAAGTGAATTATATAAATATAAAGTAGTAA
AAATTGAGCCATTAGGAGTAGCACCCACCAAGGCAAGGAGAAGAGTGGTGCAGAGAGAAAAAAGAGCAGTGAC
ACTAGGAGCTATGTTCCTTGGGTTCTTGGGAGCAGCAGGAAGCACTATGGGCGCAGCGTCACTAACGCTGACG
GTACAGGCCAGACAATTATTGTCTGGTATAGTGCAGCAGCAGAACAATTTGCTGAGGGCTATTGAGGCGCAAC
AACATATGTTGCAACTCACAGTCTGGGGCATC
>KT427797
TTTTATAGTAACTGGACTACAAATTCAACTAACGAAGGGAACTGGACTACAAATTCAACTAACAATGGGAATA
ACACCATCACACTCCCATGCAGAATAAAACAAATTGTAAACATGTGGCAGGAAGTAGGAAAAGCAATGTATGC
CCCTCCCATCAGTGGAATAATTAAATGTTCATCAAATATTACAGGGATACTGTTAACAAGAGATGGTGGTAAT
AATAGTACGAACGAGACCTTCAGACCAGCAGGAGGAGATATGAGGGACAATTGGAGAAGTGAATTATATAAAT
ATAAAGTAGTAAAAATTGAACCATTAGGATTAGCACCTACCAAGGCAAGGAGAAGAGTGGTGCAGAGAGAAAA
AAGAGCAGTGGGAACACTAGGAGCTATGTTCCTTGGGTTCTTGGGAGCAGCGGGAAGCACTATGGGCGCAGCG
TCAATGGCGCTGACGGTACAGGCCAGACTATTATTGTCTGGTATAGTGCAACAGCAAAATAATTTGCTGAAGG
CTATTGAGGCGCAACAACACCTGTTGCAACTCACGGTCTGGGGCATT
>KX181894
AGTTATTGGCTGAATAATACTTGGGTGAATACGACTCTGGAAATCAATGGTACTATCACACTCCCATGCAGAA
TAAAACAGATTATAAACAGGTGGCAAGAAGTAGGAAAAGCAATGTATGCCCCCCCCATCAGAGGACACATTAA
TTGTTTATCAAACATTACAGGGCTGATATTAGCAAGGGATGGTGGTATTAATGTGACCAACGATAGCGAGACC
TTCAGACCTGGAGGAGGAGACATGAGGGACAATTGGAGAAGTGAATTATACAAATATAAAGTAGTAAAAATTG
AGCCATTAGGAGTAGCACCCACCAAGGCAAAGAGAAGAGTGGTGCAAAGAGAAAAAAGAGCAGTGGGAACGCT

AGGAGCCATGTTCCTTGGGTTCTTGGGAGCAGCAGGAAGCACTATGGGCGCAGCGGCAACAACGCTGACGGTA
CAGGCCAGACTATTGTTGTCTGGTATAGTGCAACAGCAGAACAATCTGCTGAGGGCTATTGAGGCGCAACAGC
ATATGTTGCAACTCACAGTCTGGGGCATC
>KX181903
ATTTACGGATGGAATGGAACAATCCCCGCTAACATCACACTTCCATGTAGAATAAAACAAATTATAAACAGGT
GGCAGGAAGTAGGAAAAGCAATGTATGCCCCTCCCGTCAGCGGGCTAATTAACTGTACATCAAATATTACAGG
ACTACTATTAACAAGAGATGGTGGTATCAATGAAAATAATATATCTGAAACCTTCAGACCTGGAGGAGGAGAT
ATGAGGGACAATTGGAGAAGTGAATTATATAAATATAAAGTAGTAAAAATTGAACCATTAGGAATAGCACCCT
CCAAGGCACAGAGAAGAGTGGTGCAGAGAGAAAAAAGAGCAGTGGGAACGCTGGGAGCCATGTTCCTTGGGTT
CTTGGGAGCAGCAGGAAGCACTATGGGCGCAGCGGCAGCAACGCTGACGGTACAAGCCAGACTATTGTTGTCT
GGTATAGTGCAGCAGCAGAACAATCTGCTGAGGGCTATTGAGGCGCAACAGCATATGTTGCAACTCACAGTCT
GGGGCATC
>KX181914
AATCAGACGTCTAATGGGAATAGGACCATTACACTCCCATGCAGAATAAAACAAATCATAAACAGATGGCAAG
AAGTAGGACAAGCAATGTATGCCCCTCCCATTAGAGGACAGATTAGATGTACATCAAATATTACAGGGTTACT
ATTAACAAGAGATGGTGGTAACAGGAACACCACAGACAACAATACTGAGGTTTTCAGACCTGGAGGAGGAGAT
ATGAGGGACAATTGGAGAAGTGAATTATATAAATATAAAGTAGTAAAAATTGAGCCATTAGGAATAGCACCCA
CTAGGGCAAAGAGGAGAGTGGTGCAAAGAGAGAAAAGAGCAGCGGGACTAGGAGCTTTGTTCATTGGGTTCTT
GGGAGCAGCAGGAAGCACTATGGGCGCAGCGTCAGTGACGCTGACGGTACAGGCCAGACAATTATTGTCTGGA
ATAGTGCAACAGCAGAACAATTTGCTGAGAGCTATTGAGGCGCAACAACATCTGTTGCAACTCACAGTCTGGG
GCATC
>MG571982
ACTCATAAAACTTGGGAAAATGGTAATGATAATGAAACTATCACACTCCCATGCAGAATAAAACAAATTATAA
ACAGGTGGCAGGAAGTAGGAAGAGCAATGTATGCCCCTCCCATTGCAGGACAAATTAACTGTACATCAAATAT
AACAGGGATGATATTAACAAGAGATGGGGGTAATGAGAGCGCACCTGAAGAAATCTTCAGGCCTGGGGGAGGA
GATATGAGGGACAATTGGAGAAGTGAATTATATAAATATAAGGTAGTAAAAATTGAGCCATTAGGAGTAGCAC
CCACCAAGGCAAAGAGAAGAGTGGTGCAGAGAGAAAAAAGAGCGATAACGCTGGGAGCTATGTTCCTTGGGTT
CTTGGGAGCAGCAGGAAGCACTATGGGCGCAGCGTCGCTGACGCTGACGGTACAGGCCAGACTATTATTGTCT
GGTATAGTGCAGCAGCAGAACAATCTGCTGAGAGCTATTGAGGCGCAACAGCATATATTGCAACTCACAGTCT
GGGGCATT
>MG571991
AATTATACAGGGTCTAATGACACCATCACACTCCCATGCAAAATAAAACAAATTGTAAACAAGTGGCAGGAAG
TAGGAAAAGCAATATACGCCCCTCCCATCAGTGGAAACATTAGCTGTTCATCAAATATTACAGGGCTGATATT
AACAAGAGATGGAGGCGATAATGAGTCTGAAACTTTCAGACCTGGAGGAGGAAATATGAAAGACAATTGGAGA
AGTGAATTATATAAATATAAAGTAGTAAAAATTGAGCCATTAGGAGTAGCACCCACCAAGGCAAAGAGAAGAG
TGGTGCAGAGAGAAAAAAGAGCAGTGGGAACAATAGGAGCTATGTTCCTTGGGTTCTTGGGAGCAGCAGGAAG
CACTATGGGCGCAGCGGCAGTAACGCTGACGGTACAGGCCAGACAATTATTGTCTGGTATAGTGCAACAGCAG
AACAACCTGCTGAGGGCTATTGAGGCGCAGCAGCATATGTTGCAGCTCACAGTCTGGGGCATC
>FJ653506
GATCAAAGTAATAGTACTTGGCATACTAATAATACTTGGAATAATACTGAAGGCGTTATCACACTCCACTGCA
GAATAAAACAAGTTATAAACCTGTGGCAGGAAGTAGGAAAAGCAATGTATGCCCCTCCCATCCAAGGACAAAT
TAACTGTACATCAAATATTACAGGACTGCTTTTAACAAGAGATGGTGGGAATAACAATGGTAGTAATAACAGT
GAGACCGAGACCTTTAGACCTGGAGGAGGAGATATGAGGGACAATTGGAGAAGTGAATTATATAAATATAAAG
TAGTAAGAATTGAACCATTAGGAATAGCACCCACCAAGGCAAAAAGAAGAGTGGTGCAAAGAGAGAAAAGAGC
AGTGGGACTGGGAGCTATATTCCTTGGGTTCTTGGGAGCAGCCGGAAGCACTATGGGCGCAGCGTCGATAACG
CTGACGGTACAGGCCAGACAAGTATTGTCTGGTATAGTGCAACAGCAGAACAATTTGCTGAAGGCTATTGAGG
CGCAACAGCATTTGCTGCAACTCACAGTCTGGGGCATT
>EU023932
ACTCATTTGAGTAATACTACGGAGTCAAATGAAAATGACACAATCACACTCCAATGTAGAATAAAACAAATTA
TAAACATGTGGCAGGAAGTAGGAAAAGCAATGTATGCCCCTCCCATTGCAGGACAAATTAACTGTTCATCAAA
AATTACAGGGCTACTTTTAACAAGAGATGGTAGTAGCACAAATGAGACAAATGGGACTGAGATCTTCAGACCT
GGAGGAGGAGATATGAGGGACAATTGGAGAAGTGAATTATATAAATATAAAGTAGTAGGAATTGAACCAATAG
GAATAGCACCCACCAAGGCAAAGAGAAGAGTGGTGCAGAGAGAAAAAAGAGCAGTGGGAACATTAGGAGCTAT
GTTCCTTGGGTTCTTGGGAGCGGCAGGAAGCACTATGGGCGCAGCGTCAATGACGCTGACGGTACAGGCCAGA
CAATTATTGTCTGGTATAGTGCAGCAGCAAAATAATTTGCTGAGGGCTATTGAGGCGCAACAACATGTGTTGC
AACTCACAGTCTGGGGCATC
>JF932469
AATTATACTTGGGAAGTCACTAATACTTGGAATGATACTGCAGGAAATGACAATATCACGCTCCCTTGCAGAA
TAAAACAAATTGTAAATATGTGGCAGGAAGTAGGGAGAGCAATGTATGCCCCCCCATTGAAGGACAAATTAG
ATGTTCATCAAATATTACAGGGTTGCTATTAACAAGAGATGGTGGTAATACTACGAACGGGACCGAGATCTTC

AGACCTGGAGGAGGAAATATGAGGGACAATTGGAGAAGTGAATTATATAAATATAAAGTAGTAAAAATTGAAC
CATTAGGAGTAGCACCCACCAAGGCAAAGAGAAGAGTGGTGCAGAGAGAAAAAAGAGCAGTGGGAACGATAGG
AGCTATGTTCCTTGGGTTCTTGGGAGCAGCAGGAAGCACTATGGGCGCAGCGTCAATAACGCTGACGGTACAG
GCCAGACAATTGTTGTCTGGTATAGTGCAACAGCAGAGAAATTTGCTGAGGGCTATTGAGGCGCAACAGCAGC
TGTTGCAACTCACAGTCTGGGGCATC
>KM218138
AATTATACTTGGAATAATGGCACCTGGCTAGCGCCAAATATCACTGGAAATAACATCACCATCCATTGCAGAA
TAAAACAAATTATAAACAGGTGGCAGGAAGTAGGAAAAGCAATGTATGCCCCTCCCATCGCGGGACAACTTAA
TTGCACATCAAATATTACAGGGCTGCTATTAGTAAGAGATGGTGGTAACACCACCAACACCATCGAGAACAAC
ACCGAAATCTTCAGACCTGGAGGAGGAGATATGAGGGACAATTGGAGAAGTGAACTATATAAATATAAAGTAG
TAAAAATTGAGCCATTAGGACTAGCACCCACCAAGGCAAAGAGAAGAGTGGTGCAGAGAGAAAAAAGAGCAGT
GGGAACAATAGGAGCTATGTTCCTTGGGTTCTTGGGAGCAGCAGGAAGCACTATGGGCGCAGCGTCACTCACG
CTGACGGTACAGGCCAGACTATTACTGTCTGGGATAGTGCAACAGCAAAACAATTTGCTGAAGGCTATTGAGG
CGCAACAGCACCTGTTGCAACTCACAGTCTGGGGCATC
>KM218195
AATCATACTTGGCTGGCTAATAATACTTGGCAAGAGTCTTGGCAAGGGTCAGATACTATTACTCTCCAATGCA
GAATAAAACAAATTATAAACAGATGGCAGGAAGTAGGAAAAGCAATGTATAACCCTCCCATCAGGGGACAAAT
TAGATGTTCATCAAATATTACAGGGCTTCTGTTAACAAGAGATGGTGGTAACACGAATGGGTCCAGAAATGGG
CCTGAAACCTTCAGACCTGGAGGAGGAGATATGAGAGACAATTGGAGAAGTGAATTATATAAATATAAAGTAG
TAAAAATTGAGCCACTAGGAATAGCACCCACCAAGGCAAGGAGAAGAGTGGTGCAGAGAGAAAAAAGAGCAGT
GGGAACAATAGGAGCTATGTTCCTTGGGTTCTTGGGAGCAGCAGGAAGCACTATGGGCGCAGCGTCAGTGACG
CTGACGGTACAGGCCAGACTATTACTGTCTGGTATAGTGCAACAGCAGAACAATCTGCTGAAAGCTATTGAGG
CGCAACAGCACCTGTTGCAACTCACAGTCTGGGGCATC
>JF932478
ACTTATAATGGTACTTGGGAAGGTACTACAGGAAATGACACTATCACGCTCCCTTGCAGAATAAAACAAATTA
TAAACATGTGGCAGGAAGTAGGGAAAGCAATGTATGCCCCTCCCATTAGAGGACAAATTAGATGTTCATCAAA
TATTACAGGGTTACTATTAACAAGAGATGGTGGTACTAATGAGACCAATACCAACGAGACCTTCAGACCTGGA
GGAGGAGATATGAGAGACAATTGGAGAAGTGAATTATATAAATATAAAGTAGTACAAATTGAACCATTAGGAG
TAGCACCCACCAAGGCAAAAAGGAGAGTGGTGCAGAGAGAAAAAAGAGCAGTGGGAACAATAGGAGCTATGTT
CCTTGGGTTCTTGGGAGCAGCAGGAAGCACAATGGGCGCAGCGTCAATAACGCTGACGGTACAGGCCAGACAA
TTGTTGTCTGGCATAGTGCAACAGCAGAGAAATTTGCTGAGGGCTATTGAGGCGCAACAGCATCTGTTGCAAC
TCACAGTCTGGGGCATC
>JF346918
ACTTATGCTTGGAATGATACTAGGAATACAGTAAATGACACTATCACGCTCCCTTGCAGAATAAAGCAAATTA
TAAACATGTGGCAGGGAGTAGGGAAAGCAATGTATGCCCCTCCCATTAGAGGACAAATTAGGTGTGTATCAAA
TATTACAGGGTTGCTATTAACAAGAGATGGTGGTATTAATAAGAACAGCACCCCCGAGACCTTCAGACCTGGA
GGAGGAGATATGAGGGACAATTGGAGAAGTGAATTATATAAATATAAAGTAGTAAGAATTGAACCATTAGGAG
TAGCACCCACCAAGGCAAAGAGAAGAGTGGTGCAGAGAGAAAAAAGAGCAGTGGGAACGTTCGGAGCTATGTT
CCTTGGGTTCTTGGGAGCAGCAGGAAGCACTATGGGCGCAGCGTCACTAACGCTGACGGTACAGGCCAGACAA
TTGTTGTCTGGTATAGTGCAACAGCAGAGAAATCTGCTGAGGGCTATTGAGGCGCAACAGCACATGTTGCAAC
TCACAGTCTGGGGCATC
>KC596066
ACTTATCTGGCTAATGATACTAGATCAGAGAAGAATACTACTGGTGTCATCACACTCCCATGCAGAATAAAAC
AAATTATAAACAGGTGGCAAGAAGTAGGGAGAGCAATGTATGCCCCTCCCATCATGGGAAAAATTAACTGTTC
ATCAAATATTACAGGGATACTATTAACAAGAGATGGTGGTGAAACAAATGAGTCCAGAAATGGGTCCGAAACC
TTCAGACCGGGAGGAGGAAATATGAAAGATAATTGGAGAAGTGAATTATATAAATATAAAGTAGTAAAAATTG
AGCCACTAGGAATAGCACCCACCAAGGCAAGGAGAAGAGTGGTGCAGAGAGAAAAAAGAGCAGTAACACTAGG
AGCTGTATTTCTCGGGTTCTTGGGAGCAGCAGGAAGCACTATGGGCGCAGCGTCAATGACGCTGACGGTACAG
GCCAGACTATTACTGTCTGGTATAGTGCAACAGCAGAACAATCTGCTGAGAGCTATTGAGGCGCAACAGCACA
TGTTGCAACTCACAGTCTGGGGCATC
>KX693556
CCTCAGATTGCTACTACAGGAAATGACACTATCACGCTCCCTTGCAGAATAAGACAAATTGTAAACATGTGGC
AGCAAGTAGGGAAAGCAATGTATGCCCCTCCCATAGCAGGACAAATTAGATGTTCATCAAATATTACAGGGGT
GCTATTAACAAGAGATGGTGGTAATAATGAGAGCAAGGCCAACGCCAACGAGACCTTCAGACCTGCGGGAGGA
GATATGAGAGACAATTGGAGAAGTGAATTATATAAATATAAAGTAGTAAAAATTGAACCATTAGGAGTAGCAC
CCACCAAGGCAAAGAGAAGAGTGGTGCAGAGAGAAAAAAGAGCAGTGGGAACACTAGGAGCTATGTTCCTTGG
GTTCTTGGGAGCAGCAGGAAGCACTATGGGCGCAGCGTCAATAACGCTGACGGTACAGGCCAGACAATTGTTG
TCTGGTATAGTGCAGCAGCAGAGAAATTTGCTGAGAGCTATTGAGGCGCAACAGCATCTGTTGCAACTCACAG
TCTGGGGCATC
>KX693136

ACTCAGACAGGTACTACAGGAAACAGCACTATCACGCTCCCTTGCAGAATAAAACAAATTGTAAACATGTGGC
AGGAAGTAGGGAAAGCAATGTATGCCCCTCCCATTGCAGGAAAAATTAGATGTTCATCAAATATTACAGGGCT
GCTATTAACAAGAGATGGTGGTAATGAGAGCAAGACCACCAACACAGAAACCTTCAGACCTGGAGGAGGAGAT
ATGAGAGACAATTGGAGAAGTGAATTATATAAATATAAAGTAGTAAAAATTGAACCATTAGGAGTAGCACCCA
CCAAGGCAAAGAGAAGAGTGGTGCAGAGAGAAAAAAGAGCAGTGGGAACAATAGGAGCTATGTTCCTTGGGTT
CTTGGGAGCAGCAGGAAGCACTATGGGCGCAGCGTCAATAACGCTGACGGTACAGGCCAGACAATTGTTGTCT
GGTATCGTGCAACAGCAGAGAAATTTGCTGAGAGCTATTGAGGCGCAACAGCATCTGTTGCAACTCACAGTCT
GGGGCATC
>KX693292
AATTATACTAAAGAAGATGACAATCTCACGCTCCCTTGCAGAATAAAACAAATTGTAAACATGTGGCAGGGAG
TAGGGAGAGCAATGTATGCCCCTCCCATTAGAGGACAAATTAGATGTTCATCAAATATTACAGGGTTGATATT
AACAAGAGATGGTGGCAAAAATGGTAAAAATGAGAGCAACACCAATGAGACCTTCAGACCTATAGGAGGAAAT
ATGAAAGACAATTGGAGAAGTGAATTATATAAATATAAAATAGTAAAAATTGAACCATTAGGAGTAGCACCCA
CCAAGGCAAAGAGAAGAGTGGTGCAGAGAGAAAAAAGAGCAGTGGGAACGCTGGGAGCTATGTTCCTTGGGTT
CTTGGGAACAGCAGGAAGCACTATGGGCGCAGCGTCACTAACGCTGACGGTACAGGCCAGACAATTGTTGTCT
GGTATAGTGCAACAGCAGAGAAATCTGCTGAGGGCTATTGAGGCGCAACAGCATATGTTGCAACTCACAGTCT
GGGGCATC
>KP109511
AATTATACTCTCCTGGCTAATGGTACTTGGCAAGGGCCAAACAGTACTAATGGAAATGGCACACTCACACTCC
CATGCAGAATAAAACAAATCATAAACAGGTGGCAGGAAGTAGGAAAAGCAATGTATGCCCCTCCCATCGCGGG
AAACATTAGCTGTTTATCAAATATTACAGGGCTGCTATTAACAAGAGATGGTGGTATCAACATGACGAATAAT
GGGTCCGAAATCTTCAGACCTGAAGGAGGAGATATGAGGAACAATTGGAGAAGTGAATTATATAAATATAAAG
TAGTAAAAGTTGAGCCACTAGGAATAGCACCCACCAGGGCAAAGAGAAGAGTGGTGCAGAGAGATAAAAGAGC
AGTGGGAACAATAGGAGCTATGTTCCTCGGGTTCTTGGGAGCAGCAGGAAGCACTATGGGCGCAGCGTCAGTG
ACGCTGACGGTACAGGCCAGACTATTACTGTCTGGTATAGTGCAACAGCAGAACAATCTGCTGAGAGCTATTG
AGGCGCAACAGCACATGTTGCAACTCACAGTCTGGGGCATC
>KU724105
AATTATACTTTGTTTAATAATGGTACTTGGAAAGAGGGTACTAACAAAAGTGTCATCACACTCCCATGCAGAA
TAAAACAAATTATAAACAGGTGGCAGGAAGTAGGAAAAGCAATGTATGCCCCTCCCATCAGGGGACAAATTAA
CTGTACATCAAATATTACAGGGCTGCTATTAACAAGAGATGGTGGTAATAGTGGTACTGGGTCCAAAAATGGG
AACGAAACCTTCAGACCTGGAGGGGGAAATATGAGAGACAATTGGAGAAGTGAATTATATAAATATAAAGTAG
TAAAAATTGAGCCACTAGGAATAGCCCCCACCACGGCAAGGAGAAGAGTGGTGCAGAGAGAAAGAAGAGCAGT
GGGAACATTAGGAGCTATGTTCCTCGGGTTCTTGGGAGCAGCAGGAAGCACTATGGGCGCAGCGTCGCTGACG
CTGACGGTACAGGCCAGACTATTACTGTCTGGTATAGTGCAACAGCAGAACAATCTGCTGAGAGCTATTGAGG
CGCAACAGCATATGTTGCAACTCACAGTCTGGGGCATC
>AY561238
ACTCAGTGGAAAGGAAATAGCACACTCCCAGAAAATGTTACACTCCCATGCAGAATAAAACAATTTGTAAACA
TGTGGCAGGAAGTAGGAAAAGCAATGTATGCCCCTCCCATCAGCGGACAAATTAGATGCTCATCAAATATTAC
AGGGCTGCTATTAACAAGAGATGGTGGTGCCAATGGTACCACTGATATAGACGAGATCTTCAGGCCTGGAGGA
GGAGATATGAGGGACAATTGGAGAAGTGAATTATATAAATATAAAGTAGTAAAAATTGAACCATTAGGAATAG
CACCCACCAAGGCAAAGAGAAAAGTGGTGCAAAGAGAAAAAAGAGCAGTGGGACTAGGAGCTTTGTTCCTTAG
GTTCTTAGGAGCAGCAGGAAGCACTATGGGCGCAGCGTCAGTGACGCTGACGGTACAGGCCAGACAATTATTG
TCTGGTATAGTGCAACAGCAGAACAATCTGCTGAGAGCTATTGATGCGCAACAGCATCTGTTGCAACTCACAG
TCTGGGGCATT
>FJ388940
TTTCAATATAATAGTACTTGGAATGATACTGGAGAGATGAATGGCACTATCATGCTCCCATGCAGAATAAGAC
AAATCATAAACCTGTGGCAGGAAGTAGGAAAAGCAATGTATGCCCCTCCCATCAAAGGACTAATCAGATGTTC
ATCAAATATTACAGGACTGCTATTACTAAGAGATGGTGGTAATAATAGCAGTGGGAGTAATAACACCGAGATC
TTCAGACCTGGAGGAGGAGATATGAGGGACAATTGGAGAAGTGAATTATATAAATATAAAGTAGTAAAAATTG
AACCATTAGGAATAGCACCTACCAAGGCAAAGAGAAGAGTGGTGCAGAGAGAAAAAAGAGCAGTGGGATTCGG
AGCCGTGTTCCTTGGGTTCTTGGGAGCAGCAGGAAGCACTATGGGCGCAGCGTCAATAACGCTGACGGTACAG
GCCAGACAATTATTGTCTGGTATAGTGCAGCAGCAAAATAACTTGCTGAGGGCTATTGAGGCGCAGCAGCATC
TGTTACAACTCACAGTTTGGGGCATT
>JF683749
AATCACACGGGAAATAATGATAATGATACAATCACACTCCAATGCAGAATAAAGCAAATTATAAACATGTGGC
AGGAAGTAGGAAAAGCAATGTATGCCCCTCCCATCCAAGGAATAATTAGCTGTTCATCAAACATTACAGGGCT
GCTATTAACAAGAGATGGTGGTGATGACAATAGCAGCAGTACAGAGATCTTCAGACCTGGAGGAGGGGATATG
AGGGACAATTGGAGAAGCGAATTATATAAATATAAAGTAGTAAGAATTGAACCATTAGGGGTAGCACCCACCA
AGGCAAAGAGAAGAGTGGTGCAGAGAGAAAAAAGAGCAATAGGAATAGGAGCTTTGTTCCTCGGGTTCTTGGG
AACAGCAGGAAGCACTATGGGCGCAGCGTCATTGACGCTGACGGTACAGGCCAGACAATTATTGTCTGGTATA

GTGCAACAGCAGAACAATTTGCTGAGGGCTATTGAGGCGCAACAGCATATGTTGCAACTCACAGTCTGGGGCA
TC
>JF683765
AATCATACTTGGAATGGTACTGCAATGACTGCTTGGAATGGTACTGTAGTGACTACTTGGAATGGTACTGCAG
TGATTAATAGTACTACGGAGTCAAATATCACACTCCAATGCAGGATAAAACAAATAGTAAACATGTGGCAGGA
AGTAGGAAAAGCAATGTATGCCCCTCCCATCAGAGGACAGATTAGATGTTCATCAAATATTACAGGCCTATTA
TTAACAAGAGATGGTGGTCTTAACAGCAGCAATATCAACGAGACTTTCAGACCAGGAGGAGGAGATATGAGGG
ACAATTGGAGAAGTGAATTATATAAATATAAAGTAGTAAAAATTGAACCATTAGGGGTAGCACCCACCAGGGC
AAGGAGAAGAGTGGTGCAGAGAGAAAAAAGAGCAATAGGACTAGGAGCTGTGTTCCTTGGGTTCTTGGGAGCA
GCAGGAAGCACTATGGGCGCAGCGTCAATAACGCTGACGGCACAGGCCAGGCAATTGATGTCTGGTATAGTGC
AACAGCAGAACAATCTGCTGAGAGCTATTGAGGCGCAACAGCATCTGTTGCAACTCACAGTCTGGGGCATC
>JF683784
ACTCATAATGATACTAAAGGAATATATATCACACTCCCATGCAGAATAAGACAAATTGTAAATCTGTGGCAGG
AAGTAGGGAAAGCAATGTATGCCCCTCCCATCAGGGGACAAATTAACTGTTCCTCAAATATTACAGGGCTGAT
ATTAACAAGAGATGGTGGTCTGAATAATGATACTAATGAGATCTTCAGACCTGGAGGAGGAGATATGAGGGAT
AATTGGAGAAGTGAATTATATAAATATAAAGTAGTAAAAATTGAACCATTAGGAGTAGCACCCACCAAGGCAA
AGAGAAGAGTGGTGCAAAGAGAGAGAAGAGCAGTGGGAATAGGAGCTCTGTTCCTTGGGTTCTTGGGAGCAGC
AGGAAGCACTATGGGCGCAGCATCAATAACGCTGACGGTACAGGCCAGACAATTACTGTCTGGTATAGTGCAA
CAGCAGAACAATCTGCTGAGGGCTATTGAGGCGCAACAAAATCTGTTGCAACTCACAGTCTGGGGCATT
>JF683793
AATCATACTGGTGCTGAAAGCAATATCACACTCCCATGCAGAATAAGACAAATTATAAATATGTGGCAGAGAG
TAGGAAAAGCAATGTATGCCCCTCCCATCGAAGGAGAAATTAGATGCTCATCAAAAATTACAGGGCTGCTATT
AACAAGAGATGGTGGTAGTAGCGGCAACAACACCAACGACACAGAGATCTTCAGACCTGCAGGAGGAGATATG
AGGGACAATTGGAGAAGTGAATTATATAAATATAAAGTAGTAAGAATTGAACCTTTAGGAATAGCACCCACTA
AGGCAAAGAGAAGAGTGGTGCAAAAAGAGAAAAGAGCAGTGGGAATAGGAGCTCTATTCCTTGGGTTCTTGGG
AGCAGCAGGAAGCACTATGGGCGCAGCGTCATTGACGCTGACGGTACAGGCCAGACAGTTAATGTCTGGTATA
GTGCAACAGCAGAACAATTTGCTGAGAGCTATTGAGGCGCAACAACATATGTTGCAACTCACAGTCTGGGGCA
TC
>JF683796
ACTCACAATAGCAATAACATCACACTCTCATGCAGAATAAGACTAGTTATAAACAGGTGCCGGGAACTAGGAA
TAGCAATGTCTGCCCCTCCCCTCAAAGGCATAGTCAGCTGCTCATCCAATATTACAGGTCTAGTATTAACAAG
GGCTGGTGGCAGTAACAGTGAGTCCGACTCCAACGAGACCTTCAGACCTGGAGGGGGAGACATGAGAGATAAT
TGGAGAAGTGAACTATATAAATATAAAGTAGTAAAAATTGAGCCATTAGGAATAGCACCCACCACGGCAAAGA
GAAGAGTGGTGCAGAGAGAAAAAAGAGCAATAACGCTAGGAGCTATGTTCCTTGGGTTCTTGGGAACAGCAGG
AAGCACTATGGGCGCAGCGTCACTGACGCTGACGGTACAGGCCAGACAATTATTGTCTGGTATAGTGCAGCAG
CAGAGCAATCTGCTGAGGGCTATTGAGGCGCAACAGCATATGTTGCAACTCACAGTCTGGGGCATC
>JF683805
ACTCAAGAAGAGACCATCATACTCCCATGTAGAATAAGGCAAATTGTAAACTTGTGGCAGGAAGTAGGAAAGG
CAATGTATGCCCCTCCCATAAAGGGAAACATTAGCTGTCCATCAAACATTACAGGGCTGCTATTAATAAGAGA
TGGTGAAGATGAGAGTAATAATAGCGAGATCTTCAGACCTGGAGGAGGAGATATGAGGGATAATTGGAGAAGT
GAATTATATAAATATAAAGTAGTAAAAATTGAACCATTAGGGATAGCACCCACCAAGGCAAAGAGAAGAGTGG
TGCAGAGAGAAAAAAGAGCAGTGGGAGCACTAGGAGCTATGTTCCTTGGGTTCTTGGGAGCAGCAGGAAGCAC
TATGGGCGCAGCGTCAATGACACTGACGGTACAGGCCAGACAATTATTGTCTGGTATAGTGCAACAGCAGAAC
AATTTGCTGAAGGCTATTGAGGCGCAACAGCATCTGTTGCAACTCACAGTCTGGGGCATC
>KT124762
ACTCACAACAATGAGAGTACCCCTGAGAATATCACACTCCAATGCAGAATAAAACAAATTATAAACATGTGGC
AGGGAGTAGGAAAAGCAATGTATGCCCCTCCCATCAGCGGGCTAATTAGATGTTCATCAAATATTACAGGGCT
GTTACTAACAAGGGATGGTGGTACGAATAGCACAGATGAGATCTTCAGGCCCGGAGGAGGGGATATGAAGGAT
AATTGGAGAAGTGAATTATACAAATATAAAGTAGTAAAAATTGAGCCATTAGGAGTAGCACCCACCAAGGCAA
AGAGAAGAGTGGTGCAGAGAGAAAAAAGAGCAGTGGGATTCGGAGCTTTGTTCCTTGGGTTCTTGGGAGCAGC
AGGAAGCACTATGGGCGCAGCGTCATTGACGCTGACGGTACAGGCCAGACAATTATTGTCTGGTATAGTGCAA
CAGCAGAACAATTTGCTGAGAGCTATTGATGCGCAACAGCATCTGTTGCAGCTCACAGTCTGGGGCATC
>KT124788
AGTTATAATAGTACTGAGAATAATGGTACAATCACACTCCCATGCAGAATAAAACAAATTATAAACATGTGGC
AGGAAGTAGGAAAAGCAATGTACGCCCCTCCCATCAGAGGACAGATTAGTTGTTCATCAAATATCACAGGGCT
GTTATTAACAAGAGATGGCGGTAAAGGGAATAATGGCAGCAATACTGAGATCTTCAGACCTGGAGGAGGAAAT
ATAAAGGACAATTGGAGGAGTGAATTATATAAATATAAAGTAGTAAAAATTGAACCATTAGGAGTAGCACCCA
CCAAGGCAAAGAGAAGAGTGGTGCAGAGAGAAAAAAGAGCAATAACATTAGGAGCTGTGTTCCTGGGGTTCTT
GGGAGCAGCAGGAAGCACTATGGGCGCAGCGTCAATAACGCTGACGGTACAGGCCAGACAATTATTGTCTGGT

ATAGTGCAACAGCAAAACAACTTGCTGAAGGCTATTGAGGCACAACAACATCTGTTGCAACTCACAGTCTGGG
GCATT
>KT124782
AATTATACTAATAACAGCGAAAATATCACACTCCCATGCAGAATAAAGCAAATTATAAACCTATGGCAGGGAG
TAGGAAAAGCAATGTATGCCAATCCAGTCAAAGGACTAATTAACTGTCTATCAAATATTACAGGACTGATATT
AACAAGAGATGGTGGTGTTAATAATAGCAACAATGAGACCTTTAGGCCTGGGGGAGGAGATATGAGGGACAAT
TGGAGGAGTGAATTATATAAATATAAAGTAGTAAAAATTGAACCATTAGGATTGGCACCCACTACGGCAAAGA
GAAGAGTGGTGCAAAGAGAAAAAAGAGCAGTGGGAATAGGAGCTGTGTTCCTTGGGTTCTTGGGAGCAGCAGG
AAGCACTATGGGCGCAGCGTCAATGACGCTGACGGTACAGGCCAGACTATTACTGTCTGGTATAGTGCAGCAG
CAAAACAATTTGCTGAGGGCTATTGAGGCGCAACAGCATCTGTTGCAACTCACAGTCTGGGGCATC
>KT124760
AATTATACTAGCACAACTAGGCTCCCATGCAGGATAAAACAAATTGTAAACATGTGGCAGGAAGTAGGAAAAG
CAATGTATGCCCCTCCCATCAGAGGGCGAATCAATTGTTTATCAAATATCACCGGGTTGCTATTAACAAGAGA
TGGTGGTAACAGTAACAGCAGCAACGAGACTTTCAGACCAGGAGGAGGAAATATGAAGGATAATTGGAGAAGT
GAGTTATATAAATACAAAGTAGTAAAACTTGAACCATTAGGAATAGCACCCACCACGGCAAAAAGAAGAGTGG
TGCAGAGAGAAAAAAGAGCAGTGGGAATAGGAGCCATGCTCTTTGGGTTCTTGGGAGCAGCAGGAAGCACTAT
GGGCGCAGCATCAATATCGCTGACGGTACAAGCCAGGCAATTATTGTCTGGTATAGTGCAACAGCAGAGTAAT
CTGCTGAGGGCTATTGAGGCGCAACAACATATGTTGCAACTCACAGTCTGGGGCATC
>KT124780
TATCATAATAGTACTTGGAATAATACTAGGAATAATACTGAAGGGTCAAATGATATAATCACACTCCCATGCA
GAATAAAACAAATTATAAACATGTGGCAGGAAGTAGGAAAAGCAATGTATGCCCCTCCTATCAAAGGACGAAT
TAGATGTTCATCAAATATTACAGGGATGATATTAACAAGAGATGGTGGTAATAATGGGACTGACACAGAGATA
GAGATCTTCAGACCCCAAGGAGGAAATATGAAGGACAATTGGAGAAGTGAATTGTTTAGATATAAAGTAGTAA
GAGTTGAACCATTAGGAGTAGCTCCCACTAAGGCAAAGAGAAGGGTGGTGCAGAGAGAAAAAAGAGCAGTAGG
AATGGGAGCCTTGTTCCTTGGGTTCTTGGGAGCAGCAGGAAGCACTATGGGCGCAGCGTCAATGACGCTGACG
GTACAGGCCAGGCAATTATTGTCTGGTATAGTGCAACAGCAGAACAATCTGCTGAGGGCTATTGAGGCGCAGC
AGCACCTGTTGCAACTCACAGTCTGGGGCATC
>AY173959
AATTACAATACAAGTGGAGTAAATATCACACTCCCATGTAGAATAAAACAAATCATAAACATGTGGCAGGAAG
TAGGAAAAGCAATGTATGCCCCTCCCATTAGAGGACAAATTAGATGCATATCAAATATTACAGGACTGCTATT
AACAAGAGATGGTGGTGCTAACCAGACCAATGGGACCGAAATCTTCAGACCTGCAGGGGGAGATATGAGGGAC
AATTGGAGAAGTGAACTATATAAATATAAAGTAGTAAAAATTGAACCATTAGGAGTAGCACCCACCAAGGCAA
AAAGAAGAGTGGTGCAGAGAGAAAAAAGAGCAGTGGGGTTAGGAGCTGTGTTCCTTGGGTTCTTGTCAGCAGC
AGGAAGCACTATGGGCGCAGCGTCAATGACGCTGACGGTACAGGCCAGGCAATTATTGTCTGGTATAGTGCAA
CAGCAGAACAATCTGCTGAGGGCTATTGAGGCGCAACAGCATCTGTTGCAACTCACAGTCTGGGGCATT
>KC595166
ACTCAAGAAAGTGACACATTAATCACACTCCCATGCAGAATAAAACAAATTGTAAGAATGTGGCAGGAAGTAG
GAAAAGCAATGTATGCCCCTCCCATCAGAGGACAAATTAGATGTTCATCAAATATTACAGGGCTGATATTAAC
AAGAGATGGAGGTAATGACACGGACGAGAACGGGACCGAGATCTTTAGACCTGGAGGAGGAGATATGAGGGAC
AATTGGAGAAGTGAATTATATAAATATAAAGTAATACAAATTGAACCATTAGGAGTAGCACCCACCAAGGCAA
AGAGAAGAGTGGTGCAAAGAGAAAAAAGAGCAGCAATAATAGGAACTGTGTTCCTTGGGTTCTTGGGAGCAGC
AGGAAGCACTATGGGCGCAGCGTCAATAACGCTGACGGCACAGGCCAGACAATTATTGTCTGGTATAGTGCAA
CAGCAGAGCAATTTGCTGAAGGCCATTGAGGCGCAACAGCATCTGTTGCAACTCACAGTCTGGGGCATC
>JN054257
CCCTATACTAATGCAAGTATAGAAAATATCACACTCACACTCCCATGTAGAATAAAACAAATCATAAACATGT
GGCAGGAAGTAGGAAAAGCAATGTATGCCCCTCCCATCCCGGGAATAATCCAATGTACATCAAATATTACAGG
ACTACTATTAACAAGAGATGGGGGTAACCAAACCAATGGGATTGAGATCTTCAGACCTGCAGGGGGAGATATG
AAGGACAATTGGAGAAGTGAATTATATAAATATAAAGTAGTAAAAATTGAACCATTAGGAATAGCACCCACCA
AGGCAAAAAGAAGAGCGGTGCAAAGAGAAAAAAGAGGAATAGGAGCTGTGATCTTTGGGTTCTTGGGAGCAGC
AGGAAGCACTATGGGCGCAGCGTCGGTAACGCTGACGGTACAGGCCAGACAATTATTGTCTGGTATAGTGCAA
CAGCAGAGCAACTTGCTGAGAGCTATTGAGGCGCAACAGAATCTGTTGCAGCTCACAGTCTGGGGCATT
>KT200348
AGTCATGATGCTTGGAATTGGACTTGGAGTAATGGTACTTGGGGGGGTAGTACTAAAGAAACAAATAAAACTG
AAGGAAATGTCACCCTCACCCTCCAATGTAGAATAAAACAAATTATAAACATGTGGCAGGAAGTAGGGAAAGC
AATGTATGCCCCTCCCATCAGTGGACCAATTAGATGTAAATCAAACATTACAGGGATGATATTAACAAGAGAT
GGGGGTAATGATGACGGTACTGACAACAGCAGTGAGACCTTCAGACCTGGGGGAGGAGATATGAGGGATAATT
GGAGAAGTGAATTATACAAATATAAAGTAGTAAAAATTGAACCATTAGGAGTAGCACCCACCAAGGCAAAAAG
AAGAGTGGTGCAGAGAGAAAAAAGAGATTTAGGATTTGGAGCTTTGTTCCTTGGGTTCTTGGGAGCAGCAGGA
AGCACTATGGGCGCAGCGTCAATAACGCTGACGGCACAGGCCAGACAATTATTGTCTGGTATAGTGCAACAGC
AAAGTAATTTGCTGATGGCTGTTGAGGCGCAACAGAAGCTGTTGCAACTCACAGTCTGGGGCATC

>KC699038
ACTCAGAATAACATTACAGAAAATATCACACTCCCATGCAGAATAAAACAGTTTATAAACATGTGGCAGGAAG
TAGGAAAAGCAATGTATGCCCCTCCCATCAAAGGACAAATTAGATGTTCATCAAATATTACAGGGCTGATATT
AACAAGAGATGGTGGTAATAATGATAACACAACAGAGACCTTCAGACCTGGAGGAGGAAATATGAAGGACAAT
TGGAGAAGTGAATTATATAAATATAAAGTAGTAAAAATTGAACCATTAGGAGTAGCACCCACCAAGGCAAAGA
GAAGAGTGGTGCAGAGAGAAAAAAGAGCAGCGATAGGAGCTGTGTTCCTTGGGTTCTTGGGAGCAGCAGGAAG
CACTATGGGCGCAGCGTCAGTGACGCTGACGGTACAGGCCAGACAATTATTGTCTGGTATAGTGCAACAGCAA
AACAACTTGCTGAAGGCTATTGATGCGCAACAGCATATGTTGCAACTCACAGTCTGGGGCATC
>HQ595780
TCTCATAATAACACTGAAGGGGTAAGAAATAATAACACTGAAGGGGAAAAAAATATCATCCTCCCATGCAGAA
TAAAACAAGTTATAAACATGTGGCAGGAAGTAGGAAAAGCAATGTATGCCCCTCCCATCAGAGGACAAATCAG
ATGTTCATCAAATATCACAGGGCTGGTATTAACAAGAGATGGTGGCACTAATAATGGCACAAACCAGGCCGAG
GAGGTCTTCAGACCTGCAGGAGGAAATATGAGGGACAATTGGAGAAGTGAATTATATAGATATAAGGTAGTCA
AAATTGAACCATTAGGAGTAGCACCCACCAAGGCAAAGAGAAGAGTGGTGCAGAGAGAAAAAAGAGCAGTGGG
AATAGGAGCTGTATTCCTTGGGTTCTTGGGAGCAGCAGGAAGCACTATGGGCGCAGCGTCAATAACGCTGACG
GTACAGGCCAGACAATTACTGTCTGGTATAGTGCAACAGCAGAACAATTTGCTAAGGGCTATTGACGCGCAAC
AGCATCTGTTGCAGCTCACAGTCTGGGGCATC
>HQ595781
TTTCATAGTACTTGGGTTAATGGTAATTGGAGTAATGGCACTTGGAATGGTACTGAAGAAATTATCACACTTC
CATGCAGAATAAAACAAATTATAAACATGTGGCAGGAAGTAGGAAAAGCAATGTATGCTCCTCCCATCAAAGG
ACAAATTAACTGTACATCAAATATTACAGGGCTGCTATTAACAAGAGATGGTGGTAACATAACAAACAACAAC
AACACCGAGACCTTCAGACCTGGAGGAGGAGATATGAGGGACAATTGGAGAAGTGAATTATATAAATATAAAG
TAGTAAAAATTGAACCACTAGGAATAGCACCCAATAAGGCACAGAGAAGAGTGGTGCAGAGAGGAAAAAGAGC
AGTGGGATTAGGAGCTATGTTCCTTGGGTTCTTGGGAGCAGCAGGAAGCACTATGGGCGCAGCGTCAATAACG
CTGACGGTACAGGCCAGGCGATTATTGTCTGGTATAGTGCAACAGCAGAACAATTTGCTGAAAGCTATTGAGG
CGCAACAGCATCTGTTGCAACTCACAGTCTGGGGCATC
>HQ595805
AATCATACTTGGACAGGTCCAAATAGAACTGACACAAATATCACACTCCAATGCAAAATAAAGCAAATTATAA
ATATGTGGCAGAAAGTAGGAAAAGCAATGTATGCCCCTCCCATCAGAGGACAAATTAAATGTTCATCAAATAT
TACAGGGCTGCTATTAACAAGAGATGGTGGAAATGGTGATAATAGTACAAACGGGACTGAGACCTTCAGACCT
GGAGGAGGAGATATGAGGGACAATTGGAGAAGTGAATTATACAAATACAAAGTAGTAAAAATTGAACCATTAG
GAGTAGCACCCACCGAGGCAAAAAGAAGAGTGGTGCAGAGAGAAAAAAGAGCAGTGGGATTTGGAGCTCTGTT
CCTTGGTTTCTTGGGAGCAGCAGGAAGCACTATGGGCGCAGCATCAATAACGCTGACGGTACAGGCCAGACAA
TTATTGTCTGGTATAGTGCAACAGCAAAACAATTTGCTGAGGGCTATTGAGGCGCAACAGCATCTGTTGCAAC
TCACAGTCTGGGGCATT
>AJ535618
TTTCATAGTACTTGGGTGTTTAATAGAACTGGGGGGAATGATACTGAATACACTGAAAAAAATATCACACTTC
CATGCGGAATAAAACAAATTATAAACATGTGGCAGGGAGTAGGAAAAGCAATGTATGCCCCTCCCATCCAAGG
ACAAATTAATTGCACATCAAATATTACAGGGCTGTTATTAACAAGAGATGGTGGTAATGGTGATAACACAACT
GAGATCTTCAGACCTGGAGGAGGAGATATGAGGGACAATTGGAGAAGTGAATTATATAAATATAAAGTAGTAA
AAATTGAGCCATTAGGAGTAGCACCCACCAAGGCAAAGAGAAGAGTGGTGCAGAGGGAAAAAAGAGAAGTGGG
CCTAGGAGCTTTGTTCATTGGGTTCTTGGGAGCAGCAGGAAGCACTATGGGCGCAGCGTCAGTGACGCTGACG
GTACAGGCCAGACAATTATTGTCTGGTATAGTGCAACAGCAGAACAATTTGCTGAGAGCTATTGATGCGCAAC
AGCATCTGTTGCAACTCACAGTCTGGGGCATC
>U36877
AGTCAGGACGGTACTGAAGGATATAACATTATCACACTCCCATGCAGAATAAAACAAATTATAAACATGTGGC
AGGAAGTAGGAAAAGCAATGTATGCCCCTCCCATCAAAGGACAAATTAGATGTTCATCAAATATTACAGGACT
GCTATTAACAAGAGATGGTGGTAATAGTAGTAGCACCACCGAGACCTTCAGACCTGGAGGAGGAGACATGAGG
GACAATTGGAGAAGTGAATTATATAAATATAAAGTAGTAAAAATTGAACCATTAGGAGTAGCACCCACCAAGG
CAAAGAGAAGAGTGGTGCAAAGAGAAAAAAGAGCAGTGGGAATAGGAGCTTTGTTCCTTGGGTTCTTGGGAGC
AGCAGGAAGCACTATGGGCGCAGCATCAATGACGCTGACGGTACAGGCCAGACTATTATTGTCTGGTATAGTG
CAACAGCAGAACAATTTGCTGAGAGCTATTGAGGCGCAACAGCATCTGTTGCAACTCACAGTCTGGGGCATC
>FJ460501
AGTTATATTTTGAATGGTACTAGCTGGAGTAACAATACTAATGGGGTAAATGATATAATCACACTCCCATGCA
GAATAAGACAAATTATAAACATGTGGCAAGAAGTAGGAAAAGCAATGTATGCCCCGCCCATCATGGGACAAAT
TAACTGTTCATCAAATATTACAGGGCTGCTATTAACAAGAGATGGTGGTAATAACACAGACACAAACCAAACA
GAGATCTTCAGACCTGGAGGAGGAGATATGAGGGACAATTGGAGAAGTGAATTATATAAATATAAAGTAGTAA
GAATTGAACCATTAGGAATAGCACCCACCAAGGCAAGAAGAAGAGTGGTGCAGAGAGAGAAAAGAGCAGTGGG
AATAGGAGCTCTGTTCCTTGGATTCTTGGGAGCAGCAGGAAGCACTATGGGCGCAGCGTCACTGACACTGACG

GTACAAGCCAGACAATTATTGTCTGGTATAGTGCAACAGCAGAATAATCTGCTGAGAGCTATTGAGGCGCAAC
AGCATATGTTGCAACTCACAGTCTGGGGCATT
>EU839602
ATTTACCCTTGGAATAAGACTGAAAATATCACACTCCCATGCAGAATAAAACAAATTATCAATATGTGGCAGG
GAGTAGGAAAAGCAATGTATGCCCCTCCCATCAGCGGACCAATAAGATGTGAATCAAATATTACAGGGCTGCT
ATTAACAAGAGATGGTGGTGACCAGAACGGGACGGACGCTACCAATGAGACCTTTAGACCTGGAGGAGGAGAT
ATGAGGGACAATTGGAGAAGTGAACTATATAAATATAAAGTAGTAAGAATTGAACCATTAGGAGTAGCACCCA
CCGAGGCAAAGAGAAGAGTGGTGCAGAGAGAAAAAAGAGCAGTGGGACTAGGAGCTATGTTCCTTGGGTTCTT
AGGAGCAGCAGGAAGCACTATGGGCGCAGCGTCAATAGCGCTGACGGCACAGGCCAGGCAATTGTTGTCTGGT
ATAGTGCAGCAGCAGAACAATCTGCTGAGGGCTATTGAGGCGCAACAGCATCTGTTGCAACTCACGGTCTGGG
GCATC
>EU839604
GATTATAATGACACTATCATACTCCAATGTCGGATAAGACAAATTATAAACATGTGGCAGAAAGTAGGAAAAG
CAATGTATGCCCCTCCCATAAGTGGAATAATTAAATGTACATCAAATATTACAGGGCTACTATTAACAAGAGA
TGGTGGTAATAATACAGGAGGGAATGAGACCTTCAGACCTACAGGAGGAAATATGAAGGACAATTGGAGAAGT
GAATTATATAAATATAAAGTAGTACAAATTGACCCAATAGGAGTAGCACCCACCAAGGCAAAAAGGAGAGTGG
TGCAGAGAGAAAAAAGAGCAGTGGGAGTAGTAGGAGCTATGTTCCTTGGTTTCTTGGGAGCAGCAGGAAGCAC
TATGGGCGCAGCGTCAGTGACGCTGACGGTACAGGCCAGACAATTATTGTCTGGTATAGTGCAACAGCAGAAT
AATCTGCTGAGAGCTATTGAGGCGCAACAGCATCTGTTGCAACTCACAGTCTGGGGCATC
>HM030560
ACTCAGGATGATACTAATGACACTATCACACTCCCATGCAGAATAAAACAAATTGTAAACATGTGGCAGGAAG
TAGGCAAAGCAATATATGCCCCTCCCATTAGTGGACTAATTAGATGTAACTCAAGTATTACAGGGCTAATGTT
AACAAGAGATGGTGGTGGTGGGAATACTAGTAATAATGAGACCTTCAGACCGGGAGGAGGAAATATGAAGGAC
AATTGGAGAAGTGAATTATATAAATATAAAGTAGTGAAAATTGAACCATTAGGAGTAGCACCCACCAGGGCAA
GGAGAAGGGTGGTGCAAAGAGAAAAAAGAGCAGTGGGAGTAATAGGAGCTATGTTCCTTGGGTTCTTGGGAAC
AGCAGGAAGCACTATGGGCGCAGCGTCAGTGACGCTGACGGTACAGGCCAGACAATTATTGTCTGGTATAGTG
CAACAGCAAAACAATTTGCTGAGGGCTATTGAGGCGCAACAGCATCTGTTGCAACTCACAGTCTGGGGCATC
>AB588243
AATCAGTCTGCTTGGAAGAGAAATGATACTGAAGGGTTAAATAATACTGAGGACAAAATTACACTCCCATGCA
AAATAAAACAAATCATAAACATGTGGCAGAAAGTAGGAAAAGCAATATATGCCCCTCCCATCAGAGGAGTAAT
TAGATGTGCATCAAATATTACAGGGCTGCTATTAACAAGAGATGGTGGTGGTGATGAGAACAGAACCACCGAG
ACCTTCAGACCTGGAGGAGGGGATATGAGGGACAATTGGAGAAGTGAATTATATAAATATAAAGTAGTAAAAA
TTGAACCATTAGGAGTAGCACCCACTAAGGCAAAGAGAAGAGTGGTGCAGAGAGAAAAAAGAGCAGTGGGATT
TGGAGCTGTATTCCTTGGGTTCTTGGGAGCAGCAGGAAGCACTATGGGCGCAGCGTCAGTGACGCTGACGGTA
CAAGCCAGACAATTGCTGTCTGGTATAGTGCAACAGCAGAGCAATCTGCTGAGAGCTATTGAGGCGCAGCAAC
AAATGTTGAAACTCACAGTCTGGGGCATC
>AB588255
AATCATGGAGAGTCAAATGACACTATCACACTCCCATGTAGAATAAAACAAATTGTAAACATGTGGCAGGAAG
TAGGAAAAGCAATGTATAACCCTCCCATCGAAGGACAAATTAGATGTTCATCAAATATTACAGGACTATTATT
AATAAGAGATGGTGGTAACAGTAACAACACAAACACCACCGAAATCTTCAGACCCGGGGGAGGAAATATGAAG
GACAATTGGAGGAGTGAATTATATAAATATAAAATAGTAAAAATTGAACCATTAGGAGTAGCACCCACCAAGG
CAAAGAGAAGAGTGGTGCAAAGAGAAAAAAGAGCAGTGGGAATAGGAGCTGTGCTCTTTGGATTCTTGGGAGC
AGCAGGAAGCACTATGGGCGCAGCGTCAATAGCGCTGACGGCACAGGCCAGACAATTATTGTCTGGTATAGTG
CAACAGCAGAACAATATGCTGAGAGCCATTGAGGCGCAACAGCACCTGTTGCAACTCACAGTCTGGGGCATC
>AB588271
CTTTATACTTTGAATAGCTCTGAAGATAAAAATATCACACTCCAATGCAGAATAAAACAGATTATAAACATGT
GGCAGGAAGTAGGAAAAGCAACGTATGCCCCTCCTATCAAAGGACAGATTAGATGTTCATCAAACATTACAGG
GCTGCTATTAACAAGAGATGGTGGTACTAATAAAACAAGCGATCCCGAGATCTTCAGACCTACAGGAGGTAAT
ATGAAGGACAATTGGAGAAGTGAATTATATAAATATAAAGTAGTAAAAATTGAACCATTAGGAGTAGCACCTA
CCAGGGCAAAGAGAAGAGTGGTGCAGAGAGAAAAAAGAGCAGTGGGACTAGGAGCTGTGTTCCTTGGGTTCTT
GGGAGCAGCAGGAAGCACTATGGGCGCAGCGTCATTAACGCTGACGGTACAGGCCAGACAATTGTTGTCTGGT
ATAGTGCAACAGCAAAACAATTTGCTGAGAGCTATTGAGGCGCAACAGAATCTGTTGCAACTCACAGTCTGGG
GCATC
>AB262961
AGTTATACTTGGAATGGTACAGGACTGTCTAATAATACTGAGAATAGTTCAAATAATACTATCACACTCCAAT
GCAGAATAAAGCAAATTATAAACATGTGGCAGGAAGTAGGAAAAGCAATGTATGCCCCTCCCATCAGCGGACA
AATTAGATGTTCATCAAATATTACAGGGCTGCTATTAACAAGAGATGGTGGTAATGGCACGAACGGGACCGAG
ACCTTCAGACCTGGAGGAGGAGATATGAGGGACAATTGGAGAAGTGAATTATATAAATATAAAGTAGTAAAAA
TTGAACCATTAGGAGTAGCACCCACTAGGGCAAGGAGAAGAGTGGTGCAGAGAGAAAAAAGAGCAGTGGGAAT
AGGAGCTTTGTTCCTTGGGTTCTTGGGAGCAGCAGGAAGCACTATGGGCGCAGCGTCAATGACGCTGACGGTA

CAGGCCAGACAATTATTGTCTGGTATAGTGCAACAGCAAAACAATTTGCTGAGGGCTATTGAGGCGCAACAGC
ATCTGTTGCAACTCACAGTCTGGGGCATT
>KJ140264
TGTTATACAACAAAACTGTTTAATAGTACTTGGAATGATACAACACTGTTTAATAGTACCTGGAATAGTAGTA
GTAAAGGAACTATCACACTCCGATGCAGAATACAACATATTATCAACAGGTGGCAGGAAGTAGGAAAAGCGAT
GTATGCCCCTCCCATTGGAGGAAAAATTAGATGTTCATCAAATATTACAGGGCTGCTAATAACAAGAGATGGG
GGGACTGAGACTGGGACTACTGAGACCTTCAGACCTGGGGGAGGAAATATGAAGGACAATTGGAGAAGTGAAT
TATATAAATATAAAGTAGTAAGAATTGAGCCATTAGGGATAGCACCCACCAAGGCAAAGAGAAGAGTGGTGCA
GAGAGAAAAAAGAGCAGTGGGGACGATAGGAGCTGTGTTCCTTGGGTTCTTGGGAGCAGCAGGAAGCACTATG
GGCGCAGCGTCATTGACGCTGACGGTACAGGCCAGACAATTATTGTCTGGTATAGTGCAACAGCAGAATAATT
TGCTGAAAGCTATTGATGCGCAACAGCATATGTTGCAACTCACAGTCTGGGGCATC
>KJ140265
ACTCATACTGGTACTGAAGGAGTAAATGGAACATATTTCACACTCCCATGCAGAATAAGACAAATTGTAAACA
GGTGGCAGGAAGTAGGAAAAGCAATGTATGCCCCTCCCATCGGAGGACGAATTAGATGTTCATCAAATATTAC
AGGGCTGCTATTAACAAGAGATGGGGGGAATGAGAATAACGAGACTGAGACGACTGAGACCTTCAGACCTGGG
GGAGGAAATATGAAGGACAATTGGAGAAGTGAATTATATAAATATAAAGTAGTAAAAATTGAGCCATTAGGGA
TAGCACCCACCGAGGCAAGGAGAAGAGTGGTGCAGAGAGAAAAAAGAGCAGTGGGAGTGATAGGAGCTATGTT
CCTTGGGTTCTTGGGAGCAGCAGGAAGCACTATGGGCGCAGCGTCAATGGCGCTGACGGTACAGACCAGACAA
TTATTGTCTGGTATAGTGCAACAGCAGAATAATTTGCTGAGAGCCATTGAGGCGCAACAGCATATATTGCAAC
TCACAGTCTGGGGCATC
>MN043600
TTTCATAGTACTTGGAATGCTACGTTGAATGATACTGAAAGGGTAAATAATAGTGCAACAAAAAACATTACAC
TCCAATGCAGAATAAAACAAATTATAAACAGGTGGCAGGAAGTGGGACAAGCAATGTATGCCCCTCCCATCAG
CGGACTAATTAGATGTTCATCAAATATTACAGGGCTGATATTAACAAGAGACGGGGGGGTTAACGAGACTACT
GAGACCTTCAGACCTGGAGGAGGCGATATGAGGGACAATTGGAGAAGTGAATTATATAAATATAAAGTAGTAA
GGATTGAGCCATTAGGGATAGCACCCACCAAGGCAAGGAGAAGAGTGGTGCAGAGAGAAAGAAGAGCAGTGGG
AATGTTAGGAGCTATGTTCCTTGGGTTCTTGGGAGCAGCAGGAAGCACTATGGGCGCAGCGTCAATGACGCTG
ACGGTACAGGCCAGACAATTATTGTCTGGTATAGTGCAACAGCAGAATAATCTGCTGAAAGCTATTGAGGCGC
AACAGCATCTGTTGCAACTCACAGTCTGGGGCATC
>KJ140260
AATCATACTAGAGCGAACATTCGGAATAATCCTGGAAATGGAACTATTATCCTCCCATGCAGAATAAAACAAA
TTATAAACAGGTGGCAGGAAGTAGGAAAAGCAATGTATGCCCCTCCCATCGAAGGACAAATTAGATGTACATC
AAATATTACAGGGTTGCTAATAACAAGAGATGGGGGGATAAATGAGACCAACGGGACTGAGACCTTCAGACCT
GGGGGAGGAGATATGAGGGACAATTGGAGAAGTGAATTATATAAATATAAAGTAATAAAAATTGAGCCATTAG
GGATAGCACCCACCAAGGCAAAGAGAAGAGTGGTGCAGAGAGAAAAAAGAGCAGTGGGAGCGATAGGAGCTAT
GTTCCTTGGGTTCTTGGGAGCAGCAGGAAGCACTATGGGCGCAGCGTCATTGACGCTGACGGTACAGGCCAGA
CAATTATTGTCTGGTATAGTGCAACAGCAGAATAATTTGCTGAGAGCTATTGAGGCGCAACAGCATCTATTGC
AACTCACAGTCTGGGGCATC
>KT878035
ACTTACAATGACACTGGAGTTAATGAAACTATCACCCTCCCTTGCAGAATAAGACAAATTGTAAACAGGTGGC
AGGAAGTGGGAAAGGCAATGTATGCCACTCCCATCAGTGGACTAATTAGATGTTCATCAAATATTACAGGGTT
ACTATTAACAAGAGATGGGGGGACAGGGAACAATAAAACTGGGAATGGAACTGAGACCTTCAGACCTGGGGGA
GGAGACATGAAGGACAATTGGAGGAGTGAATTATACAAATATAAAGTAGTAAAGATTGAGCCATTAGGAGTAG
CACCCACCAAGGCAAGGAGAAGAGTGGTGCAGAGAGAAAAAAGAGCAGTGGGAACAATAGGAGCTATGTTCCT
TGGGTTCTTGGGAGCAGCAGGAAGCACTATGGGCGCAGCGTCACTGACGCTGACGGTACAGGCCAGACTATTA
TTGTCTGGTATAGTGCAACAGCAACAGAACTTGCTGAGAGCTATTGAGGCGCAACAGCATCTGTTGCAACTCA
CAGTCTGGGGCATC
>KT878026
ACTCATTGGAATGAGACTTGGGATAATGAAACAATCATACTCCCATGCAGAATAAAACAAATTATAAACAGAT
GGCAGGAAGTAGGAAAGGCAATGTATGCCCCTCCCATCAGAGGAAATATTAGTTGTACATCAAATATTACAGG
GCTGCTATTATCAAGAAATGGAGGTGGCAACGGGACTAACGGGACTGAGACCTTTAGACCTGGGGGAGGAGAT
ATGAGGGACAATTGGAGAAGTGAATTATATAAATATAAGGTAATAAGAATTGAGCCATTAGGATTAGCACCCA
CCAGGGCAAGGAGAAGAGTGGTGCAGAGAGAAAAAAGAGCAGTAACGATAGGAGCTATGTTCCTTGGGTTCTT
GGGAGCAGCAGGAAGCACTATGGGCGCAGCATCACTGACGCTGACGGTACAGGCCAGGCTACTATTGTCTGGT
ATAGTGCAACAGCAGAATAATTTGCTAAGAGCTATTGAGGCGCAACAGCATCTGTTGCGACTCACAGTCTGGG
GCATT
>KT878028
AATCAAAATGGTACCAGGAATGATACTAGTACTAATGCAACTATCACACTTCCATGCAGAATAAAACAATTTA
TAAACAGGTGGCAGGAAGTAGGAAGAGCAATGTATGCCCCTCCCATCGAAGGACAAATCAGATGCTCATCAAA
TATTACAGGGCTGCTATTAACAAGAGATGGGGGGAATAATGAAACTGAGACCTTCAGACCTGGGGGAGGAAAT

ATGAAAGATAATTGGAGAAGTGAATTATATAAATATAAAGTAGTAAAAATTGAGCCATTAGGGATAGCACCCA
CCAAGGCAAGGAGAAGAGTGGTGCAGAGAGAAAAAAGAGCAATAGGAACAATAGGAGCTCTGTTCCTTGGGTT
CTTGGGAGCAGCAGGAAGCACTATGGGCGCAGCGGCAATGACGCTGACGGTACAGACCAGACAATTGTTGTCT
GGTATAGTGCAACAGCAGAATAATCTGCTGAGAGCTGTTGAGGCGCAACAGCATCTGTTGCAACTCACAGTCT
GGGGCATC
>AJ417422
ACTCAACTGTTTAATAGTACTTGGGGGGAACATGATGTTGAAGCAAGTAACACTGAAAATAATGAAAATATCA
CACTCCCATGCAGAATAAAGCAAATTGTAACCATGTGGCAGAAAGTAGGAAAAGCAATGTATGCCCCTCCCAT
CGAAGGAAAAATTAGCTGTTTATCAAATATTACAGGGCTACTGTTAATGAGAGATGGTGGTAATATAAACAAC
AAAACAAATGGGACCACCCCCCTTGAGATCTTCAGACCTGGAGGAGGAAATATGAAGGATAATTGGAGAAGTG
AATTATATAAATATAAAGTAATAGAAATTGAACCATTAGGAGTAGCACCCACCAGAGCAAAGAGAAGAGTGGT
GCAAAGAGAAAAAAGAGCGGGAATAGGAGCTGTGCTCGTTGGGTTCTTGGGAGCAGCAGGAAGCACTATGGGC
GCAGTGTCAGTGGCGCTGACGGTACAGGCCAGAAAGTTAATGTCTGGTATAGTGCAACAGCAAAACAACTTGC
TGATGGCTATTGAGGCGCAACAGCATATGTTGCAACTCACTGTCTGGGGCATC
>JQ316129
ACTCAAAGGTTAGATGAAATTAATAAAAATAGCACAATTATCACACTTCCATGCAGAATAAAACAAATTATAA
ACAGGTGGCAGGAAGTAGGAAAAGCAACGTATGCCCCTCCCATCAGAGGACAAATTACATGTTCATCAAATAT
TACAGGGTTGCTATTAACAAGAGATGGAGGGAATGAGAGCAGCAGTACTGAGACCTTCAGACCTGGGGGAGGA
GACATGAGGGACAATTGGAGAAGTGAATTATATAAATATAAAGTAGTAAAAATTGAGCCATTAGGGGTAGCAC
CCACCAAGGCAAGGAGAAGAGTGGTGCAGAGAGAAAAAAGAGCAGTGGGAACAATAGGAGCTATGTTCCTTGG
GTTCTTGGGAGCAGCAGGAAGCACTATGGGCGCAGCGTCACTGACGCTGACGGTACAGGCCAGACAATTGTTG
TCTGGTATAGTGCAACAGCAGAATAATCTGCTGAGAGCTATTGAGGCGCAACAGCATCTGTTGCAACTCACAG
TCTGGGGCATC
>HQ386198
AATCATAATGGGACTTGGAGTACAAATAGTACTGGGAATGATACCAACAACACAACTGACACTATCACACTCC
CATGCAGAATAAAGCAAATTATAAACATGTGGCAGGAAGTAGGGAAAGCAATGTATGCCCCTCCCATCAGTGG
ACAAATTAACTGTTCATCAAATATTACAGGGCTGATATTAACAAGAGATGGTGGTAATAAGACCGAGAATGAG
AATGGGACCGAGACCTTCAGACCTGGAGGAGGAGATATGAGGGATAATTGGAGAAGTGAATTATATAAATATA
AAGTAGTAAAAATTGAACCATTAGGACTAGCACCCACCAGGGCAAAGAGAAGAGTGGTGCAAAGAGAAAAAAG
AGCAGTGGGAATGCTAGGAGCTATGTTCCTTGGGTTCTTGGGAGCAGCAGGAAGCACTATGGGCGCAGCGTCG
ATGGCGCTGACGGTACAGACCAGACAATTAATGTCTGGGATAGTGCAACAGCAGGACAGTCTGCTGAGGGCTA
TTGAGGCGCAACAGCATCTGTTGCAACTCACAGTCTGGGGCATC
>JF320191
GATCAGGATAAAAATGATACTATTACCCTTCCATGCAGGATAAAACAAATTATAAACATGTGGCAGGAAGTAG
GAAAAGCAATGTATGCCCCTCCCATCCAAGGACAAATTAGTTGTTCATCAAATATTACAGGGCTACTCTTAAC
AAGAGATGGTGGTAATAGCAGTAACACTAATGGAACTGAGATCTTCAGACCTGGAGGAGGAGATATGAGAGAC
AATTGGAGAAGTGAATTATATAAGTACAAAGTAGTAAAAATTGAACCATTAGGAATAGCACCCACCAAGGCAA
AGAGAAGAGTGGTACAAAGAGAAAAAAGAGCAGTGGGAACGCTGGGAGCTGTGTTCCTTGGGTTCTTGGGAGC
AGCAGGAAGCACTATGGGCGCAGCGTCAATAACGCTGACGGCACAGGCCAGACAATTACTGTCTGGTATAGTG
CAACAGCAGAACAATATGCTGAAGGCTATTGAGGCGCAACAGCATCTGTTGCAACTCACAGTCTGGGGCATT
>MH078554
AATTATAATTGGAATAGTAATAATAGCACTGATAGCAGAAATATCACGCTCCCATGTAGAATAAGACAAATTA
TAAACATGTGGCAGAGAGTAGGAAAAGCTATGTATGCCTCTCCCATCAAAGGACCAATTAACTGTACATCAAA
CATTACAGGGCTGCTATTGACAAGAGATGGGGGTAATGGCAAAAGCAGCAGTAATGAGACTGAGACCTTCAGA
CCTATAGGAGGAGACATGAAGGACAATTGGAGAAGTGAATTATATAAATATAAAGTAGTACAAATCAAACCGT
TAGGAATAGCACCCACCAGGGCAAAAAGAAGAGTGGTGCAGAGAGAAAAAAGAGCAGTAACACTAGGAGCTAT
GTTCCTTGGGTTCTTGGGAGCAGCAGGAAGCACTATGGGCGCAGCGTCAATAGCGCTGACGGCACAGGCCAGA
CAATTATTGTCTGGTATAGTGCAGCAGCAGAACAATCTGCTGAGGGCTATTGAGGCGCAACAACATCTGTTGC
AACTCACAGTCTGGGGCATC
>EF210725
AACTATACAACAAATGGAACTATCACTCTCCCATGTAGAATAAAACAAGTTATAAACATGTGGCAGGAAGTAG
GAAAAGCAATGTATGCCCCTCCCATCAAAGGACAAATTAGTTGTTTGTCAAATATCACAGGGTTGCTATTAAC
AAGAGATGGAGGTAAGCGCAATGACAGTAATGAGACAGAGGTCTTTAGACCTGGAGGAGGAGATATGAGGGAC
AATTGGAGAAGTGAATTATATAAATATAAAGTAGTAAAAATTGAACCAATAGGAATAGCACCCACCAAGGCAA
AGAGAAGAGTGGTGCAAAGAGAAAAAAGAGAGCTGGGAACGCTAGGAGCTATGTTCATTGGGTTCTTGGGAGC
AGCAGGAAGCACTATGGGCGCAGCGTCAGTGACGCTGACGGTACAAGCCAGACAATTATTGTCTGGTATAGTG
CAACAGCAGAACAATCTGCTGAAGGCTATTGATGCGCAACAGCATCTGTTGCAACTCACAGTCTGGGGCATC
>EF210734
ACTCAAGAAAATGAAAATATCACACTCCCATGTAGAATAAAACAAATTATAAACATGTGGCAGGAGGTAGGAA
AAGCAATGTATGCCCCTCCCATCAAAGGAATAATTAAATGTTCATCAAACATTACAGGACTGCTCCTAATAAG

AGATGGTGGTAATAGTAATGGTAATGGAAGTAACAATAGCGAGACCTTCAGACCTGGAGGAGGAGATATGAAG
GACAATTGGAGAAGTGAATTATATAAATATAAAGTAGTAAAAGTTGAACCAGTAGGACTAGCACCCACCAAGG
CAAAGAGAAGAGTGGTGCAGAGAGAAAAAAGAGCAGTGACACTAGGAGCTGTGTTCCTTGGGTTCTTGGGAGC
AGCAGGAAGCACTATGGGCGCAGCGTCAGTGACGCTGACGGTACAGGCCAGACAATTACTGTCTGGTATAGTG
CAACAGCAGAGCAATATGCTGAAGGCTATTGAGGCGCAACAACATCTGTTGCAACTCACAGTCTGGGGCATC
>KY658689
AATTACACAAATGGAACTATCACGCTCCCATGCAGAATAAAACAAATTATAAACATGTGGCAGAGAGTAGGAA
AAGCAATGTATGCCCCTCCCATTGCAGGACAACTTAACTGTTCATCAAATATTACGGGGATACTATTAACAAG
AGATGGGGGTAATGGTAATGTGACAAATGACACAGAGATCTTCAGACCTGCAGGAGGAGATATGAGGGACAAT
TGGAGAAATGAACTATACAAATATAAAGTAGTAAAGATTGAACCATTAGGAATAGCACCCACCAAGGCAAGGA
GAAGAGTGGTGCAGAGAGAAAAAAGAGCAGTGACACTGGGAGCTCTGTTCCTTGGGTTCTTGGGAGCTGCAGG
AAGCACTATGGGCGCAGCGTCAGTGACGCTGACGGTACAGGCCAGGCTATTATTGGCTGGTATAGTGCAACAG
CAGAACAATTTGCTGAGAGCTATTGAGGTGCAACAGCACATGTTGCAACTCACAGTCTGGGGCATT
>KP411827
ACTCAAAAGGCAAGTAACACAGAAAATGAAAATATCACTCTCCCATGCAAAATAAAACAAGTTATAAATATGT
GGCAGGAAGTAGGAAAAGCAATGTATGCCCCTCCCATCGAAGGACAGATTAAATGTTCATCAAATATTACAGG
GCTACTATTAACAAGAGATGGTGGTAAACATAACAGTAGCGATACTAACAAAACTGAGATCTTCAGACCTGGA
GGAGGAGATATGAGGGACAATTGGAGAAGTGAATTATATAAATATAAGGTAGTAAAAATTGAACCATTAGGAA
TAGCACCCACCAGGGCAAAGAGAAGAGTGGTGCAGAGAGAAAAAAGAGCAGTGGGATTAGGAGCTATGTTCCT
TGGGTTCTTGGGAGCAGCAGGAAGCACTATGGGCGCAGCATCATTGACGCTGACGGTACAGGCCAGACAATTA
ATGTCTGGTATAGTGCAACAGCAAAACAACTTGCTAAGAGCTGTTGAGGCGCAACAGCATATGTTGCAACTCA
CAGTCTGGGGCATC
>MF373174
AATCAAACAACTACCGGTAATGATACCATCCCACTCCAATGCAGGATAAAGCAAATTATAAACATGTGGCAGG
AAGTGGGAAAAGCAATGTATGCCCCTCCCATTAAAGGACCAATTAATTGTTTATCAAATATTACAGGGTTGTT
ATTAACAAGAGATGGTGGAAATAATAATACCAATGAAACAGAGACCTTCAGACCTGGAGGAGGAGACATGAGA
GACAATTGGAGAAGTGAATTATATAAATACAAAGTAGTAAAAATTGAACCATTAGGAGTAGCACCCACCAAGG
CAAGGAGAAGAGTGGTGCAGAGAGAAAAAAGAGCAGTGGGACTAGGAGCTTTGTTCCTTGGGTTCTTAGGAGC
AGCAGGAAGCACTATGGGCGCAGCGTCAATGACGCTGACGGTACAGGCCAGACAATTATTGTCTGGTATAGTG
CAACAGCAGAACAACTTGCTGAGGGCTATTGAGGCGCAACAGCACCTGTTGAAACTCACGGTCTGGGGCATT
>JN860769
AATCATACGTTTAATAATACTGAAGGCGAAAATATAACTGAAAGCATAAATACAACAATTATCAAGCTCCCAT
GCAGAATAAAACAAATTATAAACATGTGGCAGGAAGTAGGAAAAGCAATGTATGCCCCTCCCATCAGTGGACA
AATTAGCTGTTCATCAAATATTACAGGAATACTATTAACAAGAGATGGTGGTGGTAATAATACGGGAAATGAG
ACCTTCAGACCTGGGGGAGGAAATATGAAAGACAATTGGAGAAGTGAATTATATAAATATAAAGTAGTAAAAA
TTGAGCCATTAGGAATAGCACCCACCAAGGCAAAGAGAAGAGTGGTGCAGAGAGAAAAAAGAGCAGTGGGAGT
GATAGGAGCTATGTTCCTTGGGTTCTTGGGAGCAGCAGGAAGCACTATGGGCGCAGCGTCACTGACGCTGACG
GTACAGGCCAGACAATTGTTGTCTGGTATAGTGCAACAGCAGAACAATTTGCTGAGAGCTATTGAGGCGCAAC
AGCATCTGTTGCAACTCACAGTCTGGGGCATC
>MG989583
AATTATAATATTGAAAAGTCAAATGACACTATTGTACTCCCGTGCAGAATAAAACAAATTATAAACAGGTGGC
AGGAAGTAGGAAGAGCAATGTATGCCCCTCCCATCAGAGGACAACTTAACTGTACATCAAATATTACAGGGCT
ACTATTATTAAGAGATGGTGGGAATAACATTACGAACGAAACCTTCAGACCTGGAGGAGGAAATATGAAGGAC
AATTGGAGAAGTGAATTATATAAATATAAGGTAGTAAAAATAGAGCCATTAGGACTAGCACCCACCAAGGCAA
AGAGAAGAGTGGTGCAGAGAGAAAAAAGAGCAGCACTCGGAGCTATGTTCCTTGGGTTCTTGGCAACAGCAGG
AAGCACTATGGGCGCAGCATCGGTGACGCTGACGGTACAGGCCAGACAATTGTTGTCTGGTATAGTGCAACAG
CAAAACAACTTGCTGAGGGCTATTGAGGCGCAACAGCATATGTTGCAACTCACAGTCTGGGGCATC
>AF277065
TTTCATGGTAATTGGACAAGGTCAAATTTCACAGAATCAAATAGCACAACTATCACACTCCCATGTAGAATAA
AACAAATTGTAAACATGTGGCAGGAAGTAGGCAAAGCAATGTATGCCCCTCCCATCAGAGGACAAATTAGATG
TTCATCAAATATCACGGGGCTGCTATTAACAAGAGATGGTGGCGTGAACGGGACCCGCGAGACCTTTAGACCT
GGAGGAGGAGATATGAGGGACAATTGGAGAAGTGAATTATATAAATATAAAGTGGTAAAAATTGAGCCATTAG
GAGTAGCACCCACCAAGGCAAAGAGAAGAGTGGTGCAGAGAGAAAAAAGAGCAGTGGGAACAATAGGAGCTAT
GTTCCTTGGGTTCTTGGGAGCAGCAGGAAGCACTATGGGCGCAGCGTCAATAACGCTGACGGTACAGGCCAGA
CAATTATTGTCTGGTATAGTGCAACAGCAAAACAATTTGCTGAGAGCTATTGAGGCGCAACAGCATATGTTGC
AACTCACGGTCTGGGGCATC
>AF277067
AATCATACTGGGAGTAATAATATAAGATCAGAAGACAATGACAATATCACACTTCCATGCAGAATAAAACAAA
TAGTAAACATGTGGCAGGAAGTAGGCAAAGCAATGTATGCCCCTCCCATTAGAGGACAAATTAGATGTTCATC
AAATATTACAGGATTACTATTAACAAGAGATGGTGGTAATGACACGAGCGACACCGAGATCTTCAGACCTGGA

GGAGGAAATATGAGGGACAATTGGAGAAGTGAATTATATAAATATAAAGTAGTAAGAATTGAACCATTAGGAG
TAGCACCCACCAAGGCAAAGAGAAGAGTGGTGCAGAGAGAAAAAAGAGCAGTGGGAACATTAGGAGCTATGTT
CCTTGGGTTCTTGGGAACAGCAGGAAGCACTATGGGCGCAGCGTCACTGACGCTGACGGTACAGGCCAGACAA
TTATTGTCTGGTATAGTGCAACAGCAAAACAATTTGCTGAGAGCTATTGAGGCGCAACAGCATCTGTTGCAAC
TCACGGTCTGGGGCATC
>JN786692
ACTCAGAATGTTACTGGAGGAAATGAGACAATCATACTCCCGTGCAGAATAAAACAAATTATAAACATGTGGC
AGCAAGTAGGAAAAGCAATGTATGCCCCTCCCATCAGTGGACTACTTAATTGTACATCAAATATTACAGGACT
ACTAATAACAAGAGATGGTGGTACTGGGAACAGGACCAATGAGACCTTCAGACCTGGAGGAGGAGATATGAGG
GACAATTGGAGAAGTGAATTATATAAATATAAAGTAGTAAGAATTGAACCAATAGGCATAGCACCCACCGAGG
CAAAGAGAAGAGTGGTGCAGAGAGAAAAAAGAGCAGTGGGAATAGGAGCTTTGTTCCTTGGGTTCTTGGGAGC
AGCAGGAAGCACTATGGGCGCAGCGTCGATAACGCTGACGGTACAGGCCAGACAATTATTGTCTGGTATAGTG
CAACAGCAGAACAACTTGCTGAGGGCTATTGAGGCGCAACAGCATCTGTTGCAACTCACAGTCTGGGGCATC
>DQ869028
ACTTATACTAATGGAACTATCACAATCCCATGCAGAATAAGACAGATTGTAAACAGGTGGCAGGAAGTAGGAA
AAGCAATGTATGCCCCTCCCATCAGAGGATACATTAAATGTTCATCAAATATTACAGGGCTGCTATTAACGAG
AGATGGTGGTAGTAATGCCACGAATGAGACCTTCAGACCTGGAGGAGGAGATATGAGGGACAATTGGAGAAGT
GAATTATATAAATATAAAGTAGTAAAAATTGAACCATTAGGAGTAGCACCCACCAAGGCAAAGAGAAGAGTGG
TGCAGAGAGAAAAAAGAGCAATAGGGGGATTAGGAGCTTTGTTCCTTGGGTTCTTGGGAGCAGCAGGAAGCAC
TATGGGCGCAGCGTCATTGACGCTGACGGTACAGGCCAGACAATTATTGTCTGGTATAGTGCAACAGCAGAAC
AATTTGCTGAGAGCTATTGAGGCGCAACAGCATCTGTTGCAACTCACAGTCTGGGGCATC
>JN944911
ACTCAAGGGCCAAGTGAAAATAACACAATCATACTCCCATGCAGAATAAAACAAATTATAAACATGTGGCAGA
GAGTAGGAAAAGCAATATATGCCCCTCCCATCAGTGGACAAATTAGATGTTTATCAAATATTACAGGGCTGAT
ATTAACAAGAGATGGTGGTAATAGTAGCCTAAGCAGCCCCGAGATCTTCAGACCTGGAGGAGGAGATATGAGG
GACAACTGGAGAAGTGAATTATATAAATATAAAGTAGTACAAATTGAACCATTAGGAATAGCACCCACCAGGG
CAAAGAGAAGAGCGGTGCAGAGGGAAAAAAGAGCAGTGGGAATAGGAGCTCTGTTCCTTGGGTTTCTGGGAGC
AGCAGGAAGCACTATGGGCGCAGCGTCAGTGACGCTGACGGTACAGGCCAGACAGTTGTTGTCTGGTATAGTG
CAACAGCAGAGCAATCTGCTGAGAGCTATTGAGGCGCAACAGCATCTGTTGCAACTCACAGTCTGGGGCATT
>FJ469751
AGTCATAATACTGCTATGAATGTTACTGCAGGGAATGATAATATCACACTCCCGTGCAGAATAAAACAAATTA
TAAACAGGTGGCAGGAAGTAGGAAAAGCAATGTATGCCCCTCCCATCAGTGGATTCATTAACTGCTCATCAAA
TATTACAGGGATAATGTTAATTAGAGATGGTATTAATGAAACTGATGGGAACGAGACCTTCAGACCAGAAGGA
GGAAATATGAAGGACAATTGGAGAAATGAATTATACAAATATAAAGTAGTAAAAATAGAGCCCTTAGGAGTAG
CACCCACCAAGGCAAAGAGAAGAGTGGTGCAGAGAGAAAAAAGAGCAGTGGGAATAGGAGCTCTGTTCCTTGG
GTTCTTGGGAGCAGCAGGAAGCACTATGGGCGCAGCGTCGATCACGCTGACGGTACAGGCCAGACAATTATTG
TCTGGTATAGTGCAACAGCAGAGCAATTTGCTGAGAGCTATTGAGGCGCAACAGCATCTGTTGCAACTCACAG
TCTGGGGCATC
>DQ869032
ACTTATAACACAACAGATAATAGTACTGATAATGGGAATGAAATTATCACAATCCCATGCAGAATAAAACAGA
TTATAAACATGTGGCAGGAGGTAGGAAAAGCAATGTATGCCCCTCCCATTAGAGGACTAATTAGATGTACATC
AAATATTACAGGGCTACTATTAGTAAGAGATGGTGGTACTACTAATACTAGTACTAATGAGACCTTCAGACCT
GGAGGAGGAGATATGAGGGACAATTGGAGAAGTGAATTATATAAATATAAAGTAGTAAAAATTGAACCATTAG
GAGTAGCACCCACCAAGGCAAAGAGAAGAGTGGTGCAGAGAGAAAAAAGAGCAATAGGATTTGGAGCTATGTT
CCTTGGGTTCTTGGGAGCAGCCGGAAGCACTATGGGCGCAGCGTCACTGACGCTGACGGTACAGGCCAGACAA
TTATTGACTGGTATAGTGCAACAGCAAAACAATTTGCTGAGGGCTATTGAGGCGCAACAGCACTTGTTGCAAC
TCACAGTCTGGGGCATC
>FJ469742
ACTCAGAATGGTACTACATGGTCAAATGACAGTGACCCAATCATCATACTCCCATGCAGAATAAAACAAATTG
TAAATAGGTGGCAGGAAGTAGGAAAAGCAATGTATGCCCCTCCCATCAGTGGACAAATTAGATGCTCATCAAA
TATAACAGGACTGCTATTAACAAGAGATGGTGGTAACACAAATAGCACTATTGAAACCTTCAGACCTGGAGGA
GGAGATATGAGGGACAATTGGAGAAGTGAACTATATAAATATAAGGTAGTACAAATTGAACCATTAGGAGTAG
CACCCACCAAGGCAAAGAGAAGGGTGGTGCAGAGAGAAAAAAGAGCAGTAGTAGGACTAGGAGCTTTGGTCCT
TGGGTTCCTGGGAGCAGCAGGAAGCACTATGGGCGCAGCGTCACTGGCGTTGACGGTACAGACCAGACAATTA
TTGTCTGGTATAGTGCAACAGCAGAATAATATGCTGAGAGCTATTGAGGCGCAACAACACCTGTTGCAACTCA
CAGTCTGGGGCATC
>FJ469764
AATTACACAGAAAATGGAAATGACACACTCACAATCCCATGCAGAATAAAACAAATTATAAACATGTGGCAGG
AAGTAGGAAAGGCAATGTATGCCCCTCCCATCAGAGGACAAATTAAATGTTCATCAAATATTACAGGGCTGCT
ATTAACAAGAGATGGTGGTAATAGCAACAACACTAGCAATGAGACCTTCAGACCTGTAGGGGGAGATATGAGG

GACAATTGGAGAAGTGAATTATATAAATATAAAGTGGTAAAAATTGAACCATTAGGAGTAGCGCCCACCAGGG
CAAAGAGAAGAGTGGTGCAAAGAGAAAAAAGAGCAGTGGGAATAGGAGCTCTGTTCCTTGGGTTCTTGGGAGC
AGCAGGAAGCACTATGGGCGCAGCATCACTGACGCTGACGGTACAAGCCAGACAATTATTGTCTGGTATAGTG
CAACAGCAGAACAATCTGCTGAGGGCTATTGAGGCGCAACAGCATCTGTTGCAACTCACAGTCTGGGGCATC
>EF593298
TATTACAATGGTACCTGGAATAATACTGAAGAGTCAAATGAAACTATCACACTCCCATGCAGAATAAAACAAA
TTATAAACATGTGGCAGGAAGTAGGAAGAGCAATGTATGCCCCTCCCATCAGAGGAATAATTAGATGTTCATC
AAATATAACAGGGCTGCTATTAACAAGAGATGGTACTCCTACTAATAGCACAGAAAGCAATGACACAGAAATC
TTCAGACCGGGAGGAGGAAATATGAGAGACAATTGGAGAAGTGAATTATATAAATATAAAGTAGTACAAATTG
AACCAATAGGACTAGCACCCACAAAGGCAAAGAGAAGAGTGGTGCAGAGAGAAAAAAGAGCAGTGGGAACAAT
AGGAGCTATGTTCCTTGGGTTCTTGGGAACAGCAGGAAGCACTATGGGCGCAGCGTCAATAACGCTGACGGTA
CAGGCCAGACAATTATTGTCTGGTATAGTGCAACAGCAGAACAATCTGCTGAAGGCTATTGAGGCGCAACAAC
ATCTGTTGCAACTCACAGTCTGGGGCATC
>HQ216485
CATTACAAGGAAAAAAATGACACAATCACACTCCCATGCAGAATAAAACAAATTATAAACAGGTGGCAGGAGG
TAGGAAAAGCAATGTATGCCCCTCCCATCAGAGGACAAATTAACTGTTTATCAAATATTACAGGGCTTCTATT
ATTAAGAGATGGTGGACATGATAGTGGAAATAATAGTACAACGAACCAGAACGAGACCTTCAGGCCTGGGGGG
GGAAATATGAAGGACAATTGGAGAAGTGAATTATATAAATATAAAGTAGTACAAATTGAACCACTAGGAGTAG
CACCCACCAAGGCAAAGAGAAGAGTGGTGCAGAGAGAAAAAAGAGCAATAGGGGAACTAGGAGCTGTGTTCCT
TGGGTTCTTGGGAGCAGCAGGAAGCACTATGGGCGCAGCATCAGTGATGCTGACGGTACAGGCCAGACAATTA
CTGTCTGGAATAGTGCAACAGCAGAACAATTTGCTGAGGGCTATTGAGGCGCAACAACATTTGTTGCAACTCA
CAGTCTGGGGCATC
>JX863970
CACTACACCTGGGATGGTACTAATGGTACTTGGGGGAATAATGCATCGAAGGGCAATATCACACTCCACTGCA
GAATAAAACAAATTGTAAACATGTGGCAGAGAGTAGGAAGAGCAATGTATGCCCCCCCTATCAGTGGACCAAT
TAGCTGTTCATCAAATATTACAGGGTTACTATTAACAAGAGATGGGGGTGGTATCAACGAGACCAACACCACT
GAGACCTTTAGACCTGGAGGAGGAGATATGAAGGACAATTGGAGAAGTGAATTATATAAATATAAAGTAGTAC
AAATTGAACCAATAGGAATAGCACCCACCAAGGCAAAAAGAAGAGTGGTGCAGAGAGAGAAAAGAGCAGTGGG
AGTATTTGGAGCTATGTTCCTTGGGTTCTTGGGAGCAGCAGGAAGCACTATGGGCGCAGCATCAATAACGCTG
ACGGCACAGGCCAGACAATTATTGTCTGGTATAGTGCAACAGCAGAGCAATCTGCTGAGGGCTATTGAGGCGC
AACAACACATGTTGCAACTCACAGTCTGGGGCATC
>EU578047
AATTACACAGAAGGAAATGGCACAATCACACTCCCATGCAGAATAAAACAAATTATAAACATGTGGCAGGAAG
TAGGAAAAGCAATGTATGCCCCTCCCATCAGAGGACCAATTAATTGTACATCAAATATTACAGGGTTACTATT
AACAAGAGATGGTGGTATACAGAATGGCACTGAGAACACCACTGAGACCTTCAGACCTGGAGGAGGAGATATG
AGGGACAATTGGAGAAGTGAATTATATAAATATAAAGTAGTAAGAATTGAACCATTAGGAGTAGCACCCACCA
AGGCAATGAGAAGAGTGGTGCAAAGAGAAAAAAGAGCAGTGGGAATAGGAGCTTTGTTCCTTGGGTTCTTAGG
AGCAGCAGGAAGCACTATGGGCGCAGCGTCGCTGACGCTGACGGTACAAGCCAGACAATTATTGTCTGGTATA
GTGCAACAGCAGAACAATTTGCTGAGAGCTATTGAGGCGCAACAGCATCTGTTGCAACTCACAGTCTGGGGCA
TC
>MH060746
CCTCAGAATGAAAGTAGGCCAGAAAATGACACTATCACACTCCCATGCAAAATAAGACAAATTGTAAACCTGT
GGCAGGAAGTAGGAAAAGCAATGTATGCCCCTCCCATCAGAGGACAAATTAGATGTTCATCAAACATTACAGG
GCTGTTATTAACAAGGGATGGTGGTATGAACGTGAGCAACACCAACGAGACCTTCAGACCTGGAGGGGGGGAT
ATGAGGGACAATTGGAGAAGTGAATTATATAAATATAAAGTAGTAAAAATAGAACCATTAGGAGTAGCACCCA
CCAAGGCAAAGAGAAGAGTGGTGCAGAGAGAAAAAAGAGCAGTGACACTAGGAGCTATGTTCCTTGGGTTCTT
GGGAGCAGCAGGAAGCACTATGGGCGCAGCGTCAATGGCGCTGACGGTACAGGCCAGACAATTATTGTCTGGT
ATAGTGCAACAGCAGAACAATTTGCTGAGAGCTATTGAGGCGCAACAGCATCTGTTGCAACTCACAGTCTGGG
GCATC
>FJ469695
ACTCAGAATAATACTGACACAATTATCACACTCCCATGCAGAATAAAACAGATAATAAACATGTGGCAAGAAG
TAGGAAAAGCAATGTATGCCCCTCCCATCCAAGGACAAATTAGATGTTCATCAAATATTACAGGGTTACTATT
AACAAGAGATGGTGCTGGTGGTAATAAAAGCGAGAATGAGACCTTCAGACCTGGAGGAGGAAATATGAGGGAC
AATTGGAGAAGTGAATTATATAAATATAAAGTAGTAAAAATTGAACCATTAGGAGTAGCACCCACCAAGGCAA
GAAGAAGAGTGGTGCAGAGAGAAAAAAGAGCACTAGTAGGACTAGGAGCTGTGTTCCTTGGGTTCTTGGGAGC
AGCAGGAAGCACTATGGGCGCAGCGTCAATGGCGCTGACGGTACAGGCCAGACAATTATTGTCTGGTATAGTG
CAACAGCAGAACAATTTGCTGAGAGCTATTGAGGCGCAACAGCATATGTTGCAACTCACAGTCTGGGGCATC
>FJ469732
AATCAAACTACTAATGAATGGAATGGTACTAAAGGGTTAGATGACAGGGGAGAAAATGACACAATCACACTCC
CATGCAGAATAAAACAAATTGTAAACATGTGGCAGGGAGTAGGAAGAGCAATGTATGCCCCTCCCATCAGTGG

ACAAATTAGATGCTTATCAAATATTACAGGGCTGCTATTAACAAGAGATGGTGGTAATACAAATGGCACGAAT
GGGACTGAAACCTTCAGACCTGGAGGAGGAGATATGAGGGACAATTGGAGAAGTGAATTATATAAATATAAAG
TAGTAAAAATTAAACCATTAGGAGTAGCACCCACCAAGGCAAAGAGAAGAGTGGTGCAGAGAGAAAAAAGAGC
AGTGGGAATAGGAGCTGTGTTCCTTGGATTCTTGGGAGCAGCAGGAAGCACTATGGGCGCAGCGTCAGTGACG
CTGACGGTACAGGCCAGACAATTATTGTCTGGTATAGTGCAACAGCAGAACAATTTGCTGAGGGCTATTGAGG
CGCAACAACATCTGTTGCAACTCACAGTCTGGGGCATA
>DQ410403
AATTACACTAGCACTATCACACTCCCATGCAGAATAAAACAAGTTATAAACATGTGGCAGGAAGTAGGAAAAG
CAATGTATGCCCCTCCCATCAGAGGACAAATTAAATGTTCATCAAATATTACAGGGCTGATACTAACAAGAGA
TGGTGGTACAAACAACACCAACACCACCGAGACCTTTAGACCTGGAGGAGGAGATATGAGGGACAATTGGAGA
AGTGAATTATATAAATACAAAGTAGTAAAAATTGAACCAATAGGAATAGCACCCACCAAGGCAAAGAGAAGAA
CGGTGCAGAGAGAAAGAAGAGCAATAGGAATAGGAGCTGTGTTCCTTGGGTTCTTGGGAACAGCTGGAAGCAC
TATGGGCGCAGCGTCACTGACGCTGACGGTACAGGCCAGACAATTATTGTCTGGTATAGTGCAACAGCAAAAC
AATTTGCTGAAGGCTATTGAGGCGCAACAGCATCTGTTGCGACTCACAGTCTGGGGCATC
>EU547186
AGTCAACTTTTTAATAGTACTTGGAATGGTACCGACGGGTCAGATAACACAATGGACAAAGATATCTCACTTC
CATGCAGAATAAGACAAATTGTAAACATGTGGCAGGAAGTAGGAAAAGCAATGTATGCCCCTCCTATCCAAGG
ACAAATTAGATGTTCATCAAATATTACAGGGCTACTATTAGTAAGAGATGGTGGTAGTAATACAACCAATAAT
GAGACCTTCAGACCTGGAGGAGGAGACATGAGGGACAACTGGAGAAGTGAATTATATAAATATAAAGTAGTAA
AAATTGAACCAGTAGGAATAGCACCCACCAAGGCAAAGAGAAGAGTGGTGCAGAGAGAAAAAAGAGCAGTGGG
AACACTAGGAGCTATGTTCCTTGGGTTCTTGGGAACAGCAGGAAGCACTATGGGCGCAGCGTCAGTGACGCTG
ACGGTACAGGCCAGACAATTATTGTCTGGTATAGTGCAACAGCAAAGCAATATGCTGAGGGCTATTGAGGCGC
AACAGCATCTGTTGCAACTCACAGTCTGGGGCATC
>JF689852
AATCACACTAAAGGAAATGGCACAATCACAATTCCATGCAGAATAAAACAAATTATAAACATGTGGCAGGAAG
TAGGAAAAGCAATGTATGCCCCTCCCATCAGAGGACAAATTAGATGTTCATCAAATATTACAGGGCTGTTATT
AACAAGAGATGGTGGCAATGACGGGGACAATACCACCGAAACCTTCAGACCTGGAGGAGGAGATATGAGGGAC
AATTGGAGAAGTGAATTATATAAATATAAAGTAGTAAAAATTGAACCATTAGGAGTAGCACCTACCAAGGCAA
AGAGAAGAGTGGTGCAGAGAGGAAAAAGAGCAGTAGGAATAGGAGCTGTGTTCCTTGGGTTCTTGGGAGCAGC
AGGAAGCACTATGGGCGCAGCGTCATTAACGCTGACGGTACAGGCCAGACTATTATTGTCTGGTATAGTGCAA
CAGCAGAACAATTTGCTGAGGGCTATTGAGGCACAACAGCATCTGTTGCAACTCACAGTCTGGGGCATC
>HQ216634
GATTACACTACAGCAGAGAATGATACTAAAGAAGAAAATATCACACTCCCATGCAGAATAAAACAAATTATAA
ACATGTGGCAGAAAGTAGGAAAAGCAATGTATGCCCCTCCCATCAGAGGACAGATTAGATGTTCATCAAATAT
TACAGGGCTGCTATTAACAAGAGATGGTGGTAATGGTGATACCACAAACAATGAGACCTTCAGACCTGGAGGA
GGAGATATGAGGGATAATTGGAGAAGTGAATTATATAGATATAAAGTAGTAAGAATTGAACCATTAGGAATAG
CACCCACTAAGGCAAAGAGAAGAGTGGTGCAGAGAGAAAAAAGAGCAATAGGGGGAATAGGAGCTATGTTCCT
TGGGATCTTAGGAGTAGCAGGAAGCACTATGGGCGCAGCGTCATTGACGCTGACGGTACAGGCCAGACAATTA
TTGTCTGGTATAGTGCAACAGCAGAACAATTTGCTGAGGGCTATTGAGGCGCAACAGCATCTGTTGCAACTCA
CAGTCTGGGGCATC
>HQ216874
GTTCATAATAATAATACTGAAAGGTTAAATACCACTAACAGTACTATCATACTCCCATGCAAAATAAAACAAA
TTATAAACATGTGGCAGGGAGTAGGAAAAGCAATGTATGCCCCTCCCATTGCAGGAATAATTAATTGTGTATC
AAATATTACAGGGCTGCTATTAACAAGAGATGGTGGTGGTAGTGGTAATGGCACAAGAAGGGATAACGAAACC
TTCAGACCTGAAGGGGGAAATATGAAGGATAATTGGAGAAGTGAATTATACAAATACAAAGTAGTAAAAATTG
AACCATTAGGAGTAGCACCCACTAGGGCAAAGAGAAGAGTGGTGCAGAGAGAAAAAAGAGCGATAGGAGGACT
AGGAGCTTTGTTCCTTGGGTTCTTGGGAATGGCAGGAAGCACTATGGGCGCAGCGTCGCTGACGCTGACGGTA
CAGGCCAGGCTGTTATTGTCTGGTATAGTGCAACAGCAAAACAATTTGCTGAGGGCTATTGAGGCGCAACAGC
ACCTGTTGCAACTCACAGTCTGGGGCATC
>FJ653571
TCTCATTGGTCTGATAGTACTTGGAATGATACTACAGGGCCAAATAACACTACAGAAACAATCATACTCCCAT
GTAGACTAAAACAAATTATAAACATGTGGCAAGGAGTAGGAAGAGCAATGTATGCCCCTCCCATCAGAGGACA
AATTAGATGTTCATCAAATATTACAGGGCTGTTATTAACAAGAGATGGTGGTGGTAGTAATAGCAGCGGCAGT
AACGAGACCTTCAGACCAGAAGGAGGAAATATGAAGGACAATTGGAGAAGTGAACTATATAAATATAAAGTAG
TAAAAATTGAACCATTAGGAATAGCACCCACTAAGGCAAAAAGAAGAGTGGTGCAAAGAGAAAAAAGAGCAGT
GGGACTAGGAGCTCTGTTCCTTGGGTTCTTGGGAGCAGCAGGAAGCACTATGGGCGCAGCGGCAGTAACGCTG
ACGGTACAGGCCAGACAATTATTGTCTGGTATAGTGCAACAGCAAAACAATTTGCTGAGAGCTATTGAGGCGC
AACAGCATATGTTGCAACTCACAGTCTGGGGCATC
>KC312435

GATCATATTACTGAGGAGAACACAACTATCACACTCCAATGCAGAATAAAACAAATTATAAACATGTGGCAAA
AAGTAGGAAAAGCAATGTATGCCCCTCCCATCAAAGGACAAATTAGCTGTGTATCAAATATTACAGGGCTGCT
ATTAACAAGAGATGGTGGTCTAAACCAGTCAGGTAACAGTTCCGAGACCTTCAGACCTGGAGGAGGAGATATG
AGGGACAACTGGAGAAGTGAATTATATAAATATAAAGTAGTAAGAATTGAACCATTAGGAGTAGCACCCACCA
AGGCAAAGAGAAGAGTGGTGCAGAGAGAAAAAAGAGCAGTGGGAGTTCTAGGAGCTATGTTCCTTGGGTTCTT
GGGAGCAGCAGGAAGCACTATGGGCGCAGCGTCAATGACGCTGACGGTACAAGCCAGACAATTATTGTCTGGT
ATAGTGCAACAGCAGAACAATCTGCTGATGGCTATTGAGGCGCAACAGCATATATTGCGACTCACAGTCTGGG
GCATC
>HQ216949
AATTACACTGATGGAACTATCACACTCCCATGCAGAATAAGACAAATTATAAACATGTGGCAGGAAGTAGGAA
AAGCAATGTATGCCCCTCCCATCGGAGGGCAAATTAGATGTTCATCAGATATTACAGGGCTACTCTTAACAAG
AGATGGTGGCAGAGGAAATAACAATAATGAAACTGAGATCTTCAGACCTGGAGGAGGAGATATGAGGGACAAT
TGGAGAAGTGAATTATATAAATACAAAATAGTAAAAATTGAACCATTAGGAATAGCACCCACCAAGGCGAAGA
GAAGAGTGGTGCAGAGAAAGAAAAGAGCAGTGGGAATAGGAGCTGTGTTCCTTGGGTTCTTGGGAGCAGCAGG
AAGCACGATGGGCGCAGCGTCAGTGACGCTGACGGTACAGGCCAGACAATTACTGTCTGGTATAGTGCAACAG
CAGAACAATCTGCTGAGGGCTATTGAGGCGCAACAACATCTGTTGCAACTCACAGTCTGGGGCATC
>JX863989
CATCAGAATAATACTAATATTACTACAGAGTCAAATGGCATCATCACACTCCCATGCAGAATAAAACAAATTA
TAAACAGGTGGCAGGAAGTAGGAAAAGCAATGTATGCCCCTCCCATCAGAGGACCAATCAGGTGTAGATCAAA
TATTACAGGGGTGCTATTAACAAGAGATGGTGGTAGTACTAATGCCACCAACGAGACCTTCAGACCTGGAGGA
GGAGATATGAGGGATAATTGGAGAAGTGAATTATATAAATATAAAGTAGTAAAAATTGAGCCATTAGGAATAG
CACCCACCAAGGCACAGAGAAGAGTGGTGCAGAGAGAAAAAAGAGCAGCAATTGGAGCTATGCTCATTGGGTT
CTTGGGAATGGCAGGAAGCACTATGGGCGCAGCGTCAGTGACGCTGACGGTACAAGCCAGGCTATTACTGTCT
GGTATAGTGCAGCAGCAGAGAAATTTGCTGAGAGCTATTGAGGTGCAACAACATATGTTGCAACTCACAGTCT
GGGGCATC
>JX863993
AATTACAACATTACAGGAAATATCACACTCCCATGCAGAATAAAACAAATTATAAACATGTGGCAGGAAGTAG
GAAAAGCAATGTATGCTCCTCCCATCGCAGGACTACTTAGATGTTCATCAAATATTACAGGGCTGCTATTAAC
ACGAGATGGAGGTCTACACCCGGACAATGGACCGAGATTTTCAGACCTGGAGGAGGAGATATGAGGGACAAT
TGGAGAAGTGAATTATATAAATATAAAGTAGTAAAAATTGAACCATTAGGAATAGCACCCACCAAGGCAAAGA
GAAGAGTGGTGCAGAGAGAAAAAAGAGCAATAGGACTAGGAGCTTTGTTCTTTGGGTTCTTGGGAGCAGCAGG
AAGCACTATGGGCGCAGCGTCAGTGACGCTGACGGTACAGGCCAGACAGTTACTGTCTGGTATAGTGCAACAG
CAGAACAATTTGCTGAGAGCTATTGAGGCGCAACAACATATGTTGCAACTCACAGTCTGGGGCATC
>JF320263
AATTATACTGATCCTTTAAATGATACTTTGAATAATGATACTGAAGAAAATATCACACTCCAATGCAGAATAA
AACAATTTATAAACATGTGGCAGAAAGTAGGAAAAGCAATGTATGCCCCTCCCATTAGTGGACAAATTAACTG
TTCATCAAATATTACAGGGCTGCTATTAATGAGAGATGGTGGTATTAACCAGAGCGAGAGCAGCGAGACCTTC
AGACCTGGAGGAGGAGATATGAGGGACAATTGGAGAAGTGAATTATATAAATATAAAGTAGTAAAAATTGAAC
CTTTAGGAGTAGCACCCACTAAAGCAAAGAGAAGAGTGGTGCAGAGAGAAAAAAGAGCAGCAATAGGAGCTCT
GGTCCTTGGGTTCTTGGGAGCAGCAGGAAGCACTATGGGCGCAGCGTCACTGACGCTGACGGTACAGGCCAGA
CTATTATTGTCTGGTATAGTGCAGCAGCAAAACAATTTGCTGAGAGCTATTGAGGCGCAACAGCATATGTTGC
AACTCACAGTCTGGGGCATC
>JF320011
AATCATACTTGGAATAGTACTAATGATACTTGGAATGGTACTGAGACCATAACACTTCCATGCAGAATAAAAC
AAATTATAAACATGTGGCAAGAAGTAGGAAAAGCAATGTATGCCCCTCCCATCAGAGGACAGATTAACTGCTC
GTCAAATATTACAGGGCTGCTATTAACAAGAGATGGTGGTAACACCACAAACACCACCGAGATCTTCAGACCT
GGAGGGGGAAATATGAAGGACAATTGGAGAAGTGAATTATATAAATACAAAGTAGTAAAAATTGAACCATTAG
GAGTAGCACCCACCAAGGCAAAGAGGAGAGTGGTGCAGAGAGAAAAAAGAGCAGCAATAGGAGCTTTGTTCCT
TGGGTTCTTGGGAGCAGCAGGAAGCACTATGGGCGCAGCGTCAGTGACGCTGACGGTACAGGCCAGACTATTA
TTGTCTGGTATAGTGCAACAGCAGAGCAATCTGCTGAGGGCTATTGAGGCGCAACAGCATCTGTTGCAACTCA
CAGTCTGGGGCATC
>JF320126
AGTCATAATGATACCAGGCATAATAGTACTAAAGGGACAGATAATATCATACTCCCATGTAAAATAAGACAAA
TTGTAAACATGTGGCAGGAAGTAGGAAAGGCAATGTATGCCCCTCCCATTGCAGGACAAATTAAATGTTCATC
AAATATTACAGGGCTGATATTAACAAGAGATGGTGGTAATAATAATAGCACAAACGAGACTGAGACATTCAGA
CCTGGAGGAGGAAATATGAGGGACAATTGGAGAAGTGAATTATATAAATACAAAGTAGTAGAAATTGAACCAT
TAGGAATAGCACCCACCAAGGCAAAGAGGAGAGTGGTGCAAAGAGAAAAAAGAGCAGTGGGAACGATAGGAGC
TATGTTCCTTGGGTTCTTGGGAGCAGCAGGAAGCACTATGGGCGCAGCGTCGATAACGCTGACGGTACAGGCC
AGACAATTATTGTCTGGTATAGTGCAGCAGCAGAACAATCTGCTGAGGGCTATTGAGGCGCAACAACATCTGT
TGCAACTCACGGTCTGGGGCATC

>FJ469723
ACTCAGCTGTTTAATAGTACTAATAATAATACTGCAGAAAACATCACACTCCCATGCAGAATAAGACAAATTA
TAAACAGGTGGCAAGAAGTAGGAAAAGCAATGTATGCCCCTCCCATCAGCGGACTAATTAACTGCTTATCAAA
TATTACAGGGCTGCTATTAACAAGAGATGGAGGGAACAATACAGAGAGTAAGACTGAGACCTTCAGACCTGGA
GGAGGGGATATGAGGGACAATTGGAGAAGTGAATTATACAAGTATAAAGTAGTAAAAATTGAACCATTAGGAG
TAGCACCCACCAGGGCAAGGAGAAGAGTGGTGCAAAGAGAAAAAAGAGCAGTGGGAGTGATAGGAGCTATGTT
CCTTGGATTCTTGGGAGCAGCAGGAAGCACTATGGGCGCAGCGGCAGTGACGCTGACGGTACAGGCCAGACAA
TTATTGTCTGGTATAGTGCAACAGCAAAACAATTTGCTGCAGGCTATTGAGGCGCAACAACATCTGTTGCAAC
TCACAGTCTGGGGCATT
>JQ403097
ACTCATTGGAATGATACTAATGACACCAATAATGACACCATTACACTCCCATGCAGAATAAAACAAATTATAA
ACTTGTGGCAGGAAGTAGGAAAAGCAATGTATGCCCCTCCCATCAAAGGACAAATTAGATGTTCATCAAATAT
TACAGGGCTGCTATTAACAAGAGATGGTGGTTGTAACACGACCAATGGGACTGAGGTCTTCAGACCTGGAGGA
GGAGATATGAGGGACAATTGGAGAAGTGAATTATATAAATATAAAGTAGTAAAAATTGAACCATTAGGAGTAG
CACCCACCAAGGCAAAGAGAAGAGTGGTGCAGAGAGAAAAAAGAGCAGTGGGAGTAATAGGAGCTATGTTCCT
TGGGTTCTTGGGAGCAGCAGGAAGCACTATGGGCGCAGCGTCAGTGACGCTGACGGTACAGGCCAGACAATTA
TTGTCTGGTATAGTGCAACAGCAGAACAATTTGCTGAGAGCTATTGAGGCGCAACAGCATCTGTTGCAACTCA
CAGTCTGGGGCATC
>EU578223
AATTACACAGGAGAAAATGACACAATTACACTCCCATGCAGAATAAAACAAATCATAAATATGTGGCAGGAAG
TAGGAAAAGCAATGTATGCCCCTCCCATCAGAGGAATAATTAGGTGTTCATCTAATATTACAGGGTTGTTATT
AACAAGAGATGGGGGTAGCGGTAACGAGAGCGACGCCGAGATCTTCAGACCTGGGGGAGGGAATATGAAGGAT
AATTGGAGAAGTGAATTATATAAATATAAAGTAGTAAAAATTAAACCATTAGGGATAGCACCCACCAAGGCAA
AAAGAAGAGTGGTGCAGAGAGAAAAAAGAGCAGTGGGACTAGGAGCTTTGTTCCTTGGGTTCTTGGGAGCAGC
GGGAAGCACTATGGGCGCAGCGTCAGTGACGCTGACGGTACAGGCCAGAAACTTATTGTCTGGTATAGTGCAA
CAGCAGAACAATCTGCTGAGGGCTATTGAGGCGCAACAGCACCTGTTGCAACTCACAGTCTGGGGCATA
>JF689892
AATTACACTGAAGGAAATGATACAATCACACTCCCATGCAAAATAAAACAAATTGTAAACATGTGGCGGGAGG
TAGGAAAGGCAATGTATGCCCCTCCTATCAGAGGACTAATTAGATGCTCATCAAATATTACAGGACTGATCTT
AACAAGAGATGGTGGTAATATTAATAATGGTACCCACGGGGAACAGGACTGAGATCTTTAGACCTGGAGGAGGA
GATATGAGGGACAATTGGAGAAGTGAATTATATAAATATAAGGTAGTAAGAATTGAACCATTAGGAATAGCAC
CCACCAGGGCAAAGAGAAGAGTGGTGCAAAGAGAAAAACGAGCAGTGGGAGTAGGAGCTCTGTTCCTTGGGTT
CTTGGGAGCAGCAGGAAGCACTATGGGCGCAGCGTCAATAACGCTGACGGTACAGGCCAGACAATTATTGTCT
GGTATAGTGCAACAGCAGAACAATCTGCTGAAGGCAATTGATGCGCAACAGCATCTGTTGCAACTCACAGTCT
GGGGCATT
>JF320315
ACTCAAGAAAGTAACACTGAAGGAAATGACACAATTATACTACCATGCAGAATAAAACAAATTGTAAACATGT
GGCAGGAAGTAGGAAAAGCAATGTATGCCCCTCCCATTAGTGGACAAATTAACTGTTCATCAAATATTACAGG
GCTAATATTAGCAAGAGATGGTGGTAATGAGACATACAGGAACGAGACCTTCAGACCTATAGGAGGAGATATG
AGGGACAATTGGAGAAGTGAATTATATAAATATAAAGTAGTAAAAATTGAACCATTAGGAGTAGCACCCACCA
AGGCAAAGAGAAGAGTGGTGCAAAGAGAAAAAAGAGCAGTGGGATTAGGAGCTATGTTCCTTGGGTTCTTGGG
AGCAGCAGGAAGCACTATGGGCGCGGCGTCAGTGACGCTGACGGTACAAGCCAGACAATTATTGTCTGGTATA
GTGCAACAGCAGAACAATCTGCTGAGAGCTATTGAGGCGCAACAGCATCTGTTGCAACTCACAGTCTGGGGCA
TC
>JF320394
ACTCAGAAGATTAAAGGGTTTGATAATAATGGCAATATCACACTCCCCTGTAGAATAAAACAAATTATAAACA
GGTGGCAGGAAGTAGGAAAAGCAATGTATGCCCCTCCCATTGCAGGACAAATTAAATGTACATCAAATATTAC
AGGGCTGCTATTAACATGGGATGGTGGTAATGATGGTGGAAACGGCACTGGAACAAAAAATAACGAAACAGAG
GTTTTTAGACCATCAGGAGGAAATATGAGGGACAATTGGAGAAGTGAATTATATAAGTATAAAGTAGTAAAAG
TTGAACCATTAGGAATAGCACCCACCAAGGCAAAGAGAAGAGTGGTGCAGAGAGAAAGAAGAGCAGTGGGAAT
AGGAGCTGTGTTCCTTGGGTTCTTGGGAGCAGCAGGAAGCACTATGGGCGCAGCGTCATTAACGCTGACGGTA
CAAGCCAGACTATTGTTGTCTGGTATAGTGCAACAGCAGAACAATCTGCTGAGAGCTATTGAGGCGCAACAGC
ATCTGCTGCAACTCACAGTCTGGGGCATC
>JF320279
ACTCAATTATTCAATAGTACTTGGCTACTCAATGGATCAAGTAACACTGAAGGAAATGACACAATCACGATCC
CATGCAGAATAAAACAAATTGTAAACATGTGGCAAAAAGTAGGAAAAGCAATGTATGCCCCTCCCATCAGAGG
AAATATTAGTTGTATATCAAATATTACAGGAATACTATTAATAAGAGATGGGGGTGTTAGCAACGAGAGTGCT
AACGAGACCAGGAACGAGACCTTCAGACCAGCAGGAGGAGATATGAGGGACAATTGGAGAAGTGAATTATATA
AATATAAAGTAGTAAAAATTGAACCATTAGGAATAGCACCCACTAAGGCAAAGAGAAGAGTGGTGCAGAGAGA
AAAAAGAGCAGTGGGACTAGGAGCTGTGTTCCTTGGGTTCTTGGGAGCAGCAGGAAGCACTATGGGCGCAGCG

TCAGTGACGCTGACGGTACAAGCCAGACAATTATTGTCTGGTATAGTGCAACAGCAGAACAATTTGCTGAAGG
CTATTGAGGCGCAACAGCACCTGTTGCAACTCACAGTCTGGGGCATC
>HQ908109
ACTCAAAATGATACTGCTAACAGTACCATTATGCTCCCATGCAGAATAAAACAAGTTATAAACAGGTGGCAGG
AAGTAGGAAAAGCAATGTATGCCCCTCCCATCAGAGGACCAATTAGCTGTTCATCAAATATTACAGGGCTGAT
ATTAACAAGGGATGGTGGTATTATTAATGCCACCAATGAGACCTTCAGACCTGGAGGAGGAGATATGAGGGAC
AATTGGAGAAGTGAATTATACAAATATAAAGTAGTAAAAATTGAACCATTAGGAGTAGCACCCACCAGGGCAA
AGAGAAGAGTGGTGCAAAGAGAAAAAAGAGCAGTGGGAATAGGAGCTCTGTTCCTTGGGTTCTTGGGAGCAGC
AGGAAGCACTATGGGCGCAGCGTCAGTGACGCTGACGGTACAGGCCAGACAATTATTGTCTGGTATAGTGCAA
CAGCAGAGCAATTTGCTGAGGGCTATTGAGGCGCAACAGCATCTGTTGCAACTCACAGTCTGGGGCATC
>FJ469713
TGTTATACATCACGACTGTTTAATAGAACTTTTGGTAATGAGACTATAAATCCTACTAAGATTAATGAAAACA
CTACAGAAGTCATCACACTCCCATGTAGAATAAGACAAATTATAAATATGTGGCAGGAAGTAGGAAAAGCAAT
GTATGCCCCTCCCATCAGAGGACAAATTAACTGTATATCAAACATTACAGGACTGTTATTAACAAGAGATGGT
GGCGATGACGATACCACCAACACTGAGACCTTCAGACCTACAGGGGGAAATATGAAAGACAATTGGAGAAGTG
AATTATATAAATATAAAGTAGTAAGAATTGAACCATTGGGACTGGCACCCTCCAAGGCAAAGAGAAGAGTGGT
GCAGAGAGAAAAAAGAGCAGTGGGACTAGGAGCTTTGTTCATTGGGTTCTTGGGAGCAGCAGGAAGCACTATG
GGCGCAGCGTCGATGACGCTGACGGTACAGGCCAGACTATTATTGTCTGGTATAGTGCAACAGCAGAACAATC
TGCTGAGAGCTATTGAGGCGCAACAACATCTGTTGCAACTCACAGTCTGGGGCATC
>JQ403071
AGTCATATAGAGTCAAATGACAATGAAGAAAATATCACACTCCCCTGTAGAATAAGACAAATTGTAAACATGT
GGCAGGAAGTAGGAAAAGCAATGTATGCCCCTCCCATCAGTGGACAAATTAGATGTGTATCAACTATTACAGG
GCTGCTATTAACAAGAGATGGTGGGAACGGGACTAACAATACTGAGACCTTTAGACCTGGAGGAGGAGATATG
AGGGACAACTGGAGAAGTGAATTATATAAATATAAAGTAGTAAGAATTGAACCATTAGGAGTAGCACCCACCA
AGGCACAGAGAAGAGTGGTGCAGAGAGAAAAAAGAGCAGTGGGAATGTTAGGAGCTATGTTCCTTGGGTTCTT
GGGAGCAGCAGGAAGCACTATGGGCGCAGCGTCGATAACGCTGACGGTACAAGCCAGACAATTAATGTCTGGT
ATAGTGCAACAGCAGAACAATCTGCTGAAGGCTATTGAGGCGCAACAACATATGTTGCAACTCACAGTCTGGG
GCATC
>KM259279
AGTTATTGGAATACTACTGAAGGGTCCAATAACACTGAAGGAAATGACACCATCACAATACCATGCAGAATAA
AACAAATTATAAACATGTGGCAGGAAGTAGGAAAAGCAATGTATGCCCCTCCCATCAAAGGAATAATTAATTG
TTCATCAAATATCACAGGGCTGTTATTAACAAGAGATGGTGGTAACACAACCAACAACAGCACAGAGACCGAG
ACCTTCAGACCTGGAGGAGGAGATATGAGGGACAATTGGAGAAGTGAATTATATAAGTATAAAGTAGTAAAAA
TTGAACCATTAGGACTAGCACCCACCAAGGCAAAGAGAAGAGTGGTGCAGAGAGAAAAAAGAGCAATAGGAAT
AGGAGCTATGTTCCTTGGGTTCTTGGGAGCAGCAGGAAGCACTATGGGCGCAGCATCATTGACGCTGACGGTA
CAGGCCAGACAATTATTGTCTGGTATAGTGCAACAGCAGAACAATTTGCTGAGAGCTATTGAGGCGCAACAGC
ATATGTTGCAACTCACAGTCTGGGGCATC
>MK383753
AGTCACACTGAAGAAAATATCACACTCCCATGCAGAATAAAACAAATTATAAACATGTGGCAGGAAGTAGGAA
AAGCAATGTATGCCCCTCCCATTAGAGGAAACATTAGCTGTTCATCAAATATTACAGGGCTGCTATTAACAAG
AGATGGTGGTAATAACGAGAGCCAGACCGAGACCTTCAGACCTGGAGGAGGAAATATGAAGGATAATTGGAGA
AGTGAATTATATAAATATAAAGTAGTAAAAATTGAACCATTAGGAGTAGCACCCACCAGGGCAAAGAGAAGAG
TGGTGCAGAGAGAAAAAAGAGCAGTGGGAATAGGAGCTGTGTTCCTTGGGTTCTTGGGAGCAGCAGGAAGCAC
TATGGGCGCAGCGTCAATGACGCTGACGGTACAGGCCAGACTATTACTGTCTGGTATAGTGCAACAGCAGAGC
AATTTGCTGAGGGCTATTGAGGCGCAACAGCGTTTGTTGCAACTCACAGTCTGGGGCATC
>HQ615981
ACTCAGGAGACTAACAGGGACAATGACACTATCACGCTCCCATGCAGAATAAAACAAATTATAAACATGTGGC
AGGAAGTAGGAAGAGCAATGTATGCCCCTCCCATCAAAGGACTAATTAGGTGTTCATCAAATATCACAGGGCT
GCTACTACTAAGAGATGGTGGAAATACCAGTAACAATAATGAGACCTTCAGACCTGGAGGAGGAGATATGAGG
GACAATTGGAGAAGTGAATTATATAAATATAAAGTAGTAAAAGTTGAACCATTAGGAATAGCACCCACCAAGG
CAAAGAGAAGAGTGGTGCAGAGAGAAAAAAGAGCAGTGGGTTTTGGAGCTATGTTCCTTGGGTTCTTGGGAGC
AGCAGGAAGCACTATGGGCGCAGCATCACTAACGCTGACGGTACAGGCCAGACAATTGTTGTCTGGTATAGTG
CAACAGCAAAACAATTTGCTGAGAGCTATTGAGGCGCAACAACATATGTTGCAACTCACAGTCTGGGGCATC
>JQ403080
ACTCAACTGTTTAATAGTACTTGGGTGGTTAATAGTAGTACTGGGGAAGTTAATAGTAGTACTGGGGGGTTTA
CAGGAAATGGCATAATCACACTTCCATGCAGGATAAAACAAATTATAAACATGTGGCAGGAAGTAGGAAAAGC
CATGTATGCCCCTCCCATCGCAGGACAAATTAACTGTTCATCAAATATTACAGGACTACTACTAATAAGAGAT
GGTGGTAAAAATGTCAGCAACACCACCGAGACCTTTAGACCTGGAGGAGGAAATATGAGGGACAATTGGAGAA
GTGAATTATATAAATATAAGGTAGTAAGACTTGAGCCATTAGGAATAGCACCCACCAAGGCAAGGAGAAGAGT
GGTGCAAAGAGAAAAAAGAGCAGTGGGAACAATAGGAGTTATGTTCCTTGGGTTCTTGGGAGCAGCAGGAAGC

ACTATGGGCGCAGCGTCAGTGACGCTGATGGTACAGGCCAGACAATTACTGTCTGGTATAGTGCAGCAGCAAA
GCAATTTGCTGAAGGCTATTGAAGCGCAACACCATCTGTTGCAGCTCACAGTCTGGGGCATC
>KM082128
CATTATAATAATGATACTGGTACTTGGAATACTACTTGGATTGGGAATGGTACTGAAGGGAATGAAAATAGCA
CCATCACCCTCCCATGCAGAATAAAACAAATAATAAACATGTGGCAGGAAGTAGGAAAGGCAATGTATGCCCC
TCCCATTAAAGGAATAATTAGCTGTTCATCAAATATTACAGGGCTGCTATTAACAAGAGATGGTGGTAACAGT
AATACCACCGAGAATGAGACCTTCAGACCTGGAGGAGGAGATATGAGGGACAATTGGAGAAGTGAATTATATA
AATATAAAGTAGTAAGAATTGAACCATTAGGAGTAGCACCCACCCATGCAAAGAGAAGAGTGGTGCAGAGAGC
AGCAAGAGCAGTGGGATTCGGAGCTTTGTTCCTTGGGTTCTTGGGAGCAGCAGGAAGCACTATGGGCGCAGCG
TCAATAACGCTGACGGCACAGGCCAGACAACTGTTGTCTGGTATAGTGCAACAGCAGAACAATCTGCTGAGGG
CTATTGAGGCGCAACAACACCTGTTGCAACTCACAGTCTGGGGCATT
>MH666207
TTTCATAGTAATTGGACTAAAACAGGGAACGTTACATACATTCCAGAAAATAACTCCACTAACATCACACTCC
AATGTAGAATAAAACAAATTATAAACATGTGGCAGAAAGTAGGCAAAGCAATGTATGCCCCTCCCATCAGTGG
ACAAATTAGATGTTCATCAAACATTACAGGGCTGCTATTAACAAGAGATGGTGGTAAGAAGAACGAATCTGAA
GGGAACGAGACTGAGGTCTTCAGACCTGGAGGAGGGGATATGAGGGACAATTGGAGAAGTGAATTATATAAAT
ATAAAGTAGTGAAAATTGAGCCATTAGGAGTAGCACCCACCAAGGCAAAGAGAAGAGTGGTGCAAAGAGAAAA
AAGAGCAGTGGGACTAGGAGCTTTGTTCCTTGGATTCTTGGGAGCAGCAGGAAGCACTATGGGCGCAGCGTCA
ATAACGCTGACGGTACAGGCCAGACAAGTATTGTCTGGTATAGTGCAGCAGCAACACAATTTGCTGAGGGCTA
TTGAGGCGCAACAGCATCTGTTGCAACTCACAGTCTGGGGCATC
>KR423423
ACTTATACTTGGAATAATACAGATATCAATGGTACTATCACACTCCAATGCAGAATAAAACAAATTGTAAATA
GGTGGCAGGAAGTAGGAAAAGCAATGTATGCCCCTCCCATCAGTGGAAAGATTAGTTGCTCATCAAATATTAC
AGGGCTGCTATTAATGAGAGATGGTGGAAGCAGCAATGAGACTAAAAATAATACTGAGATCTTCAGACCTGGA
GGAGGAAATATGAGGGACAATTGGAGAAGTGAATTATATAAATATAAAGTAGTGAGAATTGAACCATTAGGAA
TAGCACCCACCAAGGCAAAGAGAAGAGTGGTGCAGAGAGAAAAAAGAGCAGTGGGATTAGGAGCCATGTTCCT
TGGGTTCTTAGGAGCAGCAGGAAGCACTATGGGCGCAGCATCAATAACGCTGACGGTACAAGCCAGACTATTA
TTGTCTGGTATAGTGCAACAGCAAAACAATCTGCTGAGAGCTATTGAGGCGCAACAGCATCTGTTGCAACTCA
CAGTCTGGGGCATC
>KR423436
AATTACACTGATACTTGGAATAATACTGAAGAGACAAATGAAACTATCACACTCCCATGCAGAATAAAACAAA
TTATAAACATGTGGCAGGAAGTAGGAAAAGCAATGTATGCCCCTCCCATTAGAGGAGCAATTAACTGCTCATC
AAACATAACGGGGCTGCTATTAACAAGAGATGGTAGTACTGATGGCCCAGACAATACCACTGAAATCTTCAGA
CCGGGAGGAGGAGATATGAGGGACAATTGGAGAAGCGAATTATATAAATATAAAGTAGTACAAATTGAACCAA
TAGGACTAGCACCCACAAAGGCAAGGAGAAGAGTGGTGCAGAGAGAAAAAAGAGCAGTAACAATGGGAGCTAT
GTTCCTTGGGTTCTTGGGAGCAGCAGGAAGCACTATGGGCGCAGCGTCAATAACGCTGACGGTACAGGCCAGA
CAATTATTGTCTGGTATAGTGCAACAGCAGAACAATCTGCTGAAGGCTATTGAGGCGCAACAGCATCTGTTGC
AACTCACAGTCTGGGGCATC
>KR423444
TTTCAGGGTACTTGGAATGGTACTACTTGGAATACTAATGATACTTGGAGGCATTGGAGGCATAGTAATTCAT
CGAATGACACTGTCACACTCCAATGCAGAATAAGACAAATTGTAAACATGTGGCAGAGAGTAGGAAAAGCAAT
ATATGCTCCTCCTATCCAAGGAAAAATTAGATGCTCATCAAATATTACAGGGTTGCTATTAACAAGAGATGGT
GGTAATAGCAACGATACCAACAGCACTGAGACCTTCAGACCTGGAGGAGGAAATATGAAGGACAATTGGAGAA
GTGAATTATATAAATATAAAGTAGTACAAATTGAACCAGTAGGAATAGCACCCACCAGGGCAAAAAGAAGAGT
GGTGCAGAGAGAGAAAAGAGCAGTGGGAATAGGAGCTCTGTTCCTTGGGTTCTTGGGAGCAGCAGGAAGCACT
ATGGGCGCAGCATCAATAACGCTGACGGTACAGGCCAGACAATTATTGTCTGGTATAGTGCAACAGCAGAACA
ATCTGCTGAGAGCTATTGAGGCGCAACAGCATATGTTGCAACTCACAGTTTGGGGCATC
>MG197136
ACTTACAGTACTTGGCAGTTTAAAAGTACTTGGAATGGTACTAACACTTGGAATAGTACTGTTACAGGAAATG
ACACTATTACACTCCAATGCAGAATAAAACAAATTATAAACTTGTGGCAGGAAGTAGGAAAAGCAATGTATGC
CCCTCCCATCAGAGGACCAATTAGATGTTCATCAAATATTACAGGACTACTCTTAACAAGAGATGGTGGTATT
ACAAATGGGAATAATACTAATACTACTGAGAACAGTGATAATAATACTGAGATCTTCAGACCTGGAGGAGGAA
ATATGAAGGACAATTGGAGAAGTGAATTATATAAATATAAAGTAGTACAAGTTGAACCATTAGGAATAGCACC
CACCAAGGCAAAGAGAAGAGTGGTGCAAAGAGAAAAAAGAGCAGTGGGAACACTAGGAGCTATGTTCCTTGGG
TTCTTGGGAGCAGCAGGAAGCACTATGGGCGCAGCGTCATTGACGCTGACGGTACAAGCCAGACTACTATTGT
CTGGTATAGTGCAACAGCAAAACAACTTGCTGAGAGCTATTGAGGCGCAACAACATCTGTTGCAACTCACAGT
CTGGGGCATT
>KM354740
ACTCAGCAGGTGCAGTTCAACAATACTGGGAATGATACTATCATACTCCCATGCAGAATAAAACAGATTATAA
ACCAGTGGCAGGGAGTAGGAAAAGCAATGTATGCCCCTCCTATCAGAGGACCAATTAACTGCACATCAAACAT

TACAGGGCTGATATTAACAAGAGATGGTGGTAATGGTAATGGGAGTGACACGGAAGAGATCTTCAGACCTGGA
GGAGGAGATATGAGGGACAATTGGAGAAGTGAACTATATAAATATAAAGTAGTACAAATTGAACCAATAGGAG
TAGCACCCACCAAGGCAAAGAGAAGAGTGGTGCAAAGAGAAAAAAGAGCAGCGTTGGGAGCTATGTTCCTTGG
GTTCTTGGGAGCAGCAGGAAGCACTATGGGCGCAGCGTCAGTGGCGCTGACGGTACAGGCCAGACAATTGTTG
TCTGGTATAGTGCAACAGCAGAACAATCTGCTGAGGGCTATTGAGGCGCAACAACATTTGTTGCAACTCACAG
TCTGGGGCATC
>KM354821
ACTCATTGGAATGATACTGAAGACTCAAAAACCAATGACACAATCACACTCCCATGCAGAATAAAACAAATTA
TAAACCTGTGGCAGGAAGTAGGAAAAGCAATGTATGCCCCTCCCATCAGAGGACAGATTAAATGTTCATCAAA
CATTACAGGGCTGCTATTAACAAGGGATGGTGGTCCTAATAGCACGACCAACGAAACCTTTAGACCTGCAGGA
GGAGATATGAGGGATAATTGGAGAAGTGAATTGTATAAATATAAAGTAGTAGAAATTGAACCATTAGGAGTAG
CACCCACCAAGGCAAAAAGAAGAGTGGTGCAGAGAGAAAAAAGAGCAGTGGGACTAGGAGCTTTGTTCCTTGG
GTTCTTGGGAGCAGCAGGAAGCACTATGGGCGCAGCATCAATGGTGCTGACGGTACAGGCCAGGCAATTATTG
TCTGGTATAGTGCAACAGCAGAACAATTTGCTGAGAGCTATTGAGGCGCAACAGCATCTGTTGCAACTCACAG
TCTGGGGCATC
>KM082106
CATCAGAATAGTACTAGGAATGAGAGTAGTAATAACCTTATCGAACTCCCATGCAGAATAAAACAAATCATAA
ACATGTGGCAGGAAGTAGGAAAAGCAATGTATGCCCCTCCCATCAGAGGAGAAATTAAATGTGTATCAAATAT
TACAGGGCTGCTATTAACAAGAGATGGTGGCCAGTCTAATGACACCAAAAACGACACAGAGATCTTCAGACCT
GGAGGAGGAGATATGAGGGACAATTGGAGAAGTGAATTATACAAATATAAAGTAGTAAAGATTGAACCATTAG
GAATAGCACCCCACAGGGCAAAGAGAAGAGTGGTGCAAAGAGAAAAAAGAGCAGTGGGAATAGGAGCTATGAT
CTTTGGGTTCTTGGGAGCAGCAGGAAGCACTATGGGCGCAACGTCACTGACGTTGACGGTACAAGCCAGACTA
TTATTGTCTGGTATAGTGCAACAGCAGAACAATTTGCTGAGGGCTATTGAGGCGCAACAGCATATGTTGCAAC
TCACAGTCTGGGGCATA
>MK384040
AATCACACTGGAAGAAATATCACACTCATCACACTCCCATGCAGAATAAAACAAATTATAAACATGTGGCAGG
AAGTAGGAAAAGCAATGTATGCCCCTCCCATTAGAGGAAACATTAGCTGTTCATCAAATATTACAGGGCTGCT
ATTAACAAGAGACGGTGGTGATAACCAGAGCCAGACTGAGATCTTCAGACCTGGAGGAGGAAATATGAAGGAT
AATTGGAGAAGTGAATTATATAAATATAAAGTAGTAAAAATTGAACCATTAGGAGTAGCACCCACCAGGGCAA
GGAGAAGAGTGGTGCAGAGAGAAAAAAGAGCAGTGGGAATAGGAGCTGTGTTCCTTGGGTTCTTGGGAGCAGC
AGGAAGCACTATGGGCGCAGCGTCAATGACGCTGACGGTACAGGCCAGACTATTACTGTCTGGTATAGTGCAA
CAGCAGAACAATTTGCTGAGGGCTATTGAGGCGCAACAGCGTCTGTTGCAACTCACAGTCTGGGGCATC
>MG196642
TCCTATAATGATACCGGGGAACATGAGGTAGAAAATGGCACAAGTAAAAACATCACACTTCCATGCAGAATAA
AACAATTTATAAACATGTGGCAGGAAGTAGGAAAAGCAATGTATGCCCCTCCTATTAGAGGACAAATTAAATG
TAAATCAAATATTACAGGGCTGCTATTAACAAGAGATGGCGGTAAGAATACCACAAAACATAATGAGACCTTC
AGACCTGGAGGAGGAAATATGAAGGACAATTGGAGAAGTGAATTATATAAATATAAAGTAGTAAGAATTGAAC
CATTAGGAGTGGCGCCCACCAACGCTAAGAGAAGAGTGGTGCAGAGAGAGAAAAGAGCAGTGACACTAGGAGC
TCTGTTCCTTGGGTTCTTGGGAGCAGCAGGAAGCACTATGGGCGCAGCGTCAATGACGCTGACGGTACAGGCC
AGACTATTATTGTCTGGTATAGTGCAGCAGCAGAACAATCTGCTGAGAGCTATTGAGGCGCAACAGCACCTGT
TGCAACTCACGGTCTGGGGCATT
>MG196653
AATTATACTTTCAATAGTACTGCATGGTCAAATAATACTAATAGCACTGAAAAAATCACACTCCAATGTAGAA
TAAAGCAATTTATAAACATGTGGCAGGAAGTAGGAAAGGCAATATATGCCCCTCCCATCAAAGGACAAATTAG
CTGCTCATCAAATATTACAGGGCTACTACTAACAAGAGATGGTGGTAACAACAATGACACCAATGGCAACGAT
ACTGAGGTCTTCAGACCTGCAGGGGGAGATATGAGGGACAATTGGAGAAGTGAGCTATATAAATATAAAGTAG
TAAAAATTGAACCATTAGGAATAGCACCCACCAAGGCAAAGAGAAGAGTGGTGCAAAGAGAAAAAAGAGCAGT
GGGATTAGGAGCTATGTTCCTTGGGTTCTTGGGAGCAGCAGGAAGCACTATGGGCGCAGCGGCAGTGACGCTG
ACGGTACAGGCCAGGCAATTATTGTCTGGTATAGTGCAACAGCAGAACAATCTGCTGAGGGCTATTGAGGCGC
AACAGCATATGTTGCAACTCACAGTCTGGGGCATC
>MG196831
AATTATACTACAGGGAATTTTACTACAGAGTCAAATAGCTCAATAGGAAACATCACACTCCCATGCAGAATAA
AACAAATTATAAACATGTGGCAGGAAGTAGGAAAAGCAATGTATGCCCCTCCCATCAAAGGACAAATCAGCTG
CACATCAAATATTACAGGGCTGCTGATAACAAGAGATGGTGGTAATGATACCGGGAGCAAGGAGACCTTCAGA
CCTGGAGGAGGAGATATGAGGGACAATTGGAGAAGTGAATTATATAAATATAAAGTAGTAAAAATTGAACCAT
TAGGAGTAGCACCCACCAAGGCAAGGAGAAGAGTGGTGCAGAGAGAAAAAAGAGCAGTGGGAATAGGAGCTGT
GTTCCTTGGGTTCTTGGGAGCAGCAGGAAGCACTATGGGCGCAGCGTCGCTAACGCTGACGGTACAGGCCAGA
CTACTATTGTCTGGTATAGTGCAACAGCAGAACAACTTGCTGAGGGCTATTGAGGCGCAACAGCGTATGTTGC
AACTCACAGTCTGGGGCATC
>MG197199

TTTCATAGTGCTTGGCTGGTTAATGGTACTTGGAATAACACTTGGAATGACACCATTGAAACAAATGGGACAA
TTACACTCCCATGTAGAATAAAACAAATTATAAACATGTGGCAGGAAGTAGGAAAAGCAATGTATGCCCCTCC
CATCAGAGGAAGAATTAGATGTGAATCAAATATTACAGGGCTGCTACTAATAAGAGATGGGGGTGTTAACGGG
ACCAATGATACAGAGACCTTCAGACCTGGAGGAGGAAATATGAGGGACAATTGGAGAAGTGAATTATATAAAT
ATAAAGTAGTAAAAATTGAACCATTAGGAGTAGCACCTACCAAGGCAAAGAGAAGAGTGGTGCAGAGAGAAAA
AAGAGCAGTGGGACTAGGAGCTTTGTTCATTGGGTTCTTGGGAGCAGCAGGAAGCACTATGGGCGCAGCGTCA
ATGACGCTGACGGTACAAGCCAGACAATTATTGTCTGGTATAGTGCAACAGCAAAATAACTTGCTGAGAGCTA
TTGAGGCGCAACAACATCTGTTGCAACTCACAGTCTGGGGCATC
>KT124784
AACTATACTGAGCTAAACACCACTGAAAGGGAAAACATCACACTCCAATGCAGAATAAAACAAATTATAAACA
TGTGGCAAGAAGTAGGAAAAGCAATATATGCCCCTCCCATCGAAGGAATAATTAGATGCACATCAAATATTAC
AGGGCTGCTAATAACAAGAGATGGTGGTGGGAACGGAACAGGGAACAATGAAACCTTCAGACTTGCAGGAGGG
GAGATGAGGGACAATTGGAGAAGTGAGTTATATAAATACAAAGTAGTAAAAGTTGAACCATTAGGAATAGCAC
CCACCAAGGCAAAGAGAAGAGTGGTGCAGAGAGAAAAGAGAGCAGTGGGAATAGGAGCTGTGTTCCTTGGGTT
CTTGGGAGCAGCAGGAAGCACTATGGGCGCAGCGTCAATGACGCTGACGGTACAGGCCAGACTATTATTGTCT
GGTATAGTGCAACAGCAAAACAATTTGCTGAGGGCTATTGAGGCGCAGCAGCATCTGTTGCAACTCACAGTCT
GGGGCATT
>KU901858
ACTCACGTGTCAAGCGACAATGAAAATATCACACTCCCATGCAGAATAAAACAATTTATAAACATGTGGCAGG
AAGTAGGAAAAGCAATGTATGCTCCTCCCATCAGCGGGCCAATTGAGTGTTCATCAGATATCACAGGGCTGCT
ATTGGTAAGAGATGGTGGTGATAATAATGAAGGTAGGAACGAAACCGAAATCTTCAGACCTGGAGGAGGAGAT
ATGAGGGACAATTGGAGAAGTGAATTATATAAATATAAAGTAGTAAAAATTGAACCATTAGGAATAACACCCA
CCAAGGCAAGGAGAAGAGTGGTGCAGAGAGAAAAAAGAGCAGTGACTCTAGGAGCTATGTTCCTTGGGTTCTT
GGGAGCAGCAGGAAGCACTATGGGCGCAGCATCAATAACGCTGACGGTACAGGCCAGACAATTATTGTCTGGA
ATAGTGCAACAGCAAAACAATTTACTGAGAGCTATTGAGGCGCAACAGCATATGTTGCAACTCACAGTCTGGG
GCATC
>MH843742
AGTCATGATAGTACTACTGAGTCAAATGATAATATCACACTCATATGCAAAATAAGACAAATTATAAACAGGT
GGCAGGAAGTAGGAAAAGCAATGTATGCCCCTCCCATCCAAGGACAAATTAGATGTTCATCAAATATTACAGG
ACTGATATTAACAAGAGATGGTGGTGGTAATAGCACGGACGGGAACGAGACCTTCAGACCTGGAGGAGGAAAT
ATGAAGGACAATTGGAGAAGTGAATTATATAAATATAAAGTAGTAAGAATTGAGCCATTAGGAGTAGCACCCA
CCAAGGCAAAGAGAAGAGTGGTGCAGAGAGAAAAAAGAGCACTAGGAACGATAGGAGCTATGTTCCTTGGGTT
CTTGGGAGCAGCCGGAAGCACTATGGGCGCAGCGTCAATGGCGCTGACGGTACAGGCCAGACAATTATTGTCT
GGTATAGTGCAACAGCAGAACAATTTGCTGAGGGCTATTGAGGCGCAACAGCATCTGTTGCAACTCACAGTCT
GGGGCATT
>KX505589
ACTCAGTCAAATAATGGAAGTAAAACAATCACACTCCCATGCAGAATAAAACAAGTTATTAACATGTGGCAGG
GAATAGGAAGAGCAATGTATGCCCCTCCCATCGAAGGACACATTAGATGTTCATCAAATATTACTGGGCTGCT
ATTAACAAGCGATGAGGGTGACAAAGGGAACAGTTCCAATGTGACCTTCAGACCTGGAGGAGGAAATATGAAG
GACAATTGGAGAAGTGAATTATATAAATATAAAGTAGTACAAGTTGAACCATTAGGAATAGCACCCACCAAGG
CAAAAAGAAGAGTGGTGCAAAAAGAGAAAAGAGCATTAGTGGGATTCGGAGCTTTGTTCCTTGGGTTCTTGGG
AGCAGCAGGAAGCACTATGGGCGCAGCGTCAATAACGCTGACGGTACAGGCCAGACAATTATTGTCTGGTATA
GTGCAACAGCAGAACAACCTGCTGAAGGCTATTGAAGCGCAACAGCATATATTGCAACTCACAGTCTGGGGCA
TC
>KX505695
AGTTATGATTTTAATAATAGTAGTAATTCTAGTAGTAGTGTTTCAAATGGCACTATCATACTCCCATGCAGAA
TAAAGCAAATTATAAACATGTGGCAGAAGGTGGGAAGAGCAATATATGCCCCTCCCATTAGAGGACAACTTAA
TTGTTCATCAAATATTACAGGGCTGATATTAACAAGAGATGGTGATAAGCCTAATAGCACCAACGAGACCTTC
AGACCTATAGGAGGAAATATGAAGGACAATTGGAGAAGTGAATTATATAAATATAAAGTAGTAAAAATTGAAC
CATTAGGACTAGCACCCACCAAGGCAAAGAGAAGAGTGGTGCAAAGAGAAAAAAGAGCAGTGGGAATAGGAGC
TCTGTTCCTTGGGTTCTTGGGAGCAGCAGGAAGCACTATGGGCGCAGCATCAATGACGCTGACGGTACAGGCC
AGACTATTATTGTCTGGTATAGTGCAACAGCAGAACAATCTGCTGAGGGCTATTGAGGCGCAACAACATCTGT
TGCAACTCACAGTCTGGGGCATC
>KX027840
TTTTATGGGACCTTGAATGATACTGGGTGGACAAGTAAAATTGATAACAGTAACGAAAATATTACACTCCAAT
GCAGAATAAAGCAAATTATAAACATGTGGCAGAAAGTAGGAAAAGCAATGTATGCCCCTCCCATCAGCGGACA
AATTAGATGTGTATCAAATATCACAGGGCTAATATTAACAAGAGATGGGGGTAATGGTACAAACAACACTGAG
ATCTTCAGACCTGAAGGAGGAGATATGAGAGACAATTGGAGAAGTGAATTATATAAATATAAAGTAGTAAAAA
TTGAACCATTAGGAATAGCACCCACCAAGGCAAGGAGAAGAGTGGTGCAGAGAGAAAAAAGAGCAGTGGGAAT
GGGAGCTTTGTTCCTTGGGTTCTTGGGAGCAGCAGGAAGCACTATGGGCGCAGCGTCAGTGACGCTGACGGTA

CAGGCCAGACAATTATTGTCTGGTATAGTGCAACAGCAGAACAACTTGCTGAAGGCTATTGAGGCGCAACAAC
ATCTGTTGCAACTCACTGTCTGGGGCATT
>KY658693
AATTACACTGAGACAACAAATAGCACTAATGGAACTGACACAATCATACTCCCATGCAGAATAAAACAAATTA
TAAACATGTGGCAGAAAGTAGGAAAAGCAATGTATGCCCCTCCCATCAAAGGAATAATTAACTGTAATTCAAG
TATTACAGGATTGCTATTAACAAGAGATGGTGGTATTACCAACGATAGTAACAGCAATGAGACTTTTAGACCT
GGAGGAGGGAATATGAAAGATAATTGGAGAAGTGAATTATATAAATACAAAGTAGTAAGAATTGAACCATTAG
GAATAGCCCCCACCAGGGCAAAAAGAAGAGTGGTGCAGAGAGAAAAAAGAGCAATAGGAATGGGAGCTGTGTT
CATTGGGTTCTTGGCAACAGCAGGAAGCGCTATGGGCGCAGCGTCATTGACGCTGACGGTACAAGCCAGACAA
TTATTGTCTGGTATAGTGCAACAGCAGAACAATCTGCTGAGGGCTATTGAGGCGCAACAGCATTTGTTGCAAC
TCACAGTCTGGGGCATT
>KU678026
TTTTATAGTAATTGGACTAAAGAAAATGCTACTGGAGAAAATGACACTGACACAATCATACTCCCATGCAGAA
TAAAACAAATTATAAACATGTGGCAGGAAGTAGGAAAAGCAATGTATGCCCCTCCTATCAAAGGACAAATTAG
ATGTTCATCAAATATCACAGGGCTGCTGATAACAAGGGATGGTGGTATGAACAACACCAACAACGACACTGAG
ATCTTCAGACCAGGAGGAGGAGATATGAGGGACAATTGGAGAAGTGAATTATATAAATATAAAGTAGTACAAA
TTGAACCATTAGGAGTAGCACCCACCAAGGCAAAGAGAAGAGTGGTGCAGAGAGAAAAAAGAGCAGCACTAGG
GGGACTAGGAGCTTTGTTCCTTGGGTTCTTGGGAGCAGCAGGAAGCACTATGGGCGCAGCGGCAGTGACGCTG
ACGGTACAGGCCAGACAATTATTGACTGGTATAGTGCAACAGCAGAACAATTTGCTGAGGGCTATTGAGGCGC
AACAGCACCTGTTGCAACTCACAGTCTGGGGCATC
>KY323815
GCTTATGAAGAGTCAAATGACACTATCACTCTCCCATGCAGAATAAAACAAATTATAAACATGTGGCAGGAAG
TAGGAAAAGCAATGTATGCCCCTCCCATCAGAGGACAAATTAGCTGTAAATCAAATATTACAGGGCTGCTATT
AACAAGAGATGGTGGTAATGGAACTGACATGAAAGAGACCTTCAGGCCTGGAGGAGGAGATATGAGGGACAAT
TGGAGAAGTGAATTATATAAATATAAAGTAGTTAGAATTGAACCATTAGGAATAGCACCCACCAAGGCACAGA
GAAGAGTGGTGCAGAGAGAAAAAAGAGCAGTGGGAGTGCTAGGAGCTATGTTCCTCGGGTTCTTGGGAGCAGC
AGGAAGCACTATGGGCGCAGCGTCAATGGCGCTGACGGTACAGGCCAGACAATTATTGGCTGGTATAGTGCAA
CAACAGAACAATCTGTTGAGAGCTATTGAGGCGCAACAGCATATGTTGCAACTCACAGTCTGGGGCATC
>KY324245
CATCAAAAAACATCAAATGGTACATACATACTTCCATGCAGAATAAAACAAATTATAAACATGTGGCAGGAAG
TAGGAAAAGCAATGTATGCCCCTCCCATCAGAGGAAACATTAGCTGTTCATCAAATATTACAGGACTGCTATT
GGCAAGAGATGGGGGTAGTGGTAGTGATAATAATGGCACAAATAATAACACAAATGAAGAGATCTTCAGACCT
GCAGGAGGAGATATGAGGGACAATTGGAGAAGTGAATTATATAAATACAAAGTAGTAAAAATAGAGCCATTAG
GATTAGCACCCACCTCGGCAAAGAGAAGAGTGGTGCAGAGAGAAAAAAGAGCAGTGGGAATAGGAGCTCTGTT
CCTTGGGTTCTTGGGAGCAGCAGGAAGCACTATGGGCGCAGCGTCAATGACGCTGACGGTACAGGCCAGACTA
TTACTGTCTGGTATAGTGCAACAGCAAAACAATTTGCTGAGAGCTATTGAGGCGCAACAACATCTGTTGCAAC
TCACAGTCTGGGGCATT
>KY113882
TATCACAATGAGACTACGAGTGTACCGGAGGGAAATGAAACAATCACACTTCCATGCAGAATAAAACAAATTA
TAAACATGTGGCAGGAAGTAGGGAGAGCAACGTATGCCCCTCCCATCAAAGGACAAATTAGATGTTCATCAAA
TATTACAGGGCTGCTACTAACAAGGGATGGAGGTATTGGGAATGAGACTGATAATAATACTGAGATCTTCAGA
CCTGGAGGAGGAGATATGAGGGACAATTGGAGAAGTGAATTATATAAATATAAAGTAGTAAGAATTGAACCAT
TGGGAGTAGCACCCACCAAGGCAAAGAGGAGAGTGGTGCAAAGAGAAAAAAGAGCAGTGGGAATAGGAGCTAT
GTTCCTTGGGATCTTAGGAGCAGCAGGAAGCACTATGGGCGCAGCGTCATTGACGCTGACGGTACAGGCCAGA
CTATTATTGTCTGGTATAGTGCAACAGCAGAACAACTTGCTGAGGGCTATTGAGGCGCAACAGCATCTGTTGC
AACTCACTGTCTGGGGCATC
>K02007
AATCACACTGAAGGAACTAAAGGAAATGACACAATCATACTCCCATGTAGAATAAAACAAATTATAAACATGT
GGCAGGAAGTAGGAAAAGCAATGTATGCCCCTCCCATTGGAGGACAAATTAGTTGTTCATCAAATATTACAGG
GCTGCTATTAACAAGAGATGGTGGTACAAATGTAACTAATGACACCGAGGTCTTCAGACCTGGAGGAGGAGAT
ATGAGGGACAATTGGAGAAGTGAATTATATAAATATAAAGTAATAAAAATTGAACCATTAGGAATAGCACCCA
CCAAGGCAAAGAGAAGAGTGGTGCAGAGAGAAAAAAGAGCAGTGGGAATAGTAGGAGCTATGTTCCTTGGGTT
CTTGGGAGCAGCAGGAAGCACTATGGGCGCAGTGTCATTGACGCTGACGGTACAGGCCAGACAATTATTGTCT
GGTATAGTGCAACAGCAGAACAATTTGCTGAGGGCTATTGAGGCGCAACAACATCTGTTGCAACTCACAGTCT
GGGGCATC
>HQ110679
ACTCAGGGGTTAAATAACACTGGAAGAAATGACACAATCACACTCCCATGCAGAATAAAACAAATTGTAAACA
TGTGGCAGGAAGTAGGAAAAGCAATGTATGCCCCTCCCATCAAAGGAAATATTAGCTGTTCATCAAATATTAC
AGGGCTGCTATTAACAAGAGATGGTGGTAGTGATGGTGGTAATAACACGAACGGGAATGAGACCTTCAGACCT
GGAGGGGGAGATATGAGGGACAATTGGAGAAGTGAATTATATAAATATAAAGTAGTAAGAATTGAACCATTAG

GAGTAGCACCCACCAAGGCAAAGAGAAGAGTGGTGCAAAGAGAAAAAAGAGCGGTGGGACTAGGAGCTATGTT
CATTGGGTTCTTGGGAGCAGCAGGAAGCACTATGGGCGCAGCGTCAGTGACGCTGACGGTACAGGCCAGGCAA
TTATTGTCTGGTATAGTGCAACAGCAGAGCAATTTGCTGAGAGCTATTGAGGCGCAACAGCATCTGTTGCAAC
TCACAGTCTGGGGCATC
>AY713410
ACTCAAGGGAACACTGGAAATATCACACTCCCATGCAGAATAAAACAAATTATAAACAGGTGGCAGGAAGTAG
GAAAAGCAATGTATGCCCCTCCCATCGAAGGAAAAATTAGATGTTCATCAAATATTACAGGGTTACTATTAAC
AAGAGATGGTGGTAAGAATAACGAGACCAATAACACCGAGACCTTCAGACCTGGAGGAGGAGATATGAAGGAC
AATTGGAGAAGTGAATTATATAAATATAAAGTAGTAAAAATTGAGCCATTAGGAGTAGCACCCACCAAGGCAA
AGAGAAGAGTGGTGCAGAGAGAAAAAAGAGCAGTGACACTAGGAGCTATGTTCCTTGGGTTCTTGGGAGCAGC
AGGAAGCACTATGGGCGCAGCGTCAGTGACGCTGACGGTACAGTCCAGACAATTATTGTCTGGTATAGTGCAA
CAGCAAAACAATTTGCTGAGAGCTATTGAGGCGCAACAGCATCTGTTGCAACTCACAGTCTGGGGCATT
>EU575593
AATTATTTTGATAACACTAAAGATGATGGGGCTAATGGGACTGCTAATGGGACTGGAGTTATCATACTTCCAT
GCAGAATAAAACAAATTGTAAACATGTGGCAGGAAGTAGGAAAAGCAATGTATGCCCCTCCCATCAGGGGACG
AATTAAATGTTTATCAAATATTACAGGACTGATGTTAACAAGAGATGGTGGTAATAATGGTGGTAACACAACT
GAAAACGCCACCGAGACCGAGACCTTCAGACCTGGAGGAGGAGATATGAGGGACAATTGGAGAAGTGAATTAT
ATAAATATAAAGTAGTAAAAATTGAACCATTAGGAGTAGCACCTACCAAGGCAAAGAGAAGAGTGGTGCAAAG
AGAAAAAAGAGCAGTAGGAATAGGAGCTTTGTTCCTTGGGTTCTTGGGAGCAGCAGGAAGCACTATGGGCGCA
GCGTCAGTGACGCTGACGGTACAAGCCAGACAGTTATTGTCTGGTATAGTGCAACAGCAGAGCAATCTGCTGA
GGGCTATTGAGGCGCAACAGCATCTGTTGCAACTCACAGTCTGGGGCATC
>AY331289
ACTCAGACTAATAGCTGGCCAAATGCCACTATCACACTTTCATGCAGAATAAAACAGTTTATAAACATGTGGC
AGGAAGTAGGAAAAGCAATGTATGCCCCTCCCATTAGGGGACCAATAAGTTGTTCATCAAATATTACAGGGTT
GCTATTAACAAGAGATGGTGGTAACGGGAACGGCACCAACAACAACACCGAGGTCTTCAGACCTACAGGAGGA
GATATGAGGGACAATTGGAGAAGTGAATTATATAAATATAAAGTAGTAAGAATTGAACCAATAGGAGTAGCAC
CCACCAAGGCAAAGAGAAGAGTGGTGCAGAGAGAAAAAAGAGCAGTGGGAACAATAGGAGCTATGTTCCTTGG
GTTCTTGGGAGCAGCAGGAAGCACTATGGGCGCAGCGTCAATAACGCTGACGGTACAGGCCAGACTATTACTG
TCTGGTATAGTGCAACAGCAGAACAATCTGCTGAGAGCTATTGAGGCACAACAACATCTGTTGCAACTCACAG
TCTGGGGCATC
>EU575697
ACTCAACTGTTTAATAGTACTTGGAATGGTAATGATACTGAAGGGTCAAATACCACTGAAGAAAATATCACAC
TCCCATGCAGAATAAAACAAATCATAAACATGTGGCAGGAAGTAGGAAAAGCAATGTATGCCCCTCCCATCAG
AGGACAAATTAGCTGTTCATCAAATATTACAGGGCTGTTGTTAACAAGAGATGGTGGCGCAGATAGTACAAAC
GAGACCTTCAGACCTGGAGGAGGAGATATGAGGGACAATTGGAGAAGTGAATTATATAAATATAAAGTAGTAA
GAATTGAACCATTAGGAATAGCACCCTCCAAGGCAAAGAGAAGAGTGGTGCAGAGAGAAAAAAGAGCAGTGGG
AATAGGAGCTGTGTTCCTTGGGTTCTTGGGAGCAGCAGGAAGCACTATGGGCGCAGCGTCAATGGCGCTGACG
GTACAGGCCAGACAATTATTGTCTGGTATAGTGCAACAGCAGAACAATTTGCTGAGGGCTATTGAGGCGCAAC
AGCATATGTTGCAACTCACAGTCTGGGGCATC
>EU577469
ACTCAAGGAAATGACACAATCATACTCCCATGCAGAATAAAACAAATTATAAACATGTGGCAGAGAGTAGGGA
AAGCAATGTATGCCCCTCCTATCGGAGGACACCTTAATTGTTCATCAAATATTACAGGGCTGCTATTCACAAG
AGATGGTGGCAACAGTAGTGATAACAGTGAGACCTTCAGACCTGGAGGAGGAGATATGAGGGATAATTGGAGA
AGTGAATTATATAAATATAAAGTAGTAAAACTTGAACCCTTAGGAGTAGCCCCCACCAAGGCCAAGAGAAGAG
TGGTGCAAAGAGAAAGAAGAGCAGTGGGTTTAGGAGCTATGTTCCTTGGGTTCTTGGGAGCCGCAGGAAGCAC
TATGGGCGCAGCGTCAATAACGCTGACGGTACAGGCCAGACAATTATTGTCTGGTATAGTGCAACAGCAGAAC
AATCTGCTGAGGGCTATTGAGGCGCAACAGCATATGTTGCAACTCACAGTCTGGGGCATC
>MN516274
GATCAAAAATATACTGCATGGGCAAATAGCACTGAGTCAAATGACACTGAAGTAATCACACTCCCATGCAGAA
TAAAACAAATTATAAACCTGTGGCAGGAAGTAGGGAAAGCAATGTATGCCCCTCCCATCAAAGGACAGATTAG
ATGTTCATCAAATATTACAGGGCTGCTACTAACAAGAGATGGTGGTAAGAACGAGAGTAGAGAGAATGAGACC
TTCAGACCTGGAGGAGGAGATATGAGGGACAATTGGAGAAGTGAATTATATAAATATAAAGTAGTAAAAATTG
AACCATTGGGAGTAGCACCCACCAAGGCAAAGAGAAGAGTGGTGCAAAGAGAAAAAAGAGCAGTGGGAATAGG
AGCTTTGTTCCTTGGGTTCTTGGGAGCAGCAGGAAGCACTATGGGCGCAGCGTCAGTGACGCTGACGGTACAG
GCCAGACTATTATTGTCTGGTATAGTGCAACAGCAGAACAACTTGCTGAGAGCTATTGAGGCGCAACAGCATC
TGTTGCAACTCACAGTCTGGGGCATC
>GU728005
AATCAAACTGGGAGCAATGATACAATTATACTCCCATGTAGAATAAAGCAAATTATAAACCTGTGGCAGGAAG
TAGGAAAAGCAATGTATGCCCCTCCCATCAGTGGAGTAATTAATTGCACATCAAATATTACAGGGCTACTATT
GACAAGAGATGGTGGTAATAATATTACTAACGGGAGCGAGATCTTCAGACCTGGAGGAGGAGATATGAGGGAT

AATTGGAGAAGTGAATTATATAAATATAAAGTAGTAAGACTTGAACCATTAGGAGTAGCACCCACCAGGGCAA
AGAGAAGAGTGGTGCAGAGAGAAAAAAGAGCAGTGGGACTAGGAGCTTTGTTCCTTGGGTTCTTGGGAGCAGC
AGGAAGCACTATGGGCGCAGCGTCACTGACGCTGACGGTACAGGCCAGACTACTACTGTCTGGTATAGTGCAA
CAGCAGAACAATCTGCTGAGGGCTGTTGAAGCGCAACAACATCTGTTGCAACTCACAGTCTGGGGCATC
>GU367405
ACTCAAGGAAATGATACTACTATCACACTCCAATGCAAAATAAAACAAATTATAAATATGTGGCAGGAAGTAG
GAAAAGCAATGTATGCCCCTCCCATCAGAGGATCAATTAAATGCCTATCAAATATTACAGGGCTGATATTAAC
AAGAGATGGTAACGGGACCGAGAGCACAAACGAGACCGAGATCTTCAGGCCTGGAGGAGGAGATATGAGGGAC
AATTGGAGGAGTGAATTATATAAATATAAAGTAGTAAGAATTGAACCATTAGGAGTAGCACCCACCAAGGCAA
AGAGAAGAGTGGTGCAGAGAGAAAAAAGAGCAGTGGGACTAGGAGCTTTGTTCCTTGGGTTCTTGGGAGCAGC
AGGAAGCACTATGGGCGCAGCGTCGGTGACGCTGACGGTACAGGCCAGACTATTGCTGTCTGGTATAGTGCAA
CAGCAAAATAATTTGCTGAGGGCTATTGAGGCGCAACAGCATCTGTTGCAACTCACAGTCTGGGGCATC
>AY795904
ACTCAAATGTTAGATAATATCACACTCACACTCCCATGCAGAATAAAACAAATCATAAACATGTGGCAAGAAG
TAGGAAAAGCAATGTATGCCCCTCCCATTGCAGGAAAAATTAGCTGTTCATCAAATATTACAGGGATACTATT
AACAAGGGATGGTGGTAACGAGAACCAGACCACCCCCGAGGTCTTCAGACCTGCAGGAGGAGATATGAGGGAC
AATTGGAGAAGTGAATTATATAAATATAAAGTAGTAAGAGTTGAGCCATTAGGAATAGCACCCACCAAGGCAA
AGAGAAGAGTGGTGCAGAGAGAAAAAAGAGCAATAGGAATGGGAGCTGTGTTCCTTGGGTTCTTGGGAGCAGC
AGGAAGCACTATGGGCGCAGCGTCAATAACGCTGACGGTACAGGCCAGACAATTATTGTCTGGTATAGTGCAA
CAGCAAAGTAATTTGCTGAGAGCTGTTGAGGCGCAACAACATCTGTTGCAACTCACAGTCTGGGGCATC
>KF725981
AATTATACTTGGAATAATACTACTGAGAGTGATAACGGTACTATCATACTCCAGTGCAGAATAAAGCAAATTG
TAAACAGGTGGCAGGAAGTAGGAAAAGCAATGTATGCCCCTCCCATCAGTGGACAAATTAATTGTTCATCAAA
TATTACAGGGCTACTACTAACAAGAGATGGTGGTATCAGTAACACAAGTAGTGATAACACAAGTGAGACCTTT
AGACCTGCAGGAGGAAATATGAAGGACAATTGGAGAAGTGAATTATATAAATATAAAGTAGTAAAAATTGAGC
CATTAGGAATAGCACCCACCAAGGCAAAGAGAAGAGTGGTGCAGAGAGAAAAAAGAGCAGTGGGAACATTAGG
AGCTATGTTCCTTGGGTTCTTGGGAGCAGCAGGAAGCACTATGGGCGCAGCGTCATTGACGTTGACGGTACAA
GCCAGACTACTATTGTCTGGTATAGTGCAACAGCAAAACAATTTGCTGAGAGCTATTGAGGCGCAACAGCATA
TGTTGCAACTCACAGTCTGGGGCATC
>MH234639
AATCATACTAAAGGAGAAGATATCATCACACTCCCATGTAGAATAAAGCAAATTATAAACATGTGGCAGCAAG
TGGGAAAAGCAATGTATGCCCCTCCCATCAGTGGACAAATTAGATGTTCATCAAATATTACAGGGCTGCTATT
AACAAGAGATGGTGGTAATAACAATGACACCACCGAGGTCTTCAGACCTGGAGGAGGAGATATGAGGGACAAT
TGGAGAAGTGAATTATATAAATATAAAATAGTAAAAATTGAACCATTAGGAGTAGCACCCACCAGGGCAAAGA
GAAGAGTGGTGCAGAGAGAAAAAAGAGCAGTAACATTAGGAGCTATGTTCCTTGGGTTCTTGGGAGCAGCAGG
AAGCACTATGGGCGCAGCGTCAATGACGTTGACGGTACAGGCCAGACGACTATTGTCTGGTATAGTGCAACAG
CAGAACAATTTGCTGAGGGCTGTTGAGGCGCAACAGCATATGTTGCAACTCACAGTCTGGGGCATC
>MH746247
AATCATAATAATACTATCATACTCCCATGCAGAATAAAACAATTTATAAACATGTGGCAGAGAGTAGGACAAG
CAATGTATGCCCCTCCCATTGAAGGAAACATAACATGTGAATCAAATATCACAGGACTACTCTTGGTACGTGA
TGGAGGCAACACAACAGAAACTAACAAAGAGACATTCAGACCGGGAGGAGGAGATATGAGGGACAATTGGAGA
AGTGAACTATATAAATATAAAGTGGTAGAAATTAAGCCATTGGGAATAGCACCCACCAAGGCAAAAAGGAGAG
TGGTGGAGAGAGAAAAAAGAGCAGTGACACTAGGAGCTATGTTCCTTGGGTTCTTGGGAACAGCAGGAAGCAC
TATGGGCGCAGCGTCAATAACGCTGACGGTACAGGCCAGACAATTGCTGTCTGGGATAGTGCAACAGCAAAGC
AATTTGCTGAGAGCTATAGAGGCGCAACAGCATATGTTGCAACTCACGGTCTGGGGCATT
>KX181928
ACTTACTGTCCTAATAATCCTAATGCAACATATAATGCAACAGAAAATTCAACCATCACAATCCAATGCAAAA
TAAAACAAATTGTAAACATGTGGCAGGAGGTAGGACGAGCAATGTATCCCCCTCCCATTAAAGGACCAATAAC
ATGTGCATCAAATATCACAGGACTATTGCTGACACGTGATGGAGGAGCAAACTCAAATAACACAGAGACATTC
AGACCTGGAGGAGGAGATATGAAGGACAATTGGAGAAGTGAATTATATAAATATAAAGTGGTAGAAATTAAAC
CATTAGGAATAGCACCCACCAAGGCAAAAAGGAGAGTGGTGGAGAGAGAAAAAAGAGCAGTGGGAATAGGAGC
TGTGTTCCTTGGGTTTTTGGGAGCAGCCGGAAGCACTATGGGCGCAGCGTCAATAACGCTGACGGTACAGGCC
AGGCAATTATTGTTTGGGATAGTGCAACAGCAAAGCAATTTGTTGAGGGCTATAGAGGCGCAACAGCATATGT
TGCAACTCACGGTCTGGGGCATT
>KX181936
AATTATCCAAACATAGGAAACAGCACATATAATGATACAGAAAACAGAAACAGCACATATAATGATACAGAAA
ACATCACAATCCCATGCAAAATAAAACAATTTATAAACATGTGGCAGGAGGTAGGGCGAGCCATGTATGCCTC
TCCCATTGAAGGAGTGATAACATGTAAATCAAATATCACAGGATTTCTACTGACCCGGGATGGGGGCAAAACC
ACAAAGAACGAGACATTCAGGCCCGAAGGAGGAAATATGAGGGACAATTGGAGAAGTGAATTATATAAGTATA
AAGTGGTAGAAATTAAACCATTAGGAATAGCACCCACTAAAGCAAAAAGGAGAGTGGTGGAGAGAGAAAAAAG

AGCAGGAATAGGAGCTGTGTTCCTTGGGTTCTTGGGAGCAGCAGGAAGCACTATGGGCGCAGCATCAATAACG
CTGACGGTACAGGCCAGACAATTATTGTCTGGGATAGTGCAACAGCAAAGCAATTTGCTGAGGGCTATAGAGG
CGCAACAGCATATGTTGCAACTCACGGTCTGGGGCATT
>KX181937
CCTCATAGTACAAGTACAAGCAGTACTCCTGGAGCAGAAAACGACACACTCACAATCCAATGCAGAATAAAAC
AAATTGTAAACATGTGGCAGGAGGTAGGACGAGCAATGTATGCCCCTCCCATTGCAGGAAACATAACATGTAA
CTCAAGTATCACAGGGCTATTACTGACACGTGATGGGGGAGAAAATAGAACAGAGAAAGGCACAAAGGAGGAG
GAATTCAGGCCTGGGGGGGGAGATATGAGGGACAATTGGAGAAGTGAATTATATAAATACAAAGTAGTAGAAA
TTAAACCCTTTGGAATAGCCCCCCCCAAGGCAAAAAGGAGGGTGGTGGAGAGAGAAAAAAAAGCAGCATTAGG
AGCTGTGTTCCTTGGGTTTTTGGGAGCAGCAGGAAGCAATATGGGGGCAGCGTCAGTAGCGCTGACGGTACAG
GCCAGACAATTATTGTCTGGGATAGTGCAACAGCAAAGCAATTTGCTGAAGGCTATAGAGGCGCAACAGCATA
TGTTGCAACTCACAGTCTGGGGCATT
>KX181941
AATTACAAGGTTAATAGTGCAAACAAGGATACAAAAAGTAATGGAACAATCACAATCCCATGCAGAATAAAAC
AAATTATAAATATGTGGCAGGGGGTGGGACGGGCAATGTATGCCCCTCCCATTGCAGGAATAATATCATGTAA
CTCAAGTATCACAGGATTACTACTGACCCGGGATGGCGGCAACAATAACAACACAGACACAGAGATATTCAGG
CCTGGAGGAGGAAATATGAGGGACAATTGGAGAAGTGAGTTATATAAATATAAAGTAGTAGAAATTAAGCCAT
TAGGAATAGCACCCACTAAGGCAAAAAGGAGAGTGGTGGGGAGAGAAAAAAGAGCAGCGGGAATAGGAGCTGT
GTTCCTTGGGTTCTTGGGAGCAGCAGGAAGCACTATGGGCGCAGCATCAATGATGCTGACGGTACAGGCCAGA
CAATTATTGTCTGGGATAGTGCAACAGCAAAGCAATTTGCTGAGGGCTATAGAGGCGCAACAGCATATGTTGC
AACTCACGGTCTGGGGCATA
>MK041572
TGCTATACATCAGGCCTGTTTAATAGTACATACAATAGTAATAGTGCAAAAAACAGTAATGAGACAGGCAATT
CAACCATCACAATCCCATGCAGAATAAAACAAATTATAAACATGTGGCAGGAGGTAGGACGAGCAATGTATGC
CCCCCCCATTAGTGGAAACATAACATGTAGATCAAATATCACAGGATTACTGCTGACACGGGATGGAGGTAAT
ACTGGCTCGAATGAGACAGAGATATTCAGGCCTGGAGGAGGAGATATGAGGGAGAATTGGAGAAGTGAATTAT
ATAAGTATAAAGTGGTAGAAATTAAACCATTAGGAATAGCACCCACTGAGGCAAAAAGGAGAGTGGTGGAGAG
AGAAAAAAGAGCAGTGGGAATAGGAGCTGTGTTCCTTGGGTTCTTGGGAGCAGCAGGAAGCACTATGGGCGCA
GCATCAATAACGCTGACGGTACAGGCCAGACAATTATTGTCTGGGATAGTGCAACAGCAAAGCAATTTGCTGA
GGGCTATAGAGGCGCAACAGCATATGTTGCAACTCACGGTCTGGGGCATT
>AF443091
TGCTATACAACAAAACTGTTTAATAGTACATACAATAGTACATACAATGATACAGGAAGTGAGTCAAACATCA
CGATTCCATGCAGAATAAAACAAATTATAAACATGTGGCAGGGGGTAGGACAAGCAATGTATGCCCCTCCCAT
TGCAGGAAACATAACATGTAGATCAAACATCACAGGACTACTATTGACACGTGATGGAGGAATAGGATTAAAT
GAAGATGATAATAACACAGAGATATTCAGACCTGGAGGAGGAAATATGAGGGACAATTGGAGAAGTAAATTAT
ATAAATATAAAGTAGTAGAAATTAAACCATTAGGAGTAGCACCCAATAAGGCAAAGAGGAGAGTGGTGGAGAG
AGAAAAAAGAGCAGTGGGACTAGGAGCTGTGTTCCTTGGATTCTTAGGAGCAGCAGGAAGCACTATGGGCGCA
GCATCAATAACGCTGACGGTACAGGCCAGACAATTATTATCTGGTATAGTGCAACAGCAAAACAATCTGATGA
GAGCTATAGAGGCGCAACAACATATGTTGCAACTCACAGTCTGGGGCATT
>AF443102
AGTTATAATAGTACAGCAAACATCACACTCCCATGCAGACTAAAACAAATTATAAACATGTGGCAGAAGGTAG
GACGAGGAATATATGCCCCTCCCATTGAGGGAAGCATAACATGTAACTCAAATATCACAGGACTACTCTTGGT
ACGGGATGGAGGCATAAATACGAGTACAGTGGAGACATTCAGACCAGAAGGAGGAAATATGAGGGACAATTGG
AGAAGTGAACTATATAAATATAAAGTGGTAGAAATTAAGCCATTGGGAGTAGCGCCCACTGAGGCACAAAGGA
GAGTGGTGGAGAAACAAAAAAGAGCAGTGGGAATGGGAGCTGTGTTCCTTGGGTTCTTGGGAGCAGCAGGAAG
CACTATGGGCGCGGCGTCAATAACGCTGACGGTACAGGCCAGACAACTGTTGTCTGGAATAGTGCAACAGCAA
AGCAATTTGCTGAGAGCTATAGAGGCGCAACAGCATATGTTGCAACTCACAGTCTGGGGCATT
>AF443103
AGCTATTCAGAACGGCATAATAACACATCAAATTCAACCATCACACTCCCATGCAATATAAAACAAATTATAA
ACATGTGGCAGGGGGTAGGACGAGCAATGTACGCCCCTCCCATTGCAGGAAACATAACATGTACATCAAATAT
CACAGGACTAATATTGACACGTGATGGAGGAGGAGGAAATGAAACTAATGAAACAGAGACATTCAGACCTGCA
GGAGGAGATATGAGGGACAATTGGAGAAGTGAATTATATAAATATAAAGTGGTAGAAATTAAGCCATTAGGAC
TAGCACCCACTAAGGCAAAAAGGAGAGTGGTGGAGAGAGAAAAAAGAGCAGTAGGAATAGGAGCTGTGTTCCT
TGGGTTCTTGGGAGCAGCAGGAAGCACTATGGGCGCGGCGTCAATAACGCTGACGGTACAGGCCAGACAACTG
TTGTCTGGTATAGTGCAACAGCAAAGCAATTTGCTGAGGGCTATAGAGGCGCAACAGCATATGTTGCAACTCA
CGGTCTGGGGCATT
>AF443105
TACTATCCTAATGCTACAAAAAGCGATACTACAGAAACCATCACACTCCCATGCAGAATAAAACAAATTGTAA
ACATGTGGCAGGGGGTAGGACGGGCAATATATGCCCCTCCCATTGCAGGAAACATAACATGTAACTCAAGTAT
CACAGGATTACTATTGCTACGTGATGGAGGGACAGATAACGAGACAGAGAACAACAGGACAGAGACATTCAGA

CCTGGAGGAGGGGATATGAGAGATAATTGGAGAAGTGAATTATATAAATATAAAGTGGTAGAAATTAAGCCAT
TAGGAGTAGCACCCACTAAGGCAAAAAGGAGAGTGGTGGAGAGAGAAAAAAGAGCAGTAGGAATGGGAGCTGT
GATCCTTGGGTTCTTGGGAGCAGCAGGAAGCACTATGGGCGCGGCGTCAATAACGCTGACGGTACAGGCCAGA
CAACTGTTGTTTGGTATAGTGCAACAGCAAAACAATTTGCTGAGGGCTATAAAAGCGCAACAGCATCTGTTGC
AGCTCACGGTCTGGGGCATT
>AF443107
AACTACAATACAAGCTCAAACTCATCCAACAACTCAAACATCACACTCCCATGCAGAATAAAACAAATTATAA
ATATGTGGCAGGGGGTAGGAAGAGCAATATATGCCTCTCCCATTGAAGGAAGCATAACATGTAAATCAAATAT
CACAGGACTACTGTTGGTACATGATGGAGGTAACAGTAACACGAGTACAGAGATATTCAGACCTGGAGGAGGA
GATATGAGGGACAATTGGAGAAGTGAATTATATAAATATAAAGTGGTAGAAGTTAAGCCATTGGGAATAGCAC
CCACTGAGGCAAAAAGGAGAGTGGTGGAGAGAGAAAAAAGAGCAGTGGGAATAGGAGCTGTGTTCCTTGGGTT
CTTGGGAGCAGCAGGAAGCACTATGGGCGCGGCGTCAATAACGCTGACGGTACAGGCCAGACAACTGTTGTTT
GGTATAGTGCAACAGCAAAGCAATTTGCTGAGGGCTATAGAGGCGCAACAGCATATGTTGCAACTCACGGTCT
GGGGCATT
>AF443108
CCTTACAATGATAATGATACAGACTCAGACATCACACTTCCATGTAAAATAAAACAAATTATAAACATGTGGC
AGGAGGTAGGACGGGCAATATATGCCCCTCCCATTGCAGGAAACATAACATGCACATCAAACATTACAGGACT
ACTATTGACACGTGATGGAGAGCCAAACAACTCCACAGAGACATTCAGACCTGGAGGAGGAGATATGAGAGAC
AATTGGAGAAGTGAATTATATAAATATAAAGTGGTAGAAGTTAAACCATTGGGAATAGCACCCACTGGAGCAA
AAAGGAGAGTGGTGGAGAGAGAGAAAAGAGCAGTAGGAATAGGAGCGGTGTTCCTTGGGTTCTTGGGAGCAGC
AGGAAGCACTATGGGCGCGGCGTCAATAACGCTGACGATACAGGCCAGACAACTGTTGTCTGGTATAGTGCAA
CAGCAAAGCAATTTGCTGAGGGCTATAGAGGCGCAACAGCATCTGTTGCAACTCACGGTCTGGGGCATT
>KC154012
TGCTATACAACAAACTTGTTTAATACTAGTCTGATTAATACTAATAACACTGATAATGGCACAATCATACTCC
CATGCAAAATAAAACAGATTATAAACATGTGGCAAGAGGTAGGACGAGCAATGTATGCCCCTCCCATTGAAGG
AAACATAACATGCAACTCAACTATCACAGGACTACTATTGCTACGTGATGGAGGCAATGGCAATGAGACCAAA
GGGAATGAGACATTCAGACCTGGAGGAGGAAATATGAAAGATAATTGGAGAAGTGAATTATATAAATATAAAG
TGGTAGAAATTAAACCATTGGGAATAGCACCCACTGAGGCAAAAAGGAGAGTGGTGGAGAGAGAAAAAAGAGC
AGCTACAATAGGAGCTGTGCTCCTTGGGTTCTTGGGAGCGGCAGGAAGCACTATGGGCGCGGCGTCAATAACG
CTGACGGTACAGGCCAGACAATTGTTGTCTGGTATAGTGCAACAGCAAAGCAATTTGCTGAGAGCCATAGAGG
CGCAACAGCACATGTTGCAACTCACAGTCTGGGGCATT
>KC154014
AATCAGATTAACAGTACAGGAAATACTTCAGATTCAAACATCACACTCCCATGCAGAATAAAACAAATTATAA
ACATGTGGCAGGGAGTAGGACGAGCAATGTATGCCCCTCCCATTGCAGGAAACATAACATGTAAATCAAATAT
CACAGGACTAATATTGACACGTGATGGAGGAACCCATAACACTAATGATACTGATACCCCAGAGATATTCAGG
CCTGCAGGAGGAGATATGAGGGACAATTGGAGAAGTGAATTATATAAATATAAAGTGGTAGAAATTAAGCCAT
TAGGACTAGCACCCACTAAGGCAAAAAGGAGAGTGGTGGAGAGAGAAAAAAGAGCAGTGGGAATAGGAGCTGT
GTTCCTTGGGTTCTTGGGAGCAGCAGGAAGCACTATGGGCGCGGCGTCAATAACGCTGACGGTACAAGCCAGA
CAACTGTTGTCTGGTATAGTGCAACAGCAAAGCAATTTGCTGAGGGCTATAGAGGCGCAACAGCATATGTTGC
AACTCACGGTCTGGGGCATT
>KF114881
AGTTACCCTAACATTGGTGATACAGAAAAGAATTCAAGCTCAATCATCACACTCCCATGCAGAATAAAACAAA
TTATAAACATGTGGCAGGGGGTAGGACGAGCAATATATGCACCTCCCATTGCAGGAAAAATAACATGTAAATC
AAATATCACAGGACTCCTGTTGACACGTGATGGAGGAAATAGTAATGAGACAAATACTACAGAGATATTCAGA
CCTGGAGGAGGAAATATGAAAGATAATTGGAGGAGTGAATTATATAAATATAAAGTGGTAGAAATTAAACCAT
TAGGATTAGCACCCAATAAGGCAAAAAGGAGAGTGGTGGAGAGACAAAAAAGAGCAGTAGGAATAGGAGCTGT
GTTCCTTGGGTTCTTGGGAGCAGCAGGAAGCACTATGGGCGCGGCGTCAATAACGCTGACGGTACAGGCCAGA
CAATTATTGTCTGGTATAGTGCAACAGCAAAGCAATTTGCTGAGAGCTATAGAGGCGCAACAGCATCTGTTGC
AACTCACAGTCTGGGGCATT
>KR861329
CCTCAGGCAAATGTAAGTGAGTCAAATTCAACCATCACACTCCCCTGCAGAATAAAGCAAATTATAAACATGT
GGCAGAGGGTGGGAGGAGCAATATATGCCCCTCCCATTGCAGGAAACATAACATGTAAATCAAATATCACGGG
ACTGCTTTTGGTACGTGATGGAGGTACAAATAAGACAGAGCCAGAGACATTCAGACCTGGAGGAGGAGATATG
AGGGACAATTGGAGAAGTGAATTGTATAAATATAAAGTGGTAGAAATTAAGCCATTAGGAGTAGCACCCACTA
AGGCAAGAAGGAGAGTGGTGGAAAGAGAAAAAAGAGCAGTGGGAATAGGAGCTGTGTTCCTTGGGTTCTTGGG
AGCAGCAGGAAGCACTATGGGCGCGGCGTCAATAACGCTGACGGTACAAGCCAGACAGTTATTGTCTGGTATA
GTGCAACAGCAAAGCAATTTGCTGAGGGCTATAGAGGCGCAACAGCATTTGTTGCAACTCACAGTCTGGGGCA
TT
>KR861340

AGTCATAACAGTACAAACAGTAATGAATATAATGTAGATAATGGTACAGACAGAAACATCACACTCCCATGCA
GAATAAAACAAATTATAAACATGTGGCAGAAGGTAGGAAGAGCAATGTATGCCCCTCCTATTAGTGGACTAAT
AACATGTACCTCAAATATCACAGGACTACTCTTGGTACGTGATGGGAACACAAGTGTCACAAATGAGACATTC
AGACCAGGAGGAGGAGATATGAGGGACAATTGGAGAAGTGAACTATATAAATATAAAGTGGTAGAAATTAAGC
CATTGGGAGTAGCGCCCACTGGAGCTAAAAGGAGAGTGGTGGAAAGAAGAGAAAAAAGAGCAGTGGGAATAGG
AGCTGTACTCTTTGGGTTCTTGGGAGCAGCAGGAAGCACTATGGGCGCAGCGTCAATAGCGCTGACGGCACAG
GCCAGACAAGTGCTGTCTGGAATAGTGCAACAGCAAAACAATTTGCTGAGGGCTATAGAGGCGCAACAGCATA
TGTTGCAACTCACAGTCTGGGGCATT
>KR861320
GACTACTGGGATAATGGTACCATATCTGGTAACAGTTCAAACATCACCATAATCTCATGCAGAATAAAGCAAA
TTATAAACATGTGGCAGGGGGTAGGACGAGCAATGTATGCCCCTCCCATTGCAGGAAAAATAACATGTAAATC
AAATATCACAGGACTACTATTGTCACGTGATGGGGGTCCAAATGGCACAGAAGAAGTATTCAGACCTGGAGGA
GGAGATATGAGGGACAATTGGAGAAGTGAATTATATAAATATAAAGTGGTAGAAATTAAGCCATTAGGAATAG
CACCCACTACTGCAAAAAGGAGAGTGGTGGAAAGAGAAAAAAGAGCAGTGGGAATAGGAGCTGTGCTCCTTGG
GTTCTTGGGAGCAGCAGGAAGCACTATGGGCGCGGCGTCAATAACGCTGACGGTACAGGCCAGGCAATTGCTG
TCTGGCATAGTGCAACAGCAAAGCAATTTGCTGAAAGCTATAGAGGCGCAACAGCATCTGTTGCAACTCACGG
TCTGGGGCATT
>KR861321
AATTATACACACAATGCAACTGAAAGTGCTATAAATTCAACAAACATCACCACACTCCATTGCAGAATAAAAC
AAATTATAAACATGTGGCAGGGGGTAGGGCGGGCAATGTATGCCCCCCCCATTGCAGGAAACATAACATGTAG
GTCAAATATCACAGGACTAATATTGACACGTGATGGAGGTAATAAGAATAATAATGAAACAACTGGGACAGAG
ATATTCAGACCTGGAGGAGGAAATATGAGGGACAATTGGAGAAGTGAATTATATAAATATAAAGTGGTGGAGG
TTAATCCATTAGGAGTGGCACCCACTACGGCAAAAAGGAGAGTGGTGGAGAGAGAGAAAAGAGCAGTGGGAAT
AGGAGCTGTGCTCCTTGGGTTCTTGGGAGCAGCAGGAAGCACTATGGGCGCGGCATCAATAACGCTGACGGTA
CAGGCCAGACAATTATTGTCTGGTATAGTGCAACAGCAAAACAATTTGCTGAAGGCTATAGAGGCGCAACAAC
ATATGTTGCAACTCACAGTCTGGGGCATT
>KR861323
GGTCATAATAGTACAGATAATAGTACAGATAATGGAAAGTCAAACAAAACCATCACAATACAATGCAAAATAA
AACAATTCATAAACCTGTGGCAGGAGGTAGGGCGAGCAATATATGCCCCTCCCATTGAAGGAAACATAACATG
TAAATCAAATATCACAGGATTACTCTTGACATGGGATGGAGGAGATGATCCAAAAGAGTCAAATGACACAGAG
ATATTCAGACCAGGAGGAGGAGATATGAAGGACAATTGGAGAAGTGAACTATATAAATATAAAGTGGTAGAAA
TTAAGCCCTTGGGAATAGCACCCACTGAGGCAAAAAGGAGAGTGGTGCAGAGAGAAAAAAGAGCAGTGGGAAT
GGGAGCTGTGCTCCTTGGGTTCTTGGGAGCAGCTGGAAGCACTATGGGCGCAGCGTCAATAGCGCTGACGGCA
CAGGCCAGACAACTGTTGTCTGGAATAGTGCAACAGCAAAGCAATTTGCTGAGGGCTATAGAGGCGCAACAGC
ATATGTTGCAACTCACGGTCTGGGGCATT
>KR861332
ACCTACCAGGTAAATATTACAAAAGATACTTCAAGTTCAGACAGTAAAAACATCACACTCCAATGCAGAATAA
AACAGATTATAAACATGTGGCAGGGAGTAGGACGAGCAATGTATGCTCCTCCCATTGAAGGAACCATAACATG
TAAATCAAACATCACAGGACTATTATTGACACGTGATGGAGGAAAACCTAATAATAGTGAAATATTCAGACCT
GGAGGAGGGGATATGAGAGATAATTGGAGAAGTGAATTATATAAATATAAAGTGGTAGAAATTAAGCCATTAG
GGGTAGCACCCACTAAGGCAAAAAGGAGAGTGGTGGAGAGAGAAAAAAGAGCAGTGGGAATAGGAGCTGTGTT
CCTTGGGTTCTTGGGAGCAGCAGGAAGCACTATGGGCGCGGCGTCAATAACGCTGACGGTACAAGCCAGACAA
CTGCTGTCTGGTATAGTGCAGCAGCAAAGCAATTTGCTGAGGGCTATAGAGGCGCAACAGCATTTGTTGCAAC
TCACAGTCTGGGGCATT
>KR861334
ACTTACACGCAGCCTAACAGTACAGAAAATACTACAGAGCCAACCATCACACTCAAATGCAGAATAAAACAAA
TTATAAACATGTGGCAGGAGGTAGGACGAGCAATGTATGCCCCTCCCATTGCAGGAAACATAACATGTAGATC
AAATATCACAGGATTAATATTGACACGTGATGGAGGAATTAGTAATGAAGGAAATAGGACTAATGAGACATTC
AGACCTATAGGAGGAGATATGAGGGACAATTGGAGAAGTGAATTATATAAATATAAAGTAGTAGAAATTAAGC
CATTAGGACTAGCACCCACTACGGCAAAAAGGAGAGTGGTGGAAAGAGAAAAAAGAGCAGTGGGAATAGGAGC
TGTGTTCCTTGGGTTCTTGGGAGCAGCAGGAAGCACTATGGGCGCGGCGTCAATAACGCTGACGGTACAGGCC
AGACAACTGTTGTCTGGTATAGTGCAACAGCAAAGTAATTTGCTGAGGGCTATAGAGGCGCAACAGCATATGT
TGCAACTCACGGTCTGGGGCATT
>KR861339
TCCTACCCTAATGACACCATCACCCTCCCATGCAGGATAAAACAAATTATTAACCTGTGGCAGAAGGTAGGAC
GGGCAATGTATGCCCCACCCATTGCAGGAAACATAACATGTAAATCAAATATCACAGGACTACTATTGACAAG
TGATGGAGGTGTAAAAGACAATAATACTAATAATATTAATACATTCAGACCTGGAGGAGGAAATATGAGGGAC
AATTGGAGAAGTGAATTATATAAATATAAAGTGGTGGAAGTTAAGCCATTAGGAATAGCACCCACTGAGTCAA
AAAGGAGAGTGGTGGAGAGAGCAAAAAGAGCAGTGGGACTGGGAGCTGTGTTCTTTGGGTTCTTGGGAGCAGC

AGGAAGCACTATGGGCGCGGCGTCAATAACGCTGACGGTACAGGCCAGACAACTATTGTCTGGTATAGTGCAA
CAGCAAAGCAATTTGCTGAGAGCTATAGAGGCGCAACAACATATGTTGCAACTCACAGTCTGGGGCATT
>KR861342
AGTTATAATGCAACAAGCAATAGAACCATCACACTCCCATGCAGAATAAAACAAATTATAAACATGTGGCAGA
GGGTAGGACAAGCAATATATGCCCCTCCCATTGCAGGAAGAATAACATGTAACTCAAATATCACAGGAATACT
CTTGACACGTGATGGAGGAACAACTAATGACACAGGGAATGGCACAGAGATATTCAGACCAGGAGGAGGAGAC
ATGAGGGATAATTGGAGAAGTGAACTATATAAATATAAAGTAGTAGAAATTAAGCCATTGGGAGTAGCACCCA
CCAAGGCAAAAAGGAGAGTGGTGGAGAGAGAAAAAAGAGCAGTAGGAATAGGAGCTGTGCTCTTTGGGTTCTT
GGGAGCAGCAGGAAGCACTATGGGCGCAGCGTCAATAGCGCTGACGGCACAGGCCAGACAAGTGCTGTCTGGA
ATAGTGCAACAGCAAAGCAATTTGCTGAGGGCTATAGAGGCGCAACAGCATATGTTGCAACTCACAGTCTGGG
GCATT
>KR861328
AGTTATAATGATACAAGAAACATCACACTCCCATGCAGAATAAAACAAATTGTAAATATGTGGCAGGGGGTAG
GAAGGGCAATATATGCCGCTCCCATTGCAGGACAAATAATGTGCACATCAAATATTACAGGACTAATATTGAC
ACGTGATGGAGGAAATGGAAATTCTACCCATGAGACATTCAGACCTGAAGGAGGAAATATGAGGGACAATTGG
AGAAGTGAATTATATAAATATAAAGTGGTAGAAATTAAACCATTAGGAATAGCACCCACTGAGGCAAAAAGGA
GAGTGGTGGAGAGAGAAAAAAGAGCAGTAGGCCTAGGAGCTGTGTTACTTGGGTTCTTGGGAGCAGCAGGAAG
CACTATGGGCGCGGCGTCCATGACGCTGACGGTACAGGCCAGACAACTGTTGTCTGGTATAGTGCAACAGCAA
AGCAATTTGCTGAGAGCTATAGAGGCGCAACAGCATATGTTGCAACTCACAGTCTGGGGCATT
>KR861257
AATCATACGTCAAATTCAAACATCACGCTCCCATGCAGAATAAAACAAATTATAAACATGTGGCAGGAGGTAG
GACGAGCAATGTATGCCCCGCCCATTGCAGGAAACATAACATGTAAATCAAATATCACAGGACTACTATTGGT
ATATGATGGAGGCATGCATAATAGAAGCAACGAGACATTCAGACCGGGAGGAGGAAATATGAAGGACAATTGG
CGAAGTGAACTATATAAATATAAAGTGGTAGAAATTAAACCATTAGGAATAGCACCCACGAAAGCAAAAAGAA
GAGTGGTGGAGAGAGAAAAAAGAGCAGTGGGAGTAGGAGCTATGTTCCTTGGGTTCTTGGGAGCAGCAGGAAG
CACTATGGGCGCGGCGTCAATAACGCTGACGGTACAGGCCAGACAACTATTGTCTGGTATAGTGCAACAGCAA
AGCAATTTGCTGAGAGCTATAGAGGCGCAACAACATATGTTGCAACTCACAGTCTGGGGCATT
>KR861259
AATTATACAAATAGCACTACAGATAATTCAACCATCACAATCCAATGCAGAATAAAACAAATCATAAACATGT
GGCAGGAGGTAGGACGAGCAATGTATGCCCCTCCCATTGCAGGAAACATAACATGTAGATCAAATATCACAGG
ACTACTATTAGTACGTGATGGAGGAAACGGAAACGACACCAATAATACAGAGACATTCAGACCTGGAGGAGGA
AATATGAAGGACAATTGGAGAAGTGAATTATACAAATATAAAGTGGTAGAAGTAAAACCATTAGGAATAGCAC
CCACTGGGGCAAAAAGGAGAGTGGTGGAGAGAGAAAAAAGAGCAGTGGGAATAGGAGCTGTGTTCCTTGGATT
CTTGGGAGCAGCAGGAAGCACTATGGGCGCGGCGTCAATAACGCTGACGGTACAGGCCAGACAATTATTGTCT
GGTATAGTGCAACAGCAAAGCAATTTGCTGAGGGCTATAGAGGCGCAACAGCATCTGTTGCAACTCACAGTCT
GGGGCATT
>KR861264
GATTATAATGATACAGAAAGTAATGAGACCATCATACTCCAATGCAGAATAAAACAAATTATAAACATGTGGC
AGAAGGTAGGAGGAGCAATATATGCCCCTCCCATTGCAGGAAACATAACATGTAAATCAAATATCACAGGACT
ACTCTTGGTACGGGATGGAGGCAATACCACTAACACATCAGAGATATTCAGACCAGGAGGAGGAAATATGAAG
GACAATTGGAGAAGTGAACTATATAAATATAAAGTGGTAGAAATTAAGCCATTGGGACTAGCGCCCACCAAGG
CAAGAAGGAGAGTGGTGGAGAGAGAAAAAAGAGCAGTGGGAATAGGAGCTGTGTTCCTTGGGTTCTTGGGAGC
AGCAGGAAGCACTATGGGCGCAGCGTCAATAACGCTGACGGTACAGGCCAGACAATTGTTGTCCGGTATAGTG
CAACAGCAAAGTAATTTGCTGAGAGCTATAGAGGCGCAGCAGCATATGTTGCAACTCACAGTCTGGGGCATT
>KR861269
TGCTATACATCAAACCTGTTTAATGATAGCAATAGGGCAAACATGTCTGATAACAGTACAGTCATCACACTCT
CATGCAGGATAAAACAAATTATAAATATGTGGCAGAAGGTAGGACGAGCAATGTATGCCCCTCCCATTGCAGG
GAACATAACATGTAAATCAAATATCACAGGACTACTATTGGTACGTGATGGAGGAACAGGTAACAAAACAGAG
ATATTCAGACCTGGAGGGGGAGATATGAGGGACAATTGGAGAAGTGAATTATATAAATATAAAGTGGTAGAAA
TAAAGCCATTGGGAATAGCACCCACTGAGGCAAAAAGGAGAGTGGTGGAGAGAGAAAAAAGAGCAGTGGGAAT
AGGAGCTGTGTTCCTTGGGTTCTTGGGAGCAGCAGGAAGCACTATGGGCGCAGCGTCAATAACGCTGACGGTA
CAGGCCAGACAATTGTTGTCTGGAATAGTGCAACAGCAAAGCAATTTGCTGAGGGCTATAGAGGCGCAACAGC
ATATGTTGCAACTCACGGTCTGGGGCATT
>KR861276
ACTTACATCACACTCCCATGCAGAATAAAGCAAATTATAAACATGTGGCAGGGGGTAGGACAAGCAATGTATG
CCCCTCCCATTGCAGGGAACATAACATGTACCTCAAATATCACAGGAATACTGTTGACACGTGATGGAGGAAA
TGAGGAAAAGGAAGAGGAGATATTCAGACCTGGAGGAGGAGATATGAGGGACAATTGGAGAAGTGAGTTGTAT
AAATATAAAGTGGTAGAAGTTAAACCATTAGGAATAGCACCCACTGAGGCAAAAAGAAGAGTAGTGGAGAGAC
AAAAAAGAGCAGCGGGACTAGGAGCTGTGTTCCTAGGGTTCTTGGGAGCAGCAGGAAGCACTATGGGCGCGGC

GTCAATAACGCTGACGGTACAGGCCAGACAATTATTGTCTGGTATAGTGCAACAGCAAAGCAATTTGCTGAGA
GCTATAGAGGCGCAACAACATATGTTGCAACTCACAGTCTGGGGCATT
>KR861281
AATTACATCAATTCATCAGATCTAATCACCCTCCAATGCAGAATAAAACAATTATAAACTTGTGGCAGAGGG
TAGGACGAGCAATGTATGCCTCCCCCATTAACGGAATCATAAAATGTAGATCAAATATCACAGGACTACTATT
GACACGTGATGGAGGTAATACAACAAACAGTAGTGAAGAAATATTCAGACCTGAAGGAGGAAATATGAGGGAC
AATTGGAGAAGTGAATTATATAAATATAAAGTGGTAGAAATTAAGCCATTAGGGATAGCACCCACTAGGGCAA
AAAGAAGAGTGGTGGAGAGAGAAAAAAGAGCAGTGGGAATAGGAGCTGTGTTCCTTGGGTTCTTGGGAGCAGC
AGGAAGCACTATGGGCGCGGCGTCAATCACGCTGACGGTACAGGCCAGACAATTATTGTCTGGTATAGTGCAA
CAGCAAAGCAATTTGCTGAGAGCTATAGAGGCGCAACAACATATGTTGCAACTCACAGTCTGGGGCATT
>AF110973
AGTTATAGGGGTACAGAAAGTAATAGTTCAATCATCACACTCCCATGCAGAATAAAACAAATTATAAACATGT
GGCAGAAGGTAGGACGAGCAATATATGCCCCTCCCATCGAAGGGAACATAACATGTAGCTCGAGTATCACAGG
ACTACTCTTGGCACGTGATGGAGGCTTAGATAATGTCACCACAGAGATATTCAGACCACAAGGAGGAGATATG
AAGGACAATTGGAGAAATGAATTATATAAATATAAAGTAGTAGAAATTAAGCCATTGGGAGTAGCGCCCACTG
AGGCAAAAAGGAGAGTGGTGGAGAGAGAAAAACGAGCAGTGGGAATAGGAGCTGTGATCTTTGGGTTCTTGGG
AGCAGCAGGAAGCACTATGGGCGCAGCGTCAATAACGCTGACGGCACAGGCCAGACAATTGTTGTCTGGAATA
GTGCAACAGCAAAGCAATTTGCTGAGGGCTATAGAGGCGCAACAGCATATGTTGCAACTCACAGTCTGGGGCA
TT
>KY658707
ACCTACAGGTCTAATAGTACAAATGCAACCATCACACTCCCATGCAGAATAAAACAAATTATAAACATGTGGC
AAGGGGTAGGACGAGCAATGTATGCCCCTCCCATTGCAGGAAACATAACATGTAAATCAAATATCACAGGACT
ACTATTGACACGTGACGGAGGAGTTGGTGAGGATAGTGATAATGAAACAGAAATATTCAGACCTGGAGGAGGA
GATATGAGAGACAATTGGAGAAGTGAATTATATAAATATAAAGTGGTAGAAATTAAACCATTAGGAATAGCAC
CCACTGAGGCAAGAAGGAGAGTGGTGGAGAGAGAAAAAAGAGCAGTGGGAATAGGAGCTGTGTTCCTTGGGTT
CTTGGGAGCAGCAGGAAGCACTATGGGCGCAGCGTCAATAACGCTGACGGTACAAGCCAGGCAATTATTGTCT
GGTATAGTGCAACAGCAAAGCAATTTGCTGAGAGCTATAGAGGCGCAACAGCATATGTTGCAACTCACGGTCT
GGGGCATT
>AF443077
AGTCAGCCTAACAGTACAGGAAACACTCCACATTCAAACATCACACTCCCATGCAAAATAAAACAAATTATAA
ACATGTGGCAGGGGGTAGGGCGAGCAATGTATGCCCCTCCCATTGCAGGAAACATAACATGTATATCAAATAT
CACAGGACTAATATTGACACGGGATGGAGGAGTAAATAGAAGTGACACAGAAATATTCAGACCTGCAGGAGGA
GATATGAGGGATAATTGGAGAAGTGAATTATATAAATATAAAGTGGTAGAAATTAAACCATTAGGACTAGCAC
CCACTAAGGCAAAAAGGAGAGTGGTGGAGAGAGAAAAAAGAGCGGTGGGAATAGGAGCTGTGTTCCTTGGGTT
CTTGGGAGCAGCAGGAAGCACTATGGGCGCGGCGTCAATAACGCTGACGGTACAGGTCAGACAACTGTTGTCT
GGTATAGTGCAACAGCAAAGCAATTTGCTGAGGGCTATAGAGGCGCAACAGCATATGTTGCAACTCACGGTAT
GGGGCATT
>AF443081
TATTATAGTAACAAGACAAGCAGTAATATGACTACAAATGAAATCATCACAATTCCATGCAAAATAAAACAAA
TTATAAACATGTGGCAGGAGGTAGGACGAGCAATGTATGCCCCTCCCATTGCAGGAAACATAACATGTAAATC
AAATATCACAGGACTACTATTAGTACGTGATGGAGGCAACAACAATACCACAGAAACATTCAGACCTGGAGGA
GGAAATATGAAGGACAATTGGAGAAGTGAATTATATAAATATAGAGTGGTAGAAATTAAGCCATTAGGAATAG
CACCCACTGGAGCAAAAAGGAGAGTGGTGGAGAGAGAAAAAAGAGCAGTGACACTAGGAGCTATGTTCCTTGG
GTTCTTGGGAGCAGCCGGAAGCACTATGGGCGCGGCGTCAATAACGCTGACGGTACAGGCCAGACAACTGTTG
TCTGGTATAGTGCAACAGCAAAGCAATTTGCTGAGGGCTATAGAGGCGCAACAGCATATGTTGCAACTCACGG
TCTGGGGCATT
>AF443087
TACTATCCAAACATGACCAATGTCACTGATACAAAGAGTAATTTAACCATCACACTGCCATGCAGAATAAAAC
AAATTATAAACAGGTGGCAGGAGGTAGGACGAGCAATGTATGCCCCTCCCATTGCAGGAAACATAACATGTAC
GTCAAATATCACAGGACTACTATTGGTACGTGATGGAGGCCGTACAAGTGACTCTACAAAAGAGATATTCAGA
CCTGGAGGAGGAGATATGAGGGACAATTGGAGAAGTGAATTATATAAATATAAAGTGGTAGAAATTAAACCAT
TGGGAATAGCACCCACTGAAGCAAAAAGGAGAGTGGTGGAGAGAGAAAAAAGAGCAGTGACAATAGGAGCTAT
GTTCCTTGGGTTCTTATCAGCAGCAGGAAGCACTATGGGCGCAGCATCAATAACGCTGACGGTACAGGCCAGA
CAATTATTGTCTGGTATAGTGCAACAGCAAAGCAATTTGCTGAGAGCTATAGAGGCGCAACAGCATATGTTGC
AACTCACAGTTTGGGGCATT
>KR861318
GACTATAATGTAAGCAACATCAACATCACACTCCCATGCAGAATAAAACAATTTATAAACATGTGGCAGGGGG
TAGGACGAGCAATGTATGCCCCTCCCATTGCAGGAAACATAACATGTATATCAAGTATCACAGGATTAATATT
GACACGTGATGGAGGAAGAGGGAATGAGACTGAGAATAATACTGAGATATTCAGACCTGCAGGAGGAGATATG
AGGGACAATTGGAGAAGTGAATTATATAAATATAAAGTAGTAGAAATTAAGCCATTAGGACTAGCACCCACTA

TGGCAAAAAGGAGAGTGGTGGAGAGAGAAAAAAGAGCAGTGGGAATAGGAGCTGTACTCCTTGGGTTCTTGGG
AGCAGCAGGAAGCACTATGGGCGCGGCGTCAATCACGCTGACGGTACAGGCCAGACAATTGCTGTCTGGTATA
GTGCAACAGCAAAGCAATTTGCTGAGAGCTATAGAGGCGCAACAGCATATGTTGCAACTCACAGTCTGGGGCA
TT
>HQ699999
TACTATCCTAATGGTACACTAGTTAATTCAACCTTAAACATCACACTCCAATGCAGAATAAAGCAAATTATAA
ACATGTGGCAGGAGGTAGGAAAAGCAATGTATGCCCCTCCCATTGCAGGAAACATAACATGTAGATCAAATAT
CACAGGACTACTATTGGTACGTGATGGAGGGATAACAAATAATACAAACCCAAGTGAGATCAAGAATGATACA
GAGATATTCAGACCTGGAGGAGGAAACATGAAAGACAATTGTAGAAGTGAATTATATAAATATAAAGTGGTAG
AAATTAAGCCATTGGGAGTAGCACCCACTCCAGCAAAAAGGAGAGTAGTGGAGAGAGAAAAAAGAGCAGCAAT
AGGAGCTGTGTTCCTTGGGTTCTTGGGAGCAGCAGGAAGCACTATGGGCGCGGCGTCACTAACGCTGACGGTA
CAGGCCAGACAATTGTTGTCTGGTATAGTGCAGCAGCAAAGCAATTTGCTGAGAGCTATAGAGGCGCAACAGC
ATCTGTTGCAACTCACGGTCTGGGGCATT
>EF514713
TCTCAGTCTAATAATATAAGCATACTTACTAATGAAACCATCACGCTCCCATGCAGAATAAAACAAATTATAA
ACATGTGGCAGGAGGTAGGACGAGCAATGTATGCCCCTCCCATTGCAGGAAACATAACATGTAAATCAAATAT
CACAGGACTACTATTGGTACGGATGGAGGAAACCAGGAAAGGAACAACGAGACATTTAGACCTGAGGGAGGA
AATATGAAGGACAATTGGAGAAGTGAATTATATAAATATAAAGTGGTAGAAATTAAGCCATTGGGAATAGCAC
CCACTGAGGCAAAAAGGAGAGTGGTGGAGAGAGAGAAAAGAGCAGTGGGAATAGGAGCTGTACTCCTTGGGTT
CTTGGGAGCAGCCGGAAGCACTATGGGCGCGGCGTCAATAACGCTGACGGTACAGGCCAGACAAGTATTGTCT
GGTATAGTGCAACAGCAAAGCAATTTGCTGAGAGCCATAGAGGCGCAACAGCATATGTTGCAACTCACGGTCT
GGGGCATT
>KX228820
AGTTATGATTCTAATAGTACAGCAAACATCACACTCCCATGCAAAATAAAACAGATTATAAACTTGTGGCAGG
AGGTAGGACGAGCGATGTACGCTCCTCCCATTGCAGGAAACATAACATGTAAGTCAACCATCACAGGATTACT
ATTGGTGCGTGATGGAGGACAAGGAAACAACTCAAATGAGACATTCAGACCTGGAGGAGGAGATATGAGGGAT
AATTGGAGAAGTGAATTGTATAAGTATAAAGTGGTAGAAATTAAGCCATTGGGAGTAGCACCCACTGAGGCAA
AAAGGAGAGTGGTGGAGAGAGAAAAAAGAGCAGTGGGACTAGGAGCTGTGATCTTTGGGTTCTTAGGAGCAGC
AGGAAGCACTATGGGCGCGGCGTCAATAACGCTGACGGTACAGGCCAGACAACTGTTGTCTGGTATAGTGCAA
CAGCAAAGCAATTTGCTGAAGGCTATAGAGGCACAACAGCATATGTTGCAACTCACGGTCTGGGGCATT
>KU319533
ACCTATAAAGGTACAAACATGTCTGATGGTCCTGAATTCATCACACTCCAATGCAGAATAAAACAAATTATAA
ATATGTGGCAAGGGGTAGGACGAGCAATGTATGCCCCTCCCATTGCAGGAAACATAACATGTATATCAAATAT
CACAGGAATACTACTGACACGTGATGGAAAAAATGATGGTGCAGATACATTCAGACCTGGAGGAGGAGATATG
AGGGATAATTGGAGAAGTGAATTATATAAATACAAAGTAGTAGAAATTAAACCATTAGGAATAGCACTCACTA
AGGCAAAGAGGAGAGTGGTGGAGAGAGAAAAAAGAGCAGTATTGGGAGGACTAGGAGCTGTGTTCCTTGGGTT
CTTGGGGGCAGCAGGAAGCACTATGGGCGCAGCATCAATGACGCTGACGGTACAGGCCAGAAAATTGTTGTCT
GGTATAGTGCAACAGCAAGGCAATTTGCTGAGGGCTATAGAGGCGCAACAGCATATGTTGCAACTCACAGTCT
GGGGCATT
>KU319536
AATCATAGTTTTACAAACTCAACCCTGTTTAATAGTAGTAATAATGCAAACTCAACTATCACACTCCCATGCA
AAATAAAACAAATTATAAACATGTGGCAGAGGGTAGGGCGAGCAATATATGCCCCTCCCATTGAAGGAAATAT
AACATGTAAATCAGGTATCACAGGGCTACTATTGACACGTGATGGAGGAAATGACACAGAGATAAATGGCACA
GAGACATTCAGACCTGGAGGAAGAGATATGAGGGACAATTGGAGAAGTGAATTATATAGATATAGGGTGGTAG
AAATTAAGCCATTGGGAATAGCACCCACCAAGGCAAAAAGGAGAGTGGTGGAGAGAGAAAAAAGAGCAGTGGG
AATAGGAGCTGTGTTCCTTGGGTTCTTGGGAGCAGCAGGAAGCACTATGGGCGCGGCGTCAATAACGCTGACG
GTACAGGCCAGACAATTGCTGTCTGGTATAGTGCAACAGCAAAGCAATTTGCTGAGGGCTATAGAGGCGCAAC
AGCATATGTTGCAACTCACAGTTTGGGGCATT
>FJ653288
AGCTACATGTCTAATACTACAAACAGTACAATCAGTGAAAATTCAACTATCACACTTTCATGCAGAATAAAAC
AAATTATAAACATGTGGCAGGGGGTAGGACAAGCAATATATGCCCCGCCCATTGCAGGAAACATAACATGTAT
ATCAAATATCACAGGACTACTGTTAACACGTGATGGAGGAAATGAGGGTAATGATACAACAGAGACATTCAGA
CCTGGAGGGGGAGATATGAGAGACAATTGGAGAAGTGAATTATATAAATATAAAGTGGTAGAAGTTAAACCAT
TAGGAATAGCACCCAATAAGGCAAAAAGGAGAGTGGTGGAGAGAGAAAAAAGAGCAGTGGGAATAGGAGCTGT
GCTCCTTGGGTTCTTGGGAGCAGCAGGAAGCACTATGGGCGCGGCGTCAATAACGCTGACGGTACAGGCCAGA
CAATTATTGTCTGGTATAGTGCAACAGCAAAGCAATTTGCTGAGAGCTATAGAGGCGCAACAGCATTTGTTGC
AACTCACAGTCTGGGGCATT
>DQ207941
AACTACACTGAGGCAAATTCAAGCATCACACTCCCATGCAGAATAAAACAAATTATAAACATGTGGCAGGAGG
TAGGACGAGCAATGTATGCCCCTCCCATTGCAGGATACATAACATGTAATTCAAATATCACAGGACTACTCTT

GACACGTGATGGAGGCAATGGTAGTAGTAATTGCACAGAGACATTCAGACCGGGAGGAGGAGACATGAGGGAC
AATTGGAGAAGTGAATTATATAAATACAAAGTGGTAGAAATTAAGCCATTAGGAGTAGCACCCACCAAGGCAA
AAAGGAGAGTGGTGGAGAAAGAAAAAAGAGCAGTGGGAATAGGAGCTGTACTCCTTGGGTTCTTGGGAGCAGC
AGGAAGCACTATGGGCGCAGCGTCAATAACGCTGACGGTACAGGCCAGACAAGTGTTGTCTGGAATAGTGCAA
CAGCAAAATAATTTGCTGAGGGCTATAGAGGCGCAACAGCATATGTTGCAACTCACAGTCTGGGGCATT
>KF766541
AATTATACATACATGCTTAATGGTACACTTACAGGAAGTAATTCAAGCTCAGACATTATCACACTCCCATGTA
GAATAAAACAATTTATAAACATGTGGCAGGAGGTAGGACGAGCAATGTATGCTCCTCCCATTGCAGGAAACAT
AACATGTTCATCAAAAATCACAGGACTACTATTGGTACGTGATGGAGGAAATACAAATAGTACTACAGAGATA
TTTAGACCTGGAGGAGGAGACATGAGGAACAATTGGAGAAGTGAATTATATAAATACAAAGTAGTAGAAATTA
GACCATTGGGAGTAGCACCCACTACAGCAAAAAGGAGAGTGGTGGAGAGAGAAAAAAGAGCAGTGGGAATAGG
AGCTGTGTTCCTTGGGTTCTTGGGAGCAGCCGGAAGCACTATGGGCGCGGCGTCAATGACGCTGACGGTACAG
GCCAGACAATTGTTGTCTGGTATAGTGCAACAGCAAAGCAATTTGCTGAGGGCTATAGAGGCGCAACAGCATC
TGTTGCAACTCACGGTCTGGGGCATT
>MH899155
AGTCATGATGGTTTAAACTCAACCATCACACTCCCATGCAAAATAAAGCAGATTATAAATATGTGGCAGGAGG
TAGGAAGAGCAATGTATGCCCCTCCCATTGAAGGAAACATAACATGTAAATCAAATATCACAGGCATACTATT
GGTACGTGATGGAGGGAATGATAACAGCTCAAATGATACAGAAACAGAGACATTCAGACCTGGAGGGGGAGAT
ATGAGAGATAATTGGAGAAGTGAATTATACAAATATAAAGTGGTAGAAATTAAGCCACTGGGAGTAGCACCCA
CTACAGCAAAAAGGAGAGTGGTGGAGAGAGGAAAAAGAGCAGTGGGCATAGGAGCTGTGTTCCTTGGGTTCTT
GGGAGCAGCAGGAAGCACTATGGGCGCGGCGTCAATAACGCTGACGGTACAGGCCAGACAATTGTTGTCTGGT
ATAGTGCAACAGCAAAGCAATTTGCTGAGAGCTATAGAGGCGCAACAACATCTGTTGCAACTCACGGTCTGGG
GCATT
>KX069220
ACCTACGTGAATGATACCACCAACGGGGATAACTCAAACATCACTTTACCATGCCGAATAAAACAAATTATAA
ACATGTGGCAGGAGGTAGGACGAGCAATGTATGCCCCTCCCATTGCAGGAAACATAACATGTAAATCAAATAT
TACAGGACTACTATTGGTACGTGATGGAGGGAAAGAGACAAATGGCACAGAGACATTCAGACCTGGAGGAGGA
GATATGAGAGACAATTGGAGAAGTGAATTATATAAATATAAAGTGGTAGAAATTAAGCCATTGGGAGTAGCAC
CCACTAAAGCAAAAAGAAGAGTAGTGGAGAGAGAAAAAAGAGCAGCACTAGGAGCTGTGTTCCTTGGGTTCTT
AGGAGCAGCAGGAAGCACTATGGGCGCGGCGTCAATAGCGCTGACGGCACAGGCCAGACAATTGTTGTCTGGT
ATAGTGCAACAGCAAAGCAATTTGCTGAGGGCTATAGAGGCGCAACAGCATCTGTTGCAACTCACAGTCTGGG
GCATT
>KY713228
TACTACTTTAATAGTAGTAAAAACAGAGGGGGCACAGAAAGTGATGAAACCATCATCCCATGCAAAATAAAGC
AAATCATAAACATGTGGCAGGAGGTAGGAAGAGCAATGTATGCCCCTCCCATTGCAGGAAACATAACATGTAA
ATCAAACATCACAGGGCTACTATTGGTACGTGATGGAGGAATAAATACTACAGAGGCTACAGAGACATTTAGA
CCAGGAGGAGGAGATATGAGGGACAATTGGAGAAGTGAATTATATAAATATAAAGTGGTAGAAATTAAGCCAC
TGGGAGTAGCACCAACTGGAGCAAAAAGGAGAGTGGTGGAGAGAGAAAAAAGAGCAGTGGGGCTAGGAGCTGT
GTTCCTTGGGTTCTTGGGAGCAGCAGGAAGCACTATGGGCGCGGCGTCAATAACGCTGACGGTACAGGCCAGA
CAACTGTTGTCTGGTATAGTGCAACAGCAAAGTAATTTGCTGAGAGCTATAGAGGCGCAACAGCATCTGTTGC
AACTCACAGTCTGGGGCATT
>KY713233
AATTACATGGGTACATACAATGAGACAAATGGTACATACAATTCAAACTCGACCATCACAATCCCATGCAGAA
TAAAACAAATTATAAACATGTGGCAGGAGGTAGGACGAGCAATATATGCCCCTCCCATTGCAGGAAACATAAC
ATGTAAATCAAATATCACAGGACTACTATTGGTACGTGATGGAGGAAGAAACAATACACATAAAAATGAGACA
TTTAGACCTGGAGGAGGAGATATGAAAGACAATTGGAGAAGTGAATTATATAAATACAAGGTGGTGGAAATTA
AGCCATTAGGAATAGCACCTACTACAGCAAAAAGGAGAGTGGTGGAGAGAGAAAAAAGAGCAGTGGGACTAGG
AGCTATGTTCCTTGGGTTCTTGGGAGCAGCCGGGAGCACTATGGGCGCGGCGTCAATAACGCTGACGGTACAG
GCCAGACAATTGTTGTTTGGTATAGTGCAGCAGCAAAGCAATTTGCTGAGAGCTGTAGAGGCGCAACACCATC
TGTTGCAACTCACGGTCTGGGGCATT
>KY713248
AATTACACTGAGACAAATAGTACATACAATGAGGCAAACTTGACCATCACAATCCCATGCAGAATAAAACAAA
TTATAAACATGTGGCAGAAGGTAGGAAAAGCAATATATGCCCCTCCCATTGCAGGAAACATAACATGTAAATC
AAATATCACAGGACTACTACTGGTACGTGATCGTGGTGGAAACAATACAAATAAGAATGAGACATTTAGACCT
GGAGGAGGAAATATGAAAGACAATTGGAGAAGTGAATTATATAAATATAAAGTGGTGGAAATTAAGCCATTAG
GAATAGCACCTACTACAGCAAAAAGGAGAGTGGTGGAGAGAGAAAAAAGAGCAGTGGGACTAGGAGCTATGTT
CCTTGGGTTCTTGGGAGCAGCTGGGAGCACTATGGGCGCGGCGTCAATAACGCTGACGGTACAAGCCAGACAA
CTGTTGTCTGGTATAGTGCAGCAGCAAAGCAATTTGCTGAAAGCTGTAGAGGCGCAACAGCATCTGTTGCAAC
TCACTGTCTGGGGCATT
>MK076659

AGTCATGATAGTTTTCTAGACTCAACCAACAACTCAACTTCAAAAAAGATCACACTCCCATGCAGAATAAAGC
AGATTATAAATATGTGGCAGGAGGTAGGAAGAGCAATGTATGCCCCTCCCATTGCAGGAAAAATAACATGTAA
ATCAAATATCACAGGCATACTATTGACACGTGATGGAGGGCATGATAACAATGTTACAGAGACATTCAGACCT
GGAGGGGGAGATATGAGAGATAATTGGAGAAGTGAATTATACAAATATAAAGTGGTAGAAGTTAAGCCACTGG
GAGTAGCACCCACTGCAGCAAAAAGGAGATTGGTGGAAAGAAACAAAAGAGACGTGGGCCTAGGAGCTGTGTT
ACTTGGGTTCTTGGGAGCAGCAGGAAGCACTATGGGCGCGGCGTCAATAACGCTGACGGTACAGGCCAGACAA
TTGTTGTCTGGTATAGTGCAACAGCAAAGCAATTTGCTGAGAGCTATAGATGCGCAACAACATATGTTGCAAC
TCACGGTCTGGGGCATT
>AY049708
AGTTATAATGGTCCAAGCCCAAATATCACAATCCCATGCAGAATAAAACAAATTATAAACATGTGGCAGGAGG
TAGGACGAGCAATGTATGCCCCTCCCATTGCAGGAAACATAACATGTAAATCAAATATCACAGGAATACTATT
GGTACGTGATGGGGGAAAAACAAAGGATAATGACACAGAGATATTCAGACCTGGAGGAGGAGATATGAGGGAC
AATTGGAGAAGTGAATTATATAAATATAAAGTGGTAGAAATTAAGCCATTGGGAGTAGCACCCACTGAAGCAA
AAAGGAGAGTGGTGGAGAGAGAAAAAAGAGCAGCGCTAGGAGCTATGATCCTTGGGTTCTTGGGAGCAGCAGG
AAGCACTATGGGCGCAGCGTCAATAACGCTGACGGTACAGGCCAGACAATTGTTGTCTGGTATAGTGCAACAG
CAAAGCAATTTGCTGAAGGCTATAAATGCGCAACAGCATATGTTGCAACTCACGGTCTGGGGCATT
>EF694036
TTTTATAGGCCTGATGGTAATTCAAGCACAGACATCATAATCCTATGCAGAATAAAACAAATTATAAACATGT
GGCAGGAGGTAGGACAAGCAATGTATAACCCTCCCATTGCAGGAAACATAACATGTAGATCAAATATCACAGG
ACTACTATTGTTACGTGATGGAGGAACAGGAAATACTACAGATAATAAAACAGAAATATTCAGACCTGGAGGA
GGAGATATGAGGGACAATTGGAGAAGTGAATTATATAAATATAAAGTGGTAGAGATTAAACCATTGGGGATAG
CACCCACTACAGCAAAAAGGAGAGTGGTGGAGAGAGAAAAAAGAGCAGTGGGAATAGGAGCTGTGTTCCTTGG
GTTCTTGGGAGCAGCAGGAAGCACTATGGGCGCGGCGTCAATAACGCTGACGGTACAGGCCAGACAACTGTTG
TCTGGTATAGTGCAACAGCAAAGCAATTTGCTGAGGGCTATAGAGGCGCAACAGCATCTGTTGCAACTCACGG
TCTGGGGCATT
>AY945738
AACTATACAAACAATACATCAGAGAACATCACACTCCCATGCAGAATAAAACAAATTATAAATATGTGGCAGG
GGGTAGGACAAGCAATATATGCCCCTCCCATTAAAGGGAAGATAATATGTAAATCAAATATCACAGGGCTACT
GCTGACACGTGATGGAGGCAATACAAATAACACAAATAACACAGAAATATTCAGACCTGCAGGAGGAGATATG
AAGGACAATTGGAGAAGTGAATTATATAAATACAAAGTGGTAGAAATTAAACCATTAGGAGTAGCACCCACCA
AGGCAAAAAGGAGAGTGGTGGAGAGAGAAAAAAGAGCAGTGGGAATAGGAGCTGCGTTCCTTGGGTTCTTGAG
TGCAGCAGGAAGCACTATGGGCGCAGCGTCAATAACGCTGACGGTACAGGCCAGACAATTATTGTCTGGTATA
GTGCAACAGCAAAGCAATTTGCTGAGGGCTATAGAGGCGCAACAGCATATGTTGCAACTCACGGTCTGGGGCA
TT
>KC894074
TTTTACAAAACAGAGTTTAATAGTACAACAAATTCAACCATCACACTCCAATGTAGAATAAAACAAATTATAA
ACATGTGGCAAGGGGTAGGGCGGGCAATGTATGCCCCTCCCATTGAAGGAAACATAACATGTAAATCAAACAT
CACAGGACTACTATTGACACGTGATGGAGGAACAAATGACAGTATGACAGAGACATTCAGACCTGGAGGAGGA
GATATGAGGGATAATTGGAGAAGTGAATTATATAAATATAAAGTGGTAGAAATTAAGCCATTAGGAGTAGCAC
CCACTGAGGCAAAAAGGAGAGTGGTGGAGAGAGAAAAAAGAGCATTGACACTAGGAGCTTTGTTCCTTGGGTT
CTTGGGAACAGCAGGAAGCACTATGGGCGCGGCGTCAATAACGCTGACGGTACAGGCCAGACAATTGTTGTCT
GGTATAGTGCAACAGCAGAGCAATCTGCTGAAGGCTATAGAGGCGCAACAGCATCTGTTGCAACTCACGGTCT
GGGGCATA
>KC894086
AGCTACATGATTAATGATACAGAAAGCACTAATAAAAGTGCAAATTCAACCATCATACTCCCATGCAGAATAA
AACAAATTATAAACATGTGGCAGGGGGTAGGACGAGCAATGTATGCCCCTCCCATTGCAGGAACCATAACATG
CAACTCAAATATCACAGGATTACTATTGACCCATGATGGAGACCCTAATAGCAATGGCACAGAGATATTCAGA
CCTGGAGGAGGAAATATGAAGGACAATTGGAGAAGTGAATTATATAAATATAAGGTGGTAGAAATTAAGCCAT
TAGGAATAGCACCCACTAATGCAAGAAGGAGAGTGGTGGAGAGAGGAAAAAGAGCAGTGGGAATAGGAGCTGT
GTTCCTTGGGTTCTTGGGAGCAGCAGGAAGCACTATGGGCGCGGCGTCACTAACGCTGACGGTACAAGCCAGA
CAACTGTTGTCTGGTATAGTGCAACAGCAAAGCAATTTGCTGAGGGCTATAGAGGCGCAACAGCATATGTTGC
AACTCACGGTCTGGGGCATT
>KC894092
AATTATACAGCAAGTACAAATAATTCAAGTGAGACCATCACACTCCCATGCAGAATCAAACAAATTATAAGAA
TGTGGCAGGGGGTAGGGCAAGCAATGTATGCCCCTCCCATTGCAGGAAACATAACATGTACATCAAACATCAC
AGGAATATTATTGGTACGTGATGGAGGAGACAATAATAGCACAAAAGAGATATTCAGACCTGGAGGAGGAGAT
ATGAGGGACAATTGGAGAAGTGAATTATACAAATATAAAGTAGTAGAAATTAAGCCATTGGGAATAGCTCCCA
CTGGAGCAAAAAGGAGAGTGGTGGAGAGAGAAAAAAGAGCAGTAGGAATAGGAGCTGTGCTCCTTGGGTTCTT
GGGAGCAGCAGGAAGCACTATGGGCGCAGCGTCAATAACGCTGACGGTACAGGCCAGACAATTGTTGTCTGGT

ATAGTGCAACAGCAAAGCAATTTGCTGAGGGCTATAGAGGCGCAACAGCACATGTTGCAACTCACTGTCTGGG
GCATT
>KC862923
AATTATACCTTTAATGGTACAGAGCTGTTGAATAGTACCTCAAATACCACAGAGATCATCACACTCCCATGCA
GAATAAAACAAATTATAAACATGTGGCAGAGGGTAGGACAAGCAATGTATGCCCCTCCCATTAAAGGAAACAT
AACATGTATATCCAATATCACAGGACTACTGCTGACACGTGATGGAGGAGGGGACAAAAATGGCAAAGAGAAT
AAAACAGAGACATTCAGACCTGGAGGAGGAAATATGAGGGATAATTGGAGAAGTGAACTATACAAATATAAAG
TGGTAGAAATTAAGCCATTAGGAATAGCACCCACTAAGGCAAAAAGGAGAGTGGTGGAGAGAGAAAAAAGAGC
AGTGGGAATAGGAGCTGTGTTCCTTGGGTTCTTGGGGGCAGCAGGAAGCACTATGGGCGCAGCATCAATAACG
CTGACGGTACAGGCCAGACAATTATTGTCTGGTATAGTGCAACAGCAAAGCAATTTGCTGAGGGCTATAGAGG
CGCAACAGCATATGTTGCAACTCACAGTCTGGGGCATA
>KC894103
TACTATCCTAATAATACAGGAAGTAATTCAACAGACAACATCATCACACTTCCATGCAGAATAAAACAAATTA
TAAATATGTGGCAGGAAGTAGGAAGAGCAATATATGCCCCTCCCATTGAAGGAAGCATAACATGCACATCAAA
TATCACAGGACTACTATTGGTACGTGATGGAGGCACTGATAATAGCACAAATAACACAGAGACATTCAGACCT
GGAGGAGGAGATATGAGGGACAATTGGAGGAGTGAATTATATAAATATAAAGTGGTAGAGATTAAGCCATTAG
GAGTAGCACCCACTGAGTCAAAAAGGAGAGTGGTGGAGAGACAAAAAAGAGCAGTGGGAATGGGAGCTGTGAT
CTTTGGGTTCTTGGGAGCAGCAGGAAGCACTATGGGCGCGGCGTCAATAACGCTGACGGTACAGGCCAGACAA
CTGTTGTCTGGTATAGTGCAACAGCAAAGTAATTTGCTGAAGGCTATAGAGGCGCAACAGCATCTGTTGCAAC
TCACGGTCTGGGGCATT
>KC894106
AACTATAATGATACAGGAGGGAATTCAAGCTCAAACATCAGACTCTCATGCAGAATAAAACAATTTATAAACA
TGTGGCAGAAAGTAGGAAGAGCAATGTATGCCCCTCCCATTGAAGGAAACATAACATGTAGATCAAATATCAC
AGGACTGCTATTGGTACGAGATGGAGGTATCAGTAGCACCACCGAGACATTCAGACCTGGAGGAGGAAATATG
AAGGACAATTGGAGAAGTGAATTATATAAATATAAAGTAGTAGAAATTAAGCCATTAGGAATAGCACCCACTA
AAGCAAAAAGGAGAGTGGTGGAGAGAGAAAAAAGAGCAGTGGGAATAGGAGCTGTGTTCCTTGGGTTCTTGGG
AGCAGCCGGAAGCACTATGGGCGCGGCGTCAATAACGCTGACGGTACAGGCCAGACAACTGTTGTCTGGTATA
GTGCAACAGCAAAGCAATTTGCTGAGGGCCATAGAGGCGCAACAGCATATGTTGCAACTCACAGTCTGGGGCA
TT
>HM070477
AATTACAGTGGGAATGGTACCATCACCATACCATGCAGAATAAAACAAATTATAAATATGTGGCAGGAGGTAG
GAAAGGCAATATATGCCCCTCCCATTGCAGGAAACATAACATGTAAATCAAATATCACAGGACTACTACTGAC
ACGTGATGGAGGACAAGGACCAGATAAAGATAACACAACAGAGACATTCAGACCTGGAGGAGGAGACATGAGG
GACAATTGGAGAAGTGAATTATATAAATATAAAGTAGTAGAAATTAAGCCATTGGGAATAGCACCCACTACGG
CAAAAAGAAGAGTGGTGGAGAGAGAAAAAAGAGCAGTGGGAATAGGAGCTGTGTTCCTTGGGTTCTTGGGAGC
AGCAGGAAGCACTATGGGCGCAGCGTCAATAACGCTGACGGTACAGGCCAGACAACTGTTGTTTGGCATAGTG
CAACAGCAAAGCAATTTGCTGAGAGCCATAGAGGCGCAACAGCATATGTTGCAACTCACAGTCTGGGGCATT
>HM070526
TTTCAGAAATCAACAGAAGATAATTCAACCAACATCATCACACTCCCATGCAAAATAAAACAAATTATAAGAA
TGTGGCAGAGGGTAGGGCAAGCAATGTATGCCCCTCCCATTGAAGGAAACATAACATGTAATTCACAAATCAC
AGGAATACTGTTGACAAGGGATGGAGGGAATAATGGGAATAATGAAGAAAGTGAGACATTCAGGCCTGGAGGA
GGAGATATGAGGGACAATTGGAGAAGTGAATTATATAAGTACAAAGTGGTAGAAGTTAAGCCATTGGGAATAG
CACCCACTGGGGCAAAAAGGAGAGTGGTGGACAGAGACAGAGAAAAAAGAGCAGTAGGAATGGGAGCTGTGTT
CCTTGGGTTCTTGGGAGCAGCAGGAAGCACTATGGGCGCGGCGTCAATAACGCTGACGGTACAGGCCAGACAA
CTGTTGTCTGGCATAGTGCAACAGCAAAGCAATTTGCTGAAGGCTATAGAGGCGCAACAGCATCTGTTGCAAC
TCACAGTCTGGGGCATT
>HQ615982
TACTATCCTAATAGTACTGATACAAACAATACAGGAAGTAACTCAAGCTTAATGATCACAATCCCATGCAGAA
TAAAACAAATTATAAACATGTGGCAGGGGGTAGGAAGAGCAATATATGCCTCTCCCGTTGCAGGAAACATAAC
GTGTGTATCAAAAATCACAGGACTCCTATTGACACGTGATGGAGGTACAGATAATAATATCACAGAGATATTC
AGACCTGGAGGAGGAGATATGAAGGACAATTGGAGGAGTGAATTATATAAATATAAAGTGGTAGAAATTAAGC
CATTAGGAATAGCACCCACTCCGGCAAAAAGGAGAGTGGTGGAGAGAGAAAAAAGAGCAGTGGGAGTAGTAGG
AGCTATGATCCTTGGGTTCTTGGGAACAGCAGGAAGCACTATGGGCGCGGCGGCCGTGACGCTGACGGTACAG
GCCAGACAACTGTTGTCTGGTATAGTGCAACAGCAAAGCAATTTGCTGAGGGCTATAGAGGCGCAACAACACA
TGTTGCAACTCACGGTCTGGGGCATT
>KC149493
AATTACACATTTAATAATATAAGTAAGAATGCAACCATCAAACTTCCATGCAGAATAAAACAAATTATTAACA
TGTGGCAGGAGGTAGGACGAGCAATGTATGCTCCTCCCATTGAAGGAAACATAACATGTAAATCAAATATCAC
AGGACTACTACTGGTACGTGATGGAGGCCCAAGTACTGAGAATGGCACAAACACAACTGCTGAGGAGATATTC
AGGCCTGGAGGAGGAAATATGAAAGACAATTGGAGAAGTGAATTATATAAATATAAAGTGGTAGAAATTAAGC

CATTGGGAATAGCACCCACTAGAGCAAAAAGGAGAGTGGTGGAGAGAGAAAAAAGAGCAGTGGGAATAGGAGC
TGTGTTCTTTGGGTTCTTGGGAGCAGCAGGAAGCACTATGGGCGCGGCGTCAATAACGCTGACGGTACAGGCC
AGACAATTGTTGTCTGGTATAGTGCAACAGCAAAGCAATTTGCTGAGGGCTATAGAGGCGCAACAGCATATGT
TGCAGCTCACGGTCTGGGGCATT
>KY112254
ACCTACAATATTACTAATAGTACTAGTGCAGATTTAAATTCAACCATCACACTCCCATGCAGAATAAAGCAAA
TTATAAACATGTGGCAGGAAGTAGGACGAGCAATGTATGCCCCTCCCATTGAAGGAAACATAACATGTAAATC
AAATATCACAGGAATACTATTGGTACGTGATGGAGGAGACAACACATCTGAGACATTCAGACCTGGAGGAGGA
GATATGAGAGATAATTGGAGAAGTGAATTATATAAATATAAAGTGGTAGAAATTAAGCCATTAGGAATAGCAC
CCACTAAGGCAAAAAGAAGAGTGGTGGAGAGGAGGGAAAAAAGAGCAGTGGGAATAGGAGCTGTGTTCCTTGG
GTTCTTGGGGGCAGCAGGAAGCACTATGGGCGCGGCGTCAATAACGCTGACGGTACAGGCCAGACAATTATTG
TCTGGTATAGTGCAACAGCAAAACAATTTGCTGAGGGCTATAGAGGCGCAACAGCATATGTTGCAACTCACAG
TCTGGGGCATT
>KY112386
ACCTATAATAGTACAGAAAGTGAGTTAGATGCAAACAGCACAAAGGAAAACATCACACTCCCATGCAGAATAA
AACAAATTATAAACATGTGGCAGGAAGTAGGAAAAGCAATGTATGCCCCTCCCATTGCAGGAAACATAACATG
TAAATCAAACATCACAGGAATACTATTGGTACGTGATGGAGGAAACACAGATAACACATCTGAGACATTCAGA
CCTGGAGGAGGAGATATGAAAGACAATTGGAGAAGTGAATTATATAAATATAAAGTGGTAGAAATTAAACCAT
TAGGACTAGCACCCACCAAGGCAAAAAGAAGAGTGGTGGAGGGGAGAGAAAAAAGAGCAGTGGGAATAGGAGC
TGTGTTCCTTGGGTTCTTGGGGGCTGCAGGAAGCACTATGGGCGCAGCGTCAATAACGCTGACGGTACAGGCC
AGACAATTATTGTCTGGTATAGTGCAACAGCAAAGCAATTTGCTGAGAGCTATAGAGGCGCAACAGCATATGT
TGCAACTCACGGTCTGGGGCATT
>HQ595814
AATCATACAGACATCATCACACTCCAATGCAGAATAAAACAATTTATAAACATGTGGCAGAAGGTAGGAAGAG
CAATGTATGCCAATCCCATTGCAGGAAACATAACATGTAGATCAAATATCACAGGACTACTATTGACACGTGA
TGGAGGTGTTAGAGGGAATACAACAAATAATGAGACTGAGACAGAGACATTCAGACCTGGAGGAGGAGATATG
AGGGATAATTGGAGAAGTGAATTGTATAAATATAAAGTGGTGGAAATTAAGCCATTGGGAATAGCACCCACTA
CGGCAAAAAGGAGAGTGGTGGAGAGAGAGAAAAGAGCAGTGGGAATAGGAGCTGTATTCCTTGGGTTCTTAGG
AGCAGCAGGAAGCACTATGGGCGCGGCGTCAATAACGCTGACGGTACAGGCCAGACAACTACTGTCTGGTATA
GTGCAACAGCAAAGCAATTTGCTGAGAGCTATAGAGGCGCAACAGCATATGTTGCAACTCACGGTCTGGGGCA
TT
>KC186976
ACCTATGGGGATAATGATACAAATGATAATGCAAATACAACTAATGCAAATAGAACTATCATACTCCAATGCA
GAATAAAACAAATTATAAACATGTGGCAAGAGGTAGGAAGGGCAATGTATGCCCCTCCCATTGCAGGAAACAT
AACATGTAAATCAAATATCACAGGACTACTATTGACATGGGATGGAGGAATTAATAATAGGACAAATGAGACA
TTCAGACCTGGAGGAGGAGATATGAGGGACAATTGGAGAAGTGAATTATATAAATATAAAGTGGTAGAAATTA
AACCATTAGGGATAGCACCCAATAAGACAAAAAGGAGAGTGGTGGAGAGAGAAAAAAGAGCAGTGGGAATAGG
AGCTGTGTTCCTTGGGTTCTTGGGAGTAGCCGGAAGCACTATGGGCGCAGCGTCAATAGCGCTGACGGGACAA
GCCAGACAATTACTGTCTGGTATAGTGCAACAGCAAAGCAATTTGCTGAGAGCTATAGAGGCGCAACAGCATA
TGTTGCAACTCACGGTCTGGGGCATT
>KC187128
AATTACACGGCTAATAGTACAGAAAGTACTTCAAATTCAACCAACATCACAATCCAATGTAGAATAAAACAGA
TTATAAACCTGTGGCAGGGGGTAGGACGAGCAATGTATGCCCCTCCCATTGCAGGAAATATAACATGTAACTC
AAACATCACAGGACTACTATTGACACGTGATGGAGGAAAAGAAGGAGAAAATAATAATACAGAGATATTCAGG
CCTGCAGGAGGAAATATGAAGGACAATTGGAGAAGTGAATTATATAAATATAAAGTGGTAGAAATTAAGCCAT
TAGGAATAGCACCCACTAGTGCATCAAGGAGAGTGGTGCAGAGAGAAAAAAGAGCAGTGGGACTGGGAGCCAT
GTTCCTTGGGTTCTTGGGAGCAGCAGGAAGCACTATGGGCGCAGCATCAATAACGCTGACGGTACAGGCCAGA
CAATTATTGTCTGGTATAGTGCAACAGCAAAGCAATTTGCTGAGGGCCATAGAGGCGCAACAGCATATGTTGC
AACTCACGGTCTGGGGCATA
>KC187665
TACTATCCTAATGGTACAAGAAGTAATTCTTCAAACTTAAACCCAAACATCACGCTTCCATGCAGAATAAAAC
AAATTATAAACAGGTGGCAGGAGGTAGGAAGAGCAATGTATGCTCCTCCCATTGCAGGAAACATAACATGTAA
CTCAAGTATCACAGGATTATTATTGACACGTGATGGAGGAAAAAATAATAATGGAACAAATGATACAGAGACA
TTCAGACCTGGAGGAGGAAATATGAGGGACAATTGGAGAAGTGAATTATATAAATATAAAGTGGTAGAAATCA
GGCCATTAGGAATAGCACCCACTGGTGCAAAAAGGAGAGTGGTGGAGAGAGAGAAAAGAGCAGTGGGAATAGG
AGCTGTGTTCCTTGGGTTCTTGGGGACAGCAGGAAGCACTATGGGCGCAGCGGCAGTGACGCTGACGGTACAG
GTCAGACAACTATTGTCTGGTATAGTGCAACAGCAAAGCAATTTGCTGAGAGCTATAGAGGCGCAACAGCATA
TGTTGCAACTCACAGTCTGGGGCATT
>KC186377

AACTACACCAACACCACACTCACACTCCAATGCAGAATAAGACAAATTGTAAACATGTGGCAAGGGGTAGGAC
GAGCAATGTATGCCCCTCCCATTGCAGGAAACATAACATGTAAATCAAATATCACAGGACTACTATTGACACG
TGATGGAGGAAAAAATAATGGTACTAATAACACAGAGATATTCAGACCTGGAGGAGGAGATATGAGGAACAAT
TGGAGAAGTGAATTATATAAATATAAAGTGGTAGAAATTAAGCCATTGGGAATAGCACCCACTAAAGCAAAAA
GGAGAGTGGTGGAGAGAGAAAAAAGAGCAGTGGGAATAGGAGCTGTGTTCCTTGGGTTCTTGGGAGCAGCAGG
AAGCACTATGGGCGCGGCGTCAATAACGCTGACGGTACAAGCCAGACAACTATTGTCTGGTATAGTGCAACAG
CAAAGCAATTTGCTGAGGGCTATAGAGGCGCAACAGCATATGTTGCAACTCACGGTCTGGGGCATT
>KC186388
AATTATACAGATAATTCAACCATCACACTCCAATGCAGAATAAAGCAAATTATAAACATGTGGCAGGAGGTAG
GAAAAGCAATGTATGCCCCTCCCATTGCAGGAAACATAACATGTGTATCAAGTATTACAGGAATACTATTGAC
ACGTGATGGAGGAGATATAAATAGTAGCATAGAGACATTCAGGCCTGGAGGAGGAGATATGAGAGACAATTGG
AGAAGTGAATTATATAAATATAAAGTGGTAGAAGTTAAGCCATTGGGAATAGCACCCACGGGAGCAAAAAGGC
GAGTGGTGGAAAGAGAGAGAAGAGCAGTGGGAATAGGAGCTGTGTTCCTTGGAATCTTGGGAGCGGCAGGAAG
CACTATGGGCGCGGCGTCAATAACGCTGACGGTACAGGCCAGACAATTGTTGTCTGGTATAGTGCAACAGCAA
AGTAATTTGCTGAGAGCTATAGAGGCGCAACAGCATATGTTGCAACTCACAGTCTGGGGCATT
>KP109525
TTTCATTCCAATGATACATTAACAGGAGGTAACTTAACCGGGAACATCACACTCCCATGTAGAATAAAACAAA
TTATAAACATGTGGCAGGAGGTAGGAAGAGCAATGTATGCCCCTCCTATTGCAGGAAACATAACATGTAAATC
AAATATCACAGGACTACTCTTGACACGTGATGGAGGAAATGGAAATGGCAGCAATAACGAGACATTTAGGCCT
GGAGGAGGAGACATGAGGGACAATTGGAGAAGTGAATTATACAAATACAAAGTGGTAGAAATTAAACCATTAG
GAATAGCACCCACTAAGGCAAAAAGGAGAGTGGTGGAGAGAGAAAAAAGAGCAGTGGGTCTAGGAGCTATGTT
CCTTGGGTTCTTGGGAGCGGCAGGAAGCACTATGGGCGCGGCGTCACTAACGCTGACGGTACAGGCCAGACAA
TTATTGTCTGGTATAGTGCAACAGCAAAGCAATTTGCTGAGGGCTATAGAGGCGCAACAGCATTTGTTGCAAC
TCACAGTCTGGGGCATT
>KC186502
AATTATACATTTAACAAGACAGAAGGTAACATCACACTCCCATGCAAAATAAGACAAATTATTAACCTGTGGC
AGGAGGTAGGACGAGCAATGTATGCCCCTCCCATTGAAGGAAACATAACATGTAGATCAGACATCACAGGATT
ACTATTGACACGTGATGGAGGAGAAAATAGTACAAGGAATGAGACATTCAGACCTGGAGGAGGAGATATGAGG
GACAATTGGAGAAGTGAATTATATAAATATAAAGTAGTAGAAATTAAGCCATTAGGAATAGCACCCACTGAGG
CAAGAAGGAGAGTGGTGGAGAGAGAAAAAAGAGCAATTGGAATAGGAGCTGTGTTCCTTGGGTTCTTGGGAGC
AGCAGGAAGCACTATGGGCGCAGCATCAATAACGCTGACGGTACAGGCCAGACAATTATTGTCTGGTATAGTG
CAACAGCAAAGCAATTTGCTGAGGGCTATAGAGGCGCAACAGCATATGTTGCAACTCACAGTCTGGGGCATT
>MF500628
AATCATACAAACAGTAATAGTACAAGTAATAGTACAAGTAATCCAAATGCAACCATCACCATCCAATGCAGAA
TAAAACAAATTATAAACATGTGGCAGGGGGTAGGACAAGCAATATATGCCCCTCCCATTAAAGGAAACATTAC
ATGTAGATCAAATATCACAGGACTACTATTGACACGTGATGGAGGTAATAATAATACTAATGAGACATTCAGA
CCTGGAGGAGGAGATATGAGAGACAATTGGAGAAGTGAATTATATAAGTATAAAGTAGTAGAAATTAAGCCAC
TAGGAGTAGCACCCACCAAGGCGAAAAGACGAGTGGTGGAGAGAGAAAAAAGAGCAGTGGGAATAGGAGCTGT
ACTCCTTGGGTTCTTGGGCGCAGCAGGAAGCACTATGGGCGCGGCGTCGCTGACGCTGACGGTACAAGCCAGA
CAATTATTGTCTGGTATAGTGCAACAGCAAAACAATTTGCTGAGGGCTATAGAGGCGCAACAGCACATGTTGC
AACTCACAGTCTGGGGCATT
>KC863183
TGCTATACATCAACACTGTTTAATAGTACATACAATGCTGAGTATAATTCAAACTCAACCAATTCAAGCTCAA
CCATCACACTCCAATGCAGAATAAAGCAAATTATAAACATGTGGCAGGGGGTAGGACGAGCAATATATGCCCC
TCCCATTGAAGGAAACATAACATGTGAGTCAAATATCACAGGACTATTATTGACACGGGATGGAGGAAAAGAT
GGAAATAACACCGAGATATTCAGACCTGGAGGAGGAGATATGAGGGACAATTGGAGAAGTGAATTATACAAAT
ATAAAGTGGTAGAAATTAAGCCATTAGGAATAGCACCCACTAAGGCCAAAAGGAGAGTGGTGGAGAGAGAAAA
AAGAGCAGTAGGAATAGGAGCTGTGTTCCTTGGGTTCTTGGGAGCAGCAGGAAGCACTATGGGCGCAGCGTCA
ATAACGCTGACGGTACAGGCCAGACAATTGTTGTCTGGTATAGTGCAACAGCAAAGCAATTTGCTGAGAGCTA
TAGAGGCGCAACAGCATATGTTGCAACTCACGGTCTGGGGCATT
>KJ158421
AATTATACAAGAAATAGTACAGGAAATGATTCAAAAGTAGTCATTACGATCCCATGCAGAATAAAACAAATTA
TAAACATGTGGCAGGGAGTAGGGCGAGCAATGTATGCCCCTCCCATTGCAGGAAACATAACTTGTAAATCCAA
TATCACAGGACTACTATTGGTACCTGATGTAGGAGAAGATGGAGGGAACACATTAAATACTACAGAAGAGACA
TTCAGACCTGTAGGAGGAAATATGAGGGACAATTGGAGAAGTGAATTATACAAATATAAAGTGGTAGAAATTA
AGCCATTGGGGGTAGCACCCACTGAAGCAAGAAGGAGAGTGGTGGAAAGAGAAAAAAGAGCAGTGGGAGTAGG
AGCTATGTTCCTTGGGTTCTTGGGAGCAGCAGGAAGCACTATGGGCGCGGCGTCACTAATGCTGACGGTACAG
GCCAGACAATTGTTGTCTGGTATAGTGCAACAGCAAAGCAATTTGCTGAGAGCTATAGAGGCGCAACAGCATC
TGTTGCAACTCACAGTCTGGGGCATT
>KJ158424

TGCTATACATCAAGACTGTTTAATGGAACATATAAGGATACTGATACCCTAAATAATACAAGCAGTAACGCAA
TATCAAACATCACAATCCCATGCAGAATAAAACAGTGTGTGAACATGTGGCAACAGGTAGGACAGGGGGGGGA
TGCCCCTCCCATTGAAGGAGCACGTGAAGCTGCTATCAAATACAAAGGACTACGATTGGTACGTGATGGAGGA
GAAGACAAAAATAATACAGACAGCAATGAGACAGAGACATTCAGACCTGGGGGAGGAGATATGAGGGACAATT
GGAGAAGTGAATTATATAAATATAAAGTGGTGGAAATTAAGCCATTGGGAGTAGCACCCACTGCAGCAAGAAG
GAGAGTGGTGGAAAGAGAAAAAAGAGCAGTGGGAATAGGAGCTGTGTTCCTTGGGTTCTTGGGAGCAGCAGGA
AGCACTATGGGCGCGGCATCAATAACGCTGACGGTACAGGCCAGACAATTGTTGTCTGGTATAGTGCAACAGC
AAAACAATTTGCTGAGGGCTATAGAGGCGCAACAGCATCTGTTGCAACTCACGGTCTGGGGCATT
>KJ158434
AATTATACAAGAAATAGTACAGGAAATGATTCAAACGTAGTCATTACGATCCCATGCAGAATAAAACAAATTA
TAAACATGTGGCAGGGAGTAGGGCGAGCAATGTATGCCCCTCCCATTGCAGGAGACATAACATGTATCTCTAA
GATCACAGGATTACTATTGGTACGTGATGGGGGAGAAAACATACAAAGAAATAATGATGCAGGGACAGAGACA
TTCAGACCTGGAGGAGGAGATATGAGGAACAATTGGAGAAGTGAATTATATAAATATAAAGTGGTAGAAATTA
AGCCATTGGGGGTAGCACCCACTAAAGCAAAAAGGAGAGTGGTGGAGAGAGAAAAAAGAGCAATGGGAATAGG
AGCTTTGTTCCTTGGGTTCTTGGGAACAGCAGGAAGCACTATGGGCGCGGCGTCAATAACGCTGACGGTACAG
GCCAGACAATTGTTGTCTGGTATAGTGCAACAGCAAAATAATTTGCTGAAGGCTATAGAGGCGCAACAGCATC
TGTTGCAACTCACGGTCTGGGGCATT
>KJ158438
GTTTATAATGGTACAGAAAGTAATAGTAACAGTAATAGTAATGCAACCATCACCCTCCCATGCAAAATAAAAC
AAATTATAAACATGTGGCAGGAGGTAGGACGGGCAATGTATGCCCCTCCTATTGAAGGAAACATAACATGTAA
CTCAAATATCACAGGATTACTCTTGGTACGTGATGGAGGCAATGAAAATAATAACAATAATAATAACTTCGCA
GAGACATTCAGACCAGGAGGAGGAGATATGAGGGACAATTGGAGAAGTGAATTATATAAATATAAAGTAGTAG
AAATTAAGCCATTGGGAGTAGCACCCACTGCAGCAAAAAGGAGAGTGGTGGAGAGAGAAAAAAGAGCGCAGCG
GCTAGGAGCTGTGTTCCTTGGGTTCTTGGGAGCAGCAGGAAGCACTATGGGCGCGGCGTCAATAACGCTGACG
GTACAAGCTAGACAATTGTTGTCTGGTATAGTGCAACAGCAAAGCAATTTGCTGAGGGCTATCGAGGCGCAAC
AGCATCTGTTGCAACTCACAGTCTGGGGCATT
>KJ158439
ACCTACATTAATGAAACAAATAGTAATGCTACATCCACCACCCCACCCTGCAGAATAAAACAAATTATAAACA
TGTGGCAAGAGGTAGGACGAGCAATGTATGCCCCTCCCATTGCAGGAAATATAACATGTAAATCAAATATCAC
AGGAATACTATTGGTACGGGATGGAGGAAACAATGATAATGATGATAATGATAATGATACAGAGACATTCAGA
CCTGGAGGAGGAGACATGAAGGACAATTGGAGAAGTGAATTATATAAATATAAAGTGGTAGAAATTAAGCCAT
TGGGATTAGCACCCACTACAGCAAAAAGGAGAGTGGTGGAGAGAGAAAAAAGAGCAGTAGGAACCTTAGGAGC
TGTGTTCCTTGGGTTCTTGTCAGCAGCCGGAAGCACTATGGGCGCGGCGTCAATAACGCTGACGGTACAGGCC
AGACAATTGTTGTCTGGTATAGTGCAACAGCAAAGCAATTTGCTGAGGGCTATAGAGGCGCAACAGCATCTGT
TGCAACTCACGGTCTGGGGCATT
>KJ541846
TTTTATGATAGTACGAGAAAGGCAAACATCACAGGAAATTCAACCATCACACTCCCATGCAGAATAAAGCAAA
TTATAAACATGTGGCAGGAGGTAGGACGAGCAATGTATGCTCCTCCCATTGCAGGAAACATAACATGTAGATC
AAATATCACAGGACTACTATTGGTACGTGATGGAGGAGAAGATAACACCAAATCAGAGACCTGGAAGACTGAA
ACATTTAGACCGGGAGGAGGAGATATGAGGGACAATTGGAGAAGTGAATTATATAAATATAAAGTGGTAGAAG
TTGAGCCAGTGGGAATAGCACCCACTAAAGCAAAAAGGAGAGTGGTGGAGAGAGAAAAAAGAGCAGTAGGAAT
GGGAGCTTTGTTCCTTGGGTTCTTGGGAGCAGCAGGAAGCACTATGGGCGCGGCGTCAATAACGCTGACGGTA
CAGGCCAGACAATTGCTGTCTGGTATAGTGCAACAGCAAAGCAATTTGCTGAGAGCTATAGAGGCGCAACAGC
ATCTGTTGCAACTCACAGTCTGGGGCATT
>KP411831
TCTTATAATAGTACCTCTAATACTACTTGCTCGACTAATAATACTACAACTTCAACCTTTACCCTCCAATGCA
GAAGAATACATATTATAATCATGTGCCTTGAACCAGGACTAGCAATAGATGCCTCTCCCACTGCCGGAGAAAT
AATCAGTAAATCCAATACCACTGACCTACTATTGACACGTGCAGGAGGATCGAATAGAACAGAGATATTCAGA
CCTGGAGGAGGAGATATGAGGGATAATTGGAGAAGTGAGTTATATAAATATAAAGTGGTAGAAATTAAACCAT
TAGGGATAGCACCCACTAAGGCAAAAAGGAGAGTGGTGGAGAGAGAAAAAAGAGCAGTGGGAATAGGAGCTGT
GTTCCTTGGGTTCTTGGGAGTAGCAGGAAGCACTATGGGCGCAGCATCAATAACGCTGACGGTACAGGCCAGG
CAATTGTTGTCTGGTATAGTGCAACAGCAAAGCAATTTGCTGAGGGCTATAGAGGCGCAACAGCATATGTTGC
AACTCACGGTCTGGGGCATT
>MF373158
ACCTACATGTCTAACAGTACTAACAGTATTAATAGTACTGAATCCATCACACTCAAATGCAAAATAAAACAAA
TTATAAACATGTGGCAAGGGGTAGGACGAGCAATGTATGCCCCTCCCGTTGAAGGAAACATAACATGTACCTC
AAATATTACAGGACTACTATTGACACGTGATGGAGGAGGAAATGGCACAGAGGAGATATTCAGACCGGGAGGA
GGAAATATGAGGGACAATTGGAGAAGTGAATTATATAAATATAAAGTGGTAGAAGTTAAACCATTAGGAGTAG
CACCCACTAAAGCAAAAAGGAGAGTGGTGGAAAGAGAAAAAAGAACAGTACTAGGAGCTGCATTCCTTGGGTT
CTTGGGAACAGCAGGAAGCACTATGGGCGCAGCGTCACTAACGCTGACGGTACAGGCCAGAAAATTGTTGTCT

GGTATAGTGCAACAGCAAAGTAATTTGCTGAGGGCTATAGAGGCGCAACAGCATATGTTGCAACTCACGGTCT
GGGGCATT
>MF373164
AACTACACAATAATCCCATGTAGAATAAAACAAATTATAAATATGTGGCAGGCGGTAGGACGAGCAATGTATG
CCCCTCCCATTGCAGGAAACATAACGTGTGCATCAAACATCACAGGACTACTATTGTCACGTGATGGAGGAAA
CACAAATACAAATGGCACAAGTAACACAGAGACTTTCAGACCTGGAGGAGGAGATATGAGAGACAATTGGAGA
AGTGAATTATATAAATATAAGGTGGTAGAAATTAAACCATTAGGAATAGCACCCACTGGGGCAAAAAGGAGAG
TGGTGGAGAGAGAAAAAAGAGCAGTGGGAATAGGAGCTGTGTTCCTTGGGTTCTTGGGAGCAGCAGGAAGCAC
TATGGGCGCGGCGTCAGTGACGCTGACGGTACAGGCCAGACAGCTGTTGTCTGGTATAGTGCAACAGCAAAAC
AACTTGCTGAAAGCTATAGAGGCGCAACAGCATATGTTGCAACTCACGGTCTGGGGCATT
>MF373197
AGTTATTCCGCCAATGGCAGCAATGGAAGCATTCCCCTCATATGCCGAATTATACAAATTTTTAACATGTGGC
AGCGGGTGGTACGAGCAATATATGCTGCTCGTATTGTAGGAAGTATAACATGTATCTCATGTATCACAGGACT
ACTGTTGACACGTGCTGGAGGAACAAATAATGATACCACAAGAGAAATATTCAGACCTGGAGGAGGAAATATG
AAGGACAATTGGAGAAGTGAATTATACAAATATAAAGTGGTAGAAGTTAAACCATTAGGAATAGCACCTACTG
AGGCAAAAAGGAGAGTGGTGGGGAGAGACAAAAGAGCAGTGGGAATAGGAGCTGTGTTCCTTGGGTTCTTGGG
AGCAGCAGGAAGCACTATGGGCGCGGCGTCAATAACGCTGACGGTACAGGCCAGACAATTACTATCTGGTATA
GTGCAGCAGCAAAGCAATTTGCTGAGAGCTATAGAGGCGCAACAGCATATGTTGCAACTCACAGTCTGGGGCA
TT
>AY734556
AGCTATACAGGAAAAGATTCAAACTCAATCATCAGAATAAGACAATTTATAAACACATGGCAGAAGGTAGGAC
GAGCAATGTATGCCCCTCCCATAGCAGGAAACATAACATGTAACTCAAGCATTACAGGACTACTATTGACATG
GGATGGAGGCAGTAGTAAAGAAACAAGCAACACCCACAGAGACATTCAGACCTTTAGGAGGAGACATGAGGGAT
AATTGGAGACGTGAATTATATAAATATAAAGTGGTAGAAATTAAGCCATTGGGAATAGCACCCACTGGGGCAA
AAAGGAGAGTGGTGGAGAGAGAAAAAAGAGCAGTGGGACTAGGAGCTATGTTCCTTGGGTTCTTGGGAGCAGC
AGGAAGCACTATGGGCGCAGCGTCGATAACGCTGACGGCACAGGCCAGACAAGTGCTGTCTGGTATAGTGCAA
CAGCAAAGCAATTTGCTGAGGGCCATAGAGGCGCAACAGCATCTGTTGCAACTCACGGTCTGGGGCATT
>KX907339
AATTATACTATAAATTCAACCATCACACTCCCATGCAGAATAAAACAAATTATAAATATGTGGCAGGGGGTAG
GACGAGCAATGTATGCCCCTCCCATTGCTGGAAACATAACATGCAGATCAAATATCACAGGAATACTATTGAC
ACGTGATGGTGGTGAAACAAATGATGGTAATACAAATGAAACAGGGACAAAGACATTCAAATCTAATGAAACA
GAGACAGAGATATTCAGACCTGGAGGAGGAGATATGAGGGACAATTGGAGAAGTGAATTATATAAATATAAAG
TGGTAGAAATTAAGCCATTAGGAATAGCACCCACCAAGGCAAAAAGGAGAGTGGTGGAGAGAGAGAAAAGAGC
AGTGGGAATAGGAGCTATGTTCCTTGGGATCTTGGGAGCAGCAGGAAGCACTATGGGCGCGGCGTCAATAACG
CTGACGGTACAGGCCAGACAATTATTGTCTGGTATAGTGCAACAGCAAAGCAATTTGCTGAGAGCTATAGAGG
CGCAACAGCATATGTTGCAACTCACGGTCTGGGGCATT
>KX907433
TACTATCCTAATGGTACAAATTCAACCCTCATCATCCCATGTAGAATTAAACAAATTATAAACATGTGGCAGG
GAGTAGGAAAAGCAATATATGCCTCTCCCATTGCAGGAAACATAACATGTAGATCAAATATCACAGGACTACT
ATTGACACGTGATGGAGGAGACACAAATGATACAGAGATATTCAGACCGGCAGGAGGAGATATGAGGGATAAT
TGGAGAAGTGAATTATATAAATATAAAATAGTAGAAATTAAACCATTAGGAGTAGCACCCACTGAGGCAAAAA
GGAGAGTGGTGAAGAGAGAAAAAAGAGCAGTGACAATAGGAGCTGTATTCCTTGGGTTCTTGGGAGCAGCAGG
AAGCACTATGGGCGCGGCGTCAATAACGCTGACGGTACAGGCCAGACAATTACTGTCTGGTATAGTGCAGCAG
CAAAGTAATTTGCTGAGAGCTATAGAGGCGCAACAGCATATGTTGCAACTCACAGTCTGGGGCATT
>KX907382
ATTCAAAATGGAACCACCATACTCCCATGCAGAATAAAACAAATTATAAACATGTGGCAGGAGGTAGGACGAG
CAATGTATGCCCCTCCCATTGCAGGAAACATAACATGTACATCAAACATCACAGGACTACTATTGACACGAGA
TGGAGGCAAAAATAACACAGGAGGCAAAAATAACACAAATGGCATAGAGATATTCAGACCTGAAGGAGGAAAT
ATGAGGGATAATTGGAGAAGTGAATTATATAAATACAAGGTGGTAGAAATTAAGCCATTGGGAATAGCACCCA
CTGAGGCAAAAAGGAGAGTGGTGGAGAGAGAGAAAAGAGCAGTGGGAATAGGAGCTGTGTTCCTTGGGTTCTT
GGGAGCAGCAGGAAGCACTATGGGCGCGGCGTCAATAACGCTGACGGTACAGGCCAGACAACTGTTGTCTGGT
ATAGTGCAACAGCAAAGCAATTTGCTAAGAGCTATAGAGGCGCAACAGCATCTGTTGCAACTCACGGTTTGGG
GCATT
>KX907385
AACTATAATGGTACATCAAATGACACCATCACACTCCGGTGTAGAATAAAACAAATTATAAACATGTGGCAGG
GGGTAGGACAAGCAATGTATGCCCCTCCCATTGCAGGAAACATAATATGTGCATCAAATATCACAGGACTACT
ATTGACACGTGATGGAGGAGACGAAAATAGGACAGATGAGATATTCAGGCCGGAAGGAGGAAATATGAGAGAT
AATTGGAGAAGTGAATTATATAAATATAAAGTGGTAGAAATTAAGCCATTAGGGATAGCACCCACTAAGGCAA
AAAGGAGAGTGGTGGAGAGAGAAAAAAGAGCAGTAGGAATAGGAGCTGTGTTCCTTGGGTTCTTAGGAGCAGC

AGGAAGCACTATGGGCGCAGCATCAATAACGCTGACGGTACAGGCCAGACAATTATTGTCTGGTATAGTGCAA
CAGCAAAGCAATTTGCTGAGGGCTATAGAGGCGCAACAGCATCTGTTGCAACTCACAGTCTGGGGCATT
>KX907368
AACTATACAGAGTTTAAAAATATCTCAACAAACGGTACTTCGAATTCAAACATCACACTCCCATGCAGAATAA
AACAAATTATAAACATGTGGCAAGGGGTAGGACGAGCAATGTATGCCCCTCCCAGGGAAGGAAACATAACATG
TGAATCGAACATCACAGGACTACTATTGACACGAGATGGAGGAAATAGTACAAAAAAGGAGATATTCAGACCT
GGAGGAGGAGATATGAGGGATAATTGGAGAAGTGAATTATATAAGTATAAAGTGGTAGAAATTAAGCCATTAG
GAATAGCACCCACTGAGGCAAAAAGGCGAGTGGTGGAGAGAGAGAAAAGAGCAGTGGGAATAGGAGCTGTGTT
CCTTGGGTTCTTAGGAGCAGCAGGAAGCACTATGGGCGCAGCGTCAATAACGCTGACGGTACAGGCCAGACAA
TTATTGTCTGGTATAGTGCAACAGCAAAGCAATTTGCTGAGGGCTATAGAGGCGCAACAGCATATGTTGCAAC
TCACAGTCTGGGGCATT
>KT982201
ACTCATGATACAGGAAGTAATTCAACCTCAAACATCACCCTCCAATGCAAAATAAAACAAATTATCAACATGT
GGCAGGAGGTAGGACGAGCAATGTATGCCCCTCCCATTGCAGGAAACATAACATGTAGATCAAATATCACAGG
ACTACTATTGGTACGGGATGGAGGCACAGATAATAGCACAAATAAGAGTGACCCAGAGGAGACATTTAGACCT
GGAGGAGGAGATATGAGGGACAATTGGAGAAGTGAATTATATAAATATAAAGTGGTAGAAATTAAGCCATTAG
GGATAGCACCAACTGAGGCAAGGAGGAGAGTGGTGCAGAGAGAAAAAAGAGCAGTGGGAATAGGAGCTCTGTT
CCTTGGGTTCTTGGGAGCTGCAGGAAGCACTATGGGCGCGGCGTCAATAACGCTGACGGCACAGGCCAGACAA
GTATTGTCTGGTATAGTGCAACAGCAAAGCAATTTGCTGAGAGCTATAGAGGCGCAACAGCATATGTTGCAAC
TCACAGTCTGGGGCATT
>AY585266
GATTACAACACAACACTAACACTCCCATGCAGGATAAACCAGATTATAAACATGTGGCAGGAGGTAGGACGAG
CAATGTATGCTCCTCCCATTGCAGGAAACATAACATGTAACTCAAATATCACAGGACTACTATTGACACGTGA
TGGAGGAAACAATGGAGGAAACAATGAAACATTCAGACCTGGAGGAGGAAATATGAAAGACAATTGGAGAAGT
GAATTATACAAATATAAAGTGGTAGAGATTAAGCCATTGGGAATAGCACCCAATAAGGCAAAAAGGAGAGTGG
TGGAGAGAGAAAAAAGAGCAGTGGGAATAGGAGCTGTGTTCCTTGGGTTCTTGGGAGCAGCAGGAAGCACTAT
GGGCGCGGCGTCAATAACGCTGACGGCACAGGCCAGACAACTGTTGTCTGGTATAGTGCAACAGCAAAGTAAT
TTGCTGAGAGCTATAGAGGCGCAACAGCATTTGTTGCAACTCACAGTCTGGGGCATT
>AY463224
GTTCAAAGCAACGGAAGCATCATACTCCCATGCAAGATAAAACAAATTATAAACATGTGGCAGGGGGTAGGAC
GAGCAATGTATGCCCCTCCCATCGCAGGAAACATAACATGTAACTCAAGTATCACAGGACTACTATTAACGAG
GGATGGAGGCATAAGTGAGAACATGACAGAGACATTCAGACCTGGAGGGGGAAATATGAAGGACAATTGGAGA
AGTGAATTATATAAATATAAAGTAGTAGAAATTAAGCCATTGGGAATAGCACCCACTAAGGCAAAAAGGAGAG
TGGTGGAGAGAGAAAAAAGAGCAGCAATAGGAGCTATGTTCCTTGGGTTCTTGGGAGCAGCAGGAAGCACTAT
GGGCGTGGCGTCAATAACGCTGACGGTACAGGCCAGAAATCTGTTGTCTGGTATAGTGCAACAGCAAAGCAAT
TTGCTGAGAGCTGTAGAAGCGCAACAACATATGTTGCAACTCACGGTCTGGGGCATT
>JX845593
GATCAAAATGATTCAACAAACATCACACTCCCATGCAGAATAAAACAAATTATAAACATGTGGCAGGAGGTGG
GACGAGCAATGTATGCCCCTCCCATTGCAGGAAACATAACATGTACATCAAATATTACAGGACTATTATTGAC
ACGTGATGGAGGAAACGCAAACACAACGGATGGTAAGAATGACACAGAGACATTCAGACCTGGAGGAGGAAAT
ATGAAGGACAATTGGAGAAGTGAATTATATAAATATAAAGTGGTAGAAATTAAACCATTGGGAGTAGCACCCA
CAAAGGCAAAAAGGAGAGTGGTGGAGAGAGAAAAAAGAGCAGTGGGAATGGGAGCTGTGTTCCTTGGGCTCTT
GGGAGCAGCAGGAAGCACTATGGGCGCGGCGTCAATAACGCTGACGGTACAGGCCAGACAACTGCTGTCTGGT
ATAGTGCAACAGCAAAGCAATTTGCTGAGAGCTATAGAGGCGCAACAGCATATGTTGCAACTCACAGTCTGGG
GCATT
>JX845600
AATCACACTCTCAATCTCACTCTCCCATGCAGAATAAAACAAATTATAAACATGTGGCAGGAGGTAGGAAGAG
CAATGTATGCCCCTCCCATTGCAGGAAACATAACATGTAAATCAAATATCACAGGACTACTATTGACACGTGA
TGGAGGAATAAATATTACAACAGAAATATTCAGACCTGCAGGAGGAAATATGAAGGACAATTGGAGAAGTGAA
TTATATAAATATAAAGTGGTAGAAATTAAGCCATTAGGAATAGCACCTACTAAGGCAAAAAGGAGAGTGGTGG
ACAAAGAAAAAAGAGCAGTAGGAATAGGAGCTGTGTTCCTTGGGTTCTTGGGAGCAGCAGGAAGCACTATGGG
CGCGGCGTCAATAACGCTGACGGTACAGGCCAGACAACTGTTGTTTGGTATAGTGCAACAGCAAAACAATTTG
CTGAGAGCTATAGAGGCGCAACAGCATATGTTGCAACTCACGGTCTGGGGCATT
>AY228557
ACTTATAATAATACAGATAATACAGATAATCCAACCCAAATCACACTCCCATGCAGAATAAAACAAATTATCA
ACATGTGGCAGGAGGTAGGACGAGCAATATATGCCCCTCCCATTGCAGGAAACATAACATGTAAATCGAATAT
CACAGGACTACTATTAACATGGGATGGAGGTAGTGGTGAAAACAATACAGAGACATTTAGACCAGGAGGAGGA
GATATGAGGGACAATTGGAGAAGTGAATTATATAAATATAAAGTGGTAGAAATTAAGCCATTAGGAATAGCAC
CCACTGAGGCAAAAAGGAGAGTGGTGGAGAGAGAAAAAAGAGCAATAGGAATAGGAGCTGTGTTCCTTGGGTT
CTTGGGAGCAGCAGGAAGCACTATGGGCGCAGCGTCAATAACGCTGACGGTACAGGCCAGACAATTGTTGTCT

GGTATAGTGCAACAGCAAAGCAATTTGCTGAGGGCTATAGAGGCGCAACAGCATCTGTTGCAGCTCACTGTCT
GGGGCATT
>DQ275647
CTTCAAAATAATAGTACAGGAAATTCAACCATCAAAATCAAATGCAGAATAAAACAAATTATAAACATGTGGC
AGGAGGTAGGACGAGCAATGTATGCCCCTCCCATTGCAGGAAACATAACATGTAGATCAAACATCACAGGACT
ACTATTAGTACGTGATGGAGGCATAAACAACACAGAAGAAATATTCAGACCTGGAGGAGGAAATATGAAGGAC
AATTGGAGAAGTGAATTATATAAATATAAAGTAGTAGAAATTAAGCCATTGGGAATAGCACCCACTCCAGCAA
AAAGGAGAGTGGTGGAGAGAGAAAAAAGAGCAGTGGGAATAGGAGCTTTGTTCCTTGGGTTCTTGGGAGCAGC
AGGAAGCACTATGGGCGCAGCGTCAATGACGCTGACGGTACAGGCCAGACAAGTGTTGTCTGGTATAGTGCAA
CAGCAAAATAATTTGCTGAAGGCTATAGAGGCGCAACAGCGTATGTTGCAACTCACAGTCTGGGGCATT
>DQ351224
TCTTATAATGCTACAGGAGATAATACAACCATCACACTGCCATGCAAAATAAAACAAATTATAAACATGTGGC
AGACGGTAGGAAAAGCGATGTATGCCCCTCCCATTGCAGGAAACATAACATGTAAATCAAATATCACAGGACT
ACTATTAACACGTGATGGAGGCGAAACTAACAGTACGAATGACACAGAGACAGAGATATTCAGACCTGGAGGA
GGAAATATGAAGGACAATTGGAGAAGTGAATTATATAAATACAAAGTGGTAGAAATTAAGCCATTGGGAATAG
CACCCACTGAAGCAAAAAGGCGAGTGGTGGAGAGAGAAAAAAGAGCAGTGGGAATAGGAGCTGTGCTCCTTGG
GTTCTTGGGAGCAGCAGGAAGCACTATGGGCGCAGCGTCAATAACGCTGACGGTACAGGCCAGACAACTGTTG
TCTGGTATAGTGCAACAGCAAAGTAATTTGCTGAGAGCTATAGAGGCGCAACAGCATCTGTTGCAACTCACGG
TCTGGGGCATT
>DQ369977
AGTTATAATAATAGTAAGGATAATGAGACCATCACACTCCAATGCAGAATAAAACAAATTATAAACATGTGGC
AGAAAGTAGGACGAGCAATGTATGCACCTCCCATTGAAGGAAACATAACATGTAACTCAAATATCACAGGACT
ACTATTGCTACGTGATGGAGGAAAAGAGAACAATTCCACAGAAATATTCAGACCTGGAGGAGGAAATATGAAG
GACAATTGGAGAAGTGAATTATACAAATATAAAGTGGTAGAAATTAAGCCATTGGGAATAGCACCCACGGAGG
CAAAAAGGAGAGTGGTGGAGAGAGAAAAAAGAGCAGTAGGAATAGGAGCTGTGTTCCTTGGGTTCTTGGGAGC
AGCAGGAAGCACTATGGGCGCGGCGTCAATAACGCTGACGGTACAGGCCAGACAACTGTTGTCTGGTATAGTG
CAACAGCAAAGCAATTTGCTGAGAGCTATAGAGGCGCAACAACATATGTTGCAACTCACGGTCTGGGGCATT
>DQ275649
ACTCAGAGTACAAACAGTACAAATTCAACCATCACACTCCAATGCAGAATAAAACAAATTATAAACATGTGGC
AGGAGGTGGGACGAGCAATGTATGCCCCTCCCATTGCAGGAAACATAACATGTAAATCAAATATCACAGGACT
ACTATTGACACGTGATGGAGGAGGAAGAGAAGGAAATAATACAGAAGGAAATAATACAGAGACATTCAGACCT
GGAGGAGGAGATATGAGGGACAATTGGAGAAGTGAATTATATAAATATAAAGTGGTAGAAATTAAGCCATTGG
GAATAGCACCCACTGAGGCAAGAAGGAGAGTGGTGCAGAGAGAGAAAAGAGCAATAATGGGACTAGGAGCTGC
GTTCCTTGGGTTCTTGGGAGCAGCAGGAAGCACGATGGGCGCAGCGTCAATAACGCTGACGGTACAGGCCAGA
CAATTATTGTCTGGTATAGTGCAACAGCAAAGCAATTTGCTGAAGGCTATAGAGGCGCAACAGCATATGTTGC
AACTCACAGTCTGGGGCATT
>DQ396377
ATTCATAATGGGACAGGAAGTAATTCAACCATCACACTTAATTGTAAAATAAAGCAAATTATAAACATGTGGC
AGGAGGTAGGACGAGCAATGTACGCTCCTCCCATTGCAGGAAGCATAACATGTAACTCAAGTATCACAGGACT
ACTATTGACACGTGATGGAGGACAAGGCCAGGAAAAGGATAATGACACAGAGGTATTCAGGCCTGGAGGAGGA
AACATGAAGGACAATTGGAGAAGTGAATTATATAAATATAAAGTGGTAGAAGTTAAACCATTGGGAATAGCAC
CCACTAAAGCAAAAAGGAGAGTGGTGGAGAGAGAAAAAAGAGCAGTGGGAATAGGAGCTGTGTTCCTTGGGTT
CTTGGGAGTAGCAGGAAGCACTATGGGCGCGGCGTCAATGACGCTGACGGTACAGGCCAGACAACTGTTGTCT
GGTATAGTGCAACAGCAAAATAATTTGCTGAGAGCTATAGAGGCGCAACAGCATATGTTGCAACTCACGGTCT
GGGGCATT
>DQ369990
ATTCAGAATAGTTCAACCATCACGGATAATTCAACCATCACCATCCCATGCAGGATAAAACAAATTATAAACA
TGTGGCAGGAGGTGGGACGAGCAATGTATGCCCCTCCCATTGCAGGAAACATAACATGTAACTCAAGTATCAC
AGGAATACTATTGACACGTGATGGTGGCGATAACGACACAAAAGAGAATGAGACATTCAGACCGGGAGGAGGA
AATATGAAGGACAATTGGAGAAGTGAATTATATAAATATAAAGTGGTAGAAATTAAGCCATTGGGAGTAGCAC
CCACGAAGGCAAAAAGGAGAGTGGTGGAGAGAGAAAAAAGAGCAGTGGGAATAGGAGCTGTGTTCCTTGGGTT
CTTGGGAGCAGCAGGAAGCACTATGGGCGCGGCGTCAATAACGCTGACGGTACAGGCCAGACAGCTGTTGTCT
GGTATAGTGCAACAGCAGAGCAATTTGCTGAGAGCTATAGAGGCGCAACAGCATCTGTTGCAACTCACGGTCT
GGGGCATT
>DQ275658
AATTACAGTAAGTCAAACATCACCAAGGGCAGTGATCCAAACATCACCACCATCCAATGCAGGATAAAACAAT
TTATAAACATGTGGCAGGGGGTAGGACGAGCAATGTATGCCCCTCCCATTGCAGGAAACATAACATGTAACTC
AAGTATCACAGGACTACTATTGACACGTGATGGTGGGAATGAAACGAATGGCACAGAGACATTCAGACCTGGA
GGAGGAAATATGAAGGACAACTGGAGAAGTGAATTATATAAATATAAAGTGCTAGAAATTAAGCCATTAGGAG
TAGCACCCACTGGAGCAAAAAGGAGAGTGGTGGAGAGAGAAAAAAGAGCAGTGGGAATAGGAGCTGTGCTCCT

TGGGTTCTTGGGAGCAGCAGGAAGCACTATGGGCGCGGCGTCAATAACGCTGACGGTACAGGCCAGACAACTG
TTGTCTGGTATAGTGCAACAGCAAAGCAATTTGCTGAGAGCTATAGAGGCGCAACAGCATATGTTGCAACTCA
CGGTCTGGGGCATT
>DQ396391
AGTTATTTAAACATAACTGATGCAAACACAACAAAGGCAAACGAAACCATCACACTCCCATGCAGAATAAAAC
AAATTATAAACATGTGGCAGGAGGTAGGACGAGCAATGTATGCCCCTCCCATTGCAGGAAACATAGAATGTAA
ATCAAATATCGCAGGACTACTATTGGTACGTGATGGAGGACTAAATAGAACAGATAATAACACAGAGATATTC
AAACCTGAAGGAGGAAATATGAAGGACAATTGGAGAAGTGAATTATATAAATATAAAGTGGTGGAAATTAACC
CATTGGGAGTAGCACCCACGAAGGCAAAAAGGAGAGTGGTGGAGAGAGAAAAAAGAGCAGTGGGAATAGGAGC
TGTGTTCCTTGGGTTCTTGGGAGCAGCAGGAAGCACTATGGGCGCGGCGTCATTAACGCTGACGGTACAGGCC
AGACAACTGTTGTCTGGTATAGTGCAGCAGCAAAGCAATTTGCTGAGAGCTATAGAGGCGCAACAGCATATGT
TGCAACTCACGGTCTGGGGCATT
>AY878056
ACTTATAATGCTAGTGAGGGAAACAGGACCATCACCATCCTATGCAGGATAAAACAAATTATAAACATGTGGC
AGGGGGTAGGACAAGCAATGTACGCCCCTCCCATTGCAGGAAACATAACATGTAACTCAAATATCACAGGACT
ACTATTGACACGTGATGGTGGTACCAACAACAACAACACAGAGACATTCAGACCTGGAGGAGGAAATATGAAG
GACAGTTGGAGAAGTGAATTATATAAATATAAAGTGGTAGAAATTAAGCCATTGGGAGTAGCACCCACCAAGG
CAAAAAGGAGAGTGGTGGAGAGAGAAAAAAGAGCAGCGGGAATAGGAGCTGTGATCTTTGGGTTCTTGGGAGC
AGCAGGAAGCACTATGGGCGCGGCGTCATTAACGCTGACGGTACAGGCCAGACAACTGTTGTCTGGTATAGTG
CAACAGCAAAGCAATTTGCTGAGAGCTATAGAGGCGCAACAGCATATGTTGCAACTCACGGTCTGGGGCATT
>DQ164104
TTTTATAATGAAAGTAAAGATAATAGAAGTGAGAACATCACAACAATCCCATGTAAGATAAAACAAATTATAA
ACATGTGGCAGGGGGTAGGACGAGCAATGTATGCCCCTCCCATTGAAGGAAACATAACATGTAACTCAAGTAT
CACAGGACTACTATTGACACGTGATGGAGGATCCTTAGACAACAAAACAGAGATATTTAGACCTGGAGGAGGA
AATATGAAGGACAATTGGAGAAGTGAATTATATAAATATAAAGTGGTAGAAATTAAGCCATTGGGAGTAGCAC
CCACTGAGGCAAAAAGGAGAGTGGTGGAGAGAGAAAAAAGAGCAGTGGGAATAGGAGCTGTGCTCCTTGGGTT
CTTGGGAGCAGCAGGAAGCACTATGGGCGCGGCGTCAATAACGCTGACGGTACAGGCCAGACAACTGTTGTCT
GGTATAGCGCAACAGCAAAGCAATTTGCTGAGAGCTATAGAGGCGCAACAGCATATGTTGCAACTCACAGTCT
GGGGCATT
>DQ011165
AATTACAGTTCTGAATATAATAGTACAGGAAGTAATTCAACCAACAGTAGTTCAACCATCACACTTCCATGCA
GAATAAAACAAATTATAAACATGTGGCAGGAGGTAGGACGAGCAGTGTATGCCCCTCCCATTGCAGGAAACAT
AACATGTAATTCAAATGTCACAGGATTACTATTAGTACGTGATGGAGGAAACGACAGCACACCAACAGAAACA
TTCAGACCTGAAGGAGGAAATATGAAGGATAATTGGAGAAGTGAATTATATAAATATAAAGTGGTAGAAGTTA
AGCCACTAGGAATAGCTCCCACTGGAGCAAAAAGGAGAGTGGTGGAGAGAGAAAAAAGAGCAGTAGGAATAGG
AGCTGTGTTCCTTGGGTTCTTGGGATTAGCAGGAAGCACTATGGGCGCAGCGTCAATAACGCTGACGGCACAG
GCCAGACAAGTGTTGTCTGGTATAGTGCAACAGCAAAGCGATTTGCTGAAGGCTATAGAGGCGCAACAGCATA
TGTTGCAACTCACGGTCTGGGGCATT
>AY901966
ACCTACAATCCTACATACAATGGTACAAGGAATGATGAGGACACAACCATCACACTCCCATGCAAAATAAAAC
AAATTATAAATATGTGGCAGAAGGTAGGAAGAGCAATGTATGCCCCTCCCATTGCAGGAAACATAACATGTAA
GTCAAATATCACAGGACTACTATTAGTACGTGATGGAGGCAACACAAATAACATAACAGAGATATTCAGACCT
GGAGGAGGAAATATGAAAGACAATTGGAGAAGTGAATTATATAAATACAAAGTGGTAGAAATTAAGCCATTAG
GAATAGCACCCACTGAAGCAAAAAGGAGAGTGGTGAAGAAAGAGAAAAGAGCAGTGGGAATAGGAGCTGTGTT
CCTTGGGTTCTTGGGAGCAGCAGGAAGCACTATGGGCGCGGCGTCAATGACGCTGACGGTACAGGCCAGACAA
GTATTGTCTGGTATAGTGCAACAGCAAAGCAATTTGCTGAGAGCTATAGAGGCGCAACAGCATATGTTGCAAC
TCACGGTCTGGGGCATT
>AY901975
AATTATACAAATGGAATCATCACACTCCCATGCAAGATAAAACAATTTATAAACATGTGGCAAGAGGTAGGAC
GAGCAATGTATGCCCCTCCCATTGCAGGAAACATAACATGCAAATCAAATATCACAGGACTACTATTGACATG
GGATGGAGGAGACACGAATGGGACAAAAAATAATAATACATTTAGACCTGGAGGAGGAAATATGAAAGACAAT
TGGAGAAATGAATTATATAAATATAAAGTGGTAGAAATTAAGCCATTGGGAATAGCACCCACTGATTCAAAAA
GGAGAGTGGTGGAGAGAGAAAAAAGAGCAGTGGGACTGGGAGCTATGCTCGTTGGGTTCTTGGGAGCAGCAGG
AAGCACTATGGGCGCGGCGTCAACGACGCTGACGGTACAGGCCAGACAACTGTTGTCTGGTATAGTGCAACAG
CAAAGCAATTTGCTGAGAGCTATAGAGGCGCAACAGCATATGTTGCAACTCACAGTCTGGGGCATT
>AY878070
AATTATAGTAGCAGCCACAAGCAAAATACAACCATCACAATCCCATGTAAAATAAAACAAATTATAAATATGT
GGCAAGGGGTAGGACGGGCAATGTATGCCCCTCCCATTGCAGGAAACATAACATGTAAATCAAATATCACAGG
ATTACTATTGACACGTGATGGAGGACCCGATAGCAAAAGCCTAATGGGACAGAGACATTCAGACCTGGAGGA
GGAGATATGAGGGACAATTGGAGGAGTGAATTATATAAATATAAAGTGGTAGAAATCAGGCCATTAGGGATAG

CACCCACCAAGGCAAAAAGAAGAGTGGTGGAGAGAGAAAAAAGAGCAGTGACAATGGGAGCTATGTTCCTTGG
GTTCTTGGGAGCAGCAGGAAGCACTATGGGCGCAGCGTCAATAACGCTGACGGTACAGGCCAGACAACTGTTG
TCTGGTATAGTGCAACAGCAGAACAATTTGCTGAGAGCTATAGAGGTGCAACAGCATATGTTGCAGCTCACAG
TCTGGGGCATT
>DQ275664
TACTATCCTAATGGTACAAACAGTACTTCAAAGGGTACAAATGTAACCATCACACTCCAATGCAGAATAAAAC
AAATTATAAACATGTGGCAGGGGATAGGACAGGCAATGTATGCCCCTCCCATTAAGGGAAACATAACATGTAA
GTCAAATATCACAGGACTACTATTGACACGTGATGGGGGAGAAAATACAAATGGAACAGAAATCTTCAGACCT
GGAGGAGGAGATATGAGAGACAATTGGAGAAGTGAATTATATAAATATAAAGTGGTGGAAATTAAGCCATTAG
GAGTAGCACCCACTACGGCAAAAAGGAGAGTGGTGGAGAGAGAAAAAAGAGCAGTAGGAATAGGAGCTGTGTT
CCTTGGGTTCTTGGGAGCAGCAGGAAGCACTATGGGCGCAGCATCAATAACGCTGACGGTACAGGCCAGACAA
TTGTTGTCTGGTATAGTGCAACAGCAAAGCAATTTGCTGAGGGCTATAGAGGCGCAACAGCATATGTTGCAAC
TCACAGTCTGGGGCATT
>DQ093589
AGTCACCTGGAAAATACAACCATCACACTTCCATGCAGAATAAAACAAATTATAAACATGTGGCAAGGGGTAG
GACGAGCAATGTATGCCCCTCCCATTGCAGGAAACATAGAATGTAAATCAAATATCACAGGAATACTATTGGC
ACATGATGGAGGAGGAGAGCAGAATGGTACAAATGAGATATTCAGACCTGGAGGAGGAGATATGAGGGACAAT
TGGAGAAGTGAGTTATATAAATATAAAGTGGTAGAAATTAAACCATTAGGAATAGCACCTACTAAGGCAAAAA
GGAGAGTGGTGGAGAGAGAAAAAAGAGCAGCGGGAATAGGAGCTGTGTTCCTTGGGTTCTTAGGAGCAGCAGG
AAGCACTATGGGCGCGGCGTCAATAGCGCTGACGGTACAGGCCAGACAATTACTGTCTGGTATAGTGCAACAG
CAAAGCAATTTGCTGAGAGCCATAGAGGCGCAACAGCATATGTTGCAACTCACAGTCTGGGGCATT
>KC863250
AATTATCCTAATGGTACAGAAAGTCTTAATAGTACAGAAAGTAATTCAAGCTTACCAATCACGATCCCATGCA
GAATAAAACAAATTATAAACATGTGGCAGGGGGTAGGACGAGCAATGTATGCCCCGCCCATTGCAGGAAACAT
AACATGTAACTCAAGTATCACAGGACTACTATTGACACGGGATGGAGGAGTGAATACACAGGAGGGAGGAGTG
AATACAACAAGGACAGAGATATTCAGACCTGGAGGAGGAAATATGAAGGACAATTGGAGAAGTGAATTATATA
AATATAAAGTGGTAGAAATTAAGCCATTAGGAATAGCACCCACTGAGGCAAAAAGGAGAGTGGTGGAGAGAGA
AAAAAGAGCAGTGACACTAGGAGCTGTGTTCCTTGGGTTCTTGGGAGCAGCAGGAAGCACTATGGGCGCGGCG
TCAATCACGCTGACGGTACAGGCCAGACAACTGCTGTCTGGTATAGTGCAACAGCAAAGCAATTTGCTGAGAG
CTATAGAGGCGCAACAGCATATGTTGCAACTCACGGTCTGGGGCATT
>DQ275659
TTTCAGAATAATGGTACAAACAGTGAAAGTAATTCAACCATCACACTCCCATGCAGAATAAGACAAATTGTAA
ACATGTGGCAGGAGGTAGGACGAGCAATGTATGCCCCTCCCATTGAAGGAAACATAACATGTAAATCAAATAT
CACAGGGCTACTATTGCAACGTGATGGAGGCACGAGTAACAATAGCAGCAATAACATAGAGATATTCAGGCCT
GGAGGAGGAAATATGAAGGACAATTGGAGAAGTGAATTATATAAATATAAAGTGGTAGAAATTAAACCATTGG
GAATAGCACCCACAACGGCAAAAAGGAGAGTGGTGGAGAGAGAAAAAAGAGCAGTGGGAATCATAGGAGCTAC
GATCCTTGGGATCTTGGGAGCAGCAGGAAGCACTATGGGCGCAGCATCAATAACGCTGACGGTACAGGCCAGA
CACCTGCTGTCTGGTATAGTGCAACAGCAAAGCAACTTGCTGAGAGCTATAGAGGCGCAACAGCATATGTTGC
AACTCACGGTTTGGGGCATT
>DQ093602
TTTCATGATAATTCAAATTTTAATAATTCAACCATCACACTCCCATGCAGAATAAAACAATTTATAAACATGT
GGCAGGGGGTAGGACGAGCAATGTATGCCCCGCCCATTGAAGGAAACATAACATGTACATCAAATATCACAGG
ACTACTATTGACACGTGATGGAGGAGTAGGTAATGGGACAGATAATACGATAGAAATATTCAGACCTGAAGGA
GGAGACATGAGGGACAATTGGAGAAGTGAATTATATAAATATAAAGTGGTGGAAATTAAGCCATTGGGAGTAG
CACCCACTGGGGCAAAAAGGAGAGTGGTGCAGAGAGAAAAAAGAGCAGTGGGAATGGGAGCTATGATCCTTGG
GTTCTTGGCAACAGCAGGAAGCACTATGGGCGCGGCGTCAATAACGCTGACGGTACAGGCCAGACAAGTGTTG
TCTGGTATAGTGCAACAGCAAAGCAATTTGCTGAGGGCTATAGAGGCGCAACAGCATATGTTGCAACTCACGG
TCTGGGGCATT
>AY901974
ACCTACAAGGAGAAGATAAATAGTACTATCACACTCCAATGCAGAATAAAACAAATTATAAACATGTGGCAGG
GGGTAGGACAAGCAATGTATGCCCCTCCCATTGAAGGAAACATAACATGTAGATCAAATATCACAGGACTACT
ATTAACACGTGATGGAGGAAATGATAAAGGAAAAGATAATGACACAGAGATATTCAGACCTGGAGGAGGAGAT
ATGAAGGACAATTGGAGAAGTGAATTGTATAAATATAAAGTGGTAGAAATTAAACCATTAGGAGTAGCACCCA
CCAAGGCAAAAAGGAGAGTGGTGGAGAGAGAAAAAAGGACAGTGGGAATGGGAGCTGTGTTCCTTGGGTTCTT
GGGAGCAGCAGGAAGCACTATGGGCGCAGCATCAATAACGCTGACGGTACAGGCCAGACAATTGCTGTCTGGT
ATAGTGCAACAGCAAAGCAATTTGCTGAGAGCTATAGAGGCGCAACAGCATATGTTGCAACTCACGGTCTGGG
GCATT
>DQ011172
AGTTATTCAAGCACAGCCAACACAACCATCACGCTCCCATGCAGAATCAGACAGATCATAAACATGTGGCAGC
AGGTAGGACGAGCAATGTATGCCCCTCCCATTGCAGGAAACATAACATGTAAATCAAATATCACAGGACTACT

ATTGCTACGTGATGGAGGAAATGAAGACAATAGAACAGAGACATTCAGACCTGGAGGAGGAAATATGAAGGAC
AATTGGAGAAGTGAATTATATAAATATAAAGTGGTAGAACTTAAGCCTTTGGGAGTAGCACCCACTAAGGCAA
AAAGGAGAGTGGTGGAGAGAGAAAAAAGAGCAGTGGGAATAGGAGTTGCGCTCCTTGGGTTCTTGGGAGCAGC
AGGAAGCACTATGGGCGCGGCGTCAATAACGCTGACGGTACAGGCCAGACAACTGTTGTCTGGTATAGTGCAA
CAGCAAAGCAATTTGCTGAGAGCTATAGAGGCGCAACAGCATATGTTGCAACTCACAGTCTGGGGCATT
>DQ093594
CCTCAGAATGATACAAAAGATGATTTAACCATCACACTTCCATGCAAAATAAAACAAATTATAAACATGTGGC
AGGGGGTAGGACGAGCAATGTATGCCCCCCCTATTGCAGGAAACATAACATGTAACTCAAGTATCACAGGACT
ATTATTGACACGTGATGGAGGAAATAACACAACAGGGACAGAGATATTCAGACCTGGAGGAGGAAACATGAAA
GACAATTGGAGAAGTGAATTATATAAGTATAAAGTGGTAGAAATTAAGCCATTGGGAGTAGCTCCCACTGAAG
CAAAAGGGAGAGTGGTGGAGAGAGAAAAAAGAGCAGTGGGAATAGGAGCTGTACTCCTTGGGTTCTTGGGAGC
AGCAGGAAGCACTATGGGCGCGGCGTCAATAACGCTGACGGTACAGGCCAGACAACTGTTGTCTGGTATAGTG
CAACAGCAAAACAATTTGCTGAGGGCTATAGAGGCGCAACAGCATATGTTGCAACTCACGGTCTGGGGCATT
>DQ351217
AACTACACATGTACTGCTCCAGAAGGGAATGGAACCATCATACTTCCATGCAGAATAAAACAAATTGTAAACA
TGTGGCAGGAGGTAGGACGAGCAATGTATGCCCCTCCCATTAAAGGAAACATAACATGTAACTCAAACATCAC
AGGACTACTATTAGTACGGGATGGAGGCGAGAATATCACGGAGGAGACATTCAGGCCTGGAGGAGGAAATATG
AAGGACAATTGGAGAAGTGAATTATATAAATATAAAGTGGTAGAAATTAAGCCATTGGGAATAGCACCCACTA
CGGCAAAAAGGAGAGTGGTGGAGAGAGAAAAAAGAGCAGTGGGAATAGGAGCTGTACTCCTTGGGTTCTTGGG
AGCGGCAGGAAGCACTATGGGCGCGGCGTCAATAACGCTGACGGTACAGGCCAGACAACTGTTGTCTGGTATA
GTGCAACAGCAAAGCAATTTGCTGAAAGCCATAGAGGCGCAACAGCACATGTTGCAACTCACGGTCTGGGGCA
TT
>DQ093604
AGTTACAATAGTACAGGAGACATCACACTCCCATGCAGAATAAAACAAATTATAAACATGTGGCAGAAGGTAG
GACGAGCAATATATGCCCCTCCCATTGCAGGAAACATAACATGTAATTCAACTATCACTGGACTACTCTTGGT
AAGAGATGGAGGCAACTCAAATAACCTAAGTGAGACATTCAGACCAGCAGGAGGAAATATGAAGGATAATTGG
AGAAGTGAACTATATAAATATAAAGTGGTAGAAATTAAGCCATTGGGAATAGCACCCACTAAGGCAAAAAGGC
GAGTGGCGGAGAGAGAAAAAAGGGCAGTAGGAATAGGAGCTGTGTTCCTTGGGTTCTTGGGAGCAGCAGGAAG
CACTATGGGCGCAGCGTCAATAGCGCTGACGGCACAGGCCAGACAAGTGTTGTCTGGAATAGTGCAACAGCAA
AGCAATTTGCTGAGGGCTATAGAGGCGCAACAGCATATGTTGCAACTCACAGTCTGGGGCATT
>AY901973
TGTTATACAACACAGCTGTTTAATAGTACATACAATAGTACATACAGGCCTAATGGTACAGAAAGTAATTCAA
ACATCACACTCCCATGCAGAATAAAGCAAGTTATAAACATGTGGCAGCAGGTAGGACGAGCAATGTATGCTCC
TCCCATTGCAGGAAACATAACATGTAAATCAAATATCACAGGATTACTATTGGTGCGTGATGGAGGAATAAAT
AACAGTACCAAAGAGACATTCAGACCTGGAGGAGGAGATATGAAGGACAATTGGAGAAGTGAATTATACAAAT
ATAAAGTAGTAGAAATTAAACCATTGGGAATAGCACCTACTGAGGCAAAAAGGAGAGTGGTGGAGAGGGAAAA
AAGAGCAGTGGGAATAGGAGCTGTGTTCCTTGGGTTCTTGGGAGCAGCAGGAAGCACTATGGGCGCGGCGTCA
ATAACGCTGACGGTACAGGCCAGACAATTGTTGTCTGGTATAGTGCAACAGCAAAGCAATTTGCTGAGGGCCA
TAGAGGCGCAACAGCATATGTTGCAACTCACGGTCTGGGGCATT
>DQ382374
ACTCATCTGCCTAATGGTACACAGGTTAATGGGACTGATGCAAATATCACACTTCCATGCAGAATAAAACAAT
TTATAAACATGTGGCAGAAGGTAAGACTAGCAATGTATGCCCCTCCCATTGCAGGAGACATAACATGTGAATC
AGATATTACAGGACTACTGTTAACGCGGGATGGAGGAGATAGTACAAATAGTAATAATAATACAGAGACATTC
AGACCTGAAGGAGGAAACATGAAAGACAATTGGAGAAGCGAATTATATAAATATAAAGTGGTAGAAATTAAGC
CATTAGGAATAGCACCCACTACTGCAAAAAGGAGAGTGGTGGAAAGAGAGAAAAGAGCAGTGGGACTAGGAGC
TGTGTTCCTTGGGTTCTTGGGAGCAGCAGGAAGCACTATGGGCGCAGCATCAATAACGCTGACGGTACAGGCC
AGACAATTATTGTCTGGTATAGTGCAACAGCAAAGTAATTTGCTGAGAGCTATAGAGGCGCAACAGCATATGT
TGCAACTCACGGTCTGGGGCATT
>DQ382376
TCCTATCCTCCTTCCTGCCCTCCTAGTACTGCAGATAATTCTTCCATCATCCTTCCTTGCTGCATAATACAAC
TTATTAACCTGTGGCGGGAGGTAGGACGACCAGTGTTGGCCGCTCCCCTTGTTGGAAGCATCATATGTTGCTC
CTCTATCACCGGAATACTATTATCAACGGATGGAGGCCCCGACGATGACGCAGAGACATTCAGACCTGGAGGA
GGAAATATGAAGGACAATTGGAGAAGTGAATTATATAAATATAAAGTGGTAGAAGTTAAGCCATTGGGAATAG
CACCCACTAAAGCAAAAAGGAGAGTGGTGGAGAGAGAAAAAAGAGCAGTGGGAATAGGAGCTGTGTTCCTTGG
GTTCTTGGGAACAGCAGGAAGCACTATGGGCGCAGCGTCAATAACGCTGACGGTACTGGCCAGACAATTATTG
TCTGGTATAGTGCAACAGCAAAGCAATTTGCTGAGGGCTATAGAGGCGCAACAACATCTGTTGCAACTCACGG
TCTGGGGCATT
>DQ382379
TGCTATACAAATGAAACAAGCAATCCAAATAATACCATCACACTCCCATGCAGAATAAGACAAATTATAAACA
TGTGGCAGAAGGTAGGACGAGCAATGTATGCCCCTCCCATTGCAGGAAACATAAATTGTAAATCAAATATCAC

AGGAATACTGTTGACACGTGATGGAGGTAACGAAACAGAGGGTGACGGAACAAAGGTAGAGATATTCAGACCT
GGAGGAGGAAATATGAGGGACAATTGGAGAAGTGAATTATATAAATATAAAGTGGTAGAAATTAAGCCATTAG
GAGTAGCACCCACTAAGGCAAAAAGGAATGTGGTGCAGAGAGAAAAAAGAGCAGTGGGAATAGGAGTTGTGCT
CCTTGGGTTCTTGGGAGCAGCAGGAAGCACTATGGGCGCGGCGTCAATAACGCTGACGGTACAGGCCAGACAA
CTGTTGTCTGGTATAGTGCAACAGCAAAGCAATTTGCTGAGAGCCATAGAGGCGCAACAACATATGTTGCAAC
TCACGGTCTGGGGCATT
>DQ382363
GATTATAATGGTGCAGGAAATAGTTCATCAGACTCCTTCATCACCATCCTCTGCAGAATAAAACAATTTATAA
TCATGTGGCAGAGGGTAGGACGAGCAATGTATGCCCCTCCCATTGTTGGAAGCGTCACATGTTGTTCATTTAT
CACAGGGCTACTATTGACGCGTGATGGAGGAAGTAACACAACAGAGACATTCAGACCTGTAGGAGGAGATATG
AGGGACAATTGGAGAAGTGAATTATATAAATATAAAGTGGTAGAAGTTAGGCCATTAGGAATAGCACCCACTA
GGGCACAAAGGAGAGTGGTGGAGAGAGTAAAAAGAGCAGTGGGAATAGGAGCTGTGTTCCTTGGGTTCTTGGG
AGCAGCAGGAAGCACTATGGGCGCAGCATCAATAACGCTGACGGTACAGGCCAGACAATTGCTGTCTGGTATA
GTGCAACAGCAAAGCAATTTGCTGAAGGCTATAGAGGCGCAACAGCACCTGTTGCAACTCACAGTCTGGGGCA
TT
>HQ615953
TTTCATAATAATACAAATGAGACAACCATCACAAATGAGACCATCATGCTCCCATGCAGGATAAAACAAATTA
TAAACATGTGGCAGAAGGTAGGACGAGCAATGTATGCTCCTCCCATTGAGGGAAACATAACATGTAATTCAAA
TATCACAGGACTACTATTACTACGTGATGGAGGCCTCTTCAATGGGACAGCAGAAGTAGAGACATTCAGACCT
GGAGGGGGAGATATGAGAGACAATTGGAGAAGTGAATTATACAAATATAAAGTGGTAGAAGTTAAGCCATTGG
GAATAGCACCCACTGGGGCAAGAAGGATAGTGGTGAAGAGAGAAAAAAGAGCAGTGGGAATAGGAGCTGTGTT
CCTTGGGTTCTTGGGAGCAGCAGGAAGCACTATGGGCGCGGCGTCAATGACGCTGACGGTACAGGCTAGACAA
CTGTTGTCTGGTATAGTGCAACAGCAAAGCAATTTGCTGAAGGCTATAGAGGCGCAGCAACATCTGCTGCAAC
TCACGGTCTGGGGCATC
>GQ999982
AATTATACACTGAGCAATAACAATAATGAAACAATCATACTCCCATGTAGAATGAAACAAATTATAAATATGT
GGCAGGGGGTAGGACGAGCAATGTATGCCCCTCCCATTGCAGGAAACATAACATGTAACTCAAGTATCACAGG
ACTGTTATTGACGCGTGACAAAGACCCAGGGCAGAAGAACAGCAACACAGAGACATTCAGACCTACAGGAGGA
AATATGAAGGACAATTGGAGAAATGAATTATACAAATGTAAAGTGGTAGAAATTAAACCATTAGGAGTAGCAC
CCACTAATGCAAAAAGGAGAGTGGTGGAGAGAGAAAAAAGAGCAGTAGGAATAGGAGCTGTGCTCCTTGGGTT
CTTGGGAGCAGCAGGAAGCACTATGGGCGCGGCGTCAATAACGCTGACGGTACAGGCCAGACAACTGTTATCT
GGTATAGTGCAACAGCAAAGTAATTTGCTGAGAGCTATAGAGGCGCAACAGCACATGTTGCAACTCACAGTCT
GGGGCATT
>GQ999988
TTTTATGAACCTTTAAATTTAACCATCACACTCCAATGCAGAATAAAACAAATTATAAACATGTGGCAGGAGG
TGGGACGAGCAATGTATGCCCCTCCCATTGCAGGAAACATAACATGTGAATCAAAGATCACAGGGCTAATATT
GACACGTGATGGAGGAAGTGACAATGGGACAGAGACATTCAGACCTGGAGGAGGAGATATGAGGGACAACTGG
AGAAGTGAATTATATAAATATAAAGTGGTAGAAATTAAGCCATTGGGAATAGCACCCACTAATGGAAGAAGGA
GAGTGGTGCAGAGAGAGAAAAGAGCAGTGGGAATAGGAGCTGTATTCCTTGGGTTCTTGGGAGCAGCAGGAAG
CACTATGGGCGCGGCATCAATAACGCTGACGGTACAGGCCAGACAATTATTGTCTGGTATAGTGCAACAGCAA
AACAATTTGCTGAGGGCTATAGAGGCGCAACAGCATATGTTGCAACTCACGGTCTGGGGCATT
>HQ615952
AATCAGACTACAAATAATTCTACTAACATCACACTCCCATGCAGAATAAAACAAATTATCAACATGTGGCAGG
AGGTAGGACGAGCAATGTATGCCCCTCCTATTGCAGGAAACGTAACATGTAACTCGAGTATCACAGGACTACT
ACTGACCCGTGATGGGGGAAAAGGAAATGACACAGGAAATGGCACAGAGATATTCAGACCTGGAGGAGGAAAT
ATGAAAGACAATTGGAGAAGTGAATTATTTAAATATAAAGTGGTAGAAGTTAGGCCATTGGGAATAGCACCCA
CTACGGCAAAAAGGAGAGTGGTGGAGAGAGAAAAAAGAGCAGTGGGAATAGGAGCTGTACTCCTTGGGTTCTT
GGGAGCAGCAGGAAGCACTATGGGCGCGGCGTCAATAACGCTGACGGTACAGGCCAGACAACTGTTGTCTGGT
ATAGTGCAACAGCAAAGCAATTTGCTGAGGGCTATAGAGGCGCAACAGCATATGTTGCAACTCACGGTCTGGG
GCATT
>HQ595762
GCTTATAATACACCAAATGCTTCAGAAAACATAACCCTCCCATGTAGAATAAAACAAATTATAAACATGTGGC
AGAGGGTAGGACGAGCAATGTATGCCCCTCCCATTGCAGGAAACATAACATGTAAATCAAATATCACAGGACT
ACTCTTGACACGTGATGGAGGGAGCAATAGTACAGGGACAGAAATATTCAGACCTGGAGGAGGAGATATGAGG
GATAATTGGAGAAGTGAATTATATAAATATAAAGTAGTAGAAGTTAAACCATTAGGAATAGCACCCACTACTG
CAAGAAGGAGAGTGGTGCAGAGAGAAAAAAGAGCAGCATTGGGAGCTGTGCTCTTGGGGTTCTTAGGAGCAGC
AGGAAGCACTATGGGCGCGGCGTCGGTGGCGCTGACGGTACAGGCCAGGCAATTATTGTCTGGTATAGTGCAA
CAGCAAAGCAATTTGCTGAGAGCTATAGAGGCACAACACCATTTGTTGCAACTCACAGTCTGGGGCATT
>HQ595758

AATTATAATGATACAGAAAGTAATTCAACCATCACACTTCCATGCAGGATAAAACAAATTATAAACATGTGGC
AGGAGGTAGGACGAGCAATGTATGCTCCTCCCATTGCAGGAAACATAACATGTAAATCAAATATCACAGGATT
ACTATTGACGCGTGATGGAGGCACAAATGACACAAATAACACAGAAATATTCAGACCTGGAGGAGGAAATATG
AAGGACAATTGGAGAAGTGAATTGTATAAATATAAAGTGGTAGAAATTAAACCATTAGGGATAGCTCCCACTG
GAGCAAAAAGGAGAGTGGTGGAGAGAGAGAAAAGAGCAGTAGGAATAGGAGCTATGATCCTTGGGTTCTTGGG
AGCAGCAGGAAGCACTATGGGCGCAGCGTCAATAACGCTGACGGTACACGCTAGACAATTGTTGTCTGGTATA
GTGCAACAGCAAAGCAATTTGCTGAGAGCTATAGAGGCGCAACAGCATATGTTGCAACTCACGGTCTGGGGCA
TT
>KT183064
AATCATACAGACAGGTCTAATGATACAGGAAGTAATAAAACCATCACACTCCCATGTAAAATAAAACAAATTA
TAAACATGTGGCAGGAGGTAGGACGAGCAATGTATGCCCCTCCCATTGCAGGAAACATAACATGTAAATCAAA
TATCACAGGTCTACTATTAGTACGTGATGGAGGAGAAAGTAACTCAACAACGGAGATATTCAGACCTGGAGGA
GGAAATATGAAGGACAATTGGAGAAGTGAATTATATAAATATAAAGTGGTAGAAATTAAACCACTGGGAATAG
CACCCACTGAGGCAAAAAGGAGAGTGGTGGAGAGAGAAAAAAGAGCAGTTGGCATAGGAGCTGTGTTCCTTGG
GTTCTTGGGAGCAGCAGGAAGCACTATGGGCGCGGCGGCAGTGACGCTGACGGTACAGGCCAGACAATTGTTG
TCTGGTATAGTGCAACAGCAAAGCAATTTGCTGAGGGCTATAGAGGCGCAACAGCATATGTTGCAACTCACAG
TCTGGGGCATT
>KT183094
TACTATCCTAATGGTACTGTTACAACAAGTAATATTACACTCCCATGCAAAATAAAACAAATTATAAACATGT
GGCAAGGGGTGGGACGAGCAATGTATGCCCCTCCCATTGCAGGAAACATAACATGTAAATCAAACATCACAGG
ATTACTATTGCTACGTGATGGGGGAAACATGAATAACACAACAGAAATATTCAGACCTGGAGGAGGAGATATG
AAGGACAATTGGAGAAGTGAATTATATAAATATAAAGTAGTAGAAATTAAACCATTAGGAATAGCACCCACTG
GGGCAAAAAGGAGAGTGGTGGAAAGAGAAAAAAGAGCAGTGGGAATAGGAGCTGTGATCTTTGGGTTCTTGGG
AGCAGCAGGAAGCACTATGGGCGCAGCGTCAATAGCGCTGACGGTACAGGCCAGACAACTGTTGTCTGGTATA
GTGCAACAGCAAAGCAATTTGCTGAGAGCTATAGAGGCGCAACAGCATATGTTGCAACTCACGGTCTGGGGCA
TT
>KT183148
ACTTATAATGGTCCAGAAGGAAACTCAAACATCACACTCCCATGCAGAATAAAACAAATTATAAACATGTGGC
AGAAAGTAGGACGAGCAATATATGCCCCTCCCATTAGAGGAAACATAACATGTAACTCAAGTATCACAGGACT
AGTCTTGGTACGTGATGGAGGTATATATAATAATACCAAAAATGAAAATGAGACATTCAGACCTGAAAATGAG
ACATTCAGACCGGGAGGAGGAGATATGAGGGACAATTGGAGAAGTGAACTATATAAATATAAAGTGGTAGAAA
TTAAACCATTGGGAATAGCGCCCACTGAAGCAAAAAGGAGAGTGGTGCAGAGAGAAAAAAGAGCAGTGGGAAT
AGGAGCTTTGTTCCTTGGGTTCTTGGGAGCAGCAGGAAGCACTATGGGCGCAGCGTCAATAACGCTGACGGTA
CAAGTCAGACAATTGTTGTCTGGAATAGTGCAACAGCAAAGCAATTTGCTGAGAGCTATAGAGGCGCAACAGC
ATATGTTGCAACTCACAGTCTGGGGCATT
>KT183196
ATTCAAAACAGCACAAATATCACACTCCCATGCAAAATAAAACAATTTGTAAATATGTGGCAGGAGGTAGGAC
GAGCAATGTATGCCCCTCCCATTGCAGGAAACATAACATGTAGATCAAATATCACAGGACTACTATTGGTGCG
TGATGGGGGCAACCAAAGCGGAAATGATATAGAGACATTCAGACCTGGAGGAGGAAATATGAAAGACAATTGG
AGAAGTGAATTATATAAATATAAAGTGGTAGAAATTAAACCATTGGGAATAGCACCCACTAAGGCAAAAAGGA
GAGTGGTGGAGAGAGAAAAAAGAGCAGTGGGAATAGGAGCTGTGTTCCTTGGGTTCTTGGGAACAGCAGGAAG
CACTATGGGCGCGGCGTCAATAGCGCTGACGGTACAGGCCAGACAAGTATTGGCTGGTATAGTGCAACAGCAA
AGCAATTTGCTGAGGGCTATAGAGGCGCAACAGCATATGTTGCAACTCACGGTTTGGGGCATT
>KC156123
GCCTACAATAAAACAGGTGATAATTCAACCATCACAACCATCACACTCCAATGCAGAATAAAACAAGTTATAA
ACATGTGGCAGGAGGTAGGACGAGCAATATATGCCCCTCCCATTGCAGGAAACATAACATGTAGCTCAAATAT
CACAGGACTACTGTTGACACGTGATGGAGGAAATAATAATAGTAGTAATGAGACAGAGACATTCAGGCCTGGA
GGAGGAGATATGAGAGACAATTGGAGAAGTGAATTATATAAATATAAAACAGTAGAAATTAAACCATTAGGAA
TAGCACCCACTACGGCAAAAAGGAGAGTGGTGGAGAGAGAAAAAAGAGCAGTGGGAATAGGAGCTGTGTTCCT
TGGGTTCTTGGGAGCAGCAGGAAGCACTATGGGCGCGGCGTCAATAACGCTGACGGTACAGGCCAGACAATTG
TTGTCTGGTATAGTGCAACAGCAAAGCAATTTGCTGAGAGCTATAGAGGCGCAACAACATCTGTTGCAACTCA
CGGTCTGGGGCATA
>KC156125
TTTTATGCTAATAGCAGTGATCCCAATGCAACTATCACACTCCCATGCAAAATAAAACAATTTATAAACATGT
GGCAGGAGGTAGGACGAGCAATGTATGCCCCTCCCATTGAAGGAAACATAACATGTAAATCAAATATCACAGG
GCTACTATTGGAACGTGATGGAGACATAAAAGAAAACAATACAGAGACATTCAGACCTATAGGAGGAAATATG
AAGAACAATTGGAGAAGTGAATTATATAAATATAAAGTGGTAGAGATTAAACCATTGGGAGTAGCACCCACAG
AATCAAAAAGGAGAGTGGTGGAGAGAGAAAAAAGAGCAGTGGGATTAGGCGGAGCTTTGTTCCTTGGGTTCTT
GGCAACAGCAGGAAGCACTATGGGCGCGGCGTCAATAACGCTGACGGTACAGGCCAGACAACTGTTGTCTGGT

ATAGTGCAACAGCAAAACAATTTGCTGAGAGCTATAGAGGCGCAACAGCATCTGTTGCAACTCACAGTCTGGG
GCATT
>KC156127
AGTCATAATGGTACGAACATCACACTCTCATGCAGAATAAAACAAATTATAAACATGTGGCAGGAGGTAGGAA
GGGCAATATATAACCCTCCCATTGCAGGAAACATAACATGTAAATCAAACATCACAGGACTACTATTGGTACG
TGATGGAGGTATCACAAATAACAACACAGAGACATTCAGACCTGGAGGAGGAGATATGAGGGACAATTGGAGA
AGCGAATTATATAAATATAAAGTGGTAGAAATTAATCCCTTAGGAGTAGCACCCACTAAGGCAAAAAGGAGAG
TGGTGGAGAGAGAAAAAAGAGCAGTGGGAGTAGGAGCTATGTTCCTTGGGTTCTTGAGTGCAGCAGGAAGCAC
TATGGGCGCGGCGTCAATAACGCTGACGGTACAGGCCAGACAACTGTTGTCTGGCATAGTGCAACAGCAAAGC
AATTTGCTGAGGGCTATAGAGGCGCAACAGCATATGTTGCAACTCACAGTCTGGGGCATT
>JN687717
ATTCATAAGAATGAAAGTGGTTCAAATGACACCATTACAATCCCATGCAGAATAAAACAAATTATAAACATGT
GGCAGGAGGTAGGAAAAGCAATGTATGCCCCTCCCATTGCAGGAAACATAACATGTGAGTCAAATATCACAGG
ACTACTATTAGAACGTGATGGAGGCACAAACATTAACACAGGCAATAACACAGGCAATAACACCACAGGCCAT
AACACAGGCAATAGCACAGATAACAGAAATGAAACATTCAGACCTGGAGGAGGAAATATGAAGGATAATTGGA
GAAGTGAACTATATAAATATAAAGTGGTAGAAATTAAGCCATTGGGAGTAGCACCCACGAAGGCAAGAAGGAG
AGTAGTGACGGGGAAAGAAAAAAGAGCAGTGGGACTAGGAGCTGTGATCTTTGGGTTCTTGGGAGCAGCAGGA
AGCACTATGGGCGCGGCGTCAGTGGCGCTGACGGTACAGGCCAGACAACTGTTGTCTGGTATAGTGCAGCAGC
AAAGCAATTTGCTGAGAGCTATAGAAGCGCAACAGCATATGTTGCAACTCACAGTCTGGGGCATT
>KC863629
AATCATTCAAATTCAAACATCACACTCAAATGCAGAATAAAACAAATTATAAACATGTGGCAGGGGGTAGGAC
AAGCAATGTATGCCCCTCCTATTGCAGGAAACATAACATGTAACTCAACCATCACAGGACTACTATTGACACG
TGATGGAGGAAGCACAACAAATAGCTCGACAGAAATATTCAGACCTGGAGGAGGAGATATGAGGGACAATTGG
AGAAGTGAATTATATAAATATAAAGTGGTAGAAGTTAAGCCATTAGGGATAGCACCCACTGAGGCAAAAAGGA
GAGTGGTGGAGAGAGAAAAAAGAGCAGTGGGAATAGGAGCTGTGTTCCTTGGGATCTTGGGAGCAGCAGGAAG
CACTATGGGCGCAGCATCAATAACGCTGACGGTACAGGCCAGACAATTGTTGTCTGGTATAGTGCAACAGCAA
AGCAATTTGCTGAGGGCTATAGAGGCGCAACAGCATATGTTGCAACTCACGGTCTGGGGCATT
>KC863647
ACCTACAACCATAATGACACATCAAATGCAACCAGCAACAACACATCAGATATCATCACCCTCCAATGCAGAA
TAAAACAAATCATAAACATGTGGCAGGGGGTAGGAAAAGCAATATATGCCCCTCCCATTGAAGGAAATATAAC
CTGTAGATCAGAGATCACAGGACTACTATTGACAAGGGATGGAGGAGATGAAGGTAAAAATGATACAGAGATA
TTCAGACCTGGAGGAGGAGATATGAGAGACAATTGGAGAAGTGAATTATATAAATATAAAGTAGTAGAAATTA
AACCATTAGGAATAGCACCCACCAAGGCAAAAAGGAGAGTGGTGGAGAGGAGAGGAAAAAGAGCAGTAGGAAT
AGGAGCTATGTTCCTTGGGTTCTTAGGAGCAGCAGGAAGCACTATGGGCGCAGCGTCAATAACGCTGACGGTA
CAGGCCAAACAATTATTGTCTGGTATAGTGCAACAGCAAAGCAATTTGCTGAGGGCTATAGAGGCGCAACAAC
ATATGTTGCAACTCACGGTCTGGGGCATT
>HM204598
GATTATAAACCAACAAATGATACTGGTGATACCATAATACTTCCATGCAGAATAAAACAAATTATAAACATGT
GGCAGGAGGTAGGACGAGCAATGTATGCCCCTCCCATTGCAGGAAACATAACATGTAATTCAAGTATCACAGG
ACTACTATTAGAACATGATGGAGAAACAGATGGAGATAATGTGACATTCAGACCTATAGGAGGAAATATGAAG
GACAATTGGAGAAGTGAATTATATAAATATAAAGTGGTAGAAATTAAGCCATTGGGAATAGCACCCACTGAGG
CAAAAAGGAGAGTGGTGGAGAGAGAGAAAAGAGCAGCAGTGGGAATAGGAGCTGTGTTCCTTGGGTTCTTGAG
CGCAGCAGGAAGCACTATGGGCGCGGCGTCAATAACGCTGACGGTACAGGCCAGACAACTGTTGTTTGGTATA
GTGCAACAGCAAAGCAATTTGCTGAAGGCTATAGAGGCGCAACAGCATATGTTGCAACTCACAGTCTGGGGCA
TT
>HM204601
AGCTATTCAAACAACACCATCACACTCCCATGCAGGATAAAACAAATTGTAAATATGTGGCAGAAGGTAGGAC
AAGCAATGTATGCCCCTCCCATTGCAGGAAACATAACATGTAACTCAAGTATCACAGGACTAATATTGACACG
TGATGGGGGGAACACAGAGAATAACACAGGGAATAACACAGAGGTATTCAGACCTGGAGGAGGAGATATGAAG
AATAATTGGAGAAGTGAATTATATAAATATAAAGTGGTAGAAATTAAACCATTGGGAATAGCACCCACCACGG
CAAAAAGGAGAGTGGTGGAGAGAGAAAAAAGAGCAGCAATAGGAGCTGTGCTCCTTGGGTTCTTGGGAACAGC
AGGAAGCACTATGGGCGCGGCGTCGGTAGCGCTGACGGTACAGACCAGACAACTGCTGTCTGGTATAGTGCAA
CAGCAAAGCAATTTGCTGAGAGCTATAGAGGCGCAACAGCATATGTTGCAACTCACAGTCTGGGGCATT
>HM204605
AATTACTCGTTTAATGAGACAGACAATGTGACAACAAGCGAGCAAGATGCAAACATCACACTCCAATGCAGAA
TAAAACAAATTATAAACATGTGGCAGGAGGTGGGGCGAGCAATGTATGCCTCCCCCATTGCAGGAGAAATAAC
ATGTAACTCAAGTATCACAGGACTACTATTGACACGTGATGGAGGAAGAAAAGAGAATAATACAGAGAGTAAT
ACAGAAATATTCAGACCTGGAGGAGGAAATATGAGGGACAATTGGAGAAGTGAATTATATAAATATAAAGTGG
TAGAAGTTAATCCATTAGGAATAGCACCCACTAAGGCACGAAGGAGAGTGGTGCAGAAAGAGAAAAGAGCAAT
AACACTGGGAGCTATGTTCCTTGGGTTCTTGGGGGCAGCAGGAAGCACTATGGGCGCAGCATCAATAACGCTG

ACGGTACAGGCCAGACAATTATTGTCTGGTATAGTGCAACAGCAAAGCAATTTGCTGAGGGCTATAGAGGCGC
AACAGCATATGTTGCAACTCACAGTCTGGGGCATT
>HM204610
CCTCAGGGTTCTAATAGTACAAAAAACATCACAATATATTGCAGAATAAAACAAATTATAAACATGTGGCAGA
GGGTAGGACGAGCAATGTATGCCCCTCCCATTGCAGGAAACATAACATGTATATCAAATATCACAGGACTACT
ATTGGTACGTGATGATCCATCAAATGCTACATTCAGACCGGGAGGAGGAGATATGAGGGATAACTGGAGAAGT
GAATTATATAAATATAAAGTGGTAGAAATTAAGCCATTGGGAATAGCACCCTCTAAGGCAAAAAGGAGAGTGG
TGGAGAGAGAAAAAAGAGCAGCGGGACTAGGAGCTGTGTTCCTTGGGTTCTTGGGAGCAGCAGGAAGCACTAT
GGGCGCGGCGTCAATAACGCTGACGGTACAGGCCAGACAATTGCTGTCTGGTATAGTGCAACAGCAAAACAAC
TTGCTGAGGGCTATAGAGGCGCAACAGCATTTGTTGCAACTCACAGTCTGGGGCATT
>FJ444091
AACTATAATGGTACAGAAAGTAATTCAAGCATCATCACACTCCCATGCAGAATAAAACAAATTGTAAACATGT
GGCAGGAGGTAGGAAGAGCAATATATGCCCCTCCTATTGCAGGAAACATAACATGTAACTCAAGTATCACAGG
ATTACTATTGCTGCGTGATGGAGGCTTAGGCTTGAATGAAACAGGAGAGACATTTAGACCTATAGGAGGAGAT
ATGAGGAACAATTGGAGAAGTGAATTATATAAATATAAGGTGGTAGAAATTAAGCCATTGGGAGTAGCACCCA
CTAAGGCAAAAAGGAGAGTGGTGGAGAGAGAAAAAAGAGCAGTGGGACTGGGAGCTGTGTTCCTTGGGTTCTT
GGGAGCAGCAGGAAGCACTATGGGCGCGGCGTCAATAACGCTGACGGTACAGGCCAGACAACTGTTGTCTGGT
ATAGTGCAACAGCAAAGCAATTTGCTGAGGGCTATAGAGGCGCAACAGCATATGTTGCAACTCACAGTCTGGG
GCATT
>JX140664
AATTATACTTCTAATGATACAGAAAACATCACACTCACACTCCCATGCAAAATAAAACAGATTATAAACTTGT
GGCAGGAGGTAGGACGAGCGATGTACGCTCCTCCCATTGCAGGAAACATAACATGTAAGTCAACCATCACAGG
ATTACTATTGGTGCGTGATGGAGGACAAGGAAACAGCTCAAATGAGACATTCAGACCTGGAGGAGGAGATATG
AGGGATAATTGGAGAAGTGAATTGTATAAGTATAAAGTGGTAGAAATTAAGCCATTGGGAGTAGCACCCACTG
AGGCAAAAAGGAGAGTGGTGGAGAGAGAAAAAAGAGCAGTGGGACTAGGAGCTGTGATCTTTGGGTTCTTGGG
AGCAGCAGGAAGCACTATGGGCGCGGCGTCAATAACGCTGACGGTACAGGCCAGACAACTGTTGTCTGGTATA
GTGCAACAGCAAAGCAATTTGCTGAAGGCTATAGAGGCACAACAGAATATGTTGCAACTCACGGTCTGGGGCA
TT
>KT183083
ACTTATGTAAATATAAGTGAAACAAACAAAACCTTCATACTCCCATGCAGAATAAAACAAATTATAAACATGT
GGCAGGGGGTAGGACGAGCAATGTATAACCCTCCCATTGCAGGAACCATAACATGTAACTCAAGTATCACAGG
ACTACTATTGGTACGTGATGGAGGAAACGGGGATACCACAAGTAATAACAGTAATAACGAGACATTCAGACCT
GGAGGGGGAAATATGAAGGACAATTGGAGAAGTGAATTATATAAATACAAGGTGGTAGAAGTTAAGCCATTGG
GAATAGCACCCACTGAGGCAAAAAGGAGAGTGGTGGAGAGAGAAAAAAGAGCAGCAGGAATAGGAGCTGTACT
CCTTGGGTTCTTGGGAGCAGCAGGAAGCACTATGGGCGCAGCATCAATAACGCTGACGGTACAGGCCAGGCAA
TTATTGTCTGGTATAGTGCAACAGCAAAGCAATTTGCTGAGGGCTATAGAGGCGCAACAGCATATGTTGCAAC
TCACGGTCTGGGGCATT
>KT183155
GATTATAATGAAATCATCACACTCCAATGCAGGATAAAACAAATTATAAACATGTGGCAGGGGGTAGGACGAG
CAATGTATGCCCCTCCCATTGAAGGAATCATAACATGTAACTCAAATATCACAGGACTACTATTGACACGAGA
TGGAGGCAACAGCAACAACAACGACACAGAGGTATTCAGACCTGGAGGAGGAAATATGAAAGACAATTGGAGA
AGTGAATTATATAAATATAAGGTGGTAGAAATTAAGCCATTGGGAATAGCTCCCACTGAAGCAAAAAGGAGAG
TGGTGGAGAGAGAAAAAAGAGCAGTGGGAATAGGAGCTGTGCTCCTTGGGTTCTTGGGAGCAGCAGGAAGCAC
TATGGGCGTGGCGTCAATAACGCTAACGGTACAGGCCAGACAATTGTTGTCTGGTATAGTGCAACAGCAAAGC
AATTTGCTGAGAGCTATAGAGGCGCAACAACATATGTTGCAACTCACGGTCTGGGGCATT
>KT183168
ACTTATAATGATACAAAGCATAATGGAACCAAAGTCATCACACTCCCATGCAGAATAAAACAAATTATAAACA
TGTGGCAGGAGGTAGGACGAGCAATGTATGCTCCTCCCATTGCAGGCAACATAACATGTACGTCAAATATCAC
AGGACTACTATTGACAAGGGATGGAGGGAATAATAGCACAGAGACAGAGACATTCAGACCTGGAGGAGGAGAT
ATGAGGGACAATTGGAGAAGTGAATTATATAAATACAAAGTAGTAGAAATTAAGCCATTGGGAATAGCACCCA
CTGGAGCAAAAAGGAGAGTGGTGGAGAGAGAAAAAAGAGCAGTGGGAATAGGAGCTGTGTTCCTTGGGTTCTT
GGGAGCAGCAGGAAGCACTATGGGCGCGGCGTCAATAACGCTGACGGTACAGGCCAGACAACTGTTGTCTGGT
ATAGTGCAACAGCAAAGCAATTTGCTGAAGGCTATAGAGGCGCAACAACACCTGTTGCAACTCACAGTCTGGG
GCATT
>KT183188
TCTCATGAGGGGTCAGGCAACATCACAAACATCACAATCCCATGCAGGATAAAACAAATCATAAACATGTGGC
AGAAGGTAGGACGAGCAATGTATGCCCCTCCCATTGCAGGAAGCATAACATGTAACTCAAGTATCACAGGAAT
ACTGTTGACACGTGATGGAGGAAACATAACAAATAGCACAAAGGAGATATTCAGACCTGGAGGAGGAGATATG
AAGGACAATTGGAGAAGTGAATTATATAAATATAAAGTGGTAGAAATTAAGCCATTGGGAATAGCACCCACTG
AATCAAAAAGGAGAGTGGTGGAGAGAGAAAAAAGAGCAGTGGGAATAGGAGCTGTGCTCCTTGGGTTCTTGGG

AGCAGCAGGAAGCACTATGGGCGCGGCGTCAATAACGCTGACGGCACAGGCCAGACAACTGTTGTCTGGTATA
GTGCAACAGCAAAGCAATTTGCTGAGAGCCATAGAGGCGCAACAGCATATGTTGCAACTCACGGTCTGGGGCA
TT
>HM623550
AATCAGACAGGAAATGATTCATTCATCATACTTCCATGTAAAATAAAACAAATTATAAATATGTGGCAGAAGG
TAGGACGAGCAATGTATGCTCCTCCCATTGCAGGAAATATAACATGTAAGTCAAATATCACAGGAATACTATT
AGTACGTGATGGAGGCCCAGATAACAGTACAGAAACATTTAGACCTGGAGGAGGAAATATGAAGGACAATTGG
AGAAGTGAATTATATAAATATAAAGTGGTAGAAATTAAGCCATTGGGAATAGCACCCACTGAAGCAAAAAGGA
GAGTGGTGGAGAGAGAAAAAAGAGCAGTGGGAATAGGAGCTGTGTTCCTTGGGTTCTTGGGAGCTGCAGGAAG
CACTATGGGCGCGGCGTCAATAACGCTGACGGTACAGGCCAGACAACTGTTGTCTGGTATAGTGCAACAGCAA
AGCAATTTGCTGAGAGCTATAGAGGCGCAACAGCATATGTTGCAACTCACAGTCTGGGGCATT
>HM623567
TGCTATACATCAAGACTGTTTAATAGTACATACAATGGTACACTTAGTATACATAATACACATAATGATACAG
GAAACGAAACACACATCACACTTCCATGCAGAATAAAACAAATTATAAACATGTGGCAGGGGGTAGGGCGAGC
AATGTATGCCCCTCCCATTGCAGGAAACATAACATGTAATTCAAGTATCACAGGTCTACTATTGACACGGGAT
GGAGGAAACACAACACATAATGACACAGAGACATTCAGACCTGGAGGAGGAAATATGAAGGACAATTGGAGAA
GTGAATTATATAAATATAAAGTAATAGAAATTAAGCCATTGGGAATAGCTCCCACTGGGGCAAAAAGGAGAGT
GGTGGAGAGAGAAAAAAGAGCAGTGGGAATAGGAGCTGTGTTCCTTGGGTTCTTGGGAGCAGCAGGAAGCACT
ATGGGCGCAGCGTCAATAACGCTGACGGTACAGGCCAGACAACTGTTGTCTGGTATAGTGCAACAGCAAAGTA
ATTTGCTGAGGGCTATAGAGGCGCAACAGCATATGTTGCAACTCACAGTCTGGGGCATT
>HM623573
AATTATACTAATAACACCATCACACTCCCATGCAGAATAAAACAAATTATAAACATGTGGCAGGAGGTAGGAA
GAGCAATGTATGCCCCTCCCATTGCAGGAAACATAACATGTAACTCAAGTATCACAGGAATACTATTGACACG
TGATGGAGGACAAGATAATAATACAAAGAATAATAAAGAGATATTCAGACCTGGAGGAGGAGATATGAGAGAC
AATTGGAGAAGTGAATTATATAAATACAAAGTGGTAGAAATTAAGCCATTGGGAGTAGCACCCACTGATGCAA
AAAGGAGAGTGGTGGAGAGAGAAAAAAGAGCAGTAGGACTAGGAGCTGTATTCCTTGGGTTCTTGGGAGCAGC
AGGAAGCACTATGGGCGCGGCGTCAATAGCGCTGACGGTACAGGCCAGACAACTGTTGTCTGGTATAGTGCAA
CAGCAAAGCAATTTGCTGAGGGCTATAGAGGCGCAACAGCATATGTTGCAACTCACAGTCTGGGGCATT
>KC154017
TTTCATCCAGACAACTCAACCAAAGGCAAAGACAATGAAACCATCACACTCCCATGCAGGATAAAACAATTTA
TAAACATGTGGCAGGGAGTAGGACGAGCAATGTATGCCTCTCCCATTGAAGGAAACATAACATGTAACTCAAG
TATCACAGGGATAATATTGACACGTGATGGAGGAAACACCACAAACAAAACAGAGGTATTCAGACCTGAAGGA
GGAAATATGAAGGACAATTGGAGAAGTGAATTATATAAATATAAAGTGGTAGAAATTAAGCCATTGGGAGTAG
CTCCAACTGAGGCAAAAAGGAGAGTGGTGGAGAGAGGAAAAAGAGCAGTGGGAATAGGAGCTGTACTCCTTGG
GTTCTTGGGAGCAGCAGGAAGCACTATGGGCGCAGCATCAATAACGCTGACGGTACAGGCCAGACAACTGTTG
TCTGGTATAGTGCAACAGCAAAGCAATTTGCTAAGAGCTATAGAGGCGCAACAACATATGTTGCAACTCACAG
TCTGGGGCATT
>KC154019
GGTCATAATGGTACAGGAGATAATTCAACCATCACAATTTCATGCAGAATAAAACAAATTATAAACATGTGGC
AGGGGGTAGGGCGAGCAATGTATGCCCCTCCCATTGCAGGAAACATAACATGTAACTCAAGTATTACAGGACT
ACTATTAGTACGTGATGGAGGTGGAAATAGCACAGAAGAGATATTCAGACCTGGAGGAGGAGATATGAGAGAC
AATTGGAGAAGTGAATTATACAAATATAAAGTAGTAGAAATTAAGCCCTTAGGAGTAGCACCCACTAAGGCAA
AAAGGAGAGTGGTGGAGAGAGAAAAAAGAGCAGTGGGAATAGGAGCTGTGTTCCTTGGGTTCTTGGGAGCAGC
AGGAAGCACTATGGGCGCAGCGTCAATAACGCTGACGGTACAGGCCAGACAATTATTGTCTGGTATAGTGCAA
CAGCAAAGCAATTTGCTGAGGGCTATAGAGGCGCAACAGCATATGTTGCAACTCACGGTCTGGGGCATT
>KC154020
GACTATAATTCAACTAAGAACATCACACTCCCATGCAGGATAAAACAAATTATAAACATATGGCAGGGGGTAG
GACAAGCAATGTATGCCCCTCCCGTTGCAGGAAAAATAATATGTACATCAAATATCACAGGACTACTATTGAC
ACGTGATGGAGGCGACGACACAAATGACACAGAGATATTCAGACCTGGAGGAGGAAATATGAAGGACAATTGG
AGAAGTGAATTATATAAATATAAAGTAGTAGAAATTAAGCCATTAGGACTAGCACCCACTGCAGCAAGAAGGA
GAGTGGTGCAGAGAGAAAAAAGAGCAATAGGAATAGGAGCTGTGTTCCTTGGGTTCTTGGGAGCAGCAGGAAG
CACTATGGGCGCAGCATCAATAACGCTGACGGTACAGGCCAGACAATTGTTGTCTGGTATAGTGCAACAGCAA
AGCAATTTGCTGAGGGCTATAGAGGCGCAACAGCATATGTTGCAACTCACAGTCTGGGGCATT
>HM204617
TTTCATGGAAATGAGACAGAAATCATACTCCAATGCAGACTAAAACAATTTATAAATATGTGGCAGGGAGTAG
GACAAGCAATGTATGCCCCTCCTATTGAAGGAAACATAACATGTACATCAAATATCACAGGACTATTATTGAC
ACGTGATGGAGGCAAAGATGATAACACAACAGAGATATTCAGACCTATAGGAGGAGACATGAAGAACAATTGG
AGAAGTGAATTATATAAATATAAAGTGGTAGAAATTAAGCCATTGGGAATAGCACCCACTGCTGCAAAAAGGA
GAGTGGTGGACAGAGAAAAAAGAGCAGTGGGAATGGGAGCTGTGTTCCTTGGGTTCTTGGGAGCAGCAGGAAG

CACTATGGGCGCAGCGTCAATAACGCTGACGGTACAGGCCAGACAACTGTTGTCTGGTATAGTGCAGCAGCAA
AGCAATTTGCTGAGAGCTATAGAGGCGCAACAGCATCTGTTGCAACTCACGGTCTGGGGCATT
>KF114894
AATCATAATGGCACCATCACACTCCCATGTAGAATAAAACAAATTATAAACATGTGGCAGAGGGTAGGACGAG
CAATGTATGCCCCTCCCATTGCAGGAAACATAACATGTAAATCTAATATCACAGGATTACTATTGGTACGTGA
TGGAGGGAATGGGAATAATAGCAGAAATGAGACATTCAGACTTGGAGGGGGAGATATGAGAGACAATTGGAGA
AGTGAATTATATAAATATAAAGTGGTAGAAATTAAGCCATTAGGAGTAGCACCCACCGAGGCAAAAAGGAGAG
TGGTGGAGAGAGAAAAAAGAGCAGTGGGAATAGGAGCTGTGTTCCTTGGGTTCTTGGGAGCAGCAGGAAGCAC
TATGGGCGCGGCGTCAATAACGCTGACGGTACAGGCCAGACAATTGTTGTCTGGTATAGTGCAACAGCAAAAC
AATTTGCTGAGGGCTATAGAGGCGCAACAGCATCTGTTGCAACTCACAGTCTGGGGCATT
>HQ595756
TATCATGCTAATGGTACAGCAAATTCTACAGATTCAAAAAATGATACATACATCACACTCCCATGCAGAATAA
AACAAATTATAAACATGTGGCAAGGGGTGGGACAAGCAATGTATGCCCCTCCCATTGCAGGAAACATAACATG
TAACTCAAATATCACAGGACTGCTATTGCTACGTGATGGAGGTCCAGAAAACAATACAGAGACATTCAGACCT
GGAGGAGGAAATATGAAGGACAATTGGAGAAGTGAATTGTATAAATATAAAGTAGTAGAAATTAATCCATTAG
GAATAGCACCCACTAAGGCAAAAAGGAGAGTGGTGGAGAGAGAAAAAAGAGCAGTGGGACTAGGCGCTATGTT
CCTTGGGTTCTTGGGAGCAGCAGGAAGCACTATGGGCGCAGCATCAATGACGCTGACGGTACAAGCCAGACAA
TTGCTGTCTGGTATAGTGCAACAGCAAAGCAATTTGCTGAAGGCTATAGAGGCGCAACAGCATATGTTGCAAC
TCACGGTCTGGGGCATT
>HQ615941
GACTATATTAAAAACAGTACATTAAACCTGGATAATAATACAAACAGTACAGACATCATACTCCAATGCAGAA
TAAAACAAATTATAAACATGTGGCAGGGGGTAGGAAAAGCAATGTATGCCCCTCCCATTGCAGGAAATATAAC
ATGTAACTCAACTATCACAGGACTACTATTGACACGTGATGGAGGCAATGCTAAAGGCAATGATACAGACAAT
AAGACAGAGACATTCAGACCTGCAGGAGGAGATATGAGGGACAATTGGAGAAGTGAATTATATAAATACAAGG
TGGTAGAAATTAAGCCATTAGGAGTAGCACCCACTGAGGCAAAAAGGAGAGTGGTGGAGAGAGAAAAAAGAGC
AGTAGGACTAGGAGCTATGTTCCTTGGGTTCTTGGGAGCAGCAGGAAGCACTATGGGCGCAGCATCAATAACG
CTGACGGTACAAGCCAGACAATTACTGTCTGGTATAGTGCAACAGCAAAGCAATTTGCTGAGGGCTATAGAGG
CGCAACAACATATGTTGCAACTCACGGTCTGGGGCATT
>GU080194
ACCTACACGCCTAATGCTACAGAAAGTATTTCCAATAAAACCATCCCACTTTCATGCAGAATAAAACAAATAA
TAAATATGTGGCAGGAAGTAGGACGGGCAATGTATGCCCCTCCCATTGCAGGAAACATAACATGTAGATCAAA
TATCACAGGACTATTATTAGTACGTGATGGAGGCAGCAATAACAATACCAACGAGACATTCAGACCTGGAGGA
GGAAATATGAAGGACAATTGGAGAAGTGAATTATACAAATATAAAGTGGTAGAAATTAAGCCATTGGGAATAG
CACCCACGAAGGCAAAAAGGAGAGTGGTGGAGAGAGAAAAAAGAGCAGTGGGAATAGGAGCTGTGTTCCTTGG
GTTCTTGGGAGCAGCAGGAAGCACTATGGGCGCGGCGTCAATAACGCTGACGGTACAGGCCAGACAATTGTTG
TCTGGTATAGTGCAACAGCAAAGCAATTTGCTGAGGGCTATAGAGGCGCAACAGCATATGTTGCAACTCACGG
TCTGGGGCATT
>GU080199
AGTTATACAAGTATTAATAAGACAGGTGAAAACAGCACAAGTGATTCAAACAGCACACTCACACTCCCATGCA
GGATAAAACAAATTATAAACATGTGGCAGGAGGTAGGACGAGCGATGTACGCTCCTCCCATTGCAGGAAATAT
AACATGTAGATCAAATATCACAGGACTACTATTGACACGTGATGGAGGAAACACAACAAACAACACAAACAAA
GAGATATTCAGACCTGGAGGAGGAAATATGAAAGACAATTGGAGAAGTGAATTATATAAATATAAAGTGGTAG
AAATTAAGCCATTGGGAGTAGCACCCACTAAGGCAAAAAGGAGAGTGGTGGAGAGAGAAAAAAGAGCAGTACT
GGGAATGGGAGCTGTGTTCCTTGGGTTCTTGGGAGCAGCAGGAAGCACTATGGGCGCAGCGTCACTAACGCTG
ACGGTACAGGCCAGACAACTGTTGTCTGGCATAGTGCAACAGCAAAGCAATTTGCTGAGAGCTATAGAGGCGC
AACAGCATCTGTTGCAACTCACAGTCTGGGGCATT
>HM623585
AACTATACAGATAACAACCAAACAGGTACCATCACACTTCCATGCAAAATAAAACAAATTATAAACATGTGGC
AGGGGGTGGGACAAGCAATGTATGCCTCTCCCATTGAAGGAAACATAACATGTAGATCAAATATCACAGGACT
ACTATTAGTACGTGATGGAGGCAATACAAGTGAGACAAATGGAACAGAGACATTCAGACCTGCAGGAGGAGAT
ATGAGGGACAATTGGAGAAGTGAATTATATAAGTATAAAGTGGTAGAAATTAAGCCATTGGGAATAGCACCCA
CTAAAGCAAAAAGGAGAGTGGTGGAGAGAGAAAAAAGAGCAGTGGGAATAGGAGCTGTGTTCCTTGGGTTCTT
GGGAGCAGCAGGAAGCACTATGGGCGCGGCGTCAATAACGCTGACGGTACAGGCCAGACAACTGTTGTCTGGT
ATAGTGCAACAGCAAAATAATTTGCTGAGGGCTATAGAGGCGCAACAGCATATGTTGCAACTCACAGTCTGGG
GCATT
>KF725903
AATCACTCAACCGATATAACCACCATCCAATGCAGGATAAAACAATTTATAAACATGTGGCAGGAGGTAGGAC
GAGCAATGTATGCCCCTCCCATAGCAGGAAACATAACATGTAAATCAAATATCACAGGACTACTATTGACAAG
TGATTCTGGAAATAGAACTAATGGAGCAGAGATATTCAGACCTGGAGGAGGAAATATGAAGGACAATTGGAGA
AGTGAATTATATAAATATAAAGTGGTAGAAATTAAGCCATTAGGAGTAGCACCCACTGGGGCAAAAAGGAGAG

AGGTGGGGAGAGGAAAAAGAGCAGCAGGAATAGGAGCTATGATCTTTGGGTTCTTGGGAGCAGCAGGAAGCAC
TATGGGCGCGGCGTCAATAACGCTGACGGTACAGGCCAGACAACTATTGTCTGGGATAGTGCAACAGCAAAAC
AATTTGCTGAGAGCTATAGATGCGCAACAGCATATGTTGCAGCTCACAGTCTGGGGCATT
>KF725921
GATCATTCAAACTTAACCATCACACTCCCATGCAGAATAAAACAAATTATAAACCTGTGGCAGGAGGTAGGAA
GGGCAATATATGCCCCTCCCATTGCAGGAAACATAACATGTACATCAAATATCACAGGATTACTATTGCTACG
AGATGGAGGTCTCTTAAACAGCACAAACGAAACATTCAGACCTGGAGGAGGAGACATGAGGAACAATTGGAGA
AGTGAATTATATAAATATAAAGTGGTAGAAATTAAGCCATTGGGGGTAGCACCCACTAATGCAAAAAGGAGAG
TGGTGGAGAGAGAAAAAAGAGCAGTAGGAATAGGAGCTGTGTTCCTTGGGTTCTTGGGAGCAGCAGGAAGCAC
TATGGGCGCGGCGTCAATGACGCTGACGGTACAGGCCAGACAACTGTTGTCTGGTATAGTGCAACAGCAAAAC
AATTTGCTGAGGGCTATAGAGGCGCAACAGCATATGTTGCAACTCACGGTCTGGGGCATT
>JX140669
AATTATACTGAGACAACAGGTAATGGGACTATCATACTTCCATGCAGAATAAAACAAATCATAAACATGTGGC
AGAAAGTAGGACGAGCAATGTATGCCCCTCCCATTGCAGGAAACATAACATGTAGTTCAAATATCACAGGACT
ACTATTGACACGTGATGGAGGAAATACAACAGATAATACAACAAATAATAACACAGAGGTATTCAGACCTGGA
GGAGGAAATATAAAAGACAATTGGAGAAGTGAATTATATAAATATAAAGTGGTAGAAATTAAACCATTAGGAA
TAGCTCCAACTAAAGCAAAAAGGAGAGTGGTGGAGAGAGAAAAAAGAGCAGTGGGAATAGGAGCTATGTTCCT
TGGATTCTTGGGAGCAGCAGGAAGCACTATGGGCGCGGCGTCAATAACGCTGACGGTACAGGCCAGACAACTG
CTGTCTGGTATAGTGCAACAGCAAAGTAATTTGCTGAGAGCTATAGAGGCGCAACAGCATATGTTGCAACTCA
CAGTCTGGGGCATT
>KF725937
AGCTATACATACAATGGTAACTCAACCATCACACTTCCATGCAGAATAAAGCAAATTATAAACATGTGGCAGG
AGGTAGGACGAGCAATGTATGCCCCTCCCATTGCAGGAAACATAACATGTGAATCAAACATCACAGGATTACT
GTTGACACGTGATGGAGGAAACAATAGTACAAATGGAACAGAGATATTCAGACCTGAAGGAGGAGATATGAGG
GACAATTGGAGAAGTGAATTGTATAAATATAAAGTGGTGGAAATTAAGCCATTGGGAGTAGCACCCACTAAGG
CAAAAAGGAGAGTGGTGGAGAGAGAAAAAAGAACAGTGGGAATAGGAGCTGTGTTCCTTGGGTTCTTGGGAGC
AGCAGGAAGCACTATGGGCGCGGCGTCAATAACGCTGACGGTACAGGCCAGACAATTGTTGTCTGGTATAGTG
CAACAGCAAAGCAATTTGCTGAGGGCTATAGAGGCGCAACAGCATATGTTGCAACTCACGGTCTGGGGCATA
>KF725945
AACTACAATATAAGTAATATAAATTCAACCTCAAATATCACACTCCAATGCAGAATAAAACAAATTGTAAACA
TGTGGCAAGGGGTAGGACGAGCAATGTATGCTCCTCCCATTGCAGGAAACATAACATGTAGATCAAATATCAC
AGGACTACTATTGACACGTGATGGAGGAAGCAATATTACTAATACTACAGAGATATTCAGACCTGCAGGAGGA
AATATGAAGGATAATTGGAGAAGTGAGTTATATAAATATAAAGTGGTAGAAGTTAAACCATTAGGAATAGCAC
CCACTGGGGCAAAAAGGAGAGTGGTGGAGAGAGAAAAAAGAGCAGTGGGAATAGGAGCTGTGTTCCTTGGGTT
CTTGGGAGCAGCAGGAAGCACTATGGGCGCGGCGTCAATAACGCTGACGGTACAGGCCAGACAATTACTGTCT
GGTATAGTGCAACAGCAAAGCAATTTGCTGAGGGCCATAGAGGCGCAACAGCATATGTTGCAACTCACGGTCT
GGGGCATT
>KF725958
AATCATACAGACCTGTTTAATGATACAGAAAGTAATAGCACAAAAGAGGATAACGCAAACATCACACTCCCAT
GCAGAATAAAACAAATTATAAACCTGTGGCAGCAAGTGGGACGAGCAATGTATGCCCCTCCCATTGCAGGAAA
CATAACATGTACATCAAATATTACAGGGATATTATTGACACGTGATGGAGGAGAGAATACAACAGAGGGGAAG
GACACAGAGACATTCAGACCTGCAGGAGGAGACATGAGGGACAATTGGAGAAGTGAATTATATAAATATAAAG
TGGTAGAAATCAGGCCATTGGGACTAGCACCCACTGGGGCAAAAAGGAGAGTGGTGGGGAGAGAAAAAAGAGC
AGTAGGAATAGGAGCTGTGTTCCTTGGATTCTTGGGAGCTGCAGGAAGCACTATGGGCGCAGCGTCATTAACG
CTGACGGTACAAGCCAGACAATTGTTGTCTGGTATAGTGCAACAGCAAAGCAATTTGCTGAAGGCTATAGAGG
CGCAACAGCATCTGTTGCAACTCACAGTCTGGGGCATT
>KF725962
TTTTATAGTCCTAATACTACAAATGGTACAGAAGAAAATACAAACTCCTCCATCACGATACCATGCAGAATAA
AACAGATAATAAACATGTGGCAGGAAATAGGACGGGCAATGTATGCCCCTCCCGTGGAAGGAAACATAACATG
CAACTCAAACATCACAGGACTGCTACTGGTACGTGATGGAGGAGATAATAATAAAACAGATAATAATAAAACA
GAGATATTTAGACCTGGAGGAGGAGATATGAGGGATAATTGGAGAAGTAAATTATATAAATATAAAGTGGTAG
AAATTAAACCATTGGGAATAGCACCCACTAAGGCAAAAAGGAGAGTGGTGGAGAGAGAAAAAAGAGCAGTGGG
AATAGGAGCTGTGTTCCTTGGGTTCTTGGGAGCAGCAGGAAGCACTATGGGCGCAGCGTCAGTGACGCTGACG
GTACAGGCCAGACAATTGTTGTCTGGTATAGTGCAACAGCAAAGCAATTTGCTGAGGGCTATAGAGGCGCAAC
AGCACATGTTGCAGCTCACAGTCTGGGGCATT
>KF726010
AATTATACAAGAAATGCTTCAGATACAACTACAAATAAAACCATCACACTTCCATGTAGAATAAAACAAATTA
TAAATATGTGGCAGGGGGTAGGAAGAGCAATGTATGCCCCTCCCATTGCAGGAAAGATAGCATGTAACTCAAG
TATCACAGGATTACTATTATTACGTGATGGAGGTATAAGCAATGACAGTGAGACCGAAATATTCAGACCTGGA
GGAGGAAATATGAAAGACAATTGGAGGAGTGAACTATATAAATATAAAGTGGTAGAAATTAAGCCATTAGGAA

TAGCACCCACTGAGGCAAAAAGGAGAGTGGTGGAGAGACAAAAAAGAGCAGTAGGACTAGGAGCTGTGTTCCT
TGGGTTCTTGGGAGCAGCAGGAAGCACTATGGGCGCAGCGTCAATAACGCTGACGGTACAGGCCAGACAATTA
TTGTCTGGTATAGTGCAACAGCAAAGCAATTTGCTGAGGGCTATAGAGGCGCAACAGCATATGTTGCAACTCA
CGGTCTGGGGCATT
>MG901120
GGCTACCAGCAAAATAGTACAGCAAATGAGGCAAACATCACACTTCCATGCAGGATAAAACAAATTATAAACA
TGTGGCAAGGGGTAGGACGAGCAATGTATGCCCCTCCTATTGCAGGAACCATACAATGTACATCCAATATTAC
AGGACTACTATTAGAACGTGATGGAGGTCCTGATAATAATGGAACTGAAAATAATGGAACTGAAAATAATGGA
ACTGAAATATTCAGACCTACAGGAGGAAATATGAGGGACAATTGGAGAAGTGAATTATATAAATATAAAGTGG
TGGAAATTAAGCCATTAGGAATAGCACCCACTGGAGCAAAAAGGAGAGTGGTGGAGAGACAAAAAAGAGCATT
GGGAATAGGAGCTATGTTCCTTGGGTTCTTGGGAGCAGCAGGAAGCACTATGGGCGCAGCGTCAATAGCGCTG
ACGGCACAGACCAGACAACTGATGGCTGGTATAGTGCAACAGCAAAGCAATTTGCTGAGGGCTATAGAGGCGC
AACAGCATATGTTGCAACTCACGGTCTGGGGCATT
>KP109516
TACTACCTGTTTAATGGTACGGTTGTTAATACCACAGATAGTAACACAAATATCACACTACCATGCAGAATAA
AACAAATTGTAAACATGTGGCAGGGGGTAGGACGAACAATGTATGCTCCTCCCATTGCAGGAGTCATAAACTG
TACATCAAACATAACAGGATTACTATTGACACGTGATGGAGGAATAAATAGCACAGGCAATGAGACATTCAGA
CCTGGAGGAGGAGATATGAGGGACAATTGGAGAAGTGAGTTATATAAATATAAAGTGGTAGAAATTAAGCCAT
TAGGGATAGCACCCACTGAGGCAAAAAGGAGAGTGGTGGAGAGAGAGAAAAGAGCAGCGATAGGAGCTGTACT
CCTTGGGTTCTTGGGAGTGGCCGGAAGCACTATGGGCGCAGCATCAATAACGCTGACGGCACAGGCCAGACAA
TTGCTGTCTGGTATAGTGCAACAGCAAAGCAATTTGCTGAGGGCTATAGAGGCGCAACAGCATATGTTGCAGC
TTACAGTCTGGGGCATT
>MN611466
TTTTATAACACTAGCAGGTTTAATAGCAGCATGACAAACAGTACAAACAACATCACACTCCCATGCAGAATAA
AACAAATTATAAACATGTGGCAGGAGGTAGGACGAGCTATGTATGCCCCTCCCATTGCAGGAAACATAACATG
TATATCAAATATCACAGGACTACTATTGACACGTGATGGAGGAATAAACAATGGGACAAATGAGACAGGTAAT
GAGACATTCAGACCTGCAGGAGGGAATATGAAGGACAATTGGAGAAGTGAATTATATAAATATAAAGTGGTGG
AGATCAAGCCATTAGGAGTAGCACCCACTAAAGCAAAAAGGAGAGTGGTGGAGAGAGAAAAAAGAGCAGTGGG
AATAGGAGCTGTGTTCCTTGGGTTCTTGGGAGCAGCAGGAAGCACTATGGGCGCGGCGTCAATAACGCTGACG
GTACAGGCCAGACAACTGTTGTCTGGTATAGTGCAACAGCAAAGCAATTTGCTGAGGGCTATAGAGGCGCAAC
AGCATATGTTGCAACTCACAGTCTGGGGCATT
>MN611467
ACTTATGCTAATAGAACAGGCAATGGTACCTTCACACTCCCATGCAGAATAAAACAAATTATAAACATGTGGC
AGGAGGTAGGAAGAGCAATGTATGCCCCTCCCATTGCAGGAAACGTAACATGTAGATCAAATATCACAGGACT
ACTATTGACACGTGATGGAGGAAAAAACAATACTGAAGAGATATTTAGACCTGGAGGAGGAAATATGAGGGAC
AATTGGAGAAGTGAATTATATAAATATAAAGTGGTAGAAATTAAGCCATTAGGAGTAGCACCCACTAATGCAC
AAAGGAGAGTGGTGAAGAGAGAAAGAAGAGCAGTGGGAATAGGAGCTGTGTTCCTTGGGTTATTGGGAGCAGC
AGGAAGCACTATGGGCGCAGCGTCAATAACGCTGACGGTACAGGCCAGACAACTGTTGTCTGGTATAGTGCAA
CAGCAAAACAATTTGCTGAGAGCTATAGAGGCGCAACAGCATATGTTGCAACTCACAGTCTGGGGCATT
>DQ411853
TGCTATACATCAGACTTGTTTAATAGTACATACATGTCTAATAATACAGGAGGAGCAAATATCACACTCCAAT
GCAGAATAAAACAAATTATAAGAATGTGGCAGGGGGTAGGACAAGCAATGTATGCCCCTCCCATTGCAGGAAA
CATAACATGCAAATCAAATATCACAGGACTACTATTGACACGTGATGGAGGAAAGGAAAAAAATGACACAGAA
ACATTTAGACCTGGAGGGGGAGATATGAGAGACAATTGGAGAAGTGAATTATATAAATACAAGGTGGTAGAAA
TTAAGCCATTAGGAATAGCACCCGATAAGGCAAAAAGGAGAGTGGTGGAGAGAGAAAAAAGAGCAGTGGGAAT
AGGAGCTGTGTTCCTTGGGTTCTTGGGAGCAGCAGGAAGCACCATGGGCGCAGCATCAATGACGCTGACGGTA
CAGGCCAGACAATTGTTGTCTGGTATAGTGCAACAGCAAAGCAATTTGCTGAGGGCTATAGAGGCGCAACAGC
ATATGTTGCAACTCACGGTCTGGGGCATT
>AY162223
TACTACCCTAAGAATGGTACATACAAATACAATGGTAATTCAAGCTTACCCATCACACTCCAATGCAAAATAA
AACAAATTGTACGCATGTGGCAAGGGGTAGGACAAGCAATGTATGCCCCTCCCATTGCAGGAAACATAACATG
TAGATCAAACATCACAGGAATACTATTGACACGTGATGGGGGATTTAACAACACAAACAACGACACAGAGGAG
ACATTCAGACCTGGAGGAGGAGATATGAGGGATAACTGGAGAAGTGAATTATATAAATATAAAGTGGTAGAAA
TTAAGCCATTGGGAATAGCACCCACTAAGGCAAAAAGAAGAGTGGTGCAGAGAAAAAAAAGAGCAGTGGGAAT
AGGAGCTGTGTTCCTTGGGTTCTTGGGAGCAGCAGGAAGCACTATGGGCGCAGCGTCAATAACGCTGACGGTA
CAGGCCAGACAACTGTTGTCTGGTATAGTGCAACAGCAAAGCAATTTGCTGAAGGCTATAGAGGCGCAACAGC
ATATGTTGCAACTCACAGTCTGGGGCATT
>AF391235
AGTTATAATACATCAGACCTGTTTAATAGTAATAATGGTTCAGCCATCACACTCCCATGCAGAATAAAACAAA
TTGTAAACATGTGGCAGGGGGTAGGACGAGCAATATATGCCCCTCCCATTGCAGGAAACATAACATGTAACTC

AAGTATCACAGGACTACTCTTGGTACGTGATGGAGGAAACACAACCAACTCAACTGAGATATTCAGACCAGAA
GGAGGAAATATGAGGGACAATTGGAGAAGTGAATCATATAAATACAAAGTGGTAGAAATTAAGCCCTTGGGAA
TAGCGCCCACTAATGCAAAAAGGAGAGTGGTGGAGAGAGAAAAAAGAGCAGTGACACTAGGAGCTATGTTCCT
TGGGTTCTTGGGAGCAGCAGGAAGCACTATGGGCGCAGCGTCAATAACGCTGACGGCACAGGCCAGACAGTTG
TTGTCTGGAATAGTGCAACAGCAAAGCAATTTGCTGAGAGCTATAGAGACGCAACAGCATATGTTGCAACTCA
CAGTTTGGGGCATT
>AF391240
AATCATACATACCTGTTTAATGGTACAGGCGTGCCTAATAATACCACACCTTCTAATGAGACCATCATACTTC
CATGCAGAATAAAACAAATTATAAACATGTGGCAGGAGGTAGGGCGAGCAATGTATGCCCCTCCCATTGCAGG
AAACATCACATGTACATCAAACATCACAGGACTACTATTAGTACGTGATGGAGGCAACAGTGGCAAAAATACC
ACAGAAGAGATATTCAGACCTGGGGGAGGAAATATGAAGGACAATTGGAGAAGTGAATTATATAAATATAAAG
TGGTAGAAATTAAGCCATTAGGAATAGCTCCCACTGCGGCAAAAAGGAGAGTGGTGGAGAGAGAAAAAAGAGC
AGTGGGAATAGGGGCTGTGTTCCTTGGGTTCTTGGGAGCAGCAGGAAGCACTATGGGCGCGGCGTCAATAACG
CTGACGGTACAGGCCAGACAATTGTTGTCTGGTATAGTGCAACAGCAAAGCAATTTGCTGAGGGCTATAGAGG
CGCAACAGCATCTGTTGCAACTCACAGTCTGGGGCATT
>AY529668
AATTATATAGCAACAAATACTTCAGAGACTTCACCCATCATGATCCAGTGCAGAATAAAGCAAATTATAAACA
TGTGGCAGAGGGTAGGACGAGCAATGTATGCCCCTCCCATTGCTGGAAGCCTAACATGTAACTCAAGTATCAC
AGGACTAATATTGACACGTGATGGAGGAGAAAATACAAGTATAGAGACATTCAGACCTGGAGGAGGAAATATG
AAGGACAATTGGAGAAGTGAATTATATAAATATAAAGTGGTAGAAGTTAAGCCATTAGGAATAGCACCCACTG
ATGCAAAAAGGAGAGTGGTGGAGAGAGAGAAAAGAGCAGTGGGAATAGGAGCTGTGTTCCTTGGGTTCTTGGG
AGCAGCAGGAAGCACTATGGGCGCGGCGTCAATAACGCTGACGGTACAGGCCAGACAATTATTGTCTGGTATA
GTGCAACAGCAAAGCAATTTGCTGAGGGCTATAGAGGCGCAACAGCATATGTTGCAACTCACGGTCTGGGGCA
TT
>AY529670
GATTACACGCCTAATAAGACAGAAAATGATTCAAATGCAACTTCAAATATCACACTCCAATGTAGAATAAAGC
AAATTGTAAACATGTGGCAGGGGGTAGGACAAGCAATGTATGCCCCTCCCATTAAAGGATACATAGAATGTAT
ATCAAATATCACAGGACTACTATTGACACGTGATGGAGGAAAAGAAAAGAATAACACAGAGACATTCAGACCT
GGAGGAGGAGATATGAGGGACAATTGGAGAAGTGAATTATATAAATATAAAGTAGTAGAAGTTGAACCATTAG
GAATAGCACCCACTAAGGCAAAAAGGAGAGTGGTGGAGAGAGAAAAAAGAGCAGTGGGAATAGGAGCTGTGTT
CCTTGGGTTCTTGGGAGCAGCAGGAAGCACTATGGGCGCGGCATCAATAACGCTGACGGTACAGGCCAGACAA
TTGTTGTCTGGTATAGTGCAACAGCAAAGCAATTTGCTGAGGGCTATAGAGGCGCAACAGCATATGTTGCAAC
TCACGGTCTGGGGCATT
>DQ447272
AATTACAATGGTACAAAAAATAGTACAGAAAACATCACACTCCCATGCAGAATAAAACAAATTATAAACATGT
GGCAGAAGGTAGGACGAGCAATGTATGCTCCTCCCATTGAAGGAAAAATAACATGTTCCTCAAACATCACAGG
ATTACTAGTGACATATGATGGAGGAACATCAGACAACGAGACAACAAAAAACACAGAGACATTCAGACCTGGA
GGAGGAGATATGAGGGACAATTGGAGAAGTGAATTATATAAATATAAAGTGGTAGAAATTAAGCCATTGGGAA
TAGCACCCACTAAGGCAAAAAGGAGAGTGGTGGAGAGAGAAAAAAGAGCAGTGGGAATAGGAGCTGTGTTCCT
TGGGTTCTTGGGAGCAGCAGGAAGCACTATGGGCGCGGCGTCAATAACGCTGACGGTACAGGCCAGACAATTG
TTGTCTGGTATAGTGCAACAGCAAAGCAATTTGCTGAGGGCTATAGAGGCGCAACAGCATATGTTGCAACTCA
CGGTTTGGGGCATT
>AY522726
AATTACACATCAAAACAGGATAATAGTACAAAAAGAAATTCAAGCTCAATCATCACAATCCCATGCAAAATAA
AACAATTTATAAACATGTGGCAGGAGGTAGGACGAGCAATGTATGCCCCTCCCATTGAAGGAATCATAAAATG
TACATCAAACATCACAGGATTACTATTACTACGTGATGGAGGGACCAATAATGATACTAATGACACAGAGATA
TTCAGACCTGGAGGAGGGGACATGAGGGACAATTGGAGAAGTGAATTATATAAATATAAAGTAGTAGAAATTA
AGCCATTGGGAATAGCTCCCACTGAAGCAAAAAGGAGAGTGGTGGAGAGAGAAAAAAGAGCAGTAGGAATAGG
AGCTGTGCTCCTTGGGTTCTTGGGAGCAGCAGGAAGCACTATGGGCGCGGCGTCAATAACGCTGACGGTACAG
GCCAGACAAGTGTTGTCTGGTATAGTGCAACAGCAAAGCAATTTGCTGAGGGCCATAGAGGCGCAACAGCATA
TGTTGCAACTCACGGTCTGGGGCATT
>AY522728
ACTCATTTGCCTAATGATACCAATAGTGAGTCCAATATAAACCTCACGATCCAATGCAGAATAAAACAAATTA
TAAATATGTGGCAGGAGGTGGGAAGGGCAATGTATGCCCCTCCCATTGAAGGAAACATAACATGTAAATCAAA
TATCACAGGACTACTATTGACGCGTGATGGAGGAAGAAGTAATAACACAGAAGAGATATTTAGACCTGGAGGA
GGAAATATGAAGGATAATTGGAGAAGTGAATTATATAAATATAAAGTGGTAGAAATTAAGCCATTGGGAGTAG
CACCCACTGAGGCAAAAAGGAGATTGGTGGGGAAAGAAAAAAGAGCAGTGGGAATAGGAGCTGTGCTCTTTGG
GTTCTTGGGAGCAGCAGGAAGCACTATGGGCGCGGCGTCAATAGCGCTGACGGTACAGGCCAGACAATTGCTG
TCTGGTATAGTGCAACAGCAAAACAATTTGCTGAGGGCTATAGAGGCGCAACAGCATCTGTTGCAACTCACGG
TCTGGGGCATT

>AY522731
AGTTATACAAAACTAAATGGAAGTGAGTTAAACAAAACCATCACACTCCCATGCAGGATAAAACAAATTATAA
ACATGTGGCAGGAGGTAGGACGAGCAATGTATGCCCCTCCCATTGCAGGGAAAATAACATGTAACTCAAGTAT
CACAGGACTACTATTGACACGTGATGGAGGAGGAAACAAAACGAATGGCACAGAGGTATTCAGACCTGGAGGA
GGAAATATGAAGGACAATTGGAGAAGTGAATTATATAAATATAAAGTAGTAGAAATTAAGCCATTGGGAATAG
CACCCACTGAGGCAAAAAGGAGAGTGGTGGAGAGAGAAAAAAGAGCAGTGGGAATAGGAGCTGTACTCCTTGG
GTTCTTGGGAGCAGCAGGAAGCACTATGGGCGCGGCGTCAATAACGCTGACGGTACAGGCCAGACAACTGTTG
TCTGGTATAGTGCAACAGCAAAGCAATTTGCTGAGAGCTATAGAGGCGCAACAGCATATGTTGCAACTCACAG
TCTGGGGCATT
>AY522733
GCTTACAGCAACACTGACAACAACAATAGTACTGGAAACGCAACCACCATCACACTGCCATGCAGAATAAAAC
AAATTATAAACATGTGGCAGGAGGTAGGACGAGCAATGTATGCCCCTCCCATTGCAGGAAACATAACATGTAA
CTCAAGTATCACAGGACTAATATTGACACGTGATGGAGGAAGCAATATGACAGAAGTAAATACCACAGAGATA
TTCAGACCTGGAGGAGGAAATATGAAGGACAATTGGAGAAGTGAATTATATAAATATAAAGTGGTAGAAATTA
AGCCATTGGGAATAGCACCCACTGAGGCAAAAAGGAGAGTGGTGGAGAGAGAAAGAAGAGCAGTGGGACTAGG
AGCTATGTTCCTTGGGATCTTGGGAGCGGCAGGAAGCACTATGGGCGCGGCGTCAATAACGCTGACGGTACAG
ACCAGACAACTGTTGTCTGGTATAGTGCAACAGCAAAGTAATTTGCTGAAGGCTATAGAGGCGCAACAGCATC
TGTTGCAACTCACGGTCTGGGGCATT
>AY522735
TGCTATACCTCAAAGCTGTTTAATAGAACATACAGGCCAAATTCAACCAGTTCAAATTCAAATTCAAATTCAA
CCAGTTTAAATTCAACCATCACAATTCCATGCAGAATAAAACAAATTATAAACATGTGGCAGAGGGTAGGACG
AGCAATGTATGCCCCTCCCATTGCAGGAAACATAACATGTAATTCAAGTATCACAGGACTACTATTAGTACGT
GATGGAGGAGAAAACAATAGTACAGAAGAGATATTCAGACCTGGAGGAGGAGATATGAGGGACAATTGGAGAA
GTGAATTATATAAATATAAAGTGGTAGAGATTAAGCCATTGGGAATAGCACCCACTAAGGCAAAAAGGAGAGT
GGTGGAGAGAGAAAAAAGAGCAGTAGTGGGACTAGGAGCTGTGTTCTTTGGGTTCTTGGGAGCAGCAGGAAGC
ACTATGGGCGCGGCGTCAATAACGCTGACGGTACAGGCCAGACAATTGCTGTCTGGTATAGTGCAACAGCAAA
GCAATTTGCTGAGGGCTATAGAGGCGCAACAGCATATGTTGCAACTCACGGTCTGGGGCATT
>DQ447268
AATCATAATAATACATACAATACAAGTGACAACTCCAACATCACACTCCCATGCAGAATAAAACAAATTATAA
ACATGTGGCAGGAGGTAGGAAAAGCAATATATGCCCCTCCCATCAGAGGAAACATAACATGTAACTCAAGTAT
CACAGGACTACTATTGGTACGTGATGGGGGTAACTCAAATAATAACTCAAATGAGACCACAGAGATATTCAGA
CCTGGAGGAGGAGATATGAGAGATAATTGGAGAAGTGAATTATATAAATATAAAGTGGTAGAAATTAAGCCAT
TGGGAGTAGCACCCACTGGGGCAAAAAGGAGAGTGGTGGAGAGAGAAAAAAGAGCAGTGGGAATAGGAGCTGT
GTTCCTTGGGTTCTTGGGAGCAGCTGGAAGCACTATGGGCGCGGCGTCAATGACGCTGACGGTACAGGCCAGA
CAACTGTTGTCTGGCATAGTGCAACAGCAAAGCAATTTGCTGAGGGCTATAGAGGCGCAACAACATATGTTGC
AACTCACAGTCTGGGGCATT
>GU216739
AATTATACTGGCAGTGGAAAAAACATAACCACCACCATCCAATGCAAGATAAAACAAATTGTAAATCTGTGGC
AAGAGGTAGGACGAGCAATGTATGCCCCTCCCATTGCAGGAAACATAACGTGTAACTCAAGTATTACAGGACT
ACTATTGACACGTGATGGCGGTAGCAACAGCAGCAGCCCAGAGACATTCAGACCTGGAGGAGGAAATATGAAG
GACAATTGGAGAAGTGAATTATATAAATATAAAGTGGTAGAAATTAAGCCATTGGGAGTAGCACCCACTGAGG
CAAAAAGGAGAGTGGTGGAGAGAGAAAAAAGGGCAGTGGGAATAGGAGCTGTGTTCCTTGGGTTCTTGGGAGC
TGCAGGAAGCACTATGGGCGCGGCGTCAATAACGCTGACGGTACAGGCCAGACAACTGTTGTCTGGTATAGTG
CAACAGCAAAGCAATTTGCTGAGAGCCATAGAGGCGCAACAGCATATGTTGCAACTCACGGTCTGGGGCATT
>GU216844
TCTTATGATACAGAAAGTAATGGAAATATCATACTCCCATGCAGAATAAAACAAATTATAAACCTGTGGCAGG
GGGTAGGACGAGCAATATATGCACCTCCCATTGCAGGAAATATAAAATGTACATCAGATATCACAGGACTACT
ATTGACACGTGATGGAGGGAATAACACAATTAATGAGACATTCAGACCTATAGGAGGAAATATGAGGGACAAT
TGGAGAAGTGAATTGTATAAATATAAAGTGGTAGAAATCAGGCCATTGGGAGTAGCACCCACTGCAGCAAAAA
GGAGAGTGGTGGAGAGAGAAAAAAGAGCAGTGGGCATAGGAGCTGTGTTCCTTGGGTTCTTGGGAGCAGCAGG
AAGCACTATGGGCGCAGCGTCAATGACGCTGACGGTACAGGCCAGACAACTGTTGTCTGGTATAGTGCAACAG
CAAAGCAATTTGCTGAGGGCTATAGAGGCGCAACAGCATATGTTGCAACTCACGGTCTGGGGCATT
>HQ625580
ACCTATAATGTTACCAGAACAAGTAATACAACTAATAGTAGTATAATTACACTCCAATGCAGAATAAAACAGA
TTATAAACATGTGGCAAGGGGTAGGACAAGCAATGTATGTCCCTCCCATTGCAGGAAACATAATATGCAAATC
AAATATCACAGGACTACTATTGACACGTGATGGAGGCAATAATACAGAAAACAATAATACAGAGATATTCAGA
CCTGCAGGAGGAGACATGAGGGACAATTGGAGAAGTGAATTATATAGATATAAAGTGGTAGAAATTAAGCCAT
TAGGAATAGCACCCACAAAAGCAAGAAGGAAAGTGGTGCAGAGAGAAAAAAGAGCAGTGGGAATAGGAGCTGT
ACTCCTTGGGTTCTTGGGAGCAGCAGGAAGCACTATGGGCGCGGCGTCAATAACGCTGACGGTACAGGCCAGA

CAACTGTTGTCTGGTATAGTGCAGCAGCAAAACAATTTGCTGAGAGCTATAGAGGCGCAACAGCATATGTTGC
AACTCACAGTCTGGGGCATT
>HQ625591
ACCTATTTAAACACAACCAATGTAAACGAAACCGACATCACACTCCAATGCAGGATAAAGCAAATTATAAACA
TGTGGCAGGGGGTGGGACGAGCAATGTATGCCCCTCCCATTGCAGGAAACATAACATGTAACTCAAATATCAC
AGGACTACTGCTGACACGTGATGGAGGCTTGAATAACACGAATAACGAGACATTCAGACCTGGAGGGGGAAAT
ATGAAGGACAATTGGAGAAATGAATTATATAAATACAAAGTGGTAGAAATTAAGCCATTGGGAGTAGCACCCA
CTGCTGCAAAAAGGAGAGTGGTGGAGAGAGAAAAAAGGGGACTAGGAGCTGTGTTCCTTGGGTTCTTGGGAGC
AGCAGGAAGCACTATGGGCGCGGCGTCAATAACGCTGACGGTACAGGCCAGACAACTGTTGTCTGGTATAGTG
CAACAGCAAAGCAATTTGCTGAGAGCTATAGAGGCGCAACAGCATATGTTGCAACTCACAGTCTGGGGCATT
>HQ625582
CATCATAAAGGAAGTAATGAAAATGAAAGCAGCATCATACTTCCATGCAGAATAAAACAAATTATAAACATGT
GGCAGGAGGTAGGACGAGCAATGTATGCATCTCCCATTGCAGGAAGGATAACATGTACATCAAATATCACAGG
ACTATTATTAGTACGAGATGGAGGCACAGGCAATACCAGTAAACCAGAAATATTCAGACCTGGAGGAGGAAAC
ATGAAGGACAATTGGAGAAGTGAATTATATAAATATAAAGTAGTAGAAATTAAGCCATTAGGAATAGCACCCA
CTGGAGCAAAAAGGAGAGTGGTGGAGAGAGAAAAAAGAGCAGTGGGAATAGGAGCTGTACTCCTTGGGTTCTT
GGGAGCAGCAGGAAGCACTATGGGCGCGGCGTCAATAACGCTGACGGTACAGGCCAGACAACTGTTGTCTGGT
ATAGTGCAACAGCAAAGCAATTTGCTGAGGGCTATAGAGGCGCAACAGCAGATGTTGCAACTCACAGTCTGGG
GCATT
>GU939049
TTTTATAAGTTCAATAGGACAGAAAATGGTTCAAATACAGAAAATAGTTCAGGCATCATCACACTCCCATGCA
AAATAAAACAAATTATAAACATGTGGCAGGAGGTAGGACGAGCAATGTATGCCCCTCCCATTGAAGGAAACAT
AACATGTAAATCAGAGATTACAGGACTCCTATTGACACGTGACGGAAAAAAATGGAACAGAGGAGATAGTTAGA
CCTATAGGAGGAAATATGAAGGATAATTGGAGAAGTGAATTATATAAATATAAAGTGGTAGAAATTAAGCCAT
TAGGAATAGCACCCACTGAGGCAAAAAGGAGAGTGGTGGAGAGAGAAAAAAGAGCAGTAGGAATAGGAGCTGT
GCTCCTTGGGTTCTTGGGAGCAGCAGGAAGCACTATGGGCGCGGCGTCAATAACGCTGACGGTACAGGCCAGA
CAATTGTTGTCTGGTATAGTGCAACAGCAAAGCAATTTGCTGAGGGCTATAGAGGCGCAACAGCATCTGTTGC
AACTCACAGTCTGGGGCATT
>AB254146
TACTATTGGAACAGTACAACAGGGAATTCAAGCTCAAACATCACAATCCCATGCAAAATAAAACAAATTATAA
ACATGTGGCAGGAGGTAGGACGAGCAATGTATGCCCCTCCCATTGCAGGGAAAATAACATGTGCATCAAATAT
CACAGGACTACTATTGACACGTGATGGAGGAGTATCAAATAAGACAGATGCAAATAAGACAGAGACATTCAGA
CCTATAGGAGGAAATATGAGGGACAATTGGAGAAGTGAATTATATAAATACAAAGTGGTAGAAATTAATCCAT
TAGGACTGGCACCCACTAAGGCAAAAAGGAGAGTGGTGGAGAGAGAAAAAAGAGCAGTGGGACTAGGAGCTGT
GCTCTTTGGGTTCTTGGGAGCAGCAGGAAGCACTATGGGCGCAGCATCACTAACGCTGACGGTACAGGCCAGA
CAATTATTGTCTGGTATAGTGCAACAGCAAAGCAATTTGCTGAGAGCTATAGAGGCGCAACAGCATATGTTGC
AACTCACGGTCTGGGGCATT
>KY229291
AATTATACAGTAGACAATTCTACCATCACACTCCAATGCAGAATAAAACAAATTATAAACACATGGCAGGAGG
TAGGACGAGCAATATACGCCCCTCCCATTGAAGGAAACATAAAATGTACATCAAAAATCACAGGACTACTCTT
GGTACGTGATGGAGGGGGGAATGACACAAGGGAAGAGATATTCAGACCTGGAGGAGGAAATATGAGGGACAAT
TGGAGAAGTGAATTATATAAATATAAAGTGGTAGAAATTAAACCATTGGGAATAGCACCCACTAAGGCAAAAC
GGAGAGTGGTGGAGAGAGAAAAAAGAGCAGTCGGAATGGGAGCTGTGATCCTTGGGTTCTTGGGAGCAGCAGG
AAGCACTATGGGCGCGGCGTCAATAACGCTGACGGCACAGGCCAGACAAGTATTGTCTGGTATAGTGCAACAG
CAAAGCAATTTGCTGAGAGCTATAGAGGCGCAACAGCATATGTTGCAACTCACAGTCTGGGGCATT
>KY229453
AATCACACAAATAATAGTACATATAATATCACACTCCCATGCAGAATAAGACAGATTGTAAGGATGTGGCAGG
GAGTAGGACAAGCAATGTATGCCCCTCCCATTGCAGGAAACATAACATGTAGATCAAATATCACAGGACTACT
ATTGACACGTGATGGAGGAAAGGGAGATAACAACACAACAGAAACATTCAGACCTGGAGGAGGAGATATGAGG
GACAATTGGAGAAGTGAATTATATAAATATAAAGTGGTAGAGATTAAGCCATTAGGAATAGCACCCACCAAGG
CAAAAAGGAGAGTGGTGGAGAGAGAAAAAAGAGCAGTGGGAATGGGAGCTGTACTCCTTGGGTTCTTGGGAGC
TGCAGGAAGCACTATGGGCGCGGCGTCAATAACGCTGACGGTACAGGCCAGACAATTGTTGTCTGGTATAGTG
CAACAGCAAAGCAATTTGCTGAGAGCTATAGAGGCGCAACAGCATATGTTGCAACTCACAGTCTGGGGCATT
>EU166359
TTTCATGGTACATACCTGCCTAATAGTACAGTCATGCTTAATGATACTTCAAACAAAACCATCACACTGCTAT
GCAGAATAAAACAAATTATAAATATGTGGCAGGAGGTAGGGCGAGCAATGTATGCCCCACCCATTGCAGGAAG
CATAATATGCAACTCAAGTATTACAGGGCTACTATTGACACGTGATGGAGGAGATGGAAATGGAAAGAATACT
ACAGGTAAGGATACAGAGACATTCAGACCTGCAGGAGGAGATATGAGGGACAATTGGAGAAGTGAATTATATA
AATATAAGGTGGTAGAAATTAACCCATTAGGAATAGCACCCACTAAAGCAAAAAGGAGAGTGGTGGAGAGAGA
GAAAAGAGCAGTGGGAATAGGAGCTGTACTCCTTGGGTTCTTGGGAGCAGCAGGAAGCACTATGGGCGCAGCA

TCAATAACGCTGACGGTACAGGCCAGACAATTATTGTCTGGTATAGTGCAACAGCAAAGCAATTTGCTGAGGG
CTATAGAGGCGCAACAACATATGTTGCAACTCACGGTCTGGGGCATT
>EU166432
AATTATAACATTACAAACAATAATGTTACAAACATTACAGACGAGAATGTAAACATCACACTCAAATGCAGAA
TAAAACAGATTATAAACATGTGGCAGGGGGTAGGACAAGCAATATATGCCCCTCCCATTGCAGGAAACATAAC
ATGTAAATCAAATATCACAGGAATACTATTGACACGCGATGGAGGCGACGAAACAAAGAATAACACAGAAATA
TTCAGACCTGGAGGAGGAAATATGAAGAACAATTGGAGAAGTGAATTATATAAATATAAAGTGGTAGAAATTA
AGCCATTAGGAATAGCACCCACTGAGGCAAAAAGGAGAGTGGTGGAGAGAGAAAAAAGAGCAGTGGGAATAGG
AGCTGTGTTCCTTGGGTTCTTGGGAGCAGCAGGAAGCACTATGGGCGCGGCATCAATAACGCTGACGGTACAG
GCCAGACAATTGTTGTCTGGTATAGTGCAACAGCAAAGCAATTTGCTGAGAGCTATAGAGGCGCAACAGCATA
TGTTGCAACTCACTGTCTGGGGCATT
>DQ388516
ACCTACACGAATGGAAGTAATTCAAATGTAAACATCACAAGTGCAACCATCACCCTCCCATGCAGAATAAAAC
AAATTATAAACATGTGGCAAGAAGTAGGACGGGCAATGTATGCCCCTCCCATTGCAGGAAACATAACATGTAT
ATCAAATATCACAGGACTACTCCTGACAAGGGATGGAGGAAATGGAAATGATACAAATGACACAGAGACATTT
AGACCTGCAGGAGGAGATATGAGGGACAATTGGAGAAGTGAATTATATAAATATAAAGTGGTAGAAATTAAAC
CATTAGGAATAGCACCCACTAAAGCAAAAAGGAGAGTGGTGGGGAGAGAAAAAAGAGCAGTGGGAATAGGAGC
TGTGTTCCTTGGGTTCTTGGGAGCAGCAGGAAGCACTATGGGCGCGGCGTCAATAACGCTGACGGTACAGGCC
AGACAATTATTGTCTGGTATAGTGCAACAGCAAAACAATTTGCTGAGAGCTATAGAGGCGCAACAGCATCTGT
TGCAACTCACGGTCTGGGGCATT
>GU939124
AATTATACATACATGAATGAGACAAGCACTAACTCAACGATCAGACTCCCATGTAAGATAAGACAAATTATAA
ACATGTGGCAGGAGGTAGGACGAGCAATATATGCCCCTCCCATTGAAGGAAACATAACATGCAACTCAAGTAT
CACAGGACTACTATTGGTACGGGATGGAGGCAACACTAGCAGTACCGGCACAGAGATAGAGATATTTAGACCT
GGAGGGGGAGATATGAGGGACAATTGGAGAAGTGAATTATATAAATATAAAGTGGTAGAAATTAAGCCATTGG
GAGTAGCTCCCACTAAGGCAAAAAGAAGAGTGGTGGAAAGAGAAAAAAGAGCAGTGGGAATAGGAGCTGTACT
CCTTGGGTTCTTGGGAGCAGCAGGAAGCACTATGGGCGCAGCGTCAATAACGCTGACGGCACAGGCCAGACAA
GTGCTGTCTGGTATAGTGCAACAGCAAAGCAATTTGCTGAGGGCTATAGAGGCGCAACAGCACCTGTTGCAAC
TCACAGTCTGGGGCATC
>JX213407
AATTATACATTTAATGATACATTTAATGATATAGGAAGTAAGTCAAATACAGGAAATATCACACTCCAATGCA
GAATAAAACAAATTGTAAACATGTGGCAGAGGGTAGGACGAGCAATATATACCCCTCCCATTAAGGGACAGAT
AACATGTGTATCAAATATCACAGGAATACTATTGGTACGTGATGGAGGAAACAACAATGGATCAGAGAATAAC
TCAGAGATATTCAGACCTGGAGGAGGAGATATGAGGGACAATTGGAGAAATGAATTGTATAAATATAAAGTAG
TAGAAATTAAGCCATTGGGAATAGCACCCACTGGGGCAAAAAGGAGAGTGGTGGAGAGAGAAAAAAGAGCAGT
GGGAATAGGAGCTGTGTTCTTTGGGTTCTTGGGAGCAGCAGGAAGCACTATGGGCGCGGCGTCAATAACGCTG
ACGGTACAGGCCAGACAATTATTGTCTGGTATAGTGCAACAGCAAAGCAATTTGCTGAGGGCTATAGAGGCGC
AACAGCATATGTTGCAACTCACGGTCTGGGGCATT
>JX213435
AATCATACACTGGATGTTAATGGTACATTATCTCCAAATTCATCTTCTTTAAACATCACACTCCCATGCAAAA
TAAAACAAATCATAAACATGTGGCAGGGGGTGGGACGAGCAATGTATGCCCCTCCCATTGCAGGAAACATAAC
ATGTAGATCAAATATCACGGGAATAATATTGACACGTGATGGTGGAAGTGGAAGTGGATCATCTAATGACACA
GAGATATTCAGACCTGGAGGAGGAGATATGAGAGACAATTGGAGAAGTGAATTATATAAATACAAAGTGGTAG
AAATTAAGCCATTAGGAATAGCACCCAACAAGGCAAAAAGGAGAGTGGTGGAAAGAGAAAAAAGAGCAGTGGG
AATAGGAGCTGTGTTCCTTGGGTTCTTAGGAGCAGCAGGAAGCACTATGGGCGCGGCGTCAATGGCGCTGACG
GTACAGGCCAGACAAGTGTTGTCTGGTATAGTGCAACAGCAAAGCAATTTGCTGAGAGCTATAGAGGCGCAAC
AGCATATGTTGCAACTCACAGTCTGGGGCATT
>JX213454
AGTTACAAATGCATGCCTAATATGCCTAATGACACATGTAATAGCACAATCACAGATAACAGCACAGGATTCA
TCATACTCCCATGCAGAATAAAACAAATTGTAAACATGTGGCAGGGGGTAGGACGAGCAATGTATGCCGCTCC
CATTGCCGGGAACATAACATGTAGATCAAACATCACAGGACTACTATTGACACGTGATGGAGGTACCCCAAGT
AAAAATGAGACCAAGGAGACATTCAGACCTGGAGGAGGGAATATGAAGGACAATTGGAGAAGTGAATTATATA
AGTATAAAGTGGTAGAAATTAAGCCACTGGGACTAGCACCCACTGGAGCAAAAAGGAGAGTGGTGGAGAGAGA
AAAAAGAGCAGTGGGAATAGGAGCTGTGTTCCTTGGGTTCTTGGGAGCAGCAGGAAGCACTATGGGCGCGGCG
TCAATGACGCTGACGGTACAGGCTAGACAACTGTTGTCTGGTATAGTGCAACAGCAAAGCAATTTGCTGAGGG
CCATAGAGGCGCAACAGCATATGTTGCAGCTCACAGTCTGGGGCATT
>GU329195
AGTTACTCAAATGAGACAAACTCAACCATCACACTCCAGTGTAAAATAAAACAAATTATAAACATGTGGCAGG
GGGTAGGACGAGCAATGTATGCCGCCCCCATTGCAGGAAACATAACATGTATATCAAACATCACAGGAATACT
ACTGACACGGGATGGAGGAAACAGTAATAGTAATGAGAGTACAGAAACATTTAGACCAGGAGGGGGAAATATG

AAAGACAATTGGAGAAGTGAATTATATAAATATAAAGTGGTAGAGGTTAAGCCATTGGGAATAGCACCCACTG
AGGCAAAAAGGAGAGTGGTGGAGAGAGAAAAAAGAGCAGTGGGACTAGGAGCTGTGTTCCTTGGGTTCTTGGG
AGCAGCAGGAAGCACTATGGGCGCAGCATCAATAACGCTGACGGTACAGGCCAGAAATCTGTTGTCTGGCATA
GTGCAACAGCAAAACAATTTGCTGAGGGCTATAGAGGCGCAACAGCATATGTTGCAACTCACAGTCTGGGGCA
TT
>GU329205
AATCACACAGACATGTCTAACATGACTAATAATAACATCACACTCACACTCCCATGCAGAATAAAACAAATTA
TAAACATGTGGCAGGAGGTAGGACGAGCAATATATGCACCTCCCATTGTAGGAAACATAATCTGTACATCAAA
TATCACAGGACTACTATTGACACGTGATGGAGGAACAGATAACAACAGAACAGAGGAGACGTTCAGACCTGGG
GGAGGAGATATGAGAGACAATTGGAGAAGTGAACTATATAAATATAAGGTGGTAGAAATTAAGCCATTGGGCA
TAGCACCCACTAAGGCAAAAAGGAGAGTGGTGGAGAGAGAGAAAAGAGCAGTGGGAATAGGAGCTGTGTTCCT
TGGGTTCTTGGGAGCAGCAGGAAGCACTATGGGCGCGGCGTCAGTGACGCTGACGGTACAGGCCAGACAACTA
TTGTCTGGTATAGTGCAACAGCAAAGCAATTTGCTGAAGGCTATAGAGGCGCAACAGCATATGTTGCAACTCA
CGGTCTGGGGCATC
>GU329282
CACTATAGTAATGGTACAGACATATTTAATGATAATAGTACAAGCAACATCACACTCCCATGCAAAATAAAAC
AAATTATAAACATGTGGCAGAAAGTAGGACAAGCAATGTATGCCCCTCCCATTGCAGGAAACATAAAATGTGA
ATCAAATATCACAGGGCTACTATTGACACGTGATGGAGGAGGAGGCTCAGATAATACAACAGATAATACAACA
GAGACATTCAGACCTGGAGGAGGAGATATGAGGGACAATTGGAGAAGTGAATTATATAAATATAAAGTGGTAG
AAGTTAAGCCATTAGGAATAGCACCCACTGAGGCAAGAAGGAGAGTGGTGCAGAGAGAAAAAAGAGCAGTGGG
AATAGGAGCAATGTTCCTTGGGTTCTTGGGAGTAGCAGGAAGCACTATGGGCGCAGCGTCAATGGCGCTGACG
GTACAGGCCAGACAATTGTTGTCTGGTATAGTGCAACAGCAAAGTAATTTGCTGAGAGCCATAGAGGCGCAAC
AGCATATGTTGCAACTCACGGTCTGGGGCATT
>GU329289
CATTATACAAGAGAGAATGTCTCAAACATCGAGCTGCTCCCATGTAAAATAAAACAGATTATAAACATGTGGC
AGGAAGTAGGACGAGCAATGTATGCCCCTCCCATTGCAGGAAGCATAACATGTAAATCAAGTATCACAGGACT
ACTATTGACACGTGATGGAGGAAACAATAGCACCGAGACAGAAATATTCAGACCTGGAGGAGGAGATATGAGG
GACAATTGGAGAAGTGAATTATATAAATATAAGGTGGTAGAAATTAAGCCATTAGGAGTAGCACCCACTACTG
CAAAAAGGAGAGTGGTGGAGAGAGAGAAAAGAGCAGTGGGAATAGGAGCTGTGTTCCTTGGGTTCTTAGGAGC
AGCAGGAAGCACTATGGGCGCAGCATCAATAACGCTGACGGTACAGGCCAGACAACTATTGTCTGGTATAGTG
CAACAGCAAAGCAATTTGCTGAGGGCTATCGAGGCGCAACAGCATATGTTGCAACTCACAGTCTGGGGCATT
>GU329439
AACTACACGAATGGTGAATACAAGCATAACAGGAATGGTACAGAGGTGCATATTAATGAGACTAGCAAACAGA
ATATCACAATCCAATGCAGAATAAAACAAATTATAAACATGTGGCAGGAGGTAGGGCGAGCAATGTATGCCCC
GCCCATTGCAGGAAACATAACATGTGCATCAAATATCACAGGACTACTATTGACATGGGATGGAGGAAAAAAT
AGAAGCGAAACAGAGGAGATATTCAGGCCTGGAGGAGGAGATATGAGGGACAATTGGAGAAGTGAATTATACA
AATATAAAGTGGTAGAAATTAAGCCATTAGGAGTAGCACCCTCTAAGGCAAAAAGGAGAGTGGTGGAGAGAGA
AAAAAGAGCAGTGGGAATAGGAGCTGTACTCCTTGGGTTCTTGGGAGCAGCAGGAAGCACTATGGGCGCAGCA
TCATTGACGCTGACGGTACAGGCCAGACAATTATTGTCTGGTATAGTGCAACAGCAAAGCAATTTGCTGAGGG
CTATAGAGGCGCAACAGCATATGTTGCAACTCACGGTCTGGGGCATT
>KX983735
CCTCAGGATAACAATACAAAAAATGATGAGACGACCATCAGCCTGCCATGCAGAATAAAACAAATCATAAACA
TGTGGCAGGAGGTAGGACGAGCAATATATGCCCCTCCCATTGAAGGAAGCATAACATGTGAATCAAACATCAC
AGGACTACTATTGACAAGTGATGGTGGAGGAAAAGGTAAAAATGAGACATTCAGACCTGCAGGAGGAGATATG
AGGGACAATTGGAGAAGTGAATTATATAAATATAAAGTGGTAGAAATTAAACCATTAGGAGTAGCACCCACTA
GAGCAAAAAGGAGAGTGGTGGAGAGAGAAAAAAGAGCAGTGGGAATAGGAGCTGTGTTCCTTGGGTTCTTGGG
AGCAGCAGGAAGCACTATGGGCGCGGCGTCAATAACGCTGACGGCACAGGCCAGACAAGTGCTGTCTGGTATA
GTGCAACAGCAAAGCAATTTGCTGAGGGCTATAGAGGCGCAACAGCATATGTTGCAACTCACGGTTTGGGGCA
TT
>KX983891
TGCTATACATCGAAACTGTTTAATAGAACACATAATGGTACAGATGATAATGAAACAAGTAGAAACATCACCA
CTATCCCATGCAGAATAAAACAAATTATTAACATGTGGCAGACAGTGGGACGAGCAATGTATGCCCCTCCCAT
TGAAGGAAACATAACATGTAAATCAAATATCACAGGAATAATATTAACACGTGATGGAGGAATTACAGAGGAG
TCAAATAACACAGAAATATTCAGACCTGGAGGAGGAGATATGAGAGATAATTGGAGAAGTGAATTATATAAAT
ATAAAGTGGTAGAGATTAAACCATTGGGAATAGCACCCTCCAAGGCAAAAAGGCGAGTGGTGGAGAGAGAAAA
AAGAGCAGTGGGAATAGGAGCTGTGTTCCTTGGGTTCTTGGGAGCAGCAGGAAGCACTATGGGCGCGGCGTCA
CTCACGCTGACGGTACAGGCCAGACAATTGTTGTCTGGTATAGTGCAACAGCAAAACAATTTGCTGAGAGCTA
TTGAGGCGCAACAACATCTGTTGCAACTCACGGTCTGGGGCATT
>AF286224

AATTATACAGAAAATAATACAGATGGTACACCCATCACACTCCCATGCAGAATAAGACAAATTATAAATATGT
GGCAGGAAGTAGGACGAGCAATGTACGCCCCTCCCATTGAAGGAAACATAGCATGTAAATCAGATATCACAGG
GCTACTATTGGTTCGGGATGGAGGAAGCACAAATGATAGCACAAATAATAACACAGAGATATTCAGACCTGCA
GGAGGAGATATGAGGGACAATTGGAGGAGTGAATTGTATAAGTATAAAGTGGTAGAAATTAAGCCATTGGGAA
TAGCACCCACTGAGGCAAAAAGGAGAGTGGTGGAGAGAGAAAAAAGAGCAGTGGGAATAGGAGCTGTGTTCCT
TGGGTTCTTGGGAGCAGCAGGAAGCACTATGGGCGCAGCGTCAATAACGCTGACGGCACAGGCCAGACAAGTG
TTGTCTGGTATAGTGCAACAGCAAAGCAATTTGCTGAGGGCTATAGAGGCGCAACAGCATCTGTTGCAACTCA
CGGTCTGGGGCATT
>KU168262
TTTCATAGTACATACAATAGTACATACAATAGTACAGACAAGGGTAACAGCACAGACGATACCATCACACTCC
CATGCAGAATAAAACAAATTATAAACATGTGGCAGGAGGTAGGAAAAGCAATATATGCCCCTCCTATTCCAGG
AAAAATAACATGTAACTCAAGCATCACGGGGCTACTACTGACACGTGATGGAGGGCAAAATAATACAAATGAG
ACATTCAGGCCTGGAGGAGGAGATATGAGAGATAATTGGAGAAGTGAATTATACAAATATAAGGTGGTAGAAA
TTAGACCATTAGGAGTAGCACCCACTGATGCAAAAAGGAGAGTGGTGGAGAGAGAAAAAAGAGCAGTGGGAAT
AGGAGCTGTGCTCTTAGGGTTCTTGGGAGCAGCAGGAAGCACTATGGGCGCAGCGTCAGTGGCGCTGACGGTA
CAGGCCAGACAATTATTGTCGGGTATAGTGCAACAGCAAAGCAATTTGCTGAGGGCTATAGAGGCGCAACAGC
ATATGTTGCAACTCACAGTCTGGGGCATT
>JQ361079
ACTTATAATGCTTCAATAGAAAATAATACTATAATCACGCTCCCGTGCAGAATAAAACAAATTATAAACATGT
GGCAAGGAGTAGGAAAAGCAATATATGCCCCTCCCATTGAAGGACTAATTAAGTGTACATCAAATATTACAGG
AATATTATTAACAAGAGATGGTGGTAGAGATAATAGTAGTAGTAACGAGACCTTCAGACCTGGAGGAGGAGAT
ATGAGAGATAATTGGAGAAGTGAATTATACAAATATAAAGTAGTAAAAATTGAACCAATAGGTCTAGCACCCA
CCAAGGCAAAGAGAAGAGTGGTGGAAAGAGAAAAAAGAGCAATAGGACTAGGAGCTATGTTCCTTGGGTTCTT
GGGAGCAGCAGGAAGCACGATGGGCGCAGCGTCAGTGACGCTGACGGTACAGGCCAGACAGTTATTGTCTGGT
ATAGTGCAACAGCAAAATAATTTGCTGAGGGCTATAGAGGCGCAACAGCATCTGTTGCAACTCACAGTCTGGG
GCATT
>HM215270
AATTATACTGGAAATGAGAGTGTAATCAACCTACCATGCAGAATAAAACAAATTATAAACATGTGGCAGGGAG
TAGGAAAAGCAATGTATGCCCCTCCCATTGAAGGGCTAATCAACTGTTCATCAAATATTACAGGACTATTGCT
GACAAGAGATGGTGGTATAAATAGTAATACGAGTAGTTCTGAGACCTTCAGACCTGGAGGAGGAGATATGAGA
GACAATTGGAGAAGTGAATTATACAAATATAAAGTAGTAAGACTTGAACCACTAGGTCTAGCACCCACCAAGG
CAAAAAGAAGAGTGGTGGAAAGAGAAAAAAGAGCAATAGGACTAGGAGCTGTGTTCCTTGGGTTCTTGGGAGC
AGCAGGAAGCACGATGGGCGCAGCGTCACTGACGCTGACGGTACAGGCCAGACAGTTACTGTCTGGTATAGTG
CAACAGCAAAATAATTTGCTGAAGGCTATAGAGGCGCAACAGCATCTGTTGCAACTCACAGTCTGGGGCATT
>L22949
AGTTATAATACATCAGCATTAAATAGTACACAGGGAAATGAGAGTGAAAACATCACACTCCCATGCAGAATAA
AACAAATTATAAACATGTGGCAGGAAGTAGGAAAAGCAATGTATGCCCCTCCCATTGAAGGGATAATCAGATG
TTCATCAAATATTACAGGACTATTGTTGACAAGAGATGGTGGTATAACTAACAATAGTAATGAGACCTTCAGA
CCTGGAGGAGGAAATATGAGAGACAATTGGAGAAGTGAATTATACAAATATAAAGTAGTAAGAATTGAACCAC
TAGGTCTAGCACCCACCACGGCAAAGAGAAGAGTGGTGGAAAGAGAAAAAAGAGCAATAGGACTGGGAGCTCT
GTTCCTTGGGTTCTTGGGAGCAGCAGGAAGCACAATGGGCGCAGCGTCACTGACGCTGACGGTACAGGCCAGA
CAAGTATTGTCCGGCATAGTGCAACAGCAAAACAATTTGCTGAGGGCTATAGAGGCGCAACAACATCTGTTGC
AACTCACAGTCTGGGGCATT
>L22950
AATTATAATATATCAAATAGTACAGGGGGAAATGAGACAATCAGACTCCAATGCAGAATAAAACAAATTATAA
ACATGTGGCAGGAAGTAGGAAAAGCAATGTATGCCCCTCCCATTATAGGACAAATCAAATGTTCATCAAATAT
CACAGGACTATTGTTAACAAGAGATGGTGGTGTAAATAATAGTAGTAATGAGACTAGCAGTAAAAATGAGATC
TTCAGACCTGGAGGAGGAGATATGAGAGACAATTGGAGAAGTGAATTATACAAATATAAAGTAGTAAGAATTG
AACCACTAGGTCGGGCACCCACCAGGGCAAAAAGGAGAGTGGTGGAAAGAGAAAAAAGAGCAATAGGACTGGG
AGCTTTGTTCCTTGGGTTCTTGGGAACAGCAGGAAGCACGATGGGCGCAGCGTCAATGACGCTGACGGTACAG
GCCAGACAGGTATTGTCTGGTATAGTGCAACAGCAAAACAATTTGCTGAGGGCTATAAAGGCGCAACAGCATC
TGTTGCAACTCACAGTCTGGGGCATT
>AY623599
ACTTATAATACATCAGGTAGTACAGGGGTAAATAACAGTACAATCAAACTCCCATGCAGAATAAAACAAATTA
TAAACATGTGGCAGGGAGTAGGAAAAGCAATGTATGCCCCTCCCATTGAAGGACTAATCAAATGTTCATCAAA
CATTACAGGACTATTGTTGACAAGAGATGGGGGAAATAATACTAGGCAGAATGAAACCTTCAGGCCTGGAGGA
GGGGATATGAGAGACAATTGGAGAAGTGAATTATACAAATATAAAGTAGTAAGAATTGAACCACTAGGTCTAG
CACCCACTGAGGCAAAGAGAAGAGTGGTGGAAAGAGAAAAAAGAGCAATAGGACTAGGAGCTATGTTCCTTGG
GTTCTTGGGAGCAGCAGGAAGCACGATGGGCGCAGCGTCAATGACGCTGACGGTACAGGCCAGACAGTTAATG

TCTGGTATAGTGCAACAGCAAAACAATTTGCTGAGGGCTATAGAGGCGCAACAGCATCTGTTGCAACTCACAG
TCTGGGGCATT
>AY713418
AATTATAATATATCAAATACAGAGGTAATCAGACTCCCATGCAGAATAAAACAAATTATAAACATGTGGCAGG
GAGTAGGCAAAGCAATGTATGCCCCTCCCATTGAAGGAATAATCAAATGTTCATCAAATATTACAGGACTATT
GTTAACAAGGGATGGTGGTGGTAATAACACTCGGAAGAATGAGACCTTCAGGCCTGGAGGGGGAGATATGAGA
GACAATTGGAGAAGTGAATTATACAAATATAAAGTAGTAAGAATTGAACCATTAGGTCTAGCACCCACCAAGG
CAAAGAGAAGAGTGGTGGAAAGAGAAAAAAGAGCAATAGGACTGGGAGCTCTGTTCCTTGGGTTCTTGGGAGC
AGCAGGAAGCACGATGGGCGCAGCGTCAATAACGCTGACGGTACAGGCCAGACAAGTATTGTCTGGTATAGTG
CAACAGCAAAACAATTTGCTGAGGGCTATAGAGGCGCAACAACACTTGTTGCAACTCACAGTCTGGGGCATT
>EU853046
AATTATAGTAGTATGTTTAATAGTAGTACATCAAATAGTAGTATAGGGGAAAATGAAACAATCACACTCCAAT
GCAGAATAAAGCAAATTATAAACATGTGGCAGGGAGTAGGAAAAGCAATGTATGCACCTCCCGTTGCAGGACA
AATCAGATGTTCATCAAATATTACAGGCCTATTGTTGACAAGAGATGGAGGTGAAAATACTAGTGAGGAGATC
TTCAGACCTGCAGGAGGAGATATGAGAGACAATTGGAGAAGTGAATTATACAAATATAAAGTAGTAAGAATTG
AACCACTAGGTCTAGCACCCACCAAGGCAAAGAGAAGAGTGGTGGAAAGAGAAAAAAGAGCAATAGGACTAGG
AGCTGTGTTCCTTGGGTTCTTGGGAGCAGCAGGAAGCACGATGGGCGCAGCGTCACTGACGCTGACGGTACAG
GCCAGACAATTATTGTCTGGTATAGTGCAACAGCAAAACAATTTGCTGAGGGCTATAGAGGCGCAGCAGCATC
TGTTGCAACTCACAGTCTGGGGCATT
>AF484489
ACTTATGATACATCGAATGGTACAGGGAGTGAAAATATCACACTCCAATGCAGAATAAAACAAATGATAAACA
TGTGGCAAGGAGTAGGCAAAGCAATGTATGCCCCTCCCATTGAAGGAACAATCAACTGTTTATCAAATATTAC
AGGACTATTGTTGACAAGAGATGGTGGTGTAAAAAATAGTACTAATGAGACCTTCAGACCTGGAGGAGGAGAT
ATGAGAGACAATTGGAGAAGTGAATTATATAAGTATAAAGTAGTAAGACTTGAACCACTAGGCCTAGCGCCCA
CAGAGGCAAGAAGAAGAGTGGTGGAAAGAGAAAAAAGAGCAATAGGCCTACTAGGAGCTATGTTCCTTGGGTT
CTTGGGAGCAGCAGGAAGCACGATGGGCGCAGCGTCACTGACGCTGACGGTACAGGCCAGACAGTTAATGTCT
GGTATAGTGCAACAGCAAAACAATCTGCTGAGGGCTATAGAGGCGCAACAGCATATGTTGCAACTCACAGTCT
GGGGCATT
>JX140673
AGTCAGTCTGAGAAGGCGGGAAATAATGGCAGTATCTTCCTTCCATGCAGAATAAGACAAATTGTAAACATGT
GGCAAAAGGTAGGACGAGCAATGTATGCCCCTCCCATTGCTGGAGTAATTCGGTGTAACTCAAGTATCACAGG
TCTGTTATTAACAAGAGATGGTGGGGAAGATAATAGTAGCACTGACACCCTCAGACCTGGAGGGGGAGATATG
AAAGATAATTGGAGAAGTGAACTGTATAAATATAAGGTAGTAAAAATTGAACCACTAGGTGTGGCACCCACCA
GGGCAAAAAGACAAGTGGTGAACAGAGAAAAAAGAGCAGTGGGAATAGGAGCTGTGTTCCTTGGATTCTTGGG
AACAGCAGGAAGCACTATGGGCGCGGCGTCAATAACGCTGACGGTACAGGTCAGACAATTATTGTCTGGTATA
GTGCAACAGCAAAGCAATTTGCTGAGGGCTATAGAAGCGCAACAGCATATGTTGCAGCTCACAGTCTGGGGCA
TT
>AJ249236
TACTACAACACATCAGGACTGTTTAATGAAACAGAGGTGGCTAATAACACAAACGAAAATATCACTCTCCCAT
GTAGAATAAGACAATTTGTAAACATGTGGCAAAGAATAGGAAGAGCAATGTATGCTCCTCCCATTGAAGGAGA
AATCCAGTGTACCTCAAATATCACAGGTCTACTATTGACAAGAGATGGTAGTAAGGATATTGACGGAAAGAG
ATTCTCAGACCTATAGGAGGAGATATGAGAGACAATTGGAGAAGTGAACTATATAAATATAAGGTAGTAAGAA
TTGAACCAGTAGGAGTAGCACCCACCAAGGCAAAAAGACGAGTGGTGCAGAGAGCAAAAAGAGCAGTGGGAAT
GGGAGCTGTGCTCTTTGGGTTCTTGGGAGCAGCAGGAAGCACTATGGGCGCGGCGGCAATCACGCTGACGGCA
CAGGCCAGACAATTATTGTCTGGTATAGTGCAACAGCAAAGCAATCTGCTGAAGGCTATAGAAGCGCAACAGC
ATCTGTTGCAGCTCACAGTCTGGGGCATT
>MH746240
GCTCAGTCTAATAATAGTAGTGGAAATGAGACCATCAGACTCTCATGTAAAATAAAACAAATTGTGAGAATGT
GGCAGAAAGTGGGACAAGCAATGTATGCCCCTCCCATTGCAGGAGAAATTACATGTAGATCAAACATTACAGG
ACTACTGCTAACAAGAGATGGTGGAAAGAATATTAGTAAAGGAGATGAGATCTTCAGGCCTATAGGAGGAGAT
ATGAGGAACAATTGGAGAAGTGAATTATATAAGTATAAAGTAGTAAAAATCAAACCACTAGGGGTAGCACCCA
CCAAAGCAAGGAGAAGAGTGGTGGAGAGAGAAAAAAGAGCAGTTGGACTGGGAGCTGCCTTCCTTGGGTTCTT
AGGAGCAGCAGGAAGCACTATGGGCGCGGCGTCAATAACGCTGACGGTACAAGCCAGACAATTACTGTCTGGG
ATAGTGCAACAGCAAAGCAATTTGCTGAGAGCTATAGAAGCGCAGCAGCATCTGTTGCAGCTCACAGTCTGGG
GCATT
>KR051445
CTTTATGAACTCCCATGTAGAATAAAGCAAATTGTGAGAATGTGGCAGAGAGTGGGACAAGCAATGTATGCCC
CTCCCATCAAAGGAAACATTACTTGTAGATCAAACATTACAGGACTACTATTAACAAGAGATGGTGGGAGAAA
TAGGAGTAATGACAGAGAGATCTTCAGACCTACAGGAGGAGACATGAGGAACAATTGGAGAAGTGAATTATAT
AAGTATAAAATAGTAAAAATAAAACCATTAGGAGTAGCACCCACCAAGGCAAGGAGAAGAGTGGTGGGGAGAG

AAAAAAGAGCAGCTGGCCTGGGAGCTGTTTTCCTTGGGTTCTTAGGAGCAGCAGGAAGCACTATGGGCGCGGC
GTCAATAACGCTGACGGTACAGGTCAGACAACTATTGTCTGGCATAGTGCAACAGCAAAGCAATTTGCTGAGA
GCTATAGAGGCACAGCAACATCTGCTGCAACTCACAGTCTGGGGCATT
>KR051437
AGTCAAGAAAATAAAACAGTTATCACAATCCCATGTAGGATAAAACAAATTGTAAGAATGTGGCAGAGAACGG
GACAAGCAATATATGCCCCTCCCATTGCAGGAAACATTACATGTATATCAAACATTACAGGACTATTATTAAC
AAGAGATGGTGGGAATGACAATAATAACAATAATACCAATGACAATGACACTGAGGTCTTCAGACCTACAGGA
GGAGATATGAGGGACAATTGGAGAAGTGAATTATATAAGTACAAAGTAGTAAAGATTAAATCACTAGGAGTAG
CACCCACCAAGGCAAGGAGAAGAGTAGTGGGAAGAGAAAAAAGAGCAGTTGGAATAGGAGCTGTCTTTCTTGG
GTTCTTAGGAGCAGCCGGGAGCACTATGGGCGCGGCGTCACTAACGCTGACGGTACAGGTCAGACAATTATTG
TCTGGCATAGTGCAACAGCAAAGCAATTTGCTGAGAGCTATAGAGGCGCAACAGCATATGTTGCAACTCACAG
TCTGGGGCATT
>KY658701
AGTTATATTAATAGGACATCAACTTCTAATGGGACTATCACACTCCCATGTAGAATAAAACAAATTGTAAGGA
TGTGGCAGAGAGTGGCACAAGCAATGTACGCCCCTCCCATTGCAGGAAAACTTGAATGTAACTCAAGCATTAC
AGGATTAATATTAACAAGAGATGGTGGGAACAATGACAATAACACACAGAACGAGACCTTCAGACCTGGAGGA
GGAAATATAAGGGACAATTGGAGAAGTGAATTATATAAATATAAAACAGTAAAAATCAAATCATTAGGAATAG
CACCCACCAGGGCAAGGAGAAGAGTGGTGGAAAGACAAAAAAGAGCAGTGGGAATGGGAGCTGTCTTCCTTGG
GTTCTTAGGAGCAGCAGGAAGCACTATGGGCGCGGCGTCAATAACGCTGACGGTACAGGTCAGACAATTATTG
TCTGGCATAGTGCAACAGCAAAGCAATTTGCTGAGAGCTATAGAGGCGCAACAGCATATGTTGCAACTCACAG
TCTGGGGCATT
>FJ817370
AGTCATCTGTTTAATGGTAGTACTCTGTCTACTAATGGTACTATCACACTTCCATGTAGGATAAAACAGATTG
TAAGAATGTGGCAGAGAGTGGGACAAGCAATGTATGCCCCTCCTATCGCAGGAAACATTACATGTAGGTCAAA
CATTACAGGACTACTATTAACAAGAGATGGTGGAACAAATAAAGATACAAATGAGGCTGAGACTTTCAGACCT
GGAGGAGGAGATATGAGGGACAATTGGAGAAGTGAATTATATAAGTATAAAGTAGTAAAAATTAAACCATTAG
GAGTAGCACCCACCAGGGCAAGGAGAAGAGTGGTGGAGAGAGAAAAAAGAGCAATTGGACTGGGAACTGTCCT
CCTTGGGTTCTTAGGAACAGCAGGAAGCACTATGGGCGCAGCGTCAATAACGCTGACGGTACAGGTCAGACAA
TTATTGTCTGGCATAGTGCAACAGCAAAGCAATTTGCTGAGGGCTATAGAGGCGCAGCAGCATCTGTTGCAAC
TCACAGTCTGGGGCATT
>EU885764
AATCATACAGATACAAATGAAAATATCACACTCCAGTGTAGGATAAAACAAATTGTGAGAATGTGGCAGAGAG
TGGGACAAGCAATGTATGCCCCTCCCATCGCTGGAAACATTACATGTATCTCAAACATTACAGGACTGCTATT
AACAAGAGATGGTGTGAATGATACCCATGACAAGGAGAATGAGACCTTCAGACCTACAGGAGGAGATATGAGG
GATAATTGGAGAAGTGAATTATATAAGTATAAAGTAATAAAACTTAAACCACTAGGAGTAGCACCCACCAGGG
CAAGGAGAAGAGTGGTGGAGAGAGGAAAAAGAGAAGTCACACTAGGAGCTTTGTTCCTTACGTTCTTAGGAGC
AGCAGGAAGCACTATGGGCGCGGCGTCATTAACGCTGACGGTACAGGCCAGACAATTATTGTCTGGCATAGTG
CAACAGCAAAGCAATTTGCTGAGAGCTATAGAAGCGCAGCAGCATCTGTTGCAGCTCACAGTCTGGGGCATT
>EU885765
AGTTATGGGCCTAATGAGACACACATCATACTCCCATGTAGGATAAGACAAATTGTGAGAATGTGGCAGAGAG
TAGGACAAGCAATGTATGCCCCTCCCATCGCAGGAAACATAACATGTAAATCAAATATTACAGGACTACTATT
AACAAGAGATGGTGGAGAGGATGGCAATAATAGCACTGAGACCTTCAGACCTGCAGGAGGAGACATGAGGGAC
AATTGGAGAAGTGAATTATATAAGTATAAAGTAGTAAAAATTAAACCATTAGGAGTAGCACCCACCAGGGCAA
GAAGAAGAGTGGTGAGGAGAGAAAAAAGAGCAGTTGGACTGGGAGCTGTCTTCCTTGGGTTCTTAGGAGCAGC
AGGAAGCACTATGGGCGCGGCGTCATTAACGCTGACGGTACAGGCCAGACAATTATTGTCTGGCATAGTGCAA
CAGCAAAGCAATTTGCTGAGGGCTATAGAGGCGCAGCAGCATCTGCTGCAACTCACAGTCTGGGGCATT
>HQ236608
AGCTATAATAGTGGCAGTAATAATAATACCACACATAATAGTGGTAATGAGACTATCACAATTCCATGTAGGA
TAAAACAAATAGTGAGAATGTGGCAGAGAGTGGGACAAGCAATGTATGCCCCTCCCGTCGCAGGAAACATTAC
ATGTAGGTCAGAAATTACAGGACTACTATTAACAAGAGATGGTAATGCAAGTGAAAATGAAATTGTCAGACCT
GGAGGAGGAGACATGAGGGACAATTGGAGAAGTGAATTATATAAGTATAAAGTAGTAAAAATTAAACCACTGG
GAGTAGCACCCACCAGGGCAAGGAGAAGAGTGGTGGGGAGAGAAAAAAGAGCAGTTGGACTGGGAGCTGTCCT
CCTTGGGTTCTTAGGAACAGCAGGAAGCACTATGGGCGCAGCGTCAATAACGCTGACGGTACAGGTCAGACAA
TTATTGTCTGGCATAGTGCAACAGCAAAGCAATTTGCTGAGGGCTATAGAGGCGCAGCAGCATATGTTGCAAC
TCACAGTCTGGGGCATT
>KX389641
GATTATGAGAGTAATATCACACTCCCATGTAAAATAAAACAAATTGTGAGAATGTGGCAGAGAGTAGGACAAG
CAATGTATGCCCCTCCCATCGCAGGAAATATTACATGTAGATCAAACATTACAGGGCTATTATTAACAAGAGA
TGGTGGTGGGAACAATAATAGCAATGAGACCTTCAGACCTGGAGGAGGAGATATGAGGGACAACTGGAGAAGT
GAGTTATATAAGTATAAAATAGTAAAAATTAAACCACTAGGAATAGCACCCACCAAGGCAAAGAGAAGAGTGG

TGCAGAGAGAGAAAAGGGCAGTAGTTGGACTGGGAGCTGTCTTCCTTGGGTTCTTAGGAGCAGCAGGAAGCAC
TATGGGCGCGGCGTCAATAACGCTGACGGTACAGGCCAGACAATTATTGTCTGGCATAGTGCAACAGCAAAGC
AATTTGCTGAGGGCTATAGAGGCACAGCAACATCTGTTGCAACTCACAGTCTGGGGCATT
>KY953202
AGTTATATAAATGCTACAAAGGATAATACAACAGGGGATAACATCACGCTCCCATGTAGGATAAAACAGATTG
TAAGAATGTGGCAGAGAGTAGGACAAGCAATGTATGCCCCTCCCATCGCAGGAAACATTACATGTATATCAAA
CATTACAGGGATAATATTAACAAGAGATGGTGGAAATAATAATAAATCAAATAATGAAACCTTCAGACCTGGA
GGAGGAGATATGAGAGACAATTGGAGAAGTGAGTTATATAAGTATAAAATAGTTAAAATTAAACCACTAGGAA
TAGCACCCACCAGGGCAAGGAGAAGAGTGGTGCAGAGAGAAAAAAGGGCAGTTGGACTGGGAGCTTTCTTCTT
TGGGTTCTTAGGAGCAGCAGGAAGCACTATGGGCGCGGCGTCAATAACGCTGACGGTACAGGTCAGACAATTA
TTGTCTGGCATAGTGCAACAGCAAAGCAATTTGCTGAGGGCTATAGAGGCGCAACAACATATGTTGCAACTCA
CAGTCTGGGGCATT
>FR846409
AGTTATGAAAATGATACTATCACACTTCCATGTAGGATAAAACAAATTGTGAGAATGTGGCAGAGAGTGGGAC
AAGCAATGTATGCCCCTCCCATAGCAGGAAACATTACATGTGTGTCAAACATTACAGGATTACTATTAGAAAG
AGATGGTGGGAATCTTAATAATAGTATAAATACAACTGAGCCTAAAAATGAAATTTTCAGACCTGTAGGAGGA
GACATGAAGAACAATTGGAGAAGTGAATTATATAAATATAAAGTAGTAAGAATTAAACCACTAGGAGTAGCAC
CTACCAGGGCAAGGAGAAGAGTGGTGGGAAGAGAAAAAAGAGCAGGAATAGGAGCTGTCATCTTTGGGTTCTT
AGGAGCAGCAGGAAGCACTATGGGCGCGGCGTCAATAACGCTGACGGTACAGGTCAGACAATTATTGTCTGGC
ATAGTGCAACAGCAAAGCAATTTGCTGAGAGCAATAGAGGCGCAGCAGCATCTGTTGCAACTCACAGTCTGGG
GCATT
>HQ236615
AATTATACTGAGAGTAGAAATGATACTATCATCTCAATTCCATGTAGGATAAAACAAATTGTGAGAATGTGGC
AGAGAGTGGGACAAGCAATGTATGCCCCTCCCATCGCAGGAAACATTACATGTAGGTCAAATATTACAGGACT
ATTATTAGTAAGAGATGGTGGCATAAATAATGAGACAACTGAGATTTTCAGACCTGCAGGAGGAGATATGAAG
GACAATTGGAGAAGTGAATTATATAAGTATAAAGTAGTAAAAATTAAACCACTAGGAGTAGCACCCACCAGGG
CAGAGATGGTGGGGAGAGAAAGAGCAGTTGGACTGGGAGCTGCCATCCTTGGGTTCTTAGGAACAGCAGGAAG
CACTATGGGCGCAGCGTCAATAGCGCTGACGGTACAGGTCAGACAATTATTGTCTGGCATAGTGCAACAGCAA
GGCAATTTGCTGAGGGCTATAGAGGCGCAGCAGCATCTGTTGCAACTCACAGTCTGGGGCATT
>MH746234
ACTTACACAGGGAGGGGGAATGACACACAGGAGAATAACAGCACTATCATACTTCCATGCAGGATAAAACAAA
TTATAAACATGTGGCAAGGAGCAGGACAAGCAATGTATGCTCCTCCCATCAGTGGAAAAATTAATTGTGTATC
AAATATTACAGGAATACTATTGACAAGAGATGGTGGTAATAATGCTACTAATGAGACCTTCAGACCTGGAGGA
GGAAATATAAAGGACAATTGGAGAAGTGAATTATATAAATATAAAGTAGTACAAATTGAACCACTGGGAATAG
CACCCACCAAGGCAAAGAGAAGAGTGGTGGAGAGAGAAAAAAGAGCAGTGGGAATAGGAGCTATGATCTTTGG
GTTCTTAGGAGCAGCAGGAAGCACTATGGGCGCGGCGTCAATAACGCTGACGGTACAAGCCAGACAATTATTG
TCTGGTATAGTGCAACAGCAAAGCAATTTGCTGAGGGCTATAGAGGCGCAGCAGCATCTGTTGCAACTCACAG
TCTGGGGCATT
>KU600817
ACTTACACAGGAAATGGAACATGGGAGAATAATAGCACTATTAATGGCACTATCATACTTCCATGCAAGATAA
AGCAAATCATAAACATGTGGCAGGGAGCAGGACAAGCAATGTATGCTCCTCCCATCAGTGGAATAATTAATTG
TGCGTCAAATATTACAGGACTACTATTGACAAGAGATGGTGGTTTTAGTAATGCAAGTGGTGGTGGTAGTAAT
GCAAATGAAGAGATCTTCAGACCTGGAGGAGGAAATATAAAAGACAATTGGAGAAGTGAATTATATAAATATA
AAGTAGTACAAATTGAACCATTAGGAATAGCACCCACCAGGGCAAAGAGAAGAGTGGTGGAGAGAGAAAAAAG
AGCAGTGGGAATAGGAGCTATGATCTTTGGGTTCCTAGGAGCAGCAGGAAGCACTATGGGCGCGGCATCAATA
ACGCTGACGGTACAGGCCAGACAATTATTGTCTGGTATAGTGCAACAGCAAAGCAATTTGCTGAAAGCTATAG
AGGCGCAACAGCATTTGTTGCAACTCACAGTCTGGGGCATT
>JX112866
ACTTACATGGGAAATGAAACCAAGGGGAAGTATAATGGCACTATTATACTTCCATGCAGGATAAAGCAAATTA
TAAACATGTGGCAGGAAACAGGACAAGCAATGTATGCTCCTCCCGTCAGTGGAAGAATTAAGTGTAGATCAAA
TATTACAGGAATACTATTGACAAGAGATGGTGGTAATACAACTAACGGGGAAGAGACCTTCAAACCTGGAGGA
GGAGATATGAGGGACAATTGGAGAAATAAATTATATAAATATAAAGTAGTACAAATTGAACCACTAGGAATAG
CACCCACCAGGGCAAAGAGAAGAATGGTGGAGAGAGAAAAAAGAGCAGTGGGAATAGGAGCTATGATCTTTGG
GTTCTTAGGAGCAGCAGGAAGCACTATGGGCGCGGCGTCAATAACGCTGACGGTACAGGCCAGACAATTATTG
TCTGGTATAGTGCAACAGCAAAGCAATTTGCTGAGGGCTATAGAGGCGCAGCAGCATCTGTTGCAACTCACAG
TCTGGGGCATT
>GU564225
ACTTACAATGGAAATGAAACCATAGGTAATAAAACTATCATACTTCCATGCAGGATAAAGCAAATTATAAACA
TGTGGCAGGGAGTAGGACAAGCAATGTATGCTCCTCCCGTCAGTGGAAGAATTAATTGTGTATCAAATATTAC
AGGAATACTATTAACAAGAGATGGTGGTAGTAATAGTACTAATGAGACCTTCAGACCTGAAGGAGGAAATATA

AAGGACAATTGGAGAAGTGAATTACATAAATATAAAGTAGTACAAATTGAACCACTAGGAATAGCACCCACCA
GGGCAAAAAGAAGAGTGGTGGAGAGAGAAAAAAGAGCAGTGGGAATAGGAGCTATGATCTTTGGGTTCTTAGG
AGCAGCAGGAAGCACTATGGGCGCGGCGTCAATAACGCTGACGGTACAGGCCAGACAATTATTGTCTGGTATA
GTGCAACAGCAAAGCAATTTGCTGAGGGCTATAGAGGCGCAGCAGCATCTGTTGCAACTCACAGTCTGGGGCA
TT
>GQ845124
ACTTACACAGAAAATGGAACCATAGAGGGGAATAAAACTATCATACTTCCATGTAGGATAAAGCAAATTATAA
ACATGTGGCAGGGAGTAGGACAAGCAATGTATGCTCCTCCCATCAGGGGAAGAATTAATTGTATATCAAATAT
TACAGGAATACTATTAACAAGAGATGGTGGTAATAGTAATGCTACCGATAGTAATACTAACGAAATCTTCAGA
CCTGGAGGAGGAAATATAAAGGACAATTGGAGAAGTGAATTATATAAATATAAAGTAGTACAAATTGAACCAC
TAGGAGTAGCACCCACCAGGGCAAGGAGAAGAGTGGTGCAGAAAGAAAAAAGAGCAGTGGGAATAGGAGCTAT
GATCTTTGGGTTCTTAGGAGCAGCAGGAAGCACTATGGGCGCGGCGTCAATAACGCTGACGGTACAGGCCAGA
CAATTATTGTCTGGTATAGTGCAACAGCAAAGCAATTTGCTGAGGGCTATAGAGGCGCAGCAGCATCTGTTGC
AACTCACAGTCTGGGGCATT
>GQ845126
ACTCACAAGGAAAATGAAACCATAGGGAGGAGAAATGACACTATCATACTTCCATGCCGGATAAAGCAAATTA
TAAACATGTGGCAGGAAGTAGGACAAGCAATGTATGCTCCTCCCATCAGTGGAATAATTAATTGTGTATCAAA
TATTACAGGAATACTATTAACAAGAGATGGTGGTAATAATGATACTAACGAGACCTTCAGACCTGGAGGAGGA
AATATAAAGGACAATTGGAGAAGTGAATTATATAAATATAAAGTAGTACAAATTGAACCACTAGGAATAGCAC
CCACCAGGGCAAAGAGAAGAGTGGTGGAGAGAGAAAAAAGAGCAGTGGGAATAGGAGCTATGATCTTTGGGTT
CTTAGGAGCAGCAGGAAGCACTATGGGCGCAGCGTCAATAACGCTGACGGTACAGGCCAGACAATTATTGTCT
GGTATAGTGCAACAGCAAAGCAATTTGCTGAGGGCTATAGAGGCGCAGCAGCATCTGTTGCAACTCACAGTCT
GGGGCATT
>GU564229
ACTCAAACCACAGAGGGGAAAGACAATATCACACTTCCATGCAGAATAAAGCAAGTTATAAAAATGTGGCAGA
GAGTAGGACAAGCAATGTATGCTCCTCCCATCAGTGGAATAATTAATTGTGTATCAAATATTACAGGTATACT
ATTAACAAGAGATGGTGGTGATAATGATAGTACTAACGAGACCTTCAGACCTGGAGGAGGAGATATGAGGGAC
AATTGGAGAAGTGAATTATATAAATATAAAGTAGTACAAATTGAACCACTAGGAATAGCACCCACCAAGGCAA
AGAGAAGAGTGGTGGAGAGAGAAAAAAGAGCAGTGGGAATAGGAGCTTTGATCTTTGGGTTCTTAGGAGCAGC
CGGAAGCACTATGGGCGCGGCGTCAATAACGCTGACGGTACAGGCCAGACAATTGTTGTCCGGTATAGTGCAA
CAGCAAAGCAATTTGCTGAGGGCTATAGAGGCGCAGCAGCATATGTTGCAACTCACAGTCTGGGGCATT
>EF036531
ACTTACAATGGAACCCTGAACGAGGAGTACAATGGCACTATCATACTTCCATGCAGAATAAAGCAAATTATAA
ACATGTGGCAGGAAGTAGGACAAGCAATGTATGCTCCTCCCATCAGTGGAAGAATTAATTGTGTATCAAATAT
TACAGGGATACTATTGACAAGAGATGGTGGTACGAATGAGACTAACGAGACCTTCAGACCTGGAGGAGGAAAT
ATAAAGGACAATTGGAGAAATGAATTATATAAATATAAAGTAGTACAAATTGAACCACTAGGAATAGCACCCA
CCAGGGCAAAGAGAAGAGTGGTGGAAAGAGAAAAAAGAGCAGTGGGAATAGGAGCTATGATCTTTGGGTTCTT
AGGAGCAGCAGGAAGCACTATGGGCGCGGCGTCAATAACGCTGACGGTACAGGCCAGACAATTATTGTCTGGT
ATAGTGCAACAGCAAAACAATTTGCTGAGGGCTATAGAGGCGCAGCAGCCTCTGTTTCTTCTCACAGTCTGGG
GCATT
>KF835499
AATTACACAGGAAATGGAACAACGAGTGGCACTATCATACTTCCATGTAGGATAAAGCAAATTATAAACATGT
GGCAGGGAGTAGGACAAGCAATGTATGCTCCTCCCATCAGTGGAAGCATTAATTGTGTATCAGATATCACAGG
AATACTATTGACAAGAGATGGTGGTAATAATACAGGTAATAATAATAGTGCTAATACAAATGAGATCTTCAGA
CCTGGAGGAGGAAATATGAAAGACAATTGGAGAAGTGAATTATATAAATATAAAGTAGTAGAAATTGACCCAC
TAGGAGTAGCACCCACCAGGGCAAAAAGAAGAGTGGTGGAGAGAGAAAAAAGAGCAGTGGGAATAGGAGCTAT
GATCTTTGGGTTCTTAGGAGCAGCCGGGAGCACTATGGGCGCAGCGTCAATAACGCTGACGGTACAGGCCAGA
CAATTATTGTCTGGTATAGTGCAACAGCAAAGCAATTTGCTGAGGGCTATAGAGGCGCAACAACATATGTTGC
AACTCACAGTCTGGGGCATT
>KF835523
ACTCACACGGAAAATGACACCACAAAGGAAAGGATCATACTTCCATGCAGGATAAAGCAAATTATAAACATGT
GGCAGAGAGTAGGACAAGCAATATACAATCCTCCCATCAGTGGAGCAATTAATTGTCTATCAAATATTACAGG
AATAATATTGACAAGAGATGGTGGTGCTAGTACTAATAATGATACTAGTACTAACAATGAAACCTTCAGACCT
GGAGGAGGAAATATAAAGGACAATTGGAGAAGTGAATTATATAAATATAAAGTAGTACAAATTGAGCCACTAG
GGGTAGCACCCACCAGGGCAAAGAGAAGAGTGGTGGAGAGAGAGAAAAGAGCAGTGGGAATAGGAGCTATGAT
CTTTGGGTTCTTGGGAGCAGCAGGAAGCACTATGGGCGCGGCGTCAATAACGCTGACGGTACAGGCCAGACAA
TTATTGTCTGGTATAGTGCAACAGCAAAGCAATTTGCTGAGGGCTATAGAGGCGCAGCAGCACATGCTGCAAC
TCACAGTCTGGGGCATT
>GU475020

ACTTATAAAGGAAATGAAACCATAGGGAATGGAACTATCATAATTCCATGCAGGATAAAGCAAATTATAAACA
TGTGGCAGGGAGTAGGACAAGCAATGTATGCTCCTCCCATCAGTGGAATAATTAATTGTATATCAAATATTAC
AGGAATACTATTAACAAGAGATGGTGGTAGTACTAATAGCACTAATGAGACCTTCAGACCTGGAGGAGGAAAT
ATAAAGGACAATTGGAGAAGTGAATTATATAAATATAAAGTAGTACAAATTGAACCACTAGGAGTAGCACCCA
CCAGGGCAAAGAGAAGAGTGGTGGAGAGAGAAAAAAGAGCAGTGGGAATAGGAGCTATGATCTTTGGGTTCTT
AGGAGCAGCAGGAAGCACTATGGGCGCAGCGTCAATAACGCTGACGGTACAGGCCAGACAATTATTGTCTGGT
ATAGTGCAACAGCAAAGCAATTTGCTGAGGGCTATAGAGGCGCAGCAGCATATGTTGCAACTCACAGTCTGGG
GCATT
>HQ699988
ACTTATAATGGAAATGGAACTGTGACCAAGTATAATGACACTATTATACTTCCATGCAGGATAAAGCAAATTA
TAAACATGTGGCAGGAAACAGGACAAGCAATGTATGCTCCTCCCGTCAGTGGAGTAATAAATTGTAGATCAAA
TATTACAGGAATACTATTGACAAGAGATGGGGGTAATAATGGGGGTAATACAACTAACGGGGAAGAGACCTTC
AGACCTGGAGGAGGAGATATGAGGGACAATTGGAGAAATGAATTATATAAATATAAAGTAGTACAAATTGAAC
CACTAGGAATAGCACCCACCAAGGCAAAGAGAAGAGTGGTGCAGAGAGAAAAAAGAGCAGTGGGAATAGGAGC
TATGATCTTTGGGTTCTTAGGAGCAGCAGGAAGCACTATGGGCGCGGCGTCAATAACGCTGACGGTACAGGCC
AGACAATTATTGTCTGGTATAGTGCAACAGCAAAGCAATTTGCTGAGAGCTATTGAGGCGCAGCAGCATCTGT
TGCAACTCACAGTCTGGGGCATT
>JX112811
ACTTACTCAGGAAATGAAACCACAGGAAATGAAACCACAGGGAAGAATACAAGTATCATACTTCCATGCAAGA
TAAAGCAAATTATAAACATGTGGCAGGGAGTAGGACAAGCAATGTATGCTCCTCCCATCAGTGGAAAAGTTAA
TTGTATATCAAATATTACAGGAATACTATTAACAAGAGATGGTGGTGGTAATAATAGTGCTAATGAGACTTTC
AGACCGGGAGGAGGAAATATAAAAGACAATTGGAGAAGTGAATTATATAAATATAAAGTAGTACAAATTGAAC
CACTAGGAATAGCACCCACCAGGGCAAAGAGAAGAGTGGTGGAGAGAGAAAAAAGAGCAGTGGGAATAGGAGC
TATGATCTTTGGGTTCTTAGGAGCAGCAGGAAGCACTATGGGCGCGGCGTCAATAACGCTGACGGTACAGGCC
AGACAATTATTGTCTGGTATAGTGCAACAGCAAAGCAATTTGCTGATGGCTATAGAGGCGCAGCAGCATCTGC
TGCAACTCACAGTCTGGGGCATT
>JX112817
ACTTACATCAGTAAAATTAACACTACAGGGGGGAGTAATGGCACTATCATACTTCCATGCAAGATAAAGCAAA
TTATAAACATGTGGCAGGGAGTAGGACAAGCAATGTATGCTCCTCCCATCAGTGGAATAATTAATTGTGTAAC
AAATATTACAGGAATACTATTAACAAGAGATGGTGGTAATGATAATAGCATTAATGAGACCTTTAGACCTGGA
GGAGGAAATATAAAGGACAATTGGAGAAATGAATTATATAAATATAAAGTAGTACAAATTGAACCACTAGGAC
TAGCACCCACCAGGGCAAAGAGAAGAGTGGTGGAGAGAGAAAAAAGAGCAGTGGGAATAGGAGCTATGATCTT
TGGGTTCTTAGGAGCAGCAGGAAGCACTATGGGCGCGGCGTCAATAACGCTGACGGTACAGGCCAGACAATTA
TTGTCTGGTATAGTGCAACAGCAAAGCAATTTGCTGAGGGCTATAGAGGCGCAGCAGCATCTGTTGCAACTCA
CAGTCTGGGGCATT
>JX112824
ACTTATTTAAATAAAACCACAAGGGAGAGTAATGGCACTATCATACTTCCATGCAGGATAAAGCAAATTATAA
ACATGTGGCAGGGAGTAGGACAAGCAATGTATGCTCCTCCCATCAGTGGAAGCATTAATTGTGTATCAAATAT
TACAGGAATACTATTAACAAGAGATGGTGGTAATAATGGTACTAGTGATAAAGAGACCTTCAGACCTGGAGGA
GGAAATATAAAGGACAATTGGAGAAGTGAATTATATAAATATAAAGTAGTAGAAATTGAACCACTAGGAATAG
CACCCACCAGGGCAAAGAGAAGAGTGGTGGAGAGAGAAAAAAGAGCAGTGGGAATAGGAGCTATGATCTTTGG
GTTCTTAGGAGCAGCAGGAAGCACTATGGGCGCGGCGTCAATAACGCTGACGGTACAGGCCAGACAATTATTG
TCTGGTATAGTGCAACAGCAAAGCAATTTGCTGAGGGCTATAGAGGCGCAGCAGCATCTGTTGCAACTCACAG
TCTGGGGCATT
>JX112825
ACTTACAATGAAACTGGAACCACAGGGGAGAATAATGGCACTAATGGCAATATCACACTTCCATGCAGGATAA
AGCAAATTATAAACATGTGGCAGGAAGTAGGACAAGCAATGTATGCTCCTCCCATCAATGGAATAATTAATTG
TGTATCAAATATTACAGGAATACTATTAACAAGAGATGGTAGTAATGAAACTAGCCCTAACGAGACCTTCAGA
CCTGGAGGAGGAAATATAAAGGACAATTGGAGAAGTGAATTATATAAATATAAAGTAATACAAATTGAACCAC
TAGGAATAGCACCCACCAAGGCAAAGAGAAGAGTGGTGGAGAGAGAAAAAAGAGCAGTGGGAATAGGAGCTTT
GATCTTTGGGTTCTTAGGAGCAGCAGGAAGCACTATGGGCGCGGCGTCAATAACGCTGACGGTACAGGCCAGA
CAATTATTGTCTGGTATAGTGCAACAGCAAAGCAATTTGCTGAGGGCTATAGAGGCGCAGCAGCATATGTTGC
AACTCACAGTCTGGGGCATT
>JX112831
AATTACACAGGAAATAAAACAGGGGGGAATAATAACACTGAAACTATCACACTTCCATGCAACATAAAGCAAA
TTATAAACATGTGGCAGGGAGTAGGACAAGCAATGTATGCTCCTCCCGTCAGTGGAAGAATTAATTGTGTAAG
TAATATTACAGGAATACTATTAACAAGAGATGGTGGTAAAACTAGTGCTGACGAGACCTTCAGACCTGGAGGA
GGAAATATAAAGGACAATTGGAGAAGTGAATTATATAAATATAAAGTAGTACAAATTGAACCACTAGGAATAG
CACCCACCAGGGCAAAGAGAAGAGTGGTGGAGAGAGAAAAAAGAGCAGTGGGAATAGGAGCTATGATCTTTGG
GTTCTTAGGAGCAGCCGGAAGCACTATGGGCGCGGCGTCAATAACGCTGACGGCACAGGCTAGACAATTATTG

TCTGGTATAGTGCAACAGCAAAGCAATTTGCTGAGGGCTATAGAGGCGCAACAGCATCTGTTGCAACTCACAG
TCTGGGGCATT
>JX112836
ACTTACACAGAAAATGGAACCATAGGGGAAAGAAATGACACTATCACACTTCCATGCAAAATAAAGCAAATTA
TAAACATGTGGCAGGAAATAGGACAAGCAATGTATGCTCCCCCCGTCAGTGGAATAATTAATTGTGTATCAAA
TATTACAGGAATACTATTAACAAGAGATGGTGGTAATAATGGTACTAGTGCTAACGAGACCTTCAGACCTGGA
GGAGGAAATATAAAGGACAATTGGAGAAGTGAATTATATAAATATAAAGTAGTACAAATTGAACCACTAGGAA
TAGCACCCACCAGGGCAAAGAGAAGAGTGGTGGACAGAGAAAAAAGAGCAGTGGGAATAGGAGCTATGATCTT
TGGGTTCTTAGGAGCAGCAGGAAGCACTATGGGCGCGGCGTCAATAACGCTGACGGTACAGGCCAGACAATTA
TTGTCTGGTATAGTGCAACAGCAAAGCAATTTGCTGAGGGCTATAGAGGCGCAGCAGCATCTGTTGCAACTCA
CAGTCTGGGGCATT
>JX112837
TATTATGAAAATGGAACCATAGGGGGGAGTAATGGCACTATCATACTTCCATGTAGGATAAAGCAAATTATAA
ACATGTGGCAGGGAGTAGGACAAGCAATGTATGCTCCTCCCATCAGTGGAAGCATTAATTGTGTATCAAATAT
TACAGGAATACTATTAACAAGAGATGGTGGTGGTAATAATAGAACTAACGAGACCTTCAGACCTGGAGGAGGA
AATATAAAGGACAATTGGAGAAGTGAATTATATAAATATAAAGTAGTAGAAATTGAGCCACTAGGAGTAGCAC
CCACCAAGGCAAAGAGAAGAGTGGTGGAGAGAGAAAAAAGAGCAGTGGGAATAGGAGCTATGATCTTTGGGTT
CTTAGGAGCAGCAGGAAGCACTATGGGCGCGGCGTCAATAACGCTGACGGTACAGGCCAGACAATTATTGTCT
GGTATAGTGCAACAGCAAAGCAATTTGCTGAGGGCTATAGAGGCGCAGCAGCATCTGTTGCAACTCACAGTCT
GGGGCATT
>JX112838
ATTTACAATGGAAATGAAACCAATATTCCTGACAATAAAACTATCATACTTCCATGCAAGATAAAGCAAATTG
TAAACTTGTGGCAGGAAGTAGGACAAGCAATGTATGCTCCTCCCATCAGGGGAAAAATTAATTGTGTATCAAA
CATTACAGGAATACTATTAACAAGAGATGGTGGTAATATTAGTAATACTAGTACCGAGACCTTCAGACCTGCA
GGAGGAGATATGAGGGACAATTGGAGAAATGAATTATATAAATATAAAGTAGTACAAATTGAACCACTAGGAA
TAGCACCCACCAGGGCAAAAAGAAGAGTGGTGAAGAGAGAAAAAAGAGCAGTGGGAATAGGAGCTATGATCTT
TGGGTTCTTAGGAGCAGCAGGAAGCACTATGGGCGCGGCGTCAATAACGCTGACGGTACAGGCCAGACAATTA
TTGTCTGGTATAGTGCAACAGCAAAGCAATTTGCTGAGGGCTATAGAGGCGCAGCAGCATATGTTGCAACTCA
CAGTCTGGGGCATT
>JX112839
CATTACGAAAATGGAACCATAAAGGGGGGTAATAACACTAGCGAGATCACACTTCCATGCAGGATAAAGCAAA
TTATAAACATGTGGCAGGAAGTAGGACAAGCAATGTATGCTCCTCCCGTCAGTGGAAGAATTAATTGTGTATC
AAATATCACAGGAATACTATTAACAAGAGATGGTGGTAATAATAATAGTACTAATGAGACCTTCAGACCTGGA
GGAGGAGATATAAAGGACAATTGGAGAAGTGAATTATATAAATATAAAGTAGTACAAATTGAACCACTAGGAA
TAGCACCCACCAGGGCAAAGAGAAGAGTGGTGGAGAGAGAAAAAAGAGCAGTGGGAATAGGAGTTATGATCTT
TGGGTTCTTAGGAGCAGCTGGAAGCACTATGGGCGCGGCGTCAATAACGCTGACGGTACAGGCCAGACAATTA
TTGTCTGGTATAGTGCAGCAGCAAAGCAATTTGCTGAGAGCTATTGAGGCGCAACAGCATCTGTTGCAACTCA
CAGTTTGGGGCATT
>GU475031
ACTTACAAAGGAAATGAAACCATAGGGGAGAGGAATGACACTATCATACTTCCATGCAGGATAAAGCAGATTG
TAAACATGTGGCAGGAAGTAGGACAAGCAATGTATGCTCCTCCCGTCAGTGGAAGAATTAATTGTGTATCAAA
TATTACAGGAATACTATTAACAAGAGATGGTGGTAATAATAATACTAACGAGACCTTCAGACCTGGAGGAGGA
AATATAAAGGACAATTGGAGAAGTGAATTATATAAATATAAAGTAGTACAAATTGAACCACTAGGAATAGCAC
CCACCAGGGCAAAGAGAAGAGTGGTGGAGAGAGAAAAAAGAGCAGTGGGAATAGGAGCTATGATCTTTGGGTT
CTTAGGAGCAGCAGGAAGCACTATGGGCGCGGCGTCAATAACGCTGACGGTACAGGCCAGACAATTATTGTCT
GGTATAGTGCAACAGCAAAGCAATTTGCTGAGAGCTATAGAGGCGCAGCAGCACATGTTGCAACTCACAGTCT
GGGGCATT
>GU475030
ACTTACCAAGGAAATGAAACAGGGGGGAATACTAGCAGCACTATCACACTTCCATGCAAGATAAAGCAAATTA
TAAACATGTGGCAGGGAGTAGGACAAGCAATGTATGCTCCTCCCATCAGTGGAATAATTAATTGTGTATCAAA
TATTACAGGAATACTATTAACAAGAGATGGTGGTAATAATAATGCTACTAACGAGACCTTCAGACCTGGAGGA
GGAAATATAAAGGACAATTGGAGAAATGAATTATATAAGTATAAAGTAGTACAAATTGAACCACTAGGAATAG
CACCCACCAGGGCAAAGAGAAGAGTGGTGGAGAGAGAAAAAAGAGCAGTGGGAATAGGAGCTATGATCTTTGG
GTTCTTAGGAGCAGCTGGAAGCACTATGGGCGCGGCGTCAATAACGCTGACGGTACAGGCCAGACAATTATTG
TCTGGTATAGTGCAACAGCAAAGCAATTTGCTGAGGGCTATAGAGGCGCAGCAGCATCTGTTGCAACTCACAG
TCTGGGGCATT
>JX112840
ACTTACACAAGAAATGGAACCATAGGGGGGAGTAATGGCACTATCATACTGCCATGCAAGATAAAGCAAATTA
TAAACATGTGGCAGGGAGTAGGACAAGCAATGTATGCTCCTCCCATCAGTGGAAGAATTAATTGTGTATCAAA
TATCACAGGAATACTATTAACAAGAGATGGTGGTAATAGTAGTGCTAACGAGACCTTCAGACCTGGAGGAGGA

AATATAAAGGACAATTGGAGAAGTGAATTATATAAATATAAAGTAGTACAAATTGAACCACTAGGAATAGCAC
CCACCAGGGCAAAGAGAAGAGTGGTGGAGAGAGAAAAAAGAGCAGTGGGAATAGGAGCTATGATCTTTGGGTT
CTTAGGAGCAGCAGGAAGCACTATGGGCGCGGCGTCAATAACGCTGACGGTACAGGCCAGACAATTATTGTCT
GGTATAGTGCAACAGCAAAGCAATTTGCTGAGGGCTATAGAGGCGCAGCAGCATCTGTTGCAACTCACAGTCT
GGGGCATT
>JX112845
AATTACACAAGAAATGAAACCATGAGTAAGGACAAGGACACTCTCATACTTCCATGCAGGATAAAGCAAATTA
TAAACATGTGGCAGGGAGTAGGACAAGCAATGTATGCTCCTCCCATCAGGGGAGTAATTAATTGTTTATCAAA
TATTACAGGAATACTATTAACAAGAGATGGTGGTAATAATAGGACTAACGAGACCTTCAGACCTGAAGGAGGA
AATATAAAGGACAACTGGAGAAATGAATTATATAAATATAAAGTAGTGCAAATTGAACCACTAGGAATAGCAC
CTACCAGGGCAAAGAGAAGAGTGGTGGAGAGAGAAAAAAGAGCAGTGGGAATAGGAGCTATGATCTTTGGGTT
CTTAGGAGCAGCAGGAAGCACTATGGGCGCGGCGTCAATAACGCTGACGGTACAGGCCAGACAATTATTGTCT
GGTATAGTGCAACAGCAAAGCAATTTGCTGAGGGCTATAGAGGCGCAGCAGCATATGTTGCAACTCACAGTCT
GGGGCATT
>JX112851
ACTTATGGAAATGAAACCATAGGGGAGAGAAATGACAGTATCATACTTCCATGCAAGATAAAGCAAATTATAA
ACATGTGGCAGGGAGTAGGACAAGCAATGTATGCTCCTCCAATCAGTGGAAGAATTAATTGTGTATCAAATAT
TACAGGGATACTCTTAACAAGAGATGGTGGTAATAATAATAATACTAATGAGATCTTCAGACCTGGAGGAGGA
AATATAAAGGACAATTGGAGAAGTGAATTATATAAATATAAAGTAGTACAAATTGAACCACTAGGAATAGCAC
CCACCAAGGCAAAGAGAAGAGTGGTGGAGAGAGAAAAAAGAGCAGTGGGAATAGGAGCTATGATCTTTGGGTT
CTTAGGAGCAGCTGGAAGCACTATGGGCGCGGCGTCAATAACGCTGACGGTACAGGCCAGACAATTATTGTCT
GGTATAGTGCAACAGCAAAGCAATTTGCTGAGGGCTATAGAGGCGCAGCAGCATCTGTTGCAACTCACAGTCT
GGGGCATT
>GU475023
ACTTACTCAGGAAATGAAAGCACAGGGGGGAATGAGAGCACTATCATACTTCCATGCAGGATAAAGCAAATTA
TAAACATGTGGCAGGGAGTGGGACAAGCAATGTATGCTCCTCCCATCAGGGGAAGAATTAATTGTGTAACAAA
CATTACAGGAATACTATTAACAAGAGATGGTGGTAATAATACTAACGAGACCTTCAGACCTGGAGGAGGAAAT
ATAAAGGACAATTGGAGAAGTGAATTATATAAATATAAAGTAGTACAAATTGAACCACTAGGAGTAGCACCCA
CCAGGGCAAAGAGAAGAGTGGTGCAGAGAGAAAAAAGAGCAGTGGGAATAGGAGCTATGATCTTTGGGTTCTT
AGGAGCAGCAGGAAGCACTATGGGCGCGGCGTCAATAACGCTGACGGTACAGGCCAGACAATTATTGTCTGGT
ATAGTGCAACAGCAAAGCAATTTGCTGAGGGCTATAGAGGCGCAGCAGCATATGTTGCAACTCACAGTCTGGG
GCATT
>GU475027
ACTTACGGAAATGGAACCACAGGGGAGAAGAATAGCAGCACTATCATACTTCCATGCAGGATAAAGCAAATTA
TAAACATGTGGCAGGGAGTAGGACAAGCCATGTATGCTCCTCCCATCAGAGGAAACATTAATTGTGTATCAAA
TATTACAGGAATACTATTAACAAGAGATGGTGGTGGTGATAATAGTACTAACGAGATCTTCAGACCTGGAGGA
GGAAATATGAAGGACAATTGGAGAAATGAATTATATAAATATAAAGTAGTACAAATTGAACCACTAGGAATAG
CACCCACCAGGGCAAAGAGAAGAGTGGTGGAGAGACAAAAAAGAGCAGTGGGAATAGGAGCTATGATCTTTGG
GTTCTTAGGAGCAGCAGGAAGCACTATGGGCGCGGCGTCAATAACGCTGACGGTACATGCCAGACAATTATTG
TCTGGTATAGTGCAACAGCAAAGCAATTTGCTGAGGGCTATAGAGGCGCAGCAGCATCTGTTGCAACTCACAG
TCTGGGGCATT
>MG655196
ACTTATACAGAAGGAAATGAAACCATAGAGGAAAGTAATGGCAATATCACACTTCCATGCAGGATAAAGCAAA
TTATAAACATGTGGCAGGGAGTAGGACAAGCAATGTATGCTCCTCCCGTCAGTGGAAAAATTAATTGTGTATC
AAATATTACAGGAATACTATTAACAAGAGATGGTGGTGCTGGTACTGGGGATAACAATACTGAGACCTTCAGA
CCTGCAGGAGGAAATATGAAGGACAATTGGAGAAGTGAATTATATAAATATAAAGTAGTGCAAATTGAACCAC
TAGGACTAGCACCCACCAGGGCAAAGAGAAGAGTGGTGGAGAGAGAAAAAAGAGCAGTGGGAATAGGAGCTAT
GATCTTTGGGTTCTTAGGAGCAGCCGGAAGCACTATGGGCGCGGCGTCAATAACGCTGACGGTACAGGCCAGA
CAATTATTGTCTGGTATAGTGCAACAGCAAAGCAATTTGCTGAGGGCTATAGAGGCGCAGCAGCATATGTTGC
AACTCACAGTCTGGGGCATT
>MG655197
ACTTACAATGAAAGTGAAACCACAGGGGAAAGTAATGGCACTATCATACTTCCATGCAGGATAAAGCAAATTA
TAAACATGTGGCAGAGAGTAGGACAAGCAATGTATGCTCCTCCCATCAGTGGAAGAATTAATTGTATATCAAA
TATTACAGGAATACTATTAACAAGAGATGGTGGTAATAATAATAATGATACTGAGATCTTCAGACCTGGAGGA
GGAAATATGAAGGACAATTGGAGAAGTGAATTATATAAATATAAAGTAGTAGAAATTGAACCACTAGGAATAG
CACCCACCAGGGCAAAGAGAAGAGTGGTGGAGAGAGAAAAAAGAGCAGTGGGAATAGGAGCTCTGATCTTTGG
GTTCTTAGGAGCAGCAGGAAGCACTATGGGCGCAGCGTCAATAACGCTGACGGTACAGGCCAGACAATTATTG
TCTGGTATAGTGCAACAGCAAAGCAATTTGCTGAGAGCTATAGAGGCGCAGCAGCATATGTTGCAACTCACAG
TCTGGGGCATT
>MG655200

ACTTACGGAAATGAAACCATAGGAAATAAAACCGAAGGGGAGAGGAATGGTACTATCACACTTCCATGCAAGA
TAAAGCAAATTGTAAGAATGTGGCAGGAAGTAGGACAAGCAATGTATGCTCCTCCCATTAGTGGAAGCATTAA
TTGTGTATCAAATATTACAGGAATACTATTAACAAGAGATGGTGGTAATGGTACTAACGAGACCTTCGAGACC
TTCAGACCTGCAGGAGGAGATATAAAGGACAATTGGAGAAGTGAATTATATAAATATAAAGTAGTACAAATTG
AACCACTAGGAATAGCACCCACCAGGGCAAAGAGAAGAGTGGTGGAGAGAGAAAAAAGAGCAGTGGGAATAGG
AGCTATGATCTTTGGGTTCTTAGGAGCAGCAGGAAGCACTATGGGCGCGGCGTCAATAACGCTGACGGTACAG
GCCAGACAATTATTGTCTGGTATAGTGCAACAGCAAAGTAATTTGCTGAGGGCTATAGAGGCGCAGCAGCATC
TGTTGCAACTCACAGTCTGGGGCATT
>JX960603
ACTTACCAAAATGGAACCATAAATGGAGAGAAGTATAAGGAAAATATTATACTTCCATGCAGGATCAAGCAAA
TTATAAACATGTGGCAGAGAGCAGGACAAGCAATGTATGCTCCTCCCATCAAGGGAAGAATTAATTGTGTATC
AAATATTACAGGAATACTATTAACAAGAGATGGTGGTGATAATAGCACAGCTAACGAGACCTTCAGACCGGGA
GGAGGAAATATAAAAGACAATTGGAGAAATGAATTATATAAATATAAGGTAGTACAAATTGAACCACTAGGAA
TAGCACCCACCAGGGCAAAGAGAAGAGTGGTGCAGAGAGAAAAAAGAGCAGTGGGAATAGGAGCTATGATCTT
TGGGTTCTTAGGAGCAGCAGGAAGCACTATGGGCGCAGCATCAATAACGCTGACGGTACAGGCCAGACAGTTA
TTGTCTGGTATAGTGCAACAGCAAAGCAATTTGCTGAGGGCTATAGAGGCGCAGCAGCATCTGTTGCAACTCA
CAGTCTGGGGCATT
>KP109508
GATCATGGTAATGGCACTATCACACTTCCATGCAGGATAAAGCAAATTATAAACATGTGGCAAAGAGCAGGAC
AAGCAATGTATGCTCCTCCCATCAGTGGAGTAATTAATTGTGTATCAAATATTACAGGAATACTATTGACAAG
AGATGGTGGTAATACTAGTAGTAGTAACGAGACTTTCAGACCTGGAGGAGGAAATCTAAAGGACAATTGGAAA
AATGAATTATATAAATATAAAGTAGTACAAATAGAACCACTAGGAGTAGCACCCACCAAGGCAAAGCGAAGAG
TGGTGGATAGAGAAAAAAGAGCAGTGGGAATAGGAGCTATGATCTTTGGGTTCTTGGGAGCAGCAGGAAGCAC
TATGGGCGCGGCGTCAATAACGCTGACGGTACAGGCCAGGCAATTATTGTCTGGTATAGTGCAACAGCAAAGC
AATTTGCTGAGAGCTATAGAGGCGCAGCAGCATATGTTGCAACTCACAGTCTGGGGCATT
>MH327760
AATCACACCGAGGGTAATGGCACTGGCAATATCATACTTCCATGCAAGATAAAGCAAATTATAAACATGTGGC
AGGGAGTAGGACAAGCAATGTATGCTCCTCCCGTCAGGGGAGTAATTAATTGTGTATCAAACATTACAGGAAT
AATATTGACAAGGGATGGTGGGACAAATAGCACGAACGATACCGAGATCTTCAGACCTGAAGGAGGAAATATA
AAAGACAATTGGAGAAGTGAATTATATAAATATAAAGTAGTACAAATTGAACCACTAGGAGTGGCACCCACCA
GGGCAAGGAGAAGAGTGGTGGAAAGAGAAAAAAGGGCAGTGGGAATAGGAGCTATGATCTTTGGGTTCTTAGG
AGCAGCAGGAAGCACTATGGGCGCGGCGTCAATAACGCTGACGGTACAGGCCAGACAATTATTGTCTGGTATA
GTGCAACAGCAAAGCAATTTGCTGAGGGCTATAGAGGCGCAACAGCATATGTTGCAACTCACAGTCTGGGGCA
TT
>KY213720
ACTTATAATAACACTGAGTTAACTGTAGGTAATAATACTGATAATGATACTATCACGCTTCCATGCAGGATAA
AGCAAATTATAAACATGTGGCAGGGAGCAGGACAAGCAATGTATGCTCCTCCCATCAGTGGAAAAATTAATTG
TATATCAAATATTACAGGAATATTATTGACAAGAGATGGTGGTAATAATGCAACAAATGAAGAGATCTTCAGA
CCTGAAGGAGGAAATATAAAGGACAATTGGAGAAGTGAATTATATAAATATAAAGTAGTACAAATTGAACCAC
TAGGAATAGCACCCACCAGGGCAAAGAGAAGAGTGGTGGAGAGAGAAAAAAGAGCAGTGGGAATAGGAGCTAT
GATCTTTGGGTTCTTAGGAGCAGCCGGAAGCACTATGGGCGCAGCGTCAATAACGCTGACGGTACAGGCCAGA
CAATTATTGTCTGGTATAGTGCAACAGCAAAACAATTTGCTGAGGGCTATAGAGGCGCAGCAGCATATGTTGC
AACTCACAGTCTGGGGCATT
>KY213739
AATTATACAGAAAATGAAGCCACGAAGGGGGATAATACAACTATCAGACTTCCATGTAAGATAAAGCAAATTA
TAAACATGTGGCAGGGAGTAGGACAAGCAATGTATGCTCCTCCCATCAGTGGAAGCATTAGTTGTATATCAAA
TATTACAGGAATACTATTGACAAGAGATGGTGGTGATAATAATGTTAATGAGACCTTCAGACCTGGAGGAGGA
AATATAAAGGACAATTGGAGAAGTGAATTATATAAATATAAAGTAGTACAAATTGAACCACTAGGAATAGCAC
CCACCAAGGCAAAGAGAAGAGTGGTGGAGAGAGAGAAAAGAGCAGTGGGAATAGGAGCTATGATCTTTGGGTT
CTTAGGAGCAGCCGGAAGCACTATGGGCGCGGCGTCAATAACGCTGACGGTACAAGCCAGACAATTATTGTCT
GGGATAGTGCAACAGCAAAGCAATTTGCTGAGGGCTATAGAGGCGCAGCAGCATCTGTTGCAACTCACAGTCT
GGGGCATC
>KY213717
AATTACACAGCAAATGAAACTGTGTGGAATGACACTATCATACTTCCATGCAGGATAAAACAAATTGTAAACA
TGTGGCAGGGAGTAGGACAAGCAATGTATGCTCCTCCCGTCAGGGGAATAATTAGTTGTGTATCAAAGATTAC
AGGATTACTATTGACAAGAGATGGTGGTAATAATAATACTAACGAGACCTTCAGACCTGGGGGAGGAAATATG
AAGGACAATTGGAGAAGTGAATTATATAAATATAAAGTAGTACAAATTGAACCACTAGGAGTAGCACCCACCA
GGGCAAGGAGAAGAGTGGTGCAGAGAGAAAAGAGAGCAGTGGGAATAGGAGCTATGATCTTTGGGTTCTTAGG
AGCAGCAGGAAGCACTATGGGCGCAGCGTCAATAACGCTGACGGTACAGGCCAGACAATTGTTGTCTGGTATA

GTGCAACAGCAAAGCAATTTGCTGAGAGCTATTGAGGCGCAGCAGCATATGTTGCAACTCACAGTCTGGGGCA
TT
>AY945721
ACTTACAGAAAAAATGAAACCAGGGAGAGGAATGATAGCACTATCATAATTCCATGCAAGATAAAACAAATTA
TAAACATGTGGCAGGGAACAGGACAAGCAATGTATGCTCCTCCCATCAGTGGAAACATTAATTGTGTATCAAA
TATTACAGGAATACTATTGACAAGAGATGGTGGTAATAATAGTGCGGATAACGAGACTTTCAGACCTGGAGGA
GGAAATATAAAGGACAATTGGAGAAGTGAATTATATAAATATAAAGTAGTAGAAATTGAGCCACTAGGAATAG
CACCCACCAGGGCAAAGAGAAGAGTGGTGGAGAGAGAAAAAAGAGCAGTGGGAATAGGAGCTATGATCTTTGG
GTTCTTAGGAGCAGCAGGAAGCACTATGGGCGTGGCGTCAATAACGCTGACGGTACAGGCCAGACAATTATTG
TCTGGTATAGTGCAACAGCAAAGCAATTTGCTGAGGGCTATAGAGGCGCAACAGCATATGTTGCAACTCACAG
TCTGGGGCATT
>AY945722
AATCACAAGGAGTGTAATGGCACTATCACACTTCCATGCAGGATAAAGCAAATTATAAACATGTGGCAGGGAG
CAGGACAAGCAATGTATGCTCCTCCCATCAGTGGAAAAATTAATTGTGTATCAAATATTACAGGAATACTATT
GACAAGAGATGGTGGTACTAATAATAATACAAATAGCAGCGAGATATTCAGACCTGGAGGAGGAAATATAAAA
GATAATTGGAGAAGTAAATTATATAAATATAAAGTAGTACAAATTGAACCACTAGGAATAGCACCCACCAGGG
CAAAAAGAAGAGTGGTGGAGAGAGAAAAAAGAGCAGTGGGAATAGGAGCTATGATCTTTGGGTTCTTAGGAGC
AGCAGGAAGCACTATGGGCGCGGCGTCACTAACGCTGACGGTACAGGCCAGACAATTATTGTCTGGTATAGTG
CAACAGCAAAGCAATTTGCTGAGGGCTATAGAGGCGCAGCAGCATCTGTTGCAACTCACAGTCTGGGGCATT
>KJ952852
GCTTACAGTGGAAATGAAACCATGAATGAAACCATGGATAGGTATAATGACACGATCATACTTCCATGCAGGA
TAAAGCAAATTATAAACATGTGGCAGGGAGTAGGACAAGCAATGTATGCTCCTCCCATCAGTGGAAGCATTAA
TTGTGTATCAAATATTACAGGAATACTATTGACAAGAGATGGTGGTAATAATCTGAATAACAGTAACGAGACC
TTCAGACCTGGAGGAGGAAATATAAAGGATAATTGGAGAAGTGAATTATATAAATATAAAGTAGTACAAATTG
AACCACTAGGAATAGCACCCACCAGGGCAAAGAGAAGAGTGGTGGAGAGAGAAAAAAGAGCAGTGGGAATAGG
AGCTATGATCTTTGGGTTTTTAGGAGCAGCAGGAAGCACTATGGGCGCGGCGTCAATAACGCTGACGGTACAG
GCCAGACAATTATTGTCTGGTATAGTGCAACAGCAAAGCAATTTGCTGAGGGCTATAGAGGCGCAGCAGCATC
TGTTGCAACTCACAGTCTGGGGCATT
>KJ953555
ACTTACAGGGAAAATGAAACCATAGGGGGTAATGGCACTATCATACTTCCATGCAAGATAAAGCAAATTATAA
ACATGTGGCAGGGAGTAGGACAAGCAATGTATGCTCCTACCGTCCGTGGAACAATTAATTGTGTATCAAACAT
TACAGGAATGTTATTAACAAGAGATGGTGGTGTTAATAAGGATGATAATACTAGCGAGACCTTCAGACCTGGA
GGAGGAAATATAAAGGACAATTGGAGAAGTGAATTATATAAATATAAAGTAGTACAAATTGAACCACTAGGAA
TAGCACCCACCAGGGCAAAGAGAAGAGTGGTGGAGAGAGAAAAAAGAGCAGTGGGAATAGGAGCTATGATCTT
TGGGTTCTTAGGAGCAGCAGGAAGCACTATGGGCGCGGCGGCAATAACGCTGACGGTACAGGCCAGACAATTA
TTGTCTGGTATAGTGCAACAGCAAAGCAATTTGCTGAGGGCTATAGAGGCGCAGCAGCATATGTTGCAACTCA
CAGTCTGGGGCATT
>KJ953646
AGTTACACAAAAGAAAAGGGATGTAATGGCACTATCATACTTCCATGCAAGATAAAGCAAATTATAAACATGT
GGCAGGGAGTAGGACAAGCAATGTATGCTCCTCCCGTCAGAGGAGTAATTAATTGTGTATCAAATATTACAGG
AATACTATTGACAAGAGATGGTGGTGAGGAGAATAATGGGAGTGAAACCTTCAGACCTGGAGGAGGAAATATA
AAGGACAATTGGAGAAGTGAATTATATAAATATAAAGTAGTACAAATTGAACCACTAGGAGTAGCACCCACCA
AGGCAAAGAGAAGAGTGGTGGAAAGACAAAAAAGAGCAGTGGGAATAGGAGCTATGATCTTTGGGTTCTTAGG
AGCAGCAGGAAGCACTATGGGCGCGGCGTCAATAACGCTGACGGTACAGGCCAGACAATTATTGTCTGGTATA
GTGCAACAGCAAAGTAATTTGCTGAGGGCTATAGAGGCGCAGCAACATCTGCTGCAACTCACAGTCTGGGGCA
TT
>AY358052
ACTTACACAGGAAATGAAACCATGGAAGGGTGTAATGGCACTATCATACTTCCATGCAAGATAAAGCAAATTA
TAAACATGTGGCAGGGAGTAGGACAAGCAATGTATGCTCCTCCCATCAGTGGAAATATTAGTTGTGTATCAAA
TATTACAGGAATACTATTGACAAGAGATGGTGGTATTAATGAGGATAACAAGACCGAGATCTTCAGACCTGGA
GGAGGAAATATAAAGGACAATTGGAGAAGTGAATTATATAAATATAAAGTAGTACAAATTGAACCACTAGGAA
TAGCACCCACCAGGGCAAAGAGAAGAGTGGTGGAGAGAGAAAAAAGAGCAGTGGGAATAGGAGCTATGATCTT
TGGGTTCTTGGGAGCAGCAGGAAGCACTATGGGCGCGGCGTCAATAACGCTGACGGTACATGCCAGACAATTA
TTGTCTGGTATAGTGCAACAGCAAAGCAATTTGCTGAGGGCTATAGAGGCGCAGCAGCATCTGTTGCAACTCA
CAGTCTGGGGCATT
>AY945719
AATCATACTTTCAAAAATAATGAAACCACGGAGGGGTATAATGGCACTATCACAATTCCATGCAAGATAAAGC
AAATTATAAACATGTGGCAAGGAGTGGGACAAGCAATGTATGCTCCTCCCATCAGGGGAAACATTAGTTGTGT
ATCAAATATTACAGGCATACTGTTGACAAGAGATGGTGGTAGTAGAGATGCGCCTAATGAGACCTTCAGACCT
GGAGGAGGAAATATAAAGGACAATTGGAGAAATGAATTATATAAATATAAAGTAGTACAAATTGAACCACTAG

GAATAGCACCCACCAGGGCAAAGAGAAGAGTGGTGGAGAGAGAAAAAAGAGCAGTGGGAATAGGAGCTATGAT
CTTTGGGTTCTTAGGAGCAGCAGGAAGCACTATGGGCGCAGCGTCAATAACGCTGACGGTACATGCCAGACAA
TTATTGTCTGGTATAGTGCAACAGCAAAGCAATTTGCTGAGGGCTATAGAGGCGCAGCAGCATCTGTTGCAAC
TCACAGTCTGGGGCATT
>AY358038
AGTTACCTAAAAAATAAAACCCTTGACGGGAATGGCACTATCATAATCCCATGCAGAATAAAGCAAATTGTAA
ACATGTGGCAGGGAGTAGGACAAGCAATGTATGCTCCTCCCATCAATGGAATAATTAATTGTGTATCAAATAT
TACAGGAATATTATTAACAAGAGATGGTGGTAACAGTACGAATAACGAGACTTTCAGACCTGGAGGAGGAAAT
ATAAAGGATAATTGGAGAAGTGAATTATATAAATATAAAGTAGTACAAATTGAACCACTAGGAATAGCACCCA
CCAGGGCAAAGAGAAGAGTGGTGGAGAGAGAAAAAAGAGCAGTGGGAATAGGAGCTATGCTCTTTGGGTTCTT
AGGAGCAGCCGGAAGCACTATGGGCGCGGCGTCAATAACGCTGACGGTACAGGCCAGACAATTATTGTCTGGT
ATAGTGCAACAGCAAAGCAATTTGCTGAGGGCTATAGAGGCGCAGCAGCATCTGTTGCAACTCACAGTCTGGG
GCATT
>AY358064
ACTTATGGAGAAAATGAAACCATGGAGGGTAATAACAATAGCACTATCATACTTCCATGCAAGATAAAACAAA
TTGTAAACATGTGGCAGGGAGCAGGACAAGCAATGTATGCTCCTCCCATCAGTGGAACAATTAATTGTGTATC
AAATATTACAGGAATACTATTAACAAGAGATGGTGGTAATAATAATAGTACTAATGAGACCTTCAGACCTGGA
GGAGGAAATATAAAAGACAATTGGAGAAGTGAATTATATAAATATAAAGTAGTACAAATTGAACCACTAGGAC
TAGCACCCACCAGGGCAAGGAGAAGAGTGGTGGAGAGAGAAAAAAGAGCAGTGGGAATAGGAGCTATGATCTT
TGGGTTCTTAGGAGCAGCAGGAAGCACTATGGGCGCGGCGTCAATAACGCTGACGGTACAGGCCAGACAATTA
TTGTCTGGTATAGTGCAACAGCAAAGCAATTTGCTGAGGGCTATTGAGGCGCAGCAGCATCTGATGCAACTCA
CAGTCTGGGGCATT
>JN248324
ACTTACCTAAAAAATGAGACAACGTGTAATGGCACATGTAATGGCACTATCATACTTCCATGCAAGATAAAAC
AAATTATAAACATGTGGCAGGGAACAGGACAAGCAATGTATGCTCCTCCCATCAGTGGAAGAATTAATTGTGT
ATCAAATATTACAGGAATACTATTGACAAGAGATGGCGGTAATGAAAGTAGTGATACTGAGATCTTCAGACCT
GAAGGAGGAAATATAAAGGACAATTGGAGAAGTGAATTATATAAATATAAAGTAGTACAAATTGAACCACTAG
GAGTAGCACCCACCAGGGCAAAGAGAAGAGTGGTGGAGAAAGAAAAAAGAGCAGTGGGAATAGGAGCTGTGAT
CTTTGGGTTCTTAGGAGCAGCAGGAAGCACTATGGGCGCGGCATCAATAACGCTGACGGTACAGGCCAGACAA
TTATTGTCTGGTATAGTGCAACAGCAAAGCAATTTGCTGAGGGCTATAGAGGCGCAACAGCATCTGTTGCAAC
TCACAGTCTGGGGCATT
>JN248330
ACTTACATGAGAAATGGAACTGTAATAAATGAAGCTGTGAATGGCACTATCTTTCTTCCATGCAGGATAAAGC
AAATTATAAACATGTGGCAGGGAGCAGGACAAGCAATGTATGCTCCTCCCATCAGGGGAACAATTAATTGTGT
CTCAAATATTACAGGAATACTATTGACAAGAGATGGTGGTACTAATACAACTAATGAGACCTTCAGACCTGAG
GGAGGAGATATGAAGGACAATTGGAGAAGTGAATTATATAAATATAAAGTAGTAGAAATTGAACCACTAGGAA
TAGCACCCACCAAGGCAAAGAGAAGAGTGGTGGAAAGAGAAAAAAGAGCAGTGGGAATAGGAGCTATGATCTT
TGGGTTCTTAGGAGCAGCAGGAAGCACTATGGGCGCAGCGTCAATAACGCTGACGGTACAGGCCAGACAATTA
TTGTCTGGTATAGTGCAACAGCAAAGCAATTTGCTGAGAGCTATAGAGGCGCAACAGCATATGTTGCAACTCA
CAGTCTGGGGCATT
>JN248334
ACTTACTCAGGAAATGGAACCATCATACTTCCATGCCGGATAAAGCAAATTATAAACATGTGGCAGGGAGTAG
GACAGGCAATGTATGCTCCTCCCATCAGTGGAGCAATTAAGTGTGTATCAAATATTACAGGAATACTATTGAC
AAGAGATGGTGGTACTAATAGGACTAACGAGACTTTCAGACCTGGAGGAGGAAATATAAAGGACAACTGGAGA
AGTGAATTATATAAATATAAGGTAGTAGAAATTGAACCACTAGGAATAGCACCCACCAGGGCAAAGAGAAGAG
TGGTGGAAAGAGAAAAAAGAGCAGTGGGAATAGGAGCTATGATCTTTGGGTTCTTAGGAGCAGCAGGAAGCAC
TATGGGCGCGGCGTCGATAACGCTGACGGTACAGGCCAGACAATTATTGTCTGGTATAGTGCAACAGCAAAGC
AATTTGCTGAGAGCTATAGAGGCGCAGCAGCATCTGTTGCAACTCACAGTCTGGGGCATT
>JX447529
GGTCAGGAGCATAATGGCACTATCACACTTCCATGCAGGATAAAGCAAATTATAAACATGTGGCAGGGAGTAG
GACAAGCAATGTATGCTCCTCCCATCAGTGGACAAATTAATTGTGTATCGAATATTACAGGAATACTATTGAC
AAGAGATGGTGGTAAGGATAATGAAACGAATGGCACAGAGACCTTCAGACCTGGAGGAGGAAACATGAAGGAC
AATTGGAGGAGTGAATTATATAAATATAAAGTAATACAAATTGAACCACTAGGAGTAGCACCCACCAGGGCAA
GGAGAAGAGTGGTGGCGAGAGAAAAAAGAGCAGTGGGAATAGGAGCTATGATCTTTGGGTTCTTAGGAGCAGC
AGGAAGCACTATGGGCGCGGCGTCAATAACGCTGACGGTACAGGCCAGACAATTATTGACTGGTATAGTGCAA
CAGCAAAGCAATTTGCTGAGAGCTATAGAGGCGCAACAGCATCTGTTGCAACTCACAGTCTGGGGCATT
>JN248341
ACTTACACAGGAAATGAAACCGGAGAGGGGGGTAATGGTAATGGCACTATCATAATTCCATGCAGAATAAAGC
AAATTATAAACATGTGGCAGGGAGTAGGACAAGCAATGTATGCTCCTCCCATCAGTGGAAGCATTAATTGTGT
ATCAAATATTACAGGAATACTATTGACAAGAGATGGTGGTAATAACAACATGACTAACGATAACGAGACCTTC

AGACCTGGAGGAGGAGATATAAGGGACAATTGGAGAAGTGAATTGTATAAATATAAAGTAGTACAAATTGAAC
CACTAGGAGTAGCACCCACCAAGGCAAAGAGAAGAGTGGTGGAGAGAGAAAAAAGAGCAGTGGGAATAGGAGC
TATGATCTTTGGGTTCTTAGGAGCAGCAGGAAGCACTATGGGCGCAGCGTCAATAACGCTGACGGTACAGGCC
AGACAATTATTGTCTGGTATAGTGCAACAGCAAAGCAATTTGCTGAGGGCTATAGAGGCGCAGCAGCATATGT
TGCAACTCACTGTCTGGGGCATT
>JN248342
AATTATAATGGCACAAGAAATGAAACGTGTAATGACACTATCATACTACCATGCAGGATAAAGCAAATTATAA
ACATGTGGCAGGGAGTAGGACAAGCAATATATGCTCCTCCAATCAGAGGAACACTTAAGTGTGTATCAAATAT
TACAGGAATACTATTGACAAGAGATGGTGGTAGTAATGAGACTAAAAATGGGACCGAGATATTCAGACCTGGA
GGGGGAAATATAAAGGACAATTGGAGAAATGAATTATATAAATATAAAGTAGTACAAATTGAACCACTAGGAA
TAGCACCCACCAGGGCAAAGAGAAGAGTGGTGGAGAGAGAAAAAAGAGCAGTGGGAATAGGAGCTATGATCTT
TGGGTTCTTAGGAGCAGCAGGAAGCACTATGGGCGCGGCGTCAATAACGCTGACGGTACAGGCCAGACAATTA
TTGTCTGGTATAGTGCAACAGCAAAGCAATTTGCTGAGGGCTATAGAGGCGCAGCAGCATCTGTTGCAACTCA
CAGTCTGGGGCATT
>JN944654
AATCAAATCATGGGGCAGCATGATGACACTATGGTACTTCCATGCAAGATAAAGCAAATTATAAATATGTGGC
AGGGAGCAGGACAAGCAATGTATGCTCCTCCCGTCAGTGGAATAATTAATTGTAGGTCAAATATTACAGGAAT
ACTATTGACAAGAGATGGTGGTGGTGGTGCTAACAATACAAATGAGACCTTCAGACCTGGGGGAGGAAATATA
AAGGATAATTGGAGAAGTGAATTATATAAATATAAAGTAGTAAAAATTGAACCACTAGGATTAGCACCCACCA
GGGCAAAGAGAAGAGTGGTGGAGAAAGAAAAAAGAGCAGCGGGAATAGGAGCTATGATCTTTGGGTTCTTAGG
AGCAGCAGGAAGCACTATGGGCGCGGCGTCAATAACGCTGACGGTACAGGCCAGACAATTATTGTCTGGTATA
GTGCAACAGCAAAGCAATTTGCTGAGAGCTATAGAGGCGCAACAGCATCTGTTGCAACTCACAGTCTGGGGCA
TT
>JX447268
ACTTACATAGGAAATGAAACCAGAGAGATATTTAATGGAACTGATGAAACTAATGGAACTATCATACTTCCAT
GCAAAATAAAGCAAATTATAAACATGTGGCAGGGAGTAGGACAAGCAATGTATGCTCCTCCCATCAGTGGAAA
AATTAATTGTGTATCAAATATTACAGGAATACTATTGACAAGAGATGGTAGTTCTAGAAATAATACGAACGAA
ACCTTCAGACCTGGAGGGGGAAATATAAAGGATAATTGGAGAAGTGAATTATATAAATATAAAGTAGTACAAA
TTGAACCACTAGGAATAGCACCCACCAGGGCAAAGAGAAGAGTGGTGGAGAGAGAAAAAAGAGCAGTGGGAAT
AGGAGCTATGATCTTTGGGTTCTTAGGAGCAGCAGGAAGCACTATGGGCGCGGCGTCAATAACGCTGACGGTA
CAGGCCAGACAATTATTGTCTGGTATAGTGCAACAGCAAAGCAATTTGCTGAGGGCTATAGAGGCGCAGCAGC
ATCTGTTGCAACTCACAGTCTGGGGCATT
>JX447579
GTTTATAATAATGACACAAAGAATAGAACCAGGGGGAGTAATGACACTATCACAATTCCATGCAAAATAAAGC
AAATTATAAACATGTGGCAGGGGATAGGACAAGCAATGTATGCTCCTCCCATCAGTGGAACAATTAATTGTGT
ATCAAATATTACAGGAATACTATTGACAAGAGATGGTGGTGATAGTACTAACGAGACCTTCAGACCTGGAGGA
GGAAATATAAAGGACAATTGGAGAAGTGAATTATATAAATACAAAGTAGTACAAATTGAACCACTAGGAATAG
CACCCACCAGGGCAAAGAGAAGAGTGGTGGAAAGAGAAAAAAGAGCAGTGGGAATAGGAGCTATGATCTTTGG
GTTCTTAGGAGCAGCAGGAAGCACTATGGGCGCGGCGTCAATAACGCTGACGGTACAGGCCAGACAATTATTG
TCTGGTATAGTGCAACAGCAAAGCAATTTGCTGAGGGCTATAGAGGCGCAGCAGCATATGTTGCAACTCACAG
TCTGGGGCATT
>JX447592
ACTCACATGATAAATGGAACCATGAAGGGGAGTAATGGCACTAATGGCACTAATGGCACTTTCATACTTCCAT
GCAGGATAAAGCAAATTATAAACATGTGGCAGGGAGTAGGACAAGCAATGTATGCTCCTCCCATCAAGGGAAT
AATTAATTGTGTATCAAACATTACAGGAATATTATTGACAAGGGATGGTGGTAATAACAACACAAATAACGAG
ACCTTCAGACCTATAGGAGGAAATATAAAGGACAATTGGAGAAGTGAATTATATAAATATAAAGTAGTACAAA
TTGAACCACTAGGAATAGCACCCACCAAGGCAAAGAGAAGAGTGGTGGAGAGAGAAAAAAGAGCAGTGGGAAT
AGGAGCTATGATCTTTGGGTTCTTGGGAGCAGCAGGAAGCACTATGGGCGCGGCGTCAATAACGCTGACGGTA
CAGGCCAGACAATTATTGTCTGGTATAGTGCAACAGCAAAGCAATTTGCTGAGGGCTATAGAGGCGCAGCAGC
ATATGTTGCAACTCACAGTCTGGGGCATT
>JX448256
TCTTACCCAGAAAACGAGACTGAGGGGAATACTACCACTACCACTATCATACTTCCATGCAAGATAAAGCGAA
TTATAAACATGTGGCAGGGAGTAGGACAAGCAATGTATGCTCCTCCCATTAGGGGAAAAATTAAGTGTGTATC
AAATATTACAGGAATACTATTGACAAGTGATGGTGGTGGTAAGGGTAATAATAGTAATAGCGAGACCTTCAGA
CCTGGAGGAGGAAATATAAGGGACAATTGGAGAAGTGAATTATATAAATATAAAGTAGTGCAAATTGAACCAC
TAGGAATAGCACCCACCAAAGCAAAGAGAAGAGTGGTGGAGAGAGAAAAAAGAGCAGTGGGAATAGGAGCTAT
GATCTTTGGGTTCTTAGGAGCAGCAGGAAGCACTATGGGCGCGGCGTCAATAACGCTGACGGTACAGGCCAGG
CAATTATTGTCTGGTATAGTGCAACAGCAAAGCAATTTGCTGAGGGCTATAGAGGCGCAGCAGCATATGTTGC
AACTCACAGTCTGGGGCATT
>EU743792

GGCTATATCATACTTCCATGCAGAATAAAGCAAATTATAAACATGTGGCAGGGAGTAGGACAAGCAATATATG
CCCCTCCTATCAGTGGAAGAATTAGTTGTGTATCAAATATTACAGGAATCCTATTGACAAGAGATGGTGGTAA
TAATAATACCGAGACCGAGATCTTCAGACCTGGAGGAGGAAATATAAAGGACAATTGGAGAAGTGAATTATAT
AAATATAAAGTAGTACAAATTGAACCACTGGGAATAGCACCCACCAAGGCAAGGAGAAGAGTGGTGGACAGAG
AAAAAAGAGCAGTGGGAATAGGAGCTATGATCTTTGGGTTCTTGGGAGCAGCAGGAAGCACTATGGGCGCAGC
GTCACTAACGCTGACGGTACAGGCCAGACAATTATTGTCTGGTATAGTGCAACAGCAAAGCAATTTGCTGAGA
GCTATAGAGGCGCAGCAGCATCTGTTGCAACTCACAGTCTGGGGCATT
>EU743759
CATCAAAACACGACAGGGTGTAATGGAACTATCACACTCCCATGCAAGATAAAGCAAATTATAAACATGTGGC
AGGAAGTAGGACAAGCAATGTATGCTCCTCCCATCAGTGGAAAAATTAAGTGTGTATCAAATATTACAGGAAT
ACTATTGACAAGAGATGGTAATAATGAGACTGGGGCTAATGAGACCTTCAGACCTGAAGGAGGAAATATGAAG
GACAATTGGAGAAATGAATTATATAAATATAAAGTAGTGCAAATTGAACCACTAGGAATAGCACCCTCCAGGG
CAAAAAGAAGAGTGGTGGAGAGACAAAAAAGAGCAGTGGGAATAGGAGCTATGATCTTTGGGTTCTTAGGAGC
AGCTGGAAGCACTATGGGCGCGGCGTCAATAACGCTGACGGTACAGGCCAGACAATTATTGTCTGGTATAGTG
CAACAGCAAAGCAATTTGCTGAGGGCTATAGAGGCGCAGCAGCATCTGTTGCAACTCACAGTCTGGGGCATT
>EU743768
ACTTACATAAATGGAACCATAAATGAAACCATGGAGGGGCTTAATGACACTATCATACTTCCATGCAAAATAA
AGCAAATTATAAACATGTGGCAGAGAGCAGGACAAGCAATGTATGCTCCTCCCATCAGGGGAAGCATTAAATG
TGTATCAAATATTACAGGAATACTACTAACAAGGGATAGTGGTGATAATGATACTGAGACCTTCAGACCTGAA
GGAGGAAATATGAAGGACAATTGGAGAAGTGAATTATATAAATATAAAATAGTACAAATTGAACCACTAGGAA
TAGCACCCACCATGGCAAAGAGAAGAGTGGTGGAGAGAGAAAAAAGAGCAGTGGGAATAGGAGCTATGATCTT
TGGGTTCTTGGGAGCAGCAGGAAGCACTATGGGCGCGGCGTCAATAACGCTGACGGTACAGGCCAGACAATTA
TTGTCTGGTATAGTGCAACAGCAAAGCAATTTGCTGAGAGCTATAGAGGCGCAGCAGCATCTGTTGCAACTCA
CAGTCTGGGGCATT
>EU743770
AACTACACGGAAAGGGTTAATGAAACTATCATACTTCCATGCAAGATAAAGCAAATTATAAATATGTGGCAGA
GAGTAGGACAAGCAATGTATGCTCCTCCCATCAGGGGAAGAATTGGGTGTATATCAAATATTACAGGAATACT
ACTGACAAGGGATAATGGTGGTACCAATAGGAGTAGTGAGACCTTCAGACCTGGAGGAGGAGATATAAGGGAC
AATTGGAGAAGTGAATTATATAAATATAAAGTAGTAGAAATTGAACCACTAGGAATAGCACCCACCAGGGCAA
AGAGAAGAGTGGTGGAGAGAGAAAAAAGAGCAGTGGGAATAGGAGCTATGATCTTTGGGTTCTTAGGAGCAGC
AGGAAGCACTATGGGCGCGGCGTCAATAACGCTGACGGTACAGGTCAGACAATTATTGTCTGGTATAGTGCAA
CAGCAAAACAATTTGCTGAGGGCTATAGAGGCGCAGCAGCATCTGTTGCAACTCACAGTCTGGGGCATT
>EU743777
AATCAAACCATGCTTAATAAAACTACCATACTTCCATGCAGAATAAAGCAAATTATAAACATGTGGCAGGGAA
CAGGACAAGCAATGTATGCTCCTCCCATCCAGGGAAGAATTAATTGTGTATCAAATATTACAGGAATACTATT
GACAAGAGATGGAGGTAATACTAATGAGAATAATAAAACTGAAATCTTCAGACCTGGAGGAGGAAATATGAAG
GATAATTGGAGAAGTGAATTATATAAATATAAAGTAGTACAAATTGAACCATTAGGAATAGCACCCACCAGGG
CAAAGAGAAGAGTGGTGGAGAGAGAAAAAAGAGCAGTGGGAATAGGAGCTATGATCTTTGGGTTCTTGGGAGC
AGCAGGAAGCACTATGGGCGCGGCGTCAATAACGCTGACGGTACAGGCCAGACAATTATTGTCTGGTATAGTG
CAACAGCAAAGCAATTTGCTGAGGGCTATAGAGGCGCAGCAGCATCTGTTGCAACTCACAGTCTGGGGCATT
>EU743780
AATCAAACCCAGTGTAAGGGCAATATCACACTTCCATGCAGGATAAAGCAAATTATAAACATGTGGCAGGAAG
TAGGAAAAGCAATATATGCTCCTCCCGTCAGTGGAAGTATTAATTGTTTATCAAATATTACAGGGATAATACT
GACAAGAGATGGTGGTACTCCTGGTAATGTTACTAATAATGGTACTGAAACCGAGATCTTCAGACCTACAGGA
GGAAATATGGAGGACAATTGGAGAAGTGAATTATATAAATATAAAGTAGTACAAATTGAACCACTAGGAATAG
CACCCACCAAGGCAAAGAGAAGAGTGGTGGAGAGAGAAAAAAGAGCAGTGGGAATAGGAGCTATGATCTTTGG
GTTCTTAGGAGCAGCTGGAAGCACTATGGGCGCGGCGGCAGTAACGCTGACGGTACAGGCCAGACAATTATTG
TCTGGTATAGTGCAACAGCAAAGCAATTTGCTGAGAGCTATAGAGGCGCAACAGCATCTGTTGCAACTCACAG
TCTGGGGCATT
>JX446961
ACTTACAGAAATGAAACCAGTAATAGCACTATCATACTTCCATGCAGAATAAAGCAAATTATAAACATGTGGC
AGGAAGTAGGACAAGCAATGTATGCTCCTCCCATCAGGGGAAGAATAAATTGTGTATCAAATATTACAGGAAT
ACTATTGACAAGAGATGGTGGTAATAGTACAAGTAATAATGAGACCTTCAGACCTGGAGGAGGAAATATGAAG
GACAATTGGAGAAGTGAATTATATAAATATAAAGTAGTACAAATTGAACCATTAGGAATAGCACCCACCAGGG
CAAAGAGAAGAGTGGTGGACAGAGAAAAAAGAGCAGTGGGAATAGGAGCTATGATCTTTGGGTTCTTAGGAGC
AGCAGGAAGCACTATGGGCGCGGCGTCAATAACGCTGACGGTACAGGCCAGACAATTATTGTCTGGTATAGTG
CAACAGCAAAGCAATTTGCTGAGGGCTATAGAGGCGCAACAGCATCTGTTGCAACTCACAGTCTGGGGCATT
>JX447515
ACTTACACAAATACAACCATACAGGGTAATGGTACTATTATACTTCCATGCAAGATAAAGCAAATTATAAATA
TGTGGCAGGAAGTAGGACGAGCAATGTATGCTCCTCCTGTCAGTGGAAGAATTAATTGTATATCAAATATTAC

AGGAATACTATTAATAAGAGATGGTGGTAATACTAGTGAGTCTAACGAGATCTTCAGACCTGGAGGAGGAAAT
ATGAAAGACAATTGGAGAAATGAATTATATAAATATAAAGTAGTAGAAATTGAACCACTAGGAATAGCACCCA
CCAAGGCAAAAAGAAGAGTGGTGGAGAGAGAAAAAAGAGCAGTGGGAATAGGAGCTTTGATCTTTGGGTTCTT
GGGAGCAGCAGGAAGCACTATGGGCGCAGCGTCAATAACGCTGACGGTACAGGCCAGACAATTATTGTCTGGT
ATAGTGCAACAGCAAAGCAATTTGCTGAGGGCTATAGAGGCGCAGCAGCATATGTTGCAACTCACAGTCTGGG
GCATT
>JX448039
AATTATACTTGCATAAATAATACGTGCATAAATAATGCAACCATGGATGATAATGGCACTATCATACTTTCAT
GCAAAATAAAGCAAATTATAAACATGTGGCAGGGAGTAGGACAAGCAATGTATGCTCCTCCCATCAGTGGAAA
CATTAGTTGTGTATCAAATATTACTGGAATACTATTGACAAGAGATGGTGGTAATAATAGGACTACGACTACC
GAGACCTTCAGACCTGCAGGAGGAGATATGAAGGACAATTGGAGAAGTGAATTATATAAATATAAAGTAGTAC
AAATTGAACCACTAGGAATAGCACCCACCAGGGCAAAGAGAAGAGTGGTGGAGAGAGAAAAAAGGGCAGTGGG
AATAGGAGCTATGATCTTTGGGTTCTTAGGAGCAGCAGGAAGCACTATGGGCGCGGCGTCAATAACGCTGACG
GTACAGGCCAGACAATTATTGTCTGGTATAGTGCAACAGCAAAGCAATTTGCTGAGGGCTATAGAGGCGCAGC
AGCATCTGTTGCAACTCACAGTCTGGGGCATT
>JX448198
ACTTACAATGGAACCATGGGGGGATGGAATGGCACTATCAATGGCACTATCAATGACACTATCATAATTCCAT
GCAGAATAAAGCAAATTATAAATATGTGGCAGGGAGTAGGACAAGCAATATATGCTCCTCCCATCAGAGGAAT
AATTAATTGTGTATCAAATATCACAGGAATACTATTGACAAGAGATGGTGGTGATAATAATGCTACTAAAGAT
ACCGAGATCTTCAGACCTGGAGGTGGAAATATAAAGGACAATTGGAGAAGTGAATTATATAAATATAAAGTAG
TACAAATTGAACCACTAGGAATAGCACCCACCAGGGCAAAGAGAAGAGTGGTGGAAAGAGAAAAAAGAGCAGT
GGGAATAGGAGCTATGATCTTTGGGTTCTTAGGAGCAGCAGGAAGCACTATGGGCGCGGCGTCACTAACGCTG
ACGGTACAGGCCAGACAATTATTGTCTGGTATAGTGCAACAGCAAAGCAATTTGCTGAGGGCTATAGAGGCGC
AGCAGCATCTGTTGCAACTCACAGTCTGGGGCATT
>JX446877
ACTTACAAAGATAAAGCCATAGAGGGGAATAATGGCACTATCATACTTCCATGCAGGATAAAACAAATTATAA
ACATGTGGCAGGGAGTAGGACAAGCAATGTATGCTCCTCCCATCAGGGGAAACATTAGTTGTGTCTCAAATAT
TACAGGAATACTATTGACAAGAGATGGTGGTTCTGGTAATGATACAAGAAATGATACAGAGACCTTCAGACCT
GGAGGAGGGAATATAAAGGACAATTGGAGAAGTGAATTATATAAATATAAAGTAGTACAAATTGAACCACTAG
GACTAGCACCCACCAAGGCAAAGAGAAGAGTGGTGGAGAGACAAAAAAGAGCAGTGGGAATAGGAGCTATGAT
CTTTGGGTTCTTAGGAGCAGCAGGAAGCACTATGGGCGCGGCGTCAATAACGCTGACGGCACAGGCCAGACAA
TTATTGTCTGGTATAGTGCAACAGCAAAGCAATTTGCTGAGAGCTATAGAGGCGCAGCAGCATCTGTTGCAAC
TCACAGTCTGGGGCATT
>JX446918
ACTTACGTAAGAAATGAAACCAAGGAAAATGATACTATCATACTTCCATGCAAGATAAAGCAAATTATAAACA
TGTGGCAGGGAGTAGGACAAGCAATGTATGCTCCTCCCATCAGTGGAAACATTAGTTGTGTATCAAATATTAC
AGGAATAATATTGACAAGAGATGGTGGTCTTGGTAATAATAGTGGAGAGACCTTCAGACCTGGAGGAGGAAAT
ATAAAGGACATTTGGAGAAGTGAATTATATAAATATAAAGTAGTACAAATAGAACCACTAGGAATAGCACCCA
CCAGGGCAAAGAGAAGAGTGGTGGAGAGAGAAAAAAGAGCAGTGGGAATAGGAGCTATGATCTTTGGGTTCTT
AGGAGCAGCAGGAAGCACTATGGGCGCGGCGTCAATAACGCTGACGGTACAGGCCAGACAATTATTGTCTGGT
ATAGTGCAACAGCAAAGCAATTTGCTGAGGGCTATAGAGGCGCAGCAGCATATGTTGCAACTCACAGTCTGGG
GCATT
>JX447381
ACTTACCTAGGAAATGAAACCACAGGGAGGTATAATGACACTATCATAATTCCATGCAAGATAAAGCAAATTA
TAAGAATGTGGCAGGGGGTAGGACAAGCAATGTATGCTCCTCCCATCAGGGGAGTCATTAAGTGTAGATCAAA
TATTACAGGAATATTATTGACAAGAGATGGTGATAAAATTAATGCTACTGAAGAGATTTTCAGACCTGGAGGA
GGAAATATAAAGGATAATTGGAGAAGTGAATTATATAAATATAAAGTAGTACAAATTGAACCACTAGGAATAG
CACCCACCAGGGCAAAGAGAAGAGTGGTGGAGAGAGAAAAAAGAGCAGTGGGAATAGGAGCTATGATCTTTGG
GTTCTTAGGAGCAGCAGGAAGCACTATGGGCGCGGCGTCAATAACGCTGACGGTACAGGCCAGACAATTATTG
TCTGGTATAGTGCAACAGCAAAGCAATTTGCTGAGGGCTATAGAGGCGCAGCAGCATCTGTTGCAACTCACAG
TCTGGGGCATT
>JX447773
ACTCACATGCCTAATGGTACTATCATAGGAAAAGACAATATCACACTTCCATGCAGGATAAAGCAAATCATAA
ACATGTGGCAGGGAGCAGGACAAGCAATGTATGCTCCTCCCATCAGTGGAATAATTAATTGTGTATCAAATAT
TACAGGAATACTATTGACAAGAGATGGTGGTGGTAATAGTAGTAATGAGAACGAGACCTTCAGACCTGGAGGA
GGAAATATAAAGGACAATTGGAGGAGTGAATTATATAAATATAAAGTAGTACAAATTGAACCACTAGGAATAG
CACCCACCAAGGCACAGAGAAGAGTGGTGCGGATAGAAAAAAGAGCAGTGGGATTAGGAGCTATGATCTTTGG
GTTCTTAGGAGCAGCAGGAAGCACTATGGGCGCGGCGTCAATAACGCTGACGGTACAGGCCAGACAATTATTG
TCTGGTATAGTGCAACAGCAAAGCAATTTGCTGAGAGCTATAGAGGCGCAGCAGCATCTGTTGCAACTCACAG
TCTGGGGCATT

>HQ690985
ACTTACCCAGGAAATGAAACCATGGAGGACCATATCATACTTCCATGCAGGATAAAGCAAATTATAAACATGT
GGCAGGGAGCAGGACAAGCAATGTATGCTCCTCCCATCAGTGGGAGAATTAATTGTGTATCAAATATTACAGG
CATACTACTGACAAGAGATGGTGGTAATAATACTAATGAGACTAAAGAAATCTTCAGACCTGGAGGAGGAAAT
ATGAAGGACAATTGGAGAAGTGAATTATATAAATATAAAGTAGTACAAATTGAACCACTAGGAATAGCACCCA
CCAGGGCAAGGAGAAGAGTGGTGCAGAGAGAAAAAAGAGCAGTGGGAATAGGAGCTATGATCTTTGGGTTCTT
AGGAGCAGCAGGAAGCACTATGGGCGCAGCGTCAATAACGCTGACGGTACAGGCCAGACAATTATTGTCTGGT
ATAGTGCAACAGCAAAGCAATTTGCTGAGGGCTATAGAGGCGCAGCAACATCTGTTGCAACTCACAGTCTGGG
GCATT
>HQ691052
TGCTACACAACAAAACTGTTTAATGATACTTGCACAGGAAATGAAACCAGCACAGGAAATGAAACCAATAAGG
AATGTAAACTGTCTAATGGTAATGGCACTATCATACTTCCATGCAAGATAAAGCAAATTATAAACATGTGGCA
GGGAGTAGGACAAGCAATGTATGCTCCTCCCATCAGTGGAAGAATTAATTGTGTATCAAATATTACAGGAATA
CTATTGACAAGAGATGGAGGTACTAACACGATCAACAATGAGACTTTCAGACCTGGAGGGGGAAATATAAAGG
ACAATTGGAGAAGTGAATTATATAAATATAAAGTAGTACAAATTGAACCACTAGGAATAGCACCCTCCAGGGC
AAGGAGAAGAGTGGTGGAAAGAGAAAAAAGAGCAGTGGGAATAGGAGCTATGATCTTTGGGTTCTTGGGAGCA
GCAGGAAGCACTATGGGCGCGGCGTCAATAACGCTGACGGTACAGGCCAGACAATTATTGTCTGGTATAGTGC
AACAGCAAAGCAATTTGCTGAAGGCTATAGAGGCGCAGCAGCATATGTTGCAACTCACAGTCTGGGGCATT
>HQ691073
ACCTATAATGACACTAATGACCCTATCATACTTCCATGCAGGATAAAGCAAATTATAAACATGTGGCAGGGAG
TAGGACAAGCAATGTATGCTCCTCCCATCCGTGGAAGCGTTAATTGTTCATCAAATATTACAGGAATACTATT
GACAAGAGATGGGGGTAATAATAGTAATACGCAAGAGACTTTCAGACCTGGAGGAGGAAATATAATGGATAAT
TGGAGAAATGAATTATATAAATATAAAGTAGTACAAATTGAACCATTAGGAATAGCACCCACCAGGGCAAAGA
GAAGAGTGGTGGAGAGAGAAAAAAGAGCAGTGGGAATAGGAGCTATGATCTTTGGGTTCTTAGGAGCAGCAGG
AAGCACTATGGGCGCGGCGTCAATAACGCTGACGGTACAGGCCAGACAATTATTGTCTGGTATAGTGCAACAG
CAAAGCAATTTGCTGAGGGCTATAGAGGCGCAGCAGCATCTGTTGCAACTCACGGTCTGGGGCATT
>GQ916574
ACTTACACAGGAAATGAAACCACAGGAGATGAAACCAAGAATATCACACTTCCATGCAGGATAAAGCAAATTA
TAAACATGTGGCAGGAAGTAGGACAAGCAATGTATGCTCCTCCCATCAGTGGACAAATTAAGTGTGTATCAAA
TATTACAGGAATACTATTGACAAGAGATGGTGGTGGTAATGCTAATGATACTAATAATACCGAGACCTTCAGA
CCTGGAGGAGGAAATATAAAGGACAACTGGAGAAGTGAATTATATAAATATAAAGTAGTACAAATTGAACCAC
TAGGAATAGCACCCACCAAGGCAAAGAGAAGAGTGGTGGAGAGACAGAAAAGAGCAGTGGGAATAGGAGCTAT
GATCTTTGGGTTCTTAGGAGCAGCAGGAAGCACTATGGGCGCGGCGTCAATAACGCTGACGGTACAGGCCAGA
CAATTATTGTCTGGTATAGTGCAGCAGCAAAGAAATTTGCTGAGGGCTATAGAGGCGCAGCAGCATCTGTTGC
AACTCACAGTCTGGGGCATT
>JX446888
GCTTACACAGGAAATGAAACCATGAAAGGGAATAAAACCATCATACTTCCATGCAGGATAAAGCAAATTATAA
ACATGTGGCAGGGAGTAGGACAAGCAATATATGCTCCTCCCGTCAGGGGAATGATTAATTGTGTATCAAATAT
TACAGGAATATTATTGACAAGGGATGGTGGTAATGGTACGACCGAGGAGATTTTCAGACCAGGAGGAGGAAAT
ATAAAAGACAATTGGAGAAGTGAATTATATAAATATAAAGTAGTACAAATTGAACCACTAGGCATAGCACCCA
CCAGGGCAAAGAGAAGAGTGGTGGAGAGAAACAAAAGAGCAGTTGGAATAGGAGCTATGATCTTTGGGTTCTT
AGGAGCAGCAGGAAGCACTATGGGCGCGGCGTCAATAACGCTGACGGTACAGGCCAGACAATTATTGTCTGGT
ATAGTGCAACAGCAAAGCAATTTGCTGAGGGCTATAGAGGCGCAGCAGCATCTGTTGCAACTCACAGTCTGGG
GCATT
>JX448072
ACTTACATGGAAAATGGAACCATGCAGAATAGTACGCAGAATAACACGCAAAATGACACTATCATACTCCCAT
GCAGGATAAAGCAAATTATAAACATGTGGCAGGGAGCAGGACAAGCAATGTATGCTCCTCCAGTTAGGGGAAG
CATTAAGTGTGTATCAAATATTACAGGAATACTATTGACAAGAGATGGTGGTGCTAATAATACCGAGATCTTC
AGACCGGCAGGAGGAAATATAAAGGATAATTGGAGAAGTGAATTATATAAATATAAAGTAGTACAAATTGAAC
CACTAGGAATAGCACCCACCAGGGCAAAAAGAAGAGTGGTGGAGAGACAGAAAAGAGCAGTGGGAATAGGAGC
TATGATCTTTGGGTTCTTAGGAGCAGCAGGAAGCACTATGGGCGCGGCGTCAATAACGCTGACGGTACAGGCC
AGACAATTATTGTCTGGTATAGTGCAACAGCAAAGCAATTTGCTGAGGGCTATAGAGGCGCAGCAGCATATGT
TGCAACTCACAGTCTGGGGCATT
>JX448086
AATTACACAAATGGTACTTTAACAAACACCACGGAGGGGGGTAGCGACACTACTATCATCCTTCCATGCAAGA
TAAAGCAAATTATAAACATGTGGCAGGGAATAGGACAAGCAATGTATGCTCCTCCCATCAGGGGATATATTAA
TTGTACATCAAATATTACAGGAATACTATTGACAAGAGATGGTGACGGTAATATTAATATTAATGACAGCGAG
ATCTTCAGACCTGGAGGAGGAAATATGAAGGACAATTGGAGAAGTGAATTATATAAATATAAAGTAGTACAAA
TTGAACCACTAGGAATAGCACCTACCAGGGCAAAGAGAAGAGTGGTGGAGAGAGAAAAAAGAGCAGTGGGAAT
AGGAGCTATGATCTTTGGGTTCTTAGGAACAGCAGGAAGCACTATGGGCGCAGCGTCAATAACGCTGACGGTA

CAGGCCAGACAATTATTGTCTGGTATAGTGCAACAGCAAAGCAATTTGCTGAGGGCTATAGAGGCGCAGCAGC
ATCTGTTGCAACTCACAGTCTGGGGCATT
>JX448312
AATTATACTTGCTCGGACAATGGGTGTAATGGCATTATCAATGGCACTATCATACTTCCATGCAGGATAAAGC
AAATTATAAACATGTGGCAAGGAGCAGGACAAGCAATGTATGCTCCTCCCATCAGGGGAAGCATTAATTGTGT
ATCAAATATTACAGGAATATTATTGACAAGAGATGGTGGTAATGGTGATGATACTAATAATACTGAGATCTTC
AGACCTGGAGGAGGAAATATAAAGGACAATTGGAGAAGTGAATTATATAAATATAAAGTAGTACAAATTGAAC
CACTAGGAATAGCACCCTCCAGGGCAAAGAGAAGAGTGGTGGAGAGAGAAAAAAGAGCAGTGGGAATAGGAGC
TATGATCTTTGGGTTCTTAGGAGCAGCAGGAAGCACTATGGGCGCGGCGTCAATAACGCTGACGGTACAGGCC
AGACAATTATTGTCTGGTATAGTGCAACAGCAAAGCAATTTGCTGAGGGCTATAGAGGCGCAACAGCATATGT
TGCAACTCACAGTCTGGGGCATT
>JN388191
ACTCATACTACTTTTAATAAAACTATCAGACTTCCATGCAAGATAAAGCAAATTATAAACATGTGGCAGAGAA
CAGGACAAGCAATGTATGCTCCTCCCATCAGTGGAAACATTAACTGTGTATCAAATATTACAGGAATACTATT
GACAAGGGATGGTGGTAATAATACTAATGAAACCTTTAGACCTGGAGGAGGAAACATAAAAGACAATTGGAGA
AGTGAATTATATAAATATAAAGTAGTAGAAATTGAACCACTAGGAATAGCGCCCACCAGGGCAAAGAGAAGAG
TGGTGGAGAGAGAAAAAAGAGCAGTGGGAATAGGAGCTATGATCTTTGGGTTCTTAGGAGCAGCAGGAAGCAC
TATGGGCGCGGCGTCAATAACGCTGACGGTACAGGCCAGACAATTATTGTCTGGTATAGTGCAACAGCAAAGC
AATTTGCTGAGGGCTATAGAGGCGCAGCAGCATATGTTGCAACTCACAGTCTGGGGCATC
>JN388201
ACTTACAATGGAACCCTACATGATGATAATAACAGTACCAATATCACATTTCCATGCAGGATAAAGCAGATTA
TAAACATGTGGCAGGGAGTAGGACAAGCCATGTATGCTCCTCCCATCAGTGGAAGAATTAATTGTGTATCAAA
TATTACAGGAATACTATTGACAAGAGATGGTGGTGATAGTAATAAGAAAAACGAGACCTTCAGACCTGGAGGA
GGAAATATAAAGGACAATTGGAGAAATGAATTATATAAATATAAAGTAGTAGAAATTGAACCATTAGGAATAG
CACCCACCAGGGCAAAGAGAAGAGTGGTGGAGAGAGAAAAAAGAGCAGTGGGAATAGGAGCTATGATCTTTGG
GTTCTTAGGAGCAGCAGGAAGCACTATGGGCGCGGCGTCAATAACGCTGACGGTACAGGCCAGACAATTATTG
TCTGGTATAGTGCAACAGCAAAGCAATTTGCTGAGGGCTATAGAGGCGCAGCAGCATATGTTGCAACTCACAG
TCTGGGGCATT
>GQ916585
TGCTATACAACAAAACTGTTTAATAATACTTGCATGGAAAATGGAACCAAGTGTAATGACACTATCACACTTC
CATGCAAGATAAAGCAAATTATAAACATGTGGCAGGGAATAGGACAAGCAATGTATGCTCCTCCCATCAGTGG
AACAATTAATTGTGCATCAAATATTACAGGAATACTATTGACAAGAGATGGTGGTAAGAATACTTCAGTTAAC
GGGACCGAGATCTTCAGACCTGGAGGAGGAAATATGAAGGACAATTGGAGAAGTGAATTATATAAATATAAAG
TAGTACAAATTGAACCACTAGGAATAGCACCCACCAAGGCAAAGAGAAGAGTGGTGAAGAGACAAAAAAGAGC
AGTAGTAGGAATAGGAGCTATGATCTTTGGGTTCTTAGGAGCAGCAGGAAGCACTATGGGCGCAGCGTCAATA
ACGCTGACGGTACAGGCCAGACAATTATTGTCTGGTATAGTGCAACAGCAAAGCAATTTGCTGAGGGCTATAG
AGGCGCAGCAGAATATGTTGCAACTCACAGTCTGGGGCATT
>GQ916588
TGCTATAATAACACTCTCATACTTCCATGTAAGATAAAACAAATTATAAACATGTGGCAGGGAGTAGGACAAG
CAATGTATGCTCCTCCCATCAATGGAAGAATTAATTGTGTATCAAATATTACAGGAATACTATTGACAAGAGA
TGGTGGTGTTAATAATGGGACTAACGACACCGAGATCTTCAGACCTGGAGGAGGAAATATAAAGGACAATTGG
AGAAGTGAATTATATAAATATAAAGTAGTACAAATTGAACCACTAGGAATAGCACCCACCAGGGCAAAGAGAA
GAGTGGTGGCGAGAGAAAAAAGAGCAGTGGGAATAGGAGCTGTGATCTTTGGGTTCTTAGGAGCAGCAGGAAG
CACTATGGGCGCGGCGTCAATAACGCTGACGGTACAGACCAGACAATTATTGTCTGGTATAGTGCAACAGCAA
AGCAATTTGCTGAGAGCTATAGAGGCGCAGCAGCATCTGTTGCAACTCACAGTCTGGGGCATT
>GQ916592
AGTTACTTAGAAAATGGAACCATGTGGGAAAATAACAGTATCATACTTCTATGCAGGATAAAGCAAATTATAA
ATATGTGGCAGGGAGTAGGACAAGCAATGTATGCTCCTCCCATCAGTGGCTTAATTAAGTGTGTATCGAATAT
TACAGGAATACTATTGACAAGAGATGGTGGTAATAGTAATGAGACTAATAGTACTGATACTGAGATCTTCAGA
CCTACAGGAGGAAATATAAAGGACAATTGGAGAAGTGAATTATATAAATACAAAGTAGTACAAATTGACCCAC
TAGGAATAGCACCCACCAAGGCAAAGAGAAGAGTGGTGGAAAGAGAAAAAAGAGCAGTGGGACTAGGAGCTAT
GATCTTTGGGTTCTTAGGAGCAGCAGGGAGCACTATGGGCGCGGCGTCAATAGCGCTGACGGGACAGGCCAGA
CAATTATTGTCTGGTATAGTGCAACAGCAAAGCAATTTGCTGAGGGCTATAGAGGCGCAGCAGCATATGTTGC
AACTCACTGTCTGGGGCATT
>GQ916580
ACCTACAGAAATGAAAACACGGGCAGTATCATACTCCCATGCAAAATAAAGCAAATTATAAACATGTGGCAGG
GAGTAGGACGAGCAATGTATGCTCCTCCCATCAGTGGAACAATTAATTGTGTATCAAATATTACAGGAATACT
ATTGACAAGAGATGGGGGTGGTGGTGATGAGGTGAATAACGAGACCGAGATCTTTAGACCTGGAGGAGGAAAC
ATAAAGGACAATTGGAGAAATGAATTATATAAATATAAAGTAGTACAAATTGAACCACTAGGAATAGCACCCA
CCAGGGCAAAGAGAAGAGTGGTGGAGAGAGAAAAAAGAGCAGTGGGAATAGGAGCTATGATCTTTGGGTTCTT

AGGAGCAGCAGGAAGCACTATGGGCGCGGCGTCAATAACGCTGACGGTACAGGCCAGACAATTATTGTCTGGT
ATAGTGCAACAGCAAAGCAATTTGCTGAGGGCTATAGAGGCGCAGCAGCATCTGTTGCAACTCACAGTCTGGG
GCATT
>KT185994
AATTACACAGAGAATAATACCACGGAGGGGTGGAATGGCACTATCATACTTCCATGCAGGATAAAGCAAATTG
TAAACATGTGGCAGGGAGCAGGACAAGCAATGTATAATCCTCCCATCAGTGGAAAAATTAATTGTGTATCAAA
TATTACAGGAATACTATTGACAAGAGATGGTGGTAATAATAATGAGACTAATGGGACTGAGACTTTCAGACCT
GGAGGAGGAAATATAAAGGACAATTGGAGAAATGAATTATATAAGTATAAAGTAGTACAAATTGAACCACTAG
GAATAGCACCCACCAGGGCAAAGAGAAGAGTGGTGGAGAGAGAGAAAAGAGCAGTGGGAATAGGAGCTATGAT
CTTTGGGTTCTTGGGAGCAGCAGGAAGCACTATGGGCGCGGCGTCAATAACGCTGACGGTACAGGCCAGACAA
TTATTGTCTGGTATAGTGCAGCAGCAAAGCAATTTGCTGAGGGCTATAGAGGCGCAGCAGCATATGTTGCAAC
TCACAGTCTGGGGCATT
>KY580553
AATCATACTTGCGAAGGAAATGAAGGGTGTAATGGCACTAGCAGTAATGGCACTATCATACTTCCATGCAGGA
TAAAGCAAATTATAAACATGTGGCAGGGAGCAGGACAAGCAATGTATGCTCCTCCCATCAGAGGAACAATTAA
TTGTGTATCAAATATTACAGGACTACTATTGACAAGAGATGGTGGTAAAGATAATAGTACTAGTGGTAACGAG
ACCTTCAGACCTGGAGGAGGAAATATAAAAGACAATTGGAGAAGTGAATTATATAAATATAAAGTAGTACAGA
TTGAACCACTAGGAATAGCACCTACCAGGGCAAAGAGAAGAGTGGTGCAGAGAGAAAAAAGAGCAGTGGGAAT
AGGAGCTATGCTCTTTGGGTTCTTAGGAGCAGCAGGAAGCACTATGGGCGCGGCGTCAATAACGCTGACGGTA
CAGGCCAGACAATTATTGTCTGGTATAGTGCAACAGCAAAGCAATTTGCTGAGGGCTATAGAGGCGCAGCAGC
ATCTGTTGCAACTCACAGTCTGGGGCATT
>JX447238
ACTTACACAGGAAAAGAAAATGACACCAGGGAAGGGAAGAATGACAATGAAATCATTCTTCCATGCAGAATAA
AGCAAATTATAAACATGTGGCAGAGAGTAGGACAAGCAATGTATGCTCCTCCCATTAGAGGAAGAATTAATTG
TGTATCAAATATTACAGGAATACTATTGACAAGAGATGGTGGTAATAATGTAAATATGACTAATGAGACCTTC
AGGCCTGGAGGAGGACATATAAAGGACAATTGGAGAAGTGAATTGTACAAATATAAAGTAGTAGAAATTGAAC
CATTAGGAATAGCACCCACCAAGGCAAAGAGAAGAGTGGTGGAAAGACAAAAAAGAGCAGTGGGAATAGGAGC
TATGATCTTTGGGTTCTTAGGAGCAGCAGGAAGCACTATGGGCGCAGCGTCAATAACGCTGACGGTACAGGCC
AGACAATTATTGTCTGGTATAGTGCAACAGCAAAGCAATTTGCTGAGGGCTATAGAGGCGCAACAGCACATGT
TGCAACTCACAGTCTGGGGCATT
>JN388224
TTTCAGACCAATGTCTGCAATGGGACGTGTAATGGCAATATCACACTTCCATGCAGGATAAAGCAAATTATAA
ACATGTGGCAGGAAGTAGGACAAGCAATGTATAACCCTACCAAAAGAGGAGAAATTAAGTGTGTATCAAATAT
TACAGGAATACTATTGACAAGAGATGGTGGTAGTAATAATAGGTCCAACGAGACCTTCAGACCTGGAGGAGGA
AATATGAAGGACAATTGGAGAAGTGAATTATATAAATATAAAGTAGTAGAAATTGAACCACTAGGAATAGCAC
CCACCAGGGCAAAGAGAAGAGTGGTGGAGAGAGAAAAAAGAGCAATAGCAATAGGAACTATGCTCTTTGGGTT
CTTAGGAGCAGCCGGAAGCACTATGGGCGCGGCGTCACTAACGCTGACGGTACAGGCCAGACAATTATTGTCT
GGTATAGTGCAACAGCAAAGCAATTTGCTGAGGGCTATTGAGGCGCAGCAGCATCTGTTGCAACTCACAGTCT
GGGGCATT
>JX512901
ACTTACACAGGAAATGAACCTATGGGGAATAGGACCGAGGGGAATAATACCAATATCATACTTCCATGTAAGA
TAAAGCAAATTATAAACATGTGGCAGGGAGTAGGACAAGCAATGTATGCTCCTCCCATCAGAGGAGCTATTAC
ATGTGTATCAAATATTACAGGAATACTATTGACAAGAGATGGTGGTAATAGTCCTAATGAGACCTTCAGACCT
GAAGGAGGAAATATAAAGGACAATTGGAGAAGTGAATTATATAAATATAAAGTAGTACAAATTGAACCACTAG
GAGTAGCACCTACCAGGGCAAAGAGAAGAGTGGTGGAGAGAGAAAAAAGAGCAGTGGGAATAGGAGCTATGAT
CTTTGGGTTCTTAGGAGCAGCAGGAAGCACTATGGGCGCAGCGTCAATAACGCTGACGGTACAGGCCAGACAA
TTGTTGTCTGGTATAGTGCAACAGCAAAGCAATTTGCTGAGGGCTATAGAGGCGCAGCAGCATCTGTTGCAAC
TCACAGTCTGGGGCATT
>KU230434
TCTTACATAAATGAAACCACACCTGGGTATAATGGCACTATCATACTCCCATGCAGGATAAAACAAATTATAA
ACATGTGGCAGGGAGTAGGACAAGCAATGTATGCTCCTCCCATCAGGGGAATCATTAGTTGTGTATCAAATAT
TACAGGAATATTATTGACAAGAGATGGTGGTAATAATAGCACGAATGAGACTGAGACCTTCAGACCTGGAGGA
GGAAATATAAAGGACAATTGGAGAAGTGAATTATATAAATATAAGGTAGTACAAATTGAACCACTAGGACTAG
CACCCACCAAGGCAAAGAGAAGAGTGGTGGAGAGAAAAAAAAGAGCAGTGGGAATAGGAGCTATGATCTTTGG
GTTCTTAGGAGCAGCAGGAAGCACTATGGGCGCGGCGTCAATAACGCTGACGGTACAGGCCAGACAATTATTG
TCTGGTATAGTGCAACAGCAAAGCAATTTGCTGAGAGCTATAGAGGCGCAACAGCATATGTTGCAACTCACAG
TCTGGGGCATT
>KU230435
AATCATAATTGCACCGAGGAGAGGTGTAATAGCACTACCAATAGCACTAGCACTGGCAATATCACACTTCCAT
GTAGAATAAAGCAGATAATAAACATGTGGCAGGGAGTAGGACAAGCAATGTACGCTCCTCCCATTAAGGGAAA

TGTTAGTTGTCTATCAAATATCACAGGAATACTATTGACAAGAGATGGTGATAATGCCAGTGATAGTGAGACC
TTCAGACCGGGAGGAGGAAATATAAAAGACAATTGGAGAAGTGAATTATATAAATATAAAGTAGTACAAATTG
AACCACTAGGAATAGCACCCACCAGGGCAAAGAGAAGAGTGGTGGAGAGAGAAAAAAGAGCAGTGGGAATAGG
AGCTATGATCTTTGGGTTCTTGGGAGCAGCAGGAAGCACTATGGGCGCGGCGTCAATAACGCTGACGGTACAG
GCCAGACAATTATTGTCTGGTATAGTGCAACAGCAAAGCAATTTGCTGAGAGCTATAGAGGCGCAACAACATA
TGTTGCAACTCACAGTCTGGGGCATT
>KU230437
ACTTACACAGACGTGAATAATAAAACCAATAAGAATGACACAATCATAATTCCATGCAGAATAAAGCAAATTA
TAAACATGTGGCAGAGAGTAGGACAAGCAATGTATGCTCCTCCCATCAAGGGAAGGATTAGTTGTGTATCAAA
TATTACAGGAATACTCTTGACAAGAGATGGTGGTAATAATACTGATGCTAATGAGACCTTCAGACCTGAAGGA
GGAAATATGAAGGACAATTGGAGAAATGAATTATATAAATATAAAGTAGTACAAATTGAACCACTAGGAATAG
CACCCACCAGGGCAAGGAGAAGAGTGGTGGACAGAGAAAAAAGAGCAGTGGGAATAGGAGCTTTGATCTTTGG
GTTCTTAGGAGCAGCCGGAAGCACTATGGGCGCGGCGTCAATAACGCTGACGGTACAGGCCAGACAATTATTG
TCTGGTATAGTGCAACAGCAAAGCAATTTGCTGAGGGCTATAGAGGCACAGCAGCATCTGCTGCAACTCACAG
TCTGGGGCATT
>MG989575
ACTTACCTAAATGAAACAAATGAATATAATGACACTATCATACTTCCATGCAGGATAAAGCAAATGATAAACA
TGTGGCAGGGAGCAGGACAAGCAATGTATGCTCCTCCCATCAGTGGAATAATTAATTGTACATCAAATATTAC
AGGAATACTATTGACAAGAGATGGTGGTGTTAATGAAAGTAATGGGACCGAGACCTTCAGACCTGGAGGAGGA
AATATAAAGGACAATTGGAGAAATGAATTATATAAATATAAAGTAGTACAAATTGAACCACTAGGAATAGCAC
CCACCAGGGCAAAGAGAAGAGTGGTGGAGAGAGAAAAAAGAGCAGTGGGAATAGGAGCTATGCTCTTTGGGTT
CTTAGGAGCAGCAGGAAGCACTATGGGCGCGGCGTCAATAACGCTGACGGTACAAGCCAGACAATTATTGTCT
GGTATAGTGCAACAGCAAAACAATTTGCTGAGAGCTATAGAGGCGCAGCAGCATCTGCTGCAACTCACAGTCT
GGGGCATT
>MG989515
AATTACACCAAGGAAGGGTGTAATGAAACTATCACACTTCCATGCAAGATAAAGCAAATAGTACGCATGTGGC
AGGAAGCAGGACAGGCAATGTATGCTCCTCCCATCAGGGGAATGATTAATTGTGTATCAAATATTACAGGAAT
ACTACTGACAAGAGATGGTGGTAGAAATAGTAGCAATGAGACCTTCAGACCTGAGGGAGGAAATATAAAGGAC
AATTGGAGAAATGAATTGTATAAATATAAGGTAGTACAAATTGAACCACTAGGAATAGCACCCACCAGGGCAA
AGAGAAGAGTGGTGGAGAGAGAAAAAAGAGCAGTGGGAATAGGAGCTATGATCTTTGGGTTCTTAGGAGCAGC
AGGAAGCACTATGGGCGCGGCGTCAATAACGCTGACGGTACAAGCCAGACAATTATTGTCTGGTATAGTGCAA
CAGCAAAGCAATTTGCTGAGAGCTATAGAGGCGCAGCAACATCTGCTGCAACTCACAGTCTGGGGCATC
>MG989597
AATTATACAGGAAATGAAACCGAGGGGGAGTATAATGGCACTAATAATGGCACTATCACACTTCCATGCAGGA
TAAAGCAAATAATAAACATGTGGCAGAAAATAGGACAAGCAATGTATGCTCCTCCCATCAGGGGAAGTATTAA
TTGTTTATCAAATATCACAGGAATACTATTGACAAGAGATGGTGGTGATGGTGGTGCTAATGAAAATGATACA
GAGACCTTCAGACCTGGAGGAGGAAATATAAAGGACAATTGGAAAAATGAATTATATAAATATAAAGTAGTAC
AAATTGAACCACTAGGAATAGCACCCACCAGGGCAAAGAGAAGAGTGGTGGAGAGAGAAAAAAGAGCAGTGGG
AATAGGAGCCATGATCTTTGGGTTCTTAGGAGCAGCAGGAAGCACTATGGGCGCGGCGTCAATAACGCTGACG
GTACAGGCCAGACAATTATTGTCTGGCATAGTGCAACAGCAAGGTAACTTGCTGAGGGCTATAGAGGCGCAGC
AGCATCTGTTGCAACTCACAGTCTGGGGCATT
>MG989530
ATTTACGTAAGAAATGAAACGTATAATGACACTATCATACTCCCATGCAAGATAAAGCAAATTATAAACATGT
GGCAGGGAGTAGGACAAGCAATGTATGCTCCTCCCATTAGAGGAAGCATTAACTGTGCATCAAATATTACAGG
AATATTATTGACAAGAGATGGTGGTGACGAAAATAGAAGCAAAGAGATCTTCAGGCCTGAAGGAGGAAACATA
AAGGACAATTGGAGAAATGAATTATATAAATATAAAGTAGTACAAATTGAACCACTAGGGGTAGCACCCACCA
GGGCAAAGAGAAGAGTGGTGGAGAGAGAAAAAAGAGCAGTGGGAATAGGAGCTATGATCTTTGGGTTCTTAGG
AGCAGCAGGAAGCACTATGGGCGCAGCGTCACTAACGCTGACGGTACAGGCTAGACAATTATTGTCTGGTATA
GTGCAACAGCAAAGCAATTTGCTGAGGGCTATAGAGGCGCAGCAGCATCTGTTGCAACTCACAGTCTGGGGCA
TT
>MG989559
AATCATAATTGCACCGAGGAGAGGTGTAATAGCACTACCAATAGCACTAGCACTGGCAATATCACACTTCCAT
GTAGAATAAAGCAGATAATAAACATGTGGCAGGGAGTAGGACAAGCAATGTACGCTCCTCCCATTAAGGGAAA
TGTTAGTTGTCTATCAAATATCACAGGAATACTATTGACAAGAGATGGTGATAATGCCAGTGATAGTGAGACC
TTCAGACCGGGAGGAGGAAATATAAAAGACAATTGGAGAAGTGAATTATATAAATATAAAGTAGTACAAATTG
AACCACTAGGAATAGCACCCACCAGGGCAAAGAGAAGAGTGGTGGAGAGAGAAAAAAGAGCAGTGGGAATAGG
AGCTATGATCTTTGGGTTCTTGGGAGCAGCAGGAAGCACTATGGGCGCGGCGTCAATAACGCTGACGGTACAG
GCCAGACAATTATTGTCTGGTATAGTGCAACAGCAAAGCAATTTGCTGAGAGCTATAGAGGCGCAACAACATA
TGTTGCAACTCACAGTCTGGGGCATT
>MG989562

TCTTACATAAATGAAACCACACCTGGGTATAATGGCACTATCATACTCCCATGCAGGATAAAACAAATTATAA
ACATGTGGCAGGGAGTAGGACAAGCAATGTATGCTCCTCCCATCAGGGGAATCATTAGTTGTGTATCAAATAT
TACAGGAATATTATTGACAAGAGATGGTGGTAATAATAGCACGAATGAGACTGAGACCTTCAGACCTGGAGGA
GGAAATATAAAGGACAATTGGAGAAGTGAATTATATAAATATAAGGTAGTACAAATTGAACCACTAGGACTAG
CACCCACCAAGGCAAAGAGAAGAGTGGTGGAGAGAAAAAAAAGAGCAGTGGGAATAGGAGCTATGATCTTTGG
GTTCTTAGGAGCAGCAGGAAGCACTATGGGCGCGGCGTCAATAACGCTGACGGTACAGGCCAGACAATTATTG
TCTGGTATAGTGCAACAGCAAAGCAATTTGCTGAGAGCTATAGAGGCGCAACAGCATATGTTGCAACTCACAG
TCTGGGGCATT
>KT185904
ACTTACATAAATAACAATGGCACTATCAATGGCACTATCATACTTCCATGCAGGATAAAGCAAATTATAAACA
TGTGGCAGGAAGTAGGACAAGCAATGTATGCTCCCCCCATCAGGGGAAGCATTAAATGTAGCTCAAATATTAC
AGGAATATTATTAACAAGAGATGGTGGTAGCAGTAATGGCAGTAATGAGATCTTCAGACCTACAGGAGGAAAT
ATAAAAGACAATTGGAGAAATGAACTATATAAATATAAAGTAGTACAAATTGAACCACTAGGAGTAGCACCCA
CCAAGGCAAAAAGAAGAGTGGTGGAGAGAGAAAAAAGAGCAGTGGGAATAGGAGCTATGATCTTTGGGTTCTT
AGGAGCAGCAGGAAGCACTATGGGCGCGGCGTCAATAACGCTGACGGTACAAGCCAGACAATTATTGTCTGGT
ATAGTGCAACAGCAAAGCAATTTGCTGAGGGCTATAGAGGCGCAGCAGCATATGTTGCAACTCACAGTCTGGG
GCATT
>MG989527
ACTTACACCCAGGAGAATAATGGCACTATCATACTTCCATGCAAGATAAAACAAATTGTAAGAATGTGGCAAG
GAGCAGGACAAGCAATGTATGCTCCTCCCATCAAGGGAATAATTAATTGTGTATCAAATATTACAGGAATACT
ATTGACAAGAGATGGTGGGAGGGGTAACCATACTGCTGATAACGAGACCTTCAGACCTGAAGGAGGAGACATG
AAGGACAATTGGAGAAGTGAATTATATAAATATAAAGTAGTACAGATTGAACCACTAGGAATAGCACCCACCA
GGGCAAGGAGAAGAGTGGTGGAGAGAGAAAAAAGAGCAGTGGGAATAGGAGCTATGATCTTTGGGTTCTTAGG
AGCAGCAGGAAGCACTATGGGCGCGGCGTCGATAACGCTGACGGTACAGGCCAGACAATTATTGTCTGGTATA
GTGCAACAGCAAAGCAATTTGCTGAGAGCTATAGAGGCGCAGCAGCATCTGTTGCAACTCACAGTCTGGGGCA
TT
>MG989617
ACTCACAGAGGAAATGAAACCATGGAGGGGAATGGCAATATCACAATTCCATGCAGGATAAAACAAATCATAA
ACATGTGGCAGGAAACAGGACAAGCAATGTATGCTCCTCCCGTCAGGGGAGTAATTAGGTGTGTATCAAATAT
TACAGGAATACTATTGACAAGAGATGGTGGTGGTTCTAATAATGCCACTAATGAGACCTTCAGACCTGGGGGA
GGAAATATAAAAGACAACTGGAGAAGTGAATTATATAAATATAAAGTAGTACAAATTGAACCATTAGGAATAG
CACCCACCAAGGCAAAGAGGAGAGTGGTGGAGAGAGAAAAAAGAGCAGTGGGAATAGGAGCTATGATCTTTGG
GTTCTTAGGAGCAGCAGGAAGCACTATGGGCGCGGCGTCAATAACGCTGACGGTACAGGCCAGACAATTATTG
TCTGGTATAGTGCAACAGCAAAGCAATTTGCTGAGAGCTATAGAGGCGCAACAGCATATGTTGCAACTCACAG
TCTGGGGCATT
>MF957454
AATCATAGTTGCACAGAGGGACATGGGTGTAACAGTACTACCAAAGATGCTAACGGTACTATCACACTTCCAT
GCAGGATAAAGCAAATTATAAAAATGTGGCAGGGAGTAGGACAAGCAATGTATGCTCCTCCCATCAGTGGAAG
TATTAATTGTAGATCAAATATTACAGGAATATTATTGACAAGAGATGGTGGTAATAGTTCAAATGAGACCTTC
AGACCTGAAGGAGGAAATATAAAAGACAACTGGAGGAGTGAATTATATAAATATAAAGTAGTGCAAATTGAAC
CACTAGGAGTAGCACCCACCAAGGCAAAAAGAAGAGTGGTGGACAGACAAAAAAGAGCAGTGGGACTAGGAGC
TCTGATCTTTGGATTCTTAGGAGCAGCAGGAAGCACTATGGGCGCGGCGTCAATAACGCTGACGGTACAGGCC
AGACAATTATTGTCTGGTATAGTGCAACAGCAAAGCAATTTGCTGAGGGCTATAGAGGCGCAGCAGCATATGT
TGCAACTCACAGTCTGGGGCATT
>MG989509
AATTATAATTGCTCAAAGAATAATACTTGCGAGGGAAATGAGGGAAATAATCCTAATGGAACCATCATACTTC
CATGCAAGATAAAGCAAATTATAAACATGTGGCAGAGAACAGGACAAGCAATGTATGCTCCTCCCATCGGGGG
ATGGATTAATTGTACATCAAATATTACAGGAATATTACTGACAAGAGATGGGGGTGAGAATAACAAGACTAAC
GAAACCGAGACCCCCGAGACCTTCAGACCTGGAGGAGGGAATATAAAGGACAATTGGAGAAGTGAATTATATA
AATATAAAGTAGTACAAATTGAACCACTAGGAATAGCACCCACCAGGGCAAAGCGAAGAGTGGTGGACAGAGA
AAAAAGAGCAGTGGGAATAGGAGCTTTGATCTTTGGGTTCTTAGGAGCAGCAGGAAGCACTATGGGCGCGGCG
TCAATAACGCTGACGGTACAGGCCAGACAATTATTGTCTGGTATAGTGCAACAGCAAAGCAATTTGCTGAGAG
CTATAGAGGCGCAGCAGCATATGTTGCAACTCACAGTCTGGGGCATT
>MG989603
AATTATACAAATGGAACCATACAGGGGGGTAATGATACTATCACACTTCCATGCAGAATAAAACAAATAATAA
GAATGTGGCAGAGAACAGGACAAGCAATATATGCTCCTCCTGTCAGTGGAATAATTAATTGTGTATCAAATAT
TACAGGAATACTATTGACAAGAGATGGTGGTGGTAACGAGACCGAGACCTTCAGACCAGGAGGAGGAAATATG
AAGGATAATTGGAGAAATGAATTATATAAATATAAAGTAGTACGAATTGAACCACTAGGGGTAGCACCCACAA
GGGCAAAGAGAAGAGTGGTGGAGAGAGAAAAAAGAGCAGTGGGACTAGGAGCTATGATCTTTGGGTTCTTAGG
AGCAGCAGGAAGCACTATGGGCGCAGCGTCAATAACGCTGACGGTACAGGCCAGACAATTATTGTCTGGTATA

GTGCAACAGCAAAGCAATTTGCTGAGGGCTATAGAGGCGCAACAGCGTCTGTTGCAACTCACAGTCTGGGGCA
TT
>MG989564
ACTTATGGGGTTAATAATACAAATGAAACCAACAAGTATAATGACACTATCACACTCCCATGCAGGATAAAGC
AAATTATAAACATGTGGCAGAGAACAGGACAGGCAATGTATGCTCCTCCCATCAGGGGAGCAATAAATTGTGT
ATCAAATATTACAGGAATACTATTGGTAAGAGATGGTGGTCCTAACAGGACTAACGAGACTTTCAGACCTGGA
GGAGGAAACATGAAGGACAATTGGAGGAGTGAATTATATAAATATAAAGTAGTACAAATTGAACCACTAGGAA
TAGCACCCACCAGGGCAAAGAGAAGAGTGGTGGACAGAGAAAAAAGAGCAGTGGGAATAGGAGCTATGATCTT
TGGGTTCTTAGGAGCAGCAGGAAGCACTATGGGCGCGGCGTCAATAACGCTGACGGTACAGGCCAGACAATTA
TTGTCTGGTATAGTGCAACAGCAAAGCAATTTGCTGAGAGCTATAGAGGCGCAACAGCATATGTTGCAACTCA
CAGTCTGGGGCATT
>MG989572
ACTTACCCGTTAAATAGTACTAACATAGAAAATAATACCACGAAGGGGTATAATGACCCTATCATACTTCCAT
GCAGAATAAAGCAAATCATAAACTTGTGGCAGGGAGTAGGACAAGCAATGTATGCTCCTCCCATCAGGGGAAG
CATTAATTGTGTATCAAATATTACAGGACTACTATTGATAAGGGATGGTGGTATGCATAATGGGACCGATAAT
ACCAAGGAGATCTTCAGACCTGGAGGAGGAAATATGAAGGACAATTGGAGAAATAAATTATATAAATATAAAA
TAGTACAAATTGAACCGCTAGGAGTAGCACCCACCAGGGCAAAGAGAAGAGTGGTGGGAGAGAAAAAAGAGC
AGTGGGAATAGGAGCTTTGATCTTTGGGTTCTTGGGAGCAGCAGGAAGCACTATGGGCGCGGCGTCAATAACG
CTGACGGTACAGGCCAGACAATTACTGTCTGGTATAGTGCAACAGCAAAGCAATTTGCTGAGAGCTATAGAGG
CGCAGCAGCATCTGTTGCAACTCACAGTCTGGGGCATT
>MG989538
AATTATACGTGGGCTAATAATACATTTAATGGAACCACGGAAAATGGGACTATCATACTTCCATGCAGGATCA
AGCAAATTGTAAACATGTGGCAGGGAGCAGGACAAGCAATATATGCTCCTCCCATCAGGGGAAATATTAGTTG
TGCATCAAATATTACAGGAATACTATTAACAAGAGATGGTGGTAATAATAATGAAACAAACGGGACAGAGATC
TTCAGACCTGGAGGAGGAAATATAAAGGACAATTGGAGAAGGGAATTATATAAATATAAAGTAGTACAAATTG
AACCACTAGGAGTAGCACCCACCAAGGCAAAGAGAAGAGTGGTGGAGAGAGAAAAAAGAGCAGTGGGACTAGG
AACTATGATCTTTGGGTTCTTAGGAGCAGCAGGAAGCACTATGGGCGCAGCGTCAATAACGCTGACGGTACAA
GCCAGACAATTATTGTCTGGTATAGTGCAACAGCAAAGCAATTTGCTGAGGGCTATAGAGGCGCAGCAGCATA
TGTTGCAACTCACAGTCTGGGGCATT
>MG989614
ACTTACAATGGAACCCTGGTGAAAAACAATACTGGAAGTGGAAATATCACACTTCCATGTAGGATAAAGCAAA
TTATAAACATGTGGCAAGGAGCAGGACAAGCAATGTATGCTCCTCCCGTTAGTGGAAATATTAATTGTACATC
AAATATTACAGGAATACTATTAACAAGAGATGGTGGTAATAATAATACAGCTAACGAGACCTTCAGACCTGGA
GGAGGAAATATAAAGGACAATTGGAGAAGTGAATTATATAAATATAAAGTAGTACAAATTGAACCACTAGGAA
TAGCACCCACCAGGGCAAGGAGAAGAGTGGTGGAGAGAGAAAAAAGAGCAGTGGGAATAGGAACTATGATCTT
TGGGTTCTTGGGAGCAGCAGGAAGCACTATGGGCGCGGCGTCAATAACGCTGACGGTACAAGCCAGACAATTA
TTGTCTGGTATAGTGCAACAGCAAAGCAATTTGCTGAGAGCTATAGAGGCGCAGCAGCATATGTTGCAACTCA
CAGTCTGGGGCATT
>MG989496
AGCTACTTAAATAGAAACTTAAGTGAAATCGCAGTAGAGAACAAAACACTTATACTTCCATGTAGAATAAAGC
AAATTATAAACATGTGGCAGGGAGTAGGACAAGCAATGTATGCTCCTCCCATCAGTGGGCCAATTAATTGTAA
ATCAAATATTACAGGAATACTGTTAACAAGAGATGGTGGTAATGTTACTGGGAATAATGAGACCTTCAGACCT
ACAGGAGGAAACATAAAGGACAATTGGAGAAGTGAATTATATAAATACAAAGTAGTACAAATTGAACCACTAG
GAATAGCACCCACCAGGGCAAAGAGAAGAGTGGTGGAGAGAGAAAAAAGAGCAGTGGGAATAGGAGCTATGAT
CTTTGGGTTCTTAGGAGCAGCAGGAAGCACTATGGGCGCGGCGTCAATAACGCTGACGGTACAGGCCAGACAA
TTATTGTCTGGTATAGTGCAACAGCAAAGCAATTTGCTGAGAGCTATAGAGGCGCAACAGCATATGTTGCAAC
TCACAGTCTGGGGCATT
>MG989539
AATTATAATTGCACAGATGGAAAATGTAATGTCACTAAGGACAATAATGGCACTATCATACTTCCATGCAGAA
TAAAACAAATTATAAACATGTGGCAAGGAGTAGGACAAGCAATGTATGCTCCTCCCATCAGTGGAATAATTAA
TTGTGTATCAGACATTACAGGAATACTATTGACAAGAGATGGTGGTAATAATAATAATAATACTAATAGTTCG
GAAGAGATCTTCAGACCTGAAGGAGGAAACATAAAGGACAATTGGAGAAGTGAATTATATAAATATAAAGTAG
TACAAATTGAACCACTGGGAATAGCACCCACCAAGGCAAAGAGAAGAGTGGTGGAGAGAGAAAAAAGAGCAGT
GGGAATAGGAGCTATGATCTTTGGGTTCTTAGGAGCAGCAGGAAGCACTATGGGCGCGGCGTCAATAACGCTG
ACGGTACAGGCCAGACAATTATTGTCTGGTATAGTGCAACAGCAAAGCAATTTGCTGAGAGCTATAGAGGCGC
AGCAGCATATGTTGCAACTCACGGTCTGGGGCATT
>AY713424
ACTTACAGAGGAAATGAAACCATGGAGAGGAGTAATGGCACTATCATACTTCCATGCAAGATAAAGCAAATTA
TAAACATGTGGCAGGAAACAGGACAAGCAATGTATGCTCCTCCCATCAATGGAGAAATTAACTGTATATCAAA
TATTACAGGAATACTATTGACAAGAGATGGTGGTATTAATAGAACGTATAACGAGACCTTCAGACCGGGAGGA

GGAAATATGAAGGACAATTGGAGAAATGAATTATATAAATATAAAGTAGTACAAATTGAACCACTAGGAGTAG
CACCCACCAGGGCAAAGAGAAGAGTGGTGGAGAGAGAAAAAAGAGCAGTGGGAATAGGAATTATGATCTTTGG
GTTCTTAGGAGCAGCAGGAAGCACTATGGGCGCGGCGTCAATAACGCTGACGGTACAGGCCAGACAATTATTG
TCTGGTATAGTGCAACAGCAAAGCAATTTGCTGAGGGCTATAGAGGCGCAGCAGCATCTGTTGCAACTCACAG
TCTGGGGCATT
>JN944664
ACTTACAAAGGAAATGAAACCATAGAGGAGGGTGATAGCACTATTACAATTCCATGCAAGATAAAGCAAATTA
TAAACATGTGGCAGGGAACAGGACAAGCAATGTATGCTCCTCCCATCAGTGGAAGTATTAATTGTGTATCAAA
TATCACAGGAATACTATTGACAAGAGATGGTGGTAATAGTAATGATAACAAGACCGAGACCTTCAGACCTGGA
GGAGGAAATATAAAAGACAATTGGAGAAGTGAATTATATAAATATAAAGTAGTACAAATTGAACCACTAGGAG
TAGCACCCACCAAGGCAAAGAGAAGAGTGGTGGAGAGAGAAAAAAGAGCAGTGGGAATAGGAGCTATGATCTT
TGGGTTCTTAGGAGCAGCAGGAAGCACTATGGGCGCGGCGTCAATAACGCTGACGGTACAGGCCAGACAATTA
TTGTCTGGTATAGTGCAACAGCAAAGCAATTTGCTGAGGGCTATAGAGGCGCAGCAGCATCTGTTGCAACTCA
CAGTCTGGGGCATT
>KJ953503
ACTTACAATGGCACTATCATACTTCCATGCAAGATAAAGCAAATTATAAACATGTGGCAGGGAGTAGGACAAG
CAATGTATGCTCCTCCCATCAGGGGAAGAATTAATTGTGTATCAAATATTACAGGAATACTATTGACAAGAGA
TGGTGGTAAGACTAATGAGACTAACGTGACTGAGATTTTCAGACCTGGAGGAGGAAATATGAAGGATAATTGG
AGAAATGAATTATATAAATATAAAGTAGTACAAATTGAACCACTAGGAATAGCACCCACCAGGGCAAAGAGAA
GAGTGGTGGAGAGAGAAAAAAGAGCAGTGGGAATAGGAGCTATGATCTTTGGGTTCTTAGGAGCAGCAGGAAG
CACTATGGGCGCGGCGTCAATAACGCTGACGGTACAGGCCAGACAATTATTGTCTGGTATAGTGCAACAGCAA
AGCAATTTGCTGAGGGCTATAGAGGCGCAACAGCATCTGTTGCAACTCACAGTCTGGGGCATT
>KJ952288
AATCACACCATGGAGGGGTGTAACAATATCACACTTCCATGCAGGATAAAGCAGATTGTAAACATGTGGCAAG
GAGTAGGACAAGCAATGTATGCTCCTCCCATCAGTGGACTCATTAATTGTTCATCAAATATTACAGGAATACT
ATTGACAAGAGATGGTGGTATTAATAACGAGACTGAAGTGACTGAGACCTTTAGACCTGGAGGAGGAGATATA
AGGGCCAATTGGAGAAGTGAATTATATAAATATAAAGTAGTACAAATTGAACCACTAGGCATAGCACCCACCA
GGGCAAAGAGAAGAGTGGTGGAGAGAGAAAAAAGAGCAGTGGGAATAGGAGCTATGATCTTTGGGTTCTTAGG
AGCAGCAGGAAGCACTATGGGCGCGGCGTCAATAACGCTGACGGTACAGGCCAGACAATTATTGTCTGGTATA
GTGCAACAGCAAAGCAATTTGCTGAGAGCTATAGAGGCGCAGCAGCATATGTTGCAACTCACAGTCTGGGGCA
TT
>KJ952992
GATCATACTGGCATAGAAAATGAAACCATAGGGAATGACACTACTATCATACTCCCATGCAAGATAAAGCAAA
TTATAAACATGTGGCAGGGAGTAGGACAAGCAATGTATGCTCCTCCCATCAGTGGAATAATTAATTGTGTATC
AAATATTACAGGAATACTATTGACAAGAGATGGTGGAGATGGTGATACTAATAACGGGACTGAGACCTTCAGA
CCTGGAGGAGGAAATATGAAGGACAATTGGAGAAGTGAATTATATAAATATAAAGTAGTAGAAATTGAACCAC
TAGGAATAGCACCCACCAGGGCAAAGAGAAGAGTGGTGGAGAGACAAAAAAGAGCAGTGGGAATAGGAGCTAT
GATCTTTGGGTTCTTAGGAGCAGCAGGAAGCACTATGGGCGCGGCGTCAATAACGCTGACGGTACAGGCCAGA
CAATTATTGTCTGGTATAGTGCAACAGCAGAGCAATCTGCTGAGGGCTATAGAGGCGCAGCAGCATCTGCTGC
AACTCACAGTCTGGGGCATT
>KJ953319
TCTTATGACCCAGAAAATGAAACCATGAAGGAGTATAATGGCACTAATGGCAATATCACACTTCCATGCAGGA
TAAAGCAAATTATAAACATGTGGCAGAGAACAGGACAAGCAATGTACGCTCCTCCCATCAGTGGAAGTATTAA
TTGTGTATCAAATATTACAGGAATACTATTGACAAGAGATGGTGGGGATAATGATGATAACGAGACCTTCAGA
CCTGGAGGAGGAAACATAAAGGACAATTGGAGAAGTGAATTATATAAATATAAAGTAGTACAAATTGAACCAC
TAGGAATAGCACCCACCAGGGCAAAGAGAAGAGTGGTGGAGAGAGAAAAAAGAGCAGTGGGAATAGGAGCTAT
GATCTTTGGGTTCTTAGGAGCAGCAGGAAGCACTATGGGCGCGGCGTCAATAACGCTGACGGTACAGGCCAGA
CAATTATTGTCTGGTATAGTGCAACAGCAAAGCAATTTGCTGAGGGCTATAGAGGCGCAGCAGCATATGTTGC
AACTCACAGTCTGGGGCATT
>FJ185244
ACTTACCCAATAAATGGAACCATGGAGGGGTATAATGGCACTATTAATGACACTATCATACTTCCATGCAGGA
TAAAGCAAATTATAAACATGTGGCAGGAAGTAGGACAAGCAATGTATGCTCCTCCCATCAGGGGAAGAATTAA
TTGTGTATCAAATATTACAGGAATACTATTGACAAGAGATGGTGGTGCTAATAATACGAACGAGACCTTCAGA
CCTGGAGGAGGAAATATAAAGGACAATTGGAGAAGTGAATTATATAAATATAAAGTAGTACAAATTGAGCCAC
TAGGAATAGCACCCACCAGGGCAAAAAGAAGAGTGGTGGAGAGAGAAAAAAGAGCAGTGGGAATAGGAGCTGT
GATCTTTGGGTTCTTAGGAGCAGCAGGAAGCACTATGGGCGCGGCGTCAATAACGCTGACGGTACAGGCCAGA
CAATTATTGTCTGGTATAGTGCAACAGCAAAGCAATTTGCTGAGGGCTATAGAGGCGCAGCAGCATATGTTGC
AACTCACAGTCTGGGGCATT
>KX389609

AATCAATCACAGGATGGAAATATCACACTCCCATGTAGGATAAAACAAATTGTAAGAATGTGGCAGAGAGTGG
GACAGGCAATGTATGCCCCTCCCATTGCAGGAAACATTACATGTACACCAAACATTACAGGATTAATATTAAC
AAGAGATGGGGGAACAAGAACAATGAGACTGAGGTCATCAGACCAGGAGGAGGAGATATGAGGGACAACTGG
AGAAGTGAACTATATAAGTATAAAGTAGTAAAAATTAAACCACTAGGAATAGCACCCACCAAGGCAAGGAGAC
GAGTAGTGGGGAGAGAAAAAAGAGCAGTTGGAATGGGAGCTGTTCTCCTTGGGTTCTTGGGAACGGCAGGAAG
CACTATGGGCGCAGCGTCAATAACGCTGACGGTACAGGTCAGACAATTATTGTCTGGCATAGTGCAACAGCAA
GGCAACTTGCTGAGAGCTATAGAGGCGCAGCAGAATCTGCTGAAACTCACAGTCTGGGGCATT
>KY346462
ACCTACAATGGTACAAGTAATTCAAACACAACCATCACGATCCCATGTAGAATAAAGCAAATTATAAATATGT
GGCAGCAGGTAGGACGAGCAATGTATGCCCCTCCCATTGCAGGAAACATAACATGTAAATCAAATATCACAGG
ACTACTATTGGTACGTGATGGAGGAAAGGGTGAAAATGGTACAGAGATATTCAGACCTGGAGGAGGAGATATG
AGGGACAATTGGAGAAGTGAATTATATAAATATAAAGTGGTAGAAATTAAGCCATTGGGAATAGCACCCAGTA
AAGCAAAAAGGAGAGTGGTGGAGAGAGAAAAAAGAGCAGTGGGAATAGGAGCTGTGTTCCTTGGGTTCTTGGG
AGCAGCAGGAAGCACTATGGGCGCGGCGTCACTGACGCTGACGGTACAGGCCAGACAATTGTTGTCTGGTATA
GTGCAACAGCAGAGCAATTTGCTGAAGGCTATAGAGGCGCAACAGCATTTGTTGCAACTCACAGTCTGGGGCA
TT
>HQ326137
TTTCATAGTACACAAAATAATTCAACCATCACAATCCCATGCAGAATAAAGCAAATTATAAATATGTGGCAGG
AGGTAGGAAGAGCAATGTATGCCCCTCCCATTGCAGGAAATATAACATGTAGATCAAATATCACAGGACTACT
ATTGGCACGTGATGGAGGAGAAGGCAATGAGACAGAGAATGATACAGAGATATTCAGACCTGGAGGAGGAAAT
ATGAGGGACAATTGGAGAAGTGAATTATATAAATATAAAGTGGTAGAAATTAAACCATTGGGAATAGCACCCA
CTAAAGCAAAAAGGAGAGTGGTGGAGAGAGAGAAAAGAGCAGTGGGAATAGGAGCTGTGTTCCTTGGGTTCTT
GGGAGCAGCAGGAAGCACTATGGGCGCGGCGTCAATAACGCTGACGGTACAGGCCAGACAATTGCTGTCTGGT
ATAGTGCAACAGCAAAGCAATTTGCTGAGGGCTATAGAGGCGCAACAGCATCTGTTGCAACTCACAGTCTGGG
GCATT
>KP718937
AATTACACAGAGTACAATATAACTCTCCCATGCAAAATAAAGCAAATTGTAAGGATGTGGCAGAGAGTAGGAC
AAGCAATGTACGCCCCTCCTATCCAAGGAGAAATAAGGTGTGACTCAAACATTACAGGACTACTATTAACAAG
AGATGGTGGGAATAATAGTACAAATGAAACCTTCAGACCTACAGGAGGAGATATGAAGGATAATTGGAGAAGT
GAGTTATATAAGTATAAAGTGGTGGAAATTAAACCACTAGGAGTAGCACCCACCAAGGCAAAAAGAAGAGTGG
TGGAGAGAGAAAAAAGAGCAGTGGGAATAGGAGCTGTGCTCTTTGGGTTCTTGGGAGCAGCAGGAAGCACTAT
GGGCGCAGCGTCAATAACGCTGACGGTACAGGCCAGACAATTATTGTCTGGCATAGTGCAACAGCAAAGCAAT
TTGCTGAAGGCTATCGAGGCACAACAACATATGTTGAAACTCACTGTCTGGGGCATT
>AJ277823
AATTACCCAGGGTCAAATGACAATGACACTATAACTCTCCCATGCAGAATAAAGCAAATTGTACATATGTGGC
AGAGAGTAGGACAAGCAATGTATGCCCCTCCCATCCAAGGAAAAATAGAGTGTAAATCAAACATTACAGGACT
ACTATTAACAAGAGATGGTGGGAATAAAACACAAAATGAAACCTTCAGACCTACAGGAGGAGACATGAGGGAC
AATTGGAGAAGTGAATTATATAAGTATAAAGTAGTGGAAATTAGACCACTAGGAGTAGCACCCACCAAGGCAA
AAAGAAGAGTGGTGCAGAGAGAAAAAAGAGCAGTGGGAATAGGAGCTGTGCTCTTTGGGTTCTTGGGAGCAGC
AGGAAGCACTATGGGCGCGGCGTCAATAACGCTGACGGTACAAGCCAGACAATTATTGTCTGGCATAGTGCAA
CAGCAAAGCAATTTGCTGAAGGCTATCGAGGCGCAACAGCAACTGTTGAAACTCACTGTCTGGGGCATT
>JF683802
AATTACACTGACATATATAATTCAACAGATAACAGCACAAATGACGCAAATCTCACTCTCCCATGCAGAATAA
AGCAGATTGTACACATGTGGCAGAGAGTAGGGCAAGCAATATATGCCCCTCCCATCCAGGGAGTAATAAGGTG
TGAGTCAAACATTACAGGGCTACTGTTAACAAGAGATGGTGGGAATAATGTCACTAATGAGACCTTTAGACCC
ACAGGAGGAAATATGAAGGACAATTGGAGAAGTGAATTATATAAGTATAAGGTGGTGGAGATTAAACCACTAG
GAGTAGCACCCACCAAGGCAAAAAGAAGAGTGGTGGAGAGAGAAAAAAGAGCAGTGGGAATAGGAGCTGTGCT
CTTTGGGTTCTTGGGAGCAGCAGGAAGCACTATGGGCGCAGCGTCAATAACGCTGACGGTACAGGCCAGACAA
TTATTGTCTGGTATAGTGCAACAGCAAAGCAATTTGCTGAAAGCTATCGAGGCACAACAGCATCTGTTGAAAC
TCCCTGTCTGGGGCATT
>KU168270
AATTACACAGAAACAAATAGCACAAATAATGAGACTATAACTCTCTCATGCAGAATAAAGCAAATTGTAAATA
TGTGGCAGAGAGTAGGACGAGCAATGTATGCCCCTCCCATCCCAGGAGAAATAAGGTGTGACTCAAACATTAC
AGGACTATTATTAACAAGAGATGGTGGGAACAAGACCAATGACGGTGAGGTCTTCAGACCTATAGGAGGAAAT
ATAAGGGACAATTGGAGAAGTGAATTGTATAGGTATAAAGTGGTGGCAATTGAACCACTAGGAGTAGCACCCA
CCAGGGCAAAAAGAAGAGTGGTGGAGAGAGAAAAAAGAGCAGTGGGAATAGGAGCTGTGCTCCTTGGGTTCTT
GGGAGCAGCAGGAAGCACTATGGGCGCGGCGTCAATAACGCTGACGGTACAAGCCAGACAATTATTGTCTGGC
ATAGTGCAACAGCAAGGCAATTTGCTGAAGGCTATTGAGGCACAACAGCAACTGTTGAGACTCACTGTCTGGG
GCATT
>AF516184

TTTCAGAATGGTAATAGTACTTGGAATGATACTGCAGGAAATGGCACTATCACGCTCCCTTGCAGAATAAGAC
AAATTGTAAACATGTGGCAGGAAGTAGGAAGAGCAATGTATGCCCCTCCCATCAAAGGACAAATTAGATGTTC
ATCAAATATTACAGGGTTGCTATTAACAAGAGATGGTGGTAAAAATGAGAATACCACGAATACCACCGAGATC
TTCAGACCTGCAGGAGGAGATATGAGGGACAATTGGAGAAGTGAATTATATAAATATAAAATAGTAAAAATTG
AACCATTAGGAGTAGCACCCACCAAGGCAAAGAGAAGAGTGGTGCAGAGAGAAAAAAGAGCAGTGGGAACGAT
AGGAGCTATGTTCCTTGGGTTCTTGGGAACAGCAGGAAGCACTATGGGCGCAGCGTCAATAACGCTGACGGTA
CAGGCCAGGCAATTGTTGTCTGGTATAGTGCAACAGCAGAGAAATCTGCTGAGGGCTATTGAGGCACAACAGC
ATCTGTTGCAACTCACAGTCTGGGGCATC
>AF457072
AGTCAAACTAATAGTACAGAGTCAGAAAATGATACAATAATCACACTCCCATGCAGAATAAAACAAATTATAA
ATATGTGGCAAGAAGTAGGAAAAGCAATGTATGCCCCTCCCATCGCAGGATTCATTAATTGTGCATCAAATAT
TACAGGGCTACTATTGACAAGAGATGGTGGGATAACTAATAGGTCTAACGAGACCTTCAGACCTGGAGGAGGA
GATATGAGGGACAATTGGAGAAGTGAATTATATAAGTATAAGGTAGTAAGAATTGAACCACTGGGAGTAGCAC
CCACCAAGGCAAGGAGAAGAGTGGTGGAAAGAGAAAAAAGAGCAATAGGACTAGGAGCTATGTTCCTTGGGTT
CTTGGGAGCAGCAGGAAGCACGATGGGCGCAGCGTCAGTGACGCTGGCGGTACAGGCCAGACAGTTATTGTCT
GGTATAGTGCAACAGCAAAATAATTTGCTGAGGGCTATAGAGGCGCAACAACATCTGTTGCAACTCACGGTCT
GGGGCATT
>JF804812
ATCTACAATGATACTAAGACTGAGAATGGTACTATCACACTCCCATGCAGAATAAAACAATTTATAAACAGGT
GGCAGGAAGTAGGAAAAGCAATGTATGCCCCTCCCATCAGAGGACTAATTAGATGTTCATCAAACATTACAGG
GCTATTAATAACAAGAGATGGTGGTAATAATGAGAGCCATCCCCTGAACGAGACCTTCAGACCTGGGGGAGGA
GATATGAGGGACAATTGGAGAAGTGAATTATATAAATATAAAGTAGTAAAAATTGAGCCATTAGGAGTAGCAC
CCACCAAGGCAAAGAGAAGAGTGGTGCAGAGAGAAAAAAGAGCAGTGGGAACAATAGGAGCTATGTTCCTTGG
GTTCTTGGGAGCAGCAGGAAGCACTATGGGCGCAGCGTCATTGACGCTGACGGTACAAGCCAGACAATTATTG
TCTGGTATAGTGCAACAGCAAAGCAATTTGCTGAGAGCTATTGAGGCGCAACAGCATCTGTTGCAGCTCACAG
TCTGGGGCATC
>AY727526
AATTATACATATAATAGTACAGAAAACATTAATGAAACAGGAGCAAACATTACAATCCCATGCAGAATAAAAC
AATTTATAAATATGTGGCAGGAGGTAGGACGAGCAATGTATGCCCCTCCCATTAAAGGAATAATAACATGTAA
ATCAAATATCACAGGAATATTACTGACACGGGATGGAGGCAGGACTGATACTAACACAGAAACATTCAGGCCT
GGAGGAGGAGATATGAGGGACAATTGGAGAAGTGAATTATATAAATATAAAGTGGTAGAAATTAAACCATTAG
GAATAGCACCCACCAAGGCAAAAAGGAGAGTGGTGGAGAGAGAAAAAAGAGCAGTGGGAATAGGAGCTGTGTT
CCTTGGGTTCTTGGGAGCAGCAGGAAGCACTATGGGCGCAGCGTCAATAACGCTGACGGTACAGGCCAGACAA
TTATTGTCTGGGATAGTGCAACAGCAAAGCAATTTGCTGAGGGCTATAGAGGCGCAACAGCATATGTTGCAAC
TCACAGTCTGGGGCATT
>AY727527
TACTATAGTACATACAATAGTACAGTTAGTAATACAACAGCAGATGTAAACATAACAATCCCATGCAGAATAA
AACAAATTATAAATATGTGGCAGGAGGTAGGAAGAGCAATGTATGCCCCTCCCATTAAAGGAGCAATAACATG
TATATCAAACATCACAGGATTATTACTGACACGGGATGGAGACAAGAATGACACAGAGACATTCAGGCCTGGA
GGAGGAGATATGAGGGACAATTGGAGAAGTGAATTATATAAATATAAAGTGGTAGAAATTAAACCATTAGGAA
TAGCACCCACTAAGGCAAAAAGGAGAGTGGTGGAGAGAGAAAAAAGAGCAGTGGGACTAGGAGCTTTGTTCTT
TGGGTTCTTGGGAGCAGCAGGAAGCACTATGGGCGCAGCATCAATAACGCTGACGGTACAGGCCCGACAATTG
TTGTCTGGGATAGTGCAACAGCAAAGCAATTTGCTGAGAGCTATAGAGGCGCAACAGCATATGTTGCAACTCA
CGGTCTGGGGCATT
>DQ366662
ACTCACAGAGAAAATGAAACCGAGGGAAAGGATAATGACACTATCATACTTCCATGCAAAATAAAGCAAATTA
TAAACATGTGGCAGAGAACAGGACAAGCAATGTATGCTCCTCCCATTGAGGGAAAAATTAATTGTGTATCAAA
TATTACAGGAATATTATTGACAAGAGATGGTGGTGGTATTAATGATACTAGGGAAAACGAGACCTTCAGACCT
GGAGGAGGAAATATGGAGGACAATTGGAGAAGTGAATTATATAAATATAAAGTAGTACAAATTGAACCACTAG
GAATAGCACCCACCAGGGCAAAGAGAAGAGTGGTGGAGAGGGAAAAAAGAGCAGTGGGAATAGGAGCTTTGAT
CTTTGGGTTCTTAGGAGCAGCAGGAAGCACTATGGGCGCGGCGTCCATAACGCTGACGGTACAGACCAGACAA
TTATTGTCTGGGATAGTGCAACAGCAAAGCAATTTGCTGAGGGCTATAGAGGCGCAGCAGCATCTGTTGCAAC
TCACAGTCTGGGGCATT
>AB845349
ACTTACTGGATAAATGGAAGTAGGGAGGAGAGTAATGGCACTACCAATGGCAATATCACTATCCCATGCAAAA
TAAAACAAATCATAAACATGTGGCAGGGAGTAGGACAAGCAATGTATGCTCCCCCCATCAAAGGAAGACTTAA
TTGTATATCAAATATTACAGGACTACTATTAACAAGAGATGGTGGTGATGGTAAAGATAACGAGACCTTCAGA
CCTGCAGGAGGAGAGATGAAGGACAATTGGAGAAGTGAATTATATAAATATAAAGTAGTACAAATTGAACCAC
TAGGAATAGCACCCACCAGGGCAAAGAGAAGAGTGGTGGAGAGAGAAAAAAGAGCAGTGGGAATAGGAGCTAT
GATCTTTGGGTTCTTGGGAGCAGCAGGAAGCACTATGGGCGCGGCGTCAATAACGCTGACGGTACAGGCCAGA

CAATTATTGTCTGGTATAGTGCAACAGCAAAGCAATTTGCTGAGGGCTATAGAGGCGCAGCAGCATCTGTTGC
AACTCACAGTCTGGGGCATT
>KJ671533
AACTACTCAGAACAAAAATTAAATAATAAAACGTCCTCTCCCAATGACACTATCACTATCCCATGTCGAATAA
AACAAATTGTAAATATGTGGCAGGAAGTAGGACGAGCAATGTATACCGCTCCCATTGCAGGAAACATTACCTG
TAACTCAAATATTACAGGTATACTGTTGACAAGAGATGGTGGTAATATAACTAATGGGACTGAGACATTCAGA
CCTGGGGGAGGAAATATGAAAGACAATTGGAGAAGTGAATTATATAAATATAAAGTAGTAGAAATTAAACCAC
TAGGAGTAGCACCCACCAGGGCAAAAAGACAAGTGGTGAAGAGAGAAAAAAGAGCAGCAGGAATAGGAGCTTT
GTTCCTTGGGTTCTTGGGGGCAGCAGGAAGCACTATGGGCGCAGCGTCAGTGACGCTGACGGTACAGGCCAGA
CAATTATTGTCTGGTATAGTGCAACAGCAGAACAATCTGCTGAGAGCTATTGAGGCGCAACAGCATATGTTGC
AACTCACAGTCTGGGGCATT
>KU161145
TATTATAATGACACTATCAGACTTCCATGCAGGATAAAGCAGATTATAAACATGTGGCAAGGAGTAGGACAAG
CAATGTATGCCCCTCCCGTCAGGGGAAGGATTAATTGTGTATCAAACATTACAGGAATACTATTGACAAGAGA
TGGTGGTAATACTACTGCAGATGCTAGTGAGACCTTCAGACCTGCAGGAGGAAATATGAAGGACAATTGGAGA
AGTGAATTATATAAATATAAAGTAGTAGAAATTGAACCACTAGGAATAGCACCCACCAAGGCAAAAAGAAGAG
TGGTGCAGAGAGAAAAAAGAGCAGTGGGAATAGGAGCTATGATCTTTGGGTTCTTAGGAGCAGCCGGAAGCAC
TATGGGCGCGGCGTCAATAACGCTGACGGTACAAGCCAGACAGTTACTGTCTGGTATAGTGCAACAGCAAAGC
AATTTGCTGAGGGCTATAGAGGCGCAGCAGCATATGTTGCAACTCACAGTCTGGGGCATT
>KY216147
AATTATACATACAAGACTAATGATAGTAGGACTAATAATACAGCTGAGGATACTAATTCGACCGTCATCACAA
TCCCATGCAAAATAAAGCAGATTATAAATATGTGGCATGAGGTGGGACGAGCAATGTATGCTCCTCCCATTGC
AGGAAACATAACATGTAAGTCAAACATCACAGGAATACTATTAGTACGTGATGGAGGAACAAAGTCAAATAGT
ACAGAGACATTCAGGCCTGGAGGAGGAGATATGAGGAATAATTGGAGAAGTGAATTATATAAATATAAAGTGG
TAGAAATTAAGCCAGTGGGAGTAGCACCCACTGAAGCAAAAAGGAGAGTGGTGGAGAGAGAAAAAAGAGCAGT
GGGAATAGGAGCTGTGTTCCTTGGGTTCTTGGGAGTAGCAGGAAGCACTATGGGCGCGGCGTCAGTGACGCTG
ACGGTACAGGTCAGACAATTGCTGTCTGGTATAGTGCAACAGCAAAACAATTTGCTGAGGGCTATAGAGGCGC
AACAGCATATGTTGCAACTCACGGTCTGGGGCATT
>KU820831
TACTATCTTAATAGTACATACAATCCTAATGATACAGCAAACTCAAACATCACCATCCCATGCAGAATAAAAC
AGATTGTAAACATGTGGCAGGAGGTAGGACGAGCAATGTATGCTCCTCCCATTGCAGGAAAAATAACATGTAA
ATCAAATATCACAGGACTACTATTGGTACGTGATGGGGGAATTAATCTAAATACAACAAATAATACCGAAACA
TTCAGGCCTGGAGGAGGAGATATGAGGGACAATTGGAGAAGTGAATTATATAAATATAAAGTGATAGAAATTA
GGCCATTGGGAATAGCACCCACTAAAGCAAAAAGGAGAGTGGTGGAGAGAGAAAAAAGAGCAGTGGGACTAGG
AGCTGTGTTCCTTGGGGTCTTGGGAGCAGCAGGAAGCACTATGGGCGCGGCGTCAATAACGCTGACGGTACAA
GCCAGACAATTGTTGTCTGGTATAGTGCAACAGCAAAGTAATTTGCTGAGGGCTATAGAGGCGCAACAGCATA
TGTTGCAACTCACGGTCTGGGGCATC
>KU992937
TTCTATACTAATGATACAGGAGGCAGTTTAAAATCAAACATCACGATTCCATGTAGAATAAAGCAAATTATAA
ATATGTGGCAGGAGGTAGGGCGAGCAATATATGCCCCTCCCATTGAAGGAAATATAACATGTAAATCAAAGAT
CACAGGACTACTATTGGTACGTGATGGAGGAAAAGGAAATAATAATACAGAGACATTTAGACCTGGAGGAGGA
GATATGAGGGACAATTGGAGAAGTGAATTATATAAATATAAAGTGGTAGAAGTTAAGCCATTGGGAGTAGCAC
CTACTAAAGCAAAAAGGAGAGTGGTGGAGAGAGAAAAAAGAGCAGTGGGAATAGGAGCTGTGTTCCTTGGGTT
CTTGGGAGCAGCAGGAAGCACTATGGGCGCGGCGTCAATAACGCTGACGGTACAAGCCAGACAATTGTTGTCT
GGTATAGTGCAACAGCAAAGCAATTTGCTGAGAGCCATAGAGGCGCAACAGCATATGTTGCAACTCACGGTCT
GGGGCATT
>KC899012
ACCTATAATGCTACTAGTACAAAAGGTAATTCAAGCTCAGTTATCACACTCCCATGCAGAATAAAGCAAATTA
TAAACATGTGGCAGGAGGTAGGGCGAGCAATGTATGCCCCTCCCATTGCAGGAAACATAACATGTAAATCCAA
TATCACAGGAATACTATTGGAATGGGATGGAGGAAATTCAAATAGTTCTAATGCTACATTCAGACCTGGAGGA
GGAGATATGAAAAACAATTGGAGAAGTGAATTATATAAATATAAAGTGGTAGAAATAAAGCCATTAGGAATAG
CACCCACTGCAGCAAAAAGGAGAGTGGTGGAGAGAGAAAAAAGAGCAGTGGGAATAGGAGCTGTGTTCCTTGG
GTTCTTAGGCGCGGCAGGAAGCACTATGGGCGCGGCGTCAATAACGCTGACGGTACAGGCCAGACAATTGCTG
TCTGGTATAGTGCAACAGCAAAGCAATTTGCTGAGGGCCATAGAGGCGCAACAGCATATGTTGCAACTCACGG
TCTGGGGCATT
>MN044632
ACCTACATGCCTAATGGTACATTCAAGTTTAATGATACAGACCTGAATGTTACAAGTAATTCAGATTCACCCA
TCACAATACCATGCAGAATAAAGCAAATTATAAACATGTGGCAGGAGGTAGGACGAGCAATGTATGCTCCTCC
CATTGAAGGAAACATAACATGTAAATCAAATATCACAGGACTACTATTGGTACGTGATGGAGGAGAGACAGAG
ACAAACATGACAGAGACATTCAGACCTGGAGGAGGGGATATGAGAGACAATTGGAGAAATGAATTATATAAAT

ATAAAGTGGTAGAAATTAAGCCATTAGGAGTAGCACCCACTGAAGCGAAAAGGAGAGTGGTGCAGAGAGAAAA
AAGAGCAGTGGGAATAGGAGCTGTGTTCCTTGGGTTCTTGGGAGCAGCAGGAAGCACTATGGGCGTGGCGTCA
ATAACGCTGACGGTACAGGCCAGGCAACTGCTGTCTGGTATAGTGCAACAGCAAAGCAATTTGCTGAGAGCTA
TAGAGGCGCAACAACATGTGTTGCAACTCACTGTCTGGGGCATT
>MK397789
ACTTACCAAAGTGGAACCATAAATGGGGATAATGGAACTATTATAATTCCATGTAAAATCAAACAAATTATAA
ACATGTGGCAGAGAACAGGACAAGCAATATATGCTCCTCCCATCAAGGGAAAAATTAATTGTATATCAAATAT
TACAGGAATACTATTAACAAGAGATGGTGGTGTTAATAGCACAGTTAATGAGACCTTCAGACCGGGAGGAGGA
AATATAAAAGACAACTGGAGAAGTGAATTATATAAATATAAAGTAGTACAAATTGAACCACTAGGAGTAGCAC
CCACCAGGGCAAGAAGAAGAGTGGTGCAGAGAGAAAAAAGAGCAGTGGGAATAGGAGCTATGATCTTTGGGTT
CTTAGGAGCAGCAGGAAGCACTATGGGCGCGGCGTCAATAACGCTGACGGTACAGGCCAGACAATTATTGTCT
GGTATAGTGCAACAGCAAAGCAATTTGCTGAGAGCTATAGAGGCGCAGCAGCATCTGTTGCAGCTAACAGTCT
GGGGCATT
>EU131799
AATCATAATTTGACTGAGAATAATACTTGGCAAGAGAATGCTAGCGAAAGTACCATCACACTCCCATGCAGAA
TAAAACAAATTATAAACAGGTGGCAGGAAGTAGGAAGAGCAATGTATGCCCCTCCCATCAGTGGACAAATTAA
ATGCTCATCAAATATTACAGGGCTGCTATTAACAAGAGATGGTGGTAAAACGAATGGGTCCGAAAATGGGTCC
GAAATCTTCAGACCTGGAGGAGGAGATATGAGAGACAATTGGAGAAGTGAATTATATAAATATAAAGTAGTAA
AAATTGAGCCACTAGGAATAGCACCCACCAAGGCAAAAAGAAGAGTGGTGCAGAGAGAAAAAAGAGCAGTGGG
AACAATAGGAGCTATGTTCCTCGGGTTCTTGGGAGCAGCAGGAAGCACTATGGGCGCAGCGTCAATGACGCTG
ACGGTACAGGCCAGACTATTACTGTCTGGTATAGTGCAACAGCAGAACAATCTGCTGAGAGCTATTGAGGCGC
AACAGCACATGTTGCAACTCACAGTCTGGGGCATC
>KM111555
ACTTACAGAGAAAATGGAACCATGGAAGGGAGTAATGGCACTATCATACTTCCATGCAAAATAAAGCAAATAA
TAAACATGTGGCAGGGAGTAGGACAAGCAATGTATGCTCCTCCCGTCAGTGGAACAATTAATTGTGTATCAAA
TATTACAGGAATACTATTAACAAGAGATGGTGGTGGTAGTACCAATGCGACTAACGAGACCGAGATCTTCAGA
CCTGGAGGAGGAAATATAAAGGACAATTGGAGAAGTGAATTATATAAATATAAAGTAGTAGAACTTGAACCAC
TAGGAATAGCACCCACCAGGGCAAAGAGAAGAGTGGTGGCGAGAGAAAAAAGAGCAGTGGGAATAGGAGCTAT
GATCTTTGGGTTCTTAGGAGCAGCAGGAAGCACTATGGGCGCAGCGTCCATAACGCTGACGGTACAGGCCAGA
CAATTATTGTCTGGTATAGTGCAACAGCAAAGCAATTTGCTGAGGGCTATAGATGCGCAGCAGCATCTGTTGC
AACTCACAGTCTGGGGCATT
>KP418805
AATTATACTTTCCAGGCTAATGATACTAGGCAAAGGTCAAACAAAACTATCACACTCCCATGCAGAATAAAAC
AAATTATAAACAGGTGGCAGGAAGTAGGAAGAGCAATGTATGCCCCTCCCATCAGGGGACAAATTAAATGTTC
ATCAAATATTACAGGAATACTATTAACAAGAGATGGTGGTGACAAGAGTAGTACTGCAACTAATGAAACCTTC
AGACCGGGAGGAGGAGATATGAGGGACAATTGGAGAAGTGAATTGTATAAATATAAAGTAGTAAAGATTGAAC
CACTAGGAATAGCACCCACCAAGGCAAAGAGAAGAGTGGTGCAGAGAGAAAGAAGAGCAGTGGGAGTAGGAGC
TATGTTCCTCGGGTTCTTGGGAGCAGCAGGAAGCACTATGGGCGCAGCATCAATGACGCTGACGGTACAGGCC
AGACTATTACTGTCTGGTATAGTGCAACAGCAGAACAATCTGCTGAGAGCTATTGAGGCGCAACAACACATGT
TGCAACTCACAGTCTGGGGCATC
>AB097867
ACTCATAGTATTTGGAATGGTACTATTTGGAATGGTACTCGGAATGATACTAATATCACGCTCCCTTGCAGAA
TAAAACAAATTGTAAACATGTGGCAGGAAGTAGGAAAAGCAATGTATGCCCCTCCCATCAGAGGACAAATTAG
ATGTTCATCAAATATTACAGGGTTGCTATTAACAAGAGATGGTGGTAATGATAAGAACGAGACCACCGAGACC
TTCAGACCAGGAGGAGGAGATATGAGGGACAATTGGAGAAGCGAATTATATAAATATAAAATAGTAAAAATTG
AACCATTAGGAGTAGCACCCACCAAGGCAAAGAGAAGAGTGGTGCAGAGAGAAAAAAGAGCAGTGGGAACGAT
AGGAGCTATGTTCCTTGGGTTCTTGGGAGCAGCAGGAAGCACTATGGGCGCAGCGTCAATAACGCTGACGGTA
CAGGCCAGACAATTGTTGTCTGGTATAGTGCAGCAGCAGAGAAATTTGCTGATGGCTATCGAGGCGCAACAGC
ATCTGTTGCAACTCACAGTCTGGGGCATC
>KT438784
AGTTACAAAGGAGATGAAACCATCATACTTCCATGTAAAATAAAGCAAATTATAAATATGTGGCAGAGAGTAG
GACAAGCAATGTATGCTCCTACCATCAGGGGAAAAATTAATTGTGTATCAAATATTACAGGAATACTATTGAC
AAGAGATGGTGGAAATGGTAGTGGCGAGACCTTCAGACCTGGAGGAGGAGATATGAAGGACAATTGGAGAAGT
GAATTATATAAATATAAAGTAGTACAAATTGAACCACTAGGATTAGCACCCACCAAGGCAAAGAGAAGAGTGG
TGGAAAGAGAAAAAAGAGCAGTGGGAATAGGAGCTATGATCTTTGGGTTCTTAGGAGCAGCTGGAAGCACTAT
GGGCGCGGCGTCCATAACGCTGACGGTACAGGCCAGGCAATTATTGTCTGGTATAGTGCAACAGCAAAGCAAT
TTGCTGAGGGCTATAGAGGCGCAGCAGCATATGTTGCAACTCACAGTCTGGGGCATT
>JN248316
ACTTACCTAGGAAATGAAACCTTGGAGAGGTATACTGGCAATAGCACTAATGGCACTATCATACTTCCATGCA
GGATAAAGCAAATTGTAAGAATGTGGCAGGGAGTAGGACAAGCAATGTATGCTCCTCCCGTCAAGGGAATAAT

TAATTGTGTATCAAATATTACAGGAATACTATTGACAAGAGATGGTGGTAATAGAACGAAAAATGAGACCTTC
AGACCTGAAGGAGGAAATATGAAGGACAATTGGAGAAATGAATTATATAAATATAAAGTAGTACAAATTGAAC
CACTAGGAATAGCACCCACCAAGGCAAAAAGAAGAGTGGTGGAGAGAGAAAAAAGAGCAGTGGGAATAGGAGC
TATGATCTTTGGGTTCTTAGGAGCAGCAGGAAGCACTATGGGCGCGGCGTCAATAACGCTGACGGTACAGGCC
AGACAATTATTGTCTGGTATAGTGCAACAGCAAAGCAATTTGCTGAGGGCTATAGAGGCGCAGCAGCATCTGT
TGCAACTCACAGTCTGGGGCATT
>JN248320
ACTTATGACATAGGAAATGAAACCACAGGGGAGGGTAATGGCACTATTGGCAATATCACACTTCCATGCAAGA
TAAAGCAAATTATAAACATGTGGCAGGGAGCAGGGCAAGCAATGTATGCTCCTCCCATCAGTGGAGTAATTAA
TTGTGTATCAAATATTACAGGTATACTATTGACAAGAGATGGTGGTGTTAATAATACGAGTGAGGAGACCTTC
AGACCTGGAGGAGGAAATATAAAGGACAATTGGAGGAGTGAATTATATAAATATAAAGTAGTACAAATTGAAC
CACTAGGAGTAGCACCCACCAGGGCAAAGAGAAGAGTGGTGGAGAGAGAAAAAAGAGCAGTGGGAATAGGAGC
TATGATCTTTGGGTTCTTGGGAGCAGCAGGAAGCACTATGGGCGCAGCGTCAATAACGCTGACGGTACAGGCC
AGACAATTATTGTCTGGTATAGTGCAACAGCAAAGCAATTTGCTGAGGGCTATAGAGGCGCAACAGCATCTGT
TGCAACTCACAGTCTGGGGCATT
>JN248349
AATTATAATGGTACTTGGAATAATAATAATCCTTGGAATGATACTACAGAAAATGGCACTATCATACTCCCTT
GCAGAATAAGACAAATAGTAAACATGTGGCAGCAAGTAGGAAGAGCAATGTATGCCCCTCCCATTGCAGGACA
AATTACATGTGCATCAAATATTACAGGAATACTATTGACAAGAGATGGTGGTGCTAAGAATGATAGTGGCACC
GAGACCTTCAGACCTGCAGGAGGAGATATGAGGGACAATTGGAGAAGTGAATTATATAAATATAAGGTAGTAA
AAATTGAACCATTAGGAGTAGCACCCACCAAGGCAAAGAGAAGAGTGGTGCAGAGAGAAAAAAGAGCAGTGGG
AATGGGAGCTATGTTCCTTGGGTTCTTGGGAGCAGCAGGAAGCACTATGGGCGCAGCGTCAATAACGCTGACG
GTACAGGCCAGACAATTGTTGTCTGGTATAGTGCAACAGCAGAGAAATTTGCTGAGGGCTATTGAGGCGCAAC
AGCATCTGTTGCAACTCACAGTCTGGGGCATT
>EU743760
ACTCACATCAATGGCACTAGCAACAGCACTGCGAGGGAGTATAATGGCACTATCACACTTCCATGCAGGATAA
AGCAAATTATAAATATGTGGCAGGAAGCAGGACAAGCAATGTATACTCCTCCCATCAGTGGAATAATTAACTG
TATATCAAATATTACAGGAATACTATTGACAAGAGATGGTGGTGCTAATAATGGGACTATCAATGAGACCTTT
AGACCTGGAGGAGGAGATATGAGGGACAATTGGAGAAGTGAATTATATAAATATAAAGTAGTAAAAATTGAAC
CATTAGGAGTAGCACCCACCAAGGCAAGGAGAAGAGTGGTGCAGAGAGAAAAAAGAGCAATAGGAATAGGAGC
TGTGTTCCTTGGGTTCTTGGGAGCAGCAGGAAGCACTATGGGCGCGGCGTCAATAACGCTGACGGTACAAGCC
AGACAATTATTGTCTGGTATAGTGCAACAGCAGAACAACTTGCTGAGAGCTATTGAGGCGCAACAGCATATGT
TGCAACTCACTGTCTGGGGCATC
>JN860765
ACTTATAACAGTACTGGCAATATCACAATTCCATGTAGGATAAAGCAGATTATAAACATGTGGCAGGAAACAG
GACAAGCAATGTATGCTCCTCCCATTAGTGGAAAAATTAATTGTGTATCAAATATTACAGGAATACTATTGAC
AAGAGATGGAGGTACTAATAATAATAATAATGGAACTGAGACCTTCAGACCTGGAGGAGGAAATATAAAGGAC
AATTGGAGAAGTGAATTATATAAATATAAAGTAGTACAAATTGAACCACTAGGAGTAGCACCCACCAAGGCAA
AGAGAAGAGTGGTGGAGAGAGAAAAAAGAGCAGTAGGAATAGGAGCTATGATCTTTGGGTTCTTAGGAGCAGC
CGGAAGCACTATGGGCGCGGCGTCAATAACGCTGACGGCACAGGCCAGACAATTACTGTCTGGTATAGTGCAA
CAGCAAAGCAATTTGCTGAGGGCTATAGAGGCGCAGCAGCATATGTTGCAACTCACAGTCTGGGGCATT
>MG989494
AATCATACCAAGGGTGAGTGTAACAACACTATCATACTTCCATGTAAGATAAAGCAAATTATAAACATGTGGC
AGAGAGTAGGACAAGCAATGTATGCTCCTCCCATCAGGGGAAACATTACTTGTGTATCAAATATTACAGGAAT
ATTATTGACAAGAGATGGTGGTAATGGGACTAACGGGACTGAAACCTTCAGACCTGGAGGAGGAGATATAAAG
GACAATTGGAGAAATGAATTATATAAATATAAAGTAGTACAAATTGATCCACTAGGAATAGCACCCACCAGGG
CAAGAAGAAGAGTGGTGGAGAGGGAAAAAAGAGCAGTGGGAATAGGAGCTATGATCTTTGGGTTCTTAGGAGC
AGCAGGAAGCACTATGGGCGCGGCGTCAATAACGCTGACGGTACAGGCCAGACAATTATTGTCTGGGATAGTG
CAACAGCAAAGTAATTTGCTGAGGGCTATAGAGGCGCAGCAGCATCTGTTGCAACTCACAGTCTGGGGCATT
>MG989490
AATTATACCAAGGATAAGGAAAATATCACACTTCCATGCAGGATAAAGCAAATTATAAACATGTGGCAGAGAG
TAGGACAAGCAATGTATGCTCCTCCCATCAAGGGAAACATTACTTGTGCATCAAATATTACAGGAATAGTATT
GACAAGAGATGGTGGTGGTAATAATGAGAATAGAAACAACGAGACCTTCAGACCAACAGGAGGAAATATAAAG
GACAATTGGAGAAATGAATTATATAAATATAAAGTAGTACAAATTGATCCACTAGGAATAGCACCCACCAAGG
CAAAAAGAAGAGTGGTGGAGAGAGAAAAAAGAGCAGTGGGAATAGGAGCTATGATCTTTGGGTTCTTAGGAGC
AGCAGGAAGCACTATGGGCGCGGCGTCAATAACGCTGACGGTACAGGCCAGACAATTATTGTCTGGGATAGTG
CAACAGCAAAGTAATTTGCTGAGGGCTATAGAGGCGCAGCAGCATATGCTGCAACTCACAGTCTGGGGCATT
>MG989520
TTTCATAATAATGGCACAAATGGCACAAGTGGAATCATCACAATCCCATGCAGGATAAAGCAAATTATAAACA
TGTGGCAGAGAGTAGGACAAGCAATGTATGCTCCTCCCATCAGTGGGGCAATCAGTTGTGTCTCAAATATTAC

AGGAATACTATTGACAAGAGATGGTGGTGATGCAAATAACACAAATAACTCTAATACAACTGATCCTAAAGAA
ATCTTCAGACCTGCAGGAGGAGATACAAGGGACAATTGGAGAAGTGAATTATATAAATATAAAGTAGTACAAA
TTGAACCACTAGGAATAGCACCCACCAAGGCAAAGAGAAGAGTGGTGGACAGAGAAAAAAGAGCAGTGGGAAT
AGGAGCTATGATCTTTGGGTTCTTAGGAGCAGCAGGAAGCACTATGGGCGCAGCGTCAATAACGCTGACGGTA
CAGGCCAGACAATTATTGTCTGGTATAGTGCAACAGCAAAGCAATTTGCTGAGGGCTATAGAGGCGCAGCAAC
ATCTGTTGCAACTCACAGTCTGGGGCATT
>MG989573
AATTATAATGGCACCATCATACTTCCATGCAGGATAAAGCAAATTATAAACATGTGGCAGAGAGTAGGACAAG
CAATGTATGCTCCTCCCATTAGGGGAAACATTACATGTACATCAAATATTACAGGAATATTATTGACAAGAGA
TGGCAAAGGTGAAAACGATACAGGGAAAGAGATCTTCAGACCTGGAGGAGGAGATATGAAGGACAATTGGAGA
AATGAATTATATAAATATAAAGTAGTACAAATTGAGCCACTAGGAATAGCACCCACCAGGGCAAGGAGAAGAG
TGGTGCAGAGAGAAAAAAGAGCAGTAGGAATAGGAGCTATGATCTTTGGGTTCTTAGGAGCAGCAGGAAGCAC
TATGGGCGCGGCGTCAATAACGCTGACGGTACAGGCCAGACAATTATTGTCTGGTATAGTGCAACAGCAAAGC
AATTTGCTGAGGGCTATAGAGGCGCAACAGCATATGTTGCAACTCACAGTCTGGGGCATT
>MG989599
ACTTATGACAGTAATGACACTATCATAATTCCATGTAGGATAAAGCAAATTATAAACATGTGGCAGGAAACAG
GACAAGCAATGTATGCTCCTCCTGTCAGTGGAGCAATTAATTGTGTATCAAATATTACAGGAATACTATTGAC
AAGAGATGGTGGTAATAATAGAACTAATGAGACCTTCAGACCTGGAGGAGGAAATATAAAGGACAATTGGAGA
AGTGAATTATATAAATATAAAGTAGTACAAATTGAACCATTAGGAATAGCACCCACCAGGGCAAGGAGAAGAG
TGGTGGAGAGACAAAAAAGAGCGGTGGGAATAGGAACTCTGATCTTTGGGTTCTTAGGAGCAGCCGGAAGCAC
TATGGGCGTGGCGTCAATGACGCTAACGGTACAGGCCAGACAATTATTGTCTGGTATAGTGCAACAGCAAAGC
AATTTGCTGAGAGCTATAGAGGCACAGCAGCATATGTTGCAACTCACAGTCTGGGGCATT
>MK697709
TTTTACTTTAATGGTACAAAAAGAAATATATTTAATGATACAGAAAGTAATTCGACCATCACAATCCCATGCA
GGATAAAGCAAATTATAAACATGTGGCAGGAAGTGGGACGAGCAATGTATGCTCCTCCCATTGCAGGAAACAT
AACATGTAAGTCAAATATCACAGGACTGATATTAGTACGTGATGGAGGAAATAGGGCCACAAATGATACAGAA
ATATTCAGGCCTGGAGGAGGAAATATGAGGGATAATTGGAGAAGTGAATTATATAAATATAAAGTGGTAGAAA
TTAAGCCATTGGGAATAGCACCCACTACATCAAAAAGGAGAGTGGTGGAGAGAGAAAAAAGAGCAGTGGGAAT
AGGAGCTGTGTTCCTTGGGTTCTTGGGAGTAGCAGGAAGCACTATGGGCGCGGCGTCAGTGGCGCTGACGGTA
CAGGCCAGACAATTATTGTCTGGTATAGTGCAGCAGCAAAGCAATTTGCTGAGGGCTATAGAGGCGCAGCAGC
ATATGTTGCAACTCACAGTCTGGGGCATT
>MN336527
TATCATACAGAAGACAATTCGACCATCACACTCCCATGCAAAATAAAGCAAATTATAAATATGTGGCAGGAGG
TGGGAAGAGCAATGTATGCTCCTCCCATTGCAGGAAACATAACATGTAGGTCAAGTATCACAGGACTACTATT
AGTACGTGATGGAGGAAATGCAAATGATACAAATAATACAGAGATATTCAGGCCTGGAGGAGGAAATATGAGG
GATAATTGGAGAAGTGAATTATATAAATATAAAGTGGTAGAAATTAAGCCATTGGGAGTAGCACCAACTGGGG
CAAAAAGGAGAGTGGTGGAAAGAGAAAAAAGAGCAGTAGGAATAGGAGCTGTGTTCCTTGGGTTCTTGGGAGC
AGCAGGAAGCACTATGGGCGCGGCGTCAGTGACGCTGACGGTACAGGCCAGACAATTGCTGTCTGGTATAGTG
CAACAGCAAAACAATTTGCTGAGGGCTATAGAGGCGCAACAGCATATGTTGCAACTCACGGTCTGGGGCATC
>JN864056
GACTATATACTTCCATGTAAGATAAAACAAATTGTGAGAATGTGGCAGAAAGTGGGACAAGCAATGTATGCCC
CTCCCATCGAAGGAAACATTACATGTACGTCAAACATCACAGGACTAATATTAACAAGAGATGGTGGGAATAA
GAACAGTACAAATGAGACCTTTAGACCTGGGGGAGGAGATATGAGGGACAATTGGAGAAGTGAATTATATAAA
TATAAAGTAGTAAAAATTGAGCCAGTAGGAATAGCTCCCACTAAGGCAAGACGAAGAGTGGTGCAGAGAGAAA
AAAGAGCAGTTGGACTAGGAGCTGTCCTCCTTGGGTTCTTAGGAGCAGCAGGAAGCACTATGGGCGCGGCGTC
AATAACGCTGACGGTACAGGTCAGACAATTATTGTCTGGCATAGTGCAACAGCAAAGCAATTTGCTGAGGGCT
ATAGAGGCGCAGCAGCATCTGTTGCAACTCACAGTCTGGGGCATT
>KT276254
TATCAGAATGATACTGGGAATAGTGTGAATAACACTGAAGACCTAATTACACTCCCATGTAAAATAAGACAAA
TTGTAAACATGTGGCAGGAAGTAGGAAAAGCAATGTATGCCCCTCCCATCAGAGGGCTGATTAATTGTTCATC
AAATATTACAGGGTTGCTATTAACAAGAGATGGTGGTAATGATACATCTGATAAGGAGACCTTCAGACCTGGA
GGAGGAGATATGAGAGACAATTGGAGAAGTGAATTATACAAATATAAAGTAGTAAAAATTGAACCATTAGGAG
TAGCACCCACCAAGGCAAAGAGAAGAGTGGTGCAGAGAGAAAAAAGAGCAATAGGAATGGGAGCTCTGTTCCT
TGGGTTCTTGGGAGCAGCAGGAAGCACTATGGGCGCAGCGTCGGTAACGCTGACGGTACAGGCCAGACAATTA
TTGTCGGGTATAGTGCAACAGCAGAACAACTTGCTGAGAGCTATTGAGGCGCAACAGCACCTGTTGCAACTCA
CAGTCTGGGGCATT
>JN882652
AACTACTCAGGGGAAAATGACACTATAACTCTCCAATGCAAAATAAAGCAAATTATAAAAATGTGGCAGAAAG
TAGGACAAGCAATGTATGCCCCTCCCATCCAAGGAGAAATAAGGTGTAACTCAAACATTACTGGAATACTATT
AACAAGAGATGGTGGCAATAATAATGCCACAAACACGAGCAAGGCCGAGATCTTCAGACCTGGAGGAGGAGAT

ATGAGGGACAATTGGAGAAGTGAATTATATAAATATAAAGTAGTAAAAATTGAGCCATTAGGAATAGCACCCA
CCAAGGCAAAGAGAAGAGTGGTGCAGAGAGAAAAAAGAGCAGTGGGAGTAATAGGAGCTATGTTCCTTGGGTT
CTTAGGAGCAGCAGGAAGCACTATGGGCGCGGCGTCAATAACGCTGACGGTACAGGCCAGACAATTATTGTCT
GGTATAGTGCAACAGCAGAACAATTTGCTGAGAGCTATTGAGGCGCAACAGCATCTGTTGCAACTCACAGTCT
GGGGCATC
>AJ276595
AGCTATAATAGTACAGAAAGTGTTAATGAAACAACCATCATACTCCCATGTAAAATAAAACAAATTATAAACA
TGTGGCAAGAGGTAGGACGAGCAATGTATGCCAATCCCATTGCAGGAAACATAACATGTAACTCAAATATCAC
AGGATTACTACTGACACGTGATGGAGGAGTAAATGAGACAACAGAGACAGAGATATTCAGGCCTGGAGGAGGA
AATATGAAAGACAATTGGAGAAGTGAATTATATAAATATAAAGTGGTAGAAATTAAACCATTGGGAGTAGCAC
CCACTAAAGCAAAAAGAAGAGTGGTGGAGAGAGAAAAAAGAGCAGTGGGAATAGGAGCTGTGTTCCTTGGGTT
CTTGGGAGCAGCAGGAAGCACTATGGGCGCAGCGTCAATAACGCTGACGGTACAAGCCAGACAACTATTGTCG
GGTATAGTGCAACAGCAAAGCAATTTGCTGAGGGCTATAGAGGCGCAACAGCATATGTTGCAACTCACGGTCT
GGGGCATT
>HQ225812
AATTATACATACATGCCTAATGGTACCATGCATAATGATACAACAGATAATTCAACCTTAATCACAATCCCAT
GCAGAATAAAACAAATTATAAATATGTGGCAGGAGGTAGGACGAGCAATGTATGCCCCTCCCATTGAAGGAAA
CATAACATGTAAATCAAAGATCACAGGACTACTATTGGTACGTGATGGAGGCCCCAATAGGACAGAAGAGATA
TTCAGACCTGGAGGAGGAGATATGAAAAACAATTGGGGAAATGAATTATATAAATATAAAGTGGTAGAAATTA
AGCCATTGGGAGTAGCACCAACTGAAGCAAAAAGGAGAGTAGTGGAGAGAGAAAAAAGAGCAGTGGGAATAGG
AGCTGTGTTCCTTGGGTTCTTGGGAGCAGCAGGAAGCACTATGGGCGCGGCGTCAATGACGCTGACGGTACAG
GCCAGACAGTTGTTGTCTGGTATAGTGCAACAGCAAAGCAATTTGCTGAGAGCTATAGAGGCGCAACAGCATA
TGTTGCAACTCACAGTCTGGGGCATT
>KY557336
ACTTACAGTAATTGGAAGTCTAATAGTACTGGGAATGATAATATCACACTCCCATGTAGAATAAGACAAATTG
TAAACATGTGGCAAGAAGTAGGAAAAGCAATGTATGCCCCTCCTATCAGCGGACAAATTAGATGCTCATCAAA
TATTACAGGGTTACTATTGACAAGAGATGGTGGTAATAATAAGGAAAGTAATAATGCTGAGACCTTCAGACCT
GGAGGAGGAAACATGAAGGACAATTGGAGGAGTGAATTGTATAAATATAAAGTAGTAAAAATTGAACCATTAG
GAGTAGCACCCACCAAGGCAAAGAGAAGAGTGGTGCAGAGAGAAAAGAGAGCAGTGGGAATAGGAGCTTTGCT
CGTTGGGTTCTTGGGAGCAGCAGGAAGCACTATGGGCGCAGCGTCAATGACGTTGACGGTACAGGCCAGACAA
CTACTGTCTGGTATAGTGCAACAGCAGAACAATCTGCTGAGAGCTATTGAGGCGCAACAGCATATGTTGCAAC
TCACAGTCTGGGGCATA
>KM606633
AACTATAGCTCATCAAGCAATAAGGAGCCTATCACACTCCCATGTAGAATAAGACAAATTGTAAGAATGTGGC
AAAAGGTAGCACAAGCAATGTATGCCCCTCCCATTGCAGGAAACATTACATGTAGAACAAACATTACAGGACT
ACTATTAACAAGAGATGGGCCTACTACGGAGGATAATAATACTGAGGTTTTCAGACCTGGAGGAGGAAATATG
AAGGACAATTGGAGAAGTGAATTATATAAGTATAAGGTAGTAAAAATTAAACCACTAGGCATAGCACCCTCCA
AGGCAAGGAGAAGAGTAGTGGGCAGAGAGAAAAGAGGAATTGGACTAGGAGCTGTTTTCCTTGGTTTCTTAGG
AACGGCAGGAAGCACGATGGGCGCAGCGTCAATAACGCTGACGGTACAGGTCAGACAATTATTGTCTGGCATC
GTGCAACAGCAAAGCAATTTGCTGAGAGCTATAGAGGCGCAACAGCATTTGTTGCAACTCACAGTCTGGGGCA
TT
>KJ541851
AATTATACAAGAAATAGTACAGGAAATGATTCAAACGTAGTCATTACGATCCCATGCAGAATAAAACAAATTA
TAAACATGTGGCAGGGAGTAGGGCGAGCAATGTATGCCCCTCCCATTGCAGGAGAAACCGCATCTATCTCTAA
TATCACAGGATTACTATTGGTACGTGATGGAGGAGAAGACAAAAATAATGAGAATGATAATGATACAGAGACA
TTCAGACCTGGAGGAGGAGACATGAAGGACAATTGGAGAAGTGAATTATATAAATATAAAGTGGTAGAAATTA
AGCCATTGGGATTAGCACCCACTACAGCAAAAAGGAGAGTGGTGGAGAGAGAAAAAAGAGCAGTAGGAATAGG
AGCTGTGTTCCTTGGGTTCTTGTCAGCAGCCGGAAGCACTATGGGCGCGGCGTCAATAACGCTGACGGTACAG
GCCAGACAATTGTTGTCTGGTATAGTGCAACAGCAAAGCAATTTGCTGAGGGCTATAGAGGCGCAACAGCATC
TGTTGCAACTCACGGTCTGGGGCATT
>AF484510
AATTATAGTATGTCAAATAGTACAGGGGTAAATGAGAATGCAACCATCATACTCCCATGCAGAATAAAACAAA
TTATAAACATGTGGCAGGGAGTAGGAAAAGCAATGTATGCCCCTCCCATTGAAGGACTGATCAATTGTACATC
AAATATTACAGGACTATTGTTGACAAGAGATGGTGGTATAACTAATGATAGTAGGAATGGGACCTTCAGACCT
GGAGGGGGAGATATGAGAGATAATTGGAGAAGTGAATTATACAAGTATAAAGTAGTAAGAGTTGAACCACTAG
GTCTAGCACCCACCAGGGCAAAGAGAAGAGTGGTGGAAAGAGAAAAAAGAGCAATAGGACTGGGAGCTCTGTT
CCTTGGGTTCTTGGGAGCAGCAGGAAGCACGATGGGCGCAGCGTCACTGACGCTGACGGTACAGGCCAGACAA
GTATTGTCTGGTATAGTGCAACAGCAAAACAATTTGCTGAGGGCTATAGAGGCGCAACAGCATCTGTTGCAAC
TCACAGTCTGGGGCATT
>DQ208433

AATCACACTGACAGCACGTCGAGGTCAGATAGCAATATGACTGGCAATATAACTCTCCTATGCAAAATAAAAC
AAATTATAAACATGTGGCAGGAAGCAGGAAAAGCAATGTATGCCCCTCCCATTGCAGGACTAATCAATTGTTC
ATCAAATATTACAGGACTATTACTAACAAGAGATGGTGGGATAATAGCAGTACAAATGAAACCTTCAGACCT
GGAGGAGGAGATATGAGAGACAATTGGAGAAGTGAATTATACAAGTATAAAGTAGTAAGAATTGAACCACTAG
GTCTAGCACCCACCAGGGCAAAGAGAAGAGTGGTGGAAAGAGAAAAAAGAGCAATAGGACTGGGAGCTATGTT
CCTTGGGTTCTTGGGAGCAGCAGGAAGCACGATGGGCGCAGCGTCACTGGCGCTGACGGTACAGGCCAGACAG
TTATTGTCTGGTATAGTGCAACAGCAAAACAATTTGCTGAGGGCTATAGAGGCGCAACAACATCTGTTGCAAC
TCACAGTCTGGGGCATT
>GQ365651
TACTACTTTAATGGTAACAGTACATACAATGATACCAATACTGGACCAAATATCACACTCCCATGCAGAATAA
AACAAATTATAAACATGTGGCAGGAAGTAGGAAAAGCAATGTATGCCCCTCCCATCAGAGGACAAATTAGATG
TTCATCAAATATTACAGGGCTGATATTAACGAGAGATGGTGGTGTGAACAACGCTGAGAACAACACTGAGACC
TTCAGACCTGGAGGAGGAGATATGAGGGACAATTGGAGAAGTGAACTATATAAATATAAAGTGGTAGAAATAA
AACCATTAGGAATAGCACCAACTAAGGCAAAAAGGAGAGTGGTGGAGAGAGAAAAAAGAGCAGCACTAGGAGC
TATGTTCCTTGGGTTCTTGGGAGCAGCAGGAAGCACTATGGGCGCAGCGTCAATAACGCTGACGGTACAGGCC
AGACAATTACTGTCTGGGATAGTGCAACAGCAAAGCAATTTGCTGAGAGCTATAGAGGCGCAACAGCATATGT
TGCAACTCACAGTCTGGGGCATT
>KF835524
AATTACAATGAGAATTCAAGCCTCATCACAATTCCATGCAAAATTAAGCAAATTATAAACATGTGGCAGGAGG
TAGGACGAGCAATGTATGCCCCTCCCATTGCAGGAAATATAACATGTAAATCAAATATCACAGGACTACTATT
GGTGCGTGATGGAGGAGTGACAAATACAAATGAGACAGAGATATTCAGACCTGGAGGAGGAGATATGAGGAAT
AATTGGAGAAGTGAATTATATAAATATAAAGTAGTAGAAATTAAGCCATTGGGAATAGCACCCACTGAAGCAA
AAAGGAGAGTGGTGGAGAGAGAAAAAAGAGCATTAGGGGGAATAGGAGCTGCGTTCCTTGGGTTCTTGAGTTT
AGCCGGAAGCACTATGGGCGCGGCGTCACTAACGCTGACGGTACAGGCCAGACAATTGCTGTCTGGTATAGTG
CAACAGCAAAGCAATTTGCTGAGGGCTATAGAGGCGCAACAGCATATGTTGCAACTCACGGTCTGGGGCATT
>KC898982
GATCAAGATGAGTCAAACCCAACCATCATAATCCCATGCAGAATAAAGCAAATTATAAACATGTGGCAGGAGG
TAGGACGAGCAATGTATGCCCCCCCCATTGCAGGAAACATAACATGTAAATCAAATATCACAGGACTACTATT
GGTACGTGATGGAGGAGACAGAAATGACACAAATAATACAGAGAAATTCAGACCTGGAGGAGGAGATATGAGG
GACAATTGGAGAAGTGAATTATATAAATATAAAGTGGTAGAAATTAAGCCATTGGGAATAGCACCCACTGAAG
CAAAGAGGAGAGTAGTGGAGAGAGAAAAAAGAGCAGTGGGAATAGGAGCTGTGTTCCTTGGGTTCTTGGGAGC
AGCAGGAAGCACTATGGGCGCGGCGTCAATAACGCTGACGGTACAAGCCAGACAATTGTTGTCTGGTATAGTG
CAACAGCAAAGCAATTTGCTGAGGGCTATAGAGGCGCAACAGCATATGTTGCAACTCACAGTCTGGGGCATT
>HM215402
AGTCATTCAAATTCAATCATCAGGCTCCCATGCAGAATAAAACAAATTATAAACATGTGGCAGAAGGTAGGCC
GAGCAATGTATGCCCCTCCCATTGAAGGAAACATAACATGTAAATCAAACATCACAGGACTACTATTGGTACG
TGATGGAGGAGAACCTAATAATATAGAGATATTCAGACCTGGAGGAGGAGATATGAGGGACAATTGGAGAAGT
GAATTATATAAATATAAAGTAGTGAAAATTGAACCATTAGGAGTAGCACCCACCAAGGCAAAAAGAAGAGTGA
TGCAGAAAGAAAAAAGAGCAGTGGGAATAGGAGCTGTGTTCCTTGGGTTCTTGGGAGCAGCAGGGAGCACTAT
GGGCGCGGCGTCAGTAACGCTGACGGTACAAGCCAGACAATTGTTGTCTGGTATAGTGCAACAGCAAAGCAAT
TTGCTGAGGGCTATAGAGGCGCAACAGCATATGTTGCAACTCACAGTCTGGGGCATC
>KC899013
ACCTACAATGGTACAAGAAGTAATTCAAGCTCAATCATCACTATCCCATGCAGAATAAAGCAAATTATAAACA
TGTGGCAGGGGGTAGGACGAGCAATGTATGCCCCTCCCATTGAAGGAAACATAACATGTAAATCAAATATCAC
AGGACTACTCTTGGTACGTGATGGAGGAATAGGGACACATGGTAAAGAGACATTCAGACCTGGAGGAGGAAAT
ATGAGGGACAATTGGAGAAGTGAATTATATAAATACAAAGTAGTAGAAATTAAGCCATTGGGAATAGCGCCCA
CTACAGCAAAAAGGAGAGTAGTGGAGAGAGAAAAAAGAGCAGTGGGAATAGGAGCTGTGTTCCTTGGGTTCTT
GGGAGCAGCAGGAAGCACTATGGGCGCAGCGTCAATAACGCTGACGGTACAGGCCAGGCAATTGTTGTCTGGT
ATAGTGCAACAGCAAAGCAATTTGCTGAAGGCTATAGAGGCGCAACAGCATATGTTGCAACTCACGGTCTGGG
GCATT
>KC899014
AATCATAAGACTAATAGTACAGAAAGTAATTCAAGCCCAGACATCATAATCCCATGCAGAATAAAACAAATTA
TAAACATGTGGCAGGAGGTAGGACGAGCCATGTATGCCCCTCCTATTGCAGGAAACATAACATGTAAATCAAA
TATCACAGGACTACTATTAGTACGGGATGGAGGACTATATCCAAATAAGACTGAGACATTCAGACCTGGAGGA
GGAGATATGAGGGACAATTGGAGAAGTGAATTATATAAATATAAAGTGGTAGAAATTAAGCCATTGGGAGTAG
CACCCACTAAAGCAAAAAGGAGAGTGGTGGAGAGAGAAAAAAGGGGAATAGGAGCTGTGTTCCTTGGGTTCTT
GGGAGTAGCAGGAAGCACTATGGGCGCGGCGTCAATAACGCTGACGGTACAGGCCAGACAATTGTTGTCTGGT
ATAGTGCAACAGCAAAGCAATTTGCTGAGGGCTATAGAGGCGCAACAGCATATGTTGCAACTCACGGTCTGGG
GCATT
>KC807958

TACTACCGCCTTAATGGTACATACGAGCCTAATGATATAAGCAATGAGTCAGACATCACACTCCCATGCAGAA
TAAAGCAAATTATAAATATGTGGCAGGAGGTAGGACGAGCAATGTATGCCCCTCCCATTGCAGGAAACATAAC
ATGTAGATCAAATATCACAGGACTACTATTGGTACGGGATGGAGGAAATGTTACAGGAGATACAGAGATATTC
AGACCGGGAGGAGGAGATATGAGGGACAATTGGAGAAGTGAATTATATAAATATAAAGTAGTAGAAATTAAGC
CATTGGGAGTAGCACCCACTGCAGCAAAAAGGAGAGTGGTGGAGAGAGAGAGACAAAAAAGAGCAGTGGGACT
AGGAGCTGTGTTCCTTGGGATCTTGGGAGTAGCAGGAAGCACTATGGGCGCGGCGTCAGTGACGCTGACGGTA
CAGGCCAGAAAATTGCTGTCTGGTATAGTGCAACAGCAAAGCAATTTGCTGAGGGCTATAGAGGCGCAACAGC
ATCTGTTGCAACTCACGGTCTGGGGCATT
>MK792289
TACTACAATAGTACATACCATACATACATAAATGATACAAAAAGTAATGAGACCATCACGATCCCATGCAAAA
TAAAGCAAATTATAAATATGTGGCAGGAGGTGGGACGAGCAATGTATGCTCCTCCCATTGCCGGAAACATAAC
ATGTAGGTCAGATATCACAGGACTGCTATTAGTACGTGATGGAGGAAATGAGCCAAATAGTACAAATGGTGCA
GAAGAAATATTCAGGCCTGGAGGAGGAAATATGAAGGATAATTGGAGAAGTGAACTATATAAATATAAAGTAG
TAAAAATTGAACCATTAGGAGTAGCACCCACCAAGGCAAGGAGGAGAGTGGTGCAGAGAGAAAAAAGAGCAGT
GGGAACGATAGGAGCTATGTTCCTTGGGTTCTTGGGAGCAGCAGGAAGCACTATGGGCGCAGCATCAATAACG
CTGACGGTACAGGCCAGACAATTGTTGTCTGGTATAGTGCAACAGCAGAGAAATCTGCTGAGAGCTATTGAGG
CGCAACAGCATCTGTTGCAACTCACAGTCTGGGGCATC
>FJ388961
AATCATACTAGGCCAATTAACACAGGTGACACTGAAGGAGGAATTATCACCACACCTACATGCAGAATAAAAC
AAATTATAAACCTGTGGCAGGAAGTAGGAAAAGCAATGTATGCCCCTCCCATCGAAGGACAAATTAGTTGTTC
ATCAAATATTACCGGGTTGCTATTAACAACAGATGGGGGTACTAACGGAGCAACGAGACCGAGGTCTTCAGA
CCTGCAGGAGGAAATATGAAGGACAATTGGAGAAGTGAATTATACAAATATAAAGTGGTACAAATTGAACCAT
TAGGAATAGCACCCACCACGGCAAAGAGAAGAGTGGTGCAGAGAGAAAAAAGAGCAGTGGGAATAGGAGCTAT
GTTCCTTGGGTTCTTGGGAGCAGCAGGAAGCACTATGGGCGCAGCATCAGTGATGCTGACGGTACAGGCCAGA
CAATTATTGTCTGGCATAGTGCAACAGCAAAACAATTTGCTGAGGGCTATTGAGGCGCAACAACATCTGTTGC
AACTCACAGTCTGGGGCATC
>KU820850
AATTATACAACAGGTAATTCAAGCTCAGTCATCACACTCCCATGCAGAATAAAGCAAATTATAAACATGTGGC
AGGAGGTAGGACGAGCAATGTATGCCCCTCCCATTGCAGGAAACATAACATGTAAATCAAATATCACAGGATT
ACTATTGGTACGTGATGGAGGAGACACAAGTAATACAACTGAAACATTCAGACCTGGAGGAGGAAATATGAAG
GACAATTGGAGAAATGAATTATATAAATACAAAGTAGTAAAAATTGAACCATTAGGAGTAGCACCCACCAAGG
CAAAGAGAAGCGTGGTGCAGAGAGAAAAAAGAGCAGTGGGAATGGGAGCTGTGTTCCTTGGGTTCTTAGGAGC
AGCAGGAAGCACTATGGGCGCGGCGTCAATAACGCTGACGGTACAGGCCAGACAATTGCTGTCTGGTATAGTG
CAACAGCAAAGTAATTTACTGAGGGCTATAGATGCGCAACAGCATATGTTGCAACTCACAGTCTGGGGCATC
>KF725906
AGTTATAATTCAAATGAGACCATCACACTCCCATGCAGAATAAAACAAATTATAAACAGGTGGCAGGAAGTAG
GAAAAGCAATGTACGCCCCTCCCATCCAAGGAGTAATTAGGTGTGCATCAAATATTACAGGGCTGCTATTAAC
AAGAGATGGGGGCAGTAATATTAAGAACAACGGTACTGAAACCTTCAGACCTGGAGGAGGAAATATGAGGGAC
AATTGGAGGAGTGAATTATATAAATATAAAGTAGTAAGAATTGAACCATTAGGAGTAGCACCCACCAAGGCAA
AGAGAAGAGTGGTGCAGAGAGAAAAAAGAGCAGCGATAGGAGCTATGATCCTTGGGTTCTTGGGAACAGCAGG
AAGCACTATGGGCGCAGCGTCAGTGACGCTGACGGTACAGGCCAGACTATTATTGTCTGGTATAGTGCAACAG
CAAAATAATTTGCTGAGAGCTATTGAGGCGCAACAACATCTGTTGCAACTCACAGTCTGGGGCATC
>KY496623
AGTCATCTGTTTAATAGTAGTCTGTCTAATAGTAGTAATAGTAGTAAGTATATCACAATTCCATGTAAGATAA
AACAAATTGTAAGAATGTGGCAGAGAGTGGGACAAGCACTGTATGCCCCTCCCATCGAAGGAAACATTACATG
TAGCTCAAACATTACAGGACTACTATTAGTTAGAGATGGAGGAAGCAATATAACAATCAATGAGACTTTCAGA
CCTGCAGGAGGAGACATGAGGGATAATTGGAGAAGTGAATTATATAAATATAAAGTAGTAAAAATTAAACCAC
TAGGAGTAGCACCCACCAGGGCAAGGAGAAGAGTGGTGGGAGAGAAAAAAGAGCAGTTGGACTGGGAGCTGT
CCTCCTTGGGTTCTTAGGAACAGCAGGAAGCACTATGGGCGCAGCGGCAGTAACGCTGACGGTACAGGTCAGA
CAACTATTGTCTGGCATAGTGCAACAGCAAAGCAATTTGCTGAGGGCTATAGAGGCGCAGCAGCATATGTTGC
AACTCACCGTCTGGGGCATT
>DQ383755
AACTACACGAAAGAAAATGACACAATCAGACTCCCATGTAGAATAAAACAGATTATAAACATGTGGCAGGAAG
TAGGAAAAGCCATGTATGCCCCTCCCATCAGAGGACTAATTAGGTGTTCATCAAATATTACAGGGCTGCTATT
AACAAGAGATGGTGGTAATGATAACACCACGAACGAGTCTGAAATTTTCAGACCTGGAGGAGGAGATATGAGG
GACAATTGGAGAAGTGAATTATATAAATACAAGGTAGTAAGAATTGAGCCAGTAGGAGTAGCACCCACCAAGG
CAAAGAGAAGAGTGGTGCAGAGAGAAAAAAGAGCAGTGGGAGTGATAGGAGCTATGTTCCTTGGGTTCTTGGG
AGCAGCAGGAAGCACTATGGGCGCAGCGTCAATGACGCTGACGGTACAGGCCAGACAATTATTGTCTGGTATA
GTGCAACAGCAAAACAATTTGCTGAGAGCTATTGAGGCGCAACAGCATCTGTTGCAACTCACAGTCTGGGGCA
TC

>KT427679
GGTCAGTGGAATAGCACTGGGAATAATACTGAAGGGTCAAATGGAACCATCACAATCCCATGCAGAATAAAAC
AAATTATAAACAGATGGCAGGAAGTAGGAAGAGCAATGTACGCCCCTCCCATCGCAGGACAAATTAACTGTTC
ATCACAAATTACAGGGTTACTGTTAACAAGAGATGGTGGTACCAATACTACCAGTCCTGAAGAAATCTTCAGA
CCTGCAGGAGGAGATATGAGGGACAATTGGAGAAGTAAATTATATAAATATAAAGTAGTAAAAATTGAGCCAT
TAGGAATAGCACCCACCAAGGCAAAGAGAAGAGTGGTGCAGAGAGAAAAAAGAGCAATGGGAGTACTAGGAGC
TGCGGTCCTTGGGTTCCTGGGAGCAGCAGGAAGCACTATGGGCGCAGCGTCACTGACGCTGACGGTACAGGCC
AGACTATTATTGTCTGGTATAGTGCAACAGCAGAGCAATCTGTTGAGAGCTATTGAGGCGCAGCAGCATATGT
TGCAACTCACAGTCTGGGGCATC
>KT427801
AATTACAATAGCACTGAGAATATCACTCTCCCATGTCGAATAAAACAAATTGTAAACATGTGGCAGGAAGTGG
GGCGAGCAATGTATGCCGCTCCCCTTGCAGGAAACATTAACTGTAGTTCAAGTATTACAGGGCTACTATTAAC
AAGAGATGGTGGTAACAAGAAAGACAATGAAACAGAAAAAGAGACCTTCAGACCTGGAGGAGGAAATATGAAA
GATAATTGGAGAAGTGAATTATATAAATATAAAGTAGTAGAAATTAAGCCATTAGGAATAGCACCCACCAAGG
CACAGAGAAGAGTGGTGCAGAGGGAAAAAAGAGCAGTGACATTCGGAGCTATGTTCCTTGGGTTCTTGGGAGC
AGCAGGAAGCACTATGGGCGCAGTGTCAATGACGCTGACGGTACAGGCCAGACAATTATTGTCCGGTATAGTG
CAACAGCAGAACAATCTGCTGAGGGCTATTGAGGCGCAACAGCATATGTTGCAACTCACAGTCTGGGGCATC
>MG365765
ACTCATAATGACACTATCACTCTCCCATGTCGAATAAAACAAATTGTAAACCTGTGGCAGGAAGTAGGACGGG
CTATGTATGCCAATCCCATTGCAGGACAAATTAACTGTACATCAAATATTACAGGTCTACTATTGACAAGAGA
TGGTGGTAATAGTAGTAAGACTGAAATCTTCAGACCTGAGGGAGGAAATATGAAAGACAATTGGAGAAGTGAA
TTATATAAATACAAAGTAGTAGAAATTGAACCACTAGGAATAGCACCCACCAGGGCAAAAAGGCGAGTGGTGG
AGAGAGAAAAAAGAGCAGTGGGACTAGGAGCTTTGTTCCTTGGGTTCTTGGGAACAGCAGGAAGCACTATGGG
CGCAGCGTCAATAACGCTGACGGTACAAGCCAGACAATTATTGTCTGGAATAGTGCAACAGCAGAGCAATTTG
CTGAGGGCTATTGAAGCGCAACAGCATCTGTTGCAGCTCACAGTCTGGGGCATT
>DQ085870
GCTCACACTTGGAATAGAAATGGGAATGGTACTAATAGTACTATCACACTCCCATGCAGAATAAAACAAATTA
TAAACAGGTGGCAGGAAGTAGGAAAAGCAATGTATGCCCCTCCCATCCAAGGACAAATTAGATGTTCATCAAA
TATTACAGGGTTGTTATTAACAAGAGATGGAGGTAACAACAACGGGACCGAGAACAATACCGAGATCTTCAGA
CCTGGAGGAGGGAATATGAGGGATAATTGGAGAAGTGAATTATACAAATATAAAGTAGTAAGAATTGAGCCAT
TAGGAGTAGCACCCACCAAGGCAAAGAGGAGAGTGGTGCAGAGAGAAAAGAGAGCAGTGGGAATAGGAGCTGT
GTTCCTTGGGTTCTTGGGAGCAGCAGGAAGCACTATGGGCGCAGCGTCAATAACGCTGACGGTACAGGCCAGA
CAATTATTGTCTGGTATAGTGCAACAGCAGAACAATCTGCTGAGAGCTATTGAGGCGCAACAACATATGTTGC
AACTCACAGTCTGGGGCATC
>AB253430
AATTATAACAATGGGAATAATACTGGTGGTAATATCACACTCCCATGCAGAATAAAACAAATTGTAAACAGGT
GGCAGGAAGTAGGAAAAGCAATGTATGCCCCTCCCATCAGAGGAAACATTACCTGTTCATCAAATATCACAGG
GCTGCTATTAACAAGAGATGGTGGTAACAACGTTACCAACGAGTCCGAGATTTTCAGACCTGGAGGAGGAAAT
ATGAAGGACAATTGGAGAAGTGAATTATATAAATATAAAGTAGTAAAAATTGAACCATTAGGAGTAGCACCCA
CCAGGGCAAAGAGAAGAGTGGTGCAGAGAGAAAAAAGAGCAGTGGGAACAATAGGAGCTATGTTCCTTGGGTT
CTTGGGAGCAGCAGGAAGCACTATGGGCGCAGCGTCTTTGACGCTGACGGTACAAGCCAGACAATTATTGTCT
GGTATAGTGCAACAGCAAAACAATTTGCTGAGAGCTATTGAGGCGCAACAGCATCTGTTGCAACTCACAGTCT
GGGGCATC
>KC699033
AATCATACTGAGCAAAATATCACACTACAATGTAGAATAAAACAAATTGTAAACCTGTGGCAGACAGTAGGAA
GAGCAATATATGCCCCTCCCATCAAAGGACAAATTAGGTGTTTATCAAACATTACAGGGCTGCTATTAACAAG
AGATGGTGGTAACGAGAACTCCACCGACAATGACACCGAGACCTTCAGACCTATAGGAGGGAATATAAGGGAC
AATTGGAGAAGTGAATTATATAAATATAAAGTAGTAAAAATTGAACCATTAGGAGTGGCACCCACCAGGGCAA
GGAGAAGAGTGGTGCAGAGAGAAAAAAGAGCAGCAGGACTAGGAGCTGTGTTCATTGGGTTCTTAGGAGCAGC
AGGAAGCACTATGGGCGCAGCATCAATAACGCTGACGGTACAGGCCAGACAATTATTGTCTGGTATAGTGCAA
CAGCAGAACAATCTGCTGAGAGCTATTGAGGCGCAACAGCATCTGTTGCAACTCACAGTCTGGGGCATC
>KX907421
GACTATCCTAATGGTACAATCATCACACTTCCATGCAAAATAAAACAAATTATAAACATGTGGCAGGCGATAG
GAAAAGCAATATATGCCCCTCCCATTGCAGGAAACATAACATGTGTATCAAATATCACAGGACTATTATTGAC
ACGAGATGGAGGAAGTGGAAATGGTACGGACCCAGAGATATTCAGACCTGCAGGAGGAGACATGAGGGATAAT
TGGAGAAGTGAATTATATAAATATAAAGTAGTAGAAATTAAGCCATTAGGAGTAGCACCCACTAAGGCACAAA
GGAGAGTGGTGAAGAGAGAGAGAGAAGAGCAGTGGGAATAGGAGCTGTGCTCTTTGGGTTCTTGGGAGCAGCAGG
AAGCACTATGGGCGCAGCGTCAATAACGCTGACGGTACAGGCCAGACAATTATTGTCTGGTATAGTGCAACAG
CAAAGCAATTTGCTGAGAGCTATAGAGGCGCAACAGCATATGTTGCAACTCACAGTCTGGGGCATT
>KX907359

AATTATACATGGAATAATAGTACAGGGCTAGGTAATATCACAAGTAATATCACACTACCATGTAGAATAAAAC
AAATTATAAACATGTGGCAGGGAGTAGGAAAAGCAATGTATGCCCCTCCCATTGAAGGACAAATTAAATGTTC
ATCAAATATTACAGGGCTAATATTGACAAGAGATGGTGGTAATACAAGTGATGATCCTGAGACCTTCAGACCT
GGAGGAGGAAATATGAAGGACAATTGGAGAAGTGAATTATATAAATATAAGGTAGTAAGAATTGAACCAATAG
GCCTAGCACCCACCAAGGCAAAGAGAAGAGTGGTGGAAAGAGAAAAAAGAGCAATAGGACTAGGAGCTATGTT
CCTTGGGTTCTTGGGAGCAGCAGGAAGCACGATGGGCGCAGCGTCAGTGGCGCTGACGGCACAGGCCAGACAG
TTATTGTCTGGTATAGTGCAACAGCAAAACAATTTGCTGAGGGCTATAGAGGCGCAACAGCATCTGTTGCAAC
TCACGGTCTGGGGCATT
>DQ054367
AATCAGACAGGGTTAAATAATAGTACAATCACACTCCAATGCAGAATAAAACAAATTATAAACATGTGGCAAG
AAGTAGGAAGAGCAATGTATGCCCCTCCCATTGAAGGAGTAATCAAATGTTCATCAAACATTACAGGGCTAAT
ATTGACAAGAGATGGTGCAAATAATAATACGAATGAGACTTTCAGACCTGGAGGAGGAAATATGAAAGACAAT
TGGAGAAGTGAACTATATAAATATAAAGTAGTACAAGTTGAACCAATAGGACTAGCACCTACCAAGGCAAAGA
GAAGAGTGGTGGAAAGGGAAAAAAGAGCAATAGGGCTAGGAGCTATGTTCCTTGGGTTCTTGGGAGCAGCAGG
AAGCACAATGGGCGCAGCGTCAGTGACGCTGACGGTACAGGCCAGACAGTTATTGTCTGGTATAGTGCAACAG
CAAAACAATTTGCTGAGGGCTATAGAGGCGCAACAGCATCTGTTGCAACTCACGGTCTGGGGCATT
>AY352654
AGCTATGACATAAGTAACACAACAGATAAGAATATTACACTGCCATGTAGAATAAAACAAATTGTACACATGT
GGCAGAGAGTAGGACAAGCAATGTATGCCCCTCCCATTGAAGGAAAGATTACATGTACATCAAATATTACAGG
ACTAATATTGACAATTGACCATGTTAATCTGACAGGAAACAATGATAATGTTACCCTCAGACCTGGAGGAGGA
GATATGAGGGACAATTGGAGAAGTGAACTATATAAATATAAAGTAGTAAAAATTGAACCACTAGGAATAGCAC
CCTCCGAGGCAAGGAGAAGAGTGGTGCAGAGAGAAAAAAGAGCAGTGGCACTGGGAGCTATGATCTTTGGGTT
CTTGGCAGCGGCAGGAAGCACTATGGGCGCGGCGTCATTAACGCTGACGGTACAGGCCAGACAATTATTGTCT
GGTATAGTGCAACAGCAAAGCAATTTGCTGAGAGCTATACAAGCGCAACAGCATATGTTGCAGCTTACTGTCT
GGGGCATT
>AY231154
AATTATAGGAGTTTGATTAAGGCTAATATCATACTCTCATGTAGAATAAAACAAATTGTAAGAATGTGGCAGA
GGGTAGGACAAGCAATATATGCCCCTCCCATCGCAGGGAACCTTACATGTACATCAAATATTACAGGAATGAT
ACTAACATGGGATGGTGGTAGTAATAATAGTAGTAAAAATGAATCTGAGACCTTCAGACCTGGAGGAGGAGAC
ATGAAGGACAATTGGAGAAGTGAATTGTATAAATATAAAGTAGTAAAGATTGAACCACTAGGAATAGCACCCA
CCAGGGCAAGAAGAAGAGTGGTGCAGAGAGAAAAAAGAGCAGTGGGAATAGGAGCTTTGTTCCTTGGGTTCTT
GGGAACAGCAGGAAGCACTATGGGCGCGGCGTCAATAACGCTGACGGTACAAGCCAGACAATTATTGTCTGGC
ATAGTGCAACAGCAAAGCAATTTGCTGAGAGCTATAGAAGCGCAACAACAACTGTTGCAGCTCACGGTCTGGG
GCATT
>MN617383
TTTCATAAGAGAACTTGGTTGTCTAATAATACTGGGCAAGATACAGAGAACATCACACTCCAATGTAGAATAA
AACAAGTTATAAACATGTGGCAGGAAGTAGGAAAAGCAATGTATGCCCCTCCCATCAGTGGACAAATTAGCTG
TTCATCAAATATTACAGGGCTGCTATTAACAAGAGATGGTGGTAACAAGAACGAGTCCAATGGAACTGAAACC
TTCAGACCTGGAGGAGGAGATATGAGGGACAATTGGAGAAGTGAATTATATAAATATAAAATAGTAAAAATTG
AGCCATTAGGACTAGCACCCACCAAGGCAAGGAGAAGAGTGGTGCAGAGGGAAAAAAGAGCAGTGGGAGCATT
AGGAGCTATGTTCCTTGGGTTCTTGGGAACAGCAGGAAGCACTATGGGCGCAGCGTCACTCACGCTGACGGTA
CAAGCCAGAATACTACTGTCTGGTATAGTGCAACAACAGAACAATTTGCTGAAAGCTATTGAGGCGCAACAGC
GTCTGTTGCAACTCACAGTCTGGGGCATC
>MN617384
AATCAACTGAATAGCAGTGAAACTGAAGGCAATGACACAATCACACTCCCATGTAGAATAAAGCAAATTGTAA
ACATGTGGCAGGAAGTAGGAAAAGCAATGTATGCCCCTCCCATCAAAGGACAACTTAACTGCTCATCACAGAT
TACAGGGCTAATATTAGTAAGAGATGGTGGTAGTAGTGGCAATAATACCACTGAGATCTTCAGACCTGGAGGA
GGAGACATGAGGGACAATTGGAGAAGTGAATTATATAAATATAAAGTAGTAAAAATTGAACCGTTAGGAATAG
CACCCACCAAGGCAAAAAGAAGAGTGGTGCAAAGAGAAAAAAGAGCAGCGGGAATAGGAGCTCTGTTCCTTGG
GTTCTTGGGAGCAGCAGGAAGCACTATGGGCGCAGCATCGATGACGCTGACGGTACAGGCCAGACAATTATTG
TCTGGTATAGTGCAACAGCAAAACAACTTGCTGAGGGCTATTGAGGCGCAACAACATATGTTGCAACTCACAG
TCTGGGGCATC
>MN617386
AATCAGACCGTCAATGGAACTGATGTCATCACACTCCCATGCAGAATAAAACAAATTATAAACAGATGGCAGG
AAGTAGGAAAAGCAATGTATGCTCCTCCCATCAGGGGACAAATTAACTGTACATCAAATATTACAGGGCTGCT
ATTAACAAGAGATGGTGGTAACGGTAACGAGACCGGCTCCAACAGGTCCGAAATCTTCAGACCTGGAGGAGGA
GATATGAGGGACAATTGGAGAAGTGAATTATATAAATATAAAGTAGTAAAAATTGAGCCATTAGGAATAGCAC
CCACCAAGGCTAAGAGAAGAGTGGTGCAGAGAGAAAAAAGAGCAGTGGGAACTATAGGAGCTATGTTCCTTGG
GTTCTTGGGAGCAGCAGGAAGCACTATGGGCGCAGCGTCACTCACGCTGACGGTACAGGCCAGACTATTACTG

TCTGGTATAGTGCAACAGCAGAACAATTTGCTGAAAGCTATTGAGGCGCAACAGCACCTGTTGCAACTCACAG
TCTGGGGCATT
>MN617388
AATTATAATTTGACTAATGGTACTTGGCAATGGTCAAATAACGAAAGTGTCATCACACTCCCATGCAGAATAA
AACAAATTATAAACAGGTGGCAGGAAGTAGGAAAAGCAATGTATGCCCCTCCCATCAGGGGACAAATTAACTG
TACATCAAATATTACAGGGCTGCTATTAACAAGAGATGGTGGTGGTAATGAGTCCAGTGAGTTCGAAATCTTC
AGACCTGGAGGGGGAGATATGAGAGACAATTGGAGAAGTGAATTATATAAATATAAAGTAGTAAAAATTGAGC
CACTAGGGATAGCCCCCACCAAGGCAAAGAGAAGAGTGGTGCAGAGAGAAAGAAGAGCAGTGGGAACAATAGG
AGCTATGTTCCTCGGGTTCTTGGGAGCAGCAGGAAGCACTATGGGCGCAGCGTCGATGACGCTGACGGTACAG
GCCAGACTATTACTGTCTGGTATAGTGCAACAGCAGAACAATCTGCTGAGAGCTATTGAGGCGCAACAGCAAC
TGTTGCAACTCACAGTCTGGGGCATC
>AJ401043
GATCACTCTGGAAAGTCAAATGAAACAAATTCAAATGACACTATTGTACTCCCATGTAAAATAAAACAAATTG
TAAGAATGTGGCAGGGAGTAGGACAAGCAATGTATGCCCCTCCAATTAAAGGAAAGATTTCCTGTAACTCAAA
TATTACAGGACTAATATTGACAAGGGATGGTGGTAATACTACTAATACTATTGAGACCTTTAGACCTGGAGGA
GGAGATATGAGGGACAATTGGAGAAGTGAACTATATAAATATAAGGTAGTAAGAATTGAACCACTAGGAATAG
CACCCACTAAGGCAAGGAGACGAGTGGTGGAGAGAGAAAAAATTAGACAAAAAAGAGCAGTGGGAATGGGAGC
CTTGTTCCTTGGGTTCTTGGGAGCAGCAGGAAGCACTATGGGCGCGGCGTCACTGACGCTGACGGTACATGAT
AGACAAGTATTGTCTGGTATAGTGCAACAGCAAAACAATTTGCTGAGAGCTATAGAGGCGCAACAGCATCTGT
TGCAACTCACAGTCTGGGGCATT
>HQ595767
TTCTATTGTAACACATCAGCTCTGTTTAATGGCACTTGGGGGGACAATACCACCATGCAGGGAAATAGCACAG
AGTTAAATGGAACTATAACTCTCCCATGCAGAATAAAGCAAATCATAAATATGTGGCAGAAAACAGGAAAAGC
AATGTATGCCCCTCCCATCCAAGGAGTAATAAGGTGTGAGTCAAACATTACAGGACTTATATTAACAAGAGAT
GGTGGGGATACTAACAGAACAAATGAAACCTTTAGACCTGAAGGAGGAAATATGAAGGACAATTGGAGAAGTG
AATTATATAAGTATAAAGTAGTAAAAATTGAACCACTAGGAGTAGCACCTACCGAGGCAAGGAGGAGAGTGGT
GCAGAGAGAAAAAAGAGCATTTGGACTGGGAGCTGTTTTCATTGGGTTCTTAGGAGCAGCAGGAAGCACTATG
GGCGCGGCGTCAATGACGCTGACGGTACAGGCTAGACAATTATTGTCTGGCATA
>MF373125
TTCTACTGTAATACATCAAAACTGTTTAACAATACTTGGTTTAATGGTACTTGGGTTAATGATACTTGGGATA
CTGAGGGTGATAATATCATACTCCCTTGTAGAATAAAACAAATTATAAACATGTGGCAGGAAGTAGGAAAAGC
AATGTACGCCCCTCCCATTAGAGGACAAATTAGTTGTTCATCAAATATTACAGGGCTACTATTAACAAGAGAT
GGTGGTAACCACAGCGAGAGTAATAATACCTCAGAGATCTTCAGACCTGGAGGAGGAGATATGAGGGACAATT
GGAGAAGTGAATTATATAAATATAAAGTAGTAAAAATTGAACCATTAGGAATAGCACCCACCAAGGCAAAGAG
AAGAGTGGTGCAGAGAGAAAAAAGAGCGGTGACACTAGGAGCTATGTTCCTTGGGTTCTTGGGAGCAGCAGGA
AGCACTATGGGCGCAGCGTCGATGACGCTGACGGTACAGGCCAGACTATTATTGTCTGGTATAGTGCAACAGC
AAAACAATTTGCTGAGAGCTATTGAGGCGCAGCAACACATG
>JN024344
TTCTACTGTAATGCATCACAACTGTTTAATAGTAGTTGGAGTGGGAATGACTCTACTTGGAATATAGAGGCAA
ATGAAACTATCACACTCCGATGTAATATAAGACAAATTATAAACATGTGGCAGGAAGTAGGGAAAGCAATGTA
TGCCCCTCCCATCAGAGGACTAATTAGATGTTCATCAAACATTACAGGGCTGTTATTAACAAGAGATGGTGGT
AATGGTAATAACACCAATAATGACACCGAAATCTTTAGACCTGGAGGAGGAGATATGAGGGACAATTGGAGAA
GTGAATTATATAAATATAAAGTAGTAAGAATTGAACCATTAGGAATAGCACCCACTAAGGCAAAGAGAAGAGT
GGTGCAGAGAGAAAAAAGAGCAGTGGGACTAGGAGCTATGTTCCTTGGGTTCTTGGGAGCAGCAGGAAGCACT
ATGGGCGCAGCATCAATAACGCTGACGGTACAGGCCAGACTATTATTGTCTGGTATAGTGCAACAGCAGAACA
ATCTGCTGAGGGCTACTGAGGCGCAACAGCATCTGTTGCAACTCACAGTCTGGGGCATC
>KC894082
TTCTATTGCAATACATCAGAACTGTTTAATGGTACATACAATGGTACATCAGAAAATAATACTAACGAGGTGA
TCACACTCCAATGCAAAATAAAACAATTTATAAACATGTGGCAGGAGATAGGAAGAGCAATGTATGCCCCTCC
CATTGAAGGAAACATAACATGTAAATCAAACATCACAGGGCTACTATTGACACGTGATGGAGGAGGGGACACA
AACGAGACTGAGACATTCAGACCTGGAGGAGGAGATATGAGGGACAATTGGAGAAGTGAATTATATAAATATA
AAGTGGTAGAAATTAAGCCATTAGGAGTAGCACCCACTAATGCAAAAAGGAGAGTGGTACAGAGAGAGAAAAG
AGCAGTGGGATTAGGAGCTGTGTTCCTTGGGTTCTTGGGAGTGGCAGGAAGCACTATGGGCGCGGCGTCAATA
ACGCTGACGGTACAGGCCAGACAATTATTGTCTGGTATAGTGCAACAGCAAAGTAATTTGCTGAGGGCCATA
>FJ444500
TTCTATTGCAATACATCAAGACTGTTTAATGGTAATAGCACATCCATGAATGAGACATACACAAAAAGTGTGG
ACAAAAGTGAGGACATCACACTCCAATGCAGAATAAAACAAATTATCAATATGTGGCAGGGGGTAGGACGAGC
AATGTATGCCCCTCCCATTGAAGGAAACATAATATGTAACTCAAGTATCACAGGACTATTATTGACACGTGAT
GGAGGAATAGCAGAAAAGAATGGCACAGAGACATTTAGACCTGGAGGAGGAGATATGAGGGATAATTGGAGAA
GTGAATTATATAAATATAAAGTGGTAGAAATTAAGCCATTGGGAATAGCACCCACTAGGGCAAAAAGGAGAGT

GGTGGAGAGAGAGAAAAGAGCAGTGGGAATAGGAGCTGTGTTCCTTGGGTTCTTGGGAGCAGCAGGAAGCACT
ATGGGCGCAGCGTCACTAGCGCTGACGGGACAGGCCAGACAACTGTTGTCTGGTATAGTGCAACAGCAAAGCA
ACTTGCTGAAGGCCATA
>KC894101
TTCTATTGCAATACAACAAAGCTGTTTAATAGTCCTAATGATACTAGTCCTAATGGTACTAACTCCACCATCA
CACTCCCATGCAGAATAAAACAAATTATAAACATGTGGCAGGAGGTGGGAAGAGCAATGTATGCCCCTCCCAT
TGCAGGAAACATAACATGCACGTCAAATATTACAGGACTGCTATTGACACGTGATGGAGGTTTACAGGTCACA
AATGACTCAGAGACATTCAGACCTGGAGGAGGAAATATGAAGGACAATTGGAGAAGTGAATTATATAAATATA
AAGTGGTGGAAATTAAGCCATTGGGGATAGCACCCACTGAGGCAAAAAGGAGAGTGGTGGAGAGAGAAAAAAG
AGCAGTGGGAATAGGAGCTGTGTTCCTTGGGTTCTTGGGAGCGGCAGGAAGCACTATGGGCGCGGCGTCAATG
ACGCTGACGGTACAGGCCAGACAACTGTTGTCTGGCATA
>KP411834
TTCTATTGTTGTACATCATACCTGTTGAATAATACACTAGACAATAATACTATAAATGATACTACTAATAATG
GCGGCACAAATGATAGCATCATACTGCAATGCAGAATAAAGCAAATTATCAACGTGGGGCAGGAAGTAGGACG
AGCAATGTATGCCCCTCCAATAAAAGGCAACATAACATGTAGATCAAATATCACAGGAATATTGCTGGCAGAA
GATGGAGGACCTATCAACGAAACGGAAACATTCAGACGTGGAGGAGGAGATATGAGAGACAATTGGAGAAGTG
AATTATATAAATATAAAGTGGTAGAAATTAAGCCATTAGGAATAGCACCCACCAAGGCAAAAAGGAGAGTGGT
GGGGAGAGAAAAAAGAGCGGCACTAGGAGCTATGTTCCTTGGGTTCTTGGGAGCAGCCGGAAGCACTATGGGC
GCAGCGTCAATAACGCTGACGGTACAGGCCAGACAATTATTGTCTGGTATAGTGCAACAGCAAAGCAATTTGC
TGAGGGCCATA
>HM215317
TTCTACTGCAATACATCAAACCTGTTTACATACAATGGTACATACAATGGTACATACAATGGTACTAAAAATT
CAACCATCACACTCCAATGCAGAATAAAACAAATTATAAACATGTGGCAGGAGGTAGGGCGAGCAATGTATGC
CCCTCCCATTGCAGGAAACATAACATGTAAATCAAATATCACAGGACTACTATTGCTACGTGATGGAGGAAAC
ACAACAGGAGGAAACGAAACAGAAGAAAAAGAGATATTCAGACCTGGAGGAGGAAATATGAGGGACAATTGGA
GAAGTGAATTATATAAATATAAAGTGGTGGAGATTAAGCCATTGGGACTAGCACCCACTAAGGCAAAAAGGAG
AGTGGTGGAGAGAGAGAAAAGAGCAGTGGGAATAGGAGCTGTGTTCCTTGGGTTCTTGGGAGCAGCAGGAAGC
ACTATGGGCGCAGCGTCAATAACGCTGACGGTACAGGCCAGACAACTGTTGTCTGGTATAGTGCAACAGCAAA
GCAATTTGCTGAGAGCCATA
>AY253321
TTCTATTGCAATACATCAAAACTGTTTAATGGTACATACAATGGTACAGACACGACAGGAAGTACTCCAAATT
CAAACATCACACTCCAATGCAGAATAAAACAAATTATAAACAGGTGGCAGGGGGTAGGGCAAGCAATGTATGC
CCCTCCCATTGCAGGAAACATAACATGTAAGTCAAATATCACAGGACTCCTATTGACGCGTGATAGAGAAAAC
ACAACCGGCAACGAGACATTCAGACCTGGAGGAGGAGATATGAGGGACAATTGGAGAAGTGAATTATATAAAT
ATAAAGTGGTAGAAATTAAGCCATTGGGACTAGCACCCACAGAGGCAAAAAGGAGAGTGGTGGAGAGAGAGAA
AAGAGCAGTGGGAATAGGAGCTGTGTTCCTTGGGTTCTTGGGAGCAGCAGGAAGCACTATGGGCGCAGCGTCA
ATAACGCTGACGGTACAGGCCAGACAACTGTTGTCTGGTATAGTGCAACAGCAAAGCAATTTGCTGAGGGCCA
TA
>HQ615958
TTCTATTGCAATACATCAAGCCTGTTTAATGGTACATATAATAACACAGGAAGTAATGCAAATTCAAACATCA
CACTCCAATGCAGAATAAAACAAATTATAAACATGTGGCAGGAGGTGGGACGAGCAATGTATGCTCCGCCCAT
TGCAGGAGAGATAACATGTAAATCAAATATCACAGGACTACTATTGACACGGGATGGGGGAGAAAAAGACAAT
GAGACAAATAATGCAGAGGTATTCAGACCTGCAGGAGGAGATATGAGGGACAATTGGAGAAGTGAATTATATA
AATATAAAGTAGTAGAAATTAAACCATTAGGATTAGCACCCACTAAAGCAAGAAGGAGAGTGGTGCAGAGGGA
GAAAAGAGCAGTGGGACTAGGAGCTGTGCTCTTTGGGTTCTTGGGAGCAGCAGGAAGCACTATGGGCGCAGCA
TCAATAACGCTGACGGTACAGGCCAGACAATTACTGTCTGGTATAGTGCAACAGCAGAGCAATTTGCTGAGGG
CTATAGAGGCGCAACAGCACATG
>KT183336
TTCTATTGCAATACATCAGGGCTGTTTAATGGGACATACAATCCTAATGCTACAAACAGTAATTCAAGTACTG
TAAATTCAACCATCACACTCCAGTGCAGAATAAAACAAATTATAAACATGTGGCAGGGAGTAGGACAAGCAAT
GTATGCCCCTCCCGTTGCAGGTAACATAACATGTAAATCAAATATCACAGGAATACTATTGGTAAGGGATGGG
GGTAATGACAATAACAGTACCACAGAGACATTCAGACCTGGAGGAGGAGATATGAGGGACAATTGGAGAAGTG
AATTGTATAAATATAAAGTGGTAGAGATTAGGCCATTAGGAGTAGCACCCACCAAGGCAAAAAGGAGAGTGGT
GGAGAGAGAAAAAAGAGCAGTGGGAATAGGAGCTGTACTCCTTGGGTTCTTGGGAGCAGCCGGAAGCACTATG
GGCGCAGCATCAATAACGCTGACGGTACAGGCCAGACAATTGTTGTCTGGTATAGTGCAGCAGCAAAGCAATT
TGCTGAGGGCTATAGAGGCGCAGCAACACATG
>JN687734
TTCTATTGCAATACAACAAAACTGTTTAATGAAACACTGTTTAAGTCAGACAATGGTTCAGACATCATACTCC
CATGCAGAATAAAACAAATCATAAACATGTGGCAGGGGGTAGGACGAGCAATGTATGCCCCTCCCATTGCAGG
AAACATAACATGCAACTCAAATATCACAGGACTATTATTGACACGTGATGGAGGAGGTGGCAACAGCACAGAA

ACATTCAGACCTGGAGGAGGAAATATGAAAGATAATTGGAGAAGTGAATTATATAAATATAAAGTGGTAGAAA
TTAAGCCATTGGGAGTAGCACCCACCAAGGCAAAAAGGAGAGTGGTGGAGAGAGAAAAAAGAGCAGTGGGAAT
AGGAGCTGTACTCCTTGGGTTCTTGGGAGCAGCAGGAAGCACTATGGGCGCGGCGTCAATAACGCTGACGGTA
CAGGCCAGACAACTGTTGTCTGGTATAGTGCAACAGCAAAGTAATTTGCTGAGAGCTATAGAGGCGCAACAGC
ACATG
>AY529669
TTCTATTGCAATACATCAAAGCTGTTCAATGATACAAGCCTGCTTAATTATACAGAAGGGAATGCAAACAATA
ATGAGACCATCATACTCCCATGCAAAATAAAACAAATTATAAACAGGTGGCAAGGGGTAGGACAAGCAATATA
TGCCCCTCCCATTGCAGGAAACATAACATGTAAATCAAAGATCACAGGACTACTATTGACACGTGATGGAGGT
AATACCACAAATAACACAGAGACATTCAGACCAGGAGGAGGCGATATGAGGGACAATTGGAGAAGTGAATTAT
ATAAATATAAAGTGGTAGAAATTAAGCCATTGGGAGTAGCACCCACTGAGGCAAAAAGGAGAGTGGTGGAGAG
AGAAAAAAGAGCAGTAGGAATAGGAGCTGTGTTCCTTGGGATCTTGGGAGCAGCAGGAAGCACTATGGGCGCA
GCATCAATAACGCTGACGGTACAGGCCAGACAATTGCTGTCTGGAATAGTGCAACAGCAAAGTAATTTGCTGA
GGGCCATA
>JX213443
TTCTATTGCAATACATCAAAACTGTTTAATGGTACATACGAGCAGCCTAACAGTAGTGTAGGATCAGATTCAA
CCATCACACTCCCATGCAGAATAAAACAAATTATAAACTTGTGGCAGGAGGTAGGACGAGCCATGTATGCCCC
TCCCATTGCAGGAACCATAACATGTAACTCAAGTATCACAGGACTAATATTGACACGTGATGGAGGAAACGAA
ACTAAGCGCAAGGACACAGAGATATTCAGACCTATAGGAGGAGATATGAGGATTAATTGGAGAAGTGAATTAT
ATAAATATAAAGTGGTAGAAATTAAACCATTAGGATTAGCACCCACTACGGCAAAAAGGAGAGTGGTGGAGAG
AGAAAAAAGAGCAGTGGGAATAGGAGCTGTGTTCCTTGGGTTCTTAGGAGCAGCAGGAAGCACTATGGGCGCA
GCATCAATAACGCTGACGGTACAGGCCAGACAACTGTTGTCTGGTATAGTGCAACAGCAAAGTAATTTGCTGA
GAGCCATA
>KF716480
TTCTACTGCAATACATCAGAACTGTTTAATAGTATATGGCTTAATAATACTGGTAACTGGACAGGGAATACAA
CAAACAGTACAGAGAAAAATAATGGTACAATCACACTTCCATGCAGAATAAAACAAATTATAAACATGTGGCA
GGGAGTAGGAAAAGCAATGTATGCCCCTCCCATTGAAGGAGAAATCAAATGTATATCAAATATTACAGGACTA
TTGTTGACAAGGGATGGTGGTAATAATAATGCTACCAATGAGACCTTCAGACCTGGAGGAGGAGATATGAGAG
ACAATTGGAGAAGTGAATTATACAAATATAAAGTAGTAACAATTGAACCACTGGGTATAGCACCCACCAAGGC
AAAGAGAGGAGTGGTGGAAAGAGAAAAAAAGAGCAATAGGACTAGGAGCTATGTTCCTTGGGTTCTTGGGAGCA
GCAGGAAGCACGATGGGCGCAGTGTCATTGACGCTGACGGTACAGGTCAGACAGCTATTGTCTGGCATA
>KP411843
TTCTATTGTAATACTTCAGGCCTGTTTAACAGCACTTGGAATGGCACGAAATCTAAAGCGTCAAATGACAATG
ACACTATCCTTCGCCCGAGAAAAAAAAATAAAATTATGTGGAAGAGACAAAGACTAGGACTACAAGCACCTGC
CCCTCCTAGCCCAAGAACGAGAAGGTGTAGCACTACCAGTACAGGATTACTATTAGATGGAGATGGTACGAAT
ACTAATAATGAGACCTTCACGCCGGGACGAGGAGATATAAATATGAATTGCAGAAGTGAACTAGATCTATATA
AATTAGTAGTAATTGAACTTGTACCAGTAGGAGCCACCCCCTCCCGATCAAGAGTGGTGCTGATGCAAAAAAA
AGCAGTTGCAATGGGAGCTTCGCTCGTTTTCTTCTTATCAGCAGCAGCAGGAAGTAATGACGCGGCGTCGACG
ACACTGACGGTGCAGGTCAGGCCATTATTGTCTGGTACAGTGCTACTGCAGCAGCATCTGCTGATGCCCATA
>HM215265
TTCTATTGTAATACATCAGGACTGTTTAATGGTACTATATCAAATAGTACTAATATCACACTCCCATGTAAGA
TAAAACAAATTGTAAGAATGTGGCAAAAAGTGGGACAAGCAATGTATGCCCCTCCCATTGCAGGAAACATTAC
ATGTACATCAAACATTACAGGACTACTATTAACAAGAGATGGGGGGAGTAATGGAACCCATGATGATGAGGTC
TTCAGACCTGGAGGAGGAGATATGAGGGACAATTGGAGAAGTGAATTATATAAGTATAAAGTAGTAAAAATTA
AACCACTAGGAATAGCACCCACCAAGGCAAGGAGAAGAGTAGTGGGAAGAGAGAAAAGAGCAGTCGGATTTGG
AGCTGTTCTCCTTGGGTTCTTAGGAACAGCAGGAAGCACTATGGGCGCGGCGTCAATAACGCTGACGGTACAG
GTCAGACAATTATTGTCTGGCATA
>KT022403
TTCTATTGTAATACAACAAGCCTGTTTAATAGCACGTGGGGAAATGACACTGTTAACACACCAAAGAATGCCA
CGGGTTCAAATGACACTATAACAATCCTATGCAGAATAAAACAAATTGTAAATATGTGGCAAAGAGTAGGAAG
AGCAATGTATGCCCCTCCCATTGAAGGAAAAATACAGTGTATATCAAACATTACAGGTATGATATTAACAAGA
GATGGTGGGAATGAGAATATCAGTGAGATCTTCAGACCTGGAGGAGGAAATATGAGGGACAATTGGAGAAGTG
AATTATATAAGTATAAAGTAGTAAAAATTGAACCACTAGGAGTAGCACCCACCCAGGCAAGGAGAAGAGTGGT
GGAGAGAGGAAAAAGGGCAGTTGGACTGGGAGCTGTTTTCCTTGGGTTCTTGGGAGCAGCAGGAAGCACTATG
GGCGCGGCGTCACTGACGCTGACGGTACAGGCCAGAAACTTATTGTCTGGCATA
>DQ020274
TTCTATTGTAATACATCAGGACTGTTTAATAGTAGTAGTGAAAATAAGACTAGTAGTGAAAATAAGACTAATA
TCACACTCCCATGCAGAATCAAACAGATTGTGAGAATGTGGCAGAGAGTAGGACAAGCAATGTATGCCCCTCC
CATCGCAGGAAAAATTACATGCATATCAAACATTACAGGACTAATACTAACAAGAGATGGTGGGACTAATAGT
ACTAATGAGACCTTCAGACCTGCAGGGGGAGACATGAGGGACAATTGGAGAAGTGAATTATATAAGTATAAAG

TAGTAAAGATTAAACCACTAGGAGTAGCACCCACCAAGGCAAGGAGAAGAGTGGTGCAGAGAGAGAAAAGAGC
AGTTGGAATAGGAGCTGTCCTCCTTGGGTTCTTAGGAGCAGCAGGAAGCACTATGGGCGCGGCGTCAATAACG
CTGACGGTACAGGCCAGGCAATTATTGTCTGGCATA
>FM877777
TTCTATTGTAATACAACAAAACTGTTTAATAGCACTTGGCTTAATAGCACTTGGAATGACACGACCCAGGCAA
ATAATACAAATGAAAACATAACACTCCCATGCAGGATAAAACAAATTGTAAACATGTGGCAGGGAGTAGGACA
AGCAATGTATGCCCCTCCTATCGCAGGAATGATACAGTGTGTATCAAATATTACAGGACTGATATTGACAAGA
GATGGGGGAGACAATAGTAGTAATAATGAGACCTTCAGGCCTGGAGGAGGAAATATGAAGGACAACTGGAGAA
GTGAATTATATAAATATAAAGTAGTACAAATTGAACCACTAGGGGTAGCACCCAACAAGGCAAAGAGAAGAGT
GGTGGAGAGAGAAAAAAGAGCAGTTGGAATAGGAGCTATGTTCCTTGGGTTCTTAGGAACAGCAGGAAGCACT
ATGGGCGCGGCGTCAATAACGCTGACGGTACAGGCCAGGCAATTATTGTCTGGCATA
>KJ849769
TTCTACTGTAACTCAACACAACTGTTTCATAGTACTTGGGGGAATAATCAGACTAAATCAGATAGCAATGGAA
CAAGTAATGGAACAATAACACTCCCATGCAGAATAAAACAAATTATAAATATGTGGCAGGGAGTAGGAAAAGC
AATGTATGCCCCTCCCATCAAAGGACAAATTAGCTGTTCATCAAACATTACAGGACTGTTATTAGTAAGAGAT
GGTGGTAATAGTACCAGCAATGAAGAGATCTTCAGACCTACAGGAGGAAATATGAAGGACAATTGGAGAAGTG
AATTATATAAATATAAAGTAGTAAAAATTGAACCCTTAGGAGTAGCACCCACCAAGGCAAAGAGAAGAGTGGT
GCAGAGAGAAAAAAGAGCAGTAGGAATAGGAACTATGTTCCTTGGGTTCTTGGGAGCAGCAGGAAGCACTATG
GGCGCAGCGTCAATGACGCTGACGGTACAGGCCAGACAATTATTGTCTGGTATAGTGCAACAGCAAAATAATT
TGCTGAGGGCCATT
>MG989549
TTCTATTGTAATACAACAAAACTGTTTAATAGTACTTGGACAGGAAATGAAACCAGTGAAGGGAATAATAGCA
CTATCATACTTCCCTGTAAGATAAAGCAAATCATAAACATGTGGCAGGAAGTAGGACAAGCAATGTATGCTCC
TCCCATCAGTGGAAGCATTAATTGTGTATCAAATATTACAGGAATACTATTGACAAGAGATGGTGGTGTTAAC
AATACGGGAAATGAGACCTTCAGACCTGGAGGAGGAAATACAAAAGACAATTGGAGAAGTGAATTATATAAAT
ATAAAGTAGTAGAAATTGAACCACTAGGAGTAGCACCCACCAGGGCAAAGAGGAGAGTGGTGGAAAGAGAAAA
AAGAGCAGTGGGAATAGGAGCTATGATCTTTGGGTTCTTAGGAGCAGCAGGAAGCACTATGGGCGCGGCGTCA
ATAACGCTGACGGTACAGGCCAGACAATTATTGTCTGGTATAGTGCAACAGCAAAGCAATTTGCTGAGGGCTA
TAGAGGCGCAGCAGCACATG
>MF373150
TTCTATTGCAATACATCAGCACTGTTTAATAGAACTTGGAATAGCAATAGCACTTGGAATAGCACTGGGGAGA
ATATCACTGATATACATCTCCCATGCAGAATAAAGCAAATTGTAAATATGTGGCAGAGAGTAGGACAAGCCAT
GTATGCCCCTCCTATCCCAGGAATAATAAGGTGTGATTCAAACATTACAGGAATACTATTAACAAGAGATGGA
GGGAAAAACAATAGTAGTAATGAAACCTTCAGACCTGGAGGAGGAGACATGAGGGATAATTGGAGAAGTGAAT
TATATAAGTATAAAGTAGTAGAAATTGAACCACTAGGTGTAGCACCCGCCAGGGCAAGATCAACAGTGGTGGA
GAGAGAGACCATAGCAGTCGGACTGGTAGCTGTCGTCCTTGGGGTCTTAGGAGCAGCAGGAAGAACTATGGGC
GCAGCATCACTAACGCTGACGGTACGAGCCATACTATCAGTGTCTGGGATACTGCAGCAGCAGATC
>KU921769
TTCTATTGCAATACAACAGGCCTGTTTAATCACACGTTTAATAGCACGTGGGGAAATGCCACTATTACAGAGA
ATGCAAATGAAACTATAATTCTTCCATGCAGAATAAAACAAATTATAAATATGTGGCAGGAAGTAGGACAAGC
AATGTATGCCCCTCCCATTGAAGGAGTAATAAAGTGTATATCAAACATTACAGGAATAATATTGACAAGAGAT
GGTGGGCACATGAGCAATGGCAGCAATGACAGTATTGAGACCTTCAGACCTGGGGGAGGAAATATGAGGGACA
ATTGGAGAAGTGAATTATATAAGTATAAAGTAGTAAAACTTGAACCACTAGGAGTAGCACCAACCAGGGCAAG
GAGAAGAGTGGTAGAGAGAGAGAAAAAAGAGCAGTTGGACTGGGAGCTGTCTTCCTTGGGTTCTTGGGAGCAGCA
GGAAGCACTATGGGCGCAGCGTCAATAACGCTGACGGTACAGGCCAGACAATTATTGTCTGGCATA
>KF725943
TTCTATTGCGATACATCAGACTTGTTTAATAGTACATACGATCTTACTAATAATTCTACTAATATAAGCAAAG
AGTCAAACGAAACAATCACGCTCAAATGCAGAATAAAACAGATCATAAACATGTGGCAGGAGGTAGGAAGAGC
AATATATGCCCCTCCCATTGCAGGAAACATAACATGTAAATCAAACATCACAGGACTACTATTAGTACGTGAT
GGAGGTAATAGCCATGGCACAAATGACACAAATAGCACAAATGAAGAGATATTCAGACCTGAAGGAGGAGATA
TGAGGGACAATTGGAGAAGTGAATTATATAAATACAAAGTAGTAAAACTTGAACCACTAGGATTAGCACCCAC
CAGGGCAAGGAGAAGAGTGGTGGAGAGAGAAAAACGAGCAGTTGGACTGGGAGCTGTCTTCCTTGGGTTCTTG
GGAACAGCAGGAAGCACTATGGGCGCGGCGTCAGTGGCGCTGACGGTACAGGCCAGACAATTATTGTCTGGCA
TA
>JX236675
TTCTATTGTAATACATCAGACCTGTTTAATAACATTTGGATAGGAAATGAGACTACAGAAACAAATGAGACTG
AAACAAATTCAAGTAAGCCTATAACGCTCCAGTGCAGAATAAAGCAAATTATAAACATGTGGCAGAGAGTAGG
GCAAGCAATATATGCCCCTCCCATCCAAGGAAAATTAGAATGTAATTCAAACATTACAGGACTGCTATTAACA
AGAGATGGTGGAAAAAATAATGACAGTAAAGAAATCTTCAGACCTGGAGGAGGAGATATGAGGGATAATTGGA
GAAGTGAATTATATAAGTACAAAGTAGTAAAAATTGAACCACTAGGAGTAGCACCCACCAGGGCACAGAGAAG

AGTGGTAAAGAGAGAAAAAAGAGCAGTCATTGGAATGGGAGCGATGTTCCTTGGGTTCTTAAGTGCAGCAGGA
AGCACTATGGGCGCAGCGTCACTAACGCTGACGGTACAGGCCAGACAATTATTGTCTGGCATA
>JN054260
TTCTATTGTAATACATCAGGACTGTTTAATAATAGTGATAATAGTAGTGGTAGTAATAATAGTAGTAGGAATG
GGAATATCATACTCCCATGCAAGCTCAAACAGATTGTGAGAATGTGGCAGAGAGTAGGACAAGCAATGTATGC
CCCTCCCATCGCAGGAAAACTCACATGTATATCAAACATTACAGGACTATTAATAACAAGAGATGGTGGAACT
AACAGTACTAATGGGACTGAGATCTTCAGACCTGCAGGGGGAGACATGAGGGACAATTGGAGAAGTGAATTAT
ATAAGTATAAAGTAGTAAAAATTAAACCACTAGGAGTAGCACCCACCAGGGCAAGGAGAAGAGTGGTGGAGAG
AGAAAAAAGAGCAGTTGGAATAGGAGCTGTTTTCTTTGGGTTCTTAGCAGCAGCAGGAAGCACTATGGGCGCG
GCGTCAATAACGCTGACGGTACAGGTCAGGCAATTATTGTCTGGCATA
>KT124783
AGTTACTCAAATGAAACGGAAGACGATAATATCACACTCCCATGCAGAATAAAACAAATTATAAACATGTGGC
AGGAAGTAGGAAAAGCAATGTATGCTCCTCCCATCAGAGGACAAATTAGATGTTCATCAAATATTACAGGGAT
ACTATTAACAAGGGATGGTGGTAAGAACGAGAACAACAACACTGAGATCTTCAGACCTGGAGGAGGAAATATG
AGGGACAATTGGAGAAGTGAATTATATAAATATAAAGTAGTAAAGATTGAGCCATTAGGAGTAGCACCCACCA
AGGCAAAACGAAGAGTGGTGCAGAGAGAAAAAAGAGCAGTAGGACTAGGAGCTGTGTTCCTTGGGTTCTTGGG
AGCAGCAGGAAGCACTATGGGCGCAGCGGCAGTGACGCTGACGGCACAGGCCAGACAATTATTGTCTGGGATA
GTGCAACAGCAGAACAATCTGCTAAGAGCTATTGAGGCGCAACAGCATCTGTTGCAACTCACAGTCTGGGGCA
TC
>KJ698288
AATTATAATAGTACTGAGAATAATACTGAAACGTCAAATGAGCCTATTATACTCCCATGCAGAATAAAACAAA
TTATAAACATGTGGCAGGAAGTAGGAAAAGCAATGTATGCCCCTCCCATTAAAGGACAAATTAGATGCTCATC
AAATATTACAGGGCTGCTATTAACAAGAGATGGTGGAAACAACAACACGAACGAGACCTTCAGACCTGGAGGA
GGAGATATGAGGGACAATTGGAGAAGTGAATTATATAAATACAAAATAGTAAAAATAGAACCATTAGGTGTAG
CACCCACCAGGGCAAAGAGAAGAGTGGTGCAGAGAGAAAAAAGAGCAGTGGGAATAGGAGCTGTGCTCTTTGG
GTTCTTGGGGGCAGCAGGAAGCACTATGGGCGCAGCGTCAATGACGTTGACGGTACAGGCCAGACAATTAATG
TCTGGTATAGTGCAACAGCAGCAGAATTTGCTGAGGGCTATTGAGGCGCAGCAGCATCTGTTGCAACTCACAG
TCTGGGGCATC
>KU901765
TACTATAGTAGTAATAGTAGTGGGAGTGGTACAAATAATAACACAATCATACTCCAATGCAGAATAAAACAAA
TTATAAACATGTGGCAGACAGTAGGAAAAGCAATGTATGCCCCCCCCATCAGAGGAAAGATTAGCTGTGTATC
AAATATCACAGGGCTATTATTAGTCAGAGATGGAGGTGATAACAGGAGTAACAAAGAGATCTTCAGACCTGCA
GGAGGAGATATGAGAGACAATTGGAGAAGTGAATTATATAAATATAAAGTAGTAAAAATTGAACCATTAGGAA
TAGCACCCACTAAGGCAAGGAGAAGAGTGGTGCAAAGAGAAAAAAGAGCAGTGGGAATAGGAGCTTTGTTCCT
TGGGTTCTTGGGAGCAGCAGGAAGCACTATGGGCGCAGCGTCAATGGCGCTGACGGTACAGGCTAGACAATTA
TTGTCTGGTATAGTGCAACAGCAGAATAATTTGCTGAGAGCTATTGAGGCGCAACAGCATATGTTGCAACTCA
CAGTCTGGGGCATC
>EU576268
TATCAGAATAATACTGAAGGGTCAAATAACACTAAAGCAAATAGCACAATCCTACTCCTATGCAGAATAAAAC
AAATTATAAACATGTGGCAGGAAGTAGGAAAAGCAATGTATGCCCCTCCCATCAGAGGCAAAATCAGATGTTT
ATCAAATATTACAGGGCTGCTACTAACAAGAGATGGTGGTGAGCCTAATAACAAAACCAACGATACCTTCAGA
CCAGGAGGAGGAGATATGAGGGACAATTGGAGAAGTGAATTATATAAATATAAAGTAGTACAAATTGAGCCAA
TAGGACTAGCACCCACCAAGGCAAAGAGAAGAGTGGTGCAAAGAGAAAAAAGAGCAGTGGGCATAGGAGCTTT
GTTCCTTGGGTTCTTGGGAGCAGCAGGAAGCACTATGGGCGCAGCGTCATTGACGCTGACGGTACAGGCCAGA
CAATTATTGTCTGGTATAGTGCAACAGCAGAACAACTTGCTGAGAGCTATTGAGGCGCAACAGCATCTGTTGC
AACTCACAGTCTGGGGCATC
>HQ699991
GACTACATGCCTAATGGTACAGCAAGTAATTCAAGCTCAAACATCACACTCCCATGCAGAATAAAGCAAATTA
TAAACATGTGGCAGGAGGTAGGACGAGCAATGTATGCCCCTCCCATTGAAGGAAACATAACATGTAAATCAAA
TATCACAGGTCTACTATTGGTCCGTGATGGAGGAAGTACAAATAATACAGAAGAGACATTCAGACCTATAGGA
GGAAATATGAAGGACAATTGGAGAAATGAATTATATAAGTATAAAGTAGTGGAAATTAAGCCATTGGGACTAG
CACCCACTGGAGCAAAAAGAAGAGTAGTGGAGAGAGAAAAAAGAGCGGGAATAGGAGCTGTGTTCCTTGGGTT
CTTAGGAGCAGCCGGAAGCACTATGGGCGCGGCGTCAATAACGCTGACGGTACAGGCCAGACAATTGTTGTCT
GGTATAGTGCAACAGCAAAGCAATTTGCTGAAGGCTATAGAGGCGCAACAGCATATGTTGCAACTCACGGTCT
GGGGCATT
>KU319545
TGTCATAATAATCCAACATCCGAACCGTCTAAAAATCGTGCTGCAAATCCAACATTCATAATCCCATGCAGGG
TGAAGCAAGTTCTAAGCATGCGCCTCGTATTGCGGGAAGCAATGTATGCCCATCCTATATCAGGAGACGTACT
ATTGACATCGGATGTCGGAGGACTGATATCCACACGAGATAACGCTAATAGCGCGAACGAGATATTTAGACCT
GGAGGAGGAGATATGAGGGACAATTGGAGAAGTGAATTATACAAATATAAAGTGGTAGAAATTAAACCATTAG

GAATAGCACCCACCAAGGCAAAAAGGAGAGTGGTGGAGAGAGAAAAAAGAGCAGTGGGAATAGGAGCTGTATT
CCTTGGGTTCTTGGGAACAGCCGGAAGCACTATGGGCGCAGCATCAATAACGCTGACGGTACAGGCCAGGCAA
TTGTTGTCTGGTATAGTGCAACAGCAAAGCAATTTGCTGAAGGCTATAGAGGCGCAACAGCATATGTTGCAAC
TCACGGTCTGGGGCATT
>KJ158423
TGTTACCTGTTTAATAGATTATACACTGCAGCCAATACAAAGAGTAATTCATGGATTGATATCAACTGCACAG
TTGCAATCCCATGCAGAATAAAGCAAATTATAAACATGTGGCAGGAGGTAGGGCGAGCAATGTATGCCAATCC
CATTGAAGGAAATATAACATGTAAATCAGATATCACAGGGATGCTATTAGAACGTGATTGTGGAAAAGAACGT
GATAAAACGATATCAACTACGAAAGAGATATTCAGACCTATAGGAGGAAATATGAGGGACAATTGGAGAAGTG
AATTATACAAATATAAAGTGGTAGAAATTAAGCCATTAGGAGTAGCACCCACGGAAGCAAAAAGGAGAGTGGT
GGAGAGAGAAAAAAGAGTAGCGGGACTAGGAGCTGTGTTCCTTGGGTTCTTGGGAGCAGCAGGAAGCACTATG
GGCGCGGCGTCAATAACGCTGACGGTACAGGCCAGACAATTGTTGTCTGGTATAGTGCAACAGCAAAGCAATT
TGCTGAGAGCTATAGAGGCGCAACAGCATCTGTTGCAACTCACGGTCTGGGGCATT
>MF373165
GCTCAAGATAAAACGACTGGGGGTAGTGGTAATAATGAAACCATTCCCCTCCCCTGCAGGATAAAACAAATTG
TCAACATGTGGCAAGTAGTAGGACCAGCAATGTATGCTCCCCTCATTGCAGGAAACATAACAAGTAACTCATC
TATCACAGGACTACTATTGACACGTGATGGAGGAATCGTAACCAATGCCACCGACACAGAGACATTTAGACCT
GGAGGAGGAAATATGAAAGACAATTGGAGAAGTGAATTATATAAATATAAAGTGGTAGAAATTAAGCCATTAG
GAGTAGCACCCACTAAGGCACAGAGGAAAGTGGTGAAGAGAGAAAGAGAAAAAAGAGCAGTGGGAATGGGAGC
TGTTCTCCTTGGGTTCTTGGGAGCAGCAGGAAGCACTATGGGCGCAGCGTCAATAACGCTGACGGTACAGGCC
AGACAACTGTTGTCTGGTATAGTGCAACAGCAAAGCAATTTGCTGAAAGCTATAGAGGCGCAACAACATCTGT
TGCAACTCACAGTCTGGGGCATT
>GU080195
AATCATGATAGTGGGACAGAAGATAATTCAACCATCACACTTCCATGCAGAATAAAACAAATTGTAAACATGT
GGCAGGAGGTAGGACGAGCAATGTACGCTCCTCCCATTGAAGGAAACATAACATGTATCTCAAATATCACAGG
ATTACTATTGACACGTGATGGGGGAGAAACAAACAGAACAAATGACACATTCAGACCTGGAGGAGGAAATATG
AAGGACAATTGGAGAAGTGAATTATATAAATATAAAGTGGTAGAAATTAAGCCATTGGGAATAGCACCCACTG
GAGCAAAAAGGAGAGTGGTGGAGAGAGAAAAAAGAGCAGTGGGACTAGGAGCTGTGATCTTTGGGTTCTTGGG
AGCAGCAGGAAGCACTATGGGCGCAGCGTCAATAACGCCGACGGTACAGGCCAGACAACTGTTGTCTGGTATA
GTGCAACAGCAAAATAATTTGCTGAGGGCTATAGAGGCGCAACAGTATATGTTGCAACTCACGGTCTGGGGCA
TT
>MF285011
AATTATACAGATAATAGTACAACTGATGATACCCAGACAAACATCACACTCCAATGTAGAATAAAACAAATTA
TAAACATGTGGCAGGAGGTAGGACGAGCAATGTATGCTCCTCCCATTGCAGGAAACATAACATGCAACTCAAA
CATCACAGGATTACTATTGACATATGATGGAGGTGAAACAGGTAATGGTACACCAGAGATATTCAGGCCTGGA
GGAGGAAATATGAAGGACAATTGGAGAAGTGAATTATATAAATATAAAGTGGTAGAAATTAAGCCATTGGGAA
TAGCACCCACTGGAGCAAAAAGGAGAGTGGTGGAGAGAGAAAAAAGAGCAGTAGGAATAGGAGCTGTGTTCCT
TGGGTTCTTGGGAGCAGCAGGAAGCACTATGGGCGCGGCGTCAATAACGCTGACGGTACAGGCCAGACAACTG
TTGTCTGGTATAGTGCAACAGCAAAGCAATTTGCTGAGAGCTATAGAGGCGCAACAGCATATGTTGCAACTCA
CGGTCTGGGGCATT
>KF725969
ACCTATCTTAGTAATGAAACCTTTAGACTCCAATGCAAAATAAAACAAATTATAAACATGTGGCAGACGGTAG
GACGAGCAATGTATGCCCCTCCCATTGCAGGAAACATAACATGTAATTCAAGCATCACAGGATTACTATTGAC
ACGGGATGGAGGTTCCACAGATAATAATAAAACAGAAACGTTCAGACCTGGAGGAGGAGATATGAGAGACAAT
TGGAGAAGTGAATTATATAAATATAAAGTGGTGGAAATTAAGCCATTGGGAATAGCACCCACTAAAGCAAAAA
GGAGAGTGGTGCAGAGAGAAAAAAGAGCAGCAATAGGAGCTATGATCCTTGGGTTCTTGGGAGCAGCAGGAAG
CACTATGGGCGCGGCGTCAATGGCGCTGACGGTACAGGTCAGACAATTGTTGTCTGGTATAGTGCAACAGCAA
AGTAATTTGCTGAGGGCCATAGAGGCGCAACAGCATATGTTGCAACTCACGGTCTGGGGCATT
>AY322187
TATTATACTGGATGCACCACCAACGTACAACAGAAAACAATGCTTCCCATCAGACTACAACTCATAATAAGTA
ATGTATGCCCTTGTCCATGGCAAGGAGTGTCAGCAGCAACATCTGCTGCTACCATCCTATTATCAACAATAAC
AGGAGGATCATTGACAACAAGAGGAGTGTTCACAACTGGTGGAGGTGATACCTTCAGACCTGGAGGAGGAGAT
ATGAGGGACAATTGGAGAAGTGAATTATACAAATATAAAGTAGTAAGAATTGAACCACTAGGTTTAGCACCCA
CCAGGGCAAAGAGAAGAGTGGTGGAAAGAGAGAAAAGAGCAATAGGACTAGGAGCTATGTTCCTTGGGTTCTT
GGGAGCAGCAGGAAGCACGATGGGCGCAGCGTCACTGACGCTGACGGTACAGGCCAGACAGTTATTGTCTGGT
ATAGTGCAACAGCAAAACAATTTGCTGAGGGCTATAGAGGCGCAACAGCATCTGTTGCAACTCACAGTCTGGG
GCATT
>KX587434
TTCTAATCAAACTGAGAGTAACACTACTGAAGGTAATGACACAGTAATTACACTCCCATGCAGAATAAAGCAA
ATTATAAACATGTGGCAGCAAGTAGGAAAAGCAATGTATGCCCCTCCCATCGCAGGGCAACTTAATTGCTCAT

CAAACATTACAGGGATATTATTAACAAGAGATGGTGGTAGCGAGAACCATACCAAGGGTAATGAGACCTTCAG
ACCAGTAGGAGGAGACATGAGGGACAATTGGAGAAGTGAATTATATAAATATAAAATAGTAAAAATTGAACCC
TTAGGAATAGCACCCACCAATGCAAAAAGAAGAGTGGTGCAGAGAGAAAAAAGAGCAGTGGGACTAGGAGCCT
TGTTCCTTGGGTTCTTGGGAATGGCAGGAAGCACTATGGGCGCAGCGTCAGTGACGCTGACGGTACAGGCCAG
ACAATTACTGTCTGGTATAGTGCAGCAGCAAAACAACTTGCTGAGGGCTATTGAGGCGCAACAACACATGTTG
CAGCTCACAGTCTGGGGCATC
