# Supplementary material for: Extending the Coding Potential of Viral Genomes with Overlapping Antisense ORFs: A Case for the De Novo Creation of the Gene Encoding the Antisense Protein ASP of HIV-1
Source: Viruses. 2022 Jan 14;14(1):146. doi: 10.3390/v14010146 (PMC8781085; doi:10.3390/v14010146)
Supplement: Supplementary file 1 [file viruses-14-00146-s001.zip › Supplementary File S4.pdf]

Supplementary File S4

Supplementary File S4 contains a control set of 2260 spurious *env*/*asp* overlaps. The great majority of them (2183 cases) belong to the group M of HIV-1, while the remaining ones belong to the groups N, O, and P of HIV-1 and to SIV infecting chimpanzee or gorilla. A spurious overlap consists of an *env* region which overlaps with an anti-sense ORF interrupted prematurely by one or more stop codons and having a length ≤450 nt. The nucleotide sequence of overlap is reported both in the alignment format with gaps and in the format without gaps.

```
>MH705158
TTCTATTGCAATACATCAGGCCTGTTTAATAGTAGTTGGAACGAAAGTGACACT-------------------
-------------------------------------------------------------------------
----------------------------
GCAAATGACACTATAACGCTCCCGTGCAAAATAAAACAAATTGTAAGGATGTGGCAGAGAGTAGGACAAGCAA
TGTATGCCCCTCCCATCCAAGGGGTAATGAGGTGTGAATCTAAC---
ATTACAGGACTACTATTAACAAGAGATGGTGGGAATGAGGGCAAT----------------------------
-------------------------------------------------------------------------
----------
GAAAGTAGTGAGACGTGCAGACCTGGAGGAGGAGATATGAGGGATAATTGGAGAAGTGAACTATATAAATATA
AAGTAGTAAAAATTGAACCACTA---GGAGTAGCACCCACCAGGGCAAAGAGAAGAGTGGTG-----------
----------------------------GAGAGAGAAAAAAGAGCA---GTTGGA---CTGGGAGCT------
---GTCTTCATTGGGTTCTTAGGAGCA---GCAGGAAGCACTATG---GGCGCGGCGTCA---
GTGACGCTGACGGTACAG---GCCAGACAA---TTATTG---TCTGGCATAGTGCAACAGCAAAAC---
AATTTG---CTGAGG---GCTATAGAGGCTCAACAGCATCTGTTGAAACTCACGGTCTGGGGCATT
>MH705151
TTCTATTGCAATACATCAGACCTGTTTAATAGCACTTGGCGGAAACTGGCAAATGGCACTTGGAGCAACATAA
ATAATGCAACG--------------------------------------------------------------
----------------------------
TCAAATGACACTATAACTCTCCAATGCAAAATAAAGCAAATTGTAAACATGTGGCAGAGAGTAGGACAAGCAA
TGTATGCCCCTCCCATCCAAGGAGTAATACAGTGTGTATCAAAC---
ATTACAGGACTACTCTTAACAAGAGATGGTGGGAGTAGTAACAGG----------------------------
-------------------------------------------------------------------------
-------------
GCAAATGAGACCTTCAGACCTGGAGGAGGAGATATGAGGGACAATTGGAGAAGTGAATTATATAAGTATAAAG
TAGTAAAAATTGAACCACTA---GGAGTAGCACCCACCAGGGCAAGGAGAAGAGTGGTG--------------
-------------------------GAGAGAGAAAAAAGAGCA---ATTGGA---ATGGGAGCT---------
CTTTTCCTTGGGTTCTTAGGAACA---GCAGGAAGTACTATG---GGCGCGGCGTCA---
ATAACGCTGACGGTACAG---GCCAGACAA---TTATTG---TCTGGTATAGTACAACAGCAGAGC---
AATCTG---CTGAGG---GCTATAGAGGCTCAACAGCATCTGTTGAAACTCACAGTCTGGGGCATT
>MH705157
TTCTATTGTAATACATCAAGCCTGTTTAATAGCACTTGGAAAGAGGAGAATGGCACTGTCAGCATGCAGAATG
ACACGGAG-----------------------------------------------------------------
----------------------------
TCAACTGACCCTATAACTCTCCAATGTAGAATAAAGCAAGTTATAAACATGTGGCAGAGAGTAGGACAAGCAA
TATATGCCCCTCCCATCCAAGGAGTAATAAGATGTGTATCAAAC---
ATTACAGGACTACTATTAACAAGAGATGGTGGGAGTAATAATAATACT-------------------------
-------------------------------------------------------------------------
-------------
TCAAATGAAACCTTCAGACCTGGAGGAGGAGATATGAGGGACAATTGGAGAAGTGAATTATATAAGTATAAAG
TAGTAAAAATTGAACCACTA---GGAGTAGCACCCACCAAGGCAAAGAGAAGAGTGGTG--------------
-------------------------GAGAGAGAAAAAAGAGCA---GTTGGA---CTGGGAGCT---------
GTCTTCCTTGGGTTCTTAGGAGCA---GCAGGAAGTACGATG---GGCGCGGCGTCA---
ATAACGCTGACGGTACAG---GCCAGACAA---TTATTG---TCTGGCATAGTGCAACAGCAGAGC---
AATCTG---CTGAGG---GCTATAGAGGCTCAACAGCAACTGTTGAGACTCACGGTCTGGGGCATT
>MH705153
TTCTATTGCGATACATCAGGCCTGTTTAATGGCACTTGGAGCAATACATCAGGCCTGATTAATAGCACTTGGA
GC-----------------------------------------------------------------------
----------------------------
GAGAATGACACTATAACTCTCCCATGTAGAATAAAGCAAATTGTAAATATGTGGCAGAGAGTAGGACAAGCAA
```

TGTATGCCCCTCCCATCCAAGGAATAATAAATTGTACATCAAAC---
ATTACAGGACTACTGTTAACAAGAGATGGTGGGAATAATAACAGTATA-------------------------
----

AATGAGCCTGAGACCTTCAGACCTGGAGGAGGAGATATGAGGGACAATTGGAGAAGTGAACTATATAAGTATA
AAGTAGTAAAAATTCAACCACTA---GGAGTAGCACCCACCAGGGCAAAGAGAAGAGTGGTG-----------
----------------------------GAGAGAGAAAAAAGAGCA---GTTGGC---ATGGGAGCT------
---CTTCTCCTTGGGTTCTTAGGAGCA---GCAGGAAGCACGATG---GGCGCGGCGTCA---
ATAACGCTGACGGTACAG---GCCAGACAA---TTATTG---TCTGGTATAGTGCAACAGCAGAGC---
AATCTG---CTGAGG---GCTATAGAGGCTCAACAGCATCTGTTGAAACTCACGGTCTGGGGCATT
>MH705133
TTCTATTGCAATACGTCAGGCCTGTTTAATAGCACTTGGAAGAATGGCACTAAAGAGGGAAATGACACTGCAA
AG---

TTAAGTGACACTATAACTCTCCCATGCAGAATAAAGCAAATTGTAAGGATGTGGCAGAGAACAGGACAAGCAA
TGTATGCCCCTCCCATCCCAGGAGTAATAAATTGTGTCTCTAAC---
ATTACAGGGATACTATTAACAAGGGATGGTGGGAATAGTAGTAAT----------------------------
----

GCCAATGAAACCTTCAGACCTGGAGGAGGAAATATGAAGGACAATTGGAGAAGTGAACTATATAAGTATAAAG
TAGTAAAAATTGAACCAATA---GGAGTAGCACCCACCAGGGCAAAGAAAAGAGTGGTG--------------
-------------------------GAGAGAGAAAAAAGAGCA---GTTGGA---CTGGGAGCT---------
GTCTTCCTTGGGTTCTTAGGAGCA---GCAGGAAGTACTATG---GGCGCGGCGTCA---
ATAACGCTGACGGTACAG---GCCAGACAA---TTATTG---TCTGGCATAGTACAACAGCAAAGC---
AATTTG---CTGAGG---GCTATAGAGGCTCAACAGCATATGTTGAGACTCACAGTCTGGGGCATT
>JQ403028
TTCTACTGCAATACAACAGAACTGTTTAATAGCACGTGGAATGACACGAGCAACACATCGCAGGGGACAAAT-
----

AACAATAGTACTATTACTCTCCCATGCAGAATAAAGCAATTTGTAAATATGTGGCAGAGAGTAGGACAAGCAA
TGTATGCCCCTCCCATCAAAGGAAAAATACAGTGCATATCAAAT---
ATTACAGGTCTACTATTAACAAGAGATGGGGGTAAGGGTAAAAGC----------------------------
----

AATATTACAGAGATCTTCAGACCTGAAGGAGGAAATATGAAGGACAACTGGAGAAGTGAATTATATAAATATA
AAGTAGTAAAAATTGAACCACTA---GGAGTAGCACCCACCAAGGCAAAGAGAAGAGTGGTG-----------
----------------------------GAGAGAGAAAAAAGAGCA---GTTGGA---CTGGGAGCT------
---GTGTTCCTTGGGTTCTTAGGAGCA---GCCGGAAGTACTATG---GGCGCGGCGTCA---
ATAACGCTGACGGCACAG---GCCAGGCAA---TTATTG---TCTGGTATAGTACAACAGCAGAGC---
AATCTG---CTGAGG---GCTATAGAGGCTCAACAGCATCTGTTGAAACTCACGGTCTGGGGCATT
>KX389622
TTCTATTGCAATACATCAGACTTGTTTAATAGCAATTGGACAAATGGGAATTGGACAAATGGGATAGGCAGCA
CGGAG--

ACTAATGACACTATAACTCTTCCATGCAGAATAAAGCAGATTATAAGAATGTGGCAGAGAGTAGGACAAGCAA
TGTATGCCCCTCCCATCAAAGGGGTAATAGAGTGTAAATCAAAT---
ATTACAGGAATAATATTAGTAAGAGATGGTGGTAAGAATAACACA----------------------------
----

GACAAGGAGACATTTAGACCTGGAGGAGGAGATATGAGGGACAATTGGAGAAGTGAACTATACAAATATAAAG
TAGTAAAAATTGAACCATTA---GGAGTAGCACCCACCAGGGCAAAAAGAAGAGTGGTG--------------
-------------------------GAGAGAGAAAAAAGAGCA---GTTGGA---ATAGGAGCT---------
GTCTTCCTTGGGTTCTTAGGAGCG---GCAGGAAGCACTATG---GGCGCAGCGTCA---
ATAACGCTGACGGTACAG---GCCAGACAA---TTATTG---TCTGGCATAGTGCAACAGCAGAGC---
AATCTG---CTGAGG---GCTATAGAGGCTCAACAACATCTGTTGAAACTCACAGTCTGGGGCATT
>DQ396400
TTCTATTGCAATACATCAGGCCTGTTTAACAGAACTTGGAATGACACCGCTTGGAATGAAACAACTTGGAATA
ACATA--

AATTCAAATGGAAGTATAACTCTCCAATGTAGAATAAAGCAAATTTTAAATATGTGGCAGAGAGTAGGACAGG

CGATGTATGCCCCTCCCATCCCAGGAGAAATAAGGTGTGAATCAAAC---
ATTACAGGACTAATCTTAACAAGAGACGGGGGGCTTGAGCTTAACAAT----------------------
----

ACGAATAATGAGACCTTCAGGCCTGGAGGAGGAGATATGAGGGACAATTGGAGAAGTGAATTGTATAAATACA
AAGTAATAAAAATTGAACCACTA---GGAGTAGCACCCACCAGGGCAAAAAGAAGAGTGGTG----------
---------------------------GAGAGAGAAAAAAGAGCA---GTTGGA---CTGGGAGCT------
---GTTTTCCTTGGGTTCTTAGGGGCA---GCAGGAAGCACTATG---GGCGCAGCGTCA---
ATAACGCTGACGGTACAG---GCTAGGCAA---TTATTG---TCTGGCATAGTGCAACAGCAAAGC---
AATTTG---CTGAGG---GCTATAGAGGCTCAACAACATTTGTTGAAACTCACAGTCTGGGGCATT
>FM165645
TTCTACTGTAACACATCAGGCCTGTTTAATAGCAGTTGGGAAAATGACACTGGCAGCACGCAGGAGTCAAATA
GAACA---

AAGTCAAATGACACTATAACTCTCCCATGCAGAATAAAGCAAATTATAAATATGTGGCAGAGGGCAGGACAAG
CAATATATGCCCCTCCCATCCCAGGGGTAATACAGTGTCAGTCAAAC---
ATTACAGGACTACTATTAACAAGAGATGGTGGGAATAACAATACA--------------------------
----

ACTAATGAAACCTTCAGACCTGGAGGAGGAAATATGAGGGATAATTGGAGAAGTGAACTATATAAATATAAAG
TAGTAAAAATTGAACCACTA---GGAGTAGCACCCACCACGGCAAGGAGAAGAGTGGTG-------------
-------------------------GAGAGAGAAAAAAGAGCA---GTGGGA---CTGGGAGCT--------
CTGTTCATTGGGTTCTTAGGAGCC---GCAGGAAGCACTATG---GGCGCGGCGTCA---
GTGACGCTGACGGTACAG---GCCAGACAA---TTATTG---TCTGGCATAGTGCAACAGCAAAAC---
AATTTG---CTGAGG---GCTATTGAGGCTCAACAGCATCTGTTGAAACTCACGGTCTGGGGCATT
>FM165628
TTCTACTGCAACACATCAAGACTGTTTAATAGCAGTTGGGAACCATTAGGCCAGTATGATGACAGTCAGAGC-
----

AATGACACAAATATAACTCTCCCATGCAGAATAAAGCAAATTATAAGGATGTGGCAGAGGACAGGACAAGCAA
TATATGCCCCTCCCATCCAAGGGGAAATAACGTGTAGATCAAAT---
ATTACAGGACTACTATTAACAAGAGATGGTGGAAATAAAACTAAT---------------------------
----

GAAACCTCCGAAACCTTCAGACCTGGAGGAGGAGATATGAGAGATAATTGGAGAAGTGAACTATATAAATATA
AAGTAGTAAAAATTGAACCACTA---GGAGTAGCACCCACCAAGGCAAAAAGAAGAGTGGTG----------
---------------------------GAGAGAGAAAAAAGAGCA---GTGGGA---CTGGGAGCT------
---GTTCTCATTGGGTTCTTAGGAACA---GCAGGAAGCACTATG---GGCGCGGCGTCA---
GTGACGCTGACGGTACAG---GCCAGACAA---TTATTG---TCTGGCATAGTGCAACAGCAAAAC---
AATTTG---CTGAGG---GCTATAGAGGCTCAACAGCATCTGTTGAAACTCACGGTCTGGGGCATT
>EU191615
TTCTACTGCAACACATCAACACTGTTTAATAGCAGTTGGCCATTAAACCAGTCTAACAGTTGGAGCAATGGCG
GA--

AATGACACAAATATAACTCTCCCATGCAGAATAAAGCAAATTATAAGGATGTGGCAGAGGACAGGACAAGCAA
TATATGCCCCTCCCATCGAAGGGGTAATAATGTGTAGGTCAAAT---
ATTACAGGACTCCTATTAACAAGAGATGGTGGAAATGAAGCTAATAAAACT---------------------
----

AATGAAACTGCTGAAATCTTCAGACCTGGAGGAGGGGATATGAGAGATAATTGGAGAAGTGAACTATATAAAT
ATAAAGTAGTAAAAATTGAACCACTA---GGAGTAGCACCCACCAGGGCAAAAAGAAGAGTGGTG-------
------------------------------GAGAGAGAAAAAAGAGCA---GTGGGA---CTGGGAGCT---
------GTTCTCATTGGGTTCTTAGGAACA---GCAGGAAGCACTATG---GGCGCGGCGTCA---
GTGACGCTGACGGTACAG---GCCAGACAA---TTATTG---TCTGGCATAGTGCAACAGCAAAAC---
AATTTG---CTGAGG---GCTATAGAGGCTCAACAGCATCTGTTGAAACTCACGGTCTGGGGCATT
>MH746258
TTCTATTGTAACACATCAGGCCTGTTTAATAGCACATGGTCTAAAAATGGCACACAGCAGGTAGACAAC----
----

ATAACCAATCCTATAATTCTCCAATGCAGAATAAAGCAGATTATAAATATGTGGCAGAGAGCAGGACAAGCAA

TATATGCCCCTCCCATCCAAGGACTAATAAGGTGTGAATCAAAC---
ATTACAGGACTAATATTAACAAGAGATGGTGGAAATGGAATACA-----------------------------
----

ACAAACAATGAAACCGAAACCTTCAGACCTGGAGGAGGAGATATGAGGGACAATTGGAGAAGTGAATTATATA
AGTATAAGGTAGTACAAGTTGAACCACTA---GGAGTAGCACCCACCAAGGCAAAAAGAAGAGTGGTG-----
-----------------------------------GAGAGAGAAAAAAGAGCA---GTTGGA---
CTAGGAGCT---------GTATTCATTGGATTCTTAGGAGCA---GCAGGAAGCACTATG---
GGCGCGGCGTCT---GTGACGCTGACGGTACAG---GCCAGGCAA---TTATTG---
TCTGGTATAGTGCAACAGCAAAGC---AATTTG---CTGAGG---
GCTATAGAGGCTCAACAGCATTTGTTGAAACTCACAGTCTGGGGCATT
>MH746253
TTCTATTGTAATACATCAGCCTTATTTAATAGCACTTGGAATGATACCAATAATACGGGATCAAATACTGACA
ATAACACCACG--

GGGTCAAATGAAGTTATAACTCTCCCTTGCAGAATAAAACAAATTATACGTATGTGGCAGAGAACAGGACAAG
CAATGTATGCCCCTCCCATTTCAGGAATAATAAATTGTACATCAAAC---
ATTACAGGACTAATATTGACAAGAGATGGTAGTAATGGTGATAAT-----------------------------
----

AACAGTGAAACCTTCAGACCTGGAGGAGGAGATATGAGGGACAATTGGAGAAGTGAATTATATAAGTATAAAG
TAGTAAAAATTGAACCATTA---GGGGTAGCACCCACCAGGGCAAGGAGAAGAGTGGTG--------------
--------------------------CAGAGAGAAAAAAGAGCA---GTTGGA---ATAGGAGCT---------
GTCTTCCTTGGGTTCTTAGGAGCA---GCAGGAAGCACTATG---GGCGCAGCGTCA---
ATAACGCTGACGGTACAG---GCCAGACAA---TTATTG---TCTGGCATAGTGCAACAGCAAAAC---
AATTTG---CTGAGG---GCTATAGAGGCTCAACAACAGCTGTTGAAACTCACAGTCTGGGGCATT
>MH746251
TTCTATTGCAATACATCAGCCCTGTTCAATAGTACTTGGAATGGCACTGTCACAAATGGCACACAA-------
----

GCAAATGGAACTATAATTCTACCATGCAGAATAAAGCAAATTATAAGAATGTGGCAGAGAGTAGGACAAGCAA
TGTATGCCCCTCCTATCAGAGGAGTGATAAGGTGTGAATCAAAC---
ATTACAGGACTAATACTAACAAGAGATGGTGGAGGTGGAACTAAT-----------------------------
----

AGCAGCAATGAGACCTTCAGGCCTACAGGAGGAGATATGAGGGACAATTGGAGAAGTGAACTATATAAGTATA
AAGTAGTAAAAATTGAACCAATA---GGATTAGCACCTACCGAGGCAAGGAGGAGAGTGGTG-----------
-----------------------------GAGAGAGAAAAAAGAGCA---ATTGGA---CTGGGAGTT------
---GCCTTCCTTGGGTTCTTAGGAGCA---GCAGGAAGTACTATG---GGCGCGGCGTCA---
TTGACGCTGACGGCACAA---GCCAGACAA---TTATTG---TCTGGTATAGTGCAACAGCAAAGC---
AATTTG---CTGAGG---GCTATAGAGGCTCAGCAGCATCTGCTGAAACTCACAGTCTGGGGCATT
>KU168256
TTCTATTGCAATACATCAGGCCTGTTTAATAGCACTTGGCATGTCAATAATACATCAAGCCTGGTTAAT----
----

AGCACTGGGAACATAACTCTCCCATGCAGAATAAAGCAAATTATAAATATGTGGCAGAGAGTAGGACAAGCAA
TGTATGCCCCTCCTATTCCAGGAATAATAAAGTGTGAGTCAAAC---
ATTACAGGACTACTATTGATAAGAGATGGTGGAATTAATAATAAC-----------------------------
----

AGTATGAATGAGACCTTCAGACCTGGAGGAGGAGATATGAGAGACAATTGGAGAAGTGAATTATATAAATATA
AAGTAGTACAAATTGAACCACTA---GGAGTAGCACCTACCAAGGCAAAGAGAAGAGTGGTG-----------
-----------------------------GAGAGAGAAAAAAGAGCA---GTTGGA---TTTGGAGCT------
---GTCTTCCTTGGGTTCTTAGGAGCA---GCAGGAAGCACTATG---GGCGCGGCGTCA---
ATAACGCTGACGGCACAG---GCCAGACAA---TTATTG---TTTGGCATAGTGCAACAGCAAAGC---
AATTTG---CTGAGG---GCTATAGAGGCTCAACAGCATCTGTTGAAACTCACAGTCTGGGGCATT
>KU168305
TTCTATTGTAATACATCAGGCCTGTTTAATAGCACTTGGAAGACTAATGGCACTAGCACTTGGAAGGAGACTA
ATGGCACT---
-------GGGAGCCAGGAA------

AATAGCAATGACAATATAACTCTCCCATGCAGAATAAAGCAAATTATAAATATGTGGCAGAGAGCAGGACAAG
CAATGTATGCCCCTCCCATCGCAGGAGTAATAAAGTGTAACTCAAAC---
ATTACAGGACTACTATTAACAAGAGATGGGGGAAGTAATAACAAT----------------------------
----

AGCCAAGAGGAGACTTTCAGACCTGGAGGAGGAAATATGAAGGATAACTGGAGAAGTGAATTATATAGGTACA
AAGTAGTACAAATTGAACCCCTA---GGAATAGCACCCACCAGGGCAAGGAGAAGAGTGGTG-----------
----------------------------GGGAGAGAAAAAAGAGCA---GTTGGA---CTGGGAGCT------
---GTCTTCATTGGGTTCTTAGGAGCA---GCTGGAAGCACTATG---GGCGCAGCGTCA---
ATAACGCTGACGGCACAG---GCCAGACAA---TTATTG---TCTGGCATAGTGCAACAGCAAAGC---
AATTTG---CTGAGG---GCTATAGAGGCTCAACAGCATATGTTGAAACTCACGGTCTGGGGCATT
>KP718918
TTCTATTGTAATACATCAGGCCTGTTTAATAGCACTTGGTTTAGTAATGGGAGCAGGGGCAATGAGAGCATGG
AAGATGACACC--

AATATAACTCATATAACTCTCCCATGCAGAATAAGGCAAATTGTGAATATGTGGCAGAGAGCAGGACAAGCAA
TGTATGCCCCTCCCATCCAAGGAGTAATAAAGTGTACATCAAAT---
ATTACAGGAATACTATTAACAAGAGATGGTGGATATAATATTAGCAAT-------------------------
----

AACGACACCGAGATTTTCAGACCTGGAGGAGGAAATATGAAGGACAATTGGAGAAGTGAATTATATAAATATA
AAGTAGTAAAAGTTGAACCACTA---GGAATAGCACCCACTAGGGCAAAGAGAAGAGTGGTG-----------
----------------------------GAGAGAGAAAAAAGAGCA---GTTGGA---ATAGGAGCT------
---GTGTTCATTGGGTTCCTAGGAGCA---GCAGGAAGCACTATG---GGCGCAGCGTCA---
ATAACGCTGACGGCACAG---GCCAGACAG---TTATTG---TCTGGAATAGTGCAACAGCAAAGC---
AATTTG---CTGAGG---GCTATAGAGGCTCAACAGCATTTGTTGAAACTCACGGTCTGGGGCATT
>KP718928
TTCTATTGTAATACATCAGGCCTGTTTAATAGCACTTGGCATAGTAATGGCACTGCAAATGGCACT-------
----

GAAAATGCCACTATAACTCTCCCATGCAGGATAAAGCAGATTATAAATCTATGGCAGAGAGTAGGGCAAGCAA
TGTATGCCCCTCCTATCCCAGGAATAATAAATTGTACATCAAAC---
ATTACAGGACTACTATTAACAAGAGATGGTGGGAATGCTAGTGAG----------------------------
----

GAAACTGAGACCTTCAGACCTGGAGGAGGAAATATGAAGGACAATTGGAGGAGTGAATTATATAAATATAAAG
TAGTAAAGATTGAACCACTA---GGAGTAGCACCCACCAGGGCAAAGAGAAGAGTGGTG--------------
-------------------------GAGAGAGAAAAAAGAGCA---GTCGGA---ATAGGAGCT---------
GTGTTCATTGGGTTCTTAGGAGCA---GCAGGAAGCACTATG---GGCGCAGCGTCA---
ATAACGCTGACGGCACAG---GCCAGACAG---TTACTG---TCTGGTATAGTGCAACAGCAAAGC---
AATTTG---CTGAGG---GCTATAGAGGCTCAACAGCACATGTTGAAACTCACAGTCTGGGGCATT
>AM279343
TTCTATTGTAATACATCAGGCCTGTTTAATAGCACTTGGTTTAATAGCACTTGGAATGACACTATCAGCAATA
ATAAC--
-------------------ACA---
GGGTCAAATGACACCATATATCTCCCATGCAGAATAAAGCAAATTATAAATATGTGGCAAAGGACAGGACAAG
CAATGTATGCCCCTCCCATCCGAGGAATAATACAGTGTAATTCAACC---
ATTACAGGACTACTATTAACAAGAGATGGTGGGATTAACAACAAC----------------------------
----

AGTACAAATGAAACCTTCAGACCTGGAGGAGGAAATATGAGGGATAATTGGAGAAGTGAACTATATAAATATA
AAGTAGTAAAAATTGAACCACTA---GGAGTAGCACCCACCAAGGCAAGGAGAAGAGTGGTG-----------
----------------------------GAGAGAGAAAAAAGAGCA---GTTGGA---CTGGGAGCT------
---GTATTCATTGGGTTCTTAGGAGCA---GCAGGAAGCACTATG---GGCGCGGCGTCA---
ATAACGCTGACGGTACAG---GCCAGACAA---TTATTG---TCTGGCATAGTGCAACAGCAAAAC---
AATTTG---CTGAGG---GCTATAGAGGCTCAACAGCATCTGTTGAAACTCACGGTCTGGGGCATT
>AM279348
TTCTATTGCAATACATCAGGCCTGTTTGCTCAGGAGTTAAAAAATGACACAAAT-------------------
----

```
GACAATAACACAATAATTCTCCTATGCAAAATAAAGCAAATTATAAATATGTGGCAGAGAGTAGGACAAGCAA
TGTATGCCCCTCCCAGAGCAGGAGTAATAAGGTGTGTATCAAAC---
ATTACAGGACTACTGTTAACAAGAGATGGTGGAAAAAATGACAATGGA-------------------------
-------------------------------------------------------------------------
-------
ACTAATGGAACTGAGATATTCAGGCCTGGAGGAGGAGATATGAGGGACAATTGGAGAAGTGAATTATATAAGT
ATAAAGTAGTAAGAATTGAACCAGTA---GGGGTAGCACCCACCAGGGCAAAGAGAAGAGTGGTG--------
-------------------------------GAGAGAGAGAAAAGAGCA---GTTGGA---CTGGGAGCT---
------GTCTTCCTTGGGTTCTTAGGAGCA---GCAGGAAGCACTATG---GGCGCGGCGTCA---
CTGACGCTGACGGTACAG---GCCAGGCAA---TTATTG---TCTGGCATAGTGCAACAGCAGAGC---
AATTTG---CTGAGG---GCTATAGAGGCTCAACAACATATGTTGAGACTCACAGTCTGGGGCATC
>FJ388894
TTCTATTGTAACACATCAAGCCTGTTTAATAGCACCTGGAATATTAATACTATTTGGAATAACACTATGTCAA
ACAGC--------------------------------------------------------------------
-------------------ACT---
GAGTCAAACGGCACTATAGTGCTCCCATGCAGAATAAAGCAAATTGTAAATGTGTGGCAGAGAGCAGGACAAG
CAATATACGCCCCTCCCATCCAAGGAATAATACAGTGTGTCTCAAAC---
ATTACAGGACTAATATTAACAAGAGATGGTGGGAATAATAATAACACA-------------------------
-------------------------------------------------------------------------
----------
AATAATACTGAAATCTTCAGACCTGGAGGAGGAGATATGAGGGACAATTGGAGAAGTGAATTATATAAATATA
AGGTAGTAAAAATTGAACCATTA---GGAGTAGCACCCACCAAGGCAAGGAGAAGAGTGGTG-----------
----------------------------GGAGAGAAAAAAGAGCA---GTTGGA---CTGGGAGCT-------
---GTCTTCCTTGGGTTCTTAGGAACA---GCAGGAAGCACTATG---GGCGCGGCGTCA---
ATAACGCTGACGGTACAG---GCCAGGCAA---TTATTG---TCTGGCATAGTGCAACAGCAAAGC---
AATTTG---CTGAGG---GCTATAGAGGCTCAACAACATCTGTTGAAACTCACTGTCTGGGGCATT
>FJ388903
TTCTATTGTAACACATCGGGCCTGTTTAATAGCCAATATAACACATCAGGCCTGGTTAATAACACGCAGGAGT
CAGGAAGCACA------------------------------------------------------
GAGTCAAATGACACTGAGTCA------------
ATTGTCACAGACAATATAACTCTCCAAATCAGAATAAAGCAAATTATAAATCAGTGGCAAAGAGTAGCTCAAG
CAACATATGCCCCTCCCATCCCAGGAGTAATAAAATGTGTATCAAAC---
ATTACAGGACTAATATTAACTAGAGATGGTGGGAGTAGTAGCGGA----------------------------
-------------------------------------------------------------------------
----------------
AATGAAACCTTCAGACCTGGAGGAGGAGATATGAGGGACAATTGGAGAAGTGAATTATATAAGTATAAAGTAG
TAAAAATTGAACCACTA---GGAGTAGCACCCACCAGGGCAAAGAGAAGAGTGGTG-----------------
----------------------GAGAGAGAAAAAAGAGCA---GTTGGA---ATAGGAGCT---------
GTCTTCATTGGGTTCTTAGGAGCA---GCAGGAAGCACTATG---GGCGCGGCGTCA---
ATGACGCTGACGGTACAG---GCCAGGCAA---TTATTG---TCTGGCATAGTGCAACAGCAAAGT---
AATTTG---CTGAGG---GCTATAGAGGCTCAACAGCATCTGTTGAAACTCACGGTTTGGGGCATT
>FJ388909
TTCTATTGTAATACATCAGGCCTGTTTAATAGCACTTGGAATAATGACACCTGGAATGGCAATGACACACAAG
AGTCAAAT-----------------------------------------------------------------
-------------GAAACC------
TCGTCAAATGGCACCATAATTCCACCATGCAAAATAAAACAAATTGTAAATATGTGGCAGAGAGTACAACAAG
CAATGTATCCCCCTCTCGTCGAAGGAGTAATAAGGTGTGAATCCATC---
ATTACAGGACTACTATCAACAAGAGATGGTGGGATTAACAGCAGG----------------------------
-------------------------------------------------------------------------
-------------
ACAAATGAAACCTTCAGACCTGGGGGAGGAGATATGAGGGACAATTGGAGAAGTGAACTATATAAATATAAGG
TAGTAAAAATTGAACCACTA---GGAGTAGCACCCACCACGGCAAAGAGAAGAGTGGTG--------------
-------------------------GAGAGAGAAAAAAGAGCA---GCTGGA---CTGGGAGTT---------
ATGTTCCTTGGGTTCTTAGGAGCA---GCCGGAAGCACTATG---GGCGCGGCGTCA---
ATAACGCTGACGGTACAG---GCCAGACAG---TTATTA---TCTGGTATAGTGCAACAGCAAAGC---
AATTTG---CTGAAG---GCTATAGAGGCTCAACAGCACCTGTTGAGACTCACGGTCTGGGGCATT
>FJ388925
TTCTATTGTAACACATCAGGCTTGTTTAATAGCACTTGGAATGCCACTGCCAGCATGCAGGAGTCAAATAGCA
CAGAG--------------------------------------------------------------------
-------------------------
```

TCAAACGACACTACTATAACTCTCCCATGCAGAATAAAGCAAATTATAAATATGTGGCAGAGAGCAGGACAAG
CAATATATGCCCCTCCCATCCGAGGAGTAATAAGGTGTGAATCAAAC---
ATTACAGGACTAATATTAACAAGAGATGGAGGGAATAGTAACAGC----------------------------
----

ACAAATGAAACCTTCAGACCTGGAGGAGGAGATATGAGGGATAATTGGAGAAGTGAATTATATAAGTATAAAG
TAGTAAAAATTGAACCACTA---GGAGTAGCACCCACCAGGGCAAGGAGAAGAGTGGTG--------------
-------------------------GAGAGAGAAAAAAGAGCA---GTTGGA---ATAGGAGCT---------
GTCTTCATTGGGTTCTTAGGAGCA---GCAGGAAGCACTATG---GGCGCGGCGTCA---
ATAGCGCTGACGGTACAG---GCCAGGCAA---TTATTG---TCTGGCATAGTGCAACAGCAAAGC---
AATTTG---CTGAGG---GCTATAGAGGCTCAACAGCATATGTTGAAACTCACGGTCTGGGGCATT
>FJ388932
CTCTATTGTAACACATCAGGCCTGTTTAATAGCACTTGGCCTGCCAATAACAGTACGCAGGGGTCAAATAGCA
CA---

GAGTCAAACGACACCGTAATTCTCCAATGCAGAATAAAGCAAATTATAAATATGTGGCAAAGAGTAGGACAAG
CAATATATGCCCCTCCCATACCAGGAGAAATAAAGTGTGTATCAAAC---
ATTACAGGACTAATATTAACAAGAGATGGTGGGAGTAGCAATAAT----------------------------
----

ACAGAAAATGAAACCTTCAGACCAGGAGGAGGAGATATGAGGGACAATTGGAGAAGTGAATTATATAAGTATA
AAGTAGTAAAAATTGAACCACTA---GGAGTAGCACCCACCAGGGCAAAGAGAAGAGTGGTG-----------
----------------------------CAGAGAGAAAAAAGAGCAGTATTAGGTGGACTGGGAGCT------
---GTCTTCGTTGGGTTCTTGGGAGCA---GCAGGAAGCACTATG---GGCGCGGCGTCA---
GTGACGCTGACGGTACAG---GCCAGGAAA---TTATTG---ACTGGCATAGTGCAACAGCAAAGT---
AATTTG---CTGAGG---GCTATCGAGGCTCAACAGCATCTGTTGAAACTCACGGTCTGGGGCATT
>FJ388938
TTCTATTGTAACACATCAGGCCTGTTTAATAGCACTTGGAATGGCACTACCATGCAGGAGTCAAATAACACAG
AGTCAAAC---
-------------GACACT------
GAGTCAAACGACATTATAACTCTCCGATGCAGAATAAGGCAAATTATAAGAATGTGGCAGAGAGCAGGACAAG
CAATGTATGCCCCACCCATCCAAGGAATAATAAAGTGTGAATCAAAC---
ATTACAGGACTAATATTAGCAAGAGATGGTGGGAATAGAAATGAA----------------------------
----

ACTGAAACCTTCAGACCTGGAGGAGGAGACATGAGGGACAATTGGAGAAGTGAATTATATAAGTATAAAGTAG
TAAAAATTGAACCATTA---GGAGTAGCACCCACCAAGGCAAGGAGAAGAGTGGTG-----------------
----------------------CAGAGAGAAAAAAGAGCA---GTTGGA---ATAGGAGCT---------
GTCTTCATTGGGTTCTTAGGAGCA---GCAGGAAGCACTATG---GGCGCGGCGTCA---
ATAACGCTGACGGTACAG---GCCAGGCAA---CTATTG---TCTGGCATAGTGCAACAGCAAAGT---
AATTTG---CTGAAG---GCTATAGAGGCTCAACAACATCTGTTGAAACTCACGGTTTGGGGCATT
>FJ388942
TTCTATTGTAACACATCAGGCCTGTTTAATAGCTCTTGGCTGAAGTCAAATAGAACAGGGCCTAGCACAGAGC
CAAACAAC---
-------------ACTGAG------
CCAAACAACACTATTATAACTCTCCCATGCAAAATAAAGCAAATTATAAATATGTGGCAGAGAACAGGACAAG
CAATGTATGCCCCTCCCATCCCAGGAGTAATAAGGTGTGAATCAAAC---
ATTACAGGACTAATATTAACAAGAGATGGTGGGAATAGTAGCAAT----------------------------
----

AGCAATGAAACCTTCAGACCTGGAGGAGGAAATATGAGGGACAATTGGAGAAGTGAATTATATAAGTATAAAG
TAGTAAAAATTGAACCACTA---GGAGTAGCACCCACCAGGGCAAGGAGAAGAGTGGTG--------------
-------------------------GAGAGAGAAAAAAGAGCA---GTTGGA---ATAGGAGCT---------
GTCTTCATTGGGTTCTTAGGAGCA---GCAGGAAGCACTATG---GGCGCGGCGTCA---
ATGACGCTGACGGTACAG---GCCAGGCAA---TTATTG---TCTGGCATAGTGCAACAGCAAAGC---
AATTTG---CTGAGG---GCTATAGAGGCTCAACAGCATATGTTGAAACTCACGGTCTGGGGCATT
>FJ388946
TTTTATTGTAATACATCAGGCCTGTTTAATAGAACTTGGTCTACCAATACCAGCATGCAGGTGTCAAATAGCA
CA---

GAGTTAAATGGCCCTATAATTCTCCAATGCAGAATAAAGCAAATTATAAATATGTGGCAAAGAGTAGGACAAG
CAATATATGCCCCTCCCATCCCAGGAGTAATAAGGTGTGAATCAAAC---
ATTACAGGACTAATATTAACAAGAGATGGTGGAAATAATAATACC----------------------------
----

GGAAATCAAACCTTCAGACCTGTAGGAGGAGATATGAAGGACAATTGGAGAAGTGAATTATATAAGTATAAAG
TAGTAAAAATTGAACCACTA---GGAGTAGCACCCTCCAAGGCAAAGAGAAGAGTGGTG--------------
-------------------------GAGAGAGAAAAAAGAGCA---ATTGGA---ATAGGAGCT---------
GTCTTCATTGGGTTCTTAGGAGCA---GCAGGAAGCACTATG---GGCGCGGCGTCA---
GTGACGCTGACGGTACAG---GCCAGGCAA---TTATTG---TCTGGCATAGTGCAACAGCAAAGT---
AATTTG---CTGAGG---GCTATAGAGGCTCAACAGCATCTGTTGAACCTCACGGTCTGGGGCATT
>JF683737
TTCTATTGTAACACATCAGGCCTGTTTAATAGCACGTGGAATGCTAGTGACATCACGCAGGGG----------
----

TTAAATGACACAATAACTCTCCAATGCAGAATAAAGCAAATTATAAATATGTGGCAGAGAGTAGGGCAGGCAA
TGTATGCCCCTCCCATCCAGGGAGAAATAAGGTGTGAATCAAAC---
ATTACAGGACTAATATTAACAAGAGATGGTGGGAATGGGAATGGA----------------------------
----

ACAAATGAAACCTTCAGACCTGGAGGAGGAGATATGAGGGACAATTGGAGAAGTGAATTATATAAGTATAAAG
TAGTAAAAATTGAACCACTA---GGAGTAGCACCCACCAGGGCAAAGAGAAGAGTGGTG--------------
-------------------------GAGAGAGAAAAAAGAGCA---GTTGGA---CTGGGAGCT---------
GTATTCATTGGGTTCTTAGGAGCA---GCAGGAAGCACTATG---GGCGCGGCGTCA---
GTGACGCTGACGGTACAG---GCCAGGCAA---TTATTG---TCTGGCATAGTGCAACAGCAAAGC---
AATTTG---CTGAGG---GCTATAGAGGCTCAACAGCATTTGTTGAAACTCACAGTCTGGGGCATT
>JF683759
TTCTATTGTGACACATCAGGCCTGTTTAATAGCACTTGGAATACCACTGACACCATGCACGAGGAGACTAATA
CA---

GAGGCAAATAGTACTATTACTCTCCCATGCAGAATAAAGCAAATTATAAATATGTGGCAGAGAGCAGGACAAG
CAATATATGCCCCTCCCATCCCAGGAGTAATACAGTGTAAATCAAAC---
ATTACAGGACTAATATTAACAAGAGATGGTGGGAGTAATAGCAGC----------------------------
----

ACAAATGAAACCTTCAGACCTGGAGGAGGAGATATGAGGGACAATTGGAGAAGTGAATTATATAAGTATAAAG
TAGTAAAAATTGAACCACTA---GGAGTAGCACCCACCAAGGCAAGGAGAAGAGTGGTG--------------
-------------------------CAGAGAGAAAAAAGAGCA---GTAGGA---CTGGGAGCG---------
GTATTCATTGGGTTCTTAGGAGCA---GCTGGAAGCACCATG---GGCGCGGCGTCA---
ATAACGCTGACGGTACAG---GCCAGGCAA---TTATTG---TCTGGTATAGTGCAACAGCAAAGC---
AATTTG---CTGAGG---GCTATAGAGGCTCAACAGCATCTGTTGAAACTCACAGTCTGGGGCATT
>JF683760
TTCTATTGTAACACCTCAAAGTTGTTTAATAGCACTTGGGATCCTAATAATACCAGCACGCAGGAGTCAAATA
GC---

ACAAATGATAATATAATTCTCCCATGCAGAATAAGGCAAATTATAAATATGTGGCAGAGAGCAGGACAAGCAA
TATATGCCCCTCCCATCCAAGGAATAATAAACTGTGTATCAAAC---
ATTACAGGATTAATATTAACAAGAGATGGGGGTGTAGGTAACAAC----------------------------
----

GCAAAGGAAGTCTTCAGACCTGGAGGAGGAGATATGAGGGACAATTGGAGAAGTGAATTATATAAGTATAAAG
TAGTAAAAATTGAGCCACTA---GGAGTAGCACCCACTGGGGCAAGGAGAAGAGTGGTG--------------
-------------------------CAGAGAGAAAAAAGAGCA---GTTGGA---CTGGGAGCT---------
GTTTTCATTGGGTTCTTGGGAGCA---GCAGGAAGCACTATG---GGCGCGGCGTCA---
ATGACGCTGACGGTACAG---GCCAGACAA---TTATTG---TCTGGCATAGTGCAACAGCAAAGC---
AATTTG---CTGAGG---GCTATAGAGGCTCAACAGCATATGTTGAGACTCACGGTCTGGGGCATT
>JF683761
TTCTATTGTAACACCTCAAAGCTGTTTAATAGCACTTGGAATAATACCAGCACGCAGGAGTCAAATAGCACTA
AT---

CCAAATGATAATATAACTCTCCCATGCAGAATAAAGCAAATTATAAATATGTGGCAAAGAGCAGGACAAGCAA
TATATGCCCCTCCCATCCAAGGAAAAATAAACTGTGTATCAAAC---
ATTACAGGATTAATATTAACAAGAGATGGGGGGGTAACAGCACA-----------------------------
----

GAGGAAGTCTTCAGACCTGGAGGAGGAGATATGAGGGACAATTGGAGAAGTGAATTATATAAGTATAAAGTAG
TAAAAATTGAGCCAATA---GGAGTAGCACCCACTGAGGCAAGGAGAAGAGTGGTG-----------------
----------------------CAGAGAGAAAAAAGAGCG---GTTGGA---CTGGGAGCT---------
GTTTTCATTGGGTTCTTGGGAGCA---GCAGGAAGCACTATG---GGCGGGGCGTCA---
ATGACGCGGACGGTACAG---GCCAGACAA---TTATTG---TCTGGCATAGTGCAACAGCAAAGC---
AATTTG---CTGAGG---GCTATAGAGGCTCAACAGCATATGTTGAGACTCACGGTCTGGGGCATT
>JF683767
TTCTATTGTAATACATCAGGTCTGTTCAATAGCTCTTGGAATATCAATGGCAGCAGGTCAAATAGCACAAGG-
----

TCAAATGGCAATATAACTCTCCCATGCAGAATAAAGCAAATTATAAATATGTGGCAAAGAACAGGACAGGCAA
TGTATGCCCCTCCCATCCAAGGAGTAATAAGGTGTGTATCAAAC---
ATTACAGGACTACTGTTAACAAGAGATGGAGGGGGTAACAATACA----------------------------
----

AATGAAACTGAAATTTTCAGACCTGGAGGAGGAGATATGAGGGGCAATTGGAGAAGTGAATTATATAAGTATA
AAGTAGTAAAAATTGACCCACCA---GGAGTAGCACCCAACACAGCAAGGAGAAGAGTGGTG-----------
----------------------------GGGAGAGAAAAAAGAGCA---GCTGGAATAATAGGAGCT------
---GTCTTCCTTGGGTTCTTAGGAGCA---GCAGGAAGCACTATG---GGCGCGGCGTCA---
GTGACGCTGACGGTACAG---GCCAGACAA---TTATTG---TCTGGTATAGTGCAACAGCAAAGC---
AATTTG---CTGAGG---GCTATAGAGGCTCAACAACACCTGTTGAAACTCACGGTCTGGGGCATT
>JF683779
TTCTACTGTAACACATCAGGCCTGTTTAATAGCACTTGGTATACCAATAACAGCATGCAGCCAAAT-------
----

TACACAAAAACTATAACTTTGCCATGCAGAATAAAGCAAGTTATAAATATGTGGCAAAGAGTAGGACAAGCAA
TGTATGCCCCTCCCATCCCAGGAATAATACAATGTGTATCAAAC---
ATTACAGGACTAATATTAACAAGAGATGGTGGGCATAATAATAGT----------------------------
----

GCAAATGAGACCTTCAGACCTGGAGGAGGAGATATGAGGGACAATTGGAGAAGTGAATTATATAAGTATAAAG
TAGTGAAAGTTGAACCATTA---GGAGTAGCACCCACCAGGGCAAGGAGAAGAGTGGTG--------------
-------------------------GAGAGAGAAAAAAGAGCA---GTTGGA---ATAGGAGCT---------
GTCTTCCTTGGGTTCTTAGGAGCA---GCAGGAAGCACTATG---GGCGCGGCGTCA---
GTGACGCTGACGGTACAG---GCCAGGCAA---TTATTG---TCTGGCATAGTGCAACAGCAAAGC---
AATTTG---CTGAGG---GCTATAGAGGCTCAACAACATATGTTGAAACTCACGGTCTGGGGCATT
>JF683782
TTCTATTGTAATACATCAAAATTGTTTAATACAACTTGGAATGCCAATGGCAGCGTACAGGAGTCAGTCAACA
CT---

GCAGGCAACACTACTATATTTCTCCCATGCAGAATAAAGCAAATTATAAGGATGTGGCAGAGAGCAGGACAAG
CAATATATGCCCCTCCCATCCAGGGAAGAATAAGGTGTGAATCAAAC---
ATTACAGGACTACTGTTAACAAGAGATGGTGGAGGTAATGCAAAC----------------------------
----

AACACAGAAATCTTTAGACCTGGAGGAGGAGATATGAGGGACAATTGGAGAAGTGAATTATATAAGTACAAGG
TAGTAAAAATTGAACCACTA---GGAGTAGCACCCACCAAGGCAAGGAGAAGAGTGGTG--------------
-------------------------CAGAGAGAAAAAAGAGCA---GTTGGA---CTAGGAGCT---------
GTCTTCCTTGGGTTCTTAGGAGCA---GCAGGAAGCACTATG---GGCGCGGCGTCA---
ATAACGCTGACGGTACAG---GCCAGGCAA---TTATTG---TCTGGCATAGTGCAACAGCAAAGC---
AATTTG---CTGAGG---GCTATTGAGGCTCAACAGCAGATGTTGAGACTCACGGTCTGGGGCATT
>JF683783
TTCTATTGTAATACATCAAAATTGTTTAATACAACTTGGAATGTCACTGACAGCATAGAAGAGTCA-------
----

GGCAACACTATTATAGTCCTCCCATGCAAAATAAAGCAAATTATAAATATGTGGCAGAGAGCAGGACAAGCAA
TATATCCCCCTCCCATCCGGGGAATAATAAGGTGTGAATCCAAC---
ATTACAGGACTATTATTAACAAGAGATGGTGGAGATACTGAAAAC----------------------------
----

ACCACAGAAATCTTTAGACCTGGAGGAGGAGATATGAGGGACAATTGGAGAAGTGAATTATATAAGTATAAAG
TAGTAAAAATTGAACCACTA---GGAGTAGCACCCACCGAGGCAAGGAGAAGAGTGGTG--------------
-------------------------CAGAGAGAAAAAAGAGCA---GTTGGA---ATAGGAGCT---------
GTCTTCCTTGGGTTCTTAGGAGCA---GCAGGAAGCACTATG---GGCGCGGCGTCA---
ATAACGCTGACGGTACAG---GCCAGGCAA---TTATTG---TCTGGCATAGTGCAACAGCAAAGC---
AATTTG---CTGAGG---GCTATAGAGGCTCAACAGCAGATGTTGAGACTCACGGTCTGGGGCATT
>JF683789
TTCTATTGTAACACCTCAAAGCTGTTTAATAGCATTTGGCATCCTAATGATACCATGCAGGAGGAGACTAATA
CA---

AATCCAAATGCTAATATAACTCTCCCATGCAGAATAAAGCAAATTATAAATCTGTGGCAAAGAGTAGGGAAAG
CAATATATGCCCCTCCCATCCGAGGAAGAATAGAGTGTGTATCAAAC---
ATTACAGGATTAATAATAACAAGAGATGGTGGAAATAACACAAAT----------------------------
----

AACACTGAAATCTTCAGACCTGCAGGAGGAGACATGAGGGACAATTGGAGAAGTGAATTATATAAATATAAAG
TAGTACAAATTGAGCCACTA---GGAATAGCACCCACTGAGGCAAGGAGAAGAGTGGTG--------------
-------------------------CAGAGAGAAAAAAGAGCA---ATTGGA---CTAGGAGCT---------
ATGTTCATTGGGTTCTTAGGAGCA---GCAGGAAGCACTATG---GGCGCGGCGTCA---
GTGACGCTGACGGTACAG---GCCAGGCAA---TTATTG---TCTGGCATAGTGCAACAGCAAAGC---
AATTTG---CTGATG---GCTATAGAGGCTCAACAGCATCTGTTGAAACTCACAGTCTGGGGCATT
>FJ670523
TTCTATTGTAACACATCAGGCCTGTTTAATAGCACTTGGCCTAATAGCACTTGGAATGCCAATACCAGCATAC
AGGAG--
-------------------TCA---
AATAGCACAGACAATATAACTCTCCAATGCAGAATAAAGCAAATTATAAATCTGTGGCAGAGAGCAGGAAAAG
CAATATATGCCCCTCCCATCCAAGGAGCAATACAGTGTATATCAAAC---
ATTACAGGACTAATATTAACAAGAGATGGAGGTAATAATGGAACT----------------------------
----

AATAACACTGAGATCTTCAGACCTGGAGGAGGAGATATGAGAGATAATTGGAGAAGTGAATTATATAAGTATA
AGGTAGTAAAAATTGAACCATTA---GGAGTAGCACCCTCCAGGGCAAAGAGAAGAGTAGTG-----------
----------------------------GGGAGAGAAAAAAGAGCA---GTTACA---ATAGGAGCT------
---GTCTTCCTTGGGTTCTTAGGAGCA---GCAGGAAGCACTATG---GGCGCGGCGTCA---
CTGACGCTGACGGTACAG---GCCAGGCAA---TTATTG---TCTGGCATAGTGCAACAGCAAAGC---
AATTTG---CTGCAG---GCTATAGAGGCTCAACAGCATCTGTTGAAACTCACGGTCTGGGGCATT
>AF219261
TTCTATTGTAATACATCAGGCCTGTTTAATAGCACTTGGGTGTCAAACAACACTGGGAAATATTATAGTAATA
GCACT--

AGGGAAAATCCAAATATAACTCTCCCATGCAGAATAAAACAAATTATAAATATGTGGCAGAGAGCAGGACAAG
CAATATATGCCCCTCCCATCGAAGGAGTAATAAGGTGTGAATCAAAC---
ATTACAGGACTACTATTAACAAGAGATGGTGGGGATAAGAACAAC----------------------------
----

ACAAGTGAAATCTTCAGACCTGGAGGAGGAAATATGAAGGACAACTGGAGAAGTGAATTATATAAGTATAAAG
TAGTAAAAATTGAACCATTA---GGAGTAGCACCCACCCCGGCAAGGAGAAGAGTGGTG--------------
-------------------------CAGAGAGAAAAAAGAGCA---GTATTTGGGCTGGGAGCT---------
GTCTTTCTTGGGTTCTTAGGAGCA---GCAGGAAGCACTATG---GGCGCGGCGTCG---
ATAACGCTGACGGTACAG---GCCAGACAA---TTATTG---TCTGGCATAGTGCAACAGCAAAGT---
AATTTG---CTGAGG---GCTATAGAGGCTCAACAGCATTTGTTGAAACTCACGGTCTGGGGCATT
>AF219265
TTCTATTGTAATACATCAGGCCTGTTTAATAGCACTTGGAGTAATAGCACTTGGAGTTCCAATGCCAGCACGC
AGGGG--

TCAGAAAATGACACTATAACTCTCCCATGCAGAATAAAACAAATTATAAATATGTGGCAGAGAGCAGGACAAG
CAATGTATGCCCCTCCCATTCAAGGAGTAATAAGGTGTGATTCAAAT---
ATTACAGGACTAATATTAACAAGAGATGGTGGGGTTACAAATGAA----------------------------
----

ACCGAAACCTCCAGACCTGGAGGAGGAGATATGGGGGACAATTGGAGAAGTGAATTATATAAATATAAAGTAG
TGAAAATTGAACCACTA---GGAGTAGCACCCACCAGGGCAAAAAGAGTAGTGGTG-----------------
----------------------GGGAGAGAAAAAAGAGCA---ATTGGA---ATAGGAGCT---------
GTTTTCATTGGGTTCCTAGGAGCA---GCAGGAAGCACTATG---GGCGCGGCGTCA---
ATGACGCTGACGGTACAG---GCCAGACAA---TTATTG---TCTGGCATAGTGCAACAGCAAAGC---
AATTTG---CTGAGG---GCTATAGAGGCTCAACAACATCTGTTGAAACTCACGGTCTGGGGCATT
>HM204635
TTCTATTGTAATACATCACACCTGTTTAATAGCGCTTGGTCTGTCAATGACACCGTGCAGGAGTCAAATAGCA
CG---

GAGTCAAATGACACTATAACTCTCCAATGCAGAATAAAGCAAATTATAAATATGTGGCAGAGAACAGGACAAG
CAATGTATGCCCCTCCCATCCCAGGAGTAATAAGATGTAACTCAAAC---
ATTACAGGACTAATATTAACAAGAGATGGTGGGAATAATAATAGTTCA-------------------------
----

AATGAAAATGAAACCTTCAGACCTGGAGGAGGAGATATGAGAGACAATTGGAGAAGTGAATTATATAAGTATA
AAGTAGTAAAAATTGAACCACTA---GGAGTAGCACCCACCAAGGCAAGGAGAAGAGTGGTG-----------
----------------------------CAGAGAGAAAAAAGAGCA---GTTGGA---CTGGGAGCT------
---GTCTTCATTGGGTTCTTAGGAGCA---GCAGGAAGCACTATG---GGCGCGGCGTCA---
GTGACGCTGACGGTACAG---GCCAGACAA---TTATTG---TCTGGCATAGTGCAACAGCAAAGC---
AATTTG---CTGAGG---GCTATAGAGGCTCAACAACATCTGTTGAAACTCACAGTCTGGGGCATT
>HM204648
TTTTATTGTAACACATCAGGCCTGTTTACTGGCACTTGGAATCAGACTAATAGTACTTGGAATAGTGGCACTT
GGACT--

AACACTACTGGCAATATAACTCTCCAATGCAGAATAAAGCAGGTCATAAGAATGTGGCAGAGAGTAGGACAAG
CAATGTATGCCCCTCCCATCCCAGGAGTAATAAGGTGTGAATCAAAC---
ATTACAGGACTAATATTAACAAGAGATGGGGAAGGTAACAGTAGC----------------------------
----

ATAAAAGATGAAACCTTCAGACCTGGAGGAGGGAATATGAAGGACAATTGGAGAAGTGAATTATATAAATATA
AAGTAGTAAAAATTGAACCAATA---GGAGTAGCACCCACCAGGGCAAAGAGAAGAGTGGTG-----------
----------------------------GAGAGAGAAAAAAGAGCA---GTTGGA---CTAGGAGCT------
---GTCTTCATTGGGTTCTTAGGAGCA---GCAGGAAGCACTATG---GGCGCAGCGTCA---
ATAACGCTGACGGTACAG---GCCAGGCAA---TTGTTG---TCTGGCATAGTGCAGCAGCAAAGC---
AATTTG---CTGAAG---GCTATTGAGGCTCAACAACAGTTGTTGAGACTCACGGTCTGGGGCATT
>HQ595782
TTCTATTGCAATACGTCTAAACTGTTTAATAGCACGTGGAAAAATGAAAGCAGGCAGGGG-------------
----

TCAAATGACATTATAACTCTCCCATGCAGAATAAAGCAAATTATAAGGATGTGGCAAAGAGTAGGGCAAGCAA
TGTATGCCCCTCCCATCCCAGGAGTAATAGAGTGTAATTCAAAC---
ATTACAGGGCTACTATTGACAAGAGATGGTGGTAATAGAACCGAA----------------------------
----

AATGAGACCTTCAGACCTGAGGGAGGAGATATGAGAAACAATTGGAGAAGTGAATTATATAAGTATAAAGTAG
TAAGAATTGAACCATTG---GGAGTAGCACCCACCAGGGCAAAAAGAAGAGTGGTG-----------------
----------------------GAGAGAGAAAAAAGAGCA---GCTGGA---TTGGGAGCT---------
GTCTTCCTTGGGTTCTTAGCAGCA---GCAGGAAGTACTATG---GGCGCAGCGTCA---
ATAACGCTGACGGCACAG---GCCAGACAA---TTATTG---TCTGGCATAGTACAACAGCAAAGC---
AATTTG---CTGAGG---GCTATAGAGGCTCAACAGCAACTGTTGAGGCTCACGGTCTGGGGCATT
>HQ595784
TTCTATTGCAACACAGCAGGTCTGTTTAATAGCAGCTGGGGGAACAATGACACTGACAGCAACCAGACA----
----

GCAAAGATAAATATAACTCTCCAATGCAGAATAAAGCAAATTGTAAGGATGTGGCAGAGAGTAGGACAAGCAA
TGTATGCCCCTCCCATCGCAGGAACGATAAACTGTTTATCAAAT---
ATTACAGGACTACTATTAACAAGAGATGGGGGGACAAATAAGACGTGG-------------------------
----

TCTCACAATGAGACCTTCAGACCTGAAGGAGGAAATATGAAGGACAATTGGAGAAGTGAATTATATAGGTATA
AAGTAGTAAAAATTGAACCACTA---GGAGTAGCACCCACCGAGGCAAGGAGAAGAGTGGTG-----------
----------------------------GAGAGAGGAAAAAGAGCA---GTTGGA---ATAGGAGCT------
---GTTCTCTTTGGGTTCTTAGGAGCA---GCAGGAAGCACTATG---GGCGCGGCGTCA---
CTAACGCTGACGGTACAG---GCCAGACAA---TTACTG---TCTGGCATAGTGCAACAGCAGAGC---
AATCTG---CTGAGG---GCTATAGAGGCTCAACAGCATCTGTTGAAGCTCACAGTCTGGGGCATT
>Y13718
TTCTATTGTAACACATCAAGACTGTTTAATAGCACTTGGAATGACACTACCAGTATGCTAAATGACACGAAG-
----

CCAAACGACACTATAACTCTCCCATGCAGAATAAAGCAAATTATAAATATGTGGCAAAGAGCAGGACAAGCAA
TATATGCCCCTCCCATCCAAGGAGTAATAAGGTGTGAATCAAAC---
ATTACAGGACTAATATTAACAAGAGATGGTGGGGGTAATAGCAGT----------------------------
----

GTAAGTAGTGAAACTTTCAGACCTGGAGGAGGAGATATGAGAGACAATTGGAGAAGTGAATTATATAAGTATA
AAGTAGTAAAAATTGAACCACTA---GGAGTAGCACCCACCAAGGCAAAGAGAAGAGTGGTG-----------
----------------------------GAGAGAGAAAAAAGAGCA---GTTGGA---CTAGGAGCT------
---GTTTTCATTGGGTTCTTAGGAGCA---GCAGGAAGCACTATG---GGCGCGGCGTCA---
ATAACGCTGACGGTACAG---GCCAGACAA---TTATTG---TCTGGCATAGTGCAACAGCAAAGC---
AATTTG---CTGAGG---GCTATAGAGGCTCAACAGCATCTGTTGAAACTCACGGTCTGGGGCATT
>Y13717
TTCTATTGTAACACATCAGGCCTGTTTAATAGCACTTGGGATAGCAGTGCCAGCATGCAAGAGTCAAAT----
----

AGCACGGCCAATATAACTCTCCCATGCAGAATAAGGCAAATTATAAATCTGTGGCAGAGAACAGGACAAGCAA
TATATGCCCCTCCCATCCAAGGAGTAATAAGGTGTGTATCAGAC---
ATCACAGGACTAATATTAACAAGAGATGGTGGGGGTAATGGTACTGAA-------------------------
----

AGTAAAAAAGAAACTTTCAGACCTGGAGGAGGAGATATGAGGGACAATTGGAGAAGTGAATTATATAAGTATA
AAGTAGTAAAAATTGAACCACTA---GGAGTAGCACCCACCAAGGCAAAGAGAAGAGTGGTG-----------
----------------------------GAGAGAGAAAAAAGAGCA---GTTGGA---ATGGGAGCT------
---GTCCTCCTGGGGTTCTTAGGAGCA---GCAGGAAGCACTATG---GGCGCGGCGTCA---
ATAACGCTGACGGTACAA---GCCAGACAA---TTATTG---TCTGGCATAGTGCAACAGCAAAGC---
AATTTG---CTGAGG---GCTATAGAGGCTCAACAGCAGGTGTTGAGACTCACGGTCTGGGGCATT
>HQ385459
TTCTATTGCGATACATCAGGCCTGTTTAATAGTATTTGGAATAGCACTGCCATCAGCCAGAATGTCGAGGAG-
----

TCAAATGATACTATAACTCTCCCATGCAAAATAAGACAAATTGTAAATATGTGGCAGAGAGTAGGACAAGCAA
TATATGCCCGTCCCATTCAAGGAATAATAAACTGTACATCAAAC---
ATTACAGGGCTACTGCTAACAAGAGATGGTGGGGATAATGATAAT----------------------------
----

AAAACTGAGACTTTCAGACCTGGAGGAGGAAATATGAAGGACAATTGGAGAAGTGAGTTATATAGATATAAAG
TAGTAAAAATTGAACCACTA---GGAGTAGCACCCACCAAGGCAAAGAGAAGAGTGGTG--------------
-------------------------GAGAGAGAAAAAAGAGCA---GTTGGA---CTAGGAGCT---------
GTCATCTTTGGGTTCCTGGGAGCA---GCCGGAAGTACTATG---GGCGCGGCGTCA---
ATAACGCTGACGGCACAG---GCCACACAA---TTATTG---TCTGGTATAGTACAACAGCAAAGC---
AATCTG---CTGAGG---GCTATAGAGGCTCAACAGCACCTGTTGAAACTCACGGTCTGGGGCATT
>HQ385452
TTCTATTGCAATACATCAGGCCTGTTTTCTGGCTCTTGGTATAATGGCACTTGGGCTAATGACTCTTGGAGTG
CTGGT--
-------------------CCT---

GGGACCAATGATAATATAACTCTCCCATGCAAAATAAAGCAAATCATAAGAATGTGGCAGAGAGTGGGACAAG
CAATGTATGCCCCTCCCATCCAAGGAGAAATAAATTGTGTATCAAAC---
ATCACAGGACTATTACTAACAAGAGATGGTGGTAATAGTAGTGAA----------------------------
----

GAGATCTTCAGACCTGGAGGAGGAGATATAAGAGACAATTGGAGGAGTGAATTATATAAATATAAAGTGGTAA
AAATTGAACCATTA---GGAGTAGCGCCCACCAGGGCAAGGAGAAGAGTGGTG--------------------
-------------------GGAAGAGAAAAAAGAGCA---GTTGGA---CTGGGAGCT---------
GTCTTCCTTGGGTTCTTAGGAGCA---GCAGGAAGCACTATG---GGCGCGGCGTCA---
ATCACGCTGACGGTACAG---GCCAGGCAA---TTATTG---TCTGGCATAGTGCAACAGCAGAGC---
AATCTG---CTGAGG---GCTATAGAGGCTCAACACCATCTGTTGAAACTCACGGTCTGGGGCATT
>HQ385453
TTCTACTGCGACACAACAGGCCTGTTTAATAACACAGAGAAA-------------------------------
----

AATGACACTATAATGCTCCCATGCAAAATAAGACAAATTGTACGTATGTGGCAGAGAGTAGGACAAGCAATGT
ATGCCCCTCCCATTCCAGGAGTAATAAACTGTCTATCAAAC---
ATTACAGGGCTACTGCTGACAAGAGATGGTGGGGATAGTACTAAT----------------------------
----

AATACTGAGAAATTCAGACCAGGAGGAGGAGATATAAGGGACAATTGGAGAAGTGAATTATATAAATATAAAG
TAGTAAAAATTGAACCACTA---GGAGTAGCACCTACCAAGGCAAAGAGAAGAGTGGTG--------------
-------------------------GAGAGAAGAAAAAGAGCA---GGA------CTAGCAGCT---------
GTCTTCCTTGGGTTCTTAGGAGCA---GCAGGAAGCACTATG---GGCGCAGCGTCA---
ATAACGCTGACGGTACAG---GCCAGACAA---TTATTG---TCTGGTATAGTGCAACAGCAAAAC---
AATTTG---CTGAAG---GCTATAGAGGCTCAACAACAGATGTTGAGACTCACGGTCTGGGGCATT
>KT152846
TTCTATTGTAATACATCAAACCTGTTTAATAGCACTTGGACGAAAAATGACAGCATGCAGGGGTTAAATCACA
CGGAG--

TCAAATGACAATATAACTCTCCCATGCAGACTAAGGCAAATTATAAAAATGTGGCAGAGAGTAGGACAAGCAA
TGTATGCCCCTCCCATCCAAGGAATAATAAAGTGTGAATCAAAC---
ATTACAGGGCTAATATTAACAAGAGATGGTGGGAATAACAACAGT----------------------------
----

GCAGAGGAAACCTTCAGACCTGGAGGAGGAGATATGAGGGACAATTGGAGAAGTGAATTATATAAGTATAAAG
TAGTACAAATTGAACCACTA---GGGGTAGCACCCACCAAGGCAAGGAGAAGAACGGTG--------------
-------------------------GGGAGAGAAAAAAGAGCA---GTTGGA---ATGGGAGCT---------
GTTTTCCTTGGGTTCTTAGGAGCA---GCAGGAAGCACTATG---GGCGCAGCGTCA---
ATAACGCTGACGGTACAG---GCCTGGCAA---TTATTG---TCTGGCATAGTGCAACAGCAAAGC---
AATTTG---CTGAGG---GCTATAGAGGCTCAACAACATCTGTTGAAACTCACGGTCTGGGGCATT
>KT152841
TTCTATTGTGATACATCAGCCCTGTTTAATGGCACTTGGAATAATACCAGCATGAAGGAGTCAAATAGCACGG
AA---

TCAAATGACACTATAACTCTCCCATGCAGAATAAAGCAAATTATAAATATGTGGCAGAGAGTAGGACAAGCAA
TGTATGCCCCTCCCATCCAAGGAATAATAAAGTGTGAATCAAAC---
ATTACAGGACTACTATTAACAAGAGATGGTGGTGGGGATAACAGTACA-------------------------
----

AATGAGGAAACCTTCAGACCTGGAGGAGGAGATATGAGGGACAATTGGAGAAGTGAATTATATAGGTATAAAG
TAGTAAAAATTGAACCACTA---GGAGTAGCACCCACCAGGGCAAGGAGAAGAACGGTG--------------
-------------------------GGGAGAGAAAAAAGAGCA---GTGGAA---ATAGGAGCT---------
GTCTTCCTTGGGTTCTTAGGAGCA---GCAGGAAGCACTATG---GGCGCAGCGTCA---
ATAACGCTGACGGTACAG---GCCAGACAA---TTATTG---TCTGGCATAGTGCAACAGCAAAAC---
AATTTG---CTGAGG---GAA---GAGGCTCAACAACATTTGTTGAAACTCACGGTCTGGGGCATA
>KT152842
TTCTATTGTAATACATCAGCTCTGTTTAATGGCACTTGGGGGCAAAATGCCAGCATGGAGTCAAATGGCACT-
----

ATAAATGACACTATAATTCTCCCATGCAGACTAAAGCAAATTGTAAGGATGTGGCAGAGAGTAGGACAAGCAA
TGTATGCCCCTCCCATCCAAGGAGAAATAAATTGTGTATCAAAC---
ATTACAGGACTAATATTAACAAGAGATGGTGGGAATAATACAGAG----------------------------
----

GAAATCTTCAGACCTGGAGGAGGAGATATGAGGGACAATTGGAGAAGTGAATTATATAAGTATAAAGTAGTAA
AAATTGAACCACTA---GGAGTAGCACCCACCAGTGCAAGGAGAAGAACGGTG--------------------
-------------------GGGAGAGAAAAAAGAGCA---GTTGGA---ATAGGAGCT---------
GTCTTCCTTGGGTTCTTAGGAGCA---GCAGGAAGCACTATG---GGCGCGGCGTCA---
ATAACGCTGACGGTACAG---GCCAGACAA---TTATTG---TCTGGCATAGTGCAACAGCAAAGC---
AATTTG---CTGAGG---GCTATAGAGGCTCAACAACATCTGTTGAAACTCACAGTCTGGGGCATT
>KT152844
TTCTACTGTAATACATCAGCCCTGCTTAATGGCACTTGGCAAAATGCCAGCATGCAGGAGTCAAATAGCACAG
AG---

TCAAATGACACCATAACTCTCCCATGCAGAATAAAGCAAGTTATAAATATGTGGCAGAGAGTAGGACAAGCAA
TGTATGCCCCTCCCATCCAAGGAATAATAAAGTGCGTATCAAAC---
ATCACAGGACTAATATTAACAAGAGATGGTGGGAATAGTAACAGT----------------------------
----

AGTACAGTTGAAACCTTCAGGCCTGGAGGAGGAGATATGAGGGACAATTGGAGAAGTGAATTATATAAGTATA
AAGTAGTAAAAATTGAACCACTA---GGAGTAGCACCCACCAGGGCAAGGAGAAGAACGGTG-----------
----------------------------GAGAGAGAAAAAAGAGCA---GTTGGA---ATAGGAGCT------
---GTCTTCCTTGGGTTCTTAGGAGCA---GCAGGAAGCACTATG---GGCGCAGCGTCA---
ATAACGCTGACGGTACAG---GCCAGACAA---TTATTG---TCTGGCATAGTGCAACAGCAAAGT---
AATTTG---CTGAGG---GAAATAGAGGCTCAACAACATCTGTTGAAACTCACGGTCTGGGGCATT
>AY736809
TTCTATTGTAATACATCAGGCCTGTTCAATAGCACTTGGCCTGTCAATGCCAGTGAGCAGGAGTCAAATAGCA
CA---

GAGTCAGATGGCATTATAACTCTCCGATGCAGAATAAAGCAAATCATAAATATGTGGCAGAGAACAGGACAAG
CAATGTATGCCCCTCCCATTCAAGGAATAATAAAGTGTGTATCAAAC---
ATTACAGGACTACTGTTAACAAGAGATGGTGGGAATAGTACTGTC----------------------------
----

AATGAAACTTTCAGACCTGGAGGAGGAGATATGAGGGACAATTGGAGAAGTGAATTATATAAGTATAAAGTAG
TAAAAATTGAACCACTA---GGAGTAGCACCCACCAAGGCAAGGAGAAGAGTGGTG-----------------
----------------------GGGAGAGAAAAAAGAGCA---GTTGGA---ATAGGAGCT---------
GTTTTCCTTGGGTTCTTAGGAGCA---GCTGGAAGCACTATG---GGCGCGGCGTCA---
ATAACGCTGACGGTACAG---GCCAGACAA---TTACTG---TCTGGCATAGTGCAACAGCAAAAC---
AATTTG---CTGAGG---GCTATAGAGGCTCAACAACAACTGTTGAAACTCACGGTCTGGGGCATT
>AY736810
TTTTATTGTAATACATCAGACCTGTTTAATAGTACTTGGAACATTGAAGGCACTGCCAGCATAAATGGCACGG
AG---

TCAAATGACAATATAACTCTCCCATGCAGAATAAAGCAAATTATAAATATGTGGCAGAGAGTAGGACAAGCAA
TGTATGCCCCTCCCATCCAAGGAGTAATAAGGTGTCAATCAAAC---
ATTACAGGAATACTATTAACAAGAGATGGTGGGAATACTGGAATAAT--------------------------
----

AGCAGGACAAATGAAACCTTTAGACCTGGAGGAGGAGATATGAGGGACAATTGGAGAAGTGAATTATATAAGT
ATAAAGTAGTAAAAATTGAACCACTG---GGAGTAGCACCCACCAAGGCAAAGAGAAGAGTGGTG--------
-------------------------------GAGAGAGAAAAAAGAGCA---GTTGGA---CTGGGAGCT---
------GTATTCATTGGGTTCTTAGGAGCA---GCCGGAAGCACTATG---GGCGCGGCGTCA---
ATAACGCTGACGGTACAG---GCCAGACAA---TTATTG---TCTGGCATAGTGCAACAGCAAAGC---
AATTTG---CTGAGG---GCTATAGAGGCTCAACAGCATTTGTTGAAACTCACGGTCTGGGGCATT
>AY736812
TTCTATTGTAATACATCAGGTCTGTTCAATAGCACCTGGAAGGCCAACAATGGCACCTGGAAGGCCAACATAT
CAGAG--

TCAAATAACACGGAGATAACTCTCCAATGCAGAATAAAGCAAATTATAAATATGTGGCAGAGAACAGGACAAG
CAATATATGCCCCTCCCATCCAGGGAGTGATAAGGTGTGAATCAAAC---
ATCACAGGACTACTGTTAACAAGAGATGGTGGGGAGGGGAACAAT------------------------------
----

GAAAGTGAGATCTTCAGACCTGGAGGAGGAGATATGAGGGACAACTGGAGAAGTGAATTATATAAGTATAAAG
TAGTAAAAATTGAACCACTA---GGAGTAGCACCCACCAGGGCAAGGAGAAGAGTGGTG----------------
-------------------------GGAAGAGAAAAAAGAGCA---GTTGGA---ATAGGAGCT---------
GTTTTCCTTGGGTTCTTAGGAGCA---GCAGGAAGCACTATG---GGCGCGGCGTCA---
ATAACGCTGACGGTACAG---GCCAGGCAA---TTATTG---TCTGGCATAGTGCAACAGCAAAGC---
AATTTG---CTGAGG---GCTATAGAGGCTCAACAACATATGTTGAAACTCACGGTCTGGGGCATT
>AY736815
TTCTATTGTAATACATCAGGTCTGTTTAATAGCACCTGGGACAACAACACCGACCCAACCAACACAACG----
----

TCAAATGACACTATCACTCTCCAATGCAGAATAAAGCAATTTATAAATATGTGGCAGAGAACAGGGCAAGCAA
TGTATGCCCCTCCCATCCAAGGGGTAATAAGGTGTGATTCCAAC---
ATTACAGGACTACTATTAACAAGAGATGGTGGAGTAGGTAACAAT------------------------------
----

ACAACTGAAACCTTCAGACCTGGAGGAGGAGATATGAGGGACAATTGGAGAAGTGAATTATATAAGTATAAAG
TAGTAAAAATTGAACCACTA---GGAGTAGCACCCACCGGGGCAAAGAGAAGAGTGGTG----------------
-------------------------GAGAGAGAAAAAAGAGCA---GTTGGA---ATAGGAGCT---------
GTTTTCATTGGGTTCTTAGGAGCA---GCAGGAAGCACTATG---GGCGCGGCGTCA---
GTAACGCTGACGGTACAG---GCCAGACAA---TTATTG---TCTGGCATAGTGCAACAGCAAAGC---
AATTTG---CTGAGG---GCTATAGAGGCTCAACAGCATCTGTTGAAACTCACGGTCTGGGGCATT
>AY736813
TTCTATTGTGATACATCAGGCCTGTTTAATAGCAGTTGGAATAATAACACTTGGAAGGAGTCAAATAGCACGG
GG---

TCAAATGACACTATAACTCTCCTATGCAGAATAAAACAAATTATAAATATGTGGCAGAGAACAGGACAAGCAA
TGTATGCCCCTCCCATCCCAGGGTTAATAAGCTGTAAATCAAAC---
ATTACAGGTATAATATTGACAAGAGATGGTGGGAATGGTAACAGT------------------------------
----

ACAAATGAAACCTTCAGACCTGGAGGAGGAGATATGAGGGACAATTGGAGAAGTGAACTATATAGGTATAAAG
TAGTACAAATTGAACCACTA---GGAGTAGCACCCACCAGGGCAAGGAGAAGAGTGGTG----------------
-------------------------CAGAGAGAAAAAAGAGCA---GTAGGA---ATAGGAGCT---------
GTCTTCCTTGGGTTCTTAGGAGCA---GCAGGAAGCACTATG---GGCGCAGCGTCA---
ATAACGCTGACGGTACAG---GCCAGACAA---TTATTG---TCTGGCATAGTGCAACAGCAAAGC---
AATTTG---CTGAGG---GCTATAGAGGCTCAACAACATCTGTTGAAACTCACGGTCTGGGGCATT
>AY736816
TTCTATTGTGACACATCAGGCCTGTTTACTTGGAATAGCACTTGGAATGGCACTGAGTCAAACAACACAAAG-
----

TCAAACGACACTATAACTCTCCAATGCAGAATAAAGCAAATTATAAATATGTGGCAGAGAGTAGGACAAGCAA
TATATGCCCCTCCCATCAAAGGAGTAATAAGGTGTGAATCAGAC---
ATTACAGGACTTATACTAACAAGAGATGGTGGGAATACTAACAGT------------------------------
----

ACAGAGGAAACCTTCAGACCTGGAGGAGGAGATATGAGGGACAATTGGAGAAGTGAATTATATAAGTATAAAG
TAGTAAAAATTGAACCACTA---GGAGTAGCACCCACCAAGGCAAAGAGAAGAGTGGTG----------------
-------------------------GGGAGAGGAAAAAGAGCA---GTTGGA---ATAGGAGCT---------
GTCTTCCTTGGGTTCTTAGGAGCA---GCCGGAAGCACTATG---GGCGCGGCGTCA---
ATAACGCTGACGGTACAG---GCCAGACAA---TTATTG---TCTGGCATAGTGCAACAGCAAAGC---
AATTTG---CTAAGG---GCTATAGAGGCTCAACAGCATCTGTTGAAACTCACGGTCTGGGGCATT
>AF457053
TTCTATTGTAATACATCAGGCCTGTTTAACAGCACCTGGCACTATAACAGCAATGGTACTTGGCACAATAACA
GCACAAAT---
-------------AGCATG------

GACTCAAATGAAACTATAACTCTCACATGCAGAATAAAGCAAATTATAAATATGTGGCAGAGAGTAGGAAAAG
CAATGTATGCTAATCCCATCCAAGGAATAATAAAGTGTGAATCAAAC---
ATTACAGGACTACTCTTAACAAGAGATGGTGGGAATACTAGCAGT----------------------------
----

ACAAATGAAACCTTCAGACCTGGAGGAGGAGATATGAGGGACAATTGGAGAAGTGAATTATACAAATATAAAG
TAGTAAAGATTGAGCCACTA---GGAGTAGCACCCACCAGGGCACAGAGAAGAGTGGTG--------------
-------------------------GGGAGAGAAAAAAGAGCA---ATTGGA---ATAGGAGCT---------
GTCTTCCTTGGGTTCTTAGGAGCA---GCAGGAAGCACTATG---GGCGCGGCGTCA---
ATGACGCTGACGGTACAG---GCCAGACAA---TTATTG---TCTGGCATAGTGCAACAGCAAAGC---
AATTTG---CTGAGG---GCTATAGAGGCTCAACAACAACTGTTGAAACTCACGGTCTGGGGCATT
>AF457055
TTCTATTGTAATACAACAAACCTGTTTAATAGCACCTGGAGAGGAAATAAAACTGTCAGCAATAGCACAGAGT
TA---

GATGAAAATGGCACTATAACTCTCCCATGCAGAATAAAGCAAATTATAAATATGTGGCAGAGAACAGGACAAG
CAATGTATGCCCCTCCCATCCAAGGAGTAATAAAGTGTGTATCAAAC---
ATTACAGGACTACTATTAACAAGAGATGGTGGAAGTGATAACAAT----------------------------
----

ATAAGTAGTGAAACCTTCAGACCAGGAGGAGGAGATATGAGGGACAATTGGAGAAGTGAATTATATAAGTATA
AAGTAGTGAAACTTGAACCACTA---GGAGTAGCACCCAACAAGGCAAGGAGAAGAGTGGTG-----------
----------------------------GAGAGAGAAAAAAGAGCA---GTTGGA---CTGGGAGCT------
---GTATTCATTGGGTTCTTAGGAGCA---GCAGGAAGCACTATG---GGCGCGGCGTCA---
ATAACGCTGACGGCACAG---GCCAGACAA---TTATTG---TCTGGCATAGTGCAACAGCAAAGC---
AATTTG---CTGAAG---GCTATAGAGGCTCAACAGCATCTGTTGAAACTCACGGTCTGGGGCATT
>AF457070
TTCTATTGTAATACATCAGGCCTGTTTAATAGCACTTGGAATAGCACTGGGAATGACACTTGGAGTGGCAATG
CCAGCACG---
-------------------CAG---
GAGTCAAATGACACTATAACTCTCCCATGCAGAATAAAGCAAATTGTAAATATGTGGCAGAGAATAGGACAAG
CAATGTATGCCCCTCCCATCCAAGGAGTAATAAGGTGTGAATCAAAC---
ATTACAGGACTACTATTAACAAGAGATGGTGGGAACAATGAAAGT----------------------------
----

GAAACCTTCAGACCTGGAGGAGGAAATATGAGGGACAATTGGAGAAGTGAATTATATAGGTACAAAGTAGTGA
AAATTGAACCACTA---GGAGTAGCACCCACCAGGGCAAAAAGAAGAGTGGTG--------------------
-------------------GGGAGAGAAAAAAGAGCA---GTTGGA---ATAGGAGCT---------
GTGTTCCTTGGGTTCTTAGGAGCA---GCAGGAAGCACTATG---GGCGCGGCGTCA---
GTAACGCTGACGGTACAG---GCCAGACAA---TTATTG---TCTGGCATAGTGCAACAGCAAAGC---
AACTTG---CTGAGG---GCTATAGAGGCTCAACAACATCTGTTGAAACTCACGGTCTGGGGCATT
>AF457077
TTCTATTGTAATACATCAGGCCTGTTTAATAGCACTTGGTCTTGGAATGACACTGCAAATGGCACTGCAATGG
AG---

CCAAATAGCACTATAACTCTCCCATGCAGAATAAAGCAAATTATAAATATGTGGCAGAGAGTAGGACAAGCAA
TGTATGCCCCTCCCATCCCAGGAGTAATAAGTTGTGTATCAAAC---
ATTACAGGACTAATATTAGTAAGAGATGGAGGGAATAATGTTACA----------------------------
----

AATGAAACCTTCAGACCTGGAGGAGGAGATATGAGGGACAATTGGAGAAGTGAATTATATAAGTATAAGGTAG
TAAAAATTGAACCACTA---GGAGTAGCACCCACCAAGGCAAAGAGAAGAGTGGTG-----------------
----------------------GAGAGAGAAAAAAGAGCA---GTTGGA---ATAGGAGCT---------
GTCTTCCTTGGGTTCTTAGGAGCA---GCAGGAAGCACTATG---GGCGCGGCGTCA---
ATAACGCTGACGGTACAG---GCCAGACAA---TTATTG---TCTGGTATAGTGCAACAGCAAAGC---
AATTTG---CTGAGG---GCTATAGAGGCTCAACAACATCTGTTGAAACTCACAGTCTGGGGCATT
>AF457080
TTCTATTGTAATACATCGGGCCTGTTTAATAGCACCTACAATGGCACTGACATCGCTGAAAATAGCACGGAG-
----

TCAAATGACACTATAGTTCTCCCATGCAGAATAAAGCAAATTATAAGGATGTGGCAGCGAGTAGGACAAGCAA
TGTATGCCCCTCCCATCCCAGGAATAATAAGATGTGACTCAAAC---
ATTACAGGATTACTATTAACAAGAGATGGTGGGGATGATAATGAA----------------------------
----

ACTGAAACCTTTAGACCTGGAGGAGGAGATATGAGGGATAATTGGAGAAGTGAATTATATAAGTATAAAGTAG
TAAAAATTGAACCACTA---GGAGTAGCACCCACCAGGGCAAAAAGAAGAGTGGTG-----------------
----------------------GAGAGAGAAAAAAGAGCA---GTTGGA---ATAGGGGCT---------
TTTGTCCTTGGGTTCTTAGGAGCA---GCAGGAAGCACTATG---GGCGCAGCATCA---
ATAACGCTGACGGTACAG---GCCAGACAA---TTATTG---TCTGGCATAGTGCAACAGCAAAGC---
AATTTG---CTGAGG---GCTATAGAGGCTCAACAACAAATATTGAGACTCACGGTCTGGGGCATT
>AF457081
TTCTATTGTAATACATCAGGCCTGTTTAATAGCACTTGGACAAATGACAGTATCAGCACAAATAGCACAAAG-
----

TCAAATGACACTATAACTCTCCCATGCAGAATAAAGCAAATTATAAATATATGGCAGAGAGTGGGACAAGCAA
TGTATGCCCCTCCCATCCAAGGAGTAATACAGTGTGTATCAAAC---
ATTACAGGACTACTATTAACAAGAGATGGTGGGGATAACAGTAGT----------------------------
----

ACGAATGAAACCTTCAGACCTGGAGGAGGAGATATGAGGGACAATTGGAGAAGTGAATTATATAAGTATAAAG
TAGTAAAAATTGAACCACTA---GGAGTAGCACCCACCAGGGCAAGGAGAAGAGTGGTG--------------
-------------------------GAGAGAGAGAAAAGAGCA---GTTGGA---CTGGGAGCT---------
GTTTTCATTGGGTTCTTGGGAGCA---GCAGGGAGCACTATG---GGCGCAGCGTCA---
ATAACGCTGACGGTACAG---GCCAGACAA---TTATTG---TCTGGCATAGTGCAACAGCAAAGC---
AATTTG---CTGAGG---GCTATAGAGGCTCAACAACATCTGTTGAAACTCACGGTCTGGGGCATT
>AF457083
TTCTATTGTAATACATCAAGTCTGTTTAATAGTACTTGGAATAACAGTACTTGGAATACCAGCAGGCAGGAGG
CAAATAAT---
-------------AGCCCG------
GGGTCAAACGACACTATAACTCTCAAATGCAGAATAAAGCAAATTATAAATATGTGGCAGAGAGCGGGACAAG
CAATATATGCCCCTCCCATCCAAGGAGTAATAAGGTGTAACTCAAAC---
ATTACAGGACTAATATTAACAAGAGATGGTGGGGGTAATAACAAT----------------------------
----

ACAAATGAAACCTTCAGACCTGGAGGAGGAGATATGAGGGACAATTGGAGAAGTGAATTATATAAGTACAAAG
TAGTAAAAATTGAACCACTA---GGAGTAGCACCCACCAGGGCAAAGAGAAGAGTGGTG--------------
-------------------------GAGAGAGGAAAAAGAGCA---GTTGGA---ATAGGAGCT---------
GTCCTCATTGGGTTCTTAGGAGCA---GCAGGAAGCACTATG---GGCGCGGCGTCA---
ATAACGCTGACGGCACAG---GCCAGACAA---TTATTG---TCTGGCATAGTGCAACAGCAAAAC---
AATTTG---CTGAGG---GCTATAGAGGCTCAACAACATCTGTTGAGACTCACGGTCTGGGGCATT
>AF457084
TTCTATTGTGACACATCAGGCCTGTTTAATAGCAGTTGGACTGCTAGCAATGATAGCATGCAGGAGGCACATA
GC---

ACAGAGTCAAATATAACTCTCCAATGCAGAATAAAGCAAATTATAAATATGTGGCAGAGAGCAGGACAAGCAA
TGTATGCCCCTCCCATTCCAGGAATAATAAGGTGTGAATCAAAC---
ATTACAGGACTAATATTAACAAGAGATGGTGGTGAGGGTAATAAC----------------------------
----

AGTACAAATGAAACCTTCAGACCAGTAGGAGGAAATATGAGGGACAATTGGAGAAGTGAATTATATAAGTATA
AAGTAGTAAAAGTTGAACCACTA---GGAGTAGCACCCACCAAGTCAAGGAGGAGAGTGGTG-----------
----------------------------GAGAGAGAAAAAAGAGCA---GTTGGA---CTAGGAGCT------
---GTGTTCATTGGGTTCTTAGGAGCA---GCAGGAAGCACTATG---GGCGCGGCGTCA---
ATGACGCTGACGGTACAG---GCCAGACAA---TTATTG---TCTGGCATAGTGCAACAGCAAAGC---
AATTTG---CTGAGG---GCTATAGAGGCTCAACAGCATCTGTTGAAACTCACGGTTTGGGGCATT
>AF457086
TTCTATTGTAATACAACAAAACTGTTTAATAGCACTTGGAGTCATAATGACAGCACGCAAGAGTCAAATAGCA
CA---

AAGTCAAATGACACTATAACTCTCCCATGCAGAATAAAGCAAATTATAAATATGTGGCAGAGAGTAGGACAAG
CAATATATGCCCCTCCCATACAAGGAATAATAAGGTGTGAATCAAAT---
ATTACAGGACTAATATTAACAAGAGATGGAGGTACCAATAGTACA----------------------------
----

AATGAAACCTTCAGACCTGGAGGAGGAGATATGAGGGACAATTGGAGAAGTGAATTATATCAGTATAAAGTAG
TAAAAATTGAACCACTA---GGAGTAGCACCTACCAGGGCAAAGAGAAGAGTGGTG-----------------
----------------------GAGAGAGCAAAAAGAGCA---GTTGGA---ATAGGAGCT---------
GTCTTCCTTGGGTTCTTAGGAGCA---GCAGGAAGCACTATG---GGCGCGGCGTCA---
ATAACGCTGACGGTACAG---GCCAGACAA---TTATTG---TCTGGCATAGTGCAACAGCAAAGT---
AATTTG---CTGAGG---GCTATAGAGGCTCAACAGCATCTGTTGAAACTCACGGTCTGGGGCATT
>AF457089
TTTTATTGCAATACATCAGATCTGTTTAATAGTACTTGGCCTGTCAATGCCAGCATGGAGTCAAATAGCACGG
AG---

TCAAATGACAATATAACACTCCAATGCAGAATAAAGCAAATTATAAATATGTGGCAGAAAGTAGGACAAGCAA
TGTATGCCCCTCCCATACAAGGAGTAATAAGGTGTGAATCAAAC---
ATTACAGGACTACTATTAACAAGAGATGGTGGATCTAACAGTACA----------------------------
----

GAAGAAACCTTCAGACCTGGAGGAGGAGATATGAGGGACAATTGGAGAAGTGAATTATATAAGTATAAAGTAG
TGAAAATTGAGCCATTA---GGGGTAGCACCCACCAGGGCAAAGAGAAGAGTGGTG-----------------
----------------------GAGAGAGAAAAAAGAGCG---GTTACA---CTAGGAGCT---------
GTATTCATTGGGTTCTTAGGAGCA---GCAGGAAGCACTATG---GGCGCAGCGTCA---
GTAACGCTGACGGTACAG---GCCAGACAA---TTACTG---TCTGGCATAGTGCAACAGCAAAGC---
AATTTG---CTGAGG---GCTATAGAGGCTCAACAGCATATGTTGAAACTCACGGTCTGGGGCATT
>EU110088
TTCTATTGTAACACATCACGCCTGTTTAATAGCACCTGGTGGGCCAATGGCACCAGGACGGGCTACAACAGC-
----

ACTGATGGCAATATAACACTCCCATGCAGAATAAAGCAAATTATAAATATGTGGCAGAGAGTAGGACAAGCAA
TGTATGCCCCTCCCATCCCAGGAAGAATAAGTTGTGAATCAAAC---
ATTACAGGACTAATATTAACAAGAGATGGTGGGAGTGATAATACA----------------------------
----

ACTGAAACCTTCAGACCTGGAGGAGGAGATATGAGGGACAATTGGAGAAGTGAATTATATAAGTATAAAGTAG
TAGAAGTTGAACCACTA---GGAGTAGCACCCACAGAGGCAAGGAGAAGAGTGGTG-----------------
----------------------CAGAGAGAAAAAAGAGCA---GTCGGA---CTGGGAGCT---------
GTCTTCATTGGGTTCTTGGGAGCA---GCAGGAAGCACTATG---GGCGCGGCGTCA---
GTGACGCTGACGGTACAG---GCCAGACAA---TTATTG---TCTGGTATAGTGCAACAGCAAAGC---
AATTTG---CTGAGG---GCTATAGAGGCTCAACAACATCTGTTGAAACTCACGGTCTGGGGCATT
>EU110092
TTCTATTGTGATACATCAGCTCTGTTCAATAGCACTTGGAATGCCACTGAGTCAAATGACACAAACTCCACAG
AATCA--

AACTCAAATGACACTATAACTCTCCCATGCAGAATAAAACAAATTATAAATATGTGGCAGAGAATAGGACAAG
CAATGTATAACCCTCCCATCCAAGGAAAAATAAAGTGTGAATCAAAC---
ATTACAGGACTATTGTTAACAAGAGATGGTGGAGAAAATAGAGAAAT--------------------------
----

AATGACAGTAGTGAAATCTTCAGACCAGGAGGAGGAGATATGAAGGACAATTGGAGAAGTGAATTGTATAAGT
ATAAAGTAGTAAAAATTGAACCACTA---GGAGTAGCACCCACCAGGGCAAGGAGAAGAGTGGTG--------
-------------------------------GGGAGAGAAAAAAGAGCA---GTTGGA---ATAGGAGCT---
------GTCTTCCTTGGGTTCTTAGGAGCA---GCAGGAAGCACTATG---GGCGCGGCGTCA---
ATAACGCTGACGGTACAG---GCCAGACAA---TTATTG---TCTGGCATAGTGCAACAGCAAAGC---
AATTTG---CTGAGG---GCTATAGAGGCTCAACAACATCTGTTGAAACTCACAGTCTGGGGCATT
>EU110094
TTCTATTGTAATACATCAGGCCTGTTTAGTAGCACTTGGAGTTGGAATGACACTACCAAAACAATGGAATCAA
ATAGC--
-------------------ACG---

GTGTCAGATGGCACTATAAATCTCCCATGCAGAATAAAGCAAATTATAAATATGTGGCAGAGAGTAGGGCAAG
CAATGTATGCCCCTCCCATCCAAGGAGCAATAAGGTGTACATCAAAC---
ATTACAGGACTAATATTAACAAGAGATGGTGGGAAAGATGACAGG---------------------------
----

AATAATGAAACCTTCAGACCTGGAGGAGGAGATATGAGGGACAATTGGAGAAGTGAATTATATAAATATAAAG
TAGTAAAAATTGAACCACTA---GGAGTAGCACCCACCAGGGCAAAGAGAAGAGTGGTG-------------
-----------------------GAGAGAGAAAAAAGAGCA---GTTGGA---ATAGGAGCT---------
GTCTTCCTTGGGTTCTTAGGAGCA---GCAGGAAGCACTATG---GGCGCGGCGTCA---
ATAACGCTGACGGTACAG---GCCAGACAA---TTATTG---GCTGGCATAGTGCAACAGCAAAGC---
AATTTG---CTGAGG---GCTATAGAGGCTCAACAACATCTGTTGAAACTCACGGTCTGGGGCATT
>KT022360
TTCTATTGTAACACATCAGGCTTGTTTAATAACACTTGGGTGTTTAATAGCATTTGGGATAATAGCACTCAGG
AGTCCAAT---
-------------AGCACA------
GAGTCAAATAAAACTATAATTCTCCAATGCAGAATAAAGCAAATTATAAATATGTGGCAGAGAGCAGGACAAG
CAATATATGCTCCTCCCATTCCAGGAGTAATAAGGTGTGAATCAAAC---
ATTACAGGACTAATATTAACAAGAGATGGTGGAAATAATAACACG---------------------------
----

ATAAATGAAACATTTAGACCTGGAGGAGGAGACATGAGGGACAATTGGAGAAGTGAATTATATAAGTACAAAG
TAGTAAAAATTGAACCACTA---GGAGTAGCACCTACCAAGGCACGGAGAAGAGTGGTG-------------
-----------------------GAGAGAGAAAAAAGAGCA---GTTACA---ATAGGAGCT---------
ATGTTCATTGGGTTCTTAGGAGCA---GCAGGAAGCACTATG---GGCGCGGCGTCA---
ATGACGCTGACGGTACAG---GCCAGACAA---TTATTG---TCTGGCATAGTGCAACAGCAAAGC---
AATTTG---CTGAGG---GCTATAGAGGCTCAACAGCATCTGTTGAAACTCACGGTCTGGGGCATC
>KT022361
TTCTATTGTAATACATCAGGCCTGTTTAATTACAGCTACAGCATGCAGGAGTCAAATGATACGGAG-------
----

GCAAATGGCACTATAACTCTCCAATGCAAAATAAAACAAATTATAAATATGTGGCAGAGAGTAGGACAAGCAA
TGTACGCCCCTCCCATCCAAGGAGTAATACAGTGTAAATCAAAC---
ATTACAGGACTAATATTAACAAGAGATGGTGGAAATAGTACTAAT---------------------------
----

GGTACAGAAATCTTCAGACCTGGAGGAGGAGATATGAGAGACAATTGGAGAAGTGAGTTATATAAGTATAAAG
TAGTACAACTTGAACCACTA---GGAGTAGCACCCACCAAGGCAAAGAGAAGAGTGGTG-------------
-----------------------GAAAGAGAAAAAAGAGCA---GTTGGA---ATAGGAGCT---------
GTCTTCCTTGGGTTCTTAGGAACA---GCAGGAAGCACTATG---GGCGCGGCGTCA---
CTGACGCTGACGGTACAA---GCCAGACTA---TTATTG---TCTGGCATAGTGCAACAGCAAAGC---
AATTTG---CTGAGG---GCTATAGAGGCTCAACAATATCTGTTGAAACTCACGGTCTGGGGCATT
>KT022363
TTCTATTGTGACACCTCGAAGCTGTTTAATAGCTCTTGGAATGAGAGCACTTGGGATGAAAATAGCACGAAGC
CTAAC--

AACAATGAAACTATAATTCTCCCATGCAGAATAAAGCAAATCATAAATATGTGGCAGAGAACAGGACAAGCAA
TATATGCCCCTCCCATTGAAGGAATAATAAAGTGTGACTCAAAC---
ATTACAGGACTAATATTAACAAGAGATGGTGGGAGGATTAACACA---------------------------
----

ACTAATGAAACTTTCAGACCTGGAGGAGGAGATATAAGGGACAATTGGAGAAGTGAATTATATAAATATAAAG
TAGTAAAAATTGAGCCACTA---GGAGTAGCACCCTCCAGGGCAAGGAGAAGAGTGGTG-------------
-----------------------GGGAGAGAAAAAAGAGCA---GTTGGA---ATAGGAGCT---------
GTCTTCCTTGGGTTCTTAGGAGCA---GCAGGAAGCACTATG---GGCGCGGCGTCA---
CTAACGCTGACGGTACAG---GCCAGGCAA---TTATTG---TCTGGCATAGTGCAACAGCAAAGC---
AATTTG---CTGAGG---GCTATAGAGGCTCAACAGCATATGTTGAAACTCACGGTCTGGGGCATT
>KT022364
TTTTATTGTGATACCTCAGGCTTGTTTAATAGTACTTGGGAAATTGAAAACAATACTGCCAGAATAAAGCCAA
ATAAC--

ACGGGGTTAAATATAACGCTCCCGTGCAGAATAAAGCAAATTATAAATATGTGGCAAAGGGTAGGACAAGCAA
TGTATGCCCCTCCCATCAAAGGAGTAATAAGGTGTGTATCAAAC---
ATCACAGGACTACTGCTAACAAGAGATGGTGGGAGTAAAAATAGA----------------------------
----

AGTAATGAAACCTTCAGACCTGGAGGAGGAGATATGAGGGACAATTGGAGAAGTGAATTATATAAATATAAAG
TAGTAAAAATTGAACCACTA---GGAGTAGCACCCTCCAGGGCAAAGAGGAGAGTGGTG--------------
-------------------------GAGAGAGAGAAAAGAGCA---GTTGGA---ATAGGAGCT---------
GTATTCCTTGGGTTCTTAGGAGCA---GCAGGAAGCACTATG---GGCGCGGCGTCA---
CTGACGCTGACGGTACAG---GCCAGACAA---TTATTG---TCTGGCATAGTGCAACAGCAAAGC---
AATTTG---CTGAGG---GCTATAGAAGCTCAACAGCATCTGTTGAAGCTTACAGTCTGGGGCATT
>KT022365
TTCTATTGTGATACGTCAGGCTTATTCAGTAGCACCTGGCAATTCAATGGGAGTACCTGGAACAACACAGATT
CAAACAGCACA--

GGGTCAGATGACACTATAGAACTCCCATGCAGAATAAAGCAAATTATAAATATGTGGCAGAGAGTAGGACGAG
CAATGTATGCCCCTCCCATCCAAGGAGTAATAAGTTGTGTATCAAAC---
ATCACAGGACTAATATTAACAAGAGATAGT---AATAACAGAACT----------------------------
----

AATGAAACCTTCAGACCTGGAGGAGGAGACATGAGAGACAATTGGAGAAGTGAATTATATAAGTATAAAGTAG
TAAAAATTGAACCACTA---GGAGTAGCACCCACCAAGGCAAAAAGAAGAGTGGTG-----------------
----------------------GAGAGAGAAAAAAGAGCA---GTTGGA---ATAGGAGCT---------
GTCTTCCTTGGGTTCTTAGGAGCA---GCAGGAAGCACTATG---GGCGCGGCGTCA---
ATAACGCTGACGGTACAG---GCCAGACAA---TTATTG---TCTGGCATAGTGCAACAGCAAAGC---
AATTTG---CTGAGG---GCTATAGAGGCTCAACAACATCTGTTGAAACTCACAGTCTGGGGCATT
>KT022367
TTCTATTGTAATACATCAGGTCTATTTAATAGCAGTTGGAATGATACTGAAATCGGGCGGGAGATAAATAGGA
CGGAG--

TCAAATGACACTATAATTCTCCCATGCAGAATAAAGCAAATTATAAATATGTGGCAGAGAGTAGGACAAGCAA
TATATGCCCCTCCCATCCAAGGAGTAATAAGGTGTGAATCAAAC---
ATTACAGGACTAATATTAACAAGGGATGGTGGGGGTAATAACAGA----------------------------
----

AAAAATGAAACCTTCAGACCTGGAGGAGGAGATATGAGGGACAATTGGAGAAGTGAATTATATAAGTATAAAG
TAGTAAAAATTGAACCAATA---GGAATAGCACCCACCAGGGCAAAGAGAAGAGTGGTG--------------
-------------------------GAGAGAGAAAAAAGAGCA---ATTGGA---ATGGGAGCT---------
GTATTCATTGGGTTCTTAGGAGCA---GCAGGAAGCACTATG---GGCGCGGCGTCA---
ATAACGCTGACGGTACAG---GCCAGACAA---TTGTTG---TCTGGCATAGTGCAACAGCAAAAC---
AATTTG---CTGAGG---GCTATAGAGGCTCAACAACATCTGTTGAAGCTCACGGTCTGGGGCATT
>KT022368
TTCTATTGTAATACAACAGACCTGTTTGATAGCATTTGGCTTTACAATGCCAGCATGCATAAT----------
----

GCAAATAGCACGATAACTCTCCAATGCAGAATAAAGCAAATTATCAATATGTGGCAGAGAGTAGGACAAGCAA
TGTATGCCCCTCCCGTCGAAGGAAAAATACAGTGCAAATCAAAC---
ATTACAGGACTAATATTAACAAGAGATGGTGGGGTTAACGATACA----------------------------
----

AATGGAACCGAAATCTTCAGACCTGGAGGAGGGGATATGAGGGACAATTGGAGAAGTGAATTATATAAGTATA
AAGTAGTAAAAATTGAACCACTA---GGAGTAGCACCCACCAGGGCAAGGAGAAGAGTGGTG-----------
----------------------------CAGAGAGAAAAAAGAGCA---GTTGGA---ATAGGAGCT------
---GTATTCCTTGGGTTCTTAGGAGCA---GCAGGAAGCACTATG---GGCGCAGCGTCA---
GTGACGCTGACGGTACAG---GCCAGAAGA---TTATTG---TCTGGCATAGTGCAACAGCAAAGC---
AATTTG---CTGAAG---GCTATAGAAGCTCAACAGCATCTGTTGAAGCTCACGGTCTGGGGCATT
>KT022369
TTCTATTGTAATACATCAGGCCTGTTTAATAGCACTTGGAATTATACTAATAGCATGCAGCAGTCAAATAGCA
CAGAG--

TCAAACGAAACTATAACTCTCCAATGCAGAATAAAGCAAATTATAAATATGTGGCAGAGAGTGGGGCAAGCAA
TATATGCCCCTCCCATCCCAGGGGTAATAAGGTGTGAATCAAAC---
ATTACAGGACTAATATTGACAAGAGATGGTGGGAGTAATAACAGT----------------------------
----

ACAAATGAAACCTTCAGACCTGGAGGAGGGAATGTGAGGGACAATTGGAGAAGTGAATTATATAAGTATAAAG
TAGTAAAAATTGAACCACTA---GGAGTAGCACCCACCAGGGCAAAGAGAAGAGTGGTG--------------
-------------------------GAGAGAGAAAAAAGAGCA---GTTGGA---CTAGGAGCT---------
GTCTTCATTGGGTTCTTGGGAACA---GCAGGAAGCACTATG---GGCGCGGCGTCA---
ATAACGCTGACGGTACAG---GCCAGACAA---TTATTG---TCTGGTATAGTGCAACAGCAAGGC---
AATTTG---CTGAGA---GCTATAGAAGCTCAACAACAACTGTTGAGACTCACGGTCTGGGGCATT
>FJ866117
TTTTATTGTAATACATCAGGCCTGTTTAATAGTACTTGGTCACCTGACAATAGTACTGGGACTAGTACTGGGA
ATAATGAC---
-------------------ACT---
GCCAGCAATGGCACTATAACTCTCCCATGTAGAATAAAGCAGATTATAAATATGTGGCAGAGAGTAGGACAAG
CAATGTATGCCCCTCCCATCCAAGGAGTAATAAGGTGTGACTCAAAC---
ATTACAGGACTACTATTAACAAGAGATGGTGGGAGTGGGAATAAT----------------------------
----

ACCACAGAGATCTTCAGACCTGGAGGAGGAGATATGAGGGACAATTGGAGAAGTGAATTATATAAGTATAAAG
TAGTAAGAATCGAACCTCTA---GGAGTAGCACCCACCAGGGCAAAGAGAAGAGTGGTG--------------
-------------------------GAGAGAGAAAAAAGAGCA---GTTGGA---ATAGGAGCT---------
GTATTCATTGGGTTCTTAGGAGCA---GCAGGAAGCACTATG---GGCGCAGCGTCA---
ATAACGCTGACGGTACAG---GCCAGACAA---TTATTG---TCTGGCATAGTGCAACAGCAAAGC---
AATTTG---CTGAGG---GCTATAGAGGCTCAACAGCATTTGTTGAAGCTCACAGTCTGGGGCATT
>KT022370
TTCTATTGTAATACATCAGGCCTGTTTAATAGAGCTTACACTAACAGCACTTACAATAACAGCACGCAGGACA
ATGACAAT---
----------AACAGCACG------
CAGGACAATGCAACTATAACTCTCCCATGCAGAATAAGGCAAATTATAAATATGTGGCAGAGAGCAGGGAAAG
CAATGTATGCCCCTCCCATCCAAGGAGTAATAAACTGTGTATCAAAC---
ATTACAGGACTAATTTTAACAAGAGATGGTGGAAAGAGTAACAGTACG-------------------------
----

GAGGGGGAAATCTTCAGACCTGGGGGAGGAGATATGAGGGATAATTGGAGAAGTGAATTATATAAGTATAAAG
TAGTAAAAATTGAACCACTA---GGAGTAGCACCTACCAAGGCAAGGAGAAGAGTGGTG--------------
-------------------------GGGAGAGAGAAAAGAGCA---GTTGGA---CTAGGAGCT---------
GTCTTCATTGGGTTCTTAGGAGCA---GCCGGAAGCACTATG---GGCGCGGCGTCA---
GTGACGCTGACGGTACAG---GCCAGGCAA---TTATTG---TCTGGCATAGTGCAACAGCAAAGC---
AATTTG---CTGAGG---GCTATAGAGGCTCAACAACATCTGTTGAAACTCACGGTCTGGGGCATT
>KT022372
TTCTATTGTGATACAGCAGGCCTGTTTAATAGCACTTGGAGTCACAATGACAGCATACAGGGGACAAATAGCG
CAGAG--

TCAAATGAAACTATAACTCTCCCATGCAGAATAAAGCAAATTATAAATATGTGGCAGAGAGCAGGACAAGCAA
TGTATGCCCCTCCCATCCCAGGAGTAATAAAGTGTGTATCAAAC---
ATTACAGGACTAATATTAACAAGAGATGGTGGGAAGAACAATACA----------------------------
----

GAGGAAGTCTTCAGACCTGGAGGAGGAGATATGAGGGATAATTGGAGAAGTGAATTATATAAGTATAAAGTAG
TAAAAATTGAACCACTA---GGAGTAGCACCCACCAAGGCAAGGAGAAGAGTGGTG-----------------
----------------------GGGAGAGAGAAAAGAGCA---GTTGGA---GTAGGAGCT---------
GTCTTCATTGGGTTCTTAGGAGCA---GCCGGAAGCACTATG---GGCGCGGCGTCA---
GTGACGCTGACGGTACAG---GCCAGACGA---TTATTG---TCTGGCATAGTGCAACAGCAAAGC---
AATTTG---CTGAAG---GCTATAGAGGCTCAACAACACCTGTTGAAACTCACGGTCTGGGGCATT
>KT022373
TTCTATTGTAACACATCAAGCTTGTTTAGGGGCGCTTGGAATAGTACAACTGCAACACTTAATAGCACTGGGA
AT---

AGCAATGACACTATAACTCTTCCATGCAGAATAAAGCAAATTATAAATATGTGGCAGAGAGCAGGACAAGCAA
TATATGCCCCTCCCATAAAAGGAGTAATAAGGTGTAACTCAAAT---
ATTACAGGACTAATATTAACAAGAGATGGTGGGGTAGTGATAAT-----------------------------
----

AGAACTGAAACTTTTAGACCTGGAGGAGGAGATATGAGAGACAATTGGAGAAGTGAATTATATAAGTATAAAG
TAGTTAAAATTGAACCACTA---GGAGTAGCACCCACCAGGGCAAAGAGAAGAGTGGTG--------------
-------------------------GAGAGAGAAAAAAGAGCA---GTTGGA---ATAGGAGCT---------
GTCTTCATTGGGTTCTTAGGAGCA---GCAGGAAGCACTATG---GGCGCGGCGTCG---
ATAACGCTGACGGTACAG---GCCAGACAA---TTATTG---TCTGGCATAGTGCAACAGCAAAGA---
AATTTG---CTGAGG---GCTATAGAGGCTCAACAACATCTGTTGAAACTCACGGTCTGGGGCATT
>KT022374
TTCTATTGTAATACAACAAAGCTGTTCAATAGCTCTTGGTCTGCCAATGCCAGTGAGTCAAATAGTACAGATG
GCAATAGT---

ACAGATGGCACTATAACTCTCCCATGCAAAATAAAGCAAATTATAAATATGTGGCAGAGAGTAGGACGAGCAA
TGTATGCCAATCCCATCCAAGGAAAAATAGAGTGTGCATCAAAC---
ATTACAGGACTAATATTAACAAGAGATGGTGGGAATGTGAGCAGG----------------------------
----

GAAAATGAAACCTTCAGACCTGGAGGAGGAGATATGAGGGACAATTGGAGAAGTGAATTATATAAGTATAAAG
TAGTAAAGATTGAACCACTA---GGAGTAGCACCCCATACGGCAAGGAGAAGAGTGGTG--------------
-------------------------GGGAGAGAAAAAAGAGCA---GTTGGA---ATGGGAGCT---------
GTCTTCCTTGGGTTCTTAGGAGCA---GCAGGGAGCACTATG---GGCGCAGCGTCA---
CTAACGCTGACGGTACAG---GCCAGACAA---TTATTG---TCTGGCATAGTGCAACAGCAAAGC---
AATTTG---CTGAGG---GCTATAGAGGCTCAACAACATCTGTTGAAACTCACAGTCTGGGGCATT
>KT022375
TTCTATTGTAATACATCAAAACTGTTTAATAGCACTTGGCCACAGTCAAACCACACGGAA-------------
----

GACAATGGAAATCTAACTCTCCCATGCAGAATAAAACAACTTATAAACCTGTGGCAAAGAGCAGGACAAGCAA
TGTATGCCCCTCCCATACCAGGAAATATAAGTTGTGTATCAAAC---
ATTACAGGACTACTATTGACAAGAGATGGTGGGAACACTGGGAAC----------------------------
----
AATACAGAGGGAAAT---
AAAACTGAAATCTTCAGACCTGGAGGAGGAAATATGAGGGACAATTGGAGAAGTGAATTATATAAGTATAAAG
TAGTGAAAATTGAACCAATA---GGAATAGCACCCACCAAGGCAAAGAGAAGAGTGGTG--------------
-------------------------GAGAGAGAAAAAAGAGCAGCACTTGCT------GGAGCT---------
TTCTTTCTTGGGTTCTTAGGAACA---GCAGGAAGCACTATG---GGCGCGGCGTCA---
ATAGCGCTGACGGTACAG---GCCAGGCAA---TTATTG---TCTGGCATAGTGCAACAGCAAAGC---
AATTTG---CTGAGG---GCTATAGAGGCTCAACAGCAATTGTTGAGACTCACAGTCTGGGGCATT
>KT022376
TTCTATTGTAATACATCAGGCCTGTTTGATAGCACTTGGTCGTTTAATAGCACACAGGAGTCAAATAACACGG
GG---

GAAAATGATACTATAATTCTCCCATGCAGAATAAAGCAAATTATAAACATGTGGCAGAGAGTAGGACAAGCAA
TGTATGCCCCTCCCATCCCAGGAGTAATACAGTGTAACTCAACC---
ATTACAGGGCTAATATTAACAAGAGATGGTGGGGAGGATAATAAT----------------------------
----

AACACAGAAACCTTCAGACCTGCAGGAGGAGATATGAGGGACAATTGGAGAAGTGAATTATATAAATATAAAG
TAGTAAAACTTGAACCACTA---GGAGTAGCGCCCACCAAGGCAAAGAGAAGAGTGGTG--------------
-------------------------GAGAGAGAAAAAAGAGCA---GTTGGA---CTAGGAGCT---------
GTCTTCCTTGGGTTCTTAGGAGCA---GCAGGAAGCACTATG---GGCGCGGCGTCA---
ATAACGCTGACGGTACAG---GCCAGGCAA---TTATTG---TCTGGCATAGTGCAACAGCAAAGC---
AATTTG---CTGAGG---GCTATAGAGGCTCAACAACATATGTTGAAACTCACAGTCTGGGGCATT
>KT022377
TTCTATTGTAATACATCAGGGCTGTTCAATAGTACTTGGAATACCACGAAGGTAAATAGCACAGAG-------
----

TCAAATGGCACTATAACTCTCCCATGCAGAATAAAGCAAATTATAAATATGTGGCAAAGAACAGGACAAGCAA
TGTATGCCCCTCCCATCAAAGGAATAATAAATTGTGCATCAAAT---
ATTACAGGACTACTATTAACAAGAGATGGTGGGGATAATAACAGT----------------------------
----

ACAAATGAAACCTTCAGACCTGGAGGAGGAGATATGAGGGACAATTGGAGAAGTGAATTATATAAGTATAAAG
TAGTAAAAATTGAACCACTA---GGGGTAGCACCCACCGAGGCAAGGAGAAGAGTGGTG--------------
-------------------------GAGAGAGAAAAAAGAGCATTTGTTGGA---ATAGGAGCT---------
GTCTTCCTTGGGTTCTTAGGAGCA---GCCGGCAGCACTATG---GGCGCGGCGTCA---
TTAACGCTGACGGTACAG---GCCAGACAA---TTATTG---TCTGGTATAGTGCAACAGCAAAAC---
AATTTG---CTGAGG---GCTATAGAGGCTCAACAACATCTGTTGAAACTCACGGTCTGGGGCATT
>FJ866116
TTCTACTGTGATACATCAGGACTGTTTAATAGCTCTTGGAATAATACATCGGGCCAGTTTAATAGCACTTGGA
CTGATGGC---
-------------TACAAT------
AGAAAGAGCGAGAATATAACTCTCCAATGCAGAATAAAGCAATTTATAAATATGTGGCAGAGAACAGGGCAAG
CAATGTATGCCCCTCCCATCCCAGGAATAATAAAATGTGAATCAAAC---
ATTACAGGACTAATACTAACAAGAGATGGTGGGCAGGCTAACAAA----------------------------
----

ACAGAGGAAACCTTCAGACCTGGAGGAGGAGATATGAGGGACAATTGGAGAAGTGAATTATTTAGGTATAAAG
TAGTAAAAATTGAACCACTA---GGAGTAGCACCCACCAAGGCAAAGAGAAGAGTGGTG--------------
-------------------------GAGAGAGAGAAAAGAGCA---GTAATTGGCATGGGAGCT---------
GTTTTCCTTGGGTTCTTAGGAGCA---GCAGGAAGCACTATG---GGCGCGGCGTCA---
CTGACGCTGACGGTACAG---GCCAGACAA---TTATTG---TCTGGCATAGTGCAACAGCAAGGC---
AATTTG---CTGAGG---GCTATAGAGGCTCAACAACATCTGTTGAGACTCACGGTCTGGGGCATT
>FJ866118
TTCTATTGTAATACAACAGGACTGTTCAATCTCACTTGGAATGGCACTGACAGCACAAATAGAAAT-------
----

GAGTCAGGCAATATAACTCTCCCATGCAGAATAAAGCAAATTATAAATATGTGGCAAAGAGTAGGACGAGCAA
TATATGCCCCTCCCATCCCAGGAGTAGTAAAGTGTAAATCAAAT---
ATTACAGGATTAATATTAACAAGGGATGGTGGAGACGACGAGAAT----------------------------
----

AACACTGAAACCTTCAGACCTGGAGGAGGAAATATGAGGGACAATTGGAGAAGTGAATTATATAAATATAAAG
TAGTAAAAATTGAACCACTA---GGGGTAGCACCCACCAAGGCAAGGAGGAGAACGGTG--------------
-------------------------GAGAGAGAAAAAAGAGCA---GTTGGA---ATAGGAGCT---------
GTCTTCCTTGGGTTCTTAGGAGCA---GCAGGAAGCACTATG---GGCGCGGCGTCA---
ATAACGCTGACGGTACAG---GCCAGACAA---TTATTG---GCTGGCATAGTGCAACAGCAAAGC---
AATTTG---CTGAGG---GCTATAGAGGCTCAACAACAGATGTTGAGACTCACAGTCTGGGGCATT
>FJ866120
TTCTATTGTAATACATCACTCCTGTTTGAAAGCATTTGGTATGGCAATGAAAGCATAAATGCCACAGAAGAGG
CA---

AATGACACTATAATAACTCTCCGATGCAGAATAAAGCAATTTATAAAAATGTGGCAGAGAGTAGGACAAGCAA
TGTATGCCCCTCCCATCCCAGGAGCAATCAGGTGTAATTCAAAC---
ATTACAGGACTAATATTAACAAGAGATGGAGGGCTTAATGACAATGGAGCT----------------------
----

GATGGAAGAAATGAAACCTTCAGACCTGGAGGAGGAGATATGAGGGACAATTGGAGAAGTGAATTATATAAGT
ATAAAGTGGTAGAAATTGAACCACTA---GGAGTAGCCCCCACTCAGGCAAGAAGAAGAGTGGTG--------
-------------------------------CAGAGAGAAAAAAGAGCA---GTTGGA---ATAGGAGCT---
------GTCTTCATTGGGTTCTTAGGAGCA---GCAGGAAGCACTATG---GGCGCAGCGTCA---
ATAGCGCTGACGGCACAG---GCCAGACAA---TTATTG---TCTGGCATAGTGCAACAGCAAAGC---
AATTTG---CTGAGG---GCTATAGAGGTTCAACAACAGTTGTTGAAACTCACGGTCTGGGGCATT
>FJ866121
TTCTACTGTAACACATCAAAACTGTTTAATAGTACTTGGCATTACAATAACAGCACTGGGTATAATAATGGCA
GCCTACAG---
-------------------GAG---

GAAAATAACACAGAGATAGTTCTCCTATGCAGAATAAAGCAAATTATAAATCTGTGGCAGAGGGTAGGAAAGG
CAATGTATGCCCCTCCCATCCAAGGAAGCATAAAGTGTAACTCAAAC---
ATTACAGGACTACTATTAATCAGGGATGGTGGGGTTAATACTACT----------------------------
----

AATGAAACCTTCAGACCTGGAGGAGGAGATATGAGGGACAATTGGAGAAGTGAATTATATAAATATAAAGTAA
TAAAAATTGAACCACTA---GGAATAGCACCCACCAGGGCAAAGAGAAGAGTGGTG-----------------
----------------------GAGAGAGAAAAAAGAGCA---GTTGGA---CTAGGAGCT---------
GTTTTCGTTGGGTTCTTAGGAGCA---GCAGGAAGCACTATG---GGCGCAGCGTCA---
GTGACGCTGACGGTACAG---GCCAGACAG---TTATTG---TCTGGTATAGTGCAACAGCAAAGC---
AATTTG---CTGAGG---GCTATAGAGGCTCAACAACATATGTTGAAACTCACGGTCTGGGGCATT
>KT022380
TTCTATTGTAATACATCAGGCTTGTTTAATAGGACTTGGAAAAAGGGGATAATCAAGGGCACTGCCAATAACA
CG---

GAGTCAGACCCTATAGTGCTCCAATGCAGAATAAAGCAAATTATAAATATGTGGCAGAGAGTAGGACGAGCAA
TGTATGCCCCTCCCATCCCAGGAATAATAAAATGTGTATCAAAC---
ATTACAGGACTAATATTAACAAGAGATGGTGGGAATAGTAATGGA----------------------------
----

GATAATGAAACCTTCAGACCTGGAGGAGGAGATATGAGGGACAATTGGAGAAGTGAATTATATAAGTATAAAG
TAGTAAAAGTTGAGCCATTA---GGAGTAGCACCCACCAGGGCAAGGAGGAGAGTGGTG--------------
-------------------------GCGAGAGAAAAAAGAGCAGTAGTTGGA---CTGGGAGCT---------
GTTTTCATTGGGTTCTTAGGAGCA---GCAGGAAGCACTATG---GGCGCGGCGTCA---
ATAACGCTGACGGTACAG---GCCAGACAA---TTATTG---TCTGGCATAGTGCAACAGCAAAGC---
AATTTG---CTGAGG---GCTATAGAGGCTCAACAACATCTGTTGAAACTCACGGTCTGGGGCATT
>KT022381
TTCTATTGTAATACATCAGGCCTGTTTAATTACACCTATCCATCAAATCACAGTGATAAGTCAAATAACACT-
----

ATAAATGAGACTATAACTCTCCCATGCAGAATAAAACAAATTATAAGAATGTGGCAGAGAACAGGACAAGCAA
TGTATGCCCCTCCCATCCCAGGAATAATTAAGTGTGAATCAAAC---
ATTACAGGACTAATATTAACAAGAGATGGT---TTTAATGGCACG----------------------------
----

AATGAAACCTTCAGGCCTGTAGGAGGAGATATGAGGGACAATTGGAGAAGTGAATTATATAAGTATAAAGTAG
TAAGAATTGAACCACTA---GGAGTAGCACCCACCAGGGCAAAGAGAAGAGTGGTG-----------------
----------------------GGAAGAGAGAAAAGAGCA---GTTGGA---ATGGGAGCT---------
GTATTCCTTGGGTTCTTAGGAGCA---GCAGGAAGCACTATG---GGCGCGGCGTCA---
ATAACGCTGACGGTACAG---GCCAGACAA---TTATTG---TCTGGCATAGTGCAACAGCAAAGC---
AATTTG---CTGAAG---GCAATAGAGGCTCAACAACATCTGCTGAGACTCACGGTCTGGGGCATC
>KT022382
TTCTATTGTAATACATCAGGCCTGTTCAATAGTAGCTGGGGTCCAAATGACACGAAATCAGATAAC-------
----

TCAACTACAAATTACACTCTCCCATGCAGAATAAAGCAAATTGTAAGGATGTGGCAGAAAATAGGACAAGCAA
TGTATGCCCCTCCAATCGAGGGAGAAATAAATTGTGTATCAAAC---
ATTACAGGACTATTATTAACAAGAGATGGTGGGCATAACAGTATT----------------------------
----

AACAATGAAACTGAAACATTCAGACCTGGAGGAGGAGATATGAAGGACAATTGGAGAAGTGAATTATATAAGT
ATAAAGTAGTAAAAATTGAACCACTA---GGAGTAGCACCCACCAGGGCAAAGAGAAGAGTGGTG--------
-------------------------------GGGAGAGAAAAAAGAGCA---GTTGGA---ATAGGAGCT---
------GTTTTCCTTGGGTTCTTGGGAGCA---GCAGGAAGCACTATG---GGCGCGGCGTCA---
ATAACGCTGACGGTACAG---GCCAGACAA---TTATTG---TCTGGCATAGTGCAGCAGCAAAAC---
AATTTG---CTGAGG---GCTATAGAGGCTCAACAACATCTGTTGAAACTCACGGTCTGGGGCATT
>KT022383
TTCTATTGTAATACATCAGGCCTGTTTAATAGCACTTGGAACAGCCCTTGGAATAACACTGGGGAA-------
----

CAAGAAGGCAATATAACTCTCCCATGCAGAATAAAGCAAATTATAAATATGTGGCAGAGAGTAGGACAAGCAA
TATATGCCCCTCCCATCCCAGGAGTAATAAAGTGTGAATCGAAC---
ATTACAGGGCTACTATTAACAAGAGATGGTGGGAACACTAACAGT----------------------------
----

AGTACTGAAACCTTCAGACCTGGAGGAGGAGATATGAGGGACAATTGGAGAAGTGAATTATATAAATATAAAG
TAGTAAAAGTTGAACCACTA---GGAGTAGCACCCACCAGGGCAAGGAGAAGAGTGGTG--------------
-------------------------GGGAGAGAAAAAAGAGCA---GTTGGA---ATAGGAGCT---------
GTCTTCCTTGGGTTCTTAGGAGCA---GCAGGAAGCACTATG---GGCGCGGCGTCA---
ATAACGCTGACGGTACAG---GCCAGACAA---TTATTG---TCTGGCATAGTGCAACAGCAAAGC---
AATTTG---CTGAGG---GCTATAGAGGCTCAACAACATATGTTGAAACTCACGGTCTGGGGCATT
>FJ623487
TTCTATTGTAATACATCAAGACTGTTTAATAGCACTTGGAACAATGGCACTTGGAACAATACCAACATGCAGG
AGTCAAATAGC--
-------------ACGAAG------
TCAGAAAATGGCACTATAACTCTCCCATGCAGAATAAAGCAAATTATAAATATGTGGCAGAGAACAGGACAAG
CAATGTATGCCCCTCCCATCCGAGGAGTAATAAGATGTGAATCAAAC---
ATTACAGGACTAATATTAACAAGAGATAGTGTGAATAATAGCAAA----------------------------
----

GACAATGAAACCTTCAGACCAGGAGGAGGAGATATGAGGGACAATTGGAGAAGTGAATTATATAAGTATAAAG
TAGTAAAAGTTGAACCACTA---GGAGTAGCACCCACCACGGCAAGGAGAAGAGTGGTG--------------
-------------------------GGAAGAGAAAAAAAGAGCA---GTGGGA---ATAGGAGCT--------
GTATTCCTTGGGTTCTTGGGAGCA---GCAGGAAGCACGATG---GGCGCGGCGTCA---
ATGACGCTGACGGTACAG---GCCAGACAA---TTATTG---TCTGGTATAGTGCAACAGCAAAGC---
AATTTG---CTGAGG---GCTATAGAGGCTCAACAACACCTGTTGAAACTCACGGTCTGGGGCATT
>FJ623481
TTTTATTGTAATACATCAGGCCTGTTTAATAGCACTTGGAATCACACTGACATAAAGGGGTCAAATAGCACGG
AG---

TCAAGTGAAACTATAACTCTCACATGCAGAATAAAGCAAATTATAAATATGTGGCAGAGAGTAGGGCAAGCAA
TGTATGCCCCTCCCATACCAGGAGCAATAAGGTGTGAATCAAAC---
ATTACAGGAATACTCTTAACAAGAGATGGTGGGAATAGCAAGAAC----------------------------
----

AGCACAACAGAAATCTTCAGACCTGGAGGAGGAAACATGCGGGACAATTGGAGAAGTGAATTATATAAGTATA
AAGTAGTACAAATTGAACCACTA---GGAGTAGCACCTACCAGGGCAAAGAGAAGAGTGGTG-----------
----------------------------GAGAGAGAAAAAAGAGCA---ATTGGA---ATAGGAGCT------
---GTATTCATTGGGTTCTTAGGAGCA---GCAGGAAGCACTATG---GGCGCGGCGTCA---
ATAACGCTGACGGTACAG---GCCAGACAA---TTATTG---ACTGGCATAGTGCGACAGCAAAGC---
AATTTG---CTGAGG---GCTATAGAGGCTCAACAACATCTGTTGAAACTCACGGTCTGGGGCATT
>FJ623475
TTTTACTGTGATACATCAGGCCTGTTTAATAGCACTTGGGGAGCCACTGACAGCATGCATGGGGCAAATGACA
CG---

GAGTCAAATGACACTATAACTCTCCCATGTAGGATAAAACAAATTATAAATAGGTGGCAGAGAGTAGGACAAG
CAATGTATGCCCCTCCCATCCAAGGAGTAATAAAGTGCAAATCAGAC---
ATTACAGGAATAATATTAACAAGAGATGGTGGGAATAATAATGAC----------------------------
----

AGTAGAAATGAAACCTTCAGACCTGGAGGAGGAGATATGAGGGACAATTGGAGAAGTGAATTGTATAAGTATA
AGGTGGTAAAGATTGAACCACTA---GGAGTAGCACCCACCAGGGCAAAGAGAAGAGTGGTG-----------
----------------------------GAGAGAGGAAAAAGAGCA---GTTGGG---ATAGGAGCT------
---GTTTTCCTTGGATTCTTAGGAGCA---GCCGGAAGCACTATG---GGCGCGGCGTCA---
ATAACGCTGACGGTACAG---GCCAGGCAA---TTATTG---TCTGGCATAGTGCAACAGCAAAGC---
AATTTG---CTGAGG---GCTATAGAGGCTCAACAACATATGTTGAAACTCACGGTCTGGGGCATA
>FJ623476
TTCTATTGTAATACATCAGGCTTGTTTAATAGCACTTGGAATATCACTGACAATCTCAACATAACTCAG----
----

TCAAATGAGAACATAACTCTCCCATGCAAAATAAAGCAAATTATAAATATGTGGCAGAGAGTAGGACAAGCAA
TATATGCCCCTCCCATCCCAGGAAGGGTAAGTTGTGTCTCAAAC---
ATTACAGGACTAATATTAACAAGAGATGGTGGGAACAATAACAGT-------------------------
----

ACAAATGAAACCTTCAGGCCTGGAGGAGGAGATATGAGGGACAATTGGAGAAGTGAATTATATAAGTATAAAG
TAGTAAGAATTGAACCACTA---GGAGTAGCACCTACCCAGGCAAAGAGAAGAGTGGTG-----------
------------------------GGAAGAGAAAAAAGAGCA---ATTGGA---ATAGGAGCT--------
GTCTTCCTTGGGTTCTTAGGAGCA---GCAGGAAGCACTATG---GGCGCGGCGTCA---
ATAACGCTGACGGTACAG---GCCAGACAG---TTATTG---TCTGGCATAGTGCAACAGCAAAAC---
AATTTG---CTGAGG---GCTATAGAGGCTCAACAACAACTGTTGAAACTCACGGTCTGGGGCATT
>FJ623485
TTTTATTGTAATACATCAGGCCTGTTTAATAGCACTTGGTATCACAATAGCACTTGGAATGATCAAACTGACG
AG--

AGGTCAAATGACACTATAACTCTCCAATGCAGAATAAAGCAAATTATAAATATGTGGCAGAGAGTAGGACAAG
CAATATATGCCCCTCCTATCCAAGGAACAATAAGGTGTGAATCAAAC---
ATTACAGGACTAATATTAACAAGAGATGGTGGGGAGGATAGCAAT-------------------------
----

ACAACAACTGAAACCTTCAGACCTAGCGGAGGAGATATGAAGGACAATTGGAGAAGTGAATTATATAAATATA
AAGTAGTAAAAATTGAACCACTA---GGAGTAGCACCCTCCAGGGCAAGGAGAAGAGTGGTG--------
---------------------------GGAAGAGAAAAAAGAGCA---GTTGGA---ATAGGAGCT-----
---GTCTTCCTTGGGTTCTTAGGAGCA---GCAGGAAGCACTATG---GGCGCAGCGTCA---
ATAACGCTGACGGTACAG---GCCAGACAA---TTACTG---TCTGGCATAGTGCAACAGCAAAGC---
AATTTG---CTGAGG---GCTATAGAGGCTCAACAACATATGTTGAGACTCACGGTCTGGGGCATT
>FJ623488
TTCTATTGTAACACATCAGGTCTGTTTAATAGCACTTGGGATAACAATACTGGCAATTGGTCTAGCAATAGCA
GCAGCATGCAG---
---------GGGTCAAAT------
AGCACAGATGATACTATAACTCTCCCATGCAGAATAAAGCAAATTATAAATATGTGGCAGAGAACAGGACAAG
CAATGTATGCCCCCCCCATCCAAGGGATAATAAGGTGTGAATCAAAC---
ATCACAGGGATAATATTAACAAGAGATGGTGGGGGTACTGAGGAT-------------------------
----

AATACTACAGAAGTCTTCAGACCTGGAGGAGGAGATATGAGGGACAATTGGAGAAGTGAATTATATAAATATA
AAGTAGTACAAGTTGAACCAGTA---GGAATAGCACCCTCCAAGGCAAGGAGAAGAGTGGTG--------
---------------------------CAGAGAGAAAAAAGAGCA---ATTGGA---ATGGGAGCT-----
---GTCTTCCTTGGGTTCTTAGGAGCA---GCAGGAAGCACTATG---GGCGCGGCGTCA---
ATGACGCTGACGGTACAG---GCCAGAAAT---TTATTG---TCTGGCATAGTGCAACAGCAGAGT---
AATTTG---CTGAGG---GCTGTAGAGGCTCAACAACAACTGTTGAGACTCACGGTCTGGGGCATT
>FJ623478
TTCTATTGTAATACATCAGGCCTGTTTAATCACACTTACCTGCCTAAGAGCACTTACCTGCCTAATAGCACTT
GGAATAAC--
------------AGTACG------
GGATCAAATGACAGTATAACTCTTCCATGTAGAATAAAGCAAATTATAAATATGTGGCAGAGAGTAGGACAAG
CAATGTATGCCCCTCCTATCCAAGGAGTAATAAAGTGTAAATCAGAT---
ATTACAGGACTAATATTGACAAGAGATGGTGGGAATAGTAACAGT-------------------------
----

GATAATGAAACCTTCAGACCTGGAGGAGGAGATATGAGGGACAATTGGAGAAGTGAATTATATAAGTATAAAG
TAGTAAAAATTGAACCACTA---GGAGTAGCACCCACCAGGGCAAAGAGAAGAGTGGTG-----------
------------------------GAGAGAGAAAAAAGAGCA---GTTGGA---ATAGGAGCT--------
GTTTTCATTGGGTTCTTAGGAGCA---GCAGGAAGCACTATG---GGCGCGGCGTCA---
ATAACGCTGACGGTACAG---GCTAGACAA---TTATTG---TCTGGCATAGTGCAACAGCAAAGC---
AATTTG---CTGAGG---GCTATAGAGGCTCAACAACATATGTTGAAACTCACGGTCTGGGGCATT
>FJ623482
TTCTATTGTAATACATCAGACCTGTTTAATGGCAATAGCACTAGGAATGGCACTGGGAATAGCAACAGCACGC
CG--

GTAACTGGCAATATAACTCTCCCATGCAGAATAAAGCAAATTATAAATATGTGGCAGAGAGTAGGACAAGCAA
TGTATGCCCCGCCCATCCAAGGGGTAATAAGGTGTGTATCAAAC---
ATTACAGGATTAATATTGACAAGAGATGGTGGGAAACAGAATAATAATAGTAAT------------------
----
-
AGTAATAGTAGTGATATTGAAGTCTTCAGACCTGGAGGAGGAGATATGAGGGACAATTGGAGAAGTGAATTAT
ATAAGTATAAAGTAGTAAAAATTGAACCACTA---GGAATAGCACCTACCAAGGCAAAAAGAAGAGTGGTG--
------------------------------------GAGAGAGAAAAAAGAGCA---ATTGGA---
ATAGGAGCT---------GTCCTCCTTGGGTTCTTAGGAACA---GCAGGAAGCACTATG---
GGCGCGGCGTCA---ATAGCGCTGACGGTACAG---GCCAGACAA---TTAATG---
TCTGGCATAGTGCAACAGCAAAGC---AATTTG---CTGAGG---
GCTATAGAGGCTCAACAGCATATGTTGAAACTCACAGTCTGGGGCATT
>FJ623477
TTCTATTGTGATACATCAGGCCTGTTTAATAGCACCTGGAATAATAGCACCTGGAATAATAATGACACTGCCC
AG--

TCAAATGACACAATAACTCTCCCATGCAGAATAAAACAAATTGTAAATATGTGGCAGAGAACAGAACAAGCAA
TGTATGCCCCTCCCATCCAAGGAGTAATAAGGTGTGAATCAAAC---
ATCACAGGACTACTATTAACAAGAGATGGTGGGATTAATAACACA---------------------------
----

AATGAAACCTTCAGACCTGGAGGAGGAGATATGAGGGACAATTGGAGAAGTGAACTATATAAGTATAAAGTAG
TAAAAATTGAACCACTG---GGAGTAGCACCCTCCAGGGCAAGGAGAAGAGTGGTG----------------
----------------------GGGAGAGAAAAAAGAGCA---GTTGGA---ATAGGAGCT---------
TTATTCCTTGGATTCTTGGGCGCA---GCAGGAAGCACTATG---GGCGCGGCGTCA---
ATGACGCTGACGGTACAG---GCCAGACAA---TTATTG---TCTGGCATAGTGCAACAGCAAAGC---
AATTTG---CTGAAG---GCTATAGAGGCTCAACAACATCTGTTGAGACTCACGGTCTGGGGCATT
>FJ623486
TTCTATTGTAATACATCAGAACTATTTAATAGCACTTGGAATCTTAATAGCACTTGGAATCACACTGGCAGCA
TACCGGAGTCA---
----------AATAGCACG------
GAGTCAAATGGCACTATAACCCTCAAATGCAGAATAAAGCAAATTATAAATATGTGGCAGAGAGTAGGGCAAG
CAATGTATGCCCCTCCCATCAAAGGGACAATAAGGTGTAAATCAAAC---
ATCACAGGACTAATATTAACAAGAGATGGTGGGATAATAACAGT----------------------------
----

ACAAAGGAAACCTTCAGGCCTGGAGGAGGAGATATGAGGGACAATTGGAGAAGTGAATTATATCAGTATAAAG
TAGTAAAACTTGAACCACTA---GGAGTAGCACCCACCAGGGCAAGGAGAAGAGTGGTG-------------
-------------------------GGGAGAAAAAAAAGAGCA---GTTGGA---CTAGGAGCT--------
GTTTTCATTGGGTTCTTGGGAGCA---GCAGGAAGCACTATG---GGCGCGGCGTCA---
ATGACGCTGACGGTACAA---GCTAGACAA---TTATTG---TCTGGCATAGTGCAACAGCAAAGC---
AATTTG---CTGAGG---GCTATAGAGGCTCAACAACATCTGTTGAAACTCACGGTCTGGGGCATT
>FJ623479
TTCTATTGTAATACATCAGACCTGTTTAATGGTACTTGGAATAATAGCACTTTGAATGGCACTGCCAAGCAGG
AG--

GTAAATGACAGCACTATAACGATCTCATGCAGGATAAAGCAATTTATAAATATGTGGCAGAGAACAGGACGAG
CAATGTATGCCCCTCCCATTCCAGGAGTAATAAGTTGTAACTCAAGC---
ATTACAGGATTAATATTAACAAGAGATGGTGGGATGATAACAAT----------------------------
----

AATAATGAAACATTCAGACCTGGAGGAGGAGATATGAGGGACAATTGGAGAAGTGAATTATATAAGTATAAAG
TAGTAAAAATTGAACCACTA---GGAGTAGCACCCACCAAGGCAAGGAGAAGAGTGGTG-------------
-------------------------GAGAGAGAAAAAAGAGCA---GTTGGA---ATAGGAGCT--------
GTACTCCTTGGGTTCTTAGGAGCA---GCAGGAAGCACTATG---GGCGCGGCGTCA---
ATAACGCTGACGGTACAG---GCCAGACAA---TTATTG---TCTGGCATAGTGCAACAGCAAAGC---
AATTTG---CTGAAG---GCTATAGAGGCTCAACAACAACTGTTGAAACTCACAGTCTGGGGCATT
>HM215275
TTCTATTGTAATACATCAGGTCTGTTCAATAGCACTTGGGAGGCCAATAGTACTGCCAATACC---------
----

AGCACGACAAATATAACTCTCCCATGCAAAATAAAGCAACTTGTAAGAATGTGGCAGAGGACAGGACAAGCAT
TGTATGCCCCTCCCATCGAAGGAGTAATAAAATGTGTATCAAAC---
ATTACAGGACTAATATTAACAAGAGATGGTGGGGGTAATAACAGT---------------------------
----

GCAACAAATGAAACCTTCAGACCTGGAGGAGGAGATATGAGGGACAATTGGAGAAGTGAATTATATAAGTATA
AAGTAGTACAAATTGAACCACTA---GGAGTTGCACCCACCGAGGCAAGGAGAAGAGTGGTG----------
---------------------------AAGAGAGAAAAAAGAGCA---GTTCCT------GGACTG------
---TTTTTCCTTGGGTTCTTAGCAGCA---GCAGGAAGCACTATG---GGCGCGGCGTCA---
ATAACGCTGACGGTACAG---GCCAGACAA---TTATTG---TCTGGCATAGTGCAACAGCAAAGC---
AATTTG---CTGAGG---GCTATAGAGGCTCAACAGCATCTGTTGAAACTCACGGTCTGGGGCATT
>KT252544
TTCTATTGTAACACATCAGGCCTGTTTAATAGTACTTGGGATGGGAATAGCACTGAGGCAAACATTACGCAG-
----

TCAAATGAGACTATAACTCTCCCATGCAAAATAAAGCAAATTGTAAATATGTGGCAGAGAGTGGGACGAGCAA
TGTATGCCCCTCCTATCCCAGGAGTAATAAAGTGTGAATCAAAC---
ATTACAGGGCTAATACTGACAAGAGATGGTGGGAATGAAACAAAT---------------------------
----

GGAACAGAAATCTTCAGACCTGTAGGAGGAGACATGAGGGACAATTGGAGAAGTGAATTATATAAGTATAAAG
TAGTACAAATTAAACCACTA---GGAGTAGCACCCACCAAGGCAAAGAGAAGAGTGGTG-------------
------------------------CAGAGAGAAAAAAGAGCA---GTTGGA---ATGGGAGCT---------
GTTTTCTTTGGGTTCTTAGGAGCA---GCTGGAAGCACTATG---GGCGCGGCGTCA---
ATAACGCTGACGGTACAG---GCCAGACAA---TTATTG---ACTGGCATAGTGCAACAGCAAAGC---
AATTTG---CTGAGG---GCTATAGAGGCTCAACAGCATCTGTTGAAACTCACTGTCTGGGGCATT
>HQ540689
TTCTACTGTAATACAACAAACCTGTTTAATAGTAGCATTCCCTTCAACGCCAGCGAGAGGGCAAATAACACCA
AT--

AGCACAGATGACATTATAACTCTCCAATGCAGAATAAAGCAAATTGTAAGGATGTGGCAAAGAGTAGGACAAG
CAATGTATGCCCCTCCCATCCCAGGAGTAATAAGGTGTGAATCAAAC---
ATTACAGGACTTATGTTAACATGGGATGGTGGGAGTAAAAATATTACTGAG---------------------
----

GGCAATAGAACTGAAACCTTTAGACCTGGAGGAGGAGACATGAGGGACAATTGGAGAAGTGAATTATATAAAT
ATAAAGTAGTAAAAATTGAACCACTA---GGAGTTGCACCCACCAAGGCAAGGAGAAGAGTGGTG-------
-------------------------------GGAAGAGAAAAAAGAGCA---GTTGGA---ATAGGAGCT---
------GTTTTCCTTGGGTTCTTAGGAGCA---GCAGGAAGCACTATG---GGCGCGGCGTCA---
GTGACGCTGACGGTACAG---GCCAGACAA---TTATTG---TCTGGTATAGTGCAACAGCAAAGC---
AATTTG---CTGAGA---GCTATAGAGGCTCAACAACAGTTGTTGAAACTCACGGTCTGGGGCATT
>KU921708
TTCTATTGTAATACAGTAAATCTGTTCAATAGCTCTTGGGGTGCCAACGCCACTGAGTCAAATAGCACAGGGT
TA--

AATAATCAAACTATACATCTCCCATGCAGAATAAAGCAAATTATAAATATGTGGCAGAGAACAGGACAAGCAA
TGTATGCCCCTCCCGTCGAAGGAATAATAAGTTGTGTATCAGAC---
ATTACAGGACTACTGTTAACAAGAGACGGAGGAGAAAATACAACA---------------------------
----

CATGAAATTTTCAGACCTGGAGGAGGAGATATGAGGGACAATTGGAGAAGTGAATTATATAAGTATAAAGTAG
TAAAAATTGAACCCCTA---GGAGTAGCACCCACCAAGGCAAGGAGAAGAGTGGTG----------------
----------------------CAGAGAGAAAAAAGAGCA---GTTGGA---ATGGGAGCT---------
GTCTTCCTTGGGTTCTTAGGAGCA---GCAGGAAGCACTATG---GGCGCAGCGTCA---
ATAACGCTGACGGTACAG---GCCAGACAA---TTATTG---TCTGGTATAGTGCAACAGCAAAGC---
AATTTG---CTGAGG---GCTATAGAGGCTCAACAGCATCTGTTGAAACTCACTGTCTGGGGCATT
>KU921712
TTCTATTGTAATACATCAAAGCTGTCTAACAGCACATTTCCACTTAATAACACTCAGAATAACAGCACGCAG-
----

ACAAACAGTACTATAACTCTCCCATGCAGAATAAAGCAAATTATAAATATGTGGCAGAGAACAGGAAAAGCAA
TATATGCCCCTCCCATCTCAGGACCAATAAAGTGTACATCAAAC---
ATTACAGGACTAATATTAACAAGAGATGGTGGAAATGATAATAATAATGAT----------------------
----

ACCAATGGAAATGAAACCTTCAGACCTGGAGGAGGAGATATGAGGGACAATTGGAGAAGTGAATTATATAAGT
ATAAAGTAGTAAAACTTGAACCACTA---GGAGTAGCACCCACCAGGGCAAGAAGAAGAGTGGTG--------
-------------------------------CAGAGAGAGAAAAGAGCA---ATTGGA---CTGGGAGCT---
------GTATTCATTGGGTTCTTAGGAGCA---GCAGGGAGCACTATG---GGCGCGGCGTCA---
GTGACGCTGACGGTACAG---GCCAGAGCC---TTATTG---TCTGGCATAGTGCAACAGCAAAGC---
AATTTG---CTGAGG---GCTATAGAAGCTCAACAACATCTGTTGAAGCTCACGGTCTGGGGCATT
>KU921727
TTCTATTGTGACACATCAAGCCTGTTTAATAGCACTTGGAATGAGAATGACAATGTCAGCAGAACAGAGTCAA
AT---

AGTACAGAAGATATAACTCTCCAATGCAGAATAAAACAAATTGTAAATATGTGGCAGAGAGTAGGACAAGCGA
TGTATGCCCCTCCCATCCAAGGAATAATAAAGTGTGAATCAAAC---
ATAACAGGACTAATATTAACAAGAGATGGTGGGAATCATAATAGT----------------------------
----
ACAAATGAAACCTCC---
AGAAATGAAACCTTCAGACCTGGAGGAGGAGATATGAGGGACAATTGGAGAAGTGAATTATATAAGTATAAGG
TAGTAAAAATTGAACCTCTA---GGAGTAGCACCCACCAAGGCAAAGAGAAGAGTGGTG--------------
-------------------------GAGAGAGAAAAAAGAGCA---GTTGGA---ATAGGAGCT---------
GTCTTCCTTGGATTCTTGGGAGCA---GCAGGAAGCACTATG---GGCGCGGCGTCA---
ATAACGCTGACGGCACAG---GCCAGACAA---TTATTG---TCTGGTATAGTGCAACAGCAAAGC---
AATTTG---CTGAGG---GCTATAGAGGCTCAACAGCATCTGTTGAGACTCACAGTCTGGGGCATT
>KU921732
TTCTATTGTAATACATCAGGCCTTTTTGATGACACTTGGAATGGCACGAAG----------------------
----

TCAAATGACACTATAACACTCCCATGCAGAATAAAGCAAGTTATAAATATGTGGCAGAGGACAGGACAAGCAA
TGTATGCCCCTCCCATCCAAGGAGTAATAAGGTGCGAATCAAAC---
ATCACAGGACTAATATTAACAAGAGATGGTGGGCATAACATTACA----------------------------
----

AATGAAACCTTCAGACCTACAGGAGGAAATATGAGGGACAATTGGAGAAGTGAATTATATAAGTATAAAGTAG
TAAGAATTGAACCACTA---GGAGTAGCACCCACCTCGGCAAAGAGAAGAGTGGTG-----------------
----------------------GAGAGAGAAAAAAGAGCAGTAGTTGGA---ATGGGAGCT---------
GTCTTCATTGGGTTCTTGGGAGCA---GCAGGAAGCACTATG---GGCGCGGCGTCA---
ATGACGCTGACGGTACAG---GCCAGACAA---TTATTG---TCTGGCATAGTGCAACAGCAAAGC---
AATTTG---CTGAGG---GCTATAGAGGCTCAACAGCATTTGTTGAAGCTCACGGTCTGGGGCATT
>KU921734
TTCTATTGTAACACATCAGGTCTGTTTAATAGCACGTGGAATAAGGATAATAACACCTTGAATGGCGCTGTAA
ATAAC--

ATAACAGACAATATAACCCTCCAATGCAGAATAAAACAAATTATAAATATGTGGCAGAGAGCAGGACAAGCAA
TATATGCTCCTCCCATCCCAGGAGTAATAAGGTGCACCTCAAAT---
ATTACAGGATTAATATTAACAAGAGATGGTGGGAAAGATAGCAATGAA-------------------------
----

AGTGCTGAAACCTTCAGACCTGGAGGAGGAGATATGGGGGACAACTGGAGAAGTGAATTATATAAGTATAAGG
TAGTAAAAATTGAACCACTA---GGAATAGCACCCAACAGGGCAAAGAGAAGAGTGGTG--------------
-------------------------GAGAGAGAAAAAAGAGCAATCTTGGGA---CTGGGAGCT---------
GTTTTCGTTGGGTTCTTAGGAGCA---GCAGGAAGCACTATG---GGCGCGGCGTCA---
CTGACGCTGACGGTACAG---GCCAGAAAC---TTATTG---TCTGGCATAGTGCAACAGCAAAAC---
AATTTG---CTGAAG---GCTATAGAGGCTCAACAGCATCTGTTGAAACTCACGGTCTGGGGCATT
>KU921738
TTCTATTGTAACACATCAGAGCTGTTTAATAGCACTTGGAATAGGACTGACCCTGGCACTTGGAATAACACC-
----

AGCACGCAGGAGTCAAAT---------
AGCACAGATCAAAAAATAACTCTCCAATGCAGCATAAAGCAAATTATAAATATGTGGCAAAGGGTAGGACGAG
CAATGTATGCCCCTCCCATCCCAGGACCAATACAGTGTAGATCAAAC---
ATTACAGGACTAATATTAGTGAGAGATGGTGGGAATACGAATACT----------------------------
----

AGCAAGGAAACCTTTAGACCTGCAGGAGGGGATATGAGGGACAATTGGAGAAGTGAATTATATAAATATAAAG
TAGTAAAAATTGAACCACTA---GGAGTAGCACCCACCAAGGCAAAGAGAAGAGTGGTG--------------
-------------------------GAGAGAGGAAAAAGAGCA---GTTGGA---CTAGGAGCT---------
GTCTTCATTGGGTTCTTAGGAGCA---GCAGGAAGCACTATG---GGCGCAGCGTCA---
ATAACGCTGACGGTACAG---GTCAGACAA---TTATTG---TCTGGCATAGTGCGACAGCAAAGC---
AATTTG---CTGAGG---GCTATAGAAGCTCAACAACATCTGTTGAAACTCACGGTCTGGGGCATT
>KU921746
TTCTATTGTAACACATCAGGTCTGTTCAACAGCACTTGGGATGATACCGCCATACAGGAAAATATCACGGAGA
TCACGGAG---

TCAAATGACACAATAACTCTCCAATGCAGAATAAGGCAATTTATAAGGATGTGGCAGAGGACAGGACAGGCAA
TGTATGCCCCTCCCATACCAGGAGTAATAAGGTGTGAATCAAAC---
ATTACAGGACTAATATTAACAAGAGATGGTGGGCATAGTAATAGT----------------------------
----

ACTAGTAATGAAACCTTCAGACCTGGAGGAGGAGATATGAGGGACAATTGGAGAAGTGAATTATATAAGTATA
AAGTAGTAAAAATTGAACCACTA---GGAGTAGCACCCTCCAGGGCAAGGAGAAGAGTGGTG----------
----------------------------GGAAGAGAAAAAAGAGCA---GTTACA---ATAGGAGCC------
---GTCTTCCTTGGGTTCTTAGGAGCA---GCCGGAAGCACTATG---GGCGCGGCGTCA---
ATAACGCTGACGGTACAG---GCCAGGCAA---TTATTG---TCTGGTATAGTGCAACAGCAAAGC---
AATTTG---CTGAAG---GCTATAGAGGCTCAACAACATCTGTTGAGACTCACGGTCTGGGGCATT
>KU921752
TTCTATTGTAACACATCAGGCCTGTTTAATAGCACTTGGAATGACACCAGCAATGGGAATGACACCAACATAT
CAAATAGC---
-------------------ACG---
GAGAAGAATGAGAATATAACTCTCCAATGCAGAATAAAGCAAATTATAAATATGTGGCAAAGAGTAGGAAAAG
CAATGTATGCTCCTCCCATCCCAGGAATAATAAAATGTGAATCAAAC---
ATTACAGGACTAATATTAACAAGAGATGGGGGAAAGGAGGAAAAC----------------------------
----

GAAAGTGAAACCTTCAGACCTGGAGGAGGAGATATGAGGGACAATTGGAGAAGTGAATTATATAAGTATAAAG
TAGTAAAAATTGAACCACTA---GGAGTAGCACCCACCAGGGCAAGGAGAAGAGTGGTG--------------
-------------------------GAGAGAGAAAAAAGAGCA---GTTGGA---ATGGGAGCT---------
TTGTTCATTGGGTTCTTAGGAGCA---GCTGGAAGCACTATG---GGCGCGGCGTCG---
ATGACGCTGACGGTACAG---GCTAGACAA---CTATTG---TCTGGCATAGTGCAACAGCAAAGC---
AATTTG---CTGAGG---GCTATAGAGGCTCAACAGCATCTGTTGAAACTCACAGTCTGGGGCATT
>KU921756
TTCTATTGTAACACATCAAACCTGTTTAATAGTACTTGGCTTAACAATGGCACATTAGGTAGTGACAATGGCA
TATCA--

GATAGTAAAAACATAACTCTCCCATGCAAAATAAGGCAAATTATCAGAATGTGGCAGAGAGTAGGACAAGCAA
TATATGCCCCTCCCATCCAAGGAGTAATAAGGTGTAAATCAGAC---
ATTACAGGACTAATATTAACAAGAGATGGTGGAGTTAATAACAAT----------------------------
----

ATAACTGAAACCTTCAGACCTGGAGGAGGAGATATGAGGGACAATTGGAGAAGTGAGTTATATAAATATAAAG
TAGTAAAAATTGAACCACTA---GGAGTAGCACCCACCAGGGCAAGGAGAAGAGTGGTG--------------
-------------------------GGGAGAGAAAAAAGAGCA---GTTGGA---GTAGGAGCT---------
GTATTCCTTGGGTTCTTAGGAGCA---GCAGGAAGCACTATG---GGCGCGGCGTCA---
ATAACGCTGACGGTACAG---GCCAGACAA---TTATTG---TCTGGCATAGTGCAACAGCAAAGC---
AATTTG---CTAAGG---GCTATAGAGGCTCAACAGCATCTGTTGAAACTCACAGTCTGGGGCATT
>KU921759
TTCTATTGCAGTACAAAAGGCCTGTTTAATAGCACTTGGAATGCTAATGGCACCAGGAATGACTTAGGGAGCT
CG---

```
-------------------------
GAATCAGATGGCACTATAACTCTCCAGTGCAGAATAAAACAAATTATAAATATGTGGCAGAGAACAGGACAAG
CAATGTATGCCCCTCCCATCGCAGGAGTAATAAGTTGTAATTCAACC---
ATTACAGGACTACTATTAACAAGAGATGGTGGGAACAATAGTACA----------------------------
-------------------------------------------------------------------------
----------
AGTGAAACTGAAACCTTTAGACCTGGAGGAGGAGATATGAGGGACAATTGGAGAAGTGAATTATATAAGTATA
AAGTAGTAAAAATTGAACCATTA---GGAGTAGCACCCACCAGGGCGCGGAGAAGAGCGGTG-----------
----------------------------GGGAGAGAAAAAAGAGCA---GTTGGA---CTGGGAGCT------
---GTTTTTCTTGGGTTCTTAGGAGCA---GCAGGAAGCACTATG---GGCGCGGCGGCA---
GTGACGCTGACGGTACAG---GCCAGACAA---TTATTG---TCTGGCATAGTGCAACAGCAAAGC---
AATTTG---CTGAGA---GCTATAGAGGCTCAACAACATCTGTTGAAACTCACTGTCTGGGGCATT
>KU921772
TTCTATTGTAACACATCAGGCTTGTTTAATAGCAGTTGGAATAGCACCAGCAAGGAGCTAAATAGCACCAACA
GGGAGCTA-----------------------------------------------------------------
----------------------------
AATAACATGACTGTAACTCTCCAATGTAGAATAAAACAAATTATAAATATGTGGCAGAGAGTGGGACAAGCAA
TGTATGCCCCTCCCATCCCAGGAGTAATAAAGTGTGTATCAAAC---
ATTACAGGATTAATATTAACAAGGGATGGTGGGCTTAATCCTACA----------------------------
-------------------------------------------------------------------------
-------
GAGAACGAAACCGAAACCTTCAGACCTGGAGGAGGAGATATGAGAGACAATTGGAGAAGTGAATTATATATGT
ATAAAGTAGTGAAAATTGAACCACTA---GGAGTAGCACCCACCAGGGCAAAGAGAAGAGTGGTG--------
-------------------------------GAGAGAGAAAAAAGAGCA---GTTGGA---ATAGGAGCT---
------GTTTTCCTTGGGTTCTTGGGAGCA---GCAGGAAGCACTATG---GGCGCGGCGTCA---
ATAACGCTGACGGTACAA---GCCAGACAA---TTATTG---TCTGGCATAGTGCAACAGCAAAGC---
AATTTG---CTGAGG---GCTATAGAGGCTCAACAGCATCTGTTGAAACTCACAGTCTGGGGCATT
>KU921780
TTCTATTGTAATACATCAGGCTTATTTAATGGCACTTGGACCAATGCTAGCATACAAGAAAATAGC-------
-------------------------------------------------------------------------
----------------------------
ACGGAGTCAAATATAACTCTCCAATGCAGAATAAAACAAATTATAAATATGTGGCAGACAGTAGGACGAGCAA
TGTATGCTCCTCCCATTGAAGGAGCAATAAGGTGTGTATCAAAC---
ATTACAGGACTAATATTAACCAGAGATGGTGGGGATAACACAACT----------------------------
-------------------------------------------------------------------------
-------------
ACAACTGAAATCTTCAGACCTGGAGGAGGAAATATGAAGGACAATTGGAGAAGTGAATTATATAAGTATAAAG
TAGTAAAAATTGAACCATTA---GGAGTAGCACCCACCAAGGCAAAGAGAAGAGTGGTG--------------
-------------------------GAGAGAGAAAAAAGAGCA---GTTGGA---CTGGGAGCT---------
GTCTTCCTTGGGTTCTTAGGAGCA---GCAGGAAGCACTATG---GGCGCGGCGTCA---
GTAACGCTGACGGTACAG---GCCAGACAA---TTATTG---TCTGGCATAGTGCAACAGCAAAAC---
AATTTG---CTGAGG---GCTATCGAGGCTCAACAACATCTGTTGAAACTCACGGTCTGGGGCATT
>KU921783
TTCTATTGTAATACAACAGGCCTGTTTAATAGCACTTGGGGGAATGGCACTTGGAATGCCAGCAGCCAGGGGG
AG-----------------------------------------------------------------------
----------------------------
TCAAATGACACTATACTCCTCCAATGCAAAATAAAGCAAATTATAAATATGTGGCAGAGAGTAGGACAAGCAA
TGTATGCTCCTCCCATCCCAGGAGTAATAAGGTGTGAATCAAAC---
ATTACAGGACTAATATTAACAAGAGATGGTGGGGGGGATGACAAT----------------------------
-------------------------------------------------------------------------
-------------
GGAACCGAAACCTTCAGACCTGGAGGAGGAGATATGAGGGATAATTGGAGAAGTGAATTATATAAGTATAAAG
TAGTAAAAATTGAACCACTA---GGAGTAGCACCCACCAGGGCAAGGAGAAGAGTGGTG--------------
-------------------------GAGAGAGAGAAAAGAGCA---GTTACA---ATAGGAGCT---------
GTTTTTCTTGGGTTCTTAGGAACA---GCAGGAAGCACTATG---GGCGCGGCGTCA---
ATAGCGCTGACGGCACAG---GCTAGACAA---TTATTG---TCTGGCATAGTGCAACAGCAAAGC---
AATTTG---CTGAGG---GCTATAGAGGCTCAACAACATATGTTGAGACTCACGGTCTGGGGCATT
>KU921785
TTCTATTGTAACACATCAGACCTGTTTAATAGCACTTGGTATGGCAATAACACCATGCAGGGATTAAATGGC-
-------------------------------------------------------------------------
```

ACAAATGACACTATAATTCTCAAATGCAGAATAAAGCAAATTATAAATATGTGGCAGAGAACAGGACAAGCAA
TATATGCCCCTCCCATCCCAGGAGTAATAGAGTGTGTATCAAAC---
ATAACAGGACTAATATTGACAAGAGATGGTGGGGGTAGTGAGAGC----------------------------
----

AGGACAGAGGAAACATTTAGACCTGGAGGAGGAGATACGAGGGACAATTGGAGAAGTGAATTATATAAGTATA
AAGTAGTAAAATTGAACCACTA---GGAGTAGCACCCACCAGGGCAAAAAGAAGAGTGGTG------------
----------------------------GAGAGAGAAAAAAGAGCA---ATTGGA---ATAGGAGCT------
---GTCTTCATTGGGTTCTTAGGAGCA---GCAGGAAGCACTATG---GGCGCGGCGTCA---
ATAACGCTGACGGTACAG---GCCAGACAA---TTATTG---TCTGGTATAGTGCAACAGCAAAGC---
AATTTG---CTGAGG---GCTATAGAGGCTCAACAGCATCTGTTGAAACTCACAGTCTGGGGCATT
>KU921788
TTCTATTGTGATACATCAAGCCTGTTTAACAGCACTTGGGAACCAAACAATGTTAGTAGCAAT----------
----

TTAAATGGCACTATAACTCTCCAATGCAGAATAAAGCAAGTCATAAATATGTGGCAGAGAGTAGGACAAGCAA
TGTATGCCCCTCCCATCCCAGGAAACATAACATGTAGATCAAAC---
ATTACAGGAATAATATTAACAAAAGATAAGGATGGTATAAATAAT----------------------------
----

TCAGATGAAACCTTGAGGCCAGGAGGAGGAGATATGAGGGACAATTGGAGAAGTGAATTATATAAGTATAAAG
TGGTAAAAATTGAACCACTA---GGAGTAGCACCCACCAAGGCAAGGAGAAGAGTGGTG--------------
-------------------------GAGAGAGAAAAAAGAGCA---GTTGGA---CTAGGAGCT---------
GTTTTCCTTGGGTTCTTAGGAGCA---GCAGGAAGCACTATG---GGCGCAGCGCCA---
ATAACGCTGACGGTACAG---GCCAGACAA---TTGTTG---TCTGGCATAGTGCAACAGCAAAGC---
AATTTG---CTGAGG---GCTATAGAGGCTCAACAACAGCTGTTGAAGCTCACGGTCTGGGGCATT
>KU921804
TTCTATTGTGATACATCAAGCCTGTTTACTGGTACTTGGAATGGTACTACTTGGATGAATAGCACT-------
----

GACACTGAAAATATAACTCTTCCATGCAGAATAAAGCAAATTATAAATATGTGGCAGAGGGTAGGGAGAGCAA
TGTATGCCCCTCCCATCCAAGGAAAAATAAATTGTACATCAAAC---
ATTACAGGACTACTATTAACAAGAGATGGTGGGAAAACTAATAAT----------------------------
----

TCATCTGAAACCTTCAGACCAGGAGGAGGAGATATGAGGGACAATTGGAGAAGTGAATTATATAAGTATAAAG
TAGTAAAGATTGAACCACTA---GGAGTAGCACCCACCAGGGCAAGAAGGAGAGTGGTG--------------
-------------------------GAAAGAGAAAAAAGAGCA---GTTGGA---CTGGGAGCT---------
GTATTCATTGGGTTCTTGGGAGCA---GCAGGGAGCACTATG---GGCGCGGCGTCA---
GTGACGCTGACGGTACAG---GCCAGACGA---TTACTA---TCTGGCATAGTGCAGCAGCAAAGC---
AATTTG---CTGAGG---GCTATAGAGGCTCAACAGCATCTGTTGAAACTCACGGTCTGGGGCATC
>KU921831
TTCTATTGTAATACATCACGCCTGTTTAGTTACAGGTGCACGTTCGAAAATGAGACTAATACTTACACCTGCA
CTAAC--
-ACCATGCATGAGTTC---------
AATAGCACGGCAAATATAACTCTCCAATGCAGACTAAAGCAGATCATAAGGATGTGGCAGAGGACAGGACAAG
CAATGTATGCCCCTCCCATCCCAGGGCAAATAATGTGTAGATCAAAC---
ATTACAGGGTTAATATTAACAAGAGATGGTGGGGTTGATAACAATACA-------------------------
----

GATGCTACCGAAATCTTCAGACCTGGAGGAGGAGATATGAAGGACAATTGGAGAAGTGAATTATATAAGTATA
AAGTAGTAAAATTGAACCACTA---GGAGTAGCACCCACCAGGGCAAGGAGAAGAGTGGTG------------
----------------------------GGGAGAGAAAAAAGAGCA---GTTGGA---CTGGGAGCT------
---GTTTTCATTGGGTTCTTAGGAGCA---GCAGGAAGCACTATG---GGCGCGGCGTCA---
GTGACGCTGACGGTACAG---GCCAGACAA---TTATTG---TCTGGTATAGTGCAACAGCAAAGC---
AATTTG---CTGAAG---GCTATTGAGGCTCAACAACATCTGTTGAGGCTCACGGTCTGGGGCATT
>KU921835
TTCTATTGTAACACATCAGAACTGTTTAATAGCACTTGGAGTTGGAATAACACTTGGAATAACACTCATGAG-
----

CCAAATGGCACTATAACTCTCCAATGCAGAATAAAGCAAATTATAAATATGTGGCAGAGAGTAGGACAAGCAA
TGTATGCCCCTCCCATACAAGGAGAAATAAAGTGTGAATCAAAC---
ATCACAGGACTAATATTAACAAGAGATGGT---ATGGGTAACAATGTA-------------------------
----

TCAAATGAAACCTTCAGACCTGGAGGAGGAGATATGAGGGACAACTGGAGAAGTGAGTTATATAAATATAAAG
TAGTAAAAATTGAACCATTA---GGAGTAGCACCCACTGGGGCAAGGAGAAGGGTGGTG--------------
-------------------------CAGAGAGAAAAGAGAGCG---GTTGGA---ATAGGAGCT---------
GTCTTCCTTGGGTTCTTAGGAGCA---GCAGGAAGCACTATG---GGCGCAGCGTCA---
TTAACGCTGACGGTACAG---GCCAGACAA---TTACTG---TCTGGCATAGTGCAGCAGCAAAGC---
AATTTG---CTGAGG---GCTATAGAGGCTCAACAACATATGTTGAAACTCACGGTCTGGGGCATT
>KU921841
TTCTATTGCAACACATCAGGCCTGTTTAATAGCACTGACATGAGTTCAAATAACACAGAG-------------
----

TTAAATGACACTATAAGACTCCCATGCAGAATAAAACAAATTATACGTATGTGGCAGAAAGTAGGACAAGCAA
TGTATGCCCCTCCCATTCAAGGAGTGATAAGATGTAGCTCAAAC---
ATTACAGGAATACTATTAACAAGAGATGGTGGGATAGAAAATGAA----------------------------
----

ACAGAAATCTTCAGACCTGGAGGAGGAAACATGAAGGACAATTGGAGAAGTGAATTATATAAGTATAAAGTAG
TAAAAATTGAACCACTA---GGAGTAGCACCCACCTCGGCAAGGAGAAGAGTGGTG-----------------
----------------------GAGAGAGAAAAAAGAGCA---GTTGGA---CTGGGAGCT---------
GTATTCATTGGGTTCTTAGGAGCA---GCAGGAAGCACTATG---GGCGCGGCGTCA---
ATGACGCTGACGGTACAA---GCCAGACAA---TTATTG---TCTGGCATAGTGCAACAGCAAAGC---
AATTTG---CTGAGG---GCTATAGAGGCTCAACAGCATCTGTTGAAACTCACGGTCTGGGGCATC
>KU921852
TTCTATTGTAATACATCAGGCTTGTTTAATAGCACTTGGAATGACACTGTCATGTTTAATAGCACTGGGAATG
ACACTGTC---
-------------GGCAGG------
GAGTCAAATGAAACTATAACACTCCAATGCAGGATAAAACAAATTATAAATATGTGGCAGAGAACAGGACAAG
CAATATATGCCCCTCCTATCCCAGGAGTAATAAGGTGTGTATCAAAC---
ATTACAGGACTAATATTAACAAGAGATGGTGGGAATACGGCAGGGACA-------------------------
----

GGTTCTGAAACCTTCAGACCTGGAGGAGGAGATATGAGGGACAATTGGAGAAGTGAATTATATAAGTATAAAG
TAGTAAAAGTTGAACCACTA---GGAGTAGCACCCACCAGGGCACAGAGAAGAGTGGTG--------------
-------------------------CAGAGGGAAAAAAGAGCA---GTTGGA---ATAGGAGCT---------
GTCTTCATTGGGTTCTTAGGAGCA---GCAGGAAGCACTATG---GGCGCGGCGTCA---
ATAACGCTGACGGCACAG---GCCAGACAA---TTATTG---TCTGGTATAGTGCAACAGCAAAGC---
AATTTG---CTGAGG---GCTATAGAGGCTCAACAGCATCTGTTGAAACTCACAGTCTGGGGCATC
>KF716474
TTCTATTGTGACACATCAGGCCTGTTTAATAGCACTTGGAATGGTACGGCCAGCAATGTCAGCATACCTGACA
CGGTG--

ACAAATGGCACTATAACACTCCCATGCAAAATAAAGCAAATTATAAATATGTGGCAGAGAGTAGGACAAGCAA
TGTATGCCCCTCCCATTGCAGGAGTAATAAGGTGTGATTCAAAC---
ATTACAGGGATGATATTAACAAGAGATGGTGGTGAT-------------------------------------
----

AACAGGACAGAAACCTTCAGACCTGGAGGAGGAGATATGAGGGACAATTGGAGAAGTGAATTATATAAATATA
AAGTAGTAAAAGTTGAACCACTA---GGAGTAGCACCTACCAGGGCAAGAAGAAGAGTGGTG-----------
----------------------------GAGAGAGAAAAAAGAGCA---GTAGGA---ATAGGAGCT------
---GTTTTCCTTGGGTTCTTAGGAGCA---GCAGGAAGCACTATG---GGCGCAGCGTCA---
ATAACGCTGACGGTACAG---GCCAGACAA---TTATTG---TCTGGTATAGTGCAACAGCAAAGT---
AATTTG---CTGAGG---GCTATAGAGGCTCAACAACAGCTGTTGAAACTCACAGTCTGGGGCATT
>KF716475
TTCTACTGTAATACATCAGGCCTGTTTAATAGCACTTGGTCCCTTAACAACACTTGGTCCCTTAACAGCACGC
AGTCAAAT---

-------------GACACA------
GGGATAAATGATACCATAACTCTCCCATGCAGAATAAAGCAAATTATAAATATGTGGCAGAGAGTAGGACAAG
CAATGTATGCCCCTCCCATCCAAGGAGTAATACAGTGTGTATCAAAC---
ATTACAGGACTACTGTTAACAAGAGATGGGGGAATGTGAATAGCAGT--------------------------
----

AATGGCAATGAAATCTTCAGACCTGGAGGAGGAGATATGAGGGACAATTGGAGAAGTGAATTATATAAGTATA
AAGTAGTAAAAATTGAACCACTA---GGAGTAGCACCCACCAGGGCAAAGAGAAGAGTGGTG-----------
----------------------------GGGAGAGAAAAAAGAGCA---ATTGGTGGGCTAGGAGCT------
---GTTTTCCTTGGGTTCTTAGGAATG---GCAGGAAGCACTATG---GGCGCGGCGTCA---
ATGACGCTGACGGTACAG---GCCAGAAAC---TTATTG---TCTGGCATAGTGCAACAGCAAAGC---
AATTTG---CTGAGG---GCTATAGAGGCTCAACAACATCTGTTGAAACTCACAGTCTGGGGCATT
>AY322184
TTCTATTGTAACACATCAGATCTGTTTAATGAGTCAAATAGTACGGAG-------------------------
----

TTAAACGACACTATAACTCTCCAATGCAGAATAAAGCAAATTATAAATATGTGGCAGAGAGCAGGACAAGCAA
TATATGCCCCTCCCATCCCAGGAGTAATAAGGTGTGTATCAAAC---
ATTACAGGACTATTGTTAACAAGGGATGGTGGGATTAATACCAGT----------------------------
----

ACAAGTGAAACCTTCAGACCTGGAGGAGGAGATATGAGGGACAATTGGAGAAGTGAATTATATAGGTATAAAG
TAGTAAAAATTGAACCACTA---GGAGTAGCACCCACCAGGGCAAAGAGAAGAGTGGTG--------------
-------------------------GAGAGAGAAAAAAGAGCA---GTTGGA---ATAGGAGCT---------
GTTTTCATTGGGTTCTTAGGAGCA---GCAGGAAGCACTATG---GGCGCGGCGTCA---
ATAACGCTGACGGTACAG---GCCAGACAA---TTATTG---TCTGGCATAGTGCAACAGCAAAGC---
AATTTG---CTGAGG---GCTATAGAGGCTCAACAACATCTGTTGAAACTCACGGTCTGGGGCATT
>AF539405
TTCTATTGTAACACATCAGGCCTGTTTAATAGCACTTGGAATGGCAGTGAGGCAAATAGCACG----------
----

CCACACGAGAATATAACTCTCCAATGCAGAATAAAGCAAATTATAAATATGTGGCAGAGAGCAGGACAAGCAA
TATATGCCCCTCCCATCCCAGGAGTAATAAAATGTAATTCAAAC---
ATTACAGGACTAATATTAACAAGAGATGGTGGGGTTAATAGCAGT----------------------------
----

GCAAATGAAACCTTCAGACCTGGAGGAGGAGATATGAGGGACAATTGGAGAAGTGAATTATATAAGTATAAAG
TAGTAAAAATTGAACCACTA---GGAGTAGCACCCACCAGGGCAAAGAGAAGAGTGGTG--------------
-------------------------GAGAGAGAAAAAAGAGCA---GTTGGA---ATAGGAGCT---------
GTCTTCATTGGGTTCTTAGGAGCA---GCAGGAAGCACTATG---GGCGCGGCGTCA---
ATAACGCTGACGGTACAG---GCCAGACAA---TTATTG---TCTGGCATAGTGCAACAGCAAAGC---
AATTTG---CTGAGG---GCTATAGAGGCTCAACAACATCTGTTGAAACTCACGGTCTGGGGCATT
>L22943
TTCTATTGTAATTCATCAGGCCTGTTTAATAGCACTTGGACCAACATGCAGGAGTCAAATAGCACGGAG----
----
----------------------------TCAAATGACACTATAACTCTCCCATGCAGAATA---
AAAATTATAAACATGTGGCAGAGAGCAGGACAAGCAATGTATGCCCCTCCCATCCAAGGAATAATAAAGTGTG
TATCAAAC---ATTACAGGACTAATATTAACAAGAGATGGTGGGGATAATAACAGT-----------------
----

GAAAGTGAAACCTTCAGACCTGGAGGAGGAGATATGAGGGACAATTGGAGAAGTGAATTATATAAGTATAAAG
TAGTAAAAATTGAACCACTA---GGAGTAGCACCCACCAAGGCAAAGAGAAGAGTGGTG--------------
-------------------------GAGAGAGAAAAAAGAGCA---GTTGGA---ATAGGAGCT---------
GTCTTCCTTGGGTTCTTAGGAGCA---GCAGGAAGCACTATG---GGCGCGGCGTCA---
ATAACGCTGACGGTACAG---GCCAGACAA---TTATTG---TCTGGCATAGTGCAACAGCAAAGC---
AATTTG---CTGAGG---GCTATAGAGGCTCAACAACATCTGTTGAAACTCACGGTCTGGGGCATT
>KX168075
TTCTATTGTAACACATCAGGCCTGTTTAATAGCACTTGGGAGTTTAATAGCACTTGGGAGTTTAATAGCACTT
GGGAGTTT---
AATAGCACTTGGAATAATTCA------------

AATAGCACAGAGAATATAACTCTCCAATGCAGAATAAAGCAAATTGTAAATATGTGGCAAAGAGCAGGACAAG
CAATATATGCCCCTCCCATACCAGGAGTAATAAGGTGTGAATCAAAT---
ATTACAGGACTAATATTAACAAGAGATGGTGGGATAATAATAGTACAAAT-----------------------
----

AATACAAGTGAAACCTTCAGACCTGGAGGAGGAGATATGAGGGACAATTGGAGAAGTGAATTATATAAGTATA
AAGTAGTAAAAATTGAACCAATA---GGAGTAGCACCCACCAGGGCAAAAAGAAGAGTGGTG-----------
----------------------------GAGAGAGAAAAAAGAGCA---GTTGGA---ATAGGAGCT------
---GTCTTCATTGGGTTCTTAGGAGCA---GCAGGAAGCACTATG---GGCGCAGCGTCA---
GTAACGCTGACGGTACAG---GCCAGACAG---TTATTG---TCTGGTATAGTGCAACAGCAAAGC---
AATTTG---CTGAGG---GCTATAGAGGCTCAACAGCATCTGTTGAAACTCACGGTCTGGGGCATT
>AF004885
TTCTATTGTAATACATCAGGCTTGTTTAATAGCACTTGGTATGTTAATAGCACTTGGAATGACACTGACAGCA
CTCAG--

GAGTCAAATGACACTATAACTCTCCCATGCAGAATAAAGCAAATTATAAATATGTGGCAGAGAGCAGGACAAG
CAATGTATGCCCCTCCCATCCCAGGAGTAATAAAGTGTGAATCAAAC---
ATTACAGGACTACTATTAACAAGGGATGGTGGAAAGGATAACAAT----------------------------
----

GTAAATGAAACCTTCAGACCTGGAGGAGGAGATATGAGGGACAATTGGAGAAGTGAATTATATAAGTATAAAG
TAGTAGAAATTGAACCACTA---GGAGTAGCACCTACCAGGGCAAAGAGAAGAGTGGTG--------------
-------------------------GAGAGAGAAAAAAGAGCA---GTTGGA---ATAGGAGCT---------
GTCTTCCTTGGGTTCTTAGGAGCA---GCAGGAAGCACTATG---GGCGCAACGTCA---
ATAACGCTGACGGTACAG---GCCAGACAA---TTATTG---TCTGGCATAGTGCAACAGCAAAAC---
AATTTG---CTGAGG---GCTATAGAGGCTCAACAACATCTGTTGAAACTCACGGTCTGGGGCATT
>AF407151
TTCTATTGTAACACATCAGGCCTGTTTAACAGAGCTTATGGCAATGTCAGCAAGCAAGAGCCAAAT-------
----

AGCTCCGGCAATATAACACTCCAATGCAGAATAAAGCAAGTTATAAATATGTGGCAGAGAGCAGGACAAGCAA
TATATGCCCCTCCCATACAAGGAGTAATAAGATGTGACTCAAAT---
ATTACAGGACTAATATTAACAAGAGATGGTGGGAAAGATAGTAAT----------------------------
----

ATAACTGAAACATTCAGACCTGGAGGAGGAGATATGAGGGACAATTGGAGAAGTGAATTATATAAGTATAAAG
TAGTAAAAATTGAACCACTA---GGAGTAGCACCCACCAGGGCAAAGAGAAGAGTGGTG--------------
-------------------------GAGAGAGAAAAAAGAGCA---GTTGGA---ATAGGAGCT---------
GTCTTCATTGGGTTCTTAGGAGCA---GCAGGAAGCACTATG---GGCGCGGCGTCA---
ATAACGCTGACGGCACAG---GCCAGAAAA---TTATTG---TCTGGCATAGTGCAACAGCAAAGC---
AATTTG---CTGAGG---GCTATAGAGGCTCAACAACATCTGTTGAAACTCACGGTCTGGGGCATT
>AF407162
TTCTATTGCAATACAACAAACCTGTTTAATAGCACTTGGAATCACACTGCCAGCATGAATAGCACAGAG----
----

TCAAATGACACTATAATTCTCCCATGCAGAATAAAACAAATTATAAATATGTGGCAGAGAGTAGGACAAGCAA
TGTATGCCCCTCCCATTCGAGGAGTAATAAGGTGTGAATCAAAC---
ATTACAGGACTAATATTAACAAGAGATGGTGGGAATACTACCGGT----------------------------
----

ACAAGTGAAATCTTCAGACCTGGAGGTGGAGATATGAGGGACAATTGGAGAAGTGAATTATATAAGTATAAAG
TAGTAAAAATTGAACCACTA---GGAGTAGCACCCACCAGGGCAAAGAGAAGAGTGGTG--------------
-------------------------GAGAGAGAAAAAAGAGCA---GTTGGA---ATAGGAGCT---------
GTCTTCATTGGGTTCTTAGGAGCA---GCAGGAAGCACTATG---GGCGCGGCGTCA---
ATAACGCTGACGGTACAG---GCCAGACAA---TTATTG---TCTGGCATAGTGCAACAGCAAAGC---
AATTTG---CTGAGG---GCTATAGAGGCTCAACAGCATCTGTTGAAACTCACGGTCTGGGGCATT
>AF407156
TTCTATTGCAATACATCAAAACTGTTTAATAGCAGGTGGGAGAACAATGGCACCGCCAACATGTTGAAGAATG
ACACA--
-------------------GGG---

TCAAATGAGACTACTCTGATTCTCCGATGCAGAATAAAACAAATTATAAATATGTGGCAGAGAGTAGGACAAG
CGATGTATGCCCCTCCCATCCAAGGAGTAATAAATTGTACATCAAAC---
ATCACAGGACTAATATTAACAAGAGATGGTGGTGGTGAAAACGAT----------------------------
----

ACAGAGACCTTCAGACCTGGAGGAGGAGATATGAGGGACAATTGGAGAAGTGAATTATATAAGTATAAAGTAG
TAAAACTTGAACCACTA---GGAGTAGCACCCACCATGGCAAAGAGAAGAGTGGTG-----------------
----------------------GAGAGGGAAAAAAGAGCA---GTTGGA---ATGGCAGCT---------
GTCTTCCTTGGGTTCTTGGGAACA---GCAGGGAGCACTATG---GGCGCGGCGTCA---
CTAACGCTGACGGTACAG---GCCAGACAA---TTATTG---TCTGGCATAGTGCAACAGCAAAGC---
AATTTG---CTGAAG---GCTATAGAGGCTCAACAGCATCTGTTGAGACTCACGGTCTGGGGCATT
>KT008652
TTCTATTGTAACACATCAGGCCTGTTTAATAGCACTTGGGGGCACAATGCCAGCAGGCAGGAGAATAGCACGG
AG---

TCAAGTGACACTATAACTCTCCCATGCAAAATAAAGCAAATTATAAATATGTGGCAGAGAGCAGGGCAAGCAA
TATATGCCCCTCCCATCCCAGGAGTAATAAGGTGTGAATCAAAC---
ATTACAGGACTAATATTAACAAGAGATGGTGGGAACAACAACAGT----------------------------
----

ACAAGTGAAACCTTCAGACCTGGAGGAGGAAATATGAGGGACAATTGGAGAAGCGAATTATATAAGTATAAAG
TAGTAAAAAATTGAACCACTA---GGAGTAGCACCCACCAGGGCAAAGAGAAGAGTGGTG-------------
-------------------------GGGAGAGAAAAAAGAGCA---GTTGGA---ATAGGAGCT---------
GTTTTCCTTGGGTTCTTAGGCGCA---GCAGGAAGCACTATG---GGCGCAGCGTCA---
GTAACGCTGACGGTACAG---GCCAGACAA---TTATTG---TCTGGCATAGTGCAACAGCAAAGC---
AATTTG---CTGAGG---GCTATAGAGGCTCAACAACATCTGTTGAAACTCACGGTCTGGGGCATT
>AF407157
TTCTATTGTAACACATCAGGCCTGTTTAATGGCATTTGGAATGGCACGCAGGAGCCAAATAGAACGGAG----
----

TCAAACGATACTATAACACTCCAATGCAGAATAAAGCAAATTATAAATATGTGGCAAAGAGTAGGACAAGCAA
TATATGCCCCTCCCATTCAAGGAGAAATAAGGTGTGAGTCAAAC---
ATTACAGGACTAATATTAACAAGAGATGGTGGGATAATTAACGGT----------------------------
----

ACAGAGGAGACCTTCAGACCTGGAGGAGGAGATATGAGGGACAATTGGAGAAGTGAATTATATAAGTATAAAG
TAGTAAAGATTGAACCATTA---GGAGTAGCACCCACCAAGGCAAAGAGAAGAGTGGTG--------------
-------------------------GAGAGGGAAAAAAGAGCA---GTTGGA---TTCGGAGCT---------
TTCTTCCTTGGGTTCTTAGGAGCA---GCAGGAAGCACTATG---GGCGCGGCGTCA---
ATAACGCTGACGGTACAG---GCTAGACAA---TTATTG---TCTGGCATAGTGCAACAGCAAAAC---
AATTTG---CTGAGG---GCTATAGAGGCTCAACAGCATCTGTTGAAACTCACGGTCTGGGGCATT
>FJ866112
TTCTATTGTAATACATCAGAACTATTTAATAGCACTTGGACTTGGAAAGGCAATAATGACAGCATGCAGGGGT
TAAATAGC---
-------------------ACG---
GGGTTAAACAACACTATAACTCTCCACTGCAGCATAAAGCAAATTATAAATATGTGGCAGAGAGTAGGACAAG
CAATGTATGCCCCTCCCATCCAAGGAGTAATAAAGTGTGAATCAAAC---
ATTACTGGACTACTATTAACAAGAGATGGTGGGAATAATAACATA----------------------------
----

AACGAGACCCTCAGACCTGGAGGAGGAGATATGAGGGACAATTGGAGAAGTGAATTATATAAGTATAAAGTAG
TAAAAATTGAACCACTA---GGAGTAGCACCCACCAAGGCAAAGAGAAGAGTGGTG-----------------
----------------------GAGAGAGAAAAAAGAGCA---GTTGGA---ATAGGAGCT---------
GTCTTCCTTGGGTTCTTAGGAGCA---GCAGGAAGCACTATG---GGCGCAGCGTCA---
ATAACGCTGACGGCACAG---GCCAGAATA---TTATTG---TCTGGCATAGTGCAGCAGCAAAGC---
AATTTG---CTGAAG---GCTATAGAGGCTCAACAGCATCTGTTGAGGCTCACGGTCTGGGGCATT
>AY322190
TTCTATTGTAATACATCAGGCCTGTTTAATAGCATTTGGAATCACACTAATGGCACTTGGAATAGCACGGAGT
TAAAT--
-------------------GGC---

AATATAACTCTCAATATAACTCTCCCATGCAGAATAAACCAAATTATAAATATGTGGCAGAGAGTAGGACAAG
CAATATTTGCCCCTCCCATCCAAGGAGTAATACAGTGTGTATCAAAC---
ATTACAGGACTAATATTAACAAGAGATGGAGGGAATAATAACACT----------------------------
----

ACAAATGAAACCTTCAGACCTGGAGGAGGAGATATGAGGGACAATTGGAGAAGTGAATTATATAAGTATAAAG
TAGTAAAAATTGAACCACTA---GGAGTAGCACCCACCAGGGCACAGAGAAGAGTGGTG--------------
-------------------------CAGAGAGAAAAAAGAGCA---ATTGGA---ATGGGAGCT---------
GTCTTCATTGGGTTCTTAGGAGCA---GCAGGAAGCACTATG---GGCGCGGCGTCA---
ATAACGCTGACGGTACAG---GCCAGACAA---TTATTG---TCTGGTATAGTGCAACAGCAAAGC---
AATTTG---CTGAGG---GCTATAGAGGCTCAACAGCATCTGTTGAAACTCACGGTCTGGGGCATT
>AY322193
TTCTATTGTAACACATCAAGCCTATTTAATGGCACTTGGACGGGCAATGACAGCAGGCAGGAGTCAAAT----
----

AGCAGCGACACTATAACTCTTCCATGCAGAATAAAACAAATTATAAATATGTGGCAGAGGGTAGGACAAGCAA
TATATGCCCCTCCCCTACAAGGAGTAATCAGATGTGATTCAAAT---
ATTACAGGACTAATCTTAACAAGAGATGGTGGGAGTCCGGATAAT----------------------------
----

GAAACCTTCAGACCTGTTGGAGGAGATATGAGGGACAATTGGAGAAGTGAATTATATAAGTATAAAGTAGTAA
AAATTGAACCATTA---GGAGTAGCACCCACCAGGGCAAAGAGAAGAGTGGTG--------------------
-------------------GAGAGAGAAAAAAGAGCA---GTTGGA---ATAGGAGCT---------
GTCCTCATTGGGTTCTTAGGAGCA---GCAGGAAGCACTATG---GGCGCGGCGTCA---
ATAACGCTGACGGTACAG---GCCAGACAA---TTATTG---TCTGGCATAGTGCAACAGCAAAGC---
AATTTG---CTGAGG---GCTATAGAGGCTCAACAGCATCTGTTGAAACTCACGGTCTGGGGCATT
>FJ396018
TTTTATTGTAATACATCAGGCCTGTTTAATAGTACTTGGGATGACAATAATACTAGGAATAATGAGACTGCC-
----

AGAAATAATACTATAACTCTCCCATGTAGAATAAAGCAAATTATAAATATGTGGCAGAGGGTAGGACAAGCAA
TGTATGCCCCTCCCATCAAAGGAGTAATAAGGTGTGAATCAAAC---
ATTACAGGACTAATATTAACAAGAGATGGTGGACTTAATAACAAT----------------------------
----

ACAGAGACCTTCAGACCTGGAGGAGGAGATATGAGGGACAATTGGAGAAGTGAATTATATAAGTATAAAGTAG
TAAAAATTGAACCACTA---GGAGTAGCACCCACCAGGGCAAGGAGAAGAGTGGTG-----------------
----------------------GGAAGAGAAAAAAGAGCA---GTTGGA---ATAGGAGCT---------
GTCTTCCTTGGGTTCTTAGGAGCA---GCAGGAAGCACTATG---GGCGCGGCGTCA---
ATAACGCTGACGGTACAG---GCCAGACAA---TTATTG---TCTGGCATAGTGCAACAGCAAAGC---
AATTTG---CTGAGG---GCTATAGAGGCTCAACGACATCTGTTGAAACTCACGGTCTGGGGCATT
>AY736811
TTCTATTGTAATACATCAGGCCTGTTTAATAGCACATATAGCGGGAACGAAACTGTCAGCAATAGC-------
----

ACAGAGTACAACATAACTCTCCCATGCAAAATAAAGCAAATTATAAATATGTGGCAGAGAACAGGACAAGCAA
TGTATGCCCCTCCCATCCAAGGAGTAATAAGCTGTGTGTCAAAC---
ATTACAGGACTACTATTAACAAGAGATGGTGGTAATGGTGGTAATAAC-------------------------
----

AATACAACTGAGACCTTCAGACCTGGAGGAGGAAATATGAAGGACAATTGGAGAAGTGAATTATATAAGTATA
AAGTAGTAAAAATTGAACCACTA---GGAGTAGCACCCACCAGGGCAAAGAGAAGAGTGGTG-----------
----------------------------GAGAGAGAAAAAAGAGCA---GTTGGA---CTGGGAGCT------
---GTATTCCTTGGGTTCTTAGGAGCA---GCAGGAAGCACTATG---GGCGCGGCGTCC---
ATAACGCTGACGGTACAG---GCCAGACAA---TTACTG---TCTGGCATAGTGCAACAGCAAAGC---
AATTTG---CTGAGG---GCTATAGAGGCTCAACAGCATCTGTTGAAACTCACGGTCTGGGGCATT
>AY736814
TTCTATTGCAACACATCAGGCCTGTTTAATAGCACTTGGGATGATAACATTAAGAATGATACTAACAGCACAC
AGGGG--

TCAAATAGCACGGAGATAATTCTCCCATGCAAAATAAAGCAAATTGTAAGGATGTGGCAGAGAACAGGACAAG
CAATGTATGCTCCTCCAATCCCAGGAGTAATAAGGTGTGTATCAAAC---
ATTACAGGACTAATACTAACAAGAGATGGTGGGAATACTAGCAGC----------------------------
----

ATAAATGAAACCTTTAGACCTGGAGGAGGAGATATGAGGGACAACTGGAGAAGTGAATTATATAAGTATAAAG
TAGTAAAAATTGAACCACTA---GGAGTGGCACCCACCAAGGCAAGGAGAAGAGTGGTG--------------
-------------------------GAGAGAGAAAAAAGAGCA---GTTGGA---ATAGGAGCT---------
GTCCTCTTTGGTTTCTTAGGAGCA---GCCGGAAGCACTATG---GGCGCGGCGTCA---
ATAACGCTGACGGTACAG---GCCAGACAA---TTATTG---GCTGGCATAGTGCAACAGCAAAGC---
AATTTG---CTGAGG---GCTATAGAGGCTCAACAGCAAATGTTGAGACTCACGGTCTGGGGCATT
>AF457075
TTCTATTGTGATACATCAAGCCTGTTTAATGGCACTTTAGAGGCCAATGCCACCGTGCAAAACTTCAAT----
----

AGCACAGAGCATATAATTCTCCCATGCAAAATAAAGCAAATTATAAATATGTGGCAGAGAGCAGGACAAGCAA
TGTATGCCCCTCCCATCCAAGGAGTAATAAATTGTGTGTCAAAC---
ATTACAGGACTAATATTAACAAGAGATGGTGGGGATAATAATAAT----------------------------
----

AGTACAAATGAAACATTCAGACCTGGAGGAGGAGATATGAGGGACAATTGGAGAAGTGAATTATATAAGTATA
AAGTAGTAAAAATTGAACCACTA---GGAGTAGCACCAACCAAGGCAAAAAGAAGAGTGGTG-----------
----------------------------GAGAGAGAAAAAAGAGCA---GTAATTGGACTGGGAGCT------
---GTTTTCATTGGGTTCTTAGGAGCA---GCAGGAAGCACTATG---GGCGCGGCGTCA---
ATAACGCTGACGGTACAG---GCCAGACAA---TTATTG---TCTGGTATAGTGCAACAGCAAAGC---
AATTTG---CTGAGG---GCTATAGAGGCTCAACAACATCTGTTGAAACTCACGGTCTGGGGCATT
>DQ208458
TTCTATTGTAACACATCAGGCCTGTTCAATAGCACTTGGATTAGCAATACCAGCGTGCAGGGGTCAAATAGCA
CG---

GGGTCAAATGACAGTATAACTCTCCCATGCAGAATAAAGCAAATTATAAATATGTGGCAGAGAATAGGACAAG
CAATGTATGCCCCTCCCATCCAAGGAGTAATAAGATGTGTATCAAAC---
ATTACAGGGCTAATATTAACAAGAGATGGTGGGAGTACTAATAGT----------------------------
----

ACAACTGAAACCTTCAGACCTGGAGGAGGAGATATGAGGGACAATTGGAGAAGTGAATTATATAAGTATAAAG
TAGTAAAAATTGAACCACTA---GGAGTAGCACCCACCAGGGCAAAGAGAAGAGTGGTG--------------
-------------------------GGGAGAGAAAAAAGAGCA---GTTGGA---ATAGGAGCT---------
GTCTTCCTTGGGTTCTTAGGAGCA---GCAGGAAGCACTATG---GGCGCGGCGTCA---
ATGACGCTGACGGTACAG---GCCAGAAAT---TTATTA---TCTGGCATAGTGCAACAGCAAAGC---
AATTTG---CTGAGG---GCTATAGAGGCTCAACAACATCTGTTGAAACTCACGGTCTGGGGCATT
>DQ208467
TTCTATTGTAATACATCAGGCCTGTTTAATAGCAATTGGAATTACACTAACAGCACGCAGGAGTCAAATAGCA
CG---

GAGTCAAATGGCACTATAACTCTCCAATGCAGAATAAAGCAAATTATAAATATGTGGCAGAGAGTAGGACAAG
CAATGTATGCCCCTCCTATCCCAGGAGTAATAAGGTGTGTATCAAAC---
ATTACAGGACTAATATTAACAAGAGATGGCGGGGACAACAACAGT----------------------------
----

ATAACTGAAACCTTCAGACCTGGAGGAGGAGATATGAGGGACAATTGGAGAAGTGAATTATATAAGTATAAAG
TAGTAAAAATTGAACCACTA---GGAGTAGCACCCACCAGGGCAAGGAGAAGAGTGGTG--------------
-------------------------GGAAGAGAAAAAAGAGCA---GTTGGA---ATAGGAGCT---------
GTCTTCATTGGGTTCTTAGGAGCA---GCCGGAAGCACGATG---GGCGCGGCGTCA---
ATAACGCTGACGGTACAG---GCCAGACAA---TTATTG---TCTGGCATAGTGCAACAGCAAAGC---
AATTTG---CTGAGG---GCTATAGAGGCTCAACAACATCTGTTGAAACTCACGGTCTGGGGCATT
>DQ208448
TTCTATTGTAACACATCAGACTTGTTTAATAGCACTTGGTATGGGAATGGGACCAGCATGCGGGAGTCAAATA
GCACG--

GAGTCAAACGACACTATAATTCTCCGATGCAGAATAAAGCAAATTATAAATATGTGGCAGAGAGTAGGACAAG
CAATATATGCCCCTCCCATCCCAGGAGTAATAAACTGTGTATCAAAC---
ATCACAGGACTAATATTAACAAGAGATGGTGGGAATAATAATAAT----------------------------
----

GAAACTGAAACCTTCAGACCTGGGGGAGGAGATATGAGGGACAATTGGAGAAGTGAATTATATAAGTATAAAG
TAGTAAAAATTGAACCACTA---GGAGTAGCACCCACCAGGGCAAGGAGAAGAGTGGTG--------------
-------------------------GAGAGAGAAAAAAGAGCA---GTAGTTGGAATAGGAGCT---------
GTCTTCCTTGGGTTCTTAGGAGCA---GCAGGAAGCACTATG---GGCGCGGCGTCA---
ATAACGCTGACGGTACAG---GCCAGACAA---TTATTG---TCTGGCATAGTGCAACAGCAAAGC---
AATTTG---CTGAGG---GCTATAGAGGCTCAACAACATCTGTTGAAACTCACAGTCTGGGGCATT
>KU749411
TTCTATTGTAATACAACAGGCCTATTTAATAGTACTTGGAATACAACAGGCCCATTTAATAATACTTGGCCT-
----

GATGATGTCAATATAGTACTCCCATGCAAAATAAAGCAAATTATAAATGTATGGCAGAGAGTAGGACAAGCAA
TATATGCCCCTCCCATCCAAGGACTAATACAATGTAACTCAAAT---
ATTACGGGGCTACTATTAACAAGAGATGGTGGGAATAATGGCACA----------------------------
----

CTTAATGAAACCTTCAGACCTGGAGGAGGAAATATGAAGGACAATTGGAGAAGTGAACTGTATAAATATAAAG
TAGTAAAAATTGAACCACTA---GGAGTAGCACCCACCAGGGCAAAGAGAAGAGTGGTG--------------
-------------------------GAGAGAGGAAAAAGAGCA---ATTGGA---ATAGGAGCT---------
GTGCTCATTGGGTTCTTGGGGGCA---GCCGGAAGCACTATG---GGCGCGGCGTCT---
GTAACGCTGACGGTACAG---GCCAGACAA---TTATTG---TCTGGCATAGTGCAACAGCAAAGC---
AATTTG---CTGAGG---GCTATAGAGGCTCAGCAACAGATGCTGAAACTCACAGTCTGGGGCATT
>KX232594
TTCTATTGTAATACAACAGGACTGTTTAATAGTACTTGGTATAAAAATGGAACGACTGAGGGAACAAATAGCA
CGGGG--

TCAAATGACACTATAACACTCCCATGCAGAATAAAGCAAGTTATAAATGTATGGCAGAGAGTAGGACAAGCAA
TATATGCCCCTCCCATCCCAGGGATAATAAGGTGTGAATCAAAT---
ATTACGGGGCTACTATTAACAAGAGATGGTGGAAATAATACAAGT----------------------------
----

AATGAAACCTTCAGACCTGGAGGAGGAAATATGAAGGACAATTGGAGAAGTGAACTGTATAAGTATAAAGTAG
TAAAACTTGAACCACTA---GGAGTAGCACCCACCAAAGCAAAGAGAAGAGTGGTG-----------------
----------------------GAGAGAGAAAAAAGAGCA---ATTGGA---TTTGGAGCT---------
TTCTTCCTTGGGTTCTTGGGAGCA---GCTGGAAGCACTATG---GGCGCGGCGTCC---
GTAACGCTGACGGTACAG---GCCAGACAA---TTATTG---TCTGGCATAGTGCAACAGCAAAGC---
AATTTG---CTGAGG---GCTATAGAGGCTCAGCAGCATATGCTGAAACTCACGGTCTGGGGCATT
>KX232597
TTCTATTGTAATACAACAGGTCTGTTTAATAGTACTTGGCCTAGTAATGAGACTAAGGAAGTAAATAACACGG
AG---

TCAAATGACATTATCACACTCCCATGCAGAATAAAGCAAATTGTAAATGTATGGCAAAGAGTAGGACAAGCAA
TATACGCCCCTCCCATCAAAGGACTAGTACAGTGTAATTCAAGT---
ATTACGGGGCTACTATTAAACAGAGATGGTGGGAATAATAACACA----------------------------
----

GATGAAACCTTCAGACCTGGAGGAGGAAATATGAAGGACAATTGGAGAAGTGAACTATATAAGTATAAAGTAG
TAAAAATTGAACCACTA---GGAGTAGCACCCACCAGGGCAAGGAGAAGAGTGGTG-----------------
----------------------GAGAGAGAAAAAAGAGCA---GCTGGA---CTAGGAGCT---------
TTATTCATTGGGTTCCTGGGAGCA---GCCGGAAGCACTATG---GGCGCAGCGTCT---
GTAACGCTGACGGTACAG---GCCAGACAA---TTATTG---TCTGGCATAGTGCAACAGCAAAGC---
AATTTG---CTGAGG---GCTATAGAGGCTCAGCAGCAGATGCTGAGACTCACAGTCTGGGGCATT
>KX232609
TTCTATTGTGATACAACAAACCTGTTTAATAGTACTTGGTTTGATAATGGGACTACTGAAGCAAATAGCACGG
GG---

TCAAGTGACATTATAACACTCCCATGCAGAATAAAACAAATTGTAAATGTATGGCAGAGAGTAGGACAAGCAA
TATATGCCCCTCCCATCAGAGGACTAATAAAGTGTGAATCAAAT---
ATTACGGGGCTACTATTAACCAGAGATGGTGGGCATAATAGCACAAATGCA----------------------
----

AGTAGTGAAACCTTCAGACCTGGAGGAGGAAATATGAAGGACAATTGGAGAAGTGAACTATATAAGTATAAAG
TAGTAAAAATTGAACCACTA---GGAGTAGCACCCACCAGAGCAAGGAGAAGAGTGGTG--------------
-------------------------GAGAGAGAAAAAAGAGCA---ATTGGA---ATAGGAGCT---------
GTACTCTTTGGGTTCTTGGGAGCA---GCCGGAAGCACTATG---GGCGCGGCGTCC---
ATAACGCTGACGGTACAG---GCCAGACAA---TTATTG---TCTGGCATAGTGCAACAGCAAAGC---
AATTTG---CTGAGG---GCTATAGAGGCTCAGCAGCATCTGCTGAAACTCACGGTCTGGGGCATT
>KX232613
TTCTATTGTAATACATCAGCCCTGTTTAATAGCACTTGGAGAGACAACCAGACAGATAACCAAACACATACAA
AT---

GACACTGACATTATAACTCTCCCATGCAGAATAAAGCAAGTTATAAACATGTGGCAGAGGACAGGACAAGCAA
TGTATGCCCCTCCTATCCAAGGAGTAATAAAGTGTACATCAAAT---
ATTACAGGACTATTATTAACAAGAGATGGGGGTGCTAACACAACA----------------------------
----

AATGAAACTTTCAGACCTACAGGAGGAGATATGAAAGACAATTGGAGAAGTGAATTATACAAGTATAAAGTAG
TAAAAATTGAACCACTA---GGAGTAGCACCCACCAGGGCAAAGAGAAGAGTGGTG-----------------
----------------------GAGAGAGAAAAAAGAGCA---GTTGGA---CTGGGAGCT---------
GTATTCATTGGGTTCTTGGGAGCA---GCAGGAAGCACTATG---GGCGCGGCGTCA---
ATCACGCTGACGGTACAG---GCTAGACAA---TTATTG---TCTGGCATAGTGCAACAGCAAAGC---
AATTTG---CTGAGG---GCTATAGAGGCTCAACAACATATGTTGAAGCTCACAGTCTGGGGCATT
>KX232614
TTCTATTGTAATACAACAAACCTGTTTAATAGTACTTGGCCTGAGAATAAAAATAGCACAGGGTCAAATGACA
CAGGG--

TCAAATGGCACTATAACTCTCCAGTGCAAAATAAAGCAAATTATAAATGTATGGCAGAGAGTAGGACAAGCAA
TATATGCCCCTCCCATCCCAGGAGCAATAAGATGTAACTCAAAT---
ATTACGGGGCTACTATTAACAAGAGATGGTGGGAATAATAACACA----------------------------
----

AATGAAACCTTCAGACCTGGAGGGGGAAATATGAAGGACAATTGGAGAAGTGAACTGTATAAATATAAAGTAG
TAAAACTTGAACCACTA---GGAGTAGCACCCACCAAGGCAAAGAGAAGAGTGGTG-----------------
----------------------GAGAGAGAAAAAAGAGCA---TTCGGA---CTAGGAGCT---------
GTATTCATTGGGTTCTTGGGAGCA---GCCGGAAGCACTATG---GGCGCGGCGTCT---
GTAACGCTGACGGTACAG---GCCAGACAA---TTATTG---TTTGGCATAGTGCAACAGCAAAGC---
AATTTG---CTGAGG---GCTATAGAGGCTCAGCAACACCTGCTGAAACTCACGGTCTGGGGCATT
>KX232618
TTCTATTGTAATACATCAGGCCTGTTTAATAGTACTTGGTCTAATAATGAGACTGAGGGAGCGGGG-------
----

TCAAATGACACTATAACACTCCCATGCAGAATAAAGCAAATTGTAAATGTATGGCAGAGAGTAGGACAAGCAA
TATATGCCCCTCCCATCGAAGGACTAATAAAATGTAACTCAAAT---
ATTACGGGGCTACTATTGACAAGAGATGGTGGGGATTATAACACATCTACA----------------------
----

AATAATGAAACCTTCAGACCTGGAGGAGGAAATATGAAGGACAATTGGAGAAGTGAACTGTATAAGTATAAAG
TAGTAAGACTTGAACCACTA---GGAGTAGCACCCACCAAAGCGAAGAGAAGAGTGGTG--------------
-------------------------GAGAGACAAAAAAGAGCA---ATTGGA---ATAGGAGCT---------
GTGTTCATTGGGTTCTTGGGAGCA---GCTGGAAGCACTATG---GGCGCGGCGTCC---
GTAACGCTGACGGTACAG---GCCAGACAA---TTATTG---TCTGGCATAGTGCAACAGCAAAGC---
AATTTG---CTGCAG---GCTATAGAGGCTCAGCAACATCTGCTGAAACTCACAGTCTGGGGCATT
>KX232620
TTCTATTGTAATACAACAAGCCTGTTTAATAGTACTTGGCCTCATAATGAGACGAAAATAAATAGCACGGAAT
CAAAT--

GACAATGGCACTATAACACTCCCATGCAGAATAAAGCAAATTGTAAATATATGGCAGAGAGTAGGACAAGCAA
TGTATGCCCCTCCCATCAAAGGACTAATAAGGTGTAATTCAACT---
ATTACGGGGCTACTATTAACAAGAGATGGGGGTGAGACTAATGGC---------------------------
----

ACAAATGAAACCTTTAGACCTGGAGGAGGAGATATGAGGGATAATTGGAGAAGTGAACTGTATAAGTATAAAG
TAGTAAAAATTGAACCACTA---GGAATAGCACCCACCAAAGCAAGGAGAAGAGTGGTG-------------
------------------------GAGAGAGAAAAAAGAGCA---ATTGGA---CTAGGAGCT---------
GTATTCATTGGGTTCTTGGGAGCA---GCCGGAAGCACTATG---GGCGCGGCGTCT---
GTAACGCTGACGGTACAG---GCCAGACAA---TTATTG---TCTGGCATAGTGCAACAGCAAAGC---
AATTTG---CTGAAG---GCTATAGAGGCTCAGCAGCAACTGCTGAAACTCACGGTCTGGGGCATT
>KX232624
TTCTATTGTAATACAACAGGCCTGTTTAATAGTACTTGGTATAATAATGGAACTGAGGAAGCAAATAACACGG
GG--

TCAAATGACACTATAACACTCCCATGCAGAATAAAACAAATTATAAATGTATGGCAAAGAGTAGGACAAGCAA
TATATGCCCCTCCCATCGAAGGACTAATAAGGTGTAATTCAACT---
ATTACGGGGCTACTATTAACAAGAGATGGGGGTAATAACACAAAT---------------------------
----

GACACAAGTGAAACCTTCAGACCTGGAGGAGGAGATATGAGGGATAATTGGAGAAGTGAACTGTATAAGTATA
AAGTAGTAAAAATTGAACCACTA---GGAATAGCACCCACCAAAGCAAGGAGAAGAGTGGTG----------
---------------------------GAGAGGGAAAAAAGAGCA---GTTGGA---CTAGGAGCT------
---GTATTCATTGGGTTCTTGGGAGCA---GCCGGAAGCACTATG---GGCGCGGCGTCC---
GTGACGCTGACGGTACAG---GCCAGGCAA---TTATTG---TCTGGCATAGTGCAACAGCAAAGC---
AATTTG---CTGAGG---GCTATAGAGGCTCAGCAACATCTGCTGAAACTCACAGTCTGGGGCATT
>KX232626
TTCTATTGTGATACAACAGGCCTGTTTAATAGTACTTGGCCTAACAATGAAACTGGGGAAGTAAATAGCACGG
GA--

TCAAACGATATTATAACACTCCCATGCAGAATAAAGCAAGTTGTAAATGTATGGCAGAGAGTGGGACAAGCAA
TATATGCCCCTCCCATCCAAGGACTAATAAAATGTAACTCAAAT---
ATTACGGGGATAATATTAACAAGAGATGGTGGGAATCTTAATTCT---------------------------
----

ACAAATGAAACTTTCAGACCTGGAGGAGGAAATATGAAGGACAATTGGAGAAGTGAACTGTATAAGTATAAAG
TAGTAAAACTTGAACCACTA---GGAGTAGCACCCACCAGAGCAAAGAGAAGAGTGGTG-------------
------------------------GAGAGAGAAAAAAGAGCA---ATTACC---ATAGGAGCT---------
GTGCTCATTGGGTTCTTGGGAGCA---GCCGGAAGCACTATG---GGCGCGGCGTCT---
GTAACGCTGACGGTACAG---GCCAGACAA---TTATTG---TCTGGCATAGTGCAACAGCAAAGC---
AATTTG---CTAAGG---GCTATAGAGGCTCAGCAACATCTGCTGAAACTCACAGTCTGGGGCATT
>KU749423
TTCTATTGTAATACATCAGGCCTGTTCAATAGCACCTGGTATCCCAATGGCACCAACATAACTAACCCA----
----

GGACCAGAGAATATAACCCTCCCATGCAGAATAAAGCAAATTGTAAATATGTGGCAGAGAGTAGGGAGAGCAA
TGTATGCTCCTCCCATCAAAGGAGTAATACAATGTGTATCAAAC---
ATTACAGGACTACTATTAACAAGAGATGGTGGGAATAATAATAAT---------------------------
----

ACAACTGAAACCTTCAGACCAGGAGGAGGAGATATGAGGGACAATTGGAGAAGTGAATTATATAAGTATAAAG
TAGTACAAGTTGAACCACTA---GGAATAGCACCCACTAGGGCAAGGAGAAGAGTGGTG-------------
------------------------GAGAGAGAAAAAAGAGCA---GTTGGA---ATGGGAGCT---------
CTTTTCATTGGGTTCCTAGGAGCA---GCCGGAAGCACTATG---GGCGCGGCATCA---
ATGATGCTGACGGTACAG---GCCAGACAA---TTATTG---TCTGGCATAGTGCAACAGCAAAGC---
AATTTG---CTGAGG---GCTATTGAAGCTCAACAGCATATGTTGAAGCTCACGGTCTGGGGCATT
>KX983643
TTCTATTGTAATACATCAGAACTGTTTAATAGCACTTGGGGAGAGGAAGGGCGAAAGGACAATATCACGAAG-
----

TCAAATGCCACTATAACTCTCCCATGTAGAATAAAGCAAGTCATCAATATGTGGCAGAGAGCAGGACGAGCAA
TGTATGCCCCTCCCATCCAAGGAGTAATAAATTGTGCATCAAAC---
ATTACAGGACTACTACTAACAAGAGACGGTGGGAATACTAATACTAGC-------------------------
----

GATCTACCTGAAATCTTCAGACCAGGAGGAGGAGATATGAGGGACAATTGGAGAAGTGAATTATATAAGTATA
AGGTAGTAAAAATTGAACCACTA---GGAGTAGCACCCACCAAGGCAAGGAGAAGAGTGGTG-----------
----------------------------GAGAGAGAAAAAAGAGCA---GTTGGA---CTGGGAGTT------
---GTCTTCCTTGGGTTCTTAGGAGCA---GCAGGAAGCACTATG---GGCGCGGCGTCA---
ATAACGCTGACGGTACAG---GCCAGACAA---TTATTG---TCTGGCATAGTGCAGCAGCAAAGC---
AATTTG---CTGAAG---GCTATAGAGGCTCAACAGCATCTGTTGAAGCTCACAGTCTGGGGCATT
>KX983542
TTCTATTGTGATACAACAGGCCTGTTTAATAGCACTTGGAAGAATGGCACTCTCAACATGCAGGAATTAACTA
TCACAGTC---
ACAAACTACACAAATCTC---------
ACAGACAGTATAAATATAACTCTTCCATGTAGAATAAAGCAAATTATAAATATGTGGCAGAGAGTAGGACAGG
CAATGTATGCTCCTCCCATCCAAGGAGTAATAAAATGTGTGTCAAAC---
ATTACGGGACTACTATTAACGAGAGATGGGGGGAAT-------------------------------------
----

AATGAGACAGAAACCTTCAGACCTGGAGGAGGAAATATGAAGGACAATTGGAGAAGTGAATTATATAAGTATA
AAGTAGTAAAAGTTGAGCCTATA---GGAGTAGCACCCACCAGGGCAAGGAGAAGAGTGGTG-----------
----------------------------GAGAGAGAAAAAAGAGCA---GTTGGA---ATAGGAGCT------
---GTTTTCATTGGGTTCTTAGGAGCA---GCAGGAAGCACTATG---GGCGCGGCGTCA---
ATAACGCTGACGGTACAG---GCCAAACAA---TTATTG---TCTGGCATAGTGCAACAGCAAAGC---
AATTTG---CTGAGG---GCTATAGAGGCTCAACAGCATCTGTTGAAACTCACGGTCTGGGGCATT
>KX983566
TTCTATTGTAATACATCAAGCCTGTTTAATAGCATTTGGGTTAATGGCACTAGGAATGACACTGTCAGC----
----

AATGGAACTTATATAACTCTCCCATGCAGAATAAAGCAAATTATAAATATGTGGCAGAGAGTAGGGCAAGCAA
TGTATGCCCCTCCCATTAGAGGAGTAATAAAGTGTGAATCAAAC---
ATTACAGGACTACTGTTAACAAGAGATGGTGGGGAGAATAATAGT----------------------------
----

ACCAATGAAACCTTCAGACCTGGAGGAGGAGATATGAGGGACAATTGGAGAAGTGAATTATATAAGTACAAAG
TAGTAAAAATTAAACCACTA---GGAGTAGCACCCACCAAGGCAAAGAGAAGAGTGGTG--------------
-------------------------GAGAGAGAAAAAAGAGCAGTAGTTGGA---CTGGGAGCT---------
GTCTTCCTTGGGTTCTTAGGAGCA---GCAGGAAGCACTATG---GGCGCAGCGTCA---
ATAACGCTGACGGTACAG---GCCAGACAA---TTATTG---TCTGGCATAGTGCAACAGCAAAGC---
AATTTG---CTGAGG---GCTATAGAGGCTCAACAACATCTGTTGAAACTCACGGTCTGGGGCATT
>JX236677
TTCTATTGTAATACATCAGCCCTGTTTGATAGCACTTGGCGGTTTAATGGCATTGGCAGTGGAAATGACACTG
TCAATGCCACT--
-------------------AGA---
AGCGAGAATGATACTATAAATCTCCCATGCAGGATAAAGCAAATTATAAATATGTGGCAGAGAGCAGGACAGG
CAATGTATGCCCCTCCCATCCAAGGAGTAATAAGGTGTGACTCAAAC---
ATTACAGGTCTATTATTAACAAGAGATGGTGGGAGGGGGAATGAT----------------------------
----

AATAACACTGAGGTCTTCAGACCTGGAGGAGGAAATATGAAGGACAATTGGAGGAGTGAATTATATAAGTACA
AAGTAGTAAAAATTGAACCATTA---GGAGTAGCACCCACCAGGGCAAGGAGAAGAGTGGTG-----------
----------------------------GAGAGAGGAAAAAGAGCA---GTTGGA---ATAGGAGCT------
---GTTTTCATTGGGTTCTTAGGAGCA---GCAGGAAGCACTATG---GGCGCAGCGTCA---
ATAACGCTGACGGTACAG---GCCAGGCAA---TTATTG---TCTGGCATAGTGCAACAGCAAAGC---
AATTTG---CTGAGG---GCTATAGAGGCTCAACAGCATCTGTTGAGACTCACAGTCTGGGGCATT
>JX236678
TTCTATTGTAACACATCAACCCTGTTTAGTAGCACTTGGGAGAACGGTACGGAACTAAACAGCACGAAG----
----

TCAAATGACACTATAACTCTCCCATGCAGAATAAAGCAAATTATAAATATGTGGCAGAGAGTAGGACAAGCAA
TATATGCTCCTCCAATCGAAGGAGAAATAAGGTGTCAATCAAAT---
ATTACAGGACTAATATTAACAAGAGATGGTGGTGAGGATATTAAC----------------------------
----

AGTACAAGTGAAACCTTCAGACCTGGAGGAGGAGATATGAGGGACAATTGGAGAAGTGAATTATATAAGTATA
AGGTAGTAAAAATTGAACCACTA---GGAGTAGCACCCACCAGGGCAAGAAGAAGAGTGGTG-----------
----------------------------GAGAGAGAAAAAAGAGCA---GTTGGA---ATAGGAGCT------
---GTCTTCATTGGGTTCTTAGGAGCA---GCAGGAAGCACTATG---GGCGCGGCGTCA---
ATAACGCTGACGGTACAG---GCCAGACAA---TTATTG---TCTGGCATAGTGCAACAGCAAAGC---
AATTTG---CTGAGG---GCTATAGAGGCTCAACAACATCTGTTGAAGCTCACGGTCTGGGGCATT
>KU749424
TTTTATTGTAATACATCAGAACTGTTTAATAGCACTTGGGTATACAATGCATCAGAACTTAATGGCACTTGGG
TAAACAATGCC--
AGCATGCCAGGATCAAT---------
AGCACAAGTGAAAATATAACTCTCCACTGCAGAATAAAGCAAATTATAAATATGTGGCAGAGAGCAGGAAAAG
CAATGTACGCCCCTCCCATCTCAGGAAACATAACGTGTGTTTCAAAC---
ATTACAGGACTCATATTAACAAGAGATGGTGGGAATACCAATACA----------------------------
----

ACTGAAATCTTCAGACCTGAAGGAGGAGATATGAGAGACAATTGGAGAAGTGAATTATATAAGTATAAAGTAG
TAAAAATTGAACCACTA---GGAGTAGCACCCACCAAGGCAAGGAGGAGAGTGGTG-----------------
----------------------GAGAGAGAAAAAAGAGCA---ATTGGA---ATAGGAGCT---------
GTCCTCTTTGGGTTCTTAGGAGCA---GCAGGAAGCACTATG---GGCGCGGCGTCA---
ATAACGCTGACGGTACAG---GCCAGACAA---TTATTG---TCTGGCATAGTGCAACAGCAAAGC---
AATTTG---CTGAGG---GCTATAGAGGCTCAACAACATCTGTTGAAACTCACAGTCTGGGGCATT
>MF565934
TTCTATTGCAATACATCAAGCCTGTTTAATAGTACTTGGGATGGGAATAGCACTACTAACAGCACGCAGAAG-
----

CCAAATGGAACTATAACTCTCCCATGCAGGATAAAGCAAATTATAAATATGTGGCAGAGAACAGGGCAAGCAA
TGTATGCCCCTCCCATCCCAGGAAAAATAAGGTGTGACTCAAAC---
ATCACAGGACTAATATTAACAAGAGATGGTGAAAATAATAACACT----------------------------
----

GAAAGTGAAACCTTTAGACCTGAAGGAGGAGACATGAGGAACAATTGGAGAAGTGAGTTATATAAGTATAAAG
TAGTAAAGATTGACCCACTA---GGAGTAGCACCCACCGGGGCAAAGAGAAGAGTGGTG--------------
-------------------------GAGAGAGAAAAAAGAGCA---GTTGGA---ATAGGAGCT---------
GTCCTCTTTGGGTTCTTAGGAGCA---GCAGGAAGCACTATG---GGCGCAGCGTCT---
CTAACGCTGACGGTACAG---GCCAGACAA---TTATTG---TCTGGCATAGTGCAACAGCAAAGC---
AATTTG---CTGAGG---GCTATAGAGGCTCAACAACATCTGTTGAGACTCACGGTCTGGGGCATT
>KX983624
TTCTACTGTAATACATCAGGCCTGTTTAATAGCCTCTGGATAAATGGCACCATGCTGAATGGTACCCTGAAGA
ATTCACCC---

AGCACAAATGACACCATAACTCTCCCATGCAGAATAAAGCAAATCATAAACATGTGGCAGAAAGTAGGACAAG
CAATGTATGCCCCTCCCATCCCAGGAAGCATAACGTGTGTATCAAAC---
ATTACAGGACTAATATTAACAAGAGATGGTGGGAATACTAACAGG----------------------------
----

ACAGATGAAATCTTCAGACCTGAAGGGGGAAATATGAGGGACAATTGGAGAAGTGAATTATATAAGTATAAAG
TAGTAAAAATTGAACCACTA---GGAGTAGCACCCACTAGGGCAAAGAGAAGAGTGGTG--------------
-------------------------GAGAGAGAAAAAAGAGCA---GTTGGA---CTGGGAGCT---------
GTCTTCATTGGGTTCTTAGGAGCA---GCAGGAAGCACTATG---GGCGCGGCGTCA---
ATAACGCTGACGGTACAG---GCCAGAAAC---TTATTG---TCTGGCATAGTGCAACAGCAAAGC---
AATTTG---CTGAGG---GCTATTGAGGCTCAACAGCATCTGTTGAAACTCACAGTCTGGGGCATT
>KF716472
TTCTATTGTAACACAACAGGTCTGTTTAATAGCACTTGGAGTCGGAACACAACAGATAATAACACTTGGTTTA
GGAACAATGCT--
-------AGCAGTAGCACA------

GGGTTAAATGGCACAATAACTCTCCAATGCAAAATAAAGCAAATTATAAATATGTGGCAGAGAACAGGACGAG
CAATGTATGCCCCTCCCATACCAGGAACAATAATGTGTAACTCAAGC---
ATCACAGGACTGCTATTAACAAGAGATGGTGGGAATAGTGGGAAC-------------------------
----

AACAGTAATGAAACCTTCAGGCCTGGAGGAGGAGATATGAAAGACAATTGGAGAAGTGAATTATATAAGTATA
AGGTAGTAAAAATTGAACCACTA---GGAGTAGCACCCACCAGGGCAAAGAGAAGAGTGGTG-----------
---------------------------GAGAGAGAAAAAAGAGCA---GTTGGA---ATGGGAGCT------
---CTTTTCATTGGGTTCTTGGGAGCA---GCAGGAAGCACTATG---GGCGCGGCGTCA---
GTGACGCTGACGGTACAG---GCCAGACAA---TTATTG---TCTGGTATAGTGCAACAGCAAAGC---
AATTTG---CTGAAG---GCTATAGAGGCTCAACAACATCTGTTGAGACTCACAGTCTGGGGCATT
>AB253421
TTTTATTGTGATTCATCAGGCCTGTTTAATAGCACTTGGGATAACACTAACATCACGCAGCCAAATAGCACAG
GG--

TCAAATGACACCATAACTCTCCAATGCAGAATAAAGCAAATTATAAATATGTGGCAGAGAGCAGGACAGGCAA
TGTATGCCCCTCCCATCCCAGGAGTAATAAGTTGTGTATCAAAC---
ATTACAGGATTACTATTAACAAGAGATGGTGGGATTACCAGTGCA-------------------------
----

AATGAAACCTTCAGACCTGGAGGAGGAGATATGAGGGACAATTGGAGAAGTGAATTATATAAGTATAAGGTAG
TAAAACTTGAACCACTA---GGAGTAGCACCCACCAGGGCAAGGAGAAGAGTGGTG--------------
---------------------GAGAGAGAAAAAAGAGCA---GTTGGA---ATAGGAGCT---------
GTATTCATTGGTTTCTTAGGAGCA---GCAGGAAGCACTATG---GGCGCGGCGTCA---
ATGACGCTGACGGTACAG---GCCAGACAA---TTATTG---TCTGGCATAGTGCAACAGCAAAGC---
AATTTG---CTGAGG---GCTATAGAGGCTCAACAACATCTGTTGAAACTCACGGTCTGGGGCATT
>AB287377
TTCTATTGTAATACATCAGGCCTGTTTAATAGCACCTGGAATAATAATAATACCAGGGAGAAGATAAATGGTA
CA--

GAGTCAAATAGCACTATAACTCTCCATTGCAGAATAAAGCAAATTATAAATAGGTGGCAGGAAGTAGGACAAG
CAATGTATGCCCCTCCCATCCCAGGAGTAATAAATTGTAGATCAAAC---
ATTACAGGACTAATATTAACAAGAGATGGTGGGGATGGGGATAAC-------------------------
----

AATACGGAAATCTTCAGACCTGGAGGAGGAAATATGAAGGACAATTGGAGAAGTGAATTATATAAGTATAAAG
TAGTAAAAATTGAACCACTG---GGAGTAGCACCCACCAGGGCTAAGAGAAGAGTGGTG-------------
------------------------GAGAGAGCAAAAAGAGCA---GTTGGA---ATAGGAGCT---------
GTTTTCCTTGGGTTCTTAGGAGCA---GCAGGAAGCACTATG---GGCGCGGCGTCA---
ATAACGCTGACGGTACAG---GCCAGACAA---TTATTG---TCTGGCATAGTGCAACAGCAAAGC---
AATTTG---CTGAGG---GCTATAGAGGCTCAACAACATCTGTTGAAACTCACGGTCTGGGGCATT
>AB287379
TTCTATTGTAATACATCAGGCCTGTTTAATAGCACTTGGACCAATGAAAGCATGCAAGTAAATAGCACAGAA-
----

TCAAATGACACTATAACTCTCCCATGCAGAATAAAGCAAATTATAAATATGTGGCAGAGGGCAGGACAAGCAA
TATATGCCCCTCCCATCGAAGGAGTAATAAGGTGTAGCTCAAAC---
ATTACAGGACTACTATTAACAAGAGATGGTGGGGTTAACAATACA-------------------------
----

GTCAATGAAACCTTCAGACCTGGAGGAGGAAATATGAGGGATAATTGGAGAAGTGAACTATATAAATATAAAG
TAGTAAAAATTGAACCACTA---GGAGTAGCACCCACCAGGGCAAAAAGAAGAGTGGTG-------------
------------------------GAGAGAGAAAAAAGAGCA---GTTGGA---CTGGGAGCT---------
GTCTTCATTGGGTTCTTAGGAGCA---GCAGGAAGCACTATG---GGCGCGGCGTCA---
ATAACGCTGACGGTACAG---GCCAGACAA---TTATTG---TCTGGCATAGTGCAACAGCAAAAC---
AATTTG---CTGAGG---GCTATAGAGGCTCAACAACATCTGTTGAAACTCACGGTCTGGGGCATT
>AY713406
TTCTATTGTAATACATCAAGCCTGTTTAATAGCACTTGGAATAACACTGCCAGCATGCAGAAGTCAAATAGCA
CG--

```
GGGTCAAATGACACTATAACTCTCCAATGCAGAATAAAGCAAATTATAAGAATGTGGCAAAGAACAGGACAGG
CAATGTATGCCCCTCCCATCAAAGGAGATATAAGGTGTGAATCAAAC---
ATTACAGGACTAATATTAACAAGAGATGGTGGGAATGATAGCAGT----------------------------
-------------------------------------------------------------------------
-------------
CCAGATGAAATCTTCAGACCTGGAGGAGGAGATATGAGGGACAATTGGAGAAGTGAATTATATAAGTATAAAG
TAGTACAAATTGAACCTCTA---GGAGTAGCACCCACCAAGGCAAGAAGAAGAGTGGTG--------------
-------------------------CAGAGAGAGAAAAGAGCA---ATTGGA---ATAGGAGCT---------
GTCTTCCTTGGGTTCTTAGGAGCA---GCAGGAAGCACTATG---GGCGCGGCGTCA---
ATAACGCTGACGGTACAG---GCCAGACAA---TTATTG---TCTGGCATAGTGCAACAGCAAAGC---
AATTTG---CTGAGG---GCTATAGAGGCTCAACAACATCTGTTGAAACTCACGGTCTGGGGCATT
>L07082
TTCTATTGTAATACAACAGGCCTGTTTAATAGCACCTGGAAAAATGTAAATGGCACCATGCAGGAGTCAAGCA
GCACA--------------------------------------------------------------------
-------------------------
AAGTTAAATGACACTATAACACTCACATGCAGAATAAAGCAAATTATAAATATGTGGCAGAGAGCAGGACAGG
CAATGTATGCCCCTCCCATCGAAGGAATAATAAGGTGTGACTCAAAC---
ATTACAGGACTACTATTAACAAGAGATGGTGGGGTTGGGGATAACAGT-------------------------
-------------------------------------------------------------------------
-------
ACAAATGGAACTGAAACATTCAGACCTGGAGGAGGAAATATGAGGGACAATTGGAGAAGTGAATTATATAAGT
ATAAAGTAGTAAAAATTGAACCACTA---GGAGTAGCACCCACCACGGCAAAAAGAAAAGTGGTG--------
-------------------------------GAGAGAGAAAAAAGAGCA---ATTGGA---ATAGGAGCT---
------GTCTTCCTTGGGTTCTTAGGAGCA---GCAGGAAGCACTATG---GGCGCGGCGTCA---
ATAACGCTGACGGTACAG---GCCAGACAA---TTATTG---TCTGGCATAGTGCAACAGCAAAGC---
AATTTG---CTGAGG---GCTATAGAGGCTCAACAGCATCTGTTGAAACTCACAGTCTGGGGCATT
>MF373167
TTCTATTGTAATACATCAGGCCTGTTTAATAGCACTTGGAATGAAACTACCATGCAGGAGTCAAATGGAACAG
AG-----------------------------------------------------------------------
----------------------------
TCAAACGACAGCATAACTCTCCCATGCAGAATAAAGCAGATTGTAAATATGTGGCAGAGAACAGGACAAGCAA
TGTATGCCCCTCCCATCCAAGGAGTAATACAGTGTGTATCAAAT---
ATTACAGGACTAATATTAATGAGAGATGGTGGGAATAGTAGTAGG----------------------------
-------------------------------------------------------------------------
-------------
GAAAATGAAACCTTCAGACCTGGAGGAGGAGATATGAGGGACAATTGGAGAAGTGAATTATATAAGTATAAAG
TAGTAAAAATTGAACCACTA---GGAGTAGCACCCACCAGGGCAAGGAGAAGAGTGGTG--------------
-------------------------CAGAGAGAAAAAAGAGCG---GTTGGA---CTGGGAGCT---------
GTTTTCATTGGGTTCTTAGGAGCA---GCAGGAAGCACTATG---GGCGCAGCGTCA---
GTGACGCTGACGGTACAG---GCCAGGCAA---TTATTG---TCTGGCATAGTGCAACAGCAAAGC---
AATTTG---CTGAGG---GCTATAGAGGCTCAACAGCATCTGTTGAGACTCACGGTCTGGGGCATT
>MF373168
TTCTATTGTGATACATCAGACCTGTTTAATAGCATTTGGCCGTTTAATAGCACTCGGGGACACAATGAGACTG
AAAGATCA-----------------------------------------------------------------
-------AATAGCACAGAG------
ACAAATAACACTATTATAAAACTTCCATGTAGAATAAAACAAATTATAAATATGTGGCAGAGAGTAGGACAAG
CAATATTTGCCCCTCCCATCCAAGGAGTAATACAGTGTGTATCAAAC---
ATTACAGGACTAATATTAACAAGAGATGGAGGGAATAATAACACT----------------------------
-------------------------------------------------------------------------
-------------
ACAAATGAAACCTTCAGACCTGGAGGAGGAGATATGAGGGACAATTGGAGAAGTGAATTATATAAGTATAAAG
TAGTAAAAATTGAACCACTA---GGAGTAGCACCCACCAGGGCACAGAGAAGAGTGGTG--------------
-------------------------CAGAGAGAAAAAAGAGCA---ATTGGA---ATGGGAGCT---------
GTCTTCATTGGGTTCTTAGGAGCA---GCAGGAAGCACTATG---GGCGCGGCGTCA---
ATAACGCTGACGGTACAG---GCCAGACAA---TTATTG---TCTGGTATAGTGCAACAGCAAAGC---
AATTTG---CTGAGG---GCTATAGAGGCTCAACAGCATCTGTTGAAACTCACGGTCTGGGGCATT
>MF373181
TTCTACTGTAATACATCAGGCCTGTTTAACAGTAGTTGGGGAAATGGCACTAACAACAGCAGCACT-------
-------------------------------------------------------------------------
----------------------------
```

GGGGGGCAAACTATAACTCTCCCATGCAAAATAAAGCAAATTGTAAATATGTGGCAGAGGACAGGACAAGCAA
TGTATGCCCCTCCTATCGAAGGAGTAATAAGGTGTGTATCAAAC---
ATTACAGGAATACTATTAACTAGAGATGGTGGGCCTAATAATAGT----------------------------
----

ACAAATGAAACCTTCAGACCGGGAGGAGGAAATATGAAGGACAATTGGAGAAGTGAATTATATAACTATAAAG
TAGTACAAATTAACCCATTA---GGAATAGCACCCACCAAGGCAAAGAGAAGAGTGGTG--------------
-------------------------GAGAGAGAAAAAAGAGCAGTAGTTGGA---CTGGGAGCT---------
TTGTTCATTGGGTTCCTAGGAGCT---GCAGGAAGCACTATG---GGCGCGGCGTCA---
ATGACGCTGACGGTACAG---GCCAGACAA---TTATTG---TCTGGCATAGTGCAACAGCAAAGC---
AATTTG---CTGAGA---GCTATAGAGGCTCAACAGCATGTGTTGAGACTCACGGTCTGGGGCATT
>MF373182
TTCTATTGTAATACATCAGGCCTGTTTAATAGCAGTTGGGGAGTAAACAACACTGATGGCAATATA-------
----

ACTAATGGCACGATAACTCTCCCCTGCAAAATAAAACAATTTATAAATATGTGGCAGAGAGTAGGACAAGCAA
TGTATGCCCCTCCCATCGAAGGGGCAATAAGTTGTAATTCAACC---
ATCACAGGACTACTATTAACAAGAGATGGTGGGTACAACAACAGCACT-------------------------
----

AACCAAATCGACAAAATCTTCAGACCTGCAGGAGGAGATATGCAGGACAACTGGAGAAGTGAATTATATAAGT
ATAAAGTAGTAAAAATTGAACCATTA---GGAGTAGCACCCACCTGGGCAAGGAGAAGAGTGGTG--------
-------------------------------CAGAGAGAAAAAAGAGCA---GTTGGA---CTGGGAGCT---
------GTCTTTCTTGGGTTCTTAGGAGCA---GCAGGAAGCACTATG---GGCGCGGCGTCG---
ATAACGCTGACGGTACAG---GCCAGACAA---TTATTG---TCTGGCATAGTGCAACAGCAAAGC---
AATTTG---CTGATG---GCTATAGAGGCTCAACAGCATTTGTTGAAACTCACAGTCTGGGGCATT
>AF069670
TTCTATTGTAATACATCAGGCCTGTTTAATAGCACTTGGAGCCAAAATGACACTGGGGTATCAAATAGCACGG
AG---

TCAAATGACACTATAATTCTTCCATGCAGAATAAAACAAATTATAAATATGTGGCAGAGAGCAGGACAAGCAA
TATATGCCCCTCCCATCCCAGGAATAATAAGGTGTGAATCAAAC---
ATTACAGGGCTACTGTTAACAAGAGATGGTGGGGTTGTTAACAGT----------------------------
----

ACAAATGAAACCTTCAGACCTGGAGGAGGAAATATGAAGGACAATTGGAGAAGTGAATTATATAAGTATAAAG
TAGTAAAAATTGAACCACTA---GGAGTAGCACCCACCAGGGCAAGGAGAAGAGTGGTG--------------
-------------------------CAGAGAGAAAAAAGAGCA---GTTGGA---CTGGGAGCT---------
TTATTCATTGGGTTCTTAGGAGCA---GCAGGAAGCACTATG---GGCGCGGCGTCA---
ATAACGCTGACGGTACAG---GCCAGACAA---TTATTA---TCTGGCATAGTGCAACAGCAAAGT---
AATTTG---CTGAGG---GCTATAGAGGCTCAACAGCATCTGTTGAAACTCACTGTCTGGGGCATT
>AF069671
TTCTATTGTAATACATCAGGCCTGTTTAATAGCACTATTTTGAATAGCACTAAGATGAATGACAATGCCAGCA
GGGAG--

TCGTATGATGACACTATAACCCTCCAGTGCAGAATAAAGCAAATTATAAATATGTGGCAGAGAGTAGGACAAG
CAATGTATGCCCCTCCCATCCGAGGAGCAATAAGATGTAAATCAAAC---
ATTACAGGACTATTATTAACAAGAGATGGTGGAAATAGTAACAGT----------------------------
----

AGTACAAATGAAACCTTCAGACCTGGAGGAGGAGATATGAGAGATAATTGGAGAAGTGAATTATATAAATATA
AAGTAGTAAAAATTGAACCACTA---GGAGTAGCACCCACCAAGGCAAAGAGAAGAGTGGTG-----------
----------------------------GAGAGAGAAAAAAGAGCA---GTTGGA---ATAGGAGCT------
---GTCTTCCTTGGGTTCTTAGGAGCA---GCAGGAAGCACTATG---GGCGCGGCGTCA---
ATAACGCTGACGGTACAG---GCCAGACAA---TTATTG---TCTGGCATAGTGCAACAGCAAAGC---
AATTTG---CTGAGG---GCTATAGAGGTTCAACAACATCTGTTGAAACTCACGGTCTGGGGCATT
>AF069673
TTCTATTGTAATACATCAGGCCTGTTTAATAGTACTTGGAATGGCACTGACAGCATGCAGAAGTTAAAT----
----

AGCACGGGCAATATAACTCTCCCATGCAGAATAAAACAAATTATAAATATGTGGCAGAGAGCAGGACAAGCAA
TATATGCCCCTCCCATCCAAGGAGTAATAAGGTGTGAATCAAAC---
ATTACAGGACTAATATTAACAAGAGATGGTGGGAATGATAACAAT----------------------------
----

GAAAGTGAAATCTTCAGGCCTGGAGGAGGAGACATGAGGGACAATTGGAGAAGTGAATTATATAAGTATAAGG
TAGTAAAAATTGAACCACTA---GGAGTAGCACCCACCAAGGCAAAAAGAAGAGTGGTG--------------
-------------------------GAGAGAGAAAAAAGAGCA---ATTGGA---ATAGGAGCT---------
GTCTTCATTGGGTTCTTGGGAGCA---GCAGGAAGCACTATG---GGCGCAGCGTCA---
ATAACGCTGACGGTACAG---GCCAGGCAA---TTATTG---TCTGGCATAGTGCAACAGCAAAGC---
AATTTG---CTGATG---GCTATAGAGGCTCAACAGCATCTGTTGAAACTCACGGTCTGGGGCATT
>AF107771
TTCTATTGTAATACATCAGGTCTGTTTAATAGTAGTTGGAATGAAAATGACACCAAAGTAAATTACAACACTG
AG---

TCAAATGACACTATAACTCTCCAATGCAGAATAAAGCAAATTATAAATATGTGGCAGAGAACAGGACAAGCAA
CGTATGCCCCTCCCATCCCAGGGGTAATACAGTGTAGATCAAAC---
ATTACAGGACTACTATTAACAAGGGATGGTGGGGTAACTAACAAT----------------------------
----

ACAAATAATGAAACCTTCAGACCTGGAGGAGGAGATATGAGGGACAATTGGAGAAGTGAATTATACAAGTATA
AAGTAGTAAAACTTGAACCACTA---GGAGTAGCACCCACCAAGGCACAGAAGAGTGGTG-------------
----------------------------AAGAGAGAAAAAAGAGCA---GTTGGA---CTGGCAGCT------
---GTATTCTTTGGGTTCTTAGGAGCA---GCAGGAAGCACTATG---GGCGCGGCGTCA---
ATAACGCTGACGGTACAG---GCCAGACAA---TTATTG---TCTGGTATAGTGCAACAGCAAAAC---
AATTTG---CTGAGG---GCTATAGAGGCTCAACAGCATATGTTGAGACTCACGGTCTGGGGCATT
>HQ659604
TTTTATTGTGATACATCAGGCCTGTTTAATAGCACATGGGGGGAAATAGCACTGACAGCATACAGGAGTCAA
ATAGC--
-------------------ACA---
GAGTCAAATGACACTATAATTCTCCCATGCAGAATAAAGCAAATTATAAATATGTGGCAGAGAGTAGGACAAG
CAATGTATGCCCCTCCCATCCAAGGAGTAATAAAGTGTATATCAAAT---
ATTACAGGACTTCTATTAACAAGAGATGGTGGGGATAATAACAGT----------------------------
----

GCAAATGAAACCTTCAGACCTACAGGAGGAAATATGAGGGACAATTGGAGAAGTGAATTATATAAGTATAAAG
TAGTAAAAATTGAACCACTA---GGAGTAGCACCCACCCCGGCAAAGAGAAGAGTGGTG--------------
-------------------------CAGAGAGAAAAAAGAGCA---GTTGGA---ATAGGAGCT---------
GTATTCATTGGGTTCTTAGGAGCA---GCCGGAAGCACTATG---GGCGCGGCGTCA---
ATAACGCTGACGGTACAG---GCTAGACAA---TTATTG---TCTGGCATAGTGCAACAGCAAAGC---
AATTTG---CTGAGG---GCTATAGAGGCTCAACAACATCTGTTGAAACTCACGGTCTGGGGCATT
>HQ659597
TTCTATTGCAACACATCAGAATTGTTTAATAGCACTTGGGATAATAGCACTTGGGATAATAGCACTTGGGATA
ATAACATG---
----------CATGAGTCG------
AATAGCACGGAGAATATAACTCTCCAATGTAGGATAAAGCAAATTATAAATATGTGGCAGAGAGCAGGACAAG
CAATATATGCCCCTCCCATCAAAGGAGTAATAGGGTGTAAATCAAAC---
ATTACAGGACTAATATTAACAAGAGATGGTGGGGAGAAGGGTAATAAC-------------------------
----

AGTATCAATGAAACCTTCAGACCTGGAGGAGGAGATATGAGGGACAATTGGAGAAGTGAATTATACAAGTATA
AAGTAGTAAAAATTGAACCACTA---GGAGTAGCACCCACCAAGGCAAAGAGAAGAGTGGTG-------------
----------------------------GAGAGAGAAAAAAGAGCA---GTTGGA---ATAGGAGCT------
---GTCTTCCTTGGATTCTTAGGAACA---GCAGGAAGCACTATG---GGCGCGGCGTCA---
GTAACGCTGACGGTACAG---GCCAGACAA---TTATTG---TCTGGCATAGTGCAACAGCAAAGC---
AATTTG---CTGAGG---GCTATAGAGGCTCAACAGCATCTGTTGAAACTCACAGTCTGGGGCATT
>HQ697994
TTCTATTGCTACACATCAGGCCTGTTTAATTATACCTTTAATGATACCAGCACAAATAGCACGGAGTCGAAG-
----

TCAAATGACACTATAACTCTCCAATGCAGAATAAAGCAAATTATAAATATGTGGCAGAGAGCAGGACAAGCAG
TATATGCCCCTCCCATACCAGGGATAATAAGGTGTGAATCAAAC---
ATTACAGGACTAATATTAACAAGAGATGGTGGGAATAATAATAGT---------------------------
----

AATACAAATGAAACCTTCAGACCTGGAGGAGGAGATATGAGGGACAATTGGAGAAGTGAATTATATCGGTATA
AAGTAGTAAAAATTGAACCAATA---GGAGTAGCACCTACCACGGCAAAGAGAAGAGTGGTG----------
---------------------------GAGAGAGAAAAAAGAGCA---GTTGGA---ATAGGAGCT------
---GTCTTCCTTGGGTTCTTAGGAGCA---GCAGGAAGCACTATG---GGCGCGGCGTCA---
ATAACGCTGACGGTACAG---GCCAGACAA---TTATTG---TCTGGCATAGTGCAACAGCAAAGC---
AATTTG---CTGAGG---GCTATAGAGGCTCAACAGCATCTGTTGAAACTCACGGTCTGGGGCATT
>HQ698029
TTCTATTGCAACACGGCAGGCCTGTTTGATAGCATTTGGAATGGTAGCATTGGGAATAACACTGACAATAGTA
GCATGGAG--
-------------TTAAAT------
GACGATAATGACACTATAACTCTCCCATGCAGAATAAAGCAAATTATAAATATGTGGCAGAGAGTAGGACAAG
CAATATATGCCCCTCCCATCCAAGGAGTAATAAGGTGTGACTCAAAT---
ATTACAGGGTTAATATTAACAAGAGATGGTGGGGATAATAATAGT---------------------------
----

GCAAATGAAACCTTCAGACCTGTAGGAGGAGATATGAGGGACAATTGGAGAAGTGAATTATATAAGTATAAAG
TAGTAAAAATTGAGCCACTA---GGAGTAGCACCCACCAGGGCAAAGAGAAGAGTGGTG-------------
------------------------GGGAGAGAAAAAAGAGCA---GTTGGA---CTAGGAGCT---------
GTCTTCATTGGGTTCTTAGGAGCA---GCAGGAAGCACTATG---GGCGCAGCGTCA---
GTAACGCTGACGGTACAG---GCCAGACAA---TTATTG---TCTGGCATAGTGCAACAGCAAAGC---
AATTTG---CTGAAG---GCTATAGAGGCTCAACAGCATCTGTTGAGACTCACAGTCTGGGGCATT
>AY253305
TTCTATTGTAACACATCAGGCCTGTTTAATGGCCTGTTTAATGGCACCTGGAATGGCAATCACACAAACAGCA
CG--

GAGTTAAATAGCACTATAATTCTCCAATGCAAAATAAAGCAAATTATAAATATGTGGCAGAGAGCAGGACAAG
CAATATATGCCCCTCCCATCCAAGGAGTAATAAAGTGTGTATCAAAT---
ATTACAGGACTAATATTAACAAGAGATGGTGAGAGGAACAATAGT---------------------------
----

CTAAATGAAACCTTCAGACCTGGAGGAGGAGATATGAGGGACAATTGGAGAAGTGAATTATATAAGTATAAAG
TAGTAAAAATTGAACCACTA---GGAGTAGCACCTACCAGGGCAAAGAGAAGAGTGGTG-------------
------------------------GAGAGAGAAAAAAGAGCA---GTCGGA---CTGGGAGCT---------
GTCTTCATTGGGTTCTTAGGAGCA---GCAGGAAGCACTATG---GGCGCGGCGTCA---
GTAACGCTGACGGTACAG---GCCAGACAA---TTATTG---TCTGGCATAGTGCAACAGCAAAGC---
AATTTG---CTGAGG---GCTATAGAGGCTCAACAGCATCTGTTGAAACTCACGGTCTGGGGCATT
>AY253314
TTCTATTGTAATACATCAGGCCTGTTTAATAGCACTTGGACTACCAACAACATGCAGGAGTCAAATGACACT-
----

AGCAATGGCACTATAACTCTCCCATGCAGAATAAAGCAAATTATAAGGATGTGGCAGAGAGTAGGACAAGCAA
TGTATGCCCCTCCCATTGAAGGAGTAATAAGATGTGAATCAAAC---
ATTACAGGACTAATACTAACAAGAGATGGCGGGAATAATAACAGT---------------------------
----

GCAAATGAAACCTTTAGACCTGGAGGAGGAGATATAAGGGACAATTGGAGAAGTGAATTATATAAGTATAAAG
TAGTAAAAATTGAACCACTA---GGAGTAGCACCCACCAGGGCAAAGAGAAGAGTGGTG-------------
------------------------GAGAGAGAGAAAAGAGCA---GTTGGA---ATAGGAGCT---------
GTCTTCCTTGGGTTCTTAGGAGCA---GCAGGAAGCACTATG---GGCGCGGCGTCA---
ATAACGCTGACGGTACAA---GCCAGACAA---TTATTG---TCTGGCATAGTGCAGCAGCAAAGC---
AATTTG---CTGAGG---GCTATAGAGGCTCAACAACAGCTGTTGAAACTCACGGTCTGGGGCATT
>KX907352
TTCTATTGTAATACATCAGGCCTGTTTAATAGCACTTGGCTATTTAACAAGTCAAATGAGAATAAGTCAAATG
AG--

TCAGATGTCAATATAACACTCCCATGCAGAATAAAGCAAATTATAAATATGTGGCAGAGAGCAGGACAAGCAA
TGTATGCCCCTCCCATCCCAGGAGTAATAAAGTGTGAATCAAAC---
ATTACAGGACTACTATTAACAAGAGATGGTGGGAATAACAACAT-----------------------------
----

TCAAATGAGACCTTCAGACCTGGAGGAGGAGATATGAGGGACAATTGGAGAAGTGAATTATATAAGTATAAAG
TAGTCAAAATTGAACCACTA---GGAGTAGCACCCACCAGGGCAAGGAGAAGAGTGGTG--------------
-------------------------GGAAGAGAAAAAAGAGCA---GTTGGA---ATAGGAGCT---------
GTTTTCCTTGGGTTCTTAGGAGCA---GCAGGAAGCACTATG---GGCGCGGCGTCA---
ATAACGCTGACGGTACAG---GCCAGACAA---TTATTG---TCTGGCATAGTGCAACAGCAAAGC---
AATTTG---CTGAGG---GCTATAGAGGCTCAACAACATCTGTTGAAACTCACAGTCTGGGGCATT
>KX907348
TTCTATTGTAACACATCAGGTCTGTTTAATAGCACTTGGTATGGCAATGGCAGCAAGCTGGAGTCAAATAGCA
CGGGG--

TCAGACGACAATATAACTCTCCACTGTAGAATAAAGCAAATTATAAATATGTGGCAAAGAGCAGGACAAGCAA
TATATGCCCCTCCCATCCCAGGAGTAATAAACTGTCTATCAAAC---
ATTACAGGACTAATATTAACAAGAGATGGTGGGGACAATAACAGT----------------------------
----

ACAAATGAAACCTTCAGACCTGGAGGAGGAGATATGAGGGACAATTGGAGAAGTGAATTATATAAGTATAAAG
TAGTAAAAATTGAACCATTA---GGAGTAGCACCCACCAGGGCAAAGAGAAGAGTGGTG--------------
-------------------------GAAAGAGAAAAAAGAGCA---GTTGGA---CTGGGAACT---------
GTTCTCTTTGGGTTCTTAGGAGCA---GCAGGAAGCACTATG---GGCGCGGCGTCA---
GTAACGCTGACGGTACAG---GCCAGAAAG---TTACTA---TCTGGCATAGTGCAACAGCAAAGC---
AATTTG---CTGAGG---GCTATAGAGGCTCAACAGCATCTGTTGAAACTCACAGTCTGGGGCATT
>KX907336
TTCTATTGTAATACATCAGGCCTGTTTAATAGCACTTGGAACAGCACTGGGTCAAGAAAT-------------
----

GACAGTGGCAATATAACTCTCCCATGCAGAATAAAGCAAATTGTAAATATGTGGCAGAGGACAGGACAAGCAA
TATACGCCCCTCCCATCCAAGGAAGAATACAGTGTGTGTCAAAT---
ATTACAGGACTACTATTGACAAGAGATGGAGGGATTAACAACAGT----------------------------
----

GAGGAAACCTTCAGACCTGGAGGAGGAAATATGAGAGATAATTGGAGAAGTGAACTATATAAATATAAAGTAG
TAAAAATTGAACCACTA---GGAGTAGCACCCACCAGGGCAAAGAGAAGAGTGGTG-----------------
----------------------GAGAGAGAAAAAAGAGCAGTAGTTGGA---CTGGGAGCT---------
GTCTTCCTTGGGTTCTTAGGAGCA---GCAGGAAGCACTATG---GGCGCGGCGTCA---
CTGACGCTGACGGTACAG---GCCAGACAT---TTATTG---TCTGGCATAGTGCAACAGCAAAAC---
AATTTG---CTGAGG---GCTATAGAGGCTCAACAGCATCTGTTGAAACTCACGGTCTGGGGCATT
>KX907372
TTCTATTGTAATACATCAGGCCTGTTTAATAGCAGTTGGAACATTAATGGCAGTCGGGAGTCAAATAAAACAC
AG---

TCAAATGGCACTATAACTCTCCCATGCAGAATAAAGCAAATTATAAATATGTGGCAGAGAGCAGGACAAGCAA
TATATGCTCCTCCCATCCAAGGAGTAATAAAGTGTGTATCAAAC---
ATTACAGGACTAATATTAACAAGAGATGGTGGGAATAATAATAAC----------------------------
----

AGTTCAAATGAAACTTTCAGACCTGGAGGAGGAGATATGAGGGACAATTGGAGAAGTGAATTGTATAAGTATA
AGGTAGTAAAAATTGAACCACTA---GGGGTAGCACCCACCAGGGCAAAGAGAAGAGTGGTG-----------
----------------------------GAGAGAGAAAAAAGAGCA---GTTGGA---ATAGGAGCT------
---GTTTTCCTTGGGTTCTTAGGAGCA---GCAGGAAGCACTATG---GGCGCGGCGTCA---
ATAACGCTGACGGTACAG---GCCAGACAA---TTACTG---TCTGGCATAGTGCAACAGCAGAGC---
AATTTG---CTGAGG---GCTATAGAGGCTCAACAACAGTTGTTGAAACTCACGGTCTGGGGCATT
>KX907383
TTCTATTGTGACACATCAAGCCTGTTTAATATCACTTGGGATAGAAGTAACGTCACGCAGGAGTTAAATAACA
CAAAG--

TTAAATGACACTATAATTCTCCAATGCAGGATAAAGCAAATTATAAATATGTGGCAGAGAGCAGGACAAGCAA
TGTATGCCCCTCCCATCCCAGGAATAATAAAGTGTGAATCAAAC---
ATTACAGGACTAATATTAACAAGAGATGGGAGT--

GAGAATGAAACTGAAACCTTCAGACCTGGAGGAGGAGACATGAGGGACAATTGGAGAAGTGAGTTATATAAGT
ATAAAGTGGTAAAAATTGAACCACTA---GGAGTAGCACCCACCAAGGCAAGGAGAAGAGTGGTG--------
-------------------------------GAGAGAGAGAAAAGAGCA---GTCGGA---CTAGGAGCT---
------GTCTTCCTTGGGTTCTTAGGAGCA---GCAGGAAGCACTATG---GGCGCAGCGTCA---
ATAACGCTGACGGTACAG---GTCAGACAA---TTGTTG---TCTGGCATAGTGCAACAGCAAAAC---
AATTTG---CTGAGG---GCTATAGAAGCTCAACAGCATCTGTTGAAGCTCACGGTCTGGGGCATT
>KX907389
TTCTATTGTAACACATCAATCCTGTTTAATAGCACTTGGAATAGCACTAGCACTTGGAATATCTCTGAGAGTG
TAAATAGC---
----------AGCAGTCAG------
GAGTCAAATGACACTATAACTCTCCAATGCAGAATAAAGCAAATTATAAATATGTGGCAGAGAACAGGACAAG
CAATGTATGCCCCTCCCATCCAAGGAGTAATAAAGTGCATATCAAAT---
ATTACAGGACTAATATTAACAAGAGATGGTGGGGGTGATGACAAT----------------------------
----

ACAACTGAAACCTTCAGACCTGGAGGAGGAGATATGAGGGACAATTGGAGAAGTGAATTATATAAGTATAAAG
TAGTAAAAGTTGAACCACTA---GGAATAGCACCCACCAGGGCAAGGAGAAGAGTGGTG--------------
-------------------------GAGAGAGAAAAAAGAGCA---GTTGGA---ATAGGAGCT---------
GTTTTCATTGGGTTCTTAGGAGCA---GCAGGAAGCACTATG---GGCGCGGCGTCA---
ATAACGCTGACGGCACAG---GCCAGACAA---CTATTG---GCTGGCATAGTGCAACAGCAAAGC---
AATTTG---CTGAGG---GCTATAGAGGCTCAACAGCATCTGTTGAAACTCACAGTCTGGGGCATT
>KX907341
TTCTATTGTAATACATCAGGCCTGTTTAATAGTACTTGGGATGACACCTCGAATCATAATACAAGTGGCACGG
AG---

TCAAATGCCAATATAACTCTCCAGTGTAGAATAAAGCAAATTATAAATATGTGGCAGAGAGCAGGACAAGCAA
TATATGCCCCTCCCATCCAAGGAATAATAAAGTGTGAATCAAAC---
ATTACAGGACTGATATTAACAAGAGATGGTGGAGATAACAATAAC----------------------------
----

ACTACCACCGAAACCTTCAGACCTGGAGGAGGAGATATGAGGGACAATTGGAGAAGTGAATTATATAAGTATA
AAGTAGTAAAAATTGAACCACTA---GGAGTAGCACCCACCAAGGCAAGGAGACGAGTGGTG-----------
----------------------------CAGAGAGAAAAAAGAGCA---GTTGGA---ATGGCAGCT------
---TTATTCTTTGGGTTCTTAGGAGCA---GCAGGAAGCACTATG---GGCGCGGCGTCC---
ATAACGCTGACGGTACAG---GCCAGACAA---TTATTG---TCTGGCATAGTGCAACAGCAAAGC---
AATTTG---CTGAGG---GCTATAGAGGCTCAACAGCATATGTTGAGACTCACGGTCTGGGGCATT
>KX907347
TTCTATTGTAATACATCAGGCCTGTTTAATAGCACTTGGCTGTCTAATAGTACTAACAGCACGGATAACACGG
GG---

ACAAATGACACTATAACTCTCCAATGCAGAATAAAGCAAATTATAAATATGTGGCAAAGAGTAGGACAAGCAA
TGTATGCCCCTCCCATCCCAGGAGTAATAAGGTGTGAATCAAAC---
ATTACAGGACTAATGTTAACAAGAGATGGTGGGAATAATGCTACC----------------------------
----

AATGAAACCTTTAGACCTGGAGGAGGAGATATGAGGGACAATTGGAGAAGTGAATTATATAAGTATAAAGTAG
TAAAAATTGAACCACTA---GGAGTAGCACCCACCAAGGCAAAGAGAAGAGTGGTG-----------------
----------------------GAGAGAGAAAAAAGAGCA---GTTGGA---ATAGGAGCT---------
GTCTTCCTTGGGTTCTTAGGAGCA---GCAGGAAGCACTATG---GGCGCGGCGTCA---
ATAACGCTGACGGTACAG---GCCAGACAA---TTATTG---TCTGGCATAGTGCAACAGCAAGGC---
AACTTG---CTGAGG---GCTATAGAGGCTCAACAACATCTGTTGAAACTCACAGTCTGGGGCATT
>KX907364
TTCTATTGTAACACATCAGCCCTGTTCAATAGCAGTTGGAGTAGTGTAAATGACAGCGGGAACATTAGCCCAG
AG---

CCAAACAAAAATATAACCCTCCGATGCAGAATAAAGCAAATTATAAATATGTGGCAGAGAGTAGGACAAGCAA
TATATGCCCCTCCCATCCAAGGAGTAATAAGGTGTGACTCAAAC---
ATTACGGGATTAATATTAACAAGCGATTATGGAGGGACTAACAGT----------------------------
----

ACACATGAAATCTTCAGACCTGCAGGAGGAAATATGAGGGACAATTGGAGAAGTGAATTATATAAGTATAAAG
TAGTACAAATTGAACCAATA---GGAGTAGCACCCATCAGGGCAAAGAGAAGAGTGGTG--------------
-------------------------GAGAGAGAAAAAAGAGCA---GTCGGA---CTGGGAGCT---------
GTCTTCCTTGGTTTCTTAGGAGCA---GCAGGAAGCACTATG---GGCGCGGCGTCA---
ATGACGCTGACGGTACAG---GCCAGACAA---TTATTG---TCTGGTATAGTGCAACAGCAAAGC---
AATTTG---CTGAGG---GCTATAGAGGCTCAACAGCATCTGTTGAAACTCACGGTCTGGGGCATT
>KX907412
TTCTATTGTAACACATCAGGCCTGTTTCATAGCACTTGGTTTAATAGCACTTGGTTTAATAGCACTTGGGAAG
ACAATCAGGCA---AAT--

AGCACAAATGACACTATAGTACTCCCATGCAGAATAAAGCAAATTATTAATATGTGGCAGAGAGCAGGACAAG
CAATATATGCCCCTCCCATCCCAGGGGAAATAAGGTGTGAATCAAAC---
ATTACAGGGCTAATGTTAACAAGAGATGGTGGGAATAATAACAATAACAGGACA-------------------
----

AATGAAACCAAAAATGAAACCTTCAGACCTGGAGGAGGAGATATGAGGGACAATTGGAGAAGTGAATTATATA
AGTATAAAGTAGTAAAAATTGAACCACTA---GGACTAGCACCCACCGGGGCAAAGAGAAGAGTGGTG-----
----------------------------------GAGAGAGAAAAAAGAGCA---GTTGGA---
ATAGGAGCT---------GTCTTCCTTGGGTTCTTAGGAGCA---GCAGGAAGCACTATG---
GGCGCGGCGTCA---ATAACGCTGACGGTACAG---GCCAGACAA---TTATTG---
TCTGGCATAGTGCAACAGCAAAGC---AATTTG---CTGAAG---
GCTATAGAGGCTCAACAACATCTGTTGAAACTCACGGTCTGGGGCATT
>KX907414
TTCTATTGTAACACATCAGGCCTGTTTAATAGCACTTGGAGTAACACTACCACTTGGGAAAACATTACCGAAA
GGCAGGAACCA--
----------AGCAGCAAC------
AAGACAAACGCAACTATAACTCTCCAATGCAGAATAAAGCAAATTATAAATATGTGGCAGAGAACAGGACGAG
CAATGTATGCCCCTCCCATCCCAGGAATAATAAGTTGTAACTCAAAT---
ATTACAGGACTAATATTAACAAGAGATGGTGGAAATAACAGTACA----------------------------
----

AATGAAACCTTCAGACCTGGAGGAGGAGACATGAGGGACAATTGGAGAAGTGAATTATATAGGTATAAAGTAG
TAAAAATTGAACCACTA---GGAGTAGCACCCACTGAGGCAAAGAGAAGAGTGGTG-----------------
----------------------GAGAGAGGAAAAAGAGCA---ATTGGA---ATAGGAGCT---------
GTTTTCATTGGGTTCTTAGGAGCA---GCAGGAAGCACTATG---GGCGCGGCGTCA---
ATAACGCTGACGGTACAG---GCCAGACAA---TTATTG---TCTGGCATAGTGCAACAGCAAAAC---
AATTTG---CTGAGG---GCTATAGAGGCTCAACAGCATCTGTTGAAACTCACGGTCTGGGGCATT
>KX907423
TTCTATTGTAACACATCAGGCCTGTTTAATAGCATTTGGGTTAAGGGCCAGGAAAGCAATCAGTCAAATAGC-
----

GCAAATGACACTATAGTACTCCCATGCAGAATAAAGCAAATTATAAATATGTGGCAGAGAGCAGGACAAGCAA
TATATGCCCCTCCCATCCCAGGGGAAATAAAGTGTGTATCAAAC---
ATTACAGGGCTAATATTAACAAGAGATGGTGGGAATAACGGGATA----------------------------
----

AATGAAACCTTCAGACCTGGAGGAGGAGATATGAGGGACAATTGGAGAAGTGAATTATATAAGTATAAAGTAG
TAAAAATTGAACCACTA---GGAGTAGCACCCACCAGGGCAAAGAGAAGAGTGGTG-----------------
----------------------GAGAGAGAAAAAAGAGCA---GTTGGA---ATAGGAGCT---------
GTCTTCCTTGGGTTCTTAGGAGCA---GCAGGAAGCACTATG---GGCGCGGCGTCA---
ATAACGCTGACGGTACAG---GCCAGACAA---TTATTG---TCTGGCATAGTGCAACAGCAAAGC---
AATTTG---CTGAAG---GCTATAGAGGCTCAACAACATCTGTTGAGACTCACGGTCTGGGGCATT
>KX907431
TTCTATTGTAACACAACAGACCTGTTTAATAGCACCTGGAATGAGACCAGCATACAGGAAAATAGCACAGAGA
CA---

AACAACGAGACTATAACTCTCCAATGCAGAATAAAGCAAATTATAAATATGTGGCAGAGAGCAGGACAAGCAA
TATATGCCCCTCCCGTACCAGGAGTAATAAGGTGTGACTCAAAT---
ATTACAGGGCTAATATTAACAAGAGATGGTGGGACTAATAATACA----------------------------
----

AATGAAACATTCAGGCCTGTAGGAGGAGATATGAAGGACAATTGGAGAAGTGAATTATATAAGTATAAAGTAG
TAAAAATTGAACCACTA---GGGGTAGCACCCACCAGGGCAAAGAGAAGAGTGGTG-----------------
----------------------GAGAGAGAAAAAAGAGCA---GTTGGA---ATAGGAGCT---------
GTCTTCCTTGGGTTCTTAGGAGCA---GCAGGAAGCACTATG---GGCGCAGCATCA---
ATAACGCTGACGGTACAG---GCCAGACAA---TTATTG---TCTGGCATAGTGCAACAGCAAAGC---
AATTTG---CTGAGG---GCTATAGAGGCTCAACAACAACTGTTGAAACTCACAGTCTGGGGCATT
>HQ615968
TTCTATTGCAACACATCAGACCTATTTAATAGCACTTGGGAAAATAACCCTGTCAGCCAGGAGTCAAATAGCA
CA--

AGGTCAAACGACACTATAACTCTCCAATGCAAAATAAGGCAAATTATAAGAATGTGGCAGAGAGTAGGACAAG
CAATGTATGCCCCTCCCATCCAAGGAGTAATAAACTGTACATCAAAT---
ATTACAGGACTAATATTAACAAGAGATGGTGGGAATAATAATAAC----------------------------
----

AGTGGAAATGAAACCTTCAGACCTGCAGGAGGAGATATGAGGGACAATTGGAGAAGTGAATTATATAAGTATA
AAGTAGTAAAAATTGAACCACTA---GGAGTAGCACCCACCGGGGCAAAGAGAAGAGTGGTG-----------
----------------------------GAGAGAGAAAAAAGAGCA---GTTGGA---ATAGGAGCT------
---GTCTTCATTGGGTTCTTAGGAGCA---GCAGGAAGCACTATG---GGCGCAGCGTCA---
ATAACGCTGACGGTACAG---GCCAGACAA---TTATTG---TCTGGCATAGTGCGGCAGCAAAGC---
AATTTG---CTGCAG---GCTATAGAGGCTCAACAGCATCTGTTGAAACTCACGGTCTGGGGCATT
>HQ615970
TTCTATTGTAATACATCAAGCCTGTTTAATAGTACTTGGAATTGGAATCACACAAACAGCTTGCAGGATTCAA
ATAGC---
-------------------ACA---
GAGACAAATGGCACTATAATTCTCAAATGCAGAATAAAACAAATTATAAGGATGTGGCAGAGAGTAGGACAAG
CAATGTATGCCCCTCCTATCCCAGGAGTAATAAGGTGTGTATCAAAC---
ATTACAGGATTAATATTAACAAGCGATGGCGGGAATAAGACCAGT----------------------------
----

ACAACAGAGACCTTCAGACCTGGGGGAGGAGATATGAGGGACAATTGGAGAAGTGAATTATATAAGTATAAAG
TAGTCAAAATTGAACCACTA---GGAGTAGCACCCACCAGGGCAAAGAGAAGAGTGGTG--------------
-------------------------GAGAGAGAAAAAAGAGCA---GTTGGA---ATAGGAGCT---------
GTCTTCCTTGGGTTCTTAGGAGCA---GCAGGAAGCACTATG---GGCGTGGCGTCA---
ATGACGCTGACGGTACAG---GCCAGGCAA---TTATTG---TCTGGCATAGTGCAACAGCAAAGC---
AATTTG---CTGAGG---GCTATAGAGGCTCAACAGCATCTGTTGAAACTCACAGTCTGGGGCATT
>HQ615971
TTTTATTGTAATACATCAAAACTGTTTAATAGCAGTATATCCTACAATGGCAGTACAAAGTCAGTAGAGTCAA
TA--

AACAGTAACGAGACAATAAGTCTCCCATGCAGAATAAAGCAAATTATAAATATGTGGCAGAGAGCAGGACAAG
CAATATATGCCCCTCCCATCCGAGGAGTAATAAAGTGTAAGTCAGAC---
ATTACAGGACTACTATTAACAAGAGATGGTGGGGTTACCAACACA----------------------------
----

AATGAGACCTTTAGACCTGGAGGAGGAAATATGAGGGATAATTGGAAAAGTGAACTATATAAATATAAAGTAG
TAAAAATTGAACCATTA---GGAGTGGCACCCTCTCCTGCAAAGAGAAGAGTGGTG-----------------
----------------------CAGAGAGAAAAAAGAGCA---GTAGGA---CTGGGAGCT---------
GTTTTCATTGGGTTCTTAGGAGCA---GCAGGAACCACTATG---GGCGCGGCGTCA---
ATGACGCTGACGGTACAG---GCCAGACAA---TTATTG---TCTGGCATAGTGCAACAGCAAGGC---
AATTTG---CTGAGG---GCTATAGAGGCTCAACAGCATCTGTTGAAACTCACGGTCTGGGGCATT
>HM204620
TTCTATTGCAACACATCAGGATTGTTTAATAGCAGATGGGAAAATGACACGCAACCAAATAACACGCAG----
----

CCAAACGGCACTATAACTCTCCCATGCAGAATAAAGCAAATTATAAATATGTGGCAGAGAACAGGACAAGCAA
TATATGCCCCTCCCATCAGAGGAGTAATAAGTTGTCAATCAAAC---
ATTACAGGACTACTACTAACAAGAGATGGGGGGGGGAAGAATACT----------------------------
----

AGTGATAATGAAACCTTCAGACCTGGAGGAGGAGATATGAGGGACAATTGGAGAAGTGAATTATATAAGTATA
AAGTAGTAAAAATTGAACCATTA---GGAGTAGCACCCAACAGGGCAAGGAGAAGAGTGGTG-----------
----------------------------GAGAGAGAAAAAAGAGCA---GTTGGA---ATAGGAGCT------
---GTGTTCCTTGGGTTCTTGGGAGCA---GCAGGAAGCACTATG---GGCGCAGCGTCA---
ATGACGCTGACGGTACAG---GCTAGACAA---TTATTG---TCTGGTATAGTGCAACAGCAAAGC---
AATTTG---CTGAGG---GCTATAGAGGCTCAACAGCATCTGTTGAAACTCACAGTCTGGGGCATT
>HM204621
TTCTATTGTAATACATCAAACCTGTTTAATAGCACTTGGGGTAATGGCAGTTACAGCACCAATGACACAGGGG
AC---

GCAAATTCAAATATAACTATCCAATGCAGAATAAAGCAAATTGTAAGGATGTGGCAGAGGACAGGACAAGCAA
TGTATGCCCCTCCCATCAAAGGAATAATAAGATGTATGTCAAAT---
ATTACAGGCCTACTATTAACAAGAGATGGTGGGATTAATAGGACG----------------------------
----

AATGAAACCTTCAGACCTATAGGAGGAGATATGATGGATAATTGGAGAAGTGAGCTATATAAATATAAAGTAG
TAAGAATTGAACCAATA---GGAGTAGCACCCAACAGGGCAAAGAGAAGAGTGGTG-----------------
----------------------GAGAGAGAAAAAAGAGCA---GTCTTTGGAATGGGAGCT---------
GTCTTCCTTGGGTTCTTAGGAGCA---GCCGGAAGCACTATG---GGCGCAGCGTCG---
ATAACGCTGACGTTACAG---GCCAGACAA---TTGTTG---TCTGGTATAGTGCAACAGCAAAGC---
AATTTG---CTGAGG---GCTATAGAGGCTCAACAGCATCTGTTGAAACTCACGGTCTGGGGCATT
>KY658695
TTCTATTGTAACACATCAAGTTTGTTTAAAGGCACTTGGAATAATGGCACTTGGAATGATAGCATGTCAGAGG
TAAT---
-------------AGCACA------
GAGTCAAATGACAACATAACTCTCCCATGCAGAATAAAGCAAATTATAAATATGTGGCAGAAAGTAGGGCAAG
CAATGTATGCCCCACCCATCCAGGGAGTAATAAGGTGTGAATCAAAC---
ATTACTGGACTAATATTAACAAGAGATGGTGGGAATAGTAGCGGT----------------------------
----

CAGAATGAAACCTTTAGACCTGGAGGAGGAGATATGAGGGACAATTGGAGAAGTGAATTATATAAGTATAAAG
TAGTAAAAATTGAACCACTA---GGAGTAGCACCCACCAAGGCAAGAAGAAGAGTGGTG--------------
-------------------------CAGAGAGAAAAAAGAGCA---GTTGGA---ATGGGAGCA---------
CTTTTCATTGGGTTCTTAGGAGCA---GCAGGAAGCACTATG---GGCGCGGCGTCA---
ATAACGCTGACGGTACAG---GCCAGACAA---TTATTG---TCTGGCATAGTGCAACAGCAAAGC---
AATTTG---CTGAAG---GCTATAGAGGCTCAACAGCATCTGTTGAGACTCACTGTCTGGGGCATT
>KY658696
TTCTACTGTAATACATCAGGCCTGTTTAATAGCAATAGCACAGCAAATAGCACAGCAAAGTTTAATAGCAATA
GCACAGCA---

AATAGCACAGCAAATATAACTCTCCCATGCAGAATAAAACAAATTGTAAATATGTGGCAGAGGCCAGGACAAG
CAATGTATGCCCCTCCAATCCAAGGGCCAATAAGTTGTAGGTCAAAC---
ATTACAGGATTACTAATAACAAGAGATGGTGGAGAAAACAATGGA----------------------------
----

ACGAATGAAACCTTCAGACCTGGAGGAGGAGAGATGAGGGATATTTGGAGAAGTGAACTATATAAATATAAAG
TAGTGAAAATTGAACCATTA---GGAGTAGCACCCACCAGGGCAAGGAGAAGAGTGGTG--------------
-------------------------GAGAGAGAAAAAAGAGCA---GTTGGA---CTGGGAGCT---------
GTCTTCATTGGGTTCTTAGGAGCA---GCCGGAAGCACTATG---GGCGTGGCGTCA---
ATAACGCTGACGGTACAG---GCCAGACAA---TTATTG---TCTGGTATAGTGCAGCAGCAAAAC---
AATTTG---CTGAGG---GCTATAGAGGCTCAACAGCATATGTTGAAACTCACAGTCTGGGGCATT
>AF361872
TTCTATTGTAATACAACAAGCCTGTTTAATAGCACCTGGTATCACAAGAACAGCACGCAGGGGTCAAATGACA
CG---

GGGTCAGATGACACTATAACTCTCCCATGCAAAATAAAACAAATTGTAAGGATGTGGCAGAGAGCAGGACAAG
CAATGTATGCCCCTCCCATTCCAGGAGTAATAAGGTGTGAATCAAAC---
ATTACAGGACTAATATTAACAAGAGATGGGGGGAGTGAGGACAAT----------------------------
----

ACCACTGAAATCTTCAGACCTGGAGGAGGAAATATGAGGGACAATTGGAGAAGTGAATTATATAAGTATAAAG
TAGTAAAAATTGAACCACTA---GGAGTAGCACCCACCAAGGCAAAGAGAAGAGTGGTG--------------
-------------------------GGAAGAGAAAAAAGAGCA---GTTGGA---CTGGGAGCT---------
GTCTTCCTTGGGTTCTTAGGAGCA---GCCGGAAGCACTATG---GGCGCGGCGTCA---
GTAACGCTGACGGTACAG---GCCAGACAA---TTATTG---TCTGGCATAGTGCAACAGCAGAAC---
AATTTG---CTGAGG---GCTATAGAGGCTCAACAACATCTGTTGAAACTCACAGTCTGGGGCATC
>AF361873
TTCTATTGTAACACATCAGGCCTGTTTAACAGCACTTGGCATAGCAATGCCAGCATACAAGAGCCAAACAGCA
CAGAG--

TCAAATGACACTATTATAACTCTCCAATGTAGAATAAAACAAATTATAAATATGTGGCAGAGAACAGGAAAAG
CAATATATGCTCCTCCCATCCAAGGAATAATAAGGTGTGAATCAAAC---
ATTACAGGACTAATATTAACAAGAGATGGTGGGAGTAATAATAGC----------------------------
----

ACCAATGAAACCTTCAGACCTGGAGGAGGAGATATGAGGGACAATTGGAGAAGTGAATTATATAAGTATAAAG
TAGTAAAAATTGAACCACTA---GGAGTAGCACCCTCCTGGGCAAGGAGAAGAGTGGTG--------------
-------------------------CAGAGAAAAAAAAGAGCA---GTTGGA---ATAGGAGCT---------
GTCTTCCTTGGGTTCTTAGGAGCA---GCTGGAAGCACTATG---GGCGCGGCGTCA---
ATAACGCTGACGGTACAG---GCCAGACAA---TTATTG---TCTGGCATAGTGCAACAGCAAAGC---
AATTTG---CTGAGG---GCTATAGAGGCTCAACAGCATCTGTTGAAACTCACGGTCTGGGGCATT
>HM027846
TTCTATTGTAACACATCAGGTCTGTTTAATAGCACGTGGAATACTACCAACATGTGGGAGCAAAATAGCACGG
AG---

TCAAACAATAATACTATATATCTTCAATGCAGAATAAAGCAAATTATCAATATGTGGCAGAGAGTAGGACAAG
CAATATATGCCCCTCCCATCCCAGGAATGATAAGGTGTGTCTCAAAT---
ATTACAGGACTAATATTAACAAGGGATGGTGGGAATATTAGTGAGAAT-------------------------
----

AATGCCAGTGAAATCTTCAGACCAGGAGGAGGAGATATGAGGGACAATTGGAGAAGTGAATTATACAAGTATA
AAGTAGTAAAAATTGAACCACTA---GGAGTAGCACCAACCCCGGCAAGGAGAAGAGTGGTG-----------
----------------------------GAGAGAGAAAAAAGAGCA---GTCGGA---ATAGGAGCT------
---GTCTTCCTTGGGTTCTTAGGAGCA---GCAGGAAGCACTATG---GGCGCGGCGTCA---
ATAACGCTGACGGCACAG---GCCAGACAA---TTATTG---TCTGGTATAGTGCAACAGCAAAGC---
AATTTG---CTGAGG---GCTATAGAGGCTCAACAGCATCTGTTGAAACTCACGGTCTGGGGCATT
>JX877522
TTCTATTGTGACACATCAGAACTATTTAATAGCACTTGGAATGACGCTGCCATTATGAGGGAATTAAATAGAA
CGGAG--

TCAAACGACACCATAACTCTCCAATGCAAAATAAAGCAAATTATAAATATGTGGCAGAGAGCAGGACGAGCAA
TGTATGCCCCTCCCATCCGAGGAGTAATAAGATGTGAGTCAAAT---
ATTACAGGACTAATATTAACAAGAGATGGTGGGAGGGATGATAAC----------------------------
----

AATACAACTGAAACCTTCAGACCTGGAGGAGGAGATATGAGGGACAATTGGAGAAGTGAATTATATAAGTATA
AAGTAGTAAAACTTGAGCCACTA---GGAATAGCACCCACCAAGGCAAAGAGAAGAGTGGTG-----------
----------------------------GAGAGAGAAAAAAGAGCA---GTTGGA---CTGGGAGCT------
---GTTTTCATTGGGTTCTTAGGAGCA---GCAGGAAGCACTATG---GGCGCGGCGTCA---
ATAACGCTGACGGTACAG---GCCAGACAA---TTATTG---TTTGGCATAGTGCAACAGCAAAGC---
AATTTG---CTGAGG---GCTATAGAGGCTCAACAGCATATGTTGAAACTCACAGTCTGGGGCATT
>HM215272
TTCTATTGTAATACATCAAGCCTATTTAATAGTACTTGGTGGAGCAACAACAGCAACAACACTGACAGTGAGG
AGTCAAAT---

```
-------------AGCATG------
GGGTTAAATGACACTATAACTCTCCAATGCAGAATAAAGCAAATTATAAATATGTGGCAGAGAGTAGGACAAG
CAATGTATGCCCCTCCCATCCAAGGAGTAATAAGGTGTGAGTCAAAC---
ATCACAGGAATACTATTAACAAGAGATGGTGGGGATAATAACAAT----------------------------
-------------------------------------------------------------------------
-------------
GAAAGTGAAACCTTCAGACCTGTAGGAGGAGATATGAGGGACAATTGGAGAAGTGAATTATATAAATATAAAG
TAGTAAAAATTGAACCATTA---GGAGTAGCACCCACAGAGGCAAGGAGAAGAGTGGTG--------------
-------------------------GGAAGAGGAAAAAGAGCAGTTGTTGGA---ATGGGAGCT---------
ATGTTCCTTGGGTTTTTGGGAGCA---GCTGGGAGCACTATG---GGCGCGGCGTCA---
ATAACGCTGACGGTACAG---GCCAGACAA---TTATTG---TCTGGCATAGTGCAACAGCAAAGC---
AATTTG---CTGAGG---GCTATAGAGGCTCAACAGCATCTGTTGAAACTCACTGTCTGGGGCATT
>HM027824
TTCTATTGTAACACATCAGGTCTGTTTAATAGCACGTGGAATGGTACCCAAATGATGGAGCCAAATAGCACAG
AA-----------------------------------------------------------------------
-------------------------
AACATTAATGATACTATAACTCTTAAATGCAGAATAAAGCAAATTATACATATGTGGCAGAGAGTAGGACAAG
CAATATATGCCCCTCCCATCAAAGGAGTGATAAGATGTAACTCAAAC---
ATTACAGGACTAATATTAACAAGAGATGGTGGGACTGAAGGGAAT----------------------------
-------------------------------------------------------------------------
-------------
AGCAGTGAAACCTTCAGACCGGGAGGAGGTGATATGAGGGACAATTGGAGAAGTGAATTATATAAGTATAAAG
TAGTAAAAATTGAACCACTA---GGAGTAGCACCTACCAAGGCAAGGAGAAGAGTGGTG--------------
-------------------------GAGAGAGAAAAAAGAGCA---ATCGGA---CTGGGAGCT---------
GTCTTCCTTGGGTTCTTAGGAGCA---GCAGGAAGCACTATG---GGCGCGGCGTCA---
ATAACGCTGACGGCACAG---GCCAGACAA---TTATTG---TCTGGCATAGTGCAACAGCAAAGC---
AATTTG---CTGCAG---GCTATAGAGGCTCAACAGCATCTGTTGAAACTCACAGTCTGGGGCATT
>JX236669
TTCTACTGTAATACATCAGGCCTGTTTAATAGCACTTGGAACATTGCTGGGAACAGGACAAATGACACGAAG-
-------------------------------------------------------------------------
----------------------------
TCAAATGAAACTATAACTCTCCCATGCAGAATAAAGCAAATTGTAAATGTGTGGCAGAGAGTAGGACAAGCAA
TATATGCCCCTCCCATCGCAGGAGTAATAAGATGTAACTCAAAC---
ATTACAGGATTACTATTAGTAAGAGATGGTGGAGCTACTAACAAT----------------------------
-------------------------------------------------------------------------
-------------
ACTGATGAAACCTTCAGGCCTGGAGGAGGAAATATGAGGGACAATTGGAGAAGTGAATTATATAAGTATAAAG
TAGTAAAAATTGAACCACTA---GGAGTAGCACCCACCAGGGCAAGGAGAAGAGTGGTG--------------
-------------------------GAGAGAGAAAAAAGAGCA---GTTGGA---CTGGGAGCT---------
GTATTCATTGGGTTCTTAGGAGCA---GCAGGAAGCACTATG---GGCGCGGCGTCA---
GTGACGCTGACGGTACAG---GCCAGACAA---TTATTG---TCTGGCATAGTGCAACAGCAAAGC---
AATTTG---CTGAGG---GCTATAGAGGCTCAACAGCATCTGTTGAAGCTCACGGTCTGGGGCATT
>JX236671
TTCTATTGTAATACAGCAGGTCTGTTCAATAGAAATTGGACTTGGAGTAAGGAGAATGGGACCAGAATGCAGA
ATAAAACGGAT--------------------------------------------------------------
----------------------------
TCAAATGGCACTATAACTCTCCAATGCAGAATAAAGCAAATTATAAATATGTGGCAGAGAACAGGACAGGCAA
TGTATGCCCCTCCCATCCAAGGAGTAATAAAATGTGCATCAAAC---
ATTACAGGACTACTGTTAACAAGGGATGGTGGAGATAACAAAAACAATAGTACC------
AATAGTACCAATCAAAAT-------------------------------------------------------
--------------------------
AACACTGAAATCTTTAGACCTATAGGAGGAGATATGAGGAATAATTGGAGAAGTGAATTATATAAGTATAAAG
TAGTAAAAATTGAACCACTA---GGAGTAGCACCTACCACGGCAAGGAGAAGAGTGGTG--------------
-------------------------GGAAGAGAAAAAAGAGCAGTACTAGGTGGACTAGGAGCT---------
GTATTCCTTGGGTTCTTAGGAGCA---GCCGGAAGCACTATG---GGCGCAGCGTCA---
ATAACGCTGACGGTACAG---GCCAGGCAA---TTATTG---TCTGGCATAGTGCAACAGCAAAGC---
AATTTG---CTGAGG---GCTATAGAGGCTCAACAACATCTGTTGAAACTCACGGTCTGGGGCATT
>JX236676
TTCTATTGTAACACATCAAGCCTATTTAATAGCATTTGGAATGGCAGTAAATCAAATAACACGGAG-------
-------------------------------------------------------------------------
```

CCAGATAGCACCATAACTCTCCAATGCAGAATAAAGCAAATTATAAATATGTGGCAGAGAGTAGGACAAGCAA
TGTATGCCCCTCCCATCCAAGGAGTAATAAGGTGTGAGTCAAAC---
ATTACAGGGCTAATATTAACAAGAGATGGTGGGAGTAATAGCAGT------------------------------
----

AGTACAAATGAAACCTTCAGACCTGGAGGAGGAGATATGAGAGACAATTGGAGAAGTGAATTATATAAGTACA
AAGTAGTAAAAATTGAACCACTA---GGAGTAGCACCCACCAAGGCAAAGAGAAGAGTGGTG-------------
------------------------------GAAAGAGAAAAAAGAGCA---GTTGGA---ATAGGAGCT------
---GTTTTCCTTGGGTTCTTAGGAGCA---GCAGGAAGCACTATG---GGCGCAGCGTCA---
ATAACGCTGACGGTACAG---GCCAGACAA---TTATTG---TTTGGCATAGTGCAACAGCAAAGT---
AATTTG---CTGAGG---GCTATAGAGGCTCAACAGCATCTGTTGAAACTCACGGTCTGGGGCATT
>KF716478
TTCTATTGTAATACATCAAGCCTGTTTAATAGTACTTGGGTATATGGTAATGGAAGCTGGGGGTCAAATAGCA
CGGGGTTAAAT---
AGTACAATGATAAATAATACT------------
ATTTTCAGTGATACCATAATTCTTCAATGCAGAATAAAGCAAATTATAAATATGTGGCAGAGAGCAGGACAAG
CAATATATGCCCCTCCCATCAAAGGAGTAATAAGCTGTAGATCAAAC---
ATCACAGGACTAATATTAACAAGAGATGGTGGGAGTAACAGCAATGCAAGT------------------------
----

ACAAATGGAAGTGAAACCTTCAGACCTGGAGGAGGAGATATGAGGGACAATTGGAGAAGTGAATTATATAAGT
ATAAAGTAGTAAAAGTTGAACCACTA---GGAATAGCACCCTCCAAGGCAAGGAGAAGAGTGGTG----------
---------------------------------GCGAGAGAAAAAAGAGCA---GTTGGA---ATAGGAGCT---
------GTACTCCTTGGGTTCTTAGGAACA---GCAGGAAGCACTATG---GGCGCAGCGTCA---
ATAACGCTGACGGTACAG---GCCAGAAAA---TTATTG---TCTGGCATAGTGCAACAGCAAAGC---
AATTTG---CTGAGG---GCTATAGAGGCTCAACAGCATCTGTTGAAACTCACTGTCTGGGGCATT
>KP109490
TTCTACTGTAATACATCAGGCCTGTTCAATAGCACTTGGAATAACACCTCGCAGGCAAATAGCTCGGAG----
----

TTAAAGGGCAATATAACTCTCCCATGCAAAATAAAACAAATTATCAGGATGTGGCAAAGAACAGGACAAGCAA
TGTATGCCCCTCCCATCCAAGGAGTAATAAATTGTGTATCAAAC---
ATTACAGGACTACTATTAACAAGAGATGGTGGGGAGGAGGACAAT------------------------------
----

AGTACTGAAACCTTCAGACCTGGAGGAGGAGATATGAGGGATAATTGGAGAAGTGAATTATATAAGTATAAAG
TAGTAAAAATTGAACCAGTA---GGAGTAGCACCCACCAAGGCAAGGAGAAGGGTGGTG----------------
-------------------------GAGAGAGAAAAAAGAGCA---GTTGGA---ATAGGAGCT---------
GTCCTCCTTGGATTCTTAGGAGCA---GCAGGAAGCACTATG---GGCGCGGCGTCA---
ATAACGCTGACGGTACAG---GCCAGACAG---TTATTG---TCTGGTATAGTGCAGCAGCAAAGC---
AATTTG---CTGAGG---GCTATAGAGGCTCAACAACAACTGTTGAAACTCACGGTCTGGGGCATT
>JF680921
TTCTATTGTAACACATCAGGCCTGTTTAATAGCACTTGGACTAAGAATAGCAGCACTAACACTTGGAATAGTA
CTGACATCATG--
-------TGGGGGGTAAAT------
AACAGTGACCATAATATAACTCTCCAATGCAAAATAAAGCAAATTATAACCATGTGGCAGAGGACAGGGCAGG
CAATGTACGCCCCTCCCATCCCAGGAATAATAAACTGTGTGTCAAAC---
ATTACAGGACTACTATTAACAAGAGATGGTGGTGAGGAGAGTAACAGT---------------------------
----

AGAGATAATGAAACCTTCAGACCTATAGGAGGAAATATGAAGGATAATTGGAGAAGTGAACTATATAAATATA
AAGTAGTAAAAATTGAACCACTA---GGAGTAGCACCCACCAAGGCAAAGAGAAGAGTGGTG-------------
------------------------------CAGAGAGAAAAAAGAGCA---GTTGGA---CTGGGAGCT------
---GTCTTCATTGGGTTCTTAGGAGCA---GCCGGAAGCACTATG---GGCGCGGCGTCA---
ATAACGCTGACGGTACAG---GCCAGACAA---TTATTG---TCTGGCATAGTGCAACAGCAAAAC---
AATTTG---CTGAGG---GCTATAGACGCTCAACAGCATCTGTTGAAGCTCACGGTCTGGGGCATT
>KF859745
TTCTATTGTAACACATCAGGCTTGTTTAATAGCACTTGGAACAATAGCACTCAAGAGTCTAAT------------
----

AGCACGGAGACTATAACTCTCCCATGCCGAATAAAGCAAATTGTAAATATGTGGCAGAGAGCAGGACAAGCCA
TATATGCCCCTCCCATCAAAGGAATAATAAATTGTAGATCAAAC---
ATTACAGGACTAATATTAACAAGAGATGGTGGACATAATAATACA----------------------------
----

AATGAAACCTTCAGGCCTGGAGGAGGAGATATGAGGGACAATTGGAGAAGTGAATTATATAAGTATAAAGTAG
TAAAAGTTGAACCACTA---GGAGTAGCACCCACCACGGCAAGGAGAAGAGTGGTG-----------------
----------------------CAGAGAGAAAAAAGAGCAGTATTTGGA---CTGGGAGCT---------
GTCTTCATTGGGTTCTTAGGAGCA---GCAGGAAGCACTATG---GGCGCGGCGTCA---
ATAACGCTGACGGTACAG---GCCAGACAA---TTATTG---TCTGGCATAGTGCAACAGCAAAGC---
AATTTG---CTGAGG---GCTATAGAGGCTCAACAGCATCTGTTGAAACTCACGGTCTGGGGCATT
>KF716486
TTCTATTGTAATACATCAAGTCTGTTTAATAGTACTTGGACCTTAATGCTACTAGGGAAGAT----------
----

AGCACGGAGAACATAACTCTCCCATGCAGAATAAAGCAAATCATAAATATGTGGCAAAGAGTAGGACAAGCAA
TGTATGCCCCTCCCATCGAAGGAATAATAAGATGTGACTCAAAC---
ATTACAGGACTACTCTTAACAAGAGATGGTGGGAATAGTAGTACAAATGAA----------------------
----

ACTCAGGAAATCTTCAGACCTATAGGAGGGGATATGAAAGATAATTGGAGAAATGAATTATACAAGTATAAAG
TAGTAAAAATTGAACCTCTG---GGAGTAGCACCCTCCTGGGCAAGGAGAAGAGTGGTG--------------
-------------------------GAGAGAGGAAAAAGAGCA---GTTGGA---ATAGGAGCT---------
GTATTCCTTGGGTTCTTAGGAGCA---GCAGGGAGCACTATG---GGCGCAGCGTCA---
ATAACGCTGACGGTACAG---GCCAGGCAA---TTATTG---TCTGGCATAGTGCAACAGCAAAGC---
AATTTG---CTGAAG---GCTATAGAAGCTCAACAACAACTGTTGAGACTCACGGTCTGGGGCATT
>M62320
TTCTATTGCAACACATCAGGCCTGTTTAATAGCATTTGGAATGGCAGCATGTCAAATGACATGGGG-------
----

CCAAATGGCACTATAACTCTCCAATGTAGAATAAAGCAAATTATAAATATGTGGCAGAGAGTAGGACAAGCAA
TGTATGCCCCTCCCATCCAAGGAGTAATAAGGTGTGAATCAAAC---
ATTACAGGACTACTATTAACAAGAGATGGGGGTACTAACAATACA----------------------------
----

AAAAATGAGACCTTCAGACCTGGAGGAGGAGATATGAGGGACAATTGGAAAAGTGAATTATATAAGTATAAAG
TAGTAAAAATTGAACCACTA---GGAGTAGCACCCACCAGGGCAAAGAGAAGAGTGGTG--------------
-------------------------GAGAGAGAAAAAAGAGCA---GTTGGA---CTAGGAGCT---------
ATCTTCCTTGGGTTCTTAGGAGCA---GCTGGAAGCACAATG---GGCGCGGCGTCA---
ATAACGCTGACGGTACAG---GCCAGACAA---TTATTG---TCTGGTATAGTGCAACAGCAGAGC---
AATCTG---CTGAGG---GCTATAGAGGCTCAACAGCATCTGTTGAAACTCACTGTCTGGGGCATT
>L22957
TTCTATTGTAATACAGCAGGCCTGTTTAATAGTACTTGGAATAAAAATGACAGCATAAAGTCAAATGACACAA
AG---

TCAAATGACATTATAACTCTTCAATGCAGAATAAAGCAAATTATAAATATGTGGCAGAGAGTAGGACGAGCAA
TGTATGCCCCTCCCATCAAAGGAGTAATAAGGTGTGAATCAAAC---
ATTACAGGACTACTATTAACAAGAGATGGTGGAAATGGGAGCAAT----------------------------
----

ACAAATGAAACCTTCAGACCAGGAGGAGGAGATATGAGGGACAATTGGAGAAATGAATTATATAAGTATAAAG
TAGTACAAATTGAACCATTA---GGAGTAGCACCTACCCCAGCAAAGAGAAGAGTGGTG--------------
-------------------------CAGAGAGAAAAAAGAGCA---GTTGGA---CTAGGAGCT---------
GTATTCATTGGGTTCTTAGGAGCA---GCAGGAAGCACTATG---GGCGCGGCGTCA---
ATAACGCTGACGGTACAG---GCCAGACAA---TTATTG---TCTGGCATAGTGCAACAGCAAAGC---
AATTTG---CTGAGA---GCTATAGAGGCTCAACAGCATCTGTTGAAACTCACAGTCTGGGGCATT
>L22951
TTCTATTGTGACACATCAGGCCTGTTTAATAGCACTTGGAATGGCAATACCAGCAAGCAGGAGTCAAGTAGCA
CA---

GAGTCAAATGGCAATATAACTCTCCAATGCAGAATAAAGCAAATTATAAATATGTGGCAGAGAGCAGGAAAAG
CAATATATGCCCCTCCTATCAAAGGAGTAATAAGGTGTGTATCAAAC---
ATTACAGGACTAATATTAACAAGAGATGGTGGGGGTAATAACAGT---------------------------
----

GAAAATGAAACCTTCAGACCTGGAGGAAGAGATATGAGGGACAATTGGAGAAGTGAATTATATAAGTATAAAG
TAGTAAAAATTGAACCACTA---GGAGTAGCACCCACCAGGGCAAGGAGGAGAGTGGTG-------------
-------------------------GAGAGAGAAAAAAGAGCA---GTTGGA---GTAGGAGCT--------
GTCTTCCTTGGGTTCTTAGGAGCA---GCAGGAAGCACTATG---GGCGCGGCGTCA---
ATAACGCTGACGGTACAG---GCCAGACAA---TTATTG---TCTGGCATAGTGCAACAGCAAAGC---
AATTTG---CTGAAG---GCTATAGAGGCTCAACAGCATCTGTTGAAACTCACGGTCTGGGGCATT
>AB253429
TTCTATTGTAATACATCAGGCCTGTTTAATAGTACTTGGGTAAATGGCACTACCAGCAGCATG---------
----

TCAAATGGCACTATAACTCTCCCATGCAGAATAAAGCAAATTATAAATATGTGGCAGAGAGTAGGACAAGCAA
TGTATGCCCCTCCCATCCAAGGAGTAATAAAGTGTGAATCAAAC---
ATTACAGGACTAATATTAACAAGAGATGGTGGGGTTAATAGCAGT---------------------------
----

GACAGTGAAACCTTCAGACCTGGAGGAGGAGATATGAGGGATAATTGGAGAAGTGAATTATATAAGTATAAGG
TAGTAAAAATTGAACCACTA---GGAGTAGCACCCACCAAGGCAAGGAGAAGAGTGGTG-------------
-------------------------GAGAGAGAAAAAAGAGCA---GTTACA---CTGGGAGCT--------
GTATTCATTGGGTTCTTAGGAACA---GCTGGAAGCACAATG---GGCGCGGCGTCA---
ATAACGCTGACGGTACAG---GCCAGAAAA---TTATTG---TCTGGCATAGTGCAACAGCAAAGC---
AATTTG---CTGAGG---GCTATAGAGGCTCAACAGCATCTGTTGAAACTCACTGTCTGGGGCATT
>AY494973
TTCTATTGTAATACATCAGGCCTGTTTAATAGCACTTGGCCTTGGAATGAGACAAATAGCGAGACAAATAAC-
----

ACGAACGCTACTATAGTGCTCCCATGCAGAATAAAACAAATTGTAAGAATGTGGCAGAGAGTAGGACAAGCAA
TGTATGCCCCTCCCATCCAAGGAGTAATAAAGTGTACATCAAAC---
ATTACAGGACTACTATTAATAAGAGATGGTGGGGTTGGTAACAATACA------------------------
----

AATGGAACTGAAACCTTCAGACCTGGAGGAGGAAATATGAGGGACAATTGGAGAAGTGAATTATATAAGTATA
AAGTAGTAAAAATTGAACCACTA---GGAGTAGCACCCACCGGGGCAAAAAGAAGAGTGGTG----------
---------------------------GAGAGAGAAAAAAGAGCA---GTTGGA---ATAGGAGCT------
---GTGTTCATTGGGTTCTTAGGAGCA---GCTGGAAGCACTATG---GGCGCAGCATCA---
GTAACGCTGACGGTACAG---GCCAGACAA---TTGTTG---TCTGGCATAGTGCAACAGCAAAGC---
AATTTG---CTGAGG---GCTATAGAGGCTCAACAGCATATGTTGAAACTCACGGTCTGGGGCATT
>EU853062
TTCTATTGTAATACATCAGACCTGTTTAATAGCACTTGGTACTTAAATGGCACTAGCACTTGGTACAATGGCA
CTGCAAAT--
-------------AACGCA------
CCGTTAAATGGTAATATAACTCTCCCATGCAGAATAAAGCAAATCATAAGGATGTGGCAGAGAACAGGACAAG
CAATGTATGCCCCTCCCATTCAAGGAAAAATAGAATGTGTATCAAAC---
ATTACAGGACTCCTATTAGTAAGAGATGGTGGGAATGATGAGAATAACAACACA-----------AGA----
----
-
AATAACACCCTCAAAGATGAAAAATTCAGACCTGGAGGAGGAGATATGAAGGACAATTGGAGAAATGAATTAT
ATAGGTATAAAGTAGTAAGAGTTGAACCACTA---GGAGTAGCACCCACCAAGGCAAGGAGAAGAGTGGTG--
------------------------------------GGGAGAGAAAAAAGAGCA---GTTGGA---
ATAGGAGCT---------GTGTTCCTTGGGTTCTTAGGAGCA---GCAGGAAGCACTATG---
GGCGCGGCGTCA---ATAACGCTGACGGAACAG---GCCAGACAA---TTATTG---
TCTGGTATAGTGCAACAGCAAAGC---AATTTG---CTGAGG---
GCTATAGAGGCTCAACAGCAACTGTTGAAACTCACAGTCTGGGGCATT
>EU852958

TTCTATTGTAATACATCAGGCCTGTTTAATAGCACCTGGGATAAAAATACCACCGTGCAGGAGTCAAATAGCA
CG---

GGGTCAAATGACACTATAACTCTCCAATGTAGAATAAAGCAAATTATAAATATGTGGCAGGAAGTAGGACGAG
CAATGTATGCTCCTCCCATCAAAGGAGTAATAAGGTGTGAATCAAAC---
ATTACAGGAATGATATTAACAAGAGATGGGGGTAATACTACCGGG----------------------------
----

AAAAATGAGATCTTTAGGCCTGGAGGAGGAGATATGAGGGATAATTGGAGAAGTGAATTGTATAAGTATAAAG
TAGTAAAAATTGAACCACTA---GGAGTAGCACCCACCGAGGCAAAGAGAAGAGTGGTG--------------
-------------------------GAGAGAGAAAAAAGAGCA---ATTGGTGGAATAGGAGCT---------
GTCTTCCTTGGGTTCTTAGGAGCA---GCAGGAAGCACTATG---GGCGCAGCGTCA---
ATAACGCTGACGGCACAG---GCCAGACAA---TTATTG---TCTGGTATAGTGCAACAGCAAAGC---
AATTTG---CTGAGG---GCTATAGAGGCTCAACAGCATCTGTTGAAACTCACGGTCTGGGGCATT
>EU852954
TTCTATTGTAACACATCAGGCCTGTTTAATAGCACCTGGTATACAAATAATACCAGCGTGCAGGAGACAAATA
GCACG--

GGGACAAATGACACTATAATTCTCCAATGTAGAATAAAGCAAATTATAAATATGTGGCAGGAAGTAGGACGAG
CCATGTATGCTCCTCCCATCAGGGGAGTAATAAGGTGTGAATCAAAC---
ATTACAGGAATGATATTAACAAGAGATGGGGGTGTGAGTGTTAACAAG-------------------------
----

ACAACTGAGACTGAGGTCTTCAGACCTGGAGGAGGAGATATGAGGGATAATTGGAGAAGTGAATTGTATAAGT
ATAAAGTAGTAAAAATTGAACCACTA---GGAGTAGCACCTACCAAGGCAAAGAGAAGAGTGGTG--------
-------------------------------GAGAGAGAAAAAAGAGCA---ATTACTGGAATAGGAGCT---
------GTCTTCCTTGGGCTCTTAGGAGCA---GCAGGAAGCACTATG---GGCGCAGCGTCA---
ATAACGCTGACGGCACAG---GCCAGACAA---TTATTG---TCTGGTATAGTGCAACAGCAAAGC---
AATTTG---CTGAGG---GCTATAGAGGCTCAACAACATCTGTTGAAACTCACGGTCTGGGGCATT
>AF484507
TTCTATTGTAATACATCACAATTGTTTAATAGCATTTGGAATGTCACTAACAGCACAGAGTCAGAGAATAGCA
CG---

GGGTCAGATGACACTATAACTCTTCCATGCAGAATAAAGCAAATTATAAAGATGTGGCAGAGAACAGGACAAG
CAATATATGCCCCCCCCATCCAAGGAGTAATAAGGTGTGAATCAAAC---
ATTACAGGACTATTATTAACAAGAGATGGTGGGAATAACAATACA----------------------------
----

GGTAATGAAACCTTCAGACCTGCAGGAGGAGATATGAAGGACAATTGGAGAAGTGAATTATATAAGTACAAAG
TAGTAAAAATTGAACCACTA---GGAGTAGCACCCACCAAGGCAAGAAGGAGAGTGGTG--------------
-------------------------GAGAGAGAAAAAAGAGCA---GTTGGA---ATAGGAGCT---------
GTATTCCTTGGGTTCTTAGGAACA---GCAGGAAGCACTATG---GGCGCGGCGTCA---
ATAACGCTGACGGTACAG---GTCAGACAA---TTATTG---TCTGGCATAGTGCAACAGCAAGGC---
AATTTG---CTGAGG---GCTATAGAGGCTCAACAACAGCTGTTGGAACTCACGGTCTGGGGCATT
>AF484508
TTCTACTGTAACACATCAAGCCTGTTTAATGGCACTTGGAATGGCACTGAGAACATAACGGAGTCAAATAGC-
----

ATGAACGACTCTATAATACTCCAATGCAGAATAAAGCAAATTATAAATATGTGGCAGAGAGCAGGACAAGCAA
TATATGCCCCTCCCATCCCAGGAGTAATAAGGTGTCAATCAAAC---
ATTACAGGACTAATATTAACAAGAGATGGTGGAAATAACAGTACA----------------------------
----

AATGAAACCTCCAGACCTGGAGGGGGAGATATGAGGGACAACTGGAGAAGTGAATTATATAAGTATAAAGTAG
TAAAACTTGAACCACTA---GGAGTAGCACCCACCAGGGCAAAGAGAAGAGTGGTG-----------------
----------------------GAGAGAGAGAAAAGAGCA---GTTGGA---CTAGGAGCT---------
GTTTTCTTTGGGTTCTTAGGAGCA---GCAGGAAGCACTATG---GGCGCGGCGTCA---
ATAACGCTGACGGTACAG---GCCAGACAA---TTATTG---TCTGGTATAGTGCAACAGCAAAGC---
AATTTG---CTGAGG---GCTATAGAGGCTCAACAGCATCTGTTGAAACTCACGGTCTGGGGCATT
>AF484509

TTCTATTGTGACACATCAGGCCTGTTTAATAGCACTTGGTATAATACTTGGAATAGCACTGAATCAAAAAGCA
CG---

GAGTCAAACGACATTACTCTCCAATGCAGAATAAAGCAAATTATAAATATGTGGCAGAGAGCAGGACAAGCAA
TATATGCCCCTCCCATCCAAGGAGTAATAATGTGTAAATCAAT---
ATTACAGGACTAATATTAACAAGAGATGGTGGAGGTAATAGCAGT----------------------------
----

GAACCTGAAACCTTCAGACCTGGAGGAGGAGACATGAGGGACAATTGGAGAAGTGAATTGTATAAGTATAAAG
TAGTAAAAATTGAACCACTA---GGAGTAGCACCCACCAAGGCAAGGAGAAGAGTGGTG--------------
-------------------------CAGAGAGAAAAAAGAACA---GTTGGA---ATAGGAGCT---------
GTGTTCCTTGGGTTCTTAGGAGCA---GCAGGAAGCACTATG---GGCGCGGCGTCA---
ATAACGCTGACGGCACAG---GCCAGAGCA---TTACTG---TCTGGCATAGTGCAACAGCAAAGC---
AATTTG---CTGAGG---GCTATAGAGGCTCAACAACATCTGTTGAGACTCACGGTCTGGGGCATT
>AF484512
TTCTATTGTGATACAACAGGACTGTTTAATAGCAATTGGACTTGGAATGACACTGCCAGCATAATGGAGTCAA
ATAGC--
-------------------ATG---
GAGTCAACTCAAAATATAACACTCCAATGCAGAATAAAGCAGATTATAAATATGTGGCAGAGAGCAGGTAAAG
CAATGTATGCCCCTCCCATCCAAGGAACAATAAGGTGTGAATCAAAC---
ATTACAGGACTACTGTTAACAAGAGATGGTGGGGATAATAGAGAT----------------------------
----

AATAGAACTGAAACCTTCAGACCTGGAGGAGGAGATATGAGGGATAATTGGAGAAGTGAATTATATAAGTATA
AAGTAGTCAAAATTGAACCACTA---GGAGTAGCACGCACCAGGGCAAAGAGAAGAGTGGTG-----------
----------------------------GAGAGAGAAAAAAGAGCA---GTTGGA---ATAGGAGCT------
---GTCTTCCTTGGGTTCTTAGGAGCA---GCAGGAAGCACTATG---GGCGCGGCGTCA---
ATAACGCTGACGGTACAG---GCCAGACTA---TTATTG---TCTGGTATAGTGCAACAGCAAAGC---
AATTTG---CTGAGG---GCTATAGAGGCTCAACAACATTTGTTGAAGCTCACGGTCTGGGGCATT
>EU853030
CTCTATTGTAATACATCAGGCCTGTTTAATAGCACTTGGAATAACACTGTCAGCACGGAGTTAAATAGCAAGG
AG---

GCAAATAACAATATAACTCTCCCATGCAAAATAAAGCAAATTATAAACATGTGGCAAAGAACAGGACAAGCAA
TGTATGCCCCTCCCATCCGAGGAGTAATAAATTGTGTGTCAAAC---
ATTACAGGACTAATATTAACAAGAGATGGTGGGCCTAACGATACA----------------------------
----

CATGAAAATGAAACCTTCAGACCTGGAGGAGGAAATATGAGGGACAATTGGAGAAGTGAATTATATAAATATA
AAGTAGTAAAAATTGAACCATTA---GGAGTAGCACCCACCAGGGCAAAGAGAAGAGTGGTG-----------
----------------------------GAAAGAGAAAAAGGAGCA---GTTGGA---ATAGGAGCT------
---GTCTTCCTTGGGTTCTTAGGAGCA---GCAGGAAGCACTATG---GGCGCGGCGTCA---
ATAACGCTGACGGTACAG---GCCAGACAA---TTGTTG---TCTGGCATAGTGCAACAGCAAAGC---
AATTTG---CTGAGG---GCTATAGAGGCTCAACAACATCTGTTGAAACTCACGGTCTGGGGCATT
>AF484478
TTCTATTGTAACACAACAAACCTGTTTAATAACATTTGGAGTAATGCCAGCATGCAGAAGGCAAATAGC----
----

ATAAACGACACTATAACTCTCCAATGCAGAATAAAGCAAATTATAAATATGTGGCAGAGAGTAGGACAAGCAA
TATATGCCCCTCCCATCCAAGGAGTGATAAAGTGTGAATCAAAC---
ATTACAGGACTAATATTAACAAGAGATGGTGGGGGTAATAACAAT----------------------------
----

ACAAATGAAACCTTCAGACCTGGAGGAGGAGATATGAGGGACAATTGGAGAAGTGAATTATATAAGTATAAAG
TAGTAAAAATTGAACCACTA---GGAGTAGCACCTACCAGGGCAAAGAGAAGAGTGGTG--------------
-------------------------GAGAGAGAAAAAAGAGCA---GTTGGA---ATGGGAGCT---------
GTCCTCATTGGGTTCTTAGGAGCA---GCAGGAAGCACTATG---GGCGCGGCGTCA---
ATAACGCTGACGGTACAG---GCCAGACAA---TTATTG---TCTGGCATAGTGCAACAGCAAAGC---
AATTTG---CTGAGG---GCTATAGAGGCTCAACAGCATCTGTTGAAACTCACGGTCTGGGGCATT
>AF484493

TTCTATTGTAATACATCAGGCCTGTTTAATAATACTTACACTACTGACAGCATAGAGTCAAATGACACGGAG-
----

CCAGATGCAAATATAACTCTTCCATGCAGAATAAAGCAAATTATAAATATGTGGCAGAGAGTAGGACAAGCAA
TATATGCCCCTCCCATCCAAGGAGTAATAAAGTGTGAGTCAAAC---
ATCACAGGACTACTATTAACAAGAGATGGTGGTACTAACAGTACT----------------------------
----

AAAAGTACTGAAACCTTCAGACCTGGAGGAGGAGATATGAGGGACAATTGGAGAAGTGAATTATATAAGTATA
AAGTAGTACAAGTTGAACCAATA---GGAGTAGCACCCACCAGGGCAAAGAGAAGAGTGGTG-----------
----------------------------GAGAGAGAAAAAAGAGCA---GTTGGA---ATAGGAGCT------
---GTACTCCTTGGGTTCTTAGGAGCA---GCTGGAAGCACAATG---GGCGCGGCGTCA---
ATAACGCTGACGGTACAG---GCCAGACAG---TTATTG---TCTGGCATAGTGCAGCAGCAAAGC---
AATTTG---CTGAGG---GCTATAGAGGCTCAACAGCAACTGTTGAGACTCACTGTCTGGGGCATT
>AB098330
TTCTATTGTAATACATCAGGCCTGTTCAATGGCACCTGGAAGCCAAACAGCACAGAGTCAAACAACACAACG-
----

CCAAATGACACTATAACTCTTACATGCAGAATAAAGCAAATTATAAATATGTGGCAGAAAGTAGGACAAGCAA
TGTATGCCCCTCCCATTCAAGGAGTAATAAGGTGTGAATCAAAC---
ATTACAGGACTACTATTAACAAGAGATGGAGGGATTAACAGTATA----------------------------
----

AATGAAACCTTCAGACCTGGAGGAGGAAATATGAGGGACAATTGGAGAAGTGAATTATATAAGTATAAAGTAG
TAAAAATTGAACCACTA---GGAGTAGCACCCTCCAGGGCAAAGAGAAGAGTGGTG-----------------
----------------------GAGAGAGAAAAAAGAGCA---GTTGGA---ATAGGAGCT---------
GTCTTCCTTGGGTTCTTAGGAGCA---GCTGGAAGCACTATG---GGCGCGGCGTCA---
ATAACGCTGACGGCACAG---GCCAGACAA---TTATTG---TCTGGTATAGTGCAACAGCAAAGC---
AATTTG---CTGAGG---GCTATAAAGGCTCAACAGCATATGTTGAAGCTCACGGTCTGGGGCATT
>GU367412
TTCTATTGTGATACATCAGGCCTGTTTAATAGTACTTGGAATGACACTGCCACCAATCAGACTTTTGAT----
----

GCAAATAGCAATATAACTCTTCAATGTAAAATAAGACAAATTGTACGTATGTGGCAGAGAATAGGACAAGCAT
TGTATGCCCCTCCCATTCAAGGAGTAATAAGCTGTAAATCAAAC---
ATTACAGGGCTGCTACTAACAAGAGATGGT---ATGAATGAGAGT----------------------------
----

AGTGAGACGTTCAGACCTCAAGGAGGAAATATGAAGGACAATTGGAGAAGTGAATTATATAAATATAAAGTAG
TAAAAATTGAACCACTA---GGAATAGCACCCACCAGGGCAAAGAGAAGAGTGGTG-----------------
----------------------GAGAGAGAAAAAAGAGCA---GTTGGA---CTGGGAGCT---------
GTCTTCTTTGGGTTCTTGGGAGCA---GCAGGAAGTACTATG---GGCGCGGCGTCA---
ATAACGCTGACGGCACAG---GCCAGACAA---TTATTG---TCTGGTATAGTACAACAGCAGAGC---
AATCTG---CTGAGG---GCTATAGAGGCTCAACAGCAACTGTTGAAACTCACAGTCTGGGGCATT
>FJ647148
TTCTATTGTAATACATCAGGTCTGTTTAGTAGCAGGTGGTTCACTAATGGCACTAACAGCACGGAGTCAAAT-
----

GGCACAGGCAATATAACTCTCCAATGCAGGATAAAGCAAATTATAAATATGTGGCAGAGAGTAGGACAAGCAA
CGTACACCCCTCCCATCCAAGGAGAAATAAGGTGTAGATCAAAC---
ATTACAGGACTACTATTAACAAGAGATGGTGGGATTAACACAACA----------------------------
----

GAGGAAATCTTCAGACCTGGAGGGGGAAATATGAAGGACAATTGGAGAAGTGAATTATATAAGTATAAAGTAG
TAAAAATTGAACCACTA---GGAGTAGCACCATCCAAGGCAAAGAGAAGAGTGGTG-----------------
----------------------GGAAGAGAAAAAAGAGCA---GTTGGA---CTGGGAGCT---------
GTATTCATTGGGTTCTTGGGAGCA---GCAGGAAGCACTATG---GGCGCGGCGTCA---
GTGACGCTGACGGTACAG---GCCAGACAA---TTATTG---TCTGGCATAGTGCAGCAGCAAAGC---
AATTTG---CTGAGG---GCTATAGAGGCTCAACAGCATCTGTTGAAACTCACAGTCTGGGGCATT
>KT183312

```
TTCTATTGTGACACATCAGGTCTGTTTAATGGCAGTTGGAGTGAAAATAGCACTTGGAATAGCACTATGGAG-
-------------------------------------------------------------------------
----------------------------
GCAAATAGCACAATTATACTCCCATGTAGAATAAAGCAAATTATAAATATGTGGCAGAGAACAGGACGAGCAA
TGTATGCCCCTCCCATCCAAGGAACAATACAGTGTACATCAAAC---
ATCACAGGATTAATATTAACAAGAGATGGTGGTGAGAAAAACAAT----------------------------
-------------------------------------------------------------------------
----------
AGCACTGAAGAAATCTTCAGACCTGGAGGAGGAGATATGAGGGACAATTGGAGGAGTGAATTATATAAATATA
AAGTAGTAAAAATTGAACCACTA---GGAGTAGCACCCACCAGGGCAAAGAGAAGAGTGGTG-----------
----------------------------GAGAGAGAAAAAAGAGCA---GTTACA---CTAGGAGCT------
---GTATTCATTGGGTTCTTAGGAGCA---GCAGGAAGCACTATG---GGCGCGGCGTCA---
GTGACGCTGACGGTACAG---GCCAGACAA---TTATTG---TCTGGCATAGTGCAACAGCAAAGC---
AATTTG---CTGAGG---GCTATAGAGGCTCAACAGCATATGTTGAAACTCACGGTCTGGGGCATT
>KC863311
TTCTATTGTAATACAACAGACCTGTTTAAGGGTACTTGGCATCCTGATAATAAAACTTGGAGTGATCCTTGGA
ATGCCAGC-----------------------------------------------------------------
-ATGAAGTCAAATAAC---------
ACATCAAATGTCAATATAACTATCCTATGCAAGATAAAGCAAATTGTAAGGATGTGGCAGAGAGTAGAACAAG
CAATGTATGCCCCTCCCATCCAAGGAGTAATAAGCTGTTCATCAAAC---
ATTACAGGACTACTATTAACAAGCGATGGTGGTAGGGATAATAGCAATAATACTGGTAAGGATAATAGCAATA
AAAAT--------------------------------------------------------------------
-------------
AATACTGAAACCTTCAGACCTGGAGGAGGAAATATGAGGGACAATTGGAGAAGTGAATTATATAAGTATAAAG
TAGTAAAAATTGAACCACTA---GGAGTAGCACCTACCCCGGCAAGGAGAAGAGTGGTG--------------
-------------------------CAGAGAGAAAAAAGAGCA---GTTGGC---CTAGGAGCT---------
GTCTTCATTGGGTTCTTAGGAGCA---GCAGGAAGCACTATG---GGCGCGGCGTCA---
GTGACGCTGACGGTACAG---GCCAGACAA---TTATTG---ACTGGTATAGTGCGACAGCAAAGC---
AATTTG---CTGAAG---GCTATAGAGGCTCAACAACATCTGTTGAGACTCACAGTCTGGGGCATT
>HM623589
TTCTACTGCAACACATCAAGCTTGTTTAATAGGACATTTCCGGCCAACTACACGTATAATAGGACTGCTGACA
GCACA--------------------------------------------------------------------
-------------------------
CAGGGGAATACAACTATAACTATCCCATGCAGAATAAAACAAATTATAAACATGTGGCAGAGAGTAGGACAAG
CAATGTATGCCCCTCCCATTGAAGGAGTAATAAGATGTGAATCAAAC---
ATTACAGGAATACTATTGACAAGAGATGGTGGGAATGAGACAAAT----------------------------
-------------------------------------------------------------------------
----------------
GTAGAAGTCTTCAGACCAGAGGGAGGAAATATGAGGGACAATTGGAGAAGTGAATTATATAATTACAAGGTAG
TAAGAATTGAACCACTA---GGAGTAGCACCCACCAGGGCAAGGAGGAGAGTGGTG-----------------
----------------------GAGAGAGAAAAAAGAGCA---GTTGGA---CTGGGAGCT---------
GTCTTCCTTGGGTTCTTAGGAGCA---GCAGGAAGCACTATG---GGCGCGGCGTCA---
ATAACGCTGACGGTACAG---GCCAGGCAA---TTATTG---GCTGGCATAGTGCAACAGCAAAGC---
AATTTG---CTGCAA---GCTATAGAGGCTCAACAGCATCTGTTGAAACTCACTGTCTGGGGCATT
>MH705163
TTCTATTGCAATACAACAGCCCTGTTTAATAGCACGTGGGAGAATGAGAATGACACGGCCAACATACAGAAGA
AT-----------------------------------------------------------------------
-------------GGCACG------
GAGTCAAATGACACTATAACACTCCCATGCAGAATAAAACAAATTATAAACATGTGGCAGAGAGTAGGACGAG
CAATGTATGCCCCTCCCATTGCAGGAGTAATAAAGTGTACATCAACC---
ATTACAGGAATAATATTGACAAGAGATGGTGGGGATAGTGGGAACAAT-------------------------
-------------------------------------------------------------------------
----------
ACCAGGGAGGAGATCTTCAGACCTGGAGGAGGAGATATGAGGGACAATTGGAGAAGTGAATTATATAAGTATA
AAGTAGTAAAACTTGAACCACTA---GGAGTAGCACCCACCAAGGCAAGGAGAAGAGTGGTG-----------
----------------------------GAGAGAGAAAAAAGAGCA---GTTGGA---CTGGGAGCT------
---GTCTTCCTTGGGTTCTTGGGAGCA---GCAGGAAGCACTATG---GGCGCAGCGTCA---
ATAACGCTGACGGTACAA---GCCAGACAA---TTATTG---TCTGGCATAGTGCAACAGCAAGGC---
AATTTG---CTGAAG---GCTATAGAGGCTCAACAGCATCTGTTGAAACTCACGGTCTGGGGCATT
>AF286241
```

TTCTATTGTAATACAACAAGCCTGTTTAATAGCACATGGAAGAATGGCGCCACCATACAGGAGAATAGCACGG
AG---

ACAAATGGAATTATGACTCTCCCATGCAGAATAAAACAAATTGTAGACATGTGGCAGGAAGTAGGACAAGCAA
TGTATGCCCCTCCCATTGCAGGAGTAATATATTGTACATCAAAC---
ATTACAGGAATAATATTGACAAGAGATGGTGGGAGCAGTAACACC----------------------------
----

AATAGTGAGATCTTTAGGCCTGGAGGAGGAGATATGAGGGACAATTGGAGAAGTGAATTATATAAGTATAAAG
TAGTAAAAATTGAACCACTA---GGAGTAGCACCCTCCAGGGCAAAGAGAAGAGTGGTG--------------
-------------------------GAGAGAGAAAAAAGAGCA---GTGGGA---ATAGGAGCT---------
GTTTTCCTTGGGTTCTTGGGAGCT---GCAGGAAGCACTATG---GGCGCGGCGTCA---
ATAACGCTGACGGTACAG---GCCAGACAG---TTATTA---TCTGGCATAGTGCAACAGCAAAGC---
AATTTG---CTGAAG---GCTATAGAGGCTCAACAGCATCTGTTGAAACTCACAGTCTGGGGCATT
>AF286238
TTCTATTGCAATACAACAGGCCTGTTTAATAGCACATGGAAAATGGCACTAACAAACAGAATTACACGGAG-
----

TCAAATGACACTATAACGCTCCAATGCAGAATAAAACAAATTATAAACATGTGGCAGAGAGTAGGCCGAGCAA
TGTATGCCCCTCCCATTGCAGGAGTAATAAAGTGTACATCAAAC---
ATTACAGGAATGATATTGACAAGAGACGGTGGGAAGAACAGTATT----------------------------
----

AATGAGACCTTCAGACCTGGAGGAGGAGATATGAGGGACAATTGGAGAAGTGAATTATATAAGTATAAAGTAG
TAAAAATTGAACCACTA---GGAATAGCACCCACAGAGGCAAGGAGAAGAGTGGTG-----------------
----------------------CAGAGAGAAAAAAGAGCA---GTTGGA---TTGGGAGCT---------
GTTTTCCTTGGGTTCTTGGGAGCA---GCAGGAAGCACTATG---GGCGCGGCGTCA---
ATAACGCTGACGGTACAG---GCCAGACAA---TTATTG---ACTGGCATAGTGCAACAGCAAAGC---
AATTTG---CTGAAG---GCTATAGAGGCTCAACAACAGATGTTGAGGCTCACAGTCTGGGGCATT
>AF286237
TTCTATTGCAATACAACAGGCCTGTTTAATGGTACGTGGTGGAACAATGGTACGTGGAACGGGCCCTACACAC
CTAAT--

AACACCAATGGAAGTATAATCCTCCCATGCAGAATAAAACAAATTATAAACATGTGGCAGAGAGTAGGAAGAG
CAATGTATGCCCCTCCCATTGCAGGAATAATAAAGTGTACATCAAAC---
ATTACAGGAATAATATTGACAAGAGATGGTGGTAACAATGGGACT----------------------------
----

AATGAGACCTTCAGACCTGGAGGAGGAGATATGAGGGACAATTGGAGAAGTGAATTATATAAATATAAAGTAG
TAAAACTTGAACCACTA---GGAGTAGCACCTACCAGGGCAAAAAGAAGAGTGGTG-----------------
----------------------GAGAGAGAAAAAAGAGCA---GTTGGA---CTGGGAGCT---------
GTCTTCCTTGGGTTCTTGGGAGCA---GCAGGAAGCACTATG---GGCGCGGCGTCA---
CTAACGCTGACGGTACAG---GCCAGACAA---TTATTG---TCTGGTATAGTGCAACAGCAAAGC---
AATTTG---CTGCAG---GCTATAGAAGCTCAACAGCATCTGTTGAAACTCACAGTCTGGGGCATT
>AY521629
TTCTATTGCAATACATCAGGTCTGTTTAATAGTACTTGGAATGACACGGCCATCAATGACACTATCAACGTGC
AG---

AATTCAACTTACAATATAACTCTCCCATGCAGAATAAGACAAATTGTACATATGTGGCAGAGAGTAGGACAAG
CAATGTATGCCCCTCCCATTCAAGGGGTAATAAACTGTACATCAAAC---
ATTACAGGGCTACTGTTAACAAGGGATGGTGGGGATAATAGTAGT----------------------------
----

ACAAATGAGACATTCAGACCTGGAGGAGGAAATATGAAGGACAATTGGAGAAGTGAATTATATAAATATAAAG
TAGTAAAAATTGAACCACTA---GGAGTAGCACCCACCAAGGCAAAGAGGAGAGTGGTG--------------
-------------------------GAGAGAGAAAAAAGAGCA---GTTGGA---CTAGGAGCT---------
GTCTTCCTTGGGTTCTTAGGAGCA---GCAGGAAGTACTATG---GGCGCGGCGTCA---
ATGACGCTGACGGTACAG---GCCAGACAA---TTATTG---TCTGGCATAGTACAACAGCAGAGC---
AATCTG---CTGAGG---GCTATAGAGGCTCAACAGCACATGTTGAAACTCACAGTCTGGGGCATT
>AY521631

TTCTATTGCAATACATCAGGCCTGTTTAATAGTACTTGGTATAATAGTACTTGGAATAACACTGACAGCACAC
AGAAA--
-------------------TCA---
AATGGCACGGAGAAAATAACTCTCCCATGCAGAATAAAACAAGTTATAAAAATGTGGCAAAGAGTAGGACAAG
CAATGTATGCCCCTCCCATTCAAGGAGTAATAAAATGTGTATCAAAC---
ATTACAGGGATACTGCTAACAAGAGATGGGGGAACCTTAATAGC-----------------------------
----

TCATTTGAGACGTTCAGACCTGGAGGAGGAAATATGAAGGACAATTGGAGAAGTGAATTATATAAATATAAAG
TAGTAAAAATTGAACCACTA---GGAGTAGCACCCACCAAGGCAAAGAGAAGAGTGGTG--------------
-------------------------GAGAGAGAAAAAAGAGCG---GTCGGA---CTGGGAGCT---------
GTCTTCCTTGGGTTCTTAGGAGCA---GCAGGAAGTACTATG---GGCGCGGCGTCA---
ATAACGCTGACGGCACAG---GCCAGACAA---TTATTG---TCTGGTATAGTACAACAGCAGAGC---
AATCTG---CTGAGG---GCTATAGAGGCTCAACAGCAAATGTTGAAACTCACGGTCTGGGGCATT
>AY521630
TTCTATTGCAATACATCAGGTCTGTTTAATAGTACTTGGAATAGCACTACCATCAAGCAGAATTTAACTAACA
AT---

GACACGGGGGTAATAAATCTCCCATGCAGAATAAGACAAATTGTACATATGTGGCAGAGAGTAGGACAAGCAA
TGTATGCCCCTCCCATTAAAGGGGTAATAAACTGTACATCAAAC---
ATTACAGGGCTACTGCTAACAAGGGATGGTGGGGATAATAATAGT----------------------------
----

ACAGATGAGAAGTTCAGACCTGGAGGAGGAGATATGAGGGACAATTGGAGAAGTGAATTATATAAATATAAAG
TAGTAAAAATTGAACCACTA---GGAGTAGCACCCACCAGGGCAAAGAGAAGAGTGGTG--------------
-------------------------CAGAGAGAAAAAAGAGCA---GTTGGA---CTAGGAGCT---------
GTCTTCTTTGGGTTCTTAGGAGCA---GCAGGAAGTACTATG---GGCGCGGCGTCA---
ATAACGCTGACGGCACAG---GCCAGACAA---TTATTG---TCTGGCATAGTACAACAGCAGAGC---
AATCTG---CTGAGG---GCTATAGAGGCTCAACAGCACTTGTTGAAACTCACAGTCTGGGGCATT
>AM000053
TTCTATTGCAATACATCAGGCCTATTTAATAGCAGTTGGGCGCCCAACGGCAGTTGGATCAACAACATTGGCA
GT---

AGGGAAAATGACACTATGACTCTGCCATGCAGAATAAAACAAATTATAAATATGTGGCAGAGAGTAGAACAAG
CAATGTATGCCCCTCCTATCAAAGGAAACATAACTTGTGTATCAAAC---
ATTACAGGACTACTGTTAACAAGAGATGGTGGAACTAATGAAACT----------------------------
----

AGGGAGAATGAGACCTTCAGACCTATAGGAGGAGAGATGAGAGACAATTGGAGAAGTGAATTATATAAGTATA
AAGTAGTAAAAATTGAACCACTA---GGAGTAGCACCCACCAAGGCAAAGAGAAGAGTGGTGGAG--------
----------------------------GGGAGAGAAAAAAGAGCA---GTTGGA---ATGGGAGCT------
---TTCTTCCTTGGGTTCTTAGGAGCA---GCAGGAAGCACTATG---GGCGCGGCGTCA---
ATAACGCTGACGGTACAG---GCCAGACAA---TTATTG---GCTGGTATAGTGCAACAGCAAAGC---
AATTTG---CTGAAG---GCTATAGAGGCTCAACAACATCTGTTGAGACTCACAGTCTGGGGCATT
>AM000054
TTCTATTGTAATACATCAAGCCTGTTTAATAGCTCTTGGCCCATTAACAGCACTACCAGCAATAACAGCACTG
CC---

AGTAATGAGACCATAATTCTGCCATGTAGAATAATACAAATTATAAATATGTGGCAGAGACTAGGACAGGCAA
TGTATGCCCCTCCCATCAAAGGATTAATAAATTGTGTATCAAAC---
ATTACAGGACTACTGTTGACAAGGGATGGTGGAGATAGGAACAGT----------------------------
----

AGTTATGAGACCTTCAGACCTATAGGAGGAAATATGAAGGACAATTGGAGAAGTGAACTGTATAAGTATAAAG
TAGTAAAAATTGAACCACTA---GGAGTAGCACCTACCAAGGCAAGGAGGAGAGTGGTG--------------
-------------------------GAGAGAGAAAAAAGAGCA---GTTGGA---ATAGGAGCT---------
TTCTTCCTTGGGATCTTAGGAGCA---GCAGGAAGCACTATG---GGCGCGGCGTCA---
ATAACGCTGACGGTACAA---GCCAGGCAA---GTATTG---TCTGGTATAGTGCAACAGCAGAGC---
AATTTG---CTGAAG---GCTATAGAGGCTCAACAACATTTGTTGAAACTCACAGTCTGGGGCATC
>FJ388892

TTCTATTGCAATACAACAGACCTGTTTAATAGCACTTGGTACACTAATGACACTGGCACAAATAGCACAGGGG
CCAATGAA---
-------------------AAT---
GGGGCCAATGAAAATATAACTTTACCATGCAGAATAAAGCAAATTATAAATATGTGGCAGAGAGTAGGGCAAG
CAATGTATGCCCCTCCTATCAAAGGAAGTATAAGGTGTGAATCAAAC---
ATTACAGGACTACTACTAACAAGAGATGGTGGAAGTGGAAATGAT----------------------------
----

AGCTACAATGAGACCTTCAGACCTATAGGAGGAAATATGAGGGATAATTGGAGAAGTGAACTATATAAGTATA
AAGTAGTAGAAATTGAACCAATA---GGAGTAGCACCTACCAAGGCAAGGAGAAGAGTGGTG-----------
----------------------------GGGAGAGAAAAAAGAGCA---ATTGGA---CTGGGAGCT------
---GCCTTCCTTGGGTTCTTAGGAGCA---GCAGGAAGCACTATG---GGCGCGGCGTCA---
ATAACGCTGACGGTACAG---GCCAGACAA---TTATTG---TCTGGCATAGTGCAACAGCAAAGC---
AATTTG---CTGAGG---GCTATAGAGGCTCAGCAGCATCTGCTGAAACTCACAGTCTGGGGCATT
>JF683763
TTCTATTGCAATACAACAGACCTGTTCAGTAGCACTTGGGACGAAGATGACACTGACATCACTCTCGCCCAG-
----

GACAATGGAACTATAACTCTGCCATGCAGAATAAAGCAAATTATAAATATGTGGCAGAGAGTAGGACAAGCAA
TGTATGCCCCTCCTATCAAAGGAAGTATAAGGTGTGAATCAAAC---
ATTACAGGACTACTACTAACAAGAGATGGTGGAGCTAATAGCAGT----------------------------
----

AATGAGACCTTCAGACCTATAGGAGGAGATATGAGAGACAATTGGAGAAGTGAACTATATAAATATAAAGTGG
TACAAATTGAACCAATA---GGAATAGCACCTACCAGGGCAAGGAGAAGAGTGGTG-----------------
----------------------GAGAGAGAAAAAAGAGCA---ATTGGA---CTGGGAGCT---------
GCCTTCCTTGGGTTCTTAGGAGCA---GCAGGAAGTACTATG---GGCGCGGCGTCA---
CTGACGCTGACGGTACAG---GCCAGAAAA---TTATTG---TCTGGCATAGTGCAACAGCAAAAC---
AATTTG---CTGAGG---GCTATAGAGGCTCAGCAGCATCTGCTGAAACTCACGGTCTGGGGCATT
>JF683798
TTCTATTGCGATACAACAAACCTGTTCAATAGCACTTGGGACGGAAATAGCACTGTCACAAATATCACACAG-
----

ACCAATGAAAATATAACTTTACCATGCAAAATAAGGCAAATTATAAGGATGTGGCAGAGAGTAGGACAAGCAA
TGTATGCCCCTCCTATCAAAGGAATTATAAGGTGTGAATCAAAC---
ATTACAGGACTACTGCTAACAAGAGATGGTGGAAATAAAACCAAT----------------------------
----

AGCAACAATGAGACCTTCAGACCTATAGGAGGAGATATGAGGGACAATTGGAGAAGTGAACTATATAAGTATA
AAGTAGTAAAAGTGGAACCAATA---GGAGTAGCACCTACCAGAGCAAGGAGAAGAGTGGTG-----------
----------------------------GAGAGAGAAAAAAGAGCA---ATAATTGGACTGGGAGCT------
---GCCTTCATTGGGTTCTTAGGAGCA---GCAGGAAGCACTATG---GGCGCGGCGTCA---
ATAACGCTGACGGTACAG---GCCAGACAA---TTATTG---TCTGGCATAGTGCAACAGCAAAGC---
AATTTG---CTGAGG---GCTATAGAGGCTCAGCAGCATCTGCTGAAACTCACGGTCTGGGGCATT
>EF545108
TTCTATTGCAATACAACAGGCCTGTTCAATAATGCCAGCAACAATGGTACAAAC-------------------
----

CGCACAGACCCTATAATTCTCCCATGCAGAATAAAGCAGATTATAAATATGTGGCAAAGAGTAGGGCAGGCAA
TGTATGCTCCTCCTATCAAAGGAATCATAAGGTGTGAATCAAAC---
ATTACAGGACTACTATTAACAAGAGATGGTGGGAATAAAAATGGAAGC-------------------------
----

AACGGGACCGAGACCTTCAGACCTATAGGAGGAGATATGAGGGACAATTGGAGAAGTGAATTATATAAGTATA
AAGTAGTAAAAATTGAACCAATA---GGAGTAGCACCTACCAGGGCAAAGAGAAGAGTGGTG-----------
----------------------------GAGAGAGAAAAGAGAGCA---ATTGGA---CTGGGAGCT------
---GCCTTCCTTGGGTTCTTAGGAGCA---GCAGGAAGTACTATG---GGCGCGGCGTCA---
ATAACGCTGACGGTACAG---GCCAGACAA---TTACTG---TCTGGCATAGTACAACAGCAAAGC---
AATTTG---CTGAGG---GCTATAGAGGCTCAGCAGCATCTGCTGAAGCTCACGGTCTGGGGCATT
>GU481566

TTCTATTGCAATACAACAGCCCTGTTCAATAGCACTTGGGATGAAAATAGCACTGTCACAAATAGCACAACG-
----

GCCAATGAAAATATAACTCTGCCATGCAGAATAAAGCAAATTATAAATATGTGGCAGAGAGTAGGACAAGCAA
TGTATGCCCCTCCTATCGCAGGAATTATAAGGTGTGAATCAAAC---
ATTACAGGACTACTAATAACAAGAGATGGTGGAACTAATAGCAGC----------------------------
----

AATGAGACTGAGACCTTCAGACCTACAGGAGGAAATATGAGGGACAATTGGAGAAGTGAACTATATAAGTATA
AAGTAGTACAAATTGAACCAATA---GGAGTAGCACCTACCAAGGCAAAGAGAAGAGTGGTG-----------
----------------------------GAGAGAGAAAAAAGAGCA---ATTGGA---CTGGGAGCC------
---GCCTTCCTTGGGTTCTTAGGAGCA---GCAGGAAGTACTATG---GGCGCGGCGTCA---
CTGACGCTGACGGTACAG---GCCAGACAA---TTATTG---TCTGGCATAGTACAACAGCAAAGC---
AATTTG---CTGAGG---GCTATAGAGGCTCAGCAGCATCTGCTGAAACTCACGGTCTGGGGCATT
>GU481615
TTCTATTGCAATACAACAGAACTGTTCAATAGCACTTGGAACACAAGCAGCACTGTCACAAATAGCACACAG-
----

GCCAATGAAACTATAAGTCTACCATGCAAAATAAAGCAAATTATAAGGATGTGGCAGAGAGTAGGACAAGCAA
TGTATGCCCCTCCTATCAACGGAACTATAAGGTGTGAATCAAAC---
ATTACAGGACTACTACTAACAAGAGATGGTGGAGGTGAAAATAAT----------------------------
----

AGCAGCAATGAGACCTTCAGACCTGCAGGAGGAGATATGAAGGACAATTGGAGAAGTGAACTATATAAGTATA
AAGTAGTAGAAATTGAACCAATA---GGATTAGCACCTACCAAGGCAAGGAGAAGAGTGGTG-----------
----------------------------GAGAGAGAAAAAAGAGCA---ATTGGA---CTGGGAGCT------
---GCCTTCCTTGGGTTCTTAGGAGCA---GCAGGAAGTACTATG---GGCGCGGCGTCA---
TTGACGCTGACGGTACAG---GCCAGACAA---TTATTG---TCTGGCATAGTGCAACAGCAAAGC---
AATTTG---CTGAGG---GCTATAGAGGCTCAGCAGCATCTGCTGAAACTCACAGTCTGGGGCATT
>HQ896489
TTCTATTGCAATACAACAGACCTGTTCACAAATAGCACTCTCACAGATAACACAGAG----------------
----

ACCAATGAAACTATAACTCTGCCATGCAGAATAAAGCAAATCATAAATATGTGGCAGAGAGTAGGACAAGCAA
TGTATGCCCCTCCTATCAGAGGAAGTATAAGGTGTAATTCAAAC---
ATTACAGGACTACTGCTAATAAGAGATGGTGGAGCTAATAGCAGC----------------------------
----

AATGAGACCTTCAGACCTATAGGAGGAAATATGAGGGACAATTGGAGAAGTGAACTATATAAGTATAAAGTAG
TAAAAATTGAACCAATA---GGAGTAGCACCTACCAAGGCAAAGAGAAGAGTGGTG-----------------
----------------------GAGAGAGAAAAAAGAGCA---ATTGGA---CTGGGAGCC---------
GCCTTCCTTGGGTTCTTAGGAACA---GCAGGAAGCACTATG---GGCGCGGCGTCA---
CTGACGCTGACGGTACAA---GCCAGACAA---TTATTG---TCTGGCATAGTGCAACAGCAAAGC---
AATTTG---CTGAGG---GCTATAGAGGCTCAGCAACATCTGCTGAAACTCACGGTCTGGGGCATT
>JQ292897
TTCTATTGCGATACAACAGCCCTGTTCAATAGCACTTGGGAAAATGGCACTGTTATAAATAGCACACAG----
----

GCCAATGAGACTATAACTCTACCATGCAGAATAAAGCAAATTATAAATATGTGGCAGAGAGTAGGACGAGCAA
TGTATGCCCCTCCTATCAGAGGAATTATAAGGTGTGAATCAAAC---
ATTACAGGACTACTACTAACAAGAGATGGTGGAGGTGGAAATAAT----------------------------
----

AGCAGCAATGAGACCTTCAGACCTATAGGAGGAAATATGGAGGACAATTGGAGAAGTGAACTATATAAGTATA
AAGTAGTAGAAATTGAACCAATA---GGAGTAGCACCTACCAAGGCAAAGAGAAGAGTGGTG-----------
----------------------------GAGAGGGAAAAAAGAGCA---ATTGGA---CTGGGAGCT------
---GCCTTCCTTGGGTTCTTGGGAGCA---GCAGGAAGCACTATG---GGCGCGGCGTCA---
CTAACGCTGACGGTACAG---GCCAGACAA---TTATTG---TCTGGCATAGTGCAACAGCAAAGC---
AATTTG---CTGAGG---GCTATAGAGGCTCAGCAGCATCTGCTGAAACTCACGGTCTGGGGCATT
>JQ292899

```
TTCTATTGCAATACAACAGACCTGTTCAATTGGGACGGAAATGGCAATGTTACAAACAGCACACAG-------
-------------------------------------------------------------------------
----------------------------
GCCGATGGAAATATAACTCTACCATGCAGAATAAAGCAAATTATAAATATGTGGCAGAGAGTAGGACAAGCAA
TGTATGCCCCTCCTATCAAAGGAAATATAACGTGTGTATCAAAC---
ATTACAGGACTACTATTAACAAGAGATGGTGGAGGTGGAATTAAT----------------------------
-------------------------------------------------------------------------
----------
AGTAGCAATGAGACCTTCAGACCTATAGGAGGAGATATGAGGGACAATTGGAGAAGTGAACTATATAAGTATA
AAGTAGTAAAAATTGAACCAATA---GGAGTAGCACCTACCAAGGCAAGGAGAAGAGTGGTG-----------
----------------------------GAGAGAGAAAAAAGAGCA---ATTGGA---CTGGGAGCT------
---GCCTTCCTTGGGTTCTTGGGAGCA---GCAGGAAGTACTATG---GGCGCGGCGTCA---
ATAACGCTGACGGTACAG---GCCAGACAA---TTATTG---TCTGGCATAGTGCAACAGCAAAGC---
AATTTG---CTGAGG---GCTATAGAGGCTCAGCAGCATATGCTGAAACTCACGGTCTGGGGCATT
>HQ385841
TTCTATTGCTATACAACAAAACTGTTCAATAGCACTTGGAACGGAAATGACACTGTTACAAATAGCACACAG-
-------------------------------------------------------------------------
----------------------------
GCCAATGAAAATATAACTATACCATGCAGAATAAAGCAAATTATAAATATGTGGCAGAGAGTAGGACAAGCAA
TGTATGCCCCTCCTATCAAAGGAAATATAAGCTGTACATCAAAC---
ATTACAGGACTATTACTAACAAGAGATGGTGGAACTAATAGCAGT----------------------------
-------------------------------------------------------------------------
----------------
AATGAGACCTTCAGACCTATAGGAGGAGATATGAGGGACAATTGGAGAAGTGAACTATATAAGTATAAAGTAG
TACAAATTGAACCAATA---GGAGTAGCACCTACCAGGGCAAAGAGAAGAGTGGTG-----------------
----------------------GAGAGAGAAAAAAGAGCA---ATTGGA---CTGGGAGCT---------
GCCTTCCTTGGGTTCTTGGGAGCA---GCAGGAAGTACTATG---GGCGCGGCGTCA---
ATAACGCTGACGGTACAG---GCCAGACAA---TTATTG---TCTGGCATAGTACAACAGCAAAGC---
AATTTG---CTGAGG---GCTATAGAGGCTCAGCAGCATCTGCTGAAACTCACGGTCTGGGGCATT
>HQ385843
TTCTATTGCAATACAACAGCCCTGTTCAATAGTACTTGGCACGAAAATAGCACTGATACAAATAGCACACAG-
-------------------------------------------------------------------------
----------------------------
GACAATGAAACTATAACTCTACCATGCAGAATAAAGCAAATTATAAATATGTGGCAGAGAGTAGGACAAGCAA
TGTATGCCCCTCCTATCGAAGGAAATATAAGCTGTACATCAAAC---
ATTACAGGACTACTACTAACAAGGGATGGTGGAGCTAATAGCAGC----------------------------
-------------------------------------------------------------------------
----------------
AATGAGACCTTCAGACCTATAGGAGGAGATATGAGGGACAATTGGAGAAGTGAACTATATAAGTATAAAGTAG
TACAAATTGAACCAATA---GGAGTAGCACCTACCAAGGCAAAGAGAAGAGTGGTG-----------------
----------------------GAGAGAGAAAAAAGAGCA---ATTGGA---CTGGGAGCT---------
GCCTTCCTTGGGTTCTTGGGAGCA---GCAGGAAGCACTATG---GGCGCGGCGTCA---
ATAACGCTGACGGTACAG---GCCAGACAA---TTATTG---TCTGGCATAGTGCAACAGCAAAGC---
AATTTG---CTGAGG---GCTATAGAGGCTCAGCAGCATCTGCTGAAACTCACGGTCTGGGGCATT
>HQ385846
TTCTATTGCAATACAACAGAACTGTTCAATAGCACTTGGGGCAATGGCACTGTCACAAATAGCACAGGG----
-------------------------------------------------------------------------
----------------------------
GACAATGAAAATATAATTCTACCATGCAGAATAAAGCAAATCATAAGGATGTGGCAGAGAGTAGGACAAGCAA
TGTATGCCCCTCCTATCAAAGGAATTATAAAGTGTAAATCAAAC---
ATTACAGGACTACTACTAACAAGAGATGGTGGAGGTGGAAATAGCAGC-------------------------
-------------------------------------------------------------------------
-------
AATGAGACCAGTGAAACCTTCAGGCCTATAGGAGGAGATATGAGGGACAATTGGAGAAGTGAACTATACAAGT
ATAAAGTAGTACAAATTGAACCAATA---GGAGTAGCACCTACCAGGGCAAAGAGAAGAGTGGTG--------
-------------------------------GAGAGAGAAAAAAGAGCA---ATTGGA---CTGGGAGCT---
------GCCTTCCTTGGGTTCTTAGGAGCA---GCAGGAAGCACTATG---GGCGCGGCGTCA---
ATAACGCTGACGGTACAG---GCCAGACAA---TTATTG---TCTGGCATAGTGCAACAGCAAAGC---
AATTTG---CTGAGA---GCTATAGAGGCTCAGCAGCATCTGCTGAAACTCACGGTCTGGGGCATT
>GU481434
```

TTCTATTGCTATACAACAAACCTGTTCAATAGCACTTGGGGCGAAAATAGCACTGATATAAATAGCACACAG-
----

GCCAATGAAAATATAACTCTACCATGCAGAATAAAGCAAATTATAAATATGTGGCAGAGAGTAGGACAAGCAA
TGTATGCCCCTCCTATCAAAGGAAGTATAATGTGTACATCAAAC---
ATTACAGGGCTATTACTAACAAGAGATGGTGGAGGTGGAGCTAAT----------------------------
----

AGCAGCAATGAGACCTTCAGACCTATAGGAGGAAATATGAGGGACAATTGGAGAAGTGAACTATATAAGTATA
AAGTAGTAAAAATTGAACCAATA---GGAGTAGCACCTACCACGGCAAGGAGAAGAGTGGTG----------
----------------------------GAGAGAGAAAAAAGGGCA---ATTGGA---CTGGGAGCT------
---GCCTTCCTTGGGTTCTTGGGAGCA---GCAGGAAGCACTATG---GGCGCGGCGTCA---
CTAACGCTGACGGTACAG---GCCAGACAA---TTATTG---TCTGGCATAGTGCAGCAGCAAAGC---
AATTTG---CTGAAG---GCTATAGAGGCTCAGCAGCATCTGCTGAAACTCACGGTCTGGGGCATT
>GU481486
TTCTATTGCAATACAACAGACCTGTTCAATAGCACTTGGAACGAAACTGACATTGATGCACAGGCCAATGAA-
----

GTCAATGAAACTATAACTCTACCATGCAGAATAAAGCAAATTATAAATATGTGGCAGAGAGTAGGGCAAGCAA
TGTATGCCCCTCCTATCAAAGGAAGTATAAGGTGTGAATCAAAC---
ATTACAGGACTACTACTAACAAGAGATGGTGGAAGACTTAATGGC----------------------------
----

AGCAATGAGACCTTTAGACCTATAGGAGGAGATATGAGGGACAATTGGAGAAGTGAACTATATAAGTATAAAG
TAGTACAAATTGAACCAATA---GGAGTAGCACCTACCAAGGCAAAGAGAAGAGTGGTG--------------
-------------------------GAGAGAGAAAAAAGAGCA---ATTGGA---CTGGGAGCT---------
GCCTTCCTTGGGTTCTTGGGAGCA---GCAGGAAGTACTATG---GGCGCGGCGTCA---
ATAACGCTGACGGTACAG---GCCAGACAA---TTATTG---TCTGGCATAGTGCAACAGCAAAGC---
AATTTG---CTGAGG---GCTATAGAGGCTCAGCAGCATCTGCTGAAACTCACGGTCTGGGGCATT
>GU481644
TTCTATTGCAATACAACAAACCTGTTCAATAGCACTTGGAATGAAACAGACATTGTTACAAATATCACACAG-
----

GGCAATGGAACTATAACTCTACCATGCAGAATAAAGCAAATTATAAATATGTGGCAGAGAGTAGGACAAGCAA
TGTATGCCCCTCCTATCAAAGGAAATATAACGTGTACATCAAAC---
ATTACAGGACTACTACTAACAAGAGATGGTGGAACTAATAGCAGC----------------------------
----

AATGAGACCTTCAGACCTATAGGAGGAGATATGAGGGACAATTGGAGAAGTGAACTATATAAATATAAAGTAG
TACAAATTGAACCAATA---GGAGTAGCACCTACCAGGGCAAGGAGAAGAGTGGTG-----------------
----------------------GAGAGAGAAAAAAGAGCA---ATTGGA---CTGGGAGCT---------
GCCTTCCTTGGGTTCTTGGGAGCA---GCAGGAAGCACTATG---GGCGCGGCGTCA---
CTAACGCTGACGGTACAG---GCCAGACAA---TTACTG---TCTGGCATAGTGCAACAGCAAAGC---
AATTTG---CTGAGG---GCTATAGAGGCTCAGCAGCATCTGCTGAAACTCACGGTCTGGGGCATT
>JQ292891
TTCTATTGCAATACAACAGACCTGTTCAATAGCACTTGGGACAATAGCACTGTCACAAATAGCACAGGG----
----

ACCAATGAAACTATAACTCTGCCATGTAGAATAAAGCAAATTATAAATATGCGGCAGAGAGTAGGACAAGCAA
TGTATGCCCCTCCTATTAGAGGAAGTATAAGGTGTGAATCAAAC---
ATTACAGGACTACTACTAATAAGAGATGGTGGAGCTAATAGCAGC----------------------------
----

AATGAGACCTTCAGACCTATAGGAGGAAATATGAGGGACAATTGGAGAAGTGAACTATATAAGTATAAAGTAG
TAAAAATTGAACCAATA---GGAGTAGCACCTACCAAGGCAAGGAGAAGAGTGGTG-----------------
----------------------GAAAGAGAAAAAAGAGCA---ATTGGA---CTGGGAGCC---------
GCCTTCCTTGGGTTCTTAGGAGCA---GCAGGAAGCACTATG---GGCGCGGCGTCA---
ATGACGCTGACGGTACAG---GCCAGACAA---TTATTG---TCTGGCATAGTGCAACAGCAAAGC---
AATTTG---CTGAGG---GCTATAGAGGCTCAGCAGCATCTGCTGAAACTCACGGTCTGGGGCATT
>HQ385820

TTCTATTGCAATACAACAAACCTGTTCAATAGCACTTGGGACACAAATATCACAGAGGCCAATATCACAGAG-
----

GCCAATGAAACTATAATTCTACCATGCAAAATAAAGCAAATTATAAATATGTGGCAGAGAGTAGGAAAAGCAA
TGTATGCCCCTCCTATCAAAGGAAGTATAAGGTGTGAATCAAAC---
ATTACAGGACTACTACTAACAAGAGATGGTGGAACTAATAGCAGC----------------------------
----

AATAGCAATGAGACCTTCAGACCTATAGGAGGAAATATGAGGGACAATTGGAGAAGTGAACTATATAAATATA
AAGTAGTAAAAATTGAACCAATA---GGAGTAGCACCTACCAAGGCAAAGAGAAGAGTGGTG-----------
----------------------------GAGAGAGAAAAAAGAGCA---ATTGGG---TTGGGAGCT------
---GCCTTCCTTGGGTTCTTAGGAGCA---GCAGGAAGCACTATG---GCCGGCGGGTCA---
ATAACGCTGACGGTACAG---GCCAGACAA---TTATTG---TCTGGCATAGTGCAACAGCAAAGC---
AATTTG---CTGAGG---GCCATAGAGGCTCAGCAACATATGCTGAAACTCACGGTCTGGGGCATT
>JF952018
TTTATTGCAATACAACAGCCCTGTTCAATAGCACTTGGGACAATAGTAGCATTGCCGAAAATAGCACAGAG-
----

GACAATGCAACTATAACTCTACCATGCAGAATAAAGCAAATTATAAATATGTGGCAGAGAGTAGGGCAAGCAA
TGTATGCCCCTCCTATCAAAGGAAGTATAAGGTGTGAATCAAAT---
ATTACAGGACTATTACTAACAAGAGATGGTGGAGATGCCGATGGAAATGGAACT-------------------
----

AATAGCAACAATGAAACCTTCAGACCTACAGGAGGAGATATGAGGAACAATTGGAGAAGTGAACTATATAAAT
ATAAAGTAGTACAAATTGAACCAATA---GGGGTAGCACCTACCACGGCAAAGAGAAGAGTGGTG--------
-------------------------------GAGAGAGAAAAAAGAGCA---ATTGGA---CTGGGAGCT---
------GCCTTCCTTGGGTTCTTAGGAGCA---GCAGGAAGCACTATG---GGCGCGGCGTCA---
ATAACGCTGACGGTACAG---GCCAGACAA---TTATTG---TCTGGCATAGTGCAACAGCAAAGC---
AATTTG---CTGAGG---GCTATAGAGGCTCAGCAGCATCTGCTGAAACTCACGGTCTGGGGCATT
>HQ338112
TTCTATTGCAATACAACAGAACTGTTCAATAGCACTTGGGACAATAGCACTTGGGACAAAAATGACACTAGCA
CA---

AATATCACACAGGTCATAACTCTGCCATGCAGAATAAAACAAATTATAAATATGTGGCAGAGAGTAGGACAAG
CAATGTATGCCCCTCCTATTAAAGGAGTTATGAGGTGTGAATCAAAC---
ATTACAGGACTACTACTAACAAGAGATGGTGGAGCTAATAGCAGC----------------------------
----

AATGAGACCTTCAGACCTATAGGAGGAGATATGAGGGACAATTGGAGAAGTGAACTATATAAGTATAAAGTAG
TAGAAATTGAACCAATA---GGAGTAGCACCTACCAGGGCAAAGAGAAGAGTGGTG-----------------
----------------------GAGAGAGAAAAAAGAGCA---ATTACA---CTGGGAGCT---------
GCCTTCCTTGGGTTCTTAGGAGCA---GCAGGAAGCACTATG---GGCGCGGCGTCA---
TTGACGCTGACGGTACAG---GCCAGACAA---TTATTG---TCTGGCATAGTGCAACAGCAAAGC---
AATTTG---CTGAGG---GCTATAGAGGCTCAGCAGCATCTGCTGAAACTCACGGTCTGGGGCATT
>GU481277
TTCTATTGCAATACAGCAGACCTGTTCAATAGCACTTGGGACACAAATAGCACACAGACCAATAGAAATATAA
CT---

GCCAGTGAAAATATAACTCTACCATGCAGAATAAAGCAAATTATAAATATGTGGCAGAGAGTAGGACAAGCAA
TGTATGCCCCTCCTATCAAAGGAATTATAATGTGTACATCAAAC---
ATTACAGGACTACTCCTAACAAGAGATGGTGGAGGTGAAACTAAT----------------------------
----

AGCAGCAATGAGACCTTCAGACCTATAGGAGGAGACATGAGGGACAATTGGAGAAGTGAACTATATAAATATA
AAGTAGTAAAAATTGAACCAATA---GGGGTAGCACCTACCAGGGCAAAGAGAAGAGTGGTG-----------
----------------------------GAGAGAGAAAAAAGAGCA---ATTGGA---CTGGGAGCT------
---GCCTTCCTTGGGTTCTTGGGAGCG---GCAGGAAGTACTATG---GGCGCGGCGTCA---
ATAACGCTGACGGTACAG---GCCAGACAA---TTATTG---TCTGGCATAGTACAACAGCAAAGC---
AATTTG---CTGAGG---GCTATAGAGGCTCAGCAGCATCTGTTGAAGCTCACGGTCTGGGGCATT
>GU481544

TTCTATTGCAATACAACAAAACTGTTCAATAGCTCTTGGGGCGAAAATGGCACAAATAGCATCGAG-------
----

ACCAATGAAACTATAATTCTACCATGCAGAATAAAGCAAATTATAAATATGTGGCAGAGAGTAGGACAAGCAA
TGTATGCCCCTCCTATCAAAGGAATTATAAAGTGTGAATCAAAC---
ATTACAGGACTACTACTAATCAGAGATGGTGGAACTAACAGCAGT----------------------------
----

AATGAGACCTTCAGACCTATAGGAGGAGATATGAGGGACAATTGGAGAAGTGAACTATATAAGTATAAAGTAG
TAAAAATTGAACCAATA---GGAGTAGCACCTACCAAGGCAAGGAGAAGAGTGGTG-----------------
----------------------GAGAGAGAAAAAAGAGCA---ATTGGA---CTGGGAGCT---------
GCCTTCCTTGGGTTCTTAGGAGCA---GCAGGAAGCACTATG---GGCGCGGCATCA---
ATAACGCTGACGGTACAG---GCCAGACAA---TTATTG---TCTGGCATAGTGCAACAGCAAAGC---
AATTTG---CTGAGG---GCTATAGAGGCTCAGCAACATCTGCTGAAACTCACGGTCTGGGGCATT
>GU481661
TTCTATTGCGATACAACAAACCTGTTCAATAGCACTTGGGAAGAAAAAAGCACTGTTACAAATAGC-------
----

ACCAATGACACTATAACTCTACCATGCAGAATAAAGCAAATTATAAATATGTGGCAGAGAGTAGGACAAGCAA
TGTATGCCCCTCCTATCCAAGGAAATATAAGTTGTGAATCAAAC---
ATTACAGGACTACTACTAACAAGAGATGGTGGAAGTGAAAATAAT----------------------------
----

GAGACCTTCAGACCTATAGGAGGAGATATGAGGGACAATTGGAGAAGTGAATTATACAAGTATAAAGTAGTAA
AAATTGAACCAATA---GGAGTAGCACCTACCAGGGCAAGGAGAATAGTGGTG--------------------
-------------------AAGAGAGAAAAAAGAGCA---ATTGGA---CTGGGAGTT---------
GCCTTCCTTGGGTTCTTAGGAGCA---GCAGGAAGCACTATG---GGCGCAGCGTCA---
CTAACGCTGACGGTACAG---GCCAGACAA---TTATTG---TCTGGCATAGTGCAACAGCAAAGC---
AATTTG---CTGAGG---GCTATAGAGGCTCAGCAACATCTGCTGAAACTCACGGTCTGGGGCATT
>KF716491
TTCTATTGCAATACAACAAACCTGTTCGATAGCACTTGGAATGACACTGACACAAATAGCACTGACACAAATA
GCACAGGG---

GCCAATGAAATTATAACTCTACCATGCAGAATAAGGCAAATTATAAATATGTGGCAGAGAGTAGGACAAGCAA
TGTATGCCCCTCCTATCAAAGGAGTTATAAGGTGTGAATCAAAC---
ATTACAGGACTACTACTAACAAGAGATGGTGGAACTAATAGCAGC----------------------------
----

AATGAGAATGAGATCTTCAGACCTATAGGAGGAGATATGAGGGACAATTGGAGAAGTGAACTATATAAGTATA
AAGTAGTAGAAGTTGAACCAATA---GGAGTAGCGCCTACCAAGGCAAGGAGAAGAGTGGTG-----------
----------------------------GAGAGAGAAAAAAGAGCA---ATTGGA---CTGGGAGCT------
---GCCTTCCTTGGGTTCTTAGGAGCA---GCAGGAAGTACTATG---GGCGCGGCGTCA---
ATAACGCTGACGGTACAG---GCCAGACAA---TTATTG---TCTGGCATAGTGCAACAGCAAAGC---
AATTTG---CTGAAA---GCTATAGAGGCTCAGCAGCATCTGCTGAAACTCACGGTCTGGGGCATT
>KF716492
TTCTATTGCAATACAACAGAACTGTTCAATAGCACTTGGAAAGAAAATAGCACTATCACAAATAACACAACA-
----

GCCAATGACACTATAACTCTACCATGCAGAATAAAGCAAATTATAAATATGTGGCAGAGAGTAGGACAAGCAA
TGTATGCCCCTCCTATCAAAGGAAGTATAAGGTGTAAATCAAAC---
ATTACAGGACTACTACTAACAAGAGATGGTGGAAGTGAACCTAAT----------------------------
----

AGCAGTGAGGAAATCTTCAGACCTATAGGAGGAAATATGAGGGACAATTGGAGAAGTGAACTATATAAGTATA
AAGTAGTAAAAATTGAACCAATA---GGAGTAGCACCTACCAAGGCAAAGAGAAGAGTGGTG-----------
----------------------------GAGAGAGAAAAAAGAGCAATAATTGGA---CTGGGAGCT------
---GCCTTCCTTGGGTTCTTAGGAGCA---GCAGGAAGCACTATG---GGCGCGGCGTCA---
CTGACGCTGACGGTACAG---GCCAGACAA---TTATTG---TCTGGCATAGTGCAACAGCAAAGC---
AATTTG---CTGAGG---GCTATAGAGGCTCAGCAGCATCTGCTGAAACTCACGGTCTGGGGCATT
>FJ864679

TTCTATTGTAATACAACAGAACTGTTCAATAGCACTTGGTACAAAAATGGCACTAACACAACTAGCACACAG-
----

ACCAGTGAAAACATAACTCTGCCATGCAGAATAAAGCAAATTGTAAGGATGTGGCAGAGAGTAGGACAAGCAA
TGTATGCCCCTCCTATCAGTGGAATTATAAAGTGTGAATCAAAC---
ATTACAGGATTAATACTAACAAGAGATGGTGGAGATAATAGTAGC----------------------------
----

AGCAATGAGACCTTCAGACCTGAAGGAGGAGACATAAGGGACAATTGGAGAAATGAACTATATAAGTATAAAG
TAGTAGAAATTGAACCAATA---GGAGTAGCACCTACTGAGGCAAGGAGAAGAGTGGTG--------------
-------------------------CAGAGAGAAAAAAGAGCA---ATTGGA---CTGGGAGCT---------
GCCTTCATTGGGTTCTTAGGAGCA---GCAGGAAGCACTATG---GGCGCAGCGTCA---
CTGACGCTGACGGTACAG---GCCAGAAAA---TTATTG---TCTGGCATAGTGCAACAGCAAAGC---
AATTTG---CTGAGG---GCTATAGAGGCTCAGCAGCATCTGCTGAAACTCACGGTCTGGGGCATT
>JQ292893
TTCTATTGCAATACCACAGACCTGTTCAATAGCACTTGGGACAAAAATGACACTATCACAAATACCCCAGAGG
TC---

AATGGAACTACTATAACTCTGCCATGCAGAATAAAGCAAATTGTAAATATGTGGCAGAGAGTAGGACAAACAA
TGTATGCCCCTCCTATCAAAGGAAGTATAAGGTGTGAATCAAAC---
ATTACAGGACTACTGCTAATAAGAGATGGTGGAGAAACTAATAGC----------------------------
----

AGCAGTGAGACCTTCAGACCTGTAGGAGGAGATATGAGGGATAATTGGAGAAGTGAACTATATAAGTATAAAG
TAGTAAAAATTGAACCAATA---GGAGTAGCACCTACCAGGGCAAAGAGAAGAGTGGTG--------------
-------------------------GAGAGAGAAAAAAGAGCA---ATTGGA---CTGGGAGCC---------
GCCTTCCTTGGGTTCTTAGGAGCA---GCAGGAAGTACTATG---GGCGCGGCGTCA---
CTGACGCTGACGGTACAG---GCCAGACAA---TTATTG---TCTGGCATAGTGCAACAGCAAAGC---
AATTTG---CTGAGG---GCTATAGAGGCTCAGCAGCATCTGCTGAAACTCACGGTCTGGGGCATT
>JF430898
TTCTATTGCAATACAACAGACCTGTTCAATAGCACTTGGAGGAATGACACTGTCGCAAATATCACG-------
----

GCCAATGGAACTATAACTCTGCCATGCAGAATAAAGCAAATTATAAATATGTGGCAGAGAGTAGGACAAGCAA
TGTATGCCCCTCCTATCGAAGGAAATATAAGTTGTGAATCAAAC---
ATTACAGGGCTACTACTAATAAGAGATGGTGGAACTGATGACAGC----------------------------
----

AATGAGACTGAGTCCTTCAGACCCATAGGAGGAGATATGAGGGACAATTGGAGAAGTGAACTATATAAATATA
AAGTGGTACAAATTGAACCAATA---GGAGTAGCACCTACCAAGGCAAGGAGAAGAGTGGTG-----------
----------------------------GAGAGAGAAAAAAGAGCA---ATTGGA---CTGGGAGCT------
---GCCTTCCTTGGGTTCTTAGGAGCA---GCAGGAAGCACTATG---GGCGCGGCGTCA---
TTGACGCTGACGGTACAG---GCCAGACAA---TTATTG---TCTGGCATAGTGCAACAGCAAAAC---
AATTTG---CTGAGG---GCTATAGAGGCTCAGCAGCATCTGCTGAAACTCACGGTCTGGGGCATT
>HQ834967
TTTTATTGCAATACAACAGGCCTGTTCAATAGCACTTGGGGCAATAGCACTGACGCAAAT-------------
----

AGCAATGAAAATATAACTCTACCATGCAGAATAAAGCAAATTATAAATATGTGGCAGAGAGTAGGACAAGCAA
TGTATGCCCCTCCTATCAGCGGAAATATAAGTTGTGTATCAAAC---
GTTACAGGACTACTACTAGTAAGAGATGGTGGAGGTGGAAATAGT----------------------------
----

AGCAACAATGAGACCTTCAGACCTATAGGAGGAGATATGAGGGACAATTGGAAAAGTGAACTATATAAGTATA
AAGTAGTAGAAATTGAACCAATA---GGAGTAGCACCTACCAAGGCAAAGAGAAGGGTGGTG-----------
----------------------------CAGAGAGAAAAAAGAGCA---ATTGGAGGACTGGGAGCT------
---GCCCTCCTTGGATTCTTAGGAGCA---GCAGGAAGCACTATG---GGCGCGGCGTCA---
CTGACGCTGACGGTACAG---GCCAGACAA---TTATTG---TCTGGCATAGTGCAACAGCAAAGC---
AATTTG---CTGAGG---GCTATAGAGGCTCAGCAGCATCTGCTGAAACTCACCGTCTGGGGCATT
>JX500696

TTCTATTGTAATACAACAAAACTGTTCAATAGCACTTGGGACAAAAATAGTACTTGGAATGAAAATAGCACT-
----

GCCAATGAAAATATAACTCTGCCATGCAGGATAAAGCAAATTATAAATATGTGGCAGAGAGTAGGACAAGCAA
TGTATGCCCCTCCTATCAAAGGAGTTATAATGTGTAAATCAAAC---
ATTACAGGTCTACTCCTAACAAGAGATGGTGGAGGTGGAGCTAAT----------------------------
----

AGCAGCAATGAGACTTTCAGACCTATAGGAGGAAATATGAGGGACAATTGGAGAAGTGAACTATATAAGTATA
AAGTAGTACAAATTGAACCAATA---GGAGTAGCACCTACCAGGGCAAAGAGAAGAGTGGTG-----------
----------------------------GAGAGAGAAAAAAGAGCA---ATTGGA---CTGGGAGCC------
---GCTTTCCTTGGGTTCTTAGGAGCA---GCAGGAAGCACTATG---GGCGCGGCGTCA---
CTGACGCTGACGGTACAG---GCCAGACAA---TTATTG---TCTGGCATAGTGCAACAGCAAAGC---
AATTTG---CTGAGG---GCTATAGAGGCTCAGCAGCAGCTGCTGAAACTCACGGTCTGGGGCATT
>JX500695
TTCTATTGCAATACAACAGGCCTGTTCAATAGCACTTGGGACACAAATGGCACTGTCACAAGTAGCACAAATG
GA---

ACAAATAAAACTATAACTCTGCCATGCAGAATAAAACAAATTATAAATATGTGGCAGAGAGTAGGACAAGCAA
TGTACGCCCCTCCTATCAAAGGAAGTATAAGGTGTGAATCAAAC---
ATTACAGGTCTACTACTAATAAGAGATGGTGAAAGGAATAGCAGC----------------------------
----

AATGAGACCTTTAGACCTATAGGAGGAAATATGAGGGACAATTGGAGGAGTGAACTATATAAGTATAAAGTAG
TACAAATTGAACCAATA---GGACTAGCACCTACCAGGGCAAAGAGAAGAGTGGTG-----------------
----------------------GAGAGAGAAAAAAGAGCA---ATTGGA---CTGGGAGCC---------
GCCCTCCTTGGGTTCTTAGGAGCA---GCAGGAAGCACTATG---GGCGCGGCGTCA---
ATGACGCTGACGGTACAG---GCCAGACAA---TTATTG---TCTGGCATAGTGCAACAGCAAAGC---
AATTTG---CTGAGG---GCTATAGAGGCTCAGCAGCATCTGCTGAAACTCACGGTCTGGGGCATT
>JX500694
TTCTATTGCAATACAACAGAACTGTTCAATAGCACTTGGGACGGAAATGGCACTGCCACAAATAAC-------
----

ACAGAGGACAATGTAACTCTGCCATGCAGAATAAAGCAAATTATAAATATGTGGCAGAGAGTAGGACAAGCAA
TGTATGCCCCTCCTATCAGAGGAAGTATAAAGTGTGTATCAAAC---
ATTACAGGACTACTACTAATAAGAGATGGTGGAGGTGGAAATAAT----------------------------
----

AGCAACAATGAGACCTTCAGACCTATAGGAGGAAATATGAGGGACAATTGGAGAAGTGAACTATATAAGTATA
AGGTAGTACAAATTGAACCAATA---GGAATAGCACCTACCACAGCAAAAAGAAGAGTGGTG-----------
----------------------------GAGAGAGAAAAAAGAGCA---ATTGGA---CTGGGAGCT------
---GCCTTCCTTGGGTTCTTAGGAGCA---GCAGGAAGTACTATG---GGCGCGGCGTCA---
ATAACGCTGACGGTACAG---GCCAGACAA---TTATTG---TCTGGTATAGTACAACAGCAAAGC---
AATTTG---CTGAGG---GCTATAGAGGCTCAGCAGCATCTGCTGAAACTCACGGTCTGGGGCATT
>MH603967
TTCTATTGCAATACAACACAACTGTTCGATAGCACGTGGGAAGAGAATGCCAATATCACAAATACAAATAATA
CACAG--

AATAATGAAAATATAACTCTACCATGCAGAATAAAGCAAATTATAAGGATGTGGCAGGGGGTAGGACAAGCAA
TGTATGCCCCTCCTATAAGAGGAGAAATAAGGTGTGAATCAAAC---
ATTACAGGATTACTACTAACAAGAGATGGTGGAGGTACAAATAGC----------------------------
----

AGCAACAATGAGACCTTCAGACCTATAGGAGGAGACATGAGGGACAATTGGAGAAGTGAACTATATAAGTATA
AAGTAGTACAAATTGAACCCATA---GGAGTAGCACCTACCAAGGCAAAGAGAAGAGTGGTG-----------
----------------------------GAGAGAGAAAAAAGAGCA---ATTGGA---CTGGGAGCT------
---GCCTTCCTTGGGTTCTTAGGAGCA---GCAGGAAGCACTATG---GGCGCGGCGTCA---
ATAACGCTGACGGTACAG---GCCAGACAA---TTATTG---TCTGGCATAGTGCAACAGCAAAGC---
AATTTG---CTGAGG---GCTATAGAGGCTCAGCAGCATCTGCTGAAACTCACGGTCTGGGGCATT
>MH604120

TTTTATTGTAATACAACAGACCTGTTCAATAGCGCTTGGGAAAATGGCAATGCTACAAATATCACACAGGCCA
AT---

GACAATGAAACTATAATTCTACCATGCAGAATAAGGCAAATTATAAATATGTGGCAAAGAGTAGGACAGGCAA
TGTATGCCCCTCCTATCAGAGGAAATATAACGTGTATATCAAAT---
ATTACAGGACTACTACTAACAAGAGATGGTGGAGGTGGAAATAAT----------------------------
----

AGCAACAATGAGACCTTCAGACCTATAGGAGGAGATATGAGGGACAATTGGAGAAGTGAACTATATAAATATA
AAGTAGTACAAATTGAACCAATA---GGAATAGCACCTAACAGGGCAAAGAGAAGAGTGGTG-----------
----------------------------GAGAGAGAAAAAAGAGCA---ATTGGA---CTGGGAGCT------
---GCCTTCCTTGGGTTCTTGGGAGCA---GCAGGAAGTACTATG---GGCGCGGCGTCG---
CTAACGCTGACGGTACAG---GCCAGAAAA---TTATTG---TCTGGCATAGTGCAACAGCAAAAC---
AATTTG---CTGAAG---GCTATAGAGGCTCAGCAGCATCTGCTGAGACTCACGGTCTGGGGCATT
>MH604308
TTCTATTGCAATACAACAGACCTGTTCAATAGCACTTGGGACGGAAATAGCACTAGAGATGAAAATGGCAATG
ATACAGAT---
-------------AGCACA------
CAGAACAATAGACCTATAACTCTGCCATGCAGAATAAAGCAAATTATAAATATGTGGCAGAGAGTAGGACAAG
CAATGTATGCCCCTCCTATAAAAGGAATTATAAGGTGTACATCAAAC---
ATTACGGGACTACTACTAATAAGAGATGGTGGAGGCGGAGCTAAT----------------------------
----

AGCAGCAATGAGACCTTCAGACCTATAGGAGGAGATATAAGGGACAATTGGAGAAGTGAACTATTTAAGTATA
AAGTAGTACAAATTGAACCAATA---GGAGTAGCACCTACCAGGGCAAAGAGAAGAGTGGTG-----------
----------------------------GAGAGAGAAAAAAGAGCA---ATTGGA---CTGGGAGCT------
---GCCTTCCTTGGGTTCTTGGGAGCA---GCAGGAAGTACTATG---GGCGCGGCGTCA---
ATAACGCTGACGGTACAG---GCCAGACAA---TTATTG---TCTGGCATAGTACAACAGCAAAGC---
AATTTG---CTGAGG---GCTATAGAGGCTCAGCAGCATCTGCTGAAACTCACGGTCTGGGGCATT
>MH604056
TTCTATTGCAATACAACAGCCCTGTTCAATTGGAACGGCAATGAAACTGTTACAAATAGCACACAG-------
----

GCCAATGAAACTATAACTTTACCATGCAGAATAAAGCAAATTATAAATATGTGGCAGAGAGTAGGACAAGCAA
TGTATGCCCCTCCTATCAAAGGAAGTATAAGGTGTACATCAAAC---
ATTACAGGACTGCTGCTAACAAGAGATGGTGGAACTAACAGCAGC----------------------------
----

AACAGCACTGAGATCTTCAGACCTATAGGAGGAAATATGAAGGACAATTGGAGAAGTGAACTATATAAATATA
AAGTAGTAAAAATTGAACCAATA---GGAGTAGCACCTACCAAGGCAAGGAGAAGAGTGGTG-----------
----------------------------GAGAGAGAAAAAAGAGCA---ATTGGA---CTGGGAGCT------
---GCCTTCCTTGGGTTCTTGGGAGCA---GCAGGAAGCACTATG---GGCGCGGCGTCA---
ATAACGCTGACGGTACAG---GCCAGACAA---TTATTG---TCCGGCATAGTGCAACAGCAAAGC---
AATTTG---CTGAGG---GCTATAGAGGCTCAGCAGCATCTGCTGAAACTCACGGTCTGGGGCATT
>MH604408
TTCTATTGCAATACAACAAACCTGTTCAATAGCACGTGGAACGAAACTGACAGTGTTACAAATAGCACACAGG
CC---

AATAATGAAACTATAATTTTACCATGCAGGATAAAGCAAATTATAAGGACGTGGCAGAGAGTAGGACAAGCAA
TGTATGCCCCTCCTATCAAAGGAAGGATAAGGTGTGAATCAAAC---
ATTACAGGACTACTACTAACAAGAGATGGTGGAGGTAAAAGTAATAATGAGACA-------------------
----

AGTAATAATGAGACCTTCAGACCTATAGGAGGAGATATGAGGGACAATTGGAGAAGTGAACTATATAAATATA
AAGTAGTAGAAATTGAACCAATA---GGAGTAGCACCTACCAAGGCAAAGAGAAGAGTGGTG-----------
----------------------------GAGAGACAAAAAAGAGCA---ATTGGA---CTGGGAGCT------
---GCCTTCCTTGGGTTCTTAGGAGCA---GCAGGAAGCACTATG---GGCGCGGCGTCA---
CTAACGCTGACGGTACAG---GCCAGACAA---TTATTG---TCTGGCATAGTGCAACAGCAAAGC---
AATTTG---CTGAGG---GCTATAGAGGCTCAGCAGCATCTGCTGAAACTCACGGTCTGGGGCATT
>MH604109

TTCTACTGCAGTACAGCAGCCCTGTTCAATAGCACTTGGGACGGAAATACCACTATTGCAAATAACACACAG-
----

AACAATGAAAATATAACTATACCATGCAGAATAAAGCAAATTATCAATATGTGGCAGAGAGTAGGACAAGCAA
TGTATGCCCCTCCTATACAAGGAGTTATAACTTGTGAATCAAAC---
ATTACAGGACTACTACTAACAAGAGATGGTGGAGTTAATAGCAGC----------------------------
----

AATGAAACCTTCAGGCCTATAGGAGGAGATATGAGGGACAATTGGAGAAGTGAATTATATAAGTATAAAGTAG
TAAAAGTTGAACCAATA---GGAGTAGCACCTACCACGGCAAGGAGAAGAGTGGTG-----------------
----------------------GGGAGAGAAAAAAGAGCA---ATTGGA---CTGGGAGCT---------
GCCCTCCTTGGGTTCTTGGGAGCA---GCAGGAAGCACTATG---GGCGCGGCGTCA---
ATAACGCTGACGGTACAG---GCCAGACAA---TTATTG---TCTGGCATAGTGCAACAGCAAAGC---
AATTTG---CTAAGG---GCCATAGAGGCTCAGCAGCATCTGCTGAAACTCACGGTCTGGGGCATT
>MH604508
TTCTATTGCTATACAACAGGGCTGTTCAATAGCACTTGGGACGAAAATAGCACTAGCACAAATATCACACAG-
----

AAAAATGAAAATATAACTATACCATGCAGAATAAAGCAAATTATAAATATGTGGCAGAGAGTAGGACAAGCAA
TGTATGCCCCTCCTATCAGAGGAAATATAAGATGTGTATCAAAC---
ATTACAGGACTACTACTAACAAGAGATGGTGGAGATGGAGATAAT----------------------------
----

AGCAGTGAGACCTTCAGACCTATAGGAGGAGATATGAGGGACAATTGGAGAAGTGAACTATATAAATATAAAG
TAGTACAAATTGAACCCATA---GGAGTAGCACCTACCAAGGCAAGGAGAAGAGTGGTG--------------
-------------------------GAGAGAGAAAAAAGAGCA---ATTGGA---CTGGGAGCT---------
GCCTTCCTTGGGTTCTTAGGAGCG---GCAGGAAGCACTATG---GGCGCGGCGTCA---
ATAACGCTGACGGTACAG---GCCAGACAA---TTATTG---TCTGGCATAGTGCAACAGCAAAGC---
AATTTG---CTGAGG---GCTATAGAGGCTCAGCAGCATCTGCTGAAACTCACGGTCTGGGGCATT
>MH604524
TTCTATTGCAATACAACAAACCTGTTCAATAGTGCTTGGAAAAATGGCAGTGCTACAAATAGCACACAAGCC-
----

AATGGAACAACTATAACTCTACCATGCAGAATAAAGCAAATTATAAATATGTGGCAGAGAGTAGGACAAGCAA
TGTATGCCCCTCCTATCAAAGGAAGTATAAGGTGTGAATCAAAC---
ATTACAGGACTACTACTAACAAGAGATGGTGGAGGTGAAACTAATAGC-------------------------
----

AGCGATGAAACCGAAACCTTCAGACCTATAGGAGGAGATATGAGGAATAATTGGAGAAGTGAATTATATAAGT
ATAAAGTAGTAAAAATTGAACCAATA---GGAGTAGCACCTACCAGGGCAAGGAGAAGAGTGGTG--------
-------------------------------GAGAGAGAAAAAAGAGCA---GTTGGA---CTGGGAGCT---
------GCCTTCCTTGGGTTCTTGGGAGCA---GCAGGAAGCACTATG---GGCGCGGCGTCA---
ATGACGCTGACGGTACAG---GCCAGAAAA---TTATTG---TCTGGCATAGTGCAACAGCAAAGC---
AATTTG---CTGAGG---GCTATAGAGGCTCAGCAGCATCTGCTGAAACTCACGGTCTGGGGCATT
>MH604541
TTCTATTGCAATACAACAAAGCTGTTCAATAGCACTTGGGAAAATAGCACTTGGGAAAATGGCACTGTTGCAA
AT---
-------------AGCACA------
GGGGACAATGACACTATAACTATACCATGCAGAATAAAGCAAATTATAAATATGTGGCAAAGAGTAGGACAAG
CAATGTATGCCCCTCCTATCAAAGGAATTATAAGGTGTGAATCAAAC---
ATTACAGGACTACTACTAACAAGAGATGGTGGAGGTGGAACTAAT----------------------------
----

AGCAGCAATGAGACCTTCAGACCTATAGGAGGAGACATGAGGGACAATTGGAGAAGTGAACTATATAAGTATA
AAGTAGTAAAAATTGAACCAATA---GGAGTAGCACCTACCAGGGCAAAGAGAAGAGTGGTG-----------
----------------------------GAGAGAGAAAAAAGGGCA---TTTGGA---CTGGGAGCT------
---GCCTTCCTTACGTTCTTGGGAGCA---GCAGGAAGCACTATG---GGCGCGGCGTCA---
ATAACGCTGACGGTACAG---GCCAGACAA---TTATTG---TCTGGCATAGTGCAACAGCAAAGC---
AATTTG---CTGAGG---GCTATAGAGGCTCAGCAGCATCTGCTGAAACTCACGGTCTGGGGCATT
>MH330347

TTCTATTGCAATACCACATACCTGTTCAATAGCACTTGGGATGGAAATAGCACTGTAAATAGCACAGGG----
----

GGCGATAGAAATATAACTTTACCATGCAGAATAAAGCAAATTATAAATATGTGGCAGAGAGTAGGACAAGCAA
TGTATGCCCCTCCTATCCGAGGAAACATAAGTTGTGAATCAAAC---
ATCACAGGACTACTCCTAATAAGAGATGGTGGAGGTGGAAATAAT----------------------------
----

AAGACTAATGAGACCTTCAGACCTATAGGAGGAGATATGAGGGACAATTGGAGAAGTGAACTATATAAGTATA
AAGTAGTAAAAATTGAACCAATA---GGGGTAGCACCTACCCTGGCCAGGAGAAGAGTGGTG-----------
----------------------------GAGAGAGAAAAAAGAGCAATAATTGGA---CTGGGAGCT------
---GCCTTCATTGGGTTCTTAGGAGCA---GCAGGAAGCACTATG---GGCGCGGCGTCA---
CTGACGCTGACGGTACAG---GCCAGACAA---TTATTG---TCTGGCATAGTGCAACAGCAAAGC---
AATTTG---CTGAGG---GCTATAGAGGCTCAGCAGCATCTGCTGAAACTCACGGTCTGGGGCATT
>MH330348
TTCTATTGCAATACAACAAACCTGTTCAACATAAATAGCACTGACACAAATATCACACAG-------------
----

GACAATGAAACTATAACTCTACCATGCAGAATAAAGCAAATTATAAATATGTGGCAGAGAGTAGGACAAGCAA
TGTATGCCCCTCCTATCAAAGGAAGTATAATGTGTGAATCAAAC---
ATTACAGGACTACTACTAACAAGAGATGGTGGAGGTGACAGAAAT----------------------------
----

GATACTAATAGTACTGAGACCTTCAGACCTATAGGAGGAGATATGAGGGACAATTGGAGAAGTGAACTATATA
AGTATAAAGTAGTAAAAATTGAACCAATA---GGAGTAGCACCTACCAGGGCAAAGAGAAGAGTGGTG-----
----------------------------------GGGAGAGAAAAAAGAGCA---ATTGGA---
CTGGGAGCC---------GCCTTCCTTGGGTTCTTAGGAGCA---GCAGGAAGCACTATG---
GGCGCGGCGTCA---CTGACGCTGACGGTACAG---GCCAGACAA---TTATTG---
TCTGGCATAGTGCAACAGCAAAGC---AATTTG---CTGAGG---
GCTATAGAGGCTCAGCAGTATATGCTGAAACTCACGGTCTGGGGCATT
>MH330349
TTCTATTGCAATACAACAGCCCTGTTCAATAGCACTTGGAGTGAAAATGGCACTGCCATAAATAGCACAGAG-
----

GCCAATGGAACTATAACTCTGCCATGCAGAATAAGGCAAATTATAAATATGTGGCAGAGAGTAGGACAAGCAA
TGTATGCCCCTCCTATCAGAGGAATTATAAGGTGTGAATCAAAC---
ATTACAGGACTACTGCTAACACGAGATGGTGTAGCTAATAGCAGC----------------------------
----

AATGAGACCTTCAGACCTATAGGAGGAGATATGAGGAACAATTGGAGAAGTGAACTATATAAGTATAAAGTAG
TAAAAATTGAACCAATA---GGAGTAGCACCTACCAGGGCAAAGAGAAGAGTGGTG-----------------
----------------------GAGAGAGAAAAAAGAGCA---ATTGGA---CTGGGAGCC---------
GCCTTCCTTGGGTTCTTAGGAGCA---GCAGGAAGCACTATG---GGCGCGGCGTCA---
CTGACGCTGACGGTACAG---GCCAGACAA---CTCTTG---TCTGGCATAGTGCAACAGCAAAGC---
AATTTG---CTGAGG---GCTATAGAGGCTCAGCAGCATCTGCTGAAACTCACGGTCTGGGGCATT
>MH330350
TTTATTGTAATACAACAGAACTGTTCAATAGTACTTGGGACGGAAATAACACTGTTACAAATAGCACACAG-
----

GACAATGGCACTATAACTCTACCATGCAGAATAAGGCAAATTATAAATATGTGGCAGAGAGTAGGACAAGCAA
TGTATGCCCCTCCTATCAGAGGAAATATAAGTTGTGAATCAAAC---
ATTACAGGACTACTACTAACAAGAGATGGTGGAGTTAATACCAGC----------------------------
----

AATGAGACCTTCAGACCTACAGGAGGAGATATGAGGAACAATTGGAGAAGTGAACTATATAAGTATAAAGTAG
TACAAATTGAACCAATA---GGAGTAGCACCTACCAAGGCAAAGAGAAGAGTGGTG-----------------
----------------------GAGAGAGGAAAAAGGGCA---ATTGGA---CTGGGAGCT---------
GCCTTCCTTGGGTTCTTGGGAGCA---GCAGGAAGTACTATG---GGCGCGGCGTCA---
ATAACGCTGACGGTACAG---GCCAGACAA---TTATTG---TCTGGCATAGTACAACAGCAAAGC---
AATTTG---CTGATG---GCTATAGAGGCTCAGCAGCATCTGCTGAAACTCACGGTCTGGGGCATT

>MH330351
TTCTATTGCAATACAACAAACCTGTTCAATAGCACTTGGGACAAAAATAGTACTGTCACAAATAGCACAAAC-
----

AGCACAGAAACTATAACTCTACCATGCAGAATAAAGCAAATTATAAATATGTGGCAGAGAGTAGGACAAGCAA
TGTATGCCCCTCCTATCAAAGGAAGTATAAGGTGTGAATCAAAC---
ATTACAGGACTAATATTAATAAGAGATGGTGGAAATAATAGCAGC----------------------------
----

AATGAGACCTTCAGACCTATAGGAGGAAATATGAGGGACAATTGGAGAAGTGAACTATATAAATATAAAGTAG
TAGAGATTGAACCAATA---GGAATAGCACCTACCAAGGCAAGAAGGAGAGTGGTG-----------------
----------------------GAGAGAGAAAAAAGAGCA---ATTGGA---CTGGGAGCT---------
GCCTTCCTTGGGTTCTTAGGAGCA---GCAGGAAGCACTATG---GGCGCGGCGTCA---
ATAACGCTGACGGTACAG---GCCAGACAA---TTATTG---TCTGGCATAGTGCAACAGCAAAGC---
AATTTG---CTGAGG---GCTATAGAAGCTCAGCAGCATCTGCTGAAGCTCACGGTCTGGGGCATT
>MH330352
TTCTATTGCAATACAACACAACTGTTCAATAGCACTTGGCCTTGGAATGACACTGTCACAAATAGCACAGAG-
----

ACCAGTGAAAATATAACTCTACCATGCAGAATAAAGCAAATTATAAATATGTGGCAGAGAGTAGGACAAGCAA
TGTATGCCCCTCCTATCAGAGGAAGTATAAGGTGTGAATCAAAC---
ATTACAGGACTACTACTAATAAGAGATGGTGGAACTAATAGCACC----------------------------
----

AATGAGACCTTCAGACCTATAGGAGGAGATATGAGGGACAATTGGAGAAGTGAACTATATAAGTATAAAGTAG
TACAAATTGAACCAATA---GGAGTAGCACCTACCAAGGCAAAGAGAAGAGTGGTG-----------------
----------------------GAGAGAGAAAAAAGAGCA---ATTGGA---CTGGGAGCT---------
GCCTTCCTTGGGTTCTTAGGAGCA---GCAGGAAGCACTATG---GGCGCGGCGTCA---
ATAACGCTGACGGTACAG---GCCAGACAA---TTATTG---TCTGGCATAGTGCAACAGCAAAGC---
AATTTG---CTGAGG---GCTATAGAGGCTCAGCAGCATCTGCTGAAACTCACGGTCTGGGGCATT
>MH330353
TTCTATTGCAATACAACAGACCTGTTCGATAGCACTTGGGATGGAAATAACACAGATAACACTGTCACAAATA
TCACG--

GGCAATGGAACTATGACTCTGCCATGCAGAATAAGGCAAATTGTAAGGATGTGGCAGAGAGTAGGACAAGCAA
TGTATGCCCCTCCTATTGAAGGGAAAATAAGGTGTGAATCAAAC---
ATTACAGGACTACTACTACTAAGAGATGGTGGAATTAATAGCAGC----------------------------
----

AATGAGACCTTCAGACCTATAGGAGGAGATATGAGGGACAATTGGAGAAGTGAGCTATATAAGTATAAAGTAG
TAAAAATTGAACCAATA---GGAGTAGCACCTACCCAGGCAAGGAGAAGAGTGGTG-----------------
----------------------GCGAGAGAAAAAAGAGCA---ATTGGA---TTAGGAGCT---------
GCCTTCCTTGGGTTCTTAGGAGCA---GCAGGAAGCACTATG---GGCGCGGCGTCA---
ATAACGCTGACGGTACAG---GCCAGACAA---TTATTG---TCTGGCATAGTGCAACAGCAAAGC---
AATTTG---CTGAAG---GCTATAGAGGCTCAGCAGCATCTGCTGAAACTCACAGTCTGGGGCATT
>MH330354
TTCTATTGCAATACAACAAGCCTGTTCAATAGCACTTGGGATGAAAATAGCACTGTCACAAACACAGAG----
----

GCCAATGGAACTATAATTCTGCCATGCAGAATAAAGCAAATTATAAATATGTGGCAAAGAGTAGGACAAGCAA
TGTATGCCCCTCCTATCAGAGGAAGGATAAGGTGTGAATCAAAC---
ATTACAGGACTACTAATAACAAGAGATGGTGGAGGTGGAAATAAT----------------------------
----

AGCACCAATGAAACCTTCAGACCTATAGGAGGAGATATGAGGGACAATTGGAGAAGTGAACTATATAAGTATA
AAGTAGTAAAAATTGAACCAATA---GGAGTAGCACCTACCAGGGCAAAGAGAAGAGTGGTG-----------
----------------------------GAGAGAGAAAAAAGAGCA---ATTGGA---CTGGGAGCC------
---GCCTTCCTTGGGTTCTTAGGAGCA---GCAGGAAGCACTATG---GGCGCAGCGTCA---
ATGACGCTGACGGTACAG---GCCAGACAA---TTATTG---TCTGGCATAGTGCAACAGCAAAGC---
AATTTG---CTGAGG---GCTATAGAGGCTCAGCAGCATCTGCTGAAACTCACGGTCTGGGGCATT

>MH330355
TTCTATTGCAATACAACAAGGCTGTTCAATAGCTCTTGGGGCGAAAATAGCACTGTCACAAATAGCACAGAT-
----

GTCAATGGAACTATAATTCTGCCATGCAGAATAAAACAAATTATAAATATGTGGCAGAGAGTAGGACAAGCAA
TGTATGCCCCTCCTATCCCAGGAAATATAACATGTATATCAAAC---
ATTACAGGACTACTACTAGTAAGAGATGGTGGACAGAACAGTAGT----------------------------
----

AACACCAGTGAAACCTTCAGACCCATAGGAGGAGATATGAAGCACAATTGGAGAAGTGAACTATATAAGTATA
AAGTAGTAGAAATTGAACCAATA---GGAGTAGCACCTACCAAGGCAAAGAGAAGAGTGGTG-----------
----------------------------GAGAGAGAAAAAAGAGCAATTATTGGA---CTGGGAGCT------
---GCCTTCATTGGGTTCTTAGGAGCA---GCAGGAAGCACTATG---GGCGCGGCGTCA---
ATAACGCTGACGGTACAG---GCCAGACAA---TTACTG---TCTGGCATAGTGCAACAGCAAAGC---
AATTTG---CTGAGG---GCTATAGAGGCTCAGCAGCATCTGCTGAAACTCACGGTCTGGGGCATT
>MH330356
TTCTATTGCAATACAACAGACCTGTTTAATAGCACTTGGGAGGAAAATAGCACTGTTACAAATAACACAAAT-
----

GCCAATGAAAATATAACTCTGCCATGCAGGATAAAGCAAATTATAAATTTGTGGCAGAGAGTAGGGCGAGCAA
TGTATGCCCCTCCTATCAGAGGAATTATAAGGTGTAACTCAACT---
ATTACAGGACTACTACTAACAAGAGATGGTGGAAATGGAAATAGTAGC-------------------------
----

AACAATGAGACTGAGACCTTCAGACCTATAGGAGGAAATATGAGGGACAATTGGAGAAGTGAACTATATAAGT
ATAAAGTAGTAGAAATTGAACCAATA---GGAGTAGCACCTACCAGGGCAAGGAGAAGAGTGGTG--------
-------------------------------CAGAGAGAAAAAAGAGCA---GTTGGA---CTGGGAGCC---
------GCCTTCCTTGGGTTCTTAGGAGCA---GCAGGAAGCACTATG---GGCGCGGCGTCA---
CTGACGCTGACGGTACAG---GCCAGACAA---TTATTG---TCTGGCATAGTGCAACAGCAAAGT---
AATTTG---CTGAGG---GCTATAGAGGCTCAGCAGCATCTGCTGAAACTCACGGTCTGGGGCATT
>MG902950
TTCTATTGCAATACAACAAACCTGTTCAGTAGCACTTGGAACGACACTATCACAAATTTCACAGAG-------
----

ACCAATGAAACTATAATTCTGCCATGCAGAATAAGGCAATTTATAAAGATGTGGCAGAGAGTAGGACAAGCAA
TGTATGCCCCTCCTATCAAAGGGAGCATAAGGTGTGAATCAAAC---
ATTACAGGACTACTACTAACAAGAGATGGTGGAAATGGAACTACT----------------------------
----

GACAGCAATGAGACCTTCAGACCTATAGGAGGAGATATGAGGGACAATTGGAGAAGTGAACTATATAAGTATA
AAGTAGTACAAATTGAACCAATA---GGAATAGCACCTACCAAGGCAAAGAGAAGAGTGGTG-----------
----------------------------GAGAGAGAAAAAAGAGCA---ATTGGA---CTGGGAGCC------
---GCTTTCCTTGGGTTCTTAGGAGCA---GCAGGAAGTACTATG---GGCGCGGCGTCA---
ATAACGCTGACGGCACAG---GCCAGACAA---TTATTG---TCTGGTATAGTACAACAGCAAAGC---
AATTTG---CTGAGG---GCTATAGAGGCTCAGCAGCATCTGCTGAAACTCACGGTCTGGGGCATT
>MG902951
TTCTATTGCAATACAACAGACCTGTTCAATAGCACTTGGGGAAATAACACAAATAGCACAGGG----------
----

ACCAATGAAACTATAACTCTGCCATGCAGAATAAAGCAAATTATAAATATGTGGCAGAGAGTAGGACAAGCAA
TGTATGCCCCTCCTATCCGAGGAATTATAAAGTGTGAATCAAAC---
ATTACAGGACTACTACTAACAAGAGATGGTGGAGGTGAAGCTAATAAT-------------------------
----

AGCGAGAGCGAGACCTTCAGACCTATAGGAGGAGATATGAGGGACAATTGGAGAAGTGAACTATATAAGTATA
AAGTAGTAGAAATTGAACCAATA---GGAGTAGCACCTACCAGGGCAAAGAGAAGAGTGGTG-----------
----------------------------CAGAGAGAAAAAAGAGCA---ATTGGA---CTGGGAGCC------
---GCCCTCCTTGGGTTCTTAGGAGCA---GCAGGAAGCACTATG---GGCGCGGCGTCA---
ATAACGCTGACGGTACAG---GCCAGACAA---TTATTG---TCTGGTATAGTGCAACAGCAAAGC---
AATTTG---CTGGAT---GCTATAGAGGCTCAGCAGCATCTGCTGAGACTCACGGTCTGGGGCATT

>MH330337
TTCTATTGCAATACAACAGACCTGTTCAATAGCACTTGGGACGAAAATGACACTGTTACAAATAGCACACAG-
----

GCCAATGAAACTATAATTCTACCATGCAGGATAAAGCAAATTATAAATATGTGGCAGAGAGTAGGACAAGCAA
TGTATGCCCCTCCTATCAAAGGAAGTATAAGGTGTGAATCAAAC---
ATTACAGGACTAATACTAACAAGAGATGGTGGAACTAATAGCAGC----------------------------
----

AATGAGACCTTCAGACCTATAGGAGGAAATATGAGGGACAATTGGAGAAGTGAACTATATAAGTATAAAGTAG
TAAAAATTGAACCAATA---GGAGTAGCACCTACCAAGGCAAAGAGAAGAGTGGTG-----------------
----------------------GAAAGAGAAAAAAGAGCA---ATTGGA---CTGGGAGCT---------
GCCTTCCTTGGGTTCTTGGGAGCA---GCAGGAAGTACTATG---GGCGCGGCGTCA---
ATAACGCTGACGGTACAG---GTCAGACAA---TTATTG---TCTGGCATAGTGCAACAGCAAAGC---
AATTTG---CTGAGG---GCTATAGAGGCTCAGCAGCATCTGCTGAAACTCACGGTCTGGGGCATT
>MH330338
TTCTATTGCGATACAACAAAACTGTTCAATAGCACTTGGAACGAAAATGGCACTAGGACAGATACAGATACCA
CA---

CAGGATGAAAATATAACTATACCATGCAGAATAAAGCAAATTATAAATATGTGGCAGAGAGTAGGACAAGCAA
TGTATGCCCCTCCCATCAGAGGAACTATAATGTGTATATCAAAC---
ATTACAGGGCTACTCCTAACAAGAGATGGTGGAGCTAATAACAGC----------------------------
----

AATGAAACCTTCAGACCTATAGGAGGAGATATGAGAGACAATTGGAGAAGTGAACTATATAAGTATAAAGTAG
TAGAAATTGAACCAATA---GGGGTAGCACCTACCAAGGCAAAGAGAAGAGTGGTG-----------------
----------------------GAGAGAGAAAAAAGAGCA---GTTGGA---CTGGGAGTT---------
GCCTTCCTCGGGTTCTTAGGAGCA---GCAGGAAGTACTATG---GGCGCGGCGTCA---
CTGACGCTGACGGTACAG---GCCAGACAA---TTATTG---TCTGGCATAGTGGAACAGCAAAGC---
AATTTG---CTGAGG---GCTATAGAGGCTCAGCAGCATCTGCTGAAACTCACGGTCTGGGGCATT
>MH330339
TTCTATTGCAATACAACAGCACTGTTCAATAGCACTTGGGGAGGAAATAACACACAG----------------
----

GGCAATGAAAGTATAACTCTACCATGCAGAATAAAGCAAATTATAAGGATGTGGCAGAGAGTAGGACAAGCAA
TGTATGCCCCTCCTATCCAAGGAAGGATAAGGTGTGAATCAAAT---
ATTACAGGACTACTATTAACAAGAGATGGTGGAGTAACTAATAGCAGC-------------------------
----

AATGGGACTGAGACCTTCAGACCTACAGGAGGAGATATGAGGGACAATTGGAGAAGTGAACTATATAAGTATA
AAGTGGTAGAAATTGAACCAATA---GGAATAGCACCTACCAGGGCAAGGAGAAGAGTGGTG-----------
----------------------------CAGAGAGAAAAAAGAGCA---ATTACA---CTGGGAGCT------
---GCCTTCCTTGGGTTCTTGGGAGCA---GCAGGAAGTACTATG---GGCGCGGCGTCA---
ATAACGCTGACGGTACAG---GCCAGGCAA---TTATTG---TCTGGTATAGTACAACAGCAAAGC---
AATTTG---CTAAAG---GCTATAGAGGCTCAGCAGCATCTGCTGAAACTCACGGTCTGGGGCATT
>MH330340
TTCTATTGCAATACAACAGACCTGTTCAATAGCATTTGGGAAAACGGCAATGTCACAAATAGCACAGAG----
----

ACCAATGAAACTATAACTCTACAATGCAGAATAAAGCAAATTATAAATATGTGGCAAAGAGTAGGACAAGCAA
TGTATGCCCCTCCTATCAGTGGAAGCATAAGGTGTGAATCAAAC---
ATTACAGGACTAATACTAATAAGAGATGGTGGATATAATAGCAGC----------------------------
----

AATGAGACCTTCAGACCTATAGGAGGAAATATGAAGGACAATTGGAGAAGTGAACTATATAAGTATAAAGTAG
TAAAAATTGAACCAATA---GGAGTAGCACCTACCAGGGCAAAGAGAAGAGTGGTG-----------------
----------------------GAGAGAGAAAAAAGAGCA---ATCGGA---CTGGGAGCT---------
GCCTTCCTTGGGTTCTTAGGAGCA---GCAGGAAGTACTATG---GGCGCGGCGTCA---
CTGACGCTGACGGTACAA---GCCAGACAA---TTATTG---TCTGGCATAGTGCAACAGCAAAGC---
AATTTG---CTGAGG---GCTATAGAGGCTCAGCAACATCTGCTGAAACTCACGGTCTGGGGCATT

>MH330341
TTCTATTGCAATACAACAGCCCTGTTCAATAGCACTTGGCACGAAAATGACACTGTTACAAATAGCACACAG-
----

AACAATGAAACTATAATTCTACCATGCAGAATAAAGCAAATTATAAATATGTGGCAGAGAGTAGGACAAGCAA
TGTATGCCCCTCCGATCAGAGGAAGTATAAGGTGTGAATCAAAC---
ATTACAGGACTACTACTAACAAGAGATGGTGGGGCTAATAGCAGC----------------------------
----

AATGAGACCTTCAGACCTATAGGAGGAGATATGAGGGACAATTGGAGAAGTGAACTATATAAGTATAAAGTAG
TAAAAATTGAACCAATA---GGAGTAGCACCTACCAGGGCAAAGAGAAGAGTGGTG-----------------
----------------------GAAAGAGAAAAAAGAGCA---ATTGGA---CTGGGAGTT---------
GCCTTCCTTGGGTTCTTGGGAGCA---GCAGGAAGCACTATG---GGCGCGGCGTCA---
ATAACGCTGACGGTACAG---GCCAGACAA---TTATTG---TCTGGCATAGTGCAACAGCAAAGC---
AATTTG---CTGAGG---GCTATAGAGGCTCAGCAGCATCTGCTGAAACTCACGGTCTGGGGCATT
>MH330342
TTCTATTGCAATACAACAGCCCTGTTCAATAACACTGACACAAATAGCACAGAG-------------------
----

ACCAGTGGAAATATAACTATACCATGTAGAATAAAGCAAATTATAAGAATGTGGCAGAGAGTAGGACAAGCAA
TGTATGCCCCTCCTATCGAAGGAAATATAACGTGTGTATCAAAC---
ATTACAGGTCTACTACTAACAAGAGATGGTGGGGTAAATAACACC----------------------------
----

AATGAGACCTTCAGACCCATAGGAGGAGATATGAGGGACAATTGGAGAAGTGAACTATATAAGTATAAAGTAG
TAGAAATTGAACCAATA---GGAGTAGCACCTACCAGGGCAAAGAGAAGAGTGGTG-----------------
----------------------GAGAGAGAAAAAAGAGCA---ATTGGA---CTGGGAGCT---------
GCCTTCCTTGGGTTCTTAGGAGCA---GCAGGAAGCACTATG---GGCGCGGCGTCA---
ATGACGCTGACGGTACAG---GCCAGACAA---TTATTG---TCTGGCATAGTGCAGCAGCAAAGC---
AATTTG---CTGCAG---GCTATAGAGGCTCAGCAGCATCTGCTGAGACTCACGGTCTGGGGCATT
>MH330344
TTCTATTGTAATACAACAAAACTGTTCAATAGCACTTGGGACAAAAATAGCACAAGTACAAATATCACACAG-
----

ACAAATGGAAATATAACTCTACCATGCAGAATAAAGCAAATTATAAATATGTGGCAGAGAGTAGGACAAGCAA
TATATGCCCCTCCTATCAAAGGAAATATAATGTGTGTATCAAAC---
ATTACAGGACTACTACTAATAAGAGATGGGGGAGGTGGAAATAAT----------------------------
----

AGCATCAATGAAACTTTCAGACCTATAGGAGGAGATATGAGGGACAATTGGAGAAGTGAACTATATAAGTATA
AAGTAGTACAAATTGAACCAATA---GGGGTAGCACCTACCAGGGCAAGGAGAAGAGTGGTG-----------
----------------------------CAGAGAGAAAAAAGAGCA---ATTGGA---CTGGGAGCC------
---GCCTTCCTCGGGTTCTTAGGAGCA---GCAGGAAGCACTATG---GGCGCAGCGTCA---
CTGACGCTGACGGTACAG---GCCAGACAA---TTATTG---TCTGGCATAGTGCAACAGCAAAGC---
AATTTG---CTGAGG---GCTATAGAGGCTCAGCAGCATCTGCTGAAACTCACGGTCTGGGGCATT
>MH330345
TTCTATTGCAATACAACAGCCCTGTTCAATAGCATTTGGAACGGAAATGACAATGGCACAAATAGTACAGAG-
----

GACACTGACCCTATAATTCTACCATGCAGAATAAAGCAAATTGTAAATATGTGGCAGAGAGTAGGACAAGCAA
TGTATGCCCCTCCTATCCCAGGAAATATAAGTTGTACATCAAAC---
ATTACAGGACTACTACTAACAAGAGATGGA---ATTAATGGCAGC----------------------------
----

AATGAGACCTTCCGACCTATAGGAGGAGACATGAGGGACAATTGGAGAAGTGAACTATATAAGTATAAAGTAG
TAAAAATTGAACCAATA---GGAGTAGCACCTACCAAGGCAAAGAGAAGAGTGGTG-----------------
----------------------GAGAGAGAAAAAAGAGCA---ATTGGA---CTGGGAGCT---------
GCCTTCCTTGGGTTCTTAGGAGCA---GCAGGAAGCACTATG---GGCGCGGCGTCA---
ATAACGCTGACGGTACAG---GCCAGACAA---TTATTG---TCTGGCATAGTGCAACAGCAAAGC---
AATTTG---CTGAGG---GCTATAGAGGCTCAGCAGCATCTGCTGAAACTCACGGTCTGGGGCATT

>MH330346
TTCTATTGCAATACATCAGACCTGTTC------ACACATAATATCACAGGGGCCAATGAAACAGGG-------
----

GCCAATGGAACTATAACTATACCATGCAAAATAAAACAAATTATAAATATGTGGCAGAGAGTAGGACAAGCAA
TGTATGCCCCTCCTATCAGAGGAATTATAAGGTGTGAATCAAAC---
ATTACAGGACTATTACTAACAAGAGATGGTGGAAAG-------------------------------------
----

AGCGAGAATGAGACCTTCAGACCTATAGGGGGAGATATGAGGGACAATTGGAGAAGTGAACTATATAAGTATA
AAGTAGTAGAAATTGGACCAATA---GGAGTAGCACCTACCAGGGCACAGAGGAAAGTGGTG-----------
----------------------------AAGAGAGAAAAAAGAGCAATTATTGGA---CTGGGAGCT------
---GCCTTCCTTGGGTTCTTAGGAGCA---GCAGGAAGCACTATG---GGCGCGGCGTCA---
TTGACGCTGACGGTACAG---GCCAGACAA---TTATTG---TCTGGCATAGTGCAACAGCAAAGC---
AATTTG---CTGAGG---GCTATAGAGGCTCAGCAGCATCTGCTGAAACTCACGGTCTGGGGCATT
>HQ616082
TTCTATTGCAATACAACAAACCTGTTCAATGGCACTTGGTCAAAAAGCACAACGGCCAATGCAACT-------
----

GCCAATGAAACTATAACTCTACCGTGCAGAATAAAGCAAATTATAAATATGTGGCAGAGAGTAGGACAAGCAA
TGTATGCCCCTCCTATCATAGGCAATATAAGCTGTGAATCAAAC---
ATTACAGGACTGCTACTGACAAGAGATGGTGGAACAACTAATAGC----------------------------
----

AGCGATGAGACCTTCAGACCTATAGGAGGAAATATGAGGGACAATTGGAGAAGTGAACTATATAAGTATAAAG
TAGTAATAATTGATCCAATA---GGAGTAGCACCTACCAGGGCAAAGAGAAGAGTGGTG--------------
-------------------------GAGAGGGAAAAAAGAGCA---ATTGGA---CTGGGAGTT---------
GCCTTCCTTGGGTTCTTAGGAGCA---GCAGGAAGCACTATG---GGCGCGGCGTCA---
CTGACGCTGACGGTACAG---GCCAGACAA---TTATTG---TCTGGTATAGTGCAACAGCAAAGC---
AATTTG---CTGAGG---GCTATAGAGGCTCAGCAGCATCTGCTGAAACTCACGGTCTGGGGCATT
>DQ823357
TTCTATTGCAATACAACAGAACTGTTCAATAGCACTTGGGGCGAAAATGGCACTATCACAAATAGCACAGAG-
----

ACCAGTGAAAATATAACTCTACCATGCAGAATAAAGCAAATTATAAATATGTGGCAGAGAGTAGGACAAGCAA
TGTATGCCCCTCCTATCAGAGGAAATATAAGTTGTGTATCAAAC---
ATTACAGGACTACTACTAACAAGAGATGGTGGAACTAATAGCAGC----------------------------
----

ACTGAGACCTTCAGACCTACAGGAGGAGATATGAGGGACAATTGGAGAAGTGAACTATATAAGTATAAAGTAG
TAGAAATTGAACCAATA---GGAGTAGCACCTACCAAGGCAAAGAGAAGAGTGGTG-----------------
----------------------GAGAGAGAAAAAAGAGCA---ATAATTGGACTGGGAGCT---------
GCCTTCCTTGGGTTCTTAAGTGCA---GCAGGAAGCACTATG---GGCGCAGCGTCA---
CTGACGCTGACGGTACAG---GCCAGACAA---TTATTG---TCTGGCATAGTGCAACAGCAAAGC---
AATTTG---CTGAGG---GCTATAGAGGCTCAGCAGCATTTGCTGAGACTCACGGTCTGGGGCATT
>KU749399
TTCTATTGCAATACAACAGGCCTGTTCAATAGTACTTGGGACAATAGTACTTGGGACAAAAATAGCACTGTCA
CAAAT--

GGCACCGAGGACAATATAACTCTACAATGCAAAATAAAGCAAATTATAAGGATGTGGCAGAGAGTAGGACAAG
CAATGTATGCCCCTCCTATCAGAGGAAATATAAAGTGTACATCAAAC---
ATTACAGGACTACTACTAATAAGAGATGGTGGAAATGGAAATAAT----------------------------
----

AGCAACAATGAGACCTTCAGACCGACAGGAGGAGATATGAGAAATAATTGGAGAAGTGAACTATATAAGTATA
AAGTAGTAGAAGTTGAACCAATA---GGAATAGCACCTACCAAGGCAAAGAGAAGAGTGGTG-----------
----------------------------GAGAGAGAAAAAAGAGCAATAGTTGGA---CTGGGAGCT------
---GCCTTCCTTGGGTTCTTAGGAGCA---GCAGGAAGTACTATG---GGCGCGGCGTCA---
ATAACGCTGACGGTACAG---GCCAGACAA---TTACTA---TCTGGCATAGTACAACAGCAGAGC---
AATCTG---CTGAGG---GCTATAGAGGCTCAGCAGCATCTGCTGAAACTCACGGTCTGGGGCATT

>KU749401
TTCTATTGCAATACAACAGACCTGTTCAATAGCACTTGGAACGGCAGCAATAGCACTGCCATGAATAGCTCA-
----

GGCAATGAAAATATAACCATACACTGCAGAATAAAGCAAATTATAAATATGTGGCAGAGAGTAGGACAAGCAA
TGTATGCCCCTCCTATCAGAGGAAATATAACATGTGTATCAAAC---
ATTACAGGGCTACTACTAATAAGAGATGGGGAAATGGAAATAAT-----------------------------
----

AGCAACAATGAGACCTTCAGACCTATAGGAGGAGATATGAGGGATAATTGGAGAAGTGAACTATATAAATATA
AAGTAGTAAAAATTGAACCAATA---GGAGTAGCACCTACCAAGGCAAGGAGAAGAGTGGTG-----------
----------------------------GGGAGAGAAAAAAGAGCA---ATTGGA---CTGGGAGCT------
---GCCTTCCTTGGGTTCTTAGGAGCA---GCAGGAAGCACTATG---GGCGCGGCGTCA---
CTGACGCTGACGGTACAG---GCCAGAAAA---TTGCTA---TCTGGCATAGTGCAACAGCAAAGC---
AATTTG---CTGAGG---GCTATAGAGGCTCAGCAGCAACTGCTGAGACTCACAGTCTGGGGCATT
>KY658681
TTCTATTGCAATACAACAGCCCTGTTCAATAGCACTTGGAACACCACTTGGAACGAAAATAGCACTGTTGAAA
GTAGTACA---

GGGGACAATGAAAATATAACTATACCATGCAGAATAAAGCAAATTATAAATATGTGGCAGAGAGTAGGACAAG
CAATGTATGCCCCTCCTATCAAAGGAATTATAAAGTGTGAATCAAAC---
ATTACAGGACTACTACTGGTAAGAGATGGTGGAAACACTAATAGC----------------------------
----

AGCAATGAGACCTTCAGACCTATAGGAGGAGATATGAGGGACAATTGGAGAAGTGAACTATATAAGTATAAGG
TAGTAAAAATTGAACCAATA---GGAGTAGCACCAACCAAGGCACAGAGAAGAGTGGTG--------------
-------------------------GGGAGAGAAAAAAGAGCA---ATTGGA---CTGGGAGTT---------
GCCTTCCTTGGGTTCTTAGGAGCA---GCAGGAAGCACTATG---GGCGCAGCGTCA---
ATAACGCTGACGGTACAG---GCCAGACAA---TTACTG---TCTGGCATAGTGCAACAGCAAAGC---
AATTTG---CTGAAG---GCTATAGAGGCTCAGCAGCATCTGCTGAAACTCACAGTCTGGGGCATT
>KU749402
TTCTATTGCAATACATCAAAACTGTTCAATAGCACTTGGGACGAAAATAGCACTGCCATAAATAGTACAGAG-
----

GACAGTGGAATTATAACTATACCATGCAGAATAAGGCAAGTTATAAATATGTGGCAGAGAGTAGGACAAGCAA
TGTATGCCCCTCCTATCAGAGGAAGTATAAGGTGTGAATCAAAC---
ATTACAGGATTAATACTAACAAGAGATGGTGGAAATATTAGTAGC----------------------------
----

ACCAATGAGACCTTCAGACCTACAGGAGGAGATATGAGGGACAATTGGAGAAGTGAACTATACAAGTATAAAG
TAGTAAGCATTGAACCAATA---GGAGTAGCACCTACCAAGGCAAGGAGAAGAGTGGTG--------------
-------------------------CAGAGAGAAAAAAGAGCA---GTTGGA---CTGGGAGCT---------
GCCTTCCTTGGGTTCTTAGGAGCA---GCAGGAAGCACTATG---GGCGCGGCGTCA---
TTGACGCTGACGGTACAG---GCCAGAAAA---TTATTG---TCTGGCATAGTGCAACAGCAAAAC---
AATTTG---CTGAAG---GCTATAGAGGCTCAGCAGCATCTGCTGAAACTCACAGTCTGGGGCATT
>KU749403
TTCTATTGTAATACAACAGACCTGTTCAATAGCACTTGGGACAGAAATGACACTGATGCAAATAGTACACAGG
TTGATAGTACA---CAG--

GTTGATGTAACTATAACTCTACCATGCAGAATAAAGCAAATTGTAAGGATGTGGCAGAGAGTAGGACAAGCAA
TGTATGCCCCTCCTATCGGAGGAGTTATAAGGTGTGAATCAAAC---
ATTACAGGACTACTACTAACAAGGGATGGTGGAGCTAATAATAGC----------------------------
----

ACTAATGAGACCTTTAGACCTCTAGGAGGAGATATGAGGGACAATTGGAGAAGTGAACTATATAAGTATAAAG
TAATACAAGTTGAACCAATA---GGAGTAGCACCTACCCCAGCAAAGAGAAGAGTGGTG--------------
-------------------------GAGAGACAAAAAAGAGCA---ATTGGA---CTGGGAGCT---------
GCCTTCCTTGGGTTCTTAGGAGCA---GCAGGAAGTACTATG---GGCGCGGCGTCA---
ATAACGCTGACGGTACAG---GCCAGACAA---TTATTG---TCTGGCATAGTACAACAGCAAAGT---
AATTTG---CTGAGA---GCTATAGAGGCTCAGCAGCATCTGCTGAAACTCACAGTCTGGGGCATT

>KU749404
TTCTATTGCAATACAACAGAGCTGTTCAATAGCACTGAGGACAAAAATGGCACTGTCACAAAT----------
----

AGCAATGAAAATATAACTCTACCATGCAGGATAAAGCAAATTATAAATATGTGGCAGAGAGTAGGACAAGCAA
TGTATGCCCCTCCTATCAGAGGAAATATAACTTGTGAATCAAAC---
ATTACAGGACTACTACTAACAAGAGATGGAGATGATAATAGCAGT----------------------------
----

GAGACCTTCAGACCTATAGGAGGAAATATGAGGGACAATTGGAGAAGTGAACTATATAAATATAAAGTAGTCC
AAATTGAACCAATA---GGAGTAGCACCTACCAAGGCAAGGAGAAGAGTGGTG--------------------
-------------------GGGAGAGAAAAAAGAGCA---ATTGGA---CTGGGAGCT---------
GCCTTCCTTGGGTTCTTAGGAGCA---GCAGGAAGCACTATG---GGCGCGGCGTCA---
CTGACGCTGACGGTACAG---GCCAGACAA---TTATTG---TCTGGTATAGTGCAACAGCAAAGC---
AATTTG---TTGAGG---GCTATAGAGGCTCAGCAGCATCTGCTGAAACTCACAGTCTGGGGCATT
>KU749405
TTCTATTGCAATACAACAGACTTGTTCAATTGGGATAGAAATAGCACTGTCCCAAATAAC-------------
----

ACAAGTAGCAATATAACTCTGCCATGCAGAATAAAGCAAATTATAAATATGTGGCAGAGAGTAGGACAATCAA
TGTATGCCCCTCCTATCAGCGGAAAGATAAGGTGTGAATCAAAC---
ATTACAGGACTACTACTAGTAAGAGATGGTGGAGATAATAATAGC----------------------------
----

ACCAATGAGACCTTCAGACCTATAGGAGGAGAAATGAGGCACAATTGGAGAAGTGAACTATATAAGTATAAAG
TAGTAGAAATTGAACCAATA---GGAGTAGCACCTACCAAGGCAAGGAGAAGAGTGGTG--------------
-------------------------GGAAGAGAAAAAAGAGCAGCCTTTGGA---CTGGGAGCT---------
GTCTTCCTTGGGTTCTTAGGAGCA---GCAGGAAGCACTATG---GGCGCGGCGTCA---
CTGACGCTGACGGTACAG---GCCAGGCAA---TTATTG---GCTGGCATAGTGCAACAGCAAAGC---
AATTTG---CTGAGG---GCTATAGAGGCTCAGCAGCATCTGCTGAAACTCACAGTCTGGGGCATT
>KY658682
TTCTATTGCAATACAACAGAACTGTTCAATAGCACTTGGAATGGCACTGACACAAACAGCACAGAGGAC----
----

AATAATGTAAATATAACTATACCATGCAGAATAAAGCAAATTATAAATATGTGGCAGAGAGTAGGACGAGCAA
TGTATGCCCCTCCTATCAGAGGAAATATAACTTGTGAATCAAAC---
ATTACAGGACTACTACTAACAAGAGATGGTGGTAAAGAGAAAAAT----------------------------
----

GACAACGAAACCTTTAGACCTATAGGAGGAAATATGAAGGACAATTGGAGAAGTGAACTATATAAGTATAAAG
TAGTAGAAATTAACCCAATA---GGAGTAGCACCTACCCCGGCAAAGAGAAGAGTGGTG--------------
-------------------------GAGAGAGAAAAAAGAGCA---GTTAGC---CTAGGAGTT---------
GCCTTCCTTGGGTTCTTAGGAGCA---GCAGGAAGTACTATG---GGCGCGGCGTCA---
CTGACGCTGACGGTACAG---GCCAGACAA---CTATTG---TCTGGCATAGTACAACAGCAAAGC---
AATTTG---CTGAGG---GCTATAGAGGCTCAGCAGCAATTGCTGAAACTCACGGTCTGGGGCATT
>KU749407
TTCTATTGCAATACAACAGAACTGTTCAATAGCACTTGGGACGACAATAGCACTGTTGCAAATATCACAAAG-
----

ACCAATGAAAATATAACTCTACCATGCAGACTAAGGCAAATCATAAAGATGTGGCAGAGAGTAGGACAAGCAA
TGTATGCCCCTCCTATCCAAGGAGTTATAAAGTGTGAATCAAAC---
ATTACAGGACTACTACTAACAAGAGATGGTGGAGATGGAACTAAT----------------------------
----

AACAGCAATAATGAGATTTTCAGACCTATAGGAGGAAATATGAGGGACAATTGGAGAAGTGAACTATATAAGT
ATAAAGTAGTAGAAATTGAACCAATA---GGAGTAGCACCTACCAAGGCAAAGAGAAGAGTGGTG--------
-------------------------------CAGAGAGAAAAAAGAGCAGTTATTGGA---CTGGGAGCT---
------GCCTTCCTTGGGTTCTTAGGAGCA---GCAGGAAGCACTATG---GGCGCGGCGTCA---
ATAACGCTGACGGTACAG---GCCAGACAA---TTACTG---TCTGGCATAGTGCAACAGCAAAGC---
AATTTG---CTGAGG---GCTATAGAGGCTCAGCAGCATCTGCTGAAACTCACGGTCTGGGGCATT

>AF082486
TTCTATTGCAATACAACAGACCTGTTCAATAGCACTTGGGACGAACATGGCACTGTCACAAATAGCACAATG-
----

GCCAATGGAACTATAACTCTACCATGCAGAATAAAGCAAATTATAAATATGTGGCAGAGAGTAGGACAAGCAA
TGTATGCCCCTCCTATTAAAGGAAGTATAAGGTGTGAATCAAAC---
ATTACAGGACTACTATTAACAAGAGATGGTGGAAGTGGAACTAAT----------------------------
----

AGCAGCAATGAGACCTTCAGACCTATAGGAGGAAATATGAGGGATAATTGGAGAAGTGAACTATATAAGTATA
AAGTAGTAAAAATTGAACCCATA---GGATTAGCACGTACCAGGGCAAAGAGAAGAGTGGTG-----------
----------------------------GAGAGAGAAAAAAGAGCC---ATTGGA---CTGGGAGCT------
---GCCTTCCTTGGGTTCTTAGGAGCA---GCAGGAAGTACTATG---GGCGCGGCGTCA---
ATGACGCTGACGGTACAG---GCCAGACAA---TTATTG---TCTGGCATAGTGCAACAGCAAAGC---
AATTTG---CTGAGG---GCTATAAAGGCTCAGCAGCATCTGCTGAAACTCACGGTCTGGGGCATT
>KY968402
TTCTACTGTAACACAACAAAACTGTTTAATAGTACTTGGTCTAATAATGATACTGGGGAGGGTACTGCAGATT
CA---

AATAACGAAACTATCACAATCCCATGCAGAATAAAACAAATTATAAACATGTGGCAACAAGTAGGAAAAGCAA
TGTATGCCCCTCCCATCAGTGGAAACATTAGTTGCTCATCAAAT---
ATTACAGGGTTACTGTTAACAAGAGATGGTGGAGATAATGATGAC----------------------------
----

CTAAACGGGACCGAGACCTTCAGACCTCAAGGAGGAAACATGAAGGACAATTGGAGAAGTGAATTATATAAAT
ATAAAGTAGTAAAAATTGAACCATTA---GGAGTAGCACCCACCAAGGCAAAGAGAAGAGTGTTG--------
-------------------------------CAGAGAGACAAAAGAGCA---GTGGGA---CTAGGAGCT---
------ATGTTCCTTGGGTTCTTGGGAGCA---GCAGGAAGCACTATG---GGCGCAGCGTCA---
ATAGCGCTGACGGTACAG---ACCAGACAA---TTATTG---TCTGGTATAGTGCAACAGCAGAAC---
AATATG---CTGAGG---GCTATTGAGGCGCAACAGCATATGTTGCAACTCACTGTCTGGGGCATC
>KY968404
TTCTACTGTAATACAACACAACTGTTTTATAGTACTTGGTTGTCTAATGGTAGTAGTTTGACAAATAACACTG
AA---

GGAAATGACACTATCACACTCCCATGCAGAATAAAACAGATTATAAATATGTGGCAGCAAGTAGGAAAAGCAA
TGTATGCCCCTCCCATCAAAGGACTAATTAGATGCTCATCGAAT---
ATTACAGGACTGCTACTAACAAGAGATGGTGGTAGTCATAACGAGAGC-------------------------
----

AATGAGACTGAGACATTCAGACCTGGGGGAGGAGATATGAGGGACAATTGGAGAAGTGAATTATACAAATATA
AAGTAGTACAAATTGAACCATTA---GGAATAGCACCCACCAAGGCAAAGAGAAGAGTGGTG-----------
----------------------------CAGAGAGAAAAAAGAGCA---GTGGGA---CTGGGAGCT------
---TTGTTCCTTGGGTTCTTGGGAACA---GCAGGAAGCACTATG---GGCGCAGCGTCA---
TTGACGCTGACGGTACAG---GCCAGACAA---TTATTG---TCTGGTATAGTGCAACAGCAGAAT---
AATCTG---CTGAGA---GCTATTGAGGCGCAACAACATCTGTTGCAGCTCACAGTCTGGGGCATC
>KY968408
TTCTACTGTAATACAACAAAACTGTTTAATAAGACTTGGAATGGTACTTGGCTTAATAATGAGAAT-------
----

ACTACACAGAATATCACACTCCCATGCAGAATAAAACAAATTATAAACAGGTGGCAGGAAGTAGGAAAAGCAA
TGTATGCCCCTCCCATCAAAGGACAAATTAGCTGCTCATCAAAT---
ATTACAGGGCTGCTATTAACAAGAGATGGTGATAACGAGACTGGG----------------------------
----

TCGGAGACCTTCAGACCTGGAGGAGGAGATATGAGGGACAATTGGAGAAGTGAATTATATAAATATAAAGTAG
TAAAAATTCAGCCATTA---GGAGTAGCACCCACCAAGGCAAAGAGAAGAGTGGTG-----------------
----------------------CAAAGAGAAAAAAGAGCA---GTGGGA---ATAGGAGCT---------
GTGTTCCTTGGGTTCTTGGGAGCA---GCAGGAAGCACTATG---GGCGCAGCGTCA---
GTAACGCTGACGGTACAG---GCCAGACAA---TTATTG---TCTGGTATAGTGCAGCAGCAGAAC---
AATTTG---CTGAGG---GCTATTGAGGCGCAACAGCATCTGTTGCAACTCACAGTCTGGGGCATC

```
>DQ383749
TTCTACTGTAATACATCACAACTGTTTAATAGTACTTGGCCTTGGAATGGGAATGCGACTACAGATAATACT-
-------------------------------------------------------------------------
----------------------------
ACAGGAGTAAATATCACACTCCCATGCAGAATAAAACAAATTATAAACATGTGGCAGGAAGTAGGAAAAGCAA
TGTATGCCCCTCCTATTAAAGGACAAATTAGATGTTCATCAAAT---
ATTACAGGGCTGCTATTAACAAGAGATGGTGGTCATAACGAGACC----------------------------
-------------------------------------------------------------------------
----------
AACGACACTGAGACCTTCAGACCTGGAGGAGGAAATATGAAGGATAATTGGAGAAGTGAATTATATAAATATA
AAGTAGTAAAAATTGAACCATTA---GGAATAGCACCCACCACGGCAAAGAGAAGAGTGGTG-----------
----------------------------CAGAGAGAGAAAAGAGCA---ATAACA---ATAGGAGCT------
---ATGTTACTTGGTTTCTTGGGAACA---GCAGGAAGCACTATG---GGCGCAGCGTCA---
ATCACGCTGACGGTACAG---GCCAGACAA---TTATTG---TCTGGTATAGTGCAACAGCAGAAC---
AATCTG---CTGAGG---GCTATTGAGGCGCAACAGCATCTGTTGCAACTCACAGTCTGGGGCATC
>MH078532
TTCTACTGTAACACATCAAAGCTGTTTAACAGTACTTGGCATAGGAATGGTACTTGGAATGGTACTAGTACT-
-------------------------------------------------------------------------
----------------------------
GAGAATAATACAATCACAATCCCATGCAGAATAAAACAAATTATAAACAGGTGGCAGGAAGTAGGAAAAGCAA
TGTATGCCCCTCCCATCAGAGGAAATATTACATGTACATCTAAT---
ATTACAGGACTGTTATTAATAAGAGATGGTGGTAACATTAACACCACG-------------------------
-------------------------------------------------------------------------
----------
AATGAGACCGAGATCTTCAGACCTGGAGGAGGAGACATGAGGGATAATTGGAGAAGTGAATTATATAAATATA
AAGTAGTAAGAATTCAGCCATTA---GGAATAGCACCCACCGAGGCAAAGAGAAGAGTGGTG-----------
----------------------------CAAAGAGAAAAGAGAGCG---GTGGGAACACTAGGAGCT------
---GTGTTCCTTGGGTTCTTGGGAGCA---GCAGGAAGCACTATG---GGCGCAGCGGCA---
ACAACGCTGACGGTACAG---GCCAGACAG---TTACTG---TCTGGCATAGTGCAACAGCAAAAC---
AATTTG---CTGAGA---GCTATTGAGGCGCAACAGCATACGTTGCAACTCACAGTCTGGGGCATT
>AF042102
TTCTACTGTAATTCAACACAACTGTTTAATGGTACTTGGAATGGTACTTGGATTAATGGTACTTGGAAGAGTA
GTTAT--------------------------------------------------------------------
-------------------GGG---
AATGATACTACAAATATCACACTCCCATGCAGAATAAAGCAAATTATAAACATGTGGCAGGAAGTAGGAAAAG
CAATGTATGCCCCTCCCATCAGAGGACAAATTAAATGTACATCAAAT---
ATTACAGGGCTGTTATTAACAAGAGATGGTGGTAACAGTAACACCACG-------------------------
-------------------------------------------------------------------------
-------
GACAACAGCACAGAGACCTTCAGACCTGGAGGAGGAGATATGAGGGACAATTGGAGAAGTGAATTATATAGAT
ATAAAGTAGTACAAGTTGAACCATTA---GGAATAGCACCCACCAGGGCAAAGAGAAGAGTGGTG--------
-------------------------------CAAAGAGAAAAAAGAGCA---GTGGGA---ATAGGAGCT---
------ATGTTCCTTGGGTTCTTAGGAGCA---GCAGGAAGCACTATG---GGCGCAGCGTCA---
GTGACGCTGACGGTACAA---GCCAGACAA---TTATTG---TCTGGTATAGTGCAGCAGCAGAAC---
AATCTG---CTGAGG---GCTATTGAGGCGCAACAGCATATGTTGCAACTCACAGTCTGGGGCATC
>AF538307
TTCTACTGTGATTCAACACAACTGTTTAATAGTACTTGGCATAATAGTACTTGGAATAATAATAAAGAGTCAA
AT-----------------------------------------------------------------------
----------------------------
AGCACTGATCCAATCACACTTCCATGCAGAATAAAACAAAATATAAACATGTGGCAGGAAGTAGGAAAAGCAA
TGTATGCCCCTCCCATCAGAGGACAAATTAAATGTTCATCAAAC---
ATTACAGGGCTAATATTAACAAGAGATGGTGGTAATAACACAGACAAT-------------------------
-------------------------------------------------------------------------
----------
AACAACACCGAAACCTTCAGACCTGGAGGAGGAAATATGAAGGACAATTGGAGAAGTGAATTATATAAATATA
AAGTAGTAAAAATTGAACCATTA---GGAGTAGCACCCACCAAGGCAAGGAGAAGAGTGGTG-----------
----------------------------CAGAGAGAGAAAAGAGAA---GTAGGA---CTAGGAGTT------
---ATGTTCCTTGGGTTCTTGGGAGCA---GCAGGAAGCACTATG---GGCGCAGCGTCA---
CTGAGGCTGACGGTACAG---GCCAGACAA---TTATTG---TATGATATAGTGCAACAGCAAAAC---
AATTTG---CTGAGA---GCTATTGAGGCGCAACATCATCTGTTGCAACTCACAGTCTGGGGCATC
```

```
>GU367398
TTCTACTGTAATACAACACAACTGTTTAATAGTACTTGGTCCTCTAATGATACTTTGAATGGTACTTGGTCCT
CTAATTATAAT-------------------------------------------------------------
-TTGAATGATACTGAA---------
GGATCAGGGGACAACATCACACTCCCATGCAGAATAAAACAAATTATAAACATGTGGCAGGGAGTAGGAAGAG
CAATGTATGCCCCTCCCATCAGAGGACAAATTAGTTGTTCATCAAAT---
ATTACAGGGTTATTATTAGTGAGAGACGGGGTAATATCCAGAAC----------------------------
------------------------------------------------------------------------
----------------
AATGAGACCTTCAGACCTGCAGGAGGAAATATGAAAGACAATTGGAGAAGTGAATTATATAAGTATAAAGTAG
TAAGAATTGAACCATTA---GGAATAGCACCCACCAAGGCAAAGAGAAGAGTGGTG----------------
---------------------CAGAGAGAGAAAAGAGCA---ATAGGAACGCTAGGAGCT---------
ATGTTCCTTGGGTTCTTGGGAGCA---GCAGGAAGCACTATG---GGCGCAGCGTCA---
GTGACGCTGACGGTACAG---GCCAGACAA---TTATTG---TCTGGTATAGTGCAACAGCAGAAC---
AATCTG---CTGAAG---GCTATTGAGGCGCAACAGCATATGTTGCAACTCACAGTCTGGGGCATT
>GU367404
TTCTATTGTAATACAACACAACTGTTTAACAGTACTTGGAGAAAGGAGTCAAATAACACTGGAGGAAAT----
------------------------------------------------------------------------
----------------------------
ATCACACGAAATATCACGCTCCCATGTAGAATAAGACAGATTGTAAACATGTGGCAGGAAGTAGGAAAAGCAA
TATATGCCCCTCCCATTGAAGGACTAATTAGATGTTTATCAAAT---
ATTACAGGGCTGCTATTAACAAGAGACGGTGGTAATCAGAATGGGACT-------------------------
------------------------------------------------------------------------
----------
AATGACACTGAAATCTTCAGACCTGGAGGGGGAGATATGAGGGACAATTGGAGAAGTGAATTATATAAATATA
AAGTAGTAAAAATTGAACCCTTA---GGAATAGCACCCACCAAGGCAAAGAGAAGAGTGGTG----------
---------------------------CAAAGAGAAAAAAGAGCA---GTGGGAGCATTAGGAGCT------
---ATGTTCCTTGGGTTCTTGGGAGCA---GCAGGAAGCACTATG---GGCGCAGCGTCA---
GTAACGCTGACGGTACAG---GCCAGACAA---TTATTG---TCTGGTATAGTGCAACAGCAGAAT---
AATCTG---CTGAAG---GCTATTGAGGCGCAACAGCATCTGTTGCAACTCACAGTCTGGGGCATC
>AF146728
TTCTACTGTAATTCAACACAACTGTTTAATAGTACTTGGTTTAATAGTACTGGGAATGATACTGAAAGGGCAA
CT----------------------------------------------------------------------
-------------------------
AACAATACAGAAAATATCACACTCCCATGTAGAATAAAACAAATTATAAACATGTGGCAGAAAGTAGGAAAAG
CAATGTATGCCCCTCCCATCAACGGACAGATTAGATGTTCATCAAAT---
ATTACAGGGCTGATACTAACAAGAGATGGTGGTAATCAAGAGAAC---------------------------
------------------------------------------------------------------------
-------------
AAGACCGAGATCTTCAGACCTGGAGGAGGAGATATGAGGGACAATTGGAGAAGTGAATTATATAAATATAAAG
TAGTAAGAATTGAACCATTA---GGAGTAGCACCCACCAAGGCAAAGAGAAGAGTGGTG-------------
------------------------CAGAGAGAAAAAAGAGCA---GTGGGAATGATAGGGGCA---------
ATGATCCTTGGGTTCTTGGGAGCA---GCAGGAAGCACTATG---GGCGCAGCGTCA---
CTGGCGCTGACGGTACAG---ACCAGGCAA---TTGTTG---TCTGGTATAGTGCAACAGCAGAAC---
AATCTG---CTGAGG---GCTATTGAGGCGCAACAGCATCTGTTGCAACTCACAGTCTGGGGCATC
>MN485974
TTCTATTGTAATACAACAAAACTGTTTAATGGTACTTGGAATGGTACTTGGCTATCGGGGAACTGG------
------------------------------------------------------------------------
----------------------------
CCAAATGAAAATATCACACTCCCATGTAGAATAAAACAAATTGTAAACATGTGGCAAGAAGTAGGAAAAGCAA
TGTATGCCCCTCCCATCCAAGGACAGATTAGATGTGACTCAAAT---
ATTACAGGGCTGCTATTAACAAGAGATGGTGGTTCCGGGAATGGAACTGAG---------------------
------------------------------------------------------------------------
----------
AATAACACTGAGATCTTCAGACCTGGAGGAGGAAATATGAGAGACAATTGGAGAAGTGAATTATATAAATATA
AAGTAGTAAAAATTGCACCATTA---GGAATAGCACCCACCAAGGCAAAGAGAAGAGTGGTG----------
---------------------------CAGAGAGAAAAAAGA------ATAGGAACGCTAGGAGCT------
---GTGTTCCTTGGGTTCTTGGGAACA---GCAGGAAGCACTATG---GGCGCAGCGTCA---
CTGACGCTGACGGTACAG---GCCAGACAA---TTATTG---TCTGGTATAGTGCAACAGCAGAAC---
AATTTG---CTGAGG---GCTATTGAGGCGCAACATCATCTGTTGCAACTCACAGTCTGGGGCATC
```

>MN485990
TTCTACTGTGATTCAACAGAACTATTTAATAGTACTTGGAATTTTAATAATAATACTTGGTATGAGAACTCAA
ATAACACAAGT--

GACAAAGAAAACATCACACTCCCATGTAGAATAAAACAAATTAGAAACTTGTGGCAGGAAGTAGGAAAAGCAA
TGTATGCCCCTCCCATCCAAGGGCTAATTACATGTCAATCAAAT---
ATTACTGGGGTGCTACTAACAAGAGATGGTGGTATTAATAACACC----------------------------
----

AGGAACGAGACCTTTAGACCTGGAGGAGGAAATATGAAAGACAATTGGAGAAGTGAACTATACAAATATAAAG
TAATAAAAATTGAACCATTA---GGAGTGGCACCCACCAAGGCAAAGAGAAGAGTGGTG--------------
-------------------------CAGAGAGAAAAAAGAGCA---GTGGGAACAATAGGAGCT---------
GTGTTCCTTGGTTTCTTGGGAACA---GCAGGAAGCACTATG---GGCGCAGCGTCA---
ATAGCGCTGACGGCACAG---GCCAGACAA---TTATTG---TCTGGTATAGTGCAACAGCAAAAC---
AATTTG---CTGAGA---GCTATTGAGGCGCAACAACATCTGTTGCAACTCACAGTCTGGGGCATC
>MN485995
TTCTACTGTAATACAACACAACTGTTTAATAGTACTTGGTGGTCTAATAGTACTGGGAATATTACTGGA----
----

CAGCCAGAAAATATCACTCTCCCATGCAGAATAAAACAAATCATAAACTTGTGGCAAGAGGTAGGAAAAGCAA
TGTATGCCCCTCCCATCCAAGGACTAATTAGATGTTCATCAAAT---
ATTACAGGGTTACTATTAACAAGAGATGGTGATAATCAAACTAAA----------------------------
----

GAGACTGAGATCTTTAGACCGGGAGGAGGAGATATGAGGGACAATTGGAGAAGTGAATTATATAAATATAAAA
TAGTAAAAATTGAGCCATTG---GGAATAGCACCCACCAGGGCAAAGAGAAGAGTGGTG--------------
-------------------------CAAAGAGAAAAAAGAGCA---GTGGGA---ATAGGAGCT---------
CTGTTCCTTGGGTTCTTGGGAGCT---GCAGGAAGCACTATG---GGCGCAGCGTCG---
GTGACGCTGACGGTACAG---GCCAGACAA---TTATTG---TCTGGTATAGTGCAACAGCAAAAC---
AACTTG---CTGAGA---GCTGTTGAGGCGCAACAACATATGTTGCAACTCACAGTCTGGGGCATC
>MN486028
TTCTACTGTAATACAACAGACCTGTTTTGGGGTATTTGGGACCCAAATAATAATACTTGGAATGGTAGTACAG
TGGATAAC---

ATTCAATGCAGAATAAAACAAATTATAAACAGGTGGCAGGAAGTAGGAAAAGCAATGTATGCCCCTCCCATTG
AAGGAAATATTAGCTGTACATCAAAC---
ATTACAGGACTACTATTGACAAGAGATGGTGGTGGTGAAGGG----------------------------
----

CCTAATGATACCGAGATCTTCAGACCTACAGGAGGAGAGATGAGGGACAATTGGAGAAGTGAATTATATAAGT
ATAAAGTAGTACAAATTGAGCCAGTA---GGGATAGCACCTACCAAGGCAAAGAGAAGAGTGGTG--------
-------------------------------CAGAGAGAAAAAAGAGCA---GTGGGA---ATGACAGCT---
------GTGTTACTTGGGTTCTTGGGCACA---GCAGGAAGCACTATG---GGCGCAGCGTCT---
CTAACGCTGACGGTACAA---GCCAGACAA---TTATTG---TCTGGTATAGTGCAACAGCAGAAC---
AATCTG---CTGAAG---GCTATTGAAGCGCAACAGCATCTGTTGCAGCTCACAGTCTGGGGCATC
>MN486034
TTCTACTGTAATACAACAGAGCTATTTAATAGGAATATTACTGAAGAGTCAAACACTAATGGAAATAGCAGT-
----

AGCGCAGGAAATATCACACTCCAATGCAGAATAAAACAGATTATAAACATGTGGCAGGAAGTAGGAAAAGCAA
TGTATGCCCCTCCCATCAGAGGACAAATTCAATGTATATCAAAT---
ATTACAGGACTAATATTAACAAGAGATGGTGGTAAGACAAATAAT----------------------------
----

GAGACAAATGGGACTGAGATTTTCAGACCTGGAGGAGGAAATATGAGAGACAATTGGAGAAGTGAATTATATA
AATATAAGGTAGTAAAAATTGAACCATTA---GGAGTAGCACCCACCAGGGCAAAGAGAAGAGTGGTG-----
----------------------------------CAGAGAGAAAAAAGAGCA---GTGGGA---
ATAGGAGCT---------GTGTTCCTTGGGTTCTTGGGAGCA---GCAGGAAGCACTATG---
GGCGCAGCGTCA---ATAACGCTGACGGTACAG---GCCAGACAA---TTATTG---

TCTGGTATAGTGCAACAGCAAAAC---AATTTG---CTGAGA---
GCTATTGAGGCGCAACAGCATCTGTTGCAACTCACAGTCTGGGGCATC
>AY037270
TTCTACTGTAATACAACAGAACTGTTTAATAGTACTTGGAATTTTACTAGTACCTGGAATATGACTAGTGGGT
CAAATTAC---
-------------------TCT---
GCAGAAAGAAATATCATCACACTCTCATGCAGAATAAAACAAATTATAAACATGTGGCAGGAAGTAGGAAAAG
CAATGTATGCCCCTCCTATCAGAGGACAAATTAAATGTTCATCAAAT---
ATTACAGGGCTGCTATTAACAAGAGATGGTGGGGATAATAACAGA----------------------------
----

AGCACCGAGACCTTTAGACCTGGAGGAGGAAATATGAGAGACAATTGGAGAAGTGAATTATATAAATATAAAG
TAGTAAAAATTGAACCATTA---GGAGTAGCACCCACCAGGGCAAGGAGAAGAGTGGTG--------------
-------------------------CAAAGAGAAAAAAGAGCA---GTGGGAACGATAGGAGCT---------
ATGTTCCTTGGGTTCTTGGGAGCA---GCAGGAAGCACTATG---GGCGCAGCGTCA---
GTGACGCTGACGGTACAA---GCCAGACAA---TTATTG---TCTGGTATAGTGCAGCAGCAGAAC---
AATCTG---CTGAGG---GCTATTGAGGCGCAACAGCATATGTTGCAACTCACAGTCTGGGGCATC
>DQ358805
TTCTACTGTAATACAACGAAGCTGTTTACTGGTACTTTGAATAGCACTGGGACTTGGCATACAGAAAAC----
----

CAGACTGATACTATCACACTCCCATGCAAAATAAAACAAATTATAAACAGGTGGCAGGAAGTAGGAAAAGCAA
TGTATGCCCCTCCCATTAAAGGACAAATTAGCTGTTCATCAAAT---
ATTACAGGGATACTATTAACACGAGATGGTGGTGGGAATGGGTCC----------------------------
----

GACACCACCGCCTCCTTAACACCAGGAGGAGGAGATATGAGGAACAGTTGGAGAAATGAATTATATAATTATG
AGGTGGTAAAAATTCCACCATTA---GGAGTACCACCCACCGAGGCGAGGAGAAGAGTGGTG-----------
----------------------------CAGAGAGAAAAAAGAGCA---GTGGGGGCACTAGGAGCT------
---ATGTTCCTTGGGTTCTTGGGAACA---GCAGGAAGCACTATG---GGCGCAGCGTCA---
CTGACGCTGACGGTACAG---GCCAGACAA---TTATTG---TCTGGTATAGTGCAACAGCAGAAT---
AATTTG---CTGAGA---GCTATTAAGGCGCAACAGCATATGTTGCAACTCACAGTCTGGGGCATC
>JN692432
TTCTACTGTAATACATCACAACTGTTTAATAGAACTTTTAATGGTACTGCAAATAAATTCAATACT-------
----

ACAAATGGAACTATCACACTTCAATGTAGAATAAAACAAATCATAAACAGATGGCAGGAAGTAGGAAAAGCAA
TATACAACCCTCCCATCAGAGGAAACATTAGTTGTTCATCAAAT---
ATTACAGGACTACTATTAACAAGAGATGGTGGTAACAATGGGAGC----------------------------
----

GAGCCCGAGACCTTCAGACCTGGAGGAGGAGAAATGAGGGACAATTGGAGAAGTGAATTATATAAATATAAAG
TAGTAAAAATTGAGCCATTA---GGAATAGCACCCACCAAGGCAAAAAGAAGAGTGGTG--------------
-------------------------CAAAGAGAAAAGAGAGCA---GTGGGAGTGGTAGGAGCT---------
ATGTTCCTTGGGTTCTTGGGAGCA---GCAGGAAGCACTATG---GGCGCAGCGTCA---
GTAACGCTGACGGTACAG---GCCAGACTA---TTATTG---TCTGGTATAGTGCAACAGCAGAAC---
AATTTG---CTGAAG---GCTATTGAGGCGCAACAACATCTGTTGCAACTCACAGTCTGGGGCATC
>JN692439
TTCTACTGTGATACCTCACAAATATTTAATAGTAATTGGAATAGTTCTTCAAATTTTAGTCATCATGAAAAT-
----

TCAAATGAAAATATCACACTCCAATGCAGAATAAAACAAATTATAAACAGGTGGCAGGAAGTAGGAAGAGCAA
TGTATGCCCCTCCCATCCGAGGACTAATTAGATGTTCATCAAAT---
ATTACAGGGCTGCTATTAACAAGAGATGGTGGTAAGAATGATAACAATGGG----------------------
----

ACTGAGAATGGGACTGAGACCTTCAGACCTGGAGGGGGAGATATGAGGGACAATTGGAGAAGTGAATTATATA
AATATAAAGTAGTTCAAATTGAGCCAGTA---GGAATAGCACCCACCAAGGCAAAGAGAAGAGTGGTG-----
----------------------------------CAAAGAGAAAAAAGAGCA---GTAGGA---
GTAGGAGCT---------GTGTTCCTTGGGTTCTTGGGAACA---GCAGGAAGCACTATG---

GGCGCAGCGTCA---CTCACGCTGACGGTACAG---GCCAGACAA---TTGTTG---
TCTGGTATAGTGCAACAGCAGAAC---AATCTG---CTGAAG---
GCTATTGATGCGCAACAGCATCTGTTGCAGCTCACAGTCTGGGGCATC
>JN692433
TTCTACTGTAATACAACACAGCTGTTTAACAGTACTTGGTGGGTTAATGGTACTTGGAATGATACTACAAAGT
CAAAT--

GCAACTCAGGAACTCATCACACTCCCATGCAGAATAAAACAAATTATAAACAAGTGGCAGGAAGTAGGAGTAG
CAATGTATGCCCCTCCCATCAGAGGAAACATTAGCTGTGTATCAAAT---
ATTACAGGGCTGCTATTAACAAGAGATGGTGGTAAAAATGAGAGT----------------------------
----

AGAACAAACGAAACCTTCAGACCTGGAGGAGGAAATATGAAGGATAATTGGAGAAGTGAATTATATAAATATA
AGGTAGTAAAAATTGAACCATTA---GGAATAGCACCCACCAAGGCAAGGAGAAGAGTGGTG-----------
----------------------------CAGAGAGAAAAAAGAGCA---GTGGGA---CTAGGAGCT------
---ATGTTCCTTGGGTTCTTGGGAGCA---GCAGGAAGCACTATG---GGCGCAGCGTCA---
GTGACGCTGACGGTACAG---GCCAGACAA---TTATTG---TCTGGTATAGTGCAACAGCAGAAC---
AATTTG---CTGAGG---GCTATTGAGGCGCAACAGCATATGTTGCAACTCACAGTCTGGGGCATC
>EF637049
TTCTACTGTAACACAACACAGCTGTTTAATAGTACTATGTTAAATGATACTAAAAGGCCAGATGAC-------
----

ACAAATCAAAATATCACACTCCCATGCAGAATAAAACAAATTATAAACAGGTGGCAGGAAGTAGGAAAAGCAA
TGTATGCCCCTCCCATCAGTGGAATAATTAGATGTATATCAAAT---
ATTACAGGGCTACTACTAACAAGAGATGGTGGTAATGGTAATGGTACTGAG----------------------
----

TGCGGTAACAATACCGAGTGCTTCAGACCTGGAGGAGGAGATATGAGGGACAATTGGAGAAGTGAACTATATA
AATATAAAGTAGTAAGAATTGAGCCATTA---GGAATAGCACCCACCAAGGCAAAGAGAAGAGTGGTG-----
----------------------------------CAAAGAGAAAAAAGAGCA---
GTGGGAACAATAGGAGCT---------ATGTTCCTTGGGTTCTTGGGAGCA---GCCGGAAGCACTATG---
GGCGCAGCGTCA---CTGACGCTGACGGTACAA---GCCAGACAA---TTATTG---
TCTGGTATAGTGCAACAGCAGAAC---AATTTG---CTGAGG---
GCTATTGAGGCGCAACAGCATATGTTGCAACTCACAGTCTGGGGCATT
>JN692462
TTCTACTGTAATACATCACCACTGTTTAATAGTACTTGGAAAATTGGAACTAATAAGTCTATA----------
----

GAAAATGGAACTATCACACTCCCATGCAGAATAAAACAAATTATAAACAGGTGGCAGGAAGTAGGAAAAGCAA
TGTATGCCCCTCCCATCGAAGGACCAATTAGATGTTCATCAAAT---
ATTACAGGGCTGCTATTAACAAGAGATGGTGGTGAAGACAACAATGGGTCC----------------------
----
-
GAGACCTCCAATGAGACCTACACCTCTGGACCAGGAGATATGAATATGAATTGCAATTGTGAATTATATTTAT
ATAAAGATATGGAAATGGAGCCTGTACCATTA---GCACCCACCCCGACCAAGGCAAAGAGAAGAGTGGTG--
-------------------------------------CAGAGAGAAAAAAGAGCA---GTGGGA---
GTAGGAGCT---------GTGTTCCTTGGGTTCTTGGGAGCA---GCAGGAAGCACTATG---
GGCGCAGCGTCA---GTGGCGCTGACGGCACAA---GCCAGACAA---TTATTG---
TCTGGTATAGTGCAACAGCAGGAC---AATCTG---CTGAGG---
GCTATTGAGGCGCAACAGCATCTGTTGCAACTCACAGTCTGGGGCATC
>JN692465
TTCTATTGTAATACAACAGAGCTGTTTAAGGGTACTTGGAATGGTACTTGGAATGGTACTACTTGGGCTAATA
GTACTTGGGCT--
-------------AATGGT------
ACTGGGAATATGAACATCACACTCCCATGCAGAATAAGACAAATTATAAACATGTGGCAGGAGGTAGGAAAAG
CAATGTATGCCCCTCCCATCAGCGGACCCATTAACTGTACATCAAAT---
ATTACAGGGATGATATTAACAAGAGATGGTGGTGATGGTAACGGG----------------------------
----

AGTCAGGAGATCTTCAGACCTGGAGGAGGAGATATGAGGGACAATTGGAGAAGTGAATTATATAAATATAAAG

TAATAAAAATTGAACCATTA---GGAGTAGCACCCACCAGGGCAAAGAGAAGAGTGGTG--------------
-------------------------CAGAGAGAAAAAAGAGCA---GCT------ATAGGAGCT---------
GTGCTCATTGGGTTCTTGGGAGCA---GCAGGAAGCACTATG---GGCGCAGCGTCA---
ATAACGCTGACGGTACAA---GCCAGGCAA---TTATTG---TCTGGTATAGTGCAACAGCAGAGC---
AATCTG---CTGAGA---GCTATTGAGGCGCAACAACACCTGTTGCAACTCACTGTCTGGGGCATC
>JN692473
TTCTACTGTAATACATCAAAGCTGTTTAATAGGAATTGGACTGATAATAGTAATAATACTGGAAATGATACT-
----

GGGAATAATACTATCACACTCCCATGCAGAATAAAACAAATTATAAACAGATGGCAGGAAGTAGGAAAGGCAA
TGTATGCCCCTCCCATTCGAGGACTAATTAGATGTTCATCAAAT---
ATTACAGGGCTGATATTAACAAGAGATGGTGGTAATCATAACAATAGT-------------------------
----

AACGGTAGTGAAATCTTCAGACCTGGGGGAGGAGATATGAGGGACAATTGGAGAAGTGAATTATATAAATATA
AAGTAATAAAAATTGAGCCATTA---GGAATAGCACCCACCAAGGCAAAGAGAAGAGTGGTG-----------
----------------------------CAGAGAGAAAAAAGAGCA---GTGGGAACGATAGGAGCT------
---ATGTTCCTTGGGTTCTTGGGAGCA---GCAGGAAGCACTATG---GGCGCAGCGGCA---
GTGACGCTGACGGTACAG---GCCAGACAA---TTATTG---TCTGGTATAGTGCAACAGCAGAAC---
AATCTG---CTGAGA---GCTATTGAGGCGCAACAGCATATGTTGCAACTCACAGTCTGGGGCATC
>JN692474
TTTTACTGTAATACAACACAGCTGTTTAATAGCACTTGGCTGTTTAACAGCACTTGGAATCAAAGTGCTAAT-
----

GATGCAGACAATATCACACTCCCATGCAGAATAAAACAAATAGTAAACAGATGGCAGGAAGTAGGAAAAGCAA
TGTATGCCCCTCCCATCAAAGGACAAATTAGATGTTCATCAAAT---
ATTACAGGGCTGCTCTTAACAAGAGATGGTGATAATAACACCAAC----------------------------
----

AACAACGAGACCTTTAGACCTGGAGGAGGAGATATGAGGGACAATTGGAGAAGTGAATTATATAAATATAAAG
TAGTAAAAATTGAGCCATTA---GGAATAGCACCCACCAAGGCAAAAAGAAGAGTGGTG--------------
-------------------------CAGAGAGAAAAAAGAGCA---GTGGGAACAATAGGAGCT---------
GTGTTCCTTGGGTTCTTGGGAGCA---GCAGGAAGCACTATG---GGCGCAGCGTCA---
GTGACGCTGACGGTACAG---GCCAGACTA---TTATTG---TCTGGTATAGTGCAACAGCAGAAC---
AATCTG---CTGAAG---GCTATTGAAGCGCAACATCATCTGTTGCAGCTCACAGTCTGGGGCATC
>JN692480
TTCTACTGTAACACAGCACAGCTGTTTAATAATACTTGGCTGTCCAATAATACCTGGCTACAGAATAATACTT
GG---

ATTAATGGCACTATCACACTCCCATGCAGAATAAGACAAATTGTAAACAGGTGGCAGGAAGTAGGGAGAGCGA
TGTATGCTCCTCCCATCGCAGGAATAATCAACTGTGTATCAAAC---
ATTACAGGGCTGATATTAACAAGAGATGGAAACATAACAACAAATAGTACCTAC-------------------
----

TCTGAAAACAATACTGAAATCTTCAGACCTGGAGGAGGAGATATGAGGGACAATTGGAGAAGTGAATTATATA
AATATAAAGTGGTAACAATTGAGCCAGTG---GGAATAGCACCCACCGAGGCAAGGAGAAGAGTGGTG-----
----------------------------------CAGAGAGAAAAAAGAGCA---
GTGGGAACGATAGGAGCT---------ATGTTCCTTGGGTTCTTGGGAGCA---GCAGGAAGCACTATG---
GGCGCAGCGTCA---CTAACGCTGACGGTACAG---GCCAGAATA---TTATTG---
TCTGGTATAGTGCAACAGCAAAAC---AATTTG---CTGAGA---
GCTATTGAGGCGCAACAACATATGTTGCAACTCACAGTCTGGGGCATC
>HQ377423
TTCTACTGTGATACAAAACCACTGTTCAATAGTACTTGGAATGATACTACAGGGTCAATTCAC----------
----

ATTAATGGAACTATCACACTCCGATGCAGAATAAAACAAATTATAAACATGTGGCAGGGAGTAGGAAAAGCAA
TGTATGCCCCTCCCATCAGAGGACTAATTAACTGTACATCAAAT---
ATTACAGGGCTGCTATTAACAAGAGATGGTGGTAAAGAGAACAGG----------------------------
----

ACCACCGAGACCTTTAGACCTCAAGGAGGAAATATGAAAGACAATTGGAGAAGTGAACTATATAAATATAAAG
TAGTAAAAATTGAACCATTA---GGAATAGCACCCACCAAGGCAAAGCGAAGAGTGGTA-------------
-------------------------CAAAGAGAAAAAAGAGCA---ATAGGGGTAATAGGAGCT---------
ATGTTCCTTGGGTTCTTGGGAACA---GCAGGAAGCACTATG---GGCGCAGCGTCA---
CTGACGCTGACGGTACAG---GCCAGACAA---TTATTG---TCTGGTATAGTGCAACAGCAGAAC---
AATTTG---CTGAGA---GCTATTGAGGCGCAACAACATCTGTTGCAACTCACAGTCTGGGGCATC
>HQ377459
TTCTACTGCAATACAACACGCCTGTTTGATAGCACTTGGAATGGTACTGCAGGAAATTGGACTGATATTACAG
GGTCAAAT--
-------------------TAC---
ACTGATGAAGATAATATTACACTCCCATGCAGAATAAAACAAATTATAAACAGGTGGCAGGAAGTAGGAAAAG
CAATGTATGCCCCCCCATTGCAGGACAAATCAGATGTTCATCAAAT---
ATTACAGGGCTGCTATTAACAAGAGATGGTGGGGCCAATAGATCC----------------------------
----

GACACCAACGAGACCTTCAGACCTGGAGGAGGAAATATGAAGGACAATTGGAGAAGTGAATTATACAAATATA
AAGTAGTAAAAATTGAGCCATTA---GGATTAGCACCCACCAAGGCAAAGAGAAGAGTGGTG-----------
----------------------------CAGAGAGAAAAAAGAGCA---GTAGGAACACTAGGAGCT------
---ATGTTCCTTGGGTTCTTGGGAGCA---GCAGGAAGCACTATG---GGCGCAGCGTCA---
ATGACGCTGACGGTACAG---GCCAGACAA---TTATTG---TCTGGTATAGTGCAACAGCAGAAC---
AATTTG---CTGAAG---GCTATTGAGGCGCAACAGCATTTGTTGCAACTCACAGTCTGGGGCATT
>KT427681
TTCTACTGTAATACATCAACGCTGTTTAATTGGACTGAAAATTCAAATAACAAT-------------------
----

GCAAGTGACAATATGACAATCCCATGCAGAATAAGACAAATTGTAAACAGGTGGCAGGAAGTAGGAAAAGCAA
TGTATGCCCCTCCCATCAGCGGACAGATTAGCTGTTTATCAAAT---
ATCACAGGGCTGATATTAACAAGAGATGGTGGTATCAACAGCAGCAGCAGC---------------------
----

AATGAGACCTATGAGACCTTCAGACCTGCAGGAGGAAATATGAAAGATAATTGGAGAAGTGAATTATATAAAT
ATAAAGTAGTAAAAGTTGAGCCATTA---GGAGTAGCACCCACCAAGGCAAAGAGAAGAGTGGTG--------
-------------------------------CAGAGAGAAAAAAGAGCA---ATAACA---CTAGGAGCC---
------ATGTTCCTTGGGTTCTTGGGAGCA---GCAGGAAGCACTATG---GGCGCAGCGTCG---
ATGACGCTGACGGTACAG---GCCAGACTA---TTATTG---TCTGGTATAGTGCAACAGCAGAAC---
AACCTG---CTGAGA---GCTATTGAGGCGCAACAACATCTGTTGCAACTCACAGTCTGGGGCATT
>KT427673
TTCTACTGTAATACAACAAAGCTGTTTAATAGTACCTGGAATACAACAGAGTTGTTTAATAGTACCAGGTGGA
CTAATCAA--
-------------------ACT---
AGGAATGATACTGACATCATACTCCCATGTAGAATAAAACAAATTGTAAACAGGTGGCAGGAAGTAGGAAAAG
CAATGTATGCCCCTCCCATCAGAGGACAAATTAAATGTACATCAAAT---
ATTACAGGGCTGCTATTAACAAGAGATGGTAAGATAAGGAACGAGACG------------------------
----

AAAAGGAACGAGACCTTCAGACCTGGAGGAGGAGATATGAGGGACAATTGGAGAAGTGAATTATATAAATATA
AAGTAGTAAAAATTGAGCCATTA---GGAGTAGCACCCACCACGGCAAAGAGAAGAGTGGTG-----------
----------------------------CAAAGAGAAAAAAGAGCA---GTAGGAACAATAGGAGCT------
---ATGTTCCTTAGGTTCTTGGGGGCA---GCAGGAAGCACTATG---GGCGCAGCGTCA---
ATAACGCTGACGGTACAG---GCCAGACAA---TTATTG---TCTGGTATAGTGCAGCAGCAGAAC---
AATCTG---CTGAGG---GCTATTGATGCGCAACAGCATCTGTTGCAACTCACAGTCTGGGGCATC
>KT427744
TTCTACTGTAATACAACACAGCTATTTAATCATACTTGGGATGGTACTAACTGGACTGATCCTAACTGGTCGT
CAAAT---

ACAACTAACAATATCACACTTCAATGCAGAATAAGACAAATTATAAACAAGTGGCAGGAAGTAGGAAGAGCAA
TATATGCCCCTCCCATCAAAGGACAAATTAGATGCTCATCAAAA---
ATTACAGGGCTGCTACTGACAAGGGATGGTGGCAAAACGAATAGCTCT------------------------
----

AACTCCTCTGAGGTCTTCAGACCTGGAGGAGGAGATATGAGAGATAATTGGAGAAGTGAGCTATATAAATATA
AGGTAGTAAAAATTGAGCCAGTA---GGAATAGCACCCACCAAGGCAAAGAGAAGAGTGGTG-----------
----------------------------CAGAGAGAAAAAAGAGCA---GTGACA---CTAGGAGCT------
---ATGTTCCTTGGGTTCTTGGGAGCA---GCAGGAAGCACTATG---GGCGCAGCGTCA---
GTGACGCTGACGGTACAG---GCCAGACTA---TTATTG---TCTGGTATAGTGCAACAGCAAAAC---
AATTTG---CTGAGA---GCTATTGAGGCGCAACAGCATATGTTGCAACTCACAGTCTGGGGCATC
>KT427732
TTCTACTGTAAGACAACACAGCTGTTTAATAGCACTTGGTTAAATACTAGTATTGAGTATAATAATACTGAAG
GGGCT--

GGAGTACCAAATATCACACTCCAATGCAGAATAAAACAAATTATAAACAGATGGCAGGGAGTAGAAAGAGCAA
TATATGCCCCTCCCATCCCGGGACAAATTAGATGTACATCAAAT---
ATTACAGGGCTGATATTGACACGGGATGGT---AATGGTAATCAAACAGAT----------------------
----

AGCACTAACGAAACCTTCAGACCAATAGGAGGAAATATGCAGGACAATTGGAGAAGTGAATTATATAAATATA
AAGTGGTAAAAATTGAGCCAGTA---GGAATAGCACCCACCAAGGCAAAGAGAAGAGTGGTG-----------
----------------------------CAGAGAGAAAAAAGAGCA---GTGGGAACACTAGGAGCT------
---ATGTTCCTTGGGTTCTTGGGAGCA---GCAGGAAGCACTATG---GGCGCAGCGTCA---
GTGACGCTGACGGTACAG---GCCAGACTA---TTATTG---TCTGGTATAGTGCAACAGCAGAAC---
AACCTG---CTGAAG---GCTATTGAGGCGCAACAGCATTTGTTGCAACTCACAGTCTGGGGCATC
>KT427721
TTCTACTGTAATACAACACAACTGTTTAATAGTACTTGGAATGCAAATGAAAATAGTACTGGGGGAGAGTCAA
AT---

AGAACTGAAGACTATGTACTCCCATGCAGAATAAAACAGTTTATAAACATGTGGCAGGAGGTAGGAAAAGCAA
TGTATGCCCCTCCCATCAGTGGAAATATTACTTGTTCATCAAAT---
ATTACAGGGCTGCTATTAACAAGAGATGGTGGTAGT-------------------------------------
----

AATGACACCGAGACCTTTAGACCTGGAGGAGGAGATATGAGGGACAATTGGAGAAGTGAATTATACAAATATA
AAGTAGTAAAAATTGAACCAATA---GGAATAGCACCCACCAAGGCAAAGAGAAGAGTGGTG-----------
----------------------------CAGAGAGAAAAAAGAGCA---GTGGGA---CTAGGAGCT------
---CTGTTCCTTGGGTTCTTGGGAGCA---GCAGGAAGCACTATG---GGCGCAGCGTCA---
ACAGCGCTGACGGTACAG---GCCAGACAA---GTATTG---TCTGGTATAGTGCAACAGCAAAAC---
AATTTG---CTGAGG---GCTATTGAGGCGCAACAGCATATGTTGCAACTCACAGTCTGGGGCATC
>KT427717
TTCTACTGTAATTCAACACAACTATTTAATAGTACTTGGAATAATACTGCTTGGAATGAGACTATTGAT----
----

AAAAATGGAACTATCATACTTCAATGCAGAATAAAACAAATTATAAACAAGTGGCAGGAAGTAGGAAAAGCAA
TATATGCCCCTCCTATTGCAGGAAATATTACATGTTCTTCAAAC---
ATTACAGGGATTTTGTTAACAAGAGATGGTGGGATGAACGTAAGC----------------------------
----

GATACCACCGAGACCTTTAGACCTGCAGGAGGAGATATGAAAGACAATTGGAGAAGTGAATTATATAAATATA
AAGTAGTAAGACTTGAGCCATTA---GGGATAGCACCCACCAAGTCAAGGAGAATAGTGGTG-----------
----------------------------CAGAGAGAAAAAAGAGCA---GTGGGGACATTAGGAGCT------
---ATGTTCCTTGGGTTCTTGGGAGCA---GCAGGAAGCACTATG---GGCGCAGCGTCA---
ATGACGCTGACGGTACAG---GCCAGACGA---CTATTG---TCTGGTATAGTGCAACAGCAGAAC---
AATATG---CTGAAG---GCTATTGAGGCGCAACAACATATGTTGCAACTCACAGTCTGGGGCATC
>KT427713
TTCTATTGTAATACAACAAACCTGTTCAACAGTACTTGGAATGACACTACTGAAGGAAATAACACAGGA----
----

AATATAGGAAATATCACACTCCCGTGCAGAATAAAACAAATTATAAACATGTGGCAGCAAGTAGGAAAAGCAA
TGTATGCCCCTCCCATCGCAGGACAAATTAGCTGTTCATCAAAT---
ATTACAGGGTTGTTACTAACAAGAGATGGTGGTAATAATGATTCT----------------------------
----

AATAATTATGAAATCTTCAGGCCTGGAGGAGGAGATATGAGGGACAATTGGAGAAGTGAATTATACAAATATA
AAGTAGTAAAAATTGAACCATTA---GGAGTAGCACCCACTAAGGCAAAGAGAAGAGTGGTG-----------
----------------------------CAGAGAGAAAAAAGAGCG---GCA------CTAGGAGCT------
---TTGTTCCTTGGGTTCTTGGGAGCA---GCAGGGAGCACTATG---GGCGCAGCGGCA---
GTAACGCTGACGGTACAG---GCCAGAACA---TTATTG---TCTGGTATAGTGCAGCAGCAAAGG---
AATTTG---CTGAGA---GCTATTGAGGCGCAACAACATCTGTTGCAACTCACAGTCTGGGGCATC
>KT427870
TTCTACTGTAATACATCAGGACTGTTTAATAGTACTTGGAATAGGGATGGTAATTGGTCTCAGGATGATACTA
AT---

GGGTCAAATGAAACAATCACACTCCCATGCAGAATAAAACAAGTTATAAACAGGTGGCAGGAAGTAGGAAAAG
CAATGTATGCCCCTCCCATCAGCGGAAGGATTTTCTGTTCATCAAAC---
ATTACAGGGCTACTCTTAGTAAGAGATGGTGGTAACCAGAGCCAC----------------------------
----

GAGAGTGAGACCTTCCGACCTGGAGGAGGAGATATGAGGGACAATTGGAGAAGTGAATTATATAAATATAAAG
TAGTAAAAATTGAGCCATTA---GGAATAGCACCCACCAAGGCAAAGAGAAGAGTGGTG--------------
-------------------------CAGAGAGAAAAAAGAGCA---GTGGGAACGCTAGGAGCT---------
ATGTTCCTTGGGTTCTTGGGAACA---GCAGGAAGCACTATG---GGCGCAGCGTCA---
CTGACGCTGACGGTACAG---GCCAGACAA---TTATTG---TCTGGTATAGTGCAACAGCAGAAC---
AATCTG---CTGAAG---GCTATTGAGGCGCAACATCATCTGTTGCGACTCACAGTCTGGGGCATA
>KT427707
TTCTACTGTAATACAACAGGGCTGTTTAATAGTAATAATACTTGGAATAATACTAATAATAATAGTAGG----
----

AATAATGAAACATATACCATACCATGCAGAATAAAGCAAATTATAAACAGGTGGCAAGAAGTAGGAAAAGCAA
TGTACGCCCCTCCCATCAAAGGAATAATTCACTGTACATCAAAT---
ATTACAGGGCTGCTATTAACAAGAGATGGTGGGAATAGTTCAGAA----------------------------
----

AATGAAACTTTCAGACCTGCAGGAGGAGATATGAGGGACAATTGGAGAAGCGAATTATATAAATATAAAGTAG
TAAAAATTGAGCCAATA---GGACTAGCACCTACCAAGGCACAGAGAAGAGTGGTG-----------------
----------------------CAGAGAGAAAAAAGAGCA---GTGACA---CTGGGAGCT---------
ATGTTCCTTGGGTTCTTAGGAGCA---GCAGGAAGCACTATG---GGCGCAGCGTCA---
ATAACGCTGACGGTACAG---GCCAGACAA---TTGCTG---TCTGGTATAGTGCAACAGCAGAGT---
AATCTG---CTGAGG---GCTATTGAGGCGCAACAGCATATGTTGCAACTCACAGTCTGGGGCATC
>KT427706
TTCTACTGTAATACATCACTGATATTTAATGATACTTGGCTTAACAGGTCTGAAGAG----------------
----

TCAAATAAAACTATCACACTTCAATGCAGAATAAAACAAATTATAAACAGGTGGCAGGAAGTAGGAAAAGCAA
TGTATGCCCCTCCTATCAAAGGACAAATTAGATGCTCATCAAAT---
ATTACAGGGCTATTATTAACAAGAGATGGTGGTAACATAAACGAG----------------------------
----

ACTGGCAATGAGACCTTCAGACCTGGAGGAGGAGATATGAGGGACAATTGGAGAAGTGAACTATATAAATATA
AAGTAGTAAAAATTGAGCCATTA---GGAATAGCACCCACCAAGGCAAAGAGAAGAGTGGTG-----------
----------------------------CAGAGAGAAAAAAGAGCA---GTGGGAACAATAGGAGCT------
---ATGTTCCTTGGGTTCTTGGGAGCA---GCAGGAAGCACTATG---GGCGCAGCGTCA---
ATGACGCTGACGGTACAG---GCCAGACTA---TTATTG---TCTGGTATAGTGCAACAGCAGAAC---
AATTTG---CTGAGA---GCTATTGAGGCGCAACAGCATATGTTGCAACTCACAGTCTGGGGCATC
>KT427703
TTCTACTGTAATACAACAAAGCTATTTAATAGTATTTGGAATAATACACAGCTAGTTAATGGTACTTGGAAT-
----
GATACTTACTGGAAGTCTAAA------------
GTGCCAAATGAAACTATCACACTTCCATGCAGAATAAAACAAATTATAAACACATGGCAGGAAGTAGGAAAAG
CAATATATGCCCCTCCCATCAAAGGGGAAATTAAATGCTCATCAGAT---
ATTACAGGGTTGCTACTGACACGGGATGGTGGTAACATGAGCAACAGCTCTAAC-------------------
----

GACGGCTTTGAAGTCTTCAGACCTGGAGGAGGAGATATGAGAGACAATTGGAGAAGTGAACTATATAAATATA
AGGTAGTGAGAATTGAGCCATTA---GGAATAGCACCCACCAAGGCAAAGAGAAGGGCGGTG-----------
----------------------------CAGAGAGAAAAAAGAGCA---GTGGGGACAATAGGAGCT------
---ATGTTCCTTGGGTTCTTGGGAGCA---GCAGGAAGCACTATG---GGCGCAGCGTCA---
GTGACGCTGACGGTACAG---GCCAGACTA---TTATTG---TCTGGTATAGTGCAACAGCAAAGC---
AATTTG---CTGAGA---GCTATTGAGGCGCAACAGCATATATTGCAACTCACAGTCTGGGGCATC
>KT427873
TTCTACTGTAATTCAACACAACTGTTTAATAGTACCTGGGATACTAATGCAACCTTGAATGCTACAGACAGCC
AAGATGAA---

CTTAACAAAACTATCACACTCCCATGTAGAATAAAACAAATTATAAACATGTGGCAGGAAGTAGGAAAAGCAA
TGTATGCCCCTCCCATTAGAGGGCTAATTAGATGTACAACAAAT---
ATTACAGGGCTACTATTAACAAGAGATGGTGGCACAGGCAATAAGACT-------------------------
----

CAAAATAACACTGAGGTCTTCAGACCTGGAGGAGGAGATATGAAGGATAATTGGAGGAGTGAGTTATATAAAT
ATAAAGTAGTAAAAATTGAACCATTA---GGAGTAGCACCTACCAAGGCCAAGAGAAGAGTGGTG--------
-------------------------------CAGAGAGAAAAAAGAGCA---GTGGGAACAATAGGAGCT---
------ATGTTCCTTGGGTTCTTGGGAGCA---GCAGGAAGCACTATG---GGCGCAGCGTCA---
ATAACGCTGACGGCACAG---GCCAGACAA---TTATTG---TCTGGTATAGTGCAGCAGCAGAAT---
AATCTG---CTGAGG---GCTATTGAGGCGCAACAACATCTGTTGCAACTCACAGTCTGGGGCATC
>KT427695
TTCTACTGCAATACATCGGTGCTGTTTAATGATACTTGGCTGAATAATAATAAGACT----------------
----

GAACACCAAAATATCACGCTCCCATGCAAAATAAAACAGATTATAAACATGTGGCAGGAAGTAGGAAAAGCAA
TGTATGCCCCTCCCATCAGTGGACCAATTAGGTGTTCATCAAAT---
ATCACAGGGCTGCTATTAACAAGAGATGGTGGTAATAGCACAAAT----------------------------
----

AATAACGAGACCTTCAGACCTGGAGGAGGAGATATGAGGGACAATTGGAGAAGTGAATTATATAAATATAAAG
TAGTAAAACTTGAGCCATTA---GGATTAGCACCCACCAAGGCAAAAAGGAGAGTGGTG--------------
-------------------------CAGAGAGAAAAAAGAGCA---GTGGGAACGCTAGGAGCT---------
ATGTTCCTTGGGTTCTTGGGAACA---GCAGGAAGCACTATG---GGCGCAGCATCA---
CTAACGCTGACGGTACAG---GCCAGACTA---TTATTG---TCTGGCATAGTGCAACAGCAGAGC---
AATTTG---CTGAGG---GCTATTGAGGCGCATCAGCATATGTTGCAACTCACAGTCTGGGGCATC
>KT427861
TTTTACTGTAATACAACACCGCTGTTTAATAGTACATGGTCGCTTAATGGTACTACTTGGCAGCTTAATGGTA
CTCAGAAT---

AATGAGTCAGGAAATATCACACTCCAATGTAGAATAAAACAAATCATAAACATGTGGCAGGGAGTAGGAAAAG
CAATGTATGCCCCTCCCATCAGCGGACTCATAAAATGTTCATCAAAT---
ATTACAGGGCTGATATTAACAAGAGATGGTGGTAATAACAATGAAACGAAG----------------------
----

AACAACTCCGAAACCTTCAGACCTGGAGGGGGAGATATGAGGGACAATTGGAGGAGTGAATTATATAAATATA
AAGTAGTAAAAATTAAGCCATTA---GGAATAGCACCTACCAAGGCAAAGAGAAGAGTGGTG-----------
----------------------------CAGAAAGAAAAAAGAGCA---GTGGGAGCAATAGGAGCT------
---ATGTTCCTTGGGTTCTTGGGAGCA---GCAGGAAGCACTATG---GGCGCAGCGGCA---
ACGACGCTGACGGTACAG---GCCAGACAA---TTATTG---TCTGGTATAGTGCAACAGCAGAAC---
AATCTG---CTGAGA---GCTATTGAGGCGCAACAACATATGTTGCAACTCACAGTCTGGGGCATC
>KT427785
TTTTACTGTAATACAACACAACTATTTAATAGTACTTGGGTTAATGATACTTTTAGTACTTGGGTTAATAATA
CTTGGGTT---AATGGT---
ACTGTAAAGTCAATTAGTAAT---------------
AAAACAAATATTAATATTACACTTCCATGCAGAATAAAGCAAATTATAAACATGTGGCAGGAAGTAGGAAAAG
CAATGTATGCCCCTCCCATCAAGGGACCAATTAGCTGTTCATCAAAT---
ATTACAGGACTGCTATTAACAAGAGATGGGGGAAATAGTGATAATAGTAGC----------------------
----
AAAAATGAGACAAAT---

GAGACCGAGACTTTTAGACCTGGAGGAGGAAATATGAAAGACAATTGGAGAAGTGAATTATATAAATATAAAG
TAGTGCAAATTGAACCATTA---GGAATAGCACCCACCAGGGCAAAAAGAAGAGTGGTG--------------
-------------------------CAAAGAGAGAAAAGAGCA---GTGGGA---ATAGGAGCA---------
TTGTTCATTGGGTTCTTGGGAGCA---GCAGGAAGCACTATG---GGCGCAGCGTCA---
ATGACGCTGACGGTACAG---GCCAGACAA---TTATTG---TCTGGTATAGTGCAACAGCAGAAC---
AACCTG---CTGAGG---GCTATTCAGGCGCAACAGCATTTGTTGCAGCTCACAGTCTGGGGCATC
>KJ849814
TTCTACTGCAATACAACACAGCTATTTAATTGGACAATTGGCAAGTCA-------------------------
----
-------AATATAACCCAA------
AATGATAGTGAAATTATTACAATCCAATGCAGAATAAAACAGATTATAAATAAGTGGCAGGAAGTAGGAAAAT
CAATATATGCCCCTCCTATCAGAGGAAATATTAGCTGTTCATCAAAT---
ATTACAGGACTGATATTAACAAGAGATGGTGGTAAT-------------------------------------
----
CACAATGAAACTAAAAACAAGACT---------
AAGACTGAGACTTTCAGACCTGGAGGAGGAGATATGAGGGACAATTGGAGAAGTGAATTATATAAATATAAGG
TAGTGAAAATTCAGCCATTA---GGAATAGCACCCACCAGGGCAAGGCGAAGAGTGGTG--------------
-------------------------CAGAGAGAAAAAAGAGCA---GTGGGAACACTAGGAGCT---------
ATGTTCCTTGGGTTCTTGGGAGCA---GCAGGAAGCACTATG---GGCGCAGCGTCA---
ATAACGCTGACGGTACAG---GCCAGACAA---TTGTTG---TCTGGTATAGTGCAGCAGCAGAAC---
AATCTG---CTGAAA---GCTATTGAGGCGCAACAGCATCTGTTGCAACTCACAGTCTGGGGCATC
>KT427771
TTCTACTGTAATACAACACAACTGTTTAATAGTACTTGGCAAAGTAATAGTACTGGAAATAGTACTGAGAAT-
----

AATAGCACAATAATCACACTCCCCTGTAGGATAAAACAAGTTATAAACATGTGGCAGGAGGTAGGAAAAGCAA
TGTATGCCCCTCCCATCAGTGGACTAATTCAATGTACATCAAAT---
ATTACAGGGCTGCTGTTAACAAGAGATGGTGGGTCTGATGGTGAATCTAATAAA-------------------
----

ACAGAGAGTGAGATCTTCAGACCTGGAGGAGGAGATATGAGGGACAATTGGAGAAGTGAATTATATAAATATA
AAATAGTAAAAATTGAACCATTA---GGAGTAGCGCCCACCAAGGCAAAGAGAAGAGTGGTG-----------
----------------------------CAGAGAGAAAAGAGAGCACTAGGGGGA---CTAGGAGCT------
---GTATTCCTTGGGTTCTTGGGAGCA---GCAGGAAGCACTATG---GGCGCAGCGTCA---
ATAACGCTGACGGTACAG---GCCAGACAA---TTATTG---TCTGGTATAGTGCAGCAGCAGAAC---
AATCTG---CTGAGG---GCTATTGATGCGCAACGGCACATGTTGCAACTCACAGTCTGGGGCATC
>KT427753
TTCTACTGTAATACAACAAAACTGTTCAATAGTACTTGGAAGTGGAATAATGGTACTTGGAATAGGAATGATA
CT---
-------GAAGGACAAGAT------
AACACTGGAGAAACTATTACACTTCCATGCAGAATAAAACAAATCATAAACATGTGGCAGGAAGTAGGAAAAG
CAATGTATGCCCCTCCCATCAGAGGACAAATTAATTGCACATCAAAT---
ATTACAGGACTGCTATTAACAAGGGATGGTGGTAATAATAATAATAGC-------------------------
----

AAGAATAACGAGACTTTCAGACCTGGAGGAGGAAATATGAAGGACAATTGGAGAAGTGAATTATATAAATATA
AAGTAGTACAAATTGAACCATTA---GGAATAGCACCCACCAAGGCAAAAAGGAGAACGGTG-----------
----------------------------CAGAGAGAAAAAAGAGCA---GTGGGA---ATAGGAGCT------
---GTGCTCTTTGGGTTCTTGGGAGCA---GCAGGAAGCACTATG---GGCGCAGCGTCA---
GTAACGCTGACGGTACAG---GCCAGACAA---TTGTTG---TCTGGTATAGTGCAACAGCAGAGC---
AATCTG---CTGAAG---GCTATTCAGGCGCAACAGCATCTGTTGCAGCTCACAGTCTGGGGCATC
>KT427749
TTCTACTGTAATACAACAGAACTGTTCTATAGTAATTGGACGTGGAATGGTACTTGGAATAGGAATGATACTG
AAGGATTA---

CAAAATAAAACTATTATACTTCCATGCAGAATAAAACAAATCATAAACATGTGGCAGGAAGTAGGAAAAGCAA
TGTATGCCCCTCCCATCAGAGGACAAATTAATTGCACATCAAAT---
ATTACAGGACTGCTATTAATAAGAGATGGTGGTAATGATACTGGAACT-------------------------
----

AGTGGGACTGAAATTTTCAGACCTCAAGGAGGAAACATGAAAGACAATTGGAGAAGTGAATTATATAAATATA
AAGTAGTACAAATTGAACCATTA---GGAATAGCACCCACCAAGGCAAAGAGAAGAACGGTG-----------
----------------------------CAGAGAGAAAAAAGAGCA---GTGGGA---TTTGGAGCT------
---GTGATCTTTGGGTTCTTGGGAGCA---GCAGGAAGCACTATG---GGCGCAGCGTCA---
ATAACGCTGACGGTACAG---GCCAGACAA---TTGTTG---TCTGGTATAGTGCAACAGCAGAGC---
AATTTG---CTGAAG---GCTATTCAAGCGCAACAGCATCTGTTGCAGCTCACAGTCTGGGGCATC
>KT427826
TTTTACTGTAATACAACAAAACTGTTTAATACTACGTGGAATATTACTGGAGAAGGAAACAACAATCTC----
----

ACTAATGAAACTATCACACTCCCATGCAGAATAAAACAGTTCATAAACATGTGGCAGGAAGTAGGAAAAGCAA
TGTATGCCCCTCCTATCAGGGGACAAATTCACTGCTCATCAAAT---
ATTACAGGGCTACTATTAACAAGAGATGGTGGTAATAGTAATAAT----------------------------
----

GAGTCGGAGACCTTCAGACCGGGAGGAGGAGATATGAGGGACAATTGGAGGAGTGAATTATATAAATATAAAG
TAGTAAAAATTGAACCATTA---GGAATAGCACCCACCAAGGCAAGAAGGAGAGTGGTG--------------
-------------------------CAAAGAGAAAAAAGAGCA---ATAGGAGGAATAGGAGTT---------
GTGATCCTTGGGTTCTTGGGAGCA---GCAGGAAGCACTATG---GGCGCAGCGTCG---
CTAACGCTGACGGTACAG---GCCAGACAA---TTATTG---TCTGGTATAGTGCAACAGCAAAGC---
AATTTG---CTGAGA---GCTATTGAGGCGCAACAGCGTTTGTTGCAACTCACTGTCTGGGGCATC
>KJ849821
TTCTACTGTGATACAGCAAAGCTGTTTAACAGTACTTGGGAGAGGCGTAATGACACGAAC-------------
----
ACTTGGGAGTCGAACAATACA------------
ATACCAAATGAGAATATCACACTCCCATGCAGAATAAAACAAATTATAAACAGGTGGCAGGAAGTAGGAAAAG
CAATGTATGCCCCTCCCATTGCAGGACGAATTGAATGTACATCAAAT---
ATTACAGGGCTACTATTAACAAGAGATGGTGGT--

-
TCTAACAATAGTAGTAACGATACCTTCAGACCAGGAGGAGGAGATATGAAGGACAATTGGAGAAGTGAATTAT
ATAAATATAAAGTAGTACAAATTCAGCCATTA---GGAGTAGCACCCACCAGGTCAAGGAGAAGAGTGGTG--
-------------------------------------CAGAGAGAAAAAAGAGCA---GTAACA---
CTGGGAGCT---------ATGTTCCTTGGGTTCTTGGGAGCA---GCAGGAAGCACTATG---
GGCGCAGCGTCA---GTGACGCTGACGGTACAG---GCCAGACAA---TTATTG---
TCTGGTATAGTGCAGCAGCAAAGC---AATTTG---CTGAGG---
GCTATTGATGCGCAACAGCACATGTTGCAACTCACAGTCTGGGGCATC
>KT427811
TTTTACTGTAATACATCAAGACTGTTTAACAGTATTTGGAACAGTACCTGGCAGGATAATACAGCTGAAGAGT
CA---

AATAATACTGACATCATACTCCATTGCAGAATAAAACAAATTATAAACAGATGGCAGGAAGTAGGAAAAGCAA
TGTATGCCCCTCCCATCAAGGGAAAAATTCAATGTTCATCAAAT---
ATTACAGGGCTGCTATTAGTAAGAGATGGTGGTAACGAGAACACT----------------------------
----

AGTAACACCGAGACCTTCAGACCTGGAGGAGGAGACATGAGGGACAATTGGAGAAGTGAATTATATAAATATA
AAGTAGTGCAAATTGAGCCAGTA---GGAATAGCACCCACCAAGGCAAGAAGAAGAGTGGTG-----------
----------------------------CAGAGAGAAAAAAGAGCA---GTAACA---ATAGGAGCT------
---ATGTTCCTTGGGTTCTTGGGAGCA---GCAGGAAGCACTATG---GGCGCAGCGTCA---
ATCGCGCTGACGGGACAG---GCCAGACAA---TTATTG---TCTGGTATAGTGCAACAGCAGAAT---
AATCTG---CTGAAG---GCTATTGAGGCGCAACAACATATGTTGCAACTCACAGTCTGGGGCATC
>KT427796
TTCTATTGTAATACATCACAGCTGTTCAATAGTACTTGGAATTTCACTAGTGCTAGGAATGAGACTGAAGAG-
----

TCAAATGACACTATCACACTCCCATGTAGGATAAAACAAATTATAAATAAGTGGCAAGAAGTAGGAAAAGCAA
TATATGCTCCTCCTATCCAAGGACGAATTTACTGTTCATCAAAC---
ATTACAGGGTTGCTATTAACAAGAGATGGGGGTGAAAACAACCTG----------------------------
----

ACCAATGAGACTTTTAGACCTGGAGGAGGAGATATGAGGGACAATTGGAGAAGTGAACTGTATAAATATAAAG
TAGTAAAAATTGAGCCATTA---GGACTAGCACCCACCAAGGCAAAGAGAAGAGTGGTG--------------
-------------------------CAGAGAGAAAAAAGAGCA---GTGGGAACGCTGGGAGCA---------
ATGTTCCTTGGGTTCTTGGGAACA---GCAGGAAGCACTATG---GGCGCAGCGTCG---
CTGACGCTGACGGTACAG---GCCAGACAA---TTATTG---TCTGGTATAGTGCAACAGCAAAAC---
AATTTG---CTGAAG---GCTATTGAGGCGCAACAGCATCTGTTGCAACTCACAGTCTGGGGCATT
>KX181905
TTCTATTGTAATACAACAAAATTGTTTGATTGGAATGATACAACAAAAGTGTTTAATTGGAATAAATCA----
----
-------TGGACGTTTACA------
GAAGGCAATGACACTATTCATATCCCATGCAGAATAAAACAAATTATAAACAGGTGGCAGGAAGTAGGAAAAG
CAATGTATGCCCCTCCCATTGGAGGGCTGATTAGATGTTCATCAAAT---
ATTACAGGGCTGCTATTAACAAGAGATGGTGGTAATGAGACAAGG----------------------------
----

GATAACGACACTTTCAGACCTGGAGGAGGAGATATGAGGGACAATTGGAGAAGTGAATTATATAAATATAAAG
TAGTAAGAATTGAACCATTA---GGGGTAGCACCCACCAAGGCAAAGAGAAGAGTGGTA--------------
-------------------------CAAAGAGAAAAAAGAGCA---GTGGGAGCAATAGGAGCT---------
ATGTTCCTTGGGTTCTTGGGAGCA---GCAGGAAGCACTATG---GGCGCAGCGGCA---
GTGACGCTGACGGTACAG---GCCAGACAA---TTGTTG---TCTGGTATAGTGCAACAGCAGAAC---
AATCTG---CTGAGA---GCTATTGAGGCGCAACAGCATTTGTTGCAGCTCACAGTCTGGGGCATC
>KX181909
TTCTACTGTAATACAGCAGAGCTGTTTAGTAATACTTGGTTAAATAAGAGTGGCAAT----------------
----

AACGATGGAACTATCACACTTCCATGCAGAATAAAACAAATTATAAACAGATGGCAGGAAGTAGGAAAAGCAA
TGTATGCCCCTCCCATCAGGGGATTAATTGAATGTTCATCAAAT---
ATTACAGGGCTGATACTAACAAGAGATGGTGGTAATAATAGTGCA----------------------------
----

AACAATGCTAGTGAAACCTTCAGACCCGGAGGGGGAAATATGAAGGACAATTGGGGAAGTGAATTATATAAAT
ATAAAGTAGTAAAAATTCAGCCCTTA---GGAATAGCACCCACCAGGGCAAAAAGAAGAGTGGTG--------
-------------------------------CAAAGAGAAAAAAAAGCA---GTAACG---CTAGGAGCT---
------ATGTTCCTTGGGTTCTTGGGAACA---GCAGGAAGCACTATG---GGCGCAGCGTCA---
GTGACGCTGACGGTACAG---GCCAGACAG---TTACTG---TCTGGTATAGTGCAACAGCAGAGC---
AATCTG---CTGAAG---GCTATTGAGGCGCAACAGCATCTGTTGCAACTCACAGTCTGGGGCATC
>KX181908
TTTACTGTGATACAACAAAGCTGTTTAATAGTACTTGGAATAATACTGGAGAGTCAAATTACACGGAG----
----

GGAAATGCCACTATCATACTTCCATGCAGAATAAAACAAATTATAAACAGATGGCAGGAAGTAGGAAAAGCAA
TGTATGCCCCTCCCCTCAGTGGAAATATTAGCTGTTTATCAAAT---
ATTACAGGGGTACTATTAACAAGAGATGGGGGTAATGAAACAAAT----------------------------
----

GCCCCTGAGACCTTCAGACCTCAAGGGGGAAATATAAAGGATAATTGGGGAAGTGAATTATATAAATATAAAG
TAGTAAAAATTGAACCCTTA---GGAGTAGCCCCCCCCAAGGCAAAGAGAAGAGTGGGG--------------
-------------------------CAAAGAGAAAAAAGAGCA---GTGGGA---ATAGGAGCT---------
ATGTTCCTTGGGTTCTTGGGAGCA---GCAGGAAGCAATATG---GGCGCAGCATCA---
CTAACGCTGACGGTACAG---GCCAGACTA---TTATTG---TCTGGTATAGTGCAACAGCAAAAC---
AATTTG---CTGAGA---GCTATTGAGGCGCAACAGCATCTGTTGCAGCTCACAGTCTGGGGCATC
>KX181898
TTCTACTGTAATACATCACAGCTGTTTAATTGGACTGAAAGAAATAGAAATAGCTCAGATGGGACAGGGTCAA
AT---

GAGACTGAGACCATTACACTCCCATGCAGAATAAAACAAATTATAAACAGATGGCAGGAAGTAGGAAAAGCAA
TGTATGCCCCTCCCATCAAAGGAAATATTAGCTGTTCATCAAAT---
ATTACTGGGCTGCTATTAACAAGAGATGGTGGTACAAACCTGACT----------------------------
----

GGGTCTAACGGGACCGAGACCTTCAGACCTCAAGGAGGAAATATGAAGGATAATTGGAGAAGTGAATTATATA
AATATAAAGTAGTAAGAATTGAGCCAGTA---GGAATAGCACCCACCGAGGCAAAGAGAAGAGTGGTG-----
----------------------------------CAAAGAGAAAAAAGAGCA---
GTGGGAACACTAGGAGCT---------ATGTTCCTTGGGTTCTTGGGAGCA---GCAGGAAGCACTATG---
GGCGCAGCGTCA---ATGACGCTGACGGTACAG---GCCAGACAA---TTATTG---
TCTGGTATAGTGCAACAGCAAAAC---AATCTG---CTGAAA---
GCTATTGAGGCGCAACATCATCTGTTGCAACTCACAGTCTGGGGCATC
>KX181899
TTCTACTGTAATACATCACAGCTGTTTAATAGTACTTGGAATGGTATTGAGAATATTTCGAATGATACTGAGA
ATTAT--

ATCACACTCCCATGCAGAATAAAACAAATTATAAATAGGTGGCAGGAAGTAGGAAAAGCAATGTATGCCCCTC
CCATTAAAGGAAATATTAGCTGCACATCAAAC---
ATTACAGGGCTGATACTAACAAGAGATGGTGGTAACAATGAGAGC----------------------------
----

AACGAGACTGAGACTTTCAGACCTGGAGGAGGAGATATGAGGGACAATTGGAGAAGTGAATTATATAAATATA
AGGTAGTAAAAATTGAGCCATTA---GGAATAGCACCCACCAAGGCAAAGAGGAGAGTGGTG-----------
----------------------------CAGAGAGAAAAAAGAGCA---GTGGGAACGCTGGGAGCC------
---ATGTTCCTTGGGTTCTTGGGAGCA---GCAGGAAGCACTATG---GGCGCAGCGTCA---
ATAACGCTGACGGTACAG---GCCAGACAA---GTATTG---TCTGGTATAGTGCAACAGCAGAAC---
AATCTG---CTGAGA---GCTATTGAGATGCAACAGCATCTGTTGCAACTCACAGTCTGGGGCATC
>KX181913
TTTTACTGCAATACAACACCACTGTTTAATTGGACTGAAGGGTCAAATAACACTCAATGG-------------
----

CCAAAGGACAATATTACACTTCAATGCAGAATAAAACAAATTGTAAACAGGTGGCAGGAAGTAGGAAAAGCAA
TGTATGCCCCTCCTATCAGTGGACTGATTAACTGTTTATCAAAT---
ATTACAGGGTTGCTATTAACAAGAGATGGTGGTAATAAGTCCGGGAAAGAT----------------------
----

ACTGGTCCCGAAATTTTTAGACCAGCAGGGGGAAATATGAAGGGCAATTGGGGAAGTGAATTATATAAATACA
AAGTAGTAAAAATTGAACCCTTA---GGAATAGCCCCCCCAGGGCAAAGAGAAGAGTGGTG-----------
----------------------------CAGAGAGAAAAAAGAGCA---GTGGGAGTAATAGGAGCC------
---ATGTTCCTTGGATTTTTGGGAGCA---GCAGGAAGCACTATG---GGCGCAGCGGCA---
ATGACGCTGACGGTACAG---ACCAGACAA---TTATTG---TCTGGTATAGTGCAGCAGCAGAAC---
AATTTG---CTGAAA---GCTATTGAGGCGCAACAGCATCTGTTGCAACTCACAGTCTGGGGCATC
>MK041573
TTCTACTGTAATACAACAAAACTATTTAATAGTACTTGGGATGGGTCAAACATCATAGGAAATGATACA----
----

AATGACACAGTGATCACACTCCCATGCAGAATAAAACAAATTATAAACATGTGGCAGCGAGTAGGAAAAGCAA
TGTATGCCCCTCCCATCAGAGGACAAATTAGATGTTCATCAAAT---
ATTACAGGGCTGCTGTTAACAAGAGATGGTGGAAATCATACAAGT----------------------------
----

GGGAATGAGACCTTCAGACCAGGAGGAGGAGATATGAGGGACAATTGGAGAAGTGAATTATATAAATATAAAG
TAGTAAAAATTGAACCATTG---GGAGTAGCACCCACCAGGGCAAAGAGAAGGGTGGTG--------------
-------------------------CAGAGAGAAAAAAGAGCA---GTAGGA---CTAGGAGCT---------
TTGTTCCTTGGGTTCTTGGGAGCA---GCAGGAAGCACTATG---GGCGCAGCGTCA---
CTAACGCTGACGGTACAG---GCCAGACAG---TTATTG---TCTGGTATAGTGCAACAGCAGAAC---
AACTTG---CTGAGG---GCTATTGAGGCGCAACAGCACATGTTGCAACTCACAGTCTGGGGCATC
>MK041575
TTCTACTGTAATACAACACAGCTGTTTAATAATACTTGGAATGATACTGCTTGGAATGATACTAGCTGG----
----

TCAAATGGCACTATCACACTCCCATGCAGAATAAAACAAATTATAAACAGGTGGCAGGAAGTAGGAAAAGCAA
TGTATGCCCCTCCCATCAGCGGACAAATTAATTGTTCATCAAAT---
ATTACAGGACTGTTATTAACAAGAGATGGTGGTAATGACAATGACACGAATACG-------------------

----

AATGAGACCGAGATCTTCAGACCTGCAGGAGGAGATATGAGGGACAATTGGAGAAGTGAATTATATAAATATA
AAGTAGTAAAAATTGAACCATTA---GGAATAGCACCCACCAGGGCAAAGAGAAGAGTGGTG-----------
----------------------------CAGAGAGAAAAAAGAGCA---GTGACA---TTAGGAGCT------
---GTGTTCCTTGGGTTCTTGGGAGCA---GCAGGAAGCACTATG---GGCGCAGCGTCA---
GTGACGCTGACGGTACAG---GCCAGACTA---TTATTG---TCTGGTATAGTGCAACAGCAGAGC---
AATTTG---CTGAGA---GCCATTCAGGCGCAACAGCATCTGCTGCAACTCACAGTCTGGGGCATC
>MG571983
TTCTACGGTAATACATCAAACCTGTTTAATAATAATATTACAGGGATTATA----------------------
----

AAGAATGGGACTATCATACTCCCATGCAGAATAAAACAAATTATAAACAGGTGGCAGGAAGTAGGAAGAGCAA
TGTATGCCCCTCCCATCAGAGGACTAATCAACTGTACATCAAAT---
ATTACAGGGCTGCTATTAACAAGAGATGGTGGTGGTGGTAATGAG----------------------------
----

AGTCATAACGAGACCTTCAGACCTGGAGGAGGAGATATGAGGGACAATTGGAGAAGTGAATTATATAAATATA
AAGTAGTAAGAATTGAGCCATTA---GGAATAGCACCCACCAAGGCACAGAGAAGAGTGGTG-----------
----------------------------CAAAGAGAAAAAAGAGCA---GTGGGAGCACTAGGAGCT------
---ATGTTCCTTGGGTTCTTGGGAGCA---GCAGGAAGCACTATG---GGCGCAGCGTCA---
ATAACGCTGACGGCACAG---GCCAGACAA---GTATTG---TCTGGTATAGTGCAACAGCAAAAC---
AATTTG---CTGAGG---GCTATTGAGGCGCAACAGCATTTGTTGCAACTCACAGTCTGGGGCATC
>MG571987
TTCTACTGTAATACAACATCACTGTTTAATAGTACTTGGCTGCTTAATGATACTGGAAATGTTACTGTGCCAA
ACAAA--

ACGGACGAAATTATCATACTCCCATGCAGAATAAAACAAATTATAAATATGTGGCAAAAAGTAGGAAGAGCAA
TGTATGCCCCTCCCATCAGAGGTTATATTAACTGTTCATCAAAT---
ATTACAGGGCTGCTATTAGTAAGGGATGGTGGTAATAATAATCAGTCT-------------------------
----

AATCAGGCTGAAACCTTCAGACCTGGAGGAGGAGATATGAGGGACAATTGGAGAAGTGAATTATATAAATATA
AAGTAGTAAAAATTGAGCCATTA---GGAATAGCACCCACCAAGGCAAAGAGAAGAGTGGTG-----------
----------------------------CAGAGAGAAAAAAGAGCA---GTGGGGACACTAGGAGCT------
---GTGTTCCTTGGGTTCTTGGGAGCA---GCAGGGAGCACTATG---GGCGCAGCGTCA---
CTAACGCTGACGGTACAG---GCTAGACTA---TTATTG---TCTGGTATAGTGCAACAGCAGAAC---
AATTTG---CTGAGA---GCTATTGAGGCGCAACAGCATATATTGCAACTCACAGTCTGGGGCATC
>MG571990
TTCTACTGTAATACAACAAAACTATTTAATAGTACTTGGGATGGGTCAAACATCATAGGAAATGATACA----
----

AATGACACAGTGATCACACTCCCATGCAGAATAAAACAAATTATAAACATGTGGCAGCGAGTAGGAAAAGCAA
TGTATGCCCCTCCCATCAGAGGACAAATTAGATGTTCATCAAAT---
ATTACAGGGCTGCTGTTAACAAGAGATGGTGGAAATCATACAAGT----------------------------
----

GGGAATGAGACCTTCAGACCAGGAGGAGGAGATATGAGGGACAATTGGAGAAGTGAATTATATAAATATAAAG
TAGTAAAAATTGAACCATTG---GGAGTAGCACCCACCAGGGCAAAGAGAAGGGTGGTG--------------
-------------------------CAGAGAGAAAAAAGAGCA---GTAGGA---CTAGGAGCT---------
TTGTTCCTTGGGTTCTTGGGAGCA---GCAGGAAGCACTATG---GGCGCAGCGTCA---
CTAACGCTGACGGTACAG---GCCAGACAG---TTATTG---TCTGGTATAGTGCAACAGCAGAAC---
AACTTG---CTGAGG---GCTATTGAGGCGCAACAGCACATGTTGCAACTCACAGTCTGGGGCATC
>MG572002
TTCTATTGTAATTCATCACAGCTGTTTAATACAACTTGGATAAATGGCACTACAGGAAATAGAACTAATGAA-
----

AATGACACACTTATCACACTCCCATGCAGAATAAAACAAATTATAAACAGATGGCAGGAAGTAGGAACAGCAA
TATATGCCCCTCCCATTGAAGGCATGATTAAATGTTCATCAAAT---
ATTACGGGGGTGCTATTAACAAGAGATGGTGGTTATGATAACAATAGCACG----------------------

----

CGCCAGAACGAGACCTTCAGACCTGGAGGGGGAAATATGAAGGACAATTGGAGAAGTGAATTATATAAATATA
AAGTAGTAAAAGTTGAGCCATTA---GGACTAGCACCCACCAAGGCAAAGAGAAGAGTGGTG-----------
----------------------------CAAAGAGAAAAAAGAGCG---GTAACA---TTAGGAGCT------
---ATGTTCCTTGGGTTCTTGGGAGCA---GCAGGAAGCACTATG---GGCGCAGCGTCA---
ATGACGCTGACGGTACAG---GCCAGACAA---TTGTTG---TCTGGTATAGTGCAACAGCAGAAC---
AATCTG---CTGAGA---GCTATTGAGGCGCAACAGCATATGTTGCAACTCACAGTCTGGGGCATA
>JF320427
TTCTACTGCAATACAACAGCACTGTTTAATAGTACATGGAACGGTACAGAGGGGATAAGTAAT----------
----

GCTACAGGAAATATCACACTCCCATGCAGAATAAAACAAGTTATAAACAGATGGCAGGAAGTAGGAAAAGCAA
TGTATGCCCCTCCCATTAAAGGAGTAATTAGATGTGTATCAAAT---
ATCACAGGGATACTATTAACGAGAGATGGGGGTAATAATAATAATACT-------------------------
----

AATCACACCGAGATATTCAGACCTGGAGGAGGAGATATGAGAGACAATTGGAGAGGTGAATTATATAAATATA
AGGTAGTAAAAAATTGAACCATTA---GGAGTAGCACCCACCAAGGCAAAGAGAAGAGTGGTG----------
----------------------------CAGAGAGAAAAAAGAGCA---GTGGGA---ATAGGAGCT------
---TTGTTCCTTGGGTTCTTGGGAGCA---GCAGGAAGCACGATG---GGCGCAGCGTCA---
GTGACGCTGACGGTACAG---GCCAGACAA---TTATTG---TCTGGTATAGTGCAACAGCAGAGC---
AATTTG---CTGAGG---GCTATTGAGGCGCAACAACATATGTTGCAACTCACAGTCTGGGGCATC
>JF320424
TTTTACTGTAGTACAACATTATTGTTTAATAGTACTTGGGGGTCTAATGAGACTAGGAATGAGACTATA----
----

GACAATAGCACTATCACAATTCAATGTAAAATAAAACAACTTATAAACATGTGGCAGGGGGTAGGAAGGGCAA
TGTATGCCCCTCCCATCAGGGGAAACATTAGCTGTTCATCAAAT---
ATTACAGGACTACTATTAACAAGAGATGGTGGAAACAATGGGACA----------------------------
----

AATGGTACTGAGACCTTCAGACCTGAAGGAGGAAATATGAAGGATAATTGGAGAAATGAATTATATAAATATA
AAGTAGTAAAAATTGAACCATTA---GGAATAGCACCCACCAAGGCAAGGAGAAGAGTGGTG-----------
----------------------------CAGAGAGAAAGAAGAGCA---GCAGGA---CTGGGAGCT------
---TTGTTCATTGGGTTCTTGTCAGCA---GCAGGAAGCACTATG---GGCGCAGCATCA---
CTAACGCTGACGGTACAA---GCCAGACAA---TTATTG---TCTGGTATAGTGCAACAGCAGAAC---
AATTTG---CTGAGA---GCTATTGAGGCGCAACAGCATATGTTGCAACTCACAGTCTGGGGCATC
>GU562236
TTCTACTGCAATACAACGCAGCTGTTTAATAGTACTTGGTATAGTAATGGTACTTGGTCTTGGAATGGTACTA
CTTGGAATGGT---ACT---
AGAGAGACAAATAGTACGGAA---------------
GGAAACGACACAATGATCACACTCCCATGCAGAATAAAACAATTTATAAACATGTGGCAGGCAGTAGGAAAAG
CAATATATGCCCCTCCCATCAGTGGACTAATCAAATGTACATCAAAT---
ATTACAGGGCTGTTAATAACAAGAGATGGGGGTCATGAAAGTAATAAAAGTAAC------TATGGT-------
----
TATGAAAGTAGTAAAAGT---
GAGAATGAGACCTTCAGACCTGGAGGAGGAAATATGAGGGATAATTGGAGAAGTGAATTGTATAAATATAAAA
TAGTAAGAATTGAACCATTA---GGACTAGCACCCACCAAGGCAAAGAGAAGAGTGGTG--------------
-------------------------CAGAGAGAAAAGAGAGCC---GCA------CTAGGAGCC---------
ATGTTCCTTGGGTTCTTGGGAGCA---GCAGGAAGCACTATG---GGCGCAGCGTCA---
CTGACGCTGACGGTACAG---GCCAGACAA---TTATTG---TCTGGTATAGTGCAACAGCAGAGC---
AATTTG---CTGAGG---GCTATTGAGGCGCAACAGCATATGTTGCAACTCACGGTCTGGGGCATC
>JQ403024
TTCTACTGTAATACAACACAACTGTTTAATAGAGATTGGAATGATACTAGTACTATTAACAGTACT-------
----

GAAAATAGAAATATCACACTCCCATGTAGAATAAAACAAATTATAAACATGTGGCAGGAAGTAGGAAAAGCAA
TGTATGCCCCTCCCATCCAAGGACAAATTAGATGTTCATCAAAT---
ATTACAGGGTTGCTATTAACAAGAGATGGTGGTAATGACAATACCACT-------------------------

----

AACACCACTGAGATCTTCAGACCTGTAGGAGGAAATATGAGGGACAATTGGAGAAGTGAATTATACAAATATA
AAGTAGTACAAATTGAACCATTA---GGATTAGCACCCACCAAGGCAAAGAGAAGAGTGGTG-----------
-----------------------------CAGAGAGAAAAAAGAGCA---GTAACG---TTAGGAGCT------
---ATGTTCCTTGGGTTCTTGGGAGCA---GCAGGAAGCACTATG---GGCGCAGCGTCA---
ATGACGCTGACGGTACAG---GCCAGACAA---TTATTG---TCTGGTATAGTGCAGCAGCAAAAT---
AATTTG---CTGAAG---GCTATTGAGGCGCAACATCATCTGTTGCAACTCACAGTCTGGGGCATC
>JQ403041
TTCTACTGTAATACAACACGACTGTTTAATAGTACTTGGAATAGTACCGAATCAAATAATACTATA-------
----

GGAAATGGCACAATCACACTCCCATGCAGAATAAAACAAATTGTAAACATGTGGCAGGAAGTAGGAAAAGCAA
TGTATGCCCCTCCCATCAGAGGACAAATTAGATGTTCATCAAAT---
ATTACAGGGCTGATATTAACAAGAGATGGTGGTCATGGTGGTCATAATAAGAAT------------------
----

ACCACCAACACCACAGAGATCTTCAGACCTGGAGGAGGAGATATGAGGGACAATTGGAGAAGTGAATTATATA
AATATAAAGTAGTAAAACTTGAACCATTA---GGAATAGCCCCCACCTCGGCAAGGAGAAGAGTGGTG-----
-----------------------------------CAGAGAGAAAAAAGAGCA---GTGGGA---
ATAGGAGCT---------ATGTTCCTTGGGTTCTTGGGAGCA---GCAGGAAGCACTATG---
GGCGCAGCGTCA---GTGACGCTGACGGTACAA---GCCAGACTA---TTATTG---
TCTGGTATAGTGCAACAGCAGAAC---AATTTG---CTGAAG---
GCTATTGAGGCGCAACAGCATATGTTGCAACTCACAGTCTGGGGCATC
>KC797225
TTCTACTGTAATACATCACAATTGTTTAATAGTACTTGGAATGCTACTGGTACTCAGAATACTACTGGTACTC
AGAATGAGACT--

AAAATAGGCAATATCACACTCCCATGTAAAATAAAACAAATTATAAACAGGTGGCAAGAAGTAGGAAAAGCAA
TGTATGCCCCTCCCATCAAAGGACTAATTAACTGTGTATCAAAT---
ATTACAGGGATACTATTAACAAGAGATGGTGGTCATTCTAAGAATACAAATAGT------------------
----

ACAGATAATGATACCGAGGTCTTCAGACCTGGAGGAGGAGATATGAGGGACAATTGGAGAAGTGAATTATATA
AATATAAAGTGGTAAAAATTGAGCCATTA---GGAGTAGCACCCACCAAGGCAAAGAGAAGAGTGGTG-----
-----------------------------------CAGAGAGAAAAAAGAGCA---GTGACG---
CTAGGAGCC---------ATGTTCCTTGGGTTCTTGGGAGCA---GCAGGAAGCACTATG---
GGCGCAGCGTCG---CTGACGCTGACGGTACAG---GCCAGACAA---TTATTG---
TCTGGTATAGTGCAACAGCAGAAC---AACCTG---CTGAGG---
GCTATTGAGGCGCAACAGCATATGTTGCAACTCACAGTCTGGGGCATC
>HQ699978
TTCTACTGTAATACATCACAACTGTTTAATAGTACTTATAATGGTACTTGGAGTGGTACTACA----------
----

GGAAATGATACTATCACGCTCCCTTGCAGAATAAAACAAATTGTAAACAGGTGGCAGGAAGTAGGGAAAGCAA
TGTATGCCCCTCCCATTCAAGGAATAATTAGATGTTCATCAAAT---
ATTACAGGGTTGCTATTAACAAGAGATGGTGGTAAGAATGAGAGCGAT-------------------------
----

AGCAATACCACCGAGACCTTCAGACCTGGAGGAGGAAATATGAGAGACAATTGGAGGAGTGAATTATATAAAT
ATAAAGTAGTAAAAATTGAACCATTA---GGAGTAGCACCCACCAAGGCAAAAAGAAGAGTGGTG--------
--------------------------------CAGAGAGAAAAAAGAGCA---GTGGGAACGATAGGAGCT---
------ATGTTCCTTGGGTTCTTGGGAGCA---GCAGGAAGCACTATG---GGCGCAGCGTCA---
ATAACGCTGACGGTACAG---GCCAGACAA---TTGTTG---TCTGGTATAGTGCAACAGCAGAGA---
AATTTG---CTGAGG---GCTATTGAGGCGCAACAGCATCTGTTGCAACTCACAGTCTGGGGCATC
>EU131792
TTCTACTGTAATACATCAGAGCTGTTTAAAAGTACTTGGAATAATACTAGTACT-------------------
----

GGGAATGATCCTATCACGCTCCCTTGCAGAATAAAGCAAATTGTAAACATGTGGCAAGAAGTAGGGAAAGCAA

```
TGTATGCTCCTCCCATTGAAGGACAAATTAGATGTTTATCAAAT---
ATTACAGGATTGCTATTAACAAGGGATGGTGGTCATAATGGGAGC----------------------------
-------------------------------------------------------------------------
----------
GAGACCAACGAGACCTTCAGACCTGCAGGAGGAAATATGAGAGACAATTGGAGAAGTGAATTATATAAATATA
AAGTAGTAAAAATTGAACCATTA---GGAGTAGCACCCACCAAGGCAAAGAGAAGAGTGGTG-----------
----------------------------CAGAGAGAAAAAAGAGCA---GTGGGAACAATAGGAGCT------
---ATGTTCCTTGGGTTCTTGGGAGCA---GCAGGAAGCACTATG---GGCGCAGCGTCA---
ATGACGCTGACGGTACAG---GCCAGACAA---ATGTTG---TCTGGTACAGTGCAACAGCAAAGA---
AATTTG---CTGAGG---GCTATTGAGGCGCAACAGCATCTGTTGCAACTCACAGTCTGGGGCATC
>EU131803
TTCTACTGTAATACATCAAAATTATTTAATAGTACTTGGTATAATAATGGTACTTGGAATAATGCTAGTACT-
-------------------------------------------------------------------------
----------------------------
GGGAATGATACTATCACGCTCCCTTGCAGAATAAAACAAATTGTAAACATGTGGCAGGAAGTAGGGAAAGCAA
TGTATGCCCCTCCCATTGCAGGACAAATTAGATGTTCATCAAAG---
ATTACAGGGTTGCTATTAACAAGAGATGGTGGTAATGAGAACAAGACTAAGGAC-------------------
-------------------------------------------------------------------------
----
AACAACACCGAGACTGAGACCTTTAGACCTGGGGGAGGAGATATGAGAGACTATTGGAGAAGTGAATTATATA
AATATAAAGTAGTAAAAATTGAACCATTA---GGAGTAGCACCCACCAAGGCAAAGAGAAGAGTGGTG-----
----------------------------------CAGAGAGAAAAAAGAGCA---
GTGGGAACAATAGGGGCT---------ATGTTCCTTGGGTTCTTGGGAGCA---GCAGGAAGCACTATG---
GGCGCAGCGTCA---ATAACGCTGACGGTACAG---GCCAGACAA---TTGTTG---
TCTGGTATAGTGCAACAGCAGAGA---AATTTG---CTGATG---
GCTATTGAGGCGCAACAACATTTGTTGCAACTCACAGTCTGGGGCATC
>EU363829
TTCTACTGTAATACAACAAAACTGTTTAATAGTATTTGGTATAATAATGGTACTTCGAGTGGTACTACA----
-------------------------------------------------------------------------
----------------------------
GGAAATGAAACTATCATACTCCCTTGCAGAATAAAACAAATTGTAAACATGTGGCAGAAAGTAGGGAGAGCAA
TGTATGCCCCTCCCATTGAAGGAAAAATTAGCTGTGTATCAAAT---
ATCACAGGGTTGCTATTAACAAGAGATGGTGGTCAGAATGAGAAC----------------------------
-------------------------------------------------------------------------
-------------
AATACCGAAACCTTCAGACCTGAAGGAGGAAATATGAAAGACAATTGGAGAAGTGAATTATATAAATATAAAG
TAGTAAAAATTGAACCATTA---GGAGTAGCACCCACCAAGGCAAAGAGAAGAGTGGTG--------------
-------------------------CAAAGAGAAAAAAGAGCA---GTGGGAGCAATAGGAGCT---------
ATGTTCCTTGGGTTCTTAGGAGCA---GCAGGAAGCACTATG---GGCGCAGCGTCA---
ATAACGCTGACGGTACAG---GCCAGACAA---TTGTTG---TCTGGCATTGTGCAACAGCAGAGA---
AATTTG---CTAAGG---GCTATTGAGGCGCAACAGCATCTGTTGCAACTCACAGTCTGGGGCATT
>HM215413
TTCTACTGTAATACAGCACAACTGTTTAATAGTACTTGGAATACTACTAAGATTTGGGGGAATACTACAGAAG
GT-----------------------------------------------------------------------
----------------------------
AACAATAGCACTATCATGCTCCCTTGCAAAATAAAGCAAATTATAAACCTGTGGCAGGAAGTAGGAAAAGCAA
TATATGCCCCTCCCATCGCAGGGCGAATTAGATGTGTATCAAAT---
ATTACAGGGTTGCTATTAACAAGAGATGGTGGTCATAATGGGAACGGG-------------------------
-------------------------------------------------------------------------
-------
TCCAACGAGACCGAGACCTTCAGACCTGCAGGAGGAGATATGAGGGACAATTGGAGAAGTGAATTATATAAAT
ATAAAGTAGTAAAAATTGAACCAATA---GGATTAGCACCCACCAAGGCAAAGAGAAGAGTGGTG--------
-------------------------------CAGAGAGAAAAAAGAGCA---ATGGGA---ATAGGAGCT---
------ATGTTCCTTGGGTTCTTGGGAGCA---GCAGGAAGCACTATG---GGCGCAGCGTCA---
ATAACGCTGACGGTGCAG---GCCAGACGA---TTATTG---TCTGGTATAGTGCAACAGCAAAGA---
AATTTG---CTGAGG---GCTATTGAGGCGCAACAACATCTGTTGCAACTCACCGTCTGGGGCATC
>EU131802
TTCTACTGTAATACATCACAACTGTTTAATAGTACTTGGAATGATACTAGTACTTGGAATGGTAAT-------
-------------------------------------------------------------------------
----------------------------
```

ACAGGAACTACTGTCACGCTCCCTTGCAGAATAAAACAAATTGTAAACATGTGGCAGAAAGTAGGGAAAGCCA
TGTACGCCCCTCCCATTCAAGGACAAATTAGATGTTCATCAAAT---
ATTACAGGGTTGCTATTAACAAGAGATGGTGGTAATAATGAGAGC----------------------------
----

GAGAATACCGAGACCTTCAGACCTGCAGGAGGAAATATGAGAGATAATTGGAGAAGTGAATTATATAAATATA
AAGTAGTAAAAATTGAACCATTA---GGAGTAGCCCCACCAAGGCAAAGAGAAGAGTGGTG------------
----------------------------CAGAGAGAAAAAAGAGCA---GTGGGATTGATAGGAGCT------
---ATGTTCCTTGGGTTCTTGGGAGCA---GCAGGAAGCACTATG---GGCGCAGCGTCA---
ATAACGCTGACGGTACAG---GCCAGGCAA---TTATTG---TCTGGTATAGTGCAACAGCAGAGA---
AATTTG---CTGATG---GCTATTGAGGCGCAACAGAATATGTTGCGACTCACAGTCTGGGGCATC
>EU131804
TTCTACTGTAACACATCACAACTGTTTAATAGTACTTGGTATAGTAATGGTACTAGGAAGGATCCTACA----
----

GGAAATGGCACTATCACGCTCCCTTGCAGAATAAAACAAATTATAAACATGTGGCAGCAAGTAGGGAAAGCAA
TGTATGCACCTCCCATTAAAGGACAAATTAGATGTTCATCAAAT---
ATTACAGGGTCGCTATTAACAAGAGATGGTGGTTATAATGAGAGC----------------------------
----

AACACCACCGAGACCTTCAGACCTGGAGGAGGAGATATGAGAGACAATTGGAGAAGTGAATTATATAAATATA
AAGTAGTAAAAATTGAACCATTA---GGAGTAGCACCCACCAAGGCAAAGAGAAGAGTGGTG------------
----------------------------CAGAGAGAAAAAAGAGCA---GTAGGA---CTAGGAGCT------
---ATGTTCCTTGGGTTCTTGGGAGCA---GCAGGAGGCACCATG---GGCGCAGCGTCA---
ATAACGCTGACGGTACAG---GCCAGACAA---TTGTTG---TCTGGTATAGTGCAACAGCAGAGA---
AATTTG---CTGAGT---GCTATTGAGGCGCAACAACGTTTGTTGCAACTCACAGTCTGGGGCATC
>JF932472
TTCTACTGTAATACAGCACAACTGTTTAATAGTACTTGGACAGGAAATGATACTAGCATTTGGAATGATACTA
CA---

GGAAATGACACTATCACACTCCCTTGCAGAATAAAACAAATTATAAACATGTGGCAGGAAGTAGGGAAAGCAA
TGTATGCCCCTCCCATTGCAGGACAAATTAGATGTTCATCAAAT---
ATTACAGGGTTGCTATTAACAAGAGATGGTGGTAATCATGAGAAC----------------------------
----

AACACTGAGACCTTCAGACCTGGAGGAGGAGATATGAGAGACAATTGGAGAAGTGAATTATATAGATATAAAG
TAGTAAAAATTGAACCATTA---GGAGTAGCACCCACCAAGGCAAAGAGAAGAGTGGTG---------------
-------------------------CAGAGAGAAAAAAGAGCA---GTGGGAACGCTAGGAGCT---------
ATGTTCCTTGGGTTCTTGGGAGCA---GCAGGAAGCACTATG---GGCGCAGCGTCA---
ATAACGCTGACGGTACAG---GCCAGACAA---TTGTTG---TCTGGTATAGTGCAACAGCAGAGA---
AATTTG---CTGAGG---GCTATTGAGGCGCAACAGCATCTGTTGCAACTCACAGTCTGGGGCATC
>JF932474
TTCTACTGTAATACATCACAACTGTTTAATAGTACTTGGCCTTGGAATAATGATACTAGTGTTTGGGATGATA
CTACA--

GAAAATGACATTATCACGCTCCCTTGCAGAATAAAACAAATTATAAACATGTGGCAGGAAGTAGGGAAAGCAA
TGTATGCCCCTCCCATTCAAGGACAACTTAACTGTTCATCAAAT---
ATTACAGGGTTGCTATTAACAAGAGATGGTGGTTATCATAATGAGAGC-------------------------
----

AATAACACCGAGACCTTCAGACCTGGAGGAGGAGATATGAGAGACAATTGGAGAAGTGAATTATATAAATATA
AAGTAGTAAAAATTGAACCATTA---GGAGTAGCACCCACCAAGGCAAAGAGAAGAGTGGTG------------
----------------------------CAAAGAGAAAAAAGAGCA---GTGGGAGCGATAGGAGCT------
---ATGTTCCTTGGGTTCTTGGGAGCA---GCAGGAAGCACTATG---GGCGCAGCGTCA---
ATAACGCTGACGGTACAG---GCCAGACAA---CTGTTG---TCTGGTATAGTGCAACAGCAGAGA---
AATTTG---CTGAGG---GCTATTGAGGCGCAACAGCATCTGTTGCAACTCACAGTCTGGGGCATT
>HQ215554
TTCTACTGTAATACAACAGGGCTGTTTAATAGTACTTTGACTAATGATACCTGGAATGATAATCGGTCATATA
AC---

AATGGAACAGGTAACATCACACTCCACTGCAGAATAAAACAAATTATAAACAGGTGGCAGGAAGTAGGAAAAG
CAATGTATGCCCCTCCCATCAGTGGACCAATTAGCTGTTTATCAAAT---
ATTACAGGACTGATATTAACAAGAGATGGAGGTAACAATACCGGGAAT-------------------------
----

CAATCTGATACTGAAACCTTCAGACCTGGGGGAGGGATATGAAGGACAATTGGAGAAGTGAATTATATAAAT
ATAAAGTAGTACAAATTGAGCCAATA---GGATTAGCACCCACCAGGGCAAGGAGAAGAGTGGTG--------
-------------------------------CAGAGAGAAAAAAGAGCA---GTGGGAACATTAGGAGCT---
------ATGTTCCTTGGGTTCTTGGGAGCA---GCAGGAAGCACTATG---GGCGCAGCGTCA---
GTCACGCTGACGGTACAG---GCCAGACAA---TTACTG---TCTGGTATAGTGCAACAGCAGAAC---
AATTTG---CTGAAA---GCTATTGAGGCGCAACAACATATGTTGCAACTCACAGTCTGGGGCATC
>JF346917
TTCTACTGTAATACATCACAACTGTTTAATAGTACTTGGGATATTAATAGTAATGGACTAATAATACAAAT-
----

AGCAATATCAATATCACACTCCCTTGCAAAATAAAACAAATTGTAAACATGTGGCAGGAAGTAGGGAAAGCAA
TGTACGCCCCTCCCATTAGAGGACAAATTAGATGTTCATCACAA---
ATTACAGGGTTGTTATTAACAAGAGATGGTGGTACTAATAATAAT----------------------------
----

CATACCAACGAAACCTTCAGACCTGCAGGGGGAGACATGAGAGACAATTGGAGAAGTGAATTATATAAATATA
AAGTAGTAAAAATTGAACCATTA---GGAATAGCACCCACCAAGGCAAAGAGAAGAGTGGTG-----------
----------------------------CAGAGAGAAAAAAGAGCA---GTGGGAACAATAGGAGCT------
---ATGTTCCTTGGGTTCTTAGGAGCA---GCAGGAAGCACTATG---GGCGCAGCGTCA---
GTAACGCTGACGGTACAG---GCCAGACAA---TTATTG---TCTGGTATAGTGCAACAGCAGAGA---
AATCTG---CTGAGG---GCTATTGAGGCGCAACAGCGTGTGTTGCAACTCACAGTCTGGGGCATC
>JX960599
TTCTACTGCAATACAACACAACTGTTTAATAGTACTTGGAATACTTGGGATAATATTACTGGGAATAGTAATG
AGAATAAT---

ACACAGAACAATATCACACTTCCATGTAGAATAAAACAAATTATAAACATGTGGCAGGGAGTAGGAAAAGCAA
TGTACGCCCCTCCCATCGCAGGACGAATTAAATGTTCATCAGAT---
ATTACAGGACTATTATTAGTGAGAGATGGT---AATAACAATAACAAT-------------------------
----

ACAGATAACAAGACAGAAACTTTCAGACCTCAGGGAGGAAACATGAAGGACAATTGGAGAAGTGAATTATATA
AATATAAAGTAGTAAAAATTGAACCAATA---GGAATAGCACCCACTACGGCAAGGAGAAGAGTGGTG-----
----------------------------------CAGAGAGAAAAAAGAGCA---GTGACA---
CTAGGAGCT---------ATGTTCCTTGGGTTCTTAGGAGCA---GCAGGAAGCACAATG---
GGCGCAGCGTCA---ATAACGCTGACGGTACAG---GCCAGACAA---TTACTG---
TCTGGTATAGTGCAACAGCAGAAC---AATTTG---CTGAGG---
GCCATTGAGGCGCAACAGCATATGTTGCAACTCACAGTCTGGGGCATC
>JF932497
TTCTACTGTAATACATCACGACTGTTTAGTAGTACATGGTATACTAATGATACTCAGAATGGTACTACA----
----

GGAAATGACACTATCACACTCCCTTGCAGAATAAAACAAATTGTAAACATGTGGCAGGAAGTAGGAAAAGCAA
TGTATGCCCCTCCCGTTGAAGGACTAATTAGATGTTCATCAAAT---
ATTACAGGGTTGCTATTAACAAGAGATGGTGGTTATAATGTAAGC----------------------------
----

AACAACACCGAGATCTTCAGACCTGGAGGAGGAGATATGAGGGACAATTGGAGAAGTGAATTATATAAATATA
AAGTAATAAAAATTGAACCATTA---GGAGTAGCACCCACCAAGGCAAAGAGAAGAGTGGTG-----------
----------------------------CAAAGAGAAAAAAGAGCA---GTGGGA---ATAGGAGCT------
---ATGTTCCTTGGGTTCTTGGGAGCA---GCAGGAACCACTATG---GGCGCAGCGTCA---
ATGACGCTGACGGTACAG---GCCAGACAA---TTGTTG---TCTGGTATAGTGCAACAGCAGGGA---
AATCTG---CTGAGG---GCTATTGAGGCGCAACAGCATATGTTGCAACTCACAGTCTGGGGCATT
>KX693642
TTCTACTGTAATACATCACAACTGTTTAATAGTACTTGGGATGTTAATGACACTAGTAATGATACTACA----
----

GGAAATGAAACTATCACGCTCCCTTGCAGAATAAAACAAATTATAAACATGTGGCAGGGAGTAGGGAAAGCAA
TGTATGCCCCTCCCATTAAAGGAAAAATTAGATGTTCATCAAAT---
ATTACAGGGTTGATATTAACAAGAGATGGTGGTGTAAATGAGAGC----------------------------
----

GAGTTCAATGAGACCTTCAGACCTCAAGGAGGAAATATGAAAGACAATTGGAGAAGTGAATTGTATAAATATA
AAGTAGTAAAAATTGAACCATTA---GGAGTAGCACCCACCAAGGCAAAGAGAAGAGTGGTG-----------
----------------------------CAGAGAGAAAAAAGAGCA------GGA---ATAGGAGCT------
---CTGTTCCTTGGGTTCTTGGGAGCA---GCAGGAAGCACTATG---GGCGCAGCGTCA---
ATAACGCTGACGGTACAG---GCCAGACAA---TTGATG---TCTGGTATAGTGCAACAGCAGAGA---
AATTTG---CTGAGA---GCTATTGAGGCGCAACAGCATCTGTTGCAACTCACAGTCTGGGGCATC
>KX693171
TTTTACTGTAATACATCACAACTGTTTGACAGTATTTGGAATGCTACTACTGGTACTTGGAATGGTACTACA-
----

GGAGGTGACAATATCACGCTCCCTTGCAGGATAAAACAAATTGTAAACATGTGGCAGGAAGTAGGGAAAGCAA
TGTATGCCCCTCCCATTAGAGGACAAATTTATTGTTCATCAAGT---
ATTACAGGGTTGCTATTAACAAGAGATGGTGGTACGAGTAAGAAT----------------------------
----

GAGAGCAATACCACCGAGACCTTCAGACCTGGAGGAGGAGATATGAGGGACAATTGGAGAAGTGAATTATATA
AATACAAAGTAGTAAAACTTGAACCACTA---GGAGTAGCACCCACCGAGGCAAAGAGAAGAGTGGTG-----
----------------------------------CAGAGAGAAAAAAGAGCA---
GTGGGAGCGATAGGAGCT---------ATGTTCCTTGGGTTCTTGGGAGCA---GCAGGAAGCACTATG---
GGCGCAGCGTCA---ATAACGCTGACGGTACAG---GCCAGACAA---TTATTG---
TCTGGTATAGTGCAACAGCAAAGA---AATTTG---CTGAGG---
GCTATTGAGGCGCAACAGCATATGTTGCAACTCACAGTCTGGGGCATC
>KX693309
TTCTACTGTAATACATCAAAACTGTTTAATAGTACTTGGAGTAAGACTAGTACTTGGAATGATACTACA----
----

GGAAATGACACTATCACGCTCCCTTGCAGAATAAAACAAATTGTAAACATGTGGCAGGAAGTAGGGAAAGCAA
TGTATGCCCCTCCCATTCAAGGACTAATTAACTGTTCATCAAAT---
ATTACCGGGTTGCTATTAACAAGAGATGGTGGTAATAATACTGAGAGC-------------------------
----

AACACCACCGAGACCTTCAGACCTACAGGAGGAAATATGAGAGACAATTGGAGAAGTGAATTATATAAATATA
AAGTAGTACAAATTGAACCATTA---GGAGTAGCACCCACCAAGGCAAAGAGAAGAGTGGTG-----------
----------------------------CAGAGAGAAAAAAGAGCA---GTGGGAACAATAGGAGCT------
---ATGTTCCTTGGGTTCTTGGGAGCA---GCAGGAAGCACTATG---GGCGCAGCGTCG---
GTAACGCTGACGGTACAG---GCCAGACAA---TTGTTG---TCTGGTATAGTGCAACAGCAGAGA---
AATTTG---CTGAGG---GCTATTGAGGCGCAACAGCATCTGTTGCAACTCACAGTCTGGGGCATT
>KX693362
TTCTACTGTAATACATCAAAACTGTTTAATAGTACTTGGAATGATAATAGTACTTGGAATGATAATGATAAGA
CA---

GGAGATGACAATCTCACGCTCCCTTGTAGAATAAAACAAATTGTAAACATGTGGCAGGAAGTAGGGAAAGCAA
TGTATGCCCCTCCCATTAAAGGACAAATTAGATGTTCATCAAAT---
ATTACAGGGTTGCTATTAACAAGAGATGGTGGTCAGAATGTGAGC----------------------------
----

AATACCACCGAGACCTTCAGACCTGCAGGAGGAGATATGAGAGACAATTGGAGAAGTGAATTATATAAATATA
AAGTAGTAAAAATTGAACCATTA---GGAGTAGCACCTACCAAGGCAAAGAGAAGAGTGGTG-----------
----------------------------CAGAGAGAAAAAAGAGCA---GTGGGAACGATAGGAGCT------
---ATGTTCCTTGGGTTCTTGGGAGCA---GCAGGAAGCACGATG---GGCGCAGCGTCA---
ATAACGCTGACGGTACAG---GCCAGACAA---TTGTTG---TCTGGTATAGTGCAACAGCAGAGA---
AATTTG---CTGAGG---GCTATTGAGGCGCAACAGCATCTGTTGCAACTCACAGTCTGGGGCATC
>KX693411

TTTTACTGTAATACATCAAAACTGTTTAATAGTACTTGGAGTAATAATAATCCTTGGAATACTACTAGTATT-
----

CAAAATGATACTATCACGCTCCCTTGCAGAATAAAACAAATTGTAAACATGTGGCAGGAAGTAGGGAAAGCAA
TGTATGCCCCTCCCATTGCAGGACCAATTAATTGTTCATCAAAT---
ATTACGGGGTTGCTATTAACAAGAGATGGTGGTAAAGGTGGGAT-----------------------------
----

GTGAGCAACTACACCGAGACCTTCAGACCTGCAGGGGGAAATATGAGAGACAATTGGAGAAGTGAATTATATA
GATATAAAGTAGTAAAAATTGAACCATTA---GGAGTAGCACCCACCAAGGCAAAGAGAAGAGTGGTG-----
----------------------------------CAGAGAGAAAAAAGAGCA---
GTGGGAGCAATAGGAGCT---------ATGTTCCTTGGGTTCTTGGGAGCA---GCAGGAAGCACTATG---
GGCGCAGCGTCA---ATAACGCTGACGGTACAG---GCCAGACAA---TTGTTG---
TCTGGTATAGTGCAACAGCAGAGA---AATTTG---CTGAGG---
GCTATTGAGGCGCAACAGCATCTGTTGCAACTCACAGTCTGGGGCATC
>AY561237
TTCTACTGTAATTCAACACAACTGTTTAATAGTACCTGGCCTGCTAATGATACTTGGCATGATACTAACGGGA
CAAGTTAT---
-------------ACTAAC------
GGGACAAGTGGAACTATCACACTCCCATGCAAAATAAAACAAATTATAAACTTATGGCAGGAAGTAGGAAAAG
CAATGTATGCCCCTCCCATCGCAGGAGCACTTAATTGTTCATCAAAT---
ATTACAGGGCTGTTATTAACAAGAGATGGTGGTAATGACAAGAACAGCAAC----------------------
----

AATAGCAACAGCAACGAGACCTTCAGACCGGGAGGAGGAGATATGAGGGACAATTGGAGAAGTGAATTATATA
GATATAAAGTAGTAAAAATTGAACCATTA---GGAGTAGCACCCACCGAGGCAAGGAGAAGGGTGGTG-----
----------------------------------CAAAGAGAAAAAAGAGCA---GTGGGA---
TTAGGAGCT---------GTGTTCCTTGGGTTCTTGGGAGCA---GCAGGAAGCACTATG---
GGCGCAGCATCA---GTGATGCTGACGGTACAG---GCCAGGCAA---TTATTG---
TCTGGTATAGTGCAACAGCAGAAC---AATCTG---CTGAGG---
GCTATTGAGGCGCAACAACATCTGTTGCAGCTCACAGTCTGGGGCATC
>KR914676
TTCTACTGTAACACAACACCACTGTTTAATAGTAGTTGGTACAAGTCAAATTGGAAAAGTCAAAGGACAAAGG
AGATTGGA---

GGAAGGGAGACAATCACACTCCAATGTAGAATAAAACAAATTGTAAACATGTGGCAGGAAGTAGGACGAGCAA
TGTATGCCCCTCCCATCAAAGGACAAATTAGATGTTCATCAAAT---
ATTACAGGGGTGCTATTAACAAGAGATGGTGGTAATACCACCAAT----------------------------
----

CACTCCGAGATCTTCAGACCTGGAGGAGGAAATATGAAGGACAATTGGAGAAGTGAATTATATAAATATAAAG
TAGTACAAATTGAACCATTA---GGAATAGCACCCACCAAGGCAAAGAGAAGAGTGGTG--------------
-------------------------CAGAGAGAAAAAAGAGCA---ATAGGA---TTCGGAGCT---------
GTGTTCCTTGGGTTCTTGGGAGCA---GCAGGAAGCACTATG---GGCGCAGCGTCA---
GTAACGCTGACGGTACAA---GCCAGACAA---TTATTG---TCTGGTATAGTGCAACAGCAGAGC---
AATTTG---CTGAAT---GCTATTCAGGCGCAACAGCATCTGTTGCAACTCACAGTCTGGGGCATT
>FJ388899
TTCTACTGCAATACAACAGAACTGTTTAATAGTACTTGGAATAGTACTACTTGGAATAGTAATAGTACTACAA
ATGCAAAT---
-------------------GCA---
ACAAATACAGCTAACCTAGTACTCCCATGCAGAATAAAACAGATTGTAAACATGTGGCAGAAAGTAGGAAAAG
CAATGTATGCCCCTCCCATCAAAGGAATAATTAGATGTTCATCAAAT---
ATTACAGGGATAATATTAACAAGAGATGGTGGTAATAGTAATCAGACC-------------------------
----

AACACCACAGAGATTTTCAGACCTGTAGGAGGAAATATGAAGGACAATTGGAGAAGTGAATTATATAAATATA
AAGTAGTACAAATTGAACCATTA---GGAGTAGCACCCACCAAGGCAAAAAGAAGAGTGGTG-----------
----------------------------CAGAGAGAAAAAAGAGCA---GTGACA---TTAGGAGCT------
---ATGTTCCTTGGGTTCTTGGGAGCA---GCAGGAAGCACTATG---GGCGCAGCGTCA---

ATAACGCTGACGGTACAG---GCCAGACAA---TTACTG---TCTGGTATAGTGCAACAGCAGAGC---
AATCTG---CTGAGG---GCTATTGAGGCGCAACAGCACCTGTTGCAACTCACAGTCTGGGGCATC
>FJ388916
TTCTACTGTAATACAACAGGGCTGTATAATAGTACTTGGCAGTTTAATAGTACTTGGAATACTACTTGGAATC
ATACTGAC---
-------------------CCT---
AATCATACTGACCCTATCACACTACCATGCAGAATAAGACAAATTGTAAACAGGTGGCAGGAAGTAGGAAAAG
CAATGTATGCCCCTCCCATCAGCGGACAAATTAGCTGTTCATCAAAC---
ATTACAGGGCTACTATTAACAAGAGATGGTGGTAGTAATGAGAGC----------------------------
----

AGAACCAACGAGACCTTTAGACCTGGAGGAGGAGATATGAGGGACAATTGGAGAAGTGAATTATATAAATATA
AAGTAGTAAGAATTCAACCATTA---GGAATAGCACCCACCAAGGCAAAAAGAAGAGTGGTG-----------
----------------------------CAAAGAGAAAAAAGAGCA---GTGGGAACGCTGGGAGCT------
---ATGTTCCTTGGGTTCTTGGGAGCA---GCAGGAAGCACTATG---GGCGCAGCGTCA---
CTGACGCTGACGGTACAG---GCCAGACAA---TTATTG---ACTGGTATAGTGCAACAGCAGAAC---
AATCTG---CTGAGA---GCTATTGAGGCGCAACAGCATATGTTGCAACTCACAGTCTGGGGCATC
>JF683738
TTCTACTGTGATACTACAAAGCTGTTTAATAGTACTTGGACTGGGAATGAAACTTGGACTGGGAATGAAACTG
AAGAG--

TCAAATAATAGTACTATCACACTCCCATGCAGAATAAAACAAATTATAAACAGATGGCAGGAAGTAGGAAAAG
CAATGTATGCCCCTCCCATCAGAGGAGAAATTAAATGTACATCAAAT---
ATTACAGGGCTACTATTAACAAGAGATGGTGGTAATGGTAATGGGACT-------------------------
----

CAGAATCAGACCGAGATCTTCAGACCTGGAGGAGGAGACATGAAGGACAATTGGAGAAGTGAATTATATAAAT
ATAAAGTAGTAAAAATTGAGCCATTA---GGAATAGCACCTACCAAGGCAAAAAGAAGAGTGGTG--------
-------------------------------CAGAGAGAAAAAAGAGCA---ATAGGA---CTGGGAGCT---
------GTGTTCATTGGGTTCTTGGGAGCA---GCAGGAAGCACTATG---GGCGCAGCGTCG---
GTGACGCTGACGGTACAG---GCCAGACAG---TTATTG---TCTGGTATAGTGCAACAGCAAAAC---
AATTTG---CTAAAA---GCTATTGAGGCGCAACAGCATCTGTTGCAACTCACAGTCTGGGGCATC
>JF683756
TTCTACTGTAATACATCAAAGCTGTTTAATAGTACTTGGACTAATAGTAGTAGTACTTGGAATAATACTGAAG
AG---

TCAAATGACACTATCACACTCCCATGTAAAATAAAACAAATTATAAACATGTGGCAGGAAGTAGGAAGAGCAA
TGTATGCCCCTCCCATCAGTGGCAAATTAGGTGCTCATCAAAT---
ATTACAGGGCTACTATTAACAAGAGATGGTGGTCATAACGGAACCAAT-------------------------
----

AACGAGACCGAGATCTTCAGACCTGCAGGAGGAAATATGAAGGACAATTGGAGAAGTGAATTATATAAATATA
AAGTAGTGAAAATTGAGCCATTA---GGAGTAGCACCCACCAGGGCAAAAAGAAGAGTGGTG-----------
----------------------------CAGAGAGAAAAAAGAGCA---GTGGGA---ATGGGAGCT------
---TTGCTCCTTGGGTTCTTGGGAGCA---GCAGGAAGCACTATG---GGCGCAGCATCG---
GTGATGCTGACGGTACAG---GCCAGACAA---TTATTG---TCTGGTATAGTGCAACAGCAGAAC---
AATCTG---CTGAGA---GCTATTGAGGCGCAACAACATATGTTGCAACTCACAGTCTGGGGCATT
>JF683764
TTCTACTGTGATACAACACAACTGTTTAATAGTACTTGGTTTATGAATGGGACTAATACAACTACC-------
----

CTTACCGCAAATGCCACAATCCCAAGCCGAATAAAACAAATTATACACAGGAGGCAGGAAGTAGGAAGAGCAA
TGTATGTCCCTGCCCTCAGAGGACCAATTACATGTATATCAAAT---
ATGACAGGGACGTTATTAACAAGAGATGGTGGTCATAACAATGGCATT-------------------------
----

AATGACATTGAGACCTTCAGACCAGGAGGAGGAGATATGAGGGACAATTGGAGAAGTGAATTATATAAATATA
AAGTAGTAAGAGTTGAACCATTA---GGAATAGCACCCACCAGGGCAAAGAGAAGAGTGGTG-----------
----------------------------CAGAGAGAAAAAAGAGCA---GCA------CTAGGAGCT------
---ATGTTCCTTGGGTTCTTGGGAGCA---GCAGGAAGCACTATG---GGCGCAGCGTCA---

ATAACGCTGACGGTACAG---GCCAGACAA---TTGTTG---TCTGGTATAGTGCAACAGCAAAAC---
AATTTG---CTGAGA---GCTATTGAGGCGCAACAACACATGTTGCAACTCACAGTCTGGGGCATC
>JF683773
TTCTACTGTAATACATCACAACTGTTTAATAGTACTTGGACACCTAATGGTACTTGGACATTTAATGCTGCTA
GTGGGGCT---
-------------------TCA---
GGATTCAAGGGCACTATCACACTCCCATGTAGAATAAAACAAATTATAAACATGTGGCAGGAAGTAGGAAAAG
CAATGTATGCCCCTCCCATCAGTGGACTAATTAGATGCTCATCAAAT---
ATTACAGGACTGCTATTAACAAGAGATGGTGGTATAAACAATACC----------------------------
----

AATACCACCGAGATCTTCAGACCTGTAGGAGGAAATATGAAGGACAATTGGAGAAGTGAATTATATAAATATA
AAGTAGTACAAGTTGAACCAATA---GGAATTGCACCCACCAAGGCAAAGAGAAGAGTGGTG-----------
----------------------------CAGAGAGAAAAAAGAGCA---GTTACA---CTAGGAGCT------
---GTGTTCCTTGGGTTCTTGGGAGCA---GCAGGAAGCACTATG---GGCGCAGCGTCA---
ATGACGCTGACGGTACAG---GCCAGGCAA---TTATTG---TCTGGTATAGTGCAACAGCAGAAC---
AATCTG---CTGAGA---GCTATTCAGGCGCAACAGCATCTGTTGCAACTCACAGTCTGGGGCATC
>JQ416158
TTCTACTGTAATACATCACAACTGTTTAATAGTATTTGGCCTTGGAATAGTACTGCAAACAACACTGGA----
----

GGAAATGACACAATCACACTCCCATGCAGGATAAGACAAATTGTAAATAGGTGGCAGGAAGTAGGAAAAGCAA
TGTATGCCCCTCCCATCAGTGGACAAATTAGATGTTCATCAAAT---
ATTACAGGGCTGCTATTAACAAGAGATGGAGGTCAGACTAACAACCAG-------------------------
----

ACTGGGAACCAGACCGAGATTTTCAGACCTGGAGGAGGAGATATAAAAGATAATTGGAGAAGTGAATTATACA
AATATAAAGTAGTACAAATTGAACCATTA---GGAGTAGCTCCCACTAAGGCAAGGAGAAGAGTGGTG-----
----------------------------------CAGAGAGAAAAAAGAGCA---CTAACA---
CTGGGAGCT---------GTGTTCCTTGGGTTCTTGGGAACA---GCAGGAAGCACTATG---
GGCGCAGCGTCA---CTAACGCTGACGGTACAG---GCCAGACAA---TTATTG---
TCTGGTATAGTGCAGCAGCAGAAC---AATCTG---CTGAGA---
GCTATTGAGGCGCAACAACATTTGTTGCAACTCACAGTCTGGGGCATC
>JQ403037
TTCTACTGTAATACAACACAGCTGTTTAATAGTACTTGGAATAGTACTTGGACTTTGAATGATACTAGAGGGT
CAAATAAC---
-------------------ACA---
AATGGAACTGGTTATATCATACTCCCATGCAGATTAAAACAATTTATAAACATGTGGCAGAGAGTAGGAAAAG
CAATGTATGCCCCTCCCATCAGTGGACAAATTAACTGTACATCACAC---
ATTACAGGGCTGCTATTAACAAGAGATGGTGGTAATAACAATAGC----------------------------
----

GAACCCACCCAGATCTTCAGACCTGGAGGAGGAGATATGAGAGACAATTGGAGAAGTGAATTATATAAATATA
AAGTAGTAAAAATTGAACCATTA---GGAGTAGCACCCACAAAGGCAAAAAGAAGAGTGGTG-----------
----------------------------CAGAGAGAAAAAAGAGCA---GTAGGA---CTGGGAGCT------
---ATGTTCCTTGGGTTCTTGGGAGCA---GCAGGAAGCACTATG---GGCGCAGCATCA---
GTGATGCTGACGGTACAG---GCCAGACAG---CTATTG---TCTGGCATAGTGCAACAGCAAAAC---
AATTTG---CTGAGA---GCTATTGAGGCGCAACAGCATCTGTTGCAACTCACAGTCTGGGGCATC
>KT124803
TTCTACTGTAATACAACAAGCCTGTTTAATAGTACTTGGTATGCAAATGGTACAAATAGCACTTGGTCTGAT-
----

GAGTCAGATGTTATCACACTTCCATGCAGAATAAGACAAATTGTAAACATGTGGCAGGGGGTAGGAAAAGCAA
TGTATGCCCCTCCCATCAGAGGACAGATTAATTGTACATCAAAT---
ATTACAGGGCTAATATTAACAAGAGATGGTGGGAACAAGAATGGCACTAATGAG-------------------
----

ACCTATGAGACCTTCAGACCTGCAGGAGGAAATATGAAGGACAATTGGAGAAGTGAATTATATAAATATAAAG
TAGTAAAGATTGAACCATTA---GGAATAGCACCCACCAAGGCAAAGAGAAGAGTGGTG--------------
-------------------------CAAAGAGAAAAAAGAGCA---ATAGGA---ATGGGAGCC---------

TTGTTCCTTGGGTTCTTGGGAGCA---GCAGGAAGCACTATG---GGCGCAGCGTCA---
ATGACGCTGACGGTACAG---GCCAGACAG---TTATTG---TCTGGTATAGTGCAACAGCAAAAC---
AATTTG---CTGAGA---GCTATTGAGGCGCAACAACACCTGTTGCAACTCACAGTCTGGGGCATC
>KT124751
TTCTACTGTAATACAACACAACTGTTTAATAGTACTTGGCCGTTTAATAGTACTTGGGATAGTACTGAAGGGT
CAAATTACACT---

GTAGAAAATGAAAATATCACACTCCCATGCAGAATAAAACAAATTGTAAACCTGTGGCAGGAAGTAGGAAAAG
CAATGTATGCCCCTCCCATCAAAGGACAAATTAGATGTTCATCAAAT---
ATTACAGGGCTGCTATTAACAAGAGATGGTGGCAATGCTGGCAATACTAGT---------------------
----

AGCAAGAACGAGACCTTCAGACCTGGGGGAGGAAATATGAAGGACAATTGGAGAAGTGAATTATATAAATATA
AAGTAGTAAAAGTTGAACCATTA---GGAATAGCACCCACTAAGGCAAAAAGAAGAGTGGTG----------
---------------------------CAGAGAGAAAAAAGAGAT---TTGGGA---CTAGGAGCC------
---TTGTTCCTTGGGTTCTTGGGAGCA---GCAGGAAGCACTATG---GGCGCAGCGTCA---
GTAACGCTGACGGTACAG---GCCAGACAA---TTATTG---TCTGGTATAGTGCAACAGCAGAAC---
AATTTG---CTGAGG---GCTATTGAGGCGCAACAGCATATGTTGCAACTCACAGTCTGGGGCATC
>KT124789
TTCTACTGTAATACAACAGAGCTGTTTAACGGTACTTGGAATGGTACTTTCTTGAATGTTACTACA-------
----

GGGCGAGAGAATATCACACTCCCATGCAGAATAAAACAGATTATAAACAGGTGGCAGGAAGTAGGAAAAGCAA
TGTACGCCCCTCCCATCAGAGGACCAATTAGATGCTCATCAAAT---
ATTACAGGGCTGATATTAACAAGAGATGGTGGTCAGAATGCGAGTGCG------------------------
----

ACCAACGAAACCTTCAGACCGGGAGGAGGAGATATGAGGGACAATTGGAGAAGTGAATTATATAAATATAAAG
TAGTAAAAATTGAGCCATTA---GGAATAGCACCCACCAAGGCAAGGAGGAGAGTGGTG-------------
------------------------CAGAGAGAAAAAAGAGCA---GTGACA---CTAGGAGCT---------
ATGTTCCTTGGGTTCTTGGGAGCA---GCAGGAAGCACTATG---GGCGCAGCTTCA---
TTGACGCTGACGGTACAG---GCCAGACTA---TTATTG---TCTGGTATAGTGCAACAGCAAAAC---
AACTTG---CTGAGG---GCTATTGAGGCGCAGCAGCATCTGCTGCAACTCACAGTCTGGGGCATC
>KT124805
TTTTACTGTGATACAACACAACTGTTCAACAGTACGTGGGATACAAATAATACTGGGGGAGTGAATGAT----
----

ACTGACGAGCCAATTACACTCCCATGCAGGATAAAACAATTTATAAACATGTGGCAGGAAGTAGGGAAAGCAA
TGTATGCCCCTCCCATCCAAGGAATAATTCACTGTTCATCAAAT---
ATTACAGGACTACTATTAACAAGAGATGGTGGTAAGACAGAGAACGGGACAGAG------------------
----

AATGTTACTGAGATCTTTAGACCTGGAGGAGGAGACATGAGGGACAATTGGAGAAGTGAATTATATAAATATA
AAGTAGTAAAAATTGAACCATTA---GGAGTAGCACCCACCAGGGCAAAGAGAAGAGTGGTG----------
---------------------------CAGAGAGAAAAAAGAGCA---GTGACA---CTGGGAGCT------
---GTGTTCCTTGGGTTCTTGGGAGCA---GCCGGAAGCACTATG---GGCGCAGCGTCA---
CTGACGCTGACGGTACAG---GCCAGACAA---TTATTG---TCTGGTATAGTGCAACAGCAGAAC---
AATTTG---CTGAGA---GCTATTGAGGCGCAACACCATCTGTTGCAACTCACAGTCTGGGGCATT
>KT124763
TTCTACTGTAATTCATCGACACTGTTTAATAGTACTTGGAATGAAACTATTGCAGGGATTACCAAT------
----

GCAAATGAAACTATCACACTCCCATGCAGAATAAGGCAAATTATAAACATGTGGCAGGAAGTAGGAAAAGCAA
TGTATGCCCCTCCCATTAGTGGAATAATTAGATGTTCATCAAAT---
ATTACAGGGCTGCTATTAACAAGAGATGGTGGTAATAATGGTAAT--------------------------
----
CATAGCGAGAATAAT---
ACCATTGAGATCTTCAGACCTGGAGGAGGAGATATGAGGGATAATTGGAGAAGTGAATTATATAAATATAAAG
TAGTACAAATTGAACCAGTA---GGAGTAGCACCTACCAAGGCAAAGAGAAGAGTGGTG-------------
------------------------CAGAGAGAAAAAAGAGCA---GTGGGA---CTAGGAGCT---------

TTGTTCCTTGGGTTCTTGGGAGCA---GCAGGAAGCACTATG---GGCGCAGCGTCA---
ATAACGCTGACGGTACAG---GCCAGACAA---TTATTG---TTTGGTATAGTGCAACAGCAGAAC---
AATCTG---CTGAGA---GCTATTGAGGCGCAACAGCACCTGTTGCAACTCACAGTCTGGGGCATC
>KT124779
TTCTACTGTGATACAACACAACTGTTTAATAGTACTTGGAGTAATGCTACTTGGAATGAGACTACAGGT----
----

TCAAATGACACGATTACACTCCAATGTAAAATAAAACAAATTATAAACAGGTGGCAGGAAGTAGGAAAAGCAA
TGTATGCCCCTCCCATCCGAGGACAAATAAAGTGTTCATCAAAT---
ATTACAGGGTTGCTATTAACAAGAGATGGTGGTAACGAGACTAAC----------------------------
----

GGGACCGAGGTCTTCAGACCTATAGGAGGAGATATGAAAGACAATTGGAGAAGTGAATTATATAAATATAAAG
TAGTTCAAATTGAACCATTA---GGAATAGCACCCACCAAGGCAAAGAGAAGAGTGGTG--------------
-------------------------CAGAGAGAAAAAAGAGCG---GTGACA---CTGGGAGCT---------
ATGTTCCTTGGGTTCTTGGGAGCA---GCGGGAAGCACTATG---GGCGCAGCGTCA---
GTGGCGCTGACGGTACAG---GCCAGACAA---TTATTG---TCTGGTATAGTGCAACAGCAGAAC---
AATATG---CTGAGG---GCTATTGAGGCACAACAGCATATGTTGCAACTCACAGTCTGGGGCATC
>KT124787
TTCTACTGTGATACAACACAGCTGTTTAATAGCAATAGCACTTGGCTGTTTAATAACACTTGGAATAGTACTG
AGAATAATAAT---ACT--

GAGAATAATAATATCACACTCCAGTGCAGAATAAGACAAATTGTAAACATGTGGCAGAAAGTAGGAAAAGCAA
TGTATGCCCCTCCCATCAGAGGACAAATTTACTGTGCATCACAA---
ATAACAGGGCTGCTATTAACAAGAGATGGTGGTAGTAGTGATCAG----------------------------
----

AACAACACCGAGATCTTCAGACCTGCAGGAGGAAATATGAGGGACAATTGGAGAAGTGAATTATATAAATATA
AAGTAGTAAAAATTAAACCATTA---GGAGTAGCACCCACCAAGGCAAAGAGAAGAGTGGTG-----------
----------------------------CAGAGAGAAAAAAGAGCA---GTAACG---CTAGGAGCT------
---ATGTTCCTTGGGTTCTTGGGAGCA---GCGGGAAGCACTATG---GGCGCAGCGGCA---
ATGACGCTGACGGTACAG---GCCAGACAA---TTATTG---TCTGGTATAGTGCAACAGCAGAAT---
AATCTG---CTGAGA---GCTATTGAGGCGCAACAGCATCTGTTGCAACTCACAGTCTGGGGCATC
>KC596067
TTCTACTGTAATACAACACAATTGTTTAACAGTACTTGGAATACTACTGAAGATTTAAATAAAACTAGAACTG
AA---

GTAAATGACACAATCACACTCCCATGTAGAATAAGACAAATTATAAACAGATGGCAGGAAGTGGGAAAAGCAA
TGTATGCCCCTCCCATCAGCGGAAACATTAGTTGTTCATCAAAC---
ATCACAGGGCTGCTATTAACAAGAGATGGTGGTCAGAATAATGTGAGC-------------------------
----

AACACCACAGAGATCTTCAGACCTGGAGGAGGAGATATGAGGGACAATTGGAGAAGTGAATTATATAAATATA
AAATAATAAAAATCGAACCATTA---GGAATAGCACCCACCAAGGCAAGGAGAAGAGTGGTG-----------
----------------------------CAGAGAGAGAAAAGAGCA---GCA------CTAGGAGCT------
---ATGTTCCTTGGGTTCTTAGGAGCA---GCAGGAAGCACTATG---GGCGCAGCGTCA---
GTGACGCTGACGGTACAG---GCCAGACAA---TTATTG---TCTGGTATAGTGCAACAGCAAAGT---
AACTTG---CTGAAA---GCTATTGAGGCGCAACAGCATCTGTTGCAACTCACAGTCTGGGGCATC
>EF514700
TTTTACTGTAATACGTCAAAACTGTTTGATAGTACCTGGAATGAAACTAGTATTTGGAATTATACCACTACTA
ATGAT--
-------------------ACC---
ACAAAAAATGACACACTCACACTCCCATGTAAAATAAAACAAATTATAAACATGTGGCAGGAAGTAGGAAAAG
CAATGTATGCCCCTCCCATCAGAGGACAAATCAGATGCTCATCAAAT---
ATTACAGGGCTGTTATTAACAAGAGATGGTGGTCAGGGTAACGGGACC-------------------------
----

GATAACACCACCGAGATCTTCAGACCTGCAGGAGGAGATATGAGGGACAATTGGAGAAGTGAATTATATAAAT
ATAAAGTAGTAAGAATTGAACCATTA---GGAGTAGCACCCACCAAGGCAAAGAGAAGAGTGGTG--------
-------------------------------CAGAGAGAAAAAAGAGCA---GTGGGA---CTGGGAGCT---

------TTGTTCCTTGGGTTCTTGGGAACA---GCAGGAAGCACTATG---GGCGCAGCATCA---
CTAACGCTGACGGTACAG---GCCAGACAA---TTGTTG---TCTGGTATAGTGCAACAGCAGAAC---
AATCTG---CTGAGA---GCTATTGAGGCGCAACAACATCTGTTGCAACTCACAGTCTGGGGCATT
>EU839598
TTCTACTGTAATACAACAAAACTGTTTAACAATACTGGACAATTAGATAAAGAAGAAGATGAAGGA-------
----

GAAGAAGGCAATATCACACTTCCATGCAGAATAAGGCAATTCGTAAACAGGTGGCAAGAAGTAGGAAAAGCAA
TGTATGCCCCTCCCATCAGTGGACAAATTAGATGTTCATCAAAT---
ATTACAGGGCTACTATTAACAAGAGATGGTGGTGGTAGTGGTAAT----------------------------
----

AGTGAGAATGAGACCTTCAGACCTGGGGGAGGAGATATGAAAGACAATTGGAGAAGTGAGTTATATAAATATA
AAGTAGTAAAAATTCAGCCATTA---GGAATAGCACCCAGCAAGGCAAAGAGAAGAGTGGTG-----------
----------------------------CAAAGAGAAAAAAGAGCA---GTGACA---CTAGGAGCT------
---ATGTTCCTTGGGTTCTTAGGAGCA---GCAGGAAGCACTATG---GGCGCAGCGTCA---
GTAACGCTGACGGTACAG---GCCAGACTA---TTATTG---TCTGGTATAGTGCAACAGCAGAAC---
AATCTG---CTGAGG---GCTATTGAGGCGCAACAACATCTGTTGCAACTCACGGTCTGGGGCATC
>KC595164
TTCTACTGTAATTCAACAAAACTGTTTAATAGTACTTGGAATGGTACTGAAGGGTCAAATAACAGTGGCACAA
GGGCAAAT---

AACAGTCACATACTCACACTCCCATGCAGAATAAAGCAAATTATAAACATGTGGCAGGAAGTAGGAAAAGCGA
TGTATGCCCCTCCCATCAGAGGACAAATTAGATGCTCATCAAAT---
ATTACAGGGTTGCTATTAACAAGAGATGGTGGTAACAACAATAGC----------------------------
----

GGGACCGAGATCTTCAGACCTGGAGGAGGAGATATGAGGGACAATTGGAGAAGTGAATTATATAAATATAAAG
TAGTAAAAATTGAACCATTA---GGAGTAGCACCCACCAAGGCAAAGAGAAGAGTGGTG--------------
-------------------------CAGAGAGAAAAAAGAGCA---GTGGGA---ATAGGAGCT---------
GTGTTCCTTGGGTTCTTGGGAGCA---GCAGGGAGCACTATG---GGCGCAGCGTCA---
ATGACGTTGACGGTACAG---GCCAGACTA---TTATTG---TCTGGTATAGTGCAACAGCAGAAC---
AATTTG---CTGAGG---GCTATTGAGGCGCAACAGCATCTGTTGCAACTCACAGTCTGGGGCATC
>KC595194
TTTTACTGTAATTCAACACAACTGTTTAACAGTGTTTGGAAGGCTAACAGTACTTGGAATAGTACTGAAGGGT
CAAATAAC---

AATGCAGGAAATATCACACTCCCATGCAGAATAAAACAAATTATAAACATGTGGCAGGAAGTAGGAAAAGCAA
TGTATGCCCCTCCCATCAGAGGCCAAATTAGCTGTTCATCAAAT---
ATTACAGGGCTACTATTAACAAGAGATGGTGGTAACATCACTAAC----------------------------
----

GGGACCGAGATCTTCAGACCTGGAGGAGGAGATATGAGGGACAATTGGAGAAGTGAATTATATAAATATAAAG
TAGTAAAAATTGAACCATTA---GGAATAGCACCCACCAAGGCAAAGAGAAGAGTGGTG--------------
-------------------------CAGAGAGAGAAAAGAGCA---GTGGGA---ATAGGAGCT---------
ATGTTCCTTGGGTTCTTGGGAGCA---GCAGGAAGCACTATG---GGCGCGGCGTCA---
ATGACGTTGACGGTACAG---GCCAGACTA---TTATTG---TCTGGTATAGTGCAACAGCAGAAC---
AATTTG---CTAAGG---GCTATTGAGGCGCAACATCATCTGTTGCAACTCACAGTCTGGGGCATC
>KC595151
TTCTACTGTGATACAACAAAATTGTTTAATAGTACTTGGGATATTAATGATACTTGGAATGGTACTAACGGG-
----

CCAGATAACACTATCATACTCCCATGCAGAATAAAACAAATTATAAACATGTGGCAGGAAGTAGGAAAAGCAA
TGTATGCCCCTCCCATCAGAGGACGAATTAGGTGTGAATCAAAT---
ATTACAGGGCTGCTATTAACAAGAGATGGTGGTGATGATAGCAGT----------------------------
----

AACCAGACCGAGATCTTCAGACCTGGAGGAGGAGATATGAGGGACAATTGGAGAAGTGAATTATATAAATATA
AAGTAGTAAAAATTCAACCATTA---GGAATAGCACCCAACAAGGCAAAGAGAAGAGTGGTG-----------
----------------------------CAGAGAGAAAAAAGAGCA---GTGGGA---ATAGGAGCT------

---TTGTTCCTTGGGTTCTTGGGAGCA---GCAGGAAGCACTATG---GGCGCAGCGTCA---
ATGACGTTGACGGTACAG---GCCAGACAA---TTATTG---TCTGGTATAGTGCAACAGCAGAAC---
AATCTG---CTGAGG---GCTATTGAGGCGCAACAGCATCTGTTGCAACTCACAGTCTGGGGCATC
>KT200350
TTCTACTGTAATTTAACAAAACTGTTTAATAGTACCTGGAATATGAATGCTACCACAGGGAACTGGACT----
----
TATATTAAAGGGACAGAT---------
TACACTGAAGGAAACATCACACTCCCATGCAAAATAAAACAGATTATAAACATGTGGCAGGAAGTAGGAAAAG
CAATGTATGCCCCTCCCATCAGTGGACAAATTAGATGTTCATCAAAT---
ATTACAGGGCTGATAATAACAAGAGATGGTGGTATTAATAATACTACCAACGAG------------------
----

ACCATCGAGACCTTCAGACCTGGAGGAGGAGATATGAGGGACAATTGGAGAAGTGAATTATATAAATATAAAG
TAGTAAGAATTGAACCATTA---GGAATAGCACCCACCAAGGCAAAGAGAAGAGTGGTG-------------
-------------------------CAGAGAGAGAAAAGAGCA---ATAGGA---TTTGGAGCT---------
TTGTTCCTTGGGTTCTTGGGAGCA---GCAGGAAGCACTATG---GGCGCAGCGTCA---
CTGACGCTGACGGTACAG---GCCAGACAA---TTATTG---TCTGGTATAGTGCAACAGCAGAAC---
AATTTG---CTGAGG---GCTATTGAGGCGCAGCAGCATATGTTGCAACTCACAGTCTGGGGCATC
>EU786679
TTCTACTGTAATACAACACAGCTGTTTAATAGTACTTGGGGTATGAATACGACTAAAGGGACAAATACC----
----

ACTGAAGGAGATATCATACTCCCATGCAGAATAAAACAAATTATAAACATGTGGCAGGAAGTAGGAAAAGCAA
TGTATGCCCCTCCCATCAGCGGGCCAATTACATGTACATCAAAT---
ATTACAGGGCTGCTACTAACAAGAGATGGGGGTCAGAGCAAGAACGAA------------------------
----

AGTAACGAGACAGAGATCTTCAGACCAGGAGGAGGAGACATGAAGGACAATTGGAGAAGTGAATTATATAAAT
ATAAAGTAGTAAAAATTGAACCATTA---GGAATAGCACCCACCAAGGCAAAAAGAAGAGTGGTG--------
-------------------------------CAAAGAGAAAAAAGAGCA---GTGGGA---CTAGGAGCT---
------GTGTTCCTTGGGTTCTTGGGAGCA---GCAGGAAGCACTATG---GGCGCAGCGTCA---
ATAACGCTGACGGTACAG---GCCAGACAA---TTATTG---TCTGGTATAGTGCAACAGCAGAAC---
AACTTG---CTGAGG---GCTATTGATGCGCAACAGCATATGTTGCAGCTCACAGTCTGGGGCATC
>KC473841
TTCTACTGTAATACATCACAACTGTTTAGTAGTACTTGGAATAATACTGTAGGGTTAAATACTACA-------
----

GGAAATGATACTATCACACTCCACTGCAGAATAAGACAAATTATAAACCTGTGGCAGAGAGTAGGAAAAGCAA
TGTATGCCCCTCCCATTAGAGGACAAATTAGTTGTTCATCAAAC---
ATTACAGGGTTGCTATTAACAAGAGATGGTGGTAACAACAATCAGAGC------------------------
----

AATAACACCGAAATCTTCAGACCTGGAGGAGGAGATATGAGGGACAATTGGAGAAGTGAATTATATAAATATA
AAGTAGTAAGAATTGAACCACTA---GGAGTAGCACCCACCAAGGCAAAGAGAAGAGTGGTG----------
---------------------------CAGAGAGAAAAAAGAGCA---GTGGGA---CTAGGAGCT------
---GTGCTCTTTGGGTTCTTGGGAGCA---GCAGGAAGCACTATG---GGCGCAGCGTCA---
GTGACGCTGACGGTACAG---GCCAGACAA---TTATTG---TCTGGTATAGTGCAACAGCAAAAT---
AATTTG---CTGAAA---GCTATTGAGGCGCAACAGCATCTGTTGCAACTCACAGTCTGGGGCATC
>KT200357
TTCTACTGTAATACAACACAGCTGTTCAATAGTACTTGGGAGCCAGGGAAGAATACTACTACAGAG-------
----

GGAAATGACACTATCACGCTCCCATGCAGAATAAAACAGATTATAAACAGGTGGCAGGAAGTAGGAAAAGCAA
TGTATGCCCCTCCCATCAGAGGACGAATTAATTGTACATCAAAT---
ATTACAGGGCTGTTATTAACAAGAGATGGTGGTAACAATAATAAC---------------------------
----

CAGACAAATAGGACAGAGACCTTTAGACCTCAAGGAGGGAATATGAAGGACAATTGGAGAAGTGAATTATATA
AATATAAAGTAGTACAAGTTGAGCCATTA---GGAATAGCACCCACCAAGGCAAGGAGAAGAGTGGTG-----
---------------------------------CAGAGAGAAAAAAGAGCA---GTGGGA---

CTAGGAGCT---------GTGTTCCTTGGGTTCTTAGGAGCA---GCAGGAAGCACTATG---
GGCGCAGCGTCA---ATAACGCTGACGGTACAG---GCCAGACAA---TTGTTG---
TCTGGTATAGTGCAACAGCAGAAC---AACCTG---CTGAGG---
GCTATTGAAGCGCAACAACATATGTTGCAGCTCACAGTCTGGGGCATC
>KP109518
TTCTACTGTAATACAACACAACTGTTTAATAGTACCTGGAATGATACTAGTATTAGTGAAAGGTCAAAT----
----

ACTACTAAAAACATCACGCTTCCATGCAGAATAAAACAAATTATAAACATGTGGCAAGAAGTAGGGAAAGCAA
TGTATGCCCCTCCCATCCCAGGACCAATTCAATGTAGATCAAAT---
ATTACAGGGATATTATTAACAAGAGATGGTGGTAATACTAGTGAT----------------------------
----

AGCCAGAATGAAACCTTCAGACCTGGAGGAGGAAATATGAAGGACAATTGGAGAAGTGAATTATATAAATATA
AAGTAGTAGAAATTGAACCATTA---GGAGTGGCACCCACCAGGGCAAGGAGAAGAGTGGTG-----------
----------------------------CAGAGAGAAAAAAGAGCA---GTGGGAGTAGTAGGAGCT------
---ATGTTCCTTGGGTTCTTGGGAGCA---GCAGGAAGCACTATG---GGCGCAGCGTCA---
ATGACGTTGACGGTACAG---GCCAGACTA---TTACTG---TCTGGTATAGTGCAGCAGCAGAAC---
AATCTG---CTGAGA---GCTATTGAAGCGCAACAGCATCTGTTGCAGCTCACAGTCTGGGGCATT
>KY465969
TTCTACTGTAATACAACACAGCTGTTTAATAGCACTTGGAATAAAACTGTAGTTGAGAATAATAATTATGGTT
CT---

GAGAATGGTACTATCACACTCCAGTGCAGAATAAGACAAATTGTAAACAGGTGGCAGGAAGTAGGAAAAGCAA
TGTATGCCCCTCCCATCAGAGGAGTAATTAACTGTTCATCAAGT---
ATTACAGGGCTGTTATTAACAAGGGATGGTGGTAATAGTAAGAACACC-------------------------
----

AACAGGTCATCTGAGATCTTCAGACCTGGAGGAGGAGATATGAGGGACAATTGGAGAAGTGAATTATACAAAT
ATAAAGTAGTAAAGATTGAACCATTA---GGAGTAGCACCCACCAAGGCAAAGAGGAGAGTGGTG--------
-------------------------------CAGAGAGAAAAAAGAGCA---GTGGGAGCATTAGGAGCT---
------ATGTTCCTTGGGTTCTTGGGAACA---GCAGGAAGCACTATG---GGCGCAGCGTCA---
CTGACGCTGACGGTACAA---GCCAGACAA---CTATTG---TCTGGTATAGTGCAACAGCAGAAC---
AATCTG---CTGCAG---GCTATCGAGGCGCAACATCATCTGTTGCAACTCACCGTCTGGGGCATC
>KC595204
TTCTACTGTAACTCAACACAGCTGTTTAATAGTACTTGGAATAGTACTGGGAATCAGAATAATATTGAAGGG-
----

TTAAATGACACAATCACACTCCCATGCAGAATAAAACAAATTATAAACATGTGGCAGGAAGTAGGAAGAGCAA
TGTATGCCCCTCCCATTAGAGGACAAATTAGATGTTCATCAAAT---
ATTACAGGGCTGTTATTAACAAGAGATGGTGGTCATAACACGACA----------------------------
----

GAGACCGAGATCTTCAGACCTGGAGGAGGAGATATGAGGGACAATTGGAGAAGTGAATTATATAAATATAAAG
TAGTAAAAATTGAACCATTA---GGAGTAGCACCCACCAGAGCAAAGAGAAGAGTGGTG--------------
-------------------------CAGAGAGAAAAAAGAGCA---GTGGGA---ATAGGAGCT---------
GTGTTCCTTGGGTTCTTGGGAGCA---GCAGGAAGCACTATG---GGCGCAGCGTCA---
GTGACGCTGACGGTACAG---GCCAGACTA---TTATTG---TCTGGTATAGTGCAACAGCAGAAT---
AATTTG---CTGAAG---GCTATTGAGGCGCAACAGCATCTGTTGCAACTCACAGTCTGGGGCATC
>DQ141342
TTCTACTGTAATACAACACAACTGTTTAATAGTACTTGGCCTAAGAATGGTACCAGCGAGTTAAAT-------
----

ACCACTGACCCATTCATACTCCCATGTAAAATAAAACAAATTATAAACATGTGGCAGAAAGTAGGAAAAGCAA
TGTATGCCCCTCCCATCCGTGGACAGATTAGATGCTCATCAAAT---
ATTACAGGGCTGCTATTACCAAGAGATGGTGGTAACAACAACAATGGTAGT----------------------
----

AACAACCGCAGTCCTGAGATCTTCAGACCTGGAGGAGGAGATATGAGGGATAATTGGAGAAGTGAATTATACA
AATATAAAGTAGTAAAAATTCAACCATTA---GGAGTAGCACCCACCAAGGCAAAGAGAAGAGCGGTG-----

----------------------------------CATAGAGACAAAAGAGCA---GTGGGA---
CTCGGAGCT---------GTGTTCCTTGGATTCTTGGGAGCA---GCAGGAAGCACTATG---
GGCGCAGCGTCA---CTGACGTTGACGGTACAG---GCCAGACAA---TTATTG---
TCTGGTATAGTGCAACAGCAAAAC---AACTTG---CTGAGG---
GCTATTGAGGCGCAACAGCATCTGTTGCAACTCACAGTCTGGGGCATC
>DQ141343
TTCTACTGTGATACATCACAACTATTTAATAGTACTTGGAATAGTAGTACTTTGAATAATTTTACTAACGGGT
CAAAT--

ATAACTAATGAAACTTTCACACTCCCATGCAGAATAAAACAATTTATAAACATGTGGCAGGAAGTAGGAAAAG
CAATGTATGCCCCTCCTATCAGAGGAGTAATTAACTGTTTATCAAAT---
ATTACAGGGCTGCTATTAACAAGAGATGGTGGTAATAATCATAGTGAC-------------------------
----

ACCAATCAGGAGATCTTCAGGCCTGCAGGAGGAGATATGAGGGACAATTGGAGAGGTGAATTATATAAGTATA
AAGTAGTAAGAGTAGAACCATTA---GGGATAGCACCCACCAAGGCAAAGAGAAGAGTGGTG-----------
----------------------------CAGAGAGAAAAAAGAGCA---GTGGGA---CTAGGAGCT------
---TTGTTCCTTGGGTTCTTGGGAGCA---GCAGGAAGCACTATG---GGCGCAGCATCA---
ATGACGCTGACGGTACAG---GCCAGACAA---TTATTG---TCTGGTATAGTGCAACAGCAGAAC---
AACCTG---CTGAGG---GCTATTGAGGCGCAACAGCATATGTTGCAACTCACAGTCTGGGGCATC
>DQ448819
TTCTACTGTAATACAACACAACTGTTTAATAGTACTTGGTATGCTAGTAATAATACTTGGAAGGGTACTAAC-
----

ATTACAGGCAATATCACACTCCCATGCAAAATAAAACAAATTATAAACATGTGGCAGGAAGTAGGAAAAGCAA
TGTATGCCCCTCCCATCAGAGGACAAATTAGATGCTCATCAAAT---
ATTACAGGGTTACTATTAACAAGAGATGGGGGTCAGGGAAACAACACC-------------------------
----

GACAACGACACTGAGATCTTCAGACCTGGAGGAGGAAATATGAGGGACAATTGGAGAAGTGAATTATATAAAT
ATAAAGTAGTAAGAATTGAACCATTA---GGACTAGCACCCACCAGGGCAAAGAGAAGAGTGGTG--------
-------------------------------CAGAGAGAGAAAAGAGCA---GTGGGA---CTAGGAGCG---
------TTGTTCATTGGGTTCTTGGGAGCA---GCAGGAAGCACTATG---GGCGCAGCGTCA---
GTGACGTTGACGGTACAG---GCCAGACAA---TTACTG---TCTGGTATAGTGCAACAGCAGAAT---
AATTTG---CTGATG---GCTATTGAGGCGCAACAGCATATGTTGAAACTCACGGTCTGGGGCATT
>EF531331
TTTTACTGTAATACAACACAACTATTTAATAGTACTTGGAATATAACAAATCTGTTTAATAGTACTTGGAATA
GTACTGAAGGG--
-------TCAAATAACACT------
AAAAGAAATGGCACAATCATACACCCATGCAGAATAAAACAGATTATAAACATGTGGCAGAAAGTAGGAAAAG
CAATGTATGCCCCTCCCATCGAAGGAATAATTAGATGTGAATCAAAA---
ATTACAGGGATGATACTAACAAGAGATGGTGGTCTTGGTCGTAACATT-------------------------
----

AGCAACGAGACCGAGATCTTCAGACCTGGAGGAGGAGATATGAGGGACAATTGGAGAAGTGAATTGTATAAAT
ATAAAGTAGTAAAAATAGAACCCTTA---GGAATAGCACCCTCCAAGGCAAAGAGAAGAGTGGTG--------
-------------------------------CAGAGAGAAAAAAGAGCA---ATAGGA---TTCGGAGCT---
------ATGTTCCTTGGGTTCTTGGGAGCA---GCAGGAAGCACTATG---GGCGCAGCGTCA---
CTAACGCTGACGGTACAG---GCCAGACAA---TTACTG---TCTGGTATAGTGCAACAGCAGAAC---
AATTTG---CTGAGG---GCTATTGAGGCGCAACATCATCTGTTGCAACTCACAGTCTGGGGCATC
>MH000288
TTCTATTGTAATACAACACAACTGTTTGATAGTACTTGGAATGAT----------------------------
----

ACTACAGACACAATCACACTCCCATGCAAAATAAGACAAATTGTAAACTTGTGGCAGGAAGTAGGAAAAGCAA
TGTATGCCCCTCCCATCAGTGGAATAATTCAGTGTACATCAAAT---
ATTATAGGGTTGCTATTAACAAGAGACGGTGGT--

ACCGAAATCTTCAGACCAGGAGGAGGAGATATGAGGGACAATTGGAGAAGTGAATTATATAAATATAAAGTAG

TACAAGTTGAACCATTA---GGAATAGCACCCACCAAGGCAAAGAGAAGAGTGGTG-----------------
----------------------CAGAGAGAAAAAAGAGCA---GTGGGAACGCTAGGAGCT---------
GTATTCCTCGGGTTCTTGGGAGCA---GCAGGAAGCACTATG---GGCGCAGCGTCA---
ACAGCGCTGACGGTACAG---GCCAGACAA---TTATTG---TCTGGTATAGTGCAACAGCAGAAC---
AATCTG---CTGAAG---GCTATTGAGGCGCAACAGCATTTGTTGCAGCTCACAGTCTGGGGCATC
>KC699040
TTCTATTGTGATACAGCACAACTGTTTAATAGTACTTGGCCTAAGAATAGCACCAGTAGCACCAATAGCACCG
GTAAAGGG---

GGGCCAACAACTATCACACTCCCATGCAGAATAAGACAAATTGTAAACATGTGGCAGGAAGTAGGAAAAGCAA
TGTATGCTCCTCCCATCAGCGGAAACATCACATGTTCATCAAAT---
ATTACAGGGCTGCTATTAACAAGAGATGGGGGTAATAAGAATGAG----------------------------
----

ACGACCAACAGGACTCAGACCTTCAGACCTATAGGAGGAGATATGAGGGACAATTGGAGAAGTGAATTATATA
AATATAAAGTAGTAAAAATTGAACCATTA---GGAATAGCACCCACCAAGGCAAAGAGAAGAGTGGTG-----
----------------------------------CAGAGAGAAAAAAGAGCA---GTGGGA---
GTAGGAGCT---------TTGTTCCTTGGGTTCTTGGGAGCA---GCAGGAAGCACTATG---
GGCGCAGCGTCA---GTGACGTTGACGGTACAA---GCCAGACAA---TTATTG---
TCTGGTATAGTGCAACAGCAGAAC---AATCTG---CTGAGA---
GCTATTGAGGCGCAACAGCATATGTTGCAACTCACAGTCTGGGGCATC
>KC699032
TTTTACTGTAATACAACACAGCTGTTTAATAGTACTTGGAATGCTAATGGTACTAGTCGGGATGGTACTACA-
----

GGGAATGATACTATCACAATCCAATGCAGAATAAAGCAAATTATAAACAGGTGGCAGGAGGTAGGAAAAGCAA
TGTATGCCCCTCCCATCCAAGGACAAATTTACTGTTCATCAAAT---
ATTACAGGGCTTCTATTAACAAGAGATGGGGGTAATGACAATAACACT-------------------------
----

AGCACTAATGAGACCTTCAGACCTGCAGGAGGAAATATGAAGGACAATTGGAGAAGTGAATTATATAAATATA
AAGTAGTAAAAATTGAGCCATTA---GGACTGGCACCCACCAGGGCAAGGAGAAGAGTGGTG-----------
----------------------------CAGAGAGAAAAAAGAGCA---GTGGGA---CTAGGAGCT------
---ATGTTCCTTGGGTTCTTGGGAGCA---GCAGGAAGCACTATG---GGCGCAGCGTCA---
TTGACGCTGACGGTACAG---GCCAGGCAA---TTATTG---TCTGGTATAGTGCAACAGCAAAAC---
AATTTG---CTAAGA---GCTATTGAGGCGCAACAGCATATGTTGCAACTCACAGTCTGGGGCATC
>KC699037
TTTTATTGTAATACATCACAACTGTTTAATAGTAGTTGGACCAATTACACTAATGGTACTTGGGACAATGACA
CCATAATA---
----------GATAACACT------
ACCACTGGAGTAAATATCACACTCCCATGCAGAATAAAACAAATTATAAACATGTGGCAGAAAGTAGGAAAAG
CCATGTATGCCCCTCCCATCAGAGGACTAATTCAATGTACATCAAAT---
ATTACAGGAATACTATTAACAAGAGATGGAGGTAATAGGAATGAGAGCAAT----------------------
----

GGCAAAAGCAATGAGACCTTCAGACCTGTAGGGGGAGATATGAGGGACAATTGGAGAAGTGAATTATATAAAT
ATAAAGTAGTAAAAATTAAACCATTA---GGAATAGCACCCACCAGGGCAAAGAGAAGAGTGGTA--------
-------------------------------CAGAGAGAAAAAAGAGCA---GTGGGAACGTTGGGAGCC---
------ATGTTCCTTGGGTTCTTGGGAGCA---GCAGGAAGCACTATG---GGCGCAGCGTCA---
CTAACGCTGACGGTACAA---GCCAGACAA---TTATTG---TCTGGTATAGTGCAACAGCAAAGC---
AATTTG---CTGAGG---GCTATTGAGGCGCAGCAGCACATGTTGCAACTCACAGTCTGGGGCATT
>MH000292
TTCTATTGTAACACAACACAGCTATTTAATAGTACTTGGATGCCT----------------------------
----

AATGGTGGCACTATCACACTCCCATGCAGAATAAAACAAATTATAAACAGGTGGCAGGAAGTAGGAAAAGCAA
TGTATGCCCCTCCCATTAGTGGACAAATTAACTGCTCATCAAAC---
ATTACAGGGTTACTATTAATAAGAGATGGTGGT--

AACGAGACCTTCAGACCTGGAGGAGGAAATATGAAAGACAATTGGAGAAGTGAATTATATAAATATAAAGTAG
TACAAATTCAGCCATTA---GGAGTAGCACCCACCAAGGCAAAGAGAAGAGTGGTG-----------------
----------------------CAGAGAGAAAAAAGAGCA---GTGGGAACGCTAGGAGCT---------
ATGTTCCTTGGGTTCTTGGGAGCA---GCAGGAAGCACTATG---GGCGCAGCGTCA---
GTGACGCTGACGGTACAG---GCCAGACAA---TTATTG---TCTGGTATAGTGCAACAGCAAAAC---
AACTTG---CTGAAA---GCTATTGAGGCGCAACACCATCTGTTGCAACTCACAGTCTGGGGCATT
>KC699006
TTCTACTGTAATACAACACAACTGTTTAATAGTACTTGGAATAGTACTTGGGATATTAATAGTACTATAGAGT
CAAATAAC---

ACTGGAGAATATATCACACTCCCATGTAGAATAAAACAAATTATAAACATGTGGCAGGAAGTAGGAAAAGCAA
TGTATGCCCCTCCCATCAAAGGACAAATTAGATGTTCATCAAAC---
ATTACAGGGCTGCTATTAACAAGAGATGGTGGTAATAACAATAGTAGC-------------------------
----

AACACCACCGAGACCTTCAGACCTGGAGGAGGAGATATGAGGGACAATTGGAGAAGTGAATTATATAAATATA
AAGTAGTAAAAATTGAACCATTA---GGAGTAGCACCCACCAAGGCAAAGAGAAGAGTGGTG-----------
----------------------------CAGAGAGAAAAAAGAGCA---GTGGGA---ATAGGAGCC------
---GTGTTCCTTGGGTTCTTGGGAGCA---GCAGGAAGCACTATG---GGCGCAGCGTCA---
ATAACGCTGACGGTACAA---GCCAGACTA---TTATTG---TCTGGTATAGTGCAACAGCAGAAC---
AATCTG---CTGAGG---GCCATTGAGGCGCAACATCATCTGTTGCAACTCACAGTCTGGGGCATC
>KC699007
TTCTACTGTAATTCAACACAACTGTTTAATAGTACTTGGAGTGAAGGGTCAAATAACACTGAAGGAAATAACA
CTGAA--

GGAAATGACACAATCACACTCCCATGCAGAATAAAACAAATTATAAACATGTGGCAGAACGTAGGAAAAGCAA
TGTATGCCCCTCCCATCAGCGGACAAATTAGATGTTCATCAAAT---
ATTACAGGGCTGATATTAACAAGAGATGGTGGTAATAACCCAAAC----------------------------
----

GGGACCGAGATCTTCAGACCTGGAGGAGGAGATATGAGGGACAATTGGAGAAGTGAATTATATAAATATAAAG
TAGTAAAAATTGAACCATTA---GGAGTAGCACCTACCAAGGCAAAGAGAAGAGTGGTG--------------
-------------------------CAGAGAGAAAAAAGAGCA---GCGGGA---CTAGGAGCT---------
GTGTTCCTTGGGTTCTTGGGAGCA---GCAGGAAGCACTATG---GGCGCAGCGTCA---
ATAACGCTGACGGTACAG---GCCAGACGA---TTATTG---TCTGGTATAGTGCAACAGCAGAAC---
AATTTG---CTGAGG---GCTATTGAGGCGCAACATCATCTATTGCAACTCACAGTCTGGGGCATC
>KC699025
TTCTACTGTAATACAACAAAACTGTTTAATAGTACTTGGAAGGAAAATGGCACCATTGAAGACGATGGTACA-
----

AAGGATACTAACATCACACTCCCATGCAAAATAAAACAAATTATAAACAGGTGGCAGGCAGTAGGAAAAGCAA
TGTATGCCCCTCCCATCAGAGGACAAATTAAATGTACATCAAAT---
ATTACAGGGTTGCTGTTAGTAAGAGATGGTGGTCATAACACTGAC----------------------------
----

AACGAGACCTTCAGACCTGGAGGGGGAGACATGAAGGACAATTGGAGAAGTGAACTATATAAATATAAAGTAG
TAAAAGTTGAACCATTA---GGAATAGCACCCACCAAGGCAAAGAGAAGAGTGGTG-----------------
----------------------CAGAGAGACAAAAGAGCA---GTGGGAACACTAGGAGCA---------
ATGTTCCTTGGGTTCTTGGGAACA---GCAGGAAGCACTATG---GGCGCAGCGTCA---
GTGGCGCTGACGGTACAG---GCCAGACAA---TTACTG---TCTGGTATAGTGCAACAGCAAAAC---
AATTTG---CTGAAG---GCTATTGAGGCGCAACAGCATCTGTTGCAACTCACAGTCTGGGGCATC
>JF706474
TTCTACTGTAATTCAGCACCACTGTTTAATAGTACTTGGAATAGTATTGAGGGTAGTACTGAGGGTAAGAATG
AA---

AGCAGTGGAGGAAATATCACACTCCTATGTAGAATAAAACAAATTATAAACATGTGGCAGGAAGTAGGAAAAG
CAATGTATGCCCCTCCTATCAGAGGAGTAATTCAATGCTCATCAAAT---
ATTACAGGGCTGCTATTAACAAGAGATGGTGGTAGTGGTAATAGTAGCAAT----------------------
----

GAGAACGAGACCGAGACCTTCAGACCTGGGGGGGGAAATATGAAGGACAATTGGAGAAGTGAATTATATAAAT
ATAAAGTAGTACAAATTGAACCATTA---GGAATAGCACCCACCAAGGCAAAGAGAAGAGTGGTG--------
-------------------------------CAGAGAGAAAAAAGAGCG---GTGGGAACACTAGGAGCT---
------GTGTTCCTTGGGTTCTTGGGAGCA---GCAGGAAGCACTATG---GGCGCAGCGTCG---
GTAACGCTGACGGTACAG---GCCAGACAA---TTATTG---TCTGGTATAGTGCAACAGCAGAAC---
AATTTG---CTGAAG---GCTATTGAGGCGCAACAGCATCTGTTGCAACTCACAGTCTGGGGCATC
>HM204630
TTCTACTGTAATACAACAAAACTGTTTAATAGTACTTGGGGGAATAATACTGCAGGACAA-------------
----

GGTAATAACACTATCACACTCCCATGCAGAATAAAACAAATTATAAACTTGTGGCAGGAAGTAGGAAAAGCAA
TGTATGCCCCTCCCATTAGTGGACACATTTACTGTTCATCAAAT---
ATTACAGGGCTACTATTAACAAGAGATGGTGGTACTACCAACATAACC-------------------------
----

AATGAAACTGAGATCTTCAGACCTGGAGGGGGAGATATGAGGGACAATTGGAGAAGTGAATTATATAAATATA
AAGTAGTAAAAGTTGAACCATTA---GGAATAGCACCCACCAAGGCGAAGAGAAGAGTGGTG-----------
----------------------------CAGAGAGAAAAAAGAGCA---GTGGGA---ATAGGAGCT------
---GTGTTCCTTGGGTTCTTGGGAGCA---GCAGGAAGCACTATG---GGCGCAGCGTCA---
CTGACGCTGACGGTACAG---GCCAGACAA---TTATTG---TCTGGTATAGTGCAACAGCAGAAC---
AATTTG---CTGAGA---GCTATTGAGGCGCAACAGCATCTGTTGCAACTCACAGTCTGGGGCATC
>HM204631
TTTTACTGTAATTCATCACAACTGTTTAATAGTACTTGGAATGCCACTAGTACTGGGAATAACAGTATAGGG-
----

TTAAATGGCACTATCACACTCCCATGTAAAATAAAACAGATTATAAACAGGTGGCAGGAAGTAGGAAGAGCAA
TGTATGCCCCTCCCATTCAAGGAATAATTAGATGTGAATCAAAT---
ATTACAGGGCTACTGTTAACAAGAGATGGTGGTAATATAACTAGT----------------------------
----

AATGGGAACGAGACCTTCAGACCCATAGGAGGAAATATGAAGGACAATTGGAGAAGTGAACTCTATAAATATA
AAGTAGTACAAATTGAACCCTTA---GGAATAGCACCCACCAAGGCAAAGAGAAGAGTGGTG-----------
----------------------------CAGAGAGAAAAAAGAGCA---GTGGGAACAATAGGAGCT------
---ATGTTCCTTGGGTTCTTGGGAGCA---GCAGGAAGCACTATG---GGCGCAGCGTCG---
GTGGCGCTGACGGTACAG---GCCAGACAA---TTATTG---TCTGGTATAGTGCAGCAGCAGAAC---
AACCTG---CTGAGG---GCTATTGAGGCGCAACAGCACATGTTGCAACTCACAGTCTGGGGCATT
>HM204636
TTCTATTGTAAAACAACAGAGCTGTTTAATAGTACTTGGCCAGGGAATAGTACTGTGAATAATGCTACAGAAT
CA---

AATGACAATGACACTATCACACTCCCATGCAGAATAAAACAAATTATAAACAGGTGGCAGGAAGTAGGAAAAG
CAATGTATGCCCCTCCCATCCAAGGAAACATTAGCTGTTCATCAAAC---
ATTACAGGACTGCTATTAACAAGAGATGGTGGTAAAGATCATAAC----------------------------
----

TCCACCGAGACCTTTAGACCTGGAGGAGGAGATATGAGAGACAATTGGAGAAGTGAATTATACAAATATAAAG
TAGTAAGAATTGAGCCATTA---GGAGTAGCACCCACCAAGGCAAGGAGAAGAGTGGTG--------------
-------------------------CAGAGAGAAAAAAGAGCA---GCACTG------GGAGCT---------
GTGTTCCTTGGATTCTTGGGAGCA---GCAGGAAGCACTATG---GGCGCAGCGTCA---
GTGGCGCTGACGGTACAG---GCCAGACAA---TTATTG---TCTGGTATAGTGCAACAGCAAAAC---
AATTTG---CTGAGG---GCTATTGAGGCGCAACAACATCTGTTGCAACTCACAGTCTGGGGCATC
>HM204637
TTCTACTGTAATACAACACAGTTGTTTAATAATACACAGTGGGTTAATAGTAATAGTACAAAT----------
----

AGCAATGACACCATCACACTCCCATGCAGAATAAAACAAATTATAAACAGATGGCAGGAAGTAGGAAAAGCAA
TGTATGCCCCTCCCATCGCGGACAAATTCACTGTTCATCAAAT---
ATTACAGGGCTAATATTAACAAGAGATGGTGGTAATAAAAATGACACC-------------------------
----

AACGGGACTGAGACTTTCAGGCCTGGAGGAGGAGATATGAGGGACAATTGGAGAAGTGAATTATATAAATATA
AAGTAGTAAAAATTGAACCATTA---GGAATAGCACCCACCAAGGCAAGGAGAAGAGTGGTG-----------
----------------------------CAGAGAGAAAAAAGAGCA---GTAACT---TTAGGAGCT------
---ATGTTCCTTGGGTTCTTGGGAGCA---GCAGGAAGCACTATG---GGCGCAGCGTCA---
ATGACGCTGACGGTACAA---GCCAGACAA---TTATTG---TCTGGTATAGTGCAACAGCAAAAC---
AATTTG---CTGAGA---GCTATTGAGGCGCAACAGCACCTGTTGCAACTCACAGTCTGGGGCATC
>HM204644
TTCTACTGCAACACATCAAAACTGTTTAATAGTAGTTGGAGTACTAATGGAACATTGTTAAATGGAACGTGGG
TAAATGAG---
-------------------ACT---
AGAATAAATGACACAATCATACTCCCATGCAAAATAAAACAAATTATAAACATGTGGCAGAAAGTAGGAAAAG
CAATGTATGCTCCCCCATCCAAGGAATAATTAACTGCTCATCAAAT---
ATTACAGGACTGCTATTAACAAGAGATGGTGGTCATAATCAAAATAGC-------------------------
----

ACCAATGACACAGAGACCTTCAGACCAGGAGGAGGAGATATGAGGGACAATTGGAGAAGTGAATTATATAAAT
ATAAAGTAGTAAGAATTGAACCATTA---GGAATAGCACCCACCAAGGCAAAGAGAAGAGTGGTG--------
-------------------------------CAGAGAGAAAAAAGAGCA---GTGGGA---TTCGGAGCT---
------ATGTTCCTTGGGTTCTTGGGAGCA---GCAGGAAGCACTATG---GGCGCAGCGTCA---
ATAACGCTGACGGTACAG---GCCAGACAA---TTGCTG---TCTGGTATAGTGCAACAGCAAAAC---
AATTTG---CTGAGG---GCTATTGAGGCGCAACAGCATATGTTGCAACTCACAGTCTGGGGCATC
>JF680923
TTCTACTGTAACACAACAAAACTGTTTAATAGTACCTGGGAGAATAATAGTACTTGGGGGAATAAT-------
----

ACTACAGGTACTATCACACTCCCATGCAGAATAAAACAAATTGTAAACATGTGGCAAGAAGTAGGAAAAGCAA
TGTATGCCCCTCCCATCCAAGGACAAATTAGATGCTCATCAAAT---
ATTACAGGACTGTTATTAACAAGAGATGGTGGTAATAATAATAGCACA-------------------------
----

AATCAGAGCGAGACCTTCAGACCTGGAGGAGGAAATATGAAGGACAATTGGAGAAGTGAATTATATAAATATA
AAGTAGTAAAAATTGAGCCATTA---GGAATAGCACCCACCAGGGCAAAAAGAAGAGTGGTG-----------
----------------------------CAGAGAGAAAAAAGAGCA---GCC------ATAGGAGCT------
---GTGTTCCTTGGGTTCTTGGGAGCA---GCAGGAAGCACTATG---GGCGCAGCGTCA---
GTGACGCTGACGGTACAG---GCCAGACAA---TTATTG---TCTGGTATAGTGCAACAGCAGAAC---
AATTTG---CTGAAG---GCTATTGATGCGCAACAGCATCTGTTGCAGCTCACAGTCTGGGGCATC
>JF680924
TTCTACTGCAATACAACATTACTGTTCAATAGTGATTGGTATCCTAATGATACTAGCACAGGTACTCCAGGGT
CA---

GATGGGAATGACTCACTCATACTCCCATGCAGAATAAGACAAATTGTAAACATGTGGCAGGAAGTAGGAAAAG
CAATGTATGCTCCTCCCATCAAAGGACAAATTAGGTGTCATTCAAAT---
ATTACAGGGATACTACTAACAAGAGATGGTGGGATTGACAACAAC----------------------------
----

AGGACTAATGAGACCTTTAGACCTGGAGGAGGAGATATGAGGGACAATTGGAGAAGTGAATTATATAAATATA
AAGTAGTAAAACTTGAACCATTA---GGATTAGCACCCACCAAGGCAAAGAGAAGAGTGGTG-----------
----------------------------CAGAGAGAAAAAAGAGCA---GTGGGA---CTAGGAGCT------
---ATGTTCCTTGGGTTCTTGGGAGCA---GCAGGAAGCACTATG---GGCGCAGCGTCA---
ATGACGCTGACGGTACAG---GCCAGACAA---TTATTG---TCTGGTATAGTGCAACAGCAGAAC---
AATCTG---CTGAAG---GCTATTGAGGCGCAACAGCATATGTTGCAACTCACAGTCTGGGGCATC
>JF680933
TTCTATTGCAATACAACACAACTGTTTAATACTACTTGGAATATTACTAATAATATTAATGTTACTAGGAATG
AT---

AGTGACAACAGCATTATCACACTCCCATGCAGAATAAAACAAATTATAAACATGTGGCAGGAAGTAGGAAAAG
CAATGTACGCCCCTCCCATCAGAGGACAGATTAGATGTTCATCAAAA---
ATTACAGGGCTACTATTAACAAGAGATGGTGGTAATACAAGCATTGCG-------------------------
----

```
AACGGGTCTGAGATCTTCAGACCTGGAGGAGGAAATATGAAGGACAATTGGAGAAGTGAATTATACAAATATA
AAGTAGTAAAAGTTCAACCATTA---GGAATAGCACCCACCAAGGCAAAAAGAAGAGTGGTC-----------
----------------------------CAGAGAGAAAAAAGAGCA---ATAACA---CTAGGAGCT------
---GTTTTCCTTGGGTTCTTGGGAGCA---GCAGGAAGCACTATG---GGCGCAGCGTCA---
ATAACGCTGACGGTACAG---GCCAGACAA---TTATTG---TCTGGTATAGTGCAGCAGCAGAGC---
AACTTG---CTGAGG---GCTATTGAGGCGCAACAACATATGTTGCAACTCACAGTCTGGGGCATC
>HQ595797
TTCTACTGTAATTCAACACAACTGTTTAGTAGTACTTGGATGGCTAATGGTACTTCAAATGGTACCGTA----
-------------------------------------------------------------------------
----------------------------
GGGAATGACACTATCATACTCCCATGTAAAATAAAACAATTTGTAAACATGTGGCAGCGAATAGGACAAGCAG
TGTATGCCCCTCCCATTAGAGGATACATTAATTGTTCATCAAAT---
ATTACAGGACTGCTATTAACAATAGATGGTGACAGGAATGACACC----------------------------
-------------------------------------------------------------------------
-------------
CAGAGTGAGACCTTCAGACCTGGAGGAGGAGATATGAGGGACAATTGGAGAAGTGAATTATATAAATATAAAG
TAGTAAAAATTGAACCATTA---GGAATAGCACCCACTAAGGCAAAGAGAAGAGTGGTG--------------
-------------------------CAGAGAGAAAAAAGAGCA---GTAACG---CTGGGAGCT---------
ATGTTCCTTGGATTCTTGGGAGCA---GCAGGAAGCACTATG---GGCGCAGCGTCA---
ATGACGCTGACGGTACAG---GCCAGACTA---TTACTG---TCTGGTATAGTGCAACAGCAGAGC---
AATCTG---CTGAGG---GCTATTCAGGCGCAACAGCATCTGTTGCAACTTACAGTCTGGGGCATC
>AJ535611
TTCTACTGTAATACAACACAGCTGTTTAATAGTACTTGGAATATTACACAGCTGTTGAATAGTACTGAGTCAA
AT-----------------------------------------------------------------------
----------------------------
AACACTGGCAATATCACACTCCCATGCAGAATAAGACAAATTGTAAACAGGTGGCAGGAAGTAGGAAAAGCAA
TGTATGCCCCTCCCATCAGCGGACAAATTAGTTGTTCATCAAAT---
ATTACAGGGTTGCTATTAACAAGAGATGGAGGTAACAGTAGCTAC----------------------------
-------------------------------------------------------------------------
----------
AATGAGACCGAGACCTTCAGACCAGGAGGAGGAAATATGAGGGACAATTGGAGAAGTGAATTATATAAATATA
AAGTAGTAAGAATTGAACCAATA---GGAGTAGCACCCACCAGGGCAAAGAGAAGAGTGGTA-----------
----------------------------CAGAGAGAAAAAAGAGCA---ATAACG---CTGGGAGCT------
---GTGCTCCTTGGGTTCTTGGGAGCA---GCAGGAAGCACTATG---GGCGCAGCGTCA---
ATAACGCTGACGGTACAG---GCCAGACAG---TTATTG---TCTGGTATAGTGCAGCAGCAGAAC---
AATCTG---CTGAGG---GCTATTGAGGCGCAACAGCACCTGTTGCAACTCACAGTCTGGGGCATC
>AJ286339
TTCTACTGTAGTACAACAGAACTGTTTAATGACACTTGGGATAAGACTAGGAATAATACTGGAGAGGCA----
-------------------------------------------------------------------------
----------------------------
GATAACAGCACAATCACACTCCCCTGCAGAATAAAACAAATTATAAACAAGTGGCAAGAAGTAGGAAAAGCAA
TGTATGCCCCTCCCATCAGAGGACAAATTAAGTGTACATCAAAT---
ATTACAGGGCTAGTCTTAACAAGAGATGGTGGTAATGAGACTCAT----------------------------
-------------------------------------------------------------------------
----------
AATGAGACTGAGATCTTCAGACCTGGAGGAGGAGATATGAGGGACAATTGGAGAAGTGAATTATACAAATATA
AAGTAGTAAAAATTGAGCCATTA---GGAGTAGCACCCACCAAGGCAAGGAGAAGAGTGGTG-----------
----------------------------CAGAGAGAAAAAAGAGCA---GTGGGAACAATAGGAGCT------
---ATGTTCCTTGGGTTCTTGGGAGCA---GCAGGAAGCACTATG---GGCGCAGCGCCA---
ATGACGCTGACGGTACAG---GCCAGACAA---TTATTG---TCTGGTATAGTGCAGCAGCAGAAC---
AATCTG---CTGAAG---GCTATTGAGGCGCAACAGCATCTGTTGCAACTCACAGTCTGGGGCATC
>DQ207942
TTCTATTGTAATACAACACAGCTGTTCAATAATACTTGGCTGAATAATGATACTGGGAATAGCACTAAG----
-------------------------------------------------------------------------
----------------------------
AATAATGCTACAATCACACTCCCGTGCAGAATAAAACAAATTATAAACAGATGGCAGGAAGTAGGAAAAGCAA
TGTATGCCCCTCCCATCAGAGGACTAATTAGATGTTCCTCAAAT---
ATTACAGGGCTACTATTAGTAAGAGATGGTGGTAATAGTAGTGCT----------------------------
-------------------------------------------------------------------------
-------------
```

GATCAGGAGATCTTCAGACCTGGGGGAGGAAATATAAAGGACAATTGGAGAAGTGAACTATATAAATATAAAG
TAGTAAAACTTGAGCCATTA---GGAGTAGCACCCACCAAGGCAAAGAGAAGAGTGGTG-------------
-------------------------CAGAGAGAAAAAAAGAGCA---GTGGGAGTGTTTGGAGCC---------
ATGTTCCTTGGGTTCTTGGGAGCA---GCAGGAAGCACTATG---GGCGCAGCGTCA---
ATAACGCTGACGGTACAG---GCCAGACAA---TTATTG---TCTGGTATAGTGCAACAGCAGAGC---
AATTTG---CTGAGA---GCTATTGAGGCGCAACAGCATCTGTTGCAACTCACAGTCTGGGGCATT
>MH078546
TTCTACTGTGATTCAACACAACTGTTCAATAGTACTTGGTGGACTAATGGTACTGAGAACGGTACTAGAGGG-
----

ACAATTGGCACCATCACACTCCAATGCAGAATAAAACAAATTATAAACATGTGGCAGAAAATAGGAAAAGCAA
TGTATGCCCCTCCCATCAAAGGACAAATTTATTGTTCATCAAAT---
ATTACAGGGATAATATTAACAAGAGATGGTGGTAGGGACAATAATACT------------------------
----

AATGGCACTGAGACCTTCAGACCTGGAGGAGGAAATATGAAAGACAATTGGAGAAATGAATTATATAAATATA
AAGTAGTAAAACTTAAGCCATTA---GGAATAGCACCCACCAAGGCACGGAGAAGAGTGGTG----------
----------------------------CAAAGAGAAAAAAAGAGCA---GTGGGA---ATAGGAGCT------
---CTGTTCCTTGGGTTCTTGGGAGCA---GCAGGAAGCACTATG---GGCGCAGCGTCA---
GTGACGTTGACGGTACAG---GCCAGACAA---TTGTTG---TCTGGTATAGTGCAACAGCAACAC---
AACTTG---CTGAAT---GCTATTGAGGCGCAACAACACCTGTTGCAACTCACAGTCTGGGGCATC
>EU839600
TTCTACTGTAATACATCAAAGCTATTTAACAATACTTGGAATACTACTAATACTAAGAAT------------
----

TGGACTGGTTTTATCATACTCCCATGCAGAATAAAACAAATTATAAATATGTGGCAAAAAGTAGGCAAAGCAA
TGTATGCCCCTCCCATCAGGGGACAAATCAAATGTATATCAGCT---
ATTACAGGGATGCTATTAACAAGAGATGGTGGGAACAACAACGAGAATAAGACT------------------
----

GTAGGGAATAGGACCGAGATCTTCAGACCTACAGGAGGAAATATGAAAGATAATTGGAGAAGTGAATTATACA
GCTATAAAGTAGTAAAAATTGAACCATTA---GGATTAGCACCCACCAGGGCGAAAAGAAGAGTGGTG-----
----------------------------------CAGAGAGAAAAAAAGAGCA---
GTGGGAGCACTGGGAGCT---------ATGTTCCTTGGGTTCTTGGGAGCA---GCAGGAAGCACTATG---
GGCGCAGCGTCA---TTGACGCTGACGGTACAG---GCCAGACAA---TTACTG---
TCTGGTATAGTGCAACAGCAAAAT---AATTTG---CTAAGG---
GCTATTGAGGCGCAACAGCATCTGTTGCAACTCACAGTCTGGGGCATC
>MK303349
TTCTACTGTGATACAACAGAACTGTTTAATAGTACTTGGAATAATAGTGTTGGAGGGTTAAATAACAGT----
----

AACAGTAATACTATCACACTCCCATGTAGAATAAAACAAATGGTAAACATGTGGCAGAGAGTAGGAAGAGCAA
TGTATGCCCCTCCCATCAAAGGACTAATTAGCTGTTCATCAGAT---
ATTACAGGGCTGCTATTAACAAGAGATGGTGGTAACTATAGTGATAACAGT---------------------
----

AATAACACTGAGACCTTCAGACCAGGAGGAGGAGATATGAAAGACAATTGGAGAAGTGAATTATATAAATATA
AAGTAGTAGAAATTAAACCACTA---GGAGTAGCGCCCACCAAGGCAAAAAGAAGAGTGGTG----------
----------------------------CAAAGAGAAAAAAAGAGCA---GTGGGA---ATAGGAGCC------
---TTGTTCCTTGGGTTCTTGGGAGCA---GCAGGAAGCACTATG---GGCGCAGCGTCA---
ATAACGCTGACGGCACAG---GCCAGACAA---TTATTG---TCTGGTATAGTGCAACAGCAGAAC---
AATCTG---CTGAGG---GCTATTGAGGCGCAACAGCAGCTATTGCAACTCACAGTCTGGGGCATC
>EF694037
TTCTACTGTATTACAACACAACTGTTTAATAGTACTTGGTGGAATGATACTGAAAGGATAAATAACATTGAAG
AA--

AACACAGAAAATATCACACTCCCATGCAGAATAAGGCAGATTGTAAACCTGTGGCAGACAGTAGGAAAAGCAA
TGTATGCCCCTCCCATCAGAGGACAAATTAGATGTTCATCAAAT---
ATTACGGGGCTGCTATTAACAAGAGATGGTGGTAATCCCAATACC---------------------------
----

ACGGAAGAGAGCTTCAGACCTGGAGGAGGAGATATGAGGGACTATTGGAGAAGTGAATTATATAAATATAAAG
TAGTAAAAATTGAACCATTA---GGAGTAGCACCCACCAAGGCAAAGAGAAGAGTGGTG--------------
-------------------------CAAAGAGAAAAAAGAGCA---GTAGGA---ATAGGAGCT---------
GTGTTCCTTGGGTTCTTGGGAGTA---GCAGGAAGCACTATG---GGCGCAGCGTCA---
ATGACGCTGACGGTACAG---GCCAGACTA---TTATTG---TCTGGTATAGTGCAACAGCAGAAT---
AACCTG---CTGAGG---GCTATTGAGGCGCAACAGCATCTGTTGCAACTCACAGCCTGGGGCATC
>JF680930
TTCTATTGTAATACAACGAAACTGTTTAGCAGTAGTTGGAATGAAACTACAAATAGCACTGAAAATGGCACT-
----

GAAAATGACACAATCACACTTCAATGCAGAATAAAACAAATTATAAACATGTGGCAGGAAGTAGGAAAAGCAA
TGTATGCCCCTCCCATCAAAGGTCCAATTAGATGCTCATCAAAT---
ATTACAGGGCTAATATTAACAAGAGATGGTGGTCATAACAATAACACG-------------------------
----

AACGAGACTGAGATATTCAGACCTGGAGGAGGAGATATGAGGGACAATTGGAGAAGTGAATTATACAAATATA
AAGTAGTAAGAATTGAACCCTTG---GGAGTAGCACCCACCAAGGCAAAGAGAAGAGTGGTG-----------
----------------------------CAGAGAGATAAAAGAGCA---GTGGGAGTATTCGGAGCT------
---ATGTTCCTTGGGTTCTTGGGAGCA---GCAGGAAGCACTATG---GGCGCAGCGTCA---
GTGGCGTTGACGGTACAG---GCCAGACAA---TTATTG---TCTGGTATAGTGCAACAGCAGAAT---
AATCTG---CTGAGA---GCTATTGAGGCGCAACAACATCTGTTGCAACTCACAGTCTGGGGCATC
>AB287372
TTCTACTGTAATTCAACACCACTGTTTAGTAGTAATTGGACTAAGAATACTACTACCGGGTCTTGGATTGGCA
GATATGGA---
-------------TCAAAT------
GAGACTGATGCAAATATTACACTCATATGCAGAATAAAACAAATTATAAACATGTGGCAGGGGGTAGGAAAAG
CAATGTATGCCCCTCCCATCAGTGGACCAATCAGATGTTCATCAAAT---
ATTACAGGGCTGCTATTAACAAGAGATGGTGGTGAGGATAAGAAC----------------------------
----

AGCACTAAAGAGACCTTCAGACCCCTAGGAGGAAATATGAAAGACAATTGGAGAAGTGAATTATATCAATATA
AAGTAGTAGGAATTGAACCATTA---GGAGTAGCACCCACCAAGGCAAGGAGAAGAGTGGTG-----------
----------------------------CAGAGAGAAAAAAGAGCA---GTGGGAACGCTGGGAGCT------
---ATGTTCCTTGGGTTCTTGGGAGCA---GCAGGAAGCACTATG---GGCGCAGCGTCA---
ATGGCGCTGACGGTACAG---GCCAGACAA---TTATTG---TCTGGTATAGTGCAACAGCAGAAC---
AATTTG---CTGAGG---GCTATTGAGGCGCAACAACATTTGTTGCAACTCACAGTCTGGGGCATC
>AB480693
TTCTACTGCAATACATCACAACTGTTTAATAGTATTTGGATTAATGGTACTGGGAATAATACTTGGAATAGTA
CTAAGGGT---
-------------CATGAA------
AGGTCAGATAACATTACCACACTCCAATGTAGAATAAAACAAATTATAAACATGTGGCAGACAGTAGGAAAAG
CAATGTATGCCCCTCCCATTGAAGGACGAATTAGATGTTCATCAAAA---
ATTACAGGGCTCTTATTAGTGAGAGATGGTGGTACGAATAGGACG----------------------------
----

GCCAACAACGAAACCTTCAGACCTGGAGGAGGAAATATGAGGGACAATTGGAGAAGTGAATTATATAAATATA
AAGTAGTAAAAATTGAACCAATA---GGAGTAGCACCCACCGAGGCAAAGAGAAGAGTGGTG-----------
----------------------------CAGAGAGAGAAAAGAGCA---GTAGGA---ATAGGAGCT------
---GTGTTCCTTGGGTTCTTGGGAGCA---GCAGGAAGCACTATG---GGCGCAGCGTCA---
GTGACGCTGACGGTACAG---GCCAGACAA---TTATTG---TCTGGTATAGTGCAACAGCAAAAC---
AATTTG---CTGAGA---GCCATTGAGGCGCAACAGCATATGTTGCAACTCACAGTCTGGGGCATC
>AB588264
TTCTACTGCAATACATCACAACTGTTTAATAGTACTTGGAATAGTACTGGGAATAGTAGTACTGAGAATAGTA
TG---

TCAAATGACACTATCACACTCCCATGTAGGATAAAACAAATTATAAATATGTGGCAGGAAGTAGGAAAAGCAA
TGTATGCCCCTCCCATCGCAGGACTAATTAGATGTTCATCAAAT---
ATTACAGGGCTATTATTAGTGAGAGATGGTGGTGGTAATCAGACT----------------------------
----

AACGGGACAGAAATCTTCAGACCTGGGGGAGGAAATATGAAGGACAATTGGAGAAGTGAATTATATAAATATA
AAGTAGTAAAAATTGAACCAGTA---GGAATAGCACCCGACAGGGCAAAGAGAAGAGTGGTG-----------
----------------------------CAGAGAGAAAAAAGGGCA---GTGGGA---ATAGGAGCT------
---GTGTTCCTTGGGTTCTTGGGAGCA---GCAGGAAGCACTATG---GGCGCAGCGTCA---
ATAACGCTGACGGCACAG---GCCAGACAA---TTATTG---TCTGGTATAGTGCAACAGCAGAAC---
AATCTG---CTGAGG---GCCATTGAGGCGCAACAGCATATGTTGCAACTCACAGTCTGGGGCATC
>AB731669
TTCTACTGTAATACATCAGCACTGTTTAATTGGAGTATTACTGGGTCAAATATTACAAAAGAC----------
----

ATTAATAGGTCAAATACTATCCCATGCAGAATAAAACAAATTATCAACATGTGGCAGAAAGTAGGAAAAGCAA
TATATGCCCCTCCCATCAGCGGGCAAATTAATTGTTCATCAAAT---
ATTACAGGGTTGCTATTAACAAGAGATGGTGGTAAAAGTAATCAGACT-------------------------
----

AATAACACTGAGGTTTTCAGACCTACAGGAGGAAATATGAGAGATAATTGGAGGAGTGAATTATATAAATATA
AAGTAGTAAGGATTGAACCATTA---GGAGTAGCACCCACCAAGGCAAGGAGAAGAGTGGTG-----------
----------------------------CAAAGAGAAAAAAGAGCA---GTGGGAACAATAGGAGCT------
---ATGTTCCTTGGGTTCTTGGGAGCA---GCAGGAAGCACTATG---GGCGCAGCGTCA---
ATAACGCTGACGGTACAA---GCCAGACTA---TTATTG---TCTGGTATAGTGCAACAGCAAAAC---
AATTTG---CTGAGA---GCTATTGAGGCGCAACAACATCTGTTGCAACTCACAGTCTGGGGCATC
>AB565496
TTCTACTGCAATACAAGACAACTGTTTAATAGTACTTGGGATAATAGTACTTGGAATGATAGTACTAGGAATA
GT---

ATTCATGACGAGAATATCACACTCCCATGTAGAATAAAACAAATTATAAACATGTGGCAAACAGTGGGAAAAG
CAATGTATGCCCCTCCCATCAAAGGACAAATTAGATGTTCATCAAAT---
ATTACAGGGCTATTATTAGAGAGAGATGGTGGTAACAATACGGAC----------------------------
----

ACCGAAACCTTCAGACCTGGAGGAGGAAATATGAAGGACAATTGGAGAAGTGAATTATATAAATATAAAGTAG
TAAAAATTGAACCAGTA---GGAATAGCACCCACCAGGGCAAAGAGAAGAGTGGTG-----------------
----------------------CAGAGAGAAAGAAGAGCA---GTGGGA---ATAGGAGCT---------
GTGTTCCTTGGGTTCTTGGGAGCA---GCAGGAAGCACTATG---GGCGCAGCGTCA---
ATAACGCTGACGGTACAG---GCCAGACAA---TTATTG---TCTGGTATAGTGCAACAGCAGAAC---
AATTTG---CTGAGA---GCTATTGAGGCGCAACAACATATGTTGCAACTCACAGTCTGGGGCATC
>AB565478
TTCTACTGTAATACAACAAAGCTGTTTAATAGTACTTGGTATACTAATGGGAATAAGACTGAAGAATCAAAT-
----

AACAATGACACTATTACACTCCCATGCAGAATAAAACAAATTATAAACAGATGGCAGGAAGTAGGAAAAGCAA
TGTATGCCCCTCCCATCCAAGGACGAATTTACTGCTCATCAAAT---
ATTACAGGACTGTTATTAATAAGAGATGGTGGTAACAGTACTGAT----------------------------
----

AACGAGTCTGAGATCTTCAGACCTGGAGGAGGAGATATGAGGGACAATTGGAGAAGTGAATTATATAAATATA
AAGTAGTACAAATTGAGCCATTA---GGAGTAGCACCCACCAAGGCAAAGAGAAGGGTGGTG-----------
----------------------------CAGAGAGAAAAAAGAGCA---GTGGGA---ATAGGAGCT------
---ATGTTCCTTGGGTTCTTGGGAGCA---GCAGGAAGCACTATG---GGCGCAGCGTCA---
CTAACGCTGACGGTACAG---GCCAGACGA---TTATTG---TCTGGTATAGTGCAACAGCAGAAC---
AATCTG---CTGAGA---GCTATTGAGGCGCAACAGCATATGTTGCAACTCACAGTCTGGGGCATC
>JQ316131
TTCTACTGTAATACATCAAAGCTGTTTAATAGTACTTGGGCTGTGAATATGACTAACGGGTCAAATATAACTA
AA---

GAAGGAAATACCACAACAATCCCATGCAGAATAAAACATATTATAAACAGGTGGCAAGAAGTGGGAAAAGCAA
TGTATGCCCCTCCCATCGAGGGACAAATTAGATGTACATCAAAT---
ATTACGGGGCTACTATTAACAAGAGATGGGGGGACTCAGAAAAAT----------------------------
----

GGGAGTAACAATGGGACTCACAACAACACTGAGATCTTCAGACCTGGGGGAGGAAATATGAAGGACAATTGGA
GAAGTGAATTATATAAATATAAAGTAGTAAAAATTGAGCCATTA---
GGAATAGCACCCACCAAGGCAAGGAGAAGAGTGGTG-------------------------------------
--CAGAGAGAAAAAAGAGCA---GTGGGAACAATAGGAGCT---------
ATGTTCCTTGGGTTCTTGGGAACA---GCAGGAAGCACTATG---GGCGCAGCGTCA---
CTGACGCTGACGGTACAG---GCCAGACTA---TTATTG---TCTGGTATAGTGCAACAGCAAAAT---
AATTTG---CTGAGA---GCTATTGAGGCGCAACAGCATCTGTTGCAACTCACAGTCTGGGGCATC
>JQ316133
TTCTACTGTAATACAACAAAACTGTTTGATAGTACTTGGAATGGTACTAAAGAGTGGAATGATACTAAAGAG-
----

TCAGATAAAAACATTACACTCCAATGCAGAATAAAACAAATTATAAACAGGTGGCAGGAAGTAGGACAAGCAA
TGTATGCCCCTCCCATCAGCGGACTAATTAGATGTTCGTCAAAT---
ATTACAGGGCTAATATTAACAAGAGACGGGGGGGCTAACGAGACT----------------------------
----

CACACCAACACTGAAACCTTCAGACCTGCAGGAGGAGATATGAAGGACAATTGGAGAAGTGAATTATATAAAT
ATAAAGTAGTAAGAATTGAGCCATTA---GGGATAGCACCCACCAAGGCAAGGAGAAGAGTGGTG--------
-------------------------------CAGAGAGAAAAAAGAGCA---GTGGGAATATTAGGAGCT---
------ATGTTCCTTGGGTTCTTGGGAGCA---GCAGGAAGCACTATG---GGCGCAGCGTCA---
ATGACGCTGACGGTACAG---GCCAGACAA---TTATTG---TCTGGTATAGTGCAACAGCAGAAT---
AATTTG---CTGAGA---GCTATTGATGCGCAACAACATCTGTTGCGACTCACAGTCTGGGGCATT
>DQ295192
TTCTACTGTGATACAACACAACTATTTAATAGCACTTGGACATGGAATGGTACTTGGATTAACGGGTCAACTA
AA---

GCTGAGAATGACACAATTGTCCTCCCATGCAGAATAAAACAAATTATAAACAGATGGCAGGAAGTAGGAAAAG
CAATGTATGCCCCTCCCATCAGCGGACACATTAATTGCTCATCAAAG---
ATTACAGGACTGCTATTAACAAGAGATGGGGGGATTAAAAACAGCACGAAC----------------------
----

GGGACTCAGACTGAGATCTTCAGACCTGGGGGAGGAGATATGAGGGACAATTGGAGAAGTGAATTATATAAAT
ATAAAGTAGTAAAAATTGAGCCATTA---GGGATAGCACCCACCAAGGCAAGGAGAAGAGTGGTG--------
-------------------------------CAGAGAGAAAAAAGAGCA---GTGGGAACAATAGGAGCT---
------ATGTTCCTTGGGTTCTTGGGAGCA---GCAGGAAGCACTATG---GGCGCAGCGTCA---
CTGACGCTGACGGTACAG---GCCAGACTA---CTATTG---TCTGGTATAGTGCAACAGCAGAAT---
AATTTG---CTGAGA---GCTATTGAGGCGCAACAGCATCTGTTGCAACTCACAGTCTGGGGCATC
>KT878039
TTCTACTGTAATACAACAAAACTGTTTAATAGTACTTGGTATAGTAATGGTACTAGGAAAGATCAAAATGAA-
----

GGAGATGACACAATTACACTCCAGTGCAGGATAAAACAAATTATAAACAGGTGGCAAGAAGTAGGAAGGGCAA
TGTACGCCCCTCCCATCAGAGGAATAATTAAATGTTCATCAAAC---
ATTACAGGGCTGCTATTAACAAGAGATGGGGGGAACGGGACTAAT----------------------------
----

GGGACTGAGACCTTCAGACCTGGGGGAGGAAATATGAAAGACAATTGGAGAAGCGAATTATATAAATATAAAG
TAGTAAAAATTGAGCCATTA---GGGATAGCACCCACCAAGGCAAGGAGAAGAGTGGTG--------------
-------------------------CAGAGAGAAAAAAGAGCA---GTGGGGACGCTAGGAGCT---------
GTGTTCCTTGGGTTCTTGGGAGCA---GCAGGAAGCACTATG---GGCGCAGCGTCA---
GTGGCGCTGACGGGACAG---GCCAGACAA---TTATTG---TCTGGTATAGTGCAACAGCAGAAT---
AATTTG---CTGAGA---GCTATTGAGGCGCAACAGCATCTGTTTCAACTCACAGTCTGGGGCATC
>KT878021
TTCTACTGTAATACAACACAGTTGTTCAATAGTACTTGGTTTCATAATGGTACTTGGAGTGGTGCTAAT----
----

GGGGATGAGACTATGACACTCCCATGCAGAATAAAACAAATTATAAACAGATGGCAAGAAGTAGGAAAAGCAA
TGTATGCCCCTCCCATCGCAGGATCAATTAATTGCTCATCAAAT---
ATTACAGGACTGTTATTAACAAGAGATGGTGGTATTACCAGCAACAAG-------------------------
----

ACTAAGAACAGCACCGAGACTGAGACCTTTAGACCGGGGGGAGGAGATATGAGGGACAATTGGAGAAGTGAAT
TATACAAATATAAAGTAGTAAAAATTGAACCACTA---
GGAGTAGCACCAACCAAGGCAAAGAGAAGAGTGGTG-------------------------------------
--CGGAGAGAAAAAAGAGCA---GTAACA---CTGGGAGCT---------
GTGTTCCTTGGGTTCTTGGGAGCA---GCAGGAAGCACTATG---GGCGCAGCGTCA---
GTGACGCTGACGGTACAG---GCCAGACTA---TTATTG---TCTGGTATAGTGCAGCAGCAGAAC---
AATCTG---CTGAGG---GCTATTCAGGCGCAACAGCATCTGTTGCAGCTCACAGTCTGGGGCATT
>KT878029
TTCTACTGTAATACAACAAGACTGTTTAATAGTACTTGGCATACTAATACTACAGGGTTAAATGAAACTGAAG
GA---

AATAGCACAACTATCACACTCCCATGCAGAATAAAACAAATTATAAACAGATGGCAGGAAGTAGGAACAGCAA
TGTATGCCCCTCCCATCAGCGGACAAATTAGATGTTCATCAAAT---
ATTACAGGGCTGCTATTAACAAGAGATGGGGGGAATAACACTCAT----------------------------
----

GGGAATGGGACTGAGATCTTCAGACCTCTGGGAGGAGATATGAAGGACAATTGGAGAAGTGAATTATATAAAT
ATAAAGTAGTGAGAATTGAGCCATTA---GGGATAGCACCCACCAGGGCAAGGAGAAGAGTGGTG--------
-------------------------------CAGAGAGAAAAAAGAGCA---GTGGGAACGATAGGAGCT---
------ATGTTCCTTGGGTTCTTGGGAGCA---GCAGGAAGCACTATG---GGCGCAGCGTCA---
TTGACGCTGACGGTACAG---GCCAGACTA---TTATTG---TCTGGTATAGTGCAACAGCAGAAT---
AATTTG---CTGAGA---GCTATTGAGGCGCAACAGCATCTGTTGCAACTCACAGTCTGGGGCATC
>KF561441
TTCTACTGTAATACAACACCACTGTTTAATAGTATTTGGTATAGTAATAATACTGAAAAGTCATATAATACTG
CA---

GAAAATGACACAATTACCCTCCAATGCAGAATAAGACAAATTATAAAAAGGTGGCAGGAGGTAGGAAAAGCAA
GGTATGCCCCTCTCATCACCTGCCTAATTAGATGCTATTCAAAT---
ATTACAGGGCTGTTATTGGCAGGTGATGGAGGTGAGGCAGGGAAGGATAAC----------------------
----

GGGAATAAGAATGAGACCTTCAGGCCTGGGGGAGGAGATATGAGGGACAATTGGAGAAGTGAATTGTATAAAT
ATAAAGTAGTAGAAATTGAACCATTA---AGGATAGCACCCACCAGGGCAAGGAGAATAGTGGTG--------
-------------------------------CAGAGAGAAAAAAGAGCA---GTGGGAACATTAGGAGCC---
------ATGTTCCTTGGGTTCTTGGGAGCA---GCAGGAAGCACTATG---GGCGCAGCGTCA---
CTGACGCTGACGGTACAG---GCCAAGCAA---GTATTG---TCTGGTATAGTGCGACAGCAGAAT---
AATTTG---CTGAGA---GCTATTGAGGCGCAACAGCGTCTGTTGCAACTCACAGTCTGGGGCATC
>MN043577
TTCTACTGTAATACAACACAACTGTTTAATAGTACTTGGTATAGTCCTTGGAATAGTACTAACGGGTCAAATA
TAACT--

GAAAGAAATATCACAACACTCCCATGCAGAATAAAACAAATTATAAACAGATGGCAGGAAGTAGGAAAAGCAA
TGTATGCCCCTCCCATCAGTGGACAAATTAGATGTTCATCAAAT---
ATTACAGGGCTGCTATTAACAAGAGATGGGGGGACTCAGAACAAT----------------------------
----

GGGACTGAGATCTTCAGACCTGGGGGAGGAAATATGAAGGACAATTGGAGAAGTGAATTATATAAATATAAAG
TAGTAAAAATTGAGCCATTA---GGGATAGCACCCACCGGGGCAAAGAGAAGAGTGGTG--------------
-------------------------CAGAGAGAAAAA---ACA---GTGGGAACGATAGGAGCT---------
ATGTTCCTTGGGTTCTTGGGAGCA---GCAGGAAGCACTATG---GGCGTAGCGTCA---
ATGACGCTGACGGTACAG---GCCAGACTA---TTATTG---TCTGGTATAGTGCAACAGCAGAAT---
AATTTG---CTGAGA---GCTATTGAGGCGCAACAGCATCTGTTGCAACTCACAGTCTGGGGCATC
>MN043581
TTCTACTGTAATACAACACAACTGTTTAATAGTACTTGGATGATTACTAATAATTGCAATAGTACTGAAGGAT
ACAATAACACT--

GCACGAAATAGCACAATTACCCTCCCATGCAGAATAAAACAAATTATAAACAGGTGGCAGGAAGTAGGAAAAG
CAATGTATGCCCCTCCCATCAGAGGACAAATTAGATGTTCATCAAAT---
ATTACAGGGCTACTATCAACAAGAGATGGGGGGACTGGAACAAC----------------------------
----

GAGACTGAGATCTTCAGACCTGGGGGAAGAAATATGAGGGACAATTGGAGAAGTGAATTATATATATATAAAG
TAGTAACAATTGAGCCATTA---GGATAGCACCCACCGGGGCAAAGAGAAGAATGGTG---------------
-------------------------CAAAGAGAAAAAAGAGCA---GTGGGAACGATGGGAGCT---------
ATGTTCCTTGGGTTCTTGGGAGCA---GCAGGAAGCACTATG---GGCGCGACATCA---
ATGACGCTGACGGTACAG---GCCAGACTA---TTATTG---TCTGGTATACTGCATCATCAGAAT---
AATCTG---CTGAAA---GCTATTGAGGCGCAACAGCATCTGTTGCAACTCACAGTCTGGGGCATC
>HQ386180
TTCTATTGTAACACAGCACAACTGTTTAATAGTACTTGGAATGCAACAGAAATGCTTAATAGTACAGAATCAA
ATGAC--
-------------------ACA---
AATAGCAACATCAACATCACACTCCAATGCAGAATAAGACAAATTATAAACATGTGGCAGGAAGTAGGAAAAG
CAATGTATGCCCCTCCCATCGAAGGACAAATTAGATGTTCATCAAAT---
ATTACAGGGATACTATTAACAAGAGATGGTGGTCATAGCACGAACGGGACT----------------------
----

GAGAACGGGAACGAGACCTTCAGACCTGGAGGAGGAAACATGAGGGACAATTGGAGAAGTGAATTATATAAAT
ATAAAGTAGTAAAACTTGAACCATTA---GGAATAGCACCCACCAAGGCAAAGAGAAGAGTGGTG--------
-------------------------------CAGAGAGAAAAAAGAGCG---GTGGGAATGCTAGGAGCT---
------ATGTTCCTTGGGTTCTTGGGAGCA---GCAGGAAGCACTATG---GGCGCAGCGTCA---
CTAACGCTGACGGTACAG---ACCAGACAT---TTATTG---GCTGATATAGTGCAACAGCAGAAC---
AATTTG---CTGAGA---GCTATTGAGGCGCAGCAGCATCTGTTGCAACTCACAGTCTGGGGCATC
>EU744081
TTCTACTGTAATACAACACAACTGTTTAATAGTACTTGGATGTTTAATGGTACTTGGGAGTCAAATAGCACTG
AGGGA--

AATCCCACAGAAAATATCACACTCCCATGCAGAATAAAACAAATTATAAACCTGTGGCAGGAAGTAGGAAAGG
CAATGTATGCCCCTCCTATCAGGGGACTAATTCAATGTGTATCAAAT---
ATTACAGGGTTGCTATTAACAAGAGATGGTGGGAACAACACGACTGAG-------------------------
----

AATGGGACCGAGATTTTCAGACCTGGAGGAGGAGATATGAGGGACAATTGGAGAAGTGAATTATATAAATATA
AAGTAGTAAAAATTGAACCATTA---GGGGTAGCACCCACCAGGGCAAAGAGAAGAGTGGTG-----------
----------------------------CAGAGAGAAAAAAGAGCA---GTGGGAGTGATAGGAGCT------
---ATGTTCCTTGGGTTCTTGGGAGCA---GCAGGAAGCACTATG---GGCGCAGCGTCA---
ATGACGCTGACGGTACAG---GCCAGACAA---TTACTG---TCTGGTATAGTGCAGCAGCAGAAC---
AATCTG---CTGAGG---GCTATTGAGGCGCAACAGCATCTGTTGCAACTCACAGTCTGGGGCATC
>JF320244
TTCTACTGTAATACATCACAACTGTTTAATGGAAGTTGGCAACCACCGCTTAATGAAACTATATTTCAATACA
ATGCTACA---
-------------TGGGCA------
AAAAATGACACTAATATCACACTCCCATGCAGAATAAAACAATTTGTAAACATGTGGCAGGAAGTAGGAAAAG
CAATGTATGCCCCTCCCATCAGCGGACCAATCAAATGTGTATCAAAT---
ATTACAGGGTTGCTAATAACAAGAGATGGGGGTTACAACAGTAGTAGC-------------------------
----

AATGAGACCAATGAGACCTTCAGACCTGCAGGAGGAGAAATGAGGGACAATTGGAGAAGTGAATTATATAAAT
ATAAAGTAGTACAAGTTGAACCATTA---GGATTAGCACCCACCAAGGCAAAGAGAAGAGTGGTG--------
-------------------------------CAGAGAGAAAAAAGAGCA---GTGACG---CTGGGAGCT---
------ATGTTCCTTGGGTTCTTGGGAGCA---GCAGGAAGCACTATG---GGCGCAGCGTCA---
ATAACGCTGACGGTACAG---GCCAGACAG---TTATTG---TCTGGTATAGTGCAACAGCAGAAC---
AATCTG---CTGAGG---GCTATTGAGGCGCAACATCATCTGTTGCAACTCACAGTCTGGGGCATA
>JF320226
TTCTACTGTAATACCACACCACTGTTTAATAGTACCTGGAATGCCACACAACTGGTTAATATTACTGGGAATA
TCACAGGG---
-------------CTTAGT------
AATAGAACTGAAAATATTACACTCCCATGTAGAATAAAACAAATTATAAACATGTGGCAGGAAGTAGGAAAAG
CAATGTATGCTCCTCCCATCGCAGGACGAATTAAATGTTTATCAAAT---
ATTACAGGTTTACTATTAGTAAGAGATGGTGGCAATAGCAATGAC----------------------------
----

ACGGAGACCTTCAGACCTCAAGGAGGAAATATGAAGGACAATTGGAGAAGTGAATTATATAAATATAAAGTAG
TAAAAATTGAACCATTA---GGAGTAGCACCCACCAAGGCAAAGAGAAGAGTGGTG-----------------
----------------------CAGAGAGAAAAAAGAGCA---GTGGGAGTACTGGGAGCT---------
ATGTTCCTTGGGTTCTTGGGAGCA---GCAGGAAGCACTATG---GGCGCAGCGTCA---
ATAACGCTGACGGCACAG---GCCAGACAA---TTATTG---TCTGGTATAGTGCAACAGCAGAAC---
AATCTG---CTGAGG---GCTATTGAGGCGCAACAGCACCTGTTGCAACTCACAGTCTGGGGCATC
>JF320189
TTCTACTGTAATTCATCAGAACTGTTTAATAGTACCTGGACCTGGAATGAGACTGAAGGGTCTATTACTGAAG
GGTCA--

ATTACTAATGGGACAATCACACTCCCATGTAGAATAAGACAAATTATAAACATGTGGCAGAGAGTAGGAAAAG
CAATGTATGCCCCTCCCATCCAAGGACAAATTAGATGTTCATCAAAT---
ATTACGGGGCTGCTATTAACAAGAGATGGTGGTGAAGGTAACACCACC-------------------------
----

AATAACAACGAAACCTTCAGACCTGGAGGAGGTGATATGAGGGACAATTGGAGAAATGAATTATATAAATATA
AAGTAGTACAAATTGAACCATTA---GGAATAGCACCCACCAAGGCAAGGAGAAGAGTGGTG-----------
----------------------------CAGAGAGAAAAACGAGCA---GTGGGA---CTAGGAGCT------
---GTGTTCCTTGGGTTCTTGGGAGCA---GCAGGAAGCACTATG---GGCGCAGCGTCA---
GTGACGCTGACGGTACAG---GCCAGACAA---TTATTG---TCTGGTATAGTGCAACAGCAGAAC---
AATCTG---CTAAAG---GCTATTGAGGCGCAACATCATCTGTTGCAACTCACAGTCTGGGGCATC
>EF210727
TTCTACTGTAACTCTACACAGCTGTTTAATAGTACTTGGAACAGCAGCAATTGGAATTGGAATGATACTGAAA
AG---

ACAAATAAAAGTATCACACTCCCATGCAGAATAAAACAAATTATAAACATGTGGCAGGAAGTAGGAAAAGCAA
TGTATGCCCCTCCCATCAGAGGACAAATTAGATGTTCATCAAAT---
ATTACAGGGCTGATGCTAACAAGGGATGGTAGTAATGAGAGTCAC----------------------------
----

AGTGAGATCTTCAGACCAGGAGGAGGAAATATGAGGGATAATTGGAGAAGTGAATTATATAAATATAAAGTAG
TAAAAATTGAACCATTA---GGAGTAGCACCCACCAAGGCAAAGAGAAGAGTGGTG-----------------
----------------------CAGAGAGAAAAAAGAGCA---GTAGTGGGGATAGGAGCT---------
GTGTTCCTTGGGTTCTTGGGAGCA---GCAGGAAGCACTATG---GGCGCAGCGTCA---
ATGATGCTGACGGTACAG---GCCAGACAA---TTATTG---TCTGGTATAGTGCAACAGCAGAAC---
AATTTG---CTGAGG---GCTATTGAGGCGCAACAGCATATGTTGCAACTCACAGTCTGGGGCATC
>EF210729
TTTTATTGTAATACAACACGGCTGTTTAATAGTACTTGGAAGAATAATACTTGGACTAAGAATCATATTGAGA
AC---

GAGACTGAAACAATCACACTCCCATGCAAAATAAAACAAATTATAAACAGGTGGCAGGAAGTAGGACAAGCAA
TGTATGCCCCTCCCATCCCAGGATGGATTAACTGTTCATCAAAT---
ATTACAGGGCTACTATTAACAAGAGATGGTGGTGAAAACGGAACC----------------------------
----

ACCGAGACCGAGACCTTCAGACCTGGAGGAGGAGATATGAGGGACAATTGGAGAAGTGAATTATATAAATATA
AAGTAGTTGAAATTCAACCATTA---GGAGTAGCACCCACCACAGCAAAGAGAAGAGTGGTG-----------
----------------------------CAGAGAGAAAAAAGGGCA---GTAACA---CTAGGAGCT------
---ATGTTCCTTGGGTTCTTGGGAACA---GCAGGAAGCACTATG---GGCGCAGCGTCG---
CTGACGCTGACGGTACAG---GCCAGACAA---TTATTG---ACTGGTATAGTGCAACAGCAGAAC---
AATCTG---CTGAGG---GCTATTGAGGCGCAACAGCATATGTTGCAACTCACAGTCTGGGGCATT
>EF210731
TTCTACTGTAATCTATCAAAATTGTTTAATAGTACATGGAATGATACTACTTGGAACACGACTGATGAGTCA-
----

AATGACACTTACATCCTGCTCCCATGTAGGATAAAACAAATTATAAACATGTGGCAGACAGTAGGAAAAGCAA
TGTACGCCCCTCCCATCAAAGGAAAAATTAGTTGTTCATCAAAT---
ATTACAGGGCTGCTATTAACAAGAGATGGAGGGAATGACAACAGGACT-------------------------
----

```
----------
CAGGGGGTCGAGATCTTCAGACCTGGAGGAGGAGATATGAGAGATAATTGGAGAAGTGAATTATATAAATATA
AAGTAGTAAAAATTGAACCATTA---GGAATAGCACCCACCGAGGCAAAAAGAAGAGTGGTG-----------
----------------------------CAGAGAGAAAAAAGAGCA---GTGGGA---ATAGGAGCT------
---GTGTTCCTTGGGTTCCTGGGAGCA---GCAGGAAGCACTATG---GGCGCAGCGGCA---
GTGACGCTGACGGTACAG---GCCAGACAA---TTATTG---TCTGGTATAGTGCAACAGCAGAAC---
AATCTG---CTAAAG---GCCATTGAGGCGCAACAGCATCTGTTGCAACTCACAGTCTGGGGCATC
>EF210735
TTCTACTGTAACACATCACACCTATTTAATAGTACTTGGAATGATACTAGTAGTTGGGATAATAGC-------
-------------------------------------------------------------------------
----------------------------
ACTGAAGGAAATATCACACTCCAATGTAGAATAAAACAAATCATAAACAGGTGGCAGGAAGTAGGAAAAGCAA
TGTATGCCCCTCCCATTGCAGGACAAATTAGATGCTCATCAAAT---
ATTACAGGGCTGCTATTAACAAGAGATGGTGGTCAGGGCAGTAAC----------------------------
-------------------------------------------------------------------------
-------------
GAGACCGAGGTCTTTAGACCTGGAGGAGGAAATATGAAGGACAATTGGAGAAGTGAACTATATAAATATAAAG
TAGTAAAAGTTGAACCATTA---GGAATAGCACCCACCAAGGCAAGGAGAAGAGTGGTG--------------
-------------------------CAGAAAGAAAAAAGAGCA---ATAACA---CTAGGAGCT---------
ATGTTCCTTGGTTTCTTGGGAGCA---GCAGGAAGCACTATG---GGCGCAGCGTCG---
ATAACGCTGACGGTACAG---GCCAGACAA---TTATTG---TCTGGTATAGTGCAGCAGCAGAAT---
AATTTG---CTGAGG---GCTATTGAGGCGCAACAGCATATGTTGCAACTCACAGTCTGGGGCATC
>MH327746
TTCTACTGCAATACAACAAAACTGTTTAACAGTAGTTGGCCAAATAGTACTAGGAATAATACTGAAGAGTCA-
-------------------------------------------------------------------------
----------------------------
AATAGCACTGTTATCACACTCCCATGTAGAATAAAACAAATTATAAACATGTGGCAGGAAGTAGGAAAAGCAA
TGTATGCCCCTCCCATCAGGGGACGAATTAATTGTACATCAAAC---
ATTACAGGGCTACTATTAACAAGAGATGGGGGCAATGGAAATAGC----------------------------
-------------------------------------------------------------------------
-------------
ACGAATGAAACCTTCAGACCTCAAGGAGGAAATATAAGGGACAATTGGAGAAGTGAACTATATAAATATAAAG
TAGTAAAAAATTGAACCATTA---GGAGTAGCACCTACCAAGGCAAAGAGAAGAGTGGTG-------------
-------------------------CAAAGAGAAAAGAGAGCA---GTGGGA---ATGGGAGCT---------
TGGTTCCTTGGGTTCTTGGCAGCA---GCAGGAAGCACTATG---GGCGCAGCGTCA---
GTGACGTTGACGGTACAG---GCCAGACAA---TTATTG---TCTGGTATAGTGCAGCAGCAAAAT---
AATTTG---CTGCAA---GCTATTGAGGCGCAACAGCATTTGTTGCAACTCACAGTCTGGGGCATC
>KY658690
TTCTACTGCAATACAACAAAACTGTTTAACAGTAGTTGGCCAAATAGTACTAGGAATAATACTGAAGAGTCA-
-------------------------------------------------------------------------
----------------------------
AATAGCACTGTTATCACACTCCCATGTAGAATAAAACAAATTATAAACATGTGGCAGGAAGTAGGAAAAGCAA
TGTATGCCCCTCCCATCAGGGGACGAATTAATTGTACATCAAAC---
ATTACAGGGCTACTATTAACAAGAGATGGGGGCAATGGAAATAGC----------------------------
-------------------------------------------------------------------------
-------------
ACGAATGAAACCTTCAGACCTCAAGGAGGAAATATAAGGGACAATTGGAGAAGTGAACTATATAAATATAAAG
TAGTAAAAAATTGAACCATTA---GGAGTAGCACCTACCAAGGCAAAGAGAAGAGTGGTG-------------
-------------------------CAAAGAGAAAAGAGAGCA---GTGGGA---ATGGGAGCT---------
TGGTTCCTTGGGTTCTTGGCAGCA---GCAGGAAGCACTATG---GGCGCAGCGTCA---
GTGACGTTGACGGTACAG---GCCAGACAA---TTATTG---TCTGGTATAGTGCAGCAGCAAAAT---
AATTTG---CTGCAA---GCTATTGAGGCGCAACAGCATTTGTTGCAACTCACAGTCTGGGGCATC
>MF373127
TTCTACTGTAATACAACAAGACTGTTTAATACTACTTGGAATAACACTGAGAAATCAAATCCCACTGAA----
-------------------------------------------------------------------------
----------------------------
GGAAATGGCACAATCACACTCCAATGCAGAATAAAACAAATTATAAACCTGTGGCAGGAAGTAGGAAAAGCAA
TGTATGCCCCTCCCATCAGAGGACAAATTAACTGTTCATCCAAC---
ATTACAGGACTGCTATTAACAACAGATGGTGGTATGAACACCAGT----------------------------
-------------------------------------------------------------------------
```

AATAACAAGACCTTTATACCTGCAGGAGGAGATATGAGAGACTATTGGAGGAGTGGAGTATTGAAATATGATG
TAGTAATAGTTGAGCCATTA---GGAATAGCACCAACCACGGATATGAGAAGAGTGGTG--------------
-------------------------CAGATACAAAAAAGAGCA---GAGCTA---CTACGAGCT---------
CTGTTGCTTGTGTTCTTGGGAGCA---GCAGGAAGGACGATG---AGCGCAGCGTCG---
ATGACGCTGACGGTACAG---GCCAGACAA---TTATTG---TCTGGTATAGTGCAACAGCAAAAT---
AATTTG---CTGCAG---GCTATTGAGGCGCAACAGCATATGTTGAAACTCACAGTCTGGGGCATC
>MF373129
TTCTACTGTAATACAGCAGAACTGTTTAACAGTACTTGGAGGAATGACACTGAAAGGTCAAATACCACTACA-
----

GGAAATGACACAATCACACTTCAATGCACAATAAAACGAATTGTAAACATGTGGCAGGAAGTAGGAAAAGCAA
TGTATGCCCCTCCCATCACAGGACAAATTAATTGTTCATCAAAA---
ATTACAGGGCTACTGTTAACAAGAGATGGTGGTGGGAACCAGAAC----------------------------
----

ACCAGCGAGACTTTCAGACCTGGAGGGGGAAATATGAAAGACAATTGGAGAAGTGAATTATATAAATATAAAG
TAATAAAAATTGAACCATTA---GGAGTAGCACCCACCAAGGCAAAGAGAAGAGTGGTG--------------
-------------------------CAGAGAGAAAAAAGAGCA---GTGGGA---ATAGGAGCT---------
GTGTTCCTTGGGTTCTTGGGAGCA---GCAGGAAGCACTATG---GGCGCAACGTCG---
ATGACGCTGACGGTACAG---GCCAGACAA---TTATTG---TCTGGTATAGTGCAACAGCAGAAT---
AATTTG---CTGAAG---GCTATTGAGGCGCATCAGCATATGTTGCAACTCACAGTCTGGGGCATC
>KP411829
TGCTATTGCAAACCATCACTTCTGTTTACTGGCACATACAAGAGTAACATTAATATTTCAAATATCACCCAAT
CCTCACAA---
-------------------TCC---GATGAAAGGTATATTTTAATTCTGGCTTGTATGACG---------
ATCAATGTGTGGCAGGTAGTAGGAAAAGCGATCTATTCCCCTCCCATCAGTGGACAAATTAGATGTTCATCAA
AT---ATTACAGGGCTGCTATTAACAAGAGATGGTGGTAGCAACAGCAGTAACGAG-----------------
----

ACCGAGACTGAAATCTTCAGACCTGTGGGAGGAAATATGAGGGACAATTGGAGAAGTGAATTATATAAATATA
AAGTAGTACAAATTGAACCATTA---GGAGTAGCACCCACCAAGGCAAAGAGAAGAGTGGTG-----------
----------------------------CAAAGAGAAAAAAGAGCA---GTGGGA---TTAGGAGCA------
---ATGTTCCTTGGGTTCTTGGGAGCA---GCAGGAAGCACTATG---GGCGCAGCGTCA---
ATAACGCTGACGGTACAG---GCCAGACAA---TTATTG---TCTGGTATAGTGCAACAGCAGAAT---
AATTTG---CTGAGA---GCTATTGAGGCGCAACAGCATCTGTTGCAACTCACAGTCTGGGGCATC
>MF373151
CTCTACTGTAATTCCACACAACTATTTAATAGTACTTGGAATGGTACTGAAAGATCAAACTATTCCACTGAG-
----

ACTAATGAAACTATCACACTCCCATGCAGAATAAAACAAATTATAAACATGTGGCAGAGAGTAGGAAGAGCAA
TGTATGCCCCTCCCATCCAAGGACTAATTAGATGTTCATCAAAT---
ATTACAGGGCTGCTGCTAACAAGAGATGGAGGTCAGAATAGTAGT----------------------------
----

AATAGCACTGAGACCTTCAGACCGGGAGGAGGAAACATGAAGGATAATTGGAGAAGTGAATTATATAAATATA
AAGTAATAAAGATTGAACCATTA---GGAGTAGCACCCACCAAGGCAAAGAGAAGAGTGGTG-----------
----------------------------CAAAGAGAAAAAAGAGCA---ATAACA---GTAGGAGCT------
---ATGTTCCTTGGGTTCTTGGGAGCA---GCAGGAAGCACTATG---GGCGCAGCGTCA---
ATGGCGCTGACGGTACAG---GCCAGACAA---TTGTTG---TCTGGTATAGTGCAACAGCAGAAC---
AATCTG---CTGAGG---GCTATTGAGGCGCAACAGCATCTGTTGCAACTCACAGTCTGGGGCATC
>KP411823
TTCTACTGTAATACAACACAACTGTTTAATAGTACTTGGAATATCACTGGAGGGTTAAATAACACTGAA----
----

GGAAATGGCACAATCACACTCCAATGCAAAATAAAACAATTTATAAACATGTGGCATGAAGTAGGGAAAGCAA
TGTATGCCCCTCCCATCAGAGGACAGATTACATGTTCATCAAAC---
ATTACAGGGCTGCTATTAACAAGAGATGGTGGTGAGAACCCGGGC----------------------------
----

AACGACACTGATACTTTCACACCTGGAGGGGGAGATATGAAAGACAATTGGAGTAGTGAATTAGATAAATATA
GAGTAATAGGAATAGCACCACTA---CCAGTAGCACCCACCAAGGCAAAGACAAGATTGTTG-----------
----------------------------CAGAGAGATAAAAGAGCA---GTTGGA---ATAGGATCT------
---GTGTTCCTTGTCTTCCTGGTAGCG---GCAGGAAGCACTATG---TCTGCAATGTCA---
ATGACGATGACGCTACAA---GCTCAAGAA---TTACTG---TATGTGACCGAGCGAATGCAAAAA---
AATTTG---CTGAAG---GCTATTGAGGCGCAACAGCATCTGTTGCAACTCACAGTCTGGGGCATC
>MF373162
TTCTATTGTAATACAACACAGTTATTTAATAGTACTTGGGAGGCTCATCAGCTTAATCAGACTTGGAATGAGC
CCCTAGGG---

CCAAATGTTACTAATATCACACTCCCATGCAGAATAAAACAAATTATAAACAGGTGGCAGGAAGTGGGAAAAG
CAATGTTTGCCGCTCCCATCAGCGGACGAATTCAATGCAAATCAAAT---
ATTACAGGGTTGTTATTAACAAGAGATGGTGGTACTAACACCAGC----------------------------
----

GACCCCAACAGAACTGAGACCTTCAGACCTGGAGGAGGAGATATGAGAGACAATTGGAGAAGTGAATTATATA
AGTATAAAGTAGTACAAATTGAGCCATTA---GGAATAGCACCCACCAAGGCAAAGAGAGTAGTGGTA-----
----------------------------------AAGAGAGAAAAAAGAGCA---
GTGGGAGTGCTAGGAGCT---------ATGTTCCTTGGGTTCTTGGGAACA---GCAGGAAGCGTTATG---
GGCGCAGCGTCA---ATGACGCTGACGGTACAG---GCCAGACAA---TTATTG---
TCTGGTATAGTGCAACAGCAGAAC---AATCTG---CTGAGA---
GCTATTGAGGCGCAACAACATATGTTGCAACTCACAGTCTGGGGCATC
>KP411824
TTCTACTGCAATTCAACACCACTGTTTAATAGCACCTGGGATACTAATGGGACTTGCAAGGGCTCTAATAGT-
----

GCTGATTGGAATGTTACTGTCCCATGCATTAGCATCCCAATTACTACAATGGGGCAGAAACTAGAACAAGCAA
TGGACGTGCGTCCCATTGCAGGAAAAGCAATATGTTCATCAAGT---
TTGTCCAAAGGTATCCTTTTAGCCAATTCCCATAATAGCATTATTGTG-------------------------
----

CCCCGGCTGGTTATGCCTAAAGGTAAAGATAAGAAGTTCAATTGGAACAGGAAAATGTATCAATGTCAAGTAC
AATGTCAAATTGAACCATTA---GGAGTAGCACCCACCATGGCAAAGAGAAGAGTGGTG--------------
-------------------------CAAAGAGAGAAAAGAGAT---TTGGGA---CTAGGAGCT---------
CTGTTCCTTGGGTTCTTGGGAGCA---GCAGGAAGCACTATG---GGCGCAGCGTCA---
ATAACGCTGACGGTACAG---GCCAGACAA---TTATTG---TCTGGTATAGTGCAACAGCAGAAC---
AACTTG---CTGAGG---GCTATTGAGGCGCAACAGCATCTGTTGCAACTCACAGTCTGGGGCATT
>MF373185
TTCTACTGTAATTCAACACCACTGTTTAATAGTACTTGGTATGTTAATGGAACTAGGAATAATGATACTGATA
TT---

GGAAAGATAAACATCACACTCTCATGTAGAATAAAACAAATTATAAACATGTGGCAGGAAGTAGGAAAAGCAA
TGTATGCCCCTCCCATCCCAGGAAAAATTCAATGTATATCAAAT---
ATTACAGGGCTGCTATTAACAAGAGATGGAGGTAATACCAGTGAGACCAGG----------------------
----

AACGGGACCGAGATTTTCAGACCTGGAGGAGGAAATATGAGGGACAATTGGAGAAGTGAATTATATAAATACA
AAGTAGTACAAGTTGAACCATTA---GGAATAGCACCCACCAAGGCAAGGAGAAGAGTGGTG-----------
----------------------------CAGAGAGAAAAAAGAGCT---CTGGGA---CTAGGAGCT------
---TTGTTTCTTGGGTTCTTGGGAGCA---GCAGGAAGCACTATG---GGCGCAGCGTCA---
GTGACGCTGACGGTACAG---GCCAGACAA---TTATTG---TCTGGTATAGTGCAGCAGCAGAAC---
AATCTG---CTGAGA---GCTATTGAGGCGCAACAACACATGTTGCAACTCACAGTCTGGGGCATC
>MF373186
TTCTACTGCAACACAACACAACTGTTTAATAGTACTTGGAATGATACACAACTGTCTGATAGCACTTGGAATA
ATACAAGA---

GGGATAAATGACACTATCACACTCCAATGTAGAATAAGACAAATCATAAACATGTGGCAGAAGGTAGGAAAAG
CAATGTATGCCCCTCCCATCAGTGGACAAATTAGATGTACATCAAAT---
ATTACAGGGCTGATATTAACAAGAGATGGTGGTAATAGTGACAACAAC-------------------------
----

ACCACCCAGCCCGAGATCTTCAGACCTCAAGGAGGAAATATGAAAGACAATTGGAGAAGTGAATTATATAAAT
ATAAGGTAGTAGCAATTGAACCATTA---GGACTAGCACCCACCAAGGCAAAGAGAAGAGTGGTG--------
-------------------------------CAGAGAGACAAAAGAGCA---GTAGGA---CTGGGAGCT---
------CTGTTCCTTGGGTTCTTGGGAGCA---GCAGGAAGCACTATG---GGCGCAGCGTCA---
ATGGCGCTGACGGTACAA---GCCAGACAA---TTATTG---TCTGGTATAGTGCAACAGCAGAAC---
AATTTG---CTGAGG---GCTATTGAGGCGCAACAGCATCTGTTGCAGCTCACAGTCTGGGGCATT
>KY213743
TTTTACTGTAATACATCAAAACTGTTTAATAGTACTTGGCTGAATAATAATACAGGGTCAAT-----------
----

GCCACTGAACCTATCATACTCCCATGCAGGATAAAACAAATTGTAAACATGTGGCAGGAAGTAGGAAAAGCAA
TGTATGCCCCTCCCATCAGAGGACAAATTAGATGCATATCAAAT---
ATTACAGGGCTCCTATTAACAAGAGATGGTGGTCAAGAGAACAAAACCGAG----------------------
----

AACCACACCGAGGTCTTTAGACCCACGGGAGGAAATATGAAGGACAATTGGAGAAGTGAATTATATAAATATA
AAGTAGTAAAAATTGAACCATTA---GGAATAGCACCCACCAAGGCAAAAAGAAGAGTGGTG-----------
----------------------------CAGAGAGAAAAAAGAGCA---GTGGGA---ATAGGAGCG------
---ATGTTCCTTGGGTTCTTGAGCGCA---GCAGGAAGCACTATG---GGCGCAGCGTCA---
ATAACGCTGACGGTACAG---GCCAGACAG---TTACTG---TCTGGTATAGTGCAACAGCAAAAC---
AATTTG---CTGAGA---GCTATTGAGGCGCAACAACATATGTTGCAACTCACAGTCTGGGGCATC
>KY213745
TTCTACTGCAATACAACACAACTATTTAATAGTTCTTGGAATAATAGTAGTTTGAATAGTACTGAGGGTAATG
GAGGG--

TCAAATGACACTATCACACTCCCGTGTAGAATAAAACAAATTATAAACATGTGGCAGGAAGTAGGAAAAGCAA
TGTATGCCCCTCCCATTCAAGGACAAATTAGATGCTCCTCAAAT---
ATTACAGGACTATTATTAGTAAGAGATGGTGGTAATAACAGGACG----------------------------
----

GGCGGCAGTGAGACCTTCAGACCTGGAGGAGGAAATATGAAGGACAATTGGAGAAGTGAATTATATAAATATA
AAGTAGTAAAAATTGAACCATTA---GGAATAGCACCCACCAGGGCAAGGAGAAGAGTGGTG-----------
----------------------------CAGAGAGAAAAAAGAGCA---GTGGGA---CTAGGAGCT------
---TTGTTCCTTGGGTTCCTGGGAGCA---GCAGGAAGCACTATG---GGCGCAGCGTCA---
ATGACGCTGACGGTACAG---GCCAGACAA---TTATTG---TCTGGTATAGTGCAACAGCAGAAC---
AATCTG---CTGAAG---GCCATTGAGGCGCAACAGCATATGTTGCAACTCACAGTCTGGGGCATC
>JN248346
TTCTACTGTGATTCAACACAACTGTTTAATAGTAGTTGGAATAGTAATTGGTCTAATAATACTGAA-------
----

GGGTCAGACAACATTACACTTCAATGCAGAATAAAACAAATTATAAACATGTGGCAGCAAACAGGAAAGGCAA
TGTATGCCCCTCCCATCAGTGGAATAATTAGCTGTTCATCAAAT---
ATTACAGGGCTGTTATTAACAAGAGATGGTGGTAACAATCAGACT----------------------------
----

AATAATAATGAGACCTTCAGACCTGGAGGAGGAAATATGAAGGACAATTGGAGAAGTGAATTATATAGATATA
AAGTAGTACAAATTGAACCATTA---GGAGTAGCACCCACCATGGCAAAGAGAAGAGTGGTG-----------
----------------------------CAGAGAGAAAAAAGAGCA---GTGGGA---ATAGGAGCT------
---CTATTCCTTGGGTTCTTGGGAGCA---GCAGGAAGCACTATG---GGCGCAGCATCA---
ATAACGCTGACGGTACAG---GCCAGACAA---TTATTG---TCTGGTATAGTGCAACAGCAGAAC---
AATTTG---CTGAGA---GCTATTGAGGCGCAACAGCATATGTTGCAACTCACAGTCTGGGGCATA
>KJ769147
TTCTACTGTAACACATCACAACTGTTTAATAGTACTTGGTATAGTAATGGT----------------------
----
-ACTCGAAATGATACT---------
GTAGGAAATGGCAATATCACGCTCCCTTGCAGAATAAAACAAATTGTAAACATGTGGCAGGAAGTAGGAAAAG
CAATGTATGCCCCTCCCATTGGAGGACAAATTAGATGTTCATCAAAT---
ATTACAGGGTTGCTATTAACAAGAGATGGTGGT--

AACAATCAGAGCAACATC---
ACCACCGAGACCTTCAGACCAGGAGGAGGAGATATGAGGGACAATTGGAGAAGTGAACTATATAAATATAAAG
TGGTAAAAATTGAACCATTA---GGAATAGCACCCACCAAGGCAAAGAGAAGAGTGGTG--------------
-------------------------CAGAGAGAAAAAAGAGCA---GTGGGAATGATAGGAGCT---------
ATGTTCCTTGGGTTCTTGGGAGCA---GCAGGAAGCACTATG---GGCGCAGCGTCA---
ATAACGCTGACGGTACAG---GCCAGGCAA---TTGTTG---TCTGGTATAGTGCAACAGCAGAGT---
AATCTG---CTGAGA---GCTATTGAGGCGCAACAGCATATGTTGCAACTCACAGTCTGGGGCATC
>KP109514
TTCTACTGTAATACAACACAGCTATTTAATAATACTTGGCTTACTTGGAAGGCCAATGCAAGTGATCCTGAAA
AAAAT--

AACACAAAAAATATCACACTCCCATGCAGAATAAAACAAATTATAAACATGTGGCAGGAAGTAGGAAAAGCAA
TGTATGCCCCTCCCATCGCAGGACAAATTAAATGTGTATCAAAT---
ATTACAGGGTTACTATTAACAAGAGATGGGGGTCAGGACAATAACACT-------------------------
----

AACGGTAACGAGACCTTCAGACCTGGAGGAGGAGATATGAGGGACAATTGGAGGAGTGAATTATATAAATATA
AAGTAGTAAAAATTGAACCATTA---GGAATAGCACCCACTAAGGCAAAGAGAAGAGTGGTG-----------
----------------------------CAGAGAGAAAAAAGAGCA---GCA------CTAGGAGCT------
---ATGTTCCTTGGGTTCTTGGGAATG---GCAGGAAGCACTATG---GGCGCAGCGTCA---
ATAACGCTGACGGCACAG---GCCAGACAA---TTGTTG---TCTGGTATAGTGCAACAGCAGAAC---
AATCTG---CTGAGG---GCTATTGAGGCGCAACAACATTTGTTGCAACTCACAGTCTGGGGCATC
>EU578539
TTCTACTGTAATGCAACACAACTGTTTAATAGTACTTGGCCCATTAACGGTACTTGGGAAGGATCA-------
----

AATAATACTAATGTCACACTACCATGCAGAATAAAACAAATTATAAACATGTGGCAGAGAGTAGGCAAAGCAA
TGTATGCCCCTCCCATCAGAGGACAAATTAGATGTTCATCAAAT---
ATCACAGGGCTGCTATTAACAAGAGATGGTGGTGGTAGTAACCAA----------------------------
----

AGCAACGAGACCTTTAGACCTGGAGGAGGAGACATGAAAGACAATTGGAGAAGTGAATTATATAAGTATAAAG
TAGTAAGAATTAAACCATTA---GGAGTAGCCCCCACCAAGGCAAAGAGAAGAGTGGTG--------------
-------------------------CAGAGAGAAAAAAGAGCA---GTGGGAACAATAGGAGCT---------
ATGTTCCTTGGGTTCTTGGGAGCA---GCAGGAAGCACTATG---GGCGCAGCGTCA---
GTGACGCTGACGGTACAG---GCCAGACAA---TTATTG---TCTGGTATAGTGCAACAGCAGAAC---
AATTTG---CTGAGG---GCTATTCAGGCGCAACAGCATCTGTTGCAACTTACGGTCTGGGGCATC
>HQ217760
TTCTATTGTAATACAACAGAGCTGTTTAATAGTACTTGGGATGTTAATAGTACTTTGAATGTTAATAATACTT
GGAATAAT---
-------------ACTGAA------
GGAGCAAGTGGCAATATCACACTCCCATGCAGAATAAAACAAATTATAAATATGTGGCAGAAAGTAGGCAAGG
CAATGTATGCCCCTCCCATCAGAGGACAAATTAGATGTACATCAAAG---
ATCACAGGGCTGCTATTAACAAGAGATGGT---TATAATAATAAT----------------------------
----

AACAACACCGAGACTTTCAGACCTGGAGGAGGAAATATGAAGGACAATTGGAGAAGTGAGTTATATAAGTATA
AAGTAGTAAAAATTGAACCATTA---GGAGTAGCACCCACCATGGCAAGGAGGAGAGTGGTG-----------
----------------------------CAGAGAGAAAAAAGAGCA---GTGGGAACAATAGGAGCT------
---ATGTTCCTTGGGTTCTTGGGAGCA---GCAGGAAGCACTATG---GGCGCAGCGTCA---
CTGACGCTGACGGTACAG---GCCAGACTA---TTATTG---TCTGGTATAGTGCAACAGCAGAAC---
AATCTG---CTGAGG---GCTATTGAGGCGCAACAGCATCTGTTGCAACTCACGGTCTGGGGCATC
>AF277071
TTCTACTGTAATTCAACACAGCTGTTTAATAGTACTTGGAATATTAATGATACTAGGAATGAT----------
----

ACTACAGGGAATATCACACTCCCATGCAGAATAAGACAAATTGTGAACAGGTGGCAGGAAGTAGGCAAAGCAA
TGTATGCCCCTCCCATCAGAGGACAAATTAGCTGTTCATCAAAT---
ATTACAGGGGTGCTATTAACAAGAGATGGTGGTAATCACACGAGC----------------------------
----

GAAACTGAGATCTTCAGACCTGGAGGAGGAGATATGAGGGACAATTGGAGAAGTGAATTATATAAATATAAAG
TAGTAAAAATTGAACCATTA---GGAAAAGCACCCACCAAGGCAAGGAGAAGAGTGGTG-------------
-------------------------CAAAGAGAAAAAAGAGCA---GTGACA---ATGGGAGCT---------
ATGTTCCTTGGGTTCTTAGGAGCA---GCAGGAAGCACTATG---GGCGCAGCATCC---
ATGACGCTGACGGTACAG---GCCAGGCTA---TTATTG---CCTGGTATAGTGCAACAGCAGAGC---
AATCTG---CTGAGG---GCTATCGAGGCGCAACAACATCTGTTGCAACTCACAGTCTGGGGCATC
>EF593267
TTCTACTGTAATACAACAAAGCTGTTTAATAGTACTTGGCATGCTAATGGCACTCGGAATGATACTGGAGGG-
----

TCAAATGACACTATCATACTACCATGCAGAATAAAACAAATTATAAACATGTGGCAGAAAGTAGGCAAAGCAA
TCTATGCCCCTCCCATCAGAGGACAAATTAGATGTGAATCAAAG---
ATCACAGGGCTGCTATTAACAAGAGATGGTGGTGATGATAACAGA---------------------------
----

ACCACCGAGATCTTCAGACCTGGAGGAGGAGATATGAGGGACAATTGGAGAAGTGAATTATATAAATATAAAG
TAGTAAAAATTGAACCATTA---GGAATAGCACCCACCAAGGCAAGGAGAAGAGTGGTG-------------
-------------------------CAGAGAGAAAAAAGAGCA---GTGGGA---ATAGGAGCT---------
GTGTTCCTTGGGTTCTTGGGAGCA---GCAGGAAGCACTATG---GGCGCAGCGTCA---
ATGACGCTGACGGTACAG---GCCAGACTA---TTATTG---TCTGGTATAGTGCAACAGCAGAAC---
AATTTG---CTGCAG---GCTATTGAGGCGCAACAGCATCTGTTGCAACTCACGGTCTGGGGCATC
>DQ853436
TTCTACTGTAATTCAACACGATTGTTTAGTAGTAAGTGGCATGCTAATGATAGTTGGAGTAGTAGTACTTGGA
ACGATACT---
-------------------GGA---
GAGTCAAATGACACTATCACACTCCCATGTAGAATAAAACAAATTATAAACATGTGGCAGGAAGTAGGAAAAG
CAATGTATGCCCCTCCCATCAAAGGACAAATTCAATGTTCATCAAAT---
ATTACAGGGCTAATATTAACAAGAGATGGTGGTGCAAACACGAAC---------------------------
----

ACGAGCGAGGAGGTCTTCAGACCTGCAGGAGGAAATATGGGGGACAATTGGAGAAGTGAATTATATAAATATA
AAGTAGTAAAAATTGAACCAATA---GGAATAGCACCCACCAAGGCAAAAAGAAGAGTGGTG----------
----------------------------CAGAGAGAAAAAAGAGCA---GTGACA---CTGGGAGCT------
---GTGTTCCTTGGGTTCTTGGGAGCA---GCAGGAAGCACTATG---GGCGCAGCGTCA---
ATAACGCTGACGGTACAG---GCCAGACAA---TTATTG---TCTGGTATAGTGCAGCAGCAGAAC---
AATCTG---CTGAGG---GCTATTGAGGCGCAACAACATCTGTTGCAACTCACAGTCTGGGGCATC
>KT124806
TTCTACTGTGATACAACAGAACTGTTTAATAGTACTTGGAGTATTAATGGTACTAGGAATACTACTGAAGGGT
CA---

AATAGCACAGACATCACACTCCAATGTAGAATAAAACAAATTATAAACATGTGGCAGGAAGTAGGGAAAGCAA
TGTATGCCCCTCCCATTAGAGGACAAATTAGATGTTCATCAAAT---
ATTACAGGGCTGCTATTAACAAGAGATGGTGGTAGCGGGAGTGAGACT------------------------
----

AAAAACGAGACTGAAACCTTCAGACCTGGAGGAGGAAATATGAAGGATAATTGGAGAAGTGAATTATATCAAT
ATAAAGTAGTAAAAATTGAACCATTA---GGAGTAGCACCCACCAGGGCAAAGAGAAGAGTGGTG--------
-------------------------------CAGAGAGAAAAAAGAGCA---GTCACA---CTAGGAGCT---
------GTGTTCCTTGGTTTCTTGGGAGCA---GCAGGAAGCACTATG---GGCGCAGCGTCA---
ATAACGCTGACGGTACAG---GCCAGACAA---TTATTG---TCTGGAATAGTGCAACAGCAAAGC---
AATCTG---CTGAGA---GCTATTGAGGCGCAACAGCATCTGTTGCAACTCACAGTCTGGGGCATC
>EF593290
TTTTACTGTGATACAACAGAGCTGTTTAATAGTACTTGGAATAATACTGATAGTACTCGGAATGATACT----
----

GAGTCAGACAAAATCACCCTCCAATGTAGAATAAAACAAATTATAAACATGTGGCAGGAAGTAGGAAAAGCAA
TGTATGCCCCTCCCATCCAAGGACAAATTCAATGTAACTCAAGT---
ATTACAGGGCTACTATTAACAAGAGATGGTGGGAACAACTCCACT---------------------------
----

AATTCCACCGAGATATTCAGACCTGGAGGAGGAGACATGAGGGATAATTGGAGAAGTGAATTATATAAATATA
AAGTAGTAAAAATTGAACCATTA---GGAGTGGCACCCACCAAGGCAAAGCGAAGAGTGGTA----------
----------------------------CAGAGAGAAAAAAGAGCA---ATAACG---ATGGGAGCT------
---ATGTTCCTTGGGTTCTTGGGAGCA---GCAGGAAGCACTATG---GGCGCAGCGTCA---
ATAGCGCTGACGGCACAG---GCCAGACTA---TTATTG---TCTGGTATAGTGCAACAGCAGAAC---
AATCTG---CTGAGA---GCTATTGAGGCGCAACAGCATATGTTGCAACTCACAGTCTGGGGCATC
>EU577629
TTTTACTGTAATACAACACAACTGTTTAATAGTACCTGGTGGAGGAATGGTACTGAAGTGTTTAATAGTACCT
GGTGGAGGAAT---GGT---
ACTGAAGTGTCAAATAACACT--------------
CAAGGGATTGACACAATCACCCTCCCATGCAGAATAAAACAATTTGTAAATATGTGGCAGTCAGTAGGAAAAG
CAATGTATGCCCCTCCCATCAGGGGACGAATTAATTGTACATCAAAT---
ATTACAGGGCTGTTATTAACAAGTGATAGAGGTGGGCTTAATAAT--------------------------
----

GCTACAAATGAGACCTTCAGACCTGGAGGAGGAGATATGAGGGACAATTGGAGAAGTGAATTATATAAATATA
AGGTAGTAAGAATTGAACCATTA---GGAATAGCACCCACTAGTGCAAAGAGAAGAGTGGTG----------
----------------------------CAAAGAGAAAAAAGAGCA---GTGGGAACAATAGGAGCT------
---ATGTTCCTTGGGTTCTTGGGAGCA---GCAGGAAGCACTATG---GGCGCAGCGTCA---
ATGACGCTGACGGTACAG---GCCAGACTA---TTATTG---TCTGGTATAGTGCAACAGCAAAAC---
AATTTG---CTGAAG---GCTATTGATGCGCAACAGCATATGTTGCAGCTCACGGTGTGGGGCATC
>JX863967
TTCTACTGTAATACAACACCACTGTTTAATAGCACTTGGAATGAAACTCAACTGTTTAATAGTACTTGGAATG
AAACTGAG--

GCAAATGGAACAATTACACTCCCATGCAGAATAAAACAAATTATAAACATGTGGCAGGAAGTAGGAAAAGCAA
TGTATGCCCCTCCCATCGAAGGACAAATTTACTGTTCTTCAAAC---
ATTACAGGGCTACTGTTATTAAGGGATGGTGGTAATACCACAAAC--------------------------
----

GGGACTGAGATCTTCAGACCTGGAGGAGGAGACATGAGGGACAATTGGAGAAGTGAATTATATAAATATAAAG
TAGTAAAAATTGAACCATTA---GGAGTAGCACCCACTAGGGCAAAGAGAAGAGTGGTG-------------
------------------------CAGAGAGAAAAAAGAGCA---GTGGGA---ATAGGAGCT---------
ATGTTCCTTGGGTTCTTGGGAATG---GCAGGAAGCACTATG---GGCGCAGCGTCA---
CTAACGCTGACGGTACAG---GCCAGACAA---TTGTTG---TCTGGTATAGTGCAACAGCAGAAT---
AATTTG---CTGAGA---GCTATTGAGGCGCAACAGCACCTGTTGCAACTCACAGTCTGGGGCATC
>KT124764
TTCTACTGTAATACCACACAACTGTTTAATAGTACTTGGTATGCAAACAATACAAATAGTACTGAAGGGGGA-
----

AACAGTGGGAATATCACACTCCCATGCAGAATAAAACAATTTATAAACATGTGGCAGGAAGTAGGAAAAGCAA
TGTATGCCCCTCCCATCAGTGGACAGATTAAATGTACATCAAAT---
ATTACAGGGCTGCTATTAACAAGAGATGGTGGTCAAGGCAATCAGACC-----------------------
----

AACAACCAGACTGAGATCTTCAGACCTGTAGGAGGAGATATGAAGGACAATTGGAGAAGTGAATTATATAAAT
ATAAAGTAGTAAGAATTGAACCATTA---GGAATAGCACCCACCAGGGCAAAAAGGAGAGTGGTG--------
-------------------------------CAGAGAGAAAAAAGAGCA---GTGGGA---ATAGGAGCG---
------TTGTTCCTTGGTTTCTTGGGAGCA---GCAGGAAGCACTATG---GGCGCAGCGTCA---
ATGACGCTGACGGTACAG---GCCAGACTA---TTATTG---TCTGGTATAGTGCAACAGCAGAAC---
AATCTG---CTGAGA---GCTATTGAGGCGCAACAGCATCTGTTGCAACTCACAGTCTGGGGCATC
>KT124814
TTCTACTGTAATACAACACCATTATTTAATAGTACTTGGAATAATACTAGAAATATCAGTGGTCTTGAAGGGT
CAAAG---

GACAATGGAAATATCACCCTCCAATGCAGAATAAAACAAATTATAAACATGTGGCAGGAAGTAGGAAAAGCAA
TGTATGCCCCTCCCATCAGAGGACAAATTCAATGCTCATCAAAT---
ATTACAGGACTCCTATTAACAAGAGATGGTGGTACTAATGTTACT--------------------------
----

GATAACACCACTGAGATCTTCAGACCTGGAGGAGAAGATATGAGGGACAATTGGAGAAGTGAATTATATAAAT
ATAAAGTAGTACAAATTGAACCATTA---GGAGTAGCACCCACCAAGGCAAGGAGAAGAGTGGTG--------
-------------------------------CAGAGAGAAAAAAGAGCA---GTGGGA---ATAGGAGCT---
------TTGTTCCTTGGGTTCTTGGGAGCA---GCAGGAAGCACTATG---GGCGCAGCGTCA---
GTGACGCTGACGGTACAA---GCCAGACAA---TTATTG---TCTGGTATAGTGCAGCAGCAGAAC---
AATCTG---CTGAGG---GCTATTGAGGCGCAACAGCATATGTTGCAACTCACAGTCTGGGGCATC
>GU331497
TTCTACTGTAATACATCACAACTGTTTAATAGTACTTGGGAGTTTAATAGTACTGGGAATGCTACTACTAAAG
AG---

TTAAATAGCACTTATACACTCCCATGCAGAATAAAACAAATTATAAACATGTGGCAGGAAGTAGGAAAAGCAA
TGTATGCCCCTCCCATCAGTGGACAAATTAGATGTTCATCAAAT---
ATTACAGGGCTGCTCTTAATGAGAGATGGTGGTAATGAGACTGATAAA-------------------------
----

AATAACACTGAGATCTTCAGACCTGGAGGAGGAGATATGAGGGACAATTGGAGAAGTGAATTATACAAATATA
AAGTAGTAAGAATTGAACCATTA---GGAGTAGCACCCACCAAGGCAAAAAGAAGAGTGGTG-----------
----------------------------CAAAGAGAAAAAAGAGCA---GTGGGAACGTTAGGAGCT------
---ATGTTCCTTGGGTTCTTGGGAGCA---GCAGGAAGCACTATG---GGCGCAGCGTCA---
ATGGCGCTGACGGTACAG---GCCAGACTA---TTATTG---TCTGGTATAGTGCAACAGCAGAAC---
AATTTG---CTGAGA---GCTATTGAGGCGCAACAGCATATGTTGCAACTCACAGTCTGGGGCATC
>DQ869023
TTCTACTGTAATACATCAAAACTGTTTAATAGTACTTGGCATGCTAATGGTACTCGGATTAATGGTACTAATG
TTTATGCTAAT--
----------GGTACTAAT------
GCAACCAACACAATTATCACACTCCCATGCAGAATAAGACAAATTATAAACATGTGGCAGGAAGTAGGAAAAG
CAATGTATGCCCCTCCCATCAAAGGACAAATTAAATGCTTATCAAAC---
ATTACAGGGCTACTATTAGTAAGAGATGGTGGAAATAACACAAAT----------------------------
----

GGGAGCGAGATCTTCAGACCTGGAGGAGGGGATATGAGGGACAATTGGAGAAGTGAACTGTATAAATATAAAG
TAGTAAAAATTGAACCATTA---GGAGTAGCACCCACTAAGGCAAAAAGAAGAGTGGTG--------------
-------------------------CAGAGAGAAAAAAGAGCA---GTGGGA---TTCGGAGCT---------
TTGTTCCTTGGGTTCTTGGGAGCA---GCAGGAAGCACTATG---GGCGCAGCGTCA---
GTGACGCTGACGGTACAG---GCCAGACAA---TTGTTG---TCTGGTATAGTGCAACAGCAGAAC---
AACCTG---CTGAGG---GCTATTGAGGCGCAACAGCGTATGTTGCAACTCACAGTCTGGGGCATC
>EU578104
TTCTACTGTAACACAGCACAACTGTTTAATACTATTTGGAATAATACTTGGAATAGTAATGGGAATGATACTG
AA---

GGGGATAATAAAACAATCATACTCCCGTGCAGAATAAAACAAATTGTAAACAGGTGGCAGGAAGTAGGAAAAG
CAATGTATGCCCCTCCCATCAGCGGAATCATTAACTGTTTATCAAAC---
ATTACGGGGCTGCTATTAACAAGAGATGGTGGAAATAACAGTAGT----------------------------
----

ACCAACGAGACCTTCAGACCTGGAGGAGGAGATATGAGGGACAATTGGAGAAGTGAACTCTATAAATATAAAG
TAGTAAAAATTGAGCCATTA---GGAGTAGCACCCACCAAGGCAAAAAGAAGAGTGGTG--------------
-------------------------CAGAGAGAAAAAAGAGCA---GTGGGAACGTTAGGAGCG---------
ATGTTCCTTGGGTTCTTGGGAGCA---GCAGGAAGCACTATG---GGCGCAGCGTCA---
GTGACGCTGACGGTACAG---GCCAGACAA---TTATTG---TCTGGTATAGTGCAACAGCAGAAT---
AATCTG---TTGAAG---GCTATTGAGGCGCAACAGCATCTGTTGCAGCTCACAGTCTGGGGCATC
>EU578122
TTCTACTGTAATACAACACAACTGTTTAATAGTACGTGGAACGGTACTGATAGTAGTCCCGGGAATAAT----
----

ACTGATCACAATATCACACTCCCATGCAGAATAAAACAAATTGTAAACATGTGGCAAGAAGTAGGAAAAGCAA
TGTATGCCCCTCCCATCCAAGGAAAAATTGTATGTAGATCAAAT---
ATTACAGGACTGTTATTAACAAGAGATGGTGGTAATGGGACAGGG----------------------------
----

ACAGAGAAAGAAACCTTCAGACCTGGAGGAGGAAATATGAGGGACAATTGGAGAAGTGAATTATATAAATATA
AAGTAGTAAGAGTTGAACCATTA---GGAGTAGCACCCACCGAGGCAAGGAGAAGAGTGGTG----------
----------------------------CAGAGAGAAAAAAGGGCA---GCAGTGACACTGGGAGCT------
---ATGTTCCTTGGGTTCTTGGGAGCA---GCAGGAAGCACTATG---GGCGCAGCGTCA---
CTGACGCTGACGGTACAG---GCCAGACTA---TTATTG---TCTGGTATAGTGCAACAGCAGAAC---
AACTTG---CTGAGA---GCTATTGAGGCGCAACAGCATATGTTGCAACTCACAGTCTGGGGCATC
>JQ403105
TTCTACTGTAATACAACAAAACTGTTTAATAGTACTTGGTATGAAAATGGTACAAATAGTACTTGGTATGCAA
ATGGTACAAAT--
-------AGTACTGGAGAG------
GGAAACAGTGATATTATCACAATCCCATGCAGAATAAAACAATTTATAAACATGTGGCAGGAAGTAGGAAAAG
CAATGTATGCCCCTCCCATCAGTGGACAGATTAGATGTTCATCAAAC---
ATTACAGGGCTGCTATTAACAAGAGATGGTGGTGAGGGCAATCAAACC------------------------
----

AACAACCAGGAGATCTTCAGACCTGTAGGAGGAGATATGAAAAACAATTGGAGAAGTGAATTATATAAATATA
AAGTAGTAAGAATTGAACCATTA---GGAATAGCACCCACCAAGGCAAAAAGGAGAGTGGTG----------
----------------------------CAGAGAGAAAAAAGAGCA---GTGGGA---ATAGGAGCG------
---TTGTTCCTTGGGTTCTTGGGAGCA---GCAGGAAGCACTATG---GGCGCAGCGTCA---
ATGACGCTGACGGTACAG---GCCAGACTA---TTATTG---TCTGGTATAGTGCAACAGCAGAAC---
AATCTG---CTGAGA---GCTATTGAGGCGCAACAGCATCTGTTGCAACTCACAGTCTGGGGCATC
>FJ469749
TTCTACTGTAATACAACAAAGTTGTTTAATAGTACTTGGCATGCGAATGGTACTATTAAAGGGACAGATAATA
CT---

GGAGGGAATGACACTCACACACTCCCTTGCAGAATAAAACAAATTGTAAACATGTGGCAAGAAGTACGAAAAG
CAATGTATGCCCCTCCCATCAGAGGACAAATTATGGGCGTATCAAAT---
ATTACAGGGATACTATTAACAAGAGATGCTGGTAATAGCGGGAACAAGACC---------------------
----

AAGAACGAGACCGAGATTTTCAGACCTGGAGGAGGAGATATGAGGGATAATTGGAGAAGTGAATTATATAAAT
ATAAAGTAGTAAAAATTGAACCATTA---GGAGTAGCACCTACCAAGGCAAAGAGAAGAGTGGTG--------
-------------------------------CAGAGAGAAAAAAGAGCA---GTGGGA---ATAGGAGCT---
------ATGTTCATTGGGTTCTTGGGAGCA---GCAGGAAGCACTATG---GGCGCAGCGTCA---
ATAACGCTGACGGTACAG---GCCAGACAA---TTATTG---TCTGGTATAGTGCAACAGCAGAAC---
AATCTG---CTGAGA---GCTATTGAGGCGCAACAGCATCTGTTGCAACTCACAGTCTGGGGCATC
>EU578230
TTCTACTGTAATACATCAGGACTGTTTAATGGTACTTGGAATGGTACTAGCACTTGGAATGATACTGCAAATT
AT---

ACTGCAAATGAGAATATCACACTCCCATGCAGAATAAAACAAATTATAAACATGTGGCAGGAAGTAGGAAAAG
CAATGTATGCCCCTCCCATCAGAGGACAAATTAACTGTTCATCAAAA---
ATTACAGGGCTGCTATTAACAAGAGATGGTGGCCATCACAACGGCAGC------------------------
----

AACAACGACACAGAAATCTTCAGACCTGGAGGAGGAGATATGAGGGACAATTGGAGAAGTGAATTATATAAAT
ATAAAGTAGTAAAAATTGAACCATTA---GGAGTAGCACCCACCAGGGCAAAAAGAAGAGTGGTG--------
-------------------------------CAGAGAGAAAAAAGAGCA---GTGGGA---ATGGGAGCT---
------TTGTTCCTTGGGTTCTTGGGAGCA---GCAGGAAGCACTATG---GGCGCAGCGTCA---
CTAACGCTGACGGTACAG---GCCAGACAA---TTATTG---TCTGGTATAGTGCAACAGCAGAAC---
AATCTG---CTGAGG---GCTATTGATGCGCAACAACATCTGTTGCAACTCACAGTCTGGGGCATT
>FJ469754
TTCTACTGTAATACAACAAGACTGTTTAATAGTAATTGGAATAGCAGTACTTGGAATAACACTGAAGGGTTAA
ATACCACT---
-------------AACCTT------
GGAGAAAATGACACAATCATACTCCCATGCAGAATAAAACAGATTATAAACACGTGGCAGAAAGTAGGAAAAG
CAATATATGCCCCTCCCATCGAAGGACAAATTAAATGTATATCAAAT---
ATTACAGGGCTGCTATTATTAAGAGATGGTGGTATAAATGTGAGT---------------------------
----

GAGACCAACGAAACCTTCAGACCTGGAGGAGGAGATATGAAGGATATTTGGAGAAGTGAATTATATAAATATA
AAGTAGTAAAAATTGAACCATTA---GGAATAGCACCCAACAAGGCAAAGAGAAGAGTGGTG-----------
----------------------------CAGAGAGAAAAAAGAGCA---GTGGGA---ATAGGAGCT------
---GTGTTCCTTGGGTTCTTGGGAGCA---GCCGGAAGCACTATG---GGCGCAGCGTCA---
ATGACGCTGACGGTACAG---GCCAGACAA---TTATTG---TCTGGTATAGTGCAACAGCAAAGC---
AATTTG---CTGAGG---GCTATTGAGGCTCAACAACATCTGTTGAAACTCACAGTCTGGGGCATC
>FJ469757
TTCTACTGTAATTCCTCACAACTGTTCAGTAGTACTTGGAATAGGACTAACGGGACTTGGAATGATGTTGAAG
GGTTAAAT---
-------------TACACT------
AATGGAAATGACACAATCACACTCCCATGCAGAATAAAACAATATATAAACATGTGGCAGGAAGTAGGAAAAG
CAATGTATGCCCCTCCCATCAGAGGGCAACTTAATTGTTCATCAAAG---
ATTACAGGGTTGCTATTAACAAGAGATGGTGTGAACGACACCGAC----------------------------
----

AAAAATGAGACTTTCAGACCTCAAGGAGGGGATATGAAGGACAATTGGAGAAGTGAATTATATAAATATAAAG
TAGTAAAAATTGAACCGTTA---GGGATAGCACCCACCAGGGCAAAGAGAAGAGTGGTG--------------
-------------------------CAAAGAGAAAAAAGAGCA---GTGGGA---TTAGGAGCT---------
ATGTTCCTTGGGTTCTTGGGAGCA---GCAGGAAGCACTATG---GGCGCAGCATCA---
ATAACGCTGACGGTACAG---GCCAGACAA---TTACTG---TCTGGTATAGTGCAACAGCAGAAC---
AATCTG---CTGAGG---GCTATTGAGGCGCAACACCATCTGTTGCAACTCACAGTCTGGGGCATC
>GU330538
TTCTACTGTAATACAACAAAACTGTTTTATAGTAATTGGACTATTAATGGGACTATTGTGAGTAATTGGACAT
ATGAAAATGAC--
-------------------ACT---
GGAACAAATGACACTATCATACTCCCATGTAGAATAAAACAAATTATAAACATGTGGCAGGAAGTAGGAAAAG
CAATGTATGCCCCTCCCATCAAAGGACCAATTACATGTAACTCAAGT---
ATTACAGGGTTGCTATTAACAAGAGATGGTGGTATTAACAGGACCAAT-------------------------
----

AATGAGACCGAGATCTTCAGACCTGTAGGGGGAGATATGAGGGACAATTGGAGAAGTGAATTATATAAATATA
AAGTAGTAGAAATTCAACCATTA---GGAATAGCACCCACCAAGGCAAAGAGAAGAGTGGTG-----------
----------------------------CAGAGAGAAAAGAGAGCG---GTAACA---CTAGGAGCT------
---ATGTTCCTTGGGTTCTTGGGAGCA---GCAGGAAGCACTATG---GGCGCAGCGTCA---
ATGGCGCTGACGGTACAG---GCCAGACAA---TTATTG---TCTGGTATAGTGCAACAGCAGAAC---
AATCTG---CTGAGG---GCTATTGAGGCGCAACAGCACATGTTGCAACTCACAGTCTGGGGCATT
>GU330550
TTCTACTGTAATTTAACACAACTGTTTAATAGTACTTGGAATATTACTGACAGATTAAATAATAATGAAACTC
AA---

GGAAATGACACAACAATTCACCTCCCGTGCAGAATAAAACAAATTATAAACATGTGGCAAGAAGTAGGAAAAG
CAATGTATGCCCCTCCCATCAGGGGGATAATTAGATGTTCATCAAAT---
ATTACAGGGCTGCTATTAACAAGAGATGGTGGTACAAATACCACTGAC-------------------------
----

AATGACACTGAGGTCTTCAGACCTGGAGGAGGAGACATGAGGGACAATTGGAGAAGTGAATTGTATAAATACA
AAGTAATACAAATTGAACCATTA---GGAGTAGCACCCACTAAGGCAAAGAGAAGAGTGGTG-----------
----------------------------CAGAGAGAAAAAAGAGCA---GTGGGA---ATAGGAGCT------
---GTGTTCCTTGGGTTCTTGGGAGCA---GCAGGAAGCACTATG---GGCGCAGCGTCA---
GTGACGCTGACGGTACAG---GCCAGACAA---TTATTG---TCTGGTATAGTGCAGCAGCAGAGC---
AATCTG---CTGAGG---GCTATTGAGGCGCATCAACATCTGTTGCAACTCACAGTCTGGGGCATC
>KT124781
TTCTACTGTAATTCAACACCACTGTTTAATAGTGAATGGTCAGATAATAATACTTGGAATAGTGCTGAAGAA-
----

GGAAATGCCACAATCACACTCCAATGCAGAATAAAACAAATTGTAAACATGTGGCAGGAAGTAGGAAGAGCAA
TGTATGCACCTCCCATCAAAGGAGTAATTAGCTGTACATCAAAT---
ATTACAGGACTGATATTAACAAGAGATCATAGTAAGGATAATGTA----------------------------
----

ACCTTCACCTTCAGACCTGGAGGAGGAAATATGAAGGACAATTGGAGAAGTGAATTATATAAATATAAAGTAG
TAAAAATTGAACCATTA---GGAGTAGCACCCACTACAGCCAAGAGAAGAGTGGTG----------------
----------------------CAGAGAGAAAGAAGAGCA---GTGGGA---ATAGGAGCT---------
GTGTTCCTTGGGTTCTTGGGAGCA---GCAGGAAGCACTATG---GGCGCAGCGTCA---
ATAACGCTGACGGTACAG---GCCAGACTA---TTATTG---TCTGGTATAGTGCAACAGCAGAAC---
AATCTG---CTGAGA---GCTATTGAGGCGCAACAGCACCTGTTGCAACTCACAGTCTGGGGCATC
>MH060766
TTCTACTGTAATACAACACAGCTGTTTAATAGTACTTGGCCAGCTAATAATACTGAAAATGAGACTAATGAG-
----

GCAGATAGCAATATTACACTTCAGTGCAGAATAAAACAAATTATAAACAGGTGGCAGGAAGTAGGAAAAGCAA
TGTATGCCCCTCCCATCAAAGGACAAATTAGCTGTTCATCAAAT---
ATTACAGGGCTACTATTAACAAGAGATGGAGGTAGTAGCAGTAATGGG------------------------
----

ACAGAGAATGGGACAGAGATCTTCAGACCTGGAGGAGGAGATATGAGGGACAATTGGAGAAGTGAATTATATA
AATATAAAATAGTAAAAATTACACCATTA---GGAGTAGCACCCACCAAGGCAAAGAGAAGAGTGGTG-----
----------------------------------CAAAGAGAAAAAAGAGCA---GTGACA---
CTAGGAGCT---------ATGTTCCTTGGGTTCTTGGGAGCA---GCAGGAAGCACTATG---
GGCGCAGCGTCG---ATAACGCTGACGGTACAG---GCCAGACAA---TTATTG---
TCTGGTATAGTGCAACAGCAGAGC---AATCTG---CTGAGG---
GCTATTGAAGCGCAACAGCATATGTTGCAACTCACAGTCTGGGGCATC
>MH060965
TTCTACTGTAATTCAACACAACTGTTTAATAGTACTTGGTTTACTGAGGATAATGCGACCGGCACTACC----
----

GGAAATGAAACAATCATACTCCCATGCAGAATAAAACAAATTATAAACATGTGGCAGGAAGTAGGAAGAGCAA
TGTATGCCCCTCCCATCAGAGGACAAATTAACTGTACATCAAAT---
ATTACAGGACTGATATTAACAAGAGATGGTGGTAATAACAATCACACT------------------------
----

AATGAGAACGAAACCTTCAGACCTGGAGGAGGAGATATGAGGGACAATTGGAGAAGTGAATTATATAAATATA
AAGTAGTAAAAGTTGAACCATTA---GGAATAGCACCCTCTAAGGCAAGGAGAAGAGTGGTG----------
----------------------------CAGAGAGAAAAAAGAGCA---GCAGGA---CTAGGAGCT------
---TTGCTCCTTGGGTTCTTGAGCGCA---GCAGGAAGCACTATG---GGCGCAGCGTCA---
CTGACGCTGACGGTACAG---GCCAGGCAA---TTATTG---TCTGGTATAGTGCAACAGCAAAAC---
AATTTG---CTGAGG---GCTATTGAGGCGCAACAGCATATGTTGCAACTCACAGTCTGGGGCATT
>FJ469686
TTCTACTGTAATTCAACAGCACTGTTTAATAGCAGTTGGAATAAGACTGGAGGGGGTTGGACTAAGGAGACAA
AT--

CACACAAACAGAACTATCACACTCCCATGCAGAATAAAACAAATTATAAACATGTGGCAGCAAGTAGGAAAAG
CAATGTATGCCCCTCCCATCAGAGGACCAATTAACTGTACATCAAGT---
ATTACAGGGCTGCTATTAACAAGAGATGGTGGGAGTAATAGCAGC---------------------------
----

GCTACAGAGATCTTCAGACCTGGAGGAGGAAATATGAGAGACAATTGGAGAAATGAATTATACAAATATAAAG
TAGTAAAAATTGAGCCATTA---GGAGTAGCACCCACCAAGGCAAAGAGGAGAGTGGTG-------------
-------------------------CAGAGAGAAAAAAGAGCA---ATAGGGGCAATAGGAGCT---------
ATGTTCCTTGGGTTCTTGGGAGCA---GCAGGAAGCACTATG---GGCGCAGCATCA---
GTGACGCTGACGGTACAA---GCCAGGCAA---TTATTG---TCTGGTATAGTGCAACAGCAGAAC---
AATCTG---CTGAGG---GCTATTGAGGCGCAACAGCATCTGTTGCAACTCACAGTCTGGGGCATC
>FJ469731
TTCTACTGCAATTCAACACCACTGTTTAATAAGACTTGGAATAAGAATGATATTAGGAATAATACTACTGGGA
AT--

GATAATGACACTATCACACTCCAATGCAGAATAAAACAAATTATAAACATGTGGCAGGAAGTAGGAAAAGCAA
TGTATGCCCCTCCCATTAGAGGACTAATCAGCTGTTCATCAAAT---
ATTACAGGGCTACTATTAACAAGAGATGGAGGTCATAACACAAGT---------------------------

----

AGGGCAAATGAGACCTTCAGGCCTGGAGGAGGAAATATGAAGGACAATTGGAGAAGTGAATTATATAAATATA
AAGTAGTAAAAATTGAACCATTA---GGAGTAGCACCCACCAGGGCAAAGAGAAGAGTGGTG-----------
----------------------------CAGAGAGAAAAAAGAGCA---GTGGGA---CTAGGAGCT------
---TTATTCCTTGGGTTCTTGGGAGCA---GCAGGAAGCACTATG---GGCGCAGCGTCG---
ATAACGCTGACGGTACAG---GCCAGACAA---TTATTG---TCTGGTATAGTGCAACAGCAGAAC---
AATTTG---CTGAGA---GCTATTGAGGCGCAACAGCATCTGTTGCAGCTCACAGTCTGGGGCATC
>HQ216397
TTCTACTGTAATTCAACACTGCTGTTTAATAGTAGTTGGAAGAATGAGACCAATGCTGACGGACAGAG----
----

GGCAATGATACTATTACACTCCAATGCAGAATAAAACAAATCATAAACAGATGGCAGGAAGTAGGAAAAGCAA
TGTATGCCCCTCCCATCGCAGGACAAATTAACTGTTCATCAAAA---
ATTACAGGATTGCTATTAACAAGAGATGGTGGTAATCATACCGCC----------------------------
----

AACACCGAGACCTTCAGACCTGGAGGAGGAGATATGAGAGACAATTGGAGAAGTGAATTATATAAATACAAAG
TAGTAAAAATTGAGCCATTA---GGAATAGCGCCCACCAAGGCAAGGAGAAGAGTGGTG--------------
-------------------------CAAAGAGAAAGAAGAGCA---GTGGGAACATTTGGAGCT---------
TTGTTCCTTGGGTTCTTGGGAGCA---GCAGGAAGCACTATG---GGCGCAGCATCA---
GTGACGCTGACGGTACAA---GCCAGACAA---TTATTG---TCTGGTATAGTGCAACAGCAGAAC---
AATCTG---CTGAAG---GCTATTGAGGCGCAACAACAGCTGTTGCAACTCACAGTCTGGGGCATC
>DQ410636
TTCTATTGTAATTCAACACAACTGTTTAATAGTACTTGGGCTAAGAATGGTAGTAGGTTAAATAACAATGGTA
GTGAGTTTAAT--
-------CACACCGAAGAA------
AAAGGAAATGACACAATCATACTCCCATGCAGAATAAAACAAATTATAAACATGTGGCAGGGAGTAGGAAAAG
CAATGTATGCCCCTCCTATCAAAGGACTAATTAGATGTTCATCAAAT---
ATCACAGGGCTGCTAATAACAAGAGATGGGGGTACGGGAAACAAGAGCGAG----------------------
----

GAGAACAGGAACGAGACCTTCAGACCTGTAGGAGGAAATATGAGGGACAATTGGAGAAGTGAATTATATAAAT
ATAAAGTAGTAAAAATTGAACCATTA---GGAGTAGCACCCACCAAGGCAAAGAGAAGAGTGGTG--------
-------------------------------CAGAGAGAAAAAAGAGCA---GTGGGA---CTCGGAGCT---
------ATGTTCATTGGGTTCTTGGGAGCA---GCAGGAAGCACTATG---GGCGCAGCGTCA---
GTGACGCTGACGGTACAG---GCCAGACAA---TTATTG---TCTGGTATAGTGCAACAGCAAAAC---
AATTTG---CTGAGA---GCTATTGAGGCGCAACAGCATCTGTTGCAACTCACAGTCTGGGGCATC
>KT124758
TTCTACTGCAACACAACACAACTGTTTAATAGTACTTGGAATGATACACAACTGTCTAATAGCACTTGGAATA
ATACAAGA---

GGGATAAATGACACTATCACACTCCAATGTAAAATAAAACAAATCATAAACATGTGGCAGAAGGTAGGAAAAG
CAATGTATGCCCCTCCCATCAGTGGACAAATTAGATGTTCATCAAAT---
ATTACAGGGCTGATATTAACAAGAGATGGTGGTAATACTGACAACACC-------------------------
----

ACCCAGCCCGAGATCTTCAGACCTCAAGGGGGAAATATGAAGGACAATTGGAGAAGTGAATTATATAAATATA
AGGTAGTAGCAATTGAACCATTA---GGAATAGCACCCACCAAGGCAAAGAGAAGAGTGGTG-----------
----------------------------CAGAGAGACAAAAGAGCA---GTGGGA---ATGGGAGCT------
---CTGTTCCTTGGGTTCTTGGGAGCA---GCAGGAAGCACTATG---GGCGCAGCGTCA---
ATAACGCTGACGGCACAG---GCCAGACAA---TTATTG---TCTGGTATAGTGCAACAGCAGAAC---
AATTTG---CTGAGG---GCTATTGAGGCGCAACAGCATCTGTTGCAGCTCACAGTCTGGGGCATT
>JX863968
TTCTATTGTAATTCAACATCACTGTTTAATAGTACTTGGAGGACAAATGAAACTGCAGACTTAAAT-------
----

ACTACAGAAATTATCACACTCCCATGCAGAATAAAACAAATTATAAACATGTGGCAAGAAGTAGGAAAAGCAA
TATATGCCCCTCCCATCGAAGGAAACATTACATGTGTATCAAAT---
ATTACAGGGCTGCTATTAACAAGAGATGGTGGTAACAATAATCAGAGTAAT----------------------

```
-------------------------------------------------------------------------
----------
GAGACGACTGAGATCTTTAGACCTGGAGGAGGAGATATGAGGGACAATTGGAGAAGTGAATTATATAAATATA
AAGTAGTAAAAATTGAACCACTA---GGAGTAGCACCCACCAAGGCAAAGAGAAGAGTGGTG-----------
----------------------------CAGAGAGAAAAAAGAGCA---GCGGGA---CTAGGAGCT------
---GTGTTTCTTGGGTTCTTGGGAGCA---GCAGGAAGCACTATG---GGCGCAGCGTCA---
ATAACGCTGACGGCACAG---GCCAGGCAA---TTATTG---TCTGGTATAGTGCAACAGCAAAAT---
AACTTG---CTGAGA---GCTATTGAGGCGCAACAACATCTGTTGCAACTCACAGTCTGGGGCATC
>EU577792
TTCTACTGTGATTCAACACAACTGTTTAATAGTACTTGGAATAGTACTAAGTTAAATAGTACTGAGTTAAGTA
GGACTGAGTCA------------------------------------------------------
AATAACACTGCAGGCACAAAT------------
GGCACTCAAGACCCTATCATGCTCCCATGCAGAATAAAACAAATTATAAACATGTGGCAGAAAGTAGGAAAAG
CAATGTATGCCCCTCCCATCAGCGGACAAATTAGATGTTCATCAAGT---
ATTACAGGTCTGCTATTAACAAGAGATGGTGGTACTAATAATACC----------------------------
-------------------------------------------------------------------------
----------------
AACGAGACCTTCAGACCTGGAGGAGGAGATATGAGGGACAACTGGAGAAGTGAATTATATAAATATAAAGTAG
TAAAAATTAAACCATTA---GGAGTAGCACCCACCAGGGCAAAGAGAAGAGTGGTG-----------------
----------------------CAGAGAGAAAAAAGGGGA------------TTAGGAGCT---------
GTGTTCCTTGGGTTCTTGGGAGCA---GCAGGAAGCACTATG---GGCGCAGCGTCA---
GTGACGCTGACGGTACAG---GCCAGACTA---TTATTG---TCTGGTATAGTGCAACAGCAAAGC---
AATTTG---CTGAGA---GCTATTGAGGCGCAACAGCATCTGTTGCGACTCACAGTCTGGGGCATC
>EF593190
TTCTACTGTAATACAACACGATTGTTTAATAGTACTTGGGAGGCTAATGGTAATTGGACTGGAACTGAAGGAA
AT-----------------------------------------------------------------------
----------------------------
GATAATGGCACAGTCACACTTCCATGCAGAATAAAACAAATTATAAACATGTGGCAACAAGTAGGAAAAGCAA
TGTATGCCCCTCCCATTGCAGGAAAGATTACATGTAACTCAAGT---
ATTACAGGAATACTATTAACAAGAGATGGAGGTATCACTAACAACACC-------------------------
-------------------------------------------------------------------------
-------
GAGACCCACACCGAGACCTTCAGACCTGGAGGAGGAGATATGAGGGACAATTGGAGAAGTGAATTATATAAAT
ATAAAGTAGTAAAAATTCAACCATTA---GGAGTAGCACCCACCAAGGCAAGAAGAAGAGTGGTG--------
-------------------------------CAGAGAGAAAAAAGAGCA---GTGGGA---TTAGGAGCT---
------ATGTTCCTTGGGTTCTTGGGAGCA---GCAGGAAGCACTATG---GGCGCAGCGTCA---
GTAACGCTGACGGTACAG---GCCAGACAA---TTATTG---TCTGGTATAGTGCAACAGCAGAAC---
AACTTG---CTGAGG---GCTATTGAGGCGCAACAGCATCTGTTGCAACTCACAGTCTGGGGCATC
>MH060681
TTCTACTGTAATTCAACACCACTATTTAATAGTACACACAACAGTACTACTACAAGGTCAAAT----------
-------------------------------------------------------------------------
----------------------------
GAAACTGAACCAATCATACTCCAATGTAGAATAAAACAAATTGTAAACATGTGGCAGGAGGTAGGAAAAGCAA
TGTATGCCCCTCCCATTGCAGGACAAATTAACTGTACATCAAAT---
ATTACAGGGCTACTGTTAACAAGAGATGGTGGCAATAATCAGAGTAGAGAA----------------------
-------------------------------------------------------------------------
----------
AATAATACCGAGATCTTCAGACCTGGAGGAGGAAATATGAAGGACAATTGGAGAAGTGAATTATATAAATATA
AAGTAGTAAAAGTTGAACCATTA---GGAGTAGCACCCACCAAGGCAAAAAGAAGAGTGGTG-----------
----------------------------CAAAGAGAAAAAAGAGCA---GTGGGA---TTCGGAGCT------
---TTGTTCCTTGGGTTCTTAGGAGCA---GCAGGAAGCACTATG---GGCGCAGCGTCA---
ATGACGCTGACGGTACAG---GCCAGACAA---TTATTG---TCTGGTATAGTGCAACAGCAGAAC---
AATCTG---CTGAGG---GCTATTGAGGCGCAACAACATCTGTTGCAACTCACAGTCTGGGGCATC
>MH060990
TTCTACTGTAATACAACAAAACTGTTTAATAGTACTTGGAATAGTACTGAGAATTTTGAGGGAGGG-------
-------------------------------------------------------------------------
----------------------------
TCAGATAACAATATCACACTCCCATGCAGGATAAAGCAAATTATAAACATGTGGCAGGAAGTAGGAAAAGCAA
TGTATGCCCCTCCCATCGCAGGACAAATTAGATGTTCATCAAAC---
ATTACAGGGCTGATATTAACAAGAGATGGTGGTAGTCATAATGGTAGT-------------------------
```


AATAATAGTGAGACCTTCAGACCTGGAGGAGGAGATATGAGGGACAATTGGAGAAGTGAACTTTATAAATATA
AAGTAGTAAAAATTGAACCATTA---GGAGTAGCACCCACTAAGGCAAAAAGAAGAGTGGTG-----------
----------------------------CAGAGAGAAAAAAGAGCA---GCA------ATAGGAGCT------
---TTGTTCCTTGGGTTCTTGGGAGCA---GCAGGAAGCACTATG---GGCGCAGCGTCA---
GTAACGCTGACGGTACAG---GCCAGACTA---TTGTTG---TCTGGTATAGTGCAACAGCAGAGC---
AATCTG---CTGAAA---GCTATTGAGGCGCAACAGCATCTGTTGCAACTCACAGTCTGGGGCATC
>DQ410526
TTCTATTGTAATACAACACCACTGTTTAATAATACTTGGTTTAATAGTACTAATAATTTAAATGAAACAAAGG
AC---

AACAACAACAACACAATCATACTCCAATGCAGAATAAAACAAATTATAAACATGTGGCAGGAAGTAGGAAAAG
CAATGTATGCCCCTCCCATCAGGGGAAACATTAGCTGTTCATCAAAT---
ATTACAGGACTATTATTAACAAGAGACGGTGGTCATAATGTGAGC----------------------------
----

AACACGAACGAGACTTTCAGACCTGGAGGAGGAGATATGAGGGACAATTGGAGAAGTGAATTATATAAATATA
AAGTAGTAAGAATTGAACCATTA---GGAATAGCACCCACCAAAGCAAAGAGAAGAGTGGTG-----------
----------------------------CAAAGAGAAAAAAGAGCA---GTGGGA---TTAGGAGCT------
---GTGTTCATTGGGTTTCTGGGAGCA---GCAGGAAGCACTATG---GGCGCAGCGTCA---
ATGACGCTGACGGTACAG---GCCAGACAA---TTATTG---TCTGGTATAGTGCAGCAGCAGAAC---
AATCTG---CTGAGA---GCTATTGAGGCGCAACAGCATCTGTTGCAACTCACAGTCTGGGGCATT
>EF363127
TTCTACTGTAATACAACAGACTTGTTTACTTGGAATGGTACTTGGAATGGTACTGCAGGGGCA----------
----

GATAACAATACAATCACACTCCAATGCAGAATAAGACAAATTATAAACATGTGGCAGGAAGTAGGAAAAGCAA
TGTATGCCCCTCCCATCCCAGGATACCTTAACTGTTCATCAAAT---
ATTACAGGACTGCTATTAACAAGGGATGGTGGTCATAACACGAAT----------------------------
----

ACTACTGAGATCTTCAGACCTGGAGGAGGAGATATGAGGGACAATTGGAGAAGTGAATTATATAAATATAAAG
TAGTAAAAATTGAACCATTA---GGAATAGCACCCACCAAGGCAAGGAGAAGAGTGGTG--------------
-------------------------CAGAGAGAAAAAAGAGCA---GTGGGA---ATAGGAGCT---------
GTGTTCCTTGGGTTCTTGGGAGCA---GCAGGAAGCACTATG---GGCGCAGCGTCA---
ATAACGCTGACGGTACAG---GCCAGGCTA---TTATTG---TCTGGTATAGTGCAACAGCAAAAC---
AACTTG---CTGAGA---GCTATTGAGGCGCAACAGCATCTGTTGCAACTCACAGTCTGGGGCATC
>DQ410205
TTCTACTGTAATACATCACAACTGTTTAATAGTAATTGGACTTGGCCTGATACTGAAGGGTCAAATGACACTA
AC---

ACTAACGAAAATATCACACTCCCATGCAGAATAAAACAAATTATAAACATGTGGCAGGAGGTAGGAAAAGCAA
TGTATGCCCCTCCCATCAGAGGACAAATTAGCTGTAAATCAAAT---
ATTACAGGACTGCTATTGACAAGAGATGGTGGTCAGGACGAGAAC----------------------------
----

GGGACCGAGACCTTCAGACCTGGAGGAGGAGATATGAGGGACAATTGGAGAAGTGAATTATATAAATATAAAG
TAGTAAAAATTGAACCATTA---GGAGTAGCACCCACCAAGGCAAAGAGAAGAGTGGTG--------------
-------------------------CAGAGAGAAAAAAGAGCA---TTAGGA---ATAGGAGCC---------
GTGTTCCTTGGGTTCTTGGGAGCA---GCAGGAAGCACTATG---GGCGCAGCGTCA---
ATGACGCTGACGGTACAA---GCCAGACTG---CTATTG---TCTGGTATAGTGCAACAGCAGAAC---
AATTTG---CTGAGG---GCTATTGAGGCGCAACAGCATCTGTTGCAACTCACAGTCTGGGGCATC
>FJ469733
TTCTACTGTAATTCAACACAACTGTTTAATAGTACTTGGTATACTAATAATACTATGGAGGGGTCACATGGTA
AT---

GAGACAAATGGCACTATCACTCTCCCATGCAAAATAAGACAAATTGTAAACAAGTGGCAAGAAGTAGGAAAAG
CAATGTATGCCCCTCCCATTAGAGGACTAATTAGATGTTCATCAAAT---
ATTACAGGGCTGCTATTAACAAGAGATGGTGGTCATAATAATAATAAT-------------------------

----

AATACTAATGAGACCTTCAGACCTGGAGGAGGAGATATGAGGGACAATTGGAGAAGTGAATTATATAAATACA
AAGTAGTAAGAATTGAGCCATTA---GGAGTAGCACCCACCAAGGCACAGAGAAGAGTGGTG-----------
----------------------------CAAAGAGAAAAAAGAGCA---GTGGGA---CTAGGAGCT------
---GTGTTCCTTGGGTTCTTGGGAGCA---GCAGGAAGCACTATG---GGCGCAGCGTCA---
ATAACGCTGACGGCACAG---GCCAGACAA---TTATTG---TCTGGTATAGTGCAACAGCAGAAC---
AACCTG---CTGAGA---GCTATTGAGGCGCAACAGCATATGTTGCAACTCACAGTCTGGGGCATA
>JQ403107
TTCTACTGTGATACAACACAACTGTTTAATAGCAGCTGGGAGGGGAATGGTACTAAAGATACTATAGAGTCAA
ATGAC--

ACTGGAGGAAATATCACACTCCCATGTAGAATAAAACAGATTATAAACATGTGGCAGGAAGTAGGAAAAGCAA
TGTATGCCCCTCCCATCAGTGGACCAATTAGCTGTTCATCAAAC---
ATTACAGGGCTACTATTAACAAGAGATGGGGGTCATAACAATGACACT-------------------------
----

AACGGGAACGAGACCTTCAGACCTGGAGGAGGAAATATGAAGGACAATTGGAGAAATGAATTATATAAATATA
AAGTAGTAAAAATTGAACCACTA---GGAGTAGCACCCACCAAGGCAAAGAGAAGAGTGGTG-----------
----------------------------CAGAGAGAAAAAAGAGCA---ATGGGA---CTAGGAGCT------
---ATGTTCATTGGGTTCTTGGGAGCA---GCAGGAAGCACTATG---GGCGCAGCGTCA---
GTGACGCTGACGGTACAG---GCCAGACAA---TTACTG---TCTGGTATAGTGCGCCAGCAGAAC---
AATCTG---CTGAGA---GCTATTGAGGCGCAACAGCATATGTTGCAACTCACAGTCTGGGGCATC
>KM259452
TTCTACTGTAATACAACAAAACTGTTTAACAGTACGTGGAATAATACTAGT----------------------
----
-ACTTGGAATTATACA---------
GATGAACCCGAAAATATCACCCTCCCATGCAGAATAAAGCAAATTATAAACATGTGGCAGGAAGTAGGAAAAG
CAATGTATGCCCCTCCCATCAGTGGAATAATTAGATGCACATCAAAG---
ATTACTGGGCTGCTATTAACAAGAGATGGTGGT--

AATCGTAATGACAGCAAT---
ACGACCGAGGTCTTCAGACCTGGAGGAGGAGATATGAGGGACAACTGGAGAAGTGAATTGTATAAATATAAAG
TAGTAAAAATTGAACCAGTA---GGACTAGCACCCACCAAGGCAAAGAGAAGAGCGGTG--------------
-------------------------CAGAGAGAAAAAAGAGCA---GTGGGA---ATAGGAGCT---------
GTGTTCCTTGGGTTCTTGGGAGCA---GCAGGAAGCACTATG---GGCGCAGCGTCA---
TTAACGCTGACGGTACAG---GCCAGACTG---TTATTG---TCTGGAATAGTGCAACAGCAGAAC---
AATCTG---CTGAGA---GCTATTGAGGCGCAACAGCATATGTTGCAACTCACAGTCTGGGGCATC
>EF593275
TTCTACTGTAATTCAACACCACTGTTTAATAGTACTTGGTATAGTAATGGTACTGGGAGTGTTTGGAATAACA
CT---

CAAGCAAATGGCACTATCATACTCCCATGCAGAATAAAGCAAATCATAAACATGTGGCAGCAAGTAGGAAAAG
CAATGTATGCCCCTCCCATCCAAGGAATAATTAATTGTTTATCAAAT---
ATTACAGGGCTGTTACTAACAAGAGATGGAGGTAGTGGTAATAATACA-------------------------
----

GACAATAACACCGAGACCTTCAGACCTGGAGGAGGAGATATGAGGGACAATTGGAGAAGTGAATTATATAAAT
ATAAAGTAGTAGAACTTAAACCATTA---GGAGTAGCACCCACCAAGGCAAAGAGGAGAGTGGTG--------
-------------------------------CAAAGAGAAAAAAGAGCA---GTGGGA---ATAGGAGCT---
------CTTCTCCTTGGGTTCTTGGGAACA---GCAGGAAGCGCTATG---GGCGCAGCGTCA---
CTGACGCTGACGGTACAG---GCCAGACAT---TTATTG---TCTGGTATAGTGCAACAGCAAAAT---
AATTTG---CTGAGG---GCTATTGAGGCGCAACAGCATATGTTGCAACTCACAGTCTGGGGCATC
>JN024100
TTCTACTGTAATACAACAAAACTGTTTAATAGTACTTGGAATAGTACTGAGAATGTGGAGGAAGGG-------
----

CCAGATAACAATATCACACTCCCATGCAGGATAAAGCAAATTATAAACATGTGGCAGGAAGTAGGAAAAGCAA
TGTATGCCCCTCCCATCGCAGGACAAATTAGATGTTCATCAAAT---
ATTACAGGGCTGATATTAACAAGAGATGGTGGTGAGAATAACAAC----------------------------

----

GAGACCGAGACCTTCAGACCTGGAGGAGGAGATATGAGGGACAATTGGAGAAGTGAACTTTATAAATATAAAG
TAGTAAAAATTGAACCATTA---GGAGTAGCACCCACTAAGGCAAAAAGAAGAGTGGTG--------------
-------------------------CAGAGAGAAAAAAGAGCA---GCA------ATAGGAGCT---------
TTGTTCCTTGGTTTCTTGGGAGCA---GCAGGAAGCACTATG---GGCGCAGCGTCA---
ATAACGCTGACGGTACAG---GCCAGACTA---TTGTTG---TCTGGTATAGTGCAACAGCAGAGC---
AATCTG---CTGAAA---GCTATTGAGGCGCAACAGCATCTGTTGCAACTCACAGTCTGGGGCATC
>HQ216601
TTCTACTGTGATACAACACAGCTGTTTAATAGTACTTGGAATATTGAGAATGATACGAACAGGTCAAAT----
----

GAAAGTGAAACAATTATACTTCCATGCAAAATAAAGCAAATTATAAACCTGTGGCAGGAAGTAGGAAAAGCAA
TGTATGCCCCTCCCATCAGAGATCAAATTAGATGCTCATCAAAT---
ATTACAGGGCTGCTATTAACAAGAGATGGTGGTAATAATACAACTGAG-------------------------
----

AACAATAACGAGACCTTCAGACCTGGAGGAGGAGATATGAGGGACAATTGGAGAAGTGAACTTTATAAATATA
AAGTAGTAAAAATTGAACCATTA---GGAATAGCACCCACTACTGCAAAAAGAAGAGTGGTG-----------
----------------------------CAGAGAGAAAAAAGAGCG---GCA------CTAGGAGCC------
---TTGTTCCTTGGGTTCTTGGGAGCA---GCAGGAAGCACTATG---GGCGCAGCGTCA---
ATAACGCTGACGGTACAG---GCCAGACTA---TTGTTG---TCTGGTATAGTGCAACAGCAGAAC---
AATCTG---CTGAAG---GCTATTGAGGCGCAACAGCATCTGTTGCAACTCACAGTCTGGGGCATC
>HQ216795
TTCTACTGTAACACATCACAGCTGTTTGATAGTACTTGGCTGTTTAATAAGACTGGGGATAATAATACTGAAG
GG---

TCAAATGACACTATCATACTTCCATGCAGAATAAAACAAATTATAAACAGGTGGCAGGAAGTAGGAAAGGCAA
TGTATGCCCCTCCCATCGCAGGACAAATTAGATGTCAATCAAAT---
ATTACAGGGGTGCTACTAACAAGAGATGGGGGTAAGAGTGGGAACGGT-------------------------
----

ACTAATGAGACCGAAACATTCAGACCTGTTGGAGGAAATATGAAGGACAATTGGAGAAGTGAATTGTATAAAT
ATAAGGTAGTAAAGATTGAACCATTA---GGAGTAGCACCCACCAAGGCAAAGAGGCTAGTGGTG--------
-------------------------------CAGAGAGAAAAAAGAGCA---GTGACG---CTGGGAGCT---
------ATGTTCATTGGGTTCTTGGGAGCA---GCAGGAAGCACTATG---GGCGCAGCGTCA---
CTGACGCTGACGGTACAG---GCCAGACAA---TTACTG---TCTGGTATAGTGCAACAGCAGAAC---
AATCTG---CTGAGA---GCTATTGAGGCGCAACAGCATCTGTTGCAACTCACAGTTTGGGGCATT
>JX863965
TTCTACTGTAATTCAGCACAACTGTTTAATAGTACTTGGGATAATACTGCTGGGAATAATACAGCTGGA----
----

GAAAATGACACAATCACACTCCCATGCAGAATAAAACAAATTGTAAATAGGTGGCAAGAAGTAGGAAAAGCAA
TGTACGCCCCTCCCATCAGCGGACCAATTAAATGTTCATCAAAT---
ATTACAGGACTGCTATTAACAAGAGATGGTGGTCAAAATACAACT----------------------------
----

AATAACACCGAAATTTTCAGACCAGGAGGAGGAGATATGAGGGACAATTGGAGAAGTGAATTATATAAATATA
AAGTAGTAAAAATTGAACCATTA---GGAGTAGCACCCACTAAGGCAAAGAGAAGAGTGGTG-----------
----------------------------CAGAGAGAAAAAAGAGCA---GTGGGA---ATAGGAGCT------
---ATGTTCCTTGGGTTCTTGGGAGCA---GCAGGAAGCACTATG---GGCGCAGCATCA---
ATAACGCTGACGGTACAG---GCCAGACAA---TTATTG---TCTGGTATAGTGCAACAGCAGAAC---
AATCTG---CTGAGA---GCTATTGAGGCGCAACAGCATCTGTTGCAACTCACAGTCTGGGGCATT
>JF320043
TTTTACTGTAATACAACAAAACTATTTAATAGCACTTGGGAGCCTAATAATGTAACTGTCACTGGT-------
----

GATAATGACACAATCACACTCCCATGCAGAATAAAACAAATTGTAAACTTGTGGCAGGAAGTAGGAAGAGCAA
TGTATGCCGCTCCCATCAGAGGACTGATCAGATGTTCATCAAAT---
ATTACAGGGCTACTATTAACAAGAGATGGTGGTGTTGATAACAATCAGACCAAT-------------------

----

GTGACTAATAACACAGAAACCTTCAGACCAGGAGGAGGAGATATGAGGGACAATTGGAGAAGTGAATTATATA
AATATAAAGTAGTAAAAATTGAACCATTA---GGAGTAGCACCCACCAAGGCAAAGAGAAGAGTGGTG-----
----------------------------------CAGAGAGAAAAAAGAGCA---ATGGGA---
TTCGGAGCT---------TTTTTCCTTGGGTTCTTGGGAGCA---GCAGGAAGCACTATG---
GGCGCAGCGTCA---ATAACGCTGACGGCACAG---GCCAGACAA---TTATTG---
TCTGGTATAGTGCAACAGCAAAAC---AATTTG---CTGAGG---
GCTATTGAGGCGCAACAACACCTGTTGCAACTCACAGTCTGGGGCATC
>JF320484
TTCTACTGTAATTTATCAAAACTGTTTAATAGTACTTGGACTAGTAATAGTACTTGGAATGATACTGGTACTT
GGAATGAG---
-------------------ACT---
GGAGAAAATGATACTATAACACTTCAATGTAGAATAAAACAAATTATAAACATGTGGCAGGAAGTAGGAAGAG
CAATGTATGCCCCTCCCATCCAGGGACAAATTAAATGTATATCAAAT---
ATCACAGGGCTGCTATTAACAAGAGATGGTGGTAATAGTAATGACACA-------------------------
----

AAAGATACAGAAATCTTCAGACCTGCAGGAGGAGATATGAGGGACAATTGGAGAAGTGAATTATATAAATATA
AAGTAGTAAAAATTGAACCATTA---GGAATAGCACCCTCCAAGGCAAAGAGAAGAGTGGTG-----------
----------------------------CAGAGAGAAAGAAGAGCA---GTAACG---CTGGGAGCA------
---ATGTTCCTTGGGTTCTTGGGAACA---GCAGGAAGCACTATG---GGCGCAGCGTCG---
ATGGCGCTGACGGTACAG---GCCAGACAA---TTATTG---TCTGGTATAGTGCAGCAGCAGAGC---
AATCTG---CTGAGG---GCTATTGAGGCGCAACAGCATCTGTTGCAACTCACAGTCTGGGGCATC
>EU577873
TTCTACTGTAATACAACAAAACTGTTTAATAGTACTTGGAATACTACTAGTATTTGGAATAATACTGCAGAGA
ATAATACTACA--
-------------GGGTCA------
AATGAAACTGAACCAATCACACTCCCATGCAGAATAAAACAAATTATAAATATGTGGCAGGAAGTAGGAAAAG
CAATGTATGCCCCTCCCATTAAAGGAAACATTAGCTGTTCATCAAAT---
ATTACAGGATTGATATTAACAAGAGATGGTGGTAACGTTAACGGGACC-------------------------
----

AATGGAACTGAAATCTTTAGACCTGCAGGAGGAAATATGAAGGACAATTGGAGGAGTGAATTATATAAATATA
AAGTAGTAAAAATTGAGCCATTA---GGAGTAGCACCCACCAAGGCAAAGAGAAGAGTGGTG-----------
----------------------------CAGAGAGAAAAAAGAGCA---GTGGGA---TTCGGAGCT------
---GTGTTCCTTGGGTTCTTGGGAGCA---GCAGGAAGCACTATG---GGCGCAGCGTCG---
GTGACGCTGACGGTACAG---GCCAGACAA---CTATTG---TCTGGTATAGTGCAACAGCAAAAC---
AATTTG---CTGAGA---GCTATTGAGGCGCAACATCATCTGTTGCAACTCACCGTTTGGGGCATC
>KT124778
TTCTACTGTAATACGTCACAACTGTTTAATAGTACTTGGCTGGGGAATGACACTGAAAGGTTAAACAGAACTG
AAGGGTCA---

GACAGCACTGAAGTAATCACACTCCAGTGTAGAATAAAACAAATTATAAACATGTGGCAGGAAGTAGGAAAAG
CAATGTATGCCCCTCCCATCGCAGGACAAATTAACTGTTCATCAAAT---
ATTACGGGGCTACTATTAACAAGAGATGGTGGTTACAATGATACT----------------------------
----

AGCCAGAACGAGACCTTCAGACCTGGGGGAGGAGATATGAGGGACAATTGGAGAAGTGAATTATATAAATATA
AAGTAGTAAGAATTGAACCATTA---GGAGTAGCCCCCACCAAGGCAAAGAGAAGAGTGGTG-----------
----------------------------CAAAGAGAAAGAAGAGCA---GTGGGA---TTAGGAGCT------
---GTGTTCCTTGGGTTCTTGGGAGCA---GCAGGAAGCACTATG---GGCGCAGCGTCA---
GTGACGCTGACGGTACAG---GCCAGACAA---TTATTG---TCTGGTATAGTGCAGCAGCAAAAC---
AATTTG---CTGAAG---GCTATTGAGGCGCAACACCATCTGTTGCAACTCACAGTCTGGGGCATC
>DQ410562
TTCTACTGTAATACAACACTGCTGTTTGATAGTACTTGGGATGATAATAATAGAAGTTTGAATTTGAATAAT-
----

ACTAATAGAAACATCACACTCCCATGCAAGATAAAACAAATTATAAACAGGTGGCAGGAAGTGGGAAAAGCAA
TGTATGCCCCTCCCATTCAAGGAGTAATTCAATGTTCATCAAAT---

ATTACAGGGCTGTTATTAGTAAGAGATGGTGGCAACACTGCTAACAATACT----------------------
----

GCTAACAAAACTGAGACCTTCAGACCTGGAGGAGGAAATATGAAGGACAATTGGAGAAGTGAATTATATAAAT
ATAAAGTAGTAAAAATTGAACCATTA---GGAGTGGCACCCACCAAGGCAAAGAGGAGAGTGGTG--------
-------------------------------CAGAGGGAAAAAAGAGCA---GTGGGA---ATAGGAGCT---
------ATGTTCCTTGGATTCTTAGGAGCA---GCAGGAAGCACTATG---GGCGCAGCGTCA---
ATAACGCTGACGGTACAG---GCCAGGCAA---TTATTG---TCTGGTATAGTGCAACAGCAGAAC---
AATCTG---CTGAGA---GCTATTGAGGCGCAACAGCATCTGTTGCAACTCACAGTCTGGGGCATC
>FJ469717
TTCTACTGTAATACAACACAGCTGTTTAATAGTACTTGGTTTACTAATGGCACTTGGATTAATGGTACTGTGT
TAAATACA---
-------------AATGAT------
ACTGACAAGGGAAACATCACACTCCCATGTAAAATAAAACAAATTATAAACATGTGGCAGGAAGTAGGAAAAG
CAATATATGCCCCTCCCATCAAAGGACCAATTAACTGTACATCAAAT---
ATTACAGGGATAATAATAACAAGAGATGGTGGTAATAACACGGGCGAAAATAAA-------------------
----

AATAAAACTTTCACTGAAACTTTCAGACCTGGAGGAGGAAATATGAGGGATAATTGGAAAAGTGAACTATATA
AATATAAAGTAGTACAGATTCAACCAATA---GAAATAGCACCCACCAGGGCAAAGAGAAGAGTGGTG-----
----------------------------------CAGAGAGAAAAAAGAGCA---
GTGGGAGCGCTGGGAGCT---------GTGTTCCTTGGGTTCTTGGGAGCA---GCAGGAAGCACTATG---
GGCGCAGCGTCA---ATAACGCTGACGGTACAG---GCCAGACAA---TTATTG---
TCTGGTATAGTGCAACAGCAGAAC---AATCTG---CTGAAG---
GCTATTGAGGCGCAACAGCATCTGTTGCAACTCACAGTCTGGGGCATC
>FJ469724
TTCTACTGTAATACAACACAACTGTTTAATAGTACTTGGAATAATGATACTTGGAATGATAATAAAACTGAA-
----

GGAAATGGCACAATTACACTCCAATGCAGAATAAAACAATTTATAAACCTGTGGCAGAAAGTAGGAAAAGCAA
TATATGCCCCTCCCATCCAAGGAAATATTAGCTGTTCATCAAAT---
ATTACAGGGCTGCTATTAACAAGGGATGGTGGTGTTAACGGGAGC----------------------------
----

GAGCACAACGAGACACTCAGACCTGGAGGAGGAGATATAAAAGACAATTGGAGAAGTGAATTATATCAATATA
AAGTAGTAAAAATTGAACCATTA---GGAATAGCACCCACTGCAGCAAAGAGAAGAGTGGTG-----------
----------------------------CAGAGAGAAAAAAGAGCA---GCAGGA---ATAGGAGCT------
---CTGCTCCTTGGGTTCTTGTCAGCA---GCAGGAAGCACTATG---GGCGCAGTGTCA---
GTGACGCTGACGGTACAG---GCCAGACAA---TTATTG---TCTGGTATAGTGCAACAGCAAAAC---
AATTTG---CTGAGA---GCTATTGAGGCGCAACAGCATCTGTTGCAACTCACGGTCTGGGGCATT
>FJ469747
TTCTACTGTAACTCAACACCACTGTTTAATAGTACTTGGATTGATGAATCATCAGGTATCGCTGAA-------
----

GGAGATGAGAACATCACACTTCAATGCAGAATAAAACAAATTATAAACATGTGGCAGGAAGTAGGAAAAGCAA
TGTATGCCCCTCCCATCAGAGGACAAATTAGATGTTCATCAAAT---
ATTACAGGGCTGCTATTAACAAGAGATGGTGGTCCTAGTAGTAATGAA-------------------------
----

ACCAACAGGAACGAGACCTTCAGACCGGGAGGAGGAGATATGAGGGACAATTGGAGAAGTGAATTATATAAAT
ATAAAGTAGTAAAAGTTGAACCATTA---GGAGTAGCACCCACCAGGGCAAAGAGAAGAGTGGTG--------
-------------------------------CAGAGAGAAAAAAGAGCA---GTGGGA---CTAGGAGCT---
------GTGTTCCTTGGGTTCTTGGGAGCA---GCAGGAAGCACTATG---GGCGCAGCGTCA---
CTGACGCTGACGGTACAG---GCCAGACAA---TTATTG---TCTGGGATAGTGCAACAGCAGAAT---
AATCTG---CTGAGA---GCTGTTGAGGCGCAACAGCATCTGTTGCAACTCACAGTCTGGGGCATC
>FJ653159
TTCTATTGTAATACAACACAACTGTTTAATAGTAATTGGAATGAGAGTAGTATTAATAGTACTTGGAATAGTA
CTTGGAAG---
-------------TCAAAT------
GACCCAAATGACACCATCACACTCCCATGCAGAATAAAACAAATTATAAACATGTGGCAAGAAGTGGGAAAAG

CAATGTATGCCCCTCCCATCAAAGGTGTAATTCAGTGTTCATCAAAT---
ATTACAGGGCTGCTATTAACAAGAGATGGTGGGAGTAATGAGACT----------------------------
----

AATACCACCGAGACTTTCAGACCTGGGGGAGGAGATATGAGGGACAATTGGAGAAGTGAACTATACAAATATA
AAGTAGTAAAAATTGAACCATTA---GGAGTAGCACCCACCAAGGCAAAAAGAAGAGTGGTG-----------
----------------------------CAAAGAGAAAAAAGAGCA---GTGGGAACAATAGGAGCT------
---ATGTTCCTTGGGTTCTTGGGAGCA---GCAGGAAGCACTATG---GGCGCAGCGTCA---
GTGACGCTGACGGTACAA---GCCAGACAA---TTATTG---TCTGGTATAGTGCAGCAGCAGAGC---
AATTTG---CTGCAG---GCTATTAATGCACAACAACATTTGCTGCAACTAACAGTCTGGGGCATC
>FJ653573
TTTTACTGTAATACAACACAACTGTTTAATAGTACTTGGAATATGACTGGTACCTGGAATGATACCTGGAATG
GTACTAAA---
-------------AGGTCA------
AATGAAACTGACAATATCACACTCCCATGCAGAATAAAACAAATAGTAAACAGGTGGCAGGAAGTAGGCAAAG
CAATGTATGCCCCTCCCATCAGAGGAAGCATTAGATGTTCATCAAAT---
ATTACAGGGCTGCTATTAACAAGAGATGGGGGGATTCATAATAGCACTGAG----------------------
----

GCAAATAACACTGAGATTTTCAGACCTGGAGGAGGAGACATGAGGGACAATTGGAGAAGTGAATTATATAAGT
ATAAAGTAGTGAAAATTGAACCATTA---GGAGTAGCACCCACCAAGGCAAGGAGAAGAGTGGTG--------
-------------------------------CAGAGAGAAAAAAGAGCA---GTGGGAGCACTAGGAGCT---
------ATGTTCCTTGGGTTCTTGGGAGCA---GCAGGAAGCACTATG---GGCGCAGCGTCA---
CTGACGCTGACGGTACAG---GCCAGACAA---TTGTTG---TCTGGTATAGTGCAACAGCAGAAC---
AACCTG---CTGAGA---GCTATTGAGGCGCAACAGCATCTGTTGCAACTCACAGTCTGGGGCATC
>FJ653360
TTCTACTGTAATACAACAGCATTGTTTAATAGTACTTGGAATAATGCTGGAGTATCAAATAGTACTTGGAATA
ATGCTGGAGTA--
-------TCAAATCACAGT------
ACAGGAAATGAAACAATCACACTCCAATGCAGAATAAAACAAATTATAAATATGTGGCAGGAAGTAGGAAAAG
CAATGTATGCCCCTCCCATTAGTGGACAAATTAAATGTTCATCAAAT---
ATTACAGGGCTGCTATTAACAAGGGATGGTGGTATTGATAATACAGGG-------------------------
----

AGCAACGAAACCGAGGTCTTCAGACCTGGAGGAGGAAATATGAAGGACAATTGGAGAAGTGAATTATATAAAT
ATAAAGTAATAAAAATTGAACCAGTA---GGAATAGCACCCACCAAGGCAAGGAGAAGAGTGGTG--------
-------------------------------CAGAGAGAAAAAAGAGCA---GCG------CTAGGAGCT---
------CTGTTCCTTGGGTTCTTGGGAATG---GCAGGAAGCACTATG---GGCGCAGCGTCA---
GTGACGCTGACGGTACAG---GCCAGGCTA---TTACTG---TCTGGTATAGTGCAACAGCAGAAC---
AATCTG---CTGAGG---GCTATTGAGGCGCAACAGCATCTGTTGCAACTCACAGTCTGGGGCATC
>FJ653426
TTCTACTGTAATACATCACCACTGTTTAATAGTACTTGGAATATTACTGAAGAGACAGATAATATTACTGGAA
AGACAGATGAC--
-------------AATAAT------
AAAAATGACACAATAATCACACTTCAATGCAGAATAAAACAAATTGTAAACATGTGGCAAAAAGTAGGAAGAG
CAATGTATGCCCCTCCCATCAAGGGACAAATTAGGTGTGCATCAAAC---
ATTACAGGGCTGCTATTAACAAGAGATGGTGGTAGTAACAAGAACGAT-------------------------
----

AGTGACACCGAGATCTTCAGACCTGGAGGTGGAGATATGAGGGATAATTGGAGAAGTGAATTATATAAATATA
AAGTAGTAGAGATTAAACCATTA---GGAATAGCACCCACCAAGGCAAAGAGAAGAGTGGTG-----------
----------------------------CAAAGAGAAAAAAGAGCA---GTGGGA---CTAGGAGCT------
---ATGTTCCTTGGGTTCTTAGGAGCA---GCAGGAAGCACTATG---GGCGCAGCGTCA---
GTGACGCTGACGGTACAG---GCCAGAAAT---TTATTG---TCTGGTATAGTGCAACAGCAGAAC---
AATTTG---CTGAAG---GCTATTGAGGCGCAACAACACCTGTTGCAACTCACAGTCTGGGGCATC
>EU578629
TTCTACTGCAATACAACAAAACTGTTCAGTAGTACTTGGAATAGTACTACTTGGGATAGTTGGAATAGTACTG
AAACA--
-------------------AAT---
AGCACTGACAGAAATATCACACTCCCATGCAGAATAAAACAAATTATAAACATGTGGCAGGAAGTAGGAAGAG

CAATGTATGCCCCTCCCATCAGAGGACAAATTAGATGCTCATCAAAT---
ATTACAGGGCTGATATTAACAAGAGATGGTGGTAACAATACAAAT----------------------------
----

ACAAACGAGACCTTCAGACCTCAAGGAGGAAACATGAAGGACAATTGGAGAAGTGAATTATATAAATATAAAG
TAGTAAAAATTGAACCACTA---GGAATAGCACCCACCAAGGCAAAGAGAAGAGTGGTG--------------
-------------------------CAGAGAGAAAAAAGAGCA---GTGGGA---ATGGGAGCT---------
TTGTTCCTTGGGTTCTTGGGAGCA---GCAGGAAGCACTATG---GGCGCAGTGTCG---
ATGACGCTGACGGTACAG---GCCAGACAA---TTATTG---TCTGGTATAGTGCAACAGCAGAAT---
AATCTG---CTGAGA---GCTATTGAGGCGCAACAGCATCTGTTGCAACTCACAGTCTGGGGCATC
>HQ217860
TTCTACTGTAATACAACAAAACTATTTAATAGTACTTGGGATATAGAAACAATACTTAAGCATACTAAAAATA
GTAAT--

GACACAAACTCAACTATTACACTCCAATGCAGAATAAAACAAATTGTAAACATGTGGCAGGAAGTAGGAAAAG
CAATGTACGCCCCTCCCATCAGAGGAATAATTAACTGTACATCAAAT---
ATTACAGGGCTGCTATTAACAAGAGATGGGGGTCATAACAACAGC----------------------------
----

ACCAACGAGACCTTCAGACCGGGAGGAGGAGATATGAGAGACAATTGGAGAAGTGAATTATACAAATATAAAG
TAGTAAAAGTTGAACCATTA---GGAATAGCACCCACCAAGGCAAAAAGAAGAGTGGTG--------------
-------------------------CAAAGAGAGAAAAGAGCA---GTGGGA---ATAGGAGCT---------
GTGTTCCTTGGGTTCTTGGGAGCA---GCAGGAAGCACTATG---GGCGCAGCGACA---
GTGACGCTGACGGTACAG---GCCAGACAA---TTATTG---TCTGGTATAGTGCAACAGCAGAGC---
AATTTG---CTGAGA---GCTATTGAGGCGCAACAGCATCTGTTGCAGCTCACAGTCTGGGGCATC
>JF689865
TTCTACTGTAATACAACAAAACTGTTTAATAGTACTTGGCCTTGGAATGTTACTGGGTCAAATGTTACTGGG-
----

TTAAATGACACAATCACAATTCCATGCAGAATAAAACAAATTATAAACATGTGGCAGGAAGTAGGAAAAGCAA
TGTATGCCCCTCCCATCCAAGGACAAATTAGATGTTCATCAAAT---
ATTACAGGGCTACTATTAACCAGAGATGGTGGTAATACAAATGAGAGC-------------------------
----

AATACCACCGAGACCTTCAGACCTGGAGGAGGAGATATGAGGGACAATTGGAGAAGTGAATTATATAAATATA
AAGTAGTAAAAATTGAACCATTA---GGAGTAGCACCCACCAAGGCAAAGAGAAGAGTGGTG-----------
----------------------------CAGAGAGAAAAAAGAGCA---GTGGGA---CTAGGAGCT------
---CTGTTCCTTGGGTTCTTGGGAGCA---GCAGGAAGCACTATG---GGCGCAGCGTCA---
ATGACGCTGACGGTACAA---GCCAGGCAA---TTATTG---TCTGGTATAGTGCAACAGCAAAAC---
AATTTG---CTGAGG---GCTATTGAAGCGCAACATCACCTGTTGCAACTCACAGTCTGGGGCATT
>JF689874
TTCTACTGTAATACAACAAAGCTGTTTACAAAGCGGTTTGATTGGAATAGGACTGAAGGGTTAAATGACACTG
CAGGAAAT---
-------------------AAC---
ACTGGAGGAAATAACATCATAATCCCATGCAGAATAAAACAAATTATAAACATGTGGCAAGAAGTAGGAAAAG
CAATATATGCCCCTCCCATCAGTGGACGAATTAACTGTACATCAAAC---
ATTACTGGGCTGCTATTAATAAGAGATGGTGGGATAGGCCCGGGGACA-------------------------
----

GACAATACTAGTGAAACCTTCAGACCTCAAGGAGGGAATATAAGAGATAATTGGAGAAGTGAATTATATAAAT
ATAAAGTAGTAAAAATTGAACCATTA---GGACTAGCGCCCACCAAGGCAAGGAGAAGAGTGGTG--------
-------------------------------CAGAGAGAAAAAAGAGCA---ATGGGA---CTAGGAGCT---
------TTGTTCCTTGGGTTCTTGGGAGCA---GCAGGAAGCACTATG---GGCGCAGCGTCA---
CTGACGCTGACGGTACAG---GCCAGACAA---TTATTG---TCTGGTATAGTGCAACAGCAGAAC---
AATTTG---CTGAGA---GCTATTGAGGCGCAACAGCATATGTTGCAACTCACAGTCTGGGGCATC
>JF689875
TTCTACTGTAATACAACACAACTGTTTAATAGTACTTGGAATGAGAATAGTACTAAAGAAGGAAATGGA----
----

AATGACACAATAACCACACTCCCATGCAGAATAAAACAAATTATAAACATGTGGCAAGAAGTAGGAAAAGCAA

TGTATGCCCCTCCCATCAGAGGAAACATTAGCTGTACATCAAAT---
ATTACAGGGCTGCTATTAACAAGAGATGGTGGGAATAAT-------------------------------
----

GAGACCGAGACCTTCAGACCTGGAGGAGGAAATATGAAGGATAATTGGAGAAGTGAATTATATAAATATAAAG
TAGTAAAAATTCAACCATTA---GGAGTAGCACCCACCAAGGCAAAGAGAAGAGTGGTG-------------
------------------------CAGAGAGAAAAAAGAGCA---GTGGGA---CTAGGAGCT---------
GTGTTCCTTGGGTTCTTGGGAGCA---GCAGGAAGCACTATG---GGCGCAGCGTCA---
ATAACGCTGACGGTACAG---GCCAGACAA---TTATTG---TCTGGTATAGTGCAACAGCAGAAC---
AATTTG---CTGAGG---GCTATTGAGGCGCAACAGCATCTGTTGCAACTCACAGTCTGGGGCATT
>JX863990
TTCTACTGTAATACAACACAACTGTTTAATAGTACTTGGAATTCTACTACTTTGAATTCTACTAGTACT----
----

GGGAATGGGACCATCACACTCTCATGTAGAATAAAACAAATTATAAACAGGTGGCAGGAAGTAGGAAAAGCAA
TGTATGCTCCTCCCATCAGAGGACAAATTAAGTGTTCATCAAAT---
ATTACAGGGCTACTGTTAACAAGAGATGGTGGTCACACAAACAACACC------------------------
----

AACAAAACTGAGACCTTTAGACCTGGAGGAGGAGACATGAGGGACAATTGGAGAAGTGAATTATACAAATATA
AAGTAGTAAAAATTGAACCATTA---GGAATAGCACCCACCAAGGCAAGGAGAAGAGTGGTG----------
---------------------------CAGAGAGAAAAAAGAGCA---ATAGGA---ATAGGAGCT------
---GTGTTCCTTGGGTTCTTGGGAACA---GCAGGAAGCACTATG---GGCGCAGCGGCA---
GTGACGCTGACGGTACAA---GCCAGACAA---TTACTG---TCTGGTATAGTGCAACAGCAAAAC---
AATTTG---CTGAGG---GCTATTGAGGCGCAGCAGCATCTGTTGCAACTCACAGTCTGGGGCATC
>JF320363
TTCTACTGTAACACAGCAAAACTGTTTGATACATCACAACTACAACTGTCTAATAGCAGCACTGAA-------
----

GAAAATGACACACTCACAATTCCATGCAGAATAAAACAAGTTATAAACAGGTGGCAGGAAGTAGGAAAAGCAA
TGTATGCCCCTCCCATCAGAGGACAAATTAAATGTGTATCAAAC---
ATTACAGGGCTGCTATTAACAAGAGATGGTGGAAAGACTGAGGATAATACT---------------------
----

ACTAATACTACTGAGGTCTTCAGACCTCAAGGAGGAAATATGAAAGATAATTGGAGAAGTGAATTATATAAAT
ATAAGGTAGTAAGAACTGAACCATTA---GGAGTAGCACCCACCAAGGCAAAGAGAAGAGTGGTG--------
------------------------------CAGAGAGAAAAAAGAGCA---ATAGGGGGATTAGGAGCG---
------TTGTTCCTTGGGTTCTTGGGAGCA---GCAGGAAGCACTATG---GGCGCAGCGTCA---
ATAACGCTGACGGCACAG---GCCAGACAA---ATATTG---TCTGGTATAGTGCAACAGCAGAAT---
AATTTG---CTGAGA---GCTATTGAGGCGCAACAACATCTGTTGCAACTCACAGTCTGGGGCATC
>JF320036
TTCTACTGTAATACAACACAGCTGTTTAATATTAGTAGTACACAACTGTTTAATAGTACTTGGAATAGTACCG
AA--
------------------GGG---
CTAAACACAAAAAATATCACACTCCAATGCAGAATAAAACAAATTATAAACATGTGGCAGCAAGTAGGAAAAG
CAATGTATGCCCCTCCCATCAGAGGACCAATTAAATGTATATCAAAT---
ATTACAGGGCTGCTGTTAACAAGAGATGGTGGGAAGATAAATGAGAGTAGG---------------------
----

GCAAATGAGACTGAGACCTTCAGACCTGGAGGAGGAGATATGAGGGACAATTGGAGAAGTGAACTCTATAAAT
ATAAAGTAGTAAAAATTAAACCATTA---GGAGTAGCACCCACTAAGGCAAAAAGAAGAGTGGTG--------
------------------------------CAGAGAGAAAAAAGAGCA---GTGGGA---CTAGGAGCA---
------TTGTTCCTTGGGTTCTTGGGAGCA---GCAGGAAGCACTATG---GGCGCAGCGTCA---
ATAACGCTGACGGTACAG---GCCAGACAA---TTATTG---TCTGGTATAGTGCAACAGCAGAAC---
AATCTG---CTGAGG---GCTATTGAGGCGCAACAGCACCTGTTGCAACTCACAGTCTGGGGCATC
>JF320502
TTCTACTGTAATACAACAAAACTGTTTAATAGCATTTGGAATGAAACACAACTGTTTAATAGTACTTGGAATG
AGACT---

GAAAGAGGAGTCAATATCACACTCCCATGTAGAATAAAACAAATTATAAATATGTGGCAGGAAGTAGGAAAAG

CAATGTATGCCCCTCCCATCAGAGGAATAATTCAATGTACATCAAAC---
ATTACAGGACTGATATTAACAAGAGATGGTGGTAAAAAAAATAATGGGAGTGAA-------------------
----

ACCTCCAATGGGAATGAAACCTTCAGACCTGGAGGAGGAGATATGAGAGACAATTGGAGAAGTGAATTATATA
AATATAAAGTAGTAAAAGTTGAACCATTA---GGAATAGCACCCACTAGGGCAAGGAGAAGAGTGGTG-----
----------------------------------CAGAGAGAGAAAAGAGCA---GTGGGA---
ATAGGAGCT---------GTGTTCCTTGGGTTCTTGGGAGCA---GCAGGAAGCACTATG---
GGCGCAGCGTCA---ATAACGCTGACGGTACAG---GCCAGACAA---TTATTG---
TCTGGTATAGTGCAACAGCAGAAC---AATCTG---CTGAAA---
GCTATTGATGCGCAACAGCATCTGTTGCAACTCACAGTCTGGGGCATC
>JF320151
TTCTACTGTAATACAACAGAGCTGTTTAATGATACTTGGCTGAGTAATAATACTTGGCTGTCAAATTATACTG
AGAGTAAT---
-------------------AAT---
AATACTGCTAAAAATATCGTACTCCCATGCAGAATAAAACAAATTATAAACAGATGGCAGGAAGTAGGAAAAG
CAATGTATGCCCCTCCCATCAGTGGACTAATTAGATGCTCATCAAAT---
ATTACAGGGCTGCTCTTAACAAGAGATGGTGGTAATCATAACACTAGT-------------------------
----

AATGACACCGAGACTTTCAGACCAGGAGGAGGAGATATGAGGGATAATTGGAGAAGTGAATTATATAAATATA
AAGTAGTAAAAATTGAGCCATTA---GGAGTAGCACCCACCAAGGCAAGGAGAAGAGTGGTG-----------
----------------------------CAGAGAGAAAAAAGAGCA---GTGGGA---CTAGGAGCT------
---TTGTTCCTTGGGTTCTTGGGAGCA---GCAGGAAGCACTATG---GGCGCAGCATCA---
GTGACGCTGACGGTACAA---GCCAGACAA---TTATTG---TCTGGTATAGTGCAACAGCAAAAC---
AATTTG---CTGAGG---GCTATTGAGGCGCAACAGCATATGTTGCAACTCACAGTCTGGGGCATT
>JF320145
TTCTACTGTAATTCAACAAAACTGTTTAGCAGTGCTTGGTATACTAATAATACTAATAGTAGTAATACTGAAG
GG---

TCAAATGACACTGTTATCATACTTCCATGCAAAATAAAACAAATTATAAACATGTGGCAGGAAGTAGGAAGAG
CAATGTATGCCCCTCCCATCGCAGGAAACATTAGCTGTTCATCAAAT---
ATTACAGGACTGCTGTTAACAAGAGATGGTGGTCACACGGAAACTGCC-------------------------
----

AACACCACAGAAATCTTCAGACCTGGAGGAGGAGATATGAGGGACAATTGGAGAAGTGAATTATATAAATATA
AAGTAGTAAAAATTGAACCATTA---GGAGTAGCACCCACCAAGGCAAGGAGAAGAGTGGTG-----------
----------------------------CAGAGAGAAAAAAGAGCA---GTAACA---GTGGGAGCT------
---GTGTTCCTTGGGTTCTTGAGTGCA---GCAGGAAGCACTATG---GGCGCAGCGTCA---
CTGACGCTGACGGTACAG---GCCAGACTA---CTGTTG---TCTGGTATAGTGCAGCAGCAGAGC---
AATCTG---CTGAGG---GCTATTGAGGCGCAACAACAACTGTTGCAACTCACAGTCTGGGGCATC
>HQ908232
TTCTACTGTAATACAACAAGGCTGTTTAATAGTACTTGGCAGTCCAATAGTACGGGGAATGATACTGGAGGGT
CA---

AATAACACTGAACCTATCACACTCCTATGCAGAATAAAACAAATTGTAAACAGGTGGCAAGAAGTAGGAAAAG
CAATGTATGCCCCTCCCATCAGAGGACAAATTTACTGTAGATCAAAT---
ATTACAGGGCTGCTATTAACAAGGGATGGTGGTAATGTCCAGAAC----------------------------
----

AACATGACTGAGACCTTCAGACCAGGAGGAGGAGATATGAGGGACAATTGGAGAAGTGAATTATATAAATATA
AAGTAGTAAAAATTCAGCCATTA---GGAATAGCACCCACCGGGGCAAAGAGAAGAGTGGTG-----------
----------------------------CAGAGAGAAAAAAGAGCG---GTGACA---CTGGGAGCT------
---ATGTTCCTTGGGTTCTTGGGAGCA---GCAGGAAGCACTATG---GGCGCAGCGTCA---
ATAACGCTGACGGTACAG---GCCAGACAA---TTACTG---TCTGGTATAGTGCAACAGCAGAAC---
AATCTG---CTGAAG---GCTATTGATGCGCAGCAGCATATGTTGCAACTCACCGTCTGGGGCATC
>KM353665
TTCTACTGTAATACAACAAAATTGTTTAATAGTACTTGGTATAATAATAGT----------------------
----
-ACTTGGAATAATACT---------

GGAGGAAATGACACATTCACACTCCCATGTAGAATAAAACAAATTATAAACATGTGGCAGACAGTAGGAAGAG
CAATGTATACCCCTCCCATCAGAGGATTAATTAAGTGTACATCAAAT---
ATTACAGGGCTGATATTAACAAGAGATGGTGGG--

GGTGGTAACGAGAGTAATCAT---------
ACCACAGAGGTCTTCAGACCTGCAGGAGGAGATATGAGGGACAATTGGAGGAGTGAATTATATAAATACAAAG
TAGTAAAAATTGAACCATTA---GGAATAGCACCCACCAGGGCAAAAAGAAGAGTGGTG--------------
-------------------------CAGAGAGAAAAAAGAGCA---GTGGGG---ATGGGAGCT---------
CTGTTCCTTGGGTTCTTAGGAGCA---GCAGGAAGCACTATG---GGCGCAGCGTCA---
ATAACGCTGACGGTACAG---GCCAGACAA---TTATTG---TCTGGTATAGTGCAACAGCAGAGC---
AATTTG---CTGAAG---GCTATTGAGGCGCAACAGCATCTGTTGCAACTCACAGTCTGGGGCATC
>JN687750
TTCTACTGTGATACAAAAAAGCTGTTTAATAGTACTTGGTGGGTTAATAATACTTGGAAAGGCACTAAAGGGG
ATGAG--

ACTAGGAATGGCACAATCGAACTCCCATGCAGAATAAAACAAATTATAAACATGTGGCAGGAAGTAGGAAAAG
CAATGTATGCCCCTCCCATCAGAGGACAAATTCAGTGTTCATCAAAT---
ATTACAGGGCTGCTATTAACAAGAGATGGGGGTAATAATGAAACG----------------------------
----

AACGGAACAGAAACCTTCAGACCTGGAGGAGGAGATATGAGGGACAATTGGAGAAGTGAATTATATAAATATA
AAGTAGTAAAAATTGAGCCATTA---GGAATAGCACCCGGCAGGGCAAAGAGAAGAGTGGTG-----------
----------------------------CAGAGAGAAAAAAGAGCA---GTGGGA---CTAGGAGCT------
---ATGTTCCTTGGGTTCTTGGGAGCA---GCAGGAAGCACTATG---GGCGCAGCGTCA---
ATAACGCTGACGGTACAG---GCCAGACAA---TTACTG---TCTGGTATAGTGCAACAGCAGAAC---
AACTTG---CTGAGA---GCTATTGAGGCGCAGCAGCATATGTTGCAACTCACAGTCTGGGGCATC
>HM204582
TTTATTGTAATACATCAGAACTGTTTAAAAGTACCTGGAATAGTACTGATACCTTCAACAGTACTGGAGAGA
GA---

GGGAATGACACAATCATTCTCCCATGCAAAATAAGGCAAGTTGTAAACATGTGGCAGGAAGTAGGAAAAGCAA
TGTATGCCCCTCCTATCAGAGGTCAAATTAACTGTTCATCAAAT---
ATTACAGGGCTGCTATTAACAAGAGATGGTGGTGATAAAAATAGT----------------------------
----

ACAAACGAGACCTTCAGACCTGGAGGAGGAGATATGAGGGACAATTGGAGAAGTGAATTATATAAATATAAGG
TAGTAAAAATTGAACCATTA---GGAATAGCACCCACCAAGGCAAAGAGAAGAGTGGTG--------------
-------------------------CAAAGAGAAAAAAGAGCA---GTGGGA---ATAGGAGCT---------
TTGTTCCTTGGGTTCTTGGGAGCA---GCAGGAAGCACTATG---GGCGCAGCGTCA---
CTGACGCTGACGGTACAA---GCCAGACTA---TTATTG---TCTGGTATAGTGCAACAGCAGAAC---
AATTTG---CTGAGG---GCTATTGAGGCGCAACAGCATCTGTTGCAACTCACGGTCTGGGGCATC
>HM204589
TTCTACTGTAATTCAACACAATTGTTTAATAGTACTTGGGATAAGAATGGTAGTAGAATAAATGGAACTGAAG
GA---

GAAAGTGGTACCATCACACTCCCATGCAGAATAAAACAAATTATAAACATGTGGCAGGAAGTAGGAAAAGCAA
TGTATGCCCCTCCCATCAGAGGACAAATTTATTGTTCATCAAAT---
ATTACAGGGATGCTATTAACAAGAGATGGTGGTACTAATGATACTGAC-------------------------
----

AAAAACGACACCGAGACCTTCAGACCTGGAGGAGGAGATATGAGGGATAATTGGAGAAGTGAATTATATAAAT
ATAAAGTAGTAAAAATTGAGCCATTA---GGAGTAGCACCCACCAAGGCAAAGAGAAGAGTGGTG--------
-------------------------------CAGAGAGAAAAAAGAGCA---GTGGGA---TTGGGAGCT---
------CTGTTCCTTGGGTTCTTGGGAGCA---GCAGGAAGCACTATG---GGCGCAGCGTCA---
GTGACGCTGACGGTACAG---GCCAGACAA---TTATTG---TCTGGTATAGTGCAACAGCAGAAC---
AATCTG---CTGAGG---GCTATTGAGGCGCAACAGCATCTGTTGCAACTCACAGTCTGGGGCATC
>KY112061
TTCTACTGTAATTCAACACAGCTGTTTAATAGTGCTTGGCCTTGGAATGATACTGAAGGGTCAAATGACGCT-
----

```
AAAAAAGATAATATCACACTCCCATGCAAAATAAAACAAATTATAAACATGTGGCAGGGAGTAGGAAAAGCAA
TGTATGCCCCTCCCATCGAAGGAAAAATTAAATGCTCATCAAAT---
ATTACAGGACTGCTATTAACAAGAGATGGTGGTTATGAGAACAAT----------------------------
-------------------------------------------------------------------------
-------
GAGACCAAGCCCGAGATCTTCAGACCTGGAGGAGGAGATATGAGGGACAATTGGAGGAGTGAATTATATAAAT
ATAAAGTAGTAAAAATTGAACCATTG---GGAGTAGCACCTACCAAGGCAAAGAGAAGAGTGGTG--------
-------------------------------CAGAGAGAAAAAAGAGCA---TTTGGA---CTAGGAGCT---
------GTGTTCCTTGGGTTCTTGGGAGCA---GCAGGAAGCACTATG---GGCGCAGCATCA---
ATAACGCTGACGGTACAG---GCCAGACAA---TTATTG---TCTGGTATAGTGCAACAGCAGAAC---
AATTTG---CTGAGG---GCTATTGAGGCGCAACAGCATCTGTTGCAACTCACAGTCTGGGGCATC
>EU576345
TTCTACTGTGATACAACAAAACTGTTTAATAGTACTTGGGGGAATAGTACTTGGGGGTATAAAATGACAAATG
AC-----------------------------------------------------------------------
-------------------------
ACTGCAAAAGACAACATCACACTCCCATGTAGAATAAAACAAATTATAAACATGTGGCAGGAAGTAGGAAAAG
CAATGTATGCCCCTCCCATCAAAGGACAAATTAGATGTACATCAAAT---
ATTACAGGGTTGGTGTTAACAAGAGATGGTGGTGGTGGTAGTGGTACA-------------------------
-------------------------------------------------------------------------
-------
AATAACACCACAGAGATCTTCAGACCTGGAGGAGGAGATATGAGGGACAATTGGAGAAGTGAATTATATAAAT
ATAAAGTAGTAAGAATTGAACCATTA---GGAATAGCACCCACCAAGGCAAAGAGAAGAGTGGTG--------
-------------------------------CAGAGAGAAAAAAGAGCA---GCA------ATAGGAGCT---
------ATGTTCCTTGGGTTCTTGGGAGCA---GCAGGAAGCACTATG---GGCGCAGCGTCA---
ATAACGCTGACGGTACAG---GCCAGACTA---TTATTG---TCTGGTATAGTGCAACAGCAGAAC---
AATCTG---CTGAGG---GCTATTGAGGCGCAACATCAACTGTTGCAACTCACAGTTTGGGGCATC
>EU576383
TTCTACTGTAATTCAACACAACTGTTTAACAGTACTTGGCATAGTAACAGTACTTGGAATGATACTGGAAAGT
CAGAA--------------------------------------------------------------------
-------------------------
AATAATACAAAAATCATCACACTCCCATGCAGAATAAAACAAATTATAAACATGTGGCAGGAAGTAGGAAAAG
CAATGTATGCCCCTCCTATCAGAGGACAAATTAGATGTTCATCAAAT---
ATTACAGGGCTGATACTAACAAGGGATGGAGGTCATGGAGGTAATGACAAT----------------------
-------------------------------------------------------------------------
-------
AACCAGACCACCGAGACCTTCAGACCTGGAGGAGGAAATATGAAGGACAATTGGAGAAGTGAATTATATAAAT
ATAAAGTAGTAAAAATTGAACCATTA---GGAGTAGCACCCACCCCGGCAAAAAGAAGAGTGGTG--------
-------------------------------CAGAGAGAAAAAAGAGCA---TTAGTGGGATTCGGAGCT---
------CTGTTCCTTGGGTTCTTGGGAGCA---GCAGGAAGCACTATG---GGCGCAGCGTCA---
ATAACGCTGACGGTACAG---GCCAGACAA---TTACTG---TCTGGTATAGTGCAACAGCAAAAC---
AATTTG---CTGAAG---GCTATTGAGGCGCAACAGCATCTGCTGCAACTCACAGTCTGGGGCATC
>KX129209
TTCTACTGTAATTCAACACAACTGTTTAATAGTACGTGGATTGGGAATAGCACTGAAGAGTCAAAT-------
-------------------------------------------------------------------------
----------------------------
GGCACTGAAAACATCACACTCCCATGTAGAATAAAACAATTTGTAAATCTGTGGCAGGAAGTAGGAAGAGCAA
TGTATGCCCCTCCCATCAGAGGACTAATTAAATGTTCATCAAAT---
ATTACTGGGCTGTTGTTAACGAGAGATGGTGGTCAGTCTGACAATACT-------------------------
-------------------------------------------------------------------------
-------
ACCAACGGGACCGAGACCTTTAGACCTGGAGGAGGAAATATGAAGGACAATTGGAGAAGTGAATTATATAAAT
ATAAAGTAGTACAAATTGAACCATTA---GGAATAGCACCCACCAAGGCAAAGAGAAGAGTGGTG--------
-------------------------------CAGAGAGAAAAAAGAGCA---GTGGGA---CTAGGAGCT---
------TTGTTCCTTGGGTTCTTGGGAGCA---GCAGGAAGCACTATG---GGCGCAGCGTCA---
GTGACGCTGACGGTACAG---GCCAGACAA---TTATTG---TCTGGTATAGTGCAGCAGCAGAAC---
AACTTG---CTGAGA---GCTATTGAGGCGCAACAACATATGTTGCAACTCACAGTCTGGGGCATC
>KM259534
TTCTACTGTAATACAACAAAACTGTTTAATAGTACTTGGAATGGGACTAATATT-------------------
-------------------------------------------------------------------------
-GATATTGGAAACAGT---------
```

ACGAAAGATGACAATATCACACTCCCATGCAGAATAAAACAAATTATAAATAGGTGGCAAGAGGTAGGAAAAG
CAATGTATGCCCCTCCCATCAGAGGACAAATTAGATGTTCATCAAAC---
ATTACAGGGCTGCTATTAACAAGAGATGGTGGT--

AATAGCAATAACACCAACGAG---------
ACCACCGAGACCTTCAGACCTACAGGAGGAAATATGGAGGACAATTGGAGAAGTGAATTATATAAATATAAAG
TAGTGCAAATTCAACCATTA---GGAGTAGCACCCACCGAGGCAAAGAGAAGAGTGGTG--------------
-------------------------CAGAGAGAAAAAAGAGCA---GTGGGA---TTAGGAGCT---------
ATGTTCCTTGGGTTCTTGGGAGCA---GCAGGTAGCACTATG---GGCGCAGCGTCA---
ATAACGCTGACGGTACAG---GCCAGACAA---TTACTG---TCTGGTATAGTGCAACAGCAGAAC---
AATTTG---CTGAGG---GCTATTGAGGCGCAACAGCATATGTTGCAACTCACAGTCTGGGGCATC
>HQ217957
TTCTACTGTAATACATCAGAACTGTTTAATAGTACCTGGAATATAACACACTTTAATAGTACCTGGAATGATA
CT---

GAAGGATCAAATAACATCACACTCCCATGCAGAATAAAACAAATTATAAATATGTGGCAACAAGTAGGAAAAG
CAATGTATGCCCCTCCCATCCAAGGATTAATTCAATGTTCATCAAAT---
ATTACAGGGCTGCTATTAACAAGAGATGGAGGTAATAATAGTACT----------------------------
----

AACGAGATCTTCAGACCTGGAGGAGGGGATATGAGGGACAATTGGAGAAGTGAATTATATAGATATAAAGTAG
TAAGAATTGAACCATTA---GGAGTAGCACCCACCAAGGCAAGGAGAAGAGTGGTG-----------------
----------------------CAAAGAGAAAAAAGAGCA---GTGGGAACAATAGGAGCT---------
ATGTTCCTTGGGTTCTTGGGAGCA---GCAGGAAGCACTATG---GGCGCAGCGTCA---
GTGACGCTGACGGTACAG---GCCAGACAA---TTATTG---TCTGGAATAGTGCAACAGCAAAAC---
AATTTG---CTGAAG---GCTATTGAGGCGCAACAACACCTGTTGCAACTCACAGTCTGGGGCATC
>HQ217984
TTCTACTGTAATTCAACACAACTGTTTAATAGTACTTGGACTAGGGAAAATGGTAGTTGGACTAGGAATGATA
CCGCT--
-------------------GCA---
AATGAGACTGAGATTATCACACTCCCATGCAGAATAAAACAAATTATAAACATGTGGCAGGAAGTAGGAAAAG
CAATGTATGCCCCTCCCATCAAAGGACTTATTCAATGTTCATCAAAT---
ATTACAGGATTGTTATTAACAAGAGATGGTGGTAATAATAGCAAC----------------------------
----

ATCAATGAGATCTTTAGACCTGGAGGAGGAGATATGAGGGACAATTGGAGAAGTGAATTGTACAAATATAAGG
TAGTAAAAATTGAACCATTA---GGAGTAGCACCCACCAAGGCAAGGAGGAGAGTGGTG--------------
-------------------------CAAAGAGAGAAAAGAGCA---GTGGGA---CTAGGAGCT---------
CTGTTCCTTGGGTTCTTGGGAGCA---GCAGGAAGCACTATG---GGCGCAGCGTCA---
TTGACGCTGACGGTACAG---GCCAGACAA---TTATTG---TCTGGTATAGTGCAACAGCAGAAC---
AATATG---CTGAGG---GCTATTGAGGCGCAGCAGCACCTGTTGCAACTCACAGTCTGGGGCATC
>JF689886
TTCTACTGTAATTCAACACAACTGTTTAATAGTACTTGGACTAAATATGATAATGGAACTTGGGTTAAGAAGA
ATGGCACT---
-------------------GAA---
TGGGTGAATGATACAATCACACTCCCATGCAGAATAAAACAAATTATAAACATGTGGCAGGAAGTAGGAAAAG
CAATGTATGCCCCTCCCATCAGGGGACTAATTAATTGTATATCAAAT---
ATTACAGGGATACTGCTAACAAGAGATGGTGGTCAGGTTAATGGT----------------------------
----

ACCAATGAGACCTTCAGACCAGGAGGAGGAGATATAAAGGATAATTGGAGAAGTGAATTATATAAATATAAAG
TAGTAAAAATTGAACCATTA---GGAGTAGCACCCACCAAGGCAAGGAGAAGAGTGGTG--------------
-------------------------CAGAGGGAAAAAAGAGCA---ATTGGA---ATAGGAGCT---------
TTGTTCCTTGGGTTCTTGGGAGCA---GCAGGAAGCACTATG---GGCGCAGCGTCA---
GTGACGCTGACGGTACAA---GCCAGACAA---TTATTG---TCTGGTATAGTGCAACAGCAGAAC---
AATTTG---CTGAGG---GCTATTGAGGCGCAACAACATCTGTTGCAACTCACAGTCTGGGGCATC
>JF320385
TTCTACTGTAATACAACACAACTGTTTAATAGTACTTGGATTCGGAATGATACTAAATGGTCAAGTAACTCTA
CA---

GGAAATGGCACCATTATACTCCCATGCAGAATAAAACAAATTATAAACATGTGGCAGGAAGTAGGAAAAGCAA
TGTATGCCCCTCCTATCAGAGGAAACATTAGCTGTTCATCAAAT---
ATTACAGGGCTGCTATTAACAAGAGATGGGGGCAATAGTAATCAAACGACC----------------------
----

CAGACCAAAGAAACAGAAACCTTCAGACCTGGAGGAGGAGATATGAGGGACAATTGGAGAAGTGAATTATATA
AATATAAAGTAGTAAAAATTGAACCATTA---GGAGTAGCACCCACCAAGGCAAAGAGAAGAGTGGTG-----
----------------------------------CAGAGAGAAAAAAGAGCA---GTGGGA---
ATAGGAGCA---------ATGTTCCTTGGATTCTTGGGAGCA---GCAGGAAGCACTATG---
GGCGCAGCGTCA---CTAACGCTGACGGTACAG---GCCAGACAA---TTATTG---
TCTGGTATAGTGCAGCAGCAGAAC---AATCTG---CTGAGA---
GCTATTGAGGCGCAACAGCATCTGTTGCAACTCACAGTCTGGGGCATC
>JF320150
TTCTACTGTAATACAACACAACTGTTTAATAGTACTTGGAATACTACTGGGAGTGTGACTAATGGGTCAAATA
ACAGT--
-------------------CAG---
GGAGAAAATGGTACTATCACACTCCCGTGCAGAATAAAACAAATTATAAACATGTGGCAGGAAGTAGGAAAAG
CAATGTATGCCCCTCCAATCAGTGGAGAAATTAAATGTACATCAAAT---
ATTACAGGACTGCTATTAACAAGAGATGGTGGTAATAGTAGCGAC----------------------------
----

AACGAGACCTTCAGACCTGGAGGAGGAGATATGAGGGACAATTGGAGAAGTGAATTATATAGATATAAAGTAG
TAAAAATTGAACCATTA---GGAATAGCACCCACCAAGGCACAAAGAAGAGTGGTG-----------------
----------------------CAGAGAGAAAAAAGAGCA---GCGGGA---CTAGGAGCG---------
TTGTTCCTTGGGTTCTTGGGAGCA---GCAGGAAGCACTATG---GGCGCAGCATCA---
ATAATGCTGACGGTACAG---GCCAGACAA---TTGTTG---TCTGGTATAGTGCAACAGCAGAAC---
AATCTG---CTGAGG---GCTATTGAGGCGCAACAGCATCTGTTGCAACTCACAGTCTGGGGCATC
>JF320539
TTCTACTGTAATACAACACAATTGTTCAATAATACTTGGAAAGGTACTGAAGAAGAGAATAGTACTAGGAAT-
----

AGTAATGACACCATCACACTCCCATGCAGAATAAAACAAATTGTAAACATGTGGCAGGAAGTAGGAAAAGCAA
TGTATGCCCCTCCCATCAGAGGACTAATTCAATGTACATCAAAC---
ATTACAGGGATAATATTAACAAGAGATGGTGGTAATAACACGAAT----------------------------
----

ACCACTGAGATCTTCAGACCAGGAGGAGGAGACATGAGGGACAATTGGAGAAGTGAATTATATAAATATAAAG
TAGTAAAAATTGAACCATTA---GGAATAGCACCCACTAGGGCAAAGAGAAGAGTGGTG--------------
-------------------------CAGAGAGAAAAAAGAGCA---GTGGGA---ATAGGAGCT---------
TTGTTCCTTGGGTTCTTGGGAGCA---GCAGGAAGCACTATG---GGCGCAGCGTCA---
ATGACGCTGACGGTACAG---GCCAGACTA---TTATTG---TCTGGTATAGTGCAACAGCAGAAC---
AATCTG---CTGCAG---GCTATTGAGGCGCAACAGCGTCTGTTGCAACTCACAGTCTGGGGCATC
>JF320131
TTCTATTGTAATACAACACAACTGTTCAATAGTACTTGGGAGAGCAATATTACTACTGGGAATTGGAGCAAAA
CTGAA--
-------------------GGA---
TCAAATAACACTAACATCACACTCCCATGTAGAATAAGACAAATTGTAAACATGTGGCAGAAAGTAGGAAAAG
CAATGTATGCCCCTCCCATCAGGGGACCAATTCAATGTATATCAGAT---
ATCACAGGGCTGCTATTAACAAGAGATGGAGGTAATAACGGGGACAAT-------------------------
----

AACAACACAGAGACCTTCAGACCTGGAGGAGGAGATATGAGGGACAATTGGAGAAGTGAATTATATAAATATA
AAGTAGTACAAATTGAACCAATA---GGAATAGCACCCACCAAGGCGCAGAGAAGAGTGGTG-----------
----------------------------CAGAGAGAAAAAAGAGCA---GTGGGA---GTAGGAGCT------
---TTTCTCTTTGGGTTCTTGGCAACA---GCAGGAAGCACTATG---GGCGCAGCGTCA---
ATAACGCTGACGGTACAA---GCCAGACAA---TTATTG---TCTGGTATAGTGCAACAGCAGAGC---
AATTTG---CTGAGG---GCTATTGAGGCGCAACAGCATATGTTGCAACTCACAGTCTGGGGCATC
>HM638503
TTCTACTGTAATACAACAGGGCTGTTTAGTAGTACTTGGAATGAGAGTACTACTTGGGAAAGGTCAAAT----
----

```
----------------------------
GACACTGAAATAATCACTCTCCCATGCAGAATAAAACAAATTATAAACATGTGGCAGGCAGTAGGAAAAGCAA
TATATGCCCCTCCCATCAGTGGACAAATTAATTGTTCATCAAAT---
ATTACAGGGCTGCTATTAACAAGAGATGGTGGTTACAATGGTACGAAT-------------------------
-------------------------------------------------------------------------
-------
GACACCCAGACCGAGACCTTCAGACCTCAGGGAGGAAATATGAAAGACAATTGGAGAAGTGAATTATATAAAT
ATAAAGTAGTAAAAATTGAACCATTA---GGACTAGCACCCACCAAGGCAAAGAGAAGAGTGGTG--------
-------------------------------CAGAGAGAAAAAAGAGCA---GTGGGA---ATAGGAGCT---
------ATGTTCTTTGGGCTCTTGGGAGCA---GCAGGAAGCACCATG---GGCGCAGCGTCA---
GTGACGCTGACGGTACAG---GCCAGAAAG---TTGTTG---TCTGGTATAGTGCAACAGCAGAAC---
AATCTG---CTGAGG---GCTATTGAGGCGCAACAGCATATGTTGCAACTCACAGTCTGGGGCATC
>KT124801
TTCTACTGTAATACAACCGAACTGTTTAATAGTACTTGGACTAGGAATAATGGTACCGGGTATGATGGGTCTA
ATGGCACT-----------------------------------------------------------------
-------------------------
GAAGGAAATGACACAATCACACTCCCATGCAGAATAAAACAAATTATAAACATGTGGCAGGAAGTAGGAAAAG
CAATGTATGCCCCTCCTATTAGAGGACAAATTAGATGCTCATCAAAT---
ATTACAGGACTGATATTAACAAGAGATGGGGGTCAAAGAAACGGG----------------------------
-------------------------------------------------------------------------
----------
AGTACTAATGAGACCTTCAGACCAGGAGGAGGAAATATGAGGGACAATTGGAGAAGTGAATTATATAAGTATA
AAGTAATAAAAATTGAACCACTA---GGAGTAGCACCCACCAGGGCAAAGAGAAGAGTGGTG-----------
----------------------------CAGAGAGAAAAAAGAGCA---GTGGGA---ATAGGAGCT------
---GTGTTCCTTGGGTTCTTGGGAGCA---GCAGGAAGCACTATG---GGCGCAGCGTCA---
CTAACGCTGACGGTACAG---GCCAGACAA---TTATTG---TCTGGTATAGTGCAACAGCAAAAC---
AATTTG---CTGAGA---GCTATTGAGGCGCAACAACATATGTTGCAACTCACAGTCTGGGGCATC
>KM354092
TTCTACTGTGATACAACACAACTATTTAATAGTACTTGGAAAAATGGTAGCACT-------------------
-------------------------------------------------------------------------
-GGGAATGATCATGAA---------
GAGTCAAATGGAAATATCACACTCCCATGCAGAATAAAACAAATTATAAACATGTGGCAGGAAGTAGGAAAGG
CAATGTATGCCCCTCCCATTAGAGGATACATTAATTGTTCATCAAAT---
ATTACAGGGCTGCTATTAACAAGAGATGGTGGT----------------------------------------
--------------------------------------------------------------------
GATAATAGCACAAAT---
GGGACCGAGATATTCAGACCTCTAGGAGGAGATATGAAGGACAATTGGAGAAGTGAATTATATAAATATAAAG
TTGTGAGAATTGAACCATTA---GGAATAGCACCCACCAAGGCAAAGAGAAGAGTGGTG--------------
-------------------------CAAAGAGAAAAAAGAGCA---GTGGGA---TTTGGAGCT---------
CTGTTCCTTGGGTTCTTGGGAGCA---GCAGGAAGCACTATG---GGCGCAGCGTCA---
ATAACGCTGACGGTACAG---GCCAGACAA---TTATTG---ACTGGTATAGTGCAGCAGCAGAAC---
AATCTG---CTGAGA---GCTATTGAGGCGCAACAACATCTGTTGCAACTCACAGTCTGGGGCATC
>JN687763
TTCTACTGCAATACAACACAACTGTTTAATAGTACTTGGAATACTAATGGTACTTGGACAGGAAGTAATACT-
-------------------------------------------------------------------------
----------------------------
AATAGCAGTAACATTACACTCCCATGCAGAATAAAACAAATTGTAAACATGTGGCAGGAAGTAGGAAAAGCAA
TGTATGCCCCTCCCATCAGCGGACTAATTCACTGTTCATCAAAT---
ATTACAGGGCTGATATTAACAAGGGATGGTGGTAGTGGTAATGACACC-------------------------
-------------------------------------------------------------------------
-------
AACAACAACACTGAGACCTTCAGACCTGCAGGAGGAGATATGAGGGACAATTGGAGAAGTGAATTATACAAAT
ATAAAGTAGTAAAAATTGAACCATTA---GGAGTAGCACCCACCAGGGCAAAGAGAAGAGTGGTG--------
-------------------------------CAAAGAGAGAAAAGAGCA---GTGGGA---ATGGGAGCT---
------CTGTTCCTTGGGTTCTTGGGAGCA---GCAGGAAGCACTATG---GGCGCAGCGTCA---
ATGACGCTGACGGTACAG---GCCAGACAA---TTATTG---TCTGGTATAGTGCAACAGCAGAGC---
AATTTG---CTGAGG---GCTATTGAGGCGCAACAGCATCTGTTGCAACTCACAGTCTGGGGCATC
>KM258901
TTCTACTGTAATACAACACAGTTATTTAATGGTACTTGGAATGGTACTTGGAATGGTACTTGGACTGAT----
-----------------------------------------------ACTTGGAATGAT---
```

ACTTGGAATGAGACTCACCCA---------------
ACTAGCAACGACACTATCACACTCCCATGTAGGATAAAGCAAATTATAAACAGATGGCAGGAAGTAGGAAAAG
CAATGTATGCCCCTCCCATCAGAGGAAACATTACCTGTTCCTCAAAT---
ATTACAGGGCTGCTATTAACAAGAGATGGTGGT--

AACCGGAATGAGACCAAT---
AGCACAGAGACCTTCAGACCTGGAGGAGGAGATATGAGGGACAATTGGAGAAGTGAATTATATAAATATAAAG
TAGTAAAAATTGAGCCACTA---GGAGTAGCACCCACCAAGGCAAAAAGAAGAGTGGTG--------------
-------------------------CAGAGAGAAAAAAGAGCG---GTGGGAATGCTAGGAGCT---------
ATGTTCCTTGGGTTCTTAGGAGCA---GCAGGAAGCACTATG---GGCGCAGCGTCA---
ATAACGCTGACGGTACAG---GCCAGACAA---TTATTG---TCTGGTATAGTGCAACAGCAAAAC---
AATTTG---CTGAGA---GCTATTGAGGCGCAACAGCATATGTTGCAACTCACAGTCTGGGGCATC
>JQ403073
TTCTACTGTAATACAACACAACTGTTTGATGATAATTGGCCTAATGGAACTTGGATGTATAATAGCACT----
----

AGAAATGACACTATCATACTCCCATGCAGGATAAAACAAATTGTAAACATGTGGCAGGAAGTAGGAAAAGCAA
TGTATGCCCCTCCCATCAAAGGACTAATTCACTGTAATTCAAGT---
ATTACAGGGCTGCTATTAACAAGAGATGGTGGCACTAATAACACC----------------------------
----

AACGAGACCTTCAGACCGGGAGGAGGAGACATGAGGGACAATTGGAGAAGTGAATTATATAAATATAAAGTAG
TAAAAATTGAACCATTA---GGAGTAGCACCCACCAAGGCAAAGAGAAGAGTGGTG-----------------
----------------------CAGAGAGAGAAAAGAGCA---GTGGGA---CTAGGAGCT---------
CTGTTCCTTGGGTTCTTGGGAGCA---GCAGGAAGCACTATG---GGCGCAGCGTCA---
ATGGCGCTGACGGTACAG---ACCAGACAA---TTATTG---TCTGGTATAGTGCAACAGCAGAAC---
AATTTG---CTGAGG---GCTATTGAGGCGCAACAGCATCTGTTGCAACTCACAGTCTGGGGCATC
>JQ403078
TTCTATTGTAATACAACACAGCTGTTTAATAGTACTTGGAATGGTACTGAGACTGAGAATAATACTACAGGGT
CAAAT--

AACACTGATAATATCAGACTCCCATGCAGAATAAAACAAATTGTAAACAGGTGGCAGGAAGTAGGAAAAGCAA
TGTATGCCCCTCCCATCGAGGGCATAATTAGTTGTTCATCAAAT---
ATTACAGGGCTTTTATTAACCAGAGATGGTGGTAACGGGAGCAGT----------------------------
----

AACGCCGAGATCTTCAGACCTCAAGGAGGAGATATAAGGGACAATTGGAGAAGTGAATTATATAAATATAAAG
TAGTAAAAATTGAGCCACTA---GGAGTAGCACCCACCAAGGCAAAGAGAAGAGTGGTG--------------
-------------------------CAGAGAGAAAAAAGAGCA---GTGGGA---ATAGGAGCT---------
GTGTTCCTTGGGTTCTTGGGAGCA---GCAGGAAGCACTATG---GGCGCAGCGTCA---
ATAACGCTGACGGTACAG---GCCAGACAA---TTATTG---TCTGGTATAGTGCAACAGCAGAAC---
AATCTG---CTGAGG---GCTATTGAGGCGCAACAACATCTGTTGCAACTCACAGTCTGGGGCATC
>JQ610064
TTCTATTGCAATACAACAGAACTGTTTACTTGGAATTTAACACAACTGTCTGAGAATTCAACTGGG-------
----

AATGATGCTAATATCACACTCCCATGTAGAATAAAACAAATTATAAACATGTGGCAGGAAGTAGGAAAAGCAA
TGTATGCCCCTCCCATCAGCGGACCAATTAATTGCACATCAAAT---
ATTACAGGACTACTATTAACAAGAGATGGTGGTAATAACAATGACAGGACTAAC------
AATGGCAGTGATAGTAAT---AACAGTACT---

CAGACTGAGATCTTCAGACCTGGAGGAGGAGATATGAAGGACAATTGGAGAAGTGAATTATATAAATATAAAG
TAGTAAGAATTGAACCATTA---GGAGTAGCACCCACCAAGGCAAAGAGAAGAGTGGTG--------------
-------------------------CAAAGAGAAAAAAGAGCG---GTGGGAACGCTAGGAGCT---------
TTGTTCCTTGGGTTCTTGGGAGCA---GCAGGAAGCACTATG---GGCGCAGCGTCA---
ATAACGCTGACGGTACAA---GCCAGACAA---TTATTG---TCTGGTATAGTGCAACAGCAAAGC---
AATTTG---CTGAGA---GCTATTGAGGCGCAACAGCATCTGTTGCAACTCACAGTCTGGGGCATC
>KM259406
TTCTACTGTAATACAACAAAACTGTTTAATAGTACTTGGGATAAAAATGGCACTTTG----------------
----

AGTGAAAATGGCACTCAG---------
AATGAAACTGAACCAATCACACTCCCATGCAGAATAAAACAAATTATAAACATGTGGCAGGAAGTAGGGAGAG
CAATGTATGCCCCTCCCATCAGAGGAAACATTAGCTGTTCATCAAAT---
ATTACAGGACTGTTGTTAACAAGAGATGGTGTT--

-
AACAAGACCTGGGGCAACGACACCTTCAGGCCTATAGGAGGAAATATGAAAGACAATTGGAGGAGTGAATTAT
ATAAATATAAAGTAGTAAAAATTGAACCATTA---GGAATAGCACCCAACACGGCAAAGAGAAGAGTGGTG--
-------------------------------------CAGAGAGAAAAAAGAGCA---GTGGGC---
ATAGGAGCT---------CTGTTCCTTGGGTTCCTGGGAGCA---GCAGGAAGCACTATG---
GGCGCAGCGTCA---ATGACGCTGACGGTACAG---GCCAGACAA---TTATTG---
TCTGGTATAGTGCAACAGCAGAAC---AATTTG---CTGAGG---
GCTATTGAGGCGCAACAGCATCTGTTGCAACTCACAGTCTGGGGCATT
>HQ217713
TTCTACTGTGATTCAACACCACTGTTTAATAGGACCTGGATGGCTAATGAGACTTTGAATAGTACTACTTTGA
ATAGTACT---
-------------------GGA---
GAGTCAAATAAAAATATCACACTCGCATGCAGAATAAAACAAATTATAAACATGTGGCAGCGAGTAGGGAAAG
CAATGTATGCCCCTCCTATCAGAGGACAAATTAGATGTTCATCAAAT---
ATTACAGGGCTGCTGTTAACAAGAGATGGGGGTCACAACAACGAGACTGGA----------------------
----

GATAACAGGACTGAGATCTTCAGACCGGGAGGAGGAGATATGAGGGACAATTGGAGAAGTAAATTATATAAAT
ATAAAGTAGTAAAAATTGAACCATTA---GGAGTAGCACCCACCAAGGCAAAGAGAAGAGTGGTG--------
-------------------------------CAGAGAGAAAAAAGAGTA---GCGGGA---TTCGGAGCT---
------TTGTTCCTTGGGTTCTTGGGAGCA---GCAGGAAGCACTATG---GGCGCAGCGTCA---
GTGACGCTGACGGTACAG---GCCAGACAA---TTACTG---TCTGGTATAGTGCAGCAGCAGAAC---
AATTTG---CTGAGG---GCTATTGAGGCGCAACAGCATCTGTTGCAACTCACAGTCTGGGGCATC
>MK383397
TTCTACTGTAATTCAACACCACTGTTTAATAGTACTTGGAATGGTACTGACGAGTCAAATCACCATGGAGAA-
----

AATGACACAATCATCACACTCCGCTGCAGAATAAAACAAATTATAAACATGTGGCAGGAAGTAGGAAGAGCGA
TGTATGCCCCTCCCATTAAAGGAAACATTAGCTGTTCATCAAAT---
ATTACAGGGCTGCTATTAACAAGAGATGGTGGTAATGGTAACCGG----------------------------
----

ACTGACAACAACACCGAGATCTTCAGACCTCAGGGAGGAAATATGAAAGATAATTGGAGAAGTGAATTATATA
AATATAAAGTAGTAAGAATTGAACCATTA---GGAATAGCACCCACCAAGGCAAGGAGAAGAGTGGTG-----
----------------------------------CAGAGAGAAAAGAGAGTA---GTGGGA---
ATGGGAGCT---------TTGTTCCTTGGGTTCTTGGGAGCA---GCAGGAAGCACTATG---
GGCGCAGCGTCA---GTGACGCTGACGGTACAA---GCCAGACTA---TTACTG---
TCTGGTATAGTGCAGCAGCAGAAC---AATTTG---CTGAGG---
GCTATTGAGGCGCAACAGCGTCTGTTGCAACTCACAGTCTGGGGCATC
>KT124761
TTCTACTGTAATACAACAAAGCTATTTAATAGTACTTGGGATCGGAATGGAACACAGATAAATGACACTATC-
----

ACAAATGACACTATCATACTCCCATGTAGAATAAAACAATTCATAAACAGGTGGCAAGAAGTAGGAAAAGCAA
TGTATGCCCCTCCCATTAGAGGAGACATTAACTGTTCATCACAG---
ATTACAGGGCTGCTATTAACAAGAGATGGTGGTCAGAGCAATGACAGC-------------------------
----

AGTATGAATGAGATCTTCAGACCTGGAGGAGGAGATATGAGGGACAATTGGAGAAGTGAATTATATAAATATA
AAGTAGTAAGAATTGAGCCATTA---GGAGTAGCACCCACCAAGGCAAAAAGAAGGGTGGTG-----------
----------------------------CAGAGAGAAAAAAGAGCA---GTAACA---CTAGGAGCT------
---GTGTTCCTTGGGTTCTTGGGAGCA---GCAGGAAGCACTATG---GGCGCAGCGTCA---
ATAACGCTGACGGCACAG---GCCAGACAA---TTATTG---TCTGGTATAGTGCAACAGCAGAGC---
AATCTG---CTGAAA---GCTATTGAGGCGCAACAGCGTCTGTTGCAACTCACAGTCTGGGGCATT
>KM353846

TTCTACTGTAATACATCAAAGTTGTTTAACAGTACTTGGAATGGCACT-------------------------
----
-------AAGGAATACCAG------
GGAGTGAATGGCACACTCACACTCCCATGCAGAATAAAACAAATCATAAACATGTGGCAGAAAGTAGGAAAAG
CAATGTATGCCCCTCCCATCCAGGGAAATATTAATTGCTCATCAAAT---
ATTACAGGGCTGCTATTAATGAGAGATGGTGGTAAC-------------------------------------
----
AAGGGGAACGAAACCGATAAT---------
ACTACCGAGATCTTTAGACCTGGAGGAGGAAATATGAGGGACAATTGGAGAAGTGAATTATATAAATATAAAG
TAGTGAGAATTGAACCATTA---GGAGTAGCACCCACCAAGGCAAAAAGAAGAGTGGTG--------------
-------------------------CAGAGAGAAAAAAAGAGCG---GTAACA---GTGGGAGCT--------
ATGTTCCTTGGGTTCTTGGGAGCA---GCAGGAACCACTATG---GGCGCAGCATCA---
TTGACGCTGACGGTACAG---GCCAGACAA---TTATTG---TCTGGTATAGTGCAACAGCAAAGC---
AATTTG---CTGAGG---GCTATTCAGGCGCAACAGCATCTGTTGCAGCTCACAGTCTGGGGCATC
>KY112135
TTCTACTGTAATACAACAGAACTGTTTAATAGTACTTGGGATAAGGATGGGACAAGGCTTAATGACACTGGA-
----

GGAGATGGCAACATCACACTCCCATGTAGAATAAAACAAATTATAAACATGTGGCAGGAAGTAGGAAAAGCAA
TGTATGCCCCTCCCATCAGAGGACAAATTAGCTGTGTATCAAAT---
ATCACAGGGCTACTATTAACAAGAGATGGGGGTGTAGACGATACT----------------------------
----

CAAAACGAAACCTTCAGACCTGGAGGAGGAGATATGAGGGACAATTGGAGAAGTGAATTATATAAATATAAGG
TAGTAAAAATTGAACCATTA---GGAGTAGCACCTAGCAAGGCAAAGAGAAGAGTGGTG--------------
-------------------------CAAAGAGAAAAAAAGAGCA---GTGGGAACGTTAGGAGCC--------
ATGTTCCTTGGGTTCTTGGGAGCA---GCAGGAAGCACTATG---GGCGCAGCGTCA---
CTGACGCTGACGGTACAG---GCCAGACAA---TTATTG---TCTGGTATAGTGCAACAGCAGAAC---
AATCTG---CTGAGA---GCTATTGAGGCGCAACAGCATCTGTTGCAACTCACAGTCTGGGGCATC
>JQ403070
TTCTACTGTAATACAACACAACTGTTTAATAGTACTTGGCTGTTTAATGATACTTTGAATGATACTCAAGAGG
AGTATGGT---

GAGAATAACGGGACTATCACACTCCCATGCAGAATAAAACAAATTATAAACAGATGGCAGGAAGTAGGAAAAG
CAACATATGCCCCTCCCATCAGAGGACAAATTAAATGTTCATCAAAT---
ATTACAGGGATACTATTAACAAGAGATGGTAGTCAAGGCAACGGG----------------------------
----

AGTGAGACCTTCAGACCAGGAGGAGGAGATATGAGGGACAATTGGAGAAGTGAATTATATAAATATAAAGTAG
TAAAAATTGAGCCATTA---GGAGTAGCACCCACCAAGGCAAGGAGAAGAGTGGTG-----------------
----------------------CAAAGGGAAAAAAGAGCA---GTGGGAACGCTAGGAGCC---------
ATGTTCCTTGGGTTCTTGGGAGCA---GCAGGAAGCACTATG---GGCGCAGCGTCA---
GTGACGCTGACGGTACAG---GCCAGACAA---CTATTG---TCTGGTATAGTGCAACAGCAGAAC---
AACCTG---CTGAGA---GCTATTGAGGCGCAACAGCATCTGTTGCAACTCACAGTCTGGGGCATC
>JQ403031
TTCTACTGCAATACAACAAAACTATTTAATAGTACTTGGAATAGTAGTAGTAGTAGTTGGGTTATTGAGGGTC
AGGAAGAG---

TCAAATGACAATATCACACTCCCATGTAGAATAAAACAAATTATAAACATGTGGCAGGGAGTAGGAAAAGCAA
TGTATGCCCCTCCCATCCAAGGAAAAATTAGATGTTCATCAAAC---
ATTACAGGGCTATTATTAGTGAGAGATGGTGGTATTAACAAGACG----------------------------
----

AATGACACTGAAACCTTCAGACCTCAAGGAGGAAATATGATGGACAATTGGAGAAGTGAATTATATAAATATA
AAGTAGTAAAAATTGAACCATTA---GGAATAGCACCCACCAAGGCAAAGAGAAGAGTGGTG-----------
----------------------------CAGAGAGAAAAAAAGAGCA---GTGGGA---ATGGGAGCT------
---TTGTTCCTTGGGTTCTTGGGAGCA---GCAGGAAGCACTATG---GGCGCAGCGTCA---
ATGGCGCTGACGGTACAG---GCCAGACAA---TTATTG---TCTGGTATAGTGCAACAGCAGAAC---
AATCTG---CTGAGA---GCCATTGAGGCGCAACAGCATATGTTGCAACTCACAGTCTGGGGCATC
>JQ403085

TTCTACTGTAATACATCACAACTGTTTAAGGGTACCTGGTTGACTAATGATACCTGGGCTAATGGTACCTGGA
CAAAT--
-------------GACACT------
AGCAAAAAATGACAACATCACACTCCCATGCCGAATAAAACAAATTGTAAACTTGTGGCAGGAAGTAGGAAGAG
CAATGTATGCCCCTCCCATCAGAGGAGAAATTCATTGTATATCAAAT---
ATTACAGGGCTGTTATTAACAAGAGATGGTGGCCAGAATGCAACC----------------------------
----

AACGAGACCTTCAGACCTGTAGGAGGAGATATGAGGGACAATTGGAGAAGTGAATTATATAAATATAAAGTAG
TAAAAATTGAACCATTA---GGAGTAGCACCCACCAAGGCAAAGAGAAGAGTGGTG-----------------
----------------------CAGAGAGAAAAAAGAGCA---GTGGGA---CTAGGAGCT---------
GTGTTCCTTGGGTTCTTGGGAGCA---GCAGGAAGCACTATG---GGCGCAGCGTCA---
CTGACGCTGACGGTACAG---GCCAGGCAA---TTATTG---TCTGGTATAGTGCAACAGCAAAAC---
AACTTG---CTGAGA---GCTATTGAGGCGCAACAGCACCTGTTGCAACTCACAGTCTGGGGCATC
>JQ403094
TTCTACTGTAATACATCACGACTATTTAATAGTACTTGGAATACTAGTAGTATTTGGAATACTACTGGAAGGC
CAAATATT---
-------------------ACA---
GGGAGCACTGACATTATCATACTCCCATGCAGAATAAGACAAATTATAAACATGTGGCAGGAAGTAGGAAAAG
CAATGTATGCCCCTCCCATCAGAGGACGAATTCAGTGTAGCTCAAAT---
ATTACAGGGCTGCTATTAACAAGAGATGGTGGTAATACTAATAACTCA-------------------------
----

GACGCACATGAGATCTTCAGACCTGGAGGAGGAGATATGAGGGACAATTGGAGAAGTGAATTATATAAATATA
AAGTAGTAAAAATTGAACCATTA---GGAATAGCGCCCACCAAGGCAAAGAGAAGAGTGGTG-----------
----------------------------CAGAGAGAAAAAAGAGCA---GTGGGA---ATGGGAGCT------
---CTGTTCCTTGGGTTCTTGGGAACA---GCAGGAAGCACTATG---GGCGCAGCGTCA---
GTGACGCTGACGGTACAG---GCCAGACAA---TTATTG---TCTGGTATAGTGCAACAGCAGAGC---
AATCTG---CTGAGG---GCTATTGATGCGCAACAGCATCTGTTGCAGCTCACAGTCTGGGGCATC
>MH666195
TTCTACTGTAATACAACAAAACTGTTTAATAGTACTTGGTTTGGGAATAGTACTGATAATACTCACAATATTA
CTCAGAAT---

GGTAATGGTACTATCACACTTCCATGCAGAATAAAGCAAATTATAAACATGTGGCAGGAAGTAGGAAAAGCAA
TGTATGCCCCTCCCATCAGTGGAATAATTAGATGTAACTCAAAT---
ATTACAGGGCTGTTATTAACAAGAGATGGTGGTAATAACAATGGCAGC-------------------------
----

AACACTACTGAGGAGATTTTTAGACCTACAGGAGGAAATATGAAAGACAATTGGAGAAGCGAATTATATAAAT
ATAAAGTAGTAGAAATTGAACCAATA---GGATTAGCCCCCACCAAGGCAAAGAGAAGAGTGGTG--------
-------------------------------CAGAGAGAAAAAAGAGCA---GTGGGA---ATAGGAGCT---
------ATGTTCCTTGGGTTCTTGGGAGCA---GCAGGAAGCACTATG---GGCGCAGCGTCA---
ATAACGCTGACGGTACAA---GCCAGACAA---TTATTG---ACTGGTATAGTGCAACAGCAAAGC---
AATTTG---CTGAAG---GCTATTGAGGCGCAACAACATCTGTTGCAACTCACAGTCTGGGGCATC
>KR423482
TTCTACTGTAATACAACGAAACTGTTTAATAGTAGTTGGAATCAGAATGCTACTAATCAGAATGCTACT----
----
-------------AACAAT------
GGAGGAAATGACACAATTACACTCCCATGTAGAATAAAACAGATTATAAACATGTGGCAGGAAGTAGGAAAGG
CAATGTATGCCCCTCCCATAAAAGGATTAATTAGATGTTCATCAAAT---
ATTACAGGGCTGATATTAACAAGAGATGGTGGTAATGACAATAGCACA-------------------------
----

AACAACAACACTGAAATCTTCAGACCGGGAGGAGGAGATATGAGGGACAATTGGAGAAGTGAATTATATAAAT
ACAAAGTAGTAAGAGTTGAACCATTA---GGGATAGCACCCAACAAGGCAAAGAGGAGAGTGGTG--------
-------------------------------CAGAGAGAAAAAAGAGCA---GTGGGG---ATAGGAGCT---
------TTGTTCCTTGGGTTCTTGGGAGCA---GCAGGAAGCACTATG---GGCGCAGCGTCA---
GTGACGCTGACGGTACAA---GCCAGACAA---TTATTG---TCTGGTATAGTGCAGCAGCAGAAC---
AATCTG---CTGAGG---GCTATTCAGGCGCAACAGCAACTGTTGCAGCTCACAGTCTGGGGCATC
>HM638568

```
TTCTACTGTAATACATCACAACTATTTAATAGTACTTGGTATGGTAATGAGACTAAAAATGGGACTGAAAATG
GA-----------------------------------------------------------------------
----------------------------
ACTGACACAATAATTCAACTCCCATGCAGAATAAAACAAATTATAAACATGTGGCAGCAAGTAGGAAAAGCAA
TGTATGCCCCTCCCATCGAAGGACTAATTAGCTGCTCGTCAAAC---
ATTACAGGACTGCTGTTAACAAGAGATGGTGGTGATAATAATAGC----------------------------
-------------------------------------------------------------------------
-------------
ATGAACGAGATTTTCAGACCTGAAGGAGGAAATATGAAGGACAATTGGAGAAGTGAATTATATAAATATAAAG
TAGTAAAAGTTGAACCATTA---GGAATAGCACCCACCAAGGCAAGGAGAAGAGTGGTG--------------
-------------------------CAGAGAGAAAAAAGAGCA---GTAGGA---ATAGGAGCT---------
TTGTTCCTTGGGTTCTTGGGAGCA---GCAGGAAGCACTATG---GGCGCAGCGTCA---
CTGACGCTGACGGTACAG---GCCAGACAA---TTATTG---TCTGGTATAGTGCAACAGCAGAAC---
AATTTG---CTGAGG---GCTATTGAGGCGCAACAGCATTTGTTGCAACTCACAGTCTGGGGCATC
>GU733713
TTCTACTGTAATACAACTAGACTGTTCAATAGTACTTGGAATGTTAGTACTTGGAATATTAGTCAGACTGAAG
GGTCA--------------------------------------------------------------------
-------------------TAC---
AACACTGAAGAAAATATCACACTCCCATGCAGAATAAAACAGATTGTAAACATGTGGCAGGAAGTAGGAAAAG
CAATGTATGCCCCTCCCATCAGAGGAATAATTAGATGTTCATCAAAT---
ATTACAGGGATAATGTTAACAAGAGATGGTGGTAAGAATAGGACAGAG-------------------------
-------------------------------------------------------------------------
----------
AACACCACTGAGACTTTCAGACCGGGAGGAGGAAATATGATGGACAATTGGAGAAGTGAACTATATAACTATA
AAGTAGTAAAAATTGAACCATTA---GGGGTAGCACCCACCAAGGCAAAGAGAAGAGTGGTG-----------
----------------------------CAGAGAGAAAAAAGAGCA---GCGGGA---CTAGGAGCT------
---TTGTTCCTTGGGTTCTTGGGAGCA---GCAGGAAGCACTATG---GGCGCAGCGTCA---
CTGACGCTGACGGTACAG---GCCAGACAA---TTATTG---TCTGGTATAGTGCAACAGCAGAAC---
AATTTG---CTGAGA---GCTATTGAGGCGCAACAGCACCTGTTGCAACTCACAGTCTGGGGCATC
>JX140657
TTCTACTGTAATACAACACCACTGTTTAATAGTACTTGGGGGAATGACACTTTGACTAATGGAGGATCAAAT-
-------------------------------------------------------------------------
----------------------------
AGCACTGAAAATATCACACTTCAATGCAGAATAAAACAAATTATAAACATGTGGCAGGAAGTAGGAAAAGCAA
TGTATGCCCCTCCCATCGCAGGAAACATTAGCTGTTCATCAAAT---
ATTACAGGGCTGCTATTAACAAGAGATGGTGGTAATGGCACAAAC----------------------------
-------------------------------------------------------------------------
-------------
GAGACAGAGATCTTCAGACCCGAAGGAGGGAATATGAAGGACAATTGGAGAAGTGAATTATATAAATATAAAG
TAGTACAAATTGAACCAGTA---GGAATAGCACCCACAAAGGCAAAGAGAAGAGTGGTG--------------
-------------------------CAGAGAGAAAAAAGAGCA---GTGGGA---CTAGGAGCT---------
GTGCTCTTTGGGTTCTTGGGAGCA---GCAGGAAGCACTATG---GGCGCAGCGTCA---
CTGACGCTGACGGTACAA---GCCAGGCAA---TTATTG---TCTGGTATAGTGCAACAGCAAAGC---
AATTTG---CTGAGG---GCTATTGAGGCGCAACAGCACCTGTTGCAACTCACAGTCTGGGGCATC
>MK164668
TTCTACTGTAATACAACAGAACTGTTTAATAGTACTTGGGGTAATAGTAATAATACTAACAATAATACTGGA-
-------------------------------------------------------------------------
----------------------------
GGGGAAGAAACTATCACTCTCCCATGCAGAATCAAACAAATTATAAACATGTGGCAGGAAGTAGGAAAAGCAA
TGTATGCCCCTCCCATCAGAGGACAAATTAGGTGTTCATCAAAG---
ATTACAGGGCTAATATTAACAAGAGATGGTGGCAACGAGAACAACGACAGTGAG-------------------
-------------------------------------------------------------------------
-------------
ATCTTCGAGATCTTCAGACCTACAGGAGGAAATATGAGGGACAATTGGAGAAGTGAATTATACAAATATAAAG
TAGTAAAAATTGAACCATTA---GGAATAGCACCCACCAAGGCAAAAAGAAGAGTGGTG--------------
-------------------------CAGAGAGAAAAAAGAGCA---GTGGGAATACTAGGAGCT---------
ATGTTCCTTGGGTTCTTGGGCGCA---GCAGGAAGCACTATG---GGCGCAGCGTCA---
CTGACGCTGACGGTACAG---GCCAGACAA---CTCTTG---TCTGGTATAGTGCAACAGCAGAAC---
AACCTG---CTGAGG---GCTATTGAGGCGCAACATCATCTGTTGCAACTCACAGTCTGGGGCATC
>KC935959
```

TTCTACTGTAATACAACAAAACTGTTTAGTAGTACTTGGAAGTGGGATAATGGTACTTGGAATATTAGTGGGT
TAGATAACAGT--

ACAGAACAAAATATCACACTCCCATGCAGAATAAAACAAATCATAAATATGTGGCAGACAGTAGGAAAAGCAA
TGTATGCCCCTCCCATTAAGGGACAAATTCAATGTATATCAAAT---
ATTACAGGGCTGCTATTAACAAGAGATGGTGGTAATGAAAGTGATACA-------------------------
----

ACCAGGAACAACGAGACCTTCAGACCGGGAGGAGGAGATATGAGGGACAATTGGAGAAGTGAATTATATAAAT
ATAAAGTAGTAGAAGTTGAACCATTA---GGAATAGCACCCACCAAGGCAAAGAGAAGAGTGGTG--------
-------------------------------CAGAGAGAAAAAAGAGCA---GTGACA---CTAGGAGCT---
------CTGTTCCTTGGGTTCTTGGGAGCA---GCAGGAAGCACTATG---GGCGCAGCATCA---
ATGACGCTGACGGTACAG---GCCAGACAA---TTATTG---TCTGGTATAGTGCAACAGCAGAAT---
AATCTG---CTGAGG---GCTATTGAGGCGCAACAGCATCTGTTGCAGCTCACAGTCTGGGGCATC
>KM082006
TTCTACTATAACACAACCCAGCTGTTTGATAGTACGTGGTTAACTAATGGTACTAGGATAAGACCAAATACTA
CTGATGGA---

ACTAATGGAAATATCACACTTCCATGCAGAATAAAACAAATTATAAACAGGTGGCAAGAAGTAGGAAAAGCAA
TGTATGCCCCTCCCATCAGAGGACAAATTAGATGTACATCAAAT---
ATTACAGGGTTAATATTAACAAGAGATGGTGGTCATGACGGGAAC----------------------------
----

GAGACAAATGAGAAGGAGACCTTCAGACCTGGAGGAGGAGATATGAGGGACAATTGGAGAAGTGAATTATATA
AATATAAAGTAGTAAAAATTGAGCCAATA---GGAATAGCACCCACCATGGCAAAGAGAAGGGTGGTG-----
----------------------------------CAGAGAGAAAAAAGAGCA---GTAACG---
CTGGGAGCT---------ATGTTCCTTGGGTTCTTGGGAGCA---GCAGGAAGCACTATG---
GGCGCAGCGTCG---ATGACGCTGACGGTACAG---GCCAGACTA---TTATTG---
TCTGGTATAGTGCAACAGCAGAGC---AATCTG---CTGAGA---
GCTATTGAGGCGCAACAGCAACTGTTGCAACTCACAGTCTGGGGCATC
>MG196775
TTCTATTGTAATACATCAGAACTGTTTAATAGTGAGTGGCATACTAATTATACATGGAATAGTACTTACAGTA
CAATAAAT---------------------------------GGTACTTGGAATAGTACT---
GACATTGAGGGTGTA---------------------
AACGCTACTGAAAAGATCACACTCCCATGTAGAATAAAACAAATTATAAACAGGTGGCAGGAAGTAGGAAAAG
CAATGTATGCCCCTCCCATCGCAGGAACAATTAAATGTGTATCAAAT---
ATTACGGGGCTGCTGTTAACCAGAGACGGTGGCAACAATGAAACC----------------------------
----

AAAGCCACTGAGGTCTTCAGACCTCAAGGAGGAAATATGAAGGACAATTGGAGAAGCGAATTATATAAATATA
AAGTAGTACAAATTGAACCATTA---GGAGTAGCACCCAACAAGGCAAAGAGAAGAGTGGTG-----------
----------------------------CAGAGAGAAAAAAGAGCA---ATAACA---ATAGGAGCT------
---ATGTTCCTTGGGTTCTTGGGAGCA---GCAGGAAGCACTATG---GGCGCAGCGTCA---
ATGACGCTGACGGTACAG---GCCAGACAA---TTACTG---TCTGGTATAGTGCAACAGCAGAGC---
AATTTG---CTGAGA---GCTATTGAGGCGCAACAGCATCTGTTGCAACTCACAGTCTGGGGCATC
>JQ182826
TTCTACTGCAATACATCAGCACTGTTTAATAGTACTTGGAGTACTAAGAATGGTACTCAGGTGTTGAATATAA
CTAATGAC---

ACCAGCACCAACATCACACTCCCATGCAGAATAAAACAAATTATAAACATGTGGCAGGAAGTAGGAAAAGCAA
TGTATGCCCCTCCCATCAGAGGACTAATTAGGTGTACATCAAAC---
ATTACAGGACTACTATTAACAAGAGATGGTGGTAATGGTAATGGCAATACCACG-------------------
----

AATGGACAGAGACCTTCAGACCTGTAGGAGGAGATATGAAGAATAATTGGAGAAGTGAATTATATAAATATA
AAGTAGTACAAATTCAACCATTA---GGAGTAGCACCCACCAAGGCAAAGAGAAGAGTGGTG-----------
----------------------------CAGAGAGAGAAAAGGGAT---TTGGGA---CTAGGAGCT------
---TTGTTCATTGGGTTCTTGGGAGCA---GCAGGAAGCACTATG---GGCGCAGCGTCA---
ATAACGCTGACGGCACAG---GCCAGACAA---TTGCTG---TCTGGTATAGTGCAACAGCAGAGC---
GATCTG---ATGAAG---GCTATTGAGGCGCAGCAGCATCTGTTGCAACTCACAGTCTGGGGCATC

>MK385523
TTCTACTGTAATTCAACACCACTGTTTAATAGTACTTGGTATGGTAATAGTACTTGGAATGGTACTGAAGGGT
CAAATTAC---

ACTGAAAAAAAATATCATACTCTCATGCAGAATAAAACAGATTATAAACATGTGGCAGGAAATAGGAAAAGCAA
TGTATGCCCCTCCCATTAGAGGAAACATTAGCTGTTCATCAAAT---
ATTACAGGGCTGCTATTAACAAGAGATGGTAGTAGTAATAACGAG----------------------------
----

AGCCAGCCCGAGATCTTCAGACCTGGAGGAGGAAATATGAAGGATAATTGGAGAAGTGAATTATATAAATATA
AAGTAGTAAGAATTGAACCATTA---GGAGTAGCACCCACCAAGGCAAGGAGAAGAGTGGTG-----------
----------------------------CAGAGAGAAAAGAGAGCA---GTGGGA---ATAGGAGCT------
---GTGTTCCTTGGGTTCTTGGGAGCA---GCAGGAAGCACTATG---GGCGCAGCGTCA---
ATGACGCTGACGGTACAG---GCCAGACTA---TTACTG---TCTGGTATAGTGCAACAGCAGAAC---
AATTTG---CTGAGG---GCTATTGAGGCGCAACAGCGTCTGTTGCAACTCACAGTCTGGGGCATC
>KM353922
TTCTATTGCGATACAACACAACTGTTTAATAGTAGTTGGCCTTGGAATGGTACA-------------------
----
GAAGAGTCAAATAACACT---------
GAAGGAAATGACACAATCACCCTCCCATGCAAAATAAAACAAATTATAAACCTGTGGCAGGAAGTAGGAAAAG
CAATGTATGCCCCTCCCATCAGAGGACAAATTAATTGCACATCAAAT---
ATTACAGGGTTACTATTAACAAGAGATGGTGGT--

-
AGTACCAGCAATGAGACCGAGATCTTTAGACCTGCAGGAGGAGACATGAGAGATAATTGGAGAAGTGAATTAT
ATAAATACAAAGTAGTAAAAATTGAACCAATA---GGAGTAGCACCCACCAGGGCAAAGAGAAGAGTGGTG--
-------------------------------------CAGAGAGAAAAAAGAGCA---GTGGGA---
TTAGGAGCT---------ATGTTCCTTGGGTTCTTGGGAGCA---GCAGGAAGCACTATG---
GGCGCAGCGTCA---GTGACGCTGACGGTACAG---GCCAGACAA---TTACTG---
TCTGGTATAGTGCAACAGCAGAAC---AATTTG---CTGAGG---
GCTATTGAGGCGCAACATCATCTGTTGCAACTCACAGTCTGGGGCATC
>KM354862
TTCTACTGTAATACAACACAACTATTTAATAGTAATTGGACTGCTACTGGA----------------------
----
-AATACAAATAACACT---------
GGAGGAAATGCCACAATCACACTCCCTTGCAGAATAAAACAACTTATAAACATGTGGCAGGGAGTAGGAAAAG
CAATGTATGCCCCTCCAATAAGAGGACTAATTAGATGTTCATCAAAC---
ATTACAGGGCTACTGTTAACAAGAGATGGTGGT--

GGTAGCAATGGAACTAAT---
GGAACTGAGGTCTTCAGACCTCATGGAGGGGATATAAGGGACAATTGGAGAAGTGAATTATATAAATACAAAG
TAGTAAAAATTGAACCATTA---GGAGTAGCACCCAACAAGGCAGAGAGAAGAGTGGTG--------------
-------------------------CAGAGAGAAAAAAGAGCA---GTGACA---ATAGGAGCT---------
GTGTTCCTTGGGTTCTTGGGAGCA---GCAGGAAGCACTATG---GGCGCAGCGTCA---
ATGACGCTGACGGTACAG---GCCAGACAA---CTATTG---TCTGGTATAGTGCAACAGCAAAGC---
AATTTG---CTGAGG---GCTATTGAGGCGCAACAGCACCTGTTGCAACTCACAGTCTGGGGCATC
>KX587037
TTCTACTGTAATACAACACCACTGTTTAATAATAGTCTGCTTGGCAATGGTATCAATGGGACAAAT-------
----

AACACAGAACCAATCGTACTTCATTGCAGAATAAAACAAATTATAAACATGTGGCAGAAAGTAGGAAAAGCAA
TGTATGCCCCTCCTATCAAAGGACCGATTACATGCTCATCAAAT---
ATAACAGGACTGATATTAACAAGAGATGGTGGTACAAATAGGACC----------------------------
----

AATGAGACCTTCAGACCTGGAGGAGGAGATATGAGGGACAATTGGAGAAGTGAATTATATAAATATAAAGTAG
TAAAAATTGAACCATTA---GGAGTAGCACCCACTAAGGCAAAGAGAAGAGTGGTG-----------------
----------------------CAGAGAGAAAAAAGAGCA---GTGGGA---CTTGGAGCT---------
TTGTTCCTTGGGTTCTTAGGAGCA---GCAGGAAGCACTATG---GGCGCAGCGTCA---

ATGACGCTGACGGTACAA---GCCAGACAA---TTATTG---TCTGGTATAGTGCAACAGCAGAAC---
AATCTG---CTGAGG---GCTATTGAGGCGCAACAACATTTGTTGCAACTCACAGTCTGGGGCATC
>KX587241
TTCTACTGTAATACGACACAACTGTTTAATAATACTTGGAATGGTACTTGGGGTGAGAATATT----------
----

ACCGGGAATATTATCACACTCCCATGCAGAATAAAACAAATTATAAACATGTGGCAGGAAGTAGGAAAAGCAA
TGTATGCCCCTCCCATCAGAGGAAATATTAGCTGTTCATCAGAT---
ATTACAGGGCTAATATTAACAAGAGATGGTGGTCATGACAATGAGACC------------------------
----

AACAACACAGCGATCTTCAGACCAGGAGGAGGAAATATGAAGGACAATTGGAGAAGTGAATTATATAAATATA
AAGTAGTAAAAATTGAACCAATA---GGAGTAGCACCCACCAAGGCAAAGAGAAGAGTGGTG-----------
----------------------------CAGAGAGAAAAAAGAGCA---GTGGGA---GTAGGAGCT------
---GTGCTCCTTGGGTTCTTGTCAGCA---GCAGGAAGCACTATG---GGCGCAGCGTCA---
ATAACGCTGACGGCACAG---GCCAGACAA---TTACTG---TCTGGTATAGTGCAACAGCAAAAC---
AATTTG---CTGAGA---GCTATTGAGGCGCAACAACATATGTTGCAACTCACAGTCTGGGGCATC
>KX587299
TTCTACTGTAATACAACAAAGCTGTTTAATAGTACTTGGGATAATACAACTGCAGGGTTAAATACC-------
----

ACTGAAAAAATTATCACACTCCCATGCAGAATAAGACAAATTATAAATATGTGGCAGCAGGTAGGAAAAGCAA
TGTATGCCCCTCCTATCAGAGGAAAAATCCAATGCACATCAAAC---
ATTACAGGGTTATTATTAACAAGAGATGGTGGTAACGAGAACGAGACC------------------------
----

TATAACAATGAGACTTTCAGACCTGGAGGAGGAGATATGAAGGACAATTGGAGAAGTGAATTATATAAATATA
AAGTAGTAAGAATTGAACCATTA---GGAATAGCACCCACCAAGGCAAAAAGAAGAGTGGTG-----------
----------------------------CAGAGAGAAAAAAGAGCA---ATAGGA---ATGGGAGCC------
---TTCTTCCTTGGGTTCTTGGGAGCG---GCCGGAAGCACTATG---GGCGCAGCGTCA---
ATGGCGCTGACGGTACAG---GCCAGACAA---TTATTG---TCTGGTATAGTGCAGCAGCAAAAC---
AACTTG---CTGAGG---GCTATTGAGGCGCAACAGCATCTGTTGCAACTCACAGTCTGGGGCATC
>MG900412
TTCTACTGTAATTCAACAAAGCTGTTTAATAGTACTTGGCCTTGGAATGATACTCAAGGGTCACATGAC----
----

ACTAATGGCACACTCACACTCCCATGCAGAATAAAACAAATTATAAACATGTGGCAGGAAGTAGGAAAAGCAA
TGTATGCCCCTCCCATCGAAGGAATAATTAAATGCTCATCAAAC---
ATTACAGGACTGTTATTAACAAGAGATGGTGGTTACGAGAGCAAT---------------------------
----

AAGACTGATGAGATCTTCAGACCTGGAGGAGGAGATATGAGGGACAATTGGAGGAGTGAATTATATAAATATA
AAGTAGTAAAAATTGAACCATTG---GGAGTAGCACCTACCAAGGCAAAGAGGAGAGTGGTG-----------
----------------------------CAGAGGGAAAAAAGAGCA---TTTGGA---CTAGGAGCT------
---GTGTTCCTTGGGTTCCTGGGAGCA---GCAGGAAGCACTATG---GGCGCAGCATCA---
ATAACGCTGACGGTACAG---GCCAGACAA---TTATTG---TCTGGTATAGTGCAACAGCAGAAC---
AATTTG---CTGAGG---GCTATTGAGGCGCAACAGCATCTGTTGCAACTCACAGTCTGGGGCATT
>KC473826
TTCTACTGTAATTCAACACAGCTGTTTAGTAGTAATTGGACTAGGGATAATACGTCTAGTAATTGGACTAGGA
AGGATAAC--
----------GATACAGAG------
CCTAATAAGACTGTGATCACACTCCCATGCAGAATAAAACAAATTATAAACATGTGGCAGGAAGTAGGAAAAG
CAATGTATGCCCCTCCTATCAAAGGACTTATTCAATGTTCATCAAAT---
ATTACAGGATTGTTATTAACAAGAGATGGTGGCGACAACAATAGC---------------------------
----

AACACTGAAATCTTTAGACCTGGAGGAGGAGATATGAGGGACAATTGGAGAAGTGAATTATACAAATATAAAG
TAGTAAAAATTGAACCATTA---GGAGTAGCACCCACCAAGGCAAGGAGGAGAGTGGTG--------------
-------------------------CAAAGAGAGAAAAGAGCA---TTGGGA---ATGGGAGCT---------
CTGTTCCTTGGGTTCTTGGGAGCA---GCAGGAAGCACTATG---GGCGCAGCGTCA---

TTGATGCTGACGGTACAG---GCCAGACAA---TTATTG---TCTGGTATAGTGCAACAGCAGAAC---
AATATG---CTGAGG---GCTATTGAGGCGCAGCAGCATCTGCTGCAACTCACAGTCTGGGGCATC
>KC473828
TTCTACTGTAATACAACAAAGCTGTTTAATAGCACTCTTAATGACACTTGGAATTTTTGGAAT----------
----

GAAACTGGGGATATCATACTCCCGTGCAGAATAAAACAAATTATAAATCTGTGGCAGAAAGTAGGAAAAGCAA
TGTATGCCCCTCCCATTAAAGGACAGATTAACTGCACATCAAAT---
ATTACAGGGCTACTCTTAATAAGAGATGGCGGTGATAACGGGACT----------------------------
----

GCCAGTGAGACCTTCAGACCTCAAGGAGGAAATATGAAGGACAATTGGAGAAGTGAATTATATAAATATAAAG
TAGCACAAGTTGAGCCAATA---GGAATAGCACCCACCAAGGCAAAGAGAAGAGTGGTG--------------
-------------------------CAGAGAGAAAAAAGAGCA---GTGACT---ATGGGAGCT---------
TTGTTCCTTGGGTTCTTGGGAACA---GCAGGAAGCACTATG---GGCGCAGCGTCG---
ATAACGCTGACGGTACAG---GCCAGACAA---TTACTG---TCTGGTATAGTGCAACAGCAGAGC---
AATCTG---CTGAGA---GCTATTGAGGCGCAACAGCATCTGTTGCAACTCACAGTCTGGGGCATC
>KC473829
TTCTACTGTAATACGACAGAACTGTTTAATAGTTCTTGGTATTGGAATGATACGAATGATACTTTAAAGAGC-
----

AATGAAGAAAATATCACACTTCTGTGCAGAATAAAACAAATTGTAAACATGTGGCAGGAAGTAGGAAAAGCAA
TGTATGCCCCTCCCATTAGAGGACCAATTAGCTGTACATCAAAT---
ATTACAGGGATACTCTTAACAAGAGATGGAGGTCAGGGTAATAATACT-------------------------
----

AATGATACTGAAATCTTCAGACCAGGAGGAGGAGATATGAGGGACAATTGGAGAAGTGAATTATATAAATATA
AAGTAGTAAAAATTGAACCATTA---GGAGTAGCACCCACCAGGGCAAAAAGAAGAGTGGTA-----------
----------------------------CAGAGAGAAAAAAGAGCA---ATAGGGGGATTAGGAGCG------
---TTGTTCCTTGGGTTCTTGGGAGCA---GCAGGAAGCACTATG---GGCGCAGCGTCA---
ATGACGCTGACGGTACAA---GCCAGGCTA---TTATTG---ACTGGTATAGTGCAACAGCAAAAT---
AATTTG---CTGAAG---GCTATTGAGGCGCAACAACATCTGTTGCAACTCACAGTCTGGGGCATT
>KC473830
TTCTACTGTGATTCAACACAACTGTTTAGTAGTAATTGGACTAGGAATGATAGTAGTAGTAATTGGACTAGGA
GTGATAATGAT--
-------------ACAGAG------
TCTAACAATACTGTGATCACACTCCCATGCAGAATAAGACAAATTATAAACATGTGGCAGGAAGTAGGAAAAG
CAATGTATGCCCCTCCTATCAAAGGACTTATTCAATGTACATCAAAT---
ATTACAGGATTATTATTAACAAGAGATGGTGGTAATGGTAATAAA----------------------------
----

ACGAACGACACTCTGGAGATCTTTAGACCTGGAGGAGGAGATATGAGGGACAATTGGAGAAGTGAATTGTACA
AATATAAAGTAGTAAAAATTGAACCATTA---GGAGTAGCACCCACCAAGGCAAGGAGGAGAGTGGTG-----
----------------------------------CAAAGAGAGAAAAGAGCA---GTGGGA---
CTAGGAGCT---------CTGTTCCTTGGGTTCTTGTCAGCA---GCAGGAAGCACTATG---
GGCGCAGCATCA---TTGACGCTGACGGTACAG---GCCAGACAA---TTACTG---
TCTGGTATAGTGCAACAGCAGAAC---AATATG---CTGAGG---
GCTATTGAGGCGCAACAGCATCTGTTGCAACTCACAGTCTGGGGCATC
>KU677990
TTCTACTGTAATACATCAAGACTGTTTAATAGTACTTTGTATAGTAATAATACTTGGAGTAGTAATATTACAG
AA---

TTAAATGGCATTATCACACTCCCATGCAGAATAAAACAAATTGTAAACATGTGGCAGGAAGTAGGAAAAGCAA
TGTATGCCCCTCCCATCAGTGGACAAATTAACTGTTCATCAAAC---
ATTACAGGACTATTATTAACAAGAGATGGTGGTGATGAGGAGAAC----------------------------
----

AAGACTGATGAGATTTTCAGACCTGGAGGAGGAGATATGAGGGACAATTGGAGAAGTGAACTATATAAATATA
AAGTAGTAAAAATTGAACCATTA---GGAGTAGCACCCTCCAGGGCAAAGAGAAGAGTGGTG-----------
----------------------------CAGAGAGAAAAAAGAGCA---GTGGGA---CTAGGAGCT------

---TTGTTCATTGGGTTCTTGGGAGCA---GCAGGAAGCACTATG---GGCGCAGCGTCA---
GTGACGCTGACGGTACAG---GCCAGACAA---TTATTG---TCTGGTATAGTGCAACAGCAGAAC---
AATTTG---CTGAGG---GCTATTGAGGCGCAACATCATCTGTTGCAACTCACAGTCTGGGGCATC
>MG196672
TTCTACTGTAATTCATCATCACTGTTTAACAGTACTTGGAATAATACTAGTTTAAATGGAAGTATAAGT----
----

TGGAATGGAAGTATCACACTTCAATGCAGAATAAAACAAATTATAAACATGTGGCAGGAAGTAGGAAAAGCAA
TGTATGCCCCTCCTATCAGAGGACAAATTAGGTGTAGATCAAAT---
ATTACAGGGCTGCTACTACTAAGAGATGGGGGTGAGGGTAATACTACT-------------------------
----

GACAACACCTCCGAGACCTTCAGACCTGGAGGAGGAGATATGAGGGACAATTGGAGAAGTGAATTATATAAAT
ACAAAGTAGTACAAATTGAACCATTA---GGAGTAGCACCCACCAAGGCAAAGAGAAGAGTGGTG--------
-------------------------------CAGAGAGAAAAAAGAGCA---ATAACG---CTAGGAGCT---
------ATGTTCCTTGGGTTCTTGGGAACA---GCAGGAAGCACTATG---GGCGCAGCGTCA---
ATAACGCTGACGGCACAG---GCCAGGCAA---TTATTG---TCTGGTATAGTGCAACAGCAGAAC---
AACTTG---CTGAGA---GCTATTGAGGCGCAACAACATCTGTTGCAACTCACAGTCTGGGGCATT
>MG196679
TTCTACTGTAATACAACACAACTGTTTAATAGTACTTGGAAGGATAATAATACTGGGTTAAATACCAATGATA
CTAATACT---
GGG---ATAAATACCTAT---------
GGCACTAATGGAACATTCATACTCCCATGTAGAATAAAACAAATTATAAACATGTGGCAGGAAGTAGGAAGAG
CAATGTATGCCCCTCCCATCAGAGGACTAATTAGATGTAAATCAAAT---
ATAACAGGGCTGATACTAACAAGAGATGGTGGTAATAATAATAGT----------------------------
----

ACAAATGAGACCTTCAGACCTGGAGGGGGAGATATGAAGGACAATTGGAGAAGTGAATTATATAAATATAAAG
TAGTAAAAATTGAACCATTA---GGAGTAGCACCTTCCAAGGCAAAGAGAAGAGTGGTG--------------
-------------------------CAGAGAGAGAAAAGAGCA---ATAGGGGGATTAGGAGCT---------
CTGTTCCTTGGGTTCTTGGGAGCA---GCAGGAAGCACTATG---GGCGCAGCGTCA---
CTGACGCTGACGGTACAG---GCCAGACTA---TTATTG---TCTGGTATAGTGCACCAGCAGAAC---
AACTTG---CTGAGA---GCTATTGAGGCGCAACAGCATCTGTTGCAGCTCACAGTCTGGGGCATC
>KF384800
TTCTACTGTAATACAACAGGACTGTTTAATAGTACTTGGAATAGTACTAGTACTGAGCTAAATGAC-------
----

ACTACAGGAAATATCAAACTCCCATGCAGAATAAAACAAATTATAAACATGTGGCAGAAAGTAGGAAAAGCAA
TGTATGCCCCTCCCATAGAAGGAGTAATTCAATGTTCATCAAAT---
ATTACAGGGCTAATATTAACAAGAGATGGTGGTGGTGGTAACGATACGATG----------------------
----

GGGAACGAGACCTTCAGACCGGGAGGGGGAGATATGAGGGACAATTGGAGGAGTGAACTATATAGATATAAAG
TAGTAAAAATTGAACCATTA---GGAATAGCACCCACCAAGGCAAAGAGAAGAGTGGTG--------------
-------------------------CAGAGAGAAAAAAGAGCA---GTG------ATAGGAGCG---------
ATGTTCCTTGGGTTCTTGGGAGCA---GCAGGAAGCACTATG---GGCGCAGCGGCA---
GTGACGCTGACGGTACAG---GCCAGACTA---TTATTG---TCTGGTATAGTGCAACAGCAAAAC---
AACTTG---CTGAGA---GCTATTGAGGCGCAACAGCATATGTTGCAACTCACAGTCTGGGGCATT
>KF384801
TTTTACTGTAATACATCACAACTGTTTAAGAGTACTTGGTGGACAAATAATACTCGGAATATACCA-------
----

CTACAAGGAAATGTCACACTCCCATGCAAAATAAAGCAAATTATAAACATGTGGCAAGAAGTAGGAAAAGCAA
TGTATGCCCCTCCCATCGGAGGACTAATTAATTGTACATCAAAT---
ATTACAGGGCTGCTATTAACAAGAGATGGTGGTATAACGAATGGG----------------------------
----

ACTGAGACCTTCAGACCTGGAGGGGGAGATATGAGGGACAATTGGAGAAGTGAATTATATAAATATAAAGTAA
TAAAAATTCAACCATTA---GGGATAGCACCCACCAAGGCAAAGAGAAGAGTGGTG-----------------
----------------------CAGAGAGAAAAAAGAGCA---GTGGGA---GTGGGAGCT---------

```
TTGTTCCTTGGGTTCTTGGGAGCA---GCAGGAAGCACTATG---GGCGCAGCGTCC---
GTAACGCTGACGGTACAG---GCCAGACAA---TTATTG---TCTGGTATAGTGCAACAGCAGAGC---
AATCTG---CTGAGG---GCTATTGAGGCGCAACAACATCTGTTGCAACTCACAGTCTGGGGCATT
>KF384803
TTCTACTGTAATTCAACACCACTGTTTAATAGTACCTGGGAAAATGGTACTTGGACGAATGAGAGTGGTATTG
GA-----------------------------------------------------------------------
----------------------------
GGATCAGATAATATCACACTTCCATGCAGAATAAAACAAATTATAAACCTGTGGCAGGAAGTAGGAAAAGCAA
TGTATGCTCCTCCCATCGAAGGACAAATTAGATGTTCATCAAAT---
ATTACAGGGCTGTTATTAACAAGAGATGGTGGTTCTAATCAGACT----------------------------
-------------------------------------------------------------------------
----------------
AACGAGACCTTCAGACCTGGAGGAGGAGATATGAGGGACAATTGGAGAAGTGAATTATATAAATATAAAGTAG
TAAAAATTGAACCATTA---GGATTAGCACCCACCAAGGCAAAGAGAAGAGTGGTG-----------------
----------------------CAGAGAGAAAAAAGAGCA---GCAGGA---CTGGGAGCT---------
CTGTTCCTTGGGTTCTTGGGAGCA---GCAGGAAGCACTATG---GGCGCAGCGTCA---
ATGACGCTGACGGTACAG---GCCAGACTA---TTATTG---TCTGGTATAGTGCAACAGCAGAAC---
AATTTG---CTGAGA---GCTATTGAGGCGCAACAGCATCTGTTGCAACTCACAGTCTGGGGCATT
>KF384804
TTCTATTGTAATACAACACAACTGTTTAATAGTACTTGGAATGTTACTGATCCTTGGAATTCTCCTGGAGGGT
CAAAT--------------------------------------------------------------------
----------------------------
AACACTGGAGATATCACACTCCCATGCAGAATAAAACAAATTGTGAACAGGTGGCAGGAAGTAGGAAAAGCAA
TGTATGCCCCTCCAATCTCAGGATATATTAACTGTTCATCAAAA---
ATTACAGGGCTGATATTAACAAGAGATGGTGGTTATAACACCACC----------------------------
-------------------------------------------------------------------------
----------
AATGAGACTGAGATCTTCAGACCTGGAGGAGGAGATATGAGGGACAATTGGAGAAGTGAATTATATAAATATA
AAGTAGTAAGAATTGAACCATTA---GGAATAGCACCTACCACGGCAAAGAGAAGGGTGGTG-----------
----------------------------CAGAGAGAAAAAAGAGCA---GTGGGA---ATGGGAGCG------
---TTGTTCCTTGGGTTCTTGGGAGCA---GCAGGAAGCACTATG---GGCGCAGCGTCA---
ATGATGCTGACGGTACAG---GCCAGACAA---TTATTG---TCTGGTATAGTGCAACAGCAGAAC---
AATCTG---CTGAGG---GCTATTGAGGCGCAACAACATCTGTTGCAACTCACAGTCTGGGGCATC
>KF384807
TTCTACTGTAACACAACAACACTGTTCAATAGTACTTGGGATGGGAATGATACTACTGAAAGGTCAAAGAGTA
CTGAAGAA-----------------------------------------------------------------
----------------------------
AATGCAACAATCATCACAATCCCATGCAGAATAAAACAAATTATAAACATGTGGCAGAAAGTAGGAAAAGCAA
TATATGCCCCTCCCATCAAAGGAAACATTAGTTGTTCATCAAAT---
ATTACAGGGCTGCTATTAACAAGAGATGGTGGGAATAACGAGGAC----------------------------
-------------------------------------------------------------------------
----------
AGCACCAATGAGACCTTCAGACCTCAAGGAGGAGATATGAGGGACAATTGGAGAAGTGAGCTATATAAATACA
AAGTAGTAAGAATTGAACCATTA---GGAATAGCACCCACCAAGGCAAGGAGAAGAGTGGTG-----------
----------------------------CAGAGAGAAAAAAGAGCA---GTGGGA---ATAGGAGCT------
---TTGTTCCTTGGGTTCTTGGGAGCA---GCAGGAAGCACTATG---GGCGCAGCGTCG---
CTGACGCTGACGGTACAG---GCCAGACAA---TTATTG---TCTGGTATAGTGCAACAGCAGAAC---
AATCTG---CTGAGA---GCTATTGAGGCGCAACAGCATATGTTGCAACTCACAGTCTGGGGCATC
>KC473835
TTCTACTGTAATACAACACCACTATTTAATAGTACTTGGCATGGGAATGAGAGTGATAGTATAGAATCAAATA
ACAGT--------------------------------------------------------------------
----------------------------
ACAGAAGAAACCATCACAATTCAATGCAGAATAAAACAAATTATAAACATGTGGCAAGAGGTGGGAAAAGCAA
TGTATGCCCCTCCCATTAGAGGACAGATTAGATGTTCATCAAAC---
ATCACAGGACTGCTATTAACAAGAGATGGTGGTAATAATAACAAG----------------------------
-------------------------------------------------------------------------
----------------
AGTGAGACCTTCAGACCGGGAGGAGGAAATATGAAGGACAATTGGAGAAGTGAACTATATAAATATAAAGTAG
TAAAAATTGAACCATTA---GGATTAGCACCCACCAGGGCAAAGAGAAGAGTGGTG-----------------
----------------------CAGAGAGAAAAAAGAGCA---GTGGGA---TTCGGAGCT---------
```

TTGTTCCTTGGGTTCTTGGGAGCA---GCAGGAAGCACTATG---GGCGCAGCGTCA---
GTGACGCTGACGGTACAG---GCCAGACAA---TTATTG---TCTGGTATAGTGCAACAGCAGAGC---
AATCTG---CTGAGG---GCTATTGAGGCGCAACAGCATCTGTTGCAACTCACAGTCTGGGGCATC
>KF384808
TTCTACTGTAATACATCAAAACTGTTTAATAGTACTTGGAATAATAATAGTACTTGGAATAATAACAGTATTT
GGAATGCTACT--

CAAAATGAAACTATCACACTTCCATGCAGAATAAAACAAATTATAAACATGTGGCAGGAAGTAGGAAAAGCAA
TGTATGCCCCTCCCATCAGAGGAGAAATTAGATGTGCATCAAAC---
ATTACTGGACTGCTATTAACAAGAGATGGTGGTAACAATGGGAGT----------------------------
----

GAGAGTGAGACCTTCAGACCTGGAGGAGGAGATATGAGGGACAATTGGAGAAGTGAATTATATAAATACAAAG
TAGTAAAAATTAAACCATTA---GGAGTAGCACCCACCAAGGCAAAGAGAAGAGTGGTG--------------
-------------------------CAGAGAGAAAAAAGAGCA---GTGGGA---ATAGGAGCT---------
GTGTTCCTTGGGTTCTTGGGAGCA---GCAGGAAGCACTATG---GGCGCAGCGTCA---
ATAACGCTGACGGTACAG---GCCAGAAAG---TTACTG---TCTGGTATAGTGCAACAGCAGAAC---
AATCTG---CTGAGG---GCTATTGATGCGCAACAGCATCTGTTGCAACTCACAGTCTGGGGCATC
>KF384811
TTCTACTGTAATTCATCAAAACTGTTTAATAATACTTGGAATGGTACTAACTGGACAGATACATGGTATAATA
GC---

ACAAATGAAACTATCATACTCCCGTGCAGAATAAAACAAGTTATAAACATGTGGCAGGAAGTAGGAAAAGCAA
TGTATGCCCCTCCCATCAGAGGAAAAATTAAATGTAAATCAAAC---
ATTACAGGGCTGTTGTTAACAAGAGATGGCGGTGGTGGGAGCAATGAA-------------------------
----

ACCACCGAGACCTTCAGACCTGGAGGAGGAAATATGAGGGACAATTGGAGAAGTGAATTATATAAATATAAAG
TAGTAAAAATTCAACCAGTA---GGCTTGGCACCCACCAGGGCAAAAAGAAGAGTGGTG--------------
-------------------------CAGAGAGAAAAAAGAGCA---GTAACA---CTGGGAGCT---------
GTGTTCCTTGGGTTCTTGGGAGCA---GCAGGAAGCACTATG---GGCGCAGCGTCA---
ATGACGCTGACGGTACAG---GCCAGACAA---TTATTG---TCTGGTATAGTGCAACAGCAGAGC---
AATTTG---CTGAAA---GCTATTGAGGCGCAACAGCATCTGTTGCAACTCACAGTCTGGGGCATT
>KX156409
TTTTACTGTAATACAACAAAACTGTTCAATAGTACTTGGAATAGTAATAGTACTAGCAACAACACTAAAGGA-
----

AATGAGACTATTATCGAAATACCATGCAGGATAAGACAAATTATAAACATGTGGCAGCAAGTAGGAAGAGCAA
TGTATGCCCCTCCCATCAGAGGACTAATTAGATGTTCATCAAAC---
ATTACAGGACTAATATTAACAAGAGATGGTGGTCATAATCAGGCC----------------------------
----

AATGACAGTGAGATCTTCAGACCTGGAGGAGGAGATATGAGGGACAATTGGAGAAGTGAATTATATAAATATA
AAGTAGTAAGAATTGAACCATTA---GGAATAGCACCCACCAAGGCAAAAAGAAGAGTGGTG-----------
----------------------------CAGAGAGAAAAAAGAGCA---GTGGGA---CTAGGAGCT------
---ATGTTCCTGGGGTTCTTAGGAGCA---GCAGGAAGCACTATG---GGCGCAGCGTCA---
GTGACGCTGACGGTACAA---GCCAGACAA---TTATTG---TCTGGTATAGTGCAACAGCAGAAC---
AATTTG---CTGAGA---GCTATTGAGGCGCAACAGCATATGTTGCAACTCACAGTCTGGGGCATT
>KY778615
TTCTACTGTAATACATCAAAGTTGTTTAACAGTACTTGGAATGGCACTGAGGAAAACCAGGGA----------
----

GTGAATGGCACACTCACACTCCCATGCAGAATAAAACAAATCATAAACATGTGGCAGAAAGTAGGAAAAGCAA
TGTATGCCCCTCCCATCCAGGGAAATATTAATTGCTCATCAAAT---
ATTACAGGGCTGCTATTAATGAGAGATGGTGGTAACAAGGGGAACGGAACC----------------------
----

GATAATACTACCGAGATCTTTAGACCTGGAGGAGGAAATATGAGGGACAATTGGAGAAGTGAATTATATAAAT
ATAAAGTAGTGAGAATTGAACCATTA---GGAGTAGCACCCACCAAGGCAAAAAGAAGAGTGGTG--------
-------------------------------CAGAGAGAAAAAAGAGCG---GTAACA---GTGGGAGCT---

------ATGTTCCTTGGGTTCTTGGGAGCA---GCAGGAAGCACTATG---GGCGCAGCATCA---
TTGACGCTGACGGTACAG---GCCAGACAA---TTATTG---TCTGGTATAGTGCAACAGCAAAGC---
AATTTG---CTGAAG---GCTATTCAGGCGCAACAGCATCTGTTGCAGCTCACAGTCTGGGGCATC
>KT124768
TTCTACTGTAATACATCAGAACTGTTTAATAGTACTTGGTATAACAGTACATACAATGAGACTGCACCA----
----

GGCAATGAAACAATCACACTCCCATGCAAAATAAGGCAGTTTGTAAACAGGTGGCAGGAAGTAGGAAAAGCAA
TGTATGCCCCTCCCATCAGAGGAATAATTAGATGTTTATCAAAT---
ATTACAGGATTGATATTAACAAGAGATGGGGGTATCTATAATAAC----------------------------
----
ACGAATGGGACCATT---
GGGAATGAGACTTTCAGACCTGGAGGGGGAGATATGAGGGACAATTGGAGAAGTGAATTATATAAATATAAAG
TAGTAAAAATTGAACCATTA---GGAGTAGCACCCACCAAAGCAAGGAGAAGAGTGGTG--------------
-------------------------CAGAGAGAAAAAAGAGCA---GTGGGA---TTCGGAGCT---------
ATGTTCCTTGGGTTCTTGGGAGCA---GCAGGAAGCACTATG---GGCGCAGCGTCA---
GTGACGCTGACGGTACAG---GCCAGGCAA---TTATTG---TCTGGTATAGTGCAGCAGCAAAAC---
AACTTG---CTGAGG---GCTATTGAGGCGCAACAGCATATGTTGCAACTCACAGTCTGGGGCATT
>MG196879
TTCTACTGTAATACAACGCAACTGTTTAGGGGTAATGGTACTGGTAATGGTACTGGAGAG-------------
----

TCAAGTGACATTGTCACACTCCCATGCAGAATAAAACAAATTGTAAACATGTGGCAGAAAGTAGGAAAAGCTA
TGTATGCCCCTCCCATCAAAGGACTAATTAGATGTTCATCAAAT---
ATTACAGGGCTAATATTAACAAGAGATGGTAGTAATAAAAGTAAA----------------------------
----

AGTGATCCAGAGATCTTCAGACCTGCAGGAGGAGATATGAGGGACAATTGGAGAAGTGAATTATATAAATATA
AAGTAGTAAAAATTCAACCATTA---GGAGTAGCACCCACCAAGGCAAAGAGAAGAGTGGTG-----------
----------------------------CAGAGAGAAAAAAGAGCA---GTGGGA---ATAGGAGCT------
---GTGTTCCTTGGGTTCTTAGGAGCA---GCAGGAAGCACTATG---GGCGCAGCGTCA---
ATAACGCTGACGGTACAG---GCCAGACAA---TTATTG---TCTGGTATAGTGCAACAGCAGAAC---
AATCTG---CTGAGA---GCTATTGAGGCGCAACAGCATCTGTTGCAACTCACAGTCTGGGGCATC
>MG197013
TTCTACTGTAATACAACACAGCTGTTTAATAGCACTTGGAATAGCACGACTAATAATGACACTGAAGGATCA-
----

AGTAATGCTACGATCACACTCCCATGTAGAATAAGACAAATTATAAACATGTGGCAGACAGTAGGAAAAGCAA
TGTATGCCCCTCCCATCAGAGGAGAGATTAGCTGTTCATCAAAT---
ATTACAGGGCTGCTGTTAACAAGAGATGGT---AATACCAATCACACT-------------------------
----

GATGCCACTGAGACCTTTAGACCTGGAGGAGGAGATATGAGGGACAATTGGAGAAGTGAATTATATAAATATA
AAGTGATACAACTTGAACCAATA---GGAGTAGCACCCACCAAGGCAAAGAGAAGAGTGGTG-----------
----------------------------CAAAGAGAAAAAAGAGCA---GTGGGGGCAATAGGAGCT------
---ATGTTCCTTGGGTTCTTGGGAGCA---GCAGGAAGCACTATG---GGCGCAGCGTCA---
ATAACGCTGACGGTACAG---GCCAGACTA---TTATTG---TCTGGTATTGTGCAACAGCAAAAC---
AATTTG---CTGAGG---GCTATTGAGGCGCAACAGCATCTGTTGCAACTCACAGTCTGGGGCATC
>MG197046
TTCTACTGTAATACCTCAAAACTGTTTAACAGTACATGGCCAAATAATACTAAAAATGCTAATATC-------
----

ACTGAAGGAACTATCACACTCCCGTGCAGAATAAAACAAATTATAAACATGTGGCAGGAAGTAGGAAAGGCAA
TGTATGCCCCTCCCATCAGAGGACAACTTAACTGTTCATCAAAT---
ATTACAGGGCTGCTATTAACAAGAGATGGGGGTTATAATCAGGCG----------------------------
----

AACAATAGCGAGACCTTCAGACCGGGAGGAGGAGATATGAGGAACAATTGGAGAAGTGAATTATATAAATATA
AAGTAGTAAGAGTTGAACCATTA---GGAGTAGCACCCACCAAGGCAAAGAGAAGAGTGGTG-----------
----------------------------CAAAGAGAAAAGAGAGCA---GTGGGA---ATGGGAGCT------

---TTGTTCCTTGGGTTCTTGGGAGCA---GCAGGAAGCACTATG---GGCGCAGCGTCA---
CTAACGCTGACGGTACAA---GCCAGACAA---TTATTG---TCTGGTATAGTGCAACAGCAAAAC---
AATTTG---CTGAGA---GCTATTGAGGCGCAGCAGCATATGTTGCAACTCACAGTCTGGGGCATC
>MG197064
TTCTACTGTAATACATCAAAACTGTTTAACAATACCTGGACTAAGGGTACTTGGAATAGTACATGGTCATGGG
CA---

AATGAAACTAACATCACACTCCCATGCAGAATAAAACAGATTATAAACATGTGGCAGGAAGTAGGAAAAGCAA
TGTATGCCCCTCCCATCGAAGGAATAATTAGATGTGCCTCAAAC---
ATTACAGGGCTGCTATTAACAAGAGATGGTGGTAAACATGATAATATTACC----------------------
----
ATTAATGGTAATGTCATTGACTATAAC------
ACCACCGAGGTCTTCAGACCTGGAGGGGGAGATATGAGGGACAATTGGAGAAGTGAATTATATAAATACAAAG
TAGTACAAATTGAACCATTA---GGAGTAGCACCCACTAAGGCAAAAAGAAGAGTGGTG--------------
-------------------------CAGAGAGAAAAAAGAGCA---GTGGTA---CTAGGAGCT---------
TTGTTCCTTGGGTTCTTGGGAGCA---GCAGGAAGCACTATG---GGCGCAGCGTCA---
ATGACGCTGACGGTACAG---GCCAGACTA---TTATTG---TCTGGTATAGTGCAACAGCAGAAC---
AATTTG---CTGAGG---GCTATTGAGGCGCAACAACATCTGTTGCAACTCACAGTCTGGGGCATT
>MG197113
TTCTACTGTAACACATCAAAGTTGTTTAATAGCACTTGGAACTATAATAATGCTACAGGAAACACTGAAGGAA
ATAGCACACAA--

GGAAATATCACACTCATCACACTCCCATGCAGAATAAAACAGATTATAAACAGGTGGCAGGAAGTAGGAAAAG
CAATGTATGCCCCTCCCATCAGCGGACAAATTAACTGTTCATCAAAT---
ATTACAGGACTACTATTAACAAGAGATGGTGGTCATAATAACAAT----------------------------
----

AGCACAATTGAGACCTTTAGACCTACAGGAGGAGATATGAGGAACAATTGGAGAAGTGAATTATATAAATATA
AAGTAGTAAAAATTGAGCCATTA---GGAATAGCACCCACCAAGGCAAAAAGAAGAGTGGTG-----------
----------------------------CAGAGAGAAAAAAGAGCA---GTAGGAACGCTGGGAGCT------
---ATGTTCCTTGGGTTCTTAGGAGCA---GCAGGAAGCACTATG---GGCGCAGCGTCA---
GTGACGTTGACGGTACAG---GCCAGACTA---TTATTG---TCTGGTATAGTGCAGCAGCAGAGC---
AAGCTG---CTGAGG---GCTATTGAGGCGCAACAGCATATGTTGCAACTCACAGTCTGGGGCATC
>KM355159
TTCTATTGTAATTTAACAAAACTGTTTAATAGTACTTGGAATAAGACTGAAGGGCCTTGG-------------
----
AATGTTACTGAAGAGTATGGG------------
AATATTACTGACCCTATCATACTCCCATGCAGAATAAAACAAATTATAAACATGTGGCAGAGAGTAGGAAGGG
CAATGTATGCCCCTCCCATCAAAGGCAAACTTCAATGTACATCAAAT---
ATTACAGGGCTCCTATTAACAAGAGATGGTGGT--

-
AAGGTGAATAATGAGACTGAGATCTTTAGGCCTGCAGGAGGAGATATGAGGGACAATTGGAGAAGCGAATTAT
ATAAATATAAAGTAGTAAAAATTGAACCATTA---GGAATAGCACCCACCAGGGCGAAGAGAAGAGTGGTG--
-------------------------------------CAGAGAGAAAAAAGAGCA---GTGGGA---
TTAGGAGCT---------ATGTTCCTTGGGTTCTTGGGAGCA---GCAGGAAGCACTATG---
GGCGCAGCGGCA---GTGACGCTGACGGTACAG---GCCAGACAA---TTATTG---
TCTGGTATAGTGCAGCAGCAGAAC---AATCTG---CTGAAG---
GCTATTGAGGCGCAGCAGCATCTGTTGCAACTCACAGTCTGGGGCATC
>MG196745
TTCTACTGTAATTCAACACCACTGTTTAATGGGACTTGGCAGAATATGACGAATATTACT-------------
----

GAAAACTTAAATATCACACTCCCATGCAGAATAAAGCAGATTATAAACATGTGGCAGAAAGTAGGAAAAGCAA
TGTATGCCCCTCCCATCAGTGGAAGTATTCAATGTTCATCAAAC---
ATTACAGGGCTACTGTTAACAAGAGATGGTGGCATCACTAATAACACTAATAGC-------------------
----

AGTAATGATGAGACCTTCAGACCTGGAGGGGGAGATATGAGGGACAATTGGAGAAGTGAATTATATAAATATA
AAGTAGTAAAAATTGAACCTTTA---GGATTAGCACCCACCAGGGCAAGGAGACTAGTGGTG-----------

----------------------------CAAAGAGACAAAAGAGCA---GTGGGA---ATGGGAGCT------
---ATGTTCCTTGGGTTCTTGGGAGCA---GCAGGAAGCACTATG---GGCGCGGCGTCA---
ATGACGCTGACGGTACAG---GCCAGACAA---TTATTG---TCTGGTATAGTGCAACAGCAAAAC---
AACTTG---CTGAAG---GCTATTGAGGCGCAACAGCACCTGTTGCGACTCACAGTCTGGGGCATC
>MG196964
TTCTACTGTAATACAACAAAATTGTTTAATAGTACTTGGACTGAGAATAGTACTGAAGGGTCAAAC-------
----

AGCACTGAAATAATCACACTCCCATGCAGGATAAAACAAATTATAAACATGTGGCAGAGAGTAGGGAGAGCAA
TGTATGCCCCTCCCATCAAAGGACCAATTAGATGTTCATCAAAT---
ATTACAGGGCTGCTATTAACAAGAGATGGTGGTCATAATATGACT----------------------------
----

AATGATAGTGAGACCTTCAGACCTGTGGGAGGAGATATGAAGAACAATTGGAGAAGTGAATTATATAAATATA
AAGTAGTAAAAGTCGAACCATTA---GGAGTAGCACCCACCAAGGCAAGGAGAAGAGTGGTG-----------
----------------------------CAGAGAGAGAAAAGAGCA---GTGGGA---ATAGGAGCT------
---GTGTTCCTTGGGTTCTTGGGAGCA---GCAGGAAGCACTATG---GGCGCAGCGTCA---
ATGACGCTGACGGTACAG---GCCAGACAA---CTATTG---TCTGGTATAGTGCAACAGCAGAAC---
AACTTG---CTAAGA---GCTATTGAAGCGCAACAGCATCTGTTGCAGCTCACAGTCTGGGGCATT
>MG197035
TTCTACTGTGATACAACACAACTGTTCAATATTACTTGGGATACTAACAACACTAGTAATAGCAATGGTACT-
----

GACGGTGATAAAATCACACTCCCATGCAGAATAAAACAAATTATAAATCTGTGGCAGGGAGTAGGAAGAGCAA
TGTATGCCCCTCCCATCAGCGGACTAATTAGCTGTTCATCAAAT---
ATTACAGGTCTGCTGTTAGTAAGTGATAGTGGTAATCAAACTAAC----------------------------
----

GAGACTGAGACCTTCAGACCTATAGGAGGAGAGATGAGGGACATTTGGAGAAGTGAATTATACAAGTATAAAG
TAGTACAAATTGAACCATTA---GGAGTAGCACCCACCAAGGCAAAGAGAAGAGTGGTG--------------
-------------------------CAGAGAGAAAAAAGAGAG---CTGACA---TTAGGAGCT---------
TTGTTCATTGGGTTCTTGGGGGCA---GCAGGAAGCACTATG---GGCGCAGCGTCG---
ATGACGCTGACGGTACAG---GCCAGACTA---TTATTG---TCTGGTATAGTGCAACAGCAGAAC---
AATCTG---CTGAGA---GCTATTGAGGCGCAACAGCATCTGTTGCAACTCACAGTCTGGGGCATC
>MK214316
TTCTACTGTAATACCGCACAACTGTTTAATAGTAGTTGGACTACTACTGAAAAGACAAATAACACTGAAGGAG
GA---

AATGACACAATAATCATGCTCCCTTGCAGAATAAAACAATTTATAAACAGGTGGCAGGAAGTAGGAAAAGCAA
TGTATGCCCCTCCAATCAGCGGACTAATTAGATGTTCATCAAAC---
ATTACAGGGCTACTGTTAACAAGAGATGGTGGTAGCAGCAATAAAACT-------------------------
----

AATGAAACTGAGATCTTCAGACCTCATGGAGGGGATATGAGGGACAATTGGAGAAGTGAATTATATAAATATA
AAGTAGTAAAAATTGAACCATTA---GGAATAGCACCTAGCAAGGCAAAGAGAAGAGTGGTG-----------
----------------------------CAGAGAGAAAAAAGAGCG---GTGACA---ATAGGAGCT------
---GTGTTCCTTGGGTTCTTGGGAGCA---GCAGGAAGCACTATG---GGCGCAGCGTCA---
CTGACGCTGACGGTACAG---GCCAGACAA---CTATTG---TCTGGTATAGTGCAACAACAAAGC---
AATTTG---TTGAGG---GCTATTGAGGCGCAACAGCACCTGTTGCAACTCACAGTCTGGGGCATC
>KX505616
TTCTACTGTAATACAACACAACTGTTTAATAGTACTTGGAAGGAAAATAGTACTGGGAATAATACTGAAGAG-
----

TTAAATGACACTATCACACTCCCATGTAGAATAAAACAAATTATAAACATGTGGCAGGAAGTAGGAAAAGCAA
TGTATGCCCCTCCCATCCAAGGACAAATTAGATGTTCATCAAAT---
ATTACAGGGCTAGTATTAACAAGAGATGGTGGTCATACCAATAAGACTGAG----------------------
----

AACAAGACTGAGGTCTTCAGACCTGTAGGAGGGAATATGAAGGACAATTGGAGAAGTGAATTATATAAATATA
AAGTAGTAAAAATTGAACCATTA---GGAGTAGCACCCACTAAGGCAAAGAGAAGAGTGGTG-----------

----------------------------CAGAGAGAAAAAAGAGCA---GCA------ATAGGAGCT------
---GTGTTCCTTGGGTTCTTGGGAGCA---GCAGGAAGCACTATG---GGCGCAGCGTCG---
ATAACGCTGACGGTACAG---GCCAGACTA---TTATTG---TCTGGTATAGTGCAACAGCAGAGC---
AATCTG---CTGAGG---GCTATTGAGGCGCAACAGCAGCTGTTGCAGCTTACAGTCTGGGGCATC
>KR182192
TTCTACTGCGATACAACACAGCTGTTTAACAGTACTTGGTGGACTAATGAAACTTGGAAGGATATCGAAGGGT
CA---

AATAACACTAACATCACACTTCCATGCAGGATAAAACAAATTATAAACAGGTGGCAGGGAGTAGGAAAAGCAA
TGTATGCCCCTCCCATCAAAGGACAAATTAGCTGTTCATCAAAT---
ATTACAGGGCTAATATTAACAAGAGATGGTGGTACTCACGAGGAC----------------------------
----

GAGACCCCTGAGATCTTCAGACCTGGAGGAGGAGATATGAGGGACAATTGGAGAAGTGAATTATATAAATATA
AAGTAGTAAAAATTGAGCCATTA---GGAGTAGCACCCACCAAGGCAAAGAGGAGAGTGGTG-----------
----------------------------CAGAGAGAAAGAAGAGCA---GTGGGAACGCTGGGAGCT------
---ATGTTCCTTGGGTTCTTGGGAGCA---GCAGGAAGCACTATG---GGCGCAGCATCG---
ATAACGCTGACGGTACAA---GCCAGACAA---TTATTG---TCTGGTATAGTGCAACAGCAGAAC---
AACTTG---CTGAAG---GCTATTGAGGCGCAACAGCACCTGTTGCAACTCACAGTCTGGGGCATC
>KR182242
TTCTATTGTAATACAACACCACTGTTTAACAGTACTTGGGTGCTTAACAGTACTTTGAATAGTACTGAAGGGT
CCAATAGT---

ACTGGACAAAATATCACACTCCAATGTAGAATAAAGCAAATTATAAATATGTGGCAGGAGGTAGGAAAAGCAA
TGTTTGCCCCTCCCATCAGAGGATCAATTCAATGTTCATCCAAC---
ATTACAGGGCTGCTACTAACAAGAGATGGTGGTAATGACAATAAC----------------------------
----

AAGACTGAGATCTTTAGGCCTTGGGGAGGAGATATGAAGGACAATTGGAGAAGTGAATTATATAAATATAAAG
TAATAAAAATTGAACCATTA---GGAGTAGCACCCACCAAGGCAAAGAGAAGAGTGGTG--------------
-------------------------CAGAGAGAAAAAAGAGCG---GCAGGA---CTGGGAGCT---------
CTGCTCATTGGGTTCTTGGGAGCA---GCAGGAAGCACAATG---GGCGCAGCGTCA---
GTGACGCTGACGGTACAG---GCCAGACTA---TTATTG---TCTGGTATTGTGCAACAGCAGAGC---
AATCTG---CTGAGA---GCTATTGAGGCGCAACAGCATATGTTGAAGCTCACAGTCTGGGGCATC
>KR182343
TTCTACTGTGATACAACAAAACTGTTTAATAGTACTTGGCCTTGGCCTGCTAATAAAACAGATGATGGCACAA
ATGCA--
-------------------ACA---
GCATCAGACAAAAATATCACACTCCCATGCAGAATAAAACAAATTATAAACATGTGGCAGAAAGTGGGAAGAG
CAATATATGCCCCTCCCATCAAAGGACCAATTGAATGTGTATCAAAT---
ATTACAGGGATAATATTAACAAGGGATGGTGATAAGAATAATACC----------------------------
----

TACAATGAGACCTTCAGACCTACAGGAGGAGAAATGAAGGACAATTGGAGAAGTGAATTATATAAATATAAAG
TAATAAAAATTGAACCATTA---GGAATAGCACCTACCAGGGCAAAGAGAAGAGTGGTG--------------
-------------------------CAGAGAGAAAAAAGAGCA---GTGGGA---ATAGGAGCT---------
CTGCTTCTTGGGTTCTTGGGAGCA---GCAGGAAGCACTATG---GGCGCAGCGTCA---
ATGACGCTGACGGTACAA---GCCAGGCAA---TTACTG---TCTGGCATAGTGCAACAGCAGAAC---
AATCTG---CTGAAA---GCTATTGAGGCGCAACAGCAGCTGTTGCAACTCACAGTCTGGGGCATC
>KT986555
TTCTACTGTAATACTACACAACTGTTTAATAGTACTTGGAATAAGAATAAAACTAATAATACTTGGGAAAGGA
ATGACACA---
-------------------AAT---
AGTAATGAAACAACAATCACAATCCCATGCAGAATAAAACAAATTATAAACATGTGGCAGGGAGTAGGCAAGG
CAATGTATGCCCCTCCCATCAGAGGATTAATCAGATGTTCATCAAAC---
ATTACAGGACTGATATTAACAAGAGATGGTGGTCAGAGTAATGAAACAGGG----------------------
----

AACAACACCGAGATATTCAGACCTGGAGGAGGAGACATGAGGGACAATTGGAGAAGTGAATTATATAGATATA
AAGTAGTAGAAATTGAACCATTA---GGAATAGCACCCACCAAGGCGAAGAGAAGAGTGGTG-----------

----------------------------CAGAGAGAAAAAAGAGCG---GTCACA---CTAGGAGCT------
---GTGTTCCTTGGGTTCTTGGGAGCA---GCAGGAAGCACTATG---GGCGCAGCGTCA---
ATGACGTTGACGGTACAA---GCCAGACAG---TTATTG---TCTGGTATAGTGCAACAGCAGAAC---
AATCTG---CTAAGA---GCTATTGAGGCGCAACAGCACCTGTTGCAACTCACGGTCTGGGGCATC
>KX505508
TTCTATTGTAATTTAACAAAACTGTTTAATAGTACTTGGAATAAGACTGAAGGGCCTTGGAATGTTACTGAAG
AGTATGGG---

AATATTACTGACCCTATCATACTCCCATGCAGAATAAAACAAATTATAAACATGTGGCAGAGAGTAGGAAGGG
CAATGTATGCCCCTCCCATCAAAGGCAAACTTCAATGTACATCAAAT---
ATTACAGGGCTCCTATTAACAAGAGATGGTGGTAAGGTGAATAAT----------------------------
----

GAGACTGAGATCTTTAGGCCTGCAGGAGGAGATATGAGGGACAATTGGAGAAGTGAATTATATAAATATAAAG
TAGTAAAAATTGAACCATTA---GGAATAGCACCCACCAGGGCGAAGAGAAGAGTGGTG--------------
-------------------------CAGAGAGAAAAAAGAGCA---GTGGGA---TTAGGAGCT---------
ATGTTCCTTGGGTTCTTGGGAGCA---GCAGGAAGCACTATG---GGCGCAGCGGCA---
GTGACGCTGACGGTACAG---GCCAGACAA---TTATTG---TCTGGTATAGTGCAGCAGCAGAAC---
AATCTG---CTGAAG---GCTATTGAGGCGCAGCAGCATCTGTTGCAACTCACAGTCTGGGGCATC
>KX027971
TTCTACTGTAATACAACACGACTGTTCGAAAGTACTTGGCATGGAAATGAGACTTGGTATGGGAATGAG----
----
-ACTATAGAGCCAGAG---------
GCAAATGACACAATAATCGAACTCCCATGCAGAATAAAACAAATTATAAACATGTGGCAGGAAGTAGGAAAAG
CAATGTACGCCCCTCCCATCAGAGGACCAATTAAATGTTCATCAAAT---
ATTACAGGGCTACTATTAACAAGAGATGGTGGTGATGATCAGTACAGTAAGAAT------
GAGACCTCCTGCAATAAT---

ACTACTGAGACCTTCAGACCTGGAGGAGGGAATATGAAGGACAATTGGAGAAGTGAATTATATAAATATAAAG
TAGTAAAAATAGAACCATTA---GGAGTAGCACCCACCAAGGCAAAAAGAAGAGTGGTG--------------
-------------------------CAGAGAGAAAAAAGAGCA---GTAGGA---ATAGGAGCT---------
ATGTTCCTTGGGTTCTTGGGAGCA---GCAGGAAGCACTATG---GGCGCAGCATCA---
ATAACGCTGACGGTACAG---GCCAGACAA---TTATTG---TCTGGTATAGTGCAACAGCAGAAC---
AATCTG---CTAAGG---GCTATTGAGGCGCAACAACATCTGTTGCAACTCACAGTCTGGGGCATC
>KX505446
TTCTACTGCAATACAACACAACTATTTAATAGTACTTGGTCAAAAAATAATACTTGGGAGAAGAATGAAGACA
ATTCA--

ATTAATAGTACTATCACACTCCCATGCAGAATAAAACAAATTATAAACATGTGGCAGGAAGTAGGAAAAGCAA
TGTATACCTCCCCAATCAAAGGACAGATTAGCTGTAACTCAAGT---
ATTACAGGGATACTATTAACAAGAGATGGTGGAAGAAATAATAGT----------------------------
----

GACAATGAGACCTTCAGACCTGGAGGAGGAGATATGAGGGACAATTGGAGAAGTGAATTATATAAATATAAAG
TAGTAAAAATTGTACCATTA---GGAATAGCACCCACTGCGGCAAAAAGAAGAGTGGTG--------------
-------------------------CAGAGAGAAAAAAGAGCA---GCA------CTAGGAGCT---------
ATGTTCCTTGGGTTCTTGGGAGCA---GCAGGAAGCACTATG---GGCGCAGCATCA---
GTGACGCTGACGGTACAG---GCCAGACTA---TTATTG---TCTGGTATAGTGCAACAGCAGAAC---
AATCTG---CTGAGG---GCTATTGAGGCGCAACATCATCTGTTGCAACTCACAGTCTGGGGCATC
>KT986947
TTCTACTGTGATACAACAAAACTATTTAATAGTACTTGGAATAGTACTTGGGACAATAATAGTACTCGGAATA
AT---
AACAGATCAGAGGACAGATCA------------
GAGAGCGATGACACAATCACACTACCATGCAGAATAAGACAAATTATAAACAGATGGCAAGAAGTAGGAAAAG
CAATGTATGCCCCTCCCATCAAAGGACTAATTGAATGTAACTCAACA---
ATTACAGGGCTGCTCTTAGCAAGAGATGGTGGTATCAACAGCACA----------------------------
----

GGGAATGAGACCTTCAGACCTCAAGGAGGAAATATGAGGGACAATTGGAGAAGTGAATTATATAAGTATAAAA
TAGTAAAAATTGAACCATTA---GGAATAGCACCCACTAAGGCAAAAAGAAGAGTGGTG--------------

------------------------CAGAGAGAAAAAAGAGGA---GCGGGA---CTAGGAGCT---------
TTGTTCCTTGGATTCTTGGGAGCA---GCAGGAAGCACTATG---GGCGCAGCGTCA---
GTGACGCTGACGGTACAA---GCCAGACAA---TTATTG---TCTGGTATAGTGCAACAGCAGAAC---
AATCTG---CTGAGG---GCTATTGAGGCGCAGCAGCATATGTTGCAACTCACAGTCTGGGGCATC
>MH264238
TTCTACTGTAATTCAACACAGCTGTTTAATAGTACTTGGTATCTTAATGGTACTTGGAATGCAACTGAAGGGT
TAAATAAC--

ACTGAAGGAAATATCACACTCCCATGCAGAATAAAACAAATTATAAACATGTGGCAGCAGGTAGGAAAAGCAA
TGTATGCCCCTCCTATCAGAGGACTAATTAGATGCACATCAAAC---
ATTACAGGGATAATATTAACAAGAGATGGTGGTTACGAGAACAAGACCGATAAC------------------
----

AATACTAATACTGAGATCTTCAGACCTGGAGGAGGAGATATGAGGGACAATTGGAGAAGTGAATTATATAAAT
ATAAAGTAGTAAGAATTGAACCATTA---GGAGTAGCACCCACCAAGGCAAAGAGAAGAGTGGTG-------
------------------------------CAGAGAGAAAAAAGAGCA---GTGGGA---ATAGGAGCC---
------TTGTTCCTTGGGTTCTTGGGAGCG---GCAGGAAGCACTATG---GGCGCAGCGTCA---
ATGACGCTGACGGTACAG---GCCAGACAA---TTATTG---TCTGGTATAGTGCAACAGCAAAAC---
AACTTG---CTGAGG---GCTATTGAGGCGCAACAGCATCTGTTGCAACTCACAGTCTGGGGCATC
>KX587339
TTCTATTGTAATACAACAAACCTGTTTAATAGTACATGGCATACTAACGGAACAAATAGCACTGACAAT----
----

GGTACTGACACAATCACAATCCAATGCAGAATAAAACAAATAATAAACACGTGGCAGGAAGTAGGAAAAGCAA
TGTATGCCCCTCCCATCCAAGGACAACTTAACTGTTCATCAAAT---
ATTACAGGGCTGCTATTAACAAGAGATGGTGGTAATGATAGTAATACT------------------------
----

ACCAATAACACTGAGATCTTCAGACCTCAAGGAGGAAATATGAGAGACAATTGGAGAAGTGAATTATATAAAT
ATAAAGTAGTAAAAATTGAACCATTA---GGAGTAGCACCCACCGAGGCAAAGAGAAGAGTGGTG-------
------------------------------CAGAGAGAAAGAAGAGCA---GTGGGA---CTAGGAGCT---
------ATGTTCCTTGGGTTCTTAGGAGCA---GCAGGAAGCACTATG---GGCGCAGCGTCA---
TTGACGCTGACGGTACAG---GCCAGACAA---TTATTG---TCTGGTATAGTGCAACAGCAGAAC---
AATTTG---CTGAGA---GCTATTGAGGCGCAACAGCATCTGTTGCAACTCACAGTCTGGGGCATT
>KX505536
TTCTACTGTAATACAACACAGCTGTTCAATAGTACATGGGATTGTACTGGAACTGCATGTACATTGAATGGA-
----
-ACTGTTCTTAATGGG---------
ATAAATAACACCAATATTACTCTCCCATGCAGGATAAAACAAATTATAAACATGTGGCAAAAAGTAGGAAAAG
CAATGTACAACCCTCCCATCAAAGGAGTAATTCAATGTACATCAAAT---
ATTACAGGGATAATATTAACAAGCGATAATGGTGGGATTGGAAAT---------------------------
----

AATGAAAATGAAACCTTCAGACCTGCAGGAGGAGATATGAGGGACAATTGGAGAAGTGAATTATATAAATATA
AAGTAGTAAGAATTGAGCCATTA---GGAATAGCACCCACCAAGGCAAAAAGGAGAGTGGTG----------
---------------------------CAGAGAGAAAAAAGAGCA---GTAACA---TTAGGAGCT------
---ATGTTCATTGGGTTCTTGGGAGCA---GCAGGAAGCACTATG---GGCGCAGCGTCA---
GTGACGCTGACGGTACAG---GCCAGACAA---TTGCTG---TCTGGTATAGTGCAACAGCAGAGC---
AATCTG---CTGAAG---GCTATTGAGGCGCAACAGCATCTGTTGCAACTCACAGTCTGGGGCATC
>MG196412
TTCTACTGTAATTCATCAAAACTGTTTAATAGTACTTGGGGAAGTACTGACGGGTCAAATAGCACTGAA----
----

ACAAATGGCACAATCATACTCCCATGCAGAATAAGACAAATTATAAACAAGTGGCAGCAAGTAGGAAAAGCAA
TATACGCCCCTCCCATCCAAGGACGAATTAAATGTTTATCAAAT---
ATTACAGGGCTGCTATTAACAAGAGATGGTGGTCATAACAACAGTAGCAAT---------------------
----

GAGAGTAGGAGTGAGATCTTCAGACCTGGAGGAGGAGACATGAGGGACAATTGGAGAAGTGAATTATATAAAT
ATAAAGTAGTAAGAATTGAACCAGTA---GGAATAGCACCCACCAAGGCAAAGAGAAGAGTGGTG-------

-------------------------------CAGAGAGAAAAAAGAGCA---GTGGGAACAATAGGAGCT---
------ATGTTCCTTGGGTTCTTGGGAGCA---GCAGGAAGCACTATG---GGCGCAGCGTCA---
TTGACGCTGACGGTACAG---GCCAGACAA---TTATTG---TCTGGTATAGTGCAACAGCAGAAC---
AATCTG---CTGAAG---GCTATTGAGGCGCAACAGCACCTGTTGCAACTCACAGTCTGGGGCATC
>MK115401
TTCTATTGTAATTCATCATCACTGTTTAATAGTACCTGGACTAAAAATGGTACTGGTAGTTGGCAGTCTAATG
ATACT--

CAGAATGGTAATATCACACTCCAATGCAGAATAAAACAAATTATAAACCTGTGGCAGGGAGTAGGAAAAGCAA
TGTATGCCCCTCCCATCAGTGGACAAATTAACTGTACATCAAAT---
ATTACAGGGCTGGTTTTAACAAGAGATGGGGGGAAGGTGATTAAT----------------------------
----

GAAACTGAGACCTTTAGACCTGGAGGAGGAAATATGAAGGATAATTGGAGAAGTGAATTATATAAATATAAAG
TAGTAAGAATTGAACCATTA---GGAATAGCACCCACCAAGGCAAAAAGAAGAGTGGTG--------------
-------------------------CAAAGAGAGAAAAGAGCA---GTAGGA---CTGGGAGCT---------
ATGTTCCTTGGGTTCTTGGGAGCA---GCCGGAAGCACTATG---GGCGCAGCGTCA---
ATAGCGCTGACGGAACAG---GCCAGACGA---GTATTG---TCTGGTATAGTGCAACAGCAGAAC---
AATTTG---CTGAGG---GCTATTGAGGCGCAACAGCATTTGTTGCAACTCACAGTCTGGGGCATT
>MK169417
TTCTACTGTAATACAACACAATTGTTTAATAGTAGTTGGATTCGGAATGGTACTGATTGGACTTGGAATAGTA
CCACA--

CAAAATGATACTATCATACTCCCATGCAAAATAAAACAAATTATAAACATGTGGCAGGAAGTAGGAAAAGCGA
TGTATGCCCCTCCTATCGAAGGAATAATTAGGTGTGTATCAAAT---
ATTACTGGGTTGCTATTAACAAGAGATGGTGGTCACAACAGCAGC----------------------------
----

AACGGTACTGAGACCTTCAGACCTGCAGGAGGAAATATGAAGGACAATTGGAGAAGTGAATTATACAAATATA
AAGTAGTAAAAGTTGAACCATTA---GGAGTAGCACCCACCAAGGCAAAGAGAAGAGTGGTG-----------
----------------------------CAGAGAGAAAAAAGAGCA---ATAGGAGGACTAGGAGCT------
---GTGTTCCTTGGGTTCTTGGGAGCA---GCAGGAAGCACTATG---GGCGCAGCATCA---
ATGACGCTGACGGTACAG---GCCAGACAA---TTATTG---TCTGGTATAGTGCAGCAGCAGAAC---
AATCTG---CTGAAG---GCTATTGAGGCGCAACAGCATATGTTGCAACTCACAGTCTGGGGCATC
>MK169834
TTCTACTGTAATACAACACCACTGTTTAATCCAAATGGAATAAGAAAT-------------------------
----

GACACTGGAAATATCACACTCCAATGCAGAATAAAACAAATTATAAACATGTGGCAGGAAGTGGGAAAAGCAA
TGTATGCCCCTCCCATCCCAGGTCAAATTAGGTGTGTATCAAAT---
ATTACAGGGCTGATATTAACAAGAGATGGTGGTGATAATAACGAG----------------------------
----

ACCGAGGTCTTCAGACCTGGAGGAGGAGATATGAGGGACAATTGGAGAAGTGAATTATATAAATATAAAGTAG
TACAAATTGAACCATTA---GGAGTAGCACCCACCACGGCAAGAAGAAGAGTGGTG-----------------
----------------------CAGAGAGAAAAAAGAGCA---GTAACT---TTTGGAGCT---------
CTGTTCCTTGGGTTCTTGGGAGCA---GCAGGAAGCACTATG---GGCGCAGCGTCA---
ATGACGCTGACGGTACAG---GCCAGGCAA---TTATTG---TCTGGTATAGTGCAGCAGCAGAAC---
AATCTG---CTGAGA---GCTATTGAGGCGCAACAACATATGTTGCAACTCACAGTCTGGGGCATT
>AY736819
TTCTACTGTAATACATCACCACTGTTTAATAGTACTTGGAATGGTAATAATACTTGGAATAATACTACAGGG-
----

TCAAATAACAATATCACACTTCAATGCAAAATAAAACAAATTATAAACATGTGGCAGAAAGTAGGAAAAGCAA
TGTATGCCCCTCCCATTGAAGGACAAATTAGATGTTCATCAAAT---
ATTACAGGGCTACTATTAACAAGAGATGGTGGTAAGGACACGGACACG-------------------------
----

AACGACACCGAGATCTTCAGACCTGGAGGAGGAGATATGAGGGACAATTGGAGAAGTGAATTATATAAATATA
AAGTAGTAACAATTGAACCATTA---GGAGTAGCACCCACCAAGGCAAAGAGAAGAGTGGTG-----------

----------------------------CAGAGAGAAAAAAGAGCA---GCG------ATAGGAGCT------
---CTGTTCCTTGGGTTCTTAGGAGCA---GCAGGAAGCACTATG---GGCGCAGCGTCA---
GTGACGCTGACGGTACAG---GCCAGACTA---TTATTG---TCTGGTATAGTGCAACAGCAGAAC---
AATTTG---CTGAGG---GCCATTGAGTCGCAACAGCATATGTTGCAACTCACAGTCTGGGGCATC
>AF025753
TTCTACTGTAATACAACAAAACTGTTTAATAGTACTTGGAATAGTACTTGGAATGATACTGAAGGGTCAATTG
CCACTGAA---
-------------GGAAAT------
ACCACTGAAGAAAATATCACACTCCCATGCAGAATAAAACAAATTATAAACATGTGGCAGGAAGTAGGAAAAG
CAATGTATGCCCCTCCCATCGCAGGACAAATTAGATGTTCATCAAAT---
ATTACAGGGCTGCTATTAACAAGAGATGGTGGTAATGGTAATAGCAGC-------------------------
----

AACTACACTTCCGAGATCTTCAGACCGGGAGGAGGAGATATGAGGGACAATTGGAGAAGTGAATTATATAAAT
ATAAAGTAGTAAAAATTGAACCATTA---GGAGTAGCACCCACCAAGGCAAAGAGAAGAGTGGTG--------
-------------------------------CAGAGAGAAAAGAGAGCA---GTGGGA---ATAGGAGCG---
------TTGTTCCTCGGGTTCTTGGGAGCA---GCAGGAAGCACTATG---GGCGCAGCGTCA---
CTGACGCTGACGGCACAG---GCCAGACTA---TTATTG---TCTGGTATAGTGCAACAGCAGAAC---
AATCTG---CTGAGG---GCTATTGAGGCGCAACAGCATCTGTTGCAACTCACAGTCTGGGGCATT
>MH012806
TTCTACTGTAATTCAACACAACTGTTTAATAGTACTTGGAGTAGTAATAGTACTGGGAGTAATAGCACTGAA-
----

GAAGAAAGAAATATCACACTCCCATGCAGAATAAAGCAAATTATAAACATGTGGCAGGAAGTAGGAAAAGCAA
TGTATACCCCTCCCATCGGAGGACAAATTCAATGTTCATCAAAT---
ATTACAGGGCTACTATTAACAAGAGATGGTGGTCATAGTGGGAACGAGTCC----------------------
----

GAGAACAGGCCCGAGATCTTCAGACCAGGAGGAGGAGATATGAGGGATAATTGGAGAAGTGAATTATATAAAT
ATAAAGTAGTAAAAGTTGAACCATTA---GGAATAGCACCCACCAAGGCAAAGAGAAGAGTGGTG--------
-------------------------------CAGAGAGAAAAGAGAGCA---GTGGGA---ATAGGAGCT---
------ATGTTCCTTGGGTTCTTGGGAGCA---GCAGGAAGCACTATG---GGCGCAGCGTCG---
GTGACGCTGACGGTACAG---GCCAGACAA---TTATTG---TCTGGTATAGTGCAACAGCAGAAC---
AATCTG---CTGAAG---GCTATTGAGGCGCAACAGCATCTGTTGCAACTCACAGTCTGGGGCATC
>MH012971
TTCTACTGTAATTCAACACAACTGTTTAAGAGTATTTGGCATGGGAATGAAACCACGTGGAATACAAAT----
----

AACACTGAAAATATCACACTCCCGTGCAGAATAAAACAATTTATAAACATGTGGCAGGAAGTAGGGAAAGCAA
TGTATGCCCCTCCCATCAGTGGACAAATTAGATGTTCATCAAAT---
ATTACAGGGCTGCTATTAACAAGAGATGGTGGTGCTAAGGAGAAC----------------------------
----

GAGACCACCTACAATGAGACCTTCAGACCTGGAGGAGGAGATATGAGGGACAATTGGAGAAGTGAATTATATA
AATATAAAGTAGTAAGAATTGAACCATTA---GGAGTAGCACCCACCAAGGCAAAGAGAAGAGTGGTG-----
----------------------------------CAGAGAGAAAAAAGAGCA---GTGGGA---
ATAGGAGCT---------CTGTTCCTTGGGTTCTTGGGAACA---GCAGGAAGCACTATG---
GGCGCAGCGTCA---CTGACGCTGACGGTACAG---GCCAGACAA---TTATTG---
ACTGGTATAGTGCAACAGCAGAAC---AACCTG---CTGAGA---
GCTATTGAGGCGCAACAGCATCTGTTGCAACTCACAGTCTGGGGCATC
>MH012542
TTCTACTGTAATACAACACAACTGTTTAATAGTACTTGGACTTGGAATGATACTAAAGGGTCAAATGACACTG
GA---

GGAAATGGCACAATCACACTCCCATGCAGAATAAAACAAATTATAAACATGTGGCAGGAAGTAGGAAAAGCAA
TGTATGCCCCTCCCATCCCAGGACAAATTAGATGTTCATCAAAT---
ATTACAGGGCTGCTATTAACAAGAGATGGTGGTAACAATAATCAGAGCAGCGAG-------------------
----

ACCACCGAGGTCTTCAGACCTGGAGGAGGAGATATGAGGGACAATTGGAGAAGTGAATTATATAAATATAAAG

TAGTAAAAATTGAACCATTA---GGAGTAGCACCCACCAAGGCAAAGAGAAGAGTGGTG--------------
-------------------------CAGAGAGAAAAAAGAGCA---GTGGGA---ATAGGAGCT---------
GTGTTCCTTGGGTTCTTGGGAGCA---GCAGGAAGCACTATG---GGCGCAGCGTCA---
ATGACGCTGACGGTACAG---GCCAGACTA---TTATTG---TCTGGTATAGTGCAACAGCAGAAC---
AATTTG---CTGAGG---GCTATTGAGGCGCAACAGCATCTGTTGCAACTCACAGTCTGGGGCATC
>AY835773
TTCTACTGTAATACAACACAACTGTTTAATAGTACTTGGAAGACAGTTAATATTTGGAATGTTACTGAAAGGT
CAAATTAC---
-------------------ACT---
GAAGGAAATGACACACTCATACTCCCATGCAGAATAAAACAAATTATAAACATGTGGCAGGAAGTAGGAAAAG
CAATGTATGCCCCTCCCATCAGTGGACAAATTAGATGTTTATCAAAT---
ATTACAGGGCTACTGTTAACAATAGATGGTGGTGATAATAGCACG----------------------------
----

ACCGAGACCTTCAGACCTGGAGGAGGAGATATGAGGGACAATTGGAGAAGTGAATTATATAAATATAAAGTAG
TAAAAATTGAACCATTA---GGAGTAGCGCCCACCAAGGCGAAGAGAAGAGTGGTG-----------------
----------------------CAAAGAGAGAAAAGAGCA---GTGGGA---CTAGGAGCT---------
ATGTTCCTTGGGTTCTTGGGAGCA---GCAGGAAGCACTATG---GGCGCAGCGTCA---
CTGACGCTGACGGTACAG---GCCAGACAA---TTATTG---TCTGGTATAGTGCAACAGCAGAGC---
AATTTG---CTGAGA---GCTATTGAGGCGCAACAGCATCTGTTGCAACTCACAGTCTGGGGCATC
>AY835435
TTCTACTGTAATACAACACAACTGTTTAATAGTACTTGGTTTAATAATGCTACTTTGAATGTTAATAGTAATG
TTACT--

GAAGGGTCAGAAAATATCACACTCCCATGCAGAATAAGACAAATTGTGAACATGTGGCAAGAAGTAGGAAAAG
CAATGTATGCCCCTCCCATCCAAGGGCAAATTAGATGTTCATCAAAT---
ATTACAGGGCTGCTATTAACAAGAGATGGTGGTGGTAGTAACAGTAGT-------------------------
----

AACACGAGCGAGGAGGTCTTCAGACCTGGAGGAGGAAATATGAGGGACAATTGGAGAAGTGAATTATATAAAT
ATAAAGTAGTAAAAATTGAACCATTA---GGAATAGCACCCACCAAGGCAAAGAGAAGAGTGGTG--------
-------------------------------CAGAGAGAAAAAAGGACA---GTGGGA---ATAGGAGCT---
------TTGTTCCTTGGGTTCTTGGGAGCA---GCAGGAAGCACTATG---GGCGCAGCGTCA---
ATGACGCTGACGGTACAG---GCCAGACAA---TTACTG---TCTGGTATAGTGCAACAGCAGAAC---
AATCTG---CTGAGG---GCTATTCAGGCGCAACAGCATCTGTTGCAGCTCACAGTCTGGGGCATT
>AY331292
TTCTACTGTGATACAACAAAACTGTTTAATAGTACTTGGCATGGTAATGAAACATCTTGGAATAATACTGAAA
AG---

TTAAATGACACTATCATACTCCCATGCAGAATAAAACAAATTATAAACATGTGGCAGGAAGTAGGAAAAGCAA
TGTATGCCCCTCCAATCGAAGGACTAATTAACTGTTCATCAAGT---
ATTACGGGGCTGCTATTAACAAGAGATGGTGGTAAGAGCAATAAT----------------------------
----

CAGACTGAGATCTTCAGACCTGGAGGAGGAGATATGAGGGACAATTGGAGAAGTGAACTATATAAATATAAAG
TAGTAAAAATTGAACCAGTA---GGAGTAGCACCCACCAAGGCAAAGAGAAGAGTGGTG--------------
-------------------------CAGAGAGACAAAAGAGCA---GTGGGA---CTAGGAGCG---------
TTGTTCATTGGGTTCTTGGGAGCA---GCAGGAAGCACTATG---GGCGCAGCGTCA---
ATGACACTGACGGTACAG---GCCAGACAA---TTATTG---TCTGGTATAGTGCAACAGCAGAAC---
AACTTG---CTGAGG---GCTATTGAGGCGCAACAACATCTGTTGCAACTCACAGTCTGGGGCATC
>EU289191
TTCTACTGTAATACATCACAACTGTTTAATAGTACTTGGAATGGTACTGAAGGGTTTAATAGTACT-------
----

GGGAGTGAAAATATCACACTCCCATGCAGAATAAGACAAGTTGTAAACCTGTGGCAGGAAGTAGGAAAAGCAA
TGTATGCCCCTCCCATCAGGGGAGTAATTAAATGTTCATCAAAC---
ATTACAGGGGTACTATTAACAAGAGATGGTGGTTATGATGATAATAATACC----------------------
----

GCAAACACCAACGAGACCTTCAGACCAGGAGGAGGAGATATGAGGGACAATTGGAGAAGTGAATTATATAAAT

ATAAAGTAGTAAGAATTGAACCATTA---GGAGTAGCACCCACCAGGGCAAAGAGAAGAGTGGTG--------
-------------------------------CAGAGAGAAAAAAGAGCA---GTGACA---CTAGGAGCT---
------TTGTTCCTTGGGTTCTTGGGAGCA---GCAGGAAGCACTATG---GGCGCAGCGTCA---
CTGACGCTGACGGTACAG---GCCAGACTC---TTGTTG---TCTGGTATAGTGCAGCAGCAGAAC---
AATCTG---CTGAGG---GCTATTGAGGCGCAACAACATCTGTTGCAACTCACCGTCTGGGGCATC
>KT124744
TTCTACTGTAATACAACACCATTATTTAATAGTACTTGGGGAGCTAATAGTAATTGGACTTGGAATAATACTC
AAGAGTTA---
-------------------AAT---
GGCACTCAAGGAAATATCACACTCCAATGCAGAATAAAACAAATTATAAACATGTGGCAGGAGGTAGGAAAAG
CCATGTATGCCCCTCCCATCAGAGGACTAATTAGTTGTTCATCAAAT---
ATTACAGGGTTGCTATTAACAAGAGATGGTGGTAATAACAACGCCACC-------------------------
----

AACAATGGCACAGAGATCTTCAGACCTGGAGGGGGAGATATGAGGGACAATTGGAGAAGTGAATTATATAAAT
ATAAAGTAGTAAAAATTGAACCAGTA---GGATTAGCACCAACCAAGGCAAAGAGAAGAGTGGTG--------
-------------------------------CAGAGAGAAAAAAGAGCA---GTGGGA---CTAGGAGCG---
------CTTTTCCTTGGGTTCTTGGGAGCA---GCAGGAAGCACTATG---GGCGCAGCGTCA---
ATGACGCTGACGGTACAG---GCCAGACAA---TTATTG---TCTGGTATAGTGCAACAGCAGAAC---
AATTTG---CTGAGA---GCTATTGAGGCGCAACAGCATATGTTGCAACTCACAGTCTGGGGCATC
>KT124770
TTCTACTGTAATACATCAAAACTGTTTAATAGTACCTGGGAGTGGAATAATAGTACCAGAGATTGGAATAATA
CT---
GAAATGTCAAATAACACT---------
GAAGGAAATGGAACTATCACTCTCCAATGCAGAATAAAACAAATTGTAAATATGTGGCAGGAAGTAGGAAAAG
CAATGTATGCCCCTCCCATCAGAGGACAAATTAGATGTTCATCAAAT---
ATTACCGGGTTGCTATTAACAAGAGATGGTGGTTACAACATCAGC----------------------------
----

AATAACAACGAGACCTTCAGACCTGGAGGAGGAGATATGAGGGACAATTGGAGAAGTGAATTATACAGATATA
AAGTAGTAAAAATTGAACCATTA---GGAATAGCACCCACCAAGGCAAGGAGAAGAGTGGTG-----------
----------------------------CAAAGAGAAAAAAGAGCA---GTGGGA---TTAGGAGCT------
---ATGTTCCTTGGGTTCTTGGGAGCA---GCAGGAAGCACTATG---GGCGCAGCATCA---
ATGATGCTGACGGTACAG---GCCAGACAA---TTATTG---TCTGGTATAGTGCAACAGCAGAGC---
AATCTG---CTGCAG---GCTATTGAGGCACAACAGCATCTGTTGAAACTCACAGTCTGGGGCATT
>EU575670
TTCTACTGTAATACAACAAAACTGTTTAATAGTACTTGGGTTAATGGTACTAGGAATGATACTGAAGAGTCA-
----

AATAGCACTGAAATCACACTCCCATGCAGAATAAAACAAATCATAAACATGTGGCAGGAAGTAGGAAAAGCAA
TGTATGCCCCTCCTATCAGAGGACAAATTAGATGTTCATCAAAT---
ATCACAGGGCTGCTATTAACAAGAGATGGGGGTGAGGGAGTTGAGAAA-------------------------
----

AACGATACCGAGATCTTTAGACCTGAAGGAGGAGATATGAAGAACAATTGGAGAAGTGAATTATATAAATATA
AAGTAGTACAAATTGAACCATTA---GGAGTAGCACCCACCAGGGCAAAGAGAAGAGTGGTG-----------
----------------------------CAGAGAGAAAAAAGAGCA---GTGGGA---ATAGGAGCT------
---ATGTTCCTTGGGTTCTTGGGAGCA---GCAGGAAGCACTATG---GGCGCAGCGTCA---
ATGGCGCTGACGGTACAA---TCCAGACAA---TTATTG---TCTGGTATAGTGCAACAGCAAAAC---
AACTTG---CTGAGA---GCTATTGAGGCGCAACATCATCTGTTGCAACTCACAGTCTGGGGCATT
>EU575927
TTCTACTGTAATACCTCACAACTGTTTAATAGCTCTTGGTATTCAAATGGTACTCGGAATGGTACTGAAGAA-
----

TCAAATGACCTTATCACACTCCCATGCAGAATTAAACAAATTGTAAACATGTGGCAGGAAGTAGGAAAAGCAA
TGTATGCCCCTCCCATTAGAGGACACATTAACTGTACATCAAAT---
ATTACAGGGCTGTTATTAACAAGAGATGGGGGTCAGAGTAATGAAACC-------------------------
----

AAGGACGAGGAGATCTTCAGACCTGGAGGAGGAGATATGAGGGACAATTGGAGAAGTGAATTATATAAATATA

AAGTAGTAAGAATTGAACCATTA---GGAATAGCACCCACCAGGGCAAAGAGAAGAGTGGTG-----------
----------------------------CAGAGAGAAAAAAGAGCA---GTGGGA---ATAGGAGCT------
---CTGTTCCTTGGGTTCTTGGGAGCA---GCAGGAAGCACTATG---GGCGCAGCGTCA---
ATAACGCTGACGGTACAG---GCCAGGCTA---TTAATG---TCTGGTATAGTGCAACAGCAAAAC---
AATCTG---CTGAGA---GCTATTGAGGCGCAACAGCATCTGTTGCAACTCACAGTCTGGGGCATC
>U84854
TTCTACTGTAACACAACACAACTGTTCAATATTACTAATACTCAAAAC-------------------------
----

ACTACAGGAAATCACACACTCCCCTGCAGAATAAAACAAATTATAAACATGTGGCAGGAAGTAGGAAAAGCAA
TGTATGCCCCTCCCATCAGAGGACAAATTAGATGTAAATCAAAT---
ATTACAGGGCTGTTATTAACAAGAGATGGTGGTGAAAACACAAACCACACA----------------------
----

GACAATAATACTGAGATCTTCAGACCTGGAGGAGGAGATATGAGGGACAATTGGAGAAGTGAATTATATAAAT
ATAAAGTAGTAAAAATTGAACCATTA---GGAGTAGCACCCACCAAGGCAAAGAGAAGAGTGGTG--------
-------------------------------CAGAGAGAAAAAAGAGCA---GTGGGA---ATAGGAGCT---
------ATGTTCCTTGGGTTCTTGGGAGCG---GCAGGAAGCACTATG---GGCGCAGCGTCA---
GTGACGCTGACGGTACAG---GCCAGACTA---TTATTG---TCTGGAATAGTGCAACAGCAGAGC---
AATTTG---CTGAGG---GCTATTGAGGCGCAACAGCATCTGTTGCAACTCACAGTCTGGGGCATC
>AY835442
TTCTACTGTAATACAACACAACTGTTTAATAATACTTGGAATGGTACTGGATGGTCAGATAACTCAGAGAACA
CT---

ACAGGAAACGGAACTATCACACTCCCATGCAGAATAAAACAAATTGTTAACCTGTGGCAGGAAGTAGGAAAAG
CAATGTATGCCCCTCCCATCAGAGGACTAATTAGATGTTCATCAAAT---
ATTACAGGGCTGCTATTAACAAGAGATGGAGGTAATAATACCACCACGAAC----------------------
----

GGGACTCAGACTGAGATATTCAGACCTGGAGGAGGAGATATGAGGGACAATTGGAGAAGTGAATTATATAAAT
ATAAAGTAGTAAAAATTGAACCATTA---GGAGTAGCACCCACTAGGGCAAAGAGAAGAGTGGTG--------
-------------------------------CGGAGAGAAAGAAGAGCA---GCA------CTAGGAGCT---
------TTGTTCCTTGGGTTCTTGGGAGCA---GCAGGAAGCACTATG---GGCGCAGCGTCA---
ATGACGCTGACGGTACAG---GCCAGACTA---TTATTG---TCTGGTATAGTGCAACAGCAGAAT---
AATTTG---CTGAGG---GCTATTGAGGCGCAACAGCATCTGTTGCAACTCACAGTCTGGGGCATC
>EU289186
TTCTACTGTAATACAGCACAACTGTTTAATAGTACTTGGGAAGCTAATAGTACTTGGGAAAATGATAATGAAA
GGGTA--
-------------------GGT---
CACAGTAACAAGACTATCATACTACAATGCAGAATAAAACAAATTATAAACATGTGGCAGGAAGTAGGAAAAG
CAATGTATGCCCCTCCCATCAGCGGACAGATTAGATGTTCATCAAAT---
ATTACGGGGCTGCTATTAACAAGAGATGGTGGTAACGGTAACGAGACC-------------------------
----

AACCGGACCGAGGTCTTCAGACCTGGAGGAGGAAATATGAAAGATAACTGGAGAAGTGAATTATATAAATATA
AAGTAGTAAAAATTGAACCATTA---GGAGTAGCACCCACCAGGGCAAAGAGAAGAGTGGTG-----------
----------------------------CAAAGAGAAAAAAGAGCA---GTGGGA---ATGGGAGCT------
---TTGTTCATTGGGTTCTTGTCAGCA---GCAGGAAGCACTATG---GGCGCAGCGTCA---
ATGACGCTGACGGTACAG---GCCAGACAA---TTATTG---TCTGGTATAGTGCAACAGCAGAGC---
AATTTG---CTGAGA---GCTATTGAGGCGCAACAACATCTGTTGCAACTCACAGTCTGGGGCATC
>DQ853463
TTCTACTGTAATACAACACAATTGTTTAATGGTATGTGGCATGCTAATGGTACTTGGAATAGTACTTGGAATG
ATACT--
-------------------GGA---
GGGTCAAATGACACTATCAGACTCCCATGTAGAATAAAACAAATTGTAAACATGTGGCAGGAAGTAGGAAAAG
CAATGTATGCCCCTCCCATCAAAGGACAAATTCAATGTTCATCAAAT---
ATTACAGGGCTGATATTAACAAGAGATGGTGGTAACAGCACGAAC----------------------------
----

GAGACCGGGGAGGTCTTCAGACCTGGAGGAGGAAATATGAAGGACAATTGGAGAAGTGAATTATATAAATATA

```
AAGTAGTAGAAATTGAACCATTA---GGAGTAGCACCCACCAAGGCAAAGAGAAGAGTGGTG-----------
----------------------------CAGAGGGAAAAAAGAGCA---GTGGGATTAATAGGAGCT------
---GTGTTCCTTGGGTTCTTGGGAGCA---GCAGGAAGCACTATG---GGCGCAGCGTCA---
ATAGCGCTGACGGTACAG---ACCAGACAC---TTATTG---TCTGGTATAGTGCAACAGCAGAAC---
AATCTG---CTGAGG---GCTATTGAGGCGCAACAACATCTGTTGCAACTCACAGTCTGGGGCATC
>KT124769
TTCTACTGTAATACAGCAGAACTGTTTAATAGTACTTGGTATGCAAATGGTACTTGGTATGCAAATGGTACA-
-------------------------------------------------------------------------
-AATAGTACTGGAGGG---------
GGAAACAAGACTAATATCACACTCCCATGCAGAATAAAACAATTTATAAACATGTGGCAGGAAGTAGGAAAAG
CAATGTATGCCCCTCCCATCAGTGGACAGATTAGATGTGCATCAAAT---
ATTACAGGACTGCTATTAACAAGAGATGGTGGTCAGGGCAATCAGACC-------------------------
-------------------------------------------------------------------------
-------
GACAACCAGACTGAGATCTTCAGACCTGTAGGAGGAGATATGAAGAACAATTGGAGAAGTGAATTATATAAAT
ATAAAGTAGTAAGAATTGAACCATTA---GGAATAGCACCCACCAGGGCAAAAAGGAGAGTGGTG--------
-------------------------------CAGAGAGAAAAAAGAGCA---GTGGGA---ATAGGAGCT---
------TTGTTCCTTGGGTTCTTGGGAGCA---GCAGGAAGCACTATG---GGCGCAGCGTCA---
ATGACGCTGACGGTACAG---GCCAGACTA---TTATTG---TCTGGTATAGTGCAACAGCAGAAC---
AATCTG---CTGAGG---GCTATTGAGGCGCAACAACATCTGTTGCAACTCACAGTCTGGGGCATC
>EU289196
TTCTACTGTAATACATCAAAACTGTTTAATAGTACTTGGAATAGTACTGGAGGGTCCATTTCTGAA-------
-------------------------------------------------------------------------
----------------------------
GACTCAGAAAATATCACACTCCCATGCAGAATAAAACAAATTGTAAACATGTGGCAGGAGGTAGGAAAAGCAA
TGTATGCCCCTCCCATCAGAGGACAGATCAGATGTTCATCAAAT---
ATTACAGGGCTGCTATTAACAAGAGATGGTGGTATAAATCAGAGC----------------------------
-------------------------------------------------------------------------
-------------
ATCAGCGAGACCTTCAGACCGGGAGGAGGAGATATGAGGGACAATTGGAGAAGTGAATTATACAAATATAAAG
TAGTAAAAATTGAACCATTA---GGAATAGCACCCACCAAGGCAAGGAGAAGAGTGGTG--------------
-------------------------CAAAGAGAAAAAAGAGCA---GTGGGA---ATAGGAGCT---------
GTGTTCCTTGGGTTCTTGGGAGCA---GCAGGAAGCACTATG---GGCGCAGCGTCA---
CTGACGCTGACGGTACAG---GCCAGACTA---TTATTG---TCTGGTATAGTGCAACAGCAGAAC---
AATCTG---CTGAGG---GCTATTGAGGCGCAACAGCATATGTTGCAACTCACAGTCTGGGGCATC
>EU576141
TTCTACTGTAATACAACACCACTGTTTAATAGTACTTGGAATAATACTCGGACTGATGATTATCCTACTGGG-
-------------------------------------------------------------------------
----------------------------
GGGGAACAAAATATCACACTCCAATGCAGAATAAAACAAATTATAAACATGTGGCAGGGAGTAGGAAAAGCAA
TGTATGCTCCTCCCATCAGAGGACAAATTAGATGTTCATCAAAT---
ATTACAGGGCTGCTATTAACAAGAGATGGTGGTAGAGATCAGAAT----------------------------
-------------------------------------------------------------------------
-------------
GGTACTGAGACCTTCAGACCTGGAGGAGGAAATATGAGGGACAATTGGAGAAGTGAATTATATAAATATAAAG
TAGTAAAAATTGAACCATTA---GGAATAGCACCCACCGCGGCAAAGAGAAGAGTGGTG--------------
-------------------------CAGAGAGAAAAAAGAGCA---GTGGGA---CTAGGAGCT---------
TTTATCCTTGGGTTCTTGGGAGCA---GCAGGAAGCACTATG---GGCGCAGCGTCA---
ATGGCGCTGACGGTACAG---GCCAGACTA---TTATTG---TCTGGTATAGTGCAACAGCAAAAC---
AATTTG---CTGAGA---GCTATTGAGGCGCAACAGCATATGTTGCAACTCACAGTCTGGGGCATC
>AY835446
TTCTACTGTAATACAGCAGAACTGTTTAATAGTACTTGGTATGCAAATGGCACAATTAGTATTGGAGGGGGA-
-------------------------------------------------------------------------
----------------------------
AACAAGACTAATATCATACTCCCATGCAGAATAAAACAATTTATAAACATGTGGCAGGAAGTAGGAAAAGCAA
TGTATGCCCCTCCCATCAGTGGACAGATTAGATGTTCATCAAAT---
ATTACAGGACTGCTATTAACAAGAGATGGTGGTAGGGGCAATCAGACC-------------------------
-------------------------------------------------------------------------
-------
GACAACCAGACTGAGATCTTCAGACCTGTAGGAGGAGATATGAAAAACAATTGGAGAAGTGAATTATATAAAT
```

```
ATAAAGTAGTAAGAATTGAACCATTA---GGAATAGCACCCACCAGGGCAAAAAGGAGAGTGGTG--------
-------------------------------CAGAGAGAAAAAAGAGCA---GTGGGA---ATAGGAGCT---
------TTGTTCCTTGGGTTCTTGGGAGCA---GCAGGAAGCACTATG---GGCGCAGCGTCA---
ATGACGCTGACGGTACAG---GCCAGACTA---TTATTG---TCTGGTATAGTGCAACAGCAGAAC---
AATCTG---CTGAGG---GCTATTGAGGCGCAACAGCATCTGTTGCAACTCACAGTCTGGGGCATC
>EF593282
TTCTACTGTAATTCAACACCACTGTTTAGTAGTACTTGGTATACTGGGAATAGTACCAATAGTACTAAAGGGT
TAAAT--------------------------------------------------------------------
-------------------------
AACGCTACAGAAATTATCACACTCCTATGTAGAATAAAACAAATTATAAACATGTGGCAGGAAGTAGGAAAAG
CAATGTATGCCCCTCCCATCAGAGGACAAATTAGATGCTCATCAAAT---
ATTACAGGGCTGCTATTAACAAGAGATGGTGGTAATAGCAGCAGC----------------------------
-------------------------------------------------------------------------
-------------
AATGAAGAGATTTTCAGACCTGCAGGAGGAGATATGAGGGACAATTGGAGAAGTGAATTATATAAATATAAAG
TAGTAAAAATTGAACCATTA---GGAGTAGCACCCACCAAGGCAAAGAGAAGAGTGGTG--------------
-------------------------CAGAGAGAAAAAAGAGCA---GTGGGA---ATAGGAGCT---------
GTGTTCCTTGGGTTCTTGGGAGCA---GCAGGAAGCACTATG---GGCGCAGCGTCA---
GTGACGCTGACGGTACAG---GCCAGACAA---TTATTG---TCTGGAATAGTGCAGCAGCAGAAC---
AATCTG---CTGAGG---GCTATTCAGGCGCAACAGCATCTGTTGCAACTCACAGTCTGGGGCATC
>JQ610043
TTCTATTGTAATACAACACCGCTGTTTAGCAGCAGGTGGGGGCCTGACGGCACTTGGAATAGCACTTGGAACA
GTACAAGT-----------------------------------------------------------------
-------------------ACA---
ACTAACAACGACACTATCACACTCCAATGCAGAATAAAACAAATTATAAACAGGTGGCAGGAAGTAGGAAGAG
CAATGTATGCCCCTCCCATCCAAGGACAAATTAGATGCTCATCAAAT---
ATTACAGGGCTGCTATTAACAAGAGATGGTGGTCATGGTAATGAC----------------------------
-------------------------------------------------------------------------
----
ACGGATAATACCACCGAGACCTTCAGACCTGGAGGAGGAAATATGAGGGACAATTGGAGAAGTGAACTGTATA
AATATAAAGTAGTAAAAATTGAGCCATTA---GGATTAGCACCCACCAAGGCAAAGAGAAGAGTGGTG-----
----------------------------------CAAAGAGAAAAAAGAGCA---
GTGGGAACGCTAGGAGCT---------ATGTTCCTTGGGTTCTTGGGAGCA---GCAGGAAGCACTATG---
GGCGCAGCGGCA---GTGACGCTGACGGTACAG---GCCAGACAA---TTATTG---
TCTGGTATAGTGCAACAGCAGAAC---AATTTG---CTGAAG---
GCTATTGAGGCGCAACAGCATATGTTGCAACTCACAGTCTGGGGCATC
>JN786752
TTCTACTGTAATACAACAAAACTGTTTAATAGTACTTGGGATGCTAATAGTACTTGGGATGGTAATAGTACTA
TGGATAAAGGG--------------------------------------------------------------
-------------TCAAAT------
CACACTAACAATACTATCACACTCCCATGCAGAATAAAACAAATTATAAACATGTGGCAGGGAGTAGGAAAAG
CAATGTATGCCCCTCCCATCAGTGGACTTATTAGCTGTACATCAAAT---
ATTACAGGGCTGATATTAACAAGAGATGGTGGTAATAATAACAAGACAGAG----------------------
-------------------------------------------------------------------------
----
AACGAAAACAAGACTGAGATCTTCAGACCTGGAGAAGGAGATATGAGGGATAATTGGAGAAGTGAATTATACA
AATATAGAGTAGTAAAAATTGAACCATTA---GGAGTAGCACCTACCGAGGCAAAGAGAAGAGTGGTG-----
----------------------------------CAAAGAGAAAAAAGAGCA---GTGGGA---
TTTGGAGCT---------TTGTTCCTTGGGTTCTTGAGTGCA---GCAGGAAGCACTATG---
GGCGCAGCGTCA---GTGACGCTGACGGTACAG---GCCAGACAA---TTATTG---
TCTGGTATAGTGCAACAGCAGAAC---AATCTG---CTGAGG---
GCTATTGAGGCGCAACAACATCTGTTGCAACTCACAGTCTGGGGCATC
>EF593212
TTCTACTGTAATACAACACCACTGTTTAATAGTACTTGGAATAATACTAATGAAGGGTCAGGTAAC-------
-------------------------------------------------------------------------
----------------------------
TATGAAGGAAATATCACACTCCAATGCAGAATAAAACAAATTATAAATATGTGGCAGCAAGTAGGAAAAGCAA
TGTATGCCCCTCCCATCCAAGGACAAATTTACTGTTCATCAAAT---
ATTACAGGGCTGCTATTAACAAGAGATGGTGGTGGTAATAACGAG----------------------------
-------------------------------------------------------------------------
```

AGCAGCGAGACCTTCAGACCTGGAGGAGGAGATATGAGGGACAATTGGAGAAGTGAATTATATAAATATAAAG
TAGTAAAACTTGAACCATTA---GGAATAGCACCCACCAGGGCAAAGAGAAGAGTGGTG--------------
-------------------------CAGAGAGAAAGAAGAGCA---GCGGGA---CTAGGAGTT----------
ATGTTCCTTGGGTTCTTGGGAGCA---GCAGGAAGCACTATG---GGCGCAGCGTCA---
ATGGCGCTGACGGTACAA---GCCAGACAA---TTATTG---TCTGGTATAGTGCAACAGCAGAGC---
AATCTG---CTGAGG---GCTATTGAGGCGCAACAACATATGTTGCAACTCACAGTCTGGGGCATC
>EF593300
TTCTACTGTAATACAACACAGTTGTTTAATAGTACTTGGAATAGTACTGGGAATAATAATAACAGG-------
----

TCAGGAGAAAATATCACACTCCCATGCAGAATAAAACAATTCATAAACCTGTGGCAAGAAGTAGGAAAAGCAA
TGTATGCTCCTCCCATCAAAGGAGAAATTCAATGCCGATCAAAT---
ATTACAGGGCTAATAATAACAAGAGATGGTGGTAACACTACTGAA----------------------------
----

GAGATCTTCAGACCTGGAGGAGGAGATATGAGGGACAATTGGAGAAGTGAATTATATAAATATAAAGTAGTAA
GAATTGAACCATTA---GGAATAGCACCCACCAAGGCAAAGAGAAGAGTGGTG--------------------
-------------------CAAAGAGAAAAAAGAGCA---GTGGGA---TTCGGAGCT---------
ATGTTCCTTGGGTTCTTGGGAGCA---GCAGGAAGCACTATG---GGCGCAGCGTCA---
ATAGCGCTGACGGCACAG---GCCAGACAA---TTATTG---TCTGGTATAGTGCAACAGCAAAAC---
AATTTG---CTGAGG---GCTATTGAGGCGCAACAGCATATGTTGCAACTCACAGTCTGGGGCATT
>GQ153937
TTCTACTGTGATACAACACAACTGTTTAATAGTACTTGGGATAATACTAGTACTTGGAATAATAGTAACACTC
AGAAT--

AAAAATGATAGGAATATCACACTCCAATGCAGGATAAAACAAATTATAAACATGTGGCAGGAAGTAGGAAAAG
CAATGTATGCCCCTCCCATTATGGGACAAATTAGATGTGTATCAAAT---
ATTACAGGGCTGCTATTAACACGGGATGGTGGTAACGGTAGCGAGGCC-------------------------
----

AAAAACGACACGGAGATCTTCAGACCTGGAGGAGGAGATATGAGGGACAATTGGAGAAGCGAATTATATAAAT
ATAAAGTAGTAAAAATTGAACCATTA---GGAGTAGCACCCACCAAGGCAAAGAGAAGAGTGGTG--------
-------------------------------CAGAGAGAAAAAAGAGCA---GTGGGAACGATAGGAGCT---
------ATGTTCCTTGGGTTCTTGGGAGCA---GCAGGAAGCACTATG---GGCGCAGCGTCA---
TTGACGCTGACGGTACAG---GCCAGACAA---TTGTTG---TCTGGTATAGTGCAGCAGCAGAAC---
AATCTG---CTGAGA---GCCATTCAGGCGCAACAGCACCTGTTGCAGCTCACAGTCTGGGGCATT
>MH263121
TTCTACTGTAACACAACACAACTGTTTAATAGTACTTGGAATAATAATAGTACTTGGAATAATACTGAAGGG-
----

ACAGATCACAATATCACACTCCCATGCAGAATAAAACAATTTATAAATCTGTGGCAGGAAGTAGGAAAAGCAA
TGTATGCTCCTCCCATCAGGGGACAAATTAGATGCTCATCAAAT---
ATTACAGGGCTGCTATTAACAAGAGATGGTGGTAATAATAATAACACG-------------------------
----

GACCACTCTGAAACCTTCAGACCTGGAGGAGGAGACATGAGGGACAATTGGAGAAGTGAACTATATAAATATA
AAGTAGTAAAAATTGAACCATTA---GGAATAGCACCCACCAGGGCAAAGAGAAGAGTGGTG-----------
----------------------------CAGAGAGAAAAAAGAGCA---GTGGGA---ATAGGAGCT------
---TTGTTCCTTGGGTTCTTGGGAGCA---GCAGGAAGCACTATG---GGCGCAGCGTCA---
GTGACGCTGACGGTACAG---GCCAGACAA---TTATTG---TCTGGTATAGTGCAACAGCAAAGC---
AATTTG---CTGAAG---GCTATTGAGGCGCAACAGCATCTGTTGCAACTCACAGTCTGGGGCATC
>MH263194
TTCTATTGCAACACAACCCAGCTGTTTAATAGTACTTGGTATAATAATGAGACTACTTGGTTTAATAATGGGA
CTCAT--

CCTAATGAGACCATCATTCTCCCATGCAAAATAAAACAAATTGTAAACCTGTGGCAGCAAGTAGGAAAAGCAA
TGTATGCCCCTCCCATCAGTGGAAGAATTATGTGTAATTCAACT---
ATTACAGGGCTGCTATTAACAAGAGATGGTGGTAATAATAGTCCC----------------------------
----

AACGAGACCTTCAGACCTGGAGGAGGAGATATGAGAGACAATTGGAGAAGTGAATTATATAAATATAAAGTAG
TTAGGATTGAACCATTA---GGAATAGCACCCACCAGGGCACAGAGAAGAGTGGTG-----------------
----------------------CAAAGAGAAAAAAGAGCA---GTGGGAGTGCTTGGAGCT---------
ATGTTCCTTGGGTTCTTGGGAACA---GCAGGAAGCACTATG---GGCGCAGCGTCA---
ATAACGCTGACGGTACAG---GCCAGACAA---TTATTG---GCTGGTATAGTGCAACAACAGAAC---
AATCTG---TTGAGA---GCTATTGAGGCGCAACAGCATATGTTGCAACTCACAGTCTGGGGCATC
>MH263520
TTCTACTGTAATACAGCAAAGCTATTTAATAGTACATGGTATAGTGCAGGGAATAGCACTGATGGGGATAAAG
GA---

AATAATAACATAATCACACTCCCATGCAGAATAAAACAAATTGTAAACATGTGGCAGGAAGTAGGAAAAGCAA
TGTATGCCCCTCCCATCAGTGGACAAATTAGATGCTCATCAAAT---
ATTACAGGGCTGCTATTAACAAGAGATGGTGGTCATGACAATGACACA-------------------------
----

AACGGGACAGAGATCTTCAGACCTGGAGGAGGGAATATGAAGGACAATTGGAGAAGTGAATTATATAAATATA
AAGTAGTAAGAATTGAACCATTA---GGAGTAGCACCCACCAGGGCAAAGAGAAGAGTGGTG-----------
----------------------------CAGAGAGACAAAAGAGCA---GTGGGA---ATAGGAGCT------
---CTGTTCCTTGGGATCTTGGGAGCA---GCAGGAAGCACTATG---GGCGCAGCGTCA---
ATAACGCTGACGGTACAG---ACCAGACAA---TTATTG---TCTGGTATAGTGCAACAGCAGAAC---
AATTTG---CTGAGG---GCTATTGAGGCGCAACAGCACATGTTGCAACTCACAGTCTGGGGCATT
>KX984437
TTCTACTGTAATACATCAAAATTGTTTAGTAGTACTTGGAATGGTACTACCACACTGCCTAGTAGTACTTGGA
ATACTACT---

CAGAATGATACTATCACACTCCCATGCAGAATAAAACAAATTATAAACCTGTGGCAGGAAGTAGGAAAAGCAA
TGTATGCCCCTCCCATCAAAGGACAGATTAAATGTTCATCAAAT---
ATTACAGGGCTGTTATTAACAAGAGATGGTGGTATTACCAACAAC----------------------------
----

AGCATGAATCAGACCTTCAGACCTGGAGGAGGAGATATGAAGGACAATTGGAGAAGTGAATTATATAAATATA
AAGTAGTAAAAATTGAACCATTA---GGAATAGCGCCTACCAAGGCAAAGAGAAGAGTGGTG-----------
----------------------------CAGAGAGAAAAAAGAGCA---GTGGGA---ATAGGAGCT------
---TTGTTCCTTGGGTTCTTGGGAGCA---GCAGGAAGCACTATG---GGCGCAGCGTCA---
ATAACGCTGACGGTACAG---GCCAGACTA---TTATTG---TCTGGTATAGTGCAACAGCAGAAC---
AATTTG---CTGAGG---GCTATTGAGGCGCAACAGCATATGTTGCAACTCACAGTCTGGGGCATC
>KX984699
TTCTACTGTAATACAACAAAACTGTTTAGCAGTACTTGGTATGATAATGGAACGTCAAAAAGCACTGAAAGA-
----

TTAAATGACACTATCATACTCCCATGCAGGATAAAACAGATTATAAACATGTGGCAGGAAGTAGGAAAAGCAA
TGTATGCCCCTCCCATCAGAGGACAAATTAACTGTTCATCAAAT---
ATTTCAGGGCTGCTATTAACAAGAGATGGTGGTAATGATAATAACACG-------------------------
----

AACGACATCGAGACATTCAGACCGGGAGGAGGAGATATGAGGGACAATTGGAGAAGTGAATTATATAATTATA
AAGTGGTAAAAATTGAACCATTA---GGAATAGCACCCACTAAGGCAAAGAGAAGAGTGGTG-----------
----------------------------CAAAGAGAAAAAAGAGCG------GGA---ATAGGAGCT------
---TTGTTCCTTGGGTTCTTGGGATTA---GCAGGAAGCACTATG---GGCGCAGCGTCA---
GTGACGCTGACGGTACAA---GCCAGACTA---TTATTG---TCTGGTATAGTGCAACAGCAGAAC---
AATCTG---CTGAGG---GCTATTGAGGCGCAACAGCATCTGTTGCAACTCACGGTCTGGGGCATC
>MH632763
TTCTACTGTGATACAACAAAATTGTTTAATAGTACTTGGAATGTTAGTAGTATTTGGAATGTTACT-------
----

GAGGATAAAAATATCACACTCACATGTAGAATAAGACAAATTATAAACAGGTGGCAAGAAGTAGGAAAAGCAA
TGTATGCCCCTCCCATCAGAGGACAAATTAGATGTTCATCAAAT---
ATTACAGGACTGTTATTAACAAGAGATGGTGGTCAGAACACAACA----------------------------
----

AGCGAGACCTTTAGACCTGGAGGAGGAGATATGAGGGACAATTGGAGAAGTGAATTATATAAATATAAAGTAG
TACAAATTAAACCATTA---GGAATAGCACCCACCAGGGCACGGAGAAGAGTGGTG-----------------
----------------------CAGAGAGAGAAAAGAGCA---GTAGGA---CTGGGAGCA---------
ATGTTCCTTGGGTTCTTGGGAGCA---GCAGGAAGCACTATG---GGCGCAGCGTCA---
GTGACGCTGACGGTACAG---GCCAGACAA---TTACTG---TCTGGTATAGTGCAACAGCAGAAC---
AATCTG---CTGAGG---GCTATTGATGCGCAACAGCACCTGTTGCAACTCACAGTCTGGGGCATC
>MH576107
TTCTACTGTAATACAACACAACTGTTT------ACTTGGGGGAGTAATAGTACTAACATTACAGGA-------
----

AGTAATAGCACACTCACACTCCCATGCAGAATAAAACAAATTATAAATATGTGGCAGAGAGTAGGAAGAGCAA
TGTATACCCCTCCTATCAGAGGACTAATTCAATGCTCATCAAAT---
ATTACAGGACTACTATTAACAAGAGATGGTGGGTATGGCAATAACAAT-------------------------
----

ACCAATGATACTGAGATCTTCAGACCTGGAGGAGGAGACATGAGGGATAATTGGAGAAGTGAATTATATAAAT
ATAAAGTGGTAAAAATTGAACCATTA---GGAGTAGCACCCACCAAGGCAAGGAGAAGAGTGGTG--------
-------------------------------CAAAGAGAAAAAAGAGCA---GTAGGA---ATAGGAGCT---
------GTGTTCCTTGGGTTCTTGGGAGCA---GCAGGAAGCACTATG---GGCGCAGCGTCA---
ATAACGCTGACGGTACAG---GCCAGACAA---TTACTG---TCTGGTATAGTGCAACAGCAGAAC---
AATTTG---CTGAGG---GCTATTGAGGCGCAACAGCACCTGTTGCAACTCACAGTCTGGGGCATC
>MH576084
TTCTATTGTAATTCATCATCACTGTTTAATAGTACCTGGACTAAAAATGATACTGGTAGTTGGCAGTCTAATG
ATACT--

CAGAATGGTAATATCACACTCCAATGCAGAATAAAACAAATTATAAACCTGTGGCAGGGAGTAGGAAAAGCAA
TGTATGCCCCTCCCATCAGTGGACAAATTAACTGTACATCAAAT---
ATTACAGGGCTGATTTTAACAAGAGATGGGGGGGAGATGATTAAT----------------------------
----

GAAACTGAGACCTTTAGACCTGGAGGAGGAAATATGAAGGATAATTGGAGAAGTGAATTATATAAATATAAAG
TAGTAAGAATTGAACCATTA---GGAATAGCACCCACCAAGGCAAAAAGAAGAGTGGTG--------------
-------------------------CAAAGAGAGAAAAGAGCA---GTAGGA---CTGGGAGCT---------
ATGTTCCTTGGGTTCTTGGGAGCA---GCCGGAAGCACTATG---GGCGCAGCGTCA---
ATAGCGCTGACGGAACAG---GCCAGACGA---GTATTG---TCTGGTATAGTGCAACAGCAGAAC---
AATTTG---CTGAGG---GCTATTGAGGCGCAACAGCATTTGTTGCAACTCACAGTCTGGGGCATT
>MH633199
TTCTACTGTAACACAACACAATTGTTTAATAGTACTTGGAATATTACTAGTAACTCTTGGAATAGTACTAGTA
ATTAT--

ACTAATGAAAATATCACACTCCCGTGCAGAATAAAACAAATTATAAACAGGTGGCAGGAAGTAGGAAAAGCAA
TGTATGCCCCTCCCATCAGTGGACCAATAAGATGTTCATCAAAT---
ATTACAGGGCTACTACTAACAAGAGATGGTGGTAACAATAATGATACC-------------------------
----

AGCACCGAAGTCTTCAGACCTGGGGGAGGAGACATGAAAGACAATTGGAGAAGTGAATTATATAAATATAAAG
TAGTACAAATTGAGCCATTA---GGAGTAGCACCCACCAAGGCAAAGAGAAGAGTGGTG--------------
-------------------------CAGAGAGAAAAAAGAGCA---GTGGGA---ATAGGAGCG---------
ATATTCCTTGGGTTCTTGTCAGCA---GCAGGAAGCACTATG---GGCGCAGCGTCG---
ATGACGCTGACGGTACAG---GCCAGACAA---TTATTG---TCTGGTATAGTGCAGCAGCAGAAC---
AATCTG---CTGAGG---GCTATTGAGGCGCAACAACATCTGTTGCAACTCACAGTCTGGGGCATC
>EU616649
TTCTACTGCAATACATCAGGACTGTTTAATAGTTCTTGGGATATTAATGGTTCTTGGTATGGTAATAGAGGG-
----

TCAAATGACACAATTACACTCCCATGCAGAATAAAACAATTTATAAACATGTGGCAGGAAGTAGGAAAAGCAA
TGTATGCCCCTCCTATCCAAGGACTAATTAACTGTTTATCAAAT---
ATTACAGGGCTGCTATTAACAAGAGATGGTGGCAATAGCAGTCATAGT-------------------------
----

AATGGACAGAGACCTTTAGACCTGGAGGAGGAGATATGAGAGATAATTGGAGAAGTGAATTATATAAATATA
AAGTAGTAAAATTGAACCATTA---GGAGTAGCACCCACCAAGGCAAAGAGAAGAGTGGTG-----------
----------------------------CAGAGAGAAAAAAGAGCA---GTGGGA---ATAGGAGCT------
---TTATTCCTTGGGTTCTTGTCAGCA---GCAGGAAGCACTATG---GGCGCAGCGTCA---
ATGACGCTGACGGTACAG---GCCAGACAA---TTATTG---TCTGGTATAGTGCAACAGCAGAAC---
AATTTG---CTGAGA---GCTATTGAGGCGCAACAGCATATGTTGCAACTCACAGTCTGGGGCATC
>FJ469704
TTCTACTGTAACTTAACAAAACTGTTTAATAGTACTTGGGCTTGGAATAGTACTTGGAATAGTACTGAAGGAA
AT--

GAAAATAATACTATCACACTCCCATGTAGAATAAAACAAATTATAAACATGTGGCAGACAGTAGGAAAAGCAA
TGTATGCCCCTCCTATCAGAGGACAAATTACATGCTCATCACAT---
ATTACAGGGATACTATTAACAAGAGATGGTGGTAACAGTGATGACACA-------------------------
----

AACTATACTGAGATCTTCAGACCTGCAGGAGGAGATATGAGGGACAATTGGAGAAGTGAATTATATAAATATA
AAGTAGTAAAATTGAACCATTA---GGAGTAGCACCTACCAAGGCAAAGAGAAGAGTGGTG-----------
----------------------------CAGAGAGAAAAAAGAGCA---GTGGGA---TTAGGAGCT------
---ATGTTCCTTGGGTTCTTGGGAGCA---GCAGGAAGCACTATG---GGCGCAGCGTCG---
CTGACGCTGACGGTACAG---GCCAGGCAG---TTATTG---TCTGGTATAGTGCAACAGCAGAAC---
AATCTG---TTGAGG---GCTATTGAGGCGCAACAGCATCTGTTGCAACTCACAGTCTGGGGCATC
>FJ469725
TTCTACTGTAATTCATCAAAACTGTTTAAGGGTACCTGGAATGGTACTACATTGTCAAATACCACTAAAGGAA
TAAATAAT--
-------------------ACT---
CAAGGAGAGAAAATAATCACAATCCAGTGCAAAATAAAACAATTTATAAACAGGTGGCAGGAAGTAGGAAAAG
CAATGTATGCCCCTCCCATCAGCGGACCAATTATATGTCCATCAAAT---
ATTACAGGGCTGCTATTAACAAGAGATGGTGGTAATGGCACCAAT---------------------------
----

AGGACGACCGAGACCTTCAGGCCTGAAGGAGGAGATATGAGGGACAATTGGAGAAGTGAATTATATAAATATA
AAGTAGTAAAATTGAACCATTA---GGATTAGCACCCACCAAGGCAAGGAGAAGAGTGGTG-----------
----------------------------CAGAGAGAAAAAAGAACA---GTGGGA---ATAGGAGCT------
---TTGTTCCTTGGGTTCTTGGGAGCA---GCAGGAAGCACTATG---GGCGCAGCGTCA---
ATGACGCTGACGGTACAG---GCCAGGCTA---TTATTG---TCTGGTATAGTGCAACAGCAAAAC---
AACTTG---CTGAGG---GCTATTGAGGCGCAACAGCATCTGTTGCAACTCACAGTCTGGGGCATC
>MN515543
TTCTACTGTAATTCAACATCACTGTTTAATAGTACTTGGATGTTTAATAGTACTTGGAATGGCACTGATGGGA
ATGATGCT--

GACTCTAGAGAAAATATCACACTCCAATGCAGAATAAAACAAATTATAAACATGTGGCAGGAAGTAGGAAAAG
CAATGTATGCCCCTCCCATCCAAGGGCTAATTAGATGTTCATCAAAT---
ATTACTGGGCTGCTATTAACAAGAGATGGTGGTAATCACAATAACAGT------------------------
----

AATGGACAGAGACCTTTAGACCTGGAGGAGGAGATATGAGGGACAATTGGAGAAGTGAATTATATAAATATA
AAGTAGTAAAATTGAACCATTG---GGAGTAGCACCCACCAAGGCAAAGAGAAGAGTGGTG-----------
----------------------------CAGAGAGAAAAAAGAGCA---CTAGGGGGACTAGGAGCT------
---TTGTTCCTTGGGTTCTTGGGAGCA---GCAGGAAGCACTATG---GGCGCAGCGTCC---
CTGACGCTGACGGTACAG---GCCAGACTA---TTATTG---TCTGGTATAGTGCAACAGCAGAAC---
AATCTG---CTGAGA---GCTATTGAGGTGCAACAGCATCTGTTGCAACTCACAGTCTGGGGCATC
>MN515731
TTCTATTGTAATACCGCAAAACTGTTTAGTAGTACTTGGCATGCTAATGGTACGTGGAATGGTACTTGGAATA
ATACT---

ACAGAGCAAAATATTACAATTCTATGCAGAATAAAACAAATTATAAACATGTGGCAGGAAGTAGGAAAAGCAA
TGTATGCCCCTCCCATCGGAGGAAAAATTAGATGCTCATCAAAT---
ATCACAGGGCTACTATTAGTGAGAGATGGTGGCAACACCACCATC---------------------------
----

GGGAACACCACTGAGATTTTCAGACCTCAAGGAGGAAATATGAAGGACAATTGGAGAAGTGAATTATATAAAT
ATAAAGTAGTACAAATTGAACCAATA---GGAGTAGCACCCACCAAGGCAAAAAGAAGAGTGGTG--------
-------------------------------CAGAGAGAAAAAAGAGCA---GTGGGA---ATAGGAGCT---
------CTGCTCCTTGGGTTCTTGGGAGCA---GCAGGAAGCACTATG---GGCGCAGCGTCA---
ATAACGCTGACGGTACAG---GCCAGACAA---TTATTG---TCTGGTATAGTGCAACAGCAAAAC---
AATTTG---CTGAGA---GCTATTGAGGCGCAACAGCATCTGTTGCAACTCACAGTCTGGGGCATC
>GU728412
TTCTACTGTAATACAACACAACTGTTTAATAGTACTTGGGATATAAAGAGTAATATGACAAGGTCAAAT----
----

AACACTGAACCAATCACCCTCCAATGCAGAATAAAGCAAATTATAAACATGTGGCAGGAAGTAGGAAAAGCAA
TGTATGCCCCTCCCATCAGAGGACAAATTAAATGTTCATCAAAT---
ATTACAGGGCTGCTATTAACAAGAGATGGTGGTAATGAGACAGGGAAAGAG---------------------
----

AATGATACAGAGAAAGAGACCTTCAGACCTGGAGGAGGAGATATGAGGGACAATTGGAGAAGTGAACTTTATA
AATATAAAGTAGTAAAAATTAAACCATTA---GGAGTAGCACCCACTGCAGCAAAAAGAAGAGTGGTG-----
----------------------------------CAGAGAGAAAAAAGAGCG---GCACTA------
GGAGCC---------TTGTTCCTTGGGTTCTTGGGAGCA---GCAGGAAGCACTATG---GGCGCAGCGTCA-
--GTAACGCTGACGGTACAG---GCCAGACTA---TTATTG---TCTGGTATAGTGCAACAGCAGAAC---
AATCTG---CTGAGA---GCTATTGAGGCGCAACAGCATCTGTTGCAACTCACAGTCTGGGGCATC
>GU727880
TTCTACTGTAATACAACACAACTGTTTAATAGTACTTGGAATGGTACACAACTGCCTAATCTTACT-------
----

GGGAATGAAAATATCACACTCCAATGCAGAATAAAGCAAATTATAAATATGTGGCAGGAAGTAGGAAAAGCAA
TATATAACCCTCCCATCCAAGGACTAATTAGATGTTCATCAAAT---
ATTACAGGGCTGTTATTAACAAGAGATGGTGGTCATGGGAATAAT----------------------------
----

GACACCGAGATCTTCAGACCAGGAGGAGGAGATATGAGGGACAATTGGAGAAGTGAATTATATAAATATAAAG
TAGTAAAAATTGAACCATTA---GGACTAGCACCCACCACGGCGAAGAGAAGAGTGGTG-------------
-------------------------CAGAGAGAAAAAAGAGCA---GTGGGA---ATAGGAGCA---------
TTGTTCCTTGGGTTCTTGGGAGCA---GCAGGAAGCACTATG---GGCGCAGCGTCA---
ATGACGCTGACGGTACAA---GCCAGACAA---TTATTG---TCTGGTATAGTGCAACAGCAGAAC---
AATCTG---CTGAGG---GCTATTGAGGCGCAACAACATGTATTGCAGCTCACAGTCTGGGGCATC
>DQ886031
TTCTACTGTAATACATCACGGCTGTTTAATAGTACTTGGAATATTACTGAAGGGTCAAATAGCACTGCAGGC-
----

TCTAACAAGAATATCACACTCCCATGCAGGATAAAACAAATTATAAACATGTGGCAGGAAGTAGGAAAAGCAA
TGTATGCCCCTCCCATCAGCGGACTAATTAGATGTACATCAAAT---
ATTACAGGGCTGTTACTAACAAGAGATGGTGGTCATGATAATAAAACA------------------------
----

AATGACACCGAGATCTTCAGACCTGGAGGAGGAAACATGAGGGACAATTGGAGAAGTGAATTATATAAATATA
AAGTAGTAAAAATTGAACCATTA---GGAATAGCACCCACCAAGGCAAGGAGAAGAGTGGTG----------
---------------------------CAAAGAGACAAAAGAGCA---GTGGGA---ATAGGAGCT------
---ATGTTCCTTGGGATCTTAGGAGCA---GCAGGAAGCACTATG---GGCGCAGCGTCA---
ATAACGCTGACGGTACAG---GCCAGACAA---CTATTG---TCTGGTATAGTGCAACAGCAGAAC---
AATTTG---CTGAGG---GCCATTGAGGCGCAACAGCATCTGTTGCAACTCACAGTCTGGGGCATA
>DQ886032
TTCTACTGTAACACAACAAAACTTTTTAATAATAATACTGAAGGGTCAAAAAACACTGAA------------
----

GACAATACCACACTCACACTCCCATGCAGAATAAAACAGATTATAAACATGTGGCAGGAAGTAGGAAGAGCAA
TGTATGCCCCTCCTATCAAAGGAATAATTAGATGTACATCAAAT---
ATTACAGGGCTGATATTAACAAGAGATGGTGGTCATAACATGAATAACACG---------------------
----

AATGAGACCACCGAGACCTTCAGACCTGGAGGAGGAGATATGAGGGACAATTGGAGAAGTGAATTATATAAAT
ATAAAGTAGTAAGAATTGAACCATTA---GGAATAGCACCTTCCAGGGCAAAGAGAAGAGTGGTG--------
-------------------------------CAGAGAGAAAAAAGAGCA---GTGGGA---ATAGGAGCT---
------GTGTTCCTTGGGTTCTTGGGAGCA---GCAGGAAGCACTATG---GGCGCAGCGTCA---
CTGACGCTGACGGTACAG---GCCAGACAA---TTATTG---TCTGGTATAGTGCAACAGCAGAAC---
AATTTG---CTGAGA---GCTATTGAGGCGCAACAGCATCTGTTGCAGCTCACAGTCTGGGGCATC
>GU367399
TTCTACTGCAATACAACAGGACTGTTTAATAGTTCTTGGAATAATGATACTATCTGGAATGATACTAGCACTG
AAGGA--

AGGAGAAATGACACAATCATACTCCCATGCAGAATAAGACAAATTGTAAACATGTGGCAGGAAGTAGGAAAAG
CAATGTATGCCCCTCCTATCCAAGGACTAATTAACTGTTCATCAAAT---
ATTACAGGGCTGCTATTAACAAGAGATGGTGGTAATAGCAACAAT----------------------------
----

AGCTATGAGACCTTTAGACCTGGAGGAGGAGATATGAGGGACAATTGGAGAAGTGAACTATATAAATATAAAG
TAGTAAAAATTGAACCAATA---GGAGTAGCACCCACCAAGGCAAAGAGACGAGTGGTG--------------
-------------------------CAGAGAGAAAAAAGAGGG---GTCGGG---ATCGGAGCT---------
TTGTTCCTTGGGTTCTTGGGAGCA---GCAGGAAGCACTATG---GGCGCAGCGTCA---
ATAACGCTGACGGTACAG---GCCAGACAA---CTATTG---TCTGGTATAGTGCAACAGCAAAAC---
AACTTG---CTGAGG---GCTATTGAGGCGCAACAACATCTGTTGCAACTCACAGTCTGGGGCATC
>GU367403
TTCTACTGTAATACATCAAAACTGTTTAATAGTACTTGGGGGTGGAATGTCACTGAAAATGGCACTGAAGTGT
CAAAT--

AACACTGAAGGAAATATCACACTTCCATGCAGAATAAAACAAATTATAAACATGTGGCAAAAAGTAGGAAAAG
CTATATATGCCCCTCCTATCAGAGGACAAATTAGCTGTTTATCAAAT---
ATTACAGGGCTGCTATTAACAAGAGATGGTGGTCATAACGAGGGTAAC-------------------------
----

GAGACTAAGACTGAGGTCTTCAGACCTGGAGGAGGAGATATGAGGGACAACTGGAGGAGTGAATTATATAAAT
ATAAAGTAGTAAAAATTGAACCATTA---GGAGTAGCACCCACCAAGGCAAAAAGAAGAGTGGTG--------
-------------------------------CAAAGAGAAAAAAGAGCA---GTAGGA---ATAGGAGCT---
------GTGTTCCTTGGGTTCTTGTCAGCA---GCAGGAAGCACTATG---GGCGCAGCGTCA---
CTGACGCTGACGGTACAG---GCCAGACAA---TTATTG---TCTGGTATAGTGCAACAGCAGAAC---
AATCTG---CTGAGG---GCTATTGAGGCGCAACAGCATCTGTTGCAACTCACAGTCTGGGGCATC
>KP754467
TTCTATTGTAATACAACACCGCTGTTTAATAGTAGTTGGAATGAAACTACTCTTCAGAAGGGAAATAATAGTA
CTCAG--

GATAATGAAACTATCACAATCCAATGCAGAATAAAACAAATTATAAACAGGTGGCAGGAAGTAGGAAAAGCAA
TGTATGCCCCTCCCATCCAAGGACTAATTATGTGTGCATCAAAT---
ATTACAGGGATACTATTAACAAGAGATGGTGGTGATGGTAATGGTACA-------------------------
----

AACAATACCACCGAGATCTTCAGACCTGGAGGAGGAGACATGAGGGACAATTGGAGAAATGAATTATATAAAT
ATAAAGTAGTACAAATTGAGCCATTA---GGAGTAGCACCCAGCAAGGCAAAGAGAAGAGTGGTG--------
-------------------------------CAAAGAGAAAAAAGAGCA---GTAGGA---CTAGGAGCT---
------TTGTTCCTTGGGTTCTTGGGAGCA---GCAGGAAGCACTATG---GGCGCAGCGTCG---
ATGACGCTGACGGTACAG---GCCAGACAA---TTATTG---TCTGGTATAGTGCAACAGCAGAAC---
AATCTG---CTGAGG---GCTATTGAGGCGCAACAACAGCTGTTGCAACTCACAGTCTGGGGCATT
>KP754469
TTCTACTGTGATACATCAAAACTGTTTAATAGTACTTGGGATAAGGGTAATTGGAGTACAGGGACAATTGCCA
CTAAC--

GGAAATGACACTATCATACTCCAATGCAGAATAAGACAAATTATAAACATGTGGCAGGAAGTAGGAAAAGCAA
TCTATGCCCCTCCTATCAGTGGATACGTTAACTGTACATCAAAT---
ATTACAGGGTTGATATTAACAAGAGATGGTGGTAATGATAATGGTACA-------------------------
----

AAAAATGGTACAAAC---
TCCACCGAGACTTTTAGACCTGGAGGAGGAGATATGAGGGACAATTGGAGAAGTGAATTATATAAATATAAAG
TAGTAAAAATTGAACCATTA---GGAATAGCACCCACCAAGGCAAAGAGAAGAGTGGTG--------------
-------------------------CAGAGAGAAAAAAGAGCA---GTGGGG---ATGGGAGCT---------
TTGTTCCTTGGGTTCTTGGGAGCA---GCAGGAAGCACTATG---GGCGCAGCGTCA---
ATAACGCTGACGGTACAA---GCCAGACAA---TTGTTG---TCTGGTATAGTGCAACAGCAGAAC---
AATCTG---CTGAAG---GCTATTGAGGCGCAACAGCATCTGTTGCAACTCACAGTCTGGGGCATC
>KP754471
TTCTACTGTAACACATCACAACTGTTTAATAGTACGTGGAATGATACTGAAAGGCCAAATAGCAATGGAGAA-
----

AATGACCAAAATATTACACTCCCTTGCAGAATAAGACAAATTGTAAACATGTGGCAGGAGGTAGGAAAAGCAA
TGTATGCCCCTCCCATCAGAGGACCAATTCAATGCTCATCAAAC---
ATTACAGGGATGATATTAACAAGAGATGGTGGCAATAACAGTGCC----------------------------
----

CAGAACGAGACCTTCAGACCTGGAGGAGGAGACATGAGGGACAATTGGAGAAGTGAATTATATAAATATAAAG
TAGTAAAAATTGAACCATTA---GGAGTAGCACCCACCAAGGCAAAAAGAAGAGTGGTG--------------
-------------------------CAGAGAGAAAAAAGAGCA---GTGGGA---ATAGGAGCT---------
TTGTTCATCGGGTTCTTGGGAGCA---GCAGGAAGCACTATG---GGCGCAGCGTCA---
ATGACGCTGACGGTACAG---GCCAGACAG---TTACTG---TCAGGTATAGTGCAACAGCAAAAC---
AATTTG---CTGAGA---GCTATTGAGGCGCAACAGCATATGTTGCAACTCACAGTCTGGGGCATC
>FJ469744
TTCTACTGTAATTCAACACAACTGTTTGATAGTACTTGGGATAATGGAACAAAAACAAATACCACTGAGGGAA
GAAATAGCGAT--
-------------GGAGGA------
AATAGCAGCGAACCCATCATACTCCCATGCAGAATAAAACAAATTATAAACATGTGGCAGGAAGTAGGAAAAG
CAATGTATGCCCCTCCCATCAGTGGAAACATTACATGTTCATCCAAT---
ATTACAGGGTTGCTATTAACAAGAGATGGTGGCAAGGGCAATGAGACTCAGGGC-------------------
----
-
TATGGGACTCAGGGCACTGAGATCTTCAGACCTGGAGGAGGAGATATGAGAGACAACTGGAGAAGTGAATTAT
ATAAATATAAAGTAGTAAGAATTGAACCATTA---GGAGTAGCACCCACCAAGCCAAAGAGAAGAGTGGTG--
-------------------------------------CAGAGAGAAAAAAGAGCA---GTGGGA---
TTAGGAGCT---------CTGTTCCTTGGGTTCTTGGGAGCA---GCAGGAAGCACTATG---
GGCGCAGCGTCA---ATGACGCTGACGGTACAG---GCCAGACTA---TTATTG---
TCTGGTATAGTGCAGCAGCAGAAC---AATCTG---CTGAGG---
GCTATTGAGGCGCAACAGCATCTGTTGCAACTCACAGTCTGGGGCATC
>GU728345
TTCTACTGTAAAACATCAAGACTATTTAATAGTACTTGGAATGGTACTTGGAATGGTACTGAAGGACCAATCT
GGAATGGTACT------------------------------------GAAAGGTCAGAA---
GGACCAATCTATGATGACCCT---------------
AAAAGAAAAGACACAATCACACTCCAATGCAGAATAAAACAAATTATAAACATGTGGCAGAGAGTAGGAAAAG
CAATGTATGCCCCTCCCATCAGTGGAATAATTAGATGTTCATCAAAT---
ATTACAGGGCTGATATTAACAAGAGATGGTGGAAACAATAACGAGACTAAT----------------------
----

ACCCCTGAGGTCTTCGAGACTAGACCTGGAGGAGGAAATATGAGGGATAATTGGAGAAATGAATTATATAAAT
ATAAAGTAGTAAAAATTGAACCATTA---GGAGTAGCACCCACCAAGGCAAAGAGAAGAGTGGTG--------
-------------------------------CAGAGGGAAAAAAGAGCA---GTGACA---CTAGGAGCT---
------GTGTTCCTTGGGTTCTTGGGAGCA---GCAGGAAGCACTATG---GGCGCAGCATCA---
ATAACGCTGACGGTACAG---GCCAGACAA---TTATTG---TCTGGTATAGTGCAACAGCAGAGC---
AATCTG---CTGAGG---GCTATTGAGGCGCAACAGCATATGTTGCAACTCACAGTCTGGGGCATC
>FJ687543
TTCTACTGTGACTCAACAAAACTGTTTAATTTTACTTGGAATGGTACTGAAACAAATAACACTGGA-------
----

GGAAGTGATAAAATCATACTCCCATGCAGAATAAAACAAATTATAAAAATGTGGCAGAAAGTAGGACAAGCAA
TGTATGCCCCTCCCATCCAAGGAAAAATTTACTGCTCATCAAAT---
ATTACAGGGCTACTATTAACAAGAGATGGTGGCATCAACAATGAACCT-------------------------

----

AACAATACTGAGACCTTCAGACCTGGAGGAGGAAATATGAAGGATAATTGGAGAAGTGAATTATATAAATATA
AAGTAGTACAAATTGAACCATTA---GGAGTAGCACCCACCAAGGCAAAAAGAAGAGTGGTG-----------
---------------------------CAGAGAGAAAAAAGAGCA---GTGGGC---ATAGGAGCT------
---TTGTTCCTTGGGTTCTTGGGAGCA---GCAGGAAGCACTATG---GGCGCAGCGTCA---
ATAACGCTGACGGTACAG---GCCAGACAA---TTATTG---TCTGGTATAGTGCAACAGCAGAAC---
AATCTG---CTGAGG---GCTATTGAGGCGCAACAGCATATGTTGCAACTCACAGTCTGGGGCATC
>GU727919
TTCTACTGTAATACAACACAACTGTTTAATAGTATTTGGAGGTTTAATAGTATTTGGAGGTTTAATGATACTA
AGGAGAACAAT--
----------CAAACTGAA------
GGAAATGACACAGTTATCACACTCCCATGTAAAATAAAACAAATTATAAACTTGTGGCAAGAAGTAGGAAAGG
CAATGTATGCCCCTCCCATCAGAGGACAAATTAACTGTACATCAAAT---
ATTACAGGGATACTATTAATAAGAGATGGTGGTAAGAGTAATGAGACT------------------------
----

AACAACACCGAGACCTTCAGACCTGGGGGAGGAAATATGAAGGACAATTGGAGAAGTGAATTATATAAATACA
AAGTAGTAAGAATTGAACCATTG---GGAATAGCACCCACCACGGCAAAGAGAAGAGTGGTG----------
---------------------------CAAAGAGAAAAAAGAGCA---GTGGGAATGCTAGGAGCT------
---ATGTTCCTTGGGTTCTTGGGAGCA---GCAGGAAGCACTATG---GGCGCAGCGTCA---
ATAACGCTGACGGTACAA---GCCAGACAA---TTATTG---TCTGGTATAGTGCAACAGCAGAGC---
AATCTG---CTGAGG---GCTATTGAGGCGCAACAGCATATGTTGCAACTCACAGTCTGGGGCATC
>KF725982
TTCTACTGTAACACAACACCACTGTTTAACAAAACACACCTGGATAGCCTGAATAGCACT------------
----

ACAGACGGTAATATTACACTTCAATGCAGAATAAAACAAATTATAAACATGTGGCAGGAAGTAGGAAAAGCAA
TGTATGCCCCTCCCATCAGAGGACAAATTAGATGTTCATCAAAT---
ATTACAGGGCTGTTATTAACAAGAGATGGTGGTAATGGTAATGAGAGC------------------------
----

AGCAACCATGAGACCTTCAGACCTGGGGGAGGAGATATGAGGGACAATTGGAGAAGTGAATTATATAAATATA
AAGTAGTACAAATTGAACCATTA---GGAATAGCACCCACCAAGGCAAAGAGAAGAGTGGTG----------
---------------------------CAGAGAGAAAAAAGAGCA---GTGGGA---ATAGGAGCT------
---TTGTTCCTTGGGTTCTTGGGAGCA---GCAGGAAGCACTATG---GGCGCAGCGTCA---
GTGACGCTGACGGTACAA---GCCAGACAA---TTACTG---TCTGGTATAGTGCAACAGCAGAAC---
AATTTG---CTGAGG---GCTATTCAGGCGCAACAACATTTGTTGCAACTTACAGTCTGGGGCATC
>KF725986
TTCTACTGTAATACAGCACCACTGTTTAATAGTACTTGGAATGATACCAGTGCCTGGAATATAACT-------
----

GATACTAGCCCACTCACAATCCAGTGCAGAATAAGACAAATTATAAATAGGTGGCAGGAAGTAGGAAAATCGA
TGTATGCCCCTCCCATCAGTGGACAAATTAACTGTTCATCAAAT---
ATTACAGGGCTGCTATTAACAAGAGATGGTGGTGGAAACAGGTCC----------------------------
----

CAGGAAAACAGGTCCGAGACCTTCAGACCTGGAGGAGGAGACATGAGGGACAATTGGAGGAGTGAATTATATA
AATATAAAGTAGTAAAAATTGAGCCATTA---GGGATAGCACCCACCAAGGCAAGGAGGAGAGTGGTG-----
----------------------------------CAAAGAGAAAAAAGAGCA---
GTGGGAGCGATAGGAGCT---------ATGTTCCTTGGATTCTTGGGGACA---GCAGGAAGCACTATG---
GGCGCAGCGGCA---TTGACGCTGACGGTACAG---GCCAGACAA---TTATTG---
TCTGGTATAGTGCAACAGCAAAAC---AATTTG---CTGAGG---
GCTATTGAGGCGCAACATCATCTGTTGCAACTCACAGTCTGGGGCATC
>KF725988
TTCTACTGTGATACAAAAAAACTGTTTAATAGTACATGGAATTTTAATAATACTTGGAATTCTACTCAAACA-
----

GGCAATGACACAATCACACTCCCATGCAGAATAAAACAAATTATAAACATGTGGCAAGGAGTAGGAAAAGCAA
TGTATGCCCCTCCCATTAGTGGACAAATCAACTGTTTATCAAAT---

ATTACAGGGCTGATATTAACAAGAGATGGTGGTCAGAACGATAGC----------------------------
----

AGTAAGGAGACCTTCAGACCTGCAGGAGGAAATATGAAGGACAATTGGAGAAGTGAATTATATAAATATAAAG
TAGTACAAATTGAACCATTA---GGAGTAGCACCCACCAAGGCAAAGAGAAGAGTGGTG--------------
-------------------------CAAAGAGACAAAAGAGCA---GTGGGA---ATAGGAGCA---------
TTGTTCCTTGGGTTCTTGGGAGCA---GCAGGAAGCACTATG---GGCGCAGCGTCA---
ATGACGCTGACGGTACAG---GCCAGGCAA---TTATTG---TCTGGTATAGTGCAGCAGCAGAAC---
AATCTG---CTGAGG---GCTATTGAGGCGCAGCAGCACCTGCTGCAACTCACAGTCTGGGGCATC
>KF726003
TTCTACTGTGATACAACACAGCTGTTTAATAGTACTTGGGTTAATGGTACTATAAAGAATAGTACTGAAGAA-
----

AATAACACATTAATTCAGCTCCCATGTAGAATAAAACAAATTATAAACAGATGGCAGGAAGTAGGAAAAGCAA
TGTATGCCCCTCCTATCAAAGGAGTAATTAGCTGTTCATCAAAT---
ATTACAGGGCTGATCTTAACAAGAGATGGTGGAAATAATAATAGTGCA-------------------------
----

GCTAATGAGACCTTTAGACCTGCAGGAGGAGATATGAAGGACAATTGGAGAAGTGAACTATATAAATATAAAG
TAGTACAGATTGAACCATTA---GGAGTAGCACCCACCCAGGCAAAGAGAAGAGTGGTG--------------
-------------------------CAGAGAGAAAAAAGAGCA---GTGGGAACAATAGGAGCT---------
ATGTTCCTTGGGTTCTTGGGAGCA---GCAGGAAGCACTATG---GGCGCAGCGTCA---
ATAACGCTGACGGTACAG---GCCAGACAA---TTATTG---TCTGGTATAGTGCAGCAGCAGCAC---
AATTTG---CTGAGA---GCTATTGAGGCGCAACAGCATCTGTTGCAACTCACAGTCTGGGGCATC
>KU168260
TTCTACTGTAATACAACACAACTGTTTAATAGTATTTGGGAAGGTAATAAGACTCAGACAGGTCATAGTACTC
ATAAT--

GCTAGGAATGACACTATCACGCTCCCATGTAGAATAAAACAAATTATAAACATGTGGCAGGAAGTAGGAAAAG
CAATGTATGCCCCTCCCATCCATGGACAAATTAGATGTATATCAAAT---
ATCACAGGTCTGCTATTAACAAGAGACGGTGGTATACACAACAAAACC-------------------------
----

CACATGACTGAGGTCTTCAGACCTGGAGGAGGAAATATGAAGGACAATTGGAGAAGTGAATTATACAAATATA
AAGTAGTACAAATTGAACCATTA---GGGCTGGCACCCACCAAGGCAAAGAGAAGAGTGGTG-----------
----------------------------CAGAGAGAAAAAAGAGCA---GTGGGAGCAATAGGAGCT------
---TTGTTCCTTGGGTTCTTGGGAGCA---GCAGGAAGCACTATG---GGCGCAGCGTCA---
CTGGCGCTGACGGTACAG---ACCAGACAA---TTACTG---TCTGGTATAGTGCAACATCAGAAC---
AATCTG---CTGAGG---GCTATTCAAGCGCAACAGCATCTGTTGCAGCTTACAGTCTGGGGCATC
>KT452481
TTCTACTGTAATACAACAAAACTGTTTAACAGTACTTGGATTTGGAATAGTACTGAAGGGTCAAACTCC----
----

AGTGAAGGAAACACAACACTCCAATGCAGAATAAAACAAATTGTAAACATGTGGCAGGAAGTAGGAAAAGCAA
TGTATGCTCCTCCCATCAAAGGAGTAATCAGATGTTCATCAAAT---
ATTACAGGGCTGTTATTAACAAGAGATGGTGGTAGCAAGAACAGTAGCGAGAAC-------------------
----

ACCACCACTAAGGAGACCTTCAGACCTGGAGGAGGAGATATGAGGGATAATTGGAGAAGTGAATTATATAAAT
ATAAAGTAGTAAAAATTGAACCATTA---GGAATAGCACCCACCAAGGCAAGGAGAAGAGTGGTG--------
-------------------------------CAGAGAGAAAAAAGAGCC---GTGGGA---ATAGGAGCA---
------TTGTTCCTTGGGTTCTTGGGAGCA---GCAGGAAGCACTATG---GGCGCAGCGTCA---
CTAACGCTGACGGTACAG---GCCAGACAA---TTATTG---TCTGGTATAGTGCAGCAGCAAAAC---
GATTTG---CTGAGA---GCTATTGAGGCGCAACATCATCTGTTGCAACTCACAGTCTGGGGCATC
>KT452508
TTCTACTGTGATACAACAAAACTGTTTGATAGCTCTTGGAATAAAACTGGAAGAATAGATAAAAGGAATAATA
CAGAAGGG---

TCAAATGACACAATCACACTCCCATGCAGAATAAAACAAATTATAAACATGTGGCAGGAAGTAGGAAAAGCAA
TGTATGCTCCTCCCATCAGTGGACGGATTAACTGTACATCAAAT---

ATTACAGGGCTGATATTAACAAGAGATGGTGGTCATAATGTTACA---------------------------
----

ACTAAAGGGGACACCTTCAGACCTGGAGGAGGAAATATGAAGGACAATTGGAGAAGTGAATTATATAAATATA
AAGTAGTAAAAATTGAACCATTA---GGAATAGCACCCACCAAAGCAAGGAGAAGAGTGGTG----------
---------------------------CAGAGAGAAAAAAGAGCC---GTGGGA---TTAGGAGCT------
---TTGTTCCTTGGGTTCTTGGGAGCA---GCAGGAAGCACTATG---GGCGCAGCGTCA---
ATGGCGCTGACGGTACAG---GCCAGACAA---TTATTG---TCTGGTATAGTGCAACAGCAGAAC---
AATCTG---CTGAGG---GCCATTGAGGCGCAACAGCATCTGTTGCAACTCACAGTCTGGGGCATC
>KT452155
TTCTACTGCAATACATCACGACTATTTAATAGTACTTGGTTTAAGAATGATAGTACTTGGTTTAGGAATGATA
CTGGGAAT---
-------------GATACA------
GAAAAGTCAGAAAAGATCATACTTCCATGCAGAATAAAACAAATTATAAATATGTGGCAGGAAGTAGGAAAAG
CAATGTATGCCCCTCCCATCCGAGGACTAATTAGTTGTCCATCAAAT---
ATTACAGGACTGTTATTAACAAGAGATGGTGGTGGAGATAATAACAGA------------------------
----

ACTAATGAGACTTTCAGACCTCAAGGAGGAGATATGAGGGACAATTGGAGAAGTGAATTATATAAATATAAAG
TAGTAAGAATTGAACCATTA---GGAATAGCACCCACCAAGGCAAGGAGGAGAGTGGTG-------------
------------------------CAAAGAGAAAAAAGAGCA---GTGGGAACATTAGGAGCT---------
ATGTTCCTTGGGTTCTTGGGAACA---GCCGGAAGCACTATG---GGCGCAGCGTCA---
ATAACGCTGACGGTACAG---GCCAGACAA---TTATTG---TCTGGTATAGTGCAACAGCAAAAT---
AACTTG---CTGAGA---GCTATTGAGGCGCAACAGCATCTGTTGCAACTCACAGTCTGGGGCATC
>U39243
TTCTATTGCAATACAACAAAACTGTTTAGTAGTACATATATAGTTAATAATACACATGACAGTAAT-------
----

ACAACAAAAATTTTAAACATCACATGCAGAATAAAACAAATTATAAACATGTGGCAAGGGGTAGGACGAGCAA
TGTATGCCCCTCCCATTGCAGGAAACATAACATGTAAATCAAAT---
ATCACGGGACTACTACTGACACGTGATGGAGGAAATAAGACAGAAAGC------------------------
----

AATGACACAGAGACATTCAGGCCTCAGGGAGGAGATATGAGGGACAATTGGAGAAATGAATTATATAAATATA
AAGTGGTAGAAGTTAACCCATTA---GGAATAGCACCCACTAAGGCAAAAAGGAGAGTGGTG----------
----------------------------GACAGAGAAAAAAGAGCA---GTAGTGGGACTAGGAGCT------
---GTGTTCCTTGGGGTCTTGACACCC---GCAGGAAGCACTATG---GGCGCAGCATCA---
ATAACGCTGACGGTACAG---GCCAGACAA---TTATTG---TCGGGCATAGTGCAACAGCAAAAC---
AATTTG---CTGAGG---GCTATAGAGGCGCAACAGCATATGTTGCAACTCACGGTCTGGGGCATT
>U39240
TTCTATTGCAATACATCAATACTGTTTAATACAACATACCTGGTTAATAATAATTTGACTGAG---------
----

AAAAATTCAACCATTATGCTCCCATGCAGAATAAAACAAATTATAAACATGTGGCAGGAGGTAGGACGAGCAA
TGTATGCCCCTCCCATTGCAGGACTCATACAATGTAACTCAAGT---
ATCACAGGACTGCTACTGACACGTGATGGAGGAGTGAGAAATGACACAAAT---------------------
----

GAGACAGAGACAGAGACATTCAGACCTGCAGGAGGAGATATGAGGGACAATTGGAGAAGTGAATTATATAAAT
ATAAAGTGGTAGAAATTCAGCCATTA---GGAATAGCACCCACTAAGGCAAAAAGGAGAGTGGTG--------
-------------------------------GAGAGAGAAAAAAGAGCA---GCGGGA---ATAGGAGCT---
------TTGTTCCTTGGGTTCTTGGGAGCA---GCAGGAAGCACTATG---GGCGCAGCGTCG---
ATAACGCTGACGGTACAG---GCCAGACAA---TTATTG---TCGGGTATAGTGCAACAGCAAAAC---
AATTTG---CTGAGA---GCTATAGAGGCGCAACAGCATATGTTACAACTCACAGTCTGGGGCATT
>AF443090
TTCTATTGCAATACATCACGCCTGTTTAATAGTACATACAATCCTAATAGTACATACAATAATAGTATAGAAG
GT---

AGGTCAAATGCAACCATTACACTCCAATGCAGAATAAAGCAAATTATAAACCTCTGGCAGGAGGTAGGACGAG
CAATATATGCCCCTCCCATTGCAGGAAACATAACATGTAAATCAAAT---

ATCACAGGATTACTCTTGACACGTGATGGAGGTAATAATTCAACG----------------------------
----

ACAGAGATATTCAGACCTCAAGGAGGGAATATGAAGGACAATTGGAGAAGTGAATTATATAAATATAAAGTGG
TAGAAGTTAAGCCATTG---GGGGTAGCACCCACTAAGGCAAAAAGGAGAGTGGTG-----------------
----------------------GAGAGAGAAAAAAGAGCA---GTAGGA---ATAGGAGCT---------
GTGTTCCTTGGGTTCTTGGGAGCA---GCAGGAAGCACTATG---GGCGCAGCGTCA---
ATGACGCTGACGGTACAA---GCCAGACAA---CTGTTG---TCTGGTATAGTGCAACAGCAAAGC---
AATTTG---CTGAGG---GCTATAGAGGCGCAACAGCATATGTTGCAACTCACAGTCTGGGGCATT
>AF443097
TTCTATTGCAATACATCAGAGCTGTTTAATGGTACATACAACAGTACAGGAAATGATTCA-------------
----

AATTCAAACCTCATCACACTCCAATGCAGAATAAAACAAATTATAAACATGTGGCAGAAGGTAGGACGAGCCA
TGTATGCCCCCCCCATTGAAGGAAACATAACATGTATATCAAAT---
ATCACAGGACTATTATTAACACGTGATGGAGGATATGAGGCAAAT----------------------------
----

CATACTGAGATATTCAGACCTATAGGAGGAGATATGAGGGACAATTGGAGAAGTGAATTATATAAATATAAAG
TGGTAGAAATTAAGCCATTA---GGACTAGCACCCACTGAGTCAAAAAGGAGAGTGGTG--------------
-------------------------GAGAGAGAAAAAAGAGCA---GTGGGA---ATAGGAGCT---------
GTGTTCCTTGGGTTCTTGGGAGCA---GCAGGAAGCACTATG---GGCGCGGCGTCA---
ATAACGCTGACGGTACAG---GCCAGACAA---CTGTTG---TCTGGTATAGTGCAACAGCAAAGC---
AATTTG---CTGAGA---GCTATAGAGGCGCAACAACATATGCTGCAACTCACGGTCTGGGGCATT
>AF443109
TTCTATTGCAATACATCACTACTGTTTAATAGCAGTTATAATGGTAATAGTAGTTATAATGATACAGGA----
----

AGCAATTCAACCATCACACTCCAATGCAGAATAAAACAAGTTATAAACATGTGGCAGAGGGTAGGACAAGCAA
TATATGCCCCTCCCATTGAAGGAATAATAACATGTAACTCAAGT---
ATCACAGGATTACTCTTAGTGAGGGATGGAGACAATCAAACAAGT----------------------------
----

GATACAGAGACATTCAGACCACAAGGAGGAGAAATGAGGGACAATTGGAGAAGTGAGTTATATAAATATAAAG
TGGTAGAAATTAAGCCATTG---GGAGTAGCACCCACTACGGCAAAAAGGAGAGTGGTG--------------
-------------------------GAGAGAGAAAAAAGAGCT---GTGGGA---ATGGGAGCT---------
ATGATCCTTGGGTTCTTGAGTGCA---GCAGGAAGCACTATG---GGCGCAGCGTCA---
ATAACGCTGACGGTACAG---GCCAGACAA---TTGTTG---TCTGGAATAGTGCAACAGCAAAGC---
AATTTG---CTGAGG---GCTATAGAGGCGCAACAGCATATGTTGCAACTCACAGTCTGGGGCATT
>AF443114
TTCTATTGCAACACATCAAGCCTGTTTAATAATACATACCGGCCTACATACTGGCCTAATGGTACAGAA----
----

AGTAATTCAACCATCACACTTCAATGCAGAATAAAACAAATTATAAACATGTGGCAAAAAGTAGGACGAGCAA
TATATGCCCCTCCCATTGCAGGAAAAATAACATGTAAATCAAAC---
ATCACAGGACTACTATTAGTACGTGATGGAGGTAATGGAGGCAACAAT-------------------------
----

ACCGCAACAGAAATATTCAGACCTGGAGGAGGAAATATGAAGGACAATTGGAGAAGTGAATTATATAAATATA
AAGTGGTAGAAATTAAGCCATTG---GGAATAGCACCCACTGGAGCAAAAAGGAGAGTGGTG-----------
----------------------------GGGAGAGAAAAAAGAGCA---GTGGGA---ATAGGAGCT------
---GTGTTCCTCGGGTTCTTGGGAGCA---GCAGGAAGCACTATG---GGCGCGGCGTCA---
ATAACGCTGACGGTACAG---GCCAGACAA---CTGTTG---TCTGGTATAGTGCAACAGCAAAGC---
AATTTG---CTGAAG---GCTATAGAGGCGCAACATCATCTGTTGCAACTCACGGTCTGGGGCATT
>KR861331
TTCTATTGCAATACATCAGGCCTGTTTAATGAAACATACTTGTTTAATGGTACAAATTTGGATAATGATAAT-
----

AGTACAAGCAACATCACACTTCAATGCAAAATAAAACAAATTATAAACATGTGGCAGGGGGTAGGACGAGCAA
TATATGCCCCTCCCATTGCAGGAAACATAACATGTAAATCAAAT---

ATCACAGGACTACTATTGACACGTGATGGAGGTATCAATACTACA----------------------------
----

AGTAACCAGATATTCAGGCCTGGAGGAGGAGATATGAGGGACAATTGGAGAAGTGAATTATATAAATATAAAG
TAGTAGAAGTTAAGCCATTG---GGAATAGCACCCACTAAAGCAAGAAGGAGAGTGGTG--------------
-------------------------CAGAGAGAAAGAAGAGCA---GTGGGA---ATAGGAGCT---------
GTGTTCCTTGGGTTCTTGGGAGCA---GCAGGAAGCACTATG---GGCGCAGCGTCA---
ATAACGCTGACGGTACAG---GCCAGACAA---CTGTTG---TCTGGAATAGTGCAACAGCAGAGC---
AATTTG---CTGAGA---GCTATAGAGGCGCAACAGCATATGTTGCAACTCACAGTCTGGGGCATT
>KR861314
TTCTATTGTAATACATCAAGACTGTTTGATAGTAATGGCACTTATAATAATACAGAA----------------
----

TCAAACAGCACAATCACACTCCCATGCAGAATAAAACAAATTATAAACATGTGGCAGAAAGTGGGACGAGCAA
TGTATGCCCCTCCCATTGAAGGAATGCTAACATGTAAATCAAAT---
ATCACTGGACTACTATTGACACGAGATGGAGGAGAAGGAAATCAA----------------------------
----

GGGTCAGAGATATTCAGACCTGGAGGAGGAAATATGAGGGACAATTGGAGAAGTGAATTATATAAATATAAAG
TGGTAGAGATTAAACCATTG---GGAGTAGCACCCACTGGGGCAAAAAGGAGAGTGGTG--------------
-------------------------GAGAGAGAAAAAAGAGCA---GTGGGA---ATAGGAGCT---------
GTGCTCCTTGGGTTCTTGGGAGCA---GCAGGAAGCACTATG---GGCGCGGCGTCA---
CTCACGCTGACGGTACAG---GCCAGACAA---TTATTG---TCCGGTATAGTGCAACAGCAAAGC---
AATTTG---CTGAGG---GCCATAGAGGCGCAACAGCATATGTTGCAACTCACAGTCTGGGGCATT
>KR861341
TTCTATTGCAATACATCAGGACTGTTTAATGGTACATACCATCCTCAATATAAT-------------------
----
-GATACACAGAATAAT---------
TACACAGACTCAAACATCACACTCCCATGCAGAATAAAACAAATTGTAAATATGTGGCAGGGGGTAGGAAGGG
CAATATATGCCGCTCCCATTGAAGGAAAAATAGTATGTACATCAAAT---
ATCACAGGACTAATATTGACAAGGGATGGAGGAGAAAATAATAGA----------------------------
----

ACAGAAACATTTAGACCTGAAGGAGGAAATATGAGGGACAATTGGAGAAGTGAATTATATAAATATAAAGTGG
TAGAAATTCAGCCATTG---GGAGTGGCACCCACTGAGGCAAAAAGGAGAGTGGTG-----------------
----------------------GAGAGAGAAAAAAGAGCA---GTGGGA---ATAGGAGCT---------
GTGTTCCTTGGGTTCTTGGGAGCA---GCAGGAAGCACTATG---GGCGCGGCGTCA---
ATAACGCTGACGGTACAG---GCCAGACAA---CTGTTG---TCTGGTATAGTGCAACAGCAAAGC---
AATTTG---CTGAGG---GCTATAGAGGCGCAACAGCATATGTTGCAACTCACAGTCTGGGGCATT
>KR861270
TTCTATTGCAATACATCAAGACTGTTTACAAACATAGATAATAATAATACAGAAACTGAT-------------
----

AATGATACCACCATCACCATCCCATGCAAAATAAAACAAATTATAAACATGTGGCAGAAGGTAGGACGAGCAA
TGTATGCCCCCCCCATTGAAGGAAACATAACATGTAAATCAAAT---
ATCACAGGACTACTATTGACACGTGATGGAGGTAATGAAACAAAC----------------------------
----

TACAACACTGAGATATTCAGACCTGGAGGAGGAGATATGAGGGACAATTGGAGAAGTGAGTTATATAAATATA
AAGTGGTGGAGGTTAGGCCATTA---GGAATAGCACCCACTAAAGCAAAAAGGAGAGTGGTG-----------
----------------------------GAGAGAGAGAAAAGAGCA---GTGGGA---ATAGGAGCT------
---GTGTTCCTTGGGTTCTTGGGAGCA---GCAGGAAGCACTATG---GGCGCGGCATCA---
CTAACGCTGACGGTACAG---GCCAGACAA---TTATTG---TCTGGTATAGTGCAACAGCAAAGC---
AATTTG---CTGAGG---GCGATAGAGGCGCAACAACATATGTTGCAACTCACAGTCTGGGGCATT
>KR861273
TTCTATTGCAATACATCTGAGCTGTTTAATAAGCAGTTTAACAATACAAACAGTACAGAAAGTGAGTCAAAT-
----

TCAAATTCAATCATCACACTCCCATGTAGAATAAAACAAATTATAAACATGTGGCAGGAGGTAGGACGAGCGA
TGTATGCCCCTCCCATTGCAGGAAACATAACATGTAAATCAAAT---

ATCACAGGACTACTATTGACACGCGATGGAGGACCTGGAAATAGA---------------------------
----

AGTAACGAGACATTCAGACCCTTAGGAGGAGATATGAAGGACAATTGGAGAAGTGAATTATATAAGTATAAAG
TGGTAGAGATTCAGCCATTA---GGACTAGCACCCACTAGGGCAAAAAGGAGAGTGGTG-------------
------------------------GAGAGAGAGAAAAGAGCA---GTGGGA---ATAGGAGCT--------
GTGTTCCTTGGGTTCTTGGGAGCA---GCAGGAAGCACTATG---GGCGCAGCATCA---
ATAACGCTGACGGTACAG---GCCAGACAA---CTGTTG---TCTGGTATAGTGCAACAGCAAAAC---
AATTTG---CTGAGG---GCTATAGAGGCGCAACAACATTTGTTGCAACTCACGGTTTGGGGCATT
>KR861280
TTCTATTGCAATACATCAAAATTGTTCAATGGTATATATAACAGTACAGGAAATAACAGTACAGGCAATAGTT
CAAGT--

CAAAATAAGACCATCACCATACCATGCAGAATAAAACAAATTATAAATATGTGGCAGAAAGTAGGACGAGCAA
TGTATGCCCCTCCCATTGCAGGAAACATAACATGTAACTCAAGT---
ATCACAGGACTACTATTAGTACGTGATGGAGGACAAGATAACAATACC------------------------
----

AATGGAACAGAAACATTCAGACCTGGAGGAGGAAATATGAAGGACAATTGGAGAAGTGAATTATATAAATATA
AAGTGGTAGAAATTAAGCCATTA---GGAATAGCACCAACTGGAGCAAAAAGGAGAGTGGTG-----------
---------------------------GGTAGAGAAAAAAGAGCA---GTAGGA---ATAGGAGCT------
---GTGGTCCTTGGGATCTTGGGAGTA---GCCGGAAGCACTATG---GGCGCGGCGTCA---
TTGGCGCTGACGGGACAG---ACCAGACAA---CTGTTG---TCTGGTATAGTGCAACAGCAAAGT---
AATTTG---CTGAGG---GCTATAGAGGCGCAACAGCATGTGTTGCAACTCACGGTCTGGGGCATT
>KR861308
TTCTATTGCAATACATCAAATCTGTTCAATAGTACATACCTGTCTAATGGTACACACTTGTTTAATAATACAA
ACATTACAGAC---
-------------------TAT---
ACAGACAATTCAACCATCACACTCCAATGCAGAATAAAGCAAATTATAAACATGTGGCAGGAGGTAGGACAAG
CAATATATGCCCCTCCCATTGCAGGAACAATAACATGTAAATCAAAG---
ATCACAGGACTACTATTGACACGTGATGGAGGATCAGATCCAGATAAT------------------------
----

AAGACTGAGGAGACATTCAGACCTGGAGGAGGAGATATGAGAGACAATTGGAGAAGTAAATTATATAAATATA
AAGTAGTAGAAATTAAGCCATTG---GAAGTAGCACCCACTGGGGCAAAAAGGAGAGTGGTG-----------
---------------------------GAGAGAGAAAAAAGAGCA---GTGGGA---ATAGGAGCT------
---GTGTTCCTTGGGTTCTTGGGAGCA---GCAGGAAGCACTATG---GGCGCAGCGTCA---
ATAACGCTGACGGTACAG---GCCAGACAA---CTGTTG---TCTGGAATAGTGCAACAGCAAAGC---
AATTTG---CTGAGA---GCTATAGAGGCGCAACAGCATATGTTGCAACTCACGGTCTGGGGCATT
>KY658708
TTCTATTGCAACACATCACAACTGTTTAATAGTACATACAGTCCCAGTTTTAATGGTACAGAAGGTAAA----
----

TTAAATGGGACCATCACAATCCCATGTAGAATAAAACAAATTATAAACATGTGGCAGAAAGTAGGACAAGCAA
TGTATGCCCCTCCCATTGCAGGAAACCTAACATGTGAATCAAAT---
ATCACAGGATTACTATTGACACGTGATGGAGGAAAAACAGGTTCA---------------------------
----

AATCACCCAGAGATATTCAGACCTGGAGGAGGGGATATGAGGGATAACTGGAGAAGTGAATTATATAAATATA
AAGTGGTAGAAATTAAGCCATTG---GGAGTAGCACCCACTGAGGCAAAAAGGAGAGTGGTG-----------
---------------------------GAGAGAGAAAAAAGAGCA---GTGGGA---ATAGGAGCT------
---GTGTTCCTTGGGTTCTTGGGAGCA---GCTGGAAGCACTATG---GGCGCAGCGTCA---
ATAACGCTGACGGTACAG---GCCAGACAA---TTGTTG---TCTGGTATAGTGCAGCAGCAAAGC---
AATTTG---CTGAGG---GCTATAGAGGCGCAACAGCATCTGTTGCAACTCACAGTCTGGGGCATT
>AF443084
TTTTATTGCAATACATCAAAACTGTTTACATACCAGTCTAATAGCACATACGTG------------------
----

GCTAATAGTACCATCACGCTCCCATGCAAAATAAAACAAATTATAAACATGTGGCAGGAGGTGGGACGAGCAA
TGTATGCACCTCCCATTGCAGGAAACATAACATGTCAATCAAAT---

ATCACAGGACTACTGTTGACACGTGACGGAGGCACAGAGACAGAC----------------------------
----

AATAAGACAGAGACATTCAGACCTGGAGGAGGAGATATGAGGGACAGTTGGAGAAGTGAATTATATAAATATA
AAGTGGTAGAAATTAAGCCATTA---GGAGTAGCACCCACTAAGGCAAAAAGAAGAGTGGTG-----------
----------------------------GAAAGAGAAAAAAGAGCA---GTGGGA---ATAGGAGCT------
---GTACTCCTTGGGTTCTTGGGAGCA---GCTGGAAGCACTATG---GGCGCAGCGTCA---
ATAGCGCTGACGGCACAG---GCCAGACAA---GTGTTG---TCTGGTATAGTGCAACAGCAAAGC---
AATTTG---CTGAGG---GCTATAGAGGCGCAACAGCATATGTTGCAACTCACAGTCTGGGGCATT
>KC899004
TTCTATTGTAATACATCAGGCCTGTTTAATGGTACATACAGGCCTAATGGTAATAATAATGGTACTAATGATA
CAATG--

AGTAATGCAACCATCACAATCCCATGCAGAATAAAGCAAATTATAAACATGTGGCAGGAGGTAGGACGAGCAA
TGTATGCCCCTCCTATTGCAGGGAACATAACATGTAAATCAAAT---
ATCACAGGATACTATTGGTACGTGATGGAGGAGATAATCAGACA----------------------------
----

GAAGAGATATTCAGACCTGGAGGAGGAGATATGAGGAACAATTGGAGAAGTGAATTATATAAATATAAAGTGG
TAGAAATTAAGCCATTG---GGAATAGCACCCACTGGAGCAAAAAGGAGAGTGGTG-----------------
----------------------GAGAGAGAAAAAAGAGCAGTAGTGGGA---ATAGGAGCT---------
GCGCTCCTTGGGTTCTTGAGTACA---GCCGGAAGCACTATG---GGCGCGGCGTCA---
ATAGCGCTGACGGTACAG---GCCAGACAA---TTGCTG---TCTGGTATAGTGCAACAGCAAAGT---
AATTTG---CTGAGG---GCTATAGAGGCGCAGCAGCATATGTTGCAACTCACGGTCTGGGGCATT
>KF835522
TTCTATTGTAATACATCAAGACTGTTTAATAGTACATACAATCCTAAGAGTACAGGAAGTAAT----------
----

TCAAGCTCAATCATCACAATCCCATGCAGAATAAAGCAAATTATAAACATGTGGCAGGAGGTAGGAAGAGCAA
TGTATGCCCCTCCCATTGAAGGAAACATAACATGTAGATCAAAG---
ATCACAGGACTACTATTGGTACGTGATGGAGGAAGCCCAGTGCCAAACAGT----------------------
----

ACAGAGACAGAGACAGAGATATTCAGACCTGGAGGAGGAGATATGAGGGACAATTGGAGAAGTGAATTATATA
AATATAAAGTGGTAGAAATTCAGCCATTG---GGAATAGCACCCACTAAAGCAAAAAGGAGAGTGGTG-----
----------------------------------GAGAGAGAAAAAAGAGCA---GCA------
CTAGGAGCT---------TTGTTCCTTGGGTTCTTGGGAGCA---GCAGGAAGCACTATG---
GGCGCGGCGGCA---GTAACGCTGACGGTACAG---GCCAGACAA---TTATTG---
TCTGGTATAGTGCAACAGCAAAGC---AATTTG---CTGAGG---
GCTATAGAGGCGCAACAGCATATGTTGCAACTCACGGTCTGGGGCATT
>HM215403
TTCTATTGTAATACATCAAGCCTGTTTAATGGCACATACAATGGCACATACATGACTAATATGGCTAAT----
----

GATTCATCAGAAATCACAATCCCATGCAGAATAAAGCAAATTGTAAACATGTGGCAGGAGGTAGGACGAGCAA
TGTATGCCCCTCCCATTGCAGGAAACATAACATGTAGATCAAAT---
ATCACAGGACTATTATTGGTACGTGATGGAGGACGATCAGAGGTGTCA-------------------------
----

AATACTACAGAGACATTCAGACCTCAAGGGGGAGATATGAGGGACAATTGGAGAAGTGAATTATATAAATATA
AAGTGGTAGAAATTAAGCCATTA---GGAATAGCACCCACTGGAGCGAAAAGGAGAGTAGTG-----------
----------------------------GAGAGAGAAAAAAGAGCA---GCGGGA---ATAGGAGCT------
---GTGTTCCTTGGGTTCTTGAGTGCA---GCCGGAAGCACTATG---GGCGCGGCGTCA---
TTAACGCTGACGGTACAG---GCCAGACAA---TTGCTG---TCTGGTATAGTGCAACAGCAAAGC---
AATTTG---CTGAGG---GCTATAGAGGCGCAACAGCATATGTTGCAACTCACAGTCTGGGGCATT
>HM215408
TTCTATTGTAATACATCAAGACTGTTTAATAGTACATACAGCAATGGTACACTCAGGTTTAATGGTACAGACT
TTAATGGTACA--
-------------ATGGAT------
AATTCAAGCTCAACCATCACAATCCCATGCAGAATAAAGCAATTTATAAACATGTGGCAGGAAGTAGGAAGAG

CAATGTATGCCCCTCCCATTGAAGGAAACATAACATGTAGATCAAAT---
ATCACAGGACTACTATTGGTACGTGATGGAGGAACAAATTTAACAGCAGAA----------------------
----

TTAAATCAGACAGAGATATTTAGACCTGAAGGAGGAAATATGAGGGACAATTGGAAAAATGAATTATATAAAT
ATAAAGTGGTAGAAATTAAGCCATTG---GGACTAGCACCCACTGGAGCAAAAAGGAGAGTGGTG--------
-------------------------------GAGAGACAAAAAAGAGCA---GTGGGA---ATAGGAGCT---
------GTAATCTTTGGGTTCTTGGGAGCA---GCCGGAAGCACTATG---GGCGCGGCGTCA---
ATGGCGCTGACGGTACAG---GCCAGACAA---TTGTTG---TCTGGTATAGTGCAACAGCAAAGC---
AATTTG---CTGAGG---GCTATAGAGGCGCAACAGCATCTGTTGCAACTCACGGTCTGGGGCATT
>HQ699950
TTCTATTGTAATACATCAGGCCTGTTTAATAGTACATACATGCCTAATGGTACCTACATGTCTAATGGTACCA
AT---

AGTAATGCAACCATCACAATCCCATGCAGAATAAAGCAAATTATAAACATGTGGCAGGAGGTAGGACGAGCAA
TATATGCCCCTCCCATTGCAGGAAACATAACATGTAAATCAAAT---
ATCACAGGGCTACTATTGGTACGTGATGGAGGACCAGATAATCAG----------------------------
----

ACAGAGATATTCAGACCTGAAGGAGGAGATATGAGGAACAATTGGAGAAGTGAATTATATAAATATAAAGTGG
TAGAAATTAAGCCATTG---GGACTAGCACCCACTGGAGCAAAAAGGAGAGTGGTG-----------------
----------------------GAGAGAGAAAAAAGAGCA---GTGGGA---ATAGGAGCT---------
GTGTTCCTTGGGTTCTTGAGTGCA---GCCGGAAGCACTATG---GGCGCGGCGTCA---
ATAACGCTGACGGTACAG---GCCAGACAA---TTGCTG---TCTGGTATAGTGCAACAGCAAAGT---
AATTTG---CTGAGG---GCTATAGAGGCGCAGCAGCATATGTTGCAACTCACGGTCTGGGGCATT
>HQ699981
TTCTATTGTAACACATCAGGCCTGTTTAATAGTACATACATGCCTAGTGGTACATACATAACTAATGATACAA
AT---

AGTAGTTCAGATACAATCACAATCCCATGCAAAATAAAGCAAATTATAAACATGTGGCAGGAGGTAGGACGAG
CAATGTATGCCTCTCCCATTGCAGGAAACATAACATGTACATCAAAT---
ATCACGGGACTACTATTGGTACGTGATGGAGACAAAACAAGTAAT----------------------------
----

ACTACAGAGACATTCAGACCTCAAGGAGGAGACATGAGGGACAATTGGAGGAGTGAATTATATAAATATAAAG
TGGTAGAAATTAAGCCATTG---GGAATAGCACCCACTAAAGCAAAAAGGAGAGTGGTG--------------
-------------------------GAGAGAGAAGAAAGAGCAGCGGGAATAGGAATAGGAGCT---------
TTGTTCCTTGGGTTCTTGAGTGCA---GCCGGAAGCACTATG---GGCGCGGCGTCA---
ATAACGCTGACGGTACAG---GCCAGACAA---TTGTTG---TCCGGTATAGTGCAACAGCAAAGC---
AATTTG---CTGAGG---GCTATAGAGGCGCAACAGCATATGTTGCAACTCACGGTCTGGGGCATT
>KC807928
TTCTATTGTAATACAACAAACCTGTTTAATAGTACATACAATAGTACAACAGGCCTGTTTACTTATACATACA
ATGGTACT--ACA---
TACAATGGTAGTGTTACAGAC---------------
AATGATAGCATCCCAATCCAAATCCAATGCAGAATAAAGCAAATTATAAACATGTGGCAGGAGGTAGGACGAG
CAATGTATGCCCCTCCCATTGCAGGGAACATAACATGTAGCTCAAAT---
ATCACAGGACTACTATTGACACGTGATGGAGGAAATACAACTGTGACA-------------------------
----

CAAAAAGAGATATTCAGACCTCAAGGAGGAAATATGAGAGACAATTGGAGAAATGAATTATATAAATATAAAG
TGGTAGAAGTTAAGCCATTG---GGACTAGCACCCACTAAAGCAAAAAGGAGAGTGGTG--------------
-------------------------GAAAGAGAAAAAAGAGCA---GTGGGA---CTAGGAGCT---------
GTGTTCCTTGGGTTCTTGGGAGCA---GCAGGAAGCACTATG---GGCGCGGCGTCA---
ATGACGCTGACGGTACAG---GCCAGACAA---TTGTTG---TCTGGTATAGTGCAACAGCAAAGC---
AATTTG---CTGAGA---GCTATAGAGGCGCAACAGCATATGTTGCAACTCACGGTCTGGGGCATT
>JF683757
TTCTATTGCAACACATCAGTACTGTCTAATAGTACCCATAATAGTGGTACTGGATGGCACCTACAATCAAGTG
ATATT--

AATTCTTATGCAACCATCGCGGTTTCATATAGAATAGGACAAATTATAAACATGTGGCGGCAGGTAGGACGAG

CAATCTATGGCCCTCCGGTGGCACGAAAAATGTCAGCCCATCCAAGT---
ACCACAGGCCTATTATTGGTATGTGATGGGGGTGAGAAGAGCTTAAATAGCACA------
AATGGCACAGGTATCACA---AAT---

GCCACAGAGATATTTAGACCTCAAGGAGGAGATATGAAGGACAATTGGAGAAGTGAATTATATAAGTATAAAG
TGGTAGAAATTAAGCCATTG---GGAATAGCACCCACTGAAGCAAGAAGGAGAGTGGTG------------
------------------------GAGAGAGAAAAAAGAGCA---GTGGGA---ATAGGAGCT--------
GTGATCCTTGGGTTCTTGGGAGCA---GCAGGAAGCACTATG---GGCGCGGCGTCA---
ATAGCGCTGACGGCACAG---GCCAGAACA---GTAATG---GCTGGTATAGTGCAACAGCAAAGC---
AATTTG---CTGAGG---GCTATAGATGCGCAACAGCATATGTTGCAGCTCACAGTCTGGGGCATT
>JF683770
TTCTATTGCAATACAACAAAACTGTTTAATAATACATACTGGTCTAATATTACACTGAATGGTCCA-------
----

CCGAACAAAACAATCATAATCCCATGCAGGATAAAACAAATTATAAACATGTGGCAGAGGGTAGGACAAGCAA
TGTATTACCCTCCCATTGCAGGAAGCATAACATGCAAATCAAAT---
ATCACAGGACTACTATTAACAAGAGATGGAGGAATACATAAAACAAATAGCAGC------
ATAAATGACACAAGCACAAATAACACA--

ACTGACCCAAACATAGAGATATTCAGACCTGGAGGAGGAGATATGAGAGATAATTGGAGAAGTGAATTATATA
AATATAAAGTGGTAGAAATTAAGCCTTTA---GGAGTAGCACCCACTGAGGCAAAAAGGAGAGTGGTG-----
---------------------------------GAGAGAGAAAAAAGAGCA---GTGGGA---
ATAGGAGCT---------GTGTTCCTTGGGTTCTTGGGAGTA---GCAGGAAGCACTATG---
GGCGCGGCGTCA---ATAACGCTGACGGTACAG---GCCAGACAA---TTGTTG---
TCTGGTATAGTGCAACAGCAAAGC---AATTTG---CTGAAG---
GCTATAGAGGCGCAACAACAGCTGTTGCGACTCACAGTCTGGGGCATT
>L22940
TTCTACTGCAATACATCAGACCTGTTTAATAATCAGAGTATC-----------------------------
----

ACAAATGAAAACATCACACTTCAATGCAGAATAAAACAAATTATAAACATGTGGCAGGAGGTAGGACGAGCAA
TGTATGCCCCTCCCATTAAAGGAATAATAACATGTAAATCAAAT---
GTCACAGGACTGCTACTGACACGTGATGGAGGGACAAATACCACA---------------------------
----

ACAGAAGGAGAGATATTCAGACCTGGAGGAGGAGATATGAGGGACAATTGGAGAAGTGAATTATATAAATACA
AAGTGGTAGAAATTAAGCCAGTA---GGAATAGCACCCACTAAGGCAAAAAGGAGAGTGGTG----------
---------------------------GGGAGAGAAAAAAGAGCA---GCA------CTAGGAGCT------
---ATGTTCCTTGGGTTCTTGGGAGCA---GCCGGAAGCACTATG---GGCGCAGCATCA---
ATAACGCTGACGGTACAG---GCCAGACAA---TTATTG---TCTGGTATAGTGCAACAGCAAAGC---
AATTTG---CTGAGG---GCTATAGAGGCGCAGCAGCATATGTTGCAACTCACGGTCTGGGGCATT
>L23065
TTCTATTGTAATACATCAGCCTTGTTTAATGATTCAATTACAAATGCACCCATTAATAATTCGAGT------
----

ACAGATGCAAACATCACACTTCAATGCAGAATAAAACAAATTATAAACATGTGGCAGGAGGTAGGACGAGCAA
TGTATGCCCCTCCCATTCAAGGAATAATAGCATGTAAATCAAAT---
ATCACAGGACTGCTACTGACACGTGATGGAGGGACAAATAGCACA---------------------------
----

GACGAGACATTCAGACCTGGAGGAGGAGATATGAGGGACAATTGGAGAAGTGAATTATATAAATACAAAGTGG
TAGAAATTAAGCCATTA---GGACTAGCACCCACTAAGGCAAAAAGGAGAGTGGTG----------------
---------------------GAGAGAGAAAAAAGAGCA---GCA------CTAGCAGCT---------
GTGTTCCTTGGGTTCTTGGGAGCA---GCCGGAAGCACTATG---GGCGCAGCATCA---
ATAACGCTGACGGTACAG---GCCAGACAA---TTATTG---TCTGGTATAGTGCAACAGCAAAGC---
AATTTG---CTGAAG---GCTATAGAGGTGCATCAGCATATGTTGCAACTCACAGTCTGGGGCATT
>EU786681
TTCTACTGCAATACAACAAAACTGTTTAATGGCACATACAGTAATGGCACATACAGTCCGGGATATAATTCAA
GCTCA--

CACTCAAACAACTCCATCATAATTCAATGCAGAATAAAACAAATTATAAACATGTGGCAGAAGGTAGGACAAG
CAATGTATGCCCCTCCTATTGCAGGAAACATAACATGTAACTCAAAT---
ATCACAGGACTACTATTGACACGTGATGGAGGAAGCAGTAATGAC-------------------------
----

ACTAAAGAGATATTCAGACCTACAGGAGGAAACATGAAAGACAATTGGAGAAGTGAATTATATAAATATAAGG
TGGTAGAAATTAAGCCATTA---GGAATAGCACCCACTAAGGCAAAAAGGAGAGTGGTGCAG--------
---------------------AGAGAAAGAGAAAAAAGAGCAGTAGTGGGA---CTAGGAGCT-------
GTGTTCCTTGGGTTCTTGGGAGCA---GCAGGAAGCACTATG---GGCGCAGCATCA---
ATAACGCTGACGGTACAG---GCCAGACAA---TTATTG---TCTGGTATAGTGCAACAGCAAAAC---
AATTTG---CTGAGG---GCTATAGAGGCGCAACAGCATCTGTTGCAACTCACGGTCTGGGGCATT
>EF531335
TTCTATTGCAACACATCAGATCTGTTTAATAGTACATATTTTAATAATACATATTTTAATGGTGTATATAATG
GTACA---

AGAGATAATGGTACCATCACACTCCCTTGCAGAATAAAACAATTTATAAACATGTGGCAGAGGGTAGGAAGAG
CAATGTACGCCCCTCCCATTCAAGGACTTATAACATGTAGGTCAAAT---
ATCACAGGACTACTATTGACACGTGATGGAGGAAACACTATTAACAATACA-------------------
----

AATAACAATACAGAGATATTCAGACCTGGAGGAGGAGATATGAGGGACAATTGGAGAAGTGAATTATATAAAT
ATAAAGTGGTAGAAATTAGGCCATTA---GGAGTAGCACCTACTGATGCAAGAAGGAGAGTGGTG-----
------------------------------GGGAGAGAAAAAAGAGCA---GTA------ATGGGAGCT---
------TTGTTCCTTGGGTTCTTGGGAGCA---GCAGGAAGCACTATG---GGCGCAGCGTCA---
ATGGCGCTGACGGTACAG---ACCAGACAA---TTATTG---TCTGGTATAGTGCAACAGCAAAGC---
AATTTG---CTGAGG---GCCATAGAGGCGCAACAGCATATGTTGCAACTCACGGTCTGGGGCATC
>KU319529
TTCTATTGCGATACATCAAATCTGTTTAATATGACATACTTGTTTAATGATTGGAGTTACGAACATAGTACTG
AC--

TCAGATTTCATCATCATGATCCGCTGCAGAATAACACATATAATAAACATGTGGCAGGTAGTGAGAGCAGTAC
TGTATGCCCCTGCCCTTCGCAGGCAACAGTTAAGCAAGCAATCCATC---
TACGCAGTACTACTATGGACTCGTGATGCAGCAAGTAGTTACTGG-------------------------
----

CGCGAGAAAGAGATATTCAGACCTGCAGGAGGAGATATGAGGGACAATTGGAGAAGTGAATTATATAAATATA
AAGTGGTAGAGATTAAACCATTA---GGAATAGCACCCACTGAGGCAAAAAGGAGAGTGGTG--------
---------------------------GAGAGAGAAAAAAGAGCA---GTGGGA---ATAGGAGCT------
---GTGTTCCTTGGGTTCTTGGGAGCA---GCAGGAAGCACTATG---GGCGCAGCGTCA---
CTGACGCTGACGGTACAG---GCCAGGCAA---TTGCTG---TCTGGTATAGTGCAACAGCAAAAC---
AATTTG---CTGAGA---GCTATAGAGGCGCAACAGCATATGTTGCAACTCACAGTCTGGGGCATA
>KU319530
TTCTATTGCAATACATCAAGACTGTTTAATGGTGCATACATGTATAACAGTACTAATAGTACT-------
----

TCAAATGCAACCATCACACTCCAATGCAGAATAAAGCAAATTATAAACATGTGGCAGGGGGTAGGACGAGCAA
TGTATGCCCCTCCCATTGAAGGAAACATTACATGTAAATCAATA---
TCACAGGATTCTATTGACCCTGATGGAGGATTGGGC----------------------------------
----
------------------------CAAAAACCCAAGGTTTCCAACTGGGAGAGGAACTTAGGGACA------
---------------TTGGAAATGGATCCTTTG---GGTTACCCCCTG------GCCAAAAAGGAAGGGGAG-
------------------------------------AAAAGAAAAAAAAGGACA---GGGGTT---
TTACGAAAA---------AACTGG--------------------GCGCAGAAGCCTATG---
GGCGCAGCATCA---ATAACGCTGACGGTACAG---GCCAGACAA---TTGTTG---
TCTGGTATAGTGCAACAGCAAAGT---AATTTG---CTGAGG---
GCTATAGAGGCGCAACAGCATATGTTGCAACTCACGGTCTGGGGCATT
>KU319538
TTCTATTGCAATACAACCAGATTGTTTAACAGTACAATAAAATACAACATTGGT----------------
----

AATTACTTAATCATTATACTCCCATGTAGAATAAGCCAAATGTTAGGAATGGGGCAGAGGGTAGGCCAAGCAA
TGTATGCCCTTCCCATTGCAGGAAACATAACATGTATTTCAAAT---
ATCACAGGACTACTATTGACACGTGATGGAGCCGAGGTATCACAT----------------------------
----

AGCACAAATGAGACATTCAGACCAGGAGGAGGAGATATGAGGGACAATTGGAGAAGTGAATTATATAAATATA
AAGTGGTAGAAGTTAAACCATTA---GGAGTAGCACCCACTGAGGCAAAAAGGAGAGTGGTG-----------
----------------------------GAGAGAGAAAAAAGAGCA---GTGACA---ATAGGAGCT------
---ATGTTCCTTGGGTTCTTGGGAGTA---GCAGGAAGCACTATG---GGCGCAGCGTCA---
ATGGCGCTGACGGTACAG---GCCAGGCAA---TTGTTG---TCTGGTATAGTGCAACAGCAAAGC---
AATTTG---CTGAGA---GCTATAGAGGCGCAACAGCATATGTTGCAACTCACAGTCTGGGGCATT
>KU319539
TTCTATTGTAATACATCAAAACTGTTTAATGGTACATACAATAAGAACAATACTAATAGGACTAATACTAAT-
----

AGTACTTCAACCATCACACTTCAATGCAGAATAAAACAAATTATAAACATGTGGCAGGGGGTAGGACAAGCAA
TGTCTGCCCCTCCCATTCAAGGAAACATAACATGTGAATCAAAT---
ATCACAGGACTACTATTGACACGTGATGGAGGAACAACTAATAATAACGAAACAAATAATAACACAGAAGCAA
ATAAT--

AACACAGAAACATTCAGACCTGGAGGAGGAGATATGAGGGACAATTGGAGAAATGGAGGTGAGTTATATAAAT
ATAAAGTGGTAGAAGTTAATCCATTA---GGAATAGCACGCACTGAGGCAAAAAGGAGAGTGGTG--------
-------------------------------CAGAGAGAAAAAAGAGCA---GTGGGA---ATAGGAGCT---
------GTGCTCCTTGGGTTCTTGGGAGCA---GCTGGAAGCACTATG---GGCGCAGCGTCA---
GTAACGCTGACGGTACAG---GCCAGACAA---TTGCTG---TTTGGTATAGTGCAACAGCAAAGC---
AATTTG---CTGAAG---GCTATAGAGGCGCAACAGCATATGTTGCAACTCACGGTCTGGGGCATT
>KU319540
TTCTATTGCAATACATCAGGCCTGTTTACTAGCACAAAACTA-------------------------------
---GAACTG---
TTTAATAGCAGTACAAATTTA---------------
AACATCACACTCCAATGCAGGATAAGAGATCAACTTGTACAACATGTGAGGCTAAAGAAGTCAGTAATGATGC
CAATGTACGCCCCTCCTATCAATGGAGATATAGCATGTAGATCAAAC---
ATCACAGGTCTACTATTGACACGTGATGGAGGCAACACCAACACA----------------------------
----

TCTGAGATATTCAGACCTGGGGGAGGAGATATGAGGGACAATTGGAGAAGTGAATTATATAAATATAAAGTAG
TAGAAATTAAGCCATTA---GGAATAGCACCCACTGAGGCGAAAGGAGAGTGGTG------------------
----------------------GAGAGAGAAAAAAGAGCA---GCA------CTAGGAGCT---------
TTGTTCCTTGGGTTCTTGGGAGCA---GCAGGAAGCACTATG---GGCGCAGCATCA---
ATAACGCTGACGGTACAG---GCCAGACAA---TTATTG---TCTGGTATAGTGCAACAGCAAAGT---
AATTTG---CTGAGA---GCTATAGAAGCGCAACAGCATATGTTGCAACTCACGGTCTGGGGCATT
>KU319542
TTCTATTGCAATACAACAAAACTGTTTAATAGTACATACAATGCTAATAGTACAAACAGTACT----------
----

TCAAATTCAACCATCACAATCCAATGCAGAATAAAACAAATTATAAACATGTGGCAGGAGGTAGGACGAGCAA
TGTATGCCCCTCCCATTGCAGGAAACATAACATGTAACTCAACT---
ATTACAGGAATACTATTAACACGTGATGGAGGGTATAATATTACC----------------------------
----

AACGACACAAAAACATTCTTACCTGGAGGAGGAGATATGATGAACAATTGGAGAAATGAATTATATAATTATA
AAGTGGTAGAAAATAAACCATTA---GGAATAGCTCCTACTAGTGCAAAGAGGAGAGTGGTG-----------
----------------------------GAGAAAAAAAAAAGAGCA---ATATGGGGAATAGGAGCT------
---GCGCTCCTTGGTTTCTTACGAGCA---GCAGGAAGCACTATG---GGCGCAGCGTCA---
TTGACGCTGACGGTACAG---GCCAGGCAA---TTGTTG---TCTGGTATAGTGCAACAGCAAAGC---
AATTTG---CTGAGG---GCTATAGAGGCGCAACAACATATGTTGCAACTCACGGTCTGGGGCATT
>KU319543
TTCTACTGCAATACATCAAATTTGTTTAATAGAACTTACCTGATTAATGGTACCTATAATAATAATAAAACT-
----

TCCATAAGCACCATCACACTCCACTGCAAAATAAAACAAATTATAAATATGTGTCAGGGGGTAGGACAAGCAA
TGTATGCCCCTGCCACTGCAGGAATCATATCATGTATATCAACT---
ATCGCAGTACTACTACTTGCTGGTGATGGAGGCAATGATACAAGT----------------------------
----

GATACAGAGACATTCAGACCTGGAGGAGGAGATATGAGGGACAATTGGAGAAGTGAATTATATAAATATAAAG
TGGTAGAAGTTCACCCATTA---GGAGTAGCACCCACTAGAGCAAAAAGGAGAGTGGTG--------------
-------------------------GAGAGAGAAAAAAGAGCA---GCA------CTAGGAGCT---------
ATGATCATTGGGTTCTTGGGAGCA---GCAGGAAGCACTATG---GGCGCAGCATCA---
ATAACGCTGACGGTACAG---GCCAGGCAA---TTGTTG---TCTGGTATAGTGCAACAGCAAAGC---
AATTTG---CTGAGG---GCTATAGAGGCGCAACAGCATATGTTGCAACTCACAGTCTGGGGCATA
>KU319547
TTCTACTGTGTTTCAATTCAAATATGTAATGGGGTTTACTTTACTGACACATGGACTACCTACAACTCTAATG
ATGCT--

GATTACACAGACATCCTCACTCTGACATGCAGAATAAAACTAATTGTAAACTTGAGTCAGGAGCTGGGGCGAG
CAAGGTCTGCCCCTCCCATTGACGGATCCGTAAAATGTACATCTAAT---
TTTACAGGACTAGTATTGACACGTGCTGGAGGAAATGCCACAGGG----------------------------
----
GATGTTACCGCGGAGAAT---
AACACAGAGATATTCAGACCTGGAGGAGGAGAGATGAGGGACAATTGGAGAAGTGAATTATATAAATATAAAG
TGGTAGAAATTAAACCATTA---GGAGTAGCACCCACTGAGGCAAAAAGGAGAGTGGTG--------------
-------------------------GAGAGAGAAAAAAGAGCA---GTGGGA---ATAGGAGCT---------
GTGTTCCTTGGGTTCTTGGGAGCA---GCAGGAAGCACTATG---GGCGCAGCATCA---
ATAACGCTGACGGTACAG---GCCAGACAA---TTGTTG---TCTGGTATAGTGCAACAGCAAAGC---
AATTTG---CTGAGG---GCTATAGAGGCGCAGCAGCATATGTTGCAACTCACAGTCTGGGGCATT
>KU319548
TTCTATTGCAATACATCACAACTGTTTAATAGGACATACATGGAAACTACTATTAGCGCTGTGTCA-------
----

ACAAATACAAAAATCACCCTCCTATGCAAAATAAAACAAATTATTCAAATGCTTCAGGGAGTAGGACGAGCAA
TGTATGCCCCTCGCAATGAAAGAAACATAACATGTCTTTCAAAT---
ATCACAGGATTACTATTGACACGTGATGGAGGAATTAACAGTAGC----------------------------
----

ACAACTGAGATATTCAGACCTGGGGGAGGAGATATGAGGGACAATTGGAGAAGTGAATTATATAAATATAAAG
TGGTAGAAATCAAACCATTA---GGAGTAGCACCCACTAAGGCAAAAAGGAGAGTGGTG--------------
-------------------------GAGAGAGAAAAAAGAGCA---GTGGGA---ATAGGAGCT---------
GTGCTCCTTGGGTTCTTGGGAGCA---GCAGGAAGCACTATG---GGCGCAGCATCA---
ATAACGCTGACGGTACAG---GCCAGGCAA---TTGTTG---TCTGGTATAGTGCAACAGCAAAGC---
AATTTG---CTGAGG---GCTATAGAGGCGCAACAGCATATGTTGCAACTCACAGTCTGGGGCATT
>KU319549
TTCTATTGCAAAACATCAAAACTGTTTATTAAGACTTGGAAAAGTTGGAACCATCGGAAT-------------
----

GATGGAACCATCATCACGATCCCATGCAGAATAAAACAAATTATACATATGTGGCAGGGAGTAGGACGAGCAA
TGTATGCCCCTCCCATCAGTGGACCAATAACATGTAGATCAAAT---
ATCACAGGACTACTACTGACACGGGATGGAGGCATCAGTGACAATAACAGT----------------------
----

ACCAATACCACAGAGATATTCAGACGTCAAGGAGGAGATATGAAAGACAATTGGAGAAGTGAATTATATAAAT
ATAAAGTGGTAGAAATTAAGCCATTA---GGAATAGCACCCACTAAGGCAAAAAGGAGAATGGTG--------
-------------------------------CAGAGAGAGAGAGGAGAA---AAGAGG---CACGGAGCT---
------ATGTTCCTTGGGTTCTTGGGAGCA---GCCGGAAGCACTATG---GGCGCAGCATCA---
ATAACGCTGACGGTACAG---GCCAGACAA---TTATTG---TCAGGTATAGTGCAACAGCAAAGC---
AATTTG---CTGAGG---GCTATAGAGGCGCAACAGCATATGTTGCAACTCACGGTCTGGGGCATT
>AF219266
TTCTATTGCTATACTTCAAGACTGTTTAACAGTACATACCTGTTTAATGGTACTTACCTGGTTAATGATATAA
ATAGT--

```
ACAGGAAATGCAACCATCATACTCCCATGCAAAATAGAACAAATTATAAACATGTGGCAGGAGGTAGGGCGAG
CAATGTATGCCCCTCCCATTGAAGGAAACATAACATGTAGCTCAAAC---
ATCACAGGACTATTATTGGTACGTGATGGAGGAAAAGATTCAAAT----------------------------
-------------------------------------------------------------------------
-------------
GGCACAGAGATATTCAGACCTGGAGGAGGAGATATGAGGGACAATTGGAGAAGTGAATTATATAAATATAAAG
TGGTAGAAATTAAGCCATTG---GGAATAGCACCCACTAAGGCAAAAAGGAGAGTGGTG--------------
-------------------------GAGAGAGAAAAAAGAGCA---GTAGGA---ATAGGAGCT---------
GTGTTCCTTGGGTTCTTGGGAGCA---GCAGGAAGCACTATG---GGCGCGGCGTCA---
ATAACGCTGACGGTACAG---GCCAGACAA---GTGTTG---TCTGGTATAGTGCAACAGCAAAGC---
AATTTG---CTGAGG---GCTATAGAGGCTCAACAGCATATGTTGCAACTCACGGTCTGGGGCATT
>FJ977095
TTCTATTGCAATACATCAGAACTGTTTAAGAATGGTACAAATAATGGTACCAATAGTACAGACTCAACA----
-------------------------------------------------------------------------
----------------------------
AATTCAACATCCATCACAATCCAATGCAGAATAAAACAAATTATAAATATGTGGCAGGGGGTAGGTCGAGCAA
TATATGCCCCTCCCATTAAAGGAAACATAACATGTAACTCAAAT---
ATCACAGGACTACTGCTGACACGTGATGGAGGAAAAGGAGAAGAAAGT-------------------------
-------------------------------------------------------------------------
-------
GATAATAAGACAGAGATATTCAGACCTGCAGGAGGAGATATGAGGGACAATTGGAGAAGTGAATTATATAAAT
ACAAAGTGGTAGAAATTCAGCCATTA---GGAGTAGCACCCACTGAGGCGAAAAGGAGAGTGGTG--------
-------------------------------GAGAGAGAAAAAAGAGCA---GTGGGA---ATAGGAGCT---
------GTGTTTCTTGGGTTCTTGGGAGCA---GCAGGAAGCACTATG---GGCGCAGCGTCA---
ATAACGCTGACGGTACAG---GCCAGACAA---TTATTG---TCAGGCATAGTGCAACAGCAAAGT---
AATTTG---CTGAGA---GCTATAGAGGCGCAACAGCATATGTTGCAACTCACGGTCTGGGGCATT
>HQ595787
TTCTATTGCAATACATCAAATTTGTTTAATAGGACATACAATATAACAAATTTGCTTAATGGGACATACAATA
TATCAAATTCG--------------------------------------------------------------
----------TCTGATGGG------
ACAAACAATACAATCATCAAACTTCAATGCAGAATAAAACAAATTATAAACATGTGGCAGGGGGTCGGGCAAG
CAATGTATGCCCCTCCCATTGCGGGAAACATAACATGTAAATCAAAT---
ATCACAGGACTACTGATAACACGAGATGGAGGGACTGAAGGTAACACT-------------------------
-------------------------------------------------------------------------
----------
ACAGAGATAGAGATATTCAGACCTGGAGGAGGAGACATGAAGGACAATTGGAGAAGTGAATTATACAAATATA
AAGTGGTAGAAATTAAGCCATTA---GGAGTAGCACCCACTGGGGCAAAAAGGAGAGTGGTG-----------
----------------------------GAGAGAGAAAAAAGAGCA---GTGGGA---ATGGGAGCT------
---GTGTTCCTTGGGTTCTTGGGAGCA---GCAGGAAGCACTATG---GGCGCGGCGTCA---
ATAACGCTGACGGTACAG---GCCAGACAA---TTATTG---TCTGGTATAGTGCAACAGCAAAGC---
AATTTG---CTGAGA---GCTATAGAGGCGCAACAGCATATGTTGCAACTCACGGTCTGGGGCATT
>HQ595800
TTCTATTGCAATACATCAGGATTGTTTAATGGCGTCTACATTGAGGGCAACTACACGCATAATATTAAGAAC-
-------------------------------------------------------------------------
----------------------------
TCAAATGAAAACATCACACTTCAATGCAGAATAAAACAAATTATAAACATGTGGCAGGAGGTAGGACGAGCAA
TATATGCCCCGCCCATTGCAGGAAATATAACATGTGTATCAAAT---
ATCACAGGACTACTGTTGACACGTGATGGAGGAGCCAATGTTTCA----------------------------
-------------------------------------------------------------------------
----------------
GAAGAGACATTCAGACCTGGAGGAGGAGATATGAGGGACAATTGGAGAAGTGAATTATATAAATATAAAGTGG
TAGAAATTAAACCATTA---GGAATAGCACCCACCGAGGCAAAAAGGAGAGTGGTG-----------------
----------------------AGGAGAGAAAAAAGAGCA---GTG------ATAGGAGCT---------
GTGTTCCTTGGGTTCTTGGGAGCA---GCAGGAAGCACTATG---GGCGCGGCGTCA---
ATAGCGCTGACGGCACAG---GCCAGGCAA---TTACTG---TCTGGTATAGTGCAACAGCAAAGC---
AATTTG---CTGAGG---GCTATAGAGGCGCAACAGCATATGTTGCAACTCACAGTCTGGGGCATT
>AY255823
TTCTATTGCAATACATCAGCCCTGTTTACAAATTCAACCATC-------------------------------
-------------------------------------------------------------------------
----------------------------
```

CCAAATACAACCATCACACTCCCATGCAAAATAAAACAAATTATAAATATGTGGCAGGAGGTAGGACGAGCAA
TGTATGCCCCTCCCATTAAAGGAGAGATAACATGTAATTCAACT---
ATCACAGGAATACTACTGACACGTGATGGAGGCAACACCACAAAC---------------------------
----

GAAATCGAAACATTCAGACCTGGAGGAGGAGATATGAGGGACAATTGGAGAAGTGAATTATATAAATACAAAG
TGGTAGAAATTAAGCCATTA---GGAATAGCACCCACGGAGGCAAAAAGGAGAGTGGTG-------------
------------------------GAGAGAGAAAAAAGAGCA---GCA------CTAGGAGCT---------
GTGTTCCTTGGGTTCTTGGGAGCA---GCCGGAAGCACTATG---GGCGCAGCATCA---
ATGACGCTGACGTTACAG---GCCAGACAA---TTACTG---TCGGGTATAGTGCAACAGCAAAGC---
AATTTG---CTGAGG---GCTATAGAGGCGCATCAGCATATGCTGCAACTCACAGTCTGGGGCATT
>KP109480
TTCTATTGCAACACATCAAGCCTGTTTAAGGGTACATATAGGCCTAATGGTACAAGTAATTCCACT------
----

TCAGGCTCTATCATCACACTCCCATGCTACATAAAACAAGTTATAAACTTGTGGCAGGAGGTAGGACGAGCAA
TATATGCCCCTCCCATTGAAGGAAACATAACATGTATATCAAAT---
ATCACAGGACTACTATTGGTAAGAGATGGAGGAAATCATGAAGAG---------------------------
----

GCAAATACGACAGAGACATTCAGACCTGGAGGAGGAAATATGAGAGACAATTGGAGAAGTGAATTATATAAAT
ATAAAGTAGTAGAAATTAAGCCATTG---GGAGTAGCACCCACTAAAGCAAGAAGGAGAGTGGTG-------
------------------------------GAGAGAGAAAAAAGAGCA---GTGGGAGTAGGAGCTGTT---
------ATGTTCCTTGGGTTCTTGGGAGCA---GCAGGAAGCACTATG---GGCGCGGCGTCA---
ATAACGCTGACGGTACAG---GCCAGACAA---TTGCTG---TCTGGTATAGTGCAACAGCAAAGC---
AATTTG---CTGAAG---GCTATAGAGGCGCAACAGCATCTGTTGCAACTCACGGTCTGGGGCATT
>KP109483
TTCTATTGTAATACATCAAGCCTGTTTAATAGTACATTTAATAGTACATACATGACTAATGATACAGACATGA
AT--

TCAAACTCAACCATCTCAATCCCATGCCGCATAAAACAAATTATAAACATGTGGCAGGAGGTAGGACGAGCAA
TGTATGCCCCTCCCATTGCAGGAAACATAACATGTAAATCAAAT---
ATCACAGGACTACTATTGGTACGAGATGGAGGAAACTCAAATGATACA------------------------
----

AATGAGCCAGAGATATTCCGACCTCAAGGAGGAGATATGAGGGACAATTGGAGAAGTGAATTATATAAATATA
AAGTGGTAGAAATTAAGCCATTG---GGAGTAGCACCCACTGCAGCAAAAAGGAGAGTGGTG----------
---------------------------GGAAGAGAAAAAAGAGCA---GTGGGA---CTAGGAGCT------
---ATGATCCTTGGGTTCTTGGGAGCA---GCCGGAAGCACTATG---GGCGCGGCGTCA---
ATAACGCTGACGGTACAG---GCCAGACAA---TTGTTG---TCTGGTATAGTGCAACAGCAAAGC---
AATTTG---CTGAGG---GCTATAGAGGCGCAACAGCATATGTTGCAACTCACGGTCTGGGGCATT
>KY929363
TTCTATTGCAATACATCAAAACTGTTCAACAGTACATACTGGCAGAATGGTACATACTCATACTGGAATAATG
GG---
TCATTCCTAGAATGGAATGATACACAAAGCACT-----------
CCAAATCGAAGCATCACAATTCCATGCAGAATAAAACAAATTATAAACATGTGGCAGGAGGTAGGACGAGCAA
TGTATGCCCCTCCCATTGCAGGAAAAATAATGTGTAAATCCAAT---
ATCACAGGAATACTATTGGAACGTGATGGAGGAAATACAAATGAGACA------------------------
----

AATAAGACAGAGACATTCAGACCAATAGGAGGAGATATGAGGAATAATTGGAGAAGTGAATTATATAAATATA
AAGTGGTAGAAATTAAGCCATTG---GGGGTAGCGCCCACTAAGGCAAGAAGGAGAGTGGTG----------
---------------------------GAGAGAGAAAAAAGAGCA---ATAGGGGGAATAGGAGCT------
---GTATTCCTTGGGTTCTTAGGAGCA---GCAGGAAGCACTATG---GGCGCGGCGTCA---
ATAACGCTGACGGTACAG---GCCAGACAA---TTGTTG---TCTGGTATAGTGCAACAGCAAAGC---
AATTTG---CTGAAG---GCTATAGAGGCGCAACATCATCTGTTGCAACTCACGGTCTGGGGCATT
>KY929365
TTCTATTGCAATACATCAGGCCTGTTTAATGCTACATACAATAGTACATCAGGCCTGTTTAATGCTACTAATC
AC--

ACAAATAACACCATCACCATCCCCTGCAGAATAAAACAATTTATAAACATGTGGCAGGGGGTAGGAAAAGCAA
TGTATGCCCCTCCCATTGCAGGGAACATAACATGTACATCAAAT---
ATCACAGGAATACTATTGACATGGGATGGAGGAGAAAATAGAACA----------------------------
----

GATAATGATACAGAGACATTCAGACCTGCAGGAGGAGATATGAGGGACAATTGGAGAAGTGAATTATACAAAT
ATAAAGTGGTAGAAATTCAGCCATTG---GGGGTAGCACCCACTAAAGCAAAAAGGAGAGTGGTG--------
-------------------------------GAGAGAGAAAAAAGAGCA---GTGGGA---CTAGGAGCT---
------GTGTTCCTTGGGTTCTTGGGAGCA---GCAGGAAGCACTATG---GGCGCAGCGTCA---
ATGGCGCTGACGGTACAG---GCCAGACAA---TTGTTG---TCTGGTATAGTGCAACAGCAAAGC---
AATTTG---CTGAGG---GCTATAGAGGCGCAACAACACATGTTGCAACTCACGGTCTGGGGCATT
>KY929369
TTCTATTGTAATACATCAGGCCTGTTTAATAGTAATAATGAGACAAACAATAATTCAAGCTTA----------
----

ATCACAATTCCATGCAGAATAAAACAAGTTGTAAACTTGTGGCAGGGGGTAGGACGAGCAATGTATGCTCCTC
CCATAGCAGGAAACATAACATGTAATTCAAAT---
ATCACAGGACTACTATTGGTACGTGATGGAGGAACAGGAAATAAC----------------------------
----

ACAATAGAGAAATTCAGACCTCAAGGAGGAGATATGAGAGACAATTGGAGGAACGAACTATATAAATATAAAG
TGGTAGAAATTAAACCATTG---GGAGTAGCACCCACTAAAGCAAAAAGGAGAGTGGTG--------------
-------------------------GAGAGAGAAAAAAGAGCAATAGCGGGA---CTAGGAGCT---------
GTGTTCCTTGGGTTCTTGGGAGCA---GCAGGAAGCACTATG---GGCGCGGCGTCA---
ATAACGCTGACGGTACAG---GCCAGACAA---TTGTTG---TCTGGTATAGTGCAACAGCAAAGT---
AATTTG---CTGAGG---GCTATAGAGGCGCAACAGCATCTGTTGCAACTCACGGTCTGGGGCATT
>KY929370
TTCTATTGTAATACATCAGGCCTGTTTAATGGTACATACATGTCTAATGGTACTTATAATGAGACAAACAATA
AT---

TCAAGCTCAAACATCACAATTCCATGCAGAATAAAACAAGTTATAAACTTGTGGCAGGGGGTAGGACGAGCAA
TGTATGCTCCTCCCATTGCAGGAAAAATAACATGTAACTCAAAT---
ATCACAGGACTACTATTGGTACGTGATGGAGGAACAGGTAATCAGACA-------------------------
----

AATGATATAGAGACATTCAGACCTCAAGGAGGAGATATGAGAGACAATTGGAGGAGCGAATTATATAAATATA
AGGTGGTAGAAATTAAACCATTG---GGAATAGCACCCACTGAAGCAAAAAGGAGAGTGGTG-----------
----------------------------GGAAGAGAAAAAAGAGCA---GTGGGA---CTAGGAGCT------
---GTGCTCTTTGGGTTCTTGGGAGCA---GCAGGAAGCACTATG---GGCGCGGCGTCA---
ATAACGCTGACGGTACAG---GCCAGACAA---TTGTTG---TCTGGTATAGTGCAACAGCAAAGC---
AATTTG---CTGATG---GCTATAGATGCGCAACAGCATCTGTTGCAACTCACAGTCTGGGGCATT
>KX756609
TTCTATTGCAATACCTCAAAACTGTTTAATATATCAAACCTGTCTTCTAATGATACA----------------
----

ACAGATAATGGCACAATCATGATCCCATGCAGAATAAAACAAATTATAAACATGTGGCAGGAGGTAGGACGAG
CAATGTATGCTCCTCCCATTGCAGGAAACATAACGTGTAGTTCAAAC---
ATCACCGGACTACTGTTGGAACGTGATGGAGGAAATTCAACTAATTCAACTAAT------------TCA----
----

AATAGGACAGAGACATTCAGGCCTCAAGGAGGAAATATGAGGAACAATTGGAGAAGTGAATTATATAAATATA
AGGTGGTAGAAATACAACCAGTG---GGAATAGCGCCCACTACAGCAAAAAGGAGAGTGGTG-----------
----------------------------GAGAGAGAAAAAAGAGCA---GTGGGA---ATAGGAGCT------
---ATGTTCCTTGGGTTCTTGGGAGCA---GCAGGAAGCACTATG---GGCGCAGCGTCG---
ATAACGCTGACGGTACAG---GCCAGACAA---CTGTTG---TCTGGTATAGTGCAACAGCAAAGC---
AATTTG---CTGAGA---GCTATAGAGGCGCAACAACATCTGTTGCAACTCACAGTCTGGGGCATT
>MK076687
TTCTATGGTAATACAGCAGGCCTGTTTAATAATACATACAATATTACAACAGGCACGCTTACATACAATGGTA
AATCCCTTAAT--

GATTTAAACTCAACCATCACACTCCCATGCAGAATAAAGCAGTTTAAAAATATGTGGCAGGGGGTAGGAAGAG
CAATGTATGCCCCTCCCATTGCAGGAAACATAACATGTACATCAAAT---
ATCACAGGCATAATATTGACACGTGATGGAGGGAATGAGAGTAGTACTGAA----------------------
----

GTTCACAATAATACAGAGACATTCAGACCTCAAGGGGGAAATATGAGAGATAATTGGAGAAGTGAATTATACA
AATATAAAGTGGTAGAAATTAAGCCATTG---GGAGTAGCACCCACTGCAGCAAAAAGGAGAGTGGTG-----
----------------------------------GAAAAAGAAAAAAGG---------GGC---
CTAGGAGCT---------ATACTCATTGGGTTCTTGGGAACA---GCAGGAAGCACTATG---
GGCGTGGCGTCA---ATAACGCTGACGGTACAG---GCCAGACAA---TTGTTG---
TCTGGTATAGTGCAACAGCAAAGC---AATTTG---CTGAGA---
GCTGTAGAGGCGCAACAACATCTGTTGCAACTCACGGTCTGGGGCATT
>KX069225
TTCTATTGCAATACATCAGGACTGTTTAATGGTACATATAATGGTACAGACAGTGGTACATACAATAGT----
----

ACAGGCAAAAACATCACACTTCAATGCAGAATAAAACAAATTATAAACATGTGGCAGGAGGTAGGGCGAGCAA
TGTATGCTCCTCCCATTAAAGGAAATATAACATGTATATCAAAT---
ATCACAGGGATACTACTGGTACGTGATGGAGGACTAAACACTAAT----------------------------
----

GATAGTGATGAGACATTCAGACCTGGAGGAGGAAACATGAGGGACAATTGGAGAAGTGAATTATATAAATACA
AAGTGGTAGAAATTAAGCCATTG---GGAGTAGCACCCACTGAAGCAAGAAGGAGAGTGGTG-----------
----------------------------GAGAGAGAAAAAAGAGCA---GTGGGA---CTAGGAGCT------
---GTGCTCCTTGGGTTCTTGGGAGCA---GCAGGAAGCACTATG---GGCGCGGCGTCA---
ATAACGCTGACGGTACAG---GCCAGACAA---TTGTTG---TCTGGTATAGTGCAACAGCAAAAC---
AATTTG---CTGAGA---GCCATAGAGGCGCAACAGCATATGTTGCAACTCACGGTCTGGGGCATT
>KY713231
TTCTATTGCAATACCTCAAGCTTGTTTAGCAGTATAAGCCCTAATAGTACA----------------------
----

AATAATACTGTAATAACAATCCCATGCAGAATAAAACAAATTATAAACATGTGGCAAGGGGTAGGACGAGCAA
TATACGCCCCTCCTATTGAAGGAAACATAACATGTAAATCAAAT---
ATCACAGGAATACTATTGGTACGTGATGGAGGCACAAATACTACA----------------------------
----
-
GAAGAAGGAAAAGAAATAGAAATATTTAGACCTCAAGGAGGAAATATGAAGGACAATTGGAGAAGTGAATTAT
ATAAATATAAAGTGGTAGAAATTAAGCCATTA---GGAGTAGCACCCACTAAAGCAAAAAGGAGAGTGGTG--
-------------------------------------GAGAGAGAAAAAAGAGCAGTAGTGGGG---
CTAGGAGCT---------GCGTTCCTTGGGTTCTTGTCAGCT---GCAGGAAGCACTATG---
GGCGCGGCGTCA---ATAACGCTGACGGTACAA---GCCAGACAA---TTGTTG---
TCTGGTATAGTGCAACAGCAAAGC---AATTTG---CTGAGA---
GCTATAGAGGCGCAACAGCATATGTTGCAACTCACAGTCTGGGGCATT
>KY713235
TTCTATAGGAGACAGTCAAAATTGTTTAATCACACATACATGCAGAATGGTACACACAATGGTACAATAAAT-
----

GGTTCAGACCCAATCATAATTCCATGCAGAATAAAGCAAATTATAAACATGTGGCAGGAGGTAGGAAGAGCAA
TGTATGCCTCTCCCATTGCAGGAAACATAACCTGTAAATCAAAT---
ATCACAGGACTACTATTGACACGAGATGGAGGACTCAAAAATGATAGTAGTAAT-------------------
----

GAGACAAAGATAGAGATATTCAGACCTGGAGGAGGAAACATGAAAGACAATTGGAGAAGTGAATTATACAAAT
ATAAAGTGGTAGAAATTAAGCCATTG---GGAATAGCACCCACTGAAGCAAGAAGAAGAGTAGTG--------
-------------------------------CAGAGAGAAAAAAGAGCA---GTGACA---CTAGGAGCT---
------ATGTTCCTTGGGTTCTTGGGAGCA---GCAGGAAGCACTATG---GGCGCGGCGTCA---
ATAACGCTGACGGTACAG---GCCAGACAA---TTGTTG---TCTGGTATAGTGCAACAGCAAAGC---
AATTTG---CTGAGG---GCTATAGAGGCGCAACATCATCTGTTGCAACTCACAGTTTGGGGCATT
>MN703343

TTCTATTGTAATACATCAAGACTGTTTAATGGTACATATAATACAACAGAGGAG-------------------
----

TCAAACTCAACCATCACAATCCAATGCAGAATAAAACAAATTATAAACATGTGGCAGGAGGTAGGACGAGCAA
TGTATGCCCCTCCCATTGCAGGAAACATAACATGTGAATCAAAC---
ATCACAGGACTAATATTGGTACGTGATGGAGGAACTCATGAAAATAGT-------------------------
----

AATACTACAGAGATATTCAGACCTGGAGGAGGAGATATGAGGGACAATTGGAGAAGTGAACTATATAAATATA
AAGTGGTAGAAGTTAGGCCTTTG---GGAATAGCACCCACTAAAGCAAAAAGGAGAGTGCTG-----------
----------------------------GAGAGACAAAAAAGAGCA---GTAGGA---ATGGGAGCT------
---GTGTTCCTTGGGATCTTGGGAGCA---GCAGGAAGCACTATG---GGCGCGGCGTCA---
ATAACGCTGACGGTACAG---GCTAGACAA---TTGTTG---TCTGGTATAGTGCAACAGCAAAAC---
AATTTG---CTGAGG---GCTATAGAGGCGCAACAGCACATGTTGCAACTCGCAGTCTGGGGCATT
>MN703361
TTCTATTGTAATACATCAGGCCTGTTTAATGAAAGCCGGCTTAACGAAACAGAAGGTGAT-------------
----

TCAAGTAAAAACATCACACTTCCATGTAGAATAAAGCAAATTATAAACATGTGGCAGGCGGTAGGAAAAGCAA
TATATGCCCCTCCCATTGCAGGAAACATAACATGTAAATCAAAT---
ATCACAGGATTACTATTGGAACGTGATGGAGGCAAAGGCAATGCA----------------------------
----

ACAGAGGAAATATTCAGACCTGGAGGAGGAAATATGGGGGACAATTGGAGAAGTGAATTATATAAATATAAAG
TGGTAGAAATTCAACCATTG---GGAATAGCACCTACTGGAGCAAAAAGGAGAGTAGTG--------------
-------------------------GAGAGAGAAAAAAGAGCAGTAGTGGGA---ATAGGAGCT---------
GTGTTCCTTGGGATCTTGAGTGCA---GCAGGAAGCACTATG---GGCGCGGCGGCA---
ATAACGCTGACGGTACAA---GCCAGACAA---TTATTG---TCTGGTATAGTGCAACAGCAAAGC---
AATTTG---CTGAAG---GCTATAGAGGCGCAACAGCATATGTTGCAGCTCACGGTCTGGGGCATT
>AF067159
TTCTATTGTAATACATCAGGCCTGTTTAATAGTACATACATGAGTGGTACATACATGAATTCAAGTGCAGACA
TG---

AATTCAAGCTACATCACAATCCCATGCAGAATAAAGCAAATTATAAACATGTGGCAGGAGGTAGGACGAGCAA
TGTATGCCCCTCCCATTGCAGGAAACATAACATGTAAATCAAAT---
ATCACAGGAATACTATTGGAACGTGATGGAGGGTCAGGGTCAAAT----------------------------
----

GGCACAGAGACATTCAGACCTGGAGGAGGAGATATGAGGAACAATTGGAGAAGTGAATTATATAAATATAAAG
TGGTAGAAATTCAGCCATTG---GGAGTAGCACCCACGGAAGCAAAAAGGAGAGTGGTG--------------
-------------------------GAGAGAGGAAAAAGAGCA---GTGGGA---ATAGGAGCT---------
GTGTTCCTTGGGTTCTTGGGAGCA---GCAGGAAGCACTATG---GGCGCGGCGTCA---
ATAACGCTGACGGTACAG---GCCAGACAA---TTGTTG---TCTGGTATAGTGCAACAGCAAAGC---
AATTTG---CTGAGG---GCTATAGAGGCGCAACAGCATCTGTTGCAACTCACGGTCTGGGGCATT
>FJ866133
TTCTATTGCAATACATCAGGACTGTTTAATAATAATATTACA-------------------------------
----

AATGCAACCACCACCCTCCCATGCAGAATAAAACAAATTGTAAACATGTGGCAGGGGGTAGGACGAGCAATGT
ATGCCCCTCCCATTGAAGGAAAAATAACATGTAACTCAAAT---
ATCACAGGACTGCTCCTGACACGTGATGGAGGGGTGAATAATGAA----------------------------
----

ACGACAGAGACATTCAGACCTGGAGGAGGAGATATGAAGGACAATTGGAGAAATGAATTATATAAATATAAAG
TGGTAGAAATTCAGCCATTA---GGAATAGCACCCACTGGAGCAAAAAGGAGAGTGGTG--------------
-------------------------GAGAGAGAAAAAAGAGCG---GTGGGA---CTAGGAGCT---------
GTGTTCCTTGGGTTCTTGGGAGCC---GCAGGAAGCACTATG---GGCGCAGCATCA---
ATAACGCTGACGGTACAA---GCCAGACAA---TTATTG---TCGGGTATAGTGCAACAGCAAAAC---
AACTTG---CTGAGA---GCTATAGAGGCGCAACAACATATGTTGCAACTCACAGTCTGGGGCATT
>AB097871

TTCTATTGTAATACATCAGGCCTGTTTAATGGGACATACAATGATACATACAGGTCTAATGATACAGAT----
----

AGTAATTCAACCATCACACTCCCATGCAGAATAAAGCAAATTATAAACATGTGGCAGGAGGTAGGACGAGCAA
TGTATGCCCCTCCTATTGCAGGAAACATAACATGTAAATCAAAC---
ATCACAGGACTACTATTGGTACGTGATGGAGGAAATGGTACACAG----------------------------
----

ACATTCAGACCTGGAGGAGGAAATATGAAGGATAATTGGAGAAGTGAATTATATAAATATAAAGTGGTAGAAA
TTCAGCCATTA---GGAATAGCACCCACTGGAGCAAAAAGGAGAGTGGTG-----------------------
-----------------GAGAGAGAAAAAAGAGCA---GCA------ATAGGAGCT---------
ATGTTCCTTGGGTTCTTGGGAGCA---GCAGGAAGCACTATG---GGCGCGGCGTCG---
ATGGCGCTGACGGTACAG---GCCAGACAA---TTGTTG---TCTGGTATAGTGCAACAGCAAAGC---
AATTTG---CTGAGG---GCTATAGAGGCGCAACAGCATATGTTGCAACTCACAGTCTGGGGCATT
>KC894076
TTCTATTGCAATACATCAGGACTGTTTAACAGTACATACAATAGTACATCAGGAATGTTTAACAGTACATACA
ATAGTTACAAT--
-------AGTGCAGGAAAT------
AATTCAAATTCAATCATCACACTCCAATGCAGAATAAAACAAATTATAAACATGTGGCAGGGGGTAGGACAAG
CAATGTATGCTCCTCCCATTAAAGGAAACATAACATGTAAATCAAAT---
ATCACAGGACTACTATTGACACGTGATGGAGGAAGGGGAAATGATACT-------------------------
----

AATGGAACAGAGACATTCAGACCTGGAGGAGGAGATATGAGGGACAATTGGAGAAGTGAATTGTATAAATATA
AAGTGGTAGAAATTAAGCCATTG---GGAATAGCACCCACTGAGGCAAAAAGGAGGATGGTG-----------
----------------------------GAGAGAGAAAAAAGAGCA---GTTGGA---ATAGGAGCT------
---GTGTTCCTTGGGTTCTTGGGAGCC---GCAGGAAGCACTATG---GGCGCAGCATCA---
CTAACGCTGACGGTACAG---GCCAGGCAA---CTGTTG---TCTGGTATAGTGCAACAGCAAAGC---
AATTTG---CTGAGG---GCCATCGAGGCGCAACAGCATATGTTGCAACTCACGGTCTGGGGCATC
>KC894073
TTCTATTGCAATACATCAAAACTGTTTAATGGTACATACAATGGTACATACACAAATAATGAT----------
----

ACAAACAGCACAATCATACTCCCATGCAGAATAAAACAAATTATAAACATGTGGCAGGAGGTAGGACAAGCAA
TGTATGCCCCTCCCATTGAAGGAATCATAGCATGTAATTCAACT---
ATCACAGGATTATTATTGACACGTGATGGAGGAGACAAGAATGGG----------------------------
----

AGTAAGCCAGAAATATTCAGACCTGGAGGAGGAGATATGAGGGACAATTGGAGGAGTGAATTATATAAATATA
AAGTGGTAGAAATTAAGCCATTG---GGAATAGCACCCACTAAGGCAAAAAGGAGAGTGGTGGAG---AAA--
----------------------GAAAAAACAATTCAAAAAAGAGCA---GTAGGA---ATAGGAGCT------
---GTGTTCCTTGGGTTCTTGGGAGCA---GCAGGAAGCACTATG---GGCGCGGCGTCA---
ATAACGCTGACGGTACAG---GCCAGACAA---TTGTTG---TCTGGTATAGTGCAACAGCAAAGC---
AATTTG---CTGAGG---GCTATAGAGGCGCAACAGCATATGTTGCAACTCACAGTCTGGGGCATT
>FJ444207
TTCTATTGCAATACATCAAAACTGTTTACAGAAAGTAATTCAACCTCAAACAGCACC----------------
----

TCAAACACCGTACTCACACTCCCATGCAGAATAAAACAAATTATAAACATGTGGCAGGGGGTAGGACGAGCAA
TGTATGCCCCTCCCATTGAGGGAAATATAACATGTGAATCAAAC---
ATCACAGGATTACTATTGACGCGTGATAGA---ACAGACAATAAA----------------------------
----

ACAGAGACATTTAGACCTGGAGGAGGAGACATGAGGGACAATTGGAGAAGTGAATTATACAAATATAAAGTGG
TAGAAATTCAGCCATTA---GGAGTAGCACCCACTAAGGCAAAAAGGAGAGTGGTG-----------------
----------------------GAGAGAGAAAAAAGAGCA---GTGGGA---ATAGGAGCT---------
GTGTTCCTTGGGTTCTTGGGAGCA---GCAGGAAGCACTATG---GGCGCAGCATCA---
ATAACGCTGACGGTACAG---GCCAGACAA---TTGTTG---TCTGGTATAGTGCAGCAGCAGAGC---
AATTTG---CTGAGG---GCTATAGAGGCGCAACAGCATATGTTGCAACTCACGGTCTGGGGCATT
>KC894078

TTCTATTGTAATACATCAAACCTGTTTAATATTAATATTACAGGAAATATTACAGGAAATATGACA-------
----

GGAAATGAAAACATCACACTCCCATGCAGAATAAAACAAATTATAAACATGTGGCAAAGGGTAGGGCAAGCTA
TATATGCCCCTCCCATTGCAGGAAACATAACATGTAACTCAAGT---
ATCACAGGACTACTGTTGACACGTAATGGAGGCAATGATAACAGAACAGGG---------------------
----

AATAATAATACAGAGACATTCAGACCTGGAGGAGGAGATATGAGGGATAATTGGAGAAGTGAATTATATAAAT
ATAAAGTGGTAGAAGTTCAGCCATTG---GGAGTAGCACCCACTGGAGCAAAAAGGAGAGTGGTG--------
------------------------------GAGAGAGAAAAAAGAGCA---GTA------ATGGGAGCT---
------ATGTTCCTTGGGTTCTTGGGAGCA---GCAGGAAGCACTATG---GGCGCGGCGTCA---
GTGGCGCTGACGGTACAG---GCCAGACAA---CTATTG---TCTGGTATAGTGCAACAGCAAAGC---
AATTTG---CTGAGG---GCTATAGAGGCGCAACAGCATCTGTTGCAACTCACGGTCTGGGGCATT
>KC894079
TTCTATTGCAATACATCAGACCTGTTTAATGGTACATACAGAAATGGTACATACAATCATACAGGAAGAAGT-
----

TCAAATGGAACCATCACCCTCCAATGCAAAATAAAACAAATTATAAACATGTGGCAGGAGGTAGGAAGAGCAA
TATATGCCCCTCCCATTGAAGGAGAAATAACATGTAACTCAAAT---
ATCACAGGACTACTATTGCTACGTGATGGAGGTCAATCAAATGAAACA------------------------
----

AATGACACAGAGACATTCAGACCTGGAGGAGGAGATATGAGGGACAATTGGAGAAGTGAATTATATAAATATA
AAGTAGTAGAAATTAAACCATTG---GGAGTAGCACCCACTGAGGCAAAAAGGAGAGTGGTG-----------
---------------------------GAGAGAGAAAAAAGAGCA---GTGGGA---ATAGGAGCT------
---GTGTTCCTTGGGTTCTTGGGAGCA---GCCGGAAGCACTATG---GGCGCAGCATCA---
ATGACGCTGACGGTACAG---GCCAGGCAA---TTATTG---TCTGGTATAGTGCAACAGCAAAGC---
AATTTG---CTGAGG---GCTATAGAGGCGCAACAGCATATGTTGCAACTCACGGTCTGGGGCATT
>KC894081
TTCTATTGTAATACATCAGGACTGTTTAATGGTACATACCACCCTAATGGTACATACAATGAGACAGCAGTTA
AT--

TCAAGCGACACAATCACACTCCAATGCAGAATAAAACAAATTATAAACATGTGGCAAGAGGTAGGAAGAGCAA
TGTATGCTCCTCCCATTGCAGGAAATATAACATGTAATTCAACT---
ATCACAGGACTATTATTGACACGTGATGGAGGAATAAATCAGACA---------------------------
----

GGAGAAGAGATATTCAGACCTGGAGGAGGAGATATGAGGGATAATTGGAGAAATGAATTATATAAATATAAAG
TGGTGGAGATTAAGCCATTA---GGAATAGCACCCACTAAGGCAAAAAGGAGAGTGGTG-------------
------------------------GAGAGAGAAAAAAGAGCA---GTGGGA---ATAGGAGCT---------
GTGTTCCTTGGGTTCTTGGGAGCA---GCAGGAAGCACTATG---GGCGCGGCGTCA---
ATAACGCTGACGGTACAG---GCCAGACAA---CTGTTG---TCTGGTATAGTGCAACAGCAAAGC---
AATTTG---CTGAGG---GCCATAGAGGCGCAACAGCATATGTTGCAACTCACGGTCTGGGGCATT
>FJ444522
TTCTATTGCAATACATCAGGCCTGTTTAATAGTACATATAATCCAAATAGTACATATAATAATTCAAGC----
----

TCAGACACACCACTTAGACTCTCATGTAGAATAAAACAAATTATAAATATGTGGCAGGGGGTAGGAAGAGCAA
TATATGCCCCTCCCATTGAAGGAAGCATAACATGTAACTCAAGT---
ATCACAGGACTACTATTGCTACGTGATGGAGGTCATGACGGTGACTCA------------------------
----

AATAACACAGAGACATTTAGACCGGGAGGAGGAGATATGAGGGACAACTGGAGAAGTGAATTATATAAATATA
AAGTGGTAGAAATTAAGCCATTG---GGAGTAGCACCCACTGACGCAAAAAGAAGAGTGGTG-----------
---------------------------GAAAGAGAAAAAAGAGCA---GTAGTGGGAATAGGAGCT------
---GTGCTCCTTGGGTTCTTGGGAGCA---GCCGGAAGCACTATG---GGCGCGGCGTCA---
CTAACGCTGACGGTACAG---GCCAGACAA---CTGCTG---TCTGGTATAGTGCAACAGCAAAAC---
AATTTG---CTGAGG---GCTATAGAGGCGCAACAGCATATGTTGCAACTCACGGTCTGGGGCATT
>KC894089

TTTTATTGCAATACATCAGCACTGTTTAATGGTACATACAATTCTACATACAATTCTACTAGTAAC-------
----

CCAAACATCACAATCACAATCCAATGCAGAATAAAACAATTATAAACATGTGGCAGGAGGTGGGACGAGCAA
TGTATGCCGCTCCCATTGCAGGAGACATAACATGTGAATCAAAC---
ATCACAGGACTACTATTGACACGTGATGGAGGAAATACTGCAAAC---------------------------
----

GGGACAGAGATATTCAGACCTGGAGGAGGAGATATGAGGGATAATTGGAGAAGTGAATTATATAAATATAAAG
TGGTAGAAGTTCAGCCATTG---GGAGTAGCACCTACCAAGGCAAAAAGAAGAGTGGTG-------------
-------------------------GGTAGAGAAAAAAGAGCA---GTA------CTGGGAGCT---------
GTGTTCCTTGGGTTCTTGGGAGCA---GCAGGAAGCACTATG---GGCGCGGCGTCA---
ATGGCGCTGACGGTACAG---GCCAGACAA---TTGTTG---TCTGGTATAGTGCAACAGCAAAGT---
AATTTG---CTGAGG---GCTATAGAGGCGCAACAGCATATGTTGCAACTCACAGTCTGGGGCATT
>KC894097
TTCTATTGCAACACATCACAACTGTTTAATGGTACATACTTTAATAGTACATACTTTAATGGTACATACAATG
GTACATACCCA---

AATAATACAAACATCACACTTCAATGCAGAATAAAACAAATTATAAACATGTGGCAGGGGGTAGGAAGAGCAA
TGTATGCCCCTCCCATTGCAGGAAACATAACATGTGAATCAAAT---
ATCACAGGACTATTATTGACATGGGATGGAGGAAATAATGGAGAAAATAACACA------
AGAAATGGTACAGGAAAT--

GGTACAGAGACATTCAGACCTGGAGGAGGAGATATGAGGGACAATTGGAGGAGTGAATTATATAAATATAAAG
TGGTAGAAATTAAGCCATTG---GGAATAGCACCCACTGAGGCAAAAAGGAGAGTGGTG-------------
-------------------------GAGAGAGAAAAAAGAGCA---GTGGGA---CTAGGAGCT---------
GTGTTCCTTGGATTCTTGGGAGCC---GCAGGAAGCACTATG---GGCGCAGCGTCA---
ATGACGCTGACGGTACAA---GCCAGACAA---TTGCTG---TCTGGTATAGTGCAACAGCAAAGC---
AATTTG---CTGAGA---GCTATAGAGGCGCAACAGCATATGTTGCAACTCACAGTCTGGGGCATT
>KC894084
TTCTATTGCAATACATCAGAACTGTTTAATAATACATACTGGCCTAATAGTACTGTTACAAATGGAACT----
----

TTAAATGGAACCTTCACACTCCCATGCAAAATAAAACAAATTATAAACATGTGGCAGGAGATAGGAAGAGCAA
TATATGCCCCTCCCATTGCAGGAAACATAACATGTAAATCAAAT---
ATCACAGGACTACTATTAACACGTGATGGAGGTCATAACCCAGAG---------------------------
----

AAGAATGACACAGAGACATTCAGACCTGGAGGAGGAGATATGAGGGATAATTGGAGAAGTGAATTATACAAAT
ATAAAGTAGTAGAAGTCAAGCCATTA---GGAATAGCACCCACGGGGGCAAAAAGGAGAGTGGTG--------
-------------------------------GAGAGAGAAAAAAGAGCA---GTGGGA---ATAGGAGCT---
------GTGTTCCTTGGGTTCTTGGGAGCA---GCAGGAAGCACTATG---GGCGCGGCGTCA---
ATAACGCTGACGGTACAG---GCCAGACAA---TTATTG---TCTGGTATAGTGCAACAGCAAAGC---
AATTTG---CTGAGG---GCTATAGAGGCGCAGCAGCATATGCTGCAACTCACGGTCTGGGGCATT
>KC156114
TTCTATTGCAACACATCAAGATTGTTTAATCATACATTTAATGAGACA------------------------
----

TCAGACGAAGACATCACACTCCAATGTAGGATAAAACAAATTATAAACATGTGGCAGGGGGTAGGACGAGCAA
TATATGCCCCTCCCATTGCAGGAAACATAACATGTAACTCAAAT---
ATCACAGGACTACTATTGACACGTGATGGAGGGAAGAGTAATAGTAGT------------------------
----

AATCAGACAGAGATATTCAGACCTGCAGGAGGAAATATGAGGGACAATTGGAGAAGTGAATTATATAAATATA
AAGTGGTAGAAATTAAACCATTA---GGAATAGCACCCACTAAGGCGAAAAGGAGAGTGGTG----------
----------------------------GAGAGAGAAAAAAGAGCG---GCA------ATAGGAGCT------
---ATGTTCCTTGGGTTCTTGGGAGCA---GCAGGAAGCACTATG---GGCGCGGCGTCA---
ATAACGCTGACGGTACAG---GCCAGACAA---TTATTG---TCTGGTATAGTGCAACAGCAAAGC---
AATTTG---CTGAGA---GCTATAGAGGCGCAACAGCATATGTTGCAACTCACGGTCTGGGGCATT
>KC156120

TTTTATTGCAATACATCAATACTGTGGGATAGTGATAAGAGT-------------------------------
----

AATGACACAAACATCACACTCCCATGCAGAATAAAACAAATTATAAATATGTGGCAGGAGGTAGGACGAGCAA
TGTATGCCCCTCCCATTGCAGGAAGCATAACATGTAAATCAAAA---
ATCACAGGACTACTATTGGTACGTGATGGAGGAATAAATAGCAGC----------------------------
----

ACAGAGACATTCAGACCTGGAGGAGGAAATATGAAGGACAATTGGAGAAGTGAATTATATAAATATAAAGTGG
TAGAAATTCAGCCATTA---GGAGTTGCACCCACTGGAGCAAAAAGGAGAGTGGTG-----------------
----------------------GAGAGGGAGAAAAGAGCA---GTGGGA---ATAGGAGCT---------
GTGTTCCTTGGGTTCTTGGGAGCA---GCTGGAAGCACTATG---GGCGCAGCGTCA---
ATAACGCTGACGGTACAG---GCCAAACAA---CTGTTG---TCTGGTATAGTGCAACAGCAGAGT---
AATTTG---CTGAGA---GCTATAGAGGCGCAACAGCATATGCTGCAACTCACAGTCTGGGGCATA
>HM204588
TTCTATTGCAACACATCAAAACTGTTTAATAGTACGTGGAATAGTACAGAAAATACTAATATA----------
----

CCAAATGCAACCATCTACATCCAATGCAGAATAAAACAAATTATAAACATGTGGCAGGGGGTAGGACGAGCAA
TATATGCCCCTCCCATTGAAGGAAACATAACATGCAACTCAATT---
ATCACAGGAATACTATTGACACGTGATGGAGGAAGGGACGATAACAATAACCAA-------------------
----
-
ACAAACCAAACAGAGATAGAGATATTCAGACCTGCAGGAGGAGATATGAGGGACAATTGGAGAAGTGAATTAT
ATAAATATAAAGTGATAGAAATTAAGCCATTA---GGAATAGCACCCACTAAGGCAAAAAGGAGAGTGGTG--
-------------------------------------GAGAGAGAAAAAAGAGCA---GTGGGA---
ATAGGAGCT---------GTGTTCCTTGGGTTCTTGGGAGCA---GCAGGAAGCACTATG---
GGCGCAGCATCA---ATAACGCTGACGGTACAG---GCCAGACAA---TTGTTG---
TCTGGTATAGTGCAACAGCAAAAC---AATTTG---CTGAGG---
GCTATAGAGGCGCAACAACATATGTTGCAACTCACAGTCTGGGGCATT
>HQ615984
TTCTATTGCAATACATCAGGCCTGTTTAATGGTACATTTAATGGTACATACATGCCAAATAGTACAGAAAGTA
CT---

TCAAATACAAACATCACACTCCAATGCAGAATAAAACAAATCATAAACATGTGGCAGCAGGTAGGACGAGCAA
TGTATGCCCCGCCCATTGAAGGAAACATAACATGTAGATCAAAT---
ATCACAGGACTACTATTGACACGTGACGGAGGCGGAAGAGCAAAT----------------------------
----

GATACTGAAGAGATATTCAGGCCTGGAGGAGGAGATATGAGGGACAATTGGAGAAGTGAATTATACAAATATA
AAGTGGTAGAGGTTCAGCCATTG---GGAATAGCACCCACTAAGGCAAAAAGGAGAGTGGTG-----------
----------------------------GAGAGAGAAAAAAGAGCA---GTGGGA---ATGGGAGCT------
---GTGTTCCTTGGGTTCTTGGGAGCA---GCAGGAAGCACTATG---GGCGCGGCGTCA---
ATAACGCTGACGGTACAG---GCCAGACAA---TTGTTG---TCTGGTATAGTGCAACAGCAAAGC---
AATTTG---CTGAGG---GCCATAGAGGCGCAACAGCATATGTTGCAACTCACGGTCTGGGGCATT
>KC156216
TTCTATTGCAATACATCAAAACTGTTTAATAGTATACACAATAGTACAGATATGTATAATAGTACAGTT----
----

AGTAATTCAACCATCACACTCCAATGCAAAATAAAACAAATTATAAATATGTGGCAGGGGGTAGGACGAGCAA
TGTATGCCCCTCCCATAGCAGGAAACATAACATGTAAATCAAAT---
ATCACAGGACTACTATTAACACGGGATGGAGGAAACGTGAGTGGA----------------------------
----

ACAACAGAGACATTCAGACCTGGAGGAGGAAATATGAAGGACAATTGGAGAAGTGAATTATATAAATATAAAG
TGGTGGAAATTCAGCCATTA---GGAATAGCACCCACTAAGGCAAAAAGGAGAGTGGTG--------------
-------------------------GAAAGAGAAAAAAGAGCA---GTGGGA---ATAGGAGCT---------
GTGTTCCTTGGGTTCTTGGGAGTA---GCCGGAAGCACTATG---GGCGCAGCATCA---
ATGACGCTGACGGTACAG---GCCAGACAA---TTGTTG---TCTGGTATAGTGCAACAGCAAAGC---
AATTTG---CTGAGG---GCTATAGAGGCGCAACAGCATATGTTGCAGCTCACGGTCTGGGGCATT

>KC156219
TTCTATTGCTATACATCAGAACTGTTTAATGAGACAGACAAACTGCTTAATAAGACAAAAGAGAAA-------
----

GACAATTCAACCATCATACTCCCATGCAGAATAAAACAAATTATAAACATGTGGCAGGGGGTAGGACGAGCAA
TATATGCCCCTCCCATTGAAGGAAATATAACATGTAAGTCAAAT---
ATCACAGGATTACTATTGACACGTGATGGAGGAGAGGACATTAATGGAACT----------------------
----

AATAGAGAAGAGATATTCAGACCTGGAGGAGGAGATATGAAGGATAATTGGAGAAGTGAATTATATAAATATA
AAGTAGTAGAAATTCAGCCATTA---GGAATAGCACCCACTAAGGCAAAAAGGAAAGTGGTG-----------
----------------------------GAGAGAGAAAAAAGAGCA---GCA------ATAGGAGCT------
---GTGTTCCTTGGGTTCTTGGGGGCA---GCAGGAAGCACTATG---GGCGCGGCGTCA---
ATAACGCTGACGGTACAA---GCCAGACAA---TTATTG---TCTGGTATAGTGCAACAGCAAAGC---
AATTTG---CTGAGA---GCTATAGAGGCGCAACAGCATATGTTGCAACTCACGGTCTGGGGCATT
>HQ615963
TTCTATTGCAATACATCAAGCCTGTTTAATAGGACATATAATAGTACAGATATGGCCAATAGTACAGAA----
----

GCTAACAGTACCATCACAATCCACTGCAGAATAAAACAAATTATAAACATGTGGCAGGAGGTGGGACGAGCAA
TGTATGCCCCTCCCATTAAAGGAAACATAACATGTATATCAAAT---
ATCACAGGACTACTATTAACAAGGGATGGAGGAGATAATAATGAA----------------------------
----

ACGGAGACATTCAGACCTGGAGGAGGAAATCTGAGGGACAATTGGAGAAGTGAATTATATAAATATAAAGTGG
TAGAAGTTCAGCCATTG---GGAGTAGCACCCACTAATTCAAGAAGGAGAGTGGTG-----------------
----------------------GAGAGAGAAAAAAGAGCA---GTGGGA---CTAGGAGCT---------
GTGTTCCTTGGGTTCTTGGGGGCA---GCAGGAAGCACTATG---GGCGCAGCATCA---
ATAACGCTGACGGTACAG---GCCAGACAA---TTATTG---TCTGGTATAGTGCAACAGCAAAGC---
AATTTG---CTGAAA---GCTATAGAGGCTCAACAGCATATGTTGAAACTCACGGTCTGGGGCATT
>HQ615964
TTCTACTGCAATACATCAAACCTGTTTAACAGTACATATAACAGTAACAGTACATATAACAGTACAGATACTC
CT---

GAGGATAAAAACATCACACTCCAATGCAGAATAAAACAAATTATAAACATGTGGCAGGGGGTAGGACAAGCAA
TGTATGCCCCTCCCATTGCAGGAAACATAACATGTAAATCAAAT---
ATCACAGGATTATTATTAACACGTGATGGAGGAACCAACACAAAC----------------------------
----

AACACAACAGAAATATTCAGACCTGGAGGAGGAGATATGAGGGACAATTGGAGAAGTGAATTATATAAATATA
AAGTGGTAGAAATTCAACCATTA---GGAGTAGCACCTACTCAGGCAAAAAGGAGAGTGGTG-----------
----------------------------GAGAGAGAAAAGAGG---------GGA---ATAGGAGCT------
---GTGTTCCTTGGGTTCTTGGGAGCA---GCAGGAAGCACTATG---GGCGCAGCATCA---
ATGACGCTGACGGTACAG---GCCAGACAA---TTGTTG---TCTGGTATAGTGCAACAGCAAAGC---
AATTTG---CTGAGG---GCTATAGAGGCGCAACAGCATATGTTGCAACTCACGGTCTGGGGCATT
>HM204613
TTCTATTGCGATACATCAAAACTGTTTAATAGTACATACCTGGCTAATGGCACACGCTGGATTAATGATAGTA
CAGAA--
-------------------CAG---
AATACAAGCAAAGTCATCTCACTCCCATGTAGAATAAAACAAATTATAAACATGTGGCAGGAGGTAGGACGAG
CAATGTATGCTCCTCCCATTGAAGGAAACATAAGATGTGAATCAAAT---
ATCACAGGATTGTTATTGACGCGTGATGGAGGAATAGATAACAAC----------------------------
----

AAGAGCGAGATATTCAGACCTGGAGGAGGAGATATGAGGGACAATTGGAGAAGCGAATTATATAAATATAAAG
TGGTAGAAATTAAACCATTG---GGAATAGCACCCACTAAGGCAAAAAGGAGAGTGGTG--------------
-------------------------GAGAGAGAGAAAAGAGCA---GTGGGA---ATAGGAGCT---------
CTGTTCCTTGGGTTCTTGGGAGCA---GCAGGAAGCACTATG---GGCGCGGCGTCA---
ATGACGCTGACGGTTCAG---GCCAGACAA---TTGTTG---TCTGGTATAGTGCAACAGCAAAGC---
AATTTG---CTGAGG---GCTATAGAGGCGCAACAACATATGTTGCAACTCACGGTCTGGGGCATT

>KC186690
TTCTATTGCAACACAACAGGATTATTTAATTATATGTACAATGCTATTAATAATAGTATACAGTTTATTAATA
GTACAAACCTT--
-------------ACAGAA------
CCAAGTGACAACAACATCACACTTCAATGTAGAATAAAACAAATTATAAACATGTGGCAGGGAGTAGGAAGAG
CAATGTATGCTCCTCCCATTGCAGGAAACATAACATGTAGATCTAAT---
ATCACAGGAATACTATTGACACGTGATGGGGGCAATAGTGATGAGAAGAATAAC------TCAACTAAT----
----

GACACTGAGACATTTAGACCTGGAGGAGGAGACATGAGGGACAATTGGAGAAGTGAATTATATAAATATAAAG
TGGTAGAAATTAGACCATTG---
GGAATAGCACCCACTAAGGCAAAAAGGAGAGTGGTGGAGATGGAGATGCCAGGGATCAAACCCACCTCACGCT
CAGCAAGGAGAAGAAGAGCA---GTGGGA---ATAGGAGCT---------
TTGTTCCTTGGGTTCTTGGGAGCA---GCAGGAAGCACTATG---GGCGCGGCGTCA---
ATAACGCTGACGGTACAG---GCCAGACAA---CTGCTG---TCTGGTATAGTGCAACAGCAAAAT---
AATTTG---CTGATG---GCTATAGAGGCGCAACAGCATATGTTGCAACTCACAGTCTGGGGCATT
>KC187578
TTCTATTGCAATACATCAGGACTGTTTAATAGTACATACAATAATGGTACATACACTAGTGCATACAATATT-
----
-------------------ACA---
GAAAATAATTCAAGTATTACACTCCCATGCAGAATAAAGCAGATTATTAACATGTGGCAGGGGGTAGGACGAG
CAATGTATGCCCCTCCCATTGCAGGAAACATAACATGCAGATCAAAT---
ATCACAGGACTACTATTGACACGGGATGGTGGCAATGGAATCAATGTG-------------------------
----

ACCACAGAGATATTCAGACCTGAAGGGGGAAATATGAAGGACAATTGGAGAAGTGAATTATACAAATACAAAG
TGGTAGAAATTCAGCCATTG---GGAATAGCACCCACCAAGGCAAAAAGGAGAGTGGTG--------------
-------------------------GAGAGAGAAAAAAGAGCA---GTGGGA---ATAGGAGCT---------
GTGTTCCTTGGGTTCTTGGGAGCA---GCAGGAAGCACTATG---GGCGCGGCGTCA---
ATAACGCTGACGGTACAG---GCCAGACAA---TTATTG---TCTGGTATAGTGCAACAGCAAAGC---
AATTTG---CTGAGA---GCTATAGAGGCGCAACAGCATATGTTGCAACTCACGGTCTGGGGCATT
>KC187323
TTCTATTGTAATACATCAAAACTGTTTAATAATACATTTGATGGTACAAACATGATGAATAACAAT-------
----

ACAGATGAAACCATCACACTCCAATGCAGAATAAAACAAATTATAAACATGTGGCAGGGGGTAGGACGAGCAA
TGTATGCCCCCCCCATTAAGGGAATCATAACATGTAAATCAAAT---
ATCACAGGACTACTATTGACACGTGATGGAGGAAATCACAATGAGACT-------------------------
----

AATAACACAGAGACATTCAGACCTGCAGGAGGAAATATGAAGGACAATTGGAGAAGTGAATTATATAAATATA
AAGTGGTAGAAATTAAGCCATTA---GGAATAGCACCCACTACGGCAAAAAGGAGAGTGGTG-----------
----------------------------GAGAGAGAAAAAAGAGCA---GTGGGA---ATAGGAGCT------
---GTGTTCCTTGGGTTCTTGGGAGCA---GCAGGAAGCACTATG---GGCGCAGCATCA---
ATAACGCTGACGGTACAG---GCCAGACAA---TTGTTG---TCTGGTATAGTGCAACAGCAAAGC---
AATTTG---TTGAGA---GCTATAGAGGCGCAACAGCATATGTTGCAACTCACAGTCTGGGGCATT
>KC186955
TTCTATTGCAATACATCGCAACTGTTTAATAGTACATACCCTAATGAGACATATAATATTAATGTTAATGATA
CA---

AATTCAACAGAGATTGTACTCCCATGCAGGATAAAACAAATTATAAACATGTGGCAGGAGGTAGGACGAGCAA
TGTATGCTCCTCCCATTGCAGGAAACATAACATGTAGATCAAAT---
ATCACAGGATTACTATTGACGCGAGATGGAGGACCTAATCAGACC----------------------------
----

ACAGAAACATTCAGACCTGCAGGAGGAGATATGAGGGACAATTGGAGAAGTGAATTATACAAATATAAAGTGG
TAGAAATTAAGCCATTG---GGAATAGCACCCACTGGGGCAAAAAGGAGAGTGGTG-----------------
----------------------GAGAGAGGAAAAAGAGCA---GTGGGA---ATAGGAGCT---------
GTGTTCCTTGGGTTCTTGGGAGCA---GCAGGAAGCACTATG---GGCGCGGCGTCA---

CTAACGCTGACGGTACAG---GCCAGACAA---TTGTTG---TCTGGTATAGTGCAACAGCAAAGC---
AATTTG---CTGAGG---GCCATAGAGGCGCAACAGCATCTGTTGCAACTCACAGTCTGGGGCATT
>KC187164
TTCTATTGCAATACATCAGGACTGTTTAAAAATAGGATATACAGTAGTACATACAATGATACAGTAAATAATT
CAAGC--

TCAGAGAACATCACAATCACACTCCCATGCAGAATAAAACAAATTATAAACATGTGGCAGAGGGTAGGACGAG
CAATGTATGCTCCTCCCATTAAAGGAAGCTTAACATGTAACTCAAGT---
ATCACAGGGTTACTATTGACGAGGGATGGAGGCAGTGGAGACAACACA-------------------------
----

AGTACAGAGATCTTCAGACCTGGAGGAGGAGATATGAGGGACAATTGGAGAAGTGAATTATACAAATATAAAG
TGGTAGAAGTTCAACCATTA---GGAATAGCACCCACTAAGGCAAAAAGGAGAGTGGTG--------------
-------------------------GAGAGAGAAAAAAGAGCA---GTGGGA---ATAGGAGCT---------
GTGTTCCTTGGGTTCTTGGGAGCA---GCCGGAAGCACTATG---GGCGCGGTGTCA---
ATAACGCTGACGGTACAG---GCCAGACAA---TTGCTG---TCTGGTATAGTGCAGCAGCAAAGC---
AATTTG---CTGAAG---GCTATAGAGGCGCAACAGCATATGTTGCGACTCACGGTCTGGGGCATT
>KC186730
TTCTACTGCAATACATCAAGACTGTTTAATGGTACATACTATAATGGTATATACAACCATACAGAGGGTAATT
CAACCATA---CACTAT---
AATGGTACATACAACCATACA---------------
GAAGGTAATTCAAACATCACACTCCCATGCAGAATAAAACAATTTATAAACCTGTGGCAGAAGGTGGGAAGAG
CAATGTATGCCCCTCCCATTGCAGGAAGAGTAACATGTAAATCAAAT---
ATCACAGGACTACTATTGTTACGTGATGGAGGTAAAACAAATAACACA-------------------------
----

AATACAGAGATATTCAGGCCTGGAGGAGGAGATATGAGGGACAATTGGAGAAGTGAATTATACAAATATAAGG
TGGTAGAAATTAAACCATTG---GGAGTAGCTCCCACCAAGGCAAAAAGGAGAGTGGTG--------------
-------------------------GAGAGAGAAAAAAGAGCA---GTGGGA---ATAGGAGCT---------
GTGTTCCTTGGGTTCTTAGGAGCA---GCAGGAAGCACTATG---GGCGCAGCGTCA---
ATAACGCTGACGGTACAG---GCCAGACAA---CTGTTG---TCTGGGATAGTGCAGCAGCAAAAC---
AATTTG---CTGAGA---GCTATAGAGGCGCAACAGCATCTGTTGCAACTCACAGTCTGGGGCATT
>KC187620
TTCTATTGCAATACATCAGGACTATTTAATGGTACATATACAAACATGAGCAGCACAGGAAAT----------
----

GCAAATGCAAACATCACACTTCAATGCAAAATAAAACAAATTATAAACATGTGGCAACAGGTGGGAAAAGCAA
TGTATGCTGCTCCCATTGCAGGAAACATAACATGTAAATCAAAT---
ATCACAGGACTTCTATTGACACGTGATGGAGGAGTAGGAAACAATGAC-------------------------
----

ACAAATGGCACAGAGACATTCAGACCTGGAGGAGGAGATATGAGGGACAATTGGAGAAGTGAATTATACAAAT
ATAAAGTGGTAGAAATTCAGCCATTA---GGAATAGCACCCACTAAGGCAAAAAGGAGAGTGGTG--------
-------------------------------GAGAGAGAAAAAAGAGCA---GTAGGA---ATAGGAGCT---
------TTGTTCCTTGGGTTCTTAGGAGCA---GCCGGAAGCACTATG---GGCGCAGCGTCA---
ATAACGCTGACGGTACAG---GCCAGACAA---TTGTTG---TCTGGTATAGTGCAACAGCAAAGC---
AATTTG---CTGAGA---GCTATAGAGGCGCAACAGCATATGTTGCAACTCACAGTCTGGGGCATT
>KY111994
TTCTATTGCAATACATCAAATCTGTTTAATGGTACATACAATGGTACATACATAAGTACAAAC----------
----

AGTACTTCAAACATCACGCTTCAATGCAGAATAAAACAAATTATAAACATGTGGCAGGGGGTAGGAAGAGCAA
TGTATGCTCCTCCCATTGCAGGAAACATAACATGTAAATCAAAT---
ATCACAGGGCTACTATTGACACGTGATGGAGGGACCAAAAATAATAGCAACGAA-------------------
----

ACAGAGGAGACATTCAGGCCTGCAGGAGGAGATATGAGGGATAATTGGAGAAGTGAATTATATAAATATAAAG
TAGTAGAAATTCAGCCATTA---GGAATAGCACCAACTGGTGCAAAAAGGAGAGTGGTG--------------
-------------------------GAGAGAGAAAAAAGAGCA---GCAGGA---CTAGGAGCT---------
TTGTTCCTTGGGTTCTTGGGAGCA---GCAGGAAGCACTATG---GGCGCAGCATCA---

ATAACGCTGACGGTACAG---GCCAGACAA---TTGTTG---TCTGGTATAGTGCAACAGCAAAGC---
AATTTG---CTGAGG---GCTATAGAGGCGCAACAGCATATGTTGCAACTCACGGTCTGGGGCATC
>KC187280
TTCTATTGTAATACATCACAATTGTTTAATAGTACACACAAGAGTACAGCCGATCCTACAAGGAGTGAGTCAA
AT---

GAAAATGAAACCATTATACTCCCATGCAAAATAAAACAAATTATAAACATGTGGCAGGAGGTAGGACGAGCAA
TGTATGCCCCTCCCATTGCAGGAAACATAACATGTAAATCAAAT---
ATCACAGGGCTATTATTGACACGTGATGGAGAGTATAATGGGACA----------------------------
----

AACAAAAATGAAACATTCAGACCTGCAGGAGGAGACATGAGGGACAACTGGAGAAGTGAATTATATAAATATA
AAGTGGTAGAAATTCAACCATTG---GGAGTGGCACCCACTAAGGCAAAAAGGAGAGTGGTG-----------
----------------------------GAGAGAGAAAAAAGAGCA---ATAACA---ATGGGAGCT------
---GTGTTCCTTGGGTTCTTAGGAGCA---GCAGGAAGCACTATG---GGCGCAGCGGCA---
GTAACGCTGACGGCACAG---GCCAGACAA---CTGTTG---TCTGGTATAGTGCAACAGCAAAGC---
AATTTG---CTGAGG---GCTATAGAGGCACAACAGCATATGTTGCAACTCACAGTCTGGGGCATT
>KC187249
TTCTATTGCAATACATCACAACTGTTTAATGGAACATACTATGCAAATGGGACATCCACGTCTAATGATACAG
CAGGTAAT---

TCAAGCGACATCATCATACTCCCATGCAGAATAAAACAAATTATAAATATGTGGCAGAAGGTAGGACGGGCAA
TGTATGCTCCTCCCATTGCAGGAAACATAACATGTCACTCAAAC---
ATCACAGGATTACTATTGACAAGGGATGGTGGAAACAGGAATGGC----------------------------
----

ACAAATGAGACATTCAGACCTGCAGGAGGAGATATGAGGGACAATTGGAGAAGTGAATTATACAAATACAAAG
TGGTAGAAGTTAATCCATTG---GGAGTAGCACCCACTAGGGCAAAAAGGAGAGTGGTG--------------
-------------------------GAGAGAGAAAAAAGAGCA---GTGGGA---ATAGGAGCT---------
ATGTTCCTTGGGTTCTTGGGAGCA---GCAGGAAGCACTATG---GGCGCGGCGTCA---
ATAACGCTGACGGTACAG---GCCAGACAG---TTGTTG---TCTGGTATAGTGCAACAGCAAAGC---
AACCTG---CTGAGG---GCCATAGAGGCGCAACAGCATATGTTGCAACTCACGGTCTGGGGCATT
>MF502153
TTCTATTGCAATACATCACAACTGTTCAATAGTAGCAGTAATTCAAGCTCAACCAGCAAT-------------
----

TCAAACTCAACCATCACCCTCCCATGCAGAATAAAACAAATTATAAACATGTGGCAGGGGGTAGGACGGGCAA
TGTATGCCCCTCCCATTCAAGGAAAAATAACATGTAATTCAAGT---
ATCACAGGATTACTATTGACACGTGATGGGGGACCTCGGAATGGC----------------------------
----

ACAGAGACATTCAGACCTGGAGGAGGAGATATGAGGGACAATTGGAGAAGTGAATTATATAAATATAAAGTGG
TAGAAGTTAAACCATTG---GGAATAGCACCCACTGGAGCAAAAAGGAGAGTGGTG-----------------
----------------------GAGAGAGAAAAAAGAGCA---GTGGGA---ATGGGAGCT---------
ATGTTCCTTGGGTTCTTGGCAGCA---GCAGGAAGCACTATG---GGCGTGGCGTCA---
ATAACGCTGACGGTACAG---GCCAGACAA---TTGCTG---TCTGGTATAGTGCAACAGCAAAGT---
AATTTG---CTGAGA---GCCATAGAGGCGCAACAGCATATGTTGCAACTCACAGTCTGGGGCATT
>MF353063
TTCTATTGCAATACATCAAGCCTGTTTAATAGGACATATATGGCTAATAGTACAGATATGGCTAATAGTACAG
AAACTAAC---

AGTAACGGAACCATCACAATCCACTGCAGAATAAAACAAATTATAAACATGTGGCAAGGGGTGGGACGAGCAA
TGTATGCCCCTCCCATTGCAGGAAACATAACATGTATATCAAAT---
ATCACAGGACTACTATTGACAAGGGATGGAGGAAACACCAGTATCAGTGAG----------------------
----

AAGACAGAGACATTCAGACCTGAAGGAGGAAATATGAAGGACAATTGGAAAAGTGAATTATATAAATATAAAG
TGGTAGAAGTTAAGCCATTA---GGAGTAGCACCCACTAATGCAAGAAGGAGAGTGGTG--------------
-------------------------GAGAGAGAAAAAAGAGCAGTAGTGGGA---ATGGGAGCT---------
GTGTTCCTTGGGTTCTTGGGAGCG---GCAGGAAGCACTATG---GGCGCAGCATCA---

ATAACGCTGACGGTACAG---GCCAGACAA---TTATTG---TCTGGTATAGTGCAACAGCAAAGC---
AATTTG---CTGAAG---GCTATAGAGGCTCAACAGCATATGTTGAGACTCACGGTCTGGGGCATT
>HM638803
TTCTATTGCAATACTGCAAGCCTGTTTAATAGTACATACAATCCTAATCCTAATGGTACAGAAGACAATTCAA
ATTCA--

ACTTCAAATTCAAGCATCACAATCAAATGCAGAATAAAGCAAATTATAAACATGTGGCAGGAGGTAGGAAAAG
CAATGTATGCCCCTCCCGTTGCAGGAAATATAACATGTATATCCAAT---
ATCACAGGACTACTATTGACACGTGATGGAGGAAAGGGAGGTGGCACA-------------------------
----

AGTAATGACACAGAGATATTCAGACCTCAAGGAGGAAATATGAAGGACAATTGGAGAAGTGAATTATATAAAT
ATAAAGTGGTAGAAATTAAACCATTA---GGAGTAGCACCAACTAAGGCAAAAAGGAGAGTGGTG--------
-------------------------------GAGAGAGAAAAAAGAGCA---GTGGGA---CTAGGAGCT---
------GTGCTCTTTGGGTTCTTGGGAGCA---GCAGGAAGCACTATG---GGCGCAGCATCA---
ATAACGCTGACGGTACAG---GCCAGACAA---TTGTTG---TCTGGTATAGTGCAACAGCAAAGC---
AATTTG---CTGAGA---GCTATAGAGGCGCAACAGCATATGTTGCAACTCACGGTCTGGGGCATT
>KC894127
TTCTATTGCAATACATCAAGATTGTTTGATCCTAAAGGTACTAATGGTACA----------------------
----

GATAATTCAACCATCCAACTCCCATGCAGAATAAAACAAATTGTAAACATGTGGCAGGGGGTAGGAAGAGCAA
TGTATGCCCCTCCCATTGCAGGAAACATAACATGTAGATCAAAT---
ATCACAGGACTACTATTGGTACGTGATGGAGGCAGAGACGCAAATACC-------------------------
----

ACAGAGCCAGAGATCTTCAGACCTGGAGGAGGAGATATGAGGGACAATTGGAGAAGTGAATTATATAAATATA
AAGTGGTAGAAATTCAGCCATTG---GGAGTAGCACCCACTAGGGCAAAAAGGAGAGTGGTG-----------
----------------------------GAGAGAGAAAAAAGAGCA---GTGGGA---ATAGGAGCT------
---GTGTTCCTTGGGTTCTTGGGAGCA---GCAGGAAGCACTATG---GGCGCGGCGTCA---
ATGACGCTGACGGTACAG---GCCAGACAA---CTGCTG---TCTGGTATAGTGCAACAGCAAAGC---
AATTTG---CTGAGA---GCTATAGAGGCGCAACAGCATATGTTGCAACTCACAGTCTGGGGCATT
>HM639260
TTCTATTGCAATACATCAGGACTGTTTAATGAGACATACAATCCTAATGGTACAGAAAGTAAT----------
----

TCAAGCGCAGTCATCACACTCCCATGCAGAATAAAACAAATTATAAACATGTGGCAGGAAGTAGGAAAAGCAA
TATATGCCCCTCCCATTCAAGGAAACATAACATGTAAATCAAAT---
ATCACAGGACTACTATTGGTACGTGATGGAGGAAACTCAAATGGCACA-------------------------
----

AATAACACAGAGATATTCAGACCTGGAGGAGGAGATATGAGGGACAATTGGAGAAGTGAATTATATAAATATA
AAGTGGTAGAAATTAAACCATTG---GGAATAGCACCCACTAGTGCAAAAAGGAGAGTGGTG-----------
----------------------------GAGAGAGAAAAAAGAGCA---GTTGGA---ATAGGAGCT------
---GTGTTCCTTGGGTTCTTGGGAGCA---GCAGGAAGCACTATG---GGCGCGGCGTCA---
ATAACGCTGACGGTACAG---GCCAGACAA---CTGTTG---TCTGGTATAGTGCAACAGCAAAGC---
AATTTG---CTGAGA---GCTATAGAGGCGCAACAGCATATGTTGCAACTCACGGTTTGGGGCATT
>KJ541837
TTCTATTGTAATACATCAGGCCTGTTTAATGGTTCATACAATTATAATGCTACAGCA----------------
----
AGTAATTCCAGCTCAGAC---------
AATTCCAGCTCAGTCATCAGAATCCCATGCAGAATAAAGCAAATTATAAACATGTGGCAGGAGGTAGGACGAG
CAATGTATGCCCCTCCCATTGCAGGAAACATAACATGTAAATCAAAT---
ATCACAGGAATACTATTGTATCACAGGACTACTGTTGGAGGA-------------------------------
----
AAGGAAAATGATAAATAC---
TGGAAAGAGACATTCAGACCTACAGGAGGAGATATGAGGGACAATTGGAGAAGTGAATTATATAAATATAAAG
TGGTAGAAATTAAACCATTG---GGGATAGCACCCACTGAAGCAAGAAGGAGAGTGGTG--------------
-------------------------GAAAGAGAAAAAAGAGCA---GTGGGA---GTAGGAGCT---------
ATGTTCCTTGGGTTCTTGGGAGCA---GCAGGAAGCACTATG---GGCGCGGCGTCA---

ATAACGCTGACGGTACAG---GCCAGACAA---TTGTTG---TCTGGTATAGTGCAACAGCAAAGC---
AATTTG---CTGAGG---GCTATAGAGGCGCAACAGCATCTGTTGCAACTCACAGTCTGGGGCATT
>KJ158428
TTCTATTGTAATACATCAGGTCTGTTTAATGTTACATACATGCCTAATGGTACA-------------------
----
GAGAGTAATTCAAATAGT---------
CAGTTAAACAATATCACAAATATCCCATCCATCACAATCCAAAAAAAAAAGATGTGGCAGAAGGTAGGACAAG
CAATGTATGCTCCTCCCATTGCAGGAAACATAACATGTAAATCCAAT---
ATCACAGGAATTATATTGGTACGTGATGGAAGAGAA-------------------------------------
----
GACAAGGTTAATATAGAGACAAAT---------
AAGACTGAGACATTCAGACCTGGAGGAGGAGATATGAGAGACAACTGGAGAAGTGAATTATATAAATATAAAG
TGGTAGAAATTAAGCCATTG---GGGATAGCACCCACCGAAGCAAAAAGGAGAGTGGTG--------------
-------------------------GAGAGAGAAAAAAGAGCA---GTGGGA---ATAGGAGCT---------
GTGTTCCTTGGGTTCTTGGGAGCA---GCAGGAAGCACTATG---GGCGCAGCGTCA---
CTAACGCTGACGGTACAG---GCCAGACAA---TTGTTG---TCTGGTATAGTGCAACAGCAAAGC---
AATTTG---CTGAGA---GCTATAGAGGCGCAACAGCATATGTTGCAACTCACGGTCTGGGGCATT
>KJ541839
TTCTATTGTAATACATCAAAACTGTTTAATGGTACATACATTCCTAATGGTACA-------------------
----
GGAAGTTACTTAAGCAGT---------
AATTCAAGCTCAAACATCACAATTCCATGCAGAATAAAGCAAATTATAAACATGTGGCAGGAGGTAGGACGAG
CAATATATGCCCCTCCCATTGAAGGAAACATAACATGTAGATCAAAT---
ATCACAGGAATAATATTGACACGTGATGGAGGAAAAATCCAAAATCAA-------------------------
----
AATGAGACAGACAAT---ACAACAGAAGACTATTCTAGCGTGGAAGGA---
ATGAAGGACAATTGGAGAAGTGAATTATATAAATATAAAGTGGTAGAAATTCAGCCATTG---
GGAATAGCACCCACTGGAGCAAAAAGGAGAGTGGTG-------------------------------------
--GAGAGAGAAAAAAGGGAT---GTGACA---CTAGGAGCT---------
TTGTTCCTTGGGTTCTTGGGAGCG---GCAGGAAGCACTATG---GGCGCGGCGTCA---
ATAACGCTGACGGTACAG---GCCAGACAA---TTGTTG---TCTGGTATAGTGCGACAGCAAAGC---
AATTTG---CTGAGA---GCTATAGAGGCGCAACAGCATCTGTTGCAACTCACAGTCTGGGGCATT
>KJ541841
TTCTATTGTAATACATCAAAACTGTTTAATGGTACATACATGCTTAATGGTACA-------------------
----
GGAAGTTATTTAATCAGT---------
AATTCAAGCTCAAACATCACAATTCCATGCAGAATAAAGCAAATTATAAACATGTGGCAGGAGGTAGGACGAG
CAATATATGCCCCTCCCATTGAAGGAAACATAACATGTAGATCAAAT---
ATCACAGGACTACTATTGACACGTGATGGAGGACAA-------------------------------------
----
TCCAAAATCAAATTAGACAGTCAA---------
TCCACAGAGATATTTAGACCTGGAGGAGGAGATATGAAGGACAATTGGAGAAGTGAATTATATAAATATAAAG
TGGTAGAAATTCAGCCATTG---GGAATAGCACCCACTGGAGCAAAAAGGAGAGTGGTG--------------
-------------------------GAGAGAGAAAAAAGGGAT---GTGACA---CTAGGAGCT---------
TTGTTCCTTGGGTTCTTGGGAGCG---GCAGGAAGCACTATG---GGCGCGGCGTCA---
ATAACGCTGACGGTACAG---GCCAGACAA---TTGTTG---TCTGGTATAGTGCAACAGCAAAGC---
AATTTG---CTGAGA---GCTATAGAGGCGCAACAGCATCTGTTGCAACTCACAGTCTGGGGCATT
>KJ541844
TTCTATTGTAATACATCAGACCTGTTTAAGGCGGCAGGTCCGTGGCCGTCTAAC-------------------
----
ACACCGGATCAGGATACA---------
AGTACAAATGAAAACATCACACTCCAATGCAGAATAAAGCAAATTATAAACATGTGGCAGGAGGTAGGACGAG
CAATGTATGCTCCTCCCATTGCAGGGAACATAATATGTAGATCAAAT---
ATCACAGGACTTCTATTAGTACGTGATGGAGGAGAA-------------------------------------
----
GACAAACATAATACAGGGACAAAT---------
GGGACAGAGACATTCAGACCTGAAGGAGGAGATATGAGGGACAATTGGAGAAGCGAATTATATAAATATAAAG
TGGTAGAACTTAAACCATTA---GGAGTAGCACCCACTGGATCCAAAAGGAGAGTGGTG--------------
-------------------------GAGAGAGAAAAAAGAGCA---GTGGGA---CTAGGAGCT---------
GTGTTCCTTGGGTTCTTGGGAGCA---GCAGGAAGCACTATG---GGCGCGGCGTCA---

ATGACGCTGACGGTACAG---GCCAGACAA---TTGTTG---TCTGGTATAGTGCAACAGCAAAGC---
AATTTG---CTGAGG---GCTATAGAGGCGCAACAGCATCTGTTGCAGCTCACGGTCTGGGGCATT
>KJ541848
TTCTATTGCAACACATCAAAACTGTTTAATAGTACATACATGCCTAAACATGCA-------------------
----
AAGTGCCATACAATCACA---------
AAGGCACACGCAATCATCACAATTCCATGCAGAATAAAACAAATTATAAATATGTGGCAGGAGGTAGGACGAG
CAATGTATGCTCCTCCCATTAAAGGAAAAATAACATGTAATTCAAGT---
ATCACAGGACTGCTATTGACACGTGACGGAAGGGAA-------------------------------------
----
AACAAAAAGAATTCAAAGGGGGGT---------
GATACAGAAACTTTCAGACCTGAAGGAGGAAATATGAAAGACAATTGGAGAAGTGAATTATATAAATATAAGG
TGGTAGAAATTAAGCCATTG---GGAATAGCACCCACTGGAGCAAAAAGGAGAGTGGTG--------------
-------------------------GAGAGAGAAAAAAGAGCA---GTGGGA---CTGGGAGCT---------
CTGTTCCTTGGGTTCTTGGGAGCA---GCAGGAAGCACTATG---GGCGCAGCGTCA---
ATGACGCTGACGGTACAG---GCCAGACAA---TTGTTG---TCTGGTATAGTGCAGCAGCAAAAC---
AATTTG---CTGAGA---GCTATAGAGGCGCAACATCAGTTGTTGCAACTCACGGTCTGGGGCATT
>MF373132
TTCTATTGCAATACATCAAACCTGTTTAATAGTAGTAAAACTAATATAACAGCAAAT----------------
----

GAAAATGGAACCATCACACTCCCATGCAGAATAAAACCAATTGAAACCTTGGGGCAGGGGGTAGGAAAAGCAA
TTATTGCCCCCTCCATTAAAGGAAAATTACCTGGTACTTCCAAA---
TTCCCAGGCTTACCACTGGCCCCGAGTAGGGGTGAAAAAATAGGAACCTAT---------------
GGAAATTTGGCA---AAAAGAGAA---

TTTTCCCCGGGGGGGGGAGATATGAGTGACAATTGGAGAAGTGAATTATATAAATATAAAGTGGTAGAAATTA
ATCCATTA---GGAGTAGCACCCACTAAGGCAAAAAGGAGAGTGGTG--------------------------
-------------GAGAGAGAAAAAAGAGCA---GTGGGA---ATAGGAGCT---------
GTGTTCCTTGGGTTCTTGAGTGCA---GCAGGAAGCACTATG---GGCGCAGCGTCA---
ATAACGCTGACGGTACAA---GCCAGACAA---TTGTTG---TCTGGTATAGTGCAACAGCAAAAC---
AATTTG---CTGAGA---GCTATAGAGGCGCAACAACATATGTTGAAACTCACAGTCTGGGGCATT
>MF373137
TTCTATTGTAATACATCAGGCTTGTTTAATACATCAGGCCCATTTAATAATAGTGAAACAAATAACACA----
----

GCAAATGTAAACATCACACTCCCATGCAGAATAAAACAAATTATAAACATGTGGCAGGGGGTAGGACAAGCAA
TGTATGCCCCTCCTGTTGCAGGAAACATAACATGTAGATCAAAT---
ATCACAGGAATACTACTGACACGTGATGGAGGAATAAATACCACAACTAAT----------------------
----

AACACATACGAACTATTCAGGCCTACAAGAAGAGACAATAGGGACAATTGGAGAAGTGAATTATATAAATATA
AAGTAGTAGAAATTCAGCCATTA---GGAGTAGCACCCACTGAGGCAAGAAGGAGAGTGGTG-----------
----------------------------GAGAGAGAAAAAAGAGCA---GTGGGA---ATAGGAGCT------
---GTGTTCCTTGGGTTCTTGGGAGCA---GCAGGAAGCACTATG---GGCGCAGCATCC---
ATAACGCTGACGGTACAG---GCCAGACAA---TTATTG---TCTGGTATAGTGCAACAGCAGAAC---
AATCTG---CTGCGG---GCTATAGAGGCGCAACAGCATATGTTGCAACTCACAGTCTGGGGCATT
>MF373138
TTCTATTGCAGTACATCATCCCTGCTTAATTTTACTTGGCGAAGTTTATTTTGCCATGCCCAAGCCCCGTTCC
AGGAAACTCAT---AAT--

AATAGTACAAATAACATCCCCTTCCCATGCAGAATAAAACAATTTATAAACATGTGGCAAACGGTAGGGCGAG
CAATGTATGCTCCTCCCATTGCAGGAGTAATAACATGTAATTCAAAC---
ATCACAGGACTACTACTGACACGAGATGGAGGAAATCCAACAAATAGCACTGAA--------------------
----

GGAAATAATGAAACTGAGATATTCAGACCTGGAGGAGGAGATATGAGAGACAATTGGAGAAGTGAATTATATA
AATACAAAGTGGTAGAAATTAAGCCATTA---GGAGTAGTACCCACTAAGGCAAAAAGGAGAGTGGTG-----
----------------------------------GAGAGAGAAAAAAGAGCA---GCA------
CTAGGAGCT---------ATGTTCCTTGGGTTCTTGGGAGCA---GCCGGAAGCACTATG---

GGCGCAGCGTCA---ATAGCGCTGACGGTACAA---GCCAGACAA---TTGTTG---
TCTGGTATAGTGCAGCAGCAAAAC---AATTTG---CTGAGG---
GCTATAGAGGCGCAACAGCATATGTTGCAACTCACGGTCTGGGGCATT
>MF373139
TTCTATTGCAATACATCGGGCCTGTTTAGTATAAATTATACAGAAAATAATACAGATAATATTCAGAGTATGG
AC---

ATCACACTCCCCTGCCCGATCAGACAAATCATAATCATGTGGCAGGGGCTAGAGGTAGCAATATCTACCCCTC
CCCCTAACGGTACAGTATCATGTACTTCTAAT---
TTCACTGGCGTAGTCCTGCTACGTGCTGGAGGGCGTCATCGGGAAATGAA-----------------------
----

AGTGGGACAGAGACATTCAGACCTGCAGGAGGAGATATGAAGGACAATTGGAGAAGTGAATTATATAAATATA
AGGTAGTAGAAATTAAGCCATTA---GGAGTAGCACCCACTAAGGCAAAAAGGAGAGTGGTG-----------
----------------------------GAGAGAGAAAAAAGAGCA---GTGGGA---CTAGGAGCT------
---GTGTTCCTTGGGTTCTTGGGAGCG---GCAGGAAGCACTATG---GGCGCAGCATCA---
ATAACGCTGACGGTACAG---GCCAGACAA---TTATTG---TCTGGTATAGTGCAACAGCAAAAC---
AATTTG---CTGAGG---GCTATAGAGGCGCAACAGCATATGTTGCAACTCACAGTCTGGGGCATT
>KP411839
TTCTACTGCAATACATCAAAGCTGTTTAATAGTACATACCCGCCTAACAGTACTGATACGTACATGAACAGTG
CT---

AATACTTCAAACATCATACTTCAATGCAGAATAAAACAAATCATAAACATGTGGCAGGGGGTAGGACAAGCAA
TGTATGCCCCTCCTATTGCAGGGAACATAACATGTGAATCAAAT---
ATCACAGGACTGCTGTTAACACGTGATGGAGGAAACAATCAAACA----------------------------
----

GAGGAGGAGACATTTACACCTGGAGGAGGAGATATGAGAGACAATTGGAGAAGTGAATTATATAAATACAAAG
TGGTAGAAATTAAGCCATTA---GGAATAGCACCCACCAAGGCAAAAAGGAGAGTGGTG--------------
-------------------------GAGAGAGAAAAAAGAGCA---GTGGGA---ATAGGAGCT---------
GTGTTCCTTGGGGTCTTGGGAGCA---GCAGGAAGCACTATG---GGCGCAGCATCA---
ATAACGCTGACGGTACAG---GCCAGACAA---TTGTTG---TCTGGTATAGTGCAGCAGCAAAGC---
AATCTG---CTGAGG---GCTATAGAGGCGCAACAGCATATGTTGCAACTCACAGTCTGGGGCATT
>MF373148
TTCTATTGCAATACATCAGAACTGTTTAACGGTACATACAGCAACGGTACATACACGCCTAATAATAATGGGA
GTGAC--

TCAAATTTGACCATCACCATCCCGTGCAGAATAAAACAAGTTGTAAATCTGTGGCAGGGAGTAGGACAAGCAA
TGGATGCCCCTCCCATTGCAGGAGCATATCGTGGGGATCAATAT---CCGAGGACTACTATTGCCCGT---
GGAGGAGGACCGAACCCCAGT--

ACCGCGGAGATATTCAGACCTGGAGGAGGAGATATGAGGGACAATTGGAGAAGTGAATTATATAAATATAAAG
TGGTGGAAGTTAACCCATTA---GGAATAGCACCCACTAGAGCAAAAAGGAGAGTGGTG--------------
-------------------------GAGAGAGAAAAAAGAGCA---GTGGGA---ATAGGAGCT---------
GTGTTCCTTGGGTTCTTAGGAGCA---GCAGGAAGCACTATG---GGCGCAGCATCA---
GTAACGCTGACGGTACAA---GCCAGGCAA---TTGTTG---TCTGGTATAGTGCAACAGCAAAGC---
AATTTG---CTGAGG---GCTATAGAGGCGCAACAGCATATGTTGCAACTCACAGTCTGGGGCATT
>KP411836
TTCTATTGCAATACATCAAACTTGTTTAATAGTACATACAATAGTACT-------------------------
----

AATGATACCATCACACTCCAATGTAAGATAAAACAAATCATAAACATGTGGCAGGGGGTAGGACGAGCAGCAG
TATTCTCCTCCCCATCGCAGGCAAGACATCACATCTTATACTAC---
TATTACAGCAGTACTGCTGACGACCTGATGGAGGACGATGAAACTAATAAT----------------------
----

ACTGTAGAGACATTCAGACCTGGAGGAGGGGATATGAGAGACAATTGGAGAAGTGAATTGTATAAATATAAAG
TGGTAGAAGTTAAAACATTA---GGAATAGCTCCATCTCATTCAAAAAGGAGAGTGGTG--------------
-------------------------CAGAGAGAAAAAAGAGCA---GTGGGC---CTGGGAGCT---------
ATGTTCCTTGGGTTCTTGGGAGCC---GCAGGAAGCACTATG---GGCGTGGCGTCA---

TTGACGCTGACGGTACAG---GCCAGACAA---TTATTG---TCTGGTATAGTGCAGCAGCAAGGC---
AACTTG---CTGAGG---GCTATAGAGGCGCAACAGCATCTGTTGCAACTCACAGTCTGGGGCATT
>MF373152
TTCTATTGCACTACATCACAACTGTTTACTAGTACATACATGGCTACATACATGTCTATAGCACTAATAGTAC
TTCAATTGAACCATGCACACTCGATGCAGGAAAAGACACAA-------------------------------
----
ATTAACATGTGGCAGGGGGTAGGCCAAGCAATGTATGCCCCTCCCATTAAAGGAAGTATAACATGTAGATCAA
AT---ATCACGGAATACTACTGACACGAGATGGAGGAACCAACTCCACA------------------------
----

AATGAAACATTCAGACCTGGAGGAGGAGATATGAGGGACAATTGGAGAAGTGAATTATATAAATATAAAGTGG
TAGAAATTAAGCCATTG---GGAATAGCACCCACTAAGGCAAAAAGGAGAGTGGTG-----------------
----------------------GAGAGAGAAAAAAGAGCG---GCA------TTAGGAGCT---------
ATGTTCCTTGGGTTCTTGGGAGCA---GCTGGAAGCACTATG---GGCGCAGCATCA---
ATTACGCTGACGGTACAG---GCCAGACAA---TTGTTG---TCTGGTATAGTGCAACAGCAAAGC---
AATTTG---CTGAGG---GCTATAGAGGCGCAACAGCATATGTTGCAACTCACGGTCTGGGGCATT
>MF373159
TTCTATTGCAATACATCAGGGCTGTTTCGAATTAATATTTCTGATGCACATAATGATGCACATAAT-------
----

GACACAAATGTAAACATCACAATTCCATGCAGAATAAAGCAAATTATAAATATGTGGCAGGGGGTAGGACGAG
CAATGTATGCTCCTCCCATTAAAGGAATAATACAATGTAGATCAAAT---
ATCACAGGACTGCTACTGACACGTGATGGTGGTAATGGTGGTAAT----------------------------
----

GAAACAAATTCAGAGACGTTTAGACCTTTAGGAGGAGATATGAAGGACAATTGGAGAAGTGAATTATATAAAT
ACAAAGTAGTAGAAATTCAACCAGTA---GGAATAGCACCCACTAGAGCAAGAAGGAGAGTGGTG--------
-------------------------------GAGAGAGAAAAAAGAGCA---GCA------CTAGGAGCT---
------ATGTTCCTTGGGTTCTTAGGAGCA---GCCGGAAGCACTATG---GGCGCAGCATCA---
ATAACGCTGACGGTACAG---GCCAGACAA---TTATTG---TCTGGTATAGTGCAACAGCAAAAC---
AACTTG---CTGAGA---GCTATAGAGGCGCAACAGCATATGTTGCAACTCACGGTCTGGGGCATT
>MF373166
TTCTATTGCAATACATCAAAACTGTTTAATAGTACATTTAATAATAATACATTTATATTTAATGGTACAGGGA
GGAAC---TAT---
ACAGGAAATGGGACTATAACC---------------
ACAGGAAATGAGACTATAACCATCCCATGCAGAATAAAACCAATTATAAACATGTGGCAGGGGGTAGGACAAG
CAATGTATGCCGCCCCCATTAAAGGAGTTATACAGTGTACTTCAAAT---
ATCACAGGAATATTACTGACACGTGATGGAGGAGGAAATGGAACA----------------------------
----

GAGGAGACATTCAGGCCTGGAGGAGGAAATATGAAGGACAATTGGAGAAGTGAATTATATAAATATAAAGTGG
TAGAAATTAAACCATTA---GGAGTAGCACCCACTAAGGCAAAAAGGAGAGTGGTG-----------------
----------------------GAGAGAGAAAAAAGAGCA---GTGGGA---ATAGGAGCT---------
ATGTTCCTTGGTTTCTTGGGAGCA---GCTGGAAGCACTATG---GGCGCAGCTTCA---
ATAACGCTGACGGTACAA---GCCAGACAA---TTATTG---TCTGGTATAGTGCAACAGCAAAGC---
AATTTG---CTAAGG---GCTATAGAGGCGCAACAGCATATGTTGCAACTCACAGTCTGGGGCATT
>MF373169
TTCTACTGCAATACATCAGGCCTGTTTAATAGTACATACAATAATGCATACAATAGTACTAATAGTACT----
----

ACAAATGACACCATCACACTTCAATGCAGAATAAAACAAATTATAAATATGTGGCAGGGGGTAGGAAAAGCAA
TATATGCTCCTCCCATTGCAGGAAACATAACATGTGAATCAAAT---
ATCACAGGACTACTATTGACACGTGATGGAGGAAATGATAAAACAGGG-------------------------
----

AACACCACAGAGACATTCAGACCTGGAGGAGGAAATATGAGGGACAATTGGAGAAGTGAATTATATAAATATA
AAGTGGTAGAAATTAAACCATTA---GGAATAGCACCCACCAAGGCAAAAAGGAGAGTGGTG-----------
----------------------------GAGAGAGAAAAAAGAGCA---GTGGGA---ATAGGAGCT------
---GTACTCCTTGGGTTCTTGGGAGCA---GCAGGAAGCACGATG---GGCGCAGCGTCA---

ATAACGCTGACGGTACAG---GCCAGGCAA---TTGTTG---TCTGGTATAGTGCAACAGCAAAGC---
AATTTG---CTGAGG---GCTATAGAGGCGCAACAGCATATGTTGCAACTCACGGTCTGGGGCATT
>MF373175
TTTTATTGCAATACATCCAACCTGTTTAATAATAGTATGATA-------------------------------
----

AATCCAAATGTTACCATCACACTCCCATGCAGAATAAAACAGATTATAAACATGTGGCAGCAGGTAGGAAAGG
CAATGTATGCCCCTCCCATTGCAGGAAGAATAACATGTAACTCAAAT---
ATCACAGGACTGCTACTGACACGTGATGGGGGAACAAATCAGACA----------------------------
----

AACAGCACAGAGATATTCAGACCTGGAGGAGGAGACATGAGAGACAATTGGAGAAGTGAATTATATAAATATA
AGGTGGTAGAAATTCAGCCATTA---GGAGTAGCACCCACTAGGGCAAAAAGGAGAGTAGTG-----------
----------------------------GGCAGAGAGAAAAGAGCA---GTGGGA---CTAGGAGCT------
---ATGTTCCTTGGGTTCTTGAGTGCA---GCAGGAAGCACTATG---GGCGCAGCATCA---
CTAACGCTGACGGTACAG---GCCAGACAA---TTGTTG---TCTGGTATAGTGCAACAGCAAAGA---
AATTTG---CTGAGG---GCTATAGAGGTGCATCAGCATATGTTGCAACTCACGGTCTGGGGCATT
>MF373179
GTCTATTGCGATACATCAGACCTGCTCACTACATCAGTTAGTGTTCAAAAAGTACTAGTTATCACT-------
----

AGTACAGATGCCATCATACTCCGCATGCCAAATACAACACATTATAAGCATGTGGCAGTGAGTACGACGAGCA
TTGTATGCCCCCCTCCTATGAAAGGCATGCGTAGCAGTACATGCACT---
ATCACAGGTACTACTGACCTCAGGGATGGAAGGACTGACTATCAC----------------------------
----

AGCATGTACATAGTCAGACCTGGAGGAGGAGATATGAGGGACAATTGGAGAAGTGAATTATATAAATATAAAG
TGGTAGAAGTTAAGCCAATA---GGAATAGCACCCACTGGGGCAAAAAGGAGAGTGGTG--------------
-------------------------GAGAGAGAAAAAAAGAGCG---GCA------CTAGGAGCT--------
GTGTTCCTTGGGTTCTTGGGAGCA---GCCGGAAGCACTATG---GGCGCAGCATCA---
GTAACGCTGACGGTACAG---GCCAGACAA---TTATTG---TCGGGTATAGTGCAACAGCAGAAC---
AATTTG---CTGAGG---GCTATAGAGGCGCAACAGCATATGTTGCAACTCACGGTCTGGGGCATT
>MF373192
TTCTATTGCAATACAACAGCCTTGTTTCATAATAATATT----------------------------------
----

ACAAATTCAACCATCATGCTCCCATGCAGAATAAAACAATTTATAAACCTGTGGCAGGGGGTAGGACGAGCAA
TGTATGCTCCTCCCATTAAAGGACTAATAACATGTAATTCAAGT---
ATCACAGGAATACTACTGACACGAGATGGAGGAGATGTCAATCAAACA-------------------------
----

GCAAATGAAACAGAGACATTCAGACCTGGAGGAGGAGACATGAGGGACAATTGGAGAAGTGAATTATATAAAT
ACAAAGTGGTAGAAATTAAACCATTA---GGAATAGCACCTACCCCGGCAAAAAGGAGAGTGGTG--------
-------------------------------GAGAGAGAAAAAAGAGCA---GCA------CTAGGAGTT---
------ATGTTCCTTGGGTTCTTGGGAGCA---GCTGGAAGCACTATG---GGCGCAGCATCA---
TTAACGCTGACGGTACAG---GCCAGACAA---TTATTG---TCAGGTATAGTGCAACAGCAAAAC---
AATTTG---CTGAGA---GCTATAGAGGCGCAACAGCATATGTTGCAACTCACAGTCTGGGGCATT
>KP411835
TTCTATTGCGATACATCAAACCTGTTTAATGAGACAAAATTTAATAGTAGT----------------------
----

ACAAATGAAAACATCACACTCCGATGCAGAATAAAACAAATTATAAACATGTGGCAGAAGGTAGGACGGGCAA
TGTATGCCCCTCCCATTCAAGGAACAATAAAATGTGACTCAAAT---
ATCACAGGATTACTACTAACACGAGATGGAGGCAAAAATGAAAATGAA-------------------------
----

ACACAAGAGATATTCAGACCTGCAGGAGGAGATATGAGGGACAATTGGAGAAGTGAATTATATAAATATAAAG
TGGTAGAAATTAAGCCATTA---GGAATAGCACCCACTGCTGCAAAAAGGAGAGTGGTG--------------
--------------------------GGGAGAGAGAAAAGAGCA---GCA------CTAGGAGCT--------
ATGTTCCTTGGGTTCTTGGGAGCA---GCAGGAAGCACTATG---GGCGCAGCATCA---

ATAACGCTGACGGTACAG---GCCAGACAA---TTATTG---TCTGGTATAGTGCAACAGCAAAGC---
AATTTG---CTGAGA---GCTATAGAGGCGCAACAACATATGTTGCAACTCACAGTCTGGGGCATT
>MF373198
TTCTATTGCAATACATCAGGCCTGTTTAATGAGACAAATGGTACATATGCTACA-------------------
----

AATGGTACAACCATCACACTTCCATGCAGAATAAAGCAAATTATAAGGATGTGGCAGGGGGTAGGACAAGCAA
TGTATGCCCCTCCCATTGCAGGAGTAATATCATGTAATTCAAAT---
ATCACAGGACTGCTACTAACCCGTGATGGAGGTAGTGGTCACAAT----------------------------
----

AATAACACAGAGATATTCAGACCTGAAGGAGGAGATATGAGGGATAATTGGAGAAGTGAATTATATAAATACA
AAGTGGTAGAAATTAAGCCATTA---GGGATAGCACCCACTAAGGCAAGAAGGATGGTGGTG-----------
----------------------------CAGAGAGAAAAAAGAGCA---GCA------CTGGGAGCT------
---GTATTCCTTGGGTTCTTGGGAGCA---GCTGGAAGCACTATG---GGCGCAGCATCA---
ATAACGCTGACGGTACAG---GCCAGACAA---TTATTG---TCTGGCATAGTGCAACAGCAAAGC---
AATTTG---CTGAAG---GCTATAGAGGCGCATCAGCATATGCTGCAACTCACAGTTTGGGGCATT
>HM215318
TTTTATTGCAATACATCCAACCTGTTTAATAGTACATACATAAACAGCACA----------------------
----

AACATCACATACATCACAATCCCATGCAGAATAAAACAAATAATAAACATGTGGCAGAGGGTAGGACAAGCAA
TGTATGCCCCTCCCATTGCAGGAAACATAACATGTAACTCAAGT---
ATCACAGGACTAATATTGACACGGGATGGAGGAAAAAACAACACA----------------------------
----

AATGAAACATTCAGACCTGGAGGAGGAGATATGAGGGACAATTGGAGAAGTGAATTATATAAATATAAGGTGG
TAGAAATTCAACCATTG---GGAATAGCACCCACCAAGGCAAAAAGGAGAGTGGTG-----------------
----------------------GAGAGAGAAAAAAGAGCA---GTTGGA---CTAGGAGCT---------
TTGTTCCTTGGGTTCTTGGGAGCA---GCAGGAAGCACTATG---GGCGCGGCGGCA---
ATAACGCTGACGGCACAG---GCCAGACAA---GTGTTG---TCTGGCATAGTGCAACAGCAGAGC---
AATTTG---CTGAGA---GCTATAGAGGCGCAACAGCATATGTTGCAACTCACGGTCTGGGGCATT
>KX907415
TTCTATTGCAATACATCAGGCTTGTTTAATGAAACAGGCTTGTTTAATGAAACACACAATAGTACC-------
----

ATGAATGATAACATCACACTCCCATGCAGAATAAAACAAATTATAAACATGTGGCAGGAGGTAGGACGAGCAA
TCTATGCCCCTCCCATTGCAGGAAACATAGCCTGTAACTCAAAT---
ATCACAGGACTACTATTGACACGTGATGGAGGCAGCATTAATGGAAGCGAG----------------------
----

GATGAGAATAACACAGAAATATTCAGACCTGGAGGAGGAAACATGAAGGACAATTGGAGAAGTGAATTATATA
AATATAAAGTGGTAGAAATTAAGCCATTG---GGAATAGCACCCACTGCGGCAAAAAGGAGAGTGGTG-----
----------------------------------GAGAGAGAAAAGAGAGCA---GTGGGA---
CTAGGAGCT---------GTGTTCCTTGGGTTCTTGGGAGCA---GCTGGAAGCACTATG---
GGCGCGGCGTCA---ATAACGCTGACGGCACAA---GCCAGACAA---GTGTTG---
TCTGGTATAGTGCAACAGCAAAGC---AATTTG---CTGAGG---
GCTATAGAGGCGCAACAGCATATGTTGCAACTCACGGTTTGGGGCATC
>KX907398
TTCTATTGCAATACATCAAAGCTGTTTGATAGTAGTCTGTTTAATAGTACTGAAAGTAATAGTACTGGTAAT-
----

AATTATACTGACATCAAACTCCCATGCAGAATAAAACAAATTATAAACATGTGGCAGGGGGTAGGACGAGCAA
TATATGCCCCTCCCATTGCAGGAAACATAACATGTAAATCAAAT---
ATCACAGGAATATTATTAACACGTGATGGAGGCTATGACAACGCTACA-------------------------
----

AATGGCACGGAGACCTTCAGACCTGGAGGAGGAGATATGAGGGACAATTGGAGAAGTGAATTATATAAATATA
AAGTGGTAGAAATTAAGCCATTG---GGAATAGCACCCACTAAGGCAAAAAGGAGAGTGGTG-----------
----------------------------GAGAGAGAAAAAAGAGCA---GCGGGA---ATAGGAGCT------

---GTGTTCCTTGGGTTCTTGGGAGCT---GCAGGAAGCACTATG---GGCGCGGCGTCA---
ATAACGCTGACGGTACAA---GCCAGACAA---TTGTTG---TCTGGTATAGTGCAACAGCAAAGT---
AATTTG---CTGAAG---GCTATAGAGGCGCAACAACATCTGTTGCAACTCACGGTTTGGGGCATT
>KX907425
TTCTATTGCAATACAGTAGGCCTGTTTAATAAAACATACAATAGTACC-------------------------
----

ATGAATGGTACCATCACACTCCCATGCAGAATAAAACAAATTATAAACATGTGGCAGGAGGTAGGACGAGCAA
TCTATGCCCCTCCCATTGAAGGAAACATAACATGTAACTCAAGT---
ATCACAGGACTACTATTGACACGTGATGGAGGCAACACTAATGAA----------------------------
----

AGTGAAGAGATATTCAGACCTGGAGGAGGAAACATGAGGGACAATTGGAGAAGTGAATTATATAAATATAAAG
TGGTAGAAATTAAGCCATTG---GGAGTAGCACCCACTGAGGCAAAAAGGAGAGTGGTG--------------
-------------------------GAGAGAGAAAAGAGAGCA---GTAGGA---CTAGGAGCT---------
GTGTTCCTTGGGTTCTTGGGAGCA---GCTGGAAGCACTATG---GGCGCGGCGTCA---
ATAACGCTGACGGCACAA---GCCAGACAA---TTGTTG---TCTGGTATAGTGCAACAGCAAAGC---
AATTTG---CTGAGG---GCTATAGAGGCGCAACAACATATGTTGCAACTCACGGTTTGGGGCATT
>KX907428
TTCTATTGCAACACATCAGGCCTGTTTAATAAAACATACAATGATACC-------------------------
----

ATGAATAGTACCATCACACTCCCATGTAGGATAAAACAAATTGTAAACATGTGGCAGGAGGTAGGACGAGCAA
TCTATGCCCCTCCTATTGCAGGAAACATAACATGTAACTCAAAT---
ATCACAGGACTACTACTGACACGTGATGGAGGCACGAATGTGACA----------------------------
----

AATGGCACACAGATTTTCAGACCTGGGGGAGGAGACATGAGGGACAATTGGAGAAGTGAATTATATAAATATA
AAGTGGTAGAAATTAAACCATTG---GGAATAGCACCCACTACGGCACAAAGGAGAGTGGTG-----------
----------------------------GCGAGAGAAAAAAGAGCA---GTGGGA---CTAGGAGCT------
---GTGTTCCTTGGGTTCTTGGGAGCA---GCTGGAAGCACTATG---GGCGCGGCGTCA---
ATAACGCTGACGGCACAA---GCCAGACAA---TTGCTG---TCTGGTATAGTGCAACAGCAAAGC---
AATTTG---CTGAGA---GCTATAGAGGCGCAACAACATATGTTGCAACTCACGGTTTGGGGCATC
>HQ615976
TTCTATTGCAATACATCAAAAATATTTAATAGTATATACCAGGAGAATTACACTAACAGTGCTAATAGTACAG
AA---

AACAACGGCACACTCACACTCCCATGCAGAATAAAACAAATTGTAAACATGTGGCAGGGGGTAGGACGGGCAG
TGTATGCCCCTCCCATTCAAGGGAACATAACATGTAAATCAAAT---
ATCACAGGACTGCTACTGACACGTGATGGAGGAATAAATGAGACA----------------------------
----

GATGAAACATTCAGGCCTATAGGGGGAAATATGAGGGACAATTGGAGAAGTGAATTATATAAATATAAGGTGG
TAGAAATTAAACCATTA---GGAATAGCACCCACTAAGGCAAAAAGGAGAGTGGTG-----------------
----------------------GAGAGAGAAAAAAGAGCG---GTAGGA---ATAGGAGCT---------
GTGCTCTTTGGGTTCTTGGGAGCA---GCAGGAAGCACTATG---GGCGCAGCGTCA---
ATAACGCTGACGGTACAA---GCCAGACAA---TTATTG---TCTGGTATAGTGCAACAGCAAAAC---
AATTTG---CTGAGG---GCTATAGAGGCGCAACAGCATATGTTGCAACTCACGGTCTGGGGCATT
>KF526226
TTCTATTGCCATACATCAACCCTGTTTAATAATAATATTACAAATTCA-------------------------
----

ACCAATTCAACCATCACACTCCCATGCAGGATAAAACAAATTATAAACATGTGGCAGGAGGTAGGACGAGCAA
TGTATGCCCCGCCCATTCAAGGAATAATAAGATGTAGATCAAAT---
ATCACAGGACTACTACTAACACGTGATGGAGGCACAGGGACAAATGAC-------------------------
----

ACAAACACAACCGAGACATTTAGACCTGGAGGAGGAGATATGAGGGACAATTGGAGAAGTGAATTATATAAAT
ATAAAGTGGTAGAAATTAAACCATTA---GGAATAGCACCCACTAAGGCAAAAAGGAGAGTGGTG--------
-------------------------------GAGAGAGAAAAAAGAGCA---GCA------CTAGGAGCT---

------TTGTTCCTTGGGTTCTTGGGAGCA---GCAGGAAGCACTATG---GGCGCAGCATCA---
ATAACGCTGACGGTACAG---GCCAGACAA---TTATTG---TCGGGTATAGTGCAACAGCAAAGC---
AATTTG---CTGAGA---GCTATAGAAGCGCAACAGCATATGTTGCAACTCACGGTCTGGGGCATT
>AY838567
TTTTATTGCAATACATCAAGCCTGTTTAATAGTACACACAATACATCAAGCCTGTTTAATGGTGCATACAATA
ATGAA--

GGAACAAATCAAACCATCACACTCCAATGCAGAATAAAACAAATTATAAACATGTGGCAGGAGGTGGGACGAG
CAATGTATGCCCCTCCCATTGAAGGAAACATACCATGTAAATCAAAT---
ATCACAGGACTACTATTGACACGTGATGGAGGAACAGAAGAAGAA----------------------------
----

AATGATAAAGAGACATTCAGACCTGGAGGAGGAGATATGAGGGACAATTGGAGAAGTGAATTATATAAATATA
AAGTGGTAGAAATTAAGCCATTA---GGAATAGCACCCACTACTGCAAAAAGGAGAGTGGTG-----------
----------------------------CAGAGAGAGAAAAGAGCA---GTGGGC---ATAGGAGCT------
---GTGTTCCTTGGGTTCTTGGGAGCA---GCAGGAAGCACTATG---GGCGCGGCGTCA---
ATAACGCTGACGGTACAG---GCCAGACAA---TTATTG---TCTGGTATAGTGCAACAGCAAAGC---
AATTTG---CTGAGG---GCTATAGAGGCGCAACAGCATATGTTGCAACTCACGGTCTGGGGCATT
>AY463229
TTCTATTGCTATACATCAGAATTGTTTAATAACACATACATACCTACATACATGAATAATAAAACAGAT----
----

GATAATTCTACCATCACACTTCCATGCAGAATAAAACAAATTATAAACATGTGGCAGGAGGTAGGACGAGCAA
TGTATGCCCCTCCCATTGCAGGAAACATAACATGTAACTCAAGT---
ATCACAGGACTACTATTGACACGTGATGGAGGCAATAATAATAAT----------------------------
----

GATACTGAAATATTCAGACCTGGAGGAGGAAATATGAAGGATAATTGGAGAAGTGAATTATATAAATATAAAG
TGGTAGAAATTCAGCCACTG---GGAGTAGCACCCACTGGGGCAAAAAGGAGAGTGGTG--------------
-------------------------GAGAGAGAAAAAAGAGCA---GTGGGA---ATAGGAGCT---------
GTGTTCCTTGGGTTCTTGGGAGCA---GCAGGAAGCACTATG---GGCGCGGCGTCA---
ATAACGCTGACGGTACAG---GCCAGACAA---CTGCTG---TCTGGTATAGTGCAACAGCAAAGC---
AATTTG---CTGAGA---GCTATAGAGGCGCAACAACACGTGTTGCAACTCACAGTCTGGGGCATT
>JX845587
TTCTATTGCAATACAACACAACTGTTTTCAAACAAAAGTGTT-------------------------------
----

CCAACCAACAAAACAATCATACTCCTATGCAGGATAAAACAAATTATAAACATGTGGCAGGGGGTAGGACGAG
CAATGTATGCCCCTCCCATTGCAGGAAACATAACATGTAACTCAAAT---
ATCACAGGACTACTATTGACACGTGATGGAGGAAAAAACAACGCAACAGGG----------------------
----

GGCAACGAAACAGAGATATTCAGACCTGGAGGAGGAAATATGAAGGACAATTGGAGAAGTGAATTATACAAAT
ATAAAGTGGTAGAAATTAAGCCATTA---GGAGTAGCACCCACTAAGGCACAAAGGAGAGTGGTG--------
-------------------------------CAGAGAGAAAAAAGAGCA---GTGGGA---ATAGGAGCT---
------GTGCTCCTTGGGTTCTTGGGAGCA---GCAGGAAGCACTATG---GGCGCGGCGTCA---
ATAACGCTGACGGTACAG---GCCAGACAG---CTGTTG---TCTGGTATAGTGCAGCAGCAAAGC---
AATTTG---CTGAGA---GCTATAGAGGCGCAACATCATATGTTGCAACTCACGGTCTGGGGCATT
>AY463235
TTCTATTGCAATACAACGCAACTGTTTAATAGTACATACTATAATAGTACTAATAGTACAGAGGAGACA----
----

GGAAATTCAAACATCACACTTCAATGCAGAATAAAACAAATTATAAACATGTGGCAAGGGGTAGGACAAGCAA
TGTATGCCCCTCCCATTCAAGGAATAATAACATGTAGATCAAAC---
ATCACAGGAATACTACTGGCACGTGATGGAGGAGCCTCAAATAGCACAGAA----------------------
----

AACTCAACTAACACAGAGATATTCAGGCCTGGAGGAGGAGATATGAGGGACAATTGGAGAAATGAATTATATA
AATATAAAGTGGTAGAAGTTAAACCATTA---GGAGTAGCACCTACTAGAGCAAAAAGGAGAGTGGTG-----
----------------------------------GAGAGAGAAAAAAGAGCA---GTGGGA---

ATAGGAGCT---------GTGTTCCTTGGGTTCTTGGGAGCC---GCAGGGAGCACTATG---
GGCGCAGCATCA---ATAACGCTGACGGTACAG---GCCAGACAA---TTATTG---
TCGGGTATAGTGCAACAGCAAAGC---AATTTG---CTGAGG---
GCTATAGAGGCGCAACAGCATATGTTGCAACTCACGGTCTGGGGCATT
>DQ275648
TTCTATTGCAATACATCAGCACTGTTTAATAGTAATATTACAAAAGATAATGATACAAAAGATAAT-------
----

GATACAGGAGATATTACACTTCAATGCAAAATAAAACAAATTATAAACATGTGGCAGGAGGTAGGACGAGCAA
TGTATGCCCCTCCCATTGAAGGAAGCATAACATGTGAATCAATT---
ATCACAGGACTACTACTAGTCCGTGATGGAGGCACAAATACTACA----------------------------
----

AAAGAAATATTCAGACCTGGAGGAGGAGATATGAAGGACAATTGGAGAAGTGAATTATATAAATATAAAGTGA
TAGAAATTAAACCATTG---GGAATAGCACCCACTGGAGCAAAAAGGAGAGTGGTG-----------------
----------------------GAGAGAGAAAAGAGAGCA---GTAGGA---ATAGGAGCT---------
GTGATCCTTGGGTTCTTGGGAGCA---GCAGGAAGCACTATG---GGCGCGGCGTCA---
ATAACGCTGACGGTACAG---GCCAGACAA---CTGTTG---TCCGGTATAGTGCAACAGCAAAGT---
AATTTG---CTGAGG---GCTATAGAGGCGCAACAGCATATGTTGCAACTCACGGTCTGGGGCATT
>DQ351220
TTCTATTGTAATACATCAGGACTGTTTAATAATACAGAAAAC-------------------------------
----

AACACCATCACACTTCAATGCAGAATAAAACAAATTATAATCATGTGGCAGGGGGTAGGGCGAGCAATATATG
CCCCTCCCATTGAAGGAAACATAAACTGTAATTCAAAT---
ATCACAGGATTACTATTGCTACGTGACGGAGGAATAACGAATACA----------------------------
----

ACAGAGGAGATATTCAGACCTGGAGGAGGAGATATGAGAGATAACTGGAGAAGTGAATTATATAAATATAAAG
TGGTAAAAATTGAGCCATTA---GGAATAGCACCCACTGGGGCAAAAAGGAGAGTGGTG--------------
-------------------------GAGAGAGAAAAAAGAGCA---GTGGGA---ATAGGAGCT---------
GTGTTCCTTGGGTTCTTGGGAGCA---GCAGGAAGCACTATG---GGCGCGGCGTCA---
ATGACGCTGACGGTACAG---GCCAGACAA---CTGTTG---TCTGGTATAGTGCAGCAGCAAAGC---
AATTTG---CTGAGG---GCTATAGAGGCGCAACAGCATATGTTGCAACTCACAGTCTGGGGCATT
>DQ369995
TTCTATTGCGACACATCACAACTATTTAATAAGACATACAATGTTACAGATGATTCAAACACC----------
----

ACAGACACAAACAACACAATTCAATGCAGAATAAAGCAGATTATAAACATGTGGCAGGAAGTAGGACGAGCAA
TGTATGCCCCTCCCATCGCAGGAAGCATAACATGTACCTCAAAT---
ATTACAGGACTACTATTGACACGTGATGGAGGGGGAGGCACAAATAGCACA----------------------
----

AGTAATACGGAAGAGATATTCAGACCCGGAGGAGGAAATATGAAGGATAATTGGAGAAGTGAATTATATAAAT
ATAAAGTGGTAGAAGTTCAGCCATTA---GGAATAGCTCCCACTAAAGCAAAAAGGAGAGTGGTG--------
-------------------------------GAGAGAGAAAAAAGAGCA---ATAGGA---ATAGGAGCT---
------GTGTTCCTTGGGTTCTTGGGAGCA---GCAGGAAGCACTATG---GGCGCAGCGTCA---
ATAACGCTGACGGTACAG---GCCAGACAA---CTATTG---TCTGGTATAGTGCAACAGCAAAGC---
AACTTG---CTGAGG---GCTATAGAGGCGCAACAGCATATGTTGCAACTCACGGTCTGGGGCATC
>DQ351223
TTCTATTGCAATACAACAGGACTGTTTGCTAGTAAAAATGATTCAAACACAACCATCAGT-------------
----

ACAAACGCAACCATCACACTCCCATGTAGAATAAAACAAATTATAAACATGTGGCAGGGGGTAGGACGAGCGA
TGTATGCCCCTCCCATTGCAGGGAACATAACATGTAGATCAAAT---
ATCACAGGATTGTTATTGCTACGTGATGGGGGAGACAATCAGACC----------------------------
----

ACAGAGACATTCAGGCCTGGAGGAGGAAATATGAAGGACAACTGGAGAAGTGAATTATATAAATATAAAGTAG
TAGAAATTAAGCCATTG---GGAATAGCACCCACGAAGGCAAAAAGGAGAGTGGTG-----------------

----------------------GAGAGAGAAAAAAGAGCA---GCGGGA---ATAGGAGCG---------
ATGATCTTTGGGTTCTTGGGAGCA---GCAGGAAGCACTATG---GGCGCGGCGTCA---
ATAACGCTGACGGTACAG---GCCAGACAG---CTGTTG---TCTGGTATAGTGCAGCAGCAAAGC---
AATTTG---CTGAGA---GCTATAGAGGCGCAACAGCATATGTTGCAACTCACGGTCTGGGGCATT
>DQ351227
TTCTATTGCAATACATCAGGACTGTTTAATGGTATATACAATCCTAATGGTACAGAGAGTAAT----------
----

TCAAGCTCAAGCATCACACTTCAATGCAGAATAAAACAAATTATAAACATGTGGCAGGAGATAGGACGAGCAA
TGTATGCCCCTCCCATTGAAGGAAACATAACATGTAAATCAAAT---
ATCACAGGAATATTATTGACACGTGATGGAGGAAACGATAGTAGT----------------------------
----

ACAGAAGAAACATTCAGACCTGGGGGAGGAGATATGAGGGACAATTGGAGAAGTGAATTATACAAATATAAAG
TGGTAGAAATTAAACCATTG---GGAATAGCACCCACTGGGGCAAAAAGGAGAGTGGTG--------------
-------------------------GAGAGAGAAAAAAGAGCA---GTGGGA---ATAGGAGCT---------
GTGTTCCTTGGGTTCTTGGGAGCA---GCAGGAAGCACTATG---GGCGCGGCGTCA---
ATAACGCTGACGGTACAG---GCCAGACAA---CTGTTG---TCTGGTATAGTGCAACAGCAAAAC---
AATTTG---CTGAGG---GCTATAGAGGCGCAACAGCATATGTTGCAACTCACAGTCTGGGGCATT
>DQ396388
TTCTATTGCAATACATCAGGACTGTTTAATAGTACATACATGCCTAATGGTACAGAAAGCAATTCAACCATC-
----

ACAAAGTCAACCATCACACTCCTATGCAGAATAAAACAAATTATAAACATGTGGCAGCAAGTAGGAAGAGCAA
TGTATGCCCCTCCCATTGCAGGAAACATAACATGTACATCAAAC---
ATCACAGGAATATTATTAGTACGTGATGGAGGCTACAGTAACAAT----------------------------
----

ACTACAGAGATATTCAGACCTGGAGGAGGAAATATGAAGGACAATTGGAGAAGTGAATTATATAAATACAAAG
TGGTAGAAGTTAAGCCATTG---GGAATAGCACCCACTAAGGCAAAAAGGAGAGTGGTG--------------
-------------------------GAGAGAGAAAAAAGAGCA---GCA------CTAGGAGCT---------
ATGTTCCTTGGGTTCTTGGGAGCA---GCAGGAAGCACTATG---GGCGCGGCGTCA---
ATAACGCTGACGGTACAG---GCCAGACAA---TTGTTG---TCTGGTATAGTGCAACAGCAAAGC---
AATTTG---CTGAGG---GCTATAGAGGCGCAACAGCATATGTTGCAACTCACGGTCTGGGGCATT
>DQ351228
TTTTATTGCAACACATCAAAACTATTTAATGGTACATACGAGAATAGTACAGACAAGGAGTCAAACAGC----
----

ACAAATTCAATCATCACAATTCCATGCAGAATAAAACAAATTATAAACATGTGGCAGGAGGTGGGACGAGCAA
TGTATAACCCTCCCATTGCAGGAAACATAACATGTAAATCAAAT---
ATCACAGGACTACTATTAGTACGTGATGGA---GACAACAATAGT----------------------------
----

ACAGAAACATTCAGACCTCAAGGAGGAAATATGAAGGACAATTGGAGGAGTGAATTATATAAATATAAAGTGG
TGGAAATTAAGCCATTG---GGAATAGCACCCACTGAAGCAAAAAGGAGAGTGGTG-----------------
----------------------GAGAGAGAAAAAAGAGCA---GTAGGA---ATAGGAGCG---------
GTACTCCTTGGGTTCTTGGGAGCA---GCAGGAAGCACTATG---GGCGCGGCGTCA---
ATAACGCTGACGGTACAG---GCCAGACAA---CTGTTG---TCTGGTATAGTGCAACAGCAAAGC---
AATTTG---CTGAGA---GCTATAGAGGCGCAACAGCATATGTTGCAACTCACAGTCTGGGGCATT
>DQ093592
TTCTATTGCAATACAACAAACCTGTTTATTAATACTACTGCTAAT----------------------------
----

AACAGTGATCACATCACACTCCCATGCAAAATAAAACAAATTATAAACATGTGGCAGGAGGTAGGACGAGCAA
TGTATGCCCCTCCCATTGCAGGAAACATAATATGTAACTCAAGT---
ATCACAGGACTACTATTGCTACGTGATGGAGGATACAATGTTAGC----------------------------
----

AATACCACAGAGACATTCAGACCTGGAGGAGGAAATATGAAAGACAATTGGAGAAGTGAATTATATAAGTATA
AAGTGGTAGAAATTAAGCCATTG---GGAATAGCTCCCACTGGAGCAAAAAGGAGAGTGGTG-----------

----------------------------GAGAGAGAAAAAAGAGCA---GTGGGA---ATAGGAGCT------
---GTGTTCCTTGGGTTCTTGGGAGCA---GCAGGAAGCACTATG---GGCGTGGCGTCA---
ATAACGCTGACGGTACAG---GCCAGACAA---CTGTTG---TCTGGTATAGTGCAACAGCAAAGC---
AATTTG---CTAAGA---GCTATAGAGGCGCAACAGCATATGTTGCAACTCACAGTCTGGGGCATT
>DQ275652
TTCTACTGCAATACATCTAAACTGTTTAATAATATATACAATCCTACATATGTGAATAATGGGTCAAAA----
----

ATTAATGAAACCATCAGACTTCAATGCAGAATAAAACAAATTATAAACATGTGGCAGGGGGTAGGACAAGCAA
TGTACGCTCCTCCCATTGAAGGAAACATAACATGTAAATCAAAT---
ATCACAGGACTACTATTGACACGTGATGGAGGAAACACAAACACG----------------------------
----

AATGACACAGAGATATTCAGACCTGGAGGAGGAAATATGAAGGACAATTGGAGAAATGAATTATATAAATATA
AAGTGGTAGAAATTAAGCCATTG---GGAATAGCACCCACTGAGGCACGAAGGAGAGTGGTG-----------
----------------------------GAGAGAGAAAAAAGAGCA---GTGGGA---ATAGGAGCT------
---GTACTCCTTGGGTTCTTGGGAGCA---GCAGGAAGCACTATG---GGCGCGGCGTCG---
CTAACGCTGACGGTACAG---GCCAGACAA---CTGTTG---TCTGGTATAGTGCAACAGCAAAAT---
AATTTG---CTGAGG---GCTATAGAGGCGCAACAGCATATGTTGCAACTCACGGTCTGGGGCATT
>DQ275655
TTCTATTGCAATACAACACAACTGTTTCAGTTTAATGAAAGTTATTCAAACACAACT----------------
----

GATTATGAAAACATCACACTCCCCTGCAGAATAAGACAAATTATAAACATGTGGCAGGAGGTAGGACGAGCAA
TGTATAACCCTCCCATTGCAGGAAATATAACATGTAACTCAAGT---
ATCACAGGACTACTATTGACACGTGATGGAGGCAAAACAAATGGCACCAACAGC-------------------
----

ACAGAAGAGACATCAGAGACATTCAGACCTGGAGGAGGAAATATGAAGGACAATTGGAGAAGTGAATTATATA
AATATAAAGTGGTAGAAATTAAGCCATTG---GGAATAGCACCCACTAAGGCAAAAAGGAGAGTGGTG-----
----------------------------------GAGAGAGAAAAAAGAGCA---GTAGGA---
ATAGGAGCT---------GTGCTCTTTGGGTTCTTGGGAGCG---GCAGGAAGCACTATG---
GGCGCAGCGTCA---ATAACGCTGACGGTACAG---GCCAGACAA---CTGTTG---
TCTGGTATAGTGCAACAGCAAAGC---AATTTG---CTGAGA---
GCTATAGAGGCGCAACAGCATATGTTGCAACTCACAGTCTGGGGCATT
>DQ011175
TTCTATTGCAATACATCAAAACTATTTAATAGTACATACAATCCTGACCATAATAGTACAGGAAAT-------
----

ATCACAGGGGTCATCACAATTCCATGCAGAATAAAACAAATTATAAACATGTGGCAGGAGGTGGGACGAGCTA
TGTATGCTCCTCCCATTAAAGGAAACATAACATGTAACTCAAGT---
ATCACAGGACTACTATTAACACGTGATGGAGGCAAGTCAAATGACACAATA----------------------
----

GAAAACAAAAATGAAACATTTAGACCTCAAGGAGGAAATATGAAGGACAATTGGAGAAGTGAATTATATAAAT
ATAAGGTGGTACAAATTGAGCCATTG---GGAATAGCACCCACTAAAGCAAAAAGGAGAGTGGTG--------
-------------------------------GAGAGAGAAAAAAGAGCA---GTGGGA---ATAGGAGCT---
------GTGTTCCTTGGGTTCTTGGGAGCA---GCAGGAAGCACTATG---GGCGCGGCGTCA---
ATGGCGCTGACGGTACAG---GCCAGACAA---GTGCTG---TCTGGTATAGTGCAACAGCAAAGC---
AATTTG---CTGAGA---GCTATAGAGGCGCAACAGCATATGTTGCAGCTCACGGTCTGGGGCATT
>AY901971
TTCTATTGCAATACATCAAGACTATTTAATAATACATTTAATTATACAGACACAACAGAACTTAACAGCACAG
ACAGC--
-------------------ACA---
GACTCAAACGAGACCATCACAATTCCATGCAGAATAAAACAAATTATAAACATGTGGCAGCAGGTAGGACGAG
CAATGTATGCTCCTCCCATTGCAGGGAACATAACATGTACATCAAAT---
ATCACAGGATTACTATTGGTACGTGATGGAGGCAAGGATAATACA----------------------------
----

GGTACAAATGAGACATTCAGACCTCAAGGAGGAAATATGAAGGACAATTGGAGAAGTGAATTATACAAATATA

AAGTGGTAGAAATTAAACCATTG---GGAATAGCACCTACTGGGGCAAAAAGGAGAGTGGTG-----------
----------------------------GAAAGGGAAAAAAGAGCA---GCG------CTAGGAGCT------
---ATGTTCCTTGGGTTCTTGGGAGCA---GCAGGAAGCACTATG---GGCGCAGCGTCA---
ATAACGCTGACGGTACAG---GCCAGACAA---CTGTTG---TCTGGTATAGTGCAACAGCAAAAC---
AATTTG---CTGAGA---GCCATAGAGGCGCAACAGCATATGTTGCAACTCACAGTCTGGGGCATA
>DQ164106
TTCTATTGCAATACATCAGGACTATTTAACAGTACATTTAATGATACATACGCACCAAACAACACAGAC----
----

ACAAACGAGACCATCACAATTCAATGCAGAATAAAACAAATTATAAACATGTGGCAGCAGGTAGGACGAGCAA
TGTATGCTCCTCCCATTGCAGGGAACATAACATGTAACTCAAGT---
ATCACAGGATTACTATTGGTGCGTGATGGAGGCACAAAGACAGATGAT-------------------------
----

AACACAACAGAGACATTCAGACCTGAAGGAGGAAATATGAGGGACAATTGGAGAAGTGAATTATACAAATATA
AAGTAGTAGAAATTAAACCATTG---GGAATAGCACCTACTGAGGCAAAAAGGAGAGTGGTG-----------
----------------------------CAGAGAGAAAAAAGAGCA---GTGGGA---ATAGGAGCT------
---GTGTTCCTTGGGTTCTTGGGAGCA---GCAGGAAGCACTATG---GGCGCGGCGTCA---
ATAACGCTGACGGTACAG---GCCAGACAA---CTGTTG---TCTGGTATAGTGCAACAGCAAAAC---
AATTTG---CTGAGA---GCCATAGAGGCGCAACAACATATGTTGCAACTCACGGTCTGGGGCATA
>DQ093597
TTCTATTGCAATACATCAAGACTATTTAATAGTACATACTGGGAAGGATCTAATAGTACAGGA----------
----

GGTGACTCAACCATCACAATTCCATGCAGAATAAAACAAATTATAAACATGTGGCAGGAGGTAGGACGAGCAA
TGTATGCCCCTCCCATAGCAGGAAACATAACATGTAACTCAAGT---
ATCACAGGATTACTATTAGTACGTGATGGAGGCCTATCCAAAAATAAC-------------------------
----

ACAAATCAAAATGAAACATTCAGACCTGGAGGAGGAGATATGAAAGACAATTGGAGGAGTGAATTATATAAAT
ACAAAGTGGTAGAAATTAAGCCATTG---GGAATAGCACCCACTAAAGCAAAAAGGAGAGTGGTG--------
-------------------------------GAGAGAGAAAAAAGAGCA---GTGGGA---ATAGGAGCT---
------GTGCTCCTTGGGTTCTTGGGAGCA---GCAGGAAGCACTATG---GGCGCAGCGTCA---
ATAGCGCTGACGGCACAA---GCCAGACAA---GTGTTG---TCTGGTATAGTGCAACAGCAAAGT---
AATTTG---CTGAGG---GCTATAGAGGCGCAACAGCATATGTTGCAACTCACGGTCTGGGGCATT
>AY901972
TTCTATTGCAATACATCACAATTATTTAATAGTACATACTATGATGCATACAATGTTACAGGCAATGTTACAA
AC---

AATGATAATTCAACCATCACAATTCAATGCAGAATAAGACAATTTATAAACATGTGGCAGAAGGTAGGAAAAG
CAATGTATGCCCCTCCCATTGCAGGAAACATAACATGTGAATCAAAT---
ATCACAGGACTACTATTAGTACGGGATGGAGGCCCAAATAACGCA----------------------------
----

GCAGAAATATTCAGACCTGGAGGAGGCAATATGAAGGACAATTGGAGAAGTGAATTATATAAATATAAAGTGG
TGGAAATTAAGCCATTG---GGAATAGCACCCACTGAGGCAAAAAGGCGAGTGGTG-----------------
----------------------GAGAGAGAAAAAAGAGCA---GTGGGA---ATAGGAGCT---------
TTGTTCCTTGGGTTCTTGGGAGCA---GCAGGAAGCACTATG---GGCGCAGCGTCA---
CTGACGCTGACGGTACAG---GCCAGACAA---CTGTTG---TCTGGTATAGTGCAACAGCAAAGC---
AATTTG---CTGCAA---GCTATAGAGGCGCAACAGCATTTGTTGCAACTCACGGTCTGGGGCATT
>AY772692
TTCTATTGCAATACAACAAAACTGTTTAATGAAAATGAAACAAGTAAG-------------------------
----

GCAAATGACACAATCATACTCCCATGCAGAATTAAACAAATCATAAACATGTGGCAGACGGTAGGACGAGCAA
TGTATGCTCCTCCCATTGAAGGATACATAAATTGTACATCAAAT---
ATCACAGGAATACTATTGACACGTGATGGAGGAAATAATAAAACAAAT-------------------------
----

AACACAAAAGAGATATTCAGACCTGCAGGAGGAGATATGAGGAACAATTGGAGAAGTGAATTATACAAATATA

AAGTAGTAGAAATTCAGCCATTG---GGAATAGCACCCACTACTGCAAAAAGGAGAGTGGTG-----------
----------------------------GAGAGAGAAAAAAGAGCA---GCA------CTAGGAGCT------
---ATATTCCTTGGGTTCTTGGGAGCA---GCAGGAAGCACTATG---GGCGCGGCGGCA---
ATAACGCTGACGGTACAG---GCCAGACAA---CTGTTG---TCTGGTATAGTGCAACAGCAAAGC---
AATTTG---CTGAGA---GCTATAGAGGCGCAACAGCATCTGTTGCAACTCACGGTCTGGGGCATT
>DQ351232
TTCTATTGCAATACATCAGAGTTGTTTACATACAATTTTACAGGGAGTAGT----------------------
----

TCAAGCAAAAACATCACACTTCAATGCAGAATAAAACAATTTATAAACATGTGGCAGGAGGTAGGACGGGCAG
TGTATGCCCCTCCCATTGAAGGGAGCATAACATGTAACTCAAAT---
ATCACAGGACTACTATTAACACGTGATGGAGGCGACAATAACACA----------------------------
----

AGTATAGAGATATTCAGACCTGGAGGAGGAAATATGAAGGACAATTGGAGAAGTGAATTATATAAATACAAAG
TAGTAGAAATTAAACCATTG---GGAATAGCACCCAACACAGCAAAAAGGAGAGTAGTG--------------
-------------------------GGGAGAGAAAAGAGAGCA---GTGGGA---ATGGGAGCT---------
GTGATCTTTGGGTTCTTGGGAGCA---GCAGGAAGCACTATG---GGCGCAGCGTCA---
GTAACGCTGACGGTACAG---GCCAGACTA---TTGTTG---TCTGGTATAGTGCAACAGCAAAAC---
AATTTG---CTGAGA---GCTATAGAGGCGCAACAGCATCTGTTGCAACTCACGGTCTGGGGCATT
>DQ164107
TTCTATTGCAATACATCGGCACTATTTCATAGTCCTACATACAATGAGACAAAAGGT----------------
----

AATTCAACAACCATCACACTTCCATGTAGAATAAAACAAATTATAAACATGTGGCAGGGGGTAGGACGAGCTA
TGTATGCTCCTCCTATTGCAGGAGAAATAACATGTAACTCAAGC---
ATCACAGGACTACTATTGACACGTGATGGAGGAGAAGGAGAGAAT----------------------------
----

CACACAGAGATATTCAGGCCTGGAGGAGGAAATATGAAGGACAATTGGAGAAGTGAATTATATAAATATAAAG
TGGTAGAAATTAAGCCATTG---GGAGTAGCACCCACTAAGGCGGAAAGGAGAGTGGTG--------------
-------------------------AAAAAAGAAAAAAGAGCA---GTGGGA---ATAGGAGCG---------
TTGTTCCTTGGGATCTTGGGAGCA---GCAGGAAGCACTATG---GGCGCGGCGTCA---
ATAACGCTGACGGTACAG---GCCAGACAA---CTGTTG---TCGGGTATAGTGCAACAGCAAAAT---
AATTTG---CTGAGG---GCTATAGATGCGCAACAGCATATGTTGCAACTCACGGTCTGGGGCATT
>DQ369997
TTCTATTGTAACACTTCAGAACTGTTTAATAGTACATACAATCCTACATACAATGGGACAAAT----------
----

AGTACAAACATCACCACAATTCAATGCAGAATAAAACAAATTATAAACATGTGGCAAGGGGTAGGACGAGCAA
TGTATGCCCCTCCCATTGCAGGAAACATAACATGCAAATCAAAA---
ATCACAGGACTACTATTGACACGTGATGGAGGAAGAAATAATAGT----------------------------
----

ACAGGGACATTCAGACCCGGAGGAGGAGATATGAGGGACAATTGGAGAAGTGAATTATATAAATATAAAGTGG
TAGAAATTAAACCATTA---GGAATAGCACCCACTGAGGCAAAACGGAGAGTGGTG-----------------
----------------------GAGAGAGAAAAAAGAGCA---GTGGGA---ATAGGAGCT---------
GTGTTCCTTGGGTTCTTGGGAGCA---GCAGGAAGCACTATG---GGCGCAGCGTCA---
ATGACGCTGACGGTACAG---GCCAGACAA---TTACTG---TCTGGTATAGTGCAACAGCAGAGC---
AATTTG---CTGAGA---GCTATAGAGGCGCAACAGCATATGTTGCAACTCACGGTCTGGGGCATT
>DQ093600
TTCTATTGCAATACAACAAAACTGTTTAATGAAAGTGAAACACAGTTTGCGGCAAACAACAGT----------
----

GCCAATAGTAACATCACACTCCCATGCAAGATAAAACAAATTATAAACATGTGGCAGCAGGTGGGACGAGCAA
TGTATGCCCCTCCTGTTCAAGGAAACATAATATGTAACTCTAGT---
ATCACAGGACTACTATTGACACGTGATGGAGGAGTTAACAATACT----------------------------
----

AATGAGACATTCAGACCTGGAGGAGGAAATATGAAAGACAATTGGAGAAATGAATTATATAAATATAAAGTGG

TAGAAATTAAGCCATTA---GGAATAGCACCCACTAGGGCAAAAAGGAGAGTGGTG-----------------
----------------------GAGAGAGAAAAAAGAGCA---GTGGGA---CTAGGAGCT---------
GTGATCTTTGGGTTCTTGGGAGCA---GCAGGAAGCACTATG---GGCGCGGCGTCA---
ATAACGCTGACGGTACAG---GCCAAACAA---CTGTTG---TCTGGTATAGTGCAACAGCAAAGC---
AATTTG---CTGAGA---GCTATAGAGGCGCAACAGCATATGTTGCAACTCACGGTCTGGGGCATT
>DQ011166
TTCTATTGTAATACAACAGATCTGTTTAATAGTACATTTAATAGTGAATACAATAGTACAGACAGGCATAAT-
----

AATAATACCAATATCACACTCCAATGCAGGATAAAACAAGTGATAAACATGTGGCAGCAGGTAGGACGAGCAA
TGTATGCTCCTCCCATTGCAGGAAACATAACATGTATCTCAAAT---
ATCACAGGATTACTATTGGTGCGTGATGGAGGAGGAAAAAATAACACA-------------------------
----

AATCAAACAGAGGAGATATTCAGACCTGGAGGAGGAGATATGAGGGACAATTGGAGAAGTGAATTATACAAAT
ATAAGGTGGTAGAACTCAAACCATTG---GGAATAGCACCTACTGAGGCAAAGAGGAGAGTGGTG--------
-------------------------------GAGAGAGAAAAAAGAGCA---GTGGGA---ATAGGAGCT---
------GTGTTCCTTGGGTTCTTGAGTGCA---GCAGGAAGCACTATG---GGCGCGGCGTCA---
ATGACGCTGACGGTACAG---GCCAGACAA---TTGTTG---TCTGGTATAGTGCAACAGCAAAAC---
AATTTG---CTGAAG---GCCATAGAGGCGCAACAACATCTGTTGCAACTCACGGTCTGGGGCATT
>DQ011167
TTCTATTGCAATACATCAGGACTGTTTAATAAGAGTAATACAGACATGACTAATAAAACAGAA----------
----

GATAATTCAACCATCACACTCCCATGTAGAATAAAACAGATTATAAACATGTGGCAGAACGTAGGACGAGCAA
TATATGCCCCTCCCGTTCAAGGAAACATAACATGTAACTCAAGT---
ATCACAGGACTACTATTGACACGTGATGGAGGTAACGGAAATGACACT-------------------------
----

AATGGCACAGAGAGATATTCAGACCTGGAGGAGGAGATATGAGGGACAATTGGAGAAGTGAATTATACAAATATA
AAGTGGTAGAAATTAAGCCATTG---GGAATAGCACCCACTAAGGCAAAAAGGAGAGTGGTG-----------
-----------------------------GAGAGAGAAAAAAGAGCA---GTGGGA---ATAGGAGCT------
---GTGTTCCTTGGGTTCTTGGGAGCA---GCAGGAAGCACTATG---GGCGCAGCATCA---
ATAACGCTGACGGTACAG---GCCAGACAA---TTGTTG---TCTGGAATAGTGCAACAGCAAAGC---
AATTTG---CTGAGG---GCTATAGAGGCGCAACAGCATATGTTGCAACTCACGGTCTGGGGCATT
>DQ093590
TTCTATTGTAATACATCAAAACTGTTTAATAGTACATACAATCCTAATAATACAGAAAGTAAT----------
----

TCAAGCGCAGTAATCACACTCCCATGCAAGATAAAGCAAATTATAAACATGTGGCAGCAGGTAGGGCGAGCAA
TGTATGCTCCTCCCATTGCAGGAAACATAACATGTAACTCAAAT---
ATCACAGGATTACTACTGGTGCGTGATGGGGGAACAATTGGCAATGGC-------------------------
----

ACAAATAACGAGACATTCAGACCTGGAGGAGGAGATATGAGGGACAATTGGAGAAGTGAATTATACAAATATA
AAGTGGTAGAAGTTAAACCATTG---GGAATAGCACCTACTCAGGCAAAAAGGAGAGTGGTG-----------
-----------------------------GAGAGAGAAAAAAGAGCA---GTGGGA---CTAGGAGCT------
---GTGTTCCTTGGGTTCTTGGGAGCA---GCAGGAAGTACTATG---GGCGCGGCGTCA---
ATAACGCTGACGGTACAG---GCCAGACAA---CTGTTG---TCTGGCATAGTGCAACAGCAAAGC---
AATTTG---CTGAGG---GCCATAGAGGCGCAACAGCATATGTTGCAACTCACGGTCTGGGGCATT
>DQ011178
TTCTATTGCAACACATCAGGCCTGTTTAATACTACAGACCTGTTTAATAATACAGACCTGTCTAATAGTACAG
GA--

AGTAATGAAAACATCACGCTTCCATGCAGAATAAAACAATTTGTGAATATGTGGCAGGAGGTAGGGCGAGCAA
TGTATGCCCCTCCCATTCAAGGAATCATAAATTGTATATCAAAT---
ATCACAGGAATACTATTGCTACGTGATGGAGGAAACACGAGTGCTAAT-------------------------
----

GGAACATTAACAGAGACATTCAGACCTGGAGGAGGAAATATGAAGGACAATTGGAGAAGTGAATTATATAAAT

ATAAGGTGGTAGAAATTAGGCCATTA---GGAGTAGCACCCACCAAGGCAAAAAGGAGAGTGGTG--------
-------------------------------GAGAGAGAAAAAAGAGCA---GTGGGA---ATAGGAGCT---
------GTGCTCTTTGGGTTCTTGGGAGCA---GCAGGAAGCACTATG---GGCGCGGCGTCA---
ATAACGCTGACGGTACAG---GCCAGACAA---CTGCTG---TCTGGTATAGTGCAACAGCAAAGC---
AATTTG---CTGAGG---GCTATAGAGGCGCAACAGCATATGTTGCAACTCACGGTCTGGGGCATT
>DQ056405
TTCTATTGCAATACATCACAGTTGTTTAATACATCATATGTGTTTAATAATACTGCTAATAATACAGAG----
----

GCAAATCAAAACATCACACTGCCATGCAGAATAAAACAAATTATAAACATGTGGCAGGAGGTGGGACGAGCAA
TATATGCCCCTCCCATTGCAGGAAAAATAACATGTAAATCAAAT---
ATCACAGGGTTACTATTGACACGTGATGGAGGAAATTCAAACGATACT-------------------------
----

AATAACACAGAGATATTCAGACCTGCAGGAGGAGATATGAGGGACAATTGGAGAAGTGAACTATATAAATATA
AAGTGGTAGAAATTAAGCCGCTA---GGAGTAGCACCCACAGAGGCAAAAAGGGGAGTGGTG-----------
----------------------------GAGAGAGAAAAAAGAGCA---GTAGGA---ATAGGAGCT------
---GTGTTCCTTGGGTTCTTGGGAGCA---GCAGGAAGCACTATG---GGCGCAGCATCA---
ATAACGCTGACGGTACAG---GCCAGACAA---TTGTTG---TCTGGAATAGTGCAACAGCAAAGC---
AATTTG---CTGAGG---GCTATAGAGGCGCAACAGCATCTGCTGCAACTCACAGTCTGGGGCATT
>AY901980
TTTATTGTAATACATCACAACTGTTTAATAGTACATACAATCCTGCATACAGTCATAATGAGACAGAA----
----

AGTAATTCAACCATCATGCTTCCATGCAGAATAAAACAAATTATAAACATGTGGCAGGAGGTAGGAAAAGCAA
TGTATGCCCCTCCCATTGCAGGAAACATAATATGTAACTCAAGT---
ATCACAGGACTACTATTGACACGTGATGGAGGAAACAATAACACG----------------------------
----

AATACAGAAGAAATATTCAGACCTGGAGGAGGAAATATGAAGGACAATTGGAGAAATGAATTATATAAATATA
AAGTGGTAGAAATTCAGCCATTG---GGAGTAGCACCCACCGAGGCAAAAAGGAGAGTGGTG-----------
----------------------------GAGAGAGAAAAAAGAGCA---GTGGGA---CTGGGAGCT------
---GTGTTCCCTGGGGTCTTGAGTTTG---GCAGGAAGCACTATG---GGCGCAGCGTCA---
ATAACGCTGACGGTACAG---GCCAGGCAA---CTGTTT---GCTGGTATAGTGCAACAGCAAAAT---
AATTTG---CTGAGG---GCTATAGAGGCGCAACAGCATATGTTGCAACTCACAGTCTGGGGCATT
>AY878059
TTCTATTGCAATACAACAAAACTGTTTAACATAACCTATAATAACAGTAATTCA-------------------
----

GACAACAATACCATCACGCTCCCCTGCAGAATCAAACAATTTATAAACCTGTGGCAGGAGGTAGGGCGAGCAA
TGTATGCCCCTCCCATTGAAGGAAAAATAACATGTCACTCAAGT---
ATTACAGGACTACTATTGACACGTGATGGAGGAACGAGTGCAAACAATGATACA------------CAA----
----
GCAAGTACAAACAAT---
AATACAGAGACATTCAGACCTGGAGGGGGAGATATGAAAAACAATTGGAGAAATGAATTATATAAATATAAAG
TGGTAGAAATTAAGCCCTTA---GGAGTAGCACCCACTGGAGCAAAAAGGAGAGTGGTG--------------
-------------------------GAGAGAGAAAAAAGAGCA---GTAGGA---ATAGGAGCT---------
GTACTCTTTGGGTTCTTGGGAGCA---GCAGGAAGCACTATG---GGCGCGGCGTCA---
ATAACGCTGACGGTACAG---GCCAGACAA---TTGTTG---TCTGGTATAGTGCAACAGCAAAGC---
AATTTG---CTGAGA---GCTATAGAGGTGCAACAGCATATGTTGCAACTCACGGTTTGGGGCATT
>DQ011174
TTCTATTGCAATACGACAGAACTGTTTAATGATAGTAGCTCA-------------------------------
----

GAAAACATCACACTCCCATGCAGAATAAAACAAATTGTAAACATGTGGCAGGGGGTAGGACGAGCAATGTATG
CCCCTCCCATTGAAGGAAGCATAACATGTAATTCAAGT---
ATCACAGGACTACTATTAGAACGTGATGGAGGGGAAAGCTATAAT----------------------------
----

AAAACAGAGATATTCAGGCCTGGAGGAGGAAATATGAAGGACAATTGGAGAAGTGAATTATATAAATATAAAG

TGGTAGAAATTAAGCCATTA---GGAGTAGCACCCACAAGGGCAAAAAGGAGAGTGGTG--------------
-------------------------GAGAGAGAAAAAAGAGCA---GTGGGA---ATAGGAGCT---------
GTGTTCCTTGGGTTCTTGGGGGCA---GCAGGAAGCACTATG---GGCGCGGCGTCA---
ATAACGCTGACGGTACAG---GCCAGACAA---CTGCTG---TCTGGTATAGTGCAACAGCAAAGC---
AATTTG---CTGAGA---GCTATAGAGGCGCAACAGCATATGTTGCAACTCACGGTCTGGGGCATT
>DQ382373
TTCTATTGCAATACATCAAACCTGTTTAATGGAAACTGGTATAGTAATGGTTCATTCCTGTTCAATGGAAGTT
GGGATAATAAT--
----------AATACAGAC------
ACAGAGAATTCAACCATCACACTTCAATGCAGAATAAAACAAATTATAAATATGTGGCAGGAGGTGGGAAGGG
CAATGTATGCCCCTCCCATCGAAGGAAACATAACATGTAAATCAAAT---
ATCACAGGACTACTATTGGTACGTGATGGAGGAACAGGCAATGACACA-------------------------
----

GAAAATGACACAGAAATATTCAGACCTGGAGGAGGAAATATGAGGGACAATTGGAGAAATGAATTATATAAAT
ATAAAGTGGTAGAAATTAGGCCATTG---GGAATAGCACCCACTGAGGCAAAAAGGAGAGTGGTG--------
-------------------------------GAGAGAGAAAAAAGAGCA---GTGGGA---ATAGGAGCT---
------GTGTTCCTTGGGTTCTTAGGAGCA---GCAGGAAGCACTATG---GGCGCAGCATCA---
ATAACGCTGACGGTACAG---GCCAGACAA---GTATTG---TCTGGAATAGTGCAACAGCAAAGC---
AATTTG---CTGAGA---GCTATAGAGGCGCAACAGCATATGTTGCAACTCACAGTCTGGGGCATT
>DQ369991
TTCTATTGCAATACTACAAACCTGTTTAATGAAAATATAACAAGTGTAAATATAACAAGTAAAAATGAT----
----

TCAACAGACAGCATCACACTTCAATGCAGGATAAAACAAATTATAAACATGTGGCAGGAGGTAGGACGAGCAA
TGTATGCCCCTCCCATTGCAGGAAACATAACATGTAATTCAACT---
ATCACAGGACTACTATTAACACGTGATGGAGGAAACATCACGAAT----------------------------
----

AACACAGAGGAGATATTTAGACCTGGAGGAGGAAATATGAAGGATAATTGGAGAAGTGAATTATATAAATACA
AGGTGGTAGAAATTAAACCATTA---GGAGTAGCACCCACCAAGGCAAAAAGGAGAGTGGTG-----------
----------------------------GAGAGAGAAAAAAGAGCA---GTAGGA---CTAGGAGCT------
---CTGTTCCTTGGGTTCTTGGGAGCA---GCAGGAAGCACTATG---GGCGCAGCATCA---
ATAACGCTGACGGTACAG---GCCAGACAA---GTGTTG---TCTGGTATAGTGCAACAGCAAAGC---
AATTTG---CTGAGG---GCTATAGAGGCGCAACAGCATATGTTGCAACTCACGGTCTGGGGCATT
>GQ999986
TTCTATTGCAATACATCAAAACTTTTTAATAATACATACTATAATGGTACAGGAAATGCTAACAGT-------
----

ACGCATGAAAACATCACACTCCCATGCAGAATAAAACAAATTATAAGGATGTGGCAGAAGGTAGGACAAGCAA
TGTACGCCCCTCCCATTGCAGGAAACATAACATGTACATCAAAT---
ATCACAGGACTACTACTGGTACGTGATGGAGGAGAGGCAAATGGCACA-------------------------
----

AACAACATAGAGACATTCAGACCTGGAGGAGGAGATATGAGGGACAATTGGAGAAGTGAATTATATAAATATA
AAGTGGTAGAAATTCAGCCATTG---GGAATAGCACCCACCAAGGCAAAAAGGAGAGTGGTG-----------
----------------------------GAGAGAGAAAAAAGAGCA---GTGGGA---ATAGGAGCT------
---ATGTTCCTTGGGTTCTTGGGAGCA---GCAGGAAGCACTATG---GGCGCGGCGTCA---
ATGGCGCTGACGGTACAG---TCCAGACAA---CTGTTG---TCTGGTATAGTGCAACAGCAAAGC---
AATTTG---CTGAGG---GCTATAGAGGCGCAACAGCATATGTTGCAACTCACGGTCTGGGGCATT
>GQ999987
TTCTACTGCAATACAACAGGACTGTTTAATCATAGTGGGGTAAACACCAAAGAACTGCTTAATAATAATACTG
AG---

TCAAACACAACCATCACACTCCCATGCAAAATAAAACAAATTATAAACATGTGGCAGGAGGTAGGACGGGCAA
TGTATGCCCCTCCCATTGCAGGAAACATAACATGTAACTCAAGT---
ATCACAGGACTACTATTAACACGTGATGGAGGTAATAATAAAAATAAAACAGAA-------------------
----

AATGACACAAAGATAGAGATATTCAGACCTGGAGGAGGAAATATGAAGGACAATTGGAGAAGTGAATTATATA

AATATAAAGTGGTAGAAATTCAACCACTG---GGAGTAGCACCCACGGAGGCAAAAAGGAGAGTGGTG-----
----------------------------------GAGAGAGAAAAAAGAGCA---GTAGGA---
ATAGGAGCT---------GTACTCTTTGGGTTCTTGGGAGCA---GCAGGAAGCACTATG---
GGCGCGGCGTCA---ATAACGCTGACGGTACAG---GCCAGACAA---CTATTG---
TCTGGTATAGTGCAACAGCAAAGC---AATTTG---CTGAGA---
GCTATAGAGGCGCAACAGCATATGTTGCAACTCACAGTCTGGGGCATT
>GQ999977
TTCTATTGCAATACATCAGGACTGTTTAATGGTACATACAATGGTACGGGAAATAAT----------------
----

TCAACAGAAACCATCACACTTCAATGCAGAATAAAACAAATTATAAATATGTGGCAGGAGGTAGGACGAGCAA
TGTATGCCCCTCCCATTGCAGGAAACATAACATGTAACTCAAAG---
ATCACAGGACTACTACTAGTACGTGATGGAGGCACATATGGAGAT----------------------------
----

AACAAAACAGAGACATTCAGACCTGGAGGAGGAAATATGAAGAACAATTGGAGAAGTGAATTATATAACTATA
AAGTGGTAGAAATTAAACCATTA---GGAATAGCACCCACTGAGGCAAAAAGGAGAGTGGTG-----------
----------------------------GAGAGAGAAAAAAGAGCA---GTAGGA---ATAGGAGCT------
---GTGTTCCTTGGGTTCTTGGGAGTA---GCAGGAAGCACTATG---GGCGCAGCGTCA---
ATAACGCTGACGGCACAG---GCCAGACAA---CTATTG---TCTGGTATAGTGCAACAGCAAAGC---
AATTTG---CTGAGA---GCTATAGAGGCGCAACAGCATATGTTGCAACTCACGGTCTGGGGCATT
>FJ443986
TTCTATTGCAATACATCAAAGCTGTTTACAGACATGCTTAATGACACA-------------------------
----

AAAGATAATCAAACCACACTCCCCTGCAAAATAAAACAAATTATAAATATGTGGCAGGGGGTAGGACGAGCAA
TGTATGCCCCTCCCATTGAGGGAAGCATAACATGTAACTCAAGT---
ATCACAGGACTACTATTAACACGTGATGGAGGAAAACCTAATGCCACGGCTAAT-------------------
----

GCCACGGTAGAGGTAGAGGTATTCAGACCTGGAGGAGGAAATATGAAGGACAATTGGAGAAGTGAATTGTATA
AATATAAAGTGGTAGAAATTAAGCCATTA---GGAATAGCACCCACTAAAGCAAAAAGGAGAGTGGTG-----
----------------------------------GAGAGAGGGAAAAGAGCA---GTGGGA---
ATAGGAGCT---------GTGCTCCTTGGGTTCTTGGGAGCA---GCAGGAAGCACTATG---
GGCGCAGCGTCA---ATAACGCTGACGGTACAG---GCCAGACAA---TTGTTA---
TCTGGTATAGTGCAACAGCAAAAC---AATTTG---CTGAGA---
GCTATAGAGGCGCAACAGCATATGTTGCAACTCACGGTCTGGGGCATT
>HQ615951
TTCTATTGCAATACATCAAAACTGTTTAATGGTACATACCCAGCAAATAGTACAAACAGT-------------
----

ACTTCAAACTACATCACACTCAAATGCAGAATAAAACAAATTATAAACATGTGGCAGGGAGTAGGACGAGCAA
TGTATGCCCCCCCCATTGCAGGAAACATAACATGTGAATCAAAT---
ATCACAGGACTACTGTTGACAAGGGATGGAGGAAACAGTAGTACT----------------------------
----

AATGAGACATTCAGACCTGCAGGAGGAGATATGAGGGACAATTGGAGAAGTGAATTATATAAATATAAAGTAG
TAGAAATTAGGCCATTA---GGAATAGCACCCACTGGGGCAAAAAGGAGAGTGGTG-----------------
----------------------GAGAGAGAAAAAAGAGCA---GTGGGA---TTCGGAGCT---------
GTGTTCCTTGGGTTCTTGGGAGCA---GCAGGAAGCACTATG---GGCGCAGCATCA---
ATAACGCTGACGGTACAG---GCCAGACAA---TTGTTG---TCTGGTATAGTGCAACAGCAAAGC---
AATTTG---CTGAGG---GCTATAGAGGCGCAACAGCATATGTTGCAACTCACGGTCTGGGGCATC
>JN687821
TTCTATTGCAATACATCACACCTGTTTAGTAATAATGGTACA-------------------------------
----

AATTCTATCATCACACTCCCATGCAGGATAAAACAAATAATAAACATGTGGCAGGGGGTGGACGAGCAATGT
ATGCCCCTCCTATTCAAGGAGACATAACATGTAAATCAAAC---
ATAACAGGAATACTATTGACACGTGATGGAGGAAGAAATGGCAAC----------------------------
----

ACAACAGAGATATTCAGACCTGGAGGAGGAGATATGAGGGACAATTGGAGAAGTGAATTATATAAATATAAAG
TGGTAGAAATTAAGCCATTG---GGAATAGCACCCACTGAAGCTAAAAGGAGAGTGGTG--------------
-------------------------GAGAGAGAAAAGAGAGCA---GTGGGA---ATAGGAGCT---------
GTGTTCCTTGGGTTCTTGGGAGCA---GCAGGAAGCACTATG---GGCGCGGCGTCG---
ATAACGCTGACGGTACAG---GCCAGACAA---CTGTTG---TCTGGTATAGTGCAACAGCAAAGC---
AATTTG---CTGAGA---GCCATAGAAGCGCAACAGCATATGTTGCAACTCACAGTCTGGGGCATT
>KC863367
TTCTATTGCAATACATCAGGCCTGTTTAATGGTACACTTAATGATACATACACGGCTAATAGTACAAGT----
----

GATAACTCAACCATCACACTCCAATGCAGAATAAAACAAATTATAAACATGTGGCAAGGGGTAGGACAAGCAA
TGTATGCCCCTCCCATTGAGGGAAACATAACATGTAAATCAAAT---
ATCACAGGGCTACTATTGACCCGTGATGGAGGAACAAATACTAGT----------------------------
----

GAGGAGGAGACATTCAGACCTGGAGGAGGAGATATGAGGGACAATTGGAGAAGTGAATTATACAAATATAAAG
TGGTAGAGATTAAGCCATTG---GGAATAGCACCCACTCAGGCAAAAAGGAGAGTGGTG--------------
-------------------------GAGAGAGAAAAAAGAGCA---GTGGGA---ATAGGAGCT---------
GTGTTCCTTGGGTTCTTGGGAGCA---GCCGGAAGCACTATG---GGCGCGGCGTCA---
ATAACGCTGACGGTACAG---GCCAGACAA---TTATTG---GCTGGTATAGTGCAACAGCAAAGC---
AATTTG---CTGAGG---GCTATAGAGGCGCAACAGCATATGTTGCAACTCACAGTCTGGGGCATT
>KC894137
TTCTATTGCAATACATCAAACCTGTTTAATGATACACATACAGCAAACATCACA-------------------
----

GCAAACATCACACTCACACTCCCATGCAGAATAAAACAAATTATAAACATGTGGCAGGAGGTAGGAAAAGCAA
TGTATGCCCCTCCCATTGCAGGAAACATAACATGTACATCAAAT---
ATCACAGGATTGCTATTGGTACGTGATGGAGGTACAAATAGAACT----------------------------
----

AATGAAACATTCAGACCTGGAGGAGGAGATATGAGGGACAATTGGAGAAGTGAATTATATAAATATAAAGTGG
TAGAAATTCAGCCATTG---GGAATAGCACCCACTAAGGCAAAAAGGAGAGTGGTG-----------------
----------------------GAGAGAGAAAAAAGAGCA---GTGGGA---ATTGGAGCT---------
GTGCTCCTTGGATTCTTGGGAGCA---GCAGGAAGCACTATG---GGCGCGGCGTCA---
ATAACGCTGACGGTACAG---GCCAGACAA---TTGTTG---TCTGGTATAGTGCAACAGCAAAGC---
AATTTG---CTGAAG---GCTATAGAGGCGCAACAGCATCTGTTGCAACTCACAGTCTGGGGCATT
>JN681229
TTCTATTGCGATACAACAAAACTGTTTAATACAACAAGAGGGGAGGAT-------------------------
----

CTTCATGAAAATACCACAATTCCATGCAAGATAAAGCAAATTATAAACATGTGGCAAGAGGTAGGACGAGCAA
TGTATGCCCCTCCCATTGCAGGAAACATAAATTGTACATCAAAC---
ATCACAGGACTACTATTGACACATGATGGAGGAAACAACCCGAAT----------------------------
----

AGCAGCACGATATTCAGACCTGGAGGGGGGAATATGAAGGACAACTGGAGAAGTGAATTATATAAATATAAAG
TGGTAGAAATTAAACCATTG---GGAATAGCACCCACTGCATCAAAAAGGAGAGTGGTG--------------
-------------------------GAGAGAGAAAAAAGAGCA---GTAGGA---ATAGGAGCT---------
GTGATCCTTGGGTTCTTGGGAACA---GCAGGAAGCACTATG---GGCGCGGCGTCA---
ATAACGCTGACGGTACAG---GCCAGACAA---CTGTTG---TCCGGTATAGTGCAACAGCAAAGC---
AATTTG---CTGAGA---GCCATAGAGGCGCAACAGCATATGTTGCAACTCACGGTCTGGGGCATT
>FJ443911
TTCTATTGCAATACAACACAACTGTTTAACAGTACATACAATACCAATGAT----------------------
----

ACAAGGGTAAACATCACACTTCCATGCAGAATAAAACAGATTATCAACATGTGGCAAAGGGTAGGACGAGCAA
TGTATGCTCCTCCCATTGCAGGAAACATAACATGTATATCAAAC---
ATCACAGGACTACTGTTAGTACGTGATGGAGGCAATAGCAGTGAC----------------------------
----

ACAACAGAAACATTCAGACCTGGAGGAGGAAATATGAAGGACAATTGGAGAAGTGAACTATATAAATATAAAG
TGGTAGAAATTCAGCCATTA---GGAGTAGCACCCACTAAAGCAAAAAGGAGAGTGGTG--------------
-------------------------GAGAGAGAAAAAAGAGCA---GTGGGA---ATAGGAGCT---------
GTGTTCCTTGGGTTCTTGGCAACA---GCAGGAGGCACTATG---GGCGCGGCGTCA---
ATAACGCTGACGGTACAG---ACCAGACAA---TTGTTG---TCTGGTATAGTGCAACAGCAAAGC---
AATTTG---CTGAAG---GCTATAGACGCGCAACAGCATCTGTTGCAACTCACAGTCTGGGGCATT
>KT183265
TTCTATTGTAATACATCAGGACTGTTTAATGGTACATACAATAATATATACAATACAGACAATGATACAACA-
----

GACAATGGAACCATCACACTCCCATGCAAAATAAAACAAATTATAAACATGTGGCAGGAGGTAGGAAGAGCAA
TATACGCCCCTCCCATTGCAGGAAATCTAACATGTATGTCAAAC---
ATCACAGGACTTCTCTTGGTACGGGATGGAGGTAATCAAAATAAT----------------------------
----

AGCACAGAGGTATTCAGACCTGGAGGAGGAGATATGAGGGACAATTGGAGAAGTGAATTATATAAATATAAAG
TGGTAGAAATTGAGCCATTG---GGAATAGCACCCACTAAGGCAAAAAGGAGAGTGGTG--------------
-------------------------GAGAGAGAAAAAAGAGCA---GTGGGA---ATAGGAGCT---------
GTACTCCTTGGGTTCTTGGGAGCA---GCAGGAAGCACTATG---GGCGCGGCGTCA---
ATAACGCTGACGGTACAG---GCCAGGCAA---CTGTTG---TCTGGTATAGTGCAACAGCAAAAT---
AATTTG---CTGAGA---GCTATAGAGGCGCAACAGCATATGTTGCAGCTCACGGTCTGGGGCATT
>KC894114
TTCTATTGTGATACATCAAGACTATTTAATGGTACATACAATGGTACAGATAGTAATCCAAACAATGGTACAG
ATAGT--
-------------GATTCA------
AACTCAAATTCAAACATCACACTTCCATGCAGAATAAAACAGATTATAAACATGTGGCAGGAGGTAGGAAGAG
CAATGTATGCTCCTCCCATTGCAGGAAGCATAACATGTAAATCAAAC---
ATCACAGGATTATTATTGACGCGTGATGGAGGCAAAGAGGAAAATAGC-------------------------
----

ACAACTAACAGAATAGAGATATTCAGACCTGGAGGAGGAAATATGAAGGACAATTGGAGAAGTGAATTATATA
AATATAAAGTAGTAGAAATCAAACCATTG---GGAATAGCACCCACTGGAGCAAAAAGGAGAGTGGTG-----
----------------------------------GAGAGAGAAAAAAGAGCA---GTGGGA---
ATAGGAGCT---------GTGTTCCTTGGGTTCCTGGGAGCG---GCAGGAAGCACTATG---
GGCGCGGCGTCA---ATAACGCTGACGGTACAG---GCCAGACAA---TTGTTG---
TCTGGTATAGTGCAACAGCAAAGC---AATTTG---CTGAAG---
GCTATAGAGGCACAACATCATCTGTTGCAACTCACAGTCTGGGGCATT
>KC894117
TTCTATTGCAATACAACAAGGTTGTTTAATGGCACCTACAATAGCACCTCAAGTGATAAGTCAAACTCAAGT-
----

GATCAGTTAAACATCACACTCCCATGCAGAATAAAACAAATTATAAACATGTGGCAGGAGGTAGGACGAGCAA
TATATGCCCCTCCCATTGCAGGATACATAAAATGTAGTTCAAAT---
ATCACAGGACTACTGTTGACACGTGATGGAGGTAATGGAGGTAATAATACT----------------------
----

GAAAATAATACAGAGACATTCAGGCCTGGAGGAGGAGATATGAGAGACAATTGGAGAAGTGAATTATATAAAT
ATAAAATAGTAGAAATTAAACCATTA---GGAATAGCACCCACTACGGCAAAAAGGAGAGTGGTG--------
-------------------------------GAGAGAGAAAAAAGAGCA---GTGGGA---ATAGGAGCT---
------GTGTTCCTTGGGTTCTTGGGAGCA---GCAGGAAGCACTATG---GGCGCGGCGTCA---
ATAACGCTGACGGTACAG---GCCAGACAA---TTGTTG---TCTGGTATAGTGCAACAGCAAAGT---
AATTTG---CTGAGA---GCTATAGAGGCGCAACAACATCTGTTGCAACTCACGGTCTGGGGCATA
>KC894118
TTCTATTGTAACACATCAGACCTGTTTAATAGAGTATACAATACTACAGGCACGTATAATAGTACAGAAAGA-
----

AGAAATTCAACCATCACAATTCAATGCAGAATAAAACAAATTATAAACATGTGGCAGAGGGTAGGACAAGCAA
TGTATGCCCCTCCCATTGCAGGAAACATAACATGTAAATCAAAC---
ATCACAGGATTACTATTGACACGAGATGGAGGACAAAATATAAAAAT-------------------------

----

GAAACAAATAAAGAGACATTCAGACCTGGAGGAGGAGATATGAGGGATAACTGGAGAAGTGAATTATATAAAT
ACAAAGTGGTAGAAATTAAACCATTG---GGAGTAGCTCCAACTGGGGCAAAAAGGAGAGTGGTG--------
-------------------------------GGGAGAGAAAAAAGAGCA---TTGGGA---ATGGGAGCT---
------ATGATCTTTGGGTTCTTGGGAGCA---GCAGGAAGCACTATG---GGCGCGGCGTCA---
ATAACGCTGACGGTACAG---GCCAGAAAT---TTGTTA---TCTGGTATAGTGCAACAGCAAAGC---
AATTTG---CTGAAG---GCTATAGAGGCGCAACAGCATCTGTTGCAACTCACAGTTTGGGGCATA
>KC894120
TTCTATTGCAATACATCAAACCTGTTTAATAATATTAGTACAGGCAAT-------------------------
----

TCTGGTAACATCACACTCCCATGCCAAATAAAACAAATTGTAAGAATGTGGCAGGGAGTGGGACAAGCAATGT
ATGCCCCTCCCATTGCAGGAAGCATAACATGTAGATCAAAT---
ATCACAGGACTATTATTGACACGTGATGGAGGCAAAACAAATTACACA-------------------------
----

GACCTAGAGATATTCAGACCAGGAGGAGGTGATATGAGGGACAATTGGAGAAGTGAATTATATAAATATAAAG
TAGTAGAAATTAAGCCATTA---GGAATAGCACCAACTGGGGCAAAAAGGAGAGTGGTG--------------
-------------------------GAGAGAGAAAAAAGAGCA---GTGGGA---ATAGGAGCT---------
GTACTCCTTGGGTTCTTGGGAGCA---GCAGGAAGCACTATG---GGCGCGGCGTCA---
ATAACGCTGACGGTACAG---GCCAGACAA---TTGTTG---TCTGGTATAGTGCAACAGCAAAGC---
AATTTG---CTGAGA---GCCATAGAGGCGCAACAGCATATGTTGCAACTCACAGTCTGGGGCATT
>KC156115
TTCTATTGCAATACATCAAAACTGTTTAATGGTACCATAGTTGATGGTACTACCCTGGTTAATAGTACCATAT
TTAAT--
-------------GATACC------
ATGTCTAATGGAACCATCACACTCCCATGTAGAATAAAACAAATTATAAATATGTGGCAGGAGGTAGGACGAG
CAATCTATGCCCCTCCCATTGCAGGAAACATCACATGTATCTCTAAT---
ATCACAGGACTACTATTGACACGTGATGGTGGAGGTGCCGGGGATGGG-------------------------
----

TCAAATGACACAGAAGAAATATTCAGACCTGGAGGAGGAGATATGAGGGACAATTGGAGAAGTGAATTATATA
AATATAAAGTGGTAGAAATTAAGCCGTTG---GGAATAGCACCCACTAAGGCAAAAAGGAGAGTGGTG-----
----------------------------------GAGAGAGAAAAAAGAGCA---GTGGGA---
ATGGGAGCT---------GTGTTCCTTGGGTTCTTGGGAGCA---GCAGGAAGCACTATG---
GGCGCAGCGGCA---GTAACGCTGACGGTACAG---GCCAGACAA---TTGTTG---
TCTGGAATAGTGCAACAGCAAAGC---AATTTG---CTGAGA---
GCTATAGAGGCGCAACAGCATATGTTGAAACTCACGGTCTGGGGCATT
>KC894124
TTTTACTGTAATACAACAAAACTGTTTAATACAACAGAATTTAATGAAAATGAT-------------------
----

TCAAATGATACCATCACACTCCCATGTAGAATAAAACAATTTATAAACATGTGGCAGGGAGTAGGAAGAGCAA
TGTATGCCCCTCCCATTGCAGGAAACATAACCTGTAACTCAAGT---
ATCACAGGAATATTATTGATACATGAAGAAAGGGGTGATAATCAGACAAGG----------------------
----

ATATTCAAACCTATAGGAGGAGATATGAAGAATAATTGGAGAAGTGAACTATACAAATATAAAGTGGTAGAAA
TTAAGCCATTG---GGAATAGCACCCACTGATGCAAAAAGGAGAGTGGTG-----------------------
----------------GAGAGAGAAAAAAGAGCA---GTGGGA---ATAGGAGCT---------
TTGTTCCTTGGGTTCTTGGGAGCA---GCAGGAAGCACTATG---GGCGCGGCGTCA---
GTGACGCTGACGGTACAG---GCCAGACAG---TTGTTG---TCTGGTATAGTGCAACAGCAAAGC---
AATTTG---CTGAGG---GCTATAGAGGCGCAACAGCATATGTTGCAACTCACAGTCTGGGGCATT
>HM204615
TTCTATTGCAATACATCAGGCCTGTTTAATGGTACATACAATAGTACAAACAATAGTACAGGA----------
----

GACAATAAGACCATCACACTCCCATGCAGAATAAAACAAATTATAAACATGTGGCAGGGGGTAGGACGAGCAA
TGTATGCCCCTCCCATTGCAGGAAACATAACATGTAAATCAAAT---

```
ATCACAGGATTACTATTGACACGTGATGGAGGAAATACTCATAAT----------------------------
-------------------------------------------------------------------------
-------------
AATACAGAGATATTCAGACCTGGAGGAGGCAATATGAAAGACAATTGGAGAAATGAATTGTATAAATATAAAG
TGGTAGAAATTAAGCCATTA---GGAATAGCACCCACTAGTGCAAAAAGGAGAGTGGTG--------------
-------------------------GCAAGAGAAAAAAGAGCA---GTGGGA---ATAGGAGCT---------
GTGTTCCTTGGGTTCTTGGGAGCA---GCAGGAAGCACTATG---GGCGCGGCGTCA---
ATAACGCTGACGGTACAG---GCCAGACAA---TTATTA---ACTGGTATAGTGCAACAGCAAAGT---
AATTTG---CTGAGG---GCTATAGAGGCGCAACAGCATATGTTGCAACTCACGGTCTGGGGCATT
>FJ443547
TTCTATTGCAATACAACAGAACTGTTCAATAATACTAATAAA-------------------------------
-------------------------------------------------------------------------
-------------------------------------
ACTGGCATACTCCCATGCAAAATAAAACAAATTATAAACATGTGGCAGGGGGTAGGACGAGCAATGTATGCCC
CTCCCATTGAAGGAAACATAACATGTAGGTCAATT---
ATCACAGGACTACTATTGGTACGTGATGGAGGAGAAGATAATAGC----------------------------
-------------------------------------------------------------------------
-------------
ACAGAGGAGGTATTCAGACCTGGAGGAGGAAATATAAAGGACAATTGGAGAAGTGAATTATATCAATATAAAG
TGGTAGCAATTAAGCCGTTG---GGAATAGCACCCACGCCGGCAAAAAGGAGAGTGGTGGAC---AGG-----
----------------GAAGTGGTGAAGAGAGGAAAAAGAGCA---GTGGGA---ATAGGAGCT---------
GTGTTCCTTGGGTTCTTGGGAGCA---GCAGGAAGCACTATG---GGCGCGGCGTCA---
ATAACGCTGACGGTACAG---GCCAGACAA---CTGTTG---TCTGGTATAGTGCAACAGCAAAGC---
AATTTG---CTGAGA---GCTATAGAGGCGCAACAGCATATGTTGCAACTCACGGTCTGGGGCATT
>FJ443838
TTCTATTGCAATACATCAAAACTGTTTAATGGTATATACAATAATACATACCATAAT----------------
-------------------------------------------------------------------------
----------------------------
ACATCTAGTAATATCATACTCAAATGCAGAATAAAACAAATTATAAACATGTGGCAAAAGGTAGGACGAGCAA
TGTATGCCCCTCCCATTGCAGGAAACATAACATGTACATCAAAT---
ATCACAGGACTACTATTGACACGTGATGGAGATAATAGGACTGAG----------------------------
-------------------------------------------------------------------------
----------------
TCAGAGACATTCAGACCTGGAGGAGGAGACATGAGGGACAATTGGAGAAGTGAATTATATAAATACAAGGTGG
TAGAAATTCAACCATTA---GGAGTAGCACCCACTGAGGCAAGAAGGAGAGTGGTG-----------------
----------------------GGGAAAGAAAAAAGAGCAGTAGTAGGA---ATAGGAGCT---------
GTGCTCCTTGGGTTCTTGAGTGCA---GCAGGAAGCACTATG---GGCGCAGCGTCA---
ATAACGCTGACGGTACAG---GCCAGACAA---TTGTTG---TCTGGTATAGTGCAACAGCAAAGC---
AATTTG---CTGAGA---GCTATAGAGGCGCAACAGCATATGTTGCAACTCACGGTCTGGGGCATT
>FJ443865
TTCTATTGCAATACATCAAACCTGTTTAATGATAGTACAAAA-------------------------------
-------------------------------------------------------------------------
----------------------------
AGTAATTCAAGCATCACACTCCCATGCAGAATAAAACAAATTATAAACATGTGGCAGAAGGTAGGACGAGCAA
TGTATGCCCCTCCCATTGCAGGAAACATAACATGTAGATCAGAG---
ATCACAGGACTACTATTGGTACATGATGGAGGAACAAATAGCAGT----------------------------
-------------------------------------------------------------------------
----------------
AATGAGACATTCAGGCCTGGAGGAGGAGATATGAGGGATAATTGGAGAAGTGAATTATATAAATATAAAGTGG
TAGAAATTAAGCCATTA---GGAGTAGCACCCACTCAGGCAAAAAGGAGAGTGGTG-----------------
----------------------GAGAGAGAAAAAAGAGCA---GTGGGA---ATAGGAGCT---------
GTGTTCCTTGGGTTCTTGGGAGCA---GCAGGAAGCACTATG---GGCGCGGCGTCA---
ATAACGCTGACGGTACAG---GCCAGACAA---TTGTTG---TCTGGTATAGTGCAGCAGCAAAGC---
AATTTG---CTGAGG---GCTATAGAGGCGCAACAGCATATGTTGCAACTCACGGTCTGGGGCATT
>MN202472
TTCTATTGTAACACATCAGACCTGTTTAATAGAGTATACAATACTACAGGCACGTATAATAGTACAGAAAGA-
-------------------------------------------------------------------------
----------------------------
AGAAATTCAACCATCACAATTCAATGCAGAATAAAACAAATTATAAACATGTGGCAGAGGGTAGGACAAGCAA
TGTATGCCCCTCCCATTGCAGGAAACATAACATGTAAATCAAAC---
```

ATCACAGGATTACTATTGACACGAGATGGAGGACAAAATATAAAAAATGAAACA-------------------
----

AATAAAGAGACATTCAGACCTGGAGGAGGAGATATGAGGGATAACTGGAGAAGTGAATTATATAAATACAAAG
TGGTAGAAATTAAACCATTG---GGAGTAGCTCCAACTGGGGCAAAAAGGAGAGTGGTG--------------
-------------------------GGGAGAGAAAAAAGAGCA---TTGGGA---ATGGGAGCT---------
ATGATCTTTGGGTTCTTGGGAGCA---GCAGGAAGCACTATG---GGCGCGGCGTCA---
ATAACGCTGACGGTACAG---GCCAGAAAT---TTGTTA---TCTGGTATAGTGCAACAGCAAAGC---
AATTTG---CTGAAG---GCTATAGAGGCGCAACAGCATCTGTTGCAACTCACAGTTTGGGGCATA
>KT183218
TTCTATTGCAATACAACAAAGCTATTCACAAACACAACC----------------------------------
----

ACAAATACAACCATCCTAATCCCATGCAGAATAAAACAATTTGTAAACATGTGGCAGGAGGTAGGGCGAGCAA
TGTATGCCCCTCCCATTGCAGGAAACATAACATGTAACTCAAGT---
ATCACAGGACTACTATTGGTACGTGATGGAGGAATTTCAAATGACACAAAT----------------------
----

AACACAACTGAAACATTCAGGCCTGGAGGAGGAAACATGAAGGACAATTGGAGAAGTGAATTATATAGCTATA
AAGTGGTAGAACTTAAGCCACTA---GGAGTAGCACCCACTGGAGCGAAAAGGAGAGTGGTGGAG--------
-------------------------ATGGAAAGATCAAAAAGAGCA---GTGGGA---ATAGGAGCT------
---GCACTCCTTGGGTTCTTGGGAGCA---GCAGGAAGCACTATG---GGCGCGGCGTCA---
ATGGCGCTGACGGTACAG---GCCAGACAA---CTGCTG---TCTGGTATAGTGCAACAGCAAAGC---
AATTTG---CTGAGA---GCCATAGAGGCGCAACAGCATATGTTGCAACTCACGGTCTGGGGCATT
>HM623548
TTCTATTGCAATACAACAAAACTGTTTAAGGAAGGTGAGTCAAATGCAACAGAT-------------------
----

TTAAATGCAACAATCATACTCCCATGCAGGATAAAACAAATTATAAACATGTGGCAGGGGGTAGGACAAGCAA
TGTATGCCCCTCCCATTGCAGGAAACATAACATGTATCTCAAAT---
ATCACAGGACTACTATTGACACGTGATGGAGGAAGAGACTCCAATAAC-------------------------
----

ACAAATGACACAGAGACATTCAGACCTGGAGGAGGAAATATGAAGGACAATTGGAGAAGTGAATTATATAAAT
ATAAAGTGGTAGAAATTCAGCCATTG---GGAGTAGCTCCCACTGGATCAAAAAGGAGAGTGGTG--------
-------------------------------GAGAGAGAAAAAAGAGCA---GTGGGA---CTGGGAGCT---
------GTACTCCTTGGGTTCTTGGGAGCA---GCAGGAAGCACTATG---GGCGCGGCGTCA---
ATAGCGCTGACGGTACAG---GCCAGACAA---CTGTTG---TCTGGTATAGTGCAACAGCAAAAC---
AATTTG---CTGAGA---GCTATAGAGGCGCAACAGCATATGTTGCAACTCACAGTCTGGGGCATT
>HM623549
TTCTATTGCAATACATCAGGACTATTTAAGGGTACATTTAATGGTACAGAAGTTGGTACATTTAATGGTACAA
AAGTTAAT---
-------------------AGT---
ACAGACACAAAAAACATCACACTTCAATGCAGAATAAAACAAATTATAAACATGTGGCAGGAGGTAGGACGAG
CAATATATGCCCCTCCCATTGCAGGAAACATAATATGTATATCAAAT---
ATCACAGGACTACTACTAGTACGTGATGGAGGCAATTTAAATAGC----------------------------
----

ACAAATGACACATTCAGACCTGGAGGAGGAAATATGAAGGACAATTGGAGAAGTGAATTATATAAATATAAAG
TGGTAGAACTTAAGCCATTG---GGAATAGCACCAACTGAAGCAAAAAGGAGAGTGGTG--------------
-------------------------GAGAGAGAAAAAAGAGCA---GTGGGA---ATAGGAGCT---------
TTGTTCCTTGGGTTCTTGGGAGTA---GCAGGAAGCACTATG---GGCGCAGTGTCA---
ATGACGCTGACGGTACAG---GCCAGACAA---CTGTTG---TCTGGTATAGTGCAACAGCAAAAC---
AACTTG---CTGAAA---GCTATAGAGGCGCAACAGCGTATGTTGCAACTCACAGTCTGGGGCATT
>HM623552
TTCTATTGCAATACCTCACAACTGTTTAAGGGTACATACAATGGTACAGACAATACATACAATGGGACA----
----

AAAGAAGCAAACATCACACTTCAATGCAGAATAAAACAAATTGTAAACATGTGGCAGGAGGTAGGAAAAGCAA
TGTATGCTCCTCCCATTGCAGGCCAAATAACATGTAAATCAAAT---

ATCACAGGACTACTATTAGTACGTGATGGAGGTAAGGTAACAAAAACAAATAGT-------------------
----
-
ACCACAGATGATAAAATAGAGATATTCAGACCTTCAGGAGGAGATATGAGGGACAATTGGAGAAGTGAATTAT
ATAAATATAAAGTGGTAGAAATTAAGCCATTA---GGAATAGCACCCACTGGAGCAAAAAGGAGAGTGGTG--
-------------------------------------CAGAGAGAAAAAAGAGCA---GTGGGA---
CTAGGAGCT---------ATGTTCCTTGGGTTCTTGGGAGCA---GCAGGAAGCACTATG---
GGCGCAGCGTCA---ATAACGCTGACGGTACAG---GCCAAACAA---CTGTTG---
TCTGGTATAGTGCAACAGCAAAAT---AATTTG---CTGAGA---
GCTATAGAGGCGCAACAGCATATGTTGCAACTCACAGTCTGGGGCATT
>HM623555
TTCTATTGCAATACAACAAACCTGTTTTATTATAATGGAAGTGAGAAG-------------------------
----

AATGACACAACCATCACCATCCAATGCAAAATAAAACAAATTATAAACATGTGGCAAGAGGTAGGACGAGCAA
TGTATGCCCCTCCCATTGCAGGAAACATAACATGTAGATCAAAT---
ATCACAGGATTACTATTGACACGTGATGGAGGAAACACGAATAGT----------------------------
----

ACAGAGGAGATATTCAGACCTGGAGGAGGAAATATGAAGGACAATTGGAGAAGTGAATTATATAAATATAAGG
TGGTAGAAATTCAGCCATTG---GGAATAGCACCCAATAAGGCAAAAAGGAGAGTGGTG--------------
-------------------------GAGAGAGAAAAAAGAGCA---GTAGCA---ATAGGAGCT---------
GTGCTCCTTGGGTTCTTGGGAGTA---GCCGGAAGCACTATG---GGCGCGGCGGCA---
CTAACGCTGACGGTACAG---GCCAGACAA---CTGTTG---TCTGGTATAGTGCAACAGCAAAGC---
AATTTG---CTGAGA---GCTATAGAGGCGCAACAGCATATGTTGCAACTCACAGTCTGGGGCATT
>HM623562
TTCTATTGCAATACAACAAAACTGTTTAATGCAAGTGAGACGGCTAGAAATATTAGTAAC-------------
----

CCAAATGAAAATATCACACTCCCATGCAGGATAAAACAAATTATAAACATGTGGCAGGAGGTAGGACGAGCAA
TGTATGCCCCTCCCATTGCAGGAAACATAACATGTAACTCAAAT---
ATCACAGGACTACTACTGACAAGGGATGGAGGAAACTATAGTATCACA-------------------------
----

AATAACACAGAGACATTCAGACCTGGAGGAGGAAATATGAAGGACAATTGGAGAAGTGAATTATATAAATATA
AAGTAGTAGAAGTTAAGCCATTG---GGAGTAGCACCCACTAAAGCAAAAAGGAGAGTGGTG-----------
----------------------------GAGAGAGAAAAAAGAGCA---ATAGGA---ATGGGAGCT------
---GTACTCCTTGGGTTCTTGGGAGCG---GCAGGAAGCACTATG---GGCGCAGCGTCA---
ATAACGCTGACGGTACAG---GCCAGACAA---TTGTTG---TCTGGTATAGTGCAACAGCAAAGC---
AATTTG---CTGAGA---GCCATAGAGGCGCAACAGCGCATGTTGCAACTCACGGTCTGGGGCATT
>HM623568
TTCTACTGCAATACATCAAACCTGTTTAATTCAACAAGACTAAAACTGTTTAATTCAAGTGATCCA-------
----

GGCGACAAAACCATCACACTCCAATGCAGAATAAAACAAATTATAAACATGTGGCAGCAGGTAGGACGAGCCA
TGTATGCCCCTCCCATTGAAGGAAAAATATCATGTAACTCAAGT---
ATCACAGGATTACTATTGACACGTGATGGAGGACATAGAAACACAACA-------------------------
----

AACAACACAGAGGTATTCAGACCTGGAGGAGGAGATATGAGGGACAATTGGAGAAGTGAATTATATAAATATA
AAGTGGTAGAAATTCAACCATTG---GGAGTAGCTCCCACCAAGGCAAAAAGGAGAGTGGTG-----------
----------------------------GAGAGAGAAAAAAGAGCA---TTGGGA---TTCGGAGCT------
---GTGCTCATTGGGTTCTTGGGAGCA---GCAGGAAGCACTATG---GGCGCGGCGTCA---
ATAACGCTGACGGTACAG---GCCAGACAA---CTGCTG---TCTGGTATAGTGCAACAGCAAAGC---
AATTTG---CTGAGA---GCTATAGAGGCGCAACAGCATATGTTGCAACTCACAGTCTGGGGCATT
>HM623571
TTCTATTGCAACACATCAAGACTGTTTAATGGTACATTTAATGAGACAAAGTTTAATAATACTTCAAACAACA
CC---

ACTCCTCAAAACATCACTCTCCAATGCAGAATAAAACAAATCATAAACACGTGGCAGGGGGTAGGACAAGCAA

TATATGCCCCTCCCATTGCAGGAAACATAACATGTAATTCAAGT---
ATCACAGGAATACTATTAACACGTGATGGAGGCCAAGACACAAATGGCACAGAT-------------------
----
-
GAGACCAAAAACGACACAGAGACATTCAGACCTATAGGAGGAGATATGAGGGACAATTGGAGAAGTGAATTAT
ATAAGTATAAAGTGGTAGAAATTCAGCCATTA---GGAATAGCACCCACTAAGGCAAGAAGGAGAGTGGTG--
-------------------------------------GAAAGAGTAAGAAGAGCA---GCA------
CTGGGAGCT---------GTGTTCCTTGGGTTCTTGGGAGCG---GCAGGAAGCACTATG---
GGCGCAGCGTCA---ATAACGCTGACGGTACAG---GCCAGACAA---TTATTG---
TCTGGTATAGTGCAACAGCAGAGT---AATCTG---CTGAGA---
GCTATAGAGGCGCAACAGCATATGTTGCAACTCACGGTCTGGGGCATT
>HM623577
TTCTATTGCAACACATCAGGACTGTTTAAGGATACAAACGATACAAACATGTCTAATGATACAGGA-------
----

AAGTCTAATGAGACAACACTTCACTGCAGAATAAAACAAATAATAAACATGTGGCAGGAGGTAGGACGAGCAA
TATATGCCCCTCCCATTGCAGGAGAAATAAGATGTGTATCAAAC---
ATCACAGGACTACTATTGACACGTGATGGAGGACAGTTAGAAAAC----------------------------
----

AGCACAGAAGAGATATTCAGACCTGGAGGAGGAAATATGAGGGACAATTGGAGAAGTGAATTATATAAATATA
AAGTGGTAGAAATCAAACCGCTA---GGAATAGCACCCACTGGTGCAAAAAGGAGAGTGGTG-----------
----------------------------GAGAGAGAAAAAAGAGCA---GTGGGA---ATAGGGGCT------
---GTGTTCATTGGGTTCTTAGGAGCA---GCCGGAAGCACTATG---GGCGCAGCGTCA---
ATAACGCTGACGGTACAG---GCCAGACAA---TTGTTG---TCTGGAATAGTGCAACAGCAAAGC---
AATTTG---CTGAGG---GCTATAGAGGCGCAACAGCATCTGTTGCAACTCACAGTCTGGGGCATT
>JN687726
TTCTATTGCAATACATCAAATCTGTTTAATACTACAGGACCTACA----------------------------
----

ACAAATGCAACTATCACACTCCCATGCAGAATAAAACAGATTATAAACATGTGGCAGGGGGTAGGACAAGCAA
TGTATGCCTCTCCCATTGCAGGAAATATAACATGTAACTCAAAT---
ATCACAGGATTACTATTGACACGTGATGGAGGTCATAATGGTACA----------------------------
----

TATGGTAATGAGACATTCAGACCTGGAGGAGGAGATATGAGGGACAATTGGAGAAGTGAATTATATAAATATA
AGGTGGTGGAAATTAAACCATTA---GGAATAGCACCCACTGAGGCAAAAAGAAGAGTGGTG-----------
----------------------------CAGAGAGAGAAAAGAGCA---GTGGGA---CTAGGAGCT------
---GTGTTCCTTGGGTTCTTGGGAGCA---GCAGGAAGCACTATG---GGCGCGGCGTCA---
GTGGCGCTGACGGTACAG---GCCAGACAA---CTGTTG---TCTGGTATAGTGCAACAGCAAAGC---
AATTTG---CTGAAA---GCTATAGAGGCGCAACAACATATGTTGCAACTCACGGTCTGGGGCATT
>KC154023
TTCTATTGCAATACAACAGGACTGTTTAATGCAACAAACCTCAATGCAACAAAT-------------------
----

CCCAATGAAAACATCACACTCCCATGCAGAATAAAGCAATTTGTAAACATGTGGCAGGGGGTAGGACGAGCAA
TGTATGCCCCTCCTGTTGAAGGAATAATAACATGTAACTCAAGT---
ATCACAGGACTACTGTTGACACGTGATGGGGGAAACACAGACAAT----------------------------
----

AACACAACAGAGACATTCAGACCTGGAGGAGGAAATATGAAGGACAATTGGAGAAGTGAATTATATAAATATA
AAGTAGTAGAAATTCAACCATTG---GGAATAGCACCCACAAACGCAAAAAGGAGAGTGGTG-----------
----------------------------GAGAGAGAAAAAAGAGCA---GTGGGA---ATAGGAGCT------
---GTATTCCTTGGGTTCTTGGGAGCA---GCAGGAAGCACTATG---GGCGCGGCGTCA---
ATAACGCTGACGGTACAG---GCCAGACTA---CTGTTG---TCTGGTATAGTGCAACAGCAAAGC---
AATTTG---CTGAGA---GCTATAGAGGCGCAACAACATATGTTGCAACTCACGGTCTGGGGCATT
>HM204619
TTCTATTGCAATACAACACAATTGTTTAATAGTACATATAATGTTACAGATCCGTTTAATAGTACAGAACTG-
----

GGTAATGATACCATCACACTCCCATGCAGAATAAAGCAAATTATAAACATGTGGCAGAGGGTAGGACGAGCAA
TGTATGCTCCTCCCATTGCAGGAAACATAACATGTAGATCAAAT---
ATCACAGGATTGCTATTGGTGCGTGATGGAGGAAATAATAATCAG----------------------------
----

ACAGGGAGAGAGATATTCAGACCTGCAGGAGGGAATATGAAAGATAATTGGAGAAGTGAATTACATAAATATA
AAGTGGTAGAAATTAAGCCATTG---GGAATAGCACCCACTAAGGCAAAAAGGAGAGTGGTG-----------
----------------------------GAGAGAGAAAAAAGAGCA---GTGGGA---CTGGGAGCT------
---GTGTTCTTTGGGTTCTTAGGCGCA---GCAGGAAGCACTATG---GGCGCGGCGTCA---
ATAGCGCTGACGGCACAG---GCCAGACAA---GTGTTG---TCTGGTATAGTGCAACAGCAAAGC---
AATTTG---CTGAGG---GCTATAGAGGCGCAACAACATATGTTGCAACTCACGGTCTGGGGCATT
>JX140666
TTTATTGCAATACAACAAATCTGTCTAATACAAGTCTGTTTAATGACAGTAGT--------------------
----

TCAAACAAAACCATCACGCTCCCATGTAGGATAAAACAAATTATAAACATGTGGCAGGGGGTAGGACGAGCAA
TGTATGCCCCTCCCATTGCAGGGAACATAACATGCAACTCAAGC---
ATCACAGGACTATTATTAACACGTGATGGAGGAAACATGAGTGGCACTGATGAAGGAAAAAACAACACGACTA
AC--
GGGAATAACACGACTAACGGGATTGAAATATTTAGACCTGGAGGAGGAAATATGAAGGACAATTGGAGAAGTG
AATTATATAAATATAAAGTGGTAGAAATTCAGCCATTG---
GGAGTAGCACCCACGAAGGCAAAAAGGAGAGTGGTG-------------------------------------
--GAGAGAGAAAAAAGAGCA---GTGGGA---ATAGGAGCT---------
GTACTCCTTGGGTTCTTGGGAACA---GCAGGAAGCACTATG---GGCGCGGCGTCA---
ATAACGCTGACGGTACAG---GCCAGACAA---CTGTTG---TCTGGTATAGTGCAACAGCAAAGC---
AATTTG---CTGAGA---GCTATAGAGGCGCAACAGCATATGTTGCAACTCACAGTCTGGGGCATT
>KF114893
TTCTATTGCAATACAACAGCACTGTTTAATAGGACTACATACAATACTACATACAATGGAACAGATACAACA-
----
-------------GACAAT------
GGGACAGATACAAACATCACACTTCAGTGCAGAATAAAACAAATTATAAACATGTGGCAGGGGGTAGGACAAG
CAATGTACGCCCCTCCAGTTGAAGGAATCATAACATGTAACTCAAAT---
ATCACAGGAATACTATTGACACGTGATGGAGGTACAGGCGAAGAC----------------------------
----

AATAAAACAGAGATATTTAGACCTGGAGGAGGAAATATGAAGGACAATTGGAGAAGTGAATTATATAAATATA
AAGTGGTAGAAATTAAGCCATTG---GGAATAGCACCCACTGAGGCAAGAAGGAGAGTGGTG-----------
----------------------------GAGAGAGAAAAAAGAGCA---GTGGGA---ATAGGAGCT------
---GTGATCATTGGGTTCTTGGGAGCA---GCAGGAAGCACAATG---GGCGCGGCGTCG---
ATGACGCTGACGGTACAG---GCCAGACAA---CTGTTG---TCTGGTATAGTGCAACAGCAAAAT---
AATTTG---CTGAGG---GCTATAGAGGCGCAACAACATATGTTGCAACTCACGGTCTGGGGCATC
>JN681253
TTCTATTGCAATACAACAAATCTGTTTAATGGAACAAAGCTGTTTAATGAAAGTGGTTCAAGTGGTTCAAAC-
----

ATAAGTAACTATATCATACTCCCATGCAGAATGAAACAAATTATAAACATGTGGCAAGGGGTAGGACGAGCAA
TGTACGCCCCTCCTATTGCAGGAAACATAACATGTAGATCAAAC---
ATCACAGGACTACTATTGACACGTGATGGA---AACAACAGCAAC----------------------------
----

ACTGAGCAAACATTCAGACCTGGAGGGGGAAATATGAAGGACAATTGGAGAAGTGAATTATATAAATATAAAG
TGGTAGAAATTAAGCCATTG---GGAATAGCTCCCACTGGAGCAAAAAGGCGAGTGGTG--------------
-------------------------GAGAGAGAAAAAAGAGCA---ATAGGA---ATAGGAGCT---------
GTGTTCCTTGGGTTCTTGGGAGTA---GCAGGAAGCACTATG---GGCGCGGCGTCA---
ATGACGCTGACGGTACAG---GCCAGACAA---CTGTTG---TCTGGTATAGTGCAACAGCAAAGT---
AATTTG---CTGAGA---GCAATAGAGGCGCAACAGCATATGTTGCAACTCACGGTCTGGGGCATT
>JN681255
TTCTATTGCAATACATCACAACTGTTTACTGGAAATGGTACAGCTAATAGTACAGGC----------------
----

AATACAACCATCACCACACTCCCATGCAGAATAAAACAAATTATAAACATGTGGCAGGGGGTAGGACGAGCAA
TGTATGCCCCTCCCATTGAAGGAAACATAACATGTAAATCAAAC---
ATCACAGGACTACTATTGACACGTGATGGAGGCAACGACAACAATCAA-------------------------
----

AGTAGAACAGAGACATTTAGACCTGGAGGAGGAAATATGAAGGACAATTGGAGAAGTGAATTATATAAATATA
AAGTGGTAGAAATTAAGCCATTA---GGAATAGCACCCACTGAAGCAAAAAGGAGAGTGGTG-----------
----------------------------GAGAGAGAAAAAAGAGCA---GTGGGA---CTAGGAGCT------
---GTAATCTTTGGGTTCTTGGGAGCA---GCAGGAAGCACTATG---GGCGCGGCGTCA---
ATAACGCTGACGGTACAG---GCCAGACAA---TTGTTG---TCTGGTATAGTGCAACAGCAAAGC---
AATTTG---CTGAAA---GCTATAGAGGCGCAGCAGCATATGTTGCAACTCACAGTCTGGGGCATT
>HQ595752
TTCTATTGTAATACATCACAGCTGTTTAATAGGACTGATACAGACATGCAGCAAGACAACACA----------
----

GGAAATGATACAATCACACTCCAATGCAGAATAAAACAAATTATAAACATGTGGCAGGAGGTAGGACGAGCAA
TGTATGCCTCTCCCATTGCAGGAGACATAACATGCAAATCAAAT---
ATCACAGGATTAATATTGACACGTGATGGAGGTAATAGCAGCACAGGGAATGAT-------------------
----

AGTAATAGCCGCACAGAGATATTCAGACCTGCAGGAGGAGATATGAGGGACAATTGGAGAAGTGAATTATATA
AATATAAAGTGGTGGAGATTAAGCCATTA---GGAGTAGCACCCACTCATTCAAAAAGGAGAGTGGTG-----
----------------------------------GAAAGAGAAAAAAGAGGA------------
ATAGGAGCT---------GTGCTCCTTGGGTTCTTGGGAGCA---GCAGGAAGCACTATG---
GGCGCAGCGTCA---ATAGCGCTGACGGTACAG---GCCAGACAA---CTGTTG---
TCTGGTATAGTGCAACAGCAAAGC---AATTTG---CTGAGA---
GCTATAGAGGCGCAACAGCATATGTTGCAACTCACGGTCTGGGGCATT
>HQ615957
TTCTATTGCAATACATCAGCACTGTTTAATGGCAATGGCACATCTGTTAATGAT-------------------
----

ACAACTTCAACCATCACACTTCCATGCAGAATAAAACAAATTATAAACATGTGGCAGGAGGTAGGACGAGCAA
TGTATGCCCCTCCCATTGCAGGAAACATAGCATGTGAATCAAAT---
ATCACAGGACTACTATTAGAACGTGATGGAGGTGGAGGCAATGAC----------------------------
----

ACAGAAGAGATATTCAGACCTGGAGGAGGAAATATGAGGGACAATTGGAGAAGTGAACTGTATAAATATAAAG
TGGTAGAAATTCAACCATTA---GGAGTAGCACCCACTAAGACAAAAAGGAGAGTGGTG--------------
-------------------------GAGAGAGAGAAAAGAGCA---GTGGGA---ATAGGAGCT---------
GTGTTCCTTGGGTTCTTGGGAGCA---GCAGGAAGCACTATG---GGCGCAGCATCA---
ATAACGCTGACGGTACAC---GCCAGACAA---TTATTG---TCTGGTATAGTGCAACAGCAAAGC---
AATTTG---CTGAGG---GCTATAGAGGCGCAACAGCATCTGTTGCAACTCACGGTCTGGGGCATT
>HQ595760
TTCTATTGTAATACAACAAGACTGTTTAATAATACAGAAAGTAACTCAACCATCACCGGGAAT----------
----

CATTCAGACACTATCACACTCCCATGTAGAATAAAACAATTTATAAACATGTGGCAGGAGGTAGGACGAGCAA
TGTATGCCCCTCCCATTGCAGGAACCATAACATGTATCTCAAAT---
ATCACAGGACTACTATTGGTACGGGATGGAGGCATACCAAATGAG----------------------------
----

AACACAGAAATATTCAGACCTCAAGGAGGAAATATGAAGGACAATTGGAGAAGTGAATTATATAAATATAAAG
TGGTAGAAATTAAGCCATTA---GGAATAGCACCCACTACGGCAAAGAGAAGAGTGGTG--------------
-------------------------CAGAGAGAAAAAAGAGCA---GTGGGA---ATAGGAGCT---------
GTGCTCCTTGGGTTCTTGGGAGCA---GCAGGAAGCACTATG---GGCGCGGCGTCA---
ATAACGCTGACGGTACAG---GCCAGACAT---CTGTTG---TCTGGTATAGTGCAACAGCAAAGC---
AATTTG---CTGAGA---GCTATAGAGGCGCAACAGCATATGTTGCAACTCACAGTTTGGGGCATT
>KT183289
TTCTATTGTAATACAACAAAATTGTTTAATAGTACATACAATAGTACATACTATAAGAATAATAATACTAATA
GTACAGAA---

GAAAACTCTACCATCACGCTCACATGCAGAATAAAGCAATTTATAAACATGTGGCAGGAGGTAGGACGGGCAA
TGTATGCCCCTCCCATTGAAGGAAACATAACATGTAGATCAAAC---
ATCACAGGACTACTACTGACGCGTGATGGAGGAAATTCAACAGAT----------------------------
----

ACTCAAACGGAGACATTCAGGCCTGAGGGAGGAAATATGAAGAACAATTGGAGAAGTGAATTATATAAATACA
AAGTGGTAGAAATTAAACCATTA---GGAATAGCACCCACTAGAGCAAAAAGGAGAGTGGTG-----------
----------------------------CAGAGGGAAAAAAGAGCA---GTGGGA---ATAGGAGCT------
---GTGTTCCTTGGGTTCTTGGGAGCA---GCAGGAAGCACTATG---GGCGCAGCATCA---
ATAACGCTGACGGTACAA---GCCAGACAA---CTATTG---TCGGGTATAGTGCAACAGCAAAGC---
AATTTG---CTGAGA---GCTATAGAGGCGCAACAGCATATGTTGCAACTCACGGTCTGGGGCATT
>GU080183
TTCTATTGCGATACATCAGCACTGTTTAATAGTACATACAATTCTGCATACAATAAGGAGACTAATAGTACAT
ACAATAAT---
-------------GGGACA------
GTAAGTAATTCAACTATCACACTTCCATGCAGAATAAAGCAAATTATAAACATGTGGCAAGAGGTAGGACGAG
CAATGTATGCCCCCCCCATTGCAGGAAATATAACATGTAAATCAAAT---
ATCACAGGATTACTATTAGTACGTGATGGAGGAGGCACAAATACA----------------------------
----

ACAAATGAGACATTCAGACCTCAAGGAGGAAATATGAAGGACAATTGGAGAAGTGAACTATATAAATATAAAG
TGGTAGAAATTGAGCCATTG---GGAATAGCACCCGATAAGGCAAGAAGGAGAGTGGTG--------------
-------------------------AAGAGAGAAAAAAGAGCA---GTGGGA---ATAGGAGCT---------
TTGTTCCTTGGGTTCTTGGGAGCA---GCAGGAAGCACTATG---GGCGCAGCATCA---
TTAATGCTGACGGTACAG---GCCAGACAA---CTGTTG---TCTGGTATAGTGCAACAGCAAAGC---
AATTTG---CTGAGA---GCTATAGAGGCGCAACAGCATATGTTGCAACTCACGGTCTGGGGCATT
>GU080187
TTCTACTGCAATACATCAAGCCTATTCAATATTACATACCATAACAATGGAACAAACACTAGTACATACACAG
AC---

AGCAACAGCATAATTACAATTCCATGCAGAATAAAACAAATTATAAACATGTGGCAGGGGGTAGGACGAGCAA
TGTATGCCTCCCCCATTGCAGGAAACATAACATGTAAATCAAAT---
ATCACAGGAATACTATTAACACGTGATGGAGGCTATCTAGAGAACAATACA----------------------
----

AATAATGACAAAGAAATATTCAGACCTGGAGGAGGAAATATGAAAGACAATTGGAGAAGTGAACTATATAAAT
ATAAAGTGGTAGAAATTAGACCATTA---GGAATAGCACCCACTAAAGCAAAAAGGCTAGTGGTG--------
-------------------------------AAGAGAGAAAAAAGAGCA---GTAACA---CTAGGAGCT---
------CTGTTCCTTGGGTTCTTGGGAACA---GCTGGAAGCGCTATG---GGCGCGGCGTCA---
CTGACGCTGACGGTACAG---GCCAGACAA---TTGTTG---TCTGGTATAGTGCAACAGCAAAAT---
AATTTG---CTGAGG---GCTATAGAGGCGCAACAGCATATGTTGCAACTCACAGTCTGGGGCATT
>HM623590
TTCTATTGTAATACATCAAAACTGTTTAATAGGACAGAAAATAATTCAATAGAAAGTAATTCCACC-------
----

AGTAATGCCAACATCACACTTCAATGCAGGATAAAACAAATTATAAACATGTGGCAGGGGGTAGGACGAGCAA
TGTATGCCCCTCCCATTGCAGGAAACATAACCTGTAACTCAAAT---
ATCACAGGAATACTATTGACACGTGATGGAGGAAGTAAAAATAAC----------------------------
----

AGTACAGAGACATTCAGACCTGGAGGAGGAAATATGAAGGACAATTGGAGAAGTGAATTATATAAATATAAAG
TGGTAGAAATTAAGCCATTG---GGAATAGCACCCACTAAGGCAAAAAGGAGAGTGGTG--------------
-------------------------GAGAGAGAAAAAAGAGCA---GTGGGA---ATAGGAGCT---------
GTGTTCCTTGGGTTCTTGGGAGCA---GCAGGAAGCACTATG---GGCGCGGCGTCA---
ATAACGCTGACGGTACAG---GCCAGACAA---CTGTTG---TCTGGTATAGTGCAACAGCAGAAT---
AATTTG---CTGAGG---GCTATAGAGGCGCAACAGCATATGTTGCAACTCACGGTCTGGGGCATT
>HM623598
TTCTATTGCAATACCACAAAATTGTTTAATAAGACTATTGCA-------------------------------
----

AACGGAACCATCACACTCCCATGCAGAATAAAACAAATTATAAACATGTGGCAGGAGGTAGGACGAGCAATGT
ATGCCCCTCCCATTGCAGGAAACATAACATGTGAATCAAAT---
ATCACAGGACTATTATTGCTACGTGATGGAGGAAGGATTAATGACACT-------------------------
----

AATAACACAGAAGAGATATTCAGACCTGGAGGAGGAAATATGAAGGACAATTGGAGAAGTGAATTATATAAAT
ATAAAGTGGTAGAAGTTCAGCCATTG---GGAATAGCTCCTACTGAATCAAAAAGGAGAGTGGTG--------
-------------------------------GAGAGAGAAAAAAGAGCA---GTGGGA---ATGGGAGCT---
------GTACTCCTTGGGTTCTTGGGAGCT---GCAGGAAGCACTATG---GGCGCGGCGTCA---
ATAACGCTGACGGCACAG---GCCAGACAA---CTGTTG---TCTGGTATAGTGCAACAGCAAAGC---
AATTTG---CTGAGA---GCTATAGAGGCGCAACAGCATATGTTGCAACTCACGGTCTGGGGCATT
>HM623604
TTCTATTGCAATACATCAGGACTGTTTAATAATAGCAAAAGT-------------------------------
----

GATTCAAACATAACCACACTCCCATGCAAAATAAAACAAATTATAAACATGTGGCAGAAGGTAGGACGAGCAA
TGTATGCCCCTCCCATTGCAGGAAACATAACATGTAACTCAAAT---
ATCACAGGACTACTATTGGTACGCGATGGGGGATACAATGTAGACAATCAG----------------------
----

ACAGACAATAAGACAGAGATTTTTAGACCTGGAGGAGGAAATATGAAGGATAATTGGAGAAGTGAATTATATA
AATATAAAGTGGTTGAAATTAAGCCATTA---GGAATAGCACCCACTAAGGCAAAAAGGAGAGTGGTG-----
----------------------------------GAGAGAGAAAAAAGAGCA---GTGGGA---
CTAGGAGCT---------GTGTTCCTTGGGTTCTTGGGAGCA---GCAGGAAGCACTATG---
GGCGCGGCATCA---ATAACGCTGACGGTACAG---GCCAGACAA---CTGTTG---
TCTGGTATAGTGCAACAGCAAAGC---AATTTG---CTGAGA---
GCTATAGAGGCGCAACAGCATATGTTGCAACTCACGGTCTGGGGCATT
>HM623606
TTCTATTGCAATACAACAAGGCTGTTTACAAATCTGAGTGCAAGT----------------------------
----

AGTGATTATACAATCACACTCCCATGCAGAATAAAACAAATTATAAACATGTGGCAGGAGGTAGGACGAGCAA
TGTATGCCCCTCCCATTGCAGGAGAAATAAAATGTAGCTCTAAT---
ATCACAGGACTACTATTGACGCGTGATGGAGGAATAGGAGACAAC----------------------------
----

ACAACAGAGATATTCAGACCTGGAGGAGGAAATATGAAAGATAATTGGAGAAGTGAATTATATAAATATAAAG
TGGTAGAAATTAAGCCATTA---GGAATAGCACCCACTGGGGCAAAAAGGAGAGTGGTG--------------
-------------------------GAGAGAGAAAAAAGAGCA---GCA------CTAGGAGCT---------
ATGTTCCTTGGGTTCTTGGGAGCA---GCAGGAAGCACTATG---GGCGCGGCGTCA---
ATAACGCTGACGGTACAA---GCCAGACAA---CTGTTG---TCTGGGATAGTGCAACAGCAAAGC---
AATTTG---CTGAGA---GCTATAGAGGCGCAACAGCACATGTTGCAACTCACAGTCTGGGGCATT
>HM623584
TTCTATTGCAATACATCAGGCCTGTTTAATGGTACATACAATAGTACAAGAAGTAATGCAAGT----------
----

TCAAGTCAAATCATCACACTCCAATGCAGAATAAAACAGATTATAAACAGGTGGCAAGAGGTGGGACGAGCAA
TGTATGCCCCTCCCATTGCAGGAAAAATAACATGTAACTCAAGT---
ATCACAGGACTACTATTGACACGGGATGGAGGAACAAGTGATAAT----------------------------
----

GGAACAGAGACATTCAGACCTGGAGGAGGAAATATAAGGGACAATTGGAGAAGTGAATTATATAAATATAAAG
TGGTAGAAATTAATCCATTG---GGAGTAGCACCCACCAAGGCAAAAAGGAGAGTGGTG--------------
-------------------------GAGAGAGAAAAAAGAGCA---GTGGGA---ATAGGAGCT---------
GTGCTCCTTGGGTTCTTGGGAGCG---GCAGGAAGCACTATG---GGCGCAGCGTCA---
ATAACGCTGACGGTACAG---GCCAGACAA---CTGTTG---TCTGGTATAGTGCAACAGCAAAGC---
AATTTG---CTGAGA---GCTATAGAGGCGCAACAGCATATGTTGCAACTCACAGTCTGGGGCATT
>HM623587

TTCTATTGCAATACAACAAACCTGTTCAATTTTAATGAAACACAACTTAATGAAATACAACTTAATAATGCAA
CAGAA--
-------------------AAC---
AACACAAGCACCAATATCACACTCACATGCAGAATAAAACAATTTGTAAACATGTGGCAAAGGGTAGGACGAG
CAATGTATGCCCCTCCCATTGCAGGAAATATAACATGTCATTCAAAC---
ATCACAGGACTGCTATTGACACATGATGAAGAAGAAATGAATAACGCAACT----------------------
----

GGGGCAAATAAAACAGAGGTATTCAGACCTATAGGAGGCAATATGAAGGACAATTGGAGAAGTGAATTATACA
AATATAAAGTAGTAGAAATTAAACCATTG---GGAGTAGCACCCACAGAGGCAAAACGGAGAGTGGTG-----
----------------------------------GAGAGAGAAAAAAGAGCA---GCA------
ATAGGAGCT---------ATGTTCCTTGGGTTCCTGGGAGCG---GCAGGAAGCACTATG---
GGCGCGGTGTCA---TTGACGCTGACGGTACAG---GCCAGACAA---CTGCTG---
TCTGGTATAGTGCAACAGCAAAGC---AATTTG---CTGAGA---
GCTATAGAGGCGCAACAACATCTGTTGCAACTCACGGTCTGGGGCATT
>KF114886
TTCTATTGCAATACATCAAACCTGTTTAATGGCACATACAATGGCACATATCAG-------------------
----

GGTAATGATACAATAACACTTCAATGCAGAATAAAACAAATTATAAACAGGTGGCAGGAGGTAGGAAGTGCAA
TGTATGCCCCTCCCATTAAAGGAAACATAACATGTAAATCAAAT---
ATCACAGGACTACTATTGGTACGTGATGGAGGCTCAGAAAACAAC----------------------------
----

ACAACCGAGACATTCAGACCTGAAGGAGGAAATATGAAAGACAATTGGAGAAGTGAATTATATAAATATAAGG
TGGTAGAAGTTAAGCCATTA---GGAATAGCACCCACTGACGCAAGAAGGAGAGTGGTG--------------
-------------------------GAGAGAGAAAAAAGAGCA---GTGGGA---ATAGGAGCT---------
GTGTTCCTTGGATTCTTAGGAGCA---GCAGGAAGCACTATG---GGCGCAGCAGCA---
GTGACGCTGACGGTACAG---GCCAGACAA---TTATTG---TCTGGTATAGTGCAACAGCAAAGC---
AATTTG---CTGAGA---GCTATAGAGGCGCAACAGCATATGTTGCAACTCACAGTCTGGGGCATT
>KF114887
TTCTACTGCAATACAACATACCTGTTTAATAACAGTGCA----------------------------------
----

TCAGAGGGAAACATCACAATCCCATGCAGAATAAAACAGATTATAAATATGTGGCAGGAGGTAGGACGAGCAA
TGTATGCCCCGCCCGTTGCAGGAAACATAACATGTAAATCAAAT---
ATCACAGGATTACTATTAGTACGTGATGGTGGAAATGAGGACAACAGCACAGAT-------------------
----

GGGAATAAGACAGAGATATTCAGACCTCAAGGAGGAAATATGAAGGACAATTGGAGAAGTGAATTATATAAAT
ATAAAGTGGTAGAAGTTAAGCCATTA---GGAATAGCACCCACTACAGCAAAAAGGAGAGTGGTG--------
-------------------------------GAAAGAGAAAAAAGAGCA---GTCGGA---ATAGGAGCT---
------GTGTTCCTTGGGTTCTTGGGAGCA---GCAGGAAGCACTATG---GGCGCAGCGTCA---
GTGGCGCTGACGGTACAA---GCCAGACAA---TTGTTG---TCTGGTATAGTGCAGCAGCAAAAT---
AATTTG---CTGAGA---GCTATAGAGGCGCAACAGCATATGTTGCAACTCACAGTCTGGGGCATT
>MF284967
TTTATTGCAATACATCAAGCCTGTTTAATAGTACATACTATCCTAATGATACAGAGAGTAACTCAAGCAAC-
----

TCAAGCAACACCATCACACTTCAATGCAGAATAAAGCAAATTGTAAACATGTGGCAGGAGGTAGGACAAGCAA
TGTATGCCCCTCCAGTTGCAGGAAAAATAACATGTAACTCAAAT---
ATCACAGGACTACTACTGACACGTGATGGAGGAAAGGGTGGTGGTACAGAA----------------------
----

AATAACACAGAGATATTCAGACCTGGAGGAGGAGATATGAGGGACAATTGGAGAAGTGAATTATATAAATATA
AAGTTGTAGAGATTAAACCATTG---GGAATAGCACCCACTGATGCAAAAAGGAGAGTGGTG-----------
----------------------------GAAAGAGAAAAAAGAGCA---GCA------ATAGGAGCT------
---GTACTCCTTGGGTTCTTGGGAGCA---GCCGGAAGCACTATG---GGCGCGGCGTCA---
ATAACGCTGACGGTACAG---GCCAGACAA---TTGTTG---TCTGGTATAGTGCAACAGCAAAGC---
AATTTG---CTGAGA---GCTATAGAGGCGCAACAGCATATGTTGCAACTCACAGTCTGGGGCATT

>KF725897
TTCTATTGCAACACATCAGGCCTGTTTAATGGAACATACAATCCTAATGGAACACACAGTTTTAATAATGGT-
----

ACCAATGGTACCATCACACTTCAATGCAGAATAAAACAAATTATAAACATGTGGCAGAAAGTAGGAAGAGCAA
TGTATGCCCCTCCCATTGCAGGAAGAATAACATGTAAATCAAAT---
ATCACAGGACTACTATTGACACGTGATGGAGGAAGCAACAATACT----------------------------
----

ACAGAGATATTCAGACCTGCAGGAGGAGATATGAGGGACAATTGGAGAAGTGAATTATATAAATATAAAGTGG
TAGAAATTAAGCCATTA---GGAGTAGCACCCACTGGGGCAAAAAGGAGAGTGGTG-----------------
----------------------GAGAGAGAAAAAAGAGCA---GTAGGA---ATAGGAGCT---------
GTGTTCCTTGGGTTCTTGGGAGCA---GCAGGAAGCACTATG---GGCGCAGCATCA---
ATAACGCTGACGGTACAG---GCCAGACAA---CTGTTG---TCTGGTATAGTGCAACAGCAAAGC---
AATTTG---CTGAGA---GCTATAGAGGCGCAACAGCATATGTTGCAACTCACGGTCTGGGGCATT
>KF725917
TTCTATTGCAACACATCACAATTGTTTAATAGTACAGTCAATGGTACAATATCTAATAATACAGGCAAT----
----

AGTACAGACATCATCACACTCCCATGCAGGATAAAACAAATTATAAACATGTGGCAGGGGGTAGGACGAGCAA
TGTATGCCCCTCCCATTGCAGGAAACATAACATGTAAGTCAAAC---
ATCACAGGATTACTATTGACACGTGATGGAGGATATAACACAAGTGGG-------------------------
----

GATAACGAGACATTCAGACCTGCAGGAGGAGATATGAGAGACAATTGGAGAAGTGAATTGTATAAATATAAAG
TAGTAGAAATTCAGCCATTA---GGAATAGCACCCACAGGAGCAAGAAGGAGAGTGGTG--------------
-------------------------GAGAGGCAAAAAAGAGCA---GTAGGA---ATAGGAGCT---------
GTGTTCCTTGGGTTCTTGGGAGTA---GCCGGAAGCACTATG---GGCGCGGCGTCA---
ATGGCGCTGACGGTACAG---GCCAGACAA---TTGTTG---TCTGGTATAGTGCAACAGCAAGGC---
AATTTG---CTGAGA---GCTATAGAGGCGCAACAACACATGTTGCAACTCACAGTCTGGGGCATT
>JN681257
TTCTATTGTAATACATCAAAACTGTTTAATGAGACAGAAACGAAGTCA-------------------------
----

AACATCTCAAACATCACACTTCCATGCAGAATAAAACAAATTATAAACATGTGGCAGGGGGTAGGACGAGCAA
TGTATGCCCCTCCTATTGCAGGAAGCATAACATGTAACTCAAAT---
ATCACAGGACTACTATTGACACGTGATGGAGGAGGAGGAAATGAAACT-------------------------
----

AATCACACAGAAACATTCAGACCTACAGGAGGAAATATGAAGGACAATTGGAGAAGTGAATTATATAAATATA
AAGTGGTAGAAATTAAGCCATTG---GGAATAGCACCCACTAGAGCAAAAAGGAGAGTGGTG-----------
----------------------------GAGAAAGAAAAAAGAGCA---GTGGGA---ATAGGAGCT------
---GTGTTCCTTGGGTTCTTGGGGGCA---GCAGGAAGCACTATG---GGCGCGGCGTCA---
ATAACGCTGACGGTACAG---GCCAGACAA---CTGTTG---TCTGGTATAGTGCAACAGCAAAAT---
AATTTG---CTGAGG---GCTATAGAGGCGCAACAGCATATGTTGCAACTCACGGTCTGGGGCATT
>JN681259
TTCTATTGCAATACAACAAAACTGTTTAATGAAAGTGAACTAAGTAAG-------------------------
----

TCAAACGAAACAATCACACTCCCATGCAGAATAAAACAAATTATAAACATGTGGCAGGAGGTAGGACGAGCAA
TGTATAACCCTCCCATTGAAGGAAACATAACATGTAACTCAAGT---
ATCACAGGACTATTATTGACATGGGATGGAGGAAATAACACAAAT----------------------------
----

AAAAATCAAACATTCAGACCTGGAGGAGGAGATATGAGGGACAATTGGAGAAGTGAATTATATAAATATAAAG
TGGTAGAAATTAAGCCATTG---GGAGTAGCACCCACTGAGGCAAAAAGGAGAGTGGTG--------------
-------------------------GAGAGAGAAAAAAGAGCA---GTGGGA---ATAGGAGCT---------
GTGCTCTTTGGGTTCTTGGGAGCA---GCAGGAAGCACTATG---GGCGCGGCGTCA---
ATAACGCTGACGGTACAT---GCCAGACAA---CTGTTG---AATGGTATAGTGCAACAGCAAAAC---
AATTTG---CTGAGA---GCTATAGAGGCGCAACAGCATATGTTGCAACTCACGGTCTGGGGCATT

>KC154028
TTCTATTGTAACACAACAGAACTGTTTTATAATGATTTTAATAATACAGGA------------------------
----

AAGAATGTAAACATCACACTCCAGTGCAAAATAAAACAAATTATAAACATGTGGCAGCAGGTAGGACGAGCAA
TGTATGCCCCTCCCATTGAAGGAAACATAACATGTACATCAAAT---
ATTACAGGACTACTATTGACATGGGATGGAAACATAACAAATGAAGATAATACA-----------TCT----
----

AGTACAAATCATACATTCAGACCTGCAGGAGGAGATATGAGGGACAATTGGAGAAGTGAACTATATAAATATA
AAGTGGTAGAAGTTAAGCCATTG---GGAGTAGCACCCACTAAGGCACAAAGGAGAGTGGTG----------
----------------------------GAGAGGAAAAAAAGAGCA---TTGGGA---ATAGGGGCT------
---ATGTTCCTTGGGATCTTGGGAACA---GCAGGAAGCACTATG---GGCGCGGCGTCA---
ATAACGCTGACGGTACAG---GCCAGACAA---TTATTG---TCTGGTATAGTGCAACAGCAAAGC---
AATTTG---CTGAGA---GCTATAGAGGCGCAACAGCATCTGTTGCAACTCACGGTCTGGGGCATT
>KF725924
TTCTATTGCGATACATCAAATCTGTTCAATGAAAGTAAAAGTTGGTATTCAAACAACAGTACA----------
----

GACGACATAAACATCACACTCCCCTGTAGGATAAAACAAATTGTAAACATGTGGCAGAGAGTAGGACAAGCAA
TATATGCCCCTCCCATTGCAGGAAGCATAACATGTAAATCAAAT---ATCACAGGACTACTATTGGCA-----
----CGCATAACAGATAACACA---

AATGGGACAGAGAAATTTAAACCTGAAGGAGGAAATATGAAGAATAATTGGAGAAGTGAATTATATAAATATA
AAGTGGTAGAAATTCAACCATTA---GGAATAGCCCCCACTGCATCAAAAAGGAGAGTGGTA----------
----------------------------GAGAGAGAAAAAAGAGCA---GTGGGA---ATAGGAGCT------
---GTGCTCCTTGGGTTCTTGGGAGCA---GCAGGAAGCACTATG---GGCGCGGCGTCA---
ATAACGCTGACGGTACAG---GCCAGACAA---CTGTTG---TCTGGTATAGTGCAACAGCAAAGC---
AATTTG---CTGAGA---GCTATAGAGGCGCAACAGCATATGTTGCAGCTCACAGTCTGGGGCATT
>KF725926
TTCTATTGCTATACATCACAACTGTTTAATAGTACATACAAGACATACAATCATAGTTGGACAACA-------
----

GGTAATGCAACCATCACACTTCCATGCAGAATAAAACAAATTATAAACATGTGGCAGGGAGTAGGACAAGCAA
TGTATGCCCCTCCCATTCAAGGAAATATAACATGTAAATCAAAT---
ATCACAGGAATACTATTAGTACGTGATGGAGACAAAAATGATACA----------------------------
----

GAGACATTCAGACCTCAAGGAGGAAATATGAAAGACAATTGGAGAAGTGAATTATATAAATATAAAGTGGTAG
AAATTAAGCCATTA---GGAATTGCACCTACTGGAGCAAAAAGGAGAGTGGTG-------------------
-------------------GAGAGAGAAAAAAGAGCA---GTGGGA---ATAGGAGCT---------
GTACTCCTTGGGTTCTTGGGAGCA---GCAGGAAGCACTATG---GGCGCAGCGTCA---
ATAACGCTGACGGTACAG---GCCAGACAA---TTATTG---TCTGGTATAGTGCAACAGCAGGAC---
AATCTG---CTGAGG---GCTATAGAGGCGCAACAGCATATGTTGCAACTCACGGTCTGGGGCATT
>KF725934
TTCTATTGCAATACATCAACCTTGTTTAATAGTACATATGGGTTTAATGGTACATACAATGGGACAACA----
----

AACAATGATACATACACACTCCAATGCAGAATAAAACAAATTATAAACATGTGGCAAAAGGTAGGACAAGCAA
TGTATGCCCCTCCTATTCAAGGAGTAATACAATGTAAATCAAAT---
ATCACAGGACTACTATTGACACGGGATGGAGGTGAAGGAAATAATACAAAT---------------------
----

AATGGTACAGAGACATTCAGACCTGGAGGAGGAGATATGAGGGATAACTGGAGAAGTGAATTATATAAATACA
AGGTGGTAGAAGTTCAGCCATTA---GGAGTAGCACCCACTGCTGCAAGAAGGAGAGTGGTG----------
----------------------------CAGAGAGAAAAAAGAGCA---GTAACA---CTAGGAGCT------
---ATGATCCTTGGATTCTTGGGAACA---GCAGGAAGCACTATG---GGCGCGGCGTCA---
ATGGCGCTGACGGTACAG---GCCAAACAA---TTGTTG---TCTGGTATAGTGCAACAGCAAAGC---
AATTTG---CTGAAG---GCTATAGAGGCGCAACAGCATATGTTGCAACTCACAGTCTGGGGCATA
>KF725961

TTCTATTGTAATACAACAAAGCTGTTTAATAGTACATACTTTCCTAATGGTACATACATGCCTAATGTTACAG
AA--

GATAATGGCACAATCATACTCCCATGCAGGATAAAGCAAATTATAAACATGTGGCAGGCGGTAGGACAAGCAA
TATATGCTCCTCCCATCGCAGGGAACATAACATGTATCTCAAAT---
ATCACAGGATTACTATTGGAGCGTGATGGAGGAAATGAAAGTAACACAGAG---------------------
----

GAGATATTCAGACCTCAAGGAGGAAACATGAGGGACAATTGGAGAAGTGAATTATACAAATATAAAGTGGTAG
AAGTTAAACCATTA---GGAATAGCACCTACTAAGGCAAAAAGGAGAGTGGTG-------------------
------------------GAGAGAGAAAAAAGAGCA---GTGGGA---CTAGGAGCT---------
GTGTTCCTTGGGTTCTTGGGAGCA---GCAGGAAGCACTATG---GGCGCGGCGTCA---
ATAACGCTGACGGTACAG---GCCAGACAA---TTGCTG---TCTGGTATAGTGCAACAGCAAAAC---
AATTTG---CTGAGA---GCCATAGAGGCGCAACAGCATATGTTGCAACTCACGGTCTGGGGCATT
>KF725984
TTCTATTGCAATACAACAAAACTGTTTAATAGGATATATTATCCTAATGGTACATACAATGGTACA-------
----

GAAAATGATACCATCATACTCCCATGTAGAATAAAACAAATTATAAACATGTGGCAAGGGGTAGGACGAGCAA
TGTATGCCCCTCCCATTGCAGGAGAAATAACATGTAACTCAAGT---
ATCACAGGACTACTATTGACACGTGATGGAGGACGTGATGGAGGCAACAATAGC------------------
----

GCAGAGACAGAGACATTCAGACCTAGCGGAGGAAATATGAGGGACAACTGGAGAAGTGAGTTATATAAATATA
AAGTGGTAGAAATTCAGCCATTA---GGAGTAGCACCCACTAAGGCAAAAAGAAGAGTGGTG----------
---------------------------GAGAGAGAAAAAAGAGCA---GTGGGG---CTAGGAGCT------
---GTATTCCTTGGGTTCTTGGGAGCA---GCAGGAAGCACTATG---GGCGCGGCGTCA---
ACAGCGCTGACGGTACAG---GCCAGACAA---CTGTTG---TCTGGTATAGTGCAACAGCAGAGC---
AATCTG---CTGAGA---GCCATAGAGGCGCAACAGCATATGTTGCAACTCACGGTCTGGGGCATT
>KF725994
TTCTATTGCAATACATCAAAACTGTTTAATAGTAATTTTACAGAGAATTTTACAGAGACTTCTAATAGT----
----

ACAGAGGGTGACATCACACTCCAATGCAGAATAAAACAAATTATTAACATGTGGCAGGGGGTAGGACGAGCAA
TGTATGCCCCTCCCATTGCAGGAACCATAACATGTAACTCAAGC---
ATCACAGGAATACTATTGACACATGATGGT---AACCATTCAGAAGAA------------------------
----

GAGGTGGTGGTATTCAGACCTGGAGGAGGAGATATGAGGGACAACTGGAGAAGTGAATTATATAAATATAAAG
TGGTAGAAGTTAAACCACTA---GGAATAGCACCCACTCAGGCAAAAAGAAGAGTGGTG-------------
------------------------GAGAGAGAAAAAAGAGCA---GTGGGA---CTAGGAGCT---------
GTGTTCCTTGGGTTCTTGGGAGCG---GCAGGAAGCACTATG---GGCGCAGCGTCA---
ATAACGCTGACGGTACAG---GCCAGACAA---CTGCTG---TCTGGTATAGTGCAACAGCAAAGC---
AATTTG---CTGAAG---GCTATAGAGGCGCAACAGCATATGTTGCAACTCACGGTCTGGGGCATT
>KF725998
TTCTATTGTAATACATCAAACCTGTTTAATGATACAGGGAGTAATTCA------------------------
----

ACTAATTCAACCATCATACTCCCATGCAGAATAAGACAAATTATAAACATGTGGCAGGGGGTAGGACGAGCAA
TGTATGCCCCTCCCATTAAAGGAAACATAACATGCAACTCAAGC---
ATCACAGGAATACTATTGACACGTGATGGAGGAAAGGACAACAGTACA------------------------
----

AATGAAACAGAGACATTCAGACCTATAGGAGGAAATATGAGGGACAACTGGAGAAGTGAATTATATAAATATA
AAGTGGTAGAAATCAAGCCATTG---GGAATAGCACCCACTCAGGCAAAAAGAAGAGTGGTG----------
---------------------------GAGAGAGAAAAAAGAGCA---GCGGGA---GTAGGAGCT------
---GTGCTCTTTGGGTTCTTGGGAGCA---GCCGGAAGCACTATG---GGCGCAGCGTCA---
ATGGCGCTGACGGTACAG---GCCAGACAA---CTGTTG---TCTGGTATAGTGCAACAGCAAAGC---
AATTTG---CTGAAG---GCTATAGAGGCGCAACAACATATGTTGCAACTCACGGTCTGGGGCATT
>KP109517

TTCTATTGCAATACATCAAAACTGTTTAATAGTAATGATACAGAGAGTGATTCA-------------------
----

ACCACAAACATCACCACACTTCAATGCAGAATAAAACAAATTATAAACATGTGGCAAAAGGTAGGACGAGCAA
TGTATGCCCCTCCCATTGCAGGAAACATAACATGTAATTCAAAC---
ATCACGGGACTGCTATTGACACGTGATGGAGGAAATAACAGCACT----------------------------
----

AACAACGAGGAGATATTCAGACCTGCAGGAGGAGATATGAGGGACAACTGGAGAAGTGAATTATATAAATATA
AAGTGGTAGAAGTTAAGCCATTG---GGAATAGCACCCACCAAGGCAAAAAGAAGAGTGGTG-----------
----------------------------GAACGAGAAAAAAGAGCA---GTGGGA---ATAGGAGCT------
---TTGTTCCTTGGGTTCTTGGGAGCA---GCAGGAAGCACTATG---GGCGCAGCGTCA---
CTAACGCTGACGGTACAG---GCTAGACAA---CTGTTG---TCTGGTATAGTGCAACAGCAAAGT---
AATTTG---CTGCAA---GCTATAGAGGCGCAACAGCATATGTTGCAACTCACAGTCTGGGGCATT
>MH933706
TTCTATTGCAACACATCAAGCTTGTTTAATGGAACATACAGTAATTACACTGATATAAACAGTTCTAGTATT-
----

ATCACACTCCCATGCAGGATAAAACAAATTATAAACATGTGGCAGGCGGTAGGACGAGCAATATATGCCCCTC
CCATCGAAGGGAACATAACATGTAAATCAAAT---
ATCACAGGACTACTATTGACACGTGATGGAGGAAAAGACAATAAT----------------------------
----

AACACAGAGGTATTCAGACCTGGAGGAGGAGAGATGAGGGACAACTGGAGAAATGAATTATATAAATATAAAG
TGGTAGAAATTAAGCCATTA---GGAATAGCACCCACTCATGCAAAAAGGAGAGTGGTG--------------
-------------------------GAGAGAGAAAAAAGAGCA---GTGGGA---ATAGGAGCT---------
TTGTTCCTTGGGTTCTTGGGAGCA---GCAGGAAGCACTATG---GGCGCGGCGTCA---
ATAACGCTGACGGTACAG---GCCAGACAA---CTGTTG---TCTGGTATAGTGCAACAGCAAAGC---
AACTTG---CTGAGG---GCTATAGAGGCGCAACAGCATATGTTGCAACTCACAGTCTGGGGCATT
>MK643550
TTCTATTGCAATACATCACAACTGTTTAATGGTACATATACAGGCAATAACACAGAGAACAAT----------
----

TCAAGCGAAACAATCACACTCCCATGCAGGATAAAACAAATTATAAACATGTGGCAGGAGGTAGGACGAGCAA
TGTATGCTCCTCCCATTGAAGGAAAAATAACATGTAACTCAAGC---
ATCACAGGACTACTATTGACAAGTGACAAAGGGGGTATAGGAAAT----------------------------
----

AATCAGACAGAGACATTCAGACCTGGGGGAGGCAATATGAAAGACAATTGGAGCAATGAATTATATAAATATA
AAGTAGTGGAAATTAGACCATTA---GGAGTAGCACCAACTGCTGCAAAGAGGAGAGTGGTG-----------
----------------------------GAGAGAGAAAAAAGAGCA---GTAGGA---ATAGGAGCT------
---GTACTCTTTGGGTTCTTGGGAGCC---GCAGGAAGCACTATG---GGCGCGGCGTCG---
ATAACGCTGACGGTACAA---GCCAGACAA---CTGTTG---TCTGGTATAGTGCAACAGCAAAGC---
AATTTG---CTGAGA---GCTATAGAGGCGCAACAGCATATGTTGCAACTCACAGTTTGGGGCATT
>MN611462
TTCTATTGTGATACAACACAACTGTTTAATCAAACACTGTTTAATGGAAGTGATTCAAACAAAACAAACATT-
----

GTCCTCCCCTACATTGTCCTCCCATGCAGAATAAAACAGATTATAAACATGTGGCAGAAGGTAGGACGAGCAA
TATATAATCCTCCCATTGCAGGAAACCTAACATGTAACTCAAGT---
ATCACAGGGCTACTATTGACACGTGATGGAGGAGAGAACATGACA----------------------------
----

CAGGGAGCGGAAACATTCAGACCTACAGGAGGAAATATGAAGGACAATTGGAGAAGTGAATTATATAAATATA
AAGTGGTAGAAATTCAGCCATTG---GGAGTAGCACCCACTGAGGCAAAAAGGAGAGTGATG-----------
----------------------------CAGAGAGAAAGAAGAGCAGTAGTGGGA---ATGGGAGCT------
---GTGCTCCTTGGGTTCTTGGGAGCA---GCAGGAAGCACTATG---GGCGCGGCGTCA---
ATAACGCTGACGGTACAG---GCCAGACAA---CTGTTG---TCTGGTATAGTGCAACAGCAAAGC---
AATTTG---CTGAGA---GCTATAGAGGCGCAACAGCATCTGTTGCAACTCACGGTCTGGGGCATT
>MN097669

TTCTATTGTAATACAACACAGTTGTTTAATGATAGCATCTCAAACGCCACTAACCTCAACATCTCAAACGCC-
----

ACTAACATCACACTCACACTCCCATGCAGAATCAAACAAATTATAAACATGTGGCAGGAGGTAGGACGAGCAA
TGTATGCCCCTCCCATCGAAGGAAACATAACATGTGTATCAAAT---
ATCACAGGACTACTACTGCTACGTGATGGCGGAAACACCAATAACACC-------------------------
----

ACTCAGCCAGAGATATTCAGACCTGGAGGAGGAAATATGAAGGATAATTGGAGAAGTGAATTATATAAATATA
AAGTGGTAGAAATTAAGCCATTA---GGAATAGCTCCCACGGAGGCAAAAAGGAGAGTGGTG-----------
----------------------------GAAAGAGAAAAAAGAGCA---GTA------CTAGGAGCT------
---TTGCTCCTTGGGTTCTTGGGAGCA---GCAGGAAGCACTATG---GGCGCGGCGTCA---
ATAGCGCTGACGGTACAG---GCCAGAAAT---CTGTTG---TCTGGTATAGTGCAGCAGCAAAGC---
AATTTG---CTGAGA---GCTATAGAGGCGCAACAGCATATGTTGCAGCTCACGGTCTGGGGCATT
>MN097680
TTCTATTGCAATACATCAAACTTGTTTAATAGTACATACAACGGTACAGACATGGCTAATGGTACAGGAAAT-
----

ACTTCAGAGTACATCACACTCCAATGCAGAATAAAACAAATTATAAACATGTGGCAGGAGGTGGGGCGAGCAA
TGTATGCCCCTCCCATTGCAGGAAACATAACCTGTGAATCAAAT---
ATCACAGGACTACTATTGACACGAGATGGAGGAAAAGAAAATAAT----------------------------
----

ACAGAGACATTCAGACCTGAAGGAGGAGATATGAGGAATAATTGGAGAAGTGAATTATATAAATATAAAGTAG
TAGAAATTCAACCATTA---GGAATAGCACCCACTAAGGCAAGAAGGAGAGTGGTG-----------------
----------------------CAGAAAGAGAAAAGAGCA---GTGGGA---ATGGGAGCG---------
TTGTTCCTTGGGTTCTTGGGGGCA---GCAGGAAGCACTATG---GGCGCAGCATCA---
ATAACGCTGACGGTACAG---GCCAGACAA---TTATTG---TCTGGTATAGTGCAACAGCAAAGC---
AATTTG---CTGAGG---GCTATAGAGGCGCAACAGCATATGTTGCAACTCACGGTCTGGGGCATT
>MN097584
TTCTATTGCAATACATCAAAGCTGTTT--

----------CTTAATAGC------
ACAAATGACAATCACACCATAATCCCCTGCAGAATAAAACAAATTATAAATATGTGGCAGGAGGTAGGACGAG
CAATGTATGCCCCTCCCATTGCGGGAAACATAACATGTAACTCAAGT---
ATCACAGGACTACTATTAACACGTGATGGAGGAAAAAATAATGACACGGAA----------------------
----

GAGGAAGAGGTATTCAGACCTGGAGGAGGAAATATGAAGGACAATTGGAGAAGTGAATTATATAAATATAAAG
TGGTAGAAATTAAGCCATTA---GGAATAGCACCCACTAAAGCAAAAAGGAGAGTGGTG--------------
-------------------------GAGAGAGAGAAAAGAGCA---TTGGGA---ATAGGAGCT---------
GTACTCCTTGGGTTCTTGGGAGCA---GCAGGAAGCACTATG---GGCGCAGCGTCA---
ATAACGCTGACGGTACAG---GCCAGACAA---TTGTTA---TCTGGTATAGTGCAACAGCAAAAC---
AATTTG---CTGAGA---GCTATAGAGGCGCAACAGCATATGTTGCAACTCACGGTCTGGGGCATT
>AY162225
TTCTATTGCAATACATCTGTACTGTTTAATGGCACATACAGTAATGGCACAAACAGTACA-------------
----

AATACAACAGTCATCACACTCCCATGCAGAATAAAACAAATTATAAACATGTGGCAGGGGGTAGGACGAGCAA
TGTATGCCCCTCCCATTGCAGGAAACATAACATGTAGATCAAAC---
ATCACAGGACTAATATTGACACGTGATGGAGGGCAGGGAGAGAAT----------------------------
----

GACACAAATGAGATATTTAGACCTGCAGGAGGAGATATGAGGGACAATTGGAGAAGTGAATTATACAAATATA
AAGTGGTAGAAATTCAGCCATTA---GGAGTAGCACCCACTAAGGCAAAAAGGAGAGTGGTG-----------
----------------------------GAGAGAGAAAAAAGAGCA---GCT------TTGGGAGCT------
---GTGTTCCTTGGGTTCTTGGGAGCA---GCAGGAAGCACTATG---GGCGCGGCATCA---
ATAATGCTGACGGTACAG---GCCAGACAA---CTGTTG---TCTGGTATAGTGCAACAGCAAAGC---
AATTTG---CTGAGA---GCTGTAGAGGCGCAACAGCATATGTTGCAACTCACGGTCTGGGGCATT
>AF391249

TTCTATTGCAATACATCAGAACTGTTTAATAGTACATACATGCCTAATGGTACAGAAAGTAAT----------
----

ACAAGCAAAACCATCATACTCCCATGCAGAATAAAACAAATTATAAATATGTGGCAGGGGGTAGGACGAGCAA
TGTATGCCCCTCCCATTGCAGGAAACATAACATGTCAATCAAAT---
ATCACAGGAATACTATTGACCCGTGATGGAGGAGAAGAGTCAAAGTCA-------------------------
----

AATGGAACAGAGATATTCAGGCCTGCAGGAGGGGATATGAAGGACAATTGGAGAAGTGAATTATATAGATATA
AAGTGGTAGAAATTAAACCATTA---GGAGTAGCACCCACTGAGGCAAAAAGGAGAGTGGTG-----------
----------------------------GAGAGAGAAAAAAGAGCA---GTGGGA---ATAGGAGCT------
---GTGTTCCTTGGGTTCTTGGGAGCA---GCAGGAAGCACTATG---GGCGCGGCGTCA---
ATAACGCTGACGGTACAG---GCCAGACAA---CCGTTT---TCTGGTATAGTGCAACAGCAAAGC---
AATTTG---CTGAGG---GCTATAGAGGCGCAACAGCATATGTTGCAACTCACAGTCTGGGGCATT
>EU293444
TTCTATTGCAATACATCAAAATTATTTAATGGTACATACAATGGTACAGAAAGTAATGGTACAAAAAGTAAT-
----

GATACAGAAATCATCACACTTCAATGCAAAATAAAACAAATTGTAAATATGTGGCAAGGGGTAGGACGAGCAA
TGTATGCCCCTCCCATTGCAGGAAATATAACATGTAAATCAAAT---
ATCACAGGATTACTATTGACACGTGATGGAGGAAATAATAACAAT----------------------------
----

AATAAGACAGAGACATTCAGACCTGGAGGAGGAGATATGAGAGACAATTGGAGAAGTGAATTATATAAATATA
AAGTGGTAGAAATTAAACCATTA---GGAATAGCACCCACTAATGCAAAAAGGAGAGTGGTG-----------
----------------------------GAGAGAGAAAAAAGAGCA---GTGGGA---ATAGGAGCT------
---GTGTTCCTTGGGTTCTTGGGAGCA---GCAGGAAGCACTATG---GGCGCAGCGTCA---
ATAACGCTGACGGTACAG---GCCAGACAA---CTGTTG---TCTGGTATAGTGCAACAGCAAAGC---
AATCTG---CTGAGA---GCTATAGAGGCGCAACAGCATATGTTGCAACTCACGGTCTGGGGCATT
>AY522721
TTCTATTGCAACACATCAAGGCTGTTTAATGAGACAGTAAAA-------------------------------
----

AATAATGCAACCATCACAATCCCATGCAAAATAAAACAATTTATAAACATGTGGCAAAAGGTAGGACAAGCAA
TGTATGCCCCTCCCATTAAAGGAAACATAACATGTACATCAAAT---
ATCACAGGACTACTATTGACACGTGATGGAGGGAATCACAACAATAGC-------------------------
----

CATAGCACAGAGATATTCAGACCTGGAGGAGGAGACATGAAAGATAACTGGAGAAGTGAATTATATAAATATA
AAGTGGTAGAAATTAAGCCATTG---GGCATAGCACCCACTGGGGCAAAAAGGAGAGTGGTG-----------
----------------------------GAGAGAGAAAAAAGAGCA---GTGGGA---ATAGGAGCT------
---GTGTTCCTTGGGTTCTTGGGAGCA---GCAGGAAGCACTATG---GGCGCAGCGTCA---
GTGACGCTGACGGTACAG---GCCAGACAA---TTGTTG---TCTGGTATAGTGCAACAGCAAAAC---
AATTTG---CTGAGA---GCTATAGAGGCGCAACAACATCTGTTGCAACTCACGGTCTGGGGCATA
>AY522732
TTCTATTGCAATACATCAATCCTGTTCAATAAGATGTACCATCCTGATGGTATAGGAAATACTTCAAATTGGA
GT---

AATTCAAGTTCAAACATCACCCTTCAATGCAGAATAAAACAAATTATAATCATGTGGCAGGGGGTAGGACAAG
CAATGTATGCCCCTCCCATTGCAGGAAACATAACATGTAAATCAAAT---
ATCACAGGACTACTATTGGTGCGTGATGGAGGCAGTAATGACACA----------------------------
----

GACCCAGAGACATTCAGACCTGGAGGAGGAGATATGAGGGACAATTGGAGAAGTGAATTGTATAAATATAAAG
TAGTAGAAATTAAACCATTA---GGAGTAGCACCCACCAAGGCAAAAAGGAGAGTGGTG--------------
-------------------------GAGAGAGAAAAAAGAGCA---GTGGGA---ATAGGAGCT---------
GTGTTCCTTGGGTTCTTGGGAGCA---GCAGGAAGCACTATG---GGCGCAGCATCA---
ATAACGCTGACGGTACAG---GCCAGACAA---TTGCTG---TCTGGTATAGTGCAACAGCAAAGC---
AATTTG---CTGAGG---GCTATAGAGGCGCAACAGCATATGTTGCAACTCACAGTCTGGGGCATT
>AY522734

TTCTACTGTAATACATCAAATCTGTTTAATGGCACATACCCAGATAATGGTACAACAGGA------------
----

AGTCATTCAACCATCATACTCCAATGCAGAATAAAACAATTTATAAACATGTGGCAGGAGGTAGGAAGAGCAA
TGTATGCCCCTCCCATTGAAGGAAACATAACATGTAACTCAAGT---
ATCACAGGACTACTGCTGACGCGGGATGGAGGAAACAGAAACGAA---------------------------
----

ACCAATGGCGAGATATTCAGACCTGGAGGAGGAGATATGAGGGACAATTGGAGAAGTGAATTATATAAATATA
AGGTGGTAGAAATTAAGCCATTG---GGAGTAGCACCCACGGCGGCAAAAAGGAGAGTGGTG----------
---------------------------GAGAGAGAAAAAAGAGCA---GCG------ATAGGAGCT------
---TTGTTCCTTGGGTTCTTAGGAGCA---GCCGGAAGCACTATG---GGCGCGGCGTCA---
ATAACGCTGACGGCACAG---GCCAGACGA---TTGCTG---TCTGGTATAGTGCAACAGCAAAGC---
AATTTG---CTGAGG---GCTATAGAGGCGCAACAGCATATGTTGCAACTCACGGTCTGGGGCATC
>DQ447267
TTCTACTGCAATACATCAGGACTGTTTAATAGAAGGTATAATGGTACAGAAAATAATACTTATGATGATACA-
----

GATAATTCAACCATCACAATTCCATGCAGAATAAAACAATTTATAAACATGTGGCAGCAGGTAGGACGGGCAA
TATATGCCCCTCCCATTGCAGGAAACATAACATGTAATTCAAGT---
ATCACAGGATTACTATTGACACGCGATGGAGGAAATCAGACTGAGGAGGAC---------------------
----

AATATCACAAAGACAGAGATATTCAGACCAGGAGGAGGAGATATGAGGGATAATTGGAGAAGTGAACTATATA
AATATAAAGCGGTAGAAATTAAGCCATTA---GGAGTAGCGCCCACTGGAGCAAAAAGGAGAGTGGTG-----
---------------------------------GAGAGAGAAAAAAGAGCA---GTGGGA---
ATAGGAGCT---------GTACTCCTTGGGTTCTTGGGAGCA---GCAGGAAGCACTATG---
GGCGCAGCGTCA---ATAGCGCTGACGGCACAG---GCCAGACAA---GTGTTG---
TCTGGAATAGTGCAACAGCAAAAC---AATTTG---CTGAGA---
GCTATAGAGGCGCAACAGCATATGTTGCAACTCACAGTCTGGGGCATT
>JX905394
TTCTACTGCAATACATCAAAACTGTTTAATGGTACATATAATGGTACATATAATGGTACA------------
----

GAAAATAGCACCATTACCCTCCCATGCAAAATAAAACAAATTATAAACATGTGGCAAGGGGTAGGAAGAGCAA
TGTATGCCCCGCCCATTCAAGGAAATATAACATGTGAATCAAAC---
ATCACAGGACTACTATTGACACGTGATGGAGGCACAAATAATACT---------------------------
----

AATAGTACAGAGACATTCAGACCTGGAGGAGGAAATATGAAGGACAATTGGAGAAGCGAATTATATAAATATA
AAGTGGTAGAAATTAAGCCATTA---GGACTAGCACCCACTAATGCAAAAAGGAGAGTGGTG----------
---------------------------GAGAGAGAAAAAAGAGCA---GTGGGA---ATAGGAGCT------
---GTGTTCCTTGGGTTCTTGGGAGCA---GCAGGAAGCACTATG---GGCGCAGCATCA---
ATAACGCTGACGGTACAG---GCCAGACAA---TTATTG---TCTGGTATAGTGCAACAGCAAAGC---
AATTTG---CTGAGA---GCTATAGAGGCGCAACAGCATATGTTGCAACTCACGGTCTGGGGCATT
>HQ625577
TTCTATTGCAATACATCAGGGCTGTTTAATAGAACATACCTGTCTAATGAAACATACCTGTCTAATGATACAC
TGACTAATGGT---

ACAAATAGGAATATAACACTCCCATGCAAAATAAAACAAATTATAAACATGTGGCAGGGGGTAGGAAAAGCAA
TGTATGCCCCTCCCATTGAAGGAAACATAACATGTAGATCAAAT---
ATCACAGGAATACTATTAACACGTGATGGAGGGAAAAGTGAGGGA---------------------------
----

ACGAAAAAATGGTACAGAGATATTCAGACCTGGAGGAGGAGATATGAAGGACAATTGGAGAAATGAATTATATA
AATATAAAGTGGTAGAAATTCAGCCATTA---GGAGTAGCACCCACTAAGGCAAAAAGGAGAGTGGTG-----
---------------------------------GAGAGAGAAAAAAGAGCA---GTGGGA---
CTGGGAGCT---------GTGCTCTTTGGGTTCTTGGGAGCA---GCAGGAAGCACTATG---
GGCGCAGCGTCA---ATAACGCTGACGGTACAG---GCCAGACAA---TTGCTG---

TCTGGTATAGTGCAACAGCAAAGC---AATTTG---CTGAGA---
GCTATAGAGGCGCAACAGCATATGTTGCAACTCACGGTCTGGGGCATT
>HQ625581
TTCTATTGCAATACATCAAAACTGTTTTCAAATCGCATGTTTAACTTTACAAACAGTTCAGACACAAACACAA
ACAGT--

TCAGGCGGCTACATCACACTCCCATGTAGGATAAAACAAATTATAAACATGTGGCAGAGGGTAGGACGAGCAA
TGTATAACCCTCCCATTGATGGAGACATAACATGTAACTCAACT---
ATCACAGGACTACTATTGTCACGTGATGGTGGTAGTGGTAACAACTCAGGG----------------------
----

ACAAACGAGACATTCAGACCTGGAGGAGGAGACATGAAGGACAATTGGAGAAATGAATTATATAAATACAAAG
TGATAGAAATTAAGCCATTA---GGAGTAGCTCCCACTGGTTCCAGAAGGAGAGTGGTG--------------
-------------------------GAGAGAGAAAAAAGAGCA---GTGGGC---ATAGGAGCT---------
GTGCTCCTTGGGTTCTTGGGAGCA---GCAGGAAGCACTATG---GGCGCGGCGTCA---
ATAACGCTGACGGTACAG---GCCAGACAA---CTGTTG---TCTGGTATAGTGCAACAGCAAAGC---
AATTTG---CTAAGA---GCTATAGAGGCGCAACAGCATATGTTGCAACTCACAGTCTGGGGCATT
>HQ625568
TTCTATTGCAATACAACAAATCTGTTTAATACAACAGACCTGTTGAGTAACAATAGTACGTTAGAAAAC----
----

AAAACAAAAACCATCACACTCCCATGCAAAATAAAACAAATTATAAACATGTGGCAGGAAGTAGGACGAGCAA
TGTATGCCCCGCCCATTGCAGGAAACATAACATGTAGATCAAAT---
ATCACAGGACTACTATTGACCCGTGATGGAGGGAGCTATAGTGATAGA-------------------------
----
GGGAATAGTAGTGCAAATGAAACA---------
GAAAAAGAAATATTCAGACCAGGAGGAGGAAATATGAAGGACAATTGGAGAAGTGAATTATATAAATATAAGG
TGGTAGAAGTTAAGCCATTG---GGAGTAGCACCCACTGAGGCAAAAAGGAGAGTGGTG--------------
-------------------------GAGAGACAAAAAAGAGCA---GTGGGA---CTAGGAGCT---------
ATGTTCCTTGGGATCTTGGGAATA---GCAGGAAGCACTATG---GGCGCGGCGTCA---
ATCACGCTGACGGTACAG---ACCAGACAA---CTGTTG---TCTGGTATAGTGCAACAGCAAAGC---
AATTTG---CTGAGA---GCTATAGAGGCGCAACAGCATATGTTGCAACTCACGGTCTGGGGCATT
>HQ625588
TTCTATTGTAATACATCACAACTGTTTAATGGTGTGTATAATACTTCAACCAGCAGTAATTCAACCGAT----
----

AGTAAGAAAAACATCACACTCCCATGCAGAATAAAACAAATTATAAACATGTGGCAAGAGGTAGGACGAGCAA
TGTATGCCCCTCCCATTGCAGGAAACATAACATGTATCTCAAAC---
ATCACAGGATTACTATTGACACGGGATGGAGGAACAAACAATGAGACT-------------------------
----

AATGGTACAGAGACATTCAGGCCTGGAGGAGGAAATATGAAGGACAATTGGAGAAGTGAATTATATAAGTATA
AGGTAGTGGAAATTCAACCATTA---GGAATAGCACCCACTGATGCAAAAAGGAGAGTGGTG-----------
----------------------------GAGAGACAGAAAAGAGCAGTAATAGGA---CTAGGAGCT------
---ATGTTCCTTGGGATCTTAGGAGTA---GCAGGAAGCACTATG---GGCGCAGCATCA---
ATAACGCTGACGGTACAG---GCCAGACAA---TTGTTG---TCTGGTATAGTGCAACAGCAAAGT---
AATTTG---CTGAAG---GCTATAGAGGCGCAACAGCATATGTTGCAACTCACAGTCTGGGGCATT
>KY229671
TTCTATTGCAATACATCAAACCTGTTTAATAGTACATACAGGCCTAATAGTACATACGGGTCTAATAGTACTT
CC---

ATCACACTTCAATGCAGAATAAAACAAATTATAAACATGTGGCAGGAGGTAGGGCGAGCAATGTATGCCCCTC
CCATTGCAGGAAACATAACATGTAAATCAAAT---
ATCACAGGACTACTATTAACACGTGATGGAGGGCACAATACAACA----------------------------
----

AACAAAACAGAGATATTCAGACCTGGAGGAGGAGATATGAGAAACAATTGGAGAAGTGAATTATATAAATATA
AAGTGGTAGAAATTAAGCCATTA---GGAGTAGCTCCCACTAGGGCAAAAAGGAGAGTGGTG-----------
----------------------------GAGAGAGAAAAAAGAGCA---GTAGGA---ATAGGAGCT------
---GTGTTCCTTGGGTTCTTGGGAGCA---GCAGGAAGCACTATG---GGCGCAGCATCA---

ATAACGCTGACGGTACAG---GCCAGACAA---TTGTTG---TCTGGTATAGTGCAGCAGCAAAGC---
AATTTG---CTGAGA---GCTATAGAGGCGCAACAGCATATGTTGCAACTCACGGTCTGGGGCATT
>AB254148
TTCTATTGCAATACATCAAAACTGTTTAATGGTACATACATGTCTAATAGTACAAAT---------------
----

AGTACTTCAAACATCACACTTCAATGCAGAATAAAACAAATTATAAACATGTGGCAGGGGGTAGGACAAGCAA
TGTATGCCCCTCCTATTGCAGGAAACATAACATGTAAATCAAAT---
ATCACAGGACTACTATTGACACGTGATGGAGGAGGTAATGACACAACAGGG---------------------
----

CAGAATAAGACAGAGACATTCAGACCTGGAGGAGGAGATATGAGGGACAATTGGAGAAGTGAGTTATATAAAT
ATAAAGTAGTAGAAATTAAGCCATTA---GGAATAGCTCCCACTCCGGCAAAAGGAGAGTGGTG--------
------------------------------GAGAGAGAAAAAAGAGCA---GTGGGA---ATAGGAGCT---
------GTGTTCCTTGGGTTCTTGGGAGCA---GCAGGAAGCACTATG---GGCGCAGCGTCA---
GTGACGCTGACGGTACAA---GCCAGACAA---TTGTTG---TCTGGTATAGTGCAACAGCAAAGC---
AATTTG---CTGAGG---GCTATAGAGGCGCAACAACATATGTTGCAACTCACGGTCTGGGGCATT
>AB254156
TTCTATTGCGACACATCAAACCTGTTTAACAGCACATTTAATGTTAACAGCACATTTAATGGTACAGATAATG
CA--

AATAGCACACAGAACATCACACTCCAATGCAGAATAAAACAAATTATAAACATGTGGCAGGAGGTAGGACGAG
CAATGTATGCCCCTCCCATTGCAGGAAACATAACATGTATTTCAAAT---
ATCACAGGAATACTATTAACACGTGATGGA---AATGATAATTAC---------------------------
----

ACAGAGACATTCAGACCTATAGGAGGAAATATGAAGGACAATTGGAGAAGTGAATTATACAAATATAAAGTGG
TAGAAATTAAGCCATTA---GGAGTAGCACCCACCAAGGCAAAAGGAGAGTGGTG-----------------
----------------------GAGAGACAAAAAAGAGCA---GTGGGA---CTAGGAGCT---------
GTGTTCCTTGGGTTCTTAGGAGCA---GCAGGAAGCACTATG---GGCGCAGCGTCA---
ATAACGCTGACGGTACAG---GCCAGACAA---TTGTTG---TCTGGTATAGTGCAACAGCAAAGC---
AATTTG---CTGAGA---GCTATAGAGGCGCAACAGCACATGTTGCAACTCACAGTCTGGGGCATA
>KY229510
TTCTATTGCAAGACATCACGACTATTTAATGGCACATACAGAGGTCCTAATGATACAAAAAGCAAT------
----

TTAAGTGAGACTATCACACTCCAATGCAAAATAAAACAGATTATAAACACGTGGCAGGGGGTAGGACGAGCAA
TATATGCCCCTCCCATTGCGGGAAACATAACATGTAAATCAAAT---
ATCACAGGACTACTCTTGGTAAGGGATGGAGGGAATAATACAAAT---------------------------
----

GAAGAAATATTTAGACCTGGAGGAGGAGATATGAGGGACAATTGGAGAAGTGAATTATATAAATATAAAGTAG
TAGAAATTAAGCCATTG---GGAATAGCACCCACCAAGGCAAAAGGAGAGTGGTG-----------------
----------------------GAGAGAGAAAAAAGAGCA---GTGGGA---ATAGGAGCT---------
GTACTCCTTGGGTTCTTGGGAACA---GCAGGAAGCACTATG---GGCGCAGCGTCA---
ATAACGCTGACGGTACAG---GCCAGACAA---GTGTTG---TCTGGTATAGTGCAACAGCAAAGC---
AATTTG---CTGAGG---GCTATAGAGGCGCAGCATCATATGTTGCAACTCACGGTCTGGGGCATT
>GU939143
TTCTATTGTAATACATCAAAACTGTTTAATAGTACATACAATCCTAATTACAATGGTACAGAAAATAAT----
----

TTAAACTACACCATAACACTCAATTGCAGAATAAAACAAATTATAAACATGTGGCAGGAGGTAGGACGAGCAA
TATATGCCCCTCCCATTGAAGGAAACATAATGTGTAATTCAAGT---
ATCACAGGACTACTGTTGCTGCGTGATGGAGGAATAAGTGAGAGTGAG------------------------
----

CCAAATGACACAGAGACATTCAGACCTGGAGGAGGAGATATGAAGGACAATTGGAGAAGTGAATTATATAAAT
ATAAAGTGGTAGAAATTAAGCCACTG---GGATTAGCACCCACCACAGCAAAAGGAGAGTGGTG--------
------------------------------GAGAGAGAAAAAAGAGCA---GTGGGA---ATAGGAGCT---
------GTGTTCCTTGGGTTCTTGGGAGCA---GCAGGAAGCACTATG---GGCGCGGCGTCG---

ATAACGCTGACGGTACAG---GCCAGACAA---CTGCTG---TCTGGTATAGTGCAACAGCAAAGC---
AATTTG---CTGAGG---GCTATAGAGGCGCAACAGCATATGTTGCAACTCACAGTCTGGGGCATT
>HM036864
TTCTATTGTAATACATCAGGCCTGTTTAATGGTACATTTAATGGAACATACCCTAATAGTACAGACCCTAAT-
----

GGAAATGATACCATCACACTCCCATGCAGAATAAAACAGTTTATAAACATGTGGCAGAAGGTAGGACGAGCAA
TGTATGCCCCTCCCATTAAAGGAAACATAACATGTAACTCAAAT---
ATCACAGGATTACTGTTGACGAGTGATTATGGAAGAAATGCAAGT----------------------------
----

AAAAACGAGACATTCAGACCTGGAGGAGGAGATATGAGGGACAATTGGAGAAGTGAATTATATAAATATAAAG
TGGTAGAAATTAAACCATTG---GGAGTAGCACCCACTGGAGCAAAAAGGAGAGTGGTG--------------
-------------------------GAGAGAGAAAAAAGAGCA---GTGGGA---ATAGGAGCT---------
GTTCTCCTTGGGTTCTTGGGAGCA---GCAGGAAGCACTATG---GGCGCAGCGTCA---
ATAACGCTGACGGTACAG---GCCAGACAA---TTGTTG---TCTGGTATAGTGCAACAGCAAAGC---
AATTTG---CTGAGG---GCTATAGAGGCGCAACAGCATATGTTGCAACTCACGGTCTGGGGCATT
>DQ422948
TTCTATTGCAATACAACAAAACTGTTTCATGAGGGAAGCAGGAAAAAT-------------------------
----

GATAATGATACAATCACACTTCCATGCAGAATAAAACAATTTATAAACATGTGGCAGGAGGTAGGACGAGCAA
TGTATGCACCTCCCATTGCAGGAAACATAACATGTAATTCAAGC---
ATCACAGGAATACTATTGGTACGTGATGGAGGTACCAATAACACA----------------------------
----

AATGAGACATTCAGACCTGGAGGAGGAGATATGAGGGACAATTGGAGAAGTGAATTATATAAATATAAAGTGG
TAGAAATTCAACCATTG---GGAATAGCACCCACTAAGGCAAAAAGGAGAGTGGTG-----------------
----------------------GAGAGAGAAAAAAGAGCA---GTGGGA---CTAGGAGCT---------
GTGTTCCTTGGGTTCTTGGGAGCA---GCGGGAAGCACTATG---GGCGCGGCGTCA---
ATAACGCTGACGGCACAG---GCCAGACAA---TTGCTG---TCTGGTATAGTGCAACAGCAAAGC---
AATTTG---CTGAGA---GCTATAGAGGCGCAACAGCATATGTTGCAACTCACAGTCTGGGGCATT
>EU166653
TTCTATTGCAATACATCAAGACTGTTTAATAGTACACACTGGGCTAATGGTACTACAGACATGTCTAAT----
----

GATACTAAGAATATCACCCTCCCATGCAAAATAAAACAAATTATAAACATGTGGCAGGGGGTAGGACGAGCAA
TATATGCCCCTCCCATTGAAGGAAGCATAACATGTAATTCAAGT---
ATCACAGGACTACTATTGACACGTGATGGAGGAAACAGTCATAAT----------------------------
----

ACTACAGAGACATTCAGACCTGGAGGAGGAGATATGAGAGACAATTGGAGAAGTGAATTATATAAATATAAGG
TGGTAGAAATTAAGCCATTA---GGACTAGCACCCACTGAGGCAAAAAGGAGAGTGGTG--------------
-------------------------CAGAGAGAAAAAAGAGCA---GTGGGG---ATAGGAGCT---------
GCATTCCTTGGGTTCTTGGGATTA---GCAGGAAGCACTATG---GGCGCGGCATCA---
ATAACGCTGACGGTACAG---GCCAGACAA---TTATTG---TCTGGTATAGTGCAACAGCAAAGC---
AATTTG---CTGAGA---GCTATAGAGGCGCAACAGCATATGTTGCAACTCACAGTCTGGGGCATT
>HM036843
TTCTATTGCAATACATCGAACCTGTTTAATAGGCTATACCTGTTTAATAGTACATACAACTCTAATAGCACAG
AAAATTAT---
-------------AATAGC------
ACAGGAAATGAGACCATCACACTCCAATGCAGAATAAAACAAATTATAAACATGTGGCAGGGGGTAGGAAAAG
CAATGTATGCCCCTCCCATTGCAGGAAACATAACATGTAATTCAAAT---
ATCACAGGACTAATATTAACGCGTGATGGAGGAACGCATGAGAACAAAAGT----------------------
----

TCCTCAGAGACAGAGACATTCAGACCTGGAGGAGGAGATATGAGGGACAATTGGAGAAGTGAATTATATAAAT
ATAAAGTGGTAGAAATCAAACCATTA---GGAATAGCACCCACTAAGGCAAAAAGGAGAGTGGTG--------
-------------------------------GAGAGAGAAAAAAGAGCAGTAGTGGGA---CTAGGAGCT---
------TTGTTCCTTGGGTTCTTGGGAGCA---GCAGGAAGCACTATG---GGCGCAGCATCA---

ATAACGCTGACGGTACAG---GCCAGACAA---TTGTTG---TCTGGTATAGTGCAACAGCAAAGT---
AATTTG---CTGAGG---GCTATAGAGGCGCAACAGCATATGTTGCAACTCACAGTCTGGGACATT
>KY229617
TTCTATTGCGAAACATCAGACCTGTTTAATAGTACATTTAGATTTAATGGTACAGACATACAGACCACATTTA
ATGATACA---

TTGAATGATACACAGATCATCATCAAATGCAGAATAAAGCAAATTATAAACATGTGGCAAGGGGTAGGGCGAG
CAATATATGCCCCTCCCATTGAAGGAAATATAACATGTGAATCAAAT---
ATCACAGGATTAATATTGACACGGGATGGAGGGAATACCACAGAT----------------------------
----

CCAGAGACATTCAGACCTGCAGGAGGAGATATGAGGGACAATTGGAGAAGTGAATTATATAAATATAAAGTGG
TAGAAATTCAGCCATTA---GGAGTAGCACCCACAAAGGCAAAAAGGAGAGTGGTGGAG--------------
----------------------AGAAGAGAAAAAAGAGCA---GTGGGA---ATAGGAGCT---------
GTGTTCCTTGGGTTCTTGGGAGCA---GCAGGAAGTACTATG---GGCGCAGCATCA---
ATAACGCTGACGGTACAG---GCCAGACAA---CTGTTG---TCTGGTATAGTGCAACAGCAGAGC---
AATCTG---CTGAGG---GCTATAGAGGCGCAACAGCATATGTTGCAACTCACGGTCTGGGGCATT
>HM036739
TTCTATTGCAATACATCAGGCCTGTTTAATTATACATTTACAGCTAATGATACAAAGATACAGATCACATCTA
ATGAC--

ACACTTAATGATACACTGACCATCAAATGCAGAATAAAGCAAATTATAAACATGTGGCAGGGGGTAGGACGAG
CAATGTATGCCCCTCCCATTGAAGGAATAATAGCATGTAATTCAACT---
ATCACAGGATTAATATTGACACGGGATGGAGGGAAGAATACCAGTAAC-------------------------
----

AATGACACAGAGACATTCAGACCTGCAGGAGGAGATATGAGGGACAATTGGAGAAGTGAATTATATAAATATA
AAGTGGTAGAAATTCAGCCATTA---GGAGTAGCACCCACAAAGGCAAAAAGGAGAGTGGTGGAG--------
----------------------------AGAAGAGAAAAAAGAGCA---GTGGGA---ATAGGAGCT------
---GTGTTCCTTGGGTTCTTGGGAGCA---GCAGGAAGCACTATG---GGCGCAGCATCA---
ATAACGCTGACGGTACAG---GCCAGACAA---CTGTTG---TCTGGTATAGTGCAACAGCAGAGC---
AATCTG---CTGAGG---GCTATAGAGGCGCAACAGCATATGTTGCAACTCACGGTCTGGGGCATT
>GU329051
TTCTATTGCGATACATCAGGCCTGTTTGATTCAAGCCTGTTGAATAAGACAGAAAATAGTACAAAGACAGAA-
----

AATAATACAAACATCATACTCCAATGCAGAATAAAACAAATTATAAACATGTGGCAAGAGGTAGGAAAAGCAA
TATATGCCCCTCCCATTGAAGGAAACATAACATGTATATCAAAC---
ATCACAGGAATACTATTGACGAGGGATGGAGGAAACAATAACAAT----------------------------
----

GACACAGAGATATTCAGACCTGGAGGAGGGAATATGAAAGACAATTGGAGAAGTGAATTATATAAATATAAAG
TAGTAGAAATTCAGCCATTG---GGAATAGCACCTACCGAGGCAAAGAGAAGAGTGGTG--------------
-------------------------GAGAGAGAAAAAAGAGCA---GTGGGA---ATAGGAGCT---------
GTGTTCCTTGGGTTCTTGGGAGCA---GCAGGAAGCACTATG---GGCGCAGCGTCA---
ATGACGCTGACGGTACAG---GCCAGACAA---TTGTTG---TCTGGTATAGTGCAACAGCAAAGC---
AATTTG---CTGAGA---GCCATAGAGGCGCAACAGCATATGTTGCAACTCACAGTCTGGGGCATT
>GU329105
TTCTACTGCAATACATCAGACCTGTTTAAGGGTGAATTTAATGGTACGCGTAATCTAGGAAATGAT-------
----

ACTTCAGGAAACATCACACTCAACTGCAGAATAAAACAAATTATAAACATGTGGCAGGAGGTAGGGCGAGCAA
TGTATGCCCCTCCCATTGCAGGAAATATAACATGTAACTCAAGT---
ATCACAGGACTACTATTGACACGTGATGGAGGAGAAACTAATGAA----------------------------
----

ACTGAGAGTGAGACATTTAGACCTGGAGGAGGAGATATGAGGGACAATTGGAGAAGTGAATTATATAAATATA
AAGTGGTAGAAGTTAATCCATTA---GGAATAGCACCCACTAATGCAAGAAGGAGAGTGGTGACA--------
-------------------AAAGAAGAAGTGAGAAAGAAAAGAGCA---GTGGGA---CTAGGAGCT------
---GTGTTCCTTGGGTTCTTGGGAGCA---GCAGGAAGCACTATG---GGCGCGGCGTCA---

ATAACGCTGACGGTACAG---GCCAGACAA---TTACTG---TCTGGTATAGTGCAACAGCAAAAC---
AATTTG---CTGAGA---GCTATAGAGGCGCAACATCATATGTTGCAACTCACAGTCTGGGGCATC
>GU329116
TTCTATTGCAATACATCAGGCCTGTTTAATAAGACATACAATGATACA-------------------------
----

AGTGATAAAAACATCACACTCCCATGCAGGATAAAACAAATTATAAACATGTGGCAGAAGGTAGGACAGGCAA
TGTATGCCCCTCCCATTAGAGGAAACATAACATGTCACTCAAAT---
ATCACAGGGCTATTATTGGTACGTGATGGGGGAAACACAAACAATACTGACACA------------AAT----
----
AATACTGATTCAAAT---
AGAACTGAGATATTTAGACCTGGAGGAGGAGATATGAGAGACAATTGGAGAAGTGAATTGTACAAATATAAAG
TGGTAGAAATTAAGCCATTG---GGAATAGCACCCACTGGAGCAAAAAGGAGAGTGGTG--------------
-------------------------GGGAGAGAGAAAAGAGCA---GCGGGA---ATAGGAGCT---------
TTACTCCTTGGGTTCTTGGGAGCT---GCAGGAAGCACTATG---GGCGCGGCGTCA---
ATAACGCTGACGGTACAG---GCCAGACAA---TTATTG---TCTGGTATAGTGCAACAGCAAAGC---
AATTTG---CTGAGA---GCCATAGATGCGCAACAGCATATGTTGCAACTCACGGTCTGGGGCATT
>GU329220
TTCTATTGCAATACATCAAACTTATTCAATAGATCACTGTCTAATGATACAGAAGAAGATACAGAC-------
----

AGTAATAGAACCATCACACTCCCATGTAGAATAAAACAGATTATAAACATGTGGCAGGAAGTAGGAAGAGCAA
TGTATGCCCCTCCCATTGCAGGAAACATAACATGTGTATCAAAT---
ATCACAGGACTACTCCTGGTGCGGGATGGAGGCCATCAAAATAGCACAGAG----------------------
----

AATAATAACACAGAGATATTCAGACCTGGAGGAGGAGATATGAGGGACAATTGGAGAAGTGAATTATATAAAT
ACAAAGTAGTAGAAATTAAACCATTA---GGAATAGCACCTACCAAGGCACGGAGGAGAGTGGTG--------
-------------------------------CAGAGAGAAAAAAGAGCA---GTGGGA---ATAGGAGCT---
------GTACTCCTTGGGTTCTTGGGAACA---GCAGGAAGCACTATG---GGCGCAGCGTCA---
ATAACGCTGACGGTACAG---GCCAGACAA---TTGCTG---TCTGGTATAGTGCAACAGCAAAGC---
AATTTG---CTGAGA---GCTATTGAGGCGCAACAGCATATGTTGCAACTCACAGTCTGGGGCATT
>GU329240
TTCTATTGCAATACATCAGATCTGTTTAATTCATCAAAACTGTTTAATACAAACAATACTGAG----------
----

AACTTAACAATAATCACCATTCCATGCAGAATAAAACAAATCATAAACATGTGGCAGGAGGTAGGACGAGCAA
TGTATGCTCCTCCCATTGCAGGAAACATAACATGTACATCAGAC---
ATCACAGGATTACTATTGGTACGTGATGGAGGCACAGAAAGTAGC----------------------------
----

ACAAAAGAGATATTCAGACCTGGAGGAGGAGATATGAGGGATAATTGGAGAAGTGAATTATACAAATATAAAG
TGGTAGAAATTAAGCCATTG---GGAATAGCACCCACCGAGGCAAAAAGGAGAGTGGTG--------------
-------------------------GAGAGAGAAAAAAGAGCA---GTGGGA---CTAGGAGCT---------
TTGTTCCTTGGGTTCTTGGGAGTA---GCAGGAAGCACTATG---GGCGCGGCGTCA---
ATAACGCTGACGGTACAG---GCCAGACAA---CTGTTG---TCTGGTATAGTGCAGCAGCAAAGC---
AATTTG---CTGCAG---GCTATAGAGGCTCAACAGCATATGTTGAAACTCACGGTCTGGGGCATT
>GU329324
TTCTATTGCAATACATCAGGGTTGTTTAATGGTACATACAATAGTACAGACAGTAAT----------------
----

TCAAACTCAACCATCACAATTCCATGCAGAATAAAACAAATTATAAACATGTGGCAGGAAGTAGGACGAGCAA
TATATGCCCCTCCCATTGCAGGAAACATAACATGTAACTCAAGT---
ATCACAGGACTACTATTGGTACGTGATGGGGGCAGCAACGCCACT----------------------------
----

AATGAAACATTCAGACCTCAAGGAGGAAATATGAAAGACAATTGGAGAAGTGAATTATATAAATATAAAGTGG
TAGAAGTTAAGCCATTA---GGAATAGCACCAACTGAAGCAAAAAGGAGAGTGGTG-----------------
----------------------GAGAGAGAAAAAAGAGCA---GTAGGA---ATAGGAGCT---------
GTGTTCCTTGGGTTCTTGGGAGCA---GCAGGAAGCACTATG---GGCGCAGCGTCA---

ATAACGCTGACGGTACAG---GCCAGACAA---CTGCTG---TCTGGTATAGTGCAACAGCAAAGC---
AATTTG---CTGAGG---GCTATAGAGGCGCAACAGTATATGTTGCAACTCACAGTCTGGGGCATC
>GU329333
TTCTATTGCAATACATCAAATCTGTTTAATAGACACCTGCTCAATAGTAAAGGTATGTCTAATAATACAGAA-
----

AGTAATTCAACAATCATACTCCCATGCAAAATAAAACAAATTGTAAACATGTGGCAGGAGGTAGGACGAGCAA
TGTATGCCTCTCCCATTGCAGGAAACATAACATGTAAATCAAAT---
ATCACAGGACTATTATTGGTACGTGATGGGGGAAGTAGCATAAAT----------------------------
----

CAAACAGAGATATTTAGGCCTCAAGGAGGAGACATGAGGGACAACTGGAGAAGTGAGTTATATAAATATAAAG
TGGTAGAAATTAAACCATTG---GGAGTAGCACCCACTGAGGCAAAAAGGAGAGTGATG--------------
-------------------------CAGAGAGAAAAAAGAGCA---GTGGGA---ATGGCAGCT---------
GTGTTCCTTGGGTTCTTGGGAGCA---GCAGGAAGCACTATG---GGCGCGGCGTCA---
ATAACGCTGACGGTACAG---GCCAGACAA---TTATTG---TCTGGTATAGTGCAACAGCAAAGC---
AATTTG---CTGAGG---GCTATAGAGGCGCAACAGCATCTGTTGCAACTCACGGTCTGGGGCATT
>GU329368
TTCTATTGCAATACAACACCACTGTTTAATTACAGTGCAACAGAGCTGTTTAGTAATAGTAGTAAAAACATTA
GT---

GAAAAGGACACCATCACACTCCAGTGCAGAATAAAACAAATTATAAACATGTGGCAGAAAGTAGGACGAGCAA
TGTATGCCCCTCCCATTGCAGGAAACATAACATGTACATCAAAC---
ATCACAGGACTACTATTGGTCCGGGATGGAGGCGTCAACAGAACA----------------------------
----

AATCACAATGAGACATTTAGACCTGGAGGGGGAGACATGAGAGACAATTGGAGAAGTGAATTATATAAATATA
AAGTGGTAGAGATAAAGCCATTG---GGAATAGCACCAACTGAGGCAAAAAGGAGAGTGGTG-----------
----------------------------GAGAGACAAAAAAGAGCA---GTGGGA---CTGGGAGCT------
---ATGTTCCTTGGGTTCTTGGGAGCA---GCAGGAAGCACTATG---GGCGCGGCGTCA---
ATAGCGCTGACGGCACAG---GCCAGACAA---TTGTTG---TCTGGTATAGTGCAGCAGCAAAAC---
AATTTG---CTGCAG---GCCATAGAGGCGCAACAGCATCTGTTGCAACTCACTGTCTGGGGCATT
>GU329512
TTCTATTGCAATACAACGAACCTGTTTAAGGGTGTGTATAATGGTACTTATAATGATACTTCAACTAATACTA
AT---

GGAACAAGCAAAAACATCACACTTCAATGCAGAATAAAACAAATTATAAACATGTGGCAGGAGGTAGGACGAG
CAATGTATGCCCCTCCCATTGCAGGAAACATCCAATGTATATCAAAT---
ATCACAGGACTACTATTGACACGGGATGGAGGCAGCACAAATAGC----------------------------
----

TCAAATGAGACATTCAGACCTGGAGGAGGAAATATGAAGGACAATTGGAGAAGTGAATTATATAAATATAAAG
TGGTAGAAATTAAGCCATTA---GGAATAGCACCCGATAAGGCAAAAAGGAGAGTGGTG--------------
-------------------------GAGAGAGAAAAAAGAGCA---GTAGGA---ATAGGAGCT---------
GTGTTCCTTGGGTTCTTGGGAGCA---GCCGGAAGCACTATG---GGCGCAGCGTCA---
ATAACGCTGACGGTACAG---GCCAGACAA---TTGTTG---TCTGGTATAGTGCAACAGCAAAGC---
AATTTG---CTGAGG---GCTATAGAGGCGCAACAGCATATGTTGCAACTCACAGTCTGGGGCATT
>MK749242
TTCTATTGCAACACATCAGGGCTGTTTAAGGGAATGTACCTGGATAATAGCACAACCACAGAGAATTCA----
----

ACTTCGCCAAACATCACACTCCACTGCAAAATAAAACAATTTATAAACATGTGGCAGGGGGTAGGTCAAGCAA
TGTATGCCCCTCCCATTGCAGGAAACATAAGATGTGAATCAAAT---
ATCACAGGAATACTGTTAACACGTGATGGAGGAGGGGAAAATAAT----------------------------
----

AACACAAAAGAAACCTTCAGACCTGGAGGAGGAGATATGAGGGACATATGGAGAAGTGAGTTATATAAATATA
AAGTGGTAGAAATTAAGCCATTA---GGAGTAGCACCCACTGAGGCAAAAAGGAGAGTTGTG-----------
----------------------------GAGAGAGGAAAAAGAGCA---GTGGGA---GTAGGAGCT------
---GTGATCTTTGGGTTCTTGGGAGCA---GCAGGAAGCACTATG---GGCGCAGCGTCA---

TTAACGCTGACGGTACAG---GCCAGACAA---TTGCTG---TCTGGTATAGTGCAACAGCAAAGC---
AATTTG---CTGAGA---GCCATAGAGGCGCAACAGCATATGTTGCAACTCACGGTCTGGGGCATT
>KU749426
TTCTATTGCAACACATCAGGACTGTTTAATGGTATATACAATACTACAAGTAATTCAAGTGAT----------
----

ACAAATAAAAACATCACACTCCAATGCAGGATAAGACAATTTATAAACTTGTGGCAGGAGGTAGGAAAGGCAA
TGTATGCACCTCCCATTGCAGGAAACATCACTTGTAAATCAAAT---
ATCACAGGAATACTATTAACACGTGATGGAGGAGAAGGAAAGAAT---------------------------
----
-
CAAACAAACAGTAGTCAAGAGATATTCAGACCTGGAGGAGGAAATATAAAGGATAATTGGAGAAGTGAACTAT
ACAAATATAAAGTGGTAGAAATTAAACCATTA---GGACTAGCACCCACTCCGGCAAAAAGGAGAGTGGTG--
------------------------------------GAGAGAGAAAAAAGAGCA---GTGGGA---
ATAGGAGCT---------GTGTTCCTTGGGTTCTTGGGAGCA---GCCGGAAGCACTATG---
GGCGCGGCGTCA---ATAACGCTGACGGTACAG---GCCAGACAA---CTATTG---
TCTGGTATAGTGCAACAGCAAAGC---AATTTG---CTGAGG---
GCTATAGAGGCGCAACAGCATATGTTGCAACTCACGGTCTGGGGCATT
>KR820394
TTCTATTGCAATACATCAAGACTATTCAATGATGATGATAATGATACAGCACTGGCTAGTGATACAGCTTTAA
AT--

AAAAATAAAACCATCACACTCCCATGCAAAATAAAACAGATTATAAACATGTGGCAGGAAGTAGGACGAGCAA
TGTATGCCCCTCCCATTGCAGGGAACATAACATGTAACTCAAAC---
ATCACAGGAATACTATTAACACGTGATGGAGGAAATGGAAATCGC---------------------------
----
ACAGGAAATCAGACAGAGGCCAATCGCAAT---------
CGCACAGAGATATTTAGACCTGCAGGAGGAAATATGAAGGACAATTGGAGAAGTGAATTATATAAATATAAAG
TGGTAGAAATTAAGCCATTA---GGAATAGCACCAACTGAAGCAAAAAGGAGAGTGGTG--------------
------------------------GAGAGAGAAAAAAGAGCA---GTGGGA---ATAGGAGCT---------
GTGTTCCTTGGGTTCTTGGGAGCA---GCAGGAAGCACTATG---GGCGCGGCGTCA---
ATAACGCTGACGGTACAG---GCCAGACAA---CTGCTG---TCTGGTATAGTGCAACAGCAAAGT---
AATTTG---CTGAGG---GCTATAGAGGCGCAACAGCATCTGTTGCAACTCACGGTCTGGGGCATT
>AF286225
TTCTATTGCAATACATCAAAACTGTTTAATGGTACGTTTAATGGTACAAACACTTCTAATGATAGA-------
----

AGTAATTCGACCATTACGCTTCAATGCAGAATAAAACAAATTACAAACATGTGGCAGGGGGTAGGACAAGCAA
TGTATGCTCCTCCAATTAAAGGAAACATAACATGTAAATCAAAT---
ATCACAGGACTACTATTAACACGTGATGGAGGGACAAATGACACA---------------------------
----

GAGACACCAGAGACATTCAGACCTGGAGGAGGAGACATGAAGGACAATTGGAGAAGTGAATTATATAAATATA
AAGTGGTAGAAATTAAGCCATTA---GGAGTAGCACCCACTAAGGCACGAAGGAGAGTGGTG-----------
---------------------------GAGAGAGAAAAAAGAGCA---GTAGGA---ATAGGAGCT------
---GTGTTCCTTGGGTTCTTGGGAGCA---GCAGGAAGCACTATG---GGCGCAGCATCA---
ATAACGCTGACGGTACAG---GTCAGACAA---TTATTG---TCTGGTATAGTGCAACAGCAAAGC---
AATTTG---CTGAGG---GCTATAGAGGCGCAACAGCACATGTTGCAACTCACAGTCTGGGGCATT
>KJ787684
TTCTACTGTAATACATCAGGACTGTTTAATAATACATGGAATGTTACTGCAAAGGAA----------------
----
TGGGAGAATAACAGTACATGG------------
AATCAGACAAATGTAGTCAAAATCCCATGCAGAATAAAACAGATTGTAAACCTGTGGCAAACAGTAGGAACAG
CAATGTATGCCCCTCCCAGAGAAGGACTAATTAGATGTTCATCAAAC---
ATTACAGGGCTAATATTGACAAGAGATGGTGGT---------------------------------------
----

AAGAATAGCTCTACTGAGATCTTCAGACCTGGAGGAGGAGATATGAGAGACAATTGGAGAAGTGAACTATATA
AATACAAAGTAGTGCAAATTGAACCAATA---GGAATAGCACCCACCGGGGCAAAGAGAAGAGTGGTA-----
---------------------------------CAAAGGAAAGAAGAGCA---ATAGGA---

CTGGGAGTT---------ATGTTCCTTGGGTTCTTGGGAGCA---GCAGGAAGCACGATG---
GGCGCAGCGTCA---GTGACGCTGACGGTACAG---GCCAGACAG---CTACTG---
TCTGGTATAGTGCAACAGCAAAAC---AATTTG---CTGAGG---
GCTATAGAGGCGCAACAGCATATGTTGCAACTCACAGTCTGGGGCATT
>U88822
TTCTACTGCAATACATCAGGACTGTTTAATAGTGCATGGAATATATCAGGACATAGTACAGGGTTA-------
----

AATGATACAATCATAACCATCCCATGCAGAATAAAACAAATTATAAACATGTGGCAGGAAGTAGGAAAAGCAA
TGTATGCCCCTCCCATTGAAGGGCAAATTAATTGTTCATCAAAT---
ATTACAGGGCTACTATTGACAAGAGATGGTGGTGCTAATAATACT----------------------------
----

CAGAATGACACCTTTAGACCTGGAGGAGGAGATATGAGAGACAACTGGAGAAGTGAATTATATAAATATAAAG
TAGTAAAAATTGAACCACTA---GGAGTAGCACCCACCAAGGCAAAGAGAAGAGTGGTG--------------
-------------------------GAAAGAGAAAAAAGAGCA---ATAGGA---CTAGGAGCT---------
ATGTTCCTTGGGTTCTTGGGAGCA---GCAGGAAGCACGATG---GGCGCAGCGTCA---
ATGACGCTGACGGTACAG---GCCAGACAA---GTATTG---TCTGGTATAGTGCAACAGCAAAAC---
AATTTG---CTGAGG---GCTATAGAGGCGCAACAGCATCTGTTGCAACTCACGGTCTGGGGCATT
>AJ277820
TTCTACTGCAATACATCAAAACTATTTAATAGTACATGGAGTATAGAGAGTAATATTACAGAGTCA-------
----

AATGATAGTACAATCATACTTCCATGCAGAATAAAACAGATTGTAAACATGTGGCAGAGAGTAGGAAAAGCAA
TGTATGCCCCTCCCATTGAAGGAATAATCAGATGTTCATCAAAT---
ATTACAGGGCTACTATTGCTAAGAGATGGTGGGGTAAATAATCAT----------------------------
----

AGTAATGAGACCTTCAGACCTGGAGGAGGAGATATAAGGGACAATTGGAGAAGTGAATTATATAAATATAAAG
TAGTAAGACTTGAACCACTA---GGAGTAGCACCCACCAGGGCAAAGAGAAGAGTGGTG--------------
-------------------------GAAAGAGAAAAAAGAGCA---ATAGGA---TTAGGAGCT---------
ATGTTCCTTGGGTTCTTGGGAGCA---GCAGGAAGCACGATG---GGCGCAGTGTCA---
ATGACGCTGACGGTACAG---GCCAGACTG---TTACTG---TCTGGTATAGTGCAACAGCAAAAC---
AATTTG---CTGAGG---GCTATAGAGGCGCAACAGCATCTGTTGCAACTCACGGTCTGGGGCATT
>AY371156
TTCTACTGCAATACATCAGGACTGTTTAATAGAAATATTAGTACAGAGAGTACTGAAAATAGTACAGGA----
----

GTAAATGATACAATAACCATCCCATGTAAAATAAGACAAATTATAAACATGTGGCAGGGAGTAGGAAAAGCAA
TATATGCCCCTCCCATTGAAGGACAAATTAAATGTTCATCAAAT---
ATTACAGGGCTACTATTGACAAGAGATGGTGGTAATACTTCTTAT----------------------------
----

GAGGAGACCTTCAGGCCTGGAGGAGGAAATATGAAGGACAATTGGAGAAGTGAATTATATAAATATAAAGTAG
TAAGAATTGAACCACTA---GGAGTAGCACCCACCAGGGCAAAGAGAAGAGTGGTG-----------------
----------------------GAAAGAGAAAAAAGAGCA---ATAGGA---TTAGGAGCT---------
ATGTTCCTTGGGTTCTTGGGAGCA---GCAGGAAGCACGATG---GGCGCAGCGTCA---
GTGACGCTGACGGTACAG---GCCAGACAA---TTATTG---TCTGGTATAGTGCAACAGCAAAAT---
AATTTG---CTGAGG---GCTATAGAGGCGCAACAGCATCTGTTGCAACTCACGGTCTGGGGCATT
>FJ388945
TTCTACTGTAATACATCAAAACTGTTTAATACAACATGGTCTAGTGAAACATGGGAGAATAGTACGGGAAATA
ATACC--

GAATTAAATGGTACGATAACCATCCCATGTACAATAAAACAAATTATAATCATGTGCCAGGGAGTGGGAGTAG
CAATGTATACCCCTCCCCCTGCAGGAATAATTCAGTGTAAATCAAAT---
ATTACAATTACAGGGCTGACAAGAGATGGTGGTAATAATAGTAAT----------------------------
----

TGTAATGAGACCTTCAGGCCTGGAGGAGGAGATATGAGGGACAATTGGAGAAGTGAATTGTATAAATATAAAG
TAGTAAGAATTGAACCATTA---GGAATAGCACCCACCAGAGCAAAGAGAAGAGTGGTG--------------

-------------------------GAAAGAGAAAAAAGAGCA---ATAGGA---TTAGGAGCT---------
ATGTTCCTTGGGTTCTTGGGAGCA---GCAGGAAGCACGATG---GGCGCAGCGTCA---
ATAGCGCTGACGGCACAG---GCCAGACAA---GTGTTG---TCCGGTATAGTGCAACAGCAAAAT---
AATTTG---CTGAGG---GCGATAGAGGCGCAACAACATCTGTTGCAACTCACGGTCTGGGGCATT
>AF219271
TTCTACTGTAATACATCAAAACTGTTTAATAGTAATATTAGTACATCAGAAGGGGCTAATAGTACAAAGTCA-
----

AATAATAGAACAATCACACTCCAATGCAGAATAAAACAAATTATAAATATGTGGCAAGGAGTAGGAAAAGCAA
TGTATGCCCCTCCCATTGAAGGACTAATTAGATGTTCATCAAAT---
ATTACAGGGCTACTATTGACAAGAGATGGTGGTTCAAATACTAGT----------------------------
----

TCTCATGAGACCTTCAGACCTGGAGGAGGAAATATGAAGGACAATTGGAGAAGTGAATTATATAAATATAAGG
TAGTAAAAATTGAACCACTA---GAAGTAGCACCCACCAGGGCAAAGAGAAGAGTGGTG--------------
-------------------------GAAAGAGAAAAAAGAGCA---ATAGGA---CTAGGAGCT---------
ATGTTCCTTGGGTTCTTGGGAGCA---GCAGGAAGCACGATG---GGCGCAGCGTCA---
GTGACGCTGACGGTACAG---GCCAGACAG---TTATTG---TCTGGTATAGTGCAACAGCAAAAT---
AATTTG---CTGAGG---GCTATAGAGGCGCAACAACATCTGTTGCAACTCACAGTCTGGGGCATT
>AF219272
TTCTACTGCAATACATCAGGACTGTTTAATAGTACATGGGGTTCAGGACTGTCTAAT----------------
----

GGTACTGAGAATATCACCATCCCATGCAGAATAAAACAAATTATAAACATGTGGCAGGGAGTAGGAAAAGCAA
TGTATGCCCCTCCCATTGCAGGAATAATCAGCTGTTCATCAAAT---
ATTACAGGGCTACTATTGACAAGAGATGGAGGTACAAATAGTAGT----------------------------
----
-------------CATAACGAGACCTTC---AGACCT---
GGAGATATGAGAGACAATTGGAGAAGTGAATTATATAAATATAAAGTAGTAAGAATTGAACCCCTA---
GGAGTAGCACCCACCAGGGCAAAAAGAAGAGTGGTG-------------------------------------
--GAAAGAGAAAAAAGAGCA---ATTGGA---TTAGGAGCT---------
TTGTTCCTTGGGTTCTTGGGAGCA---GCAGGAAGCACAATG---GGCGCAGCGTCA---
TTGACGCTGACGGTACAG---GCCAGACAA---TTATTG---GCTGGTATAGTGCAACAGCAAAAC---
AATTTG---CTGAGG---GCTATAGAGGCGCAACAGCATCTGTTGCAACTCACGGTCTGGGGCATT
>HQ595791
TTCTACTGCGATACATCAAGACTATTTAATAGTACCTGGACGAATAGTACCTGGGGGAACGATACTAGCTCAA
AT---

AGGAATGATACAATCACACTCCCATGCAGGATAAAACAAATTATAAACATGTGGCAGGAAGTAGGAAAAGCAA
TGTATGCCCCTCCCATTCGAGGACTAATCAGATGCTCATCACGC---
ATTACAGGACTATTGTTGACAAGAGATGGTGGTATCTATAATAGTAGT-------------------------
----

CAGGAAGAAGAGACCTTCAGACCGGGAGGAGGAGATATGAGGGACAATTGGAGAAGTGAATTATACAAATATA
AGGTAGTAAGAATTGAACCACTA---GGTCTAGCACCCACCAGGGCAAAGAGAAGAGTGGTG-----------
----------------------------GAAAGAGAAAAAAGAGCA---ATAGGA---CTGGGAGCT------
---GTGTTCCTTGGGTTCTTAGGAGCA---GCAGGAAGCACAATG---GGCGCAGCGTCA---
CTGACGCTGACGGTACAG---GCCAGACAG---TTACTG---TCTGGTATAGTGCAACAGCAAAAC---
AATTTG---CTGAGG---GCTATAGAGGCGCAACAGCATCTGTTGCAACTCACAGTCTGGGGCATT
>KF716476
TTCTACTGCAATACATCAAAATTGTTTACAACAAATATAACAAGTAACACT----------------------
----

AGTGAAGGGAACATCACACTCCCATGCAGAATAAAACAAATTATAAACATGTGGCAGGGAGTAGGAGGAGCAA
TGTATGCCCCTCCTAGGGAAGGATTGATCACATGTTCATCAAAT---
ATCACAGGACTACTGTTGACAAGAGATCAAGAGGGCTCAAATAAC----------------------------
----

ATTAATGAGACCTTCAGACCTGGAGGAGGAAATATGAAAGACAATTGGAGAAGTGAATTATACAAATATAAAG
TAGTAAGAATTGAACCACTA---GGTCTAGCACCCACCAGGGCAAAAAGAAGAGTGGTG--------------

-------------------------GAAAGAGAAAAAAGAGCA---ATAGGC---CTGGGAGCT---------
ATGTTCCTTGGGTTCTTGGGAGCA---GCAGGAAGCACAATG---GGCGCAGCGTCA---
CTGACGCTGACGGTACAG---GCCAGACAG---TTACTG---TCTGGTATAGTGCAACAGCAAAAC---
AATTTG---CTGAGA---GCTATAGAGGCGCAACAGCATCTGTTGCAACTCACAGTCTGGGGCATT
>FJ866137
TTCTACTGCAATACATCAGAACTGTTTAATAATACATGGTTAAATAATACAGGGAACTGGTCATCAGAT----
----

AGTACAGGGATAATCATGCTCCCATGCAGAATAAAACAAATTATAAACATGTGGCAGGGAGTAGGAAAAGCAA
TGTATGCCCCTCCCATTGCAGGACTAATCAATTGTTCATCAAAT---
ATTACAGGACTATTGTTGACAAGAGATGGTGGTGCAAATAGTAGT----------------------------
----

CAGAATGAGACCTTCAGACCGGGAGGAGGAGATATGAGAGACAATTGGAGAAGTGAATTATACAAATATAAAG
TAGTAAAAATTGAACCACTA---GGATTAGCACCCACCAAGGCAAGGAGAAGAGTGGTG--------------
-------------------------GAAAGAGAAAAAAGAGCA---ATAGGA---CTAGGAGCT---------
ATGTTCCTTGGGTTCTTGGGAGCA---GCAGGAAGCACGATG---GGCGCAGCGTCA---
CTGACGCTGACGGTACAG---GCCAGACAG---TTATTG---TCTGGTATAGTGCAACAGCAAAAC---
AATTTG---CTGAGG---GCTATAGAGGCGCAACAGCATCTGTTGCAACTCACAGTCTGGGGCATT
>FJ866135
TTCTACTGCAACACATCAAGACTGTTTAATAATAGTGAATGGAAATCAAATAGTAGTACAGGGGGA-------
----

AATGAAAGTATAATCATACTCCCATGCAGAATAAAACAAATTATAAACATGTGGCAGGGAGTAGGAAGAGCAA
TGTATGCCCCTCCCATTGAAGGACACATCAATTGTTCATCAAAT---
ATTACAGGACTATTGTTGACAAGAGATGGTGGTGTAACTAATCAG----------------------------
----

AGTTCGAATGAGACCTTCAGACCTGGGGGAGGAGACATGAGAGACAATTGGAGAAGTGAATTATACAAATATA
AAGTAGTAAGAATTGAACCACTA---GGTCTAGCACCCACCAGGGCAAAGAGAAGAGTGGTG-----------
----------------------------GAAAGAGAAAAAAGAGCA---ATAGGA---CTGGGAGCT------
---ATGTTCCTTGGGTTCTTGGGAGCA---GCAGGAAGCACGATG---GGCGCAGCGTCG---
CTGACGCTGACGGTACAG---GCCAGACAG---TTATTG---TCTGGTATAGTGCAACAGCAAAAC---
AATTTG---CTGAGG---GCTATAGAGGCGCAACAGCATCTGTTGCAACTCACAGTCTGGGGCATT
>AY322189
TTCTACTGCAACACATCAAACCTGTTTAATAGCATTTGGAATGTTACTAATGGTACTAGCCAGCTGGAGTCAA
ATAGC--
-------------------ACA---
GAGTTAGCCGGAAATATAACTCTCCCATGCAGAATAAAGCAAATTATAAACATGTGGCAGGGAGTAGGAATAG
CAATGTATGCCCCTCCCATTGCAGGAATAATCAAATGTTCATCAAAT---
ATTACAGGACTATTGTTAACAAGAGATGGTGGTGCAGATAATAGT----------------------------
----

AGTCAGAATGAGACCTTCAGACCAGGAGGAGGAGATATGAGAGACAATTGGAGAAGTGAATTATACAAATATA
AAGTAGTAAGAGTTGAACCACTA---GGTATAGCACCCACCAAGGCAAAGAGAAGAGTGGTG-----------
----------------------------GAAAGAGAAAAAAGAGCA---ATAGGA---CTGGGAGCT------
---ATGTTCCTTGGGTTCTTGGGAGCA---GCAGGAAGCACGATG---GGCGCAGCGTCA---
GTGACGCTGACGGTACAG---GCCAGACAG---TTACTG---TCTGGTATAGTGCAACAGCAAAAC---
AATTTG---CTGAGA---GCTATAGAGGCGCAACAGCATCTGTTGCAACTCACAGTCTGGGGCATT
>FJ866138
TTCTACTGCAGTACATCAGAACTGTTTAATAGTACATGGAATACATCAATTAGTAGTACAAGGAATACATCAA
AT---

AGTACAAGGGATATCAGACTCCCATGCAGAATAAAACAAATTATAAACATGTGGCAGGGAGTAGGAAAAGCAA
TGTATGCCCCTCCCATAGAAGGACTAATCAAATGTTCATCAAAT---
ATTACAGGACTATTGTTGGCAAGAGATGGTGATGTAAACAATAAT----------------------------
----

AGTCAGGAGACCCTTAGACCTGGAGGAGGAGATATGAGAGACAATTGGAGAAGTGAATTATACAAATATAAAG
TAGTAAGACTTGAACCACTA---GGTCTAGCACCCACCAGGGCAAAGAGAAGAGTGGTG--------------

-------------------------GAAAGAGAAAAAAGAGCA---ATAGGA---CTAGGAGCT---------
ATGTTCCTTGGGTTCTTGGGAGCA---GCAGGAAGCACGATG---GGCGCAGCGTCA---
CTGACGCTGACGGTACAG---GCCAGACAG---TTATTG---TCTGGTATAGTGCAACAGCAAAAC---
AATTTG---CTGAGG---GCTATAGAGGCGCAACAGCATCTGTTGCAACTCACAGTCTGGGGCATT
>FJ866141
TTCTACTGCAATACATCAAGACTGTTTAATAGTACATGGGGGATAATAATACATCAAGTGATACA--------
----

GAGGAAGGTAACATCACCATCCCATGTAGAATAAAACAAATTATAAACATGTGGCAAGGAGTAGGAAAAGCAA
TGTATGCCCCTCCCATTGAAGGACTAATCAGATGTTCATCAAAT---
ATTACAGGATTACTGTTAACATATGATGGGGGTGTAAATAATAAT----------------------------
----

AGTCAGAGTGAGATCTTCAGACCTGGAGGAGGAGATATGAGAGACAATTGGAGAAGTGAATTATACAAATATA
AAGTAGTAAGACTTGAACCACTA---GGTCTAGCACCCACCAAGGCAAAAAGAAGAGTGGTG-----------
----------------------------GAAAGAGAAAAAAGAGCA---ATAGGC---CTAGGAGCT------
---ATGTTCCTTGGGTTCTTGGGAGCA---GCAGGAAGCACGATG---GGCGCAGCGTCA---
TTGACGCTGACGGTACAG---GCCAGACAA---TTATTG---TCTGGTATAGTGCAGCAGCAAAAC---
AATCTG---CTGATG---GCTATAGAGGCGCAACAGCATCTGTTGCAACTCACAGTCTGGGGCATT
>GU204938
TTCTACTGCAATACAACTGGACTGTTTAATAATAGTGTATGGAAGAATGATAGTGATACAGCAAAT-------
----

AGTACAGAGGTAATCAAACTCCCATGCAGAATAAAACAAATTATAAACATGTGGCAGGGAGTAGGCAAAGCAA
TGTATGCCCCTCCCATTGAAGGACAAATCGAATGTACATCAAAT---
ATCACAGGACTATTGCTGACAAGAGATGGTGGTGTAAGTAATAAT----------------------------
----

AGTCAGAATGAAACCTTTAGGCCTGGAGGAGGAGATATGAGAGACAACTGGAGGAGTGAACTATACAAATATA
AGGTAGTAAGAATTGAACCACTA---GGTCTAGCACCCACCAGGGCAAGGAGAAGAGTGGTG-----------
----------------------------GAAAGAGAAAAAAGAGCA---ATAGGA---CTAGGAGCT------
---ATGTTCCTTGGGTTCTTGGGAGCA---GCAGGAAGCACGATG---GGCGCAGCGTCA---
TTGACGCTGACGGTACAG---GCCAGACAG---TTAATG---TCTGGTATAGTGCAACAGCAAAAC---
AATTTG---CTGAGA---GCTATAGAGGCGCAGCAGCATCTGTTGCAACTCACAGTCTGGGGCATT
>HQ659623
TTCTACTGCAATACATCAGGACTGTTTAATAATAGTGAGTGGACAGATACGACACCAAATACGACA-------
----

TCAAATGGTACCATCATACTCCCATGCAGAATAAAACAATTTATAAACCTGTGGCAGGGAGTAGGAAGAGCAA
TGTATGCCCCTCCCATTCAAGGACTAATCAGATGTTCATCAAAT---
ATTACAGGACTATTGTTGACAAGAGATGGTGGAAATAATAATAGT----------------------------
----

AGTCTGAATGAGACCTTCAGACCTGGAGGAGGAGATATGAGAGACAATTGGAGAAGTGAATTATACAAATATA
AAGTAGTAAGAATTGAACCACTA---GGTCTAGCACCCACCAGTGCAAAGAGAAGAGTGGTG-----------
----------------------------GAAAGAGAAAAAAGAGCA---ATAGGA---CTGGGAGCT------
---GTGTTCCTTGGGTTCTTGGGAGCA---GCAGGAAGCACGATG---GGCGCAGCATCA---
CTGATGCTGACGGTACAG---GCCAAACAG---CTATTG---TCTGGTATAGTGCAACAGCAAAAC---
AATTTG---CTGAGA---GCTATAGAGGCGCAGCAACATCTGTTGCAACTCACAGTCTGGGGCATT
>KX907406
TTCTACTGTAATACATCAGGACTGTTCAATAGTACATGGGATATTACGAATACTAGTAGCCCAAACAAC----
----

ACAGACCCAATTATCACACTACAATGCAGAATAAAACAAATTATAAACATGTGGCAGGGAGTAGGAAGAGCAA
TATATGCCCCTCCCATTGCAGGGCAAATTCTATGTTCATCAAAT---
ATTACAGGGCTACTATTGACAAGAGATGGTGGTGCAGATAATAGC----------------------------
----

AGTCATAATGAGACCTTCAGACCTGGAGGAGGAAATATGAAGGACAATTGGAGAAGTGAATTGTATAAATATA
AAGTAGTAAAAATTGAACCACTA---GGAATAGCACCCACCAAGGCAAGGAGAAGAGTGGTG-----------

----------------------------GAAAGGGAAAAAAGAGCA---ATAGGA---CTAGGAGCT------
---ATGTTCCTTGGGTTCTTGGGAGCA---GCAGGAAGCACGATG---GGCGCAGCGTCA---
GTGACGCTGACGGTACAG---GCCAGACAG---TTACTG---TCTGGTATAGTGCAGCAGCAAAGC---
AATTTG---CTGAGG---GCTATACAGGCGCAACAGCATCTGTTGCAGCTCACTGTCTGGGGCATT
>HQ615975
TTCTACTGTAATACAACAAAACTGTTTAATACAAGTGAATCAAATAGGACAGAG-------------------
----

GTAAATGGAACAATCACACTCCCATGCAGAATAAAACAAATTATAAACATGTGGCAGGGAGTAGGGAAAGCAA
TCTATGCCCCTCCCATTGAAGGACACATCAACTGTTTATCAAAT---
ATTACAGGACTATTGTTGACAAGAGATGGTGTTGCAAACAATAAG----------------------------
----

AGTCAGAATGAGACCTTCAGACCTGGAGGAGGAGATATGAGAGACAATTGGAGAAGTGAATTATACAAATATA
AAGTAGTAAGAATTGAACCACTA---GGTCTAGCACCCACCAAGGCAAAGAGAAGAGTGGTG-----------
----------------------------GAAAGAGAAAAAAGAGCA---ATAGGA---CTAGGAGCT------
---TTGTTCCTTGGGTTCTTGGGAGTA---GCAGGAAGCACAATG---GGCGCAGCGTCG---
ATGACGCTGACGGTACAG---GCCAGGCAA---TTATTG---TCTGGTATAGTGCAACAGCAAAAC---
AATTTG---CTGAGG---GCTATAGAGGCGCAACAGCATCTGTTGCAACTCACAGTCTGGGGCATT
>JX512899
TTCTACTGCAATACAACAAGACTGTTCAATAGTACATGGAATACAACTACATCAAATAGTACAGAACAA----
----

AATGATACAAGAATACTATTCCCATGCAGAATAAAACAAATTATAAACATGTGGCAGGGAGTAGGAAAAGCAA
TGTATGCTCCTCCCATTGAAGGACTAATCAGATGTACATCAAAT---
ATTACAGGACTATTGTTGGCAAGAGATGGTGGTGCAAGCAATAAT----------------------------
----

AGTCAGACTGAGACCTTCAGACCTGGAGGAGGAGATATGAGAGACAATTGGAGAAGTGAATTGTACAAATATA
AAGTAGTAAGAATTGAGCCACTA---GGTCTAGCACCCACCAGGGCAAAGAGAAGAGTGGTG-----------
----------------------------GAAAGAGAAAAAAGAGCA---ATAGGA---CTGGGAGCT------
---ATGTTCCTTGGGTTCTTGGGAGCA---GCAGGAAGCACGATG---GGCGCAGCGTCA---
CTGACGCTGACGGTACAG---GCCAGACAG---TTATTG---TCTGGTATAGTGCAACAGCAAAAC---
AATTTG---CTGAGG---GCTATAGAGGCGCAACAGCATCTGTTGCAACTCACAGTCTGGGGCATT
>KF986106
TTCTACTGCAATACAACAAAACTGTTTAACAGTACATGGCATAACAGTACATGGAAG----------------
----
AATGATACTGCATTAAATACA------------
ACAGGGGATAAGATAATCACACTCCCATGTAGAATAAAACAAATTATAAACATGTGGCAGGGAGTAGGCAAAG
CAATGTATGCCCCTCCCATTGCAGGACTAATCAGATGTTCATCAAAT---
ATTACAGGACTATTGTTAACAAGAGATGGTGGTAAAACTAAT-------------------------------
----
AATCATAGTGAGACC---
CTTAATGAGACCTTCAGACCTGGAGGAGGAGATATGAGAGACAATTGGAGAAGTGAATTATACAAATATAAAG
TAGTAAGACTTGAACCACTA---GGTCTAGCGCCCACCAAGGCAAAGAGAAGAGTGGTG--------------
-------------------------CAGAGAGAAAAAAGAGCA---ATAGGA---CTAGGAGCT---------
ATGTTCCTTGGATTCTTGGGAGCA---GCAGGAAGCACAATG---GGCGCAACGTCA---
CTGACGTTGACGGTACAG---GCCAGACAG---TTATTG---TCTGGTATAGTGCAACAGCAAAAC---
AATCTG---CTGAGG---GCTATAGAGGCGCAACAGCATCTGTTGCAACTCACAGTCTGGGGCATT
>KF986033
TTCTACTGTAATACAACAAAACTGTTTAATAACAATAAT----------------------------------
----
-------------------AGT---
ACTAATGAGACAGTAATCACACTCCCATGCAGAATAAAACAAATTATAAACATGTGGCAGGGAGTAGGAAAAG
CAATGTATGCCCCTCCCATTGCAGGAAGCATTCGCTGTTCATCCAAT---
ATTACAGGACTATTGTTGACAAGAGATGGTGGG--

-
AATAAGACTGCTCAGAATGAAACCTTCAGACCTGGAGGAGGAGATATGAGAGACAATTGGAGAAGTGAATTGT
ACAAATATAAAGTAGTAAAAATTGAACCACTA---GGTCTAGCACCCACCAAGGCAAAAAGAAGAGTGGTG--

-------------------------------------GAAAGAGAAAAAAGAGCA---GTAGGA---
CTAGGAGCT---------ATGTTCCTTGGGTTCTTGGGAGCA---GCAGGAAGCACAATG---
GGCGCAGTGTCA---ATGACGCTGACGGTACAG---GCCAGACAG---GTGTTG---
TCTGGTATAGTGCAACAGCAAAAC---AATTTG---CTGAGG---
GCTATAGAGGCGCAACAGCATCTGTTGCAACTCACAGTCTGGGGCATT
>KF716479
TTCTACTGCAATACATCAGGACTGTTTAATAGTACATGGCAGGAAAATAGTACTCTATTAAATAAAACAAATT
AT---
-------------------ACA---
GAAGAAAATAAAACAATCACACTCCCATGCAGAATAAAACAAATTATAAATATGTGGCAGAGAATAGGAAAAG
CAATGTATGCCCCTCCCATTGAAGGACTAATCAAATGTTCCTCAAAT---
ATTACAGGACTATTGTTGGAAAGAGATGGTAGTGTTGAAAATAAT----------------------------
----

AGTCAGGAGACTGTCAGACCTGTAGGAGGAGAGATGAGAAACAATTGGAGAAGTGAATTATACAAATATAAAG
TAGTAAGAATTAAACCAATA---GGTCTAGCACCCACCAGGGCAAAGAGAAGAGTGGTG--------------
-------------------------GAAAAACAAAAAAGAGCA---ATAGGA---CTAGGAGCT---------
ATGTTCCTTGGGTTCTTGGGAGCA---GCGGGAAGCACGATG---GGCGCAGCGTCA---
ATGGCGCTGACGGTACAG---GCCAGACAA---GTACTG---TCTGGTATAGTGCAACAGCAAAAC---
AATTTG---CTGAGG---GCTATAGAGGCGCAACAGCATCTGTTGCAACTCACAGTCTGGGGCATT
>AB485651
TTCTACTGTAATACATCAAGATTGTTTAATAGTGAATGGAGATACAATACAACAAGTAAGGAATGGAGTTCTA
ATACAACA---
-------------AATAAT------
ACAGGGGTAAATGAAATCAAACTCCCATGCAGAATAAAACAGCTTATAAACATGTGGCAGAGAGTAGGAAAAG
CAATGTATGCCCCTCCCATTGAAGCACTAATTAACTGTTCATCAAAC---
ATTACAGGACTATTGTTGTCAAGAGATGGTGGTGCAAATAATAAT----------------------------
----

AGTCAGAATGAGACCTTCAGACCTGGAGGAGGAGATATGAGAGACAATTGGAGAAGTGAATTGTACAAATATA
AAGTAATAAGAATTGAACCAATA---GGTCTAGCACCCACCAAGGCAAAAAGAAGAGTGGTG-----------
----------------------------GAAAGGGAAAAAAGAGCA---ATAGGA---CTAGGAGCT------
---ATGTTCCTTGGGTTCTTGGGAGCA---GCAGGAAGCACGATG---GGCGCAGCGTCA---
TTGACGCTGACGGTACAG---GCCAGACAG---TTAATG---TCTGGTATAGTGCAACAGCAAAAT---
AATTTG---CTGAGG---GCTATAGAGGCGCAACAACATCTGTTGCAACTCACAGTCTGGGGCATT
>AJ320484
TTCTACTGCAATACATCAAAACTGTTTAACAGTGCATGGAATGACAGTACATGGAATATAGGGAATAATAATA
CAGGG--

TCAGATAATGAGACAATCATTATCCCATGCAGAATAAAACAAATTATAAACATGTGGCAGGGAGTAGGAAAAG
CAATGTATGCCCCTCCCATCGAAGGATGGATCAATTGTGCATCAAAT---
ATTACAGGGCTCTTACTGGTAAGGATGGTGGTGGTGCAAATGAT-----------------------------
----

AGTCAGAACGAGACCTTCAGACCTCAAGGAGGAGATATGAGAGACAATTGGAGAAGTGAATTATACAAGTATA
AAGTAGTAAAAATTGAACCACTA---GGAATAGCACCCACCAAGGCAAAGAGAAGAGTGGTG-----------
----------------------------GAAAGAGAAAAAAGAGCA---ATAGGA---CTAGGAGCT------
---ATGTTCCTTGGGTTCTTGGGAGCA---GCAGGAAGCACGATG---GGCGCAGCGTCA---
TTGACGCTGACGGTACAG---GCCAGACAA---TTATTG---TCTGGTATAGTGCAACATCAAAAC---
AATTTG---CTGATG---GCTATAGAGGCGCAACAGCATCTGTTGCAACTCACAGTCTGGGGCATT
>U08805
TTCTACTGCAATACATCAAAATTGTTTAATCATAGTGTAGGGGAGAATAATACTCTATCAATTAGCAACGGG-
----

GTAAATACTAACATCACACTCCCATGCAAAATAAAACAAATTGTAAACATGTGCCAGGGAGTAGGAAAAGCAA
TGTATGCCCCTCCCATTCAAGGACTAATCAGATGTTCATCAAAT---
ATTACAGGACTATTGTTGACAAGAGATGGTGGTAATACTAGTCAG----------------------------
----

AATGAGACCTTCAGACCTGGAGGAGGAGATATGAGAGACAATTGGAGAAGTGAATTATACAAATATAAAGTAA

TAAAAATTGAACCACTA---GGTCTAGCACCCACCAGGGCAAAGAGAAGAGTGGTG-----------------
----------------------GCAAGAGAAAAAAGAGCA---ATAGGA---CTAGGAGCT---------
GTGTTCCTTGGGTTCTTGGGAGCA---GCAGGAAGCACAATG---GGCGCAGCGTCA---
CTGACGCTGACGGTACAG---GCCAGACAG---TTAATG---TCTGGTATAGTGCAACAGCAAAAC---
AATTTG---CTGAGG---GCTATAGAGGCGCAACAGCATCTGTTGCAACTCACAGTCTGGGGCATT
>EU852998
TTCTACTGCAATACATCAGGACTGTTTAACACTAATGTGACATCAAATAGTACAGCAAAT-------------
----

CCAAATGGGAACATCACACTCCCATGCAGAATAAAACAAATTGTAAACATGTGGCAGGGAGTAGGAAAAGCAA
TATATGCCCCTCCCATTGAAGGACTAATCAACTGCTCATCAAAT---
ATTACAGGACTATTGTTGGCAAGAGATGGTGGTGCAAATAATAAT----------------------------
----

AGTCAGAATGAGACCTTCAGACCTGCAGGAGGAGATATGAGAGACAATTGGAGAAGTGAATTATACAAATATA
AAGTAGTAAGAATTGAGCCAATA---GGTCTAGCACCTGCCAGGGCAAAGAGAAGAGTGGTG-----------
----------------------------GAAAGAGAAAAAAGAGCA---ATAGGA---CTGGGAGCT------
---ATGTTCCTTGGGTTCTTGGGAGCA---GCAGGAAGCACGATG---GGCGCAGCGTCA---
ATAACGCTGACGGTACAG---GCCAGACAA---GTACTG---TCTGGTATAGTGCAACAGCAAAAC---
AATTTG---CTGAGG---GCTATAGAGGCGCAACAGCATCTGTTGCAACTCACAGTCTGGGGCATT
>JX658585
TTCTACTGCAATACATCAGGACTGTTTAATAGTAGTGGTATAACAAATAGTACAGGAATGTTGAATAATAGT-
----

GATATATCAAATATCACAATCCACTGCAGAATAAGACAAATTATAAACATGTGGCAGGGAGTAGGAAAAGCAA
TGTATGCCCCTCCCATTAAAGGACTAATCTACTGTTCATCAAAT---
ATTACAGGACTATTGTTAACAAGAGATGGTGCAGATAATAATAGT----------------------------
----

CCGAATGAAGCCTTCAGACCTGAAGGAGGAAATATGAAAGACAATTGGAGGAGTGAATTATACAAATATAAAG
TAGTAAGAATTGAGCCATTA---GGTCTAGCACCCACCAGGGCAAAGAGAAGAGTGGTG--------------
-------------------------GAAAGAGAAAAAAGAGCA---ATAGGA---CTAGGAGCT---------
ATGTTCCTTGGGTTCTTGGGAACA---GCAGGAAGCACGATG---GGCGCAGCGTCA---
CTGACGCTGACGGTACAG---GCCAGACAA---TTATTG---TCTGGTATAGTGCAACAGCAAAAC---
AATTTG---CTGAGG---GCTATAGAGGCGCAACAGCATCTGTTGCAACTCACAGTCTGGGGCATT
>EU852934
TTCTACTGCAATACATCACAGCTGTTTAATAGTACATGGGAAGCCAATACGACAGATAAG-------------
----

CTAAATGAGACAATCACACTCCCATGCAGAATAAAACAAATTATAAACATGTGGCAGGGAGTAGGAAAAGCAA
TGTATGCCCCTCCCATTGAAGGACAAATCACATGTTCATCAAAT---
ATTACAGGGCTTTTATTGACAAGAGATGGTGGTACAAATAATAGT----------------------------
----

AGTCAGAATGAGACCTTCAGACCTGGAGGAGGAGATATGAGAGATAATTGGAGAAGTGAATTATACAAATATA
AAGTAGTAAGAATTGAACCACTA---GGTCTAGCACCCACCAGGGCAAAGAGAAGAGTGGTG-----------
----------------------------GAAAGAGAAAAAAGAGCA---ATAGGA---CTGGGAGCT------
---ATGTTCCTTGGGTTCTTGGGAGCA---GCAGGAAGCACGATG---GGCGCAGCGTCA---
CTGACGCTGACGGTACAG---GCCAGACAA---TTATTG---TCTGGTATAGTGCAACAGCAAAAC---
AATTTG---CTGAGA---GCTATAGAGGCGCAACAGCATCTGTTGCAACTCACAGTCTGGGGCATT
>EU852982
TTCTACTGCAATACATCAGGACTGTTTAACAATACGTTTAAATGGGAGAATGGCACTTTATTAAATACTACAG
TAAATATT---
-------------ACAGAG------
AATAGTACAGAGAATATCACACTCCCATGCAGAATAAAACAAATTATAAACATGTGGCAGGGAGTAGGAAAAG
CAATGTATGCCCCTCCCATCGCAGGACTAATTAAATGTTCATCAAAT---
ATTACAGGACTATTATTGACAAGGGATGGTGGTACAAATAATAGT----------------------------
----

CAGGATGATGAGACCTTTAGACCTGGAGGAGGAGATATGAGGGACAATTGGAGAAGTGAATTATATAAATATA

AAGTAGTAAAAATTGAACCACTA---GGTCTAGCACCCACCAAGGCAAAGAGAAGAGTGGTG-----------
----------------------------GAAAGAGAAAAAAGAGCA---ATAGGA---CTGGGAGCT------
---TTGTTCCTTGGGTTCTTGGGAGCA---GCAGGAAGCACGATG---GGCGCAGCGTCA---
ATAACGCTGACGGTACAG---GCCAGACAG---TTATTG---TCTGGTATAGTGCAGCAGCAAAAT---
AATTTG---CTGAGA---GCTATAGAGGCGCAACAGCATCTGTTGCAACTCACAGTCTGGGGCATT
>EU853014
TTCTACTGCAATACATCAGGACTGTTCAATCATAGTGTATGGAGCAATAATGGTACATGGAATACTACATCAA
TT---

AATAAAACAGAGAATATCATACTCCCATGCAGAATAAAACAAATTATAAACATGTGGCAAAGAGTAGGAAAAG
CAATGTATGCCCCTCCCATTGCAGGAGTAATCAATTGTGTATCAAAT---
ATTACAGGACTATTGTTGATAAGAGATGGTGGTAATACTAATACTAGTCAT----------------------
----

AATAATGGGACTGAGACCTTCAGACCTGGAGGAGGAGATATGAGAGACAATTGGAGAAGTGAATTATACAAAT
ATAAAGTAGTAAGACTTGAGCCACTA---GGTCTAGCACCCACCAGGGCAAAGAGAAGAGTGGTG--------
-------------------------------GAAAGAGAAAAAAGAGCA---GTAGGA---CTAGGAGCT---
------ATGTTCCTTGGGTTCTTGGGAGCA---GCAGGAAGCACGATG---GGCGCAGCGTCA---
CTGACGCTGACGGTACAG---GCCAGACTG---TTATTG---TCTGGTATAGTGCAACAGCAAAAT---
AATTTG---CTGAGG---GCTATAGAGGCGCAACAGCATCTGTTGCAACTCACAGTCTGGGGCATT
>AF484502
TTCTACTGCAATACAGCAGGACTGTTTAATAGTACATGGAACAGGACAAATAGTGAATGGATAAATAGTAAAT
GGACAAAT---
-------------------AAG---
ACAGAAGATGTAAATATCACACTTCAATGCAGAATAAAACAAATTATAAACACGTGGCAGGGAGTAGGAAAAG
CAATGTATGCCCCTCCCGTTAGTGGAATAATCCGATGTTCATCAAAT---
ATTACAGGACTGTTGCTGACAAGAGATGGTGGTGGTGCAGATAATAAT-------------------------
----

AGGCAGAATGAGACCTTCAGACCTGGGGGAGGAGATATGAGAGACAATTGGAGAAGTGAATTATACAAATATA
AAGTAGTAAGAATTGAACCACTA---GGTATAGCGCCCACCAAGGCAAGGAGAAGAGTGGTG-----------
----------------------------GAAAGAGAAAAAAGAGCA---ATAGGA---CTGGGAGCC------
---TTGTTCCTTGGGTTCTTGGGAACA---GCAGGAAGCCCGATG---GGCGCAGTGTCA---
ATGACGCTGACGGTACAG---GCCAGACAA---GTATTG---TCTGGTATAGTGCAACAGCAAAAC---
AATCTG---CTGAGG---GCTATAGAGGCGCAACAGCATCTGTTGCAACTCACAGTCTGGGGCATT
>AF484506
TTCTACTGCAATACATCAGGACTGTTTAATAGTACATGGGAAAATGGTACATTAAATAGTGCGGAG-------
----

GGAAATGATACAATCACACTCCCATGCAGAATAAAACAAATTATAAACATGTGGCAGGAAGTAGGAAAAGCAA
TGTATGCCCCTCCCATTGAAGGACTAATCCAATGTTCATCAAAT---
ATTACAGGACTATTGTTGACAAGAGATGGTGGTGTAAATAATAGT----------------------------
----

CAGAATGAGACCTTCAGGCCTGGAGGGGGAGATATGAGAGACAATTGGAGAAGTGAATTATACAAATATAAAG
TAGTAAAAATTGAACCACTA---GGTATAGCACCCACCAAGGCAAAGAGAAGAGTGGTG--------------
-------------------------GAAAAAGAAAAAAGAGCA---ATAGGC---CTGGGAGCT---------
ATGTTCCTTGGGTTCTTGGGAGCA---GCAGGAAGCACGATG---GGCGCAGCGTCA---
CTGACGCTGACGGTACAG---GCCAGACAG---TTATTG---TCTGGTATAGTGCAACAGCAAAAC---
AATTTG---CTGAGG---GCTATAGAGGCGCAACAGCATCTGTTGCAACTCACAGTCTGGGGCATT
>AF484513
TTCTACTGCAATACATCAGAACTGTTTAATAGTACATGGGGGAATTCTAGTACATCAACTAGTACAGGGGTA-
----

AATAAGAGTACAATCACTATCCCATGCAGAATAAAACAAATTATAAACATGTGGCAGGAAGTAGGAAAAGCAA
TGTATGCCCCTCCCATTCGAGGACTAATCAAATGTTCCTCAAAT---
ATTACAGGACTATTGTTGGTAAGAGATGGTGGTGCAACTAATAAT----------------------------
----

AGTCAGAATGAGACCTTCAGACCTGGAGGAGGAGATATGAGAGACAATTGGAGAAGTGAATTATACAAATATA

AAGTAGTAAGACTTGAACCACTA---GGTCTAGCACCCACCAAGGCAAAGAGAAGAGTGGTG-----------
----------------------------GAAAGAGAAAAAAGAGCA---ATAGGA---CTGGGAGCT------
---ATGTTCCTTGGGTTCTTGGGAACA---GCAGGAAGCACGATG---GGCGCAGCGTCA---
CTGACGCTGACGGTACAG---GCCAGACAG---TTATTG---TCTGGTATAGTGCAACAGCAAAAC---
AATTTG---CTGAGG---GCTATAGAGGCGCAACAGCATCTGTTGCAACTCACAGTCTGGGGCATT
>AF484518
TTCTACTGCAATACAACAGTACTGTTTAATAGCACATGGGAAAATAATAGTACATGGGGTAATGAGAGT----
----

GCAATGGGTAATGTCACACTCCCATGCAGAATAAAACAAATTATAAACATGTGGCAGGGAGTAGGAAAAGCAA
TGTATGCCCCTCCCATTGAAGGACTAATCAGCTGCTCATCAAAT---
ATTACAGGATTACTGTTGACAAGAGATGGTGGTGGAAATGAAAGT----------------------------
----

AGTCAGAATGAGACCTTCAGACCTGGAGGGGGAGATATGAGAGACAATTGGAGAAGTGAATTATATAAATATA
AAGTAGTAAGAATTGAACCATTA---GGTCTAGCACCCTCCAAGGCAAAAAGAAGAGTAGTA-----------
----------------------------GAAAGAGAGAAAAGAGCA---ATTGGA---CTAGGAGCT------
---ATGTTCCTTGGGTTCTTGGGAGCA---GCAGGAAGCACGATG---GGCGCAGCGTCA---
CTGACGCTGACGGTACAG---GCCAGACAG---CTACTG---TCTGGTATAGTGCAACAGCAAAAC---
AATTTG---CTGAGG---GCTATAGAGGCGCAACAGCACCTGTTGCAACTCACAGTCTGGGGCATT
>AY304496
TTCTACTGCAATACATCAGGACTGTTTAATGGTGTATGGAAGAATAATACATCAGATAATGAAACGGTAAAT-
----

GGAAGTGAGACAATAACAATCCCATGCAGAATAAAACAAATTATAAACACGTGGCAAGGAGTAGGAAGAGCAA
TGTATGCCCCTCCCATTAGAGGACTAATCCGATGTTCATCAAAC---
ATTACAGGACTATTGTTAACAAGAGATGGTGGTACAAATGGTGCT----------------------------
----

AGTCAGAAGGAGACCTTCAGACCCGGAGGAGGAGATATGAGAGACAATTGGAGAAGTGAATTATACAAATATA
AAGTAGTAAAAATTGAACCATTA---GGTCTAGCACCCACCAGGGCTAAAAGAAGAGTGGTG-----------
----------------------------GAAAGAGAAAAAAGAGCA---ATAGGA---CTAGGAGCT------
---ATGTTCCTTGGGTTCTTGGGAACA---GCAGGAAGCACGATG---GGCGCAGTGTCA---
CTGACGCTGACGGTACAG---GCCAGACAG---GTACTG---TCTGGTATAGTGCAGCAGCAAAAC---
AATTTG---CTGAGG---GCCATAGAGGCGCAACAGCATCTGTTGCAACTCACAGTCTGGGGCATT
>AF484480
TTCTACTGCAATACATCAAAACTGTTTAATAATACATTTAATACTAGTGATTGGAGGACTTATAATACATCAA
ATAGT--

ACAGGGAATGAGACAATCACACTCCCATGCAGAATAAAACAAATTATAAACATGTGGCAGGGAGTAGGAAGAG
CAATGTATGCCCCTCCCATTGAGGGACTAATCAGATGTTCATCAAAT---
ATTACAGGACTATTGTTGACAAGAGATGGGGGTAATGCAAATAATACT-------------------------
----

AATCAAAGTGAGACCTTCAGACCTGGAGGAGGAGATATGAGAGACAATTGGAGAAGTGAATTATACAAATATA
AAGTAGTAAGAATTGAGCCAATA---GGTCTAGCACCCACCAAGGCAAGGAGAAGAGTGGTG-----------
----------------------------GAAAGAGAAAAAAGAGCA---ATAGGA---CTAGGAGCT------
---ATGTTCCTTGGGTTCTTGGGAGCA---GCAGGAAGCACGATG---GGCGCAGCGTCA---
CTGACGCTGACGGTACAG---GCCAGACAG---GTGTTG---TCTGGTATAGTGCAACAGCAAAAC---
AACTTG---CTGAAG---GCTATAGAGGCGCAACAGCATCTGTTGCAACTCACAGTCTGGGGCATT
>AF484485
TTCTACTGCAATACATCAGAATTGTTTAATAATAGTGTATGGACATCAAATAATACAGGGATGTCAAATAGT-
----

ACAAAGGCAAATATCACACTCCCGTGCAGAATAAAACAAATTATAAACATGTGGCAGGGAGTAGGAAAAGCAA
TGTATGCCCCTCCCATTGCAGGACAAATCAGATGCTCGTCAAAT---
ATTACAGGATTATTGTTGACAAGAGATGGTGGTGCAAATAATAAT----------------------------
----

AGTCAGAATGAGACCTTCAGACCTGGAGGAGGAGATATGAAAGACAATTGGAGAAGTGAATTATACAAATATA

AAGTAGTAAGACTTGAACCACTA---GGTCTAGCACCCACCAAGGCAAAGAGAAGAGTGGTG-----------
----------------------------GAAAGAGAAAAAAGAGCA---ATAGGA---CTGGGAGCT------
---ATGTTCCTTGGGTTCTTGGGAGCA---GCAGGAAGCACGATG---GGCGCAGCGTCG---
CTGACGCTGACGGTACAG---GCCAGACTA---TTATTG---TCTGGTATAGTGCAACAGCAAAAC---
AATTTG---CTGAGG---GCTATAGAGGCGCAACAGCATCTGTTGCAACTCACAGTCTGGGGCATT
>AF484486
TTCTATTGCAATACATCAGGACTGTTTAATAGTACATGGAGGAATGATACATCAAGTAGTACATGGAGGAATG
ATACATCAAGT--
-------------AGTACA------
AAGCCAGATGAGACACTCACACTCCCATGCAGGATAAAACAAATTATAAACCTGTGGCAGGAAGTAGGAAGAG
CAATGTATGCCCCTCCCATTGCAGGAAACATCACATGTTCATCAAAT---
GTTACAGGACTATTGTTGACAAGAGATGGTGGTACACATAATAATAAT-------------------------
----

AGTCAGACTGAGACCATCAGGCCTGGAGGAGGAGATATGAGAAACAATTGGAGAAGTGAATTATACAAATATA
AAGTAGTAAGAATTGAGCCAATA---GGCCTAGCACCCACCAAGGCAAAGAGAAGAGTGGTG-----------
----------------------------GAAAGAGAAAAAAGAGCA---ATAGGC---CTGGGAGCT------
---ATGTTCCTTGGGTTCTTGGGAGCA---GCAGGAAGCACGATG---GGCGCAGCGTCA---
CTGACGCTGACGGTACAG---GCCAGACAA---GTATTG---TCTGGTATAGTGCAACAGCAAAAT---
AATTTG---CTGAGG---GCTATAGAGGCGCAACAGCACCTGTTGCAACTCACAGTCTGGGGCATT
>AF484487
TTCTACTGTGATACAACAGGACTGTTTAATGGTACAAATAGTACAGGGGGA----------------------
----

GATAATGAGACAATCACACTCCAATGCAGAATAAAACAAATTATAAACATGTGGCAGGGAGTAGGAAGAGCAA
TGTATGCCCCTCCCATTGCAGGGCAAATCTATTGCTCATCAAAT---
ATTACAGGACTATTGCTGACAAGAGATGGTGGTAACAATGATACG----------------------------
----

GGGGAGGAGATTTTCAGACCTGTAGGAGGAGATATGAGAGACAATTGGAGAAGTGAATTATACAAATATAAAG
TAGTAAGAATTGAACCACTA---GGTCTAGCACCCTCCAAGGCAAGGAGAAGAGTGGTG--------------
-------------------------GAAAGAGAAAAAAGAGCA---ATAGGA---CTAGGAGCT---------
GTGTTCCTTGGGTTCTTGGGAGCA---GCAGGAAGCACGATG---GGCGCAGCGTCG---
CTGACGCTGACGGTACAG---GCCAGACAG---TTATTG---TCTGGTATAGTGCAACAGCAAAAC---
AATTTG---CTGCAG---GCTATAGAGGCGCAACAGCATCTGTTGCAACTCACAGTCTGGGGCATT
>AF484519
TTCTATTGCAACACATCAGGACTGTTTAATAGTACATGGACATCAAATAGTACATCAAATAGTACAAAGGTAA
AT---

AGTACTGAGCCAATCACACTCCAATGCAGAATAAAACAAATTATAAATATGTGGCAGGGAGTAGGAAGAGCAA
TGTATGCCCCGCCCATTGAAGGACTAATTCATTGTTCATCAAAT---
ATTACAGGACTACTGTTGACAAGAGATGGTGGTACAAATAATACT----------------------------
----

AGTCAGAATGAGACTTTCAGACCTGGAGGAGGAAATATGAGAGACAATTGGAGAAGTGAATTATACAAATATA
AAGTAGTAAGAATTGAACCAATA---GGTCTAGCACCCACCAAGGCAAAGAGGAGAGTGGTG-----------
----------------------------GAAAGAGAAAAAAGAGCA---ATAGGA---CTAGGAGCT------
---ATGTTCCTTGGGTTCTTGGGAACA---GCAGGAAGCACGATG---GGCGCAGCGTCA---
ATAACGCTGACGGTACAG---GCCAGACAG---TTACTG---TCTGGTATAGTGCAGCAGCAAAAC---
AATTTG---CTGAGG---GCTATAGAGGCGCAACAGCATCTGTTGCAACTCACAGTCTGGGGCATT
>AF484494
TTCTACTGCAATACATCAGGACTGTTTAATAATAGTGTATGGAATACTAGTGTATGGAGAAATGATAGTACAT
TA---

GATAATGAGACTATCACACTCCCATGCAGAATAAAACAAATTATAAACATGTGGCAAGGAGTAGGAAAAGCAA
TGTATGCCCCTCCCATTGAAGGACGAATCAGTTGTTCATCAAAT---
ATTACAGGACTATTGTTGACAAGAGATGGTGGTGCAAATAATAAT----------------------------
----

AGTCAGAATGAGACCTTCAGACCTGGAGGAGGAGATATGAGAGATAATTGGAGAAGTGAATTATACAAATACA

AAGTAATAAGAATTGAACCACTA---GGTCTAGCACCCACCAAGGCAAAAAGAAGAGTGGTG-----------
----------------------------GAAAGAGAAAAAAGAGCA---ATAGGA---CTAGGAGCT------
---ATGTTCCTTGGGTTCTTGGGAGCA---GCAGGAAGCACGATG---GGCGCAGCGTCA---
CTGACGCTGACGGTACAG---GCCAGACAG---TTATTG---TCTGGTATAGTGCAGCAGCAAAAC---
AATTTG---CTGAGG---GCTATAGAGGCGCAACAACATCTGTTGCAACTCACAGTCTGGGGCATT
>AF484495
TTCTACTGCAACACAACAGGATTGTTTAATCATAGTAAATGGAATAATAATAATACATCAGAATGGAATAAT-
----

ACAGATAAGATAATCACACTCCCATGCAGACTAAAACAAATTATAAACATGTGGCAGGGAGTAGGAAGAGCTA
TGTATGCCCCTCCCATTGAAGGAGAAATTAAATGTTCATCAAAT---
ATTACAGGACTATTGTTAACAAGAGATGGAGGTACAAATAGTACT----------------------------
----

CAGAATGAGACCTTCAGACCTGGAGGGGGAGATATGAGAGACAATTGGAGAAGTGAATTATACAAATATAAAG
TAGTAAGAATTGAACCACTA---GGTCTAGCACCCACCAGAGCAAAGAGAAGAGTGGTG--------------
-------------------------CAAAGAGAAAAAAGAGCA---ATAGGA---CTGGGAGCT---------
ATGTTCCTTGGGTTCTTGGGAGCA---GCAGGAAGCACGATG---GGCGCAACGTCG---
ATGGCGTTGACGGTACAG---GCCAGACAA---TTGTTG---TCTGGTATAGTGCAACAGCAAAAC---
AATTTG---CTGAGG---GCTATAGAGGCGCAACAGCATCTGTTGCAACTCACAGTCTGGGGCATT
>AF484497
TTCTACTGCAATACATCAGGACTGTTTAATAGTGAATGGACATCGTCTAAAGGTGAATGGACAGGGGTAGAAA
AGAAT--

AGTACAAGCAGCAACAACAACCTCCCATGCAGAATAAAACAAATTATAAACATGTGGCAGGGAGTAGGAAAAG
CTATGTATGCCCCTCCCATTAAAGGACAAATCAGATGTTCATCAAAT---
ATTACAGGACTATTGTTGACAAGAGATGGTGGTGTAAATAATAAT----------------------------
----

AGTCAGGAGACCTTCAGACCTGGAGGAGGAGATATGAGAGACAATTGGAGAAGTGAATTATACAAATATAAAG
TAGTAAGAATTGAACCACTA---GGCCTAGCACCCACCAAGGCAAAAAGAAGAGTGGTG--------------
-------------------------GAAAGAGAAAAAAGAGCA---ATAGGA---CTAGGAGCT---------
TTGTTCCTTGGGTTCTTGGGAGCA---GCAGGAAGCACAATG---GGCGCAGCGTCA---
CTGACGCTGACGGTACAG---GCCAGACAG---GTATTG---GCTGGTATAGTGCAACAGCAAAAC---
AATTTG---CTGAGG---GCTATAGAGGCGCAACAGCATCTGTTGCAACTCACAGTCTGGGGCATT
>AF484498
TTCTACTGCAATACAGCAAGATTGTTTAATTATGAATGGAGTAATAATACATTAGCTAGTACAGGGAATGACA
GT---

AAAGGGAATGACAGTATCACACTCCAATGCAGAATAAAACAAATTATAAACATGTGGCAGGGAGTAGGAAAAG
CAATGTATGCCCCTCCCATTGAGGGACTAATCACCTGTTCATCAAAT---
ATTACAGGACTATTGTTGACAAGAGATGGTGGTGCAAATAATAAT----------------------------
----

AGTCAGAATGAGACCTTCAGACCTGGAGGAGGAGATATAAGGGACAATTGGAGAAGTGAATTATACAAATATA
AAGTAGTAAAACTTGAACCACTA---GGTCTAGCACCCACCAGGGCAAAGAGAAGAGTGGTG-----------
----------------------------GAAAGAGAAAAAAGAGCA---ATAGGA---CTAGGAGCT------
---ATGTTCCTTGGGTTCTTGGGAGCA---GCAGGAAGCACGATG---GGCGCAGCGTCC---
CTGACGCTGACGGTACAG---GCCAGACAG---TTATTG---TCTGGTATAGTGCAACAGCAAAAT---
AATTTG---CTAATG---GCTATAGAGGCGCAACAGCATCTGTTGCAACTCACAGTCTGGGGCATT
>AF484499
TTCTACTGCAATACATCAAAGCTGTTTAATAGTACATGGACAGGGGGTGGAAATATCACAAATATCACA----
----

AGTATCACAAATATCACACTCCCATGCAGAATAAAACAAATTATAAACATGTGGCAGGGAGTAGGCAAAGCAA
TGTATGCCCCTCCCATTGAAGGACTAATCCAATGTTCATCAAAT---
ATTACAGGACTATTATTGACAAGAGATGGTGGTGCAACTAATGAT----------------------------
----

AGTCAGAGTGAGACCTTTAGACCTGCAGGAGGAGATATGAGAGATAATTGGAGAAGTGAATTATACAAATATA

AAGTAGTAAGAATTGAACCACTA---GGTCTAGCACCCACCAAGGCAAAGAGAAGAACAGTG-----------
----------------------------GAAAGAGAAAAAAGAGCA---ATAGGA---CTAGGAGCT------
---ATGTTCCTTGGGTTCTTGGGAGCA---GCAGGAAGCACGATG---GGCGCAGCGTCA---
CTGACGCTGACGGTACAG---GCCAGACTG---TTATTG---TCTGGTATAGTGCAACAGCAAAAC---
AATTTG---CTGAGG---GCTATAGAGGCGCAACAGCATCTGTTGCAACTCACAGTCTGGGGCATT
>U36871
TCCTACTGCAATACATCAAAACTGTTTAATAGTACATGGAGGAATAATAATACATCAAATAGTACAGGGGTAA
ATAGT--
-------------------ACA---
GGGGAAAATGAGATAATCAAACTCCCATGCAGAATAAAACAAATTGTAAATATGTGGCAGGGAGTAGGAAAAG
CAATGTATGCCCCTCCCATTGAAGGGCTAATCAATTGTTCATCAAAT---
ATTACAGGACTATTGTTGACAAGAGATGGTGGTGCCAATAATAGT----------------------------
----

CAGAATGAGACCTTCAGACCTGGAGGAGGAGATATGAGGGACAATTGGAGAAGTGAATTATACAAATATAAAG
TAGTAAAACTTGAACCACTA---GGTCTAGCACCCACCAGGGCAAAGAGAAGAGTGGTG--------------
-------------------------GAAAGAGAAAAAAGAGCA---ATAGGA---CTAGGAGCT---------
ATGTTCCTTGGGTTCTTGGGAGCA---GCAGGAAGCACGATG---GGCGCAGCGTCA---
CTGACGCTGACGGTACAG---GCCAGACAG---TTAATG---TCTGGTATAGTGCAACAGCAAGAC---
AATTTG---CTGAGG---GCTATAGAGGCGCAACAGCATCTGTTGCAACTCACAGTCTGGGGCATT
>EF575364
TTCTACTGCAACACATCAGGACTATTTAATGGTACATGGAGAAATGCTACATCAAATAGTACACAG-------
----

CTGAATGGTACAATCACGCTCCCATGCAGAATAAAACAAATTATAAACATGTGGCAAGAAGTAGGAAAAGCAA
TGTATGCCCCTCCCATTGAAGGATTCATCAATTGTTCATCAAAT---
ATTACAGGACTATTGTTGGTAAGAGATGGTGGTAGTAATGATGAT----------------------------
----

AGTCAGAATGAGACCTTCAGACCTGGAGGAGGAGATATGAGAGACAATTGGAGAAGTGAATTATACAAATATA
AGGTAGTAAGAATTGAACCACTA---GGTCTAGCACCCACCAAGGCAAAAAGAAGAGTGGTG-----------
----------------------------GAAAGAGAAAAAAGAGCA---ATAGGA---CTGGGAGCT------
---ATGTTCCTTGGGTTCTTGGGAGCA---GCAGGAAGCACGATG---GGCGCAGCGTCA---
ATAACGCTGACGGTACAG---GCCAGACAG---TTACTG---TCTGGTATAGTGCAACAGCAAAAC---
AATTTG---CTGAGG---GCTATAGAGGCGCAACAGCATCTGTTGCAACTCACAGTCTGGGGCATT
>EF575370
TTCTACTGCAATACATCAAAACTGTTTAATAGTACATGGGAGAATGTGAATGTTACTACATCAAATAATACAG
GG---

CCAGATAGTACAATCACACTCCCATGCAGAATAAAACAAATTATAAACATGTGGCAGGGAGTAGGAAAAGCAA
TGTATGCCCCTCCCATTGAAGGGCTAATCAAATGTTCATCAAAT---
ATTACAGGACTATTGTTACTAAGAGATGGTGGTCTAAATACTAGT----------------------------
----

CAGAGTGAAACCTTCAGACCTGGAGGAGGAGATATGAGGGACAATTGGAGAAGTGAATTATACAAATATAAAG
TAGTAAAAATTGAACCAATA---GGTCTAGCACCTACCAAGGCAAGGAGAAGAGTGATG--------------
-------------------------GAAAGAGAAAAGAGAGCA---ATAGGA---CTGGGAGCT---------
GTGTTCCTTGGGTTCTTGGGAGCA---GCGGGAAGCACAATG---GGCGCAGCGTCA---
CTAACGCTGACGGTACAA---GCCAGACAA---GTATTG---TTTGGTATAGTGCAACAGCAAAAC---
AATTTG---CTGAGG---GCTATAGAGGCGCAGCAGCATCTGCTGCAACTCACAGTCTGGGGCATT
>EF575429
TTCTACTGCAATACATCAGGACTGTTTAATAATACATTTAATACTAAGAATAGTACAGAGGTAAATAGTAAT-
----

GGGACAGAGGTAATCATACTCCCATGCAGAATAAAACAAATTACAAACATGTGGCAGAAAGTAGGAAAAGCAA
TGTATGCCCCTCCTATTGAAGGACTAATCAGCTGTTCATCAAAT---
ATCACAGGACTATTGTTGACAAGAGATGGTGGTAATATAACTAGT----------------------------
----

CAGAATGAGACCTTCAGGCCTGCAGGAGGAGATATGAGAGATAATTGGAGAAGTGAATTATACAAATATAAAG

TAGTAAGAATCGAACCACTA---GGTCTAGCACCCACCAAGGCAAGGAGAAGAGTGGTG--------------
-------------------------GAAAGGGAAAAAAGAGCA---ATAGGA---CTGGGAGCT---------
TTGTTCCTTGGGTTCTTGGGAACA---GCAGGAAGCACGATG---GGCGCAGCGTCA---
CTGACGCTGACGGTACAG---GCCAGACAG---TTATTG---TCTGGTATAGTGCAACAGCAAAAC---
AATTTG---CTGAGG---GCTATAGAGGCGCAACAGCATCTGTTGCAACTCACAGTCTGGGGCATT
>EF575482
TTCTACTGCAATACATCACGCCTGTTTAATAGTACATGGAATAATAATACAGGGGATATAGGGAGTAGTGAT-
----

ACAAATAAGAATATCACAATCCCATGCAGAATAAAACAAATTATAAACATGTGGCAGGGAGTAGGAAAAGCAA
TGTATGCCCCTCCCATCGAAGGAAAAATCCAGTGTAGATCAAAT---
ATTACAGGGCTCTTACTGACAAGAGATGGTGGTGCAGATGCTAAT----------------------------
----

CAGAATGAGACCATCAGACCTGGAGGAGGAGATATGAGAGACAATTGGAGAAGTGAATTATACAAATATAAGG
TAGTAAGAATTGAACCACTA---GGAATAGCACCCACCAAGGCAAAGAGAAGAGTGGTG--------------
-------------------------GAAAGAGAAAAGAGAGCA---ATAGGA---CTAGGAGCT---------
GTGTTCCTTGGGTTCTTGGGAGCA---GCAGGAAGCACGATG---GGCGCAGTGTCA---
TTAACGCTGACGGTACAA---GCCAGACAG---TTATTG---TCTGGTATAGTGCAACAGCAAAGC---
AATTTG---CTGAAG---GCTATAGAGGCGCAACAGCGTCTGTTGCAACTCACAGTCTGGGGCATT
>JQ403079
TTCTACTGCAATACATCAGGACTGTTTAATAATATATGGAATGGCACTTGGAATGGCACTTTAAATAGTACA-
----

GGGAGAGAGAATATCACACTCCGATGCAGAATAAAACAAATTATAAACATGTGGCAGGGAGTAGGAAAAGCAA
TGTATGCCCCTCCCATTGAAGGAGTAATTAATTGTACATCAAAT---
ATTACAGGACTAATATTAACAAGAGATGGTGGTGCTAATGAGAGT----------------------------
----

CAGAATAATAATGAGACCTTCAGACCTGGAGGAGGAGATATGAGGGACAATTGGAGAAGTGAGTTATACAAAT
ATAAAGTAGTAAAAATTGAACCATTA---GGTCTAGCACCCACCAGGGCAAAAAGGAGAGTGGTG--------
-------------------------------GAAAGAGAAAAAAGAGCA---ATAGGA---CTGGGAGCT---
------TTGTTCCTTGGGTTCTTGGGAGCA---GCAGGAAGCACGATG---GGCGCAGCGTCA---
ATGGCGCTGACGGTACAG---GCCAGACAA---GTATTG---TCTGGTATAGTGCAACAGCAAAAC---
AATTTG---CTGAGG---GCTATAGAGGCGCAACAGCATTTGTTGCAACTCACAGTCTGGGGCATT
>AY773338
TTCTACTGTAATACATCAAGGCTGTTTAATAATACATGGAATGGTACAACATGGTCAAATAAGACAGAC----
----

ACCAATGGGACAGTCACACTCCCATGCAGAATAAAACAAATTATAAACATGTGGCAGGAAGTAGGAAAAGCAA
TGTATGCCCCCCCCATAGAAGGACTACTTAGATGTTCATCAAAT---
ATTACAGGGTATATATTGACAAGAGATGGTGGTTATACCAGTTCT----------------------------
----

GGCAATGCGACCTTCAGACCTGGCGGAGGAGATATGAGGGACAATTGGAGAAGAGAATTATATACATACAAAG
TAGTACAAATTGGACCAATA---GGAGTAGTGCCCACCAGGGCAAAGAGAAGAGTGGTG--------------
-------------------------GAAAGGGAAAAAAGAGGG---GTT------------------------
------------TTCTTGGGAGCA---GCAGGAAGCACGATG---GGCGCAGCGTCA---
TTGTCGCTGCCGGTACAG---GCCAGACAG---GTATTG---TCTGGTACAGTGCAACAGCAAAGC---
AATTTG---CTCAGG---GCTATATCGGCGCAACAGCATCTGTTGCAACTCACGGTCTGGGGCATT
>AY773340
TTCTACTGTAATACATCAAGGCTGTTTAGTAATACATGGATGGTACATGGGATAATAATACATGGT-------
----

TCAAATCAGACAGTCAGACTCCCATGCAGAATAAAACAAATTATAAACATGTGGCAGGAAGTAGGAAAAGCAA
TGTATGCCCCTCCCATAGAAGGAACAATTAGGTGTTCATCAAAT---
ATTACAGGGCTAATATTGACAAGAGATGGTGGTAATAATAGTTCT----------------------------
----

AACAACGAGACCTTCAGACCTGGCGGAGGAGATATGAGGGACAATTGGAGAAGTGAATTATATAAATACAAAG

TAATACAAATTGAACCAATA---GGAGTAGCGCCCACCAAGGCAAAGAGAAGAGTGGTG--------------
-------------------------GAAAGGGAAAAAAGAGCA---ATAGGA---CTAGGAGCT---------
ATGTTCCTTGGGTTCTTGGGAGCA---GCAGGAAGCACAATG---GGCGCAGCGTCA---
GTGACGCTGACGGTACAG---GCCAGACAG---GTATTG---TCTGGTAGAGTGCAACAGCAAAAC---
AATTTG---GCCAGG---GCTATAGAGGCGCAACAGCATCTGTTGCAACTCACGGTCTGGGGCATT
>AY773341
TTCTACTGTAATACATCAGGGCTGTTTAATAATACATGGGATAATAGTAATAGGACATGGTCAAATAAGGGAG
CA---

TGGTCAAATCAGACAGTCACACTCCCATGCAGAATACGACAAATTATATACATGTGGCAGAAAGTTGGAAAAG
CAATGTATGCCCCTCCCATACAAGGAACACTTAGATGCTCATCAAAT---
ATTACAGGACTACTATTCACAAGAGATGGTGGTAATAATAGTTCT----------------------------
----

AACAACGAGACCTTCAGACCTGGCGGAGGAGATACGAGGGACAATTGGAGAAGTGAATTATATAAATACAAAG
TACTACAAATTGAACCAAGA---GGAGCAGCGCCCACCAAGGCAAAGAGAAGAGTGGTG--------------
-------------------------GAAAGGGAAAAAAGAGCA---ATACGA---CTCGGAGCT---------
ATGTTCCTTGGGTTCTTGGGAGCA---GCAGGAAGCACAATG---GGCGCAGCGTCA---
GAGACGCGGACGGTACAG---GCCAGACAG---GTATTG---TCTGGTATACTGCAACAGCAAAAC---
AATTTG---CTCAGG---GCTATCGAGGCGCAACAGCATCTGTTGCAACTCACGGTCTGGGGCATT
>EF633445
TTCTACTGTAATACATCAGGGCTGTTTAATAGTACATGGAATAGGACAGGGAATAATAGTAGTAGTACAGGG-
----

TCAAATGAGACAATCACACTTCCATGCAGAATAAGACAAATTATAAACATGTGGCAGGGAGTAGGAAAAGCAA
TGTATGCCCCTCCCATAGAAGGACTAATTAGATGTTCATCAAAT---
ATTACAGGGCTAATATTGACAAGGGATGGTGGTGATAATAGTAGT----------------------------
----

TCTCACAATGAGACCTTCAGACCTGGAGGAGGAGATATGAGGGACAATTGGAGAAGTGAATTATATAAATACA
AAGTAGTAAGAATTGAACCAATA---GGATTAGCACCCACCAGGGCAAAGAGAAGAGTGGTG-----------
----------------------------GCAAGGGAAAAAAGAGCA---ATAGGA---CTAGCGAGC------
---TGGTTCCTTGGGTTCTTGGGAGCA---GCAGGAAGCACAATG---GGCGCAGCGTCT---
GTGACGCTGACGGTACAG---GCCAGACAG---TTATTG---TCTGGTATAGTGCAACAGCAAAAC---
AATTTG---CTGAGA---GCTATAGAGGCGCAACAGCATCTGTTGCAACTCACAGTCTGGGGCATT
>FJ900266
TTCTACTGCAATACATCAGGATTGTTTAATAACAGTGATACC-------------------------------
----

TCAAACAGAACTATTACTCTCCCATGTAGAATAAAACAAATTGTAAACATGTGGCAGGGAGTAGGACAAGCAA
TGTATGCCGCTCCTGTTGCAGGAAAAATTACCTGTAACTCAAAC---
ATTACAGGCCTACTATTGACAAGAGATGGTGGTAATAATAGTCAT----------------------------
----

ACCGAGACCTTCAGACCTGGGGGAGGAAATATGAAAGACAATTGGAGAAGTGAACTATATAAATATAAAGTAG
TAGAACTTGAACCATTA---GGAGTAGCACCCACCAAGGCAAGAAGAAGAGTGGTGGCG--------------
-------------------AGAGGGAGAGAAAAAAGAGCA---GTGGGA---ATAGGAGCT---------
TTGTTCTTTGGGTTCTTGGGAGCA---GCAGGAAGCACTATG---GGCGCAGCGTCA---
ATAACGCTGACGGTACAG---GCCAGACAA---TTATTG---TCTGGAATAGTGCAACAGCAGAAC---
AATCTG---CTGAGA---GCTATTGAAGCGCAGCAGCATATGTTGCAGCTCACAGTCTGGGGCATT
>FJ900267
TTCTACTGCAATACATCAAAATTGTTTAATAATAACACAGAA-------------------------------
----

CCCAATGGCACTATCACTCTTCCATGTAAAATAAAACAAATTGTAAACATGTGGCAGGGAGTAGGACAAGCAA
TGTATGCCGCTCCTATTGCAGGAAACATTACCTGTAACTCAAAT---
ATTACAGGCTTACTATTGACAAGAGATGGTGGTACGAATCAGAGT----------------------------
----

GCTACTGAGACATTCAGACCTGGGGGAGGAAATATGAAAGACAATTGGAGAAGTGAGCTATATAAATATAAAG

TAGTACAAATTGAACCACTA---GGAGTAGCACCCACCAAGGCAAAAAGACGAGTGGTGGAG-----------
----------------------AAAGAGAGACCAAAGAGAGCA---GTGGGA---ATAGGAGCT---------
CTGTTCCTTGGGTTCTTGGGAGCA---GCAGGAAGCACTATG---GGCGCAGCATCA---
ATAACGCTGACGGCACAG---GCCAGACAA---TTATTG---TCTGGAATAGTGCAACAGCAGAAC---
AATCTG---CTGAGG---GCTATTGAAGCGCAGCAGCATCTGTTGCAGCTCACAGTCTGGGGCATT
>DQ189088
TTCTACTGCAATACATCAGGACTGTTTAATGACACAGATGAC-------------------------------
----

AATGGCACTATCACTCTCCCATGTCGAATAAAACAAATTGTAAACATGTGGCAGGAAGTAGGACGAGCAAACA
ATCACCCTCCCCATGCAGGAAACATTACCTGTGGTTCAAAT---
ATTACAGGTCTACTATTGACAAGAGATGGTGGTACTGGGAATTGCACTGATGGT-------------------
----
-
GGTACTGGGAATTGCACTGAGACCTTCAGACCTGGGGGAGGAAATATGAAAGATAATTGGAGAAGTGAATTAT
ATAAATACAAAGTAGTAGAAATTGAACCACTA---GGAGTAGCACCCACCAAAGCAAGAAGACAAGTGGTA--
-------------------------------------AAGAGAGAAAAAAGAGCA---GTGGGA---
ATGGGAGCT---------TTGTTCATTGGGTTCTTGGCAGCA---GCAGGAGGCACTATG---
GGCGCGGCGTCA---ATAACGCTGACGGTACAG---GCCAGACAG---TTACTG---
TCTGGAATAGTGCAACAGCAAAAC---AATTTG---CTGAGA---
GCTATTGAAGCGCAACAGCATATGTTGCAGCTCACAGTCTGGGGCATT
>MH746263
TTCTACTGCGATACATCACAACTGTTTAATGAGTCA-------------------------------------
----
-------------GCAGGT------
CAATTCAATGGAACTATCATTCTCCCATGTAAAATAAAACAAATTATAAACATGTGGCAGGAAGTAGGACGAG
CAATGTATGCTGCTCCTGTTGCAGGAAACATTACCTGTAACTCAAAT---
ATCACAGGCCTATTGTTAACAAGAGATAGACATGGTGAGGCTAAT----------------------------
----

ACTACTGAGACCTTCAGACCTGGAGGAGGAAATATGAGAGACAATTGGAGAAGTGAACTATATAAATATAAAG
TAGTAAGAATTGAGCCACTA---GGGGTAGCACCTACCAGGGCAAAAAGACGAACGGTG--------------
-------------------------CAGAGAGAAAAAAGAGCA---GTAGGA---ATGGGAGCC---------
TTGTTCCTTGGGTTCTTGGGAGCA---GCAGGAAGCACTATG---GGCGCAGCATCA---
ATAACGCTGACGGTACAG---GCCAGACAA---TTGCTG---TCTGGAATAGTGCAACAGCAAAAC---
AATTTG---CTGATG---ACTATTGAAGCGCAACAACACCTGTTGCAGCTCACAGTCTGGGGCATT
>MH746246
TTCTACTGCGATACATCAAAATTGTTTAAT---

-AATAATACAAAAGCATCA------
GCATCCAATGGCACTATCACTATCCCATGTAAAATAAAACAAATTATAAACATGTGGCAGGGAGTAGGACAAG
CAATGTATGCCCCTCCCATTGCAGGAAACATTACCTGTCGTTCAAAC---
ATTACAGGCCTACTATTGATAAGAGATGGTGGTAGTAATCAGAAT----------------------------
----

AATTCTGAGGAGATCTTTAGACCTGGGGGAGGAAATATGAAAGACAATTGGAGAAGTGAACTATATAAATATA
AAGTAGTAGAAATTGAGCCGCTA---GGAGTAGCACCCACCAAGGCAAGAAGACCAGTGGTG-----------
----------------------------AGCAGAGAAAAAAGAGCA---GCAGGA---ATAGCAGCG------
---TTGTTCCTTGGGTTCTTGGGAGCA---GCAGGAAGCACTATG---GGCGCAGCGTCA---
ATAACGCTGACGGTACAG---GCCAGACAA---TTATTG---TCTGGAATAGTGCAACAGCAAAAC---
AATTTG---CTGAGG---GCTATTGAAGCGCAACAGCATATGTTGCAGCTCACAGTCTGGGGCATT
>FJ771006
TTCTACTGCAATACATCAGGACTGTTTAATGACACAGTATCC-------------------------------
----

AATGACACTATCACTCTCCCATGTCAAATAAAACAAATTGTAAATATGTGGCAGGAAGTGGGACGAGCAATGT
ATGCCGCTCCCATTGAAGGGAACATTACCTGTAACTCCAAT---
ATTACAGGTCTACTATTGACAAGAGATGGTGGTAAGAATAATGATACTGAG----------------------
----

AATAAAGATACTGAGATCTTCAGACCTGGGGGAGGAGATATGAGAGACAATTGGAGAAGTGAATTATATAAAT
ATAAAGTAGTAGAAATTGAACCACTA---GGAGTAGCACCCACCAGGGCAAAAAGACAAGTGGTG--------
-------------------------------AAGAGAGAAAAAAGAGCA---GTAGGA---ATGGGAGCT---
------GTTTTCCTTGGGTTCTTGGGAGCA---GCAGGAAGCACTATG---GGCGCAGCGTCA---
ATAACGCTGACGGTACAG---GCCAGACAA---TTATTG---TCTGGCATAGTGCAACAGCAAAAC---
AATTTG---CTGAGG---GCTATTAAAGCGCAACAGCATCTGTTGCAGCTCACAGTCTGGGGCATT
>FJ771008
TTCTACTGTAATACATCAGGACTGTTTAAATTCAATGCCTCATCCGGTGAG----------------------
----

AATAGTACAAATATCACTCTCCCATGTAGAATAAAACAAGTTATAAACATGTGGCAGGAAGTAGGACGAGCAA
TGTATGCCGCTCCTATTGAAGGAAAAATTTATTGTAGCTCAAAT---
ATTACAGGTCTACTATTGACTAGAGATGGTGGTACTAATGATACT----------------------------
----

AATGGTACTGAGACCTTCAGACCTGGGGGAGGAGATATGAGAGACAATTGGAGAAGTGAATTATATAAATATA
AGGTAGTAAGAATTGAACCACTA---GGAGTAGCACCCACCAAGGCAAAAAGACAAGTGGTG-----------
----------------------------CAGAGAGAAAAAAGAGCA---GTGGGA---CTAGGAGCT------
---GTGTTCCTTGGGTTCTTGGGAGCA---GCAGGAAGCACTATG---GGCGCAGCGTCA---
ATAACGCTGACGGTACAG---GCCAGACAA---TTATTG---TTTGGAATAGTGCAACAGCAGAAC---
AATCTG---CTGAGG---GCTATTGAAGCACAACAGCATATGTTGCAGCTCACAGTCTGGGGCATT
>KJ849782
TTCTACTGCAATACATCAGAACTGTTTAATGACACAGGCAAT-------------------------------
----
-------------------GAC---
ACTAGTGCCGCTACTATCACTCTCCCATGTAAAATAAAACAAGTTATAAACATGTGGCAGGAAGTAGGAAAAG
CAGTGTATACTGCTCCCATTGCAGGAAACCTTGCCTGTAACTCAACT---
ATTACAGGTCTACTATTGACAAGAGATGGTGGT--

CAGGACAATAATAGT---
ACTACTGAGATCTTCAGACCTGGGGGAGGAAATATGAAAGACAATTGGAGAAGTGAGTTATATAAATATAAAG
TAGTAGAAATTGAACCACTA---GGAGTAGCACCCACCAGGGCAAAAAGACAAGTGGTG--------------
-------------------------CAGAAGCAAAAAAGAGCA---GTGGGG---CTGGGAGCT---------
ATGTTCCTTGGGTTCTTGGGAGCA---GCAGGAAGCACTATG---GGCGCAGCGTCA---
ATAACGCTGACGGCACAG---GCCAGACAA---GTATTG---TTTGGAATAGTGCAACAGCAGAGC---
AATTTG---CTGAGG---GCTATTGAAGCGCAACAGCACATGTTGCAGCTCACAGTCTGGGGCATT
>KT427774
TTCTACTGCAATACATCAAAACTGTTTAATGACAATAGCACTCAAAAATCC----------------------
----

AATAATGACACTATCACTCTCCCATGTCGAATAAAACAAATGGTAAACATGTGGCAGGAAGTGGGGCGAGCAA
TGTATGCCGCTCCCATTGCAGGGAACATTACCTGTAATTCAAAT---
ATTACAGGTCTACTATTGGTAAGAGATGGTGGAAATGGTGGAAATAAT-------------------------
----

ACTAATAAGACTGAGATCTTCAGACCTGGGGGAGGAAATATGAAAGACAACTGGAGAAGTGAATTATATAAAT
ATAAAGTAGTAGAAATTCAGCCACTA---GGAGTAGCACCCACCAGGGCAAAAAGACAAGTGGTG--------
-------------------------------AAGAGAGAAAAAAGAGCA---GTGGGA---CTGGGAGCT---
------TTGTTCCTTGGGTTCTTGGGAGCA---GCAGGAAGCACTATG---GGCGCAGCGTCA---
ATAACGCTGACGGTACAG---GCCAGACAA---TTATTG---TCTGGTATAGTGCAACAGCAGAAT---
AATCTG---CTGAGG---GCTATTGAAGCGCAACAGCATCTGTTGCAGCTTACAGTCTGGGGCATT
>MG365768
TTCTACTGCAATACAGCAGGACTGTTTAACAACACTGAT----------------------------------
----

AAGAATATCACTATCACTCTCCCATGTCAAATAAAACAGATCATAAACATGTGGCAGGAAGTGGGACGAGCAA
TGTATGCCGCTCCCATTGCAGGAAGCATTACCTGTAACTCAAAT---
ATTACAGGTCTGATATTGACAAGAGATGGTGGCAATAGAAATGATACT-------------------------
----

AATTGCACTGAGACCTTCAGACCTGGGGGAGGAAATATGAAAGACAATTGGAGAAGTGAATTGTATAAATATA
AAGTAGTAGAAATTGAACCATTA---GGAATAGCACCCACCACGGCAAAAAGACAAGTGGTG-----------
----------------------------AAGAGAGACAAAAGAGCA---GTCGGA---ATAGGAGCT------
---GTGCTCATTGGATTCTTGGGAGCA---GCAGGAAGCACTAGG---GGCTTAGCCTCA---
GTGGCGCTGACGGTACAG---GCCAGACAA---TTATTG---TCTGGAATAGTGCAACAGCAGAAC---
AATTTG---CTGAGG---GCCATTGAAGCGCAACAGCATATGTTGCAGCTCACAGTCTGGGGCATT
>KU749396
TTCTACTGCAATACAACAAAACTGTTTAATAATACAAGATAC-------------------------------
----

AATGATACTATCACTCTCCCTCTCCCATGTCAAATAAAACAAATAGTGAACATGTGGCAGGAAGTAGGAAGAG
CAATGTATACCTCTCCCATTGCAGGAAACATTACTTGTAACTCAAAT---
ATTACAGGTCTACTATTGACAAGAGATGGTGGTAATAATACTGATAAT-------------------------
----

AATAATACTGAGATCTTCAGACCTGGGGGAGGAGATATGAAAGACAATTGGAGAAGTGAATTATATAAATATA
AAGTAGTAGAAATTGAACCACTA---GGAATAGCACCCACCAGGGCAAAAAGACGAGTGGTG-----------
----------------------------GAGAGACAAAAAAGAGCA---GTAGGA---ATGGGAGCT------
---ATGTTCCTTGGGTTCTTGGCAGCA---GCAGGAAGCACTATG---GGCGCAGCGTCA---
ATAACGCTGACGGCACAG---GCCAGACAA---TTATTG---TTTGGAATAGTGCAACAGCAAAGC---
AATTTG---CTGAGG---GCTATTGAAGCGCAACAGCATATGTTGCAGCTCACAGTCTGGGGCATT
>MG365764
TTCTACTGTAATACATCAGGACTGTTTAATAATAAC-------------------------------------
----
-------------------ACA---
GAATCCAATGACACTATCACTCTCCCATGTCGAATAAGACAAATTGTAAACATGTGGCAGGAAGTAGGAAAAG
CAATGTATGCCGCTCCCATTGCAGGAAACCTTGCCTGCAGCTCAAAC---
ATTACAGGTCTACTATTGACAAGAGATGGTGGTCAGAGTAATGGGACTAGTAAT-------------------
----

GGGACTACTGAGACCTTCAGACCTGGGGGAGGAAATATGAAAGACAATTGGAGAAGTGAATTATATAAATATA
AAGTGGTAGAAATTGAACCACTA---GGAGTAGCACCCACCAAGGCAAGAAGACCAGTGGTG-----------
----------------------------AAGAGAGAAAAAAGAGCA---GTGGGAATAATAGGAGCT------
---TTGTTCCTTGGGTTCTTGGGAGCA---GCAGGAAGCACTATG---GGCGCAGCGTCA---
ATAACGCTGACGGTACAG---GCCAGACAA---TTATTG---TCTGGAATAGTGCAACAGCAGAGC---
AATTTG---CTGAGG---GCTATTGAAGCGCAACAGCATATGTTGCAGCTCACAGTCTGGGGCATT
>KX181923
TTCTACTGCAATACATCAAGACTGTTTAATGACACAAAA----------------------------------
----

TTCAATGACACTATCGTTCTCCCATGTCGAATAAGACAACTGGTAAGACTGTGGCAGGAAGTAGGACGAGCAA
TGTATGCCGTTCCCATTGCAGGAAGCATTACCTGTAACTCAACT---
ATTACAGGTCTACTATTGACAAGAGATGGTGGTCAGCATAATGAAACT-------------------------
----

AATAAAACTGAGATCTTCAGACCTGGGGGAGGAAATATGAAAGACAATTGGAGAAGTGAATTATATAAATATA
AAGTAGTAGAAATTGAACCACTA---GGAGTAGCACCCACTGGGGCAAGGAGACAAGTGGTG-----------
----------------------------AAGAGAGAAAAAAGAGCAGTAGTGGGA---ATAGGAGCT------
---GTGCTCATTGGGTTCTTGGGAGCA---GCAGGAAGCACTATG---GGCGCGGCGTCA---
GTGACGCTGACGGTACAG---GCCAGACAA---TTATTG---TCTGGAATAGTGCAACAGCAGAAC---
AATTTG---CTGAAG---GCTATTGACGCGCAACAACATATGTTGCAGCTCACAGTCTGGGGCATT
>KX181926
TTTTACTGCAATACATCAGGCCTGTTTAATGACTCAATAATA-------------------------------
----

AATGGCACTGAGAGTATCACTCTCCCATGTCGAATAAAACAAATTGTAAACATGTGGCAGGAAGTAGGACGAG
CAATGTATGCCGTTCCTATTGCAGGAAACATTACCTGTATTTCAAAT---
ATTACAGGTCTGCTATTAACAAGAGATGGTGGTCTGAACAATGATACT-------------------------
----

AATCAGACTGAGACCTTCAGACCTGGGGGGGGAAATATGAAGGACAATTGGAGAAGTGAATTATATAAATATA
AAGTAGTAGAAATTGAACCCCTA---GGGGTAGCCCCCCCCAGGGCAAAAAGACAAGTGGTA-----------
----------------------------AAGAGAGAAAAAAGAGCA---GTGGGA---ATAGGAGCT------
---TTGTTCCTTGGGTTTTTGGGAGCA---GCAGGAAGCAATATG---GGCGCGGGGTCA---
ATGACGCTGACGGTACAG---GCCAGACAA---TTGTTG---TCTGGGATAGTGCAACAGCAAAAC---
AATTTG---CTGAGG---GCTATTGAAGCGCAACAGCATTTGTTGCAGCTCACAGTCTGGGGCATT
>KX181925
TTCTACTGCAATACATCAGCTCTGTTTAATAATGAAACAGGATCCAATGACACTACAGGA-------------
----

TCCAATAGCACTATCACTCTCCCATGTAGAATAAAACAAGTTGTAAACATGTGGCAGGAAGTAGGGCGAGCAA
TGTATACCGCTCCCATTGCAGGAAGCATTACCTGTAACTCAAAT---
ATTACAGGTCTACTATTGACAAGAGATGGTGGTTATGAAAATAAT----------------------------
----

CAGACTGAGACCTTCAGACCTGGGGGAGGAAATATGAAAGACAATTGGAGAAGTGAATTATATAAATATAAAG
TAGTAGAAATTCAGCCACTA---GGAGTAGCACCCACCAGGGCAAAAAGACCAGTGGTG--------------
-------------------------AGGAGAGAGAAAAGAGCAGCAGTGGGA---ATAGGAGCT---------
ATGTTCCTTGGGTTCTTGGGAGCA---GCAGGAAGCACTATG---GGCGCGGCGTCA---
ATAGCGCTGACGGTACAG---GCCAGACAA---TTATTG---TCTGGCATAGTGCAACAGCAGAAC---
AATCTG---CTGAGG---GCTATTGAAGCGCAACAGCATCTGTTGCAGCTCACAGTCTGGGGCATT
>MK041565
TTCTACTGCAATACAACAAATCTATTTGGTGACACAATGGTAAATAAC-------------------------
----

ACAGATGACACAATCACACTCCCATGTAGAATAAAACAAATTGTAAACATGTGGCAGGAAGTGGGACGAGCAA
TGTATGCCAATCCTGTTGCAGGAAACATTACCTGTAACTCAAAT---
ATTACAGGTCTACTATTGACAAGAGATGGTGGTACTAATAAAACA----------------------------
----

GATGAAACTGAGACCTTTAGACCAGGGGGAGGAAATATGAAAGACAATTGGAGAAGTGAATTATATAAATATA
AAGTAGTAAAAATTGAACCACTA---GGAATAGCACCCACCAGGGCAAAAAGACTAGTGGTG-----------
----------------------------CAGAGAGAAAAAAGAGCA---GTAGGA---CTGGGAACT------
---CTGTTCCTTGGGTTCTTGGGAGCA---GCCGGAAGCACTATG---GGCGCAGCGTCA---
ATGACGCTGACGGTACAG---GCCAGACAA---TTATTG---TCTGGAATAGTGCAACAGCAGAGC---
AATCTG---CTGAGG---GCTATTGAAGCGCAACATCACATGTTGCAGCTCACAGTCTGGGGCATT
>AY173958
TTCTACTGCAATACATCAGGACTGTTTAATGACACAGGATCC-------------------------------
----

AATGGCACTATCACTCTCCTATGTCGAATAAAACAAATGGTAAACATGTGGCAGGAAGTGGGACGAGCAATGT
ATGCCGCTCCCATCTCAGGAAACATTACCTGTAACTCGAAT---
ATTACAGGTCTACTATTGACAAGAGATGGTGGTCAGAATAATAAT----------------------------
----

CAGACTGAGACCTTCAGACCTGGGGGAGGAAATATGAAAGACAATTGGAGAAGTGAATTATATAAGTATAAAG
TAGTAGAAATTGAACCACTA---GGAGTAGCACCCACCAGGGCAAAAAGACAAGTGGTG--------------
-------------------------AGGAGAGAAAAAAGAGCA---GTGGGA---ATGGGAGCT---------
TTGTTCCTTGGGTTCTTGAGCGCG---GCAGGAAGCACTATG---GGCGCGGCGTCA---
ATAACGCTGACGGTACAG---GCCAGACAA---TTATTG---TCTGGAATAGTGCAACAGCAGAGT---
AATCTG---CTGAGG---GCTATTGAAGCGCAACAGCATCTGTTGCAGCTCACAGTCTGGGGCATT
>JF683771
TTCTATTGTAATACATCAGAATTGTTTAATAATAGTAACTCA-------------------------------
----

AACAATGGCACTATCACTCTCCCATGTAAAATAAAACAAATTATAAACATGTGGCAGGGAGTAGGGCAAGCAA
TGTATGCCCCTCCCATTGCAGGAAAAATTACCTGTAACTCAAGT---
ATTACAGGCCTACTATTGACAAGAGATGGTGGTCAAAGTAATAAGAGTAAA----------------------
----

GATAATGGGAGTGAGATCTTCAGACCTGGGAGAGGAAATATAAAAGACAATTGGAGAAGTGAACTATATAAAT
ACAAAGTAGTAGAAATTGAACCACTA---GGAATAGCACCCACCAAGGCAAAAAGACAAGTGGTA--------
------------------------------AAGAGAGAAAAAAGAGCA---GTGGAA---ATAGGAGCT---
------GTGTTCCTTACGTTCTTGGGAGCA---GCAGGAAGCACTATG---GGCGCAGCGTCA---
CTAACGCTGACGGTACAG---GCCAGACAA---TTATTG---TCTGGGATAGTGCAGCAGCAAAAC---
AATTTG---CTGAAG---GCTATTGAAGCGCAACAGCATCTGTTGCAGCTCACAGTCTGGGGCATT
>HM068554
TTCTACTGCAATACATCAAAACTGTTTAACAACACAGAATTCAAT---------------------------
----

GTCAGTGACACTATCATTCTCCCATGTCGAATAAAGCAAATTGTAAACATGTGGCAGGAAGTAGGACAAGCAA
TGTATGCTAATCCTATTGCAGGAAACATCACCTGTAACTCAAGT---
ATTACAGGTATACTATTGACAAGAGATGGTGGTAACGGGAATGAAACTGAG---------------------
----

AATAATGATACTGAAATCTTCAGACCTGGGGGAGGAAATATGAAAGACAATTGGAGAAGTGAATTATATAAAT
ATAAAGTAGTAGAAATTGAACCACTA---GGAGTAGCACCCACCAGGGCAAAAAGACAAGTGGTG--------
------------------------------AAGAGAGAAAAGAGAGCA---GCGGGA---ATGGCAGCT---
------TTGCTCCTTGGGTTCTTGGGCGCA---GCAGGAAGCACTATG---GGCGCAGCGTCA---
ATAACGCTGACGGTACAG---GCCAGACAA---TTATTG---TCTGGAATAGTGCAGCAGCAGAAC---
AATCTG---CTGAGG---GCTATTGAAGCTCAACAGCATATGTTGAAGCTCACAGTCTGGGGCATT
>JX140671
TTCTACTGTAATACATCAAAACTGTTTAATGAAACAGAATCCGGTAACACA---------------------
----

CAGAATGACACTATCATACTCCCATGTAGAATAAAACAAATTATAAACCTGTGGCAGCAAGTAGGGCGAGCAA
TGTATGCCGCTCCCATTGCAGGACACATTACATGTAATTCAAGT---
ATTACAGGGCTACTACTAATAAGAGATGGTGGTCAGCATAATAGTAGT------------------------
----

AATGATGCTGAGATCTTCAGACCTGGGGGAGGAGATATGAGAGACAATTGGAGAAGTGAATTATACAAATATA
AAGTAGTAGAAATTGAACCACTA---GGAGTAGCACCCACCAAGGCAAAAAGACAAGTGGTG-----------
---------------------------AGGAGAGAAAAAAGAGCA---GTGGGA---CTAGGAGCT------
---CTGTTCCTTGGGTTCTTGAGTACA---GCAGGAAGCACTATG---GGCGCAGCGTCA---
ATAACGCTGACGGTACAG---GCCAGACAA---TTATTG---TCTGGAATAGTGCAACAGCAAAAC---
AATTTG---CTGAGG---GCTATTGAAGCGCAACAGCATATGTTGCAGCTCACAGTCTGGGGCATT
>KJ883138
TTCTACTGTAATACATCAAAACTGTTTAATGAAAAAGAACTCAATAACACA---------------------
----

CAGAATGACACTATCATACTCCCATGTAGAATAAAACAAGTTATAAACCTGTGGCAGGAAGTAGGGCGAGCAA
TGTATGCCGCTCCCATTGCAGGAAACATTACATGTCATTCAAAT---
ATTACAGGGCTACTATTGACAAGAGATGGTGGTCAAAGGAATGAGACT------------------------
----

AATACTACTGAGATCTTCAGGCCTGGGGGAGGAGATATGAGAGACAATTGGAGAAGTGAATTATATAAATATA
AAGTAGTAGAAATTGAACCACTA---GGAGTAGCACCCACCAAGGCAAAAAGACAAGTGGTG-----------
---------------------------AGGAGAGAAAAAAGAGCA---GTGGGA---CTAGGAGCT------
---CTGTTCCTTGGGTTCTTGAGCGCA---GCAGGAAGCACTATG---GGCGCGGCGTCA---
ATGACGCTGACGGTACAG---GCCAGACAA---TTATTG---TCTGGAATAGTGCAACAGCAAAAC---
AATTTG---CTGAGG---GCTATTGAAGCGCAACAGCATATGTTGCAGCTCACAGTCTGGGGCATT
>DQ979023
TTCTACTGCAATACATCAAAATTGTTTACAAATAACATAAGC------------------------------
----

TCCAATGGGACTATCACTCTCCCATGTAGAATAAAACAGATTATAAACATGTGGCAGGGAGTAGGACAAGCAA
TGTATACCGCCCCCATTGCAGGAAACATTACATGTAACTCAAAT---
ATTACAGGCTTACTCATAACAAGAGATGGTGGTCAGGAGGGGCGTAAT------------------------
----

GATACTGAAGAAATATTCAGACCTGGGGGAGGAAATATGAAAGACAATTGGAGAAGTGAACTATATAAATATA
AAGTAGTAGAAATTAAACCACTA---GGAATAGCACCCACCGAGGCAAGGAGACAAGTGGTG-----------
----------------------------AAGAGAGAAAAAAGAGCA---GCAGGA---CTAGGACTT------
---TTGTTCCTTGGGTTCTTGGGAGCA---GCAGGAAGCACTATG---GGCGCAGCGTCA---
ATAACGCTGACGGTACAG---GCCAGACAT---TTATTG---TCTGGAATAGTGCAACAGCAAAAC---
AATTTG---CTGAAG---GCTATTGAAGCACAACAGCATATGTTGCAGCTCACAGTCTGGGGCATT
>DQ979024
TTCTATTGCAATACAACAAAATTGTTTAATGGTAGTGGAAACAATGACATTATC-------------------
----

ACTAATGGCACTATCACTATCCCATGTAGAATAAAACAAATTGTAAACATGTGGCAGGGAGTAGGACAAGCAA
TGTATGCCGCTCCCATTGCAGGACAAATTACCTGTAACTCAAAT---
ATTACAGGCATAATATTGACAAGAGATGGTGGTAACAAAAATGAGACTAAT----------------------
----

AAAAATGGGACTGAGATCTTCAGACCTGCAGGAGGAAATATGAAAGACAATTGGAGAAGTGAACTCTATAAAT
ATAAAGTAGTAGAAATTGAACCACTA---GGAGTAGCACCTACCAAGGCAAAAAGACGAGTGGTG--------
-------------------------------ACGAGAGAAAAAAGAGCA---TTGGGA---ATGGGAGCC---
------TTGTTCCTTGGGTTCTTGGGAGCA---GCAGGAAGCACTATG---GGCGCAGCGTCA---
ATAACGCTGACGGTACAG---GCCAGACAA---TTATTG---TCTGGAATAGTGCAACAGCAAAAC---
AATCTG---CTGAAG---GCCATTGAAGCGCAACAGCATCTGTTGCAGCTCACAGTCTGGGGCATT
>AF075703
TTCTACTGCAATACATCACTACTGTTTAATAATACAGTACCT-------------------------------
----

AATAATGGCACTATCACTCTCCCATGTCGAATAAAACAATTTGTAAACATGTGGCAGGAAGTAGGACGAGCAA
TGTATGCCGCTCCCATTGCAGGAAACATTACCTGTAACTCAAAT---
ATTACAGGTCTGCTATTGACAAGAGATGGTGGTCAGAGTAATAAT----------------------------
----

AGTGATTCTGAGACCTTCAGACCTGGGGGAGGAGATATGAAAGACAATTGGAGAAGTGAACTATATAAATATA
AAGTAGTAGAAATTGAACCACTA---GGAGTAGCACCCACCAGGCCCAAAAGACCAGTGGTG-----------
----------------------------AGGAGAGAAAGAAGAGCA---GTGGCA---ATAGGAGCT------
---GTATTCCTTGGGTTCTTGAGCGCA---GCAGGAAGCACTATG---GGCGCGGCGTCA---
CTAACGCTGACGGTACAG---GCCAGACAA---TTATTG---TCTGGAATAGTGCAACAGCAGAAC---
AATCTG---CTGCAG---GCTATTGAAGCGCAACAGCATATGTTGCAGCTCACAGTCTGGGGCATT
>KU168276
TTCTACTGCAATACATCAGGGCTGTTTAATAATACAGGAACT-------------------------------
----

AATGAAACTATCACTCTCCCATGTCAAATAAAACAAATTGTAAACATGTGGCAGGGAGTAGGACGAGCAATGT
ATGCTGCTCCCATTGCAGGAAACATTACCTGTAGATCAAAC---
ATTACAGGTCTACTATTGACAAGAGATGGTGGTCAGAGTACTAAT----------------------------
----

GGTAGTATTGAGACCTTCAGACCTGGGGGAGGAGATATGAAAGACAATTGGAGAAGTGAACTATATAAATACA
AAGTAGTAGAAATTCAACCACTA---GGAATAGCACCCACCGAGGCAAAAAGACAAGTGGTG-----------
----------------------------AAGAGAGAAAAAAGAGCA---GTGGGA---ATAGGAGCT------
---TTGTTCCTTGGGTTCTTGGGAGCA---GCAGGAAGCACTATG---GGCGCGGCGGCA---
ACAACGCTGACGGTACAG---GCCAGACAA---TTATTG---TCTGGAATAGTGCAACAGCAAAAC---
AATTTG---CTGAGG---GCTATTGAAGCGCAACAGCATATGTTGCAGCTCACAGTCTGGGGCATT
>AJ249238
TTCTACTGCGATACATCAGGATTGTTTAATGAAAGTGAAAAG-------------------------------
----

TACAATGGCACTATCATTCTCCCATGTAAAATAAAACAAATTATAAACATGTGGCAGGGAGTAGGACAAGCAA
TGTATTCCGCTCCCATTGCAGGACGCATTAACTGTAACTCAACT---
ATTACAGGCCTACTATTAACAAGAGATGGTGGTCAGAGTAATGATACT-------------------------
----

AATAGGACTGAGACTTTCAGACCTGAGGGAGGAAACATGAAAGACAATTGGAGAAATGAACTATATAAATATA
AAGTAGTAGAAATTGAACCACTA---GGAGTAGCACCCACCAAGGCAAGAAGACGAGTGGTG-----------
----------------------------CAGAGAGAAAGAAGAGCA---GTGGGA---ATAGGAGCT------
---CTGTTCCTTAGATTCTTGGGAGCA---GCAGGAAGCAACATA---GGCGCAGCGTCA---
ATAACGCTGACGGTACAG---GCCAGACAA---TTACTG---TCTGGAATAGTGCAGCAGCAAAAC---
AATTTG---CTGAGG---GCTATTGAAGCGCAACAGCATCTGTTGCAGCTCACAGTCTGGGGCATT
>AY231157
TTCTACTGCGATACATCACAGCTGTTTGATAGTACAGTACTT-------------------------------
----

AATGGCACCATCACTCTCCCATGTCGAATAAAACAAATTGTAAACATGTGGCAGGGAGTAGGAAAAGCAATGT
ATGCTGCGCCCATTGCAGGAAGCATTACCCGTCATTCAAAT---
ATTACAGGTCTACTATTGACAAGAGATGGTGGTCAGAATAGTAGG----------------------------
----

AATGAGACCTTCAGACCTGGGGGAGGAGATATGAAAGACAATTGGAGAAGTGAACTATATAAATATAAAGTAG
TAGAAATTGAACCACTA---GGAATAGCACCCACCAGGGCAAAAAGACCAGTGGTG-----------------
----------------------AAGAGAGAAAAAAGAGCG---TTGGGA---ATGGGAGCT---------
TTGTTTCTTGGGTTCTTGAGCGCA---GCAGGAAGCACTATG---GGCGCAGCGTCA---
ATAACGCTGACGGCACAG---GCCAGACAA---TTATTG---TCTGGAATAGTGCAACAGCAGAAC---
AATCTG---CTGAGG---GCTATTGAAGCGCAACAGCATCTGTTGCAGCTCACAGTCTGGGGCATT
>KU168274
TTCTATTGCAATACATCAGAGTTGTTTAAAACTAATATA----------------------------------
----

ACCAATAACACTATCACTCTCCCATGTAGAATAAAACAGATTGTAAAAATGTGGCAGGGAGTAGGACAAGCAA
TGTATGCCGCTCCCATTGCAGGAAGCATTACCTGTAACTCAAAT---
ATTACAGGATTACTATTGACAAGAGATGGTGGTCAGGATAATAAAAATGGG----------------------
----

ACTAATAGGACTGAGACCTTCAGACCTGGGGGAGGAAATATGAAAGACAATTGGAGAAGTGAACTATATAAAT
ATAAAGTAGTAGAAATTGAACCACTA---GGAATAGCACCCACCAGGGCAAAAAGACAAGTGGTG--------
-------------------------------CAGAGAGAAAAAAGAGCA---GCGGGA---ATAGGACTT---
------TTGTTCCTTGGGTTCTTGGGAGCA---GCAGGAAGCACTATG---GGCGCAGCGTCG---
ATAACGCTGACGGTACAG---ACCAGACAA---TTATTG---TCTGGAATAGTGCAACAGCAAAAC---
AATTTG---CTGAAG---GCTATTGAAGCGCAACAGCATATGTTGCAGCTCACAGTCTGGGGCATT
>AB485659
TTCTACTGCAATACATCAGGACTGTTTAATAGTAGCACGAGCCCCGAAGGAGTGTTTAACACGAGC-------
----

CACAAAGGAAATATCACTCTCCTATGTAGAATAAAACAGATTATAAACATGTGGCAGGGAGTAGGCCAAGCAA
TGTATGCCGCTCCCATTGCAGGAAGCATTACCTGTAACTCAAAT---
ATTACAGGCTTACTATTGACAAGAGATGGTGGTCAGAATAATGAG----------------------------
----

ACTGAGACCTTCAGACCTGGGGGAGGAAATATGAAAGACAATTGGAGAAGTGAACTATATAAATATAAAGTAG
TAGAAATTGAACCACTA---GGAATAGCACCCACCAGGGCACAAAGACAAGTGGTG-----------------
----------------------AAGAGAGAAAAACGAGCA---GCGGGA---TTAGGACTT---------
TTGCTCCTTGGGTTCTTGGGAGCA---GCAGGAAGCACTATG---GGCGCGGCGTCA---
ATAACGCTGACGGTACAG---GCCAGACAA---TTATTG---TCTGGAATAGTGCAACAGCAAAAC---
AATTTG---CTGAAG---GCTATTGAAGCGCAACAGCATCTGTTGCAGCTCACAGTCTGGGGCATT
>HQ236605
TTCTACTGCAATACAACACAACTGTTTAACTACACAAGATATTCCAATGGCACTATCATTTTCGGT-------
----

TCCAATGGCACTATCACTCTCCCATGCAGAATAAAACAAATTGTAAACATGTGGCAGGAAGTAGGACGAGCAA
TATATGCCGCTCCAATTGCAGGGAACATCACCTGTCACTCAAAT---
ATTACAGGCCTACTATTGACAAGAGATGGTGGTAGTGGCAATGGTACT-------------------------
----

GACAATAGAAGTGAGATCTTCAGACCTGGAGGGGGAAACATGAAAGACAATTGGAGAAATGAATTATATAAAT
ATAAAGTAGTAAGAATTGAACCACTA---GGAATAGCACCCACCAGGGCACACAGACGAGTGGTG--------
-------------------------------AAGAGACAAAAAAGAGCA---CTCGGA---ATGGGAGCC---
------ATGTTCATTGGGTTCTTGGGAGCA---GCAGGAAGCACTATG---GGCGCAGCGTCA---
GTGACGCTGACGGTACAG---GCCAGACAA---TTGATG---TCTGGAATAGTGCAACAGCAAAAT---
AATTTG---CTGATG---GCCATTAAAGCGCAACAGCATCTGTTGCAGCTCACAGTCTGGGGCATT
>KU168275
TTCTACTGCAATACATCAGGGCTGTTTAATAATACAGTACTC-------------------------------
----

AATAACACTACTATCACTCTCCCATGTCGAATAAAACAAATTGTAAACATGTGGCAGGGAGTAGGACAAGCAA
TGTATGCCGCTCCCATTGCAGGAAACATTACCTGTAGATCAAAT---
ATTACAGGTCTATTATTGACAAGAGATGGTGGTGAGAATAATGAT----------------------------
----

ACTGAGACCTTCAGACCTGGGGGAGGAGATATGAAAGACAATTGGAGAAGTGAACTATATAAATACAAAGTAG
TAGAAATTCAACCACTA---GGAATAGCACCCACCAAGGCAAAAAGACAAGTGGTG-----------------
----------------------AAGAGGGAAAAAAGAGCA---GTTGGA---ATGGGAGCC---------
TTGTTCCTTGGGTTCTTGGGAGCA---GCAGGAAGCACTATG---GGCGCGGCGTCA---
GTAACGCTGACGGTACAG---GCCAGACAA---TTATTG---TCTGGAATAGTGCAACAGCAAAGC---
AATTTG---CTGAGG---GCTATTGAAGCGCAACAGCATCTGTTGCAGCTCACAGTCTGGGGCATT
>MH705144
TTCTACTGCAACACAAGTAAGCTGTTTAACACAACTTGGCTAAATAATAAAACGGAACACAATGGCATTATCA
ACAGC--

AGCACTGAAATTATGGTGCTTCCGTGTAGAATAAGACAATTTGTAAACATGTGGCAAAGAGTAGGAAGAGCAA
TGTATGCCCCTCCCATTGCAGGGGTAATTCAGTGTAAATCAGAT---
ATCACAGGTCTACTATTGACAAGAGATGGTGGTAATACTGGTAATAAT-------------------------
----
ACAGGAGGTGATGGTAAT------------
AATACCGACATCCTCAGACCTACAGGGGGAGATATGAGAGACAATTGGAGAAGTGAACTATATAAATATAAGG
TAGTAAAACTTGAACCACTA---GGAGTAGCACCCACCAGGGCAAAAAGACAAGTGGTGAAA-----------
-------------------GGAAAAGAAAGAGAAAAAAGAGCA---GTGGGA---CTGGGAGCT---------
ATGTTCCTTGGGTTCTTGGGAGCA---GCAGGAAGCACTATG---GGCGCAGCGTCA---
ATAACGCTGACGGTACAG---GCTAGACTA---TTATTG---TCTGGTATAGTGCAACAGCAAAGC---
AATTTG---CTGAGG---GCTATAGAAGCGCAACAGCATATGTTGCAGCTCACAGTCTGGGGCATT
>AY371158
TTCTACTGCGACACAACAGCACTGTTTAACACAACATTACTTAACACAACAATGGAT----------------
----

AATAATGGTACTATCATTATTCCATGTAGAATAAAACAAATTGTAAACGTGTGGCAAAGAGTAGGGCGAGCAA
TGTATGCCCCTCCCATTGCAGGAAAAATTCAGTGCAACTCAAAT---
ATCACAGGTCTACTATTGACAAGAGATGGTGGAAGTAAGGCAAAT----------------------------
----

AACACAGACATCCTCAGACCTATAGGGGGAGAGATGAGAGACAATTGGAGAAGTGAATTATATAAATATAAGG
TAGTACAAATTCAACCACTA---GGAATAGCACCCTCCAGGGCAAAAAGACAAGTGGTG--------------
-------------------------AAGAGAGAAAGAAGAGCA---GTAGGA---ATAGGAGCT---------
GTGTTCCTTGGGTTCTTGGGAGCA---GCAGGAAGCACTATG---GGCGCAGCGTCA---
ATAACGCTGACGGTACAG---GCCAGACAA---TTATTG---TCTGGTATAGTGCAACAGCAAAAC---
AATTTG---CTGAAG---GCTATAGAAGCGCAACAGCATCTGTTGCAGCTCACAGTCTGGGGCATT
>JX140672
TTCTACTGCAATACATCAGCTCTGTTTAATACAGAAGCTCTGAAAAATAACACAGGT----------------
----

CAAAATGGCACTACCACCATTTATTGTAGAATAAAACAAATTATAAATATGTGGCAAAGAGTAGGAAAAGCAA
TGTATGCCCCTCCCATTGCAGGAAAAATTCAGTGTAACTCAAGT---
ATCACAGGTCTAATATTAACAATAGATGGT---AATAGAACAAAT----------------------------
----

AATAAGAGTGAGACCCTCAGACCTGGAGGGGGAGATATGAAAGACAATTGGAGAAGTGAACTGTATAAATATA
AGGTAGTGAAAATTGAACCATTA---GGAGTAGCACCCACCAGGGCAAAAAGACGAGTGGTG----------
---------------------------AGCAGAGAGAAAAGAGCA---GTGGGA---CTAGGAGCT------
---ATGTTCCTTGGGTTCTTGGGAGCA---GCAGGAAGCACTATG---GGCGCGGCGTCA---
ATAACGCTGACGGTACAG---GCCAGACAA---TTGTTG---TCTGGTATAGTGCAACAGCAAAGC---
AATTTG---CTGAAG---GCTATTGAAGCGCAGCAGCACTTGTTGCAGCTCACAGTCTGGGGCATT
>KU749420
TTCTACTGTAACACAAGCAGACTGTTTAATAACACACTGTTTAATAACACACAGCTTAATAACACA-------
----

GAAGGAGGGAACATTACTCTTCCATGTAGAATAAAACAATTTGTAAACATGTGGCAAAGAGTAGGAAAAGCAA
TGTATGCCCCTCCCATTGCTGGAAGAATTCAGTGTATCTCAAAT---
ATCACAGGTCTACTATTGACAAGAGATGGTGGTACTAATGATACGTCT------------------------
----

ACAAATGAGACCTTCAGACCTGGAGGGGGAGACATGAGAGATAATTGGAGAAGTGAACTGTATAAATATAAGG
TAGTAAAAATTGAGCCACTA---GGAGTAGCCCCCACTAGGGCAAAAAGGCGAGTGGTG-------------
------------------------CAGAGAGAAAAAAGAGCT---GTGGGA---CTAGGAGCT---------
CTGTTCCTTGGGTTCTTGGGAGCA---GCAGGAAGCACTATG---GGCGCGGCGTCA---
ATAACGCTGACGGTACAG---GCCAGAAAC---TTATTG---TCTGGTATAGTGCAACAGCAGAAT---
AATCTG---CTGAAG---GCTATAGAAGCGCAACAGCATCTGTTGCAGCTCACAGTCTGGGGCATT
>AJ277824
TTCTACTGCAACACAGATGAGCTGTTTAATAACAGCTTGTTGAATAACACA---------------------
----

AAAAAAGAAAATATCACTCTTCCATGTAGAATAAAACAATTTGTAAACATGTGGCAAAGGGTAGGACAAGCAA
TGTATGCCCCTCCTATAGCCGGACAAATTCAGTGCAACTCAAGT---
ATCACAGGTCTACTATTGACAAGAGATGGT---AACAAAAATGGG---------------------------
----

TCCGAGACCCTCAGACCTGGAAGGGGAGATATGAGAGACAATTGGAGAAGTGAACTATATAAATATAAGGTAG
TAAAAATTGAACCCCTA---GGAGTAGCACCCACCAGGGCAAAAAGACAAGTGGTG----------------
---------------------AAGAGAGAAAGAAGAGCA---GTGGGA---ATAGGAGCT---------
GTGTTCCTTGGGTTCTTGGGAGCA---GCAGGAAGCACTATG---GGCGCGGCGTCA---
ATAACGCTGACGGTACAG---GCCAGACAA---TTATTG---TCTGGTATAGTGCAACAGCAAAGC---
AATTTG---CTGAAG---GCTATAGAAGCGCAACAGCATCTGTTGCAGCTCACAGTCTGGGGCATT
>AJ249237
TTCTACTGCAACACAACTATTCTGTTTAATCACACAAGAGTAAATGACATTCTGTCTAATAATCACACGAGA-
----

GAAAATGACACTATCACTCTTCCATGTAGAATAAAACAAATTGTAAACATGTGGCAAAGAGTAGGACAGGCAA
TGTATGCCCCTCCCATTGCCGGAAAAAATTCAGTGTAACTCAAAT---
ATCACAGGTCTACTATTAACAATAGATGGTGGGGAGGGAAATGAG---------------------------
----

TCCGAGACCCTCAGACCTGGAGGGGGAGATATGAGAGACAATTGGAGAAGTGAACTATATAAATATAAGGTAG
TAAAAATTGAACCACTA---GGAGTAGCACCCACCAAGGCAAAAAGACAAGTGGTG----------------
----------------------CAGAGAGAAAAAAGAGCA---GTGGGA---ATGGGAGCT---------
ATGTTCCTTGGGTTCTTGGGAGCA---GCCGGAAGCACTATG---GGCGCGGCGTCA---
ATAACGCTGACGGTACAG---GCCAGAAAC---TTATTG---TCTGGTATAGTGCAGCAGCAAAGC---
AATTTG---CTGAAA---GCTATTGAAGCGCAACAGCATCTGTTGCAGCTCACAGTCTGGGGCATT
>AF377956
TTCTACTGCAACACAGTAGCTCTGTTTAATGCAACTAACATGACTAATGCAATGAACAGG------------
----

TCCAATGGCATTATCACTCTTCCATGTAGAATAAGACAAATTGTAAACATGTGGCAAAGAGTAGGACGAGCAA
TGTATGCCGCTCCCATTGCTGGACAAATTCAGTGTAACTCAAGC---
ATCACAGGTCTAATATTGACAAGAGATGGTGGGAAAAATAATACC---------------------------
----

AATAATGACACCCTCAGACCTGGAGGGGGAGATATGAGAGACAATTGGAGAAGTGAACTGTATAAATATAAGG
TAGTAAAAATTGAACCACTA---GGAGTAGCACCCACCAAGGCAAAAAGACAAGTGGTGAAG-----------
----------------------AGAGAAAGAGAAAAAAGAGCA---GTGGGA---ATAGGAGCT---------
GTGCTCCTTGGGTTCTTGGGAGCA---GCAGGAAGCACTATG---GGCGCGGCGTCA---
ATGACGCTGACGGTACAG---GCCAGACAA---TTATTG---TCTGGTATAGTGCAACAGCAAAAC---
AATTTG---CTGAAG---GCTATAGAAGCGCAACAGCATCTGTTGCAGCTCACAGTCTGGGGCATT
>JN054264
TTCTACTGTAACACAACAAAGCTGTTTAATTCAAGTCTGCTTAACAGTACAAGTACAAATGAAAGTATACCTG
AG---

ATTATTCTTCCATGTAGAATAAAACAAATTATAAACATGTGGCAAAGAGTAGGAAAAGCAGTGTATTCCTCCC
CCATTGCAGGGAAAATTCAGTGTATCTCAAAT---
ATCACAGGTCTATTATTGGTGAGGGATGGTGGTAATACATCCCAC----------------------------
----

AATGACAATGACACCCTCAGACCTATGGGGGGAGATATGAAAGCCAATTGGAGAAGTGAACTGTATAAATATA
AGGTAGTAAAAATTGAGCCACTA---GGAATAGCACCCTCCAGGGCAAAAAGACGAGTGGTG-----------
----------------------------CAGAGAGAGAAAAGAGCT---GTGGGA---ATAGGAGCT------
---TTGCTCTTTGGGTTCTTGGGAGCA---GCAGGAAGCACTATG---GGCGCAGCGTCA---
CTAACGCTGACGGTACAG---GCCAGACAA---TTATTG---TCTGGTATAGTGCAACAGCAAAGC---
AATTTG---CTGAAG---GCTATAGAAGCGCAACAGCATCTGTTGCAGCTCACAGTCTGGGGCATT
>KF725967
TTCTACTGTAATACAACAGATCTGTTTAATATAACAGCTCTGGTTAATAAC----------------------
----

ACAGGGAACAATATCACTATCCCATGTAGAATAAAACAAATTATAAACATGTGGCAAAGAGTAGGACGAGCAA
TGTATGCCCCTCCCATAGCCGGAAGAATTCAGTGTAACTCAACT---
ATCACAGGTCTACTATTAACAAGAGATGGTAGAAATGATACAAAT----------------------------
----

GAGACCCTCAGACCTGGAGGGGGAGATATGAGAGACAATTGGAGAAGTGAGCTATATAAATATAAGGTAGTGA
AAATTGAACCACTA---GGAGTAGCACCCACTGAGGCAAGAAGACAAGTGGTA--------------------
-------------------AAGAGAGAAAAAAGAGCA---GTGGGA---ATGGGAGCT---------
GTGCTCTTTGGGTTCTTGGGAACA---GCAGGAAGCACTATG---GGCGCGGCGTCA---
ATAACGCTGACGGTACAG---GCCAGACAA---TTATTG---TCTGGTATAGTGCAGCAGCAAAGC---
AATTTG---CTGAAG---GCTATAGACGCGCAACAGCATCTGTTGCAGCTTACAGTCTGGGGCATT
>AY371121
TTCTATTGTAATACATCAGAACTGTTTAATATTAATATATCAAATAGTAGTACAAATAGTACT----------
----

GAGAATGAAACTATCACACTCCCATGTAAAATAAAACAAATTGTAAGAATGTGGCAGAGAGTGGGACAAGCAA
TGTATGCCCCTCCTATCGCCGGAAACATTACATGTAAATCAAAC---
ATTACAGGACTAATATTAACAAGAGATGGTGGAAATACTAGGGAT----------------------------
----

AATACCTCAGAGATCTTCAGACCTGGAGGAGGAGAAATGAGAGACAATTGGAGAAATGAATTATATAATTATA
AAACAGTAAAAATCAAATCATTA---GGAGTAGCACCCACCAGGGCAAGGAGAAGAGTGGTG-----------
----------------------------GAGAGAGAAAAAAGAGCA---GTTGGA---ATAGGAGCT------
---GTCTTCCTTGGGTTCTTAGGAGCA---GCAGGAAGCACTATG---GGCGCGGCGTCA---
ATAACGCTGACGGTACAG---GTCAGACAA---TTATTG---TCTGGCATAGTGCAACAGCAAAAC---
AATTTG---CTGAGG---GCTATAGAGGCGCAGCAGCATCTGTTGCAACTCACAGTCTGGGGCATT
>FJ389367
TTCTATTGTAATACATCAGGCCTGTTTAATATATCATATAATAGTACAGAGAATAGTACTTATAATGAA----
----

GGGAATGAGACTTATACACTCCCATGTAAAATAAAACAAATTGTGAGAATGTGGCAAAGAGTGGGACAAGCAA
TGTATGCCCCTCCCATCAAAGGAAACATTACATGTAGATCAAAC---
ATTACAGGACTACTATTAACAAGAGATGGTGGGGAGAATGATAATAAT-------------------------
----

AACAGTAGTGAGGTCTTCAGACCTGCAGGAGGAGACATGAGGGACAATTGGAGAAGTGAATTATATAAGTATA
AAATAGTAAAAATAAAACCATTA---GGAGTAGCACCCACCAAGGCAAGGAGAAGAGTGGTG-----------
----------------------------GGGAGAGAAAAAAGAGCA---GTAGTTGGACTAGGAGCT------
---GTTTTCCTTGGGTTCTTAGGAGCA---GCAGGAAGCACTATG---GGCGCGGCGTCA---
ATAACGCTGACGGTACAG---GTCAGACAA---TTATTG---TCTGGCATAGTGCAACAGCAAAGC---
AATTTG---CTGAGA---GCTATAGAGGCACAGCATCATCTGTTGCAACTCACAGTCTGGGGCATT
>FJ389365
TTCTATTGTAATACATCAGACTTGTTTAATATCAGTACAACAAATAGT-------------------------
----

ACTAATGATACTATCACACTCCCATGTAAGATAAAACAAATTGTGAGAATGTGGCAGAGAGTGGGACAAGCAA
TGTATGCCCCTCCCATTGCAGGAGAAATTACATGCAGGTCAAAT---
ATTACAGGACTACTATTAACAAGAGATGGTGGGGGTGGAGGTAAT----------------------------
----

CAAACAAATGAAACCTTCAGACTTGCAGGAGGAAATATGAGGGACAATTGGAGAAGTGAATTATATAAGTATA
AAGTAGTAAAGATTAAACCACTA---GGAATAGCACCCACCAAGTCAAGGAGAAGAGTGGTG-----------
----------------------------GAGAGAGAAAAAAGAGCA---GTTGGA---ATAGGAGCT------
---GCCTTCCTTGGGTTCTTAGGAGCA---GCAGGAAGCACTATG---GGCGCGGCGTCA---
ATAACGCTGACGGTACAG---GTCAGACAA---TTATTG---TCTGGCATAGTGCAACAGCAAAGC---
AATTTG---CTGAGA---GCTATAGAGGCGCAGCAACATCTGCTGCAACTCACAGTCTGGGGCATT
>AM279346
TTCTATTGTAATACATCAAAACTGTTTAATCCTAATGGTACAGAG----------------------------
----

AATGAGACTCATATCACAATCCCATGTAAAATAAAACAAATTGTGAGAATGTGGCAGAGAGTGGGACAAGCAA
TGTATGCCCCTCCCATCGCAGGAAACATTACATGTAGATCAAAC---
ATTACAGGACTATTATTAACAAGAGATGGTGACAGTAGGAGTAAT----------------------------
----

GACAGTGAGATCGTCAGACCTGCAGGAGGAGACATGAGGGACAATTGGAGAAGTGAATTATATAAGTATAAAG
TAGTAAAAATAAAACCATTA---GGAATAGCACCCACCAGGGCAAGGAGAAGAGTGGTG--------------
-------------------------GAGAGAGAAAAAAGAGCAGTAGTTGGA---ATGGGAGCT---------
TTTCTCCTTGGGTTCTTAGGAGCA---GCAGGAAGCACTATG---GGCGCGGCGTCA---
ATAACGCTGACGGTACAG---GTCAGACAA---TTACTG---TCTGGCATAGTGCAACAGCAAAGC---
AATTTG---CTGAGG---GCTATAGAGGCGCAGCAGCACCTGCTGCAACTCACAGTCTGGGGCATT
>JF327806
TTCTATTGTAATACATCAAAATTATTTACATCAAACCTGACA-------------------------------
----

TCAAATAATGACACTATCATGCTCCCATGTCAGATAAAACAAATTGTGAGAATGTGGCAGGGAGTGGGACAAG
CAATGTATGCCCCTCCCATCGCAGGAGAAATTACATGTAGATCAAGT---
ATTACAGGAATACTATTAACAAGAGATGGTGGGAATGATAATAGCACA-------------------------
----

ATTGGAAATGAAACCTTCAGACCTGGAGGAGGAGATATGAGGGACAATTGGAGAAGTGAATTATATAAGTATA
AAATAGTAAAAATTAAACCACTA---GGAATAGCATCCACTAGGGCAAGGAGAAGAGTAGTG-----------
----------------------------GAGAGAGAAAAAAGAGCA---GCA------TTTGGAGCT------
---ATCTTCCTTGGGTTCTTAGGAACA---GCCGGAAGCACTATG---GGCGCAGCGTCA---
ATAACGCTGACGGTACAG---GTCAGACAA---TTATTG---TCTGGCATAGTGCAACAACAAAGC---
AATTTG---CTGAGG---GCTATAGAGGCGCAGCAACATCTGTTGCAACTCACAGTCTGGGGCATT
>AY231155
TTCTATTGTAATACATCAAGTCTGTTTACTAATTGGGAGAATATGACA-------------------------
----

AATCAGACTAATCACACAATCCCATGTAAGATAAAACAAATTGTAAGAATGTGGCAGAGAGTGGGACAAGCAA
TGTATGCCCCTCCCATCGCAGGAAACATTGAATGTAGCTCAAAC---
ATTACAGGACTACTGTCGACAAGAGATGGTGGGAAGAATGATAATGGA-------------------------
----

AGTGAGAATTTTGAGATTTTCAGACCTGGAGGAGGAGATATGAGGGACAATTGGAGAAGTGAATTGTATATGT
ATAAAACAGTAAAAATCAAATCACTA---GGAGTAGCACCCACCAGGGCACGAAGAAGAGTGGTG--------
------------------------------GAGAGAGAAAAAAGAGCA---GTTGGA---CTTGGAGCT---
------GTCTTCCTTGGGTTCTTAGGAGCA---GCAGGAAGCACTATG---GGCGCAGCGTCA---
ATAACGCTGACGGTACAG---GTCAGACAA---TTATTG---TCTGGCATAGTGCAACAGCAAAGC---
AATTTG---CTGAAG---GCTATAGAGGCGCAGCAGCATCTGTTGCAACTCACAGTCTGGGGCATT
>AY231156
TTCTATTGTAATACATCAGGACTGTTTAATAGTAGTAATCTGTCTAATAGTAGTAACAAT------------
----

GAGACCGATATTATAACGCTCCCATGTAGAATAAAACAAATTGTAAGAATGTGGCAGAGAGTGGGACAAGCAA
TGTATGCCCCTCCCATAGAAGGAAACATTACATGTATATCAAAT---
ATTACAGGACTATTATTAACAAGGGATGGTGGGAAGACTAACAGT---------------------------
----

AGTAATGAGACCTTCAGACCTGCAGGAGGAGACATGAGGGACAATTGGAGAAGTGAATTATATAAGTATAAAG
TAGTAAAAATTAAACCACTA---GGAATAGCACCCACCAAGGCAAGGAGAAGAGTGGTG--------------
------------------------GGGAGAGAAAAAAGAGCA---GTTGGA---ATAGGAGCT---------
GTCCTCCTTGGGTTCTTAGGAACA---GCAGGAAGCACTATG---GGCGCAGCGGCA---
ATAACGCTGACGGTACAG---GTCAGACAA---TTATTG---TCTGGCATAGTGCAACAGCAAAGC---
AATTTG---CTGAGG---GCTATAGAGGCTCAGCAACATATGTTGCAGCTCACAGTCTGGGGCATT
>EF033660
TTCTATTGTAATACATCAGGATTGTTTAATAGTAGTAACCTTAAAAGTAGTAACCTGACT------------
----

GGTAATGAGACCATCACACTCCCATGTAGGATAAAACAAATTGTGAGAATGTGGCAGAGAGTGGGACAAGCAG
TGTATGCCCCTCCCATTGCAGGAAACATTACATGTATATCAAAC---
ATTACAGGACTACTAATAACAAGAGATGGTGGGAATAATAGCAGC---------------------------
----

CAAAATGAGACCTTTAGACCTGCAGGAGGAGACATGAGGGACAATTGGAGAAGTGAATTATATAAGTATAAAG
TAGTAAAAATTAAACCACTA---GGAGTAGCACCCACCAGGGCAAGGAGAAGAGTGGTG--------------
------------------------GGGAGAGAAAAAAGAGCA---GTTGGA---CTGGGAGCT---------
GTCCTCCTTGGGTTCTTAGGAACA---GCAGGAAGCACTATG---GGCGCAGCGGCA---
GTAACGCTGACGGTACAG---GTCAGACAA---CTATTG---TCTGGCATAGTGCAACAGCAAAGC---
AATTTG---CTGAGG---GCTATAGAGGCTCAGCTACATCTGTTGCAACTCACAGTCTGGGGCATT
>KT022379
TTCTATTGTAATACATCTAGATTGTTTAATGATAATATTACACATAATAATAGTGCATCTAATAATAAT----
----

ACTAAGGGAAACATCACACTCCCATGTAAAATAAAACAAATTGTGAGAATGTGGCAAAGAGTGGGACAAGCAA
TGTATGCCCCTCCTATCGCAGGAGAACTTACATGTAAATCAAAC---
ATTACAGGACTATTATTAACAAGAGATGGTGGGACTCATAAAAAT---------------------------
----

AATAAAAGTGAGATCTTCAGACCTGCAGGAGGAGATATGAAGGACAATTGGAGAAGTAAATTATATAAGTATA
AAGTAGTAAAAATTAAACCACTA---GGAGTAGCACCCACCAAAGCAAGGAGAAGAGTGGTG-----------
---------------------------GGAAGAAAAAAAAAGAGCA---GTTGGA---CTGGGAGCT------
---GTCTTCCTTGGGTTCTTGGGAGCA---GCAGGAAGCACTATG---GGCGCAGCGTCG---
ATAACGCTGACGGTACAG---GCCAGACAA---TTATTG---TCTGGCATAGTGCAACAGCAAAAC---
AATTTG---CTGAGA---GCTATAGAAGCGCAGCAGCATTTGTTGCAGCTCACAGTCTGGGGCATT
>JN248582
TTCTATTGTAACACAACAGGACTGTTTAATAATAGTACATTGGAGCTTAATAACACT---------------
----

AGTAATGGCACTATCACACTCCCATGTAAGATAAAACAAATTGTGAGAATGTGGCAGAGAACTGGACAAGCAA
TGTATGCCCCTCCCATCGCAGGAAATATTACATGTAGATCAAAC---
ATTACAGGACTATTATTAACAAGAGATGGTGGGAATAATGATACT---------------------------
----

CATGAGACCTTCAGACCTGGAGGAGGAGATATGAGGGACAATTGGAGAAGCGAACTATATAAATATAAAATAG
TAAAAATTAAACCACTA---GGAATAGCACCCACCAAGGCAAGGAGAAGAGTGGTG----------------
----------------------CAGAGGAAAAAAGAGCAGTAGTTGGA---CTAGGAGCT---------
GTCTTCCTTGGGTTCTTGGGAGCA---GCAGGAAGCACTATG---GGCGCAGCGTCA---
ATAACGCTGACGGTACAG---GTCAGACAA---TTATTG---TCTGGCATAGTGCAACAGCAAAGC---
AATTTG---CTGAGG---GCTATAGAGGCACAGCAACATCTGTTGCAACTCACAGTCTGGGGCATT
>AF069947
TTCTATTGTAATACATCAGGATTGTTTAATGATAGTGCATCA------------------------------
----

AATGACACTATCACACTCCCATGTAAGATAAAACAAATAGTGAGAATGTGGCAAAGAGTGGGACAAGCAATGT
ATGCCCCTCCCATCGCAGGAAACATTACATGTAGATCAAAT---
ATTACAGGGCTACTATTAACAAGAGATGGTGGAAATGATGGGGCTAATAGC---------------------
----

ACAAATCAAAATGAAACCTTCAGACCTGGAGGAGGAGATATGAGGGACAATTGGAGAAGTGAATTATATAAGT
ATAAAATAGTAAGAATTAAACCACTA---GGAATAGCACCCACCAGGGCAAGAAGAAGAGTGGTG-------
------------------------------GAGAGAGAAAAAAGGGCA---GTTGGA---CTGGGAGCT---
------GTCCTCCTTGGGTTCTTAGGAACA---GCAGGAAGCACTATG---GGCGCGGCGTCA---
ATAACGCTGACGGTACAG---GTCAGACAA---TTATTG---TCTGGCATAGTGCAACAGCAAAGC---
AATTTG---CTGAGG---GCTATAGAGGCGCAGCAACATCTGTTGCAACTCACAGTCTGGGGCATA
>KY229519
TTCTATTGTAATACATCAGAACTGTTTAATCCTAATACCCTGAATAGTAATAGTAGT---------------
----

GATCAGTCTAACATCACCCTCCCATGTAAAATAAAACAAATTGTGAGAATGTGGCAGAAAGTGGGACAAGCGA
TGTATGCCCCTCCCATTGCAGGAGAGATTACATGTAGATCAAAC---
ATTACAGGAATACTATTAACAAGAGATGGTGGAAATCACCAGAACATT------------------------
----

AGTGGAAATGAAACCTTCAGACCTGCAGGAGGAGACATGAGGGACAATTGGAGAAGTGAATTATATAAGTATA
AAGTAGTAAAAATTAAACCACTA---GGAATAGCACCCACCAAGGCAAAAAGAAGAGCGGTG----------
---------------------------GAGAGAGAAAGAAGAGCA---GTTGGA---ATGGGAGCT------
---TTACTCCTTGGGTTCTTAGGAACA---GCAGGAAGCACTATG---GGCGCAGCGTCA---
ATAACGCTGACGGTACAG---ATCAGACAG---TTGTTG---TCTGGTATAGTGCAACAGCAAAGC---
AATTTG---CTGAGA---GCTATAGAGGCGCAGCAGCATCTGTTGCAGCTTACAGTCTGGGGCATT
>KY229535
TTCTATTGTAATACATCAGGACTGTTTATGAATAACCTGACTAGTGGTAGT---------------------
----

GATCAGGATAACATCACCCTCCCATGTAAAATAAAACAAATTGTGAGAATGTGGCAGAGAGTGGGACAAGCGA
TGTATGCCCCTCCCATTGCAGGAGAGATTACATGTAGATCAAAC---
ATTACAGGAATACTATTAACAAGAGATGGTGGAAGTAACAATAACAAT------------------------
----
GATTCCAATAACAAT---
GGAAATGAAACCTTCAGACCTGCAGGAGGAGACATGAGGGACAATTGGAGAAGTGAATTATATAAGTATAAAG
TAGTAAAAATTAATCCACTA---GGAATAGCACCCACCAGGGCAAGAAGAAGAGTGGTGGGG----------
---------------------AAAGAAAGAGAAAGAAGAGCA---GTTGGA---ATGGGAGCT---------
TTCCTCCTTGGGTTCTTAGGAGCA---GCAGGAAGCACTATG---GGCGCAGCGTCA---
ATAACGCTGACGGTACAG---GTCAGACAA---TTGTTG---TCTGGCATAGTGCAACAGCAAAGC---
AATTTG---CTGAGA---GCTATAGAGGCGCAGCAGCATCTGCTGCAGCTTACAGTCTGGGGCATT
>KU168273
TTCTATTGCAATACATCAAAACTGTTTAATAGTATCTGGTATGTTAACGGGACAAATGGGACCGTACTAAATG
GAACAATTACT---
-------------GGGACC------
GGACTAAATGGAACAATTACACTGCCATGCAGAATAAAACAAATTGTAAACATGTGGCAGAGAGTAGGACAAG
CAATGTATGCCCCTCCCATTAGGGGAGATATTACATGCGAATCAAAT---
ATTACAGGACTAATATTAACAAGTGACTATGTTAATACAACAGATACA------------------------
----

AATGGAAGTGTTACCCTCAGACCTACAGGAGGAGACATGAGGGACAATTGGAGAAGTGAATTATATAAATATA
AAGTAGTAAAAATTGAGCCACTA---GGAATAGCACCCTCCAGGGCAAGGAGAAGAGTGGTG-----------
----------------------------GAAAGAGAAAAAAGAGCT---GTGGGA---ATGGGAGCT------
---TTTTTCCTTGGGTTCTTGGGAGCA---GCAGGAAGCACTATG---GGCGCGGCGTCC---
ATGGCGCTGACGGTACAG---GCCAGACAA---TTATTG---TCTGGTATAGTGCAACAGCAAAGT---
AATCTG---CTGAGA---GCTATAGAGGCGCAACAGCATATGTTGCAGCTCACAGTCTGGGGCATT
>AF005496
TTCTATTGCAATACATCAGGGCTGTTTAATAGTAGTTGGGAAATGCATACTAATTACACATCAAATGACACAA
AG---

GGAAACGAAAACATTACACTGCCATGCAGAATAAAACAAATTGTAAACATGTGGCAGAGAGTAGGACGAGCAA
TGTATGCCCCTCCCATCCAAGGAAACATTATGTGTGTATCAAAT---
ATTACAGGACTAATATTGACAATTGACGAGGGTAACGCGTCTGCA----------------------------
----

GAAAATTATACCTTCAGACCTGGAGGAGGAGATATGAGGGACAATTGGAGAAGTGAATTGTATAAATATAAAG
TAGTAAAAATTGAACCACTG---GGAATAGCACCCACCAAGACAAGGAGAAGAGTGGTG--------------
-------------------------GAGAGAGAAAAAAGAGCA---GTGGGA---ATGGGAGCT---------
TCTTTCCTTGGGTTCTTGGGAGCA---GCAGGAAGCACTATG---GGCGCGGCGTCA---
ATAACGCTGACGGTACAG---GCCAGGCAA---TTATTG---TCTGGTATAGTGCAGCAGCAAAGC---
AATTTG---CTGAGA---GCTATACAGGCGCGACAGCATATGTTGCAGCTCACGGTCTGGGGCATT
>KY392776
TTCTATTGTAATACATCAAAACTGTTCAATAGTAGTGAAAAT-------------------------------
----

TCCAATGCAAACATCACACTCCCATGTAGAATAAGGCAAATTGTAAGGATGTGGCAGAGAGTAGGACAAGCAA
TATATGCCCCTCCCATCGCAGGAAACATTACATGCATGTCAAAT---
ATTACAGGATTAATATTGACACGTGATGGGGGAGTTAACAATAACAATGACAGT-------------------
----

ACTAATAATACAGAAATCTTCAGACCTACAGGAGGAAATATGAAGGACAATTGGAGAAGTGAGCTATATAAAT
ATAAAGTGGTAAAAATTGAGCCACTA---GGAGTAGCACCTACCAGGGCAAAAAGAAGAGTGGTG--------
-------------------------------GAGAGAGAAAAAAGAGCA---GTGGGA---ATGGGAGCT---
------GTGTTCCTTGGGTTCTTGGGAACA---GCAGGAAGCACTATG---GGCGCAGCGTCA---
ATAACGCTGACGGTACAG---GTCAGACAA---TTGTTG---TCTGGCATAGTGCAACAGCAAAGC---
AATTTG---CTAAAG---GCTATAGAGGCTCAACAGCATCTGTTGAGACTCACTGTCTGGGGCATT
>KU168280
TTCTATTGCAATACATCACAACTGTTCAATAATAGTTGGAATGGAAGTAGTTGGAATAACAATGCT-------
----

ACAAATGACACTATCACAATTCCATGTAAAATAAGACAAATTGTGAGAATGTGGCAGAGAGTAGGACAAGCCA
TATACGCCCCTCCCATCGCAGGAAACATTACATGTGTATCAAAT---
ATTACAGGATTAATATTAACACGTGATGGGGGACAGGGAGGAAAT----------------------------
----
GAGACAAACAAGACT---
GAGGCTGAGACCTTCAGACCTACAGGAGGAGATATGAGGGACAATTGGAGAAGTGAATTATATAAATATAAAG
TAGTGGAGGTTGAGCCACTA---GGAGTAGCACCCACCAGGGCAAAAAGAAGAGTGGTG--------------
-------------------------GGGAGAGAAAAAAGAGCG------GGA---ATAGGAGCT---------
CTGTTCCTTGGGTTCTTGGGAGCA---GCAGGAAGCACTATG---GGCGCAGCGTCA---
ATAACGCTGACGGTACAG---GCCAGACAA---TTGCTG---TCTGGCATAGTGCAACAGCAAAGC---
AATTTG---CTGAAT---GCTATAGAGGCTCAACAGCATCTGTTGAAGCTCACTGTCTGGGGCATT
>GU237072
TTCTATTGTAATACATCAAGCCTGTTCAATAGTAGCTGGAGTAGTAAGAATGATACAAATAGC----------
----

ACAGCAAATTACACAATCATCCCATGTAAAATAAGACAAATAGTGAGAATGTGGCAGAAAGTAGGACAAGCAA
TATATGCCCCTCCCATTGAAGGAAACATTACATGCTCATCAAAT---
ATTACAGGATTAATATTGACACGTGATGGAGGAGACAAGAACAGCACTAGC----------------------
----

```
AACAACAGTACTGAGATCTTTAGACCCACAGGAGGAGATATGAAAGACAATTGGAGAAGTGAATTATATAAAT
ATAAAGTAGTAGAGATTGAGCCACTA---GGAGTAGCACCCACCAGGGCAAAAAGAAGAGTGGTG--------
-------------------------------GAGAGAGAAAAAAGAGCA---CTGGGA---ATAGGAGCT---
------GTGTTCCTTGGGTTCTTGGGAACA---GCAGGAAGCACTATG---GGCGCAGCGTCA---
ATAACGCTGACGGTACAG---GTCAGACAA---TTGTTG---TCTGGCATAGTGCAACAGCAAAGC---
AATTTG---CTGAAG---GCTATAGAGGCTCAACAGCATCTGTTGAAGCTCACTGTCTGGGGCATT
>AJ249239
TTCTACTGCAATACAACAAAACTGTTTAATGAGACAGGGGAG-------------------------------
-------------------------------------------------------------------------
-------------------------------
AATGGTACTATCACACTCCCATGTAGAATAAAGCAGATTATAAACATGTGGCAGAAAGTGGGAAAAGCAATAT
ATGCCCCTCCCATTGCAGGAAGTATTAACTGTAGCTCAAAT---
ATTACAGGAATGATATTGACAAGAGATGGTGGTAATAATACTCAT----------------------------
-------------------------------------------------------------------------
----------------
AATGAGACCTTCAGACCTGGAGGAGGAGACATGAGGGACAATTGGAGAAGTGAACTGTATAAATATAAAGTAG
TACAGATTGAACCACTA---GGAATAGCACCCACCAGGGCCAGGAGAAGAGTGGTG-----------------
----------------------CAGAGAGAAAAAAGAGCA---GTAGGA---TTAGGAGCT---------
GTGTTCTTTGGATTCTTGGGAGCA---GCAGGAAGCACTATG---GGCGCGGCGTCA---
ATAACGCTGACGGTACAG---GCCAGACAA---TTATTG---TCTGGTATAGTGCAACAGCAAAGC---
AATTTG---CTGAGA---GCTATAGAAGCGCAACAACATCTGTTACAGCTCACGGTCTGGGGCATT
>MH746262
TTCTATTGCAATACAACACAACTGTTTAATGGC----------------------------------------
-------------------------------------------------------------------------
----------------------------
ACGATTGGCAATATCACACTGCCATGTAGGATAAAACAAATTATAAACATGTGGCAAGGAGTAGGACAAGCAA
TGTATGCTCCTCCCATCAATGGAATACTTAATTGTGTATCAAAT---
ATTACAGGAATACTATTGACAAGAGATGGTGGTAATCATACGAAT----------------------------
-------------------------------------------------------------------------
-------------
GAAACTGAGACCTTCAGACCGGGAGGAGGAAATATAAAGGACAATTGGAGAAGTGAATTATATAAATATAAAG
TAGTAGAAATTGAACCACTA---GGAATAGCACCCACCAAGGCAAAGAGAAGAGTGGTG--------------
-------------------------GAGAGAGAAAAAAGAGCA---GTGGGA---ATAGGAGCT---------
ATGATCTTTGGGTTCTTAGGAGCA---GCCGGAAGCACGATG---GGCGCGGCGTCA---
ATAACGCTGACGGTACAG---GCCAGACAA---TTATTG---TCTGGTATAGTGCAGCAGCAAAGC---
AATTTG---CTGAGG---GCTATAGAGGCGCAGCAGCATATGTTGCAACTCACAGTCTGGGGCATT
>U51188
TTCTATTGCAATACAACAGCACTGTTTAATAGTACTTGGATAAATGGAACCATGCAGGAGGTTAATGGCACA-
-------------------------------------------------------------------------
----------------------------
AACTCAGGCAATATCACACTTCCATGCAGGATAAAGCAAATTGTAAACATGTGGCAGGAAGTAGGACGAGCAA
TGTATGCTCCTCCCATCAGTGAAGTAATTAATTGTGTATCAAAT---
ATTACAGGAATACTATTGACAAGAGATGGGGGTATTAATCAAAATCAG-------------------------
-------------------------------------------------------------------------
-------
ACTAACAAAAATGAGACCTTCAGACCTGGAGGAGGAAATATAAAGGACAATTGGAGAAGTGAATTATACAAAT
ATAAAGTAGTACAAATTGAACCACTA---GGAATAGCACCCACCAAGGCAAGGAGAAGAGTGGTG--------
-------------------------------GAGAGAGAAAAAAGAGCA---GTGGGA---ATAGGAGCT---
------ATGATCTTTGGGTTCTTAGGAGCA---GCAGGAAGCACTATG---GGCGCGGCGTCA---
ATAACGCTGACGGTACAG---GCCAGACAA---TTATTG---TCTGGTATAGTGCAACAGCAAAGC---
AATTTG---CTGAGG---GCTATAGAGGCGCAGCAGCATCTGTTGCAACTCACAGTCTGGGGCATT
>JX112863
TTCTATTGCAATACAACAAAACTGTTTAATAATACTTGCATAAATGGAACCACGAAGGGG-------------
-------------------------------------------------------------------------
----------------------------
TGTAATGGTACTATCATAATTCTATGCAAGATAAAGCAAATTGTAAACATGTGGCAGGGAGTAGGACAAGCAA
TGTATGCTCCTCCCGTCAGAGGAACAATTAATTGTGTATCAAAT---
ATTACAGGAATAATATTGACAAGAGATGGTGGAGTTAATAGGACA----------------------------
-------------------------------------------------------------------------
-------------
```

CATAACGAGACCTTCAGACCTGGAGGAGGAAATATGAAAGATAATTGGAGAAATGAATTATATAAATATAAAA
CAGTACTAATTGAACCACTA---GGAATAGCACCCACCAGGGCAAAGAGAAGAGTGGTG--------------
-------------------------GAGAGAGAAAAAAGAGCA---GTGGGA---ATAGGAGCT---------
ATGATCTTTGGGTTCTTGGGAGCA---GCAGGAAGCACTATG---GGCGCAGCGTCA---
ATAACGCTGACGGTACAG---GCCAGACAA---TTATTG---TCTGGTATAGTGCAACAGCAAAGC---
AATTTG---CTGAGG---GCTATAGAGGCGCAACATCATCTGTTGCAACTCACAGTCTGGGGCATT
>EF036528
TTCTATTGCAATACAACAAGATTGTTTAATAATACTTGCATAAACAATGAAACCATAGAG-------------
----

GGGTGTCAGAACATCACAATTCCATGTAAGATAAAGCAAATTATAAACATGTGGCAGGAAACAGGACAAGCAA
TGTATGCTCCTCCCATCAGGGGAAAAATTAGTTGTATATCAAAT---
ATTACAGGAATACTATTGACAAGAGATGGTGGTGCTAATAAGACG----------------------------
----

AATGAGGAGATCTTCAGACCTGGAGGAGGAAATATAAGGGACAATTGGAGAAGTGAGTTATACAAATATAAAG
TAGTACAAATTGAACCACTA---GGAGTAGCACCCACCAGGGCAAAGAGAAGAGTGGTG--------------
-------------------------GAAAGAGAAAAAAGAGCA---GTGGGA---ATAGGAGCT---------
ATGATCTTTGGGTTCTTAGGAGCA---GCAGGAAGCACTATG---GGCGCGGCGTCA---
ATAACGCTGACGGTACAG---GCCAGACAA---TTATTG---TCTGGTATAGTGCAACAGCAAAGC---
AATTTG---CTGAGG---GCTATAGAGGCGCAACAGCATATGTTGCAACTCACCGTCTGGGGCATT
>HM215415
TTCTATTGCAATACAACAAAATTGTTTAATAATACTTGCATAGAGGAGTGCAATGAAACCATTTGC-------
----

TACAATGAAACCACTTATCTTCCATGTAGGATAAAGCAAATTATAAACATGTGGCAGGGAGTAGGGCAAGCAA
TGTATGCTCCTCCTATTGAGGGAAAAATTAGTTGTGTATCAAAT---
ATTACAGGAATACTATTAACAAGAGATGGTGGTAATAATAATAGT----------------------------
----

ACAGGTAATGAGACCTTCAGACCTGGAGGAGGAAATATAAAGGACAATTGGAGAAATGAATTATATAAATATA
AAGTAGTACAAATTGAACCACTA---GGAATAGCACCCACCAGGGCAAGGAGAAGAGTGGTG-----------
----------------------------GAGAGAGAAAAAAGAGCA---GTGGGA---ATAGGAGCT------
---ATGATCTTTGGGTTCTTAGGAGCA---GCAGGAAGCACTATG---GGCGCGGCGTCA---
ATAACGCTGACGGTACAG---GCCAGACAA---TTATTG---TCTGGTATAGTGCAACAGCAAAGC---
AATTTG---CTGAGG---GCTATAGAGGCGCAGCAGCATTTGTTGCAACTCACAGTCTGGGGCATT
>KF835503
TTCTATTGTAATACATCACAACTGTTTAATACAACACAACTTAAACGGTTTAATAATGGTGGG----------
----

GGTAGTGACACTATCATACTTCCATGTAAGATAAAACAAATTATAAACATGTGGCAGGGAGCAGGACAAGCAA
TGTATGCTCCTCCCATCAGTGGAGCAATTAATTGTTCATCAAAT---
ATTACAGGAATACTATTAACAAGAGATGGTGGTGTTCATAATGGGAGT-------------------------
----

AACGAGACCGAGATCTTCAGACCTGGAGGAGGAAATATAAAGGACAATTGGAGAAGTGAACTATATAAATATA
AAGTAGTAGAAATTGAACCATTA---GGAATAGCACCCACCAAGGCAAGGAGAAGAGTGGTG-----------
----------------------------GAGAGAGAAAAAAGAGCA---GTGGGA---ATAGGAGCT------
---ATGATCTTTGGGTTCTTAGGAGCA---GCAGGAAGCACTATG---GGCGCGGCGTCA---
ATAACGCTGACGGTACAG---GCCAGACAA---TTATTG---TCTGGTATAGTGCAACAGCAAAGC---
AATTTG---CTGAGG---GCTATTGAGGCGCAACAGCATATGTTGCAACTCACAGTCTGGGGCATT
>KF835505
TTCTATTGCAATACATCACAACTGTTTACTTGGAAAGAAAATGAAACCATAAATGGG----------------
----

GGTAATGGTAGTATCACACTTCAATGCAAGATAAAGCAAATTATAAACATGTGGCAGGGAGTGGGACAAGCAA
TGTATGCTCCTCCCGTCAATGGAACTATTAATTGTGTATCAAAT---
ATTACAGGAATACTATTGACAAGAGATGGTGGTAATAATAATGAGAGT-------------------------
----

AATACAAATGAGACCTTCAGACCTGGAGGAGGAGATATGAGGGACAATTGGAGAAATGAATTATATAAATATA
AAGTAGTAGAAATTGAACCACTA---GGAATAGCACCCACCAGGGCAAAGAGAAGAGTGGTG-----------
----------------------------GAGAGAGAAAAAAGAGCA---GTGGGA---ATAGGAGCT------
---ATGATCTTTGGGTTCTTGGGAGCA---GCTGGAAGCACTATG---GGCGCAGCGTCA---
ATAACGCTGACGGTACAG---GCCAGACAA---TTATTG---TCTGGTATAGTGCAACAGCAAAGC---
AATTTG---CTGAGG---GCTATTGAGGCGCAGCAGCATATGTTGCAACTCACAGTCTGGGGCATT
>KF835527
TTCTATTGCAACACATCAAACCTGTTTAATACAACATACCTTAATAGTAGCACA-------------------
----

GAAAATAACAATATCACACTTCCATGCAGAATAAAGCAAATTATAAACATGTGGCAGGGAACAGGACAAGCAA
TGTATGCTCCTCCCATCAGTGGAAACATTAGCTGTGTATCAAAT---
ATTACAGGAATACTATTGACAAGAGATGGTGGTGGGAATGGTACAGAGGGG----------------------
----

ACGAATGAGACCTTCAGACCTGGAGGAGGAAATATGAAGGACAATTGGAGAAATGAATTATATAAATATAAAG
TAGTACAAATTGAACCACTA---GGAATAGCACCCACCAGGGCAAGGAGAAGAGTGGTG--------------
-------------------------GAGAGAGAAAAAAGAGCA---GTGGGA---ATAGGAGCT---------
ATGATCTTTGGATTCTTGGGAGCA---GCAGGAAGCACTATG---GGCGCGGCGTCA---
ATAACGCTGACGGTACAG---GCCAGACAA---TTATTG---TCTGGTATAGTGCAACAGCAAAGC---
AATTTG---CTGAGG---GCTATAGAGGCGCAGCATCATTTGCTGCAACTCACAGTCTGGGGCATT
>JX112809
TTCTATTGCAATACAAGCCAACTGTTTAATGCTACTTACGGAACAAATGAAACCACGCGTACG----------
----

CGTAATGACACTATCATACTTCCATGCAGGATAAAGCAAATTATAAACATGTGGCAGGGAGTAGGACAAGCAA
TGTATGCTCCTCCCATCAGTGGAAGAATTAATTGTGTATCAAAT---
ATTACAGGAATACTATTAACAAGAGATGGTGGTGATAATAATAGT----------------------------
----

ACTAACGAGACCTTCAGACCTGGAGGGGGAGATATAAAGGACAATTGGAGAAGTGAATTATATAAATATAAAG
TAGTACAAATTGAACCACTA---GGAGTAGCACCCACCAGGGCAAAAAGAAGAGTGGTG--------------
-------------------------GAGAGAGAAAAAAGAGCA---GTGGGA---ATAGGAGCT---------
ATGATCTTTGGGTTCTTAGGAGCA---GCAGGAAGCACTATG---GGCGCGGCGTCA---
ATAACGCTGACGGTACAG---GCCAGACAA---TTATTG---TCTGGTATAGTGCAACAGCAAAGC---
AATTTG---CTGAGG---GCTATAGAGGCGCAGCATCATCTGTTGCAACTCACAGTCTGGGGCATT
>JX112813
TTCTATTGCAATACAACAAAACTGTTTAACAGTACTTGGACAGGAAATGAAACCATAAATGAGAAT-------
----

GGGACTACTAAAATCATAATTCCATGCAGGATAAAGCAAATTATAAACATGTGGCAAGGAGCAGGACAAGCAA
TATATGCTCCTCCCATCAGTGGAGCAATTAATTGTGTATCAAAT---
ATTACAGGAATACTATTGACAAGAGATGGTGGTGATGGTAATCAT----------------------------
----

ATAAACGAGACCTTCAGACCTGCAGGAGGAAATATGAGGGACAATTGGAGAAGTGAATTATATAAATATAAAG
TAGTACAAATTGAACCATTA---GGAATAGCACCCACCAGGGCAAAGAGAAGAGTGGTG--------------
-------------------------GAGAGACAAAAAAGAGCA---GTGGGA---ATAGGAGCT---------
ATGATCTTTGGGTTCTTAGGAGCA---GCAGGAAGCACTATG---GGCGCGGCGTCA---
ATAACGCTGACGGTACAG---GCCAGACAA---TTATTG---TCTGGTATAGTGCAACAGCAAAGC---
AATTTG---CTGAGG---GCTATAGAGGTGCAGCAGCATATGTTGCAACTCACAGTCTGGGGCATT
>JX112814
TTCTATTGCAATACAAGCCAACTGTTTAATAAAACTTACTATGGAAATGAAACTACTTCCATAGGGAATACC-
----

ACGAATAACACTATCGTACTTCCATGCAAGATAAAGCAAATTATAAACATGTGGCAGGGAGTAGGACAAGCAA
TGTATGCTCCTCCCATCAGTGGAAACATTAGTTGTGTATCAAAT---
ATTACAGGAATACTATTAACAAGAGATGGTGGTAATAATAAGTCT----------------------------
----

CAGGTGAACGAGACCTTCAGACCTGGAGGAGGAAATATGAAGGACAACTGGAGAAGTGAATTATATAAATATA
AAGTAGTACAAATTGAACCATTA---GGAATAGCACCCACCAGGGCAAAGAGAAGAGTGGTG-----------
----------------------------GAGAGAGAAAAAAGAGCA---GTGGGA---ATAGGAGCT------
---ATGATCTTTGGGTTCTTAGGAGCA---GCAGGAAGCACTATG---GGCGCGGCGTCA---
ATAACGCTGACGGTACAG---GCCAGACAA---TTATTG---TCTGGTATAGTGCAACAGCAAAGC---
AATTTG---CTGAGG---GCTATAGAGGCGCAGCAGCATCTGCTGCAACTCACAGTCTGGGGCATT
>JX112820
TTCTATTGCAATACATCAGGACTGTTTAATAGTACTTGGGAAAGAAATGGAACCATGAGGGGGCATAATAGC-
----

ACTAATGGCACTATCATACTTCCATGCAGGATAAAGCAAATTATAAACATGTGGCAGGGAGTAGGACAAGCAA
TGTATGCTCCTCCCATCGCAGGAAGTATTAATTGTACATCAAAT---
ATTACAGGGATACTATTGACAAGAGATGGTGGTTATGATGCGGAGAAT-------------------------
----

AATGAGACTGAGACTTTCAGACCTGGAGGAGGAAATATAAAAGACAATTGGAGAAGTGAATTATATAAATATA
AAGTAGTACAAATTGAACCACTA---GGAATAGCACCCACCATGGCAAAGAGAAGAGTGGTG-----------
----------------------------GAAAGAGAAAAAAGAGCA---GTGGGA---ATAGGAGTT------
---ATGATCTTTGGGTTCTTAGGAGCA---GCCGGAAGCACTATG---GGCGCAGCGTCA---
CTAACGCTGACGGTACAG---GCCAGACAA---TTATTG---TCTGGTATAGTGCAACAGCAAAGC---
AATTTG---CTGAGG---GCTATAGAGGCGCAGCAGCATATGTTGCAACTCACAGTCTGGGGCATT
>JX112823
TTCTATTGCAATACAAGCCAACTGTTTAATGATACAGGAAATGAAACCATAGGGAAT----------------
----

AATAGTAACACTATCATACTTCCATGCAGGATAAAGCAAATTATAAACATGTGGCAGGGAGTAGGACAAGCAA
TGTATGCTCCTCCCATCAGTGGAACAATTAATTGTGTATCAAAT---
ATTACAGGAATACTATTAACAAGAGATGGCGGTACTAATGGTAATAATACTAAT------------AAT----
----

ACCACTAATAATACCGAGACCTTCAGACCTGGGGGAGGAAATATAAAGGACAATTGGAGAAGTGAATTATATA
AATATAAAGTAGTACAAATTGAACCACTA---GGAATAGCACCCACCAGGGCAAAGAGAAGAGTGGTG-----
----------------------------------GACAGAGAAAAAAGAGCA---GTGGGA---
ATAGGAGCT---------ATGATCTTTGGGTTCTTAGGAGCA---GCTGGAAGCACTATG---
GGCGCGGCGTCA---ATAACGCTGACGGTACAG---GCTAGACAA---TTATTG---
TCTGGTATAGTGCAACAGCAAAGC---AATTTG---CTGAGG---
GCTATAGAGGCGCAGCATCATTTGTTGCAACTCACAGTCTGGGGCATT
>JX112826
TTCTATTGCAATACAAGCAAACTGTTTAATTTTACTTTCCCAAGAAATGAAACAGGGGAG-------------
----

AATGATACAACTATCATACTTCCATGCAGGATAAAGCAAATTATAAACATGTGGCAGGGAGTAGGACAAGCAA
TGTATGCTCCTCCCATCAGTGGGATAATTAATTGTGTATCAAAT---
ATTACAGGAATACTATTAACAAGAGATGGTGGTCATAATAATAATACT-------------------------
----

ATTACTAATGAGACCTTCAGACCTGGAGGAGGAAATATAAAGGACAATTGGAGAAGTGAATTATACAAATATA
AAGTAGTACAAATTGAACCACTA---GGAATAGCACCCACCAGGGCAAAGAGAAGAGTGGTG-----------
----------------------------GAGAGAGAAAAAAGAGCA---GTGGGA---ATAGGAGCT------
---ATGATCTTTGGGTTCTTAGGAGCA---GCAGGAAGCACTATG---GGCGCGGCGTCA---
ATAACGCTGACGGTACAG---GCCAGACAA---TTATTG---TCTGGTATAGTGCAACAGCAAAGC---
AATTTG---CTGAGG---GCTATAGAGGCGCAGCAGCATATGTTGCAACTCACAGTCTGGGGCATT
>JX112835
TTCTATTGCAATACAAACAAACTGTTTAATGTAACTTACAATGAAAAGAATACCACAGGGGGG----------
----

AGTAATGGCACTATCATACTCCTATGCAAGATAAAGCAAATTATAAACATGTGGCAGGAAGTAGGACAAGCAA
TGTATGCTCCTCCCATCAGTGGAAGAATTAATTGTGTATCAAAT---
ATTACAGGAATACTACTAACAAGAGATGGTGGTGGTACTAATAGG----------------------------
----

ACTAATGAGACCTTCAGACCTGGAGGAGGAAATATAAAGGACAATTGGAGAAGTGAATTATATAAATATAAAG
TAGTACAACTTGAACCACTA---GGAATAGCACCCACCAGGGCAAAGAGAAGAGTGGTG--------------
-------------------------GAGAGAGAAAAAAGAGCA---GTGGGA---ATAGGAGCT---------
ATGATCTTTGGGTTCTTGGGAGCA---GCAGGAAGCACTATG---GGCGCGGCGTCA---
ATAACGCTGACGGTACAG---GCCAGACAA---TTATTG---TCTGGTATAGTGCAACAGCAAAGC---
AATTTG---CTGAGG---GCTATAGAGGCGCAGCATCATCTGTTGCAACTCACAGTCTGGGGCATT
>JX112844
TTCTATTGCAATACAACAAACCTGTTTAACAGTACTTGGGAAAATGGAACCACGGAGGGTAAT----------
----

GGCACCGAGAAGATCATACTTCCATGCAGGATAAAGCAAATTATAAACATGTGGCAGGGAGCAGGACAAGCAA
TGTATGCTCCTCCCATCAGTGGAACAATTAATTGTGTATCAAAT---
ATTACAGGAATACTATTAACAAGAGATGGTGGTTATAATAATGCG----------------------------
----

GATAACGAGACCTTCAGACCTGGAGGAGGAAATATAAAGGACAATTGGAGAAGTGAATTATATAAATATAAAG
TAGTAGAAATTGAACCACTA---GGAATAGCACCCACCAGGGCAAAGAGAAGAGTGGTG--------------
-------------------------GAGAGAGAAAAAAGAGCA---GTGGGA---ATAGGAGCT---------
ATGATCTTTGGGTTCTTGGGAGCA---GCAGGAAGCACTATG---GGCGCGGCGTCA---
ATAACGCTGACGGTACAG---GCCAGACAA---TTATTG---TCTGGTATAGTGCAACAGCAAAGC---
AATTTG---CTGAGG---GCTATAGAGGCGCAGCAGCATATGCTGCAACTCACAGTCTGGGGCATT
>GU475036
TTCTATTGCAATACAACAAAACTGTTTAATAGGAATAATAAC-------------------------------
----

TATAGCACTATCATACTTCCATGCAAGATAAAGCAAATTGTAAGAATGTGGCAGGGAGTAGGACAAGCAATGT
ATGCTCCTCCCATCAGTGGAAGCATAAATTGTGTATCAAAT---
ATTACAGGAATACTATTGACAAGAGATGGTGGTATTCATAATAGT----------------------------
----

AGTGATGAGACCTTCAGACCTGGAGGAGGAAATATAAAGGACAATTGGAGAAGTGAATTATATAAATATAAGG
TAGTAGAAATTGAACCACTA---GGAATAGCACCCACCAAGGCAAAGAGAAGAGTGGTG--------------
-------------------------GAGAGAGAAAAAAGAGCA---GTGGGA---ATAGGAGCT---------
ATGATCTTTGGGTTCTTAGGAGCA---GCAGGAAGCACTATG---GGCGCGGCGTCA---
ATAACGCTGACGGTACAG---GCCAGACAA---TTATTG---TCTGGTATAGTGCAACAGCAAAGC---
AATTTG---CTGAGG---GCTATAGAGGCGCAACAGCATATGTTGCAACTCACAGTCTGGGGCATT
>GU475019
TTCTATTGCAATACAACAAATCTGTTTAATAATACTTGCATAAACAATGCAAGCACAAAAGGG----------
----

TGTAATGAAAATATCATACTTCCATGCAGGATAAAACAAATTATAAACATGTGGCAAGGAGTAGGACAAGCAA
TGTATGCTCCTCCCATCAGTGGAAAAATTACTTGTAGATCAAAT---
ATTACAGGAATACTATTGACAAGAGATGGTGGTAATCATAGCACA----------------------------
----

GGTAATGAGACCTTCAGACCTGAAGGAGGAAATATAAAGGACAATTGGAGAAATGAATTATATAAATATAAAG
TAGTAGAAATTGAACCACTA---GGAATAGCACCCACCAGGGCAAAGAGAAGAGTGGTG--------------
-------------------------GAGAGAGAAAAAAGAGCA---GTGGGA---ATAGGAGCT---------
ATGATCTTTGGGTTCTTAGGAGCA---GCAGGAAGCACTATG---GGCGCAGCGTCA---
ATAACGCTGACGGTACAG---GCCAGACAA---TTATTG---TCTGGTATAGTGCAACAGCAAAGC---
AATTTG---CTGAGG---GCTATAGAGGCGCAGCAGCATCTGTTGCAACTCACAGTCTGGGGCATT
>JX960627
TTCTATTGCAATACAACAAACCTGTTCAATAGTACTTGGAAAAATGAGACCATGGAGGGAGGTAATGAT----
----

AATTACACTAAAATCATACTTCCATGCAGGATAAAGCAAGTTATAAACATGTGGCAAGGAGCAGGACAAGCAA
TGTATGCTCCTCCCATCAGTGGAATAATTAACTGTGTATCAAAT---
ATTACAGGAATACTATTGACAAGAGATGGTGGTAATAATATAACT----------------------------
----

GAAGAGACTTTCAGACCTGGAGGAGGAGATATAAAGAACAATTGGAGAAGTGAATTATATAAATATAAAGTAG
TACAAATAGAACCACTA---GGAGTAGCACCCACCAGGGCAAAGAGAAGAGTGGTG-----------------
----------------------GATAGAGAAAAAAGAGCA---GTGGGA---ATAGGAGCT---------
ATGATCTTTGGGTTCTTGGGAGCA---GCAGGAAGCACTATG---GGCGCGGCGTCA---
ATAACGCTGACGGTACAG---GCCAGACAA---TTATTG---TCTGGTATAGTGCAACAGCAAAGC---
AATTTG---CTGAGG---GCTATAGAGGCGCAGCAGCATATGTTGCAACTCACAGTCTGGGGCATT
>JX960615
TTCTATTGCAATACAACAAAGCTGTTCAATAGTACTTGGATAGGAAATGAAACCACGGAGGGAGGTAAT----
----

GATAATGACACTATCATACTTCCATGCAGGATAAAGCAAATTATAAACATGTGGCAAGGAGCGGGACAAGCAA
TGTATGCTCCTCCCATCAGTGGAATAATTAAATGTGTATCAAAT---
ATTACAGGAATACTATTGACAAGAGATGGTGGTGATTATAATAAT----------------------------
----

ACAAATGAGACCTTCAGACCTGGAGGAGGAAATATAAAGGACAATTGGAGAAGTGAATTATATAAATATAAGG
TAGTACAAATAGAACCACTA---GGAGTAGCACCTACCAGGGCAAGGAGAAGAGTGGTG--------------
-------------------------GAGAGAGAAAAAAGAGCA---GTGGGA---ATAGGAGCT---------
ATGATCTTTGGGTTCTTAGGAGCA---GCAGGAAGCACTATG---GGCGCGGCGTCA---
ATAACGCTGACGGTACAG---GCCAGACAA---TTATTG---TCTGGTATAGTGCAACAGCAAAGC---
AATTTG---CTGAGG---GCTATAGAGGCGCAACAGCATATGTTGCAACTCACAGTCTGGGGCATT
>JX960639
TTCTATTGCAATACAACAAAATTGTTTAATAGTACAGAAAATGGAACCATGGAGGGG----------------
----

CTTAATGGCACTATTATACTCCCATGCAGGATAAAACAAATTGTAAACCTGTGGCAGGGAGCAGGACAAGCAA
TGTATGCTCCTCCCGTCAGTGGAAAAATTAATTGTGCATCAAAT---
ATTACAGGAATACTATTAACAAGAGATGGTGGTAATAATGCAACA----------------------------
----

ACTAACGAGACCTTCAGACCGGGAGGAGGAAATATAAAGGACAATTGGAGAAGTGAATTATATAAATATAAAG
TAGTACAAATTGAACCACTA---GGAGTAGCACCCACCAGGGCAAAGAGAAGAGTGGTG--------------
-------------------------GATAAAGAAAAAAGAGCA---GTGGGA---ATAGGAGCT---------
ATGATCTTTGGGTTCTTAGGAGCA---GCAGGAAGCACTATG---GGCGCGGCGTCA---
ATAACGCTGACGGTACAG---GCCAGACAA---TTATTG---TCTGGTATAGTGCAACAGCAAAGC---
AATTTG---CTGAGA---GCTATAGAGGCGCAGCATCATCTGTTGCAACTCACAGTCTGGGGCATT
>JX960632
TTCTATTGCAATACAACAAGGCTGTTCAATAGTACTTGGATAGGAAATGAGACCATAGGAAATGAGACCATGG
GGGAAGGTAAT--

AATAATAACACTATCATACTTCCATGCAGGATAAAGCAAATTATAAACATGTGGCAAGGAGCAGGACAAGCAA
TATATGCTCCTCCCATCAGTGGAATAGTTAATTGTGTATCAAAT---
ATTACAGGGATACTATTGACAAGAGATGGTGGTCAAGAAGATAAT----------------------------
----

GGAACAAATAGCGAGATTTTCAGACCTGGAGGAGGAAATATAAAGGACAATTGGAGAAGTGAATTATATAAAT
ATAAAGTAGTAGAAATAGAACCACTA---GGAGTAGCACCCACCAGGGCAAAGAGAAGAGTGGTA--------
-------------------------------AATAGAGAAAAAAGAGCA---GTGGGA---ATAGGAGCT---
------ATGATCTTTGGGTTCTTGGGAGCA---GCAGGAAGCACTATG---GGCGCGGCGTCA---
ATAACGCTGACGGTACAG---GCCAGACAA---TTATTG---TCTGGTATAGTGCAACAGCAAAGC---
AATTTG---CTGAGG---GCTATAGAGGCGCAGCAGCATATGTTGCAACTCACAGTCTGGGGCATT
>MH672757
TTCTATTGCAATACAACAAAGCTGTTCAATGGTAATGGCACTAAAAATGGTAGTGAGACA-------------
----

AACAGTAGCACTATCATACTTCCATGCAGGATAAAGCAAATTATAAACATGTGGCAAGGAGCAGGACAAGCAA
TGTATGCTCCTCCCATCAGTGGAATAATTAATTGTGTATCAAAT---
ATTACAGGAATACTATTAACAAGAGATGGTGGTAATCATAATAAT----------------------------
----

ACTACCGAGATTTTCAGACCTGGAGGAGGAAATATGGAGGACAATTGGAGAAGTGAATTATATAAATATAAAG
TAGTACAAATAGAACCACTA---GGAGTAGCACCCACCAAGGCAAAGAGAAGAGTGGTG--------------
-------------------------GATAGAGAAAAAAGAGCA---GTGGGA---ATAGGAGCT---------
ATGATCTTTGGGTTCTTGGGAGCA---GCAGGAAGCACTATG---GGCGCGGCGTCA---
ATAACGCTGACGGTACAG---GCCAGACAG---TTATTG---TCTGGTATAGTGCAGCAGCAAAGC---
AATTTG---CTGAGG---GCTATAGAGGCGCAACAGCATATGTTGCAACTCACAGTCTGGGGCATT
>JX112798
TTCTATTGCAATACAACAAAACTGTTCAATAATGAAACCATG-------------------------------
----

AAAGAGGACACTATCATACTTCCATGCAGGATAAAGCAAATTATAAACTTGTGGCAAGGAGTGGGACAAGCAA
TGTATGCTCCTCCCATCAGTGGAATAATTCAATGTGTATCAAAT---
ATTACAGGAATAATATTAACAAGGGATGGTGGTAATCAAAGTAATAGTAATGTA-------------------
----

ACTAGAGAGATCTTCAGACCTGGAGGAGGAAATATGAAGGACAATTGGAGAAGTGAATTATATAAATATAAAG
TAGTACAAATTGAACCACTA---GGAGTAGCACCCACCAGGGCAAAGAGAAGAGTGGTG--------------
-------------------------GATAGACAAAAAAGAGCA---GTGGGA---ATAGGAGCT---------
ATGATCTTTGGGTTCTTAGGAGCA---GCAGGAAGCACTATG---GGCGCGGCGTCA---
ATAACGCTGACGGTACAG---GCCAGACAA---TTATTG---TCTGGTATAGTGCAACAGCAAAGC---
AATTTG---CTGAGA---GCTATAGAGGCGCAGCAGCATATGTTGCAACTCACAGTCTGGGGCATT
>JX112799
TTCTATTGTAATACAACAAAATTGTTTAATAAGACAGAGAATGGAACCATGGAGGGG----------------
----

TTTAATACCACTATCATACTTCCATGCAGGATAAAGCAAATTGTAAGGATGTGGCAGGGAGTAGAACAAGCAA
TGTATGCTCCTCCCATCAGTGGAAACATTACTTGTAGATCAAAT---
ATTACAGGAATACTATTAACAAGAGATGGTGGTGGTTATGATACA----------------------------
----

TCTCCCGAAACCTTCAGACCAGGAGGAGGAAATATGAAGGACAATTGGAGAAGTGAATTATATAAATATAAAG
TAGTACAAATTGAACCACTA---GGAATAGCACCCTCCAGGGCAAAGAGAAGAGTGGTG--------------
-------------------------GATAGACAAAAAAGAGCA---GTGGGA---ATAGGAGCT---------
ATGATCTTTGGGTTCTTAGGAGCA---GCAGGAAGCACTATG---GGCGCAGCGTCA---
ATAACGCTGACGGTACAA---GCCAGACAA---TTATTG---TCTGGTATAGTGCAACAGCAAAGC---
AATTTG---CTGAGG---GCTATAGAGGCGCAGCAGCATCTGTTGCAACTCACAGTCTGGGGCATT
>KC870041
TTCTATTGCAATACAACAAAACTGTTTAATAAGACTTTCGATAAGAACATAGAA-------------------
----
-AATGACACCATGGAT---------
GGCAGTAATAACACTATCATACTTCCATGCAGGATAAAGCAAATTATAAACATGTGGCAGGGAGTAGGACAAG
CAATGTATGCCCCTCCCATTAGGGGAAAAATTCATTGTGTATCAAAT---
ATTACAGGAATACTATTGACAAGAGATGGTGGT--

ACTGATAATAACGAGACCTTCAGACCTGGAGGGGGAAACATAAAAGACAATTGGAGAAGTGAATTATATAAAT
ATAAAGTAGTACAAATTGAACCACTA---GGGGTAGCACCTACCAGGGCAAAGAGAAGAGTGGTG--------
-------------------------------GAAAGAGAAAAAAGAGCA---GTGGGA---ATAGGAGCT---
------ATGATCCTTGGGTTCCTAGGAGCA---GCAGGAAGCACTATG---GGCGCGGCGTCA---
ATAACGCTGACGGTACAG---GCCAGACAA---TTATTG---TCTGGTATAGTGCAACAGCAAAGC---
AATTTG---CTGCGG---GCTATAGAGGCGCAACAGCATATGTTGCAACTCACAGTCTGGGGCATT
>MH672923
TTCTATTGCAATACAACAAAATTGTTTAATAGTACAGAAAATGGAACCATGGAGGGG----------------
----

TTTAATACCACTATCATAATTCCATGCAAGATAAAGCAAATTGTAAATATGTGGCAGGGAGTAGGACAAGCAA
TATATGCTCCTCCCATTAGTGGAGTAATTAAGTGTCATTCAAAT---
ATTACAGGAATACTATTAACAAGAGATGGTGGTAATAATAATGCA----------------------------
----

AATAACGAAACCTTCAGACCGGGAGGAGGAAATATAAAGGACAATTGGAGAAATGAATTATATAAATATAAAG
TAGTACAAATTGAACCACTA---GGAATAGCACCCACCAAGGCAAAAAGAAGAGTGGTG-------------
-------------------------GAGAGAGAAAAAAGAGCA---GTGGGA---ATAGGAGCT---------
ATGATCTTTGGGTTCTTAGGAGCA---GCAGGAAGCACTATG---GGCGCAGCGTCA---
GTAACGCTGACGGTACAG---GCCAGACAA---TTATTG---TCTGGTATAGTGCAACAGCAAAGC---
AATTTG---CTGAGA---GCTATAGAGGCGCAACAGCACCTGTTGCAACTCACAGTCTGGGGCATT
>MG655206
TTCTATTGCAATACTAGCAGACTGTTTAATGCTACTTATTATAAAAATGGAACCACAGAGAATGGAACCACAG
AGGGG--

AGTAATCACACTATCATACTTCCATGCAGAATAAAGCAAATTATAAACATGTGGCAGGGAGTAGGACAAGCAA
TGTATGCTCCTCCCATCAGTGGAAGAATTAATTGTGTATCAAAT---
ATTACAGGAATACTATTAACAAGAGATGGTGGTAGTAATGCTACA----------------------------
----

AATGAGACCTTCAGACCTGAAGGAGGGAATATAAAGAACAATTGGAGAAGTGAATTATATAAATATAAAGTAG
TACAAATTGAACCACTA---GGAGTAGCACCCACCAGGGCAAAGAGAAGAGTGGTG----------------
----------------------GAGAGAGAAAAAAGAGCA---GTGGGA---ATAGGAGCT---------
ATGATCTTTGGGTTCTTGGGAGCA---GCTGGAAGCACTATG---GGCGCGGCGTCA---
ATAACGCTGACGGCACAG---GCCAGACAA---TTATTG---TCTGGTATAGTGCAACAGCAAAGC---
AATTTG---CTGAGG---GCTATAGAGGCGCAGCAACATCTGCTGCAACTCACAGTCTGGGGCATT
>MG655209
TTCTATTGCGATACAACAAAACTGTTTAATAGTACAGGAAATGGAACCATGGAGGGG----------------
----

CTTAATGGCACCATTATACTTCCATGCAAGATAAAGCAAATTATAAACATGTGGCAGGGAGCAGGACAAGCAA
TATATGCTCCTCCCATCAGTGGAAGAATTGAGTGTAGATCAAAT---
ATTACAGGAATACTATTAACAAGAGATGGTGGTCATAATATAACT----------------------------
----

AACGAGACCTTCAGACCAGGAGGAGGAAATTTAAAGGACAATTGGAGAAATGAATTATATAAATATAAAGTAG
TACAAATTGAACCACTA---GGAATAGCACCCACCAAGGCAAAGAGAAGAGTGGTG----------------
----------------------AATAGAGAAAAAAGAGCA---GTGGGA---ATAGGAGCT---------
ATGATCTTTGGGTTCTTAGGAGCA---GCAGGAAGCACTATG---GGCGCGGCGTCA---
ATAACGCTGACGGTACAG---GCCAGACAA---TTATTG---TCTGGTATAGTGCAACAGCAAAGC---
AATTTG---CTGAGA---GCTATAGAGGCGCAGCAGCATCTGTTGCAACTCACAGTCTGGGGCATT
>MG655214
TTCTATTGCAATACAACAAAGTTATTTAATAGTACAGAAAATGGAACCAGGGAAGAGGGT-------------
----

CATAATGGCACTATTATACTCCCATGCAGGATAAAGCAAATTGTAAACATGTGGCAGGGAGCAGGCCAAGCAA
TGTATGCTCCTCCCGTCAGAGGAAACATTAATTGTGTATCAAAT---
ATTACAGGACTACTATTAACAAGAGATGGTGGTATTAAGGGAACT----------------------------
----

CACAACGAGACCTTCAGACCGGGAGGAGGAAATATAAAGGACAATTGGAGAAGTGAATTATATAAATATAAAG
TAGTACAAATTGAACCACTA---GGAGTAGCACCCACCAAGGCAAAAAGAAGAGTGGTG-------------
-------------------------GATAGAGAAAAAAGAGCA---GTAGGA---ATAGGAGCT---------
ATGATCTTTGGGTTCTTAGGAGCA---GCAGGAAGCACTATG---GGCGCGGCGTCA---
ATAACGCTGACGGTACAG---GCCAGACAA---TTATTG---TCTGGTATAGTGCAACAGCAAAGC---
AATTTG---CTGAGA---GCTATAGAGGCGCAGCAGCATATGCTGCAACTCACAGTCTGGGGCATT
>MG655215
TTCTATTGCAATACAACACAATTGTTTAATAATAATTCCACG-------------------------------
----

TTTAATACCACTGTCATACTTCCATGCAGGATAAAGCAAATTGTAAATATGTGGCAGGGGGTAGGACGAGCAA
TGTATGCTCCTCCCATCAGTGGAATGATTAATTGTGTATCAAAT---
ATTACAGGAATAATATTAACAAGAGATGGTGGTTATAGTAATACA----------------------------
----

ACTAACGAAACCTTCAGACCGGGAGGAGGAAATATAAAGGACAATTGGAGAAGTGAACTATATAAATATAAAG
TAGTACAAATTGAACCACTA---GGAATAGCACCCACCAGGGCAAAGAGAAGAGTGGTG--------------
-------------------------ACTAGAGAAAAAAGAGCAGTAGTGGGA---ATAGGAGCT---------
ATGATCTTTGGGTTCTTAGGAGCA---GCAGGAAGCACTATG---GGCGCAGCGTCA---
ATAACGCTGACGGTACAG---GCCAGACAA---TTATTG---TCTGGTATAGTGCAACAGCAAAGC---
AATTTG---CTGAGA---GCTATAGAGGCGCAGCAGCATCTGCTGCAACTCACAGTCTGGGGCATT
>DQ234790
TTCTATTGCAATACAACACAACTGTTTGATAGTAGTTGGAATACAACAACAACCAATAGGGAG----------
----

AACCGTAGTAATTTCATACTTCCATGCAGGATAAAACAAATTATAAACATGTGGCAGGAAACAGGAAAAGCAA
TGTATGCTCCTCCCATCAGGGGAAGCATTCAGTGTGTATCAAAT---
ATTACAGGAATACTATTGACAAGAGATGGTGGTAATAATAATGGGTCT-------------------------
----

AACGAGACCGAGACCTTTAGACCTGGAGGAGGAGATATAAGAGACAATTGGAGAAGTGAATTATATAAATATA
AAGTAGTACAAATTGAACCACTA---GGAGTAGCACCCACCAGGGCAAAGAGAAGAGTGGTG-----------
----------------------------GAGAGAGAAAAAAGAGCAGTAGTGGGA---ATAGGAGCT------
---ATGATCTTTGGGTTCTTAGGAGCA---GCAGGAAGCACTATG---GGCGCGGCGTCA---
TTAACGCTGACGGTACAG---GCCAGACAA---TTACTG---TCTGGTATAGTGCAACAGCAAAGC---
AATTTG---CTGAGG---GCTATAGAGGCGCAACAGCATATGTTGCAACTCACAGTCTGGGGCATT
>KF859741
TTCTATTGCAATACAACAAAATTATTTAATCACACAAACAATGACACCATGCAG-------------------
----

TATAATGACACTATCACACTTCCATGCAGGATAAAGCAAATTATAAACATGTGGCAGAGAGTAGGACAAGCAA
TGTATGCTCCTCCCGTCAGGGGAAGAATTAATTGTGTATCAAAC---
ATTACAGGAATACTATTGACAAGGGATGGTGGTGGGACAAACAGT----------------------------
----

ACGAACGAGATCTTCAGACCTCAAGGAGGAAATATAAAGGACAATTGGAGAAGTAAATTATATAAATATAAAG
TAGTACAAATTGAACCACTA---GGAGTGGCACCCACCAGGGCAAGGAGAAGAGTGGTG--------------
-------------------------GAGAGAGAAAAAAGAGCA---GTGGGA---ATAGGAGCT---------
CTGATCTTTGGGTTCTTAGGAGCA---GCAGGAAGCACTATG---GGCGCGGCGTCA---
ATAACGCTGACGGTACAG---GCCAGACAA---TTATTG---TCTGGTATAGTGCAACAGCAAAGC---
AATTTG---CTGAGG---GCTATAGAGGCGCAGCAGCATCTGTTGCAACTCACAGTCTGGGGCATT
>MH327759
TTCTATTGCAATACAACACAATTATTTAATAAC--

ACCAATGGTACTATCATACTTCCATGCAAGATAAAACAAATTATAAACATGTGGCAGGGAGTAGGACAAGCAA
TGTATGCTCCTCCCGTCAGGGGAAAAATTAATTGTGTATCAAAC---
ATTACAGGAATACTATTGACAAGGGATGGTGGGGCAAATAGCACG----------------------------
----

AATGAGACCTTCAGACTTCAAGGAGGAAATATAAAGGACAATTGGAGAAATGAATTATATAAATATAAAGTAG
TACAAATTGAACCACTA---GGAATAGCACCCACCAGGGCACGGCGAAGAGTGGTG-----------------
----------------------GAGAGAGAAAAAAGAGCA---GTGGGA---ATAGGAGCT---------
ATGATCTTTGGGTTCTTAGGAGCA---GCAGGAAGCACTATG---GGCGCGGCGTCA---
ATAACGCTGACGGTACAG---GCCAGACAA---TTATTG---TCTGGTATAGTGCAACAGCAAAGC---
AATTTG---CTGAGG---GCTATAGAGGCGCAGCAGCATCTGTTGCAACTCACAGTCTGGGGCATT
>MH078562
TTCTATTGCAATACAACACGATTATTTAATTGCACAAACAATAATAACACG----------------------
----

TGTAATGAAACTATCATACTTCCATGCAAGATAAAGCAAATTATAAACATGTGGCAGGGAGTAGGACAAGCAA
TGTATGCTCCTCCCATCAGGGGAAGAATTAATTGTGTATCAAAC---
ATTACAGGAATACTATTAACAAGGGATGGTGGTCAGACAAACGATACG-------------------------
----

AACTACGAAACCTTCAGACCTGCAGGAGGAAATATAAAGGACAATTGGAGAAATGAATTATATAAATATAAAG
TAATACAAATTGAACCACTA---GGATTGGCACCCACCAGGGCAAGGCGAAGAGTGGTG-------------
-------------------------GAGAGAGAAAAAAGAGCA---GTGGGA---ATAGGAGCT---------
ATGATCTTTGGGTTCTTAGGAGCA---GCAGGAAGCACTATG---GGCGCGGCGTCA---
ATAACGCTGACGGTACAG---GCCAGACAA---TTATTG---TCTGGTATAGTGCAACAGCAAAGC---
AATTTG---CTGAGG---GCTATAGAGGCGCAGCAGCATATGTTGCAACTCACAGTCTGGGGCATT
>MF373160
TTCTATTGCAATATAACAAAACTGTTTAATAGTACTTGGGGGAGTAATGGCACTTGGAATGGCACTGAA----
----

TACAATAACGATACCATACTCCCATGCAGGATAAAGCAAATTATAAACATGTGGCAACAACCAGGACAAGCAA
TGTATGCTCCTCCCGTTCAGGGAGTAATTAATTGTATATCAAAT---
ATTACAGGACTAATATTGACAAGAGATGGTGGTATTAATACTACA----------------------------
----

ATTAATGAAACCTTCAGACCTGGAGGAGGAAATATAAAGGACAATTGGAGAAGTGAATTATATAAATATAAAG
TAGTACAAATTGAACCACTA---GGAATAGCACCCACCAAGGCAAAGAGAAGAGTGGTG-------------
-------------------------GAGAGAGAGAAAAGAGCA---GTGGGA---ATAGGAGCT---------
ATGATCTTTGGGTTCTTAGGAGCA---GCCGGAAGCACTATG---GGCGCGGCGTCA---
ATAACGCTGACGGTACAG---GCCAGACAA---TTATTG---ACTGGTATAGTGCAGCAGCAAAGC---
AATTTG---CTGAGA---GCTATAGAGGCGCAACAGCATCTGTTGCAACTCACAGTCTGGGGCATT
>KP411840
TTCTATTGCAATACATCAAGGCTGTTTAATAGTACTTACAATGGAACTGAAACCGGGAAAGAG----------
----

GGTAATGGCACTATTATACTTCCATGCAGGATAAAGCAAATTATAAACATGTGGCAGGGAGTGGGACGAGCAA
TGTATGCTCCTCCCATCAGTGGAAGCATTAATTGTACATCAAAT---
ATTACAGGAATACTATTGACAAGCGATTATGGTGGTAATAATACA----------------------------
----

CAGAATGAGACCTTCAGACCTGCAGGAGGAAATATAAAGGACAATTGGAGAAGTGAATTATATAAATATAAAG
TAGTACAAATTGAACCACTA---GGAGTAGCACCCACCAAGGCAAAGAGAAGAGTGGTG-------------
-------------------------GAGAGACAAAAAAGAGCA---GTGGGA---ATAGGAGCT---------
ATGATCTTTGGGTTCTTGGGAGCA---GCAGGAAGCACTATG---GGCGCGGCGTCA---
ATAACGCTGACGGTACAG---GCCAGACAA---TTATTG---ACTGGTATAGTGCAACAGCAAAGC---
AATTTG---CTGAGG---GCTATAGAGGCGCAGCAGCATCTGTTGCAACTCACAGTCTGGGGCATT
>KP411841
TGCTACTGCAACCCCCTGCAACTGTTTAATAATAAGACTCATACTAATGGT----------------------
----

ACTAATGGCGCTATTGTACTTCTACGGATAATTAAGATAATGAAAATGATGAACCTGGGAGGAGGAGCAGCA-
--CAGGCTCCGCCTGCTCCTCGAAGTAGTATATGTGTTATTTCA---
GTTTCAAGAATAACACTGATACTAGTGGGTGGTGATGATGATAATAATAAT----------------------
----

AATAACCGCCAGAGCTTCGGACGAGCGGGAGGATGGATGAGGTACGATTATGGATATGAAGTATTACAATATG
AACTAGTACGAATAGTACCA------------CCACCAACAACAACAACCATGGTGGAGATG-----------
-------------------AGAAAAAGAGAAAAAGGAAAAAGAGCA---GTGGTA---ATAAGAGTT------
---TTGATCATCGGAGGAATAGGAACA---GCA------------------GCGGCACCA---
TTATCGCTGACGGCGATG---GCCCAGCAG---ATATTG---TCATGGATATTGAGACAGCGGAGG---
CTGCTG---CTGATG---AGTATTAAGTTGATGGAGCATCAGCAGCAGCTCACCTGCTGGGCCATC
>KP411842
TTCTATTGCAATACATCAAGGCTGTTTAATAGTACTTACAATGGAACTGAAAGTACCGAAGGGTCAAATGAG-
----

GGTAATGGCACTATTATACTTCCATGCAGGATAAAGCAAATTATAAACATGTGGCAGGGAGCAGGACGAGCAA
TGTATGCTCCTCCCATCAGTGGAAGCATTAATTGTACATCAAAT---
ATTACAGGAATACTATTGACAAGCGATTATGGTGGTAGTAATACA----------------------------
----

CAGAATGAGACCTTCAGACCTGCAGGAGGAAATATAAAGGACAATTGGAGAAGTGAATTATATAAATATAAAG
TAGTACAAATTGAACCACTA---GGAGTAGCACCCACCAAGGCAAAGAGAAGAGTGGTG--------------
-------------------------GAGAGACAAAAAAGAGCA---GTGGGA---ATAGGAGCT---------
ATGATCTTTGGGTTCTTGGGAGCA---GCAGGAAGCACTATG---GGCGCGGCGTCA---
ATAACGCTGACGGTACAG---GCCAGACAA---TTATTG---ACTGGTATAGTGCAACAGCAAAGC---
AATTTG---CTGAGG---GCTATAGAGGCGCAGCAGCATCTGTTGCAACTCACAGTCTGGGGCATT
>KY213735
TTCTATTGCAATACAACAAAACTGTTTAATAGTCCTTGCCTGAAAAATGAAGAAAATGGAACA----------
----

TGTAATAGCACTATCATACTTCCATGTAAGATAAAGCGAATTGTAAGATTGTGGCAGGAAGTAGGTCAAGCAA
TGTATGCTCCTCCCATCAGCGGAATCATTAGGTGTAACTCAACT---
ATTACAGGAATACTATTGACAAGAGATGGTGGTAACCTTAATAAC----------------------------
----

AGTACTAATGAAACCCTCAGACCTGGAGGAGGAAATATAAAGGACAATTGGAGAAGTGAATTATATAAATATA
AAGTAGTACAAATTGAACCACTA---GGAATAGCACCCACCAAGGCAAAAAGAAGAGTGGTG-----------
----------------------------GAGAGAGAAAAAAGAGCA---GTGGGA---ATAGGAGCT------
---ATGATCTTTGGGTTCTTAGGAGCA---GCAGGAAGCACTATG---GGCGCGGCGTCA---
ATAACGCTGACGGTACAG---GCCAGACAA---TTATTG---TCTGGTATAGTGCAACAGCAAAAC---
AATTTG---CTGAGG---GCTATAGAGGCGCAGCAGCATCTGTTGCAACTCACAGTCTGGGGCATT
>KY213733
TTCTATTGCAATACAACAAAACTGTTTAATAACACTTGCATAAATGGAACAATGGAAAATGAG----------
----

AGTAATAGCACTATCATACTTCCATGCAAGATTAAACAAATTATAAACATGTGGCAGGGAGCAGGACAAGCAA
TGTATGCTCCTCCCATCAGTGGAAGAATTAATTGTATATCAAAT---
ATTACAGGAATACTATTAACAAGAGATGGTCAG--

CCGAATGATACGGAGATCTTCAGACCTGGAGGAGGAGATATGAAGGACAATTGGAGAAATGAATTATATAAAT
ATAAAGTAGTACAAATTGAACCACTA---GGAATAGCACCCACCAGGGCAAAGAGAAGAGTGGTG--------
-------------------------------GAGAGAGAAAAAAGAGCA---GTGGGA---ATAGGAGCG---
------ATGATCTTTGGGTTCTTAGGAGCA---GCAGGAAGCACTATG---GGCGCGGCGTCA---
ATAACGCTGACGGTACAG---GCCAGACAG---TTATTG---TCTGGTATAGTGCAACAGCAAAGC---
AATTTG---CTGAGG---GCTATAGAGGCGCAGCAGCATCTGCTGCAACTCACAGTCTGGGGCATT
>KY213722
TTCTATTGCAATACAACAAAACTGTTTAATAGTACTTGGGGAGAAAATGAAAACATAACCACGGGGGGGTCTA
AT---

AACACTCACACTGTCATTCCATGCAGGATAAAGCAAATTATAAACATGTGGCAGGGAACAGGACAAGCAATGT
ATGCTCCTCCCATCAGGGGAAACATTACTTGTGTATCAAAC---
ATTACAGGAATACTATTGACAAGAGATGGTGGTGATAATAATAGT----------------------------
----

ATTAACGAGACCTTCAGACCTGGAGGAGGAAATATAAAGGACAATTGGAGAAGTGAATTATATAAATATAAAG
TAGTACAAATTGAACCACTA---GGAGTAGCACCCTCCAAGGCAAGGAGAAGAGTGGTG--------------
-------------------------GAGAGAGAAAAAAGAGCA---GTTGGA---ATAGGAGCT---------
ATGATCTTTGGGTTCTTAGGAGCA---GCAGGAAGCACTATG---GGCGCGGCGTCG---
CTAACGCTGACGGTACAG---GCCAGACAA---TTATTG---TCTGGTATAGTGCAACAGCAAAGC---
AATTTG---CTGAGG---GCTATAGAGGCGCAGCAGCATATGTTGCAACTCACAGTCTGGGGCATT
>KJ952667
TTCTATTGCAATACAACAAAACTGTTTAATAATACGTGCATAGGAAATGAAACCATAGAGGGG----------
----

TGTAATGACACTATCATACTTCCATGCAAGATAAAGCAAATTGTGAACATGTGGCAGGGAGCAGGACAAGCAA
TGTATGCTCCTCCCATCAGTGGAATAATTAATTGTGTATCAAAT---
ATTACAGGAATACTATTGACAAGAGATGGTGGTTATAATAATAATACT-------------------------
----

AAGGCTAACGAGACCTTCAGACCTGAAGGAGGAAATATAAAGGACAATTGGAGAAATGAATTATATAAATATA
AAGTAGTAGAAATTGAACCACTA---GGAATAGCACCCACCAGGGCAAAGAGAAGAGTGGTG-----------
----------------------------GAGAGACAAAAAAGAGCA---GTGGGA---ATAGGAGCT------
---ATGATCTTTGGGTTTTTGGGAGCA---GCAGGAAGCACTATG---GGCGCGGCGTCA---
ATAACGCTGACGGTACAG---GCCAGACAA---TTATTG---TCTGGTATAGTGCAACAGCAAAGC---
AATTTG---CTGAGG---GCTATAGAGGCGCAGCAGCATATGTTGCAACTCACAGTCTGGGGCATT
>KJ953089
TTCTATTGCAATACATCACAACTGTTTAATAGTACTTGGATAGGAAATGAAACCAAGGAAGAGTGT-------
----
-AATAAATTCAAGAAA---------
GAGTGTAATAGCACTATCATACTTCCATGCAAGATAAAGCAAATTATAAACATGTGGCAGGGAGTAGGACAAG
CAATGTATGCTCCTCCCATTAGAGGAAGAATTAATTGTGTATCAAAT---
ATCACAGGAATACTATTGACAAGAGATGGTGGTAGTATTAATAGGAAT-------------------------
----

TATGAGAATGAGACCTTCAGACCTGGAGGAGGAAATATAAAGGACAATTGGAGAAGTGAATTATATAAATATA
AAGTAGTAGAAATTGAACCACTA---GGAGTAGCACCCACCAAGGCAAAGAGGAGAGTGGTG-----------
----------------------------GAGAGAGAAAAAAGAGCA---GTGGGA---ATAGGAGCT------
---ATGATCTTTGGGTTCTTAGGAGCA---GCAGGAAGCACTATG---GGCGCGGCGTCA---
ATAACGCTGACGGTACAG---GCCAGACAA---TTATTG---TCTGGTATAGTGCAACAGCAAAGC---
AATTTG---CTGAAG---GCTATAGAGGCGCAACAGCATCTGTTGCAGCTCACAGTCTGGGGCATT
>KJ953475
TTCTATTGTAATACAACAAAATTGTTTAATAGTACTTGGACAGAAAATGAAACCGAGGAGGGG----------
----

AATAATAACACTATCATACTTCCATGCAAGATAAAGCAAATTATAAACATGTGGCAGGGAGTAGGACAAGCAA
TGTATGCTCCTCCCATCAGTGGAAAAATTAATTGTGTATCAAAT---
ATTACAGGAATACTATTGACAAGAGATGGTGGTAATAATGCGATGAATGAG----------------------
----

ACCTATGAGACCTTTAGACCTGGAGGAGGAAATATGAAGGACAATTGGAGAAGTGAATTATATAAATATAAAG
TGGTACAAATTGAACCACTA---GGAATAGCACCCACCAGGGCAAGGAGAAGAGTGGTG--------------
-------------------------GAGAGAGAAAAAAGAGCA---GTGGGA---ATAGGAGCT---------
ATGATCTTTGGGTTCTTAGGAGCA---GCAGGAAGCACTATG---GGCGCGGCGTCA---
ATAACGCTGACGGTACAG---GCCAGACAA---TTATTG---TCTGGTATAGTGCAACAGCAAAGC---
AATTTG---CTGAGG---GCTATAGAGGCGCAGCAGCATCTGTTGCAACTCACAGTCTGGGGCATT
>KU168264
TTCTATTGCAATACAACAGACCTGTTTAATAAAACTTACAGAGGAAATGAAACTGGGGAAGAGAGT-------
----

AATGGCACTAATATCATACTTCATTGCAGGATAAAGCAAATTATAAACATGTGGCAGGGAGTAGGACAAGCAA
TGTATGCTCCTCCCATCAGTGGAAGCATTAATTGTGCATCAAAT---
ATTACAGGAATACTATTGACAAGAGATGGTGGGTTGGGTAATACA----------------------------
----

AGTAACAATGAGACCTTCAGACCTGGAGGAGGAAATATAAAAGACAATTGGAGAAGTGAATTATATAAATATA
AAGTAGTACAAATTGAACCACTA---GGAATAGCACCCACCAAGGCAAAGAGAAGAGTGGTG-----------
----------------------------GACAGAGAAAAAAGAGCA---GTGGGA---ATAGGAGCT------
---ATGATCTTTGGGTTCTTAGGAGCA---GCAGGAAGCACTATG---GGCGCGGCGTCA---
ATAACGCTGACGGTACAG---GCCAGACAA---TTATTG---TCTGGTATAGTGCAACAGCAAAGC---
AATTTG---CTGAGA---GCTATAGAGGCGCAGCAGCATCTGCTGCAACTCACAGTCTGGGGCATT
>AY358059
TTCTATTGCGATACAACAAAACTGTTTAATAATACTATAAAA-------------------------------
----

AATGGAACCATAACGCTTCCATGCAAGATAAAGCAAGTTGTAAACATGTGGCAGGGAGCAGGACAGGCAATGT
ATGCTCCTCCCATCAAGGGAAGCATTAATTGTGTATCAAAT---
ATTACAGGAATACTATTGACAAGAGATGGTGGTAATCAAAACGCG----------------------------
----

ACTGAGATTTTCAGACCTGGAGGAGGAAATATAAGGGACAATTGGAGAAGTGAATTATATAAATATAAAGTGG
TACAAATTGAACCACTA---GGAATAGCACCCACCAGGGCAAAGAGAAGAGTGGTG-----------------
----------------------GAGAGAGAAAAAAGAGCA---GTGGGA---ATAGGAGCT---------
ATGATCTTTGGGTTCTTAGGAGCA---GCAGGAAGCACTATG---GGCGCGGCGTCA---
ATAACGCTGACGGTACAG---GCCAGACAA---TTATTG---TCTGGTATAGTGCAACAGCAAAGC---
AATTTG---CTGAGG---GCTATAGAGGCGCAGCAGCATCTGTTGCAACTCACAGTCTGGGGCATT
>AY358068
TTCTATTGCAATACAACAAAATTGTTTAATAGTACTTGGACAGAAAATGGAAACATAGGGGAGTATAATTACA
CGGGGTAT---
-------------------AAT---
TACACGGGTAATAATACCATACTTCCATGCAGGATAAAGCAAATTATAAACATGTGGCAGGGAGCAGGACAAG
CAATGTATGCTCCTCCCATCAGTGGAAACATTAGTTGTGTATCAAAT---
ATTACAGGAATACTATTGACAAGAGATGGTGGTAATACTACGGAT----------------------------
----

AACGAGACCTTCAGACCTGGAGGAGGAAATATAAAGGACAATTGGAGAAATGAATTATATAAATATAAAGTAG
TACAAATTGAACCACTA---GGAGTAGCACCCACCAGGGCAAAGAGAAGAGTGGTG-----------------
----------------------GAGAGAGAAAAAAGAGCA---GTGGGA---ATAGGAGCT---------
GTGATCTTTGGGTTCTTAGGAGCA---GCAGGAAGCACTATG---GGCGCGGCGTCA---
ATAACGCTGACGGTACAG---GCCAGACAA---TTATTG---TCTGGTATAGTGCAACAGCAAAGC---
AATTTG---CTGAGG---GCTATAGAGGCGCAGCAGCATCTGTTGCAACTCACAGTCTGGGGCATT
>DQ314732
TTCTATTGCAATACAACACAACTGTTTAATAGTACTTGGGTAGGAAATGAAACCAAAAAGGGGGATAATGGCA
CTATAAGAAAT--
-------GAAACCAAAAAG------
GGGGATAATGGCACTATCATACTTCCATGCAGGATAAAGCAAATTATAAACATGTGGCAGGAAACAGGACAAG
CAATGTATGCTCCTCCCATCAGTGGAACAATTAATTGTGTATCAAAT---
ATTACAGGACTACTATTGACAAGAGATGGTGGAGGTAATAATGACTCG-------------------------
----

AGTCAGAACGAGACCTTCAGGCCTGGAGGGGGAAATATAAAGGACAATTGGAGAAGTGAATTATATAAATATA
AAGTAGTAGAAATTGAACCACTA---GGAATAGCACCCACCAGGGCAAAGAGAAGAGTGGTG-----------
----------------------------GAGAGAGAAAAAAGAGCA---GTGGGA---ATAGGAGCT------
---ATGATCTTTGGGTTCTTAGGAGCG---GCAGGAAGCACTATG---GGCGCGGCGTCA---
ATAACGCTGACGGTACAG---GCCAGACAA---TTATTG---TCTGGTATAGTGCAACAGCAAAGC---
AATTTG---CTGAGG---GCTATAGAGGCGCAGCAGCATCTGTTGCAACTCACAGTCTGGGGCATT
>JX447561
TTCTATTGCAATACATCAAACCTGTTTGATAGAAATGAAACCACGAGTAAT----------------------
----

GGCACTGGCACTATCATACTTCCTTGCAAGATAAAGCAAATTGTACGCATGTGGCAGAGAACAGGACAAGCAA
TGTATGCTCCTCCCATCAGTGGAAGCATCAATTGTGTATCAAAT---
ATTACAGGAATTCTGTTGACAAGAGATGGTGGTAATGGTAATAATACT-------------------------
----

AATCAGACAGAGATCTTCAGACCTGAGGGAGGAAATATAAAGGACAATTGGAGAAGTGAATTATATAAATATA
AAGTGGTACAAATTGAACCACTA---GGAATAGCACCCACCAGGGCAAGAAGAAGAGTGGTG-----------
----------------------------CAGAGAGAAAAAAGAGCA---GTGGGA---ATAGGAGCT------
---ATGATCTTTGGGTTCTTAGGAGTA---GCAGGAAGCACTATG---GGCGCGGCGTCA---
ATAACGCTGACGGTACAG---GCCAGACAA---TTATTG---TCTGGTATAGTGCAACAGCAAAGC---
AATTTG---CTGAGG---GCTATAGAGGCGCAGCAGCATCTGTTGCAACTCACAGTCTGGGGCATC
>JX447855
TTCTATTGCAATACAACACAACTGTTTAATAGTACTTACGGAGAAAATGGAGAAAATGAAACCATGGGGGGG-
----

AATAATGGCACTATCATACTTCCATGTAAGATAAAGCAGATTATAAACATGTGGCAGGGAGTAGGACAAGCAA
TGTACGCTCCTCCCATCAGTGGAAGAATTAATTGTAGATCACAA---
ATTACAGGAATACTATTGATAAGAGATGGTGGTTATGATCGTGTT----------------------------
----

AACGAGACCTTCAGACCTGAAGGAGGAAATATGAAGGACAATTGGAGAAGTGAATTATATAAATATAAAGTAG
TACAAATTGAACCACTA---GGAGTAGCACCCACCAGGGCAAAGAGAAGAGTGGTG-----------------
----------------------GAGAGAGAAAAAAGAGCA---GTGGGA---ATAGGAGCC---------
ATGATCTTTGGGTTCTTAGGAGCA---GCAGGAAGCACTATG---GGCGCGGCGTCA---
ATAACGCTGACGGTACAG---GCCAGACAA---TTATTG---TCTGGTATAGTGCAACAGCAAAGC---
AATTTG---CTGAGG---GCTATAGAGGCGCAGCAGCATCTGTTGCAACTTACAGTCTGGGGCATT
>JX448022
TTCTATTGCAATACAACATCACTGTTTAATGATACTTGCATAGGAAATGAAACCAAGGAAGGG----------
----

TGTAATACAACTATCATACTTCCATGCAGGATAAAACAAATTGTAAACATGTGGCAGGGAGTAGGACAGGCAA
TGTATGCTCCTCCCATCAGTGGAAGAATTAATTGTGTATCAAAT---
ATTACAGGAATACTATTGACAAGAGATGGTGGTGTTAATAATGAT----------------------------
----

TCTAGTGAGATCTTCAGACCTGGAGGAGGGGATATAAGAGACAAATGGAGAAGTGAATTATATAAATATAAAG
TAGTACAAATTGAACCATTA---GGAGTAGCACCCACCAGGGCAAAGAGAAGAGTGGTG--------------
-------------------------GAGAGACCAAAAAGAGCA---GTGGGA---ATAGGAGCT---------
ATGATCTTTGGGTTCTTAGGAGCA---GCAGGAAGCACTATG---GGCGCGGCGTCA---
ATAACGCTGACGGTACAG---GCCAGACAA---TTATTG---TCTGGTATAGTGCAACAGCAAAGC---
AATTTG---CTGAGG---GCTATAGAGGCGCAACATCATCTGTTGCAACTCACAGTCTGGGGCATT
>EU743764
TTCTATTGCAATACAACACAACTGTTTAATCTTAATAATGCTACTGACAGA----------------------
----

GAAAATAAAAATATCACACTTCCATGCAGGATAAAGCAAATTATAAAAATGTGGCAGAGAGTAGGACAAGCAA
TGTATGCTCCTCCCATCAGGGGAAAAATTAATTGTGTCTCCAAT---
ATTACAGGAATACTATTAACAAGAGATGGTGGTAGTCACAATGATACT-------------------------
----

AATAATAATACTGAGACCTTTAGACCCGCAGGAGGAAATATAAAAGACAATTGGAGAAGTGAATTATATAAAT
ATAAAGTAGTAAGAATTGAACCACTG---GGAATAGCACCCACCAGGGCAAGGAGAAGAGTGGTG--------
-------------------------------GACAGACCGAAAAGAGCA---GTGGGA---ATAGGAGCT---
------ATGATCTTTGGGTTCTTAGGAGCA---GCAGGAAGCACTATG---GGCGCGGCGTCA---
CTAACGCTGACGGTACAG---GCCAGACAA---TTACTG---TCTGGTATAGTGCAACAGCAAAGC---
AATTTG---CTGAGG---GCTATAGAGGCGCAGCAGCATCTGTTGCAACTCACAGTCTGGGGCATT
>EU743781
TTCTATTGCGATACAACAAAACTGTTTAATAATAATTGCACACAAAATGCAACCGATCAGGATGAAAAAAAG-
----

TGTAATGGCACTCTCATACTTCCATGCAGGATAAAGCAAATTATAAACATGTGGCAGGGAGTAGGACAAGCAA
TATATGCTCCTCCCGTCCGTGGAAGTATTACTTGTGTATCAAAT---
ATTACAGGAATAATACTGACAAGAGATGGTGGTACTCATAAGAATGGTACT----------------------
----

GCTGATAATACAACCGAGATCTTCAGACCTACAGGAGGAAATATGAAGGACAATTGGAGAAGTGAATTATATA
AATATAAAGTAGTACAAATTGAACCACTA---GGAATAGCACCCACCAGGGCAAAGAGAAGAGTGGTG-----
----------------------------------GAGAGAGAAAAAAGAGCA---GTGGGA---
CTAGGAGCT---------ATGATCTTTGGGTTCTTAGGAGCA---GCTGGAAGCACTATG---
GGCGCGGCGGCA---GTAACGCTGACGGTACAG---GCCAGACAA---TTATTG---
TCTGGTATAGTGCAACAGCAAAGC---AATTTG---CTGAGA---
GCTATAGAGGCGCAACAACATCTGTTGCAACTCACAGTCTGGGGCATT
>JX446699
TTCTATTGCAATACATCAAGACTGTTTAATAGCACTTGCTTCAAAAATCATAACACCACTGAGGCCACTCAGG
AGTGTAAT---
----------GACACTATC------
ACAACAGATGGCAAGATCATACTTCACTGCAGGATAAAGCAAATTATAAACATGTGGCAGGGAGCAGGACAAG
CAATGTATGCTCCTCCCATCAGTGGAAGAATTAATTGTGTATCAAAC---
ATTACAGGAATACTATTGACAAGAGATGGTGGTGATACTGGTAAAAATTATACA------------AAT----

----

GGTACCAGGAAAGAGATCTTCAGACCTGGAGGAGGAAATATGAAGGACAATTGGAGAAGTGAATTATATAAAT
ATAAAGTAGTACAAATTGAACCACTA---GGAATAGCACCCACCAGGGCAAAGAGAAGAGTGGTG--------
-------------------------------GAGAGAGAAAAAAGAGCA---GTGGGA---ATAGGAGCT---
------ATGATCTTTGGGTTCTTAGGAGCA---GCCGGAAGCACTATG---GGCGCAGCGTCA---
ATAACGCTGACGGTACAA---GCCAGACAA---TTATTG---TCTGGTATAGTGCAACAGCAAAGC---
AATTTG---CTGAGG---GCTATAGAGGCGCAGCAGCATATGTTGCAACTCACAGTCTGGGGCATT
>JX446736
TTCTATTGCAATACAACAAAACTGTTTAATAATGCTTGCATAAAAAATGAAACCAATGGGAAC----------
----

TGTAATGGTACTATCTATCTTCCTTGCAAGATAAAGCAAATTATAAACATGTGGCAGGGAGCAGGACAAGCAA
TGTATGCTCCTCCCATCAGTGGAAGAATTAATTGTGAATCAAAT---
ATTACAGGAATACTATTGACAAGTGATGGGGGTAACATGACCGGA----------------------------
----

AACAACAAGACCTTCAGACCTGCAGGGGGAAATATGAAAGACAATTGGAGAAGTGAATTATATAAATATAAAG
TAGTGCAAATTGAACCACTA---GGAATAGCACCCACCAGGGCAAGGAGAAGAGTGGTG--------------
-------------------------GAGAGAGGAAAAAGAGCA---GTGGGA---ATAGGGGCT---------
ATGATCTTTGGGTTCTTAGGAGCA---GCAGGAAGCACTATG---GGCGCAGCGTCA---
ATAACGCTGACGGTACAG---GCCAGACAA---TTATTG---TCTGGTATAGTGCAACAGCAAAGC---
AATTTG---CTGAGA---GCTATAGAGGCGCAGCAGCATCTGTTGCAACTCACAGTCTGGGGCATT
>JX447312
TTCTATTGCAACACAACAAAACTGTTTAATTGGACAGAAGGTGAAACCATAGAGAAT----------------
----

AGCACGGAGAATATCATGCTGCCATGCAAGATAAAGCAAATTATAAACATGTGGCAGGAAACAGGACAAGCAA
TGTATGCTCCTCCCATCAGTGGAATGATTAATTGTGTATCAAAT---
ATTACAGGAATACTATTGACAAGAGATGGTGGTCATGATGGTAATAATACAAAT------------AAT----
----

ACAAATAATAATACCGAGACCTTCAGACCTGGAGGAGGAAATATAAAGGACAATTGGAGAAGTGAATTATATA
AATATAAAGTAGTACAAATTGAACCACTA---GGAATAGCACCCACCAGGGCAAAGAGAAGAGTGGTG-----
----------------------------------GAGAGAGAAAAAAGAGCA---GTGGGA---
ATAGGAGCT---------ATGATCTTTGGGTTCTTGGGAGCA---GCAGGAAGCACTATG---
GGCGCAGCGTCA---ATAACGCTGACGGTACAG---GCCAGACAA---TTATTG---
TCTGGTATAGTGCAACAGCAAAGC---AATTTG---CTGAGA---
GCTATAGAGGCGCAGCAGCATCTGTTGCAACTCACAGTCTGGGGCATT
>JX447646
TTCTATTGCAATACAACAAACCTGTTTAATAATACTTGCTTAAATGAAACCAAG-------------------
----

TGTAATGGCACTGTCACACTTCCATGCAGGATAAAGCAAATTATAAACATGTGGCAGGGAGTAGGACAAGCAA
TGTATGCTCCTCCCATCAGGGGAAATATTACTTGTGTATCAAAT---
ATTACAGGAATACTATTGACAAGAGATGGTGGTAATGGGGGGAAC----------------------------
----

GAGACCTTCAGACCTGAAGGAGGAAATATGAAGGACAATTGGAGAAATGAATTATATAAATATAAAGTAGTAC
AAATTGAACCACTA---GGATTAGCACCCACCAAGGCAAAAAGAAGAGTGGTG--------------------
-------------------GAGAGAAAAAAAAGAGCA---GTGGGA---ATAGGAGCT---------
ATGATCTTTGGGTTCTTAGGAGCA---GCAGGAAGCACTATG---GGCGCGGCGTCA---
ATAACGCTGACGGTACAG---GCCAGACAA---TTATTG---TCTGGTATAGTGCAACAGCAAAGC---
AATTTG---CTGAGG---GCTATAGAAGCGCAGCATCATCTGTTGCAACTCACAGTCTGGGGCATT
>JN944659
TTCTATTGTAATACAACAAAACTGTTTAATAGTACTTGGAGAGAAAATGAAACCATAGAGGAGCATAATGGC-
----

ACTAATGGCACTATCATACTTCCATGTAGGATAAAGCAAATTATAAACATGTGGCAGGGAGTAGGACAAGCAA
TGTATGCTCCTCCCATCAGTGGAAAAATTAATTGTATATCAAAT---

ATTACAGGAATACTATTGACAAGAGATGGAGGTCATAATACGAGT---------------------------
----

AACGAGACCTTCAGACCTGGAGGAGGAAATATAAAGGACAATTGGAGAAGTGAATTATATAAATATAAAGTAG
TACAAATTGAACCACTA---GGAATAGCACCCACCAGGGCAAAGAGAAGAGTGGTG-----------------
----------------------GAGAGAGAAAAAAGAGCA---GTGGGA---ATAGGAGCT---------
ATGATCTTTGGGTTCTTAGGAGCA---GCAGGAAGCACTATG---GGCGCGGCGTCA---
ATAACGCTGACGGTACAG---GCCAGACAA---TTACTG---TCTGGTATAGTGCAACAGCAAAGC---
AATTTG---CTGAGG---GCTATAGAGGCGCAACAGCATCTGTTGCAACTCACAGTCTGGGGCATT
>HQ691090
TTCTATTGCAATACAACAAAACTGTTTAATAATACCTGCATAAATGAAACCATGAGGGGAAGCAATGAAACCA
GG---

GAGTGTAATAACACTATTACACTTCCATGCAGGATAAAGCAAATTATAAACATGTGGCAGGGAGTAGGACAAG
CAATGTATGCTCCTCCCATCAGTGGAATAATTAGTTGTGTATCAAAT---
ATTACAGGAATACTATTGACAAGAGATGGTGGTAATAATACTGGG----------------------------
----

AATGACGAGATTTTCAGACCTGCAGGAGGAAATATAAGGGACAATTGGAGAAATGAATTATATAAGTATAAAG
TAGTACAAATTGAACCACTA---GGAGTAGCACCCACCAAGGCAAAGAGAAGAGTGGTG--------------
-------------------------GAGAGACAAAAAAGAGCA---GTGGGA---ATAGGAGCT---------
ATGATCTTTGGGTTCTTAGGAGCA---GCAGGAAGCACTATG---GGCGCGGCGTCA---
ATAACGCTGACGGTACAG---GCCAGACAA---CTATTG---TCTGGTATAGTGCAACAGCAGAGC---
AATCTG---CTGAGG---GCTATAGAGGCGCAACATCATCTGTTGCAACTCACAGTCTGGGGCATT
>JX446728
TTCTATTGCAATACAACAAAATTGTTTCATAGTAATTGGACAACAACAAATGGCACTATAGAAAGGTACAAT-
----

GACAATGAAACTATCATACTTCCATGCAAGATAAAGCAAATTATAAATATGTGGCAGGGAGTAGGACAAGCAA
TGTATGCTCCTCCCATCAGAGGAATAATTAATTGTACATCAAAT---
ATTACAGGAATATTATTGACAAGAGATGGTGGTCTTCATAATGGGACT-------------------------
----

AACGATACAGAGACATTCAGGCCTGAAGGAGGAAATATAAAGGACAATTGGAGAAGTGAATTATATAAATATA
AAGTAGTACAGATTGAACCATTA---GGAGTAGCACCTACCAGGGCAAGGAGAAGAGTGGTG-----------
----------------------------GAGAGAGAAAAAAGAGCA---GTGGGA---ATAGGAGCT------
---ATGATCTTTGGGTTCCTAGGAGCA---GCAGGAAGCACTATG---GGCGCGGCGTCA---
ATAACGCTGACGGTACAG---GCCAGACAA---TTATTG---TCTGGTATAGTGCAACAGCAAAGC---
AATTTG---CTGAGA---GCTATAGAGGCGCAGCAGCATCTGTTGCAACTCACAGTCTGGGGCATT
>JX447013
TTCTATTGCAATACAACAAAACTATTTAATGGTAATGGGACA-------------------------------
----

AATGAAACCATCTATCTTCCATGCAGGATAAAGCAAATTATAAATATGTGGCAGGAAGTAGGACAAGCAATGT
ATGCTCCTCCCATCAGAGGATGCATTAATTGTGTATCAAAT---
ATTACAGGGATACTATTGACAAGAGATGGTGGTGGTGGTAATAAT----------------------------
----

AGTAACAATGAGACCTTCAGACCTGGAGGAGGAAATATAAAGGACAATTGGAGAAGTGAATTATATAAATATA
AAGTAGTACAAATTGAGCCACTA---GGACTAGCTCCCACCAGGGCAAAGAGAAGAGTGGTG-----------
----------------------------GATAGAGAAAAAAGAGCA---GTGGGA---ATAGGAGCT------
---ATGATCTTTGGGTTCTTAGGAGCA---GCAGGAAGCACTATG---GGCGCGGCGTCA---
ATAACGCTGACGGTACAG---GCCAGACAA---TTATTG---TCCGGTATAGTGCAACAGCAAAGC---
AATTTG---CTGAGG---GCTATAGAGGCGCAACAGCATATGTTGCAACTCACAGTCTGGGGCATT
>JX447057
TTCTATTGCAATACAACACGACTGTTTAATCATTCTCACAATGAAACCTGGAAGGAGAAG-------------
----

TATAATGGCACTATCATACTTCCATGCAAGATAAAGCAAATTATAAACATGTGGCAGGGAGTAGGACAAGCAA
TGTATGCTCCTCCCATCAGTGGAAAGATTGAGTGTGTATCAAAT---

ATTACAGGAATACTATTGACAAGAGATGGTGGTGTTAATCAAACT----------------------------
----

AATGGGACCGAGATCTTCAAACCTGGAGGAGGAAATATAAAGGACAATTGGAGAAGTGAATTATATAAATACA
AAGTAGTACAAATTGAACCACTA---GGATTGGCACCTACCAAGGCAAAGAGAAGAGTGGTG-----------
----------------------------GAGAGAGAAAAAAGAGCA---GTGGGA---ATAGGAGCT------
---ATGATCTTTGGGTTCTTAGGAGCA---GCAGGAAGCACTATG---GGCGCGGCGTCA---
ATAACGCTGACGGTACAG---GCCAGACAA---TTATTG---TCTGGTATAGTGCAACAGCAAAGA---
AATTTG---CTGAGG---GCTATAGAGGCGCAGCAGCATCTGTTGCAACTCACAGTCTGGGGCATT
>JX447480
TTCTATTGCAACACAACAAAACTGTTTAATAGTACTTGGAGAGGAAATGAAACCATAGCGGGCTATGAT----
----

AATAACAGCAATATCATACTTCCATGCAGGATAAAGCAAATTATAAACATGTGGCAGGAAGTAGGACAAGCAA
TGTATACTTCTCCCGTCAGTGGAAGAATTAATTGTATCTCAAAT---
ATCACAGGAATACTATTAACAAGAGATGGTGGTGGTAATCATAGC----------------------------
----

AATGAGACCTTTAGACCTGGAGGAGGAAATATGAAGGACAATTGGAGAAGTGAACTATATAAATATAAAGTAG
TACAACTTGAACCACTA---GGAATAGCACCCACCAAGGCAAAGAGAAGAGTGGTG-----------------
----------------------GAGAGAGAAAAAAGAGCA---GTGGGA---ATAGGAGCT---------
ATGATCTTTGGGTTCTTAGGAGCA---GCCGGAAGCACTATG---GGCGCAGCGTCA---
ATAACGCTGACGGTACAG---GCCAGACAA---TTATTG---TCTGGTATAGTGCAACAGCAAAGC---
AATTTG---CTGAGG---GCTATAGAGGCGCAGCATCGTCTGTTGCAACTCACAGTCTGGGGCATT
>HQ691006
TTCTATTGCAATACAACACAACTGTTTAATAATAATTGTACAAATGAAACCATGAATGGG-------------
----

TGTGATGGCAATATCACACTTCCATGCAAGATAAAGCAAATTGTAAACATGTGGCAGGGAGTGGGACAAGCAA
TGTATGCTCCTCCCATCAGAGGACAAATTAGTTGTGTGTCAAAT---
ATTACAGGGATACTACTGACAAGAGATGGTGGTCATAATGGTAGT----------------------------
----

ACAAATAATGAGACCTTCAGACCTGGAGGAGGAAATATAAAGGACAATTGGAGAAGTGAATTATATAAATATA
AAGTAGTAGAAATTGAACCACTA---GGGATAGCACCTTCCTGGGCAAGGAGAAGAGTGGTG-----------
----------------------------GAGAGACAAAAAAGAGCA---GTGGGA---ATAGGAGCT------
---ATGATCTTTGGGTTCTTAGGAGCA---GCAGGAAGCACTATG---GGCGCGGCGTCA---
GTAACGCTGACGGTACAG---GCCAGACAA---TTATTG---TCTGGTATAGTGCAACAGCAAAGC---
AATTTG---CTGAGG---GCTATAGAGGCGCAGCAGCATCTGTTGCAACTCACAGTCTGGGGCATT
>GQ916573
TTCTATTGCAATACAACAACACTGTTTAATAATACTTGCATAAAAAATGAAACCGCGGAGGGG----------
----

TGTAATAAAACTATCACACTTCAATGCAGGATAAAGCAAATTATAAATATGTGGCAGGGAGTAGGACAAGCAA
TGTATGCTCCTCCCATCAGTGGAAGCATCAGTTGTGAATCTAAT---
ATTACAGGATTACTACTGACAAGAGATGGTGGAAGCAATATTAATACT-------------------------
----

ATTAATAAGAATGAGATCTTCAGACCTGGAGGAGGAAATATGAAGGATAATTGGAGAAATGAATTATATAAAT
ATAAAGTAGTAAAAATTGAACCACTA---GGAATAGCACCCACCAGGGCAAAGAGAAGAGTGGTG--------
-------------------------------GAGAGAGAAAAAAGAGCA---GTGGGA---ATAGGAGCT---
------TTGATCTTTGGGTTCTTAGGAGCA---GCAGGAAGCACTATG---GGCGCGGCGTCA---
ATAACGCTGACGGTACAG---GCCAGACAA---TTATTG---TCTGGTATAGTGCAACAGCAAAGC---
AATTTG---CTGAGG---GCTATAGAGGCGCAGCAGCATATGTTGCAACTCACAGTCTGGGGCATT
>JX447074
TTCTATTGCAACATAACAACACTGTTCAATACTACCTACTTA-------------------------------
----
-------------------AAT---
CAAACCACAGGAAAGTATACACTTCCATGTAAGATAAAGCAAATTATAAACATGTGGCAGGGAGTAGGGCAAG
CAATGTATGCTCCTCCCATCAGGGGAACAATTAATTGTATATCAAAT---

ATTACAGGAATATTATTGACAAGAGATGGTGGTATGAATATAAAT----------------------------
----

AGCACTAACGAGACCTTCAGACCTGGAGGAGGAAATATAAAGGACAATTGGAGAAATGAATTATATAAATATA
AAGTAGTACAAATTGAACCACTA---GGAGTAGCACCCACCAAGGCAAAGAGAAGAGTGGTG-----------
----------------------------GAGAGAGAAAAAAGAGCA---GTGGGA---ATAGGAGCT------
---CTGATCTTTGGGTTCTTAGGAGCA---GCAGGAAGCACTATG---GGCGCGGCGTCA---
ATAACGCTGACGGTACAG---GCCAGACAA---TTATTG---TCTGGTATAGTGCAACAGCAAAGC---
AATTTG---CTGAGG---GCTATAGAGGCGCAACAGCATATGTTGCAACTCACAGTCTGGGGCATT
>JX447303
TTCTATTGCAATACATCACAACTGTTTAATTACACAACAGATGCAGACATGAGGAACAAGACAGCCATA----
----

AAAAATAGCACTATCATACTTCCATGCAGGATAAAGCAAATTATAAACATGTGGCAGGGAGTAGGACAAGCAA
TGTATGCTCCTCCCATCAGTGGAATAATTAATTGTGTATCAAAT---
ATTACAGGAATATTATTGACAAGAGATGGTGGTGAGCAGAAGAATGAGAGT----------------------
----

AGGCGAAATGAGACCTTCAGACCTGAAGGAGGAAATATGAAGGACAATTGGAGAAGTGAATTATATAAATATA
AAGTAGTACAAATTGAACCACTA---GGAGTAGCACCCACTAAGGCAAGGAGAAGAGTGGTG-----------
----------------------------GAGAAACAAAAAAGAGCA---GTGGGA---ATAGGAGCT------
---ATGATCTTTGGGTTCTTAGGAGCA---GCAGGAAGCACTATG---GGCGCGGCGTCA---
ATAACGCTGACGGTACAG---GCCAGACAA---TTATTG---TCTGGTATAGTGCAACAGCAAAGC---
AATTTG---CTGAGA---GCTATAGAGGCGCAACATCATATGTTGCAACTCACAGTCTGGGGCATT
>JX447335
TTCTATTGCAATACATCACAACTGTTTAATAGTACTGTTAATGGCACTATCACACTTCCTAAT----------
----

GGCACACTTCCATGCAAGATAAAGCAAATTATAAAAATGTGGCAAGGGGTAGGACAAGCAATGTATGCTCCTC
CCATCAATGGAAGCATTAATTGTGTATCAAAC---
ATTACGGGACTACTATTAACAAGGGATGGTGGTAATCAGGGTAATCAT-------------------------
----

ACTAATGATACTGAGACCTTCAGACCTGGAGGAGGAAACATAAAGGACAATTGGAGAAGTGAATTATATAAAT
ATAAGGTGGTACAAATTGAACCACTA---GGAATAGCACCCACCAGGGCAAAGAGAAGAGTGGTG--------
-------------------------------GAAAGAGAAAAAAGAGCA---GTGGGA---ATAGGAGCT---
------ATGATCTTTGGGTTCTTAGGAGCA---GCAGGAAGCACTATG---GGCGCGGCGTCA---
ATAACGCTGACGGTACAG---GCCAGACAA---TTATTG---TCTGGTATAGTGCAACAGCAAAGC---
AATTTG---CTGAGG---GCTATAGAGGCGCAGCAGCATCTGTTGCAACTCACAGTCTGGGGCATT
>JN388161
TTCTATTGCAATACAACACAACTGTTTAATAGTACTTGCATGGGAGATATGCCCAAGGATGGGTGTAATGGCA
CTTTC--

AATGGCACCGGCACTATCATACTTCCATGCAGAATAAAGCAATTTATAAACATGTGGCAGGGAGTAGGACAAG
CAATGTATGCTCCTCCCATCAAGGGAAACATTAGTTGTGTATCAAAT---
ATTACAGGAATACTATTAACAAGAGATGGTGGTAATAGGACTCATAAC-------------------------
----

GGGACTAAGGAAGAGATCTTCAGACCTGCAGGAGGAAATATGAAGGACAATTGGAGAAGTGAATTATATAAAT
ATAAAGTAGTACAAATTGAACCACTA---GGAGTAGCACCTACCAAGGCAAAGAGAAGAGTGGTG--------
-------------------------------GATAGACAAAAAAGAGCA---GTGGGA---ATAGGAGCT---
------ATGATCTTTGGGTTCTTGGGAGCA---GCAGGAAGCACTATG---GGCGCAGCGTCA---
ATAACGCTGACGGTACAG---GCCAGACAA---TTATTG---TCTGGTATAGTGCAACAGCAAAGC---
AATTTG---CTGAGA---GCTATAGAGGCGCAACAACATCTGTTGCAACTCACAGTCTGGGGCATT
>GQ916591
TTCTATTGCAATACAACAAAACTGTTTAATGAGACTTACCTGAATGGGTCTACTTACCTGAATGAG-------
----

TCTACTGGCTATATCATACTTCCATGCAAGATAAAGCAAATTATAAACATGTGGCAGGGAGCAGGACAAGCAA
TGTATGCCCCTCCCATCAGTGGAAAAATTAATTGTGTATCAAAT---

ATTACAGGAATACTATTGACAAGAGATGGTGGGACTAATAATACA---------------------------
----

AACGAGACCTTCAGACCTGGAGGAGGAAATATGAAGGACAATTGGAGGAGTGAATTATATAAATATAAAGTAG
TACAAATTGAACCACTA---GGAGTAGCACCCACCAAGGCAAGGAGAAGAGTGGTG-----------------
----------------------GAGAGAGAAAAAAGAGCA---GTGGGA---ATAGGAGCT---------
ATGATCTTTGGTTTCTTAGGAGCA---GCAGGAAGCACTATG---GGCGCGGCGTCA---
ATAACGCTGACGGTACAG---GCCAGACAA---TTATTA---TCTGGTATAGTGCAACAGCAAAGC---
AATTTG---CTGAGG---GCTATAGAGGCGCAGCAGCATCTGTTGCAACTCACAGTCTGGGGCATT
>GQ916581
TTCTATTGCAATACAACAAAACTGTTTAATAGTACCTGGGCCAAGGAGGGGTGGAATGGCACTAATTATAATG
ACACTTATTAT--
----------AATGGCACT------
AATTATACTGGCAATATCACACTTCCATGCAGGATAAAGCAAATTGTAAACATGTGGCAGGGAATAGGACAAG
CAATGTATGCTCCTCCCATCAGTGGAACGATTCAGTGTGTATCAGAA---
ATTACAGGAATACTATTGACAAGAGATGGTGATATTACTAATGCG---------------------------
----

GCTAACGAGACTTTCAGACCTGGAGGAGGAAATATAAAGGACAATTGGAGAAGTGAATTATATAAATATAAAG
TAGTGCAAATTGAACCACTA---GGAATAGCACCCACCAGGGCAAAGAGAAGAGTGGTG--------------
-------------------------GAGAGAGAAAAAAGAGCA---GTGGGA---ATAGGAGCG--------
ATGATCTTTGGGTTCTTAGGAGCA---GCAGGAAGCACTATG---GGCGCGGCGTCA---
ATAACGCTGACGGTACAG---GCCAGGCAA---TTATTG---TCTGGTATAGTGCAACAGCAAAGC---
AATTTG---CTGAGG---GCTATAGAGGCGCAGCAGCATCTGTTGCAACTCACAGTCTGGGGCATT
>JF680932
TTCTATTGCAATACAACAAAGATGTTTAATGCGACAGAAAATGGAACCAGGGAAGGG---------------
----

AATAACAATACGATCATAATTCCATGCAGGATAAAGCAAATTGTAAACATGTGGCAGAAAACAGGACAAGCAA
TGTATGCTCCTCCCATCAGGGGAATAATTAATTGTGTATCAAAT---
ATTACAGGAATATTATTAACAAGAGATGGTGGAAATGAGGATAATCAT------------------------
----

AGTAAGGACAACGAGACCTTCAGACCTGCAGGAGGAAATATAAAGGACAATTGGAGAAGTGAATTATATAAAT
ATAAAGTAGTACAAATTGAACCACTA---GGAATAGCACCCACCAAGGCAAAGAGAAGAGTGGTG--------
-------------------------------GAGAGAGAAAAAAGAGCA---GTGGGA---ATAGGAGCT---
------ATGATCTTTGGGTTCTTAGGAGCA---GCAGGAAGCACTATG---GGCGCGGCGTCA---
ATAACGCTGACGGTACAG---GCCAGACAA---TTATTG---TCTGGTATAGTGCAACAGCAAAGC---
AATTTG---CTGAGG---GCTATAGAGGCGCAACAGCATATGTTGCAACTCACAGTCTGGGGCATT
>KT185951
TTCTATTGCAATACAACAAAACTGTTTAATGGTACTTACCCGAAAAATGGAACAGATGGAACA---------
----

AATAATGACACTATCATACTCCCATGTAGGATAAAGCAAATTGTAAGATTGTGGCAGGAAGTAGGTCAAGCAA
TGTATGCTCCTCCCATCAGTGGAAGCATTGAGTGCTACTCAAAT---
ATTACAGGAATACTATTGACAAGAGATGGTGGTAATACTAGCAATAGT------------------------
----
GATGGTGGTAATATTAGC---
AATAGTGAAACCTTCAGACCTGGAGGAGGAAATATAAAGGACAATTGGAGAAGTGAATTATATAAATATAAAG
TAGTACAAATTGAACCACTA---GGAATAGCACCCACCAAGGCAAAAAGAAGAGTGGTG--------------
-------------------------GAGAGAGAAAAAAGAGCA---GTAGGA---ATAGGAGCT---------
ATGATCTTTGGGTTCTTAGGAGCA---GCAGGAAGCACTATG---GGCGCGGCATCA---
ATAACGCTGACGGTACAG---GCCAGACAA---TTATTG---TCTGGTATAGTGCAACAGCAAAGC---
AATTTG---CTGAGG---GCTATAGAGGCGCAGCAGCATCTGTTGCAACTCACAGTCTGGGGCATT
>KT185973
TTCTATTGCAATACATCAAAACTGTTCAACAATACTTGCAGAGAAGATAGCACGAAGGAGGGGTGTAATGAT-
----

ACCATACTGCCATGCAAAATAAAGCAAATTATAAGGATGTGGCAGGGAGTAGGACAAGCAATGTATGCTCCTC
CCATCAGTGGGAGTATCAACTGTATATCAAAT---

ATTACAGGAATACTATTGACAAGAGATGGTGGTTACAACAATAAGAGTAATGGG-------------------
----

AACGAGACTGAGACTTTCAGACCTGGAGGAGGAAATATGAAGGACAATTGGAGAAATGAATTATATAAATATA
AAGTAGTACAAATTGAACCACTA---GGAATAGCACCCACCAAGGCAAAGAGAAGAGTGGTG-----------
----------------------------GAGAGAGAAAAAAGAGCA---GTGGGA---ATAGGAGCT------
---ATGATCTTTGGGTTCTTAGGAGCA---GCAGGAAGCACTATG---GGCGCGGCGTCA---
ATAACGCTGACGGTACAG---GCCAGACAA---TTATTG---TCTGGTATAGTGCAACAGCAAAGC---
AATTTG---CTGAGG---GCTATAGAGGCGCAACAGCATCTGTTGCAACTCACAGTCTGGGGCATT
>KT185837
TTCTATTGCAATACAACAAAACTGTTTAATAGTACAGGGAAC-------------------------------
----

TATAATGACACTATCATACTTCCATGCAGGATAAAGCAAATTATAAACATGTGGCAGAGAGTAGGACAAGCAA
TGTATGCTCCTCCCATCAGGGGCAACATTACTTGTGTATCAAAT---
ATTACAGGAATATTATTGACAAGAGATGGTGGTGCGAATCAGACTAATGAG----------------------
----

ACCGATGAGACCTTCAGACCGGGAGGAGGAGATATAAAGGACAATTGGAGAAATGAATTATATAAATATAAAG
TAGTACAAATTGATCCATTA---GGAATAGCACCCACCAAGGCAAAGAGAAGAGTGGTG--------------
-------------------------GAGAGAGAAAAAAGAGCA---GTGGGA---ATAGGAGCT---------
ATGATCTTTGGGTTCTTAGGAGCA---GCAGGAAGCACTATG---GGCGCGGCGTCG---
ATAACGCTGACGGTACAG---GCCAGACAA---TTATTG---TCTGGGATAGTGCAACAGCAAAGT---
AATTTG---CTGAGG---GCTATAGAGGTGCAGCAGCATATGTTGCAACTCACAGTCTGGGGCATT
>MG989500
TTCTATTGCAATACAACAAAACTGTTTAATAGGACTTACCCAGGAAATGATACCACTGAGGGG----------
----

CAGAATGGCACTATCATACTCCCATGCAGAATAAAGCAAATTATAAACATGTGGCAGGAAGTAGGACAAGCAA
TGTATGCTCCTCCCATCAGAGGACCGATTAACTGCTCATCAATT---
ATTACAGGAATACTATTGACAAGGGATGGTGGTAGTAGTACTCAAAACGAG----------------------
----

ACTCAAAACGGGACCGAGATCTTCAGACCTGGAGGAGGAAATATAAAGGACAATTGGAGAAGTGAATTATATA
AATATAAAGTAGTACAAATTGAACCACTA---GGAATAGCACCCACCAGGGCAAAGAGAAGAGTGGTG-----
----------------------------------GAGAGGGAAAAAAGAGCA---GTGGGA---
ATAGGAGCT---------ATGATCTTTGGGTTCTTGGGAGCA---GCAGGAAGCACTATG---
GGCGCGGCGTCA---ATAACGCTGACGGTACAG---GCCAGACAG---TTACTG---
TCTGGTATAGTGCAACAGCAAAGC---AATTTG---CTGAGA---
GCTATAGAGGCGCAGCAGCATATGCTGCAACTCACAGTCTGGGGCATA
>MG989502
TTCTATTGCAATACAACAAAACTGTTTAATGGTACTTACCCGAAAAATGGAACAGATGGAACA----------
----

AATAATGACACTATCATACTCCCATGTAGGATAAAGCAAATTGTAAGATTGTGGCAGGAAGTAGGTCAAGCAA
TGTATGCTCCTCCCATCAGTGGAAGCATTGAGTGCTACTCAAAT---
ATTACAGGAATACTATTGACAAGAGATGGTGGTAATACTAGCAATAGTGATGGT-------------------
----
-
GGTAATATTAGCAATAGTGAAACCTTCAGACCTGGAGGAGGAAATATAAAGGACAATTGGAGAAGTGAATTAT
ATAAATATAAAGTAGTACAAATTGAACCACTA---GGAATAGCACCCACCAAGGCAAAAAGAAGAGTGGTG--
-------------------------------------GAGAGAGAAAAAAGAGCA---GTAGGA---
ATAGGAGCT---------ATGATCTTTGGGTTCTTAGGAGCA---GCAGGAAGCACTATG---
GGCGCGGCATCA---ATAACGCTGACGGTACAG---GCCAGACAA---TTATTG---
TCTGGTATAGTGCAACAGCAAAGC---AATTTG---CTGAGG---
GCTATAGAGGCGCAGCAGCATCTGTTGCAACTCACAGTCTGGGGCATT
>MG989521
TTCTATTGCAATACATCAAAACTGTTCAACAATACTTGCAGAGAAGATAGCACGAAGGAGGGGTGTAATGAT-
----

ACCATACTGCCATGCAAAATAAAGCAAATTATAAGGATGTGGCAGGGAGTAGGACAAGCAATGTATGCTCCTC
CCATCAGTGGGAGTATCAACTGTATATCAAAT---
ATTACAGGAATACTATTGACAAGAGATGGTGGTTACAACAATAAGAGTAATGGG-------------------
----

AACGAGACTGAGACTTTCAGACCTGGAGGAGGAAATATGAAGGACAATTGGAGAAATGAATTATATAAATATA
AAGTAGTACAAATTGAACCACTA---GGAATAGCACCCACCAAGGCAAAGAGAAGAGTGGTG-----------
----------------------------GAGAGAGAAAAAAGAGCA---GTGGGA---ATAGGAGCT------
---ATGATCTTTGGGTTCTTAGGAGCA---GCAGGAAGCACTATG---GGCGCGGCGTCA---
ATAACGCTGACGGTACAG---GCCAGACAA---TTATTG---TCTGGTATAGTGCAACAGCAAAGC---
AATTTG---CTGAGG---GCTATAGAGGCGCAACAGCATCTGTTGCAACTCACAGTCTGGGGCATT
>KU230418
TTCTATTGCAATACAACAAGCTTGTTTAATAAAACTTACATAGGAAATGAAACCAGAAATGAAACCCATGATG
GG---

GGGAGTAATAAAACTATTACACTTCCATGCAAGATAAAGCAAATTGTAAGGATGTGGCAGGGAGTAGGACAAG
CAATGTATGCTCCTCCCATCAGTGGAGTAATTAACTGTGTATCAAAT---
ATTACAGGAATACTATTAACAAGAGATGGTGGTCAGGGTGGTCATAATAAT----------------------
----

ACAACTGAGACCGAGACCTTCAGACCTGGAGGAGGAAATATAAAGGATAATTGGAGAAGTGAATTATATAAAT
ATAAAGTAGTACAAATTGAACCACTA---GGAATAGCACCCACCAGGGCAAAGAGAAGAGTGGTG--------
-------------------------------GAGAGAGAAAAAAGAGCA---GTGGGA---ATAGGAGCT---
------ATGATCTTTGGGTTCTTAGGAGCA---GCAGGAAGCACTATG---GGCGCAGCGTCA---
ATAACGCTGACGGTACAG---GCCAGACAA---TTATTG---TCTGGTATAGTGCAGCAGCAAAGC---
AATTTG---CTGAGG---GCTATAGAGGCGCAACAGCATCTGTTGCAACTCACAGTCTGGGGCATT
>MG989579
TTCTATTGCAATACAACAAAATTGTTTAATGGAAACCATAGCAAGAGT-------------------------
----

GGTAATGACAGCATCGTACTTCCATGTAAGATAAAGCAGATTATAAACCTGTGGCAGGGAGTAGGACAAGCAA
TGTATGCTCCTCCCATCAGGGGGATAATTAACTGTGTATCAAAT---
ATTACAGGAATACTATTGACAAGAGATGGTGGAGCTCACAATGGGAGT-------------------------
----

AACGAGACTGAAATCTTTAGACCTGGAGGAGGAAATATGAAGGACAATTGGAGAAGTGAACTATATAAATATA
AAGTAGTACAAATTGAACCTCTA---GGAATAGCACCCACCAGGGCAAAGAGAAGAGTGGTG-----------
----------------------------GAGAAAGAAAAAAGAGCA---GTGGGA---ATAGGAGCT------
---ATGATCTTTGGGTTCTTAGGAGCA---GCAGGAAGCACTATG---GGCGCGGCGTCA---
CTAACGCTGACGGTACAG---GCCAGACAA---TTATTA---TCTGGTATAGTGCAACAGCAAAGC---
AATTTG---CTGAGG---GCTATAGAGGCGCAGCAGCATCTGTTGCAACTCACAGTCTGGGGCATT
>MG989561
TTCTATTGCAATACAACAAAACTATTTAATAATACTTGCATAAATGAAACCACGGAGGGG-------------
----

TGTAATAAAACTATCTATCTTCCATGCAGAATAAAACAAATTATAAACATGTGGCAGGGAGCAGGACAAGCAA
TGTATGCTCCTCCCATCAGGGGAAGGATTACTTGTGTATCAAAT---
ATTACAGGAATACTATTGACAAGAGATGGTGGTGTTGATAATGCTACG-------------------------
----

AACGGGACCGAGATCTTCAGACCCGGAGGAGGAAATATAAAGGACAATTGGAGAAGTGAATTGTTTAAATATA
AAGTAGTACAAATTGAACCACTA---GGACTAGCACCCACCAAGGCAAAGAGAAGAGTGGTG-----------
----------------------------GAGAGAGAAAAAAGAGCA---GTGGGA---ATAGGAGCT------
---ATGATCTTTGGGTTCTTAGGAGCA---GCAGGAAGCACTATG---GGCGCGGCGTCA---
ATAACGCTGACGGTACAG---GCCAGACAA---TTACTG---TCTGGTATAGTGCAACAGCAAAGC---
AATTTG---CTGAGG---GCTATAGAGGCGCAGCAGCATCTGTTGCAACTCACGGTCTGGGGCATT
>MF957493
TTCTATTGCAATACAACAAAATTATTTAATGGTACAGAAAATGGAACCATGGAGGGGAATACTGAC-------
----

ACTAATGGCACTATCATCCTTCCATGCAGGATAAAGCAAATTGTAAGAATGTGGCAGGGAGTAGGACAAGCAA
TATATGCTCCTCCCATCAGTGGAAACATTAGTTGTGAATCAAAC---
ATTACAGGAATACTATTGACAAGAGATGGTGGTAGTGGTAGTTATACTAATGAT------------------
----
-
AGTGATGCTAATAATACAGAAATCTTCAGACCTGAGGGAGGAAATATAAAGGACAATTGGAGAAGTGAATTAT
ACAAATATAAAGTAGTACAAATTGAACCACTA---GGAGTAGCACCCACCAAGGCAAGGAGAAGAGTGGTG--
------------------------------------GAGAGAGAAAAAAGAGCA---GTGGGA---
ATAGGAGCT---------ATGATCTTTGGGTTCTTAGGAGCA---GCAGGAAGCACTATG---
GGCGCGGCGTCA---ATAACGCTGACGGTACAG---GCCAGACAA---TTATTG---
TCTGGTATAGTGCAACAGCAAAGC---AATTTG---CTGAGG---
GCTATAGAGGCGCAGCAGCATCTGTTGCAACTCACAGTCTGGGGCATT
>MG989524
TTCTATTGCAATACAACACAACTTTTTAATAGTACTAGTACTAACAAC------------------------
----

TCAGAAGGGAATATCATAATTCCATGCAGGATAAAGCAAATTATAAACATGTGGCAGGGAGTAGGGCAAGTAA
TGTATGCTCCTCCTGTCCAAGGGCGAATTAATTGTGCATCAAAT---
ATTACAGGAATACTATTAACAAGAGATGGTGGTCATAACGGGACATCC------------------------
----

CAGAACGAGACATTTCGACCTGGAGGAGGAAATATAAAGGACAATTGGAGGAGTGAATTATATAAATATAAAG
TAGTACAAATTGAACCACTA---GGAATAGCACCCACCAGGGCAAAGAGAAGAGTGGTA-------------
-------------------------AAGAGAGAAAAAAAGAGCA---GTGGGC---ATAGGAGCT---------
ATGATCTTTGGGTTCTTAGGAGCA---GCAGGAAGCACTATG---GGCGCGGCGTCA---
ATAACGCTGACGGTACAG---GCCAGACAA---TTATTG---TCTGGTATAGTGCAACAGCAAAGC---
AATTTG---CTGAGG---GCTATAGAGGCGCAGCAGCATATGTTGCAACTCACAGTCTGGGGCATT
>MG989535
TTCTATTGCAATACAACAAAACTGCTTAATAATACTTGTATAAAAAATCACACCCAGAAGGGG----------
----

TGTAATGACAGTATCATACTTCCATGCAGAATAAAGCAGTTCATCAACATGTGGCAGGGAGTAGGACAAGCAA
TGTATGCTCCTCCCATCAGGGGAATCATTAATTGTGTATCAAAT---
ATTACAGGAATACTATTGACAAGAGATGGTGGTAATGTAACTAAT---------------------------
----

ACTAGTGAAATCTTCAGACCAGGGGGAGGAAATATAAAGGACAATTGGAGAAGTGAATTATATAAATATAAAG
TAGTACAAATTGAACCACTA---GGAATAGCACCCACCAGGGCAAAGAGAAGAGTGGTG-------------
-------------------------GAGAGAGAAAAAAAGAGCA---GTGGGA---ATAGGAGCT---------
ATGATCTTTGGGTTCTTAGGAGCA---GCAGGAAGCACTATG---GGCGCAGCGTCA---
ATAACGCTGACGGCACAG---GCCAGACAA---TTATTG---TCTGGTATAGTGCAACAGCAAAGC---
AATTTG---CTGAGA---GCTATAGAGGCGCAGCATCATCTGTTGCAACTCACAGTCTGGGGCATT
>MG989548
TTCTACTGCAATACAACACAACTATTTAATGGTACTGGT---------------------------------
----

ACTAATAGCACTATCACACTTCCATGCAGGATAAAGCAAATTATAAACATGTGGCAGGGAGTAGGACAAGCAA
TGTATGCTCCTCCCATCAGTGGAACAATCAATTGTATATCAAAT---
ATTACAGGAATATTATTGACAAGAGATGGTGGTCATAATAATGGT---------------------------
----

ACTAATGAGACCTTCAGACCTGGAGGAGGAAATATAAAGGACAATTGGAGAAGTGAATTATATAAATATAAAG
TAGTAGAAATTGAACCACTA---GGAATAGCACCCACCAAGGCAAGGAGAAGAGTGGTG-------------
-------------------------GAGAGAGAAAAAAAGAGCA---GTAGGA---ATAGGGGCT---------
ATGATCTTTGGGTTCTTAGGAGCA---GCAGGAAGCACTATG---GGCGCGGCGTCA---
ATAACGCTGACGGTACAG---GCCAGACAA---TTATTG---TCTGGTATAGTGCAACAGCAAAGC---
AATTTG---CTGAGG---GCTATAGAGGCGCAGCAGCATCTGTTGCAACTCACAGTCTGGGGCATT
>MG989534
TTCTATTGCAATACATCAAAACTGTTTAATAGAACATATGAGTTTAATAGAACACATGAGGGAAATGATACCA
TG--

AATGATACCACTATCATAATTCCATGCAAAATAAAGCAAATAATAAGAATGTGGCAGGGAGCAGGACAAGCAA
TGTATGCTCCTCCCGTCAGGGGAAAAATTCAGTGTGTATCAAAT---
ATTACAGGAATATTATTGACAAGAGATGGTGGTGGGAATACT-------------------------------
----

AGTAATAATGAGACCTTCAGACCTGGAGGAGGAAACATAAAGGACAATTGGAGAAGTGAATTATACAAATATA
AAGTAGTACAAATTGAACCATTA---GGGATAGCACCCACCAGGGCAAAAAGAAGAGTGGTG-----------
----------------------------GAGAAACACAAAAGAGCA---GTGGGA---ATAGGAGCT------
---GTGATCTTTGGGTTCTTAGGAGCA---GCCGGAAGCACTATG---GGCGCGGCGTCA---
ATATCGCTGACGGTACAG---GCCAGACAA---TTATTG---TCTGGTATAGTGCAACAGCAAAGC---
AATTTG---CTGAGA---GCTATAGAGGCGCAACAGCATCTGTTGCAACTCACAGTCTGGGGCATC
>MG989537
TTCTATTGCAATACAACAAAACTGTTT--

-GATAATGAAACCACAGGG------
CCGTGTAATGACACCATTATACTTCCATGCAGAATAAAGCAAATTATAAGAATGTGGCAGGGAGTAGGACAAG
CAATGTATGCTCCTCCCATCAGTGGAAGTATTAATTGTGTATCAAAT---
ATTACAGGAATACTATTGACAAGAGATGGTGGTAATCATAATGGG----------------------------
----

AACAATAGTACTGAGACCTTCAGACCTGGAGGAGGAAATATAAAAGACAATTGGAGAAGTGAATTGTTTAAAT
ATAAAGTAGTAGAAATTGAACCACTA---GGAATAGCACCCACCAGGGCAAAGAGAAGAGTGGTG--------
-------------------------------GAAAGAGAAAAAAGAGCA---GTGGGA---CTAGGAGCT---
------ATGATCTTTGGGTTCTTAGGAGCA---GCAGGAAGCACTATG---GGCGCAGCGTCA---
ATAACGCTGACGGTACAT---GCCAGACAA---TTGTTG---TCTGGTATAGTGCAACAGCAAAGC---
AATTTG---CTGAGG---GCTATAGAGGCGCAGCAGCATATGCTGCAACTCACAGTCTGGGGCATT
>MG989554
TTCTATTGCAATACAACACAACTGTTTAATAGTAATGAAACT-------------------------------
----

ACCTATAATAAAACTATCATACTTCCATGCAGGATAAAGCAGATTATAAGATTGTGGCAGGGAATAGGACAAG
CAATGTATGCTCCTCCCATTAGTGGAACAATTAATTGTGTATCAAAT---
ATCACAGGAATACTATTGACAAGAGATGGTGGAGATGGTGATAATACTAGT----------------------
----

AGTAAAAATGAGACCTTCAGACCTGGAGGAGGAAATATAAAAGACAATTGGAGAAATGAATTATATAAATATA
AAGTAGTAGAAATTGAACCACTA---GGAATAGCACCCTCCAAGGCACGGAGAAGAGTGGTG-----------
----------------------------GACAGACAAAAAAGAGCA---GTGGGA---ATAGGAGCT------
---ATGATCTTTGGGTTCTTAGGAGCA---GCAGGAAGCACTATG---GGCGCGGCGTCA---
ATAACGCTGACGGTACAG---GCCAGACAA---TTATTG---TCTGGTATAGTGCAACAGCAAAGC---
AATTTG---CTGAGA---GCCATAGAGGCGCAGCAGCATATGTTGCAACTCACAGTCTGGGGCATT
>JX848346
TTCTATTGCAATACAACACAACTGTTTAATAAGACTGACATAGAAAATGAAACCAAGGAGGCTAAT-------
----

GCTAATGGCACTATCATACTTCCATGCAAGATAAAGCAAATTATAAACATGTGGCAGGGAGTAGGACAAGCAA
TGTATGCTCCTCCCATCAGTGGACAAATTAGTTGTGTATCAAAT---
ATTACAGGAATACTATTGACAAGAGATGGTGGTAGTCAGAACGAGAACGGG----------------------
----

ACCACCGAGACCTTCAGACCTGGAGGAGGAAATATAAAGGACAATTGGAGAAGTGAATTATATAAATATAAAG
TAGTACAAATTGAACCACTA---GGAGTAGCACCCACCAGGGCAAAGAGAAGAGTGGTG--------------
-------------------------GAGAGAGAAAAAAGAGCA---GTGGGA---ATAGGAGCT---------
ATGATCTTTGGGTTCTTAGGAGTA---GCAGGAAGCACTATG---GGCGCAGCGTCA---
ATAACGCTGACGGTACAG---GCCAGACAA---TTATTG---TCTGGTATAGTGCAACAGCAAAGC---
AATTTG---CTGAGA---GCTATAGAGGCGCAACAACATCTGTTGCAACTCACAGTCTGGGGCATT
>KJ952820
TTCTATTGCAATACAACAGGACTGTTTAATAATTGCACAGGAAATGAAACC----------------------
----

TGTAATGACACTATCATACTTCCATGCAGGATAAAGCAAATTATAAACATGTGGCAGGGAGCAGGACAAGCAA
TGTATGCTCCTCCCATCAGGGGAACAATTAATTGTCTATCAAAT---
ATTACAGGAATAATATTGACAAGAGATGGTGGTAATAATATTAAGAAT-------------------------
----

AACGAGACGGAGATCTTCAGACCTGGAGGAGGAGATATGAAGGACAATTGGAGAAATGAATTATATAAATATA
AAGTAGTACAGATTGAACCACTA---GGAGTAGCACCCACCAGGGCAAAGAGAAGAGTGGTG-----------
----------------------------GAGAGAGAAAAAAGAGCA---GTGGGA---ATAGGAGCT------
---ATGATCTTTGGGTTCTTAGGAGCA---GCAGGAAGCACTATG---GGCGCAGCGTCA---
ATAACGCTGACGGTACAG---GCCAGACAA---TTATTG---TCTGGTATAGTGCAACAGCAAAGC---
AATTTG---CTGAGG---GCTATAGAGGCGCAGCATCATCTGTTGCAACTCACAGTCTGGGGCATT
>HM215432
TTCTATTGCAATACATCAAAACTATTTAATAGTACAAAAAATAGTACTTGTGAGGGG----------------
----

TGTAATGGTACTATCATACTTCCATGTAAGATAAAGCAATTTATAAACATGTGGCAGAACCCAGGACAAGCAA
TGTATGCTCCTCCCATCGAGGGAAAAATTAATTGTGTATCAAAT---
ATTACAGGAATACTATTGACAAGAGATGGTGGTGTTAATAGTACA----------------------------
----

AATAACGAGACCTTCAGACCTCAAGGAGGAAATATGAAGGACAATTGGAGAAGTGAATTATATAAATATAAAG
TAGTACAAATTGAACCACTG---GGAATAGCTCCCACCAGGGCAAGGAGAAGAGTGGTG--------------
-------------------------GAGAGAGAAAAAAGAGCA---GTGGGA---ATAGGAGCT---------
ATGATCTTTGGGTTCTTAGGAGCA---GCAGGAAGCACTATG---GGCGCGGCGTCA---
ATAACGCTGACGGTACAG---GCCAGACAA---TTATTG---TCTGGTATAGTGCAACAGCAAAGC---
AATTTG---CTGAGG---GCTATAGAGGCGCAGCAGCATCTGTTGCAACTCACAGTCTGGGGCATT
>AY444805
TTTTATTGCAATACAACACAGTTGTTTAATAGTACTTGGAGAATAAATGAAACTATGGGGGAGTCTAATGAC-
----

ATAAACTCAAATATCACACTTCCATGCAGGATAAGGCAAATTGTAAAAATGTGGCAGGGAGTAGGACAAGCAA
TGTATGCTCCTCCCATCGATGAAGCAATTCAGTGTGTATCAAAT---
ATTACAGGAATACTATTGACAAGAGATGGTGGTCATAATGTGAGT----------------------------
----

AATGATACTGAGACCTTCAGACCTGGAGGAGGAGATATAAGGGACAATTGGAGAAGTGAATTATATAAATATA
AAGTAGTAGAAATTGAACCACTA---GGAATAGCACCCACCAGGGCAAGGAGAAGAGTGGTG-----------
----------------------------GAGAGAGAAAAAAGAGCA---GTGGGA---ATAGGAGCT------
---ATGATCTTTGGGTTCTTAGGAGCA---GCAGGAAGCACTATG---GGCGCGGCGTCA---
ATAACGCTGACGGTGCAG---GCCAGACAA---TTATTG---TCTGGTATAGTGCAACAGCAAAGC---
AATTTG---CTGAGA---GCTATAGAGGCACAACAGCATCTGTTGCAACTCACAGTCTGGGGCATT
>FJ185251
TTCTATTGCAATACAACAAGACTGTTTAATAATACTTGCATAAAAAATGAAACCACGGAGGGG----------
----

TGTGATGGCACTATCACACTGCCATGCAGGATAAAGCAAATTATAAACATGTGGCAGGGAGTAGGACAAGCAA
TATATGCTCCTCCCATCAGAGGAAAAATTAATTGTGTATCAAAT---
ATTACAGGAATACTATTGACAAGAGATGGTGGTGCTCATAATACG----------------------------
----

ACGACTAATGAGACCTTCAGACCTGGGGGAGGAAATATGAGGGACAATTGGGAAAGTGAATTATATAAATATA
AAGTAGTAGAAATTGAACCACTA---GGAATAGCACCCACCAGGGCAAAGAGAAGAGTGGTG-----------
----------------------------GAGATAGAAAAAAGAGCA---GTGGGA---ATAGGAGCT------
---ATGATCTTTGGGTTCTTAGGAGCA---GCAGGAAGCACTATG---GGCGCGGCGTCA---
ATAACGCTGACGGTACAG---GCCAGACAA---TTATTG---TCTGGTATAGTGCAACAGCAAAGC---
AATTTG---CTGAGG---GCTATAGAGGCGCAGCAGCATCTGTTGCAACTCACAGTCTGGGGCATT
>FN432728
TTCTATTGCAATACATCAAAACTGTTTAATAGCACTTGGGACAGCAATAGCACTGTTAGCTCAGATGACAGTG
ATACT--

AATGACAATGTCAATATAACTCTCCCATGCAGAATAAAGCAAATTATAAATATGTGGCAGAGAGTAGGACAAG
CAATATATGCCCCTCCCATCGAAGGAGTAATACAGTGTGAATCAAAC---
ATCACAGGACTACTATTAACAAGAGATGGAGGGAATGAGAATAATACAAAC----------------------
----

ACAACTAAAGGAGAAGTTTTCAGGCCTGGAGGAGGAAATATGAGGGACAATTGGAGAAGTGAACTATACAAGT
ATAAAGTAGTAAAAATTGAACCACTA---GGTGTAGCACCCTCACCTGCAAGAAGAAGAGTGGTG--------
-------------------------------CAGAGAGAAAAAAGAGCA---GTCGGC---CTAGGAGCT---
------TTCTTCCTTGGGTTCTTAGGAGCA---GCAGGAAGCACTATG---GGCGCAGCGTCA---
ATAACGCTGACGGTACAG---GCCAGACAG---TTACTG---TCTGGGATAGTGCAACAGCAAAGC---
AATTTG---CTGAGG---GCTATAGAGGCTCAACAACATATGTTGAAACTCACGGTCTGGGGCATT
>HQ912710
TTCTATTGCAATACATCAGAACTGTTTAATAGCACTTGGAATAACAATAGCATTGCCAACTCAACAGAT----
----

AGCAAGAGCACCATAACTCTCCAATGCAAAATAAAGCAAATTGTAAATATGTGGCAGAAAGTAGGACAAGCAA
TGTATGCCCCTCCCATCCAAGGAGTAATAAGCTGTAATTCAAGC---
ATTACAGGATTACTATTAACAAGAGATGGAGGGAATAATAATAGT----------------------------
----

ACAAATGAGACCTTCAGGCCTGGAGGAGGAGATATGAGGGACAATTGGAGGAGTGAATTATATAAGTATAAAG
TAGTAAAAATTGAACCAATA---GGTGTAGCACCCACCCATGCAAAAAGAAGGGTGGTG--------------
-------------------------CAGAGAGAAAAAAGAGCA---GTTGGA---CTAGGAGCT---------
GTCCTCTTTGGGTTCTTAGGAGCA---GCAGGAAGCACTATG---GGCGCGGCGTCA---
ATAACGCTGACGGTACAG---GCCAGACAA---TTATTG---TCCGGTATAGTGCAACAGCAGGGC---
AATCTG---CTGAGA---GCTATAGAGGCTCAACAACATCTGTTGAAACTCACGGTCTGGGGCATT
>DQ313242
TTCTATTGCAATACACCGATACTGTTTAATAGCACCTGGGAGTACAATAGCACTTGGGGTAACTATAGCTCAA
ATTAC--
-------------------ACA---
GGGTCAAATGACATTATAACTCTCCAATGCAAAATAAAGCAAATTGTAAATATGTGGCAGAAAGTAGGACAAG
CAATGTATGCCCCTCCCATCCCAGGAGAGTTAAGGTGTGAATCAAAC---
ATTACAGGATTATTATTAACAAGGGATGGAGGGACTAATAGTACAAAT-------------------------
----

GAGACTTTCGAGACTTTTAGGCCTGGAGGAGGAGACATGAGGGACAATTGGAGAAGTGAATTATATAAGTATA
AGGTAGTAAAAATTGAACCACTA---GGTGTGGCACCCACCCATGCAAAAAGAAGAGTGGTG-----------
----------------------------CAGAGAGAAAAAAGAGCA---GTTGGA---CTGGGAGCT------
---GTCTTCCTTGGGTTCTTAGGAGCA---GCAGGAAGCACTATG---GGCGCGGCGTCA---
ATAACGCTGACGGTACAG---GCCAGACAA---TTATTG---TCCGGTATAGTGCAACAGCAGAAC---
AATTTG---CTGAGG---GCTATAGAGGCTCAACAACATCTGTTGAAACTCACGGTCTGGGGCATT
>MH746242
TTCTATTGCAATACATCAGCACTATTTAATAGTAGTTGGAATAGCACTACTTGGAATAGCACTCGAAATACCA
ATGCTAGCTCA--

AATGACACAGAACCTATAACTCTCCAATGCAGAATAAAACAAATTGTAAATATGTGGCAGAGGATAGGACAAG
CAATGTATGCCCCTCCCATCAAAGGAGAAATAAGGTGTGAATCAAAC---
ATTACAGGACTACTATTAACAAGAGATGGAGGGACAAATAATAGT----------------------------
----

ACAAATGAAACATTCAGACCTGGAGGAGGAGATATGAGGGACAATTGGAGAAGTGAATTATATAAGTATAAGG
TAGTACAAATTGAACCAATA---GGTGTGGCACCCACCCATGCAAAAAGAAGAGTGGTG--------------
-------------------------GAGAGAGAAAAAAGAGCA---GTTGGA---CTGGGAGCT---------
GTCTTCCTTGGGTTCTTAGGAGCA---GCAGGAAGCACTATG---GGCGCGGCGTCA---
ATAACGCTGACGGTACAG---GCCAGACAA---TTATTG---TCTGGTATAGTGCAACAGCAGAGC---
AATTTG---CTGAGG---GCTATAGAGGCTCAACAACATATGTTGAGACTCACGGTCTGGGGCATT
>MH746250
TTCTATTGCAATACATCAGGACTATTTAATAGCACTTGGAAATGGAATACTACCACTAGGAATTGGATTGACA
TCTCA--

```
-AATAACACAGACACTGTC------
TCAAATAACACAAATATAACTCTCCAGTGCAGAATAAAACAATTTGTAAATATGTGGCAGAGGGTAGGACAAG
CAATGTATGCCCCTCCCATCCCAGGAGAGATAAGATGTAACTCAAAC---
ATTACAGGACTACTATTAACAAGAGATGGAGGGAATAACAGTGGT----------------------------
-------------------------------------------------------------------------
----------
GATGCAAATGAGACATTCAGACCTGGAGGAGGAGATATGAGGGACAATTGGAGAAGTGAATTATATAAGTATA
AGGTAGTACAAATTGAACCAATA---GGTCTGGCACCCACCCCTGCAAAACGAAGAGTGGTG-----------
----------------------------GAGAGAGAAAAAAGAGCA---ATTGGA---ATGGGAGCT------
---TTCTTCCTTGGGTTCTTGGCAACA---GCAGGAAGCACTATG---GGCGCAGCGTCA---
ATAACGCTGACGGTACAA---GCCAGACAA---TTATTG---TCTGGTATAGTGCAACAGCAAAGC---
AATTTG---CTGAGG---GCTATAGAGGCTCAACAACATATGTTGAAACTCACGGTCTGGGGCATT
>MH078536
CTCTATTGCAATACATCAGGCTTGTTTAATAGCACTTGGGAAAACAATACTAACATA----------------
-------------------------------------------------------------------------
----------------------------
ACCAACTCCAATATAACGCTCAAATGCCAAATAAAGCAAATTATAAGGATGTGGCAGAGAGTAGGACAAGCAA
TGTATGCCCCTCCCATTAAAGGAAATATAACATGTGTATCAAAC---
ATTACTGGACTACTATTAACAAGAGATGGAGGGGGGAATAATACT----------------------------
-------------------------------------------------------------------------
----------------
AATGAGACCTTCAGACCTGGAGGAGGAGATATGAGGGACAATTGGAGAAGTGAATTATATAAGTATAAAGTAG
TAAAAATTGAACCACTA---GGTGTAGCACCCACCCATGCGAAGAGAAGAGTGGTG-----------------
----------------------GAGAGAGAAAAAAGAGCA---GTTGGA---CTGGGAGCT---------
GTCTTTCTTGGGTTCTTAGGGGCA---GCAGGAAGCACTATG---GGCGCAGCGTCA---
ATAACGCTGACGGTACAG---GCCAGACAA---TTATTG---TCTGGTATAGTGCAACAGCAGAGC---
AATCTG---CTGAAG---GCTATAGAGGCTCAACAACAGCTGTTGAGACTCACAGTCTGGGGCATT
>MH078539
TTCTATTGCGATACATCAGAGCTGTTCAATAGGACTTGGGTGTGGAATGACACTGTTGACAATAACACAGTTA
ACAAT--------------------------------------------------------------------
-------------AGCACA------
GATGACAATAGCACAATAACTCTCCCATGCAAAATAAAGCAAATTGTAAGGATGTGGCAGAAAGTAGGACAAG
CAATGTATGCCCCTCCCATCCCAGGAGTAATAAGGTGTGACTCAAAC---
ATTACAGGACTAATATTAACAAGAGATGGAGGGAATAGTAATAGTAGTAATAAT-------------------
-------------------------------------------------------------------------
-------
GGCACAAGGGAAGAAACTTTCAGGCCTGGAGGGGGAGACATGAGGGACAATTGGAGAAGTGAATTATATAAGT
ATAAGGTAATACAAATTGAACCATTA---GGAGTAGCACCCACCAAGGCAAGGAGAAGAGTGGTG--------
-------------------------------GAGAGAGAAAAAAGAGCA---GTTGGA---ATGGGAGCT---
------CTTTTCCTTGGGTTCTTAGGAGCA---GCAGGAAGCACTATG---GGCGCGGCGTCA---
ATAACGCTGACGGTACAG---GCCAGACAA---TTATTG---TCTGGTATAGTGCAACAGCAGAGC---
AATCTG---CTGAAA---GCTATAGAGGCTCAACAACAGCTGTTGAGACTCACAGTCTGGGGCATT
>HQ912707
TTCTATTGCAATACATCAACACTGTTTAATAGCACTTGGAATGGCAATGACACTGCCAGCTCAAATTACACAG
GA-----------------------------------------------------------------------
----------------------------
TCAAATGATACTATAACTCTCCAATGCAGAATAAAGCAAATTGTAAACATGTGGCAGAGAGTAGGACAAGCCA
TGTATGCCCCTCCCATCAAAGGAAAAATAAAGTGTGAATCAAAC---
ATTACAGGACTACTATTAACAAGAGATGGAGGAAAGAATAGTAGT----------------------------
-------------------------------------------------------------------------
----------
AATACAAGTGAGACCTTCAGACCTGGAGGAGGAGATATGAGGGACAATTGGAGAAGTGAATTATATAAGTATA
AAGTAGTCAAAATTGAACCACTA---GGTGTAGCACCTAACCGTGCAAAAAGAAGAGTGGTG-----------
----------------------------GAGAGAGAAAAAAGAGCAGTTGGAGTTGGACTGGGAGCA------
---GTCTTCCTTGGGTTCTTAGGAGCA---GCAGGAAGCACTATG---GGCGCGGCGTCA---
CTAACGCTGACGGTACAG---GCCAGACAA---TTATTG---TCCGGTATAGTGCAACAGCAGAGC---
AATTTG---CTGAGG---GCTATAAAGGCTCAACAACATCTGTTGAAACTCACGGTCTGGGGCATT
>EU513199
TTCTATTGCAATACAACAAAGCTGTTTAATAGCACTTGGAATAATGATAACTACAATGTCAGCGCAAACCTCA
CAGAG--------------------------------------------------------------------
```

-------------------GAA---
AACAGAAAGGAAAACATAACTCTCCACTGCAGAATAAAGCAAATTGTAAATATGTGGCAGAGAGTAGGACAAG
CAATATATGCCCCTCCCATCCCAGGAAACATAACTTGTGGATCAAAC---
ATTACTGGGCTACTATTAACAAGAGATGGAGGGAATAATGGTACA---------------------------
----

AATGATACTGAGACCTTCAGGCCTGGAGGAGGAGATATGAGGGACAATTGGAGAAGTGAATTATATAAATATA
AAGTAGTAAAAATTGAACCACTA---GGTGTAGCACCAACCCCTGCAAAAAGAAGAGTGGTG----------
----------------------------GAAAGAGAAAAAAGAGCA---GTTGGA---ATGGGAGCT------
---TTGATCTTTGGGTTCTTAGGAGCA---GCAGGAAGCACTATG---GGCGCGGCGTCA---
ATGGCGCTGACGGTACAG---GCCAGACAA---TTATTG---TCTGGTATAGTGCAACAGCAGAGC---
AATCTG---CTGAAG---GCTATAGAGGCTCAACAACATCTGTTGAGACTCACGGTCTGGGGCATT
>KU168265
TTCTATTGTTATACATCAAAATTATTTAATAGCATTTGGAGGAGCAATGACACTTGGAGGAGCGATGGCAATG
ACCAT---

ACAAAGGAAACCATAACTCTCCAATGCAGAATAAAGCAAATTGTAAATATGTGGCAGAGAGTAGGACAAGCAA
TGTATGCCCCTCCCATCCCAGGAGTAATAAGGTGTGAATCAAAC---
ATTACAGGACTACTATTAACAAGAGATGGAGTAGGGAATAATATG---------------------------
----

ACAAATGAAACCTTCAGGCCTGGGGGAGGAAATATGAAGGACAATTGGAGAAGTGAACTATATAAGTATAAAG
TAGTAAAAATTGAACCACTA---GGTGTAGCACCCTCCTATGCAAGGAGAAGAGTGGTG-------------
-------------------------GAAAGAGAAAAAAGAGCA---GTTGGA---ATAGGAGCT---------
GTCTTCCTTGGGTTCTTAGGAGCA---GCAGGAAGCACTATG---GGCGCGGCGTCA---
CTAACGCTGACGGTACAG---GCCAGAAAA---TTATTG---TCTGGTATAGTGCAACAGCAGAGC---
AATCTG---CTGCAG---GCTATAGAGGCTCAACAACAGCTGTTGAGGCTCACGGTCTGGGGCATT
>MK086123
TTCTATTGCAATACAACAGACCTATTT------TCTTGGAATAGTAGTAACCCATGGAAGAACATAACG----
----

TCAAATGACAGTATAACTCTCCAATGCAGAATAAAGCAAATTGTAAGGATGTGGCAGCAAGTAGGACAAGCAA
TGTATGCCCCTCCTATACCAGGAATAATAAGGTGTGAATCAAAC---
ATTACAGGAATACTGTTAACAAGAGATGGAGGAAATAATAATACA---------------------------
----

AATAACACAGAGGTATTCAGGCCTGGAGGAGGAGATATGAGGGACAATTGGAGAAGTGAATTATATAAATATA
AAGTAGTAAAAATTGAACCATTA---GGTGTAGCACCCTCCCGTGCAAGAAGAAGAGTGGTG----------
----------------------------GAGAGAGAAAAAAGAGCA---GTTGGC---CTGGGAGCT------
---TTCTTCCTTGGGTTCTTAGGAGCA---GCAGGAAGCACTATG---GGCGCGGCGTCA---
ATAACGCTGACGGTACAG---GCCAGACAA---TTATTG---TCTGGTATAGTGCAGCAGCAGAGC---
AATCTG---CTGCGG---GCCATAGAGGCTCAACAACATCTGTTGAAACTCACGGTCTGGGGCATT
>AY371122
TTCTATTGCAATACAACAAGCCTGTTTAATAGCACTTGGCATGACAATGACACTGCCAGCTCAAACCAC----
----

ACAAATGGCACTATAACTCTCCAATGCAGAATAAAGCAAATTATAAATATGTGGCAGAGAGTAGGACAAGCAA
TATATGCCCCTCCTATCCCAGGAGAAATAAGGTGTGACTCAAAC---
ATTACAGGACTACTATTAACAAGAGATGGAGGGAATAATAATGTGACG------------------------
----

AATGGGACTGAGACTTTCAGGCCTGAAGGAGGAGATATGAGAAACAATTGGAGAAGTGAATTATATAAGTATA
AAGTAGTAAAAATTGAACCAATA---GGTGTAGCACCTACCCCTGCAAAAAGAAGAGTGGTG----------
----------------------------GGGAGAGAAAAAAGAGCA---CTG------GGAGCTGCT------
---GTCTTCCTTGGGTTCTTAGGAGCA---GCAGGAAGCACTATG---GGCGCGGCGTCA---
ATAACGCTGACGGTACAG---GCCAGACAA---CTATTG---TCCGGTATAGTGCAACAGCAGAGC---
AATTTG---CTGAGG---GCTATAGAGGCTCAACAACACCTGTTGAGACTCACGGTCTGGGGCATT
>AY371123
TTCTATTGCAATACAACAGACCTATTTAATAGCACTTGGAACGAAACCACAGAGTCAAAT------------
----

GGCAATATGAATCTAACAATCCCATGCAGAATAAAACAATTTGTGAATTTGTGGCAGAAAGTAGGACAAGCAA
TGTATGCCCCTCCCATCCAAGGAGAAATAAGGTGTGAATCAAAC---
ATTACAGGATTACTATTAACAAGAGATGGAGGGGTAAATAATAAT----------------------------
----

AATAGTACGGAGATATTCAGGCCTGGAGGAGGAGATATGAGGGACAATTGGAGAAGTGAATTATATAAGTATA
AGGTAGTTCAAATTAAACCACTA---GGTGTGGCACCTACCCATGCAAAACGAAGAGTGGTG-----------
----------------------------GAGAGAGAAAAAAGAGCA---GTTGGA---ATAGGAGCT------
---GTTTTCCTTGGGTTCTTAGGAGCA---GCAGGAAGCACTATG---GGCGCGGCGTCA---
ATAACGCTGACGGCACAG---GCCAGACAA---TTATTG---TCTGGTATAGTGCAACAGCAGAGC---
AATCTG---CTGAGG---GCTATAGAGGCTCAACAACATCTGTTGAAACTCACGGTCTGGGGCATT
>AY371124
TTCTATTGCAATACATCACAACTGTTTAATAGAACTTGGGGTAATAATAGCACCTGGGGAAACGAGACAGGGG
AG---

GGCAATATAACTATCACTCTCCCATGCAGAATAAAGCAAATTGTAAATATGTGGCAAAGAGTAGGACGAGCAA
TGTATGCCCCTCCCATCCAAGGAGAAATAAGGTGTAACTCAAAC---
ATTACAGGACTACTATTAACAAGAGATGGAGGGAATAATACTAAT----------------------------
----

AGTACAGATGAGATCTTCAGGCCTGGAGGAGGAGATATGAGGGACAATTGGAGAAGTGAATTATATAAGTATA
AGGTAGTAAAAATTGAACCACTA---GGTGTAGCACCCACCCATGCAAAAAGAAGAGTGGTG-----------
----------------------------GAGAGAGAAAAAAGAGCA---GTTGGA---CTGGGAGCT------
---GTTTTCCTTGGGTTCCTAGGAGCA---GCAGGAAGCACTATG---GGCGCGGCGTCA---
ATAACGCTGACGGTACAG---GCCAGACAA---TTATTG---TCTGGTATAGTGCAACAGCAGAGC---
AATTTG---CTGAAG---GCTATAGAGGCTCAACAACATCTGTTGAGACTCACAGTCTGGGGCATT
>AY371131
TTCTATTGCGATACATCAGGACTGTTTAATAGCACTTGGGACAGCACTTGGGGCAATAACAATGACAGCTCAG
AG---

GCAAATGGCACTATAACTCTCCAATGCAGAATAAAGCAAATTGTAAATATGTGGCAGAAAGTAGGACAAGCAA
TGTATGCCCCTCCCATCCAAGGAGTAATAAGGTGTGAATCAAAC---
ATTACAGGACTAATATTAACAAGGGATGGAGGGCCTGATAGTCCA----------------------------
----

AATGAGATATTCAGGCCTGGAGGAGGAGATATGAGGGACAATTGGAGAAGTGAATTATATAAGTATAAGGTAG
TACAAATAGAACCAATA---GGTGTGGCACCCACTTATGCAAAGAGAAGAGTGGTG-----------------
----------------------GAGAGAGAAAAAAGAGCA---TTTGGA---GTGGGAGCT---------
GTCTTCCTTGGGTTCTTAGGAGCA---GCAGGAAGCACTATG---GGCGCGGCGTCA---
ATAACGCTGACGGTACAG---GCCAGACAA---TTATTG---TCCGGTATAGTGCAACAGCAAAGC---
AATTTG---CTGAGG---GCTATAGAGGCTCAACAACATCTGTTGAAACTCACGGTCTGGGGCATT
>GU201498
TTCTATTGCAATACATCACAACTATTTAATAGCACTTGGGATGGTCCATTTAATAGCACTTGGAATGACAATA
GCACAGAC---
-------------------ACA---
GAGTTAAATGACACTATAACTCTCCAATGCAGAATAAAGCAAATTGTAAATATGTGGCAGAGAGTAGGACAAG
CAATGTATGCCCCTCCCATCCAAGGAGTAATAAGGTGTGAATCAAAC---
ATTACAGGACTGCTATTAACAAGGGATGGAGGGAATAATAGCACA----------------------------
----

AATGAGACATTCAGGCCTGGAGGAGGAGATATGAGGGACAATTGGAGAAGTGAACTATATAAGTATAAGGTAG
TACAAATTGAACCACTA---GGTGTGGCACCCACCCGGGCAAAAAGAAGAGTGGTG-----------------
----------------------GAGAGAGGAAAAAGAGCA---GTAGGA---CTGGGAGCT---------
GTCTTCTTTGGGTTCTTAGGAGCA---GCAGGAAGCACTATG---GGCGCGGCGTCA---
ATAACGCTGACGGTACAG---GCCAGACAA---TTATTG---TCCGGTATAGTGCAACAGCAGAGC---
AATCTG---CTGAGG---GCTATAGAGGCTCAACAACATCTGTTGAAACTCACGGTCTGGGGCATC
>GU201499
TTCTATTGCAATACATCAAAACTGTTTAATAGCACTTGGATTAATGGCACTAGCAATTGGATTAATGACACTT
GGACTAAT---

-------------GGCAAT------
GAGGATGATAATAAAATAACTCTCCAATGCAGAATAAAGCAAATTATAAATATGTGGCAGAGAGTAGGACAAG
CAATGTATGCCCCTCCCATCCGAGGAGAAATAAGGTGTCATTCAAAT---
ATTACAGGTCTACTATTAACAAGAGATGGAGGGAATAATAATAGT----------------------------
----

GCTGCAAATGAAACCTTCAGGCCTACAGGAGGAGACATGAGGGACAATTGGAGAAGTGAATTATATAAATATA
AAGTAGTAAAAATTGAACCACTA---GGTGTAGCACCAAACAAGGCAAAAAGAAGAGTGGTG-----------
----------------------------GAGAGAGAAAAAAGAGCA---GTTGGA---ATGGGAGCT------
---TTCTTCCTTGGGTTCTTAGGAGCA---GCAGGAAGCACTATG---GGCGCGGCGTCA---
ATAACGCTGACGGTACAG---GCCAGACAA---TTATTG---TCTGGTATAGTGCAACAGCAGAGA---
AATCTG---CTGCAG---GCTATAGAGGCTCAACAACATCTGTTGAGACTCACGGTCTGGGGCATT
>AY371137
TTCTATTGCAATACATCACAACTGTTTAATAGTACTTGGAATAGTACTTATAGCAATAATGCCAACTTA----
----

ACAAATGGCACTATAACTCTCCAATGCAGAATAAAGCAAATTGTACAGATGTGGCAGAGAGTAGGACAAGCAA
TGTATGCCCCTCCCATCCAAGGAGTAATAAGGTGTGAGTCAAAC---
ATAACAGGATTACTATTAACAAGAGATGGAGGGACAAATAATACA----------------------------
----

AATAATACCGAGACATTCAGGCCTGGAGGAGGAGATATGAGGGACAATTGGAGAAGTGAATTATATAAGTATA
AGGTAGTACAAATTGAACCAGTG---GGTGTGGCACCCACCCATGCAAAAAGAAGAGTGGTG-----------
----------------------------GAGAGAGAAAAAAGAGCA---GTTGGA---CTGGGAGTT------
---GCGTTCCTTGGGTTCTTAGGAGCA---GCAGGAAGCACTATG---GGCGCGGCGTCA---
ATAACGCTGACGGTACAG---GCCAGACAA---TTATTG---TCTGGTATAGTGCAACAGCAGAGC---
AATTTG---CTGAAG---GCTATAGAGGCTCAACAACATATGTTGAAACTCACGGTCTGGGGCATT
>AY371132
TTCTATTGCAGTACATCAAGACTATTTAATAGCACGTGGGATAATAGCACGTGGAATAACAATACCACT----
----

GACACAGATGACATAACTCTCCCATGCAGAATAAAGCAATTTGTAAATATGTGGCAGAGAGTAGGACAAGCAA
TGTATGCCCCTCCTATCCGAGGAGAAATAAGGTGTGAGTCAAAC---
ATTACAGGACTACTATTAACAAGGGATGGAGGAAATAATAGCACA----------------------------
----

AATGAGACATTCAGGCCTGGAGGAGGAGATATGAGGGACAACTGGAGAAGTGAATTATATAAGTATAAGGTAG
TACAAATTGAACCACTA---GGTGTGGCACCCACCCGTGCAAGGAGAAGAGTGGTG-----------------
----------------------GAGAGAGAAAAAAGAACA---GTAGGA---CTGGGAGCT---------
GTCTTCCTTGGGTTCTTAGGAGCA---GCAGGAAGCACTATG---GGCGCGGCGTCA---
ATAACGCTGACGGTACAG---GCCAGACAA---TTATTG---TCCGGTATAGTGCAACAGCAGAGC---
AATCTG---CTGAGG---GCTATAGAGGCTCAACAACATCTGTTGAAACTCACGGTCTGGGGCATT
>GU201500
TTCTATTGCAATACATCAAAACTGTTTAATAGCATTTGGTCTAACAATACATCAAAATCGTTTAATAGCACTT
GGCTTAACAGT--
-------------AGCATT------
GGGAATGACAGTAGCATAACTCTCCAATGCAGAATAAAGCAATTTGTAAGTATGTGGCAGAGAGTAGGACAAG
CAATGTATGCTCCTCCCATCCCAGGAGAACTAAGGTGCGAATCAAAC---
ATTACAGGACTACTATTAACAAGAGATGGAGGAGGTAATAATAGG----------------------------
----

GAAAATGAGACCTTCAGGCCTGGAGGAGGAGATATGAGGGACAATTGGAGAAGTGAATTATATAAGTATAAAG
TAGTAAAAATTGAACCACTA---GGTGTAGCACCCTCCCAGGCAAAAAGAAGAGTGGTG--------------
-------------------------AATAGAGAAAAAAGAGCA---GTTGGA---ATAGGAGCT---------
CTCTTCCTTGGGTTCTTAGGAGCA---GCAGGAAGCACTATG---GGCGCGGCGTCA---
ATGACGCTGACGGTACAG---GCCAGACAA---TTATTG---TCTGGTATAGTGCAACAGCAGAGC---
AATCTG---CTGAAG---GCTATAGAGGCTCAACAACATCTGTTGAGACTCACGGTCTGGGGCATT
>AY371134
TTCTATTGCAATACATCACAACTGTTTAATAGCACTTGGAAATCTAATAGCACTTGGAACAGCACTAGGAACA
AT---

AACACTGACATTATAACTCTCCAATGCAGAATAAAGCAAATTGTAAATATGTGGCAGAAAATAGGACAAGCAA
TGTATGCCCCTCCCATCCAAGGAGTAATAGAGTGTAAATCAAAC---
ATTACAGGACTACTATTAACAAGGGATGGAGGGCGAGGGCCTGAT----------------------------
----

AATACAACTGAGATATTCAGGCCTGGAGGAGGAGATATGAGGGACAATTGGAGAAGTGAATTATATAAGTATA
AGGTAGTACAAATAGAGCCAATA---GGTGTGGCACCCACCCATGCAAAAAGAAGAGTTGTG-----------
----------------------------GAGAGAGAAAAAAGAGCA---GTTGGA---CTGGGAGCT------
---GTCTTCCTTGGTTTCTTAGGAGCA---GCAGGAAGCACTATG---GGCGCGGCGTCA---
ATAACGCTGACGGTACAG---GCCAGACAA---TTACTG---TCTGGTATAGTGCAACAGCAGAGC---
AATCTG---CTGAGG---GCTATAGAGGCTCAACAGCATCTGTTGAAACTCACAGTCTGGGGCATT
>AY371136
TTCTATTGCAATACATCAAAGCTATTTAATAGCACTTGGACTTGGACCAATGACACTGCCAGCTCAAATGACA
CAAAG--

GGGTTAAATGACACTATAACGCTCCAATGCAGAGTAAGGCAAATTGTAAGAATGTGGCAGAAAGTAGGACAAG
CAATGTATGCCCCTCCCATCCGAGGAGTAATAAGATGTGAGTCAAAC---
ATTACAGGACTACTATTAACAAGGGATGGAGGGACAAATAATAAA----------------------------
----

AATAATGAGACATTTAGGCCTGGAGGAGGAAATATGAGGGACAATTGGAGAAGTGAATTATATAAGTATAAGG
TAGTACAAATTGAACCACTA---GGTGTGGCACCCACCCATGCAAGAAGAAGAGTGGTG--------------
-------------------------CAGAGAGAAAAAAGAGCA---GTTGGA---CTGGGAGCT---------
GTCTTCCTTGGGTTCTTAGGAGCA---GCAGGAAGCACTATG---GGCGCGGCGTCA---
ATAACGCTGACGGTACAG---GCCAGACAA---TTATTG---TCCGGTATAGTGCAACAGCAGAGC---
AATCTG---CTGAGG---GCTATAGAGGCTCAACAGCATCTGTTGAAACTCACGGTCTGGGGCATT
>AY371138
TTCTATTGCAATACATCAGAACTATTTAATAGCACTTGGGCACATGACACAAATAACACAGGGTTG-------
----

GATAATGGCAATCTAACTCTCCAATGCAGAATAAAGCAAATTGTAAGAATGTGGCAGAGGACAGGACAAGCAA
TGTTTGCCCCTCCCATCCAAGGAGAAATAAAGTGTGAATCAAAC---
ATTACAGGATTACTATTAACAAGAGATGGAGGGAACAATGGTAGT----------------------------
----

GCAAATGAGACATTCAGGCCTGGAGGAGGAGATATGAGGGACAATTGGAGAAGTGAATTATATAAGTATAAGG
TAGTACAAATTGAACCAATA---GGTGTGGCACCCACCCATGCAAAAAGAAGAGTGGTG--------------
-------------------------GAGAGAGGAAAAAGAGCAGTAGTTGGA---CTGGGAGCT---------
GTCTTCCTTGGGTTCTTAGGAGCA---GCAGGAAGCACTATG---GGCGCAGCGTCA---
ATAACGCTGACGGTACAA---GCCAGACAA---TTATTG---TCTGGTATAGTGCAGCAGCAGAGC---
AATTTG---CTGAGG---GCCATAGAGGCTCAACAACAACTGTTGAAACTCACGGTCTGGGGCATT
>AY371142
TTCTATTGCAATACATCAAAACTGTTTAATAGCACTTGGGAAAATGGAACTTTAACCAGCTCAAACCACACAA
GG---

TCAAATGGCACCATAACCCTCCAATGCAAAATAAGGCAAATTGTAAGAATGTGGCAGAGAGTAGGACAAGCAA
TGTATGCCCCTCCCATCGACGGGGACATAAGGTGCGAATCAAAC---
ATTACAGGACTACTATTAACAAGAGATGGAGGGAGTAAAAATAGC----------------------------
----

ACAAATAATGAGACTTTCAGGCCTGGAGGAGGAGATATGAGGGACAATTGGAGAAGTGAATTATATAAGTATA
AAGTAGTAGAAGTTGAACCAATA---GGTGTAGCACCCACCTATGCAAAAAGAAGAGTGGTG-----------
----------------------------GAGAGAGAAAAAAGAGCA---GTTGGA---CTGGGAGCT------
---GTCTTCCTTGGGTTCTTAGGAGCA---GCAGGAAGCACTATG---GGCGCGGCGTCA---
ATAACGCTGACGGTACAG---GCCAGACAA---TTGTTG---TCTGGTATAGTGCAACAGCAGAGC---
AATTTG---CTGAAG---GCTATAGAGGCTCAACAACATCTGTTGAGACTCACGGTCTGGGGCATT
>AY371125
TTCTATTGCAATACATCAGGACTATTTAATAGCACTTGGACTTGGAATAGTGATAATAACACTGGATATAGCA
AC---

AACACTAGTGAACTTATAACTCTCCAATGTAGAATAAAGCAGATTATAAATATGTGGCAGAGAGTAGGACAAG
CAATGTATGCCCCTCCCATTAGAGGAATAATAAAATGTGAATCAAAC---
ATTACAGGGCTATTATTAACAAGAGATGGAGGAAATAATAGTACA----------------------------
----

AATGAGACATTCAGGCCTGGAGGAGGAGATATGAGGGACAATTGGAGAAGTGAATTATATAAATATAAGGTAA
TACAAATTGAACCATTA---GGTGTGGCACCCAACAAGGCAAAAAGAAGAGTGGTG-----------------
----------------------GAGAGAGAAAAAAGAGCA---GTGGGA---ATTGGAGCT---------
GTCCTCTTTGGGTTCTTAGGAGCA---GCAGGAAGCACTATG---GGCGCAGCGTCA---
ATAACGCTGACGGTACAG---GCCAGACAA---TTATTG---TCTGGTATAGTGCAACAGCAGAGC---
AATCTG---CTGAGG---GCTATAGAGGCTCAACAACATCTGTTGCAACTCACGGTCTGGGGCATT
>AY371126
TTCTATTGCAATACATCAAACCTGTTTAATAGCACTTGGATTAGCAATAGCACTTGGAATAGCAGTAGCAGTA
CA---

GGGTCAGACATTATAACTCTCCAATGCAGAATAAAGCAAATTGTAAATATGTGGCAGAAAGTAGGACAAGCAA
TGTATGCCCCTCCCATCCGAGGAGAAATAAGATGTAACTCGAAC---
ATTACAGGACTACTATTAACAAGGGATGGAGGGGATAGAGGGAATAGTGGT----------------------
----

GGTGCAAATGGGACCGAAACTTTCAGGCCTGGAAGAGAAAATATGAAGGACAATTGGAGAAGTGAATTATATA
AGTATAAGGTAGTAAAAATTGAACCACTA---GGTGTAGCACCCTCCCATGCAAAAAGAAGAGTAGTG-----
----------------------------------GAGAGAGAAAAAAGAGCA---GTTGGA---
ATGGGAGCT---------GTCTTCCTTGGGTTCTTAGGAGCA---GCAGGAAGCACTATG---
GGCGCAGCGTCA---ATAACGCTGACGGTACAG---GCCAGACAA---TTATTG---
TCCGGTATAGTGCAACAGCAGAAC---AATCTG---CTGAGA---
GCTATAGAGGCTCAACAACATCTGTTGAAACTCACGGTCTGGGGCATT
>AY371127
TTCTATTGCAACACAACAGACTTATTTAATAGCACTTGGGATAACAGTAGCATTGAGACTAACTATAGCACTG
GG---

AATAACAATGAAACTATAACTCTCCAATGCAGAATAAAACAAATTGTAAATATGTGGCAGAAAGTAGGACAAG
CAATGTATGCCCCTCCCATCCCAGGAGTAATAAGGTGTGAATCAAAC---
ATTACAGGGTTACTATTAACAAGAGATGGAGGGAACAATACTGAGGCA-------------------------
----

AATGAGAATGAGACATTCAGGCCTGGAGGAGGAGATATGAGGGACAATTGGAGAAGTGAATTATATAAGTATA
AGGTAGTACAAATTGAGCCACTA---GGTGTGGCACCCACCCAGGCAAAGAGAAGAGTGGTG-----------
----------------------------CAGAGAGAAAAGAGAGCA---GTTGGA---ATGGGAGCT------
---TTCTTCCTTGGGTTCTTAGGAGCA---GCAGGAAGCACTATG---GGCGCGGCGTCA---
ATAACGCTGACGGTACAG---GCCAGACAA---TTATTG---TCTGGTATAGTGCAACAGCAGAGC---
AATCTG---CTGAGG---GCTATAGAGGCTCAACAACATCTGTTGAAACTCACGGTCTGGGGCATT
>AY371139
TTCTATTGCAATACATCAAGCCTGTTTAATAGCATTTGGAGTACCAATAGTACTGGGAATAACAATAGCACT-
----

GGGGATGGCAATATAACTCTCCAATGCAGAATAAAGCAAATTATAAAGATGTGGCAGAGAGTAGGACAAGCAA
TGTATGCCCCTCCCATCCCAGGAGAAATAAGGTGCAACTCAAAC---
ATTACAGGACTACTATTAACAAGAGATGGAGGGGTTAATAATAGT----------------------------
----

ATAAATGAGACCTTCAGGCCTGGAGGAGGAGATATGAGGGACAACTGGAGAAGTGAATTATATAAGTATAAAG
TAGTAAAGATTGAACCACTA---GGTGTAGCACCCACCCGTGCAAAAAGAAGAGTGGTG--------------
-------------------------GGGAGAGAAAAAAGAGCAGTTGTTGGA---CTGGGAGCT---------
GTCTTCCTTGGGTTCTTAGGAGCG---GCAGGAAGCACTATG---GGCGCGGCGTCA---
ATAACGCTGACGGTACAG---GCCAGACAA---TTATTG---TCTGGTATAGTGCAACAGCAGAGC---
AATTTG---CTGAGG---GCTATAGAGGCTCAACAACATCTGTTGAGACTCACGGTCTGGGGCATT
>AY371140

TTCTATTGCAATACATCAAAACTGTTTAATAGCACTTGGAGTAACAATGACACTGCCAGCTCAAACCACACAG
GG---

TTAAATAACACTATAACTCTCCAATGCAGAATAAAGCAAATTATAAATATGTGGCAGAAAGTAGGACAAGCAA
TGTATGCCCCTCCCATCCGAGGAGAAATAAGGTGTGAATCAAAC---
ATTACAGGACTACTACTAACAAGAGATGGAGGGAGTAATAATAGT----------------------------
----

ACAAATGAGACTTTCAGGCCTGGAGGAGGAGATATGAGGGACAATTGGAGAAGTGAATTGTATAAGTATAAAG
TAGTAAAAATTGAACCACTA---GGTGTAGCCCCCACCAAGGCAAAAAGAAGAGTGGTG--------------
-------------------------GGGAGAGAAAAAAGAGCA---GTTGTTGGACTGGGAGCT---------
GTCTTCCTTGGGTTCTTAGGAGCA---GCAGGAAGCACTATG---GGCGCGGCGTCA---
ATGACGCTGACGGTACAG---GCCAGACAA---TTGTTG---TCCGGTATAGTGCAACAGCAGAGC---
AATTTG---CTGAGG---GCTATAGAGGCTCAACAACATCTGTTGAAACTCACGGTCTGGGGCATT
>AY371146
TTCTATTGCAATACAACAGCACTATTTAATAGCACTTGGTTTGGCAATGAGACTGACAGCTCAAACCATACAA
GG---

TCAAATGACACTATAACTCTCCAATGCAGAATAAAGCAAATTGTAAGGATGTGGCAGAAAGTAGGACAAGCAA
TGTATGCCCCTCCCATCAAAGGAAACATAACGTGTAAATCAAAT---
ATTACAGGAATACTATTAACAAGAGATGGAGGAACTAATAGAACA----------------------------
----

AATGAGACATTCAGGCCTGGAGGAGGAGATATGAGAGACAATTGGAGAAGTGAATTATATAAGTATAAGGTAG
TACAAATTGAACCACTA---GGTGTGGCACCTACCCCTGCAAAAAGAAGAGTGGTG-----------------
----------------------CAGAGAGAAAAAAGAGCA---GTTGGA---ATGGGAGCT---------
TTCTTCCTTGGGTTCTTAGGAGCA---GCAGGAAGCACTATG---GGCGCGGCGTCC---
CTGACGCTGACGGTACAG---GCCAGACAA---TTGTTG---TCTGGTATAGTGCAACAGCAGAGC---
AATTTG---CTGAGG---GCTATAGAGGCTCAACAACATCTGTTGAAACTCACAGTCTGGGGCATT
>AY371128
TTCTATTGCAATACATCAAAACTGTTCAATAGCATTTGGACTTTGGAGAATGGCAATTGGACTAACAATAGCA
CAGAA--

AATGGAAATGACACTATAACTCTCCAATGCAGAATAAAGCAAATTATAAATATGTGGCAGAGAGTAGGACAAG
CAATGTATGCCCCTCCCATCCCAGGAGTAATAATGTGTAATTCAAAT---
ATCACAGGACTACTATTAACAAGAGATGGAGGGAACAGTGGTGGT----------------------------
----

GCAAATGAGACCTTCAGGCCTGGAGGAGGAGATATGAGAGACAATTGGAGAAGTGAATTATATAAGTATAAAG
TGGTGAAGATTAACCCACTA---GGAGTAGCACCCACCAATGCAAAAAGAAGAGTGGTG--------------
-------------------------GGGAGAGAGAAAAGAGCA---GTTGGA---CTGGGAGCT---------
GTCTTCCTTGGGTTCTTAGGAGCA---GCAGGAAGCACTATG---GGCGCGGCGTCA---
ATAACGCTGACGGTACAG---GCCAGACTA---TTATTG---TCTGGTATAGTGCAACAGCAAAGC---
AATTTG---CTGAAG---GCTATAGAGGCTCAACAACATCTGTTGAGACTCACGGTCTGGGGCATT
>AY371129
TTCTATTGCAATACATCAGAACTATTTAATAGCACTTGGGGTAATAGCACTTGGGATAATAGCACTTGGGATA
ACAGC--

TCAAATTATGACAATATAACTCTCCAATGCAGAATAAAGCAAATTATAAATATGTGGCAGAAAGTAGGACAAG
CAATGTATGCCCCTCCCATCCCAGGAGTAATAAGGTGTGAATCAAAC---
ATTACAGGACTACTATTAACAAGGGATGGAGGGAGCAGTAATGGTACA-------------------------
----

AATGAGACAAGTGAGACATTCAGGCCTGGAGGAGGAGATATGAGAGACAATTGGAGAAGTGAATTATATAAGT
ACAAGGTAGTACAAATTGAACCATTA---GGTGTGGCACCCTCCCATGCAAAAAGAAGAGTGGTG--------
-------------------------------GAGAGAGGAAAAAGAGCA---GTTGGA---ATGGGAGCT---
------TTCTTCCTTGGGTTCTTAGGAGCA---GCAGGAAGCACTATG---GGCGCAGCGTCA---
CTGACGCTGACGGTACAA---GCCAGACAA---TTATTG---TCTGGTATAGTGCAGCAGCAGAGC---
AATTTG---CTGAAG---GCTATAGAGGCTCAACAACATCTGTTGAAACTCACGGTCTGGGGCATT
>AY371130

TTCTATTGTAATACAACAAAACTATTTAACACTGCCAGCTTAAATGACACAGAG-------------------
----

ACAAATGGCACTATAACTCTCCCATGCAGAATAAAACAAATTGTAAATATGTGGCAGAAAGTAGGACAGGCAA
TGTATGCCCCTCCCATCCAAGGAGTAATAAGGTGTGAATCAAAC---
ATTACAGGACTACTATTAACAAGAGATGGAGGGAACAATAGGAGT----------------------------
----

ACAGAAACCTTTAGGCCTGGAGGAGGAGATATGAGGGACAATTGGAGAAGTGAATTATATAAGTATAAAGTAG
TAAAAATTGAACCACTA---GGTGTGGCACCCACCCGTGCAAAAAGAAGAGTGGTG-----------------
----------------------GAGAGAGAAAAAAGAGCA---GTTGGA---ATGGCAGCT---------
GTCTTCTTTGGGTTCTTAGGAGCA---GCAGGAAGCACTATG---GGCGCGGCGTCA---
ATAACGCTGACGGTACAG---GCCAGACAA---TTATTG---TCCGGTATAGTGCAACAGCAGAGC---
AATTTG---CTGAAA---GCTATAGAGGCTCAACAACATATGTTGAGACTCACGGTCTGGGGCATT
>GU201513
TTCTATTGCAATACATCAAGACTATTTAATAGTACTTGGACTAGCAACTCTACAACAAATTCAACT-------
----

GCTAATGAAATTATAACTCTCCAATGTAGAATAAAGCAAATTGTAAATATGTGGCAGACGGTAGGACAAGCAA
TGTATGCCCCTCCCATCCCAGGAATAATAAGGTGTGACTCAAAC---
ATTACAGGACTACTATTAACAAGAGATGGAGGGGATACAAATAGA----------------------------
----

ACAAATGAAATATTCAGACCTGGAGGAGGAGATATGAGGGACAATTGGAGAAGTGAGTTATATAAGTATAAGG
TAGTAAAAATTGAACCAATA---GGTGTGGCACCCACCCCTGCAAAAAGAAGAGTGGTG--------------
-------------------------GAAAGAGGAAAAAGAGCA---GTTGGA---CTGGGAGCT---------
GTCTTCCTTGGGTTCTTAGGAGCA---GCAGGAAGCACTATG---GGCGCGGCGTCA---
ATAACGCTGACGGTACAG---GCCAGACAA---TTATTG---TCCGGTATAGTGCAACAGCAGAGC---
AATTTG---CTGAGG---GCTATAGAGGCTCAACAACATCTGTTGAAACTCACGGTCTGGGGCATT
>GU201514
TTCTATTGCAATACATCAGAGTTGTTTAATAGCATTTGGAATAACAATGGCAGCATTTGGTATAACAATGACA
CTGCTATCAAT--
----------AACACAGAG------
CCAAAGAATAAGACTCTAACTCTCCAATGCAGAATAAAGCAGGTTATAAAGATGTGGCAGAGAGTAGGACAAG
CAATGTATGCCCCTCCCATCCGAGGAGAAATAAGGTGTGATTCAAAC---
ATTACAGGACTATTATTAACAAGAGATGGAGGGAATAATAGGACA----------------------------
----

GATGAGACCTTCAGGCCTGGAGGAGGAGATATGAGGGACAATTGGAGAAGTGAATTATATAAGTATAAAGTAG
TAAAAATTGAACCACTA---GGTGTAGCACCCACCTATGCAAAAAGAAGAGTGGTG-----------------
----------------------GAGAGAGAAAAAAGAGCA---GTTGGA---ATAGGAGCT---------
GTCCTCCTTGGGTTCTTAGGAGCA---GCAGGAAGCACTATG---GGCGCGGCGTCA---
ATAACGCTGACGGTACAG---GCCAGACAA---TTATTG---TCTGGTATAGTGCAACAGCAGAGC---
AATTTG---CTGAAG---GCTATAGAGGCTCAACAACATCTGTTGAGACTCACGGTCTGGGGCATT
>AY371141
TTCTATTGCAATACATCAGGACTGTTTAATAGCAGTTGGGATAGCAATAGCACTGAGGAAACCAATAATTCAG
AA---

AATAATGAAACTATAACTCTCCAATGCAGAATAAAGCAAATTGTAAACATGTGGCAGAGAGTAGGACAAGGAA
TGTATGCCCCTCCCATCCAGGGAATAATAAGGTGTGAATCAAAC---
ATTACAGGACTACTATTAACAAGAGATGGAGGGGAAAATAATAGT----------------------------
----

ACAAATCAGACCTTCAGGCCTGGAGGAGGAGATATGAGGGACAATTGGAGAAGTGAATTATATAAGTATAAAG
TAGTAAAAATTGAACCAATA---GGTGTAGCACCCACCCATGCAAAAAGAAGAGTGGTG--------------
-------------------------GAGAGAGGAAAAAGAGCA---GTTGGA---CTGGGAGCT---------
GTCTTCCTTGGGTTCTTAGGAGCA---GCAGGAAGCACTATG---GGCGCGGCGTCA---
ATAACGCTGACGGTACAG---GCCAGACAA---TTATTG---TCTGGTATAGTGCAACAGCAGAGC---
AATCTG---CTGAAA---GCTATAGAGGCTCAACAACATCTGTTGAAACTCACGGTCTGGGGCATT
>DQ313247

TTCTATTGTAATACAACAGATCTGTTTAATAGCACTTGGGCTAATAATAGCAATAAGTGGGACTCAACAGCCC
CA---

GAGAATGGCACTATAACTCTCAAATGCAGAATAAAGCAAATTGTAAATATGTGGCAGAGAGTAGGACAAGCAA
TATATGCCCCTCCCATCGAAGGAGACATAAGGTGTGAATCAAAT---
ATTACAGGACTACTATTAACAAGAGATGGAGGGGAAAATAGTAGT----------------------------
----

GAGCCCAGTGAGACCTTCAGGCCTGGAGGGGGAGATATGAGGGATAATTGGAGAAGTGAATTATATAAGTATA
AAGTAGTAAAAATTGAACCACTA---GGTGTAGCACCTACCCATGCAAAAAGAAGAGTGGTG-----------
----------------------------GAGAGAGGAAAAAGAGCA---GTTGGA---CTGGGAGCT------
---TTCTTCTTTGGGTTCTTAGGAGCA---GCAGGAAGCACTATG---GGCGCGGCGTCA---
ATAACGCTGACGGTACAG---GCTAGACAA---TTATTG---TCTGGTATAGTGCAACAGCAGAGC---
AATTTG---CTGAGA---GCTATAGAGGCTCAACAACATATGTTGAAACTCACGGTCTGGGGCATT
>MK086121
TTCTATTGCAATACATCAAATTTATTCAATAGCACTTGGAATAGCACTTGGGGTAATAACACTTGGAATAGCA
GTAGC--

ACTAATATTAATATAACGCTTCAATGCAGAATAAAGCAAATTGTAAATATGTGGCAGACAGTAGGACAAGCAA
TGTATGCCCCTCCCATCCAAGGAGAAATAAGGTGTGAATCAAAC---
ATTACAGGATTACTATTGACAAGAGATGGAGGGGGGACAGAGACC----------------------------
----

AATAATACAGAGATCTTCAGGCCTGGAGGAGGAGATATGAGGGACAATTGGAGAAGTGAATTATATAAGTATA
AAGTAGTAAAAATTGAACCACTA---GGTGTAGCACCCACCCATGCAAAAAGAAGAGTGGTG-----------
----------------------------GAGAGAGAAAAAAGAGCA---GTTGGA---CTGGGAGCT------
---GTCTTCCTTGGGTTCTTAGGAGCA---GCAGGAAGCACTATG---GGCGCGGCGTCA---
ATAACGCTGACGGTACAG---GCCAGACAA---TTATTG---TCTGGTATAGTGCAACAGCAGAGC---
AATTTG---CTGAAG---GCTATAGAGGCTCAACAACAGATGTTGAGACTCACGGTCTGGGGCATT
>EU513187
TTCTATTGCAATACAACAGCACTATTTAATAGCACTTGGGATAACAATAGTACTAGGGAAGACAATAACTCAA
CA---

GGGAATGACACTATAACTCTCCAATGCAGAATAAAGCAAATTGTGAGGATGTGGCAAAGATCAGGACAAGCAA
TGTATGCCCCTCCCATCCAAGGAGTAATAAAGTGTAAATCAAAT---
ATCACAGGACTACTATTAACAAGGGATGGGGGGAATAATGAAAGT----------------------------
----

ACAAATGAGACCTTCAGACCTGGAGGAGGAGATATGAGGGACAATTGGAGAAGTGAATTATATAAGTATAAAG
TAGTAAAAATTGAACCACTA---GGTGTAGCACCCACCCATGCAAAAAGAAGAGTGGTG--------------
-------------------------CAGAGAGAAAAAAGAGCA---GTAGGA---CTGGGAGCT---------
GTCTTCTTTGGGTTCTTAGGAGCA---GCAGGAAGCACTATG---GGCGCGGCGTCA---
ATAACGCTGACGGTACAG---GCCAGACAA---TTATTG---TCTGGTATAGTGCAACAGCAGAGC---
AATTTG---CTGAAG---GCTATAGAGGCTCAACAGCATCTGTTGAAACTCACGGTCTGGGGCATT
>EU513195
TTCTATTGCAATACATCAAGACTGTTTAATAGCACTTGGGGTAGCAATAACACTTGGGATAACAGC-------
----

ACGGAGCAAAATATAACTCTCAGATGCAAAATAAAGCAGATTATAAATATGTGGCAGAGAGTAGGACAAGCAA
TGTATGCCCCTCCCATCCGAGGAGAAATAAGATGTGAATCAAAC---
ATTACAGGACTACTATTAACAAGGGATGGAGGGGGTAATAGTAGT----------------------------
----

AGTGCAAATGAGACCTTCAGACCTACAGGAGGAAATATGAAGGACAATTGGAGAAGTGAATTATATAAGTATA
AAGTAGTAAAAATTGAACCACTA---GGTGTAGCACCCACCCGTGCAAAAAGAAGAGTGGTG-----------
----------------------------GGGAGAGAAAAAAGAGCA---GCA------CTGGGAGCT------
---GTCTTCCTTGGGTTCTTAGGAGCA---GCAGGAAGCACTATG---GGCGCGGCGTCA---
ATAACGCTGACGGTACAG---GCCAGACAA---TTATTG---TCCGGTATAGTGCAACAGCAGAGC---
AATTTG---CTGAAG---GCTATAGAGGCTCAACAACATCTGTTGAAACTCACGGTCTGGGGCATT
>EU513189

TTCTATTGCAATACATCAGGACTATTTAATAGCACTTGGCACAATAGCACTTGGACCAATAGCACCACTGGCT
CAAATGGC---
-------------------ACA---
GAGTCAAATGACACTATAACTCTCCAATGCGAAATAAAGCAATTTATAAATATGTGGCAGAGAGTAGGACGAG
CAATGTATGCCCCTCCCATCCCAGGAGTGATAAGGTGTGAATCAGAC---
ATTACAGGACTACTATTAACAAGAGATGGACCGAATAGTACACAA----------------------------
----

AATGAGACATTCAGGCCTGGAGGAGGAGATATGAGAGACAATTGGAGAAGTGAATTATATAAGTATAAAGTAG
TACAAATTGAACCACTA---GGTGTGGCACCCACCCATGCAAAAAGAAGAGTGGTG-----------------
----------------------GAGAGAGAAAAAAGAGCA---GTTGGA---CTGGGAGCT---------
GTCTTCTTTGGGTTCTTGGGAGCG---GCAGGAAGCACTATG---GGCGCGGCGTCA---
ATAACGCTGACGGTACAG---GCCAGACAA---TTATTG---TCTGGTATAGTGCAACAGCAGAGC---
AATTGG---CTGAAA---GCTATAGAGGCTCAACAACATCTGTTGAGACTCACGGTCTGGGGCATT
>EU513196
TTCTATTGCAATACATCAGGCCTATTTAATAGCACTTGGTATAGCAATAGCACTAACAGCTCAAATCACACAA
AT---

GGCACAGATGATATAACTCTCCAATGCAGAATAAAGCAAATTGTAAATATGTGGCAGAGAGTAGGACAAGCAA
TGTATGCCCCTCCCATCCGAGGAGAAATAAGGTGTGAATCAAAC---
ATTACAGGAATACTATTAACAAGAGATGGAGGGAATAATGGTAGT----------------------------
----

AAAGCAAATGAGACCTTCAGACCTGGAGGAGGAGACATGAGGGACAATTGGAGAAGTGAATTATATAAATACA
AAGTAGTAAAAATTGAACCAATA---GGTATAGCACCCACCCATGCAAAAAGGAGAGTGGTG-----------
----------------------------GAGAGAGAAAAAAGAGCA---GTCGGA---CTGGGAGCT------
---TTCTTCCTTGGGTTCTTAGGAGTA---GCAGGAAGCACTATG---GGCGCGGCGTCA---
ATGACGCTGACGGTACAG---GCCAGACAT---TTATTG---TCTGGTATAGTGCAACAGCAGAGC---
AATCTG---CTGAAG---GCTATAGAGGCTCAACAACAGCTGTTGAGACTCACAGTCTGGGGCATT
>EU513191
TTCTATTGCAATACATCAAAACTGTTTAGTAGCACTTGGGATAATAGCACTTGGGGTAATAACACTTGGGGTA
ACAGCACT---
-------------------GGG---
GACAACAATGAGACCATAACCCTCCAATGCAAAATAAAACAAATTATAAATATGTGGCAGCAAGTAGGACGAG
CAATGTATGCCCCTCCCATCCAAGGAGAGATAAGGTGTGAGTCAAAC---
ATTACAGGACTACTATTAACAAGAGATGGAGGGGCTAATAGCAGT----------------------------
----

ACAAATGAGACCTTCAGGCCTGGAGGAGGAGATATGAGGGATAATTGGAGGAGTGAATTATATAAGTATAAAG
TTGTAAAAATTGAACCACTA---GGTGTAGCACCCACCCGTGCAAAAAGAAGAGTGGTG------------
-------------------------CAGAGAGAAAAAAGAGCG---GTTGGA---CTGGGAGCT---------
GTCTTCATTGGGTTCTTAGGAGCA---GCAGGAAGCACTATG---GGCGCAGCGTCA---
ATAACGCTGACGGTACAG---GCCAGACAA---TTATTG---TCCGGTATAGTGCAACAGCAGAGC---
AATTTG---CTGAAG---GCTATAGAGGCTCAACAACATCTGTTGAGACTCACGGTCTGGGGCATT
>EU513184
TTCTATTGCAATACATCAAAACTATTTGATAGCATTTGGAATAGAACTAATGGGTCAAATTACACAAGGTTAG
GTGATGAC---
-------------------ACA---
GAGACAAATGACCCTATAACTCTCCAATGCAGAATAAAGCAAATTGTAAGAATGTGGCAGACAGTAGGACAAG
CAATGTATGCCCCTCCCATCCGGGGAGAAATAAAATGTGAATCAAAC---
ATTACAGGACTGCTATTAACACGAGATGGAGGAAATAATAATAGT----------------------------
----

ATGAATGAGACATTCAGGCCTGGAGGAGGAGATATGAGGGACAATTGGAGAAGTGAATTATATAAGTATAAGG
TAGTACAAATTGAACCACTA---GGTGTGGCACCCACCTTTGCAAAAAGAAGAGTGGTG------------
-------------------------GAGAGAGGAAAAAGAGCA---GTTGGA---CTGGGAGCT---------
GTTTTCCTTGGGTTCTTAGGAGCT---GCAGGAAGCACTATG---GGCGCGGCGTCA---
ATAACGCTGACGGTACAG---GCCAGACAA---TTACTG---TCTGGTATAGTGCAACAGCAAAGC---
AATTTG---CTGAGG---GCTATAGAGGCTCAACAACATCTGTTGAAACTCACGGTCTGGGGCATA
>EU513193

TTCTATTGCAATACATCAGGACTGTTTAATAGCACCTGGTGGGATAGCAACAATAGCACTGAGAATATAACTC
AC--

AGAAAAGAAAATATAACTCTCCAATGCAGAATAAAGCAATTTATAAATATGTGGCAGAGAGTGGGACGAGCAA
TGTATGCCCCTCCCATCCAAGGAGTAATAAGGTGTGAATCAAAC---
ATTACAGGAATACTATTAACAAGAGATGGTGGGGTTAATAATAATAAC--------------------------
----

ACAAACAAAACTGAGACCTTCAGGCCTGGAGGAGGAGATATGAGGGACAATTGGAGAAGTGAATTATATAAGT
ATAAAGTAGTGAAAATTGAACCACTA---GGTGTAGCACCCACCCATGCAAAAAGAAGAGTGGTG---------
--------------------------------GAGAGAGAAAAAAGAGCA---GTTGGA---CTGGGAGCT---
------GTCTTCCTTGGGTTCTTAGGAGCA---GCAGGAAGCACTATG---GGCGCGGCGTCA---
ATAACGCTGACGGTACAG---GCCAGACAG---TTATTG---TCTGGTATAGTGCAACAGCAGAGC---
AATTTG---CTGAAG---GCTATAGAGGCTCAACAACAGCTGTTGAGACTCACAGTCTGGGGCATT
>EU513186
TTCTATTGTAATACATCAAGCCTATTTAATTATACATTTAGCAATAGCACTGAGGATAACAACCCA--------
----

AGTAATGCCACAATAACTCTCCAATGCAGAATAAGGCAAGTTGTAAATATGTGGCAGAGAGTAGGACTAGCAA
TGTATGCCCCTCCCATCAAAGGAAAACTAAGATGTGAATCAAAC---
ATTACAGGACTACTATTAACAAGAGATGGAGGGAATAATAATAAT-----------------------------
----

GCAACTGAGACATTCAGGCCTGGAGGAGGAGATATGAGGGACAACTGGAGAAGTGAATTATATAAATATAAGG
TAGTACAAATTGAACCCCTA---GGTGTGGCACCCACCTATGCAAAAAGAAGAGTGGTG---------------
-------------------------GAAAGAGGAAAAAGAGCAGTAGTTGGA---CTAGGAGCT----------
GTCTTCCTTGGGTTCTTAGGAGCA---GCAGGAAGCACTATG---GGCGCGGCGTCA---
ATAACGCTGACGGTACAG---GCCAGACAA---TTATTG---TCTGGTATAGTGCAACAGCAGAGC---
AATCTG---CTGAGG---GCTATAGAGGCTCAACAACAGCTGTTGAAACTCACGGTCTGGGGCATT
>MH705136
TTCTATTGCAATACCTCAAATCTATTTGATAGCGCTTGGGAAGCAAACTCAAATCACACAGAGTCAAATGAC-
----

ACAAGGGCAAATATAACTCTCCAATGCAGAATAAGGCAAATTGTAAGGATGTGGCAGAGAGTAGGACAAGCAA
TGTATGCCCCTCCCATCCGAGGAGAAATAAGGTGTGAGTCAAAC---
ATTACAGGACTACTATTAACAAGAGATGGAGGGGATGGGAACAATGCA--------------------------
----

ACTAATGAGACATTCAGGCCTGGAGGAGGAGATATGAGAGACAATTGGAGAAGTGAACTATATAAATATAAGG
TAGTACAAATTGAACCACTA---GGTGTAGCACCCACCCATGCAAAAAGAAGAGTGGTG---------------
-------------------------GAGAGAGAAAAAAGAGCA---GTGGGA---ATAGGAGCT----------
GTCTTCCTTGGGTTCCTAGGGGCA---GCAGGAAGCACTATG---GGCGCGGCGTCA---
ATAACGCTGACGGTACAG---GCCAGACAA---TTATTG---TCCGGTATAGTGCAACAGCAGAGC---
AATTTG---CTGAGG---GCTATAGAGGCTCAACAACAACTGTTGAAACTCACAGTCTGGGGCATT
>MH460467
TTCTATTGTAATACATCAGGACTATTTAATTATAGTTGGAATACATCAGAACCAACCAATAATTGGAATAGTA
GTGCTCAG--

GATAACGATACCATAACTCTCCAATGCAGAATAAAGCAAATAGTAAATATGTGGCAGGAAGTAGGACAAGCAA
TGTATGCCCCTCCCATCCAAGGAATAATAAGGTGTGAATCAAAC---
ATTACAGGACTACTATTAACAAGAGATGGAGGGACAAATAATAGT-----------------------------
----

ACAAATGAGACATTCAGACCTGGGGGAGGAGATATGAGGGACAATTGGAGAAGTGAATTATATAAGTATAAGG
TAGTACAAATTGAACCAATA---GGTTTGGCACCCACCCATGCAAAAAGAAGAGTGGTG---------------
-------------------------CAGAGAGAAAAAAGAGCAGTAATTGGA---ATGGGAGCT----------
GTCTTCCTTGGGTTCTTAGGAGCA---GCAGGAAGCACTATG---GGCGCGGCGTCA---
ATAACGCTGACGGTACAG---GCCAGACAA---TTATTG---TCCGGTATAGTGCAACAGCAGAGC---
AATTTG---CTGAGG---GCTATAGAAGCTCAACAACATCTGTTGAAACTCACGGTCTGGGGCATT
>EU513198

TTCTATTGCAATACCTCAAACCTGTTTAATGGCACTTTGTATAACAACACCTGGATGTTTAATGGCACTTTGT
ATAATAATACT--
----------AACGAGAGC------
TCAACATCAGGTACTATAACTATCAAGTGCAGAATAAAGCAAATCATAAATATGTGGCAGACAGTAGGACAAG
CAATGTATGCCCCTCCCATCCCAGGAGAAATAAGGTGTGAATCAAAC---
ATTACAGGATTGCTATTAACAAGAGATGGAGAGGCTGGAAAAAGC----------------------------
----

ACAAATGAGACCTTCAGGCCTATAGGAGGAAATATGAGAGACAATTGGAGAAGTGAATTATACAAATATAAAG
TAGTAAAAATTGAACCACTA---GGTGTAGCACCCACACCTGCAAAAAGAAGAGTGGTG--------------
-------------------------GAGAGAGAAAAAAGAGCA---GTGGGA---CTGGGAGCT---------
GTCTTCCTTGGGTTCTTAGGAGCA---GCAGGAAGCACTATG---GGCGCAGCGTCA---
ATAACGCTGACGGTACAG---GCTAGACAA---TTATTG---TCTGGTATAGTGCAACAGCAAAGC---
AATTTG---CTGAAG---GCTATAGAGGCTCAACAACATCTGTTGAAACTCACGGTCTGGGGCATT
>JF706407
TTCTATTGTGATACATCAAGACTGTTTAATAGCACTTGGCCTAATAGCACATGGGACAATAATACTCAAAAGA
ATGAG--

AATGACACAGGGTACTATACTCTCCAATGCAGAATAAAGCAAATTATAAATATGTGGCAGAGAGTAGGACAAG
CAATGTATGCCCCTCCTATCCCAGGAGAAATAAGTTGTGAATCAAAC---
ATTACAGGACTACTATTAACAAGAGATGGAGGGAATAATAATAAT----------------------------
----

AGTATAAATGAGACCTTCAGGCCTGGTGGAGGAGATATGAGGGACAATTGGAGAAGTGAATTATATAAGTACA
AAGTGGTACAAATTGAGCCAATA---GGAATAGCACCCACCCCTGCAAAAAGAAGAGTGGTG-----------
----------------------------GAGAGAGAGAAAAGAGCAGTAGTTGGA---CTAGGAGCT------
---GTCTTCCTTGGGTTCTTAGGAGCA---GCAGGAAGCACTATG---GGCGCAGCGTCA---
ATAACGCTGACGGTACAG---GCCAGACAA---TTATTG---TCTGGTATAGTGCAACAGCAGAGC---
AATCTG---CTGCAG---GCTATAGAGGCTCAACAACATCTGTTGAGACTCACGGTCTGGGGCATT
>JF706428
TTCTATTGCAATACATCAGGACTATTTAATGGCACTTTGAATGGCACTGAAACTTGGAATAACACTTGGGGTG
ACAGTAGC---
-------------AATGAG------
AATGAAAATGAGAATATAACTATCCAATGCAGAATAAAACAAATTGTAAATATGTGGCAGAGAGTAGGACGAG
CAATGTATGCCCCTCCCATCAAAGGAGTAATAAAGTGTGAATCAAAC---
ATTACAGGACTACTATTAACAAGAGATGGAGAGGGTAATAATAGG----------------------------
----

ACAAATGAGACCTTCAGGCCTGGAGGAGGAGATATGAGGGATAATTGGAGAAGTGAATTATATAAATATAAAG
TAGTAAAAATTGAACCACTA---GGTATAGCACCCACCCATGCAAAAAGAAGAGTGGTG--------------
-------------------------CAGAGAGAAAAAAGAGCA---GTTGGA---CTGGGAGCT---------
GTCTTCCTTGGGTTCTTAGGAGCA---GCAGGAAGCACTATG---GGCGCGGCGTCA---
ATAACGCTGACGGTACAG---GCCAGACAA---TTATTG---TCTGGTATAGTGCAACAGCAGAGT---
AATCTG---CTGAAG---GCTATAGAGGCTCAACAACATCTGTTGAGACTCACAGTCTGGGGCATT
>KU168310
TTCTATTGCAATACATCACAACTATTTAATAGCACTTGGAACAACACCAATATCAGCTTAAATGAC-------
----

ACAAATGAAAATATAACTCTCCCATGCAGAATAAAGCAAGTTATAAATATGTGGCAGAGAGTAGGACAAGCAA
TGTATGCCCCTCCCATCGCAGGAACACTAAAGTGTAAATCAAAC---
ATTACAGGATTACTATTAACAAGAGATGGAGGGGAGAGCAATAATACA-------------------------
----

AATACAAATGAGACCTTCAGGCCTGGAGGAGGAGATATGAGGGACAATTGGAGAAGTGAATTATATAAGTATA
AAGTAGTAAAAATTGAACCACTA---GGTGTAGCACCTACCCATGCAAAAAGAAGAGTGGTG-----------
----------------------------GAGAGAGAAAAAAGAGCAGTGTTTGGA---CTGGGAGCT------
---GTCTTCCTTGGGTTCTTAGGAGCA---GCAGGAAGCACTATG---GGCGCGGCGTCA---
ATAACGCTGACGGTACAG---GCCAGACAA---CTATTG---TCTGGTATAGTGCAGCAGCAGAGC---
AATTTG---CTGAGG---GCTATAGAGGCTCAACAACATCTGTTGAAACTCACGGTCTGGGGCATT
>JN687776

TTCTATTGTAATACAACAAAACTATTTAATAGCACTTGGACTAATGAGACAAATCACACAGCTAGC-------
----

ATAGATAGAAATATAACTCTCCAATGCAGAATTAAGCAAATTGTAAATATGTGGCAAAGAGTAGGACAAGCAA
TATATGCCCCTCCCATCCCAGGAGTAATAAGGTGTGAATCAAAC---
ATTACAGGACTACTACTAACAAGAGATGGAGGGACAAATAGTTCA----------------------------
----

AATAATGAGACATTCAGGCCTGGAGGAGGAGATATGAGGGACAATTGGAGAAGTGAATTATATAAGTATAAGG
TAGTACAAATTGAACCAATA---GGTGTGGCACCCACCCCTGCAAAAAGAAGAGTGGTG--------------
-------------------------GAGAGAGAAAAAAGAGCA---GTTGGA---GTAGGAGCT---------
TTTATCCTTGGGTTCTTAGGAGCA---GCAGGAAGCACTATG---GGCGCGGCGTCA---
ATAACGCTGACGGTACAA---GCCAGACAA---TTATTG---TCTGGTATAGTGCAACAGCAGAGC---
AATTTG---CTGAAG---GCTATAGAGGCTCAACAACATCTGTTGAAACTCACGGTCTGGGGCATT
>JX140646
TTCTATTGCAATACAACAGAATTATTTAATAAGACTTTTAATGATACTGATCTAAAGTTAAATAGCAGTAGCA
TTAAGGAG---

TCAAATGATACTATAACTCTCCAATGTAGAATAAAGCAATTTGTAAGGATGTGGCAGAGAGTAGGACAAGCAA
TGTATGCCTCTCCCATCCCAGGAGTAATAAGGTGTGAATCAAAC---
ATTACAGGTCTACTATTAACAAGAGATGGAGGGGGTAATGAAAGCACA-------------------------
----

AGAGATAATGAGACATTCAGGCCTGGAGGAGGAGATATGAGAGACAATTGGAGAAGTGAATTATATAAGTATA
AGGTAGTACAAATTGAACCAATA---GGAGTGGCACCCACCTATGCAAAAAGAAGAGTGGTG-----------
----------------------------GAGAGAGAAAAAAGAGCA---GTTGGA---CTGGGAGCT------
---GTCTTCCTTGGGTTCTTAGGGGCA---GCAGGAAGCACTATG---GGCGCGGCGTCA---
ATAACGCTGACGGTACAG---GCCAGGCAA---TTATTG---TCTGGTATAGTGCAACAGCAGGGC---
AATCTG---CTGAGG---GCTATAGAGGCTCAACAACACCTGTTGAAACTCACAGTCTGGGGCATT
>JX140647
TTCTATTGTAATACATCAGGACTATTTAATAGTAGTTGGACCGCGAACGACATTTTGAATAACAGTACTGCTA
GCTCAAAT---

AACACAGAAGTTATAACTCTCCAATGCAGAATAAAGCAAATTATAAATATGTGGCAGACAGTAGGACAAGCAA
TGTATGCCCCTCCCATCCGAGGAGAAATAAGGTGTGAGTCAAAT---
ATTACTGGACTACTATTAACAAGAGATGGAGGGAATAATATGACA----------------------------
----

AATGAAACCTTCAGGCCTGGAGGAGGGGATATGAGGGACAATTGGAGAAGTGAATTATATAAGTATAAAGTAG
TACAAATTGAACCACTA---GGTGTAGCACCCACCCATGCAAAAAGAAGAGTGGTG-----------------
----------------------GAGAGAGAAAAAAGAGCA---GTTGGA---ATAGGAGCT---------
GTCTTCCTTGGGTTCTTAGGAGCA---GCAGGAAGCACTATG---GGCGCGGCGTCA---
ATAACGCTGACGGTACAA---GCCAGACAA---TTATTG---TCTGGTATAGTGCAACAGCAGAGC---
AATTTG---CTGATG---GCTATAGAGGCTCAACAACATATGTTGAAACTCACTGTCTGGGGCATT
>KF859739
TTCTATTGCAGTACATCAGCACTGTTTAATAGCACTTGGCTGTTTAATGAAACTTATAGCACTTGGCATAGCT
CTTGGAACAGT--

AGCGCTGAGAATATAACTCTCCAATGCAGAATAAAGCAATTTGTGAATATGTGGCAGAGAGTAGGGCAAGCAA
TGTATGCCCCTCCCATTCGAGGAATGATAAGGTGTGAATCAAAC---
ATTACAGGACTACTATTAACAAGAGATGGAGGGAATAAAAATACT----------------------------
----

AGTGCAAATGAGACCTTCAGGCCTGGAGGAGGAGATATGAAGGACAATTGGAGAAGTGAATTATATAAGTATA
AAGTAATAAAAATAGAACCACTA---GGTGTGGCACCCACCCCTGCAAAAAGAAGAGTGGTG-----------
----------------------------GGAAGAGAAAAAAGAGCA---GTTGGA---CTAGGAGCT------
---GTCTTCTTTGGGTTCTTAGGAGCA---GCAGGAAGCACTATG---GGCGCAGCGTCA---
ATAACGCTGACGGTACAG---GCCAGACAA---TTATTG---TCTGGTATAGTGCAACAGCAGAGC---
AATTTG---CTGAAG---GCTATAGAGGCTCAACAACATCTGTTGAGACTCACGGTCTGGGGCATT
>KU749419

TTCTATTGCAGTACATCAAGCCTGTTTAATAGCACTTGGAATAATAGCATTGGGGATAATAGCACTAGGGAT-
----

GACAATAGCACTATTACTCTCCCATGCAGAATAAAGCAAATTGTAAATATGTGGCAGGGAGTAGGACAAGCAA
TGTATGCCCCTCCCATCCCAGGAGAAATAAAGTGTGAATCAAAT---
ATTACAGGACTATTATTGACAAGAGATGGTGGGAGAAATAGCACA----------------------------
----

AATGAGACCTTCAGGCCTGGAGGAGGAGATATGAGGGACAATTGGAGAAATGAATTATATAAGTATAAAGTAG
TAAAAATTGAACCACTA---GGTGTGGCACCCACCCATGCAAAAAGAAGGGTGGTG-----------------
----------------------GAGAGAGAAAAAAGAGCA---GTTGGA---CTAGGAGCT---------
GTCTTCCTTGGGTTCTTAGGAGCA---GCAGGAAGCACTATG---GGCGCGGCGTCA---
ATAACGCTGACGGTACAG---GCCAGACAA---TTATTG---TCCGGTATAGTGCAACAGCAGAGC---
AATTTG---CTGAGG---GCTATAGAGGCTCAACAACAGGTGTTGAGACTCACGGTCTGGGGCATT
>MK086109
TTCTATTGCAATACATCTAACCTATTTAATAGCACTTGGGGGAGCAATACCACTACCAGCTGGAATACCAGCT
CAATTGGC---

ACAAATGAAACTATAACTATCCAGTGCAGAATAAAGCAGATTGTAAGAATGTGGCAGAGAGTAGGACAAGCAA
TGTATGCCCCTCCCATCCCAGGAAAAATAAGGTGTAACTCAAGC---
ATTACAGGACTGCTATTAACAAGAGATGGAGGGAATAATAGTAGT----------------------------
----

GACAATGAGACCTTCAGGCCTGGAGGAGGAGATATGAGGGACAATTGGAGAAGTGAATTATATAAGTATAAAG
TAGTAAAAATTGAACCACTG---GGTGTAGCACCCACCCATGCAAGGAGAAGAGTGGTG--------------
-------------------------GAGAGAGAAAAAAGAGCA---GTTGGA---CTGGGAGCT---------
GTCTTCCTTGGGGTCTTAGGAGCA---GCAGGAAGCACTATG---GGCGCGGCGTCA---
ATAACGCTGACGGTACAG---GCCAGACAA---TTATTG---TCTGGTATAGTGCAACAGCAAAGT---
AATTTG---CTGAGG---GCTATAGAGGCTCAACAGCATATGTTGAGACTCACGGTCTGGGGCATT
>MK086113
TTCTATTGCAATACAACAAAACTATTTAATAGCATTTGGAGTAAGAATAGTGGGAATTGGAGTAGTACAAATG
GG---

ACAAATGACACTATAACTCTCCAATGCAGAATAAAACAAATTGTAAGAATGTGGCAGAGAGTAGGACAAGCAA
TGTACGCCCCTCCCATCCGAGGAAACATAACGTGTGGATCAAAC---
ATTACAGGATTACTATTAACAAGAGATGGAGATGGAGGTGCACAT----------------------------
----

AGTCCCAATGAAACATTCAGGCCTGGAGGAGGAAATATGAAGGACAATTGGAGAAGTGAATTATACAAGTATA
AGGTAGTACAAATTAAACCATTA---GGTGTAGCACCCACCCAGGCAAAAAGAAGAGTGGTG-----------
----------------------------GAGAGAGAAAAAAGAGCA---GTTGGA---CTGGGAGCT------
---GTCTTCCTTGGGTTCTTAGGAGCA---GCAGGAAGCACTATG---GGCGCGGCGTCA---
CTGACGCTGACGGTACAG---GCCAGACAA---TTATTG---TCTGGTATAGTGCAGCAGCAGAGC---
AATCTG---CTGCGG---GCCATAGAGGCTCAACAACATCTGTTGAAACTCACGGTCTGGGGCATT
>MK086125
TTCTATTGCAATACATCAGGCCTATTCAATAGCACTTGGCACAATAGCACTTGGGATAGTAATGAAACTGCCA
GTGCA--

ACAAATGACACTATAACTCTCCGATGCAGAATAAAGCAATTTGTAAATATGTGGCAGAAAGTAGGACGAGCAA
TGTATGCCCCTCCCATTCCAGGAGTAATAAGATGTGAGTCAAAC---
ATTACAGGATTACTATTAACAAGAGATGGAGGAAATAATAATAAT----------------------------
----

AGTGTAAATGAGACATTCAGGCCGGGAGGAGGAGATATGAGGGACAATTGGAGAAGTGAATTATATAAGTATA
AAGTAGTACAAATTGAGCCACTA---GGTGTAGCACCCACCCATGCAAGAAGAAGAGTGGTA-----------
----------------------------CAGAGGGAAAAAAGAGCA---GTTGGA---CTGGGAGCT------
---GTCTTCCTTGGGTTCTTAGGAGCA---GCAGGAAGCACTATG---GGCGCGGCGTCA---
ATAACGCTGACGGTACAG---GCCAGACAA---TTATTG---TCTGGTATAGTGCAGCAGCAGAGT---
AATTTG---CTGCGG---GCTATAGAGGCTCAACAACATCTGTTGAAACTCACGGTCTGGGGCATT
>HQ912706

TTCTATTGCAATACAACAGAACTATTTAATAGCACTTGGAACGTAACTGCCAGCTCAAACTACACAGAGTCAA
AC---

TCAAATGACAGTATAACTCTCCCATGCAGAATAAAACAAATTGTGAATTTATGGCAGAAAGTAGGACAAGCAA
TGTATGCCCCTCCCATCCAAGGAGAAATAAGGTGTGAATCAAAC---
ATTACAGGATTACTATTAACAAGAGATGGAGGGAATAATAGTACA----------------------------
----

AATGAGACATTCAGGCCTGGAGGAGGAGATATGAGGGACAATTGGAGAAGTGAATTATATAAGTATAAGGTAG
TACAAATTGAACCACTA---GGTGTGGCACCCACCCAGGCAAAAAGAAGAGTGGTG-----------------
----------------------GAGAGAGAAAAAAGAGCA---GTTGGA---ATAGGAGCT---------
GTCTTCCTTGGGTTCTTAGGAGCA---GCAGGAAGCACTATG---GGCGCGGCGTCA---
CTAACGCTGACGGTACAG---GCCAGACAA---TTATTG---TCTGGTATAGTGCAACAGCAGAGC---
AATCTG---CTGAGG---GCTATAGAGGCTCAACAACATCTGTTGAAACTCACGGTCTGGGGCATT
>AM279349
TTCTATTGCAATACATCAGGCCTGTTTAATAACACTTTGAATAATAACACTTGGACTTGGGATAATAGCACA-
----

AAGGATGACAATATAACTCTCCGATGCAGGATAAAGCAAATTGTAAATATGTGGCAGAGAGTAGGACAAGCAA
TGTATGCCCCTCCCATCCAAGGAGTGATAAGGTGTGAATCAAAC---
ATTACAGGATTACTATTAACAAGAGATGGAGGGAATAATAATAGC----------------------------
----

ACAACAAATGAGACCTTCAGGCCTGGAGGAGGAGATATGAGGGACAATTGGAGAAGTGAATTATATAAATATA
AGGTAGTAAAAATTGAACCACTA---GGTGTAGCGCCTACCCATGCAAAAAGAAGAGTGGTG-----------
----------------------------GAGAGAGAAAAAAGAGCA---GTTGGA---CTGGGAGCT------
---GTCTTCCTTGGGTTCTTAGGAGCA---GCAGGAAGCACTATG---GGCGCGGCGTCA---
ATAACGCTGACGGTACAG---GCCAGACAA---TTATTG---TCCGGTATAGTGCAACAGCAGAGC---
AATCTG---CTGAAG---GCTATAGAGGCTCAACAACATCTGTTGAGACTCACGGTCTGGGGCATT
>AM279361
TTCTATTGCAATACATCAGCACTGTTCAATAGCACTTATGACAATAGCACTTGGAATGACAGTACAAATACCA
GCTCA--

GAGACAAATGACACTATAACTCTCCAATGCAAAATAAAGCAAATTGTAAGAATGTGGCAGAGAGTAGGACAAG
CAATGTATGCCCCTCCCATCCCAGGAATAATAAGGTGTGAATCAAAC---
ATTACAGGACTAATATTAACAAGAGATGGAGGGAATAATAATAGT----------------------------
----

GAAAATCAGACTTTCAGGCCTGGAGGAGGAGATATGAGGGACAATTGGAGAAGTGAATTATATAAGTATAAAG
TAGTAAAAATTGAACCACTA---GGTATAGCACCCACCCATGCAAAAAGAAGAGTGGTG--------------
-------------------------CAGAGAGAAAAAAGAGCA---GTTGGA---ATAGGAGCT---------
GTCTTCCTTGGGTTCTTAGGAGCA---GCGGGAAGCACTATG---GGCGCGGCGTCA---
ATAACGCTGACGGTACAG---GCCAGACAA---TTATTG---TCCGGTATAGTGCAACAGCAGAAC---
AATTTG---CTGAAG---GCTATAGAGGCTCAACAACAGTTGTTGAAACTCACAGTCTGGGGCATT
>AM279360
TTCTATTGCAATACAACAAAACTGTTTAATAGCACGTGGAATAGCAGTACTGGGAATAACAATGTCAGC----
----

TCAAATGACACTATAACCCTCCAATGCAAAATAAAGCAAATTGTAAATATGTGGCAAAAAGTAGGACAAGTAA
TGTATGCCCCTCCCATCCCAGGAATAATAAGGTGTGAATCAAAT---
ATTACAGGACTATTATTAACAAGAGATGGAGGAAATAATAATGAAACA-------------------------
----

GAGAATGGGACTGAGACCTTCAGGCCTGGAGGAGGAGATATGAGGGACAATTGGAGAAGTGAATTGTATAAGT
ATAAAGTAGTAAAAATTGAACCACTA---GGTGTAGCACCCACCCATGCAAAAAGAAGAGTGGTG---GAG--
-------------------------------AGGAGAGAAAAAAGAGCA---GTTGGA---CTGGGAGCT---
------GTCTTCTTTGGGTTCTTAGGAGCA---GCAGGAAGCACTATG---GGCGCGGCGTCA---
CTAACGCTGACGGTACAG---GCCAGACAA---TTATTG---TCTGGTATAGTGCAACAGCAGAGC---
AATTTG---CTGAAG---GCTATAGAGGCTCAACAACATCTGTTGAAACTCACGGTCTGGGGCATT
>AM279358

TTCTACTGCAATACATCAGCACTGTTTAATAGCACTTGGAATAACACTTTGGATACTAACAATAGCACTCAG-
----

GATAATGACACTATAACTCTCCAATGCAGAATAAAGCAAATTATAAGGATGTGGCAGACAGTAGGACAAGCAA
TGTATGCCCCTCCCATCCAAGGAAAAATAAGGTGTGAATCAAAC---
ATTACAGGACTACTGTTAACAAGAGATGGAGGGAACAGTGATGGT----------------------------
----

GCAAATGAGACCTTCAGGCCTGGAGGAGGAGATATGAGGGACAATTGGAGAAGTGAATTATATAAGTATAAAG
TAGTAAAAATTGAACCACTA---GGTGTAGCACCCACCCGAGCAAAAAGAAGAGTGGTG--------------
-------------------------GAGAGAGAAAAAAGAGCAGTAGTTGGA---CTGGGAGCT---------
GTCTTCCTTGGGTTCTTAGGAGCA---GCAGGAAGCACTATG---GGCGCGGCGTCA---
CTAACGCTGACGGTACAG---GCCAGACAA---TTATTG---TCCGGTATAGTGCAACAGCAGAGC---
AATCTG---CTGAGG---GCTATAGAGGCTCAACAACATATGTTGAAACTCACAGTCTGGGGCATT
>AJ286133
TTCTATTGTAATACATCAGAACTATTTAATAGCATTTGGAATATTAATAGCACTTGGAATAACAGTGACACTG
CCAGC--
-------------------TCG---
AAGCCAAATGACACTATAACTCTACAATGCAGAATAAGGCAAATTGTAAGAATGTGGCAGAAAGTAGGACAAG
CAATATATGCCCCTCCTATCCCAGGAGTAATAAGGTGTGAATCAAAC---
ATTACAGGACTACTATTAACAAGAGATGGAGGGGAAGATAATAAT----------------------------
----

GCAAGTGAGACATTCAGGCCTGGAGGAGGAGATATGAGGGACAATTGGAGAAGTGAATTATATAAGTATAAGG
TAGTACAAATTGAACCACTA---GGCGTGGCACCCACCTGGGCAAAAAGAAGAGTGGTG--------------
-------------------------GAGAGAGGAAAAAGAGCA---GTTGGA---CTGGGAGCT---------
GTCTTCCTTGGGTTTTTAGGAGCA---GCAGGAAGCACTATG---GGC---CGGTCA---
ATAACGCTGACGGTACAG---GCCAGACAA---TTATTG---TCCGGTATAGTGCAACAGCAGAGC---
AATTTG---CTGAGG---GCTATAGAGGCTCAACAACATCTGTTGAAACTCACGGTCTGGGGCATT
>AF377955
TTCTATTGTAATACATCAGCACTGTTTAATAGTACTTGGTTTAGCAATAGTACTGGGGATAACAATAGTTCAA
CA---

GGGAATGAAACTATAACTCTCCAATGCAGAATAAAGCAAATTGTAAATATGTGGCAGAGAGTAGGACAAGCAA
TGTATGCCCCTCCCATCCAAGGAGTAATAAGGTGTGAATCAAAC---
ATTACAGGATTACTATTGACAAGAGATGGAGGGAATAATAATAGC----------------------------
----

ACAAATGAGACCTTCAGACCTGGAGGAGGAGATATGAGGGACAATTGGAGAAGTGAATTATATAAGTATAAAG
TAGTAAAACTTGAGCCACTG---GGTGTAGCACCCACCCGTGCAAAAAGAAGAGTGGTG--------------
-------------------------GAGAGAGAAAAAAGAGCA---GTTGGA---CTGGGAGCT---------
GTCTTCTTTGGGTTCTTAGGAGCA---GCAGGAAGCACTATG---GGCGCGGCGTCA---
ATAACGCTGACGGTACAG---GCCAGACTA---TTATTG---TCTGGTATAGTGCAACAGCAGAGC---
AATTTG---CTGAAA---GCTATAGAGGCTCAACAGCATCTGTTGAAACTCACGGTCTGGGGCATT
>AM279352
TTCTATTGCAATACATCAGGACTCTTTAATAGCACTTGGAATAGCAATGACACTGACAGATCAAACCACACAG
GA---

CCACATAACAATATAACTCTCCAATGCAGAATAAAGCAAATTGTAAATATGTGGCAGAGAGTAGGACAAGCAA
TGTATGCCCCTCCCATCCAAGGCGAAATAAGGTGTGTATCAAAC---
ATTACAGGATTACTACTAACAAGAGATGGAGGGATCAGTAATAAG----------------------------
----

ACAGAGGAGATATTCAGGCCTGGAGGAGGAGATATGAGGGACAATTGGAGAAGTGAATTATATAAGTATAAGG
TAGTACAAATTAAACCACTA---GGTGTGGCACCAGACCGGGCAAAAAGAAGAGTGGTG--------------
-------------------------GGAAGAGAAAAAAGAGCA---GTTGGA---ATGGGAGCT---------
CTTTTCCTTGGGTTCTTAGGAGCA---GCAGGAAGCACTATG---GGCGCGGCGGCA---
ATGACGCTGACGGTACAG---GCCAGACAA---TTATTG---TCTGGTATAGTGCAACAGCAGAGC---
AATTTG---CTGAGG---GCTATAGAGGCTCAACAACATCTGTTGAAACTCACGGTCTGGGGCATT
>AY271690

TTCTATTGCAATACATCAGGACTATTTAATAGCACTTGGAGTAGGAATAGCACTGATAACAGCACTTGGAATA
TCAATGACACT--
----------GTCAGCTCA------
AATCACACAGGAAATATAACTCTCCAATGCAGAATAAGGCAAGTTGTAAGAATGTGGCAGAGGGTAGGACAAG
CAATGTATGCCCCTCCCATCCCAGGAGAAATTAAGTGTAAATCAAAC---
ATTACAGGAATACTATTGACAAGAGATGGAGGGACAAAAAATCAGAATGCAAGT-------------------
----
-
GAGGAATTTAATACAACTGAGGTATTCAGACCTGGAGGAGGAGACATGAGGGACAATTGGAGAAGTGAATTAT
ATAAATATAAGGTAGTACAAATTGAACCAATA---GGTGTGGCACCCACCCGTGCAAAAAGAAGAGTGGTG--
-------------------------------------CAGAAAGAAAAAAGAGCA---GTTGGA---
CTGGGAGCT---------TTCTTCCTTGGGTTCTTAGGAGTT---GCAGGAAGCACTATG---
GGCGCAGCGTCA---ATAACGCTGACGGTACAG---GCCAGACAA---TTATTG---
TCTGGTATAGTGCAACAGCAGAGC---AATCTG---CTGAAG---
GCTATAGAGGCTCAACAACATATATTGAGACTCACGGTCTGGGGCATT
>HQ700002
TTTTATTGCAATACATCAAAGCTGTTTAATAGCACCTGGCCTGGGAATATCACTGGGAATAACTATACCAGCT
CA---

AACTCAAGTGACAATATAGCTCTCCAATGCAGAATAAGACAAATTGTAAATATGTGGCAGAAAGTAGGAAAAG
CAATATATGCTCCTCCCATCCCAGGAGAAATAAAGTGTGAATCAAAC---
ATTACTGGACTACTACTAACAAGAGATGGAGGAAACAATAATGCA----------------------------
----

AGTAATGAGACCTTCAGGCCTGGAGGAGGAGATATGAGGGACAATTGGAGAAGTGAATTATATAAGTATAAAG
TAGTGAGAATTAACCCACTA---GGTATAGCACCCACCCACGCAAAAAGAAGAGTGGTG--------------
-------------------------GAGAGAGAAAAAAGAGCA---GTTGGA---CTGGGAGCT---------
GTCTTCCTTGGGTTCTTAGGAGCA---GCAGGAAGCACTATG---GGCGCGGCGTCA---
ATAACGCTGACGGTACAA---GCCAGACAA---TTATTG---TCTGGTATAGTGCAACAGCAGAGC---
AATCTG---CTGAAG---GCTATAGAGGCTCAACAGCATCTGTTGAAGTTCACAGTCTGGGGCATT
>JF683758
TTCTATTGCAATACATCAGGACTGTTTAATAGCATTTGGGATAATAACAGCACTTGGAATGGGAGC-------
----

AACTCAGGCAATATAACTCTCCCATGCAGAATAAGGCAAATTGTAAATATGTGGCAGAGAGTAGGAAAAGCAA
TGTATGCCCCTCCCATCCAAGGAGAAATAAGGTGTGACTCAAAC---
ATTACTGGACTACTATTAGCAAGAGATGGAGGGGATGGTACTGAG----------------------------
----

ACAAATGAGACTTTCAGGCCTATAGGAGGAGATATGAGAGACAATTGGAGAAGTGAATTATATAAGTATAAAG
TAGTAAAAATTGAACCATTA---GGCATAGCACCCACCCGGGCAAAAAGAAGAGTGGTG--------------
-------------------------AGCAGAGAGAAAAGAGCA---GTTGGA---ATGGCAGCT---------
ATCTTCCTTGGGTTCTTAGGAGCA---GCAGGAAGCACTATG---GGCGCGGCGTCA---
ATAACGCTGACGGTACAG---GCCAGACAG---TTATTG---TCTGGTATAGTGCAACAGCAGAGC---
AATCTG---CTGCAG---GCTATAGAGGCTCAACAGCACATGTTGAGACTCACGGTCTGGGGCATT
>JF683786
TTCTATTGCAATACATCAAAGTTGTTTAATAGCAGTTGGGGAAACAACACTGCCAGTAACCATACAGAGGCAA
GT---

GGAGAAAATGACACTATAACTATACAATGCAGAATAAAGCAAATTATAAATCTATGGCAGAGAGTAGGACAAG
CAATGTATGCCCCTCCTATCCAAGGAATAATAAGGTGTGAATCAAAC---
ATTACAGGATTACTATTAACAAGAGATGGAGGGAATAATAGTAGT----------------------------
----

GGTGCAGATGAGACTTTCAGGCCTGGAGGAGGAGATATGAGAGACAATTGGAGAAGTGAATTATATAAGTATA
AAGTAGTAAAAATTGAACCACTA---GGTGTTGCACCCACCCGTGCAAAAAGAAGAGTGGTG-----------
----------------------------GAAAGACAAAAAAGAGCA---ATTGGA---ATGGGAGCT------
---GTCTTCCTTGGGTTCTTAGGAGCA---GCAGGAAGCACTATG---GGCGCAGCGTCA---
CTATCGCTGACGGTACAG---GCCAGACAA---TTATTG---TCCGGTATAGTGCAACAGCAGAGC---
AATTTG---CTGAAG---GCTATAGAGGCTCAACAACATATGTTGAGACTCACGGTCTGGGGCATT

>JF683792
TTCTACTGCAATACAACAAACCTGTTTAATAGCACTTGGAATAGCACTTGGTATAATAATGGGAGTAGCGCCA
AC---

AATATAACTCACACTATAACTCTCCCATGCAGAATAAGGCAAATTGTAAATATGTGGCAGAGAGTAGGACAAG
CAATGTATGCCAATCCCATCCCAGGAGTAATAAGGTGTGAATCAAAC---
ATTACAGGACTACTACTAACAAGAGATGGAGGGAATAATAACTCA----------------------------
----

AATGAAGAGACCTTCAGGCCTGGAGGAGGAGATATGAGGGACAATTGGAGAAGTGAATTATATAAGTATAAAG
TAGTAAAAAATTGAACCACTA---GGTCTAGCACCCACCCATGCAAAAAGAAGAGTGGTG-------------
------------------------GAAAGAGAAAAAAGAGCAGTTGTTGGA---CTGGGAGCT---------
GTCTTCCTTGGGTTCTTAGGAGCA---GCAGGAAGCACTATG---GGCGCAGCGTCA---
ATAACGCTGACGGTACAG---GCCAGACAA---TTATTG---TCTGGTATAGTGCAACAGCAGAGC---
AATTTG---CTGAAA---GCTATAGAGGCTCAACAACAGCTGTTGAGACTCACAGTCTGGGGCATT
>JF683795
TTCTATTGCAATACATCAGGACTATTTAATAGCACTTGGGATAATAGCACTTGGAATACCACTGCAAATTACA
CA---

GAGTCAAGTGACAATATAACTCTCCAATGCAGAATAAAGCAAATTGTAAATATGTGGCAGAGAGCAGGACAAG
CAATATATGCCCCTCCCATCCCAGGAGTAATAAGGTGTGAATCAAAC---
ATTACAGGACTACTATTAACAAGAGATGGAGGAAATGACAGTAGT----------------------------
----

ACAGAGGAGACCTTCAGGCCTGGAGGAGGAGATATGAGGGACAATTGGAGAAGTGAACTATATAAGTATAAAG
TGGTAAAAAATTGAACCACTA---GGTGTAGCACCCACCCGTGCAAAAAGAAGAGTGGTG-------------
------------------------GAGAGAGGAAAAAGAGCAGTAGTTGGA---CTGGGAGCT---------
GTCTTCCTTGGGTTCTTAGGAGCA---GCAGGAAGCACTATG---GGCGCGGCGTCA---
ATAACGCTGACGGTACAG---GCCAGACAA---TTATTG---TCCGGTATAGTGCAACAGCAGAGC---
AATTTG---CTGAAG---GCTATAGAGGCTCAACAACATATGTTGAAACTCACAGTCTGGGGCATT
>JF683799
TTTTATTGCGATACATCAAGACTGTTTAATAGCACTTGGGATACTAGCACTGGGTATGACAATAGCAGCAGA-
----

GACAATGACACTATAACTCTCAAATGCAAAATAAGGCAAGTTGTAAGGATGTGGCAAAGAGTAGCACAAGCAA
TGTATGCCCCTCCCATCCAAGGAGAAATAAGGTGTAACTCAACA---
ATTACAGGACTACTATTAACAAGAGATGGAGGGAATAGTAGTGGT----------------------------
----

GCAAATGAGACCTTCAGGCCTGGAGGAGGAGATATGAGGGACAATTGGAGAAGTGAATTATATAAGTATAAAG
TAGTAAAGATTGAACCACTA---GGTGTAGCACCCACCCATGCAAAAAGAAGAGTGGTG-------------
------------------------GAGAGAGAAAAAAGAGCA---GTTGGA---ATAGGAGCT---------
GTCCTCCTTGGGTTCTTAGGAGCA---GCAGGAAGCACTATG---GGCGCGGCGTCA---
ATAACGCTGACGGTACAG---ACCAGACAA---TTATTG---TCTGGTATAGTGCAGCAGCAGAGC---
AATTTG---CTGAAG---GCTATAGAGGCTCAACAACAGCTGTTGAGACTCACGGTCTGGGGCATT
>KT124792
TTCTATTGCAATACATCAGGACTGTTTAATAGCACTTGGATCAATGGGAGCTGGGTCAATAATGATACCTGGG
CCAAT--
-------------------AAT---
GATACCTCAAAAAATATAACTCTCCAATGCAGAATAAAGCAAATTATAAATATGTGGCAGAGAGTAGGGCAAG
CAATGTATGCCCCTCCCATCAAAGGAGAAATAAGGTGTAGATCAAAC---
ATTACTGGACTACTATTAACAAGAGATGGAGGGAATAATAGTAGT----------------------------
----

GGAAATGAGACATTCAGGCCGGCAGGAGGAGATATGAGAGACAATTGGAGAAGTGAATTATATAAGTATAAGG
TAGTAAAAAATTGAACCACTA---GGTGTAGCACCCACACATGCAAGGAGACAAGTAGTG-------------
------------------------GGGAGAGAAAAAAGAGCA---GTTGGA---CTGGGAGCT---------
GTCTTCCTTGGGTTCTTAGGAGCA---GCAGGAAGCACTATG---GGCGCGGCGTCA---
ATAACGCTGACGGTACAG---GCCAGACAA---TTATTG---TCTGGTATAGTGCAGCAGCAGAGC---
AATCTG---CTGAAG---GCTATAGAGGCTCAACAACATATGTTGAGACTCACAGTCTGGGGCATT

>L22939
TTCTATTGCAATACATCAAGTCTGTTTAATAGCACTTGGAATAGCAGCTCAATTCACACAAATTACAGCTCAA
AT---

GACACAGACAATATAACTCTCCAATGCAGAATAAAGCAAATTGTAAATATGTGGCAGAAAGTAGGAAAAGCAA
TATATGCCCCTCCCATCTCAGGAGAAATAAGGTGTGAATCAAAC---
ATTACAGGACTAATATTAACAAGAGATGGAGGGAGTAATAATAGT----------------------------
----

ACAAATGAGATCTTCAGGCCTGGAGGAGGAGATATGAGGGATAATTGGAGAAGTGAATTATATAAGTATAAAG
TAGTAAAAATTGAACCACTA---GGTGTAGCACCCACCCGTGCAAAAAGAAGAGTGGTG--------------
-------------------------GAGAGAGAAAAAAGAGCA---GTTGGA---CTGGGAGCT---------
TTCTTCCTTGGGTTCTTAGGAGCT---GCAGGAAGCACTATG---GGCGCGGCGTCA---
ATAACGCTGACGGTACAG---GCCAGACAA---TTATTG---TCCGGTATAGTGCAACAGCAGAGC---
AATTTG---CTGAGG---GCTATAGAGGCTCAACAACATCTGTTGAAACTCACGGTCTGGGGCATT
>AY151002
TTCTATTGCAATACAGCAGGCCTGTTTAATAGCACTTGGGATGATAACAATAGCACAGGGTCAAACACAAAG-
----

TCAAATGACATTATAACTCTCCAATGTAGAATAAAGCAACTTATAAATATGTGGCAGAGAGTAGGACAGGCAA
TGTATGCCCCTCCCATCCGAGGAATAATAAGGTGTAACTCAAAC---
ATTACTGGATTACTATTAACAAGAGATGGAGGGAATAATAGTACA----------------------------
----

AATGAGACCTTCAGGCCTGGAGGAGGAGATATGAGGGACAATTGGAGGAGTGAATTATATAAATATAAAGTAG
TAAAAATTGAACCACTA---GGTGTAGCACCCACCCCTGCAAAAAGAAGAGTGGTG-----------------
----------------------GAGAGAGAAAAAAGAGCA---GCTGGA---CTGGGAGCT---------
GTCTTCCTTGGGTTCTTAGGAGCA---GCAGGAAGCACTATG---GGCGCGGCGTCA---
ATAACGCTGACGGTACAG---GCCAGACAG---TTATTG---TCCGGTATAGTGCAACAGCAAAGC---
AATTTG---CTGAAG---GCTATAGAGGCTCAACAACATATGTTGAGACTCACGGTCTGGGGCATT
>MK177825
TTCTATTGCAATACATCACAACTGTTTAATAGCACCTGGAATGAACATAACATTACCAACTCAACCTTTACAG
AG---

GACAATACAACTCTCACTCTCACATGTAGAATAAAGCAATTTATAAATATGTGGCAGAGAGTAGGACAAGCAA
TGTATGCCCCTCCCATCCAAGGAGAAATAAAATGTAATTCAAAC---
ATTACTGGATTACTGTTAACACGGGATGGAGGGAAGAACACTGAG----------------------------
----

AGCAATGAGACCTTCAGGCCTGGAGGAGGGGATATGAGGAACAATTGGAGAAGTGAATTATATAAGTATAAGG
TAGTAGAAATTAAACCACTA---GGTGTAGCACCCTCACCTGCAAAAAGAAGAGTGGTG--------------
-------------------------GAGAGAGAAAAAAGAGCA---GCA------CTGGGAGCT---------
GTCTTCCTTGGGTTCTTAGGAGCA---GCAGGAAGCACTATG---GGCGCGGCGTCA---
ATAACGCTGACGGTACAG---GCCAGAAAG---TTATTG---TCTGGTATAGTGCACCAGCAAAGC---
AATTTG---CTGAAG---GCTATAGAGGCTCAACAACATATGTTGAGACTCACGGTCTGGGGCATT
>MK177826
TTCTATTGCAATACATCAAAACTGTTTAATAGCACCTGGGAGAATGAAAATAACACTACCAACTCAAACTCTG
CA---

GAGAATATAACTCTCACTCTCCCATGTAGAATAAAGCAAGTCATAAATATGTGGCAGAGAGTAGGACAAGCAA
TGTATGCCCCTCCCATCCAAGGAATAATAAAATGTAATTCAAAC---
ATTACTGGATTACTGCTAACACGGGATGGAGGGAATAACAATACA----------------------------
----

ACGAATGAGACCTTCAGGCCTGGAGGAGGGGATATGAGGGACAATTGGAGAAGTGAATTATACAAGTATAAAG
TAGTAAAAATTGAACCACTA---GGTGTAGCACCCTCCTATGCAAAAAGAAGAGTGGTG--------------
-------------------------GAGAGAGAAAAAAGAGCA---GTTGGA---CTAGGAGCT---------
GTCTTCCTTGGGTTCTTAGGAGCA---GCAGGAAGCACTATG---GGCGCGGCGTCA---
ATAACGCTGACGGTACAG---GCCAGACAA---TTATTG---TCTGGTATAGTGCAACAGCAGAGC---
AATCTG---CTGAAG---GCTATAGAGGCTCAACAACAAATGTTGAGACTCACGGTCTGGGGCATT

>KY989950
TTCTATTGTAATACATCAAAATTGTTTAATAGCACTTGGAATACCAGCCACACAGAG----------------
----

TCAGGTGACAATATAACTCTCCCATGCCGAATAAAACAAATTGTAAGGATGTGGCAGAATGTAGGACAAGCAA
TGTATGCCCCTCCCATCCCAGGAGAAATAAGGTGTGACTCAAAC---
ATTACTGGACTACTATTAACAAGAGATGGAGGGGAGGAAAATAGGAATGGGACA------------
AACGGGAATGGG---ACA---

AAGGAGGAGACCTTCAGGCCTGGAGGAGGAGATATGAGGGACAACTGGAGAAGTGAATTATACAAGTATAAAG
TAGTAAAAATTGAACCAGTA---GGTGTAGCACCTACTCATGCAAAAAGAAGAGTGGTG--------------
-------------------------GAGAGAGAAAAAAGAGCA---GTA------CTTGGAGCT---------
GTCTTCCTTGGGTTCTTGGGAGCA---GCAGGAAGCACTATG---GGCGCGGCGTCA---
ATAACGCTGACGGTACAG---GCCAGACAA---TTGTTG---TCTGGTATAGTGCAACAGCAAAGC---
AATTTG---CTGAAG---GCTATAGAGGCTCAGCAACAACTGTTGAGGCTCACGGTCTGGGGCATT
>MF157740
TTCTATTGCAGTACATCAGAACTGTTTAATAGCACTTGGAAACGGGTTAATAACACTACTACTTGGAATAGCA
CTGCCAAC---

TCAACAAATGACCCTATAACTCTCCGGTGTAGAATAAAGCAAATTGTAAGGATGTGGCAGAGAGTAGAACAAG
CAATGTATGCCCCTCCCATCCGAGGAGAAATAAGGTGTGAGTCAAAC---
ATTACAGGATTACTATTAACAAGAGATGGAGGGCATGATGATGGGACAGAT----------------------
----

GAGACAGAGGAGACCTTCAGGCCTGGAGGAGGAGATATGAGGGACAATTGGAGAAGTGAATTATATAAGTATA
AAGTAGTAAAAATTGAACCACTA---GGTGTAGCACCCACCCATGCAAAAAGAAGAGTGGTG-----------
----------------------------GAGAGAGAAAAAAGAGCA---GTTGGA---CTGGGAGCT------
---GTCTTCCTTGGGTTCTTAGGAGCA---GCAGGAAGCACTATG---GGCGCGGCGTCA---
ATAACGCTGACGGTACAG---GCCAGACAA---TTATTG---TCTGGTATAGTGCAACAGCAGAGC---
AATTTG---CTGAGA---GCTATAGAGGCTCAACAACATCTGTTGAGACTCACGGTCTGGGGCATT
>MH742763
TTCTATTGCAATACATCAGAATTATTTAACAATAACACAGAATTAAACAATGGCACTGCCGGC----------
----

GTAAATGACACTATAAAACTCCCATGCAGGGTAAAGCAAATTGTAAGAATGTGGCAGAAAGTAGGGCAAGCAA
TGTATGCCCCTCCCATCAAAGGAGTAATAAGGTGTAACTCAAAC---
ATTACAGGACTACTGTTAACAAGGGATGGAGGGAATACTAGCACA----------------------------
----

GAGGAGGAGATATTTAGGCCTGGAGGAGGAGATATGAGGGACAACTGGAGAAGTGAATTATATAAGTATAAGG
TAGTACAAATTGAACCATTA---GGTGTGGCACCCACCCATGCAAAAAGAAGAGTGGTG--------------
-------------------------GAGAGAGAAAAAAGAGCA---CTTGGA---ATAGGAGCT---------
GTCTTCATTGGGTTCTTAGGAGCA---GCAGGAAGCACTATG---GGCGCGGCGTCA---
ATAACGCTGACGGTACAG---GCCAGACAA---TTATTG---TCCGGTATAGTGCAACAGCAGAGC---
AATCTG---CTGAGG---GCTATAGAGGCTCAACAACATCTGTTGAAACTCACGGTCTGGGGCATT
>MH742765
TTCTATTGCAATACATCAGCACTGTTTAATAGCACTTGGAATAACAGTACTGGCACTTGGACTGACACTAACA
ATAGCAGTTCA--

GAGTCAAATGGCACTATCACTCTCAAATGCAAAATAAAGCAAATTGTAAATATGTGGCAGAGAGTAGGGCAAG
CAATGTATGCCCCTCCCATCGAAGGAATAATAAGGTGTGAATCAAAC---
ATTACAGGACTACTATTAACAAGAGATGGAGGGAATAATAATGCA----------------------------
----

AATGAGACTTTCAGGCCTGGAGGAGGAGATATGAGGGATAATTGGAGAAGTGAATTATATAAATATAAAGTAG
TAAAAATTGAACCACTA---GGTGTAGCACCCACCTATGCAAAAAGAAGAGTGGTG-----------------
----------------------GAGAGAGAAAAAAGAGCA---GTTGGA---CTGGGAGCT---------
GTTCTCTTTGGGTTTCTAGGAGCA---GCAGGAAGCACTATG---GGCGCGGCGTCA---
ATAACGCTGACGGTACAG---GCCAGACAA---TTATTG---TCCGGTATAGTGCAACAGCAGAGC---
AATTTG---CTGAGA---GCTATAGAGGCTCAACAACATCTGTTGAAACTCACGGTCTGGGGCATT

>MH742766
TTCTACTGCAATACGACAGACCTGTTTAACAGCCAGTGGCCCACTAACAACTCAGAG----------------
----

TCAAATGACACTATAACTCTCCAATGCAGAATAAAGCAATTTATAAATATGTGGCAGAGAGTAGGACAAGCAA
TGTATGCCCCTCCCATCCAAGGAAATATAAGTTGTAGATCAAAC---
ATTACAGGACTACTATTAACAAGAGATGGAGGGAATAATAATAGT----------------------------
----

AAGACAAATGAGACTTTTAGGCCTGGAGGAGGAGATATGAGGGACAATTGGAGAAGTGAATTATATAAGTATA
AAGTAGTAAAAATTGAACCAATA---GGTGTAGCACCCACCCATGCAAAAAGAAGAGTGGTG----------
----------------------------GAGAGAGAAAAAAGAGCA---GCTGGA---CTGGGAGCT------
---GTCTTCCTTGGGTTCTTAGGAGCA---GCAGGAAGCACTATG---GGCGCGGCGTCA---
ATAACGCTGACGGTACAG---GCCAGACAA---TTATTG---TCTGGTATAGTGCAACAGCAGAGC---
AATCTG---CTGAGG---GCTATAGAGGCTCAACAGCATCTGTTGAAACTCACGGTCTGGGGCATT
>MH742768
TTCTATTGCAATACAACAAAACTGTTTAATAGCACTTGGTTTAACAATGGCACAAACGCCACAAACAACGAAG
CA---

ATGAATGACACTCTAACTCTCCAATGCAGAATAAAGCAAATTGTGAGAATGTGGCAGACAGTAGGACAAGCAA
TGTATGCCCCTCCCATCTCAGGAGAGATAAGGTGTAATTCAAAC---
ATTACTGGGTTGCTATTAACAAGGGATGGAGGGGAGGATAATAGTACAAAAAAT-------------------
----

GAGACAAAGGAGACTTTCAGGCCTGGAGGAGGAGATATGAGGGACAATTGGAGAAGTGAATTATATAAATATA
AAGTAGTAAAAATTGAACCTCTA---GGTGTAGCACCCACCCGTGCAAAGAGAAGAGTGGTG----------
----------------------------GAGAGAGAAAAAAGAGCA---GTTGGA---CTGGGAGCT------
---GTCTTCCTTGGGTTCTTAGGAGCA---GCAGGAAGCACTATG---GGCGCGGCGTCA---
ATAACGCTGACGGTACAG---GCCAGACAA---TTATTG---TCTGGTATAGTGCAACAGCAGAGC---
AATCTG---CTGAAG---GCTATAGAGGCTCAACAACAGCTGTTGAGACTAACAGTCTGGGGCATT
>MH742770
TTCTATTGTGATACATCAGGTCTGTTTAATAGCGTTTGGGATAGGAATGGCACTACTTGGAATAGCAACACCA
CAAGT--

GACACTGATGATATAATTCTCTCATGCAGAATAAGACAAATTATAAATATGTGGCAGAGAGTAGGACAAGCAA
TGTATGCCCCTCCCATTAAAGGAGTGATAAACTGTGAATCAAAC---
ATTACAGGGCTACTGCTAACAAGAGATGGAGGGACTAATAGGGGT----------------------------
----

AGTACAAATGAGACTTTCAGGCCTGTAGGAGGAGATATGAGAGACAATTGGAGAAGTGAATTATATAAGTATA
AAGTAGTAAAAATTGAGCCACTA---GGAGTAGCACCCACCAGGGCAAAAAGAAGAGTGGTG----------
----------------------------GAGAGAGAAAAAAGAGCA---GTTGGA---CTGGGAGCT------
---GTCTTCTTTGGGTTCTTAGGAGCA---GCAGGAAGCACTATG---GGCGCGGCGTCA---
ATAACGCTGACGGTACAA---GCCAGACAA---TTATTG---TCTGGTATAGTGCAACAGCAAAGC---
AATTTG---CTGAAG---GCTATAGAGGCTCAACAACATCTGTTGAGACTCACGGTCTGGGGCATT
>MH742762
TTCTATTGCAATACATCAGACCTGTTTAATGCATCAAGCACT-------------------------------
----

TGGGGTAACAATATAACTCTCCCATGCAGAATAAAGCAAATTGTAAATATGTGGCAGAAAGTAGGACGAGCAA
TGTATGCCCCTCCCATTCCAGGAGAAATAAGGTGTGAATCAAAC---
ATAACAGGACTACTATTAACAAGAGATGGAGGGAATAATAATAAT----------------------------
----

ACAAATGAGACCTTCAGACCTGGAGGAGGAGATATGAGGGACAATTGGAGAAGTGAATTATATAAGTATAAAG
TAGTAAAAATTGAACCACTA---GGTGTAGCACCCACCCATGCAAAAAGAAGAGTGGTG--------------
-------------------------CAGAGAGAAAAAAGAGCA---GTTGGA---CTGGGAGCT---------
GTCTTCCTTGGGTTCTTAGGAGCA---GCAGGAAGCACTATG---GGCGCGGCGTCA---
ATAACGCTGACGGTACAG---GCCAGACAA---TTGCTA---TCTGGTATAGTGCAACAGCAGAGC---
AATTTG---CTGAAG---GCTATAGAGGCTCAACAACATCTGTTGAGACTCACGGTCTGGGGCATT

>MH000309
TTCTATTGCAATACGACAGACCTGTTTAACAGCAAGTGGTTCAGT----------------------------
----

AACAACGGCATTATAACTCTCCAATGCAGAATAAAGCAATTCATAAATATGTGGCAGAGAGTAGGACAAGCAA
TGTATGCCCCTCCCATCTCAGGAATTATAAATTGTACATCAAAC---
ATTACAGGACTATTATTAACCAGAGATGGAGGA--

ACTGAGACTTTTAGGCCTGGAGGAGGAGATATGAGGGACAATTGGAGAAGTGAATTATATAAGTATAAAGTAG
TAAAAATTGAACCAATA---GGAATAGCACCCACCCATGCAAAAAGAAGAGTGGTG-----------------
----------------------GAGAGAGAAAAAAGAGCA---GTTGGA---CTGGGAGCT---------
GTCTTCCTTGGGTTCTTAGGAGCA---GCAGGAAGCACTATG---GGCGCGGCGTCA---
ATAACGCTGACGGTACAG---GCCAGACAA---TTATTG---TCTGGTATAGTGCAACAGCAGAGC---
AATCTG---CTGAGG---GCTATAGAGGCTCAACAACAGCTGTTGAAACTCACAGTCTGGGGCATT
>MH742757
TTCTACTGCAATACAACAAACTTGTTTAACATCAGCCTGGGGAACAGTACCACTAACAGCTCAGAG-------
----

TCAAATGACAATATAACTCTCCAATGCAGAATAAAGCAAATTATAAGGATGTGGCAGAGAGTAGGACAAGCAA
TGTATGCCCCTCCCATCCAAGGAGATATAAGTTGTAGATCAAAC---
ATTACAGGACTACTATTAACAAGAGATGGAGGGACAAGAGATGGAGGGAAT----------------------
----

GTGACTAATAATACTGAGATTTTTAGGCCTGAAGGAGGAAATATGAAGGACAATTGGAGAAGTGAATTATATA
AGTATAAAGTAGTAAGAATTGAACCAATA---GGTGTAGCACCCACCCGTGCAAAAAGAAGAACGGTG-----
----------------------------------GGGAGAGAGAAAAGAGCA---GTTGGA---
CTGGGAGCT---------GTCTTCCTTGGGTTCTTAGGAGCA---GCAGGAAGCACTATG---
GGCGCGGCGTCA---ATAACGCTGACGGTACAG---GCCAGACAA---TTATTG---
TCTGGTATAGTGCAACAGCAGAGC---AATCTG---CTGAGG---
GCTATTGAGGCTCAACAGCATCTGTTGAAACTCACAGTCTGGGGCATT
>AY231152
TTCTATTGCAATACATCAATACTGTTTAATAGCACTTGGAATACCAATGACACTTGGAATATCAATAACACTG
ACACA--

GAGTCAAATGACACTATAACTATCCAATGCAGAATAAGGCAAGTTGTAAAGATGTGGCAGAAAGTAGGACAAG
CAATGTATGCCCCTCCCATCGAAGGAGTAATAAGTTGTAAATCAAAC---
ATTACAGGACTACTATTAACAAGAGATGGAGGGAATCATAATAAT----------------------------
----

ACAAATGAGACTTTCAGGCCTGGAGGAGGAGATATGAGGGACAATTGGAGAAGTGAATTATATAAGTATAAAG
TAGTAAAAATTGAGCCACTA---GGTGTAGCACCCACCCGTGCAAAAAGAAGAGTGGTG--------------
-------------------------GAGAGAGAAAAAAGAGCAGTATTTGGA---CTGGGAGCT---------
GTTTTCCTTGGGTTCTTAGGAGCA---GCAGGAAGCACTATG---GGCGCGGCGTCA---
ATAACGCTGACGGTACAG---GCCAGACAA---TTATTG---TCCGGTATAGTGCAACAGCGGAGC---
AATTTG---CTGAGG---GCTATAGAGGCTCAACAACATCTGTTGAAACTCACAGTCTGGGGCATT
>AY231153
TTCTATTGCAATACATCAAAACTGTTTGATAGCACTTGGTATAGCAATGATAGCACTTGGTATAGCAATGACA
CCAGCTTAAAC--
-------------CACACA------
GAGTCAAATGGCACTATAACTCTCCAATGCAGAATAAAGCAAATTATAAATATGTGGCAAAAGGTAGGGCGAG
CAATGTATGCCCCTCCCATCCAAGGAGTAATAAGGTGTGATTCAAAC---
ATTACAGGACTACTATTAACAAGAGATGGAGGGGATAATAATACT----------------------------
----

AAAAATGAGACCTTCAGGCCTGGAGGAGGAGATATGAGGGACAATTGGAGAAGTGAATTATATAAGTATAAAG
TAGTAAAAATTGAACCACTA---GGTGTAGCACCCACCCATGCAAAAAGAAGAGTGGTG--------------
-------------------------GAGAGAGAAAAAAGAGCA---GTTGGA---CTGGGAGCT---------
GCCTTCCTTGGGTTCTTAGGAGCA---GCAGGAAGCACTATG---GGCGCGGCGTCA---

ATAACGCTGACGGTACAA---GCCAGACAA---TTATTG---TCCGGTATAGTGCAACAGCAGAGC---
AATCTG---CTGAAA---GCTATAGAGGCTCAACAACAGATGTTGAGACTCACGGTCTGGGGCATT
>MH742764
TTCTATTGCAATACATCAATACTGTTTAATAGCACTTGGAATAATAGCACTTGGGATAATAACACTAGAGATA
ACAAT---
-------------AACAAT------
GCCACAAATGATACTATGACTCTCCAATGCAGAATAAAGCAAATTGTAAGAATGTGGCAGACAGTAGGACAAG
CAATGTATGCCCCTCCCATCCAAGGAACAATAAAGTGTGAGTCAAAC---
ATTACAGGACTACTATTAACAAGAGATGGAGGAAATAATAATAAT----------------------------
----

ACAAATGAGACTTTCAGGCCTGGAGGAGGAGATATGAGGGACAATTGGAGAAGTGAATTATATAAGTATAAAG
TAGTAAAACTTAAACCACTA---GGTGTAGCACCCACCCCTGCAAAAAGAAGAGTGGTG--------------
------------------------ATGAGAGAAAAAAGAGCA---GCA------CTGGGAGCT---------
ATTTTCCTTGGGTTCTTAGGAGCA---GCAGGAAGCACTATG---GGCGCGGCGTCA---
ATAACGCTGACGGTACAG---GCCAGACAA---TTATTG---TCCGGTATAGTGCAACAGCAGAGC---
AATTTG---CTGAGA---GCTATAGAGGCTCAACAACATATGTTGAAACTCACGGTCTGGGGCATT
>AF321079
TTCTATTGCAATACAGCATCACTGTTTAATAGCACTTGGTATAGCAATGGCAGCTCAAACTACGCAGGG----
----

TCAAATGGGAATATAACTCTCCAATGCAGAATAAAGCAAATTGTAAATATGTGGCAGAGAGTAGGACAAGCAA
TGTATGCCCCTCCCATCCAAGGAGTAATAAGGTGTGTATCAAAC---
ATTACTGGACTACTATTAACAAGAGATGGAGGGAATAATAATAGT----------------------------
----

AGTACAAATGAGACCTTTAGGCCTGGAGGAGGAGACATGAGAGACAATTGGAGAAGTGAATTATATAAGTATA
AAGTAGTAAAAATTGAACCACTA---GGTGTAGCACCCTCCCGTGCAAAAAGAAGAGTGGTG-----------
-----------------------------GAGAGAGAAAAAAGAGCA---ATCGGA---CTGGGAGCT------
---GTTTTCCTTGGGTTCTTAGGAGCA---GCAGGAAGCACTATG---GGCGCGGCGTCA---
ATAACGCTGACGGTACAG---GCCAGACAA---TTATTG---TCCGGTATAGTGCAACAGCAGAGC---
AATCTG---CTGAAG---GCTATAGAGGCTCAACAACATATGTTGAAACTCACGGTCTGGGGCATT
>AB286856
TTCTATTGCTATACATCAGACCTGTTTAATAGCACTTGGCCCAATAACACTGCAGAGTCAAATAGCACAGGGT
CAATA---

AGGTCAAATGAGACTATAACTCTCAAATGCAGAATAAAGCAAATTATAAATATGTGGCAGAGAGTAGGACAAG
CAATATATGCCCCTCCCATCAGAGGAGAAATAAGGTGTGATTCAAAC---
ATTACAGGACTAATATTAACAAGAGATGGAGGGAATAATAGTGAGAGT-------------------------
----

GCAAATGGAACTGAAATTTTCAGGCCTGGAGGAGGAGATATGAGGGACAATTGGAGAAGTGAATTATATAAGT
ATAAAGTAGTAAAAATTGAACCACTA---GGATTAGCACCCACTAGAGCAAAAAGAAGAGTGGTG--------
------------------------------CAGAGAGAAAGAAGAGCA---GTTGGC---CTGGGAGCT---
------TTATTCATTGGGTTCCTAGGAGCA---GCAGGAAGCACTATG---GGCGCGGCGTCA---
TTAACGCTGACGGTACAG---GCCAGACAA---TTACTG---TCTGGTATAGTGCAACAGCAAAGC---
AATTTG---CTGAGG---GCTATAGAGGCTCAACAACATCTGTTGAAACTCACAGTCTGGGGCATT
>AB286857
TTCTATTGCAATACATCAGGACTGTTTAATAGCACTTGGGAGAATAATACTAGCACAGCCAGCTCAACAAATG
TTAGCTCA--
-------------------ATA---
AATGACACTGACATTATAGAACTCAAATGCAGAATAAAGCAAATTGTAAATATGTGGCAGAAAGTAGGACAAG
CAATGTATGCTCCTCCCATTCCAGGAGTAATAAGGTGTGATTCGAAC---
ATTACAGGACTACTATTAACAAGAGATGGAGGGAAGAATAGTAGT----------------------------
----

GCTACAAATGAGACCTTCAGGCCTGGAGGGGGAGATATGAGAGACAATTGGAGAAGTGAATTATATAAATATA
AAGTAGTAAAAATTGAACCACTG---GGTGTAGCACCCACCCATGCAAAAAGAAGAGTGGTG-----------
----------------------------GAGAGAGAAAAAAGAGCA---GTTGGA---CTAGGAGCT------
---GTACTCCTTGGGTTCTTAGGAGCA---GCAGGAAGCACTATG---GGCGCGGCGTCA---

ATAACGCTGACGGTACAG---GCCAGACAA---TTATTG---TCTGGTATAGTGCAACAGCAGAGC---
AATCTG---CTGAGA---GCTATAGAGGCTCAACAACAGCTGTTGAGACTCACGGTCTGGGGCATT
>AB286859
TTCTATTGTAATACAACAACACTGTTTAATAGCACTTGGGATGACAATAACACTGCCAGCTCAAACTACACAG
GG---

TCAAATGGCACTATAACTCTCCAATGCAGAATAAAGCAAATTATAAATATGTGGCAGAAAGTAGGACAAGCAA
TGTATGCCCCTCCCATCCAAGGAGTAATAAGGTGTAACTCAAGC---
ATTACTGGACTACTATTAACAAGAGATGGAGGAAGTAATAGTAGT----------------------------
----

ACAAATGAAACTTTCAGGCCTGGAGGAGGAGACATGAGGGACAATTGGAGAAGTGAATTATATAAGTATAAAG
TAGTAAAAATTGAACCACTG---GGTGTAGCACCCACCCATGCAAAAAGAAGAGTGGTG--------------
-------------------------GAGAGAGAAAAAAGAGCA---GTTGGA---CTGGGAGCT---------
GTCTTCCTTGGGTTCTTAGGAGCA---GCAGGAAGCACTATG---GGCGCAGCGTCA---
CTAACGCTGACGGTACAG---GCCAGACAA---TTATTG---TCTGGTATAGTGCAACAGCAAAGC---
AATTTG---CTGAAG---GCTATAGAGGCTCAACAACATCTGTTGAAACTCACAGTCTGGGGCATT
>AB286862
TTCTATTGCAATACAACAAATTTGTTTAATAGCACTTGGGAAAAGAAAAAGGGCACTTGGAATGATACCACTG
CCAAC--

CCACCAAATGGCACTATAACTCTCCAATGCAGAATAAAGCAAATTGTAAATATGTGGCAGAGAGTAGGACAAG
CAATATATGCCCCTCCCATCCCAGGAATAATAAGTTGTAATTCAAGC---
ATCACAGGATTACTATTAACAAGAGATGGGGGTGAGGGGAATGAAACT-------------------------
----

AGGAAAAATGAGACCTTCAGACCTGGAGGAGGAGATATGAGGGACAATTGGAGAAGTGAATTATATAAGTATA
AAGTGGTAAAAATTGAACCACTA---GGTGTGGCACCTACCTATGCAAAAAGAAGAGTGGTG-----------
----------------------------GAGAGAGAAAAAAGAGCA---GTTGGA---ATGGGAGCT------
---GTCTTACTTGGGTTCTTAGGAGCA---GCAGGAAGCACTATG---GGCGCAGCGTCA---
ATAACGCTGACGGTACAG---GCCAGACAA---TTATTG---TCCGGTATAGTGCAACAGCAGAGC---
AATCTG---CTGAAA---GCTATAGAGGCTCAACAACAACTGTTGAGACTCACGGTCTGGGGCATT
>AB286863
TTCTATTGCTATACATCAGAACTGTTTAATAGCACTTGGAATAATAATACTGGCAGCCCAAACAGC-------
----

ACAAATGACACTATAACTCTTCAATGCAAAATAAAGCAAATTGTAAATATGTGGCAGAGGGTAGGGCAAGCGA
TGTATGCCCCTCCCATCCCAGGAGAGCTAAGGTGTGACTCAAAC---
ATTACTGGACTACTATTAACAAGAGATGGAGGGAATACTAATAATACA-------------------------
----

AATACAACTGAAACCTTCAGGCCGGGAGGGGGAGATATGAGGGACAATTGGAGAAGCGAACTATACAAGTATA
AAGTAGTACAAATTGAACCAATA---GGAGTAGCACCCACCCCGGCAAAAAGAAGAGTGGTG-----------
----------------------------GAGAGAGAAAAAAGAGCA---GTTGGA---CTGGGAGCT------
---GTCTTCCTTGGGTTCTTAGGAGCA---GCAGGAAGCACTATG---GGCGCAGCGTCA---
ATAACGCTGACGGTACAG---GCCAGACAA---TTATTG---TCCGGTATAGTGCAACAGCAGAGC---
AATTTG---CTGAGA---GCTATAGAGGCTCAACAACATCTGTTGAAACTCACGGTCTGGGGCATT
>AB231895
TTCTATTGCAATACATCAGGACTGTTTAATAGTACTTGGTATAGCGATACCACTGCAAACCAAACAGAG----
----

TCAAATGACACCATAACTCTACAATGCAGAATAAAGCAAATTATAAATATGTGGCAGACAGTAGGACAGGCAA
TGTATGCCCCTCCCATCCAAGGAGTAATAAGGTGTGAATCAAAC---
ATCACTGGATTACTATTAACAAGAGATGGAGGGAATAAGAATCAT----------------------------
----

ACAAATGAGACCTTCAGGCCTGGAGGAGGAGATATGAGAGACAATTGGAGAAGTGAATTATATAAGTATAAAG
TAGTAAAGATTGAACCAATA---GGTGTAGCACCCTCTCCTGCAAAAAGAAGAGTGGTG--------------
-------------------------GGAAGAGAAAAAAGAGCA---GTCGGA---CTGGGAGCT---------
GTCTTCCTTGGGTTCTTAGGAGCA---GCAGGAAGCACTATG---GGCGCGGCGTCA---

ATGACGCTGACGGTACAA---GCCAGACAA---TTATTG---TCTGGTATAGTGCAACAGCAGAGC---
AATTTG---CTGAAG---GCTATAGAGGCTCAACAACATCTGTTGAAACTCACGGTTTGGGGCATT
>AB231896
TTCTATTGCGATACATCAGGGCTGTTTAATAGCACTTGGGATAACATTACCAGTACAAATGGCACAAAGGTAA
AT---

GAAACTGAAACTATAACTCTCCCATGCAGAATAAAACAAATTGTAAGAATGTGGCAGAGAGTAGGACAAGCAA
TGTATGCCCCTCCTATCCAAGGAGAACTACGGTGTGAATCAAAT---
ATTACAGGACTACTATTAACAAGAGATGGAGGGAATAATAATCGT----------------------------
----

ACAAATGAGACCTTCAGGCCTATAGGAGGAGATATGAAGGACAATTGGAGAAGTGAATTATATAAATATAAAG
TGGTAAAAATTGAACCACTA---GGTATAGCACCCTCCAGGGCAAAAAGGAGAGTGGTG--------------
-------------------------GAGAGAGAAAAAAGAGCA---GTTGGA---CTGGGAGCT---------
GTCTTCCTTGGGTTCTTAGGAGCA---GCAGGAAGCACTATG---GGCGCGGCGTCA---
TTAACGCTGACGGTACAG---GCCAGACAA---TTATTG---TCCGGTATAGTGCAACAGCAAAGC---
AATTTG---CTGAGG---GCTATAGAGGCTCAACAACATCTGTTGAAACTCACAGTCTGGGGCATT
>AB231898
TTCTATTGCAATACATCAAGACTGTTTAATAGCAATTGGAAAAACGGTACTGCCAGCTCAAATGGCACT----
----

GCAAATGACATTATAACTCTCCAATGCAGAATAAGGCAAATTATAAATATGTGGCAGAAAGTAGGAAAAGCAA
TGTATGCCCCTCCCATCCCAGGAGTAATAAGGTGTGAGTCAAAC---
ATTACAGGACTACTATTAACAAGAGATGGAGGGAAAAATACTAGT----------------------------
----

GGTGTAAATGAGACTTTCAGACCTGAAGGAGGAAATATGAAAGACAATTGGAGAAGTGAATTATATAAGTATA
AAGTAATAAAAATTGAACCACTA---GGTGTAGCACCCACCCGTGCAAGAAGAAGAGTGGTG-----------
----------------------------GGAAGAGAAAAAAGAGCA---ATAGGTGGACTGGGAGCT------
---GCCCTCCTTGGGTTCCTAGGAGCA---GCAGGAAGCACTATG---GGCGCGGCGTCA---
ATAACGCTGACGGTACAG---GCCAGACAA---TTATTG---TCTGGTATAGTGCAACAGCAGAGC---
AATCTG---CTGAGG---GCTATAAAGGCTCAACAAGAACTGTTGAGACTCACGGTCTGGGGCATT
>AB049811
TTCTATTGCAATACATCAGAACTGTTTAATAGGAATTACAATAGGTCTAGCAATAGCACTTGGAATTACAATA
GTTCTAGCAAG--
-------------GGCACA------
GAGTCAAATGGCACTATAATTCTCCAATGCAGAATAAAGCAAATTGTAAATATGTGGCAGAGAGTAGGACAAG
CAATATATGCCCCTCCCATCCCAGGAGTAGTAAGGTGTGTCTCAAAC---
ATTACAGGACTACTATTAACAAGAGATGGAGGGAATGGTAATAGT----------------------------
----

ACAAATGAGACTTTCAGACCGGGAGGAGGAGATATGAGGGACAATTGGAGAAGTGAATTATATAAGTATAAAG
TAGTAAAAATTGAACCACTA---GGTGTAGCACCCACCCATGCAAAAAGAAGAGTGGTG--------------
-------------------------GGGAGAGAAAAAAGAGCA---GTTGGA---CTGGGAGCT---------
GTCTTCCTTGGGTTCTTAGGAGCT---GCAGGAAGCACTATG---GGCGCAGCGTCA---
ATAACGCTGACGGTACAG---GCCAGACAA---TTATTG---TCTGGTATAGTGCAACAGCAGAGC---
AATCTG---CTGAGG---GCTATAGAAGCTCAACAACATATGTTGAAGCTCACGGTCTGGGGCATT
>AB485633
TTCTATTGTAAGACATCGAGACTGTTTAATAGCACTTGGCAGTTTAATAGCACTTGGAAGAGCATTCGGGATG
GC---

AATGACAATGACACTATAACTCTCCCATGCAGAATAAGACAAATTGTACATATGTGGCAGAGAGTAGGACAAG
CAATGTATGCCCCTCCCATTCGAGGAGTAATAAACTGTCTATCAAGC---
ATTACAGGACTACTGCTAACAAGAGATGGTGGTGGGAATGGGAAT----------------------------
----

AGTACAAAGGAGATCTTCAGGCCTGGAGGAGGAGATATGAGGGACAATTGGAGAAGTGAATTATATAAGTATA
AGGTAGTGAAAATTGAACCACTA---GGTGTAGCACCCACCCATGCAAAAAAAAGAGTGGTG-----------
----------------------------GAGAGAGAAAAGAGAGCA---GTTGGA---CTGGGAGCT------
---GTCTTCCTTGGGTTCTTAGGAGCA---GCAGGAAGCACTATG---GGCGCGGCGTCA---

ATAACGCTGACGGTACAG---GCCAGACAA---TTATTG---TCCGGTATAGTGCAACAGCAGAGC---
AATCTG---CTGAAG---GCTATAGAGGCTCAACAACATCTGTTGAGACTCACGGTCTGGGGCATT
>HQ385450
TTCTATTGCAATACATCAGAACTGTTTACTAGCACTTGGCCCAATGAGAATAATAGCACCGAGACCAATAATA
CTGCCAAC---
-------------------TTA---
AATAATACAGACACTATAACTCTCCAATGCAGAATAAAGCAAATTGTAAGAATGTGGCAGACAGTGGGACAAG
CAATGTATGCCCCTCCCATCAAAGGGGTAATAAATTGTAAATCAAAC---
ATTACAGGACTATTATTAATAAGAGATGGAGGGAAAAATAATAGT----------------------------
----

GAAAATGAGACTTTCAGGCCTGGAGGAGGAGATATGAGGGACAATTGGAGAAGTGAATTATATAAGTATAAAG
TAGTAAAGATTGAACCAATA---GGTGTAGCACCCACCCATGCAAAAAGAAGAGTGGTG--------------
-------------------------GAGAGAGAAAAAAGAGCA---GTTGGA---ATGGGAGTG---------
TTTATCCTTGGGTTCTTAGGAGCA---GCAGGAAGCACTATG---GGCGCGGCGTCA---
ATAACGCTGACGGTACAG---GCCAGACAA---TTATTG---TCTGGTATAGTGCAACAGCAGAGC---
AATTTG---CTGAGG---GCTATAGAGGCTCAACAACATCTGTTGAAACTCACAGTCTGGGGCATC
>HQ385451
TTCTATTGCAATACATCAGGACTGTTTAATAGCACCTGGGTGAATAACAGTAGCACTGAGGGTGCCAGTAACA
ATGCCAGC---
-------------------TCA---
GATGCAAATGACACTATAACTCTCCAATGCAGGATAAAGCAAATTATAAATCTGTGGCAGAGAGTAGGAAAAG
CAATGTATGCCCCTCCCATCCCAGGAATAATAAGGTGTGACTCAAAC---
ATTACAGGACTATTATTAACAAGAGATGGAGGGTATAATAATAAT----------------------------
----

AGTAGAAATGAGACTTTCAGACCTGGAGGAGGAGATATGAGGGACAATTGGAGAAGTGAATTATATAAGTATA
AAGTAGTAAAAATTGAACCACTA---GGTATAGCACCCACCAAGGCAAGGAGAAGAGTGGTG-----------
----------------------------GAGAGAGAAAAAAGAGCA---GTTGGC---ATGGGAGCT------
---CTCTTCCTTGGGTTCTTAGGAGCA---GCAGGAAGCACTATG---GGCGCGGCGTCA---
ATAACGCTGACGGTACAG---GCCAGACAA---TTATTG---TCTGGTATAGTGCAACAGCAGAGC---
AATTTG---CTGAGA---GCTATAGAGGCTCAACAACATCTGTTGAAACTCACGGTCTGGGGCATT
>HQ385460
TTCTACTGCAATTCATCAGCACTGTTTGATACAACATGGAACGCCACTACATGGAATAATACCATAGAGTCAA
ATAATACC---
-------------------ACA---
GGGCCAAACGATACAATCACACTCCACTGCAGGATAAAACAGATTATAAACATGTGGCAGACAGTAGGACAAG
CAATGTACGCCCCTCCCATCCGAGGAGAAATAAGGTGTGAATCAAAC---
ATTACAGGACTACTATTAACAAGAGATGGAGGGAATGGTAATAGTAGT-------------------------
----

GGTGGTGCAAATGAGACTTTTAGGCCTGGAGGAGGAGATATGAGGGACAATTGGAGGAGTGAATTATATAAGT
ATAAAGTAGTAAAAATTGAACCACTA---GGTGTAGCACCCACCCATGCAAAGAGAAGAGTGGTG--------
-------------------------------GAGAGAGAAAAAAGAGCA---ATTGGA---CTGGGAGCT---
------TTCTTCCTTGGGTTCTTAGGAACA---GCAGGAAGCACTATG---GGCGCAGCGTCA---
ATAACGCTGACGGTACAG---GCCAGACAA---TTATTG---TCTGGTATAGTGCAACAGCAAAGC---
AATTTG---CTGATG---GCTATAGAGGCTCAACAACATATGTTGAAACTCACTGTCTGGGGCATT
>HQ385446
TTCTATTGCAATACATCAGGACTGTTTAACATTTGGGAGAAC-------------------------------
----

AATGACAATATAACTCTCCCATGCAGAATAAAGCAAATTATAAATATGTGGCAGAAAGTAGGACAAGCAATGT
ATGCCCCTCCCATTGCAGGAAAAATAGAGTGTGAGTCAAAC---
ATTACAGGACTACTGCTAACAAGAGATGGAGGAAATAATAATAGTACA-------------------------
----

AATAAAAGTGAGACTTTCAGGCCTGGAGGAGGAGACATGAGGGACAATTGGAGAAGTGAATTATATAAGTATA
AGATAGTAAAAATTGAACCACTA---GGTGTGGCACCCACTCATGCAAAAAGAAGAGTGGTG-----------
----------------------------GGGAGAGAAAAAAGAGCA---GTTGGA---CTGGGAGCT------
---GTTCTCTTTGGGTTCTTAGGAGCA---GCAGGAAGCACTATG---GGCGCGGCGTCA---

ATAACGCTGACGGTACAG---GCCAGACAA---CTATTG---TCTGGTATAGTGCAACAGCAGAGC---
AATCTG---CTGAAA---GCTATAGAGGCTCAACAACATCTGTTGAAACTCACAGTCTGGGGCATT
>HQ385447
TTCTATTGCAATACAACAGCACTGTTTATGGTACATTGTATAACAATGACACTTGGGATAGAAATAGCACTT
GGGAT---
-------------------AAC---
AACAACTCAACAAATATAACTCTCCAATGCAAAATAAAGCAAATTATAAGAATGTGGCAGAGAGTAGGGCAAG
CAATGTATGCCCCTCCCATCCGAGGAAAAATATGGTGTGAATCAAAC---
ATTACAGGAATACTATTAACAAGAGATGGGGGGAATGATAATGCA-----------------------------
----

AATGAGACCTTCAGGCCTGGAGGAGGAGATATGAGGGACAATTGGAGAAGTGAATTATATAAATATAAAGTAG
TAAAAATTGAGCCACTA---GGTGTAGCACCCACCCATGCAAAAAGAAGAGCGGTG-----------------
----------------------GATAGAGAAAAAAGAGCA---GTTGGA---CTGGGAGCT---------
TTTTTCCTTGGGTTCTTAGGAGCA---GCAGGAAGCACTATG---GGCGCGGCGTCA---
ATAACGCTGACGGTACAG---GCCAGACAA---TTATTG---TCTGGTATAGTGCAACAGCAGAGC---
AATCTG---CTGAAG---GCTATAGAGGCTCAACAACATCTGTTGAGACTCACGGTCTGGGGCATT
>HQ385462
TTCTATTGCAATACATCAGGACTGTTTAATAGCACTTGGCCAGAGGCAAATGCCACACAGTCAAATACCACAC
AG--

TCAAATAGCACTATAACTCTCCCATGCAGAATAAAGCAAATTGTAAATATGTGGCAGAAAGTAGGACAAGCAA
TGTATGCCCCTCCCATCCAAGGAATAATAAAGTGTGAATCAAAC---
ATTACAGGGTTATTGTTAACAAGAGATGGGGGAAATAATAGTGGAAAT------------------------
----

AATGGTGAACCTGATATTTTCAGGCCCGAAGGAGGAGATATGAGAGATAATTGGAGAAGTGAATTATATAAGT
ATAAAGTAGTAAAAATTGAACCACTG---GGTATAGCACCCAGCCATGCAAAAAGAAGAGTGGTG--------
-------------------------------GAGAGAGAAAAAAGAGCA---GTTGGA---CTGGGAGCT---
------GTCTTCCTTGGGTTCTTAGGAGCA---GCAGGAAGCACTATG---GGCGCGGCGTCA---
ATAACGCTGACGGCACAC---GCCAAACAA---TTACTG---TCTGGTATAGTGCAACAGCAGAGT---
AATCTG---CTGAGG---GCTATAGAGGCTCAACAACATCTGTTGAAACTCACGGTCTGGGGCATT
>HQ385455
TTCTATTGCAATACAACAGCCCTGTTTAACACAACTTGGATCAATGGCACTGACAGCTCAAACAAC-------
----

AGCACAGAGTCTATAACGATCCAATGCAGAATAAAGCAAATTATAAACATGTGGCAGAAAGTAGGACAAGCAA
TATATGCCCCTCCTATCCCAGGAACAATAAGGTGTGAATCAAAC---
ATTACAGGACTATTATTAACAAGAGATGGAGGAAAGAATAGAACA-----------------------------
----

AATAATGAGACTTTCAGGCCTGAAGGAGGAGATATGAGGGACAATTGGAGAAGTGAATTATATAAGTATAAAG
TAGTAAAAATTGAACCACTA---GGTATAGCACCTACCTATGCAAAAAGAAGAGTGGTG-------------
-------------------------GAGAGAGAAAAAAGAGCA---GTTGGA---CTGGGAGCT---------
GTCTTCCTTGGGTTCCTAGGAGCA---GCAGGAAGCACTATG---GGCGCGGCGTCA---
GTGACGCTGACGGTACAG---GCCAGACAA---TTATTG---TCTGGTATAGTGCAGCAGCAAAGC---
AATTTG---CTGAAG---GCTATAGAGGCTCAACAACATCTGTTGAGACTCACAGTCTGGGGCATT
>HQ385457
TTCTATTGCAATACATCAGAACTGTTTAATAGTACTTGGAATAACAATAGCACTGGGGATAACAAT-------
----

AGCTCAACACCAATAATTCTCCAATGCAAAATAAAGCAAATTATAAGAATGTGGCAGAAAGTAGGACAAGCAA
TGTATGCCCCTCCCATCCCAGGAGAAATAAGGTGTGAATCAAAC---
ATTACTGGACTACTATTAACAAGAGATGGAGGGAATAATAATAAT-----------------------------
----

ACAAATGAAACATTCAGGCCTGGGGGAGGAGATATGAGAGACAATTGGAGAAGTGAATTATATAAGTATAAAG
TAGTAAAAATTGAACCACTA---GGTGTAGCACCTACCCGTGCAAAAAGAAGAGTGGTG-------------
-------------------------GAGAGAGAAAAAAGAGCA---GTTGGA---ATGGGAGCT---------
TTATTCCTTGGGTTCTTAGGAGCA---GCAGGAAGCACTATG---GGCGCGGCGTCA---

ATAACGCTGACGGTACAG---GCCAGACAA---TTATTG---TCCGGTATAGTGCAACAGCAGAGC---
AATTTG---CTGAAG---GCTATAGAGGCTCAACAGCATCTGTTGAGACTCACGGTCTGGGGCATT
>HQ385449
TTCTACTGCAATACATCAAGACTGTTTAATAGCACTTGGAATAACATTAATAGCACTGGGTATAACCTCACAG
GG---

TCACAGGAAAATATAACTCTCCAATGCAGAATAAAGCAAATTGTAAGAATGTGGCAGAGAGTAGGACAAGCAA
TGTATGCCCCTCCCATCCGAGGAGAAATAAGGTGTAACTCAAAC---
ATTACTGGACTACTATTAACAAGAGATGGAGGGAATGAAAATAAT----------------------------
----

AATACAAACGAGACCTTCAGGCCTGGAGGAGGAGATATGAGGGACAATTGGAGAAGTGAATTATATAAGTATA
AAGTAGTAAAAATTGAACCACTA---GGTGTAGCACCCACCCGTGCAAGAAGAAGAGTGGTG-----------
----------------------------GAGAGAGAAAAAAGAGCA---GTTGGA---CTGGGAGCT------
---GTCTTCCTTGGGTTCTTAGGAGCA---GCAGGAAGCACTATG---GGCGCGGCGTCA---
ATAACGCTGACGGTACAG---GCCAGACAA---TTATTG---TCCGGTATAGTGCAACAGCAAAGC---
AATTTG---CTGAAG---GCTATAGAGGCTCAACAACATCTGTTGAGACTCACGGTCTGGGGCATT
>HQ385454
TTCTATTGCTACACATCAAACCTGTTTAACAGTACTTGGGATGACAACAGTACTTGGGATAACAATACTACTT
GGGATAAC---
-------------AGCACG------
ACAGAGGACACCAACATAACTCTCCAATGCAGGATAAAGCAAATTGTAAATATGTGGCAGAGAGTAGGACAAG
CAATATATGCCCCTCCCATCCAAGGAGACATAAAGTGTGAATCAAAC---
ATTACTGGATTATTGTTAACAAGAGATGGAGAGAATAATACTATG----------------------------
----

AATGAGACTTTCAGGCCTGGAGGAGGAGACATGAGGGACAATTGGAGAAGTGAATTATATAAATATAAAGTAG
TAAAAATTGAACCACTA---GGTGTAGCACCCACCCATGCAAAAAGAAGAGTGGTG-----------------
----------------------GAGAGAGAAAAAAGAGCA---GTTGGA---CTAGGAGCT---------
GTCCTCTTTGGGTTCTTAGGAGCA---GCAGGAAGCACTATG---GGCGCGGCGTCA---
ATAACGCTGACGGTACAG---GCCAGACAA---TTATTG---TCTGGTATAGTGCAACAGCAGAGC---
AATTTG---CTGAAG---GCTATAGAGGCTCAACAACATCTGTTGAAACTCACGGTCTGGGGCATT
>MH078541
TTCTACTGTAATACATCAGCACTGTTTAATAGCACTTGGAATTGGAATAATAACACTTGGGGGACCAATGAGA
GCAGCTCACAG--
-------------GCAAAT------
GACACTGGTGAACCTATAACTCTCCAATGCAGAATAAAGCAAATTATAAATATGTGGCAGAAAGTAGGACGAG
CAATGTATGCCCCTCCCATCCCAGGAGTCATAAGGTGTGAGTCAAAC---
ATTACAGGCCTACTGCTAACAAGAGATGGAGGAAGTACTAATAGT----------------------------
----

ACAAATGAGACTTTCAGGCCTGGAGGAGGAGACATGAGGGATAATTGGAGGAGTGAATTATATAAGTATAAAG
TAGTAAAAATTGAACCACTA---GGTGTAGCACCTACTCGTGCAAGAAGAAGAGTGGTG--------------
-------------------------GGAAGAGAAAAAAGAGCA---GTTGGA---CTGGGAGCT---------
GTTTTCCTTGGGTTCTTAGGAGCA---GCAGGAAGCACTATG---GGCGCAGCGTCA---
CTAACGCTGACGGTACAG---GCCAGACAA---TTATTG---TCTGGTATAGTGCAACAGCAGAGT---
AATCTG---CTGAGG---GCTATAGAGGCTCAACAACAGATGTTGAGGCTCACGGTCTGGGGCATT
>MH078542
TTCTATTGCAATACATCAAAATTGTTTAATAGTACTTGGAATGAGAATAGTACTTGGAATAGTAGTCAG----
----

AGTAATGAGAATATAACCATCCCATGCAGAATAAGACAAATTGTACATCTGTGGCAGAGAGTAGGACAAGCAA
TGTATGCCCCTCCCATTCCGGGAGTAATAACCTGTACATCAAAT---
ATTACAGGGCTACTGCTAACAAGAGATGGTGGGAATAATAATAGT----------------------------
----

ACAAATGAGACCATCAGGCCTCAAGGAGGAAATATGAAGGACAATTGGAGGAGTGAATTATACAAATATAAAG
TAGTAAAAATTGAACCAATA---GGAGTAGCACCCACCAAGGCAAAGAGAAGAGTGGTG--------------
-------------------------GAGAGAGAAAAAAGAGCA---GTTGGA---CTGGGAGCT---------
GTCTTCCTTGGGTTCTTAGGAGCA---GCAGGAAGCACTATG---GGCGCAGCGTCA---

ATAACGCTGACGGTACAG---GCCAGACAA---TTATTG---TCTGGTATAGTGCAACAGCAAAGC---
AATTTG---CTGAAG---GCTATAGAGGCTCAACAGCAGCTGTTGAGACTCACAGTCTGGGGCATT
>MH078544
TTCTATTGTAATACAACAAAATTGTTTAGTAGCACTTGGGCTAATAATAGCACTTTGGGTAATAGCACTGAGG
ATAAC--

AACAATACTACTATAACTCTCCAATGTAGAATAAAGCAAATTGTGAATATGTGGCAGAGAGTAGGACAAGCAA
TGTATGCCCCTCCCATTCCAGGAGAAATAAGTTGTGTATCGAAC---
ATTACTGGACTTTTATTAACAAGAGATGGAGAGGGTAGTAATAGG----------------------------
----

ACAAATGAGACCTTCAGGCCTGGAGGAGGGGATATGAGGGACAATTGGAGAAGTGAATTATATAAGTATAAAA
TAGTAAAAATTGAACCACTA---GGTATAGCACCTACTCATGCAAGAAGGAGAGTGGTG--------------
-------------------------GAGAGAGAAAAAAGAGCA---GTTGGA---CTGGGAGCT---------
TTCTTCCTTGGGTTCTTAGGAGCA---GCAGGAAGCACTATG---GGCGCGGCGTCA---
ATAACGCTGACGGTACAA---GCCAGACAA---TTATTG---TCTGGTATAGTGCAACAGCAGAGC---
AATCTG---CTGAAG---GCTATAGAGGCTCAACAACATCTGTTGAAACTCACGGTCTGGGGCATT
>MK397970
TTCTATTGCAATACATCACAGTTGTTTAATAGCACTTGGGAGAACGGGGAATTAAATGGCAGTATA-------
----

TCAGATGGCAATATAACGATCCCCTGCAGAATAAAGCAAATTATAAATATGTGGCAGAGAGTAGGACAAGCAA
TGTATGCCCCTCCCATCCAAGGAGAGATAAGGTGTCAATCAAAC---
ATTACTGGACTAATATTAACAAGAGATGGAGGTAAAAATGAGACA----------------------------
----

CATACTGAGACCTTCAGGCCTGGAGGAGGAGATATAAGGGACAATTGGAGAAGTGAATTATATAAATATAAAG
TAGTAAAAATTGAACCACTA---GGAGTAGCACCCACCAGGGCAAAGAGAAGAGTGGTG--------------
-------------------------GAAAGAGAAAAAAGAGCA---GTTGGA---CTGGGAGCT---------
GTCTTCCTTGGGTTCTTAGGAGCA---GCAGGAAGCACTATG---GGCGCAGCGTCA---
ATAACGCTGACGGTACAA---GCCAGACAA---CTATTG---TCCGGTATAGTGCAACAGCAGAAC---
AATTTG---CTGAAG---GCCATAGAGGCTCAACAACAGATGTTGAGACTCACGGTATGGGGCATT
>KF561435
TTCTATTGCAATACATCAAAATTGTTTAATAGCACTTGGGACAACAATAGCACTTGGGACAACAATAGCACTT
GGGAAAACAAT--
----------AGCAATACC------
AACTCAAGTGGCACTATAACTTTCCAATGCAAAATAAAGCAAATTGTAAATATGTGGCAGAGAGTAGGACAAG
CAATGTATGCCCTTCCCATCCAAGGAGTAATAAGGTGTGAGTCAAAT---
ATTACAGGATTATTGTTAACAAGAGATGGAGGGAATAAGAATAAT----------------------------
----

AGTAATAATGAGACTTTCAGACCTGGAGGAGGAGATATGAGGGATAATTGGAGAAGTGTATTATATAAGTATA
AAGTAGTAAAAATTGAACCACTG---GGTGTAGCACCCACCCATGCAAGAAGAAGAGTGGTG-----------
----------------------------GGGAGAGAAAAAAGAGCA---GTCGGA---CTGGGAGCT------
---GTCCTCTTGGGATTCTTAGGAGCA---GCAGGAAGCACTATG---GGCGCGGCGTCA---
CTAACGCTGACGGTACAG---GCCAGACAA---TTATTG---TCCGGTATAGTGCAACAGCAGAGC---
AATTTG---CTAAGG---GCTATAGAGGCTCAACAACATCTGTTGAAACTCACGGTCTGGGGCATT
>DQ168577
TTCTATTGCAATACATCAAAACTGTTTAATAGCACTGGGGATACCAATACCACTGCCAAC-------------
----

TCAACAGGAACTATAGAGCTCCAATGCAGAATAAAGCAAATTGTAAATATGTGGCAGAAAGTAGGACAAGCAA
TATATGCCCCTCCCATCCAAGGAATAATAAGGTGTGATTCAAAC---
ATTACAGGACTACTATTAACAAGAGATGGAGGGATTGAGAATGCA----------------------------
----

GATAATGAGACCTTCAGGCCGGGAGGAGGAGATATGAGGGACAATTGGAGAAGCGAATTATATAAGTATAAAG
TAGTAAAAATTGAACCACTA---GGTGTAGCACCCACAAAAGCAAAAAGAAGAGTGGTG--------------
-------------------------GAGAGAGAAAAAAGAGCA---GTC------GTTGGAGCT---------
CTCTTCCTTGGGTTCTTAGGAGCA---GCAGGAAGCACTATG---GGCGCGGCGTCA---

ATAACGCTGACGGTACAG---GCCAGACAA---TTATTG---TCCGGTATAGTGCAACAGCAGAGC---
AATTTG---CTGAGA---GCTATAGAGGCTCAACAACATCTGTTGAAACTCACGGTCTGGGGCATT
>DQ168578
TTCTATTGTAATACATCAAACCTGTTTAATAGCACTTGGAATACCAGCTCAATCCACACAGAGCAAAATGCA-
----

ACTAATGAAACTATAACTCTCCAATGCAAAATAAAGCAAATTGTAAATATGTGGCAGAGAGCAGGACAAGCAA
TATATGCCCCTCCCATCCAAGGAGAAATAAGGTGTGACTCAAAC---
ATTACAGGAATATTATTAACAAGAGATGGAGGAAATAATAATAGT----------------------------
----

ACAAATGAGACCTTCAGGCCTGGAGGAGGAGATATGAGAGACAATTGGAGAAGTGAATTATATAAGTACAAAG
TAGTAAAACTTGAACCACTA---GGTATAGCACCCACCCATGCAAAAAGAAGAGTGGTG--------------
-------------------------GAGAGAGAAAAAAGAGCA---GTA------CTGGGAGCT---------
GTCTTCATTGGGTTCTTAGGAGCA---GCAGGAAGCACTATG---GGCGCGGCGTCA---
ATAACGCTGACGGTACAG---GCCAGACAA---TTATTG---TCCGGTATAGTGCAACAGCAAAGC---
AATTTG---CTGCAG---GCTATAGAGGCTCAACAACATCTGTTGAGACTCACGGTCTGGGGCATT
>JN248580
TTCTACTGCAATACATCAGCACTGTTTAACAACAGCTTAACA-------------------------------
----

AATGGAACTATAACTCTCCCATGCAGAATAAAGCAAATTGTAAATATGTGGCAGAGAGTAGGACTAGCAATGT
ATGCCCCTCCCATCCAAGGAAACATAACATGTAATTCAAAC---
ATTACAGGACTACTATTAACAAGAGATGGAGGGAATAATAGGAATAATGAG----------------------
----

AATAGTAGTGAACCTGAGACCTTCAGGCCGGGAGGAGGAGATATGAGGGATAATTGGAGAAGTGAATTATATA
AGTATAAAGTAGTAAAAATCGAACCACTA---GGTGTAGCACCTACTAAAGCAAAAAGAAGAGTGGTG-----
----------------------------------GAGAGAGAAAAAAGAGCA---GCCGGA---
CTGGGAGCT---------TTTTTCCTTGGGTTCTTAGGAGCA---GCAGGAAGCACTATG---
GGCGCGGCGTCA---ATAACGCTGACGGTACAG---GCCAGACAA---TTATTG---
TCTGGTATAGTGCAACAGCAGAGC---AATCTG---CTGAGA---
GCTATAGAGGCTCAACAACATCTGTTGAAACTCACGGTCTGGGGCATT
>KX389630
TTCTATTGCAATACATCATCACTGTTTAACGGCACCTGGAGGGCCAATGGCACTGACAATAACACTTTCAAC-
----

TTAAATGACACTATAATTATCCCATGCAGAATAAAACAAATTGTAAACATGTGGCAGAAAGTAGGACAAGCAA
TGTATGCCCCTCCCATCAAAGGAGTAATAAGTTGTATTTCAAAC---
ATTACGGGATTACTATTAACAAGAGATGGAGGGGAGCGTAACGAT----------------------------
----

AGTACAAATGAGACCTTCAGGCCTGGCGGAGGAGATATGAGGGACAATTGGAGAAGTGAATTATATAAGTATA
AAGTAGTAAAAATTGAACCATTA---GGTGTAGCACCCACCTCTGCAAAAAGAAGAGTGGTG-----------
----------------------------GAGAGAGAAAAAAGAGCA---GCTGGA---CTAGGAGCT------
---TTCTTCCTTGGGTTCTTAGGAGCA---GCAGGAAGCACTATG---GGCGCGGCGTCG---
ATGACGCTGACGGTACAA---GCCAGACAA---TTATTG---TCTGGTATAGTGCAACAGCAAAGT---
AATTTG---CTGAAA---GCTATAGAGGCTCAACAACAGATGTTGAGACTCACAGTCTGGGGCATT
>KX389629
TTCTATTGCAACACATCAGGACTATTTAATAGCAGTTGGCCTAACAATAGCACTTGGAATAATATA-------
----

ACAAATGACAATATAACTCTCCAATGCAGAATAAAGCAATTTGTCAATATGTGGCAGAGAGTAGGACAAGCAA
TGTATGCCCCTCCCATCCAAGGAGTAATAAGGTGTGAATCAAAC---
ATTACAGGACTACTATTAACAAGAGATGGAGGGTATGATAATAGTACC-------------------------
----

AATAACACAGAAACATTCAGGCCTGCAGGAGGAGATATGAGAGATAATTGGAGAAGTGAATTGTATAAGTATA
AAGTAGTAAAAATTGAACCGCTG---GGTGTAGCACCCACCCATGCAAAAAGAAGAGTGGTG-----------
----------------------------GAGAGAGAAAAAAGAGCA---GTAGGA---CTGGGAGCT------

---GTCTTCCTTGGGTTCTTAGGAGCA---GCAGGAAGCACTATG---GGCGCGGCGTCA---
ATAACGCTGACGGTACAG---GCCAGACAA---TTATTG---TCTGGTATAGTGCAACAGCAAGGC---
AATTTG---CTGAAG---GCCATAGAGGCTCAACAACAGCTGTTGAGACTCACGGTCTGGGGCATT
>JN248585
TTCTATTGCAATACATCAGGACTGTTTAATAGTAATTGGCATAGAAATACTACTAATGATACTTGGTATACTA
ACGAC--
-------------------ACA---
GCAAACAGCAGCACTATAACTATCCAATGCAGAATAAAACAAATTGTAAACATGTGGCAGAGAGTAGGACAAG
CAATATATGCTCCTCCCATTGCAGGAAAAATAGAGTGTAAATCAAAC---
ATTACTGGACTATTATTAATAAGAGATGGGGGAATACGAATACTAGC--------------------------
----

AGTACAAATGAGACTTTCAGACCTGTAGGAGGAGAGATGAGAGACAATTGGAGAAGTGAATTATATAAGTATA
AAGTAGTAAAAATTGAACCACTA---GGTGTAGCACCCACCTATGCAAAAAGAAGAGTGGTG-----------
----------------------------GAGAGAGAAAAAAGAGCA---GTTGGA---ATGGGAGCT------
---TTCTTCTTTGGGTTCTTAGCAGCA---GCAGGAAGCACCATG---GGCGCGGCGTCA---
ATAACGCTGACGGTACAG---GCCAGACAA---TTATTG---ACTGGTATAGTGCAACAGCAGAGC---
AATCTG---CTGAAG---GCTATAGAGGCTCAACAACAGCTGTTGAGACTCACAGTCTGGGGCATT
>JN248589
TTCTATTGCAATACATCAGACCTGTTTAATAGCAGTTGGGATAATAACAATAACACTGATAGCTCAAACCACA
CAGGG--

TCAGGTAACGATACCATAACTCTCCAATGCAGAATAAAGCAAATTATAAATATGTGGCAGAGAGTAGGACAAG
CAATATATGCCCCTCCCATCCAAGGAGTAATAAACTGTGAATCAAAC---
ATTACAGGGCTAATATTAACAAGAGATGGAGAGAATAATACTAAG----------------------------
----

GCAAATGAAACCTTCAGGCCTGGAGGAGGAGATATGAGGGACAATTGGAGAAGTGAATTGTATAAGTACAAGG
TAGTAAAAATTGAACCACTA---GGTGTAGCACCCACCCGTGCAAGAAGAAGAGTGGTG--------------
-------------------------GAGAGAGAAAAAAGAGCG---GTTGGA---CTGGGAGCT---------
GTCTTCCTTGGGTTCTTAGGAGCA---GCAGGAAGCACTATG---GGCGCGGCGTCA---
CTGACGCTGACGGTACAG---ACCAGACAA---TTATTG---TCTGGTATAGTGCAACAGCAGAGC---
AATTTG---CTGAGA---GCTATAGAGGCTCAACAGCATCTGTTGAAACTCACGGTCTGGGGCATT
>JN248590
TTCTATTGCAACACATCAAAACTGTTTAATAGCACTTGGTTGTCTAATAGCACTTGGAACACTACTGGCAAC-
----

CAAAATGAAAATATAACACTCCAATGTAGGATAAAGCAAATTATAAATATGTGGCAGAGAGTAGGACAAGCAA
TATATGCCCCTCCTATCCAAGGAGAAATAAAATGTGACTCAAAC---
ATTACTGGACTACTATTAACAAGAGACGGAGGGGAGAGTAACAGT----------------------------
----

ACAGAGGAGACCTTCAGGCCTGGAGGAGGAGATATGAGGGACAATTGGAGAAGTGAATTATATAAGTATAAAG
TAGTAAAAATTGAACCACTA---GGTATAGCACCCACCCATGCAAAAAGAAGAGTGGTG--------------
-------------------------GAGAGAGAAAAAAGAGCA---GTTGGA---CTGGGAGCT---------
GTCTTCCTTGGGTTCCTAGGAGCA---GCAGGAAGCACTATG---GGCGCGGCGTCA---
ATAACGCTGACGGTACAA---GCCAGACAA---TTATTG---TCCGGCATAGTGCAACAGCAAAGC---
AATTTG---CTGAAG---GCTATAGAGGCTCAACAACATCTGTTGAAACTCACGGTCTGGGGCATT
>JN248592
TTCTATTGCAATACAACACCACTGTTTAAGAGCGTTTGGAATAAAAATGGGAGCACTTGGAACAGCAATACCA
CTAACAGT---
-------------------ACA---
GAGTCAAATAACACTATAACCCTCCAGTGCAAAATAAAGCAAATTATAAATCTGTGGCAGAGAGTAGGACAAG
CAATATATGCCCCCCCCATCCCAGGAGTAATAAGGTGTGAATCAAAT---
ATTACAGGACTACTATTAACAAGAGATGGGGGAAATATTGATGAT----------------------------
----

GATGCAAATGAGACTTTCAGGCCGGGAGGAGGAGATATGAGGGACAATTGGAGAAGTGAATTATATAAGTATA
AAGTAGTAAAAATTGAACCACTA---GGTGTAGCACCCACCCGTGCAAGAAGAAGAGTGGTG-----------
----------------------------GGGAGAGAAAAAAGAGCA---GTTGGA---CTAGGAGCT------

---GTCTTCCTTGGGTTCTTAGGAGCA---GCAGGAAGCACTATG---GGCGCAGCGTCA---
GTAACGCTGACGGTACAA---GCCAGACAA---TTATTG---TCTGGTATAGTGCAACAGCAAAGC---
AATTTG---CTGAGG---GCGATAGAGGCTCAACAACAGTTGTTGAAACTCACGGTCTGGGGCATT
>KX389617
TTCTATTGCAATACAACAGGACTGTTTAATAGCACGTGGACTAGCAGTGGCCATGGCACTTATAACATCACTG
ACAGCTCA---
-------------------AAA---
AACCTCACAGAGGACATTACACTCCAATGCAGAATAAAGCAAATTATAAATCTGTGGCAGAAAGTAGGGCAAG
CAATATATGCCCCTCCCATCCCAGGAGTAATAAGTTGCAACTCAAAC---
ATTACTGGACTACTATTGACAAGAGATGGAGGGAATAATAATGAT----------------------------
----

TCAACAGAGACCTTCAGACCTGGAGGAGGAGACATGAGGGACAATTGGAGAAGTGAATTATATAAGTATAAAG
TAGTAAAAATTGAACCACTA---GGTATAGCACCTACCCATGCAAAAAGAAGAGTGGTG--------------
-------------------------GAAAGAGAAAAAAGAGGA---TTTGGA---ATGGGAGCT---------
GTCTTCCTTGGGTTCTTAGGAGCA---GCAGGAAGCACTATG---GGCGCAGCGTCA---
ATAACGCTGACGGTACAG---GCCAGACAA---TTATTG---TCCGGTATAGTGCAACAGCAGAGC---
AATCTG---CTGAAG---GCTATTGAGGCTCAACAGCATCTGTTGAAACTCACAGTCTGGGGCATT
>KX389614
TTCTATTGCAATACATCAAAACTGTTTGATAGCACTTGGCCTACAAATAGCACT-------------------
----

TCAAATGGCACTATAACTCTTCAATGCAGAATAAAGCAAATAATCAATATGTGGCAGAGAGTAGGACAGGCAA
TGTATGCCCCTCCCATCAAAGGAGCACTAAATTGTTCTTCAAAC---
ATTACAGGACTACTATTAACAAGAGATGGAGGGATAAGGAATGATACA-------------------------
----

GATAAGAATGAGACTTTCAGGCCGGGAGGAGGAGATATGAGGGACAATTGGAGAAGTGAATTATATAAATATA
AAGTAGTAAAAATTGAACCACTA---GGTGTAGCACCCACCCCTGCAAGAAGAAGAGTGGTG-----------
----------------------------GAGAGAGGAAAAAGAGCA---GTCGGA---ATGGGAGCT------
---GTCTTCCTTGGGTTCTTGGGAGCA---GCAGGAAGCACTATG---GGCGCAGCGTCA---
ATAACGCTGACGGTACAG---GCCAGACAA---TTATTG---TCTGGTATAGTGCAACAGCAGAGC---
AATCTG---CTGAGA---GCTATAGAGGCTCAACAACATCTGTTGAAACTCACGGTCTGGGGCATT
>KX389610
TTCTATTGCAATACATCAAAACTGTTTAATAGCAGTTGGAGTAACGACAGTAACACTGACAACAATGACAATA
ACACTGAC---

AACTCTAGCATTATAACTCTCCAATGCAGAATAAGGCAAATTGTAAATATGTGGCAGAGAGTAGGACAAGCAA
TGTATGCCCCTCCCATCCCAGGAGTAATTAGTTGTAATTCAAGC---
ATTACAGGACTATTATTAACAAGAGATGGGGGAAATAAGAGTGAA----------------------------
----

AACGAGACCTTCAGGCCTGGAGGAGGAAATATGAGGGACAATTGGAGAAGTGAATTATATAAGTATAAGGTAG
TAAGAATTGAACCATTA---GGTGTAGCACCCACCCATGCAAAAAGAAGAGTGGTG-----------------
----------------------GAGAGACAAAAAAGAGCA---GTTGGA---CTGGGAGCT---------
GTCCTCTTTGGGTTCTTAGGAGCG---GCAGGAAGCACTATG---GGCGCGGCGTCA---
ATAACGCTGACGGTACAG---GCCAGACAA---TTATTG---TCCGGTATAGTGCAACAGCAGAGT---
AATCTG---CTGAAA---GCTATAGAGGCTCAACAACATCTGTTGAGACTCACGGTCTGGGGCATT
>KX389643
TTCTATTGCAACACATCGAACCTGTTTAATAGCAGTTGGGAGAACGGTACCAGATTAAACAACACAAAG----
----

TCAAATGGCACTATAATTCTCCAATGTAGAATAAAGCAAATTGTAAATATGTGGCAGAAAGTGGGACAAGCAA
TGTATGCCCCTCCTATCCCAGGAATAATAAGGTGTGAGTCAAAC---
ATCACTGGACTATTATTAACAAGAGATGGAGGGGAAAATAGTTCA----------------------------
----

ACTGAGACTTTCAGACCTGAAGGAGGAAATATGAAGGACAATTGGAGAAGTGAATTATATAAGTATAAAGTTG
TAAAAATTGAACCACTA---GGTGTAGCACCCTCCCGTGCAAAAAGAAGAGTGGTG-----------------
----------------------GAGAGAGAAAAAAGAGCA---GTTGGA---ATAGGAGCT---------

GTCTTCCTTGGGTTCTTAGGAGCA---GCAGGAAGCACTATG---GGCGCGGCGTCA---
ATAACGCTGACGGTACAG---GCCAGACAA---TTATTG---TCTGGTATAGTGCAACAGCAGAAT---
AATTTG---CTGAAA---GCTATAGAGGCTCAACAACAGATGTTGAGACTCACGGTCTGGGGCATT
>KX389644
TTCTATTGCAATACATCAGGACTGTTTAATAACACGTTTAATCATGAAACGTTTGATAGCAATATGACGACTG
ATAAC--

AGCACAGGAAATATAACTCTCCCATGTAGAATAAGACAAATTGTAAATATGTGGCAGAGAGTAGGACAAGCAA
TGTATGCCCCTCCCATCAGCGGAGTAATAAGGTGTAATTCAAAT---
ATTACTGGACTAATATTAACAAGAGATGGAGGGAATAACAGTACA----------------------------
----

AATGAGACCTTTAGGCCTGGAGGAGGGGATATGAGGGACAATTGGAGAAGTGAATTATATAAATATAAGGTAG
TAAAAATTGAACCAATA---GGTGTGGCACCCACCTATGCAAGAAGAAGAGTGGTG-----------------
----------------------CAGAGAGAAAAAAGAACA---GTTGGA---CTGGGAGCT---------
GTCCTCTTTGGGTTCTTAGGAGCA---GCAGGAAGCACTATG---GGCGCGGCGTCA---
ATAACGCTGACGGTACAG---GCCAGACAA---TTATTG---TCTGGTATAGTGCAACAGCAGAGC---
AATCTG---CTGAGG---GCTATAGAGGCTCAACAACATATGTTGAAACTCACAGTCTGGGGCATT
>KY953197
TTCTATTGCAACACAACACAACTGTTTAATAGTACTTGGGGACTGATTAATAATACTATTTGGGGAAGCTACA
ATAGTAGCTAC---AAT--
-------AGTAGCCAGATA------
AAGGAAAATGATACCATAATTCTCCCATGCAGACTAAAGCAAATTGTAAGAATGTGGCAGAGAGTAGGACAAG
CAATGTATGCCCCTCCTATCCAGGGAGTAATAAAGTGTTATTCAAAC---
ATTACAGGATTACTGTTAACGAGCGATGGAGGGTTTAGGAATACA----------------------------
----

AGTGAGATCGTCAGGCCTGGAGGAGGAGACATGAGGGACAATTGGAGAAGTGAATTATATAAATACAAAGTAG
TAAAAATTATGCCACTA---GGTGTAGCACCCTCCTATGCAAAAAGAAGAGTGGTG-----------------
----------------------CAGAGAGAAAAAAGAGCA---GTTGGA---ATGGGAGCT---------
ATGTTCGTTGGGTTCTTAGGAGCT---GCAGGAAGCACTATG---GGCGCGGCGTCA---
ATAACGCTGACGGTACAG---GTCAGGCAA---TTATTG---TCTGGCATAGTGCAACAGCAGAGA---
AATTTG---CTGAAT---GCTATAGAGGCTCAACAACAGCTGTTGAGACTCACGGTCTGGGGCATT
>KY953198
TTCTATTGTGATACATCAAACCTGTTTAATAGCACTTGGGATAATATCACTAGGAACAATAGCACTTGGGACA
AGAGC--
-------------AATACA------
GAGTCAAATGACACTATAACACTCCAATGCAAAATAAAACAGATTGTAAATATGTGGCAGAGAGTAGGACAAG
CAATATATGCCCCTCCCATCGCAGGAGAAATAAGGTGTGAATCAAAT---
ATTACTGGATTATTATTAACAAGAGATGGAGGGGATATAAATGAG----------------------------
----

ACTGAGATCTTCAGGCCTGGAGGGGGAGATATGAGGGACAATTGGAGAAGTGAATTATATAAGTATAAAGTAG
TAAAAATTGAACCCCTA---GGTATAGCACCTACTCATGCAAAAAGAAGAGTGGTG-----------------
----------------------GGGAGAGAAAAAAGAGCA---GTTGGA---CTGGGAGCT---------
GTCTTCTTTGGGTTCTTAGGAGCA---GCAGGAAGCACTATG---GGCGCGGCGTCA---
ATAACGCTGACGGTACAG---GCCAGACAA---TTATTG---TCTGGTATAGTGCAACAGCAGAGC---
AATCTG---CTGAAG---GCTATAGAGGCTCAACAGCATCTGTTGAAACTCACGGTCTGGGGCATT
>KX389647
TTCTATTGCAATACATCAGGACTGTTTAATAGCGCTTGGAGGATTAATGGCACTGTTAACCATACAGAATCAA
ATACCAACCAT--

ACAAATAACACCATAATTCTCCCATGCAGAATAAAGCAAATTGTAAATATGTGGCAAAGGGTAGGACAAGCAA
TATATGCCCCTCCCATCCGAGGAATCATAAGGTGTGTATCAAAC---
ATTACAGGACTATTATTAACAAGAGATGGAGGGAATGAGGCAAAT----------------------------
----

AATAGTGAGACCTTCAGACCTGGAGGAGGGGATATGAGGGACAATTGGAGAAGTGAATTATATAAGTATAAAG
TAGTAAAAATTGAACCACTG---GGTGTAGCACCCACCCATGCAAAAAGAAGAGTGGTG--------------
-------------------------GAGAGAGAAAAAAGAGCA---GTTGGA---ATGGGAGCT---------

CTTCTCTTTGGGTTCTTAGGAGCA---GCAGGAAGCACTATG---GGCGCGGCGTCA---
ATAACGCTGACGGTACAA---GCCAGACAA---TTATTG---TCCGGTATAGTGCAACAGCAGAGC---
AATTTG---CTGAGA---GCTATAGAGGCTCAACAACATCTGTTGAAACTCACAGTCTGGGGCATT
>KX389649
TTCTATTGCAATACATCAGGACTGTTTAGTAGCGTTTGGAAGAACGAT-------------------------
----

ACCAATGCAACGATAACTCTCCAATGCAGAATAAAGCAAATTATCAATATGTGGCAGAGAGTAGGACAAGCAA
TGTATGCCCCTCCCATCCCAGGAGCAATAAGGTGTGACTCAAAC---
ATTACAGGACTACTATTACTAAGAGATGGAGGAGATAATAATAGT----------------------------
----

ACAAATGAGACTTTCAGGCCTGGAGGAGGAAATATGAAGGACAATTGGAGAAGTGAGTTATATAAGTATAAAG
TAGTAAAAGTTGAACCACTA---GGTATAGCACCTACCCGTGCAAAAAGAAGAGTGGTG--------------
-------------------------ATGAGAGAAAAAAGAGCA---GTTGGA---CTGGGAGCT---------
GCCTTCCTTGGGTTCTTAGGAGCA---GCAGGAAGCACTATG---GGCGCGGCGTCA---
ATAACGCTGACGGCACAG---GCCAGACAA---TTACTG---TCCGGTATAGTGCAACAGCAGAGC---
AATCTG---CTGCAA---GCTATAGAGGCTCAACAGCATCTGTTGAAACTCACTGTCTGGGGCATT
>AF069933
TTCTATTGCAGTACATCAAAACTTTTTAATAGCACTTGGAATAACAGTAACATTAGCAGCTCAAATAACACAG
AT---

TCAAATGACACTATAACTATCCAATGCAGAATAAAGCAAATTATAAATATGTGGCAGAGAGTAGGACAGGCAA
TGTATGCCCCTCCCATCCGAGGAGAAATAAGGTGTGAATCAAAC---
ATTACAGGACTACTATTAACAGGAGATGGAGGGGAAAATAATGGGACA-------------------------
----

AACGGGACAGAGACATTCAGGCCTGGAGGAGGAGATATGAGGGACAATTGGAGAAGTGAATTATATAAATATA
AGGTAGCACAAATTGAACCAATA---GGTGTGGCACCCACCCATGCAAAAAGAAGAGTGGTG-----------
----------------------------GAGAGAGAAAAAAGAGCA---GTTGGA---CTGGGAGTT------
---GCCTTCCTTGGGTTCTTAGGAGCA---GCAGGAAGCACTATG---GGCGCAGCGTCA---
ATAACGCTGACGGTACAG---GCCAGACAA---TTATTG---TCTGGTATAGTGCAACAGCAGAGC---
AATTTG---CTGAAG---GCTATAGAGGCTCAACAACATCTGTTGAAACTCACGGTCTGGGGCATT
>AF069941
TTCTATTGCAATACATCAGGACTGTTTAATAGCACTTGGAATAATAACAATTGGAATACCACTAGCAATAACA
GCTCA--

AAGTCAAATGAAATTATAAAACTCCAATGCAGAATAAGGCAAATTGTAAATATGTGGCAGAGAGTAGGACAAG
CAATGTATGCCCCTCCCACCCTAGGAGTAATAAGGTGTGAATCAAAC---
ATTACAGGACTACTATTAACAAGAGATGGAGGGAAGCATAATAGT----------------------------
----

ACAAATGAGACTTTCAGGCCTGGAGGAGGAGATATGAGGGACAATTGGAGAAGTGAATTATATAAGTATAAAG
TAGTAAAAATTGAAGCACTA---GGTGTAGCACCTACCTATGCAAAAAGAAGAGTGGTG--------------
-------------------------GAGAGAGAAAAAAGAGCA---GTTGGA---CTGGGAGCT---------
GTCTTCCTTGGGTTCCTAGGAGCA---GCAGGAAGCACTATG---GGCGCGGCGTCA---
ATAACGCTGACGGTACAG---GCCAGACAA---TTATTG---TCCGGTATAGTGCAACAGCAGAGC---
AATCTG---CTGAGG---GCTATAGAGGCTCAACAACATCTGTTGAAACTCACAGTCTGGGGCATT
>L39106
TTCTATTGCAATACATCAAAACTGTTTAATAGCACTTGGGATAATAGCAATAGCACTGCCAACCACACAGGG-
----

TCAAATGACACTATAACTCTCCAATGCAGAATAAAGCAAATTGTAAATATGTGGCAGAAAGTAGGACAAGCAA
TGTATGCCCCTCCCATCCAAGGAATAATAAGATGTGATTCAAAC---
ATTACAGGACTACTATTAACAAGAGATGGAGGGAATAATAGTACA----------------------------
----

AATGAAACCTTCAGGCCTGGAGGAGGAGATATGAGGGACAATTGGAGAAGTGAATTATATAAGTATAAAGTAG
TAAAAATTGAACCACTA---GGTGTAGCACCCACCCGTGCAAAAAGAAGAGTGGTG-----------------
----------------------GAGAGAGAAAAAAGAGCG---GTTGGA---CTGGGAGCT---------

GTCTTCCTTGGGTTCTTAGGAGCA---GCAGGAAGCACTATG---GGCGCGCGGTCA---
ATAACGCTGACGGTACAG---GCCAGACAA---TTATTG---TCCGGCATAGTGCAACAGCAGAGC---
AATTTG---CTGAAA---GCTATAGAGGCTCAACAACATCTGTTGAAACTCACGGTCTGGGGCATT
>KX232598
TTCTATTGTAATACAACAGGACTATTTAATAGCGCTTGGCAATTTAATAGCGCTTGGCAGGAGAATGACACTG
AA---

ACAGAGGGAAATATAACTCTTCCATGCAAAATAAAGCAAATTATAAATATGTGGCAGAGGGTAGGACAAGCAA
TTTATGCCCCTCCCATCCAAGGAGTAGTAAAGTGTGAGTCAAAC---
ATTACTGGACTACTATTGACAAGAGATGGGGGAATTAAGAATAGT----------------------------
----

ACGAATGAAACTTTCAGGCCTGGAGGAGGAGATATGAGGGACAATTGGAGAAGTGAATTATATAAGTATAAAG
TAGTAAAAATTGAACCATTA---GGTGTAGCACCCACCCGTGCAAAAAGAAGAGTGGTG--------------
-------------------------GAGAGAGAAAAAAGAGCA---ATTGGTGGACTGGGAGCT---------
GCATTCCTTGGGTTCTTAGGAGCA---GCAGGAAGCACTATG---GGCGCGGCGTCA---
ATAACGCTGACGGTACAG---GCCAGACAA---TTATTG---TCTGGTATAGTGCAACAGCAGAGG---
AATTTG---CTGAAG---GCTATTGAGGCTCAACAACAGATGTTGAGACTCACGGTCTGGGGCATT
>KY658712
TTCTATTGTAATACAACAAAACTATTTAATAGCACTTGGAATGCAACAAATCCATCTAATAGCAGCTAT----
----

GGCAACGAAACTATAACTCTTCCATGCAGAATAAAGCAAATTATAAATATGTGGCAGAGGGTAGGACAAGCAA
TTTATGCCCCTCCCATCCAAGGAATAATAAAGTGTGAGTCAAAC---
ATTACTGGACTACTATTGACAAGAGATGGGGGAACAACTAATAGCTCG-------------------------
----

AAGAATGAAACTTTCAGGCCTGGAGGAGGAGACATGAGGGATAATTGGAGAAGTGAATTATATAAGTATAAAG
TAGTAAAAATTGAACCATTA---GGTATAGCACCCACCCCTGCAAGAAGGAGAGTGGTG--------------
-------------------------GAGAGAGAAAAAAGAGCA---ATTGGT---GTGGGAGCT---------
GTATTCCTTGGGTTCTTATCAGCA---GCAGGAAGCACTATG---GGCGCGGCGTCA---
ATAACGCTGACGGTACAG---GCCAGACAA---TTATTG---TCTGGTATAGTGCAACAGCAAAGT---
AATTTG---CTGAAG---GCTATTGAGGCTCAACAACAGATGTTGAGACTCACGGTCTGGGGCATT
>KX232616
TTCTATTGTGATACAACACAACTATTTAATAGCACTTGGAATACAACACAACTCAACAATGACAACGAGACAG
AG---

GACAATACAACTCTTACTCTTCCATGCAGAATAAAGCAAATTGTAAATATGTGGCAGAGGGTAGGACAAGCAA
TTTATGCCCCTCCCATCCCAGGAATAATAGAGTGTGAATCAAAC---
ATTACTGGACTACTGTTGACAAGAGATGGGGGAAATAGTGATAGC----------------------------
----

TCGAATGAAACTTTTAGGCCTGGAGGAGGAGACATGAGAGACAATTGGAGAAGTGAATTATATAAATATAAAG
TAGTAAAAATTGAACCACTA---GGAGTAGCACCCACCAGGGCAAAGAGAAGAGTGGTG--------------
-------------------------GAGAGAGAAAAAAGAGCA---GTTGGA---CTAGGAGCT---------
TTGTTCATTGGGTTCTTAGGAGCA---GCAGGAAGCACTATG---GGCGCGGCGTCA---
GTGACGCTGACGGTACAG---GCCAGACAA---TTATTG---TCTGGTATAGTGCAACAGCAAAGC---
AATTTG---CTGAGG---GCTATTGAGGCTCAACAACAGATGTTGAGACTCACGGTCTGGGGCATT
>KX232622
TTCTATTGTAATACAACAAAACTATTTAATAGCACTTGGAATAGCAATGGCACTTGGAATAGCACTGGGAAGG
ACAATAGCACT--
-------------------GGA---
ACAGGGAACGATACAATAACTCTTCCATGCAAAATAAAGCAAATTATAAATATGTGGCAGAGGGTAGGACAAG
CAATTTATGCCCCTCCTATCCCAGGAGTAATACAGTGTGAGTCAAAC---
ATTACTGGACTACTATTGACAAGAGATGGGGGAGTTAATAGTACT----------------------------
----

ACAAATGAAACTTTCAGGCCTGGAGGAGGAGACATGAGGGACAATTGGAGAAGTGAATTATATAAGTATAAAG
TAGTAAAAATTGAACCAGTA---GGTGTAGCACCCAACCGTGCAAGAAGGAGAGTGGTG--------------
-------------------------GAGAGAGAAAAAAGAGCA---ATTGGA---ATGGGAGCT---------

TTTTTCCTTGGGTTCTTAGGAGCA---GCAGGAAGCACTATG---GGCGCGGCGTCA---
ATAACGCTGACGGTACAC---GCCAGACAA---TTATTG---TCTGGTATAGTGCAACAGCAAAGT---
AATTTG---CTGAAG---GCTATTGAGGCTCAACAACAGATGTTGAGACTCACGGTCTGGGGCATT
>KP411845
TTCTATTGTAATACTTCAGGCCTGTTTAACAGCACTTGGGATGGCACAGCACAAAGCAACACAGAT-------
----

GATGATGACACTATCCTTCTCCCGCGAAAAATAAATATAATTATAAATATGAGGAGAAGAGTAGGACTATCAA
TATCTGCTCCTCCTAGACGAGGAACAAGGAGGTGTACTTCTATT---
ATTACAGTACTACTATTAACAGGAGGTGGGAGTACTACTAATACT----------------------------
----
-------------AATGATGACATCTTG---
CCTGCAGGAGGAGAAAAGATGAACTACTGTAGAAGAGAACAATATATATATAAAGTAGTAATAATAGCAGCAT
TA---GTAGCAGCACCCCCCCCCTCCTCAAGAAGAGTAGTG--------------------------------
-------CTGAGAAAAAAAAGAGCA---GCAGTA---GTAGTAGCA---------
GTCGTCTTTGTCGTCTTAAGAGCA---GCAGGAAGCAGTATG---
ATCGCGTCGTCGATAATGATGATGACAGTAGAC---GCATACCAG---TGTTTG---
TCTGGTATAGTGCAACAGCAGTAC---AATTTG---CTGTAT---
GCTAATGAGGTTCAACAACATCTGTTGAAACTCTCATTTTGGGGCATT
>MF373200
TTCTATTGCAATACATCAGCATTGTTTAATAGCACTTGGGTTAATAACACTTGGAATAACGAGATCAACGAG-
----

ACCAACAAGACCATAACTCTCCAATGCAGGATAAAGCAAATTATAAATATGTGGCAGAGAGTAGGACAAGCAA
TGTATGCCCCTCCCATCCCAGGAGCAATAAAATGTGAATCAAAC---
ATTACAGGACTACTACTGACAAGAGATGGAGGAAATAATAATACA----------------------------
----

ACTGAGACTTTTAGGCCTGGAGGAGGAGACATGAGGGACAATTGGAGAAGTGAATTATATAAGTATAAAGTAG
TAAAAATTGAACCACTA---GGTGTAGCACCCAGTCGTGCAAAAAGAAGAGTGGTG-----------------
----------------------GAGAGAGAAAAAAGAGCAGTTGTTGGA---ATGGCAGCT---------
GTTTTCCTTGGTTTCTTAGGAGCA---GCAGGAAGCACTATG---GGCGCGGCGTCA---
ATAACGCTGACGGTACAG---GCCAGACAA---TTATTG---TCTGGTATAGTGCAACAGCAGAGC---
AATCTG---CTGAAG---GCTATAGAGGCTCAACAACATATGTTGAGACTCACGGTCTGGGGCATT
>AF107770
TTCTATTGCAATACATCAAATCTGTTTAATAGAACTTGGAATCATAATGGCACTTGGAATGCACCAGGACCGT
TTAATGACACT--
GAGGATAAAACAATAAATGGC------------
ACTGAGGATAAAACAATAACTCTCCAATGCAGAATAAAGCAAATTGTGCGTATGTGGCAGAAAGTAGGACAAG
CAATGTATGCCCCTCCCATCCCAGGAGAAATAAGGTGTGAATCAAAC---
ATTACAGGACTACTATTAACAAGAGATGGAGGGAATGATAATAAT----------------------------
----

AATACAGAGACCTTCAGGCCTGGAGGAGGAGATATGAGGGACAATTGGAGAAGTGAATTATATAAATATAAAG
TAGTAAAAATTGAACCACTA---GGTGTAGCACCCTCCCATGCAAAAAGAAGAGTGGTG--------------
-------------------------GAGAGAGAAAAAAGAGCACTTGTTGGA---CTGGGAGCT---------
TTCTTCTTTGGGTTCTTAGGAGCA---GCAGGAAGCACTATG---GGCGCGGCGTCA---
ATAACGCTGACGGTACAG---GCCAGACAA---TTATTG---TCTGGTATAGTGCAACAGCAGAGC---
AATCTG---CTGAAG---GCTATAGAGGCTCAACAACATCTGTTGAGACTCACGGTCTGGGGCATT
>HM215281
TTCTATTGCAATACAACACCACTGTTTAATAGCACTTGGTACAGCAATAACACTGCCAATAGCTCAAACAGCA
CA---

GAGTTAAATGGAACTATAACTCTCCAATGCAGAATAAAGCAAATTGTAACTATGTGGCAAAGGGTAGGACAGG
CAATGTATGCCCCTCCCATCCCAGGAGAAATAAGGTGTGAATCAAAC---
ATTACTGGACTACTATTAACAAGAGATGGAGGGGGGAATAATACA----------------------------
----

AATACAAATGAGACCTTTAGGCCTGGAGGAGGAGATATGAGGGACAATTGGAGAAGTGAATTATATAAGTATA
AAGTAGTAAAAATTGAACCACTA---GGTGTAGCACCCACCCCTGCAAAAAGAAGAGTGGTG-----------

----------------------------GAGAGAGAAAAAAGAGCA---GTTGGA---CTGGGAGCT------
---GTCTTCTTTGGGTTCTTAGGAGCA---GCAGGAAGCACTATG---GGCGCGGCGTCA---
TTAACGCTGACGGTACAG---GCCAGACAA---TTATTG---TCCGGTATAGTGCAACAGCAAAGT---
AATTTG---CTGAAG---GCTATAGAGGCTCAACAACATCTGTTGAAACTCACAGTCTGGGGCATT
>HM215292
TTCTATTGCGATACATCAAAACTGTTTAATAGCACTTGGACCAAAAATAGCACTTGGAACACTACAGAA----
----

TTAGGTGGCAATATAACGCTCCAGTGCAAAATAAAGCAAATTATAAATACGTGGCAAAAAGTAGGACTAGCAA
TGTATGCCCCTCCCATCCAAGGAATAATAAGGTGTGATTCAAAC---
ATTACAGGACTACTATTAACAAGAGATGGAGGGAATAATAATGCA----------------------------
----

AGTGAGACTGAGACCTTCAGGCCTGGAGGAGGAGATATGAGGGACAATTGGAGAAGTGAATTATATAAATACA
AAGTAGTAAAAATTAAACCACTA---GGTGTCGCACCCACCCATGCAAAAAGAAGAGTGGTG-----------
----------------------------GAGAGAGAAAAAAGAGCA---GTTGGA---CTGGGAGCT------
---GTTTTCCTTGGGTTCTTAGGAGCA---GCAGGAAGCACTATG---GGCGCGGCGTCA---
ATAACGCTGACGGTACAG---GCCAGACAA---TTATTG---TCCGGTATAGTGCAACAGCAGAGC---
AATTTG---CTGCAA---GCTATAGAGGCTCAACAACATCTGTTGAAACTCACGGTCTGGGGCATT
>KT223759
TTCTATTGCAATACATCAAAGCTGTTTAATAGCACTTGGAATGCCACTGACATCAATAGCAATGTCAGCACAG
ACTCAGGG---

AAAAATGACACTATAACTCTCCCATGCAGAATAAAGCAAATTATAAGGATGTGGCAGAGCGTAGGGCAAGCAA
TGTATGCCCCTCCTATCCAAGGAATAATAAAGTGTGAATCAAAC---
ATTACAGGACTACTATTAACAAGAGATGGAGGGAAGGAAAACAAT----------------------------
----

AGTACAAATGAGACTTACAGGCCAGGAGGAGGAGACATGAGGGACAATTGGAGAAGTGAATTATATAAGTATA
AAGTAGTGAAAATTGAACCATTT---GGTATAGCACCCACCCTTGCAAGGAGAAGAGTGGTG-----------
----------------------------GAGAGAGAAAAAAGAGCA---GTTGGA---CTGGGAGCT------
---GTCTTCCTTGGGTTCTTAGGAGCA---GCAGGAAGCACTATG---GGCGCGGCGTCA---
GTGACGCTGACGGTACAA---GCCAGACAA---TTGTTG---TCTGGTATAGTGCAACAGCAGAGC---
AATCTG---CTGAAG---GCTATAGAGGCTCAACAACACTTGTTGAGACTCACAGTCTGGGGCATT
>KT223760
TTCTATTGCAACACATTAGGACTGTTTAATAGCACTTGGAACTGGGAATGGCACTTGGATAACAATAGCTCAA
TT---

AGCTATGCTCGTGTAACTCTCCAATGCAAAATAAAGCAAATTGTAAATATGTGGCAGAGAGTAGGACAAGCAA
TGTATGCCCCTCCCATCCCAGGGGAACTAAGGTGTGATTCAAAT---
ATTACAGGACTATTATTAACAAGAGATGGGGGA--

-
AAAATAATACTAATGAATGAGACCTTCAGGCCTGGAGGAGGAGATATGAGGGACAATTGGAGAAGTGAATTAT
ATAAGTATAAAGTAGTAAAATTTGAACCACTA---GGTGTAGCACCTACCCATGCAAAAAGAAGAGTGGTG--
-------------------------------------CAGAGAGAAAAAAGAGCA---GTTGGA---
ATGGGAGCT---------CTCTTCCTTGGGTTCTTAGGAGCA---GCAGGAAGCACTATG---
GGCGCGGCGTCA---ATAACGCTGACGGTACAG---GCCAGACAA---TTATTG---
TCTGGTATAGTGCAACAGCAGAGT---AATCTG---CTGAAG---
GCTATAGAGGCTCAACAACAGCTGTTGAGACTCACAGTCTGGGGCATT
>AJ251056
TTCTATTGCAATACAGCAGAACTGTTTAATAGCACTTGGGCTAGCAATACTAATGGCATTTGGGCTAGCAATA
TAAATGCTAGC--
----------AATAACAAG------
GACGCAAATGACACTATAACTCTCAAATGCAAAATAAAACAAATTATAAATATGTGGCAGAAAGTAGGACAAG
CAATATATGCCCCTCCCATCGAAGGAGTAATAAGGTGTGATTCAAAC---
ATTACAGGAATACTATTAACAAGAGATGGAGGGGATAATACTAAT----------------------------
----

GGTGATGAGACTTTCAGGCCTGGAGGAGGAGATATGAGGGACAACTGGAGAAGTGAACTATATAAGTATAAAG

TAGTAAAAATTGAACCACTA---GGTGTAGCACCCTCCCATGCAAAAAGAAGAGTGGTG--------------
-------------------------GAGAGAGAAAAAAGAGCA---GTTGGA---CTGGGAGCT---------
GTCTTCCTTGGGTTCTTAGGAGCA---GCAGGAAGCACTATG---GGCGCAGCGTCA---
ATAACGCTGACGGTACAG---GCCAGACAA---TTATTG---TCTGGTATAGTGCAACAGCAGAAC---
AATCTG---CTGAAG---GCTATAGAGGCTCAACAACATTTGTTGAAACTCACGGTCTGGGGCATT
>AJ251057
TTCTATTGCAATACATCAGGACTGTTTAATGGCACTTGGAATGGCACTAGCAGCTCAAACCACACATGG----
----

GTAAATGACACTATAACTCTCCAATGCAGAATAAGGCAAATTGTAAATATGTGGCAGAGAGTAGGACGAGCAA
TGTATGCCCCTCCCATCCAGGGAGAAATAAAGTGTGTATCAAAC---
ATTACTGGACTACTATTAACAAGAGATGGAGGGGATAATAATAGT----------------------------
----

ACAAATGAGACTTTCAGGCCTGGAGGAGGAGATATGAGGGACAATTGGAGAAGTGAATTATATAAATATAAAG
TAGTAAAAATTGAACCACTG---GGTGTAGCACCCTACCGTGCAAAAAGGAGAGTGGTG--------------
-------------------------GAGAGAGAAAAAAGAGCA---GTTGGA---ATAGGAGCT---------
GTCCTCCTTGGGTTCTTAGGAGCA---GCAGGAAGCACTATG---GGCGCGGCGTCA---
ATGACGCTGACGGTACAG---GCCAGACAA---TTATTG---TCTGGTATAGTGCAACAGCAGAGC---
AATCTG---CTGAGG---GCTATAGAGGCTCAACAACATCTGTTGAAACTCACAGTCTGGGGCATT
>HM215264
TTCTATTGCAATACATCAGAACTGTTTAATAGCACTTGGACTAGCAATAACACTTGGGGTAGCAGTAGCTTA-
----

AATCACACAAATATAACTCTCCAATGCAGAATAAAGCAAATTATAAGAATGTGGCAGAGAGTAGGACAAGCAA
TGTATGCCCCTCCTATCAAAGGGGAAATAAGGTGTGAATCAAAC---
ATTACTGGACTACTATTAATAAGAGATGGA---ATTGTTAATAGT----------------------------
----

ACAAATGAGACCTTCAGGCCTGGAGGAGGAGATATGAGGGACAATTGGAGAAGTGAATTATATAAATATAAAG
TAGTAAAAATTGAACCACTA---GGGGTAGCACCTAACCGTGCAAAAAGAAGAGTGGTG--------------
-------------------------GAGAGAGAAAAAAGAGCA---GTTGGA---CTGGGAGCT---------
TTCTTTCTTGGGTTCTTAGGAGCA---GCAGGAAGCACTATG---GGCGCGGCGTCA---
ATGACGCTGACGGTACAG---GCCAGACAA---TTATTG---TCCGGTATAGTGCAACAGCAGAGC---
AATTTG---CTGAAG---GCTATAGAGGCTCAACAACATCTGTTGAGACTCACGGTCTGGGGCATT
>HM215290
TTCTATTGTAATACATCAGCACTGTTTAATAGCACTTGGAATGGCACTGACAGTGCAAACAAGACAAAG----
----

TCAAATGAAACTATAACTCTCCAATGCAAAATAAAGCAAATTATAAATATGTGGCAGAGAGTAGGACAAGCAA
TGTATGCCCCTCCCATCCAAGGAGAAATAAAGTGTACATCAAAC---
ATTACAGGACTACTACTAACAAGAGATGGAGGGAATACTAGTGGT----------------------------
----

GCAAATGAGACTTTCAGGCCTGGAGGAGGAGACATGAGGGACAATTGGAGAAGTGAGTTATATAAATATAAAG
TAGTAAAAATTGAGCCACTA---GGTGTAGCACCCACCCCTGCAAAAAGAAGAGTGGTG--------------
-------------------------GGGAGAGAAAAAAGAGCA---GTTGGA---CTGGGAGCT---------
GTCTTCCTTGGGTTCTTAGGAGCA---GCAGGAAGCACTATG---GGCGCGGCGTCA---
ATAACGCTGACGGTACAG---GCCAGACAA---TTATTG---TCTGGCATAGTGCGACAGCAGAGC---
AATCTG---CTGAGG---GCTATAGAGGCTCAACAACATCTGTTGAAACTCACGGTCTGGGGCATT
>HM215291
TTCTATTGCAATACATCAAAACTATTTAATAGCACTTGGAATAGCACTGAAAATAACAATACCAGTGAC----
----

ACAAATGGTATTATAACTCTCCAATGCAGAATAAAGCAAATTGTAAATATATGGCAGAGAGTAGGACAAGCAA
TGTATGCCCCTCCTATCCAAGGAGTAATAAGGTGTGAATCAAAC---
ATTACAGGACTACTATTAACAAGAGATGGAGGGGATAATGATAGT----------------------------
----

ACAAATGAGACCTTCAGGCCTGGAGGAGGAGATATGAGGGACAATTGGAGAAGTGAATTATATAAGTATAAGG

TAGTACAAGTTGAACCACTA---GGTGTGGCACCCACCCATGCAAGAAGAAGAGTGGTG--------------
-------------------------GAGAGAGGAAAAAGAGCA---GTTGGA---GTGGGAGCT---------
GTTTTCCTTGGGTTCTTAGGAGCA---GCAGGAAGCACTATG---GGCGCGGCGTCA---
ATAACGCTGACGGTACAG---GCCAGACAA---TTATTG---TCTGGTATAGTGCAACAGCAGAGC---
AATTTG---CTGAAG---GCTATAGAGGCTCAACAACATCTGTTGAAACTCACGGTCTGGGGCATT
>HM215293
TTCTATTGCAATACATCAAAGCTGTTTAATAGCACTTGGGGTAACAATACCACTAACAGCTCATCAGAATCAA
AT---

AACAATGACACTCTAATTCTCCAATGCAGAATAAGACAAATTGTAAGGATGTGGCAGAGAGTAGGACAAGCAA
TGTATGCCCCTCCCATCAGAGGAGAAATAAGGTGTGAGTCAAAC---
ATTACAGGGCTACTATTAACAAGAGATGGAGGGATTAATAATACA----------------------------
----

AATAATGAGACCTTCAGGCCTGGAGGAGGAGATATGAGGGACAATTGGAGAAGTGAATTATATAAGTATAAAG
TAGTAAAAATTGAACCACTA---GGTGTAGCACCTACCCATGCAAAAAGAAGAGTGGTG--------------
-------------------------GAGAGAGAAAAAAGAGCA---GTTGGA---CTGGGAGCT---------
TTCTTCCTTGGGTTCTTAGGAGCA---GCAGGAAGCACTATG---GGCGCGGCGTCA---
ATAACGCTGACGGTACAA---GCCAGACAA---TTACTG---TCTGGTATAGTGCAACAGCAGAGC---
AATCTG---CTGAGA---GCAATTGAGGCTCAACAACATCTGTTGAGACTCACGGTCTGGGGCATT
>JX447154
TTCTATTGCAATACATCAGGACTGTTTAATAGCACTTGGAATCACAGGAGCACTTGGAATAGCACTGATCACA
ATATCTCT---
-------------------GAC---
AGCTCAAACCCCAATATAACTCTCCAATGCAGAATAAAGCAAGTTATAAATATGTGGCAGAGAGTAGGACAAG
CAATGTATGCCCCTCCCATCCCAGGAGTAATAAGGTGTGAATCAAAC---
ATTACTGGACTGTTATTAACAAGAGATGGAGGAAATGAGAGTAGT----------------------------
----

ACAAATGAGACTTTCAGACCTGGAGGAGGAGACATGAGGGACAATTGGAGAAGTGAATTATATAAGTATAAAG
TAGTAAAAATTGAACCAGTG---GGTGTAGCACCCTCCCGTGCAAAAAGAAGAGTGGTG--------------
-------------------------GAGAGAGAAAAAAGAGCA---GTTGGA---CTGGGAGCT---------
GTCTTCCTTGGGTTCTTAGGAGCA---GCAGGAAGCACTATG---GGCGCGGCGTCG---
GTGACGCTGACGGTACAG---GCCAGACAG---TTATTG---TCTGGTATAGTGCAACAGCAGAGC---
AATTTG---CTGAGG---GCTATAGAGGCTCAACAACATTTGTTGAAACTCACGGTCTGGGGCATT
>KF716488
TTCTATTGCAACACCTCAGGACTGTTTAATAGCAGCTTCAATAGTAGCAGCATCAATGACACAGACACAGAG-
----

TTAACTGGCAATATAACTCTTCCATGCAGAATAAAACAGATTGTAAATATGTGGCAGAAGGTAGGAAAAGCAA
TGTATGCCCCCCCTATTGAAGGAATAATAAGGTGTGAGTCAAAC---
ATTACAGGACTGATATTAACAAGAGATGGAGGGGGCAATACAACA----------------------------
----

GAGGAGACGGAGACCTTCAGGCCTGGAGGAGGAGATATGAGGGACAATTGGAGAAATGAATTATATAAATATA
AGGTAGTGAGAATTGACCCAATA---GGTGTAGCACCCACTAAGGCAAAAAGAAGAGTGGTG-----------
----------------------------GAGAGAGGAAAAAGAGCA---GTTGGA---CTGGGAGTT------
---GCCTTCCTTGGGTTCTTAGGAGCA---GCAGGAAGCACTATG---GGCGCGGCGTCA---
ATAACGCTGACGGTACAG---GCCAGACAA---TTATTG---TCTGGTATAGTGCAACAGCAGAGC---
AATCTG---TTGAGA---GCTATAGAGGCTCAACAACATCTGTTGAAACTCACAGTCTGGGGCATT
>AY444811
TTCTATTGCAATACATCGGGACTGTTTAATAGTACTTTTAATAACACTCAGGCCAACTCAAGTTACACCTCAG
GG---

ACAAATGATACTATACTTCTCCAATGCAGAATAAGGCAAATTATAAGAATGTGGCAGAGAGTAGGACAAGCAA
TGTATGCCCCTCCCATCCAAGGAGAAATAAGGTGTGAATCAAAC---
ATTACTGGACTACTATTAACAAGAGATGGAGGGGATACTAGTGAT----------------------------
----

AATAGTACCGAGACCTTCAGGCCTGGAGGAGGAGATATGAGGGACAATTGGAGAAGTGAATTATATAAGTATA

AAGTAGTAAAAATTGAACCAATA---GGTATAGCACCCACCAAGGCAAAAAGAAGAGTGGTG----------
----------------------------CAGAGAGAAAAAAGAGCA---GTTGGA---ATAGGAGCT-----
---GTCCTCTTTGGGTTCTTGGGAGCA---GCAGGAAGCACTATG---GGCGCAGCGTCA---
ATAACGCTGACGGTACAG---GCCAGACAA---TTATTG---TCCGGTATAGTGCAACAGCAAAGC---
AATTTG---CTGAAG---GCCATAGAGGCTCAACAACATCTGTTGAGACTCACAGTCTGGGGCATT
>DQ869018
TTCTACTGCAATACAACAGCACTGTTTAATAGCACTTGGGATAGCAATACCACTGCCAGCGAAGAGTCAAATA
GC--

ACCATAGATGACATTATAACTCTCCAATGCAGAATAAAGCAAGTTGTAAACATGTGGCAGAAAGTAGGACAAG
CAATGTATGCCCCTCCCATTGCAGGAGAAATAAGGTGTGAATCAAAC---
ATTACAGGACTACTATTAACAAGAGATGGAGGGGATAATAATAAC---------------------------
----

AGTGAGACCTTCAGACCTGGAGGAGGAGATATGAGGGACAATTGGAGAAGTGAATTATATAAATATAAAGTAG
TAAAAATTGAACCGCTA---GGAGTAGCACCCTCCAGGGCAAAAAGAAGAGTGGTG----------------
----------------------GAGAGAGAAAAAAGAGCA---GTTGGA---CTGGGAGCT--------
GTCTTCCTTGGGTTCTTAGGAACA---GCAGGAAGCACTATG---GGCGCGGCGTCA---
ATAACGCTGACGGTACAG---GCCAGACAA---TTATTG---TCCGGTATAGTGCAACAGCAGAGC---
AATCTG---CTGAAG---GCCATAGAGGCTCAACAGCAACTGTTGAGACTCACGGTCTGGGGCATT
>JF320297
TTCTATTGCAATACATCAGAACTATTTAATAGCAGTTGGAATAATGACACTGCCAGCTCAAATAACACAGGG-
----

TCAAATGACACTATAACTCTCCAATGCAGAATAAAGCAAATTGTAAATATGTGGCAGAAGGTAGGACAAGCAA
TGTATGCCCCCCCCATCAAAGGAGTAATAAGGTGTGAATCAAAC---
ATTACAGGACTAATATTAACAAGAGATGGAGGGGATAATAATAATAAT------------------------
----

AGTGTAAATGAGACCTTCAGGCCTGGAGGAGGAGATATGAGGGACAATTGGAGAAGTGAATTATATAAGTATA
AAGTAGTAAAAATTGAACCACTA---GGTATAGCGCCCACCCATGCAAGAAGAAGAGTGGTG----------
----------------------------CAGAGAGAAAAAAGAGCA---GTTGGA---CTAGGAGCT-----
---CTCTTCCTTGGGTTCTTAGGAGCA---GCAGGAAGCACTATG---GGCGCGGCGTCA---
ATAACGCTGACGGTACAG---GCCAGACAA---TTATTG---TCCGGTATAGTGCAACAGCAGAGC---
AATCTG---CTGAAA---GCTATAGAGGCTCAACAACATCTGTTGAAACTCACGGTCTGGGGCATT
>AY736840
TTCTATTGCAATACATCAATACTGTTTAATAGCACTTGGGACAATAGCACTGTCAACACAAATAATATCACAG
AG--

CCAAATGATACTATAACCCTCCAATGCAGAATAAAGCAAATTATAAATATGTGGCAGAAAGTAGGGCAAGCAA
TGTATGCCCCTCCCATCAAAGGAATCATAAGGTGTGATTCAAAC---
ATTACTGGATTACTATTAACAAGAGATGGAGGGAATAATAATAGT---------------------------
----

ACAAATGAGACCTTCAGACCTGGAGGAGGAGATATGAGGGACAATTGGAGAAGTGAATTATATAAGTATAAAG
TAGTAAAAATTGATCCAATA---GGGGTGGCTCCCACCCATGCAAAAAGAAGAGTGGTG-------------
-------------------------GAGAAAGAAAAAAGAGCA---GTTGGA---CTGGGAGCT--------
GTCTTCATTGGGTTCTTAGGAGCT---GCAGGAAGCACTATG---GGCGCGGCGTCA---
ATAACGCTGACGGTACAA---GCCAGACAA---TTATTG---TCTGGTATAGTGCAACAGCAAAGC---
AATTTG---CTGAAG---GCTATAGAGGCTCAACAACATCTGTTGAGACTCACAGTCTGGGGCATT
>KU168266
TTCTATTGCAATACATCAGGCCTGTTTAATAGTACTTGGAATGTGACCACCAGCCTCAACCAGAGTGAA----
----

GCGGACGTAAATATAACTCTCCCATGCAAAATAAAACAAATTGTACGTATGTGGCAGGGAGTAGGACAAGCAA
TGTATGCCCCTCCCATTCAAGGAGTAATTAACTGTACATCAAAC---
ATCACAGGGCTACTGCTAACAAGAGACGGGGGGAGGGATAATGGT---------------------------
----

GCAAATGAGACGTTCAGACCCGGAGGAGGAAATATAAAGGACAATTGGAGAAGTGAATTATATAAATATAAGG

TAGTAAAAATTGAACCACTA---GGTGTAGCACCCACCCGTGCAAAAAGAAGAGTGGTG-------------
-------------------------GAGAGAGAAAAAAGAGCA---GTTGGA---CTGGGAGCT--------
GTCCTCTTTGGGTTCTTAGGAGCA---GCAGGAAGCACTATG---GGCGCGGCGTCA---
ATAACGCTGACGGTACAG---GCCAGACAA---TTATTG---TCTGGTATAGTGCAACAGCAGAAC---
AATCTG---CTGAGG---GCTATAGAGGCTCAACAGCAAATGTTGAAACTTACGGTCTGGGGCATT
>KU168267
TTCTATTGCAATACATCAGATCTGTTCGATAGCACTTGGACTTCAAACCACACCTCAAACCACACAGGGTCAG
ATGAGACC---
------------------ATA---
ACGTCAAATGACACTATAATTCTCCAATGTAGAATAAAGCAAATTGTAAGGATGTGGCAGACAGTAGGACAAG
CAATGTATGCCCCTCCCATCCCAGGAGAAATAAGGTGTGAATCAAAC---
ATTACTGGACTACTATTAACAAGAGATGGAGGGCAAACAGAAAAT---------------------------
----

AATAATGAGACCTTCAGGCCTGGAGGAGGAAATATGAAGGACAATTGGAGAAGTGAATTATATAAGTATAAAG
TAGTAAAAATTGAACCACTA---GGTGTAGCACCCACCCCTGCAAGAAGAAGAGTGGTG-------------
-------------------------CAGAGAGAAAAAAGAGCA---GTTGGACTACTGGGAGCT--------
GTCTTCCTTGGGTTCTTAGGAGCA---GCAGGAAGCACTATG---GGCGCGGCGTCA---
ATAACGCTGACGGTACAG---GCCAGACAA---TTACTG---TCTGGTATAGTGCAACAGCAAAGC---
AATTTG---CTGAAA---GCTATAGAGGCTCAACAACATCTGTTGAGACTCACGGTCTGGGGCATT
>MF510462
TTCTATTGCAATACATCAGACCTGTTTAATAGCACTTGGGATAACAATACCAACAGTTCAAACTTCACA----
----

GGTAATGACACTATAACTCTCCAATGCAGAATAAAGCAATTTGTAAATATGTGGCAGAGGGTAGGACAAGCAA
TGTATGCCCCTCCCATCGAAGGAAGAATAAGATGTGAATCAAAT---
ATTACTGGACTACTATTAACAAGAGATGGAGGAGAAGGTAATAAT---------------------------
----

AGGACAAATGAAACCTTCAGGCCTGGAGGAGGAGATATGAGGGACAATTGGAGAAGTGAATTATATAAGTATA
AAGTAGTAAAAATTGAACCACTA---GGTGTAGCACCCACCCATGCAAAAAGAAGAGTGGTG----------
----------------------------CAGAGAGAAAAAAGAGCA---GTGGGA---CTGGGAGCT------
---GTCTTCCTTGGGTTCTTAGGAGCG---GCAGGAAGCACTATG---GGCGCGGCGTCA---
ATAACGCTGACGGTACAG---GCCAGACAA---TTATTG---TCTGGTATAGTGCAACAGCAGAGC---
AATTTG---CTGAAG---GCTATAGAGGCTCAACAACATCTGTTGAAACTCACAGTCTGGGGCATT
>AF082485
TTCTACTGTAATACAACAAAACTGTTTGATAGCACTTGGAATGGTACTGAAGAGTTAGATAACACT-------
----

GAAGGAGATATAGTCACACTCCCATGCAGAATAAAACAAATTATAAACATGTGGCAGGAAGTAGGAAAAGCAA
TGTATGCCCCTCCCATCGCGGGACAAATTAGATGTTCCTCAAAT---
ATTACAGGACTGCTATTAACAAGAGATGGTGGTTACCAGAGCAAT---------------------------
----

GTCACCGAGATCTTCAGACCTGGAGGAGGAGATATGAGGGACAATTGGAGAAGTGAATTATATAAATATAAAG
TAGTAAAAATTGAACCATTA---GGAGTAGCGCCCACCAGGGCAAAGAGAAGAGTGGTG-------------
-------------------------CAGAGAGAGAAAAGAGCA---GTGGGA---ATAGGAGCT--------
GTGTTCCTTGGGTTCTTGGGAGCA---GCAGGAAGCACTATG---GGCGCGGCGTCA---
ATAACGCTGACGGTACAG---GCCAGACAA---TTATTG---TCTGGTATAGTGCAACAGCAGAAT---
AATCTG---CTGAGG---GCTATTGAGGCGCAACAACATCTGTTGCAACTCACAGTCTGGGGCATC
>FJ388917
TTCTATTGCAATACAACAAAACTGTTTAATAGTACACTGAAAAATGGTACATTCATTCCGGGTACAAAT----
----

TCTAATTCAAACATCACACTCCAATGCAGACTAAAACAATTTGTAAGGATGTGGCAGGAAGTGGGACAAGCAA
TGTACTCCTCCCCAATGGAAGGGAAAATTTACTGCAGCTCAAAT---
ATTACAGGACTAATATTAACAAGAGATGGTGGTAATAATGGTGATGAC------------------------
----

AATGGTACTGAGACCTTCAGACCTGGAGGAGGAGACATGAGGGACAATTGGAGAAGTGAACTATATAAATATA

AAGTAGTAAGGATTGAACCAATA---GGAGTAGCACCCACTGAAGCAAGGAGACGGGTGGTG-----------
----------------------------CAGAGAGAAAAACGAGCA---GTGGGA---ATAGGAGCC------
---GTGTTCCTTGGGTTCTCGGGAGCA---GCAGGAAGCACTATG---GGCGCAGCGTCA---
TTGACGCTGACGGTACAG---GCCAGACAA---TTATTG---TCTGGTATAGTGCAGCAGCAGAGC---
AATCTG---CTGAGG---GCTATAGAGGCTCAACAACATCTGTTGAGACTCACAGTCTGGGGCATT
>JN687742
TTCTATTGTAGCACAACAGCACTGTTTAATAGTACCTGGACGTATAATGGTACCTGGACTACCAATGGCAGCA
CTGAG--

ACAGATGACACAATCATTACACTGCAATGCAGAATAAGACAAATTGTAAACCTGTGGCAGGCAGTGGGACAAG
CAATGTACGCCCCCCCCATTGCAGGAAACATTACCTGCAGCTCAAAT---
ATTACAGGAATGATATTGACAAGAGATGGTACTAATAGTACTAAT----------------------------
----

AATGATACCTTCAGACCTGGAGGAGGAGACATGAGGGACAATTGGAGAAGTGAACTATATAAATATAAAGTAG
TAAAGATTGAACCAATA---GGAGTAGCACCCACTAGGGCAAGGAGACGAGTGGTG-----------------
----------------------CAGAGAGAAAAACGAGCA---GTAGGA---CTGGGAGCT---------
GTGTTCCTTGGGTTCTTGGGAGCA---GCAGGAAGCACTATG---GGCGCAGCGTCA---
ATAACGCTGACGGTACAG---GCCAGACAA---TTATTG---TCTGGCATAGTGCAACAGCAGAGC---
AATCTG---CTAAGG---GCTATAGAGGCTCAACAACATCTGTTGAAACTCACAGTCTGGGGCATT
>JX140648
TTCTATTGCAACACAACAGAACTGTTTAATAGTACCTGGACACTGAATAATAGTACCAATAGCAGCATTGAGA
CA---

AATGGCACAACTATTATACTGCCATGCAGAATAAGACAATTTATAAACATGTGGCAGGAAGTGGGACAAGCAA
TGTACGCCCCCCCCATTGAAGGAAGCATTAAATGCAGCTCAAAT---
ATTACAGGAATGATACTGACAAGAGATGGTGGTAATAGTACTACT----------------------------
----

AATGAGACCTTCAGACCTGGAGGAGGAAACATGAGGGACAATTGGAGAAGTGAACTATATAAATATAAAGTAG
TAAAGATTGAACCAGTA---GGAGTAGCACCCACTAAGGCAAGGAGACGAGTGGTG-----------------
----------------------CAGAGAGAAAAACGAGCA---GTAGGA---CTGGGAGTG---------
ATGTTCCTTGGGTTCTTGGGAGCA---GCAGGAAGCACTATG---GGCGCAGCGTCA---
ATGACGCTGACGGTACAG---GCCAGACAA---TTATTG---TCTGGCATAGTGCAACAGCAGAGC---
AATTTG---CTAAGG---GCTATAGAGGCTCAACAACATCTGTTGAAACTCACAGTCTGGGGCATT
>JN687748
TTCTATTGCAACACATCACAGCTGTTTTTAGGTAATGGTACAAATAGTACAAACACAAATAAT----------
----

ACAAATTCAACTATTGAACTCAAATGCAGAATAAGACAAATTGTAAACATGTGGCAGGGAGTGGGACAAGCAA
TGTACGCCCCACCCATTGCAGGGAGCATTAGCTGCAGCTCAAAT---
ATTACAGGATTAATATTGACAAGAGATGGTGGTAATGATACTGGG----------------------------
----

ACGAATGAGACCTTCAGACCTGGAGGAGGAGACATGAGGGACAATTGGAGAAGTGAACTATATAAATATAAAA
TAGTAAAGATTGAACCAGTA---GGAGTAGCACCCACTAGGGCCAGGAGACGAGTGGTG--------------
-------------------------CAGAGAGAAAAACGAGCA---GTGGGA---ATAGGAGCT---------
GTGCTCCTTGGGTTCTTGGGAGCA---GCAGGAAGCACTATG---GGCGCAGCGTCA---
ATAACGCTGACGGTACAG---GCCAGACAA---TTATTG---TCTGGCATAGTGCAACAGCAAGGC---
AATTTG---CTGAGG---GCTATAGAAGCTCAACAACATCTGTTGAGGCTTACGGTCTGGGGCATT
>AF119820
TTCTACTGCAATACATCAGGACTGTTTAATAGTACATACATGTTTAATAGTACAAATAGGACAAACACAACCA
AT---

GGAACAAACTCAACCATCACGCTCCCATGCAGAATAAAACAAATTGTAAGAATGTGGCAGGGAGTAGGACAAG
CAATGTACGCCCCTCCCATTGCAGGGAGCATTAACTGCAGCTCAGAT---
ATTACAGGAATAATATTGACAAGAGATGGTGGTATAAGTAATAATAATGAG----------------------
----

ACTAATGATAATGAGACCCTCAGACCTGGAGGAGGAGACATGAGGGACAATTGGAGAAGTGTACTATATAAAT

ATAAAGTAGTAAAGATTGAACCAGTG---GGAATAGCACCCACGGGGGCAAGGAGACGAGTGGTG--------
-------------------------------CAGAAAGAAAAACGAGCA---GTGGGA---CTAGGAGCT---
------TTGTTCCTTGGGTTCTTGGGAGCA---GCAGGAAGCACTATG---GGCGCAGCGTCA---
CTAACGCTGACGGTACAG---GCCAGACAA---TTATTG---TCTGGTATAGTGCAACAGCAGAGC---
AATCTG---CTGAGG---GCTATAGAGGCTCAGCAACATCTGCTGAAACTCACGGTCTGGGGCATT
>KU168269
TTCTATTGTAATACATCCAATCTGTTTAATAGTAATATAACAGAGAATGATACAACAAAT-------------
----

AGTAATGACAATATCACACTCCCATGTAGGATAAAACAAATTGTAAGAATGTGGCAGAGAGTGGGACAAGCAA
TGTATGCCCCTCCCATTGCAGGAAACATTACATGTAGATCAAAC---
ATTACAGGAATACTATTAACAAGAGATGGTAATGGTGGGAATGATACC-------------------------
----

AATAGCAATGAGACTTTCAGACCTGGAGGAGGAGATATGAGGGACAATTGGAGAAGTGAATTATACAACTATA
AAGTAGTAAAAATTAAACCACTA---GGAGTAGCACCCACCCATGCAAGAAGAAGAGTGGTG-----------
----------------------------GGGAGAGAGAAAAGAGCA---GTTGGA---CTAGGAGCT------
---CTTCTCCTTGGGTTCTTGGGAACA---GCAGGAAGCACTATG---GGCGCAGCGTCA---
ATAACGCTGACGGTACAG---GCCAGACAA---TTATTG---TCTGGTATAGTGCAACAGCAAAGT---
AATTTG---CTGCAA---GCTATAGAGGCGCAACAGCATCTGTTGCAGCTCACAGTCTGGGGCATT
>AB286852
TTCTATTGTAATACATCAAAACTATTTAATAGTACTTTTTCTAATGATAATTCCACAGATAATGATACCTCTT
TA---

GAAAATAATGGTACCATCACACTCCCATGTAAGATAAAACAAATTGTAAGAATGTGGCAGAGAGTGGGACAGG
CAATGTATGCCCCTCCCATTGAAGGAAACATTACATGTATATCAAAC---
ATTACAGGACTAATATTAACAAGAGATGGGCACAATGGGCACAATCAG-------------------------
----

AGTAATAATGAGACCTTCAGACCTACAGGAGGAGATATGAGGGACAATTGGAGAAGTGAATTATATAAGTATA
AAGTAGTAAGAATTAAACCACTA---GGAATAGCACCCACCAAGGCAAGAAGAAGAGTAGTG-----------
----------------------------GGCAGAGAGAAAAGAGCA---GTTGGA---CTGGGAGCT------
---GTTTTCCTTGGGTTCTTAGGAGCG---GCAGGAAGCACTATG---GGCGCAGCGTCA---
ATAACGCTGACGGTACAG---GTCAGACAA---TTATTG---TCTGGCATAGTGCAGCAGCAAAGC---
AATTTG---CTGAGA---GCTATAGAAGCGCAGCAGCATCTGTTGCAGCTCACAGTCTGGGGCATT
>AF069934
TTTTATTGTAATACATCAGAGCTGTTTACTAATACATCAACTAAGAATGGG----------------------
----

ACCAATGAAACACCCACACTCCCATGTAAGATAAAACAAATTGTAAGAATGTGGCAGAGAGTGAGACAAGCAA
TGTATGCCCTTCCCATTGCAGGAAACATTACGTGTACATCAAAC---
ATTACAGGACTACTATTAACAAGAGATGGG---AATAATGACAGT----------------------------
----

CACACTGAGACCTTCAGACCTGGAGGGGGAGATATGAGGGACAATTGGAGAAGTGAATTATATAAGTATAAAG
TAGTAAAAATTAAACCACTA---GGAATAGCACCCACCTGGGCGAGGAGAAGAGTAGTG--------------
-------------------------GGGAGAGAGAAAAGAGCA---GTTGGA---CTGGGAGCT---------
GTTTTCCTTGGGTTCTTAGGGACA---GCAGGAAGCACTATG---GGCGCAGCGTCA---
ATAACGCTGACGGTACAG---GTCAGACAA---TTATTG---TCTGGCATAGTGCACCAGCAAAGC---
AATTTG---CTGAGA---GCTATAGAGGCGCAGCAGCATCTGTTGCAACTCACAGTCTGGGGCATT
>AJ288981
TTTTATTGTAATACATCACAACTGTTTAACAGTAGTAACCTTAATAATAATAGCTCAGAT-------------
----

AATAATGGGACCATCACACTCCCATGTAGGATAAATCAAATTATAAGAATGTGGCAAAGAGTGGGACAAGCAA
TGTATGCCCCTCCCATTGCAGGAAACATTACATGTACATCAAAC---
ATTACAGGACTACTATTAACAAGAGATGGGCATAATGACACTGAA----------------------------
----

ATCTTCAGACCTGGAGGAGGAAATATGAAGGACAATTGGAGAAGTGAATTATATAAGTATAAAGTAGTAAAAA

TTAAACCACTA---GGGATAGCACCCACCAAGGCAAGGAGAAGAGTAGTG-----------------------
----------------GGGAGAGAGAAAAGAGCAGTTGTTGGA---CTGGGAGCT---------
GTTTTCCTTGGGTTCTTAGGAACG---GCAGGAAGCACTATG---GGCGCAGCGTCA---
ATAACGCTGACGGTACAG---GTCAGACAA---TTATTG---TCTGGCATAGTGCACCAGCAAAGC---
AATTTG---CTGAGA---GCTATAGAGGCGCAGCAGCATCTGTTGCAACTCACAGTCTGGGGCATT
>EU363837
TTCTATTGTAATACATCAGGCCTGTTTAATGGTACATACATGTCTAATGGTACACAAAGTAAT----------
----

TCAAGCTCAATCATCACGATTCCATGCAGAATAAAGCAAATTATAAACATGTGGCAGGAGGTAGGACGGGCAA
TGTATGCCCCTCCCATTGAAGGAAACATAACATGTAAATCAAAT---
ATCACAGGACTACTATTGGTACGTGATGGAGGAACGACAAATACTATA-------------------------
----

AATGATACAGAGATATTCAGACCTGGAGGAGGAGACATGAGGGACAATTGGAGAAGTGAATTATATAAATATA
AAGTGGTAGAAATTAAGCCATTA---GGAGTAGCACCCACTGCAGCAAAAAGGAGAGTGGTG-----------
----------------------------GAGAGAGAAAAAAGAGCA---GTGGGA---CTAGGAGCT------
---GTGTTCCTTGGGTTCTTGGGTGCA---GCAGGAAGCACTATG---GGCGCGGCGTCA---
ATAACGCTGACGGTACAA---GCCAGACAA---TTATTG---TCTGGTATAGTGCAACAGCAAAGC---
AATTTG---CTGAGA---GCTATAGAGGCGCAACATCATCTGTTGCAACTCACAGTCTGGGGCATT
>KC492737
TTCTATTGTAATACATCAAGACTGTTTAATGGTACATACATGTTTAATGGTACAAGGGGTAATTCAAGC----
----

TCAAACTCAACCATCACAATCCCATGCAGAATAAAGCAAATTATAAATATGTGGCAGCAGGTAGGACGAGCAA
TGTATGCCCCTCCCATTGAAGGAAACCTAACATGTAGATCAAAT---
ATCACAGGATTACTATTGGTACGTGATGGAGGAGACAACACAAAT----------------------------
----

AAAACAGAGATATTCAGACCTCAAGGGGGAGATATGAGGGACAATTGGAGAAGTGAATTATATAAATATAAAG
TAGTAGAAATTAAGCCATTG---GGAGTAGCACCCACTGCAGCAAAAAGGAGAGTGGTG--------------
-------------------------GAGAGAGAAAAAAGAGCA---GTGGGA---ATAGGAGCT---------
GTGTTCCTTGGGTTCTTGGGAGCA---GCAGGAAGCACTATG---GGCGCGGCGTCA---
ATAACGCTGACGGTACAG---GCCAGACAA---TTGCTG---TCTGGTATAGTGCAACAGCAAAGC---
AATTTG---CTGAGG---GCTATAGAGGCGCAACAACATCTGTTGCAACTCACGGTCTGGGGCATT
>JX392379
TTCTATTGTAATACATCAGGCCTGTTTAATGGTACATACAGGAATGGTACATACATGCCCAATGGTACCACAA
AT---

AATTCAAGCTACATCACAATCCCATGCAGAATAAAGCAAATTATAAATATGTGGCAGGAGGTAGGACGAGCAA
TGTATGCCCCTCCCATTGCAGGAAACATAACATGTAGATCAAAT---
ATCACAGGACTACTATTGGTACGTGATGGAGGAAAAACAAATGATACA-------------------------
----

AATAATACAGAGATATTCAGACCTGGAGGAGGAGATATGAGGGACAATTGGAGAAGTGAATTATATAAATATA
AAGTGGTAGAAATTAAGCCATTG---GGAGTAGCACCCACTGCAGCAAAAAGGAGAGTGGTG-----------
----------------------------GAGAGAGAAAAAAGAGCA---GTGGGA---CTAGGAGCT------
---GTGTTCCTTGGGTTCTTGGGAGCA---GCAGGAAGCACTATG---GGCGCGGCGTCA---
ATAACGCTGACGGTACAG---GCCAGACAA---TTGCTG---TCTGGTATAGTGCAACAGCAAAGC---
AATTTG---CTGAAG---GCTATAGAGGCGCAACAGCATCTGTTGCAACTCACGGTCTGGGGCATT
>KF835494
TTCTATTGTAATACATCAGGCCTGTTTAATAGTACATACATGTTCAATAATACAGACATATTTAATGGTACAG
GAAAT--

AGTTCAAAAGTCATCACGATTCCATGCAGAATAAAGCAAATTATAAATATGTGGCAGGAGGTAGGACGAGCAA
TGTATGCCCCTCCCATTGAAGGAAACATAACATGTAACTCAACT---
ATCACAGGACTACTATTGGTACGTGATGGAGGAAAGCATAATAATAGT-------------------------
----

AATACTACAGAAACATTCAGACCTGGAGGAGGAGATATGAAGGACAATTGGAGAAGTGAATTATACAAATATA

AAGTGGTAGAAATTCAGCCATTG---GGAGTAGCACCCACTGGAGCAAAAAGGAGAGTGGTG-----------
----------------------------GAGAGAGAGAAAAGAGCA---GCAGGA---CTAGGAGCT------
---GTGTTCCTTGGGTTCTTGGGAGCA---GCAGGAAGCACTATG---GGCGCGGCGTCA---
TTAACGCTGACGGTACAG---GCCAGACAA---TTGCTG---TCTGGTATAGTGCAACAGCAAAGC---
AATTTG---CTGAAG---GCTATAGAGGCGCAACAGCATCTGTTGCAACTCACAGTCTGGGGCATT
>KF835511
TTCTATTGTAATACATCAGACCTGTTTAATGGTACATATAATGGTACATACATACATAATGATACACAAGGT-
----

AATTCAACTGTCTTTACAATCCAATGCAGAATAAAGCAAATTATAAATATGTGGCAGGAGGTAGGACGAGCAA
TGTATGCTCCTCCCATTGCAGGAAATATAACATGTAAATCAAAT---
ATCACAGGACTACTATTGGTACGTGATGGAGGAAATACTAGTACA----------------------------
----

GAAGAGATATTCAGACCTGGAGGAGGAGATATGAAAAACAATTGGAGAAGTGAATTATATAAATATAAAGTGG
TAGAAATTAAGCCATTG---GGAATAGCACCCACTGCAGCAAAAAGGAGAGTGGTG-----------------
----------------------GAGAGAGAAAAAAGAGCAGTAATGGGA---ATAGGAGCT---------
GTGTTCCTTGGGTTCTTGGGAGCA---GCAGGAAGCACTATG---GGCGCGGCGTCA---
ATAACGCTGACGGTACAG---GCCAGACAA---TTGCTG---TCTGGTATAGTGCAACAGCAAAGC---
AATTTG---CTGAAG---GCTATAGAGGCGCAACATCATCTGTTGCAACTCACGGTCTGGGGCATT
>JF346907
TTCTATTGTAATACATCAGGCCTGTTCAATAGTACATACCTGTTCAATAGTACATACATGTCTAATGGTAGTT
CAAACTCA---
-------------------AAC---
AACACAAACTCAACCATCACAATCCCATGCAGAATAAAGCAAATTATAAATATGTGGCAGGAGGTAGGACGAG
CAATGTATGCCCCTCCCATTGCAGGAAACATAACATGTAAATCAAAT---
ATCACAGGACTACTATTGGTCCGTGATGGAGGAGATCAAAAATCAAATGAGACA------------GAG----
----
-
AATGGGACAGAGACTGAGGAGACATTTAGACCTGGAGGAGGAGATATGAGGGACAATTGGAGAAGTGAATTAT
ATAAATATAAAGTGGTAGAAATTAAGCCATTG---GGAATAGCACCCACTAAAGCAAAAAGGAGAGTGGTG--
-------------------------------------GAGAGAGAAAAAAGGGGA------------
CTAGGAGCT---------GTGTTCCTTGGGTTCTTAGGAGCA---GCAGGAAGCACTATG---
GGCGCGGCGTCA---ATAACGCTGACGGTACAG---GCCAGACAA---TTGCTG---
TCTGGTATAGTGCAACAGCAAAGC---AATTTG---CTGAAG---
GCTATAGAGGCGCAACAGCATCTGTTGCAACTCACGGTCTGGGGCATC
>JF346913
TTCTATTGTAATACAACAGGCCTGTTTAAGGGTACATACAACCCTAATTACACAAACCCTAATGGTACAGGGA
GT---

AATTCAAGCTCAACCATCACAATACCATGCAAAATAAAGCAAATTATAAATATGTGGCAGGAGGTAGGACGAG
CAATGTATGCCCCTCCCATTGCAGGAAACATAACATGTAAATCAAAT---
ATCACAGGACTGCTATTGGTACGTGATGGAGGATACACAAATAAGACA-------------------------
----

AATCATACAGAGATATTCAGGCCGGGAGGAGGAGATATGAAGGATAATTGGAGAAGTGAATTATATAAATATA
AAGTGGTAGAAATCAAGCCATTG---GGAGTAGCACCCACTAAAGCAAAAAGGAGAGTGGTG-----------
----------------------------GAGAGAGAAAAAAGAGCA---GCA------TTAGGAGCT------
---GTGTTCCTTGGGTTCTTGGGAGCA---GCAGGAAGCACTATG---GGCGCGGCGTCA---
ATGGCGCTGACGGTACAG---GCCAGACAA---TTGCTG---TCTGGTATAGTGCAACAGCAAAGC---
AATTTG---CTGAGG---GCTATAGAGGCGCAACAACATCTGTTGCAACTCACGGTATGGGGCATT
>EU363847
TTCTATTGTAATACCTCAAAACTGTTTAATGGTACATACAACAATGGTACATACAATGGTACATACGGT----
----

ACGGAAAGTAATATCACAATCCCATGCAGAATAAAGCAAATTATAAACATGTGGCAGGAGGTAGGACGAGCAA
TGTATGCTCCTCCCATTGCAGGAAACATAACATGTAACTCAACT---ATCACAGGCTACTAT---
GTACGTGATGGAGGAACAGGAAATGGGACA---

GATAATGGTACAGAGATATTCAGACCTGGAGGAGGAGATATGAGAAACAATTGGAGAAGTGAATTATATAAAT

ATAAAGTGGTAGAAATTAAGCCATTG---GGAGTAGCACCCACTGAAGCAAAAAGGAGAGTGGTG--------
-------------------------------GAGAGAGAAAAAAGAGCA---GTGGGA---CTAGGAGCT---
------GTGTTCCTTGGGTTCTTGGGAGCA---GCAGGAAGCACTATG---GGCGCGGCGTCA---
ATAACGCTGACGGTACAA---GCCAGACAA---TTGTTG---TCTGGTATAGTGCAACAGCAAAGC---
AATTTG---CTGAGA---GCTATAGAGGCGCAACAGCATCTGTTGCAACTCACAGTCTGGGGCATT
>KF835500
TTCTATTGTAATACATCAGGCATATTTAATAGTACATACAATGAGACATACCAAGGTAACTCA----------
----

ACTAACTTAAACATCACAATTCCATGCAAAATAAAGCAAATTATAAACATGTGGCAGGGGGTAGGACGAGCAA
TGTATGCCCCTCCCATTAAAGGAAACATAACATGTAGATCAAAT---
ATCACAGGACTACTACTGACACGTGATGGAGGAATAAATGTAACAAAT-------------------------
----

AGAACAGATCAAGAGATATTCAGACCTGCAGGAGGAGATATGAGGGACAATTGGAGAAGTGAATTATATAAAT
ATAAAGTGGTAGAAATTAAGCCATTG---GGGGTAGCACCCACTACAGCAAAAAGGAGAGTGGTG--------
-------------------------------GAGAGAGAAAAAAGAGCA---GTGGGA---CTGGGAGCC---
------GTGTTCCTTGGGTTCTTGGGAGCA---GCAGGAAGCACTATG---GGCGCAGCGTCA---
ATAACGCTGACGGTACAA---GCCAGACAA---TTGTTG---TCTGGTATAGTGCAACAGCAAAGC---
AATTTG---CTGAAA---GCTATAGAGGCGCAACAACATCTGTTGCAACTCACGGTCTGGGGCATT
>KF835507
TTCTATTGTAATACATCAGGCCTGTTTAATGGTACATACAGACAGCCTAATGGTACATACGTGTTTAAAGGAG
ATTCA--
-------------------AAC---
TCAAACATTACAACCATTACACTCCCATGCAGAATAAAGCAAATTATAAACATGTGGCAGGAGGTAGGACGAG
CAATGTATGCCCCTCCCATTGCAGGAAAAATAACATGTAAATCAAAT---
ATCACAGGACTACTGTTGGTACGTGATGGAGGGTCAAATGATACA----------------------------
----

GAGACAAATAAAACAGAGACATTTAGACCTCAAGGAGGAGATATGAGGGACAATTGGAGAAGTGAATTATATA
AATATAAAGTGGTAGAAATTAAGCCATTG---GGAATAGCACCCACTGGGGCAAAAAGGAGAGTGGTG-----
----------------------------------GAGAGAGAAAAAAGAGCA---GTGGGA---
CTAGGAGCT---------GTGTTCCTTGGGTTCTTGGGAGCA---GCAGGAAGCACTATG---
GGCGCGGCATCA---ATAACGCTGACGGTACAG---GCCAGACAA---CTGTTG---
TCTGGTATAGTGCAACAGCAAAGC---AATTTG---CTGAAA---
GCTATAGATGCGCAACAGCATCTGTTGCAACTCACGGTCTGGGGCATT
>KF835509
TTCTATTGTAATACATCAGGCCTGTTTAACAGTACATACAATCCTAATGGTACAAATTATAAT----------
----

TCAAACTCAACCATCACAATCCCATGCAGAATAAAGCAAATTATAAACATGTGGCAGGGGGTAGGACGAGCAA
TGTATGCCCCTCCCATTGCAGGAAACATAACATGTAGATCAAAT---
ATCACAGGACTACTATTGTTACGTGATGGAGGAACTTCAGATTCAAGT-------------------------
----

AATACAACAGAGATATTCAGACCTGGAGGAGGGGATATGAGGGACAATTGGAGAAATGAATTATATAAATATA
AAGTGGTAGAAATTCAGCCATTG---GGAATAGCACCCACTGGAGCAAAAAGGAGAGTGGTG-----------
----------------------------GAGAGAGAAAAAAGAGCA---GTGGGA---CTAGGAGCT------
---ATGTTCATTGGGTTCTTGGGAGCA---GCAGGAAGCACTATG---GGCGCGGCGTCA---
ATAACGCTGACGGTACAA---GCCAGACAA---TTGTTG---TCTGGTATAGTGCAACAGCAAAGC---
AATTTG---CTGAGA---GCTATAGAGGCGCAACAGCATCTGTTGCAACTCACTGTCTGGGGCATT
>KF835534
TTCTATTGTAATACATCAGGCCTGTTTAATAGTACATACATGTCTAATGGTACATACATGCCTAATGGT----
----

ACAGGAACAAACATCACAATTCCATGCAGAATAAAACAAATTATAAACATGTGGCAGGAGGTAGGACGAGCAA
TGTATGCCCCTCCCATTGCAGGAAACATAACATGTAGGTCAAAT---
ATCACAGGATTACTCTTGGTACGTGATGGAGGAAAGAGAAATGGTACA-------------------------
----

AATAATACAGAGATATTCAGACCTGGAGGAGGAGATATGAGAGACAATTGGAGAAATGAATTATATAAATATA
AAGTGGTAGAAATTCAGCCATTG---GGAGTAGCACCCACTAGAGCAAAAAGGAGAGTGGTG----------
----------------------------GAGAGAGAAAAAAGAGCAGTAGTAGGA---CTAGGAGCT------
---GTGTTCCTTGGGTTCTTGGGAGCA---GCAGGAAGCACTATG---GGCGCGGCGTCA---
ATAACGCTGACGGTACAG---GCCAGACAA---CTGTTG---TCTGGTATAGTGCAACAGCAAAGC---
AATTTG---CTGAAA---GCTATAGAGGCGCAACAGCATCTGTTGCAACTCACGGTCTGGGGCATT
>KF835545
TTCTATTGTGATACGTCAGACCTGTTTAATGATACATACAATGACAAGTTTAATGGTACAACAGGTGAT----
----

TCAAACAAAACCATCACAATCCAATGCAGAATAAAGCAAATTATAAACATGTGGCAGGAGGTAGGACGAGCAA
TGTATGCCCCTCCCATTGCAGGAAACATAACATGTAGATCAAAT---
ATCACAGGACTACTATTGGTACGTGATGGAGGTAATGGGACAGAGACA------------------------
----

AATGGCACAGAAACATTCAGACCTGGAGGAGGAGATATGAGGGACAATTGGAGAAATGAACTATATAAATATA
AAGTGGTAGAAATTCAGCCATTG---GGAGTAGCACCCACTGCAGCAAAAAGGAGAGTTGTG----------
----------------------------GAGAGAGAAAAAAGAGCA---ATAGGA---TTTGGAGCT------
---ATGTTCCTTGGGTTCTTGGGAGCA---GCAGGAAGCACTATG---GGCGCGGCGTCA---
ATAACGCTGACGGTACAA---GCCAGACAA---TTGCTG---TCTGGTATAGTGCAACAGCAAAGT---
AATTTG---CTAAGA---GCTATAGAGGCGCAACAGCATCTGTTGCAACTCACTGTCTGGGGCATT
>HQ699961
TTCTATTGCAATACATCAGGCCTGTTTAATGGTAATATTACAAAAGATAACTCAAGCTCAGATAAT-------
----

TCAAGCTCAGTCATCATACTCCCATGCAAAATAAAGCAAATTATAAATATGTGGCAGGAGGTAGGACGAGCAA
TGTATGCCCCTCCCATTGAAGGAAACATAACATGTAAATCAAAT---
ATCACAGGACTACTCTTAGTACGTGATGGGGGATACACAAATGGCACA------------------------
----

GAGAGTAATAACGAGACATTCAGACCAGGAGGAGGAGATATGAGAGACAATTGGAGAAGTGAATTATATAAAT
ATAAGGTGGTAGAAATTAAGCCATTG---GGAATAGCACCCACTAAATCAAAAAGGAGAGTGGTG--------
-------------------------------GAGAAAGAAAAAAGAGCA---GTGGGA---CTGGGAGCT---
------GTGTTCCTTGGGTTCTTGGGAGCT---GCAGGAAGCACTATG---GGCGCGGCGTCA---
ATAACGCTGACGGTACAA---GCCAGACAA---TTGCTG---TCTGGTATAGTGCAACAGCAAAGC---
AATTTG---CTGAGA---GCTATGGAGGCGCAGCATCATCTGTTGCAACTCACAGTCTGGGGCATT
>HM215416
TTCTATTGTAATACATCAGGCCTGTTTAATAGTACGTACAATGGTACATACAGGGGTACAGGAGATAAT----
----

TCAAGCTCAATCATCACAATCCCATGCAGAATAAGGCAAATTATAAACATGTGGCAGAAGGTAGGACGAGCAA
TGTATGCCCCTCCCATTGAAGGAAACATAGCATGTAACTCAAGT---
ATCACAGGACTACTGTTGGTACGTGATGGAGGAAGCCCAAATAATCCAGAG---------------------
----

ATTCCAAATAATACAGAGATATTCAGACCTGGAGGAGGAGACATGAAGGACAATTGGAGAAATGAATTATATA
AATATAAAGTGGTAGAAATTCAACCATTG---GGAGTAGCACCCACTGAAGCAAAAAGGAGAGTGGTG-----
------------------------------------CAGAGAGAAAAAAGAGCA---GTGGGA---
ATAGGAGCT---------GTGTTCCTTGGGTTCTTGGGAGCA---GCAGGAAGCATTATG---
GGCGCGGCGTCA---ATAACGCTGACGGTACAA---GCCAGACAA---CTGTTG---
TCTGGTATAGTGCAACAGCAAAGC---AATTTG---CTGAGA---
GCTATAGAGGCGCAACAGCACATGTTGCAACTCACGGTCTGGGGCATT
>HQ700001
TTCTATTGTAATACATCAGGCCTGTTTAATAGTACATACATGGTTAATGGTACATACATGTTTAATGGTACAG
AGATGTTTAAT---
AGTACAACAGGTAATTATTCA------------
GGCTCAAACTCAAACATCACAATCCCATGCAGAATAAAACAAATTGTAAACATGTGGCAGGAGGTAGGACGAG
CAATGTATGCCCCTCCCATTGCAGGAAACATAACATGTAAATCAGAT---
ATCACAGGATTACTATTGGTACGTGATGGAGGAAGAGAGCCAAAT---------------------------
----

AATGATACAGAGACATTCAGACCTGGAGGAGGAGATATGAAGGACAATTGGAGAAGTGAATTATATAAATATA
AAGTGGTACAAATTAAGCCATTG---GGAGTAGCACCCACTGAAGCAAAAAGGAGAGTGGTG-----------
----------------------------GAGAGAGAAAAAAGAGCAGTACTGGGAGGACTAGGAGCT------
---GTGTTCCTTGGGTTCTTGGGAGCA---GCAGGAAGCACTATG---GGCGCGGCGTCA---
ATAACGCTGACGGTACAA---GCCAGAAAA---TTGTTG---TCTGGTATAGTGCAACAGCAAAGC---
AATTTG---CTGAAA---GCTATAGAGGCGCAACAGCATCTGTTGCAACTCACGGTCTGGGGCATT
>KF250369
TTCTATTGTAATACATCAAGACTGTTTAATAGTACATACAATAGTACATTAGACCTGTCTAGT----------
--AGT---
ACATACAATAGTACAAAAGAT---------------
AATTCAAGCTCCCCCATCACAATCTCATGCAGAATAAAGCAAATTATAAACATGTGGCAGGAGGTAGGACGAG
CAATGTATGCCCCTCCCATTGCAGGAAGCATAACATGTAAATCAAAT---
ATCACAGGACTACTATTGGTGCGTGATGGAGGA--

-
ATAGGACCAAAAGATACAGAGACATTCAGACCTGGAGGAGGAGATATGAGAGACAATTGGAGAAATGAATTAT
ATAAATATAAAGTGGTAGAAATTCAGCCATTG---GGAGTAGCACCCACTGAAGCAAAAAGGAGAGTGGTG--
-------------------------------------GAGAGAGAAAAAAGAGCA---GTGGGA---
CTAGGAGCT---------GTGTTCCTTGGGTTCTTGGGAGCA---GCAGGAAGCACTATG---
GGCGCGGCGTCA---ATAACGCTGACGGTACAG---GCCAGACAA---TTGTTG---
TCTGGTATAGTGCAACAGCAAAGC---AATTTG---CTGAGA---
GCTATAGAGGCGCAACAACATATGTTGCAACTCACGGTCTGGGGCATT
>KU820846
TTCTATTGTAATACATCAGGCCTGTTTAATGGTACATACAATGATACATACGAGCTAAATGGTACAGAAAGT-
----

AATTCAAGCTTAATCACAATCCCATGCAGAATAAAGCAAATTATAAACATGTGGCAAAAGGTGGGACGAGCAA
TGTATGCCCCTCCCATTCAAGGAAACATTACATGTAAATCAAAT---
ATCACAGGACTACTATTAGTACGTGATGGAGGAGCAAATCAGACA----------------------------
----

AATGATACAGAGATATTCAGGCCTGGAGGAGGAAATATGAAGGACAATTGGAGAAGTGAATTATATAAATATA
AAGTGGTAGAAGTTAAGCCAGTA---GGAATAGCCCCCACTAAAGCAAAAAGGAGAGTGGTG-----------
----------------------------GAGAGAGAAAAAAGAGCA---GCG------CTAGGAGCT------
---GTGTTCCTTGGGTTCTTGGGAGCA---GCAGGAAGCACTATG---GGCGCGGCGTCA---
ATAACGCTGACGGTACAA---GCCAGACAA---TTGTTG---TCTGGTATAGTGCAACAGCAAAGC---
AATTTG---CTGAGA---GCTATAGAGGCGCAACAGCATATGTTGCAACTCACGGTCTGGGGCATT
>AJ866553
TTCTATTGCAATACATCAGACCTGTTTAATAGCATTTGGAATAGCACTAGGGAAAATACAAATAACACAAATG
CA---

AATTACACAGATTACATTATCCCATGCAGAATAAAGCAAATTGTGAGAATGTGGCAGAGAGTAGGACAAGCAA
TGTATGCTCCTCCCATCCCAGGAGTAATAAGCTGTACATCCAAT---
ATTACAGGACTAATAATAACAAGAGATGGTGGGAGTAATAACACT----------------------------
----

GGGAGTGAAACCTTCAGACCTACAGGAGGAAATATGAAAAATAATTGGAGAAGTGAATTATATAAGTATAAAG
TAGTAGAAATTGAACCACTA---GGAGTAGCACCCACCAGGGCAAGGAGAAGAGTGGTG--------------
-------------------------CAGAGAGAAAAAAGAGCA---GTTGGA---ATGGGAGCT---------
TTGTTCCTTGGGTTCCTAGGAGCA---GCAGGAAGCACTATG---GGCGCAGCGTCA---
ATAACGCTGACGGTACAG---GCCAGACAA---TTACTG---TCTGGTATAGTGCAGCAGCAGAGC---
AATCTG---CTGAAG---GCTATAGAGGCTCAACAGCATATGTTGAGACTCACAGTCTGGGGCATA
>AY093603
TTCTATTGCAATACAACAAACCTATTTAATTGGAATAACACTAAGGATATCATGCCAAAT-------------
----

GGCACAAAGAATTACTTTATCCAATGCAAAATAAAGCAAATTGTGAGAATGTGGCAGAGAGTAGGACAAGCAA
TGTATGCCCCTCCTATCCCAGGAATAATAGAGTGTGTATCAAAC---
ATTACAGGACTAGTATTAACAAGAGATGGTGGGACTGGCAATACA----------------------------

----

AGTGAGACCTTCAGACCTACAGGAGGAAATATGAAGGACAATTGGAGAAGTGAATTATATAAGTATAAAGTAG
TAAAAATTGAACCACTA---GGAGTGGCACCCACCAGGGCAAAGAGAAGAGTGGTG-----------------
----------------------GAGAGAGAAAAAAGAGCA---GTTGGA---ATGGGAGCT---------
CTTTTCCTTGGGTTCTTAGGAGCA---GCAGGAAGCACTATG---GGCGCGGCGTCA---
ATGACGCTGACGGTACAG---GCCAGACAA---TTATTG---TCTGGTATAGTGCAACAGCAGAGC---
AATCTG---CTGAAG---GCTATAGAGGCTCAACAGCATCTGTTAAAACTCACAGTCTGGGGCATT
>AY093604
TTCTATTGCGATACATCAGGCCTGTTTAATAGCACGTGGAAAAATGGCACTAATATACAGGGGTCAAATGGCA
CT---

GGATCAAATTACACACAGATCCAGTGCAAAATAAAGCAAATTGTGAGAATGTGGCAGAGAGTAGGACAAGCAA
TGTATGCCCCTCCCATCCCAGGAGTAATAGGGTGTATATCAAAC---
ATTACAGGACTAATATTAACAAGAGATGGTGGGGACAATACAGGT----------------------------
----

GATAATGAAACCTTCAGACCTATAGGAGGAAATATGAAGGACAATTGGAGAAGTGAATTATATAAGTATAAAG
TAGTAAAACTTGAACCACTA---GGAGTAGCACCCACCCCGGCAAAGAGAAGAGTGGTG--------------
-------------------------GAGAGAGAAAAAAGAGCA---GTTGGA---ATGGGAGCT---------
CTTTTCCTTGGGTTCTTAGGAGCA---GCAGGAAGCACTATG---GGCGCAGCGTCA---
ATAACGCTGACGGTACAG---GCCAGACAA---TTATTG---TCTGGCATAGTGCAACAGCAGAGC---
AATCTG---CTGAAG---GCTATAGAGGCTCAACAGCATTTGTTGAAACTCACGGTCTGGGGCATT
>AY093607
TTCTATTGCAATACATCAAGCCTATTTAATAGCACTTGGGATGAAAATAACACTAAAAGTGCACAGGGGCCAA
AT---

GGCACGGCCAATATCACAATCCCATGTAAAATAAGACAAATTGTGAGAATGTGGCAGAGAGTAGGACAAGCAA
TGTATGCCCCTCCCATCCCAGGAGTAATAAATTGTATATCAAAC---
ATTACAGGACTAATATTAATAAGAGATGGGGGGAATGAGAGCGCT----------------------------
----

ACAAATGAGACCTTCAGACCTATAGGAGGAGATATGAAGAATAATTGGAGAAGTGAATTATATAAGTATAAAG
TAGTAAAAATTGAACCACTA---GGAGTGGCACCCACCAGGGCAAGAAGAAGAGTGGTG--------------
-------------------------CAGAGAGAAAAAAGAGCA---GTTGGA---ATGGGAGCT---------
CTGTTCCTTGGGTTCTTAGGAGCA---GCAGGAAGCACTATG---GGCGCAGCGTCA---
ATAACGCTGACGGTACAG---GCTAGACAA---TTATTG---TCTGGTATAGTGCAACAGCAGAGC---
AATCTG---TTGAAG---GCTATAGAGGCTCAACAGCATCTGTTGAAACTCACGGTCTGGGGCATT
>MN178644
TTCTATTGCAATACAACAAAGCTGTTCAATAGTAGTTGGATAGGAAATGAGACTAGGAATGAGACCATAGAGG
AAGGT--

CATGATAACACTATCATACTTCCATGCAAAATAAAGCAAATTATAAATATGTGGCAAAGAACAGGACAAGCAA
TGTATGCTCCTCCAGTCAGAGGAATAATTAATTGTGCATCAAAT---
ATTACAGGAATACTATTGACAAGAGATGGTGGGAATGATAGGACT----------------------------
----

ACAAACGAGACTTTCAGACCTGGGGGAGGAAATATGAAGGACAATTGGAGAAGTGAATTATATAAATATAAAG
TAGTACAAATAGAACCACTA---GGAGTAGCACCCACCAGGGCAAAGAGAAGAGTGGTG--------------
-------------------------AGTAGAGAAAAAAGAGCA---GTGGGA---ATAGGAGCT---------
ATGATCTTTGGGTTCTTGGGAGCA---GCAGGAAGCACTATG---GGCGCAGCGTCA---
ATAACGCTGACGGTACAG---GCCAGACAA---TTATTG---TCTGGTATAGTGCAACAGCAAAGC---
AATTTG---CTGAGG---GCTATAGATGCGCAACAGCATATGTTGCAACTCACAGTCTGGGGCATT
>AF289548
TTCTACTGCAATACATCAGAACTGTTTAATAGTACATGGACATCAAATAATACAAGTACAGGGGTA-------
----

AATGGGAGTACAATCACACTCCCATGCAGAATAAGACAAATTATAAATATGTGGCAGGGAGTAGGAAAAGCAA
TATATGCCCCTCCCATTGCAGGACTGATCAATTGTTCATCAAAT---
ATTACAGGACTAGTGTTGACAAGAGATGGTGGTGCGAATAATAAT----------------------------

----

AGTCAGAATGAGACCTTCAGACCTGGAGGAGGAGATATGAGGGACAATTGGAGAAGTGAATTATACAAATATA
AAGTAGTAAAAATTGAACCAATA---GGTCTAGCACCCACCACGGCAAAGAGAAGAGTGGTG-----------
----------------------------GAAAGAGAAAAAAGAGCA---ATAGGA---CTGGGAGCT------
---GTGTTCCTTGGGTTCTTGGGAGCA---GCAGGAAGCACAATG---GGCGCGGCGTCG---
CTGACGCTGACGGTACAG---GCCAGACAA---GTATTG---TCTGGTATAGTGCAGCAACAAAAC---
AATTTG---CTGAGG---GCTATAGAGGCGCAACAGCATCTGTTGCAACTCACAGTCTGGGGCATT
>AF289549
TTCTACTGCAATACAACAATACTGTTTAATAATAGTGAATGGACATCAAATAATACAGGTAATACAAGTTCAG
GG---

GTAAATGATAGTACAATCATAATCCCATGCAGAATAAAACAAATTGTAAACATGTGGCAGAAAGTAGGAAAAG
CAATATATGCCCCTCCCATTGCAGGACTAATCAGCTGTTCATCAAAC---
ATAACAGGACTATTGTTGACAAGAGATAGAGATGGTGGTGCAAATAAT-------------------------
----

ACTAGTCAGAATGAGACCTTCAGACCTGGAGGAGGAGATATGAGAGACAATTGGAGAAGTGAATTATACAAAT
ACAAAGTAGTAAAAATTGAACCACTA---GGTCTAGCACCCACCAAGGCAAAGAGAAGAGTGGTG--------
-------------------------------GAAAGAGAAAAAAGAGCA---ATAGGA---CTGGGAGCT---
------ATGTTCCTTGGGTTCTTGGGAGCA---GCAGGAAGCACGATG---GGCGCAGCGTCG---
CTGACGCTGACGGTACAG---GCCAGACAG---TTATTG---TCTGGTATAGTGCAACAGCAAAAC---
AATTTG---CTGAAG---GCTATAGAGGCGCAACAGCATCTGTTGCAACTCACAGTCTGGGGCATT
>AF289550
TTCTACTGCAATACATCAAAACTGTTTAATAGCAGTATAGAGATATCAAATACAAGTATAGGT----------
----

AATACAAGTATAATCAAACTCCCATGCAAAATAAAACAAATTATAAACATGTGGCAGGGAGTAGGAAAAGCAA
TATATGCCCCTCCCATTGCAGGATTAATCAACTGTTCATCAAAT---
ATTACAGGACTATTGTTAACAAGAGATGGTGGTGCAAATAATAGTAAT-------------------------
----

CAGAGTGGGGAGATCTTCAGACCTGGAGGAGGAGATATGAGAGACAATTGGAGAAGTGAATTATATAAATATA
AAGTAGTAAGGATTGAACCACTA---GGTCTAGCACCCACCAAGGCAAAGAGAAGAGTGGTG-----------
----------------------------GAAAGAGAAAAAAGAGCA---ATAGGA---CTGGGAGCT------
---GTGTTCCTTGGGTTCTTGGGAGCA---GCAGGAAGCACAATG---GGCGCAGCGTCG---
TTGACGCTGACGGTACAG---GCCAGGCAG---TTATTG---TCTGGTATAGTGCAACAGCAAAAC---
AATTTG---CTGAGG---GCTATAGAGGCGCAACAGCATATGTTGCAACTCACAGTCTGGGGCATT
>AY371149
TTCTATTGTAATACATCAGGCCTGTTTAATAGCAGTTGGAGTAGGGAGAATGGCAATGGCACAGACAATTGGA
ATATCACAACA--
----------AACCACACG------
AGCTCCAATGACACTATAACTCTCCCATGCAAAATAAAGCAAATTGTACATATGTGGCAGAGAGTAGGACAAG
CAATGTATGCCCCTCCCATCCGAGGAGAAATAAGGTGTGTGTCAAAC---
ATTACAGGAATACTATTAACAAGAGATGGTGGGAGAGATACAAAT----------------------------
----

CATACTACCGATACCTTCAGACCTACAGGAGGAGATATGAGGGACAATTGGAGAAGTGAATTATATAGGTATA
AAGTAGTGGCAATTGAACCACTA---GGAGTAGCACCCACCAGGGCAAGAAGAAGAGTGGTG-----------
----------------------------GAGAGAGAAAAAAGAGCA---GTAGGG---ATAGGAGCT------
---GTGCTCCTTGGGTTCTTGGGAACA---GCAGGAAGCACTATG---GGCGCAGCGTCA---
ATAACGCTGACGGTACAA---GCCAGACAA---TTATTG---TCTGGCATAGTGCAACAGCAAGGC---
AATTTG---CTGAAG---GCTATCGAGGCTCAACAGCAACTGTTGAGACTCACTGTCTGGGGCATT
>KP718935
TTCTATTGCAATACATCAAACCTGTTTAATAGCAGTTGGAAAATTGGAAATAGCACTGCAAATAACACTGGC-
----

ACAAATGACACTATAACTCTCCAATGTAGAATAAAGCAAATTGTACATATGTGGCAGAGAGTAGGACAAGCAA
TGTATGCCCCTCCCATTCCAGGAAAAATAAGGTGTGAATCAAAC---
ATTACAGGACTACTATTAACAAGAGATGGTGGGAATAATAGTGCA----------------------------

----

AATGAAACCTTCAGACCTACAGGAGGAGATATGAAGGACATTTGGAGAAGTGAATTATATAAGTATAAAGTAG
TGGAAATTAAACCACTA---GGAGTAGCACCCACCAGGGCAAAAAGAAGAGTGGTG-----------------
----------------------GAGAGAGAAAAAAGAGCA---GTTGGA---CTAGGAGCT---------
GTACTCCTTGGGTTCTTGGGAACA---GCAGGAAGCACTATG---GGCGCGGCGTCA---
ATAACGCTGACGGTACAG---GCCAGACAA---TTATTG---TCTGGTATAGTGCAACAGCAAAGC---
AATTTG---CTGAAA---GCTATTGAGGCTCAACAGCAACTGTTGAAACTCACTGTCTGGGGCATT
>KP718938
TTCTATTGCAATACATCACAACTGTTTAATAGCACTTGGGATAACAATACAACTTGGGATAACAATACAACAC
AGAATAAT---

AGCAGTAAGAATATAACTCTTCCATGCAAAATAAAGCAAATTGTAAGGATGTGGCAGAGAGTAGGACAAGCAA
TGTATGCCCCTCCCATCCAAGGAGAAATAAAGTGTGAATCAAAC---
ATTACAGGACTAATATTAACAACAGATGGT---ACGGGAAATAGC----------------------------
----

ACAAATGAGACCGTCAGACCTGCAGGAGGAGATATGAGGGACAATTGGAGAAGTGAGCTATATAAGTATAAAG
TAGTGGAGATTAAACCACTA---GGAGTAGCACCCACCAGGGCAAAAAGAAGAGTGGTG--------------
-------------------------GAGAGAGAAAAAAGAGCA---GTGGGA---CTAGGAACT---------
TTGCTCCTTGGGTTCTTGGGAACA---GCAGGAAGCACTATG---GGCGCAGCGTCA---
ATAACGCTGACGGTACAG---GCCAGACAA---TTATTG---TCTGGCATAGTGCAACAGCAAAGC---
AATTTG---CTGAAG---GCTATTGAAGCTCAACAGCAACTGTTGAAGCTCACTGTCTGGGGCATT
>KY658700
TTCTATTGCAACACAACAAAGCTGTTTAATAGCACTTGGCAGTTTAATGATACTAATGGTACTTGGCGGGAAA
ATGGCACT---
-------------------GAA---
GACAAAAATGACACTATATATCTTCCATGCAGAATAAAGCAGATTGTAAGACTATGGCAGACAGTAGGACAAG
CAATGTATGCCCCTCCCATCCGAGGAGAAATAAGGTGTAATTCAAAT---
ATTACAGGGTTGCTATTAACAAGAGATGGTGGACAGTCAAATGAGTCA-------------------------
----

AAGGTTAATGAGACCTTCAGACCTACAGGAGGAAATATGAAAGACAATTGGAGAAGTGAATTATATAGGTATA
AGGTGGTGGAAATTAAACCACTA---GGAGTAGCACCCACCAAGGCAAGAAGAAGAGTGGTG-----------
----------------------------CAGAGAGAAAAAAGAGCA---GTGGGA---ATGGGAGCT------
---GTACTCCTTGGGTTCTTGGGAGCA---GCAGGAAGCACTATG---GGCGTAGCGTCA---
ATAACGCTGACGGTACAA---GCCAGACAA---TTATTG---TCTGGTATAGTGCAACAGCAAAGC---
AATTTG---CTGAGG---GCTATTGAGGCTCAACAGCAATTGTTGAGACTCACTGTCTGGGGCATT
>AF069945
TTCTATTGTAATACATCAAGTCTGTTTAATAGCACTTGGGAAAATGAAAATACAACTAGCAATGGGACTGAG-
----

GAAAATGGCACTATAACTCTCCCATGCAGAATAAAGCAAATTGTAAATCTGTGGCAGAGAGTAGGACAAGCAA
TGTATGCCCCTCCCATCCAAGGAAACTTAACATGTAACTCAAAC---
ATTACAGGGCTACTATTAACAAGAGATGGTGGGAAAAATAACACT----------------------------
----

CATAATGAGACCTTCAGACCTACAGGAGGAGATATGAAGGACAATTGGAGAAGTGAATTATATAAATATAAAG
TGGTAGAAATTAAACCACTA---GGAGTAGCACCCACCAGGGCAAAAAGAAGAGTGGTG--------------
-------------------------GAGAGAGAAAAAAGAGCA---CTGGGA---ATAGGAGCT---------
GTGCTCCTTGGGTTCTTGGGGACA---GCAGGAAGCACTATG---GGCGCGGCGTCA---
ATAACGCTGACGGTACAG---GCCAGACAA---TTATTG---TCTGGCATAGTGCAACAGCAAAGC---
GATTTG---CTGAAG---GCTATAGATGCACAACAGCAACTGCTAAAACTCACTGTCTGGGGCATA
>AF408631
TTCTACTGCAATACATCAAAACTGTTTAATGACACAACAACC-------------------------------
----

AATGACACTATTATTCTCCCATGCAAAATAAAACAAATTGTAAATATGTGGCAGGAAGTGGGACGAGCAATGT
ATGCCGCTCCCATCGCAGGAAGCATTACCTGTAGATCAAAT---
ATTACAGGTCTACTATTGACAAGAGATGGTGGTCAGACTAATATTACTAATCAG------------ACT----

----
-
GAGACTAATCAGACTACTGAGATCTTCAGACCTGGGGGAGGAGATATGAGAGACAATTGGAGAAGTGAATTAT
ATAAATATAAAGTAGTAGAAATTGAGCCACTA---GGAGTAGCACCCACCAGGGCAAGAAGACAAGTGGTG--
-------------------------------------AGAAGAGAAAAAAGAGCA---GTGGGA---
ATGACAGCT---------GTGTTCTTTGGGTTCTTGGCAGCA---GCAGGAAGCACTATG---
GGCGCAGCGTCA---ATAACGCTGACGGCACAG---GCCAGACAA---TTATTG---
TCTGGAATAGTGCAACAGCAGAAC---AATCTG---CTGAGG---
GCTATTGAAGCGCAACAGCATCTGTTGCAGCTCACAGTCTGGGGCATT
>AF408629
TTCTACTGCAATACATCAAAACTGTTTGATGAAGGACTGTTTAATACAACAGAT-------------------
----

GACAATGACACTATTATTCTCCCATGTCGAATAAAACAAATTGTAAACATGTGGCAGGAAGTGGGACGAGCAA
TGTATGCCGCTCCCATTGCAGGAAACATTAACTGTAACTCAACT---
ATTACAGGTCTAATATTGACAAGAGATGGTGGGAAAAATACTACT----------------------------
----

GGGAATGAGACCTTCAGACCTGGGGGAGGAAATATGAAAGACAATTGGAGAAGTGAATTATATAAATATAAGG
TGGTAGAAATTCAACCACTA---GGAGTAGCACCCACCACGGCAAAGAGACAAGTGGTG--------------
-------------------------CAGAGAGAAAGAAGAGCA---GTGGGA---ATGGGAGCT---------
TTGTTCCTTGGGTTCTTGGGAGCA---GCAGGAAGCACTATG---GGCGCGGCGTCA---
ATAACGCTGACGGTACAG---GCCAGACAA---TTATTG---TCTGGAATAGTGCAACAGCAAAAC---
AATTTG---CTGAGG---GCTATTGAAGCGCAACAGCATCTGTTGCAGCTCACAGTCTGGGGCATT
>AY037266
TTCTACTGTGATACATCAAAACTGTTTCATGACACGCTGATTAATACCAATACATCCGATGACACT-------
----

GCTAATGGCACTATAACTCTCCCATGTAGAATAAAACTAATTGTAAACCTGTGGCAGGAAGTGGCACGAGCAA
TGTATGCCGCTCCCATTGCAGGAAGCGTTACCTGTAACTCAAGT---
ATTACAGGTCTACTATTGACAAGAGATGGTGGTCAGAAGAATAGT----------------------------
----

ACTAATGACACCTTCAGACCTGGGGGAGGAAATATGAGGGACAATTGGAGAAGTGAATTATATAAATATAAAG
TAGTAGAAATTAAACCATTA---GGAATAGCTCCCACCAAGGCAAAAAGACAAGTGGTG--------------
-------------------------AAGAGAGAAAGAAGAGCA---GTGGGA---ATAGGAGCG---------
TTGTTCCTTGGGTTCTTGGGAACA---GCAGGAAGCACTATG---GGCGCGGCGTCA---
ATAACGCTGACGGTACAG---GCCAGACAA---TTATTG---TCTGGAATAGTGCAACAGCAGAAC---
AATTTG---CTGAGG---GCTATTGAAGCGCAACAGCATCTGTTGCAGCTCACAGTCTGGGGCATT
>AF385936
TTCTACTGCAATACATCAGGACTGTTTAATGATACAATATCC-------------------------------
----

AATGATACTATTATTCTCCCATGTAAAATAAAACAAATTGTAAACCTGTGGCAGGAAGTGGGACGAGCAATGT
ATGCCGCTCCCATTGAAGGAAACATTACCTGTAACTCAAAT---
ATTACAGGTCTACTATTGACAAGAGATGGTGGTCTTAATGAGACT----------------------------
----

AATCAGACTGAAACCTTCAGACCTGGGGGAGGAAATATGAAAGACAATTGGAGAAGTGAATTATATAAATATA
AAGTGGTAGAAATTGAACCACTA---GGAGTAGCACCCACCAGGGCAAAAAGACAAGTGGTG-----------
----------------------------ACGAGAGAAAAAAGAGCA---GTGGGA---ATAGGAGCT------
---TTGTTCCTTGGGTTCTTGGGAGCA---GCAGGAAGCACTATG---GGCGCGGCGTCA---
GTGACGCTGACGGTACAG---GCCAGACAA---TTATTG---TCTGGAATAGTGCAACAGCAAAAC---
AACTTG---CTGCAG---GCTATTGAAGCGCAACAGCATCTGTTGCAGCTCACAGTCTGGGGCATT
>JN054280
TTCTACTGCAATACATCAAAACTGTTTAATAACACAAAAATT-------------------------------
----

AATGGCACTATTACTCTCCCATGTCGAATAAAACAAATTGTAAACCTGTGGCAGGAAGTGGGACGAGCAATGT
ATGCCGCTCCCATTGCAGGAAACATTACCTGTAACTCAAAT---

ATTACAGGTCTGCTATTAACAAGAGATGGGGGACAAAGTTATAATAGTACTAAT------------AAC----
----
-
AGTACTATTAATAGGACTGAGATCTTCAGACCTGGGGGAGGAAATATGAAAGACAATTGGAGAAATGAATTAT
ATAAATATAAAGTGGTAGAAATTGAACCACTA---GGAATAGCACCCACCAAGGCAAAAAGACAAGTGGTG--
-------------------------------------AAGAGAGAAAAAAGAGCA---
GTAGGAGTAATAGGAGCT---------ATGTTCCTTGGGTTCTTGGGAGCA---GCAGGAAGCACTATG---
GGCGCAGCGTCA---ATAACGCTGACGGTACAG---GCCAGACAA---TTATTG---
TCTGGAATAGTGCAACAGCAAAAC---AATTTG---CTGAGG---
GCTATTGAAGCGCAACAGCATCTGTTGCAGCTCACAGTCTGGGGCATT
>JN235963
TTCTACTGCAATACATCAGGACTGTTTAATAATGACACAGTA-------------------------------
----

TCCAATGGCACTATTACTCTCCCATGTCGAATAAAACAAATTATAAACATGTGGCAGGAAGTGGGACGAGCAA
TGTATGCCAATCCCATTGCAGGAAACATTACCTGTAACTCAAAT---
ATTACAGGTCTACTATTGACAAGAGATGGTGGTGGTCATAGTAATGAA-------------------------
----

ACTAATCAGACTGAGATCTTCAGACCTGGGGGAGGAAATATGAAAGACAATTGGAGAAGTGAATTATATAAAT
ATAAAGTAGTAGAAATTGAACCACTA---GGAGTAGCACCCACCAGGGCAAAAAGACAAGTGGTG--------
-------------------------------AAGAGAGAAAAAAGAGCA---GTGGGA---CTAGGAGCT---
------GTGTTCATTGGGTTCTTGGGAGCA---GCAGGAAGCACTATG---GGCGCAGCGTCA---
GTAACGCTGACGGTACAG---GCCAGACAA---TTATTG---TCTGGAATAGTGCAACAGCAGAGC---
AATTTG---CTGAAG---GCTATTGAAGCGCAACAGCATCTGTTGCGGCTCACAGTCTGGGGCATT
>AY037272
TTCTACTGCAATACATCAGGACTGTTTAATGAAAGCCTGATTAATGTCACA----------------------
----

CAACCCAATGACATTACTCTCCCATGTCGAATAAAACAAATTGTAAACCTGTGGCAGAAAGTGGGACGAGCAA
TGTATGCCGCTCCCATTGCAGGAAGCATTATCTGTATCTCAAAT---
ATTACAGGTCTACTATTGACAAGAGATGGTGGTCAGAATAATAGT----------------------------
----

ACTCCTGAGATCTTCAGACCTGGGGGAGGAAATATGAAAGACAATTGGAGAAGTGAATTATATAAATATAAAG
TAGTAGAAATTGAACCACTA---GGAATAGCACCCACCGGGGCAAAAAGACAAGTGGTA--------------
-------------------------AAGAGAGAAAAAAGAGCA---ATAGGA---ATGGGAGCT---------
TTGTTCCTTGGGTTCTTGGGAGCA---GCAGGAAGCACTATG---GGCGCGGCGTCA---
ACAGCGCTGACGGTACAG---GCCAGACAG---TTATTG---TCTGGAATAGTGCAACAGCAGAAC---
AATTTG---CTGAGA---GCTATTGAAGCGCAACAGCATCTGTTGCAGCTCACAGTCTGGGGCATT
>AY371154
TTCTATTGCAATACATCAGCCCTGTTTAATAGCGCTTGGAGAAATGATACTAACAGCACGCAGGTTTCGGGG-
----

TCAAATGACAATATAACTCTCCCATGCAGAATAAAGCAATTTGTAAAGCTATGGCAGAGGGTGGGACAAGCAA
TGTATGCCCCTCCCATCCAAGGAGTAATAAGGTGCGAATCAAAC---
ATTACAGGGCTACTATTAACAAGAGATGCTGAGGGTAATAATAGA----------------------------
----

ACAAATGAGACCTTCAGACCTGGAGGAGGAGATATGAGGGATAATTGGAGAAGTGAATTATATAAATATAAAG
TAGTAAAAATTGAACCACTA---GGAGTAGCACCCAGCAGAGCAAGGAGAAGAGTGGTA--------------
-------------------------GCAAGAGAAAAAAGAACA---GTTGGA---CTAGGAGCT---------
GTCTTCCTTGGGTTCTTAGGAGCA---GCAGGAAGCACTATG---GGCGCAGCGTCA---
CTGACGCTGACGGTACAG---GCCAGACAA---TTATTG---TCTGGCATAGTGCAACAACAGAAC---
AATCTG---CTGATG---GCTATAGAGGCTCAACAGCACCTGTTGAAACTCACGGTCTGGGGCATT
>DQ845388
TTCTATTGCAATACATCAGCCCTGTTTAATAGCACTTGGGAAGGGAACAATACCAGCACGCAAGGGTCAAATG
ACACA--

GGGTCAAATGACACTATAACTCTCCCATGCAGAATAAAACAATTTGTAAATATATGGCAGAGAGTAGGACAAG

CAATATATGCCCCCCCCATCCAAGGAGTAATAAGGTGTGACTCAAAC---
ATTACAGGACTACTATTAACAAGAGATGGTGGGGATAATGATACTACT------------------------
----

AGGGCAAATGAGACCTTCAGACCTGGAGGAGGAGATATGAAGGACAATTGGAGAAGTGAATTATATAAATACA
AAGTAGTAAAAATTGAACCACTA---GGAGTAGCACCCACCAAGGCAAAGAGAAGAGTGGTG----------
---------------------------GCAAGAGAAAAAAGAGCA---GCA------CTGGGAGCT------
---GTCTTCCTTGGGTTCTTAGGAGCA---GCAGGAAGCACTATG---GGCGCGGCGTCA---
ATAACGCTGACGGTACAG---GCCAGACAA---TTATTG---TCTGGCATAGTGCAACAGCAGAGC---
AATCTG---CTGATG---GCTATACAGGCTCAACAGCATATGTTGAAACTTACGGTCTGGGGCATT
>DQ845386
TTCTATTGCAATACATCAGAACTGTTTAATAGTGCATGGAGAAATGGGACCAGAGTGCGGGGGTCAACTAAC-
----

ACAAGTGAAAATATAACTCTCCCATGCAGAATAAAGCAAATTGTAAAATTATGGCAGAGAGTAGGGCAGGCAA
TATATGCCCCTCCCATCCAAGGAGTAATAAGGTGTGAATCAAAC---
ATTACAGGGCTACTATTAACAAGAGATGGTGGGAATAATAATGAGACA------------------------
----

AATAAGACTGAGACCTTCAGACCTGGAGGAGGAGATATGAAGGACAATTGGAGAAGTGAATTATATAAATATA
AAGTAGTAAAAATTGAACCACTA---GGAGTAGCACCCAGCAGAGCAAGGAGAAGAGTGGTG----------
---------------------------GCAAGAGAAAAAAGAGCA---GTTGGA---CTAGGAGCT------
---GTCTTCCTTGGGTTCTTAGGAGCA---GCAGGAAGCACTATG---GGCGCGGCGTCA---
TTAACGCTGACGGTACAG---GCCAGACAA---TTGTTG---TCTGGTATAGTGCAACAGCAGAAT---
AACCTG---CTGATG---GCTATAGAGGCTCAACAGCATATGTTGAAACTCACAGTCTGGGGCATT
>DQ845387
TTCTATTGTAATACATCAGAACTGTTTAATAGCACTTGGACAAATGACACTAACAGCACGCAGGGGTCAAAT-
----

GACACAACGCCCATTATTCTCCCATGCAGAATAAAGCAAATTATAAATATGTGGCAGAGAGTAGGACAAGCAA
TATATGCCCCTCCCATTGAAGGAGTAATAAGGTGTGAATCAAAC---
ATTACAGGACTACTATTAACAAGAGATGGGGGTAATAACAGAACA---------------------------
----

AGTGAGATCTTCAGACCTGGAGGAGGGGATATGAGGGACAACTGGAGGAGTGAATTATATAAATATAAAGTAG
TAAAAATTGAGCCACTA---GGAGTAGCACCCAGCAAGGCAAGGAGAAGAGTGGTG----------------
---------------------ACGAGAGAAAAAAGAGCA---GTTGGA---ATAGGAGCT---------
GTCTTCCTTGGGTTCTTAGGAGCA---GCAGGAAGCACTATG---GGCGCGGCGTCA---
TTAACGCTGACGGTACAG---GCCAGAAAC---TTATTG---TCTGGTATAGTGCAACAGCAGAAC---
AACCTG---CTGAGG---GCTATAGAGGCTCAACAGCAACTGTTGAAACTCACGGTCTGGGGCATT
>MH705141
TTCTACTGCAATACATCAGGCCTGTTTAATAGCAGTTGGGAAAATGACAATGCCAGCACAGGGTCAAATAACA
CAGAG---

TCAAATGCCAATATAACTCTCCCATGCAAAATAAAGCAATTTGTAAATGTATGGCAGAGAGTAGGACAAGCAA
TGTATGCCCCTCCCATTGCAGGATTAATAAGATGTGAATCAAAT---
ATTACAGGACTATTATTAACAAGAGATAATGAGGGTAATAACAGG---------------------------
----

ACAAATGAGACCTTCAGACCCGGAGGAGGAGACATGAAGGACAATTGGAGAAGTGAATTATATAAGTATAAAG
TAGTAAAAATTGAACCACTA---GGAGTAGCACCCAGCAAGGCAAGGAGAAGAGTGGTG-------------
------------------------GCAAGAGAAAAAAGAGCAGTTGTTGGA---CTAGGAGCT---------
GTCTTCATTGGGTTCTTAGGAGCA---GCAGGAAGCACTATG---GGCGCGGCGTCA---
TTAACGCTGACGGTACAG---GCCAGACAA---TTATTG---TCTGGCATAGTGCAACAACAGAAC---
AATCTG---TTGATG---GCTATAGAGGCTCAACAGCATTTGTTGAAGCTCACGGTCTGGGGCATT
>KP718924
TTCTATTGCAATACATCAGGCCTGTTTAATAGTACTTGGGAATCTAACAATACCAGCACGTCGGGGTCAAATG
ACACAGGG--

TCAAATGACACTATAACTCTCCCATGCAGAATAAAGCAATTTGTAAAGATGTGGCAGAGAGTAGGACAAGCAA

TATATGCCCCTCCCATCCCAGGAGTAATACAGTGTAATTCAAAC---
ATTACAGGACTACTATTAGCAAGAGATGGTGGGGATGATAATACA----------------------------
----

ACAAATGAGACCTTCAGACCTATAGGAGGAGATATGAAGGACAATTGGAGAAGTGAATTATATAAATATAAAG
TAGTAAAAATTGAACCATTA---GGAGTAGCACCCAGCAAGGCAAGGAGAAGAGTGGTA--------------
-------------------------GCAAGAGAAAAAAGAGCA---ATCGGAGGACTAGGGGCT---------
GTCTTCCTTGGGTTCTTAGGAGCA---GCAGGAAGCACTATG---GGCGCGGCGTCA---
CTGACGCTGACGGTACAG---GCCAGACTA---TTATTG---TCTGGCATAGTGCAACAGCAGAAC---
AATCTG---CTGAGG---GCTATAGAGGCTCAACAACATCTGTTGAAACTCACGGTCTGGGGCATT
>KP718926
TTCTATTGCAATACAACAAAACTGTTTAATAGCACTTGGGACAACTACACGCAAGGGTCAAATGAC-------
----

ACAGAGGACCCTATAGTTCTCCCATGCAGAATAAAGCAATTTGTAAATATATGGCAGAGAGTAGGACAAGCAA
TATATGCCCCTCCCATCCAAGGAGTAATAAATTGTAGCTCAAAC---
ATTACAGGACTACTATTAACAAGAGATAATGATGAGGGTAGTAAT----------------------------
----

AGGGCAAATGAGACCTTCAGACCTGGAGGAGGAGATATGAGGGACAATTGGAGAAGTGAGTTATATAAATATA
AAGTAGTAAAAATTGAACCACTA---GGAGTAGCACCCAGCAGGGCAAGGAGAAGAGTGGTG-----------
----------------------------GCAAGAGAAAAAAGAGCAATAGTTGGA---ATAGGAGCT------
---CTCTTCCTTGGGTTCTTAGGAGCA---GCAGGAAGCACTATG---GGCGCGGCGTCA---
CTAACGCTGACGGTACAG---GCCAGACAA---TTATTG---TCTGGCATAGTGCAACAGCAGAGC---
AATCTG---CTGAGG---GCTATAGAGGCTCAACAGCATCTGTTGAAACTCACGGTCTGGGGCATT
>AF460972
TTCTATTGCAATACATCAGCCCTGTTTAATAGCACTTGGGAAAATGACACTGTCAGCACGCAAGGGTCAAATG
ACACA--

GGGTCAAATGACACTATAACTCTCCCATGCAGAATAAAGCAATTTGTAAACATGTGGCAGAGAGTAGGACAAG
CAATGTATGCCCCTCCCATCCAAGGAATAATAAGGTGTGAATCAAAC---
ATTACAGGACTACTATTATCAAGAGATGGTGGTGTGGGTAATAAT----------------------------
----

AGGACAAATGAGACATTCAGACCTGGAGGAGGAAATATGAAAGACAATTGGAGAAGTGAATTATATAAGTATA
AAGTAGTAAAAATTGAACCATTA---GGAGTAGCACCCAGCAGAGCGAGGAGAAGAGTGGTG-----------
----------------------------GCAAGAGAAAAAAGAGCA---GTAGTTGGACTAGGGGCT------
---GTCTTCCTTGGGTTCTTAGGAGCA---GCAGGAAGCACTATG---GGCGTGGCGTCA---
CTAACGCTGACGGTACAG---GCCAGACAA---TTATTG---TCTGGCATAGTGCAACAACAGAAC---
AATCTG---CTGATG---GCTATAGAGGCTCAACAGCATCTGTTGAAACTCACGGTCTGGGGCATT
>AF460974
TTCTATTGCAATACATCAACCCTGTTTAATAGCACTTGGGCAAATGACACAAAGGCAAATGACACAGGG----
----

TCAAATGACACTATAACTCTCCCATGCAGAATAAAGCAATTTGTAAATAAGTGGCAAAGAGTAGGACAATCAA
TGTATGCCCCTCCCATCCAAGGAACAATAAGGTGTGACTCAAAC---
ATTACAGGACTACTATTAACAAGAGATGGGGGTGATAATAGCCTA----------------------------
----

AATGAGACCTTCAGACCTGGAGGAGGAGATATGAGGGACAATTGGAGAAGTGAATTATATAAATATAAAGTAG
TAAAAATTGAACCACTA---GGAGTAGCACCCAGCAAGGCAAGGAGAAGAGTGGTG-----------------
----------------------ACAAGAGAAAAAAGAGCA---GTGGGA---CTAGGGGCT---------
GTCTTCCTTGGGTTCTTAGGAGCA---GCAGGAAGCACTATG---GGCGCGGCGTCA---
CTAACGCTGACGGTACAG---GCCAGACAA---TTATTG---TCTGGCATAGTGCAACAACAGAAC---
AATCTG---CTGAGG---GCTATAGAGGCTCAACAGCATCTGTTGAAACTCACGGTCTGGGGCATT
>AF450097
TTCTATTGTAATACAACAAAATTGTTTAACAGTACTTGGACTGTGAATGGAACTACAAATACTACAAAT----
----

AACAATGACACAATCACACTCCCATGCAAAATAAAACAAATTATAAACATGTGGCAGGAAGTAGGAAAAGCAA

TGTATGCCCCTCCCATCAGCGGTCAAATTAGATGCTCATCAAAT---
ATTACAGGGCTGCTATTAGTAAGAGATGGTGGTAATAGTAACGAG----------------------------
----

ACCGAGATCTTCAGACCTGGAGGAGGAAATATGAAGGACAATTGGAGAAGTGAATTATATAAATATAAAGTAG
TAAAAATTGAACCATTA---GGAGTAGCACCCACCAGGGCAAGGAGAAGAGTGGTG-----------------
----------------------CAAAGAGAAAAAAGAGCA---GTGGGA---ATAGGAGCT---------
TTGCTCATTGGGTTCTTGGGAGCA---GCAGGAAGCACTATG---GGCGCAGCGTCA---
ATGACGTTGACGGTACAG---GCCAGACAA---TTACTG---TCTGGTATAGTGCAACAGCAGAAC---
AATCTG---CTGAGG---GCTATTGAGGCGCAACAGCATATGTTGCAACTCACAGTCTGGGGCATA
>JN248351
TTCTACTGTAATACATCACAACTGTTTAATAGTACATGGAATAATACTGCAGGAAATTCTAATAATGAT----
----

ACCAATACCACTATCACGCTCCCTTGCAGAATAAGACAAATTGTAAATATGTGGCAGGAAGTAGGAAGAGCAA
TGTATGCCCCTCCCATTGAAGGACAAATTAGCTGTTCATCAAAT---
ATTACAGGGTTGCTATTAACAAGAGATGGTGGTCATAACAATGTTAGC-------------------------
----

AATACCACCGAGACCTTCAGACCTGGAGGAGGAGATATGAGGGACAATTGGAGAAGTGAATTATATAAATATA
AAGTAGTAAAAATTGAACCATTA---GGAGTAGCACCCACCAAGGCAAAGAGGAGAGTGGTG-----------
----------------------------CAAAGAGAAAAAAGAGCA---GTGGGAACGATAGGAGCT------
---GTGTTCCTTGGGTTCTTGGGAGCA---GCAGGAAGCACTATG---GGCACAGCGTCA---
ATAACGCTGACGGTACAG---GCCAGACAA---TTGTTG---TCTGGTATAGTGCAACAGCAGAGA---
AATTTG---CTGAGG---GCCATTGAGGCGCAACAGCATCTGCTGCAACTCACAGTCTGGGGCATC
>KT022406
TTCTATTGCAATACATCAAGCCTGTTTAATGGCACATGGAACAGGGCTAACTACACAATGGAG----------
----

AATGACACTATTATGAATATCACATGCAGAATAAAACAAATTATAAACATGTGGCAGAGAGTAGGACAAGCAA
TGTATGCCCCTCCCATTGCAGGAGTAATAAAGTGTACATCAAAC---
ATTACAGGAATAATATTGACAAGAGATGGTGGGAACGGGAACAACAGT-------------------------
----

ACCAACAGTACAGAGACCTTCAGGCCTGGAGGAGGAGATATGAGAGACAATTGGAGAAGTGAATTATATAAGT
ATAAAGTAGTAAAAATTGAACCATTA---GGAGTAGCACCCACCAAGGCAAAGAGAAGAGTGGTG--------
-------------------------------GAGAGAGAAAAAAGAGCA---GTTGGA---CTGGGAGCT---
------GTCTTCCTTGGGTTCTTGGGAGCA---GCAGGAAGCACTATG---GGCGCAGCGTCA---
ATAACGCTGACGGTACAG---GCCAGACAA---TTATTG---TCTGGCATAGTGCAACAGCAAAGC---
AATTTG---CTGAAG---GCTATAGAGGCTCAACAACATCTGTTGAGACTCACGGTCTGGGGCATT
>AY945736
TTCTATTGCAATACATCAGACCTGTTTAATAGCACGTGGACCAACGACACGTGGGGGAAAATGGCACTGCCA
ACGAC--
-------------------TCA---
AAGTCAAATGACACTATAATGCTCCCATGCAGGATAAAACAAATTATAAACATGTGGCAGAGAGTAGGACAAG
CAATGTATGCCCCTCCCATTGCAGGAATAATAAAGTGTACATCAAAC---
ATTACAGGAATAATATTGACAAGAGATGGTGGAGAGAACGATAGT----------------------------
----

ACAGAGACCTTCAGACCTGGAGGAGGAGATATGAGAGACAATTGGAGAAGTGAATTATATAAGTATAAAGTAG
TAAAACTTGAACCACTA---GGAGTAGCACCCACCAAGGCCAGGAGAAGAGTGGTG-----------------
----------------------GAGAGAGACAAAAGAGCA---GTTGGA---CTGGGAGCT---------
TTCTTCCTTGGGTTCTTGGGAGCA---GCAGGAAGCACTATG---GGCGCAGCGTCA---
CTAACGCTGACGGTACAG---GCCAGACAA---TTATTG---TCTGGCATAGTGCAACAGCAAAGC---
AATTTG---CTGAAG---GCTATAGAGGCTCAACAACATCTGTTGAGACTCACAGTCTGGGGCATT
>AF286239
TTCTATTGCGATACATCAGGCCTGTTTAATAGCACGTGGCCTGCCAATGCATCAAGGGAGAATGAGGAG----
----

AAAGATCGCAATGTGACTCTCCCATGCAGAATAAAACAAATTGTAAACATGTGGCAGAGAGTAGGACGAGCAA

TGTATGCCCCTCCCATTAACGGAACAATAAAGTGTACATCCAAC---
ATTACAGGAATGATATTAACAAGAGATGGG---AACAGTGGGGGCAAT-------------------------
----

GCAACCAATGAGACCTTCAGACCTGGAGGAGGAGATATGAGGGACAATTGGAGAAGTGAATTATATAAGTATA
AAGTAGTAAAACTAGAACCACTA---GGAGTAGCACCTACCAGGGCAAGAAGAAGAGTGGTG-----------
----------------------------GAGAGGGAAAAAAGAGCA---GCA------GTTGGACTG------
---TTCTTCCTTGGGTTCTTGGGAGCA---GCAGGAAGCACTATG---GGCGCGGCGTCA---
GTGACGCTGACGGTACAG---GCCAGACAA---TTATTG---TCTGGCATAGTGCAACAGCAAAGC---
AATTTG---CTGAAG---GCTATAGAGGCTCAACAGCATATGTTGAGACTCACGGTCTGGGGCATT
>EU581825
TTCTACTGCAATACATCAGGATTGTTTAATAACTCAGTATTC-------------------------------
----

AATAATAACACTATCACTCTCCCATGTCGAATAAAACAAATTGTAAACATGTGGCAGGAAGTGGGACGAGCAA
TGTATGCCGCTCCCATTGCAGGAAACATTGCCTGTAGCTCAAAT---
ATTACAGGTCTACTATTGACAAGAGACGGTGGTTGTCAGAACCAGAGCAAT----------------------
----

ACTAACTGTACAGAGACCTTCAGACCTTGGGGAGGAAATATGAAAGACAATTGGAGAAGTGAATTATATAAAT
ATAAAGTAGTAGAAATTGAACCATTA---GGAATAGCACCCACCAAGGCAAAGAGAAGAGTGGTG--------
-------------------------------CAGAGAGACAAAAGAGCA---GTGGGA---TTGGGAGCT---
------ATGTTCCTTGGGTTCTTGGGAGCA---GCAGGAAGCACTATG---GGCGCAGCGTCA---
ATAACGCTGACGGTACAA---GCTAGACAA---TTATTG---TCTGGAATAGTGCAACAGCAAAAC---
AATTTG---CTGAGG---GCTATTGAAGCGCAACAGCATATGTTGCAGCTCACAGTCTGGGGCATT
>AY037281
TTCTACTGCAATACATCAAAACTGTTTAATGACTCCCGGGATAATGACACAGAA-------------------
----

TCCAATGACACTATCATTCTCCCATGTCGAATAAAACAAATTGTAAACAGGTGGCAGGAAGTAGGACAAGCAA
TGTATGCCGCTCCCATTGCAGGAAACATTGTTTGTAACTCAAAT---
ATTACAGGTCTACTATTGACAAGAGATGGTGGTCAGGGAAATGGT----------------------------
----

AGTGGACCAGAAATCTTCAGACCTGAGGGAGGAAATATGAAAGACAATTGGAGAAGTGAATTATATAAATACA
AAGTAGTAGAAATTGAACCATTA---GGAGTAGCACCCACCAAGGCAAAGAGAAGAGTGGTG-----------
----------------------------CAGAGAGAAAAAAGAGCA---GTGGGA---ATGGCAGCT------
---GTGTTCATTGGGTTCTTGGGAGCA---GCAGGAAGCACTATG---GGCGCAGCGTCA---
ATAACGCTGACGGTACAG---GCCAGACAA---TTATTG---TCTGGAATAGTGCAACAGCAGAGC---
AATCTG---CTGAGG---GCTATTGAAGCGCAACAGCATATGTTGCAGCTCACAGTCTGGGGCATT
>EU581827
TTCTACTGCAATACATCAGGATTGTTTAATGACACAGTATCT-------------------------------
----

AATGGCACTATCACTCTCCCATGTAAAATAAAACAAATTGTAAACATGTGGCAGGAAGTGGGAAGAGCAATGT
ATGCCGCTCCCATTGCAGGAAAAATTACCTGTAACTCAAGT---
ATTACAGGTCTTCTATTGACAAGAGATGGTGGTAGTAATACAACA----------------------------
----

GGCAACACAGAGACCTTCAGACCTGGGGGAGGAGATATGAGAGACAATTGGAGAAGTGAATTATATAAATATA
AAGTAGTAGAAATTCAACCAATA---GGAGTAGCACCCACCCCGGCAAAAAGAAGAGTGGTG-----------
----------------------------CAAAGAGAAAAAAGAGCA---GCA------TTAACAACT------
---ATGTTCCTTGGGTTCTTGGGAGCA---GCAGGAAGCACTATG---GGCGCAGCGTCA---
ATAGCGCTGACGGTACAG---GCCAGACAA---TTATTG---TCTGGAATAGTGCAACAGCAAAGC---
AATTTG---CTGAGA---GCTATTGAAGCGCAACAACATATGTTGCAGCTCACAGTCTGGGGCATT
>KP718931
TTCTATTGTGATACAACACAACTGTTTAATAGTACCTGGGATAAGAGTACCTGGAATAACACCAATAGCAGCT
ATGAGGGA---

AATGGAGATGATACTATTACATTGCAGTGCAAAATAAAACAAATTGTAAACATGTGGCAGAGAGTAGGACAAG

CAATGTATGCCCCTCCCATCGCGGGAAATATTACATGTGTATCAAAT---
ATTACAGGACTAATATTGACAAGTGACTTTGTTAATGCAACTACA---------------------------
----

GATGTGACCTTCAGACCTGGAGGAGGAGATATGAGGGACAATTGGAGAAGTGAATTATATAAATATAAAGTGG
TGAAAATTGAACCACTA---GGAGTAGCACCCTCTAAGGCAAAGAGAAGAGTGGTG-----------------
---------------------GAGAGAGAAAAAAGAGCA---ATTGGA---ATAGGAGCT---------
GTCTTCCTTGGGTTCTTAGGAGCA---GCAGGAAGCACTATG---GGCGCGGCGTCA---
ATAACGCTGACGGTACAG---GCTAGACAA---TTATTG---TCTGGTATAGTGCAACAGCAAAGC---
AATTTG---CTGAGG---GCTATAGAGGCTCAACAGCATCTGTTGAAACTCACGGTCTGGGGCATT
>AF377959
TTTTATTGTAATACAACAAACCTGTTTACATGGGATAGCACCACCAATGGCACCAATGGCAACTACACAGTAA
GT--

AATAATGATACTATTACACTGCAATGCAGAATAAAACAAATTATAAACATGTGGCAGAGAGTAGGACAAGCAA
TGTACGCTCCTCCCATCGAAGGAAGCATTACATGTGTATCAAAT---
GTTACAGGACTAATATTGACAAGTGACTTTGTTAATAAGTCAACA---------------------------
----

GAGGAGGTGACCTTCAGACCTGGAGGGGGAGATATGAGAGACAATTGGAGAAGTGAATTATATAAATATAAAG
TAGTGAAAATTGAACCACTA---GGAGTAGCACCCACTAAGGCAAAGAGAAGAGTGGTG-------------
-------------------------GGGAGAGAAAAAAGAGCA---GTTGGA---CTAGGAGCT---------
GTCTTCCTTGGGTTCTTAGGAGCA---GCAGGAAGCACTATG---GGCGCGGCGTCA---
ATAACGCTGACGGTACAG---GCTAGACAA---TTATTG---TCTGGTATAGTGCAACAGCAAAGC---
AATTTG---CTGAGG---GCTATAGAGGCTCAACAGCATCTGTTGAAACTCACGGTCTGGGGCATT
>EU743963
TTCTATTGCAATACATCAGGACTGTTCAATAGCACTTGGACTGACAACACGACAGAATTAAATAGCACGGATG
GC--

AATATAACTCATATAATTCTTCCGTGCAGAATAAAGCAAATTATAAATATGTGGCAGAGAGTAGGACAAGCAA
TGTATGCCCCTCCCATCCCAGGAATAATAAGGTGTGAGTCAAAC---
ATTACAGGATTACTATTGACAAGAGATGGTGGGGTTACTGATAACAGT------------------------
----

ACAGAAGATGAGACTTTCAGACCTGGAGGAGGAGATATGAGAGACAATTGGAGAAGTGAATTATATAAATATA
AAGTAGTACAAATTGAACCACTA---GGAGTAGCACCCACCAAGGCAAGGAGAAGGGTGGTG----------
----------------------------GAGAGAGAAAAAAGAGCA---GTTGCTGGACTTGGAGCT------
---GTCCTCTTTGGGTTCTTAGGAGCA---GCAGGAAGCACTATG---GGCGCGGCGTCA---
ATAACGCTGACGGTACAG---GCCAGACAA---TTATTG---TCTGGCATAGTGCAACAGCAAAGC---
AATTTG---CTGAAT---GCTATAGAGGCTCAACAGAATCTGTTGAGACTCACGGTCTGGGGCATT
>AY371165
TTCTATTGTAATACAGCAAGCCTGTTTAACAGTACTTGGGGACCAGGGTCAAATAACACGAATGACCCTAGTA
AC--

ACGAGTGACACTATAATTCTCCCGTGCAGAATAAAGCAAATTATAAATATGTGGCAGAGAGCAGGACAAGCAA
TGTATGCCCCTCCCATCCCAGGAATAATAAAGTGTGAATCAAAC---
ATTACAGGACTACTATTGACAAGAGATGGTGGGGATAATAATAGT---------------------------
----

ACAAATGAGACTTTCAGACCTGGAGGAGGAGATATGAGAGACAATTGGAGAAGTGAATTATTTAAATATAAAG
TAGTAAAAATTGAACCACTA---GGAGTAGCACCCACCAAGGCAAAGAGAAGAGTGGTG-------------
-------------------------GAAAGAGAAAAAAGAGCA---GTTGGA---TTTGGAGCT---------
GTCTTCCTTGGGTTCTTAGGAGCA---GCAGGAAGCACTATG---GGCGCGGCGTCA---
ATAACGCTGACGGTACAG---GCCAGACAA---TTATTG---TCTGGCATAGTGCAACAGCAAAGC---
AATTTG---CTGAGG---GCTATAGAGGCTCAACAGCATATGTTGAGACTCACAGTCTGGGGCATT
>JN864049
TTCTATTGCAATACATCAGGCCTGTTTAATAGCACTTGGCATAATAATGACACTGGCAGTGGGGCAGAGTACC
AGAGC---

ACGAATGATAGTATAACTCTCCCGTGCAAAATAAAGCAAATTATAAATATGTGGCAGAGAGTAGGACAAGCAA

TGTATGCTCCTCCCATCCCAGGAATAATAGAGTGTAAGTCAAAC---
ATTACAGGACTACTGTTGACACGAGATGGTGGAAATGGTAGTGAC----------------------------
----

AGTGCAAATGAGACTTTCAGACCTGGAGGAGGAGATATGAGAGACAATTGGAGAAGTGAATTATATAAATATA
AAGTAGTAAAAATTGAACCACTA---GGAGTAGCACCCACTAAGGCAAAGAGAAGGGTGGTGGAA---AGA--
-------------------AAGGTGGTGGAGAGAAAGAAAAGAGCA---ATTGGA---TTTGGAGCT------
---GTCTTCCTTGGGTTCTTAGGAGCA---GCCGGAAGCACTATG---GGCGCGGCGTCA---
ATAACGCTGACGGTACAG---GCCAGACAA---TTATTT---TCTGGCATAGTGCAGCAGCAAAGC---
AATTTG---CTGAGG---GCTATAGAGGCTCAACAACAAATGTTGAAACTCACGGTCTGGGGCATT
>JN864050
TTCTATTGCAATACATCAGGCCTGTTTAATAGCACTTGGTATCCAAATGGCAGCATGACAGGGTATACAAATG
ACACAAAT---

GACAATGGCACCATAATTCTCCCTTGCAGAATAAAGCAGGTTATAAATATGTGGCAGAGAGCAGGAAAAGCAA
TGTATGCCCCTCCCATCCCAGGAGTAATAAGGTGTGAGTCAAAC---
ATTACAGGACTACTATTAACAAGAGATGGTGGGATAAACAGTACA----------------------------
----

GAGGAGACTTTTAGACCTGGAGGAGGAGATATGAGAGACAATTGGAGAAGTGAATTATATAAATATAAGGTAG
TAAAAATTGAACCACTA---GGAGTAGCACCCACCCACTCAATGAGAAGAGTGGTG-----------------
----------------------CAGAGAGAAAAAAGAGCA---GTTGGA---TTGGGAGCT---------
GTCTTCCTTGGGTTCTTAGGAGCA---GCAGGAAGCACTATG---GGCGCGGCGTCA---
ATAACGCTGACGGCACAG---GCCAGACAA---TTATTG---TCTGGCATAGTGCAACAGCAAAGC---
AATTTG---CTGAGG---GCTATAGAGGCTCAACAGAATATGTTGAGACTCACGGTCTGGGGCATT
>JN864051
TTCTATTGCAATACATCAGGCCTGTTTAATAGCACTTGGAATTGGAATGACACTGTCAACACGACAGAGAACA
ATGAT--

ACAAACGGCACTATAACTCTCCAGTGCAGAATAAAGCAAATTATAAATATGTGGCAGAGAGTAGGACAAGCAA
TGTATGCCCCTCCCATCCCAGGAGTAATACGATGTGAGTCAAAC---
ATTACAGGAATACTATTGACAAGGGATGGTGGGACTAATGACAGT----------------------------
----

AGTCTAAATGAGACTTTCAGACCTGGGGGAGGAGATATGAAAGACAATTGGAGAAGTGAATTATATAAATATA
AAGTAGTAAAAATTGAACCACTA---GGAGTAGCGCCCACCAAGGCAAGGAGAAGGGTGGTG-----------
----------------------------CAGAGAGAAAAAAGAGCA---ATTGGA---TTTGGAGCT------
---TTCTTCCTTGGGTTCTTAGGAGCA---GCAGGTAGCACTATG---GGCGCGGCGTCA---
CTGACGCTGACGGTACAG---GCCAGACAA---TTATTA---TCTGGCATAGTGCAACAGCAAAAC---
AATTTG---CTGAGG---GCTATAGAGGCTCAACAACAGATGTTGAGACTCACAGTCTGGGGCATT
>JN864058
TTCTATTGCAATACATCAGGCCTGTTCAATAGCACTTGGAATAATAGCACTTGGAATACAACAGACATGTTTA
ATAGCACT---
-------------------GGG---
AATGAGAATGAGAACATAACTCTCCCATGCAGAATAAAGCAAATTATAAATATGTGGCAGAGAACAGGACAAG
CAATGTATGCCCCTCCCATCCCAGGAATAATAAAGTGTGAGTCAAAC---
ATTACAGGACTACTATTGACAAGAGATGGTGGGATTAATAACAAT----------------------------
----

TCAAATGAGACTTTTAGACCTGGAGGAGGAGATATGAAAGACAATTGGAGAAGTGAATTATACAAATATAAAG
TAGTAAAAATTGAACCACTA---GGAGTAGCACCCACCAGGGCAAGGAGAAGGGTGGTG--------------
-------------------------CAGAGAGAAAAAAGAGCA---GTTGGA---TTGGGAGCT---------
GTCTTCCTTGGGTTCTTAGGAGCA---GCAGGAAGCACTATG---GGCGCGGCGTCA---
CTAACGCTGACGGTACAG---GCCAGACAA---TTGTTG---TCTGGCATAGTGCAACAGCAAAAC---
AATTTG---CTGCAG---GCTATAGAGGCTCAACAGCAGATGTTGAGACTCACGGTCTGGGGCATT
>FM877780
TTCTATTGTAATACATCAGGCCTGTTTAATAGCACTTGGAAAAATGGAGTTTGGGAAAGGGGCAGGATGAACC
AGACA--
-------------------AAT---
GACACGAATGAAAACATAACACTCCCATGCAGGATAAAACAAATTGTAAACATATGGCAGGAAGTAGGACAAG

CAATGTATGCCCCTCCAGTTGCAGGAGAGATACAGTGTGTAACAAAC---
ATTACAGGAATAATATTGACAAGAGATGGGGGAATTAATAATGAG----------------------------
----

ACTACTGAGACCTTCAGACCTGGAGGAGGAAATATGAAGGACAATTGGAGAAGTGAGTTATACAAATATAAAG
TAGTAAAACTTGAACCACTA---GGAGTAGCACCCGACAAGGCAAAGAGAAGAGTGGTG--------------
-------------------------GAGAGAGAAAAAAGAGCA---GTTGGA---CTGGGAGCT---------
CTTTTCCTTGGGTTCTTAGGAACA---GCAGGAAGCACTATG---GGCGCGGCGTCA---
ATAACGCTGACGGTACAG---GCCAGACAA---TTATTG---TCTGGCATAGTGCAACAGCAAAGC---
AATTTG---CTGAGG---GCTATAGAGGCTCAACAGCATGTGTTGAAGCTCACCGTCTGGGGCATT
>FM877781
TTCTATTGTGATACATCAGGACTGTTTAATAGCAGTTGGCCAATTAATAGCAGCTGGCCAACTAATAACAGTG
AG---

ACAAATGAAACAATAACACTCCCATGCAGGATAAAACAAATTGTAAACATGTGGCAAGGAGTAGGACAAGCAA
TGTATGCCCCTCCTATCGAAGGAGAGATACAGTGTATATCAAAT---
ATTACAGGAATAATATTGACAAGAGATGGGGGAAATAACAGTAAC----------------------------
----

ACTGAAATCTTCAGACCTGGAGGAGGAAATATGAAGGACAATTGGAGAAGTGAATTATATAAGTATAAGGTAG
TTCAAATTGAACCGCTG---GGAGTAGCACCCAGCGAGGCAAGGAGAAGAGTGGTG-----------------
----------------------CAGAGAGAAAAAAGAGCA---GTAGGA---ATGGCAACT---------
CTTTTCCTTGGGTTCTTGGGAACA---GCAGGAAGCACTATG---GGCGCAGCGTCA---
ATAACGCTGACGGTACAG---GCCAGAAAA---TTATTG---TCTGGCATAGTGCAACAGCAAAGC---
AATTTG---CTGAAG---GCTATAGAAGCTCAACAGCATATGTTGAAGCTCACAGTCTGGGGCATT
>FM877782
TTCTATTGTAACACATCAGGCCTGTTTAATAGAACTTGGAAAGATGGCAATTGGACCACCCAGGAAAATGGG-
----

ACAAATGAAAACATAACACTCCCATGCAGGATAAAACAAATTGTACGCATGTGGCAAGAAGTAGGACAAGCAA
TGTATGCCCCTCCTGTCGCAGGAATGATACAGTGTATATCAAAT---
ATTACAGGATTAATATTGACAAGAGATGGGGGGAAAGATAAGAAT----------------------------
----

GATACTGAGATCTTCAGACCTGGAGGAGGAAATATGAAAGACAATTGGAGAAGTGAATTATATAAGTATAAAG
TAGTACAAATTGAGCCACTA---GGATTAGCACCTACCAAGGCAAGGAGAAGAGTGGTG--------------
-------------------------GAGAGAGAAAAAAGAGCA---GTTGGA---ATGGGAGCT---------
CTTTTCCTTGGGTTCTTGGGAGCA---GCCGGAAGCACTATG---GGCGCGGCGTCA---
ATAACGCTGACGGTACAG---GCCAGACAA---TTGCTG---TCTGGCATAGTGCAACAGCAAAGC---
AATTTG---CTGAGG---GCTATAGAAGCTCAACAGCATATGTTGAAGCTCACAGTCTGGGGCATT
>AM851091
TTCTATTGCGATACAAAAGAACTGTTTAATAGTACCTGGAATAAGAGCACCAATGGCACCTGGACTAAGAACA
GCACCACTGGC--
-------------GAGACA------
GGGAACACAGCAAATATTACACTGCAATGCAGAATAAAACAAATTGTAAACATGTGGCAGAGAACAGGACAAG
CAATATATGCCCCTCCTATTAGAGGAATAATTCAGTGTGAATCAAAT---
ATTACAGGGATACTATTGACAAGAGATAGG---CATAATCATACT----------------------------
----

AATGAGACCTTCAGACCTGGAGGAGGAAATATGAAGGACAATTGGAGAAATGAATTATATAAATATAAAGTAG
TAGAAATTGAACCACTA---GGAATAGCACCCACCAGGGCAAAAAGGAGAGTGGTG-----------------
----------------------GAGAGAGAAAAAAGAGCA---GTGGGA---ATAGGAGCT---------
TTGATCTTTGGGTTCTTAGGAGCA---GCAGGAAGCACTATG---GGCGCGGCGTCA---
ATAACGCTGACGGTACAT---GCCAGACAA---TTATTG---TCTGGTATAGTGCAACAGCAAAGC---
AATTTG---CTGAGG---GCTATAGAGGCGCAACAGCATATGTTGCAACTCACAGTCTGGGGCATT
>JF804811
TTCTACTGTAATACATCACTGCTGTTTAATACTACTTGGGAGAAGCATAATACCACTCAACCTTGGATAATGA
AAAATGAACCT--
-------------GGGTAT------
GATGTGAATAGGAATATCACACTCCACTGCAGAATAAAACAAATTATAAACAGGTGGCAGGAAGTAGGAAAAG

CAATGTATGCCCCTCCCATCAGTGGAATAATTAACTGTTTATCAAAT---
ATTACAGGGCTGTTACTAACAAGAGATGGAGGTCCAAACGGGACC----------------------------
----

CAAAATGAGACCTTCAGACCTCAAGGAGGAGATATGAGAGACAATTGGAGAAGTGTATTATATAAATATAAAG
TAGTAAAAATTGAGCCATTA---GGGGTAGCACCCACCAGGGCAAAGAGAAGAGTGGTG--------------
-------------------------CAGAGAGAAAAAAGAGCA---GTGGGA---CTGGGAGTC---------
TTGTTCCTTGGGTTCTTGGGAGCA---GCAGGAAGCACTATG---GGCGCAGCATCA---
ATAACGCTGACGGTACAG---GCCAGACAG---TTATTG---TCTGGTATAGTGCAACAGCAAAAC---
AATTTG---CTGAGG---GCTATTGAGGCGCAACAGCATCTGTTGCAGCTCACAGTCTGGGGCATC
>AY455778
TTCTACTGCAATACAACACAGCTGTTTATTAGTACTTGGCTGTATAATACTACTTGGACACGGCCGAGTCAAA
AG---

TGCTATGATACTATCACAATCCCATGCAGAATAAAACAAATTATAAACAGGTGGCAGGAAGTAGGAAAAGCAA
TGTATGCCCCTCCCATCAAAGGAAATATTAGCTGTTCATCAAAT---
ATTACAGGGTTGCTATTAACAAGAGATGGTGGAAATAACAACGACACG-------------------------
----

AACGGGACCGAGACCTTCAGACCTGGAGGAGGAGATATGAGGGACAATTGGAGAAGTGAATTATATAAATATA
AAGTAGTAAAAATTGAGCCATTA---GGAATAGCACCCAACAAGGCAAAGAGAAGAGTGGTG-----------
----------------------------CAGAGAGAAAAAAGAGCA---GTGGGA---ATAGGAGCT------
---CTGTTCCTTGGGTTCTTGGGAGCA---GCAGGAAGCACTATG---GGCGCAGCGTCA---
ATGACGCTGACGGTACAA---GCCAGACAA---TTATTG---TCTGGTATAGTGCAACAGCAAAGC---
AATTTG---CTGAGA---GCTATTGAGGCGCAACAGCATCTGTTGCAGCTCACAGTCTGGGGCATC
>EF158043
TTCTATTGTAATACATCAGGCCTGTTTAATAGCACTTGGAATATCAATGGCAGCGGAGGGTTTAATAGC----
----

ACAAATGACAACATAACTCTCCCATGTAGAATAAAGCAAATTATAAATATGTGGCAGAGAACAGGACAAGCAA
TGTATGCCCCTCCCATCAAAGGAGAAATCAGGTGTGTATCAAAC---
ATTACAGGACTATTGTTGACAAGAGATGGTGGTGGTAATGCGAGT----------------------------
----

CAGAATGAGACCTTCAGACCTGGAGGAGGAGATATGAGAGACAATTGGAGAAGTGAATTATACAAATATAAAG
TAGTAAAAATTGAACCACTA---GGAGTAGCACCCACCGGGGCAAAGAGAAGAGTGGTG--------------
-------------------------GAGAGAGAAAAAAGAGCA---GTTGGA---CTAGGAGCT---------
GTGTTCATTGGGTTTTTAGGAGCA---GCCGGAAGCACTATG---GGCGCGGCGTCA---
GTGACGCTGACGGTACAG---GCCAGGCAA---TTATTG---TCTGGCATAGTGCAACAGCAAAGC---
AATTTG---CTGAGG---GCTATAGAGGCTCAACAGCATGTGTTGAGGCTCACGGTCTGGGGCATT
>EF158040
TTCTATTGTGATACATCAGGTCTGTTTAATAGCGCTTGGAATGGCACCAATGGCAACGAAATGTCAAATATC-
----

ACAAATGGCACTATAACTCTCCAATGCAGAATAAAGCAAATTGTAAATATGTGGCAGAGAGTAGGACAGGCAA
TGTATGCCCCTCCCATCCCAGGAGTAATCAGGTGTGTATCAAAC---
ATTACCGGACTATTGTTAACAAGAGATGGTGGTCAGGATAATGGG----------------------------
----

AGTAGTGAGATCTTCAGACCTGGAGGAGGAGATATGAGAGACAATTGGAGAAGTGAATTATATAAATATAAAG
TAGTACAACTTGAACCACTA---GGAGTAGCACCCACTAAGGCAAGGAGAAGAGTGGTG--------------
-------------------------GAGAGAGAAAAAAGAGCA---GTTGGA---ATAGGAGCT---------
GTGTTCATTGGGTTCTTAGGAGCA---GCAGGAAGCACTATG---GGCGCAGCGTCA---
ATGACGCTGACGGTACAG---GCCAGACAA---TTATTG---TCTGGCATAGTGCAACAGCAAAGT---
AATTTG---CTGAGG---GCTATAGAGGCTCAACAGCATATGTTGAAACTCACGGTCTGGGGCATT
>EF158041
TTCTATTGTGATACATCAGGCCTGTTTAACAGTACCTGGAATATCACTGACATAGGAGAGCCAAATAGCACAA
AT---

AGCACAAATGACACTATAACTCTCCCATGCAGAATAAAGCAAATTATAAATATGTGGCAGAGAACAGGACAAG

CAATATATGCCCCTCCCATCCGAGGAGAAATCAGGTGTGTATCAAAC---
ATTACAGGACTATTGTTGACAAGAGATGGTGGTACTAATAACAGT----------------------------
----

AGTCTGAATGAGACCTTCAGACCTGGAGGAGGAGATATGAGAGACAACTGGAGAAGCGAATTATACAAATATA
AAGTAGTAAAACTTGAACCACTA---GGAGTAGCACCCACCAGGGCAAAGAGAAGAGTGGTG-----------
----------------------------GAGAGAGAAAAAAGAGCA---GTCGGA---CTGGGAGCT------
---ATGTTCATTGGGTTCTTAGGAGCA---GCAGGAAGCACTATG---GGCGCGGCGTCA---
ATGACGCTGACGGTACAG---GCCAGACAA---TTATTG---TCTGGCATAGTGCAACAGCAAAGC---
AATTTG---CTGAGG---GCTATAGAGGCTCAACAGCATATGTTGAAACTCACGGTCTGGGGCATT
>EF158042
TTCTATTGTAATACATCAGGACTGTTTAACAGCACTTGGAAAATCAATGCCAGCGAAGAGTTAAATAGCACAA
AT---

GACAATGTAACTATAACTCTCCCATGCAGAATAAAGCAAATTATAAATATGTGGCAGAGAACAGGACAGGCAA
TGTATGCCCCTCCCATCCGAGGAGAAATTAGGTGTGTATCAAAC---
ATTACAGGAATATTGTTGACCAGAGATGGTGGTAGAGTTAATGAA----------------------------
----

AGTCTAAATGAGACCTTCAGACCTGGAGGAGGAGATATGAGAGACAATTGGAGAAGTGAATTATACAAATATA
AAGTAGTAAAAATTGAACCACTA---GGAGTAGCACCCACCAGGGCAAAGAGAAGAGTGGTG-----------
----------------------------GAGAGAGAAAAAAGAGCA---GTTGGA---CTGGGAGCT------
---GTGTTCATTGGGTTCTTAGGAGCA---GCAGGAAGCACTATG---GGCGCGGCGTCA---
GTGACGCTGACGGTACAG---GCCAGACAA---TTATTG---TCGGGCATAGTGCAACAGCAAAGC---
AATTTG---CTGAGG---GCTATAGAGGCTCAACAGCATATGTTGAAACTCACGGTCTGGGGCATT
>GQ477443
TTCTATTGTGATACATCAGGCCTGTTTAATAGCACTTGGAATATCAGTGGCAGCGAAGAGTTAAATAGC----
----

ACAAATGACATAATAACTCTCCCATGCAGAATAAAGCAAATTATAAATCTATGGCAGAGAATAGGGCAAGCAA
TGTATGCCCCTCCCATCCGAGGAGAAATTAGGTGTGAATCAAGC---
ATTACAGGACTATTGTTGACAAGAGATGGTGGTGTTAATGGGAGT----------------------------
----

CAGAATGAAACCTTCAGACCTGGAGGAGGAGATATGAGAGACAATTGGCGAAGTGAATTATACAAATATAAAG
TAGTAGAACTTGAACCACTA---GGAGTAGCTCCCACCAGGGCAAAGAGAAGAGTGGTG--------------
-------------------------GAGAGAGAAAAAAGAGCA---GTCGGA---CTAGGAGCT---------
ATGTTCATTGGGTTCTTAGGAGCA---GCAGGAAGCACTATG---GGCGCGGCGTCA---
ATGACGCTGACGGTACAG---GCCAGACAA---TTATTG---TCTGGCATAGTGCAACAGCAAAGT---
AATTTG---CTGAGG---GCTATAGAGGCTCAACAGCATATGTTGAAACTCACGGTCTGGGGCATT
>GQ477442
TTCTATTGTAATACATCAGGCCTGTTTAATAGCACTTGGAATATCAATGGCAGTGAAGGGGTAAATAGC----
----

ACAAATGTCAATATAACTCTCCCATGCAGAATAAAGCAATTTATAAATATGTGGCAGAGAACAGGACAAGCAA
TGTATGCCCCTCCCATTCAAGGAGAAATCAGGTGTGTATCAAAC---
ATTACAGGACTATTGTTGACAAGAGATGGTGGTAATGGTAATGACAGT-------------------------
----

CAGGCTAATGAGACCTTCAGACCTGGAGGAGGAGATATGAGAGACAATTGGAGAAGTGAATTATACAAATATA
AAGTAGTAAAACTTGAACCACTA---GGAGTAGCACCCACCAGGGCAAGGAGAAGAGTGGTG-----------
----------------------------GAGAGAGAAAAAAGAGCA---GTTGGA---ATAGGAGCT------
---GTGTTCATTGGGTTCTTAGGAGCA---GCAGGAAGCACTATG---GGCGCGGCGTCA---
ATGACGCTGACGGTACAG---GCCAGACAA---TTATTG---TCTGGCATAGTGCAACAGCAAAGC---
AATTTG---CTGAAG---GCTATAGAGGCTCAACAGCATATGTTGAGACTCACGGTCTGGGGCATT
>GQ477446
TTCTATTGTAATACATCAGGCCTGTTTAACAATAGCCTGTTTAACAGCAGCGGAGAGTCA-------------
----

GATAGCACAAATATAACTCTCCCATGCAGAATAAAGCAAATTATAAATATGTGGCAGAGAACAGGACAAGCAA

TGTATGCCCCTCCCATCCGAGGAGAAATCAGGTGTGTATCAAAC---
ATTACAGGACTATTGTTGACAAGAGATGGTGGTGCTAATGAGAAT----------------------------
----

GAGAGTGAGACCTTCAGACCTGGAGGAGGAGATATGAGAGATAATTGGAGAAGTGAATTATACAAATATAAAG
TAGTAAAAATTGAACCACTA---GGAGTAGCACCCACCAAGGCAAAGAGAAGAGTGGTG--------------
-------------------------GAGAGAGAAAAAAGAGCA---GTTGGA---CTGGGAGCT---------
TTGTTCATTGGGTTCTTAGGAGCA---GCAGGAAGCACTATG---GGCGCGGCGTCG---
GTGACGCTGACGGTACAG---GCCAGACAA---TTATTG---TCTGGCATAGTGCAACAGCAAAGC---
AATTTG---CTGAAG---GCTATAGAGGCTCAACAGCATATGTTGAGACTCACGGTCTGGGGCATT
>GQ477448
TTCTATTGTAATACATCAGGCCTGTTTAACAGCACTTGGGATAATAGCACTTGGTATAACAATGGCAGCCAAA
GGTCA--
-------------------AAT---
AGCACAAGTGGCATTATAACTCTCCCATGCAGAATAAAGCAAATTATAAATATGTGGCAGAGAACAGGACAAG
CAATGTATGCCCCTCCCATTCAAGGAGAAATCAAGTGTATATCAAAC---
ATTACAGGACTATTGTTGACAAGAGATGGTGGTAATCATGATGGTAAT-------------------------
----

GAGAGTCAGGAGACCTTCAGACCTGGAGGAGGAGATATGAGAGACAATTGGAGAAGTGAATTATACAAATATA
AAGTAGTAAAAATTGAACCACTA---GGAGTAGCACCTACCAGGGCAAGGAGAAGAGTGGTG-----------
----------------------------GAGAGAGAAAAAAGAGCA---GTTGGA---CTAGGAGCT------
---GTGTTCATTGGGTTCTTAGGAGCA---GCAGGAAGCACTATG---GGCGCGGCGTCA---
GTGACGCTGACGGTACAG---GCCAGACAA---TTGCTG---TCTGGCATAGTGCAACAGCAAAGC---
AATTTG---CTAAAG---GCTATAGAGGCTCAACAGCAGATGTTGAGACTCACAGTCTGGGGCATT
>AB703608
TTCTATTGTAATACATCAGGCCTGTTTAACAGCACTTGGAATACCAATGGCAGCGAAGGGTCAACAGAC----
----

ACAAGTGGCAATATAACTCTCCAATGCAGAATAAAGCAAATTATAAATATGTGGCAGAGAACAGGACAAGCAA
TATATGCCCCTCCCATCCGAGGAGAAATCATGTGTAAATCAAAC---
ATTACAGGACTATTGTTAACAAGAGATGGTGGTGGTGGTAATCAG----------------------------
----

AGTCAGAATGAGACCTTCAGACCTGGAGGAGGAGATATGAGAGACAATTGGAGAAGTGAATTATACAAATATA
AAGTAGTAAAAATTGAACCACTA---GGAGTAGCACCCACCAAGGCAAAGAGAAGAGTGGTG-----------
----------------------------CAGAGAGAAAAAAGAGCA---GTTGGA---CTGGGAGCT------
---TTGTTCATTGGGTTCTTAGGAGCA---GCAGGAAGCACTATG---GGCGCGGCGTCA---
GTGACGCTGACGGTACAG---GCCAGACAA---TTATTG---TCTGGCATAGTGCAACAGCAAAAC---
AACTTG---CTGAGG---GCTATAGAGGCTCAACAGCATATGTTGAAACTCACGGTCTGGGGCATT
>AB703615
TTCTATTGTAATACATCAAACCTGTTTAGTAGCACTTGGTTTATCAATGGCAGCGGAGAGTCACATAATAAAA
ATGGC--

ACTAATGACACTATAACTCTCCAATGCAGAATAAAGCAAATTATAAATCTGTGGCAGAGAACAGGACAAGCAA
TGTATGCCCCTCCCATCCAAGGAGAAATTAGGTGTGTATCAAAC---
ATTACAGGACTGTTGTTGACAAGAGATGGTGGTGTTAATGTGAGC----------------------------
----

AATGAGACTGAAACCTTCAGACCTGGAGGAGGAGATATGAGAGACAATTGGAGAAATGAATTATACAAATATA
AAGTAGTAAAAATTGAACCACTA---GGAGTAGCACCCACCAGGGCAAAGAGAAGAGTGGTG-----------
----------------------------GAGAGAGAAAAAAGAGCA---GTTGGA---CTAGGAGCT------
---GTGTTCATTGGGTTCTTAGGAGCA---GCAGGAAGCACTATG---GGCGCGGCGTCA---
GTGACGCTGACGGTACAG---GCCAGACAA---TTATTG---TCTGGCATAGTGCAACAGCAAAGC---
AATTTG---CTGAGG---GCTATAGAGGCTCAACAGCATATGTTGAAACTCACGGTCTGGGGCATT
>AB703609
TTCTATTGTAATACATCAGGCCTGTTTAACAGTACTTGGAATATCAATGGCAGCGGAGGGTTAGATAAC----
----

ACAAATGATAATATAACTCTCCAATGCAGAATAAAGCAAATTATAAATATGTGGCAGAGAACAGGGCAAGCAA

TGTATGCCCCTCCCATCCGAGGAGAAATCAGGTGTGTATCAAAC---
ATTACCGGACTATTGTTGACAAGAGATGGTGGTAATAATAGTGAG----------------------------
----

AGTCAGGAGACCTTCAGACCTGGAGGAGGAGATATGAGAGACAATTGGAGAAGTGAATTATACAAATATAAAG
TAGTAAAAATTGAACCAATA---GGAGTAGCACCCACCAAGGCAAAAAGAAGAGTAGTG--------------
-------------------------GAGAGAGAAAAAAGAGCA---GTTGGA---CTGGGAGCT---------
ATGTTCATTGGGTTCTTAGGAGCA---GCAGGAAGCACTATG---GGCGCGGCGTCA---
GTGACGCTGACGGTACAG---GCCAGACAA---TTATTG---TCTGGCATAGTGCAACAGCAAGGC---
AACTTG---CTGAGG---GCCATCGAGGCTCAACAGCATATGTTGAGACTCACAGTCTGGGGCATT
>AB703611
TTCTATTGTAATACGTCAGGTCTGTTTAACAGTACTTGGAATGTCACTGGCAGCAAAGAGTCACATAGC----
----

CCAAATGATACTATAACTCTCCCATGCAGAATAAAGCAAATTATAAATATGTGGCAGAGAACAGGACAAGCAA
TGTATGCCCCTCCCATCAAAGGAGAAATCAGGTGTGTATCAAAC---
ATTACAGGACTATTGTTGACAAGAGATGGTGGGGTTAATAACAGC----------------------------
----

CAGAATGAGACCTTCAGACCTGGAGGAGGGAATATGAGAGACAATTGGAGAAGTGAATTATACAAATATAAAG
TAGTGAAAATTGAACCACTA---GGAGTAGCACCCACCAGGGCAAAGAGAAGAGTGGTG--------------
-------------------------GAGAGAGAAAAAAGAGCA---GTAGGA---CTGGGAGCT---------
GTGTTCATTGGGTTCTTAGGAGCA---GCAGGAAGCACTATG---GGCGCAGCGTCA---
ATGACGCTGACGGTACAG---GCCAGACAA---TTATTG---TCTGGCATAGTGCAACAGCAAAGT---
AATTTG---CTGAAG---GCTATAGAGGCTCAACAGCATATGTTGAAACTCACGGTCTGGGGCATT
>AB703612
TTCTATTGTAATACATCAGGCCTGTTTAATAGCACTTGGCCTATCAATGGTAGCAGCGAAGGGAAAAATAGC-
----

ACAGATGACACTATAACTCTCCCACGCAGAATAAAGCAGATTATAAATATGTGGCAGAGAACAGGACAAGCAA
TGTATGCCCCTCCCATTAAAGGAGAAATCAGATGTGTATCAAAC---
ATTACAGGAATATTGTTGACAAGAGATGGTGGTGAGGGGAATCAGACTGAGGGG------
AATCAAACTGAGGGGAAC---

CGGACCGAGACCTTCAGACCTGGAGGAGGAGATATGAGAGACAATTGGAGAAGTGAATTATACAAATATAAAG
TAGTAGAACTTGAACCACTA---GGAGTAGCACCCACCAAGGCAAAGAGAAGAGTGGTG--------------
-------------------------GAGAGAGAAAAAAGAGCA---GTTGGA---CTAGGAGCT---------
GTGTTCATTGGGTTCTTGGGAGCA---GCAGGAAGCACTATG---GGCGCGGCGTCA---
ATGACGCTGACGGTACAG---GCCAGACAA---TTATTG---TCTGGCATAGTGCAACAGCAAAGC---
AATTTG---CTGAGG---GCTATAGAGGCTCAACAGCAGACGTTGAGACTCACGGTCTGGGGCATT
>AB703613
TTCTATTGTAATACATCGAGCCTGTTTAACAGCACTTGGTATAACAATGGCAGCAAAGAGTTAAATAGC----
----

GCAAATGGCATTATAAAACTCCCATGTAGAATAAAGCAAATTATAAATACGTGGCAGAGAACAGGACAAGCAA
TGTATGCCCCTCCCATCCGAGGAGAAATTAGGTGTGAATCAAAC---
ATTACAGGACTATTGTTGACAAGAGATGGTGGAAATGATACTAAT----------------------------
----

CAGACTGAGACCTTCAGACCTGGAGGAGGAGATATGAGAGACAATTGGAGAAGTGAATTATACAAATATAAAG
TAGTAAAAATTGAACCACTA---GGAGTAGCACCCACCAAGGCAAAGAGAAGAGTGGTG--------------
-------------------------GAGAGAGAAAAAAGAGCA---TTTGGA---CTGGGAGCT---------
TTGTTCGTTGGGTTCTTAGGAGCA---GCAGGAAGCACTATG---GGCGCGGCGTCA---
GTGACGCTGACGGTACAG---GCCAGACAA---TTATTG---TCTGGCATAGTGCAACAGCAAAGC---
AATTTG---CTGAGG---GCTATAGAGGCTCAACAGCATATGTTGAAACTCACGGTCTGGGGCATT
>AB703614
TTCTATTGTAATACATCAGGCCTGTTTAACAGCGCTTGGAATATCACTGGCAGCAGCGAAGGGTTAAATAGT-
----

ACAAATGACACTATAACTCTCCCATGCAGAATAAAGCAAATTATAAATATGTGGCAGAGAATAGGACAAGCAA

TGTATGCCCCTCCCATCCAAGGAGTAATCAGGTGTGAATCAAAC---
ATTACAGGACTATTGTTGACACGAGATGGTGGTAATAATAGGAATGAGAGT----------------------
----

GAAAATGAGACTGAGACCTTCAGACCTGGAGGGGGAGATATGAAAGATAATTGGAGAAGTGAATTATACAAAT
ATAAAGTAGTAAAACTTGAACCACTA---GGAGTAGCACCCACCAGGGCAAAGAGAAGAGTGGTG--------
-------------------------------GAGAGAGAAAAAAGAGCA---GCTGGA---CTGGGAGCT---
------GTGTTCATTGGGTTCTTAGGAGCA---GCAGGAAGCACTATG---GGCGCGGCGTCA---
ATGACGCTGACGGTACAG---GCCAGACAA---TTATTG---TCTGGCATAGTGCAACAGCAAAGC---
AATTTG---CTGAGG---GCTATAGAGGCTCAACAGCATATGTTGAAACTCACGGTCTGGGGCATT
>EF087995
TTCTATAGCGATACATCAGGCATGTTTAATAGCACTTGGGATAACAATAGCACTTGGAATAACAATAGCAGCA
TGACAGGG---
-------------TCAAAT------
AACACAAATGACAATATAACACTCCCGTGTAGAATAAAGCAAGTTATAAATATGTGGCAGAGAGTAGGACAAG
CAATGTATGCCCCTCCCATCGCAGGAATAATAAGGTGTGAGTCAAAT---
ATTACAGGACTACTATTGACAAGAGATGGTGGGAATACTAGCAGT----------------------------
----

ACAAATGAGACTTTCAGACCAGGAGGAGGAGATATGAGGGACAATTGGAGAAGTGAATTATATAAGTATAAGG
TAGTACAAATTGAACCACTA---GGTGTGGCACCCACCCGTGCAAAAAGAAGGGTGGTG--------------
-------------------------GAGAGAGAAAAAAGAGCAGTTGTTGGA---CTGGGAGCT---------
GTCTTCCTTGGGTTCTTAGGAGCA---GCAGGAAGCACTATG---GGCGCGGCGTCA---
ATAACGCTGACGGTACAG---GCCAGACAA---TTACTG---TCCGGTATAGTGCAACAGCAAAGC---
AATTTG---CTGAGG---GCTATAGAGGCTCAACAACATCTGTTGAAACTCACGGTCTGGGGCATT
>EF087994
TTCTATTGCAATACATCAGGCCTGTTTAATAGCACTTGGGGGTATAACAATAGCAACGTGACAAGGTTAAATA
AC---

ACGAATGACACTATAATTCTCCCGTGTAAAATAAAGCAAATTATAAAGATGTGGCAGAGAGTAGGACAAGCAA
TGTATGCCCCTCCCATCGCAGGAAGGATAAGGTGTGAGTCAAAT---
ATTACAGGACTACTATTGACAAGAGATGGTGGGAAAAATAACAGT----------------------------
----

ACAAATGAGACTTTCAGACCAGGAGGAGGAGATATGAGGGACAATTGGAGGAGTGAATTATATAAGTATAAGG
TAGTACAAATTGAACCACTA---GGTGTGGCACCCACCCGAGCAAAGAGAAGGGTGGTG--------------
-------------------------GAGAGAGAAAAAAGAGCA---GTTGGA---CTGGGAGCT---------
GTCTTCCTTGGGTTCTTAGGAGCA---GCAGGAAGCACTATG---GGCGCGGCGTCA---
ATAACGCTGACGGTACAA---GCCAGACAA---TTACTG---TCCGGTATAGTGCAACAGCAAAGC---
AATTTG---CTGAGG---GCTATAGAGGCTCAACAACATCTGTTGAAACTCACGGTCTGGGGCATT
>EF116594
TTCTACTGCAATACATCAGGCCTGTTCAATAGTACATGGAACACTACTACCAGCACGCAGATTCCAAATAGGA
CT---

ACAGATGGGAATATAACTCTCCCTTGCAGAATAAAGCAAATTGTCAATATGTGGCAGAGAGTAGGACAAGCAA
TGTATGCCCCTCCCATCCCAGGAGTAATAAGGTGTGAGTCCAAC---
ATTACAGGACTAATGTTAACAAGAGATGGTGGGATTAACAAAACA----------------------------
----

GATAATGAGACCTTCAGACCTGGGGGAGGAGATATGAGGGATAATTGGAGAAGTGAATTATATAAGTATAAAG
TAGTAAAAATTGAACCAATA---GGAATAGCACCCACCAAGGCAAGGAGAAGAGTGGTG--------------
-------------------------GAGAGAGAAAAAAGAGCA---GTTGGA---CTGGGAGCT---------
GTCTTCCTTGGGTTCTTAGGAGCA---GCAGGAAGCACTATG---GGCGCGGCGTCT---
ATAACGCTGACGGTACAG---GCTAGACAA---TTATTG---TCTGGCATAGTGCAACAGCAGAGC---
AACCTG---CTAAGG---GCTATAGAGGCTCAACAACATCTGTTGAAACTCACGGTCTGGGGCATT
>KP718917
TTCTATTGCAATACAACAAAACTGTTCAATCACCTGTTCAATAATAGTGAGTGGGACAATGCAGCTAACAGC-
----

ACGAATGAGAGTATAACTCTCCCTTGCAGAATAAAGCAAATTATAAATATGTGGCAGAGAGTAGGACAAGCAA

TGTATGCCCCTCCCATCCCAGGAGAAATAAGGTGTAGATCTAAT---
ATTACAGGACTATTGTTAACAAGAGATGGTGGAAGCAACAATGCA----------------------------
----

ACTAATGAAACCTTCAGACCTGGAGGAGGAGACATGAGGGACAATTGGAGAAGTGAATTATATAAGTATAAGG
TAGTGAAAATTGAACCAATA---GGAGTAGCACCCACCGGGGCAAGGAGAAGAGTGGTG--------------
-------------------------CAGAGAGAAAAAAGAGCA---GTTGGC---CTAGGAATT---------
GCCTTCCTTGGGTTCTTAGGAGCA---GCAGGAAGCACTATG---GGCGCGGCGGCC---
ATAACGCTGACGGTACAG---GCCAGACAA---TTGTTG---TCTGGCATAGTGCAACAGCAGAGC---
AATCTG---CTAAGG---GCTATAGAGGCTCAACAACACATGTTGAGACTCACGGTCTGGGGCATT
>AF377957
TTCTATTGCGATACAACAGGCCTGTTCAATAGTAGTTGGAGCTACAATGGCACGAGTTGGAATAAGACCACTA
CAAAT--
-------------------GGC---
ACTACAAATGGCAATATAACTCTCCATTGCAGAATAAAACAAATTATAAATATGTGGCAGAGAGTAGGACAAG
CAATGTATGCCCCTCCCATCCGAGGAGTAATAAGGTGTGAATCTAAC---
ATTACAGGATTGCTATTAACAAGAGATGGTGGGACAAACAATAGT----------------------------
----

AACACTACTGAAACCTTCAGACCTGGAGGAGGAGATATGAGGGACAATTGGAGAAGTGAATTATATAAGTATA
AAGTAGTAAAAATTGAACCAATA---GGAGTAGCACCTACCGGGGCAAGGAGAAGAACGGTG-----------
----------------------------GAGAGAGAGAAAAGAGCA---GTTGGA---CTAGGAGCT------
---GTTTTTCTTGGATTCTTAGGAGCA---GCAGGAAGCACTATG---GGCGCGGCGTCT---
GTGACGCTGACGGTACAG---GCCAGACAA---TTATTG---TCTGGCATAGTGCAACAGCAGAGC---
AATCTG---CTGAGG---GCTATAGAGGCTCAACAACATCTGTTGAAACTCACGGTCTGGGGCATT
>JF683745
TTCTATTGCAATACAACAAACCTGTTCAATAGTGTTTGGACAGACGGGAAATGGAATAATACCATGAACAGCA
GTCAA--

TCAGATAGTAATAATATAACTCTCCATTGCAAAATAAAACAAATTGTAAGGATGTGGCAAAGAGTAGGACAAG
CAATGTATGCCCCTCCCATCCGAGGAGTAATAAACTGTATATCCAAC---
ATTACAGGACTGCTATTAACAAGAGATGGTGGGCTAAGTAATAGG----------------------------
----

ACAAATGAGACCTTCAGACCTGGAGGAGGAGATATGAGGGACAATTGGAGAAGTGAATTATATAAGTATAAAG
TAGTAAAAAATTGATCCACTA---GGAGTAGCACCCTCCAGGGCAAGGAGAAGAGTGGTG-------------
-------------------------GAGAGAGAAAAAAGAGCA---GTTGGA---CTGGGAGCT---------
ATGTTCTTTGGATTCTTAGGAGCA---GCAGGAAGCACTATG---GGCGCGGCGTCC---
TTAACGCTGACGGTACAG---GCCAGACAA---TTATTG---TCTGGCATAGTGCAACAGCAGAGC---
AATCTG---CTGAGG---GCTATAGAGGCTCAACAGCATCTGTTGAAACTCACGGTCTGGGGCATT
>FJ213783
TTCTACTGCAATACATCAAAACTGTTTAATGACACAGCTTCC-------------------------------
----

AATAATGGTACCATCACTCTCCCATGTCGAATAAAACAAATTGTAAACATGTGGCAGGAAGTGGGACGAGCAA
TGTATACCGCTCCCATTGCAGGAGACATTACCTGTAGATCAAAT---
ATTACAGGTCTACTATTGACAAGAGATGGTGGTCAGAAAAATAATGGAAGTTGT-------------------
----
-
ACAGAGGCAACTTGTACACAGATCTTCAGACCTCAGGGAGGAAATATGAAAGACAATTGGAGAAGTGAATTAT
ATAAATATAAAGTAGTAGAAATTGAACCATTA---GGAATAGCACCCACCAGGGCAAAGAGAAGAGTGGTG--
-------------------------------------CAGAGAGAAAAAAGAGCA---GTGGGA---
ATGGCAGCT---------GTGTTCATTGGGTTCTTGGGAGCA---GCAGGAAGCACTATG---
GGCGCGGCGTCA---ATAACGCTGACGGTACAG---GCCAGACAA---TTATTG---
TCTGGAATAGTGCAACAGCAGAAC---AATCTG---CTGAGG---
GCTATTGAAGCGCAACAGCATATGTTGCAGCTCACAGTCTGGGGCATT
>FJ213780
TTCTACTGCAATACATCAAAACTGTTTGATAACATAGAAAAC-------------------------------
----

AATGGAACTATCATTCTCCCATGTAAAATAAAACAAATAGTAAACATGTGGCAAGAAGTGGGACGAGCAATGT
ATGCCGCTCCCATTGCAGGAAACATTGTCTGTAACTCAACT---
ATTACAGGTCTACTCTTAACAAGAGATGGTGGTCATGGTAATGGCACA-------------------------
----

ATCAATAAGACAGAGATCTTCAGACCTGGGGGAGGAAATATGAAAGACAATTGGAGAAGTGAATTATATAAAT
ATAAAGTAGTAGAAATTGAACCATTA---GGAATAGCACCCACCAGGGCAAGGAGAAGAGTGGTG--------
-------------------------------CAGAGAGATAAAAGAGCA---GTGGGA---ATGGCAGCT---
------ATGTTCCTTGGGTTCTTGGGAGCA---GCAGGAAGCACTATG---GGCGCAGCGTCA---
ATAACGCTGACGGTACAG---GCCAGACAA---TTATTG---TCTGGAATAGTGCAACAGCAAAGC---
AATTTG---CTGAGG---GCTATTGAAGCGCAACAGCATATGTTGCAGCTCACAGTCTGGGGCATT
>EU735535
TTCTACTGCAATACATCAAATCTGTTTAATGACACGATAGGC-------------------------------
----

AATGGCACTATAATTCTCCCATGTAAAATAAGACAAATTGTAAACATGTGGCAGGAAGTGGGACGAGCAATGT
ATGCTGCTCCCATTGCAGGAAACATTGCCTGTAACTCAACT---
ATTACAGGTCTACTATTGACAAGAGATGGTGGTAGTGGAAGTAATGGT-------------------------
----

ACTAATCAAAATGAGACCTTCAGACCTGGGGGAGGAAATATGAAAGACAATTGGAGAAGTGAATTATACAAAT
ATAAAGTAGTAGAAATTGAACCACTA---GGAATAGCACCCACCAGGGCACAAAGACAAGTGGTG--------
-------------------------------AAGAGAGAAAAAAGAGCA---GCA------CTAGGAGTT---
------GTGTTCCTTGGGTTCTTGGCAGCA---GCAGGAAGCACTATG---GGCGCGGCGTCA---
ATAACGCTGACGGTACAG---GCCAGACAA---TTACTG---TCTGGAATAGTGCAACAGCAGAAT---
AATCTG---CTGAAG---GCTATTGATGCGCAACAGCATCTGTTGCAGCTCACAGTCTGGGGCATT
>EU735538
TTCTACTGCAATACATCAGGACTGTTTAATGACACAGAAATTGAAGCTAATCACACAAATGCTAAT-------
----

GATAATGACACTATCATTCTCCCATGTAAAATAAAACAGATGATAAACATGTGGCAGGAAGTAGGACGAGCAA
TGTATGCTGCCCCCGTGGCAGGAAACATTATCTGTAACTCAAAT---
ATTACAGGACTACTATTAACAAGAGATGGTGGTAATAAGACTCATAAT-------------------------
----

GATACTAGGAATGAGATCTTTAGACCTGGAGGAGGAAATATGAAGGACAATTGGAGAAGTGAATTATATAAAT
ATAAAGTAGTAAGAATTGAGCCATTA---GGAGTAGCACCCACCAGGGCAAGGAGAAGAGTGGTG--------
-------------------------------CAGAGAGAAAAAAGAGCA---GTAACA---ATGGGAGCT---
------ATGTTCCTTGGGTTCTTGGGAGCA---GCAGGAAGCACTATG---GGCGCAGCGTCA---
CTAACGCTGACGGTACAG---GCCAGACAA---TTATTG---TCTGGTATAGTGCAACAGCAGAAC---
AATCTG---CTGAAG---GCTATTGAAGCGCAACAACATCTGTTGCAGCTCACAGTCTGGGGCATT
>EU735537
TTCTACTGCAATACATCAGGATTGTTTGATAATAACACAGGGACTGAC-------------------------
----

ACTAATGACACTATCACTCTCCCATGTAAAATAAAACAAATAGTAAATATGTGGCAAGAAGTAGGACGAGCAA
TGTATGCCGCCCCCATTGCAGGAAACATTACCTGTAACTCAAGT---
ATTACAGGACTACTATTGACAAGAGATGGTGGTCAGAATAATGAGACT-------------------------
----

AATAATACTGAGACCTTCAGACCTGGGGGAGGAAATATGAAGGACAATTGGAGAAGTGAATTATATAAATATA
AAGTAGTAAAAATTGAGCCATTA---GGAGTAGCACCCACCAGGGCAAGGAGAAGAGTGGTG-----------
----------------------------CAGAGAGAGAAAAGAGCA---GTGACG---CTGGGAGCT------
---ATGTTCCTTGGGTTCTTGGGAGCA---GCAGGAAGCACTATG---GGCGCAGCGGCA---
ACAACGCTGACGGTACAG---GCCAGACAA---TTATTG---TCTGGTATAGTGCAGCAGCAAAGC---
AATTTG---CTGAGG---GCTATTGAAGCGCAACAGCATATGTTGCAGCTCACAGTCTGGGGCATT
>EU170155
TTCTACTGCAATACAACAGAACTGTTTAATGACACAGAATCC-------------------------------
----

AATAACACTATCATTACTCTCCCATGTCGAATAAAACAAATTATAAACATGTGGCAGGAAGTGGGACGAGCAA
TGTATGCTGCTCCCATTGCAGGAAACATTACCTGTTCATCAAAT---
ATTACAGGTCTACTATTGACAAGAGATGGTGGTCTGGCTAATCAGACT-------------------------
----

GGAAATCAGAATGACACCTTCAGACCTGGGGGAGGAGATATGAGAGACAATTGGAGAAGTGAATTATATAAAT
ATAAAGTAGTAGAAATTGAGCCATTA---GGAATAGCACCCACCACAGCAAAGAGAAGAGTGGTG--------
-------------------------------CAGAGAGAAAAAAGAGCA---GTG------------------
------------------------------------GGAAGCACTATG---GGCGCAGCGTCA---
GTAACGCTGACGGTACAG---GCCAGACAA---TTATTG---TCTGGTATAGTGCAACAGCAGAAC---
AATTTG---CTGAAG---GCTATTGAGGCGCAACAGCACCTGTTGCAACTCACAGTCTGGGGCATC
>EU170139
TTCTACTGTAATACAACAATACTGTTTAATGAAACAGGATTTAATGACACAAGA-------------------
----

TCCAATGACACCATTACTCTCCCATGTCGAATAAAACAGATTATAAACATGTGGCAGGAAGTGGGACGAGCAA
TGTATACTGCTCCCATCGCAGGAAACATTGCTTGTTCATCAAAT---
ATTACAGGTCTACTATTGACAAGAGATGGTGGTAAGACTACTGAC----------------------------
----

AATCAGACTGACACCTTCAGACCTGGGGGAGGAGATATGAGAGACAATTGGAGAAGTGAATTATATAAATATA
AAGTAGTAGAAATTGAGCCATTA---GGAATAGCACCCAATAAAGCAAGGAGAAGAGTGGTG-----------
----------------------------CAGAGAGAAAAAAGAGCA---GTGGGG------------------
--GGCGCAGCGTCA---
GTAACGCTGACGGTACAG---GCCAGACAA---TTATTG---TCTGGTATAGTGCAACAGCAGAAT---
AATTTG---CTGAGG---GCTATTGAGGCGCAACAGCACATGTTGCAACTCACAGTCTGGGGCATC
>FN392874
TTCTATTGCAATACATCAGGCCTATTTAATAGCACTTGGTCTGACAAAAATAATACAGACAGCAATAGCACGG
AG---

TCAAATAGCACTATAACTCTTCCATGCAGAATAAAGCAAATTATAAATATGTGGCAGAGAGTAGGACGAGCAA
TGTATGCTCCTCCCATCCGAGGAATAATAAAGTGTACTTCAAAC---
ATTACAGGACTACTATTAACAAGAGATGGTGGTGTTAATAATAGTACA-------------------------
----

AATGAGACCTTCAGACCAGGAGGAGGAGATATGAGGGACAATTGGAGAAGTGAATTATATAAGTATAAAGTAG
TAAAAATTGAACCACTA---GGAGTAGCACCCACAAAGGCAAGGAGAAGAGTGGTG-----------------
----------------------GAGAGAGAAAAAAGAGCA---GTTGGA---CTGGGAGCT---------
TTCTTCCTTGGGTTCTTAGGAGCA---GCAGGAAGCACTATG---GGCGCAGCGGCA---
GTAACGCTGACGGTACAG---GCTAGACAA---TTATTG---TCTGGCATAGTGCAACAGCAGAGC---
AATCTG---CTGAGG---GCTATAGAGGCTCAGCAGCATCTGTTGAAACTCACGGTCTGGGGCATT
>FN392876
TTCTATTGCAATACATCAGGCCTGTTTAATACCACTTGGTACAACAATGGTTCAGACACTTGGTACAATGGTA
CAAAT--

AGCACGGAGCCAATAACTCTTCAATGCAGAATAAAGCAAATTATAAATATGTGGCAGAGAGTAGGACAAGCAA
TGTATGCCCCTCCCATCCCAGGAATAATACAATGTAACTCAAGC---
ATTACAGGAATACTATTAACAAGAGATGGTGGTAACAATAGTACA----------------------------
----

AATGAGACCTTCAGACCTGGAGGAGGAGATATGAGGGACAATTGGAGAAGTGAATTATATAGGTATAAAGTAG
TACAAGTTGAACCACTA---GGAGTAGCACCCACCAGGGCAAAGAGAAGAGTGGTG-----------------
----------------------GAGAGAGAAAAAAGAGCA---GTTGGA---ATAGGGGCT---------
GTCTTCCTTGGGTTCTTAGGAGCA---GCAGGAAGCACTATG---GGCGCAGCGTCA---
ATAACGCTGACGGTACAG---GCCAGACAA---TTATTG---TCTGGCATAGTGCGACAGCAGAAC---
AATCTG---CTGAAG---GCTATAGAGGCTCAACAGCATCTGTTGAAACTCACGGTCTGGGGCATT
>EU448295
TTCTATTGTAATACATCACAATTGTTTAATAGCACTTGGAGCCACAATGATACAGGCAGCACGAAGGAG----
----

TACAATGGCACTATACAACTTCCATGCAGAATAAAGCAAATTATAAATATGTGGCAGAGAGCAGGACGAGCAA
TGTTTGCCCCTCCCATCCAAGGAGCAATAAGGTGTGAATCAAAC---
ATTACAGGACTATTATTAACAAGAGATGGTGGTAATGCAAATGAGGCT-------------------------
----

AGAAATGAGACTGAGATCTTCAGACCTGGAGGAGGAGATATGAGGGACAATTGGAGAAGTGAATTATATAAGT
ATAAAGTAGTACAAATTGAGCCACTA---GGAGTAGCACCCACCAGGGCAAGGAGAAGAGTGGTG--------
-------------------------------GAGAGAGAAAAAAGAGCAGTTGTTGGA---CTGGGAGCT---
------GTCTTCATTGGGTTCTTAGGAGCA---GCAGGAAGCACTATG---GGCGCAGCGGCA---
GTGACGCTGACGGTACAG---GCCAGGCAA---TTATTG---TCTGGCATAGTGCAACAGCAGAGC---
AATCTG---TTGAGG---GCTATAGAGGCTCAACAGCATCTGTTGAAGCTCACGGTCTGGGGCATT
>DQ358801
TTCTACTGCAATACATCAGGACTGTTTCAGTTTAATGACACAGAT----------------------------
----

TCCAATGACACTATCATTCTCCCATGTCGAATAAAACAAATTGTAAACATGTGGCAGGAAGTGGGGCGAGCAA
TGTATGCCGCTCCCATTGCAGGAAACATTACCTGTAACTCAAAT---
ATTACAGGTCTACTATTGACAAGAGATGGTGGTCAGGATAATGGGACT-------------------------
----

AATGATATTGAGACCTTCAGACCTGGGGGAGGAAATATGAAAGACAACTGGAGAAGTGAATTATATAAATATA
AAGTAGTAGAAATTAATCCATTA---GGAATAGCACCCTCCAGGGCAAAAAGACAAGTGGTG-----------
----------------------------CAGAGAGAAAAAAGAGCA---GCGGGA---ATAGCAGTT------
---ATGCTCTTTGGGTTCTTGGGAGCA---GCAGGAAGCACTATG---GGCGCGGCGTCA---
ATGGCGCTGACGGTACAG---GCCAGACAA---TTATTG---TCTGGCATAGTGCAACAGCAGAAC---
AATCTG---CTGAGA---GCTATTGAAGCGCAACAGCATCTGTTGCAGCTCACAGTCTGGGGCATT
>DQ358802
TTCTACTGCGATACATCAACACTGTTTAATGACAATGACACAGTA----------------------------
----

TCCAATGACACTATCACTCTCCCATGTAAAATAAAACAAATTGTAAACATGTGGCAGGAAGTGGGGCGAGCAA
TGTATGCCGCTCCCATTGCAGGAGAAATTACCTGTAGATCAAAT---
ATTACAGGTCTACTATTGACAAGAGATGGTGGCCGAAATATTAGTACT-------------------------
----

GATCAGCCTGAGATCTTCAGACCTGGGGGAGGAAATATGAAAGACAACTGGAGAAGTGAATTATATAAATATA
AAGTAGTAGAAATTGAGCCACTA---GGAGTAGCACCCACCGAGGCAAAAAGACAAGTGGTG-----------
----------------------------AAGAGAAAAAAAAGAGCA---GCGGGA---ATAACAGCT------
---TTGTTCCTTGGGTTCTTGGGAGCA---
GCAGGAAGCACTATGGGCGCAGCCTCAATGACGCTGACGGTACAGGACCAG---ACGAATTAT---
TGGACTTGGCCATATTGCAGTCCGCGGGAACAA---TCTGCT---GAGAGG---
ACTATTGAAGCGCAACAGCATCTGTTGCAGCTCACAGTCTGGGGCATT
>MG365771
TTCTACTGCAATACATCAAAACTGTTTAATAATGACACAGGATCCAATGACACTACAGGA-------------
----

TCCAATGACACTATCACTCTCCCATGTAGAATAAAACAAGTTGTAAACATGTGGCAGGAAGTGGGGCGAGCAA
TGTATGCCGCTCCCATTGCAGGAAGAATTACCTGTAACTCAAAT---
ATTACAGGTCTACTATTGACAAGAGATGGTGGTCACACTAGTACTAATGAGACT-------------------
----

AATCAGACTGAGACCTTCAGACCTGAGGGAGGAAATATGAAAGACAATTGGAGAAGTGAATTATATAAATATA
AAGTAGTAGAAATTCAGCCACTA---GGAGTAGCACCCTCCAGGGCAAAAAGACCAGTGGTG-----------
----------------------------CAGAGAGAAAAAAGAGCA---GTGGGA---CTAGGAGCT------
---ATGTTCCTTGGGTTCTTGGGAGCA---GCAGGAAGCACTATG---GGCGCGGCGTCA---
ATAACGCTGACGGTACAG---GCCAGACAA---TTATTG---TCTGGCATAGTGCAACAGCAGAAC---
AATCTG---CTGAGG---GCTATTGAAGCGCAACAGCATCTGTTGCAGCTCACAGTCTGGGGCATT
>HM026456
TTCTACTGCAATACATCAGGACTGTTTAATGACACAGGATCC-------------------------------
----

AACAATGAGACTATCATTCTCCCATGTAGAATAAAACAAATGATAAACATGTGGCAGGAAGTGGGGCGAGCAA
TGTATGCCGCCCCCGTTGCAGGAAACATTACCTGTAACTCGAAT---
ATTACAGGTCTACTATTGACAAGAGATGGTGGTCCTGGTAATAAG----------------------------
----

ACTCAAATCTTCAGACCTGGGGGAGGAGATATGAAAGACAACTGGAGAAGTGAATTATACAAATATAAAGTAG
TAGAAATTGAGCCACTA---GGAATAGCACCCACCAAGGCAAAAAGACAAGTGGTG-----------------
----------------------ACGAGAGAAAAAAGAGCA---GTGGGA---CTGGGAGCG---------
TTGTTCCTTGGGTTCTTGGGAGCA---GCAGGAAGCACTATG---GGCGCGGCGTCA---
ATAACGCTGACGGTACAG---GCCAGACAA---TTATTG---GCTGGCATAGTGCAACAGCAAAAC---
AATTTG---CTGAGG---GCTATTGAAGCGCAACAGCATCTGTTGCAGCTCACAGTCTGGGGCATT
>HM026458
TTCTACTGCAATACATCAGGACTGTTTGATGAATCAATATTCAATAACACAGAC-------------------
----

AACAGTGACAACATCACTCTCCCATGTCAAATAAAACAAATTGTAAACATGTGGCAGGAAGTGGGACGAGCAA
TGTATGCCGCCCCCGTTGCAGGAAGCATTACCTGCAACTCAAAT---
ATTACAGGTCTACTATTGACAAGAGATGGTGGACAGAATAATGATACT-------------------------
----

AATGAAACTGAGACCTTCAGACCTGGGGGAGGAAATATGAAAGACAATTGGAGAAGTGAATTATATAAATATA
AAGTAGTAGAAATTGAGCCACTA---GGAGTAGCACCCACCAGGGCAAAAAGACAAGTGGTG-----------
----------------------------CAGAGAGAAAAAAGAGCA---ATAGGA---ATGGGAGCT------
---TTGTTCCTTGGGTTCTTGGGAGCA---GCAGGAAGCACTATG---GGCGCAGCGTCG---
GTAGCGCTGACGGTACAG---GCCAGACAA---TTATTG---TCTGGCATAGTGCAGCAGCAAAAC---
AATTTG---CTGAGG---GCTATTGAAGCGCAACAGCATCTGTTGCAGCTCACAGTCTGGGGCATT
>HM026459
TTTTACTGCAATACATCAGGACTGTTTAATAATAATATCACACAA----------------------------
----

TCTAAGGACATTATCACCCTCCCATGTCGAATAAAACAAGTGGTAAACCTGTGGCAGGAAGTGGGGCGAGCAA
TGTATGCCGCCCCCATTGAAGGAATAATTACCTGTAACTCAAGT---
ATTACAGGTCTACTATTGACAAGAGATGGTGGTCAGGGTAATAGTACT-------------------------
----

GATAATAACACTGAGATCTTTAGACCTGGGGGGGGAAATATGAAAGACAACTGGAGAAGTGAATTATATAAAT
ATAAAGTAGTAGAAATTGAGCCACTA---GGAGTAGCACCCACCGGGGCAAAAAGACAAGTGGTG--------
-------------------------------AAGAGAGAGAAAAGAGCA---GTAGGGCTACTGGGAGCT---
------TTGTTCCTTGGGTTCTTGGGAACA---GCAGGAAGCACTATG---GGCGCAGCGTCA---
ATGGCGCTGACGGTACAG---GCCAGACAA---TTATTG---TCTGGCATAGTGCAACAGCAAAAC---
AATTTG---CTGAGG---GCTATTGAAGCGCAACAACATCTGTTGCAGCTCACAGTCTGGGGCATT
>HM026460
TTCTACTGCAACACATCAAGACTGTTTAATGACACAGGATCCAGA----------------------------
----

TCCAATGACACTATCATTCTCCCATGTCGAATAAAACAAATTGTAAACATGTGGCAGGAAGTGGGGCGAGCAA
TGTATGCCGCTCCCATTGCAGGAAATATTACCTGTAATTCAAAT---
ATTACAGGTCTACTATTGACAAGAGATGGTGGTAATCAGACTGAGAGT-------------------------
----

AATAATAATGAGATCTTCAGACCTGGGGGAGGAAATATGAAAGACAACTGGAGAAGTGAGTTATATAAATATA
AGGTAGTAGAAATTGAGCCACTA---GGAATAGCACCCACCAAGGCAAGAAGACGAGTGGTGGCGAGAGAA--
-------------AAAAGAGCAGTGGTGGCGAGAGAAAAAAGAGCA---GTAGGA---CTAGGAGCT------
---ATGTTCATTGGTATCTTGGGAGCA---GCAGGAAGCACTATG---GGCGCGGCGTCA---
ATAACGCTGACGGTACAG---GCCAGACAA---TTATTG---TCTGGCATAGTGCAACAGCAGAGT---
AATCTG---CTGAGG---GCTATTGAAGCGCAACAGCATCTGTTGCAGCTCACAGTCTGGGGCATT
>KJ849798
TTCTACTGTAATACAACAGTACTGTTTAATAGTACATGGAATGAGACAAATATT-------------------
----

-AATCAAGAGTCAAAT---------
AACTCAACTGACATAATCACACTCCGATGCAAAATAAAACAAATTATAAACATGTGGCAGGAAGTAGGAAAAG
CAATGTATGCCCCTCCCATTAGTGGATACCTTAATTGTTCATCAAAT---
ATCACAGGGCTGATATTAATAAGAGATGGTGGT--

AATAACAGCACAAAT---
GAGACTGAGATCTTCAGACCTGGAGGAGGAAATATGAAGGACAATTGGAGAAGTGAATTATATAAATATAAAG
TAGTAAAAATTGAACCATTA---GGAATAGCACCCACCAGGGCAAAGAGAAGAGTGGTG--------------
-------------------------CAGAGAGAAAAAAGAGCA---GTGGGA---TTTGGAGCT---------
TTGTTCCTTGGGTTCTTGGGAGCA---GCAGGAAGCACTATG---GGCGCAGCGTCA---
GTGGCGCTGACGGTACAG---GCCAGACAA---TTATTG---TCTGGTATAGTGCAACAGCAAAAC---
AATTTG---CTGAGG---GCTATTGAGGCGCAACAGCATATGTTGCAACTCACAGTCTGGGGCATC
>GQ175883
TTCTATTGCAATACAACAAAACTATTTAATGATACTTGCATAGAAAATAATACCAGG----------------
----

TGTAATCACGCTATCATACTTCCATGCAGGATAAAGCAGATCATAAACATGTGGCAAGGAGTAGGACAAGCAA
TGTATGCTCCTCCCATCAATGGAAAAATTAATTGTGTATCAAAT---
ATTACAGGAATACTATTAACAAGAGATGGTGGTAGAAATAGTAGT----------------------------
----

GATGGTAATGAAACCTTCAGACCTGGAGGAGGAAATATAAAGGACAATTGGAGAAGTGAATTATATAAATATA
AAGTAGTACAAATTGAACCACTG---GGAATAGCACCCACCAGGGCAAAGAGAAGAGTGGTG-----------
----------------------------GAGAGAGAAAAAAGAGCA---GTGGGC---ATAGGAGCT------
---CTGATCTTTGGGTTCTTAGGAGCA---GCAGGAAGCACTATG---GGCGCGGCGTCA---
ATAACGCTGACGGTACAG---GCCAGACAA---TTATTG---TCTGGTATAGTGCAACAGCAAAGC---
AATTTG---CTGAGG---GCTATAGAGGCGCAGCAGCATATGTTGCAACTCACAGTTTGGGGCATC
>AF192135
TTCTATTGTAATACATCAGGATTGTTTAATAAATCATTATTGAATGAGACTAGTAATGAGACTACAGATGGT-
----

GCAAATAATACTATCACACTCACATGTAGAATAAAGCAAATTGTAAGAATGTGGCAGAGAGTAGGACAAGCAA
TATATGCCCCTCCCATTGCAGGAAACATTACATGCACATCAAAT---
ATTACAGGATTGCTATTGACACGTGATGGGGGATATACAAATAATACT-------------------------
----

AATGGAACAGAGATTTTCACACCTACAGGAAGAAATATGAGGGACAATTGGAGAAGTGAGTTATATAAATATA
AAGTGGTGAAGATTGAGCCAATA---GGAGTAGCACCCACCAGGGCAAAAAGAAGAGTGGTG-----------
----------------------------GGGAGAGAAAAAAGAGCA---GTAGGAATAATGGGAGCT------
---ATGTTCCTTGGGTTCTTGGGAACA---GCAGGAAGCACTATG---GGCGCAGCGTCA---
ATAACGCTGACGGTACAG---GCCAGACAA---TTGTTG---TCTGGTATAGTGCAACAGCAAAGC---
AATTTG---CTGAAG---GCTATAGAGGCTCAACAGCACCTGTTGAGACTCACTGTCTGGGGCATT
>JN417239
TTCTATTGCAATACATCACACCTGTTTAATAGCACTTGGAATAACAGCACACAGGTGTCAAATAACACACAG-
----

TTAAATGACACTATAACTCTCCAATGCAGAATAAAACAAATTATAAACATGTGGCAGGGAGTAGGAAAAGCAA
TGTATGCCCCTCCCATTGAAGGAAAGATTACATGTTTATCAAAT---
ATTACAGGACTAATATTAACAAGAGATGGTGGTGCAAATAATAAT----------------------------
----

AGTCAGAATGAGACTTTCAGACCTGCAGGAGGAGATATGAGAGACAATTGGAGAAGTGAATTATACAAATATA
AAGTAGTAAGACTTGAACCACTA---GGTCTAGCACCCACCGGGGCAAGGAGAAGAGTGGTG-----------
----------------------------GCAAGAGAAAAAAGAGCA---ATAGGA---CTAGGAGCT------
---ATGTTCCTTGGGTTCTTGGGAGCA---GCAGGAAGCACAATG---GGCGCAGCGTCA---
CTGACGCTGACGGTACAG---GCCAGAAAA---TTATTG---TCTGGTATAGTGCAGCAGCAAAAC---
AATTTG---CTGAGG---GCTATAGAGGCGCAACAACATTTGTTGCAACTCACAGTCTGGGGCATT
>JN882655
TTCTATTGCAATACATCAGGACTGTTTAATAGTACTTGGAATGACACTAGTACTTGGAATGAC----------
----

ACTGATAGGAATGTAACTCTCCCATGCAGAATAAAGCAATTTATAAATATGTGGCAGAGAGTAGAGCGAGCAA
TGTATGCCCCTCCCATCCGAGGAGACATAAAATGTGCATCAAAC---
ATCACTGGACTACTACTAACAAGAGATGGAGGGAATAATAATAGT----------------------------
----

ACAAATGAGACCTTCAGGCCTGCAGGAGGAGATATGAGAGACAATTGGAGAAGTGAATTATATAAGTATAAGG
TAGTAAAAATTGAACCATTA---GGAATAGCACCCACCAGGGCAAAAAGGAGAGTAGTG--------------
-------------------------GGGAGAGAGAAAAGAGCA---GTTGGA---ATGGGAGCT---------
GTCTTCCTTGGGTTCTTGGGAGCA---GCAGGAAGCACTATG---GGCGCGGCGTCA---
ATAACGCTGACGGTACAG---GCCAGACAA---TTATTG---TCTGGTATAGTGCAACAGCAAAAC---
AATTTG---CTGAAG---GCTATAGAGGCTCAACAACATATGTTGAGACTCACAGTTTGGGGCATT
>KC898985
TTCTATTGTAACACATCAGGCCTGTTTAATAGTACATACATACTTAATGGTACATACATGTACAGGGCTAATG
GTACAGACAAG---AAT---
ACGACTGATGGTACAATTAGT---------------
AATCCAAACTCAACCATCACAATCCCATGTAGAATAAAGCAAATTGTAAACATGTGGCAGGAGGTGGGACAAG
CAATGTATGCCTCTCCCATTGCAGGAAACATAACATGTAAATCAAAT---
ATCACAGGACTACTATTGGTACGTGATGGAGGTGGAGAGAAAAAT----------------------------
----

GATACAGAAATCTTCAGACCTGGAGGAGGAGATATGAGGGACAATTGGAGAAGTGAATTATATAAATATAAAG
TGGTAGAAATTCAGCCATTG---GGGGTAGCACCCACTAAAGCAAAAAGGAGAGTAGTG--------------
-------------------------GAGAGAGAAAAGAGAGCA---GTGGGA---ATAGGAGCT---------
GTGTTCCTTGGGATCTTGAGTGTA---GCTGGAAGCACTATG---GGCGCGGCGTCA---
ATGGCGCTGACGGTACAG---GCCAGACAA---TTGCTG---TCTGGTATAGTGCAACAGCAGAAC---
AATTTG---CTGAGG---GCTATAGAGGCGCAACAGCATATGTTGCAACTCACGGTCTGGGGCATT
>KC899008
TTCTATTGTGATACATCAGGCCTGTTTAATGGTACATATGGTACATACGATCCTAATGGCACAGAAGGTAATT
CAAGC--
-------------TCAGAA------
GGTAATTCAAAATTCATCATACTTCCATGCAGAATAAAGCAAATTATAAACATGTGGCAGGAGGTAGGACGAG
CAATGTATGCCCCTCCCATTGAGGGAAACATAACATGTAAATCAAAT---
ATCACAGGACTACTATTGGTACGTGATGGAGGAACTCAAAATGAAACA-------------------------
----

AAAAATGAAGAGATATTCAGACCTGAAGGAGGAGATATGAGGGACAATTGGAGAAGTGAATTATATAAATATA
AAGTGGTAGAAATTAAGCCATTG---GGAGTAGCACCCACTGAAGCAAAAAGGAGAGTAGTG-----------
----------------------------GAGAGAGAAAAAAGAGCA---GTGGGA---CTAGGAGCT------
---GTGTTCCTTGGGTTCTTGGGTGCA---GCCGGAAGCACTATG---GGCGCAGCGTCA---
GTAGCGCTGACGGTACAG---GCCAGACAA---TTGCTG---TCTGGTATAGTGCAACAGCAAAAC---
AATTTG---CTGAGG---GCTATAGAGGCGCAACAGCATATGTTGCAACTCACGGTCTGGGGCATT
>JX679207
TTCTATTGTAATACATCAAGACTATTTAATAGTACATACATGTCTAATGGTACATACACATACACAATTAATA
GT---

AACATCTCAAACATCACAATTCCATGCAGAATAAAGCAAATTGTAAACATGTGGCAGGAGGTAGGACGAGCAA
TGTATGCCCCTCCCATTGCAGGAAATATAACATGTAAATCAAAT---
ATCACAGGACTACTATTGGTACGTGATGGAGGATCAAATGAGACT----------------------------
----

CAGCCAGAGATATTCAGACCTGGAGGAGGAGATATGAGGGACAATTGGAGAAGTGAATTATATAAATATAAAG
TGGTAGAAATTAAGCCATTG---GGACTAGCACCCACTACTGCAAAAAGGAGAGTAGTG--------------
-------------------------GAGAGAGAAAAAAGAGCA---GTGGGA---ATAGGAGCT---------
GTGTTCCTTGGGTTCTTGGGTGCA---GCCGGAAGCACTATG---GGCGCGGCGTCA---
ATAACGCTGACGGTACAG---GCCAGACAA---TTGTTG---TCTGGGATAGTGCAACAGCAAAGC---
AATTTG---CTGAAG---GCTATAGAGGCGCAACAGCATCTGTTGCAACTCACGGTCTGGGGCATT
>KC870044
TTCTATTGTAATACATCAGGTCTGTTTAATAGTACATACCTGCCTAATGGCACA-------------------
----

TTCATGACTAATGATACA---------
ACTTATAGCCCAAACATCACAATCCCATGCAGAATAAAACAAATTATAAACATGTGGCAGGAGGTAGGACGAG
CAATGTACGCCCCTCCCATTGCAGGAGAAATAACATGTAAATCAAAG---
ATCACAGGACTACTATTGGTAAGGGATGGAGGA--

-
CTACCAAATAAGACAAATGAGACATTCAGACCTGGAGGAGGAGATATGAGAGACAATTGGAGAAGTGAATTAT
ATAAATATAAAGTGGTAGAAATTAAGCCATTG---GGCATAGCACCCACTACTGCAAAAAGGAGAGTGGTG--
-------------------------------------GAGAGAGAAAAAAGAGCA---GTGGGA---
CTAGGAGCT---------TTGTTCCTTGGGTTCTTGGGTGCA---GCCGGAAGCACTATG---
GGCGCGGCGTCA---ATAACGCTGACGGTACAG---GCCAGACAA---TTGTTG---
TCTGGTATAGTGCAACAGCAAAAC---AATTTG---CTGAGG---
GCTATAGAGGCGCAACAGCATATGTTGCAACTCACGGTCTGGGGCATT
>JN230353
TTCTATTGCAATACATCAGCACTATTTAATAGCAGTTGGAATAGCACTGGGAATAGCACTGGGGTGAAT----
----

ATCACTGACCCTATAAGACTCCCATGCAAAATAAAGCAAATTATAAATATGTGGCAGAGAGTAGGACAAGCAA
TATATGCCCCTCCTATCCCAGGAGTAATAAGGTGTAATTCAAAC---
ATTACAGGAATACTATTAACAAGAGATGGAGGGACTGGACTAACAGT--------------------------
----

ACAAATGAGACCGAGACCTTCAGACCTGGAGGAGGAGACATGAGGGATAATTGGAGAAGTGAATTATATAAGT
ATAAAGTAGTAAAAATTGAACCACTA---GGTGTAGCACCCACCGGGGCAAAACGAAGAGTGGTG--------
-------------------------------GAGAGAGGAAAAAGAGCAGTCGTCGGA---CTGGGAGCT---
------GTCTTCCTTGGGTTCTTAGGAACA---GCAGGAAGCACTATG---GGCGCAGCAGCA---
TTAACGCTGACGGTACAG---GCCAGACAA---TTATTG---TCTGGTATAGTGCAACAGCAGAAC---
AATCTG---CTGAGG---GCTATAGAGGCTCAACAGCACATGTTGAAACTCACGGTCTGGGGCATT
>KC899009
TTCTATTGTAATACATCAGGTCTGTTTAATGGAACATACATGCCTAATGGTACAATGGATAGTCCA-------
----

AGCACAATCCCATGCAGAATAAAGCAAATTATAAACATGTGGCAGGAGGTAGGGCGAGCAATGTATGCCCCTC
CTATTGAAGGAAACATAACATGTCAATCAAAT---
ATCACAGGAATACTATTGGTACGGGATGGAGAGTTAAATAGGACA----------------------------
----

GAAGAGATATTCAGACCTGGAGGAGGAGATATGAGGAACAATTGGAGAAGTGAATTATATAAATATAAAGTGG
TAGAAATTAAGCCATTG---GGAATAGCACCCACTGAAGCAAAAAGGAGAGTGGTG-----------------
----------------------GAGAGAGAAAAAAGAGCA---GTGGGA---ATAGGAGCT---------
GTGTTCCTTGGGTTCTTGGGAGCA---GCAGGAAGCACTATG---GGCGCGGCGTCA---
ATAACGCTGACGGTACAG---GCCAGACAA---TTGTTG---TCTGGGATAGTGCAACAGCAAAGC---
AATTTG---CTGAGG---GCTATAGAGGCGCAACAGCATATGTTGCAACTCACGGTCTGGGGCATT
>KC870043
TTCTATTGTAATACATCAGGCCTGTTTAAGGGTACATACACTAATGGT-------------------------
----
----------ACATTAAGT------
AATTCAAGCTCAGACATCACAATCCCATGCAGAATAAAGCAAATTATAAACATGTGGCAGGAGGTAGGAAGAG
CAATGTATGCCCCTCCCATTGAAGGAAACATAACATGTAAATCAAAT---
ATCACAGGACTACTATTGGTACGTGATGGAGGT--

-
ACTTCTAATAATAATACAGAGGTATTTAGACCTGGAGGAGGAGATATGAGGGACAATTGGAGAAGTGAATTAT
ATAAATATAAAGTGGTAGAAATTAAGCCATTG---GGAGTAGCACCCACTGGAGCAAAAAGGAGAGTGGTG--
-------------------------------------GAGAGAGAAAAAAGAGCA---GTGGGA---
ATAGGAGCT---------GTGTTCCTTGGGTTCTTGGGAGCA---GCAGGAAGCACTATG---
GGCGCGGCGTCA---ATAACGCTGACGGTACAG---GCCAGACAA---TTGTTG---
TCTGGCATAGTGCAACAGCAAAAC---AATTTG---CTGAAG---
GCTATAGAGGCGCAACATCATCTGTTGCAACTCACAGTCTGGGGCATT
>JN251903

TTCTACTGCAATACATCAGGACTGTTTAATAACACACTGTTAAATGTAAATGAAACAGGA-------------
----

TCTAATAACACTATCACTCTCCCATGTCGAATAAAACAAATTGTAAACATGTGGCAGGAAGTAGGGCGAGCAA
TGTATGCCGCTCCCATTGCAGGAAACATTACCTGTAACTCAAAT---
ATTACAGGTCTACTATTGACAAGAGATGGTGGTCAGACTAATGAGACT-------------------------
----

AATGGGAATGAGACTGTCAGGCCTGGGGGAGGAAATATGAAAGACAATTGGAGAAATGAATTATATAAATATA
AAGTAGTAGAAATTGAACCTCTA---GGAGTAGCACCCACCAAGGCAAAAAGACAAGTGGTG-----------
----------------------------CAGAGAGAAAAAAGAGCA---ATAGGA---ATAGGAGCT------
---ATGTTCCTTGGGTTCTTGGGAGCA---GCAGGAAGCACTATG---GGCGCGGCGTCA---
ATAACGCTGACGGTACAG---GCCAGACAA---TTATTG---TCTGGAATAGTGCAACAGCAGAAC---
AATTTG---CTGCAG---GCTATTGAAGCGCAACAGCATCTGTTGCAGCTTACAGTCTGGGGCATC
>AB845347
TTCTATTGCAATACAACAAAACTGTTTAATAATACTTGCATAAGAAATAAAACCATGGAGGGG----------
----

TGTAGTGGCAATATCACTATCCCATGCAAGATAAAACAAATTATAAACATGTGGCAGGGCATAGGACAAGCAA
TGTATGCTCCTCCCATCAGTGGAAAAATTAATTGTGTATCAAAT---
ATTACAGGAATACTATTGACAAGAGATGGTGGTGCTAATAATACA----------------------------
----

AATAATGAGACCTTCAGACCTGGAGGAGGAGATATGAAGGACAATTGGAGAAGTGAATTATATAAATATAAAG
TAGTAGAAATTGAACCACTA---GGAATAGCACCCACCAGGGCAAAGAGAAGAGTGGTG--------------
-------------------------GAGAGAGAAAAAAGAGCA---GTGGGA---ATAGGAGCT---------
ATGATCTTTGGGTTCTTAGGAGCA---GCAGGAAGCACTATG---GGCGCGGCGTCA---
ATAACGCTGACGGTACAG---GCCAGACAA---TTATTG---TCTGGTATAGTGCAACAGCAAAGC---
AATTTG---CTGAGG---GCTATAGAGGCGCAGCATCATCTGTTGCAACTCACAGTCTGGGGCATC
>KU749388
TTCTACTGTGACTCCACTCCACTGTTTAATAGTACTTGGAATGGTACTGAAGAAATAAATGGCACTGAA----
----

GGAAATGACACGATCACACTCAAATGCAGAATAAAACAAATTATAAACATGTGGCAGAGAGTAGGAAGAGCAA
TGTATGCCCCTCCCATTCAAGGACAAATTAGCTGTTCATCAAAT---
ATTACAGGGATTCTATTAACAAGAGATGGTGGTAGTGGCAACGAG----------------------------
----

ACGAACAACAAGACAGAGACCTTCAGACCTGGAGGAGGAGATATGAGGGACAATTGGAGAAGTGAATTATATA
AATATAAAGTAGTAAAAATTGAACCATTA---GGAGTGGCACCAACCCATGCAAAGAGAAGAGTGGTG-----
----------------------------------CAGAGAGAAAAAAGAGCA---ATAGGA---
CTAGGAGCT---------ATGTTCCTTGGGTTCTTGGGAGCA---GCAGGAAGCACTATG---
GGCGCAGCGTCA---ATAACGCTGACGGTACAG---GCCAGACAA---TTATTG---
TCTGGTATAGTGCAGCAGCAGAAT---AATCTG---CTGAGG---
GCTATTGAAGCGCAACAGCATATGTTGCAACTCACAGTCTGGGGCATC
>DQ358811
TTCTACTGTAACTCAACACAACTGTTTAATAGTACTTGGTATGGTAATGAAACTTGGAATGGTACTGAAAAAT
TCAAT--

GAGGAAAATGACACAATCAGCCTCCCATGCAGAATAAAACAAATTATAAACATGTGGCAAGGAGTAGGAAAAG
CAATGTATGCCCCTCCCATCCAAGGACAAATTAGATGTACATCAAAT---
ATTACAGGGCTGATGTTAACAAGAGATGGTGGTCATAACAATGACACG-------------------------
----

CACCAGATGACAGAGACCTTCAGACCTGGAGGAGGAGACATGAGGGACAATTGGAGAAGTGAATTATATAAAT
ATAAAGTAGTAAGAATTGAACCATTA---GGAATAGCACCCACCAAGGCAAAGAGAAGAGTGGTG--------
-------------------------------CAGAGAGAAAAAAGAGCA---ATAGGA---ATAGGAGCT---
------ATGCTCCTTGGGTTCTTGGGAGCA---GCAGGAAGCACTATG---GGCGCAGCATCA---
ATAACGCTGACGGTACAG---GCCAGACTA---TTATTG---TCTGGTATAGTGCAACAGCAAAAC---
AATTTG---CTGAGG---GCTATTGAGGCGCAACAGCATCTGTTGCAACTCACAGTCTGGGGCATC

>KJ671535
TTTTATTGCAATACATCAAGATTGTTTACTGGCACAGTGGTCAATGGCACT----------------------
----
-CTCACTAATGCCACT---------
ATCACTAATGGCACTATCACTCTCCCCTGTCGAATAAAACAAATTGTAAACATGTGGCAAGAAGTAGGACGAG
CAATGTATGCCGCTCCTGTTGCAGGAAGCATTAACTGTACATCAAAT---
ATTACAGGGCTACTATTGACAAGAGATGGGGGT--

AAGGATAATCAGACTAAT---
AAGAATGAGATCTTTAGACCTGGAGGAGGAAATATGAAAGATAATTGGAGAAGTGAATTATATAAATATAAAG
TGGTAGAAATTGAACCACTA---GGAGTAGCACCCACCAGGGCAAAGAGGCAAGTGGTG--------------
-------------------------AAGAGAGAAAGAAGAGCA---GTGGGA---ATAGGAGCT---------
TTGTTCCTTGGGTTCTTGGGAGCA---GCAGGAAGCACTATG---GGCGCAGCGTCA---
ATGACGCTGACGGTACAA---GCTAGACTA---TTATTG---TCTGGTATAGTGCAACAGCAGAAC---
AATCTG---CTGAGA---GCTATTGAGGCACAACAGCATCTGTTGCAACTCACAGTCTGGGGCATT
>KJ671537
TTCTATTGCAATACATCAGGACTGTTTAATGACTCAAAATTCAATGACTCAAAATTC----------------
----
AATGGCAATTTCTCTTCC---------
AATGACACTAGCACTATCACTATCCCATGTAGAATAAAACAAGTCATAAACATGTGGCAGGAAGTAGGACGAG
CAATGTATGCTGCTCCTATTGCAGGAAACATTATCTGTAACTCAAAT---
ATTACAGGTCTACTATTGACAAGAGATGGTGGT--

-
CAAACAAATAATACAAACGAGACCTTCAGACCTGGGGGAGGAAATATGAAAGACAATTGGAGAAGTAAGTTAT
ATAAATATAAAGTAGTAGAAGTTGAACCACTA---GGAGTAGCACCCACCAAGGCACAAAGACGAGTGGTA--
-------------------------------------AAGAGAGACAAAAGAGCA---GTGGGA---
ATGGGAGCT---------TTGTTCATTGGGTTCTTGAGCGCA---GCAGGAAGCACTATG---
GGCGCAGCGTCA---GTGACGCTGACGGTACAG---GCCAGACAA---TTATTA---
TCTGGTATAGTGCAACAGCAGAAC---AACTTG---CTGAGA---
GCTATTGAGGCGCAACAGCATCTGTTGCAACTCACAGTCTGGGGCATT
>MK341078
TTTTATTGTAATACTTCACAACTGTTTAATAGTACTTGGATCAATAGTATTTGG-------------------
----

AGCAATGACACAATCATACTTCCATGCAGAATAAGACAAATTATAAACTTGTGGCAGGAAGTAGGAAGAGCAA
TGTATGCCCCTCCCATTAGTGGACCTATTAAATGTTCATCAAAT---
ATTACAGGTCTACTACTAACAAGGGATGGGGGTCAGAGTAATAAC----------------------------
----

GAGACTGAGACCTTCAGACCTGGGGGAGGGAATATGAAAGACAATTGGAGAAGTGAATTATATAAATATAAAG
TAGTAGAACTTAAACCACTA---GGAGTAGCACCCACCAAGGCAAAAAGACAAGTGATA--------------
-------------------------GGGAGACAAAAAAGAGGG------------CTAGGCGCT---------
GTGTTCCTTGGGTTCTTGGGAGCA---GCAGGAAGCACTATG---GGCGCAGCGTCG---
CTGACGCTGACGGTACAA---GCCAGACTA---TTATTG---TCTGGTATAGTGCAACAGCAGAAC---
AATCTG---CTGAGG---GCCATTGAGATGCAACAACATCTGTTGAAACTCACAGTCTGGGGCATT
>GU595149
TTCTATTGTAATACAGCACAACTGTTTAATAGTACTTGGACGAATGGTACTGAACAGACAAATAGCACTATA-
----

GGAAATGACACAATCACACTCCCATGCAAAATAAAACAAATTATAAACTTGTGGCAGGAAGTAGGAAAAGCAA
TGTATGCCCCTCCCATCAGTGGACTAATTAGATGTTCATCAAAT---
ATTACAGGTCTACTACTGACAAGGGATGGGGGTCAGAATAATGAT----------------------------
----

ACTAAGAATGAGACCTTCAGACCTGGGGGAGGGGATATGAAAGACAATTGGAGAAGTGAATTATATAAATATA
AAGTAGTAGAAATTAAACCACTA---GGAGTAGCACCCACCAAGGCAAAAAGACAAGTGGTG-----------
----------------------------GGGAGACAAAAAAGAGGA------------CTAGGAGCT------
---GTGTTCCTTGGGTTCTTGGGAGCA---GCAGGAAGCACTATG---GGCGCAGCGTCA---

ATAACGCTGACGGTACAG---GCCAGACTA---TTATTG---TCTGGTATAGTGCAACAGCAGAAC---
AATCTG---CTGAGG---GCTATTGAGGCGCAACAGCATCTGTTGCAACTCACAGTCTGGGGCATC
>KU992930
TTCTATTGTAATACTTCAAACCTGTTTAATAGTACATACTACTCTAATGGTACATACTCTAATGGTACAGTAG
ACAAT--

TCAAGCTCATCCATCACGATTCAATGCAGAATAAAGCAAATTATAAATATGTGGCAGGAGGTAGGACGAGCAA
TATATGCCCCTCCCATTGAAGGAAATATAACATGTAGATCAAAT---
ATCACAGGACTACTATTAGTACGTGATGGAGGAGTTGGAAATGATACAGAG---------------------
----

AATAATACAGAGATATTTAGACCTGGAGGAGGAGATATGAAGGACAATTGGAGAAGTGAATTATATAAATATA
AAGTGGTAGAAATTAAGCCATTG---GGAGCAGCACCCACTAAAGCAAAAAGGAGAGTGGTG----------
---------------------------GAGAGAGAAAAAAGAGCA---GTGGGA---ATAGGAGCT------
---GTGTTCCTTGGGTTCTTGGGAGCA---GCAGGAAGCACTATG---GGCGCGGCGTCA---
ATAACGCTGACGGTACAA---GCCAGACAA---TTGTTG---TCTGGTATAGTGCAACAGCAAAGC---
AATTTG---CTGAGA---GCTATAGAGGCGCAACAGCATATGTTGCAACTCACGGTCTGGGGCATT
>AY037271
TTCTACTGCAATACATCAAAACTGTTTAATAACAACACAGAA------------------------------
----

TTCAATAACACTATTATTCTCCCATGTCGAATAAAACAGATGGTAAACATGTGGCAGGAAGTGGGACGAGCAA
TGTATGCCGCTCCCATTGCAGGAAACATTAACTGTAGCTCAAAT---
ATTACAGGTTTACTATTGACAAGAGATGGTGGTATAAATATAACA---------------------------
----

AGTGGTCAGGAGACCTTCAGACCTGGGGGAGGAAATATGAAAGACAATTGGAGAAGTGAATTATATAAATATA
AAGTAGTAGAAATTGAACCATTA---GGAGTAGCACCCACCAAGGCAAAGAGAAGAGTGGTG----------
---------------------------CAGAGAGAAAAAAGAGCA---GTGGGA---ATGGGAGCT------
---TTGTTCCTTGGGTTCTTGGGAGCA---GCAGGAAGCACTATG---GGCGCGGCGTCA---
ATAACGCTGACGGTACAG---GCCAGACAA---TTATTG---TCTGGAATAGTGCAACAGCAGAAC---
AATTTG---CTAAGG---GCTATTGAAGCGCAACAGCATCTGTTGCAGCTCACAGTCTGGGGCATT
>MF372649
TTCTATTGCAATACATCAGCACTGTTTAATAGCACTTGGAATTCCACTTCAATTGCAAATTTCACAGAG----
----

GAAAATGGCACTATTACTATCCAATGCAGAATAAAGCAATTTATAAATATGTGGCAGAGAGTAGGAAGAGCAA
TGTATGCCCCTCCCATCAAAGGAAAGATACAATGTAACTCAACC---
ATAACAGGACTCATATTAACAAGAGATGGAGGTAATGGTAATAGC---------------------------
----

AGTGCAAATGAGACATTCAGACCTGGAGGAGGAGACATGAGGGACAATTGGAGAAGTGAATTATACAAGTACA
AAGTAGTAAAAATTGAACCATTA---GGTGTAGCACCCACCAAGGCAAAAAGAAGAGTGGTG----------
---------------------------GAGAGAGAAAAAAGAGCG---GTTGGA---CTGGGAGCT------
---GTTTTCCTTGGGTTCTTAGGAGCA---GCAGGAAGCACTATG---GGCGCGGCGTCA---
ATAACGCTGACGGTACAA---GCCAGACAA---TTATTG---TCTGGTATAGTGCAACAGCAGAGC---
AATCTG---CTGAAG---GCTATAGAGGCTCAACAACAGCTGTTGAGACTCACAGTCTGGGGCATT
>MH666154
TTCTATTGCAATACATCAGGACTGTTTGATAGTACTTGGAATACATCAAGACTGTCTAATGGTACTTGGAATG
AT---
-ACATCAAGAGAATCTAAT------
AACTCAGTCAATAACATAACTCTTCAATGCAGAATAAGGCAAATTGTAAGAATGTGGCAGAGAGTAGGGCAAG
CAATGCATGCCCCTCCCATCCGAGGAGAAATAAGATGTGTATCAAAC---
ATTACTGGACTACTATTAACAAGGGATGGAGGGACGACTAATGAGACAGAG---------------------
----

ACAGAGGAGATCTTCAGGCCTGCAGGAGGAGATATGAGAGACAATTGGAGAAGTGAATTATATAAGTATAAAG
TAGTAAAAATTGAACCACTA---GGTGTAGCACCCAACTATGGAAGAAGAAGAGTGGTG-------------
------------------------GAGAGAGAAAAAAGAGCA---GTTGGA---CTGGGAGCT--------
GTCTTCCTTGGGTTCCTAGGAGCA---GCAGGAAGCACTATG---GGCGCGGCGTCA---

ATAACGCTGACGGTACAG---GCCAGGCAG---TTATTG---TCTGGTATAGTGCAACAGCAAAGC---
AATTTG---CTGAAG---GCTATAGAGGCTCAACAACAGCTGTTGAGACTCACAGTCTGGGGCATT
>MH666157
TTCTACTGCAATACATCAGAACTGTTTAATAGTACTTGGAATGCCAATGGTAGCAATGATAGCCTAAAC----
----

CACACTGGCAACATAACTCTCAATTGCAGAATAAAGCAAATTATAAATATGTGGCAGAGAGTAGGGCAAGCAA
TTTATGCCCCTCCCATCCGAGGAGTAATAAGGTGTGAATCAAAC---
ATTACTGGAATATTATTAACAAGAGATGGAGGGAAAAGTAATGCT----------------------------
----

ACAAATGAGACCTTCAGGCCTGCAGGAGGAGATATGAGAGACAATTGGAGAAGTGAATTATATAAGTATAAAG
TAGTAAAAATTGAACCACTA---GGTGTAGCACCCACCCCTGCAAAAAGAAGAGTAGTG--------------
-------------------------GGGAGAGAAAAAAGAGCA---GTTGGA---CTGGGAGCT---------
GTCTTCCTTGGGTTCCTGGGAGCA---GCAGGAAGCACTATG---GGCGCGGCGTCA---
ATAACGCTGACGGTACAG---GCCAGACAA---TTATTG---TCTGGTATAGTGCAACAGCAGAGC---
AATTTG---CTGAAG---GCTATAGAGGCTCAACAGCATATGTTGAGACTCACAGTTTGGGGCATT
>MH666161
TTCTATTGCAATACATCAGGACTGTTTAATAGTACTTGGAACAATACTTGGAAAGATACATCAGAATAT----
----

AATGGATCTACCATAACTCTCCAATGCAGAATAAGGCAAATTGTAAGGATGTGGCAGAGAGTAGGGCAAGCAA
TGTATGCCCCTCCCATCCAAGGAGAAATAAGATGTGCATCAAAC---
ATTACTGGACTACTATTAACAAGAGATGGAGGGGATAATAGTGGG----------------------------
----

ACAAATGAGACCTTCAGGCCTGAGGGAGGAGATATGAAAGACAATTGGAGAAGTAAATTATATAAATATAAAG
TAGTGAAAATTGAACCACTA---GGTGTAGCACCCACCTATGGAAGAAGGAGAGTGGTG--------------
-------------------------GGGAGAGAAAAAAGAGCA---GTTGGA---CTGGGAGCT---------
GTCTTCTTTGGGTTCCTAGGAGCA---GCAGGAAGCACTATG---GGCGCGGCGTCA---
ATAACGCTGACGGTACAG---GCCAGACAA---TTATTG---TCTGGTATAGTGCAACAGCAAAGC---
AATTTG---CTGAAG---GCTATAGAGGCTCAACAACAACTGTTGAGACTCACAGTCTGGGGCATT
>MH666156
TTCTATTGTAATACATCAGGACTGTTTAATAGTACTTGGAGCAATACGAGCAATACATCAGGACCTAATACC-
----

TCAGACGCTGACATAACTCTCCAATGCAGAATAAGGCAAATTATAAGGATGTGGCAGAGAGTAGGGCAGGCAA
TATATGCCCCTCCCATCCAAGGAGAAATAAGGTGTGTATCAAAC---
ATTACTGGACTACTATTAACAAGGGATGGAGGGACTGATAATAAT----------------------------
----

ACAGATGAGACCTTCAGGCCTGCAGGAGGAGATATGAGAGATAATTGGAGAAGTGAATTATATAAATATAAAG
TAGTAAAAATTGAACCACTA---GGTGTAGCACCCACCCCTGCAAGAAGAAGAGTGGTG--------------
-------------------------GGGAGAGAAAAAAGAGCA---GTT------CTGGGAGCT---------
GTCTTCCTTGGGTTCCTAGGAGCA---GCAGGAAGCACTATG---GGCGCGGCGTCA---
ATAACGCTGACGGTACAG---GCCAGACAA---TTATTG---TCTGGTATAGTGCAACAGCAAAGT---
AATTTG---CTGAAG---GCTATAGAGGCTCAACAACAGCTGTTGAGACTCACAGTCTGGGGCATT
>MH666155
TTCTATTGCAATACATCAGGACTGTTTAATAGTACTTGGAATAGTACTTGGACCAATACATCAGGCATTAAT-
----

AGCTCAGAACCTATAATTCTCCAATGCAGGATAAGGCAAATTATAAGAATGTGGCAGAGAGTAGGGCAAGCAA
TGTATGCCCCTCCCATCCGAGGAGAAATAAGGTGTGAATCAAAC---
ATTACTGGACTACTATTAACAAGGGATGGAGGGAATGATAATACA----------------------------
----

AATGAGACCTTCAGGCCTGCAGGAGGAGATATGAGAGACAATTGGAGAAGTGAATTGTATAAGTATAAAGTAG
TAAAAATTGAACCACTA---GGTGTAGCACCCGGCCAGGCAAGGAGAAGAGTAGTG-----------------
----------------------GGGAGAGAAAAAAGAGCA---GTTGGA---ATAGGAGCT---------
GTCTTCCTTGGGTTCCTAGGAGCA---GCAGGAAGCACTATG---GGCGCGGCGTCA---

ATAACGCTGACGGTACAG---GCCAGGCAG---TTATTG---TCTGGGATAGTGCAACAGCAAAGC---
AATTTG---CTGAAG---GCTATAGAGGCTCAACAACATATGTTGAGACTCACAGTCTGGGGCATT
>KF850149
TTCTATTGTAATACAACAAAATTGTTTAATAGTACTTGGAAGGGAAATGAA---------------------
----
-ACCATGGAGAGGGAT---------
AATAAGACTGAGATCATCATACTTCCATGCAGGATAAAGCAAATTATAAACATGTGGCAGGGAGCAGGACAAG
CAATGTATGCTCCTCCCATCAAGGGATTAATTAATTGTACATCAAAT---
ATTACAGGAATACTATTAACAAGAGATGGTGGT---------------------------------------
----

CATAATGGTACAAATGAGACCTTCAGACCTGGAGGAGGAAATATAAAGGACAATTGGAGAAGTGAATTATATA
AATATAAAGTAGTAGAACTTGAACCACTA---GGGGTAGCACCCACCAGGGCAAAGAGAAGAGTGGTG-----
----------------------------------GAAAGAGAAAAAAGAGCA---GTGGGA---
ATAGGAGCT---------ATGATCTTTGGGTTCTTAGGAGCA---GCAGGAAGCACTATG---
GGCGCGGCGTCA---ATAACGCTGACGGTACAG---GCCAGACAA---TTATTG---
TCTGGTATAGTGCAACAGCAAAGC---AATTTG---CTGAGG---
GCTATAGAGGCGCAACAGCGTATGTTGCAACTCACAGTCTGGGGCATT
>MG518476
TTCTATTGTAATACAACAAAATTGTTTAATAGTACTTGGAAGGGAAATGAAACCATGGAGAGGAATAAT----
----

AACACTGACACTATCATACTTCCATGCAGGATAAAGCAAATTATAAACCTGTGGCAGGGAGTAGGACAAGCAA
TGTATGCTCCTCCCATCAGGGGAGCAATTAATTGTGTATCAAAT---
ATTACAGGAATACTATTAACAAGAGATGGTGGTCATAATAGTACA---------------------------
----

AATGAGACCTTCAGACCTGGAGGAGGAAATATAAAGGACAATTGGAGAAGTGAATTATATAAATATAAAGTAG
TAGAGATTGAACCACTA---GGAATAGCACCCACCAGGGCAAAGAGAAGAGTGGTG-----------------
----------------------GAGAGAGAAAAAAGAGCA---GTGGGA---ATAGGAGCT---------
ATGATCTTTGGGTTCTTAGGAGCA---GCCGGAAGCACTATG---GGCGCGGCGTCA---
CTAACGCTGACGGTACAG---GCCAGACAA---TTATTG---TCTGGTATAGTGCAACAGCAAAGC---
AATTTG---CTGAGG---GCTATAGAGGCGCAACAGCATATGTTGCAACTCACAGTCTGGGGCATT
>AY371145
TTCTATTGCAATACATCAGGACTATTTAATAGCACTTGGGAAATGAACACAACCAGCTTAGGG---------
----

TTAAATGATACTATAATTCTCCAGTGCAGAATAAAGCAAATTGTAAATATGTGGCAGAGAGTAGGACAAGCAA
TGTACGCCCCTCCCATCCCAGGAAAAATAAGGTGTCAGTCAAAC---
ATTACAGGACTACTATTGACAAGAGATGGTGGGACTAGTAACAATACAGAG---------------------
----

ACTAGTAACAGTACAGAGACTTTCAGACCTGGAGGAGGAGATATGAGAGATAATTGGAGAAGTGAATTATATA
AATATAAAGTAGTAAAAATTGAACCACTA---GGAGTAGCACCTACCAGGGCAAAGAGGAGGGTGGTG-----
----------------------------------GAGAGAGAAAAAAGAGCA---GTTGGA---
CTGGGAGCT---------GTCTTCCTTGGGTTCTTAGGAGCA---GCAGGAAGCACTATG---
GGCGCGGCGTCA---ATAACGCTGACGGTACAG---GCCAGACAA---TTATTG---
TCTGGCATAGTGCAACAGCAAAGC---AATTTG---CTGAGG---
GCTATAGAGGCTCAACAACATATGTTGAGACTCACGGTCTGGGGCATT
>KP718921
TTCTATTGTAATACAACAGAACTATTTAATAGCACTTGGGAGAGTAGCAATAGCACTAACAGCTCAAGTGCA-
----

ACAAATGGAACTATAACTCTCAAATGCAGGATAAAGCAAATTATAAATATGTGGCAGAGAGTAGGACAAGCAA
TGTATGCCCCTCCCATCCAAGGAATAATAAGGTGTGAATCAAAC---
ATTACAGGACTACTATTAACAAGAGATGGAGGAGATACTAATAGT---------------------------
----

AATAATAGTGAGATCTTCAGGCCTGGAGGAGGAGATATGAGGGACAATTGGAGAAGTGAATTATATAAGTATA
AAGTAGTAAAAATTGAACCAATA---GGTATAGCACCCACCCATGCAAAAAGAAGAGTGGTG----------

----------------------------GAGAGAGAAAAAAGAGCA---GTTGGACTACTGGGAGCT------
---GCCTTCCTTGGGTTCTTAGGAGCA---GCAGGAAGCACTATG---GGCGCAGCGTCA---
ATAACGCTGACGGTACAG---GCCAGACAA---TTATTG---TCTGGCATAGTGCAACAGCAAAGC---
AATTTG---CTGAGG---GCTATAGAGGCTCAACAACAGCTGTTGAAACTCACAGTCTGGGGCATT
>KP718922
TTCTATTGCCATACATCAAACCTATTTAATAGCACTTGGAAAGAGAACACAACAGAGTCAAGTGGCAATATA-
----

ACAAGTGGCAATATAACTCTCCAGTGCAGAATAAAGCAAATTGTAAATATGTGGCAGAGAGCAGGAAAAGCAA
TGTATGCCCCTCCCATTCCAGGAGTGATAGAGTGTAGGTCAAAC---
ATTACAGGGCTCCTATTACAAAGAGATGGAGGGAAAAATAATACAAATAAT----------------------
----

ACCTTATGGCCTGGAGGAGGAGATATGAGGGATAATTGGAGAAGTGAGTTATACAAATATAAAGTAGTGAAAA
TTGAACCACTA---GGAGTAGCACCCACCAGGGCAAGGAGAAGGGTGGTG-----------------------
----------------CAGAGAGAAAAAAGAGCA---GTTGGA---ATAGGAGCT---------
GTCTTCCTTGGGTTCTTAGGAACA---GCAGGAAGCACTATG---GGCGCAGCGGCA---
GTAACGCTGACGGTACAG---GCCAGACAA---TTATTG---TCTGGTATAGTGCAACAGCAAAGC---
AATTTG---CTGAAG---GCTATAGAGGCTCAACAACAACTGTTGAGACTCACGGTCTGGGGCATT
>JF683748
TTCTATTGTAACACATCAGGCCTGTTTAATAGCACTTGGACTGCCAATGGTGGCAGGCTGCGGGAAGATAGTG
CA---

AAGTCAAACAGCACTATAACTCTCCAATGTAGAATAAAGCAAATTATAAATATGTGGCAGAGAGCAGGACAAG
CAATATATGCCCCTCCCATCCAAGGAGTAATAAGGTGTGAATCAAAC---
ATTACAGGGCTAATATTAACAAGAGATGGTGGGGGGAGTAATGAT----------------------------
----

ACAGCGGAAACCTTCAGACCTGGAGGAGGAGATATGAGGGACAATTGGAGAAGTGAATTATATAAGTATAAAG
TAGTAAAAATTGAACCATTA---GGAGTAGCACCCACCAAGGCAAGGAGAAGAGTGGTG--------------
-------------------------GAGAGAGAAAAAAGAGCA---GTTGGA---CTGGGAGCT---------
GTCTTCATTGGGTTCTTGGGAGCA---GCAGGAAGCACTATG---GGCGCGGCGTCA---
GTGACGCTGACGGTACAG---GCCAGGCAA---TTATTG---TCTGGCATAGTGCAACAGCAAAGC---
AATTTG---CTGAAG---GCTATAGAGGCTCAACAGCAGCTGTTGAGACTCACAGTTTGGGGCATT
>MK086114
TTCTATTGCAATACAACAAACCTGTTTAACAGCATTTGGAATTACAATAGCACTGGGAATCACAGTGACAGCA
TGACAAATAAC--

ACGGATGGCAATATAATTCTCCAGTGCAGAATAAAGCAAATTATAAATATGAGGCAGAGAGCAGGAAAAGCAA
TATATGCCCCTCCCATTCGAGGAAGAATACAGTGTGAGTCAAAC---
ATTACAGGAATACTATTGACAAGAGATGGTGGGAATAATGCTAGT----------------------------
----

AATTCAAATGAGACTTTTAGACCTGGAGGAGGAGATATGAGAGACAATTGGAGAAGTGAATTATATAAATATA
AAGTAGTAAAAATTGAACCACTA---GGAGTAGCACCCACCAAGGCAAGAAGGAGAGTGGTG-----------
----------------------------GAGAGAGAAAAAAGAGCA---TTTGGA---TTGGGAGCT------
---ATGTTCCTTGGGTTCTTGGGAACA---GCAGGAAGCACTATG---GGCGCAGCGTCG---
CTAACGCTGACGGTACAG---GCCAGACAA---TTATTG---TCTGGCATAGTGCAACAGCAAAGC---
AATTTG---CTGAGA---GCTATAGAGGCTCAACAGAATATGTTGAGACTCACAGTCTGGGGCATT
>MF373389
TTCTATTGCAATACATCAGGCCTGTTTAATAGTACATACACTAATGGTACATTTAATTATAATGGTACATCTA
ATTAT--
-------------------ACA---
GAAAGTAATGTGACCATCACACTCCCATGCAGAATAAAACAAATTATAAATATGTGGCAGAAGGTGGGAAGAG
CAATGTATGCTCCTCCCATTGCAGGAAACATAACATGTAAGTCAAAT---
ATCACAGGACTACTGTTAGTACGTGATGGAGGATATGCAAAGGATGCA-------------------------
----

AATGATACAGAGATATTCAGGCCTGGAGGAGGAAATATGAAGGACAATTGGAGAAGTGAATTATATAAATATA
AAGTGGTAGAAATTAAGCCATTG---GGACTAGCACCCACTGAAGCGAAAAGGAGAGTGGTG-----------

----------------------------GAGAGAGAAAGAAGAGCA---GTAGGA---ATAGGAGCT------
---GTGTTCCTTGGGTTCTTGGGAGTA---GCAGGAAGCACTATG---GGCGCGGCGTCA---
ATGGCGCTGACGGTACAG---GCCAGACAA---TTGCTG---TCTGGTATAGTGCAACAGCAAAAC---
AATTTG---CTGAGG---GCTATAGAGGCGCAACAGCATATGTTGCAACTCACGGTCTGGGGCATT
>KX159285
TTCTACTGCGATACAACAAAATTGTTTAATGAGAGACAGAAAATGGATCACGGAAGA----------------
----

GCTATAATGGCTACTATCTTACATCCATGTAGGATAAAACAAATTGTAAACATCTGGCAGCGAGTGGGACAAG
CAACGTGCTCCTCCCATGCTGCAGCAAACATATCATGTCAGTCAAAT---
ATCACAGGACTACTATTAGTACGTGATGGAGGAAATGATACAGAG----------------------------
----

GAGACAGAGACATTCAGGCCTGGAGGAGGAGATATAAAGGACAATTGGAGAAGTGAATTATATAAATATAAAG
TAGTACAAATTGAACCATTA---GGAATAGCACCCACCAAAGCAAAGAGAAGAGTGGTA--------------
-------------------------GACAGAGAAAAAAGAGCA---GTGGGA---ATAGGAGCT---------
ATGATCTTTGGGTTCTTAGGAGCA---GCAGGAAGCACTATG---GGCGCGGCGTCA---
ATAACGCTGACGGTACAG---GCCAGACAA---TTATTG---TCTGGTATAGTGCAACAGCAAAAC---
AATTTG---CTGAGA---GCTATAGAGGCGCAACAACATCTGTTGCAACTCACAGTCTGGGGCATT
>MN452901
TTCTATTGTAATACATCAAGCCTGTTTAATGGTACATACAATAGCAGTAGGCCAAACAGTACT----------
----

TCAAACTCAATCATCATAATCCCATGCAGAATAAAGCAAATTATAAATATGTGGCAAGGAGTAGGACAAGCAA
TGTACGCTCCTCCCATCAGTGGAACAATTAATTGTGTATCAAAT---
ATTACAGGAATCTTATTGACAAGGGATGGTGGTAATGATACCGTGAGTAGG----------------------
----

GAAAATGAGACCTTCAGACCTGGAGGAGGCAATATAAAGGACAATTGGAGAAATGAATTATATAAATATAAAG
TAGTAGAAATTGAACCACTA---GGAATAGCACCCACCAGGGCAAAGAGAAGAGTGGTG--------------
-------------------------GAGAGAGAAAAAAGAGCA---GTGGGA---ATAGGAGCT---------
ATGATCTTTGGGTTCTTAGGAGCA---GCAGGAAGCACTATG---GGCGCAGCGTCA---
ATAACGCTGACGGTACAG---GCCAGACAA---TTATTG---ACTGGTATAGTGCAACAGCAAAGC---
AATTTG---CTGAGG---GCTATAGAGGCGCAACATCATCTGTTGCAACTCACAGTCTGGGGCATT
>MH682099
TTCTATTGCAATACATCAGGCCTGTTTAATAAGACATACTCGTCTAATAGTACATTTAATAATACAGAA----
----

GATAATTCAACCATCACAATCCCATGCTACATAAAGCAAATTATAAATATGTGGCAGGAGGTGGGACGAGCAA
TGTATGCTCCTCCCATTGCAGGAAACATAACATGTAGGTCAAAT---
ATCACAGGACTACTATTAGTACGTGATGGAGGACCAGAGTCAAATTGTACAAAG-------------------
---ACA---
GGATCAGAGTCAAATTGTACAGAGACATTCAGGCCTGGAGGAGGAGATATGAGGGACAATTGGAGAAGTGAAT
TATATAAATATAAAGTGGTAGAAGTTAAGCCATTG---
GGAGTAGCACCCACTGAAGCAAAAAGGAGAGTGGTG-------------------------------------
--GAGAGAGAAAAAAGAGCA---GTGGGA---ATAGGAGCT---------
GTGTTCCTTGGGTTCTTGGGAGTA---GCAGGAAGCACTATG---GGCGCGGCGTCA---
ATGACGCTGACGGTACAG---GCCAGACAA---TTGCTG---TCTGGTATAGTGCAACAGCAAAGC---
AATTTG---CTGAGG---GCTATAGAGGCGCAACAGCATATGTTGCAACTCACAGTCTGGGGCATT
>AY371167
TTCTATTGCAATACATCAGGCCTGTTTAATAGCACTTGGAATGACACTGGCAGCATGCCAGAGTCAAATAAC-
----

ACGAATGGCAATATAACTCTCCAGTGCAGAATAAAGCAGATTATAAATATGTGGCAGAGAACAGGACAAGCAA
TGTATGCCCCTCCCATCCCAGGAACAATAAGGTGTGAGTCAAAC---
ATTACAGGACTACTACTGACAAGAGATGGTGGGACTCCTAACAGT----------------------------
----

ACAAATGAAACTTTCAGACCTGGAGGAGGAGATATGAGAGACAATTGGAGAAGTGAATTATATAAATATAAAG
TAGTAAAACTTGAACCACTA---GGAGTAGCACCCACCAGGGCAAAGAGAAGAGTGGTG--------------

-------------------------CAGAGAGAAAAAAGAGCG---GTTGGA---TTGGGAGCT---------
GTCTTCCTTGGGTTCTTAGGAGCA---GCAGGAAGCACTATG---GGCGCGGCGTCA---
ATAACGCTGACGGTACAG---GCCAGACAA---TTATTG---TCTGGCATAGTGCAACAGCAAAGC---
AATTTG---CTGAAG---GCTATAGAGGCTCAACAGCATCTGTTGAGACTCACAGTCTGGGGCATT
>FJ388953
TTCTATTGTAACACATCAGGCCTGTTTAATAGCACTTGGCATGCCAATGACAGTTTGCAAGAGTCAAATGACA
CA---

ACAATAAAGAAGAATATAACTCTCCAATGCAGAATAAAGCAGATTATAAATCTGTGGCAGAGAGCAGGACAAG
CAATGTATGCCCCTCCCATCCCAGGAGTAATAAAGTGTGTATCAAAC---
ATAACAGGACTAATATTAACAAGAGATGGTGGAGGTAATAACACAGCT-------------------------
----

CGTGATAATGAAACCTTCAGACCTGGAGGAGGAGATATGAGGGACAATTGGAGAAGTGAATTATATAAGTATA
AAGTAGTAAAAATTGAGCCACTA---GGAGTAGCACCCACCAGGGCAAGGAGGAGAGTGGTG-----------
----------------------------GAGAGAGAAAAAAGAGCA---GTTGGA---ATAGGAGCT------
---GTCTTCATTGGGTTCTTGGGAGCA---GCAGGAAGCACTATG---GGCGCGGCGTCA---
GTGACGCTGACGGTACAG---GCCAGGCAA---TTATTG---TCTGGCATAGTGCAACAGCAAAGC---
AATTTG---CTGAGG---GCTATAGAGGCTCAACAACATCTGTTGAAACTCACAGTCTGGGGCATT
>KR017779
TTCTATTGCAATACATCAGGACTGTTTAATAGCACTTGGAAGATTAATAGCACTTGGAATAGC----------
----

ACTAATGGCACTATAACTCTCCTGTGCAGAATAAAGCAAATTGTAAACATGTGGCAGAGAGCAGGACAAGCAA
TGTATGCCCCTCCCATCCCAGGAGTAATAAGATGTGAATCAAAC---
ATTACAGGGATACTATTGACAAGAGATGGCGGGGAGGATTCTAAT----------------------------
----

AAGACTAATGAAACAGAAATTTTCAGACCTGGAGGAGGAGATATGAGAGACAATTGGAGAAGTGAATTATATA
AATATAAAGTAGTAAAAATTGAACCACTA---GGAGTAGCACCCACCGAGGCAAAGAGAAGGGTGGTG-----
----------------------------------GAGAGAGAAAAAAGAGCA---GTTGGA---
CTGGGAGCT---------GTTTTCCTTGGGTTCTTAGGAGCA---GCAGGAAGCACTATG---
GGCGCGGCGTCA---ATAACGCTGACGGTACAG---GCCAGACAA---TTATTG---
TCTGGTATAGTGCAACAGCAAAGC---AATTTG---CTGAGG---
GCCATAGAAGCTCAACAGCATGTGTTGAGACTCACAGTCTGGGGCATT
>U76035
TTCTATTGCAATACATCAGGCCTGTTTAATAGCACTTGGAAAAATAGCACCAGCATAAATGACACGGTG----
----

TCAAATGGCACTATAACTCTCCCATGCAGAATAAAGCAAATTGTAAATATGTGGCAAAGAGTAGGACAAGCAA
TGTATGCCCCTCCCATCAAAGGAGTAATAAAGTGTGAATCAAAC---
ATTACAGGAATACTATTAACAAGAGATGGTGTGGGTAATAACACA----------------------------
----

GCAAATGAGACCTTCAGACCTGGAGGAGGAGATATGAGGGACAATTGGAGAAGTGAATTATATAAGTATAAAG
TAGTAAAGATTGAACCACTA---GGAGTAGCACCCACCAAGGCAAAGAGAAGAGTGGTG--------------
-------------------------GCGAGAGAAAAAAGAGCA---ATTGGA---ATGGGAGCT---------
TTCTTCCTTGGGTTCTTAGGAGCA---GCAGGAAGCACTATG---GGCGCGGCGTCA---
ATAACGCTGACGGTACAT---GTCAGACAA---TTATTG---TCTGGTATAGTGCAGCAGCAGAGC---
AATCTG---CTGAGG---GCTATAGAGGCTCAACAGCATCTGTTGAAACTCACAGTCTGGGGCATT
>MH327751
TTCTATTGCAATACAACAAAATTGTTTAATTGCACAGACAATAATAACACATACACA----------------
----

TGTAATGACACTATCGTACTTCCATGCAAGATAAAGCAAATTATAAACATGTGGCAAGGAGTAGGACAAGCAA
TGTATGCTCCTCCCGTCAGGGGAATAATTAATTGTGTATCAAAC---
ATTACAGGAATACTATTGACAAGGGATGGTGGGACAAATAGCACT----------------------------
----

AATAGAAATGAGACCTTCAGACCTCAAGGAGGAAATATAAAGGACAATTGGAGAAGTGAATTATATAAATATA

AAGTAGTACAAATTGAACCACTA---GGAGTGGCACCCACTAGGGCAAGGCGAAGAGTGGTG-----------
----------------------------GAGAGAGAAAAAAGAGCA---GTGGGA---ATAGGAGCT------
---ATGATCTTTGGGTTCTTAGGAGCA---GCAGGAAGCACTATG---GGCGCGGCGTCA---
ATAACGCTGACGGTACAG---GCCAGACAA---TTATTG---TCTGGTATAGTGCAACAGCAAAGC---
AATTTG---CTGAGG---GCTATAGAGGCGCAGCAGCATCTGTTGCAACTCACAGTCTGGGGCATT
>KP411826
TTCTACTGTAACACAACAGAACTGTTTAACAGTACTTGGAATAGCACTGAAGAGTCAAGTACCCCTAAA----
----

GGAAATGACACAATTACACTCCAATGTAGAATAAAACAAATTATAAACATGTGGCAGGAAGTAGGAAAAGCAA
TGTATGCCCCTCCCATCAGAGGACAAATTAATTGTTCATCAAAC---
ATCACAGGGCTACTGTTAACAAGAGATGGCGATCAGAACGACAGT----------------------------
----

GAGACTTTCAGACCTGGAGGAGGAAATATGAAAGACAATTGGAGAAGTGAATTATATAAATATAAAGTAATAA
AAATTGAACCACTA---GGAATAGCACCCACCAGGGCAAAGAGAAGAGTGGTG--------------------
-------------------GAGAGAGAAAAAAGAGCA---GTGGGA---ATAGGAGCT---------
ATGATCTTTGGGTTCTTGGGAGCA---GCAAGAAGCACTATG---GGCGCAGCGTCA---
CTGACGCTGACGGTACAG---GCCAGACAA---TTATTG---TCTGGTATAGTGCAACAGCAGAAT---
AATTTG---CTGAAG---GCTATTGAGGCGCAACAGCATCTGTTGCAACTCACAGTCTGGGGCATT
>AY358069
TTCTATTGCAACACAACAGATCTGTTCAACACAACAAAACTAAATGAAACCACGTTAAATAATAGC-------
----

ACTAATAGCACTATCATACTTCCATGCAGAATAAAGCAAATTATAAACATGTGGCAGGGAGTAGGACAAGCAA
TGTATGCTCCTCCCATCAGAGGAAAGATTAATTGTGTATCAAAT---
ATTACAGGAATACTATTGACAAGAGATGGTGGTAATAATAATCAGACTAATAAT-------------------
----

CAGACTAACAAGACCGAGACCTTCAGACCTGGAGGAGGAAATATGAAGGACAATTGGAGAAATGAATTATATA
AATATAAAGTAGTACAAATTGAACCACTA---GGAATAGCACCCACCAAGGCAAAAAGAAGAGTGGTG-----
----------------------------------GAGAGAGAAAAAAGAGCA---GTGGGA---
ATAGGAGCT---------ATGATCTTTGGGTTCTTAGGAGCA---GCAGGAAGCACTATG---
GGCGCGGCGTCA---ATAACGCTGACGGTACAG---GCCAGACAA---TTATTG---
TCTGGTATAGTGCAACAGCAAAGC---AATTTG---CTGAGG---
GCTATAGAGGCGCAGCAGCATCTGTTGCAACTCACAGTCTGGGGCATT
>MG989522
TTCTATTGCAATACATCACGACTGTTTAATAATACTTGTGCAAAAAATGTAACCCAGGAAGGG----------
----

TGTAATGAAACTATCATACTCCCATGCAGGATAAAGCAAATAGTAAACATGTGGCAGAGAGCAGGACAAGCAA
TGTATGCTCCTCCCATCAGTGGACAAATTAATTGCATATCAAAT---
ATTACAGGAATACTGTTGACAAGAGACGGTGGTAAGGACGGTGATAATGGT----------------------
----

ACTCATAATGGGAGCGAGATCTTCAGACCTACAGGAGGAAATCTAAGGGACAATTGGAGAAATGAATTATATA
AATATAAGGTAGTGCAAATTGAACCACTA---GGAATAGCACCCACCAAGGCAAAGAGAAGAGTAGTG-----
----------------------------------GAGAGAGAAAAAAGAGCAGTAGTGGGA---
ATAGGAGCT---------ATGATCTTTGGGTTCTTAGGAGCA---GCAGGAAGCACTATG---
GGCGCGGCGTCA---ATAACGCTGACGGTACAG---GCCAGACAA---TTATTG---
TCTGGTATAGTGCAACAGCAAAGC---AATTTG---CTGAGA---
GCTATAGAGGCGCAACAGCATATGTTGCAACTCACAGTCTGGGGCATC
>MG989595
TTCTACTGTAATACAACACAACTGTTTAATAGTACTTGGTATGGGAATGATTCTGACAAGAATAATAACAATA
ATAAT--

GACAATGACACAATCATACTCCCATGTAAAATAAGACAAATTGTAAACATGTGGCAGGAAGTAGGAAAAGCAA
TATATGCTCCTCCTATCAGTGGACAAATTAACTGTTCATCAAAG---
ATTACAGGGCTGTTATTAACAAGAGATGGTGGTAATGGTAACGGTACC-------------------------
----

AGGAATGAAACCTTCAGACCTGGAGGAGGAAATATGAGGGACAATTGGAGAAGTGAATTATATAAATATAAAG
TAGTAAAAATTGAACCATTA---GGAGTAGCACCCACTAAGGCAAAGAGAAGAGTGGTG--------------
-------------------------CAGAGAGAAAAAAGAGCT---GTAACA---CTGGGAGCT---------
GTGTTCCTTGGGTTCTTGGGAGCA---GCAGGAAGCACTATG---GGCGCAGCGTCA---
TTGACACTGACGGTACAG---GCCAGACAA---TTATTG---TCTGGTATAGTGCAACAGCAGAAC---
AATCTG---CTGAGG---GCTATTGAGGCGCAACATCATCTGTTGCAACTCACAGTCTGGGGCATT
>MG989556
TTCTATTGCAATACATCAAAACTGTTTAATAGTACTTGGAATGGAACAAACAATACAGGAGGG----------
----

GATACTGGCACTATCATACTTCCATGTAGGATAAAGCAAATTATAAACATGTGGCAGCAAACAGGACAAGCAA
TGTATGCTCCTCCCATCAGTGGACCAATTAATTGTATGTCAAAT---
ATTACAGGAATACTATTGACAAGAGATGGTGGAGTTAATAATAGT----------------------------
----

CATACTAATGAAACCTTCAGACCTGGAGGAGGAGATATGAAAAATAATTGGAGAAATGAATTATATAAATATA
AAGTAGTACAAATTGACCCACTA---GGAATAGCACCCACCAAGGCAAAGAGGAGAGTGGTG-----------
----------------------------GAGAGAGAAAAAAGAGCA---GTGGGA---ATAGGAGCT------
---ATGATCTTTGGGTTCTTAGGAGCA---GCAGGAAGCACTATG---GGCGCGGCGTCA---
ATAACGCTGACGGTACAG---GCCAGACAA---TTATTG---TCTGGTATAGTGCAACAGCAAAGC---
AATTTG---CTGAGG---GCTATAGAGGCGCAGCAGCATCTGTTGCAACTCACAGTCTGGGGCATT
>MG989612
TTCTACTGTAATACAACAAAACTGTTTAATAGTACTTGGAATAATACTGGAGAGTCAAATGAGATCTCTACA-
----

GGAAATGACACCATCACACTCCCATGCAGAATAAAACAAATTATAAACATGTGGCAGCAAGTAGGGAAAGCAA
TGTATGCTCCTCCCATCAGTGGTCAAATTAACTGCTCATCAAAT---
ATTACAGGGCTACTATTATTAAGAGACGGTGGTAACCAGAACAATAGCGCC----------------------
----

AACACAGAGACATTCAGACCGGGAGGAGGAGACATGAGGGACAATTGGAGAAGTGAATTATATAAATATAAAG
TAGTAAAAATTGAACCATTA---GGAGTAGCACCAACCAAGGCAAGAAGAAGAGTGGTG--------------
-------------------------CAGAGAGACAAAAGAGCA---GCA------CTAGGAGCG---------
GTGTTCCTTGCGTTCTTGGGAGCA---GCAGGAAGCACTATG---GGCGCAGCGTCA---
ATAACGCTGACGGTACAG---GCCAGACAA---TTACTG---TCTGGTATAGTGCAACAGCAGAAC---
AATTTG---CTGAGA---GCTATTGAGGCGCAACAGCATATGTTGCAACTCACAGTCTGGGGCATA
>KF250395
TTCTATTGTAATACATCACAACTGTTTAATAGTACATGGAAGAATCACACTGGTACATATGAGACGAATGATA
CAAGTAACAAT--

CCAAACATCACAATCACAATTCCATGCAGAATAAAGCAAGTTATAAACATGTGGCAGGAGGTCGGGCGAGCAA
TGTATGCCCCTCCCATTCAAGGAACCATAACATGTAAATCAAAT---
ATCACAGGGCTACTATTGACACGTGATGGAGGAAATGGAAATATGACA-------------------------
----

AATGAGACAGAGACATTCAGACCTGGAGGAGGAGATATGAGGGACAATTGGAGAAGTGAATTATATAAATATA
AAGTAGTAGAAATTAAGCCATTG---GGAGTAGCACCCACTGGAGCAAAAAGGAGAGTGGTG-----------
----------------------------GAGAGAGAAAAAAGAGCA---GTGGGA---ATAGGAGCT------
---GTGTTCCTTGGGTTCTTGGGAGCA---GCAGGAAGCACTATG---GGCGCGGCGTCA---
ATAACGCTGACGGTACAG---GCCAGACAA---TTGTTG---TCTGGTATAGTGCAACAGCAAAGT---
AATTTG---CTGAGG---GCTATAGAGGCGCAACAACATATGTTGCAACTCACGGTCTGGGGCATT
>KF250398
TTCTATTGTAATACATCAGGCCTGTTTAATGAGACAGACATGCTGCGTATTACA-------------------
----

GAAGGTAATATCACCACAATCCCATGCAGAATAAAGCAAATTATAAACATGTGGCAGAGGGTAGGAAAAGCAA
TGTATGCCTCTCCCATCAAAGGAAACATAACATGTAAATCAAAT---
ATCACAGGACTACTATTGGTACATGATCAAGTA------AATTCA----------------------------
----

ACCAATATGACATTCAGACCTGAAGGAGGAGATATGAAGAACAATTGGAGAAGTGAATTATATAAATATAAAG
TAGTAGAAATTAATCCATTG---GGAATAGCACCCACTGCAGCAAAAGGAGAGTGGTGGAG---AGA-----
------------------GAAAATAAGAGAGAAAAAAGAGCG---GTGGGA---CTAGGAGCT---------
TTGTTCCTTGGGTTCTTGGGAGCA---GCAGGAAGCACTATG---GGCGCGGCGTCA---
ATAACGCTGACGGTACAA---GCCAGACAA---TTGCTG---TCTGGTATAGTGCAACAGCAAAAC---
AATTTG---CTGATG---GCTATAGAGGCGCAACAGCATATGTTGCAACTCACAGTCTGGGGCATT
>KY200514
TTCTATTGCAATACATCAAGCCTGTTTAATGGGACATACATGTCTGATGGTACACCTACTGGTATACTTAATG
GTACAAAC--

GATAATGAGACCATCACAATTCAATGCAAAATAAAGCAAATTATAAATATGTGGCAGAAGGTGGGACGGGCAA
TGTATGCTCCTCCCATTGCAGGAAACATAACATGTAGGTCAAAT---
ATCACAGGACTACTATTAGTACGTGATGGAGGAACAGTGTCAAATAATACA-------------------
----

GAGTCAAATAACACAGAGATATTCAGGCCTGGAGGAGGAGATATGAGGGACAATTGGAGAAGTGAATTATACA
AATATAAAGTGGTAGAAATTGAACCACTA---GGAATAGCACCTACCATGGCAAAAAGAAGAGTGGTG-----
---------------------------------GACAGACAAAAAAGAGCA---GTGGGA---
ATAGGAGCT---------ATGATCTTTGGGTTCTTAGGAGCA---GCAGGAAGCACTATG---
GGCGCAGCGTCA---ATAACGCTGACGGTACAG---GCCAGACAA---TTATTG---
TCTGGTATAGTGCAACAGCAAAGC---AATTTG---CTGAGA---
GCTATAGAGGCGCAACAGCATCTGTTGCAACTCACAGTCTGGGGCATT
>MG519330
TTCTATTGCAATACAACAAACCTGTTTAATAATGATTGCATAAACAATACCACAGCGGGGGGG---------
----

TGTAATAAGACTATCATACTTCCATGCAAGATAAAACAAATTATAAACATGTGGCAAGGAGTAGGACAAGCAA
TGTATGCTCCTCCCATCCAGGGAATAATTAATTGTGTATCAAAT---
ATTACAGGAATACTATTGACAAGAGATGGTGGTATTCATAACACAAGCGCA---------------------
----

GGTAACGAGACCTTTAGACCAGGAGGAGGAAATATGAAGGACAATTGGAGAAATGAATTATATAAATATAAAG
TAGTAGAAATTGAACCACTA---GGAATAGCACCCACCAGGGCAAAGAGAAGAGTGGTG-------------
------------------------GAAAGACAAAAAAGGGCA---GTGGGA---ATAGGAGCT---------
ATGATCTTTGGGTTCTTAGGAGCA---GCAGGAAGCACTATG---GGCGCGGCGTCA---
ATAACGCTGACGGTACAG---GCCAGACAA---TTATTG---TCTGGTATAGTGCAACAGCAAAGC---
AATCTG---CTAAGG---GCTATAGAGGCGCAGCAGCATCTGTTGCAACTCACAGTCTGGGGCATT
>MN336531
TTCTATTGCAATACATCAAAATTGTTT---AGCACCAGGGAAGGG---------------------------
----

CTTAATAGCACTATCATACTTCCATGCAGGATAAAACAAATTGTAAACATGTGGCAGGGAGCAGGACAAGCAA
TGTATGCTCCTCCCATCAAAGGAAGCATTAATTGTTATTCAAAT---
ATTACAGGAATACTATTAACAAGAGATGGTGGTAATAATAGTACG---------------------------
----

GAGACCTTCAGACCAGGAGGAGGAAATATGAAGGACAATTGGAGAAATGAATTATATAAATATAAAGTAGTAG
AAATTGAACCACTA---GGAGTAGCACCCACCAGGGCAAAGAGGAGAGTGGTG-------------------
------------------GATAGAGAAAAACGAGCA---GTGGGA---ATAGGAGCT---------
ATGATCTTTGGGTTCTTAGGAGCA---GCAGGAAGCACTATG---GGCGCAGCGTCA---
ATAACGCTGACGGTACAG---GCCAGACAA---TTATTG---TCTGGTATAGTGCAGCAGCAAAGC---
AATTTG---CTGAGA---GCTATAGAGGCGCAGCAGCATCTGTTGCAACTCACAGTCTGGGGCATT
>KU820848
TTCTATTGTAATACATCGAAACTGTTTAATGGTACATACAATACAACAAGTAATTCAAGTAATGGTACATACA
ATACAACA--
------------------AGT---
AATTCAAGTTCAGTCATCACAATCCCATGCAAAATAAAACAAATTATAAACATGTGGCAGGAGGTAGGACGAG
CAATGTATGCTCCACCCATTGAAGGAAATATAACATGTAGATCAAAT---
ATTACAGGGTTGCTATTAACAAGAGATGGTGGTAGT----------------------------------

----

AATACCACCGAGACATTCAGACCTCAAGGAGGAGATATGAGAGACAATTGGAGAAGTGAATTATATAAATATA
AAGTAGTAAAAATTGAACCATTA---GGAGTAGCACCCACCAGGGCAAAGAGAAGAGTAGTG-----------
----------------------------CAGAGAGAAAAAAGAGCA---GTGGGA---ATAGGAGCT------
---GTGTTCCTTGGGTTCTTGGGAGCA---GCAGGAAGCACTATG---GGCGCAGCGTCA---
ATAACGCTGACGGTACAG---GCCAGACAA---TTGTTG---TCTGGTATAGTGCAACAGCAAAAC---
AATTTG---CTGAGA---GCTATAGAGGCGCAACAACATATGTTGCAACTCACGGTCTGGGGCATT
>MH327758
TTTTATTGCAATACATCAGGCCTGTTTAATAAGACATACCCGTCTAATAGTACAGTTAATAATACAGAA----
----

ACTGATTCGATCATCACGATCCCATGCAAAATAAAGCAAATTATAAATATGTGGCAGGAGGTGGGACGAGCAA
TGTATGCTCCTCCCATTGCAGGAAACATAACATGTAGGTCAAAT---
ATCACAGGACTACTATTAGTACGTGATGGAGGATTAAATAATACA----------------------------
----

ACTGAGGTATTCAGGCCTCAAGGAGGAGATATGAGGGACAATTGGAGAAGTGAATTATATAAATATAAAGTGG
TAGAGATTAAGCCATTG---GGAATAGCACCCACTGGAGCAAAAAGGAGAGTGGTG-----------------
----------------------GAGAGAGAAAAAAGAGCA---GTGGGA---ATAGGAGCT---------
GCGTTCCTTGGGTTCTTGGGAGTA---GCAGGAAGCACTATG---GGCGCGGCGTCA---
GTGACGCTGACGGTACAG---GCCAGACAA---TTGCTG---TCTGGTATAGTGCAACAGCAAAGC---
AATTTG---CTGAGG---GCTATAGAGGCGCAACAGCATATGTTGCAACTCACGGTCTGGGGCATT
>KF250401
TTCTATTGTAATACATCAGGCCTGTTTAATAGTACATACTCGTCTAATAGTACATACAATGATACAGGA----
----

AGTAATGAAACCATCACAATCCCATGCAAACTAAAGCAAATTATAAACATGTGGCAGAAGGTAGGACGAGCAA
TGTATGCCCCTCCCATTGCAGGAAACATAACATGTAACTCAAAT---
ATCACAGGACTACTGTTGGTACGTGATGGAGGAGGAACAAATAAT----------------------------
----

TCAGAGGAGATATTCAGACCTCAAGGAGGAAATATGAAGGACAATTGGAGAAGTGAATTATATAAATATAAAG
TAGTAAAAATTGAACCATTA---GGATTGGCACCCACCCCTGCAAAGAGAAGCGTGGTG--------------
-------------------------CAGAGAGAAAAAAGAGCA---GTGACA---CTAGGAGCT---------
TTGTTCCTTGGGTTCTTGGGAACA---GCAGGAAGCACTATG---GGCGCGGCGTCA---
ATAACGCTGACGGTACAG---GCCAGACAA---TTGTTG---TCTGGTATAGTGCAACAGCAAAGC---
AATTTG---CTGATG---GCTATAGAGGCGCAACATCATATGTTGCAACTCACGGTCTGGGGCATT
>AJ508597
TTCTATTGTAATACATCAGGACTGTTTAATGGTACAGAACTGCTTAATAGTACATCT----------------
----

AATAATACTGCAATCACACTCCGATGTAGAATAATACGAATTATAAGGATGTGGCAGAGAGTGGGACAAGCCA
TTTACGCCCCTCCCATTGAAGGATACATTACATGTACATCAAAT---
ATAACAGGATTACTATTTACAAGAGATGGA---AATAACAATGAC----------------------------
----

AGTGAGACCTTCAGACCTGGAGGAGGAGATATGAGGGACAATTGGAGAAGTGAATTATATAAGTATAAAGTAG
TAAAAGTTAAACCACTA---GGAATAGCACCCAACAAGGCAAGGAGAAGAGTAGTG-----------------
----------------------GGGAGAGAGAAAAGAGCA---GTTGGA---CTGGGAGCT---------
GTTTTCCTTGGGTTCTTAGGAACA---GCCGGAAGCACTATG---GGCGCGGCGTCA---
ATAACGCTGACGGTACAG---GTCAGACAA---TTATTG---TCTGGCATAGTGCAACAGCAAAGC---
AATTTG---CTGAGA---GCTATAGAGGCGCAGCAGCATCTGTTGCAACTCACAGTCTGGGGCATT
>AJ508595
TTCTATTGTAATACATCAGGCCTGTTTAATATGTCAGGGAGTAGTAATATATCAGGGAAT-------------
----

AGTAATGAGACCATCACACTCCCATGTAGGATAAAACAAATTGTAAGGATGTGGCAGAGAGTGGGACAAGCCA
TTTATGCCCCTCCCATCGCAGGATACATTACATGCACATCAGAC---
ATTACAGGACTACTATTTACAAGAGACGGGGGTCATCACAGCAGT----------------------------

```
-------------------------------------------------------------------------
----------------
AATGAGACCTTCAGACCGGAAGGAGGAGATATGAGGAACAATTGGAGAAGTGAATTATATAAGTATAAAGTAG
TAAAAATTAAACCACTA---GGAATAGCACCCACCAGAGCAAGGAGAAGAGTAGTG-----------------
----------------------GGGAGAGAGAAAAGAGCA---GTTGGA---CTGGGAGCT---------
GTGTTCCTTGGGTTCTTAGGAACA---GCAGGAAGCACTATG---GGCGCAGCGTCA---
ATAACGCTGACGGTACAG---GCCAGACAA---TTATTG---TCTGGCATAGTGCAACAGCAAAGC---
AATTTG---CTGAGA---GCTATAGAGGCGCAGCAGCATCTGTTGCAACTCACAGTCTGGGGCATT
>KU168299
TTCTATTGCAATACAACAGCATTGTTTAATAGCTCTTGGGATGCCAACACACTAAGTAATAGCACT-------
-------------------------------------------------------------------------
----------------------------
GTAAATGACACTATAACTCTCCAATGCAGAATAAAGCAAATTGTAAATATGTGGCAGAAAGTAGGACAAGCAA
TGTATGCCCCTCCCATCCCAGGAATAATAAAGTGTGAATCAAAC---
ATTACAGGGTTACTATTAACAAGAGATGGAGGGAACACAGATAAT----------------------------
-------------------------------------------------------------------------
-------------
ATAACTGAGACTTTCAGGCCTGGAGGAGGAAATATGAGGGACAATTGGAGGAGTGAATTGTATAAATATAAAG
TAGTAAAAATTGAACCATTA---GGTGTAGCACCCACCCATGCAAAAAGAAGAGTGGTG--------------
-------------------------GGAAGAGAAAAAAGAGCA---GTTGGA---CTGGGAGCT---------
GTCTTCCTTGGGTTCTTAGGAGCA---GCAGGAAGCACTATG---GGCGCGGCGTCA---
ATAACGCTGACGGTACAA---GCCAGACAA---TTATTG---TCCGGTATAGTGCAACAGCAGAGC---
AATCTG---CTAAGG---GCTATAGAGGCTCAACAACATATGTTGAAGCTCACGGTCTGGGGCATT
>AJ866554
TTCTATTGCAATACAACAAAACTGTTTAATAGCACTTGGAATAGTTGGGATAATAGCACTTGGAATAGTACCA
GCTCAAACGAC--------------------------------------------------------------
-------------ACATGG------
CCAAAGGGGTCAAATATAACTCTCCAATGCAGAATAAAGCAAATTATAAATATGTGGCAGAGAGTAGGACAAG
CAATGTATGCCCCTCCCATCCCAGGAGAAATAAAGTGTGTATCAAAC---
ATTACTGGACTAATATTAACAAGAGATGGAGGGAATAATAATAGT----------------------------
-------------------------------------------------------------------------
-------------
GCAAGTGAGACCTTCAGGCCTGGAGGAGGAGATATGAGGGACAATTGGAGAAGTGAATTATATAAATATAAAG
TAGTAAAAATTGAACCACTA---GGAGTAGCACCCACTAGAGCGAAGAGAAGAGTGGTG--------------
-------------------------GAGAGAGAAAAAAGAGCA---GTTGGA---CTGGGAGCT---------
TTCTTCTTTGGGTTCCTAGGAGCA---GCAGGAAGCACTATG---GGCGCGGCGTCA---
ATAACGCTGACGGCACAG---GCCAGACAA---TTATTG---TCTGGCATAGTGCAACAGCAGAGC---
AATCTG---CTGAAG---GCTATAGAGGCTCAACAGCATCTATTGAAACTCACGGTCTGGGGCATT
>AJ866556
TTCTATTGCAATACATCAGCACTGTTTAATAGCACTTGGGTAGGGGTTAATGGCAATTGGAGCAATACCAGCT
CAAACAAC-----------------------------------------------------------------
-------------------ACA---
GGGTTAAATGACATTATAACTCTTCGATGCAGAATAAAACAAATTATAAATATGTGGCAGAGAGTAGGACAAG
CAATGTATGCCCCTCCCATCCAAGGAGAACTAAGGTGTGACTCAAAC---
ATTACTGGATTACTATTAACAAGAGATGGAGAGGGTGATACTAAG----------------------------
-------------------------------------------------------------------------
-------------
GAAAATGAGACCTTCAGGCCTGGAGGAGGAGATATGAGGGACAATTGGAGAAGTGAATTATATAAGTATAAAG
TAGTAAAAATTAAACCACTA---GGTGTAGCACCCACCCGTGCAAAAAGGAGAGTGGTG--------------
-------------------------GAGAGAGAAAAAAGAGCA---GTTGGA---CTGGGAGCT---------
GTCTTCCTTGGGTTCTTAGGAGCA---GCAGGAAGCACTATG---GGCGCGGCGTCA---
ATAACGCTGACGGTACAG---GCCAGACAA---TTATTG---TCCGGTATAGTGCAACAGCAGAAC---
AATTTG---CTGAAG---GCTATAGAGGCTCAACAACATTTGTTGAGACTCACGGTCTGGGGCATT
>AJ866558
TTCTATTGCAGTACATCAGCATTGTTTAATAGCACCTGGATGATGGTTAATGGCACCTGGAGCGACACTGCAA
ATGACACAGAG--------------------------------------------------------------
-------------GCAAAT------
AACAATATAACTCATATAACTCTCCAATGCAAAATAAAGCAAATTGTAAATATGTGGCAGAGAGTAGGACAAG
CAATATATGCCCCTCCTATCCAAGGAAAAATAAGTTGTAATTCAAGC---
ATTACAGGACTGCTATTAACAAGAGATGGAGGAGGGAATAATACT----------------------------
```


GCAAATGAAACTTTCAGGCCTGGAGGAGGAGATATGAGGGACAATTGGAGAAGTGAATTATATAAGTATAAAG
TAGTAAAAATTGAACCACTA---GGTGTGGCACCCACCCATGCAAAAAGAAGAGTGGTG--------------
-------------------------GGAAGAGAGAAAAGAGCG---GTTGGA---CTGGGAGCT---------
GTCTTCCTTGGGTTCTTAGGAGCA---GCAGGAAGCACTATG---GGCGCGGCGTCA---
ATAACGCTGACGGTACAG---GCCAGACAA---TTATTG---TCCGGTATAGTGCAACAGCAGAGT---
AATTTG---CTGAGG---GCTATAGAGGCTCAACAACAACTGTTGAAACTCACGGTCTGGGGCATT
>AJ866555
TTCTACTGCAATACAACACAACTGTTTAATAGCGCTTGGTTTAATGGCACTTGGGTATACAACAATAACACCA
ATGTC--

AGCTCAGGGTCAAATATAACTCTCCCATGCAGCATAAAGCAAATTGTGAGAATGTGGCAGAAAGTAGGACAAG
CAATGTATGCCCCTCCCATCCCAGGAATAATAAGGTGTGAGTCAAAT---
ATTACAGGACTAATCTTAACAAGAGATGGGGGGAACGATAGCAGT----------------------------
----

GTAAATGAGACCTTCAGGCCTGGAGGAGGAGATATGAGGGACAATTGGAGAAGTGAATTATATAAGTATAAAG
TAGTAAAAATTGAACCACTA---GGTGTAGCACCCACCCCTGCAAAAAGAAGAGTGGTG--------------
-------------------------CAGAGAGAAAAAAGAGCAGTAGTTGGA---ATGGGAGCT---------
CTTTTCCTTGGGTTCTTAGGAGCA---GCAGGAAGCACTATG---GGCGCAGCGTCA---
GTAACGCTGACGGTACAG---GCCAGACAA---TTATTG---TCCGGTATAGTGCAACAGCAGAGC---
AATTTG---CTGAGG---GCTATAGAGGTTCAACAACATCTGTTGAAACTCACGGTCTGGGGCATT
>AF184155
TTCTATTGCAATACATCAGGACTGTTTAATAGCACTTGGTATAAAAATAGCACTTGGTATAGCAATAGCACTG
CCAGCTCAAAC--
-------------CACACA------
GAGTTAAATAGCACTATAACTCTCCAATGCAAAATAAAGCAAATTATAAATATGTGGCAGAAAGTAGGACAAG
CAATGTATGCCCCTCCCATCCAAGGAGTAATAAGATGTGATTCAAAC---
ATTACAGGACTACTATTAACAAGAGATGGAGGGAGTAATAATAAT----------------------------
----

ACACCTGAGATCTTCAGGCCTGGAGGAGGAAATATGAGGGACAATTGGAGAAGTGAATTATATAAGTATAAAG
TAGTAAAAATTGAACCACTA---GGTGTAGCACCCACCCGTGCAAAAAGAAGAGTGGTG--------------
-------------------------GAGAGAGAAAAAAGAGCA---GTTGGA---CTGGGAGCT---------
GTCTTCCTTGGGTTCTTAGGAGCA---GCAGGAAGCACTATG---GGCGCAGCGTCA---
ATAACGCTGACGGTACAG---GCCAGACAA---TTATTG---TCCGGTATAGTGCAACAGCAGAGC---
AATTTG---CTGAGA---GCTATAGAGGCTCAACAACATCTGTTGAAACTCACGGTCTGGGGCATT
>HQ385445
TTCTATTGCAATACATCAAGCCTATTTAATAGCACTTGGAATACATCAAGCATTAGCATTGAGAATAGTACCG
AT---

TACTCAAATGAAACTATAACTCTCCAATGCAGAATAAAGCAAATTATAAATATGTGGCAGACAGTAGGACAAG
CAATATATGCCCCTCCCATCCCAGGAATAATAAGCTGTAATTCAACT---
ATTACTGGACTACTATTAACAAGAGATGGAGGAAATACTAATAGT----------------------------
----

ACGAATGAAACCTTCAGGCCTGGGGGAGGAGATATGAGGGACAATTGGAGAAGTGAATTATATAAGTATAAAG
TAGTGAAAATTGAACCACTA---GGTGTAGCACCCACTCATGCAAAAAGAAGAGTGGTG--------------
-------------------------GAGAGAGAAAAAAGAGCA---GTTGGA---CTGGGAGCT---------
GTCTTCCTTGGGTTCTTAGGAGCA---GCAGGAAGCACTATG---GGCGCGGCGTCA---
ATGGCGCTGACGGTACAG---GCCAGACAA---TTATTG---TCCGGTATAGTGCAACAGCAGAGC---
AATTTG---CTGAAG---GCTATAGAGGCTCAACAGCATCTGTTGAGACTCACGGTCTGGGGCATT
>GU201497
TTCTATTGCAATACATCAAGACTGTTTAATAGCACTTGGTATAATGACACTAGAAGGCCCAACTACACAGAG-
----

TTAAATGGCACTATAACCCTCCAATGCAGAATAAAGCAAATTATAAATATGTGGCAGAGAACAGGACAAGCAA
TATATGCCCCTCCCATCCCAGGAGAAATAAGGTGTAATTCAAAC---
ATTACAGGAATACTATTAGTAAGAGATGGAGGGGTTAATACTAGT----------------------------

----

ACAAATGAGACCTTCAGGCCTGCAGGAGGAGATATGAGGGATAATTGGAGAAGTGAATTATATAAGTATAAAG
TAGTAAAAATTGAACCACTA---GGTGTAGCACCCACCCATGCAAGAAGAAGAGTGGTG--------------
-------------------------CAGAGAGAAAAAAGAGCA---GTTGGA---CTGGGAGCT---------
GTCTTCCTTGGGTTCTTAGGAGCA---GCAGGAAGCACTATG---GGCGCGGCGTCA---
ATAACGCTGACGGTACAA---GCCAGACAA---TTATTG---TCCGGTATAGTGCAACAGCAGAGC---
AATCTG---CTGAGA---GCTATAGAGGCTCAACAACATATGTTGAGACTCACGGTCTGGGGCATT
>JN864047
TTCTATTGCAATACATCAGGCCTGTTTAATGGCACTTGGGATAAATCTTACAACATGACAGAG----------
----

TTGACTGGCACTATAATTCTCCCGTGCAGAATAAAGCAAATTATAAATATGTGGCAGAGAGTGGGACAAGCAA
TGTATGCCCCTCCCATCCCAGGAAGAATAAAGTGTGAGTCAAAT---
ATTACAGGGCTACTATTGACAAGAGATGGTGGAAGTAATAATGAT----------------------------
----

ACTAGTGAGATTTTTAGACCTGGAGGAGGAGACATGAGAGATAATTGGAGAAGTGAACTATATAAATATAAAG
TAGTAAAAATTGAACCAATA---GGAGTAGCACCCACCAAGGCAAGAAGAAGGGTGGTG--------------
-------------------------GAGAGAGAAAAAAGAGCA---ATTGGA---TTTGGAGCT---------
GCCTTCATTGGGTTCTTAGGAGCA---GCCGGAAGCACTATG---GGCGCGGCGTCA---
ATAACGCTGACGGTACAG---GCCAGACAA---TTATTG---TCTGGCATAGTGCAACAGCAAAGC---
AATTTG---CTGAAG---GCTATAGAGGCTCAACAGCATATGTTGAGACTCACGGTCTGGGGCATT
>JN864052
TTCTATTGCAATACATCACAACTATTTAATGGCACTTGGTCTAATAACACTGGGAATGGGAATGCTACCAGCT
CA---

AATGAAACTATAATAACTCTTCCATGTAGAATAAAGCAAATTATCAATATGTGGCAGAGAGTAGGACAAGCAA
TGTATGCCCCTCCCATCCGAGGAGGAATAAGGTGCGACTCAAAC---
ATTACAGGATTACTATTAACAAGAGATGGAGGAACGAATAGTACA----------------------------
----

CATCAGACATTCAGGCCTGGAGGAGGAGATATGAGAAACAATTGGAGAAGTGAATTATATAAGTATAAGGTAG
TACAAATTGAACCAATA---GGTGTGGCACCCACCCCTGCAAAAAGAAGAGTGGTG-----------------
----------------------GAAAGAGAAAAAAGAGCA---GTAGGA---CTGGGAGCT---------
GTCTTCCTTGGGTTCTTAGGAGCA---GCAGGAAGCACTATG---GGCGCAGCGTCA---
ATAACGCTGACGGTACAG---ACCAGACAA---TTATTG---TCCGGTATAGTGCAGCAGCAGAGC---
AATCTG---CTGAGG---GCTATAGAGGCTCAACAGCATCTGTTGAAACTCACAGTCTGGGGCATT
>JN864053
TTCTATTGCAATACAACAAACCTGTTTAATAGCACTTGGTATAACAATGACACTAGTGCTTGGTATAACAATG
GCACAGGACCA--
TACAATGGCACAGGACTA---------
TGGATAAATGGCACTATAATTCTCTCGTGTAGAATAAAGCAAATTGTAAACATGTGGCAAAGAGTAGGACAAG
CAATGTATGCTCCCCCCATCCGAGGAAGAATAGAGTGTCAATCAAAC---
ATTACAGGACTACTGTTGACAAGAGATGGTGGGGAGCATAATAATACTAATAAT------
AATAATACTAATAAT--

AATACTGAGATTTTTAGGCCTGGAGGAGGAGATATGAGAGACAATTGGAGAAGTGAATTATATAAATATAAAG
TAGTAAAAATTGAACCACTA---GGAATAGCACCCACCAAGGCAAGGAGAAGAGTGGTG--------------
-------------------------CAGAGAGAAAAAAGAGCA---GTTGGA---TTTGGAGCT---------
GTCTTCCTTGGGTTCTTAGGAGTA---GCCGGAAGCACTATG---GGCGCGGCGTCA---
ATGGCGCTGACGGTACAG---GCCAAACAA---TTATTG---TCTGGCATAGTGCAACAGCAAAGC---
AATTTG---CTGAGG---GCTATAGAGGCTCAACAGCAGATGTTGAGACTCACGGTCTGGGGCATT
>DQ826727
TTCTATTGCAATACATCAGGCTTATTTAATAGTAGTTGGACAGGAAATAACACAGAAAATAACACTGCA----
----

CTGAATGGCACTATAACTCTCCCGTGCAGAATAAAACAAATTGTAAACATGTGGCAGAGAGTAGGACGAGCAA
TGTATGCCCCTCCCATCCCAGGAGTAATAAGGTGCACATCAAAC---
ATTACAGGATTAATACTGACAAGGATGGTGGAAACCATGGAAATAGT--------------------------

----

AGTTATAAAATGAAACCTTCAGACCTGGAGGAGGAGACATGAGGGACAATTGGAGAAGTGAATTATATAAGT
ATAAGGTAGTACAAATTGAACCAGTA---GGTGTGGCACCCACCCATGCAAGAAGAAGAGTGGTG-------
------------------------------GAGAGAGGAAAAAGAGCA---GTTGGA---CTGGGAGCT---
------GTCTTCCTTGGGTTCTTAGGAGCA---GCAGGAAGCACTATG---GGCGCGGCGTCA---
ATAACGCTGACGGTACAG---GCCAGACAA---TTACTG---TCCGGTATAGTGCAACAGCAGAGC---
AATTTG---CTGAAG---GCTATAGAGGCTCAACAACAGCTGTTGAAACTCACGGTCTGGGGCATT
>MH078538
TTCTATTGCAATACATCAGGACTGTTCAATAAGAATTGGACATATGATAACAGCAATATCACCTCAAATAAC-
----

ACAGAATCAACTATAACTCTCCCATGCAACATAAGGCAAATTGTAAAGATGTGGCAGAGAGTAGGACGAGCAA
TGTATGCCCCTCCTATCGCAGGAAACATAAGTTGCAACTCAAAC---
ATTACTGGATTACTCTTAACAAGAGATGGAGGGAATAATACACAT---------------------------
----

AAGAATAAGACCGAGACCTTCAGGCCTGCAGGAGGAGATATGAGGGACAATTGGAGAAGTGAATTATATAAAT
ATAAAGTAGTAAAAATTGAACCATTA---GGTATAGCACCCTCCAAGGCAAAAAGAAGAGTGGTG-------
------------------------------GAGAGAGAAAAAAGAGCA---GTTGGA---ATGGGAGCT---
------GTCTTCTTTGGGTTCTTAGGAGCA---GCAGGAAGTACTATG---GGCGCGGCGTCA---
ATAACGCTGACGGCACAG---GCCAGGCAA---CTATTG---TCTGGTATAGTACAACAGCAGAGT---
AATCTG---CTAAGG---GCTATAGAGGCTCAACAAAACATGTTGAGACTCACGGTCTGGGGCATT
>AY371143
TTCTATTGCAGTACATCACCACTGTTTAATAGCACTTGGGACAGTAATAACACTGCCAGTTCAAACCACACAG
AG--

TCAAATGACATTATAACTCTCCAATGCAGAATAAAGCAAATTATAAATATGTGGCAGAGGGTGGGACAAGCAA
TGTATGCCCCTCCCATCAGTGGAAACATAACTTGTGAATCAAAC---
ATAACAGGACTACTATTAACAAGAGATGGAGGGGCTACTAATAGT---------------------------
----

ACAACAAATGAGACCTTCAGGCCTGGAGGAGGAGATATGAGGGACAATTGGAGAAGTGAATTATATAAGTATA
AAGTAGTAAAAATTGAACCACTA---GGTGTAGCACCCACCCCTGCAAGAAGAAGAGTGGTG----------
---------------------------CAGAGAGAAAAAAGAGCA---GTTGGA---CTGGGAGCT------
---GTTTTCCTTGGGTTCTTAGGAGCA---GCAGGAAGCACTATG---GGCGCGGCGTCA---
GTGACGCTGACGGTACAG---GCCAGACAA---TTATTG---TCTGGTATAGTGCAGCAGCAGCGC---
AATCTG---CTGCAG---GCTATAGAGGCTCAACAACATCTGTTGAAACTCACGGTCTGGGGCATT
>KF716465
TTCTATTGCAATACATCACAACTGTTTAATGGCACTTGGTTTAATGACACTTGGAACAACAATACCAATGACA
AC--

AGCTCAGAATTTATAACTCTCCAATGCAGAATAAAGCAAATTGTAAATATGTGGCAGAGAGTAGGACAAGCAA
TGTATGCCCCTCCCATCCCAGGAGTAATAAGTTGTAATTCAAAC---
ATTACTGGACTACTATTAATAAGAGATGGAGGGAATAATAATATT---------------------------
----

ATGAAGGAGACCTTCAGGCCTGGAGGAGGAGATATGAGGGACAATTGGAGAAGTGAATTATATAAATATAAAG
TAGTAAAAATTGAACCACTA---GGTGTAGCACCCACCCATGCAAAAAGAAGAGTGGTG-------------
------------------------GAGAGAGAAAAAAGAGCA---GTTGGA---CTGGGAGCT---------
GTCTTCTTTGGGTTATTAGGAGCA---GCAGGAAGCACTATG---GGCGCGGCGTCA---
ATCACGCTGACGGTACAG---GCCAGACAA---TTATTG---TCCGGTATAGTGCAACAGCAGAGC---
AATTTG---CTGAAG---GCTATAGAGGCTCAACAACATATGTTGAGACTCACGGTCTGGGGCATT
>FJ670515
TTTTATTGCAATACATCAAGCCTGTTTAATAGTACTTGGTACGGTAATGGCACTAACAATACAACTGACACGG
GG--

TCCAATGATACTATAAGTCTCCCATGCAGGATAAAACAAATTGTACATATGTGGCAGAGAGTAGGACAAGCAA
TGTATGCCCCTCCAATTCAAGGAATAATAAACTGTAAATCAAAC---
ATTACAGGGCTACTGCTAGTAAGAGATGGTGGGAATGGGAATGGTACA------------------------

----

AATGAGACCGAGACGTTCAGACCTGGAGGAGGAAACATGAAGGACAATTGGAGAAGTGAATTATATAAATATA
AAGTAGTAAAAATTGAACCACTA---GGAGTAGCACCCACCAGGGCAAAGAGAAGAGTGGTG-----------
-----------------------------GGGAGAGAAAAAAGAGCA---GTTGGA---CTGGGAGCT------
---GTCTTCCTTGGGTTCTTAGGAGCA---GCCGGAAGTACTATG---GGCGCGGCGTCA---
ATAACGCTGACGGCACAG---GCCAGACAA---TTATTG---TCTGGCATAGTACAACAGCAGAGC---
AATCTG---CTGAGG---GCTATAGAGGCTCAACAGCACATGTTGAGACTCACGGTCTGGGGCATT
>KU685587
TTCTATTGCAGTACATCAGGATTGTTTAATATGACTTGGTATAAGAACAGCTCAGAGTCAAATAGCACTGAC-
----

AGGCCTGGAAATATAACTCTCCAATGCAGAATAAAGCAATTTATAAATATGTGGCAGAGAGTAGGGCAAGCAA
TGTATGCCCCTCCCATCAGAGGAGAAATAAGTTGTGTGTCAAAC---
ATTACTGGAATACTATTAACAAGAGATGGAGGGAAAAATAGTACT----------------------------
----

GAAAAGGAGATCTTCAGGCCTGGAGGAGGAGACATGAGGGACAATTGGAGAAGTGAATTATACAAGTATAAAG
TAGTAGAAATTGAACCACTA---GGTGTAGCACCCACCCGTGCAAAAAGAAGAGTGGTG--------------
--------------------------GAGAGAGAAAAAAGAGCA---GCA------CTAGGAGCT---------
GTCTTCATTGGGTTCTTGGGAGCA---GCAGGAAGCACTATG---GGCGCGGCGTCA---
GTGGCGCTGACGGTACAG---GCCAGACAA---TTATTG---TCTGGTATAGTGCAACAGCAAAGC---
AATTTG---CTGAAA---GCTATAGAGGCTCAACAGCAACTGTTGAAACTCACAGTCTGGGGCATT
>KR067669
TTCTATTGTAATACATCAAAACTGTTCAATAGCACTTGGCCGGTTAATAGCACTTGGCATAATAGAACTAACA
TTGACAGT---

TCAAATGGCACAATAACTCTCCAATGCAGAATAAAGCAAGTTATAAATATGTGGCAGAAAGTAGGGCAAGCCA
TGTATGCCCCTCCCATCCAAGGAGAAATAAGGTGTGACTCAAAC---
ATTACTGGACTACTATTAACAAGAGATGGAGGGAGTAGTAATGAT----------------------------
----

ATGAGTGAGACCTTCAGGCCTGGAGGAGGAGATATGAAGGACAATTGGAGAAGTGAATTATATAAGTATAAAG
TAGTAAAAATTGAACCAATA---GGGGTGGCACCCACCCCTGCAAAAAGAAGAGTGGTG--------------
--------------------------GAGAGAGAAAAAAGAGCA---GTAGGA---CTGGGAGCT---------
GTCTTCTTTGGGTTCTTAGGAGCA---GCAGGAAGCACTATG---GGCGCGGCGTCA---
ATAACGCTGACGGTACAG---GCCAGACAG---TTATTG---TCTGGTATAGTGCAACAGCAAAGC---
AATTTG---CTGAGG---GCTATAGAGGCTCAACAACAGCTGTTGAGACTCACGGTCTGGGGCATT
>KR067670
TTCTATTGCAATACATCAAAACTGTTTAGTGGCTCTTGGAATGATAGCTCTTTGAATGATAGTAATACAAATG
GAAAC--

TCAAGTGAAACTACTATAATTCTCCCATGCAAAATAAGACAAATTGTACGTATGTGGCAGACAGTAGGACAAG
CAATGTATGCCCCTCCCATTCAAGGAGCAATAAATTGTACATCAAAC---
ATTACAGGGCTACTGCTAACGAGAGATGGTGGGGATGAACCAGAG----------------------------
----

AATGAGACGTTCAGACCTGGAGGAGGAGATATGAGGGATAATTGGAGAAGTGAATTATATAAATATAAAGTAG
TAAAAATTGAACCACTA---GGAGTGGCACCCACCAGGGCAAAGAGAAGAGTGGTG-----------------
-----------------------GGGAGAGAGAAAAGAGCA---ATTGGA---ATAGGAGCT---------
GTCTTCCTTGGGTTCTTAGGAGCA---GCAGGAAGCACTATG---GGCGCGGCGTCA---
GTAACGCTGACGGTACAG---GCCAGACAA---TTATTG---TCCGGTATAGTGCAACAGCAAAAC---
AATTTG---CTGAGG---GCTATAGAGGCTCAACAGCAGTTGTTGAAACTCACGGTCTGGGGCATT
>KR067667
TTCTATTGCAATACATCAAACTTGTTTAACAGTACTTGGTATAGTAACGGTACCAACAGTACTGAGAAAAAT-
----

AATGACACCAACATAACTCTCCAATGTAGAATAAGACAAATTGTAAATATGTGGCAGAGGGTAGGACAAGCAA
TGTATGCCCCTCCCATCAGAGGAAACATAACTTGTATATCAAAC---
ATTACAGGACTGTTGTTAACAAGAGATGGAGGGGATAATAGTACA----------------------------

----

AATGCAACTGAGACTTTCAGGCCTGGAGGAGGAGATATGAGGGACAATTGGAGAAGTGAATTATATAAATATA
AAGTAGTACAAGTTGAACCGCTA---GGAGTAGCACCCAACAGGGCAAAAAGAAGAGTGGTG-----------
----------------------------CAGAGAGAAAAAAGAGCA---GTTGGA---CCGGGAGCT------
---GTCTTCATTGGGTTCTTAGGAGCT---GCAGGAAGCACTATG---GGCGCGGCGTCA---
ATGACGCTGACGGTACAG---GCCAGACAA---TTATTG---TCTGGTATAGTGCAGCAGCAAAGT---
AATTTG---CTGAGG---GCTATAGAGGCTCAACAGCAGGTGTTGAGACTCACGGTCTGGGGCATT
>KR067671
TTCTATTGTAATACAGCAGAGTTGTTTAATAGCACTTGGAATAATGATACTATGAGCTCAAATGACACTAGC-
----

TCAAATGACACTATAACTCTCCAATGCAGAATAAGGCAAATTATAAAGATGTGGCAGAAAGTAGGACAAGCAA
TGTATGCCCCTCCCATCCCAGGAGAAATAAGGTGTGAATCAAAC---
ATTACTGGACTACTATTAACAAGGGATGGAGGGAATGGGAATAAT----------------------------
----

GATCCAAATGAGACCTTCAGGCCTGGAGGAGGAGATATGAGGGACAATTGGAGAAGTGAATTATATAAGTATA
AAGTAGTAAAAATTGAACCACTA---GGTGTAGCGCCCACCCATGCAAGAAGAAGAGTGGTG-----------
----------------------------GAAAGAGAAAAAAGAGCA---GTTGGA---CTGGGAGCT------
---GTCCTCTTTGGATTCTTAGGAGCA---GCAGGAAGCACTATG---GGCGCGGCGTCA---
ATAACGCTGACGGTACAA---GCCAGACAA---TTATTG---TCCGGTATAGTGCAGCAGCAGAGC---
AATCTG---CTGAAG---GCTATAGAGGCTCAACAACATTTGTTGAGACTCACGGTCTGGGGCATT
>MH078547
TTCTATTGCAATACATCAGCATTATTTAATAGCACTTGGAATAAGCCTTGGAATAATTCAATGAATAACACTG
AGGGAGGG---

TCAAATGACATTCTTACTCTCAAATGCAAGATAAGGCAAGTTGTAAGAATGTGGCAAAGAGTGGGACAAGCAA
TGTATGCCAATCCCATCCCAGGAGTAATAAGGTGTGAATCAAAC---
ATAACAGGACTACTATTAACAAGAGATGGAGGGAATAACAATAGT----------------------------
----

ACAAATGAAACTTTTAGGCCTGGAGGAGGAGATATGAGGGACAATTGGAGAAGTGAATTATATAAGTATAAAG
TAGTAAAAATTGAACCATTA---GGTGTAGCACCTACCCGTGCAAGAAGAAGAGTGGTG--------------
-------------------------CAGAGAGAAAAAAGAGCA---GTTGGA---ATGGCAGCT---------
GTCTTCTTTGGGTTCTTGGGAGCA---GCAGGAAGCACTATG---GGCGCGGCGTCA---
ATAACGCTGACGGTACAA---GCCAGACAA---TTATTG---TCTGGTATAGTGCAACAGCAGAGC---
AATCTG---CTGCGG---GCTATAGAGGCTCAACAACATCTGTTGAAACTCACGGTCTGGGGCATT
>MH078549
TTTTATTGCAATACATCAAGCCTGTTTAATAGCAGATGGTATAATGGAACTAACACTGCAAACAACACAGATG
AC---

AATGCTGATAATATAACACTCCCATGCAGACTAAAACAAGTGATAAGAATGTGGCAGAAAGTAGGGCAAGCAA
TGTATGCCCCTCCTATCAAAGGAGAAATAAGGTGTGTCTCAAAT---
ATTACTGGACTACTATTAACAAGAGATGGAGGGTATGGAGGGAAT----------------------------
----

AGTACAGATGAGACCTTCAGGCCTGGAGGAGGAGATATGAGGGACAATTGGAGGAGTGAATTATATAAGTATA
AAGTAGTAAAAATCGAACCACTA---GGGGTGGCACCCACCCGAGCAAAAAGAAGAGTGGTG-----------
----------------------------GAGAGAGAAAAAAGAGCA---GTTGGA---ATGGCAGCT------
---GTCTTCCTTGGGTTCTTAGGAGCA---GCAGGAAGCACTATG---GGCGCGGCGTCA---
CTGACGTTGACGGTACAA---GCCAGACAA---TTATTG---TCCGGTATAGTGCAACAGCAAAGC---
AATTTG---CTGAAG---GCTATAGAGGCTCAACAACAGATGTTGAGACTCACGGTCTGGGGCATT
>MH078550
TTCTATTGCAACACAGCAGGCCTGTTTAATGGTACTTGGGCTAATGATACTTGGGATCAAGGAAATGGCACGA
AA---

GAAAATGACACTATAACTCTCCAATGCAAAATAAAACAAGTTGTACATATGTGGCAGAGAGTAGGACAAGCAA
TGTATGCCCCTCCCATTCCAGGAGAAATAAGATGTGGATCAAAC---
ATTACAGGGCTACTGCTAACAAGAGATGGTGGGAATACTAATAAT----------------------------

----

ACAGAGGAGACGTTCAGACCTGGAGGAGGAAATATGAAAGACAATTGGAGAAGTGAATTATATAAATATAAAG
TAGTACAAATTGAACCAATA---GGAATAGCACCCACCAAGGCAAAGAGAAGAGTGGTG--------------
-------------------------GAGAGAGAAAAAAGAGCA---ATTGGA---CTGGGAGCT---------
GTCTTCCTTGGGTTCTTAGGAGCA---GCAGGAAGTACTATG---GGCGCGGCGTCA---
ATAACGCTGACGGTACAG---GCCAGACAA---TTATTG---ACTGGTATAGTACAACAGCAGAGC---
AATCTG---CTGAGG---GCTATAGAGGCTCAACAGCACATGTTGAAGCTCACAGTCTGGGGCATT
>KX389616
TTCTATTGCAACACATCAAAACTGTTTAATAGTATTTGGGATAACAATGCCACAAAAAATAATATTACTGACA
AAAATGGT---

ATTAATGATACTATAACTCTCCCATGCAGAATAAGACAAATTGTACATATGTGGCAGAGAGTAGGACAAGCAA
TGTATGCTCCTCCCATTCCAGGAGTAATAAACTGTACATCAAAC---
ATTACAGGGATATTGCTAACAAGAGATGGTGGCAATAATAGTGAT----------------------------
----

ACAAATGAGACATTCAGACCTGGAGGAGGAGATATGAGAGATAATTGGAGAAGTGAATTGTATAAATATAAAG
TAGTAAAAATTGAACCACTA---GGAGTAGCACCCACCAATGCAAAGAGAAGAGTGGTG--------------
-------------------------GAGAGAGAAAAAAGAGCA---GTTGGA---CTGGGAGCT---------
GTCTTCCTTGGGTTCTTAGGAGCA---GCAGGAAGTACTATG---GGCGCGGCGTCA---
ATGGCGCTGACGGCACAG---GCCAGACAA---CTATTG---ACTGGTATAGTACAACAGCAGAAC---
AATCTG---CTGAGG---GCTATAGAGGCTCAGCAGCAAATGTTGAGACTCACAGTCTGGGGCATT
>KU168304
TTCTATTGCAATACATCAGGACTATTTAATAGCACTTGGAGTAATCTAAATGACACAAATGGCACAAATCCAG
AGCCAAAT---

GACACTCACACTCTAACTATCCAATGCAGAATAAAGCAAATTGTAAGAATGTGGCAGAGGGTAGGACAAGCAA
TGTATGCCCCTCCCATCGAAGGAGAAATAAGGTGTGAATCAAAC---
ATTACAGGATTACTATTAACAAGAGATGGAGGAGCAAATAATAAT----------------------------
----

AATACAAAGGAGACATTCAGGCCTGGAGGAGGAGATATGAGGGACAATTGGAGAAGTGAATTATATAAGTATA
AGGTAGTACAAATTGAACCAATA---GGTGTGGCACCCACCCATGCAAAAAGAAGAGTGGTG-----------
----------------------------GAGAGAGGAAAAAGAGCA---GTTGGA---ATAGGAGCT------
---GTCTTCCTTGGGTTCTTAGGAGCA---GCAGGAAGCACTATG---GGCGCAGCATCA---
ATGACGCTGACGGTACAG---GCCAGACAA---TTATTG---TCCGGTATAGTGCAGCAGCAAAGC---
AATTTG---CTGAGG---GCTATAGAGGCTCAACAACAGCTGTTGAAGCTCACAGTCTGGGGCATT
>EU513188
TTCTATTGCAATACATCAGAACTGTTTAATAGCAATTGGACTAATACTAATAACAATACCAATAATGCCAGCT
CA---

GAGGCAAATGGCAATATAACTCTCACATGCAAAATAAAGCAAATTATAAATATGTGGCAAAGAGTGGGACAAG
CAATGTATGCCCCTCCCATCCAGGGAATAATAAGGTGTGAATCAAAC---
ATTACAGGATTATTATTAACAAGAGATGGAGGGTTTAGGAATGATACA-------------------------
----

AATGAGACCTATGAGGCCTTCAGGCCTGGAGGAGGAGATATGAGGGACAATTGGAGAAGTGAACTATATAAGT
ATAAAGTAGTAAAAATTGAACCACTA---GGTGTAGCACCCACTCATGCAAAAAGAAGAGCGGTG--------
-------------------------------CAGAGAGAAAAAAGAGCA---GTTGGG---CTGGGAGCT---
------GTCTTCCTTGGGTTCTTAGGAGCA---GCAGGAAGCACTATG---GGCGCGGCGTCA---
ATGACGCTGACGGTACAG---GCCAGACAA---TTATTG---TCCGGTATAGTGCAACAGCAGAGT---
AATCTG---CTGAGG---GCTATAGAGGCTCAACAACATCTGTTGAGACTCACGGTCTGGGGCATT
>EU513185
TTCTATTGCAGTACATCAAATCTATTTAATAGTACCTGTACAAATAATAACGGCACTGCAAATTGTACAAATA
CAAATGGG---
-------------------ACA---
GCATTAAATGAAACTATAACTCTCCAATGCAGAATAAAACAAATTGTAAGGATGTGGCAGAGAGTAGGACAAG
CAATGTATGCCCCTCCCATCCAAGGAGTAATAAGGTGTGAATCAAAC---
ATTACAGGAATAATATTAACAAGGGATGGAGGGGATAGTAATGATACA-------------------------

----

AATGGAAATGAGACATTCAGGCCTGGAGGAGGAGATATGAGGGACAATTGGAGAAGTGAATTATATAAGTATA
AAGTAGTGCAACTTGAACCATTA---GGTGTGGCACCTACCCAGTCAAGAAGAAGAGTGGTG-----------
----------------------------GAGAGAGAAAAAAAGAGCA---GTTGGA---ATGGGAGCT-----
---TTGTTCCTTGGGTTCTTAGGAGCA---GCAGGAAGCACTATG---GGCGCGGCGTCA---
GTGACGCTGACGGTACAG---GCCAGACAA---TTATTG---TCTGGTATAGTGCAACAGCAAAGC---
AATTTG---CTGAGG---GCTATAGAGGCTCAACAGCATCTGTTGAAACTCACAGTCTGGGGCATT
>EU513194
TTCTATTGCAATACATCAGGACTGTTTAACAGCACTTGGTGGAATAACAATAACAGT----------------
----

TCAAATAACATTACCACTCTCCAATGCAGAATAAAGCAGATTGTGAATCTGTGGCAGAGAGTAGGAAAAGCAA
TGTATAACCCTCCCATCCAAGGAGTAATAAGGTGCGAATCAAAC---
ATTACAGGGCTACTATTAACAAGAGATGGAGGGCTTAATGATAGT----------------------------
----

AGTACAAATGAAACCTTCAGGCCTGGAGGAGGAGATATGAGGGACAATTGGAGAAGTGAATTGTATAGGTACA
AAGTAGTAAAAATTGAACCATTA---GGAGTAGCACCCTCCAGGGCAAGGAGAAGAGTGGTG-----------
----------------------------CAGAGAGAAAAAAAGAGCA---ATTGGA---ATGGGAGCT-----
---GTCTTCCTTGGGTTCTTAGGAGCA---GCAGGAAGCACTATG---GGCGCGGCGTCA---
ATAACGCTGACGGTACAG---GCCAGACAA---TTATTG---TCTGGTATAGTGCGACAGCAAAGC---
AATTTG---CTGAAG---GCTATAGAGGCTCAACAACATCTGTTGAAACTCACGGTCTGGGGCATT
>EU513183
TTCTATTGCAATACATCAAGCCTGTTTAATAGCACTTGGAATTGGAATGGCACTAGCATACAGGGGCCAAATG
ACACG--

CAGTCAAATGGCACTATAACACTCCCATGCAGAATAAAACAAATTGTAAATATGTGGCAGAGAGCAGGACAAG
CAATGTATGCCCCTCCCATCCCAGGAATAATAAAGTGTGAGTCAAAC---
ATTACAGGACTACTATTGGTAAGAGATGGTGGGAATAATAGTGGT----------------------------
----

TCAAATGAAACCTTCAGACCTACAGGAGGAGATATGAGAGACAATTGGAGAAGTGAACTATATAAATATAAAG
TAGTAGAAATTGAACCACTA---GGAGTAGCACCCACCAGGGCAAAAAGAAGAGTGGTG--------------
-------------------------GAGAGAGAAAAAAAGAGCA---GTTGGA---TTGGGAGCT--------
GTCTTCCTTGGGTTCTTAGGAGCA---GCAGGAAGCACTATG---GGCGCGGCGTCA---
ATAACGCTGACGGTACAG---GCCAGACAA---TTATTG---TCTGGCATAGTGCAACAGCAAAGC---
AATTTG---CTGAGG---GCTATAGAGGCTCAACAACATCTGTTGAAACTCACGGTCTGGGGCATT
>KU168307
TTCTATTGCAATACATCAGACCTGTTTGATAGCACATGGCCTAACAATAGTAGTAACAATAGCACTAAC----
----

AATAGCACTACCATAACTCTCAAATGCAGAATAAAGCAAATTGTAAATATGTGGCAGAGAACAGGGCAAGCAA
TGTATGCCCCTCCCATCCCAGGACTAATAAGGTGTGAGTCAAAC---
ATTACAGGACTGCTATTGACAAGAGATGGAGGGGATAATAACAGT----------------------------
----

ACAAATGAGACTTTCAGACCTGGAGGAGGAGATATGAGAGATAATTGGAGAAGTGAATTATATAACTATAAAG
TAGTAAAAATTGAACCAGTA---GGCATAGCACCCACCAAGGCAAAAAGAAGGGTGGTG--------------
-------------------------GAGAGAGAAAAAAAGAGCA---GTTGGA---ATAGGAGCT--------
GTCTTCCTTGGGTTCTTAGGAGCA---GCAGGAAGCACTATG---GGCGCGGCGTCA---
ATAACGCTGACGGTACAG---GCCAGACAA---TTATTG---TCTGGCATAGTGCAACAGCAAAGT---
AATTTG---CTGAGA---GCTATAGAGGCTCAACAACAGTTGTTGAGACTCACAGTCTGGGGCATT
>AM279362
TTCTATTGCAATACATCAGGCCTATTTAATAGCATTTGGTCTTGGAATACTAGCACTTGGAATTATACCAACT
CAAATTCC---
-------------ACAGGG------
TTAGATAATGACACTATAACTCTCCAATGCAGAATAAAGCAAATTGTAAATATGTGGCAGAGAGTAGGACAAG
CAATGTATGCCCCTCCCATCAAAGGAATGATAAGGTGTGAATCAAAC---
ATTACAGGATTACTATTAACAAGAGATGGAGGGAAGACTAATGATACT-------------------------

----

AATACAACTGAGACATTCAGGCCTGGAGGAGGAGATATGAGGGATAATTGGAGAAGTGAATTATATAAGTATA
AGGTAGTACAAATTAAACCATTG---GGTGTGGCACCCACCCATGCAAAAAGAAGAGTGGTG-----------
----------------------------GAGAGAGAAAAAAGAGCA---GTTGGA---CTGGGAGTT------
---GCCTTCCTTGGGTTCTTAGGAGCA---GCAGGAAGCACTATG---AGCGCAGCGTCC---
ATAACGCTGACGGTACAG---GCCAGACAA---TTATTG---TCTGGTATAGTGCAGCAGCAAAGC---
AATTTG---CTGAGG---GCTATAGAGGCTCAACAACATCTGTTGAAACTCACGGTCTGGGGCATT
>AM279356
TTCTATTGCAATACATCAAAACTATTTAATAGCACTTGGTATAGCAATGGTAGTGCCAGCTTAGATCACACAA
AT---

AAAGATGAAAATATAACTCTCCTATGTAGAATAAGGCAAATTGTAAATATGTGGCAGAGAGTAGGACAAGCAA
TGTATGCCCCTCCCATCGAAGGAGTAATAAGGTGTGAATCAAAC---
ATTACAGGATTGCTATTAACAAGAGATGGAGGGAAAGATAATAGTACA-------------------------
----

AATAGTACACAGGAGATATTCAGGCCTGGAGGAGGAGATATGAGGGACAATTGGAGAAGTGAATTATATAAGT
ATAAGGTAGTACAAATTGAACCAATA---GGTGTAGCACCCACACATGCAAAAAGAAGAGTGGTG--------
-------------------------------GAGAGAGAAAAAAGAGCA---GTTGGA---CTGGGAGTT---
------TTCTTCCTTGGGTTCTTAGGAGCT---GCAGGAAGCACTATG---GGCGCGGCGTCA---
ATAACGCTGACGGCACAG---GCCAGACAA---TTATTG---TCCGGTATAGTGCAACAGCAAAGC---
AATTTG---CTGAGG---GCTATAGAGGCTCAACAACATATGTTGAAACTCACGGTCTGGGGCATT
>AM279363
TTCTATTGCAATACATCAGGATTATTTAATAGTACTTGGAAATCTAATAGCACTTGGGACAATAACACTGACA
CAGGG--

TCAAACAATATAACTCTCACCCTCCAATGCAGAATAAAGCAAATTGTAAATATGTGGCAGAGGGTAGGACAAG
CAATGTATGCCCCTCCCATCCCAGGAGTAATAAGGTGTGACTCAAAC---
ATTACAGGACTCCTATTAACAAGGGATGGAGGGAATACTAGCACA----------------------------
----

GATGAAACATTCAGGCCTGGAGGAGGAGATATGAGGGACAACTGGAGAAGTGAATTATATAAGTATAAGGTAG
TACAAATTGAACCAATA---GGTGTGGCACCCACCCATGCAAAAAGAAGAGTGGTG-----------------
----------------------GAGAGAGAAAAAAGAGCA---GTAGGA---CTGGGAGCT---------
GTCCTCATTGGGTTCTTAGGAGCA---GCAGGAAGCACTATG---GGCGCGGCGTCA---
ATAACGCTGACGGTACAG---GCCAGACAA---TTATTG---TCTGGTATAGTGCAACAGCAGAGT---
AATCTG---CTGAAG---GCTATAGAGGCTCAACAACATCTGTTGAAACTCACAGTCTGGGGCATT
>KM606636
TTCTATTGTAATACATCAAATCTGTTTAATAAAACCTTTAACCACAACTCAACAGGGTCAAATTCAACAGAG-
----

TCAGATAGGACTATAACTCTCCCATGCAGAATAAAGCAAATTATAAATATGTGGCAGAAAGTAGGACAAGCAA
TGTATGCCCCTCCCATCCGAGGAGTAATAAAGTGTGAATCAAAC---
ATTACAGGACTACTATTAACAAGAGATGGAGGGGTGAATAATGGT----------------------------
----

AGTACAAACGAGACTTTCAGGCCTGGAGGAGGAGACATGAGGGACAATTGGAGAAGTGAATTATATAAGTATA
AAGTAGTAAAAATTGAACCACTA---GGTGTGGCACCCACCCCTGCAAAAAGAAGAGTGGTG-----------
----------------------------GAGAGAGAAAAAAGAGCA---ATTGGA---ATAGGAGCT------
---GTCTTCCTTGGGTTCTTAGGAGCA---GCAGGAAGCACTATG---GGCGCAGCGTCA---
ATAACGCTGACGGTACAG---GCCAGACAA---TTACTG---TCTGGTATAGTGCAACAGCAGAGC---
AATCTG---CTGAGA---GCTATAGAGGCTCAACAACATATGTTGAAACTCACGGTCTGGGGCATT
>AB052867
TTCTATTGCAATACATCAAGACTGTTTAATAGTACTTGGAATGACAATAGCACTTGGAATGACAGTAGCTCAA
ATTAC--
-------------------ACA---
GAGTCAAATGACACTATAACTCTCCAATGCAGAATAAAGCAAATTATAAATATGTGGCAGGGAGTAGGACAAG
CAATGTATGCCCCTCCCATTAGAGGAGTGATAAGGTGTGAATCAAAT---
ATTACAGGACTACTATTAACAAGAGATGGAGGGATTAATAATAGT----------------------------

----

ACAAATGAGACCTTCAGACCTGGAGGAGGAGATATGAGAGACAATTGGAGAAGTGAATTATACAAGTATAAAG
TAGTAAAACTTGAACCACTA---GGAGTGGCACCCACCAGGGCAAGGAGAAGAGTGGTA--------------
-------------------------ACAAGAGAAAAAAGAGCA---GTTGGA---ATGGGAGCT---------
TTGTTCCTTGGGTTCTTAGGAGCA---GCAGGAAGCACTATG---GGCGCGGTGTCA---
ATGACGCTGACGGTACAG---GCCAGACAA---TTATTG---TCCGGTATAGTGCAACAGCAGAGC---
AATCTG---CTAAAA---GCTATAGAGGCTCAGCAGCATCTGTTGAAACTCACGGTCTGGGGCATT
>HQ385444
TTCTATTGCAATACATCAGACCTGTTTAATAGTACTTGGCTAAACTCAACTGTGGAA----------------
----

TCAAATGAGACTATAATTCTCCCATGCAAAATAAAACAAATTATAAATATGTGGCAGAGAGTAGGACAAGCAA
TGTATGCACCTCCCATTCAAGGAGTAATAAACTGTGTATCAAAC---
ATTACAGGGATACTGCTAGTAAGAGATGGTGGGAATAATAATAGT----------------------------
----

ACAGCTGAGACATTCAGACCTGGAGGAGGAGATATGAAGGACAATTGGAGAAGTGAATTGTATAAATATAAAG
TAGTAAAAATTGAACCACTA---GGAGTAGCACCTACCAGGGCAAAGAGACGAGTGGTG--------------
-------------------------GAGAGAGAAAAAAGAGCA---GTTGGA---CTGGGAGCT---------
GTCTTCTTTGGGTTCTTAGGAGCA---GCAGGAAGTACTATG---GGCGCAGCGTCA---
ATAACGCTGACGGTACAG---GCCAGACAA---TTATTG---TCTGGTATAGTACAACAGCAGAGT---
AATTTA---CTGAAG---GCTATAGAGGCTCAACAACATCTGTTGAAACTCACGGTCTGGGGCATT
>KR067668
TTCTATTGCAATACATCAGGACTGTTTAATAGCGCTTGGAACAGTAGCACTTGGAACAATACCCTGACCAAC-
----

AATACTGGCAATATAACGCTCCACTGCAGAATAAAGCAAATTATAAATAAGTGGCAGAGAGTAGGACAAGCAA
TGTATGCCCCCCCCATCCCAGGAGTAATAAGGTGTGAGTCAAAC---
ATCACAGGATTACTACTAACAAGAGATGGAGGAAACAATAATAGG----------------------------
----

ACAAATGAGACTTTCAGGCCTGAAGGAGGAGATATAAGGGACAATTGGAGGAGTGAATTATATAAGTATAAAG
TAGTAAAAATTGAACCAGTA---GGAGTAGCACCCACCAGGGCAAGGAGAAGAGTGGTG--------------
-------------------------GAAAGAGAGAAAAGAGCA---GTCGGA---CTGGGAGCT---------
GTCTTCCTTAGGTTCTTAGGAGCA---GCAGGAAGCACCATG---GGCGCGGCGTCA---
ATAACGCTGACGGTACAG---GCCAGACAA---TTATTG---TCTGGTATAGTGCAACAGCAAAGT---
AATTTG---CTGAAG---GCTATAGAGGCTCAACAACACCTGTTGAGACTCACGGTCTGGGGCATT
>KU749413
TTCTATTGTAATACAACAAGCCTGTTTAATAGTACTTGGTATAATAATGAGACTGAGAACACAAATAGCACGG
GA---

TCAAATGAAACCATAACACTCCCATGCAGAATAAAGCAAATTGTAAATGTATGGCAGAGAGTAGGACAAGCAA
TATATGCCCCTCCCATCCAAGGACTAATACAATGTAACTCAAAT---
ATTACGGGGATACTATTAACAAGAGATGGTGGGAATAACGGATCA----------------------------
----

TCTAATGAAACTTTCAGGCCTGGAGGAGGAGATATGAGGGACAATTGGAGAAGTGAATTATATAAGTATAAAG
TAGTGAAAATTGAACCATTA---GGTGTAGCACCCACCCATGCAAAAAGAAGAGTGGTG--------------
-------------------------CAGAGAGAAAAAAGAGCA---ATTGGA---ATAGGAGCT---------
GTGCTCATTGGGTTCTTGGGAGCA---GCCGGAAGCACTATG---GGCGCGGCGTCC---
ATAACGCTGACGGTACAG---GCCAGACAA---TTATTG---TCTGGTATAGTGCAACAGCAAAGC---
AATTTG---CTGAGG---GCTATAGAGGCTCAGCAACATATGCTGAAACTCACAGTCTGGGGCATT
>KX232596
TTCTATTGTAATACAACACAGCTATTTAATAGCACTTGGAATATAACAGGACTATCTAATAGCAATTGGACAG
GTAAT--

GACACTAGCAATATAACTCTTCCATGCAGAATAAAGCAAGTTGTAAATATGTGGCAGAGGGTAGGACAAGCAA
TTTATGCCCCTCCCATCCCAGGAATAATACAGTGTCAGTCAAAC---
ATTACTGGACTACTATTGACAAGAGATGGGGGAAATAATAATAAT----------------------------

----

AGTTCGAATGAAACTTTCAGGCCTGGAGGAGGAGACATGAGGGACAATTGGAGAAGTGAGTTATATAAGTATA
AAGTAGTAAAAATTGAGCCATTA---GGTGTAGCACCCACTCCTGCAAAAAGGAGAGTGGTG-----------
----------------------------GAGAGAGAAAAAAGAGCA---ATTGGTGGACTGGGAGCT------
---GTATTCCTTGGGTTCTTAGGAGCA---GCAGGAAGCACTATG---GGCGCGGCGTCA---
ATAACGCTGACGGTACAG---GCCAGACAA---TTATTG---TCTGGTATAGTGCAACAGCAGAGT---
AATTTG---CTAAAG---GCTATTGAGGCTCAACAACAGATGTTGAGACTCACGGTCTGGGGCATT
>KY658718
TTCTATTGTAATACAACAGGACTATTTAATAGCACTTGGATGGAAAATAGCAGCATTTGGGAAAGCAATGGCA
ATAAA--

ACAAATGAAACTATAACTCTTCCATGCAGAATAAAGCAAATTGTAAATATGTGGCAGAGAGTGGGACAAGCAA
TTTATGCCCCTCCCATCCCAGGAATAATACAGTGTGAGTCAAAC---
ATTACTGGACTACTATTGACAAGAGATGGGGAAATTAATAGTTCA----------------------------
----

AATGAAACTTTCAGGCCTGGAGGAGGAGACATGAGGGACAATTGGAGAAGTGAATTATATAAGTATAAAGTAG
TAAAAATTGAACCAGTA---GGTGTAGCACCCACCCGTGCAAGAAGGAGAGTGGTG-----------------
----------------------GAGAGAGAAAAAAGAGCA---ATTGGTGTACTGGGAGCT---------
GCATTCCTTGGGTTCTTAGGAGCA---GCAGGAAGCACTATG---GGCGCGGCGTCA---
ATAACGCTGACGGTACGG---GCCAGACAA---TTATTG---TCTGGTATAGTGCAACAGCAGAGT---
AATTTG---CTGAAG---GCTATTGAGGCTCAACAACAGATGTTGAGACTCACGGTCTGGGGCATT
>KX232617
TTCTATTGTAATACAACAAAACTATTTAATAGCACTTGGAAAGGAAATATCACTTGGGAGAGCAATGGCAATA
AA---

ACAAATGACAGTATAACTCTTCCATGCAGAATAAAGCAAATTGTAAATATGTGGCAGAGGGTAGGACAAGCAA
TTTATGCCCCTCCCATCCCAGGAGTAATAACGTGTGAGTCAAAC---
ATTACTGGACTACTATTGACAAGAGATGGGGGAAATAATAATAATAGA-------------------------
----

AATAATGAAACTTTCAGGCCTGGAGGAGGAGACATGAGGGACAATTGGAGAAGTGAATTATATAAGTATAAAG
TAGTAAAAATTGAACCATTA---GGTGTAGCACCCACCCCTGCAAGAAGGAGAGTGGTG--------------
-------------------------GAGAGAGAAAAAAGAGCA---ATTGGTGGACTGGGAGCT---------
GTATTCTTTGGGTTCTTAGGAGCA---GCAGGAAGCACTATG---GGCGCGGCGTCA---
ATAACGCTGACGGTACAG---GCCAGACAA---TTATTG---TCTGGTATAGTGCAACAGCAGAGT---
AATTTG---CTGAAG---GCTATAGAGGCTCAACATCAAATGTTGAGACTCACGGTCTGGGGCATT
>KX232623
TTCTATTGTAATACAACAGGCCTGTTTAATAGTACTTGGCCTAGGAATGAGAATGAGACTAGGGAAGCAAATA
GCACAGGG---

TCAGATGACATCATAACACTCCCATGCAGAATAAAGCAAATTATAAATGTATGGCAGAGAGTAGGGCAAGCAA
TATATGCCCCTCCTATCCCAGGACTAATAAGATGTAACTCAAGT---
ATTACGGGGCTACTATTAGTAAGAGATGGTGGAAATAATAACACA----------------------------
----

GATAATGAAACCTTCAGACCTGGAGGAGGAAATATGAAGGACAATTGGAGAAGTGAATTGTATAAGTATAAAG
TAGTAAAACTTGAACCACTA---GGAGTAGCACCCACCAGGGCAAAGAGAAGAGTGGTG--------------
-------------------------GAGAGAGAAAAAAGAGCA---TTTGGA---TTAGGAGCT---------
GTATTCATTGGGTTCTTGGGAGCA---GCCGGAAGCACTATG---GGCGCGGCGTCT---
GTAACGCTGACGGTACAG---GCCAGGCAA---TTATTG---TCTGGCATAGTGCAACAGCAAAGC---
AATTTG---CTGAGG---GCTATAGAGGCTCAGCAGCAGATGCTGAAACTCACGGTCTGGGGCATT
>KX232627
TTCTATTGTAATACAACAAAACTATTTAATAGTACTTGGGGCAATGGCAGTGAACCAGAG-------------
----

GTAAACAATACCATAACCCTGCCATGCAGAATAAAGCAATTTGTAAATATGTGGCAGAGGGTAGGACAAGCAA
TTTATGCCCCTCCCATCCCAGGAAGAATAAAGTGTGAATCAAAC---
ATTACTGGACTACTATTGACAAGGGATGGGGGACAGAATAATAGG----------------------------

----

TCAAATGAAACTTTCAGGCCTGGAGGAGGAGATATGAGGGACAATTGGAGAAGTGAATTATATAAGTATAAAA
TAGTAAAAATTGAACCATTA---GGTGTAGCACCCACCCATGCAAGAAGGAGAGTGGTG--------------
-------------------------GGGAGAGAAAAAAGAGCA---ATTGGTGGACTGGGAGCT---------
GTATTCCTTGGGTTCTTAGGAGCA---GCAGGAAGCACTATG---GGCGCAGCGTCA---
ATAACGCTGACGGTACAG---GCCAGACAA---TTATTG---TCTGGTATAGTGCAACAGCAAAGT---
AATTTG---CTGAAG---GCTATTGAGGCTCAACAACAGATGTTGAGACTCACAGTCTGGGGCATT
>HM215282
TTCTATTGTAATACATCAAGCCTGTTTAATAGCACTTGGTATAACAATAGCAATAACAACAATGCCAGCTCAA
ACCCC--
-------------------ACA---
GAGTTAAATGGCACTATAACTCTCCCATGCAGAATAAAGCAAATTGTAAATATGTGGCAGAAAGTAGGACAAG
CAATGTATGCCCCTCCCATCCCAGGAATAATAGAGTGTAAATCAAAC---
ATTACAGGATTGTTATTAACAAGAGATGGAGGGAATAGTAATGGT----------------------------
----

GCAAATGAAACTTTCAGGCCTGGAGGAGGAGATATAAGGGACAATTGGAGAAGTGAATTATATAAGTATAAAG
TAGTAAAAATTGAACCACTA---GGTGTAGCACCCACCCATGCAAGAAGAAGAGTGGTG--------------
-------------------------GGGAGAGAAAAAAGAGCA---GTAGGA---CTGGGAGCT---------
GTCATCTTTGGGTTCTTAGGAGCA---GCAGGAAGCACTATG---GGCGCGGCGTCA---
ATAACGCTGACGGTACAG---GCCAGACAA---TTATTG---TCTGGTATAGTGCAACAGCAAAGC---
AATTTG---CTGCAG---GCTATAAAGGCTCAACAACATCTGTTGAGGCTCACGGTCTGGGGCATT
>KR017777
TTCTATTGCAATACATCAAAGCTGTTTGAAGGTTGGATTTGGAATCATAGCAATGGGAGTTGGGAAAGGAATA
ATAAA---
ACAAATGATGAAAATAAA---------
ACAGAGGATGGAAATATAATTCTCCCATGCAAAATAAAGCAAATAGTAAGAATGTGGCAGAGAGCAGGACAGG
CAATGTATGCCCCTCCCATCAAAGGATTAATAAATTGTAAATCAAAC---
ATTACAGGACTACTATTAACAAGGGATGGA---ACACATAATGGG----------------------------
----

ACCAACGAGACCTTCAGGCCTATAGGAGGAGAGATGAGGGACAATTGGAGAAGTGAATTATATAAATATAAAG
TAGTGGAAATTAAACCACTA---GGAGTAGCACCCACCGAGGCAAGGAGAAGAGTGGTG--------------
-------------------------CAGAGAGAAAAAAGAGCA---GTGGGA---ATAGGAGCT---------
GTGCTCCTTGGGTTCTTAGGAGCA---GCAGGAAGCACTATG---GGCGCGGCGTCA---
ATAACGCTGACGGTACAG---GCCAGACAA---TTATTG---TCTGGTATAGTGCAACAGCAGAGC---
AATTTG---CTGAAG---GCTATAGAGGCTCAACAACATCTGTTGAGACTCACGGTCTGGGGCATT
>KU749421
TTCTATTGTAATACATCAAAGCTATTTAATAGCACCTGGAATAGCACTGGGGAGAATCAGAAAATT-------
----

AGGAATGGGACTCTCATCCTCCCATGCAGAATAAAGCAAATTGTACGGATGTGGCAGAGAGTAGAACAAGCAA
TGTATGCCCCTCCCATCCAAGGAGAAATAAGATGTCATTCAAAT---
ATTACAGGAATACTGTTAACGAGAGATGGCGGGGGTAACGAGACA----------------------------
----

AAGGAGACCTTCAGACCTGTAGGAGGAAATATGAAAGACAATTGGAGAAGTGAATTATATAAGTATAAAGTAG
TAAAAATTGAGCCAATA---GGAATAGCACCCTCCAGGGCACGAAGAATAGTGGTG-----------------
----------------------GGGAGAGAAAAAAGAGCA---GTTGGA---CTGGGAGCT---------
GTCTTCCTTGGGTTCTTAGGAGCA---GCAGGAAGCACTATG---GGCGCGGCGTCA---
GTGACGCTGACGGTACAG---GCCAGACAA---TTATTG---TCGGGCATAGTGCAACAGCAAAGC---
AATTTG---CTGATG---GCTATAGAGGCTCAACAACATATGTTGAAACTCACGGTCTGGGGCATT
>GU201504
TTCTATTGTGATACAGCAGAACTATTTAATAGTACTTGGGAAAGGGAAAATGGCACTCGGAGAAAT-------
----

AGCACTGGGAATATAAATCTCCAGTGCAGAATAAAGCAAATTGTAAATATGTGGCAGAGAACAGGACAAGCAA
TGTATGCCCCTCCCATTCCAGGAATAATAGAATGTAAGTCAAAC---
ATTACAGGACTACTATTGACAAGAGATGGTGGAGCTAATAACAGT----------------------------

----

GCAGAGGAGACTTTCAGACCTGGAGGAGGAGATATGAGAGACAATTGGAGAAGTGAATTATATAAGTATAAAG
TAGTAAAAATTGAACCACTA---GGGGTAGCACCCACCCATGCAAAAAGAAGAGTGGTG-------------
------------------------GAGAGAGAAAAAAGAGCA---GTTGGA---CTGGGAGCT---------
GTCTTCCTTGGGTTCTTAGGAGCA---GCAGGAAGCACTATG---GGCGCGGCGTCA---
ATAACGCTGACGGTACAG---GCTAGACAA---TTATTG---TCTGGCATAGTGCAACAGCAAAGC---
AATTTG---CTGAGG---GCTATAGAGGCTCAACAGCATATGTTGAGACTCACGGTCTGGGGCATT
>KR017773
TTCTATTGCAATACATCAGGCCTATTTAATAGCACTTGGAATAGCACTGGGAATAATAATAATATC-------
----

AGCTCAGACAATATCACTCTCCCATGCAGAATAAAGCAAATTGTGAGGCTATGGCAGAGAGTAGGACAAGCAA
TGTATGCCCCTCCCGTCTCAGGAGTAATAAGATGTGAATCAAAC---
ATTACAGGACTACTATTAACAAGAGATGGAGGGAAGAAAAATAAGAGT------------------------
----

AGTGACCCTGAAACCTTCAGACCTGGAGGAGGAGATATGAAGGACAATTGGAGAAGTGAATTATATAAGTATA
AAGTAGTAAAAATTGAACCATTA---GGAGTAGCACCCACCAAGGCAAAAAGAAGAGTGGTG----------
---------------------------GAGAGAGAAAAAAGAGCA---GCTGGA---CTGGGAGCT------
---GTCCTCCTTGGGTTCTTAGGAGCA---GCAGGAAGCACTATG---GGCGCGGCGTCA---
ATAACGCTGACGGTACAG---GCCAGACAA---TTATTG---TCTGGCATAGTGCAACAGCAAAGC---
AATTTG---CTGAGA---GCTATAGAGGCTCAACAGCAGATGTTGAGACTCACGGTCTGGGGCATT
>KR017772
TTCTATTGCGATACATCAGATCTGTTTAATAGCACTTGGGACAATAATACTGTCAGCCCAAACGACACAGAG-
----

GCAAAAGACAATATAACCCTCCAGTGCAGAATAAAGCAGATTGTAAATATGTGGCAGAGAGTAGGACAAGCAA
TATATGCCCCTCCCATCCGAGGAGTAATAAGGTGTGAATCAAAC---
ATTACTGGACTACTATTAGTAAGAGATGGAGGAATTGATAATAGG---------------------------
----

ACAAATGAGACCTTCAGGCCTGGAGGAGGAGATATGAGGGACAATTGGAGAAGTGAATTATATAAGTATAAAG
TAGTAAAAATTGAACCACTA---GGTGTAGCACCCACTAGGGCAAAAAGAAGAGTGGTG-------------
------------------------GAGAGAGAAAAAAGAGCA---GTTGGA---ATAGGAGCT---------
GTCCTCCTCGGGTTCTTAGGAGCA---GCAGGAAGCACTATG---GGCGCGGCGTCA---
CTAACGCTGACGGTACAG---GCCAGACAA---TTATTG---TCTGGTATAGTGCAACAGCAAAGC---
AATTTG---CTGAAG---GCTATAGAGGCTCAACAACATCTGTTGAAACTCACGGTCTGGGGCATT
>GQ372986
TTCTATTGCAATACAACAGATCTGTTTAATAGCGCCTGGAATGCCAGCTCAACCTGGCCCAAC---------
----

TCAACAGGTAATATAACTCTCCAATGCAGAATAAAGCAAATTATAAATATGTGGCAGAGGGTAGGACAAGCAA
TGTATGCCCCTCCCATCCCAGGAGAAATAAGGTGTGAATCAAAC---
ATTACAGGACTACTCTTAACAAGAGATGGAGGGACTAACACTAACAGT------------------------
----

AGTGGTGCAAACGAGACTTTCAGGCCTGGAGGAGGAGATATGAGAGACAATTGGAGAAGTGAATTATATAAAT
ATAAAGTAGTAAAAATTGAACCACTA---GGAGTAGCACCCTCCAAGGCAAAGAGAAGAGTGGTG--------
-------------------------------GAGAGAGAAAAAAGAGCA---GTTGGA---TTTGGAGCT---
------GTCTTCCTTGGGTTCTTGGGAGCA---GCAGGAAGTACTATG---GGCGCGGCGTCA---
ATAACGCTGACGGTACAG---GCCAGACAA---TTATTG---TCTGGGATAGTACAACAGCAGAGC---
AATCTG---CTGAGG---GCTATAGAGGCTCAACAGCAACTGTTGAAACTCACGGTCTGGGGCATT
>AB231894
TTCTACTGCAATACAGATGAACTGTTTAATAGCACTTGGAATAGTAGCACTTGGAATAGTAGCAGTACTACC-
----

GATAGCTCAAATATAACTCTCCACTGCAGAATAAAACAAATTATAAATATGTGGCAGACAGTAGGACGAGCAA
TGTATGCCCCTCCCATCCCAGGAATAATACAGTGTAACTCAAAC---
ATTACAGGATTGCTATTAACAAGAGATGGAGGAAAGGATAATAGTACTGAG---------------------

----

ACTAATAATACTGAGACTTTCAGGCCTGGAGGAGGAGACATGAGGGACAATTGGAGAAGTGAATTATATAAAT
ATAAAGTAGTAAAAGTTGAACCAATA---GGTGTAGCACCCACCAAGGCAAAAAGAAGAGTGGTG--------
-------------------------------GAGAGAGAAAAAAGAGCA---GTTGGA---ATAGGAGCT---
------GTCTTCCTTGGGTTCTTAGGAGCA---GCAGGAAGCACTATG---GGCGCGGCGTCA---
ATAACGCTGACGGTACAA---GCCAGACAA---TTATTG---TCTGGTATAGTGCAACAGCAGAGC---
AATTTG---CTGAGA---GCTATAGAGGCTCAACAACAGATGTTGAGACTCACGGTCTGGGGCATT
>AY521632
TTCTATTGCAATACATCAGGCCTGTTTAATAGTAGTTGGAGTTGGAATGACACTACAAATTTAACGGAA----
----

TCAAATGGTACTATAAATCTCCCATGTAAAATAAAACAAATTATACGTATGTGGCAGAGAGTAGGGCAAGCAG
TGTATGCCCCTCCCATCCAAGGAGTAATACGCTGTAAATCAAAC---
ATTACAGGGCTACTGCTAACAAGAGATGGTGGGAATGATAATGAT----------------------------
----

AATACAACTGAGAAGTTTAGACCTGGAGGAGGAAATATGAAGGACAATTGGAGAAGTGAATTATATAAATATA
AAGTAGTAAAAATTGAACCACTA---GGAGTAGCACCCACCAAGGCAAAAAGAAGAGTGGTG-----------
----------------------------GAGAGAGAAAAAAGAGCA---GTTGGA---CTGGGAGCT------
---GTCTTCCTTGGGTTCTTAGGCGCA---GCTGGAAGTACTATG---GGCGCGGCGTCA---
ATAACGCTGACGGCACAG---GCCAGACAA---TTACTG---TCTGGCATAGTACAACAGCAGAGT---
AATCTG---CTGAGA---GCTATAGAGGCTCAACAGCACCTGTTGAAACTCACGGTCTGGGGCATT
>AY521633
TTCTATTGCAATACATCAGGCCTGTTTAATAGTACTTGGATTCGGTATGCCAATGGCACCGTGCAGAACAATG
GCACG--

GAGCCAAATGGTATTATTCCTCTCCAATGCAGAATAAGACAAGTTGTACGTATGTGGCAGAAAGTACGACAAG
CAATATATGCCCCTCCCATTAGAGGAGTAATATGCTGTGTATCCATC---
ATTGCAGGGCTACTACTAACAAGAGATGGTGGGGATAATAATACT----------------------------
----

ACCGAGAAGTTCAGACCTGGAGGAGGAGATATGAGGGACAATTGGAGAAGTGAATTATATAAATATAAAGTAG
TAAAAATTGAACCACTA---GGAGTAGCACCCACCAAGGCAAAAAGAAGAGTGGTG-----------------
----------------------GGGAGAGAAAAGAGAGCA---GTTGGA---CTGGGAGCT---------
GTCCTCTTTGGGTTCTTAGGAGCA---GCAGGAAGTACTATG---GGCGCGGCGTCA---
ATAACGCTGACGGCACAG---GCCAGACAA---TTATTG---TCTGGCATAGTACAACAGCAGAGC---
AATCTG---CTGAGG---GCTATAGAGGCTCAACAGCACCTGCTGAAACTCACAGTCTGGGGCATT
>JN864055
TTCTATTGCGATACAACAGAATTATTTAATAGTACTTGGGAGACTAATAACACTGCCAGCTCCAATACC----
----

ACAAATGGAACTATAACTCTCCAATGCAGAATAAAGCAAATTATAAATATGTGGCAGAGAGTAGGACAAGCAA
TGTATGCCCCTCCCATCCGAGGAGTAATAAGGTGTGAATCAAAC---
ATTACAGGACTACTATTAACAAGAGATGGAGGGACAACTGAGACA----------------------------
----

AATAATAAAGAGACATTCAGGCCTGCAGGAGGAGATATGAGGGACAATTGGAGAAGTGAATTATATAAGTATA
AGGTAGTACAAATTGAACCACTA---GGTGTGGCACCCACCCATGCAAAAAGAAGAGTGGTG-----------
----------------------------GAGAGAGGAAAAAGAGCA---GTTGGA---ATAGGAGCT------
---GCTTTCCTTGGGTTCTTATCAGCA---GCAGGAAGCACTATG---GGCGCGGCGTCA---
ATAACGCTGACGGTACAG---GCCAGACAA---TTATTG---TCTGGTATAGTGCAACAGCAAAGC---
AATTTG---CTGAAG---GCTATAGAGGCTCAACAACATCTGTTGAAACTCACAGTCTGGGGCATT
>FJ388896
TTCTATTGCAATACATCAGGCCTGTTTAATAGCTCTTGGTATAACAATGGCACTGCCATCTCAAACCACACAG
GGTCAAAT---
-------------GGCACT------
GGGTCAAATGGCACTATCACTCTCCAATGCAGAATAAAGCAAGTTATAAATATGTGGCAGAAAGTAGGACAGG
CAATGTATGCCCCTCCCATCCCAGGAATAATAAGATGTGACTCAAAC---
ATTACTGGACTACTATTAACAAGAGATGGAGGGAGTAATAATAATACAAAT----------------------

----

GAGACTAAGACTAAGATCTTCAGGCCTGGAGGAGGAGATATGAGGGACAATTGGAGAAGTGAATTATACAAGT
ATAAAGTAGTAGAAATTGAACCACTA---GGTGTAGCACCCACCCCTGCAAGAAGAAGAGTGGTG--------
-------------------------------CAGAGAGAAAAAAGAGCA---GTTGGA---ATGGGAGCT---
------TTGCTCTTTGGGTTCTTAGGAGCA---GCAGGAAGCACTATG---GGCGCGGCGTCA---
ATAACGCTGACGGTACAG---GCCAGACAA---TTACTG---TCTGGTATAGTGCAACAGCAGAGC---
AATCTG---CTGAAG---GCTATAGAGGCTCAACAACAGATGTTGAGACTCACGGTCTGGGGCATT
>DQ926899
TTCTATTGCAATACATCAGAACTGTTTAATAGCACTTGGGGCAATAACACCTGGGACAATAACACTGCC----
----

AGCTCAGAAAATATAACTCTCCAATGCAGAATAAAACAATTTATAAATATGTGGCAGAGAGTAGGAAGAGCAA
TGTATGCCCCTCCCATCCGAGGAATAATAAGTTGTAAATCAAAC---
ATTACTGGACTACTATTAACAAGAGATGGAGGGAAATATAATGATAGC-------------------------
----

AATGCAACTCAGATCTTCAGGCCTGGAGGAGGAGATATGAGGGACAATTGGAGAAGTGAATTATATAAGTATA
AAGTAGTAAAAATTGAACCACTA---GGTGTAGCACCCACACATGCAAGAAGAAGAGTGGTG-----------
----------------------------GGGAGAGACAAAAGAGCA---GTTGGA---CTGGGAGCT------
---TTCTTCCTTGGGTTCTTAGGAGCA---GCAGGAAGCACTATG---GGCGCAGCGTCA---
ATAACGCTGACGGTACAA---GCCAGACAA---TTATTG---TCTGGTATAGTGCAACAGCAAAGC---
AATTTG---CTGAAA---GCTATAGAGGCTCAACAACAGCTGTTGAGACTCACGGTCTGGGGCATT
>JN882651
TTCTATTGCAATACGACAAGCCTGTTTAACAACAGCCAGTGGCCATTTAATGGGAGTAGTAACCCTGTC----
----

CCTAATGGCACCATAACTCTCCAATGCAGAATAAAGCAATTCATAAATATGTGGCAGAGAGTAGGACAAGCAA
TGTATGCCCCTCCCATCCCAGGAAATATAAGTTGTATGTCAAAC---
ATTACAGGACTACTATTAATAAGAGATGGAGGGAATGGTAATAGT----------------------------
----

ACAAATGAGACTTTTAGGCCTGGAGGAGGAGATATGAGGGACAATTGGAGAAGTGAATTATATAAGTATAAAG
TAGTAAAAATTGAACCAATA---GGTATAGCACCCACCCATGCAAGAAGAAGGGTGGTG--------------
-------------------------GGGAGAGACAAAAGAGCA---GCTGGA---CTGGGAGCT---------
GTCTTCCTTGGGTTCTTAGGAGCA---GCAGGAAGCACTATG---GGCGCGGCGTCA---
ATAACGCTGACGGTACAG---GCCAGACAA---TTATTG---TCTGGTATAGTGCAACAGCAGAGC---
AATCTG---CTGAAG---GCTATTGAGGCTCAACAGCATCTGTTGAAACTCACGGTCTGGGGCATT
>MH666158
TTCTATTGCGATACATCAGGACTGTTTAATAGTTCTTGGAATGGCACTAGTCCTAGGAATGATACT-------
----

ACCAATAGCACTATAACTCTCCAATGCAGAATAAAGCAAATTATCAATATGTGGCAGAGAGTAGGGCAAGCAA
TGTATGCCCCTCCCATCCGAGGGGTAATAAGGTGTGAATCAAAC---
ATTACTGGACTATTATTAACAAGAGATGGAGGGAATAAAAATAAT----------------------------
----

AGTGATCCTGAGATCTTCAGGCCTGCAGGAGGAGATATGAGAGACAATTGGAGAAGTGAATTATATAAGTATA
AAGTAGTGAAAATTGAACCACTA---GGTATAGCACCCAACCGTGCAAAAAGAAGAGTAGTG-----------
----------------------------GGGAGAGAAAAAAGAGCA---GTTGGA---CTGGGAGCT------
---GTCTTCCTTGGGTTCCTAGGAGCA---GCAGGAAGCACTATG---GGCGCGGCGTCA---
ATAACGCTGACGGTACAG---GCCAGACAA---TTACTG---TCCGGTATAGTGCAACAGCAGAGC---
AATCTG---CTGAAG---GCTATAGAGGCTCAACAACAGCTGTTGAGACTCACAGTTTGGGGCATC
>JN882653
TTCTATTGCAATACATCAATACTGTTTAATGGTACATTTAATGGTACAGAATTTAATGATACAGAAATTAAC-
----

AATACAACCACACTCCGACTCCCATGCAGAATAAAACAGATTGTAAACATGTGGCAGGGAGTAGGAAAAGCAA
TGTATGCCCCTCCCATTGAAGGAAAAATCAACTGTTTCTCAAAT---
ATTACAGGGCTACTATTAGTAAGAGATGGAGGGACTAATAATAGT----------------------------

----

ACAAATGAGACCTTTAGACCTGGAGGAGGAGATATGAGGGACAATTGGAGAAGTGAATTATATAAGTATAAAG
TAGTAAAAATTGAACCATTA---GGAGTAGCACCCACCAGGGCAAGGAGAAGAGTGGTG--------------
-------------------------GAGAGAGAAAAAAGAGCA---ATTGGA---ATGGGAGCT---------
CTTTTCCTTGGGTTCTTAGGAGCA---GCAGGAAGCACTATG---GGCGCAGCGTCA---
ATAACGCTGACGGTACAG---GCCAGACAA---TTATTG---TCTGGCATAGTGCAACAGCAGAGC---
AATCTG---CTGAGA---GCTATAGAGGCTCAGCAGCAAATGTTGAGGCTCACGGTCTGGGGCATT
>JF683752
TTCTATTGCAATACATCAGGACTGTTTAATAGTACTTGGAATACCACTGATACTTGGAATAACACTTATAGC-
----

CACAATGACACTATAACTCTCCAATGCAGAATAAAGCAATTTGTAAATATGTGGCAGAGAGTAGGGCGGGCAA
TGTATAACCCTCCCATCCCAGGAGAAATAAAGTGTGCATCAAAC---
ATTACTGGACTACTATTAACAAGAGATGGAGGGAGTAATAATATT----------------------------
----

ACAAATGAGATCTTCAGGCCTGCAGGAGGAGATATGAGAGACAATTGGAGAAGTGAACTATATAAGTATAAAG
TAGTAAAAATTGAACCACTG---GGTGTAGCACCCACCCCTGCAAAAAAAAGAGTAGTG--------------
-------------------------GGGAGAGAGAAAAGAGCA---GTTGGA---CTGGGAGCT---------
GTCTTCCTTGGGTTCCTAGGACCA---GCAGGAAGCACTATG---GGCGCGGCGTCA---
ATAACGCTGACGGTACAG---GCCAGACAA---TTATTG---TCTGGTATAGTGCAACAGCAGAGC---
AATTTG---CTGAAG---GCTATAGAGGCTCAACAACATATGTTGAGACTCACAGTCTGGGGCATT
>MH666162
TTCTACTGCAATACATCAGGACTGTTTAATAGTACTTGGAATAGTACTTGGATCAACTTAGATAAC-------
----

TTAAATGGCAGCATAACTCTCCAATGCAAAATAAAGCAATTTATAAATATGTGGCAGAGAGTAGGGCAAGCAA
TGTATGCCCCTCCCATCAAAGGAATGATACAGTGTAGATCAAAC---
ATTACTGGACTAATATTAACAAGAGATGGAGGGAATACTGAGAATGCT-------------------------
----

ACTGATAATGATACAGAGATCTTCAGGCCTACAGGAGGAGATATGAGAGACAATTGGAGAAGTGAATTATATA
AGTATAAAGTAGTAAAAATTGAACCACTA---GGTGTAGCACCCACCCATGCAAGAAGAAGAGTGGTG-----
----------------------------------GAGAGACAAAAAAGAGCA---GTTGGA---
CTGGGAGCT---------GTCTTCCTTGGGTTTCTAGGAGCA---GCAGGAAGCACTATG---
GGCGCAGCGTCA---ATAACGCTGACGGTACAG---GCCAGACAA---TTATTG---
TCTGGTATAGTGCAACAGCAGAGA---AATTTG---CTGCAA---
GCTATAGAGGCTCAACAACATATGTTGAGACTCACAGTCTGGGGCATT
>JF683766
TTCTACTGCAATACATCAACACTATTTAATAGAACATGGAATGGTACCACATGGAAT----------------
----

AACACAGCAAATAGTACAATCCCATGTAGAATAAAACAAATTATAAACATGTGGCAGGGAGTAGGAAAAGCAA
TGTATGCCCCTCCCATTGAAGGACAAATAAATTGTACATCAAAT---
ATTACAGGGCTACTACTGACAGTAGATGGTGGTAATAATACTAAT----------------------------
----

AATTCAGAGACATTCAGACCTGGAGGAGGAGATATGAAAGACAATTGGAGAAGTGAATTATATAAGTATAAGG
TAGTACAAATTGAACCAATA---GGTGTGGCACCCACCCATGCAAAAAGAAGAGTGGTG--------------
-------------------------GAGAGAGAAAAAAGAGCA---GTTGGA---CTGGGAGCT---------
GTCTTCCTTGGGTTCTTAGGAGCA---GCAGGAAGCACTATG---GGCGCGGCGTCA---
ATAACGCTGACGGTACAG---GCCAGACAA---TTATTG---TCTGGTATAGTGCAACAGCAGAGC---
AATCTG---CTGAGG---GCTATAGAGGCTCAACAACATCTGTTGAAACTCACAGTCTGGGGCATT
>AB231897
TTCTATTGCAATACATCAACACTGTTTAATAGCACTTGGATTAGGAATAGCTCAAATAGCACTTGGAATAGCT
CTGCCTCGAAC--
-------------AGCACA------
GAGTTAAATAGCAATATAACTCTTCAGTGCAGAATCAAGCAAATTGTAAGAATGTGGCAGAGAGTAGGACAAG
CAATGTATGCCCCTCCAATCGCAGGAGACATAAGGTGTGAATCAAAC---

ATTACTGGACTACTATTAACAAGAGATGGAGGGCATGAGACTGAG----------------------------
----

AATAAGACTGAGATCTTCAGGCCTGGAGGAGGAGATATGAGGGACAATTGGAGAAGTGAATTATATAAGTATA
AAGTAGTAAAAATTAAACCATTA---GGTATAGCACCCACCCATGCAAAAAGAAGAGTGGTG-----------
----------------------------ACGAGAGAAAAAAGAGCA---GTTGGA---CTGGGAGCT------
---GTCTTCCTTGGGTTCTTAGGAACA---GCAGGAAGCACTATG---GGCGCAGCGTCA---
CTAACGCTGACGGTACAG---GCCAGACAA---TTATTG---TCCGGTATAGTGCGACAGCAAAAC---
AATTTG---CTGAAG---GCTATAGAGGCTCAACAACAGCTGTTGAGACTCACGGTCTGGGGCATT
>AB480046
TTCTATTGCAATACATCAGGCCTGTTTAATAGTACCTGGTATGGAAATGGCAATGACACCACAAATGGCACGA
TTCTA--
-------------------CAT---
GGTAATAGAACTCATATAACTCTCCCATGCAGAATAAGACAAATTGTACATATGTGGCAGAGAGTAGGACAAG
CAATGTATGCCCCTCCCATTAAAGGAATAATAAAATGTACATCAAAC---
ATTACAGGGCTACTGCTAACAAGAGATGGTGGGAATAATAATAGT----------------------------
----

ACAAATGAGACATTCAGACCTGGGGGAGGAAATATGAAGGACAATTGGAGAAGTGAATTATATAGATATAAAG
TAGTAAAAATTGAACCACTA---GGAGTAGCACCCACCAGGGCAAAAAGAAGAGTGGTG--------------
-------------------------GAGAGAGAAAAAAGAGCAATTGTTGGA---CTGGGAGCT---------
GTCTTCTTTGGGTTCTTAGGAGCA---GCAGGAAGTACTATG---GGCGCGGCGTCA---
ATAACGCTGACGGTACAG---GCCAGACAA---TTATTG---TCTGGTATAGTACAACAGCAGAAC---
AATCTG---CTAAGG---GCCATAGAGGCTCAACAGCAACTGTTGAAACTCACGGTCTGGGGCATT
>MK086119
TTCTATTGCAATACAACAGACCTATTT------TCTTGGAATAGTAGTAGCCCATGGAAGAACATAACG----
----

TCAAATGACAGTATAACTCTCCAATGCAGAATAAAGCAAATTGTAAGGATGTGGCAGCAAGTAGGACAAGCAA
TGTATGCCCCTCCTATACCAGGAATAATAAGGTGTGAATCAAAC---
ATTACAGGAATACTGTTAACAAGAGATGGAGGAAATAATAATACA----------------------------
----

AATAACACAGAGGTATTCAGGCCTGGAGGAGGAGATATGAGGGACAATTGGAGAAGTGAATTATATAAATATA
AAGTAGTAAAAATTGAACCATTA---GGTGTAGCACCCTCCCGTGCAAGAAGAAGAGTGGTG-----------
----------------------------GAGAGAGAAAAAAGAGCA---GTTGGC---CTGGGAGCT------
---TTCTTCCTTGGGTTCTTAGGAGCA---GCAGGAAGCACTATG---GGCGCGGCGTCA---
ATAACGCTGACGATACAG---GCCAGACAA---TTATTG---TCTGGTATAGTGCAGCAGCAGAGC---
AATTCTGCTGACGGGCTC---ATAGAGGCTCAACAACATCTGTTGAAACTCACGGTCTGGGGCATT
>MK086120
TTCTATTGCAATACAACAGACCTATTT------TCTTGGAATAGTAGTAGCCCATGGAAGAACATAACG----
----

TCAAATGACAGTATAACTCTCCAATGCAGAATAAAGCAAATTGTAAGGATGTGGCAGCAAGTAGGACAAGCAA
TGTATGCCCCTCCTATACCAGGAATAATAAGGTGTGAATCAAAC---
ATTACAGGAATACTGTTAACAAGAGATGGAGGAAATAATAATACA----------------------------
----

AATAACACAGAGGTATTCAGGCCTGGAGGAGGAGATATGAGGGACAATTGGAGAAGTGAATTATATAAATATA
AAGTAGTAAAAATTGAACCATTA---GGTGTAGCACCCTCCCGTGCAAGAAGAAGAGTGGTG-----------
----------------------------GAGAGAGAAAAAAGAGCA---GTTGGC---CTGGGAGCT------
---TTCTTCCTTGGGTTCTTAGGAGCA---GCAGGAAGCACTATG---GGCGCGGCGTCA---
ATAACGCTGACGGTACAG---GCCAGACAA---TTATTG---TCTGGTATAGTGCAGCAGCAGAGC---
AATCTG---CTGCGG---GCCATAGAGGCTCAGCAACATCTGTTGAAACTCGCGGTCTGGGGCATT
>KR017771
TTCTACTGTAATACAACAAATCTGTTTAATAATCTGTTTAATAATACAGAA----------------------
----

GACAATGGTACAATCACTATTCCATGTAGAATAAAGCAAATTATAAACATGTGGCAAAGAGTAGGACGAGCAA
TGTATGCCCCTCCCATAGCCGGAAAAATTCAGTGTAACTCAACT---

ATCACAGGTCTACTATTAACAAGAGATGGTGGTAATACTAATGTG----------------------------
----

ACTGTGACTGAGATCCTCAGACCTGGAGGGGGAGATATGAGAGACAATTGGAGAAGTGAACTGTATAAATATA
AGGTAGTGAAAATTGAACCACTA---GGAGTAGCACCCACCAGGGCAAAAAGACAAGTGGTA-----------
----------------------------ACCAGAGCAAAAAGAGAC---ATGGGA---ATAGGAGCT------
---ATGCTCCTTGGGTTCTTGGGAGCA---GCAGGAAGCACTATG---GGCGCGGCGTCA---
ATAACGCTGACGGTACAG---GCCAGACAA---CTATTG---TCTGGTATAGTGCAACAGCAAAAT---
AATTTG---CTGAAG---GCTATAGAAGCGCAACAGCATCTGTTGCAGCTTACAGTCTGGGGCATT
>MK086117
TTCTATTGCAATACAACAGACCTATTT------TCTTGGAATAGTAGTAGCCCATGGAAGAACATAACG----
----

TCAAATGACAGTATAACTCTCCAATGCAGAATAAAGCAAATTGTAAGGATGTGGCAGCAAGTAGGACAAGCAA
TGTATGCCCCTCCTATACCAGGAATAATAAGGTGTGAATCAAAC---
ATTACAGGAATACTGTTAACAAGAGATGGAGGAAATAATAATACA----------------------------
----

AATAACACAGAGGTATTCAGGCCTGGAGGAGGAGATATGAGGGACAATTGGAGAAGTGAATTATATAAATATA
AAGTAGTAAAAATTGAACCATTA---GGTGTAGCACCCTCCCGTGCAAGAAGAAGAGTGGTG-----------
----------------------------GAGAGAGAAAAAAGAGCA---GTTGGC---CTGGGAGCT------
---TTCTTCCTTGGGTTCTTAGGAGCA---GCAGGAAGCACTATG---GGCGCGGCGTCA---
ATAACGCTGACGATACAG---GCCAGACAA---TTATTG---TCTGGTATAGTGCAGCAGCAGAGC---
AATCTG---CTGCGG---GCCATAGAGGCTCAACAACATCTGTTGAAACTCACGGTCTGGGGCATT
>MK086118
TTCTATTGTAATACATCAGAACTATTTAATAGCACATGGAATAGTAGCACTCCTAGCACT-------------
----

GGAAATAGAACTATAACTCTCCAATGCAAAATAAAGCAAATTGTAAGAATGTGGCAAAAGGTAGGACAAGCAA
TGTATGCTCCTCCCATTAGTGGAAATATAAGTTGTAACTCAAAC---
ATTACAGGATTGCTATTAACAAGAGATGGAGTGGACAATAATAGT----------------------------
----

ACAAATGAGACATTCAGGCCTGGAGGAGGAGATATGAGGGACAATTGGAGAAGTGAATTATATAAGTATAAGG
TAGTACAAATTAAACCATTA---GGTGTGGCACCCACCCAGGCAAAAAGAAGAGTGGTG--------------
-------------------------CAGAGAGAAAAAAGAGCA---ATTGGA---CTGGGAGCT---------
GTGTTCATTGGGTTCTTAGGAGCA---GCAGGAAGCACTATG---GGCGCGGCGTCA---
ATAACGCTGACGGTACAG---GCCAGAGAA---TTATTG---TCTGGTATAGTGCGACAGCAGAGC---
AATCTG---CTGCAG---GCTATAGAGGCTCAACAGCATCTGTTGAAACTCACAGTCTGGGGCATT
>MK086115
TTCTATTGCAATACAACAAGACTATTTAATAGCATTTGGAGTAGGAATGAGACTAAGAATGACAATGACACTT
GGAGTAAT---
-ATCACTAATAGCACTGCC------
TGGACAAATGACACTATAACTCTCCAATGCAGAATAAAGCAAATTGTAAGAATGTGGCAGAGAGTAGGACAAG
CAATGTACGCCCCTCCCATCCAAGGAGAAATAAAGTGTGAGTCAAAC---
ATTACAGGATTACTATTAACAAGAGATGGAGGTGCACATGGTGAC----------------------------
----

AATGAGACATTCAGGCCTGGAGGAGGAAATATGAAGGACAATTGGAGAAGTGAATTATATAAGTATAAGGTAG
TACAAATTAAACCATTA---GGTGTAGCACCTACACATGCAAAAAGAAGAGTGGTG-----------------
----------------------GAGAGAGAAAAAAGAGCA---GTTGGA---CTGGGAGCT---------
GTCTTCCTTGGGTTCTTAGGAGCA---GCAGGAAGCACTATG---GGCGCGGCGTCA---
ATAACGCTGACGGTACAG---GCCAGACAA---TTATTG---TCTGGTATAGTGCAACAGCAGAGC---
AATTTG---CTGAGG---GCTATAGAGGCTCAACAACATCTGTTGAAACTCACAGTCTGGGGCATT
>MK086126
TTCTATTGTAATACAACACCACTATTTAATAGCACTTGGAAGAGCAATGACACTGCCAGTTCAAAC-------
----

CCAAATGACACTATAACTCTCCAATGCAGAATAAAACAAATTGTAAATATGTGGCAGAAAGTAGGACAAGCAA
TGTATGCCCCTCCCATCTCAGGAAACATAATGTGTAACTCAACC---

ATTACAGGAATACTATTAACAAGAGATGGAGGGATTAATAATACA----------------------------
----

GAGGAGGAGGAGACGTTCAGGCCTGGAGGAGGAGATATGAGGGACAATTGGAGAAGTGAATTATATAAGTATA
AGGTAGTAAAAATTGAGCCACTA---GGTGTGGCACCCACCTATGCAAAAAGAAGAGTGGTG-----------
----------------------------GAGAGAGAAAAAAGAGCA---GTTGGA---CTGGGAGCT------
---GTCTTCCTTGGGTTCTTAGGAGCA---GCAGGAAGCACTATG---GGCGCGGCGTCG---
ATAACGCTGACGGTACAG---GCCAGACAA---TTATTG---TCTGGTATAGTGCAACAGCAGAGC---
AATCTG---CTGAAG---GCTATAGAGGCTCAACAACATCTGTTGAAACTCACGGTCTGGGGCATT
>JN864057
TTCTATTGCAATACAACAAGACTGTTTAATAGCATGTGGATCAAAAATGTCAGCAGTGACACTTGGAATAACA
ACATCACTAGT--

GATAACAATGGCACTATAACTCTCCAATGCAGAATAAAGCAAATTGTAAATATGTGGCAAAGAGTAGGACAAG
CAATGTATGCCCCTCCCATCCAAGGAGAATTAAGGTGTGAATCAAAC---
ATTACAGGACTACTGTTAACAAGAGATGGAGGGGATACTAATAAT----------------------------
----

ACAGAGACCTTCAGGCCTGGAGGAGGAGATATGAAGGACAATTGGAGAAGTGAATTATACAAGTATAAAGTAG
TGAAAATTGAACCACTA---GGTATAGCACCCACCCCTGCAAAAAGAAGAGTGGTG-----------------
----------------------GAGAGAGAAAAAAGAGCA---GTTGGA---CTAGGAGCT---------
GTTTTCCTTGGGTTCTTAGGAGCA---GCAGGAAGCACTATG---GGCGCGGCGTCA---
ATAACGCTGACGGTACAG---GCCAGACTA---TTAATG---TCTGGTATAGTGCAACAGCAGAGC---
AATTTG---CTGAAG---GCTATAGAGGCTCAACAACAGCTGTTGAGACTCACGGTCTGGGGCATT
>MK086116
TTCTACTGTAATACAACAGTTCTGTTTAATACCACAGGG----------------------------------
----

TTAAAAGACAGTATCACTATTCCATGTAAAATAAAACAAATCATAAACATGTGGCAAAGAGTAGGACGAGCAA
TGTATGCCCCTCCCATTGCAGGAAAAATTCAATGTAACTCAAGT---
ATTACAGGTCTACTCTTAACAAGAGATGGTGTTAATGATACAAAA----------------------------
----

AATGAAACCCTCAGACCTGGAGGGGGAGATATGAGAGACAATTGGAGAAGTGAACTGTATAAATATAAGGTAG
TGCAAATTGAACCACTA---GGAGTAGCACCCACCAGGGCACAAAGACGAGTGGTA-----------------
----------------------CAGAGAGAAAAAAGAGCAGTAGTGGGA---CTAGGAGCT---------
ATGTTCATTGGGTTCTTGGGAGCA---GCAGGAAGCACTATG---GGCGCGGCGTCA---
ATAACGCTGACGGTACAG---GCCAGACAA---TTATTG---TATGGTATAGTGCAACAGCAGAGC---
AATCTG---CTGAGG---GCTATAGAAGCGCAACAGCATCTGTTGCAGCTCACAGTCTGGGGCATT
>AY371147
TTCTATTGCAATACAACAACACTATTTAATAGCACTTGGGATACAAATAGCACTTGGAATACCAGCATAAGCA
GCACA--

GGGTCAAATGACACCATAACTATCCCATGCAGAATAAAGCAAATTGTAAATATGTGGCAGAGAGTAGGACAAG
CAATGTATGCCCCTCCCATCCGAGGAAAGATACAGTGTAAGTCAAAC---
ATTACAGGATTACTATTGACAAGAGATGGTGGGGGTGATAACAGG----------------------------
----

ACAAATGAGACTTTTAGACCTGAAGGAGGAAATATGAAAGACAATTGGAGAAGTGAATTATATAAATATAAAG
TGGTAAAACTTGAACCACTA---GGTGTAGCACCCACACAGGCAAGAAGAAGAGTGGTG--------------
-------------------------CAGAGAGAAAAAAGAGCA---GTTGGA---ATGGGAGCT---------
TTATTCCTTGGGTTCTTAGGAGCA---GCCGGAAGCACTATG---GGCGCGGCGTCG---
CTAACGCTGACGGTACAG---GCCAGACAA---TTATTG---TCTGGTATAGTGCAACAGCAAAGC---
AATTTG---CTGAGG---GCTATAGAGGCTCAACAGCATATGTTGAGACTCACAGTCTGGGGCATT
>AB480048
TTCTATTGTAATACAACAGGACTGTTTAATATATCAAGTAATGAACTAAATAATACATTAGATAATAAT----
----

AACAATGAGAGTATCACACTCCCATGTAGAATAAAACAAATTGTAAGAATGTGGCAGAGAGTGGGACAAGCAA
TGTATGCACCTCCCATTGCAGGAAGAATTCAATGTAAATCAAAC---

ATTACAGGAATACTATTAACAAGAGATGGTGGAGAAGGAGAGAAT---------------------------
----

AGCACTAAGGAGATCTTCAGACCTGGAGGAGGAGATATGAGGGACAATTGGAGAAGTGAATTATATAAATATA
AAACAGTAAAAATCAAATCACTA---GGAATAGCACCCACCAGGGCAAGGAGACGAGTGGTG----------
---------------------------GGGAGAGAAAAAAGAGCA---GTTGGA---CTGGGAGCT------
---GTCTTCCTTGGGTTCTTAGGAGCA---GCTGGGAGCACTATG---GGCGCGGCGTCA---
ATAACGCTGACGGTACAG---GTCAGACAA---TTGTTG---TCTGGCATAGTGCAACAGCAAAGC---
AATTTG---CTGAGG---GCTATAGAGGCGCAGCAGCATCTGTTGCAACTCACAGTCTGGGGCATT
>AJ866557
TTCTATTGCAATACATCACAACTGTTTAACAGTACTTGGAGTAACAATGGCACTGCCATCCACACAGGG----
----

TCAAATGATACTATAACTCTCCAATGCAGAATAAAGCAAATTGTAAGGATGTGGCAGAGAGTAGGACAAGCAA
TGTATGCCCCTCCTATCCCAGGAATAATAAGGTGTGAATCAAAC---
ATTACAGGCCTACTATTAACAAGAGATGGAGGGAATAGTAATAATAGT------------------------
----

AATACAAATGAGACTTTCAGGCCTGGAGGAGGAGATATGAGGGACAATTGGAGAAGTGAATTATATAAGTATA
AAGTAGTAAGAATTGAACCACTA---GGTGTAGCACCCTCCCGTGCAAGAAGAAGAGTGGTG----------
---------------------------GAGAGAGAAAAAAGAGCA---GTTGGA---ATAGGAGCT------
---GTTTTCCTTGGGTTCTTAGGAGCA---GCAGGAAGCACTATA---GGCGCAGCATCA---
GTAACGCTGACGGTACAG---GCCAGACAA---CTATTG---TCTGGTATAGTGCACCAGCAGAGC---
AATCTG---CTGAAG---GCTATAGAGGCTCAACAACAGCTGTTGAGACTCACAGTCTGGGGCATT
>MH667256
TTCTATTGTAATACATCAAACCTGTTTAATAGCACTTGGAACAATAGCACTTGGGATAACAATAACACT----
----

GGGAATGAGAATATAACTCTCCAATGCAAAATAAAGCAATTTGTAAATATGTGGCAGAGGGTAGGAAGAGCAA
TGTATGCCCCTCCCATCCGAGGAGTAATAAAGTGTGTATCACAC---
ATTACAGGACTAATATTAACAAGAGATGGAGGAAATAATAATACA---------------------------
----

CAGGAGGAGATTTTTAGGCCTGGAGGAGGAGATATGAGGGACAATTGGAGAAGTGAATTATATAAATATAAAG
TAGTAAAAATTAAACCACTA---GGAGTAGCACCCACTATGGCTAAAAGAAGAGTGGTG-------------
------------------------GAGAGAGAAAAAAGAGCA---GTTGGA---ATAGGAGCT---------
GTCTTCCTTGGGTTCTTAGGAGCA---GCAGGAAGCACTATG---GGCGCGGCGTCA---
GTGACGCTGACGGTACAG---GCCAGACAA---TTATTG---TCTGGCATAGTGCAACAGCAAAGC---
AATTTG---CTGAAG---GCTATAGAGGCTCAACAACAGCTGTTGAGACTCACGGTCTGGGGCATT
>FJ388944
TTCTATTGCAATACAACATTACTATTTGATAGCACTTGGTATGGAAATGGCTCTGACATCGACAAAATAAATA
AAACA---

ATGTCAAATGAAACTATAATTCTCCCATGCAGAATAAAGCAAATTGTACGCATGTGGCAGAGAGTAGGACAAG
CAATGTACGCCCCTCCCATCCCAGGAGAAATAAGGTGTGAGTCAAAC---
ATTACAGGACTACTATTGACAAGAGATGGTGGGGTAACGGTAGC---------------------------
----

AGTACAAATGAGACCTTCAGGCCTGGAGGAGGAGATATGAGAGACAATTGGAGAAGTGAATTATATAAGTATA
AGGTAGTAAAAATTGAACCACTA---GGTGTAGCACCCAACAAGGCAAGAAGGAGAGTGGTG----------
---------------------------GAGAGAGAAAAAAGAGCA---GTTGGA---ATAGGAGCT------
---GTCTTCCTTGGGTTCTTAGGAGCG---GCAGGAAGCACTATG---GGCGCGGCGTCA---
ATAACGCTGACGGTACAG---GCCAGGCAA---TTATTG---TCTGGTATAGTGCAACAGCAGAGT---
AATTTG---CTGAAG---GCTATAGAGGCTCAACAACACTTGTTGAGACTCACGGTCTGGGGCATT
>DQ313254
TTCTATTGCAATACATCAGGTCTGTTTAATAGTACTTGGTATCGGAATGGCACTGCCATCAGGCAGAACGGCA
CG--

GGGTTAAATGATACTATAACTCTCCCATGCAGAATAAGGCAAATTGTACGTACATGGCAGAGAGTAGGACAAG
CAATGTATGCCCCTCCCATTCAAGGAGTAATAAAATGTGAATCAAAC---

```
ATTACGGGGCTACTGTTAACAAGAGATGGTGGGAATAATAGTAGTAAT-------------------------
-------------------------------------------------------------------------
----------
AATGACACTGAGACCTTCAGACCTGGAGGAGGAGATATGGAAGACAATTGGAGAAGTGAATTATACAACTATA
AGGTAGTAAAAATTAAACCATTA---GGAATAGCACCCACCAAGGCAAGGAGAAGAGTAGTG-----------
----------------------------GGGAGAGAGAAAAGAGCA---GTTGGA---CTGGGAGCT------
---GTTTTCCTTGGGTTCTTAGGGACA---GCAGGAAGCACTATG---GGCGCAGCGTCA---
ATAACGCTGACGGTACAG---GTCAGACAA---TTATTG---TCTGGCATAGTGCACCAGCAAAGC---
AATTTG---CTGAGA---GCTATAGAGGCGCAGCAGCATCTGTTGCAGCTCACAGTCTGGGGCATT
>AM279364
TTCTATTGCAATACATCAGGCCTGTTTAATAGTACTTGGAATGACACTAGCATCAATGAGAACTACACGGAT-
-------------------------------------------------------------------------
----------------------------
CCAAATGCAACTCTTATTCTCCTATGCAGAATAAAACAAATTGTGCGTATGTGGCAGAGAGTAGGGCAAGCAA
TGTATGCCCCTCCCATTCCAGGAGTAATACAGTGTGTATCAAAC---
ATTACAGGGCTACTGCTAACAAGAGATGGTGGGAATAATACTAAT----------------------------
-------------------------------------------------------------------------
----------
AGGACAGATGAGACGTTCAGACCTGGAGGAGGAGATATGAAGGACAATTGGAGAAATGAGTTATATAAGTATA
AAGTAGTAAAAATTGAACCACTA---GGAGTAGCACCCACCAAGGCAAGGAGAAGAGTGGTG-----------
----------------------------CAGAGAGAAAAAAGAGCA---ATTGGA---ATGGGAGCT------
---CTTTTCCTTGGGTTCTTAGGAACA---GCAGGAAGCACTATG---GGCGCGGCGTCA---
ATAACGCTGACGGTACAG---GCCAGGCAA---TTATTG---TCTGGCATAGTGCAACAGCAGAGC---
AATCTG---CTGATG---GCCATAGAGGCTCACCAGCATTTGTTGAAACTCACGGTCTGGGGCATT
>KR017778
TTCTATTGCAATACATCACGATTGTTTAATAGCACTTGGACTGGGAATAACACTACTTGGAGTATCAGCAGCG
AGAACGATACT--------------------------------------------------------------
-------------------------
GGGAAAGATGATCCCATAATTCTCCCATGTAGAATAAAGCAAATTATAAATATGTGGCAGAGAGTAGGACAAG
CAATATACACTCCTCCTATTCAAGGAGAACTAAGGTGTGACTCAAAC---
ATTACAGGACTATTGTTAACAAGAGATGGTGGGAATAACAATGAC----------------------------
-------------------------------------------------------------------------
----------------
ACAGAGATCTTCAGACCTATAGGAGGAAATATGAAGGACAATTGGAGAAGTGAATTATATAAGTATAAAGTGG
TGGAGATTAAGCCACTA---GGAATAGCACCCACCAAGGCAAAAAGAAGAGTGGTG-----------------
----------------------GAGAGAGAAAAAAGAGCA---GTAGGA---ATAGGAGCT---------
GTACTCCTTGGGTTCTTGGGAGCA---GCAGGAAGCACTATG---GGCGCGGCGTCA---
ATAACGCTGACAGTGCAG---GCCAGACAA---TTATTG---TCTGGCATAGTGCAACAGCAAAAC---
AACTTG---CTGAAG---GCTATAGAAGCACAACAGCAGCTGTTAAAACTCACTGTCTGGGGCATA
>EU513192
TTCTATTGTAATACATCAGACCTGTTTAATTGGGAGAATGACACCTTGCAGGGGCCAAATAAGGCTGTG----
-------------------------------------------------------------------------
----------------------------
TCAAATGATAATATAACTCTCCCATGCAGAATAAAGCAAATTATAAATATGTGGCAGAGAGTAGGACAAGCAA
TATATGCCCCTCCTATCCAAGGAGTAATAAGGTGTAACTCAAAC---
ATTACAGGACTACTATTAACAAGAGATGGTGGGAACAATAGTAAT----------------------------
-------------------------------------------------------------------------
-------------
CCGACTGAGACCTTCAGACCTGAAGGAGGGGATATGAGGGACAATTGGAGGAGTGAATTATATAAGTATAAAG
TAGTAAAAATTGAACCACTA---GGAGTAGCACCCACCAAGGCAAGGAGAAGAGTGGTG--------------
-------------------------ACGAGAGAAAAAAGAGCA---ATTGGA---ATGGGAGCT---------
GTCCTCTTTGGGTTCTTAGGAGCA---GCAGGAAGCACTATG---GGCGCGGCGTCA---
ATAACGCTGACGGTACAG---GCCAGACAA---TTATTG---TCTGGCATAGTGCAACAACAGAAC---
AACCTG---CTGAGG---GCTATAGAGGCTCAACAGCAACTGTTGAAACTCACAGTCTGGGGCATT
>AY894995
TTCTATTGTAATACAACAAAATTATTTAATGGTATCTGGAATAAAACAGGTAGTACCACATATAACTATACAA
ATAGC--------------------------------------------------------------------
-------------------------
ACAGAGAATGATACTATTACACTGCCATGCAGAATAAAACAAGTTGTAAGAATGTGGCAGAGGACAGGACAAG
CAATGTATGCCCCTCCTATCCAGGGAATAATAAGGTGTGCATCTAAC---
```

ATTACAGGAATAATATTAACAAGAGATGGTGGGAATAACAATAAT----------------------------
----

GCAACTGAGACCTTCAGACCTGGAGGAGGAAATATGAAAGACAATTGGAGAAGTGAATTATATAAATATAAGG
TAGTGAAAATTGAACCACTA---GGAGTAGCACCCACTAGGGCAAGGAGGAGAGTAGTG--------------
-------------------------GGGAGAGAAAAAAGAGCA---GTTGGA---CTAGGAGCT---------
GTCTTCCTTGGGTTCTTGGGA------------------------GGCGCAAAATCA---
ATGACGCTGACGGTACAG---GCCAGACAA---TTATTG---TCTGGTATAGTGCAACAGCAAAGC---
AATTTG---CTGAGG---GCTATAGAGGCTCAACAACAGATGTTGAGACTCACGGTCTGGGGCATT
>AY371163
TTCTATTGCAATACATCAGGCCTGTTTAATAGCACTTGGAGAAACATTAATGGCACTTGGAATAGCACTGGCA
ACACA--

GAGTTAAATAGCACTATAATTCTCCTGTGTAGGATAAAACAAATTGTAAATATGTGGCAGAGAACAGGACAAG
CAATGTATGCCCCTCCCATCCCAGGAGTGATAAGGTGTGAGTCAAAC---
ATTACAGGACTACTATTGACAAGAGATGGTGGGAATGAGAATAATATTAAT----------------------
----

ACAAATACAACTGAAACCTTCAGACCTGGAGGAGGAGATATGAGAGACAATTGGAGAAGTGAATTATATAAAT
ATAAAGTAGTAAAAATCGAACCACTA---GGAGTAGCACCCACCAAGGCAAAGAGAAGAGTGGTG--------
-------------------------------GAGAGAGAAAAAAGAGCA---GTTGGA---TTGGGAGCT---
------GTTTTCCTTGGGTTCTTAGGAGCA---GCAGGAAGCACTATG---GGCGCGGCGTCA---
ATAACGCTGACGGTACAG---GCCAGACAA---TTATTG---TCTGGCATAGTGCAACAGCAAAGC---
AATTTG---CTGAGG---GCTATAGAGGCTCAACAGCATCTGTTGAGACTCACGGTCTGGGGCATT
>FM877779
TTCTATTGTAATACATCAAGCCTGTTTAATAGCACGTACTTGAATATAACA----------------------
----

AAGGGAAACATAACAATGCTCCCATGCAGGATAAAACAAATTGTAAACATGTGGCAGGGAGTAGGACGAGCAA
TATATGCCCCTCCTATTGCAGGAAAGATACAGTGTGTATCTAAT---
ATAACAGGACTAATATTGACAAGAGATGGGGGAAATGAAGATAAT----------------------------
----

AATACTGAAACCTTCAGACCTGGAGGAGGAAATATGAAAGACAATTGGAGAAGTGAATTATATAAGTATAAAG
TAGTAAAAATTGAACCACTA---GGAATAGCACCCACCAAGGCAAAGAGAAGAGTGGTG--------------
-------------------------GAGAGAGAAAAAAGAGCA---GTTGGA---CTGGGAGCT---------
CTTTTCTTTGGGTTCTTAGGAGCA---GCAGGAAGCACTATG---GGCGCAGCGTCA---
ATAACGCTGACGGTACAG---GCCAGACAA---TTATTG---TCTGGCATAGTGCAACAGCAAAGC---
AATTTG---CTGAGG---GCTATAGAGGCTCAACAGCACATGTTGAAGCTCACAGTCTGGGGCATT
>FM877778
TTCTATTGTAATACATCAGGACTGTTTAATAGCACTTGGCTGGTTAATAGCACTAGGGAGAATGGCACGAGC-
----

ACCATGGGAAACATAACACTCCCATGCAGGATAAAACAAATTGTAAACATGTGGCAGGGAGTAGGACAAGCAA
TGTATGCCCCTCCTGTCGCAGGAATGATACAGTGTGTATCAAAT---
ATTACAGGACTAATATTAACAAGAGATGGGGGAAGTAATGATAATAGTACT----------------------
----

GATAATAATACTGAGACCTTCAGACCTACAGGAGGAAATATGAAAGACAATTGGAGAAGTGAATTATACAAAT
ATAAAGTAGTACAACTTGAACCACTA---GGATTAGCACCCACCGAGGCAAAAAGAAGAGTGGTG--------
-------------------------------GAGAGAGAAAAAAGAGCA---GTTGGA---ATGGGAGCT---
------CTTTTCCTTGGGTTTTTAGGAACA---GCAGGAAGCACTATG---GGCGCGGTGTCA---
ATGACGCTAACGGTACAG---GCCAGACAA---TTATTG---TCTGGCATAGTGCAACAGCAAAGC---
AATTTG---CTGAGG---GCTATAGAGGCTCAACAGCATATGTTGAAACTCACAGTCTGGGGCATT
>FM877783
TTCTATTGTAATACATCAGGCCTGTTTAATAACGAGTGGAATAGCACTTGGAAGAATAGCACTTGG-------
----

GAAAATGGCACCATAACACTCCCATGCAGGATAAAACAAATAGTAAATATGTGGCAGGGAGTAGGACAAGCAA
TGTATGCCCCTCCTGTCGCAGGAATGATACAGTGTGCATCAAAT---

ATTACAGGACTAATATTGACAAGAGATGGGGGAGAGAATAATAGT----------------------------
----

AGTAAGGAGACCTTCAGACCTGGAGGAGGAGATATGAAGGACAATTGGAGAAGTGAATTATATAAGTACAAAG
TAGTACAACTTGAACCATTA---GGAGTAGCACCCACCAAGGCAAAAAGAAGAGTGGTG--------------
-------------------------GAGAGAGAAAAAAGAGCA---ATGGGA---ATGGCAGCT---------
ATGTTCCTTGGGTTCTTAGGAGCA---GCAGGAAGCACTATG---GGCGCGGCGTCA---
ATAACGCTGACTGTACAG---GTCAGACAA---TTATTG---TCTGGAATAGTGCAACAGCAAAGC---
AATTTG---CTGAGA---GCTATAGAGGCTCAACAGCATATGTTGCAGCTCACAGTCTGGGGCATT
>FM877784
TTCTATTGCAATACCTCAGGTCTGTTTAATAGCACTTGGAATGGAACTACAAGTGGGTCAAATGTCAAT----
----

ACAACTGGCAATATAACACTACCATGCAAAATAAAACAAATTATAAACATGTGGCAGAGAGTAGGACAAGCAA
TGTATGCTCCTCCCATCCCAGGAATAATACAGTGTATATCAAAT---
ATTACAGGGTTATTAATAACAAGAGATGGTGGAAATAATGCAACT----------------------------
----

AATGAAACGGAGATCTTCAGACCTGGAGGAGGAGATATGAGGGACAATTGGAGAAGTGAATTATATAAATATA
AAGTAGTAGAAATTGAACCACTA---GGAGTAGCACCCACCAAAGCAAAGAGAAGGGTGGTG-----------
----------------------------CAGAGAGAAAAAAGAGCA---GTAGGA---ATAGGAGCT------
---GTGTTCCTTGGGTTCCTAGGAGCA---GCAGGAAGCACTATG---GGCGCGGCGTCA---
ATGACGCTGACGGTACAG---GCCAGACAA---CTATTG---TCGGGCATAGTGCAACAGAAAAAC---
AACTTT---CTGAAG---GCTATAGAGGCTCAACAGCACCTGTTGAAACTCACGGTCGGGGGCATT
>KT022402
TTCTATTGTAACACATCAGGCCTTTTTAATAGCAAGTGGCATAGGGATAACAATAGCAGCATTGGGTATAGCA
ATAATAGCACA--

GAGTCAAATGACACTATAACTCTCCAATGCAGAATAAAGCAATTTATAAAACTGTGGCAGAGAGTAGGACAAG
CAATGTATGCCCCTCCCATCCCAGGAATAATAAGGTGTGACTCAAAT---
ATTACAGGTCTCATATTAACAAGAGATGGGGGAAATAACAATGCA----------------------------
----

AGCAATGAAACCTTCAGACCTGGGGGAGGAGATATGAGGGACAATTGGAGAAGTGAACTATATAAGTATAAAG
TAGTAAAAATTGAACCACTA---GGGTTAGCACCCTCCAGGGCAAGGAGAAGAGTGGTG--------------
-------------------------GAGAGAGAAAAAAGAGCA---GTTGGA---CTGGGAGCT---------
GTATTCCTTGGGTTCTTGGGAGCA---GCTGGAAGCACTATG---GGCGCAGCGGCA---
ATAACGCTGACGGCACAG---GCCAGACAA---TTATTG---TCTGGTATAGTGCAGCAGCAAAGC---
AATTTG---CTGCAA---GCTATAGAGGCTCAACAGCAACTGTTGAGACTCACGGTCTGGGGCATT
>KT022407
TTCTATTGCAATACATCAAGCTTGTTTAATAGCACATGGAACGGCACTGCACGGGATAACAAAAAC-------
----

TCAAATGATACTATGACTCTCCCATGCAAAATAAAACAAATTATAAACATGTGGCAGAGAGTAGGACAAGCAA
TATATGCCCCTCCCATTGCAGGAATGATACAGTGTACATCAAAC---
ATTACAGGAATAATATTGATAAGAGATGGGGGTAATCTCAGTGCG----------------------------
----

AGCAATGAGACCTTCAGACCTGGAGGAGGAGATATGAGGGACAATTGGAGAAGCGAATTATATAAGTATAAAG
TAGTAAAAATTGAACCACTA---GGAGTGGCACCCACCAGGGCAAGGAGAAGAGTGGTG--------------
-------------------------GAGAGAGAAAAAAGAGCA---GTTGGA---ATAGGAGCT---------
GTCTTCCTTGGGTTCTTAGGAGCA---GCAGGTAGCACTATG---AGCGCGGCGTCA---
ATAACGCTGACGGTACAG---GCCAGACAA---TTATTG---TCTGGCATAGTGCAACAGCAAAGC---
AATTTG---CTGAGG---GCTATAGAGGCTCAACAGCATCTGTTGAAACTCACGGTCTGGGGCATT
>KT022386
TTTTATTGTAACACATCAGGTCTGTTCAATAGCTCTTTCAAGAGCTCTTGGAAGGTTAATGTGTCAAAT----
----

GACAATAGCACTATAACTCTCCCATGCAGAATAAAACAAATTGTAAACATGTGGCAGAGAGTAGGACGTGCAA
TGTATGCCCCTCCCATTGCAGGAGTAATAAAATGTGAGTCAAAC---

ATTACAGGACTAATATTAACAAGAGATGGAGGGAAAGTGGACAAT----------------------------
----

GACAATGACACTGAGACCTTCAGACCTGGAGGAGGAGATATGAGGGACAATTGGAGAAGTGAATTATATAAGT
ATAAAGTAGTAAAACTTGAACCACTA---GGAGTAGCACCCACCGAGGCAAGGAGAAGAGCGGTG--------
-------------------------------CAGAGAGAAAAAAGAGCA---ATTGGA---CTGGGAGCT---
------TTCTTCCTTGGGTTCTTGGGAACA---GCAGGAAGCACTATG---GGCGCGGCGTCA---
CTAACGCTGACGGTACAG---GCCAGGCAA---TTATTA---TCTGGCATAGTGCAACAGCAAAGC---
AACTTG---CTGAAG---GCTATAGAGGCTCAACAACATATGTTGAGACTCACGGTCTGGGGCATT
>KU749429
TTCTATTGTGATACATCAATCCTGTTTAATAGCACTTGGTATCACAATGGCAGTATCTTGCAGAAGTCAAAT-
----

AGCACGGATAATATAATTCTCCCATGCAGAATAAAGCAAATTATAAATATGTGGCAGAGAGCAGGACAAGCAA
TATATGCCCCTCCCATCAAAGGACCAATAGTGTGTGAATCAAAT---
ATTACAGGACTAATATTAACAAGAGACGGTGGCACGGATGACAAT----------------------------
----

ACAACTGAAACTTTCAGACCTGGAGGAGGAGATATGAGGGACAATTGGAGAAGTGAATTATATAAGTATAAAG
TAGTAAAAATTGAACCATTA---GGATTAGCACCCTCCAGGGCAAGGAGAAGAGTGGTG--------------
-------------------------GAGAGAGAAAAAAGAGCA---GCGGGG---CTAGGAGCT---------
GTCTTCATTGGGTTCTTAGGAATG---GCAGGAAGCACTATG---GGCGCGGCGTCG---
ATGACGCTGACGGTACAG---GCCAGACAA---TTATTG---TCTGGTATAGTGCAACAGCAAAGC---
AATTTG---CTGAGG---GCTATAGAGGCTCAACAGCAGATGTTGAGACTCACGGTCTGGGGCATT
>KU749427
TTTTATTGCAATACAACAGAGTTGTTTAATGGCACATGGAGTAATGGCACTGAATGGAGCAAGGGCAATGTCA
GC---

ACAAATGGCAATATAACTATCCCATGCAGAATAAAACAAATTATAAACATGTGGCAGAGAGTAGGAAAAGCAA
TGTATGCTCCTCCCATCCCAGGAGTAATAAGGTGTGTATCAAAC---
ATTACAGGACTAATATTAACAAGAGATGGTGATACTAGCAGTGCA----------------------------
----

GATGAAACCTTCAGACCTGGAGGAGGAGATATGAGAGACAATTGGAGAAGTGAATTGTATAAGTATAAAGTAG
TAAAGATTGAACCACTA---GGAGTAGCACCTACCAGGGCAAAGAGAAGAGTGGTG-----------------
----------------------CAGAGAGACAAAAGAGCA---GTGGGA---ATGGGAGCT---------
TTATTCCTTGGGTTCTTGGGAGCA---GCAGGAAGCACTATG---GGCGCAGCGTCA---
ATAACGCTGACGATACAG---GCCAGACAA---TTATTG---TCTGGCATAGTGCAACAGCAAAGC---
AATTTG---CTGAAA---GCTATAGAGGCTCAGCAGCAACTGTTGAGGCTCACGGTCTGGGGCATT
>AF457088
TTCTATTGTAACACATCAGGCCTGTTTAATAATAGTGTTTGGAATAGCACTACCGTCACGCAGGAGTCAAATA
GCACG--

GAGTCAAATGGCAATATAACTCTCAATTGCAGAATAAAGCAAATTATAAATATGTGGCAGAGAGTAGGACAAG
CAATGTATGCCCCTCCTATTCCAGGAGTAATACAGTGTGTATCAAAC---
ATTACAGGACTAATATTAACAAGAGATGGTGGGAAGAATAACAGT----------------------------
----

ACAAATGAAACCTTCAGACCTGGAGGAGGAGATATGAGGGACAATTGGAGAAGTGAACTATATAAATATAAAG
TAGTAAAAATTGAACCACTA---GGAGTAGCACCCACCAGGGCAAAGAGAAGAGTGGTG--------------
-------------------------GAGAGAGAAAAAAGAGCC---GTTGGA---ATAGGAGCT---------
GTATTCCTTGGGTTCTTGGGAGCA---GCAGGAAGCACTATG---GGCGCGGCGTCA---
ATAACGCTGACGGTACAG---GCCAGACAA---TTATTG---TCTGGCATAGTGCAACAGCAAAGC---
AATTTG---CTGAGA---GCTATAGAGGCTCAACAACATCTGTTGAAACTCACGGTCTGGGGCATT
>FJ623492
TTCTATTGCAATACATCAGGCCTGTTTAATAGCACATGGAATAGCACATGGGAGAATAACATTAATGAC----
----

ACGAAGCCAAATATAACTCTCCCATGCAGAATAAAGCAAATTATAAATATGTGGCAGAGAACAGGACAAGCAG
TATATGCTCCTCCCATCCAAGGAGTAATAAGGTGTATATCAAAC---

ATTACAGGACTAATATTAACAAGAGATGGTGGGAATAATAGTAGG-------------------------
----

ACAAATGAAATCTTCAGACCTGGAGGAGGAGATATGAGAGACAATTGGAGAAGTGAATTATATAAGTATAAAG
TAGTAAAGATTGAACCACTA---GGAGTAGCACCCACCAAGGCAAGGAGAAGAGTGGTG-----------
------------------------CAGAGAGAAAAAAGAGCA---GTTGGA---ATAGGAACT-------
GTCCTCCTTGGGTTCTTAGGAGCA---GCCGGAAGCACTATG---GGCGCGGCGTCA---
ATAACGCTGACGGTACAG---GCCAGACAA---TTATTG---TCTGGCATAGTGCAACAGCAAAGC---
AATTTG---CTGAAG---GCTATAGAGGCTCAACAGCAGATGTTGAGACTCACTGTCTGGGGCATT
>AY945739
TTCTATTGTAATACATCAGGTCTGTTCAATAGCACTTGGTATGCCAATAGCAGCATGCAAGGGTCAAATGACA
CG--

GAGTTAAATGGCACTATAACTCTCCCATGCAGAATAAAACAAATTATAAATATGTGGCAAAGAGTAGGACAAG
CAATGTATGCCCCTCCCATCCAAGGAGTAATAAGATGTAAATCAAAT---
ATCACAGGACTACTGTTAACAAGAGATGGAGGGAAGGGAAATGAC-------------------------
----

ACTGAAACTTTCAGACCCGGAGGAGGAGATATGAGGGACAATTGGAGAAGTGAATTATATAAGTACAAAGTAG
TAAAAATTGAACCATTA---GGAGTAGCACCCAACAAGGCAAAGAGAAGAGTGGTG--------------
---------------------GAGAGAGAAAAAAGAGCA---GTTGGA---ATAGGAGCT-------
GTCTTCCTTGGGTTCTTAGGAGCA---GCAGGAAGCACTATG---GGCGCAGCGTCA---
CTAACGCTGACGGTACAG---GCCAGACAA---TTATTG---TCTGGCATAGTGCAACAGCAAAGC---
AATTTG---CTGAGG---GCTATAGAGGCTCAACAACATATGTTGAAACTCACGGTCTGGGGCATT
>KC473836
TTCTACTGTAACACATCAGACCTGTTTAATAGCACTTGGAATAGCACTGAGTCAAATAGCACAGAGGCAGAGT
CA--

AACAATGACACTATAACTCTTCCATGCAGAATAAAGCAAATTATAAATATGTGGCAGAGAGTAGGACAAGCAA
TATACGCCCCTCCCATCCCAGGAGTAATAAAGTGTAAATCAAAC---
ATTACAGGACTAATATTAACAAGAGATGGGGGTATTAACAATAGT-------------------------
----

AGCAGCTCCGAGACCTTCAGACCTGGAGGGGGAGATATGAGGGACAATTGGAGAAGTGAATTATATAAGTATA
AAGTAGTACAAATTGAACCATTA---GGAGTAGCACCCTCCAGGGCAAGGAGAAGAGTGGTG--------
---------------------------CAGAGAGAAAAAAGGGCA---GTAACC---CTAGGAGCT----
---CTATTCCTTGGGTTCTTGGGAACA---GCAGGAAGCACTATG---GGCGCAGCGTCA---
GTAACGCTGACGGTACAG---GCCAGGCAA---TTATTG---TCTGGTATAGTGCAACAGCAGAGC---
AATCTG---CTGAGG---GCTATTGAGGCGCAACATCATCTGTTGCAACTCACAGTCTGGGGCATT
>EU220698
TTCTATTGTAATACATCACAACTGTTTAATAGCACTTGGGACAAAAATGGCACTTGGATCAATGGCAGCGCAG
AC--

ACAACAAATGGAACTATCACTCTCCCGTGCAGAATAAAGCAAATTATAAATATGTGGCAGCGAGTAGGAAAAG
CAATGTATGCCCCTCCCATCCAAGGAGTAATAGAATGTAGATCAGAG---
ATTACAGGACTACTATTAACAAGAGATGGTGGGAATGAGAGCAGT-------------------------
----

TCAGCAGAGACCTTCAGACCTGGAGGAGGAAATATGAAGGACAATTGGAGAAGTGAATTATATAAGTATAAAG
TAGTAAAACTAGAACCACTA---GGAGTAGCACCCACCAAGGCAAGGAGAAGAGTGGTG-----------
------------------------GAGAGAGAAAAAAGAGCA---GCT------CTAGGAGCA-------
GTCCTTTTTGGGTTCTTAGGAGCA---GCAGGAAGCACTATG---GGCGCAGCGTCA---
ATAACGCTGACGGTACAG---GCCAGAAAA---TTATTG---TCTGGCATAGTGCAACAGCAAAGC---
AATTTG---CTGATG---GCTATAGAGGCTCAACAGCATATGTTGAAACTCACGGTCTGGGGCATT
>DQ083238
TTCTATTGTAATACATCAGAACTGTTTAATAGCACTTGGAATTCCAGTACGCAGGGGACAAATAACACAGAG-
----

CTAAATGACACGATAACTCTCCCATGCAGAATAAAGCAAGTTATAAATATGTGGCAGAGAGTGGGACAAGCAA
TGTATGCCCCTCCCATCAAAGGAATAATAAAGTGTGTATCAAAC---

ATTACAGGACTAATATTAACAAGAGATGGTGGGATTAATAGTACA----------------------------
----

AATGGAACTGAAACCTTCAGACCTGGAGGAGGAGATATGAGGGACAATTGGAGAAGTGAATTATATAAATATA
AGGTAGTACAAATTGAACCACTA---GGAGTAGCACCCACCAAGGCAAGGAGAAGAACGGTG-----------
----------------------------GGGAGAGAAAAAAGAGCA---ATTGGA---CTAGGAGCT------
---GTGTTCCTTGGGTTCTTAGGAGCA---GCAGGAAGCACTATG---GGCGCAGCATCA---
ATAACGCTGACGGTACAG---GCCAGACAA---TTATTG---TCTGGTATAGTGCAACAGCAAAGC---
AATTTG---CTGAGG---GCTATAGAGGCTCAACAACAAATGTTGAGACTCACGGTCTGGGGCATT
>AF067156
TTCTATTGTAATACATCAGGCCTGTTTAATGGCACTTGGAATGCCAGCATGCAGGAGTCAAATAGCACGGAG-
----

TCAAATGAAACTATAATTCTCCCATGCAGAATAAAACAAATTATAAATATGTGGCAGAGAGTAGGACAAGCAA
TGTATGCCCCTCCCATTCAAGGAATAATAAAGTGTGTATCAAAC---
ATTACAGGACTAATATTAACAAGAGATGGT---AAAAGTAGTAAC----------------------------
----

AGTACAGATGAAATCTTCAGACCTGGAGGAGGAGATATGAGGGACAATTGGAGAAGTGAATTATATAAGTATA
AAGTAGTAAAAATTGAACCACTA---GGAGTAGCACCCACCAGGGCAAGGAGAAGAGCGGTG-----------
----------------------------GGGAGAGAAAAAAGAGCA---GTTGGA---ATAGGAGCT------
---GTCTTCCTTGGGTTCTTAGGAGCA---GCAGGAAGCACTATG---GGCGCAGCGTCA---
ATAACGCTGACGGTACAG---GCCAGACAA---TTATTG---TCTGGCATAGTGCAACAGCAAAGC---
AATTTG---CTGAGG---GCTATAGAGGCTCAACAACATCTGTTGAAACTCACGGTCTGGGGCATT
>AF457061
TTCTATTGTAATACATCAGGCCTGTTTAATAGCACTTGGGGCTGGTATAATAACACTTGGAATAATAGCACTT
GGAATAAC---
-------------AGTGGC------
AACACACAGGAGAATATAACTCTTCAATGCAGAATAAAACAAATTGTAAACATGTGGCAGGAGGTAGGAAAAG
CAATGTATGCCCCTCCCATTGCAGGAGAAATAACATGTAAATCAAAT---
ATCACAGGACTACTACTGACACGTGATGGAGGAGTAGGGACAGACAATAAG----------------------
----

ACAAATGACACAGAAATATTCCGGCCTGGAGGAGGAGATATGAGGGACAATTGGAGAAGTGAATTATATAAAT
ATAAAGTAGTAGAAATTAAACCATTA---GGAATAGCACCCACTAAGGCAAAAAGGAGAGTGGTG--------
-------------------------------GAGAGAGAGAAAAGAGCA---GTGGGA---ATAGGAGCT---
------GTGTTCCTTGGGTTCTTGGGAGCA---GCAGGAAGCACTATG---GGCGCAGCATCA---
ATAACGCTGACGGTACAG---GCCAGACAA---TTACTG---TCTGGTATAGTGCAGCAGCAAAGC---
AATTTG---CTGAGG---GCTATAGAGGCGCAACAGCATATGTTGCAACTCACGGTCTGGGGCATT
>AF457087
TTCTATTGTAACACATCAGGCCTGTTTAATAGCACTTGGAATGACACTGCCATCGAGGCTAAGTCAAATAACA
CG---

GAGACAGACGGTATTATAACTCTCCAATGCAGAATAAAGCAACTTATAAATATGTGGCAGAGGGTAGGACAAG
CAATATATGCCCCTCCCATCCCAGGATTAATAAGGTGTGAATCAAAC---
ATTACAGGGCTAATATTAACAAGAGATGGTGGGAATAATGGTGGGAATAAT----------------------
----

AGCAGTGAAAAAAATGAAACCTTCAGACCTGGAGGAGGAGATATTAGGGACAATTGGAGAAGTGAATTATATA
AGTATAAAGTAGTAAAAATTGAACCACTA---GGAGTAGCACCCACCAAGGCAAGGAGAAGAGTGGTG-----
----------------------------------CAGAGAGAAAAAAGAGCA---ATTGGA---
CTGGGAGCT---------GTCTTCATTGGGTTCTTAGGAGCA---GCAGGAAGCACTATG---
GGCGCGGCGTCA---ATAACGCTGACGGTACAG---GCCAGACAA---TTATTG---
TCTGGCATAGTGCAACAGCAAAGC---AATTTG---CTGAGG---
GCTATAGAGGCTCAACAGCATCTGTTGAAACTCACAGTCTGGGGCATT
>KT022391
TTCTATTGTGATACATCAGGCCTGTTTAATAGCGCTTGGGCAGCCAATAGCACTTGGACAGCCAATGACACTG
AC---
-AGAAATCAGGTAGAT---------
AACAATAATGACACCATAACTCTCCCATGTAAAATAAAGCAAATTATAAATATGTGGCACAGAGTAGAACAAG

CAATATATGCCCCTCCCATTCCAGGAGTAATAAGGTGTGAATCAAAC---
ATTACAGGACTACTATTAACAAGAGATGGTGGGGTTAGGGTTGAC----------------------------
----
-
AATACAAGTAATACAAGTGAAACCTTCAGACCTGGAGGAGGAGATATGAAGGATAATTGGAGAAGTGAATTAT
ATAAGTATAAAGTAGTAGAAATTGAACCACTA---GGAGTAGCACCCACCAGGGCAAAGAGAAGAGTGGTG--
-------------------------------------GAGAGAGAAAAAAGAGCA---GTTGGA---
ATAGGAGCT---------GTTTTCATTGGGTTCTTAGGAGCA---GCAGGAAGCACTATG---
GGCGCGGCGTCA---GTGACGCTGACGGTACAG---GCCAGACAA---GTATTG---
TCTGGCATAGTGCAACAGCAAAGC---AATTTG---CTAAGG---
GCTATAGAGGCTCAACAACATCTGTTGAAACTCACGGTCTGGGGCATT
>KT022393
TTTTATTGTAACACATCAGGCCTGTTTAATAGCAGTTGGGCAAATAACACAGAGTCAAACAACACAGAG----
----

TTAAACAGAACTATAACGCTCCCATGCAGAATAAAGCAAATTATAAATATGTGGCAGAGAACAGGACAAGCAA
TGTATGCCCCTCCCATCCAAGGAGTAATAAGGTGTGAATCAAAC---
ATTACAGGATTAATATTGACAAGAGATGGTGGGGATAATAGTATA----------------------------
----

AATGAAACCTTCAGACCGGGAGGAGGAGATATGAGGGATAATTGGAGAAGTGAATTATATAAGTATAAAGTAG
TAAAAATTGAACCACTA---GGAGTAGCACCCACCAGGGCAAAGAGAAGAGTGGTG-----------------
----------------------GAGAGAGAAAAAAGAGCA---GTTGGA---ATAGGAGCT---------
GTCTTCCTTGGGTTCTTAGGAGCA---GCAGGAAGTACTATG---GGCGCGGCGTCA---
ATGACGCTGACGGTACAG---GCCAGGCAA---TTATTG---TCTGGAATAGTACAACAGCAAAGT---
AATTTG---CTGAGG---GCTATAGAGGCTCAACAGCATCTGTTGAAGCTCACGGTCTGGGGCATT
>KT022395
TTTTATTGTAATACATCAGGACTGTTTAATGAGAGTTGGGTAAATGGCACTGAACCAAGTAGCAATGATACT-
----

GATATGACTCCCATAACTCTCCAATGCAGAATAAAGCAAATTATAAATATGTGGCAGAGAATAGGACAGGCAC
TGTATGCCCCTCCCATCCCAGGAGAAATAAGGTGTAACTCAAAC---
ATTACCGGACTACTATTAGTAAGAGATGGAGGGTCTCTTAACAGT----------------------------
----

ACAAATGAAACCTTCAGACCTGGAGGAGGAGACATGAGGGACAATTGGAGAAGTGAATTATATAAGTATAAAG
TAGTAAAAATTGAACCACTA---GGAGTAGCACCCAACAAGGCAAAGAGAAGAGTGGTG--------------
-------------------------GAAAGAGAAAAAAGAGCA---GCTGGA---ATAGGAGCT---------
TTGCTCATTGGGTTCTTAGGAGCA---GCAGGAAGCACTATG---GGCGCGGCGTCA---
ATAACGCTGACGGTACAG---GCCAGACAA---TTGTTG---TCTGGCATAGTGCAACAGCAAAGC---
AATTTG---CTGAGG---GCTATAGAGGCTCAACAGCATCTGTTGAAACTCACGGTCTGGGGCATT
>KT022408
TTCTATTGTAATACATCAGGCCTGTTTAATAGTACTTGGAATAGCACAGAGCGGTCTAGAAATAGCACTAACA
GCACAGGG---

GAAGGTAGAAATATAACTCTCCAATGCAGAATAAAGCAAATTATAAATATGTGGCAGAGAGTAGGACAAGCAA
TGTATGCCCCTCCCATCCAAGGAGAAATAAAGTGTGTATCAAAC---
ATTACAGGAATACTATTAACAAGAGATGGTGGGAACAATACAAGT----------------------------
----

CAAAATGAAACCTTTAGACCTGGAGGAGGAGATATGAGGGACAATTGGAGAAGTGAATTATATAAGTATAAAG
TAGTACAAATTGAACCACTA---GGAGTAGCACCCACCAGGGCAAGGAGAAGAGTGGTG--------------
-------------------------CAGAGAGAAAAAAGAGCA---GTTGGA---CTGGGAGCT---------
GTCTTCATTGGGTTCTTAGGAGCA---GCAGGAAGCACTATG---GGCGCAGCGTCA---
ATAACGCTGACGGTACAA---GCCAGACAA---TTATTG---TCTGGCATAGTGCAACAGCAAAGC---
AATTTG---CTGAGG---GCTATAGAGGCTCAACAGCATCTGTTGAAACTCACAGTCTGGGGCATT
>KT022413
TTCTATTGTAATACATCAAGCCTGTTTAATAGCGCTTGGAATCACACTCACAATGACAGCATACAGGGG----
----

TCAAATGACACTCTAACTCTCCCATGTAGAATAAAGCAAATTATAAATATGTGGCAGAGAGTAGGACAAGCAA
TGTATGCCCCTCCCATCCAAGGAATAATAAAGTGTGAATCAAAC---
ATTACAGGACTAATATTAACAAGAGATGGTGGTGGGAGTAATAAC----------------------------
----

AGTACAGATGAAATCTTCAGACCGGGAGGAGGAAATATGAGGGACAATTGGAGAAGTGAATTATATAAGTATA
AAGTAGTAAAAATTGAACCAATA---GGAGTAGCACCCACCAAGGCAAGGAGAAGGGTGGTG-----------
----------------------------GAGAGAGAAAAAAGAGCA---GTTGGA---ATAGGAGCT------
---GTTTTCCTTGGGTTCTTGGGAGCA---GCAGGAAGCACTATG---GGCGCGGCGTCA---
ATAACGCTGACGGTACAG---GCCAGACAA---TTATTG---TCTGGCATAGTGCAACAGCAAAGC---
AATTTG---CTGAGG---GCTATAGAGGCTCAACAACAGATGTTGAGACTCACGGTCTGGGGCATT
>FJ623489
TTCTATTGTGACACATCAGGCCTATTTAATAGCACTTGGTATGGGAATGGGACCAACACAACGGAACCAGATA
GC---

ACAAATGACAATATAACTCTCCCATGCAGAATAAAGCAAATTATAAATATGTGGCAGAGAGTAGGACAAGCAA
TGTACGCCCCTCCCATCCCAGGAGCAATAAGCTGTACCTCAAAC---
ATTACAGGATTATTATTAACAAGAGATGGTGGGTATAATAGCACT----------------------------
----

AATGAAACCTTCAGACCTGGTGGAGGAGATATGAGGGACAATTGGAGAAGTGAATTATTTAAGTACAAGGTAG
TACAAATTAAACCACTA---GGAGTAGCACCCACCGAGGCAAGGAGAAGAGTGGTG-----------------
----------------------CAGAGAGAAAAAAGAGCA---GTTGGA---CTAGGAGCT---------
TTCTTCATTGGGTTCTTAGGAGCA---GCTGGAAGCACTATG---GGCGCGGCGTCA---
ATAACGCTGACGGTACAG---GCCAGACAA---CTGTTG---TCTGGCATAGTGCAACAGCAAAGC---
AATTTG---CTGAGG---GCTATAGAGGCTCAACAGCATCTGTTGAAACTCACAGTCTGGGGCATT
>HM215276
TTCTATTGTGATACATCAATCCTGTTTAATAGCACTTGGTATCACAATGGCAGTATCTTGCAGAAGTCAAAT-
----

AGCACGGATAATATAATTCTCCCATGCAGAATAAAGCAAATTATAAATATGTGGCAGAGAGCAGGACAAGCAA
TATATGCCCCTCCCATCAAAGGACCAATAGTGTGTGAATCAAAT---
ATTACAGGACTAATATTAACAAGAGACGGTGGCACGGATGACAAT----------------------------
----

ACAACTGAAACTTTCAGACCTGGAGGAGGAGATATGAGGGACAATTGGAGAAGTGAATTATATAAGTATAAAG
TAGTAAAAATTGAACCATTA---GGATTAGCACCCTCCAGGGCAAGGAGAAGAGTGGTG--------------
-------------------------GAGAGAGAAAAAAGAGCA---GCGGGG---CTAGGAGCT---------
GTCTTCATTGGGTTCTTAGGAATG---GCAGGAAGCACTATG---GGCGCGGCGTCG---
ATGACGCTGACGGTACAG---GCCAGACAA---TTATTG---TCTGGTATAGTGCAACAGCAAAGC---
AATTTG---CTGAGG---GCTATAGAGGCTCAACAGCAGATGTTGAGACTCACGGTCTGGGGCATT
>KF716469
TTCTATTGTAATACATCAGGCCTGTTTAATCATACATACAATCACACGCAGGAGGCAAATGGCACGGGG----
----

TCAAATGGCAATATAACTCTCCCATGCAGAATAAAGCAAATTATAAATATGTGGCAGAGAACAGGACAAGCAA
TGTATGCCCCTCCCATCCAAGGAGTAATAAAATGTGTATCAAAT---
ATTACAGGACTACTATTAACAAGAGATGGTGGGGGAAATAACAGGACAGAG----------------------
----

GAAATCTTTAGACCTGGAGGAGGAGATATGAGGGACAATTGGAGAAGTGAATTATATAAGTATAAAGTAGTAA
AAATTGAACCACTG---GGAGTAGCACCTACCACGGCAAGGAGAAGAGTGGTG--------------------
-------------------GGAAGAGAAAAAAGAGCA---GTTGGA---CTAGGAGCT---------
GTCTTCTTTGGGTTCTTAGGAGCA---GCCGGAAGCACTATG---GGCGCGGCGTCA---
ATAACGCTGACGGCACAG---GCCAGACAA---TTATTG---TCTGGCATAGTGCAACAGCAGAGC---
AATCTG---CTGAGG---GCTATAGAGGCTCAACAACATCTGTTGAAACTCACGGTCTGGGGCATT
>KF716471
TTCTATTGTAATACATCAGGCCTGTTTAATCATACATACAATGACACGCAGGAGGCAAACAGCACGGGG----
----

TTAAATGGCAATATAACTCTCCCATGCAGAATAAAGCAAATTGTAAATATGTGGCAGAGAGCAGGACAAGCAA
TGTATGCCCCTCCCATCCAAGGAGTAATAAAATGTGTATCAAAT---
ATTACAGGACTACTATTAACAAGAGATGGTGGGGAAATAGCAGT-----------------------------
----

ACAAAGGAAATCTTTAGACCTGGAGGAGGAGATATGAGGGACAACTGGAGAAGTGAATTATATAAGTATAAAG
TAGTAAAAATTGAGCCACTG---GGGGTAGCACCTACCGAGGCACGGAGAAGAGTGGTG--------------
-------------------------GGAAGAGAAAAAAGAGCA---GTTGGA---ATAGGAGCT---------
GTCTTCCTTGGGTTCTTAGGAGCA---GCAGGAAGCACTATG---GGCGCGGCGTCA---
ATAACGCTGACGGTACAG---GCCAGACAA---TTATTG---TCTGGCATAGTGCAACAGCAGAGC---
AATCTG---CTGAGG---GCTATAGAGGCTCAACAACATCTGTTGAAACTCACGGTCTGGGGCATT
>KU921793
TTCTATTGTAACACATCAAGCCTGTTTAATAGCACTTGGGGTAATACCAGTGTGCCAGAGTTAAATAGCACGG
AGGCAAATAGC---ACG------GAG---

CAAAACAATATAACAATAACACTCCAATGCAGAATAAAGCAAATTATAAACATGTGGCAGAGAGCAGGACAAG
CAATGTATGCCCCTCCCATCCAAGGAGAGATAAGGTGTATATCAAAC---
ATTACAGGATTAATATTAACAAGAGATGGTGGGAATAATAATAAC----------------------------
----

AGCACAAATGAAACCTTCAGACCTGGAGGAGGAGATATGAGGGACAATTGGAGGAGTGAATTATATAAGTATA
AAGTGGTAAAAATTGAACCAATA---GGAATAGCACCCACCAGGGCAAAAAGAAGAGTGGTG-----------
----------------------------CAGAGAGAGAAAAGAACA---GTAGGA---CTAGGAGCT------
---GTCTTCATTGGGTTCTTAGGAGCA---GCAGGAAGCACTATG---GGCGCGGCGTCA---
GTGACGCTGACGGTACAG---GCTAGACAA---CTATTG---TCTGGCATAGTGCAACAGCAAAGC---
AATTTG---CTGAGG---GCTATAGAGGCTCAACAGCATCTGTTGAAACTCACGGTCTGGGGCATT
>KU921796
TTCTATTGTGACACATCAGGCCTGTTTAATAGCACTTGGGATAGCAATAACAGCGAGCAAGAGTCAAATAGCA
CTGAGTTA---

AACAATATAACTCTAACTCTCCACTGCAGAATAAAGCAAATTATAAATATGTGGCAGAGAGCAGGACAAGCAA
TGTATGCCCCTCCCATCCAAGGAGTGATAAGATGTGTATCAAAC---
ATTACAGGATTAATATTAACAAGAGATGGTGGGGATCATAATAAC----------------------------
----

AGCGCAAATGAAACCTTCAGACCTGGAGGAGGAGATATGAGGGACAATTGGAGGAGTGAATTATATAAGTATA
AAGTAGTAAAAATTGAACCAATA---GGAATAGCACCCACCAAGGCAAGAAGAAGAGTGGTG-----------
----------------------------CAGAGAGAGAAAAGAGCA---ATAGGA---ATGGGAGCC------
---GTCTTCCTTGGGTTCTTAGGAGCA---GCAGGAAGCACTATG---GGCGCAGCGTCA---
GTAGCGCTGACGGTACAG---GCCAAACAA---CTATTG---TCTGGCATAGTGCAACAGCAAAAC---
AATTTG---CTGAGG---GCTATAGAGGCTCAACAGCATATGTTGAAACTCACGGTCTGGGGCATT
>KU921839
TTCTATTGTAATACATCAGGACTGTTTAATAGCACTTGGAATTCCACTAACATGCAGGAGCTAGATAGCACAG
AG---

GCAAATGGTACTATAATACTCCCATGCAAAATAAAGCAAATCATAAATATGTGGCAGAGAGTAGGACAAGCAA
TGTATACCCCTCCCATACCGGGAGTAATAAATTGTGAATCAAAC---
ATCACAGGATTAATATTAACAAGGGATGGTGGCCCTAATAACGAT----------------------------
----

ACAGAAATCTTCAGACCAGGAGGAGGAGATATGAGGGACAATTGGAGAAGTGAATTATACAAGTATAAAGTAG
TAAAAATTGAACCATTA---GGAGTAGCACCCACCAAAGCAAGGAGAAGAGTGGTG-----------------
----------------------GAGAGAGAAAAAAGAGCA---GTTGGA---ATAGGAGCT---------
GTTCTCGTTGGGTTCTTGGGAGCA---GCAGGAAGCACTATG---GGCGCGGCGTCA---
ATAACGCTGACGGCACAG---GCCAGGCAA---TTATTG---TCTGGCATAGTGCAACAGCAAAGC---
AATTTG---CTGAGG---GCTATCGAGGCTCAACAACAGCTGTTGAAACTCACGGTCTGGGGCATT
>KU749431
TTCTATTGTGATACAACAAGCCTGTTTAATAGTACTCACTCCCCTAACGACACGCAGGGTGACCAGGAGTCAA
AT---

```
TCAAATGACACTATAACTCTCCCATGCAGAATAAAGCAAATTATAAATATGTGGCAGAGAACAGGACAAGCAA
TATATGCCCCTCCCATCGAAGGAGATATAAGGTGTAGCTCAAAC---
ATTACAGGATTATTATTAACAAGAGATGGTGGGATTAATGTGAGC----------------------------
-------------------------------------------------------------------------
----------
ACTACTAATGAAACCTTCAGACCTAGTGGAGGAGATATGAGGGACAATTGGAGAAGTGAATTATATAAGTACA
AGGTAGTAAGAATTAAACCACTA---GGAGTAGCACCCACCAAGGCAAGGAGAAGAGTGGTG-----------
----------------------------GAGAGAGAAAAAAGAGCA---GTTGGA---ATGGGAGCT------
---TTCTTCCTTGGGTTCTTAGGAGCA---GCAGGAAGCACTATG---GGCGCAGCGTCA---
ATAACGCTGACGGTACAG---GCCAGAAAC---TTATTG---TCTGGCATAGTGCAACAGCAAAAC---
AATTTG---CTGAGG---GCTATAGAGGCTCAACAACATCTGTTGAAACTCACAGTCTGGGGCATT
>FJ396022
TTCTATTGTAACACATCAGGCCTGTTTAATAGCACTTGGTATAATGGCAGCATGTCAAATAGCACGGAG----
-------------------------------------------------------------------------
----------------------------
TCAAATGACAATATAACACTCCAATGCAGAATAAAGCAAATTATAAATATGTGGCAGAGAGTAGGACAAGCAA
TGTATGCCCCTCCCATCCAAGGAGAAATAAAGTGTGTATCAAAC---
ATTACAGGGCTAATATTAACAAGAGATGGTGGTGACAGCACAAAT----------------------------
-------------------------------------------------------------------------
-------------------
GAAACCTTCAGACCTGGAGGAGGAGATATGAGGGACAATTGGAGAAGTGAATTATATAAATATAAAGTGGTAG
AAATTAAACCATTA---GGAGTAGCACCCACTGAGGCAAAAAGGAGAGTGGTG--------------------
-------------------GAGAGAGAAAAAAGAGCA---GTGGGA---ATAGGAGCT---------
GTGTTCCTTGGGTTCTTGGGAGCA---GCAGGAAGCACTATG---GGCGCAGCGTCA---
ATAACGCTGACGGTACAG---GCCAGACAA---TTATTG---TCTGGCATAGTGCAACAGCAAAGC---
AATTTG---CTGAGG---GCTATAGATGCTCAACAGCATATGTTGAAACTCACGGTCTGGGGCATT
>AF457064
TTCTATTGTAACACATCAGGCCTGTTTAATAGCACTTGGAATGACAGTGACAGCATGCAGGGGCCAAATAGCA
CG-----------------------------------------------------------------------
-------------------------
AAGTCAAATGGCACTATAACTCTCCCATGCAGAATAAAGCAAATTATAAATATGTGGCAGAGAGCAGGACAAG
CAATATATGCCCCTCCCATCCCAGGAATAATAAGGTGTGAATCAAAC---
ATTACAGGCCTAATATTAACAAGAGATGGTGGGGATAATAGCAGT----------------------------
-------------------------------------------------------------------------
-------------
ACAAATGAAACCTTCAGACCTGGAGGAGGAGATATGAGGGACAATTGGAGAAGTGAATTATATAAGTATAAAG
TAGTAAAAATTGAACCACTA---GGAGTAGCACCCACCGAGGCAAAGAGAAGAGTGGTG--------------
-------------------------GAGAGAGAAAAAAGAGCA---GTTGGA---ATAGGAGCT---------
GTCTTCATTGGGTTCTTAGGAGCA---GCAGGAAGCACTATG---GGCGCGGCGTCA---
ATGACGCTGACGGTACAG---GCCAGACAA---TTATTG---TCTGGCATAGTGCAACAGCAAAGC---
AATTTG---CTGAGG---GCTATAGAGGCTCAACAGCAACTGTTGAAACTCACGGTCTGGGGCATT
>KU749428
TTCTATTGCAACACATCACAGCTGTTTAATTCATCACTGCTCAATAGTACAGAAAGTATTAATGAAACAAGT-
-------------------------------------------------------------------------
----------------------------
GGTAATGAAACAATCACACTCCCATGCAGAATAAAACAGATTATAAACATGTGGCAGGAGGTAGGGCGGCAA
TGTATGCCCCTCCCATTAGAGGAATAATAACATGTAAATCAAAT---
ATCACAGGACTACTACTGACACGTGATGGAGGAAATGAGAAAAAT----------------------------
--------------------------------------------------------------------
AACTCAGAGGCAAAT---
AACACAGAGATATTCAGGCCTGGAGGAGGAAATATGAAGGACAATTGGAGAAGTGAATTATATAAGTATAAAG
TAGTAAAAGTTGAACCACTA---GGAGTAGCACCCACCAAGGCAAAGAGAAGAGTGGTG--------------
-------------------------GAGAGAGAAAAAAGAGCA---GTAGGA---ATAGGAGCT---------
GTCTTCATTGGGTTCTTAGGAGCA---GCCGGAAGCACTATG---GGCGCAGCGTCG---
GTGACGCTGACGGTACAG---GCCAGACAA---TTATTG---TCTGGCATAGTGCAACAGCAAAGT---
AATTTG---CTGAGG---GCTATAGAGGCTCAACAACATTTGTTGAAACTCACGGTCTGGGGCATT
>KX983685
TTCTATTGTAATACATCAGGCCTGTTTAATAACACATGGGCTAATAACAATTGGACTGACAGCACAAATTAC-
-------------------------------------------------------------------------
----------------------------
```

ACAAATGACAATATAACTCTCCAATGCAGAATAAAGCAAATTATAAATATGTGGCAGAGGACAGGACAAGCAA
TGTATGCCCCTCCCATCCAAGGAATAATAATGTGTAGGTCAAAC---
ATTACAGGCCTACTATTAACAAGAGATGGAGGGAATACAAATGATACA-------------------------
----

AATGCAACTGAAACCTTCAGACCTGGGGGAGGAAATATGAGGGATAATTGGAGAAGTGAGCTATATAAATATA
AAGTAGTAAAAGTTGAACCACTA---GGAGTAGCACCCACCGAGGCAAGAAGAAGAGTGGTG-----------
----------------------------CAGAGAGAAAAAAGAGCA---GTTGGA---CTGGGAGCT------
---GCCCTCCTTGGGTTCTTAGGAGCA---GCAGGAAGCACTATG---GGCGCGGCGTCA---
ATAACGCTGACGGTACAG---GCCAGACTA---TTATTG---TCTGGTATAGTGCAACAGCAAAGC---
AATTTG---CTGAGG---GCTATAGAGGCTCAACAGCATATGTTGAAGCTCACAGTCTGGGGCATT
>U88823
TTCTATTGTAATACATCAGGCTTGTTTAATAGCACCTGGAGTAAAAGAAATGGCACCTGGCAGTCAAATGGC-
----

ACAGAATTAAATATAACACTCCCATGCAGAATAAAGCAAATTATAAATATGTGGCAGAGGACAGGACAAGCAA
TGTATGCCCCTCCCATCCAAGGAGTAATAAGCTGTGTATCAAAC---
ATTACAGGACTACTATTAACAAGAGATGGTGGAAATAATAATACT----------------------------
----

ACAACTGAAACCTTCAGACCTGGAGGAGGAGATATGAGGGATAATTGGAGAAGTGAACTATATAAATATAAAG
TAGTAAAAATTGAACCACTA---GGAGTAGCACCCACCAGGGCAAAGAGGAGAGTGGTG--------------
-------------------------GAGAGAGAAAAAAGAGCA---GTTGGA---CTGGGAGCT---------
GTCTTCATTGGGTTCTTAGGAGCA---GCAGGAAGCACTATG---GGCGCGGCGTCA---
ATAACGCTGACGGTACAG---GCCAGACAA---TTATTG---TCTGGCATAGTGCAACAGCAAAGC---
AATTTG---CTGAGG---GCTATAGAGGCTCAACAGCATCTATTAAAACTCACAGTCTGGGGCATT
>MF373141
TTTTATTGTAATACATCAGTACTTTTTAACAGAAGTATAACAGGCAATATCACAAACCTAACAGAAGTA----
----

AACAGTACAAACATCACACTCCCATGCAGAATAAAACAAATTATAAACATGTGGCAGGGGGTAGGACGAGCAA
TGTATGCCCCTCCCATTCAAGGATTCATAAAATGTGAATCAAAT---
TTCACAGGACTACTCCTGACACGTGATGGAGGGAATATTAGTACA----------------------------
----

GAGAACGAGACATTCAGGCCGGAAGGAGGAAATATGAAGGACAATTGGAGAAGTGAATTATATAAATATAAAG
TGGTAGAAATTAAACCATTA---GGAATAGCACCCCATAAGGCAAAAAGAAGAGTGGTG--------------
-------------------------GAGAGAGAAAAAAGAGCA---GTGGGA---ATAGGAGCT---------
GTGTTCCTTGGGTTCTTGGGAGCA---GCAGGAAGCACTATG---GGCGCAGCATCA---
CTAACGCTGACGGTACAG---GCCAGACAA---TTATTG---TCGGGTATAGTGCAACAGCAGAGC---
AATCTG---CTGAGG---GCTATTGAGGCGCAACAGCATATGTTGCAACTCACAGTCTGGGGCATT
>MF373155
TTCTATTGTAATACAACAGGCCTGTTTAATAGCACTTGGAATGATACCACTTTGAACAGCACCACTGAGTTAA
ACAAC--

ACATATGAAAATATAACTCTTCCATGTAGAATAAAGCAAATTATAAACATGTGGCAGAGAGCAGGAAAAGCAA
TGTATGCCCCTCCCATCCCAGGAATAATAAGGTGTGAGTCAAAC---
ATTACAGGACTACTGTTAACAAGCGATGGTGGGAATAAAAGTACT----------------------------
----

ACAAATGAAACCTTCAGACCTGGAGGAGGAGATATGAGGGACAATTGGAGAAGTGAATTATATAAGTATAAAG
TAGTAAAACTTGAACCACTA---GGAGTAGCACCTGAAAGGGCAAAAAGAAGAGTGGTG--------------
-------------------------GCGAGAGAAAAAAGAGCA---GTTGGA---CTGGGAGCT---------
GTCTTCATTGGGTTCTTAGGAGCA---GCAGGAAGCACTATG---GGCGCGGCGTCA---
CTGACGCTGACGGTACAG---GCCAGACAA---TTATTG---TCTGGTATAGTGCAACAGCAAAGC---
AATTTG---CTGAAG---GCTATAGAGGCTCAACAGCACCTGTTGAGACTCACGGTCTGGGGCATT
>AF071474
TTCTATTGTGATACATCAGGGCTGTTTAATAGCACCTGGCCGTTTAATAGCACAAACAGCACAGGG-------
----

CCAAATGGCACTATAACTCTCCAATGCAGAATCAAGCAAATTATAAGGATGTGGCAGAGAACAGGACAAGCAA
TATATGCCCCTCCCATCCCAGGAGAAATAAACTGTGTATCAAAC---
ATTACAGGACTACTATTAACAAGAGATGGTGGGAATAATATTACA----------------------------
----

AATGAAACCTTCAGACCTGGAGGAGGAGATATGCAGGACAATTGGAGAAGTGAATTATATAAGTATAAGGTAG
TACAAATTGAACCACTA---GGAGTAGCACCCACCAAGGCAAGGAGAAGAGTGGTG-----------------
----------------------GAGAGAGAAAAAAGAGCA---GTTGGC---ATGGGAGCT---------
GTCTTCATTGGGTTCTTAGGAGCA---GCAGGAAGCACTATG---GGCGCAGCGTCA---
GTAACGCTGACGGTACAG---GCCAGACAA---TTATTG---TCTGGCATAGTGCGACAGCAAAGC---
AATTTG---CTGAGA---GCTATAGAGGCTCAACAACATTTGTTGAAACTCACAGTCTGGGGCATT
>AY253318
TTCTATTGTAACACATCAGGCCTGTTTAATAGCACTTGGAATAGCAATGCCAGCATGCAGACGTCAAATGGTA
CG---

GGGTTAAATGACACTATAATTCTTCTATGTTGTATAATACAACTTATTATTATGTGGCGGAGAGCAGGACGAG
CAGTATTTGCCCCTCCTATCCTACGAGGAGTAATGTGGGGATCATAC---
ATCACAGGGCTACTATTATCAACAGATGGTGGGGATGGTACACAT----------------------------
----

GAAGAACTCTTCGTACCTGGACGAGGAGATATGAAGGTCAAGGGCAGAAGCAAATTCTATTTGTATAAAGTTG
TAAAAATTGAACCTGTA---CGTGTAGCACCCACCCCTGCCAACACAAAAGTGGTG-----------------
----------------------GAGAGAGAAAAAAGAGCA---GTTGCA---ATAGGAACT---------
GGATTTGTTGGGTTTTTAGGATCA---GGAGCAAGCACTATG---GGTGCGGCGTCA---
ATAACGCTGACGGTACAG---GCCAGACAA---TTATTG---TCTGGCATAGTGCAACAGCAAAGC---
AATTTG---CTGAGG---GCTATAGAGGCTCAACAACATCTGTTGAAGCTTACGGTCTGGGGCATT
>AY253315
TTCTATTGCAACACAACAGACCTGTTTAATTGGAATAATGACACCAGGCAGAAGCGAAATAGAACAGAG----
----

TCAGATAACACTATAACTCTCCAATGCAGAATAAAGCAAATTATAAATATGTGGCAGAGAACAGGACAAGCAA
TATATGCCCCTCCCATCCAAGGAGCAATAAGGTGTGACTCAAAC---
ATTACAGGACTAATATTAGCAAGAGATGGGGGTAACACAAATGAA----------------------------
----

AGTGAAGAGGAAATCTTCAGACCTATAGGAGGAGATATGAGGGACAATTGGAGAAGTGAATTATATAAGTACA
AAGTAGTGAAAATTGAGCCACTA---GGAGTAGCACCCACCACGGCAAAGAGAAGAGTGGTG-----------
----------------------------GAGAGAGAAAAAAGAGCA---GTTGGA---CTGGGAGCT------
---GTCTTCCTTGGGTTCTTAGGAGCA---GCAGGAAGCACTATG---GGCGCAGCATCA---
CTGACGCTGACGGTACAG---GCCAGACAA---TTATTG---TCTGGCATAGTGCAACAGCAAAGC---
AATTTG---CTGAGG---GCTATAGAGGCTCAACAACATCTGTTGAGACTCACAGTCTGGGGCATT
>AY253319
TTCTATTGCAACACATCAGGCCTGTTTGATAGCATTTGGAATGACACTGACAGCATGCAGGGGTTAGATAGCA
TG---

GAGTCAGATGGCACTATAACTCTCCAATGCAGAATAAAGCAAATTATAAATATGTGGCAGAGAGCAGGACAAG
CAATATATGCCCCTCCCATCCCAGGAGTAATAGAGTGTACATCAAAC---
ATTACAGGACTAATATTAACAAGAGATGGTGGGAATAGTAGCAGT----------------------------
----

ACAAATGAAACCTTCAGACCTGGAGGAGGAGATATGAGGGACAATTGGAGAAATGAATTATATAAGTATAAGG
TAGTAAAAATTGAGCCACTA---GGAGTAGCACCCACCAAGGCAAAAAGAAAAGTGGTG--------------
-------------------------GAGAGAGAAAAAAGAGCA---GTTGGA---ATAGGAGCT---------
GTTTTCCTTGGGTTCTTGGGAGCA---GCAGGAAGCACTATG---GGCGCGGCGTCA---
ATAACGCTGACGGTACAG---GCCAGACAA---TTATTG---TCTGGCATAGTGCAACAGCAAAGC---
AATTTG---CTGAGG---GCTATAGAGGCTCAACAGCATCTGTTGAAACTCACGGTCTGGGGCATT
>AY734552
TTCTATTGTAATACATCAGGCCTGTTTAATGGCATTTGGAACAATACCAGCACGCAGGAGTCAAATAGCACGG
AA---

TCAAATGACACTATAACTATTCCATGCAGAATAAAGCAAATTATAAATATGTGGCAGAGAGTAGGACAAGCAA
TGTATGCCCCTCCCATCCAAGGAATGATAAGGTGTGAATCAAAC---
ATTACAGGACTACTGTTAACAAGAGATGGTGGGAATGAGAGCAGT----------------------------
----

ACAAATGAAACCTTCAGACCTGGAGGAGGAGATATGAGGGACAATTGGAGAAGTGAATTATATAAGTATAAAG
TAGTAAAAATTGAACCGTTA---GGAGTAGCACCCACCAGGGCAAAGAGAAGAGTGGTG--------------
-------------------------GGAAGAGAAAAAAGAGCA---GTTGGA---ATAGGAGCT---------
GTCTTCCTTGGGTTCTTAGGAGCA---GCCGGAAGCACTATG---GGCGCGGCGTCA---
ATAACGCTGACGGTACAG---GCCAGACAA---TTATTG---TCTGGTATAGTGCAACAGCAAAGC---
AATTTG---CTGAAG---GCTATAGAGGCTCAACAACATATGTTGAGACTCACAGTCTGGGGCATA
>AY734553
TTCTATTGCAACACATCAAGCCTGTTTAATAGCACTTGGAATGGCACTACTGATAATGGCACTGGCAGCACAA
AC---

AGCGCAAATAGCAATATAACTCTCCAATGCAAAATAAAGCAAATTATAAATATGTGGCAGAGAGCAGGACAAG
CAATATATGCCCCTCCCATCCCAGGAGTGATAAAGTGTGTATCAAAC---
ATTACAGGACTAATATTAACAAGAGATGGTGGGAATAATAACAGT----------------------------
----

ACAAATGAAACCTTCAGACCAGGAGGAGGAGATATGAGGGACAATTGGAGAAGTGAATTATATAAGTATAAGG
TAGTAAAAATTGAACCACTA---GGAGTAGCACCCACCAGGGCAAAGAGAAGAGTGGTG--------------
-------------------------GAGAGAGAAAAAAGAGCA---GTCGGA---ATAGGAGCT---------
GTTCTCCTTGGGTTCTTAGGAACA---GCAGGAAGCACTATG---GGCGCGGCGTCA---
ATAACGCTGACGGTACAG---GCCAGACAA---TTATTG---TCTGGCATAGTGCAACAGCAAAGC---
AATTTG---CTGAGG---GCTATAGAGGCTCAACAACACATGTTGAAACTCACAGTCTGGGGCATT
>AY734562
TTCTATTGTGATACATTTGAACTGTTTAATAGCACTTGGAATCTCACTGCCAGCATGCAGGGGTCAAATAGA-
----

ACGGAGGGCAATATAACACTCCCGTGCAGAATAAAACAAATTGTAAACATGTGGCAGGGGGTAGGACGAGCAA
TGTATGCCCCTCCCATTGCAGGAAATATAACATGTAGATCAAAC---
ATCACAGGACTATTATTGACAAGTGATCATGGTAATGGTAGTCCA----------------------------
----

ACAAATGAGACATTCAGACCTGGAGGAGGAAATATGAGGGACAATTGGAGAAATGAATTATATAAATACAAAG
TGGTAGAAATTAAGCCATTA---GGAATAGCACCCACTAAGGCAAAAAGGAGAGTGGTG--------------
-------------------------GAGAGAGAAAAAAGAGCA---GTGGGA---ATAGGAGCT---------
GTGCTCCTTGGGTTCTTGGGAGCA---GCAGGAAGCACTATG---GGCGCGGCGTCA---
ATAACGCTGACGGTACAG---GCCAGACAA---TTATTG---TCTGGTATAGTGCAACAGCAAAGC---
AATTTG---CTGAAG---GCTATAGAGGCGCAACAACATATGTTGCAACTCACAGTCTGGGGCATT
>AY734554
TTCTATTGTAACACATCAGGACTGTTTAATAGCACTTGGAATAGCAATGACAACGTGCAGGAGTCAAATAGCA
CA---

AAGTCAAACGACACTATAACTCTCCAATGCAGAATAAAACAAATTATAAATATGTGGCAGAGGGTAGGACGAG
CAATGTATGCCCCTCCCATCCCAGGAGTAATAAGGTGTAATTCAAAC---
ATTACAGGACTAATATTAACAAGAGATGGTGGGAATGAGAGCAGT----------------------------
----

ATAAATGAAACCTTCAGACCTGGAGGAGGAGATATGAGAGACAATTGGAGAAGTGAATTATATAAGTATAAAG
TAGTAAAAATTGAACCACTA---GGAGTAGCACCCACCAGGGCAAGGAGAAGAGTGGTG--------------
-------------------------GAGAGAGAAAAAAGAGCA---GTTGGA---ATAGGAGCT---------
GTCTTCATTGGGTTCTTAGGAGCA---GCAGGAAGCACTATG---GGCGCGGCGTCA---
ATAACGCTGACGGTACAG---GCCAGACAA---TTACTG---TCTGGCATAGTGCAACAGCAAAGC---
AATTTG---CTGAGG---GCTATAGAGGCTCAACAGCATCTGTTGAAACTCACGGTCTGGGGCATT
>AY734555
TTCTATTGTAACACATCAGGCCTGTTTAATAGCACTTGGAATACTAACACTTTGAATAGCAGTAACAGCATGG
AG---

GAGTCAAATGACAATATAACTCTCCAATGTAGAATAAAGCAAATTATAAATATGTGGCAGAGAGCAGGACAAG
CAATGTATGCCCCTCCCATCCCAGGAATAATAAGGTGTGAATCAAAC---
ATTACAGGACTAATATTAATGAGAGATGGTGGGAATAATAATACA----------------------------
----

AATGAGACATTCAGACCTGGAGGGGGAGATATGAGGGACAATTGGAGAAGTGAATTATACAAATATAAAGTAG
TAGAAATTAAGCCATTA---GGAATAGCACCCAACAGGGCAAAAAGGAGAGTGGTG-----------------
----------------------GAGAGAGAAAAAAGAGCA---GTTGGA---CTGGGAGCT---------
TTGTTCCTTGGGTTCTTAGGAGCA---GCAGGAAGCACTATG---GGCGCGGCGTCA---
ATAACGCTGACGGTACAG---GCCAGAAAT---TTACTG---TCTGGCATAGTGCAGCAGCAAAGC---
AATTTG---CTGAGG---GCTATAGAGGCTCAACAGCAGATGTTGAGACTCACGGTCTGGGGCATT
>KX907342
TTCTACTGCAATACATCAGGTCTGTTTAATTATACAGAAGGTACTTTAAATAGTACAATCAAT----------
----

AGTACAGAACCAATCACACTCCCATGCAGGATAAAACAAATTATAAACATGTGGCAGGGGGTAGGACGAGCAA
TGTATGCCCCGCCCATTGCAGGAACCATAACATGTAGATCAAAC---
ATTACAGGACTACTATTGACACGTGATGGAGGATGTCATAATAAT----------------------------
----

TGTACAGAGAAATTCAGACCTGGAGGAGGAGATATGAGGGACAATTGGAGAAGTGAATTATATAAATATAAAG
TGGTAGAAATCAAACCATTA---GGAATAGCACCCACTAGGGCAAAAAGGAGAGTGGTG--------------
-------------------------GAGAGAGAAAAAAGAGCA---GTGGGA---ATAGGAGCT---------
GTGTTCCTTGGGTTCTTGGGAGCA---GCAGGAAGCACTATG---GGCGCAGCATCA---
ATAACGCTGACGGTACAG---GCCAGACAA---TTACTG---TCTGGTATAGTGCAACAGCAAAGC---
AATTTG---CTGAGG---GCTATAGAGGCGCAACAGCATATGTTGCAACTCACAGTCTGGGGCATT
>KX907377
TTCTATTGCAACACCACAGAACTGTTTAATAGAACATACTGGTCAAATGAGACAGGAAGCAATTCAAACAATT
CAAGCAGT---

TCAAATGACACCATCACACTCCCATGCAGAATAAAACAAATTATAAACATGTGGCAGAGAGTAGGACAAGCAA
TGTACGCCCCTCCCATTGCAGGAAACATAACATGTGAATCAAAC---
ATTACAGGGCTACTGTTAACAAGAGATGGTGAGAAT-------------------------------------
----

AACGGTACAGAAACTTTTAGACCTGGAGGAGGAGATATGAGGGACAATTGGAGAAGTGAATTATATAAGTATA
AAGTAGTAAAAATTGAACCACTA---GGAGTGGCACCCACCAGGGCAAAAAGGAGAGTGGTG-----------
----------------------------GAGAGAGAAAAAAGAGCA---GTTGGA---ATAGGAGCT------
---GTCCTCTTTGGGTTCTTAGGAACA---GCAGGAAGCACTATG---GGCGCGGCGTCA---
ATGACGCTGACGGTACAG---GCCAGACAA---TTATTG---TCTGGCATAGTGCAACAGCAAAGC---
AATTTG---CTGAGG---GCTATAGAGGCTCAACAACATATGTTGAAACTCACGGTCTGGGGCATT
>KX907379
TTCTATTGTAATACATCAAGCCTGTTTAATAGCACTTGGGACAACAGTAGCATCACACAGAATTACACGGAG-
----

TCAAATGAGACTATAACTCTCCAGTGCAGAATAAAACAAATTGTAAACGCGTGGCAGAGAGTAGAACAAGCAA
TATATGCCCCTCCCATCCAAGGAGTAATAAGGTGTGACTCAAAC---
ATTACAGGACTACTATTAACAAGAGATGGTGGTGGTAATAACAGT----------------------------
----

AGTGAAAACGAAACCTTTAGACCTGGAGGAGGAGATATGAGGGACAATTGGAGAAGTGAATTATATAAGTATA
AAGTAGTAAAACTTGAACCACTA---GGAGTAGCACCTACCAAGGCAAAAAGAAGAGTGGTG-----------
----------------------------GAAAGAGAAAAAAGAGCA---GTCGGA---ATAGGAGCT------
---GTTTTCCTTGGGTTCTTAGGAGCA---GCAGGAAGCACTATG---GGCGCGGCGTCA---
ATGACGCTGACGGTACAG---GCCAGACAA---TTATTG---ACTGGCATAGTGCAACAGCAAAGC---
AATTTG---CTGAGG---GCTATAGAGGCTCAACAACACCTGTTGAAACTCACGGTCTGGGGCATT
>KX907409
TTCTATTGTAACACATCATCCCTGTTTAATAGTACTTGGCAGGTCAGCGGGCAGGGGTTAAATAGCACGGAG-
----

TTAGGTGACACTATAACTCTCCAATGCAGAATAAAGCAAATTATAAATATGTGGCAGAGAGCAGGACAAGCAA
TATATGCCCCTCCCATCCCAGGAGTAATAAGGTGCGAATCAAAC---
ATAACAGGACTAATATTAACAAGTGATTATGGGAATAGTAGCAGT---------------------------
----

GACAATGAAACCTTCAGACCTACAGGAGGAGATATGAGGGACAATTGGAGAAGTGAATTATATAAGTATAAAG
TAGTGAAAATTGAACCAATA---GGAGTAGCACCCACCAAAGCAAGAAGAAGAGTGGTG-------------
-------------------------CAGAGAGAAAAAAGAGCA---GTTGGA---CTGGGAGCT---------
GTCTTCATTGGGTTCTTAGGAGCA---GCAGGAAGCACTATG---GGCGCGGCGTCA---
GTGACGCTGACGGTACAG---GCCAGACAA---TTATTA---TCTGGCATAGTGCAACAGCAAAGC---
AATTTG---CTGAGG---GCTATAGAGGCTCAACAGCATATGTTGAAACTCACAGTCTGGGGCATT
>KX907362
TTCTATTGTGACACATCAGACCTGTTTAATAGCACTTGGCCTAGCAATACCAATAACAGCATACAGGAGTCAA
ATAGCACA--

GAGCCAAACGCCACTATAACACTCCCATGCAGAATAAAACAAATTATAAATATGTGGCAGAGAGCAGGACAAG
CAATGTATGCCCCTCCCATCCCAGGAATGATAAAGTGTAACTCAAAC---
ATTACAGGACTCATATTAACAAGAGATGGTGGAAATGATAGCAGT---------------------------
----

ACAGATGAAATCTTCAGACCTGGAGGAGGAGACATGAGGGACAATTGGAGAAGTGAATTATATAAGTATAAAG
TAGTACAAATTGAACCACTA---GGAGTAGCACCTACCAGGGCAAGGAGAAGAGTGGTG-------------
-------------------------CAGAGAGAAAAAAGAGCA---GTTGGA---ATGGGAGCT---------
GTGCTCATTGGGTTCTTAGGAGCA---GCAGGAAGCACTATG---GGCGCAGCGTCA---
GTAACGCTGACGGTACAG---GCCAGACAA---TTATTG---TCTGGCATAGTGCAACAGCAAAGC---
AATTTG---CTGAGG---GCTATAGAGGCTCAACAGCATCTGTTGAAACTCACAGTCTGGGGCATT
>KX907381
TTCTATTGTAATACATCAGGTCTGTTTAATAGCACTTGGTCTAACAGCACGCAGGCGTCAAATGACACAGAG-
----

TCAAATGACACTATAACTCTCCCATGCAGAATAAAGCAAATTATAAATATGTGGCAGAGAGTAGGACAAGCAA
TGTATGCCCCTCCCATCCAAGGAATAATAAACTGTATATCAAAT---
ATTACAGGACTAATATTAACAAGAGATGGTGGGAATAACAATGGA---------------------------
----

ACTGAAACCTTCAGACCTGGAGGAGGAGACATGAGGGACAATTGGAGAAGTGAATTATATAAGTATAAAGTAG
TAAAAATTGAACCACTA---GGAGTAGCACCCACCAGGGCAAAGAGAAGAGTGGTG----------------
----------------------GAGAGAGAAAAAAGAGCA---GTAGGA---ATAGGAGCT---------
GTCCTCTTTGGGTTCTTAGGAGCA---GCAGGAAGCACTATG---GGCGCGGCGTCA---
ATAACGCTGACGGTACAG---GCCAGACAA---TTATTG---TCTGGTATAGTGCAACAGCAAAGC---
AATTTG---CTGAGG---GCCATAGAGGCTCAACAACAACTGTTGAAACTCACAGTCTGGGGCATC
>KX907386
TTCTATTGTAATACATCAAACCTGTTTAATGACACTTGGCCTATGTCAAATAACACGGAG------------
----

TCAGATGACAACATAACCCTCCAATGCAGGATAAAGCAAATTATAAATATGTGGCAGAGAGCAGGACAAGCAA
TGTATGCCCCTCCCATTGCAGGAAACATAACATGTATATCAAAT---
ATTACAGGACTACTGTTAACAAGAGATGGTGGGGGGAATAGCAGT---------------------------
----

ACTGGTAATGAAACCTTCAGACCTGGAGGAGGAAATATGAAGGACAATTGGAGAAGTGAATTATATAAGTATA
AAGTAGTACAACTTGAACCCCTA---GGAGTAGCACCTACCAGGGCAAGAAGAAGAGTGGTG----------
----------------------------CAGAGAGAAAAAAGAGCAGTAGTTGGA---CTGGGAGCT------
---GTTTTCATTGGGTTCTTAGGAGCA---GCAGGAAGCACTATG---GGCGCGGCGTCA---
ATAACGCTGACGGTACAG---GCCAGACAA---TTATTG---TCTGGTATAGTGCAACAGCAAAGC---
AATTTG---CTGAGG---GCTATAGAGGCTCAACAGCATATGTTGAAACTCACAGTCTGGGGCATT
>KX907429
TTCTATTGCAACACATCAGGTTTGTTTAATAGCACTTGGGATATCAATGACACTGCCAGCGTGCAGGTGTCAA
ATGGCACG--

```
AAGTCAAACAGCACTATAACTCTCCAATGCAGAATAAAGCAAATTATAAATATGTGGCAAAGAGTAGGGCAAG
CAATATATGCCCCTCCCATCCAAGGAATGATAAGGTGTGTATCAAAT---
ATTACAGGACTAATATTAACAAGAGATGGTGGGAATGATAGCAGG----------------------------
-------------------------------------------------------------------------
-------------
ACAAATGAAACCTTCAGACCTGGAGGAGGAGATATGAGGGACAATTGGAGAAGTGAATTATATAAGTATAAGG
TAGTAAAACTTGAGCCATTA---GGAATAGCACCCAACAGGGCAAAGAGAAGAGTGGTG--------------
-------------------------GAGAGAGAAAAAAGAGCA---GTTGGA---ATAGGAGCT---------
GTCCTCTTTGGGTTCTTGGGAGCA---GCAGGAAGCACTATG---GGCGTGGCGTCA---
ATGACGCTGACGGTACAG---GCCAGACAA---TTATTG---TCTGGCATAGTGCAACAGCAAGGC---
AATTTG---CTGAGG---GCTATAGAGGCTCAACAGCATCTGTTGAAACTCACAGTCTGGGGCATT
>HQ615972
TTTTATTGTAACACATCAGGCCTGTTTAATAGCACTTGGAGTTTTAATAGCACTGGAAATAATTACAGCATGA
ATAGCACCAAT--------------------------------------------------------------
-------------GACACT------
ATAACTAACGACACTATAATTCTCCAATGTAGAATAAAGCAAATTATAAATATGTGGCAGAGAGTAGGACAGG
CAATGTATGCCCCTCCCATTCGAGGAGTAATAAGGTGTGAATCAAAC---
ATTACAGGACTAATATTAACAAGAGATGGAGGTAATAATGGGTCA----------------------------
-------------------------------------------------------------------------
----------------
AATGAAATCTTCAGACCTGGAGGAGGAGATATGAGGGACAATTGGAGAAGTGAATTATATAAATATAAAGTAG
TAAAAATTGAGCCACTA---GGAGTAGCACCCACCAAGGCAAAAAGAAGAGTGGTG-----------------
----------------------GAGAGAGCAAAAAGAACA---GTAGGA---CTGGGAGCT---------
GTCTTCCTTGGGTTCTTAGGAGCA---GCAGGAAGCACTATG---GGCGCAGCGTCA---
ATAACGCTGACGGCACAG---GCCAGACAA---TTATTG---TCTGGCATAGTGCAACAGCAAAGC---
AATTTG---CTGAGG---GCTATAGAGGCTCAACAGTATCTGTTGAAGCTCACAGTCTGGGGCATT
>HM204625
TTCTATTGTGACACATCAGGCCTGTTTGATAGCCAGGCAAATAGA----------------------------
-------------------------------------------------------------------------
----------------------------
ACAAATGACACTATAACTCTCCAATGCAGAATAAAGCAAATTATAAATATGTGGCAGAGAGTAGGACAAGCAA
TGTATGCCCCTCCCATTAAAGGAGTAATAAATTGTACATCACAT---
ATTACAGGATTAATATTAACAAGAGATGGTGGTAATGATACAACT----------------------------
-------------------------------------------------------------------------
-------------------
GAAACCTTCAGACCTGGAGGAGGAGATATGAGGGACAATTGGAGAAGTGAACTATATAAGTATAAAGTAGTAA
AAATTGAGCCACTA---GGAGTAGCACCCACCAGGGCAAGGAGAAGAGTGGTG--------------------
-------------------GGGAGAGAAAAAAGAACA---GTTGGA---CTAGGAGCT---------
GTCCTCTTTGGGTTCTTAGGAGCA---GCAGGAAGCACTATG---GGCGCGGCGTCA---
ATAACGCTGACGGTACAG---GCCAGACAA---TTATTG---TCTGGCATAGTGCAACAGCAAAGC---
AATTTG---CTGAAG---GCTATAGAGGCTCAACAGCATATGTTGAGACTCACGGTCTGGGGCATT
>KY658699
TTCTATTGTAACACATCAGGCCTGTTTGATAACAACACGCTGACAAATACA----------------------
-------------------------------------------------------------------------
----------------------------
ACGCAGGAAAATATAACTCTCCCATGCAGAATAAAGCAAATTATAAATATGTGGCAGAGAGCAGGACAAGCAA
TATATGCCCCTCCCATTAAAGGAGTAATAAAGTGTGAATCAAAT---
ATTACAGGATTAATATTAACAAGAGATGGTGGTAATACTAATGATAGCGAT----------------------
-------------------------------------------------------------------------
-------------
GAAATTGAAACCTTCAGACCTGGAGGAGGAGATATGAGGGACAATTGGAGAAGTGAACTATATAAGTATAAAG
TAGTAAAAATTGAGCCACTA---GGAGTAGCACCCACCAAGGCAAGGAGAAGAGTGGTG--------------
-------------------------GGGAGAGAAAAAAGAGCA---GTTGGA---ATAGGAGCT---------
GTCCTCTTTGGGTTCTTAGGAGCA---GCAGGAAGCACTATG---GGCGCGGCGTCA---
ATAACGCTGACGGTACAG---GCCAGACAA---TTATTG---TCTGGCATAGTGCAGCAGCAAAGC---
AATTTG---CTGCAG---GCTATAGAGGCTCAACAGCATATGTTGAGACTCACGGTCTGGGGCATT
>AF361876
TTCTATTGTAATACAACAGACCTGTTTAATAGCACCAGGTACCGGGTTAATAGCACTTGGAATATGCAGGGGT
CAAATAGT-----------------------------------------------------------------
-------------------ACG---
```

GAGTCCAATGACACTATAACTCTCCAATGCAGAATAAAGCAAATCATAAATATGTGGCAGAGAGCAGGACAAG
CAATGTATGCCCCTCCCATTGAAGGAAACATAACATGTATGTCAAAC---
ATTACAGGACTACTGTTAACAAGAGATGGTGGGAATAATAACAGT---------------------------
----

ACAAATGAAACCTTCAGACCTGAAGGAGGAGATATGAGGGACAATTGGAGAAGTGAATTATATAAGTATAAAG
TAGTAAAACTTGAACCACTA---GGAGTAGCACCTACCAGGGCAAAAAGAAGAGTGGTG-------------
------------------------GAAAGAGAAAAAAGAGCA---GTTGGA---CTGGGAGCT--------
GTCTTCCTTGGGTTCTTAGGAGCA---GCAGGAAGCACTATG---GGCGCGGCGTCA---
ATAACGCTGACGGTACAG---GCCAGAAAT---TTATTG---TCTGGTATAGTGCAACAGCAAAAC---
AATTTG---CTGAGG---GCTATAGAGGCTCAACAGCATATGTTGAAACTCACGGTCTGGGGCATT
>AF361879
TTCTATTGTAATACTTCAGGCCTGTTTAATAGCACTTGGAACATTACCAGCACACAGAAGTCAAATAGCACGG
AG--

TCAAATAATGACACTATAACTCTCCAATGCAGAATAAAGCAAATTATAAATATGTGGCAGAGAGTAGGACAAG
CAATGTATGCCCCTCCCATCCCAGGAGCAATAGGGTGTAAATCAAAC---
ATTACAGGACTATTGTTAACAAGAGATGGTGGGGAGAATAACAGT---------------------------
----

GAAAATGAAACCTTCAGACCTGGAGGAGGAAATATGAGGGACAATTGGAGAAGTGAATTATATAAGTATAAAG
TAGTAAAACTTGAACCACTA---GGAGTAGCACCTACCAAGGCAAAAAGAAGAGTGGTG-------------
------------------------GAGAGAGAAAAAAGAGCA---GTTGGA---CTAGGAGCT--------
GTCTTCATTGGGTTCTTAGGAGCA---GCAGGAAGCACTATG---GGCGCGGCGTCA---
ATAACGCTGACGGTACAG---GCCAGACAA---TTATTG---TCTGGCATAGTGCAACAGCAAAGC---
AATTTG---CTGAAG---GCTATAGAGGCTCAACAACAATTGTTGAGACTCACGGTCTGGGGCATT
>HM215279
TTCTATTGCAATACATCAGACCTATTTAATGGCACGTTTAATGAGACATCAGGCCAGTTTAAT---------
----

AGCACATTTAATAGTACACTCCAGTGCAGAATAAAACAAATTATAAACATGTGGCAGGAAGTCGGACAAGCAA
TGTATGCCCCTCCCATTGCAGGAAGCATAACATGTATATCGAAT---
ATCACAGGACTAATATTAACAAGAGATGGTGGGAATACTAACAGT---------------------------
----

ACAAAGGAAACCTTCAGACCTGGAGGAGGAAATATGAGGGACAATTGGAGAAGTGAATTATATAAGTATAAAG
TAGTAAAAATTGAACCACTA---GGAGTAGCACCCACCAAGGCAAGGAGAAGAGTGGTG-------------
------------------------GAGAGAGAAAAAAGAGCA---GTAGGA---ATAGGAGCT--------
GTCTTCATTGGGTTCTTAGGCGCA---GCAGGAAGCACTATG---GGCGCGGCGTCA---
ATAACGCTGACGGTACAG---GCCAGACAA---TTATTG---TCTGGTATAGTGCAACAGCAAAGC---
AATTTG---CTGAGG---GCTATAGAGGCTCAACAGCATCTGTTGAAACTCACGGTCTGGGGCATT
>HM215315
TTCTATTGTGATACATCAGCCCTGTTTAATAGCACTTGGAATAACAGCACGAGGAACAGCATGGAGTGG----
----

TTAAATGACACTATAACTCTCCAATGCAAAATAAAGCAAATTATAAATATGTGGCAGAGAGCAGGACAAGCTA
TGTACGCCCCTCCCATCCAAGGAACAATAAGTTGTAACTCAAAC---
ATTACAGGACTACTGTTAACAAGAGATGGGGGGAAGGACACCACA---------------------------
----

AATGAAACCTTTAGACCTGGAGGAGGAGACATGAGGAACAATTGGAGAAGTGAATTATATAAGTATAAAGTAG
TAAAACTTGAACCACTA---GGAGTAGCACCTACCAAGGCAAAAAGAAGAGTGGTG----------------
---------------------GAGAGAGAAAAAAGAGCA---GTTGGA---ATAGGAGCT---------
GTCCTCATTGGGTTCTTAGGAGCA---GCAGGAAGCACTATG---GGCGCGGCGTCA---
ATAACGCTGACGGTACAG---GCCAGACAA---TTATTG---TCTGGTATAGTGCAACAGCAAAGC---
AATTTG---CTGAGG---GCTATAGAGGCTCAACAGCAACTGTTGAAACTCACGGTCTGGGGCATT
>HM215322
TTCTATTGTGATACATCAGACCTGTTTAATAGCACTTGGAATTATACTAGTATCACGTCAAATAGCACGGAG-
----

TCAAATGACACTATAACTCTCCAATGCAGAATAAAGCAAATTATAAATATGTGGCAGAGAGAAGGACAAGCAA
TGTATGCCCCTCCCATTGAAGGAAACATAACATGTATGTCAAAC---
ATTACAGGACTACTGTTAGTAAGAGATGGTGGGATTAATAACAGG---------------------------
----

ACAGATGAAATCTTCAGACCTGGAGGAGGAAATATGAAAGACAATTGGAGAAGTGAATTATATAAGTATAAAG
TAGTAAAACTTGAACCACTA---GGAGTAGCACCTACCAGGGCAAAAAGAAGAGTGGTG-------------
------------------------GAGAGAGAAAAAAGAGCA---GTTGGA---CTGGGAGCT---------
GTCTTCATTGGGTTCTTAGGAGCA---GCAGGAAGCACTATG---GGCGCGGCGTCA---
ATAACGCTGACGGTACAG---GCCAGAAAT---TTATTG---TCTGGTATAGTGCAACAGCAAAAC---
AATTTG---CTGAGG---GCTATAGAGGCTCAACAGCATATGTTGAAACTCACAGTCTGGGGCATT
>AF484491
TTCTATTGTAATACATCAGGTTTGTTTAATAGCTCCTGGGCAAATAACACTGACAATAGCAATAACACTGTCA
AT--

AGCACGGACATTATAACTCTCCCATGCAGAATAAAGCAAATTGTAAATATGTGGCAAAGAACAGGACAAGCAA
TGTATGCCCCTCCCATCAAAGGAGTGATAAGGTGTGAATCAAAT---
ATTACAGGACTACTATTAACAAGAGATGGTGGGGCTACTAGCATT---------------------------
----

ATAAATGAAACCTTCAGACCTGGAGGAGGAAATATGAGGGACAATTGGAGAAGTGAATTATATAAGTATAAAG
TAGTACAAATTGAACCACTA---GGAGTAGCACCCACCAGGGCAAGGAGAAGAGTGGTG-------------
------------------------GAGAGGGGAAAAAGAGCA---ATTGGA---ATAGGAGCT---------
GTATTCCTTGGGTTCTTAGGAACA---GCAGGAAGCACTATG---GGCGCGGCGTCA---
ATAACGCTGACGGTACAG---GCCAGACAA---TTACTG---TCTGGTATAGTGCAACAGCAAAGC---
AATTTG---CTGAGG---GCTATAGAGGCTCAACAGCATATGTTGAAACTCACGGTCTGGGGCATT
>AF484501
TTTTATTGTAACACATCAGGCCTGTTTAATCACACTTACACTTGGTATAATGGCAGCTTGCAGGAGTCAAATA
GC--

ACGGGGTCAGACAACATAACTCTCCAATGCAGAATAAAGCAAATTATAAATATGTGGCAGAGAGTGGGACAAG
CTATGTATGCCCCTCCCATTCGAGGAGTAATACAGTGTGAATCAAAC---
ATTACAGGACTAATATTAACAAGAGATGGTGGAAATAATAATGAT---------------------------
----

AATACAACTGAAATCTTCAGACCTGGAGGAGGAGATATGAGGGACAATTGGAGAAGTGAATTATATAAGTATA
AAGTAGTAACAATTGAACCACTA---GGAGTAGCACCCACCCAGGCAAGGAGAAGAGTGGTG----------
---------------------------GAGAGAGAAAAAAGAGCA---GTGGGA---ATAGGAGCT------
---GTCCTCCTTGGGTTCTTAGGAACA---GCAGGAAGCACTATG---GGCGCGGCGTCC---
ATGACGCTGACGGTACAG---GCCAGACAA---TTATTG---TCTGGCATAGTGCAACAGCAAAGC---
AATTTG---CTGAAG---GCTATAGAGGCTCAACAGCAACTGTTGAGACTCACGGTCTGGGGCATT
>FJ647147
TTCTATTGCAATACATCAGGCCTGTTTAACAGCACTTGGGAGTTTAACAACCCTTTTAATGAAACTGAGTGGC
CA--

CAAAATAAAACTATAATTCTCCAATGCAGAATAAAGCAAATTGTAAATATGTGGCAGAGAGTAGGACAGGCGA
TGTATGCCCCTCCCATCAAAGGAGTAATAAGGTGTAATTCAACC---
ATTACAGGACTATTCTTAACAAGAGATGGTGGAAATACTAGCAGT---------------------------
----

ACAAATGAGACCTTCAGGCCTGGAGGAGGAGATATGAGGGACAATTGGAGAAGTGAATTGTATAAATATAAAG
TAATAAAAATTGAACCAATA---GGAGTAGCACCCACCAGGGCAAAAAGAAGAGTGGTG-------------
------------------------GAAAGAGAAAAAAGAGCA---GTTGGA---CTGGGAGCT---------
GTTTTCCTTGGGTTCTTAGGAGCA---GCAGGAAGCACTATG---GGCGCAGCGTCA---
ATAACGCTGACGGTACAG---GCCAGACAA---TTATTG---TCTGGCATAGTGCAACAGCAAAGC---
AATTTG---CTGAGG---GCTATAGAGGCTCAACAGCATCTGTTGAAACTCACAGTCTGGGGCATT
>KT183118
TTCTACTGCAATACATCAGGCCTGTTTAATGACACATTTATGCCCAATAATACGGCTAATAGTACTAAGAATG
AAACA---

CAGGAAAAGAATATAACTATCTCATGCAAAATAAAACAAATTATAAGAATGTGGCAAAGAGTAGGACAAGCAA
TGTATGCCCCTCCCATTGAAGGATTAATAAAATGTGAATCAAAC---
ATTACAGGAATACTATTGACAAGAGATGGTGGGAACAATACTAAC----------------------------
----

AGTACAGAGGTCTTCAGACCTGGAGGGGGAAATATGAAGGACAAGTGGAGAAGTGAATTATATAATTATAAGG
TAGTAAAAATTGAACCACTA---GGAGTAGCACCCACCGGGGCAAGGAGAAGAGTGGTG--------------
-------------------------GAGAGAGAGAAAAGAGCAGTAGTTGGA---CTGGGAGCT---------
GTCTTCCTTGGGTTCTTAGGAGCA---GCAGGAAGCACTATG---GGCGCGGCGTCA---
TTAACGCTGACGGTACAG---GCCAGGCAA---TTATTG---GCTGGCATAGTGCAACAGCAAAGC---
AATTTG---CTGCAG---GCTATAGAGGCTCAACAACATATGTTGAAACTCACTGTCTGGGGCATT
>GU201611
TTCTATTGTAACACAACAGACTTGTTTAATAGCAGTTGGGCTAACAATGGCAGCAGGCTGGGGCCAAATGGTA
CA---

GAGTCAGACATTATAACTATCCAATGCAGAATAAAGCAAATTATAAATATGTGGCAAAGAGTAGGACAAGCAA
TATATGCCCCTCCCAGAAAAGGAGTAATAAGGTGTGACTCAAAC---
ATTACAGGACTGATATTAACAAGAGATGGTGGTAGCAATAACACA----------------------------
----

AATAATGAAACCTTCAGACCTGGAGGAGGAGACATGAGGGACAATTGGAGAAGTGAATTATATAAGTATAAAG
TAGTAAAACTTGAACCACTA---GGGGTAGCACCTACCCCGGCAAAAAGAAGAGTGGTG--------------
-------------------------GAGAGAGAGAAAAGAGCA---GTTGGA---CTAGGAGCT---------
GTTTTCATTGGGTTCTTAGGAGCA---GCAGGAAGCACTATG---GGCGCGGCGTCA---
GTAACGCTGACGGTACAG---GCTAGACAA---TTATTG---TCTGGCATAGTGCAGCAGCAAAGT---
AATTTG---CTGAGG---GCTATAGAGGCTCAACAACATATGTTGAAACTCACGGTCTGGGGCATT
>KF114883
TTCTATTGCAATACATCAAATCTATTCAATAGTACATACAAT-------------------------------
----

GAAACAACCATCACAATTCACTGCAGAATAAAACAATTTATAAACATGTGGCAGGGGGTAGGACGAGCAATGT
ATGCCCCTCCCATTGAAGGAAAAATAACATGTAACTCAAAT---
ATCACAGGGCTACTACTGACCCGTGATGGAGGCATAAACACAACAGAAAACGGC------ATAAACACA---
ACA--

GGAAAAGAGATATTCAGACCTGAGGGAGGAAATATGAAAGACAATTGGAGAAATGAATTATATAAATATAAAG
TGGTGGAAATTAAGCCATTG---GGAATAGCACCCACTGAGGCGAAAAGAAGAGTGGTG--------------
-------------------------GAAAGAGAAAAAAGGGCA---GTGGGA---CTAGGAGCT---------
GTGTTCCTTGGGTTCTTGGGAGCA---GCAGGAAGCACTATG---GGCGCGGCGTCA---
ATAACGCTGACGGTACAG---GCCAGACAA---CTGTTG---TCTGGTATAGTGCAACAGCAAAAC---
AATTTG---CTGAGA---GCTATAGAGGCGCAACAGCATATGTTGCAACTCACGGTCTGGGGCATT
>GU329121
TTCTATTGCAACACATCAGGCCTGTTTAATAGCAGTTGGAAAATTGAGAATGGCAATGCCACGCTAAATAGCA
CG---

TGGCCAAATGGCACCATAACTCTTCAATGCAGAATAAAGCAAATTATAAATATGTGGCAAAGGACAGGACAAG
CAATATATGCCCCTCCCATCCAAGGAGTAATAAGGTGTGACTCAAAC---
ATTACAGGACTACTATTAACAAGAGATGGTGGGGATAATAATGGT----------------------------
----

ACAGAAACCTTCAGACCTGGAGGAGGAGATATGAGGGACAATTGGAGAAGTGAGTTATATAAATATAAAGTAG
TAAAAATTGAGCCACTA---GGAGTAGCACCCACCAGGGCAAAGAGAAGAGTGGTG-----------------
----------------------GAGAGAGAAAAAAGAGCA---GTAGGA---ATAGGAGCT---------
GTGTTCCTTGGGTTCTTAGGAGCA---GCAGGAAGCACTATG---GGCGCGGCGTCA---
ATAACGCTGACGGTACAG---GCCAGACAA---TTATTG---TCTGGCATAGTGCAACAGCAAAGC---
AATTTG---CTGAGG---GCTATAGAGGCTCAACAGCATATGTTGAAACTCACGGTCTGGGGCATT
>JF680922
TTCTATTGCAACACATCAGGACTGTTTAATAGCACTTGGTTTCAGAATGGCACTAACATTACACAGGGG----
----

TCAGATGGCAATATAACTCTTCATTGCAGAATAAAGCAAATCATAAATATGTGGCAGAGAGCAGGACAAGCAA
TATATGCCCCTCCCATCAAGGGAGTAATAAAGTGTGTATCAAAC---
ATTACAGGACTAATATTAACAAGAGATGGTGGTACCAATGTGACG----------------------------
----

AATGGAACTGAAATCTTCAGGCCTGGAGGGGGAGATATGAAGGATAATTGGAGAAGTGAATTATATAAATATA
AAGTGGTAGAAATTAAACCATTA---GGAGTAGCACCCACCAAGGCAAAAAGGAGAGTGGTG-----------
----------------------------GGAAGAGAAAAAAGAGCA---GTAGGA---ATAGGAGCT------
---GTGTTCCTTGGGTTCTTGGGAGCA---GCAGGAAGCACTATG---GGCGCAGCGTCA---
ATAACGCTGACGGTACAG---GTCAGACAA---TTATTG---TCTGGCATAGTGCAACAGCAAAAC---
AATTTG---CTGAGG---GCTATAGAGGCTCAACAGCATCTGTTGAGACTCACAGTCTGGGGCATT
>AF457056
TTCTATTGTGATACATCAGGCCTGTTTAATTACACGTTTTATCCCAATGGCAGCTTGCAGGGGGAATCAAATG
AC---

ACGCAGGAGAAGATAACTCTCCCATGCAGAATAAAGCAAATTATAAATATGTGGCAGAGAACAGGGCAAGCAA
TATATGCCCCTCCCATCCAAGGAGTAATAAGATGTGTATCAAAC---
ATTACAGGAATACTATTAACAAGAGATGGTGGAGATACTGGGGAC----------------------------
----

AACAATACTGAAACCTTCAGACCTGGAGGAGGAGATATGAGGGACAATTGGAGAAGTGAATTATATAAGTATA
AAGTAGTACAAATTGAACCACTA---GGAGTAGCACCCACCAGGGCAAGGAGAAGAGTGGTG-----------
----------------------------GGAAGAGAAAAAAGAGCA---GTTGGA---ATAGGAGCT------
---GTTTTCCTTGGGTTCTTAGGAGCA---GCAGGAAGCACTATG---GGCGCGGCGTCA---
ATAACGCTGACGGTACAG---GCCAGACAA---TTGTTG---TCTGGTATAGTGCAACAGCAAAGC---
AATTTG---CTGAGG---GCTATAGAGGCTCAACAACATCTGTTGAAACTCACGGTCTGGGGCATT
>KT022400
TTCTATTGTAACACAGCAGGCCTGTTTAATAGCACTTGGAATAGTAGCACTTATAGTAACAATGCCAGCGGGC
AGGAG--

TCAAATGACACTATAACTCTCCAATGCAGAATAAAGCAAATTATAAATATGTGGCAGAGAGCAGGACAAGCAA
TATATGCCCCTCCCATCGAAGGAGTAATAAAGTGTGTATCAAAC---
ATTACAGGACTAATATTAACAAGAGATGGTGGGAATGCTAACAGT----------------------------
----

ACAAATGAAACCTTCAGACCTGGAGGAGGAGATATGAGGGACAATTGGAGAAGTGAATTATATAAGTATAAAG
TAGTAGAACTTGAACCACTA---GGAGTAGCACCCACCAAGGCAAAGAGAAGAGTGGTG--------------
-------------------------GAGAGAGAAAAAAGAGCA---GTTGGA---CTGGGAGCT---------
GTTCTCATTGGGTTCTTAGGAGCA---GCAGGAAGCACTATG---GGCGCAGCGTCA---
GTGACGCTGACGGTACAG---GCCAGACAA---CTATTG---TCTGGCATAGTGCAACAGCAAAGC---
AATTTG---CTGATG---GCTATAGAGGCTCAACAGCAACTGTTGAAACTCACAGTCTGGGGCATT
>KT022401
TTCTATTGCAACACAAATGAATTGTTTAATAGCACTTGGTTTTTGAACACTACTAATACCAGCACGTGGGAGT
CAAATGGG---

ACGAGGTCAGAAAATATAACTCTCAAATGCAGAATAAAGCAAATTATAAATATGTGGCAGAGAGCAGGACAAG
CAATGTATGCCCCTCCCATCCGAGGAGTAATAAACTGTACATCAAAC---
ATTACAGGACTAATATTAACGAGAGATGGTGGGGTTAATAACAAT----------------------------
----

ACAAATGAAATCTTCAGACCTGGAGGAGGAGATATGAGGGACAATTGGAAAAGTGAATTATATAAATATAAGG
TAGTAAAAATTGAACCACTA---GGAGTAGCACCCACCAAGGCAAAGAGAAGAGTGGTG--------------
-------------------------GAGAGAGAAAAAAGAGCA---GTTGGA---ATAGGAGCT---------
GTATTCATTGGGTTCTTAGGAGCA---GCAGGAAGCACTATG---GGCGCGGCGTCA---
ATAACGCTGACGGTACAG---GCCAGACAA---TTATTG---TCTGGCATAGTGCAACAGCAAAGC---
AATTTG---CTGAAG---GCTATAGAGGCTCAACAGCATCTGTTGAGACTCACGGTCTGGGGCATT
>KT022414
TTCTATTGCAATACATCAAGACTGTTTAATAGCATTTGGGAGCATAATAGCACTTGGACCAATACACGGGGA-
----

```
TCAAATAGCACAATAACTCTCCCATGCAGAATAAAGCAAATTATAAATATGTGGCAGAGAGTAGGACAAGCAA
TGTATGCTCCTCCCATCGCAGGAGCAATACAGTGTGCATCAAAT---
ATTACAGGACTAATATTAACAAGAGATGGTGGAAATCATAACAGG----------------------------
-------------------------------------------------------------------------
-------------
ACAAATGAAACCTTCAGACCTGGAGGAGGAGATATGAGGGACAATTGGAGAAGTGAATTATATAAGTATAAAG
TAGTAAAAATTGAACCACTA---GGAGTAGCACCCACCAGGGCAAAGAGAAGAGTGGTG--------------
-------------------------GAGAGAGAAAAAAGAGCA---GTTGGA---CTAGGAGCT---------
GTTTTCATTGGGTTCTTAGGAGCA---GCAGGAAGCACTATG---GGCGCAGCGTCA---
GTGACGCTGACGGTACAA---GCCAGGCAA---TTACTG---TCTGGCATAGTGCAACAGCAAAGC---
AATTTG---CTGAGG---GCTATAGAGGCTCAACAGCACCTGTTGAGACTCACGGTCTGGGGCATT
>KU749430
TTCTATTGCGATACAGCAAAACTGTTTAATAGCACGTGGGAGTACAATGGCACTGAATACAACATGACCAAAG
GG-----------------------------------------------------------------------
----------------------------
TCAAATGGAAATCTGACTCTCCCATGCAGAATAAAACAAATTGTACACATGTGGCAGAGAGTAGGACAAGCAA
TGTATGCCCCTCCCATCCCAGGAATAATAAAGTGTGAATCAAAC---
ATCACAGGACTAATATTAACAAGAGATGGTGGGAAGAATACCAGT----------------------------
-------------------------------------------------------------------------
----------------
AATGAGACCTTCAGACCTGGAGGAGGAGATATGAGGGATAATTGGAGAAGTGAATTATATAAGTATAAAGTAG
TAAAACTTGAACCACTA---GGAGTAGCACCCACCGCAGCAAGGAGAAGAGTGGTG-----------------
----------------------CAGAGAGAAAAAAGAGCA---GTTGGA---ATGACAGCT---------
GTCTTCCTTGGGTTCTTAGGAGCA---GCAGGAAGCACTATG---GGCGCGGCGTCA---
ATAACGCTGACGGTACAG---GCCAGACAA---CTGTTG---TCTGGCATAGTGCAACAGCAAAGC---
AATTTG---CTGAAG---GCTATAGAGGCTCAACAGCATATGTTGAGACTCACGGTCTGGGGCATT
>KF716470
TTCTATTGTAATACATCAGGCCTATTTAATAGTACTTGGCATTGGAATAATACTGATACTAATGGCACCAATG
GT-----------------------------------------------------------------------
----------------------------
ACTAATGATACTATAACTCTCCCATGCAGAATAAAGCAAATTATAAATATGTGGCAGAGGGTAGGACAAGCAA
TATATGCCCCTCCCATCCCAGGGGTAATAAAATGTGTATCAGAC---
ATTACAGGACTCCTATTAACAAGAGATGGTGGGGTCAATAATAGC----------------------------
-------------------------------------------------------------------------
----------
AGCAATAATGAAACCTTCAGACCTGGAGGAGGGGATATGAGGGACAATTGGAGAAGTGAATTATATAAGTATA
AGGTAGTAAAAATTGAACCACTA---GGAGTAGCACCCACCAAGGCAAAGAGAAGAGTGGTG-----------
----------------------------GAGAGAGAAAAAAGAGCA---GTTGGA---CTGGGAGCT------
---GTCTTCATTGGGTTCTTGGGAGCA---GCAGGAAGCACTATG---GGCGCGGCGTCA---
ATAACGCTGACGGTACAG---GCCAGGCAA---TTATTG---TCTGGCATAGTGCAACAGCAAAGC---
AATTTG---CTGAGG---GCTATAGAGGCTCAACAACATCTGTTGAAACTCACAGTCTGGGGCATT
>KU921717
TTCTATTGTAACACATCAAGCCTGTTTAATAGCACTTGGAATAGCACTTGGAATGGCAATGACAGCAGCACA-
-------------------------------------------------------------------------
----------------------------
GAAAATGGCACTATAACTTTACAATGCAAAATAAAGCAAATCATAAATATGTGGCAGAGAGTAGGACAAGCAA
TGTATGCCCCCCCCATTCAAGGAGTCATAAATTGTAAATCAAAC---
ATTACAGGATTGATATTAACAAGGGATGGTGGAAACACTACCAGC----------------------------
-------------------------------------------------------------------------
-------------
AGTGAGGAAATTTTCAGACCTGGAGGGGGAGATATAAGGGATAATTGGAGAAGTGAATTATATAAGTATAAAG
TAGTAAAAATTGAGCCACTA---GGAGTAGCACCCACCAGGGCAAGGAGAAGAGTGGTG--------------
-------------------------AAGAGAGAAAAAAGAGCA---GTTGGA---CTGGGAGCT---------
TTTTTCCTTGGGTTCTTAGGAGCA---GCGGGAAGCACTATG---GGCGCGGCGTCA---
ATAACGCTGACGGTACAG---GCCAGGCAA---TTATTG---TCTGGCATAGTGCAACAGCAAAGC---
AATTTG---CTGAGG---GCTATAGAGGCTCAACAACATCTGTTGAGACTCACGGTCTGGGGCATT
>KU921730
TTCTATTGTAATACATCAAAACTGTTTAATAGCACTTGGTATAATTCCACTAATACCACTTGGAGGATGAC-
-------------------------------------------------------------------------
----------------------------
```

GGCACACAGAATATAACTCTCCCATGCAGAATAAAGCAAATTATAAATCTGTGGCAGAGAACAGGACGAGCAA
TGTATGCCCCTCCCATCCGAGGAGTAATACAGTGTAGGTCAAAT---
ATTACAGGGCTAATATTAACAAGAGATGGTGGGAATGATAATGATACC-------------------------
----

AGTAACAATGAAACCTTCAGACCTGGAGGTGGAAATATGAGGGACAATTGGAGAAGTGAATTATATAAGTATA
AAGTAGTACAAATTGAACCAATA---GGAATAGCACCCACCAAGGCAAGGAGGATAGTGGTA-----------
----------------------------GGGAGAGAAAAAAGAGCA---GTTGGA---CTAGGAGCT------
---GTCTTCCTTGGGTTCTTAGGAGCA---GCCGGAAGCACTATG---GGCGCAGCGTCA---
ATGACGCTGACGGTACAG---GCCAGACAG---TTATTG---TCTGGCATAGTGCAACAGCAAAGC---
AATTTG---CTGAGG---GCTATAGAGGCTCAACAGCATCTGTTGAAACTCACAGTCTGGGGCATT
>KU921766
TTCTACTGCAATACATCAAATCTGTTTAATGGTACATGGATAGTAAATAATACAAATAGTACAAATAATGCAG
ATACA--

TCAAATAGTAATATTACACTCCAATGCAGAATAAAACAAATTATAAACAGGTGGCAGGAAGTAGGAAAAGCAA
TCTATGCCCCTCCTATTGCAGGACTAATAAAATGTTCATCAAAT---
ATTACAGGAATATTGTTAACAAGAGATGGTGGTGCAAGTCATAAT----------------------------
----

AATCAGAGTGAGACCCTCAGACCTGGAGGAGGAAATATGAAAGATAATTGGAGAAGTGAATTATACAAATATA
AAGTAGTAAGGCTTGAACCAATA---GGTCTAGCACCCACCAGGGCAAAAAGAAGAGTGGTG-----------
----------------------------GAAAGAGAAAAAAGAGCA---ATAGGA---CTGGGAGCT------
---ATGTTCCTTGGGTTCTTGGGAGCA---GCAGGAAGCACAATG---GGCGCAGCGTCG---
GTGACGCTGACGGTACAG---GCCAGACAG---TTATTG---TCTGGCATAGTGCAACAGCAAAAT---
AATTTG---CTGAGG---GCTATAGAGGCGCAACAGCATATGTTGCAACTCACAGTCTGGGGCATT
>AF075702
TTCTATTGTAATACATCAGGCCTGTTTAATAGCACTTGGGTAAATGGCAGCAGGGAATCAAATAGCACA----
----

GATAATGATACTATAACTCTCCCGTGTAGAATAAAGCAAATTATAAATATGTGGCAGAGAGTAGGACAAGCAA
TGTATGCCCTTCCCATCCGAGGAGTAATAAGGTGTGAATCAAAC---
ATTACAGGATTAATATTAACAAGAGATGGTGGGAATAATACCAGT----------------------------
----

ACAAATGAAACCATCAGACCTGCAGGAGGAGATATGAGGGACAATTGGAGAAGTGAATTATATAAATATAAAG
TAGTAAAAATTGAACCACTA---GGAGTAGCACCCACCAAGGCAAGGAGAAGAGTGGTG--------------
-------------------------GAGAGAGAAAAGAGAGCA---GTTGGA---ATAGGAGCT---------
GTGTTCCTTGGGTTCTTAGGAGCA---GCAGGAAGCGCTATG---GGCGCAGCGGCA---
GCAACGCTGACGGTACAG---GCCAGGCAA---TTATTG---TCTGGCATAGTGCAACAGCAAAGC---
AATTTG---CTGAAG---GCTATAGAGGCTCAACAGCATCTGTTGAAACTCACGGTCTGGGGCATT
>HQ659612
TTTTATTGTAATACATCAGACATGTTTAATAGCACTTGGAATTGGAATAGCAGTGCCAACATGATGGAGTCAA
ATAGCACCAGC--
-GTGCAGGGGCCAAAT---------
ATAACTGAGCAAAATATAACTCTCCAATGCAGAATAAAGCAAATTATAAATATGTGGCAGAGAGCAGGACAAG
CAATATATGCCCCTCCTATCAAAGGAGTAATAAGGTGTGAATCAAAC---
ATTACAGGGCTAATATTAACAAGAGATGGGGGAAAAAGTAACAACACA-------------------------
----

AATAATACTGAAACCTTCAGACCTACAGGAGGAGATATGAGGGACAATTGGAGAAGTGAATTATACAAATATA
AAGTGGTGGAAATTAAACCATTA---GGAGTAGCACCCACTGAGGCAAAAAGGAGAGTGGTG-----------
----------------------------AAGAGAGGAAAAAGAGCA---GTAGGA---ATAGGAGCT------
---GTATTCCTTGGGTTCTTGGGAGCA---GCAGGAAGCACTATG---GGCGCAGCGTCA---
ATAACGCTGACGGTACAG---GCCAGACAA---TTATTG---TCTGGCATAGTGCAACAGCAAAGC---
AATTTG---CTGAGG---GCTATAGAGGCTCAACAGCATCTGTTGAAGCTCACAGTCTGGGGCATT
>KX907397
TTCTATTGTAATACATCAGGCCTGTTTAATAGCACGTGGAACAATAGCATGTGGAACAACATGCTGACTTGGA
ATGCC--

ACGGGGAAGTACATTATGACTCTCCCATGTAAAATAAAACAAATTATAAATATGTGGCAGGGAGTAGGAAAAG
CAATGTATGCCCCTCCCATTGAAGGACTAATTCAATGCTCATCAAAT---
ATTACTGGACTATTATTAACAAGAGATGGGGGTGTAAATAATGAT----------------------------
----

AGTCAGAGTGAGACCTTCAGACCTGGAGGGGGAGATATGAGAGACAATTGGAGAAGTGAATTATACAAATATA
AAGTAGTAAGAATTGAACCAATA---GGTCTGGCACCTACCAGGGCAAAAAGAAGAGTGGTG-----------
----------------------------GAAAGAGAAAAAAGAGCA---ATAGGA---CTGGGAGCT------
---ATGTTCCTTGGGTTCTTGGGAGCA---GCAGGAAGCACGATG---GGCGCAGCGTCA---
CTGACGCTGACGGTACAA---GCCAGAGAG---TTATTG---TCTGGTATAGTGCAACAGCAAAAT---
AATTTG---CTGAGA---GCTATAGAGGCGCAACAGCATCTGTTGCAACTCACTGTCTGGGGCATT
>KX907350
TTCTATTGCAATACATCAAACCTGTTTAACGGCACTTGGAATGGTAGCACTTGGGATAGCCCTTGGAATAGGA
ATAAC--

AGCAATGAAAATATAACACTCCAATGCAGAATAAAACAAATTATAAATATGTGGCAGAGAGCAGGACAAGCAA
TGTATGCCCCTCCCATCCAAGGAGTAATAAGGTGTGAATCAAAC---
ATCACAGGACTACTATTAACACGTGATGGAGGAGAAAATAATAAT----------------------------
----

ACTACAGAGACATTCAGGCCGGTAGGAGGAAATATGAAAGACAACTGGAGAAGTGAATTATATAAGTATAAAG
TAGTAAAAATTGAACCACTA---GGAGTAGCACCCACTAGGGCAAGGAGAAGAGTGGTG--------------
-------------------------GAGAGAGAGAAAAGAGCA---GTTGGA---ATAGGAGCT---------
GTGTTCCTTGGGTTCTTGGGAGCA---GCAGGAAGCACTATG---GGCGCAGCGTCA---
ATAACGCTGACGGTACAG---GCCAGACAA---TTATTG---ACTGGCATAGTGCAACAGCAAAGC---
AATTTG---CTGAGG---GCTATAGAGGCTCAACAGCAAATGTTGAGACTCACAGTCTGGGGCATT
>KX907351
TTTTATTGTAACACATCAGGCTTGTTTAATAGCTCTTGGTCTTGGAATGGTACCAATAACACGGAGTCAGGTA
AC---

ATAAATGTCAATATAACTCTCCCATGCAGAATAAAGCAAATTATAAATATGTGGCAGAGAGTAGGACAAGCAA
TGTATGCCCCTCCCATTCAAGGAGTGATACAGTGTGTATCAAAC---
ATTACAGGACTAATATTAACAAGAGATGGTGGGAATAATAGCAGT----------------------------
----

ACAAATGAAATCTTCAGACCTGGAGGAGGAGATATGAGGGACAATTGGAGAAGTGAATTATATAAGTATAAAG
TAGTAAGAATTGAACCATTA---GGAGTAGCACCCACTAAGGCAAAAAGGAGAGTGGTG--------------
-------------------------GAGAGAGAAAAAAGAGCA---GTGGGA---ATAGGAGCT---------
GTGTTCCTTGGGTTCTTGGGAGCA---GCAGGAAGCACTATG---GGCGCAGCGTCA---
ATAACGCTGACGGTACAG---GCCAGACAA---TTATTG---TCTGGTATAGTGCAACAGCAAAGC---
AATTTG---CTGAGG---GCTATAGAAGCGCAACAGTACCTGTTGAAGCTCACGGTCTGGGGCATT
>KX907353
TTCTATTGTAACACATCAAAGTTGTTTGACAGCAGTTGGCCAAAAACAAATAGCACGGTGCAGGAGTCAAAT-
----

AGCACAGACAATATAACGCTCCAATGTAGAATAAAGCAAATTATAAATATGTGGCAGAGAGTAGGACAAGCAA
TGTATGCCCCTCCCATTGCAGGAGTAATAAGGTGTGAATCAAAC---
ATTACAGGACTAATACTAACAAGAGATGGTGGAAGTAATACCAAT----------------------------
----

GCAAGTGAAACCTTCAGACCTGGAGGAGGAGATATGAGGGACAATTGGAGAAGTGAATTATATAAGTACAAAG
TAGTAAAAATTGAACCACTA---GGAGTAGCACCCACCAGGGCAAAGAGAAGAGTGGTG--------------
-------------------------GAGAGAGAAAAAAGAGCA---GTTGGA---ATAGGAGCT---------
GTATTCATTGGGTTCTTAGGAGCA---GCAGGAAGCACTATG---GGCGCAGCGTCA---
ATAACGCTGACGGTACAG---GCCAGACAA---TTATTG---GCTGGCATAGTGCAACAGCAAAGT---
AATTTG---CTGAGG---GCTATAGAGGCTCAACAGCATCTGTTGAAACTCACGGTCTGGGGCATT
>KX907419
TTTTATTGTGACACATCAAACCTGTTTAATAGCAATTGGACTGTGACTGACAGCGAACCGAAGCTAAATAGCA
CGGAG--

CCAAACGACAATATAACTCTCCAATGCAGAATAAAGCAAATTATAAGGATGTGGCAGAGAGTAGGACAAGCAA
TGTATGCCCCTCCCATCCCAGGAATAATAGAGTGTAAATCAAAC---
ATTACAGGACTAATACTAACAAGAGATGGTGGGCCTCCTAATACA----------------------------
----

ACTGAGACCTTCAGACCTGGAGGAGGAGATATGAGGGACAATTGGAGAAGTGAATTATATAAATATAAGGTAG
TAAAAATTGAACCAATA---GGAGTAGCACCCACCGGGGCACGGAGAAGAGTGGTG-----------------
----------------------GGGAGAGAAAAAAGAGCA---GTAGGA---ATAGGAGCT---------
GTGTTCCTTGGGTTCTTGGGAGCA---GCAGGAAGCACGATG---GGCGCAGCGTCA---
GTGACGCTGACGGTACAG---GCCAGACAG---TTACTG---TCTGGTATAGTGCAACAGCAAAGC---
AATTTG---CTGAGG---GCTATAGAGGCTCAACAGCATCTGTTGAGACTCACAGTCTGGGGCATT
>KX907370
TTCTACTGCAATACAACAAATTTGTTTAATGCAACAAAATGGTTTAATAGGAGTATAGAGGAAAGTGCAAATG
CAAATGAC---
-------------------ACA---
AGTGAGAATTCCACACTCACACTCCCATGCAGAATAAAACAAATTATAAACATGTGGCAGGGAGTAGGAAAAG
CAATGTATGCCCCTCCCATTGCAGGGCAAATCAAATGTTCATCAAAT---
ATTACAGGACTATTGTTGACAAGAGATGGT---AATAATAGTCAT----------------------------
----

GAGACCTTCAGACCTGGGGGAGGAGATATGAGAGACAATTGGAGAAGTGAATTATACAAATATAAAGTAGTAA
AAATTGAACCAATA---GGTCTAGCACCCACCAGGGCAAAGAGAAGAGTGGTG--------------------
-------------------GAAAGAGAAAAAAGAGCA---ATAGGA---CTGGGAGCT---------
GTGTTCCTTGGGTTCTTGGGGGCA---GCAGGAAGCACAATG---GGCGCAGCGTCG---
CTAACGCTGACGGTACAG---GCCAGGCAA---GTATTG---TCTGGTATAGTGCAACAGCAAAAC---
AATTTG---CTGAGG---GCTATAGAGGCGCAACAGCATCTGTTGCAACTCACAGTCTGGGGCATT
>HM215280
TTCTACTGCAATACATCACAACTGTTTAATACTACATGGAGTAAAAATGGTACATCAAATAGTACAGGGTCA-
----

AATAATAGTACAATCATACTCCCATGCAGAATAAAACAAATTATAAATATGTGGCAGAGAACAGGACAAGCAA
TGTATGCCCCTCCCATCCCAGGAGTAATAAGGTGTGTATCAAAC---
ATTACAGGACTAATTTTAACAAGAGATGGTGGAAATAATAACAAA----------------------------
----

ACAGATGAAACTCTCAGACCTGGAGGTGGAGATATGAGGGACAATTGGAGAAGTGAATTATATAAGTATAAAG
TAGTAAAACTTGAACCACTA---GGAGTAGCACCCACCAGGGCAAAGAGAAGAGTGGTG--------------
-------------------------GGGAGAGAAAAAAGAGCA---GTTGGA---ATAGGAGCT---------
GTTTTCATTGGGTTCTTAGGAGCA---GCAGGAAGCACTATG---GGCGCAGCGTCA---
GTAACGCTGACGGTACAG---GCCAGACAA---TTATTG---TCTGGCATAGTGCAACAGCAAAGC---
AATTTG---CTGAGG---GCTATAGAGGCTCAACAGCATATGTTGAAACTCACGGTCTGGGGCATT
>KF716489
TTCTATTGTAATACATCAGGCCTGTTTAATAGTACTTGGTATCCTAATAGTACTTTGAATGATAGTACTGGAA
AG---

TATAATAAGACAATAACACTCCCATGTAGAATAAAGCAAATTGTAAACATGTGGCAAAGAGTAGGACAAGCAA
TATATACCCCTCCCATTGCAGGAATAATAAAGTGTGTATCAAAC---
ATTACAGGACTATTATTAACAAGAGATGGTGGTATGAGTAACACAAAT-------------------------
----

AACACAACTGAAATCTTCAGACCTGGAGGAGGAGATATGAGGGACAATTGGAGAAGTGAATTGTATAAGTATA
AAGTAGTAGAAATTAAACCACTA---GGAATAGCCCCTAGCAAGGCAAAGAGAAGAGTGGTG-----------
----------------------------GAGAGAGAAAAAAGAGCA---GTTGGA---ATAGGAGCT------
---GTACTCTTTGGGTTCTTAGGAGCA---GCAGGAAGCACTATG---GGCGCAGCGTCA---
ATAACGCTGACGGTACAG---GCCAGACAA---TTATTG---TCTGGCATAGTGCAACAGCAAAGC---
AATTTG---CTGAGA---GCTATAGAGGCTCAACAGCATATGTTGAAACTCACAGTCTGGGGCATT
>EU110096
TTCTATTGTAACACATCAGGCTTGTTTAATAGCACTTGGAATAGCAATAGCACTTGGAATAGCACTGCCACGC
AGGAGTCAAAT--
-------------AACACG------

```
GAGTTAAATGGCAATATAACTCTCCCATGCAGAATAAAGCAAATTGTAAGAATGTGGCAGAGAGTAGGACAAG
CAATGTATGCCCCTCCCATTGCAGGAGTAATACAGTGTAAATCAAAC---
ATTACAGGAATGATATTGACAAGAGATGGTGGAAATAGTACAGAGACT-------------------------
-------------------------------------------------------------------------
-------
TCCAATAATAATGAGACTTTCAGACCTGGAGGAGGAGATATGAGAGATAATTGGAGAAGTGAATTATATAAGT
ATAAAGTAGTAAAACTTGAACCACTA---GGAGTAGCACCCACAAAAGCAAGGAGAAGAGTGGTG--------
-------------------------------GAGAGAGAGAAAAGAGCA---GTGGGA---CTGGGAGCT---
------CTCTTCCTTGGGTTCTTAGGAGCA---GCCGGAAGCACTATG---GGCGCGGCGTCA---
CTAACGCTGACGGGACAG---GCCAGACAA---TTATTG---TCTGGCATAGTGCAACAGCAAAGC---
AATTTG---CTGCAG---GCTATAGAGGCTCAACAGCATCTGTTGAAACTCACAGTCTGGGGCATT
>DQ912822
TTCTATTGTAACACATCAGGTCTGTTTAATAGCACTTGGAACAAAACTGACAGTGGCAGCATGCAGGGATCAA
ATAGC--------------------------------------------------------------------
-------------------------
ACAACAAGTGACGTTATAACTCTCCCATGCAGAATAAAACAAATTGTAAATATGTGGCAGAGAGCAGGACAAG
CAATGTATGCCCCTCCCATTCCAGGAGTAATAAGGTGTGTATCAAAC---
ATTACAGGACTACTATTAACAAGAGATGGTGGGAATGATAATACT----------------------------
-------------------------------------------------------------------------
----------------
ACCGAAATTTTCAGACCTGGAGGAGGAAATATGAGGGACAATTGGAGAAGTGAATTATATAAGTATAAAGTAG
TAAAAATTGAACCACTA---GGAGTAGCACCCACCAGGGCAAAGAGAAGAGTGGTG-----------------
----------------------GAGAGAGAAAAAAGAGCA---GTTGGA---ATAGGAGCT---------
GTCCTCATTGGGTTCTTAGGAGCA---GCAGGAAGCACTATG---GGCGCGGCGTCA---
ATAACGCTGACGGTACAG---GCCAGACAA---TTATTG---TCTGGTATAGTGCAACAGCAAAGC---
AATTTG---CTGAGA---GCTATAGAGGCTCAACAGCATATGTTGAGACTCACGGTCTGGGGCATT
>AF457058
TTCTATTGTAATACATCAGGACTGTTCAATAGTACTGTGAATACTTTGAATATTAGCACTTGGAATTACAATG
AG-----------------------------------------------------------------------
-------------------------
TCAGATAACACTACTATACAACTCCCATGCAGAATAAAACAAATTATAAACATGTGGCAGGGAGTGGGACAAG
CAATGTATGCCCCTCCCATTGCAGGAGTCATCAAATGTTCATCAAAT---
ATTACAGGACTATTGTTGACAAGAGATGGTGGTGCAAATAGTACC----------------------------
-------------------------------------------------------------------------
----------
AGTCAGAAAGAAACCTTCAGACCTGGAGGGGGAGACATGAGAGACAATTGGAGAAGTGAATTATATAAATATA
AAGTAGTAAGAATTGAACCACTA---GGTCTAGCACCCACCAGGGCAAGAAGAAGAGTGGTG-----------
----------------------------GAAAGAGAAAAAAGAGCA---ATAGGA---CTGGGAGCT------
---ATGTTCCTTGGGTTCTTGGGAGCA---GCAGGAAGCACGATG---GGCGCAGCGTCA---
CTGACGCTGACGGTACAT---GCCAGACAG---TTATTA---TCTGGTATAGTGCAACAGCAAAAC---
AATTTG---CTGAGG---GCTATAGAGGCGCAACAACATCTGTTGCAACTCACAGTCTGGGGCATT
>AF457082
TTCTATTGCAACACATCAGACCTGTTTAATAGCAATTGGACTTGGGATAGTAATAATGGCACTATGAAGGAGG
TAAATAGC-----------------------------------------------------------------
-------------------ACG---
GGGTCAAATGGCACTATAACTCTCCAATGCAGAATAAAGCAAATTATAAATATGTGGCAGAGAGCAGGACAAG
CAATATATGCCCCTCCCATCAAAGGAGTAATAAACTGTGTATCAAAT---
ATTACAGGACTAATATTAACAAGAGATGGTGGGGATAATAGTAGT----------------------------
-------------------------------------------------------------------------
----------------
ACAGAAACCTTCAGACCTGGAGGAGGAGATATGAGGGACAATTGGAGAAGTGAATTATATAAGTATAAAGTAG
TAAAAATTGAACCACTA---GGAGTAGCACCCACCAGGGCAAAGAGAAGAGTGGTG-----------------
----------------------GAGAGAGAAAAAAGAGCA---GTTGGA---ATAGGAGCT---------
GTCTTCCTTGGGTTCTTGGGAGCA---GCAGGAAGCACTATG---GGCGCGGCGTCA---
ATAACGCTGACGGTACAG---GCCAGACAA---TTATTA---TCTGGCATAGTGCAACAGCAAAGC---
AATTTG---CTGAGG---GCTATAGATGCTCAACAACATCTGTTGAAGCTCACGGTCTGGGGCATT
>KT022385
TTCTATTGTAATACAACAGCCCTGTTTAATAGCATTTGGAATGATACTGGCCTTAATAATAAAACTTGGGATA
CAAAT--------------------------------------------------------------------
-------------------------------
```

ACAAATGATACTATAATCCCATGCAGAATAAAGCAAATTATACGAATGTGGCAGAGAGTAGGACAAGCAATGT
ACGCCCCTCCCATCCCGGGAGTGATAAAGTGTATATCTAAC---
ATTACAGGGCTACTATTAACAAGAGATGGTGGACAGGGCAATAAT----------------------------
----

GAAACTGAAACCTTCAGACCTGGAGGAGGAAATATGAAGGACAATTGGAGAAGTGAATTATATAAGTATAAAG
TAGTAAAAATTGAACCACTA---GGAGTAGCACCCACCAGGGCAAGGAGAAGAGTGGTG--------------
-------------------------GAGAGAGAAAAGAGAGCA---GTTGGA---CTGGGAGCT---------
GTATTCATTGGGTTCTTAGGAGCA---GCAGGAAGCACTATG---GGCGCAGCGTCA---
ATAACGCTGACGGCACAG---GCCAGACAA---TTGTTG---TCTGGTATAGTGCAACAGCAAAGC---
AATTTG---CTGAGG---GCTATAGAGGCTCAACAGCATCTGTTGAAACTCACAGTCTGGGGCATT
>KT022387
TTCTATTGTAATACAACAGAACTGTTTAATAGCACCTGGTTTAATAGCACTTGGGACAGCACGCAGGCGCCAA
ATAGCACG---

GATTCAAATGACACTATAACCCTCCAATGCAGAATAAAACAAATTATAAATATGTGGCAGAGAGTAGGACAAG
CAATGTATGCCCCTCCCATCCCAGGAGTAATACAGTGTGTATCAAAC---
ATTACAGGATTACTATTAACAAGAGATGGTGGGGATAATAACAGT----------------------------
----

ACAAATGAAACCTTCAGACCTGGAGGAGGAGATATGAGGGACAATTGGAGAAGTGAATTATATAAGTATAAAG
TAGTAAAAATTGAACCACTA---GGAGTAGCACCCACCGGGGCACAGAGAAGAGTGGTG--------------
-------------------------GAAAGACAAAAAAGGGCA---GTTGGA---ATAGGAGCT---------
GTGTTCCTTGGGTTCTTAGGAGCA---GCAGGAAGCACTATG---GGCGCGGCGTCA---
ATAACGCTGACGGTACAG---GCCAGACTA---TTATTG---TCTGGCATAGTGCAACAGCAAAGC---
AATTTG---CTGAGG---GCTATAGAGGCTCAGCAACATCTGTTGAAACTCACAGTCTGGGGCATT
>KT022388
TTCTATTGTGACACATCCAGCCTGTTTAATAGCACTTGGGATACCGATGACACCAGGCGAGAGTTAAATAGCA
CAGAG--

TCAAATGACACTATAACACTCCAATGCAGAATAAAGCAAATTATAAATATGTGGCAGAGAGTAGGACAAGCAA
TGTATGCCCCTCCCATCCAAGGAATAATAAGGTGTAAATCAAAA---
ATTACAGGACTAATATTAACAAGAGATGGTGGTAAGAATAGCAGT----------------------------
----

ACCAATGAAATCTTCAGACCTGGAGGAGGAGATATGAGGGACAATTGGAGAAGTGAATTATATAAGTATAAAG
TAGTAAAACTTGAACCATTA---GGAGTAGCACCCACAAGGGCAAAAAGAAGAGTGGTG--------------
-------------------------GAGAGAGAAAAAAGAGCA---GTTGGA---ATAGGAGCT---------
GTCTTCCTTGGGTTCTTAGGAGCA---GCAGGAAGCACTATG---GGCGCAGCGTCA---
ATAACGCTGACGGCACAG---GCCAAACAA---TTATTG---TCTGGCATAGTGCAACAGCAAAGC---
AATTTG---CTGAGG---GCTATAGAGGCTCAACAACATCTGTTGAAACTCACAGTCTGGGGCATT
>KT022389
TTCTACTGCAATACATCAGCACTGTTTAATAGGACTTATAATATATCAGAACTGGGTAATACTAATAATATAT
CAGATACTAGT--

AATACATCAAAGGTTATCCCACTCCCATGCAGAATAAGACAAATTATAAACATGTGGCAGGAAGTAGGCAAAG
CAATATATGCCCCTCCCATTGCAGGAATAATCAGATGTTCATCAAAT---
ATTACAGGACTATTGATGACAAGGATGGTGGTGAATATAATAAT-----------------------------
----

AGTCAGAATGAAACCTTCAGACCTGGAGGGGGAGATATGAGAGACAACTGGAGAAGTGAATTATATAAATATA
AAGTAGTAAAACTCGAACCATTA---GGTCTAGCACCCACCAGGGCAAAGAGAAGAGTGGTG-----------
----------------------------GACAGAGAAAAAAGAGCA---ATAGGA---CTAGGAGCT------
---ATGTTCCTTGGGTTCTTGGGAGCA---GCAGGAAGCACGATG---GGCGCAGCGTCA---
CTGACGCTGACGGTACAG---GCCAGACAG---TTATTG---TCTGGTATAGTGCAACAGCAAAAT---
AATTTG---CTGAGA---GCTATAGAGGCGCAACAGCATATGTTGCAACTCACAGTCTGGGGCATT
>KT022390
TTCTATTGCAATACATCAGGACTGTTTAATACATTAAATAGTACAGGATA-----------------------
----

AATGAGACTACAATCACACTCCCATGCAGGATAAAACAAATTATAAACATGTGGCAGGGAGTAGGAAAAGCAA
TGTATGCCCCTCCCATTGAAGGACTAATCCACTGTTCATCAAAC---
ATTACAGGACTATTATTGACAAGAGATGGTGGTAATGATGGTGCA---------------------------
----
-
AATATTACTAATCAGAATGAGACTTTTAAACCTGAAGGAGGAGATATGAGAGACAATTGGAGAAGTGAATTAT
ACAAATATAAAGTAGTAAAAATTGAACCACTA---GGTCTAGCACCCACCAAGGCAAAAGAAGAGTGGTG--
------------------------------------GAAAGACAAAAAAGAGCA---ATAGGA---
CTAGGAGCT---------GTGTTCCTTGGGTTCTTGGGAGCA---GCAGGAAGCACAATG---
GGCGCAGCGTCA---CTGACGCTGACGGTACAG---GCCAGACAG---TTATTG---
TCTGGTATAGTGCAACAGCAAAAC---AATTTG---CTGAGG---
GCTATAGAGGCGCAACAGCATCTGTTGCAACTCACAGTCTGGGGCATT
>KT022392
TTCTATTGTAACACATCAGGCCTGTTTAATGGCACTGTCAAACAGGAGTCAAATGGCACAGAG---------
----

TCAAATGATACTATAATTCTCCAATGCAAAATAAAGCAAATTATAAATATGTGGCAGAGAGTAGGACAAGCAA
TATATGCCCCTCCCATCCCAGGAGTAATAAGGTGTGTCTCAAAC---
ATTACAGGACTAATATTAACAAGAGATGGTGTTAATAGCAGTGCA---------------------------
----

AATGAAACCTTCAGACCTGGAGGAGGGGATATGAGGGACAACTGGAGAAGTGAATTATATAAGTATAAAGTAG
TAAAAATTGAACCACTA---GGAGTAGCACCCACCAGGGCAAAAAGAAGAGTGGTG----------------
---------------------GAGAGAGAAAAAAGAGCA---GTTGGA---ATAGGAGCT---------
GTTTTCATTGGGTTCTTGGGAGCA---GCAGGAAGCACTATG---GGCGCGGCGTCA---
ATGACGCTGACGGTACAG---GCCAGACAA---TTATTG---TCTGGCATAGTGCAACAGCAAAGC---
AATTTG---CTGAGG---GCTATAGAGGCTCAACAACATCTGTTGAAACTCACGGTCTGGGGCATT
>KT022394
TTCTACTGCAATACATCAAGACTGTTCAATAGTACATGGGTATTAAATAGTACATCAAATAGTACATGGACAT
TAAATAGTACA---
----------ACGGGAGAT------
AGCAGTACATCAAATATCACAATCCCATGCAGAATAAAACAAATTATAAACAGGTGGCAGGAAACAGGAACAG
CAATATACGCCCCTCCCATTGAAGGACTAATTCACTGTTCATCAAAT---
ATTACAGGACTACTATTAACAAGAGATGGTGGGAACCCTAATGAGACAAGG---------------------
----

GAGAATGAAACCTTTAGACCAGAAGGAGGAAATATGAAAGACAATTGGAGAAGTGAATTATACAAATATAAAG
TAGTAAGACTTGAACCACTA---GGTCTAGCACCCACCAAGGCAAAGAGAAGAGTGGTG-------------
------------------------GAAAGAGAAAAAAGAGCA---ATAGGA---CTAGGAGCT---------
ATGTTCCTTGGGTTCTTGGGAGCA---GCAGGAAGCACGATG---GGCGCAGCGTCA---
CTGACGCTGACGGTACAG---GCCAGACAG---TTATTG---TCTGGTATAGTGCAACAGCAAAAC---
AATTTG---CTGAGG---GCTATAGAGGCGCAACAGCATATGTTGCAGCTCACAGTCTGGGGCATT
>KT022396
TTCTATTGCAGCACATCAGGCCTGTTTAATGGCACTTGGACTTGGACTAATGACACTGGGAGTAATAACAATG
TCAGCCAGCAA---

GAGTTAAATGACACTATAACTCTCCCATGCAAAATAAAGCAAGTTATAAATATGTGGCAAAGGGTGGGACAAG
CAATATATGCCCCTCCCATCCCAGGAGTAATAAGGTGTGAATCAAAC---
ATTACAGGACTAATATTATCAAGAGATGGTGGAGATAATAACAGT---------------------------
----

ACAAATGAAACCTTCAGACCTGGAGGAGGAGATATGAGGGACAATTGGAGAAGTGAATTATATAAGTATAAAG
TAGTAAAAATTGAACCACTA---GGAGTAGCACCCACCAGGGCAAAGAGAAGAGTGGTG-------------
------------------------GAGAGAGAAAAAAGAGCA---GTTGGA---CTGGGAGCT---------
GTTTTCATTGGGTTCTTAGGAGCA---GCAGGAAGCACTATG---GGCGCGGCGTCA---
GTGACGCTGACGGTACAG---GCCAGACAA---TTATTG---TCTGGCATAGTGCAACAGCAAAGC---
AATTTG---CTGAGG---GCTATAGAGGCTCAACAACATCTGTTGAAACTCACGGTCTGGGGCATT
>KT022399
TTCTACTGCAATACATCAAATCTGTTTAAGGGGACATTTAATGGGACATCAAATAGTACAGGGAATAAT----
----

GAGAATCAAAACATCACACTCCCATGCAGAATAAAACAAATTATAAACATGTGGCAGGAAGTGGGAAAAGCAA
TGTATGCCCCTCCCATTGCAGGAATAATTAAATGCTCATCAAAT---
ATTACAGGACTATTGTTGACAAGAGATGGTGGTACAGAAAATAAT----------------------------
----

GATAGTGAGACCATCAGGCCTGGAGGAGGAGATATGAGAGACAATTGGAGAAGTGAATTATACAAATATAAAG
TAGTAAGACTTGAACCACTA---GGTCTAGCACCCACCAGGGCAAAGAGAAGAGTGGTG--------------
-------------------------GAAAGAGAAAAAAGAGCA---GTAGGA---CTAGGAGCT---------
ATGTTCCTTGGGTTCTTGGGAGCA---GCAGGAAGCACAATG---GGCGCAGCGTCA---
CTGACGCTGACGGTACAG---GCCAGACTG---TTATTG---TCTGGTATAGTGCAGCAGCAAAAC---
AATTTG---CTGAGG---GCTATAGAGGCGCAACAACATCTGTTGCAACTCACAGTCTGGGGCATT
>KT022409
TTCTACTGTAATACAACAGGCCTGTTTAATAGCACTTGGAAGTTTAATAGCACTTGGGAGCAAGGGTCAAATA
CCACGGAG---

ACAAATGAAACTATAACTCTCCAATGCAGAATAAAGCAAATCATAAACATGTGGCAGACAGTAGGACAAGCAA
TGTATGCTCCCCCCATCCCAGGAGTAATAAAGTGTAGATCAAAC---
ATTACAGGATTAATATTAACAAGAGATGGTGGGAATAATAACAAT----------------------------
----
GGAAGTGGAAATAATAACAAT---------
GGAAGTGAAATCTTCAGACCTGGAGGAGGAGATATGAGAGACAATTGGAGAAGTGAATTATATAAGTATAAAG
TAGTAAAAATTGAACCCCTA---GGAGTAGCACCCACCAGGGCAAAGAGAAGAGTGGTG--------------
-------------------------GAGAGAGAAAAAAGAGCA---GTTGGA---ATAGGAGCT---------
GTCTTCCTTGGGTTCTTAGGAGCA---GCAGGAAGCACTATG---GGCGCGGCGTCA---
ATAACGCTGACGGTACAG---GCCAGACAA---TTATTG---TCTGGCATAGTGCAACAGCAAAGC---
AATTTG---CTGAAG---GCTATAGAGGCTCAACAACATCTGTTGAAACTCACGGTCTGGGGCATT
>KT022410
TTCTATTGTAACACATCAAGCCTGTTTAATAGCACTTGGAATTTAACTGCCAACAGGCAGGGACCAAATAGCA
CGGAG--

TCAACTGACACCATAACTCTACAGTGCAGAATAAAGCAAATTATAAATATGTGGCAGAGAACAGGACAAGCAA
TATATGCCCCTCCAATCCAGGGAGTAATAAGGTGTGCATCAAAC---
ATTACAGGACTACTATTAACAAGAGATGGTGCT--

GGCAGTGACAGTGAAACCTTCAGACCTACAGGAGGAGATATGAGAGACAATTGGAGAAGTGAATTATATAAAT
ATAAAGTAGTAAAAATTGAACCATTA---GGAGTAGCACCCACCAGGGCAAGGAGAAGAGTGGTG--------
-------------------------------GGGAGAGAAAAAAGAGCA---GTTGGA---ATAGGAGCT---
------GTATTCCTTGGGTTCCTAGGAGCA---GCAGGAAGCACTATG---GGCGCGGCGTCA---
ATAACGCTGACGGTACAG---GCCAGACAA---TTGTTG---TCTGGCATAGTGCAACAGCAAAGC---
AATTTG---CTGAAG---GCTATAGAGGCTCAACAACAGCTGTTGAGACTCACTGTCTGGGGCATT
>KT022411
TTCTACTGTAACACATCAAATCTGTTTAATAGCACATGGACTAATAGTACATGGAATATAACAGAAGAGGCTA
AT---

AGTACAGAGCCAATTATACTCCAATGCAGAATAAAGCAAATTATAAATCTGTGGCAGAGAGTAGGACAAGCAA
TGTATGCTCCTCCCATCCAAGGAGTAATAAGGTGTGAATCAAAC---
ATTACAGGACTAATATTAACAAGAGATGGTGGGCATAATAAGACA----------------------------
----

AATGAAACCTTCAGACCTGGAGGAGGAGACATGAGGGATAATTGGAGAAGTGAATTATACAAATATAAGGTGG
TAAAAATCGAGCCACTA---GGAGTAGCACCCACAAGGGCAAGGAGAAGAGTGGTG-----------------
----------------------CAGAGAGAAAAAAGAGCA---GTTGGA---ATAGGAGCT---------
GTCTTCCTTGGGTTCTTGGGAGCA---GCAGGAAGCACTATG---GGCGCAGCGTCA---
ATAACGCTGACGGTACAG---GCTAGACAA---TTATTG---TCTGGCATAGTGCAACAGCAAAGC---
AATTTG---CTGAAG---GCTATAGAGGCTCAACAACATCTGTTGAAACTCACGGTCTGGGGCATT
>FJ623491
TTCTATTGTAACACCTCAGGCCTGTTTAATAGCGCTTGGTCGGCAATACCAGTAATACCAGAACCAATAGT-
----

GCAGAAGCCAATATAACTCTCCAATGCAGGATAAAGCAAGTTATAAATATGTGGCAGAGAGTAGGACAAGCAA
TATATGCCCCTCCCATCTCAGGAGTAGTAAGGTGTAAATCAAAT---
ATTACAGGACTACTATTAACAAGAGATGGTGGGGTAATGGTACA-----------------------------
----

AATGAAACCTTTAGACCTGGAGGAGGAGATATGAGGGACAATTGGAGAAGTGAATTATATAAGTATAAGGTAG
TAAAAATTGAACCACTA---GGAGTAGCACCCACCAGGGCAAAGAGAAGAGTGGTG-----------------
----------------------GAGAGAGAAAAAAGAGCA---GTTGGA---ATAGGAGCT---------
GTTTTCATTGGGTTCTTGGGAGCA---GCAGGAAGCACTATG---GGCGCGGCGTCA---
ATAACGCTGACGGTACAG---GCTAGACAA---TTATTG---TCTGGCATAGTGCAACAGCAAAGC---
AATTTG---CTGAGG---GCTATAGAGGCTCAACAACATCTGTTGAAACTCACGGTCTGGGGCATT
>KP174771
TTCTATTGTAACACATCAGGCCTATTTAATAGCACGTGGGATGCGAATGCCACCATGAATGCCAACAGTACGG
AGTCA--

AATGACACAACTATCACTCTCCAATGCAGAATAAAGCAAATTATAAATATGTGGCAGAGAACAGGACAAGCAA
TGTATGCCCCTCCCATCCCAGGAGAAATAAGGTGTGAATCAAAC---
ATTACAGGACTAATACTAACAAGAGATGGGGGAAGTAATAATACA----------------------------
----

AATGAGACCTTCAGACCTGGAGGAGGAGATATGAGGGACAATTGGAGAAGTGAATTATATAAGTATAAAGTAG
TAAAAATTGAACCACTA---GGAGTAGCACCCACCAGGGCAAAAAGGAGAGTGGTG-----------------
----------------------GAGAGACAAAAAAGAGCA---GTTGGC---ATAGGAGCT---------
GTCTTCCTTGGGTTCTTAGGAGCA---GCAGGAAGCACTATG---GGCGCGGCGTCA---
ATAACGCTGACGGTACAG---GCCAGACAA---TTATTG---TCTGGTATAGTGCAGCAGCAAAGC---
AATTTG---CTGAGG---GCTATAGAGGCTCAACAACATCTGTTGAAACTCACAGTCTGGGGCATT
>KU921707
TTCTACTGTAATACATCGAACCTGTTTAATAGCACTTGGTACAGTAACGGTTCGATGCCAAATATC-------
----

ACTACTGGCAATATAACTCTCCCGTGCAGAATAAAACAAATTATAAAGATGTGGCAGAGAGCAGGACAAGCAA
TATATGCCCCTCCCATCAAAGGAGTAATAAGGTGTGACTCAAAC---
ATTACAGGATTACTACTAACAAGAGATGGTGGGAGTACTAACAGT----------------------------
----

ACAAATGAAACCTTCAGACCTGCAGGAGGAGACATGAGGGACAATTGGAGAAGTGAATTATATAAATATAAAG
TAGTAAAAATTGAACCACTA---GGAGTAGCACCCACCAAGGCAAAGAGAAGAGTGGTG--------------
-------------------------GAAAGAGAAAAAAGAGCAATTATTGGA---CTGGGAGCT---------
GTATTCGTTGGGTTCTTAGGAGCA---GCAGGAAGCACTATG---GGCGCGGCGTCA---
GTGACGCTGACGGTACAG---GCCAGACAA---TTATTG---TCTGGCATAGTGCAACAGCAAAGC---
AATTTG---CTGATG---GCTATAGAGGCTCAACAACATTTGTTGAAACTCACAGTCTGGGGCATT
>KU921719
TTCTACTGTAATACATCAGGCTTGTTTAATAGCACTTGGTTTAGCAATGTCAGCTTGCAAGAAAATAGCACAG
AG---

TCAAGTGGCAATATAACTCTCCACTGCAGAATAAAGCAAGTTATAAATATGTGGCAGAGAGTAGGACAAGCAA
TGTATGCCCCTCCCATCTCGGGAAAAATAAGGTGTGAATCAAAC---
ATTACAGGACTAATATTAACAAGGGATGGTGGGAATAGTGACAATACA-------------------------
----

AGTACAGATGAGACCTTCAGACCTGCAGGAGGAGATATGAGGGACAATTGGAGAAGTGAATTATACAAGTATA
AAGTAGTACAAATTGAACCACTA---GGAGTAGCACCCACCAGGGCAAAAAGAAGAGTGGTG-----------
----------------------------GAGAGAGGAAAAAGAGCA---GTTGGG---CTGGGAGCT------
---GTGTTCATTGGGTTCTTGGGAGCA---GCAGGAAGCACGATG---GGCGCAGCGTCC---
CTGACGCTGACGGTACAG---GCCAGACAA---TTATTG---TCTGGTATAGTGCAACAGCAAAGC---
AATTTG---CTGAGG---GCTATAGAGGCTCAACAAAGTCTGTTGAAACTCACAGTCTGGGGCATT
>KU921749
TTCTACTGCAATACATCAAGCCTGTTTAGGAGTACATGGGAAAATAGTACTACATTAAATAGTACAGGG----
----

GGAAATGAGACAATTACACTCCAATGCAGAATAAAACAGATTATAAACATGTGGCAGGGAGTAGGGAAGGCAA
TGTATGCCCCTCCTATTGAAGGACTAATCTCCTGTTCATCCAAT---
ATTACAGGGCTATTATTAACGAGAGATGGT---AATAAAAATAGT----------------------------
----

CAGAAGAATGAGACCTTCAGACCTGGAGGAGGAGATATGAGGGACAATTGGAGAAGTGAATTATATAAATATA
AAGTAGTAAAAATTGAACCACTA---GGAGTAGCACCCACCGGGGCACGGAGAAGAGTGGTG-----------
----------------------------GGAAGAGAAAAAAGAGCA---GTTGGA---ATAGGAGCG------
---GTCCTCCTTGGGTTCTTAGGAGCA---GCAGGAAGCACTATG---GGCGCGGCGTCA---
ATAACGCTGACGGTACAG---GCCAGAGAA---TTACTG---TCTGGCATAGTGCAACAGCAAAGT---
AATTTG---CTGAGG---GCTATAGAGGCTCAACAACAACTGTTGAAACTCACGGTCTGGGGCATT
>KU921798
TTCTATTGTAACACATCAGGCCTGTTTCATGGCAAATGGTCTAATGGCACTTGGACTAGGAATGACACT----
----

GCAAACGACACTATAAGGCTCCCATGCAGAATAAAGCAAATTATAAATATGTGGCAGAGAGCAGGACAAGCAA
TATATGCCCCTCCCATAAAAGGAGTAATAACATGTAGATCAAAT---
ATTACAGGACTACTATTAACAAGAGATGGTGGAACGAATAGTACA----------------------------
----

AATGAAACCTTCAGACCTGGAGGAGGAGATATGAGGGACAATTGGAGAAGTGAATTATATAATTATAAAGTAG
TAAAAATTGAACCACTA---GGAGTAGCACCCTCCAAGGCAAAGAGAAGAGTGGTG-----------------
----------------------GAGAGAGAAAAAAGAGCA---GTC---------GGAATG---------
GTCCTCATTGGGTTCTTAGGAGCA---GCAGGAAGCACTATG---GGCGCAGCGGCA---
GTAACGCTGACGGAACAG---GCCAGACAA---TTATTG---TCTGGCATAGTGCAACAGCAAAGC---
AATTTG---CTGAAG---GCTATAGAGGCTCAACAGCATCTGTTGAAACTCACGGTCTGGGGCATT
>KU921799
TTCTATTGTGACACATTAGGCCTGTTTAAAGGCACTTGGTCTAATGGCACTTGGTCTAATGGCAATTGGACTA
GGAATGACACT--

GGCAGCAACAACACTATAATTCTCCCATGCAGAATAAAGCAAATTATAAAGATGTGGCAGAGAACAGGACAAG
CAATATATGCCCCTCCCATAGAAGGAGTAATAAGATGTGAATCAAAT---
ATTACAGGACTAATATTAACAAGAGATGGTGGAAAGAATGAGCTA----------------------------
----

AATGAAACCTTCAGACCTGGAGGAGGAAATATGAAGGACAATTGGAGAAGTGAATTATATAAATATAAAGTAG
TAAAAATTGAACCACTA---GGAGTAGCACCCACCAGGGCAAAGAGAAGAGTGGTG-----------------
----------------------GAGAGAGAAAAAAGGGGA---GTTGGA---ATAGGAGTT---------
GTCTTCTTTGGGTTCTTAGGAGCA---GCAGGAAGCACGATG---GGCGCAGCGGCA---
CTAACGCTGACGGTACAG---GCCAGACAA---TTATTG---TCTGGCATAGTGCAACAGCAAAGC---
AATTTG---CTGAAG---GCTATAGAGGCTCAACACCATCTGTTGAAACTCACAGTCTGGGGCATT
>KU921805
TTCTATTGTGACACATCAAGACTGTTTAATGGCGCTTGGTCTGGGAATGACACTAGCATTGAACAGGAGCAAA
AT---

GGAACCGACCCTATAACTCTCCCATGCAGAATAAAGCAAATTATAAATATGTGGCAGAGAACAGGACAAGCAA
TATATGCCCCTCCCATTGCAGGAGTAATAAGATGTGAATCAAAT---
ATTACAGGATTAATATTAACAAGAGATGGGGGACTTAATAATACAAAT-------------------------
----

AAAGATAATGAAACCTTCAGACCTACAGGAGGAAATATGAGGGACAATTGGAGAAGTGAATTATATAAATATA
AGGTAGTAAAAATTGAACCACTA---GGAGTCGCACCCACCAGAGCAAGGAGAAGAGTGGTG-----------
----------------------------GAGAGAGAAAAAAGAGCA---GTTGGA---ATAGGAGCT------
---GTCTTCCTTGGGTTCTTAGGAGCA---GCAGGAAGCACTATG---GGCGCAGCGGCA---
GTGACGCTGACGGTACAG---GCCAGACAA---TTATTG---TCTGGTATAGTGCAACAGCAAAGC---
AATTTG---CTGCAG---GCTATAGAGGCTCAACAGCATCTGTTGAAACTCACAGTCTGGGGCATT
>KU921813
TTCTATTGTAATACATCAAGCTTGTTTGGTAGCACTTGGGTCCCTAATAGCACTGTGGACACCAACAGTACCA
ACAGC--

ACGCAGGGGACAACTATAACTCTCCAATGCAGAATAAAACAAATTATAAACATGTGGCAGAGAGTAGGAAGAG
CCATGTATGCCCCTCCCATTGCAGGAACAATCAGCTGTTCATCAAAT---
ATTACAGGACTATTGTTGACAAGAGATGGTGGTGCAAACAGTAGT----------------------------
----

CATAATGAGACTTTCAGACCTGGAGGGGGAGATATAAGAGACAATTGGAGAAGTGAATTATACAAATACAAAG
TAGTAAGAATTGAACCATTA---GGACTAGCACCCACCAGGGCAAAGAGAAGAGTGGTG--------------
-------------------------GAAAGAGAAAAAAGAGCA---ATAGGA---CTAGGAGCT---------
GTGTTCCTTGGGTTCTTGGGAGCA---GCAGGAAGCACGATG---GGCGCAGCGTCA---
TTGACGCTGACGGTACAG---GCCAGACAA---TTATTG---ACTGGTATAGTGCAACAGCAAAAC---
AATTTG---CTGAGG---GCTATAGAGGCGCAACAGCATTTGTTGCAACTCACAGTCTGGGGCATT
>AF407149
TTCTATTGTAATACATCAGGCCTGTTTGATAGCACTTGGAATGACACTGACAGCAGACAG-------------
----

GAAAATGGCACTATAACTCTCCCATGCAGAATAAAGCAAATTATAAATATGTGGCAGAGAACAGGACAAGCAA
TATATGCCCCTCCCATCCAAGGAGCAATAAGGTGTGTATCAAAC---
ATTACAGGACTAATATTAACAAGAGATGGTGGGAACAATAACAGT----------------------------
----

ACAAATGAAACCTTCAGACCTGGAGGAGGAGATATGAGGGACAATTGGAGAAGTGAATTATATAAGTATAAAG
TAGTAAAAATTGAACCACTA---GGAGTAGCACCCACCAAGGCAAGGAGAAGAGTGGTG--------------
-------------------------GGGAGAGAAAAAAGAGCA---GTTGGA---ATAGGAGCT---------
GTCTTCCTTGGGTTCTTAGGAGCA---GCAGGAAGCACTATG---GGCGCGGCGTCA---
ATAACGCTGACGGTACAG---GCCAGACAA---TTACTG---TCTGGCATAGTGCAACAGCAAAGC---
AATTTG---CTGAAA---GCTATAGAGGCTCAACAGCATCTGTTGAGACTCACGGTCTGGGGCATT
>AF457059
TTCTACTGTAACACATCAAGCCTGTTTAATAGCACTTGGAATAGTACTAGCACTGGGCACAATGCCAGCGGGC
AG---

GAGATAAATGACACTATAATTCTCCAATGCAGAATAAAGCAAATTATAAATATGTGGCAGAGAATAGGACAAG
CAATGTATGCCCCTCCCATCAAAGGAGCAATATGGTGTAGATCAAAC---
ATTACAGGACTAATATTAACAAGAGATGGTGGGGAGGACAATAGC----------------------------
----

AGTACTGAAACCTTCAGACCTGGAGGAGGAAACATGAAGGATAATTGGAGAAGTGAATTATATAAGTATAAAG
TAGTAAAACTTGAACCATTA---GGAGTGGCACCCACCAGGGCAAAGAGAAGAGTGGTG--------------
-------------------------GAGAGAGAAAAAAGAGCA---GCTGGA---CTGGGAGCT---------
GTTTTCATTGGGTTCTTAGGAGCA---GCCGGAAGCACTATG---GGCGCAGCGTCA---
GTAACGCTGACGGTACAG---GCCAGACAG---TTATTG---TCTGGCATAGTGCAACAGCAAAAC---
AATTTG---CTGAAG---GCTATAGAGGCTCAACAACATCTGTTGAAACTCACGGTCTGGGGCATT
>AF457073
TTCTATTGTAACACATCAGACCTGTTTAATAGCACCTGGAACAGCACCACACTGGAGTCAAACACCACAGGG-
----

TCAAATGGCACTTTAATTCTCCAATGCAGAATAAAGCAAATTATAAATATGTGGCAGAGAGTAGGACAAGCAA
TGTATGCCCCTCCCATCAAAGGAGTAATAAGGTGTAATTCAACA---
ATTACAGGGCTACTATTAACAAGGGATGGTGGAAAGGATAACAGTAGT-------------------------
----

GAGAGTAGTGAAGAAATCTTCAGACCTGAAGGAGGAAATATGAGGGACAATTGGAGAAATGAATTATATAAGT
ATAAAGTAGTAAAAATTGAACCACTA---GGAGTAGCACCCACCAGGGCAAAGAGAAGAGTGGTG--------
-------------------------------GGGAGAGAGAAAAGAGCA---GTTGGA---CTGGGAGCT---
------GTTTTCCTTGGGTTCTTAGGAGCA---GCAGGAAGCACTATG---GGCGCGGCGTCA---
ATAACGCTGACGGTACAG---GCCAGACAA---TTATTG---TCTGGCATAGTGCAACAGCAAAGC---
AATTTG---CTGAGA---GCTATAGAGGCTCAACAGCATCTGTTGAAACTCACGGTCTGGGGCATT
>DQ208501
TTCTATTGTAATACATCAGGCCTGTTTAATAGCACTTGGAGTTGGAATCACACTGTCAGCAGCATGCAGGAAG
CAAATGGC---

-------------------ACG---
GAGTCAAATGGCACTATAACCCTCCAATGCAGAATAAAGCAAATTATAAATATGTGGCAGAGAGTAGGACGAG
CAATGTATGCCCCTCCCATCGAAGGAATAATAAGGTGTGAATCAAAC---
ATTACAGGACTAATATTAACAAGAGATGGTGGGGTTAATAACAGT----------------------------
----

ACAAATGAAACCTTCAGACCTGGAGGAGGAGATATGAGGGACAATTGGAGAAGTGAATTATATAAGTATAAGG
TAGTAAAAATTGAACCACTA---GGAGTAGCACCCACCCCGGCGAAGAGAAGAGTGGTG--------------
-------------------------GAGAGAGAAAAAAGAGCA---GTCGGA---ATAGGAGCT---------
GTCTTCCTTGGTTTCTTAGGAGCA---GCAGGAAGCACTATG---GGCGCGGCGTCA---
ATAACGCTGACGGTACAG---GCCAGACAA---TTATTG---TCTGGCATAGTGCAACAACAAAGC---
AATTTG---CTGAGG---GCTATAGAGGCTCAACAGCAGATGTTGAGACTCACGGTCTGGGGCATT
>DQ187009
TTCTATTGTAATACATCAGGCCTGTTTTCCAGCACGCAGGAG-------------------------------
----

TCAAGTGATCCTATAACTCTCCCATGCAGAATAAAGCAAATTATAAATATGTGGCAGAGAGTAGGACAAGCAA
TGTATGCTCCTCCCATCCAAGGAGAAATAAGGTGTAAATCAAAC---
ATTACAGGAATAATATTAACAAGAGATGGTGGGCTTGGTACTACA----------------------------
----

AATGAAACCTTCAGACCTGGAGGAGGAGACATGAGGGACAATTGGAGAAGTGAGTTATATAAGTATAAAGTAG
TAAAAATTGAACCACTA---GGAGTAGCACCCACCAGGGCAAGGAGAAGAGTGGTG-----------------
----------------------GAGAGAGAGAAAAGAGCA---GTTACA---ATAGGAGCT---------
GTTCTCCTTGGGTTCTTGGGAACA---GCAGGAAGCACTATG---GGCGCAGCGTCA---
CTAACGCTGACGGTACAG---GCCAGACTA---TTATTG---TCTGGCATAGTGCAACAGCAAAGC---
AATTTG---CTGAGG---GCTATAGAGGCTCAACAACATCTGTTGAAACTCACAGTCTGGGGCATT
>AF075701
TTCTATTGTAACACAACAGGCCTGTTTAATAGCACGTGGAATGACACTGCCACAGAGCAGAAG----------
----

CCAAACGACACTATAAGACTCCAATGCAGAATAAAGCAAATTATAAATATGTGGCAAAGAGCAGGACGAGCAA
TATATGCCCCTCCCATCCAAGGAGTAATAAATTGTGTATCAGAC---
ATTACAGGACTAATATTAACAAGAGATGGTGGGGTTAATAATACA----------------------------
----

AATGAAACCTTCAGACCTGGAGGGGGAGATATGAGGGACAATTGGAGAAGTGAATTATATAAGTATAAAGTAG
TAAAAATTGAACCACTA---GGAGTAGCACCCACCAGGGCAAAGAGAAGAGTGGTG-----------------
----------------------GAGAGAGAAAAAAGAGTA---GCTGGA---CTAGGAGCT---------
GTCTTCATTGGGTTCTTAGGAGCA---GCAGGAAGCACTATG---GGCGCGGCGTCA---
ATAACGCTGACGGTACAG---GCCAGACAA---TTGTTG---TCTGGCATAGTGCAACAGCAAAAC---
AATTTG---CTGAAG---GCTATAGAGGCTCAACAACATCTGTTGAAACTCACGGTCTGGGGCATT
>AF071473
TTCTATTGTAATACATCAGGCCTGTTTAATAGCACTTGGAATAACACTGACAGCATGCAGGAGTCACATAGCA
CG---

GAGACAAATGACACTATAACTCTCCCATGCAGAATAAAGCAAATTATAAATATGTGGCAGAGAGTAGGACAAG
CAATGTATGCCCCTCCCATCCAAGGAGTAATAAAGTGTACATCAAAC---
ATTACAGGACTGATATTAACAAGAGATGGTGGGGGTAATAACAGC----------------------------
----

ATAAATGCAACCTTCCGACCTGGAGGAGGAGATATGAGGGACAATTGGAGAAGTGAATTATATAAGTATAAAG
TAGTAAAAATTGAACCAGTA---GGAATAGCACCCAACAGGGCAAAGAGAAGAGTGGTG--------------
-------------------------GAGAGAGAAAAAAGAGCA---GTTGGA---ATAGGAGTT---------
GTCTTCTTTGGGTTCCTGGGAGCA---GCAGGAAGCACTATG---GGCGCAGCGTCA---
ATAACGCTGACGGTACAG---GCCAGACAA---TTATTG---TCTGGCATAGTGCAACAGCAAAGC---
AATTTG---CTGAGG---GCTATAGAGGCTCAACAACATCTGTTGAAACTCACGGTCTGGGGCATT
>AY253316
TTCTACTGCAATACATCAGAACTGTTTAATAGTACATGGAGCATGAACAATAATACATCAAATAGTACAGGG-
----

GTAAATGAGAATATCACACTCCAATGCAGGATAAAACAAATTATAAACATGTGGCAGGGAGTAGGAAAAGCAA
TATATGCTCCTCCCATTGAAGGAATAATCAGCTGTTCATCAAAT---
ATTACAGGACTAATATTAACAAGAGATGGTGGGAATAATAACAGTACA-------------------------
----

AATGAAAATGAAACCTTTAGACCTGGAGGAGGAGATATGAGGGACAATTGGAGAAGTGAATTATATAAGTATA
AAGTAGTAAAAATTGAACCACTA---GGAGTAGCACCCACCAGGGCAAAGAGAAGAGTGGTG----------
----------------------------GAGAGAGAAAAAAGAGCA---GTTGGA---ATAGGAGCT------
---GTCCTCTTTGGGTTCTTAGGAGCA---GCAGGAAGCACTATG---GGCGCAGCGTCA---
ATAACGCTGACGGCACAG---GCCAGACAA---TTATTG---TCTGGTATAGTGCAACAGCAAAGC---
AATTTG---CTGAGG---GCTATAGAGGCTCAACAGCATGTGTTGAGACTCACGGTCTGGGGCATT
>KX907376
TTCTATTGTAATACATCAGGCCTGTTTAGTAGCACTTGGAATGTCACTTACAATAATAACACTTGGAATGCCA
CTCAGGTG---
-------TCAAATAGCACA------
GAGCCAAATGGCACTATAGTGATCCCATGCAGAATAAAGCAAATTATAAATATGTGGCAGAGAACAGGACAAG
CAATGTATGCCCCTCCCATTCCAGGAAAAATACAGTGCATATCAAAC---
ATTACAGGACTACTATTAACAAGAGATGGTGGGGACACTAGCAGT----------------------------
----

ATGAATGAAACCTTCAGACCTGGAGGAGGAGATATGAGGGACAATTGGAGAAGTGAATTATATAAATATAAAG
TAGTAAAAATTGAACCATTA---GGAGTAGCACCCACCAGGGCGCAGAGACGAGTGGTG--------------
-------------------------GGAAGAGAAAAAAGAGCA---GTTGGA---CTGGGAGCT---------
GTCTTCCTTGGGTTCTTAGGAGCA---GCAGGAAGCACTATG---GGCGCAGCGTCA---
CTGACGCTGACGGTACAG---GCCAGACAA---TTATTG---TCTGGCATAGTGCAGCAGCAAAAT---
AATTTG---CTGAGG---GCTATAGAGGCTCAACAACAACTGTTGAAACTCACAGTCTGGGGCATT
>KX907355
TTCTATTGTAACACATCAGGCCTGTTTAGCAGCATTAGCCGATTTAATAACAATAGCGAAATAAATAGCACAG
GG---

TTAAACGGCACTATAACTCTCCAATGCAAAATAAAGCAAATTATAAGAATGTGGCAGAGAACAGGACAAGCAA
TGTATGCCCCTCCCATCCCAGGAGTAATAAGGTGTCAATCAAAC---
ATTACAGGAATAATATTAACAAGAGATGGTGGGATTAATAACACA----------------------------
----

AATGAAACCTTCAGACCTGGAGGAGGAGATATGAGGGACAATTGGAGAAGTGAATTATACAAATATAAAGTAG
TAAAAATTGAACCAATA---GGAGTAGCACCCACCAGGGCAAAGAGAAGAGTGGTG-----------------
----------------------GAGAGAGAAAAAAGAGCA---GTTGGA---ATAGGAGCT---------
GTCTTCATTGGGTTCTTAGGAGCA---GCAGGAAGCACTATG---GGCGCGGCGTCA---
ATAACGCTGACGGTACAA---GCCAGACAA---TTATTG---TCTGGTATAGTGCAACAGCAAAGT---
AATTTG---CTGAGG---GCTATAGAAGCTCAACAGCATCTGTTGAAACTCACAGTCTGGGGCATT
>KX907361
TTCTATTGTAACACATCAAAGTTATTTAATAATGGCACTGCCAAC----------------------------
----

ATAGATGACTCTATAACTCTCCCATGCAAAATAAAGCAAATTATAAGGATGTGGCAGAGAACAGGACAAGCAA
TGTATGCCCCTCCTATCCAAGGAACAATAGAGTGTATATCAAAC---
ATTACAGGACTGCTATTAACAAGAGATGGTGGGAGTGATAGCAAT----------------------------
----

ACAAATGAAACCTTCAGACCTGGAGGAGGAGATATGAGGGACAATTGGAGAAGTGAATTATATAAGTATAAGG
TAGTAAAAATTGAACCACTA---GGAGTAGCACCCACCAAGGCAAAGAGAAGAGTGGTG--------------
-------------------------GAGAGAGAAAAAAGAGCA---GTTGGA---ATAGGAGCT---------
GTCCTCTTTGGGTTCTTAGGAGCA---GCAGGAAGCACTATG---GGCGCGGCGTCA---
TTAGCGCTGACGGAACAG---GCCAGGCAA---TTATTG---TCTGGCATAGTGCAACAGCAAAGT---
AATTTG---CTGAGG---GCTATAGAGGCTCAACAGAATATGTTGAGGCTCACAGTCTGGGGCATT
>HQ615969
TTCTACTGCAATACATCAGGACTATTTAATAGTACATGGTTAGCAAATGGGACACCAAATAGTACAGGGATA-
----

AATGAGAGTACAATTACACTCCCATGCAGAATAAAACAAATTATAAATATGTGGCAGAGAACAGGACAAGCAA
TATATGCCCCTCCCATCCAAGGAGTAATAAGGTGTGAATCAAAT---
ATTACAGGACTAATATTAACAAGAGATGGTGGGGATACTAATGGA----------------------------
----

ACAGATGAAACCTTCAGGCCTGGAGGAGGAGATATGAGAGACAATTGGAGAAGTGAATTATACAAATATAAGG
TAGTAAAAATTGAACCACTA---GGAGTAGCACCCACCAGGGCAAGGAGAAGAGTGGTG--------------
-------------------------GACAGAGAAAAAAGAGCA---GTTGGA---TTAGGAGCT---------
GTCTTCCTTGGGTTCTTAGGAGCA---GCAGGAAGCACTATG---GGCGCAGCGTCA---
ATAACGCTGACGGTACAG---GCTAGACAA---TTATTG---TCTGGCATAGTGCGGCAGCAAAGC---
AATTTG---CTGCAG---GCTATAGAGGCTCAACAGCAGCTGTTGAAACTTACGGTCTGGGGCATT
>KY658698
TTCTATTGTAATACATCAGGCCTGTTTAACAGTGCTTGGAATAACACTAACCTCTTGACAAATAGCACAGGG-
----

ACAAATGGCACTATAACTCTCCCATGCAGGATAAAGCAAATTATAAACATGTGGCAGAGAGTAGGGCAAGCAA
TGTATGCCCCTCCCATCCAAGGAGTAATAAAGTGTGTATCAAAC---
ATTACAGGACTAATATTAACAAGAGATGGAGGGGATAATAACAGT----------------------------
----

ACAAGGGAAATCTTCAGACCTGGAGGAGGAGATATGAGGGACAATTGGAGAAGTGAATTATATAAGTATAAAG
TAGTACAACTTGAACCATTA---GGAGTAGCACCCACCAGGGCAAAGAGAAGAGTGGTG--------------
-------------------------GAGAGAGAAAAAAGAGCA---GTCGGA---ATGGCAGCT---------
GTTTTCATTGGGTTCTTAGGAGCA---GCAGGAAGCACTATG---GGCGCGGCGTCA---
ATAACGCTGACGGTACAG---GCCAGACAA---TTATTG---TCTGGCATAGTGCAACAGCAAAGC---
AATTTG---CTGAGG---GCTATAGAGGCTCAACAACATCTGTTGAAACTCACAGTCTGGGGCATT
>AF442565
TTCTATTGTAACACATCAGGCCTGTTTAATAGCACTTGGGAGGGCAACAATACCAGCATATTAAATGGCACGG
GG---

TCAAACGACAATACAACTCTCCCATGCAGAATAAAGCAAATTATAAATATGTGGCAGAGAGTAGGACAAGCAA
TATATGCCCCTCCCATCCCAGGAGTAATAAAGTGTGTGTCAAAT---
ATTACAGGACTAATATTAACAAGAGATGGTGGTAATGATGGTAGT----------------------------
----

AATGAGACCTTCAGACCTGGAGGAGGAGATATGAGGGACAATTGGAGAAGTGAATTATATAAGTATAAAGTAG
TAAAAATTGAACCAATA---GGAGTAGCACCCACCAGGGCAAAGAGAAGAGTGGTG-----------------
----------------------GAGAGAGAAAAAAGAGCA---GTTGGA---CTGGGAGCT---------
GTCTTCATTGGGTTCTTAGGAGCA---GCAGGAAGCACTATG---GGCGCGGCGTCA---
ATAACGCTGACGGTACAG---GCCAGGCAA---TTATTG---TCTGGCATAGTGCAACAGCAAAGC---
AATTTG---CTGAGG---GCTATAGAGGCTCAACAGCATCTGTTGAAACTCACTGTCTGGGGCATT
>AF442570
TTCTACTGTAACACATCAAACCTGTTTAATAGCACTTGGAATAATGACAATGCCACACAGTTAAATAGCACGG
AG---

TCAAACGAAACTATAACTCTTCAATGCAAAATAAAGCAAATTATAAATATGTGGCAGAGAGTAGGACAAGCAG
TATATGCCCCTCCCATTCAAGGAGTAATAAAATGTGAATCAAAC---
ATTACAGGAATAATATTAACAAGAGATGGTGGAAATAATAGCAGT----------------------------
----

GCAGAGGAGACCTTCAGACCTGGAGGAGGAGATATGAGGGACAATTGGAGAAGTGAATTATATAAATATAAAG
TAGTAAAAATTGAACCACTA---GGAGTAGCACCCACCAAGGCAAGGAGAAGAGTGGTG--------------
-------------------------GAGAGAGAAAAAAGAGCA---GTTGGA---ATAGGAGCT---------
GTTTTCATTGGGTTCTTAGGAGCA---GCTGGAAGCACTATG---GGCGCGGCGTCA---
GTGACGCTGACGGTACAG---GCCAGACAA---TTATTG---TCTGGCATAGTGCAACAGCAAAGC---
AATTTG---CTGAGG---GCTATAGAGGCTCAACAGCAAATGTTGAAACTCACAGTCTGGGGCATT
>AF442566
TTCTATTGTAACACATCAGGCCTGTTTAATAGCACTTGGAGTAATGGCACTAGGAAGGACAATGACAGCAGGC
AGAAA--

ACAAATGACACTATAATAACTCTCCAATGCAGAATAAAGCAAATTATAAATTTGTGGCAGAGAGCAGGACAAG
CAATATATGCCCCTCCCATCGAAGGAATAATAAGATGTAACTCAAAC---
ATTACAGGACTAATATTAACAAGAGATGGGGGCGAATCTAATAACAAA------------------------
----

ACTGAAGAAACTGAAACCTTCAGACCTGGAGGAGGAGATATGAGGGACAATTGGAGAAGTGAATTATATAAGT
ATAAAGTAGTAAAAATTGAACCACTA---GGAGTAGCACCCACCAGGGCAAGGAGAAGAGTGGTG--------
-------------------------------CAGAGAGAAAAAAGAGTA---GCT------TTTGGAGCT---
------GTCTTCATTGGGTTCTTAGGAGCA---GCAGGAAGCACTATG---GGCGCAGCGTCA---
ATAGCGCTGACGGCACAG---GCCAGACAG---TTATTG---TCTGGTATAGTGCAACAGCAAAGC---
AATTTG---CTGAGG---GCTATAGAGGCTCAACAGCATATGTTGAAACTCACGGTCTGGGGCATT
>HM027851
TTCTATTGTAACACAACAAACCTGTTCAATAGCACCTGGGAGAACGGCAAGCAGACATCAAATAGCACA----
----

AACAATGACACTATAACTCTCCCATGTAGAATAAAACAAATTGTAAATATGTGGCAGAGAACAGGACAGGCAA
TATATGCCCCTCCCATCCAAGGAATAATAAGGTGTAACTCAACC---
ATTACAGGACTACTATTAACAAGAGATGGTGGGAATAATAACAAT---------------------------
----

ACAAATGACACCTTCAGACCTGGAGGAGGAGATATGAGGGACAATTGGAGAAGTGAATTATATAAATATAAAG
TAGTACGAATTGAACCATTA---GGAGTAGCACCCACCGAGGCAAGGAGAAGAGTGGTG-------------
-------------------------GGGAGAGAAAGAAGAGCA---GTTGGA---CTGGGAGTT---------
TTCTTCCTTGGGTTCTTAGGAGCA---GCAGGAAGCACTATG---GGCGCAGCGTCA---
ATAACGCTGACGGTACAG---GCCAGACAG---TTACTG---TCTGGCATAGTGCAACAGCAAAAT---
AATTTG---CTGAGA---GCTATAGAGGCTCAACAGCATCTGTTGAGACTCACAGTCTGGGGCATT
>HM027850
TTCTATTGTAACACATCAGATCTGTTTAATGGCAGTTGGAATAACACAATGCAGGGGTCAAATAACACGGGG-
----
---------------------------TCAAATGACACTATAACTCTCAAATGTAGAATA------------
----
--ATAACAGTACAA--------------------AATGAAACTAAA--------------------------
----

ACCTTTAGACCTACAGGAGGAGATATGAGGGACAATTGGAGAAGTGAATTATATAAATATAAGGTAGTAAAAA
TTGAGCCACTA---GGAGTAGCACCCACCAAGGCAAAGAGAAGAGTGGTG----------------------
----------------GAGAGAGAAAAAAGAGCA---GTTGGA---CTAGGAGCT---------
GTCTTCCTTGGGTTCTTAGGAGCA---GCAGGAAGCACTATG---GGCGCGGCGTCA---
ATGACGCTGACGGTACAG---GCCAGACAA---TTATTG---TCTGGTATAGTGCAACAGCAAAAC---
AATTTG---CTGAAG---GCTATAGAGGCTCAACAACATCTGTTGAAACTCACAGTCTGGGGCATT
>HM027864
TTCTACTGTAATACATCAGGCCTATTTAATGGCAGTTGGAGTAGAAATGATACTACAAATGACACAGAG----
----

TCAAATGACACTATAACTCTACAATGTAGAATAAAACAATTTATAAATATGTGGCAAAGAGTAGGACAAGCAA
TGTATGCCCCTCCCATTAAAGGACCAATCACGTGTAACTCAAAT---
ATAACAGGACTATTGTTGACAAGGGATGGTAGTGGTAATCAAAAT---------------------------
----

AATGATACCGAGACCTTTAGACCTGGAGGAGGAAATATGAGGGACAATTGGAGAAGTGAATTATACAAATATA
AAGTAGTAAAAATTGAACCACTA---GGAGTAGCACCCACCAGGGCAAAGAGAAGAGTGGTG----------
----------------------------GAGAGAGAAAAAAGAGCA---GTTGGA---ATAGGAGCT------
---GTCCTCTTTGGGTTCCTAGGAGCA---GCAGGAAGCACTATG---GGCGCAGCGTCA---
ATAACGCTGACGGTACAG---GCCAGACAA---TTATTG---TCTGGCATAGTGCAACAGCAAAGC---
AATTTG---CTGAGG---GCTATAGAGGCTCAACAACATATGTTGAAACTCACAGTCTGGGGCATT
>HM027870
TTCTATTGTAATACATCAGGCCTGTTTAATAGCACTTGGGATAGCAATGAGCTTAATAGCACTTTGAATGGCA
CT--

GAGTCAAATGACACTATAACTCTCCCATGCAGAATAAAACAAATTATAAATATGTGGCAGAGAGTAGGACAGG
CAATGTATGCCCCTCCCATCCCAGGAATAATAAGGTGTGAATCAAAC---
ATTACAGGACTAATATTAACAAGAGATGGTGGAAACATTACCAAC----------------------------
----

AATACAGAAATCTTCAGACCGGGGGGAGGAGATATGAGGGACAATTGGAGAAGTGAATTATATAAGTATAAAG
TAGTAAAAATTGAACCACTA---GGAGTAGCACCTACCAAAGCAAAGAGAAGAGTGGTG--------------
-------------------------GAGAGAGAAAAAAGAGCA---GTTGGA---CTAGGAGCT---------
GTCTTCATTGGATTCTTAGGAGCA---GCAGGAAGCACTATG---GGCGCGGCGTCA---
ATAACGCTGACGGTACAG---GCCAGACAA---TTATTG---TCTGGCATAGTGCAACAGCAAAGC---
AATTTG---CTGAGA---GCTATAGAGGCTCAACAACATCTGTTGAAGCTCACGGTCTGGGGCATT
>JX658625
TTCTACTGTAATACATCAAAACTGTTTAATAATAGTATAGGGAGGAATGCTACTACATCAAAT----------
----

AGTACAGAATCTATAACACTCCCATGCAAAATAAAACAAATTATAAACATGTGGCAGGGAGTAGGCAAAGCAA
TGTATGCCCCTCCCATTGAAGGACTAATCAAATGTGAATCAAAT---
ATTACAGGGCTATTGTTGACAAGAGATGGTGGTAATACTAATAAT----------------------------
----

CAGAATGAGACCTTCAGACCTGGAGGAGGAGATATGAGAGACAATTGGAGAAGTGAATTATACAAATATAAAG
TAGTAAGAATTGAACCACTA---GGTCTAGCACCCACCAGGGCAAAGAGAAGAGTGGTG--------------
-------------------------GAAAGAGAAAAAAGAGCA---ATAGGA---CTGGGAGCT---------
TTGTTCCTTGGGTTCTTGGGAGCA---GCAGGAAGCACAATG---GGCGCAGCGTCG---
CTGACGCTGACGGTACAG---GCCAGACAA---GTACTG---TCTGGTATAGTGCAGCAACAAAAT---
AATTTG---CTGAGA---GCTATTGAGGCGCAACAGCATCTGTTGCAACTCACAGTCTGGGGCATT
>HM027827
TTCTACTGTAATACATCAGGCCTGTTTAATGGCACCTGGAATAGTAATGATACTATAAATGAAACAGAG----
----

ACAAATGACATTATAATTCTCCAATGTAGAATAAAACAAATTATAAATATGTGGCAAAGAGTAGGACAAGCAA
TGTATGCCCCTCCCATTAAAGGACCAATCACGTGTAACTCAAAT---
ATAACAGGACTATTGTTGACAAGAGATGGTGGTGAAGAGAAAGAGAAA-------------------------
----

CAGAATAATGAGACCTTTAGACCTGAAGGAGGAAATATGAAGGACAATTGGAGAAGTGAATTATACAAATATA
AAGTAGTAAAAATTGAACCACTA---GGAGTAGCACCCACCAGGGCAAAGAGAAGAGTGGTG-----------
----------------------------GAGAGAGAAAAAAGAGCA---GTTGGA---ATAGGAGCT------
---GTCCTCTTTGGGTTCCTAGGAGCA---GCAGGAAGCACTATG---GGCGCAGCGTCA---
ATAACGCTGACGGTACAG---GCCAGGCAA---TTATTG---TCTGGCATAGTGCAACAGCAAAGC---
AATTTG---CTGAGG---GCTATAGAGGCTCAACAACATATGTTGAAACTCACAGTCTGGGGCATT
>HM215273
TTCTATTGTAATACATCAGGATTGTTTAATAGCACTCATCCTATCAACAGGACGACAAATAGCACAGAG----
----

AACAATGGAACTATAACTCTCCCATGCAGAATAAAACAAATTATAAATATGTGGCAGAGGGCAGGACAAGCAA
TATATGCCCCTCCCATCCCAGGAATAATAAGGTGTACATCAAAC---
ATTACAGGACTATTATTAACAAGAGATGGTGGGAATCATAATGATACT-------------------------
----

AACAATACTGAAACCTTCAGACCTGGAGGAGGAGATATGAAAGATAATTGGAGAAGTGAACTGTATAAATATA
AAGTAGTAAAAATTGAACCACTA---GGTCTAGCACCCACCAAGGCAAAAAGAAGGGTGGTG-----------
----------------------------AAAAGAGAAAAAAGAGCA---ATAGGA---CTAGGAGCT------
---TTGTTCCTTGGGTTCTTGGGAGCA---GCAGGAAGCACGATG---GGCGCAGCGTCA---
CTGACGCTGACGGTACAG---GCCAGACAA---GTACTG---TCTGGTATAGTGCAACAGCAAAAC---
AATTTG---CTGATG---GCTATAGAGGCGCAACAGCATCTGTTGCAACTCACAGTCTGGGGCATT
>HM215274
TTCTATTGTAATACATCAAGCTTGTTTAATAGCACCTGGAACAAGAACAGTGACACCTGGAACACCACCAGGA
ACGTG--

CCTAAGTTAGAACCTATAACACTCCCATGCAGAATAAAGCAAATTATAAATATGTGGCAGCGAACAGGACAAG
CAATGTATGCCCCTCCCATCCAGGGAGTAATAAGGTGTAACTCAACC---
ATTACAGGACTCCTATTAACAAGAGATGGTGGGCTTAATAATGAA----------------------------
----

ACCTATGAAACCTTCAGACCTGGAGGAGGAGATATGAGGGACAATTGGAGAAGTGAGTTATACAAATATAAAG
TAGTAAGAATTGAACCAATA---GGAATAGCACCCACCAAGGCAAAGAGAAGAGTGGTG--------------
-------------------------GAGAGAGAAAAAAGAGCA---GTTGGA---ATGGCAGCT---------
GTATTCATTGGGTTCTTGGGAGCA---GCAGGAAGCACTATG---GGCGCGGCGTCA---
ATAACGCTGACGGTACAG---GCCAGACAA---TTATTG---TCTGGCATAGTGCGACAGCAAAAC---
AAGTTG---CTGAGA---GCTATAGAGGCTCAGCAGCATCTGCTGAGACTCACGGTCTGGGGCATT
>JX236674
TTCTATTGCAACACATCAGACCTGTTTAATAGCAGTTGGAAGAACAGTACCAGCATACATAACAGTACC----
----

AGCAATGACACTATAACTATCCCATGCAGGATAAAGCAAATTATAAATATGTGGCAGAGAGCGGGAAAAGCAA
TATATGCCCCTCCCATCCAAGGAGTAATAAAGTGTGAATCCAAC---
ATTACAGGACTAATATTAACAAGAGATGGTGGAATTAGTAACAGTACA-------------------------
----

AATAAAAATGAAACCTTCAGACCTGGAGGGGGAGATATGAGGGACAATTGGAGAAGTGAATTATATAAGTATA
AAGTAGTAAAAATCGAGCCAATA---GGAATAGCACCCACCAGGGCAAGGAGAAGAGTGGTG-----------
----------------------------GAGAGAGAGAAAAGAGCA---GTTGGA---CTAGGAGCT------
---GTTTTCATTGGGTTCTTAGGAGCA---GCAGGAAGCACTATG---GGCGCGGCGTCA---
ATAACGCTGACGGTACAG---GCCAGACAA---TTATTG---TCTGGCATAGTGCAACAGCAGAGT---
AATCTG---CTGAAG---GCTATAGAGGCTCAACAGCAGCTGTTGAGACTCACGGTCTGGGGCATT
>KF859746
TTCTATTGTAATACATCAAGCCTGTTCAATAGCACGTGGCCTGCTAATGACAGCATGCAGGCAAATAGCACGG
AAAAT--

AGCACGGAGATGATAAAACTCTCATGCAACATAAAGCAAATTATAAATATGTGGCAGAGAGTAGGACAAGCAA
CATATGCCCCTCCCATCAAAGGAATAATAAAGTGTGAATCAAAC---
ATTACAGGATTAATATTAACAAGAGATGGTGGGAATAATAACAATGGA-------------------------
----

AACAATAAAAGTGAAACCTTCAGACCTGGAGGAGGAGATATGAGGGATAATTGGAGAAGTGAATTATATAAGT
ATAAGGTAGTAAGGATTGAACCATTA---GGAGTAGCACCCACCAGGGCAAAAAGACGAATGGTG--------
-------------------------------GAGAGAGAAAAAAGAGCA---GTTGGC---CTGGGAGCT---
------GTCTTCTTTGGGTTCTTAGGAGCA---GCAGGAAGCACTATG---GGCGCGGCGTCA---
ATAACGCTGACGGTACAG---GCCAGACAA---TTATTG---TCTGGCATAGTGCAACAGCAAAGC---
AATTTG---CTGAGA---GCTATAGAGGCTCAACAGCAGCTGTTGAGACTCACAGTCTGGGGCATT
>KF859747
TTCTACTGTGATACATCAGGCCTGTTTAATAGTACTTGGAATACCACTGACACTGGCAGCATAAACTCAAAT-
----

AGTACAGATAATATAACTCTCCCATGCAGAATAAAGCAAATTGTAAATATGTGGCAGAGAGTAGGACGAGCAA
TGTACGCCCCTCCCATCCAGGGAATAATAAGGTGTGAATCAAAC---
ATTACAGGATTACTATTAACAAGAGATGGTGGTAACAGTACAAAT----------------------------
----

GAAACCTTCAGACCTGGGGGAGGAGATATGAAAGACAATTGGAGAAGTGAATTATACAAATATAAAGTAGTAA
GAATTGAACCACTA---GGAATAGCACCCAACAGGGCCAGGAAAAGAGTGGTG--------------------
-------------------GGAAGAGAAAAAAGAGCA---GTTGGA---ATTGGAGCT---------
GTATTCCTTGGGTTCTTAGGAGCA---GCAGGAAGCACTATG---GGCGCGGCGTCA---
ATAACGCTGACGGTACAG---GCCAGACAA---TTATTG---TCTGGCATAGTGCAACAGCAAAGC---
AATTTG---CTGAGG---GCTATAGAGGCTCAACAGCAGATGTTGAAACTCACAGTCTGGGGCATT
>KP109491
TTCTATTGTAATACATCAGACCTGTTTAATAGCACCTGGCCCAGGATGCCTGGAATAGCAGCAACCAGACTG
GGACAAGG---

GACAATATAAATATAACTCTCCAATGTAGAATAAAGCAAATTATAAATATGTGGCAGAGAACAGGACAAGCAA
TATATGCCCCTCCCATCCCAGGAAACATAACTTGTGTATCAAAC---
ATTACAGGACTAATATTAACAAGAGATGGTGGGGCTAATAATGAA----------------------------
----

GGCAATGAAACCTTCAGACCTGGAGGAGGAGATATGAGGGACAATTGGAGAAGTGAATTATATAAGTATAAAG
TAGTAAAAATTGAACCACTA---GGAGTGGCACCCACCAAGGCAAGGAGAAGAGTGGTG--------------
-------------------------CAGAGAGAAAAAAGAGCA---GTTGGA---ATGGGAGCT---------
TTGTTCATTGGGTTCTTAGGAGCA---GCAGGGAGCACTATG---GGCGCGGCGTCA---
ATAACGCTGACGGTACAG---GCCAGACAA---TTATTG---TCTGGTATAGTGCAACAGCAAAGC---
AATTTG---CTGAGG---GCTATAGAGGCTCAACAACATCTGTTGAAGCTCACAGTCTGGGGCATT
>KF716487
TTCTATTGTAATACAACAAAATTGTTTGATAGCACTTGGAACAAAACCACTTGGAACACAACGAGCACAGGGG
AGACAAAT---
----------AACACAGGG------
GAGACAAATGACACTATAACTATCCCATGTAGAATAAAGCAAATTATAAATATGTGGCAGAGGACAGGACAAG
CAATATATGCCCCTCCCATTCAAGGAATAATAAGGTGTGACTCAAAC---
ATTACAGGACTACTATTAACAAGAGATGGTGGGCCTGGTAATACA----------------------------
----

ACTGAAATCTTCAGACCTGGAGGAGGAGATATGAGGGATAATTGGAGAAGTGAACTATATAAATATAAAGTAG
TAAAAGTTGAACCATTA---GGAGTAGCACCCACCAGGGCAAAGAGAAGAGTGGTG-----------------
----------------------GAGAGAGAAAAAAGAACA---GTTGGA---CTGGGAGCT---------
GTTTTCCTTGGGTTCTTGGGAGCA---GCAGGAAGCACTATG---GGCGCGGCGTCA---
ATAACGCTGACGGTACAG---GCCAGACAG---TTATTG---TCTGGCATAGTGCAACAGCAAAGT---
AATTTG---CTGAGG---GCTATAGAGGCTCAACAGCATCTGTTGAAACTCACGGTCTGGGGCATT
>KF716490
TTCTATTGTAATACAACAAACCTGTTTAATACTACTTGGAGTAATGGCAGCCTGGTTAATATTACGGAGTCAA
ATAAT--
-------------------GCT---
ACAAATAATGCTACGATTCTTCTCCCATGCAGAATAAAGCAAATTATAAATATGTGGCAGAGAGTAGGACAAG
CAATGTATGCCCCTCCCATCCCAGGAGTAATACAGTGTACATCAAAC---
ATTACAGGACTATTATTAACAAGAGATGGTGGGAATATTACTAAC----------------------------
----

AATACAGAGATCTTTAGACCTGGAGGGGGAGATATGAGGGACAATTGGAGAAGTGAACTATATAAATATAAAG
TAGTAAGAATTGAACCATTA---GGAGTAGCACCCACCGGGGCAAAGAGAAGAGTGGTG--------------
-------------------------GAGAGAGAAAAAAGAGCAATTGCTGGA---ATAGGAGCT---------
GTCCTCTTTGGGTTCTTAGGAGCA---GCAGGAAGCACTATG---GGCGCGGCGTCA---
CTAACGCTGACGGTACAG---GCCAGACAA---TTATTG---TCTGGCATAGTGCAACAGCAAAGC---
AATTTG---CTGCAG---GCTATAGAGGCTCAACAGCATCTGTTGAAACTCACGGTCTGGGGCATT
>KF716482
TTCTATTGTGACACATCAGGCCTGTTTAATAGCAGCTGGGATGGCAACAGCGAGGATAACAATACTCAG----
----

GGAACTGACACTATAACTCTCCAATGCAAAATAAAGCAAATTATAAGGATGTGGCAAAGAACAGGACAAGCAA
TGTATGCCCCTCCCATCCAAGGAACAATAAAGTGTCAATCAAAC---
ATTACAGGGCTATTATTGACAAGAGATGGGGGGAGTGAAAGTACA----------------------------
----

AATGAAAGTGAAACCTTCAGACCTGGAGGAGGAAATATGAGGGACAATTGGAGAAGTGAATTATATAAATATA
AAGTAGTAAAGATTGAACCACTA---GGAGTAGCACCCACCAGGGCAAAGAGAAGAGTGGTG-----------
----------------------------GAGAGAGAAAAAAGAGCA---GTTGGA---CTGGGAGCT------
---GTCTTCATTGGGTTCTTAGGAGCA---GCAGGAAGCACTATG---GGCGCAGCGTCA---
GTGACGCTGACGGTACAG---GCCAGACAA---TTATTG---TCTGGTATAGTGCAACAGCAAAAC---
AATTTG---CTGAGG---GCTATAGAGGCTCAACAGCATCTGTTGAAACTCACGGTCTGGGGCATT
>KP109493
TTCTATTGCAACACAACAAGGCTGTTTAATAGCACTTGGAGTAATGACACTTGGGATAATAGCACTTGGGATA
AA---

TCAAACAACATTATAAATCTCCAATGCAGAATAAAGCAAATCATAAATATGTGGCAGAGAACAGGACGAGCAA
TGTATGCCCCTCCCATTCAAGGAGTAATAAGGTGTGAATCAAAC---
ATTACAGGACTAATATTAACAAGAGATGGTGGGAATAGTAACGAG---------------------------
----

ACAGAAATCTTCAGACCTGGGGGAGGAGATATGAGGGACAATTGGAGAAGTGAATTATATAAGTATAAAGTAG
TAAAAATTGAACCACTA---GGAGTAGCACCCACCGAGGCAAAGAGAAGAGTGGTG----------------
---------------------GAGAGAGAAAAAAGAGCAGCTGTTGGA---CTGGGAGCG---------
TTCTTCATTGGGTTCTTAGGAGCA---GCAGGAAGCACTATG---GGCGCGGCGTCA---
ATAACGCTGACGGTACAG---GCCAGACAA---TTATTG---TCTGGCATAGTGCAACAGCAAAGC---
AATTTG---CTGAGG---GCTATAGAGGCTCAACAGCATATGTTGAAACTCACGGTCTGGGGCATT
>KY580490
TTCTATTGTGATACAGCAGAACTGTTTGATAGCGCTTGGAATAGTAGTAGCAATTGGACTATAACTGACAAGT
CA--

AATAATACCAATATAACCCTCCAGTGCAGAATAAAGCAAATTATAAATATGTGGCAGAGAGTAGGACAAGCAA
TGTATGCCCCTCCCATCCCAGGAGTAATAAGGTGTACATCAAAC---
ATTACAGGACTAATATTAACAAGAGATGGTGGGGAGACTAACAATGCA------------------------
----

ACTGAAGAAACCTTCAGACCGGGAGGAGGAGACATGAGGGACAATTGGAGAAGTGAATTATATAAATATAAAG
TAGTAAAAATTGAACCACTA---GGAGTAGCACCCACCGGGGCAAAAAGAAGAGTGGTG-------------
------------------------GAGAGAGAAAAAAGAGCA---ATTGGA---ATAGGAGCT---------
GTCTTCATTGGGTTCTTAGGAGCA---GCAGGAAGCACTATG---GGCGCAGCGTCA---
ATAACGCTGACGGTACAG---GCCAGACAA---TTATTG---TCTGGCATAGTGCAACAGCAAAGC---
AATTTG---CTGAAG---GCTATAGAGGCTCAACAACATCTGTTGAGACTCACAGTCTGGGGCATT
>KP109497
TTCTACTGTAATACATCAGGGTTGTTTAATAGTACATGGAGAAGGAACAGTACAAATGGTACATGGGAAAGTG
ATATATCAGGG---

GTAAATGAGAGTACAATCACACTCCCATGCAGAATAAAACAAATTATAAACATGTGGCAGGGGGTAGGAAGAG
CAATGTATGCCCCTCCCATTCCAGGATCAATTAATTGTTCATCAAAT---
ATTACAGGATTATTGTTGACAAGAGATGGTGGTACAAACAGTAGT---------------------------
----

CAGAATGACACCTTCAGACCTGGAGGAGGAGATATGAGGGACAATTGGAGAAGTGAATTATACAAATATAAAG
TAGTAAGAATTGAACCAATA---GGTCTGGCACCCACCAAGGCACAAAGAAGAGTGGTG-------------
------------------------GCAAGAGAAAAAAGAGCA---ATAGGA---CTGGGAGCT---------
ATGTTCCTTGGGTTCTTGGGAGCA---GCAGGAAGCACGATG---GGCGCAGCGTCA---
GTGACGCTGACGGTACAG---GCCAGACAG---TTATTG---TCTGGTATAGTGCAACAGCAAAAC---
AATTTG---CTGAGG---GCTATAGAGGCGCAACAGCATCTGTTGCAACTCACAGTCTGGGGCATT
>KF716484
TTCTATTGTAACACATCAGGTCTGTTTAATAGCACATGGGAAAGGAATATGACATCAAATAGCACAGAG----
----

TCAAATGACACCATAACTCTCCAATGCAGAATAAAGCAGATTGTAAACATGTGGCAGAGAGTAGGACAAGCAA
TATATGCCCCTCCCATTCCAGGAGAAATAAGGTGTAGATCAAAC---
ATTACAGGACTAATATTAACAAGAGATGGTGGGGGGCAGAACGATACAGGA---------------------
----

GGAGGAGGAAACGAAACCTTCAGACCTGGAGGAGGAGATATGAGGGACAATTGGAGAAGTGAATTATACAAGT
ATAAAGTAGTAAAGATTGAACCACTA---GGAGTAGCACCCACCAAGGCAAAGAGAAGAGTGGTG-------
------------------------------GCGAGAGAAAAAAGAGCA---ATTGGA---CTGGGAGCT---
------GCGTTCCTTGGGTTCTTAGGAGCA---GCAGGAAGCACTATG---GGCGCAGCGTCA---
ATAACGCTGACGGTACAG---GCCAGAGCA---TTATTG---TCTGGCATAGTGCAACAGCAAAGC---
AATTTA---CTGAAG---GCTATAGAGGCTCAACAGCAACTGTTGAAACTCACGGTCTGGGGCATT
>AY352657
TTCTACTGCAATACATCAGGACTGTTTAATAATAGTGTATGGAGGAATGATTATCCATCAAATAGTACAGGAG
TA--

```
AAGGAGAGCGTAATCACACTCCCATCCAAAATAAAACAAATTATAAACCTGTGGCAGGGAGTAGGAACAGCAA
TGTATCCCCCTCCCATTGAAGGACTAATCAAATGTTCATCAAAT---
ATTACAGGACTATTATTGACAAGAGATGGTGGTGTCAATAATAGT----------------------------
-------------------------------------------------------------------------
-------------
CAGAATGAGACCTTCAGACCTGGAGGAGGAGATATGAGGGACAATTGGAGAAGTGAATTATACAAATATAAAG
TAGTAAGACTTGAACCACTA---GGTCTGGCACCTACCAGGGCAAAGAGAAGAGTGGTG--------------
-------------------------GAAAGAGAAAAAAGAGCA---ATAGGA---CTAGGAGCT---------
ATGTTCCTTGGGTTCTTGGGAGCA---GCAGGAAGCACGATG---GGCGCAGCGTCA---
CTGACGCTGACGGTACAG---GCCAGACTG---TTATTG---TCTGGTATAGTGCAACAGCAAAAC---
AATTTG---CTGATG---GCTATAGAGGCGCAACAGCATCTATTGCAACTCACAGTCTGGGGCATT
>U36883
TTCTACTGCAATACATCAGGACTGTTTAATGGTACATGGAGTAATGGTACATGGAGTAATGATACATCAAATA
GTGCA--------------------------------------------------------------------
-------------------------
AATGGAAATGGTACAATCACTCTCCCATGTAGAATAAAACAAATTATAAACATGTGGCAGGGAGTAGGAAAAG
CAATGTATGCCCCTCCCATTAAAGGACGAATCCAATGTGAATCAAAT---
ATTACAGGACTATTGTTGACAAGAGATGGCGGTCAGACCAATGAT----------------------------
-------------------------------------------------------------------------
-------------
AGTACTGAGACCTTCAGACCTGGAGGAGGAGATATGAGGGACAATTGGAGAAGTGAATTATACAAATATAAAG
TAGTAAGAGTTGAACCACTA---GGTCTAGCACCCACCAGGGCAAAGAGAAGAGTGGTG--------------
-------------------------GAAAGAGAAAAAAGAGCA---ATAGGA---CTAGGAGCT---------
GTGTTCCTTGGGTTCTTGGGAGCA---GCAGGAAGCACGATG---GGCGCAGCGTCA---
CTAACGCTGACGGTACAG---GCCAGACAG---TTAATG---GCTGGTATAGTGCAACAGCAGAAC---
AATTTG---CTGAGG---GCTATAGAGGCGCAACAGCACCTGTTGCAACTCACAGTCTGGGGCATT
>KJ579955
TTCTATTGCAACACATCAGGCCTATTTAATAGCATTTGGGAGAATGGCACT----------------------
-------------------------------------------------------------------------
-TTGAAAAGCAATGAC---------
AGCATGTCAAACAACATAACTCTCCAATGCAGAATAAAGCAAATTATAAATATGTGGCAGAGAACAGGACAAG
CAATGTATGCCCCTCCCATCCCAGGAGTAATAAGGTGTGAGTCAAAC---
ATTACAGGATTAATATTAACAAGAGATGGTGGG----------------------------------------
-------------------------------------------------------------------------
-
AATAATAACAATGAAAGTGAAACCTTCAGACCTGGAGGAGGAGATATGAGGGACAATTGGAGAAGTGAATTAT
ATAAGTATAAAGTAGTAAAAATTGAACCACTA---GGAGTAGCACCCACCAAAGCAAGGAGAAGAGTGGTG--
-------------------------------------GAGAGAGAAAAAAGAGCAGTTGTTGGA---
ATAGGAGCT---------GTCTTCTTTGGGTTCTTAGGAGCA---GCAGGAAGCACTATG---
GGCGCGGCGTCA---CTAACGCTGACGGTACAG---GCCAAGCAA---TTATTG---
TTTGGCATAGTGCAACAGCAAAGC---AATTTG---CTGAGG---
GCTATAGAGGCTCAACAGCATCTGTTGAAACTCACGGTCTGGGGCATT
>AF484503
TTCTATTGTAACACATCAGGCCTGTTTAATAACAGTTGGAATGGCACGCAGATGTCAAATAGCATGAAG----
-------------------------------------------------------------------------
----------------------------
CCAAATGACACCATAACTCTCCAATGCAGGATAAAGCAAATTGTAAATATGTGGCAGAGAGCAGGACAAGCAA
TATATGCCCCTCCTATCCGAGGAGTAATAAGGTGTGTATCAAAC---
ATTACAGGACTGATATTAACAAGAGATGGTGCTGAGGATAAGGAC----------------------------
-------------------------------------------------------------------------
----------
AGTACAAAGGAAACCTTCAGACCTGCAGGAGGAGATATGAGGGACAATTGGAGAAGCGAATTATATAAATATA
AAGTAGTAAAAATTGAACCACTA---GGAGTAGCACCCACCAAGGCAAAGAGAAGAGTGGTG-----------
----------------------------GAGAGAGAAAAAAGAGCA---GTTGGA---ATAGGAGCT------
---GTCTTCCTTGGGTTCTTAGGAGCA---GCCGGAAGCACTATG---GGCGCAGCGTCA---
ATAACGCTGACGGTACAG---GCCAGACAA---TTGTTG---TCTGGCATAGTGCAACAGCAAAGC---
AATTTG---CTGAAG---GCTATAGAGGCTCAACAGCATCTGTTGAAACTCACGGTCTGGGGCATT
>AF484521
TTCTACTGCAATACATCACAACTATTTAATAGAACATGGAATGATAGTATATCAAATAATGCATGGAAGAATA
ATAGT--------------------------------------------------------------------
```

-------------------ACA---
GGGGAAAATGACACAATCACTCTCCCATGCAGAATAAAACAAATTATAAACATGTGGCAGGAAGTAGGAAAAG
CAATGTATGCCCCTCCCATTAAAGGTCTAATCAGATGTTCCTCAAAT---
ATTACAGGACTATTGTTAGTAAGAGATGGTGGAAATAATAGTAGT----------------------------
----

CAGAATGAAACCTTCAGACCTGGAGGAGGAGATATGAGAGACAATTGGAGAAGTGAATTATACAAATATAAAG
TAGTAAGAATTGAACCACTA---GGTCTAGCACCCACCAAGGCAAAGAGGAGAGTGGTA--------------
-------------------------GAAAGAGAAAAAAGAGCA---ATAGGA---CTGGGAGCT---------
GTATTCCTTGGGTTCTTGGGAGCA---GCGGGAAGCACGATG---GGCGCAGCGTCA---
CTGACGCTGACGGTACAG---GCCAGAAAT---CTATTG---TCTGGTATAGTGCAACAGCAAAAT---
AATTTG---CTGAGG---GCTATAGAGGCGCAACAGCATCTGTTGCAACTCACAGTCTGGGGCATT
>AF484522
TTCTACTGCAATACATCAGGACTGTTTAACAATAGTGTATGGACATCAAATAGTACGATAGGG----------
----

GCAAATGGGACAATCACACTCCCATGCAGAATAAAACAAATTATAAATATGTGGCAGGGAGTAGGAAAAGCAA
TGTATGCCCCTCCCATTGAAGGACAAATCAACTGTTCATCAACT---
ATTACAGGACTATTGTTGACAAGAGATGGTGGTGTAAAAAATAAT----------------------------
----

AGTCAGAACGAGACCTTCAGACCTGGAGGGGGAGATATGAGAGACAACTGGAGAAATGAATTATACAAATATA
AAGTAGTAAGAATTGAACCACTA---GGTCTAGCACCCACCAAGGCAAGGAGAAGAGTGGTG-----------
----------------------------GAAAGAGAAAAAAGAGCA---ATAGGA---CTGGGAGCT------
---GTGTTCCTTGGGTTCTTGGGAGCA---GCAGGAAGCACGATG---GGCGCAGCGTCG---
CTGACGCTGACGGTACAG---GCCAGACAG---TTACTG---TCTGGTATAGTGCAACAGCAAAAC---
AATTTG---CTGAGG---GCTATAGAGGCGCAACAGCATTTGTTGCAACTCACAGTTTGGGGCATT
>AF484482
TTCTACTGCAATACATCAGAACTGTTTAATAGTGAATGGAAAAATGGGACTACATCAAACAGTACA-------
----

AGAAATAGGACAATCACACTCCCATGCAGAATAAAACAAATCATCAACATGTGGCAGGGAGTAGGAAGAGCAA
TGTATGCCCCTCCCATTAAAGGACTAATCAATTGTTCATCAAAT---
ATTACAGGACTATTGTTAACAAGAGATGGTGGTACAAATCAAAGT----------------------------
----

GAGGAGACTTTCAGACCTGGGGGAGGAGATATGAGAGACAATTGGAGAAATGAATTATACAAATATAAAGTAG
TAAGAATTGAACCACTA---GGTCTAGCACCCACCAGGGCAAAAAGAAGAGTGGTG-----------------
----------------------GAAAGAGAAAAAAGAGCA---ATAGGA---CTAGGAGCT---------
TTGTTCCTTGGGTTCTTGGGAACA---GCAGGAAGCACGATG---GGCGCAGCGTCA---
CTGACGCTGACGGTACAG---ACCAGACAA---GTATTG---TCTGGTATAGTGCAACAGCAAAAC---
AATTTG---CTGAGG---GCTATAGAGGCGCAACAGCATCTGTTGCAACTCACAGTCTGGGGCATT
>AF484479
TTCTATTGTAACACATCAGGGTTGTTTAATAGCACTTGGACGGGTAACAACGCTGCCAACAGC----------
----

ACGGAGGGCAATATAACTCTCCCGTGCAGAATAAAGCAAATTATAAATATGTGGCAGAGAGTAGGACAAGCAA
TGTATGCCCCTCCCATCCAAGGAGTAATAAAATGTGTATCAAAT---
ATTACAGGAATATTATTAACAAGAGATGGTGGATCAAATGGAACT----------------------------
----

GGAAATGAAACCTTCAGACCTGGAGGAGGAGATATGAGGGACAATTGGAGAAGTGAACTATATAAGTATAAAG
TAGTAAAAGTTGAGCCACTA---GGGGTAGCACCCACCAGGGCAAGGAGAAGAGTGGTG--------------
-------------------------GAGAGAGAAAAAAGAGCA---GTTGGA---ATAGGAGCT---------
GTATTCCTTGGGTTCTTAGGAGCA---GCAGGAAGCACTATG---GGCGCGGCGTCA---
ATAACGCTGACGGTACAG---GCCAGACAA---TTATTG---TCTGGCATAGTGCAGCAGCAAAGC---
AATTTG---CTGAGG---GCTATAGAGGCTCAACAGCATTTGTTGAGACTCACAGTCTGGGGCATT
>AF484517
TTCTATTGTAACACATCAGGCCTGTTTAACAGCACTTGGACAAATGGCACTATGCAGGGGTTAAAT-------
----

GGCACAGGAAACATAACACTCTCATGCAGAATAAAACAAATTATAAACATGTGGCAGGGAGTAGGAAAAGCAA
TGTATGCCCCTCCCATTGAAGGACTAATCCAATGTTCATCAAAT---
ATTACAGGACTATTGTTGACAAGAGATGGTGGTAATAATAATAGT----------------------------
----

CAAAATGAGACCTTCAGACCTGGAGGAGGAGATATGAGAGACAATTGGAGAAGTGAATTATATAAATATAAAG
TAGTAAGAGTTGAACCAATA---GGTCTAGCACCCACCAAGGCAAAGAGAAGAGTGGTG--------------
-------------------------GAAAGAGAAAAAAGAGCA---ATTGGA---CTGGGAGCT---------
GTGTTCCTTGGGTTCTTGGGAGCA---GCAGGAAGCACGATG---GGCGCAGCGTCA---
ATGACGCTGACGGTACAG---GCCAGACAG---GTATTG---TCTGGTATAGTGCAACAGCAAAAT---
AATTTG---CTGAGG---GCTATAGAGGCGCAACAGCATCTGTTGCAACTCACAGTCTGGGGCATT
>AF484488
TTCTATTGTAATACATCAGGTCTGTTTAATAGTACTTGGAATAGTACTAAGGAAAATGATACTGACAGC----
----

GGGAGTGGCACCATAACTCTCCCATGCAAAATAAAGCAAATTATAAATATGTGGCAGAGAGTAGGACAAGCAA
TATATGCCCCTCCCATCAGAGGAGTAATAAGGTGTGATTCAAAC---
ATTACAGGACTACTGTTAACAAGAGATGGAGGGGATAGTAACAAT----------------------------
----

AATGAAACCTTCAGACCTGGAGGGGGAGATATGAGGGACAATTGGAGAAGTGAATTATATAAGTATAAAGTAG
TAAAAATTGAGCCAATA---GGAGTAGCACCCACCAGGGCAAAGAGAAGAGTGGTG-----------------
----------------------CAGAGAGAAAAAAGAGCA---GTTGGA---ATAGGAGCT---------
GTATTCCTTGGGTTCTTGGGAGCA---GCAGGAAGCACTATG---GGCGCAGCGTCA---
ATAACGCTGACGGTACAG---GCCAGACAA---TTATTG---TCTGGCATAGTGCAACAGCAAAGC---
AATTTG---CTGATG---GCTATAGAGGCTCAACAGCATCTGTTGAAACTCACGGTCTGGGGCATT
>AF484492
TTCTATTGTAACACATCAAAGTTGTTTAATAGCACTTGGGGGGAAAATAGCACTTGGGGGGAAAATAGCACTA
GCATA--

GAGTTAAATGACACTATAACTCTCCCGTGCAAAATAAAGCAAATTATAAATATGTGGCAGAGAACAGGACAAG
CAATATATGCCCCTCCCATCCAAGGAGTGATAAGGTGCGAGTCAAAT---
ATTACAGGACTATTGTTAACAAGAGATGGTGGTAAAAATAATAGT----------------------------
----

AATCAGAATGAAACCTTCAGACCTGGAGGAGGAGATATGAGGGACAACTGGAGAAGTGAGTTATATAAGTATA
AAGTAGTAAAAATTGAACCTTTG---GGAGTAGCACCCACCAAGGCAAGGAGAAGAGTGGTG-----------
----------------------------GGAAGAGAAAAAAGAGCA---GTAGGA---CTAGGAGCT------
---TTGTTCCTTGGGTTCTTGGGAGCA---GCAGGAAGCACTATG---GGCGCAGCGTCA---
ATAACGCTGACGGTACAA---GCCAGACAA---TTATTG---TCTGGCATAGTGCAACAGCAAAAC---
AATTTG---CTGAGG---GCTATAGAGGCTCAACAGCATCTGTTGAAACTCACGGTCTGGGGCATT
>AF484496
TTCTATTGTAATACATCAGGCCTGTTTAATAGCACCTGGGACAGCAAGACAAATTACACGGAGTCAAAT----
----

AGCACTACTAATATAACTCTTCCATGCAGAATAAAGCAAATTATAAATATGTGGCAGAGAGTAGGACAAGCAA
TGTATGCCCCTCCCATCCAAGGAGTAATAAATTGTGTGTCAGAC---
ATTACAGGACTACTATTAGTAAGAGATGGTGGTCCTGGAAATACA----------------------------
----

TCTGAAACCTTCAGACCTGGAGGAGGAAATATGAGGGACAATTGGAGAGGTGAATTATATAAGTATAAAGTAG
TACAAATTGAACCACTA---GGAGTAGCACCCACCAGGGCAAAGAGAAGAGTGGTG-----------------
----------------------GAGAGAGAAAAAAGAGCA---GTTGGA---ATAGGAGCT---------
GTATTCCTTGGGTTCTTGGGAGCA---GCAGGAAGCACTATG---GGCGCGGCGTCA---
ATAACGCTGACGGTACAG---GCCAGACTA---TTATTG---TCTGGTATAGTGCAACAGCAAAAT---
AATTTG---CTGAGG---GCTATAGAGGCTCAACAGCATCTGTTGAAACTCACAGTCTGGGGCATC
>KJ948659
TTCTATTGTAACACATCAGAACTGTTTAATAGCACTTGGAATGACACTAAC----------------------
----

-------TGGCAGGGATCA------
AATGACACGGAGAACATAACGCTCCCATGCAGAATAAAGCAAATTATAAATATGTGGCAGAGAACAGGACAAG
CGATATATGCCCCTCCCATCCAAGGAGTAATACAGTGTAAATCAAAC---
ATTACAGGACTAATATTAACAAGAGATGGGGGG--

-
AAGAATAACAGTAATAATGAAACCTTTAGACCTATAGGAGGAGATATGAGGGACAATTGGAGAAGTGAATTGT
ATAAGTATAAAGTAGTAAAAATTGAACCCTTA---GGAGTAGCACCCACTAAGGCAAAGAGAAGAGTGGTG--
-------------------------------------GAGAGAGAAAAAAGAGCA---GTGGGA---
CTAGGAGCT---------GTTTTCCTCGGGTTCTTAGGAGCA---GCAGGAAGCACTATG---
GGCGCGGCGTCA---ATAACGCTGACGGCACAG---GCCAGACAA---CTATTG---
TCTGGCATAGTGCAACAGCAAAGC---AATTTG---CTGAGG---
GCTATAGAGGCTCAACAGCATCTGTTGAAGCTCACGGTCTGGGGCATT
>JF683744
TTCTACTGCAATACATCACGGCTATTTAATAATAGCGCAGCATCCAAT-------------------------
----

GGTACTATCCCTATCACAATCCCATGTATCATAAACATGTGGCAGGGAGTGGGGCAGGCAATGTATCAGGCAA
TCTATGCTGGAAACATTGCAGGAAAAAATAAATAGTACATCCATT---ATT-----------
TCAGCAAGAGATGGGGCCAGAGATGGAGGCAATGAT-------------------------------------
----
AGTAACTATACTGAAACCTTCAGGCCTGGGGGAGGAAATATGAAAGACAATTGGAGAAGTGAGCTATATAAAT
ACAAAGTAGTAGAAATTAAACCACTA---GGAGTAGCGCCCACCAGGGCAAAAAGACAAGTGGTG--------
-------------------------------AGAAGAGAAAAAAGAGCG---GCA------CTAGGAGCT---
------TTGTTCCTTGGGTTCTTGGGAGCA---GCAGGAAGCACTATG---GGCGCGGCGTCA---
ATAACGCTGACGGTACAG---GCCAGACAA---TTATTG---TCTGGAATAGTGCAACAGCAAAAT---
AATTTG---CTGAGA---GCTATTGAAGCGCAACAACATCTGTTGCAGCTCACAGTCTGGGGCATT
>AY371164
TTCTATTGTAATACATCAGGCCTGTTTAATAGCACTTGGCCCAATGGCACTGGGCCTAATAATGGCACTTGGA
GC---

AGTGAAAGTGACATTATACAACTCCCATGCAAAATAAAGCAAATTATAAATGTGTGGCAGAGAACAGGACAAG
CAATATATGCCCCTCCCATCCCAGGAGTAATAAGGTGTGAATCAAAC---
ATTACAGGACTACTTTTAACAAGAGATGGTGGGAATGGTGGGAATGAT-------------------------
----

ACCAATCACACTGAGATCTTCAGACCTGGAGGAGGAAATATGAAGGACAATTGGAGAAGTGAATTATATAAAT
ATAAAGTAGTAAAAATTGAACCATTA---GGAATAGCACCCACCAGGGCAAAGAGAAGAGTGGTG--------
-------------------------------GAGAGAGAAAAGAGAGCA---GTCGGA---ATAGGAGCT---
------GTGTTCATTGGGTTCTTGGGAGCA---GCAGGAAGCACTATG---GGCGCAGCGTCA---
ATAACGCTGACGGCACAG---GCCAGACAG---TTATTG---TCTGGTATAGTGCAACAGCAAAGC---
AATTTG---CTAAGG---GCTATAGAGGCTCAACAGCAACTGTTGAAACTCACGGTCTGGGGCATT
>KP718932
TTCTATTGCAATACATCAGGCCTGTTTAATAGCACGTGGAATAACACTGGCAGCATGCAAGACAATAATAGTG
AC---

ACTACTGGCACTATAATTCTCCCGTGCAACATAAAGCAAATTGTAAACATGTGGCAGAGAGTAGGACAAGCAA
TGTATGCCCCTCCCATCCCAGGAATAATAAAATGTGACTCAAAC---
ATTACAGGGATGTTATTGACAAGGGATGGCGGTCTTAATGATAGT----------------------------
----

GCAGAGACTTTCAGACCTGGAGGAGGAGATATGAGAGACAATTGGAGAAGTGAATTATATAAATATAAAGTAG
TAAAAATTGAACCACTA---GGAGTAGCACCCACCAGGGCAAAGAGAAGGGTGGTG-----------------
----------------------GAGAGAGAAAAAAGAGCA---GTTGGA---CTGGGAGCT---------
GTTTTCCTTGGGTTCTTAGGAGCA---GCAGGAAGCACTATG---GGCGCGGCGTCA---
ATAACGCTGACGGTACAG---GCCAGACAA---TTATTG---TCTGGTATAGTGCAACAGCAAAGC---
AATTTG---CTGAGG---GCCATAGAAGCTCAACAGCATATGTTGAGACTCACAGTCTGGGGCATT
>GU332510
TTCTACTGTAACACAACCCAGCTGTTTAATAACATAAGAGGA-------------------------------
----

AATGACACTATCACCCTACCATGTAGAATAAAACAAATTGTAAATATGTGGCAGGGGGTAGGACAAGCAATGT
ATGCTTCTCCCATTGCCGGAAAAATTCAGTGTAACTCAACT---
ATCACAGGTCTCATATTGACAAGAGATGGTGGTAATACCACCAAT----------------------------
----

GGTACCGACACCCTCAGACCTGGAGGGGGAAATATGAAAGACAATTGGAGAAGTGAATTGTATAAATATAAGG
TAGTGAAAATTGAGCCAATA---GGAGTAGCACCCACCAAGGCAAAAAGACAAGTGGTG--------------
-------------------------AGGAGAGAAAAAAGGGCA---GTGGGA---ATGGGAGCT---------
GTGTTCCTTGGGTTCTTAGGAGCA---GCAGGAAGCACTATG---GGCGCAGCGTCA---
ATAACGCTGACGGTACAG---GCCAGACAA---TTATTG---TCCGGTATAGTGCAACAGCAGAGC---
AATCTG---CTGAGG---GCTATAGAGGCTCAACAACATCTGTTGAAGCTCACGGTCTGGGGCATT
>KY392772
TTCTATTGTAATACCTCAGGCCTGTTTAATAGCACTTGGGTCAAGAATGGTACAGACATGAGGGCAATTGACA
CA---
-GGATTAAATGACACA---------
GAATCAAATGGCACTATAACTCTTCAATGCAGAATAAAGCAAATTATAAGAATGTGGCAGAAAGTAGGACAAG
CAATGTATGCCCCTCCCATCCCAGGAGAAATAAGGTGTAACTCAAAC---
ATTACAGGATTACTACTAACAAGGGATGGTGATAAAAACACTAGT----------------------------
----

GAAACCTTCAGACCTGGAGGAGGGGATATGAGGGACAATTGGAGAAGTGAATTATATAAATATAAAGTAGTCA
AAATTGAACCACTA---GGAGTAGCACCTACCAGGGCAAGGAGAAGAGTGGTG--------------------
-------------------GAGAGAGAAAAAAGAGCA---GTTGGA---CTGGGAGCT---------
GTCTTCCTTGGGTTCTTAGGAGCA---GCAGGAAGCACTATG---GGCGCAGCGTCA---
ATAACGCTGACGGTACAG---GTCAGACAA---TTATTG---TCTGGCATAGTGCAACAGCAAAGC---
AATTTG---CTGAGG---GCTATAGAGGCGCAGCAGCATCTGCTGCAACTCACAGTCTGGGGCATT
>EU191618
TTCTATTGCAGTACATCAGGCCTGTTTAATAGCACTTGGGAAAATGGCACTCAGGTGGCAAATGACACGGGG-
----

TCAAATGACACTATAACTCTCCCATGCAGAATAAAGCAAATTGTAAGAATGTGGCAGAGAGTAGGACAAGCAA
TGTATGCCCCTCCCATCCAAGGAATGATAAGATGTGAATCAAAC---
ATTACAGGATTAATATTAACAAGAGATGGTGTTGGGAATAACAGT----------------------------
----

ACAAATGAAACCTTCAGACCTGGAGGAGGAGATATGAGGGACAATTGGAGAAGTGAATTATATAAGTATAAAG
TAGTAAAAATTGAACCATTA---GGAGTAGCACCCAGCAGGGCAAAGAGAAGAGTGGTG--------------
-------------------------GCGAGAGAAAAAAGAGCA---GTTGGA---CTGGGAGCT---------
GTTTTCCTTGGGTTCTTAGGAGCA---GCAGGAAGCACTATG---GGCGCAGCGTCA---
CTAACGCTGACGGTACAG---GCCAGACAA---TTATTG---TCTGGCATAGTGCAGCAGCAGAGC---
AATCTG---CTAAGG---GCTATAGAGGCTCAACATCATCTGTTGCAGCTCACTGTCTGGGGCATT
>EF025170
TTCTATTGTAATACATCAGGCCTGTTTAATAGTACTTGGCCTAATGGCACTCGGAGCGAAGAGGACAAT----
----

GAAACTGACATTATAACTCTCCCATGCAGAATAAAGCAAATTATAAATATGTGGCAGAGAACAGGACAAGCAA
TATATGCCCCTCCCATCCAAGGAGTAATAAGGTGTGAATCAAAC---
ATTACAGGACTACTTTTAACAAGAGATGGTGGGAATGATAGCAAT----------------------------
----

CACACTGAGATCTTCAGACCTGGAGGGGGAAATATGAAGGACAACTGGAGAAGTGAATTATATAAGTATAAAG
TAGTAAAAATTAACCCATTA---GGGGTAGCACCCACCAGGGCAAGAAGAAGAGTGGTG--------------
-------------------------GGAAGAGAAAAAAGAGCA---GTTGGA---CTGGGCGTC---------
GTCCTCCTTGGGTTCTTAGGAACA---GCAGGAAGCACTATG---GGCGCGGCGTCA---
ATAACGCTGACGGTACAG---GTCAGACAA---TTATTG---TCTGGCATAGTGCAACAGCAAAGC---
AATTTG---CTGAGG---GCTATAGAGGCGCAGCAACATCTGCTGCAACTCACAGTCTGGGGCATT
>GU332511
TTCTATTGTGATACATCAGAGCTGTTTAATAGTAGTCTCATTGGTGATAATCTAACTAGTAGTAATACATCT-
----

GATAATAAAACAATCACACTTCAATGTAAGATAAAACAAATTGTAAGAATGTGGCAGAGAGTGGGACAAGCAA
TGTATGCCCCTCCCATCGCAGGAAATATTACATGTAAATCAAAT---
ATTACAGGGCTACTATTAACAAGAGATGGTGGGAATAGCAGTAAA---------------------------
----

AATAATGACACCTTCAGACCTGCAGGAGGAGATATGAGGGACAATTGGAGAAGTGAATTATATAAGTATAAAG
TAGTAAAAATTAAACCACTA---GGAGTAGCACCCACCAAGGCAAGGAGAAGAGTGGTG-------------
------------------------GGGAGAGAAAAAAGAGCA---GTTGGA---GTAGGAGCT---------
GTTCTCCTTGGGTTCTTATCAGCA---GCCGGAAGCACTATG---GGCGCGGCGTCA---
ATAACGCTGACGGTACAG---GCCAGACAA---ATATTG---TCTGGCATAGTGCAACAGCAAAGC---
AATTTG---CTGAGA---GCTGTAGAGGCGCAGCAGCATCTGTTGCAACTCACAGTCTGGGGCATT
>JX422197
TTCTATTGCAATACATCAGGACTGTTTAATAGCACTTGGAAGAACAATGGCAATGGCACTTGGAGGGAAAATA
GCACTGGGGAT---

GATAATACAGGCAACATAACTCTCCAATGTAGAATAAAGCAAATTGTAAATATGTGGCAGAAAGTAGGACAAG
CAATGTATGCCCCTCCCATCCAAGGAGTAATAAGGTGTGAATCAAAC---
ATTACTGGGCTATTATTAACAAGAGATGGAGGAAATACTAATAATACA------------------------
----

ATTAATGAAACCTTCAGACCTGGAGGGGGAGATATGAGGGACAATTGGAGAAGTGAATTATATAAGTATAAAG
TAGTAAAAATTGAACCACTA---GGTGTAGCACCCACCCGTGCAAAAAGAAGAGTGGTG-------------
------------------------GAGAGAGAAAAAAGAGCA---GTAGGA---CTGGGAGCT---------
GTCTTCCTTGGGTTTCTAGGAGCA---GCAGGAAGCACTATG---GGCGCGGCGTCA---
ATAACGCTGACGGTACAG---GCTAGACAA---TTATTG---TCTGGTATAGTGCAACAGCAGAGC---
AATTTG---CTGAAG---GCTATAGAGGCTCAACAACAGCTGTTGAGACTCACAGTCTGGGGCATT
>AF457062
TTCTATTGTAATACATCAAGCCTGTTTAATAGCACCTGGCCCTGGGAGAAGTTAAACAGCACAGATAATAGTA
GC--

ACAGATGACAATATAACTCTCACATGCAGAATAAAGCAAATTATAAATATGTGGCAGAGAGTAGGACAAGCAA
TATATGCCCCTCCTATCCCAGGAGAAATAAGATGTGTATCAAAC---
ATTACAGGACTAATATTAACAAGAGATGGTGGGGATAATAGCAGT---------------------------
----

ACAAATGAAACCTTCAGACCTGGAGGAGGAAATATGAGGGACAATTGGAGAAGTGAATTATATAAGTATAAAG
TAGTAAAAATTGAACCACTA---GGAGTAGCACCCACCACGGCAAGGAGAAGAGTGGTG-------------
------------------------CAGAGAGAAAAAAGAGCA---GTTGGA---ATAGGAGCT---------
GTTTTCATTGGGTTCTTAGGAGCA---GCAGGAAGCACTATG---GGCGCAGCGTCA---
ATAACGCTGACGGTACAG---GCCAGACAA---TTATTG---TCGGGCATAGTGCAACAGCAAAGC---
AATTTG---CTGAGG---GCTATAGAGGCTCAACAGCATCTGTTGAAACTCACAGTCTGGGGCATT
>KY392771
TTCTATTGTAATACAACAAAGTTGTTTAATAGCACCTGGTATAATATCAATAGTACATGGAATACCAATAGCA
GTACT---
------------GGGAAT------
CACACCAATGATACCATTATACTGCCATGTAGAATAAAACAAATTGTAAATATGTGGCAGAGAGTAGGACAAG
CAATGTATGCCCCTCCCATTAGAGGAGAGATTAATTGCAGCTCAAAT---
ATTACAGGAATGATATTGACATTTGACCGTCTGAATAAGAGTGAG---------------------------
----

GTGAATGTGACCTTCAGACCTGGAGGAGGAAATATGAAAGACAATTGGAGAAGTGAATTATATAAATATAAAG
TAGTAAAAATTAGACCACTA---GGAGTAGCACCCACCAAGGCAAGGAGAAGAGTGGTG-------------
------------------------CAGAGAGAAAAAAGAGCA---ATAGGTGGACTGGGAGCT---------
GCCTTCCTTGGGTTCTTAGGAGCA---GCAGGAAGCACTATG---GGCGCGGCGTCA---
ATAACGCTGACGGTACAG---GTCAGACAA---TTATTG---TCTGGCATAGTGCAACAGCAAAGC---
AATTTG---CTGAGA---GCTATAGAGGCGCAACAACATCTGTTGCAACTCACAGTCTGGGGCATT
>KC186563
TTCTATTGCAACACATCAGGCCTGTTTAATAGCACTTGGAGTAATACTACTGTGGCAAATAATACGGGG----
----

GTAAATGGAACTATAATACTCCCATGCAGAATAAAACAAATTATAAATATGTGGCAGAGAGCAGGACAAGCAA
TATATGCCCCTCCCATCCAAGGATTAATAAATTGTGTATCAAAC---
ATTACAGGACTACTATTAACAAGAGATGGTCAGAAGAATGGTAGT----------------------------
----

GACAATGATACCTTTAGACCTATAGGAGGAAATATGAAGGACAATTGGAGAAGTGAATTATATAAATATAAAG
TGGTGGAGATTAGACCATTA---GGAGTAGCACCCACCAGGGCAAAAAGAAGAGTGGTG--------------
-------------------------GAGAGAGAAAAAAGAGCA---GTGGGA---ATAGGAGCT---------
GTGCTCTTTGGGTTCTTGGGAGCA---GCAGGAAGCACTATG---GGCGCGGCGTCA---
ATAACGCTGACGGTACAG---GCCAGACAA---TTGTTG---ACTGGCATAGTGCAACAGCAAAGC---
AATTTG---CTGCAG---GCTATAGAGGCACAACAGCATCTATTAAAGCTCACTGTCTGGGGCATT
>GU201508
TTCTATTGCAATACATCACTCCTGTTTAACAGCACCTGGATGGTGAATGGCACTGGGGTCACCAATGGCAATA
AT---

GTCACCAATGACATTATAACACTCCCATGCAGAATAAAGCAAATTATAAATATGTGGCAGAGGGTAGGACAAG
CAATGTACGCCCCTCCCATCCGAGGAATCATAAATTGTGTATCAAAC---
ATTACAGGACTACTCTTAACAAGAGATGGTGGTAGCAGCCAAAAC----------------------------
----

GGATCTGAGACCTTCAGACCTGGAGGAGGAGATATGAGAGACAATTGGAGAAGTGAATTATATAAGTATAAAG
TAGTAAAAATTGACCCAATA---GGAGTAGCACCCACCAGGGCAAAAAGAAGAGTGGTG--------------
-------------------------GAGAGAGAAAAAAGAGCA---CTTGTTGGACTGGGAGCT---------
GTCTTCCTTGGGTTCTTAGGAACA---GCAGGAAGCACTATG---GGCGCGGCATCA---
CTAACGCTGACGGTACAG---GCCAGACAA---TTATTG---TCTGGCATAGTGCAACAGCAGAGC---
AATCTG---CTGAGG---GCTATAGAGGCTCAACAGCATTTGTTGAGACTCACGGTCTGGGGCATT
>KP718927
TTCTATTGCAATACAACACAACTGTTTAATAGTACCTGGCAAGAGGGCACCAACAACAGCAATGCAGGT----
----

AATGATACTACTATTACACTGCCATGCAGAATAAAACAAATTGTAAACATGTGGCAGAGAGTAGGACAAGCAA
TGTATGCCCCTCCCATCCAAGGAGCGATAAAGTGTGTATCTAAC---
ATTACAGGACTAATATTGACAAGAGATGGTGGGAATAATAGGACA----------------------------
----

AATGAGACCATCAGACCTGGAGGAGGGGATATGAGGGACAATTGGAGAAGTGAATTATATAAATATAAAACAG
TGAGAATTGAACCACTA---GGAATAGCACCCACTCATGCAAGGAGAAGAATGGTG-----------------
----------------------AACAGAGAAAAAAGAGTA---GCTGGA---ATGGGAGCT---------
GTCTTCTTTGGGTTCTTAGCAGCA---GCAGGAAGCACTATG---GGCGCAGCGTCA---
ATAACGCTGACGGTACAG---GCCAGACAA---CTATTG---TCTGGTATAGTGCAACAGCAAAGC---
AATTTG---CTGAAT---GCTATAGAGGCTCAACAGCAGCTGTTGAAACTCACGGTCTGGGGCATT
>KP718919
TTCTATTGCAATACATCGGGCCTGTTTAATAGCACTTGGGGAACTGGGGACTACACTGCAGGAAATGACACTG
TA---

GGAAATGACACTATAACTCTCCGGTGCAGAATAAAGCAAATTATAAATATGTGGCAGAAAGTAGGACAAGCAA
TGTATGCCCCTCCCATCCCAGGAGTAATAAGGTGTGAGTCAAAT---
ATTACAGGACTACTCTTGACAAGAGATGGTGGGAATAATAATAGT----------------------------
----

ATAAATGAGACTTTCAGACCTGGAGGAGGAGATATGAGAGACAATTGGAGAAGTGAATTATATAAATATAAAG
TAGTAAAAATTGAACCACTA---GGAATAGCACCTACCCAGGCAAAGAGAAGGGTGGTG--------------
-------------------------GAGAGAGAAAAAAGAGCA---GTTGGA---TTAGGAGCT---------
TTCTTCCTTGGGTTCTTAGGAGCA---GCTGGAAGCACTATG---GGCGCAGCATCA---
CTAACGCTGACGGTACAG---GCCAGACAA---TTATTG---TCTGGCATAGTGCAACAGCAAAGC---
AATTTG---CTAAGG---GCTATAGAGGCTCAACAACAGATGTTGAGACTCACGGTCTGGGGCATT
>KY359380
TTTTATTGCAATACATCAGGCCTGTTTACTAATGGCACAAATATAACT-------------------------
----

ACAAATATAAATATAACTCTCCCGTGCAGAATAAAACAAATTATAAATATGTGGCAGAGAGTAGGACAAGCAA
TGTATGCCCCTCCCATCGCCGGAGTAATAAGATGTGACTCAAAC---
ATTACAGGACTACTGTTAACAAGAGATGGTGGAATTAACAATGAC---------------------------
----

AGTAATACTGAGGTCTTCAGGCCTACAGGAGGAGATATGAGGGACAATGGGAGAAGTGAATTATACAAATATA
AAGTAGTAAAACTTGAACCCCTA---GGAGTAGCACCCACCAGGGCAAGGAGAAGAGTGGTG----------
---------------------------GGAAGAGAAAAAAGAGCA---GTTGGA---CTGGGAGCT------
---GCCTTCCTTGGGTTCTTAGGAGCA---GCAGGAAGCACTATG---GGCGCAGCGTCA---
CTAACGCTGACGGTACAG---GCCAGACAA---TTATTG---TCTGGCATAGTGCAACAGCAAAGC---
AATTTG---CTGAGG---GCTATAGAGGCTCAACAACATATGTTGAAACTCACAGTCTGGGGCATT
>KJ849789
TTCTATTGCAATACATCAGGCCTGTTTAATAGCACTTGGAATAACACACAGCCA------------------
----
-GATAACACAACAGAT---------
AACACAACAGAGTTCATAACTCTGCCATGCAGAATAAAGCAAATCATAAATATGTGGCAGAGAGTAGGACAAG
CAATGTATGCCCCTCCCATTCAAGGAATAATAAATTGTACATCAAAT---
ATTACAGGGCTAATATTAACAAGAGACGGT--

-
AATCCTAATGCGAGTAGTGAAACCTTCAGACCTGGAGGAGGGGATATGAGGGACAATTGGAGAAGTGAACTAT
ATAAGTATAAAGTAGTACAAATTGAACCACTA---GGAGTAGCACCCACCTGGGCAAGGAGAAGAGTGGTG--
------------------------------------CAGAGAGAAAAAAGAGCA---GTTGGA---
CTGGGAGCT---------GTCTTCCTTGGGTTCTTGGGAGCA---GCAGGAAGCACTATG---
GGCGCAGCGTCA---ATGACGCTGACGGTACAG---GCCAGACAA---TTATTG---
TCTGGTATAGTGCAACAGCAGAGC---AATCTG---CTGAAG---
GCTATAGAGGCTCAACAACATCTGTTGAAACTCACAGTCTGGGGCATT
>FJ388929
TTCTATTGCAATACATCAGGCCTGTTTAATAGTACTTACAATGGGACAGAGGAGGCAAATGAAAATGACACTA
AG--

GCAAATGACACTATAATACTTCCATGCAGAATAAAGCAATTTATAAATATGTGGCAGAGAGTAGGAAAAGCAA
TGTATGCCCCTCCCATTCAAGGAGTAGTACAGTGTAATTCAAGC---
ATTACAGGATTATTATTAACAAGAGATGGTGGTAGTAAAAATGAG---------------------------
----

ACTGGGGAAACATTCAGACCTGGAGGAGGAAATATGAGAGACAATTGGAGAAGTGAATTATATAAGTATAAAG
TAGTAAGGATTGAACCACTA---GGAGTAGCACCCACCAAGGCAAAGAGAAGAGTGGTG-------------
------------------------GAGAGAGAAAAAAGAGCA---GTCGGA---CTGGGAGCG---------
GTTTTCCTTGGGTTCTTAGGAACA---GCAGGAAGCACTATG---GGCGCAGCGTCA---
ATAACGCTGACGGTACAG---GCCAGACAG---CTATTA---TCTGGCATAGTGCAACAGCAGAGA---
AATCTG---CTGCAG---GCTATAGAGGCTCAACAGCATCTGTTGAAACTCACAGTTTGGGGCATT
>EU191612
TTCTATTGCGATACAACAAAATTGTTTACAAACTTGTCTACAAATAGCACGGGGTTGGATGATAATAGCACGG
GG--

TCGAATGATACCATAATTCTCCCGTGCAGAATAAAACAAGTTATAAATATGTGGCAGAGAGTAGGACAAGCAA
TGTATGCCCCTCCTATCCGAGGAGTAATAAGGTGTAACTCTAAC---
ATTACAGGACTACTATTAACAAGAGATGGTGGGAATGGGAATAAC---------------------------
----

AGTACAGAGGAGATTTTCAGACCTGGAGGAGGAGATATGAGGGATAATTGGAGAAGTGAATTATATAAGTATA
AAGTAGTAAAAATTGAACCACTG---GGAGTAGCACCCACCAGGGCAAGGAGAAGAGTGGTG----------
---------------------------CAGAGAGAAAAAAGAGCA---GTTGGA---CTGGGAGCT------
---GTCTTCCTTGGATTCTTAGGAGCA---GCAGGAAGCACTATG---GGCGCGGCGTCC---
ATAACGCTGACGGTACAG---GCCAGACAA---TTATTG---TCTGGCATAGTGCAACAGCAGAGC---
AATTTG---CTGAGG---GCTATAAAGGCTCAACAACATCTGTTGAAACTCACGGTCTGGGGCATT
>AF069672

TTCTATTGTAATACATCAAGCCTGTTTAACAGCACGTGGAGTAACGATAACAACACTCAGGGGTCAAATAGCA
CA---

GAGACAAAAGGCACTATAACTCTCCCATGCAGAATAAAGCAAATTATAAATATGTGGCAGAGAGCAGGAAAAG
CAATGTATGCCCCTCCCATCCAAGGAGTAATAAGGTGTGAATCAAAC---
ATCACAGGACTAATATTAACAAGAGATGGTGGAGATGCCGGGGAA----------------------------
----

AATGAAACCTTCAGACCTGGAGGAGGAGATATGAGGGACAATTGGAGAAGTGAGTTATATAAGTATAAAGTAG
TAAAAATTGAACCACTA---GGAGTAGCACCCACCCAGGCAAGGAGAAGAGTGGTG-----------------
----------------------AAGAGAGAAAAAAGAGCA---GTTGGA---CTAGGAGCT---------
GTCTTCATTGGGTTCTTAGGAGCA---GCAGGAAGCACTATG---GGCGCGGCGTCA---
ATAACGCTGACGGTACAG---GCCAGACAA---TTATTG---TCTGGCATAGTGCGACAGCAAAGT---
AATTTG---CTGAAG---GCTATAGAGGCTCAACAACATCTGTTGAAACTCACGGTCTGGGGCATT
>AF411965
TTCTACTGCAATACATCAAGACTGTTTAATAGCACATTTGATAACACATCTCTGGTTAATGGCACGGGTAATA
GTACCAAC---
-------------ACACAG------
GGGGCAAATGACACTATATATATTCCATGCAGAATAAAACAGATTATAAACATGTGGCAAAGAGTGGGACAAG
CAATGTATGCCCCTCCCATTGCAGGAGTAATAAAATGTGAATCAAAC---
ATTACAGGAATGCTATTAACAAGAGACGGTGGGTCTGAAAGTAAC----------------------------
----

GGTACAGAGGTCTTCAGACCTGGAGGAGGAGATATGAAGGACAATTGGAGAAGTGAATTATATAATTATAAAG
TAGTAAAAATTGAACCACTA---GGAGTAGCACCCACCAGGGCAAAGAGAAGAGTGGTG--------------
-------------------------GAGAGAGAAAAAAGAGCA---GCTGGA---ATAGGAGCT---------
GTCCTCCTTGGCTTCTTAGGAGCA---GCAGGAAGCACTATG---GGCGCGGCGTCA---
ATAACGCTGACGGTACAG---GCCAGGAAA---TTATTG---ACTGGCATAGTGCGGCAGCAAAGC---
AATTTG---CTGCAG---GCTATAGAGGCTCAACAACATCTGTTGAAACTCACGGTCTGGGGCATT
>U86778
TTCTATTGCAATACATCAGGCCTGTTTAATAGTACATACAATAGGGCATACACGACTAATGGTACAGGA----
----

AATGACTCAACCATCACACTCCCATGCAGAATAAAACAAATTATAAACATGTGGCAGGAGGTAGGGCGAGCAA
TATATGCCCCTCCCATTGCAGGAAACATAACATGTATATCAAAT---
ATCACAGGACTATTATTGCTACGTGATGGAGGAAAAGAAGACAAT----------------------------
----

AAAACAGAGATGTTCAGACCTGGAGGAGGAGATATGAGGGACAATTGGAGAAGTGAATTATATAAATATAAAG
TGGTAGAAATTAAACCATTG---GGAATAGCACCCACTAAGCCACCCAGGAGAGTGGTG--------------
-------------------------GAGAGAGGTGGAAGAACA---GTGGGA---ATAGGAGCT---------
GTGTTCCTTGGGTTCTCGGGAGCA---GCAGGAAGCACTATG---GGCGCGGCGTCA---
ATAACGCTGACGGTACAG---GCCAGACAA---CTGTTG---TCTGGTATAGTGCAACAGCAAAGC---
AATTTG---CTGAAG---GCTATAGAGGCTCAACAGCATCTGTTGAAACTCACGGTCTGGGGCATT
>L22955
TTCTATTGCAATACATCAAACCTGTTTAATAGTACATACAATCCTAATAGTACAGAAAGTAAT----------
----

TCAGACTTAAACATCACACTCCCATGCAGAATAAAACAAATTATAAACATGTGGCAGAGAGTAGGACGAGCAA
TGTATGCCCCTCCCATTGCAGGAGTAATAAAGTGTACATCAAAC---
ATTACAGGAATAATATTGTCAAGAGATGGTGGGAACAGTGGGAACAGT-------------------------
----

ACCAATGGGACTGAGACCTTCAGACCTGGAGGAGGAGATATGAGGGACAATTGGAGAAGTGAATTATATAAGT
ATAAAGTAGTAAAACTAGAACCACTA---GGAGTAGCACCCACCAAGCCAAGGAGAAGAGTGGTG--------
-------------------------------GAGAGAGAAAAAAGAGCA---GTTGGA---CTGGGAGCT---
------GTCTTCCTTGGGTTCTTGGGAGCA---GCAGGAAGCACTATG---GGCGCGGCATCA---
ATAACGCTGACGGTACAG---GCCAGACAA---CTGTTG---TCTGGTATAGTGCAACAGCAAAGC---
AATTTG---CTGAAG---GCTATAGAGGCTCAACAGCATCTGTTGAAACTCACGGTCTGGGGCATT
>MK086111

TTCTATTGTAATACATCAAGCCTGTTCGATAGTGCTTGGCTTAATAATGAAACTGGCATCGGGCAAAACATCA
CAGAG--

TCAAATAATACTATAACTCTCCAATGCAGAATAAAACAATTTATAAATATGTGGCAGAGAGTAGGGCAAGCAC
TGTATGCCCCTCCTATTCCAGGAAACATAACCTGTAGATCAAAC---
ATTACAGGGCTGCTACTAACAAGAGATGGTGGGAATAATACGAGTGATAATGGT-------------------
----

ACAGTTATTGAAACATTCAGACCTGGAGGAGGAGATATGAGGGACAATTGGAGAAGTGAATTATATAAATATA
AAGTAGTAAAACTTGAACCACTA---GGAATAGCACCCACCGAGGCAAGGAGAAGAGTGGTG-----------
----------------------------GAGAGAGAAAAAAGAGCA---ATTGGA---CTAGGAGCT------
---TTTTTCCTTGGGTTCTTGGGAGCA---GCAGGAAGCACTATG---GGCGCAGCGTCA---
CTAACGCTGACGGTACAG---GCCAGACAA---TTATTG---TCTGGTATAGTGCAACAGCAAAAC---
AATTTG---CTGAAG---GCTATAGAGGCTCAGCAGCTCATGTTGAAACTCACGGTCTGGGGCATT
>HQ385818
TTCTACTGTAATACATCACAATTGTTTAATAGTACTTGGAAGAATGGTACTTTTGCCAATGGCACTGAA----
----

GGAAATGGCACAATCACACTCCCATGCAGAATAAAGCAATTTATAAACATGTGGCAACAAGTAGGAAAAGCAA
TGTATGCCCCTCCCATCAGTGGACAAATTAACTGCTCATCAAAT---
ATTACAGGGCTACTATTAACAAGAGATGGTGGTAGTGGTAACAGCACA-------------------------
----

GAGAACGAGACTGAGATCTTCAGACCTGCAGGGGGAGATATGAGGGACAATTGGAGAAGTGAATTATATAAAT
ATAAAGTAGTACAAGTTGAGCCATTA---GGATTAGCACCCACCAGGGCAAAGAGAAGAGTGGTG--------
-------------------------------CAGAGAGAAAAAAGAGCA---ATAGGC---CGGGGAGCT---
------GCCTTCCTTGGGTTCTTAGGAGCA---GCAGGAAGCACTATG---GGCGCGGCGTCA---
ATGACGCTGACGGTACAG---GCCAGACAA---TTATTG---TCTGGCATAGTGCAACAGCAAAGC---
AATTTG---CTGAGG---GCCATAGAGGCTCAGCAACATATGCTGAAACTCACGGTCTGGGGCATT
>MN485971
TTCTATTGCAATACATCAGGCCTGTTTAATAGTATTTGGAATAGTAGCACTTGGAATAATAATAGCACTGGGA
ATAACAGCACA---CAG------GAG---

TCAAATGACACTATAACTCTCCAATGCAGTATAAAACAAATTATAAACATGTGGCAGGGAGTAGGAAAAGCAA
TGTATGCCCCTCCCATTGAAGGAAAGATTGCATGTTTATCACAA---
ATTACAGGACTAATATTAACAAAAGATGGTGGTGCAAAAAATAAT----------------------------
----

AGTCAGAATGAGACTTTCAGACCTGCAGGAGGAGATATGAGAGACAATTGGAGAAGTGAATTATACAAATATA
AAGTAGTAAGACTTGAACCACTA---GGTCTAGCACCCACCAGGGCAAGGAGAAGAGTGGTG-----------
----------------------------GTAAGAGAAAAAAGAGCA---ATAGGA---CTAGGAGCT------
---ATGTTCCTTGGGTTCTTGGGAGCA---GCAGGAAGCACAATG---GGCGCAGCGTCA---
CTGACGCTGACGGTACAG---GCCAGACAA---TTACTG---TCTGGTATAGTGCAACAGCAGAAC---
AATTTG---CTGAGG---GCTATTGAGGCGCAACAGCATATGTTGCAACTCACAGTCTGGGGCATC
>AJ277825
TTCTATTGTAATACATCAGGCCTGTTTAATAGCACTTGGAGCCTTAACAGCAGCGCGCCAGATGACACAGAA-
----

TCAAATGACACTATAACTCTCCCATGCAGAATAAAGCAAATTATAAACATGTGGCAAAGGGCAGGACGAGCAA
TATATGCCCCTCCCATCCAAGGAATAATAAGGTGTAATTCAAGC---
ATTACAGGACTACTATTAACAAGAGATGGTGGGGATAACAATACG----------------------------
----

CATAATGAAACCTTCAGACCTGGAGGTGGAAATATGAGGGATAATTGGAGAAGTGAACTATATAAATATAAAG
TAGTAAAAATTGAACCACTA---GGAGTAGCACCCACCAAGGCAAAAAGAAGAGTGGTG--------------
-------------------------GAGAGAGAAAAAAGAGCA---GTTGGA---CTGGGAGCT---------
GTCTTCATTGGGTTCTTAGGAGCA---GCAGGAAGCACTATG---GGCGCGGCGTCA---
ATAACGCTGACGGTACAG---GCCAGACAA---TTATTG---TCTGGCATAGTGCGACAGCAAAGC---
AATTTG---CTGAGG---GCTATAGAGGCTCAACAGCATCTGTTGAAACTCACGGTCTGGGGCATT
>HQ595799

```
TTCTATTGTAACACATCAAGACTGTTTAATAGCACTTGGCATGATAACAGCACGCTAGAGTCAAATCAAACAG
AG-----------------------------------------------------------------------
----------------------------
TCAAACGATACTATAATACTCCCATGCAGAATAAAACAAATTATAAACATGTGGCAGAGAGCAGGACAAGCAA
TGTATGCCCCCCCCATTCAAGGAGTGATAAGGTGTGTATCAAAT---
ATTACAGGACTAATATTAACAAGAGATGGTGGGGATAATAACAGC----------------------------
-------------------------------------------------------------------------
-------------
GCAAACGAAACCTTCAGACCTGGAGGAGGAGATATGAGGGACAATTGGAGGAGTGAATTATATAAATATAAAG
TAGTAAAAATTGAACCAATA---GGAATAGCACCCACCGAGGCAAGGAGAAGAGTGGTG--------------
-------------------------CAGAGAGAGAAAAGAGCA---GTAGGA---CTAGGAGCT---------
GTTTTCATTGGGTTCTTAGGAGCA---GCAGGAAGCACTATG---GGCGCGGCGTCA---
ATGACGCTGACGGTACAG---GCCAGACAA---TTATTG---TCTGGCATAGTGCAACAGCAAAGC---
AATTTG---CTGAAG---GCTATAGAAGCTCAACAGCATCTGTTGAAACTCACGGTCTGGGGCATT
>AY322191
TTCTATTGTACTACCTCAGGCCTGTTTAATGGCACTTGGGACAGCAAGCAGAAGTCAAATAGCACGGAG----
-------------------------------------------------------------------------
----------------------------
TCAGATGACACTATAACTCTCCCATGCAAAATAAAGCAAATTATAAATATGTGGCAGAGGGTGGGACAAGCAA
TGTATGCCCCTCCTATCCAAGGAATAATAAGTTGTGTATCAAAC---
ATTACAGGACTAATATTAACAAGAGATGGTGGGGGTAATAACAGT----------------------------
-------------------------------------------------------------------------
-------------
AGCAATGAAACCTTCAGACCTGGAGGAGGAGATATGAGAGACAATTGGAGAAGTGAATTATATAAGTATAAAG
TAGTAAAAATTGAACCACTA---GGAGTAGCACCCACCAGGGCAAGGAGAAGAGTGGTG--------------
-------------------------GGGAGAGAAAAAAGAGCA---GTTGGA---ATGGGAGCT---------
GTCTTCCTTGGGTTCTTAGGAGCA---GCAGGAAGCACTATG---GGCGCGGCGTCA---
ATAACGCTGACGGTACAG---GCCAGACAA---TTATTG---TCTGGCATAGTGCAACAGCAAAGC---
AATTTG---CTGAGA---GCTATAGAGGCTCAACAACATCTGTTGAAACTCACGGTCTGGGGCATT
>JN977603
TTCTATTGCAACACATCACAGCTGTTTAATTCATCACTGCTCAATAGTACAGAAAGTATTAATGAAACAAGT-
-------------------------------------------------------------------------
----------------------------
GGTAATGAAACAATCACACTCCCATGCAGAATAAAACAGATTATAAACATGTGGCAGGAGGTAGGGCGGCAA
TGTATGCCCCTCCCATTAGAGGAATAATAACATGTAAATCAAAT---
ATCACAGGACTACTACTGACACGTGATGGAGGAAATGAGAAAAATAACTCA----------------------
-------------------------------------------------------------------------
----
GAGGCAAATAACACAGAGATATTCAGGCCTGGAGGAGGAAATATGAAGGACAATTGGAGAAGTGAATTATATA
AGTATAAAGTAGTAAAAGTTGAACCACTA---GGAGTAGCACCCACCAAGGCAAAGAGAAGAGTGGTG-----
----------------------------------GAGAGAGAAAAAAGAGCA---GTAGGA---
ATAGGAGCT---------GTCTTCATTGGGTTCTTAGGAGCA---GCCGGAAGCACTATG---
GGCGCAGCGTCG---GTGACGCTGACGGTACAG---GCCAGACAA---TTATTG---
TCTGGCATAGTGCAACAGCAAAGT---AATTTG---CTGAGG---
GCTATAGAGGCTCAACAACATTTGTTGAAACTCACGGTCTGGGGCATT
>AY887516
TTCTATTGTGATACAAAAAACCTGTTTAATAGCACTTGGAACGACAGTAACATCAACATCACACAGGAGTCAA
ATGAC--------------------------------------------------------------------
-------------------ACA---
GATTCAAAAGGCAATATAACTCTCCAGTGCAGAATAAAGCAAATTATAAATATGTGGCAGAGAGCAGGACAAG
CAATATATGCCCCTCCCATCCCGGGAGTAATAAGATGTGAGTCGAAC---
ATTACAGGAATAATATTAACAAGAGATGGTGGGAATGTTAGCAGT----------------------------
-------------------------------------------------------------------------
-------------
CAAAATGAAACCTTCAGACCTGGAGGAGGAGATATGAGGGACAATTGGAGAAGTGAATTATATAAGTATAAAG
TAGTAAAAATTGAACCACTA---GGAGTAGCACCCTCCAAGGCAAAGAGAAGAGTGGTG--------------
-------------------------GAGAGAGAAAAAAGAGCA---GTCGGA---ATAGGAGCT---------
GTTTTCCTTGGGTTCTTAGGAGCA---GCAGGAAGCACTATG---GGCGCGGCGTCA---
CTGACGCTGACGGTACAG---GCCAGACAA---TTATTG---ACTGGCATAGTGCAACAGCAAAGC---
AATTTG---CTGAGG---GCTATAGAGGCTCAACAACATCTGTTGAAACTCACAGTCTGGGGCATT
```

>HQ540690
TTCTATTGTAATACATCAGGCCTGTTTAATAGCACTTGGAACTCTACTAATGGAAATTGGACTCAAAACGAAA
CTGAC---

AATAAGACTGAAACTATAAGTCTCCCATGCAGAATAAAGCAAATTATAAACATGTGGCAGAGAGTAGGACAAG
CAATGTATGCCCCTCCCATACAAGGAGTAATAAGCTGTAACTCAAGC---
ATTACAGGACTGCTACTAACAAGAGATGGTGGTTACAATAATAGC----------------------------
----

AATAATGAAACCTTCAGACCTGTAGGAGGAAACATGAAGGACAATTGGAGAAGTGAATTATATAAGTATAAAG
TAGTAAAAATTGAGCCACTA---GGAATAGCACCCACCAGGGCAAAAAGGAGAGTGGTG--------------
-------------------------GAGAGAGAAAAAAGAGCA---GTGGGA---TTCGGAGCT---------
TTGTTCCTTGGGTTCCTGGGAGCA---GCAGGAAGCACTATG---GGCGCAGCATCA---
ATAACGCTGACGGTACAG---GCCAGACAA---TTATTG---TCGGGTATAGTGCAACAGCAAAAC---
AATTTG---CTGAGG---GCTATAGAGGCGCAACAGCATATGTTGCAACTCACAGTCTGGGGCATT
>DQ093606
TTCTACTGCAATACATCAAGATTGTTTAATAGCACATTTAAGGTCAAGGACACAGATAATAATGCTGGGGACA
AC---

ACAGAAGAGGATATAACTATCCCATGCAGAATAAAACAAATTATAAACATGTGGCAGAGAGTAGGACAAGCAA
TGTATGCCCCTCCCATTGCAGGAGTAATAAAATGTGAATCAAGC---
ATCACAGGAATACTATTGACAAGAGATGGGGGGGAGTCTGATAAC----------------------------
----

AAAACAGAAGAGGTCTTCAGACCTGAAGGAGGAAATATGAAGGACAATTGGAGAAGTGAATTATATAATTATA
AGGTAGTAAAAATTGAACCACTA---GGAGTAGCACCCACCAAGGCAAAGAGAAGAGTGGTG-----------
----------------------------GAGAGAGAAAAAAGAGCAGTAGTTGGA---CTGGGAGCT------
---GTTTTCCTTGGGTTCTTAGGAGCA---GCAGGAAGCACTATG---GGCGCAGCATCG---
ATAACGCTGACGGTACAG---GCCAGGCAA---TTATTG---ACTGGCATAGTGCGGCAGCAAGGC---
AATTTG---CTGCAG---GCTATAGAGGCTCAACAACATCTGTTGAAGCTCACTGTCTGGGGCATT
>HQ540691
TTCTACTGCAATACATCAGGCCTGTTTAAGGGCACATTTCAGGTTAATGACACGGGTAATAGTAACAGCACAC
AG---

GGGGCAAATGACACCATAACTATCCCATGCAGAATAAAACAGATTATAAATATGTGGCAAAGAGTGGGACAAG
CAATGTATGCCCCTCCCATTGAAGGAGTAATAAAATGTAAGTCAAAC---
ATTACAGGAATGCTATTGACAAGAGACGGCGGGGCTGAAAGCAAT----------------------------
----

ACAGAGGTCTTCAGACCTGGGGGAGGAGATATGAAGGACAATTGGAGAAGTGAATTATATAATTATAAAGTAG
TAAAAATTGAACCAGTA---GGAGTAGCACCCGCCAAGGCAAAGAGAAGAGTGGTG-----------------
----------------------GAGAGAGAAAAAAGAGCA---GTTGGA---ATAGGAGCT---------
GTCCTCCTTGGGTTCTTAGGAGCA---GCAGGAAGCACTATG---GGCGCAGCGTCA---
ATAACGCTGACGGTACAG---GCCAGACAA---TTATTG---ACTGGCATAGTGCGGCAGCAAAGC---
AATTTG---CTGCAG---GCTATAGAGGCTCAACAACATCTGTTGAAACTCACGGTCTGGGGCATT
>HQ595773
TTCTATTGCAATACATCAAAACTGTTTAATAGCACTTGGAAGAATGACACTTGGGAGTCAAATAGCACAGAG-
----

TCAAATGGCACTATATATCTTAATTGCAGAATAAAACAAATTATAAACATGTGGCAGGGAGTAGGAAAAGCAA
TGTATGCCCCTCCCATTGAAGGAATGATTACATGTATATCAAAT---
ATTACAGGGATATTATTAACAAGAGATGGTGGTGCAAATAATAAT----------------------------
----

AGTCAGAATGAGACTTTCAGACCTGGAGGAGGAGATATGAGAGACAATTGGAGAAGTGAATTATATAAATATA
AAGTAGTAAGGCTTGAACCACTA---GGTCTAGCACCCACCAAGGCAAGGAGAAGAGTGGTG-----------
----------------------------ACAAGAGAAAAAAGAGCA---ATAGGA---CTAGGAGCT------
---ATGTTCCTTGGGTTCTTGGGAGCA---GCAGGAAGCACAATG---GGCGCAGCGTCA---
CTGACGCTGACGGTACAG---GCCAGGCAG---TTATTG---TCTGGTATAGTGCAGCAGCAAAAC---
AATTTG---CTGATG---GCTATAGAGGCGCAACAACATTTGTTGCAACTCACAGTCTGGGGCATT

```
>HQ595793
TTCTATTGCAATACATCAGGCCTGTTTAATAGCACTTGGAATAACAGCACACAGGTGTCAAATGACACACAG-
-------------------------------------------------------------------------
----------------------------
TTAAATGACACTATAACTCTCCAATGCAGGATAAAACAAATTATAAACTTGTGGCAGGGAGTAGGAAAAGCAA
TGTATGCTCCTCCCATTGAAGGAAATATTACATGTTTATCAAAT---
ATTACAGGACTAATATTAACAAGAGATGGTGGTGCAAATACTACT----------------------------
-------------------------------------------------------------------------
----------
AGTCAGAATGAGACTCTCAGGCCTGCAGGAGGAGATATGAGAGACAATTGGAGAAGTGAATTATACAAATATA
AAGTAGTAAGACTTGAACCACTG---GGTCTAGCACCCACCAGGGCAAGGAGAAGAGTGGTG-----------
----------------------------GCAAGAGAAAAAAGAGCA---ATAGGA---CTAGGAGCT------
---GTGTTCCTTGGGTTCTTGGGAGCA---GCAGGAAGCACAATG---GGCGCAGCGTCA---
CTGACGCTGACGGTACAG---GCCAGACAA---TTATTG---TCTGGTATAGTGCAGCAGCAAAAC---
AATTTG---CTGAGG---GCTATAGAGGCGCAACAACACTTGTTGCAACTCACAGTCTGGGGCATT
>L22942
TTCTACTGCAATACATCAAAACTGTTTAATAGTGTATGGGGAAATTCAAGTAATGTGACAAAGGTAAATGGGA
CA-----------------------------------------------------------------------
-------------------------
AAGGTAAAAGAGACAATCACACTCCCATGTAAAATAAAACAAATTATAAACATGTGGCAGGAAGTAGGACGAG
CAATGTATGCCCCTCCCATTGAAGGACTCATCAACTGTTCATCAAAT---
ATTACAGGACTATTGTTGACAAGAGATGGTGGTGAAAATAATAGT----------------------------
-------------------------------------------------------------------------
----------
AGTCAGAATGAGACCTTCAGACCTGGAGGAGGAGATATGAGAGACAATTGGAGAAGTGAATTATACAAATATA
AAGTAGTAAAAATTGAACCACTA---GGTCTAGCACCCACCAGCGCAAAGAGAAGAGTGGTG-----------
----------------------------GAAAGAGAAAAAAGAGCA---ATAGGA---CTGGGAGCT------
---ATGTTCCTTGGGTTCTTGGGAGCA---GCAGGAAGCACGATG---GGCGCAGCGTCA---
ATGACGCTGACGGTACAG---GCCAGACAG---TTAATG---TCTGGTATAGTGCAACAGCAAAAC---
AATTTG---CTGAGG---GCTATAGATGCGCAACAGCATCTGTTGCAACTCACAGTCTGGGGCATT
>DQ208482
TTCTACTGTAACACATCAGAACTGTTTAATAGTGTATGGGCAAATGATAGTACAGAAAATAATAGTACATGGA
AC-----------------------------------------------------------------------
----------------------------
AATGAGAGTATAATCACACTCCCATGCAGAATAAAACAATTTATAAACATGTGGCAGGGAGTAGGACGAGCAA
TGTATGCCCCTCCCATTGAAGGATACATCAACTGTTCATCAAAT---
ATTACAGGACTATTGCTGACAAGAGATGGTGGTATAAATAATAATAGT-------------------------
-------------------------------------------------------------------------
----------
CATGATAATGAGACCTTCAGACCTGGAGGAGGAGATATGAGAGACAATTGGAGAAGTGAATTATACAAATATA
AAGTAGTAAAAATTGAACCACTA---GGTCTAGCACCCACCAAGGCAAGGAGAAGAGTGGTG-----------
----------------------------GAAAAAGAAAAAAGAGCA---ATAGGA---CTAGGAGCT------
---ATGTTCCTTGGGTTCTTGGGAGCA---GCAGGAAGCACGATG---GGCGCAGCGTCA---
CTGGCGCTGACGGGACAG---GCCAGACAG---TTAATG---TCTGGTATAGTGCAACAGCAAAAC---
AATTTG---CTGAGG---GCTATAGATGCGCAACAGCATCTGTTGCAACTCACAGTCTGGGGCATT
>U36866
TTCTACTGCAATACATCAGACCTGTTTAATAGTACATGGGAGAGTAATAATACATCAAATAGTACAGGGTCA-
-------------------------------------------------------------------------
----------------------------
AATAATGGTACAATCATACTTCCATGCAGAATAAAACAAATTATAAACCTGTGGCAGCAAGTAGGAAAAGCAA
TGTATGCCCCTCCCATCGAAGGAATAATCACGTGTAGATCAAAT---
ATTACAGGGCTATTACTGACAAGAGATGGGGGTAGTAATAATAGT----------------------------
-------------------------------------------------------------------------
-------------
CATAACGAGACCTTCAGACCTGGAGGAGGAGATATGAGAGACAATTGGAGAAGTGAATTATACAAATATAAAG
TAGTAAAAATTGAACCACTA---GAAGTAGCACCCACCAGGGCAAAGAGAAGAGTGGTG--------------
-------------------------GAGAGAGAAAAAAGAGCA---GTTGGA---ATAGGAGCT---------
GTCTTCATTGGGTTCTTAGGAGCA---GCAGGAAGCACTATG---GGCGCGGCGTCA---
ATAACGCTGACGGTACAG---GCCAGACAA---TTATTG---TCTGGCATAGTGCAACAGCAAAGT---
AATTTG---CTGAGG---GCTGTAGAGGCTCAACAGCATATGTTGAAACTCGCGGTCTGGGGCATT
```

>AY444799
TTCTATTGTGACACATCAGGCCTGTTTAATAGCACGTGGAATGTCAATACCAGCGTGCAGCAGTCAAATATCA
CGGGC--

AATATCACGGACAATATAACTCTCCCATGCAGAATAAAGCAAATTATAAATATGTGGCAGAGAGCAGGACAAG
CAATGTATGCCCCTCCCATCCAAGGAAAAATAAAGTGTGTATCAAAC---
ATTACAGGACTAATATTAACAAGAGATGGTGGAGGTAATAGCAGT----------------------------
----

GCAAATGAAACCTTTAGACCTGGAGGAGGAGATATGAGGGACAATTGGAGAAGTGAATTATATAAGTATAAAG
TAGTAAGAATTGAACCACTA---GGAGTAGCACCCACCAAGGCAAAAAGAAGAGTGGTG--------------
-------------------------CAGAGAGAAAAAAGAGCA---ATTGGA---ATAGGAGCT---------
GTCCTCATTGGGTTCTTAGGAGCA---GCCGGAAGCACTATG---GGCGCAGCGTCA---
ATAACGCTGACGGTACAG---GCCAAACAA---TTATTG---TCTGGTATAGTGCAACAGCAAAGC---
AATTTG---CTGAGG---GCTATAGAGGCTCAACAGCAGCTGTTGAAACTCACAGTCTGGGGCATT
>AY371160
TTCTATTGTAATACATCAGGCTTGTTTAACAGCACTTGGTATAAGGAGACTGCCCCTGTCAGCAGAACAGGGT
CAAAT--

GACACAAGTGACAATATAACTCTCCCGTGTAGAATAAAGCAAATTATAAATATGTGGCAGAGAACAGGACAAG
CAATGTATGCCCCTCCCATCCCAGGAATAATAAGGTGTGAGTCAAAC---
ATTACAGGACTATTATTGACAAGAGATGGTGGGATTAGCAATAAC----------------------------
----

AGTAATGAGACTTTCAGACCTACAGGAGGAGAGATGAAAGATAATTGGAGAAGTGAATTATATAAATATAAAG
TAGTAAAAATTGAACCACTG---GGAGTAGCACCCACCAAGGCAAGGAGAAGGGTGGTG--------------
-------------------------GAGAGAGAAAAAAGAGCA---GTTGGA---CTTGGAGCT---------
GTCTTCCTTGGGTTCTTAGGAGCA---GCCGGAAGCACTATG---GGCGCGGCGTCA---
ATAACGCTGACGGTACAG---GCCAGACAA---TTATTG---TCTGGCATAGTGCAACAGCAAAGT---
AATTTG---CTGAAG---GCTATAGAGGCTCAACAGAATCTGTTGAGACTCACAGTCTGGGGCATT
>MK086110
TTCTATTGCAACACATCAAACTTGTTC------AATTGGAATGGTACTTGGAATGGTAACACAACA-------
----

GGCAATGACACGATAACTCTCCAGTGCAGAATAAAGCAAATTATAAATATGTGGCAGAGGGTAGGACAAGCAA
TGTATGCCCCTCCCATTCCAGGAGAAATAAAGTGTGAGTCAAAC---
ATTACAGGACTACTATTGACAAGAGATGGTGGGAACAGTACAGAT----------------------------
----

AATGAGACTTTCAGACCTGGAGGAGGAGATATGAGAGACAATTGGAGAAGTGAATTATATAAATATAAAGTAG
TAAAAATTGAACCACTA---GGAGTAGCACCCAACAAGGCACGGAGGAGAGTGGTG-----------------
----------------------GGGAGAGAAAAAAGAGCA---GTTGGA---CTAGGAGCT---------
GTCTTCCTTGGGTTCTTGAGTGCA---GCAGGAAGCACTATG---GGCGCGGCGTCA---
CTGACGCTGACGGTACAG---GCCAGGCAA---TTATTG---TCTGGCATAGTGCAACAGCAAAAC---
AATTTG---CTGAGG---GCAATAGAGGCTCAACAGCATATGTTGAGACTCACGGTCTGGGGCATT
>AF377958
TTCTATTGCGATACATCAAAACTGTTTAATAGTACTTGGGACAAGAATGGCACGTGGGTTAATGGCCACACAG
GG---

TCAGATAATGACAATATAACTCTCCAATGCAGAATAAAACAAATTATAAATATGTGGCAGAGAGTAGGACAAG
CAATGTATGCCCCTCCCATCCCAGGAGTAATAAGGTGTGAATCTAAC---
ATTACAGGACTACTATTAACAAGAGATGGAGGGAATAATAATAAT----------------------------
----

AGTGCAAATGAGACCTTCAGGCCTGGAGGAGGAGATATGAGGGACAATTGGAGAAGTGAATTATATAAGTATA
AGGTAGTAGAAATTGAACCACTA---GGTGTAGCACCCAACCATGCAAGAAGAAGAGTGGTG-----------
----------------------------GAGAGAGAAAAAAGAGCA---GTTGGA---CTGGGAGCT------
---GTCTTCCTTGGGTTCTTAGGAGCA---GCAGGAAGCACTATG---GGCGCAGCGTCA---
ATAACGCTGACGGTACAG---GCCAGACAA---TTATTG---TCTGGTATAGTGCAACAGCAGAGC---
AATTTG---CTGAAA---GCTATAGAGGCTCAACAACATATGTTGAGACTCACGGTCTGGGGCATT

>MH705142
TTCTATTGCAATACATCAGCCCTGTTTAATAGCAAGTGGGAAAGTGAATACAATGCCAGCGAGAATGAACAAA
AG---

AGTGACACTAATATAACTATTCCATGTAGAATAAAGCAATTTGTAAATGTATGGCAGAGAGTAGGACAAGCAA
TGTATGCCCCTCCAGTCCAAGGAGAAATAAGATGTGACTCAAAC---
ATTACAGGACTACTATTAGAAAGAGAATATGGGGATCATCATAATGGG-------------------------
----

ACAAATGAGACTTTCAGACCTACAGGAGGAGACATGATGAACAATTGGAGAAGTGAATTATATAAGTATAAAG
TAGTAAAAGTTGAACCACTA---GGAGTAGCACCCAGCGGAGCAAGGAGAAGAGTGGTG--------------
-------------------------GCAAGAGAAAAAAGAGCA---CTTGGAGGACTAGGGGCT---------
GTCTTCCTTGGGTTCTTAGGAGCA---GCAGGAAGCACTATG---GGCGCGGCGTCA---
CTGACGCTGACGGTACAG---GCCAGACAA---TTACTG---TCTGGTATAGTGCAACAGCAGAGC---
AATCTG---CTAAGG---GCTATAGAGGCTCAACAACATATGTTGAAACTCACAGTCTGGGGCATT
>AY371162
TTCTATTGCAATACAACACAACTGTTTAATAGTACCTGGGAAAATGACACCCTGAAGGGAGCGGCA-------
----

GGTAATGATACTATTACACTGCCATGCAGAATAAAACAAATTGTAAACATGTGGCAGAGAGTAGGACAAGCAA
TGTATGCCCCTCCCATCCAAGGAGTAATACAGTGTGTATCAAAC---
ATTACAGGACTAATATTGACAAGAGATGGTGGGAGTAATAACAAT----------------------------
----

ACAAATGAGACCATCAGACCTGGAGGAGGGGAGATGAGGGACAATTGGAGAAGTGAATTATATAAATATAAAG
TAGTACAAATTGAACCACTA---GGAGTAGCACCCACCAGGGCAAGGAGAAGAGTGGTG--------------
-------------------------CAGAGAGAAAAAAGAGCA---GTTGGA---TTGGGAGCT---------
CTTTTCCTTGGGTTCTTAGGAGCA---GCAGGAAGCACTATG---GGCGCAGCGTCA---
ATAACGCTGACGGTACAG---GCCAGACAA---TTATTG---TCTGGCATAGTGCAACAGCAGGGC---
AATCTG---CTGAGG---GCTATAGAGGCTCAACAACATCTGTTGAAGCTCACGGTCTGGGGCATT
>MH705135
TTCTATTGTGATACATCAAGCCTGTTTAATAGCACTTGGTCCTGGGAAAATGGCAATTGGTCTTGGAATAATA
ATGACACGACC--
----------ACCATGAAC------
GAGATAAATACAAATGTGACTATCCCATGCAGAATAAAGCAAATAGTAAATATGTGGCAGAGAGTAGGACAAG
CAATGTATGCCCCTCCCATCCCAGGAATAATAAATTGTGTATCAAAC---
ATTACAGGACTATTATTGACAAGAGATGGTGGGAGTAATAACAGT----------------------------
----

ACAAATGAGACTTTTAGACCTGGAGGAGGAGATATGAGGGACAATTGGAGAAGTGAATTATATAAGTATAAAG
TAGTAAAAATTGAACCACTA---GGAGTAGCACCCACTAGGGCCAGGAGACGAGTGGTG--------------
-------------------------CAGAGAGAAAAACGAGCA---ATGGGA---ATAGGAGCT---------
GTGCTCCTTGGGTTCTTGGGAGCA---GCAGGAAGCACTATG---GGCGCGGCGTCA---
ATAACGCTGACGGTACAG---GCCAGACAA---TTATTG---TCTGGTATAGTGCAACAGCAAAGC---
AATTTG---CTGAGG---GCTATAGAGGCTCAACAGCATCTGTTGAAACTCACGGTCTGGGGCATT
>AF069932
TTCTATTGCAATACATCAGGCTTGTTTAATAGCACTTGGACAAATGGGACAAACAGCACGGGG----------
----

ACTAATGACAATATAACTCTTCCATGCAGAATAAAGCAAATTATAAATATGTGGCAGAGAGTAGGACAAGCAA
TGTATGCCCCTCCCATCAAAGGAGTAATAGAGTGTAAATCAAAT---
ATTACAGGAATACTATTAATGAGAGATGGTGGTGCTAATGATACA----------------------------
----

GCTAACAGTGAGACATTCAGACCTGGAGGAGGAGATATGAAGGACAATTGGAGAAGTGAATTATATAAGTATA
AAGTAGTAAAAATTGAACCACTA---GGAGTAGCACCCACCAGGGCAAAGAGAAGAGTGGTG-----------
----------------------------GAGAGAGAAAAAAGAGCA---GTTGGA---CTGGGGGCT------
---GTCTTCCTTGGGTTCTTAGGAGCA---GCAGGAAGCACTATG---GGCGCGGCGTCA---
TTAACGCTGACGGTACAG---GCCAGACAA---TTATTG---TCTGGCATAGTGCAACAGCAGAGC---
AATCTG---CTGAAG---GCTATAGAGGCTCAACAGCATCTGTTGAAACTCACAGTCTGGGGCATT

>AF069939
TTCTATTGCAACACATCAGGACTGTTTAATAGCACTTGGGATAACAACAACATTGCCAGCTCAAACCACACAG
GG---

TCAAATGATACTATAACTCTCCAATGCAGAATAAAGCAAATTATAAATCTGTGGCAGAAAGTAGGACAAGCAA
TGTATGCCCCTCCCATCCCAGGAATAATAAGGTGTGAATCAAAT---
ATTACTGGACTCCTATTAACAAGAGATGGAGGGAATAATAATAGT----------------------------
----

ACAAATGAGACCTTCAGACCTGGAGGAGGAGATATGAGGGACAACTGGAGAAGTGAATTATATAAGTATAAAG
TAGTAAAAATTGAACCACTA---GGTGTAGCACCCACCAAGGCAAAAAGAAGAGTGGTG--------------
-------------------------GCGAGAAAAAAAAGAGCA---GTTGGA---CTGGGAGCT---------
GTCTTCCTTGGTTTCTTAGGAGCT---GCAGGAAGCACTATG---GGCGCAGCGTCA---
ATAGCGCTGACGGTACAG---GCCAGACAA---TTATTG---TCCGGCATAGTGCAACAGCAGAGC---
AATCTG---CTGAGG---GCTATAGAGGCTCAACAGCATATGTTGAAACTCACGGTCTGGGGCATT
>KT427849
TTCTACTGCAACACAATACAGCTGTTTAATAGTACTTGGTTCAATGAGAATAGTACTTGGTTCAATGAGAGTG
GTACAGGG---

TCAAACACTGAAAATATCACACTCCCATGCAGAATAAAGCAAATTATAAATAGGTGGCAGGAAGTAGGAAAAG
CAATGTATGCCCCTCCCATCAGTGGACGAATTAACTGTTCATCAAAT---
ATTACAGGGCTGCTATTGGTAAGAGATGGTGGTGATGGGAACGCG----------------------------
----

ACAGCAGAAATCTTCAGACCTGGAGGAGGAGATATGAGGGACAATTGGAGAAGTGAATTATATAAATATAAAG
TAGTACAAATAGAACCATTA---GGAATAGCACCCACCAAGGCGAGGAGAAGAGTGGTG--------------
-------------------------CAGAGAGAAAAAAGAGCA---GTGGGAACGATAGGAGCT---------
ATGTTCCTTGGGTTCTTGGGAACA---GCAGGAAGCACTATG---GGCGCAGCGTCA---
GTAGCGCTGACGGTACAG---GCCAGACAA---TTATTG---TCTGGTATAGTGCAACAGCAAAAT---
AATTTG---CTGAAG---GCTATTGAGGCGCAACATCATCTGTTGCAACTCACAGTCTGGGGCATT
>KT427790
TTCTATTGCAATACATCAGGACTGTTTAATGGTACATACAGACCTAATATTACAGGAAACCATAGTGAAATA-
----

GAAAATTCGACCATTACAATCCCATGCAGAATAAAACAAATTATAAATATGTGGCAGGAGGTAGGAAAAGCAA
TGTATGCCCCTCCCATTCAAGGAATAATAACATGTAGATCAAAT---
ATCACAGGACTACTACTAACACGGGATGGAGGAGGCAATGTCAAT----------------------------
----

GACAGCACAGAGACATTCAGGCCTGGAGGAGGAGATATGAGGGACAATTGGAGAAGTGAATTATATAAATATA
AAGTGGTAGAAATTAAACCATTA---GGAATAGCACCCACTGCGGCAAAAAGGAGAGTGGTG-----------
----------------------------GAGAGAGAAAAAAGAGCA---GTGGGA---ATAGGAGCT------
---ATGATCTTTGGGTTCTTGGGAGCA---GCAGGAAGCACTATG---GGCGCAGCATCA---
ATAACGCTGACGGTACAA---GCCAGACAA---TTATTG---TCTGGGATAGTGCAACAGCAAAGC---
AATTTG---CTGAGA---GCTATAGAGGCGCAACAGCATATGTTGCAACTCACGGTCTGGGGCATT
>KC898981
TTCTATTGTAATACATCAGGCCTGTTTAATAGTACATACAATAATGGTACATACATGTTTAATGGTACAAACC
TGTCTAAT---
GAGACACAAGAGTATTAT---------
AATTCAAGCTCAGTCATTACAATCCAATGCAAAATAAAGCAAATTATAAATATGTGGCAGGCGGTAGGACGAG
CAATGTATGCCCCTCCCATTGCAGGAAACATAACATGTAGATCAAAT---
ATCACAGGACTACTATTGGCACGTGATGGAGGAACAAAAAATGAGACA-------------------------
----

AATAATACAGAGATATTCAGACCTGGAGGAGGAGATATGAAGGACAACTGGAGAAGTGAATTATATAAATATA
AAGTGGTAGAAATTAAACCATTA---GGAATAGCACCCACTGCAGCAAAAAGGAGAGTGGTG-----------
----------------------------GAGAGAGAAAAAAGAGCA---GTGGGA---ATAGGAGCT------
---GTGTTCTTTGGGTTCTTGGGAGCA---GCAGGAAGCACTATG---GGCGCGGCGTCA---
ATGACGCTGACGGTACAG---GCCAGACAA---TTGCTG---TCTGGTATAGTGCAGCAGCAAAGC---
AATTTG---CTGAGG---GCTATAGAGGCGCAACAACATATGTTGCAACTCACGGTCTGGGGCATT

>KF835539
TTCTATTGTAATACTTCAGGCCTGTTTAATGGTACATACCTGCCTAATGGTACATACATGCCTAATGATATAC
AAGAT--

ACAAACTCAACCATCACGATTCCATGCAGAATAAAGCAAATTATAAATATGTGGCAGGAGGTAGGACGAGCAA
TGTATGCCCCTCCCATTGAAGGAAACATAACATGTAAATCAAAT---
ATCACAGGACTACTATTGGTACGTGATGGAGGAAACCAAGAAAAAACAGGAAAT------------AAT----
----
-
ACAACAAATGATACAACAGAGACATTCAGACCTCAAGGAGGAGATATGAGGGATAATTGGAGAAGTGAATTAT
ATAAATATAAAGTGGTAGAAATTAAGCCATTG---GGAATAGCACCCACTAAAGCAAAAAGGAGAGTAGTG--
-------------------------------------GAGAGAGAAAAAAGAGCA---GTGGGA---
CTAGGAGCT---------GTGTTCCTTGGGTTCTTGGGAGCA---GCAGGAAGCACTATG---
GGCGCGGCGTCA---ATAACGCTGACGGTACAG---GCCAGACAA---TTGCTG---
TCTGGTATAGTGCAACAGCAAAGC---AATTTG---CTGCAA---
GCTATAGAGGCGCAACAGCATCTGTTGCAACTCACGGTCTGGGGCATT
>KC898983
TTCTATTGTAATACATCAAAGCTGTTTAATGGCACATACAATGGCACAGACATGACTAATATGATTAAT----
----

AGTTCAGCAGTAATCATAATCACATGCAGAATAAAGCAAATTGTAAACATGTGGCAGGAGGTAGGACGAGCAA
TGTATGCCCCTCCCATTGCAGGAAACATAACATGTAGATCAAAT---
ATCACAGGACTATTATTGGTACGTGATGGAGGAAAATCAAATACT----------------------------
----

ACAGAGACATTCAGACCTCAAGGGGGAGATATGAGGGACAATTGGAGAAGTGAATTATATAAATATAAAGTGG
TAGAAATTAAGCCATTA---GGAATAGCACCCACTGAAGCAAAAAGGAGAGTAGTG-----------------
----------------------GAGAGAGAAAAAAGAGCA---GCGGGA---ATAGGAGCT---------
GTGTTCCTTGGGTTCTTGAGTGCA---GCCGGAAGCACTATG---GGCGCGGCGTCA---
GTAACGCTGACGGTACAG---GCCAGACAA---TTGCTG---TCTGGTATAGTGCAACAGCAAAGC---
AATTTG---CTGAGG---GCTATAGAGGCGCAACAGCATATGTTGCAACTCACGGTCTGGGGCATT
>HM776938
TTCTATTGTAATACATCAGGCCTGTTTAATAGTACATACTGGCCTAATGGTACATACATGTATTATAAAACAA
AC---

AATGATTCAATCATCACAATCCCATGCAGAATAAAGCAAATTATAAACATGTGGCAGCAGGTAGGAAGAGCAA
TGTATGCCCCTCCCATTGCAGGAAGAATAACATGTAACTCAAAT---
ATCACAGGACTACTATTGGTACGTGATGGAGGCCTAGGTAATACTACA-------------------------
----

GATAATACTACAGAGACGTTCAGACCCCAAGGAGGAAATATGAGGGACAATTGGAGAAGTGAACTATATAAAT
ATAAAGTGGTAGAAATTCAGCCGTTG---GGAATAGCACCCACTAAAGCAAAGAGGAGAGTGGTG--------
-------------------------------GAGAGAGAAAAAAGAGCA---GTGGGA---ATAGGAGCT---
------GTGTTCCTTGGGTTCTTGGGAGCA---GCAGGAAGCACTATG---GGCGCGGCGTCA---
ATAACGCTGACGGTACAG---GCCAGACAA---TTGCTG---TCTGGTATAGTGCAACAGCAAAGC---
AATTTG---CTGAAG---GCTATAGAGGCGCAACAGCATATGTTGCAACTCACGGTCTGGGGCATT
>KF250381
TTCTATTGTAATACATCAGGTCTGTTTAATGGTACATACAATAGTACAAGA----------------------
----
-------TACAATAATACA------
AGGGAGAATGGAACCATCACAATCCCATGTAGAATAAAGCAAATTATAAACATGTGGCAAGAGGTAGGACGAG
CAATGTATGCCCCTCCCATTAAAGGAAACATAACATGTAAATCAAAT---
ATCACAGGACTACTATTGGTACGTGATGGAGGA--

ACAAATCAAAGTAAT---
ACAACAGAGATATTCAGACCTGGAGGAGGGGATATGAGGGACAATTGGAGAAGTGAATTATATAAATATAAAG
TGGTAGAAATTAAGCCATTG---GGAGTAGCACCCACTAAAGCAAAAAGGAGAGTGGTG--------------
-------------------------GAGAGAGAAAAAAGAGCA---GTGGGA---TTGGGAGCT---------
ATGTTCCTTGGGTTCTTGGGAGCA---GCAGGAAGCACTATG---GGCGCAGCGTCA---

ATAACGCTGACGGTACAA---GCCAGACAA---TTGTTG---TCTGGTATAGTGCAACAGCAAAGC---
AATTTG---CTGAGA---GCTATAGAGGCGCAACAGCATCTGTTGCAACTCACTGTCTGGGGCATT
>KF250382
TTCTATTGTAATACATCAGGCTTGTTTAATGGTACATACCATAATGGTACATAC-------------------
----
ACATTTAATGGTACAGAA---------
GGTAATGCAAGCTCAATCATCATTCCATGCAGAATAAAGCAAATTATAAATATGTGGCAAGAGGTAGGACGAG
CAATGTATGCCCCTCCCATTGAAGGAAAATTAGAATGTAAATCAAAT---
ATCACAGGACTACTATTGGTACGTGATGGAGGG--

ATAAATACTACAGAGGAGACATTCAGACCTGGAGGAGGAGATATGAGGGACAATTGGAGAAATGAATTATATA
AATATAAAGTGGTAGAAGTTCAGCCATTG---GGAATAGCACCCACTAAAGCAAAAAGGAGAGTGGTG-----
----------------------------------GAGAGAGAAAAAAGAGCA---GCGGGA---
CTGGGAGCT---------GTGTTCCTTGGGTTCTTGGGAGCA---GCAGGAAGCACTATG---
GGCGCGGCGTCA---ATAACGCTGACGGTACAG---GCCAGACAA---TTGTTG---
TCTGGTATAGTGCAACAGCAAAGC---AATCTG---CTGAGA---
GCTATAGAGGCGCAACAACATCTGTTGCAACTCACTGTCTGGGGCATT
>KC898988
TTCTATTGTAATACATCAAGCCTGTTTAATAGTACATACACGAATGGTACATACATGTTTAATGGTACGATAG
GTAAT--

TCAAGTTCAGACATCACAATTCAATGCAAAATAAAGCAAATTATAAACATGTGGCAGGAGGTAGGACGAGCAA
TGTATGCCCCTCCCATTAAAGGAAACATAACATGTAAATCAAAT---
ATCACAGGACTACTATTGGTACGTGATGGAGGAAATGGGACAAAT----------------------------
----

CAGACAGAGACATTCAGACCTGGAGGAGGAGATATGAGAGACAACTGGAGAAGTGAATTATATAAATATAAAG
TGGTAGAAATTAAGCCATTG---GGAGTAGCACCCACTAAGGCAAAAAGGAGAGTGGTG--------------
-------------------------GAGAGAGAAAAAAGAGCA---GTGGGA---ATAGGAGCT---------
GTGTTCCTTGGGTTCTTAGGTGCA---GCCGGGAGCACTATG---GGCGCGGCGTCA---
ATAACGCTGACGGTACAG---GCCAGACAA---TTGTTG---TCTGGTATAGTGCAACAGCAAAGC---
AATTTG---CTGAGG---GCTATAGAGGCGCAACAGCATATGTTGCAACTCACGGTCTGGGGCATT
>KC899010
TTCTACTGTAATACAACAAAACTGTTTAATAATACTTGGAATAGTACTAAACGAGACTTTGAGAAACCTTGGA
GTGATGATAAC--

AGAACAGGCATTATCACAATCCCATGCAGAATAAAACAAATTATAAACATGTGGCAGGGAGTAGGAAAAGCAA
TGTATGCCCCTCCCATTGCAGGAAACATAACATGTAACTCCACT---
ATCACAGGACTACTGTTGGTACGTGATGGAGGAAATACTACAGGGAAAAATGAT-------------------
----

ACAAATAATATAGAGATATTCAGGCCTCAAGGAGGAAATATGAAGGACAATTGGAGAAGTGAATTATATAAAT
ATAAAGTAGTAAAAATTGAACCATTG---GGAGTAGCACCCACCAAGGCAAAGAGAAGAGTGGTG--------
-------------------------------CAGAGGGAAAAAAGAGCA---GTGGGGGCGTTTGGAGCT---
------ATGTTCCTTGGGTTCTTGGGAGCA---GCAGGAAGCACTATG---GGCGCAGCGTCA---
ATAACGCTGACGGTACAG---GCCAGACAA---TTGTTG---TCTGGTATAGTGCAACAGCAGAGA---
AATTTG---CTGAGG---GCTATTGAGGCGCAACAACATCTGTTGCAACTCACAGTCTGGGGCATC
>KC807955
TTCTATTGTAATACATCAAGCCTGTTTAATAGTACATACAATGGTACATACATGTTTAATGGTACAATGGTTG
ATAATATA---
-------------AATAGG------
ACTTCAGACTTAAACTTCACAATCCCATGCAGGATAAAGCAAATTATAAATCTGTGGCAGGAGGTAGGACGAG
CAATGTATGCCCCTCCCATTGAAGGAAGAATAACATGTAAATCAAAT---
ATCACAGGACTACTATTGACACGTGATGGAGGGAATGGGACAGAGACA-------------------------
----

AATCAGACAGAGATATTCAGACCTGGAGGAGGAGATATGAGGGACAATTGGAGAAGTGAATTATATAAATATA
AAGTAGTAGAAATTAAGCCATTG---GGAGTAGCACCCACTACAGCAAAAAGGAGAGTGGTG-----------
----------------------------GAGAGAGAAAAAAGAGCA---GTGGGA---ATAGGAGCT------

---GTGTTCCTTGGGGTCTTGGGAGTA---GCCGGAAGCACTATG---GGCGCGGCGTCA---
ATAACGCTGACGGTACAG---GCCAGACAA---TTGCTG---TCTGGTATAGTGCAACAGCAAAAT---
AATTTG---CTGAGA---GCTATAGAGGCGCAACAGCATCTGTTGCAACTCACGGTCTGGGGCATT
>KC807982
TTCTATTGTAATACATCAAAACTGTTTAATAGTACATACATGCCTACATACAATGATACAGAGAATAGT----
----

TCAAATGAAAGCATCACAATCCCATGCAGAATAAAGCAAATTATAAATATGTGGCAGAAGGTAGGACGAGCAA
TGTATGCCCCTCCCATTGCAGGAATCATAACATGTAAATCAAAT---
ATCACAGGACTACTATTGGCACGTGATGGAGGAAAAGGTAATGAGACAGAT----------------------
----

AATAAGACAGAAATATTTAGACCTCAAGGAGGAAATATGAGGGACAATTGGAGAAATGAATTATATAAATATA
AAGTGGTAGAAATCAAGCCATTG---GGAATAGCACCAACTGGAGCAAAAAGGAGAGTGGTG-----------
----------------------------GACAGACAAAAAAGAGCA---GTGGGA---ATAGGAGCT------
---GTGTTCCTTGGGTTCTTGGGAGCA---GCAGGAAGCACTATG---GGCGCGGCGTCA---
ATAACGCTGACGGTACAG---GCCAGAAAA---TTGCTG---TCTGGTATAGTGCAACAGCAAAGC---
AATTTG---CTGAGA---GCTATAGAGGCGCAACAGCACATGTTGCAACTCACGGTCTGGGGCATT
>KF250399
TTCTATTGTAATACATCAAAACTGTTTAATGGTACATACAATGATACAGACATGTCTAATGGTACAGAA----
----

AGTAATTCAACCATCACAATTCCATGCAGAATAAAGCAAATTATAAACATGTGGCAGGGGGTAGGACGAGCAA
TGTATGCCCCTCCCATTGAAGGAAACATAACATGTAAATCAAAT---
ATCACAGGACTACTATTGGTACGTGATGGAGGAGAAGAGACAAAT----------------------------
----

GTTACAAATAACACAGAGACATTCAGACCTCAAGGAGGAGATATGAAGGACAATTGGAGAAGTGAATTATATA
AATATAAAGTGGTAGAAATTAAGCCATTG---GGAATAGCACCCACTGGAGCAAAAAGGAGAGTGGTG-----
----------------------------------GAGAGAGAAAAAAGAGCA---GTAGGA---
ATAGGAGCT---------GTGTTCCTTGGGTTCTTGGGAGCA---GCAGGAAGCACTATG---
GGCGCGGCGTCA---ATAACGCTGACGGTACAG---GCCAGACAA---TTGTTG---
TCTGGTATAGTGCAACAGCAAAGC---AATTTG---CTGAGA---
GCTATAGAGGCGCAACAACATCTGTTGCAACTCACGGTCTGGGGCATT
>KF250405
TTCTATTGTAATACATCAGGCCTGTTTAATGGTACATACAACAATACAGCCAATAATTCAAGC----------
----

TCAAACAGCACAATCATAATCCCATGTAGAATAAAGCAAATTATAAATATGTGGCAGGAGGTAGGACGAGCAA
TGTATGCCCCTCCCATTAAAGGAAACATAACATGTAAATCAAAT---
ATCACAGGAATATTATTGATCCGTGATGGAGGAAATGGAAATGAGACA-------------------------
----

AATGAAACAGAGACATTCAGACCTCAAGGAGGAGATATGAGGGACAATTGGAGAAGTGAATTATATAAATATA
AAGTAGTAAAAATAGAACCATTA---GGAGTAGCACCCACCAAGGCAAAAAGAAGAGTGGTG-----------
----------------------------CAGAGAGAAAAAAGAGCA---GTAGGA---ATAGGAGCT------
---GTGTTCCTTGGGTTCTTGGGAGCA---GCAGGAAGCACTATG---GGCGCGGCGTCA---
ATAACGCTGACGGTACAG---GCCAGACAA---TTGTTG---TCTGGTATAGTGCAACAGCAAAGC---
AATTTG---CTGAGG---GCTATAGATGCGCAACAACATATGTTGCAACTCACAGTCTGGGGCATC
>MK792287
TTCTATTGCAATACATCAGGCCTGTTTAATAGTACATACTGGTCTAATGGTACATACAAGATCAATGATACAT
ACAAAGTT---
-AATGATACAGAAAGT------------
AAGTCAATCATCTTCCAAATCCCATGCAGAATAAAGCAAATTGTAAACATGTGGCAGGAGGTAGGAAGAGCAT
TTTATGCCCCTCCCATTGAAGGAAACATAACATGTAAATCAAAT---
ATCACAGGACTACTATTGGTACGTGATGGAGGGCAGCCAAAAAATGGT-------------------------
----

ACAAATAGTACAGAAGAGATATTCAGACCTATAGGAGGAAATATGAAGGACAATTGGAGAAGTGAATTATACA
AGTATAAAGTGGTAGAAATTCAACCATTG---GGAGTAGCACCCACTACAGCAAAAAGGAGAGTGGTG-----

----------------------------------GGAAGAGAAAAAAGAGCAGTATTTGGA---
ATAGGAGCT---------GTGTTCCTTGGGTTCTTGGGAGCA---GCAGGAAGCACTATG---
GGCGCGGCGTCA---ATAGCGCTGACGGTACAG---GCCAGACAA---TTGCTG---
TCTGGTATAGTGCAACAGCAAAAC---AATTTG---CTGAGA---
GCTATAGAGGCGCAACAGCATATGTTGCAACTCACGGTCTGGGGCATT
>EU839599
TTCTATTGCAATACATCAAGACTATTTAATGGTACATACCCACGTATGAACAGTACAGGAGAT----------
----

AATTCAAGTGTAATCACACTTCAATGCAGAATAAAACAAATTATAAACATGTGGCAGAGGGTAGGACGAGCAA
TGTATGCCCCTCCCATTGCAGGAAACATAACATGTAACTCAAGT---
ATCACAGGACTAATACTGACAAGGGATGGTGGTAACATAACAAAT----------------------------
----

AACACAGAAGAAAAATTCAGACCTATAGGAGGAGATATGAGGGACAATTGGAGAAGTGAATTATATAAATATA
AAGTGGTAGAAGTTGAGCCATTA---GGAATAGCACCTACTAATGCAAAAAGGAGAGTGGTG-----------
----------------------------GAGAGAGAAAAAAGAGCA---GTGGGA---GTAGGAGCT------
---ATGCTCCTTGGGTTCTTGGGAGCA---GCAGGAAGCACTATG---GGCGCGGCATCA---
GTGATGCTGACGGTACAG---GCCAGACAA---CTGTTG---TCTGGTATAGTGCAACAGCAAAGC---
AATTTG---CTGAGG---GCTATAGAGGCGCAACAGCATTTGTTGCAACTCACGGTCTGGGGCATT
>EU000514
TTCTACTGTAATACATCAAAACTGTTTAATAGTACTTGGAACAGTAGTAGTACTTGGACTACT----------
----

ACAGGAAATATCACCACAATCCCATGCAGAATAAAGCAAATTGTAAATATGTGGCAGGAAGTAGGACGAGCAA
TGTATGCCCCTCCCATTGAAGGACTAATTAGATGTACATCAAAT---
ATTACAGGGTTACTATTAACAAGAGATGGTGGTTATGAGAACAAGACA-------------------------
----

GGGAACAGGACAGAGACCTTCAGACCTGCAGGAGGAAATATGAGGGACAATTGGAGAAGTGAATTATATAAAT
ATAAAGTAGTAAAAATTGAACCATTA---GGAGTAGCACCCACCAAGGCAAAGAGAAGAGTGGTG--------
-------------------------------CAAAGAGAAAAAAGAGCA---GTGGGAGCGATAGGAGCT---
------GTGTTCCTTGGGTTCTTGGGAGCA---GCAGGAAGCACTATG---GGCGCAGCGGCA---
ATAACGCTGACGGTACAG---GCCAGACAA---TTGATG---TCTGGTATAGTGCAACAGCAGAGA---
AATTTG---CTGAGA---GCTATTGAGGCGCAACAGCATCTGTTGCAACTCACAGTCTGGGGCATT
>AB097873
TTCTATTGTAATACAACACAACTGTTTAATAGTAGTTGGAATGAGACTGATATTGGGAATAATACTAGTACTG
GG---

AATGATACTACTATCATGCTCCCTTGCAGAATAAAACAAGTTGTAAACATGTGGCAGGAAGTAGGAAAAGCAA
TGTATGCCCCTCCCATCAGAGGACTAATTAAATGTTCATCAAAT---
ATTACAGGGTTACTATTGACAAGAGATGGTGGTCATAATGAGACT----------------------------
----

GAGACTGAGACCTTCAGACCTGGAGGAGGAGATATGAGGGACAATTGGAGAAGTGAATTATATAAATATAAAG
TAGTAAAAATTGAACCATTA---GGAGTAGCACCCACCAGGGCAAAGAGAAGAGTGGTG--------------
-------------------------CAGAGAGAAAAAAGAGCA---GTGGGAATGATGGGAGCT---------
ATGTTCCTTGGGTTCTTGGGAACA---GCAGGAAGCACTATG---GGCGCAGCGTCA---
ATAACGCTGACGGTACAG---GCCAGACAA---TTGTTG---TCTGGTATAGTGCAACAGCAGAGA---
AATTTG---CTGAGG---GCTATTGAGGCGCAACAGCATATATTGCAACTCACAGTCTGGGGCATC
>MF373189
TTCTACTGTAATACATCAAACCTGTTTAATACATCAAACCTGTTTAATACTGATGATAATGCAACTGGA----
----

AAAAGTGACACTATCACTCTCACATGCCCAAGAAAACTAATTATAAACATGTGGCAGGAATTAGGGAAAGGGA
AGGATGCGCCTCCCATTCCAAGCCAAAGACAATGTACATCTTCC---
ATTACCGGACTGCTATTGCTAATAGATGGAGGTTTAGACAACACC----------------------------
----

AACAACGAGACCTTCAGACCTGGAGGAGGAGATATAAGAGATAATTGGAAAAGTGGAATATATAAATATTATG

CATTAAAAATAGAACCATTA---GGAATATCACCCAACACTGCAACGAAAACAGTGATG--------------
-------------------------CTAAGGGAACAAAGAGTA---ATGAGA---ACAGGAGCT---------
GTGTTCCTTGGGTTCTTGGGAGCA---GCAGGAAGCACTATG---GGCGCAGCGTCA---
ATAACGCTGACGGTACAG---GCCAGGCTA---TTATTG---TCTGGTATAGTGCAGCAGCAGAGC---
AATTTG---CTGAGG---GCTATTGAGGCGCAACAACATCTGTTGCAACTCACAGTCTGGGGCATC
>KT183077
TTCTACTGTAATACATCAAAACTGTTTAATACTACACTGTTTAATAAGAATAGTACTACACTGACTTGGAATG
ATACTGAC---

ACTGCAGAAAATATTACAATCCCATGCAGAATAAAACAAATTATAAATATGTGGCAGGAAGTAGGAAAAGCAA
TATATGCTCCTCCCATCAGTGGACTAATTAGATGCTCATCAAAT---
ATTACAGGACTGCTATTAATAAGAGATGGGGGTAATGATAATCAG----------------------------
----

ACTGAGATCTTTAGACCTGGAGGAGGAGATATGAGGGATAACTGGAGAAGTGAATTATACAAATATAAAGTAG
TAGAAATTAAGCCATTA---GGAGTAGCACCCACCAAGGCAAAGAGAAGAGTGGTG-----------------
----------------------CAGAGAGAAAAAAGAGCA---ATAATAGGGATGGGAGCT---------
GTGCTCTTTGGGTTCTTGGGAGCA---GCAGGAAGCACTATG---GGCGCAGCGTCA---
ATAACGCTGACGGTACAG---GCCAGACAA---TTACTA---TCTGGTATAGTGCAACAGCAAAAC---
AATTTG---CTGAGA---GCTATTGAGGCGCAACAACATCTGTTGCAACTCACAGTCTGGGGCATT
>KJ849787
TTCTACTGCAATACATCAGGACTGTTTAATTACTTCAATAATAACACCAAT----------------------
----
-AATAACAAATCCTCT---------
TCTGATGACACTCATATCACTCTCCCATGTCGAATAAAACAAATTATAAACATGTGGCAGGAAGTAGGAAGAG
CAATGTATGCTAATCCCATTGCAGGAAACATTACCTGTAGATCAAAT---
ATTACAGGCCTACTATTGACTAGAGATGGTGGT--

CATAATGATAATACTACTAAT---------
CGGACTGAGATCTTTAGACCTGGGGGAGGAAATATGAAAGACAATTGGAGAAGTGAATTATATAAATACAAAG
TAGTAGAAATTGAACCACTA---GGAGTAGCACCCACAAAGGCAAAAAGACAAGTGGTG--------------
-------------------------AGCAGACACAGAAGAGCA---GTGGGA---TTTGGAGCT---------
GTGTTCCTTGGGTTCTTGTCGGCA---GCAGGAAGCACTATG---GGCGCAGCGTCA---
GTGACGCTGACGGTACAG---GCCAGGCAA---TTATTG---TCTGGTATAGTGCAACAGCAGAAC---
AATTTG---CTGAAA---GCTATTGAGGCGCAACAGCATTTGTTGCAACTCACAGTCTGGGGCATT
>KJ849800
TTCTACTGCAATACATCACAACTGTTTAATGAGACA-------------------------------------
----

ATAAGCAATGACACTATCAAACTCCCATGTCGAATAAAACAAATTGTAAACATGTGGCAAGAAGTAGGAAAAG
CAATGTATGCCACTCCCATTGAAGGAAACATTACCTGTAACTCAAAC---
ATTACAGGTCTACTATTGACAAGGGATGGTGGT--

CAGAATAATGATACCAAT---
AAGACTGAGACCTTCAGACCTGGGGGAGGAAATATGAAGGACAATTGGAGAAGTGAATTATATAAATATAAAG
TAGTAGAAATTCAACCACTA---GGAATAGCACCCACCAAGGCAAAAAGACAAGTGGTG--------------
-------------------------AAAAAAGAAAAAAGAGCA---ATAGGA---CTAGGAGCT---------
ATGTTCCTTGGGTTCTTGGGAGCA---GCAGGAAGCACTATG---GGCGCAGCGTCA---
ATAACGCTGACGGTACAG---ACCAGACAA---TTAATG---TCTGGTATAGTGCAACAGCAAAGC---
AATTTG---CTGAGG---GCTATTGAGGCGCAACAGCATCTGTTGCAACTCACGGTTTGGGGCATC
>KY968400
TTCTACTGTAATACAACACAACTATTTAATACTACTTGGAAAAATAATAATACTGAAGAGACAAATAGCACTA
AA---

GGAAATGACACTATCACACTCCCATGCAGAATAAAACAGATCATAAATATGTGGCAGGAAGTAGGAAAAGCAA
TGTATGCCCCTCCTATCAGCGGAAAAATTAGATGCTCATCAAAT---
ATTACAGGACTGCTACTAACAAGAGATGGTGGTATTCATAACAATAGC-------------------------
----

AATGGGACTGAGACCTTCAGACCTGGGGGAGGAAATATGAAGGATAATTGGAGAAGTGAATTATATAAATATA

AAGTAGTACAAATTGAACCATTA---GGAGTAGCACCCACCAGGGCAAAGCGAAGAGTGGTG-----------
----------------------------CAGAGAGAAAAAAGAGCA---GCAGGA---ATGGGAGCT------
---TTGTTCCTTGGGTTCTTGGGAGCA---GCAGGAAGCACTATG---GGCGCAGCGGCA---
GTGACGCTGACGGTACAG---GCCAGACAA---TTATTG---TCTGGTATAGTGCAACAGCAAAAC---
AATTTG---CTGATG---GCTATTGAGGCGCAACAGCATCTGTTGCAACTCACAGTCTGGGGCATC
>AY037267
TTCTACTGTAATACAACACCGCTATTTAATAGTACTTGGCCGTCTAATAATACTCAGAATAATACTGAC----
----

CCTGGAAGAAACATCACACTTCAATGCAGAATAAAACAAATTATAAACATGTGGCAGGAAGTAGGAAGAGCAA
TGTATGCCCCTCCCATTAAAGGACTAATTAGTTGTTCATCAAAT---
ATTACAGGGCTACTATTAACATTCGATGGTGGTAAACCGAATAGC----------------------------
----

ACCAACGAGACTTTCAGACCAGCAGGAGGAAATATGAGGGACAATTGGAGAAGTGAATTATATAAATATAAAG
TAGTAAAAATTGAGCCATTA---GGAGTAGCACCCAACAAGGCAAAAAGAAGAGTGGTG--------------
-------------------------CAAAGAGAAAAAAGAGCA---GTGGGAACAATAGGAGCC---------
ATGTTCCTTGGGTTCTTGGGAGCA---GCAGGAAGCACTATG---GGCGTAGCGTCA---
ATGACGCTGACGGTACAG---GCCAGACAA---TTATTG---TCTGGTATAGTGCAACAGCAGAAC---
AATCTG---CTGAGG---GCTATTGAGGCGCAACAGCATATGTTGCAACTCACAGTCTGGGGCATC
>KY968405
TTCTACTGTAATACAACACCACTGTTTAATAGCACTTGGAATAGGACTAATAGCACTTGGAATAGGAATGGGA
ATAGT--

ACTAATGGTACAATCACACTCCAATGCAGAATAAAACAAATTATAAACATGTGGCAGGAAGTAGGAAAAGCAA
TGTATGCCCCTCCCATCCAAGGACTAGTTAACTGTTCATCAAAT---
ATTACAGGTCTACTATTGACAAGAGATGGTAGTCAG-------------------------------------
----

AATGATACTGAGACCTTCAGACCTGGGGGAGGAGATATGAAAGACAATTGGAGAAGTGAATTATATAAATATA
AAGTAGTAAGAATTGAACCATTA---GGAGTAGCACCAACAAAGGCAAAGAGAAGAGTGGTG-----------
----------------------------CAGAGAGAAAAAAGAGCA---GTGGGA---CTAGGAGCT------
---ATGTTCCTTGGGTTCTTGGGAGCA---GCAGGAAGCACTATG---GGCGCAGCGTCA---
GTGACGCTGACGGTACAG---GCCAGACAA---TTATTG---TCTGGTATAGTGCAACAGCAGAAC---
AATCTG---CTGAGA---GCTATTGAGGCGCAACAACATCTGTTGCAACTCACAGTCTGGGGCATC
>MH078535
TTTTACTGCAATACAGCAGGATTGTTTAATAACATAAAA----------------------------------
----

AATAATGGAACAATCATACTCCCTTGCAGAATAAAACAAATAATAAACATGTGGCAGGAAGTAGGAAAAGCAA
TGTATGCTCCTCCCATCAGCGGACGAATCAACTGTTCATCAAAT---
ATTACAGGTCTAATATTGACAAGAGATGGTGGCTATCAAAATGATAGT-------------------------
----

AATGCTAGTGAAATCTTCAGACCTGGAGGAGGAGATATGAGGGACAATTGGAGAAGTGAATTATATAAATATA
AAGTAGTAAAAGTTGAACCATTA---GGGATAGCACCCACCAAGGCAAAGAGAAGAGTGGTG-----------
----------------------------CAGAGAGAAAGAAGAGCA---GTGGGA---CTAGGAGCT------
---GTGTTCCTTGGGTTCTTGGGAGCA---GCAGGAAGCACTATG---GGCGCAGCGTCA---
ATAACGCTGACGGTACAG---GCCAGACAA---TTATTG---TCTGGTATAGTGCAGCAGCAGAAC---
AATCTG---CTGAGG---GCTATTGAGGCGCAACAGCACCTGTTGCAACTCACAGTCTGGGGCATC
>AF408628
TTCTACTGTAACTCAACACCGCTGTTTAATAGTACTTGGCATACAAATGGTACTAGTACTTTAGAGTCAAATG
CCACT--

GAAGGAAACGACACAATCATACTCCCATGCAGAATAAAACAAATTATAAACAGGTGGCAGGAAGTAGGAAAAG
CAATGTATGCCCCTCCCATCAGAGGAATAATTAACTGTTTATCAAAT---
ATTACAGGGCTGCTGTTAATAAGAGATGGTGGTAATCAGAATCAGACA-------------------------
----

GGGGATCAGACAGAGACCTTCAGACCTGGAGGAGGAAATATAAAAGACAATTGGAGAAGTGAATTATATAAAT

ATAAAGTAGTAGAAATTGAACCATTA---GGAGTAGCACCCACCAAGGCAAAGAGAAGAGTGGTG--------
-------------------------------CAGAGAGAAAAAAGAGCA---GCG------ATAGGAGCT---
------ATATTCCTTGGGTTCTTGGGAGCA---GCAGGAAGCACTATG---GGCGCAGCGTCA---
ATAACGCTGACGGTACAG---GCCAGACTA---TTATTG---TCTGGTATAGTGCAACAGCAGAAC---
AATTTG---CTGAGG---GCTATTGAGGCACAACAACATCTGTTGCAACTCACAGTCTGGGGCATC
>AY037278
TTCTACTGCAATACATCAGGACTGTTTAATAACACAAAAGAA-------------------------------
----

AATGGCACTATTATTCTCCCATGTAGAATAAAACAAATTGTAAACATGTGGCAGGAAGTGGGACAAGCAATGT
ATGCCGCTCCCATTGCAGGAAACATTTCCTGTAACTCAAAT---
ATTACAGGTCTACTATTGACAAGAGATGGTGGTCAGAATAATGATACT-------------------------
----

AATGGAACTGAGATCTTCAGACCTGGGGGAGGAAATATGAAAGACAATTGGAGAAATGAATTATATAAATATA
AGGTAGTAGAAATTGAACCACTA---GGAATAGCACCCACCAGGGCAAAGAGACGAGTGGTG-----------
----------------------------GAGAGAGAAAAAAGAGCA---GTGGGA---ATAGGAGCT------
---TTGTTCCTTGGGTTCTTGGGAGCA---GCAGGAAGCACTATG---GGCGCAGCGTCA---
ATAACGCTGACGGTACAG---GCCAGGCAA---TTATTG---TCTGGAATAGTGCAACAGCAGAAC---
AATCTG---CTGAGA---GCTATTGAAGCGCAACAACATATGTTGCAGCTCACAGTCTGGGGCATT
>AY037283
TTCTACTGCAATACATCAAGACTGTTTAATGACACACTGTTTAATGATACAGAA-------------------
----

TCCAATGAAACTATTATTCTCCCATGTAAAATAAAACAAGTTGTAAACCTGTGGCAGGAAGTGGGACGAGCAA
TGTATGCCGCTCCCATTGCAGGAGCCATTACCTGTAACTCAACT---
ATTACAGGTCTATTATTGACAAGAGATGGTGGTCAGAGTAATGATACTAAT----------------------
----

GGTACTAAGATTGAGACCTTCAGACCTGGGGGAGGAAATATGAAAGACAATTGGAGAAGTGAATTATATAAAT
ATAAAGTAGTAGAAATTGAACCAATA---GGAGTAGCACCCACCAAGGCAAAAAGACAAGTGGTG--------
-------------------------------AGGAGAGAAAAAAGAGCA---GCA------TTGGGAGCG---
------TTGCTCCTTGGGTTCTTGGGAGCA---GCAGGAAGCACTATG---GGCGCGGCGTCA---
ATAACGCTGACGGTACAG---GCCAGACAA---TTATTG---TCTGGAATAGTGCAACAGCAAAAC---
AATTTG---CTGAGG---GCTATTGAAGCGCAACAGCATCTGTTGCAGCTCACAGTCTGGGGCATT
>DQ358803
TTCTACTGTAATACAACACAACTGTTTAATAGTACTTGGGATAGTACTGCAGGGAGCAACACTGAT-------
----
----------------------------GGAAGTGACGCCATCATACTCCTATGCCGAATA---
AACAATTATACATGTGGCAGAAGTAGGAAAAGC---
ATGTTTGCCCCTCCCATCAGCGGACAAATCAGATGCTCATCAAAT---
ATTACAGGTATACTATTGACAAGAGATGGTGGTCAGAATAATGATACT-------------------------
----

AATGAGACTGAGATCTTCAGACCTGGAGGAGGAGATATGAGAGACAATTGGAGGAGTGAATTATATAGATATA
AAGTGGTAAAAATTGAGCCATTA---GGAGTAGCACCCACCTTGGCAAAGAGAAGAGTGGTG-----------
----------------------------CAGAGAGAAAAAAGAGCA---GTGGGG---ATAGGAGCT------
---TTGTTCCTTGGGTTCTTGGGAGCA---GCAGGAAGCACTATG---GGCGCAGCGTCA---
ATAACGCTGACGGTACAG---GCCAGACAA---TTATTG---TCTGGTATAGTGCAACAGCAGAAC---
AATTTG---CTGAGG---GCTATTGAGGCGCAACAACATCTGTTGCAACTCACAGTCTGGGGCATC
>DQ358804
TTCTACTGCAATACATCAGGACTGTTTGATGAAACAAAATTTAATGACACAGAATCC----------------
----

AATAACGCCACTATCACTCTCCTATGTCGAATAAAACAAATTGTAAACATGTGGCAGGAAGTAGGACGAGCAA
TGTATGCCAATCCCATTGCAGGAAGCATTACCTGTAACTCAACT---
ATCACAGGTCTATTATTGACAAGAGATGGTGGTCAGAATACTACG----------------------------
----

AATAGTACTGAGATCTTCAGACCTGGGGGAGGAAATATGAAAGACAATTGGAGAAGTGAATTATATAAATATA

AAGTAGTAGAAATTGAACCACTA---GGAATAGCACCCTCCAGGGCAAAAAGACAAGTGGTG-----------
----------------------------AAGAGAGAAAAAAGAGCA---GTTGGA---CTAGGAGCT------
---ATGTTCATTGGGTTCTTGGGAGCA---GCAGGAAGCACTATG---GGCGCGGCGTCA---
ATAACGCTGACGGTACAG---GCCAGACAA---TTACTG---TCTGGAATAGTGCAGCAGCAAAAT---
AATTTG---CTGAGG---GCTATTGAAGCGCAACAGCATCTGTTGCAGCTCACAGTCTGGGGCATT
>DQ358806
TTCTACTGCAATACATCAGGACTGTTTAATGACACAGTACCC-------------------------------
----

AATGGCACTATCACTCTCCCATGTCGAATAAAACAAATTGTAAATATGTGGCAAGGAGTGGGGCAAGCAATGT
ATGCCGCTCCCATTGCAGGAAATATTACCTGCAGATCAAAT---
ATTACAGGTATACTATTGACAAGAGATGGTGGTGATGCTAATCAGAATGAT----------------------
----

CCCAATGGAAATGAGACCTTTAGACCTGGGGGAGGAAATATGAAAGACACCCGGAGAAGTGAATTATATAAAT
ATAAAGTAGTAAAAATTGAGCCATTA---GGAATAGCACCCACCAAGGCAAAGAGAAGAGTGGTG--------
-------------------------------CAGAGAGAAAAAAGAGCA---GTGGGCACGTTAGGAGCT---
------ATGTTCCTTGGGTTCTTGGGAGCA---GCAGGAAGCACTATG---GGCGCAGCGTCA---
ATAACGCTGACGGTACAG---GCCAGGCAA---TTATTG---TCTGGTATAGTGCAACAGCAAAAC---
AATTTG---CTGAGG---GCTGTTGAAGCGCAACAACATCTGTTGCAACTCACAGTCTGGGGCATC
>DQ358812
TTTTACTGCAATACATCAGAACTGTTTAATAACACAAAAAAC-------------------------------
----

AATGGCACCATCATTCTCCCATGTAAAATAAAACAAATTGTAAACATGTGGCAGAGAGTAGGACGAGCAATGT
ATGCCAATCCCATTGCAGGAAAAATTAACTGTAACTCAAAT---
ATTACAGGTCTATTATTGACAAGAGATGGTGGTAATCAGGCTAATGAGACTAGT------------GGT----
----
-
ATTCAGACTAATGGGACTGAGATCTTCAGACCTGGAGGAGGAAATATGAGAGACAATTGGAGAAGTGAATTAT
ATAAATATAAAGTAGTACAAATTAAACCACTA---GGAATAGCACCCACCAGGGCAAAAAGACGAGTGGTG--
-------------------------------------CAGAGCAAGAAGAGCA---GTGGGA---
ATAGGAGCT---------CTTTTCATTGGATTCTTAGGAGCA---GCAGGAAGCACTATG---
GGCGCGGCGTCA---ATAACGCTGACGGTACAG---GCCAGACAA---TTATTG---
TCTGGAATAGTGCAACAGCAAAAC---AATTTA---CTGCAG---
GCTATTGAAGCGCAACAGCATCTGTTGCAGCTCACAGTCTGGGGCATT
>MG365770
TTCTACTGCAATACATCAGGGCTGTTTAATGAAACAAAC----------------------------------
----

TTTAATGACACTATCACTCTCCCATGTCGAATAAAACAAATTGTAAACATGTGGCAGGAAGTAGGACGAGCAA
TGTATGCCAATCCCATTAAAGGAAACATTACCTGCAGCTCAAAT---
ATTACAGGTCTAATATTGACAAGAGATGGTGGTCAGAAGAATGATACT-------------------------
----

AATCAGACTGAGACCTTCAGACCTGGGGGAGGAAATATGAAAGACAATTGGAGAAGTGAATTATACAAATATA
AAGTAGTAGAAATTGAACCACTA---GGAGTAGCACCCACCAAGGCAAAAAGACAAGTGGTG-----------
----------------------------ACGAGACAAAAAAGAGCA---GCA------ATAGGAGCT------
---TTGTTCCTTGGGTTCTTGAGTGCA---GCAGGAAGCACTATG---GGCGCGGCGTCA---
ATAACGCTGACGGTACAG---GCCCGACAA---TTATTG---TCTGGGATAGTGCAACAGCAGAGC---
AATTTG---CTGAGG---GCTATTGAAGCGCAACAACATCTGTTGCAGCTCACAGTCTGGGGCATA
>JN692449
TTCTACTGTAATACAACACAACTGTTTAATAGTACTTGGCATTTGAATGCCACTGGAAAGTCTAATTACACAG
AA---

GGAAATGACACAATCACACTCCCATGCAGAATAAAACAAATTGTAAACATGTGGCAAGAAGTAGGAAAAGCAA
TGTATGCCCCTCCCATCAGAGGACAAATTAGCTGTTCATCAAAT---
ATTACAGGACTGCTATTAACAAGAGATGGTAGTAACAACACCAAC----------------------------
----

GAAAGCGAGATCTTCAGACCTGGAAGAGGAAATATAAAGAACAATGGAAGGAGTGAACTATACAAATATAAAG
TAGTAAAAATTCAGCCATTA---AGAGTAGCACCTACCAAGGCAAAGAGAAGAGTGGTG-------------
------------------------CAGAGAGAAAAAAGAGCA---GTGACC---CTAGGAGCT---------
GTGTTCCTTGGGTTCTTGGGAGCA---GCAGGAAGCACTATG---GGCGCAGCGTCG---
CTGACGCTGACGGTACAG---GCCAGACAA---TTATTG---TCTGGTATAGTGCAGCAGCAAAAC---
AATTTG---CTGAGA---GCTATTGAAGCGCAACAACATATGTTGCAGCTCACAGTCTGGGGCATC
>EF637052
TTCTACTGCAATACATCAGGACTATTTAATAACGCACTGTTTAATGACACAGGAGTCAATAATGGC-------
----

AGTGATGACAATATCATTCTCCCATGTCGAATAAAACAAATTGTAAACATGTGGCAGGAAGTAGGACGAGCAA
TGTATACTGCTCCTATTGCAGGAAACATTACCTGTAGATCAAAT---
ATTACAGGTCTTCTATTGACAAGGGATGGTGGTAATAATGGTGAT----------------------------
----

AATCAGACTCAGATTTTCAGACCTGGGGGAGGAAACATGAAAGACAATTGGAGAAGTGAATTATATAAATATA
AAGTAGTAGAGATTGAACCACTA---GGAGTAGCACCCACCAGGGCAAAAAGACAAGTGGTG-----------
---------------------------ACAAGAGAAAGAAGAGCA---GCA------GTGGGACTG------
---TTTTTCCTTGGGTTCTTGGGAGCA---GCAGGAAGCACTATG---GGCGCAGCGTCA---
CTGACGCTGACGGTACAG---GCCAGACTA---TTACTG---TCTGGTATAGTGCAGCAGCAGAGC---
AATCTG---CTGAGA---GCTATTGAGGCGCAACAGCATCTGTTGCAACTCACAGTCTGGGGCATT
>MH938678
TTCTACTGTAATACAACAAAGCTGTTTAATAGTACTTGGAATGGCACTAATAGTATTAAT-------------
----

AAGACTGGTACTATCACAATCCCATGCAGAATAAAACAAATTATAAACAGGTGGCAGGAAGTAGGAAAAGCAA
TATATGCCCCTCCCATTGGAGGACAAATTAGATGTTCATCAAAT---
ATTACAGGGCTGCTAATAACAAGAGATGGTGGTCATAATCAGACT----------------------------
----

GCTGAAACCTTCAGACCTGGAGGAGGAGATATGAAAGACAACTGGAGAAATGAATTATATAAATATAAAGTAG
TAGAAATTGAGCCACTA---GGAATAGCACCCTCCAAGGCAAAGAGAAGAGTAGTG-----------------
----------------------CAGAGAGAAAAGAGAGCA---GTGACA---TTAGGAGCT---------
ATGTTCCTTGGGTTCTTGGGAGCA---GCAGGAAGCACTATG---GGCGCAGCGTCA---
ATGACGCTGACGGTACAG---GCCAGACAA---TTATTG---TCTGGTATAGTGCAGCAGCAGAAC---
AATTTG---CTGAAG---GCTATTGAAGCGCAACAGCATATGTTGCAGCTCACAGTCTGGGGCATT
>JN692456
TTCTACTGTAATACATCAGCACTGTTTAATAGTACTTGGCCGTCTAATAGTACTTGGAATGATACTGACAATA
AA---

GGAAATGATACTATCATCATACTCCCATGCAGAATAAAACAAATTATAAACAGGTGGCAGGAAGTAGGAAAAG
CAATGTATGCCCCTCCCATCGCAGGACAAATTAGATGTTCATCAAAC---
ATTACAGGGCTGCTATTAACAAGAGATGGTGGTAACAACAGCACC----------------------------
----

AATGAAACCTTCAGACCAGGAGGAGGAGATATGAGGGACAATTGGAGAAGTGAATTATTTAAATATAAAGTAA
TAAGAATTGAACCATTA---GGAATAGCCCCCACTCAGGCAAAGAGAAGAGTGGTG-----------------
----------------------CAGAGAGAAAAAAGAGCA---GCA------CTAGGAGCT---------
ATGTTCCTTGGGTTCTTGGGAGCA---GCAGGAAGCACTATG---GGCGCAGCATCA---
ATAACGCTGACGGTACAG---GCCAGACAA---TTATTG---TCTGGTATAGTGCAACAGCAGAAC---
AATCTG---CTGAGA---GCTATTGAGGCGCAACAACATCTGTTGCAACTCACAGTCTGGGGCATC
>JF804808
TTCTACTGTAATACAACACAACTGTTTAATAGTACTTGGACTTGGAATAATAGCAATAGTACTTGGAATAGGA
ATGATAGCACT--
-------AATACTTGGGAT------
AGTGCTAATGAAAATATCACACTCCCATGCAGAATAAAACAAATTATAAACAGGTGGCAGGAAGTAGGAAAAG
CAATGTATGCCCCTCCCATCAAAGGACAAATTAGATGTTCATCAAAT---
ATTACGGGGCTGATATTAACAAGAGATGGTGGGTCCAATCACAGC----------------------------
----

```
TCCACCGAGACCTTCAGACCTGGAGGGGGAGATATGAGGGATAATTGGAGAAGTGAGTTATATAAATATAAAG
TAGTAAAAATTGAGCCATTA---GGAATAGCCCCCAACAAGGCAAAAAGAAGAGTGGTG--------------
-------------------------CAGAGAGAAAAAAGAGCA---GTGGGAGTGATAGGAGCT---------
ATGTTCCTTGGGTTCTTGGGAGCA---GCAGGAAGCACTATG---GGCGCAGCGTCA---
ATGACGCTGACGGTACAG---GCCAGACAA---CTGTTG---TCTGGTATAGTGCAACAGCAAAAT---
AATTTG---CTGAGA---GCTATTGAGGCGCAACAGCATCTGTTGCAGCTCACAGTCTGGGGCATC
>HM026455
TTCTACTGCAATACATCAGAATTGTTTACTGACAAAAAATTCAATGCC-------------------------
-------------------------------------------------------------------------
----------------------------
ACCCATGGCACTATCACTCTCCCATGTCGAATAAAACAAATTGTAAACCTATGGCAGGAAGTAGGAAGAGCAA
TATATGCCGCTCCCATTGAAGGAAACATTACCTGTAATTCAAAT---
ATTACAGGTCTACTACTAACAAGAGATGGTGGTCAGAACAGTAGT----------------------------
-------------------------------------------------------------------------
-------------
GCTAATGAGACCTTCAGACCTGGGGGAGGAGATATGAGAGACAATTGGAGAAGTGAATTATATAAATATAAAG
TAGTAGAAATTGAACCACTA---GGAATAGCACCCACCAAGGCAAAAAGAAGAGTGGTG--------------
-------------------------CAGAGAGAAAAAAGAGCA---GTGGGA---ATAGGAGCG---------
TTGTTCATTGGGTTCTTGGGAGCA---GCAGGAAGCACTATG---GGCGCAGCGTCA---
ATAACGCTGACGGTACAG---GCCAGACAA---TTATTG---TCTGGAATAGTGCAGCAGCAGAAC---
AACCTG---CTGAGG---GCTATTGAAGCGCAACAGCATCTGTTGCAGCTCACAGTCTGGGGCATT
>JN687738
TTCTACTGTGACACAACACAGCTGTTTAATAGTACTTGGAATATTACTAAAACAGATAATAGCTCTTGGAGTG
GT-----------------------------------------------------------------------
----------------------------
ACAACTCAGAATATCACACTCCCATGTAGAATAAAACAATTCATAAACAGGTGGCAGGAAGTAGGAAAAGCAA
TGTATGCCCCTCCCATCCAAGGACTAATTAAATGTACATCAAAT---
ATTACAGGGCTGCTATTAACAAGAGATGGTAGTAACGAATCCCTA----------------------------
-------------------------------------------------------------------------
----------
AAAGACATCGAGATCTTCAGACCTGTAGGGGGAAATATGAGGGACAATTGGAGAAGTGAATTATATAAATATA
AAGTAGTAAAAGTTGAGCCAATA---GGAATAGCACCCACCAAGGCAAAGAGAAGGGTGGTG-----------
----------------------------CAGAGAGAAAAAAGAGCG---GTAACG---CTGGGAGCT------
---GTGTTCCTTGGGTTCTTGGGAGCA---GCAGGAAGCACTATG---GGCGCAGCGGCA---
GTGACGCTGACGGTACAG---GCCAGACAA---TTATTG---TCTGGTATAGTGCAACAGCAGAGC---
AATCTG---CTGAGG---GCTATTGAGGCGCAACAACATATGTTGCAACTCACAGTCTGGGGCATC
>HQ236610
TTCTACTGTAATACATCAGGACTGTTTAATACATCAAGACTGATTATTAATGACACTTCCCCT----------
-------------------------------------------------------------------------
----------------------------
CTCAATGACACTATCATTCTCCCATGTAGAATAAAACAAATTGTAAACATGTGGCAGGGAGTAGGAAGAGCAA
TGTATGCTAATCCAATTGCAGGAAACATTACCTGTAACTCAAAT---
ATTACAGGGCTACTATTGACAAGAGATGGTGGAAAGAAAGATAAT----------------------------
-------------------------------------------------------------------------
----------
CAGACTGACGAGATCTTCAGACCTGGGGGAGGAAATATGAAAGACAATTGGAGAAGTGAATTATATAAATATA
AAGTAGTAGAAATTGAACCACTA---GGAATAGCTCCCACAAAGGCAAAAAGACAAGTGGTG-----------
----------------------------CGGAGAGACAAAAGAGCA---GTGGGA---TTTGGAGCT------
---TTGTTCCTTGGGTTCTTGTCAGCA---GCAGGAAGCACTATG---GGCGCAGCGTCA---
GTGACGCTGACGGTACAG---GCCAGGCAA---TTGTTG---TCTGGTATAGTGCAACAGCAGAAT---
AATTTG---CTGAAA---GCTATTGAGGCGCAACAGCATCTGTTGCAACTCACAGTCTGGGGCATT
>MG365769
TTCTACTGCAATACATCAGAACTGTTTAATGAAACACTAACAAAAACCAAT----------------------
-------------------------------------------------------------------------
----------------------------
GACAATAACACAATCACTCTCCTATGTCAAATAAAACAAATTGTAAACATGTGGCAGGGAGTAGGACGAGCAA
TGTATGCCGCTCCCATTGCAGGAAACATTACCTGTAACTCAAAT---
ATTACAGGTTTACTATTGACAAGAGATGGTGGTAGAACTACCAAG----------------------------
-------------------------------------------------------------------------
----------
```

AATGATACTGAGATCTTCAGACCTGGGGGAGGAGATATGAAAAACAATTGGAGAAGTGAACTATATAAATATA
AAGTAGTAGAAATTGAACCACTG---GGAATAGCACCCACCAAGGCAAAAAGACAAGTGGTG-----------
----------------------------AAGAGAGAAAAAAGAGCAGTAGTGGGA---ATGGGAGCT------
---TTGTTCCTTGGGTTCTTGAGCGCA---GCAGGAAGCACTATG---GGCGCGGCGTCA---
ATAACGCTGACGGTACAG---GCCAGACAA---CTACTG---TCTGGAATAGTGCAACAGCAAAAC---
AATTTG---CTGAGG---GCTATTGAAGCGCAACAGCATCTGTTGCAGCTCACAGTCTGGGGCATT
>KT427871
TTCTACTGTAACACATCAAAACTGTTTAGTAGTACTTGGAATAGTAGTACTTTGAATAGTACTGGAGAGTCAA
ATAAC--

ACTGAGGACACAATTATACTCCCATGCAGAATAAAACAAATTATAAACATGTGGCAGAGAGTAGGAAAAGCAA
TGTATGCCCCTCCCATCCAAGGACAAATTAACTGTTCATCAAAT---
ATTACAGGGATGATATTAACAAGAGATGGGGGTCATAACACGAGC----------------------------
----

GAGACGACCGAAATCTTCAGACCTGGAGGAGGAAATATGAAGGACAATTGGAGAAGTGAATTATATAAATATA
AAGTAGTACAAATTGAACCATTA---GGAATAGCACCCACCAGGGCTAAGAGAAGAGTGGTG-----------
----------------------------CAGAGAGAAAAAAGAGCA---GTAGGA---CTAGGAGCT------
---TTGTTCCTTGGGTTCTTAGGAGCA---GCGGGAAGCACTATG---GGCGCAGCGTCA---
ATAACGCTGACGGTACAG---GCCAGACAA---TTATTG---TCTGGTATAGTGCAACAGCAAAAT---
AATTTG---CTGAGG---GCTATTGAGGCGCAACAGCATATGTTGCAACTCACAGTCTGGGGCATT
>KT427779
TTCTACTGCAATACATCACAGCTGTTTAATGACACAGAATCC-------------------------------
----

AATAATAGCATTATTACTCTCCCATGTAGAATAAAACAAATAGTAAACATGTGGCAGGAAGTAGGAAAGGCAA
TGTATGCCGCTCCCATTGCAGGAAACATTACCTGTAACTCAAAT---
ATTACAGGTCTGCTACTAACAAGAGATGGTGGCCAGGATAATAATACT-------------------------
----

AATCAGAATGAGACCTTCAGACCTGGGGGAGGAGATATGAGAGATAATTGGAGAAGTGAGTTATATAAATATA
AAGTAGTAGAAATTGAACCATTA---GGAATAGCACCCACCAGGGCAAGAAGACGAGTGGTG-----------
----------------------------CAGAGAGAGAAACGAGCA---GTGGGA---CTAGGAGCT------
---GTGTTCCTTGGATTCTTAGGAGCA---GCAGGAAGCACTATG---GGCGCAGCATCA---
ATAACGCTGACGGTACAG---GCCAGACAA---TTATTG---TCTGGAATAGTGCAACAGCAGAAC---
AATTTG---CTGAGG---GCTATTGAAGCGCAACAGCATCTGTTGCAGCTCACAGTCTGGGGCATT
>KJ849813
TTCTACTGCAACACATCAAAACTGTTTAATGACACAGAA----------------------------------
----

TTCAATAATGACACTATCACTCTCCATTGTCAAATAAAACAAATTGTAAACATGTGGCAGGAAGTAGGACGAG
CAATGTATGCCGCTCCCGTTGCAGGAAACATTACCTGTAACTCAACT---
ATCACAGGTTTAATACTGACAAGAGATGGGGGT--

GGTAATGGTAATGAAACTAAT---------
TCGACTGAGACTTTCAGACCTGGGGGAGGAGATATGAGAGACAATTGGAGAAGTGAATTATATAAATATAAAG
TAGTAGAAATTGAACCACTA---GGAATAGCACCCACCAGGGCACAAAGACGAGCGGTG--------------
-------------------------CAGAGAGAAAAAAGAGCA---GTGGGA---CTAGGAGCT---------
TTGTTCCTTGGGTTCTTGGGAACA---GCAGGAAGCGCTATG---GGCGCAGCGTCA---
TTGACGCTGACGGTACAG---GCCAGACAA---TTATTG---TCTGGTATAGTGCAACAGCAGAGC---
AATTTG---CTGAGG---GCTATTGAGGCGCAACAGCATATGTTGCAACTCACAGTCTGGGGCATC
>KT427651
TTCTACTGTAACACATCCCGACTATTTGATGATAGTTGGAATGGTACTTGGTCAAAGAACACT----------
----

ACTATAGAAAATATCACACTCCCATGCAGAATAAAACAAGTTGTAAACCTGTGGCAGCAAGTAGGAAAAGCAA
TGTATGCCGCTCCCATCAAAGGACTACTTAACTGTACATCAAAT---
ATTACAGGGCTGATATTAGCAAGAGATGGTGGTTATAATGTTGAT----------------------------
----

AACAATACAGAGATCTTCAGACCTGCAGGAGGAGATATGAGGGACAATTGGAGAAGTGAATTATATAAATATA
AAGTAGTAAAAATTGAGCCATTA---GGAGTAGCACCCACCAAGGCAAAGAGAAGAGTGGTG----------
---------------------------CAGAGAGAAAAAAGAGCA---GTAACA---TTAGGAGCT------
---ATGTTCCTTGGGTTCTTGGGAGCA---GCAGGAAGCACTATG---GGCGCAGCGTCA---
ATGACGCTGACGGTACAG---GCCAGACTA---TTATTG---TCTGGTATAGTGCAGCAGCAGAAC---
AATTTG---CTGAGA---GCTATTGAGGCGCAACAGCATCTGTTGCAACTCACAGTCTGGGGCATT
>KJ849794
TTCTACTGCAATACAACACTATTGTTTAACGAAGAATTAAATAACACTGGC---------------------
----
-------ACTGATGACACT------
GGCACTAACGACACTATCATTCTCCCATGTAAAATAAAACAAATAATAAACATGTGGCAGGAAGTGGGACGAG
CTATGTACGCTGCTCCCATTGCAGGAAACATTACCTGTTACTCAAAT---
ATTACAGGTCTACTATTGGTAAGAGATGGTGGTAATACT---------------------------------
----
AATGATGGTAATACTACTAGTGGTAAT---------
AAGACCGAGATCTTCAGACCTGAGGGAGGAAATATGAAAGACAATTGGAGAAGTGAACTATATAAATATAAAG
TAATAGAAATAGAACCACTA---GGGGTAGCACCCACCAAGGCAAAAAGACAAGTGGTG-------------
------------------------AAGAGAGAAAAAAGAGCA---ATAGGA---ATGGGAGCT---------
TTGTTCCTTGGGTTCTTGGGAGCA---GCAGGAAGCACTATG---GGCGCAGCGTCA---
GTAACGCTGACGGTACAG---GCCAGACAA---TTATTG---TCTGGTATAGTGCAACAGCAGAAC---
AATCTG---CTGAGG---GCTATTGAAGCGCAACAGCATCTGTTGCAGCTCACAGTCTGGGGCATC
>KT427660
TTCTACTGTAATACAACACAGCTGTTTAATAGTACAATAGTTAATAATACAATGCAGAATAAGACT------
----

GGGAATGGTAATATCACACTCCCATGCACAATAAAACAAATTATAAATATGTGGCAGAAAGTAGGGAAAGCAA
TGTATGCCCCTCCCATCAGAGGACAAATTAGCTGTACATCAAAT---
ATTACAGGTCTACTATTGACTAGAGATGGGGATCAGAATGACAGT---------------------------
----

GAGACCTTCAGACCTCAGGGAGGAAATATGAAAGATAATTGGAGAAGTGAATTATATAAATACAAAGTAGTAA
GAATTGAGCCATTA---GGAGTAGCACCCACCAAGGCAAAGAGAAGAGTGGTG-------------------
------------------CAGAGAGAAAAAAGAGCA---GTGGGAACATTAGGAGCT---------
ATGTTCCTTGGGTTCTTGGGAGCA---GCAGGAAGCACTATG---GGCGCAGCGGCA---
CTGACGCTGACGGTACAG---GCCAGACTA---TTATTG---TCTGGTATAGTGCAACAGCAAAAC---
AATTTG---CTGAGA---GCTATTGAGGCGCAACAGCATCTGTTGCAACTCACAGTCTGGGGCATC
>KX181927
TTCTATTGCAATACATCAGGCCTGTTCAATATGTCCCATAACAATGGCAATGAC------------------
----

ACAACCACTGTCACTATCACCCTCCCGTGTCGAATAAAACAAATTGTAAACATGTGGCAGGAGGTAGGACGAG
CAATGTATGCCGCTCCCATTGCAGGAAGCATTACCTGCAAATCAAAT---
ATTACAGGTCTGCTATTGACAAGAGATGGTAGTAGCAATAATAGTACT------------------------
----

CATAATAACACTGAGACCTTCAGACCTGGGGGAAAGAATATAAAAGACAATTGGAGAAGTGAACTGTATAAAT
ATAAAGTAATAGAAATTGAACCACTA---GGAATAGCACCCACCAAGGCAAAAAGACGAGTGGTG-------
------------------------------ACGAGAGAAAAAAGAGCA---GTAGGA---ATAGGAGCT---
------TTGTTCCTTAAGTTCTTAGGAGCA---GCAGGAAGCACTATA---GGCGCAGCGTCA---
GTGACGCTGACGGTACAG---GCCAGACAA---TTATTG---TCTGGTATAGTGCAACAGCAGAAC---
AATTTG---CTGAGA---GCTATTGAGGCGCAACAGCATATGTTGCAACTCACAGTCTGGGGCATT
>MK041580
TTCTACTGCAATACATCAGGACTGTTTAATGAGACA------------------------------------
----

GAATTCAATGGTACTATCACTCTCCCATGTCGAATAAAACAAATTGTAAACATGTGGCAGGAAGTAGGAAAAG
CAATGTATGCCGCTCCCATCGCAGGAAACATTACCTGCAACTCAAAT---
ATTACAGGTCTACTATTGACAAGAGATGGTGGTCAGAATAATAAT---------------------------
----

GCTACTGAGATCTTCAGACCTGGGGGAGGAAATATGAAAGACAATTGGAGAAGTGAATTATATAAATATAAAG
TAGTAGAAATTGAACCACTA---GGAGTAGCACCCACCAGGGCAAAAAGACAAGTGGTG--------------
-------------------------AGGAGAGAAAAAAGGGCA---GTGGGA---ATAGGAGCT---------
TTGTTCCTTGGGTTCTTGGGAGCA---GCAGGAAGCACTATG---GGCGCAGCGTCA---
CTAACGCTGACGGTACAG---GCCAGACAA---TTATTG---TCTGGAATAGTGCAACAGCAGAGC---
AATTTG---CTGAGG---GCTATTGAAGCGCAACAGCATATGTTGCAGCTCACAGTCTGGGGCATT
>AF005495
TTCTACTGCAATACATCAGGACTGTTTAATGACACAGTAGAC-------------------------------
----

AATGGCACTATCACTCTCCCATGTCGAATAAAGCAAATTGTAAATATGTGGCAGGAAGTGGGGCGAGCAATGT
ATGCCGCTCCCATTGCAGGAAACATTACCTGTAGCTCAAAT---
ATTACAGGTCTACTATTGACAAGAGATGGTGGTCAGAATAATCAG----------------------------
----

ACGGAGGAGACCTTCAGACCTGGGGGAGGAAATATGAAAGACAACTGGAGAAGTGAATTATATAAATATAAAG
TAGTAGAAATTGAGCCATTA---GGAGTAGCACCCACCAAGGCAAAAAGACAAGTGGTG--------------
-------------------------AAGAGAGAAAAAAGAGCA---GTGGGA---ATGGGAGCT---------
TTGTTCCTTGGGTTCTTGGGAGCA---GCAGGAAGCACTATG---GGCGCAGCGTCA---
ATAACGCTGACGGCACAG---GCCAGACAA---TTATTG---TCTGGCATAGTGCAACAGCAGAAT---
AATTTG---CTGAGG---GCTATTGAAGCGCAACAGCATCTGTTGCAGCTCACAGTCTGGGGCATT
>AY455784
TTCTACTGCAATACATCAGGACTGTTTAATAACACAAGATCC-------------------------------
----

AATGGCACTATCACTCTCCCATGTCGAATAAAACAAATTATAAATATGTGGCAGGAAGTAGGACGAGCAATGT
ATGCCCCTCCCGTTGCAGGAAACATTACCTGTAACTCAAAT---
ATTACAGGTCTACTATTGACAAGAGATGGTGGTAAACAGAATGGTACT-------------------------
----

AATCAGACTGAGATCTTCAGACCTGGGGGAGGAGATATGAGAGACAATTGGAGAAGTGAATTATATAAATATA
AAGTAGTAGAAATTGAACCACTA---GGAGTAGCACCCACCAAGGCAAAAAGACAAGTGGTG-----------
----------------------------AAGAGAGAAAAAAGAGCA---ATAGGA---ATAGGAGCT------
---GTACTCCTTGGGTTCTTGGGAAGA---GCAGGAAGCACTATG---GGCGCGGCGTCA---
ATAACGCTGACGGCACAG---GCCAGACAA---GTATTG---TCTGGAATAGTGCAACAGCAGAAC---
AATCTG---CTGAGG---GCTATTGAAGCGCAACAGCATCTGTTGCAGCTCACAGTCTGGGGCATT
>AY455785
TTCTACTGCAATACATCAAAACTGTTTAATAACATAGGAGAC-------------------------------
----

AATGGCACTATCATTCTCCCATGTCGAATAAAACAAATTATAAACATGTGGCAGGAAGTAGGACGAGCAATGT
ATGCCGCTCCCATTGCAGGAAACATTACCTGTAACTCAAAT---
ATTACAGGTTTACTATTGACAAGAGATGGTGGTCAGACTAATGAA----------------------------
----

ACTAATACTGAGACCTTCAGACCTGGGGGAGGAAATATGAAAGACAATTGGAGAAGTGAATTATATAAATATA
AAGTAGTAGAAATTGAACCAATA---GGAATAGCACCCACCAGGGCAAAAAGACGAGTGGTG-----------
----------------------------CAGAAAGAAAAAAGAGCA---GTGGGA---ATGGGAGCT------
---TTGTTCCTTGGGTTCTTGGGAGCA---CAAGGAAGCACTATG---GGCGCGGCGTCA---
ATAGCGCTGACGGTACAG---GCCAGACAA---TTATTG---TCTGGAATAGTGCAACAGCAGAAC---
AATTTG---CTGAGG---GCTATTGAAGCGCAACAGCACCTGTTGCAGCTCACAGTCTGGGGCATT
>AY455780
TTCTATTGCGACACATCACAACTGTTTAATAACACAAAAGTC-------------------------------
----

AATGACACTATCACCCTCCCGTGTAGAATAAAACAAATTGTAAACATGTGGCAGGAAGTAGGACGAGCAATGT
ATGCCGCTCCTATTGCAGGAAACATTACCTGTAATTCAAGT---
ATTACAGGTCTACTATTGACAAGAGATGGTGGTCAGGCTAATAGG----------------------------
----

ACTGAGACCTTCAGACCTGGGGGAGGAAATATGAAAGACAATTGGAGAAGTGAATTATATAAATATAAAGTAG
TAGAAATTGAACCACTA---GGAGTAGCACCCACCGGGGCAAGAAGACAAGTGGTG-----------------
----------------------AGGAGAGAAAGAAGAGCA---GTGGGAATGCTAGGAGCT---------
ATGTTCCTTGGGTTCTTGGGAGCA---GCAGGAAGCACTATG---GGCGCAGCGTCA---
ATATCGCTGACGGTACAG---GCCAGACAA---TTATTG---TCTGGTATAGTGCAACAGCAGAAC---
AATCTG---CTGAGA---GCTATTGAGGCGCAACAGCATATGTTGCAACTCACAGTCTGGGGCATT
>AY771592
TTCTACTGTGATACAACACAACTGTTTAATAGTACTTGGAATGATACTTGGTATAATAATGTGAATGAT----
----

ACTAGTGAAAATATCACACTCCCATGCAGAATAAAACAAATTATAAACAAGTGGCAGGAAGTAGGAAAAGCAT
TTTATGCCCCTCCTATCAGCGGACAAATTAGATGTTCATCAAAT---
ATTACAGGTATACTATTGACAAGAGATGGTGGTCAGAATAGTCAG----------------------------
----

AATGAGACCTTCAGACCAGGAGGAGGAAATATGAAGGACAATTGGAGAAGTGAATTATATAAATATAAAGTAG
TAAGAGTTGAGCCATTA---GGAATAGCACCCACCAGGGCAAAGAGAAGAGTGGTG-----------------
----------------------CAGAGAGAAAGAAGAGCA---GTGGGAACACTAGGAGCT---------
ATGTTCCTTGGGTTCTTGGGAGCA---GCAGGAAGCACTATG---GGCGCAGCGTCA---
ATGACGCTGACGGTACAG---GCCAGACTA---CTATTG---TCTGGTATAGTGCAACAGCAGAAC---
AATCTG---CTGAGA---GCTATTGAGGCGCAACAGCATATGTTGCAGCTCACAGTCTGGGGCATC
>AY771593
TTCTACTGTAATTCAACTCAACTGTTCAATAGTACTTGGCTCAATAATACTTGGTATAATACTACTTGGGAAG
GAAAT--

AGCACTGAAGACAATATCACACTCCCATGCAGAATAAAACAAATTATAAACCTGTGGCAGAAAGTAGGAAAAG
CAATGTATGCCCCTCCTATCAGAGGACAAATTAGATGCTCATCAAAT---
ATTACAGGTCTAATATTGACAAGAGATGGTGGTCGTCAGAATGATACT-------------------------
----

AATGCTACTGACACCTTAACAGCTGGAGGAGGAAATATGAACGACTGTTGGAGAGGTGAATTTTATTATTATA
AAGTAGTAATAGTTGCGCCAATA---GGAGCACCCCCCCCCCAGGCAAAGAGAATAGTGGTG-----------
----------------------------CAAAAAGAAAAAAGAGCG---GTGGGAGTAATAGGAGCT------
---ATGTTCCTTGGGTTCTTGGGAGCA---GCAGGAAGCACTATG---GGCGCAGCGTCA---
ATGACGCTGACGGTACAG---GCCAGGTCA---TTATTG---TCTGGTATAGTGCAACAGCAGAAC---
AATCTG---CTGAGG---GCTATTGAGGCGCAACAGCATCTGTTGCAACTCACAGTCTGGGGCATC
>AY536233
TTCTACTGCAATACATCAGGACTGTTTAATGAAACAAGGTTTAATAACACAACA-------------------
----

TCCAATGACACCATTATTCTCCAATGTAAAATAAGACAAATAGTAAACCTGTGGCAGGAAGTGGGACGAGCAA
TGTATGCCGCTCCCATTGAAGGAAATATTACCTGTAACTCAAAT---
ATTACAGGTCTACTATTGACAAGAGATGGTGGTAATACTAGTCAGGCT-------------------------
----

AATAAGACTGAGACCTTCAGACCTGGGGGAGGAAATATGAGAGACAATTGGAGAAGTGAATTATATAAATATA
AAGTAGTAGAAATTGAACCACTA---GGAATAGCACCCACCAAGGCAAAAAGACAAGTGGTG-----------
----------------------------CAGAGAGAAAAAAGAGCAGTAGTGGGA---ATAGGAGCT------
---GTTCTCCTTGGGTTCTTGGGAGCA---GCAGGAAGCACTATG---GGCGCAGCGTCA---
ATAACGCTGACGGTACAG---GCCAGACAA---TTATTG---TCTGGAATAGTGCAACAGCAGAGC---
AATTTG---CTGAGG---GCTATTGATGCGCAACAGCATATGTTGCAGCTCACAGTCTGGGGCATT
>AY536238
TTCTACTGCAATACATCAGAACTGTTTAATAACACAGAAACCAATAGCACAAACACCAATGGCACAGAA----
----

ACCAATGGCACTATTATTCTCCCATGTAGAATAAAACAAATTGTGAACATGTGGCAGGAAGTGGGACGAGCAA
TGTATGCCAATCCCATTGCAGGAAACATTACCTGCAACTCAAAT---
ATTACAGGTCTACTATTGACAAGAGATGGTGGTAATACTAGTAAT----------------------------
----

AATACTGAGACCTTCAGACCTGGGGGAGGAGATATGAGAGACAATTGGAGAAGTGAATTATATAAATATAAGG
TAGTAGAAATTCAACCATTA---GGAATAGCACCCACCAAGGCAAGGAGACCAGTGGTG--------------
-------------------------AAGAGAGAAAAAAGAGCA---GTGGGA---CTAGGAGCT---------
TTGATCATTGGGTTCTTGGGAGCA---GCAGGAAGCACTATG---GGCGCAGCGTCA---
ATGGCGCTGACGGTACAG---GCCAGACAA---TTATTG---TCTGGAATAGTGCAACAGCAAAAC---
AATTTG---CTGAGA---GCTATTGAAGCGCAACAACATATATTGCAGCTCACAGTCTGGGGCATT
>KU749408
TTCTACTGCAATACATCAGGACTGTTTAATAACTCACTGCTCAATGACACAAAATCCAATAATGACACAGGA-
----

TCCAATAATACTATTACTCTCCCATGTAGAATAAGACAAATTGTAAACCTGTGGCAGGAAGTAGGACGAGCAA
TGTATGCCGCTCCCATTGCAGGAAACATTACCTGTAACTCAAAT---
ATTACAGGTATACTATTGACAAGAGATGGTGGTCAGAATAATAAGAATGAGACC------------TTC----
----

AAGAATGAGACCTTCAGACCTGGGGGAGGAAATATGAGAGACAATTGGAGAAGTGAATTATATAAATATAAAG
TAGTAGAAATTGAACCACTA---GGAGTAGCACCCACCAAGGCAAAAAGACAAGTGGTG--------------
-------------------------AAGAGAGAAAAAAGAGCA---GTAGGA---ATGGGAGCT---------
TTGTTCCTTGGGTTCTTGGGAGCA---GCAGGAAGCACTATG---GGCGCAGCGTCA---
GTAGCGCTGACGGTACAG---GCCAGACAA---TTATTG---TCTGGAATAGTGCAACAGCAGAAC---
AATTTG---CTGAGG---GCTATTGAAGCGCAACAGCATATGTTGCAGCTCACAGTCTGGGGCATT
>FJ853621
TTCTACTGTAATACATCAGAACTGTTTAATAACACAATATCC-------------------------------
----

AAAAATGGCACTATCATTCTCCCATGTCGAATAAAACAAATTATAAACATGTGGCAGGAAGTGGGGCGAGCAA
TGTATGCCGCTCCCATTGCAGGAAACATTACCTGTAACTCAAAT---
ATTACAGGTCTACTATTGGTAAGAGATGGTGGTAATCGTAATCAGACA-------------------------
----

AATAAGACTGAGACCTTCAGACCTGGGGGAGGAAATATGAAGGACAATTGGAGAAGTGAATTATATAAATATA
AAGTAGTAGAAATTGAACCACTA---GGAATAGCACCCACCAAGGCAAAAAGACAAGTGGTA-----------
----------------------------ACGAGAGAAAAAAGAGCA---GTGGGA---CTGGGAGCT------
---CTGTTCCTTGGGTTCTTGGGAGCA---GCAGGAAGCACTATG---GGCGCGGCGTCA---
ATAACGCTGACGGTACAG---GCCAGACAA---TTATTG---TCTGGAATAGTGCAACAGCAGAGC---
AATCTG---CTGAGA---GCTATTGAAGCGCAACAGCATATGTTGCAGCTCACAGTCTGGGGCATT
>JX140661
TTCTATTGCAATACATCAGGACTGTTTAATAACATAAAAATT-------------------------------
----

AATGGTACTATTACTCTCCCATGTCGAATAAAACAAATTGTAAACATGTGGCAGGAAGTGGGACGAGCAATGT
ATGCCAATCCCATTGCAGGAAACATTACCTGTAGCTCAATT---
ATTACCGGTCTGTTATTGACAAGAGATGGTGGTAATCAGACTGGTAATAAT----------------------
----

AGTTATATTGAGACCTTCAGACCTGGGGGAGGAAATATGAAAGACAATTGGAGAAGTGAATTATATAAATATA
AAGTAGTAGAAATTGAACCACTA---GGAGTAGCACCCACCAGGGCAAAAAGACAAGTGGTG-----------
----------------------------AAGAGAGAAAAGAGAGCA---CTAGGA---CTAGGAGCT------
---TTGTTCCTTGGGTTCTTGGGAGCA---GCAGGAAGCACTATG---GGCGCAGCGTCA---
GTGACGCTGACGGTACAG---GCCAGACAA---TTATTG---TCTGGAATAGTGCAGCAGCAGAAC---
AATTTG---CTGAAG---GCCATTGAAGCGCAGCAGCATCTGTTGCAGCTCACAGTCTGGGGCATT
>FJ904244
TTCTATTGCGATACATCTAAATTGTTTAAAGTGTTTAATGACACCAATGGCACAAAAGCCAATAAT-------
----

GACACAGACCCTATCATTCTCCCATGTCGAATAAAACAAATTGTAAACATGTGGCAAGAAGTAGGACGAGCAA
TGTATGCTAATCCCATTGAGGGAAACATCACCTGTAGATCAAAT---
ATTACAGGTCTAATATTGACAAGAGATGGTGGTAATCATAATAATGATAGT----------------------
----

CAGGATAATCAGACTGAAATCTTTAGACCTGGAGGAGGAAATATGAAAGACAATTGGAGAAGTGAATTATATA
AATATAAAGTAGTAGAAATTGAACCACTA---GGAGTAGCACCCACCAGGGCAAAAAGGCAAGTGGTG-----
----------------------------------AAGAGAGAAAAAAGAGCA---ATGGGA---
CTAGGAGCT---------TTGTTCCTTGGGTTCTTGGGAGCA---GCAGGAAGCACTATG---
GGCGCAGCGTCA---GTGACGCTGACGGTACAA---GCCAGACAA---TTACTG---
TCTGGTATAGTGCAACAGCAGAAC---AATCTG---CTGAGA---
GCTATTGAGGCACAACAGCATCTGTTGCAACTCACAGTCTGGGGCATT
>AB480301
TTCTACTGCAATACATCAGGATTGTTTAATGACACAGAAACC-------------------------------
----

AATGATACTATCATTCTCCCATGTAAAATAAAACAAATTATAAACATGTGGCAGGAAGTGGGACGAGCAATGT
ATGCCGCTCCCATTGCAGGAAACATTACCTGTAACTCAAGT---
ATTACAGGTCTACTATTGACAAGAGATGGTGGTCAGAAAAATAAT----------------------------
----

ACTAATGAGACCTTCAGACCTGGGGGAGGAAATATGAAAGACAATTGGAGAAGTGAATTATATAAATATAAAG
TAGTAGAAATTCAACCACTA---GGAGTAGCACCCACCAGGGCAAGAAGATTGGTGGTG--------------
-------------------------AAGAGAGAAAAAAGAGCA---GTGGGA---ATGGGAGCT---------
ATGTTCCTTGGGTTCTTGGGAGCA---GCAGGAAGCACTATG---GGCGCAGCATCA---
ATAACGCTGACGGTACAG---GCCAGACAA---TTATTG---TCTGGAATAGTGCAACAGCAAAAC---
AATTTG---CTGAAG---GCTATTGAAGCGCAACAGCATCTGTTGCAGCTCACAGTCTGGGGCATT
>JN251899
TTCTACTGCAATACATCAGGACTGTTTAATGACACAAGACTGTTAAATGACACA-------------------
----

TCCAATAACACTATTGTTCTACCATGTCAAATAAAACAAATTGTAAACTTATGGCAGGAAGTAGGACGAGCAA
TGTATGCCGCTCCCATTGCAGGAAATATTACCTGTAGCTCAAAT---
ATTACAGGTCTACTATTGACAAGAGATGGTGGTCACAGTAATAAT----------------------------
----

ACTAATGAGACCTTCAGACCTGGGGGAGGAAACATGAAAGACAATTGGAGAAGTGAATTATATAAATATAAAG
TAGTAGAAATTGAACCACTA---GGAGTAGCACCCACCAAGGCAAAAAGACAAGTGGTG--------------
-------------------------AAGAGAGAAAAAAGAGCA---GTGGGA---ATAGGAGCT---------
GTTCTCCTTGGGTTCTTGGGAGCA---GCAGGAAGCACTATG---GGCGCAGCGTCA---
GTAGCGCTGACGGTACAG---GCCAGACAA---TTATTG---TCTGGAATAGTGCAACAGCAGAAC---
AATCTG---CTGAGG---GCTATTGAAGCGCAACAGCATATGTTGCAGCTCACAGTCTGGGGCATT
>JN251905
TTCTACTGTGATACATCAAAACTGTTTAATGAGACAAAATTCAATGACACTAGTGAA----------------
----

GGTAGTCCTAATATTACTCTCCCATGTCGAATAAAACAAATTGTAAACATGTGGCAGGAAGTGGGGCGAGCAA
TGTATGCCGCTCCCATTGCAGGAGCCATTACCTGTAACTCAAAT---
ATTACAGGTCTCCTATTGACAAGAGATGGTGGTAATACTACTAAT----------------------------
----

CAGACTATTGAGATCTTCAGACCTGGGGGAGGAGATATGAAAGACAATTGGAGAAGTGAATTATATAAATATA
AAGTAGTAGAAATTGAACCATTA---GGAATAGCACCCACCAAGGCAAAGAGACGAGTGGTG-----------
----------------------------CAGAGAGAAAGAAGAGCA---GTGGGA---ATGGCAGCT------
---GTACTCCTTGGGTTCTTGGGAGCA---GCAGGAAGCACTATG---GGCGCAGCGTCA---
ATAACGCTGACGGTACAG---GCCAGACAA---TTATTG---TCTGGGATAGTGCAACAGCAGAGC---
AATTTG---CTGAGG---GCTATTGAAGCGCAACAGCATCTGTTGCAGCTCACAGTCTGGGGCATT
>AY781128
TTCTACTGCAATACAACACAACTGTTTAATGACGCAGTATCT-------------------------------
----

AGTGACACTATTATTCTCCCATGTAAAATAAAGCAAATTATAAACATGTGGCAGGAAGTGGGACGAGCAATGT
ATGCCGCTCCCATTGCAGGAAATATTACCTGTAACTCAAAC---
ATTACAGGTCTATTATTGGTAAGAGATGGTGGTAATAATAATCAGACT-------------------------
----

AATAATACTGAGACCTTTAGACCTGGGGGAGGAAATATGAAAGACAATTGGAGAAATGAATTATATAAATATA
AAGTAGTAGAAATTGAACCACTA---GGAGTAGCACCCACCAGGGCAAAAAGACAAGTGGTG-----------
----------------------------AAGAGAGAAAAAAGAGCA---GCAGGA---ATAGGAGCT------
---GTGCTCATTGGGTTCTTGGGAGCA---GCTGGAAGCACTATG---GGCGCGGCGTCA---
ATGACGCTGACGGTACAG---GCCAGACAA---TTATTG---TCTGGAATAGTGCAACAGCAGAGC---
AATCTG---CTGAGG---GCTATTGAAGCGCAACAGCATCTGTTGCAGCTCACAGTCTGGGGCATT
>JN235953
TTCTACTGCAATACATCAGGACTGTTTAATGACACAATATCC-------------------------------
----

AATGACACTATCATCCTCCCGTGTCGAATAAAACAAATTGTAAACATGTGGCAGGAAGTGGGACGAGCAATGT
ATGCCGCTCCCATTGCAGGAAACATTACCTGTAGCTCAAAT---
ATTACAGGTCTACTATTGACAAGAGATGGTGGTCAGAATAATGGA----------------------------
----

ACAGAGACCTTCAGACCTGGAGGAGGAGATATGAGGGACAATTGGAGAAGTGAATTATATAAATATAAAGTAG
TAGAAATTGAACCATTA---GGAATAGCACCCACCAAGGCAAAGAGAAGAGTGGTG-----------------
----------------------CAGAGAGAAAAAAGAGCAGTAATGGA---TTAGGAGCT----------
GTGTTCCTTGGGTTCTTGGGAGCA---GTAGGAAGCACTATG---GGCGCAGCATCA---
CTAACGCTGACGGTACAG---GCCAGACAA---TTATTG---TCTGGTATAGTGCAACAGCAAAAC---
AATTTG---CTGAGG---GCTATTGAGGCGCAACAGCATATGTTGCAACTCACAGTCTGGGGCATC
>KF726017
TTCTACTGTAATACAACAAAACTGTTTAATAGTACTTGGAGACTGCTTAATAGTACTTGGAATAGTACTGAAG
GGTTAAAT---
-------------GACACT------
GTAGGAAATGACACAATCACACTCCCATGCAGAATAAAACAAATTGTAAATTTGTGGCAGGAAGTAGGAAGAG
CAATGTATGCCCCCCCCATCAGTGGAAACATTAGATGTTCATCAAAT---
ATTACAGGTCTATTACTGACAAGGGATGGGGGTCAGGGTAATTCAAGTAAT----------------------
----

AGTACTACGGAGACCTTCAGACCTGGGGGAGGAAATATGAAAGACAATTGGAGAAGTGAACTATATAAATATA
AAGTAGTAGAAATCAAACCACTA---GGAGTAGCACCCACCAAGGCAAGAAGACAAGTGGTG-----------
----------------------------AGGAGAGAAAAAAGAGGC------------CTAGGAGCT------
---GTGTTCCTTGGGTTCTTAGGAGCA---GCAGGAAGCACTATG---GGCGCAGCGTCA---
ATAACGCTGACGGTACAG---GCCAGACTA---TTATTG---TCTGGTATAGTGCAACAGCAGAAC---
AATCTG---CTGAGG---GCTATTGAGGCGCAACAGCATCTGTTGCAACTCACAGTCTGGGGCATC
>MK086130
TTCTACTGTAACACAACAAGGCTGTTT------GAGGAGAATAATAATGCAACACAGAAAAAT----------
----

AATGAAACTATTATCACTCTTCCCTGTAGAATAAGACAACATGTAAACATGTGGCAAAGAGTGGGACGAGCAA
TGTATGCCCCCCCCATTGCTGGAATAATTCAGTGTAACTCAAAT---
ATCACAGGTCTACTATTAACAAGAGATGGT---AATAACACTGGA----------------------------
----

AATAATGACACCCTCAGACCTGGAGGGGGAGATATGAGAGACAATTGGAGAAGTGAACTGTATAAATATAAGG
TAGTAAAAATTGAACCACTA---GGAATAGCACCCTCCAAGGCAAAAAGACGAGTGGTG--------------
-------------------------CAGAGAGAAAAAAGAGCA---GTGGGA---ATGGGAGCT---------
ATGTTCCTTGGGTTCTTGGGAACA---GCAGGAAGCACTATG---GGCGCGGCGTCA---
ATGGCGCTGACGGTACAG---GTCAGACAA---TTATTG---TCTGGTATAGTGCAGCAGCAAAGC---
AATTTG---CTGAAG---GCTATAGAAGCGCAACAGCATCTGTTGCAGCTCACAGTCTGGGGCATT
>U39253
TTCTACTGCAATACATCAAAACTGTTTAGTACATCAAATAGTACATGGGTA----------------------
----

AACAAGACTACAATCACACTCCCATGCAGAATAAGACAAATTATAAACATGTGGCAGAAAGTAGGAAAAGCAA
TGTACGCCCCTCCCATTGAAGCACTAATCAACTGTTCATCAAAT---
ATTACAGGACTATTGTTGACAAGAGATGGTGGTGTAAATAATAGT----------------------------
----

CAGGAGGAGATCTTCAGGCCTGGAGGTGGAGATATGAGAGACAATTGGAGGAGTGAATTATACAAATATAAAG
TAATAAGAATTGAACCAATA---GGTCTAGCACCCACCAGGGCAAAGAGAAGAGTGGTG--------------
-------------------------GAAAGAGAAAAAAGAGCA---ATAGGA---CTGGGAGCT---------
GTGTTCCTTGGGTTCTTGGGAGCA---GCAGGAAGCACTATG---GGCGCAGCATCA---
ATAACGCTGACGGTACAA---GCCAGACAA---TTATTG---TCAGGTATAGTGCAACAGCAAGAT---
AATTTG---CTGAGG---GCTATAGAGGCGCAACAGCATATGTTGCAACTCACAGTCTGGGGCATT
>AY074891
TTCTACTGTAACACATCAGGACTGTTTAATAGTATAAGTATAAGTGAATCAAATAGTACAGGG----------
----

GTAAATGGTACAATCACACTCCGATGCAGAATAAAACAAATTATAAACATGTGGCAGGGAGTAGGAAAAGCAA
TATATGCCCCTCCCATTGAGGGACTAATCCAATGTTCATCAAAT---
ATTACAGGACTATTGTTGACAAGAGATGGTGGTAATAATAGTCAG----------------------------
----

ACTGAGACTGAGACCTTCAGACCTGGAGGAGGAGATATGAGAGATAATTGGAGAAGTGAATTATACAGATATA
AAGTAGTAAGAATTGAACCAATA---GGTCTAGCACCCACCAGGGCAAAAAGAAGAGTGGTG-----------
----------------------------ACAAGAGAAAAAAGAGCA---ATAGGA---CTGGGAGCT------
---ATGTTCCTTGGGTTCTTGGGAGCA---GCAGGAAGCACGATG---GGCGCAGCGTCG---
CTGACGCTGACGGTACAG---GCCAGACAG---TTATTG---TCTGGTATAGTGCAACAGCAAAAC---
AATTTG---CTGAGA---GCTATAGAGGCGCAACAGCATCTGTTGCAACTCACAGTCTGGGGCATT
>KU921741
TTCTACTGCAAGACATCAGAACTGTTTAATAGTACATGGACATTAAATAGTACAAGTGAAGGACATCAAATA
GTACAAAG---
-------------------ATA---
ACAAATAGTACAAATATAACACTCCCGTGCAGAATAAAACAAATTATTAACATGTGGCAGGGAGTAGGAAAAG
CAATGTATGCCCCTCCCATTGAAGGACTTATCAAATGCTCATCAAAT---
ATTACAGGAATATTGTTGACAAGAGATGGTGGTACAGATAATCAGACAAATGAG-------------------
----

ACCTTCAGTGAGACCTTCAGACCTGGAGGAGGAGATATGAGGGACAATTGGAGAAGTGAATTATACAAATATA
AAGTAGTAAAAATTGAACCAATA---GGTCTAGCACCCACCAGGGCAAAAAGGAGAGTGGTG-----------
----------------------------GAAAGAGAAAAAAGAGCA---ATAGGA---CTGGGAGCT------
---ATGTTCCTTGGGTTCTTGGGAGCT---GCAGGAAGCACGATG---GGCGCAGCGTCG---
GTGACGCTGACGGTACAG---GCCAGACAG---CTAATG---TCTGGTATAGTGCAACAGCAAAGC---
AATTTG---CTGAGG---GCTATAGAGGCGCAACAACATATGTTGCAACTCACAGTCTGGGGCATT
>MF373183
TTCTATTGCAATACATCAAGCCTGTTTCATGAGAACATCACTGCAAACAGCACAAACAGCACAGAT-------
----

GTAAATAAAAACATCACACTTCAATGCAAAATCAGACAAATTGTAAGAATGTGGCAAGGATAGGACAAGCAA
TGTATGCCCCTCCCATTGAAGGACTAATAAATTGTAAATCAAAT---
ATCACAGGGCTGTTGCTGACACGTGATGGAGGCCCCAACAACCGG----------------------------
----

ACTAATGAAACATTTAGACCTCAAGGGGGAAATATGAGGGACAATTGGAGAAGTGAATTATATAAATACAAAG
TGGTAGAAATTAAGCCATTA---GGACTAGCACCCACTGGGGCACAGAGGAGAGTGGTG--------------
-------------------------GGAAGAGAAAAAAGAGCA---GCAACA---CTAGGAGCT---------
TTGTTCCTTGGGTTCTTGGGAGCA---GCCGGAAGCACTATG---GGCGCAGCGTCA---
ATAACGCTGACGGTACAG---GCCAGACAA---TTATTG---TCTGGTATAGTGCAACAGCAAAGC---
AATTTG---CTGAGA---GCCATAGAGGCGCAACAGCATATGTTGCAACTCACAGTCTGGGGCATT
>AY253309
TTCTACTGCAATACATCAGGGCTATTTAATAATAGTGAGTGGACATCAAATAGTACAAATAGTACATCAAATA
GTACA--

GGGGGAGATGATGTAATCACACTCCAATGCAAAATAAAACAGATCATAAACATGTGGCAGGGAGTAGGAAAAG
CAATGTATGCCCCTCCCATTGAAGGCCAAATCAGATGTTCATCAAAT---
ATTACAGGACTATTGTTAACAAGAGATGGTGGTCAGAATAAGAGT----------------------------
----

CAGGAGGAGACCTTCAGACCTGGAGGAGGAAATATGAGAGACAATTGGAGAAGTGAATTATACAAATATAAAG
TAGTAAGGATTGAACCACTA---GGTCTAGCACCCACCAGGGCACAAAGAAGAGTGGTG--------------
-------------------------CAAAGAGAAAAAAGAGCA---ATAGGC---CTGGGAGCT---------
ATGTTCCTTGGGTTCTTGGGAGCA---GCAGGAAGCACGATG---GGCGCGGCGTCG---
CTGACGCTGACGGTACAG---GCCAGACAG---TTATTG---TCTGGTATAGTGCAACAGCAAAAC---
AATTTG---CTGAGG---GCTATAGAGGCGCAACAACATTTGTTGCAACTCACAGTCTGGGGCATT
>HQ615974
TTCTACTGCAATACAACACCACTGTTTAATAATAGTGTATGGTCAAGTAATACAAGTATAGATACAAGTACAA
ATAATACA---
-------------------GAG---
GGAAATAATGGGACAATCATACTCCAATGTAGAATAAAACAAATTGTAAACATGTGGCAAAAAGTAGGAAAAG
CAATATATGCCCCTCCCATTGCAGGACTAATCAATTGTCACTCAAAT---
ATTACAGGAATATTGTTGACAAGAGATGGTGGTAACAATATTAATCAGACTAAG------
AACAATAGTAGTCAGACTGAGAGC---

AATAGGAGTCAGACTGAGACCTTCAGACCTGGAGGAGGAGATATGAGAGACAATTGGAGAAGTGAATTATATA
AATATAAAGTAGTAAGAATTGAACCAATA---GGTATAGCACCCACCAAGGCAAAGAGAAGAGTGGTG-----
----------------------------------GAAAGAGAAAAAAGAGCA---ATAGGA---
CTGGGAGCT---------ATGTTCCTTGGGTTCTTGGGAGCA---GCAGGAAGCACAATG---
GGCGCAGCGTCG---GTGACGCTGACGGTACAG---GCCAGACAG---TTATTG---
TCTGGTATAGTGCAACAGCAGAGC---AATTTG---CTGAGG---
GCTATAGAGGCGCAACAGCATCTGTTGCAACTCACAGTCTGGGGCATT
>AF484500
TTCTACTGCAATACATCAGGGATATTTAATAGTACATGGGAGGCTAATAATACAGCAAATAGTACAGGG----
----

GTAGATAAGAATATCACACTCTCATGCAGAATAAAACAAATTATAAACATGTGGCAGGGAGTAGGAACAGCAA
TGTATGCCCCTCCCATTGAAGGACTAATTAAATGTACATCAAAT---
ATTACAGGACTATTGTTAACAAGAGATGGTGGTGCAAATAATAAT----------------------------
----

AGTCAGAATGAGACCTTCAGACCTGGAGGGGGAGATATGAGAGACAATTGGAGAAGTGAATTATACAAATATA
AAGTAGTAAGAATTGAACCACTA---GGTCTAGCACCCACCAGGGCAAAGAGAAGAGTGGTG-----------
----------------------------GAAAGAGAGAAAAGAGCA---ATAGGA---CTGGGAGCT------
---ATGTTCCTTGGGTTCTTGGGAGCA---GCAGGAAGCACGATG---GGCGCAGCGTCA---
CTGACGCTGACGGTACAT---GCCAGACAG---TTATTG---TCTGGTATAGTGCAACAGCAAAAT---
AATTTG---CTGAGG---GCTATAGAGGCGCAGCAGCATCTGTTGCAACTCACAGTCTGGGGCATT
>MF373188
TTCTACTGCAATACATCAGAGCTGTTTAATGAAACAGAAAAC-------------------------------
----

TCCAATGGCACTATTATTCTCCCATGTAGAATAAAACAAATGATAAACATGTGGCAGGAAGTGGGACGAGCAA
TGTATGCCGCTCCTATTGCAGGAAACATTGTCTGTAACTCATCT---
ATTACAGGTCTACTATTGACAAGAGATGGGGGTAATAATGATAAT----------------------------
----

AATAACACTGAGATCTTCAGGCCTGGAGGAGGAGATATGAGGGACAATTGGAGAAGTGAATTATACAAATATA
AAGTGGTAGAAATTCAACCATTA---GGAATAGCACCCACCAAGGCAAAAAGGAGAGTGGTG-----------
----------------------------GAGAGAGAAAAAAGAGCA---GCA------TTGGGAGCT------
---TTGTTCCTTGGGTTCTTGGGAGCA---GCCGGAAGCACTATG---GGCGCAGCATCA---
ATAACGCTGACGGTACAG---GCCAGACAA---TTATTG---TCTGGGATAGTGCAACAGCAGAGC---
AATCTG---CTGAGG---GCTATTGAAGCGCAACAGCATCTGTTGCAGCTCACAGTCTGGGGCATT
>EU166607
TTCTATTGCAACACATCAGAACTGTTTAATGATACATACAATAATACTACT----------------------
----

CCAAATGGGACCATCACACTCCAATGCAGAATAAAACAAATTATAAACATGTGGCAGGGAGTAGGACAAGCAA
TGTATGCCGCCCCCATTGCAGGAAACATTATCTGTAACTCAAGT---
ATTACGGGGCTACTATTGACAAGAGATGGTGGTAAAAATAAAACT----------------------------

----

GGAGAAGAGATATTCAGACCTGGAGGAGGAGATATGAGGGACAATTGGAGAAGTGAACTCTATAAATATAAAG
TAGTAGAAATTCAACCACTA---GGAGTAGCACCCACCAGGGCAAGAAGACAAGTGGTG-------------
-------------------------AGAAGAGAAAAAAGAGCA---GTGGGA---ATAGGAGCC---------
TTGTTCCTTGGGTTCTTGGGAGCA---GCAGGAAGCACTATG---GGCGCAGCATCA---
ATAACGCTGACGGTACAG---GCCAGACAA---GTGCTG---TCTGGTATAGTGCAACAGCAAAAC---
AATTTG---CTGAGA---GCTATAGAGGCGCAACAGCATATGTTGCAACTCACAGTCTGGGGCATT
>KY392768
TTCTACTGCGACACATCAGGATTATTTAATGTTACATATGAGGTCAATAGTTCAAACCAATTAATAATGCT-
----

ACTAATAATAACATCACACTTCCATGTAAAATAAAACAATTTATAAACATGTGGCAGAAGGTAGGACGAGCAA
TGTATGCCCCTCCTATTGAAGGAATAATAACATGTGTATCAAAT---
ATTACAGGTCTACTATTGACTCGTGATGGAGGTCAAAATAACTCA---------------------------
----

GACACAGAGACCTTCAGACCAACAGGGGGAAATATGAAGGACAATTGGAGAAGTGAATTATATAAATATAAAG
TAGTAGAAATTAAACCACTA---GGAGTAGCACCCACCAAGGCAAGAAGGAGAGTGGTG-------------
-------------------------GAGAGAGAAAAAAGAGCA---GCGGGA---CTGGGAGCT---------
GTGATCATTGGGTTCTTGGGAGCA---GCAGGAAGCACTATG---GGCGCAGCGTCA---
CTAACGCTGACGGTACAG---GCCAGACAA---TTATTG---TCTGGTATAGTGCAACAGCAAAAT---
AATTTG---CTGAAA---GCTATAGAGGCGCAACAGCATCTGTTGCAGCTCACAGTCTGGGGCATT
>AB286849
TTCTATTGCAATACATCAGGCCTGTTTAATAGTACATATTACAGCCTGTTTAATAGTACATATTACAATGGTA
CATAC---

AATAATAATACCAACATCACCCTCCAATGCAGGATAAGACAAATTGTAAACATGTGGCAAGAGGTAGGAAAAG
CAATGTATGCTGCTCCCATTCAAGGAAGCATAACATGTGCATCAAAC---
ATCACAGGGTTACTCTTGACACGCGACGGAGGCAATGGAGAGACA---------------------------
----

AATGGGACAGAGATTTTCAGACCTCAAGGAGGAAATATAAAGGACAATTGGAGAAGTGAATTATATAAATATA
AAGTAGTAGAAGTTGAACCACTA---GGAGTAGCACCCACTAAGGCAAAAAGGAGAGTGGTG----------
----------------------------GAGAGAGAAAAAAGAGCA---GTGGGA---CTGGGAGCT------
---GCGTTCTTGGGGTTCTTAGGAGCA---GCAGGAAGCACTATG---GGCGCAGCGTCA---
CTAGCGCTGACGGTACAG---GCCAGACAA---TTACTG---TCTGGTATAGTGCAACAGCAAAAT---
AATTTG---CTGAGA---GCTATAGAGGCGCAACAACATATGTTGCAACTCACAGTCTGGGGCATT
>AJ277819
TTCTACTACAACACAACAGATCTGTTTAATAACATGGAAAAAACT---------------------------
----

AACAATAACACTATCATTCTCCCATGTAAAATAAGACAATTTGTAAACATGTGGCAAAGAGTGGGACGAGCCA
TGTATGCCCCTCCCATTGCCGGACAAATTCAGTGTAATTCAAAG---
ATCACAGGTCTACTATTGACAAGAGATGGTGGAGAGAATACAAAT---------------------------
----

GGGTCCGAGACCCTCAGGCCTGGAGGAGGAGATATGAGAGACAATTGGAGAAGTGAACTATATAAATATAAGG
TAGTAAGAATAGAACCACTA---GGAGTAGCACCTACCAAGGCAAAAAGACAAGTGGTG-------------
-------------------------CAGAGAGGAAAAAGAGCA---GTGGGA---ATAGGAGCT---------
GTACTCTTTGGGTTCTTAGGAGCA---GCAGGAAGCACTATG---GGCGCGGCGTCA---
ATAACGCTGACGGTACAG---GCCAGACAA---TTATTG---TCTGGTATAGTGCAACAGCAGAAT---
AATTTG---CTGAAG---GCTATAGAAGCGCAACAGCATCTGTTGCAGCTCACAGTCTGGGGCATT
>MN485986
TTCTACTGCAACACCTCACAACTGTTT------ACATGGTCAGATAATAGCACAGGGATA------------
----

AATGATAGCACAATCGTACTCTCTTGTAGAATAAAACAAATTATAAATATGTGGCAGGAAGTAGGAAAAGCTA
TGTATGCCCCTCCCATTGCAGGAAAAATCAGTTGTTCATCAAAA---
ATTACAGGGCTGTTATTGACAAGGGATGGTGGTAAAGAGACTAACGAGACCTCC------------------

----

GCTCACGAGATAGAGATCTTCAGACCTGCAGGAGGAGATATGAGGGACAATTGGAGAAGTGAATTATATAAAT
ATAAAGTAGTAAGAATTGAACCCCTT---GGAATAGCACCCACTAGGGCAAAAAGAAGAGTGGTG--------
-------------------------------GAAAGAGAAAAAAGAGCA---ATAGGA---CTAGGAGCT---
------ATGTTCCTTGGGTTCTTGGGAGCA---GCAGGAAGCACTATG---GGCGCGGCGTCA---
ATAACGCTGACGGTACAG---GCCAGACAA---TTATTG---TCTGGTATAGTGCAACAGCAAAAT---
AATTTG---CTGAGA---GCTATAGAGGCGCAACAGCATCTGTTGCAACTCACGGTCTGGGGCATT
>FJ670527
TTCTACTGCAACACATCATCCCTGTTTAATGGGACATGGACTAATAATACATGGCACAATATTACA-------
----

TGGAACACAAATATGACAATCCCATGCAAAATAAGGCAAATTGTAAACATGTGGCAAGGAGTAGGAAAAGCAA
TGTATGCCCCTCCCATCGAAGGACACATCAACTGTTCATCAAAT---
ATTACAGGACTACTATTGATAAGAGATGGGGGTAAAGGACAGAATCAG------------------------
----

AGTAATCAGAGTGAGACTTTCAGACCTGGAGGAGGAGATATGAGGGACAATTGGAGAAGTGAATTATATAAAT
ATAAAGTAGTAAAAATTGAACCTCTA---GGAATAGCACCCACCAAGGCAAGGAGAAGAGTGGTG--------
-------------------------------CAAAGAGAAAAAAGAGCA---ATAGGA---CTAGGAGCT---
------ATGTTCCTTGGGTTCCTGGGAGCA---GCAGGAAGCACGATG---GGCGCAGCGTCA---
GTGACGCTGACGGTACAG---GCCAGACAA---TTGATG---TCTGGTATAGTGCAACAGCAAAAC---
AATTTG---CTGAGA---GCTATAGAGGCGCAACAGCATCTGTTGCAACTCACAGTCTGGGGCATT
>FJ388908
TTCTACTGCAATACATCAAAACTGTTTAATAGGACATGGGATACAGGGAATACTACAGAACAGGGTATTAGTA
TA---

GGGTCAAATGATATAATCATACTCCCATGCAAACTAAAACAAATTATAAACATGTGGCAGGAAGTAGGAAAAG
CAATGTATGCCCCTCCCATTGAAGGAGAAATCAAATGCTCATCAAAT---
ATTACAGGGCTACTATTAACAAGAGATGGGGGAAATATTAATGGAAGT------------------------
----

AGTAATCAGACAGAGATCTTCAGACCTGGAGGAGGAGATATGAGAGACAATTGGAGAAGTGAATTATATAAAT
ATAAAGTAGTAACAATTGAACCAATA---GGACTAGCACCCACCAAGGCAAAAAGAAGAGTGGTG--------
-------------------------------GAAAGAGAAAAAAGAGCA---ATAGGG---TTAGGAGCT---
------ATGTTCCTTGGGTTCTTGGGAGCA---GCCGGAAGCACGATG---GGCGCAGCGTCA---
GTGACGCTGACGGTACAG---GCCAGACAA---TTGTTG---TCTGGTATAGTGCAGCAGCAAAAC---
AATTTG---CTGAGG---GCTATAGAGGCGCAACAGCACTTGTTGCAACTCACGGTCTGGGGCATT
>KX579838
TTCTACTGCAATACATCAGAACTGTTTAATTGGGATAATAGTACAAAGAGTACTGTAAATGGA----------
----

ACAAAATTAAATATAACTATCCACTGTAAAATAAAACAAATTATAAACATGTGGCAGGGAGTAGGAAGAGCAA
TGTATGCCCCTCCCATCGAAGGACAAATTCAATGTATATCAAAT---
ATTACAGGGCTACTATTGACAAGAGATGGTGGTAGTGATTCTAAT---------------------------
----

GAGACCTTCAGGCCTGGAGGAGGAGATATGAGAGACAATTGGAGAAGTGAATTATATAAATATAAAGTAGTAA
GAGTTGAGCCATTA---GGAGTAGCACCCACCAGGGCAAAGAGAAGAGTGGTG--------------------
------------------GACAGAGAAAAAAGAGCA---ATAGGA---TTAGGAGCT---------
ATGTTCCTTGGGTTCTTGGGAGCA---GCAGGAAGCACTATG---GGCGCAGCGTCA---
GTGACGCTGACGGTACAG---GCCAGACAA---TTATTG---TCTGGTATAGTGCAACAGCAAAAT---
AATTTG---CTGAGG---GCTATAGAGGCGCAACAGCATCTGTTGCAACTCACGGTCTGGGGCATT
>MN337382
TTCTACTGCAATACATCAGGACTGTTTAATAGAGCATTTAATAATACAACATGGGATACTAGTACAGGGAGTG
CTAGA--

TTAAATAATACAATAACCATCCTATGTAGAATAAAACAAATTATAAACATGTGGCAGGGAGTAGGAAGAGCAA
TGTATGCCCCTCCCATTGAAGGACAAATTAAATGTTCATCAAAT---
ATTACAGGGTTACATTTGACAGCAGATTGGGTTGGAAATGAGTCTGTG------------------------

----

TCTAGAGTAATTTACAGGCCTGGAGGAGGAAATATGAAGGACAATTGGAGAAGTGAATTATATAAATATAAAG
TAGTAAAAATTGAACCACTA---GGAGTAGCACCCACCAGGGCAAGGAGAAGAGTGGTG--------------
-------------------------GAAAGAGAAAAAAGAGCA---ATAGGA---CTAGGAGCT---------
ATGTTCCTTGGGTTCTTGGGAGCA---GCAGGAAGCACGATG---GGCGCAGCGTCA---
GTGACGCTGACGGTACAA---GCCAGACAA---CTGTTG---TCTGGTATAGTGCAACAGCAAAAT---
AACTTG---CTGAGG---GCTATAGAGGCGCAACAGCATCTGTTGCAACTCACGGTCTGGGGCATT
>AY352655
TTCTATTGCAACACAACAAAACTGTTTAATAGTAGCTGGAAACATGACAATAACACCAATAACACCTGGAATA
GAACTAATGGT--
AAAATCGATGGCCGCTAT---------
ACAGAAAATGATACTATTACACTGCCATGTAGAATAAAACAAATTATAAACATGTGGCAGAGAGTGGGACAAG
CAATGTATGCCCCTCCCATCCAAGGAAATATTACATGTGTATCAAAT---
ATTACAGGACTAATATTGACAAGGGACCATCCGAGTAATAAT-------------------------------
----

ACCTTCAGACCTGGAGGAGGAGATATGAGAGACAATTGGAGAAGTGAACTATATAAATATAAAGTAGTAAAAA
TTGAACCACTA---GGAGTGGCACCCTCCGAGGCAAGGAGAAGAGTGGTG-----------------------
----------------CAGAGAGAAAAAAGAGCA---GTGGGA---CTAGGAGCC---------
ATGATCTTTGGGTTCTTGGGAGCA---GCTGGGAGCACTATG---GGCGCGGCGTCA---
TTAACGCTGACGGTACAG---GCCAGACAA---TTGCTG---TCTGGTATAGTGCAACAGCAAAGC---
AATTTG---CTGAGA---GCTATACAAGCGCAACAGCATATGTTGCAGCTTACTGTCTGGGGCATT
>KU310619
TTCTATTGTAATACATCAGAACTGTTCAATCTATCAAGATTGTTCAATGGCAGTGGGGAAGAGACTAGC----
----

AATAAGACTCATATCACACTCCCATGTAGGATAAAACAAATTGTGAGAATGTGGCAGAGAGTAGGACAAGCAA
TATATGCCCCTCCCATCGCAGGAAACATCACATGCACATCAAAT---
ATTACAGGATTATTATTGACACGTGATGGGGGACCCAGTAATGGA----------------------------
----

ACTAATGAAACCTTCAGACCTACAGGAGGAGATATGAGGGACAATTGGAGGAGTGAGTTATATAAATATAAAG
TAGTAGAGATTGAGCCAGTA---GGAATAGCACCCACCAAGGCAAAAAGAAGAGTGGTG--------------
-------------------------GAGAGAGAAAAAAGAGCA---GTGGGA---ATGGGAGCT---------
GTGTTCCTTGGGTTCTTGGGAGCA---GCAGGAAGCACTATG---GGCGCAGCGTCA---
ATAACGCTGACGGTACAG---GTCAGACAA---TTGTTG---TCTGGCATAGTGCAACAGCAAAGC---
AATTTG---CTGAAG---GCTATAGAGGCGCAACAGCATCTGTTGAAACTCACTGTCTGGGGCATT
>AF286236
TTCTATTGCAACACATCAGAGCTGTTTACTGGTATTTGGAATGGTACTTGGGATAAGAATTGCACTAGCACTG
AGAGT--

AATTGCACTGGAAATATTACACTCCCATGCAGGATAAAACAAGTGGTAAGAACATGGCAGGGAGTAGGACAAG
CAATGTATGCCCCTCCTATCGAAGGGACAATTAGGTGCTCATCAAAT---
ATTACAGGTCTACTATTGACAAGAGATGGTGGTAATGGCAATGCA----------------------------
----

ACTCAAAATGAGACCTTTAGACCTGGAGGAGGAGACATGAAAGATAATTGGAGAAGTGAATTGTATAAGTATA
AAGTAGTAAAAATTGAACCACTA---GGAGTAGCACCCACCAGGGCAAAAAGAAGAGTGGTG-----------
----------------------------GAGAGAGAAAAAAGAGCA---GTGGGG---ATGGGAGCT------
---TTGTTTCTCGGGTTCTTGGGAGCA---GCCGGAAGCACTATG---GGCGCGGCGTCA---
ATGGCGCTGACGGCACAG---GCCAGACAA---TTATTG---TCTGGTATAGTGCAGCAGCAAAAC---
AATTTG---CTGAGG---GCTATAGAGGCGCAACAGCATCTGTTGCAACTCACAGTCTGGGGCATT
>MN617377
TTCTACTGTAATACATCACAACTGTTTAGTAGTACTTGGGATAATAATGGTACTGAGATTAATACTACAGGAA
CTACA--

GGAAATGACACTATCATTCTCCCTTGCAGAATAAAACAACTTGTAAACATGTGGCAGGAAGTAGGGAAAGCAA
TGTATGCCCCTCCCATTAGTGGAATAATTAGATGTTCATCGAAT---
ATTACAGGATTGCTATTAACAAGAGATGGTGGTAACAATCAGAGCACT-------------------------

----

ATCAATGAGACTTTCAGACCTGGAGGAGGAGACATGAGAGACAATTGGAGAAGTGAATTATATAAATATAAAG
TAGTAAAAATTGAACCATTA---GGAGTAGCACCCACCAAGGCAAAGAGAAGAGTGGTG--------------
-------------------------CAAAGAGAAAAAAGAGCA---GTGGGAACTATAGGAGCT---------
ATGTTCCTTGGGTTCTTGGGAGCA---GCAGGAAGCACAATG---GGCGCAGCGTCA---
ATAACGCTGACGGTACAG---GCCAGACAA---TTGTTG---TCTGGTATAGTGCAACAGCAGAGA---
AATCTG---CTGAGG---GCTATTGAGGCGCAACAGCATCTGTTGCAACTCACAGTCTGGGGCATC
>HM215251
TTTTATTGTAATACATCAAAACTATTTAATAGTAGTTGGTGGTTTAATACTACAAATAGCATAGGAATAAATA
CAAATAGC---
-------------------ACG---
GAAATAAATGATACTATCATACTTCCATGTAGAATAAAACAAATTGTGAGAATGTGGCAGAGAGTAGGACAAG
CAATGTATGCCCCTCCCATTGCAGGAAATATTACATGTGTATCAAAC---
ATTACAGGCTTATTACTGACACGTGATGGGAAATATAGGCCAAAT----------------------------
----

AGTACTAATGAGACCTTCAGACCTACAGGAGGAGAAATGAGGGACATTTGGAGAAATGAATTATATAATTATA
AGGTAGTGGAGATTGAACCACTA---GGAGTAGCACCCACCAAGGCAAGGAGAAGAGTGGTG-----------
----------------------------CAGAGAGAGAAGAGAGCA---GTGGGA---ATAGGAGCT------
---TTGTTCCTTGGGTTCTTAGGAGCA---GCAGGAAGCACTATG---GGCGCAGCGTCA---
ATAACGCTGACGGTACAG---GCCAGACAA---TTATTG---TCTGGTATAGTGCAACAGCAAAGT---
AATTTG---CTGAGG---GCTATAGAGGCTCAACAACATCTGTTGAAACTCACTGTCTGGGGCATT
>HM215249
TTCTATTGTAATACATCAGGATTATTCAATAAGAGTTGGGGGAGTTGGGAGCCTAATACTACAGAGGTTAATA
ATGTA--
-------------------ACT---
AAAGAAAATGGTACTATCACACTTCAATGTAAAATAAAACAAATTGTGAGAATGTGGCAGAGAGTAGGACAAG
CAGTGTATGCCCCTCCCATTGCAGGAAATATTACATGTACATCAAAC---
ATTACAGGCTTATTACTGACACGTGATGGGGGTAACAATAATAGT----------------------------
----

GATAATGAGACCTTCAGACCTACAGGAGGAGATATGAGGGACATTTGGAGAAGTGAATTATATAATTATAAGA
TAGTGGAGATTGAACCACTA---GGAGTAGCACCCACCAAGGCAAGGAGAAGAGTGGTG--------------
-------------------------CAGAGAGAAAAGAGAGCA---GTGGGA---ATAGGAGCT---------
TTGTTCCTTGGGTTCTTAGGAACA---GCAGGAAGCACTATG---GGCGCAGCGTCA---
ATAACGCTGACGGTACAG---GCCAGACAA---TTATTG---TCTGGCATAGTGCAACAGCAAAGT---
AATTTG---CTGAGG---GCTATAGAGGCTCAACAACATCTGTTGAAACTCACTGTCTGGGGCATT
>KY392780
TTTTATTGTAATACATCAGGATTGTTCAATGGCACTAGGTTCAATGGCACTAGGACAAATGGC----------
----

ACAAATGAAACTATCACACTCCCATGTAAAATAAAACAAGTTGTGAGAATGTGGCAGAGAGTAGGACAAGCAA
TTTATGCCCCTCCCATCGCAGGAAACATTACATGCTCATCAAAT---ATTACAGGATTAATATTAACA-----
-------CATGAAAGTTCT--

AATGATACAGAAATCTTCAGACCTATAGGAGGAAATATGAAGGACAATTGGAGAAGTGAATTATATAAGTATA
AGGTAGTGAAGATTGAGCCACTA---GGAGTGGCACCCACCAGGGCAAAGAGAAGAGTGGTG-----------
----------------------------GAGAGAGAAAAAAGAGCAATAGCTGGA---ATAGGGGCT------
---GTCTTCCTTGGGTTCTTGGGAACA---GCAGGAAGCACTATG---GGCGCAGCGTCA---
ATAACGCTGACGGTACAG---GCCAGACAA---TTATTG---TCTGGCATAGTGCAACAGCAGAAC---
AATCTG---CTGAAG---GCTATAGAGGCTCAACAACATCTGTTGAAGCTCACAGTCTGGGGCATT
>FN392873
TTCTATTGCAATACATCAGGACTGTTTAATGACAGTTGGTCTAATGGCAGTTGGTCCAAAAACAGTACAGAC-
----

AGCACACAGAACATAACTCTTCCATGCAGAATAAAGCAAATTATAAATATGTGGCAGAGAGTAGGACAAGCAA
TATATGCCCCTCCCATTCGAGGAATAATAAACTGTAAATCAAAC---
ATTACAGGACTACTACTAACAAGAGATGGGGGAGATAATAACAGT----------------------------
----

ACAAATGAGACCTTCAGACCGGGAGGAGGAGACATGAGAGATAATTGGAGAAGTGAATTATATAAGTATAAAG
TAATAAAAATTGAACCACTA---GGGGTAGCACCCACCAAGGCAAGGAGAAGAGTGGTG--------------
-------------------------CAGAGAGAAAAAAGAGCA---GTTGGA---CTGGGGGCT---------
TTTTTCCTTGGGTTCTTGGGAGCA---GCAGGAAGCACTATG---GGCGCAGCGTCG---
CTAACGCTGACGGTACAG---GCCAGACAA---TTACTG---TCTGGCATAGTGCAACAGCAGAGC---
AATCTG---CTAAGG---GCTATAGAGGCTCAACAGCACCTGTTGAAACTCACAGTCTGGGGCATT
>KY392773
TTCTATTGCAATACAACACAGCTATTTAATAGCACCTGGGAGAAAAACACAACCAGTGAAGGCTATAGACAGA
TAAATAAC---
-------------ACAACA------
AATGATACTACTACTATTGTGCTGCCATGCAGAATAAAACAAATTGTAAACATGTGGCAGAGGGTAGGACAAG
CAATGTATGCCCCTCCCATCAGTGGAAAAATTACATGTGAGTCAAAT---
ATTACAGGACTAATATTGACAAGCGACTTTGTTAATCAGTCTAAG----------------------------
----

GATAGTGATAGTGTTACCTTCAGACCTGGAGGAGGAGATATGAGGGACAATTGGAGAAGTGAATTATATAAAT
ATAAAGTAGTAAAAATTGTACCACTA---GGTGTAGCACCCACCGAGGCAAGGAGAAGAGTGGTG--------
-------------------------------CAGAGAGAAAGAGCAGAA---GACAGTGGCAACGAGGCT---
------GTCCTCTTTGGGTTCTTGGGAGCA---GCAGGGAGCACTATG---GGCGCAGCGTCA---
ATGACGTTGACGGTACAG---GCCAGACAA---TTATTG---TCTGGTATAGTGCAACAGCAGAGC---
AATCTG---CTGAAG---GCTATAGAGGCTCAACAACATATGTTGAGACTCACAGTCTGGGGCATA
>JF706452
TTCTATTGCAATACATCAGCGCTATTTAACAGCTCTTGGGAACCCAACAAAACTAACACAACTAACACATCAA
TG---

TTAAATGGCAATATCACTCTCCCATGCAGAATAAAACAAATTGTAAACATGTGGCAAAGAGCAGGGCGAGCAA
TGTACGCCCCTCCCATTGCCGGAAAAATTCAATGTAACTCAAAT---
ATCACAGGTCTCATATTAACAAGAGATGGTGGTAATAATAGTAAGACAAGT----------------------
----

AATGAGACTGAGACTGAGACCTTCAGACCTGGAGGGGGAGACATGAGAGACAACTGGAGAAGTGAACTGTATA
AATATAAGGTAGTGAAAATTGAACCACTA---GGAGTAGCACCCACCAAGGCAAGAAGACATGTGGTG-----
----------------------------------CAGAGAGCAAAAAGAGCA---GTGGGA---
TTAGGAGCT---------GTATTCCTTGGGTTCTTGGGAGCA---GCAGGAAGCACTATG---
GGCGCAGCGTCA---ATAACGCTGACGGTACAG---GCCAGACAA---TTATTG---
TCTGGTATAGTGCAACAGCAAAGC---AATTTG---CTGAAG---
GCTATAGAAGCTCAACAGCATCTGTTGCAGCTCACAGTCTGGGGCATT
>KP718920
TTCTATTGCAATACATCAGAACTGTTTAATAGCACATGGACATATAATGATACTAAAAATAACACTGACAGCC
AGACAGGG---

TCAAATGACAACATAACTCTCCCATGTAGGATAAAGCAAATTATAAATATGTGGCAGAGAACAGGAAAAGCAA
TGTATGCCCCTCCCATCCCAGGAATAATAAGGTGTGAGTCAAAC---
ATTACAGGACTAATATTGACAAGAGATGGTGGGGTTAATAGTACA----------------------------
----

AATGAGACTTTCAGACCTGGAGGAGGAGATATGAGAGACAATTGGAGAAGTGAATTATATAAATATAAAGTAG
TAAAAATTGAACCACTA---GGAGTAGCACCCAGCAGGGCAAGGAGAAGGGTGGTG-----------------
----------------------CAGAGAGAAAAAAGAGCA---GTTGGA---ATTGGAGCT---------
GTCTTCCTTGGGTTCTTAGGAGCA---GCAGGAAGCACTATG---GGCGCGGCGTCA---
ATGACGCTGACGGTACAG---GCCAGACAA---TTATTG---TCTGGTATAGTGCAACAGCAAAGC---
AATTTG---CTGAAG---GCTATAGAGGCTCAACAGCATCTGTTGAAACTCACGGTCTGGGGCATT
>JF683772
TTCTATTGCAATACATCCAAACTGTTTAATGGTACATGGTTTAATGGCACATGGGTTAATGGCACATGGTACA
ATAATACACTA--
-------GACAGCAATGAC------
ACAGAGAATGGGACTATAACTCTCCCATGCAAAATAAGACAAATAGTAAGAATGTGGCAAAGAGTAGGACAAG
CAATGTATGCCCCTCCCATCCAAGGGGTAATACATTGTCAATCAAAC---
ATTACAGGACTACTATTAACGAGAGATGGAGACAAGAAT----------------------------------

----

GAAACTGAAACCTTCAGACCTACAGGAGGAGATATGAGGGACAATTGGAGAAGTGAATTATATAAGTATAAAG
TAGTAAAAATTGAACCACTA---GGAGTAGCACCCACCAGGGCAAGGAGAAGAGTGGTG--------------
-------------------------GAGAGAGAAAAAAGAGCA---GTTGGA---ATGGGAGCT---------
ATCTTCCTTGGTTTCTTAGGAGCA---GCAGGAAGCACTATG---GGCGCGGCGTCA---
ATAACGCTGACGGTACAG---GCCAGACAG---TTATTG---GCTGGCATAGTGCAACAGCAGAGT---
AATCTG---CTGAAA---GCTATAGAGGCCCAACAGCAACTGTTGAGGCTCACAGTCTGGGGCATT
>JX140679
TTCTATTGCAATACAACACAACTGTTTGATAGCAGTTGGGGTATCAATGACACTGTCAGCAATAGCAATGACG
CTGAC--

AGCGATGACAACATAACTCTTCAATGCCGAATAAAGCAAATTATAAAAATGTGGCAGAGAGTAGGACAAGCAA
TATATGCCCCTCCCATCCGAGGAAACATAAGTTGTCACTCAAAC---
ATTACAGGGCTACTATTAACAAGAGACGGAGGGAACAATAGTACA----------------------------
----

AATGAGACTTTCAGGCCTGGAGGAGGAGACATGAGAGATAATTGGAGAAGTGAATTATATAAGTATAAAATAG
TAAAAATTGAACCACTT---GGTGTAGCACCCACCCATGCAAAAAGAAGAGTGGTG-----------------
----------------------GGGAGAGAAAAAAGAGCA---ATTGGA---ATGGGAGCT---------
TTCTTCCTTGGGTTCTTAGGAGCA---GCAGGAAGCACTATG---GGCGCAGCGTCA---
ATAACGCTGACGGCACAG---GCCAGACAA---TTATTG---TCCGGTATAGTGCAACAGCAGAGC---
AATTTG---CTGAAG---GCTATAGAGGCTCAACAAAATCTGTTGAGACTCACGGTCTGGGGCATT
>JF680905
TTCTATTGCAATACATCAGGACTGTTTAATAGCTCTTGGAATAGCACTGACAACTCAAACCACACAGGGTTA-
----

AATAATGACACTATAATTCTCCCATGCAGAATAAAGCAAATTGTAAATATGTGGCAGAGAGTAGGACAAGCAA
TATATGCCCCTCCCATCCAAGGAGCAATAAGATGTAATTCAAAT---
ATTACAGGACTACTATTAACAAGAGATGGAGGGAATAATGGGACA----------------------------
----

AATAACACCGAAATCTTCAGACCTGAGGGAGGAAATATGAAGGACAATTGGAGAAGTGAATTATATAAATATA
AAGTAGTAAAAATTGAGCCACTA---GGTGTAGCACCCTCACGTGCAAAAAGAAGAGTGGTG-----------
----------------------------GAGAGAGAAAAAAGAGCA---GTTGGA---CTGGGAGCT------
---GTCCTCTTTGGATTCTTAGGAGCA---GCAGGAAGCACTATG---GGCGCGGCGTCA---
ATAACGCTGACGGTACAG---GCCAGACAA---TTATTG---TCCGGCATAGTGCAACAGCAGAGC---
AATCTG---CTGAGA---GCTATAGAGGCTCAACAACATATATTGAAACTCACGGTCTGGGGCATT
>MH078558
TTCTATTGCAATACATCAAATCTGTTTAATAGGACTTACAGCAGGGGGATGAACGGCACTTGGGAGACAAATG
ACACAGGG---

ATAAATGACACTAAAAGCCTTCAATGTAAAATAAAGCAAATTATAAATATGTGGCAGAGAGTAGGACGAGCAA
TGTATGCCCCTCCCATCCCAGGAGTAATAGAGTGTAAGTCAAAC---
ATTACAGGACTACTATTGACAAGAGATGGGGGTGCTAATAACAAT----------------------------
----

AGGACTGAGGAAACATTTAGACCTGGAGGAGGAGATATGAGAGATAATTGGAGAAGTGAATTATATAAGTACA
AAGTAGTAAAAATTGAACCACTA---GGAGTAGCACCCACCAAGGCAAAGAGAAGAGTGGTG-----------
----------------------------GAGAGAGGGAAAAGAGCA---GTCGGA---ATGGGGGCT------
---TTCTTCCTTGGGTTCTTAGGAGCA---GCAGGAAGCACTATG---GGCGCAGCGTCA---
ATGACGCTGACGGTACAG---GCCAGACAA---TTATTA---TCTGGCATAGTGCAACAGCAGAGT---
AATCTG---CTGAGA---GCTATAGAGGCTCAACAGCATCTGTTGAAACTCACAGTCTGGGGCATT
>MH078559
TTCTATTGCAACACATCAGATCTGTTTAATAGTACTTGGGATAAGAACACTACTTGGAATAACAACTATCAGA
AT---

GAGAATGACAATATAACTCTCCCATGCAGAATAAAACAATTTGTACATATGTGGCAGAGAGTAGGACAAGCAA
TATATGCCCCTCCCATTCAAGGAGTAATAAAATGTAAATCAAAC---
ATTACTGGAATACTATTAACAAGTGATGAAGGTTTTAAAAACACA----------------------------

----

ACTGAGATCTTCAGACCTGGAGGAGGAGATATGAGGGACAATTGGAGAAGTGAATTATATAAATATAAAGTAG
TAAAAATTGAACCACTA---GGAGTAGCACCCTCCTGGGCAAGGAGGAGAGTGGTG-----------------
----------------------CAGAGAGAAAAAAGAGCA---GTTGGA---CTGGGAGCT---------
GTCTTCCTTGGGTTCTTGGGAGCA---GCAGGAAGTACTATG---GGCGCAGCGTCA---
CTAACGCTGACGGTACAG---GCCAGACAA---TTATTG---TCTGGCATAGTACAACAGCAGAAC---
AATCTG---CTGAAG---GCTATAGAGGCTCAACAGCAACTGTTGAAACTCACGGTCTGGGGCATT
>JF680912
TTCTATTGCAATACATCAACCCTATTTAATAGCACATGGAATTGGAATGGCACTCAGAACATAACT-------
----

GGCACTGCAAATCACATTATCCCATGCAAAATAAAGCAAATTGTGAGAATGTGGCAGGGAGTAGGACAAGCAA
TGTATGCCCCTCCCATCAGCGGAATGATACAATGCAATTCAAGC---
ATTACAGGACTATTGTTAACAAGAGATGGAGGGAACAGAAACAAT----------------------------
----

TCAGACAATGAGACATTCAGGCCTGGAGGAGGAGATATGAGGGACAATTGGAGAAGTGAATTATATAAGTATA
AAGTAGTAAAAATTGAACCACTA---GGAGTAGCACCCACCAGGGCAAAGAGAAGAGTGGTG-----------
----------------------------GAGAGAGAAAAAAGAGCA---GTTGGA---ATGGGAGCT------
---TTGTTCCTTGGGTTCTTAGGAGCA---GCAGGAAGCACTATG---GGCGCAGCGTCA---
ATAACGCTGACGGTACAA---GCCAGACAA---TTATTG---TCTGGTATAGTGCAACAGCAAAGT---
AATTTG---CTGAAG---GCTATAGAGGCTCAACAACAACTGTTGAAACTCACGGTCTGGGGCATT
>KU168285
TTTTATTGTAACACAACTACGCTATTTAATCATACTTTTTCTTGTAACAATTCCACCTGTCAGAACATGTCTG
GCAGTGAC---

ACTTCTAGCAATGACACTATACCTTGCAGGTTGAAACAGATAGTAAGGTCATGGATAGGGGGAGAGTCGGGAT
TCTATGCACCTCCCATCAAGGGTGCCCTAAATTGCACCTCACAA---
ATAACTGGAATGATTTTACAACTGGACTATCCATGGAACAACACAGAGGGAAAG-------------------
----
GCCACAAATAACACAAAAAACAAT---------
ACCAACGTCATATTTAGACCAACAGGGGGAGATATGAAAGATATATGGAGGACTAAATTATTTAACTATAAGG
TAGTAAAGGTAAAACCTTTT---AGTATAGCACCTACAAAAGTTGCAAGACCAGTCATAGGAACT--------
-------------------GGCACTCATAGGGAGAAAAGGGCA---GCAGGT---ATGGCAATG---------
CTATTCTTGGGGGTTCTAAGTGCA---GCAGGAAGCACTATG---GGCGCAGCGGCA---
ACAGCGCTGACGGTACAG---ACCCGAAGT---TTAATA---AAGGGTATAGTGCAACAGCAGGAT---
AAACTG---CTGAGA---GCAATACAGGCCCAGCAACACTTGCTGAGGTTATCTGTATGGGGCATT
>KU168294
TTTTATTGCAACACATTTGAGTTGTTTAATTATACTTTTAGATGTAGAGGGTCCAACTGTACTTCTGAAAATA
GC---

ACCCAAATAAATGGAACAATACCTTGCAAGATAAAACAAGTAGTAAGGACATGGATGGGGGGAAGGTCAGGAC
TCTATGCACCTCCCATTAAAGGTGAACTAACATGTGAATCAAAC---
ATAACTGGAATGCTACTACAAAGGGATCAACCATGGAACCAAACATGTAACAGC-------------------
----

ACATTTAGACCAATAGGGGGAGATATGAGAGATATATGGAGAACTAGCTTATACAACTACAAAGTAGTAAAAA
TAAAACCTTTT---AGTGTGGCACCTACAAGAATTGCAAGGCCAGTCATAAGTCTT-----------------
----------AGATCTCCTAGAGAAAAAAGGGCA---GCAGGA---TTGGGAATG---------
CTATTCTTGGGGGTGCTAAGTGCA---GCAGGTAGCACTATG---GGCGCAGTGTCA---
ACGACACTGACGGTACAG---ACCCAAAAA---GTGTTA---AAGGGTATAGTGCAACAGCAGGAC---
AACCTG---CTGAGA---GCGATACAGGCCCAGCAACATTTGCTGAGGCTATCTGTATGGGGCATT
>KU168284
TTTTATTGCAACACATCTGGGTTGTTTAATCATACTTTTGAGTGTATAGAGCACAACTGTACTTCTAAAATTA
ATAGCACCCAT--
-------------GAAAAT------
AGCTCCCAAAGAAATGGAACAATACCGTGCAGGATAAGACAGGTAGTAAGAGCATGGATGGGGGGAAGGTCAG
GACTCTATGCACCTCCCATCAAAGGTATCCTAACATGTCAATCAAAT---
ATAACTGGAATGCTACTAGAAAGGGATGAACCATGGAACCAAACA----------------------------

----

GGCACAAATAACAGCACATTTAGACCAACAGGGGGAGATATGAGAGATATATGGAGAACTAGCTTATACAATT
ACAAAGTAGTAAGAATAAAACCTTTT---AGTGTGGCACCTACAAAAATTGCAAGACCAGTCATAAAAGCT--
------------------------------CCTAGAGAAAGAAGGGCA---GCAGGA---TTGGGAATG---
------CTATTCTTGGGGGTGCTAAGTGCA---GCAGGTAGCACTATG---GGCGCAGTGTCA---
ATGACACTGACGGTACAG---ACCCAAAAC---GTGTTA---AAGGGTATAGTGCAACAGCAGGAC---
AACCTG---CTGAGA---GCGATACAGGCCCAGCACCACTTGCTGAGGCTATCTGTATGGGGCATT
>AY532635
ATCTATTGTAATACTTCCAGATTATTCAATGAGAGCATGAATACAAATGGGACT------------------
----

AATGGGACTATTACTCTGCCCTGTAGAATAAGACAAATTGTAAATTTGTGGACAAGAGTAGGAAAAGGAATTT
ATGCACCGCCAATTCGGGGAAATCTTACCTGTAACTCCACT---
ATTACTGGACTGATTCTAGAACATAGTGGTGGCAGTAAT----------GGC--------------------
----

ACAGTATATCCCACAGGAGGAAACATGGTCAATCTCTGGAGACAAGAGTTGTATAAGTACAAAACAGTCAGTA
TAGAACCCATA---GGAGTAGCACCAGGTAAAGCTAAAAGACGCACAGTG----------------------
----------------AGTAGAGAAAAAAGAGCAGCCTTTGGA---CTAGGTGCG---------
CTGTTTCTGGGGTTTCTTGGAGCA---GCAGGGAGCACTATG---GGCGCAGCGTCA---
ATAACGCTGACGGTACAG---GCCCGGACA---TTGTTA---TCTGGGATAGTGCAACAGCAGAAT---
AATCTG---GTGAGA---GCAATAGAGGCGCAACAACATTTGTTGCAACTCTCAATCTGGGGCATT
>GQ324959
TTCTATTGTAACACTTCCAAATTATTTGATAAAAATTTACTTAACAAGACA---------------------
----

AACGAGACTATTGTTCTGCCCTGTAGAATAAAACAGATTGTAAACTTGTGGACAAGGGTAGGAAAAGGGATTT
ATGCACCACCAGTTAGGGGAAATCTTACCTGTAACTCCAAT---
ATTACTGGACTGATTCTAGAGTATAGTGGTAACAGTGGTAACGAA---------------------------
----

ATGGTGTATCCCTCAGGAGGACACATGGTTAATCTTTGGAGACAAGAGTTGCATAACTACAAAGTAGTTAGTA
TAGAACCCATA---GGAGTAGCACCAAGTAAAGCTAAAAGGCACACAGTG----------------------
----------------AGTAGAGAAAAGAGAGCAGCCTTTGGA---CTAGGTGCG---------
CTGTTTCTTGGGTTTCTTAGAGCA---GCAGGGAGCACTATG---GGCGCAGCGTCA---
ATAACGCTGACGGTACAG---GCCCGGACA---TTATTA---TCTGGGTTAGTGCAACAACAGAAT---
AATCTG---TTGAGA---GCTTTAGAGGCGCAACACCATTTGTTGCAACTCTCAGTCTGGGGCATT
>AJ006022
TTCTATTGTAACACTTCCAAATTATTTAATGAGGAATTACTTAACGAGACA---------------------
----

GGTGAGCCTATTACTCTGCCTTGTAGAATAAGACAGATTGTAAATTTGTGGACAAGGGTAGGAAAAGGAATTT
ATGCACCACCAATTCGGGGAGTTCTTAACTGTACCTCCAAT---
ATTACTGGACTGGTTCTAGAATATAGTGGTGGGCCTGACACCAAGGAA------------------------
----

ACAATAGTATATCCCTCAGGAGGAAACATGGTTAATCTCTGGAGACAAGAGTTGTATAAGTACAAAGTAGTTA
GCATAGAACCCATA---GGAGTAGCACCAGGTAAAGCTAAAAGACGCACAGTG-------------------
-------------------AGTAGAGAAAAAAGAGCAGCCTTTGGA---CTAGGTGCG---------
CTGTTTCTTGGGTTTCTTGGAGCA---GCAGGGAGCACTATG---GGCGCAGCGTCA---
ATAACGCTGACGGTACAG---GCCCGGACA---TTATTA---TCTGGGATAGTGCAACAGCAGAAT---
ATTCTG---TTGAGA---GCAATAGAGGCGCAACAACATTTGTTGCAACTCTCAATCTGGGGCATT
>HQ179987
TTTTATTGTAACACCAGCAGCCTGTTTAACTTTAGTTATACTTGGAATGACACCACCTGGCGGGCTGCAGGCA
ATCATAAT--

ACAAGTGAAAATATAACACTGTCTTGTAGACTGAAGCAAGTAGTTAATTCATGGATGAGAGTGGGATCAGGCT
TGTTTGCGACACCTATTGAGGGGGAATTAAAGTGTCAATCCAAC---
ATTACAGGTTTAATGTTAGAAAGAGAACTACCTTACAAT---GACAGC------------------------

----

AGCAAGAACACCACTTTAAGTCCTATAGGGGGAGACATGACAAATATCTGGAGAAGTGAGTTATATCCCTACA
AAGTAGTTCAAGTAAAAGCTCTG---GCCGTGGCACCTACAAAAATTTCCAGACCTACAATTATGGCT-----
-------------------CATGATGGACATAGGAAGAAAAGGGCA---GCAGGC---CTAGGAATG------
---CTATTCCTTGGGTTTCTGAGCGCA---GCAGGAAGCACTATG---GGCGCAGCGGGA---
ATCACGCTGACGGTACAG---GCCAAGCAA---TTATTG---CATGGTATAGTGCAGCAGCAGAAC---
AATATG---CTAAGA---GCTATAGAGGCACAGCAAGAATTGCTGAGACTCTCTGTGTGGGGCATA
>KY953207
TTTTATTGTAACACCAGTAGCCTGTTTAATTATAGCTATACTTGTAATACCTCGGGTGCATGTATCATTAATC
CTACAGGTAAT--
----------------CACACA---
GGTGAGAACATGATAATAACACTACCTTGTAGACTAAAGCAAGTAGTTAATTCATGGATGAAAGTGGGATCAG
GCTTGTTTGCGCCACCTATCGAGGGACAATTGCAGTGCCACTCCAAC---
ATTACAGGTTTAATATTAGACAGAGCATCACCTTATAATGTCAAC----------------------------
----

AGCAGTAACACCACTTTAAGTCCTACCGGGGGAGAAATGAGAAATATCTGGAGAAGTGAGTTATATCCCTACA
AGGTAGTTCAAGTAAAAGCTCTG---GCTGTGGCACCTACAAGGGTCTCCAGACCTACAATCATGGCT-----
-------------------CATGATGCTCATAGGAAAAAAAGGGGA---GCAGGC---CTAGGAATG------
---CTATTCCTTGGGTTCATGAGTGCA---GCAGGAAGCACTATG---GGCGCAGCGGCA---
GTTACGCTGACGGTACAG---GCCAGGCAA---TTATTG---CATGGTATAGTGCAGCAGCAGAAC---
AATATG---CTACGA---GCTATAGAGGCACAGCAAGAATTGCTGAGACTCTCTGTGTGGGGCATA
>JQ866001
TTCTATTGTGACCTCAAAAATTGGACAAACAATGGCATCGAG-------------------------------
----

ATCGATGGAATGCTGATAGCACCATGTCGACTTAGACAGATAGTAAATCATTGGGGAATAGTATCCAGGGGAA
TTTATCTCCCTCCCAGGGAGGGACAGGTCAAATGTGTATCTAGT---
ATTACAGGATTCATAATGACAGCTGAGACGAATGACACTGAT-------------------------------
----

GGCACTATCACACCCACCTTCTCTGCAAAGGTGGAAGACTACTGGAAAGTAGAAATGAGTAGATACAAAGTAG
TGGAGATACAGCCATTA---TCAGTGGCACCCACTAAAGGAAAAAGACCAGTAGTTGGTGAC-----------
----------CCAGAAGCAAAAAGTAGAAGTAAAAGAGAA---ATAGGA---ATGGGAATG---------
ATCTTTATGGGATTTCTCAGTGCA---GCAGGAAGTACTATG---GGCGCAGCGTCA---
ATAGCGCTGACGGTACAA---GCCAGAGGA---TTGCTC---TCTGGTATAGTACAGCAGCAAGCA---
AATTTG---CTGCAG---GCAATAGAGGCTCAACAACATTTGTTGCAGCTCTCGGTGTGGGGCATA
>JX178450
TTTTATTGTGAGACATCTTCATTGTTTAATGCTACAATAGATATTACACCAAATACTACACTGAATGAGACGA
CCTGGGATTAT---CCA------GCT---
-------------GAGAAC------
AATATTAACCAAGAAATGATAATCCATTGTAGAATAAAACAAATTGTATCAATGTGGCAGAGAGTGGGAAAAG
GAATATTTTCTCCTCCTATTAGAGGAACCATTCAATGTAACTCCACT---
ATTACAGGACTACTGCTAACTCGCGATGGTAATAACAAAGCAGATAGTGAGAACAAAACAAGGATTGAAACAG
GACAG--
AACAAAGCAGAAGAGATTAATAGGACTGACATTTTTAGACCTGCAGGAGGAGAAATGAGAGACAATTGGAGAA
GTGAATTATATAGATACAAGGTGGTGAAAATTGAACCACTA---
GGACTAGCACCCACCAAGGCAAAAAGAAGGACTGTA-------------------------------------
--AATAGAGAAAAAAGAGCA---CCAGGA---CTAGGACTG---------
ATGTTCCTTGGGTTCTTGGGAGCA---GCAGGGAGCACTATG---GGCGCAGCATCA---
TTAACGCTGACGGTACAG---GCCAGGCAA---TTATTG---ACTGGCATAGTGCAACAGCAAAGC---
AATTTG---CTGAGA---GCTATAGAGGCGCAACAGCACTTGTTGCAACTCTCAGTCTGGGGCATT
>EF535993
TTTTATTGTAACACCACCCCATTGTTCAATAACAGTTGGACAAATACAACTAGCTCAGATATA----------
----

AATAGCTCAAATATAATAATAAACTGTAGAATAAGGCAAATTGTAAACTCCTGGATGAGAGTGGGCAAGGGAA
TTTACGCCCCACCAATTAGAGGAACTATAAGTTGCACATCAAAT---
ATTACTGGGCTCCTGCTCGAGTTAGACAGAGGACTCCCGAGAAATGGGACTAAT------ACCAATAAC---

AAG--

TTAACACTATACCCTACAGGAGGAGAGATGAGAGATTTGTGGAGGCTGGAATTACATAAATACAAAGTAGTCA
GCATAGAACCCATA---GGAGTGGCACCAAGTAAAGCTAAAAGGCACACAGTG--------------------
-------------------ACAAGAGAAAAAAGAGCAGCCTTTGGA---CTAGGTGCG---------
CTGTTTCTTGGGTTTCTAGGAGCA---GCAGGAAGCACTATG---GGCGCAGCATCA---
CTAACGCTGACGGTACAG---GCCCGGCAA---TTATTG---TCAGGGATAGTGCAACAGCAGAAC---
AACCTG---CTGAGA---GCAATAGAGGCGCAACAGCATTTGTTGCAACTCTCAGTATGGGGCATT
>DQ373065
TTTTACTGTAATACCAGTCAATTGTTTACAAATCAAAATGGCAACACAACTGGCAAT----------------
----

ATCACCTTACAATGCAGAATAAGACAAATTGTAAATTTATGGACAAGAGTAGGAAAAGGAATTTATGCTCCGC
CAATTAAAGGACCTATTAACTGTCTCTCTAAC---
ATCACTGGGATAATACTTGACTATACAAAGTCTGGAACTGAGAAGTAC-------------------------
----

ACCATATATCCCACAGGGGGAGACATGACTAACCTCTGGAGGCAGGAGTTGTATAAGTATAAGGTGGTCAGCA
TAGAACCCATA---GGAGTAGCACCAGGAAAAGCTAAAAGACACACAGTG-----------------------
-------------ACTAGACAAAAAAGAGCAGCC---TTTGGA---CTAGGTGCG---------
CTGTTTCTTGGGTTTCTTGGAGCA---GCAGGGAGCACTATG---GGCGCAGCGTCA---
ATAACGCTGACGGTACAG---GCCCGGAAA---TTATTA---TCTGGGATAGTGCAGCAGCAGAAT---
AATCTG---CTGAGA---GCAATAGAGGCGCAACAACACTTGCTGCAACTCTCAGTTTGGGGCATT
>DQ373064
TTTTATTGTAACACCTCGGCCTTGTTCAATGACACCTTGTTACGCAATAAAACCTTGCTCAATGAAACC----
----

TTGCACAATAACTTGACACTACCTTGTAGGATTAGACAAATTGTAAACCTATGGCTAAGAGTAGGAAAGGGAA
TATTTGCTCCACCTATTAGAGGTAACATCCGCTGTAATTCCACT---
ATTACTGGGTTGATCCTAGAAAAACACACAAACACAGACAAT-------------------------------
----

ATCACCTTCCGTCCAATAGGAGGGGATATGACAGACATCTGGAGAAGTGAATTATATAACTATAAAATAGTAA
AAATTGAACCATTA---GGAGTAGCGCCTACCAGGGCGAGAAGGAGGACTGTG--------------------
-------------------GATAGAGAAAAAAGAGCA---GCAGGG---CTGGGAGTG---------
TTTTTCCTTGGGTTCTTGGGAGCA---GCAGGGAGCACTATG---GGCGCAGCATCA---
TTAACGCTGACGGTACAG---GCCAGACAA---TTATTG---ACTGGCATAGTGCAACAGCAGAGT---
AATTTG---CTGAGA---GCTATAGAGGCGCAGCAACACTTGCTGCAACTCTCAGTCTGGGGCATC
>EF535994
TTTTACTGTAATACATCAAACTTATTCAATCAATCCACTATT-------------------------------
----

AACAATATTACACACATTCCGTGCAGAATAAGACAAATTGTAAATCAGTGGCAAGGAGTAGGAAAAGGAATCT
TTGCACCTCCTATTAGAGGAACAATTCAATGTAATTCCACA---
ATCACAGGCCTACTCTTAACAAGAGATGGTAAAAATGAGACT-------------------------------
----

GAAACTTTTAGACCTACAGGAGGAGACATGAGAAATAATTGGAGAAGTGAATTATACAAATACAAGGTAGTAA
AAATTGAACCCCTA---GGATTAGCACCAACCAAGGCAAAAAGGAGAACAGTG--------------------
-------------------CAAAGAGAAAAGAGGGCA---GTAGGA---CTGGGAGTG---------
ATGTTCCTTGGATTCATGGGAGCA---GCAGGAAGCACTATG---GGCGCAGCGGCA---
CTGACGCTGACGGTACAG---GCCAGAAAT---CTACTC---TCTGGTATAGTGCAACAGCAAAGC---
AATTTG---CTGAAA---GCTATAGAGGCGCAACAGCATCTGTTGCAACTCTCAGTCTGGGGCATT
>AF115393
TTTTATTGCAATACTACTACATTGTTTAATTATACGTGGAAAAACAACAATATAACTAAGGGA----------
----

GATAACACCACCTTCTTCCCATGTAGAATAAGGCAAATTGTAAACTCCTGGATGAGAGTGGGGAAGGGAATTT
ATGCCCCACCAATTAGAGGAGTTATAAGTTGCACATCAAAC---
GTTACTGGGATTATACTTGAGACAGGGCATGGCATCAACAACAGCATCACCAAC-------------------

----

ATCACGCTATACCCTACAGGAGGAAACATGGTAGACTTGTGGAGGCTTGAATTGCATAAATACAAGGTAGTTA
GCATAGAACCCATA---GGAGTAGCACCAAGTAAAGCTAAAAGGCACACAGTA--------------------
-------------------TCAAGAGAAAAAAGAGCAGCCTTTGGA---CTGGGTGCG---------
CTGTTTCTTGGGTTCTTAGGAGCA---GCAGGAAGCACTATG---GGCGCAGCATCA---
GTGGTGCTGACGGTACAG---GCCCGGCAA---TTATTG---TCAGGGATAGTGCAACAGCAGAAC---
AACCTG---CTGCGA---GCAATAGAGGCGCAACAGCATTTGTTGCAACTCTCAGTTTGGGGCATT
>AJ271369
TTTTATTGTAATACCACTCCACTCTTCAATAAGTTTAATTATACTTGTGATAACCAAAACAACTGCACCAGAA
ATAACACAAAT--
----------GACACAAAC------
TCAGACAATAGTACACTAACAATATCATGTAGAATAAGGCAAATTGTAAACTCCTGGATGAGAGTGGGGAAAG
GAATCTATGCCCCACCAATAAGAGGGAACATAACATGCATATCAAAT---
ATAACTGGACTCCTCCTCGAAGCAGGAGGAAAATGGAAAACAATACT--------------------------
----

ATAACAGTGTACCCTTCAGGGGGAGAAATGAGAGACTTGTGGAGGCTTGAATTAAACAGGTATAAAGTAGTTA
GCATAGAACCCATA---GGAGTAGCACCAACTAAGGCTAAAAGGCACACGGTG--------------------
-------------------GCAAGAGAGAAAAGAGCAGCCTTTGGA---CTAGGAGCA---------
CTGTTCCTCGGGTTTCTAGGAGCA---GCAGGAAGCACTATG---GGCGCAGCATCA---
GTGGTGCTGACGGTACAG---GCCCGGCAC---TTGTTG---TCAGGGATAGTGCAACAGCAGAAC---
AACCTG---CTGCGA---GCAATAGAGGCGCAACAACATTTGTTGCAACTCTCAGTTTGGGGCATC
>AF382828
TTTTATTGTAATACATCAAGATTAATTACCTGTAACAGTAGTGACACAAGTGAG-------------------
----

TACATTCTCCCATGCAAAATTAGGCAAGTAGTAAATTCTTGGATGAGAGTGGGGAAAGGTATCTTTGCCCCGC
CTAGAAGAGGAACTATAACCTGTAATTCCACC---
ATTACAGGACTCCTCTTAGAAGTACAGAATGGCACGGGGAAC-------------------------------
----

AACACAGAAGTTTACCTGTCAGGGGGAGATATGAGAGATATTTGGAGGAGTGAATTATATAAATATAAAATAG
TAAAAATTGAGCCACTG---GGGGTGGCACCAACAAAAGCAAAGAGGTACACAGTGGCA---AAA--------
----------------GCACTTGATAGATCCAAAAGAGCAGCCTTTGGA---CTGGGGGCG---------
CTGTTTCTTGGATTCCTTGGAGCA---GCAGGAAGCACTATG---GGCGCAGCATCT---
GTGATGCTGACGGTACAA---GCTCGAAAC---CTGCTT---TCGGGAATAGTGCAGCAGCAAAAT---
AACTTG---CTGCGA---GCCATAGAAGCTCAGCAACACCTGCTGCAGCTATCAGTCTGGGGCATA
>AF447763
TTTTACTGTAATCTCACAAATTGGACTAATACCTGGACAGCTAATAGAACCAATAAT----------------
----

ACTCATGGTACTCTTGTTGCACCATGCAGACTGAGGCAGATAGTAAATCATTGGGGTATAGTGTCAAAAGGGG
TTTACCTTCCCCCAAGGAGGGGAACAGTAAAATGTCACTCAAAC---
ATCACAGGACTTATCATGACAGCAGAAAAAGACAAC-------------------------------------
----

AATAATAGTTATACCCCCCAATTTTCTGCTGTAGTAGAAGACTATTGGAAAGTAGAATTAGCAAGATATAAAG
TGGTGGAAATTCAGCCCTTG---TCAGTGGCTCCA---AGGCCAGGAAAAAGGCCTGAAATT---AAG-----
----------------GCCAATCATACTAGGTCAAGAAGAGAT---GTGGGC---ATAGGACTG---------
TTGTTTCTTGGATTTCTTAGTGCA---GCAGGAAGTACAATG---GGCGCAGCGTCA---
ATAGCGCTGACGGCACAG---GCCAGAGGA---TTACTC---TCTGGTATTGTACAGCAGCAACAA---
AACCTG---CTTCAG---GCCATAGAAGCGCAACAACACTTGTTGCAGCTCTCTGTATGGGGCATT
>DQ374657
TTTTATTGTAATCTTACTGGTTGGACAAATCCCTGGAACAACACAAACCAC----------------------
----

AGAAATGGAACTCTAGTAGCACCATGTAGACTTAGACAAATAGTTAATCATTGGGGGATAGTATCTAAAGGAA
TTTATCTTCCTCCAAGGAGGGGAACATTAAAGTGCAATTCAAAT---
ATTACAGGACTTATTATGACAGCAGAAAAAGGA--

GGTAATAACACAGTTCCTACATTCTCCGGTAAAGTAGAAGACTATTGGAAAGTAGAGTTAGCAAGGTATAAAG
TAGTAGAGATTCAGCCCTTG---TCAGTGGCTCCT---AGGCCAGGAAAGAGGCCTGAAATT---AAG-----
----------------GCTAACCACACTAGATCAAGAAGGGAT---GTTGGC---ATAGGACTG---------
TTGTTTCTTGGATTTCTCAGTGCA---GCAGGAAGTACAATG---GGCGCAGCGTCA---
ATAGCGCTGACGGCACAG---GCCAGAGGG---TTACTC---TCTGATATTGTACAGCAGCAACAA---
AACCTG---CTGCAG---GCCATAGAGGCGCAACAACATCTGTTGCAGCTCTCTGTGTGGGGCATC
>DQ374658
TTTTATTGTAATCTTACAACTTGGACAGGTAACTGGACACGTAACAGGACACATCAGACAAGTGCCCTG----
----

AACAAAACACATCTATTAGCACAATGTAGGCTTAGACAGATAGTTAATCATTGGGGTATAGTATCTAAAGGAA
TTTACCTCCCTCCAAGGAAGGGACATATAAAGTGCGTTTCAAAC---
ATCACAGGACTGATTATGACAGCAGAAGGAGGAAACAGCAACAGA----------------------------
----

ACCCCCACATTCTCCAGTAAGGTAGAAGACTATTGGAAAGTAGAATTAGCAAGGTATAAAGTGGTGGAGATTC
AGCCCTTG---TCAGTGGCTCCT---AGGCCAGGAAAAAGGCCTGAAATT---AAG-----------------
----GCTAATCACACTAGATCAAGAAGGGAT---GTTGGC---ATAGGACTG---------
TTGTTTCTTGGATTTCTCAGTGCA---GCAGGAAGTACAATG---GGCGCAGCGTCA---
CTAGCGCTGACGGCACAG---GCCAGAGGG---TTACTC---TCTGGTATTGTACAGCAGCAACAA---
AACCTG---CTGCAG---GCCATAGAGGCGCAACAACATCTGTTGCAGCTCTCTGTGTGGGGCATC
>JN091691
TTTTACTGCAATCTGACAAATTGGACTAGCCCAGATTACATTGGCAAGAATGGCAGC----------------
----

ATTGTAGCACAATGTAGACTTAGACAGATAGTTAATCATTGGGGTATAGTATCTAAAGGAATTTATCTTCCTC
CAAGAAGAGGAACAATAAAATGTACATCAAAC---ATTACGGGGTTAATCCTTACAGCAGAAGGCGGA-----
----

AATGAAACTATAACACCTACATTCTCCAGTAAAGTAGAAGATTATTGGAAAGTAGAATTGGCAAGATATAAAG
TAGTGGAAATTCAGCCCTTG---TCGGTGGCTCCA---AGGCCAGGGAAAAGGCCTGAGATT---AAG-----
----------------GCTAATCATACTAGGTCAAAAAGAGAT---GTTGGC---ATAGGGCTG---------
TTATTTCTTGGATTTCTCAGTGCA---GCAGGAAGTACAATG---GGCGCAGCGTCA---
ATAGCGCTGACGGCACAG---GCCAGAGGG---TTACTC---TCTGGTATTGTACAACAGCAACAA---
AACTTG---CTGCAG---GCCATAGCAGCGCAACAACATCTGTTGCAGCTCTCTGTGTGGGGCATT
>JN091690
TTCTATTGCAACCTCACTAACTGGACAAATATAACGTGGCTAGAGACGTGGCTAAAGACTAACAAT-------
----

AGTTCCCGTCCCCTTGTAGCACCATGTAAACTTAGGCAGATAGTAAACCATTGGGGTATAGTATCTAAAGGTA
TCTACCTACCTCCTAGAAGGGGAAATTTAACATGCAGATCTAAC---
ATTACAGGGTTCATTACGACATGGGATAATGAC--

ACGGAGAACGTGCTTGTTACCTTTTCAGCCAAAGTAGAAGACTATTGGAAGGTAGAAATGTCACGATACAAAG
TAGTGGAAATTCAGCCCTTG---GCCATAGCTCCT---ACTACAGGAAAAAGGCCGGAGATT---AGA-----
----------------GCCAATCACACAAGGTCAAAAAGAGAT---GTTGGC---ATAGGACTG---------
TTGTTCCTGGGATTTCTCAGTGCA---GCAGGAAGTACAATG---GGCGCAGCGTCA---
ATAGCGCTGACGGCACAG---GCCAGAGGA---TTGCTC---TCTGGTATTGTACAGCAGCAGCAC---
AACCTG---CTGCAG---GCCATAGAAGCTCAACAGCACTTGTTACAGCTTTCTGTGTGGGGCATT
>AF103818
TTTTATTGTAATACAATTCCCCTATTTAACATGACCTACAACAATACC-------------------------
----

GACAACACCACTATCACACTTAAGTGTAGAATAAGACAAATTGTAAATCAGTGGATGAGAGTAGGAAAAGGAA
TCTTTGCCCCACCAATCAAAGGTGTGCTAAGTTGTAACTCAAAT---
ATAACGGAATGATTCTTGACATAAGCATAAGCGCAGTCAATAACGATAGTAGG-------------AAT----
----

ATAACAGTGATGCCTACAGGAGGAGATATGACGGCTTTATGGAAAAATGAATTACATAAGTATAAGGTGGTCA
GCATAGAACCTATA---GGAGTGGCACCAGGTAAGGCCAAAAGGCATACAGTG-----------------
------------------AAAAGAGAAAAAAGAGCAGCCTTCGGA---CTAGGTGCA---------
CTGTTCCTGGGGTTTCTTGGAGCA---GCAGGAAGCACTATG---GGCGCAGCATCA---
GTAGTGCTGACGGTACAG---GCCCGACAA---TTATTG---TCAGGGATAGTGCAACAGCAGAAT---
AATCTG---CTAAGA---GCAATAGAGGCCCAACAGCATTTATTGCAACTATCAGTTTGGGGCATT
>FJ424866
TTCTACTGTAATACCACTAAAATGTTTAGCTACAATTGCACAACAGAGGGTTGCAATACCACTGAAACAAGCA
AT--

ACCACTGAAACAACTAAATATATTCCCTGCCATCTAAAACAGGTAGTCAGGTCCTGGATGAGAGTAGGCTCAG
GCCTATTTGCTCCGCCTATTAGGGGAACTCTAAGATGTATATCTAAC---
ATTACAGGAATAATATTACAAAGGGATGCACCCCTAAAAACTGAT-------------------------
----

GAGAACAGCACCTTAAGACCAATAGGAGGTGACATGACAAACATTTGGAGGAGTGAACTGTACCCCTACAAGG
TGGTGAGGGTCAAGGCATTG---ACTGTGGCACCAACAAAAGCAAAGAGGCCTGTTATTGGC--------
---------------------CATAACAGGGAAAAACGTGCA---GCAGGC---CTGGGAATG-------
CTGTTTCTTGGGTTCATGAGTGCA---GCAGGAAGCACGATG---GGCGCAGCGGCA---
GTAACGCTGACGGTACAG---GCCAGGCAA---GTCTTG---CATGGTATCGTGCAGCAGCAAAAC---
AACATG---CTGAGA---GCTATAGCAGCTCAGCAAGAATTGCTGAGACTCTCTGTTTGGGGCATA
>KP004990
TTTTATTGTAATACCTCTAAACTCTTCAACTATACCTATGTCTGTAATCAGACAGATCAAGGTAATGCTAATT
ATTCCTGTCAT---
------AGTACTAGTACA------
TTAGAAAATGGAACCTTAGTAATACCCTGCAAATTAAGGCAGGTAGTTAATTCATGGATGAGAGTGGGATCAG
GACTTTTTGCACCACCTGTCCCGGGAAGCCTAACGTGCCACTCCAAT---
ATCACAGGTCTAATCCTTCAAAGGGACTGGCCACTAAACAATAAT-------------------------
----

ACGACTACTATTTTGAGGCCTGAAGGAGGAGACATGAAAGACATCTGGAGAAGTGAGCTATATCCATATAAGG
TAGTCCAAGTAAAAGCTTTG---GCTGTGGCACCTACAAAAATCTCCAGACCAACAATTATGGCA--------
---------------CAGACTCATCAGAGGGAAAAAAGAGGA---GCAGGC---CTCGGAATG---------
CTATTCCTTGGGTTCATGAGTGCA---GCAGGAAGCACGATG---GGCGCAGCGGCA---
GTAACGCTGACGGTACAA---GCCAAGCAA---TTATTG---CATGGTATAGTGCAACAGCAGAAT---
AATCTG---CTCAGA---GCGATAGAGGCACAGCAAGAATTGCTGAGGCTCTCTGTGTGGGGCATA
>HQ615979
TTCTATTGTAACACAACAGCCCTGTTTAATCATAGTATCACTGCAAATAGCACAGAGTCAGATAATGGCACTA
TA--

ACTCATAATGGCACTATAACTCTCCCATGTAGAATAAAGCAAATTATAAAAATGTGGCAAAGAACAGGACAAG
CAATATATGCCCCTCCCATCCAAGGAATAATAAGGTGTGAATCAAAC---
ATTACAGGACTAATATTAACAAGAGATGGTGGGAATAACAGTACAGAT----------------------
----

AACAGCACAGAAATCTTCAGACCTGGAGGAGGAGATATGAGGGACAATTGGAGAAGTGAACTATATAAGTATA
AAGTAGTAAAACTTGAACCACTA---GGAGTAGCACCCACCAGGGCAAGGAGAAGAGTGGTG----------
---------------------------CAGAGAGAAAAAAGAGCA---GTCGGA---ATAGGAGCT------
---GTCTTCCTTGGGTTCTTAGGAACA---GCAGGAAGCACTATG---GGCGCGGCGTCA---
CTGACGCTGACGGTACAG---GCTAGACAA---CTATTG---TCTGGTATAGTGCAACAGCAAAGC---
AATTTG---CTAAGG---GCTATAGAGGCTCAACAGCATATGTTGAAACTCACAGTCTGGGGTATT
>HQ834965
TTCTATTGCAATACAACAGACCTGTTCAATAGCACTTGGGACAAAAATGGCACTGGCACAAGTACGGGG----
----

GCCAGTGACAATATAACTCTGCCATGCAAAATAAGGCAAATTATAAGAATGTGGCAGAGAGTAGGACAAGCAA
TGTATGCCCCTCCTATCAAAGGAATTATAAAGTGTGAATCAAAC---
ATTACAGGACTACTGCTAACAAGAGATGGTGGAACTAATAGCAGC-------------------------
----

```
----------------
TATGAGACCTTCAGACCTATAGGAGGAAATATGAGGGACAATTGGAGAAGTGAACTATATAAGTATAAAGTAG
TAAAAATTGAACCAATA---GGAGTAGCACCTACCGGGGCAAAGAGAAGAGTGGTG-----------------
----------------------GAGAGAGAAAAAAGAGCA---ATTGGA---CTGGGAGCT---------
GCCTTCCTTGGGTTCTTAGGAGCA---GCAGGAAGCACTATG---GGCGCGGCGTCA---
ATAACGCTGACGGTACAG---GCCAGACAA---TTATTG---TCTGGCATAGTGCAACAGCAAAGC---
AATTTG---CTGCAG---GCTATAGAGGCCCAGCAGCATCTGCTGAAACTCACGGTCTGGGGTATT
>KY658684
TTCTACTGTAATACCTCAGAACTGTTTAGTAGTACTTGGAATGAAACTAATGGATCAAGTACTTGGAATGGTA
CT-----------------------------------------------------------------------
----------------------------
CAGAATGAAACTATCACACTTCCATGCAGAATAAGACAAATTGTAAACATGTGGCAGCAAGTAGGAAGAGCAA
TATATGCCCCTCCCATCAAAGGACCAATTGTATGTGTATCAAAT---
ATTACAGGGCTGATATTGACAAGAGATGGTAGTCAGAGCAACCAGACT-------------------------
-------------------------------------------------------------------------
----------
GACCAGCCTGAGGTCTTCAGACCTACAGGAGGAAATATGAAGGACAATTGGAGAAGTGAATTATATAAATATA
AAGTAGTACAAATTGAACCAGTA---GGAATAGCACCCACTAAGGCAAGAAGAAGAGTGGTG-----------
----------------------------CAGAGAGAAAAAAGAGCA---GTGGGA---ATAGGAGCT------
---GTGTTCCTTGGGTTCTTGGGAGCA---GCAGGAAGCACTATG---GGCGCAGCGGCG---
GTGACGCTGACGGTACAG---GCCAGACAA---TTATTG---TCTGGTATAGTGCAACAGCAGAAC---
AATCTG---CTGAGG---GCTATTGAGGCGCAACAGCATATGTTGCAACTCACAGTCTGGGGTATC
>DQ358808
TTCTACTGTAATACATCAAAGCTGTTTAATAGTACTTGGTCGCGTAATAGTACTAATGGTACTTGGACGCTTA
ACAGTACT-----------------------------------------------------------------
-------------------TAT---
ACTACAGAGAATATCACAATACTCCCATGCAGAATAAAACAATTATAC---
AGGTGGCAGGAAGTAGGAAAAGCAATGTATGCCCCTCCCATCAGTGGACTAGTTAGATGTTCATCAAAT---
ATTACAGGGCTGCTATTAACAAGAGATGGTGGCAGGAACAAGTCC----------------------------
-------------------------------------------------------------------------
----------------
GACGAGACCTTCAGACCTGGAGGAGGAGACATGAGGGACAATTGGAGAAGTGAATTATATAAATATAAAGTAG
TAAAAATTGAGCCATTA---GGAGTAGCACCCACCAGGGCAAAGAGAAGAGTGGTG-----------------
----------------------CAGAGAGAAAAAAGAGCA---GTGGGAACGATGGGAGCT---------
TTGTTCCTTGGGTTCTTGGGAGCA---GCAGGAAGCACTATG---GGCGCAGCGTCA---
TTGACGCTGACGGTACAG---GCCAGACAA---TTATTG---TCTGGTATAGTGCAACAGCAAAAC---
AATTTG---CTGAGG---GCAATTCAGGCACAACAGAGCCTGTTGCAACTCACAGTCTGGGGCGTC
>JN692461
TTCTACTGTAATACAACAAAACTGTTTAATAGTACTTGGATTAAGAGTAATAATACTTGGAGCAGTAATTGGA
CT-----------------------------------------------------------------------
----------------------------
GATACAGAAAATATCACACTCCCATGCAGAATAAAACAAATTATAAACAAATGGCAGGAAGTAGGAAAAGCAA
TGTATGCCCCTCCCATCAAGGGACCAATTAGCTGTTTATCAAAC---
ATTACAGGGCTGTTATTAACAAGAGATGGCGGTAACGAGACCACTAAT-------------------------
-------------------------------------------------------------------------
----------
AAAACTAATGAAACTTTCAGACCTGCAGGAGGAGATATGAGGGACAATTGGAGAAGTGAATTATATAAATATA
AAATAGTAAAAATTGAACCATTA---GGAGTAGCACCCACCAAGGCAAAGAGAAGAGTGGTG-----------
----------------------------CAGAGAGAAAAAAGAGCA---GCAGGA---CTAGGAGCT------
---GTGTTCCTTGGGTTCTTGGGAGCA---GCAGGAAGCACTATG---GGCGCAGCGGCA---
GTGACGCTGACGGTACAG---GCCAGACAA---TTATTG---TCTGGTATAGTGCAACAGCAAAGC---
AATTTG---CTGAGA---GCTATTGAGGCGCAACAACATATGTTGCAACTCACAGTCTGGGGCGTC
>JN692471
TTCTACTGTAATACAACAAAACTGTTTAATAATACTTGGAACAATAGTGCTTGGATGCATAATGAAGGGTCA-
-------------------------------------------------------------------------
----------------------------
AATGAAACTGAAATCACAATCCTATGCAGAATAAAACAAATTATAAACAGGTGGCAGGACATAGGAAAAGCAA
TGTATGCCCCTCCCATCCAAGGACAAATTAGATGTTCATCAAAT---
ATTACAGGGCTGCTATTAACAAGAGATGGTGGTAATAACAGGAAC----------------------------
-------------------------------------------------------------------------
```

GAGAGCAGTGAGACCTTCAGACCTGGAGGAGGAGATATGAGGGACAATTGGAGAAGTAAATTATATAAATATA
AAGTAGTAAAATTGAGCCATTA---GGAGTAGCACCCTCCAAGGCAAGGAGAAGAGTGGTG------------
----------------------------CAAAGAGAAAAAAGAGCA---GTGGGA---TTTGGAGCT------
---ATGTTCCTTGGGTTCTTGGGAGCA---GCAGGAAGCACTATG---GGCGCAGCGTCA---
GTGACGCTGACGGTACAG---GCCAGACAA---TTATTG---TCTGGTATAGTGCAACAGCAGAGC---
AATCTG---CTGAGG---GCTATTGAGGCGCAACAGCACCTGTTGCAACTCACAGTCTGGGGTATC
>KT427743
TTCTATTGTAATACAACAAAGCTGTTTAATTGGACTGAAGGATCAAATAACACTGGAGAAGAC----------
----

AATACAACAACTATTACACTCCCATGCAGAATAAAACAAATTATAAACAGGTGGCAGGAAGTAGGAAGAGCAA
TGTATGCCCCTCCCATTAGAGGACAAATTAGATGCTCATCAAAT---
ATTACAGGGTTGTTATTAACAAGAGATGGAGGTAACAATAACAAT----------------------------
----

AACTCAAAGGAGATCTTCAGACCTGGAGGAGGAAATATGAAGGACAATTGGAGAAGTGAATTATATAAATATA
AAGTAGTAAGAATTGAGCCATTA---GGAGTAGCACCCAGCAAGGCAAAGAGAAGAGTGGTG------------
----------------------------CAGAGAGAAAAAAGAGCA---GTGGGAACAATAGGAGCT------
---ATGTTCCTTGGATTCTTGGGAGCA---GCAGGAAGCACTATG---GGCGCAGCGTCA---
TTAACGCTGACGGTACAG---GCCAGGCTA---TTATTG---TCTGGTATAGTGCAACAGCAAAAC---
AACTTG---CTGAGA---GCTATTGAGGCGCAACATCATCTGTTGCAACTCACAGTCTGGGGCGTC
>KJ849820
TTCTATTGTAACACATCACAGCTGTTTGATAGTACTTGGTTGAAAAATGGG----------------------
----
-ACTGGTACTATAAAT---------
AATGCTAGCAAAACTATCACACTCCCTTGCAGAATAAAACAAATTATAAACAAGTGGCAGGAAGTGGGAAAAG
CAATGTATGCTCCTCCCATCAAAGGACAAGTTAGATGTTCATCAAAT---
ATTACAGGGCTGCTATTAACAAGAGATGGAGGCAGCAGTAGC-------------------------------
----
ACAAACAATACCAAC---
GACACAGAGATCTTTAGACCTGCAGGAGGAGATATGAGGGACAATTGGAGAAGTGAATTATACAAATATAAAG
TAGTAAGAATTGAGCCATTA---GGAGTAGCACCTTCCAGGGCAAAGAGAAGAGTGGTG--------------
-------------------------CAGAGAGAAAAAAGAGCA---GTGGGAACGATAGGAGCT---------
ATGTTCCTTGGGTTCTTGGGAGCA---GCAGGAAGCACTATG---GGCGCAGCGTCA---
CTGACGCTGACGGTACAG---GCCAGACAA---TTATTG---TCTGGTATAGTGCAACAGCAAAAC---
AATTTG---CTGAGA---GCTATTGATGCGCAACAACATATGTTGCAGCTCACAGTCTGGGGTATA
>KJ849785
TTCTACTGTAATACATCAAAATTGTTTAATAGTACCTGGCCTAATAGTACTTTGGAA----------------
----
AATAGTACTGGGTCAAATAGT------------
ACTCAGAATGAGACTATTACACTCCCATGCAGAATAAAACAAATTATAAACAGGTGGCAGGAAGTAGGAAGAG
CAATATATGCCCCTCCCATCAGAGGACAAATTAAATGTACATCCAAT---
ATTACAGGGTTGATATTAACAAGAGATGGGGGT--

AATAGTAATGAGAGC---
ACAACAGAAACCTTCAGACCTGGAGGAGGAGATATGAGGGACAATTGGAGAAGTGAATTATATAAATATAAAG
TAGTAAAAATTGAACCATTA---GGACTAGCACCCACCAAGGCAAAGAGAAGAGTGGTG--------------
-------------------------CAGAGAGAAAAAAGAGCA---ATAACA---CTGGGAGCT---------
ATGTTCCTTGGGTTCTTGGGAGCA---GCAGGAAGCACTATG---GGCGCAGCGTCA---
CTGACGCTGACGGTACAG---GCCAGACTA---TTATTG---TCTGGTATAGTGCAGCAGCAGAAC---
AATCTG---CTGAGA---GCTATTGAGGCGCAACAGCATATATTGCAACTCACAGTCTGGGGCGTT
>EU023921
TTCTACTGTAATTCAACAGAGCTGTTTAATAGTACTTGGAATCAGAATGATACTAGAAATAACACTGGA----
----

CAAAATGACACGATCACGCTCCCATGCAGAATAAAACAAATTATAAACATGTGGCAGGAAGTAGGAAAAGCAA
TGTATGCCCCTCCCATCAGAGGACAAATTAGATGTTCATCAAAT---
ATTACAGGGCTACTATTAACAAGAGATGGGGGTAATGATGGTGGTAATTAC----------------------
----

ACAAACAAGACAGAGACCTTCAGACCTGGAGGTGGAAATATGAGGGATAATTGGAGAAGTGAATTATATAAAT
ATAAAGTAGTAAAAATTGATCCAATA---GGAGTAGCACCCACCAAGGCAAAGAGAAGGGTGGTG--------
-------------------------------CAGAGAGAAAAAAGAGCA---ATGGGA---CTAGGAGCT---
------ATGTTCCTTGGGTTCTTGGGAGCA---GCAGGAAGCACTATG---GGCGCAGCGTCA---
ATAACGCTGACGGCACAG---GCCAGACAA---TTATTG---TCTGGTATAGTGCAACAGCAGAAT---
AATTTG---CTGAGG---GCTATTGAGGCGCAACAGCATCTGTTGCAACTCACAGTCTGGGGTATC
>EU131798
TTCTACTGTAATACATCACAACTGTTTAATAGTACTTGGAATAAAACTAGTATTGGGTATAATATTACA----
----

GGAAATAGCATTATCACGCTCCCTTGCAGAATAAAACAAATTGTAAACAGGTGGCAGGAAGTAGGGAAAGCAA
TGTATGCCCCTCCCATTGCAGGACAAATTAGATGTTCATCAAAT---
ATTACAGGGTTGATATTAACAAGAGATGGTGGTAAGAATGAGAACAACACC----------------------
----

ACCGAGACCTCCGAGACCTTCAGACCTGGAGGAGGAAATATGAAAGACAATTGGAGAAGTGAATTATATAAAT
ATAAAGTAGTACAAATTGAACCATTA---GGAGTAGCACCCACCAGGGCAAAGAGAAGAGTGGTG--------
-------------------------------CAGAGAGAAAAAAGAGCA---CTGGGA---ATAGGAGCT---
------ATGTTCCTTGGGTTCTTGGGAGCA---GCAGGAAGCACTATG---GGCGCAGCATCA---
ATAACGCTGACGGTACAG---GCCAGACAA---TTGTTG---TCTGGTATAGTGCAACAGCAGAGA---
AATTTG---CTGAGA---GCAACTGAGGCGCAACAGCATCTGTTGCAACTCACAGTCTGGGGCGTC
>EU131810
TTCTACCGTAATACATCACAACTGTTTAATAGTACTTGGAATGTTAGTAGTACTTGGAATGATACTACA----
----

GGAAATGACAATATCACACTCTCTTGCAGAATAAAGCAAATTGTAAACATGTGGCAGGAAGTAGGGAAAGCAA
TGTATGCCCCTCCCATTAGAGGACAAATTAGCTGTTCATCAAAA---
ATTACAGGGTTACTATTAACAAGAGATGGTGGTAATAATGAGAGC----------------------------
----

GAGAACACCGAGACCTTCAGACCTGGAGGAGGAGATATGAGAGACAATTGGAGAAGTGAATTATATAAATATA
AAGTAGTAAAAATTGAACCATTA---GGAGTAGCACCCACCAAGGCAAAGAGAAGAGTGGTG----------
----------------------------CAGAGAGAAAAAAGAGCA---GTGGGAACGATAGGAGCT------
---ATGTTCCTTGGGTTCTTGGGAGCA---GCAGGAAGCACTATG---GGCGCAGCGTCA---
ATAACGCTGACGGTACAG---GCCAGACAA---CTGTTG---TCTGGTATAGTGCAACAGCAGAGA---
AATTTG---CTGAGG---GCTATTGAGGCGCAACAGCATATGTTGCAACTCACAGTCTGGGGTATC
>JF932488
TTCTACTGTGATACATCACGACTGTTTAATAGTACTTGGTCTAATAATGGTACTACGAATGGTACTACA----
----

GGAAATGACACTATCACGCTCCCTTGCAGAATAAGACAAATTGTAAACATGTGGCAGGAAGTAGGGAGAGCAA
TGTATGCCCCTCCCATTGCAGGACAAATTAACTGTTCATCAAAT---
ATTACAGGGTTGCTATTAACAAGAGATGGTGGTAATACTAATGATACT-------------------------
----

AACAATAGCACCGAGACCTTCAGACCTGGAGGAGGAGATATGAGAGACAATTGGAGAAGTGAATTATATAAAT
ATAAAATAGTAAAAATTGAACCATTA---GGAATAGCACCCACCAAGGCAAAGAGAAGAGTGGTG--------
-------------------------------CAGAGAGAAAAAAGAGCA---GTGGGAACGATAGGAGCT---
------ATGTTCCTTGGGTTCTTGGGAGCA---GCAGGAAGCACTATG---GGCGCAGCGTCA---
ATAACGCTGACGGTACAG---GCCAGACAA---TTGTTG---TCTGGTATAGTGCAACAGCAGAGA---
AATCTG---CTGAGG---GCTATTGAGGCGCAGCAGCATCTGTTGCAACTCACAGTCTGGGGTATC
>KR914678
TTCTACTGTAATACATCAAAACTGTTTAATAGCACTTGGAATAAGGATAGTTGGAATAATGAAGCTTGGAATG
AAACTGAA---

GCAAGTGATACTATCACACTTCCATGCAGAATAAAACAAATTATAAACATGTGGCAGGAAGTAGGAAAAGCAA
TGTATGCCCCGCCCATTAGTGGACAGATTAGATGTGTATCAAAT---
ATTACAGGGTTGCTGTTAACAAGAGATGGTGGTAATAGTAACAAT----------------------------
----

GAGTCCGAGACCTTCAGACCTGCAGGAGGAAATATGAAGGACAATTGGAGAAGTGAATTATATAAATATAAAG
TAGTAAAAATTGAACCATTA---GGAGTAGCACCCACCAAGGCAAAGAGAAGAGTGGTG-------------
------------------------CAAAGAGAAAAAAGAGCA---ATAACA---CTAGGAGCT---------
ATGTTCCTTGGGTTCTTGGGAGCA---GCAGGAAGCACTATG---GGCGCAACGTCA---
ATAACGTTGACGGTACAG---GCCAGACGA---TTGTTG---TCTGGTATAGTGCAACAGCAGAGC---
AATCTG---CTGAGA---GCTATTGAGGCGCAACAGCATATGTTGCAACTCACAGTCTGGGGCACA
>JF683781
TTTTATTGTAATACAACACAATTGTTTAATAGTACTTGGCCAAATGATACAACT------------------
----

ATTGAACAAAATATCACACTCCCATGCAGAATAAAACAAATTATAAACATGTGGCAGGGAGTAGGAAAAGCGA
TGTATGCCCCTCCCATCAGTGGACAAATTAGATGTTTATCAAAT---
ATTACAGGACTGCTATTAACAAGAGATGGTGGTAAGCATGAGAGC---------------------------
----

AGGGACGAGACCTTCAGACCTGGAGGAGGAGATATGAGGGACAATTGGAGAAGTGAATTATATAAATATAAAG
TAGTAAAAATTGAACCATTA---GGAGTAGCACCCACCAGGGCAAAGAGAAGAGTGGTG-------------
------------------------CAAAGAGAAAAAAGAGCG---GTGACG---CTGGGAGCC---------
TTGTTCCTTGGGTTCTTGGGAGCA---GCAGGAAGCACTATG---GGCGCAGCGTCA---
ATGACGCTGACGGTACAG---GCCAGACGA---TTATTG---TCTGGTATAGTGCAGCAGCAGAAC---
AATCTG---CTGAGG---GCTATTGAGGCGCAACAGCATCTGTTGCAACTCACAGTCTGGGGCGTT
>JF683807
TTCTACTGTAATTCAACACCACTGTTTAATAGTATTTGGAACAAGACTGATGATACTTGGAATTCAACACAAC
AATCG---

GGTGGGAATAAAACTATCACACTCTCATGCAGAATAAAACAAATTATAAACAGGTGGCAGGAGGTAGGAAAGG
CAATATATGCCCCTCCCATCAAAGGAATCATTAAATGCACATCAAAT---
ATTACAGGGTTACTATTAACAAGAGATGGTGGTAATAATAATGAAAGT------------------------
----

GACACCACCGAAACCTTCAGACCTGGAGGGGGAGATATGAGAGACAATTGGAGAAGTGAATTATATAAATATA
AAGTAGTAAGAATTGAACCATTA---GGAATAGCACCCACCAAGGCAAAGAGAAGAGTGGTG----------
---------------------------CAGAGAGAAAAAAGAGCA---GTGGGA---CTAGGAGCT------
---TTGTTCCTTGGGTTCTTGGGAGCA---GCAGGAAGCACTATG---GGCGCAGCGTCA---
CTAACGCTGACGGTACAG---GCCAGACAA---TTATTG---TCTGGTATAGTGCAACAGCAGAAC---
AATTTG---CTGAGA---GCTATTGAGGCGCAACAGCATATGTTGCAACTCACAGTCTGGGGTATA
>KY324591
TTCTACTGTAATACAACACAGCTGTTTAATAGTACTTGGAATTCTACTGATACTTGGAATGAAACAGAAGAG-
----

TCAAATGACAATATCACCATCCCATGCAGAATAAAACAAATTATAAACAAGTGGCAAAGGGTAGGACAGGCAA
TGTATGCCCCTCCCATCAGTGGACTAATTAGGTGTTCATCAAAC---
ATCACAGGGATACTATTAACAAGAGATGGTGGTAGTGGTAATAAAAACAACAGT------------------
----
ACTGAGGAAAAGAAAAGCAACAGTACTGAG---------
GAAAAGGAAATCTTCAGGCCTCAAGGAGGAGACATGAGGGATAACTGGAGAAATGAATTATATAAATATAAAG
TAGTAAAAATTGAACCATTA---GGATTAGCACCCACCAAGGCAAAGAGAAGAGTGGTG-------------
------------------------CAGAGAGAAAAAAGAGCA---GTGGGG---TTCGGAGCC---------
TTGTTCCTTGGGTTCTTGGGAGCA---GCAGGAAGCACTATG---GGCGCAGCGTCA---
GTGACGCTGACGGTACAG---GCCAGACAA---TTATTG---TCTGGTATAGTGCAACAGCAGAAC---
AACTTG---CTGAGA---GCTATTGAGGCGCAACAGCATATGTTGCAACTCACAGTCTGGGGTATT
>AF041127
TTCTATTGTAATACAACAGGACTGTTTAATAGTACTTGGAATGCTAATAGTACTGGGAATGAT---------
----

ATTAAAGAACATATCATACTCCCATGTAGAATAAAACAAATTATAAACATGTGGCAGGAAGTAGGAAAAGCAA
TGTATGCCCCTCCTAGCAGAGGAAAAATTAAATGTTCATCAAAT---
ATTACAGGGCTACTATTAACAAGAGATGGTGGTAATAATAACACA---------------------------
----

AATGAGACCTTCAGACCTGGAGGAGGAGATATAAGGGACAATTGGAGAAGTGAATTATATAAATATAAAATAG
TAAAAATCGAACCATTA---GGAGTAGCACCCACCAGGGCAAAGAGAAGAGTGGTG-----------------
----------------------CAGAGAGAAAAAAGAGCA---GTAACG---ATGGGAGCT---------
CTGTTCCTTGGGTTCTTGGGAACA---GCAGGAAGCACTATG---GGCGCAGCGTCA---
ATGATGCTGACGGTACAG---GCCAGACAA---TTATTG---TCTGGTATAGTGCAACAGCAAAGC---
AATTTG---CTGAGG---GCTATTGAGGCGCAACAGCATCTGTTGCAACTCACAGTCTGGGGTATC
>KC699023
TTCTACTGTGATACAACACAGTTGTTTAATAGTACTTGGCAATCTAATAGTACTTGGAATAATGCTACA----
----

GGCAATGAAAATATCACACTCCCATGCAGGATAAAACAAATTATAAATAGATGGCAGGAAGTAGGAAAAGCAA
TGTATGCCCCTCCCATCGCAGGACAAATTAGATGTTCATCAAAT---
ATTACAGGGATAATATTAACAAGAGATGGTGGGGTAATGATACT-----------------------------
----

AATAAGGAGACCTTCAGACCTGGAGGAGGAAATATGAAAGACAATTGGAGAAGTGAGTTATATAAATATAAAG
TAGTAAAAGTTGAGCCACTA---GGAGTAGCACCCACCAAGGCAAAGAGAAGAGTGGTG--------------
-------------------------CAGAGGGAAAAAAGAGCA---GTGGGAACGCTAGGAGCT---------
ATGTTCCTTGGGTTCTTGGGAGCA---GCAGGAAGCACTATG---GGCGCAGCGTCA---
ATGGCGCTGACGGTACAG---GCCAGACAA---TTATTG---TCTGGTATAGTGCAACAGCAAAAC---
AATTTG---CTGAGG---GCTATTGAGGCGCAACAACATATGTTGCAACTCACAGTCTGGGGCGTC
>HQ595775
TTCTACTGTAACACAGCACAGCTGTTTAATAGTAGCTGGCCTCATAATAGGACAGATAATAGTACAGAGTCAA
GT---

AATAGTAATAAAACAATCATCCTCCAATGCAGAATAAAACAAATTATAAACATGTGGCAGGGAGTAGGAAAAG
CAATGTATGCCCCTCCAATCAGTGGACCAATTAGATGTACATCAAAT---
ATTACAGGGCTGATATTGACAAGAGATGGTGGTAATAGTAGTGATATT-------------------------
----

AAGAATGATACCGAGACCTTCAGGCCTCAAGGAGGAAATATGAGGGACAATTGGAGAAGTGAATTATATAAAT
ATAAAGTAGTAAGAATTGAACCATTA---GGAGTAGCACCCACCAGGGCAAGGAGAAGAGTGGTG--------
-------------------------------CAGAGAGAAAAAAGAGCA---GTAACT---CTAGGAGCT---
------GTGTTCCTTGGGTTCTTGGGAGCA---GCAGGAAGCACTATG---GGCGCAGCGTCA---
ATAGCGCTGACGGCACAA---GCTAGACAA---TTATTG---TCTGGTATAGTGCAACAGCAGAAC---
AATCTG---CTGAGG---GCTATTGAGGCGCAACAGCATCTGTTGCAACTCACAGTCTGGGGAATC
>HQ595795
TTCTACTGTAATACATCACAACTGTTTAATAGTAGTTGGCCTGCTAATAGTAGTTGGTCTGTTCAGAATAAC-
----

ACAAATGAAACTATCACACTCCCGTGTAGAATAAAACAAATTATAAACATGTGGCAGGAAGTAGGAAAAGCAA
TGTATGCCCCTCCCATCAAAGGACAAATTAACTGTTTATCAAAT---
ATTACAGGGTTGATATTAACAAGAGATGGTGGTACAAACTCAACT----------------------------
----

AGCACCACCGAGACCTTCAGACCTGCAGGGGGAAATATGAGGGACAATTGGAGAAGTGAACTATATAAATATA
AAGTAGTAAAACTTGAACCATTA---GGAATAGCACCCACCAAGGCACAGAGAAGAGTGGTG-----------
----------------------------CAGAGAGAAAAAAGAGCA---GTGGGA---CTAGGAGCT------
---CTGTTCCTTGGGTTCTTGGGTGCA---GCAGGAAGCACTATG---GGCGCAGCGTCA---
GTAACGCTGACGGTACAG---ACCAGACAA---TTATTG---TCTGGTATAGTGCAACAGCAAAAC---
AATCTG---CTGATG---GCTATTGAGGCGCAACAGCATATGTTGCAACTCACAGTCTGGGGTATT
>MK303347
TTCTACTGTAACACAACACGACTGTTTAATAGCACTGATTGGAATAGTACTAATAATAATAAT----------
----

GAGTCAAATATTATCACAATCCAATGCAAAATAAAACAATTTATAAACATGTGGCAGAGAGTAGGAAAAGCAA
TGTATGCCCCTCCCATCAAAGGACAAATTAACTGTACATCAAAT---
ATTACAGGGCTGCTATTAACAAGAGATGGCGGAAACGAGACCAAC----------------------------
----

AACACTGAGACCTTCAGACCTGGGGGAGGAGATATGAGGGATAATTGGAGAAATGAATTATATAAATATAAAG
TAGTAAAAATTGAACCATTA---GGAATAGCACCCACCAAGGCAAAGAGAAGAGTGGTG--------------
-------------------------CAGAGAGAAAAAAGAGCA---GTGGGA---ATAGGAGCT---------
TTGTTCCTTGGGTTCTTAGGAACA---GCAGGAAGCGCTATG---GGCGCAGCGTCA---
GTGACGCTGACGGTACAG---GCCAGACAA---TTATTG---TCTGGTATAGTGCAACAGCAGAAC---
AATCTG---CTGAGG---GCTATTGAGGCGCAACATCATCTGTTGCAACTCACAGTCTGGGGCGTC
>AB588331
TTCTACTGTAATACAACAAAGCTGTTCAATAGTGCTTGGTTTAATGGTACTAGGGATAATACTGATAACTTAA
AT---

AACGCTGATACAGAAATCACACTCCCATGCAGAATAAAACAAATTATAAACAGGTGGCAAGAAGTAGGAAAAG
CAATGTATGCCCCTCCCATCCAAGGACAAATTAGCTGTTCATCAAAT---
ATTACAGGGATAATATTAACAAGAGATGGTGGTGATAACAACAAG----------------------------
----

TCCGAAACCTTTAGACCTGGAGGAGGAAATATGAAGGACAATTGGAGAAGTGAATTATATAAATATAAAGTAG
TAAGAATTGAGCCATTA---GGATTAGCACCCACCAAGGCAAAGAGAATAGTGGTG-----------------
----------------------CAAAGAGAAAAAAGAGCA---GTAACA---TTCGGAGCT---------
CTGTTCCTTGGGTTCTTAGGAGCA---GCAGGAAGCACTATG---GGCGCAGCGTCA---
ATAGCGCTGACGGGACAG---GCCAGACAA---TTATTG---TCTGGTATAGTGCAGCAGCAGAAC---
AATCTG---CTGAGA---GCTATTGAGGCGCAACAGCATATGTTGCAACTCACAGTCTGGGGCGTC
>HQ386159
TTCTACTGTGACACAACACAACTGTTTAATAGTACTTGGCCTGTTAATAGTACTGGGAATGGTACTGTGTCAA
AT---

AACACTGAAGAAATAACACTCCCATGCAGAATAAAACAAATTATAAACATGTGGCAGGAAATAGGAAGAGCAA
TGTATGCCCCTCCCATCCAAGGATACATTAACTGTTCATCAAAT---
ATTACAGGGCTGCTATTAACAAGAGATGGTGGTAATGATAATGAG----------------------------
----

AGCAAACCCGAGACATTCAGACCAGGAGGAGGAAATATGAAGGACAATTGGAGAAGTGAATTATATAAATATA
AAGTAGTAAAAATTGAACCATTA---GGACTAGCACCCACCAAGGCAAAGAGAAGAGTGGTG-----------
----------------------------CGGAGAGAAAAAAGAGCA---GTGGGA---ATAGGAGCT------
---TTGTTCCTTGGGTTCTTGGGAGCA---GCAGGAAGCACTATG---GGCGCAGCGTCA---
CTGACGCTGACGGTACAG---GCCAGACAA---TTACTG---TCTGGTATAGTGCAACAGCAGAGC---
AATTTG---CTGAGG---GCTATTGAGGCGCAACAGCATATGTTGCAACTCACAGTCTGGGGCGTC
>EU744114
TTCTACTGTGATTCAACACAATTGTTTAATAGTACTTGGATAGCTAATAGGACTTGGGAGAATGATAATAGC-
----

ACTGGAGGAAATATCATGCTCCCATGCAGAATAAAACAAATTATAAACATGTGGCAGGAAGTAGGAAAAGCAA
TGTATGCCCCTCCCATCAGAGGACAAATTAGATGTTCAACAAAT---
ATCACAGGGCTGCTATTAACAAGAGATGGTGGAGATGCTGGGGAC----------------------------
----

AACGAGACCGAGATCTTCAGACCTGGAGGAGGAAATATGAGGGACAATTGGAGAAGTGAATTATATAAATATA
AAGTAGTAAAAATTGAACCATTA---GGAGTAGCACCCACTAAGGCAAGGAGAAGAGTGGTG-----------
----------------------------CAGAGAGAAAAAAGAGCA---GTGGGA---CTAGGAGCT------
---ATGTTCCTTGGGTTCTTGGGAGCA---GCAGGAAGCACTATG---GGCGCAGCGTCA---
ATGACGCTGACGGTACAG---GCCAGACAA---TTATTG---TCTGGTATAGTGCAGCAGCAAAAC---
AACTTG---CTGAGG---GCTATTGAGGCGCAACAGCATCTGTTGCAACTCACAGTCTGGGGTATC
>KP411822
TTCTACTGTAATACAACAGGACTGTTTAATAGTACTTGGAATAGCACTGAAGGGTTAAATGACACTGAA----
----

GAAAATGACACACTCACACTCCAATGCAGAATAAAACAAATTATAAACATGTGGCAGGAAGTAGGAAAAGCAA
TGTATGCCCCTCCCATCACAGGACGAATTAGATGTACATCAAAC---
ATTACAGGGCTGCTCTTAACAAGAGATGGTGGTACCGACCAGAGT----------------------------
----

GATGCTAAGGTCTTCATACCTGGAGGAGGAGATATGAGAGACAATTGGAGAAGTGAATTATATAAATATAAAG
TAGTAAAAGTTGAGCCATTA---GGATTAGCACCCACCAAGGCAAAGAGAAGAGTGGTG--------------
-------------------------CAGAGAGAAAAAAGAGCA---GTGGGA---CTAGGAGCT---------
TTGTTCCTTGGGTTCTTGGGAGCA---GCAGGAAGCACTATG---GGCGCAGCGTCG---
ATGACGCTGACGGTACAG---GCCAGACAA---TTATTG---TCTGGTATAGTGCAACAGCAAAAT---
AATTTG---CTGCAG---GCTATTGAGGCGCAACAGCATCTGTTGAAACTCACAGTCTGGGGTATC
>KP411825
TTCTACTGGAACACAACATCCCTATTTGATAGTACTTGGAATACGAATGCTACTTGGGATAGGAATTGGAATG
ATACC--
-------------------AGT---
GGAGAAAATGACATCATCACCCTCCCATGTAGAATAAAGCAAATTATATACATGGGGCAGGAAGTAGGAAAAG
CTATGTATGCCCCTCCCATTACTGGACTAATTAGCTGCTCATCCAAC---
ATTACCGGGTTGTTATTAATAGGATGTGGTGGTGAAAGCTACAAT----------------------------
----

GATGAGATCTTCACACCTGGAGGAGGAAATATGAAAGATTACTGGAGAAGTGAATTATATAAATATGATGTAC
AAGAAATTGAACCATTA---TTAGTAGCACCCACCAAGGCAAAGAGGATAGTGGTG-----------------
----------------------CATAGAGAAAAAAGAGCA---TTTAGA---TTCGGATCT---------
ATGTTCCTTGGGTTCTTGGAAGCG---CCAGGAAGCACTATG---GGCGCAGCGTCA---
ATAACACTGACGGTACAG---GCCATACTA---TTATTG---TCTGGTATACTGCACCAGCATAGC---
AATCTG---CTGAAA---GCTATTGAGGCTCATCAGCTTCTGTTGCTTGTCACAGTCAGTCTTATT
>MF373201
TTCTACTGTAATACATCACAATTGTTTAATAGTACTTGGAAGGATGGAAATATTACTGAAGGGAAC-------
----

ATTACAGCAAATATCACACTCCCATGCAGAAGAAGACAAATAGTAAACATGTGGCAGGAAGTAGGAAAAGCAA
TGTATGCCCCTCCCATCAGAGGACAAATTAATTGTGCATCAGAT---
ATTACTGGGTTGCTATTAACAAGAGATGGTGGTTCTGAGTCCAAC----------------------------
----

CACACAGAGATATTCAGACCTGGAGGAGGAGATATGAGAGACAATTGGAGAAGTGAATTATACAAATATAAAG
TAGTGAAAATTGAACCATTA---GGAATAGCACCCACCAAGGCAAAGAGAAGAGTGGTG--------------
-------------------------CAGAGAGAGAAAAGAGCA---GTAGGA---ATAGGAGCT---------
GTGTTCCTTGGGTTCTTGGGAGCA---GCAGGAAGCACTATG---GGCGCAGCATCA---
ATGACGCTGACGGTACAG---GCCAGACAA---TTATTG---TCTGGTATAGTGCAACAGCAGAAC---
AATCTG---CTGAGA---GCTATTGAGGCGCAACAACATCTGTTGCAACTCACAGTCTGGGGTATC
>JX448103
TTTTACTGTAATACAACACCACTGTTTAATAGTACTTGGGACAATAATACATGGGACAATAATACATGGGACA
CAACACAG---
----------TCCAATGCG------
ACTGTCACTGACTCAATTATACTTCAATGCAGAATAAAACAGGTTATAAACCTGTGGCAGGAAGTAGGAAAAG
CAATGTATGCCCCTCCCATCAAAGGAAACATTAGCTGTTCATCAAAT---
ATTACAGGGTTGCTATTAACAAGAGATGGGGGCAATGACAATAGCACA-------------------------
----

AATGGGAACGAGACCTTCAGACCGGGAGGAGGAAACATGAAGGACAATTGGAGAAGTGAATTATATAAATATA
AAGTAGTACAAGTTGAACCATTA---GGAGTAGCACCCACCAAGGCAAGGAGGAGAGTGGTG-----------
----------------------------CAAAGAGAAAAAAGAGCA---GTGGGA---ATAGGAGCT------
---GTGTTCCTTGGGTTCTTGGGAGCA---GCAGGAAGCACTATG---GGCGCAGCGTCA---
ATAACGCTGACGGTACAG---GCCAGGCAA---TTATTG---TCTGGTATAGTGCAACAGCAGAAC---
AACTTG---CTGAGA---GCTATTGAGGCGCAACAACATATGTTGCAACTCACAGTCTGGGGTATC
>AF277059
TCCTACTGTAATACAACAAAGCTGTTTAATGGTACTTGGAATGATAATAGTACTTGGAATGATACTGGGGAG-
----

TCAAATAACACTATCACACTCCCATGCAGAATAAAACAAATTATAAACATGTGGCAGAAAGTAGGCAAAGCAA
TGTATGCCCCTCCCATCAGAGGACAAATTAGATGTGCATCAAAT---
ATCACAGGGCTGCTGTTAACAAGAGGTGGTGGCAGTAATAATAGC----------------------------
----

```
------------
AACAACGAGACTTTCAGACCTGGAGGAGGAGATATGAGGGACAATTGGAGAAGTGAATTATATAAATATAAAG
TAGTAAAAATTGAACCATTA---GGAATAGCACCCACCAAGGCAAAGAGAAGAGTGGTG--------------
-------------------------CAGAGAGAAAAAAGAGCA---GTGGGAACAATAGGAGCT---------
ATGTTCCTTGGGTTCTTGGGAGCA---GCAGGAAGCACTATG---GGCGCAGCGTCA---
GTGACGCTGACGGTACAG---GCCAGACTA---TTATTG---TCTGGTATAGTGCAACAGCAGAAC---
AATCTG---CTGAAG---GCTATTGAGGCGCAACGGCATCTGTTGCAACTCACGGTCTGGGGCGTC
>FJ496078
TTCTACTGTAATACAACAAAACTATTCAATAGTACTTGGAATAGTACTAGGAATGATACTGAAAGGAATAGTA
AT-----------------------------------------------------------------------
----------------------------
GAAACTGACACAATCACACTCCCATGCAGAATAAAACAAATTATAAACATGTGGCAGGGAGTAGGAAAAGCAA
TGTATGCCCCTCCTATCAGAGGACTAATTAGATGTTCATCAAAT---
ATTACAGGGCTGTTATTAACAAGGGATGGTGGTAATACTAACAGGAGCGGG----------------------
-------------------------------------------------------------------------
-------
AGCAACGGGTCTGAGACCTTCAGACCTGGAGGAGGAAATATGAAGGACAATTGGAGAAGTGAATTATATAAAT
ATAAAGTAGTAAAACTTGAACCATTA---GGAATAGCACCCACCAAGGCAAGGAGAAGAGTGGTG--------
-------------------------------CAGAGAGAAAAAAGAGCA---GTAGGA---CTAGGAGCG---
------TTGTTCATTGGGTTCTTGGGAGCA---GCAGGAAGCACTATG---GGCGCAGCGTCA---
GTAACGCTGACGGTACAG---GCCAGACAA---TTATTG---TCTGGTATAGTGCAACAGCAGAAC---
AATCTG---CTGATG---GCTATTGATGCACAACAGCATCTGTTGCAACTCACAGTCTGGGGCGTC
>FJ469758
TTCTACTGTAATACAACACAACTATTTAAGAGTACTTGGCAGGTTAATGATACTTGGCAGGGAGAGTCAAATG
ACTCT--------------------------------------------------------------------
-------------------------
AAAGGGAATGACACAATCATACTCCCATGCAAAATAAACAAATATAAACATGTG---
GTTGGAAGTAGGAAAAGCATGTATGCCCCTCCC---ACATTAACAATTAAATGTTCATCAAAT---
ATTACAGGGCTGATATTAACAAGAGATGGTGGACATGGTGGTAATAGTAGTGAT-------------------
-------------------------------------------------------------------------
-
GATGATGTGAACAAAACCGAGATATTCAGACCTGGAGGAGGAGATATGAGGGACAATTGGAGAAGTGAATTAT
ATAAATATAAAGTAGTAAGAATTGAACCATTA---GGAGTAGCACCCACCAAGGCAAAGAGAAGAGTGGTG--
-------------------------------------CAGAGAGAGAAAAGAGCA---GTGGGC---
CTAGGAGCT---------ATGTTCCTTGGGTTCTTGGGAGCA---GCAGGAAGCACTATG---
GGCGCAGCGTCA---ATAACGCTGACGGTACAG---GCCAAACAA---TTATTG---
TCTGGTATAGTGCAACAGCAGAAC---AATCTG---CTGAGG---
GCTATTGAGGCGCAACAGCATCTGTTGCAACTCACAGTCTGGGGTATC
>KX156454
TTCTACTGTAATACAACAAAGCTGTTTAATAGTACTTGGAAGTTTAATACTACTTCTAATGGTACTTGGAATT
TTAATGGTACT--------------------------------------------------------------
-TGGAATGGTACAGGA---------
CCAGGAAACGACACCATCAGGCTCCCATGCAGAATAAAACAAATTATAAACATGTGGCAGCAAGTAGGAAAAG
CAATGTATGCCCCTCCCATCGAAGGACACATTAAATGTTCATCAAAT---
ATTACAGGGCTGCTATTGATAAGAGATGGTGGTAATAATGGGAAT----------------------------
-------------------------------------------------------------------------
----------
AACGAGACCGAGACCTTCAGACCTGGAGGAGGAGATATGAGGGACAATTGGAGAAGTGAATTATATAAATATA
AAGTAGTAAAAATTGAACCCTTA---GGAGTAGCACCCACTAAGGCAAAGAGAAGAGTGGTG-----------
----------------------------CAGAGAGAAAAAAGAGCTGCATTTGGA---CTAGGAGCT------
---TTGTTCCTTGGGTTCTTGGGAGCA---GCAGGAAGCACTATG---GGCGCAGCGTCA---
ATAACGCTGACGGTACAA---GCCAGACAA---TTACTG---TCTGGTATAGTGCAGCAGCAAAAC---
AATTTG---CTGAGA---GCTATTGAGGCGCAACAGCACCTGTTGCAACTCACAGTCTGGGGTATT
>HQ217850
TTCTACTGTAACTCAACACCACTGTTTAATAGCACTTGGAATAGGACTTGGAATGAAGAATCAAATAAC----
-------------------------------------------------------------------------
----------------------------
ACTGATAAAAATATCACACTCCAATGCAGAATAAAACAAATTGTAAACATGTGGCAGCGAGTAGGAAAAGCAA
TGTATGCCCCTCCCATCAATGGACTAATTAGATGCTCATCAAAT---
ATTACGGGGCTGCTATTAACAAGAGATGGGGGTAATAAACTAAACGAG-------------------------
```


TCCGAGATCGAGATCTTCAGACCTGGAGGAGGAAATATGAGGGAAAATTGGAGAAGTGAATTATATAAATATA
AAGTAGTGCAAATTGAACCAATA---GGACTAGCACCCACAAAGGCAAAGAGAAGAGTGGTG-----------
----------------------------CAGAGAGAAAAAAGAGCA---GTGGGAATGTTAGGAGCT------
---ATGTTCCTTGGGTTCTTGGGAACA---GCAGGAAGCGCTATG---GGCGCAGCGGCA---
ATAACGCTGACGGTACAG---GCCAGACAG---TTACTG---TCTGGTATAGTGCAACAGCAAAAC---
AATTTG---CTGAGG---GCTATTGAGGCGCAACAGCATCTGTTGCAACTCACAGTCTGGGGCGTC
>JF689889
TTCTACTGTAATTCAACACCACTGTTTAATAGTACTTGGGATAATAGTACTTGGGATGATAATGGAAGGTCAA
AT---
-------------------AAC---
ACTGGAAACGACTCAATCGTACTCCTATGCAGAATAAAACAAATTATAAACATGTGGCAGGAAGTAGGAAAAG
CAATGTATGCCCCTCCCATCAGAGGACCAATTAAATGTTCATCAAAT---
ATTACAGGGCTGATATTAACAAGAGATGGTGGTCATAACACCACC----------------------------
----

AACGAGACCTTCAGACCTGGAGGAGGAGATATGAGGGACAATTGGAGAAGTGAATTATATAAATATAAAGTAG
TAAAAATTGAACCATTA---GGAATAGCACCCACCAAGGCAAAGAGAAGGGTGGTG-----------------
----------------------CAAAGAGAAAAAAGAGCA---GTGGGA---ATAGGAGCT---------
ATGTTCCTTGGGTTCTTGGGAGCA---GCAGGAAGCACTATG---GGCGCAGCGTCA---
CTAACGCTGACGGTACAG---GCCAGACTA---CTATTG---TCTGGTATAGTGCAACAGCAGAAC---
AATTTA---CTGAGA---GCTATTGAGGCGCAACAGCATCTGTTGCAACTCACAGTCTGGGGAGTC
>JX864021
TTCTACTGTAATACAACAAAACTATTTAATAGTACTTGGAGTCAAAATGGCACTAGGAGTGGTCCTGAAAGGA
TAAAT--

GACACTGACATCATCACACTCCAATGCAGAATAAAACAAGTTGTAAACATGTGGCAGGAAGTAGGAAAAGCAA
TGTATGCCCCTCCCATCAGTGGACAAATTAAATGCTCATCAAAT---
ATTACAGGGCTGCTATTAACAAGAGATGGTGGTAACACAAATGTGAGC-------------------------
----

AATGAGACCGAGACCTTCAGACCAGGAGGAGGAAATATGAGAGATAATTGGAGAAGTGAATTATATAAATATA
AAGTAGTAAAAATTGAGCCATTA---GGAATAGCACCCACCAAGGCAAAGAGAAGAGTGGTG-----------
----------------------------CAGAGAGAAAAAAGAGCA---GTGGGA---GTAGGAGCT------
---GTGCTCCTTGGGTTCTTGGCAGCA---GCAGGAAGCACTATG---GGCGCAGCGTCA---
ATAACGCTGACGGTACAG---GCCAGACAA---TTACTG---TCTGGTATAGTGCAACAGCAGAAC---
AATCTG---CTGAGG---GCTATTGAGGCGCAACAACATCTGTTGCAACTCACAGTCTGGGGAATC
>JF320467
TTCTACTGTGACTCAACAAAACTGTTTACTTGGAATAGTACTTGGAATAGTACTGAAAGATCAAATAAC----
----

ACTGCAGGAAATATCACACTCCCATGCAGAATAAAACAAATTATAAACATGTGGCAGGGAGTAGGAAGAGCAA
TGTATGCCCCTCCCATCAAAGGACAAATTAGCTGTTCATCAAAT---
ATTACAGGGCTACTACTAACAAGAGATGGTGGTAATGATGGTAACCAAACGAGA-------------------
----
-
GGGAATGAGACAAAGACAGAAATCTTCAGACCTATAGGAGGAAATATGAAAGACAATTGGAGAAGTGAACTAT
ATAAATATAAAGTAGTAAAAATTGAACCATTA---GGAATAGCACCCACCAAGGCAAAGAGAAGAGTGGTG--
-------------------------------------CAGAGAGAAAAAAGAGCA---GTGGGG---
CTAGGAGCT---------TTGTTCCTTGGGTTCTTGGGAGCA---GCAGGAAGCACTATG---
GGCGCAGCATCA---CTAACGCTGACGGTACAA---GCCAGATCA---TTATTG---
TCTGGTATAGTGCAACAGCAGAAC---AATCTG---CTGAAG---
GCTATTGAGGCGCAACAGCGTCTGTTGCAACTCACAGTCTGGGGCACC
>GU330994
TTCTACTGTAATACATCACAACTGTTTAGGGATACTTGGAATAATACTTGGAATGGTATTTGGAATAATGATA
CT---

GAAGTAAATGAAACTATTACACTCCCATGCAGAATAAAACAAATTATAAACATGTGGCAGGAAGTAGGAAAAG
CAATGTATGCCCCTCCCATCAGAGGACAAATTAATTGTTCATCAAAT---

ATTACAGGGCTGCTATTAACAAGAGATGGTGGTGAGAGTAACAAG----------------------------
----

ACCATCGAGGTCTTCAGACCTGGAGGAGGAGATATGAGGGACAATTGGAGAAGTGAATTATATAAATATAAAG
TAGTAAAAATTGAACCATTA---GGAGTAGCACCCACCAAGGCAAAGAGAAGAGTGGTG--------------
-------------------------CAGAGAGAAAAAAGAGCA---GTGGGA---CTAGGAGCT---------
CTGTTCGTTGGGTTCTTGGGAGCA---GCAGGAAGCACTATG---GGCGCAGCGTCA---
ATAACGCTGACGGTACAA---GCCAGACAA---TTATTG---TCTGGTATAGTGCAACAGCAGAAC---
AATCTG---CTGAGA---GCTATTGAGGCGCAACAGCATCTGTTGCAGCTCACAGTCTGGGGCGTC
>KC312386
TTCTACTGTAACTCAACACAACTGTTTAATAGCACTTGGAATGCTACTAAAGAGCTTAATGACACTGGGAGTA
GTACT--

GAAGGGTCAAATATCACACTCCCATGCAGAATAAAACAAATTATAAACATGTGGCAGGAAGTAGGAAAAGCAA
TGTATGCCCCTCCCATCAGAGGACAAATTACATGTGTATCAAAT---
ATTACAGGACTGCTATTAACAAGAGATGGTGGTAATGATACCAATACT-------------------------
----

AACAATGACACCGAGATCTTCAGACCTGCAGGAGGAGACATGAGGGACAATTGGAGAAGTGAATTATATAAAT
ATAAAGTAGTAAAAATTGAACCATTA---GGAATAGCACCCACCAAGGCAAAGAGAAGAGTGGTG--------
-------------------------------CAGAGAGAAAAAAGAGCA---GTGGGA---ATAGGAGCT---
------GTGTTCCTTGGGTTCTTGGGAGCA---GCAGGAAGCACTATG---GGCGCAGCGTCA---
ATGACGCTGACGGTACAA---GCCAGACTA---TTATTG---TCTGGTATAGTGCAACAGCAGAAC---
AACTTG---CTGAGA---GCTATTGAGGCGCAACAACATCTGTTGCAACTCACAGTCTGGGGTATC
>JQ403062
TTCTACTGTGATTTAGCACAACTGTTCAAGAGTAGTTGGAATGGTACTGAACAGATAAATGGC----------
----

ACTGACAAAAATATCACTCTCCCATGCAGAATAAAACAAATTATAAATATGTGGCAGGAAGTAGGCAGAGCAA
TGTATGCCCCTCCCATCAGTGGACAAATTAAATGTGCATCAAAT---
ATTACAGGGCTGCTATTAACAAGAGATGGTGGTAATAATAATAGTAGT-------------------------
----

AATGAGACTGAGACCTTCAGACCTGCAGGAGGAGATATGAGGGACAATTGGAGAAGTGAACTATATAAATATA
AAGTAGTAAAGATTGAACCATTA---GGAATAGCACCCACCAAGGCAAAGAGAAGAGTGGTG-----------
----------------------------CAGAGAGAAAAAAGAGCA---GTGGGAACATTAGGAGCT------
---ATGTTCCTTGGGTTCTTGGGAGCA---GCAGGAAGCACTATG---GGCGCAGCGTCG---
CTGACGCTGACGGTACAA---GCCAGACTA---TTATTG---TCTGGTATAGTGCAACAGCAGAAC---
AATCTG---CTGAGA---GCTATTGAGGCGCAACAGCACCTGTTGCAACTCACAGTCTGGGGAGTT
>JQ403063
TTCTACTGTAATACAACACAATTGTTTAACAGTACTTGGAATGAAAGTATCCCACTCAATTCTTCCGATGATA
ATATCCCA---
-------------------AAT---
TCTTCCAATGATAATATCATACTCCCATGTAGGATAAAACAAATTATAAACATGTGGCAGGGAGTAGGAAAAG
CAATGTATGCCCCTCCCATCAGAGGACAAATTAGATGTTCATCAAAT---
ATTACAGGGCTATTATTAACAAGAGATGGTGGTGATAGTAATAAAAGTGAG----------------------
----

GAGGTGTTTAGACCTGGGGGAGGAGATATGAAGGACAATTGGAGAAGTGAATTATATAAATATAAAGTAGTAA
AAATTGAACCATTA---GGAATAGCACCTACCAAGGCAAAGAGAAGAGTGGTG--------------------
-------------------CAGAGAGAAAAAAGAGCA---GTGGGAACAATAGGAGCT---------
ATGTTCCTTGGGTTTTTGGGAGCA---GCAGGAAGCACTATG---GGCGCAGCGTCA---
ATGACGCTGACGGTACAG---GCCAGGCAA---TTATTG---TCTGGTATAGTGCAACAGCAGAAC---
AATCTG---CTGAGA---GCTATTGAGGCGCAACAGCATATGTTGCAACTCACAGTCTGGGGTATT
>JF680915
TTCTACTGTAATTCAACACAACTGTTTAATAGTACTTGGGAAAATAATGGTACAAATGGTAATGAAGGATCA-
----

AATAGTGACAATATTACACTCCCATGCAGAATAAAACAAATTATAAACATGTGGCAGGAAGTAGGAAAAGCAA
TGTATGCCCCTCCCATCAGTGGACCAATTAGCTGTTCATCAAAT---

ATTACAGGGCTGCTATTAACAAGAGATGGTGGTAACATTACCAAT----------------------------
----

AACACTGAAACCTTCAGACCAGGAGGAGGAAATATGAGGGACAATTGGAGAAGTGAATTATATAAATATAAAA
TAGTAAGAATTGAGCCCTTA---GGTATAGCACCCACCAGGGCAAGGAGAAGAGTGGTG--------------
-------------------------CAGAGAGAAAAAAGAGCA---GTGGGA---ATAGGAGCT---------
GTGTTCCTTGGGTTCTTGGGTGCA---GCAGGAAGCACTATG---GGCGCAGCGTCG---
CTAACGCTGACGGTACAG---GCCAGACGA---TTATTG---TCTGGTATAGTGCAACAGCAGAAC---
AACTTG---CTGAGG---GCTATTGAGGCGCAACAGCATATGTTGCAGCTCACAGTCTGGGGCGTC
>MK145772
TTCTACTGTAATTCAACACGACTGTTTAATAGTACTTGGTCTAATAGTACTTGGCCTAATAATGGTACTTGGA
ATGATACA---

GGAGCAGAGATTATCACACTCCCATGTAAAATAAGACAAATTGTAAACAAGTGGCAAGAAATAGGAAAAGCAA
TATATGCCCCTCCCATCAGCGGACTGATTAGATGTTCATCAAAT---
ATTACAGGGATACTCTTAACAAGAGATGGTGGTATTAATGGGAAT----------------------------
----

AATACTACTGAGATCTTCAGACCTCAAGGAGGAGATATGAAGGACAATTGGAGAAGTGAATTATATAAATATA
AAGTGGTAGAACTTGAACCATTA---GGAATAGCACCCACCAGGGCACAGAGAAGAGTGGTG-----------
----------------------------CAGAGAGAAAAAAGAGCA---GTGGGA---CTAGGAGCT------
---TTGTTCCTTGGGTTCTTGGGAGCA---GCAGGAAGCACTATG---GGCGCAGCGTCA---
GTGGCGCTGACGGTACAA---GCCAGACAA---TTATTG---TCTGGTATAGTGCAACAGCAGAAC---
AATTTG---CTGAGA---GCTCTTGAGGCGCAACAGCATCTGTTGCAACTCACAGTCTGGGGTGTC
>KM354962
TTCTACTGTAATACAACACAACTGTTTAATAGTACTTGGAATTCAACACACCTATTTAATAGT----------
---AGTTGG---
AATATGACTAAAGAATTAAAT---------------
ATCACTAATGAGCCACTCACACTCCCATGCAGAATAAGGCAAATCATCAACATGTGGCAGGAAGTAGGAAAAG
CAATGTATGCCCCTCCCATCAGTGGAAAAATTAAATGCTCATCAAAT---
ATTACAGGGCTGCTATTAACAAGAGATGGTGGT--

AGTGGTAATAGCACAGAGAAC---------
ACAACGGAGATCTTCAGACCTGGAGGAGGAGACATGAGGGACAATTGGAGAAGTGAATTATATAAATATAAAG
TGGTAAAAATTGAACCCTTA---GGAGTAGCACCCACTAAGGCAAAGAGAAGAGTGGTG--------------
-------------------------CAGAGAGAAAAAAGAGCA---GTGGGA---ATAGGAGCT---------
GTGTTCCTTGGGTTCTTGGGAGCA---GCAGGAAGCACTATG---GGCGCAGCGTCA---
ATGATGCTGACGGTACAG---GCCAGACAA---CTATTG---TCTGGTATAGTGCAACAGCAGAAC---
AACTTG---CTGAGG---GCTATTGAGGCGCAACAGCATCTGTTGCAACTCACAGTCTGGGGTATC
>KT124809
TTCTACTGTAATACAACAAAATTGTTTAATAGTACTTGGACTAATGGTACTCGAAATGATACTGAAGAGATCC
AT---
-------GCTAAAGAGAGC------
AATCAAACTGTTAATATTACCCTTCCATGCAGAATAAAACAAATTATAAACATGTGGCAAAAAGTAGGAAGAG
CAATGTATGCCCCTCCCATCAGTGGACAAATTAAATGCGTATCAAAT---
ATTACAGGGTTGCTATTAACAAGAGATGGTGGTACTGAGAACGAG----------------------------
----

ACCGAAACCTTTAGACCTGCAGGAGGAAATATGAAGGACAATTGGAGAAGTGAATTATATAAATATAAAGTAG
TAAAGATTGAACCATTA---GGAGTAGCACCCACCAGGGCAAAGAGAAGAGTGGTG-----------------
----------------------CAGAGAGAAAAAAGAGCA---GTGGGA---ATAGGAGCT---------
GTGTTCCTTGGGTTCTTAGGAGCA---GCAGGAAGCACTATG---GGCGCAGCGGCA---
GTGACGCTGACGGTACAG---GCCAGACAA---TTATTG---TCTGGTATAGTGCAACAGCAGAAC---
AATTTG---CTGAGG---GCTATTGAGGCGCAACAGCATCTGTTGCAGCTCACAGTCTGGGGTGTC
>KU641402
TTCTACTGTAATACAACAAAACTGTTTAATAGTACTTGGAATGCAACACACATGATTAATGGTACTTGGAATG
AT---

ACTGAGGGAAATATCACCCTCCCATGCAGAATAAAACAAATTATAAACATGTGGCAGGAAGTAGGAAAAGCAA
TGTATGCCCCTCCCATCAGAGGACAAATTAGATGTTCATCCAAT---

ATTACAGGGTTGCTATTAACAAGAGATGGTGGTAAGACTAACGAGAGC-------------------------
----

AACACCACCGAGATCTTTAGACCTGGAGGAGGAGATATGAGAGACAATTGGAGAAGTGAATTATATAAATATA
AAGTAGTAAGAATTGAACCATTA---GGGGTAGCACCCACCAAGGCAAAGAGAAGAGTGGTG-----------
----------------------------CAGAGAGAAAAAAGAGCA---GTGGGA---ATAGGAGCT------
---CTGTTCCTTGGGTTCTTGGGAGCA---GCAGGAAGCACTATG---GGCGCAACGTCG---
ATGGCGTTGACGGTACAG---GCCAGACAA---TTATTG---TCTGGTATAGTGCAACAGCAGAAC---
AATTTG---CTGAGA---GCTATTGAGGCGCAACAACATTTGTTGCAACTCACAGTCTGGGGCGTC
>KC473832
TTCTACTGTAATACAACACAACTGTTTAATAGTACTTGGCACACAAATGGTACTTGGCAGGGTATTGAAGGGT
CAAATAAC---

ACTGAAGACCTTATCACACTCCCATGCAGAATAAAACAAATTATAAATATGTGGCAGGAAGTAGGAAAAGCAA
TGTATGCCCCTCCCATAAGAGGAAATATTATTTGTTCATCAAAA---
ATTACAGGGCTGTTATTAACAAGAGATGGGGGTGAGAAAAATCAG----------------------------
----

AGCAGCAACGAGACCTTCAGACCTGCAGGAGGAGATATGAGGAACAATTGGAGAAGTGAATTATATAAATATA
AAGTAGTAAAAATTGAACCATTA---GGAGTAGCACCCACCAAGGCAAGGAGAAGAGTGGTG-----------
----------------------------CAGAGAGAAAAAAGAGCA---GTGGGA---ATGGGAGCT------
---TTGTTCCTTGGGTTCTTGAGTGCA---GCAGGAAGCACTATG---GGCGCAGCATCA---
ATAACGCTGACGGTACAG---GCCAGACAA---TTACTG---TCTGGTATAGTGCAACAGCAGAAC---
AATCTG---CTGAGG---GCTATTGAGGCGCAACAGCATCTGTTGCAACTCACAGTCTGGGGCGTC
>MG197055
TTCTACTGTAATACAACACAATTGTTTAATAGTACTTGGAAAAAGGGCAAGAATGATACTTGGAGTAGGAATA
AAACTGAT---
-------------GAGACA------AATAGCACTGAG---
ATCATACTCCAATGTAGAATAAAACAAATTATAAACATGTGGCAGAAGGTAGGAAAAGCAATGTATGCCCCTC
CCATTAGAGGAAACATTAGATGTACATCAAAT---
ATTACAGGACTAATGCTAGTAAGAGATGGTGGTAGTGATACACAG----------------------------
----

GGCAATGAGACCTTCAGACCTGGAGGAGGAGACATGAGGGACAATTGGAGAAGTGAATTATATAAATATAAAG
TAGTAAAAATTGAACCATTA---GGAGTAGCACCCACCAAGGCAAGGAGAAGAGCGGTG--------------
-------------------------CAGAGAGAAAAAAGAGCA---ATAACA---CTAGGAGCT---------
ATGTTCCTTGGGTTCTTGGGAACA---GCAGGAAGCACTATG---GGCGCAGCGTCA---
GTAACGCTGACGGTACAG---GCCAGGCGA---TTATTG---TCTGGTATAGTGCAGCAGCAGAAC---
AATCTG---CTGAGG---GCTATTGAGGCGCAGCAGTATATGCTGCAACTCACAGTCTGGGGTATT
>KY778345
TTCTACTGTAATACAACACAACTATTTAATAGTAGTTGGAATGGTACTGAGAATAGTATTAACGGATCAAGT-
----

AGCAATGGAACTATCATACTCCCATGCAGAATAAAACAAATTATAAACATGTGGCAGAAAGTAGGAAAAGCAA
TGTATGCCCCTCCCATCAGAGGACAAATTAGATGTTCATCGAAT---
ATTACAGGACTGATATTAACAAGAGATGGTGGAGCCGGGGGCAAGGGC-------------------------
----

AATGACAGTGAGATCTTCAGACCTGGAGGAGGAGATATGAGGGACAATTGGAGAAGTGAATTATATAAATATA
AAGTAGTAAAAATTGAACCATTA---GGAGTAGCACCCACCAGGGCAAAGAGAAGAGTGGTG-----------
----------------------------CAAAGAGAAAAAAGAGCA---GTGGGA---ATAGGAGCT------
---ATGGTCCTTGGGTTCTTGGGAGCA---GCAGGAAGCACTATG---GGCGCAGCGTCA---
GTGACGCTGACGGTACAG---GCCAGACTA---TTATTG---TCTGGTATAGTGCAACAGCAAAAC---
AATTTG---CTGAGG---GCTATTGAGGCGCAACAGCACCTGTTGCAACTCACAGTCTGGGGTATC
>KR182306
TTCTACTGTAATACAACAGAACTGTTTAATAGTACGTGGACTAGGAATGGTACTGACAAATGGTCAAGTAACA
ATACA--

GGAAATGAAACAATTATACTCCCATGCAGAATAAGACAAATTATAAACATGTGGCAGCAAGTAGGAAAAGCAA
TGTATGCCCCTCCCATCAGCGGATACATTAATTGTACATCAAAT---

ATTACAGGGATGCTATTAACAAGAGATGGTGGTAATAACAATGAAACC-------------------------
----

AATGGGACTGAGATCTTCAGACCTGCAGGAGGAGATATGAGGGACAATTGGAGAAGTGAATTATATAAATATA
AAGTAGTACAAATTGAACCATTA---GGAATAGCACCCACCAAGGCAAGAAGAAGAGTGGTG-----------
----------------------------CAAAGAGAGAAAAGAGCA---GTGGGAGTAATAGGAGCT------
---ATGTTCCTTGGGTTCTTGGGAGCA---GCAGGAAGCACTATG---GGCGCAGCGTCA---
ATAACGCTGACGGTACAA---GCCAGACAA---TTATTG---TCTGGTATAGTGCAACAGCAGAAT---
AATTTG---CTGAGA---GCTATTGAGGCGCAACAACATCTGTTGCAACTCACAGTCTGGGGTATC
>KR182345
TTCTACTGTGATACAGCATCACTGTTTAATAGTACTTGGAATAGTACTTGGAATATTGGAAATGATACTGAAG
GGTCAGATGGC--

ACTGAAAGAAATATCAAACTCAAATGCAGAATAAAACAAATTATAAACATGTGGCAGGAAGTAGGAAGAGCAA
TGTATGCCCCTCCTATCAAAGGACTAATTAGATGTACATCAAAT---
ATTACAGGGTTAATATTAACAAGAGATGGTGGTAAAAACAACAAT----------------------------
----

GACACCGAAATCTTTAGACCTGGAGGGGGAGATATGAGAGACAATTGGAGAAGTGAATTATATAAATATAAAG
TAGTACAAATTGAACCATTA---GGAGTAGCACCCTCCAAGGCAAAGAGAAGAGTGGTG--------------
-------------------------CAGAGAGAAAAAAGAGCA---GTGGGA---ATAGGAGCT---------
GTGTTCCTTGGGCTCTTGTCAGCA---GCCGGAAGCACTATG---GGCGCAGCGTCA---
GTGACGCTGACGGTACAA---ACCAGACAA---TTATTG---GCTGGTATAGTGCAACAGCAGAAC---
AATTTG---CTGAGA---GCTATTGAGGCGCAACAGCATATGTTGCAACTCACAGTCTGGGGTATC
>AY247224
TTCTACTGTGATTCAACACAACTGTTTAATAGTACTTGGCTGTTTAATAGTACTTGGGAAAATGATACTAGAG
GG---

TCAGATAGCGAAAATATCACACTCCCATGCAGAATAAAACAAATTATAAACATGTGGCAGGAAGTAGGAAAAG
CAATGTATGCCCCTCCCATCAGAGGACAAATTAGATGTTCATCAAAT---
ATTACAGGGCTGCTATTAACAAGAGATGGTGGGAATAATAGGAAC----------------------------
----

GAGACCACCGAGATCTTCAGACCTGGAGGAGGAGATATGAGGGACAATTGGAGAAGTGAATTATATAAATATA
AAGTAGTAAAAATTGAACCATTA---GGAGTAGCACCCACCAAGGCAAAGAGAAGAGTGGTG-----------
----------------------------CAGAGAGAAAAAAGAGCA---GTGGGAACGATAGGAGCT------
---ATGTTCCTTGGGTTCTTGGGAGCA---GCAGGAAGCACTATG---GGCGCAGCGTCA---
ATGGCGCTGACGGTACAG---GCCAGACAA---TTATTG---TCTGGTATAGTGCAGCAGCAGAAC---
AACTTG---CTGAGA---GCTATTGAGGCGCAACAGCATCTGTTGCAACTCACAGTCTGGGGAATC
>AY835774
TTCTACTGTGATTCATCACGATTGTTTAACAGTACTTGGAATGGTACTTGGGAGACAGGGTGGAATGATACTG
AAAAG--

GCAGATGAGACAAATATCATACTCCCATGTAGAATAAAACAAATTATAAACATGTGGCAGGAAGTAGGAAAAG
CAATGTATGCCCCTCCCATCAGAGGACTAATTAGATGTTCATCAAAT---
ATTACAGGGCTACTATTAACAAGAGATGGTGGTAATTATAATAAAACCGGGAAT------------ATA----
----
ACCGGGAATAAGACC---
GAGATCGAAGTCTTCAGACCTGGAGGAGGAGATATGAGGGACAATTGGAGAAGTGAATTATATAAATATAAAG
TAGTAAAAATTGAACCATTA---GGAGTAGCACCCACCAAGGCAAAGAGAAGAGTGGTG--------------
-------------------------CAGAGAGAAAAAAGAGCA---GTGGGAACGTTAGGAGCT---------
ATGTTCCTTGGGTTCTTGGGAGCA---GCAGGAAGCACTATG---GGCGCAGCGTCA---
ATGACGCTGACGGTACAG---GCCAGACTA---TTATTG---TCTGGTATAGTGCAACAGCAAAAC---
AATTTG---CTGAGG---GCTATTGAGGCGCAACAGCATCTGTTGCAACTCACAGTCTGGGGTATC
>EU289185
TTCTATTGCAACTCAACACCACTGTTTAATAGTACTTGGAATGATACAAAAGGGTCAAATAACACAGTA----
----

GGAAATGACACAATCATACTTCAATGCAGGATAAAACAAATTATAAACATGTGGCAGGAAGTAGGCAAAGCAA
TGTATGCCCCTCCCATCAAAGGAAACATTAGCTGTTCATCAAAT---

```
ATTACAGGGCTGCTATTAACAAGAGATGGTGGTATTGTGGACAAT----------------------------
-------------------------------------------------------------------------
----------
AACGATACCGAGACCTTCAGACCTGGAGGAGGAGATATGAGGGACAATTGGAGAAGTGAATTATATAAATATA
AAGTAGTAAAAATTGAACCATTA---GGAATAGCACCCACCAAGGCAAAGAGAAGAGTGGTG-----------
----------------------------CAGAGAGAAAAAAGAGCA---ATAGGA---ATAGGAGCT------
---ATGTTCCTTGGGTTCTTAGGAGCA---GCAGGAAGCACTATG---GGCGCAGCGTCA---
ATGACGCTGACGGTACAG---GCCAGACAA---TTATTG---TCTGGTATAGTGCAACAGCAGAAC---
AATTTG---CTGAGG---GCTATTGAGGCGCAACAGCATCTGTTGCAACTCACAGTCTGGGGCGTC
>EF593311
TTCTACTGTGATTCAACACCACTATTTAATAGTACTTGGGAGAATAGTACTTGGGATAATAGTACTTGGAATA
CT-----------------------------------------------------------------------
-------------------------
GAAGGAAATGACACAATCACCCTCACATGTAGAATAAAACAAATTATAAACATGTGGCAAAAAGTAGGAAAAG
CAATGTATGCCCCTCCCATTAGAGGACAAATTAGATGCTCATCAAAT---
ATTACAGGGCTGCTATTAACAAGAGATGGTGGTGTTAAGAATAATGAA-------------------------
-------------------------------------------------------------------------
-------
ACCAACACCACCGAGACCTTCAGACCTGGAGGAGGAGATATGAGGGACAATTGGAGAAGCGAATTATATAAAT
ATAAGGTAGTAAAAATTGAACCACTA---GGGTTAGCCCCCACCAAGGCAAGGAGAAGAGTGGTG--------
-------------------------------CAGAGAGAAAAAAGAGCA---GTGGGA---TTAGGAGCT---
------TTGTTCCTCGGGTTCTTGGGAGCA---GCAGGAAGCACTATG---GGCGCAGCGTCA---
ATAACGCTGACGGTACAG---GCCAGACTA---TTATTG---TCTGGTATAGTGCAACAGCAGAAC---
AATCTG---CTGAAG---GCTATTGAGGCGCAACAGCGTATGTTGCAACTCACAGTCTGGGGTATC
>KT808396
TTTTACTGTAATACAACATCACTATTTAGTAGTACTTGGGATAATAATACAATATCACTATTTAATAGTACTG
GGAATAGT-----------------------------------------------------------------
-------------AATGAA------
GGATTAAATGACACAATCACCCTCACATGCAGAATAAAACAAATTATAAACATGTGGCAGGAAGTAGGAAAAG
CAATGTATGCCCCTCCCATCAGAGGACAAATTAGATGTTCATCAAAT---
ATTACAGGGCTGCTATTAACAAGAGATGGTGGTAATAATAGCTCTGATAAT----------------------
-------------------------------------------------------------------------
----------
AGCTCTGGGGAGACCTTCAGACCTGGAGGAGGAGATATGAGGGACAATTGGAGAAGTGAATTATATAAATATA
AAGTAATAAAAATTGAACCATTA---GGAGTAGCACCCACCAAGGCAAGAAGAAGAGTGGTG-----------
----------------------------CAGAGAGAAAAAAGAGCA---GTGGGA---CTAGGAGCT------
---TTGCTCTTTGGGTTCTTGGGAGCA---GCAGGAAGCACTATG---GGCGCAGTGTCA---
ATGACGCTGACGGTACAG---GCCAGACAA---TTATTG---TCTGGTATAGTGCAACAGCAGAAC---
AACCTG---CTGAGG---GCTATTGAGGCGCAACAACACCTGTTGCAACTCACAGTCTGGGGCGTC
>KP754464
TTCTACTGTGATACAACAAAACTGTTTAACAGTACTTGGCCCAATAACAGTACTTGGCCCAATAACACTGAAG
TCACA--------------------------------------------------------------------
-------------AATACC------
ACTGAAAATGGCACTATCACACTCCAATGCAAAATAAAACAAATTATAAACCTGTGGCAAGAAGTAGGAAAAG
CAATGTATGCCCCTCCCATCAATGGACCGATTAAATGTAGATCAAAT---
ATTACAGGGTTAATATTAACAAGAGATGGTGGTATAAACAACACCACC-------------------------
-------------------------------------------------------------------------
----
GAGACTAACACCACCGAGACTTTTAGACCTGGAGGAGGAGATATGAGGGACAATTGGAGAAGTGAATTATATA
AATACAAAGTAGTAAGAATTGAACCATTA---GGAATAGCACCCACCAAGGCAAAGAGAAGAGTGGTG-----
----------------------------------CAGAGAGAAAAAAGAGCA---GTGGGG---
CTGGGAGCT---------TTGTTCCTTGGGTTCTTGGGAGCA---GCAGGAAGCACTATG---
GGCGCAGCGTCA---ATGGCGCTGACGGTACAA---GCCAGACAA---TTATTG---
TCTGGTATAGTGCAGCAGCAGAAC---AATCTG---CTGAGG---
GCTATTGAGGCGCAACAGCATCTGTTGCAACTCACTGTCTGGGGTATC
>GU728348
TTCTATTGTGATACAACACAACTGTTTAATAGTACTTGGGAGAATGATAATGCTACTGCGGGGGCAACA----
-------------------------------------------------------------------------
----------------------------
GGAAATGACACAATCATACTCCCATGCAGAATAAAACAAATTATAAACATGTGGCAGAAAGTAGGAAAAGCAA
```

TGTATGCCCCTCCCATCCAGGGACAAATTAGATGCTTATCAAAT---
ATTACAGGGCTGCTATTAACAAGAGATGGTGGTCATAACAGGACC----------------------------
----

GGTAATGAGACCTTCAGACCTGGAGGAGGAGATATGAGGGACAATTGGAGAAGTGAGCTATATAAATACAAAG
TAGTAAAAATTGAACCATTG---GGAATAGCACCCACCAGGGCAAAGAGAAGAGTGGTG--------------
-------------------------CAGAGAGAAAAAAGAGCG---TTGGGCATAGGAGCTGCT---------
TTGTTCCTTGGGTTCTTGGGAGCA---GCAGGAAGCACTATG---GGCGCAGCGTCA---
GTGACGCTGACGGTACAG---GCCAGACAA---TTATTG---TCTGGTATAGTGCAACAGCAGAAC---
AATTTG---CTGATG---GCTATTGAGGCGCAACAGCATCTGTTGCAACTCACAGTCTGGGGCGTC
>GU728283
TTCTACTGTAATTCATCACAACTGTTTAGTAGTTCTTGGAATGGTAATAGTACTAGAAATAGTACTGGGTCA-
----

AAAGACAAAGACATTATACTCCCATGCAGAATAAAACAATTTATAAACATGTGGCAGAGAGTAGGAAAAGCAA
TATATGCCCCTCCCATAAAAGGACCAATTAGCTGTTTATCAAAT---
ATTACAGGGCTGTTATTAACAAGAGATGGGGGCTTAGGAAACAACAGTTCA----------------------
----

GAAAATAAGACTGAGACCTTCAGACCTGGAGGGGGAAATATGAAGGACAATTGGAGAAGTGAGTTATATAAAT
ATAAAGTAGTAAAAATTGAACCATTA---GGAGTAGCACCCACCAAGGCAAGGAGAAGAGTGGTG--------
-------------------------------CAGAGAGAAAAAAGAGCA---GTGGGA---CTAGGAGCT---
------CTGTTCCTTGGGTTCTTGGGAGCA---GCAGGAAGCACTATG---GGCGCAGCGTCA---
ATGACGCTGACGGTACAG---GCCAGACAA---TTATTG---TCTGGTATAGTGCAACAGCAAAAC---
AATTTG---CTGAGA---GCTATTGAGGCGCAACAACATCTGTTGCAACTCACAGTCTGGGGCGTC
>KY658703
TTCTACTGTAATTCAACACCACTGTTTAATGGTACTTGGAATAATACTAACAATGGA----------------
----

GAAAATAATGATACAATCACACTCCACTGCAGAATAAAACAAATTATAAACATGTGGCAAAGAACAGGCAAAG
CAATGTATGCCCCTCCCATTAGAGGACCAATTAACTGTTCATCAAAT---
ATTACAGGACTGCTATTAACAAGAGATGGTGGTAATACCAGTGAG----------------------------
----

AGCAACACTGAGATCTTCAGACCTCTAGGAGGAGATATCAGGGACAATTGGAGAAGTGAATTATACAAATATA
AAGTAATAAAAATTGAACCAGTA---GGAATAGCACCCACCAAGGCAAAGAGAAGAGTGGTG-----------
----------------------------CAGAGAGAAAAAAGAGCA---GTGGGAACAATAGGAGCT------
---ATGTTCCTTGGGTTCTTGGGAGCA---GCAGGAAGCACTATG---GGCGCAGCGTCA---
ATGACGCTGACGGTACAG---GCCAGACAA---TTGTTG---TCTGGTATAGTGCAACAGCAAAAC---
AATTTG---CTGAGG---GCTATTGAGGCGCAACAGCATCTGTTGCAGCTCACGGTCTGGGGAGTC
>KT452596
TTCTATTGTAATACAACACCATTGTTTAATGATACTTGGACTAGGAATGGTACTGATGGTACTTGGATTTCAA
ATAGTAAT---

AGGACTGGAAATATCACACTCCTATGCAGAATAAAACAAATTATAAACATGTGGCAGGAAGTAGGAAAAGCAA
CATATGCCCCTCCCATCAGAGGGCCAATTACGTGTGTATCAAAC---
ATTACAGGTCTGATGTTAACAAGAGATGGTGGTAATAATCAGACA----------------------------
----

AATAATACCGAGATCTTCAGACCTGGAGGGGGAAATATGAAGGACAATTGGAGAAGTGAATTATATAAATATA
AAGTAGTCCAAATTGAACCATTG---GGAGTAGCACCCACCAAGGCAAAGAGAAGAGTGGTG-----------
----------------------------CAGAGAGAAAAAAGAGCA---GTCACG---CTAGGAGCT------
---ATGTTCCTTGGGTTCTTGGGAGCA---GCAGGGAGCACTATG---GGCGCAGCGTCA---
ATGGCGCTGACGGTACAG---GCCAGACAA---TTATTG---TCTGGTATAGTGCATCAGCAGAAT---
AACCTG---CTGAGA---GCTATTGAGGCGCAACAACATATGTTGCAACTCACAGTCTGGGGCGTC
>KR861317
TTCTATTGCAATACATCAGACCTGTTTAATACATCAAAACTGGTTAATGAGACTCATAAA-------------
----

CGTAATGATACCATCACAATCCCATGCAGAATAAAACAAATTATAAACATGTGGCAGGAGGTAGGACGAGCAA

TGTATGCTCCTCCCATTGCAGGAAACATAACATGTACATCAAAT---
ATCACCGGACTACTATTAGAATACGATGGAAGAAAGGATAAAAAC----------------------------
----

AAAACAGAGACATTCAGACCTGGGGGAGGAGATATGAGGGATAACTGGAGAAGTGAGTTATATAAATATAAAG
TGGTAGAAATTAAGCCATTG---GGAATAGCACCCACTGGAGCAAAAAGGAGAGTGGTG--------------
-------------------------GAAAGAGGAAAAAGAGCA---GTAGGA---ATAGGAGCT---------
GTGCTCCTTGGGTTCTTGGGAGCA---GCAGGAAGCACTATG---GGCGCGGCGTCA---
ATAACGCTGACGGTACAG---GCCAGACAA---TTGTTG---TCTGGTATAGTGCAACAGCAAAGC---
AATTTG---CTGAGG---GCTATAGAGGCGCAGCAGCATCTGTTGCAACTCACAGTCTGGGGTATT
>KR861333
TTCTATTGCAATACATCAGGCCTGTTTAATGATACAAATGAT-------------------------------
----

GGTACAACAAACATCACACTCCCATGCAGAATAAAACAAATTATAAACATGTGGCAGGGGGTAGGGCGAGCAA
TGTATGCCCCCCCCATTGCAGGAAACATAAAATGTGAATCAAAC---
ATCACAGGATTACTATTGACACGTGATGGAGGAAACAATAGTAGT----------------------------
----

GAGAAAATTGAGAGATTCAGACCTGGAGGAGGAGATATGAGGGACAATTGGAGAAGTGAATTATATAAATATA
AAGTGGTAGAGATTAAGCCATTA---GGAATAGCACCCACTGAGGCAAGAAGGAGAGTGGTG-----------
----------------------------GAGAGAGAGAAAAGAGCA---GTGGGA---ATAGGAGCT------
---GTGTTCCTTGGGTTCTTGGGAGCA---GCAGGAAGCACTATG---GGCGCGGCATCA---
ATAACGCTGACGGTACAG---GCCAGACAA---TTATTG---TCTGGTATAGTGCAACAGCAGAGC---
AATTTG---CTGAGG---GCTATAGAGGCGCAACAACATTTGTTGCAACTCACGGTCTGGGGTATT
>KR861283
TTCTATTGCAATACATCAGGATTGTTTAATGGAACATACAATGGAACTAATATTACAGGAGACAGCAACATCA
CA---

CTAGACAGCAACATCACACTCCCATGCAGAATAAAACAATTTGTAAACATGTGGCAGGGGGTAGGACGAGCAA
TGTATGCCCCTCCCATTGAAGGAATCATACAATGTAACTCAAAT---
ATCACAGGACTATTGTTAACACGTGATGGAGGAGGAGGCGATAAT----------------------------
----

TCAACTGAAATATTCAGGCCTACAGGAGGAAATATGAGAGACAATTGGAGAAGTGAATTATACAAATATAAAG
TGGTAGAAATTCAACCATTG---GGAATAGCACCCACTACAGCAAGGAGGAGAGTGGTG--------------
-------------------------GAGAGAGAAAAAAGAGCA---GTAGGA---GTGGGAGCC---------
TTGTTCCTTGGGTTCTTAGGAGCA---GCAGGAAGCACTATG---GGCGCAGCATCA---
ATAACGCTGACGGTACAG---GCCAGACAA---TTGTTG---TCTGGAATAGTGCAACAGCAAAAC---
AATTTG---CTGAGA---GCTATAGAGGTGCAACAACATATGTTGCAACTCACAGTCTGGGGTATT
>KC898980
TTCTATTGTAATACATCAGGCCTGTTTAATGGTACATACATGCCTAATGGTACATACATGCCTAATGGTACAG
AAAGTAAT---

TCAAACTCAACCATCACAATACCATGCAAAATAAAGCAAATTATAAACATGTGGCAGGAAGTAGGACGAGCAA
TGTATGCCCCTCCCATTGCAGGAAATATAACATGTAAATCAAAT---
ATCACAGGACTACTATTGGTACGTGATGGAGGAAGCGAGCCAAATATT-------------------------
----

ACAAATAATGATACAGAGACATTCAGACCTGGAGGAGGAGATATGAGGGACAATTGGAGAAGTGAATTATATA
AATATAAAGTGGTAGAAATTAAACCATTG---GGAATAGCACCCACTGCAGCAAAAAGGAGAGTGGTG-----
----------------------------------GAGAGAGAAAAAAGAGCA---GTGGGA---
ATAGGAGCT---------GTGTTCCTTGGGTTCTTGGGAGCA---GCAGGAAGCACTATG---
GGCGCGGCGTCA---ATAACGCTGACGGCACAG---GCCAGACAA---TTATTG---
TCTGGTATAGTGCAACAGCAAAGC---AATTTG---CTGAGG---
GCTATAGAGGCGCAACAGCATATGTTGCAACTCACAGTCTGGGGCGTT
>KU319550
TTTCATTGCAGTACTACAGGACTGTTTACTGATACATATGGTACAAGTACATGCGGCACTAACAGTACAGGAA
ATACT--

TCAGGAATTCACACCATCACACTCCACTGCAAAATAAAACAATTTATAAACATGTGGCAGGGGGTAGGGCTAG
CAATGTATGCCCCTTCCCTTGCAGGAAACATAATATGTAAATCAAAT---
TTCACAGGACTAGTCTTGACATGTGATGGAGGAGAGGGGAATAAC----------------------------
----

GTGACTAACTTGACAGAGACATTTAGACCTGGAGGAGGAGATATGAGGGACAATTGGAGAAGTGAATTATATA
AATATAAAGTGGTAGAAATTAAACCATTA---GGAGTAGCACCTACTAAGGCAAAAAGGAGAGTGGTG-----
----------------------------------GAGAGAGAAAAAAGAGCA---GTGGGA---
ATAGGAGCT---------GTGTTCCTTGGGTTCTTGGGAGCA---GCAGGAAGCACTATG---
GGCGCAGCATCA---ATAACGCTGACGGTACAG---GCCAGGCAA---TTGTTG---
TCTGGTATAGTGCAACAGCAGAAC---AATTTG---CTGAGG---
GCTATAGAGGCGCAACAGCATATGTTGCAACTCACGGTCTGGGGCGTT
>FJ977093
TTCTATTGCAATACATCACAATTGTTTAATACATCACATTGGTTTAATAATACAACAAAT-------------
----

CAGACTGAACCCATCACACTCCAATGCAGAATAAAACAGATTGTAAACATGTGGCAGGGGGTAGGACGAGCAA
TGTATGCCCCTCCCATTAAAGGAAACATAACATGCAGATCAAAT---
ATCACAGGATTACTATTGACACGTGATGGAGGAAACAATGCAAGT----------------------------
----

GCAAGTGAGGAGACATTTAGACCTGGAGGAGGAAATATGAAAGACAATTGGAGAAGTGAATTATATAAATATA
AGGTAGTAGAAATTAACCCATTA---GGAATAGCACCCACTAAGGCAAAAAGGAGAGTGGTG-----------
----------------------------GAGAGAGAAAAAAGAGCA---GCA------ATAGGGGCT------
---GTGTTCCTTGGGTTCTTGGGAGCC---GCAGGAAGCACTATG---GGCGCGGCGTCG---
CTAACGCTGACGGTACAG---GCCAGACAA---TTATTG---TCTGGTATAGTGCAACAGCAAAGC---
AATTTG---CTGAGG---GCTATAGAGGCGCAACAGCATATGTTGCAACTCACAGTCTGGGGTATT
>KU921827
TTCTATTGTAATACATCAGGCCTGTTTAATAGTAGTTGGAAACAGGAACATAATAATAACAGTTGGAATAGAA
TTGACAAC---
-------------AGGACA------
GAATCAAATGACACTATAATTCTCCCATGCAAGATAAAACAAATTATAAACATGTGGCAGGAGGTAGGACGAG
CAATGTATGCTCCTCCCATTCAAGGAAACATAACATGCAAATCAAAT---
ATCACAGGATTATTACTAACACGTGATGGGGGACCAGACTCAAAT----------------------------
----

GGCACAGAAATATTCAGGCCTGTAGGAGGAAACATGAAGGACAATTGGAGAAGTGAATTATATAAATACAAAG
TGGTAGAAATTAAACCATTA---GGACTAGCACCCACTAGGGCAAAAAGGCGAGTGGTGGACAGA--------
-------------GAAAAAATGGTGGACAGAAGAAAAAGAGCA---GTGGGA---CTAGGAGCT---------
ATGTTCCTTGGGATCTTGGGAGTA---GCAGGAAGCACTATG---GGCGCAGCATCA---
ATGACGCTGACGGTACAG---GCCAGACTA---TTATTG---GCTGGTATAGTGCAACAGCAAAAC---
AATTTG---CTGAGA---GCTATAGAGGCGCAACAGCATATGTTGCAACTCACTGTCTGGGGCGTT
>KC894080
TTCTATTGCAATACATCAAACCTGTTTAATACTACTAATAGTACAGAAAGTACTTCAAATTCAACCTTT----
----

ATCACGCTCCAGTGCAGAATAAAACAGATTATAAACATGTGGCAGGGGGTAGGACAAGCAATGTATGCCCCTC
CCATTGCAGGAAACATAACATGTAAATCAAAT---ATCACAGGACTACTCTTGACCCGTGATGGA---
ATACCAAATGCA---

ACAAATGAGACATTCAGACCTGGAGGAGGAGATATGAGGGACAATTGGAGAAGTGAATTATATAAATATAAAG
TGGTAGAAATTAAGCCATTG---GGACTAGCACCCACTACAGCAAAAAGGAGAGTGGTG--------------
-------------------------GAGAAACAAAAAAGAGCA---GTGGGA---TTAGGAGCT---------
GTGTTCCTTGGGTTCTTGGGAGTA---GCAGGAAGCACTATG---GGCGCGGCGTCA---
ATCACGCTGACGGCACAG---GCCAGACAA---CTGTTG---TCTGGTATAGTGCAACAGCAAAAC---
AATTTG---CTGAGA---GCTATAGAGGCGCAACAGCATATGTTGCAACTCACGGTCTGGGGTATT
>HM070584
CTCTATTGCAATACATCAAAACTGTTTAATGGTATATACAATAATTATACAGAAGACAATTCA----------
----

ACAGGCCCAGACATCACACTCCCATGCAGAATAAAACAAATTATAAACATGTGGCAGGAGGTAGGAAGAGCGA
TGTATGCCCCTCCCATTGCAGGAGAAATAACATGTAACTCAAGT---
ATCACAGGACTACTATTGACACGTGATGGAGGAAATACTAGAGGAAATACT----------------------
----

ACGGGAGGAAATGAGACATTCAGACCTGGAGGAGGAGATATGAGGGACAATTGGAGAAGTGAATTATATAAAT
ATAAAGTGGTAGAAATTAGGCCATTA---GGAATAGCACCCACTGAGGCAAAAAGGAGAGTGGTG--------
-------------------------------GAGAGAGAAAAAAGAGCA---GTGGGA---ATAGGAGCC---
------TTGTTCCTTGGGTTCTTGGGAGCA---GCAGGAAGCACTATG---GGCGCGGCGTCA---
ATAACGCTGACGGTACAG---GCCAAACAA---TTGTTG---TCTGGCATAGTGCAACAGCAAAGC---
AATTTG---CTGAGG---GCTATAGAGGCGCAACAGCATCTGTTGCAACTCACAGTCTGGGGTATT
>HM204597
TTCTATTGCAATACATCAGGCCTGTTTAATAAGACATACTATAGTAATGGTACATACAATAATATTAGTACAG
ACAAT--

AGTACTGATTCTAACATCACACTCCCATGCAGAATAAAACAGATTATAAACATGTGGCAGCAGGTAGGAAGAG
CAATGTATGCCCCTCCTATTGCAGGAAACATAACATGTATATCAAAT---
ATCACAGGACTACTATTAACACGTGATGGAGGACCTGAAAATAATGAC-------------------------
----

ACAAATAGAAACGAGACATTCAGACCTGGAGGAGGAGATATGAGGGACAATTGGAGAAGTGAATTATATAAAT
ATAAAGTGGTAGAAATTAAGCCATTA---GGAATTGCTCCCACTGAGGCGAAAAGGAGAGTGGTG--------
-------------------------------GAGAGAGAAAAAAGAGCA---GTGGGA---ATAGGAGCT---
------GTGTTCCTTGGGTTCTTGGGAGCA---GCTGGAAGCACTATG---GGCGCAGCGTCA---
ATAACGCTGACGGCACAG---GCCAGACAA---CTGTTG---TCTGGTATAGTGCAACAGCAAAGC---
AATTTG---CTGAGA---GCTATAGAGGCGCAACAGCATATGTTGCAACTCACAGTCTGGGGTATT
>KC187073
TTCTATTGCGATTCATCAGCACTGTTTAATGGCCAGTATAATGGTACAAATGTCCAGTATAAT----------
----

AGTACAGAAAATATAACACTTCAATGCAGAATAAAACAAATTATCAACATGTGGCAGGGGGTAGGACGAGCAA
TATATGCCCCTCCCATTGCAGGAAACATAACATGTAAATCAAAT---
ATCACAGGACTAATATTAACACGGGATGGAGGAGAAAGTAATGATTCA-------------------------
----

AAAATTGCAGAGATATTCAGACCTGCAGGAGGAGATATGAGGGACAATTGGAGAAGTGAACTATATAAATATA
AAGTGGTAGAAATTAAGCCATTA---GGAATAGCACCCACTGAGGCAAAAAGGAGAGTGGTG-----------
----------------------------GAGAGAGAAAAAAGAGCA---GTGGGA---ATGGGAGCT------
---GTGTTCCTTGGGTTCTTGGGAGCA---GCAGGAAGCACTATG---GGCGCGGCGTCA---
ATAACGCTGACGGTACAA---GCCAGACAA---CTGTTG---TCTGGTATAGTGCAACAGCAAAGC---
AATTTG---CTGAGG---GCTATAGAGGCGCAACAGCATATGTTGCAACTCACGGTCTGGGGTATT
>KP109526
TTCTATTGCAATACATCAGGACTGTTCAATGGCACATACAATAATACAAATAGTACAGAGGGT----------
----

ATAAATAAAACCATCACACTCCCATGCAGAATAAAACAAATTATAAACATGTGGCAGAAGGTAGGACGAGCAA
TGTATGCCCCGCCCATTGCAGGAAACATAATATGTAACTCAAGT---
ATCACAGGACTACTATTGACACGTGATGGAGGAGTACATGACAAT----------------------------
----

GAGAATGAGACGGAGACATTCAGACCTGGAGGAGGAGACATGAGGGACAATTGGAGAAGTGAATTATATAAAT
ATAAAGTGGTAGAAGTGGAGCCATTG---GGAGTGGCACCCACTAAGGCAAAAAGAAGAGTGGTG--------
-------------------------------GAGAGAGAGAAAAGAGCA---GTGGGA---ATAGGAGCT---
------TTGTTCCTTGGGTTCTTGGGAGCA---GCAGGAAGCACTATG---GGCGCGGCGTCA---
ATAACGCTGACGGTACAG---GCCAGACAA---TTATTG---TCTGGTATAGTGCAACAGCAGGAC---
AATCTG---CTGAGA---GCTATAGAGGCGCAACAGCATATGTTGCAACTCACAGTCTGGGGTATT
>HM639114
TTCTATTGCAACACATCAAGCCTGTTTAATGGTACATACAATAGTACAGGAATGCCTAATAATACA-------
----

GGAGGTGCAAATATCACACTCCAATGCAAAATAAAACAAATTATAAACATGTGGCAGGAGGTGGGACGAGCAA
TGTATGCCCCTCCCATTGCAGGAAACATAACATGTAGATCAAAT---
ATCACAGGACTACTATTGACACGCGATGGAGGAAATAGTACAGAGACA---------------------------
----

AAAAATGGTACAGAGATATTCAGACCTGGAGGAGGAGATATGAGGGACAATTGGAGAAGTGAATTATATAAAT
ATAAAGTGGTAGAAATTAAGCCATTA---GGAATAGCACCCACTGAGGCAAAAAGGAGAGTGGTG--------
---------------------------------GAGAGAGAGAAAAGAGCA---GTGGGA---ATAGGAGCT---
------ATGTTCCTTGGGTTCTTGGGAGCA---GCAGGAAGCACTATG---GGCGCGGCATCA---
ATAACGCTGACGGTACAG---GCCAGACAA---TTATTG---TCTGGTATAGTGCAACAGCAAAGC---
AATTTG---CTGAAG---GCTATAGAGGCGCAACAGCATATGTTGCAACTCACGGTCTGGGGTATT
>KC894130
TTCTATTGCAATACATCAAGCCTGTTTAATGGTAGATACAATAGTACAAGAAGTACT------------------
----

TCAAATTCAACCATCACACTCCAATGCAAAATAAAACAAATTATAAACATGTGGCAGGAGGTGGGACGAGCAA
TGTATGCCCCTCCCATTGAAGGAAACATAGCATGTAGATCAAAT---
ATAACAGGACTATTATTGACACGTGATGGAGGTAATGGAGGAGAC------------------------------
----

AATACTACAGAGATATTCAGACCTGGAGGAGGAAATATGAGGGACAATTGGAGAAGTGAATTATATAAATATA
AAGTGGTGGAAATTAAGCCATTA---GGAGTAGCACCCACTGAGGCAAAAAGGAGAGTGGTG-----------
-----------------------------GAGAGAGAGAAAAGAGCA---GTGGGA---ATAGGAGCT------
---GTGTTCCTTGGGTTCTTGGGAGCA---GCAGGAAGCACTATG---GGCGCGGCATCA---
ATAACGCTGACGGTACAG---GCCAGACAA---TTATTG---TCTGGTATAGTGCAACAGCAAAGT---
AATTTG---CTGAGG---GCTATAGAGGCGCAACAGCATATGTTGCAACTCACGGTCTGGGGTATT
>KU341728
TTCTATTGTAATACCTCAGGCCTGTTTAATAGGACATACATGCCTACATACATGCCTAATGGTACAGAAAGTA
AT---

TCAAACTCAACTATCACAATTCCATGCAGAATAAAACAAATTATAAACATGTGGCAGCAGGTAGGACGAGCAA
TATATGCCCCTCCCATTGCAGGAAACATAATATGTAAATCAAAC---
ATCACAGGACTACTATTGGTACGTGATGGAGGAATAAAGGAAAAT------------------------------
----
-
GATACAGAGAATAAGACAGAGACATTCAGACCTGGAGGAGGAGATATGAGGGACAATTGGAGAAGTGAATTAT
ATAAATATAAAGTAGTAGAAATTAAGCCATTG---GGAGTAGCACCCACTAAAGCAAAAAGGAGAGTGGTG--
-------------------------------------GAGAGAGAAAAAAGAGCA---TTGGGA---
ATAGGAGCT---------GTGTTCCTTGGGTTCTTGGGAGCA---GCCGGGAGCACTATG---
GGCGCAGCGTCA---ATAACGCTGACGGTACAG---GCCAGACAA---TTGCTG---
TCTGGTATAGTGCAACAGCAAAGC---AATTTG---CTGATG---
GCTATAGAGGCGCAACAGCATCTGTTGCAACTCACAGTCTGGGGCGTT
>MF373134
TTCTACTGTAATACAACAGGCCTGTTTAATAATAATAGGATA---------------------------------
----

AATTCAACCATCACACTCCCATGCAGAATAAAGCAAATTATAAACATGTGGCAGGAAGTAGGAAGAGCAATAT
ATGCCCCTCCCGTTGAAGGACTCATAACATGTAGATCAAAT---
ATCACAGGAATACTACTAACACGTGATGGAGGCAATACTACTGCA------------------------------
----

ACAAACGAGACATTCCGGCCGGGAGGAGGAGATATGAGGGACAATTGGAGAAGTGAATTGTATAAATATAAAG
TGGTAGAGATCAAGCCATTA---GGAATAGCACCCACTGGGGCAAGGAGGAGAGTGGTG--------------
--------------------------AAGAGAGAAAAAAGAGCA---GCAACA---CTAGGAGTT---------
ATGTTCCTTGGGTTCTTGGGAGCA---GCTGGAAGCACTATG---GGCGCAGCATCA---
ATAACGCTGACGGTACAG---GCCAGACAA---TTATTG---TTGGGTATAGTGCAACAGCAAAAT---
AATTTG---GTGAGG---GCTATAGAGGCGCAACAGCATTTATTGCAACTCACAGTTTGGGGAATT
>MF373135
TTCTATTGCGATACATCAGAACTGTTTAATAGTACATACATGTCTAACAGTACTAACAGTACAAAT-------
----

TCAAGTTCAACCATCACACTCCCATGCAGGATAAAACAAATTATAAACATGTGGCAGGGGGTAGGACGAGCAA
TATATGCCCCTCCCATTGAAGGAAACATAACATGTATATCAAAC---
ATCACAGGAATACTATTGACGCGTGATGGAGGAAACAATAACACA---------------------------
----

GAGACAGAGAAATTCAGACCTGGAGGAGGAGATATGAGGGACAATTGGAGAAGTGAATTATATAAATATAAAG
TGGTGGAAATTAAACCATTA---GGAATAGCACCCACTAAGGCAAAAAGGAGAGTGGTG-------------
-------------------------GAGAGAGAAAAAAGAGCA---GTGGGA---ATAGGAGCT--------
GTGTTCCTTGGGTTCTTGGGGGCA---GCAGGAAGCACTATG---GGCGCAGCGTCA---
ATAACGCTGACGGTACAG---GCCAGACAA---TTGTTG---TCTGGTATAGTGCAACAGCAAAAC---
AATTTG---CTGAGG---GCTATAGAGGCGCAACAGCATATGTTGCAACTCACGGTCTGGGGTATT
>KP411830
TTCTATTGCAATACATCAAGACTGTTTAATACGACATCATTGTTATGTACTATGTGTACTGCAGGGTCTAATA
GTACT---
-------------AATAGT------
ACTTCAGATGAACCCATCATCCTCCCATGCAGAATAAAACAAATAGTAAACATGTGGCAGAGGGTAGGACGAG
CAATGTATGCCAATCCCATTGCAGGAAACATAACATGTAGATCAAAT---
ATCACAGGACTACTACTGACACGTGATGGAGGCAATACTAATAAC---------------------------
----

ACAACTGAAATATTCAGACCAGGAGGAGGAGATATGAGGGACAATTGGAGAAGTGAATTATATAAATACAAAG
TGATAGAGATTAAGCCATTA---GGAATAGCACCCTCAAAGGCAAAAAGGAGAGTGGTG-------------
-------------------------GAGAGAGAAAAAAGAGCA---GCA------CTAGGAGTT--------
ATGTTCCTTGGGTTCTTGGGAGCA---GCTGGAAGCACTATG---GGCGCAGCGTCT---
TTAACGCTGACGGTACAG---GCCAGACAA---TTGTTG---TCTGGTATAGTGCAACAGCAAAGC---
AATTTG---CTGAGG---GCTATAGAGGCGCAACAGCATCTGTTGCAACTCACGGTCTGGGGGATT
>MF373176
TTCTATTGTAATACATCAGGCCTGTTTAATGGTACATACATGCCTAATGGTACAAACAGTAAC---------
----

TCAAGCTCAAACATCACACTCCCATGCAGAATAAAGCAATTTATAAACATGTGGCAGGGGGTAGGACGAGCAA
TGTATGCCCCTCCCATTGAAGGAAACATAACATGTAATTCAATT---
ATCACAGGACTACTATTGGAACGTGATGGAGGGGAAACAAACAGT---------------------------
----

AGTACAGAGACATTTAGACCTGGAGGAGGAGATATGAGGAACAACTGGAGAAGTGAATTATATAGATATAAAG
TGGTAGAAATTAAGCCCTTG---GGAGTAGCACCCACTACAGCAAAAAGGAGAGTGGTG-------------
-------------------------GAGAGAGAAAAAAGAGTA---GTGGGA---ATAGGAGCT--------
GTGTTCCTTGGGTTCTTAGGAGCA---GCTGGAAGCACTATG---GGCGCGGCGTCA---
ATAACGCTGACGGCACAG---GCCAGACAA---TTGTTG---TCTGGTATAGTGCAACAGCAAAGC---
AATTTG---CTGAGA---GCTATAGAGGTGCAGCAGCATATGTTGCAACTCACGGTCTGGGGTATT
>AY253307
TTCTATTGCAATACATCAGAATTGTTTAAGTTTAATGGGACATACATAGGTAATACTACA------------
----

GACAATGAGACAATCACACTCCCATGTAGAATAAAACAAATTATAAACATGTGGCAGAGGGTAGGACAAGCGA
TGTATGCCCCTCCAATTAAAGGAAATATAACATGTAAATCAAAG---
ATCACAGGAATACTAATGACACGTGATGGTGGTATGAATGGGACA---------------------------
----

AAGGAGGAGATCTTCAGACCTGGAGGAGGAGATATGAGAGATAATTGGAGAAATGAATTATATAAATATAAAG
TGGTAGAAATCAAACCATTA---GGAGTAGCACCCACTAGGGCAAAAAGGAGAGTGGTG-------------
-------------------------GAGAGAGAAAAAAGAGCA---GTGGGA---ATAGGAGCT--------
GTGTTCCTTGGGTTCTTGGGGGCA---GCAGGAAGCACTATG---GGCGCGGCGTCA---
ATAACGCTGACGGTACAG---GCCAGACAA---TTATTG---TCTGGTATAGTGCAACAGCAAAGC---
AATTTG---CTGAAG---GCTATAGAGGCGCAGCAGCATATGTTGCAACTCACGGTCTGGGGTGTT
>AY253317
TTCTATTGCAATACATCAAAACTGTTTAATGGCACATACATGCTTAATCACACTGACACAGAAAGTAAT----
----

TCAACATCAGACATCACACTCCAATGCAGAATAAAACAAATTATAAACATGTGGCAGGAGGTAGGACGAGCAA
TGTATGCCCCTCCCATTGCAGGACACATAATATGTAACTCAAGT---
ATCACAGGACTACTATTGCTACGTGATGGAGGCAGTGGCAAGGGCATA-------------------------
----

AATAATGAAACAGAGATATTCAGACCTGGAGGAGGAGATATGAGGGATAATTGGAGAAGTGAATTATATAAAT
ATAAAGTGGTAGAAATTAAACCATTG---GGAGTAGCACCCACCGAGGCAAAAAGAATAGTGGTG--------
-------------------------------GAGAGAGAAAAAAGAGCA---GTGGGA---ATAGGAGCT---
------GTGCTCCTTGGGTTCTTGGGAGCA---GCAGGAAGCACTATG---GGCGCAGCGTCA---
ATAACGCTGACGGCACAG---GCCAGACAA---TTGTTG---ACTGGTATAGTGCGACAGCAAAGC---
AATTTG---CTAAGG---GCTATAGAGGCGCAACAGCATCTGTTGCAACTCACTGTCTGGGGTATT
>AY253320
TTCTATTGTAATACAACAGACCTATTTAATAGAACTATTACTGATGGAAACTACAAATACTTACTGATGAAA-
----

ATTCAAATATCACACTCCAATGTAGAAATAAAACAAATTATAAATATGTGGCAGGGGGTAGGACGAGCAATAT
ATGCCCCTCCAATTGAAGGAGAAATAATATGTACATCAAAC---
ATCACAGGACTGCTATTGACACGTGATGGAGGAACTGGAGAAAATAGCAGT----------------------
----

ACAAACAATACAGAGATATTCAGACCTGGAGGAGGAGATATGAGAGACAATTGGAGAAGTGAATTATATAAAT
ATAAAGTGGTAGAAATTAAGCCATTA---GGAATAGCACCCACTAGGGCAAAAAGGAGAGTGGTG--------
-------------------------------GAGAGAGAGAAAAGAGCA---GTGGGA---ATGGGAGCT---
------GTGCTCCTTGGGTTCTTGGGAGCA---GCAGGAAGCACTATG---GGCGCAGCGTCA---
ATAACGCTGACGGTACAG---GCCAGACAA---CTATTG---TCTGGTATAGTGCAACAGCAAAAC---
AATTTG---CTGAGA---GCTATAGAGGCGCAACAGCATATGTTGCAACTCACGGTCTGGGGTATT
>KX907357
TTCTATTGCAACACATCAGGCCTGTTTAATAGTACATACAACGATACATACATGCCTAATGATACAACAAGTA
CA---

AACAATACAAAAATCACACTCCCATGCAGGATAAAACAGATTATAAACATGTGGCAGGGGGTAGGACGAGCAA
TGTATGCCCCTCCCATTGCAGGAAACATAACATGTAGATCAAGT---
ATCACAGGACTAATATTAGTACGTGATGGAGGTTCTGAAACAAAT----------------------------
----

GAAACATTCAGACCTGAAGGAGGAAACATGAGGGACAATTGGAGAAGTGAATTATATAAATATAAAGTGGTAG
AAATTAAGCCATTA---GGAATAGCACCCACTGACGCAAAAAGGAGAGTGGTG--------------------
-------------------GAGAGACAAAAAAGAGCA---GTGGGA---ATAGGAGCT---------
ATGTTCCTTGGGTTCTTGGGAGCA---GCAGGAAGCACTATG---GGCGCGGCGTCA---
ATAACGCTGACGGCACAG---GCCAGAAAA---GTGTTG---TCTGGTATAGTGCAACAGCAAAGC---
AATTTG---CTGAGG---GCTATAGAGGCGCAACAGCATCTGTTGCAACTCACAGTCTGGGGGATT
>AY838566
TTCTATTGCAACACATCAAACCTGTTTTATAATGATACATACCAGTCT-------------------------
----

AATAACGGCAGCATCACACTCCCCTGCAAAATAAAACAAATTATAAATATGTGGCAGAAGGTAGGACGAGCAA
TGTATGCCCCTCCCATTAAAGGAAACATAACATGTAAATCAAGT---
ATCACAGGAATACTATTGACACGGGATGGAGGGGCAGAAAACAACACA-------------------------
----

GAGAAGCCAGAGATATTCAGGCCTGGAGGAGGGGATATGAGGGACAATTGGAGAAGTGAATTATATAAATATA
AAGTAGTAGAAATTAAGCCATTG---GGAGTAGCACCCACCGAGGCAAAAAGGAGAGTGGTG-----------
----------------------------GAGAGAGAAAAAAGAGCA---GTGGGA---ATAGGAGCT------
---GTGTTCCTTGGGTTCTTGGGAGCA---GCAGGAAGCACTATG---GGCGCGGCGTCA---
ATAACGCTGACGGTACAG---GCCAGACAA---TTGTTG---TCTGGTATAGTGCAACAGCAAAAC---
AATTTG---CTGAGA---GCTATAGAGGCGCAACAGCATATGTTGCAACTCACGGTCTGGGGAATT
>JX845611
TTCTATTGCAATACAACAAAACTGTTTAATAAAACACTGTTCAATGAGACA----------------------
----

AGTGAGAACAACATCACACTCTCATGCAGAATAAAACAGATTATAAACATGTGGCAGCAGGTAGGACGAGCAA
TGTATAACCCTCCCATTGAAGGAAGCATAACATGTAACTCAACT---
ATAACAGGACTACTCTTGTTCCGTGATGGAGGAGGCAAGAATAAC----------------------------
----

ACTGAGATATTCAGACCTGGAGGAGGGAATATGAAGGACAATTGGAGAAGTGAATTATATAAGTATAAAGTGG
TAGAAATTAAGCCATTG---GGAATAGCACCCACTGGGGCAAAAAGGAGAGTGGTG-----------------
----------------------GAGAGAGAAAAAAGAGCA---GTAGGA---ATAGGAGCT---------
GTGTTCCTTGGGTTCTTGGGTGCA---GCCGGAAGCACTATG---GGCGCGGCGTCG---
ATAACGCTGACGGCACAG---GCCAGACAA---CTGTTG---TCTGGTATAGTGCAACAGCAAAGC---
AATTTG---CTGAGA---GCTATAGAGGCGCAACAGCATCTGTTGCAACTCACAGTCTGGGGTATT
>AY529666
TTCTATTGCAATACATCAGGCCTATTTAATGGTACATTTAATGGTACATACATGTCTAATGGTACAGAG----
----

AATAATTCAAACATCACACTCCAATGTAGAATAAAACAAATTATAAAAATGTGGCAGGGGGTAGGACAAGCAA
TGTATGCCCCTCCCATTCAAGGAAACATAACATGTAAATCAAAT---
ATCACAGGACTGCTATTGACACGTGATGGAGGAACAGAAAACGGAACA-------------------------
----

GAGAGAAATAATGAGACATTTAGACCTGGAGGAGGAGATATGAAGGACAATTGGAGAAGTGAATTATACAAAT
ATAAAGTGGTAGAAATTAAGCCATTA---GGAATAGCACCCACTAGTGCAAAAAGGAGAGTGGTG--------
-------------------------------GAAAGAGAGAAAAGAGCA---GTGGGA---ATAGGAGCT---
------GTGTTCCTTGGGTTCTTGGGAGCA---GCAGGAAGCACTATG---GGCGCGGCGTCA---
ATAGCGCTGACGGTACAG---GCCAGACAA---TTATTG---TCTGGTATAGTGCAACAACAAAGC---
AATTTG---CTGAGG---GCTATAGAGGCGCAACAGCATTTGTTGCAACTCACGGTCTGGGGCGTT
>DQ447270
TTTTATTGCAATACAACAAAACTGTTTAATAATAGTAATTAT-------------------------------
----

AATAATAGTATGGTTATACTCCCATGTAGAATAAAACAAATTATAAACATGTGGCAGGAGGTAGGACGAGCAA
TGTATGCCCCTCCCATTGCAGGAAACCTAACATGTAAATCAAAT---
ATCACAGGACTGCTGTTGACACGTGATGGAGGAAAGGAAAAAAAT----------------------------
----

GGGACAGAGATATTCAGACCTGGAGGGGGAGATATGAGGGACAATTGGAGAAGTGAATTATATAAATATAAAG
TGGTAGAAATTAAACCATTA---GGAATAGCACCCACTGAGGCAAAAAGGAGAGTGGTG--------------
-------------------------GAGAGAGAAAAAAGAGCA---GTGGGA---ATAGGAGCT---------
GTGTTCCTTGGGTTCTTGGGAGCA---GCAGGAAGCACTATG---GGCGCGGCGTCA---
ATAACGCTGACGGTACAG---GCCAGACAA---CTGTTG---TCTGGTATAGTGCAACAGCAAAGC---
AATTTG---CTGAGA---GCTATAGAGGCGCAACAGCATATGTTGCAACTCACGGTCTGGGGTATT
>DQ275653
TTCTATTGCAATACATCAAGTCTGTTTAATGAAAGTAAAACAAACAGAAGTGAGTCAAAT-------------
----

CCAAATGACACAATCACACTCCCATGCAGGATAAAACAAATTATAAACATGTGGCAGGAGCCAGGGCGAGCGA
TGTATGCCCCTCCCATTGCAGGAAAAATAACATGTAACTCAAAT---
ATCACAGGACTGTTATTGACACGTGATGGAGGGAATGACACACAG----------------------------
----

GGAAGCGAAACATTCAGACCTGGAGGAGGAAATATGAAGGACAATTGGAGAAGTGAATTATATAAATATAAAG
TGGTAGAGATTAAGCCATTG---GGAATAGCACCCACTACATCAAAAAGGAGAGTGGTG--------------
-------------------------GAGAGAGAAAAAAGAGCA---GTGGGA---ATAGGAGCT---------
GTACTCCTTGGGTTCTTGGGAGCA---GCAGGAAGCACTATG---GGCGCGGCGTCA---
ATAACGCTGACGGCACAG---GCCAGACAA---CTGTTG---TCTGGTATAGTGCAACAGCAAAAC---
AATTTG---CTGAGA---GCTATAGAGGCGCAACAGCATATGTTGCAACTCACAGTCTGGGGTATT
>DQ396384
TTCTATTGCAATACCACAAAGTTGTTTAATAGTACATACAATGGAACAGAAAGC-------------------
----

AACAATGAAACTATCACACTCCCATGCAGAATAAAACAAATTATAAACATGTGGCAGGAGGTAGGACGAGCAA
TGTATGCCCCTCCCATTGCCGGTAACATAACATGTAGCTCAAAT---
ATCACAGGACTACTCCTGACACGTGATGGAGGAAATGGAACTGACACT-------------------------
----

AATGGGACAGAGACATTCAGACCTGGAGGAGGAAATATGAGGGACAATTGGAGAAGTGAATTATACAAATATA
AAGTGGTAGAAATTAAGCCATTG---GGGATAGCACCCACTGACGCAAAAAGGAGAGTGGTG-----------
----------------------------GAAAGAGAAAAAAGAGCA---GTGGGA---ATAGGAGCT------
---GTACTCCTTGGGTTCTTGGGAGCA---GCAGGAAGCACTATG---GGCGCGGCGTCA---
ATAACGCTGACGGCACAG---GCCAGACAA---GTGCTG---TCTGGTATAGTGCAACAGCAAAGC---
AATTTG---CTGAAG---GCTATAGAGGCGCAACAGCATCTGTTGCAACTCACGGTCTGGGGTATT
>DQ275661
TTCTATTGCACTACAACAGACCTGTTTAATACAACAAAAATATTTAATGATACA-------------------
----

GGTAATCAAACAATCACACTCCCATGCAGAATAAAACAGATTATAAATATGTGGCAAGAGGTAGGACGAGCAA
TGTATAACCCTCCCATTGAAGGAATCATAGCATGTAATTCAACT---
ATAACAGGATTACTCTTGCTACGTGATGGAGGAATCACTGATAATACTAAT----------------------
----

GGCACAAAGGACAAAGAGATATTCAGACCTGGAGGAGGAAATATGAAGGACAATTGGAGAAGTGAATTATATA
AGTATAAAGTAGTAGAAATTAAGCCATTG---GGAATAGCACCCACTAAGGCAAAAAGGAGAGTGGTG-----
----------------------------------GAGAGGGAAAAAAGAGCA---GTGGGC---
ATAGGAGCT---------GTGTTCCTTGGGTTCTTGGGTGCA---GCAGGAAGCACTATG---
GGCGCGGCGTCG---GTAACGCTGACGGTACAG---GCCAGACAA---CTGTTG---
TCTGGTATAGTGCAACAGCAAAGT---AATTTG---CTGAGA---
GCTATAGAGGCGCAACAGCATATGTTGCAACTCACAGTCTGGGGTATT
>AY901976
TTCTATTGCAATACAACAAATCTGTTCACAAATAATACAACAGATAAT-------------------------
----

AACAGTAGTATCATCACACTCCCATGCAGAATAAAACAAATTATAAACATGTGGCAGGAGGTAGGACGAGCAA
TGTATGCCCCTCCCATTGCAGGAAACTTAACATGTAACTCAAGT---
ATCACAGGACTACTACTGACACGTGATGGAGGAAACAAAAACGGAACA-------------------------
----

AATAACACAGAGATATTCAGGCCTGGGGGAGGAAATATGAAAGACAATTGGAGAAGTGAATTATATAAATATA
AAGTGGTAGAAATTAAGCCATTG---GGAATAGCTCCCACTAAGGCAAAAAGGAGAGTGGTG-----------
----------------------------GAGAGAGAAAAAAGAGCA---GTGGGA---ATAGGAGCT------
---GTGCTCCTTGGTTTCTTGGGAGCA---GCAGGAAGCACTATG---GGCGCGGCGTCA---
ATAACGCTGACGGTACAG---GCTAGACAA---CTGTTG---TCTGGTATAGTGCAACAGCAAAGC---
AATTTG---CTGAGA---GCTATAGAGGCGCAACAGCATTTGTTGCAACTCACGGTCTGGGGAATT
>AY878071
TTCTATTGTAATACAACAAAACTGTTTACAAATCTGACTAGTAACAATGAAACAAATCTGACTAGTAAA----
----

AATGAATCAGACATCGAACTTCCATGCAGGATAAAACAAATTATAAATATGTGGCAGAAGGTAGGACGAGCAA
TGTATGCCCCTCCCATTGCAGGAAACATAACATGTAAATCAAAT---
ATCACAGGACTACTGTTGACACGGGATGGAGGAAATACCAACAAAAGT-------------------------
----

AATAATACAGAAACTTTTAGACCTGGAGGAGGAAACATGAAAGACAATTGGAGAAGTGAATTATATAAATATA
AAGTGGTAGAAACTAAGCCATTG---GGAATAGCACCCACTGGATCAAAAAGGAGAGTGGTG-----------
----------------------------GAGAGAGAAAAAAGAGCA---GTGGGA---ATAGGAGCT------
---GTGCTCTTTGGGTTCTTGGGAGCA---GCAGGAAGCACTATG---GGCGCGGCGTCA---
ATAACGCTGACGGTACAG---GCCAGACAA---CTGTTG---TCTGGTATAGTGCAACAGCAAAGC---
AATTTG---CTGAGA---GCTATAGAGGCGCAACAGCATATGTTGCAACTCACAGTCTGGGGTATT
>DQ093587

TTCTATTGCAATACAACAAAACTGTTCAATGACAGTGCTTTAAGTGCTTTCAACAAAACC-------------
----

TCTAATGAAACCATCACACTCCCATGCAGAATAAAACAGATTATAAACATGTGGCAGGAGGTAGGACGAGCAA
TGTATGCCCCTCCCATTGAAGGGAACATAACATGTAACTCAAGT---
ATCACAGGACTATTATTGCTACGTGATGGAGGAAAAGAAAATAATGAG-------------------------
----

ACCAATAACACAGAGATATTCAGACCTGGAGGAGGAAACATGAAAGACAATTGGAGAAGTGAACTATATAAAT
ATAAAGTGGTAGAAATTAAGCCATTG---GGAATAGCACCCACGATGGCAAAAAGGAGAGTGGTG--------
-------------------------------GAGAGAGAAAAAAGAGCA---GTGGGA---ATAGGAGCT---
------GTGCTCCTTGGGTTCTTGGGAGCA---GCAGGAAGCACTATG---GGCGCGGCGTCA---
ATAACGCTGACGGTACAG---GCCAGACAA---CTGTTG---TCTGGTATAGTGCAACAGCAAAGC---
AATTTG---CTGAGA---GCTATGGAGGCGCAACAGCATATGTTGCAACTCACAGTCTGGGGCACT
>DQ382380
TTCTATTGCAATACATCAAAACTGTTTCATGGAACATACAATGGTACATACAATTCTAGTGGAGAG-------
----

GATAATTCAACCATCACACTCCAATGCAGAATAAAACAATTTATAAACATGTGGCAGGAGGTAGGAAGAGCAA
TGTATGCCCCTCCCATTGCAGGAAACATAACATGTAAATCAAAC---
ATCACAGGACTAATATTGACACGTGATGGAGGAAATGACAATGGCACAAATGAC-------------------
----
-
ACTACCACAAATAAAACAGAGATATTCAGACCTGGAGGAGGAGATATGAGGGACAACTGGAGAAGTGAATTAT
ATAAATATAAAGTGGTAGAAGTCAAGCCATTG---
GGAGTAGCACCCACCAAGGCAAAAAGGAAAGTGGTGGAG---------------------------------
AGAGAAAGAGTAAAAAGAGCA---GTGGGA---CTAGGAGCT---------
GTGTTCATTGGGTTCTTGGGAGCA---GCAGGAAGCACTATG---GGCGCAGCGTCA---
ATAACGCTGACGGCACAG---GCCAGACAA---CTGTTG---TCTGGTATAGTGCAACAGCAAAGC---
AATTTG---CTGAGA---GCTATAGAGGCGCAACAACATATGTTGCAACTCACGGTCTGGGGCGTT
>GQ999972
TTCTATTGCAATACATCAAAACTGTTTAATGAGAGCATGTTAAATAGTACAGACAATAGTACAAAA-------
----

GAGGATGTAAACATCATACTCCCATGCAGGATAAAACAAATTATAAACATGTGGCAGGAGGTAGGACGAGCAA
TGTATGCCCCTCCCATTGCAGGAAACATAATATGTACCTCAAAT---
ATCACAGGGCTACTACTGACACGTGATGGAGGGCCAGAGAATGGC----------------------------
----

ACAGATACAGTCAGACCTGTAGGAGGAGATATGAGGAACAATTGGAGAAGTGAATTATATAAATATAAGGTGG
TAGAAATTAAGCCATTG---GGAATAGCACCCACTAAGGCAAAAAGGAGAGTGGTGGAG--------------
----------------------AGGAGGGAAAAAAGAGCA---GTGGGA---ATAGGAGCT---------
GTGTTCCTTGGGTTCTTGGGAGCA---GCAGGAAGCACTATG---GGCGCAGCGTCA---
ATGACGCTGACGGTACAG---GCCAGACAA---TTGTTG---TCTGGAATAGTGCAACAGCAAAGC---
AATTTG---CTGAGG---GCTATAGAGGCGCAACAGCATATGTTGCAACTCACGGTCTGGGGAGTT
>HQ615945
TTCTATTGCAATACATCACTACTATTTAATAGCAATGATACAGAAAGTAACAATGATACAGTAAGTAGTAATT
CA---

ACCAGTAATTCAACCATCACACTTCGATGCAGAATAAAACAAATTATAAACATGTGGCAGGAGGTAGGACGAG
CAATGTATGCCCCTCCCATTGAAGGAAACATAACATGTAAATCAAAT---
ATCACAGGACTAATATTAGCACGTGATGGTGGAAACTCAAATGAC----------------------------
----

TCAAATAAAGAGATATTCAGACCTGAAGGAGGAAATATGAAGGACAATTGGAGAAGTGAATTATATAAATATA
AAGTAGTAGAAATTAAACCATTG---GGAATAGCACCCACTAAAGCACAAAGGAGAGTGGTG-----------
----------------------------AAGAGAGAAAAAAGAGCA---GTAGGA---ATAGGAGCT------
---GTGTTCCTTGGATTCTTGGGAGTA---GCCGGAAGCACTATG---GGCGCAGCGTCA---
ATAACGCTGACGGCACAG---GCCAGACAA---GTGTTG---TCTGGTATAGTGCAACAGCAAAGC---
AATTTG---CTGAGG---GCTATAGAGGCGCAACAGCATATGTTGCAACTCACGGTCTGGGGTATT

>KC156116
TTCTATTGCAATACAACAAAACTGTTTAATGATACAGAAAAATATAAC-------------------------
----

AGTACAAACATCATACTCCCATGTAGAATAAGACAAATTGTAAACATGTGGCAGGGGGTAGGACGAGCAATAT
ATGCCCCTCCCATTAAAGGAAACATAACATGTGAATCAAAT---
ATTACAGGACTACTGTTGACACGTGATGGAGGGAATAAAAGCAAC----------------------------
----

CAAACTAACCAAAATGAGACATTCAGACCTGGAGGAGGAGATATGAGGGATAATTGGAGAAGTGAATTATATA
AATATAAAGTGGTAGAAATTAAGCCATTG---GGAATAGCACCCACTGAAGCAAAAAGGAGAGTGGTG-----
----------------------------------GAGAGAGAAAAAAGAGCA---GCAGGA---
ATAGGAGCT---------GTGTTCCTTGGGTTCTTGGGAGCA---GCAGGAAGCACTATG---
GGCGCGGCGTCA---ATAACGCTGACGGTACAG---ACCAGACAA---TTGTTG---
TCTGGTATAGTGCAACAGCAAAGC---AATTTG---CTGAAG---
GCTATAGAGGCGCAACAGCACCTGTTGCAACTCACAGTCTGGGGTATT
>HM204618
TTCTATTGCAATACATCACAACTGTTTAATAGCGCTGAAAATGAAACAGATATT-------------------
----

ATAAATTCAAACATCACACTGCCATGCAGAATAAAACAAATTATAAACATGTGGCAGGAGGTAGGACGAGCAA
TGTATGCCCCTCCCATTGCAGGAAACATAATATGTAAATCAAAT---
ATCACAGGACTACTATTAGTACGTGATGGAGGCAACATCAGCGACAGC-------------------------
----

AACCAAACAGAGATATTCAGACCTGGAGGAGGAGATATGAGGGACAATTGGAGAAGTGAATTATATAAATATA
AAGTGGTAGAAATTAAGCCATTG---GGAATAGCCCCCACTGAAGCAAAAAGGAGAGTGGTG-----------
----------------------------GAGAGAGAAAGAAGAGCA---GTGGGA---ATAGGAGCT------
---GTGTTCCTTGGGTTCTTGGGAGCA---GCAGGAAGCACTATG---GGCGCAGCGTCA---
ATAACGCTGACGGTACAG---GCCAGACAA---CTGTTG---TCTGGTATAGTGCAACAGCAAAGC---
AATTTG---CTGAGG---GCTATAGAGGCGCAACAGCATATGTTGCAACTCACGGTCTGGGGTATA
>HQ615961
TTCTATTGCAATACAACATTTCTGTTTAATAACAGTGCTTCA-------------------------------
----

AACACAACTATCACTCTCCCATGCAAAATAAAACAGATAATAAACATGTGGCAGGGGGTAGGACGAGCAATGT
ATGCCCCTCCCATTCAAGGAAACATAACATGTGAATCAAAT---
ATCACAGGACTACTATTGACACGTGATGGAGAAGATAACAACAAA----------------------------
----

ACAGAGACATTCAGACCTGGAGGAGGAGATATGAAGGACAATTGGAGAAGTGAATTATATAAATATAAAGTAG
TAGAAATTAGGCCATTG---GGAGTAGCACCCACAAAGGCAAAAAGGAGAGTGGTGGAG--------------
-------------------AAAAAAGAAGAAAAAAGAGCA---GTGGGA---TTAGGAGCT---------
GTGATCCTTGGGTTCTTGGGAGCA---GCAGGAAGCACTATG---GGCGCGGCGTCA---
ATAACGCTGACGGTACAG---GCCAGACAA---CTGTTG---TCTGGTATAGTGCAACAGCAAAGT---
AATTTG---CTGAGA---GCTATAGAGGCGCAACAGCATATGTTGCAACTCACGGTCTGGGGAGTT
>HM623611
TTCTATTGCAATACATCAAAACTATTTAATAATGAGACAGAGAGGAAT-------------------------
----

TCAACCACCATCACCCTCCCATGCAGAATAAAACAAATTATAAACATGTGGCAGGAGGTAGGACGAGCGATGT
ATGCCCCTCCTGTTGCAGGAAACATAACATGTAAATCAAAT---
ATCACAGGACTACTATTGGTACGTGATGGAGGAACAACAAATGAG----------------------------
----

AGCACACAAGAAATATTCAGACCTGGAGGAGGAGACATGAGGGACAATTGGAGAAGTGAATTATATAAATATA
AAGTGGTAGAAATTAAGCCATTG---GGAGTAGCACCCACTAAGGCAAAAAGGAGAGTGGTG-----------
----------------------------GAGAGAGAAAAAAGAGCA---GCG------CTAGGAGCT------
---GTGTTCCTTGGGTTCTTGGGAGCA---GCAGGAAGCACTATG---GGCGCAGCGTCA---

```
ATAACGCTGACGGTACAG---GCCAGACAA---CTATTG---TCGGGTATAGTGCAACAGCAAAGC---
AATTTG---CTGAAG---GCTATAGAAGCGCAACAGCATCTGTTGCAGCTTACGGTCTGGGGTATT
>KF725900
TTCTATTGCGATACATCACAACTGTTTAATGGAACATACGTGAATAACACAAGCAATAATTCT----------
-------------------------------------------------------------------------
----------------------------
GACATCACCACACTCACACTCCCATGCAGAATAAAACAGATTATAAACATGTGGCAGGAGGTAGGACGAGCAA
TATATGCCCCTCCCATTGCAGGAAACATAACATGTAGCTCAAAT---
ATCACAGGAATTTTATTAGTACGGGATGGAGGCGAAGATGAAGATAGT-------------------------
-------------------------------------------------------------------------
----------
AATGACACAGAGATATTTAGACCTGGAGGAGGAGATATGAGGAACAATTGGAGAAGTGAACTATACAAATATA
AAGTGGTAGAAGTTAAACCATTA---GGAATAGCACCAACTGAAGCAAAAAGGAGAGTGGTG-----------
----------------------------GAGAGAGAAAAAAGAGCA---GTGGGA---CTAGGAGCT------
---GTGCTCTTTGGGTTCTTGGGAGCA---GCAGGAAGCACTATG---GGCGCAGCATCA---
ATGACGCTGACGGTACAG---GCTAGACAA---TTATTG---TCTGGTATAGTGCAACAGCAAAGC---
AATTTG---CTGAAG---GCTATAGAGGCGCAACAGCATATGTTGCAACTCACGGTCTGGGGAATT
>JX213387
TTCTATTGTGATACATCAAAACTATTTAACATGTATAATGGTACATATAATGATACAGAAAGAGGG-------
-------------------------------------------------------------------------
----------------------------
TCAAACTTAACCATCATACTCCAATGCAGAATAAAACAATTTATAAACATGTGGCAGGAGGTAAGACAAGCAA
TGTATGCCCCTCCCATTGAAGGAAACATAACATGTAGATCAAAT---
ATCACAGGACTACTCTTGACAAGGGATGGAGGTAAGGAAGATCCAAAC-------------------------
-------------------------------------------------------------------------
----------
AATACAGATGAGACATTCAGACCTGGAGGAGGAAATATGAAAGACAATTGGAGAAGTGAATTATATAAATATA
AAGTGGTAGAAATTAAGCCATTG---GGAGTAGCACCCACTGAGGCAAAAAGGAGAGTGGTG-----------
----------------------------GAGAGAGGAAAAAGAGCA---GTGGGA---CTGGGAGCT------
---ATGTTCCTTGGGATCTTGGGAGCA---GCAGGAAGCACTATG---GGCGCAGCGTCA---
ATCACGCTGACGGTACAA---GCCAGACAA---TTGCTG---TCTGGTATAGTGCAACAGCAAAGC---
AATTTG---CTGAGG---GCTATAGAGGCACAACAACATCTGTTGCAACTCACAGTCTGGGGTATT
>KF986058
TTCTACTGCAATACATCAAGACTGTTTAATAGTACATGGAATGATAAT-------------------------
-------------------------------------------------------------------------
-------AGAATTACAGGG------
GGTAATGACAGTACAATCACACTCCCATGCAGAATAAAACAACTTATAAACATGTGGCAGAGAGTGGGAAAAG
CAATGTATGCCCCTCCCATTGCAGGAGTAATCAGTTGTACATCAAAT---
ATTACAGGACTATTGTTGACAAGGGATGGTGGT----------------------------------------
--------------------------------------------------------------------
GTAGATAATCAGAGG---
AATAGTGAGACCTTCAGACCTGGAGGAGGAGATATGAGAGACAATTGGAGAAGTGAATTATACAAATATAAAG
TAGTAAGAATTGAACCACTA---GGTCTAGCACCCACCAAGGCAAAAAGAAGAGTGGTG--------------
-------------------------GAAAGAGAGAAAAGAGCA---ATAGGA---CTAGGAGCT---------
CTGTTCCTTGGGTTCTTGGGAGCA---GCAGGAAGCACAATG---GGCGCAGCGTCA---
CTGACGCTGACGGTACAG---GCCAGACAA---TTATTG---TCTGGTATAGTGCAACAGCAAAAC---
AATTTG---CTGAGG---GCTATAGAGGCGCAACAGCACCTGTTGCAACTCACAGTCTGGGGCGTT
>KF986041
TTCTACTGCAATACATCAGGATTGTTCAATATATCA-------------------------------------
-------------------------------------------------------------------------
-------------------------
GGAGGAAATGAGACTATAACCCTTCCATGCAGAATAAAACAAATCATAAACATGTGGCAGGGAGTAGGAAAAG
CAATGTATGCCCCTCCCATTGAAGGGCTAATCAAATGTTCATCAAAT---
ATTACAGGCTTATTGTTGACAAGAGATGGTGGC----------------------------------------
--------------------------------------------------------------------
GCAAACAATACTAGT---
CAAAGTGAGACCTTCAGACCTGGAGGAGGAGATATGAGAGACAATTGGAGAAGTGAATTATACAAATATAAAG
TAGTAAGAATTGAACCACTA---GGTCTAGCACCCACCAAGGCAAGGAGAAGAGTGGTA--------------
-------------------------GAAAGAGAAAAAAGAGCA---ATAGGA---CTGGGAGCT---------
GTGTTCCTTGGGTTCTTGGGAGCA---GCAGGAAGCACGATG---GGCGCAGCGTCA---
```

TTGACGCTGACGGTACAG---GCCAGACAA---GTGTTG---TCAGGTATAGTGCAACAGCAAAAC---
AATTTG---CTGAGG---GCTATAGAGGCGCAACAGCATCTGTTGCAACTCACAGTCTGGGGTATT
>JX236670
TTCTACTGTAATACATCAAAATTGTTTAATAGTGAATGGGGAATAATAATACATTAGATAGAACAGGGGAA-
----

AATGGGAGTACAATCACACTCCCATGCAGAATAAAACAAATTATAAACATGTGGCAGGGAGTAGGAAAAGCAA
TGTATGCCCCTCCCATTGCAGGAATAATCCAATGTTCATCAAAT---
ATTACAGGACTATTGTTGACAAGAGATGGGGGTGTAAATAATAAT----------------------------
----

AGTCAGAATGAGACCTTTAGACCGGGAGGAGGAGATATGAGAGATAATTGGAGGAGTGAATTATACAAATATA
AAGTAGTAAGACTTGAACCAATA---GGTCTAGCACCCACCAGGGCAAAGAGAAGAGTGGTG-----------
----------------------------GAAAGAGAAAAAAGAGCA---ATAGGA---ATGGGAGCT------
---ATGTTCCTTGGGTTCTTAGGAGCA---GCAGGAAGCACGATG---GGCGCAGCGTCA---
CTGACGCTGACGGTACAG---GCCAGACAA---TTATTG---TCTGGTATAGTGCAACAGCAAAAC---
AATTTG---CTGAAG---GCTATAGAGGCGCAACAGCATCTGTTGCAACTCACAGTCTGGGGTATT
>EU852966
TTCTACTGCAATACATCAGGACTGTTTACATCAAATATTACAGGG----------------------------
----

GTAAATGAGACAATCAAACTCCCATGTCGAATAAAACAAATTATAAACATGTGGCAGGGAGTAGGAAAAGCAA
TGTATGCTCCTCCCATTGCGGGAGTAATCAAGTGTACATCAAAT---
ATTACAGGACTATTGTTGACAAGAGATGGTGGTGCAGATACTCAA----------------------------
----

AATGAGACCTTCAGACCTGGAGGAGGAGATATGAGAGACAATTGGAGAAGTGAATTATATAAATATAAAGTAG
TAAGAATTGAACCACTA---GGTCTAGCACCCACCAAGGCAAAGAGAAGAGTGGTG-----------------
----------------------GAAAGAGAAAAAAGAGCA---ATAGGA---CTGGGAGCT---------
ATGTTCCTTGGGTTCTTAGGAGCA---GCAGGAAGCACAATG---GGCGCAGCGTCA---
TTGACGCTGACGGTACAA---GCCAGACAG---TTATTG---TCTGGTATAGTGCAACAGCAAAAC---
AATTTG---CTGAGG---GCTATAGAGGCGCAACAGCATCTGTTGCAACTCACAGTCTGGGGCGTT
>AF484505
TTCTACTGCAATACATCAGGACTGTTTAATAATAGTGTATGGATAGATGGTAAATTAAATAGTACAGGGGTA-
----

AATGACAGTACAATCACACTCCAATGCAGAATAAAACAAATTATAAATATGTGGCAGAGCGTAGGAAGAGCAA
TGTATGCTCCTCCCATTGCAGGACCGATCAACTGTTCATCAAAT---
ATTACAGGACTATTGTTGACAAGAGATGGTGGTAATGATACTAGA----------------------------
----

CTGAGTGAGACCTTCAGACCTGGAGGAGGAGATATGAGAGACAATTGGAGAAGTGAACTATACAAATATAAAG
TAGTAAGAATTGAACCAATA---GGTCTAGCACCCACCAAAGCAAAGAGAAGAGTGGTA--------------
-------------------------GAAAGAGAAAAAAGAGCA---ATAGGA---CTGGGAGCT---------
GTGTTCCTTGGGTTCTTGGGAGCA---GCAGGAAGCACGATG---GGCGCAGCGTCA---
GTGACGCTGTCAGTACAG---GCCAAACAG---TTAATG---TCTGGTATAGTGCAACAGCAATAC---
AATTTG---CTGAGG---GCTATAGAGGCGCAACAGCATCTGTTGCAACTCACAGTCTGGGGCAAT
>AF484516
TTCTACTGCAATACATCAGGACTGTTTAATAGTACATGGACTAATAGTACAGGGGTAAAT-------------
----

AACAACAGTACAATCACCATCCCATGCAGAATAAAACAAATTATAAACATGTGGCAGGGAGTAGGAAAAGCAA
TATATGCCCCTCCCATTGCAGGACAAATCAACTGTTCATCAAAT---
ATTACAGGACTATTGTTGACAAGAGATGGCGGTGCAAATAATAAT----------------------------
----

GGTACAAATGAGACCGTCAGACCTGGAGGAGGAGATATGAGGGACAATTGGAGAAGTGAACTATATAAATATA
AAGTAGTAAGAATTGAACCACTA---GGTATAGCACCCACCAAGGCAAAGAGAAGAGTGGTG-----------
----------------------------GAAAGAGAAAAAAGAGCA---ATAGGA---CTAGGAGCT------
---ATGTTCCTTGGGTTCTTGGGAGCA---GCAGGAAGCACGATG---GGCGCAGCGTCA---

ATGGCGCTGACGGTACAG---GCCAGACAG---TTATTG---TCTGGTATAGTGCAACAGCAAAAC---
AATTTG---CTGAGG---GCTATAGAGGCGCAACAGCATCTGTTGCAACTCACAGTCTGGGGCGTT
>AF484481
TTCTACTGCAACACATCAGGACTGTTTAATAGTACATGGGGAAAAGATACAAATAGTACAAGGGTAGAG----
----

AATAATAGTACAATCACACTCCGATGCAACATAAAACAAATTATAAACATGTGGCAGGGAGTAGGAAAAGCAA
TGTATGCCCCTCCCATTAAAGGAGTAATCAGCTGTTCATCGAAT---
ATTACAGGACTATTGTTGACAAGAGATGGTGGAGTAAAGAATAAT----------------------------
----

AGTGAGGAGACCGTCAGACCTGGAGGAGGAGATATGAGAGACAATTGGAGAAGTGAATTATACAAATATAAAG
TAGTAAAAATCGAGCCACTA---GGTCTAGCTCCCACCAGGGCAAAGAGAAGAGTGGTG--------------
-------------------------GAAAGAGAAAAAAGAGCA---GTAGGA---CTAGGAGCT---------
TTGTTCCTTGGGTTCTTGGGAGCA---GCAGGAAGCACGATG---GGCGCAGCGTCA---
CTGACGCTGACGGTACAG---GCCAGACAG---TTATTG---TCTGGTATAGTGCAACAGCAAAAC---
AATTTG---CTGAGG---GCTATAGAGGCGCAACAGCATCTGTTGCAACTCACAGTCTGGGGCGTT
>AY795903
TTCTATTGCAATACATCAGAACTGTTTAATCAAAGTGCATGGGGA----------------------------
----

AATACAAGTGAGATCACACTCCAATGTAGAATAAAACAAATCATAAACATGTGGCAGGGAGTAGGAAAAGCAA
TGTATGCCCCTCCCATTGAAGGACTAATCAGGTGTTCATCAAAC---
ATTACAGGGCTATTATTGACAAGAGATGGGGGCAATGAAAATAAT----------------------------
----

GAGACCTTCAGGCCTGGAGGAGGAGACATGAGGGACAATTGGAGAAGTGAATTATACAAATATAAAGTAGTAA
GAATTGAACCACTA---GGTCTAGCACCCACCAGGGCAAAGAGAAGAGTGGTG--------------------
-------------------GAAAGAGAAAAAAGAGCA---ATAGGA---CTAGGAGCT---------
TTGTTCCTTGGGTTCTTGGGAGCA---GCAGGAAGCACGATG---GGCGCAGCGTCA---
CTGACGCTGACGGTACAG---GCCAGACAG---TTATTG---TCTGGTATAGTGCAGCAGCAAAAT---
AATTTG---CTGAGG---GCTATAGAGGCGCAACAGCACCTGTTGCAACTCACAGTCTGGGGCGTT
>MG365767
TTCTACTGCAATACATCAGGACTGTTTAATGACACAGAA----------------------------------
----

ACCAATGACACTATCATTCTCCCATGTCGAATAAAACAAATTGTAAACATGTGGCAGGAGGTAGGAAGAGCAA
TGTATGCCGTTCCCATTGAAGGAAACATTACCTGTAGATCAAAT---
ATTACAGGTCTACTCTTAACAAGAGATGGTGGTCAGAACAATGATACT-------------------------
----

AAAGGAAATGAGACCTTCAGACCTGGGGGAGGAGATATGAGAGACAATTGGAGAAGTAAATTATATAAATATA
AAGTAGTAGAAATTGAACCACTA---GGGGTAGCACCCACCAGGGCAAAAAGACAAGTGGTG-----------
----------------------------AGAAGAGAAAAAAGAGCA---GTGGGA---ATAGGAGCT------
---TTGTTCCTTGGGTTCTTGGGAGCA---GCAGGAAGCACTATG---GGCGCGGCGTCA---
ATGACGCTGACGGTACAG---GCCAGACAA---TTATTG---TCTGGGATAGTGCAACAGCAGAAC---
AACTTG---CTGAGG---GCTATTGAAGCGCAACAGCACATGTTGCAGCTCACAGTCTGGGGTATT
>KU749395
TTCTACTGCAACACATCAGGACTGTTTAATGACACAGTGTCTAATGACACAGTATTC----------------
----

AATGATGAAACTATCACTCTCCCATGTAGAATAAAACAAATTGTAAACCTGTGGCAAGAAGTGGGGCGAGCAA
TGTATGCCGCTCCCATTGCAGGAAACATTACTTGTAACTCAAAT---
ATTACAGGTCTACTATTAACAAGAGATGGTGGTAGTGATACTAGT----------------------------
----

AACAATGAGACAGAGATCTTCAGACCTGGGGGAGGAAATATGAAAGACAATTGGAGAAGTGAATTATATAAAT
ATAAAGTAGTAGAGATTCAGCCACTA---GGAATAGCACCCACCAAGGCAAAAAGACAAGTGGTG--------
-------------------------------CAGAGAGAAAAAAGAGCA---GTGGGA---ATGGGAGCT---
------GTGCTCCTTGGGTTCTTGGGAGCA---GCAGGAAGCACTATG---GGCGCGGCGTCA---

ATAGCGCTGACGGTACAG---GCCAGACAA---TTATTG---TCTGGCATAGTGCAACAGCAGAAC---
AATCTG---CTGAAG---GCTATTGAAGCGCAACAACATCTGTTGCAGCTCACAGTCTGGGGTATT
>HQ236559
TTCTACTGTAATACAACAACACTGTTTGATGAAACAAAATGG-------------------------------
----

AATGACGACACCATCGTTCTCCCATGTAGAATAAAGCAAATTGTAAACATGTGGCAAGCAGTAGGAAGAGCAA
TGTATGCCGCTCCTATTACAGGCAACATTACCTGTAACTCAAAT---
ATTACAGGCCTATTATTGACAAGAAATGGG---AAAAAT----------------------------------
----

GACACTGAAACCTTTAGCCCTGAAGGAGGAAATATGAAAGACAATTGGAGAAGTGAACTATATAAATATAAAG
TAGTAGAAATTCAGCCACTA---GGAGTAGCACCTTCCAGGGCAAGGAGACGAGTGGTG--------------
-------------------------CAGAGAGAAAAAAGAGCA---GTAGGG---TTGGGAGCC---------
ATGTTCCTTGGGTTCTTGGGAGCA---GCAGGAAGCACTATG---GGCGCAGCGTCA---
ATAACGCTGACGGTACAG---ACCAGACAA---TTATTG---TCTGGGATAGTGCAGCAGCAGAGC---
AATCTG---CTGAGG---GCCATTGAAGCACAACAGCATATGTTGCAGCTCACAGTCTGGGGCGTT
>AF190127
TTCTATTGCAATACAACAAAATTGTTTAATAGCACCTGGACCAATAGCAGCTATACCAATGACACCTATAACA
GCAACAGC---
-------------ACAGAA------
GATATCACAGGAAATATCACACTACAATGCAAAATAAAACAAATTGTGAACATGTGGCAGAGAGTAGGACAAG
CAATGTATGCCCCTCCCATCAGAGGAAACATCACATGTATATCAAAT---
ATTACAGGACTAATATTGACATTTGACCGG---AATAATACAAAT----------------------------
----

AATGTTACCTTCAGACCTGGAGGAGGAGATATGAGGGACAATTGGAGAAGTGAATTATATAAATATAAAGTAG
TAAAAATTGAACCACTG---GGAGTAGCACCAACCGAGGCAAGGAGAAGAGTGGTG-----------------
----------------------GAGAGAGAAAAAAGAGCA---GTAGGA---ATGGGAGCT---------
TTTTTCCTTGGGTTCTTGGGAGCA---GCAGGAAGCACTATG---GGCGCAGCGTCT---
ATAACGTTGACGGTACAG---GCCAGACAA---TTATTG---TCTGGTATAGTGCAACAGCAAAGC---
AATTTG---CTGAGA---GCTATACAGGCGCAACAGCATATGTTGCAGCTCACGGTCTGGGGTATT
>AF190128
TTCTATTGCAATACATCAGGGCTGTTTAATAGTAGTTGGACTGGGGACAATATTAATATGCCAAATGAC----
----

ACAGGAAAAAATATTACACTGCCATGCAGAATAAAACAAATTGTAAATATGTGGCAGAGAGTAGGACAAGCAA
TGTATGCTCCTCCCATTAAAGGAAGCATTACATGTGTATCAAAT---
ATTACAGGATTAATCCTGACTTATGACGAAGATAAGGGAAATAAT----------------------------
----

GATAATGTTACCTTCAGACCTGGAGGAGGAGATATGAGGGACAATTGGAGAAGTGAATTATATAAATATAAAG
TAGTAAAAATTGAACCACTG---GGAGTAGCACCCACCGAGGCAAGGAGAAGAGTGGTG--------------
-------------------------GAGAGAGAAAAAAGAGCT---GTGGGA---ATGGGAGCT---------
TTTTTCCTTGGGTTCTTGGGAGCA---GCTGGAAGCACTATG---GGCGCAGCGTCA---
ATAACGCTGACGGTACAG---GCCAGGCAA---TTATTG---TCTGGTATAGTGCAGCAGCAAAGC---
AATTTG---CTAAGA---GCTATACAGGCGCAACAGCACATGTTGCAGCTCACGGTCTGGGGTGTT
>KY392777
TTCTATTGCGATACAACAGACTTGTTTAATAGCACCTGGATCAATAGCAGCTATAGCACTAACTACAATAAT-
----

AGCAACTACAATATCACACTGACATGCAAGATAAAACAAATTGTGAGAATGTGGCAGAGAGTAGGACAAGCAA
TGTATGCCCCTCCCATCAAAGGACAGATTACATGTGTATCAAAT---
ATTACAGGACTAATTTTGACAAGAGACGGTGGTAATGGGTCCAAT----------------------------
----

GTTAGTGATACCTTCAGACCAGGAGGGGGAGATATGAGGGATAATTGGAGAAGTGAATTATATAAATATAAAG
TAGTAAAAGTTGAACCACTA---GGAGTAGCACCAACTAAGGCAAGGAGAAGAGTGGTG--------------
-------------------------GAGAGAGAAAAAAGAGCT---GTGGGA---ATAGGAGCT---------
TTGTTCCTTGGTTTCTTGGGAGCA---GCAGGAAGCACTATG---GGCGCAGCGTCT---

GTAACGCTGACGGTACAG---GCCAGACAA---CTATTG---TCTGGTATAGTGCAACAGCAAAGT---
AATTTG---CTGAGA---GCTATACAAGCGCAACAGCATATGTTGCAGCTCACGGTCTGGGGTGTT
>KY392778
TTCTATTGTAATACATCAAGGCTATTTAATAGTACTTGGCTATCCAATAGTACTTGGAACTCGACTGACATGC
CAAGT---

AACACATCAAATATAACACTGCCATGCAGAATAAAACAAATTGTAAACATGTGGCAAAGAGTAGGACAAGCAA
TGTATGCCCCTCCCATCCAAGGAAAACTTCAATGTGTATCAAAT---
ATTACAGGCTTAATATTGACAGTAGATAATGCTAACATGTCTGCA---------------------------
----

GCTAACTTTACCTTCAGACCTGGAGGAGGAGATATGAAGGACAATTGGAGGAGTGAACTATATAAGTATAAAG
TAGTAAAAATTGAACCATTG---GGAATTGCACCAACCAGGGCAAGGAGAAGAGTGGTG-------------
-------------------------GAGAGAGAAAAAAGAGCT---GTGGGA---ATGGGAGCT---------
TTTTTCCTTGGGTTCTTGGGAGCA---GCAGGAAGCACTATG---GGCGCGGCGTCA---
ATAACGCTGACGGTACAG---GCCAGGCAA---TTATTG---TCTGGTATAGTGCAGCAGCAAAGC---
AATTTG---CTGAGA---GCTATACAGGCGCAACAGCATATGTTGCAGCTCACGGTCTGGGGTGTT
>KU168279
TTCTATTGCAATACATCAAAATTATTTAATAGTACCTGGGACGCCAACAAAACCTCTAGCACA---------
----

AACAATACAATGATCACACTGCCATGCAGAATAAAACAAATTATAAAGATGTGGCAGAGAGTAGGACAGGCAA
TGTATGCCCCTCCCATCAGCGGAGATATTACATGTATATCAAAT---
ATTACAGGATTAATATTGACAAATGATGAGGGTAATATGTCTGCA---------------------------
----

GAGAATTTTACTTTCAGACCTGTAGGAGGAGAGATGAGGGACAATTGGAGAAGTGAATTATATAAATATAAAG
TAGTAAAGATTGAACCACTG---GGAGTAGCACCCACCAAGGCAAGGAGAAGAGTGGTG-------------
-------------------------GAGAGAGAAAAAAGAGCT---GTGGGA---ATGGGAGCC---------
TTTTTCCTTGGGTTCTTGGGAGCA---GCAGGAAGCACTATG---GGCGCGGCGTCA---
CTAACGCTGACGGTACAG---GCCAGACAA---TTATTG---TCTGGTATAGTGCAACAGCAAAGC---
AATTTG---CTGAAA---GCTATACAGGCGCAACAGCATATGTTGCAGCTCACGGTCTGGGGTGTT
>MH327752
TTCTATTGCAATACAACACAATTATTTAATTGCACAAACAATAATACAAACAATAATGACACCGAAGTG----
----

TGTAATGACCCTATCATACTTCCATGCAAGATAAAGCAAATTATAAACATGTGGCAGGGAGTAGGACAAGCAA
TGTATGCTCCTCCCGTCAGGGGAATAATTAATTGTGTATCAAAC---
ATTACAGGAATACTGTTGACAAGGAATGGTGGGAATAATAGCACG---------------------------
----

AACGAGAGCGAGACCTTCAAACCTGAAGGAGGAAATATAAAAGACAATTGGAGAAATGAATTATATAAATATA
AAGTAGTACAAATTGAACCACTA---GGAGTGGCACCCACCAGGGCAAGGCGAAGAGTGGTG----------
----------------------------GAAAGAGAAAAAAGAGCA---GTGGGG---ATAGGAGCT------
---ATGATCTTTGGGTTCTTAGGAGCA---GCAGGAAGCACTATG---GGCGCGGCGTCA---
ATAACGCTGACGGTACAG---GCCAGACAA---TTATTG---TCTGGTATAGTGCAACAGCAAAGC---
AATTTG---CTGAGG---GCTATAGAGGCGCAACAGCATATGTTGCAACTCACAGTCTGGGGTATT
>MF373147
TTCTATTGCAATACAACAAAACTGTTTAATAATACTTGCATTAATGAAACCATAAGTGGA------------
----
-------------------AGT---
GAAGGTACCGTCACCATCCCATGTCCGAGAAGGCTAAATAAAAATATGAGGATGGGGCAAGGACTAGGACTGG
AAGCGCATGCCCCTCCCGTAACTATAAATTTTGTCTGTGAATTTAAC---
GTTACACGAATACCAATGACTAGAGATAGTGGTATTAATAATACTAATACT---------------------
----

ACCTACACACCCGCACGAGGAAGAAGAAAGGACAATGACAGATGTAAAATGGATATATATAAATATAAACTAG
TTGAACCTGTACGACTA---GGAATAACATCCGCAAAGACAAAA------ATGATA----------------
---------------------GAGATAGAGAGAAAAGAAATAGCACTGGGAGTTATAACT---------
ATTATCTTCGTGTTCTTGGCAGCA---ACCAGTATGACTACG---GCCTCGGCGTCG---

CTGACGCTGACGGCACAG---ACTTACCAG---TCAGTG---ACAGGAGTATACTTGCAAATCAAT---
ATGCTG---ATGCCT---ATAGATGAGCAGCAGCATCTAGCTCTGCTGCTACTCATAGTCTGGTGT
>EU743757
TTTTATTGCAATACATCAAAACTGTTTAATACTACTGTCCTAAAAAATGGAACT-------------------
----

ATGAGTGGTAATATCACAATTCCATGCAAGATAAAGCAAATTATAAACATGTGGCAGGGAGTAGGACAAGCAA
TGTATGCTCCTCCCATCAGTGGAACAATTAATTGTGTATCAAAT---
ATTACAGGAATACTATTGACAAGAGATGGTGGTGAAGGGACTAAGAAT------------------------
----

AATGAGACTGAGACCTTCAGACCTGGAGGAGGAAATATAAAGGACAATTGGAGAAGTGAATTATATAAATATA
AAGTAGTACAAATTGAACCACTA---GGAATAGCACCCACCAGGGCAAAGAGGAGAGTGGTG-----------
---------------------------GAGAGAGAAAAAAGAGCA---GTGGGA---ATAGGAGCT------
---ATGATCTTTGGGTTCTTAGGAGCA---GCAGGAAGCACTATG---GGCGCGGCGTCA---
ATAACGCTGACGGTACAG---GCCAGACAG---TTATTG---TCTGGTATAGTGCAACAGCAAAGC---
AATTTG---CTGAGG---GCTATAGAGGCGCAGCAGCATATGTTGCAACTCACAGTCTGGGGTATT
>KU230430
TTCTATTGCAATACATCAAGATTGTTTAATGGTACAGGAAATGGAACCATGGAGGGGCCTAATGGC-------
----

ACTAATGGCACTGTCATACTCCCATGCAGGATAAAGCAAATTGTAAACATGTGGCAGGGAGCAGGACAGGCAA
TGTATGCTCCTCCCATCAGGGGAATAATTAATTGTGTATCAAAT---
ATTACAGGGATATTATTAACAAGAGATGGTGGTAAGAATACTACG---------------------------
----

AATAACGAGACCTTCAGACCTGGAGGAGGAAATATAAAGGACAATTGGAGAAGTGAATTATATAAATATAAAG
TAGTAGAACTTGAACCACTA---GGAATAGCACCCACCAGGGCAAGGAGGAGAGTGGTG-------------
------------------------GACAGAGAAAAAAGAGCA---GTGGGA---ATAGGAGCT---------
ATGATCTTTGGGTTCTTAGGAGCA---GCAGGAAGCACTATG---GGCGCAGCATCA---
ATAACGCTGACGGTACAA---GCCAGACAA---TTATTG---TCTGGTATAGTGCAACAGCAAAGC---
AATTTG---CTGAGG---GCTATAGAGGCGCAGCAGCATCTGTTGCAACTCACAGTCTGGGGTATT
>AF015916
TTCTATTGCAACACAACACGACTGTTTAATAATACTTGCGTAGAGAATGGAACCAGGGAGGGG---------
----

TGTAATGGCAATATCACACTTCCATGCAAGATAAAGCAAATTATAAACATGTGGCAGGGAGCAGGACAAGCAA
AATATGCTCCTCCCATCAGTGGAACAATTAATTGTGTATCAAAT---
ATTACAGGAATACTATTGACAAGAGATGGTGGTGCAAATAATGCAGCGAGT---------------------
----

AACAACGGGACCGAGACCTTCAGACCTGGAGGAGGGAATATAAAAGACAATTGGAGAAGTGAATTATATAAAT
ATAAAGTAGTACAAATTGAACCACTA---GGAATAGCACCCACCAGGGCAAAGAGAAGAGTGGTG--------
------------------------------GAGAGAGAAAAAAGAGCC---GTGGGA---ATAGGAGCT---
------ATGATCTTTGGGTTCTTAGGAGCA---GCAGGAAGCACTATG---GGGGCAGCATCA---
ATAACGCTGACGGTACAG---GCCAGACAA---TTATTG---TCTGGTATAGTGCAACAGCAAAGC---
AATTTG---CTGAGG---GCTATAGAGGCACAGCAGCATCTGTTGCAACTAACAGTCTGGGGGATT
>EU513182
TTCTATTGCAATACATCAAGACTGTTTAATAGCACTTGGAATAATAGTAGCATTTGGGATAATAGCACTGGGG
ACTATAAC---
-------------AGCACT------
AAGGACAACGAGACAATAACTCTCCCATGCAGAATAAAGCAAATTATAAATATGTGGCAGAAAGTAGGACAAG
CAATGTATGCCCCTCCCATCAAAGGAATAATAAGGTGTGATTCAAAC---
ATTACAGGACTACTATTAACAAGGGATGGAGGGATTAATAATAGT---------------------------
----

ACGAGTGAGACATTCAGGCCTGGAGGAGGAGATATGAGGGACAATTGGAGAAGTGAATTATATAAGTATAAGG
TAGTACAAATTGAACCAATA---GGTTTGGCACCCACCCATGCAAAAAGGAGAGTGGTG-------------
------------------------GAGAGAGAAAAAAGAGCA---GTTGGA---CTGGGAGCT---------
CTTTTCCTTGGGTTCTTAGGAGCA---GCAGGAAGCACTATG---GGCGCGGCGTCA---

ATGACGCTGACGGTACAG---GCCAGACAA---TTATTG---TCTGGTATAGTGCAACAGCAGAGC---
AATTTG---CTGAGG---GCTATAGAGGCTCAACAACATCTGTTGAAACTCACGGTCTGGGGCGTT
>AF119819
TTCTACTGCAATACATCAGACCTGTTTAATAGGACATACATGGTTAATAAAAACGAAACAAATAGTACAAACA
CG---

ACAGATGAAAAAATTATCAGGCTTCCATGCAGAATAAAACAAATTGTAAACAGGTGGCAGGAAGTGGGACAAG
CAATATACGCCCCCCCATTGCAGGGATCATTAACTGCAACTCAAGTT---
ATTACAGGAATAATATTGACAAGAGATGGTGGTAATAGAAGTGAT----------------------------
----

ACTGGCAATGAGACCTTCAGACCTGGAGGAGGAGACATGAGGGACAATTGGAGAAGTGAACTATATAAATATA
AAGTAGTACAAATTGAACCAGTA---GGAGTAGCACCCACTAGGGCAAGGAGACGGGTGGTG-----------
----------------------------CAGAGAGAAAAACGAGCA---GTGGGAATAGGAGCTGTG------
---TTCCCTGGCTTGTTCTTGGGAGCA---GCAGGAAGCACTATG---GGCGCAGCGTCA---
ATAACGCTGACGGTACAG---GCCAGGCAA---TTGTTG---TTTGGCATAGTGCAACAGCAGAGC---
AATCTG---CTGAGG---GCTATAGAGGCTCAACAACAGCTGTTGAGACTCACGGTCTGGGGCGTT
>AF193253
TTCTACTGCGATACATCAAAATTGTTTAATGCCACAGTGTTTAATGATACAGTGTTTAATGCCACAATGTTTA
ATAAT--

GACAGTGACAAGAATATCATTCTCCCATGTAAAATAAAACAAATAGTAAGAATGTGGCAGGGAGTAGGACAAG
CAATGTATGCCGCTCCCATTGCAGGAAACATTGCCTGTAACTCAACT---
ATTACAGGGATACTATTGGCAAGAGATGGTGGAAATGGTAATGATAGC-------------------------
----

TCCAACGACACAGAGATATTCAGACCTGGAGGAGGAGATATGAGAGACAATTGGAGAAGTGAACTATATAAAT
ATAAAGTAGTAGAGATTCAACCACTA---GGAATAGCACCCACCAGGGCAAAAAGACAAGTGGTG--------
-------------------------------AAGAGAGAAAAAAGAGCA---ATGGGA---ATAGGAGCT---
------ATGTTCCTTGGGTTCTTGGCAGCA---GCAGGAAGCACTATG---GGCGCGGCGTCA---
ATAGCGCTGACGGTACAG---GCCAGACAA---TTATTG---TCTGGAATAGTGCAACAGCAAAAC---
AATTTG---CTGCAG---GCTATTGAAGCGCAACAGCATCTGTTGCAGCTCACAGTCTGGGGTATT
>JF346906
TTCTATTGTAATACATCACGCCTGTTTAATAGTACATACATGACTAATGGCACATACATGTTTAATGGTACAC
AA---

AATAATTCAGACATCACAATCCCATGCAGAATAAAGCAAATTATAAATATGTGGCAGGAGGTAGGACGAGCAA
TGTATGCCCCTCCCATTGCAGGAAACATAACATGTAACTCAAGT---
ATCACAGGACTACTATTGATACGTGATGGAGGAAATACAACTGAGTCA-------------------------
----

AATACTACAGAGATATTCAGACCTGGAGGAGGAGATATGAGGGACAATTGGAGAAATGAATTATATAAATATA
AAGTGGTAGAAATTAAGCCATTG---GGAGTAGCACCCACTGACGCAAGAAGGAGAGTGGTG-----------
----------------------------GAGAGAGAAAAAAGAGCA---GTGGGA---ATAGGAGCT------
---GTGTTCCTTGGGTTCTTGGGAGCA---GCAGGAAGCACTATG---GGCGCGGCGTCA---
ATAACGCTGACGGTACAG---GCCAGACAA---TTGCTG---TCTGGTATAGTGCAACAGCAAAGC---
AATTTG---CTGAGG---GCTATAGAGGCGCAACAGCATATGTTGCAACTCACAGTCTGGGGTATT
>JF346914
TTCTATTGTAATACATCAGGCCTGTTTAATGGTACAAGAAAGTTTAATAGTACAGGAAGTAAT----------
----

TCAAGCTCAATCATCACAATCCCATGCAGAATAAAGCAATTTATAAATATGTGGCAGGAGGTAGGACGAGCAA
TGTATGCCCCTCCCATTGCAGGAAACATAACATGTAGATCAAAT---
ATCACAGGACTACTATTGGTACGTGATGGAGGAAATTCAAGTGGA----------------------------
----

ACAGAAGAGACATTCAGACCTCAAGGAGGAGATATGAGGGACAATTGGAGAAGTGAATTATATAAATATAAAG
TGGTAGAAATTAAGCCATTG---GGAGTAGCACCCACTGCAGCAAAAAGGAGAATAGTG--------------
-------------------------GAGAGAGAAAAAAGAGCA---GCG------ATAGGAGCT---------
GTGTTCCTTGGGTTCTTGGGAGCA---GCAGGAAGCACTATG---GGCGCGGCGTCA---

ATAACGCTGACGGTACAG---GCCAGAAAA---TTGTTG---TCTGGTATAGTGCAACAGCAAAGC---
AATTTG---CTGAGA---GCTATAGAGGCGCAACAGCATCTGTTGCAACTCACGGTCTGGGGTATT
>HQ326146
TTCTATTGTAATACTTCAGGACTGTTTAATGGTACATACAGGTCTAATGATACAAAAGGTAAT----------
----

TCAAGCTCAATCATCACGATTCCATGCAGAATAAAACAAATTATAAATATGTGGCAGGAGGTAGGACGAGCAA
TGTATGCCCCTCCCATTGAAGGAAACATAACATGTAAATCAAAT---
ATCACAGGACTACTATTGGTACGTGATGGAGGAGCGACAAGTGCG----------------------------
----

GGAACAGAGACATTCAGACCTGAAGGAGGAGATATGAAGGACAATTGGAGAAGTGAATTATATAAATATAAAG
TGGTAGAAATTAAACCATTG---GGAGTAGCACCCACTGGAGCAAAAAGGAGAGTGGTG--------------
-------------------------GAGAGAGAAAAAAGAGCA---GTGGGA---CTGGGAGCT---------
GTGTTCTTTGGGTTCTTGGGAGCA---GCAGGAAGCACTATG---GGCGCGGCGTCC---
ATGACGCTGACGGTACAG---GCCAGACAA---TTGCTG---TCTGGTATAGTGCAACAGCAAAGC---
AATTTG---CTGAAG---GCTATAGAGGCGCAACAACATCTGTTGCAACTCACGGTCTGGGGCACT
>AF408627
TTCTACTGCAATACATCAAGACTGTTTAATGACACAATACCC-------------------------------
----

AATGGCACTATTATTCTCCCATGTCGAATAAAACAAATTGTAAACCTGTGGCAAGAAGTGGGACGAGCAATGT
ATGCCGCTCCCATTGCAGGAAACATTACCTGTAGCTCAAAC---
ATTACAGGTCTACTATTGACAAGAGATGGTGGTCATACTCCTAATCAGACT----------------------
----

GAGAATAGTACTGAGACCTTCAGACCTGGGGGAGGAAATATGAAAGACAATTGGAGAAGTGAATTATATAAAT
ATAAAGTAGTAGAAATTGAACCACTA---GGAGTAGCACCCACCAGGGCAAAAAGACAAGTGGTG--------
-------------------------------CAGAGAGAAAAAAGAGAAACAGTGGTGGGAATAGGAGCG---
------TTGTTCCTTGGGTTCTTGGGAGCA---GCAGGAAGCACTATG---GGCGCGGCGTCA---
ATGGCGCTGACGGTACAA---ACCAGACAA---TTATTG---TCTGGGATAGTGCAACAGCAGAAC---
AATTTG---CTGAGG---GCTATTGAAGCGCAACAGCATCTGTTGCAGCTCACAGTCTGGGGCGTT
>DQ354120
TTCTACTGTAATACATCACAACTGTTTAATAGTACTTGGAATGGTACTAGTGCTTGGAATTATACTACA----
----

GAAAATGACACTATCAGGCTCCCTTGCAGAATAAAACAAATTATAAACATGTGGCAGGAAGTAGGAAAAGCAA
TGTATGCCCCTCCCATTGCAGGACTAATTAGATGTTCATCAAAT---
ATTACAGGGTTGCTATTAACAAGAGATGGTGGTAATAATACGAAC----------------------------
----

ACCGAGACCTTCAGACCTGCAGGAGGAGATATGAGGGACAATTGGAGAAGTGAATTATATAAATATAAAGTAG
TAAAAATTGAACCATTA---GGAGTAGCACCCACCAGGGCAAAGAGAAGAGTGGTG-----------------
----------------------CAAAGAGAAAAAAGAGCA---GTGGGAACGATAGGAGCT---------
ATGTTCCTTGGGTTCTTGGGAGCA---GCAGGAAGCACTATG---GGCGCAGCGTCA---
ATAACGCTGACGGTACAG---GCCAGACAA---TTGTTG---TCTGGTATAGTGCAACAGCAGAGA---
AATTTG---CTGAAG---GCTATTGAGGCGCAACAGCATCTGTTGCAACTCACAGTCTGGGGTATT
>JF804807
TTCTACTGTAATACATCAAAGCTGTTTAATAGTACTTGGACAGGGAATGACACTGGGACTAAT----------
----

ACTACTGGTAATATCACACTCCCATGCAGAATAAAGCAAATTGTAAACATGTGGCAGGAAGTAGGAAAAGCAA
TGTATGCCCCTCCCATCACAGGACAAATTAGTTGTTCATCAAAT---
ATTACAGGGCTGCTATTAACAAGAGACGGTGGTAACTATACCAAG----------------------------
----

GACAATGAGACCTTTAGACCTGCAGGAGGAGATATGAGGGACAATTGGAGAAGTGAATTATATAAATATAAAG
TAGTAAAGATTGAGCCATTA---GGAGTAGCACCCACCAAGGCAAAGAGAAGAGTGGTG--------------
-------------------------CAGAGAGAAAAAAGAGCA---GTGGGAACATTAGGAGTT---------
CTGTTCCTTGGGTTCTTGGGAGCA---GCAGGAAGCACTATG---GGCGCAGCGTCA---

GTGGCGCTGACGGTACAG---GCCAGACAA---TTATTG---TCTGGCATAGTGCAACAGCAAAAC---
AATTTG---CTGAGA---GCTATTGAGGCGCAACAACATCTGTTGCAACTCACAGTCTGGAGCGTC
>KX907411
TTCTACTGTAATACATCAAACCTGTTTAATAATACATGGAATAGTAGCAGTTGGACAGGGGAA----------
----

AATAACTCAACAATCACAATACCATGCAGAATAAAACAAATTATAAACATGTGGCAGAGAGTAGGAAAAGCAA
TGTATGCTCCTCCCATCGAAGGAGAAATTAAATGTTCATCAAAT---
ATTACAGGGCTACTATTGACAAGAGATGGAGATGGTGGTAACAATAAT------------------------
----

AGTTCTCAGGAGACCTTCAGACCTGGAGGAGGAAATATGCAAGACAATTGGAGAAGTGAATTATATAAATATA
AGGTAGTAAAAATTGAGCCACTA---GGAATAGCACCCACCAAGGCAAAAAGAAGAGTGGTG----------
---------------------------GAAAGAGAAAAAAGAGCA---ATAGGA---TTAGGAGCT------
---ATGTTCCTTGGGTTCTTGGGAGCA---GCAGGAAGCACGATG---GGCGCAGCGTCA---
ATGGCGCTGACGGTACAG---GCCAGACAG---TTATTG---TCTGGTATAGTGCAACAGCAAAAT---
AATTTG---CTGAGG---GCTATAGAGGCGCAACAGCATCTGTTGCAACTCACGGTCTGGGGCGTT
>JN417240
TTCTATTGCAATACATCAGGCCTGTTTAATAGCATTTGGAATAGCAGCACATGGGAGTCAAATAGCACAGAGT
TAAAT--
-------------AGCACA------
GAGTTAAATGACAATATAACTCTCCAATGCAGAATAAAACAAATTATAAACATGTGGCAGGGAGTAGGAAAAG
CAATGTATGCCCCTCCCATTGAAGGAAAGATTATATGTAGATCAAAT---
ATTACAGGACTATTATTAACAAGAGATGGTGGTGCAAATAATACT---------------------------
----

AATCAGAATGAGACTCTCAGACCTGGGGGAGGAGATATGAGAGACAATTGGAGAAATGAATTATACAAATATA
AAGTAGTAAGACTTGAGCCACTA---GGTATAGCACCCACCAGGGCAAGAAGAAGAGTGGTG----------
---------------------------GCAAGAGAAAAAAGAGCA---ATAGGA---CTAGGAGCT------
---ATGTTCCTTGGGTTCTTGGGAGCA---GCAGGAAGCACAATG---GGCGCAGCGTCA---
CTGACGCTGACGGTACAG---GCCAGACAG---CTATTG---TCTGGTATAGTGCAGCAGCAAAAT---
AATTTG---CTGAGG---GCTATAGAGGCGCAACAACATCTGTTGCAACTCACAGTCTGGGGCGTT
>KC898991
TTCTATTGTAATACATCAAGGCTGTTTAATGGTACATTTAATGGTACATTCATACCTAATGGTAACATG----
----

ACTAATGAAACAATCACAATTCCATGCAGAATAAAGCAGATTATAAACATGTGGCAGAAGGTAGGACAAGCAA
TGTATGCCCCTCCCATTGCAGGAAACATAACATGTATATCAAAT---
ATCACAGGATTACTATTGGTACGTGATGGAGGAATAAATCAGAGT---------------------------
----

TCAGAGACATTCAGACCTGGAGGAGGAGATATGAGGGACAATTGGAGAAGTGAATTATATAAATATAAAGTGG
TAGAAATTAAGCCATTG---GGAGTAGCACCCACTGAAGCAAAAAGGAGAGTGGTG----------------
----------------------GAGAGAGAAAAAAGAGCAGTATTTGGAGGAATAGGAGCT---------
GTGTTCCTTGGGTTCTTGAGTGCA---GCCGGAAGCACTATG---GGCGCGGCGTCA---
ATAACGCTGACGGTACAG---GCCAGACAA---TTGCTA---TCTGGTATAGTGCAACAGCAAAGC---
AATTTG---CTGAGG---GCTATAGAGGCGCAACAGCATATGTTGCAACTCACAGTCTGGGGTATT
>KC898994
TTCTATTGTAATACATCAGGCCTGTTTAATGGTACCTACAATGATACAGAAAATAATTCAACTAATAAT----
----

TCAAACTCAACCATCACAATCCCATGCAGAATAAAGCAAATTATAAACATGTGGCAGGAGGTAGGACGAGCAA
TGTATGCCCCTCCCATTGAAGGAAATATAACATGTAAATCAAAT---
ATCACAGGACTACTATTGGTATATGATGGAGGAAGAACAGAGCGA---------------------------
----

AATACTACAGAGATATTCAGACCTGGAGGAGGAGATATGAGAGACAATTGGAGAAGTGAATTATATAAATATA
AAGTGGTAGAAATTAAGCCATTG---GGAGTAGCACCCACTGGAGCAAAAAGGAGAGTGGTG----------
---------------------------GAGAGAGAAAAAAGAGCA---GTGGGA---ATAGGAGCT------
---GTGTTCCTTGGGTTCTTGGGAGCA---GCAGGAAGCACTATG---GGCGCGGCGTCA---

ATAACGCTGACGGTACAG---GCCAGACAA---TTGTTG---TCTGGTATAGTGCAACAGCAAAGC---
AATTTG---CTGAGG---GCTATAGAGGCACAACAGCATCTGTTGCAACTCACGGTCTGGGGTATT
>MH986013
TTCTATTGTAATACATCAAAACTGTTTGATAGTACTTGGGCTCGGAATACCAGTACTGGGAATAATGCCACTT
GG--
-AATAAGACTGGGATAGC------
ACTAATGATACTCATATCATACTCCCATGCAGAATAAAACAAATTATAAATATGTGGCAGGAAGTAGGAAAAG
CAATGTATGCCCCTCCCATCAGTGGAAAAATTAGCTGTTTATCAAAT---
ATTACAGGGCTTCTATTAATAAGGGATGGTGGTGAAAACAGGACCAAT------------------------
----

AATGAGACAGAGACCTTCAGACCTGGAGGAGGAAATATGAAGGACAATTGGAGAAGTGAATTATATAAATATA
AAGTAGTAAGAATTGAGCCATTA---GGAGTAGCACCCACCAAGGCAAAGAGAAGAGTGGTG----------
---------------------------CAGAGAGAAAAAAGGGCA---GTGGGAACACTAGGAGCT------
---ATGTTCCTTGGGTTCTTGGGAGCA---GCAGGAAGCACTATG---GGCGCAGCGTCC---
TTGACGCTGACGGTACAG---GCCAGGCAA---TTATTG---TCTGGTATAGTGCAACAGCAGAAC---
AATTTG---CTGAGA---GCTATTGAGGCGCAACAACATCTGTTGCAACTCACAGTCTGGGGTATC
>MN055644
TTCTATTGTAATACATCAGGCATGTTTAACGGCACAGACAAGTTTAATGATTCAAGC---------------
----

ATTACAATCCCATGCAGAATAAAACAAATTATAAATATGTGGCAGGAGGTAGGACGAGCAATGTATGCCCCTC
CCATTGCAGGAAACATAACATGTAAATCAAAT---
ATCACAGGACTACTATTGGTACGTGATGGAGGACGGACAAATGAT---------------------------
----

ACTGAAGAGACATTCAGACCTGGAGGAGGAGATATGAGGGACAATTGGAGAAGTGAATTATACAAATATAAAG
TGGTAGAAATTAAGCCATTG---GGAATAGCACCCACTGAAGCAAAAAGAAGAGTGGTG-------------
-------------------------CTGAGAGAAAAAAGAGCA---GTGGGA---ATAGGAGCT--------
GTGTTCCTTGGGTTCTTGGGAGTA---GCAGGAAGCACTATG---GGCGCGGCGTCA---
CTGACGCTGACGGTACAG---GCCAGACAA---TTGCTG---TCTGGTATAGTGCAACAGCAAAAT---
AATTTG---CTGAGG---GCTATTGAGGCGCAACAGCATATGTTGCAACTCACAGTCTGGGGTATT
>KF716464
TTCTATTGCAATACATCAGAACTATTTAATAGTATTTGGAATAGCACTTGGAATAACACTCAGGACAACAGC-
----

ACAAGTCCAAATATAACTCTCCAATGCAGAATAAAGCAAATTGTAAGAATGTGGCAGCAAGTAGGAAAAGCAA
TGTATGCCCCTCCCATTCCAGGAATAATAAGGTGTGTATCAAAC---
ATTACAGGACTACTATTAACAAGAGACGGTGGGACTAATATGACA---------------------------
----

AATGAGACTTTCAGACCTGGAGGAGGAGATATGAGAGACAATTGGAGAAGTGAATTATATAAATATAAAGTAG
TAAAAATTGAACCACTA---GGAGTAGCACCCACCAGGGCAAAGAGAAGGGTGGTG----------------
---------------------CAGAGAGAAAAAAGGGCA---GTTGGA---CTGGGAGCT---------
GTTTTCCTTGGGTTCTTAGGAGCA---GCAGGAAGCACTATG---GGCGCAGCGTCA---
ATAACGCTGACGGTACAG---GCCAGACAA---TTATTG---TCTGGCATAGTGCAACAGCAAAGC---
AATTTG---CTGAGG---GCTATAGAGGTTCAACAGCATATGTTGAAACTCACAGTCTGGGGCGTT
>JX447830
TTCTATTGTAATACAACACAACTGTTTAATAGTACTTGGCCTTCAAATGATACTAGTGCT------------
----

AGGACTGGTACTATCACGCTCCCTTGCAAAATAAAACAAATTATAAACATGTGGCAGGGAGCAGGACAAGCAA
TGTATGCTCCTCCCATCAGGGGAAAAATTAATTGTCTATCAAAT---
ATTACAGGAATACTGTTGACAAGAGATGGTGGTAATAGTACAAGT---------------------------
----

AATGAGACCTTTAGACCTGGAGGAGGAGATATGAGGGACAATTGGAGAAGTGAATTATATAAATATAAAGTAG
TAAAAATTGAACCATTA---GGAGTAGCACCCACCAGGGCAAAGAGAAGAGTGGTG----------------
---------------------CAGAGAGAAAAAAGAGCG---GTGGGAACACTAGGAGCT---------
ATGTTCCTTGGGTTCTTGGGAACA---GCAGGAAGCACTATG---GGCGCAGCGTCA---

ATAACGCTGACGGTACAG---GCCAGACAA---TTGTTG---TCTGGTATAGTGCAACAGCAGAGA---
AATTTG---CTGAGG---GCTATCGAGGCGCAACAGCATCTGTTGCAACTCACAGTCTGGGGTATC
>AY082968
TTCTATTGCAATACAACACAAATGTTTAATAATACTTGCATAGGAAATGAAACCAAGGAGGGG----------
----

TGTAATGGCACTATCATACTTCCATGCAAGATAAAGCAAATTATAAACATGTGGCAGGGAGCAGGACAAGCAA
TGTATGCTCCTCCCATCAGGGGAAGAATTAGTTGTGTATCAAAT---
ATTACAGGAATACTATTGACAAGAGATGGTGGTGCTGATACAACA----------------------------
----

AATAATGAGACCTTCAGACCTGGAGGAGGAAATATAAAGGATAATTGGAGAAGTGAATTATATAAATATAAAG
TAGTACAAATTGAACCACTA---GGAATAGCACCCACCAGGGCAAAGAGAAGAGTGGTG--------------
-------------------------GAGAGAGAAAAAAGAGCA---GTGGGA---ATAGGAGCT---------
ATGATCTTTGGGTTCTTAGGAGCA---GCAGGAAGCACTATG---GGCGCGGCGTCA---
ATAACGCTGACGGTACAG---GCCAGACAA---TTATTG---TCTGGTATAGTGCAACAGCAAAGC---
AATTTG---CTGAGG---GCTATAGAGGCGCAGCAGCATCTGTTGCAACTCACAGTCTGGGGCAGT
>KU310618
TTCTATTGCAATACATCACAGCTATTTAATAGTACTTGGAATAGCAATAACACGTCAGATGGCACA-------
----

TCAAATATTACACTTACACTGAAATGCAGAATAAAACAAATTATAAACATGTGGCAGAGAGTAGGACAAGCAA
TGTATGCCCCTCCCATCCCGGGAAGTCTTACATGTGTATCAAAT---ATTACAGGTTTAATATTGACA-----
-------ATTGACAAGGTTAACAGGTCT---

GAGGCTAACGGAAGTCTTACCTTCAGGCCAGGAGGAGGAGATATGAGGGACAATTGGAGAAGTGAGCTATATA
AATATAAAGTAGTAAAAATTGAACCACTG---GGAATAGCACCCACCAAGGCAAGGAGAAGAGTGGTG-----
----------------------------------CAGAGAGAAAAAAGAGCT---GTGGGA---
ATGGGAGCT---------TTGTTCCTTGGGTTCTTGGGAGCA---GCAGGAAGCACTATG---
GGCGCGGCGTCA---ATAACGCTGACGGTACAG---GCCAGGCAA---TTATTG---
TCTGGTATAGTGCAACAGCAAAGC---AATTTG---CTGAAA---
GCTATACAGGCGCAACAGCATATGTTGCAGCTTACAGTCTGGGGTATT
>KX398187
TTTTATTGTGACACATCTGGGATGTTTAATTATACCTTTAATTGTACAGGAACCAGCTGCGATAACAAAACTA
GT---

ACTGAGAATAGTACCACAATATTTTGCAAGATAAAACAGGTGGTAAGATCATGGATGAGGGGAGCGTCAGGAC
TCTATGCCCCTCCCATTCGAGGTCAACTAAAATGTATGTCAAAT---
ATAACTGGGATGATTCTACAAATGGATAAGCCATGGAACAATACT----------------------------
----

GACGAGAATGCTACATTTAGACCAGGAGGGGGAAATATGGAAGATATATGGAGAACACAATTGTTCAATTATA
AAGTAGTAAGGGTAAAACCTTTT---AGTGTAGCACCTACAAAAATTGCAAGGCCAGTCATAGGC---ACT--
----------------------GGTACTCATAGAGGAAAGAGAGCA---GTAGGG---TTGGGAATG------
---CTATTCTTAGGGGTTCTAAGTGCA---GCAGGTAGCACTATG---GGCGCAGCGGCA---
ACAGCGCTGACGGTACAG---ACCCACACT---TTGATG---AAGGGTATAGTGCAACAGCAGGAC---
AACCTG---CTAAGA---GCAATACAGGCTCAGCAGCAACTGCTGAAGCTATCTGTATGGGGTATC
>AJ239083
TTCTATTGTAACACATCTGGGATGTTTAATTATACCTTTTCATGTAAAGGAACTAACTGTACCCAAGTTGGTT
CCCAAAAT---
-------------------GAA---
TATAATAATCATACAACCAAGATACCTTGCAGGATAAAACAGGTGGTAAGGTCATGGATAAGGGGAGGGTCGG
GACTCTATGCACCTCCCAGGCAAGGTCCCCTAAAATGTAGCTCAAAC---
ATAACTGGAATGATTCTACAATTGGATAAGCCATGGAACAGAAGTGGG-------------------------
----

CACAACAATGACACCACATTTAGACCAATAGGAGGAGAAATGAAAGATATATGGAGAACTGAATTGTTCAAAT
ACAAAGTAGTAAAGGTAAAACCTTTT---AGTGTGGCACCTACAAAAATTGCAAGGCCAGTCATAGGC---
ACG------------------------GGCACTCAAAGAGAAAAGAGAGCA---GTAGGA---TTGGGAATG-
--------CTATTCTTAGGGGTTCTAAGTGCA---GCAGGTAGCACTATG---GGCGCAGCGGCA---

ACAACGCTGGCGGTACAG---ACCCACACT---TTGATG---AAGGGTATAGTGCAACAGCAGGAC---
AACCTG---CTAAGA---GCAATACAGGCCCAGCAGCAATTGCTGAGGCTATCTGTATGGGGTATC
>KX907367
TTCTATTGTAATACATCAGGCCTGTTCAACAGCACTTGGAATAATAATGACTCTGCCAGCACAAGTCGCACGG
AG---

TCAAATGACACTATAACCCTCCCATGCAGAATAAAACAAATTATAAATATGTGGCAGGGGGTAGGAAGAGCAA
TGTATGCCCCTCCCATTGCAGGAAACATAACATGTAAATCAAAT---
ATTACAGGAATACTATTGACACGCGATGGGGGGTCAGATAACAAC----------------------------
----

AGTAACACAGAAATATTCAGACCTGGAGGAGGAAATATAAAGGATAATTGGAGAAGTGAATTGTACAAATATA
AAGTAGTAGAAATTAAACCATTG---GGAATAGCACCCACTACGGCAAAAAGGAGAGTGGTG-----------
----------------------------GAGAGAGAAAAAAGAGCA---GTTGGA---ATAGAAGCT------
---GTGTTCCTTGGGCTCTTGGGAGCA---GCAGGAAGCACTATG---GGCGCAGCGTCA---
ATGACGCTGACGGTACAG---GCCAGACAA---TTGCTG---TCTGGTATAGTGCAACAGCAAAGT---
AATTTG---CTGAGA---GCTATAGAGGCGCAACAGCATATGTTGCAACTCACAGTCTGGGGAATT
>KX907387
TTCTACTGCAACACAACAGGACTGTTTAATGACAGTAAGTGGCAACACAATAATAGTGAATGGGAAGAAACAA
GTAAAAGG---
-------------ATGAAT------
AGTCCAGAGGCAAATATCACACTCCAATGCAGAATAAAACAAATTGTAAACATGTGGCAGGGGGTAGGACGAG
CAATGTATGCTCCTCCCATTAGGGGAGTAATCAAATGTACATCAAAT---
ATTACAGGGCTATTGTTGACAAGGGATGGTGGTTTAAATACTAGC----------------------------
----

AATAATGAGACCTTCAGACCTGGAGGAGGAGATATGAGAGACAATTGGAGAAGTGAATTATACAAATATAAAG
TAGTAAGAATTGAACCACTA---GGTCTAGCACCTACCAAGGCAAAAAGAAGAGTGGTG--------------
-------------------------GAAAGAGAAAAAAGAGCA---ATAGGA---ATGGGAGCT---------
TTGTTCCTTGGGTTCTTGGGAACA---GCAGGAAGCACGATG---GGCGCGGCGTCA---
CTGACGCTGACGGTACAG---GCCAGACAG---TTACTG---TCTGGTATAGTGCAACAGCAAAAT---
AATTTG---CTGAGA---GCTATAGAGGCGCAACAACATCTGTTGCAACTCACAGTCTGGGGCGTT
>KP109492
TTCTATTGTAATACATCAGACCTGTTTAATAGCAAGTGGGAAAATGGCAGCATGTGGGAAAATGACACC----
----
GAAATACGGGAAAATAGT---------
ACAGAGGTGGTAAATATAACTCTTCAATGTAAAATAAAGCAAATTATAAATATGTGGCAAAGAGTAGGACAAG
CAGTATATGCCCCTCCCATCCCAGGAAAAATAAAGTGTGATTCAAAC---
ATTACAGGGCTAATATTAACAAGAGATGGTGGGAAAGTTAACAGT----------------------------
----

ACAAATGAAACCTTCAGACCTGGAGGAGGAGATATGAGGGACAATTGGAGAAGTGAATTATATAAGTATAAGG
TAGTAAAAATTGAACCAATA---GGAATAGCACCCACCAAGGCAAAGAGAAGAGTGGTG--------------
-------------------------GAGAGAGAAAAAAGAGCA---GTTGGA---ATAGGAGCT---------
GTTCTCCTTGGGTTCTTAGGAGCA---GCAGGAAGCACTATG---GGCGCGGCGTCA---
CTAACGCTGACGGTACAG---GCCAGACAA---TTATTG---TCTGGCATAGTGCAGCAGCAAAGT---
AATTTG---CTGAAG---GCTATTGAGGCTCAACAACACCTGTTGAAACTCACAGTCTGGGGTATT
>KF716485
TTCTATTGTAACACATCAAACCTGTTTAAGGGTATTTGGAATGGCAAGTGGAATGGCACAAATAGC-------
----

ACTGAGTCATACATAAATATCACATGTAGAATAAAGCAAATTATAAATATGTGGCAGAGAGCAGGAAAAGCAA
TGTATGCCCCACCCATTAAAGGAATAGTCAATTGTATATCAAAT---
ATTACAGGGTTATTGTTGACACGCGATGGTGGTGTAAATAGTACT----------------------------
----

AATGAGACCTTCAGACCTGGAGGAGGAAATATGAGAGACAATTGGAGAAGTGAATTATATAAATATAAAGTAG
TAAGACTTGAACCACTA---GGTCTAGCACCCACCAGGGCGAAGAGAAGAGTGGTG-----------------
----------------------GAAAGAGAAAAAAGAGCA---ATAGGA---CTGGGAGCT---------
ATGTTCCTTGGATTCTTGGGAGCA---GCAGGAAGCACGATG---GGCGCAGCGTCA---

CTGACGCTGACGGTACAG---GCCAGACTA---CTATTG---TCTGGTATAGTGCAACAGCAAAAC---
AATTTG---CTGAGG---GCTATAGAGGCGCAACAGCATCTGTTGCAACTCACAGTCTGGGGCGTT
>GQ365649
TTCTATTGCAATACATCAAAACTGTTTAATGGTACATATAATGGTACATATAATAGTGCTGCAACA-------
----

GAAACTTCAAACATTACAATCCCATGCAGAATAAAACAAATTATAAATATGTGGCAGAGGGTAGGAGGAGCAA
TGTATGCCCCTCCCATTGCAGGACTAATAACATGTAGATCAAAT---
ATCACAGGAATATTACTGACACGGGATGGAGGCGTGGATGACACAGAG------------------------
----

AATGGCACAGAGACATTCAGGCCTGGAGGAGGAGATATGAAAGACAATTGGAGAAGTGAATTATATAAATATA
AAGTGGTAGAAATCAAACCATTA---GGAGTAGCACCTACTAAGGCAAAAAGGAGAGTGGTG----------
---------------------------GAGAGAGAAAAAAGAGCA---GTGGGA---ATAGGAGCT------
---GTGTTCCTTGGGTTCTTGGGAGCA---GCAGGAAGCACTATG---GGCGCAGCATCA---
ATAACGCTGACGGTACAG---GCCAGACAA---TTATTG---TCTGGGATAGTGCAACAGCAAAAC---
AATTTG---CTGAGG---GCTATAGAGGCGCAACAGCATATGTTGCAACTCACGGTCTGGGGTATT
>MN659380
TTCTATTGCAATACATCAGTCCTGTTTAATAGTACATACATGTCTAATGGTACCTGGAAAAATGGTACATGGG
AA--

GGTTCGAACATCACAATCATCCCATGCAGAATAAAGCAGATTATAAACATGTGGCAGGAGGTAGGACGAGCAA
TGTATGCTCCTCCCATTGCAGGAAACATAACATGTAAGTCAAAT---
ATCACGGGACTATTAATAGTACGTGATGGAGGATCAGAGTCAAATAGCACA---------------------
----

AATGATACAGAGATATTCAGGCCTGGAGGAGGAGATATGAGGGACAATTGGAGAAGTGAATTATATAAATATA
AAGTGGTAGAAATTAAGCCATTG---GGAGTAGCACCCACTGAAGCAAAGAGGAGAGTGGTG----------
---------------------------GAGAGAGAAAAAAGAGCA---GTGGGA---ATAGGAGCT------
---GTGTTCCTTGGGTTCTTGGGAGTA---GCAGGAAGCACTATG---GGCGCAGCGTCA---
ATGACGCTGACGGTACAG---GCCAGACAA---TTGCTG---TCTGGTATAGTGCAACAGCAAAGC---
AATTTG---CTGAGG---GCTATAGAGGCGCAACAGCATATGTTGCAACTCACGGTCTGGGGTATT
>AB485665
TTCTATTGCAACACAACAGAATTGTTTGATAGTACTTGGAATGGCACCAGTGGCAACTATACGACAAATGCCA
CAACA---

AATGAAACTGATACTATTATACTACCATGCAAAATAAAACAAATTGTAAACATGTGGCAGAGAGTAGGGCAAG
CAATATATGCCCCTCCCATCAGAGGAAATATTACATGTGTATCAAAT---GTTACAGGACTAATATTGACA--
----GTTGACCATGCTGCAGAG--

AATGATAGTGTTACCTTCAGACCTGGAGGAGGAGATATGAGGGACAATTGGAGAAGTGAACTATATAAATATA
AAGTAGTAAAAATTGAACCATTA---GGAGTAGCACCCTCCCCGGCAAGAAGAAGAGTGGTG----------
---------------------------CAGAGAGAAAAAAGAGCA---GTGGGA---ATAGGAGCT------
---ATGATCTTTGGGTTCTTGGGAGCA---GCAGGAAGCACTATG---GGCGCGGCGTCA---
TTAACGCTGACGGTACAG---GCCAGACAA---TTATTG---TCTGGTATAGTGCAACAGCAAAGC---
AATTTG---CTGAGA---GCTATACAAGCGCAACAGCATCTGTTGCAGCTCACTGTCTGGGGCGTC
>MH705148
TTTTATTGTGACACATCTGGGATGTTTAATTATACCTTTTCATGTAACGGGAGCAATTGTATTAATACCCAAA
ATAACAGC--

AGCAGAAATGACACTACTATGTTTTGTAAATTGAGACAGGTAGTAAGGTCATGGATGGGGGGAGAGTCAGGAC
TCTATGCACCTCCCATCCGAGGTCACCTACAATGCAGCTCAAAC---
ATAACTGGAATGATTCTAGAACTGGATAATCCATGGAACAGCAGC---------------------------
----

CACAGCAACAACACATTTAGACCAACAGGGGGAAATATGAAAGATATATGGAGAACTGAATTGTTCAAATACA
AAGTAGTAAGGGTAAAACCTTTT---GGTGTGGCACCTACACATATTGCAAGACCAGTCATAGGCACG-----
---------------------GGCACTCGTAGAGAGAAAAGAGCA---GTAGGA---TTGGGAATG------
---CTATTCTTGGGGTTTCTAAGTGCA---GCAGGTAGCACTATG---GGCGCAGCGGCA---

GTAACGCTGACGGTACAG---ACCCACACT---GTGATA---AAGGGTATAGTGCAACAGCAGAAC---
AACCTG---CTAAGA---GCAATACAGGCCCAGCAGGAATTGCTGAGGCTATCTGTATGGGGTATC
>MH705150
TTCTATTGTGACACAACTAAGATGTTTAATTATACCTTCCGATGTAACGGAAACACCTGTGATGATGGCAATG
ATAACCAA--

AGTAACAATAAAACTCAGATACCTTGCAGGCTGAAACAGGTGGTAAGGTCATGGATACAGGGAGGATCGGGAA
TCTATGCACCTCCCATCAAAGGTAATCTAACATGTGCATCACGC---
ATAACTGGAATGATTCTACAATTAGATGTGCCATGGGAAACAAGAGCAGCAATGGACAC------AAT----
----

AGCAGCACTGCAATATTTAGACCAGTAGGGGGAGATATGAAAGACATATGGAGAACTGAATTGTTCAACTACA
AAGTAGTAAGGATAAAGCCTTTT---AGTGTGGCACCCACATATATTACAAGGCCAGTCGTAGGCACT-----
---------------------AGCACTCATAGAGGAAAAAGAGCA---GTAGGA---TTAGGAATG------
---CTATTCTTGGGGGTTCTAAGTGCA---GCAGGAAGCACTATG---GGCGCAGCGGCA---
ACAGCGCTGACGGTACAG---ACCCAAGCT---TTGATG---AAGGGTATAGTGCAACAGCAGGAC---
AACCTG---CTAAGA---GCAATACAGGCCCAGCAGCACTTGCTGAGGCTATCTGTATGGGGTATC
>L20571
TTTTATTGTAACACATCTGGGATGTTTAACTATACTTTTATCAACTGTACAAAGTCCGGATGCCAGGAGATCA
AAGGGAGCAAT---

GAGACCAATAAAAATGGTACTATACCTTGCAAGTTAAGACAGCTAGTAAGATCATGGATGAAGGGAGAGTCGA
GAATCTATGCACCTCCCATCCCCGGCAACTTAACATGTCATTCCAAC---
ATAACTGGAATGATTCTACAGTTAGATCAACCATGGAATTCCACAGGT----------------------
----

GAAAATACACTTAGACCAGTAGGGGGAGATATGAAAGATATATGGAGAACTAAATTGTACAACTACAAAGTAG
TACAGATAAAACCTTTT---AGTGTAGCACCTACAAAAATGTCAAGACCAATAATAAACATT----------
-------------CACACCCCTCACAGGGAAAAAAGAGCA---GTAGGA---TTGGGAATG---------
CTATTCTTGGGGGTGCTAAGTGCA---GCAGGTAGCACTATG---GGCGCAGCGGCA---
ACAGCGCTGACGGTACGG---ACCCACAGT---GTACTG---AAGGGTATAGTGCAACAGCAGGAC---
AACCTG---CTGAGA---GCGATACAGGCCCAGCAACACTTGCTGAGGTTATCTGTATGGGGTATT
>AB485666
TTTTATTGTGACACATCTCGGCTGTTTAACTATACTTTTAAGTGTAATGGTTCCCAATGTAATGAGACCAATA
AAACTCAGACA---AAT---

AAAACTCAGGATACTATAATACCTTGCAAGATAAGACAGGTAGTAAGATCATGGATAAAGGGAGAGTTAGGAC
TCTATGCACCTCCCATCCCAGGTGATCTAACATGTAAATCCAAC---
ATAACTGGGATGATTTTACAACTAGATACACCCTACAACTCCTCATGTGACAAT----------------
----

GTCACATTTAGACCAACAGGGGGAGATATGAGAGATATATGGAGAACTGAATTGTACAACTACAAAGTAATAC
AGGTAAAACCTTTT---AGTGTAGCACCTACAAAAATTTCAAGACCAATAATAGGCCTT-------------
----------AACACCACCCACAGAGGAAAAAGAGCA---GTAGGA---TTGGGAATG---------
CTATTCTTAGGGGTTCTAAGCGCA---GCAGGTAGCACTATG---GGCGCAGCGGCA---
ACAACGCTGGCGGTACGG---ACCCAAGGT---GTACTA---AAGGGTATAGTGCAACAGCAGGAC---
AACCTG---CTGAGA---GCGATACAGGCCCAGCAACATTTGCTGAGGTTATCTGTATGGGGTATT
>KU168293
TTTTATTGTAACACAAGTCACATGTTTAATTATACATTCAATTGTACTAACTCCAACTGTAGTAATACTACT-
----

TATGATAACAGTACAAGAATATATTGCCAGTTGAGACAGGTAGTAAGGTCATGGATGAGGGGAGGGTCAGGGC
TCTATGCACCTCCTATCAAAGGTAATCTAACCTGCAGTTCAAAC---
ATAACTGGATTGATTCTACAAATGGATACACCATGGAATAAAACC-------------------------
----

AACCACACAGTCACATTTAGACCAACAGGAGGAGATATGAAGGATATATGGAAAACCCAATTGTACAATTACA
AAGTAGTAAGGGTAAAATCTTTT---AGTGTAGCACCTACTAAGATTAGTAGACCAGTTGTAGGCACT-----
---------------------AACCATCAAAGAGAAAAAAGGGCA---GTAGGA---TTGGGAATG------
---CTATTCTTGGGGGTTCTAAGTGCA---GCAGGTAGCACTATG---GGCGCAGCGGGA---

ATAACGCTGTCGGTACGA---ACCCACTCA---TTAATA---AGGGGGATAGTGCAACAGCAGGAC---
AACCTG---CTGAGA---GCGATACAGGCCCAGCAACATCTGCTGAGGTTATCTGTATGGGGTATT
>KU168281
TTCTATTGTAACACATCTGGGATGTTTAATTATACCTTTGCATGTACAGGAATCAACTGTAATATTACTCAAG
ATAACAGT---

AGAAATAATGACACTTGGAGACTGCCTTGCAGGATAAAACAGGTGGTAAGGTCATGGATAAGGGGAGGGTCGG
GACTCTATGCACCTCCCAGGCCAGGTAACCTAACATGCAGTTCACAC---
ATAACTGGAATGATTCTGCAAGTGGATGAGCCATGGAACAAGACCAAA-------------------------
----

AACAACAGCAAAGCCATCTTTAGACCAATAGGGGGAAATATGGAAGATATATGGAGAACTGAACTGTTCAAAT
ACAAAGTAGTAAAGGTGAAACCTTTT---AGTGTGGCACCTACAAAAATTGCAAGGCCAATCATAGGCACT--
-------------------------GGCAATCAAAGAGAGAAAAGAGCA---GTAGGA---TTGGGAATG---
------CTATTCTTGGGGGTTCTAAGTGCA---GCAGGTAGCACTATG---GGCGCAGCGGCA---
ACAACGCTGGCGGTACAG---ACCCACACT---TTGATG---AAGGGTATAGTGCAACAGCAGGAC---
AACCTG---CTAAGA---GCAATACAGGCCCAGCAGCAATTGCTGAGGCTATCTGTATGGGGTATC
>KU168295
TTTTATTGTAACACAGCGGAAATGTTTAATTATACCTTTAAGTGTAAGGGCGCCACCTGTAACTCCACCAGTT
TA---

GAAAACAATGGGACTAGCAATCTAACTTGTAAGATTAGACAGGTGGTAAGGTCCTGGATGAAGCAAGGGTCGG
GACTCTATGCACCCCCCATCAGAGGTAACCTAGCATGCATGTCAAAC---
ATAACTGGAATAATTCTACAATTAGATACACCATGGAACAACAGTAAAGAA----------------------
----

AATACAACAGCCACATTTAGACCAACGGGGGGAGATATGACAGATATATGGAGAACTCAATTGTTCAACTACA
AAGTAGTAAGGGTAAAACCTTTT---AGTGTGGCACCTACAAAAATTGCAAGGCCAGTCATAGGCACT-----
----------------------GGCAATCTTAGACAGAAGAGAGCA---GTAGGA---TTGGGAATG------
---CTATTCTTGGGGGTTCTAAGTGCA---GCAGGAAGCACTATG---GGCGCAGCGGCA---
ACGGCGCTAGCGGTACAG---ACCCACACT---TTGATG---AAGGGTATAGTGCAACAGCAGGAC---
AACCTG---CTAAGA---GCAATACAGGCCCAGCAGCACCTGCTGAGGCTATCTGTATGGGGTATT
>AY169813
TTTTATTGTGACACAAGTAAGATGTTTAATTATACCTTTAATTGTAACAAGACCAAATGTAATAATAGTAAAA
ATAGTACCCAT--

AATAACACTATGACAACAATACCTTGCAGAATAAAACAGGTAGTAAGGTCATGGATAAGGGGAGAGTCGGGAC
TCTATGCACCCCCCATTAAAGGTAATTTAACCTGCATATCAAAC---
ATAACTGGAATGATTCTATTAAGGGACGAGCCAGGG---AATAACAGCAGA----------------------
----

CACACCCTTAGGCCAGTAGGGGGAGATATGAAAGATATATGGAGAACTGAATTAGTCAATTACAAAGTAGTAA
AAATAAAACCTTTT---AGTGTAGCACCTACAAAAATTACAAGGCCAGTCATAAGC-----------------
----------------ACCCATAGAGAAAAAAGAGCA---GTAGGA---TTGGGAATG---------
CTATTCTTAGGGGTTCTAAGTGCA---GCAGGTAGCACTATG---GGCGCAGCGGCA---
GTGACGCTGACGGTACAG---ACCCAAACT---TTGTTG---AAGGGTATAGTGCAACAGCAGGAC---
AACCTG---CTAAGA---GCAATACAGGCCCAGCAACAGTTGCTGAGGTTATCTGTATGGGGTATC
>AY169809
TTTTATTGTAACACAACGGGGTTGTTTAATTATACCTTTCTGTGTGAAAACTCCATCTGTAACTTCACCCATA
ACAAT--

ACTTCAGAGGAGCGCAATCTAACTTGTAGAATTAGACAGGTGGTAAGGTCATGGATGAAGACAGGGTCGGGAC
TCTATGCACCTCCCATCGAAGGTAACCTAACATGTAGGTCAAAC---
ATAACTGGAATGATTCTAGAAATGGATAAACCATGG---AACAAAAGTACAAAC-------------------
----

AATGAAACAGCCATATTTAGACCAATAGGGGGAGATATGAAAGAAATATGGAGAACTGAATTGTTCAACTACA
AAGTAGTAAGGGTAAAACCTTTT---AGTGTGGCACCTACAAAAATTGCAAGGCCAGTCATAGGCACT-----
----------------------GGCACTCCTAGACAGAAGAGAGCA---GTAGGA---TTGGGAATG------
---CTATTCTTGGGGGTTCTAAGTGCA---GCAGGAAGCACTATG---GGCGCAACGGCA---

ACAACGCTGGCGGTACAG---ACCCACACT---TTAATG---AAGGGTATAGTGCAACAGCAGGAC---
AACCTG---CTAAGA---GCAATACAGGCCCAGCAGCATTTGCTGAGGCTATCTGTATGGGGTATT
>AY169802
TTTTATTGTAACACATCGGGGATGTTTAATTATACCTTTACATGTAACAAAACCAACTGCACTAACAATAGGA
CTGAT--

AATAGCACTAATGACACAATACCTTGCAAGTTGAGACAGGTAGTGAGGTCATGGATACAGGGGGGGTCAGGAC
TCTATGCCCCTCCCATCAAAGGTACCCTAACATGTATGTCAAAC---
ATAACTGGGATGATTCTAGAAATGGAACGGTCATGGAACGGGAGTGCTACAAAG------
GACATTAATGCTACATGGAAC--

ATTAATGCTACATTCAGACCAATAGGGGGAGATATGAAAGATATATGGAGAACTCAATTATTCAACTATAAGG
TGGTAAGGGTGAAACCTTTT---AGTGTAGCACCTACAAAAATTGCAAGGCCAGTCATAGGCATG--------
-------------GGCACTGGTACTCATAGGGAAAAGAGAGCA---GTAGGA---TTGGGAATG---------
CTATTCTTAGGGGTTCTAAGTGCA---GCAGGTAGCACTATG---GGCGCAGCAGCA---
ACAGCGCTGACGGTACAG---ACCCACACT---TTGATA---AAGGGTATAGTGCAACAGCAGGAC---
AACCTG---CTGAGA---GCAATACAGGCTCAGCATCAACTGCTGAAGCTATCTGTATGGGGTATC
>AY169816
TTTTATTGTAACACATCTAATTTATTTAATTATACCTTTTCATGTAATGGAACCACCTGTAATGATAGCCAAA
GT---

ACCACCAATGACACACAGATACCTTGCAAACTGAGACAGGTAGTAAGGTCATGGATAAAAGGACAGTCAGGAT
TCTATGCACCTCCCATCAAGGGTAACCTGACATGTACATCAAAC---
ATAACAGGAATGATTCTACAAATGGATGCACCATGGAACAGCACCAGA---AAC------AACAGCGAA---
AAC--

CAAACTGCAACATTTAGACCAACAGGGGGAGAAATGAAAGATATATGGAGAACTGAGTTGTTCAACTACAAAG
TAGTAAGAGTAAAACCTTTT---AGTGTGGCACCCACATCAATTGCAAGGCCAGTTGTAGGCACT--------
-------------------AGCACTCATAGAGAAAAAAGAGCA---ATAGGA---TTAGGAATG---------
CTATTCTTGGGGGTTCTAAGTGCA---GCAGGTAGCACTATG---GGCGCAGCGGCA---
ACAGCGCTGACGGTACAG---ACCCACACT---TTGATA---AAGGGTATAGCGCAACAGCAGGAC---
AACCTG---CTAAGA---GCAATACAGGCCCAGCAGCAATTGCTGAGGCTATCTGTATGGGGTATC
>AY169807
TTTTACTGTAACACATCTCAGATGTTTAATTATACCTTTACATGTGATGGAACCACCTGTAAGAACTTAGGAT
TCAAT--

AGCACTAATAACACTCAGATACCTTGCAGGTTGAAGCAGGTAGTAAGGTCATGGATAAAAGGAGAATCAGGAC
TCTATGCACCTCCCATCCAAGGTCAATTAACATGTAGATCAAAC---
ATAACTGGAATGATTCTACAAATGGATCAGCCATGGAACGGGAGCCAGAGC----------------------
----

AATAGCAATAGCACATTTAGACCGATAGGGGGAAATATGAAAGATATATGGAGAACTGAATTGTTTAGATACA
AAGTAATAAGGATAAAACCTTTT---AGTGTGGCACCTACAAGAATTACAAGGCCAGTCATAGGCTTT-----
----------------------GGCCACCACAAGGAAAAAAGAGCA---GTGGGC---TTGGGAATG------
---CTATTCTTGGGGGTTCTAAGTGCA---GCAGGTAGCACTATG---GGCGCAGCGGCA---
ACAGCGCTGACGGTACAG---ACCCACACT---TTGATA---AAGGGTATAGTGCAACAGCAGGAC---
AACCTG---CTAAGA---GCAATACAGGCCCAGCAGCACTTGCTGAGGCTATCTGTATGGGGTATC
>AY169804
TTTTATTGTAACACATCTCAAATGTTTAACTATACTTGTAACTGTACAAGCTCAGAATGCAACTGTACAAGAC
CCGGATACAAC--

GGAACCAATGGAAATGAGACTATACCTTGCAGGTTAAGACAGGTAGTAAGATCATGGATAAAGGGAGAGTCGG
GACTCTATGCACCTCCCATCCCTGGCAACCTAACATGTCAATCCAAC---
ATAACTGGAATGATTCTGCAATTAGATGCACCATGGAATTCCACATGT-------------------------
----

ACTAAAACATTTAGACCAGTAGGGGGAGATATGAAAGATATATGGAGAACTGAATTGTACAACTACAAGGTAG
TGCAGATAAAACCTTTT---AGTGTAGCACCTACAAAAGTTTCAAGACCAATAATAAGCATT-----------
-------------AACACCAATCACAGAAGAAAAAGAGCA---GTAGGA---CTGGGAATG---------
CTGTTCTTTGGGGTACTAAGTGCA---GCAGGTAGCACTATG---GGCGCAGCGGCA---

ACAACGCTGGCGGTACGA---ACCCACAGT---GTACTG---AGGGGTATAGTGCAACAGCAGGAT---
AACCTG---CTGAGA---GCGATACAGGCCCAGCAACATTTGCTGAGGTTATCTGTATGGGGTATT
>AY169812
TTCTATTGTAACACATCTGGGATGTTTAATTATACCTTTTTATGTAATGGGACCAACTGTAAAAACCAAAGTA
CCCAT--

AGCAATAATGGCACGATACCTTGCAAGTTGAAACAGGTGGTAAGATCATGGATGAGGGGAGGGTCGGGACTCT
ATGCACCTCCCATCCCAGGTAACCTAACATGCATGTCACAC---
ATAACTGGAATGATTCTACAAATAGATGGGCCATGGAACAAC---AAC-------------------------
----

ACCACAAACAATACATTTAGACCAATAGGGGGAGATATGAAAGATATATGGAGAAATGAATTGTTCAACTACA
AAGTAGTGAGGGTAAAACCTTTT---AGTGTGGCACCTACATCAATTGCAAGGCCAGTCATAGGCACT-----
----------------------GGCACTCATAGAGAGAAAAGAGCA---GTAGGA---TTGGGAATG------
---CTATTCTTGGGGGTTCTAAGTGCA---GCAGGTAGCACTATG---GGCGCAGCGGCA---
ACAGCGCTGACGGTACAG---ACCCACTCT---CTGATA---AAGGGTATAGTGCAACAGCAGGAC---
AACCTG---CTAAGA---GCAATACAGGCCCAGCAGGAATTGCTGAGGCTATCTGTATGGGGTATC
>MH705147
TTCTATTGTAACACATCTGGGATGTTTAATTATACCTTTTCACGCAATAGGACCAACTGTAATATCCAAAGTA
AC---

AGTAGCAATAATGGCACGATACCTTGCAAGTTGAAACAGGTGGTAAGAGCATGGATGAGGGGAGGGTCGGGAC
TCTATGCACCTCCCATCAAAGGTAACCTAATATGCAGATCAAAC---
ATAACTGGAATGCTTCTAGAAATAGATGGGCCATGGAACAAAACAACCACA----------------------
----

AACAAGAGCACATTTAGACCAACAGGGGGAGAGATGAAAGATATATGGAGAACTGAATTGTTCAAATATAAAG
CAGTGAGGGTAAAACCTTTT---AGTGTGGCACCTACATCAATTGCAAGGCCAGTCATAGGCACT--------
-------------------GGCACTCATAGAGAGAAAAGAGCA---GTAGGA---TTGGGAATG---------
CTATTCTTAGGGGTTCTAAGTGCA---GCAGGTAGCACTATG---GGCGCAGCGGCA---
ACAGCGCTGACGGTACAG---ACCCACACT---GTGATA---AAGGGTATAGTGCAACAGCAGGAC---
AACCTG---CTGAGA---GCGATACAGGCCCAGCAAGAATTGCTGAGGCTATCTGTATGGGGTATC
>AY618998
TTCTATTGTAACACATCTGGGATGTTTAATTATACTTTCTCATGTAAAGGAACCAACTGCACTACTAACCAAA
GT---

AGTAATGAAACTAGTATACCTTGCAGAATAAAACAGATAGTAAGGTCATGGATGAGGGGAGAGTCGGGACTCT
ATGCACCTCCCATCAAAGGTAATCTAACATGTAGATCACGA---
ATTACTGGAATGATTCTACAAATAGATATGCCATGGAATAAAAGC----------------------------
----

GAAGCAAACGTTACATTTAGACCAGTAGGGGGAGATATGAAAGACATATGGAGAACTGAATTGTTCAAATACA
AAGTAGTGGAAATAAAACCTTTT---AGTGTGGCACCTACAAAAATTGCAAGGCCAGTCATAGGCACT-----
----------------------GACACTCATAGAGAAAAAAGAGCA---GTAGGA---TTGGGAATG------
---CTATTCTTGGGGGTTCTAAGTGCA---GCAGGTAGCACTATG---GGCGCAACGGCA---
ACAGCGTTGACGGTACAG---ACCCACACT---TTGATA---AAGGGTATAGTGCAACAGCAGGAC---
AACCTG---CTAAGA---GCAATACAGGCCCAGCAGCAATTGCTGAGACTATCTGTATGGGGTATC
>KF859742
TTTTATTGTATCACAGATCAAATGTTTAACTATACCTTCCACTGTAACAAAACCAAATGTACTGATAACAGTA
GCTAT--

ATCGACCCAAATAATTATATACCTTGCAAACTGAAACAGGTGGTAAGGTCATGGATGCGGGGAGGGTCGGGAA
TCTATGCACCTCCCATCAAAGGTAATCTAACATGTATATCAAAC---
ATAACTGGAATGATTCTACAATTAGATAGCCCATGGAACAGAAGT----------------------------
----

GAAAATGCAAACGCAACATTTAGACCAGAAGGAGGAAATATGAAAGAGATATGGAGAGCTGAATTGTTCAACT
ACAAAGTAGTAAGGGTAAAACCTTTT---AGTGTAGCACCCACACCTATTGCAAGACCAGTCATAGGCACT--
-------------------------AGCACTCATAGAGAAAAAAGAGCA---GTAGGA---TTAGGAATG---
------CTGTTCTTGGGGGTTCTAAGTGCA---GCAGGAAGCACTATG---GGCGCAGTGTCA---

ACAACGCTGACGGTACGG---ACCCACACT---TTGATA---AAGGGTATAGTGCAACAGCAGGAC---
AACCTG---CTAAGA---GCAATACAGGCCCAGCAGCATTTGCTGAGGCTAACTGTATGGGGTATC
>KX228804
TTTTATTGTAACACAAGTAATATGTTTAATTATAGCTTTGAATGTGACGGAACCACCTGTACC----------
----

ATACCTTGCAGGTTAAAACAGGTGGTAAGGTCATGGATACAGGGAGGGTCGGGACTCTATGCACCTCCCATCA
GAGGTAATCTAACATGTACATCAAAC---
ATAACTGGAATGATTCTACAAATGGATATGCCATGGAACAACAGCGGTACAGGA-------------------
----

AACAGGACTGCAACATTGAGACCAGGAGGGGGAGAGATGAAAGACATATGGAGAACTGAATTGATCAACTACA
AAGTAGTAAGGATAAAACCTTTT---AGTGTGGCACCCACACCTATTGCAAGGCCAGTCATAGGCACT-----
----------------------AGCACTCATAGAGAAAAAAGAGCA---GTAGGA---TTGGGAATG------
---CTATTCTTGGGGGTTCTAAGTGCA---GCAGGTAGCACTATG---GGCGCAGCGGCT---
ACAGCGCTGACGGTACGG---ACCCACACA---TTGATG---AAGGGTATAGTGCAACAGCAGGAC---
AACCTG---CTAAGA---GCAATACAGGCCCAGCAGCAATTGCTGAGGCTATCTGTATGGGGTATC
>KU168289
TTTTATTGTAACACATCTCAATTGTTTAATTATACCTTGTCATGTAACAAAACCACCTGTAAATTTAACCAAA
GT---

AGCACTAATAACACTCGGATACCTTGCAGGCTGAAACAGGTGGTAAGATCATGGATGAGGGGAGGGTCAGGAA
TCTATGCACCTCCCATCCCAGGTAATCTAACATGTACATCAAAC---
ATAACTGGAATGATTCTACAAAATGATGAACCATGGAACAGCAGCGTA-------------------------
----

GGACGCGCAATATTTAAACCTACAGGGGGAGATATGATGGATATATGGAGAACTGAATTGTACAACTACAAGG
TAGTAAGGGTAAAACCTTTT---AGTGTAGCACCCACACCTATTGCAAGGCCAGTCATAGGCACT--------
-------------------GGCACTCATAGAGAAAAAAGAGCA---GTAGGA---TTGGGAATG---------
CTATTCTTGGGGGTTCTAAGTGCA---GCAGGTAGCACTATG---GGCGCAGCGGCA---
ACAGCGCTGACGGTACAG---ACCCGCACT---TTGATG---AAGGGTATAGTGCAACAGCAGGAC---
AACCTG---CTGAGA---GCAATACAGGCCCAGCAGCACTTGCTGAGGCTATCTGTATGGGGTATC
>KU168286
TTCTATTGTAACACATCTAATCTATTTAATTATACCTTTTCATGTGAAGGAACCAACTGTACATGTAACGGAA
CCAACTGT---
-------------GGGACT------
AACAATAGCACAAATAGCACGATACCCTGTAGGTTGAGACAGGTGGTAAGGTCATGGATAAGGGGACAGTCGG
GACTCTATGCAGCTCCCATCCGAGGTAACCTAACATGCATATCAAAC---
ATCACTGGAATAATTTTACAAATGGATTCGCCATGGAAACACAAC----------------------------
----

AACACCAATGCCACATTTAGGCCAACAGGGGGAGATATGGAAGATATATGGAGAACTGAATTGATCAAATACA
AAGTAGTAAAGGTAAAACCTTTT---AGTGTGGCACCTACAAAAATTGCAAGGCCAGTCATAGGCACT-----
----------------------GGCACTCACAGAGGAAAAAGGGCA---GTAGGA---TTGGGAATG------
---CTATTCTTGGGGGTTCTAAGTGCA---GCAGGAAGCACTATG---GGCGCAGCGGCA---
ACAGCGCTGACGGTACAG---ACCCACACT---TTGATA---AAGGGTATAGTGCAACAGCAGGAC---
AACCTG---CTAAGA---GCAATACAGGCCCAGCAGCAATTGCTGAGGCTCTCTGTATGGGGTATC
>KU168292
TTTTATTGTAACACAACGGAGTTGTTTATTTATAACTTTAATTGTAGCAACTCCACATGCAACATCTCAGATG
CCTAT--
-AGGAAAATTAATAGT---------
GGTAGTGATGGATTGCATAATATAACTTGTAGAATTAAACAGGTGGTAAGGTCATGGATGAAGACAGGGTCGG
GACTCTATGCACCTCCCATCAGAGGTCACTTAAAATGTGAGTCAAAC---
ATAACTGGAATGATTCTACAATTGGATTACCCATGGAACAGTACA----------------------------
----

ATTAGAAATGCCACATTTAGCCCAATAGGGGGAGATATGAAAGACATATGGAGAACTCAATTGTACAACTACA
AAGTAGTAAGGGTAAAACCTTTT---AGTGTGGCACCTACAAAAATTGCAAGGCCAGTCATAGGTACT-----
----------------------GGCACCGGTAGACAGAAGAGAGCA---GTAGGA---TTGGGAATG------
---CTATTCTTGGGGGTTCTAAGTGCA---GCAGGAAGCACTATG---GGCGCAGCGGCA---

ACAGCGCTGACGGTACGG---ACCCAAACT---TTAATA---AAGGGTATAGTGCAACAGCAGGAC---
AACCTG---CTAAGA---GCAATACAGGCCCAGCAGCACTTGCTGAGGCTATCTGTATGGGGTATT
>KY112585
TTTTATTGTAACACAAGTAGCATGTTTAACTATACCTTTTACTGTAACAACTCCACCTGTAACAATGGAAGCA
ATCAG---

AATGGAAACAACATGACAATACCTTGCAGGATAAAACAGGTAGTGAGATCATGGATGAAGGGAGGGTCAGGAC
TATATGCACCTCCCATCAGAGGTCACTTACAGTGTATATCAAAT---
ATAACTGGAATGATCCTACAATTGGATCGAGAATGGAATTATACTAATAATGAC------------------
----

ACAAGAGATGCCAATGCCACATTTAGACCAACAGGGGGAGAGATGAGAGATATATGGAGAACAGAATTGTACA
ACTATAAAGTAGTAAGGGTAAAGCCTTTT---
AGTGTGGCACCTACAACAATTACAAGGCCAGTCATAAGCAAC------------------------------
--CATAGAAATAAAAGAGCA---GCAGGA---TTGGGAATG---------
CTATTCTTGGGGGTACTAAGTGCA---GCAGGAAGCACTATG---GGCGCAGCGGCA---
ACAGCGCTGACGGTACGG---ACCCAAAAC---TTAATA---AAGGGTATAGTGCAACAGCAGGAC---
AACCTG---CTGAGA---GCGATACAGGCCCAGCAACACTTGCTGAGGTTATCTGTGTGGGGTATC
>AF407418
TTTTATTGTAACACAAATCAGCTGTTTAATCATACCTTTTCCTGCAAGAAGAATATGACCTATAACAAGATCA
ATTGTACTAAT---
----------ATTAGCAAT------
AATAGCAATGGCACTCAGGCAATACCTTGCAGGTTGAGACAAGTAGTAAGGGACTGGATGAGGGGAGGATCGG
GACTTTATGCACCTCCCATCCCAGGAAACCTAGTATGCAGGTCAAAC---
ATAACTGGAATGATTCTACAATTGGACACGCCATGG---AATAAAACACAT---------------------
----

CCTAACAGCACCACCCTTAGACCAGGAGGGGGAGATATGAAAGATATATGGAGAACTCAATTGTTCAAATATA
AAGTAGTAAGAGTAAAACCTTTT---AGTGTAGCACCAACAAAAATTGCAAGGCCAACTATAGGAACT-----
----------------------AGCACTCATAGAGAGAAAAGAGCA---GCAGGT---TTGGCAATG------
---CTATTCTTGGGGATTCTAAGTGCA---GCAGGAAGCACTATG---GGCGCAGCGGCA---
ACAGCGCTGACGGTACGG---ACCCAGCAT---CTGATA---AAGGGTATAGTGCAACAGCAGGAT---
AACCTG---CTAAGA---GCAATACAGGCCCAGCAACACTTGCTGAGGCTATCTGTATGGGGTATT
>KU168288
TTTTACTGTAACACATCTTGGTTGTTTAATTATACCTTTATGTGTAATGGAACTACCTGTGAGAGCCCAAATA
TCAACAGC--

ACTAATAACACTCTGATACCTTGCAGGTTGAGGCAGGTAGTAAGGTCATGGATGAGGGGAGGATCGGGACTCT
ATGCACCTCCCATCCGAGGTAACTTAACATGTAGATCAAAC---
ATAACTGGGATGATTCTACAGATGGATCAACCATGGAACATGAGC---------------------------
----

CGCCAGAACAGCACATTTAGACCAACAGGGGGAGATATGAAAGACATATGGAGAACTGAATTGCTCAGATACA
AAGTAGTAAGGATAAAACCTTTT---AGTGTGGCACCTACAAAAATTACAAGGCCAGTCATAGGCTTT-----
----------------------GGCACCCGCAGAGAAAAAAGAGCA---GTGGGC---TTGGGAATG------
---CTATTCTTGGGGGTTCTAAGTGCA---GCAGGTAGCACTATG---GGCGCAGCGGCA---
ACAGCGCTGACGGTACGG---ACCCACACT---TTGATA---AAGGGTATAGTGCAACAGCAGGAC---
AACCTG---CTAAGA---GCAATACAGGCCCAGCAGCAATTGCTGAGGCTATCTGTATGGGGTATC
>KU168290
TTCTATTGTAATACATCTAAGATGTTTAATTATACCTTTTTATGTAAAGGAACCAACTGTACTAATACCCAAA
GTAATAGT--

ACCAGAAATGACACTGTAATATATTGCAAGATAAGACAGGTAGTAAGATCATGGATGAGGGGAGGGTCAGGAC
TCTTTGCACCGCCCATTCCAGGTAACCTAACATGCAGATCACAA---
ATAACTGGAATGATGCTACAAATGGATAAGCCATGGAATGGAAGC---------------------------
----

AACAGCAACAGCACATTTAGACCAACAGGGGGAGATATGAAAGATATATGGAGAACTGAATTGTTCAAATACA
AAGTAGTAAGGGTAAAACCTTTT---AGTGTGGCACCTACAAAAATTGCAAGGCCAGTCATAGGCATA-----
----------------GGCACTGGCACTCATAGAGAGAAAAGAGAC---ATAGGA---TTGGGAATG------

---CTATTCTTGGGGGTTCTAAGTGCA---GCAGGTAGCACTATG---GGCGCAGCGGCA---
ACAACGCTGGCGGTACAG---ACCCAAAAA---GTGATG---AAGGGTATAGTGCAACAGCAGGAC---
AACCTG---TTGAGA---GCAATACAGGCCCAGCAGCAATTGCTGAGGCTATCTGTATGGGGTATC
>KU168291
TTTTACTGTAACACATCTGCAATGTTTAATTATACCTTTTCATGTAATAGAACCACCTGTAATTTCCCCAATA
TCAAT--

AGAGATAATAACACTCTGATACCTTGCAGGTTGAGACAGGTAGTAAGGTCATGGATGAGGGGAGGATCGGGAC
TTTATGCACCTCCCATCCGAGGTAACTTAACATGTAAATCAAAC---
ATAACTGGAATGATTCTACAAATGGATCAGCCATGGAACATGAGC----------------------------
----

CGGGGGAACAGCACATTTAGACCAACAGGGGGAGATATGAAAGATATATGGAGAACTGAATTGCTCAGATACA
AAGTAGTAAGGATAAAACCTTTT---AGTGTGGCACCTACAAAAATTACAAGGCCAGTCATAGGCTAC-----
----------------------GGCACCCGCAGAGAAAAAAGAGCA---GTGGGC---TTGGGAATG------
---CTATTCTTGGGGGTTCTAAGTGCA---GCAGGTAGCACTATG---GGCGCAGCGGCA---
ACAGCGCTGACGGTACAG---ACCCACACT---TTGATA---AAGGGTATAGTGCAACAGCAGGAC---
AACCTG---CTAAGA---GCAATACAGGCCCAGCAGCACTTGCTGAGGCTATCTGTATGGGGTATC
>KU168282
TTTATTGTAACACATCTAAGATGTTTAACTATACTTTTGACTGTACAAGTGGCAACTGTACTGAGATCAATT
CCATGAAT---

AAAACTGATGAGCATAATGGACTACCTTGCAGGTTAAGACAGGTAGTAAGATCATGGATAAAGGGAGAGTCAG
GACTTTATGCACCTCCCATCAGAGGCAACCTCACATGCCAGTCCAAC---
ATAACTGGAATGATTCTACAACTAGATGAACCATATAACCGCACATCA-------------------------
----

GATGCAAATGTCACACTTAGACCAAGTGGGGGAGACATGCAAGATATATGGAGAGCTCAATTGTACAACTACA
AAGTAGTACAGATAAAACCTTTT---AGTGTAGCACCTACAAGAATCTCAAGGCCAACAATAAACCTT-----
-------------------AACACCCCTCGCAGAGAAAAAAGAGCA---GTAGGA---TTGGGAATG------
---CTGTTCTTGGGGGCGCTAAGCCTT---GCAGGTAGCACTATG---GGCGCAGTGGCA---
ACAACACTGACGGTACAG---ACCCACAGT---GTACTG---AAGGGTATAGTGCAACAGCAGGAC---
AACCTG---CTGAGA---GCGATACAGGCCCAGCAACACCTGCTGAGGCTATCTGTATGGGGTATC
>KU168296
TTTTACTGTAACACATCTCAGATGTTCAATTATACCTTTATATGTAATGAAACCACCTGTGAGAACACCTGTA
AGAGCCCA---
-------------------AAT---
TGCAGTAGCAATATCACTCAGATACCTTGCAGGTTGAAGCAGGTAGTAAGGTCCTGGATGAAGGGAGGATCAG
GACTCTATGCACCTCCCATCCGAGGTCAATTAAGATGTGAATCAAAC---
ATAACTGGAATGATTCTAGAAATGGATCAGCCATGGAACGGGAAC----------------------------
----

CAAAGCAACAGCACAAGGACATTTAGACCAACAGGGGGAAATATGAAAGATATATGGAGAACTGAATTGCTCA
GATACAAAGTAGTAAGAATAAAACCTTTT---
AGTGTGGCACCTACAAAAATTACAAGGCCAGTCATAGGCTTT---------------------------
GGCCCCCGCAGAGAAAAAAGAGCT---GTGGGC---TTGGGAATG---------
CTATTCTTGGGGGTTCTAAGTGCA---GCAGGTAGCACTATG---GGCGCAGCGGCA---
ACAGCGCTGACGGTACAG---ACCCACACT---TTGATA---AAGGGTATAGTGCAACAGCAGGAC---
AACCTG---CTAAGA---GCAATACAGGCCCAGCAGCACTTGCTGAGGCTATCTGTATGGGGTATC
>KU168297
TTTTACTGTTACACAGCTGATATGTTTAAGTATACCTTTAGATGTAATGAAACCACCTGTATCCCAGGATTCA
ATAGCTCA---

GAATCCAAGAATAACACCCGGATACCTTGCAGGATAAAGCAGGTAGTAAGGTCATGGATGAGGGGAGGATCAG
GACTCTATGCACCTCCAATCCGAGGAAACTTAACATGTGAATCAAAC---
ATAACTGGAATGATTCTACAAATGGATGAGCCATGGAACACGAGCAGA-------------------------
----

AAGAACAACACATTTAGACCAGTAGGGGGAGATATGAAAGATATATGGAGAACTGAATTGCTCAGATACAAAG
TAGTAAGGATAAAACCTTTT---AGTGTGGCACCTACAAAAGTTACAAGACCAGTCATAGGCTTT--------

-------------------GGCCCCCGCAGAGAAAAAAGAGCA---GTGGGC---TTGGGAATG---------
CTATTCTTGGGGGTTCTAAGTGCA---GCAGGTAGCACTATG---GGCGCAGCGGCA---
ACAGCGCTGACGGTACGG---ACCCACACT---TTGATA---AAGGGTATAGTGCAACAGCAGGAC---
AACCTG---CTGAGA---GCAATACAGGCCCAGCAGCACTTGCTGAGGCTATCTGTATGGGGTATC
>JX245014
TTTTATTGTAACACCACTAAGTTGTTTAATTATAACTTTACATGTAATGGAGCCACCTGTAATGAAACCATCC
ATAACCAAGT---
-------------TACCAT------
AGCTATAAGAATCAAAGTATAATACCTTGCAAATTGAAGCAGGTGGTAAGGTCATGGATACAGGGAGGGTCGG
GACTCTATGCACCTCCCATCAAAGGTAATCTAACATGTACATCAAAC---
ATAACTGGAATGCTTCTGGAAATGGATAGCCCATGGAACAAAAGT----------------------------
----

AGCACGAATGCAACTTTTAGACCAATAGGGGGAAATATGAAAGATATATGGAGAACTGAGTTGTACAACTACA
AAGTAGTAAGGGTAAAACCTTTT---AGTGTGGCACCCACATATATTACAAGGCCAGTCATTAAATCT-----
----------------------------CATAGGGAAAAAAGAGCA---GTAGGA---CTGGGAATG------
---CTATTCTTGGGGGTTCTAAGTGCA---GCAGGTAGCACTATG---GGCGCAGCGGCA---
ACAGCGCTGACGGTACAG---ACCCACACA---TTGATA---AAGGGTATAGTGCAACAGCAGGAC---
AACCTG---CTAAGA---GCAATACAGGCCCAGCAGCACTTGCTGAGGCTATCTGTATGGGGTATC
>JX245015
TTTTATTGTAACACCTCTAAGATGTTTTGTTATACTTTCAACTGTAGTAGATCCAACAACTGCACCCTTATTA
AGAACAACTGT--
----ACCATTAATAGT---------
ACTAGTAATAGTACGAGTGAGATACCTTGCAGGTTGAGACAGGTGGTAAGATCATGGATGGGGGGACAGTCAG
GACTCTATGCTCCTCCCATCAAAGGTAATCTAACATGTATATCAAAC---
ATAACTGGAATGATTCTACAACTGGATAAGCCATGGAACATCAGT----------------------------
----

AATAGGAACATCACACTTAGACCCACAGGGGGAGATATGAAAGATATATGGAGAACTGAATTGTTCCAATACA
AGGTAGTAAAGGTAAAACCTTTT---AGTGTGGCACCTACAAAAATTGCAAGGCCAGTCATAGGCACT-----
----------------------GGCACTCATAGAGAAAAAAGAGCA---GTAGGA---TTGGGAATG------
---CTATTCTTGGGGGTTCTAAGTGCA---GCAGGTAGCACTATG---GGCGCAGCGGCA---
ACAGCGCTGACGGTACAG---ACCCACACT---TTGATA---AAGGGTATAGTGCAACAGCAGGAC---
AACCTG---CTAAGA---GCGATACAGGCCCAGCAGCATTTGCTGAGGCTATCTGTATGGGGTATC
>AJ302646
TTCTATTGTAACACATCTAAGATGTTTAATTATACCTTTTCATGTATAGGAACCAACTGTACTAGTAACCAAA
ATAGCAGC---

AACAGTAATGACACTCGGATATATTGCAGGATAAAACAGGTGGTAAGGTCATGGATACAGGGAGGGTCAGGAC
TCTATGCACCTCCCAGGAAAGGTAACCTAACATGCAGCTCACTC---
ATAACTGGAATGATTCTACAATTGGATATGCCATGGAACAGCACCAAC-------------------------
----

AACAGCAACGCCACATTTAGACCAACAGGGGGAGATATGAAAGATATATGGAGAACTGAATTGTTCAAATACA
AAGTAGTAAAGGTAAAACCTTTT---AGTGTGGCACCTACAAAAATTGCAAGGCCAGTCATAGGCACT-----
----------------------GGCACTCAAAGAGAAAAAAGAGCA---GTAGGA---TTGGGAATG------
---CTATTCTTAGGGGTTCTAAGTGCA---GCAGGAAGCACTATG---GGCGCAGCGGCA---
ACAGCGCTGGCGGTACAG---ACCCAATCT---TTGATG---AAGGGTATAGTGCAACAGCAGGAC---
AACCTG---CTAAGA---GCAATACAGGCCCAGCAGCAATTGCTGAGGCTATCTGTATGGGGTATC
>AJ302647
TTCTATTGTAACACATCTCAGATGTTTAATTATACCTTTTCATGTACAAGAACCAACTGTATTAGACAAAGTA
ACAGT--

AGCATTAATGGCACAATATCTTGCAGGATAAAACAGGTGGTAAGGTCATGGATACAGGGAGGGTCGGGACTTT
ATGCACCTCCCAGGCCAGGTTACCTAACATGCAACTCATCC---
ATAACTGGAATGATTCTACAATTGGATAAGACATGGAACCGCACCAAC-------------------------
----

AACAGCGAATCCACATTTAGACCAATAGGGGGAGATATGAAAGATATATGGAGAACTGAATTGTTCAAATACA
AAGTAGTAAAGATAAAACCTTTT---AGTGTGGCACCTACAAAAATTGCAAGACCAGTCATAGGCACT-----

----------------------GGCACTCGAAGAGAAAAAAGAGCA---GTAGGA---TTGGGAATG------
---CTGTTCTTGGGGGTTCTAAGTGCA---GCAGGAAGCACTATG---GGCGCAGCGGCA---
ACAACGCTGGCGGTACAG---ACCCACACT---TTGATG---AAGGGTATAGTGCAACAGCAGGAC---
AACCTG---CTAAGA---GCAATACAGGCCCAGCAGCAATTGCTGAGGCTATCTGTATGGGGTATC
>JN571034
TTTTATTGTGACACAGGGAAAATGTTTAATTATACCCTGGAATGTAACGGAACCATCTGTGAGAATATCAGTA
GTGAGAGTAAT--
----------AGCAGTAGT------
GAGAGTAAGAATAAAACGCGGATACCTTGCAGGCTGAAACAGATGGTAAGGTCATGGGCAACAGGAGGGTCAG
GAATCTATGCACCTCCCATCCAAGGCAATCTCACATGTACATCAAAC---
ATAACTGGGATGATTCTACAAACAGATGGGGTATGGACTACCCC-----------------------------
----

CACCCCAACAATGTAACATTTAGACCAACAGGGGGAAATATGGAAGATATATGGAGAACTGAATTGTACAACT
ACAAAGTAGTAAAGGTAAAACCTTTT---AGTGTGGCACCCACAAGAATTGCAAGGCCAGTCATAGGCACT--
-------------------------GGCACTCATAGAGAAAAAAGAGCA---GCAGGA---TTGGGAATG---
------CTGTTCTTGGGGGTTCTAAGTGCA---GCAGGAAGCACTATG---GGCGCAGCGGCA---
ACAGCGCTGACGGTACAG---GCCCACACT---TTGATG---AAGGGCATAGTGCAACAGCAGGAC---
AACCTG---CTAAGA---GCAATACAGGCCCAGCAACACTTGCTGAGGCTGTCTGTATGGGGTATC
>AY169805
TTTTACTGCAACACATCTCAGATGTTTAATTATATCTTTAACTGTACCGGAACCACCTGTAATTATACCAAAA
ACATC--

CAAAATTATACCTTTAGCCAAAATACCACTAGCACTAAGATACCTTGCAGATTGAGGCAGGTAGTAAGGTCAT
GGATGAGGGGAGGATCGGGACCCTATGCACCTCCCATCCGAGGTAACTTAACATGTGGATCAAAC---
ATAACTGGAATGATTCTAGAAATGGATGAGCCATGGAACGAGAGC----------------------------
----

CACAGGGAAAGCATATTTAGACCAACAGGGGGAAATATGAAAGATATATGGAGAACTGAATTGCTCAGATACA
AAGTAGTAAGGATAAAACCTTTT---AGTGTGGCACCTACAAAAATTACAAGGCCAGTCATAGGCTTT-----
----------------------GGCACCCGCAGAGAAAAAAGAGCA---GTGGGC---TTGGGAATG------
---CTATTCTTGGGGGTTCTAAGTGCA---GCAGGTAGCACTATG---GGCGCAGCGGCA---
ACAGCGCTGACGGTACAG---ACCCACACT---TTGATA---AAGGGTATAGTGCAACAGCAGGAC---
AACCTG---CTAAGA---GCAATACAGGCCCAGCAGCACTTGCTGAGGCTATCTGTATGGGGTATC
>KF859744
TTTTACTGTGACACAACTCAGATGTTTAATTATACCTTTAACTGTACTGACACCAGGTGTGATAATCAAAGT-
----

ACCACCAGCAGTAATAATATACCTTGCAGGTTGAGGCAGGTAGTAAGGTCATGGATGAGAGGAGGATCGGGAC
TCTATGCACCTCCCATCCGAGGTAACTTAACATGTGGATCAAAC---
ATAACTGGAATGATTCTACAAATGGATCAGCCATGGAACGGGAGCCAGAGCAAC-------------------
----

ACAAACAGGAGCAGCACCTTTAGACCAATAGGGGGAGATATGAAAGATATATGGAGAACTGAATTGCTCAGAT
ACAAAGTAGTAAGGATAAAACCTTTT---AGTGTGGCACCTACAAAAATTACAAGGCCAGTCATAGGCTTT--
-------------------------GGCACCCGCAGAGAGAAAAGAGCA---GTGGGC---TTGGGAATG---
------CTATTCTTGGGGGTTCTAAGTGCA---GCAGGTAGCACTATG---GGCGCAGCGGCA---
ACAGCGCTGACGGTACGG---ACCCACACT---TTGATA---AAGGGTATAGTGCAACAGCAGGAC---
AACCTG---CTAAGA---GCAATACAGGCCCAGCAGCACTTGCTGAGGCTATCTGTATGGGGTATC
>AY169968
TTTTACTGCGATACTAGAAAAATGTTTAATGAAAGTGAACCTTTTCACGAGAAC-------------------
----

ATGACAATACCATGCAGAATTAGACAAATAGTGAATTCATGGATGAGAGTAGGAAGGGGAATCTATGCACCTC
CAATACCAGGACATATAACTTGTAACTCTCTA---
ATTACTGGGCTCATATTGACACGAGACCATGTTAATAATACTAATAAT-------------------------
----

ACATTTAGACCAATAGGAGGAGACATGAAAAACATTTGGAGGAGTGAACTCTATAAATACAAAGTGGTGCGCA
TAGAACCATTA---TCTGTGGCACCAACCAAAGCAAAAAGGCATACAGTAGGAGAA-----------------

----------------AGGAGACAGAAGAGAGCAGCCTTTGGC---CTGGGAGCA---------
CTGTTCCTTGGGTTTCTGGGTGCA---GCAGGAAGCACAATG---GGCGCAGCGGCA---
GTCACGCTGACGGTACAG---GCCCGACAG---TTACTA---TCGGGAATAGTGCAACAACAGAAC---
AACCTG---TTGAGA---GCAATAGAGGCCCAACAGCATCTATTGCAACTAACAGTCTGGGGAGTA
>DQ373066
TTCTACTGTAATACAAGTGAGATCATCAATATCACTAAGATAAACAAAACT----------------------
----

GAGAATATGACAATAATCCCTTGTAGAATAAGACAAATAGTGAATTCCTGGATGCGTGTAGGGAAAGGAATTT
TTGCACCACCAATTAGAGGTAACATTACCTGCACTTCTAAT---
ATCACAGGTATGCTACTAGAGATACATAAGAACCGAGAAGATCAGGGAGAAGAT------CAGGATCAG---
AAT--

AACACATACGTTTGTCTAACAGGGGGTAACATGAAAGATATTTGGAGAAGTGAACTATACAAATACAAAATAG
TTGAGATACAACCACTA---GGAGTAGCACCTACTAAAAGTAGAAGATATGCAGTAGAA---AAA--------
----------------CAACACCATAGAGAAAAGAGAGCC---CTTGGA---CTAGGAGCA---------
CTGTTTCTTGGGTTCCTGGGAGCA---GCAGGAAGCACTATG---GGCGCAGCATCA---
GTCGTGCTGACGGTACAA---GCCCGGCAA---TTGTTA---ACGGGCATAGTGCAGCAGCAGAAT---
AATCTG---CTAAGA---GCAATAGAAGCTCAACAACATCTGTTGCAGCTTTCTGTCTGGGGAATC
>GQ217539
TTTTACTGCAATACTACCCCTCTTTTCACAGGCAATAAGACAAATATAACA----------------------
----

ATAATACTGCCATGCAAAATTAGACAGATAGTTAATTCCTGGATGAGAGTTGGAAAGGGTATCTATGCTCCAC
CCATTAGAGGAAATCTCTCCTGTAATTCTAAC---
ATAACAGGGCTTCTATTAACAAGAGATGGACCAGACAAGAATATAAACGAGACT-------------------
----

GAAACACTAAGGCCAGGAGGAGGAGACATGAAAGACATTTGGAGAAGTGAACTTTATAAATATAAAGTAGTAA
AAATTGAACCCCTG---GCAGTAGCTCCAACTAAGGCAAGGAGATACACTATAAAT-----------------
----------ATGGAAAAACACAGAGCAAAAAGAGCA---GCCTTT---------------------------
---------------GCA---GCAGGAAGCACTATG---GGCGCAGCGGCA---GTTACGCTGACGGTACAG-
--GCCCGAAAT---TTACTT---TCGGGAATAGTGCAACAACAGAAT---AATCTG---CTAAGA---
GCCATAGAAGCTCAGCAACATCTGCTGCAGCTGTCTGTATGGGGTGTC
>X52154
TTCTATTGTAATACTAGCCAAATATTTACTGACAACATTACAAATGGCATT----------------------
----

ATAATACTGCCATGCAGAATTAGACAGATAGTTAGTTCATGGATGAGGGTAGGAAGAGGAATATATGCACCCC
CAATCAGAGGAAACATCACTTGCAATTCTAAC---
ATTACTGGACTTCTCTTAACTTCAGACACTCCTGTGACTAACAACAGTGGTAAC-------------------
----

CTGACATTTAGACCTACAGGAGGAAACATGAAAGATATTTGGAGAAGTGAGCTCTATAAGTATAAAGTAGTTC
GAATAGAGCCTTTA---TCAGTGGCACCAACAAAAGCAAGAAGGCATACAGTGGCA---AGA-----------
----------CAGAAAGACAGACAGAAAAGAGCAGCC---TTCGGT---CTGGGAGCA---------
CTGTTCCTTGGGTTCCTGGGTGCA---GCAGGAAGCACTATG---GGCGCAGCGGCA---
GTAACGCTGACGGTACAG---GCCCGACAG---TTGCTG---TCGGGTATAGTGCAGCAACAGAAT---
AATCTG---TTGAAA---GCAATAGAGGCTCAACAGCACCTATTGCAACTATCAATCTGGGGAGTA
>JQ768416
TTCTACTGCAATCTAACAAAATGGATTAACGAGACCTGGCTTAACGAGACG----------------------
----

GACCAGACCAATCTTGTAGCACCCTGTAGACTCAGACAAATAGTTAACCATTGGGGCATTGTGGCTAAAGCCA
TCTACCTCCCTCCAAGAAGAGGAACAGTAAAATGTGTGTCAAAC---
ATCACGGGTTTCCTGATGACAAACGAGGAAACGGGTGCT----------------------------------
----

ACCTTCTCCGGTAAAGTAGAGGATTATTGGAGGGTGGAATTAGCAAAGTACAAAATTGTAGAAATTCAGCCCC
TA---TCAGTGGCTCCTACCACAGGAAAAAGGCCAGAGATTAAG---GCC-----------------------

-AATCATACAAGATCAAAAAGGGAC---GTTGGC---ATAGGGCTG---------
TTGTTTCTGGGATTTCTCAGCGCA---GCAGGAAGTACAATG---GGCGCAGCGTCA---
ATAGCGCTGGTGGCACAG---GCCAGAGGG---GTCCTC---TCTGGTATTGTACAGCAGCAGCAC---
AACCTG---CTGCAG---GCAATAGAGGCGCAACAGCATTTGTTGCAGCTTACTGTATGGGGCGTC
>KU921778
TTCTTTTGTAATACATCAGAACTATTTAATAGCAGTTGGAATAATAGCAGTTGGAGTGACAATAGCAGTGAGA
ATAGGACG---

TCAAATGACACCACCATAACTCTTAAATGCAGAATAAAGCAAATTATAAATATGTGGCAGAGAACGGGAAAAG
CAATGTATGCCCCTCCCATTCAAGGAGTAATAAACTGTGTATCAAAC---
ATTACAGGACTAATATTAACAAGAGATGGGGGTGGAGATGATAGC----------------------------
----

AGTACAAATGAAACTTTCAGACCTGGAGGAGGAGATATGAGGGACAATTGGAGAAGTGAATTATATAAGTATA
AAGTAGTAAAAAATTGAACCAATA---GGAGTAGCACCCACCAAGGCAAAGAGAAGAGTGGTG----------
----------------------------GAGAGAGAAAAAAGAGCA---GTGGGA---ATAGGAGCT------
---GTATTCCTTGGGTTCTTAGGAGCA---GCAGGAAGCACGATG---GGCGCGGCGTCA---
ATAACGCTGACGGTACAG---GCCAGACAA---TTGTTG---TCTGGCATAGTGCAACAGCAAAGC---
AATTTG---CTGAGG---GCTATAGAGGCTCAACAACATCTGTTGAAACTCACAGTCTGGGGCATT
>MF373124
TTCTTTTGTGATACATCAGGCCTGTTTAATAGTACTTGGCCTTGGCAAAATGACACTGCCAGCCTATCAAATA
ACACAGAG---
TTAAATGGTAATAGCACA---------
GAGTTAACTGGTACTATAACTCGCCCATGCAGAATAAAGCAAATTGTAAATATGGGGCAAAGAGTAAGAAAAG
CAATGCATGCCCCTCCCATCCAAGGGTTAATAAGGTGGGAATCAAAC---
ATTACAGGACTACTATGGACGAGAGATGGTGGGGATAATAGCTAC----------------------------
----

AATGAAACCTTCCGACCTGGAGGAGAGGGAGATATGAGGGACAATTGGAGAAGTGAATTATATAAGTATAAAG
TAGTAAAAAATTGAACCACTA---GGAGTAGCACCTACCAGGGCGAAGAGAAGAGTGGTG-------------
-------------------------GAGAGAGAAAAAAGAGCA---GTTGGA---ATAGGAGCT---------
GTATTCATTGGGTTCTTAGGAGCA---GCAGGAAGCACTATG---GGCGCGGCGTCA---
ATAACGCTGACGGTACAG---GCCAGACAA---CTATTG---TCTGGCATAGTGCAACAGCAAAGC---
AATTTG---CTGAGG---GCTATAGAGGCTCAACAGCATCTGTTGAAACTCACAGTATGGGGCATT
>GU201516
TTCTTTTGCAATACAACAGGCTTGTTCAATAGCACATGGACGAAAGAGGGCAATAGCACATGGAAAAAGAACG
ATAGTGTCTCT--
-------------GAAAGG------
GAGTCAAATGACAATATAACACTCCAATGCAAAATAAAACAGATTATAAACATGTGGCAGAGAGTAGGACGAG
CAATGTATGCCCCTCCCATTGCAGGAGTAATAAAGTGTATATCAAAT---
ATCACAGGAATGATCTTGACAAGAGATGGTGGGAAGCGCAATGAT----------------------------
----

TCCAGTAGTGAGACCTTCAGGCCTGGAGGAGGAGATATGAGGGACAATTGGAGAAGTGAATTATATAAGTATA
AAGTAGTAAAAAATTGAACCACTA---GGAATAGCACCCACCAAGGCAAGGAGAAGAGTGGTG----------
----------------------------GAGAGAGAAAAAAGAGCA---GTTGGA---CTGGGAGCT------
---GCTTTCCTTGGGTTCTTGGGAGCT---GCAGGAAGCACTATG---GGCGCGGCGTCA---
ATAACGCTGACGGTACAG---GCCAGACAA---TTATTG---TCTGGCATAGTGCAACAGCAAAGC---
AATTTG---CTGAAG---GCTATAGAGGCTCAACAGCAGCTGTTGAAACTCACGGTCTGGGGCATT
>MN486000
TTCTTCTGTAATACATCAAAATTGTTTAATAATAATGAAACC-------------------------------
----

ATTACAGAAAATATCACACTCCCATGCAGAATAAAACAAATTGTAAACATGTGGCAGGAAGTAGGAAAAGCAA
TGTATGCCCCTCCTATTAGAGGAATAATTAGATGTGTATCAAAT---
ATTACAGGTCTACTATTAACAAGAGATGGTGGTATAAACACCACC----------------------------
----

AACAACAACACTGAAATCTTTAGACCTGGAGGAGGAGATATGAGGGACAATTGGAGAAGTGAATTATATAAAT
ATAAAGTAGTAAAAAATTGAACCGTTA---GGAATAGCACCCACCAAGGCAAAGAGAAGAGTGGTG-------

-------------------------------CAGAGAGAAAAAAGAGCA---GTAACA---TTTGGAGCT---
------CTGTTCCTTGGGTTCTTGGGAGCA---GCAGGAAGCACTATG---GGCGCAGCGTCA---
GTGACGCTGACGGTACAG---GCCAGACAA---TTGCTG---TCTGGTATAGTGCAACAGCAGAAC---
AACCTG---CTGAAG---GCTATTGAGGCGCAACAGCACCTGTTGCAACTCACAGTCTGGGGCATC
>KT427670
TTCTTTTGTAACACAACAAAACTGTTTAATAGTACCTGGCCTATTAATAGTACCTGGAATGCAACT-------
----

GGAGAGTCTATCATCACACTCCCATGCAGAATAAAACAAATTATAAACATGTGGCAGGAAGTAGGAAAAGCAA
TGTATGCCCCTCCCATCAGCGGACCCATTAATTGTACATCAAAC---
ATTACAGGGCTACTATTAACAAGAGATGGTGGGGTTGCAAACGCC----------------------------
----

ACCAACGAGACCTTCAGACCTGGAGGAGGAGATATGAGGGACAATTGGAGAAGTGAATTATATAAATATAAAG
TAGTAAAAGTTGAACCATTA---GGAGTAGCACCCACCAAGGCAAAGAGAAGAGTGGTG--------------
-------------------------CAGAGAGAAAAAAGAGCA---GTGGGA---TTCGGAGCT---------
CTGTTCCTTGGGTTCTTGGGAGCA---GCAGGAAGCACTATG---GGCGCAGCGTCA---
GTAACGCTGACGGTACAG---GCCAGACAA---TTATTG---TCTGGTATAGTGCAACAGCAGAAC---
AATCTG---CTGAGA---GCTATTGAGGCGCAACAGCACCTGTTGCAACTCACAGTCTGGGGCATC
>KT427718
TTCTTCTGTAATACATCAGGACTGTTCAATAGTACTTGGTCATCTAATAGTACGTGGAATGATACTAACGAGT
CAGAAAAT---

ATCACAGAAATTATCACACTCCCATGCAGAATAAAACAAATTATAAACATGTGGCAGAGAGTAGGAAGAGCAA
TGTATGCCCCTCCCATCAGAGGAAAAATTAGTTGTTCATCAAAT---
ATTACAGGTCTGCTATTAACAAGGGATGGTGGTGATGAGAGCAAC----------------------------
----

AATGGGACTGAGATCTTCAGACCTGGAGGAGGAGATATGAGGGACAATTGGAGAAGTGAATTATATAAATATA
AAGTAGTAAAAATTCAACCATTA---GGGATAGCACCCAACAAGGCAAAGAGAAGAGTGGTG-----------
----------------------------CAAAGAGAAAAAAGAGCA---GTAACA---TTTGGAGCT------
---ATGTTCCTTGGGTTCTTGGGAGCA---GCAGGAAGCACTATG---GGCGCAGCGTCA---
ATAACGCTGACGGTACAG---GCCAGACAG---TTATTG---TCTGGTATAGTGCAACAGCAGAAC---
AATCTG---CTGAGA---GCTATTGAGGCGCAACAGCATATATTGCAACTCACAGTCTGGGGCATC
>KX181896
TTTTTCTGCAATACAACACAGCTGTTTAATAGTACTTGGCTACTTAATGATACTTGGGATAGTACTGGGGAGT
CAAATAAC---

ACTGCAGAGAATATCACACTTCCATGCAGAATAAAACAAATTATAAACAGGTGGCAGGGGGTAGGAAAAGCAA
TGTATGCCCCTCCCATCGAAGGACTAATTAGATGTTCATCAAAT---
ATTACAGGGCTGATATTAACAAGAGATGGTGGTGGTGATAATAAC----------------------------
----

AGGTCCAAAGAAACCTTCAGACCTGGGGGGGGGGGGATGAGGGGCAATTGGGGAAGTGAATTATATAAATATA
AAGTAGTAAAAAATGAGCCCTTA---GGAATAGCCCCCCCCCCGGCAAAAAGAAGGGGGGGG-----------
----------------------------CAGAGAGAAAAAAGAGCA---GTGGGAACGATAGGAGGT------
---ATGTTCCTTGGGTTTTTGGGAACA---GCAGGAAGCACTATG---GGGCCAGCGTCA---
ATCACGCTGACGGTACAG---GCCAGACTA---GTATTG---TTTGGTATAGTGCAGCAGCAGAAC---
AATTTG---CTGAAG---GCTATTGAGGCGCAACAGCATTTGTTGCAACTCACAGTCTGGGGCATC
>JQ403021
TTCTTTTGTAACACAACACCACTGTTTAATAGTACTCGGCTGTCTAATGGTACTTGGCTGTTTAATAGTACTG
AGAATAGT---
-------------------AAT---
GACCCTAAAGCCAACATTACACTCCAATGCAGAATAAAACAAATTATAAACATGTGGCAGGAAGTTGGAAAAG
CAATGTATGCCCCTCCCATCAAGGGACTAATTAGATGTAAGTCAAAT---
ATTACAGGGCTGCTATTAACAAGAGATGGTAGTACTAACAGCACT----------------------------
----

GAGGAGATTTTCAGACCTGGAGGAGGAGATATGAGGGACAATTGGAGAAGTGAATTATATAAATATAAAGTAG
TAAAAATTGAACCATTA---GGAGTAGCACCCACCAAGGCAAAGAGAAGAGTGGTG-----------------

----------------------CAGAGAGAAAAAAGAGCA---GTAGGGGTAATAGGAGCC---------
ATGTTCCTTGGGTTCTTGGGAGCA---GCAGGAAGCACTATG---GGCGCAGCGTCA---
ATAACGCTGACGGTACAG---GCCAGACAA---TTGCTG---TCTGGTATAGTGCAACAGCAGAAC---
AATCTG---CTGAGG---GCTATTGAGGCGCAACAACATCTGTTGCAACTCACAGTCTGGGGCATC
>JQ403047
TTCTTCTGTAATACATCACAACTGTTTAGTAGTACTTGGCTGTCTAATGGTACTTGGAATGGTACTACAAGTA
AAAATGAT---

ACTACAGGAAATATCACACTCCCATGCAGAATAAAACAAATTATAAACATGTGGCAGGAAGTAGGAAAAGCAA
TGTATGCCCCTCCCATCAAAGGACCAATCAAATGTTCATCAAAT---
ATTACAGGGCTGATATTAACAAGAGATGGGGGTATCAACAATACGAGC-------------------------
----

AATGATACAGAGGTCTTTCGACCTACAGGAGGAAATATGAAGGATAATTGGAGAAGTGAACTATATAAATATA
AAGTAGTAAAAATTGAACCATTA---GGAGTAGCACCCAACAAGGCGAAGAGAAGAGTGGTG-----------
----------------------------CAAAGAGAAAAAAGAGCAGTAGTGGGA---CTAGGAGCT------
---GTGTTCCTTGGGTTCTTGGGAGCA---GCAGGAAGCACTATG---GGCGCAGCGGCA---
GTAACGCTGACGGTACAG---GCCAGACAA---TTATTG---TCTGGTATAGTGCAGCAGCAGAAC---
AATCTG---CTGAGG---GCTATTGAGGCGCAACAGCATATGTTGCAACTCACGGTCTGGGGCATC
>EU363825
TCTACTGTATACATCACACTGTTTACTAGTAACTGGAATGAGAGTGATACTAGA-------------------
----

GTAAATATCCACGCTCCTTGCAGAATAAAACAAATTGTAAACATGTGGCAGGAAGTAGGGAAAGCAATGTATG
CCCCTCCCATTGCAGGACAAATTAGATGTTCATCAAAT---
ATTACAGGATTGCTATTAACAAGAGATGGTGGTAATGAGACCACC----------------------------
----

ACCGGGAACGAGACCTTCAGACCTGGAGGAGGAGATATGAGAGACAATTGGAGAAGTGAATTATATAAATATA
AAGTAGTAAAAATTGAACCATTA---GGAGTAGCACCCACCAAGGCAAAGAGAAGAGTGGTG-----------
----------------------------CAAAGAGAAAAAAGAGCA---GTGGGAACGATAGGAGCT------
---ATGTTCCTTGGGTTCTTGGGAGCA---GCAGGAAGCACTATG---GGCGCAGCGTCA---
ATAACGCTGACGGTACAG---GCCAGACAA---ATGTTG---TCCGGTATAGTGCAACAGCAGAGA---
AATCTG---CTGAGG---GCTATTGAGGCGCAACAGCATCTGTTGCAACTCACAGTCTGGGGCATC
>AY586543
TTCTGCTGTAATACAACACAGCTGTTTAATAGTACTTGGTTTGGTAATGGTACTAGCAGGTCAAATGAC----
----

ACTGGAGAAAATATCACACTCACATGTAAAATAAAACAAATTGTAAACCTGTGGCAGGAAGTAGGAAAAGCAA
TGTATGCTCCTCCCATCAAAGGACAAATTAGATGTTCATCAAAC---
ATTACAGGACTGCTATTAACAAGAGATGGTGGTGGTAACAGCAGT----------------------------
----

AGTAGCAACGAGACCTTCAGACCTGGAGGAGGAGATATGAGGGACAATTGGAGAAGTGAATTATATAAATATA
AAGTAGTAAAAATTGAACCATTA---GGAATAGCACCCACCAAGGCAAAGAGAAGAGTGGTG-----------
----------------------------CAGAGAGAAAAAAGAGCA---GTGGGA---ATAGGAGCT------
---GTGTTCCTTGGGTTCTTGGGAGCA---GCAGGAAGCACTATG---GGCGCAGCGTCA---
GTGACGCTGACGGTACAG---GCCAGGCAA---TTATTG---TCTGGTATAGTGCAGCAGCAGAAC---
AATTTG---CTGAGG---GCTATTGAGGCGCAACAACATATGTTGCAACTCACAGTCTGGGGCATC
>FJ388904
TTCTTCTGCTGTACTACAGGGCTGTTGAATTGGAATTTTACTGGGAATAAT----------------------
----

ACTAATCATACTACCACACTACCATGCAGAATAAGACAAATTGTAAATAGGTGGCAGGAAGTAGGAAAAGCAA
TGTATGCCCCTCCCATCAGTGGACAGATTAACTGCTCATCAAAC---
ATTACAGGGCTACTATTAACAAGAGATGGTGGTGATGAGAACGGG----------------------------
----

ACCAACGAGATCTTTAGACCTGGAGGAGGAGATATGAGGGACAATTGGAGAAGTGAATTATATAAATATAAAG
TAGTAAAAATTCAACCATTA---GGAATAGCACCCACCAAGGCAAAAAGAAGAGTGGTG--------------

-------------------------CAAAGAGAAAAAAGAGCA---GTGGGAACGCTAGGAGCT---------
ATGTTCCTTGGGTTCTTGGGAGCA---GCAGGAAGCACTATG---GGCGCAGCGTCA---
CTGACGCTGACGGTACAG---GCCAGACAA---TTATTG---ACTGGTATAGTGCAACAGCAGAAC---
AATCTG---CTGAGA---GCTATTGAGGCGCAACAGCATATGTTGCAACTCACAGTTTGGGGCATC
>JF683750
TTCTTTTGTAACACAACACAACTGTTTAATAGTACTTGGAACAGTACTACTTGGAATAGTACTGAGAATGAAG
CTGGA--

GAGTCAAATGACAATATCACACTCCAGTGCAGAATAAAACAAATTATAAACATGTGGCAAGAAGTAGGAAAAG
CAATGTATGCCCCTCCCATTAGGGGACCAATTAACTGCTCGTCAAAT---
ATCACAGGGCTGCTATTAACAAGAGATGGTGGTAACAGGAATGGGAGT-------------------------
----

AATGAGACCGAGATCTTCAGACCTGGAGGAGGAGATATGAGGGACAATTGGAGAAGTGAATTATATAAATACA
AAGTAGTGCAAATTGAACCAATA---GGATTAGCACCCACCAAGGCAAAGAGAAGAGTGGTG-----------
----------------------------CAGAGAGAAAAAAGAGCA---GTGGGA---CTAGGAGCC------
---TTGTTCCTTGGGTTCTTGGGAGCA---GCAGGAAGCACTATG---GGCGCAGCGTCG---
ATATCGCTGACGGTACAA---GCCAGACAA---TTATTG---TCTGGTATAGTGCAACAGCAGAAC---
AACCTG---CTGAGG---GCTATTGAGGCGCAACAGCACATGTTGCAACTCACAGTCTGGGGCATC
>JF683797
TTCTTCTGTAATTCATCACAACTGTTTAATAGTACTTGGGCTGACGAAAATGGCACAATCACACAAGGAAATG
ACACA--

CAAGGAAATGACACAATCATACTCCCATGTAGAATAAAACAAATTATAAACATGTGGCAGCAGGTAGGAAAAG
CAATGTATGCCCCTCCCATACGAGGACTAATTAACTGTACATCAAAT---
ATTACAGGAATACTATTAACAAGAGATGGTGGGCATAATGTGAAC----------------------------
----

AATACCACAGAGACCTTCAGACCAGCAGGAGGAGATATGAGGGACAATTGGAGAAGTGAATTATATAAATATA
AAGTAGTACAAATTGAGCCATTA---GGAATAGCACCCACCAGGGCAAAGAGAAGAGTGGTG-----------
----------------------------CAGAGAGAAAAAAGAGCA---GTGGGA---ATAGGAGCT------
---TTGTTCCTTGGGTTCTTGGGAGCA---GCAGGAAGCACTATG---GGCGCAGCGTCA---
ATGACGTTGACGGTACAG---GCCAGACAA---CTATTG---TCTGGTATAGTGCAACAGCAGAAC---
AACTTG---CTGAGA---GCTATTGAGGCGCAACAGCATCTGTTGCAACTCACAGTCTGGGGCATC
>KT124808
TTCTTCTGTAACACAACAAATCTGTTTAATAGCATTTGGGATATCAATAGTACTTGGAATAGTACTGAAAGGA
CAAACAAC---

ACAGACAACACTAACATCACACTCCAATGCAGAATAAAACAAATTATAAACATGTGGCAGGGAGTAGGAAGAG
CAATGTATGCCCCTCCCATCAGAGGGCTGATTAAATGTTCATCAAAT---
ATCACAGGGCTGCTATTAACAAGGGATGGTGGTAATAGTAATGACACC-------------------------
----

AACAACACAGAGACCTTTAGACCAGGAGGGGGAGATATGAAGGACAACTGGAGAAGTGAATTATATAAATATA
AAGTAGTACAAATTAAACCATTA---GGAATAGCACCCACCAAGGCAAAGAGAAGAGTGGTG-----------
----------------------------CAGAGAGAGAAAAGAGCA---GTTACC---TTCGGAGCT------
---ATGTTCCTTGGGTTCTTGGGAGCA---GCAGGAAGCACTATG---GGCGCAGCGTCA---
CTGACGTTGACGGTACAG---GCCAGGCAA---TTATTG---TCTGGCATAGTGCAACAGCAGAAC---
AATCTG---CTGAGA---GCTATTGAGGCACAACAACATCTGTTGCAGCTCACAGTCTGGGGCATT
>KT200352
TTCTTCTGCAATTCATCACAACTGTTTAATAGTACTTGGCCAAGTAACAGTACTTGGAATATTAGCACTAATA
CTCAGGGGAAT--

AGCACTGAAGACATTATCACACTCCCATGTAGAATAAAGCAAATTATAAACATGTGGCAGGAGGTAGGAAAAG
CAATGTATGCCCCTCCCATCCAAGGACAAATTAGATGTTCATCAAAT---
ATTACAGGGCTGCTATTAACAAGAGATGGTGGTATAAACAATACA----------------------------
----

AATAATACTGAAACCTTCAGACCAGGAGGAGGAAATATGAAGGATAATTGGAGAAGCGAATTGTATAAATACA
AAGTAGTACAAATTGAACCATTA---GGAGTAGCACCCACCAAGGCAAAGAGAAGAGCGGTG-----------

----------------------------CAGAGAGAAAAAAGAGCA---ATAGGA---CTAGGAACT------
---ATGTTCCTTGGGTTCTTGGGAGCA---GCAGGAAGCACTATG---GGCGCAGCGTCA---
ATGACGCTGACGGTACAG---GCCAGACAA---TTATTG---TCTGGTATAGTGCAACAGCAGAAC---
AATCTG---CTGAGA---GCTATTGAGGCGCAACAGCATCTGTTGCAACTCACAGTCTGGGGCATT
>KC699022
TTCTTCTGTGATACAACAAAGCTGTTTAATAGTACTTGGCTGGCTAATGGTAGGAGGAATGATACTTCAAGTT
CA---

AATGACACTCTCATCATACTCCCATGCAGAATAAAACAAATTATAAACAGATGGCAAGAAGTAGGTAAAGCAA
TGTATGCCCCTCCCATCAGTGGACTAATTAAATGTTCATCAAAT---
ATTACAGGGCTGCTATTAACAAGAGATGGTGATGAGGATAATAGTAATAGCACG-------------------
----

AAAAACAACACCATAGAGACCTTCAGACCTGGAGGGGGAGATATGAGAGACAATTGGAGAAGTGAATTATACA
AATATAAAGTAGTAAGAATTGAGCCATTG---GGAGTAGCACCCACCAAGGCAAAGAGAAGAGTGGTG-----
----------------------------------CAGAGAGAAAAAAGAGCA---GTAACG---
CTCGGAGCT---------ATGTTCCTTGGGTTCTTGGGTGCA---GCAGGAAGCACTATG---
GGCGCAGCGTCA---ATAACGCTGACGGTACAG---GCCAGACAA---TTATTG---
TCTGGTATAGTGCAGCAGCAAAAC---AATTTG---CTGAGA---
GCTATTGAGGCGCAACAACATCTGTTGCAACTCACGGTCTGGGGCATC
>KC699024
TTCTTCTGTAATTCATCACAACTGTTTAATAGTACTTGGTATGGTAATGGTACTGGAAATGCCAAC-------
----

ACTACAGGCACAATCACACTCCCATGCAGAATAAAACAGATTATAAACATGTGGCAGGAGGTAGGAAAAGCAA
TGTATGCCCCTCCCATCAAAGGACAAATTAGATGTTCATCAAAT---
ATTACAGGGCTGCTATTAACTAGAGATGGTGGCAAGGATCAGACT----------------------------
----

GAGAATGAAACCGAGACCTTCAGACCTGAAGGGGGAAATATGAAGGACAATTGGAGAAGCGAATTATATAAAT
ATAAAGTAGTAAAAATTGAACCATTA---GGAATAGCACCCACCAAGGCAAAGAGAAGAGTGGTG--------
-------------------------------CAAAGAGAAAAAAGAGCA---GTGGGAGTGATAGGAGCT---
------ATGTTCCTTGGGTTCTTGGGAGCA---GCAGGAAGCACTATG---GGCGCAGCGTCA---
GTGACGCTGACGGTACAG---GCCAGACAA---TTATTG---TCTGGTATAGTGCAACAGCAGAAC---
AATTTG---CTGAGG---GCTATTGAGGCGCAACAGCATCTGTTGCAACTCACAGTCTGGGGCATT
>HQ595798
TTCTTCTGCAATACAACAGCACTGTTTAATAGTACTTGGAATAATGAAAGGTCAAATGAC-------------
----

ACTGGAGGAAATATCACACTCCCATGCAGAATAAAACAAATTATAAACATGTGGCAGGAAGTAGGAAAAGCAA
TGTATGCCCCTCCCATCAGTGGACAAATTAGATGTTCATCAAAT---
ATTACAGGGCTGCTATTAACAAGAGATGGTGGTGACAGTCCAAAT----------------------------
----

GACACTGAGACCTTCAGACCTGGAGGAGGAGATATGAGGGACAATTGGAGAAGTGAATTATATAAATATAAAG
TAGTAAGAATTGAACCATTA---GGAGTAGCACCCACCAAGGCAAAAAGAAGAGTGGTG--------------
-------------------------CAGAGAGAAAAAAGAGCA---GTGGGA---ATAGGAGCT---------
CTGTTCCTTGGGTTCTTGGGAGCA---GCGGGAAGCACTATG---GGCGCAGCGTCA---
ATGACGCTGACGGTACAG---GCCAGACTA---TTATTG---TCTGGTATAGTGCAACAGCAAAAC---
AATTTG---CTGAGG---GCTATTGAGGCGCAACAGCATCTGTTGCAACTCACAGTCTGGGGCATC
>HQ595777
TTTTTCTGTAATACAACACAACTGTTTAATAGTAGTTGGAACAGTAACAGTAACAGTTCTGTAAATGAA----
----

AGTTCTGTGAATATCACACTCCCATGTAGAATGAAACAAATTATAAACATGTGGCAGGAAGTAGGAAAAGCAA
TGTATGCCCCTCCCATCAGAGGACCAATCCAATGCATATCAAAT---
ATCACAGGGCTATTATTAACAAGAGATGGTGGTAATAATGGAACA----------------------------
----

AATGAGAGCGAGATCTTCAGACCTGCAGGAGGCAATATGAGGGACAATTGGAGAAGTGAATTATATAAATATA

AAGTAGTAAAAATTGAACCATTA---GGAATAGCACCCACCAAGGCAAGGAGAAGAGTGGTG-----------
----------------------------CAGAGAGAAAAAAGAGCA---GTGGGAACGCTAGGAGCT------
---ATGTTCCTTGGTTTCTTGGGAGCA---GCAGGAAGCACTATG---GGCGCAGCGTCA---
CTGACGCTGACGGTACAG---GCCAGACAA---CTGTTG---TCTGGTATAGTGCAACAACAGAAC---
AATCTG---TTGAGG---GCTATTGATGCGCAACAGCATATGTTGCAACTCACAGTCTGGGGCATC
>AB588226
TTCTTCTGTAATTCAACACCACTGTTTAATAGTACTTGGAATAGTACTGGATGGTTAAGTAACACAAATAAC-
----

GCTACAGGAAATATCACACTCCAATGTAGAATAAAACAAATTATAAACATGTGGCAGGAAGTAGGAAAAGCAA
TGTATGCCCCTCCCATCAGAGGACAAATTAGATGTTCATCAAAT---
ATTACAGGGCTACTATTGACAAGAGATGGTGGTAATAGCACGAGC----------------------------
----

GGGGCAGAGATCTTCAGACCTGGAGGAGGAGATATGAGGGACAATTGGAGAAGTGAATTATATAAATATAAAG
TAGTAAAAATTGAACCATTA---GGAGTAGCACCCACCACGGCAAAGAGAAGAGTGGTG--------------
-------------------------CAGAGAGAAAAAAGAGCA---GTGGGAACAATAGGAGCT---------
ATGTTCCTTGGGTTCTTGGGAGCA---GCAGGAAGCACTATG---GGCGCAGCGTCA---
ATGGCGCTGACGGTACAG---GCCAGACAA---CTATTG---TCTGGTATAGTGCAACAGCAGAAC---
AATCTG---CTGAGG---GCTATTGAGGCGCAACAGCATATGTTGCAACTCACAGTCTGGGGCATC
>AJ417428
TTCCACTGTAATTCAACACAACTGTTTAATAGTACCTGGAACGGTAATGATACTGATACTAAAGAAACAATG
AC---

ACTGAAGGAACTATCATACTCCCATGCAGAATAAAACAAATTGTAAACCTGTGGCAGGAAGTAGGAAAAGCAA
TGTATGCTCCTCCCATCAGGGGACAAATCAGATGTTCATCAAAT---
ATTACAGGGCTGCTATTAACAAGAGATGGTGGTAATATCACGAAT----------------------------
----

GAGACCGAGATCTTCAGACCTGGAGGAGGAGATATGAGGGACAATTGGAGGAGTGAATTATATAAATATAAAG
TAGTAAAAATTGAACCATTA---GGAGTAGCACCCACCAAGGCAAAGAGAAGAGTGGTG--------------
-------------------------CAGAGAGAAAAAAGAGCA---GTGGGA---CTAGGAGCT---------
TTGTTCCTTGGGTTCTTGGGAGCA---GCAGGAAGCACTATG---GGCGCAGCGTCA---
ATGACGCTGACGGTACAG---GCCAGACAA---TTATTG---TCTGGTATAGTGCAACAGCAAAAC---
AATTTG---CTGAGG---GCTATTGAGGCGCAACAGCATCTGTTGCAACTCACAGTCTGGGGCATC
>MF373203
TTC---TCCGGAACCCCACCAGGACAATCCACCATACCTGGAATCATGGGAACTCGAAAAATAACTAAGGGA-
----

CCAATTAAGGCATTAATCCCCTTCCCTGGAAGAATAAAACAAATTATAAACCTGTGGCAAGAAGTAGGAAAAG
CAATGTATGCCCCTCCCATCAGGGGAAACATTAGTTGTTCATCAAAT---
ATTACAGGACTACTATTAACAAGGGATGGTGGTAATAATGAAACT----------------------------
----

GAGACTGAGACCTTCAGACCTGGAGGAGGAGATATGAAGGACAATTGGAGAAGTGAATTATATAGATATAAAG
TAGTAAAAATTGAACCATTA---GGAATAGCACCCACCAAGGCAAGGAGACGGGTGGTG--------------
-------------------------CAGAGAGAAAAAAGAGCA---GTAACA---CTAGGAGCT---------
ATGTTCCTTGGGTTCTTGGGGGCA---GCAGGAAGCACTATG---GGCGCAGCGTCA---
CTGACGCTGACGGTACAA---GCCAGACAA---TTATTG---TCTGGTATAGTGCAGCAGCAAAAC---
AATTTG---CTGAGG---GCTATTGAGGCGCAACAACACCTGTTGCAACTCACAGTCTGGGGCATT
>AY173951
TTCTTCTGTAATTCAACAAAACTGTTTAATAGTACTTGGAATAGCACTGAAGGAAATGACGATGGA-------
----

GAGGAAAGAAATATCACACTCCCATGCAGAATAAAACAAATTGTAAACATGTGGCAGGAAGTAGGAAAAGCAA
TGTATGCCCCTCCCATCGGAGGACAAATTAGATGCACCTCAAAT---
ATTACAGGGCTGCTATTAACAAGAGATGGAGGTAACCAAAATGGGACC-------------------------
----

AACGAGACTGAAATCTTCAGACCTGGAGGAGGAAATATGAGGGACAATTGGAGAAGTGAACTATATAAATATA

AAGTAGTAAAAATTGAACCATTA---GGAGTAGCACCCACTAAGGCAAAGAGAAGAGTGGTG-----------
----------------------------CAGAGAGAAAAAAGAGCA---GTGGGA---ATAGGAGCT------
---GTGTTCCTTGGGTTCTTGGGAGCA---GCAGGAAGCACTATG---GGCGCAGCGTCA---
GTGACGCTGACGGTACAG---GCCAGACTA---TTATTG---TCTGGTATAGTGCAACAGCAGAAC---
AATCTG---CTGAGG---GCTATTGAGGCGCAACAGCATCTGTTGCAACTCACAGTCTGGGGCATC
>AF277068
TTCTTCTGTAATACAACACAATTGTTTAACAGTACTTGGAATATTAATGGTACTTGGCATGGTACTACAGTG-
----

TCAAATAAGACTATCATACTCCCATGCAGAATAAAACAAATTATAAACATGTGGCAGGAAGTAGGCAAAGCAA
TGTATGTCCCTCCTATTAGAGGACAAGTTAGATGTTCATCAAAT---
ATTACAGGATTACTATTAACGAGAGACGGTGGTAATAATAACAGC----------------------------
----

ACCACCGAGATCTTCAGACCTGGAGGAGGAGATATGAGGGACAATTGGAGAAGTGAATTATATAAATATAAAG
TAGTAAAAATTGAACCATTA---GGAATAGCACCCACCAAGGCGAGGAGAAGAGTGGTG--------------
-------------------------CAAAGAGAAAGAAGAGCA---GTGGGAACAATAGGAGCT---------
ATGTTCCTTGGGTTCTTGGGAGCA---GCAGGAAGCACTATG---GGCGCAGCATCA---
GTAACGCTGACGGTACAG---GCCAGACTA---TTATTG---TCTGGTATAGTGCAACAGCAGAAC---
AATCTG---CTGAGG---GCTATTGAGGCGCAACAGCATCTGTTGCAACTCACAGTCTGGGGCATC
>FJ469735
TTGTACTGTAATACATCACATCTGTTTAATAGTACCTGGATGTGTAATAGTACTTGGAATAGTACTAACACTG
AA---

GGGAACAACACAATCACACTCCCATGCAGAATAAAACAAATTGTAAACATGTGGCAGGAAGTAGGAAAAGCAA
TGTATGCCCCTCCCATCAAAGGACAAATTAAATGCTTATCAAAT---
ATTACAGGGCTGTTATTAACAAGAGATGGTGGTGTCGGTGATGACAGT-------------------------
----

TCAAACGACACCGAGATCTTCAGACCTGGAGGAGGAGATATGAGGGACAATTGGAGAAGTGAATTATATAAAT
ATAAAGTAGTAAGAATTGAACCATTA---GGAATAGCACCCACCAAGGCAAAGAGAAGAGTGGTG--------
-------------------------------CAGAGAGAAAAAAGAGCA---GTGGGAACACTAGGAGCT---
------ATGTTCCTTGGGTTCTTGGGAGCA---GCAGGAAGCACTATG---GGCGCAGCGTCA---
GTGACGCTGACGGTACAG---GCCAGACAA---TTATTG---TCTGGTATAGTGCAACAGCAGAAC---
AATCTG---CTGAGG---GCTATTGAGGCGCAACAGCATCTGTTGCAACTCACAGTCTGGGGCATC
>JQ403106
TTCTTCTGTAATACATCAAAACTGTTTACTTGGAAGAGTACTAACAGGTCACTTAGCATTGGA----------
----

GAAAATGACACAATCACACTCCCATGCAGAATAAAACAAATCATAAATATGTGGCAAAGTGTAGGAAAAGCAA
TGTATGCCCCTCCCATCAAGGGACAAATTAAATGTACATCAAAT---
ATTACAGGGCTGCTAATAACAAGAGATGGTGGTCAGGGTAATGAAACA-------------------------
----

GATAATAGTACTGAGATCTTCAGACCTGGAGGAGGAGATATGAGGGACAATTGGAGAAGTGAATTATATAAAT
ATAAAGTAGTAAAAATTGAACCATTA---GGAGTAGCACCCACCAAGGCAAAGAGAAGAGTGGTG--------
-------------------------------CAGAGAGAAAAAAGAGCA---GTGGGA---CTGGGAGCT---
------TTGTTCCTTGGGTTCTTGGGAGCA---GCAGGAAGCACTATG---GGCGCAGCGTCA---
GTGACGCTGACGGTACAG---GCCAGACAA---TTATTG---TCTGGTATAGTGCAACAGCAGAAC---
AATTTG---CTGAGG---GCTATTGAGGCGCAACAGCATATGTTGCAACTCACAGTCTGGGGCATC
>FJ469759
TTCTTCTGTAATACAACACAACTGTTTAATAGTACTTGGAATAGTACTGGGTCAAATAACACAGAA-------
----

GGAAATGATACAATCACCATCCCATGCAAAATAAGACAATTTATAAACATGTGGCAGAAAGTAGGAAAAGCAA
TGTATGCCCCTCCCATCAAAAGAAACATTAGTTGTACATCAAAT---
ATTACAGGGTTGCTATTAACAAGAGATGGTGGTAGTGAAGAGAGC----------------------------
----

AATACCACTGAGACCTTCAGACCTGGAGGAGGAGATATGAGGGACAATTGGAGAAGTGAATTATATAAATATA

AAGTAGTAAAACTTGAACCATTA---GGAGTAGCACCCACTAAGGCAAAGAGAAGAGTGGTG-----------
----------------------------CAGAGAGAAAAAAGAGCA---TTGGGA---CTAGGAGCT------
---CTGTTCCTTGGGTTCTTGGGAGCA---GCAGGAAGCACTATG---GGCGCAGCGTCA---
GTGACGCTGACGGTACAG---GCCAGACTA---TTAATG---TCTGGTATAGTGCAACAGCAGAAC---
AATTTG---CTGAAG---GCTATTGAGGCGCAACAGCATCTGTTGCGACTCACAGTCTGGGGCATC
>FJ469722
TTCTTCTGTAATACAACACAACTGTTTAATAGTACGTGGCCAGTTAATGGTAATAACACTGAAGGA-------
----

GATAGCACAAACATCACGATCCCATGTAGAATAAAACAGATCATAAATATGTGGCAGGAAGTAGGAAAAGCAA
TGTATGCCCCTCCCATCAGAGGATCAATTAAATGTTCATCTAAT---
ATAACAGGGCTGCTATTAACAAGAGATGGTGGTAATATCACGAAC----------------------------
----

GAGACCACCGAAATCTTCAGACCTGGAGGAGGAGATATGAGGGACAATTGGAGAAGTGAATTATATAAATATA
AAGTAGTAAAAATTGAACCCTTA---GGAATAGCACCCACTAAGGCAAAGAGAAGAGTGGTG-----------
----------------------------CAGAGAGAAAAAAGAGCA---GCA------ATAGGGGCT------
---GTGTTCCTTGGGTTCTTGGGAGCA---GCAGGAAGCACTATG---GGCGCAGCGTCA---
CTGACGCTGACGGTACAG---GCCAGACTA---CTATTG---TCTGGTATAGTGCAACAGCAGAAC---
AACTTG---CTGAGG---GCTATTGAGGCGCAACAGCATATGTTGCAACTCACAGTCTGGGGCATC
>KF990605
TTCTTCTGTAATACAACACAATTGTTTAGTAGCACTTGGAATGATACTAATAAAAATATCACAGGGTCA----
----

ACTGACACTACTATCACACTCCCATGTAGAATAAAACAAATTGTAAACTTGTGGCAGGAAGTAGGAAAAGCAA
TGTATGCCCCTCCCATCAAAGGACAAATTAACTGTGTATCAAAT---
ATTACAGGGCTGCTATTAACAAGAGATGGGGGTAAGGGGAACAACCACACTGAG-------------------
----

AACATTGAGATCTTCAGACCTGGAGGAGGAGATATGAGGGACAATTGGAGAAGTGAATTATATAAATATAAAG
TAGTAGCACTTGAACCATTA---GGAGTAGCACCCACCACGGCAAAGAGAAGAGTGGTG--------------
-------------------------CAAAGAGAGAAAAGAGCA---ATAGGA---ATAGGAGCC---------
TTGTTCCTTGGGTTCTTGGGAGCA---GCCGGAAGCACTATG---GGCGCAGCGTCA---
ATATCGCTGACGGTACAG---GCCAGACAA---TTATTG---TCTGGTATAGTGCAACAGCAGAAC---
AATTTG---CTGAGG---GCTATTGAGGCGCAACAACATATGTTGCAACTCACAGTCTGGGGCATC
>FJ653127
TTCTTCTGTAATACAACACCACTATTTAATAGTACTTGGAATATAACTGATGAAAGGGTAAATAACACTGAAG
GA---

AATAGCACTATCATCACACTCCCATGCAGAATAAAACAAATTATAAACATGTGGCAAGAAGTAGGAAAAGCAA
TGTATGCCCCTCCCATTAGAGGACTAATTAACTGTGTATCAAAT---
ATTACAGGGATAATATTAACAAGGGATGGTGGGCAGGGAAATGACACA-------------------------
----

AGTGGAGCCGAGACCTTCAGACCTGGAGGAGGAGATATGAGGGACAATTGGAGGAGTGAATTATACAAATATA
AAGTAGTACGAATTGAACCATTA---GGAATAGCACCCACCAAGGCAAGGAGAAGAGTGGTG-----------
----------------------------CAAAGAGAAAAAAGAGCA---GTGGGAACGTTGGGAGCT------
---ATGTTCCTTGGGTTCTTAGGAGCA---GCAGGAAGCACTATG---GGCGCAGCGTCA---
CTGACGCTGACGGTACGG---GCCAGACTA---TTACTG---TCTGGTATAGTGCAACAGCAAAAT---
AATTTG---CTGAGG---GCTATTGAGGCGCAGCAGCACCTGTTGCAACTCACAGTCTGGGGCATC
>JF320329
TTCTTCTGTAACACAACACAATTGTTTAACAGTACTTGGGGGCTTAATGATACTTGGGAAAGTGATACTGCTA
CTGAA--
-------------------GGA---
TCAGATAACATCACAATCACACTCCCATGCAAAATAAAGCAAATTATAAACATGTGGCAGGAAGTAGGAAAAG
CAATGTATGCCCCTCCTATCAGAGGACAAATTAAATGTTTATCAGAT---
ATTACAGGGCTGCTATTAACAAGAGATGGTGGTACTAATAATGAGACT-------------------------
----

AATGGCACTGAGACCTTCAGACCTCAAGGAGGAAATATGAAGGACAATTGGAGGAGTGAATTATATAAATATA

AAGTAGTAAAAATTGAACCATTA---GGAGTAGCACCCACCAGGGCAAAGAGAAGAGTGGTG-----------
----------------------------CAGAGAGAAAAAAGAGCA---GTGGGA---ATGGGAGCT------
---ATGTTCATTGGGTTCTTGGGAGCA---GCAGGAAGCACTATG---GGCGCAGCATCA---
ATAACGCTGACGGTACAG---GCCAGACAA---TTGTTG---TCTGGTATAGTGCAACAGCAGAAC---
AATTTG---CTGAGA---GCTATTGAGGCGCAACAACATCTGTTGCAACTCACAGTCTGGGGCATC
>FJ469710
TTGTACTGTGATACATCAAAACTGTTTAATAGTACTTGGTATAGGAATGGTACTATAGTAAAGTCAAACAACA
CT---

AGTGGAAATGACACAATCGTACTCCCATGCAGAATAAAACAAATTATAAACATGTGGCAGGAAGTAGGAAAAG
CAATGTATGCCCCTCCCATCAGAGGACCAATCATCTGTTTATCAAAT---
ATTACAGGGTTGCTATTAACAAGAGATGGTGGTAATGACAATAAAACA-------------------------
----

AATAACACTGAGACCTTCAGACCTGGAGGAGGAGATATGAGGGACAATTGGAGAAGTGAATTATATAAATATA
AAGTAGTAAAAATTGAACCATTA---GGAGTAGCACCCACCAGGGCAAAGAGAAGAGTGGTG-----------
----------------------------CAAAGAGAGAAAAGAGCA---GTGGGA---CTAGGAGCT------
---TTGTTCCTTGGGTTCTTGGGAGCA---GCAGGAAGCACTATG---GGCGCAGCGTCA---
GTGACGCTGACGGTACAG---GCCAGACAA---TTATTG---TCTGGTATAGTGCAACAGCAGAAC---
AATTTG---CTGAGG---GCTATTGAGGCGCAACAGCATATGTTGCAACTCACAGTCTGGGGCATC
>KF990608
TTCTTCTGTAATACATCAAAGCTGTTTAATAGTACTTGGCCATATAATAGTACTTGGAATGAAACTGTGCCCA
AT---

AACAATGACACTATCACACTCCCATGTAGAATAAAACAAATTGTAAACATGTGGCAGCAAGTAGGAAAAGCAA
TGTATGCTCCTCCCAGCAGAGGACAAATTAGCTGTTCATCAAAT---
ATTACAGGGTTGCTACTAACAAGAGATGGTGGTATTAACATTACCACA-------------------------
----

AACAATACTGAAACCTTCAGACCTGCAGGAGGAAATATGAAGGACAATTGGAGAAGTGAATTATATAAATATA
AAGTAGTAAGAATTGAACCACTA---GGAGTAGCACCCACCAAGGCAAGGAGAAGAGTGGTG-----------
----------------------------CAGAGAGAAAAAAGAGCA---GTAGGA---CTAGGAGCT------
---TTGTTCGTTGGGTTCTTGGGAGCA---GCAGGAAGCACTATG---GGCGCAGCGTCA---
ATAACGCTGACGGTACAG---GCCAGACAA---TTATTG---TCTGCTATAGTGCAACAGCAAAAC---
AATTTG---CTGAGA---GCTATTGAGGCGCAACAGCATCTGTTGCAACTCACAGTCTGGGGCATT
>JQ403066
TTCTTCTGTAATACAACACAACTGTTTAATAGTACTTGGAATGGTACTGAAGGGTCAAATAGCACTAGAGAA-
----

AAGAATGACACAATCATACTCCCATGCAGAATAAAACAAATTATAAACATGTGGCAGGAAGTAGGAAAAGCAA
TGTATGCCCCTCCCATCGCAGGGCAAATTAATTGTTCATCAAAT---
ATTACAGGGCTGCTATTAATAAGAGATGGTGGTGATAAGAACGAGACC-------------------------
----

ACCACCAACGAAACCTTCAGACCTATAGGAGGAAATATGAAAGACAATTGGAGGAGTGAATTATATAAATATA
AAGTAGTAAAAATTGAACCATTA---GGAGTAGCACCCACCAAGGCAAAGAGAAGAGTGGTG-----------
----------------------------CAGAGAGAAAAAAGAGCA---GTGGGA---TTAGGAGTC------
---ATGTTCCTTGGGTTCTTGGGAGCA---GCAGGAAGCACTATG---GGCGCAGCGTCA---
ATGGCGCTGACGGTACAG---GCCAGGCAA---TTATTG---TCTGGTATAGTGCAACAGCAGAAT---
AATTTG---CTAAGA---GCTATTGAGGCGCAACAGCATATGTTGCAACTCACAGTCTGGGGCATC
>JQ403075
TTCTTCTGTAATACAACACAACTGTTTAATAGTACTTGGAATATTACTGGAGGGTCAAATAACACGGAA----
----

GGAAATGATACAATCACACTCCCATGCAGAATAAAACAAGTTATAAACATGTGGCAACAAGTAGGAAAAGCAA
TGTATGCCCCTCCCATCAGAGGACAAATTAGATGTTCATCAAAT---
ATTACAGGGCTGTTATTAACAAGAGATGGTGGCCATGGTGGCAACAACAGT----------------------
----

AACGAGACCGAGACCTTCAGACCTGGAGGAGGAAATATGAGGGACAATTGGAGAAGTGAATTATATAAATATA

AAGTAGTAAAAATTGAACCATTA---GGAGTAGCACCCACCAGGGCAAAGAGAAGAGTGGTG-----------
----------------------------CAGAGAGAAAAAAGAGCA---GTGGGA---ATAGGAGCT------
---CTGTTCCTTGGGTTCTTGGGAACA---GCAGGAAGCACTATG---GGCGCAGCGTCA---
ATAACGCTGACGGTACAG---GCCAGACAA---TTATTG---TCTGGTATAGTGCAACAGCAAAGC---
AATTTG---CTGCAG---GCTATTGAGGCGCAACAGCATCTGTTGCAACTCACAGTCTGGGGCATC
>JQ403074
TTCTTCTGCAATACAACAAAACTGTTTAATAGTACTTGGACAACTAATGGTACTGAAACGACAAATAACACAA
AA---

GGAAATGAGACAATCACACTCCCATGCAAAATAAAACAAATTATAAACATGTGGCAGGAAGTAGGAAAAGCAA
TGTATGCCCCTCCCATCAGAGGACAAATTAGGTGTGCATCAAAC---
ATTACAGGGTTGTTATTAACAAGAGATGGTGGTAATAGTGGTAATAAC-------------------------
----

ACGAACAACAGCACTGAGACCTTCAGACCTGGAGGAGGGGATATGAGAGACAATTGGAGGAGTGAACTATATA
AATATAAAGTAGTAAAAATTGAACCATTA---GGAGTAGCACCCACCAAGGCAAAGAGAAGAGTGGTG-----
----------------------------------CAGAGAGAAAAAAGAGCA---GTA------
CTCGGAGCT---------CTGATCCTTGGGTTCTTGGGAGCA---GCAGGAAGCACTATG---
GGCGCAGCGTCA---ATGACGCTGACGGTACAG---GCCAGACTA---TTATTG---
TCTGGTATAGTGCAACAGCAGAGC---AATTTG---CTGAGG---
GCTATTGAGGCGCAACAGCATCTGTTGCAACTCACAGTCTGGGGCATC
>KX587391
TTCTTTTGTAATTCAACACAACTGTTTAATAGTACTTGGGATAATGCTACTTTGAATAGTAATGCTAATGCTA
CT---

GATAGTGGCAATATCACACTCCCATGCAGAATAAAACAAATTATAAACATGTGGCAACAAGTAGGAAAAGCAA
TGTATGCCAATCCCATCAGAGGAGAAATTAAATGTACATCAAAT---
ATTACAGGGCTGTTATTAACCAGAGATGGTGGTCTTAACAGTACT----------------------------
----

GCCAATGAGACCTTCAGACCTGGAGGAGGAAACATGAAGGACAATTGGAGGAGTGAATTATATAAATACAAAG
TAGTAAAAATTGAACCATTA---GGAGTAGCACCCACCGCGGCAAAGAGAAGAGTGGTG--------------
-------------------------CAGAGAGAAAAAAGAGCA---GTGGGA---TTAGGAGCT---------
ATGTTCCTTGGGTTCTTGGGAGCA---GCAGGAAGCACTATG---GGCGCAGCGTCA---
CTGACGCTGACGGTACAG---GCCAGGCAA---TTATTG---TCTGGTATAGTGCAGCAGCAGAAC---
AATTTG---CTGAGG---GCTATTGAGGCGCAACAACATCTGTTGCAACTCACAGTCTGGGGCATC
>JF680917
TTCTTTTGTAATACAACACAACTGTTTGATAGTACATGGAATAAGACAGACAATAACACGGGAAGATCAGAT-
----

AACAATGAAACAATCACACTTCCATGCAGAATAAAACAATTTATAAACATGTGGCAGAGTGTAGGAAAAGCCA
TGTATGCCCCTCCCATTAGTGGACCAATTAACTGTTCATCAAAT---
ATCACAGGGCTGCTACTAACAAGAGATGGTGGTGAGACAAAAATAAATGAGACAGGGACAGAATTAAATGAGA
CAGAG--

AAGACCGAGATCTTCAGACCTGCAGGAGGAGATATGAGGGACAATTGGAGAAGCGAGTTATATAAATATAAAG
TAGTAAGAATTGAACCATTA---GGAGTAGCACCCACCAAGGCAAAGAGAAGAGTGGTG--------------
-------------------------CAGAGAGAAAAAAGAGCA---GTAGGA---ATGGGAGCT---------
TTGTTCCTTGGGTTCTTGGGAGCA---GCAGGAAGCACTATG---GGCGCAGCGTCA---
CTGACGCTGACGGTACAG---GCCAGACAA---TTATTG---TCTGGTATAGTGCAACAGCAAAAC---
AATTTG---CTGAGG---GCTATTGAGGCGCAACAGCATCTGTTGCAGCTCACGGTCTGGGGCATC
>JQ182910
TTCTTCTGTAACACATCACAACTGTTTAATAGTACTTGGAGTAAGACTGATTGGAATAGTACTGGGACAAATA
GCACT--

GAAGCAGAAAATATGACACTCCCCTGCAGAATAAAACAAATTATAAACATGTGGCAGAGTGTAGGAAAAGCAA
TGTATGCCCCTCCCATCAAGGGACGAATTGAGTGCTCGTCAATT---
ATTACAGGGCTACTACTAACAAGAGATGGTGGTCATAAAAATGAGAATGAG----------------------
----

ACAAACCAAAACGAGACCTTCAGACCTGGAGGAGGAGATATGAGGGACAATTGGAGAAGTGAATTATATAAAT
ATAAAGTAGTCCAAATTGAACCATTA---GGAGTAGCACCCACCAGGGCAAAGAGAAGAGTGGTG--------
-------------------------------CAGAGAGAAAAAAGAGCA---GTAACA---ATAGGAGCT---
------ATGTTCCTTGGGTTCTTGGGAACA---GCAGGAAGCACTATG---GGCGCAGCGTCA---
ATAGCGCTGACGGCACAG---GCTAGACAA---TTATTG---TCTGGTATAGTGCAGCAGCAGAAC---
AATCTG---CTGAGG---GCTATTGAGGCGCAACAGCATCTGTTGCAACTCACGGTCTGGGGCATT
>KF384798
TTCTTCTGTAATACAACAAAACTGTTTAATAGTATTTGGTTTAATAAAAATGGTACTGAAGGGTCAAAAAGC-
----

CCTGACAGAAATATCACACTCCCATGTAGAATAAGACAACTTATAAATATGTGGCAGGAAGTAGGAAAAGCAA
TGTATGCCCCTCCCATCGAAGGACAAATTAGATGTTCATCAAAT---
ATTACAGGGCTGCTGTTAACAAGAGATGGGGGTATCAACACAACT----------------------------
----

AATGGGACCGAGACCTTCAGACCTACAGGAGGAGATATGAGGGACAATTGGAGGAGTGAATTATATAAATATA
AGGTAGTAAAAATTGAACCATTA---GGAGTAGCACCCACCAAGGCAAAGAGAAGAGTGGTG-----------
----------------------------CAGAGAGAAAAAAGAGCA---GTTGGAATGCTGGGAGTT------
---ATGTTCCTTGGGTTCTTGGGAGCA---GCAGGAAGCACTATG---GGCGCAGCGTCA---
GTGACGCTGACGGTACAG---GCTAGACAG---TTATTG---TCTGGTATAGTGCAACAGCAGAAC---
AATCTG---CTGAGA---GCTATTGAGGCGCAACAGCATCTGTTGCAACTCACAGTCTGGGGCATC
>KF384802
TTCTTCTGTGACACAACACAACTGTTTAATAGTACTTGGGCGCCTAATGGTACTTGGATTAATGGAACACTAT
ATAAC--

TATACAGGAAATATTAAACTCCCATGCAGATTAAAACAAATTGTAAACATGTGGCAGGAAGTAGGGAAAGCCA
TGTATGCCCCTCCCATCGAAGGACAAATTAGCTGTTCATCAAAT---
ATTACGGGGCTGCTATTAACAAGAGATGGGGGTAACAGTAATGAGACTGAG----------------------
----

AACAGGACCGAGATCTTCAGACCTGGAGGAGGAAATATGAAGGACAACTGGAGAAGTGAATTATATAAATATA
AAGTAATAAGAGTTGAACCATTA---GGAGTAGCACCCACCAAGGCAAAGAGACGAGTGGTG-----------
----------------------------CAGAGAGAAAAAAGAGCA---GTGGGA---ATAGGAGCT------
---CTGCTCGTTGGGTTCTTGGGAGCA---GCAGGAAGCACTATG---GGCGCAGCGTCT---
GTGACGCTGACGGTACAG---GCCAGACAA---TTATTG---TCTGGTATAGTGCAACAGCAGAAC---
AATCTG---CTGAGA---GCTATTGAGGCGCAACAACATCTGTTGCAACTCACAGTCTGGGGCATC
>MH897915
TTCTTCTGTAATACCACACCATTGTTTAATAGTACTTGGGTTAATGGTACTTGGCAGAATGGTACTAGAGTG-
----

TCAGGTGACACAATCACACTCCAATGCAAAATAAAACAAATTATAAACATGTGGCAGAGTGTAGGAAAAGCAA
TGTATGCCCCTCCCATCAATGGACGAATTAATTGTTCATCAAAT---
ATTACAGGACTGCTACTAACAAGAGATGGGGGTGATAAGAATGCA----------------------------
----

ACAAATGACACCTTCAGACCTGTAGGAGGAAACATGAAGGACAATTGGAGAAGTGAATTATATAAATATAAAG
TAGTAGAAATTGAACCATTA---GGAGTAGCACCCACCAAGGCAAAAAGAAGAGTGGTG--------------
-------------------------CAGAGAGAAAAAAGAGCA---GTAACA---TTTGGAGCT---------
CTGTTCCTTGGGTTCTTGGGAGCA---GCAGGAAGCACTATG---GGCGCAGCGTCA---
GTGACGCTGACGGTACAG---GCCAGGCAA---TTATTG---TCTGGTATAGTGCAACAGCAGAAC---
AATCTG---CTGAGA---GCTATTGAGGCGCAACAACATATGTTGCAACTCACGGTCTGGGGCATC
>KU901997
TTCTTTTGTGATTCAACACAACTGTTTAATAGTACTTGGGATAATGCTACTTTAAATAGTAATGCTAGTGCTA
CT---

AATAATGGCAATATCACACTCCCATGCAGAATAAAACAAATTATAAACATGTGGCAACAAGTAGGAAAAGCAA
TGTATGCCAATCCCATCAAAGGAGAAATTAAATGTACATCAAAT---
ATTACAGGGCTGTTATTAACCAGAGATGGTGATCTTAACAATACT----------------------------
----

GCCAATGAGACCTTCAGACCTGGAGGAGGAAACATGAAGGACAATTGGAGGAGTGAATTATATAAATACAAAG
TAGTAAAAATTGAACCATTA---GGAGTAGCACCCACCGCGGCAAAGAGAAGAGTGGTG--------------
-------------------------CAGAGAGAAAAAAGAGCA---GTGGGA---TTAGGAGCT---------
ATGTTCCTTGGGTTCTTGGGAGCA---GCAGGAAGCACTATG---GGCGCAGCGTCA---
CTGACACTGACGGTACAG---GCCAGGCAA---TTATTG---TCTGGTATAGTGCAGCAGCAGAAC---
AATTTG---CTGAGG---GCTATTGAGGCGCAACAACATCTGTTGCAACTCACAGTCTGGGGCATC
>KU749387
TTCTTCTGTAATACAACAAAGCTGTTTGACAGTACTTGGCTGTCTGACGGTACTCGGAATGATACTAAAGGG-
----

TCAAATGAAACTATCATACTCCCATGCAGAATCAAACAAATTATAAACAGGTGGCAGGAAGTAGGAAAAGCAA
TGTATGCCCCTCCCATCAAAGGAATAATTAGATGTTCATCAAAT---
ATTACAGGGCTGTTATTAACAAGAGATGGTGGGCAAACTAACGATACA-------------------------
----

AACGACACAGAAATCTTCAGACCGGCAGGAGGAGATATGAGGGACAATTGGAGAAGTGAATTATATAAATATA
AAGTAGTAAAAATTGAACCATTA---GGAGTAGCACCCACCAGGGCAAAGAGAAGAGTGGTG-----------
----------------------------CAGAGAGAAAAAAGAGCA---GTGGGA---ATAGGAGCT------
---CTGTTCCTTGGGTTCTTAGGAGCA---GCAGGAAGCACTATG---GGCGCAGCGTCA---
TTGACGCTGACGGTACAG---GCCAGACAA---CTATTG---TCTGGTATAGTGCAACAGCAAAAC---
AATTTG---CTGAGA---GCTATTGAGGCGCAACAGCATATGTTGCAACTCACAGTCTGGGGCATC
>KU678125
TTCTTCTGTAATACAACACAACTGTTTAATACAACATGGGGTAATGATACTGAAGTGACAAATAACACTAAA-
----

GGAAATGAAACTATCACACTCCAATGCAAAATAAAACAAATTATAAACATGTGGCAGGAGGTAGGAAAAGCAA
TGTATGCCCCTCCCATCGAAGGACCAATTAGATGTTCATCAAAT---
ATTACAGGGCTACTATTAACAAGAGATGGTGGTAATGATGGTAATAGT-------------------------
----

AGCACAAACGAGACCTTCAGACCTGGGGGAGGAGATATGAGGGACAATTGGAGAAGTGAATTGTATAAATATA
AAGTAGTAAAAATTGAACCATTG---GGAATAGCACCCACCAAGGCAAAGAGAAGAGTGGTG-----------
----------------------------CAGAGAGAAAGAAGAGCA---ATAGGA---CTAGGAGCG------
---ATGTTCATTGGGTTCTTGGGAGCG---GCAGGAAGCACTATG---GGCGCAGCGTCA---
CTGACGCTGACGGTACAG---GCCAGACAA---TTATTG---TCTGGTATAGTGCAACAGCAGAAC---
AATCTG---CTGAGG---GCTATTGAGGCGCAACAACATATGTTGCAACTCACAGTCTGGGGCATC
>KY612720
TTCTTCTGTAATACAACACAACTGTTTAATAGCACTTGGAATAGCACTTGGAATAGCAATGATACTAGAGGGT
CAAAT--

GACACTGGAGTTATCACACTCCCATGCAGAATAAAACAAATTATAAACATGTGGCAGGAAGTAGGAAAGGCAA
TGTATGCCCCTCCCATCAGGGGAAACATTAGCTGTTCATCAGAT---
ATTACAGGGCTGCTATTAACAAGAGATGGTGGTAATAATAATAACACG-------------------------
----

AACGGGACTGAGATCTTCAGACCTGCAGGAGGAGATATGAGGGACAATTGGAGAAATGAATTATATAAATATA
AAGTAGTAAAAATTGAACCATTA---GGAGTAGCACCCACCAAGGCAAAAAGAAGAGTGGTG-----------
----------------------------CAGAGAGAAAAAAGAGCA---ATAGGA---ATAGGAGCT------
---TTGTTCCTTGGGTTCTTGGGAGCA---GCAGGAAGCACTATG---GGCGCAGCGTCA---
GTGACGCTGACGGTACAG---GCCAGGCAA---TTATTG---TCTGGTATAGTGCAACAACAGAAC---
AATATG---TTGAGA---GCTATTGAGGCGCAACAGCATCTGTTGCAACTCACAGTCTGGGGCATC
>KX129293
TTCTTCTGTGATACAACACAACTGTTTAATAGTACTTGGAATAGTACTTGGAATAGTACTGGAGGGTCAAAT-
----

AACACTGACCCAATCATACTCCCATGCAGAATAAAACAAATTATAAACACGTGGCAGAGTGTAGGAAAAGCAA
TGTATGCCCCTCCCATCAGGGGACAAATTAAATGTTCATCAAAT---
ATTACAGGGCTGCTGCTAACAAGAGATGGTGGTAATAATAATAAT----------------------------
----

ACAAACGAGACCTTCAGACCTGGAGGAGGAGATATGAGGGACAATTGGAGAAGTGAATTATATAAATATAAAG
TAGTAAAAATTGAACCATTA---GGAGTAGCACCCACCAAGGCAAAGAGAAGAGTGGTG--------------
-------------------------CAGAGAGAAAAAAGAGCA---GTGGGAACAATAGGAGCT---------
ATGTTCCTTGGGTTCTTGGGAGCA---GCAGGAAGCACTATG---GGCGCAGCGTCA---
ATGACGCTGACGGTACAG---GCCAGACTG---TTATTG---TCTGGTATAGTGCAACAGCAGAAC---
AATTTG---CTGAGG---GCTATTGAGGCGCAACAACATCTGTTGCAACTCACGGTCTGGGGCATC
>U84819
TTCTTCTGTAATACAACACAACTGTTTAATAGTACTTGGACCCTGTTCAATAATACTTGGAATAGTACTGAAG
GG---

TCAAATAACACAATCACACTCCCATGCAGAATAAAACAAATTATAAACATGTGGCAGGAAGTAGGAAAAGCAA
TGTATGCCCCTCCCATCAGAGGGGAAATTAGATGCTCATCAAAT---
ATTACAGGGTTGCTATTAACAAGAGATGGAGGTAATGATAATAACGCA-------------------------
----

AACAACGGGACTGAGATCTTCAGACCTACAGGAGGAGATATGAGAGACAATTGGAGAAGTGAATTATATAAAT
ATAAAGTAGTAAGAATTGAACCATTA---GGAGTAGCACCCACCAAGGCAAAGAGAAGAGTGGTG--------
-------------------------------CAGAGAGAAAAAAGAGCA---GCA------CTAGGAGCT---
------ATGTTCCTTGGGTTCTTGGGAGCA---GCAGGAAGCACTATG---GGCGCAGCGTCA---
ATGACGCTGACGGTACAG---GCCAGACTA---TTATTG---TCTGGTATAGTGCAGCAGCAGAAC---
AATTTG---CTGAGG---GCTATTGAAGCGCAACAGCATCTGTTGCAACTCACAGTCTGGGGCATC
>EU576016
TTCTTCTGTAATTCAACACCACTATTTAATAGTACTTGGAATGTGACTGATAGTACTACTTGGATAAATGGTA
CTGAA--

AGGTCAAATGACACGGTCACACTCCAATGCAGAATAAAACAAATTATAAACATGTGGCAGGAAGTAGGAAAAG
CAATGTATGCCCCTCCCATCAGAGGACAAATTAATTGCACATCAAAT---
ATTACAGGGCTACTATTAACAAGAGATGGTGGTGGTAACGGTAGCTTG-------------------------
----

AGTGAGGACGAGACCTTCAGACCTGGGGGAGGAGATATGAGGGACAATTGGAGAAGTGAATTATATAAATATA
AAGTAGTAAGAATTGAACCATTA---GGAATAGCACCCACCAAGGCAAAGAGAAGAGTGGTG-----------
----------------------------CAAAGAGAAAAAAGAGCA---GTGGGA---ATAGGAGCT------
---GTGTTCCTTGGGTTCTTGGGAGCA---GCAGGAAGCACTATG---GGCGCAGCGTCA---
GTGGCGCTGACGGTACAG---GCCAGACAA---TTATTG---TCTGGTATAGTGCAACAGCAAAAC---
AATTTG---CTGAGA---GCTATTGAGGCGCAACAACATCTGTTGCAACTCACAGTCTGGGGCATC
>JQ085287
TTCCACTGTAATACAACAAAACTGTTTAATAGTACCTGGTATAGTCCTGAAAATTCAACACAACTGAGTAATA
ATGATACT---

GCTAATGGGACTATCACACTCCAATGCAGAATAAAACAAATTATAAACACGTGGCAGGAAGTAGGAAAAGCAA
TGTATGCCCCTCCCATCAGAGGACAAATTAGATGTTCATCAAAT---
ATTACAGGGCTGTTATTAACAAGAGATGGTGGTAATACCAATGCC----------------------------
----

ACGAATGAAACCTTCAGACCTGGAGGAGGAGATATGAGGGACAATTGGAGAAGTGAATTATATAAATATAAAG
TAGTAAAAATTGAACCATTA---GGAGTAGCACCCACCACTGCAAGGAGAAGAGTGGTG--------------
-------------------------CAGAGAGAAAAAAGAGGG---GTAGGA---CTAGGAGCT---------
TTGTTCCTTGGGTTCTTGGGAGCA---GCAGGAAGCACTATG---GGCGCAGCGTCA---
GCGACGCTGACGGTACAG---GCCAGACAA---TTATTG---TCTGGTATAGTGCAACAGCAAAAC---
AATTTG---CTGAGA---GCTATTGAGGCGCAACAGCATCTGTTGCAACTCACAGTCTGGGGCATC
>KX984753
TTCTTCTGTAATACAACACAACTGTTTAATAGTAGTTGGAATAAGAATAATACTATAAATGGGAATGATACTG
AAGAG--

TCAAATGACACTCTCACACTTCCGTGCAGAATAAAACAAATTGTAAACATGTGGCAAGAAGTAGGAAAAGCAA
TGTATGCCCCTCCCATCAGAGGACTAATTAGATGTTCATCAAAT---
ATTACAGGGCTGATATTAACAAGAGATGGTGGTAAAAATAACGGGACG-------------------------
----

AATGAGACCGAGACCTTCAGACCTGGGGGAGGAGATATGAGGGACAATTGGAGAAGTGAATTATATAAATATA
AAGTAGTAAAAATTGAACCATTA---GGAGTAGCACCCACCAAGGCAAAGAGAAGAGTGGTG-----------
----------------------------CAGAGAGAAAAAAGAGCA---ATAGGA---ATAGGAGCT------
---TTGTTCCTTGGGTTCTTGGGAGCA---GCAGGAAGCACTATG---GGCGCAGCGTCA---
GTAGCGCTGACGGCACAG---GCCAGACAA---TTAATG---TCTGGTATAGTGCAACAGCAGAGC---
AATCTG---CTGAGG---GCTATTGAGGCGCAACAGCATATGTTGCAACTCACAGTCTGGGGCATC
>M21098
TTCTTCTGTAATACAACACAACTGTTTAATAGTACTTGGTATAGGAACACTACAGGAAATATCACTGAA----
----

GGAAATAGCCCAATCACACTCCCATGCAGAATAAAACAAATTATAAACATGTGGCAGGAAGTAGGAAAAGCAA
TGTATGCCCCTCCCATCAGAGGACAAATTAAATGTTCATCAAAT---
ATTACAGGGCTGCTATTAACAAGAGATGGTGGTAATAACAACGAGACC-------------------------
----

ACCGACACCGAGATCTTCAGACCTGGAGGAGGAAATATGAGGGACAATTGGAGAAGTGAATTATATAAATATA
AAGTAGTAAAAATTGAACCATTA---GGAGTAGCACCCACCAAGGCAAAGAGAAGAGTGGTG-----------
----------------------------CAGAGAGAAAAAAGAGCA---GTGGGA---CTAGGAGCT------
---TTGTTCCTTGGGTTCTTAGGAGCA---GCAGGAAGCACTATG---GGCGCAGCGTCC---
CTGACGCTGACGGTACAG---GCCAGACTA---TTATTG---TCTGGTATAGTGCAACAGCAGAAC---
AACTTG---CTGATG---GCTATTGAGGCGCAACAGCATATGTTGGAACTTACAGTCTGGGGCATC
>GU728014
TTCTTCTGTAATACAACAAAGCTGTTTAATAGTACTTGGACAAAGGGTAATGAGTCTATTATTGATGAAGGG-
----

ATAAATGGAACGATCACACTCCCATGCAGAATAAGACAAATTATAAACATGTGGCAGAGGGTAGGAAAAGCAA
TGTATGCCCCTCCCATCCAGGGAAAAATTCAATGTTTATCAAAC---
ATTACAGGGCTACTGCTAACAAGAGATGGTGGCATTAATGCAACC----------------------------
----

AACAATGATATCTTCAGACCTGGAGGAGGAGATATGAGGGACAACTGGAGAAGTGAATTATATAACTATAAAG
TAGTAGAAATTGAACCATTA---GGAATAGCACCCACCAAGGCAATGAGAAGAGTGGTG--------------
-------------------------CAGAGAGAAAAAAGAGCA---GTGACA---CTAGGAGCT---------
ATGTTCCTTGGGTTCTTGGGAGCA---GCAGGAAGCACTATG---GGCGCAGCGTCA---
ATAACGCTGACGGCACAG---GCCAGACAA---TTATTG---TCTGGTATAGTGCAACAGCAGAGC---
AATCTG---CTGAGA---GCTATTGAGGCGCAACAACATCTGTTGCAACTCACGGTCTGGGGCATC
>EU604640
TTCTTCTGTAATTCATCACAATTGTTTAATAGTACTTGGCCTTGGAATGATACTGCAGAGTTAAATGGCAAGG
ATACTGCA---
-------------GAGTTA------
AATGGCAATGACACAATCATACTCCCATGCAGAATAAAACAAATTGTACACATGTGGCAGGAAGTAGGAAAAG
CAATGTATGCCCCTCCCATCAGTGGGCAAATTAGATGTTCATCAAAT---
ATTACAGGGCTGCTATTATCAAGAGATGGTGGTAAGAATAATGGA----------------------------
----

TCCCAGGAGACTTTCAGACCTGGAGGAGGAAATATGAAGGACAATTGGAGAAGTGAATTATATAAATATAAAG
TAGTAAAAATTGAACCATTA---GGAATAGCACCCACCAAGGCAAAGAGAAGAGTGGTG--------------
-------------------------CATAGAGAAAGAAGAGCA---GTGGGA---TTAGGAGCT---------
ATGTTCCTTGGGTTCTTGGGAGCA---GCAGGAAGCACTATG---GGCGCAGCGTCA---
ATAACGCTGACGGTACAG---GCCAGACAA---TTATTG---TCTGGTATAGTGCAACAGCAAAAC---
AATTTG---CTGAGA---GCCATTGAGGCGCAACAACATCTGTTGCAACTCACAGTCTGGGGCATT
>KT452465
TTCTTCTGTAATACAACACGACTGTTTAACAGTACTTGGAATACTACTGATACTTGGAATGTTACTAAGATTG
GGAATGAG---
----------ACGACAAAA------
GACGATAACAATACAATCACACTCCCATGCAGAATAAAACAAATTATAAATATGTGGCAGGAAGTAGGAAAGG
CTATGTATGCCCCTCCCATCAGTGGACCAATTAACTGTACATCAAAT---
ATTACAGGGCTGCTATTAACAAGAGATGGGGGTAATGGCAGTCAA----------------------------
----

GACACTGAAATCTTTAGACCTGCAGGAGGAGATATGAGGGACAATTGGAGAAGTGAATTATATAAATATAAAG
TAGTAAAAATTGAACCATTA---GGACTAGCACCCACTAAGGCAAAGAGAAGAGTGGTG-------------
-------------------------CAGAGAGAAAAAAGAGCA---GTGGGA---ATAGGAGCT---------
GTGTTCCTTGGATTCTTGGGAGCA---GCAGGAAGCACTATG---GGCGCAGCGTCA---
ATAACGCTGACGGTACAG---GCCAGACAA---TTATTG---TCTGGTATAGTGCAACAGCAGAAC---
AATCTG---CTGAGG---GCTATTGAGGCGCAACAACATCTGTTGCAACTCACAGTCTGGGGCATC
>KU319528
TTTTTATGCAATACATCAGGCCTGTTCAATAATTCACTGTTGGCTAATACGAGTAATATACCG---------
----

GACAATGACACCATCACACTTCCATGCAAAATAAAACAATATCTAAACATGTGGCAGCAGGTAGGACGAGCAA
TGTATGCCCCTCCCATTAGCGGAAGAATATCATGTAACTCAAAT---
ATCACAGGACTGCTATTGACACGTGATGGAGGAAAAGGAAACAAC----------------------------
----

AAGCAAAATGAAACATTCAGACCTATAGGAGGAGATATGAGGAACAATTGGAGAAGTGAATTATATAAATATA
AAGTAGTAGAAATTAAGCCATTA---GGACTAGCACCCACTGGAGCAAAAAGGAGAGTGGTG----------
----------------------------GGAAGAGAAAAAAGAGCA---GCA------CTAGGAGCT------
---ATGTTCCTTGGGTTCTTGGGAGCA---GCTGGAAGCACTATG---GGCGCAGCATCA---
ATAACGCTGACGGTACAG---GCCAGACAA---TTGTTG---TCTGGTATAGTGCAACAGCAAAGC---
AATTTG---CTGAGG---GCTATAGAGGCGCAGCAGCATATGTTGCAACTCACGGTCTGGGGCATT
>MF373128
TCCCAAGGCAAATCATTAAGACTGTTTAATGGGTCCACGGGTAAAGGGACCAGGGGGAATGGAAGGTCA----
----
-------AGGAACCCATCT------AAGTCAGACGTCACTATCACTTTCCCATGCAGAATAATCCAATTT---
------TTGCCAGGAGCAGGGGTAGCAATCAACGCCCTTTCCCGTACCGGAAACATTACATGTATTCTACTG-
--GTTACAGGATTACTGCTGGCAGGGGGTGGTGGTAACGATACTAAA-------------------------
----

GGGAAAGAGATCTTCAGACCTGGAGGAGGAGATATGAGGGACAATTGGAGAAGTGAATTATATAAATATAAAG
TGGTGGAAATTAAACCATTG---GGAATAGCACCCACTACAGCAAAGAGGAGAGTGGTG-------------
-------------------------GAGAGAGGAAAAAGAGCA---GTAGGA---ATAGGAGCT---------
GTATTCCTTGGGTTCTTGGGAGCA---GCAGGAAGCACTATG---GGCGCGGCGTCA---
ATAACGCTGACGGTACAG---GCCAGACAA---TTATTG---TCTGGTATAGTGCAACAGCAAAGC---
AATTTG---CTGAGA---GCCATAGAGGCGCAACAACATATGTTGCAACTCACAGTCTGGGGCATT
>MF373178
TTTTTTTGCAATACAACAAAACTGTTTGATAATAATTCAACACGGGGTTCA---------------------
----

GCCAATGGCACAATCACACTCCCATGCAGAATAAAACAGATTATAAACATGTGGCAGGAGGTAGGGCGAGCAA
TGTATGCCCCTCCCATTGCAGGAAACATAACATGTAACTCAAAT---
ATCACAGGACTACTATTGACATATGATGGAGGAAAAAACAATAAC----------------------------
----

ACTAACAACGAAACATTCAGACCTGGAGGAGGAAATATGAAGGACAATTGGAGAAGTGAATTATACAAATATA
AAGTAGTAGAAATTAAGCCATTG---GGAATAGCACCCACTGGAGCAAGAAGGAGAGTGGTG----------
----------------------------GAGAAACAAAAAAGAGCA---GTAGGA---ATGGGAGCT------
---GTGATCCTTGGGTTCTTGGGAGCA---GCAGGAAGCACTATG---GGCGCGGCGTCA---
ATAACGCTGACGGTACAG---GCCAGACAA---CTGCTG---TCTGGTATAGTGCAACAGCAAAGC---
AATTTG---CTGAGA---GCTATAGAGGCGCAACAGCATATGTTGCAACTCACAGTCTGGGGCATT
>HM623561
TTCTTTTGCAATACAACACAACTGTTTAATGAAACTAATGTT------------------------------
----

ACTGAAGATACCATTACAATCCCATGCAGAATAAAACAAATTGTAAACATGTGGCAGAGGGTAGGACGAGCAA
TTTATGCCCCTCCCATTGCAGGAAACATAACATGTAATTCAAAT---
ATCACAGGAATACTATTGCTACGTGATGGAGGCACAAACAATAAC----------------------------
----

ACAAGTAACGAAACATTCAGACCTGGAGGGGGAAATATGAAGGACAATTGGAGAAGTGAATTATATAAATATA

```
AAGTAGTAGAGATTAAGCCATTG---GGAATAGCACCCACTAATGCAAGAAGGAGAGTGGTG-----------
----------------------------GAGAGAGAAAAAAGAGCA---GTGGGA---ATAGGAGCC------
---ATGATCTTTGGGTTCTTGGGAGCA---GCAGGAAGCACTATG---GGCGCGGCGTCA---
ATAACGCTGACGGTACAG---GCCAGACAA---CTGTTG---TCTGGTATAGTGCAACAGCAAAGC---
AATTTG---CTGAGA---GCTATTGAGGCGCAACAGCATATGTTGCAACTCACGGTCTGGGGCATT
>KU749417
TTCTTTTGCAATACATCAAAACTGTTTAATAGCACATACTATCCTAATGGTACAGAAAGTTCAAATGGTACAG
AAGGT-------------------------------------------------------------------
---------------------------
TCAAATGCAACCATCACACTCCCATGCAGAATAAAGCAAATTATAAATATGTGGCAGGGGGTAGGACAAGCAA
TGTATGCCCCTCCCATTGCAGGAAACATAACATGTAAATCAAAT---
ATCACAGGACTGCTACTGACACGTGATGGAGGAGGTAATGGTAAT---------------------------
-------------------------------------------------------------------
GAAACAGAGAGTAAT---
AGGACTGAGATATTCAGACCTGCAGGAGGAGATATGAGGGACAATTGGAGAAGTGAATTATATAAATATAAAG
TGGTAGAAGTTCAGCCATTG---GGAGTAGCACCCACTAAGGCAAAAAGGAGAGTGGTG------------
------------------------GAGAGAGAAAAAAGAGCA---GTGGGA---ATAGGAGCT---------
GTGTTCCTTGGGTTCTTGGGAGCA---GCAGGAAGCACTATG---GGCGCAGCATCA---
ATAACGCTGACGGTACAG---GCCAGACAA---CTGTTG---TCTGGTATAGTGCAACAGCAAAGC---
AATTTG---CTGAGG---GCTATAGAGGCGCAACAGCATATGTTGCAACTCACTGTCTGGGGCATT
>KF986074
TTCTTCTGTAACACATCAGAACTGTTTAACAGTACATGGAATAGAACTAGTACTAATAGTACT----------
-------------------------------------------------------ACA---
TGGGATAGTACTAGTACACCC--------------
AATGAGACAGAGGATATTACACTCCAGTGCAGAATAAAACAAATTATAAACATGTGGCAGAAAGTAGGAAAAG
CAATGTATGCCCCTCCCATTGAAGGAAAAATCAGATGTTCATCAAAT---
ATTACAGGACTATTGTTGACGAGAGATGGTGAT--------------------------------------
------------------------------------------------------------------------
-
GCAAATAATACTAGTAATGAGACCTTCAGACCTGGAGGAGGAGATATGAGAGACAATTGGAGAAGTGAATTAT
ATAAATATAAAGTAGTAAGAATTGAGCCAATA---GGCTTAGCACCCACCAAGGCAAAAAGAAGAGTGGTG--
------------------------------------GAAAGGGAGAAAAGAGCA---ATAGGA---
CTAGGAGCT---------ATGTTCCTTGGGTTCTTGGGAGCA---GCCGGAAGCACGATG---
GGCGCAGCGTCA---CTGACGCTGACGGTACAG---GCCAGAAAT---TTATTG---
TCTGGCATAGTGCAACAGCAAAAC---AATCTG---CTGAGG---
GCTATTGAGGCGCAACAGCATCTGTTGCAACTCACAGTCTGGGGCATT
>AF484490
TTCTTCTGCAATACATCAGGACTGTTTAATAGTACATGGGAGAAAAATAATAATAATAGTAAATGGGAAAATA
ATAGTACC----------------------------------------------------------------
-------------AGGGGG------
AATAATAATACACTTATCACACTCCAATGCAGAATAAAACAAATTATAAACATGTGGCAGAAAGTAGGAAAAG
CAATGTATGCCCCTCCCATTGAAGGACTAATCAGATGTTCATCAAAT---
ATTACAGGACTATTGTTGACAAGAGATGGTGGTATAAATAGTACT---------------------------
------------------------------------------------------------------------
----------
AATGGTAATCAGACCTTCAGACCTGGAGGAGGAGATATGAGAGACAATTGGAGAAGTGAATTATACAAATATA
AAGTAGTAAGAATTGAACCACTA---GGTCTAGCACCCACCAGGGCAAAGAGAAGAGTGGTG----------
---------------------------GAAAGAGAAAAAAGAGCA---ATAGGA---CTAGGAGCT------
---TTGTTCCTTGGGTTCTTGGGAACA---GCAGGAAGCACGATG---GGCGCAGCGTCG---
CTGACGCTGACGGTACAG---GCCAGACAG---GTACTG---TCTGGTATAGTGCAACAGCAAAAC---
AATTTG---CTGAGG---GCTATAGAGGCGCAACAGCACCTGTTGCAACTCACAGTCTGGGGCATT
>KX181917
TTATCATTCAATACAAATAATTTATTTAATGACACAGTAATAAATGACACA---------------------
------------------------------------------------------------------------
---------------------------
ACCACTGTCAATATCACTCTCCCATGTCGAATAAAAAAAATACTAAACGTGAGGCAGGAAGTAGAAGGAGCAA
TGTATGCCAATCCCATTGGAGGAAAGATAACCTGTACCTCTATT---
ATTACAGGTATAGTATTAACAAGAGAAGGAGGTAATAATAGGGGT---------------------------
------------------------------------------------------------------------
----------
```

AATAATAATGAGACCTTCAGACCTGGGGGAGGAAATATGAAAGACAATTGGAGAAGTGAATTATATAAATATA
AAGTAGTAGAAATTGAACCACTT---GGAGTAGCACCCACCAAAGCAAAAAGACAAGTGGTG-----------
----------------------------AAGAGAGAAAAAAGAGCA---GTGGGA---ATGGGAGCT------
---TTGTTCCTTGGGTTCTTGGGAGCA---GCAGGAAGCACTATG---GGCGCGGCGTCA---
ATGACGCTGACGGTACAG---GCCAGACAA---TTATTG---TCTGGAATAGTGCAACAGCAGAAC---
AATTTG---CTGAGG---GCTATTGAAGCGCAACAGCATCTGTTGCAGCTCACAGTCTGGGGCATT
>JN248591
TTCTTTTGTAACACATCAGGACTGTTTAATGCATCAGAAAAAGTTAACGCATCA-------------------
----

GAACAGGGAAATATCACACTCCCATGTAGAATAAAACAAATTGTGAGAATGTGGCAGAGAGTGGGACAAGCAA
TGTATGCCCCTCCCATCGCAGGAGATATTACATGTCACTCAAAC---
ATTACAGGGCTTTTATTAACAAGAGATGGTGGGAATAATATTACT----------------------------
----

AATGAGACCTTCAGACCTGGAGGAGGAGATATGAGAGACAATTGGAGAAGTGAATTATATAAGTACAAAATAG
TAAAAATTAAACCACTA---GGAATAGCACCCACCAAGGCAAGGAGAAGAGTGGTG-----------------
----------------------CAGAGAGAAAAAAGGGCA---ATAGGTGGACTGGGAGCT---------
GTCTTCCTTGGGTTCTTAGGAGCA---GCAGGAAGCACTATG---GGCGCGGCGTCA---
ATAACGCTGACGGTACAG---GTCAGACAA---TTATTG---TCTGGCATAGTGCAACAGCAAGCT---
AATTTG---CTGAGA---GCTATAGAGGCACAGCAACATCTGTTGCAACTCACAGTCTGGGGCATT
>MH742769
TTCTTTTGCAATACATCAAAACTGTTTGATAGCACTTGGGCACACAATACTACCAGCGCAAACTACACAGGAT
TAAAT--
-------------AACACA------
GAATTAAATGACACTATGACTCTCCAATGCAAAATAAAGCAGTTTATAAACATGTGGCAGAAAGTAGGGCAAG
CAATGTATGCCCCTCCTATCCAAGGAGTAATAAGGTGTGAATCAAAC---
ATTACAGGACTACTATTAACAAGAGATGGAGGAAATAATAGTAGT----------------------------
----

GACCAGGAGATCTTCAGGCCTGGAGGAGGAGATATGAGGGACAATTGGAGAAGTGAATTATATAAGTATAAAG
TAGTAAAAATTGAACCACTA---GGTGTAGCACCCACCCATGCAAAAAGAAGAGTGGTG--------------
-------------------------GAGAGAGAAAAAAGAGCA---GTTGGA---CTGGGAGCT---------
TTATTCCTTGGGTTCTTAGGAGCA---GCAGGAAGCACTATG---GGCGCGGCGTCA---
ATAACGCTGACGGTACAG---GCCAGACAA---TTGTTG---TCTGGTATAGTGCAACAGCAGAGC---
AATTTG---CTGAGA---GCTATAGAGGCTCAACAACATATGTTGAGACTCACGGTCTGGGGCATT
>KF561437
TTCTCTTGTAATACATCAATACTGGTTGACAACACTTTTAACAACACTTCGAACGAGAGCAGCTCAGGACACA
CAGGGTGG---

TCAAATGAATCTCTAACTCTCCCATGCAGAATAAATCAAATTGTAAATATGTGGCAGAAGGTAGGACAAGCAA
TATATGCCCCTCCCATCAATGGAGTCATAAGGTGTGATTCAAAT---
ATTACAGGACTTCTATTCACAAGGGATGGAGGGAACAGTAGGAATGGAGGGGAC-------------------
----

AGTGGTGGTGACAATGAGACCTTCAGGCCTGGAGGAGGAGATATGAGGGATAATTGGAGAAGTGTATTATATA
GGTACAAGGTAGTAAAAATTGAACCGCTA---GGTGTAGCACCCACCCATGCAAAAAGGAGAGTGGTG-----
----------------------------------GAAAGAGAAAAAAGAGCA---GTTGGA---
CTAGGAGCT---------GTCTTCCTTGGGTTCTTAGGAGCA---GCAGGAAGCACTATG---
GGCGCGGCGTCA---ATAACGCTGACGGTACAG---GCCAGACAA---TTATTG---
TCTGGTATAGTGCAACAGCAGAGC---AATTTG---CTGAGA---
GCTATAGAGGCTCAACAACACCTGTTGAAACTCACGGTCTGGGGCATT
>KX389633
TTCTTTTGCAATACATCAGGGCTGTTTAATAGCACTTGGTATGAGAATGACACTGAAACAAATAACACGGGTT
CAGTAGAG---

GGCAATACAACTATAACTCTCCCATGTAGAATAAAACAGATTGTACGTATGTGGCAGAATGTAGGACAAGCAA
TGTATGCCCCTCCCATCCCCGGAGAAATAAGGTGTGACTCAAAC---
ATTACAGGACTACTATTAACAAGAGATGGTGGGGCACCGAACAGG----------------------------
----

ACTAATGATACCTTCAGGCCTACAGGAGGAGATATGAAGGACAATTGGAGAAGTGAATTATATCAGTATAAAG
TAGTGGAGATTAGCCCTTTA---GGAGTAGCACCCACCAGGGCAAGAAGAAGAGTGGTG--------------
--------------------------GAGAGAGAAAAAAGAGCA---GTAGGA---ATGGGAGCT---------
GTACTCCTTGGGTTCTTGGGAGCA---GCAGGAAGCACTATG---GGCGCGGCGTCA---
ATAACGCTGACGGTACAG---GCCAGACAA---TTATTG---TCTGGCATAGTGCAACAGCAAAAC---
AACTTG---CTGAAG---GCTATTGAAGCGCAACAGCATCTGTTGCAGCTCACTGTCTGGGGCATT
>DQ085872
TTATACTGTAATACAACACAGCTGTTCAATGGTACTTGGAATGGCAATGACACTTGGAATGATACTTGGAATA
GT---

ACTAATGGTATTATCACACTCCCATGCAGAATAAGACAAATTATAAACAGGTGGCAGGAAGTAGGAAAAGCAA
TGTATGCCCCTCCCATCAAAGGACAACTTAACTGTTCATCAAAG---
ATTACAGGGCTGCTACTAACAAGAGATGGGGGTGGTAACGTGAATGGCACT----------------------
----

GAGAATGACACTGAGACCTTCAGACCTGGAGGAGGAGATATGAGGGACAATTGGAGAAGTGAATTATATAAAT
ATAAAGTAGTAAGAATTGAACCATTA---GGAGTAGCACCCACCAAGGCAAAGAGAAGAGTGGTG--------
--------------------------------CAGAGAGAAAAGAGAGCA---GTGGGAACGATGGGAGCT---
------ATGTTCCTTGGGTTCTTGGGAGCA---GCAGGAAGCACTATG---GGCGCAGCGTCA---
GTGACGCTGACGGTACAG---GCCAGACTA---TTATTG---TCTGGTATAGTGCAACAGCAAAAC---
AATTTG---CTGAGA---GCTATTGAGGCGCAACAGCATTTGTTGCAGCTCACAGTCTGGGGCATC
>AY536235
TTCTTCTGCAATACAACAAGACTGTTTAATGACACAGGATTCAATGACACAAAATCCAAT-------------
----

GACACTGACACTATCACGCTCCCATGTCGAATAAAACAAATTGTAAACATGTGGCAGCAAGTGGGAAGAGCAA
TGTATGCCGCTCCCATTGCAGGAAAAATATCCTGTAGATCAAAT---
ATTACAGGTCTACTATTGACAAGAGATGGTGGTAATACTACTACAGATGGTAAT-------------------
----

AATAATACTACTACTGAAATCTTCAGACCAGGGGGAGGAAATATGAAAGACAATTGGAGAAGTGAATTATATA
AATATAAAGTGGTAGAACTTGAACCACTA---GGAGTAGCACCCACCAAGGCAAAAAGACAAGTGGTG-----
-----------------------------------CAGAGAGAAAAAAGAGCA---GTGGGA---
ATAGGAGCT---------ATGTTCCTTGGGTTCTTGGGAGCA---GCAGGAAGCACTATG---
GGCGCAGCGTCA---ATAACGCTGACGGTACAG---GCCAGACAA---TTATTG---
TCTGGAATAGTGCAACAGCAGAGA---AATTTG---CTGATG---
GCTATTGAAGCGCAACAGCATATGTTGCAGCTCACAGTCTGGGGCATT
>MH078537
[truncated: 1,337,562 more chars]
