# Supplementary material for: Extending the Coding Potential of Viral Genomes with Overlapping Antisense ORFs: A Case for the De Novo Creation of the Gene Encoding the Antisense Protein ASP of HIV-1
Source: Viruses. 2022 Jan 14;14(1):146. doi: 10.3390/v14010146 (PMC8781085; doi:10.3390/v14010146)
Supplement: Supplementary file 1 [file viruses-14-00146-s001.zip › Supplementary File S5.pdf]

Supplementary File S5.

Sample set of 124 *env* sequences assembled by selecting the coding region of *env* from multiple primate lentiviruses that is homologous to the *env/asp* overlap in the reference genome sequence HIV-1 (ac. number K03455, nucleotides 7371-7943; see Materials and Methods section *2.6 Assembly of a dataset of env sequences covering a wide phylogenetic range of primate lentiviruses*). The dataset was assembled by selecting the coding region of *env* from multiple primate lentiviruses that covers a wide range of primate lentiviruses, including the four subgroups of HIV-1 (M, N,O, and P), HIV-2, and several SIV species infecting African apes and Old-World monkeys.

```
>HIV-1-M-B-HXB2
TTCTACTGTAATTCAACACAACTGTTTAATAGTACTTGGTTTAATAGTAC
TTGGAGTACTGAAGGGTCAAATAACACTGAAGGAAGTGACACAATCACCC
TCCCATGCAGAATAAAACAAATTATAAACATGTGGCAGAAAGTAGGAAAA
GCAATGTATGCCCCTCCCATCAGTGGACAAATTAGATGTTCATCAAATAT
TACAGGGCTGCTATTAACAAGAGATGGTGGTAATAGCAACAATGAGTCCG
AGATCTTCAGACCTGGAGGAGGAGATATGAGGGACAATTGGAGAAGTGAA
TTATATAAATATAAAGTAGTAAAAATTGAACCATTAGGAGTAGCACCCAC
CAAGGCAAAGAGAAGAGTGGTGCAGAGAGAAAAAAGAGCAGTGGGAATAG
GAGCTTTGTTCCTTGGGTTCTTGGGAGCAGCAGGAAGCACTATGGGCGCA
GCCTCAATGACGCTGACGGTACAGGCCAGACAATTATTGTCTGGTATAGT
GCAGCAGCAGAACAATTTGCTGAGGGCTATTGAGGCGCAACAGCATCTGT
TGCAACTCACAGTCTGGGGCATC
>HIV-1-M-B-VH
TTCTACTGTAATTCAACACAACTGTTTAATAGTACTTGGTTTAATAGTAC
TGGGAATGATACTGAAAGGGCAACTAACAATACAGAAAATATCACACTCC
CATGTAGAATAAAACAAATTATAAACATGTGGCAGAAAGTAGGAAAAGCA
ATGTATGCCCCTCCCATCAACGGACAGATTAGATGTTCATCAAATATTAC
AGGGCTGATACTAACAAGAGATGGTGGTAATCAAGAGAACAAGACCGAGA
TCTTCAGACCTGGAGGAGGAGATATGAGGGACAATTGGAGAAGTGAATTA
TATAAATATAAAGTAGTAAGAATTGAACCATTAGGAGTAGCACCCACCAA
GGCAAAGAGAAGAGTGGTGCAGAGAGAAAAAAGAGCAGTGGGAATGATAG
GGGCAATGATCCTTGGGTTCTTGGGAGCAGCAGGAAGCACTATGGGCGCA
GCGTCACTGGCGCTGACGGTACAGACCAGGCAATTGTTGTCTGGTATAGT
GCAACAGCAGAACAATCTGCTGAGGGCTATTGAGGCGCAACAGCATCTGT
TGCAACTCACAGTCTGGGGCATC
>HIV-1-M-B-BK132
TTCTTCTGTAATTCAACAAAACTGTTTAATAGTACTTGGAATAGCACTGA
AGGAAATGACGATGGAGAGGAAAGAAATATCACACTCCCATGCAGAATAA
AACAAATTGTAAACATGTGGCAGGAAGTAGGAAAAGCAATGTATGCCCCT
CCCATCGGAGGACAAATTAGATGCACCTCAAATATTACAGGGCTGCTATT
AACAAGAGATGGAGGTAACCAAAATGGGACCAACGAGACTGAAATCTTCA
GACCTGGAGGAGGAAATATGAGGGACAATTGGAGAAGTGAACTATATAAA
TATAAAGTAGTAAAAATTGAACCATTAGGAGTAGCACCCACTAAGGCAAA
GAGAAGAGTGGTGCAGAGAGAAAAAAGAGCAGTGGGAATAGGAGCTGTGT
TCCTTGGGTTCTTGGGAGCAGCAGGAAGCACTATGGGCGCAGCGTCAGTG
ACGCTGACGGTACAGGCCAGACTATTATTGTCTGGTATAGTGCAACAGCA
GAACAATCTGCTGAGGGCTATTGAGGCGCAACAGCATCTGTTGCAACTCA
CAGTCTGGGGCATC
>HIV-1-M-B-1058-11
TTCTATTGCAACTCAACCAAACTATTTAATAGCATTTGGGATAATAATAA
AGATAGCACTAAAACAAATGAACCAAATGATGGAAAAAATATCACACTCC
CATGCAGAATAAAACAAATTATAAACATGTGGCAGGGAGTAGGAAAAGCA
ATGTATGCCCCTCCCATCAGAGGACAAATTAGATGTACATCAAATATTAC
AGGGCTACTATTAACAAGAGATGGTGGCAAGAACAATGGCACTAATGGGA
CCGAGGTCTTCAGACCTGGAGGAGGAAATATGAAGGACAATTGGAGAAGT
GAATTATATAAATATAAAGTAGTAAAAATTGAACCATTAGGAGTAGCACC
CACCACGGCAAAGAGACGAGTGGTGCAGAGAGAAAAAAGAGCAGTGACAC
```

TAGGAGCTCTGTTCCTTGGGTTCTTGGGAGCAGCAGGAAGCACTATGGGC
GCAGCGTCAATGACGCTGACGGTACAGGCCAGACTATTATTGTCTGGTAT
AGTGCAACAGCAGAACAACTTGCTGAGGGCTATTGAGGCGCAACAACATC
TGTTGCAACTCACGGTCTGGGGCATC
>HIV-1-M-B-671-00T36
TTCTACTGTAATACAACACAGCTGTTTAATAGTACTTGGGATGTGAATGC
TACTGGGAATGGTACTACAGAACCAAATAGTACTATAACACTCCCATGCA
GAATAAAACAAATTATAAACAGATGGCAGGAAGTAGGAAAAGCAATGTAT
GCCCCTCCCATTGCAGGACAAATTAGCTGTTCATCAAACATCACAGGGCT
GCTATTAACAAGGGATGGTGGTGGTGGTGAGAACAATAGCACCGAGATCT
TTAGACCTGGAGGAGGAGATATGAGAGACAATTGGAGAAGTGAATTATAT
AAATATAAAGTAGTAAAAATTGAGCCATTGGGAGTAGCACCCACCAAGGC
AAAGAGAAGAGTGGTGCAGAGAGAAAAAAGAGCAATAACGCTAGGAGCTA
TGTTCCTTGGGTTCTTGGGAGCAGCAGGAAGCACTATGGGCGCAGCGTCA
ATGGCGCTGACGGTACAGGCCAGACAATTATTGTCTGGTATAGTGCAACA
GCAAAACAATTTGCTGAGGGCTATTGAGGCGCAACAGCATCTGCTGCAAC
TCACAGTCTGGGGCATT
>HIV-1-M-D-94UG114
TTCTACTGCAATACAACAAGACTGTTTAATAGTACATGGAAAAGGAATAA
TAGTGAATGGAGGAGTGATAATACACCAGATGAGACAATCACACTCCAAT
GCAGAATAAAACAAATTATAAACATGTGGCAGGAAGTAGGAAAAGCAATG
TATGCCCCTCCCATTGAAGGATTCATCAACTGTTCATCAAATATTACAGG
ACTATTGTTGACAAGAGATGGTGGTGCAATTAATAGTAGCCAGAATGAGA
CCTTCAGACCTGGAGGAGGAGATATGAGAAACAATTGGAGAAGTGAATTA
TACAAATATAAAGTAGTAAAACTTGAACCAATAGGTCTAGCGCCCACCGC
GGCAAAGAGAAGAGTGGTAGAAAGAGAAAAAAGAGCAATAGGACTGGGAG
CTCTGTTCCTTGGGTTCTTGGGAACAGCAGGAAGCACGATGGGCGCAGTG
TCACTGACGCTGACGGTACAGGCCAGACAGGTATTGTCTGGTATAGTGCA
ACAGCAAAACAATTTGCTGAGGGCTATAGAGGCGCAACAGCATCTGTTGC
AACTCACAGTCTGGGGCATT
>HIV-1-M-D-A08483M1.vrc9
TTCTACTGCAATACATCAGGACTGTTTAATGGTGTATGGAAGAATAATAC
ATCAGATAATGAAACGGTAAATGGAAGTGAGACAATAACAATCCCATGCA
GAATAAAACAAATTATAAACACGTGGCAAGGAGTAGGAAGAGCAATGTAT
GCCCCTCCCATTAGAGGACTAATCCGATGTTCATCAAACATTACAGGACT
ATTGTTAACAAGAGATGGTGGTACCAATGGTATTAGTCAGAATGAGATCT
TCAGACCTGGAGGAGGAGATATGAGAGACAATTGGAGAAGTGAATTATAC
AAATATAAAGTAGTAAAAATTGAACCATTAGGTCTAGCACCCACCAGGGC
TAAAAGAAGAGTGGTGGAAAGAGAAAAAAGAGCAATAGGACTAGGAGCTA
TGTTCCTTGGGTTCTTGGGAACAGCAGGAAGCACGATGGGCGCAGTGTCA
CTGACGCTGACGGTACAGGCCAGACAGGTACTGTCTGGTATAGTGCAGCA
GCAAAACAATTTGCTGAGGGCCATAGAGGCGCAACAGCATCTGTTGCAAC
TCACAGTCTGGGGCATT
>HIV-1
TTCTATTGTGACACATCAGGCCTGTTTAATAGCACTTGGTATAATACTTG
GAATAGCACTGAATCAAAAAGCACGGAGTCAAACGACATTACTCTCCAAT
GCAGAATAAAGCAAATTATAAATATGTGGCAGAGAGCAGGACAAGCAATA
TATGCCCCTCCCATCCAAGGAGTAATAATGTGTAAATCAAATATTACAGG
ACTAATATTAACAAGAGATGGTGGAGGTAATAGCAGTGAACCTGAAACCT
TCAGACCTGGAGGAGGAGACATGAGGGACAATTGGAGAAGTGAATTGTAT
AAGTATAAAGTAGTAAAAATTGAACCACTAGGAGTAGCACCCACCAAGGC
AAGGAGAAGAGTGGTGCAGAGAGAAAAAAGAACAGTTGGAATAGGAGCTG
TGTTCCTTGGGTTCTTAGGAGCAGCAGGAAGCACTATGGGCGCGGCGTCA
ATAACGCTGACGGCACAGGCCAGAGCATTACTGTCTGGCATAGTGCAACA
GCAAAGCAATTTGCTGAGGGCTATAGAGGCTCAACAACATCTGTTGAGAC
TCACGGTCTGGGGCATT
>HIV-1-M-A-92UG037
TTCTATTGTAATACATCAGGCCTGTTTAATAGTACTTGGGTAAATGGCAC
TACCAGCAGCACGTCAAATGGCACTATAACTCTCCCATGCAGAATAAAGC
AAATTATAAATATGTGGCAGAGAGTAGGACAAGCAATGTATGCCCCTCCC

ATCCAAGGAGTAATAAAGTGTGAATCAAACATTACAGGACTAATATTAAC
AAGAGATGGTGGGGTTAATAGCAGTGACAGTGAAACCTTCAGACCTGGAG
GAGGAGATATGAGGGATAATTGGAGAAGTGAATTATATAAGTATAAGGTA
GTAAAAATTGAACCACTAGGAGTAGCACCCACCAAGGCAAGGAGAAGAGT
GGTGGAGAGAGAAAAAAGAGCAGTTACACTGGGAGCTGTATTCATTGGGT
TCTTAGGAACAGCTGGAAGCACAATGGGCGCGGCGTCAATAACGCTGACG
GTACAGGCCAGAAAATTATTGTCTGGCATAGTGCAACAGCAAAGCAATTT
GCTGAGGGCTATAGAGGCTCAACAGCATCTGTTGAAACTCACTGTCTGGG
GCATT
>HIV-1-M-A-SE7253
TTCTATTGTAATACATCAGGCCTGTTTAATAGCACTTGGAGCCAAAATGA
CACTGGGGTATCAAATAGCACGGAGTCAAATGACACTATAATTCTTCCAT
GCAGAATAAAACAAATTATAAATATGTGGCAGAGAGCAGGACAAGCAATA
TATGCCCCTCCCATCCCAGGAATAATAAGGTGTGAATCAAACATTACAGG
GCTACTGTTAACAAGAGATGGTGGGGTTGTTAACAGTACAAATGAAACCT
TCAGACCTGGAGGAGGAAATATGAAGGACAATTGGAGAAGTGAATTATAT
AAGTATAAAGTAGTAAAAATTGAACCACTAGGAGTAGCACCCACCAGGGC
AAGGAGAAGAGTGGTGCAGAGAGAAAAAAGAGCAGTTGGACTGGGAGCTT
TATTCATTGGGTTCTTAGGAGCAGCAGGAAGCACTATGGGCGCGGCGTCA
ATAACGCTGACGGTACAGGCCAGACAATTATTATCTGGCATAGTGCAACA
GCAAAGTAATTTGCTGAGGGCTATAGAGGCTCAACAGCATCTGTTGAAAC
TCACTGTCTGGGGCATT
>HIV-1-M-A-97CDKTB48
TTCTATTGCAATACAACAGGCCTGTTTAATAGCACATGGGAAAATGGCAC
TAACAAACAGAATTACACGGAGTCAAATGACACTATAACGCTCCAATGCA
GAATAAAACAAATTATAAACATGTGGCAGAGAGTAGGCCGAGCAATGTAT
GCCCCTCCCATTGCAGGAGTAATAAAGTGTACATCAAACATTACAGGAAT
GATATTGACAAGAGACGGTGGGAAGAACAGTATTAATGAGACCTTCAGAC
CTGGAGGAGGAGATATGAGGGACAATTGGAGAAGTGAATTATATAAGTAT
AAAGTAGTAAAAATTGAACCACTAGGAATAGCACCCACAGAGGCAAGGAG
AAGAGTGGTGCAGAGAGAAAAAAGAGCAGTTGGATTGGGAGCTGTTTTCC
TTGGGTTCTTGGGAGCAGCAGGAAGCACTATGGGCGCGGCGTCAATAACG
CTGACGGTACAGGCCAGACAATTATTGACTGGCATAGTGCAACAGCAAAG
CAATTTGCTGAAGGCTATAGAGGCTCAACAACAGATGTTGAGGCTCACAG
TCTGGGGCATT
>HIV-1-M-A-94CY017.41
TTCTATTGCAATACAACAGGCCTGTTTAATGGTACGTGGTGGAACAATGG
TACGTGGAACGGGCCCTACACACCTAATAACACCAATGGAAGTATAATCC
TCCCATGCAGAATAAAACAAATTATAAACATGTGGCAGAGAGTAGGAAGA
GCAATGTATGCCCCTCCCATTGCAGGAATAATAAAGTGTACATCAAACAT
TACAGGAATAATATTGACAAGAGATGGTGGTAACAATGGGACTAATGAGA
CCTTCAGACCTGGAGGAGGAGATATGAGGGACAATTGGAGAAGTGAATTA
TATAAATATAAAGTAGTAAAACTTGAACCACTAGGAGTAGCACCTACCAG
GGCAAAAAGAAGAGTGGTGGAGAGAGAAAAAAGAGCAGTTGGACTGGGAG
CTGTCTTCCTTGGGTTCTTGGGAGCAGCAGGAAGCACTATGGGCGCGGCG
TCACTAACGCTGACGGTACAGGCCAGACAATTATTGTCTGGTATAGTGCA
ACAGCAAAGCAATTTGCTGCAGGCTATAGAAGCTCAACAGCATCTGTTGA
AACTCACAGTCTGGGGCATT
>HIV-1-M-G-DRCBL
TTCTATTGTAATACATCAGGACTATTTAATAATAGTATATTAAAGAGTAA
TATATCAGAAAATAATGATACTATCACACTTAATTGTAAGATAAAACAAA
TTGTGAGAATGTGGCAGAGAGTGGGACAAGCAATGTATGCCCCTCCCATT
GCAGGAAACATTACATGTAGATCAAACATTACAGGACTAATATTAACAAG
AGATGGTGGGGATAACAATAGCACTAGTGAGATCTTCAGACCTGGAGGAG
GAGATATGAAAAACAATTGGAGAAGTGAATTATATAAGTATAAAACAGTA
AAAATCAAATCACTAGGAATAGCACCCACCAGGGCAAGGAGAAGAGTGGT
GGAGAGAGAAAAAAGAGCAGTTGGAGTAGGAGCTATCTTCCTTGGGTTCT
TAGGAACAGCAGGAAGCACTATGGGCGCAGCGTCAATAACGCTGACGGTA
CAGGTCAGACAATTATTGTCTGGCATAGTGCAACAGCAAAGCAATTTGCT
GAGAGCTATAGAGGCGCAGCAGCATCTGTTGCAACTCACAGTCTGGGGCA

TT
>HIV-1-M-G-PBS1191
TTCTATTGTAATACATCAAAACTATTTAATAATAGTTGGCTTAATAATAG
TAGTAATGAAGCCAATATCACACTCCCATGTAAGATAAAACAAATTGTGA
GAATGTGGCAGAGAGTGGGACAAGCAATATATGCCCCTCCCATCGCAGGA
AACATTGTGTGTAGATCAAACATTACAGGACTAATATTAACAAGAGATGG
TGGGAATAACAATACAAATGAGACCTTCAGACCTGGAGGAGGAGATATGA
GGGACAATTGGAGAAGTGAATTATATAAGTATAAAGTAGTAAAGATCAAA
TCACTAGGAGTAGCACCCACCAGGGCAAGGAGAAGAGTGGTGGGAGAGA
AAAAAGAGCAGTAGTTGGACTAGGAGCTTTCTTCCTTGGGTTCTTAGGAG
CAGCAGGAAGCACTATGGGCGCGGCGTCAATAGCGCTGACGGTACAGGCC
AGACAATTATTGTCTGGCATAGTGCAACAGCAAAGCAATTTGCTGAGGGC
TATAGAGGCGCAACAGCATCTGTTGCAACTCACAGTCTGGGGCAT
>HIV-1-M-H-90CR056
TTCTATTGCAATACATCAGGGCTGTTTAATAGTAGTTGGGAAATGCATAC
TAATTACACATCAAATGACACAAAGGGAAACGAAAACATTACACTGCCAT
GCAGAATAAAACAAATTGTAAACATGTGGCAGAGAGTAGGACGAGCAATG
TATGCCCCTCCCATCCAAGGAAACATTATGTGTGTATCAAATATTACAGG
ACTAATATTGACAATTGACGAGGGTAACGCGTCTGCAGAAAATTATACCT
TCAGACCTGGAGGAGGAGATATGAGGGACAATTGGAGAAGTGAATTGTAT
AAATATAAAGTAGTAAAAATTGAACCACTGGGAATAGCACCCACCAAGAC
AAGGAGAAGAGTGGTGGAGAGAGAAAAAAGAGCAGTGGGAATGGGAGCTT
CTTTCCTTGGGTTCTTGGGAGCAGCAGGAAGCACTATGGGCGCGGCGTCA
ATAACGCTGACGGTACAGGCCAGGCAATTATTGTCTGGTATAGTGCAGCA
GCAAAGCAATTTGCTGAGAGCTATACAGGCGCGACAGCATATGTTGCAGC
TCACGGTCTGGGGCATT
>HIV-1-M-C-B002865-A11-12
TTCTATTGCAATACATCAAAACTGTTTAATGAAACATACCTTAATGATAC
AAAGAGTGAAACAATCATACTCCCATGCAGAATAAAACAGATTATAAACA
TGTGGCAGGAGGTAGGACGAGCAATGTATGCCCCTCCCATTGCAGGAAAC
ATAACATGTAACTCAAGTATCACAGGACTACTGTTGACACGGGATGGAGG
AGAGACAAATGGCACAAATGGCACAGAAACGTTCAGACCTGGAGGAGGAG
ATATGAGGGACAATTGGAGAAGTGAATTATATAAATATAAAGTGGTAGAA
ATTAATCCATTGGGAATAGCACCCACTAAGGCAAAAAGGAGAGTGGTGGA
GAGAGAAAAAAGAGCAGTGGGAATGGGAGCTGTGTTCCTTGGGTTCTTGG
GAGCAGCAGGAAGCACTATGGGCGCAGCGTCACTAACGCTGACGGCACAG
GCCAGACAATTGTTGTCTGGAATAGTGCAACAGCAAAGCAATTTGCTGAG
GGCTATAGAGGCGCAACAGCATATGTTGCAACTCACGGTCTGGGGCATA
>HIV-1-M-C-NIRT005
TTCTATTGCAATACATCAAGACTGTTTAATGGAACATATGATGGAACATA
TAATGGTACAGCAAGTAACAATACAGGAAACATCACCCTCCCATGCAGAA
TAAAACAAATTATAAACATGTGGCAGGGGGTAGGACGAGCAATATATGCC
CCTCCCATTGCAGGAAACATAACATGTGTATCAAATATCACAGGACTATT
ATTGGTACGTGATGGAGGAACAGAGAATGGCACAAATGGCACAGAAACAT
TCAGACCTACAGGAGGAAATATGAGGGACAATTGGAGAAGTGAATTATAT
AAATATAAAGTGGTAGAAATTAAGCCATTGGGAATAGCACCCACTGCAGC
AAGAAGGAGAGTGGTGGAGAGAGGAAAAAGAGCAGTGGGAATAGGAGCTG
TGTTCCTTGGGTTCTTGGGAGCAGCAGGAAGCACTATGGGCGCGGCGTCA
ATAACGCTGACGGTACAGGCCAGACAATTGTTGTCTGGTATAGTGCAGCA
GCAAAGCAATTTGCTGAGAGCTATAGAGGCGCAACAGCATCTGTTGCAAC
TCACGGTCTGGGGCATT
>HIV-1-M-C-DEMC08MW004
TTCTATTGTAATACATCAAGATTGTTTACATACAATGGTACAGAAAGTAA
GAATTCAACAAACAAAAGTAAGAATTCAACAGACATCATACTCCCATGCA
GAATAAAGCAGATTATAAACATGTGGCAAGAGGTAGGACGAGCAATGTAT
GCTCCTCCCATTGCAGGAAACATAACATGTACGTCAAATATCACAGGACT
ACTATTGACACGAGATGGAGGAGGTAAAAATAACACAAAAGAAGAGATAT
TCAGACCTGGAGGAGGAGATATGAGGGACAATTGGAGAAGTGAATTATAC
AAGTATAAAGTGATAGAAATTAAGCCATTAGGAATAGCTCCAACTACGGC
AAAAAGGAGAGTGGTGGAGAGAGAAAAAAGAGCAGTGGGAATAGGAGCTG

TGCTCCTTGGGTTCTTGGGAGCAGCCGGAAGCACTATGGGCGCAGCATCA
ATAACGCTGACGGTACAGGCCAGACAATTGCTGTCTGGTATAGTGCAACA
GCAAAGCAATTTGTTGAGGGCTATAGAGGCGCAACAGCATATGTTGCAAC
TCACAGTCTGGGGCATT
>HIV-1-M-C-097SO
TTCTATTGCAATACGTCTAGACTGATTAATAGTACATACAGGTCTAATAG
TACTAATAGTACTTCAGATACATCCATCATACTCCCATGCAGAATAAAAC
AAATTATAAACATGTGGCAGGGGGTAGGACGAGCAATGTATGCCCCTCCC
ATTGAAGGAAACATAACATGTAAATCAAATATTACAGGACTACTATTGAC
ACGTGATGGAGGAACGGACAATGAAACAATAGAGACATTCAGACCTGGAG
GAGGAGATATGAGGGACAATTGGAGAAGTGAATTATATAAATATAAAGTG
GTAGAAATTAAACCATTAGGAATAGCACCCACTAAGGCAAAAAGGAGAGT
GGTGGAGAGAGAAAAAAGAGCAGTGGGAATAGGAGCTGTGTTCCTTGGGT
TCTTGGGAGCAGCAGGAAGCACTATGGGCGCAGCATCACTGACGCTGACG
GTACAGGCCAGGCAATTGCTGTCTGGTATAGTGCAACAGCAAAGCAATTT
GCTGAGGGCTATAGAGGCGCAACAGCATATGTTGCAACTCACGGTCTGGG
GCATT
>HIV-1-M-C-PWJ-0513.39
TTCTATTGCAATACATCAAAACTGTTTAATAGTACATACAATCCTAATGG
AACAAAAAATGATACAAACTCAAGTGCATACATCATCCTTCCATGCAGAA
TAAAACAGATTATAAACATGTGGCAGGAGGTAGGACGAGCAATGTATGCC
AATCCCATTGAAGGATACATAACATGTAGATCAAATATCACAGGACTACT
ATTAGTACGTGATGGAAGCACAAATAGTAGTAACGAGACATTCAGACCTG
GAGGAGGAAATATGAAGGATAATTGGAGAAGTGAATTATATAAATATAAA
GTGGTAGAAATTAAGCCATTGGGAATAGCACCCACTGAAGCAAAAAGGAG
AGTGGTGGAGAGAGAAAAAAGAGCAGTGGGAATAGGAGCTGTGTTCCTTG
GGTTCTTGGGAGCAGCAGGAAGCACTATGGGCGCGGCGTCAATGACGCTG
ACGGTACAGGCCAGACAATTGTTGTCTGGTATAGTGCAACAGCAAAGCAA
TTTGCTGAGGGCTATAGAGGCGCAACAGCATTTGTTGCAACTCACGGTCT
GGGGCATT
>HIV-1-M-C-03ZAPS021MB1
TTCTATTGCAATACAACACCACTGTTTAATATGTCTGCTGACAATAACAG
CACCAATAATGGCACCATCACACTCCAATGCAAGATAAAACAAATTATAA
ACATGTGGCAGGGGGTAGGACGAGCAATGTATGCCCCTCCCATTGCAGGA
AACATAACATGTGCGTCAAATATCACAGGACTACTATTGACACATGACGG
AGGCATAAAAAATGACACAGTCCCAGAGGTATTTAGACCTGGAGGAGGAA
ATATGAAGGACAATTGGAGAAGTGAATTATATGAATATAAAGTGGTAGAA
ATTAAGCCATTGGGAATAGCCCCCACTGAGTCAAGAAGGATAGTGGTGGA
GAGAGAAAAAAGAGCAGTGGGAATGGGAGCTGTGATCTTTGGGTTCTTAG
GAGCAGCAGGAAGCACTATGGGCGCGGCGTCAACGGCGCTGACGGTACAG
GCCAGACAACTGTTGTCTGGTATAGTGCAACAGCAAAGCAATTTGCTGAG
AGCTATAGAGGCGCAACAGCATATGTTGCAACTCACAGTCTGGGGCATT
>HIV-1-M-C-C.ZA.x.Du246-B3
TTCTATTGCAATACATCAAAACTGTTTAATAAGAATGGTACAGAAAAGAA
TACAACCATCACACTCCCATGCAGAATAAAACAAATTATAAACATGTGGC
AGGAGGTAGGACGAGCAATATATGCCCCTCCCATTAAAGGAAACATAACA
TGTAGCTCAAACATCACAGGACTACTATTGACACGGGATGGAGGAGAAAC
AAACATAAAAAACACAGAGACAGACAATACCACAGAGACATTCAGACCTG
AAGGAGGAGATATGAGAAATAATTGGAGAAGTGAATTGTATAAATATAAA
GTGGTAGAAGTTAAGCCACTAGGAGTAGCACCTACTGAGGCAAAAAGGAG
AGTGGTGGAGAGAGAAAAAAGAGCAGTGGGAATAGGAGCTGTGTTCCTTG
GGTTCTTGGGAGCAGCAGGAAGCACTATGGGCGCGGCGTCAATAACGCTG
ACGGTACAGGCCAGACAATTGTTGTCTGGAATAGTGCAACAGCAAAGCAA
TTTGCTGAAGGCTATAGAGGCGCAACAGCATATGTTGCAACTCACGGTCT
GGGGCATTAAGCAACTCCAGGCAAGAGTCCTGGCTATAGAAAGATACCTA
AAGGATCAACAGCTCCTAGGGTTGTGGGGCTGCTCTGGAAAACTCATCTG
CACCACTGCTGTGCCTTGGAACATCAGTTGGAGTAATAAATCTTATGAAG
ACATTTGGAATAACATG
>HIV-1-M-C-05ZASK247B1
TTCTATTGCAATACATCAAGACTGTTTACATCAAACCTGTCTAGTAATAC

AGAAGACATCATCACAATCCCATGCAGAATAAAACAGGTTATAAACATGT
GGCAGAAGGTAGGACGAGCAATGTATGCCCCTCCCATTGCAGGAAACATA
ACATGTAGATCCAACATCACAGGACTACTATTGACACGTGATGGAGGATC
AGATAACCATACCAATAACACAGAGACTTTCAGACCTGGAGGAGGAGACA
TGAGGGATAATTGGAGAAGTGAATTATATAAATATAAAGTAGTAGAAATT
AAGCCATTGGGAATAGCACCCACCAAGGCAAAAAGGAGAGTGGTGGAGAG
AGAAAAAAGAGCAGCGGGAATAGGAGCTGTGTTCCTTGGGTTCTTGGGAG
CAGCAGGAAGCACTATGGGCGCGGCGTCAATAACGCTGACGGTACAGGCC
AGACAATTGTTGTCTGGTATAGTGCAACAGCAAAGCAATTTGCTGAAGGC
TATAGAGGCGCAACAGCATCTGTTGCAACTCACGGTCTGGGGCATT
>HIV-1-M-C-704MC024F
TTCTATTGCAATACATCAAGACTGTTTAATAGAAATAGTACAGACCTGTC
AACTAATACAGGAAATAATTCAACCATCACACTCCCATGCAGAATAAAAC
AAATTATAAACATGTGGCAGGGGGTAGGACGAGCAATGTATGCCCCTCCC
ATTGAAGGAAACATAACATGTAAGTCAATTATCACAGGACTACTATTGGT
GCGTGATGGAGGCCCAAATAATAGCACAGAAACATCAGACCGGAACCTGG
GAGGGAGGGAAAATATGAAAGGGAACAAATTTGGAAGGAAAAGTGAAATT
ATTATTAAATATAAAGTGATAGAAAATTTAAGCCCATTGGGGGAAATAAG
CACCCCACTAAGGCAAAAGAGGAGAGCGGTGGAGAGAGAGAAAAGAGCAG
TGGTGGGAATAGGAGCTATGATCCTTGGGTTCTTGGGAGCAGCAGGAAGC
ACTATGGGCGCGGCGTCAATAACGCTGACGGTACACGCTAGACAATTGTT
GTCTGGTATAGTGCAACAGCAAAGTAATTTGCTGAGGGCTATAGAGGCGC
AACAGCATATGTTGCAACTCACGGTCTGGGGCATT
>HIV-1-M-C-DEMC11ZM006
TTCTATTGTGATACATCAGGACTGTTTGAGAATAGGACATACACGCTTAA
TGATACAGGAAGCACTTCAAACAACACTAATTCAACCATCACAATACAAT
GCAGAATAAAACAAATTATAAACATGTGGCAGAGGGTAGGACAAGCAATG
TATGCCCCTCCCATTGCAGGAAACATAACATGTAAATCAAATATCACAGG
ACTGCTATTGACACGTGATGGAGGAGCAAAAAAGAATGAGAGTGACCCCC
CAGAGACATTCAGACCTGGAGGAGGAGATATGAGGGACAACTGGAGAAGT
GAATTATATAAATATAAAGTGGTAGAAGTTAAACCATTGGGATTAGCACC
CACCAAGGCACAAAGAAGAGTGGTGGGAAGAGAAAAAAGAGCAGTGGGAA
TAGGAGCTGTGTTCCTTGGGTTCTTGGGAGCAGCAGGAAGCACTATGGGC
GCGGCGTCAATCACGCTGACGGTACAGGCCAGACAATTGCTATCTGGCAT
AGTGCAACAGCAAAGCAATTTGCTGAGGGCTATAGAGGCGCAACAGCATC
TGTTGCAACTCACAGTCTGGGGCATT
>HIV-1-M-C-503-06310-30
ACTTATAATGATACAAAGCATAATGGAACCAAAGTCATCACACTCCCATG
CAGAATAAAACAAATTATAAACATGTGGCAGGAGGTAGGACGAGCAATGT
ATGCTCCTCCCATTGCAGGCAACATAACATGTACGTCAAATATCACAGGA
CTACTATTGACAAGGGATGGAGGGAATAATAGCACAGAGACAGAGACATT
CAGACCTGGAGGAGGAGATATGAGGGACAATTGGAGAAGTGAATTATATA
AATACAAAGTAGTAGAAATTAAGCCATTGGGAATAGCACCCACTGGAGCA
AAAAGGAGAGTGGTGGAGAGAGAAAAAAGAGCAGTGGGAATAGGAGCTGT
GTTCCTTGGGTTCTTGGGAGCAGCAGGAAGCACTATGGGCGCGGCGTCAA
TAACGCTGACGGTACAGGCCAGACAACTGTTGTCTGGTATAGTGCAACAG
CAAAGCAATTTGCTGAAGGCTATAGAGGCGCAACAACACCTGTTGCAACT
CACAGTCTGGGGCATT
>HIV-1-M-C-00BW-1921.13
TTCTATTGCAATACATCACAACTGTTCAATGGTACATACAATGATACATA
CAATGGTACAGAAAGTAATTCAGGTAATTCAACTATCACACTCCCATGCA
GAATAAAACAAATTATAAACATGTGGCAGGGGGTAGGACGAGCAATATAT
GCCCCTCCCATTGAAGGAAACATAACATGTAAATCAAATATCACAGGACT
ACTATTGACGCGGGATGGAGGGAAAGGGAATGATACCGCAGAGATATTCA
GACCTGAAGGAGGAGATATGAAGAACAATTGGAGAAGTGAATTATATAAA
TATAAAGTAGTAGAAATTAGGCCATTGGGAGTAGCACCCACTAAAGCAAA
AAGGAGAGTGGTGGAGAGAGAAAAAAGAGCAGCACTAGGAGCTGTGCTCC
TTGGGTTCTTGGGAGCAGCAGGAAGCACTATGGGCGCAGCATCAATAACG
CTGACGGTACAGGCCAGACAATTGCTGTCTGGAATAGTGCAACAGCAAAG
CAATTTGCTGAAGGCTATAGAGGCGCAACAGCATCTGTTGCAACTCACGG

TCTGGGGCATT
>HIV-1-M-C-CNE88.U
TTCTATTGTAATACATCAGGCCTGTTTAATAGTACATACATGACTAATGG
TACATACATGTTTAATGGTACATACAGGCTTAATAATATAACAAGTAATT
CAAACATCACAATTCCATGCAGAATAAAGCAAATTATAAACACGTGGCAG
GAGGTAGGGCGAGCAATGTATGCCAATCCCATTGCAGGAAACATAACATG
TAAATCAAATATCACAGGACTACTATTGGTACGTGATGGAGGAACAAATA
ATGCTACAACAGAGACATTCAGACCTGGAGGAGGAAATATGAGGGACAAT
TGGAGAAGTGAATTATATAAATATAAGGTGGTAGAAATTAAGCCATTGGG
AATAGCACCCACTGAAGCAAAAAGGCGAGTGGTGGAGAGAGAAAAAAGAG
CAGTGGGAATAGGAGCTGTGTTCCTTGGGTTCTTGGGTGCAGCCGGAAGC
ACTATGGGCGCAGCGTCAATAACGCTGACGGTACAGGCCAGACAATTGCT
GTCTGGTATAGTGCAACAGCAAAGCAATTTGCTGAGGGCTATAGAGGCGC
AACAGCATATGTTGCAACTCACGGTCTGGGGCATT
>HIV-1-M-C-C113_E6_2S
TTCTATTGCAATACATCAGGACTGTTTAATGAGACATACAATCCTAATGG
TACAGAAAGTAATTCAAGCGCAGTCATCACACTCCCATGCAGAATAAAAC
AAATTATAAACATGTGGCAGGAAGTAGGAAAAGCAATATATGCCCCTCCC
ATTCAAGGAAACATAACATGTAAATCAAATATCACAGGACTACTATTGGT
ACGTGATGGAGGAAACTCAAATGGCACAAATAACACAGAGATATTCAGAC
CTGGAGGAGGAGATATGAGGGACAATTGGAGAAGTGAATTATATAAATAT
AAAGTGGTAGAAATTAAACCATTGGGAATAGCACCCACTAGTGCAAAAAG
GAGAGTGGTGGAGAGAGAAAAAAGAGCAGTTGGAATAGGAGCTGTGTTCC
TTGGGTTCTTGGGAGCAGCAGGAAGCACTATGGGCGCGGCGTCAATAACG
CTGACGGTACAGGCCAGACAACTGTTGTCTGGTATAGTGCAACAGCAAAG
CAATTTGCTGAGAGCTATAGAGGCGCAACAGCATATGTTGCAACTCACGG
TTTGGGGCATT
>HIV-1-M-C-04ZASK146B1
TTCTATTGCAATACATCAAATCTGTTTAATATAACTGCAAGTAATGCAAG
TGATGCAAACAACAACACCATCACACTCCCATGCAAAATAAAACAAATTA
TAAACATGTGGCAGGAGGTAGGACGAGCAATGTATGCCCCTCCCATTGCA
GGAAACATAACATGTAACTCAAGTATCACAGGACTACTATTGACACGTGA
TGGAGGCAATAACAATGACACAGGCAATAACAATGACACAGAGATATTCA
GACCTGGAGGAGGAAATATGAAGGACAATTGGAGAAGTGAGTTATATAAA
TATAAAGTGGTAGAAATTAAGCCATTAGGAATAGCACCCACTAAGGCAAA
AAGGAGAGTGGTGGAGAGAGAGAAAAGAGCAGTGGGACTAGGAGCTGTAC
TCCTTGGGTTCTTAGGAACAGCAGGAAGCACTATGGGCGCGGCGTCAATA
ACGCTGACGGTACAGGCCAGGCAACTGTTGTCTGGTATAGTGCAACAGCA
AAGCAATTTGCTGAGGGCTATAGAGGCGCAACAGCACATGTTGCAACTCA
CGGTCTGGGGCATT
>HIV-1-M-C-21068
TTCTATTGCAATACATCAGGCCTGTTTAATAGGACATACATGCCTAATGA
TACAAAGAGTAATTCAAGCAGTAATCCAAACGCAAACATCACAATCCCAT
GCAGAATAAAGCAAATTATAAACATGTGGCAGGAGGTAGGACGAGCAATG
TATGCCCCTCCCATTGAAGGAAAAATAACATGTAGATCAAATATCACAGG
ACTACTATTGGTACGTGATGGAGGAGAAGACAAAAATAATACAGAGACAA
ATAAGACGGAGACATTCAGACCTGGAGGAGGAGATATGAGGGACAATTGG
AGAAGTGAATTATATAAATACAAAGTGGTAGAAGTTAAGCCATTGGGAGT
AGCACCCACTACAGCAAAAAGGAGAGTGGTGGAGAGAGAAAAAAGAGCAG
TGGGAATAGGAGCTGTGTTCCTTGGGTTCTTGGGAGCAGCAGGAAGCACT
ATGGGCGCGGCGTCAATAACGCTGACGGTACAGGCCAGACAATTGTTGTC
TGGTATAGTGCAACAGCAAAGCAATTTGCTGAGGGCTATAGAGGCGCAAC
AGCATCTGTTGCAACTCACGGTCTGGGGCATT
>HIV-1-M-C-92BR025
TTCTATTGCAATACATCAAGCCTGTTTAATAGTACATACACACCTAATAG
TACAGAAAACATTACTGGAACAGAAAATTCAATCATCACAATCCCATGCA
GAATAAAACAAATTATAAATATGTGGCAGGGGGTCGGACGAGCAATGTAT
GCCCCTCCCATTGAAGGAATATTAACATGTAGATCAAATATCACAGGATT
ACTACTGACACGGGATGGAGGTACAGGTATGCATGACACAGAGATATTCA
GGCCTGAAGGAGGAGATATGAGGGACAATTGGAGAAGTGAATTATATAAA

TATAAAGTGGTAGAAATTAAACCATTAGGAATAGCACCCACTAAGGCGAA
AAGGAGAGTGGTGGAGAGAGAAAAAAGAGCAGTGGGAATAGGAGCTGTGT
TCCTTGGGTTCCTGGGAGCAGCAGGAAGCACTATGGGCGCAGCATCAATA
ACGCTGACGGTACAGGTCAGACAATTATTGTCTGGGATAGTGCAACAGCA
AAGCAATTTGCTGAGAGCTATAGAGGCGCAACAGCATATGTTGCAACTCA
CGGTCTGGGGCATT
>HIV-1-M-C-C2220
TTCTATTGCAATACATCAAACCTGTTTAATAGCACAAAACTAGAACTGTT
TAATAGCAGTACAAATTTAAACATCACACTCCAATGCAGAATAAAACAAA
TTATAAACATGTGGCAGGGAGTAGGACGAGCAATGTATGCCCCTCCCATT
GAAGGAATAATAATGTGTAGATCAAATATCACAGGACTACTACTGACACG
TGATGGAGCCAAAGAGCCACATAGCACAAAAGAGATATTCAGACCTGAAG
GAGGAGATATGAGGGACAATTGGAGAAGTGAATTATATAAATATAAAGTG
GTAGAAATTAAGCCACTAGGAGTAGCACCCACTAAGCCAAAAAGGAGAGT
GGTGGAGAGAGAAAAAAGAGCAGCACTAGGAGCTTTGTTCCTTGGGTTCT
TGGGAGCAGCAGGAAGCACTATGGGCGCAGCATCAATAACGCTGACGGTA
CAGGCCAGACAATTATTGTCGGGTATAGTGCAACAGCAAAGCAATTTGCT
GAAAGCTATAGAGGCGCAACAGCATATGTTGCAGCTCACGGTCTGGGGCA
TT
>HIV-1-M-K-97ZR-EQTB11
TCCTACTGTGATACTACAGACACGGTTGATGACACAGAGGAGGAAGAGGA
TACCACAATCACAATCCCATGTAGGATAAAACAGATAATAAACATGTGGC
AGAAAGTGGGGCAAGCAATATATGCCCCTCCCACCGCAGGAAACATTACC
TGTAGATCAAATATTACAGGAATGATATTGACAAGAGATGGTGGTAATGA
CAATAATACTAGGACTGAGGAGACCTTCAGACCTGGAGGAGGAGACATGA
GAGACAATTGGAGAAGTGAACTATATAAATATAAAGTAGTACAGATTGAA
CCACTAGGAATAGCACCCACCAGGGCAAGGAGAAGAGTGGTGCAGAGAGA
AAAGAGAGCAGTAGGAATAGGAGCGTTGTTCCTTGGGTTCTTGGGAGCAG
CAGGAAGCACTATGGGCGCGGCGTCAATAACGCTGACGGTACAGGCCAGA
CAATTATTGTCTGGTATAGTGCAACAGCAAAACAATTTGCTGAGAGCTAT
AGAAGCGCAACAACAAATGTTACAGCTTACGGTCTGGGGCATT
>HIV-1-M-F-CM53657
TTCTACTGCAACACAGTAGCTCTGTTTAATGCAACTAACATGACTAATGC
AATGAACAGGTCCAATGGCATTATCACTCTTCCATGTAGAATAAGACAAA
TTGTAAACATGTGGCAAAGAGTAGGACGAGCAATGTATGCCGCTCCCATT
GCTGGACAAATTCAGTGTAACTCAAGCATCACAGGTCTAATATTGACAAG
AGATGGTGGGAAAAATAATACCAATAATGACACCCTCAGACCTGGAGGGG
GAGATATGAGAGACAATTGGAGAAGTGAACTGTATAAATATAAGGTAGTA
AAAATTGAACCACTAGGAGTAGCACCCACCAAGGCAAAAAGACAAGTGGT
GAAGAGAGAAAGAGAAAAAAGAGCAGTGGGAATAGGAGCTGTGCTCCTTG
GGTTCTTGGGAGCAGCAGGAAGCACTATGGGCGCGGCGTCAATGACGCTG
ACGGTACAGGCCAGACAATTATTGTCTGGTATAGTGCAACAGCAAAACAA
TTTGCTGAAGGCTATAGAAGCGCAACAGCATCTGTTGCAGCTCACAGTCT
GGGGCATT
>HIV-1-M-J-97DC.KTB147
TTCTACTGTAATACATCAATACTGTTCAATAGTAGTTGGGATGAGAATAG
CACTGTGACTATAAATGAAACAGTCACACTCCCATGTAAAATAAGACAAA
TTGTGAGAATGTGGCAGAGAGTAGGACAAGCAATATATGCCCCTCCCATC
GCAGGAAACATCACATGCACATCAAATATTACAGGGTTACTATTGACACG
TGATGGGGGAAATACCAAGGAGAGTAATAGTAGTGAGACCTTCAGACCTA
CAGGAGGAGATATGAAGGACAATTGGAGAAATGAGTTATATAAATATAAA
ATAGTGAAGGTTGAGCCATTAGGAGTAGCACCCACCAGGGCAAAAAGAAG
AGTGGTGGAGAGAGAAAAAAGAGCAATAGTGGGAATGGGAGCTGTGTTCC
TTGGGTTCTTGGGAACAGCAGGAAGCACTATGGGCGCAGCGTCAATAGCG
CTGACGGTACAGGCCAGACAGTTGTTGTCTGGCATAGTGCAACAGCAAAG
CAATTTGCTGAAGGCTATAGAGGCGCAACAGCATCTGTTGAGACTCACTG
TCTGGGGCATT
>HIV-1-M-CRF01-AG-CAM1970-LE.EC6
TTCTATTGCAATACATCAAAACTGTTCAAGCACACTTGGGAGAATGGCAA
TTTGACTAATCATAGCACAGAAAATGGAAATGACACTATAACTCTCCAAT

GCAGAATAAAGCAAATTATAAATATGTGGCAGAGAGTAGGACAAGCAATG
TATGCCCCTCCCATCCCAGGAGAAATAAGGTGTGAATCAAACATTACAGG
GCTACTATTAACAAGAGATGGAGGGAATAATGGTGGTGCAAATGAGACCT
TCAGGCCTGGAGGAGGAGATATGAGAGACAATTGGAGAAGTGAATTATAT
AAGTATAAAGTGGTGAAGATTAACCCACTAGGAGTAGCACCCACCAATGC
AAAAAGAAGAGTGGTGGGGAGGGAGAAAAGAGCAGTTGGACTGGGAGCTG
TCTTCCTTGGGTTCTTAGGAGCAGCAGGAAGCACTATGGGCGCGGCGTCA
ATAACGCTGACGGTACAGGCCAGACTATTATTGTCTGGTATAGTGCAACA
GCAAAACAATTTGCTGAAGGCCATAGAGGCTCAACAACATCTGTTGAGAC
TCACGGTCTGGGGCATT
>HIV-1-M-CRF01-AG-DJ263.EC3
TTCTATTGCAATACATCAAAACTGTTCAAGCACACTTGGGAGAATGGCAA
TTTGACTAATCATAGCACAGAAAATGGAAATGACACTATAACTCTCCAAT
GCAGAATAAAGCAAATTATAAATATGTGGCAGAGAGTAGGACAAGCAATG
TATGCCCCTCCCATCCCAGGAGAAATAAGGTGTGAATCAAACATTACAGG
GCTACTATTAACAAGAGATGGAGGGAATAATGGTGGTGCAAATGAGACCT
TCAGGCCTGGAGGAGGAGATATGAGAGACAATTGGAGAAGTGAATTATAT
AAGTATAAAGTGGTGAAGATTAACCCACTAGGAGTAGCACCCACCAATGC
AAAAAGAAGAGTGGTGGGGAGGGAGAAAAGAGCAGTTGGACTGGGAGCTG
TCTTCCTTGGGTTCTTAGGAGCAGCAGGAAGCACTATGGGCGCGGCGTCA
ATAACGCTGACGGTACAGGCCAGACTATTATTGTCTGGTATAGTGCAACA
GCAAAACAATTTGCTGAAGGCCATAGAGGCTCAACAACATCTGTTGAGAC
TCACGGTCTGGGGCATT
>HIV-1-M-CRF01-AE-JS2011001
TTCTATTGCAATACAACAGGACTGTTTAATAGTACTTACAGAGAAAATGG
AACCATGGAAGGGAGTAATGGCACTATCATACTTCCATGCAAAATAAAGC
AAATAATAAACATGTGGCAGGGAGTAGGACAAGCAATGTATGCTCCTCCC
GTCAGTGGAACAATTAATTGTGTATCAAATATTACAGGAATACTATTAAC
AAGAGATGGTGGTGGTAGTACCAATGCGACTAACGAGACCGAGATCTTCA
GACCTGGAGGAGGAAATATAAAGGACAATTGGAGAAGTGAATTATATAAA
TATAAAGTAGTAGAACTTGAACCACTAGGAATAGCACCCACCAGGGCAAA
GAGAAGAGTGGTGGCGAGAGAAAAAAGAGCAGTGGGAATAGGAGCTATGA
TCTTTGGGTTCTTAGGAGCAGCAGGAAGCACTATGGGCGCAGCGTCCATA
ACGCTGACGGTACAGGCCAGACAATTATTGTCTGGTATAGTGCAACAGCA
AAGCAATTTGCTGAGGGCTATAGATGCGCAGCAGCATCTGTTGCAACTCA
CAGTCTGGGGCATT
>HIV-1-M-CRF01-AE-620435.c1
TCTATTGTGATACAGCACAACTGTTTAATAGTACTTGGAGAGGAAATGAA
ACCAAGGAGGAAAGAAATGGCACTAGTAATGACATTATCATACTTCCATG
CAGGATAAAGCAAATTGTAAGAATGTGGCAGGGAGTAGGACAATCAATGT
ATGCTCCTCCCATCAGTGGAATAATTAATTGTGTATCAAATATTACAGGA
ATACTATTGACAAGTGATGGTGATGGTGGTCCTACTGCAGATAACGAGAC
CTTCAGACCTGCAGGAGGAGATATGAGGGACAATTGGAGAAGTGAATTAT
ATAAATATAAAGTAGTACAAATTGAACCACTAGGAATAGCACCCACCAGG
GCAAAGAGAAGAGTGGTGGACAGAGAAAAAAGAGCAGTGGGAATAGGAGC
TATGATCTTTGGGTTCTTGGGAGCAGCAGGAAGCACTATGGGCGCAGCGT
CAATAACGCTGACGGTACAGGCCAGACAATTATTGTCTGGTATAGTGCAA
CAGCAAAGCAATTTGCTGAGAGCTATAGAGGTGCAACAG
>SIVcpz-LB715
TTTTATTGTGAGACATCTTCATTGTTTAATGCTACAATAGATATTACACC
AAATACTACACTGAATGAGACGACCTGGGATTATCCAGCTGAGAACAATA
TTAACCAAGAAATGATAATCCATTGTAGAATAAAACAAATTGTATCAATG
TGGCAGAGAGTGGGAAAAGGAATATTTTCTCCTCCTATTAGAGGAACCAT
TCAATGTAACTCCACTATTACAGGACTACTGCTAACTCGCGATGGTAATA
ACAAAGCAGATAGTGAGAACAAAACAAGGATTGAAACAGGACAGAACAAA
GCAGAAGAGATTAATAGGACTGACATTTTTAGACCTGCAGGAGGAGAAAT
GAGAGACAATTGGAGAAGTGAATTATATAGATACAAGGTGGTGAAAATTG
AACCACTAGGACTAGCACCCACCAAGGCAAAAAGAAGGACTGTAAATAGA
GAAAAAAGAGCACCAGGACTAGGACTGATGTTCCTTGGGTTCTTGGGAGC
AGCAGGGAGCACTATGGGCGCAGCATCATTAACGCTGACGGTACAGGCCA

GGCAATTATTGACTGGCATAGTGCAACAGCAAAGCAATTTGCTGAGAGCT
ATAGAGGCGCAACAGCACTTGTTGCAACTCTCAGTCTGGGGCATT
>SIVcpz-LB7
TTTTATTGTAACACCTCGGCCTTGTTCAATGACACCTTGTTACGCAATAA
AACCTTGCTCAATGAAACCTTGCACAATAACTTGACACTACCTTGTAGGA
TTAGACAAATTGTAAACCTATGGCTAAGAGTAGGAAAGGGAATATTTGCT
CCACCTATTAGAGGTAACATCCGCTGTAATTCCACTATTACTGGGTTGAT
CCTAGAAAAACACACAAACACAGACAATATCACCTTCCGTCCAATAGGAG
GGGATATGACAGACATCTGGAGAAGTGAATTATATAACTATAAAATAGTA
AAAATTGAACCATTAGGAGTAGCGCCTACCAGGGCGAGAAGGAGGACTGT
GGATAGAGAAAAAAGAGCAGCAGGGCTGGGAGTGTTTTTCCTTGGGTTCT
TGGGAGCAGCAGGGAGCACTATGGGCGCAGCATCATTAACGCTGACGGTA
CAGGCCAGACAATTATTGACTGGCATAGTGCAACAGCAGAGTAATTTGCT
GAGAGCTATAGAGGCGCAGCAACACTTGCTGCAACTCTCAGTCTGGGGCA
TC
>SIVcpz-MB66
TTTTATTGCAATACCTCCATACTCTTTAATACCTCCATCATCTTTAATGA
GACTAAGGATGATAATATCACTATTCCTTGCAGAATAAGGCAAATTGTAA
GGCTATGGCAAAGAGTAGGAAGAGGAATATTTCTTCCCCCCATTAGAGGA
ACAATAAACTGTATCTCGAATATCACAGGGATATTGTTTGCGCAGCAAAA
AACAGACAGGATGAACAAATCAGCCATGTTCACACCTGTTGGGGGAGAAA
TGAGAAATAATTGGAGAAGTGAATTATACAAATATAAAGTAGTAAGAATT
GAACCCTTAGGAGTAGCACCCACCAAGGCAAAAAGAAGAACGGTGCATAG
GGAAAAAAGAGCAGCCGTAGGATTGGGAGCTTTGTTCCTTGGGTTCTTGG
GAGCAGCAGGAAGCACTATGGGCGCAGCGTCACTGACGCTGACGGTACAG
GCCAGACAACTGCTGTCTGGTATAGTGCAGCAGCAGAGCAATCTGCTGAG
AGCCATAGAGGCGCAACAGCATCTGTTGCAACTCTCAGTCTGGGGCATT
>SIVcpz-MB897
TTTTACTGTAATACATCAAACTTATTCAATCAATCCACTATTAACAATAT
TACACACATTCCGTGCAGAATAAGACAAATTGTAAATCAGTGGCAAGGAG
TAGGAAAAGGAATCTTTGCACCTCCTATTAGAGGAACAATTCAATGTAAT
TCCACAATCACAGGCCTACTCTTAACAAGAGATGGTAAAAATGAGACTGA
AACTTTTAGACCTACAGGAGGAGACATGAGAAATAATTGGAGAAGTGAAT
TATACAAATACAAGGTAGTAAAAATTGAACCCCTAGGATTAGCACCAACC
AAGGCAAAAAGGAGAACAGTGCAAAGAGAAAAGAGGGCAGTAGGACTGGG
AGTGATGTTCCTTGGATTCATGGGAGCAGCAGGAAGCACTATGGGCGCAG
CGGCACTGACGCTGACGGTACAGGCCAGAAATCTACTCTCTGGTATAGTG
CAACAGCAAAGCAATTTGCTGAAAGCTATAGAGGCGCAACAGCATCTGTT
GCAACTCTCAGTCTGGGGCATT
>SIVcpz-GAB2
TTTTATTGTAATACATCAAGATTAATTACCTGTAACAGTAGTGACACAAG
TGAGTACATTCTCCCATGCAAAATTAGGCAAGTAGTAAATTCTTGGATGA
GAGTGGGGAAAGGTATCTTTGCCCCGCCTAGAAGAGGAACTATAACCTGT
AATTCCACCATTACAGGACTCCTCTTAGAAGTACAGAATGGCACGGGGAA
CAACACAGAAGTTTACCTGTCAGGGGGAGATATGAGAGATATTTGGAGGA
GTGAATTATATAAATATAAAATAGTAAAAATTGAGCCACTGGGGGTGGCA
CCAACAAAAGCAAAGAGGTACACAGTGGCAAAAGCACTTGATAGATCCAA
AAGAGCAGCCTTTGGACTGGGGGCGCTGTTTCTTGGATTCCTTGGAGCAG
CAGGAAGCACTATGGGCGCAGCATCTGTGATGCTGACGGTACAAGCTCGA
AACCTGCTTTCGGGAATAGTGCAGCAGCAAAATAACTTGCTGCGAGCCAT
AGAAGCTCAGCAACACCTGCTGCAGCTATCAGTCTGGGGCATA
>SIVcpz-CAM13
TTTTACTGCGATACTAGAAAAATGTTTAATGAAAGTGAACCTTTTCACGA
GAACATGACAATACCATGCAGAATTAGACAAATAGTGAATTCATGGATGA
GAGTAGGAAGGGGAATCTATGCACCTCCAATACCAGGACATATAACTTGT
AACTCTCTAATTACTGGGCTCATATTGACACGAGACCATGTTAATAATAC
TAATAATACATTTAGACCAATAGGAGGAGACATGAAAAACATTTGGAGGA
GTGAACTCTATAAATACAAAGTGGTGCGCATAGAACCATTATCTGTGGCA
CCAACCAAAGCAAAAAGGCATACAGTAGGAGAAAGGAGACAGAAGAGAGC
AGCCTTTGGCCTGGGAGCACTGTTCCTTGGGTTTCTGGGTGCAGCAGGAA

GCACAATGGGCGCAGCGGCAGTCACGCTGACGGTACAGGCCCGACAGTTA
CTATCGGGAATAGTGCAACAACAGAACAACCTGTTGAGAGCAATAGAGGC
CCAACAGCATCTATTGCAACTAACAGTCTGGGGAGTA
>SIVcpz-DP943
TTTTATTGTAACACCACCCCATTGTTCAATAACAGTTGGACAAATACAAC
TAGCTCAGATATAAATAGCTCAAATATAATAATAAACTGTAGAATAAGGC
AAATTGTAAACTCCTGGATGAGAGTGGGCAAGGGAATTTACGCCCCACCA
ATTAGAGGAACTATAAGTTGCACATCAAATATTACTGGGCTCCTGCTCGA
GTTAGACAGAGGACTCCCGAGAAATGGGACTAATACCAATAACAAGTTAA
CACTATACCCTACAGGAGGAGAGATGAGAGATTTGTGGAGGCTGGAATTA
CATAAATACAAAGTAGTCAGCATAGAACCCATAGGAGTGGCACCAAGTAA
AGCTAAAAGGCACACAGTGACAAGAGAAAAAAGAGCAGCCTTTGGACTAG
GTGCGCTGTTTCTTGGGTTTCTAGGAGCAGCAGGAAGCACTATGGGCGCA
GCATCACTAACGCTGACGGTACAGGCCCGGCAATTATTGTCAGGGATAGT
GCAACAGCAGAACAACCTGCTGAGAGCAATAGAGGCGCAACAGCATTTGT
TGCAACTCTCAGTATGGGGCATT
>SIVcpz-US.85.CPZUS
TTTTATTGTAATACAATTCCCCTATTTAACATGACCTACAACAATACCGA
CAACACCACTATCACACTTAAGTGTAGAATAAGACAAATTGTAAATCAGT
GGATGAGAGTAGGAAAAGGAATCTTTGCCCCACCAATCAAAGGTGTGCTA
AGTTGTAACTCAAATATAACGGGAATGATTCTTGACATAAGCATAAGCGC
AGTCAATAACGATAGTAGGAATATAACAGTGATGCCTACAGGAGGAGATA
TGACGGCTTTATGGAAAAATGAATTACATAAGTATAAGGTGGTCAGCATA
GAACCTATAGGAGTGGCACCAGGTAAGGCCAAAAGGCATACAGTGAAAAG
AGAAAAAAGAGCAGCCTTCGGACTAGGTGCACTGTTCCTGGGGTTTCTTG
GAGCAGCAGGAAGCACTATGGGCGCAGCATCAGTAGTGCTGACGGTACAG
GCCCGACAATTATTGTCAGGGATAGTGCAACAGCAGAATAATCTGCTAAG
AGCAATAGAGGCCCAACAGCATTTATTGCAACTATCAGTTTGGGGCATT
>SIVcpz-EK505
TTTTACTGTAATACCAGTCAATTGTTTACAAATCAAAATGGCAACACAAC
TGGCAATATCACCTTACAATGCAGAATAAGACAAATTGTAAATTTATGGA
CAAGAGTAGGAAAAGGAATTTATGCTCCGCCAATTAAAGGACCTATTAAC
TGTCTCTCTAACATCACTGGGATAATACTTGACTATACAAAGTCTGGAAC
TGAGAAGTACACCATATATCCCACAGGGGGAGACATGACTAACCTCTGGA
GGCAGGAGTTGTATAAGTATAAGGTGGTCAGCATAGAACCCATAGGAGTA
GCACCAGGAAAAGCTAAAAGACACACAGTGACTAGACAAAAAAGAGCAGC
CTTTGGACTAGGTGCGCTGTTTCTTGGGTTTCTTGGAGCAGCAGGGAGCA
CTATGGGCGCAGCGTCAATAACGCTGACGGTACAGGCCCGGAAATTATTA
TCTGGGATAGTGCAGCAGCAGAATAATCTGCTGAGAGCAATAGAGGCGCA
ACAACACTTGCTGCAACTCTCAGTTTGGGGCATT
>HIV-1-N-YBF30
TTCTATTGTAACACTTCCAAATTATTTAATGAGGAATTACTTAACGAGAC
AGGTGAGCCTATTACTCTGCCTTGTAGAATAAGACAGATTGTAAATTTGT
GGACAAGGGTAGGAAAAGGAATTTATGCACCACCAATTCGGGGAGTTCTT
AACTGTACCTCCAATATTACTGGACTGGTTCTAGAATATAGTGGTGGGCC
TGACACCAAGGAAACAATAGTATATCCCTCAGGAGGAAACATGGTTAATC
TCTGGAGACAAGAGTTGTATAAGTACAAAGTAGTTAGCATAGAACCCATA
GGAGTAGCACCAGGTAAAGCTAAAAGACGCACAGTGAGTAGAGAAAAAAG
AGCAGCCTTTGGACTAGGTGCGCTGTTTCTTGGGTTTCTTGGAGCAGCAG
GGAGCACTATGGGCGCAGCGTCAATAACGCTGACGGTACAGGCCCGGACA
TTATTATCTGGGATAGTGCAACAGCAGAATATTCTGTTGAGAGCAATAGA
GGCGCAACAACATTTGTTGCAACTCTCAATCTGGGGCATT
>HIV-1-N-02CM-DJO0131
ATCTATTGTAATACTTCCAGATTATTCAATGAGAGCATGAATACAAATGG
GACTAATGGGACTATTACTCTGCCCTGTAGAATAAGACAAATTGTAAATT
TGTGGACAAGAGTAGGAAAAGGAATTTATGCACCGCCAATTCGGGGAAAT
CTTACCTGTAACTCCACTATTACTGGACTGATTCTAGAACATAGTGGTGG
CAGTAATGGCACAGTATATCCCACAGGAGGAAACATGGTCAATCTCTGGA
GACAAGAGTTGTATAAGTACAAAACAGTCAGTATAGAACCCATAGGAGTA
GCACCAGGTAAAGCTAAAAGACGCACAGTGAGTAGAGAAAAAAGAGCAGC

CTTTGGACTAGGTGCGCTGTTTCTGGGGTTTCTTGGAGCAGCAGGGAGCA
CTATGGGCGCAGCGTCAATAACGCTGACGGTACAGGCCCGGACATTGTTA
TCTGGGATAGTGCAACAGCAGAATAATCTGGTGAGAGCAATAGAGGCGCA
ACAACATTTGTTGCAACTCTCAATCTGGGGCATT
>HIV-1-N-04CM-1015-04
TTCCTTTGTAACACTTCCAGATTATTTGATGAGAACGGGACTGTAAATGG
GACTATTATTCTGCCCTGTAGAATAAAACAAATTGTAAATTTGTGGACAA
GAGTAGGAAAAGGAATTTATGCACCACCAATTCGGGGAAATATTACCTGT
AACTCCAGTATTACTGGACTAATTCTAGAAGTTAGTGGAAATAGTACAGT
ATATCCTTCAGGAGGAAACATGGTTAATCTCTGGAGACAAGAGTTGTACA
AGTACAAAGTAGTTAGCATAGAACCCATAGGAGTAGCACCAGGTAAAGCT
AAAAGACGCACAGTGAATAGAGAAAAGAGAGCAGCCTTTGGACTAGGTGC
GCTGTTTCTTGGGTTTCTTGGTGCAGCAGGGAGCACTATGGGCGCAGCGT
CAATAACGCTGACGGTACAGGCCCGGAACTTATTATCTGGGATAGTGCAA
CAGCAGAATAATCTGTTGAGAGCAATAGAGGCGCAACAACAATTGTTGCA
ACTCTCAATCTGGGGCATT
>HIV-1-P-U14788
TTTTATTGTAACACCAGCAGCCTGTTTAACTTTAGTTATACTTGGAATGA
CACCACCTGGCGGGCTGCAGGCAATCATAATACAAGTGAAAATATAACAC
TGTCTTGTAGACTGAAGCAAGTAGTTAATTCATGGATGAGAGTGGGATCA
GGCTTGTTTGCGACACCTATTGAGGGGGAATTAAAGTGTCAATCCAACAT
TACAGGTTTAATGTTAGAAAGAGAACTACCTTACAATGACAGCAGCAAGA
ACACCACTTTAAGTCCTATAGGGGGAGACATGACAAATATCTGGAGAAGT
GAGTTATATCCCTACAAAGTAGTTCAAGTAAAAGCTCTGGCCGTGGCACC
TACAAAAATTTCCAGACCTACAATTATGGCTCATGATGGACATAGGAAGA
AAAGGGCAGCAGGCCTAGGAATGCTATTCCTTGGGTTTCTGAGCGCAGCA
GGAAGCACTATGGGCGCAGCGGGAATCACGCTGACGGTACAGGCCAAGCA
ATTATTGCATGGTATAGTGCAGCAGCAGAACAATATGCTAAGAGCTATAG
AGGCACAGCAAGAATTGCTGAGACTCTCTGTGTGGGGCATA
>SIVgor-BPID15
TTTTATTGTAATACCTCTAAACTCTTCAACTATACCTATGTCTGTAATCA
GACAGATCAAGGTAATGCTAATTATTCCTGTCATAGTACTAGTACATTAG
AAAATGGAACCTTAGTAATACCCTGCAAATTAAGGCAGGTAGTTAATTCA
TGGATGAGAGTGGGATCAGGACTTTTTGCACCACCTGTCCCGGGAAGCCT
AACGTGCCACTCCAATATCACAGGTCTAATCCTTCAAAGGGACTGGCCAC
TAAACAATAATACGACTACTATTTTGAGGCCTGAAGGAGGAGACATGAAA
GACATCTGGAGAAGTGAGCTATATCCATATAAGGTAGTCCAAGTAAAAGC
TTTGGCTGTGGCACCTACAAAAATCTCCAGACCAACAATTATGGCACAGA
CTCATCAGAGGGAAAAAAGAGGAGCAGGCCTCGGAATGCTATTCCTTGGG
TTCATGAGTGCAGCAGGAAGCACGATGGGCGCAGCGGCAGTAACGCTGAC
GGTACAAGCCAAGCAATTATTGCATGGTATAGTGCAACAGCAGAATAATC
TGCTCAGAGCGATAGAGGCACAGCAAGAATTGCTGAGGCTCTCTGTGTGG
GGCATA
>SIVgor-CP2139.287
TTCTACTGTAATACCACTAAAATGTTTAGCTACAATTGCACAACAGAGGG
TTGCAATACCACTGAAACAAGCAATACCACTGAAACAACTAAATATATTC
CCTGCCATCTAAAACAGGTAGTCAGGTCCTGGATGAGAGTAGGCTCAGGC
CTATTTGCTCCGCCTATTAGGGGAACTCTAAGATGTATATCTAACATTAC
AGGAATAATATTACAAAGGGATGCACCCCTAAAAACTGATGAGAACAGCA
CCTTAAGACCAATAGGAGGTGACATGACAAACATTTGGAGGAGTGAACTG
TACCCCTACAAGGTGGTGAGGGTCAAGGCATTGACTGTGGCACCAACAAA
AGCAAAGAGGCCTGTTATTGGCCATAACAGGGAAAAACGTGCAGCAGGCC
TGGGAATGCTGTTTCTTGGGTTCATGAGTGCAGCAGGAAGCACGATGGGC
GCAGCGGCAGTAACGCTGACGGTACAGGCCAGGCAAGTCTTGCATGGTAT
CGTGCAGCAGCAAAACAACATGCTGAGAGCTATAGCAGCTCAGCAAGAAT
TGCTGAGACTCTCTGTTTGGGGCATA
>SIVgor-BQID2
TTTTATTGCAACACCACGACAATGTTTAATTATACTTACAATTGTAGCCA
CCATAGCTGTAAAGCTCAATCAAAGAACATTTCAGAGGGCAGCGGGGACT
CGTATATTCCCTGTAAATTGAAACAAGTAGTAAATTCATGGATGAGAGTA

GGATCAGGTCTTTTTGCCCCACCTATACGGGGAACTCTCAAATGTATGTC
AAATATAACAGGTTTACTACTAGAAAGAGATGTCCCATTGAACATAACTG
CCACTAACAGAAATATAACTCTCAGACCAACGGGGGGAGAAATGAAAGAT
ATATGGAGAAGTGAACTATACCCATACAAGGTGGTGCAAGTCAAAGCATT
ATCTGTGGCACCCACAAAGATAAAAAGACCCATAATTGGTCTGAACAGAG
AAAAAAGAGGAGCAGGACTAGGAATGCTATTCCTTGGATTCATGAGTGCA
GCAGGAAGCACTATGGGCGCAGCATCTCTGACGCTGACGGTACAGGCCAA
GCAATTATTGCATGGTATAGTGCAACAGCAAAATAACATGCTGAGAGCTA
TAGCAGCCCAGCAGGAATTGCTGAGGCTCTCTGTATGGGGCATT
>HIV-1-O-MVP-5180
TTTTATTGTAACACATCTGGGATGTTTAACTATACTTTTATCAACTGTAC
AAAGTCCGGATGCCAGGAGATCAAAGGGAGCAATGAGACCAATAAAAATG
GTACTATACCTTGCAAGTTAAGACAGCTAGTAAGATCATGGATGAAGGGA
GAGTCGAGAATCTATGCACCTCCCATCCCCGGCAACTTAACATGTCATTC
CAACATAACTGGAATGATTCTACAGTTAGATCAACCATGGAATTCCACAG
GTGAAAATACACTTAGACCAGTAGGGGGAGATATGAAAGATATATGGAGA
ACTAAATTGTACAACTACAAAGTAGTACAGATAAAACCTTTTAGTGTAGC
ACCTACAAAAATGTCAAGACCAATAATAAACATTCACACCCCTCACAGGG
AAAAAAGAGCAGTAGGATTGGGAATGCTATTCTTGGGGGTGCTAAGTGCA
GCAGGTAGCACTATGGGCGCAGCGGCAACAGCGCTGACGGTACGGACCCA
CAGTGTACTGAAGGGTATAGTGCAACAGCAGGACAACCTGCTGAGAGCGA
TACAGGCCCAGCAACACTTGCTGAGGTTATCTGTATGGGGTATT
>HIV-1-O-9CMA105
TTTTATTGTAACACATCTAATTTATTTAATTATACCTTTTCATGTAATGG
AACCACCTGTAATGATAGCCAAAGTACCACCAATGACACACAGATACCTT
GCAAACTGAGACAGGTAGTAAGGTCATGGATAAAAGGACAGTCAGGATTC
TATGCACCTCCCATCAAGGGTAACCTGACATGTACATCAAACATAACAGG
AATGATTCTACAAATGGATGCACCATGGAACAGCACCAGAAACAACAGCG
AAAACCAAACTGCAACATTTAGACCAACAGGGGGAGAAATGAAAGATATA
TGGAGAACTGAGTTGTTCAACTACAAAGTAGTAAGAGTAAAACCTTTTAG
TGTGGCACCCACATCAATTGCAAGGCCAGTTGTAGGCACTAGCACTCATA
GAGAAAAAAGAGCAATAGGATTAGGAATGCTATTCTTGGGGGTTCTAAGT
GCAGCAGGTAGCACTATGGGCGCAGCGGCAACAGCGCTGACGGTACAGAC
CCACACTTTGATAAAGGGTATAGCGCAACAGCAGGACAACCTGCTAAGAG
CAATACAGGCCCAGCAGCAATTGCTGAGGCTATCTGTATGGGGTATC
>HIV-1-O-98CMU2901
TTCTATTGTAACACATCTGGGATGTTTAATTATACCTTTTTATGTAATGG
GACCAACTGTAAAAACCAAAGTACCCATAGCAATAATGGCACGATACCTT
GCAAGTTGAAACAGGTGGTAAGATCATGGATGAGGGGAGGGTCGGGACTC
TATGCACCTCCCATCCCAGGTAACCTAACATGCATGTCACACATAACTGG
AATGATTCTACAAATAGATGGGCCATGGAACAACAACACCACAAACAATA
CATTTAGACCAATAGGGGGAGATATGAAAGATATATGGAGAAATGAATTG
TTCAACTACAAAGTAGTGAGGGTAAAACCTTTTAGTGTGGCACCTACATC
AATTGCAAGGCCAGTCATAGGCACTGGCACTCATAGAGAGAAAAGAGCAG
TAGGATTGGGAATGCTATTCTTGGGGGTTCTAAGTGCAGCAGGTAGCACT
ATGGGCGCAGCGGCAACAGCGCTGACGGTACAGACCCACTCTCTGATAAA
GGGTATAGTGCAACAGCAGGACAACCTGCTAAGAGCAATACAGGCCCAGC
AGGAATTGCTGAGGCTATCTGTATGGGGTATC
>HIV-1-O-99SE-MP1299
TTCTATTGTAACACATCTAAGATGTTTAATTATACCTTTTCATGTATAGG
AACCAACTGTACTAGTAACCAAAATAGCAGCAACAGTAATGACACTCGGA
TATATTGCAGGATAAAACAGGTGGTAAGGTCATGGATACAGGGAGGGTCA
GGACTCTATGCACCTCCCAGGAAAGGTAACCTAACATGCAGCTCACTCAT
AACTGGAATGATTCTACAATTGGATATGCCATGGAACAGCACCAACAACA
GCAACGCCACATTTAGACCAACAGGGGGAGATATGAAAGATATATGGAGA
ACTGAATTGTTCAAATACAAAGTAGTAAAGGTAAAACCTTTTAGTGTGGC
ACCTACAAAAATTGCAAGGCCAGTCATAGGCACTGGCACTCAAAGAGAAA
AAAGAGCAGTAGGATTGGGAATGCTATTCTTAGGGGTTCTAAGTGCAGCA
GGAAGCACTATGGGCGCAGCGGCAACAGCGCTGGCGGTACAGACCCAATC
TTTGATGAAGGGTATAGTGCAACAGCAGGACAACCTGCTAAGAGCAATAC

AGGCCCAGCAGCAATTGCTGAGGCTATCTGTATGGGGTATC
>HIV-1-O-99SE-MP1300
TTCTATTGTAACACATCTCAGATGTTTAATTATACCTTTTCATGTACAAG
AACCAACTGTATTAGACAAAGTAACAGTAGCATTAATGGCACAATATCTT
GCAGGATAAAACAGGTGGTAAGGTCATGGATACAGGGAGGGTCGGGACTT
TATGCACCTCCCAGGCCAGGTTACCTAACATGCAACTCATCCATAACTGG
AATGATTCTACAATTGGATAAGACATGGAACCGCACCAACAACAGCGAAT
CCACATTTAGACCAATAGGGGGAGATATGAAAGATATATGGAGAACTGAA
TTGTTCAAATACAAAGTAGTAAAGATAAAACCTTTTAGTGTGGCACCTAC
AAAAATTGCAAGACCAGTCATAGGCACTGGCACTCGAAGAGAAAAAAGAG
CAGTAGGATTGGGAATGCTGTTCTTGGGGGTTCTAAGTGCAGCAGGAAGC
ACTATGGGCGCAGCGGCAACAACGCTGGCGGTACAGACCCACACTTTGAT
GAAGGGTATAGTGCAACAGCAGGACAACCTGCTAAGAGCAATACAGGCCC
AGCAGCAATTGCTGAGGCTATCTGTATGGGGTATC
>SIVcpz-BF1167
TTCTATTGTGACCTCAAAAATTGGACAAACAATGGCATCGAGATCGATGG
AATGCTGATAGCACCATGTCGACTTAGACAGATAGTAAATCATTGGGGAA
TAGTATCCAGGGGAATTTATCTCCCTCCCAGGGAGGGACAGGTCAAATGT
GTATCTAGTATTACAGGATTCATAATGACAGCTGAGACGAATGACACTGA
TGGCACTATCACACCCACCTTCTCTGCAAAGGTGGAAGACTACTGGAAAG
TAGAAATGAGTAGATACAAAGTAGTGGAGATACAGCCATTATCAGTGGCA
CCCACTAAAGGAAAAAGACCAGTAGTTGGTGACCCAGAAGCAAAAAGTAG
AAGTAAAAGAGAAATAGGAATGGGAATGATCTTTATGGGATTTCTCAGTG
CAGCAGGAAGTACTATGGGCGCAGCGTCAATAGCGCTGACGGTACAAGCC
AGAGGATTGCTCTCTGGTATAGTACAGCAGCAAGCAAATTTGCTGCAGGC
AATAGAGGCTCAACAACATTTGTTGCAGCTCTCGGTGTGGGGCATA
>SIVcpz-TAN13
TTCTACTGCAATCTAACAAAATGGATTAACGAGACCTGGCTTAACGAGAC
GGACCAGACCAATCTTGTAGCACCCTGTAGACTCAGACAAATAGTTAACC
ATTGGGGCATTGTGGCTAAAGCCATCTACCTCCCTCCAAGAAGAGGAACA
GTAAAATGTGTGTCAAACATCACGGGTTTCCTGATGACAAACGAGGAAAC
GGGTGCTACCTTCTCCGGTAAAGTAGAGGATTATTGGAGGGTGGAATTAG
CAAAGTACAAAATTGTAGAAATTCAGCCCCTATCAGTGGCTCCTACCACA
GGAAAAAGGCCAGAGATTAAGGCCAATCATACAAGATCAAAAAGGGACGT
TGGCATAGGGCTGTTGTTTCTGGGATTTCTCAGCGCAGCAGGAAGTACAA
TGGGCGCAGCGTCAATAGCGCTGGTGGCACAGGCCAGAGGGGTCCTCTCT
GGTATTGTACAGCAGCAGCACAACCTGCTGCAGGCAATAGAGGCGCAACA
GCATTTGTTGCAGCTTACTGTATGGGGCGTC
>SIVcpz-TAN1
TTTTACTGTAATCTCACAAATTGGACTAATACCTGGACAGCTAATAGAAC
CAATAATACTCATGGTACTCTTGTTGCACCATGCAGACTGAGGCAGATAG
TAAATCATTGGGGTATAGTGTCAAAAGGGGTTTACCTTCCCCCAAGGAGG
GGAACAGTAAAATGTCACTCAAACATCACAGGACTTATCATGACAGCAGA
AAAAGACAACAATAATAGTTATACCCCCCAATTTTCTGCTGTAGTAGAAG
ACTATTGGAAAGTAGAATTAGCAAGATATAAAGTGGTGGAAATTCAGCCC
TTGTCAGTGGCTCCAAGGCCAGGAAAAAGGCCTGAAATTAAGGCCAATCA
TACTAGGTCAAGAAGAGATGTGGGCATAGGACTGTTGTTTCTTGGATTTC
TTAGTGCAGCAGGAAGTACAATGGGCGCAGCGTCAATAGCGCTGACGGCA
CAGGCCAGAGGATTACTCTCTGGTATTGTACAGCAGCAACAAAACCTGCT
TCAGGCCATAGAAGCGCAACAACACTTGTTGCAGCTCTCTGTATGGGGCA
TT
>SIVcpz-UG38
TTCTATTGCAACCTCACTAACTGGACAAATATAACGTGGCTAGAGACGTG
GCTAAAGACTAACAATAGTTCCCGTCCCCTTGTAGCACCATGTAAACTTA
GGCAGATAGTAAACCATTGGGGTATAGTATCTAAAGGTATCTACCTACCT
CCTAGAAGGGGAAATTTAACATGCAGATCTAACATTACAGGGTTCATTAC
GACATGGGATAATGACACGGAGAACGTGCTTGTTACCTTTTCAGCCAAAG
TAGAAGACTATTGGAAGGTAGAAATGTCACGATACAAAGTAGTGGAAATT
CAGCCCTTGGCCATAGCTCCTACTACAGGAAAAAGGCCGGAGATTAGAGC
CAATCACACAAGGTCAAAAAGAGATGTTGGCATAGGACTGTTGTTCCTGG

GATTTCTCAGTGCAGCAGGAAGTACAATGGGCGCAGCGTCAATAGCGCTG
ACGGCACAGGCCAGAGGATTGCTCTCTGGTATTGTACAGCAGCAGCACAA
CCTGCTGCAGGCCATAGAAGCTCAACAGCACTTGTTACAGCTTTCTGTGT
GGGGCATT
>SIVdeb-04CMPF3061
TTTTATTGCAATGTCAGTAAGGCCTATCAACTTTTCACCAATGACACCAT
AACATGGAGTAACAACACTTTGATGCCCTGCAAAATTAATCAATTGGTAA
ACACCTGGTACAAGATAGGGCAGCATATATACTTGCCACCAAGAGAAGGG
GAGTTGAGGTGTGACTCACATGTTTCTGCAATAATTTTTGATGTGGCTCA
CTATGGACACATGTTGAATTTGACCCCATCATCAGATATTAAGGCAGTGT
GGAGAGCAGATCTGTATAAGTACAAAATCATAGAAGTGCAGCCAATAGGC
TTTGCACCTACTGCTGTCAGACGCTACGAGGGACCAGAGTCTGTGAGACA
AAAAAGAGCAGTGGGAATGGCTTTAGGTCTAGTGGCATTTCTCAGCACAG
CAGGTGCTGCTATGGGCGCGGCGTCCACAGCGCTGACGGTACAGTCTCGC
TCTTTGCTGAGCGGGATAGTGCAGCAGCAACAAGAGTTGCTGAAGGCTGT
TGAGGCTCACGGGCACCTGTTAACATTGACAGCGTGGGGAGTG
>SIVdeb-CM5
TTTTATTGTAATGTTTCCAAAGCATTTAATCTGCTCATGAATAAAACTAA
TAACATAACCTGGAGTAATAACACTTTGATGCCTTGTAAGATTAATCAAT
TGGTAAACACATGGTTCAAGGTAGGACAACATGTATATTTGCCTCCAAAG
GAAGGAGAATTGAAGTGTCATTCTTCTGTTTCTGCACTTGTTTTTGAGGT
AGCTCATTATGGGCAAAACATGAACATAACACCCTCCTCAGATATTAAGG
CAGTTTGGAGGGCAGATCTATACAAGTATAAGATCATAGAAGTTAAACCC
ATCGGGATGGCCCCTACAAGTATAAGAAGGTACGAGGGACCGGAGTCTGC
AAGACAAAAAAGGGCGGCGGGAATTGCTTTTGGTCTAGTGGCTTTTCTGA
GTACAGCAGGTGCTGCTATGGGCGCAGCGTCAACAGCGCTGACGGTACAG
TCCCGCTCTTTGCTGAGTGGGATAGTGCAGCAACAGCAAGAGCTGTTGAA
GGCTGTGGAGGCTCACGGGCATCTGTTGTCACTAACAGCATGGGGAGTG
>SIVdeb-CM40
TTCTATTGTAACATTTCCAAAGCCCTAGATCTCCTACTCCTACAAAATAA
CACTAGAAATAGCACTTGGTCTGACAAGTGGTTGATGCCTTGTAGGATAA
ATCAGTTAGTAAACACTTGGTATACGGTAGGGCAACATATCTATCTACCT
CCGAAAGAAGGAGAGTTGAAGTGCAGCTCACACATTTCTGCATTTGTTTT
TGATGTAGATCACTATAATGGGAGTATAACCCTTACACCATCAGCAGACA
TCAGGGCAGTATGGAGAGCAGACCTATTTAAATATAAAATCATAGAAGTA
AAACCTATAGGATTCGCTCCCTCCGCAGTGAGAAGGTACGAGGGACCGGA
GTCGGTAAGACACAAGAGGGCAGCAGGAATTGCTTTTGGTCTGGTAGCAT
TTCTCAGTACAGCAGGTGCTGCTATGGGCGCAGCGTCAACAGCGCTGACG
GTACAGTCTCGCTCTTTGCTGAGTGGGATAGTGCAGCAACAGCAAGAGCT
GTTGAAGGCTGTGGAGGCTCACGGGCAGCTGTTAACATTAACAGCCTGGG
GAGTG
>SIVtal-00CM266
TTCTATTGCAACATAAGTTCTATGTTCCTGAATACTTCCTATGAGTATAA
CTACACATCAAACCAAGGCAAATTACACCAAGGGGTCATAGGAAAGAAGC
CGAGGTCGAGTGATAGGACCAAGTTCTACATGTCATGCCAACTGAGGCAA
ATGATCAACAGGTGGAGCAGGGTGGAGAAGTTAATGTACCTCCCACCCAG
GGAAGGACACCTGCAGTGCAAAAGTAACATCACAGGATTGTTGGTGGATA
TCAACCACCACCGCGGCTCTAGTGACATAACAGTAGAGCCCTCGTCACAG
ATACGTGACTCGTGGCGAGCAGCAGTTTCACGGTACAAGGTGATAGAGAT
CGTGCCTTTTGGGTATGCGCCGACAAATATCAGAAGGTACGATGGGCCCG
CTAACAAGCAAAAACGAGCGGCCCCGCTAGCCTTAGGCTTCATAGGATTT
CTCAGCACAGCAGGCACTGCCATGGGCGCAGTGGCGACAGCCCTGACGGT
GCAGTCTCGCTCTTTGCTGAGTGGGATAGTGCAGCAGCAGGAACACCTGC
TGAGGGCAATTGAGCATCAGCAACACCTGTTACAATTAACAGTGTGGGGC
ATC
>SIVtal-01CM8023
TTCTATTGTAATATTTCTAGCATGTTCATGAAAACCAACTATACATACAA
TTACACAGCAGAAAAAGGAGTACTGCACCAGGAGAAGATAGGCCGACAAG
AGCACAGGTATAGTGACAGAACCAAATTTTTCATGTCCTGCCAGTTAAGG
CAAATGATCAATAGGTGGAGCAAAGTAGAGAAGCTAATGTATCTGCCGCC

AAGAGAGGGACACCTGAGTTGCAAAAGCAACGTCACAGGTATCCTGGTGG
ACATCAACCACTATAAGGGGAATGACATCATAAGTGTGGAACCCTCTTCA
CAAATCAGAGATGCTTGGCGAGCAGCGGTAGCACGGTACAAGGTGATAGA
GATCACACCAATTGGCTTTGCACCAACGAATATCAGAAGGTATGATGGGC
CGGCAACAAAAGAGAAAAGAGCCGCGCCTCTGGCTTTGGGATTCATAGGA
TTTCTCAGCACAGCAGGCACTGCCATGGGCGCAGTGGCGACAGCCCTGAC
GGTGCAGTCTCGTTCTTTGCTGAGCGGGATAGTGCAGCAGCAGGAGCACC
TGCTGAGGGCAATTGAGCATCAGCAACACCTGTTACAATTAACAGTGTGG
GGCATC
>SIVsyk-KE51
TTTTACTGCAATGTTTCAAAGATTTTTGCAAATGTGTCAAATGGCTATGC
TAATCCAAGCAATTATGCAAAAAATTTATATCTCTCGTGTGCCATACGAC
AGATTATAAACTATTGGGGGTATGTAACCAAGCTAATGTATTTGCCACCT
ACAGAAGGGCACATTAAGTGCACTTCTAATATTACTGCTGTATTAACAGA
TATAGAGTACTATCCAAATTCACAGTTGAATTTTGCACCTACTGCTAATG
TGGAAGATGTGTGGAGAGCTGATTTATTCAATTATAAATTGATTCGGATT
AAACCTATTGGCTTTGCGCCTACAAGTCAAAGGCGCTATGAGCTGCCTAC
GAAACAGAAAAGAGCAGCACCATTGGCACTTGGGTTCCTGGGATTACTCT
CGGCAGCGGGTACTGCAATGGGGAGCGCTGCGACAGCGCTTACACTCCAG
TCCCAGACTTTGTTGGCTGGGATAGTGCAGCAGCAGCAAAAGTTGCTGGA
GGCTGTAGAGGCACAACAGCACCTCTTAGGCTTAACGGTCTGGGGTGTG
>SIVsyk-173-1-2
TTCTACTGTAATGTATCTAAGCTCTTTGCTAACATTACGAATGGAAACGC
AAGTAAGAATAATTATGCCAGTAACCTTCGTCTGTCATGTGCCATTCGCC
AAATAATTAATGATTGGAGATATGTAAGAAAATTGATATACCTGCCCCCT
ACGGCAGGGCATATTAAATGTACTTCCAATGTCACTGCAGTATTGACGGA
CATAGAATACTACCCAGGATCTACGTTGAACTTTACTCCAACAGCAAATG
TGGAGGATGTTTGGAGAGCAGATCTGTTCAATTATAAATTGATTCAAATT
AAACCAATTGGCTTTGCGCCCACTGATCAACGTCGGTATGAGTTGCCTAA
TACCAGGGAAAAAAGGGCAGCACCATTGGCACTTGGGTTCCTGGGATTAC
TCTCGGCTGCGGGCACTGCAATGGGGGGCGCTGCGACAGCGCTAACACTC
CAGTCTCAGACTTTGTTGGCTGGGATAGTGCAGCAGCAGCAAAAGTTGCT
GGAGGCAGTTGAGGCACAGCAACACCTCTTAGGCTTAACTGTCTGGGGTG
TC
>SIVlhoest-485
TTTTTCTGCAATATAACAAGAATACTCCAAGAATGGAATAATAAAAACTC
AGATAAATGGTATCCCTGGGCCAATTGCCACATAAAATCAGTCATAGATG
ATTGGGCAACTGTAGGGAAGAAAATCTATATCCCTCCAACATCAGGCTTT
AATAACAGAATAAGATGTACTCATAGAGTAACAGAAATGTGGTTTGAACT
GGAAAAGTGGGAGCCAGAGGAAAACCTGGGGGGAAATTTGTCTGTCAGAT
TCTTGCCTCCCTCATGGGAGACAAATCAATTTGTGGCAACGGGAGCTCAT
TATAAATTAATAAGGATTAGACCAATTGGCTTTGCTCCAACAGCTGAGCA
CCAGTACGCACCCAGTAGTAAGCAGAAGAGAGCAGCACCGCTGGCTCTAG
GTGCCTTGGGACTTCTCAGTGCTGCAGGTACTGCAATGGGCTTAGTATCG
ACGATACTAACTGTCCAGGCCCAAGTAGTAATACAAGGGATATTGCAGCA
GCAGAAGCAGTTGCTGGTGCTTGTTGAGAAACAGCAAGAGCTCTTAAGAC
TCACCATATGGGGAGTG
>SIVlhoest-524
TTCTTTTGTAATGTCACAAGAATCTTCCAGGAATGGAACAATAAAAACTC
AGATAAATGGTATCCCTGGGCCAATTGCCACATAAAATCAATTATAGATG
ATTGGGCAACTGTTGGGAAGAAAATTTATCTTCCTCCAACATCAGGATTT
AACAATAGAATAAGATGTACTCACAGAGTGACAGAAATGTGGTTTGAAAT
GGAAAAAATGGGAACCACATGAAGACTTAGGAGGAAATCTGTCTATTAAGT
TTTTGCCTCCCTCATGGGAGACAAATCAATTTGTGGCAACAGGAGCTCAT
TACAAATTGATCAGACTGAGACCAATTGGCTTTGCTCCAACATCAGAGCA
TCGGTACGCACCCAGGGGAAGGCAAACAAGAGCAGCTCCACTAGCTCTAG
GTGCCTTGGGACTTCTCAGTGCTGCAGGTACTGCAATGGGCTTAGTATCG
ACGATACTAACTGTCCAGGCCCAAGTAGTAATACAAGGGATATTGCAGCA
GCAGAAGCAGTTGCTGGTGCTTGTTGAGAAACAGCAAGAGCTGCTAAGAC
TCACCATCTGGGGAGTG

>SIVlhoest-7
TTCTTTTGTAATGTAACTAGGATATTCCAAGAATGGAACAATAAAAATTC
AGATAAATGGTATCCTTGGGCCAACTGCCACATAAAAAGCATCATTGATG
ATTGGGCTACAATAGGAAAGAAGATTTATCTACCACCTACTTCAGGTTTC
AACAATAGAATCAGATGTACTCATAGAGTAACAGAAATGTTCTTTGAGAT
GGAAAAATGGGAACCACATGAGGACTTAGGAGGCAACCTAAGTATAAAGT
TCTTGCCTCCATCATGGGAAACTAATCAATTTGTGGCAGAAGGGTCTAAA
TATAAATTGATAAAATTGAATCCAATTGGGTTTGCTCCTACAGATGAGCA
CAGGTACGCACCCAGGGGAAGGCAAACCAGAGCAGCGCCTCTGGCTTTAG
GAGCCTTGGGACTTCTCAGTGCTGCAGGCACTGCAATGGGCTTAGTATCG
ACGATACTAACTGTCCAGGCCCAAGCAGTCTTGCAAGGGATATTGCAGCA
GCAGAAGCAGTTGCTGGTGCTTGTTGAGAAACAGCAAGAACTGTTAAGAC
TCACCATATGGGGAGTG
>SIVsun-L14-S2
TTCTTTTGTAATTTAACAAGAATTTTCAAAGTATGGAATGACACAACCTC
CAATAAATGGTATCCTTGGGCCAATTGCCACATAAAAAGTGTGATAGATG
ATTGGGCGTCAGTGGGCAAGAAGATTTACTTGCCCCCAACATCAGGGTTT
AATAACAGAATAAGATGTGCAAACAGAGTGACAGAAGCATGGTTCACACT
GGAAAGGGTGGAAGACTGGAAGGTAAATGGATCAAACATCTCAGTTGTAG
CCTTCCAGCCACCTACCAACACCTTAAATCAATTTGTGAGCACAGGAGCA
CATTACAAACTAGTAAGAATTAGGCCAATTGGGTTTGCTCCAACAGATGA
GCACCGGTACGCACCCAGGAAGGAAAAGAGGGCAGCACCTGTTGCCCTAG
GAGCTTTGGCACTTCTCAGTGCTGCGGGTACTGCAATGGGCTTAGTATCG
ACGATACTAACTGTCCAGGCCCAAGCAGTCTTGCAAGGGATATTGCAGCA
GCAGAAGCAGTTGCTGGTGCTTGTTGAGAAGCAGCAAGAGCTACTAAGAC
TCACCATATGGGGAGTG
>SIVolc-12CI97
TGGTTTTGTAATATCACTAAAATTCTTAAAACATGGAATAATGCGTCTGA
TCCTAAGTGGTATCCATGGTCACAGTGTAGACTGAGACAAAATGTAGATG
ATTGGATGAAAGTAGGAAGAAGAATTTATCTTCCTCCACAACCAGGATTC
AATAATAGAATCCGATGTACACAAAGAGTAACTGAAATGTTTTTGGAAAT
ACAAAAAGTAGAGAACGGAACGCAAGTGTCATTGCTTCCACCATCAGACA
ACGTGAATCAATTTGTCTCAGTGGCAAGTGATTATAAATTGGTAGAGTTG
GAATTGATTGGCTTTGCTCCTACAGAGACACAAAGGTATCAGCCTAGAAA
TAAAAGAGGAGCATTGGTGCTGGGGTTATTGGGGGTTCTCGGCCTAGCAG
GATCCGCAATGGGCACAGTATCAACGATACTGACTGTCCAGTCTTCGCAT
TTGCTTGCGGGGATAGTGCGGCAGCAGAAGCACATGCTGGAGATGATTGA
GAAGCAGCAAGAATTGCTGAAGCTCACTATCTGGGGAGTG
>SIVwrc-98CI04
TTCTTTTGCAATATTACTAAGATTGCTGATGCATGGAACAACAAGAATGA
GAGCAAATGGTACCCTTGGAGTCCATGCACGATTAGAAATATAGTGGATG
ATTGGGCACATGTAGGAGTAAAAGTATATTTGCCTCCAGTATCAGGATTC
AGGAATCATATAAGATGTACACAAAGAGTGACTGAAATTCTATTCAATTG
GGAAAATAAGCAAGTGGAATTCCAAGCTCCTTCCAACATACAGAATTCAT
TTGTGGCAGTGGGACAAAAGTATAAACTGGTGAGACTAAAAATGATAGGG
CTGGCTCCTGGTGATGGGAGAAGAGTGACACCAAAAGAACATCATAGAGA
AAAAAGAGGAGCTATTGTCTTAGGAATTCTAGGATTCCTCGGGCTGGCAG
GATCTGCAATGGGCACAGTATCAACAGTACTGACGATCCAGTCTCAGCAT
TTGCTTGCTGGGATATTGCAGCAGCAAAAGAACCTGCTGGAGCTTGTTGA
GAAGCAACAAGAATTGTTGCGACTCACCATCTGGGGGGTG
>SIVwrc-05GM-X02
TTCTTTTGTAACATTACAAAAATAGCAGATTGGTGGAATAATAAAACAGA
AAAGAGATGGTATCCTTGGGCACCTTGCACTATCAGATCAATAGTGGATG
ATTGGGCATCTATAGGGAAGAAAATATACTTGCCTCCGGTGTCTGGATTT
AACAATCACATTAGATGTACCCAAAGGGTATCAGAAGTACTCTTTAATTG
GGAAAACGGGCAGGTGGAGTTCCAAGCTCCTTCCCACATGCAAAACTCCT
TTGTGGCAGTAGGACAAAAATATAAACTGGTAAAACTAAAATTGATAGGA
GTAGCTCCCACTGATGGAAAGAGGGTGACACCTCCTGACCATCATAGAGA
AAAACGAGGAGCAATTGTTTTAGGACTTCTAGGCTTCCTCGGCCTGGCAG
GATCTGCAATGGGCACGGTATCAACAGTACTGACGATCCAGTCTCAGCAT

TTGCTTGCTGGGATAGTGCAGCAGCAGAAGAACTTGCTGGAGCTTGTAGA
GAAGCAACAAGAGCTGTTGCGACTCACCATCTGGGGGGTT
>SIVmnd1-GB1
TTCTTCTGTAATGTAACAAGAATCATGAGGGCATGGAATGATCCTAATGA
GAAGAAGTGGTATCCTTATGCCTCATGTCAAATTAGGCAAATAGTAGATG
ACTGGATGCAAGTAGGAAGAAAGATATATTTACCACCTACATCAGGATTT
AATAATCACATAAGGTGTACACATAGGGTAACAGAAATGTACTTTGAAAT
GCAAAAGATAGATAGTAATGAAACAAAAATGCAAATTAAATTCTTGCCTC
CCAGTGAAACCTCCAATCAATTTGTTGCTTATGGAGCTCATTATAAATTA
GTCAAAATAATGCCAATTGGCATAGCACCTACAGATGTGAAAAGACACAC
TTTACCTGAACATCATAAAGAGAAGAGAGGAGCAGTAATACTTGGTATCC
TTGGTCTGCTCTCGCTGGCAGGATCCGCGATGGGCTCAGTGTCGGTGGCA
CTGACTGTCCAATCTCAGTCTTTGGTGACTGGGATAGTGGAACAACAAAA
ACAGTTGTTGAAGCTCATAGAGCAACAGTCTGAACTCTTAAAACTCACCA
TATGGGGAGTA
>SIVdrl-D3
TTCTTCTGTAATATCTCTAAAATATGGAGAACATGGAATAATAGATCAAG
TAATGTGTGGTATCCATATGCATCCTGCCACATAAAACAAATAGTAGATG
ATTGGGCAAGAGTGGGCAGGAAAATTTATATGCCTCCTGTATCAGGATTT
AATAATGAAATAAGATGTACACAAGATGTCACAGAAATGTTCTTTGAGAT
CCAAAAATTGGAGACAAATGAAATCAATGATTCCAACTATCAGATTAAAT
TTGTGCCACAAGATGAAGTGCAAAATCAGTTCACAGCAATAGGAGCGCAT
TACAAATTGGTCAAGGTGGAGCCAATTGGCTTTGCTCCAACTGATGTACA
TAGATACAACCTGCCGGAACACAGGCAGAAGAGAGGTGCAGTCGTGCTTG
GGATCCTTGGCCTCTTGAGCTTGGCAGGTTCCACAATGGGCTCGGTGGCG
ACAGCCATGACTGTCCAGTCTCAGGCTTTGTTGTCTGGGATAGTGGAGCA
GCAAAAGCAACTGCTGCGCCTGGTGGAACAGCAACAGGAGTTACTCAAGC
TAACTATCTGGGGCGTA
>SIVdrl-D4
TTCTTCTGCAATGTTACTAAAATATGGAAAACATGGAATAATAGATCAAG
TAGTGTGTGGTATCCATATGCATCCTGCCACATAAAACAAATAATAGATG
ATTGGGCAAAAGTTGGCAGGAAAATTTATATGCCTCCTGTATCAGGATTT
AATAATGAGATAAGATGTACACAAGAAGTCACAGAAATGTTCTTTGAGGT
TCACCGTTTGGACACAAATGTAACCGATGATTCTCATTATCAGATTAAAT
TTATACCACAAGATGAAGTGCAAAATCAGTCCACAGCAGTAGGAGCACAT
TACAAATTGGTCAAGGTGGAGCCAATTGGCTTTGCTCCAACTGACGTGCA
TAGATACAACCTGCCGGAACACAGGCAGAAAAGAGGTGCAGTCGTGCTTG
GAATCCTTGGCCTCTTGAGCTTGGCAGGTTCCACAATGGGCTCAGTGGCG
ACGGCCATGACTGTCCAGTCCCAGGCTTTGTTGAATGGGATAGTGGAGCA
GCAAAAGCAACTGCTGCGCCTGGTGGAACAGCAACAGGAGTTACTTAAGT
TAACTATCTGGGGCGTA
>SIVmnd2-M14
TTCTTTTGTAATTGGACAAGAATTTGGAAAACCTGGAATAACCAAAACTC
AAATGTTTGGTATCCATATATGTCATGTAAAATCAGACAGATAGTGGATG
ATTGGCATAAAGTGGGAAGGAAGATTTATATGCCTCCTGTGTCTGGATTT
AATAATCATATAAGATGCACTAATGATGTCACAGAGATGTTTTTTGAGGT
CCAACTAGTTGAAGGAAAAAGATACCTAATAAAGTTTCTGCCACAAGATG
AGGTACAAAATCAATTCACAGCAGTGGGAGCACATTATAAATTGGTAAAA
GTGGATCCGATAGGATTCGCCCCTACTGATGTACACAGATATCATCTACC
AGATGCAAAGCAGAAGAGAGGAGCAGTCTTGCTTGGAATGCTCGGCCTCT
TGGGCTTGGCAGGTTCCGCGATGGGCTCAGTGGCGGTGGCCCTGACTGTC
CAGTCCCAGGCTTTGTTGAATGGGATTGTGGAGCAGCAGAAGATTCTGCT
GAGCCTGATAGATCAGCACTCCGAGTTATTAAAACTAACTATCTGGGGTG
TA
>SIVmnd2-CM-98-CM14
TTCTTTTGTAATTGGACAAAAATTTGGAGAACATGGAATGATCAAAATTC
ATCAGTATGGTATCCATGGATGTCATGTAATATTAGACAAATAATAGATG
ATTGGCATAAAGTAGGAAAGAAGATTTATATGCCTCCTGTATCTGGATTT
AATAATGAGATAAGATGTTCTAATGATGTAACAGAAATGTTCTTTGAGGT
TCAAAAGACAGAAGAGGGATACATCATCAAATTTGTGCCACAAGATGAGG

TACAAAACCGATTTACAGCGGTAGGAGCACACTATCAACTAGTAAAAGTG
GACCCAATTGGCTTTGCTCCTACAGAAGTGGCAAGATATCATCTGCCAGA
AGCTAGGCAGAAGAGGGGAGCGGTCTTGCTTGGGATGTTCGGCCTCCTAG
GTTTGGCAGGTTCCACGATGGGCTCAGTGGCGGTGGCCCTGACTGTCCAG
TCTCAGGCTTTGTTGAATGGGATTGTGGAGCAGCAGAAGGTTCTGCTGAG
CCTGATAGATCAGCACTCCGAGTTATTAAAACTAACTATCTGGGGTGTA
>SIVmnd2-5440
TTCTTTTGTAATTGGACTAAAATATGGAAGGCATGGAATAGCAAACAAAG
TTCAGTTTGGTACCCTTACATGTCTCGCAATATTAGACAAATAGTAGGTG
ATTGGCATAAAGTAGGCAAGAAAATTTATATGCCTCCTGTGTCTGGGTTT
AATAATGAAATAAGATGTACTAATGATGTGACAGAAATGTTCTTTGAGGT
CCAAAAAACTGATGATGACAATGGATATATCATAAAATTTATACCACAAG
ATTGGATTCAAAATCAATACACAGCAGTGGGGGCTCATTACAAATTGGTG
AAGGTGGATCCGATAGGTTTTGCCCCCACAGACATACACAGACATCATTT
GCCTAATACAAGGCAAAAAAGAGGAGCGGTCTTACTTGGAATGCTCGGCC
TCCTAGGTTTGGCAGGTTCCGCGATGGGCTCAGTGGCGGTGGCCCTGACT
GTCCAGTCCCAGACTTTACTGAATGGGATTGTGGAGCAGCAGAAGGTTCT
GCTGAGCCTGATAGATCAGCACTCCGAGTTATTAAAACTAACTATCTGGG
GTGTA
>SIVcol-BMC07
CTCTATTGCAATGCTAGTTCCTTGTGGAGTGGTACCACCACAGTAATGAA
CTGCACTATAAGAAAATTGGTGAATAGTTGGGTGACCCATGCAAAGATAT
TGTATGGGCCACCTCTAAAAGGAACTCTGCAGACTTATTGGGAGAAACAA
CCAGTTTTGGCCTTTATGGGTTCAGTAGAAAAAGGGGAGAATAAAACTGG
ATGTGCATACCCGGCAGCAGTGAATTGGAAGCATGCTGCCAGTACATTGG
AGTTAGGAAATTACAAATTGGTGAAAATAACTCCTACAGCATTTGTTCCC
ACTGATCACAGAAGGGGAGGAACAGCTGTGGTACGAGAGAAACGGGGAAT
CTTTGCTTTCTCCTTGATAGCTCTCTTGAGTGGGGCCGGCGCTGCAATGG
GCTCAGCGTCTGTGGCGCTGACGATCCAGGCTCAGTCCTTATTGGATGGG
CTAGTGCGGCAGCAGCGGCAGCTGCTGAAGCTGGTTGAAACACAGTCTGC
ACTGTTACAACTCACGGTATGGGGGGTT
>SIVcol-CGU1
TTTTACTGTAATGCATCTAGTTTATGGAAACATGATTCCCCAGTCATGAA
CTGTACCATTAGAAAGCTGGTGAATAGTTGGGTCACTCATGCCAGGATCC
TGTATGGGCCGCCTCCAGGAGGTCATCTACAATGTAACTGGGAGAAACAG
CCAGTTATTGCGTTCATGGGCACCATAGAAGGAGACAATGATGGTAATGG
GTGTGCTTATCCAGCAGCACCAAATTTTAAGCATGCATTAAGTACTTTGG
AATTGGGAAGATACAAATTGGTAAAAATGAGGACAACTACTTATGTCCCA
ACAGATATAAAAAGATCAGTGAATGTAAATTGGCATCATGGGAGACAAAA
GAGAGGCATCTTTGCATTCTCCATCTTAGCCCTCTTGAGTGGGGCCGGTG
CTGCAATGGGCTCAGCGTCTGTGGCGCTGACGATCCAGGCTCAATCCCTT
AATGGAAGGGCTAGTGCCAGCAGCAACCGAATGTTGCTGAAACTGGTCGA
GACGCAGTCTGCACTGCTACAGCTCACGGTCTGGGGGGTT
>SIVcol-BMC01
TTTTATTGTGATGCTTCATCATTGTGGGCAAAAAATTCATCAGTAATGAA
TTGTACCATTAGAAAATTGGTAAATAGTTGGGTGTCACATGCCAGGCTGC
TATATGGCCCTCCGCCTGATGGACATTTGCAATGTAACTGGGAAAAGCAA
CCAGTAATTGCTTTTATGGGCACAGTAGAAAAAGGGGGAAATGAGACAGG
ATGTGCTTATCCTGCAGCTCCAAATTTTAAGCATGCACTGAGTACACTTG
AATTGGGCAGATATAAATTGGTAAAGATGAGAACAACAGTGTATGCTCCT
ACAGATGTAAAAAGGCAAGTGGCTATTAATTGGCATCATGGGAGGCAAAA
AAGAGGCATTTTTGCATTCTCCATCTTAGCTCTCTTAAGTGGGGCCGGTG
CTGCAATGGGCTCAGCGTCAGTGGCGTTGACGATCCAGGCTCAATCCTTA
TTGGAAGGGCTAGTGCAGCAACAGCGGATGCTGTTGAAACTGGTCGAAAC
GCAGTCAGCACTGCTCCAACTCACGGTATGGGGGGTT
>SIVagmVer-155
TTCTACTGTAAAATGGACTGGTTTTTAAATTATCTGAATAATTTAACAGT
GGATGCTGATCATAATCATTGTAAAAACAACGCAGGGAAAGGTCGAAGTC
CAGGTCCCTGTGTACAGAGAACTTATGTTGCCTGCCATATCCGATCTGTC
ATAAATGATTGGTATACTATATCAAAGAAAACATATGCTCCACCAAGAGA

AGGACATTTGCAGTGCACGTCCACAGTTACTGGGATGACAGTAGAGCTAA
ACTATAATAACCAGAACAGGACAAATGTAACATTGAGTCCCCAGATAGAA
ACCATCTGGGCGGCAGAATTGGGCAGATACAAATTGGTAGAGATTACACC
AATTGGATTTGCACCCACAGAAGTCAGGCGATACACGGGAGGCCAAGAGA
GGCAAAAACGAGTCCCGTTCGTGCTAGGGTTCCTAGGCTTCTTGGGAGCT
GCTGGGACTGCAATGGGAGCAGCGGCGACAGCCCTGACGGTCCAGTCTCA
GCATTTACTTGCTGGGATATTGCAGCAGCAGAAGAATCTGCTGGCGGCTG
TGGGAGCTCAACAGCAGATGTTGAAGCTGACCATTTGGGGTGTG
>SIVagmVer-VSAG1003
TTCTATTGTAAGATGGATTGGTTCCTAAACTACCTTAATAACCTTACAGT
AGATGCAAATCACAATAAATGCACAAACAGCACCAAGAAGGGACACGCAC
CAGGACCTTGTGTACAGAGGACCTATGTTGCTTGCCATATCCGAACAGTT
ATTAATGATTGGTACACAGTATCAAAGAGAACATATGCCCCACCAAGAGA
AGGACACTTGGAGTGTTTATCTACAGTCACAGGGATGACAGTGGAATTGA
ATTATAATTCAAAGAACAGAACTAATGTGACATTGAGTCCCCAAATAGAA
GGCATTTGGGCAGCAGAATTGGGCAGATATAAATTAGTGGAAATAACACC
AATTGGCTTTGCACCCACGGATGTCAGGCGATATACGGGAGGACACGAAA
GACAAAAACGTGTGCCTTTCGTGCTAGGGTTCCTAGGATTCTTGGGAGCT
GCTGGTACTGCAATGGGAGCAGCGGCGACAGCCCTGACGGTCCAGTCTCA
GCATTTACTTGCTGGGATATTGCAGCAGCAGAAGAACTTGCTGGCGGCAG
TGGAGGCTCAACAGCAAATGTTGAAGCTGACCATTTGGGGAGTA
>SIVagmVer-VSAM0022
TTTTATTGTAAGATGGATTGGTTTCTTAATTATCTAAACAACCTAACAGT
AGATGCAGATCATAATAAGTGCAACAATTCGCCAAAAGGAAAAGCACCAG
GACCCTGTGTTCAGAGAACATACGTTGCCTGCCATATCCGATCTGTCATA
AACGATTGGTATACCATCTCTAAAAGGACTTATGCTCCTCCAAGAGAAGG
TCACTTGGAGTGTCTTTCCACGGTCACTGGTATGACAGTAGAACTTAATT
ATATATCCAACAATAGAACAAATGTTACCCTATCCCCTCAGATAGGGGGT
ATATGGGCAGCCGAATTGGGGAGATACAAATTAGTTGAAATTACGCCAAT
TGGTTTTGCACCCACGGACGTCAGGCGGTATACAGGAGGGCACGACAGAG
CAAAACGTGTACCTTTCGTGCTAGGATTCCTAGGCTTCTTAGGGGCTGCT
GGAACTGCAATGGGAGCAGCGGCGACAGCCCTGACGGTCCAGTCTCAGCA
TTTACTTGCTGGGATATTGCAGCAGCAGAAGAACTTGCTGGCGGCAGTGG
AGGCTCAACAGCAGATGTTGAAGCTGACCATTTGGGGTGTT
>SIVagmVer-VSAC4004
TTCTATTGTAAAATGGATTGGTTCCTTAATTACATCAACAATCAGACAGT
TACCTTTGATGGAAATACGTGTAATAATACTAAGACTAGACCGAGAAAGG
GAGCGCCTGGACCTTGTGTGCAAAGAACATATGTTGCCTGCCACATTCGA
TCTGTCGTAAATGATTGGTACAAAGTGGCAAAATTAACCTATGCTCCACC
AAGAGAAGGTCATTTGCAATGTACCTCCACGGTGACGGGTATGACAGTAG
AACTGAATTACAATTCAGGAAATCGGACAAATGTGACACTCAGCCCTCAA
ATAAGAGGCATATGGGCAGCAGAATTGGGTAGGTATAAATTAGTGGAAAT
TACTCCAATTGGCTTTGCACCAACAGATGTCAGGCGATATGAAGGACCCC
AAAGACAGAAACGGGTGCCTTTCGTGCTAGGATTCCTAGGATTCTTGGGA
GCAGCAGGAACTGCAATGGGAGCAGCGGCGACAGCCCTGACGGTCCAGTC
CCAGCATTTACTTGCGGGGATATTGCAGCAGCAGAAAAATCTGCTGGCGG
CTGTGGAGCAGCAACAACAAATGTTGAAGCTGACCATTTGGGGTGTG
>SIVagmMal-ZMB
TTCTATTGTAAAATGGATTGGTTTCTAAATTACATAAACAATGCCACAAC
TACGTTTGATGGAAACAAGTGTGGTAATAAAACAAAGAAAGGAAATGCTC
CAGGACCCTGTGTTCAAAGGACATATGTTGCCTGCCACATAAGATCAGTT
GTAAATGATTGGTATACAGTAAGCAAATTAACCTATGCCCCGCCAAGAGA
AGGGCACTTACAATGTACTTCCACGGTAACGGGTATGACAGTAGAGCTAA
ATTACAACAATAAGAACGTAACAAATGTGACCCTAAGTCCCCAAATAGAA
GGGATTTGGGCAGCAGAATTGGGTCGATACAAATTGGTGGAGATTACCCC
AATTGGCTTTGCACCCACAGAGGTCAGAAGATACACAGGGGGACATGAGA
GACAAAAAAGAGTCCCTTTCGTGCTAGGGTTCCTAGGCTTCTTAGGAGCT
GCTGGAACTGCAATGGGAGCAGCGGCGACAGCCCTGACGGTCCAGTCTCA
GCATTTACTTGCTGGGATATTGCAGCAGCAAAAGAACTTGCTGGCGGCTG
TGGAGCAGCAACAACAGATGTTGAAGCTGACCATTTGGGGTGTG

>SIVgri-677
TTCTATTGTAAGATGGACTGGTTTATCAATTATCTAAACAATCGAACAGA
AGATGCAGAAGGTACTAATAGGACCTGTGACAAAGGGAAGCCAGGACCAG
GACCATGTGTTCAGAGAACTTATGTTGCCTGCCATATACGACAAGTAGTA
AATGATTGGTACACTGTCTCTAAAAAGGTATATGCTCCACCAAGGGAAGG
TCATTTGGAGTGTAACTCATCAGTCACGGCACTATACGTGGCAATAGATT
ATAACAACAAGTCTGGCCCAATAAATGTGACCCTAAGTCCTCAGGTACGC
AGCATATGGGCGTACGAACTGGGAGACTATAAATTAGTAGAGATAACACC
AATTGGCTTTGCTCCTACAGATGTAAGAAGATATACTGGCCCCACAAGAG
AAAAAAGGGTGCCATTCGTGCTAGGGTTTCTAGGCTTCTTGGGAGCTGCT
GGAACTGCAATGGGCGCAGCGGCAACAACGCTGACAGTCCAGTCTCGGCA
TTTGCTTGCTGGGATATTGCAGCAGCAGAAGAACTTGCTGGCGGCTGTGG
AACAGCAACAACAGTTGTTGAAGCTGACCATTTGGGGTGTG
>SIVagmSab-SAB1
TTCTATTGTAAAATGGATTGGTTCCTCAACTATTTGAATAATAAGTCAGT
AGACCCAGACCATAACAACTGTGCAAAAAATAATACAAAGCCATGTTGGC
AGAGAACCTATGTTCCCTGCCACATAAGGCAAGTAGTAAATGATTGGTAC
ACCTTGTCTAAAAAGACATACGCCCCTCCCAGAGAAGGCCACTTGGAGTG
TAACTCGACAGCCACAGCGCTGTATGTAGAATTGAACTATAACAGTAAAA
ACAGGACCAATGTGACGTTATCACCTCAGATTGAAAGCATTTGGGCTAAT
GAATTGGGAGATTACAAATTAGTGGAAATCAAGCCAATTGGATTTGCTCC
AACCAAAGTAAGAAGATACACTGGCCCCGAAAGACAAAAAAGGGTGCCAT
TCGTGCTAGGGTTCCTAGGTTTCTTGGGAGCAGCTGGAGCTGCAATGGGT
GCAGCGGCGACAGCCCTGACGGTCCAGTCTCAGCAGTTACTCGCTGGGAT
ACTGCAGCAGCAGAAAAATCTGCTTGCGGCTGTAGAGCAGCAGCAGCAAA
TGCTAAAGCTCACTATTTGGGGTGTA
>SIVagmTan-TAN1
TTCTATTGTACTCCTGATTGGTTTGTTAATTGGCTGAATAATGAGTCTAA
TAGTGGAAGAAATGTAGATGTAGAAGGTAATAATTGCACCACTGGAAAGG
ATAAACGCTGCTACAAAAGGACATATGTCCCCTGCCATATTAGGTCAATT
GTCAATGACTGGTACACGCTCAGTAAGAAAACCTATGCACCACCAAGGGA
AGGACACTTAGAATGCACATCAACAGTGACATCTATGATGGTATCACTGG
ATTATAACAGCAAAGAAAGGACTAATGTGACATTGACAGCTAATTTGGAG
AACATATGGGCTTATGAATTGGGAAGATATAAGCTCATAGAAATTGAACC
AATCGGTTTCGCGCCAACAGAGATAAGAAGATATGTTGGGCCAACTCGAG
AAAGAGGGTGCCCTTCGTGTTGGGGTTCCTAGGCTTTTTGGGGGCAGCT
GGAGCTGCAATGGGTGCAACAGCGACAGCGTTGACGGTCCAGTCCCAGCA
ATTACTTGCAGGGATATTGCAGCAGCAGAAGAATCTGCTGGCGGCAGTTG
AGCAGCAGCAGCAGATGCTAAAGCTCACCATTTGGGGTGTG
>SIVrcm-GAB1
TTATATTGCAAGTTAAATTGGTTTTTAAATTTGTTAAATAATGAGACAGT
AGGGACAACAAATGAGAAGAGAAAAGCACCTTTTGTACCATGCATCACAA
AAATGATAGTCAATGATTGGTATACAGTATCGAGGAAGGTATACACGCCA
CCGAGGCCAGATGCGTTAAAGTGCAGTGCACAGGTATCCTATCTGTTGGC
AGACATAGACTATATTAATGACAGTGAGACAAACATCACCCTCTCAGCGG
AAGTGGGTGATTATTGGGCAGCAGAATTGGGGAGATATAAGGCAATAGAA
ATCAGACCAATTGGCTTTGCACCAACAGAAATAAAAAGGTACCAGACGAA
ACAGAAAAGGGTACCTTTGGTGCTGGGTTTTCTAGGTTTCCTCTCAGCAG
CAGGTACTGCAATGGGCGCAGCGGCGACAGCCCTGACTGTCCAGTCCCGG
CATTTGCTTGCAGGGATATTGCAGCAGCAAAAGAACCTGCTGGACATAGT
TAAGCGGCAGCAGAATCTGCTAAAGCTCACCGTCTGGGGAACT
>SIVagi-00CM312
CTCTATTGTAAACTCAATTGGTTTTTGAATTATTTGAACAATAAGACAGA
GGGTAATGAGAAGGAAAGAAGGCAAGCCATGTTTGTTCCTTGCGTCACGA
AAATGATTGTTAATGATTGGTACACGGTGTCAAGGAAAGTTTATACACCG
CCAAGGCAAGATGCGTTAACATGTAATGCTACAGTAAGCTATTTGTTAGC
AGACATAGAGTATATGTGAATCAAAATGAGACTAATGTGACACTGTCTGCAG
AAGTAGGAGACCTATGGGCAGCAGAATTGGGAAGATACAAAGTAGTGGAA
ATTAAACCAATTGGATATGCACCAACAGCTATAAAAAGGTACGAGACAAA
ACAGAAAAGAGTACCTTTGGTGCTGGGTTTTCTAGGTTTCCTCTCAGCAG

CAGGTACTGCAATGGGCGCAGCGGCGACAGCCCTGACAGTCCAGTCCCGG
CATTTGCTTGCTGGGATATTGCAGCAGCAGAAGCAGTTGCTGGACATAGT
TGAGCAGCAGCAACACTTGCTAAAGCTCACCGTCTGGGGGACT
>SIVrcm-02CM8081
TTATATTGCAAATTGAATTGGTTTCTCAACCTATTAAATAATAGAACTGA
TGGAAACATAAATGAGCGAAGACAAGCCATGTTTGTGCCTTGTATCACAA
AAATGGTGGTTAATGATTGGTATACAGTGTCAAAAAAGGTATACACACCA
CCAAGACCTGATGCATTGAAATGCAGGGCCACGGTCACTTACTTGTTGGC
AGACATAGATTATATACATACCAATGAAACAAATGTTACTCTGACTGCAG
AGGTAGGAGATCTATGGGCAGCAGAGTTAGGAAGATACAAAGTAGTAGAA
ATTAAACCAATTGGCTATGCACCTACAGATGTTAAAAGGTACGAGACAAG
ACAGAAAAGAGTACCTTTGGTGCTGGGTTTTCTAGGTTTCCTCTCGGCAG
CAGGTACTGCAATGGGCGCAGCGGCGACAGCCCTGACAGTCCAGTCCCGG
CATTTGCTTGCTGGGATATTGCAGCAGCAGAAGAAGTTGCTGGACATAGT
TGAGCAGCAGCAGCACTTGCTGAGGCTCACCGTCTGGGGGACT
>HIV-2-EHO
CTCTACTGTAATATGACTTTTTTCTTAAACTGGGTAGAAAATAGAACTGG
GCTCAAGAGAAATTATGCGTCATGCCACATCAGACAGATAGTCAACACGT
GGCACAAGATTGGAAGAAATGTGTATTTGCCTCCAAGAGAGGGTGAACTC
TCCTGTAATTCCACTGTTACCAGCCTCATAGCCAACATTGACTGGATAGA
TAAGAACCTTACTAATATTACTGTGAGTGCAGAAGTGTCAGAACTGTATA
AATTGGAACTGGGGGACTACAAATTAGTAGAGATAACACCAATTGGCTTT
GCACCTACAAGTATAAAAAGATATTCCTCAGTGACACCGAGGAATAAAAG
AGGTGTACTTGTGCTAGGGTTTTTGGGATTCCTTGCGACGGCAGGTTCTG
CAATGGGCGCGGCGTCCTTGACGTTGTCGGCTCAGTCCCGGACTTTACTG
GCTGGGATAGTGCAGCAGCAGCAACAGCTCGTGGACGTGGTCAAAAGACA
ACAAGAACTGTTGCGGCTGACCGTCTGGGGGACG
>HIV-2-D205
TTATACTGTAATATGACCTGGTTCTTAAACTGGGTAGAGAACAAAACAAA
TACAACTCGGAGAAATTATGCGCCATGTCATATCAGACAGATAATCAACA
CGTGGCACAAGGTTGGGAAAAATATATATTTGCCTCCAAGAGAAGGTGAG
CTCTCATGTAATTCCACTGTGACTAGCCTCATAGCCAACATCAACTCAGA
CAACAGTACCACCAATATTTCTGTGAGTGCAGAAGTGTCAGAACTGTATA
GATTGGAACTGGGGGACTATAAATTAGTAGAAATAACACCAATTGGCTTC
GCACCTACAGATGTAAGAAGATACTCCTCAGTAAAACCGAGGAATAAAAG
AGGTGTAATGGTGCTAGGATTCTTGGGATTCCTTGCAATGGCAGGTTCTG
CAATGGGCGCAACGTCTTTGACGCTGTCAGCTCAGTCCCGGACTTTACTG
GCCGGGATAGTGCAGCAGCAGCAACAGCCTGTGGACGTGGTCAAAAGACA
ACAGGAACTGTTGCGGCTGACCGTCTGGGGGACG
>HIV-2-ALI
CTCCATTGCAACATGACTTGGTTCCTCAATTGGGTGGAAAACAAAACGGG
TCAGGAACAGCACAATTATGCACCGTGCCATATAAAGCAAATAATTAATA
TCTGGCACAAAGCAGGGAAAAATGTATATTTGCCTCCTAGGGAAGGAGAG
TTGACCTGCAACTCAACAGTAACCAGCTTGATTGCTAACATTGACACGGA
TGGCAACCAGACAAATATTACCTTTAGTGCAGAGGTGGCAGAACTATACC
GATTAGAATTGGGGGATTATAAATTAGTAGAGATAACACCAATTGGCTTC
GCACCTACATCAGAAAGGAGATACTCCTCTACTCCAAGGAGGAATAAAAG
AGGTGTGTTCGTGCTAGGGTTCTTAGGTTTTCTCGCGACAGCAGGTTCTG
CAATGGGCACGGCAGCTTTAACGCTGTCTGCTCAGTCTCGGACTTTATTG
GCCGGGATAGTGCAGCAACAGCAACAGCTGTTGGACGTGGTCAAGAGACA
ACAGGAAATGTTGCGACTGACCGTCTGGGGAACG
>HIV-2-ST
CTCTACTGCAATATGACTTGGTTCCTCAATTGGGTAGAAAACAGAACGAA
TCAGACACAGCACAATTATGTGCCATGCCATATAAAGCAAATAATTAATA
CCTGGCACAAGGTAGGGAAAAATGTATATTTGCCTCCTAGGGAAGGACAG
TTAACCTGCAACTCTACAGTGACCAGCATAATTGCTAACATTGACGGAGG
AGAGAACCAGACAAATATTACCTTTAGTGCAGAGGTGGCAGAACTATACC
GATTAGAATTGGGGGATTATAAATTGATAGAAGTAACACCAATTGGCTTT
GCACCTACACCAGTAAAAAGATACTCCTCTGCTCCAGTGAGGAATAAAAG
AGGTGTATTCGTGCTAGGGTTCTTAGGTTTTCTCACGACAGCAGGAGCTG

CAATGGGCGCGGCGTCCTTGACGCTGTCGGCTCAGTCTCGGACTTTATTG
GCCGGGATAGTGCAGCAACAGCAACAGCTGTTGGACGTGGTCAAGAGACA
ACAAGAAATGTTGCGACTGACCGTCTGGGGAACA
>HIV-2-BEN
CTCTACTGTAACATGACTTGGTTCCTCAATTGGGTAGAAGACAAGAACCA
AACACGGCGCAACTATTGCCATATAAAGCAGATAATTAATACCTGGCATA
AAGTAGGGAAAAATGTATATTTGCCTCCTAGGGAAGGGGAGTTGGCCTGT
GAATCAACAGTAACCAGCATAATTGCTAACATTGACATAGATAAAAATCG
GACTCATACCAACATTACCTTTAGTGCAGAAGTGGCAGAACTGTACCGAT
TAGAACTGGGAGACTACAAATTAATAGAAATAACACCAATTGGCTTCGCA
CCTACAGATCAGAGAAGGTACTCCTCAACTCCAGTGAGGAACAAAAGAGG
TGTGTTCGTGCTAGGGTTCTTGGGTTTTCTCGCGACAGCAGGTTCTGCAA
TGGGCGCGCGGTCCCTGACGCTGTCAGCCCAGTCCCGGACTTTACTGGCC
GGGATAGTGCAGCAACAGCAACAGCTGTTGGACGTAGTCAAGAGACAACA
AGAAATGTTGCGACTGACCGTCTGGGGAACG
>HIV-2-Abt96
TTGTATTGTAAAATGACTTGGTTTCTAAATTGGATAGAGGACAGAAATAT
GACTCTCCTAAAGCCCCAGGAACGACAAAGACGCAATTATGTACCCTGCC
ATATTAGGCAGATAATTAACACATGGCACAAAGTAGGAAAAAATGTGTAC
TTGCCCCCCAGAGAAGGTGATCTGACGTGTAATTCAACAGTCACCAGCCT
AATAGCGAATATAGAAGAGAATAGGTCTAACAACCATACAAATATCATTT
TCAGTGCAGAGGTGGCAGAACTGTATCGATTAGAATTGGGGGATTATAAA
TTAGTAGAAATAACTCCAATTGGCTTTGCACCCACAAATGTAAAGAGATA
TTCCTCTGTGACACCGAAAAATAAAAGAGGTGTATTTGTGCTAGGGTTTT
TGGGATTTCTTGCAATGGCAGGTTCTGCAATGGGCACAGCGTCGTTGACG
CTGTCGGCTCAGTCCCGGACTTTGTTGGCTGGGATAGTGCAGCAACAGCA
ACAGCTGTTGGATGTGGTCAAGAGACAACAGGAAATGTTGCGACTGACCG
TCTGGGGGACG
>HIV-2-07IC-TNP3
TTGTATTGTAAGATGAATTGGTTTCTGAATTGGGTAGAAGACAGAGGTGA
AAACGACACGAAAGACCAAAACATTAGAGAGCGACACAAAAGAAACTATG
TACCTTGTCACATTAGACAGGTAATTAATACATGGAATAGAGTAGGAAAG
AATGTATATTTGCCTCCAAGGGAAGGAGATCTCACTTGTAATTCCACAGT
GACAAGTCTGATAGCCAACATAGATTGGACTGATACAAATCGAACTAATA
TCACCATGAGTGCAGAGGTGGCAGAACTGTATCGATTGGAACTGGGTGAT
TACAAACTAGTAGAGATAACACCAATTGGCTTTGCTCCCACAAGTGTGAA
AAGATATTCCTCAGCAACACCGAGGAATAAAAGGGGAGTGTTTGTGCTTG
GGTTCTTGGGTTTTCTCGCTACGGCAGGTACTGCAATGGGCACGGCGTCT
TTGACGCTGTCTGCTCAGTCTCGGACTTTATTGGCTGGGATAGTGCAGCA
ACAACAACAGTTGTTGGACGTGGTCAAAAGACAACAAGAGCTGTTGCGGC
TGACCGTCTGGGGGACT
>HIV-2-96FR12034
CTCTACTGTAAGATGACATGGTTCCTCAATTGGGTAGAAAACAGGAGCAT
GGAGGGACAAACGTCAAGGGAGAGACATAAAAGGAACTATGTGCCTTGCC
ATATTAGGCAGGTAATTAACACATGGCATAAAGTAGGGAGAAATGTGTAT
TTGCCTCCAAGAGAAGGAGATCTCACCTGCAATTCTACAGTAACTAGCAT
GATTGCAAATATTGATCGGATTAACAAAAATCAAACAAATATCTCCATGA
GCGCAGAAGTGGCAGAACTGTATAGATTAGAGTTGGGGGATTATAAATTG
GTAGAAATAAGACCAATTGGCTTTGCTCCCACACCAGTGAAAAGATATTC
CTCAGTGACACCGAGGAATAAAAGGGGAGTATTTGTGCTTGGGTTCTTGG
GTTTTCTCGCCACGGCAGGTTCTGCAATGGGTGCGGCGTCGTTGACGCTG
TCAGCTCAGTCCCGGACTTTACTGGCTGGGATAGTGCAGCAACAGCAACA
GCTGTTGGACGTGGTCAAAAGACAACAAGAAATGTTGCGGCTGACCGTCT
GGGGAACT
>SIVsmm-CI2
CTCTACTGCAAGATGACATGGTTCCTCAATTGGGTAGAGGAAAGGAACAT
GACAGAACAAAACCCGGAGGAAAAAAAGAGACATAATTATGCACCCTGTC
ATATTAGACAAATAATAAATACTTGGCATAAAGTAGGTAAAAACGTATAT
TTGCCTCCCAGAGAAGGTGATCTGACATGCAATTCAACAGTAACCAGTCT
TATAGCCAATATCAACTGGAATGACGGAAATGGCACAAATATCACCTTTA

GTGCCGAGGTGGCAGAGTTGTACAGATTGGAGTTGGGGGACTACAAATTG
GTAGAAATTACACCAATTGGCTTTGCACCCACAAATGTGAAAAGATACTC
CTCGGCAACGCCTGCCAGGAATAAAAGAGGTGTATTTGTGCTAGGGTTCT
TGGGATTTCTTGCGACGGCAGGTTCTGCAATGGGCGCAGCGTCCTTGACG
CTGTCTGCTCAGTCTCGGACTTTATTGGCTGGGATAGTGCAGCAACAGCA
ACAGCTGTTGGACGTGGTCACGAGACAACAAGAACTGTTGCGACTGGCCG
TCTGGGGAACT
>SIVsmm-FTq
CTCTATTGTAAAATGACTTGGTTCCTCAATTGGGTTGAAGACAGAAACAT
GTCTTCACAAAACAAGGCAGAACAAAAAAGACGTAACTATGTGCCTTGCC
ACATTCGGCAGATAATTAACACTTGGCACAAGGTAGGGAAAAATGTATAT
TTGCCTCCTAGGGAAGGAGAACTGACATGTAATTCAACAGTAACCAGTCT
TATTGCTAACATATATTGGATTGATGGAAACAGAACAAATATCACCTTCA
GTGCAGAGGTGGCAGAACTGTACAGATTGGAGTTGGGGGATTACAAATTA
GTAGAAATTACTCCAATTGGCTTTGCACCTACCGATGTAAAAAGATATTC
CTCTGTAACGACACCGAGGAATAAAAGAGGTGTGTTCGTGCTTGGGTTCT
TGGGATTTCTCGCAACGGCAGGTTCTGCAATGGGCGCAGCGTCGTTGACG
CTGTCCGCTCAGTCCCGGACTTTGTTGGCTGGGATAGTGCAGCAACAGCA
ACAGCTGTTGGACGTGGTCAAAAGACAACAAGAACTGTTGCGGCTGACCG
TCTGGGGGACT
>SIVsmm-SL92B
CTTTACTGTAAAATGAATTGGTTCCTCAATTGGGTAGAGGATAGGAATAC
GAGCAGCCCAAGGTGGACAACTCAAACCAAAAAGGAACAGCACAAAAGAA
ATTATGTACCTTGCCACATCAGGCAAATAATTAATACGTGGCACAGGGTA
GGAAAAAATGTCTATTTGCCTCCAAGGGAAGGAGACTTAACTTGTAACTC
CACAGTGACCAGCCTAATAGCAAATATTGATTGGATTGACAACAATGAGA
CCAATATTACCATGAGTGCAGAAGTGGCAGAATTGTATCGATTGGAATTG
GGGGATTATAAATTGGTAGAGATCACTCCAATTGGCATGGCTCCCACACA
TGTGAAAAGGTACACCACAAGTACCTCAAAGAATAAAAGAGGAGTCTTTG
TGCTAGGGTTCTTGGGTTTTCTCGCGACGGCAGGTTCTGCAATGGGCGCA
GCGTCGCTAACGCTGACTGCTCAGTCCCGGACTTTATTGGCTGGGATAGT
GCAACAACAGCAACAGCTGTTGGACGTGGTCAAGAGACAACAAGAATTGT
TGCGACTGACCGTCTGGGGAACT
>SIVsmm-E660-FL8
TTATACTGCAAAATGAATTGGTTTCTTAATTGGGTAGAGGACAGAGACCA
AAAGAGTAGCAGATGGAGACAACAAAATACGAGAGAGCGACAGAAGAAAA
ATTATGTGCCATGTCATATTAGACAAATAATCAACACGTGGCACAAAGTA
GGCAAAAATGTATATTTGCCTCCTAGGGAAGGAGACCTGACATGTAATTC
CACTGTAACTAGTCTCATAGCAGAGATAGATTGGACCAATAGCAATGAGA
CCAATATCACCATGAGTGCAGAGGTGGCAGAACTGTATCGATTGGAGTTG
GGAGATTACAAATTAGTAGAGATTACTCCAATTGGCTTGGCCCCCACAAG
TGTAAGAAGGTACACCACAACTGGTGCCTCAAGAAATAAGAGAGGGGTCT
TTGTGCTAGGGTTCTTGGGTTTTCTCGCGACAGCAGGTTCTGCAATGGGC
GCGGCGTCGCTGACGCTGTCGGCTCAGTCCCGGACTTTGTTGGCTGGGAT
AGTGCAGCAACAGCAACAGCTGTTGGATGTGGTCAAGAGACAACAAGAAT
TGTTGCGACTGACCGTCTGGGGAACT
>SIVmac-239
CTCTACTGTAAAATGAATTGGTTTCTAAATTGGGTAGAAGATAGGAATAC
AGCTAACCAGAAGCCAAAGGAACAGCATAAAAGGAATTACGTGCCATGTC
ATATTAGACAAATAATCAACACTTGGCATAAAGTAGGCAAAAATGTTTAT
TTGCCTCCAAGAGAGGGAGACCTCACGTGTAACTCCACAGTGACCAGTCT
CATAGCAAACATAGATTGGATTGATGGAAACCAAACTAATATCACCATGA
GTGCAGAGGTGGCAGAACTGTATCGATTGGAATTGGGAGATTATAAATTA
GTAGAGATCACTCCAATTGGCTTGGCCCCCACAGATGTGAAGAGGTACAC
TACTGGTGGCACCTCAAGAAATAAAAGAGGGGTCTTTGTGCTAGGGTTCT
TGGGTTTTCTCGCAACGGCAGGTTCTGCAATGGGCGCGGCGTCGTTGACG
CTGACCGCTCAGTCCCGAACTTTATTGGCTGGGATAGTGCAGCAACAGCA
ACAGCTGTTGGACGTGGTCAAGAGACAACAAGAATTGTTGCGACTGACCG
TCTGGGGAACA
>SIVmne-027

CTCTACTGTAAAATGAATTGGTTTCTAAATTGGGTAGAAGATAAGAATCT
GACTGGAACTACCCAGAAGCCACAGGAACAGCATAAAAGGAATTACGTGC
CATGTCATATTAGACAAATAATCAACACTTGGCATAAAGTAGGCAAAAAT
GTTTATTTGCCTCCAAGAGAGGGAGACCTCACGTGTAACTCCACAGTAAC
CAGTCTCATAGCAAACATAGATTGGATTGATGGAAACCAAACTAATATCA
CCATGAGTGCAGAGGTGGCAGAACTGTATCGATTGGAATTGGGAGATTAT
AAATTAGTAGAGATCACTCCAATTGGCTTGGCCCCCACAAATGTGAAGAG
GTACACTACTGGTGGCACCCCAAGAAATAAAAGAGGGGTCTTTGTGCTAG
GGTTCTTAGGTTTTCTCGCAACGGCAGGTTCTGCAATGGGCGCGGCGTCG
TTGACGCTGACCGCTCAGTCCCGGACTTTATTGGCTGGGATAGTGCAGCA
ACAGCAACAGCTGTTGGACGTGGTCAAGAGACAACAAGAATTGTTGCGAC
TGACCGTCTGGGGAACA
>SIVsmm-SL92A
CTCTATTGTAAAATGAATTGGTTTCTGAATTGGGTAGAAGACAGAAATTT
GACACTCCCAAGATGGAAAAACCAAAAGCCAGAGGAAAAAGCTAAAAGGA
ATTATGTACCTTGTCACATCAGACAAGTGATTAATACTTGGCATAGAGTA
GGCAAAAATGTTTATTTGCCTCCAAGAGAAGGAGACCTGCAATGTAACTC
CACGGTAACAAGTTTAATAGCAGATATAGATTGGATTAATGAAAATGAGA
CCAATATCACCATGAGTGCAGAAGTGGCAGAACTGTATCGATTGGAATTG
GGAGATTACAAATTGGTAGAAATCACTCCAATTGGTTTGGCCCCTACAAA
TGTGAAAAGGTACACCACCAGTACCTCAAGAAATAAGAGAGGGGTCTTTG
TGCTGGGGTTCTTGGGTTTTCTTGCGACGGCAGGTTCTGCAATGGGCGCA
GCGTCGTTGACGCTGACTGCTCAGTCCCGGACTTTATTGGCTGGGATAGT
GCAGCAACAGCGACAGCTGTTGGACGTGGTCAAGAGACAACAAGAACTGT
TGCGACTGACCGTCTGGGGAACT
>SIVsmm-CFU212
CTCTATTGTAAAATGAATTGGTTCCTAAATTGGGTAGAAGATAGAAACAG
AACGCAGATGGAGGCGAGAAACAACAACGATAAAGAGAGGAGAAAGAGGA
ACTATGTGCCATGTCACATTAGACAGGTGATCAATACATGGCACAGAGTA
GGTAAAAATGTATATTTGCCGCCTAGAGAAGGAGACCTGACATGTAACTC
CACTGTGACCAGTCTAATAGCAGAAATAGACTGGTATGATAAAAACCAAA
CTAATATCACCATGAGTGCAGAGGTGGCAGAACTGTATCGATTGGAATTG
GGGGATTACAAATTAGTAGAAATCACTCCAATTGGCATAGCCCCAACAAA
TGTAAAGAGGTACACCACTACTACTACCTCAAGAGCTAAAAGAGGGGTCT
TTGTGCTAGGGTTCTTGGGATTTCTCGCAACGGCAGGTTCTGCAATGGGC
GCGGCGTCGTTGACGCTGACTGCTCAGTCTCGGACTTTACTGGCTGGGAT
AGTGCAGCAACAGCAACAGCTGTTGGACGTGGTCAAGAGACAACAAGAAT
TGTTGCGACTGACCGTCTGGGGAACT
>SIVasc-RT08
TTTTATTGTAATTTATCTAGGATCTTTCAGAAAACCAATCTTACAAATAA
CACCTTTTACCCACAAAACATAACACAGGAAGGGGAAGCGTTGAGCAACA
TATGGTTCACATGTACCATCAGGCAAGTAGTGAACAGATGGTCACATGTA
GAAAAGTTGATGTACCTACCACCGAGAAGGGGACATGTTCAGTGTACATC
CAACATCACAGGAATCTTGGTGGAGTCAGAATACTATCCAGGAAGCCCAT
TTAACATGACCCCATCAGCCAATATCAAGGACCTGTGGAAAATAGATTTA
AGAAGATATAAGGTGGTGGAAATAGACCCAATTGGCCTAGCACCCACTCC
ACTAAAAAGGTATGAACCACCACCAAGCAAAAGCATTGTCAAAAGAGCAG
CCGCATTGACCCTGGGATTCTTGGGATTTCTCAGTACGGCAGGGGCGACA
ATGGGCTCGGTAGCGACCGCCTTGACAGTCCAGTCGCGTTCCCTCTTAGA
AGGCATAGTAGCCCAGCAAAAGGCGCTACTTGAGGCAGTTGAGCACCAAC
AACAGCTTCTACAGCTGTCTGTGTGGGGGATC
>SIVasc-RT11
TTTTATTGTAACTTATCTAGGATCTTTCAGCAAACCAATTTTACCAATGA
CACCTTCTATCCAAGAAACATAACAGAGGAAGGGGAAAGGTTGAGCAGAA
CATGGTTCACATGTACCATCAGGCAAGTAGTGAATAGGTGGTCACATGTA
GAAAAGTTGATGTACTTACCACCAAGAAGGGGACATGTTCAGTGTACATC
TAATATCACAGGAGTCTTGGTGGAATCAGAATACTATCCAGGGAGTCCAT
TTAATATGACCCCATCAGCCAACATCAAGGACCTGTGGAAAATAGATTTA
AGAAGATATAAGGTGGTGGAAATAGATCCAATTGGCTTAGCACCCACTCC
AATTAAAAGGTATGAACCACCATCAAACACCAAAAGTGTTGTCAAAAGAG

CAGCCGCATTGACCCTGGGATTCTTGGGATTTCTCAGTACGGCAGGGGCG
ACAATGGGCTCGGTGGCAACCGCCTTGACAGTCCAGTCGCGTTCCCTCTT
AGAAGGCATAGTAGCCCAGCAAAAGGCGCTACTTGAGGCAGTTGAGCACC
AACAACAGCTTCTACAGCTGTCTGTGTGGGGGATC
>SIVasc-RT03
TTTTACTGTAATTTATCTAGGATCTTTCAGAAAACCAATTTCACAAATGA
TACCTTCTATCCACGAAACATAACAAAGGAAGGGGCAGAGTTGAGCAACA
TATGGTTCACATGTACCATCAGGCAAGTAGTGAACAGATGGTCACATGTA
GAAAAGTTGATGTACTTACCACCAAGAAGGGGACATGTTCAGTGTACATC
TAATGTCACAGGAATCTTGGTGGAATCTGAATACTATCCAGGAAGTCCAT
TTAACATGACCCCATCAGCCAATATCAAGGACTTGTGGAAAATAGATTTA
AGAAGATATAAGGTGGTGGAAATAGATCCAATTGGCCTAGCACCCACTCC
AATAAAAAGATATGAACCACCACCAAGCAAAAGCGTTGTCAAAAGAGCAG
CCGCATTGACTCTGGGATTCTTGGGATTTCTCAGTACGGCAGGGGCGACA
ATGGGCTCGGTGGCAACCGCCTTGACAGTCCAGTCGCGTTCCCTCTTAGA
AGGCATAGTAGCCCAGCAAAAGGCGCTACTTGAGGCAGTTGAGCACCAAC
AACAGCTTCTACAGCTGTCTGTGTGGGGGATC
>SIVmon-99CMCML1
TTTTATTGTAATGTCTCAGCTTTATTCATTAATAGAAGAACCAACAAAAC
GGACGGGATATCTCCATTCGACGTGAATAACAAGCCCAACACCACGTATC
ATGGTGGGTGGCTAGCATGTACCATAAGACAAATGGTGACACAATGGGGA
TATGTGTCAAAGAGTATCTACCTACCTCCACGGAAAGGCCATGTGCAGTG
TACATCTAATATCACTGCCCTCCTGATTACAGGGGAGTTGTATCAAAACA
ACGTGACCCTTGTTCCCTCGGCACAGGTGAGTGACTCATGGAGATCAGAG
CTCAGTCGATATAAGGTGGTGGAAATTGACCCCTTATCTATGGCACCTAC
AACAGCGCAGAGAAGGACTGGGGTACACAGGGAAAAGAGAGCAATTACTT
TGGGTATGGCCTTTCTGGGCTTCCTCAGCACGGCAGGAGGCACAATGGGC
GCAGCGGCGACAGCCCTGACGGTACAGTCTCGGTCATTACTGGCTGGGAT
AGTGCAGCAGCAGGAGAACCTGCTGAGGGCCGTAACAGCCCAACAGAGCT
TACTACAGCTTACTGTTTGGGGAGTC
>SIVmus-01CM1239
TTTTACTGCAATCTATCAGTGCTGTTTCAATTTAACAACAATATCACACA
AATTAACGAGAGCAATATACATAATGTGACAACCAAATACCACCAAGATC
AATGGATGGTCTGTAGGATTAGACAGTTTGTCACGCAATGGGGCTATGTG
TCAAAATCCATCTACTTGCCTCCACGACGGGGACATGTGCAGTGTACTTC
TAATGTGACTGGGCTCCTTATTGATGGAGCTATGTATAAAGACACAGTCA
ACATGACTCCATCCGCGAATGTGATGGACGCCTGGCGAATGGAACTAAAA
AATTACAAGGTAGTGGAAATAGACCCGCTTTCCATGGCTCCCACAGAGGT
GCAGAGGAGGCCTGTTCCCCATGTAAAAGCAAGAGCAATATCTCTAGGCA
TAACCTTTCTGGGATTTCTCAGCGCGGCAGGAGGCACGATGGGCGCAGCG
GCGACAGCCCTGACGGTACAGTCTCGGTCATTACTGGCCGGGATAGTGCA
GCAGCAGGCAAATCTGCTGAGGGCCGTAGAAGCCCAGCAACATCTGTTAC
AGTTGTCTGTCTGGGGTATA
>SIVmus-01CM1246
TTCTACTGCAACTTGTCAGTATTATTCCAATTTGAGAATAACACCAGAGA
AATTAATGACTCAAATATACATACAGTAAAAACCAAATATCGCCATGACC
AATGGATGGTCTGCAGAATAAGGCAGTTTGTAACCCAGTGGGGTTACGTA
TCAAAGTCCATATACCTGCCCCCTCGAAAAGGTCATGTTCAATGTACATC
CCATGTAACAGGGCTATTAATAGATGGGGCCATGTACGGCAATACAATAA
ACATGACCCCCTCAGCCAATGTCTGGACGCGTGCGTCTAGACTGAGAGAT
ACAGTGGTAGAGATAGACCCTCTTTCCGTGGCACCGACAGAAGTGCGCAG
GAGACCTGTGCCCCACGTCAAGGCTAGGGCAATCAGCTTGGGAATAACCT
TCTTAGGATTCCTCAGTGCTGCGGGAGGCACAATGGGCGCAGCGGCGACA
GCCCTGACGGTACAGTCTCGGTCTTTGTTGGCTGGGATAGTGCAGCAACA
GGCTAATCTGTTGAGAGCCGTAGAAGCCCAACAACATCTACTACAGTTGT
CTGTTTGGGGCATA
>SIVmus-09GabOI81
TTCTATTGTAATCTCTCAGCCCTTTTCCAATTTGAGAATAATGTAACAGA
AATTAATTCAGGCAATATTGATAATGTGACGTCAAAGTATAAAGAAAATC
AATGGATGACTTGCAAAATAAAGCAATTTGTCACCCAATGGGGCTTTACC

TCTAAGTCAATATATCTCCCACCAAAACGAGGGCATATTCAATGCACCTC
CAATATTACTGGTTTATTGATAGAAGGGGCAATGTATCGCGACAGTATCA
ATATGACCCCCTCAGCAAATGTAATGGATGCTTGGAGAGCAGAATTGAGT
AGATACAAAGTGGTGGAGATAGACCCGCTCTCCGTGGCCCCAACGAGAGT
TCCAAGGCGAGAGGCGCCTAGACTGCATACCCGTGCGATATCACTGGGGA
TAACGTTTCTCACGTTTCTCTCAGCTGCTGGAGGCACGATGGGCTCAGCG
GCGATAGCCCTGACGGTACAGTCTCGGTCATTATTGGCTGGGATAGTGCA
GCAGCAGCAAAACCTGTTGCGAGCAGTACAGACGCAGCAACACCTGCTGC
AGTTATCTGTATGGGGTATT
>SIVgsm-99CM166
TTCTATTGCAATTTGTCAATACTCTTTCAACTTAATAATAACACCATTAA
CAGCAGCAATATTGGGAACATCACAAGCAAATACAAAGGACAATGGCTGG
CTTGCAAAATTAGGCAATTTGTCACCCAATGGGGATATGTGTCTAAATCA
ATCTACTTACCTCCACGGCAGGGTCATATAAATTGCACATCCAACATTAC
AGGTCTGTTGATAGATGGCGCTATGTATGAAAGCTCTATCAACATGACCC
CGTCGGCCGATGTGGCTGATGCGTGGAACTATGAATTGTCCAGGTACAAA
GTGGTGGAGATAGATCCGCTCTCGATGGCCCCAACTCCGGCTAAAAGGAA
AGAACACCCCGCTGTGGAGAAAAGAGCCCTTTCATTGGGAATATCCTTCT
TGACGTTTCTCAGTGCGGCAGGTACCACGATGGGCGCAGCGGCAACAGCG
CTGACGGAACAGTCCCGGTCTTTGTTGGCTGGGATAATGCAGCAGCAGGA
GAACCTGCTGAGGGCTGTAGAGGCCCAACAGAGCTTATTACAGCCATCTG
TATGGGGCATA
>SIVgsn-99CM71
TTCTATTGTAATTTGTCAGTACTCTTTCAATTCAACAACAATATTACGGC
AGTGGATGACACAAACATTAACAATGTCACAAGCAAATACAAAGGACAAT
GGATGGCTTGCAGAATTAGGCAATTTGTCACACAATGGGGATATGTGTCT
AGGTCGATCTACTTACCTCCACGGCAAGGTCATATAAATTGTACATCTAA
CATCACTGGCCTCCTAATAGACGGAGCGATGTATGGACATTCCATCAATA
TGACCCCATCGGCCGATGTAGCCGATGCATGGAAGTATGAGTTATCCAGG
TACAAGGTGGTGGAGATAGATCCACTTTCGCTGGCCCCAACCTCGGCTCA
AAGGAGACCACATCCAGGGGTGCATGTGAAAAAGAGGGCCATTTCATTGG
GCATATCCTTCTTGACGTTTCTCAGTGCGGCAGGTGCCACGATGGGCGCA
GCGGCAACAGCGCTGACGGTACAATCCCGGTCATTGTTGGCTGGGATAGT
GCAGCAGCAGGAGAACCTGCTGAGGGCTGTAGAGGCCCAACAGAGCTTAT
TACAGCTATCTGTATGGGGCATA
