# Supplementary material for: Extending the Coding Potential of Viral Genomes with Overlapping Antisense ORFs: A Case for the De Novo Creation of the Gene Encoding the Antisense Protein ASP of HIV-1
Source: Viruses. 2022 Jan 14;14(1):146. doi: 10.3390/v14010146 (PMC8781085; doi:10.3390/v14010146)
Supplement: Supplementary file 1 [file viruses-14-00146-s001.zip › Supplementary Table S1.pdf]

Position	Hydrophobic		Polar		Basic		Acidic	
	% variation	Amino acid	% variation	Amino acid	% variation	Amino acid	% variation	Amino acid
7	-39.2	Leu, Phe	39.2	Cys				
9	12.0	Ile	-5.6		-6.3			
12			40.6	Cys				
54	-9.2	Met			9.7	Lys		
55	11.1	Ile	18.0	Asn	-26.6	Lys		
56	14.3	Ile	-13.1	Thr				
59	16.9	Ile	-16.6	Ser				
61	-7.4	Ile	7.1	Thr				
67	-10.3	Pro	10.3	Cys				
70	5.7	Leu	-5.2					
78	18.4	Ile	-15.5	Thr				
80	-6.8	Ile	9.5	Asn				
82	17.9	Leu	-12.6	Ser				
84	-17.2	Phe	17.4	Ser				
100	10.1	Phe	-10.1	Ser				
106			9.7	Asn	-9.0	Lys		
110	5.6							
114	9.2	Leu						
118	-13.6	Leu			14.8	Arg		
121	-10.1	Ile	10.8	Ser				
122	7.2	Ile						
128			-17.1	Ser			13.2	Glu
129			6.3	Gln	-6.1	His		
130	8.5	Val, Leu						
132	7.1	Phe	-6.7	Thr, Ser				
134	16.4	Ala, Leu	-10.0	Gly				
142	14.2	Phe	-20.8	Cys	8.8	Arg		
144	-7.9		7.9	Thr				
145	-20.8	Leu	21.3	Ser				
150	7.6	Ile	-7.5	Thr				
157	-8.3	Trp	13.6	Gly				
158			31.0	Ser	-32.6	Arg		
162			-7.6	Ser			5.3	Glu
166	-5.7							
167	-7.5							
168	-6.7							
169	-7.7							
171	-5.1							
173	-5.4							
175	-8.9		7.1					
177			5.3					
178	7.3		-6.3					
184			17.3	Ser	-15.8	Arg		
185	-9.9	Pro	9.2	Cys				
186	16.7	Val					-16.5	Asp
187	-5.0	Val					5.2	Glu

Supplementary Table S1. Evolution of the intact and interrupted *asp* ORFs. We assembled a set of 4362 sequences containing an intact *asp* ORF able to express a protein >150 aa, and a set of 2260 control sequences where the *asp* ORF is interrupted by internal *stops*. The *asp* ORFs in both datasets were translated into proteins, and then we compared the amino acid content in accordance with four functional classes: hydrophobic (Ala, Ile, Leu, Met, Phe, Pro, Trp, Val), polar (Asn, Cys, Gln, Gly, Ser, Thr, Tyr), basic (Arg, His, Lys), and acidic (Asp, Glu) using the contingency-table χ^2 test. We selected only the amino acid positions where functional amino acids showed an enrichment higher than 5% or depletion lower than –5%. At positions with significant enrichment/depletion of a given class of amino acids, we used again the χ^2 test to identify the specific amino acid(s) of that class with enrichment above 5% or depletion below –5% in ASP encoded by intact ORFs. The table reports the variation (enrichment >5% and depletion <–5%) of the four classes of amino acids, and the specific differentially represented amino acid at each position within ASP encoded by intact ORFs compared to interrupted ORFs.
