# Supplementary material for: Extending the Coding Potential of Viral Genomes with Overlapping Antisense ORFs: A Case for the De Novo Creation of the Gene Encoding the Antisense Protein ASP of HIV-1
Source: Viruses. 2022 Jan 14;14(1):146. doi: 10.3390/v14010146 (PMC8781085; doi:10.3390/v14010146)
Supplement: Supplementary file 1 [file viruses-14-00146-s001.zip › Supplementary Table S2.pdf]

Virus species (group-clade-isolate)	NCBI acc. num.	Genome coordinates[#]	Length of ASP ORF (bp)	Codons from ATG to nearest *stop*	Internal *stop* co-dons	Canonical ATG and *stop*?[¤]
HIV-1 (M-B-HXB2)	K03455	7371-7943	570	190	0	YY
HIV-1 (M-B-VH)	AF146728	6723-7295	570	114	1	YY
HIV-1 (M-B-BK132)	AY173951	6709-7272	561	>187§	0	YN
HIV-1 (M-B-1058-11)	AY331295	6557-7132	573	191	0	YY
HIV-1 (M-B-671-00T36)	AY423387	6938-7504	564	188	0	YY
HIV-1 (M-D-94UG114)	U88824	6692-7261	567	189	0	YY
HIV-1 (M-D-A08483M1.vrc9)	HM215358	1162-1728¶	564	110	1	YY
HIV-1 (M-A-98UG57136)	AF484509	6567-7133	564	163	0†	YY
HIV-1 (M-A-92UG037)	U51190	6750-7304	552	159	0†	YY
HIV-1 (M-A-SE7253)	AF069670	6572-7138	564	163	0†	YY
HIV-1 (M-A-97CDKTB48)	AF286238	6693-7253	558	141	1†	YY
HIV-1 (M-A-94CY017.41)	AF286237	6746-7315	567	45	2	YY
HIV-1 (M-G-DRCBL)	AF084936	7331-7882	549	183	0	YY
HIV-1 (M-G-PBS1191)	MH705134	7421-7966	543	181	0	YY
HIV-1 (M-H-90CR056)	AF005496	6696-7262	564	108	2	NY
HIV-1 (M-C-03ZAPS021MB1)	DQ369978	6775-7323	546	182	0	YY
HIV-1 (M-C-503-06310-30)	KT183169	6638-7204	564	171	0@	YY
HIV-1 (M-C-B002865-A11-12)	KC154013	1144-1692¶	546	182	0	YY
HIV-1 (M-C-04ZASK146B1)	AY772699	6750-7313	561	187	0	YY
HIV-1 (M-C-21068)	AF067155	6733-7314	579	193	0	YY
HIV-1 (M-C-92BR025)	U52953	6717-7280	561	187	0	YY
HIV-1 (M-C-C2220)	U46016	6768-7319	549	183	0	YY
HIV-1 (M-C-DEMC08MW004)	KP109522	6698-7264	564	188	0	YY
HIV-1 (M-C-05ZASK247B1)	DQ369994	6823-7368	543	181	0	YY
HIV-1 (M-C-C.ZA.x.Du246-B3)	KC863229	1150-1707¶	555	185	0	YY
HIV-1 (M-C-00BW-1921.13)	AF443101	6713-7273	558	186	0	YY
HIV-1 (M-C-PWJ-0513.39)	HM215433	1159-1716¶	555	185	0	YY
HIV-1 (M-C-704MC024F)	GU080186	1141-1725¶	582	195	0	YY
HIV-1 (M-C-NIRT005)	KX069223	6497-7063	564	188	0	YY
HIV-1 (M-C-C113-E6-2S)	HM639260	1183-1743¶	558	136	1	YY
HIV-1 (M-C-CNE88.U)	HQ699992	1195-1779¶	582	194	0	YY
HIV-1 (M-C-097SO)	MF373199	6588-7142	552	184	0	YY
HIV-1 (M-C-DEMC11ZM006)	KF716467	6738-7313	573	191	0	YY
HIV-1 (M-K-97ZR-EQTB11)	AJ249235	6584-7126	540	180	0	YY
HIV-1 (M-J-97DC.KTB147)	EF614151	6586-7146	558	186	0	YY
HIV-1 (M-CRF01-AG-91DJ_263)	JQ715398	1162-1749¶	585	67	3	YY
HIV-1 (M-CRF01-AG-CAM1970 LE.EC6)	JQ715394	1105-1671¶	564	155	0†‡	YY
HIV-1 (M-F-CM53657)	AF377956	6557-7114	555	132	1	YY
HIV-1 (M-CRF01-AE-JS2011001)	KM111555	6594-7157	561	176	0‡	YY
HIV-1 (M-CRF01-AE-620435_c01)	JX512900	1141-1710¶	567	189	0	YY
SIVcpz (LB715)	KP861923	7464-8108	642	148	2	YY
SIVcpz (LB7)	DQ373064	6903-7454	549	88	1	YY
SIVcpz (MB66)	DQ373063	7019-7567	546	182	0	YY
SIVcpz (MB897)	EF535994	6930-7451	519	127	3	YY
SIVcpz (GAB2)	AF382828	6785-7327	540	96	2	YY
SIVcpz (CAM13)	AY169968	6991-7527	534	42	3	YY
SIVcpz (DP943)	EF535993	6934-7506	570	145	1	NY
SIVcpz (US.85.CPZUS)	AF103818	7400-7948	546	151	2	YY
SIVcpz (EK505)	DQ373065	6893-7426	531	117	4	YY
HIV-1 (N-YBF30)	AJ006022	6952-7491	537	139	1	YY
HIV-1 (N-02CM-DJO0131)	AY532635	6834-7367	531	136	1	YY
HIV-1 (N-04CM-1015-04)	DQ017382	6827-7345	516	134	1	YN
HIV-1 (P-U14788)	HQ179987	6902-7492	588	70	7	YY
SIVgor (BPID15)	KP004990	6770-7375	603	67	9	YY
SIVgor (CP2139.287)	FJ424866	6926-7501	573	85	3	YY
SIVgor (BQID2)	KP004991	6891-7484	591	176	3	YY
HIV-1 (N-04CM-1015-04)	DQ017382	6827-7345	591	23	7	NY
HIV-1 (O-9CMA105)	AY169816	6862-7458	594	15	4	NY
HIV-1 (O-98CMU2901)	AY169812	6851-7432	579	21	4	NY
HIV-1 (O-99SE-MP1299)	AJ302646	7457-8047	588	74	3	NY
HIV-1 (O-99SE-MP1300)	AJ302647	7436-8020	582	67	4	NY
SIVcpz (BF1167)	JQ866001	7529-8074	543	19	4	YY
SIVcpz (TAN13)	JQ768416	7033-7563	528	41	4	NY

SIVcpz (TAN1)	AF447763	7018-7569	549	17	9	YY
SIVcpz (UG38)	JN91690	6962-7519	555	54	5	YY
SIVdeb (04CMPF3061)	FJ919724	7235-7777	540	27	7	NY
SIVdeb (CM5)	AY523866	7056-7604	546	32	5	NY
SIVdeb (CM40)	AY523865	7129-7683	552	27	5	NY
SIVtal (00CM266)	AY655744	7171-7773	600	116	4	YY
SIVtal (01CM8023)	AM182197	6718-7323	603	72	4	YY
SIVsyk (KE51)	AY523867	7055-7603	546	18	7	NY
SIVsyk (173/1.2)	L06042	7359-7910	549	47	6	NY
SIVlhoest (485)	AF188115	6554-7120	564	86	5	NN
SIVlhoest (524)	AF188116	6560-7126	564	86	5	NN
SIVlhoest (7)	AF075269	7632-8198	564	94	3	NN
SIVsun (λ20L14/S2)	AF131870	7700-8266	564	85	3	NN
SIVolc (12CI97)	FM165200	7031-7570	537	49	4	NN
SIVwrc (98CI04)	AM713177	7580-8119	537	29	3	NN
SIVwrc (05GM-X02)	AM937062	6476-7015	537	29	3	NN
SIVmnd1 (GB1)	M27470	6990-7550	558	29	9	NN
SIVdrl (D3)	KM378563	7134-7700	564	53	6	NN
SIVdrl (D4)	KM378564	7090-7656	564	53	7	NN
SIVmnd2 (M14)	AF328295	7471-8022	549	0	6	NN
SIVmnd2 (CM.98.CM14)	AF367411	7545-8093	546	9	8	NN
SIVmnd2 (5440)	AY159322	7172-7726	552	36	8	NN
SIVcol (BMC07)	KF214241	6832-7359	525	0	4	NY
SIVcol (CGU1)	AF301156	6814-7353	537	19	6	NY
SIVcol (BMC01)	KF214240	6640-7176	534	19	3	NY
SIVagmVer (155)	M29975	7478-8071	591	18	7	NY
SIVagmVer (VSAG1003)	KR862363	6933-7526	591	18	6	NY
SIVagmVer (VSAM0022)	KR862356	6964-7554	588	19	9	NY
SIVagmVer (VSAC4004)	KR862336	6973-7569	594	97	6	NY
SIVagmMal (ZMB)	LC114462	7467-8060	591	29	5	NY
SIVgri (677)	M58410	7360-7950	588	85	7	NY
SIVagmSab (SAB1)	U04005	7669-8244	573	6	6	NY
SIVagmTan (TAN1)	U58991	7458-8048	588	90	4	NY
SIVrcm (GAB1)	AF382829	7088-7630	540	79	3	NN
SIVagi (00CM312)	HM803690	6872-7414	540	93	4	NY
SIVrcm (02CM8081)	HM803689	6950-7492	540	61	3	NN
HIV-2 (EHO)	U27200	7845-8378	531	42	3	NY
HIV-2 (D205)	X61240	7859-8392	531	38	4	NN
HIV-2 (ALI)	AF082339	7867-8400	531	42	4	NN
HIV-2 (ST)	M31113	7313-7846	531	42	4	NY
HIV-2 (BEN)	M30502	7886-8416	528	42	4	NY
HIV-2 (Abt96)	AF208027	7217-7777	558	54	3	NN
HIV-2 (07IC-TNP3)	KC693505	7319-7885	564	50	4	NN
HIV-2 (96FR12034)	AY530889	7343-7900	555	38	4	NY
SIVsmm (CI2)	JX860430	6954-7514	558	16	4	NY
SIVsmm (FTq)	JX860414	7086-7646	558	42	3	NY
SIVsmm (SL92b)	AF334679	7237-7809	570	34	5	NY
SIVsmm (E660-FL8)	JQ864086	7795-8370	573	50	4	NN
SIVmac (239)	M33262	8057-8617	558	34	5	NY
SIVmne (027)	U79412	7272-7838	564	34	5	NY
SIVsmm (SL92A)	JX860432	6886-7458	570	34	5	NY
SIVsmm (CFU212)	JX860407	7053-7628	573	50	4	NY
SIVasc (RT08)	KJ461715	6844-7425	579	91	4	NY
SIVasc (RT11)	KJ461714	6802-7386	582	92	4	NY
SIVasc (RT03)	KJ461716	6814-7395	579	91	4	NY
SIVmon (99CMCML1)	AY340701	7331-7906	573	9	3	NY
SIVmus (01CM1239)	EF070330	7277-7846	567	33	5	NY
SIVmus (01CM1246)	EF070329	7412-7975	561	125	3	YY
SIVmus (09GabOI81)	KF304707	6906-7475	567	0	2	NY
SIVgsn (99CM166)	AF468659	7320-7880	558	94	4	YY
SIVgsn (99CM71)	AF468658	7320-7892	570	75	4	YY

Supplementary Table S2. List of viral strains utilized for the generation of the cladogram in Figure XYZ. The cladogram was generated using the portion of the *env* sequence of each viral strain comprised between the canonical *start* and *stop* codons in the *asp* ORF of HIV-1_{HXB2}.

#, coordinates comprise the minimal number of whole *env* codons spanning from the canonical *start* to the canonical *stop* of the *asp* gene. Thus, the first coordinate corresponds to the nucleotide residue located 2 bp upstream of the third base in the ASP canonical *stop* codon, and the second coordinate corresponds to the nucleotide residue located 1 bp downstream of the first base in the ASP canonical *start* codon.

¤, the presence of *start* and *stop* codons at or near (± 10 positions) the canonical position is indicated by the first and second letter, respectively. Y, yes; N, no.

§, viral strain in which the ASP ORF contains a *start* codon at the canonical position, but it lacks a *stop* codon at the canonical position. The first *stop* codon is located at position 253 from the canonical *start*, thus expressing an ASP protein of 252 residues.

†, viral strains in which the premature *stop* codon at position 12 from the canonical *start* codon is followed by a new in-frame *start* codon after the premature *stop*. The new *start* codon re-opens the ASP reading frame, essentially nullifying the premature *stop* codon. In the case of these sequences, only premature *stop* codons downstream of the new *start* codon are counted.

¶, viral strains for which complete genomic sequence is not available. Coordinates provided in the table are relative to the *env* gene.

‡, viral strain in which the ASP ORF contains an early *stop* codon in proximity of the canonical *stop*. Despite the early *stop* codon, the ASP ORF still encodes a protein of >150 aa with intact functional domains, and therefore this sequence is classified as having zero internal *stop* codons.
